# Analysis of the circadian transcriptome of the Antarctic krill *Euphausia superba*

**DOI:** 10.1038/s41598-019-50282-1

**Published:** 2019-09-25

**Authors:** Alberto Biscontin, Paolo Martini, Rodolfo Costa, Achim Kramer, Bettina Meyer, So Kawaguchi, Mathias Teschke, Cristiano De Pittà

**Affiliations:** 1 Dipartimento di Biologia, Università degli Studi di Padova, Padova, Italy; 2 Laboratory of Chronobiology, Charité Universitätsmedizin Berlin, Berlin, Germany; 3 Section Polar Biological Oceanography, Alfred Wegener Institute Helmholtz Centre for Polar and Marine Research, Bremerhaven, Germany; 4 Institute for Chemistry and Biology of the Marine Environment, Carl von Ossietzky University of Oldenburg, Oldenburg, Germany; 5 Helmholtz Institute for Functional Marine Biodiversity (HIFMB) at the University of Oldenburg, 26111 Oldenburg, Germany; 6 Department of Environment and Heritage, Australian Antarctic Division, Kingston, Tasmania Australia

**Keywords:** Gene expression, Circadian mechanisms, Marine biology

## Abstract

Antarctic krill (*Euphausia superba*) is a high latitude pelagic organism which plays a central role in the Southern Ocean ecosystem. *E*. *superba* shows daily and seasonal rhythms in physiology and behaviour, which are synchronized with the environmental cycles of its habitat. Recently, the main components of the krill circadian machinery have been identified and characterized. However, the exact mechanisms through which the endogenous timing system operates the control and regulation of the overt rhythms remains only partially understood. Here we investigate the involvement of the circadian clock in the temporal orchestration of gene expression by using a newly developed version of a krill microarray platform. The analysis of transcriptome data from krill exposed to both light-dark cycles (LD 18:6) and constant darkness (DD), has led to the identification of 1,564 putative clock-controlled genes. A remarkably large proportion of such genes, including several clock components (*clock*, *period*, *cry2*, *vrille*, and *slimb*), show oscillatory expression patterns in DD, with a periodicity shorter than 24 hours. Energy-storage pathways appear to be regulated by the endogenous clock in accordance with their ecological relevance in daily energy managing and overwintering. Our results provide the first representation of the krill circadian transcriptome under laboratory, free-running conditions.

## Introduction

Life on earth, throughout evolution and from cyanobacteria to humans, has been confronted with the necessity of synchronizing genetic, physiological and behavioural processes with the daily oscillations in environmental conditions due to the earth’s rotation around its axis^1^. Adaptation to day-night cycles requires an endogenous timing system – a circadian clock – that allows not only the synchronization with but especially the anticipation of daily recurrent environmental changes (*Zeitgebers)*. In the marine ecosystem, photoperiod^2^, light transitions^3^, lunar phases^4^, and tides^5^ represent relevant *Zeitgebers* for the entrainment of the endogenous clock of marine organisms. While the circadian rhythms of terrestrial organisms have been the object of intense studies and are currently well understood, little is known about the circadian rhythmicity in marine organisms, apart from some laboratory studies in ecological niche species^5–9^. This is particularly true for high latitude pelagic organisms, presumably due to the inaccessibility to these regions and to the problems with rearing these organisms under controlled laboratory conditions for purposes of long-term experiments.

Due to its central position in the food web^10^, the ongoing environmental changes in its habitat^11^, and increasing commercial interest^12^, the Antarctic krill *Euphausia superba* (hereafter referred as krill) has become a model organism for the study of high latitude endogenous clock machinery and its effect on daily and seasonal life-cycle functions^13–16^.

Krill shows multiple daily and seasonal rhythms in physiology and behaviour, which are synchronized with the cyclic changes of the Southern Ocean ecosystem. Among them are the diel vertical migrations (DVM), which maximize feeding and minimize predation^2^; the daily orchestration of metabolic activity, which optimizes energy usage; and seasonal lipid storage for overwintering^17–19^. However, the molecular mechanisms underlying the control and regulation of these rhythms are not fully understood.

*Cryptochrome2* (*cry2*) was the first molecular component of the circadian clock to be identified in krill^20^. Daily changes in *cry2* expression levels under natural conditions, namely during the Antarctic Summer, represented the first hint to the existence of an endogenous time-keeper in krill^20^. Afterwards, a rhythmic expression of *cry2* both in a light-dark cycle and in constant darkness conditions was reported^13^, suggesting the presence of an endogenous circadian timing system. Furthermore, the same authors observed a bimodal oscillatory pattern in oxygen consumption and in the catalytic activity of four metabolic enzymes, which appeared to mirror cyclic locomotor activity patterns^2^ (i.e. diel vertical migration). These observations suggest a link between the endogenous clock and metabolism. De Pittà *et al*.^14^ then provided the first description of the diurnal transcriptome of krill in natural conditions during the Antarctic summer, which is characterized by limited variation in light intensity and spectral composition through the 24 hours. About 600 genes showed a daily sinusoidal expression pattern, and the majority of these (60%) exhibited bimodal oscillatory profiles. The temporal orchestration of specific biological processes (translation, proteolysis, energy and metabolic process, visual transduction and stress response), under prolonged almost continuous light conditions, also suggest that in krill an endogenous oscillator controls and synchronizes the progression of biochemical and physiological events through the 24-hour cycle.

More recently, KrillDB, the most comprehensive krill transcriptome database available^21^, has allowed identification of the main genetic components of the krill circadian clock (*clock*, *cycle*, *period*, *timeless*, and *cryptochrome1*). The functional characterization of these genes revealed that krill possesses an “ancient” circadian clock exhibiting both mammalian and insect features^22^.

To investigate in further detail the involvement of the krill circadian clock in orchestrating gene expression, we analysed the circadian transcriptome of *E*. *superba* under laboratory free-running conditions (i.e. in the complete absence of environmental cues, in continuous darkness, DD) as well as under a 16:8 LD regime. The most complete microarray platform available (named Krill 2.0), consisting of a total of 57,358 probes, was used. Gene expression profiles were obtained from individuals sampled at regular intervals (every 3 hours) over a complete 24-hour LD cycle (16:8 LD cycle) as well as on the third day after switching to a DD regime. A total of 1.564 putative clock-controlled genes, showing rhythmic daily expression in both LD and DD, were identified.

This study provides the first picture of the circadian transcriptome of the Antarctic krill under laboratory free-running conditions. A better understanding of the functioning of its “ancient” circadian clock could help to shed light on the evolution of the animal circadian machinery.

## Results and Discussion

### Gene expression data analysis

Gene expression profiling was performed on specimens collected at regular intervals throughout a 24-hour cycle in light-dark (LD) conditions and in constant darkness (DD)^13^ (Supplementary Fig. 3) using the “Krill 2.0” custom platform (Agilent Technologies) based on the krill “master” transcriptome^23^. Total RNA was extracted from the head, including brain, eyestalk, and compound eyes, which play a crucial role in the modulation of neuroendocrine and behavioural circadian rhythms. Only krill heads were selected in order to reduce background noise deriving from peripheral clocks, and to compare gene expression data with similar data previously obtained from krill heads, as described in De Pittà *et al*.^14^ and Piccolin *et al*.^24^. RAIN (Rhythmicity Analysis Incorporating Nonparametric methods) software, which allows the detection of rhythms of any period and waveform^25^, was utilised to identify genes with rhythmic expression through the 24 hours in both LD and DD.

RAIN identified 8,953 and 6,561 genes (Benjamini-Hochberg adjusted *p*-value < 0.05) showing rhythmic expression patterns in LD and DD respectively (Supplementary Table 1 and Supplementary Table 2). 1,564 of these genes showed oscillatory expression signatures both in LD and DD (Supplementary Fig. 2) and could therefore be qualified as clock or clock-controlled genes (Supplementary Table 3).

Interestingly, 32% (51 out of 159 annotated) of the genes which had previously shown sinusoidal expression patterns under natural conditions^14^ during the Antarctic Summer, showed rhythmic expression profiles also in this study, in both LD and DD. Therefore, they should be considered *bona fide* clock-controlled or clock-related genes (Supplementary Table 4). The expression levels of 10 clock-controlled genes (*6-4 photolyase*, *arrestin*, *neither inactivation nor afterpotential protein C*, *casein kinase 1 epsilon*, *slimb*, *adenylyl cyclase*, *cytochrome C oxidase subunit I*, *glycogen debranching enzyme*, *glutamine synthetase*) were successfully validated by qRT-PCR, showing expression profiles which were comparable to those obtained by microarray experiments (Pearson correlation >0.60; Supplementary Fig. 3).

**Figure 1:**
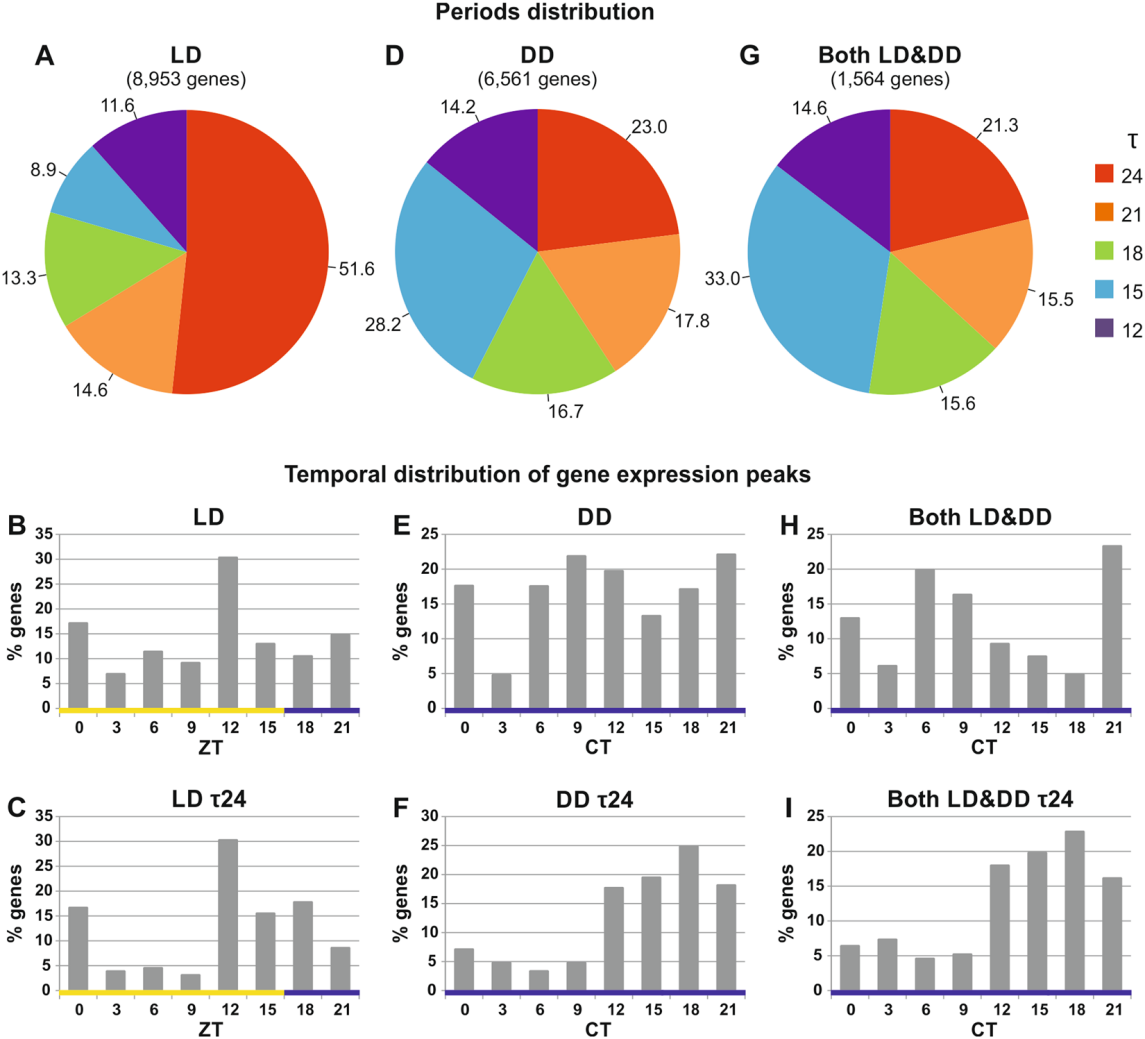
Oscillation periods and expression peaks distribution of genes with sinusoidal expression patterns in LD, DD, and in both conditions. Classification of the genes showing sinusoidal oscillatory patterns on the basis of their oscillation period (τ: 12, 15, 18, 21 and 24 h) in LD (**A**), DD (**D**), and in both LD and DD (**G**). Different colours refer to different period lengths (see legend in the figure). Percentage of genes characterized by each period length are reported on each pie chart. Distribution of expression peaks of genes showing oscillatory profiles in LD (**B**), DD (**E**), and in both LD and DD (**H**). Distribution of expression peaks of genes showing oscillatory profiles characterized by a 24-hour period in LD (**C**), DD (**F**), and in both LD and DD (**I**). Proportion of genes showing a peak of expression at each time point is reported. Yellow and blue bars refer to light and dark intervals, respectively.

In LD, about 52% (4,624 out of 8,953) of oscillating genes showed a single peak with a periodicity (τ) of about 24 hours (Fig. 1A) and the majority (63.4%) of them showed this peak late in the day, from *Zeitgeber* time (ZT) 12 to ZT18 (Fig. 1C). This broad peak of expression, encompassing the transition from late afternoon to the evening/early night, seems related to the upward migration of krill that allows to maximize food intake in the surface layers during the night, while also minimizing predator risk. Diel vertical migrations are extremely pronounced in spring and autumn under long day photoperiods, the latter being quite similar to our LD 16:8 laboratory conditions^2^. 30.3% of all oscillating genes in LD (regardless of period length) showed a peak of expression at ZT12 (Fig. 1B), in the second part of the light phase, which may be related to physiological modifications anticipating light-off transitions. The anticipation of daily environmental changes represents one of the main pieces of evidence of the presence of an endogenous time-keeping system, which tunes physiology and behaviour, leading to an improvement in the organism’s fitness.

By contrast, in DD conditions, peaks of expression were widely distributed throughout the 24-hour cycle (Fig. 1E). Moreover, a large proportion of genes (77%) showed a period of expression shorter than 24 hours, and 42.4% of them displayed a clear bimodal pattern with periods ranging between 12 and 15 hours (Fig. 1D). A reduced level of phase synchronization between transcripts oscillating in DD was observed (Fig. 1E,H). However, focusing our attention only on those genes characterized by a periodicity of 24 hours in DD (Fig. 1F), we observed a clear enrichment (80.1%) in genes with a peak of expression in the second part of the subjective day and throughout the subjective night from circadian time (CT)12 to CT21. Similar expression profiles were observed for genes oscillating with a period of 24 hours only in LD and for those oscillating with a period of 24 hours in both LD and DD (21%) (Fig. 1C,I).

### Gene expression periodicity

The harmonics theory in mammals^26^ and the interaction of different endogenous clocks in marine organisms^6^ shed light on natural occurrence of rhythms characterized by periodicities shorter than 24 hours (Fig. 1A,D,G). According to the harmonics theory, 12-hour rhythms in gene expression profile could be generated by the non-competitive binding of two circadian transcription factors oscillating in antiphase^27^. An alternative theory suggests that a 12-hour rhythm could be generated by the interaction between a circadian clock (24 hours period) and a circatidal clock (12 hours period)^28^.

Rhythmic locomotor activity with a 12-hour period is common in marine organisms from the intertidal zone and it has been also described in crustaceans apparently living beyond the influence of tidal movements^29^, including the Antarctic krill^2^. Our knowledge the krill endogenous clock suggests that 12-hour periods might be explained by both theories. However, given the lack of evidence to support the existence of a circatidal clock in krill, the harmonics hypothesis seems more likely. Concerning the other periods which we have estimated from our transcriptional data, sinusoidal expression patterns with periods ranging from 18 to 28 hours are usually considered under the control of the circadian clock. As for transcriptional oscillations with an estimated periodicity of 15 hours, we suggest that these are likely to be an approximation of a 12-hour periodicity, especially if we consider our measurement resolution, i.e. 3-hour intervals between two consecutive samples. Circadian clocks generate an endogenous molecular oscillation characterized by a specific period (free-running period, FRP). Every day, Zeitgebers (in particular light-dark cycles) entrain the phase of this oscillation to maintain the synchronization with the environment^30–32^. In order to cope with extreme photoperiods (from up to 24 hours light in summer to less than 3 hours light in winter), the strategy adopted by organisms living at high latitudes could be based on a circadian clock with a short FRP (<24 hours), which can be synchronized by photic entrainment stimuli to a wide range of periodicities^33,34^. Interestingly, short oscillatory periods, such as those we observed in LD and especially in DD (Fig. 1A,D,G), have been previously described in other krill studies performed in DD^13^ and in natural conditions, under almost continuous light, during the Antarctic summer (hereafter named LL natural condition)^14^. In accordance with the hypothesis proposed by Teschke *et al*.^13^, the occurrence of short periodicities, in particular under free-running conditions (DD and LL), could represent a feature of the krill oscillating transcriptome, and it supports the hypothesis that the krill endogenous clock might be characterized by a periodicity shorter than 24 hours. This hypothesis is not only in accordance with the inverse relationship that has been widely observed between latitude and FRP^34^ but it is also consistent with Aschoff’s second rule, which states that the free-running period of nocturnal organisms is typically shorter than 24 hours^35^.

### Functional enrichment analysis

**Figure 2:**
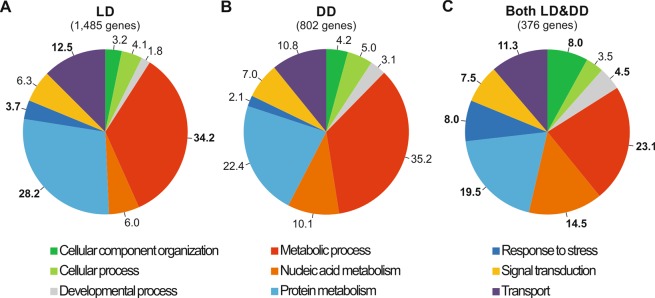
GO analysis of genes with sinusoidal expression patterns in LD, DD, and in both conditions. Classification of annotated genes with sinusoidal expression patterns throughout the 24-hour cycle in (**A)** LD (1,485 with GO annotation out of 2,743 annotated genes), (**B)** DD (802 out of 1,776 annotated genes), and **C)** in both LD and DD (376 out of 428 annotated genes) into 9 main GO terms. The diagrams show the proportion of each GO term. Percentage of genes belonging to each GO term are reported on each pie chart. Percentages of the significantly enriched GO terms are marked in bold typeface. See Supplementary Tables 4, 5, and 6 for further details.

31% (2,743 out of 8,953) and 27% (1,776 out of 6,561) of genes that showed a daily pattern of expression in LD and DD, respectively, were successfully annotated according to the krill “master” transcriptome^23^. The remaining 69% and 73% showed no or limited similarity with publicly available sequences. The functional characterization of annotated genes is shown in Fig. 2A–C. As previously observed in the fruit fly^36,37^, mouse^38^, zebrafish^39^, and krill (in nature, during the Antarctic Summer)^14^, the biological processes that are mainly influenced by environmental changes, such as “Metabolic Process”, “Protein Metabolism”, “Response to Stress”, and “Transport”, were significantly enriched in LD (Fig. 2A and Supplementary Table 5). By contrast, in DD (Fig. 2B), none of the main GO terms were significantly enriched. Nevertheless, and despite an overall decrease in the number of genes with oscillatory expression patterns in DD, we observed a significant enrichment of two GO “child” terms: “Gene Expression Regulation” (belonging to “Nucleic acid Metabolism”) and “Proteolysis” (belonging to “Protein Metabolism”) (Supplementary Table 6). It is known that transcription factors, epigenetic factors, and proteolysis pathways are circadian effectors involved in the regulation of key clock-controlled pathways such as metabolism, clock entrainment, hormone signaling, and phototransduction^40–43^. The rhythmic patterns of expression, exhibited by genes belonging to the above described GO terms in DD (mimicking Antarctic Winter conditions), could suggest a role for the molecular clock in the reprogramming of gene expression observed during the transition from the active to the quiescent state (decrease in growth rates, feeding, and swimming activity) in krill exposed to few days of DD^44^.

25.5% (399 out of 1,564) of the putative clock-controlled genes, with oscillating expression in both LD and DD, were successfully annotated (Supplementary Table 4). The key regulative role of krill endogenous timekeeping over all main biological processes of the cell was proved by the significant enrichment of almost all the main GO terms in the “Biological Process” category (Fig. 2C).

### Clock genes

**Figure 3:**
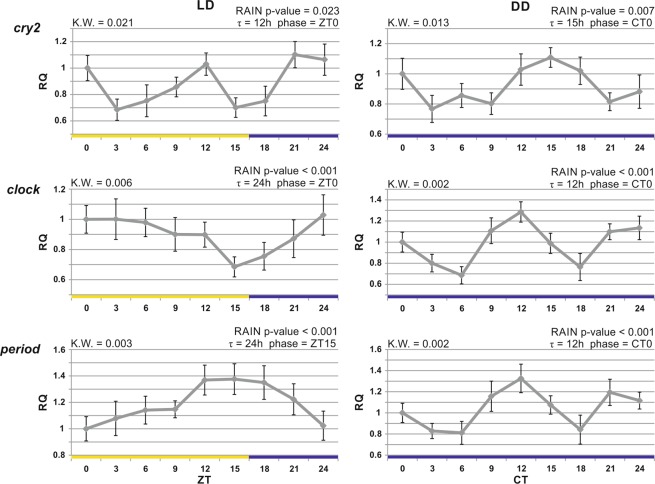
Gene expression profiles of three core components of the krill circadian clock. Temporal expression profiles of *cryptochrome 2*, *clock* and *period* by using qRT-PCR performed on heads of krill sampled every 3 hours, starting from light-on (ZT0) or subjective light-on (CT0) in LD 16:8 and after 3 days of constant darkness. Relative quantification (RQ) is represented as mean ± SD (n = 3 pools of 7 individuals). Kruskal-Wallis p-value is reported (8 degrees of freedom), as well as the adjusted p-value, putative period (τ) and phase of the oscillation estimated using the RAIN algorithm. Yellow and blue bars refer to light and dark intervals, respectively.

When we designed the microarray platform (Krill 2.0) adopted in this study, limited information was available on the molecular components of the krill circadian clock. Only the sequence of *cryptochrome 2* (c*ry2*)^20^ and a few fragments of *clock* (*clk*), *period* (*per*), *casein kinase ε* (*ckε* also known as *doubletime*), and *slimb* were available. Nevertheless, good quality probes were successfully designed for *ckε* and *slimb*, which were found among the 1,564 putative clock-controlled genes that showed oscillating expression profiles in both LD and DD (Fig. 3). By contrast, microarray probes for *clk*, *per*, and c*ry2* were not successfully designed. Considering the importance of these clock genes in interpreting the data obtained from the krill’s circadian transcriptome, we decided to quantify the expression levels of such genes by using qRT-PCR, in the same samples which were analysed with microarrays. *cry2* showed a bimodal oscillation in expression (Fig. 3), as previously observed in heads of animals sampled in LL natural conditions^22^ and in DD^13^. Similarly, *clk* and *per* showed sinusoidal expression profiles in both LD and DD, with a clear antiphase relationship in LD. By contrast, a period length reduction (from 24 to 12 hours) was observed for both genes in DD (Fig. 3). The functioning of endogenous clocks is based on a feedback loop mechanism in which the positive elements induce the expression of their own inhibitors, generating a self-sustained oscillation in their abundance. In light of this, the presence of a working molecular clock was confirmed by the rhythmic expression of a positive element (*clk*) and the two main inhibitors (*per* and *cry2*), in both LD and DD. Furthermore, the antiphase expression of *clk* and *per* is a prevalent feature of the circadian clock of several organisms and it has been observed in krill exposed to early autumn and late winter photoperiods in controlled laboratory conditions (LD 16:8 and LD 8:16, respectively)^24^. Nevertheless, our expression data showed an apparently complete loss of *clk*-*per* antiphase in DD, similarly to what has been observed in heads of krill sampled in LL natural conditions^22^ and from krill exposed to long-term extreme photoperiod simulations (LL and LD 3:21 respectively)^24^. Interestingly, the loss of *clk*-*per* antiphase we observed did not cause a significant impairment in the circadian oscillator, as suggested by the maintenance of sinusoidal expression profiles of clock and clock-controlled genes in our DD experiment, in LL natural condition^22^, and in the simulated seasonal photoperiods^24^. Unexpectedly, non-canonical expression of clock genes has already been associated with persisting circadian behaviours in other crustaceans. In *E*. *pulchra*, only *timeless-1* showed a significant rhythmic oscillation in DD^5^, and in crayfish, PER and CLK protein amounts are characterized by the same phase in LD^45^. The persistence of circadian rhythms in the presence of an apparently impaired transcriptional/translational feedback loops mechanism could be explained by a relevant role of post-translational modifications in regulating those timing mechanisms that are essential for clock functioning and seem to be conserved in krill (i.e.: the multiple predicted CK*ε* phosphorylation sites in *Es*PER and *Es*TIM1 likely involved in their activity regulation)^22^.

The short period length of the expression profiles of *clock*, *period*, and *cry2* in DD might therefore reflect a molecular clock period shorter than 24 hours. Interestingly, *ckε* and *slimb*, which also showed short periodicity in both LD and DD (*ckε* τ_LD_ = 12 h, τ_DD_ = 12 h; *slimb* τ_LD_ = 12 h, τ_DD_ = 12 h; Supplementary Fig. 3), encode two regulatory factors of the circadian clock which are involved in PER degradation^46,47^, and thus in period length definition^48^.

Castellana *et al*. observed that genes oscillating with a periodicity of about 12 hours are more likely to be evolutionary ancient and essential for cellular functions, suggesting that their short periodicity may reflect the periodicity of ancient clocks^49^. Short periodicities in the expression profile of several clock and clock-controlled genes in DD might suggest that not only the architecture but also the features of ancient endogenous clocks have been conserved in krill throughout speciation.

### Metabolic processes

**Figure 4:**
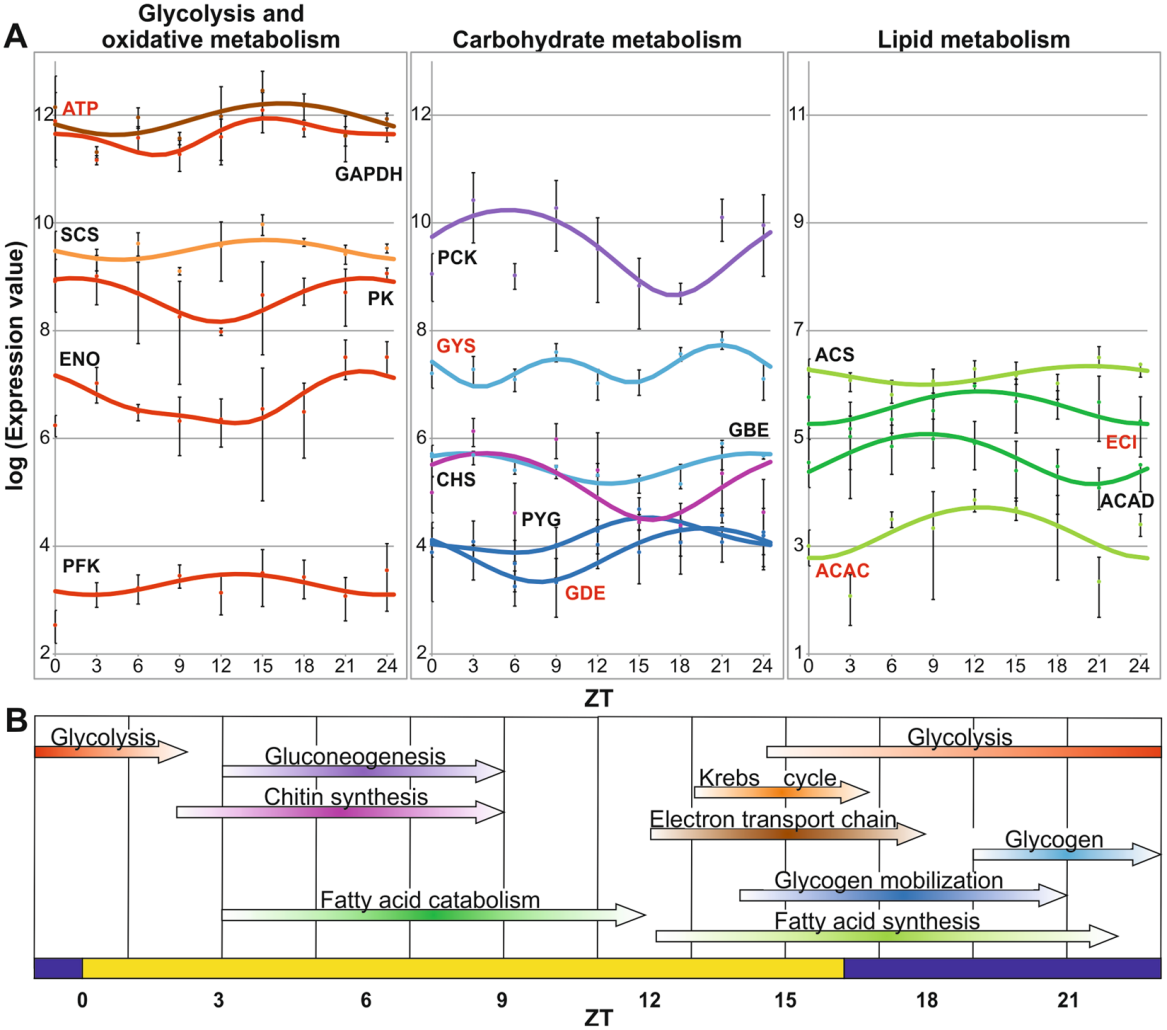
Genes with rhythmic expression patterns in LD involved in energetic and metabolic processes. (**A**) Expression profiles of most relevant genes involved in “Energetic and metabolic process” in LD. Clock-controlled genes showing a sinusoidal expression pattern in both LD and DD, are marked in red. *acyl-CoA dehydrogenase* (ACAD, ID:N19130), *acetyl-CoA carboxylase* (ACAC, ID: N29097), *acyl-CoA synthetase* (ACS, ID:M101952), *ATP synthase* (ATP, ID:M87362), *chitin synthase* (CHS, ID:N19106), *enoyl-CoA isomerase* (ECI, ID:M1878), *enolase* (ENO, ID:N20765), *glyceraldehyde-3-phosphate dehydrogenase* (GAPDH, ID:M80754), *glycogen branching enzyme* (GBE, ID:M8882), *glycogen debranching enzyme* (GDE, ID:M17951), *glycogen synthase* (GYS, ID:M2029), *pyruvate kinase* (PK, ID:N18962), *phosphoenolpyruvate carboxykinase* (PCK, ID:N18402), *phosphofructokinase* (PFK, ID:N21844), *glycogen phosphorylase* (PYG, ID:M105964), and *succinate-CoA ligase* (SCS, ID:M18729). (**B)** Schematic representation of the daily distribution of metabolic processes resulting from the transcriptional signature of several genes through the 24-hour cycle. Different metabolic processes are marked by arrows shaded with a colour gradient showing the time of day corresponding to the higher expression levels of gene groups. The length of the arrows and darker colours indicate intervals and peaks of expression, respectively. ZTs are indicated at the bottom of each panel; yellow and blue bars refer to light and dark intervals.

In LD, 508 genes were grouped under the “Metabolic Process” GO term (Supplementary Table 5). 55.1% of these genes had peaks of expression at night (from ZT18 to ZT21) corresponding to a high levels of activity and oxygen consumption^13^ when krill swim to surface layers to feed. In particular, five out of ten genes encoding proteins involved in glycolysis were up-regulated through the night, from ZT15 to ZT3 [*fructose-bisphosphate aldolase*, *glyceraldehyde 3-phosphate dehydrogenase* (GAPDH), *enolase* (ENO), and *pyruvate kinase* (PK), and the rate-limiting enzyme *phosphofructokinase* (PFK); Fig. 4A). The only exception was represented by the first enzyme of the glycolysis pathway (*hexokinase*) that showed a peak at ZT3. Since *hexokinase* is also involved in gluconeogenesis and chitin synthesis, we analysed the expression profiles of six oscillating genes [*phosphoenolpyruvate carboxykinase* (PCK), *glutamine:fructose-6-phosphate aminotransferase*, *UDP-N-acetylglucosamine pyrophosphorylase*, *chitin synthase* (CHS), and *glutamine synthetase*; Fig. 4A] related to these metabolic pathways, and all of them showed a peak of expression at ZT3. Several genes involved in oxidative metabolism (four components of *ATP synthase*, 8 subunits of Complex I, one subunit of Complex III and 5 subunits of Complex IV) and one gene encoding an enzyme of the citric acid cycle [s*uccinate-CoA ligase* (SCS); Fig. 4A] showed a peak of expression at ZT15 (near lights-off). Furthermore, we detected an over-expression of genes involved in fatty acid synthesis [*acetyl-CoA carboxylase* (ACAC), *fatty acid synthase*, *acyl-CoA synthetase* (ACS); Fig. 4A] from middle afternoon through the night (from ZT12 to ZT0), By contrast, e genes involved in mitochondrial fatty acid β-oxidation [*acyl-CoA dehydrogenase* (ACAD) and *enoyl-CoA isomerase* (ECI); Fig. 4A] had a peak of expression in the first part of the day (from ZT3 to ZT12). Finally, genes encoding proteins involved in glycogen synthesis [*glycogen synthase* (GYS) and *branching enzyme* (GBE); Fig. 4A] and glycogenolysis [*alpha glucosidase*, *glycogen phosphorylase* (PYG), *glycogen debranching enzyme* (GDE); Fig. 4A] showed peaks of expression at ZT21 and ZT15-21, respectively. The overlap between aerobic respiration (glycolysis, citric acid cycle, and oxidative metabolism) and energy-storage pathways (fatty acid and glycogen biosynthetic pathways), as well as the antiphase relationships between aerobic respiration and the main carbohydrate anabolic processes (gluconeogenesis and chitin synthesis), and between fatty acids synthesis and breakdown (Fig. 4B), are reminiscent of the profiles and relationships which have been observed in LL natural conditions^14^. However, the main difference between the gene expression signatures defined in LD (present study) and those obtained in nature during the Antarctic Summer^14^ consists of a ~12 hours phase shift of all metabolic processes. For example, aerobic respiration took place at the beginning of the night in LD and at the beginning of the day in LL (11). A robust light-dark regime (such as LD 16:8, i.e. a typical spring/autumn photoperiod) represents a strong *Zeitgeber* that is able to synchronize gene expression, aerobic respiration, feeding activity, and DVM with the beginning of the dark phase. By contrast, during the Antarctic Summer^14^, the dawn light transition – characterized by an increase in light irradiance and a specific spectral composition of light – could represent the most effective *Zeitgeber*, and thus the one responsible for the observed ~12 hour phase shift modifying the temporal orchestration of physiology and behaviour. This hypothesis is in line with the weak DVM and the loss of the daily feeding schedules shown by krill during summer and winter^50,51^.

In DD, the number of genes with an oscillatory expression profile, grouped under the “Metabolic Process” GO term, were 282 (Supplementary Table 6), compared to the 508 observed in LD. Only 92 of them showed an oscillatory expression pattern in both LD and DD (Supplementary Table 4). Among these, two subunits of Complex I, one subunit of Complex IV, and one component of *ATP synthase* (oxidative metabolism), *acetyl-CoA carboxylase* (fatty acid synthesis), *enoyl-CoA isomerase* (fatty acid β-oxidation), *glycogen synthase* (glycogenesis), *glycogen debranching enzyme* (glycogenolysis), and *glutamine synthetase* (chitin synthesis) should be considered clock-controlled genes. In DD, we observed a lower synchronization in the oscillation of genes involved in the same metabolic processes (compared to LD). This lack of synchronization could be due to the fact that the free running expression periodicity of such genes is generally variable and shorter than 24 hours, making the precise temporal localization of the metabolic process more difficult. Only fatty acid synthesis (*acetyl-CoA carboxylase* and *2 enoyl-CoA reductase* CT12) and glycogenesis (*phosphoglucomutase* and *glycogen synthase* CT3–12) seem to have a precise temporal localization in DD. Moreover, *acetyl-CoA carboxylase* and *glycogen synthase*, that are rate-limiting enzymes of these two metabolic processes, are clock-controlled. These results suggest a more effective temporal regulation of fatty acid and glycogen biosynthetic processes. This is in agreement with the pivotal ecological role that energy-storage pathways play in krill physiology, including the fine-tuned daily distribution of energy resources to sustain the DVM strategy^14^, and the sufficient accumulation of lipids to guarantee survival through the winter^19^.

Finally, none of the genes involved in glycolysis and in the citric acid cycle showed an oscillatory expression profile in DD, suggesting that the temporal localization of these processes in LD (from ZT15 to ZT3) may be defined by the photoperiod or other environmental cues, but not by the circadian clock.

This analysis showed a daily orchestration of metabolic pathways in krill heads, which is where the master clock is located. However others organs, including the stomach and digestive gland, may possess peripheral clocks that could differentially regulate local levels of gene expression of metabolic pathways and other tissue-specific functions.

### Phototransduction

The complexity of the recently described krill photoreception system^16^ suggests that photic environmental cues, such as changes in irradiance and light spectral composition, could play a relevant role as Z*eitgebers*. Several genes involved in phototransduction showed a daily oscillatory pattern in LD, such as *opsin Rh2* (peak of expression at ZT18), *opsin Rh3* (peak at ZT0), and *opsin Rh4* (peak at ZT15). Furthermore, *arrestin* (peak at ZT0), *ninaC* (*neither inactivation nor afterpotential protein C*, peak at ZT0, Supplementary Fig. 3), *opsin Rh5* (peak at ZT18), and the flavoprotein *6–4 photolyase* (peak at ZT18) showed an oscillatory expression profile in both LD and DD; suggesting that their expression is likely to be under the control of the endogenous clock. Daily oscillations of genes involved in photic signal transduction could represent a strategy aimed at optimizing entrainment at high latitudes, which are characterized by extreme changes in photoperiod, irradiance, and spectral composition of sunlight throughout the seasons.

### Neuroendocrine rhythms

Neuropeptides secreted by the crustacean eyestalk control several rhythmic biological process, such as locomotor activity, metabolism, stress response, eye sensitivity, and pigment dispersion^52^. Here we observed that the pigment-dispersing hormone (PDH) and crustacean hyperglycaemic hormone (CHH) showed rhythmic expression profiles over the 24 hours. PDH induces pigment dispersion in the retina and abdominal photophores, to protect photoreceptors from solar radiation during the light phase. Even though an involvement of the circadian clock in the rhythmic release of PDH has been observed in other crustaceans^53^ but excluded in krill^54^, we found that the putative PDH3 ortholog showed oscillatory expression profiles in both LD and DD, with a periodicity of about 24 hours. In *Crustacea*, CHH is responsible for an increase in glucose levels in the haemolymph during phases of high locomotor activity, in particular during the upward and downward vertical migrations^52^. Circadian oscillations in CHH expression and release have been observed in *Procambarus clarkii*^55^. In krill, CHH showed an oscillatory expression profile in both LD and DD, with a periodicity of 24 hours. This suggests an involvement of the circadian clock in the neuroendocrine regulation of carbohydrate metabolism.

## Conclusion

Our results provide insight into the circadian transcriptome of the Antarctic krill, and shed light on the molecular mechanisms underlying the control and regulation of metabolic, physiological, and behavioural rhythms. The existence of a functional circadian clock in the Antarctic krill *E*. *superba* is supported by: i) our observation of a daily oscillatory expression of hundreds of genes in both LD and DD, and ii) the detection of anticipatory features of the light/dark transition at the transcriptional level. Furthermore, the krill clock seems crucial for the synchronization of seasonal cycles, defining the transcriptome composition during the transition to the quiescent state and for the regulation of energy storage pathways, which are key to the overwintering processes.

## Methods

### Ethics statement

All animal work was conducted according to relevant national and international guidelines. Krill catches, welfare and experimentation were based on permission from the Department of Environment and Heritage of the Australian Government and were conducted in accordance with the Antarctic Marine Living Resources Conservation Act 1981 (permit number: 06_09_2220) and the Environment Protection and Biodiversity Conservation Act 1999 (permit number: WT2007-1480).

### Animals

Krill were caught by oblique hauls of several Rectangular Midwater Trawls, using a pelagic net (RMT 8), in the upper 200 m of the water column. Catches were made in East Antarctica (between 65°19′S, 125°37′E, 17 Sep 2007 and 64°08′S, 119°16′E, 09 Oct 2007) during the voyage V1 07/08 with RSV Aurora Australis. Krill were immediately transferred into 200 L tanks at 0 °C and dim light. Each day 50% of the water was exchanged with fresh pre-chilled seawater to ensure food and nutrients turnover. Twice a day, dead animals and moults were removed from the tanks^56^. After arrival in Hobart, Tasmania, krill were delivered directly to the Australian Antarctic Division (AAD) aquarium and kept in a 1.670 L holding tank. A detailed description of the holding tank system and of krill maintenance at AAD has been provided elsewhere^57,58^.

### Maintaining krill in the laboratory

340 krill of mixed sex (mean length ~38 mm) from the holding tank was moved into two cylindrical 100 L tanks (170 krill each in experimental tank I and II). The design of the experimental re-circulating facility guaranteed identical water quality and temperature (0.5 °C) for every experimental stock throughout the study^56^.

Each experimental tank was covered with a black lightproof plastic container with a sliding door at the front side to create a separate light compartment. Lighting was provided by fluorescent tubes (Osram L18W/640 Cool White) covered with a filter film around the outside (ARRI, Marine Blue 131). The two experimental tanks were exposed to a light:dark regime of 16 hours light and 8 hours darkness with lights-on at 6:00 h and lights-off at 22:00 h external time, and with a maximum of 100 lux light intensity at the surface of the tanks during midday.

Prior to sampling, all specimens were exposed to LD16:8 for 4 weeks, to ensure successful acclimatization. Both experimental stocks were fed daily with the same algae used in the holding tank at final densities of 3.8 × 10^4^ cells mL^−1^ for *Phaeodactylum tricornutum*, 9.2 × 10^4^ cells mL^−1^ for *Isochrysis sp*. and 6.6 × 10^4^ cells mL^−1^ for *Pavlova sp*. Animals were fed at random times during the day to avoid a feeding pattern becoming a potential entrainment cue.

### Experimental design

A first 24-hour time series was sampled in experimental tank I under LD 16:8 conditions. Krill were sampled at regular intervals (every three hours) over a full 24-hour cycle. Animals were taken at 6:00, 9:00, 12:00, 15:00, 18:00, 21:00, 24:00, 3:00 and 6:00 h day time corresponding to ZT0, 3, 6, 9, 12, 15, 18, 21, and 24. After the light regime was changed from LD to DD in experimental tank II, a second 24 hours collection series was performed on the third day of DD. Krill were sampled every 3 hours at CT0, 3, 6, 9, 12, 15, 18, 21, and 24. No feeding was conducted during sampling campaigns.

For purpose of molecular analyses, 9 animals were sampled at each time point, immediately frozen in liquid nitrogen and stored at −80 °C. Sampling in darkness was carried out under dim red light. Krill heads were cut off on a cooling element behind the eyestalks and were immediately transferred to a mortar and preground in liquid nitrogen to a homogenous powder. The powder was then stored in 1 mL TRIzol^®^ reagent (Invitrogen) and total RNA extracted according to the supplier’s instructions. Total RNA was quantified using the NanoDrop ND-1000 spectrophotometer (Peqlab Biotechnology) and RNA integrity was checked using the Agilent Bioanalyzer 2100 (RNA 6000 Nano LabChip, Agilent Technologies).

### Design of *E. superba* microarray platform

The krill “master” transcriptome, previously generated by our group, provided about 57,400 contigs longer than 300 bp^23^. One probe for each consensus sequence was designed to construct the high-density oligo DNA microarray. As described in De Pittà *et al*.^14^, probe design was carried out by using the Agilent eArray Custom Microarray Design Service, which applies proprietary prediction algorithms to design 60 mer oligo-probes. Microarrays were synthesized *in situ* using the Agilent ink-jet technology with 8 × 60 K format. Microarray custom platform, named “Krill 2.0” (eArray Design ID: 049690), showed 57,358 single probes and 1,319 default positive and negative controls. Probe sequences and other details on the microarray platform can be found in the Gene Expression Omnibus (GEO) database (http://www.ncbi.nlm.nih.gov/geo/) under accession number: GSE94757.

### Microarray labeling and hybridization

Gene expression profiling was carried out in krill sampled at different times throughout the 24 hours (day times: 6:00, 9:00, 12:00, 15:00, 18:00, 21:00, 24:00, 3:00 and 6:00) in LD and DD conditions with the Krill 2.0 custom platform (Agilent). In every entrainment condition, for each time point, a total of three biological replicates were considered, for a grand total of 54 microarrays. As described in De Pittà *et al*.^14^, equal amounts of total RNA extracted from three different specimens were mixed to prepare each biological replicate. 800 ng of total RNA were labelled with “Agilent One-Color Microarray-Based Gene Expression protocol”. Slides were scanned on an Agilent microarray scanner (model G2565CA) and Agilent Feature Extraction software version 10.5.1.1 was used for image analysis. Gene expression data are available in GEO database with the accession number: GSE94757.

### Statistical analysis of gene expression data

As described in De Pittà *et al*.^14^, inter-array normalization of expression levels was performed with quantile^59^ to correct for possible experimental distortions. Furthermore, *Feature Extraction Software* (Agilent) provided spot quality measures to evaluate the quality and the reliability of the hybridization. In particular, flag “glsFound” (set to 1 if the spot has an intensity significantly different from the local background, 0 otherwise) was used to filter out unreliable probes: flags equal to 0 were noted as “not available (NA)”. Probes with more than 26% of NA values were then removed from the dataset. After filtering, a total of 38,028 krill genes were obtained in LD, and 38,231 in DD conditions. Cluster analysis and profile similarity searches were performed by the Multi Experiment Viewer version 4.8.1 (tMev, mev.tm4.org/) of the TM4 Microarray Software Suite^60^.

The RAIN software package for R/Bioconductor (www.bioconductor.org) was used to identify genes showing rhythmicity during the 24 hours^25^. the following set of periods was tested: 12, 15, 18, 21 e 24. A gene was considered rhythmic when the regression was statistically significant: Benjamini-Hochberg adjusted *p*-value < 0.05 (Supplementary Table 7). RAIN was also used for period estimation because it has been previously demonstrated that fitting-based algorithms are more accurate than point estimation methods in most circumstances, including difficult scenarios such as short data sets, noisy data, low sampling rates, and non-sinusoidal signals^61^. Furthermore, RAIN implements all the features that a good fitting-based algorithm should have^62^, such as sinusoidal and non-sinusoidal fits, non-parametric test, multiple testing correction, and short running-time. On the basis of our sampling plan, the following periods were tested: 12, 15, 18, 21 e 24. The following parameters were set: a) 12 hours as minimum period value because an oscillation cannot be properly described by 4 points or less; b) 3-hour increments to avoid estimated periods having a lower resolution than sampling intervals; c) 24 hours as maximum period value not to exceed the 24-hour time window described in our gene expression data (9 time points).

### Annotation and functional enrichment analysis

As described in Meyer *et al*.^23^, each consensus, converted in FASTA format, was searched locally in the NCBI nucleotide and UniProtUK databases, using Blast-X and Blast-N, respectively. The annotation of rhythmic genes was further examined manually. Gene Ontology (GO) analysis of these genes was performed by BLAST2GO tool^63^ (www.blast2go.com).

### Validation of relative gene expression by quantitative RT-PCR

Validation was performed on the same RNA pools used in the microarray experiments. As described in De Pittà *et al*.^14^, 1 μg of RNA was converted into cDNA using random hexamers and SuperScript II reverse transcriptase (Life Technologies). 1 μl aliquot of 1:100 diluted first-strand cDNA was PCR amplified in 10 μl volume using the SYBR Green chemistry (GoTaq qPCR Master Mix, Promega). Gene-specific primers (Supplementary Table 8) were designed using the web-tool Primer3^64^ (primer3.ut.ee). Total RNA samples were treated with DNase I (Qiagen). Dissociation curves confirmed the specificity of the amplicons. Primer efficiencies were assessed by standard curves. *Ubiquitin carboxyl-terminal hydrolase 46* and *RNA polymerase I-specific transcription initiation factor RRN3 isoform 1* have been used as endogenous controls. PCR reactions were performed in triplicate in a 7500 Real-Time PCR System (Applied Biosystems). The 2^−ΔΔCt^ method was used to calculate the relative expression ratio^65^. 95% confidence intervals are associated to each time point. Pearson correlation was calculated to estimate the association between microarray and qRT-PCR results (Supplementary Fig. 3).

## Supplementary information

Supplementary figures and tables

Supplementary Table 1

Supplementary Table 2

Supplementary Table 3

Supplementary Table 4

Supplementary Table 5

Supplementary Table 6

## References

[1] Stillman, B. & Stewart, D. J. *Clocks and rhythms*. (Cold Spring Harbor Laboratory, 2007).

[2] Gaten E, Tarling G, Dowse H, Kyriacou C, Rosato E (2008). Is vertical migration in Antarctic krill (Euphausia superba) influenced by an underlying circadian rhythm?. J. Genet..

[3] Silverin B (2009). Persistent diel melatonin rhythmicity during the Arctic summer in free-living willow warblers. Horm. Behav..

[4] Kaiser TS (2016). The genomic basis of circadian and circalunar timing adaptations in a midge. Nature.

[5] Zhang L (2013). Dissociation of circadian and circatidal timekeeping in the marine crustacean Eurydice pulchra. Curr. Biol..

[6] Tessmar-Raible K, Raible F, Arboleda E (2011). Another place, another timer: Marine species and the rhythms of life. BioEssays.

[7] Zantke J (2013). Circadian and circalunar clock interactions in a marine annelid. Cell Rep..

[8] Tosches MA, Bucher D, Vopalensky P, Arendt D (2014). Melatonin signaling controls circadian swimming behavior in marine zooplankton. Cell.

[9] Brady AK, Willis BL, Harder LD, Vize PD (2016). Lunar phase modulates circadian gene expression cycles in the broadcast spawning coral Acropora millepora. Biol. Bull..

[10] Siegel, V. *Biology and Ecology of Antarctic Krill*. (Springer International Publishing, 2016).

[11] Atkinson A, Siegel V, Pakhomov E, Rothery P (2004). Long-term decline in krill stock and increase in salps within the Southern Ocean. Nature.

[12] Schiermeier Q (2010). Ecologists fear Antarctic krill crisis. Nature.

[13] Teschke M, Wendt S, Kawaguchi S, Kramer A, Meyer B (2011). A Circadian clock in Antarctic krill: an endogenous timing system governs metabolic output rhythms in the euphausid species Euphausia superba. PLoS One.

[14] De Pittà C (2013). The Antarctic krill Euphausia superba shows diurnal cycles of transcription under natural conditions. PLoS One.

[15] Groeneveld J (2015). How biological clocks and changing environmental conditions determine local population growth and species distribution in Antarctic krill (Euphausia superba): a conceptual model. Ecol. Modell..

[16] Biscontin A (2016). The opsin repertoire of the Antarctic krill Euphausia superba. Mar. Genomics.

[17] Kawaguchi S, Yoshida T, Finley L, Cramp P, Nicol S (2007). The krill maturity cycle: a conceptual model of the seasonal cycle in Antarctic krill. Polar Biol..

[18] Meyer B (2010). Seasonal variation in body composition, metabolic activity, feeding, and growth of adult krill Euphausia superba in the Lazarev Sea. Mar. Ecol. Prog. Ser..

[19] Meyer B (2012). The overwintering of Antarctic krill, Euphausia superba, from an ecophysiological perspective. Polar Biol..

[20] Mazzotta GM (2010). A cry from the krill. Chronobiol. Int..

[21] Sales G (2017). KrillDB: A de novo transcriptome database for the Antarctic krill (Euphausia superba). PLoS One.

[22] Biscontin A (2017). Functional characterization of the circadian clock in the Antarctic krill, Euphausia superba. Sci. Rep..

[23] Meyer B (2015). Pyrosequencing and de novo assembly of Antarctic krill (Euphausia superba) transcriptome to study the adaptability of krill to climate-induced environmental changes. Mol. Ecol. Resour..

[24] Piccolin F (2018). Photoperiodic modulation of circadian functions in Antarctic krill Euphausia superba Dana, 1850 (Euphausiacea). J. Crustac. Biol..

[25] Thaben PF, Westermark PO (2014). Detecting Rhythms in Time Series with RAIN. J. Biol. Rhythms.

[26] Hughes ME (2009). Harmonics of circadian gene transcription in mammals. PLoS Genet.

[27] Westermark PO, Herzel H (2009). Mechanism for 12 Hr Rhythm Generation by the Circadian Clock. Cell Rep..

[28] Naylor E (1958). Tidal and diurnal rhythms of locomotor activity in Carcinus maenas. J. Exp. Biol..

[29] Aguzzi J, Company JB, Abelló P (2004). Locomotor Activity Rhythms of Continental Slope Nephrops norvegicus (Decapoda: Nephropidae). J. Crustac. Biol..

[30] Zheng B (2001). Nonredundant roles of the mPer1 and mPer2 genes in the mammalian circadian clock. Cell.

[31] Czeisler CA (1999). Stability, precision, and near-24-hour period of the human circadian pacemaker. Science.

[32] Saez L, Derasmo M, Meyer P, Stieglitz J, Young MW (2011). A key temporal delay in the circadian cycle of Drosophila is mediated by a nuclear localization signal in the timeless protein. Genetics.

[33] Comas M, Beersma DGM, Spoelstra K, Daan S (2006). Phase and period responses of the circadian system of mice (Mus musculus) to light stimuli of different duration. J. Biol. Rhythms.

[34] Hut RA, Paolucci S, Dor R, Kyriacou CP, Daan S (2013). Latitudinal clines: an evolutionary view on biological rhythms. Proceedings. Biol. Sci..

[35] Aschoff J (1960). Exogenous and endogenous components in circadian rhythms. Cold Spring Harb. Symp. Quant. Biol..

[36] Ceriani MF (2002). Genome-wide expression analysis in Drosophila reveals genes controlling circadian behavior. J. Neurosci..

[37] Claridge-Chang A (2001). Circadian regulation of gene expression systems in the Drosophila head. Neuron.

[38] Panda S (2002). Coordinated transcription of key pathways in the mouse by the circadian clock. Cell.

[39] Weger BD (2011). The Light Responsive Transcriptome of the Zebrafish: Function and Regulation. PLoS One.

[40] Le Martelot G (2012). Genome-wide RNA polymerase II profiles and RNA accumulation reveal kinetics of transcription and associated epigenetic changes during diurnal cycles. PLoS Biol..

[41] Aguilar-Arnal L, Sassone-Corsi P (2013). The circadian epigenome: how metabolism talks to chromatin remodeling. Curr. Opin. Cell Biol..

[42] Doi M, Hirayama J, Sassone-Corsi P (2006). Circadian Regulator CLOCK Is a Histone Acetyltransferase. Cell.

[43] Henriques R, Jang I-C, Chua N-H (2009). Regulated proteolysis in light-related signaling pathways. Curr. Opin. Plant Biol..

[44] Seear P (2009). Effects of simulated light regimes on gene expression in Antarctic krill (Euphausia superba Dana). J. Exp. Mar. Bio. Ecol..

[45] Escamilla-Chimal EG, Velazquez-Amado RM, Fiordelisio T, Fanjul-Moles ML (2010). Putative pacemakers of crayfish show clock proteins interlocked with circadian oscillations. J. Exp. Biol..

[46] Syed S, Saez L, Young MW (2011). Kinetics of doubletime kinase-dependent degradation of the Drosophila period protein. J. Biol. Chem..

[47] Ko HW, Jiang J, Edery I (2002). Role for Slimb in the degradation of Drosophila Period protein phosphorylated by Doubletime. Nature.

[48] Muskus MJ, Preuss F, Fan J-Y, Bjes ES, Price JL (2007). Drosophila DBT lacking protein kinase activity produces long-period and arrhythmic circadian behavioral and molecular rhythms. Mol. Cell. Biol..

[49] Castellana S (2018). Systematic Analysis of Mouse Genome Reveals Distinct Evolutionary and Functional Properties Among Circadian and Ultradian Genes. Front. Physiol..

[50] Ross, R. M., Quetin, L. B. & Lascara, C. M. Foundations for Ecological Research West of the Antarctic Peninsula (American Geophysical Union, 1996).

[51] Taki K, Hayashi T (2005). Characteristics of seasonal variation in diurnal vertical migration and aggregation of Antarctic krill (Euphausia superba) in the Scotia Sea, using Japanese. Ccamlr Sci..

[52] Strauss J, Dircksen H (2010). Circadian clocks in crustaceans: identified neuronal and cellular systems. Front Biosci.

[53] Noël, P. Y. & Chassard-Bouchaud, C. Chromatophores and pigmentation. In *The Crustacea I* eds J. Forest & J. C. von Vaupel Klein (2004).

[54] Auerswald L, Freier U, Lopata A, Meyer B (2008). Physiological and morphological colour change in Antarctic krill, Euphausia superba: a field study in the Lazarev Sea. J. Exp. Biol..

[55] Loredo-Ranjel R, Fanjul-Moles ML, Escamilla-Chimal EG (2017). Crustacean hyperglycemic hormone is synthesized in the eyestalk and brain of the crayfish Procambarus clarkii. PLoS ONE.

[56] Kawaguchi S (2010). An experimental aquarium for observing the schooling behaviour of Antarctic krill (Euphausia superba). Deep Sea Res. Part II Top. Stud. Oceanogr..

[57] King R, Nicol S, Cramp P, Swadling KM (2003). Krill maintenance and experimentation at the australian antarctic division. Mar. Freshw. Behav. Physiol..

[58] Teschke M, Kawaguchi S, Meyer B (2007). Simulated light regimes affect feeding and metabolism of Antarctic krill, Euphausia superba. Limnol. Oceanogr..

[59] Bolstad BM, Irizarry RA, Astrand M, Speed TP (2003). A comparison of normalization methods for high density oligonucleotide array data based on variance and bias. Bioinformatics.

[60] Saeed AI (2006). TM4 microarray software suite. Methods enzymol..

[61] Zielinski T, Moore AM, Troup E, Halliday KJ, Millar AJ (2014). Strengths and Limitations of Period Estimation Methods for Circadian Data. PLoS ONE.

[62] Wu G (2014). Evaluation of five methods for genome-wide circadian gene identification. J. Biol. Rhythms.

[63] Conesa A (2005). Blast2GO: a universal tool for annotation, visualization and analysis in functional genomics research. Bioinformatics..

[64] Untergasser A (2012). Primer3—new capabilities and interfaces. Nucleic Acids Res..

[65] Livak KJ, Schmittgen TD (2001). Analysis of Relative Gene Expression Data Using Real-Time Quantitative PCR and the 2−ΔΔCT Method. Methods.

