# Supplementary material for: Analysis of the circadian transcriptome of the Antarctic krill Euphausia superba
Source: Sci Rep. 2019 Sep 25;9:13894. doi: 10.1038/s41598-019-50282-1 (PMC6761102; doi:10.1038/s41598-019-50282-1)
Supplement: Supplementary file 1 — Supplementary figures and tables [file 41598_2019_50282_MOESM1_ESM.pdf]

Analysis of the circadian transcriptome of the Antarctic krill *Euphausia superba*

Alberto Biscontin[1,2,*], Paolo Martini[1], Rodolfo Costa[1], Achim Kramer[2], Bettina Meyer[3,4,5], So Kawaguchi[6], Mathias Teschke[3], Cristiano De Pittà[1,*]

[1]Dipartimento di Biologia, Università degli Studi di Padova, Padova, Italy

[2]Laboratory of Chronobiology, Charité Universitätsmedizin Berlin, Berlin, Germany

[3]Section Polar Biological Oceanography, Alfred Wegener Institute Helmholtz Centre for Polar and Marine Research, Bremerhaven, Germany

[4]Institute for Chemistry and Biology of the Marine Environment, Carl von Ossietzky University of Oldenburg, Oldenburg, Germany

[5]Helmholtz Institute for Functional Marine Biodiversity (HIFMB) at the University of Oldenburg, 26111 Oldenburg, Germany

[6]Department of Environment and Heritage, Australian Antarctic Division, Kingston, Tasmania, Australia

[*]Corresponding authors:

Cristiano De Pittà, Dipartimento di Biologia, Università degli Studi di Padova, via U. Bassi 58/B 35131 Padova, Italy; Phone: +39-049-8276210; Fax: +39-049-8276209; e-mail address: cristiano.depitta@unipd.it

Alberto Biscontin, Dipartimento di Biologia, Università degli Studi di Padova, via U. Bassi 58/B 35131 Padova, Italy; Phone: +39-049-8276228; Fax: +39-049-8276209; e-mail address: alberto.biscontin@unipd.it

SUPPLEMENTARY FIGURES

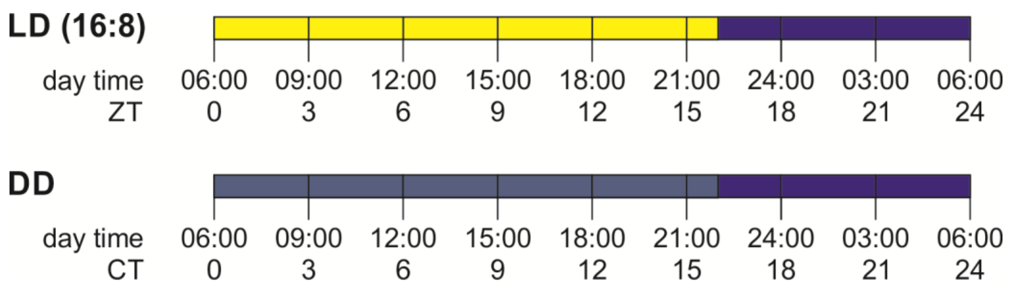

Supplementary Figure 1. Experimental design.

In order to define the gene expression profiles, specimens were collected at regular intervals throughout the 24-hour cycle in a light:dark (LD) regime of 16 hours light and 8 hours darkness followed by constant darkness (DD). Yellow and blue bars refer to light and dark periods, respectively.

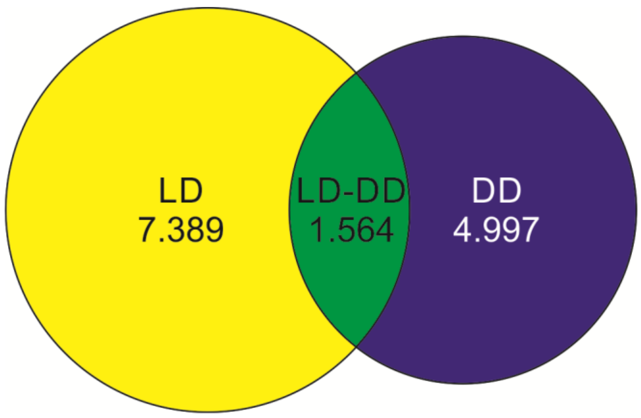

Supplementary Figure 2. Circadian expression signatures.

A weighted Venn diagram showing the relative portion of genes with sinusoidal expression patterns (RAIN) in LD and DD (see Table S1 and S2). The intersection represents putative clock-controlled genes with an oscillatory expression both in LD and DD (see Table S3).

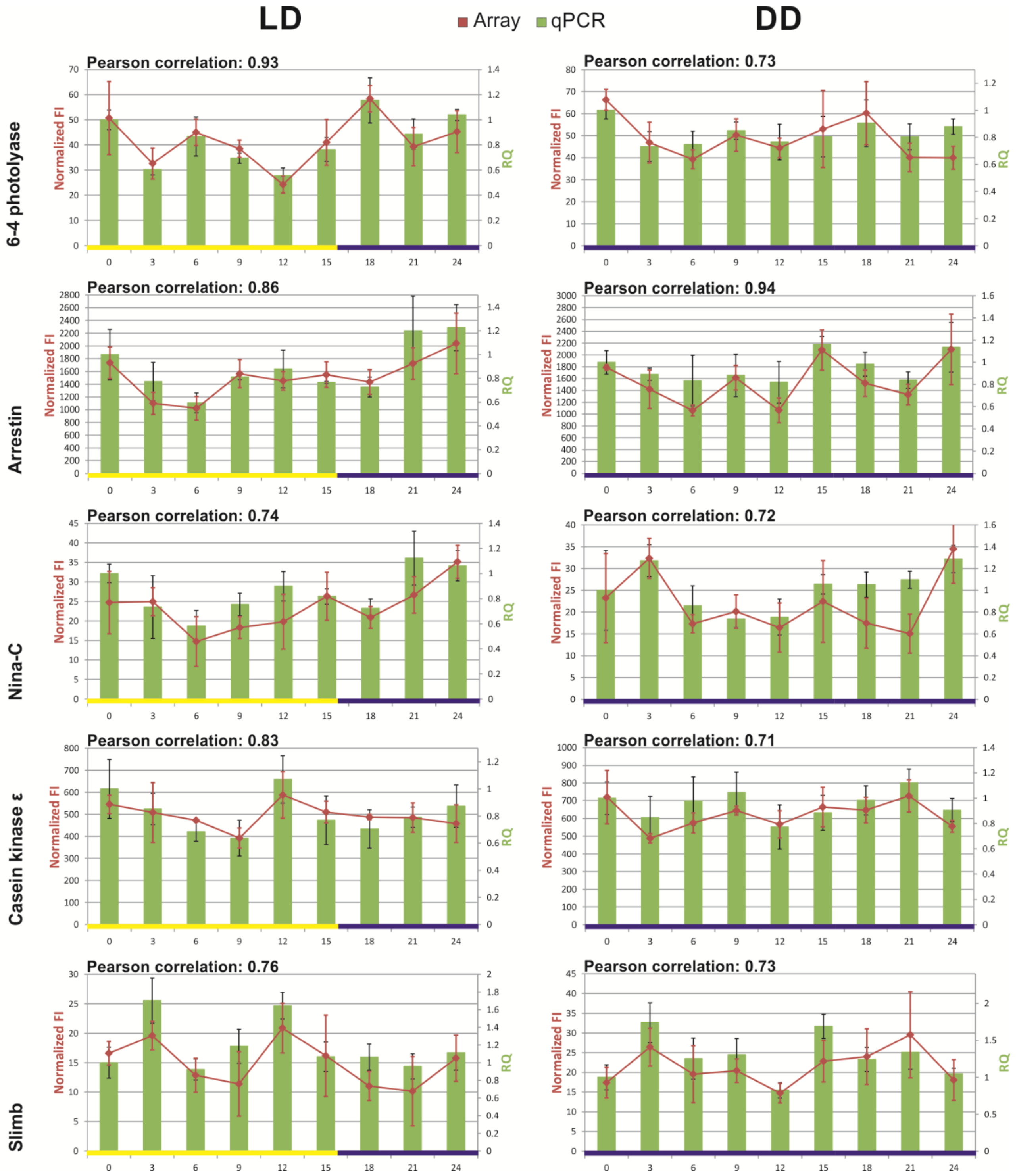
LD
Array
qPCR
DD
6-4 photolyase
Pearson correlation: 0.93
Pearson correlation: 0.73
Arrestin
Pearson correlation: 0.86
Pearson correlation: 0.94
Nina-C
Pearson correlation: 0.74
Pearson correlation: 0.72
Casein kinase ε
Pearson correlation: 0.83
Pearson correlation: 0.71
Slimb
Pearson correlation: 0.76
Pearson correlation: 0.73
Normalized FI
RQ
0
3
6
9
12
15
18
21
24

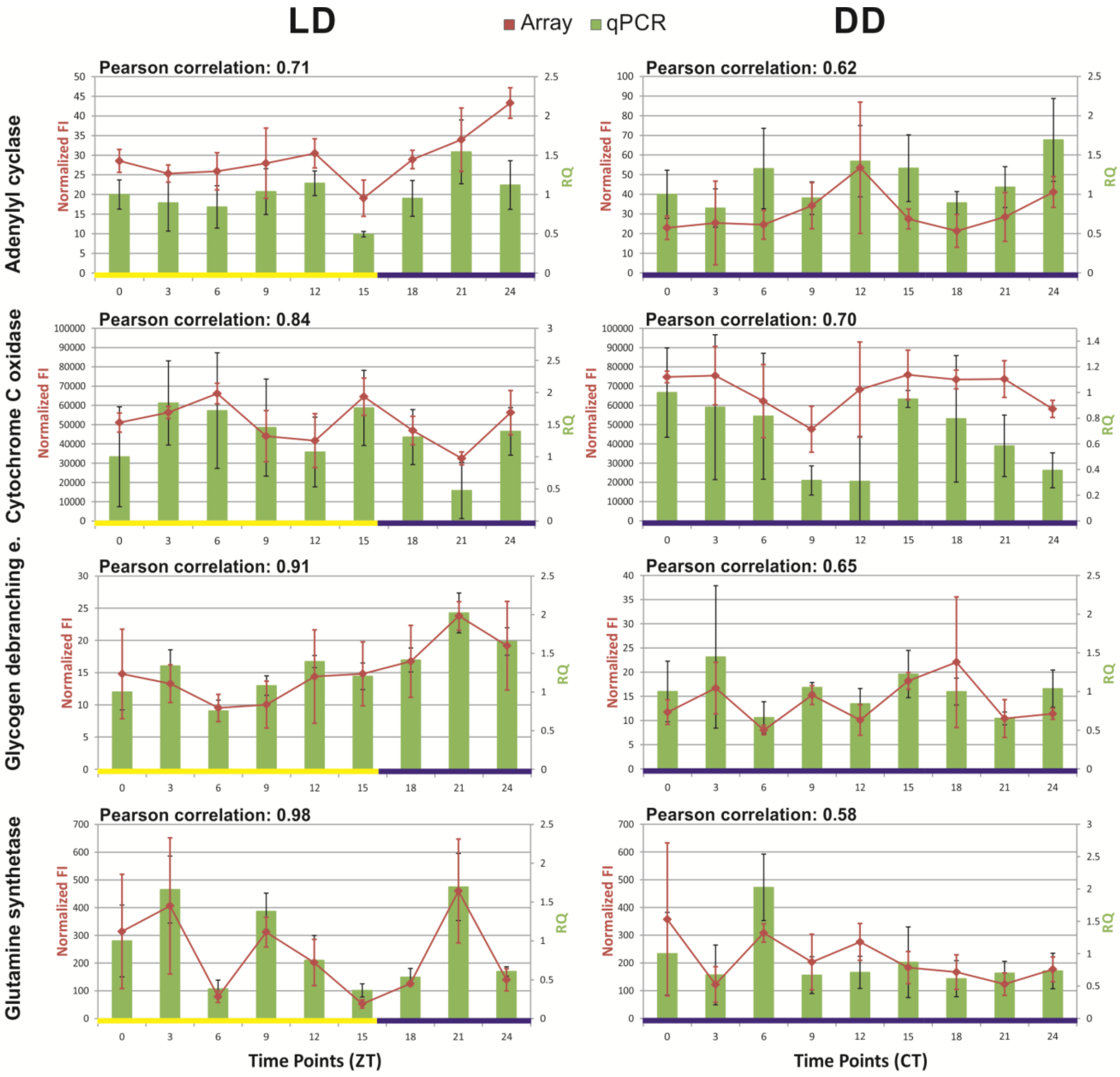

Supplementary Figure 3. Validation of microarray expression values by qRT-PCR.

mRNA expression levels are represented by histograms. Normalized qRT-PCR data are expressed as fold changes (RQ) relative to the average expression for ZT0 and CT0. Yellow and blue bars refer to light and dark periods, respectively. *Ubiquitin carboxyl-terminal hydrolase 46* and *RNA polymerase I-specific transcription initiation factor RRN3 isoform 1* were used as endogenous controls. The microarray expression profiles of each gene are shown as line graphs (red) while qRT-PCR results are reported as histograms. Pearson correlation was calculated to estimate the association between microarray data and qRT-PCR results (r > 0.6 is considered as statistically significant).

SUPPLEMENTARY TABLES

```
>require(rain)
>data<-read.table(file)
>result<-rain(data, period=18, period.delta=6, deltat=3, nr.series=3, method='independent')
```

Supplementary Table 7. List of commands used to identify genes showing rhythmic expression profiles during the 24 hours in microarray data by using RAIN.

Description	Forward Primer (5'-3')	Reverse Primer (5'-3')
6-4 photolyase	CTTGGAACGCACCACTCTCT	CACGATTCTACGTGGGTAGTCC
arrestin homolog	AGCTAGTTCACCCAGACCCA	ACGGCGGAGGTTCTTGAAAT
casein kinase 1 epsilon	TATTGTCTCGTCGGCTTGC	TGGGTATTTGGATCCTACAACAT
neither inactivation nor afterpotential protein C	AACTGAAGTTGCAATAAAGGATCA	TGCCTCCACCATATGTAGTCC
slimb	GCTGCCACAGATGCTTCA	TGCCTCCACCATATGTAGTCC
cytochrome c oxidase subunit I	GCAATAAGCGAATGGTTGACC	GATACTCAGACTACCCAGATGC
glycogen debranching enzyme	TGCAAATGAGTCTCCATGGC	TGTGCAGTAGACGATCAAGG
glutamine synthetase	TGTATCTTCCTCCAGGATGGC	ATACATGGCCTGGCATTTGC
adenylyl cyclase	AGCAACACCAGCAGTTTAGG	TTCATGGTCACCACATACGG
clock	GCTGCAGCAAATGATAATGC	TGCCATATTGGGCCATAACT
cryptochrome 2	TCATGAACCATGGACTGCAC	GGTGGACACGACTTCAACAA
period	GCAGCTATGCCCAACTTTAATC	GGACTAGCAACAGGGACATTTT
ubiquitin carboxyl-terminal hydrolase 46	AAATCGTCAGAAACGGGCTA	TTAGCGGTTATGGAACATTACG
RNA polymerase I-specific transcription initiation factor RRN3 isoform 1	CTTGGACGGGGTAGTTCCTA	TGTGATGTTTACCTCCGCAGT

Supplementary Table 8. Primers used in quantitative qRT-PCR.
