# Supplementary material for: Analysis of the circadian transcriptome of the Antarctic krill Euphausia superba
Source: Sci Rep. 2019 Sep 25;9:13894. doi: 10.1038/s41598-019-50282-1 (PMC6761102; doi:10.1038/s41598-019-50282-1)
Supplement: Supplementary file 2 — Supplementary Table 1 [file 41598_2019_50282_MOESM2_ESM.pdf]

Analysis of the circadian transcriptome of the Antarctic krill *Euphausia superba*

Alberto Biscontin[1,2,*], Paolo Martini[1], Rodolfo Costa[1], Achim Kramer[2], Bettina Meyer[3,4,5], So Kawaguchi[6], Mathias Teschke[3], Cristiano De Pittà[1,*]

[1]Dipartimento di Biologia, Università degli Studi di Padova, Padova, Italy

[2]Laboratory of Chronobiology, Charité Universitätsmedizin Berlin, Berlin, Germany

[3]Section Polar Biological Oceanography, Alfred Wegener Institute Helmholtz Centre for Polar and Marine Research, Bremerhaven, Germany

[4]Institute for Chemistry and Biology of the Marine Environment, Carl von Ossietzky University of Oldenburg, Oldenburg, Germany

[5]Helmholtz Institute for Functional Marine Biodiversity (HIFMB) at the University of Oldenburg, 26111 Oldenburg, Germany

[6]Department of Environment and Heritage, Australian Antarctic Division, Kingston, Tasmania, Australia

[*]Corresponding authors:

Cristiano De Pittà, Dipartimento di Biologia, Università degli Studi di Padova, via U. Bassi 58/B 35131 Padova, Italy; Phone: +39-049-8276210; Fax: +39-049-8276209; e-mail address: cristiano.depitta@unipd.it

Alberto Biscontin, Dipartimento di Biologia, Università degli Studi di Padova, via U. Bassi 58/B 35131 Padova, Italy; Phone: +39-049-8276228; Fax: +39-049-8276209; e-mail address: alberto.biscontin@unipd.it

Supplementary Table 1. List of 8,953 genes with a sinusoidal expression pattern in LD.

Expression levels of genes, identified by RAIN (adjusted p-value < 0.05) throughout the 24 hour- cycle in LD regime, are shown. [a]Probe ID: ID of probe sequence in "Krill 2.0" Agilent microarray platform; [b]ID: accession number of krill master transcriptome [23];
[c]p-value: RAIN Benjamini-Hochberg adjusted p-value; [d]Period: period of oscillation; [e]Phase: maximum peak of expression throughout the 24 hour-cycle; [f]EBI ID: accession number of EBI; [g]Annotation = description of the gene; [h]GO term: gene ontology biological process;
[i]GO child term: most fitting gene ontology child term to further refine the functional annotation; [l]e-value: score of GO functional annotation with BLAST2GO; [k-ak]mRNA expression levels.

Probe ID	ID	p-value	Period	Phase	EBI ID	Annotation	GO category	Go term	E-value	0	0	0	3	3	3	6	6	6	9	9	9	12	12	12	15	15	15	18	18	18	21	21	21	24	24	24
CUST_5203_PI429010886	N18394	2.47445E-11	24	18			Signal transduction	Other		1546.88	3024.414	3475.106	848.7243	1142.458	1115.579	995.515	1038.012	1194.794	2323.856	1299.503	2191.923	3197.119	3258.199	2072.212	3936.583	4467.089	2609.919	6421.864	7695.811	7248.769	6000.152	4368.987	3786.16	4185.629	2670.787	3007.18
CUST_39669_PI429010886	M42544	1.38546E-08	24	15						1235.266	1738.077	1171.488	969.8589	1002.076	1016.824	1020.366	1202.203	1268.665	1151.33	1344.214	1232.079	1251.736	1475.969	1277.638	1729.252	1894.407	2179.188	1648.551	1430.525	1766.713	1640.994	1400.583	1492.153	1380.017	1302.487	1491.134
CUST_51366_PI429010886	M93282	4.98783E-08	24	6	532759720	antimicrobial peptide type 1 pre			0.000000325	271.3104	293.568	286.926	375.9296	298.3765	297.8825	570.2732	314.7808	562.0064	461.1854	287.6916	421.8557	310.1119	331.0671	207.209	197.1967	277.2489	340.6386	105.6309	157.519	149.047	103.3894	203.8839	249.1319	169.9945	293.835	170.8814
CUST_39293_PI429010886	M37357	7.70244E-08	18	9						62.02618	78.06756	81.72083	21.1933	45.65444	8.076758	162.7602	171.0275	98.36388	181.8309	151.4662	204.1816	146.1482	118.6698	94.96672	128.0247	50.26433	137.2059	103.2488	51.29815	41.00859	60.39038	74.93498	39.75301	99.00226	121.3422	90.85891
CUST_3609_PI429010886	N14873	8.61495E-08	12	9						138.7489	188.0176	318.1116	264.4742	114.6239	80.71612	295.8069	542.7794	947.376	927.9648	1014.938	585.434	800.8587	364.6437	255.7585	26.42527	41.71225	60.69474	655.4746	478.5299	654.3402	1245.448	2304.738	1622.787	240.3937	823.2974	1335.991
CUST_46318_PI429010886	M72195	1.9209E-07	21	12						47.43656	190.289	76.02418	36.44264	65.01967	28.86811	109.1677	108.1208	127.9261	354.1831	168.0498	144.6793	339.5673	130.8473	324.1912	104.5846	320.7735	281.8133	119.3614	179.1574	269.1635	166.8118	76.00489	133.8226	63.09832	43.39176	59.17675
CUST_33410_PI429010886	M6816	2.5548E-07	21	18	321473201	hypothetical protein DAPPUDRA	Signal transduction	Other	2.35E-13	180.5859	269.1036	217.2347	174.6876	175.7096	180.9942	155.7011	181.6282	184.6034	157.4324	148.6649	145.9501	146.4666	171.5864	141.7833	267.7311	160.1694	213.7437	233.8458	293.835	293.6477	195.7159	220.5131	211.0148	184.4435	209.666	180.1324
CUST_37036_PI429010886	M17630	2.99918E-07	15	12						26.28423	28.99089	33.45999	22.1135	22.13507	17.0629	22.86182	21.85018	15.61539	27.23544	26.71901	32.05226	29.46925	31.4218	29.63277	21.27506	28.77245	23.27075	13.55428	20.58996	10.84292	21.46399	20.00439	22.905	27.08947	23.18831	29.19296
CUST_33205_PI429010886	M6266	4.68899E-07	12	3						168.4808	184.0263	141.7704	207.0821	231.8895	169.3049	177.05	153.4348	166.9595	155.7673	143.8296	129.2573	167.0371	157.4103	184.9648	182.6192	191.0167	194.0105	155.2845	131.9615	157.9287	150.8908	132.0536	133.4909	156.1362	173.4147	154.177
CUST_31797_PI429010886	M2433	5.02461E-07	24	0						NA	14.82226	15.62926	10.50491	11.52651	NA	12.1995	7.697503	8.969422	9.320269	9.775246	NA	4.819136	7.392041	5.727124	5.574105	7.742854	9.562348	4.251763	9.935802	8.847627	10.64938	8.186475	10.13728	13.37422	11.63479	13.3556
CUST_32891_PI429010886	M5397	5.83166E-07	18	0						128.5165	315.8899	394.193	88.0075	64.61049	NA	13.99541	91.45416	62.64991	7.155078	12.86873	6.174251	34.00168	35.99005	5.320114	19.1981	10.53078	28.90562	223.6033	163.4636	69.8604	48.82419	67.93845	46.90198	37.92348	NA	31.24606
CUST_49029_PI429010886	M83694	6.69567E-07	15	6						6.436561	7.628654	8.451625	10.98917	9.385701	5.829325	15.54734	14.41191	18.61955	7.648589	18.30947	13.5348	13.36386	9.752082	13.68243	NA	4.801909	4.126691	10.37109	8.858158	7.546327	24.45128	17.44773	25.60678	9.294827	10.59607	17.26425
CUST_5163_PI429010886	N18342	7.24639E-07	24	21			Cellular component org	Cytoskeleton organization		103.7192	106.6811	118.0597	88.10909	84.96211	80.35316	34.62846	31.28202	37.83455	100.783	55.96387	79.92025	98.42724	62.33316	105.2701	162.3371	72.15825	126.5408	157.6143	96.03692	110.298	233.5705	119.0449	166.5631	193.6659	84.55241	169.9945
CUST_16399_PI429010886	N36161	7.34846E-07	24	18						62.74684	93.76286	102.7497	67.71277	84.72348	60.89951	63.46562	95.62259	83.95829	77.34627	80.72426	91.22685	85.98865	91.45416	92.31393	106.1184	117.3251	108.7869	130.6255	110.0991	119.5301	121.1493	113.5376	120.4803	93.99213	96.54391	118.4588
CUST_51947_PI429010886	M95782	7.56917E-07	21	3						34.02915	44.80331	53.49503	62.93557	37.80064	48.09966	29.30404	36.67874	37.8045	28.45904	33.07741	24.27221	28.98683	27.30031	22.45228	19.34679	33.75196	19.57461	33.83378	42.19574	34.59167	47.21843	40.95208	47.41757	40.16574	52.56738	63.23529
CUST_4711_PI429010886	N17320	7.98984E-07	24	6						16.4789	44.18092	44.86243	41.02313	84.19786	53.24219	61.52728	104.2173	82.59378	47.43656	38.58331	57.94759	32.63321	28.91213	33.42156	35.27117	32.66605	42.20935	9.570901	20.84244	22.25295	25.36487	40.68971	38.15601	19.08178	45.18702	46.23699
CUST_45295_PI429010886	M68631	9.27271E-07	24	15						60.62225	49.67735	52.97194	51.85541	26.7573	38.89189	52.48031	33.46312	36.78126	41.76732	34.53535	53.4446	64.23608	41.15339	46.5849	86.15946	79.01425	80.81117	96.46623	54.68833	71.00454	64.78842	59.8943	64.06114	62.63588	55.84882	53.23278
CUST_26818_PI429010886	N54935	1.0119E-06	21	12						56.30846	51.90036	58.57419	78.21211	67.76221	71.4584	121.4663	98.1824	78.48038	79.57438	71.65566	76.14902	116.0897	92.42138	85.40783	90.60669	77.13441	75.13872	39.14498	51.86945	60.48555	57.51884	52.81957	71.76549	66.84176	69.08897	72.66933
CUST_45107_PI429010886	M68020	1.24584E-06	21	12						12.32463	28.4851	9.834143	73.93804	48.66253	65.80074	114.4742	167.708	107.9667	121.7517	318.037	164.0459	471.2164	284.4024	436.7155	27.51681	102.5834	55.2321	282.0632	6.669026	218.5351	93.92415	42.80977	84.9077	79.5268	95.54211	96.24348
CUST_9576_PI429010886	N24862	1.2767E-06	18	3						87.16322	87.54238	104.8565	131.5859	96.30356	104.5461	78.43693	101.852	66.74201	70.68343	60.0855	74.40311	63.31668	54.94337	46.92175	64.40257	92.52233	78.80045	84.1875	78.0963	78.38177	90.84788	124.3244	87.01734	76.31853	72.19272	63.72422
CUST_31297_PI429010886	M1163	1.2835E-06	24	21	156543561	PREDICTED: ubiquitin-conjugati	Cellular Process	Cell cycle	1.28E-60	32.26017	32.75067	30.46011	20.77278	27.34627	25.38551	23.60134	23.26849	21.99703	27.77009	19.50478	34.69169	34.95791	33.90697	30.46011	29.37527	30.69119	34.85212	35.30149	36.8949	36.28732	34.69476	49.4079	54.2431	23.6675	40.57158	37.14266
CUST_3012_PI429010886	N13602	1.47995E-06	12	9						48.00737	56.34222	78.64253	96.66958	63.4939	28.81777	161.691	331.7259	440.8715	372.3645	370.2353	416.3476	231.289	119.3879	71.7184	NA	10.48413	NA	201.2	133.7339	131.5353	562.2603	767.8994	668.7382	179.9541	264.9858	530.6367
CUST_37896_PI429010886	M22133	1.57017E-06	24	3						17.78243	10.46956	24.09442	34.98417	29.52107	41.98856	9.832908	18.32398	16.72797	12.88293	7.123709	13.60289	8.005861	44.44058	10.99723	3.450966	3.340805	8.367548	4.01398	6.979131	3.69546	6.398645	9.817879	7.560056	9.221685	8.606721	NA
CUST_49535_PI429010886	M86007	1.70246E-06	24	0						621.9301	1474.205	1304.861	320.4829	214.4299	98.45312	495.5669	1140.288	941.0274	166.838	283.1538	90.09793	306.565	295.8069	99.03286	328.7563	406.1674	366.0882	564.6439	968.7419	748.709	513.7138	622.3504	669.1662	1068.554	947.0352	804.0438
CUST_54390_PI429010886	M105834	1.78984E-06	18	12						8.586447	7.283509	NA	46.97174	47.82733	23.39255	48.6216	93.30975	149.73	88.12342	1486.662	45.14878	1363.035	1119.882	237.5415	21.54756	47.11505	80.12221	18.52406	28.52375	16.92315	18.55182	67.95059	69.36872	37.95648	41.7466	74.29925
CUST_47007_PI429010886	M74473	1.79384E-06	21	0						55.02895	74.49329	62.59981	54.6004	55.70355	45.7171	55.61434	56.62992	55.17016	55.4869	46.11055	52.0293	40.65621	38.75298	51.19908	35.34705	34.98836	24.99752	27.2823	37.89317	45.0273	55.73395	68.95833	65.87993	55.73019	53.12256	84.51406
CUST_2826_PI429010886	N13190	1.8769E-06	24	21						1332.694	1685.048	1519.636	1217.388	1023.228	990.2906	532.3756	939.4725	889.0282	1117.04	929.9574	728.6567	1677.845	695.3717	1243.512	1309.684	1606.909	841.6057	1824.284	1247.204	1489.353	2177.847	1867.24	1825.053	2937.545	1456.541	2110.599
CUST_12202_PI429010886	N29097	2.01695E-06	18	12	307177147	Acetyl-CoA carboxylase [Campo			6.5E-21	8.447449	9.603299	6.029956	3.190585	5.717288	3.737478	11.7387	10.0026	12.1414	4.600805	16.57137	9.075187	14.27946	14.41191	16.67597	15.05771	11.41802	12.55015	12.83426	4.405976	13.57384	3.870848	4.11877	7.227902	9.108156	10.50491	12.11843
CUST_36728_PI429010886	M16675	2.02406E-06	21	0						158.0988	46.94606	48.53255	40.98681	44.14843	43.23211	32.77954	37.14266	31.23427	30.3261	42.84965	40.83466	26.61853	33.09195	30.53244	42.66588	42.07726	43.78502	47.10201	38.57471	38.7656	44.65936	50.41623	44.37034	53.72874	48.28814	38.84896
CUST_27398_PI429010886	N55982	2.06445E-06	24	6						209.5586	193.3377	138.2385	232.6083	227.3555	162.7885	320.2208	369.0567	506.9058	228.253	156.1818	130.8147	195.1305	318.9156	181.5802	149.2223	172.9954	139.5452	156.5799	164.794	138.6464	116.8305	133.2218	86.56139	169.1505	199.908	205.5946
CUST_46818_PI429010886	M73816	2.10248E-06	18	9						4.696988	NA	2.969905	4.319683	NA	NA	NA	9.319096	NA	10.97116	9.632394	10.72088	7.667937	8.884176	9.592986	4.280426	7.241625	6.236495	4.590475	NA	4.862023	7.276912	5.567085	6.965443	8.45067	NA	4.928978
CUST_9969_PI429010886	N25485	2.13809E-06	21	0			Signal transduction	Other		182.1611	168.3482	272.6726	92.43032	47.65574	25.11048	20.51331	135.864	124.2633	50.60267	43.32608	44.36058	29.98295	23.90534	20.141	72.92411	83.15856	126.7896	94.41489	30.96219	99.80727	121.0114	167.872	146.0289	98.10696	67.72081	156.394
CUST_6952_PI429010886	N20825	2.14929E-06	24	21						711.4323	758.9827	855.307	552.9475	610.9907	466.7106	228.29	367.4169	382.7119	729.1294	475.8315	481.1553	1025.404	409.8716	783.6564	921.4706	767.3697	546.6865	1181.431	695.8214	764.2764	1682.203	925.4144	1113.188	2082.19	695.3717	1309.131
CUST_16_PI429010886	N60	2.17757E-06	24	18						115.5552	53.7918	148.0504	67.85905	19.43327	63.50827	155.5805	249.3839	217.3699	202.9059	73.3346	148.298	1020.798	498.4242	328.4033	569.3405	275.7847	544.2292	1155.769	1663.655	2589.848	348.7073	72.94835	262.4374	1049.887	414.9926	611.6853
CUST_3987_PI429010886	N15731	2.20941E-06	12	3						30.88831	63.15515	NA	62.73874	NA	61.88614	81.84916	54.39234	73.53729	29.15166	19.17216	6.005973	46.22189	45.51721	38.17039	158.0297	80.88928	87.68846	43.56758	21.7119	32.02499	7.519886	31.1591	5.061086	19.7414	38.7656	14.86874
CUST_38310_PI429010886	M25222	2.25374E-06	24	12						80.24156	96.89897	118.1715	447.5258	233.3271	337.46	439.7541	425.0628	707.3888	121.3126	1214.037	193.6334	491.0486	615.9354	243.8613	244.0894	552.2424	247.4344	172.2883	136.9653	141.5601	105.1748	175.4398	129.3215	45.9512	82.50222	62.84262
CUST_8587_PI429010886	N23265	2.26241E-06	24	21	291230131	PREDICTED: hypothetical protei	Metabolic process	Other	2.81E-09	8.189998	22.81773	16.79507	11.03537	15.25449	20.11384	6.163656	13.6951	7.069413	16.24245	17.08471	14.22807	16.75338	10.56207	24.49986	18.14784	16.63618	14.29283	25.33166	23.16703	22.92862	25.66233	28.89254	25.17932	35.84121	19.60329	23.44924
CUST_35047_PI429010886	M11425	2.30512E-06	24	15	390517062	hemocyanin alpha subunit 1 [Ca			7.22E-49	11.42158	10.53476	10.18923	4.307028	7.410322	3.909088	9.154669	9.027928	8.593041	9.102869	12.27005	13.96585	13.40748	8.009934	17.96574	15.865	12.48201	20.29057	16.48073	17.80167	11.01416	12.19654	14.96193	16.60032	11.36284	5.739438	10.02353
CUST_38542_PI429010886	M27808	2.33759E-06	24	3						108.3488	48.49422	47.2724	105.5272	179.4021	107.1382	37.13937	62.94384	77.58626	34.13826	126.6958	155.0166	23.51188	37.92065	38.38502	7.853693	3.488528	5.973561	19.96477	44.56209	13.80069	42.28696	43.34384	36.34979	18.77257	115.2129	45.25971
CUST_56222_PI429010886	M113176	2.4697E-06	24	15						72.67801	42.78489	42.06094	21.73892	9.497226	13.58218	103.5143	103.0482	100.2745	90.14554	71.1642	21.33315	84.00818	104.3062	32.8145	396.05	199.6163	279.0664	106.0881	134.2508	185.717	86.76212	NA	58.6831	115.4443	31.70729	45.9872
CUST_39737_PI429010886	M43711	2.8514E-06	24	0						1762.47	1240.074	1207.663	1777.355	841.3097	865.141	640.002	579.8505	206.98	832.328	491.9043	341.732	610.5014	170.4291	395.9506	470.8549	429.8212	80.3791	1690.112	532.2614	1099.423	1299.503	1818.148	1250.489	2258.648	1631.254	2652.405
CUST_39497_PI429010886	M40440	3.02294E-06	24	15						861.5088	905.9928	1608.753	2279.977	113.7707	1596.49	1645.987	295.8529	1230.895	3434.818	632.4923	1857.76	4012.41	3798.572	2904.283	7459.987	5489.861	8605.765	4378.72	2761.678	4008.648	2049.491	3774.541	2047.134	3924.629	2381.656	612.7708
CUST_48801_PI429010886	M82246	3.1434E-06	24	3						39.63021	44.45593	67.1207	59.38272	65.87261	79.29416	90.12112	43.57254	96.37995	43.96941	31.10352	54.89094	50.10131	57.46071	18.41787	14.5933	31.21665	22.37825	5.596537	30.14806	9.569825	30.92873	27.56589	14.01651	17.44104	49.96679	34.1983
CUST_3253_PI429010886	N14113	3.18019E-06	24	18						43.49402	38.94229	47.7665	36.95845	32.86972	26.77083	42.02831	39.11041	33.14322	33.15745	38.38927	35.43021	43.53457	66.72605	42.55235	44.65936	46.46695	43.08599	43.61117	50.01635	46.68552	41.79921	48.88764	50.73158	41.46642	40.31371	39.10716
CUST_6662_PI429010886	N20425	3.26298E-06	18	6	260787331	hypothetical protein BRAFLDRA	Response to stress	DNA repair	2.32E-62	5.644653	5.430904	9.293644	NA	NA	6.697207	9.266709	NA	10.98328	5.183821	6.039588	10.34183	8.817048	6.897646	4.419529	3.648392	3.154682	5.112944	NA	7.581791	4.353849	NA	7.791167	9.591858	11.80584	10.88207	9.497226
CUST_25885_PI429010886	N53239	3.32985E-06	24	18						17.80788	14.16203	19.50992	13.23813	NA	10.33279	10.21743	12.78096	16.19225	14.54128	17.28228	8.323564	12.49454	18.47174	15.68645	20.72247	21.89139	20.77278	22.4839	20.85338	26.16165	18.05945	19.70331	32.62382	11.66711	16.19225	18.91649
CUST_42634_PI429010886	M60808	3.54231E-06	24	15						251.2413	835.3759	288.5968	227.0582	240.6918	252.989	304.4188	306.9528	321.3471	416.3476	190.463	345.514	350.791	566.3117	544.3805	544.5236	1148.933	761.8025	412.7396	631.0985	365.4954	352.5932	262.4014	325.9381	349.3947	343.4733	401.3498
CUST_52936_PI429010886	M99519	3.66088E-06	18	15						63.74337	92.63177	79.30248	58.03388	49.68059	37.08249	103.1735	76.90215	119.2267	99.18844	85.4597	71.84826	101.4459	71.74885	99.97142	119.3614	129.7636	106.786	84.35052	78.41566	88.61521	64.79523	59.65352	62.90417	77.44274	63.77213	106.0103
CUST_17272_PI429010886	N37685	3.71093E-06	24	21						65.1725	55.27672	87.29938	49.02346	61.97033	54.93024	5.277268	34.96505	31.65179	40.80027	58.20456	NA	56.2194	22.81991	46.97174	54.37948	59.94713	18.94041	94.54537	66.9011	62.02003	126.8388	116.8939	89.01154	172.9208	96.91497	127.2375
CUST_43975_PI429010886	M64509	3.75379E-06	24	9						95.41451	135.1214	103.4142	114.8511	230.649	217.1534	179.2719	162.4604	131.0644	136.0437	185.0545	239.7853	113.622	174.3407	183.5918	213.7437	117.7279	172.8416	94.40167	92.55588	125.3435	84.46858	91.00224	82.87384	98.33769	125.2461	97.79679
CUST_39488_PI429010886	M40202	3.75717E-06	24	0						2103.097	1386.294	1399.933	2277.337	942.7053	1156.223	737.5198	718.4732	260.9147	873.9122	617.9354	478.9906	630.9023	190.9659	503.5534	499.9903	587.733	100.6344	2291.48	735.2877	1312.243	1517.515	2105.784	1479.335	2773.888	1968.185	3499.526
CUST_15158_PI429010886	N34099	4.22962E-06	24	18						79.44437	48.5088	100.8605	103.7192	60.92036	46.06605	60.11411	60.13944	46.33211	108.4858	75.73146	60.95576	382.191	134.6822	200.7515	153.7401	194.4274	241.54	301.8993	487.7778	532.8364	243.8189	531.3775	177.1027	538.1212	227.8105	413.2217
CUST_46282_PI429010886	M72061	4.26119E-06	21	9						73.97121	77.08119	65.08113	92.05562	163.0857	109.9078	92.56838	132.4149	106.2034	105.1035	163.7578	138.8656	96.79055	126.7896	118.993	116.6925	134.0586	110.4383	102.1346	100.4907	85.18385	88.67629	61.44094	89.09696	106.506	88.76092	80.93024
CUST_22664_PI429010886	N47419	4.31957E-06	21	9	284022472	cyclooxygenase [Caprella sp. KV			0.000000201	12.87704	14.49041	21.7713	22.08755	23.74587	33.746	23.12326	25.32605	31.1712	50.51216	28.35209	36.01433	25.55069	22.53853	24.27972	24.88093	26.73698	21.41224	34.63148	22.03386	20.09102	18.54665	17.59796	21.7225	26.24865	26.35385	30.19535
CUST_20466_PI429010886	N43502	4.6454E-06	21	6						157.5632	179.1302	123.4547	225.9507	222.3734	140.0219	302.345	383.478	440.7516	149.7452	100.0093	156.8206	251.0027	281.2628	74.86678	65.7688	149.578	153.409	43.05532	52.43098	62.63588	39.50565	139.9665	196.6443	65.60638	438.8282	281.8768
CUST_38028_PI429010886	M22894	4.74616E-06	12	9						10626.1	2749.137	9444.83	16288.46	4145.789	7111.995	5593.889	8042.108	12442.6	20222.93	28561.19	16760.98	27347.94	7270.382	14439.79	824.7782	317.3105	944.8425	15297.04	8669.063	13906.34	24199.32	29037.53	32295.88	14858.64	21645.19	22711.46
CUST_53741_PI429010886	M103029	4.98279E-06	12	0			Transport	Neurotransmitter transport		64.30529	61.84336	68.35388	47.05022	43.61505	35.53811	40.36067	39.38283	42.53392	53.79961	49.33322	49.78973	68.20885	65.66275	59.09232	63.88253	52.82485	45.54712	46.08569	46.61175	55.61954	55.88596	48.40188	57.58975	67.31813	50.55672	66.98556
CUST_2841_PI429010886	N13222	5.08223E-06	21	18	321469638	hypothetical protein DAPPUDRA	Metabolic process	Oxidation-reduction process	7.12E-67	NA	27.93918	22.84451	NA	6.834174	5.667368	5.386403	21.47745	9.92008	25.7544	13.42054	12.79947	NA	24.98919	32.72715	38.9642	57.13485	25.41267	30.31074	77.33923	49.41341	48.27158	20.42404	66.02134	NA	6.856268	NA
CUST_31101_PI429010886	M704	5.16508E-06	12	0						924.3063	1144.272	986.649	802.754	700.5788	589.1435	564.5167	744.9105	746.9967	745.5832	818.0478	1003.255	925.4144	808.2743	993.1872	794.3963	972.8403	918.7311	569.4689	688.6527	447.5258	686.6456	875.8259	738.0615	939.4725	944.1713	1420.628
CUST_38450_PI429010886	M26781	5.32589E-06	24	12						135.8001	165.4279	176.3993	816.0394	277.5468	695.1268	567.1816	576.4765	1000.348	196.7017	1293.42	248.4962	964.6221	951.856	302.6664	446.0553	412.0092	397.2907	263.3104	178.3834	199.421	230.3006	184.4707	259.9408	65.08782	87.72633	90.12112
CUST_39116_PI429010886	M34718	5.3371E-06	24	3						158.9219	221.1265	215.5592	372.6214	531.3775	451.2333	369.4099	327.5322	172.2883	188.656	368.8823	379.6086	296.1058	316.5315	249.5189	265.1693	149.129	219.4299	123.1444	77.9006	85.7431	127.8904	320.8212	246.1269	212.5696	256.2294	374.0444
CUST_28152_PI429010886	N57382	5.53935E-06	12	3	457871162	transcription elongation factor [	Protein metabolism	Regulation of translational elongation	1.61E-09	42.16378	45.81835	NA	81.07922	NA	105.6633	86.21825	48.12354	44.37034	46.90198	29.3303	13.50964	44.37502	69.23652	60.24608	266.6324	107.1549	113.92	109.5302	9.308623	95.46305	19.50992	29.6361	15.51127	66.31026	50.25143	NA
CUST_54935_PI429010886	M108205	5.55948E-06	24	21			Metabolic process	Carbohydrate metabolic process		384.5219	738.0615	428.5726	426.1127	465.6755	346.9643	212.3717	306.5038	331.9294	262.4813	405.9933	275.9447	521.3103	373.5014	445.0179	538.6242	667.6928	505.369	568.8248	449.1793	425.8369	910.2628	564.8143	585.2891	831.0254	376.7239	543.6971
CUST_48438_PI429010886	M80321	5.55948E-06	24	21	197940771	Euphausia superba mitochondri			2.34E-57	415.7252	592.8013	578.1548	377.1487	400.7119	313.0268	214.3618	346.2958	343.0574	424.3124	385.6874	336.6041	618.3282	382.5381	534.1168	448.2125	618.5099	332.4145	571.275	501.9234	548.9123	741.6851	666.3032	621.285	791.8334	493.1272	678.5721
CUST_34497_PI429010886	M9846	5.6336E-06	24	18						203.8839	132.6306	338.9144	185.8751	51.6582	106.4444	283.5165	407.7964	264.9542	449.9406	196.3246	253.9457	1935.604	1222.23	438.6999	1185.739	530.9393	1374.54	1743.671	3565.017	4715.833	673.5376	131.6028	499.5403	2824.276	1110.326	1122.961
CUST_14414_PI429010886	N32843	5.6336E-06	24	21						4798.856	7353.952	5322.868	3735.581	3224.848	3027.036	2335.81	4495.71	4072.42	4928.217	3478.142	2618.303	5783.496	3071.483	5476.032	7194.306	5799.539	5151.779	6125.57	5840.985	5872.99	8238.918	6799.146	6622.666	9804.488	4928.217	7861.824
CUST_22545_PI429010886	N47216	5.64673E-06	24	15						1417.404	1485.178	2010.612	1250.867	1393.298	1285.646	1044.09	1041.894	1191.068	1506.647	1001.191	1329.124	1568.389	1102.848	2039.749	3085.208	2114.8	4539.587	1858.682	2273.452	2343.924	1812.631	1182.042	1585.44	1723.472	1249.272	2679.375
CUST_17952_PI429010886	N38896	5.68872E-06	18	15						125.0755	66.87814	42.28326	32.07484	15.34805	41.65139	75.30381	46.63196	49.82313	115.8791	30.53244	58.25453	109.1371	73.9144	70.79864	624.5971	183.4518	405.8942	69.6885	69.28931	18.38792	30.5034	39.65854	23.26849	31.1591	32.57817	18.27355
CUST_2235_PI429010886	N11936	5.70773E-06	24	21	340725870	PREDICTED: arrestin homolog [B	Signal transduction	Other	2.17E-39	334.8644	368.4034	350.7074	315.2731	287.938	265.1693	196.4115	209.666	285.4272	325.7046	295.9223	249.7708	319.0038	248.554	375.2193	361.7315	374.4283	301.4906	440.6377	357.8637	257.678	527.9796	504.6417	428.2506	541.8648	312.3683	403.6726
CUST_39147_PI429010886	M35264	5.77116E-06	18	3						437.4032	215.0811	375.7494	561.8923	551.2795	488.8743	360.8837	272.0128	340.8616	295.0388	369.6716	315.6961	378.0804	370.4445	298.2569	130.5163	75.264	96.31678	332.1692	305.1178	387.3552	495.5669	460.6841	492.2464	333.3795	326.5385	504.4324
CUST_43128_PI429010886	M62133	5.85361E-06	18	3			Metabolic process	Nucleobase-containing compound met		34.54981	17.84159	56.03663	57.58975	49.26677	66.21459	14.72662	31.87855	49.95516	36.01049	36.05055	28.26373	35.15355	26.05011	27.28454	22.35262	20.67603	19.43172	42.80977	35.46629	32.77639	79.1244	44.31933	38.06618	44.635	53.48046	42.71301
CUST_16832_PI429010886	N36935	5.86201E-06	24	15						279.8449	370.5351	321.0799	301.8993	315.8311	273.134	318.037	403.0078	226.8253	349.1843	301.2626	297.8825	391.8616	334.5944	317.0103	416.251	475.7174	448.0872	373.8469	364.7211	382.4457	371.7268	358.247	375.415	345.2692	342.9765	211.3027
CUST_35953_PI429010886	M14087	6.07269E-06	24	15	196000326	hypothetical protein TRIADDRA			2.08E-12	15.52429	19.31757	NA	6.462617	7.772157	8.132185	47.05022	26.69789	69.64615	83.55746	51.29155	NA	18.1699	34.6239	7.779225	137.8653	80.44707	90.66543	31.53769	42.45213	47.19838	42.1373	NA	27.24634	40.634	9.083891	16.16791
CUST_47055_PI429010886	M74665	6.19161E-06	24	0						951.5195	2271.439	1651.623	447.759	292.0008	95.61224	739.59	1670.335	1267.497	167.3103	356.2511	88.12342	366.0882	347.8293	126.6958	400.7119	486.8888	429.4693	757.1366	1332.694	958.1681	683.6777	814.9423	697.0808	1358.461	1179.914	865.6479
CUST_5692_PI429010886	N19053	6.20184E-06	24	21						1329.875	1568.389	1715.071	1473.47	1201.304	1149.936	1193.156	1280.738	1444.952	1127.159	1271.126	1067.28	1305.464	1240.074	1360.186	1132.475	1587.121	1436.159	1363.959	1497.206	1620.506	1530.86	1770.862	1873.347	1664.278	1340.037	2096.347
CUST_51711_PI429010886	M94674	6.33612E-06	12	0						137.4234	116.2155	131.5859	76.51051	50.6629	63.1189	61.51933	52.25224	73.97753	93.31746	105.3966	119.1916	128.6276	148.107	134.4996	64.48863	95.50727	67.7294	58.30569	32.33466	36.89757	161.8917	113.0417	97.82425	106.1766	107.365	78.9339
CUST_14139_PI429010886	N32385	6.45671E-06	24	15	198432355	PREDICTED: exportin-1 [Ciona in			5.72E-62	13.87684	15.44101	19.86967	4.902653	4.844365	4.471808	18.84339	7.560056	16.03034	12.59391	11.36156	14.30044	20.98727	13.68381	19.79478	24.5024	26.61351	23.5934	22.55063	34.76036	18.45917	20.04202	17.4518	23.422	18.88565	17.82688	27.01915
CUST_7015_PI429010886	N20917	6.68905E-06	24	18						80.95294	NA	100.771	14.97194	15.37323	4.046361	NA	70.45711	63.39626	56.11745	NA	33.69474	149.0265	50.52247	80.65861	29.58249	76.5633	103.9956	196.4999	401.757	475.7174	220.0171	NA	177.9352	93.67836	41.31713	40.93434
CUST_37461_PI429010886	M19113	6.86364E-06	21	12	157118946	NADH dehydrogenase, putative	Transport	Ion transport	4.36E-30	79.81483	43.16345	53.13079	55.25246	54.69951	41.28987	63.45821	45.44669	42.42056	70.53623	78.44375	89.95321	79.78242	71.67272	98.14527	89.00153	66.74815	80.46117	62.27316	56.92501	68.68072	55.85351	49.10462	62.80714	39.14498	47.87448	51.92132
CUST_40466_PI429010886	M55209	7.3316E-06	21	0						117.8894	47.34933	138.5034	21.35681	26.53793	29.76443	11.42758	18.20374	19.44853	19.09713	26.71367	33.10821	33.32109	28.31587	43.20078	64.90539	33.64664	28.26843	46.71775	33.22829	39.02298	61.63075	29.08472	42.80111	44.85324	31.97376	35.86995
CUST_15933_PI429010886	N35371	7.38333E-06	18	3						55.08315	68.56321	62.98013	56.48171	108.7707	61.95078	38.76155	58.25453	41.96937	47.87148	52.52277	44.50237	31.3526	30.97694	13.09363	52.52723	53.18236	52.11425	55.65083	67.87655	81.67118	53.63563	69.07482	90.52573	49.93448	59.31427	60.97733
CUST_36366_PI429010886	M15719	7.49353E-06	18	12						143.7214	245.9078	204.1402	121.7366	246.4965	146.2169	153.5202	186.792	194.4885	185.937	197.088	222.6199	271.2533	279.7784	382.4457	208.8952	270.6947	249.888	175.6882	192.3094	181.6944	145.0599	168.511	129.5335	148.4065	141.5173	150.3698
CUST_34311_PI429010886	M9297	7.51635E-06	12	3						150.9062	177.4828	190.5906	181.5978	233.0946	196.9337	177.9607	186.658	172.9098	137.3382	174.6876	169.1505	178.3834	189.8628	170.4605	242.9432	233.9614	210.7377	145.0185	156.8401	131.8408	122.709	128.3485	121.2857	159.7501	195.0513	258.0601
CUST_7688_PI429010886	N21924	7.58895E-06	24	18						985.4686	777.0993	1124.051	985.8154	746.9967	771.5383	569.0422	794.9446	745.3715	830.4195	687.9587	600.669	1066.707	783.1683	768.539	835.3759	935.0962	885.1856	1441.945	1245.448	1424.355	1395.332	1223.646	1132.862	1486.662	826.1267	1578.545
CUST_33676_PI429010886	M7582	7.79659E-06	12	0	338224394	ADP-ribosylation factor GTPase-			1.45E-13	36.52621	24.31278	27.88568	21.9708	15.82041	17.02795	16.6676	12.95843	17.47317	21.10822	5.52076	24.65812	36.16824	26.05879	25.11048	23.65648	28.67614	22.35703	18.54037	19.30796	11.54107	33.26038	19.28668	17.63683	36.05055	35.89334	28.52375
CUST_27104_PI429010886	N55454	7.92038E-06	24	21						159.2473	92.13598	223.1865	NA	45.01222	38.6365	12.98896	27.15149	9.09449	113.0417	113.3132	199.908	7.473014	41.39421	21.96186	223.0141	96.7742	101.7154	213.4477	75.58915	270.6947	662.3233	386.4267	336.3009	84.11819	77.58626	75.63986
CUST_42560_PI429010886	M60585	8.01234E-06	24	0						33.71063	24.81983	40.26358	23.76937	31.4218	23.49626	22.48649	22.94786	21.29385	13.27388	13.79482	21.3729	16.0527	19.68126	16.79051	37.16734	17.12548	17.68077	36.22942	30.22988	19.60068	24.00156	27.37887	19.35535	45.84356	31.87047	29.75114
CUST_4522_PI429010886	N16960	8.27288E-06	24	3						19.29772	12.17645	104.7593	15.21906	44.84231	16.61149	7.318417	13.10406	29.79615	8.210092	14.47659	13.05124	12.14327	6.734835	3.609099	6.598734	4.632417	8.994279	9.923975	11.12081	9.538918	5.379272	11.84707	14.7781	15.42011	14.26389	17.26844
CUST_53216_PI429010886	M100760	8.35786E-06	24	3	223670745	alanine racemase [Marsupenae			1.58E-20	19.30163	23.37085	20.07697	32.46193	27.19181	27.22159	22.39833	27.97979	27.42998	22.87088	23.75071	24.485	18.77059	27.9284	17.68077	10.56207	18.97317	17.13864	14.8912	16.33804	13.81248	16.42131	23.91812	13.50148	24.29996	20.29507	15.10046
CUST_52335_PI429010886	M97104	8.71101E-06	24	21						182.2404	229.0326	254.9746	168.4808	180.1675	202.0095	162.6721	180.9942	187.5559	184.5044	158.1747	215.5226	186.1968	166.3679	198.0827	247.0114	214.2802	210.5427	228.29	216.3122	203.1024	286.5775	233.8809	290.3829	274.4544	232.4886	253.1631
CUST_1613_PI429010886	N10239	8.78425E-06	21	15	242012413	Coatomer subunit gamma-2, pu	Transport	Protein transport		102.9317	360.6127	210.991	276.7278	343.406	231.4946	297.7147	402.1648	336.7218	493.2216	511.4855	519.6084	435.2817	452.142	347.2002	502.9461	634.8724	571.9784	387.5344	393.4684	297.9948	252.401	225.4711	261.0327	339.4175	543.3224	311.1315
CUST_11408_PI429010886	N27759	9.06416E-06	24	18						2177.847	2001.707	1906.005	1803.597	1005.258	910.9934	3313.258	1636.385	2620.241	2144.939	969.8589	1127.686	4445.119	1854.805	1257.339	3020.109	2166.525	3127.267	5039.165	4685.458	5304.997	3516.69	3373.758	2577.27	2893.96	3284.996	2507.724
CUST_2639_PI429010886	N12811	9.1654E-06	24	15			Protein metabolism	Cellular protein modification process		53.081	72.77442	74.93498	37.17924	42.09646	38.14824	61.77408	57.69989	67.50363	60.0628	48.6368	68.33012	71.0111	53.28017	60.47985	77.76407	91.51354	81.04337	73.65204	62.76069	84.5309	65.11364	60.11411	80.23561	47.60538	50.7989	67.69664
CUST_53681_PI429010886	M102785	9.28354E-06	21	15	498936229	PREDICTED: ovochymase-1-like [	Protein metabolism	Proteolysis	5.62E-09	294.0637	645.7283	507.9246	319.8028	337.4051	214.0769	477.1875	469.784	564.5167	517.5058	464.4221	188.4898	466.3373	559.5956	557.036	1212.98	762.4429	941.0274	557.036	519.1671	702.1022	499.3569	308.8108	431.4309	446.7799	332.5739	369.8268
CUST_32981_PI429010886	M5626	9.29959E-06	24	21						179.0994	179.5567	215.9822	150.3467	186.3368	136.8604	92.92674	128.4222	117.6651	164.8179	169.6456	132.9821	206.0093	133.196	186.9781	195.9869	202.7406	146.7658	315.18	156.6771	164.5654	335.4857	215.2625	217.7022	337.9166	161.0268	195.1701
CUST_42252_PI429010886	M59804	9.29959E-06	24	21						113.7894	104.1208	107.9823	82.45499	91.6474	91.71339	33.33788	84.78014	67.43392	89.1801	95.27189	73.7088	131.7124	72.50711	151.9479	116.2734	120.9166	83.4815	183.4751	120.7334	138.8656	211.4099	138.2596	172.5896	265.541	105.9997	212.1398
CUST_46065_PI429010886	M71240	9.83933E-06	24	15	291232140	PREDICTED: RNA guanylyltransf	Metabolic process	Cellular amino acid metabolic process	8.57E-16	13.29205	13.08456	20.28829	12.34559	16.68416	11.70368	19.29772	17.86876	16.97405	20.49356	10.53196	20.08859	26.81517	18.72559	12.8605	21.99703	21.70516	26.91338	24.82555	19.84259	20.59384	19.81872	13.55607	17.99711	17.60139	12.46071	16.8636
CUST_47369_PI429010886	M75818	9.88042E-06	21	18	13898841	CUB-serine protease [Panulirus	Protein metabolism	Proteolysis	4.61E-09	25.62527	27.9125	39.33451	16.99266	10.76358	11.63109	24.52901	68.17964	47.19052	47.14137	38.50344	6.963509	51.98033	46.33211	39.20662	92.26014	57.73118	93.04693	71.23962	98.12003	89.95321	64.2158	11.72058	42.55235	37.73322	7.734794	29.76443
CUST_42209_PI429010886	M59694	1.01668E-05	24	18						21.8116	38.30668	39.12428	24.27972	47.95677	32.99838	27.62832	38.42576	38.78289	33.09195	28.33805	36.85694	41.85499	54.88564	40.4062	44.03643	54.11401	80.15283	71.89101	58.76888	102.5315	47.00541	52.17564	59.42381	40.0648	43.87393	39.84392
CUST_37962_PI429010886	M22486	1.02654E-05	15	9						70.80726	118.9114	144.7014	194.4561	94.52718	55.76126	147.0744	128.3309	257.2967	430.3398	309.9338	325.3527	436.3894	242.833	217.2732	30.41985	67.07909	35.56743	92.7356	66.03803	142.5195	301.5476	248.1114	295.5482	170.4605	194.8762	455.3201
CUST_44116_PI429010886	M64987	1.05133E-05	24	15						427.1681	1276.272	550.4921	350.649	302.1401	340.7195	418.5156	558.2968	480.9139	685.9099	474.5724	449.3664	557.162	455.8434	588.164	1171.1	1839.274	1256.746	872.6161	654.9488	842.6773	520.6055	813.5332	661.1838	709.2377	638.4715	761.0743
CUST_56944_PI429010886	M114968	1.1452E-05	18	9						10.22784	13.67765	12.16729	9.290836	14.49503	11.4263	11.58566	12.59938	14.25286	19.23624	22.93309	24.82555	17.21755	13.48116	12.7945	16.08831	13.16217	15.33609	8.371114	13.65208	9.504797	9.317073	6.344447	5.785239	13.97991	9.508309	14.48048
CUST_42313_PI429010886	M59979	1.17564E-05	12	3						57.56013	43.81166	36.32296	116.5601	127.5646	160.389	81.81979	64.92793	64.07605	29.72388	37.49842	27.97979	59.83108	45.53592	41.92055	149.8334	68.82621	103.7428	93.90326	27.38699	100.2134	64.52421	23.3627	54.76127	88.43455	104.203	55.78477
CUST_41879_PI429010886	M58780	1.2104E-05	24	12						88.6413	77.2068	53.5836	116.7583	124.2797	103.0482	96.94761	106.2034	121.3126	197.2216	154.7807	176.8026	240.3058	194.4561	239.2059	121.0574	170.9358	208.794	97.00725	112.0077	71.64925	135.864	115.9671	202.3086	128.2244	96.25473	83.14687
CUST_8393_PI429010886	N22976	1.28268E-05	15	6						104.9377	73.39823	70.70842	119.5986	61.61447	95.14144	499.8092	161.9628	402.2649	116.0279	78.04556	109.3354	99.04563	51.83065	91.45416	109.425	80.63078	112.2221	106.0541	107.696	117.2299	154.4454	127.5443	162.7885	117.3803	76.7295	141.2055
CUST_10273_PI429010886	N25984	1.28714E-05	21	15	195494829	GE19913 [Drosophila yakuba] >	Transport	Ion transport	3.23E-26	12.38261	19.44173	17.5341	13.0479	11.88722	19.11956	13.25033	39.00145	17.927	NA	35.62662	13.18135	31.39279	28.08439	20.89958	45.13797	31.22074	26.95753	22.83896	21.03435	30.01166	9.169725	20.4296	20.63115	5.964848	11.27831	12.2785
CUST_31663_PI429010886	M2104	1.29278E-05	24	21						1282.19	1710.003	1352.068	1228.382	1039.627	873.2461	826.9656	890.6088	971.9693	1164.205	1072.507	1180.485	1219.279	893.2381	1182.698	1347.499	1260.62	1387.245	1417.404	1186.54	1100.31	1670.335	1305.464	1520.832	1719.004	1274.879	1987.037
CUST_41260_PI429010886	M57268	1.29278E-05	24	6	530567046	PREDICTED: carbohydrate sulfot	Metabolic process	Carbohydrate metabolic process	1.59E-16	37.80064	30.65156	40.28969	87.10926	70.36116	72.26194	73.13284	112.5245	116.0138	44.73914	86.81904	45.33378	25.45483	94.39327	106.8768	39.41457	82.56422	59.75785	52.96757	53.64076	34.24084	29.53809	28.86593	28.95792	38.97889	38.24515	42.06849
CUST_38075_PI429010886	M23222	1.29367E-05	18	3	321455420	hypothetical protein DAPPUDRA	Metabolic process	Carbohydrate metabolic process	6.7E-81	81.75003	67.88505	78.5366	301.3368	109.2822	149.3381	88.98877	116.1509	56.37975	38.81388	36.71863	26.01047	69.86685	15.3089	56.2194	80.77747	82.40323	5.385232	109.3921	36.89757	61.3302	221.0635	304.4188	195.1036	218.7135	142.126	188.5963
CUST_49139_PI429010886	M84314	1.31125E-05	24	21						75.2089	96.53451	119.8767	52.83064	37.44599	56.85229	36.18918	78.43006	68.92908	108.7551	64.13867	59.16434	95.96208	77.49407	108.1394	110.5907	148.2361	93.71999	114.9073	109.561	91.16754	225.9507	113.2581	158.9424	112.8468	94.65834	90.29464

Probe ID	ID	p-value	Period	Phase	EBI ID	Annotation	GO category	Go term	E-value	0	0	0	3	3	3	6	6	6	9	9	9	12	12	12	15	15	15	18	18	18	21	21	21	24	24	24
CUST_11197_PI429010886	N27424	1.33117E-05	18	9						11.97474	13.14544	8.631384	15.48991	14.74939	19.43327	22.53853	42.25942	44.83771	25.42345	46.04292	51.8805	13.40443	13.91427	5.447639	39.48899	11.37669	37.98691	9.576311	8.247584	12.90521	30.18612	14.7781	9.406434	55.39639	43.88384	24.93287
CUST_38504_PI429010886	M27503	1.35472E-05	15	9						482.9421	602.7354	583.0039	459.4079	310.2931	377.0504	663.5871	507.3489	533.6749	681.8481	755.8283	586.5026	704.9081	627.1961	576.7675	663.1483	618.1517	554.6469	378.0804	388.382	384.2558	663.8187	444.7023	651.0737	656.4851	509.8873	752.0705
CUST_42228_PI429010886	M59736	1.38512E-05	24	21						110.8393	123.675	139.9139	111.4946	105.9717	109.3218	85.26437	103.657	112.2997	107.9218	99.51051	111.5065	109.0861	106.3835	119.5461	155.7011	119.0449	132.3978	140.4625	114.2182	119.3101	166.5929	125.4319	153.2351	131.8851	115.0038	137.7644
CUST_11325_PI429010886	N27624	1.40359E-05	24	21	307190239	BTB/POZ domain-containing pro			3.29E-51	127.6821	121.5207	111.6419	98.71772	91.98987	77.16447	92.16269	90.19219	69.79048	98.91443	87.15453	162.165	101.98	105.8805	141.3865	113.5681	106.8903	128.2527	109.0351	112.1553	131.4171	133.4052	140.3412	198.1201	109.7929	121.3825	171.7541
CUST_44183_PI429010886	M65195	1.44053E-05	24	21						267.5084	338.4518	380.6302	318.4002	252.8481	219.8411	157.1328	281.0852	268.9804	314.7154	299.2328	213.152	660.6448	292.5506	410.9137	296.7459	484.4076	312.1009	493.728	415.8814	446.7799	660.9643	475.5631	526.1263	660.4277	329.4331	469.6287
CUST_2564_PI429010886	N12639	1.45756E-05	24	15						75.12194	101.7856	134.9328	67.18486	88.4112	94.98744	102.6741	87.17685	96.80429	100.9966	90.56235	73.52981	101.3719	109.6493	103.7123	163.488	257.6419	229.0326	135.397	98.4933	136.2198	103.4142	92.53323	103.6807	94.04538	92.39145	116.1322
CUST_41715_PI429010886	M58374	1.46017E-05	21	15						66.0686	50.45124	24.97563	54.87487	27.36851	27.67843	38.40048	67.48873	65.69754	67.87655	67.19251	32.23946	80.84339	39.10716	37.39308	128.2695	68.24011	161.8663	93.08615	46.49241	65.82127	50.4056	45.58935	43.98563	25.91577	21.7829	27.03026
CUST_27765_PI429010886	N56656	1.5345E-05	12	9						76.00489	61.10664	63.45821	95.91396	48.65167	49.33322	48.95167	62.98013	65.67004	113.8069	196.7753	122.0363	115.6261	64.38614	73.24134	35.54077	31.81107	40.28969	48.81917	39.60825	39.76397	103.0416	143.9614	146.5888	61.06467	98.96283	126.7539
CUST_38261_PI429010886	M24805	1.60543E-05	24	15						243.5905	314.9328	355.5754	226.205	212.5696	238.4834	270.6292	288.1795	274.5195	319.4595	288.3603	298.2569	465.3594	238.6215	298.439	391.1906	439.9905	454.5758	387.249	310.1119	359.943	345.1067	307.1973	365.4091	368.8823	299.9819	435.1623
CUST_4608_PI429010886	N17124	1.61088E-05	24	6						536.3161	683.6777	889.8299	1098.604	836.3643	1091.098	1154.406	929.9574	1257.92	527.6644	906.4938	616.2414	877.7497	1138.567	880.5962	129.1955	104.258	208.404	450.3327	873.9122	317.9491	445.9579	301.4906	290.029	477.7276	737.2275	128.2402
CUST_38574_PI429010886	M28143	1.63127E-05	24	21	326781697	crustacyanin subunit C [Fennero	Response to stress	Defence responce	5.92E-39	495.0948	194.0917	575.2243	559.1602	293.0253	151.3747	103.8712	107.2469	183.5637	135.1101	317.1285	94.83653	312.4297	55.91239	192.8968	110.9089	212.8617	69.77877	2659.616	832.894	1495.315	4870.528	1643.622	2749.137	1774.191	325.3527	1390.116
CUST_32899_PI429010886	M5422	1.65564E-05	12	9	13624645	arrestin [Libelloides macaronius	Signal transduction	Other	1.13E-95	46.38013	67.14461	53.82263	43.83432	31.53769	45.5838	61.6196	56.98746	69.39785	103.5895	94.31617	90.14554	78.518	73.4071	74.10459	32.4397	23.5771	27.01915	47.7889	79.92025	68.27308	77.65458	76.92811	28.00193	83.08821	66.3578	72.62444
CUST_14003_PI429010886	N32159	1.66881E-05	24	18						29.83087	24.28357	22.43976	21.89139	25.8029	20.82309	17.92495	21.1277	25.90137	28.93482	18.2576	26.27536	22.11134	27.79096	32.70202	29.0511	21.7829	30.43559	39.76763	36.7	35.30149	40.55055	29.4873	37.29216	34.69802	25.12586	25.93287
CUST_11117_PI429010886	N27293	1.67067E-05	24	0						17.81749	15.77123	14.39849	29.83399	8.156982	9.370822	NA	10.35947	15.19805	27.35507	6.423762	7.717206	3.643247	3.805914	NA	6.844388	4.508663	NA	10.76826	7.259084	5.375793	9.782412	11.66289	9.853533	19.56045	16.18394	16.54899
CUST_33903_PI429010886	M8195	1.68675E-05	12	6						6.256607	24.95555	26.53793	19.32541	28.04196	26.26192	32.64017	32.44579	37.30482	23.38184	27.44734	25.71381	14.00894	18.54665	25.5482	26.20838	31.86686	32.93138	27.99056	32.85244	37.7496	21.3257	26.78263	21.54205	22.72787	26.11949	11.39401
CUST_47620_PI429010886	M76909	1.7338E-05	21	3	390333997	PREDICTED: RNA-directed DNA p			1.79E-12	17.50958	11.99747	12.41811	26.55873	19.02577	20.6404	21.83781	18.57947	19.31397	19.38111	17.50536	17.57039	23.34681	15.44101	19.88289	12.61565	17.4076	12.21233	13.1101	7.678598	14.52879	20.59384	20.45768	17.30435	35.96649	20.57756	26.346
CUST_30745_PI429010886	N62542	1.76956E-05	15	12						50.64865	53.5836	45.47674	44.1584	34.08501	51.48244	20.56044	23.82015	27.65463	28.3093	28.6389	28.53702	55.43214	53.70551	67.90691	47.39716	48.39798	40.86304	36.31158	42.53392	48.28205	44.06952	30.56155	36.91238	40.23235	35.4732	44.16632
CUST_19941_PI429010886	N42487	1.7897E-05	24	18						55.92368	5.03462	53.33823	27.54533	21.43518	NA	38.35367	128.1168	77.96514	130.3441	12.17645	76.08583	216.896	135.9051	58.57991	173.502	140.7771	189.1039	250.35	423.1563	572.5541	118.7214	NA	96.89897	311.8576	105.7248	214.3188
CUST_19924_PI429010886	N42458	1.80024E-05	12	3						52.27137	12.74627	NA	40.96379	59.25391	64.06114	31.22451	17.18131	3.860008	22.42126	29.91504	14.68427	39.61981	51.56856	33.69474	98.84501	40.86771	54.03718	36.01433	NA	39.77797	NA	13.79883	3.396718	30.59991	23.60789	NA
CUST_39965_PI429010886	M47565	1.849E-05	24	12						68.34584	178.0606	99.43494	438.2151	147.9373	259.3611	512.6794	651.9522	855.8718	160.5879	1746.391	154.7807	767.6438	801.0558	341.4287	241.8418	254.1863	308.4293	124.1767	167.1713	178.9536	108.5581	334.1482	256.9019	15.91592	111.355	103.8983
CUST_47182_PI429010886	M75089	1.86049E-05	15	6						171.722	34.87919	52.47367	501.0897	122.709	76.46737	666.6396	145.843	752.4222	212.8617	180.3948	240.3937	165.8756	287.5406	14.95707	26.86328	11.25405	25.97013	60.28486	311.8576	142.8672	689.88	587.5636	854.3715	158.132	858.7677	542.9984
CUST_31426_PI429010886	M1498	1.9226E-05	18	3						715.4355	340.4211	909.1828	935.4596	884.7471	864.5859	370.6325	531.2109	657.7333	451.9245	458.3577	456.7205	777.6219	466.3373	620.4556	370.8194	312.0575	324.2431	758.1822	562.0064	658.5111	852.2653	907.1782	745.1009	691.8963	630.7177	877.7497
CUST_54469_PI429010886	M106157	1.97833E-05	18	3						12.83426	10.17486	29.7311	42.87617	25.44551	31.19127	7.933892	13.22308	28.40807	14.49179	8.623114	5.097218	15.40011	17.04802	12.57186	9.956264	4.590859	4.079769	14.98029	12.70744	14.26253	25.74244	17.23806	17.68234	17.16032	25.46958	22.93584
CUST_22555_PI429010886	N47228	1.99463E-05	24	9						69.08095	104.7593	78.33954	115.7562	189.7104	210.8385	225.5927	142.9154	124.5835	197.1276	163.8654	138.2203	177.6996	90.47296	183.4518	150.2498	165.0676	130.4587	123.0866	129.7428	131.0463	116.2897	114.2182	138.2964	103.8305	111.6675	93.26197
CUST_37979_PI429010886	M22563	2.00472E-05	24	21						816.5697	671.5725	756.6003	625.9001	450.6028	518.0684	220.6649	381.5238	370.0497	598.0964	491.5182	377.0504	730.7886	228.3687	572.9401	532.2614	690.3228	180.9657	963.1582	543.8441	782.6486	1136.704	1102.526	855.8718	1654.651	811.6064	1366.714
CUST_48484_PI429010886	M80570	2.09064E-05	24	21						1621.358	1773.012	1678.527	1488.548	1025.404	937.9336	714.2812	974.9689	873.9122	1589.718	1047.687	682.3134	1906.005	740.8082	1521.677	1393.878	1920.18	799.983	2273.452	1445.709	1846.233	2622.032	2119.199	1945.845	3751.984	1715.071	2543.39
CUST_23787_PI429010886	N49461	2.09794E-05	15	9	321478405	hypothetical protein DAPPUDRA			1.8E-40	105.8953	122.7362	102.9823	125.4485	137.5851	108.2394	153.4511	153.7672	152.4151	175.4084	149.0265	155.0803	153.1975	133.3487	158.0057	161.3784	127.3153	128.1317	127.259	132.8754	149.6035	138.2964	153.6889	172.7187	159.6864	165.742	196.1132
CUST_32190_PI429010886	M3481	2.11928E-05	24	21						3702.472	6461.658	5525.521	3568.199	3940.028	2983.608	1789.218	3041.467	3098.696	4508.46	2996.567	2751.324	5686.353	2757.04	3310.069	4940.836	6676.906	3664.856	8321.498	5587.034	4919.057	8945.499	5966.983	5833.315	12674.82	5756.98	6913.964
CUST_13764_PI429010886	N31731	2.16455E-05	24	9						11.17863	NA	NA	8.3196	28.90326	15.35881	14.90623	9.368146	10.16018	16.03888	18.74591	22.25295	11.45197	20.33857	10.24931	5.226331	4.935572	5.996867	7.473014	9.673796	4.46262	6.285156	3.666007	4.553311	6.284027	6.970533	7.471039
CUST_10308_PI429010886	N26036	2.17656E-05	24	21						190.5205	200.6783	304.6294	170.9358	155.7673	175.8219	70.06906	120.7183	130.7209	238.9649	154.5921	194.6876	227.7726	150.4102	267.7885	354.6555	249.2581	271.8944	342.6821	191.5454	215.8263	460.6841	247.6563	328.9434	436.601	205.1165	345.1684
CUST_719_PI429010886	N6910	2.17656E-05	24	18						7157.757	7147.632	6376.466	4061.362	2551.022	2371.181	6649.946	5068.287	7558.705	5322.868	3130.289	3082.237	7950.355	5476.032	4140.108	10408.52	8669.063	9330.025	14470.28	10109.9	12807.83	7048.129	9241.731	6543.051	11429.91	10506.53	9167.401
CUST_16510_PI429010886	N36354	2.18312E-05	24	18	322782929	hypothetical protein SINV_0537	Transport	Transmembrane transport	1.78E-24	45.61423	62.53388	118.1166	53.95752	33.13254	13.10546	6.804117	50.72013	50.15592	26.80276	24.9948	14.55272	40.34223	22.55249	56.50326	51.46015	47.01945	43.41687	153.9484	123.305	143.2644	98.13418	51.08768	75.07482	134.6275	44.23244	103.7192
CUST_1638_PI429010886	N10306	2.2052E-05	24	21						183.9367	172.7187	217.909	153.1826	170.0456	148.4065	93.22902	133.2629	136.6363	189.9176	157.2999	143.647	211.8745	146.4225	192.0011	192.1306	220.5454	121.5461	314.0471	187.0484	164.3905	360.0208	203.9603	215.4758	325.1203	190.2229	208.8218
CUST_3634_PI429010886	N14933	2.21431E-05	24	18	321461243	hypothetical protein DAPPUDRA			4.67E-15	604.0998	1359.053	1228.932	600.2455	442.2313	221.9709	798.8065	591.4732	1290.575	252.0561	623.6004	269.2665	672.7456	363.9263	380.915	373.5884	1005.258	872.0312	1904.856	1556.093	2449.312	924.3063	1456.541	1807.527	1095.503	754.5011	1472.69
CUST_7824_PI429010886	N22136	2.21431E-05	24	21						100.3016	75.13872	71.30275	89.57069	54.37537	70.32352	28.16113	43.10438	35.75625	82.82852	62.7253	35.15693	47.17279	20.82309	39.21008	40.36067	50.45617	5.170512	170.2707	79.30248	103.3338	125.5586	158.0988	112.0856	148.0804	111.007	182.2041
CUST_3288_PI429010886	N14189	2.22033E-05	18	15						478.0895	287.3062	200.9666	90.98859	43.70175	52.75072	385.0272	334.6481	318.5699	342.7829	196.3457	82.49497	399.9663	322.3894	214.9341	1429.079	621.285	1296.153	300.3068	539.2249	769.5468	232.9522	31.04592	212.8617	353.2411	132.5501	190.0624
CUST_27822_PI429010886	N56757	2.23005E-05	15	0						59.70409	128.2695	135.7428	49.28723	63.83347	46.86356	19.24654	58.445	59.27468	27.76408	30.15628	40.05574	134.703	48.87698	151.1698	192.8968	74.84749	121.9377	83.1934	71.84187	69.22757	72.04345	40.5799	37.34026	30.31787	55.70902	48.28814
CUST_5618_PI429010886	N18954	2.23383E-05	24	18						35.89334	41.59688	32.47615	31.37822	31.12088	33.86827	38.47726	43.84747	41.56334	47.73425	47.87448	36.18124	40.03773	32.58484	35.77181	42.32017	48.90741	44.73116	52.44267	66.54171	47.61903	42.99816	46.99361	45.83287	41.35381	32.0098	43.39176
CUST_52479_PI429010886	M97580	2.29111E-05	24	3						74.7748	63.41449	120.8801	112.9448	123.935	98.42724	58.67768	95.47293	81.85533	58.49428	116.3062	80.03641	68.08811	91.18387	65.59134	62.23993	64.57231	60.74813	51.71141	57.50494	53.51076	67.97894	89.05929	74.09264	57.14912	92.67361	103.9393
CUST_42396_PI429010886	M60180	2.30377E-05	12	3						10.25273	28.21781	9.731693	35.59076	52.2471	41.18226	35.72862	25.74244	29.23756	24.03468	35.1142	17.82084	7.735995	10.14305	5.578787	50.64118	96.89897	33.82054	18.8882	38.4415	20.50045	15.54537	35.75247	29.85598	15.7085	34.10624	14.33104
CUST_955_PI429010886	N7968	2.31001E-05	21	18	291241039	PREDICTED: Caenorhabditis Epo	Metabolic process	Other	4.8E-56	43.73952	40.04223	54.32526	55.0756	25.87427	29.90874	18.1992	34.44149	34.97429	20.87993	30.07238	28.28634	33.80053	36.96718	21.37642	29.67188	61.06467	43.23211	58.41256	55.08315	73.49385	66.82276	58.9878	50.1087	44.88521	21.53675	36.32916
CUST_57300_PI429010886	E4642	2.38855E-05	24	9	548856974	hypothetical protein AMTR_s00	Cellular component org	Cytoskeleton organization	9.02E-08	271.2533	319.5317	295.6289	236.7562	439.7541	398.088	502.0555	385.0272	362.5008	397.3902	450.394	563.9832	215.9178	382.191	366.157	384.0543	517.7643	411.776	241.7074	226.9579	354.2621	135.4351	205.8558	225.0517	294.3919	371.5772	267.0346
CUST_17081_PI429010886	N37327	2.38901E-05	24	6						43.71813	29.19296	15.09931	63.76387	98.97699	90.21633	148.7099	81.58851	99.66557	69.83801	73.67616	123.8539	NA	100.1016	43.79783	116.9614	64.9627	68.64759	20.35931	90.87055	40.53157	NA	5.684912	3.671	50.38885	65.05732	9.723255
CUST_12694_PI429010886	N29883	2.40144E-05	24	18						232.1097	26.89377	211.2323	310.7267	240.4351	10.05212	89.36499	144.7602	211.695	69.264	8.474912	50.03259	649.7567	305.1744	165.0194	47.64701	71.29445	83.91189	1063.832	828.8502	1186.54	613.3285	968.0088	779.846	271.9604	158.518	403.1106
CUST_31743_PI429010886	M2290	2.42127E-05	24	15	91091526	PREDICTED: similar to bone mor	Protein metabolism	Proteolysis	7.68E-08	170.1972	67.05704	59.98742	17.33888	13.02032	24.33371	134.5506	150.4473	117.7425	270.8095	172.1367	32.42334	238.8236	151.3747	141.3156	867.2487	202.7719	846.6405	186.5169	150.1608	292.4745	191.4096	12.6499	161.7314	248.8552	93.04693	138.4394
CUST_15583_PI429010886	N34824	2.42127E-05	24	15						114.9878	119.2089	119.6713	101.6228	94.92219	92.59408	108.298	113.7096	93.97841	117.6651	95.92856	144.5615	98.52549	110.9429	112.6387	148.1501	143.8649	139.9464	126.0899	131.771	127.4883	114.4474	131.8408	138.8443	129.2412	111.2894	123.8699
CUST_30607_PI429010886	N62217	2.49827E-05	24	18						61.98061	102.4255	88.40609	70.89193	50.16118	60.10219	60.77638	70.48984	72.09362	76.79923	81.28557	78.44375	61.76701	74.69118	62.00499	87.71525	88.37671	94.16687	96.84289	93.20756	105.9309	92.67361	110.1773	105.2262	72.38862	72.67801	111.5466
CUST_460_PI429010886	N5425	2.50552E-05	12	9						5350.891	944.8425	4690.093	9699.55	2345.461	3336.354	2180.402	1035.674	2865.238	22092.06	42662.49	14363.02	27153.68	4594.069	11667.94	164.2936	560.1392	327.3332	5799.539	2526.145	4922.978	30141.56	33963.44	33637.86	11112.95	30352.12	31801.71
CUST_2275_PI429010886	N12009	2.53685E-05	21	6						27.03026	15.55749	15.4847	42.32505	27.37466	NA	46.06104	196.7413	61.49909	14.39152	12.96002	12.34178	46.69101	63.70746	94.16687	13.3386	7.589324	9.970616	3.5127	4.835599	5.33569	24.03275	NA	22.22815	NA	452.9256	NA
CUST_3063_PI429010886	N13719	2.53904E-05	21	3			Metabolic process	Lipid metabolic process		292.7676	71.58388	348.7073	355.0179	210.2979	182.0409	167.3313	416.251	177.2962	81.29518	54.54964	38.00456	89.36499	79.06068	65.94538	132.3978	72.62444	139.3207	275.293	236.6326	176.9139	211.695	148.1737	157.3269	311.3849	378.0804	286.926
CUST_38411_PI429010886	M26337	2.59224E-05	24	15						119.6854	267.9409	183.2542	694.4759	374.0444	496.3451	601.9971	601.5188	898.0277	151.5867	1432.11	410.4537	1547.502	1235.266	361.9725	1745.528	999.3353	1542.881	157.9135	316.5959	302.274	65.44741	205.2018	184.6852	92.99115	94.16687	147.3568
CUST_38156_PI429010886	M23744	2.61609E-05	24	12						78.07876	124.7151	98.83232	521.9217	219.3902	352.5182	490.5865	446.2058	690.0542	65.67573	1136.704	214.4299	645.8971	732.7914	234.6915	232.1999	365.9284	278.2449	170.7496	134.2073	215.6896	85.50328	197.6734	189.6492	33.91249	100.875	111.653
CUST_32817_PI429010886	M5186	2.61609E-05	24	12			Symbiosis, encompassin	Other		58.91866	48.73108	64.45499	281.9365	60.69474	157.9287	306.0384	172.6519	408.1953	99.49612	300.549	52.06198	386.8846	297.8825	118.7499	121.8844	98.76623	99.56289	70.16535	38.77522	86.73529	67.60668	41.63922	78.8865	13.91119	19.95709	37.94299
CUST_56149_PI429010886	M112964	2.61759E-05	24	6						29.13899	32.01836	36.26515	34.43964	45.47674	55.47729	50.81159	96.09055	71.72818	45.57662	30.36386	25.23652	30.29264	29.71228	14.0307	12.82717	80.5549	19.41383	17.32266	21.60932	6.954276	28.59585	17.44435	15.25766	13.82768	31.90012	14.11506
CUST_23811_PI429010886	N49513	2.6193E-05	24	0						91.12829	83.49206	118.4103	102.6964	48.30656	97.32812	46.17834	90.66543	99.31589	52.34706	61.40643	65.86088	81.4582	56.86184	69.43522	70.86476	87.89749	74.46936	100.4643	99.6269	146.4225	106.3359	111.0263	117.494	153.2351	107.6017	147.2789
CUST_16769_PI429010886	N36836	2.63505E-05	12	9						5.182985	11.51019	4.02905	252.884	188.695	NA	84.5309	76.82742	57.39735	965.4976	1691.116	871.592	33.30723	38.74481	21.08968	5.511281	15.88592	11.21773	6.25226	90.12112	112.2102	1370.424	220.7835	1011.588	9.381399	5.085084	9.026741
CUST_44782_PI429010886	M66977	2.74893E-05	18	15						155.7673	113.5034	80.3791	32.56117	18.33712	25.98862	55.67726	80.33191	74.01825	54.35078	37.1975	16.57521	195.4372	81.67118	132.3768	387.9483	192.2095	440.5339	172.2349	215.3375	344.1561	71.5149	8.937861	50.25143	124.7322	68.19401	27.28454
CUST_38920_PI429010886	M31970	2.76696E-05	24	9						6.988127	84.06095	87.41282	9.203065	78.24597	49.5489	189.6097	NA	174.8016	135.4989	1078.02	NA	103.0089	94.93084	201.5828	24.2919	12.85763	23.21635	4.9725	3.697711	3.742322	13.80235	NA	11.783	11.59928	11.43085	5.40299
CUST_6937_PI429010886	N20804	2.77404E-05	24	18						12.47186	NA	9.841307	5.262435	4.934123	6.319446	16.4789	NA	15.40374	20.84466	8.129627	14.85649	20.67603	NA	7.885832	18.40393	NA	15.91592	28.97134	43.54722	70.73344	23.25881	11.97145	27.79642	20.83109	12.54058	25.85908
CUST_10304_PI429010886	N26031	2.83099E-05	18	15						131.6028	197.7606	222.9863	162.1415	142.4683	158.5985	158.469	154.0925	129.5849	201.4635	172.4491	160.713	154.4681	172.5012	178.6434	216.9264	222.6501	203.0154	197.4586	140.7964	161.0998	137.6616	135.9631	142.8672	121.9201	121.7034	135.397
CUST_24154_PI429010886	N50128	2.84642E-05	21	6						244.5822	65.47772	73.4442	275.6746	65.5613	96.10147	912.8746	217.909	606.1742	305.7738	84.84135	108.4858	270.2762	93.35268	79.74416	135.0731	89.08902	202.5815	38.41822	57.18227	68.84252	71.02716	64.59327	93.22341	97.51431	97.70277	107.0748
CUST_40889_PI429010886	M56308	3.00282E-05	15	0						436.2064	666.4362	390.6122	323.0951	240.479	191.0479	176.3429	258.9303	213.4873	138.6664	292.4745	300.8131	199.421	148.298	252.989	337.7577	1994.973	1347.499	291.1427	250.7116	312.4297	104.0803	113.878	155.5483	85.97141	310.2931	265.5849
CUST_11490_PI429010886	N27893	3.11819E-05	18	15						62.367	70.24722	73.22433	44.34431	37.98691	41.15865	93.61951	69.53147	59.22327	95.64524	86.02033	76.29019	67.31002	54.04744	63.07317	83.76355	110.0991	97.26952	57.92587	71.64925	75.81657	53.33381	52.48812	64.46055	62.68299	58.53483	68.4765
CUST_37859_PI429010886	M21901	3.16195E-05	24	18						228.29	171.9553	163.1045	62.3082	44.57171	47.39234	179.1574	210.1175	93.03552	963.73	69.53723	726.5143	1476.878	1981.815	1095.073	115.8791	1719.004	1808.449	2564.444	1303.295	2073.458	67.51408	2455.967	33.75549	269.6374	207.6167	66.28735
CUST_31621_PI429010886	M1985	3.16933E-05	24	18						80.47316	101.361	96.9842	113.6471	104.3412	86.90266	101.522	108.0057	74.87652	82.42776	51.35849	60.94991	96.04821	72.27008	77.57132	126.1572	102.4924	135.5781	128.0468	132.4149	144.849	131.0265	106.5406	211.6596	106.3489	106.1339	111.355
CUST_19970_PI429010886	N42534	3.26254E-05	24	3						15.88272	14.47089	16.8811	16.31601	15.32206	14.07152	11.79955	13.76486	10.5374	9.974531	11.12735	7.032137	8.575367	6.668119	4.366932	7.227037	7.548659	8.373343	10.45447	9.503205	5.442961	7.174372	11.41802	13.60934	14.75483	12.70063	6.512115
CUST_17697_PI429010886	N38423	3.27705E-05	24	0						22.13777	18.14187	24.85437	21.21527	14.53951	15.36202	14.05555	15.62519	8.378958	11.06316	9.988283	10.38947	18.02767	8.94306	9.048157	12.66337	15.36685	10.36406	14.49774	13.18326	4.882539	24.48908	16.42131	16.77987	21.51875	15.865	18.61955
CUST_35663_PI429010886	M13177	3.40237E-05	24	12						338.5986	453.6305	312.9583	840.4028	333.1336	683.1382	573.6451	601.3269	816.8898	379.9317	925.9054	351.2056	1274.133	1287.523	1026.487	371.3812	661.8815	686.6456	288.3047	521.9217	553.413	211.3776	351.7434	261.9707	209.0675	242.7012	440.2791
CUST_52659_PI429010886	M98359	3.40237E-05	24	21						65.54266	71.95656	98.00625	53.24219	74.28032	59.35684	31.28202	44.61738	58.98036	58.21997	67.61972	51.23629	57.28678	47.58263	40.47822	60.41841	61.68952	61.72095	56.94521	69.86685	76.23852	90.65467	92.84934	92.04475	91.30891	66.02134	108.8896
CUST_34193_PI429010886	M9014	3.42544E-05	24	21						603.562	960.1156	854.3715	725.6388	681.2288	572.3479	570.427	614.3598	641.9649	675.2885	690.3228	679.9519	668.7382	641.7824	722.1173	695.1268	996.7263	958.49	693.5896	705.624	722.3326	873.9122	955.1294	1035.674	876.3228	756.6003	1021.91
CUST_29274_PI429010886	N59528	3.42703E-05	24	3						13.33001	18.63493	12.55197	42.0926	22.79012	33.3276	18.76907	11.95362	17.31531	23.10726	28.22804	31.84126	23.74286	20.69694	11.66013	5.303114	6.548842	6.38796	8.524647	12.43346	11.32628	8.054328	22.905	16.96814	28.51273	18.9004	18.82944
CUST_2515_PI429010886	N12540	3.44017E-05	12	6						19.65735	25.32432	38.87535	17.81281	37.87771	31.14654	55.08315	29.68907	62.1238	27.41038	34.00084	39.70866	26.7573	34.94317	11.08066	43.18058	42.23986	37.89317	51.92132	49.27688	65.87993	27.35733	44.25351	30.7358	26.45386	22.24463	24.66557
CUST_34758_PI429010886	M10567	3.48113E-05	12	0						850.8759	1248.523	1157.025	944.508	707.1593	631.8697	549.679	850.8759	768.79	823.5364	1032.084	966.9786	1028.452	949.2292	1005.258	775.6832	945.6613	1019.654	525.7914	669.9449	671.0624	875.8259	945.6613	835.7807	967.3181	923.4428	1360.696
CUST_9107_PI429010886	N24074	3.50789E-05	24	6						123.5591	50.84751	117.9157	136.0065	17.14735	123.2485	82.3809	140.0577	152.8487	122.9511	119.9872	116.9965	155.6724	56.34222	171.7541	45.42128	55.08838	25.8664	25.38297	23.36033	12.59509	42.5786	41.46154	43.43013	59.18726	43.126	62.35954
CUST_47460_PI429010886	M76216	3.51327E-05	24	21						17.33337	26.95458	26.27536	29.28939	15.98138	24.67313	18.42634	18.21058	25.20136	28.00193	19.23236	22.79379	25.50686	35.32216	22.34874	34.16334	54.65618	42.12804	36.23287	33.09951	26.2902	76.84692	102.7749	83.74447	44.84883	35.36195	22.59279
CUST_1699_PI429010886	N10470	3.558E-05	24	15						158.9424	164.4418	123.5754	107.0876	76.93465	95.2367	123.5754	141.417	113.015	189.5124	93.06282	154.8276	244.537	125.9513	191.1727	327.9636	204.7788	277.2002	182.1021	139.698	237.9355	151.4448	134.9856	209.4303	215.0483	80.64549	192.3394
CUST_1369_PI429010886	N9427	3.60069E-05	24	21	321455420	hypothetical protein DAPPUDRA	Metabolic process	Carbohydrate metabolic process	1.29E-91	9179.508	12807.83	10835.48	7950.355	6592.219	7177.873	5744.161	7122.178	7912.634	9954.878	6481.59	6385.128	10987.48	5298.376	6885.506	11667.94	13616.29	6276.978	16452.07	12584.9	11695.66	18435.82	14956.1	12939.25	25247.34	13532.16	17023.68
CUST_52637_PI429010886	M98266	3.60811E-05	24	3						33.43972	24.12932	39.70716	52.52723	62.52685	72.00199	39.03986	49.5489	45.43624	13.49255	64.85257	37.67421	40.17351	34.30109	14.62671	19.77651	21.00723	30.82525	31.07275	25.80839	27.14447	32.90163	29.66835	32.83178	28.40206	47.18539	44.03097
CUST_31834_PI429010886	M2521	3.63722E-05	24	15						603.9154	1598.489	1198.216	620.2559	286.0534	340.0191	584.1152	341.84	699.7985	1281.883	684.1238	312.2924	1061.158	579.065	754.9422	2769.46	3132.033	3360.114	1312.243	1106.912	2192.968	1288.693	712.1062	1400.583	1855.874	1016.824	1055.851
CUST_37677_PI429010886	M20539	3.65823E-05	24	15	209418846	serine proteinase [Portunus tritu	Protein metabolism	Proteolysis	1.93E-11	1682.203	947.376	1008.499	381.9964	270.1829	347.6198	4392.913	1476.878	3538.483	2853.1	936.2266	586.3741	2467.704	2214.163	1205.686	13248.98	4965.926	10142.18	2742.804	2958.733	5879.268	1405.334	147.8633	1371.826	2613.493	688.6527	1329.124
CUST_33707_PI429010886	M7663	3.65823E-05	24	18						78.18007	93.51275	122.3631	80.98106	90.41336	104.2957	66.6173	61.44094	72.96939	80.70329	85.69239	77.84495	101.4865	119.1723	77.54349	76.68447	111.007	91.72224	134.703	113.0827	129.9776	103.8305	125.5586	89.25205	89.99586	110.4251	101.3475
CUST_8565_PI429010886	N23228	3.65823E-05	24	15	478859730	glutathione S-transferases [Mac	Metabolic process	Cellular amino acid metabolic process	2.6E-79	290.2155	345.674	273.6157	306.3603	293.8829	350.791	504.7574	301.5476	321.1394	409.8716	351.0025	302.345	411.8854	402.8177	315.8899	578.5106	682.5361	837.2458	610.0117	515.6628	791.3056	533.3565	378.0804	415.0852	480.4617	343.8057	414.9089
CUST_37226_PI429010886	M18272	3.6859E-05	12	0	390368561	PREDICTED: uncharacterized prc			7.91E-10	32.76569	20.99538	37.18674	29.96081	25.62847	28.99912	18.67811	4.869797	16.47106	26.96277	25.49661	18.71237	38.64548	26.83656	27.13523	28.11335	22.30308	28.33501	24.73549	15.90885	21.96464	20.29507	8.218417	22.03549	42.72194	34.39723	41.72041
CUST_42726_PI429010886	M61082	3.71182E-05	24	12						5.323176	3.759605	NA	5.783029	NA	4.982765	5.627759	10.33279	6.073281	NA	13.65748	NA	NA	NA	41.87153	7.785469	10.85303	10.97673	14.39646	6.557583	NA	9.048157	7.515502	6.121824	6.096379	3.285598	3.653973
CUST_7934_PI429010886	N22293	3.74418E-05	15	12	156546735	PREDICTED: nicalin-1-like [Nasor			1.26E-32	33.4688	37.29216	38.51205	20.91851	20.66527	18.02378	17.28788	20.49843	16.32398	53.53717	17.10366	20.03269	44.18092	27.98453	48.77487	40.32964	29.89602	53.66051	27.24405	35.04644	23.34012	20.88553	12.46634	21.33315	28.72317	31.60721	38.5634
CUST_4141_PI429010886	N16100	3.80855E-05	21	18	402691421	serine protease like protein [Gra	Protein metabolism	Proteolysis	0.000000339	81.53976	53.83595	8.192393	NA	NA	3.927335	61.96517	31.10668	NA	48.47319	49.81991	30.19535	182.1021	3.569686	117.5411	123.1603	116.8754	NA	337.294	381.7573	636.1587	218.5351	NA	135.6294	8.755998	5.888762	NA
CUST_41692_PI429010886	M58324	3.85868E-05	24	3						57.36369	59.51724	44.35453	54.4761	102.2878	62.1017	32.43254	41.6878	13.67194	19.83817	32.92749	20.12179	24.485	18.47	17.39468	4.739737	20.78179	12.96776	26.75452	29.84354	5.271402	52.85457	13.93323	7.332239	29.91504	38.35367	14.36734
CUST_6536_PI429010886	N20230	3.85868E-05	24	21						570.427	699.296	588.6767	587.0902	556.2705	492.0502	503.6798	565.0242	703.6419	553.1321	567.871	491.6864	651.9522	495.7052	573.4698	577.8423	674.2308	623.7953	661.6594	622.9027	660.4277	723.6856	767.0668	678.9908	718.4732	605.9768	886.9505
CUST_32469_PI429010886	M4197	3.97096E-05	21	15						11.4038	13.35744	15.0661	9.257058	14.0289	7.137762	8.501072	12.24624	8.622045	10.0277	17.425	20.04202	15.34065	137.4674	18.83943	16.56168	30.46326	26.01873	24.43918	26.59448	14.9705	8.64742	20.34912	15.7815	13.39577	8.607441	9.960257
CUST_46741_PI429010886	M73569	4.00064E-05	15	12						897.3252	1219.83	1212.445	544.8319	482.0377	524.8326	620.9232	925.0688	583.3052	951.5195	1032.593	876.9516	1157.507	963.73	1017.256	815.4763	1110.326	1034.116	737.2275	650.2714	521.5155	925.0688	496.9611	657.3507	810.6462	1083.233	1088.703
CUST_38476_PI429010886	M27167	4.01179E-05	18	15	302668071	Trichophyton verrucosum HKI 0	Protein metabolism	Ribosome biogenesis	3.5E-59	116.6401	110.1161	136.0891	105.2701	119.0143	104.0674	126.3851	132.1609	116.4978	121.9035	121.6309	155.5805	116.7583	166.7642	126.2717	146.3562	134.5716	179.2719	103.1229	110.5024	122.8211	105.5064	113.3335	94.15594	108.5581	112.3711	115.6261
CUST_14711_PI429010886	N33335	4.15462E-05	24	3						129.29	158.1747	81.47133	158.3229	199.5583	162.3844	86.60823	129.1403	142.2741	85.60411	142.6928	106.4149	32.95195	80.05281	44.83298	42.78489	109.1677	38.54735	66.32665	95.74045	63.28914	41.7466	91.03605	65.91522	59.41357	135.2901	154.4269
CUST_20019_PI429010886	N42658	4.16676E-05	15	9						13.35017	34.06922	13.64514	39.22978	49.69173	23.9201	26.78523	31.5845	34.33958	50.29015	141.0115	167.261	63.53279	72.83732	79.45422	38.34449	38.17039	19.10119	41.87629	31.07275	39.48899	40.16574	51.86945	65.54992	65.26399	69.24371	91.69645
CUST_542_PI429010886	N5908	4.2195E-05	15	9						4242.57	1444.129	4900.819	11052.65	5258.496	1719.004	4445.119	6165.981	10949.85	11805.59	20320.15	8098.961	16348.66	5119.273	7459.987	284.8134	157.4566	280.6399	13017.21	7704.294	16348.66	17424.76	34173.92	39213.8	16520.01	16760.98	32639.19
CUST_543_PI429010886	N5909	4.27225E-05	15	9						963.404	263.8276	1067.28	2656.978	1032.084	530.0108	600.976	1604.089	2517.49	2613.493	7353.952	1720.155	3654.032	1107.545	1640.994	32.66921	20.83811	37.86955	2975.418	1466.746	3024.414	4275.766	9096.626	7653.847	3936.583	4270.698	9629.338
CUST_2996_PI429010886	N13567	4.30693E-05	18	0	329565704	ribosomal protein L24 [Azumape	Protein metabolism	Ribosome biogenesis	4.27E-40	193.5706	314.1063	294.9139	194.1916	182.1021	191.741	186.5755	191.0167	236.2759	118.6368	127.048	158.6925	216.7269	167.4488	185.1715	241.668	245.2392	276.7645	275.1575	228.7481	279.3607	220.1523	260.1465	213.1128	257.241	229.0326	159.5774
CUST_42473_PI429010886	M60363	4.39206E-05	12	3						353.3256	443.191	243.453	448.2125	603.766	587.8441	489.7282	469.0501	486.5784	415.4114	446.6878	147.7749	334.5246	337.6329	368.4918	678.5721	491.9043	621.285	399.6202	344.1561	408.9306	372.2167	411.4386	289.9679	314.7154	403.3152	218.3332
CUST_16667_PI429010886	N36656	4.40969E-05	12	0						14.85493	12.3024	16.58358	12.94982	6.904947	11.64898	5.811668	3.864296	4.968715	16.81278	7.789818	11.88179	14.47219	NA	13.52663	12.16427	9.939018	5.498349	9.602206	5.142937	6.948808	8.375216	12.55746	8.91225	14.71575	7.607506	15.9512
CUST_5068_PI429010886	N18219	4.55068E-05	18	15	321473948	hypothetical protein DAPPUDRA	Metabolic process	Carbohydrate metabolic process		512.3561	785.8536	463.4514	543.9588	357.9365	467.9385	771.7617	602.5729	597.1413	668.7382	504.4324	676.3457	725.3859	478.8721	575.8046	886.7077	868.2894	789.6051	674.5328	569.0422	865.141	409.6823	455.4195	483.9464	672.5292	514.4981	571.8635
CUST_2239_PI429010886	N11943	4.59681E-05	24	6	13898841	CUB-serine protease [Panulirus a	Protein metabolism	Proteolysis	5.71E-45	668.1223	555.0536	443.9249	1475.969	688.4549	1155.365	1792.472	1457.293	1485.843	548.1274	1594.801	1708.051	677.0488	699.596	548.75	903.1163	540.0952	733.9767	860.5197	71.46735	841.994	175.7096	456.5305	112.7999	684.4289	560.1392	1054.885
CUST_14169_PI429010886	N32428	4.7187E-05	21	0						25.9479	26.6898	36.55792	15.22541	8.468746	21.68449	12.49326	9.101709	9.267762	9.217287	8.052062	3.68689	7.31445	16.49157	18.56513	7.776877	7.553305	13.3386	14.55272	13.0678	7.939364	18.06541	26.85809	18.99331	21.70341	18.00324	8.667029
CUST_40729_PI429010886	M55953	4.73005E-05	24	21	383865502	PREDICTED: SET and MYND dom			1.27E-08	25.46423	19.26727	28.95548	29.37054	24.85202	32.50381	13.36765	20.96508	17.34867	23.65067	16.06042	24.06897	29.1296	17.7667	27.04259	25.7544	21.22392	27.49657	43.6037	29.83769	36.12473	49.1413	27.73284	42.0926	32.51568	23.12555	36.28088
CUST_12904_PI429010886	N30241	4.82688E-05	24	18	1.56E+09	Crustacean hyperglycemic horm	Signal transduction	Other	3.00E-06	312.9583	359.5095	264.2762	405.4699	407.1156	349.9633	355.1717	390.6122	319.8754	286.2998	232.6083	302.7771	333.8469	335.3952	305.1744	528.895	527.1701	606.7201	513.5436	622.3504	576.7675	398.7982	461.6561	443.191	381.849	543.9588	400.146
CUST_41671_PI429010886	M58281	4.87876E-05	12	3	157106769	ankyrin 2,3/unc44 [Aedes aegyp			5.98E-29	171.7821	466.3373	26.50208	896.2629	NA	751.8458	567.3781	463.096	241.4097	267.7311	223.0141	26.9108	366.7915	441.3596	294.796	1242.744	545.1995	1019.08	874.1945	66.25687	736.5204	25.55323	418.1662	20.75137	646.3558	743.9121	124.0832
CUST_6545_PI429010886	N20244	4.90995E-05	18	15			Protein metabolism	Protein folding		78.10699	100.1016	54.8814	41.98856	52.24236	56.01896	99.3998	107.2961	41.77121	103.4975	116.1822	59.85927	78.29555	30.59991	64.38614	121.0707	200.1232	116.2357	70.84605	61.56482	63.89423	29.91879	43.8296	7.124762	57.6297	78.24597	88.50381
CUST_33058_PI429010886	M5855	5.02607E-05	24	18						13.42167	13.59114	14.46435	8.447449	11.55732	12.52949	7.141342	6.041845	9.411311	12.15592	10.0998	11.79315	9.825217	13.50493	6.370812	10.33423	14.23509	13.74225	15.30076	17.74047	19.16283	12.77646	9.154669	17.56701	11.36638	18.94041	16.11449
CUST_38422_PI429010886	M26420	5.02643E-05	12	6	260796361	hypothetical protein BRAFLDRAI	Metabolic process	Cellular amino acid metabolic process	3.14E-50	10.91561	32.97743	36.90923	51.80267	40.16574	30.28156	121.0405	107.8221	127.6534	48.23305	76.6076	43.08271	29.33753	32.04295	26.38647	30.16583	44.53859	37.64453	48.23892	110.0556	170.1187	37.23401	56.98746	87.47672	7.430249	58.84073	58.79928
CUST_40824_PI429010886	M56179	5.02643E-05	12	0	321472460	hypothetical protein DAPPUDRA	Signal transduction	Other	1.85E-59	149.9295	70.69299	120.4309	105.5565	158.3229	105.8028	61.04906	55.93542	48.95729	106.6811	106.1766	109.6887	125.7971	128.7652	196.6122	90.37428	91.13599	83.14687	81.32217	61.5382	61.33441	108.3596	95.83012	88.96871	104.0183	103.2209	121.1493
CUST_13573_PI429010886	N31402	5.02643E-05	12	0						322.5903	305.1744	367.4169	372.2167	289.7381	339.6934	185.2774	239.4431	182.4362	247.5439	302.7771	319.8028	304.282	333.9851	523.8156	289.1171	247.4668	260.4457	245.8278	253.4735	222.8209	262.1593	221.7872	292.95	275.3403	267.3212	360.2879
CUST_33457_PI429010886	M6940	5.02985E-05	15	12						38.32178	59.3203	124.0147	17.03783	8.740614	16.24051	73.58526	NA	76.07226	NA	556.2705	26.23786	323.0408	166.7131	145.5507	29.73756	463.9651	128.6276	52.32559	61.16253	3.875353	NA	24.11397	NA	78.5366	158.8243	120.9342
CUST_19676_PI429010886	N41996	5.03374E-05	24	18						92.00118	108.7869	90.57569	54.06732	63.63996	68.83252	69.41702	88.50381	94.64399	88.01649	53.37075	47.1467	71.87251	70.23956	78.43693	98.14527	67.85905	96.58233	97.65071	116.5916	118.1075	97.2956	102.0887	130.586	97.40632	91.8129	109.0704
CUST_29745_PI429010886	N60463	5.03576E-05	21	0						90.93696	84.61891	84.85915	82.74445	77.73937	66.60304	69.33519	57.10009	48.81917	83.23692	43.39176	69.42506	66.17125	51.87516	59.38892	59.04201	55.47226	74.06253	64.89736	85.0591	58.41936	96.9842	77.89321	87.57759	82.32889	71.64925	73.59425
CUST_7426_PI429010886	N21529	5.06315E-05	24	21	478251559	hypothetical protein YQE_11312	Signal transduction	Other	7.67E-13	680.8236	1052.857	745.5832	472.6199	575.2243	392.1412	347.8293	376.9694	391.9617	608.1132	631.2553	725.3859	690.9865	486.4482	537.1828	707.937	837.2458	702.7679	868.9348	569.3405	540.7576	1187.041	700.1848	1202.203	1161.622	734.9802	952.9844
CUST_15639_PI429010886	N34903	5.06315E-05	24	3						25.72369	29.49099	18.84141	35.67884	31.9432	31.27544	14.69878	26.05591	27.2791	17.18732	26.23786	23.22906	21.06356	18.37247	22.55486	16.57137	21.35401	17.24881	10.07839	16.55114	18.02767	24.15844	22.97455	22.19826	20.02577	22.75502	18.84481

Probe ID	ID	p-value	Period	Phase	EBI ID	Annotation	GO category	Go term	E-value	0	0	0	3	3	3	6	6	6	9	9	9	12	12	12	15	15	15	18	18	18	21	21	21	24	24	24
CUST_9527_PI429010886	N24782	5.08487E-05	12	0						56.67374	30.40674	53.77161	43.04131	56.31298	46.59528	35.15355	27.84822	35.55837	44.03643	62.45522	49.73252	48.6073	66.93125	63.14073	41.6878	39.09234	43.73952	23.26849	37.28248	37.65275	43.70979	45.74256	45.90992	47.88919	44.67899	61.16761
CUST_1672_PI429010886	N10407	5.09419E-05	18	3						50.02722	27.22363	10.93877	100.4774	48.31614	37.68818	23.94163	32.88066	8.546932	29.6976	27.73485	4.87786	17.06953	5.211705	10.47591	23.67748	22.71469	NA	13.53805	14.74939	52.67704	57.65509	65.78378	57.18227	38.35932	56.26482	62.14833
CUST_29232_PI429010886	N59440	5.17926E-05	24	18						34.89809	32.05226	37.88664	16.9284	13.70291	18.61955	23.30722	7.885832	7.176989	103.4755	55.73019	132.8754	72.02191	105.6867	99.98511	82.30731	66.27962	35.59474	116.9098	125.2728	109.9526	150.0105	72.77442	117.5547	25.55884	14.74793	23.96712
CUST_31264_PI429010886	M1104	5.19639E-05	24	21						210.7737	269.707	280.9343	191.21	246.7787	203.1325	89.54662	193.0588	147.2411	251.4577	198.1577	223.9415	340.0191	222.0531	365.5842	246.8231	334.0615	227.7173	296.8179	297.1434	250.3956	412.9087	332.8061	305.3135	499.3569	288.5968	394.269
CUST_16432_PI429010886	N36226	5.19639E-05	24	3						275.9079	172.1631	276.2644	276.55	486.7832	358.0959	157.0815	259.1614	176.6544	226.1685	303.4454	203.2877	233.4998	168.6428	205.6562	155.9036	161.0498	134.703	121.8844	185.5286	99.2507	193.9616	196.8082	151.8271	178.3394	231.4277	156.8401
CUST_26801_PI429010886	N54904	5.20052E-05	24	9						9.516754	9.815468	5.758808	12.51386	21.82274	11.09473	21.95424	NA	6.88406	12.62841	14.57201	15.71	17.68949	9.795733	15.62171	11.96077	7.910866	8.491715	4.120124	7.009565	5.685492	6.533775	NA	4.26269	5.044156	6.076625	9.029102
CUST_39504_PI429010886	M40586	5.30099E-05	12	9						3098.696	268.4249	2436.323	3721.413	943.8069	1375.747	670.9198	341.965	736.9876	9565.255	16520.01	5736.233	9330.025	1732.526	4130.841	45.52672	167.0808	144.6342	1663.655	878.4972	1656.675	11738.35	15230	13360.37	3847.793	11378.62	15630.09
CUST_14715_PI429010886	N33340	5.32309E-05	18	15						244.537	120.4106	276.8158	168.685	109.6769	161.586	94.31617	105.8805	102.7355	147.8425	101.1701	112.683	122.2649	91.15714	148.4289	184.1934	213.4477	151.6954	136.314	148.4065	167.6224	147.1665	165.6517	178.9851	77.69968	78.26455	68.06818
CUST_36411_PI429010886	M15834	5.37852E-05	24	6						344.5549	427.5284	556.047	736.9876	537.7378	699.9941	769.0192	600.0613	878.4972	334.2355	509.999	420.5189	569.7522	774.2411	502.2054	66.28735	74.33815	115.286	288.5968	532.6369	203.6502	246.5228	150.9476	176.4771	236.1021	532.2614	67.96481
CUST_30774_PI429010886	N62641	5.40353E-05	15	12						14.55841	4.217288	14.86033	4.800498	3.558382	4.350674	9.504797	6.540338	5.659483	23.42712	12.31117	8.944558	13.80852	18.79764	10.23726	16.09693	12.23558	11.83721	8.382186	7.941808	4.480857	9.375499	6.870771	7.69611	16.13712	10.33813	13.0692
CUST_38986_PI429010886	M33013	5.42626E-05	24	12						5.958994	34.59782	18.92052	31.25004	52.5945	34.72664	30.88603	52.44267	52.10827	28.88967	36.00369	28.16926	35.0318	55.96387	68.31562	26.14998	23.51188	22.45487	17.83592	33.69474	17.07298	5.776661	6.698312	19.52695	26.50821	20.64485	16.87762
CUST_31076_PI429010886	M641	5.42626E-05	24	18						194.2319	351.343	251.5671	166.6906	133.3669	177.9352	135.864	133.7339	44.53859	110.0862	261.7871	163.2608	183.2542	364.8648	248.9012	143.5809	379.4005	269.9329	195.7919	544.6702	405.9933	175.6627	431.5244	648.9592	177.9352	270.2277	258.5627
CUST_38378_PI429010886	M26020	5.43188E-05	12	9						30577.23	9301.136	34877.59	53496.25	20172.23	21834.29	18070.71	23435.22	32639.19	56054.28	100092.3	46875.63	50283.97	22946.38	49649.01	2484.399	827.5626	2636.073	41095.52	30065.36	48051.75	64995.27	92404.68	99004.2	48847.04	62215.85	96349.38
CUST_34331_PI429010886	M9345	5.46287E-05	24	21	321468460	hypothetical protein DAPPUDRA	Protein metabolism	Cellular protein modification process	8E-83	120.4647	80.83391	131.2558	108.4356	70.68343	76.17615	77.51144	75.38714	107.4112	98.27861	70.85676	85.37067	101.7015	123.4933	111.6675	128.3075	89.85516	116.5601	167.4144	90.57569	135.6294	254.8129	139.4798	177.9607	202.9634	84.28713	182.7293
CUST_36537_PI429010886	M16168	5.46636E-05	24	21	378558198	chitinase-like protein, partial [Pa	Metabolic process	Carbohydrate metabolic process	2.62E-35	365.7666	364.2801	459.9674	414.8181	435.731	417.1463	347.2002	330.2787	359.0835	336.9544	316.7134	91.4929	407.5243	388.031	389.9629	374.2544	438.2874	433.0002	363.7634	389.8933	414.226	434.2782	442.9918	475.9252	379.2367	379.8743	421.977
CUST_1233_PI429010886	N8945	5.47857E-05	15	3						152.4523	120.6137	168.4308	194.3971	250.35	157.1328	163.8342	164.8179	187.0484	133.3192	83.43947	114.6554	113.5376	61.39194	116.8167	150.113	174.5369	103.0089	224.6812	211.8745	268.9316	174.6338	120.5552	94.94344	201.0489	141.898	171.8649
CUST_56241_PI429010886	M113241	5.54033E-05	24	12						69.24371	151.6381	79.87529	583.5463	242.2901	328.8423	544.2292	368.7015	604.3308	57.63804	1102.848	167.9403	837.7142	837.7142	343.6496	558.6463	328.1119	568.8248	80.23561	160.5879	142.6928	68.17964	151.7315	159.7718	51.99386	76.89496	83.04063
CUST_26995_PI429010886	N55249	5.54228E-05	24	18						180.3409	70.89193	227.0055	69.60581	20.17341	70.27366	16.31227	53.14759	59.41831	66.40286	106.5773	76.79264	127.7162	12.69014	163.0633	71.64167	33.2543	77.08119	466.2067	304.875	307.9833	281.8768	142.8045	134.7853	278.0706	79.38788	372.1298
CUST_38616_PI429010886	M28570	5.62821E-05	12	9						8591.806	2254.423	9345.369	12743.08	3401.586	6385.128	9872.039	8051.463	14922.71	15394.16	21758.66	11356.17	19903.59	5992.677	10683.23	493.1272	873.9122	1165.385	15367.74	13812.43	17023.68	17453.24	26740.38	23658.24	9067.463	18198.77	25555.67
CUST_37977_PI429010886	M22559	5.62867E-05	15	3						266.1986	238.4834	428.2506	835.3759	545.4629	559.1602	318.8359	425.365	420.9575	306.565	225.3861	172.4022	159.2709	311.3849	78.96469	193.1329	534.1168	420.9575	364.8648	910.719	777.0993	907.8057	473.4786	183.1939	534.593	41.72651	340.7848
CUST_37490_PI429010886	M19215	5.69366E-05	12	9						25792.96	4279.69	26491.1	37427.64	13391.14	17982.7	14794.99	18198.77	27832.29	45257.85	46193.24	33304.41	56054.28	19635.13	33749.71	1906.005	1676.387	2744.675	35862.04	26740.38	33838.77	53773.55	66134.01	71692.31	30577.23	43280.43	60302.68
CUST_36771_PI429010886	M16813	5.69413E-05	24	6						59.50711	67.10463	66.66302	77.78826	61.70924	72.9865	84.92778	99.94989	134.2321	58.08057	97.26952	54.23194	68.94498	114.3521	71.94693	61.3302	90.89393	77.98875	48.62526	54.61715	49.69782	52.60565	65.43257	58.5677	58.23269	55.12067	54.68833
CUST_1961_PI429010886	N11172	5.73488E-05	24	0						3495.947	2782.49	2767.762	1876.444	1594.029	1297.493	759.5695	1453.134	978.4034	1861.323	889.3882	1179.476	2306.791	699.1348	1879.179	1420.017	1191.902	529.2519	2940.045	1371.826	2122.683	4190.519	4058.337	2881.653	5578.907	2985.873	4628.184
CUST_56122_PI429010886	M112848	5.77005E-05	24	15						237.7241	127.5646	110.7598	38.84447	37.3089	32.24502	429.4693	241.887	334.2355	386.6278	235.6105	60.93815	233.0946	301.5476	140.9588	1067.709	504.3232	698.508	302.7771	345.514	539.0498	330.9321	12.17947	207.7685	259.1236	92.31393	128.3485
CUST_13231_PI429010886	N30821	5.81322E-05	15	9						145.6946	136.5009	151.6078	56.57986	77.42501	57.54767	62.77844	181.4338	142.5195	123.3723	209.8556	151.2383	101.0612	109.8522	145.7916	87.00868	107.6017	78.67787	82.0771	89.24382	68.28737	70.74232	109.2995	58.78776	205.143	139.7386	223.5617
CUST_37159_PI429010886	M18036	5.84597E-05	24	15						96.86942	118.8436	98.85767	106.6009	97.51431	79.03209	145.0599	158.4947	176.2281	143.9807	99.19887	106.9035	152.9408	132.0536	128.2119	264.4099	183.8614	211.4099	181.5127	192.9931	228.6197	139.9842	116.3209	159.645	164.2345	109.6887	170.4605
CUST_32775_PI429010886	M5085	5.84597E-05	24	15						88.54675	111.3836	100.875	68.31562	15.59925	76.51051	154.8085	128.8295	91.75455	62.61945	79.86465	58.78776	153.9484	164.2345	89.91214	180.4189	163.2608	138.7489	141.7174	136.0065	161.7701	122.8685	209.031	158.9628	115.5725	97.64083	130.7332
CUST_33959_PI429010886	M8365	5.88625E-05	15	9						11323.45	4097.003	12789.24	21590.74	5550.987	11528.01	13559.72	12288.42	20913.02	24199.32	26567.15	19387.17	28943.19	7785.133	12860.58	941.7984	1519.636	1918.312	16411.2	20059.08	19195.08	21113.92	28492.44	23084.2	13906.34	24199.32	28025.82
CUST_2955_PI429010886	N13469	5.88971E-05	15	0	541041787	vps33b-interacting protein [Asca			3.65E-26	14.91599	22.1135	20.08349	6.936046	15.865	12.05888	11.47889	10.80517	8.557191	14.15013	15.5828	14.1279	12.76017	14.5311	11.24696	17.83143	21.9474	20.02577	11.76947	16.72797	13.94283	11.50267	8.774182	13.48307	11.48896	4.93131	15.03094
CUST_54572_PI429010886	M106601	5.892E-05	24	0						36.1598	54.02066	53.43823	42.5482	41.25004	36.03727	41.03485	39.2713	34.25309	33.4664	41.28574	26.09025	36.1598	47.4031	29.14227	41.60531	34.50418	41.66058	40.36492	45.97159	48.7205	51.1596	53.99104	52.98141	45.27834	55.34491	57.99775
CUST_37617_PI429010886	M19874	5.892E-05	24	0						37.35922	24.24527	23.62689	13.79706	20.4011	14.62025	14.93559	12.72961	15.54337	17.927	19.64434	15.91453	15.98731	19.0017	7.1028	12.59237	8.630404	12.94826	4.183073	16.21895	7.806436	16.5534	18.08089	18.71505	24.92985	20.49356	32.34672
CUST_14879_PI429010886	N33619	5.89411E-05	21	0						30.32287	61.21971	44.23244	16.27834	16.81161	7.963679	NA	NA	6.882653	NA	8.901364	4.47227	22.47011	9.51357	21.99164	5.907624	45.74936	19.64262	23.57211	23.21635	26.6898	23.25373	22.36702	29.96804	22.81449	15.5722	21.66982
CUST_31503_PI429010886	M1680	5.9348E-05	24	18	226693233	arginine kinase 2 [Neocaridina d	Metabolic process	Cellular amino acid metabolic process	3.43E-33	98.13418	113.7707	123.0345	98.53709	111.8609	110.6039	102.5048	103.7748	111.9225	92.78173	86.42647	101.3884	109.5487	100.8962	74.93498	115.6711	121.9822	166.7131	153.2351	118.2766	135.7196	109.6769	150.2055	114.8511	110.9564	106.6278	114.6993
CUST_16726_PI429010886	N36759	5.95544E-05	12	9						24.51492	29.14227	30.96531	20.72897	15.46584	12.60041	24.12109	26.21615	19.58546	42.64535	26.13567	33.28409	75.80655	18.29235	19.46418	7.869717	24.9948	15.12028	21.88142	18.33289	26.45917	44.50237	35.71025	59.3504	23.28393	12.54489	28.57519
CUST_36227_PI429010886	M15086	5.96372E-05	24	9	195448274	GK25063 [Drosophila willistoni]	Protein metabolism	Proteolysis	6.98E-72	4.755095	7.445689	NA	10.98328	37.04769	22.88115	12.76732	NA	NA	28.44439	16.04875	50.99975	14.14657	25.80525	40.93762	10.41528	11.76845	NA	4.135548	10.27106	NA	NA	6.466135	6.688662	5.501586	NA	5.856234
CUST_4405_PI429010886	N16691	5.97056E-05	24	0						179.9541	134.2508	99.35613	20.18416	85.73084	53.41659	132.7723	78.33954	89.07659	48.49422	76.27282	33.26038	89.01154	86.89375	134.1618	96.03692	94.18113	81.84916	102.1002	114.9362	91.6033	93.31746	101.3206	95.56729	229.7888	105.1903	149.2547
CUST_13367_PI429010886	N31065	5.97787E-05	18	15	321478280	hypothetical protein DAPPUDRA	Transport	Ion transport	1.5E-24	44.23722	46.47246	72.88356	46.45178	41.31439	62.68299	27.04259	48.74993	33.67688	34.10624	33.13955	44.13896	45.04591	53.06387	47.57931	62.65711	91.11591	74.29925	36.8949	71.47305	52.16251	46.33211	64.94456	67.25975	26.89377	33.53619	21.40258
CUST_15990_PI429010886	N35468	5.99781E-05	24	21						66.19633	94.26006	87.11871	88.30662	79.66914	80.57219	46.45178	56.28495	83.27222	64.7701	55.90731	47.72449	90.28649	73.95531	66.74815	86.19309	89.96257	94.67757	85.19452	74.00845	65.15776	103.5796	90.76223	99.16406	102.5834	97.71482	92.83981
CUST_30765_PI429010886	N62607	6.0645E-05	18	3						510.7551	258.2813	338.8265	386.3361	320.6536	366.157	381.6653	329.3762	353.5795	338.993	290.7989	353.9275	313.739	219.0011	318.9156	199.982	193.5997	185.8172	342.1248	699.1348	297.492	519.7601	427.1681	445.9579	297.8139	431.7294	334.4355
CUST_16497_PI429010886	N36336	6.11918E-05	15	9						132.8564	153.9248	143.0166	131.9615	142.0823	122.6677	107.567	128.7652	147.1038	145.0834	154.6439	166.158	154.9422	128.2527	151.3747	128.2244	136.0599	146.0289	110.822	122.5288	130.9714	137.2967	141.0554	134.8769	150.4308	160.5413	190.3204
CUST_7091_PI429010886	N21026	6.15114E-05	18	15	513139111	C1q-binding protein [Litopenaeu			6.05E-91	77.19972	144.5615	110.7454	88.05908	67.45544	72.97666	86.50032	100.6757	81.39312	105.6512	90.13423	89.85516	95.02561	98.70542	89.47762	132.0886	123.0596	130.6615	104.9676	112.1712	118.1871	76.94539	96.97291	76.47464	99.88608	86.15946	98.79304
CUST_10571_PI429010886	N26445	6.16421E-05	21	18	443727166	hypothetical protein CAPTEDRAI			3.37E-21	100.0827	149.1062	172.187	76.06374	49.23962	61.2068	76.86672	112.1403	128.7173	27.9424	22.67723	35.81401	141.2799	39.4752	185.6848	42.1572	38.93757	66.1068	227.9064	159.5774	172.5896	165.3421	119.971	130.3816	71.30275	110.2867	66.31026
CUST_55495_PI429010886	M110357	6.18237E-05	12	9						10.70051	13.93951	24.08979	22.18214	24.03468	22.08061	32.55402	19.91267	22.97677	34.0139	37.50453	27.27378	19.79906	17.59422	16.2053	20.42799	27.87333	30.08709	22.23218	23.65648	19.80965	29.05862	104.437	31.2986	14.30676	20.26341	20.50908
CUST_35735_PI429010886	M13347	6.19567E-05	15	6						274.3383	174.7074	391.1173	322.8914	992.6472	437.7611	443.191	627.1961	981.9828	409.117	957.0763	544.0973	579.8505	479.8831	264.9858	41.94644	130.752	53.43212	1570.104	597.7182	856.2286	1016.824	934.3693	692.3061	493.1272	877.3403	687.9587
CUST_33217_PI429010886	M6313	6.20645E-05	12	0	321471664	hypothetical protein DAPPUDRA			9.52E-26	84.4188	87.66036	111.9766	78.70208	61.64797	69.33519	39.29429	45.42698	61.11356	70.37823	55.8366	37.22647	100.6934	64.18044	95.79812	69.17285	72.30147	69.17285	63.39626	65.04883	61.47323	97.41787	72.05007	99.89645	85.06849	73.1662	102.3977
CUST_38100_PI429010886	M23341	6.22558E-05	24	3	83628250	arthrodial cuticle protein AMP16			1.89E-16	1631.254	245.4565	1199.116	3670.22	3499.526	4913.686	175.6627	537.0249	170.3606	1159.214	2213.256	2422.76	730.3491	1801.332	2210.04	63.01997	67.22535	67.03577	90.46312	632.1075	93.08615	1021.074	329.7179	736.5204	139.8319	1333.943	229.8852
CUST_20422_PI429010886	N43410	6.23613E-05	18	9						46.31434	74.89382	64.80829	55.26895	66.75504	50.46482	74.78378	78.783	84.47977	83.44953	93.75176	93.12448	69.87542	77.91051	91.33505	65.122	80.09649	73.92515	45.77544	70.37823	74.84749	47.6242	53.49898	68.27308	59.55202	52.96326	56.60172
CUST_11077_PI429010886	N27230	6.28949E-05	12	3						29.4515	35.00336	NA	38.87196	NA	48.65633	42.16901	30.8769	29.38152	23.64554	9.981463	10.96209	14.3798	20.61709	18.17826	83.31874	38.38502	45.29856	44.97359	13.11189	38.06969	NA	17.38353	8.865478	16.0845	18.36834	3.331481
CUST_50275_PI429010886	M89277	6.29959E-05	21	18						36.72861	67.85905	27.45352	31.30932	29.21491	35.99005	26.40704	18.86307	24.99752	73.24134	13.85771	84.37355	118.8608	125.4991	93.20756	35.5206	146.1694	125.4627	197.2974	154.8735	141.8013	45.11511	170.5061	18.37064	28.4735	23.13824	18.7418
CUST_41442_PI429010886	M57743	6.32441E-05	18	15						17.33156	31.32237	19.66736	16.52701	13.9779	16.57521	15.01966	28.14643	19.21337	24.368	14.24384	23.11231	20.6613	36.23287	25.82769	65.45785	60.35053	39.31755	27.37466	14.56346	11.91707	8.554104	9.861577	14.74939	33.39115	32.29221	11.77077
CUST_16171_PI429010886	N35788	6.36998E-05	18	6	443703721	hypothetical protein CAPTEDRAI	Cellular component orga	Cytoskeleton organization	5.79E-23	4.341365	12.87353	13.3556	17.72282	18.65957	8.374367	24.08979	21.03035	19.38793	34.54667	15.39651	21.09303	23.24382	16.40332	18.15454	21.35681	12.48451	16.97648	15.78875	7.74189	13.59114	19.1752	13.72176	17.77361	26.05349	16.62059	24.57559
CUST_38134_PI429010886	M23574	6.37742E-05	24	3						194.8463	24.55645	209.1753	423.1563	847.4628	777.6219	17.55977	50.87686	22.5581	26.64465	243.8613	230.649	17.17797	716.3366	758.9827	16.44032	11.19099	6.925201	26.97175	41.6878	19.70331	33.42156	24.23412	37.35922	33.2543	49.95516	61.6196
CUST_54499_PI429010886	M106257	6.45821E-05	24	15						71.54027	45.98151	20.64485	18.52564	8.310351	9.228702	51.77472	94.12797	43.40869	49.66595	39.30254	NA	86.6454	47.86566	40.61754	191.082	134.3961	144.9581	34.70595	95.78807	53.94311	78.81902	9.905888	53.61862	73.22433	26.20838	30.41385
CUST_55614_PI429010886	M110974	6.58432E-05	18	9						28.19237	10.23726	34.36788	14.11031	17.84997	12.88584	25.29001	18.40907	28.72317	40.59839	37.03848	25.52907	23.23737	25.43012	22.18413	28.65291	22.45935	39.2895	22.17612	21.33315	23.95909	12.70744	12.8639	15.53957	31.88585	33.7495	37.44318
CUST_39236_PI429010886	M36544	6.67095E-05	18	3	149675211	cuticle proprotein proCP5.2 [Cal			2.66E-55	34173.92	919.3983	20913.02	40915.77	34620.42	44511.54	2844.645	5151.779	6292.839	32454.44	37632.98	17388.9	28858.93	18235.97	4829.887	760.24	829.47	731.069	7821.557	6326.89	10311.23	35862.04	33076.81	39389.41	8683.686	19052.86	16690.76
CUST_45270_PI429010886	M68531	6.84422E-05	18	15	460838092	strongly chitin-binding protein-1			1.81E-08	29037.53	40024.21	21113.92	19791.01	10852.86	13222.77	23016.75	21710.8	18579.76	27758.19	19855.57	21177.49	22279.42	19010.63	18198.77	41095.52	37083.68	37083.68	24426.06	24426.06	19960.25	16726.21	18476.28	14670.24	28658.52	18476.28	19387.17
CUST_42693_PI429010886	M60989	6.93346E-05	24	12						134.2073	80.06182	101.3081	75.86824	79.66104	54.91706	116.3818	105.0258	99.74451	98.80176	95.55779	122.709	156.9414	137.8428	185.653	188.7199	117.2793	138.3467	161.2869	97.48939	140.1401	131.6242	69.21158	138.0928	90.32894	74.24613	136.7679
CUST_1645_PI429010886	N10327	6.95059E-05	21	15						63.63996	30.36663	36.62239	44.15404	13.98312	31.10352	104.9484	132.4508	115.5725	136.0065	26.90354	70.94231	164.1132	183.166	35.39516	356.4497	92.2128	252.3498	91.00224	95.30674	130.8147	62.77181	10.06209	50.26083	56.80856	29.79615	24.49039
CUST_5481_PI429010886	N18760	7.10413E-05	18	15	325170046	protein disulfide isomerase A6 [I	Metabolic process	Oxidation-reduction process	1.21E-160	593.5138	1133.812	739.9176	585.1407	393.0106	477.058	1235.811	905.7144	973.9492	1079.142	790.4703	707.3888	1104.992	605.6304	653.8973	1223.105	1307.316	1267.497	854.7576	645.4936	970.7242	612.7708	477.5471	697.2436	801.5781	642.342	639.5264
CUST_30543_PI429010886	N62093	7.14152E-05	12	3	442630141	masquerade, isoform C [Drosop	Protein metabolism	Proteolysis	6.41E-14	30.20183	30.34519	NA	44.87342	NA	45.37684	57.36964	33.70156	38.88446	18.90266	18.79971	NA	30.22132	49.91114	30.23399	114.6554	46.21776	65.71042	65.15059	7.036384	45.36304	NA	17.29971	3.300133	36.19278	24.68878	NA
CUST_41909_PI429010886	M58856	7.14366E-05	21	15						977.5298	2080.932	705.624	731.2874	157.1785	445.8569	2858.309	1367.344	1363.035	3728.752	1371.826	1064.673	2715.678	2164.77	828.1272	1073.581	3786.16	2285.617	2383.479	1467.602	1778.328	496.2739	2258.648	570.0085	93.38599	251.1902	18.36667
CUST_18662_PI429010886	N40188	7.19949E-05	21	9						27.77009	27.39004	23.81554	33.63623	18.80558	43.79783	41.28987	44.53304	44.58117	54.28814	34.48395	54.98064	47.06434	10.98328	34.92495	11.96493	10.01935	15.94333	25.53483	16.65157	29.30404	21.79222	19.9833	26.22964	32.55747	16.49157	26.48347
CUST_29775_PI429010886	N60524	7.30139E-05	24	18	345494134	PREDICTED: chitotriosidase-1-lik	Metabolic process	Carbohydrate metabolic process	6.98E-08	64.47129	54.48188	82.95322	72.19272	14.88678	37.13496	42.9547	45.93201	49.014	105.8805	54.06274	76.3423	134.6275	92.13598	37.43476	180.7742	77.37879	148.9148	159.645	85.69239	172.2349	174.7345	150.4308	66.96376	78.18913	42.65681	108.9908
CUST_32635_PI429010886	M4643	7.4765E-05	24	15	212286110	centromere/kinetochore proteir	Protein metabolism	Macromolecular complex assembly	1.25E-75	16.65548	48.58376	34.99565	22.6315	35.55837	35.48221	22.74717	34.26775	31.77803	20.00439	46.10158	26.54115	52.15678	40.29385	38.65654	55.04149	65.84893	53.84528	42.24729	46.77922	42.38836	36.4809	55.01217	43.16345	31.80176	43.64769	36.16345
CUST_3245_PI429010886	N14095	7.50143E-05	24	21						5900.066	8146.369	6165.981	5238.942	3683.886	4266.005	3633.442	4825.163	4207.659	6401.205	4589.607	2937.545	6551.409	2749.137	5407.94	7621.268	8086.369	3627.136	11952.65	6265.89	8321.498	10394.61	10294.69	8631.841	13988.25	7632.364	11112.95
CUST_5506_PI429010886	N18791	7.50143E-05	24	21	321455420	hypothetical protein DAPPUDRA	Metabolic process	Carbohydrate metabolic process	7.37E-86	781.7261	757.3112	694.9037	738.2631	582.3778	478.0895	335.0262	428.1289	344.2242	617.0017	533.0556	512.7881	783.939	324.6065	656.3228	408.5695	745.3715	341.177	1095.073	628.2632	751.8458	1128.705	1008.499	829.47	1702.984	835.7807	1224.714
CUST_15356_PI429010886	N34450	7.50143E-05	24	21						23.70635	56.73963	56.65739	38.21258	49.56614	44.43314	22.89571	37.11143	48.43013	50.50174	36.91238	48.37004	36.45493	51.3632	47.81362	58.4567	46.15443	55.8366	66.38702	52.0048	51.92761	75.38714	60.93815	58.55963	58.59505	48.70868	77.21485
CUST_10474_PI429010886	N26292	7.50323E-05	12	3						34.90142	21.51658	24.22177	34.58559	38.27451	44.55279	36.5971	28.3794	29.6926	22.9911	19.43519	34.68278	30.35424	29.84142	41.89159	72.95425	40.75864	41.26235	33.24054	14.12155	34.54981	14.42653	23.58742	22.43161	27.06969	28.84319	22.74972
CUST_44760_PI429010886	M66902	7.54964E-05	18	15						492.0502	370.0497	241.5804	140.6785	109.4907	107.696	575.1095	534.1168	518.9718	477.9381	325.9381	118.7327	587.0902	371.4802	292.7159	1965.01	869.9307	1512.535	376.7778	668.297	1023.762	343.6496	71.3852	300.4795	474.3448	217.7355	264.4099
CUST_45120_PI429010886	M68052	7.58572E-05	21	15	241859217	purple acid phosphatase, putati			1.16E-31	18.03262	61.81635	27.96239	34.73799	27.61004	31.92535	50.86043	37.15987	42.88477	43.32608	36.1964	27.40129	125.4991	118.5947	103.0218	74.52512	277.7663	211.3422	144.9379	83.77516	83.6658	49.76639	23.22906	49.46595	26.36289	53.69851	23.96408
CUST_38502_PI429010886	M27437	7.59909E-05	15	3						4.414398	30.45492	14.75483	25.58795	25.89322	23.98666	9.284798	14.67376	14.83785	13.06456	5.804532	11.2795	10.72911	12.2785	12.12259	11.35899	34.14439	15.432	15.74607	38.95566	23.96712	20.90896	17.88948	13.57384	6.209536	13.51353	10.55248
CUST_5701_PI429010886	N19071	7.60145E-05	24	21						186.3368	191.5454	206.8979	109.1508	132.9821	119.4775	93.75176	109.8412	134.3589	133.7558	141.9148	174.1934	121.2857	117.2637	180.9657	155.7969	213.7796	148.956	143.9807	131.274	152.3742	226.3355	277.3623	294.9139	192.4063	160.766	224.0655
CUST_10072_PI429010886	N25649	7.61351E-05	21	15						168.7955	270.8525	275.2113	237.1374	251.7468	255.2927	388.566	322.0602	324.9732	323.2809	343.1775	278.8807	299.3538	362.1172	143.0339	404.7958	442.8695	535.748	279.3607	320.8974	422.7276	178.6979	204.2476	163.0857	264.3178	202.7406	314.254
CUST_41881_PI429010886	M58782	7.62395E-05	18	15						210.0683	249.3411	185.6261	204.2081	115.6931	182.0698	315.1053	217.3138	213.2675	323.6649	149.9295	237.5901	219.0011	195.082	214.8056	425.6386	324.2431	408.5695	230.4612	189.3839	260.9505	168.0943	176.3724	183.9367	272.5092	229.8852	208.941
CUST_37912_PI429010886	M22216	7.69319E-05	12	9						7290.969	978.9259	6401.205	10746.95	2887.827	4168.103	2484.399	1414.36	3339.651	27073.29	44511.54	16954.37	28561.19	4928.217	11112.95	238.2559	685.4537	569.4689	6913.964	2988.845	6000.152	32969.41	37632.98	38719.75	13078.51	33963.44	45257.85
CUST_14514_PI429010886	N33003	7.69319E-05	12	9						86.1454	57.73118	42.01431	117.9157	51.1228	63.37029	75.52401	57.81074	54.25064	90.3392	171.7821	85.18385	62.41935	80.72426	64.88027	53.68776	48.65633	51.81269	77.35718	74.96434	65.09533	132.3608	194.0511	152.2217	125.2342	123.2894	83.3606
CUST_7941_PI429010886	N22304	7.70147E-05	24	3	383865855	PREDICTED: hexokinase-1-like [M	Metabolic process	Carbohydrate metabolic process	2.03E-50	127.2978	60.20069	87.19961	180.4396	106.4017	176.5842	64.10142	87.57759	67.89364	70.3428	66.0686	66.81481	97.64083	64.18993	76.07871	66.168	60.93815	45.92624	63.82525	55.89295	63.90528	58.84948	56.734	75.71093	68.29523	78.82605	64.46055
CUST_20278_PI429010886	N43150	7.74469E-05	21	0						87.92365	80.78521	74.75403	36.4809	61.35249	49.39632	35.04281	50.96545	37.34896	36.63038	60.17344	48.98205	19.0538	41.85879	38.55552	65.79182	74.09844	50.87686	69.35987	41.47342	60.0766	98.27861	65.75115	95.04674	127.3678	63.74337	97.05434
CUST_42417_PI429010886	M60227	7.80149E-05	24	21						29.02823	47.89413	43.08599	25.45303	18.37064	21.89919	29.76443	25.16449	28.21177	41.91066	23.13824	32.56117	50.63563	24.91804	34.05393	34.56311	33.36729	35.62359	46.72264	41.68317	37.57177	53.99735	49.38843	51.39126	45.18209	32.04743	55.16243
CUST_47239_PI429010886	M75320	7.85394E-05	24	15	195432615	GK19768 [Drosophila willistoni]			1.07E-08	1204.7	1632.718	1075.462	1019.654	886.9505	867.6626	1013.822	1319.093	902.4975	1049.395	988.0134	934.7113	1212.98	1091.098	1165.385	1654.651	2409.846	2073.458	1002.608	1377.595	1589.718	1066.406	1550.781	1355.75	1363.959	1098.604	1256.746
CUST_24996_PI429010886	N51605	7.91721E-05	24	15						12.00032	18.94465	10.57698	12.56759	20.77726	11.66991	8.273433	22.77868	12.14327	14.05324	20.14753	15.21169	26.1001	18.05808	24.15844	32.90163	29.03394	24.91503	17.52792	12.38585	14.40144	13.87446	15.43474	15.42011	10.24666	8.671876	13.69771
CUST_55169_PI429010886	M108972	8.00153E-05	24	3						707.6592	279.5075	550.8269	1004.431	557.4698	941.0274	260.798	337.8262	223.3238	454.0605	278.0706	358.247	242.6675	572.5541	277.8871	213.3383	201.4162	150.4308	157.0068	213.853	178.5273	345.993	182.8746	228.5093	237.6854	237.5901	128.3867
CUST_19610_PI429010886	N41868	8.00153E-05	24	3	443707171	hypothetical protein CAPTEDRAI			3.42E-15	219.1432	235.4202	370.3281	601.9971	463.096	522.0737	276.8158	364.4673	363.4898	248.2992	564.8143	341.3363	154.3283	302.345	290.7989	102.4313	278.9328	230.2547	116.2567	169.6715	195.8784	141.8167	286.0534	291.6226	127.5443	245.8656	218.5686
CUST_44106_PI429010886	M64967	8.02872E-05	24	9						34.96815	52.91229	48.25642	43.89958	58.13362	35.07936	44.96721	54.41132	52.2471	53.42717	66.67393	65.54266	46.48232	53.6558	54.39842	47.92699	69.71651	52.90722	49.16853	52.46839	45.64916	42.21331	38.76155	41.25004	38.10178	33.4748	36.87147
CUST_8669_PI429010886	N23389	8.03069E-05	21	15	307169148	6-phosphofructo-2-kinase/fructo			5.66E-87	28.10987	18.66401	26.08093	31.04592	29.56314	32.84255	46.13169	38.20339	30.75559	44.61738	35.00336	40.28089	38.81388	37.11143	43.15443	46.85827	46.2714	48.3568	48.4991	19.97632	31.67678	27.81464	26.84554	35.8443	49.1314	30.20493	34.313
CUST_16237_PI429010886	N35893	8.0731E-05	12	9						26129.84	5209.792	27153.68	39875.67	15394.16	17254.51	16348.66	17729.56	17254.51	44285.99	55277.47	33637.86	54598.59	18715.71	31435.61	2251.39	1812.631	3087.565	33176.01	26567.15	35022.2	50283.97	64273.41	60302.68	34620.42	38384.01	63683.37
CUST_43503_PI429010886	M63221	8.08589E-05	12	0						91.01249	137.6616	122.9378	69.93972	62.59981	53.96355	67.3975	65.10135	74.29925	83.29367	78.64253	83.4815	89.40824	93.44995	76.55656	54.56309	112.7474	71.41406	76.27282	82.86519	67.01762	78.61195	71.5317	78.33954	90.18372	76.36789	126.5408
CUST_6772_PI429010886	N20580	8.16207E-05	15	12	260790163	hypothetical protein BRAFLDRAF			5.43E-40	13.78443	10.57304	13.71845	9.63137	10.52214	10.30511	18.05597	17.28408	21.11455	12.97087	21.64956	21.18826	19.65095	19.15316	41.28281	13.93323	14.70794	31.14331	8.770103	12.12132	15.00133	23.13174	11.57988	18.18639	28.13497	18.76106	29.23556
CUST_38077_PI429010886	M23225	8.16808E-05	12	9	156356316	predicted protein [Nematostella			3.88E-11	8146.369	436.2924	6601.274	8768.122	2086.93	5009.272	1242.354	1081.498	2053.392	14670.24	13222.77	10699.09	14032.06	2998.296	5131.503	243.453	450.1126	377.789	4198.712	2369.531	5435.742	17345.96	19195.08	21334.13	5671.62	4700.854	11356.17
CUST_39172_PI429010886	M35591	8.16808E-05	12	9						8869.924	344.8027	7130.037	8857.18	2765.552	4253.847	1739.873	883.4797	2105.784	16981.94	13301.65	11763.32	18522.28	3786.16	10532.72	332.0817	422.0714	508.1759	5558.806	2833.623	6601.274	16690.76	17131.85	22092.06	5407.94	9272.221	15798.02
CUST_33465_PI429010886	M6960	8.16808E-05	12	9						6183.806	709.2377	5593.889	12378.85	4928.217	3053.693	4275.766	6041.324	9643.272	13503.69	17453.24	10485.81	23739.57	7762.077	11643.13	522.4996	149.047	546.3173	14885.24	10193.49	14670.24	25611.3	29219.06	28943.19	13193.16	18070.71	27153.68
CUST_52499_PI429010886	M97666	8.19541E-05	24	15						49.54311	92.06463	60.67275	45.3219	40.22045	49.67166	72.00199	83.4815	68.77617	52.41365	53.04738	27.96239	85.51486	58.08057	38.64131	94.36986	119.0143	91.05781	90.57569	71.71217	94.62002	73.71889	68.0002	65.78378	50.6873	48.15989	53.84528
CUST_34704_PI429010886	M10403	8.20157E-05	24	21	242024511	ankyrin-1, putative [Pediculus hu	Metabolic process	Lipid metabolic process	3.09E-100	185.2774	167.3552	205.3015	186.762	189.6492	173.1737	143.3502	152.6002	156.8206	199.5249	165.8756	164.8664	199.2487	170.8363	155.9594	199.2156	210.0683	212.0165	179.4641	168.564	191.6851	237.6854	212.0999	227.3167	199.6427	176.6314	251.8474
CUST_38451_PI429010886	M26784	8.26306E-05	12	9	395536939	PREDICTED: keratin-associated p			9.26E-15	13281.09	1289.52	14526.85	28743.72	6601.274	11695.66	4971.086	4750.447	9855.129	29647.7	20762.38	20020.97	27918.92	10053.8	9923.978	361.9725	124.7912	448.6833	16372.71	9272.221	13154.56	29219.06	31967.8	32639.19	15461.72	16372.71	16491.53
CUST_54108_PI429010886	M104603	8.30924E-05	24	15						2440.627	3838.854	2831.186	2164.77	1781.913	1988.355	3098.696	2571.491	3441.376	2685.938	2010.612	2043.437	2926.153	3272.412	2646.596	4536.055	3460.858	3983.53	2886.102	4203.047	3956.525	2937.545	2552.554	3379.597	2685.938	2631.802	3838.854
CUST_39385_PI429010886	M38675	8.31599E-05	24	6	260815865	hypothetical protein BRAFLDRAF			2.46E-08	113.4417	95.64524	68.74828	1019.08	69.22757	735.8335	982.8111	171.0604	532.6369	148.2715	55.97133	84.6858	547.8118	419.0196	61.07789	45.5101	71.71217	60.50212	33.16469	66.51317	53.29749	59.97997	55.04149	47.11911	63.07768	199.0877	191.3144
CUST_1476_PI429010886	N9745	8.31599E-05	24	15			Metabolic process	Carbohydrate metabolic process		522.4996	194.7103	278.8215	225.2142	38.47726	126.9491	395.2192	513.2014	358.6508	1168.934	574.9115	122.1097	550.3617	384.8278	261.1993	1980.694	678.9908	1337.377	1101.918	919.0444	1734.409	548.1274	47.98232	447.4189	766.3188	259.4124	434.7194
CUST_1408_PI429010886	N9543	8.36325E-05	24	15						107.4552	85.95066	31.32237	36.71092	95.27189	69.40758	212.2553	105.3837	195.2973	169.2478	153.8104	81.55186	280.9343	156.6771	172.9496	684.1238	334.8644	461.1854	77.82741	270.8525	337.294	185.3999	15.46338	148.9789	116.6229	55.72212	86.62987
CUST_55852_PI429010886	M111890	8.42306E-05	24	15						77.67075	45.5101	50.16118	19.1841	8.62661	12.87704	107.8039	100.9118	102.222	100.4391	59.94713	12.49687	69.20307	115.984	34.4378	369.6716	187.5751	249.7708	92.90072	128.3485	184.2949	93.93436	8.051031	59.30659	100.6757	28.0193	38.43
CUST_15633_PI429010886	N34893	8.43034E-05	21	9						33.89153	24.38317	10.78644	255.6336	28.86811	177.5447	226.205	208.3716	150.1836	834.7743	NA	540.2756	52.8068	46.08569	43.88384	317.1285	37.35922	288.9896	54.30655	58.79368	99.07571	26.44115	NA	20.25356	36.09076	14.19869	40.27654
CUST_55578_PI429010886	M110815	8.4638E-05	15	9						13.54908	11.49555	28.17546	12.17947	18.32237	14.29143	12.78681	24.93951	23.74587	36.40347	36.63348	19.6201	19.60068	12.04777	24.368	5.891833	6.450227	10.58169	28.29802	17.20923	26.12743	25.77932	19.62318	27.80576	29.65588	28.2205	31.5845
CUST_51932_PI429010886	M95731	8.4638E-05	15	9						96.5246	79.95529	109.9078	228.7822	156.7032	96.94761	67.92426	130.174	102.2993	296.9431	833.7381	193.1768	378.7986	205.079	105.1338	48.72562	74.91262	57.39387	150.113	97.10574	87.38427	383.2384	582.785	251.0873	136.8604	476.9732	386.0289
CUST_1936_PI429010886	N11120	8.49326E-05	12	9						954.4926	NA	1028.813	1181.431	256.2719	636.1587	33.3337	46.78844	86.73529	1938.89	5533.093	1252.458	3475.106	311.5873	1977.598	4.870551	5.600819	18.57773	287.8256	445.7269	472.8686	3995.862	4754.165	4727.546	750.0607	1437.647	2377.165
CUST_20115_PI429010886	N42837	8.57758E-05	18	3						81.33153	50.42215	99.60174	94.58629	57.40907	82.1262	28.04985	45.59558	44.10093	43.94355	42.10224	36.51861	42.47062	46.34771	51.9164	35.91186	57.18227	57.47217	41.07397	50.97241	57.7947	114.5979	55.53662	107.9064	54.22249	54.80156	64.35808
CUST_1220_PI429010886	N8901	8.57997E-05	18	15	197210409	60S ribosomal protein L30 [Hapl	Protein metabolism	Ribosome biogenesis	4.79E-34	171.0708	168.7741	138.7489	158.8008	104.5179	116.2155	149.578	129.2573	107.6151	119.8257	90.04305	99.18844	110.1773	126.9826	130.6083	199.4867	191.8796	188.4419	164.8664	138.4615	170.3275	120.6137	172.4755	116.0279	104.5461	120.4106	143.902
CUST_6581_PI429010886	N20300	8.62471E-05	18	3						NA	10.12975	NA	16.41161	10.58169	11.30587	NA	4.484845	5.730283	9.14743	4.467522	7.146978	4.987484	9.921498	NA	NA	4.258711	5.574105	6.046947	8.820637	4.955878	17.30435	16.0336	12.4284	9.960257	11.15725	14.0733
CUST_18712_PI429010886	N40274	8.64297E-05	12	3	321463102	hypothetical protein DAPPUDRA			1.09E-37	114.6686	96.62834	40.48248	65.61458	131.8408	143.647	128.179	141.417	106.4444	50.62618	43.15923	69.45139	82.26609	61.44094	80.26815	205.6925	139.2203	188.767	55.21231	87.03898	81.44134	38.29632	76.03311	54.30068	61.58	133.5115	68.82621
CUST_35786_PI429010886	M13510	8.65071E-05	24	21						493.3336	539.0498	510.4694	379.6985	406.254	306.2147	361.4833	672.5292	546.6865	202.9634	117.9412	239.9273	274.5195	185.4321	385.4238	237.3958	678.5721	372.5479	421.2021	267.8251	367.7282	1003.255	871.592	589.1435	520.31	418.2862	714.0141
CUST_39494_PI429010886	M40390	8.69778E-05	24	0						162.9278	273.2919	290.7067	119.7663	66.54894	26.71059	136.3336	213.7108	269.8306	70.5683	91.47922	64.55985	153.0619	91.96611	47.84164	115.8122	102.0603	162.2856	199.5249	194.1257	285.8344	166.3679	165.0428	230.2547	257.5954	187.3503	335.5615
CUST_11311_PI429010886	N27605	8.76454E-05	24	3						220.4205	100.0919	197.961	169.7886	426.7646	348.2785	106.4806	67.66335	97.72445	105.1207	208.8952	306.279	99.90955	290.7067	348.7951	82.04507	45.35253	70.26755	82.54419	91.4929	70.15908	107.424	92.35614	98.00625	112.3846	103.1963	126.1993
CUST_52397_PI429010886	M97294	8.77566E-05	15	12	156550245	PREDICTED: serine/threonine-pr	Protein metabolism	Cellular protein modification process	3.75E-62	23.30722	19.8869	19.48546	11.91014	NA	8.891795	13.3386	16.06184	15.5351	19.85199	15.66225	20.05879	24.8137	17.23806	21.06356	12.66822	21.36623	24.48777	13.12104	12.35829	9.710211	25.87427	14.03646	13.26729	22.6585	15.46338	22.71026
CUST_36520_PI429010886	M16117	8.80211E-05	15	9						6.220036	7.401064	10.54823	19.27054	17.12239	NA	11.55337	14.62238	17.73766	19.6697	40.27208	25.38297	43.41187	15.7815	23.75071	6.183844	12.71213	6.361012	11.60747	12.66963	28.74405	31.93086	52.90159	49.20954	22.41168	33.47807	52.44267
CUST_11218_PI429010886	N27464	8.8672E-05	21	15						25.17677	25.90662	28.90914	19.91041	19.78273	13.50622	11.74409	17.66664	17.74472	23.63826	23.4544	32.41501	21.15511	23.06634	21.41005	24.57834	37.42766	30.46326	28.42785	26.0084	28.6788	22.44233	21.82274	28.63092	26.58261	13.6932	20.25781
CUST_53184_PI429010886	M100602	8.87836E-05	24	15	241564915	ribosomal protein S25, putative			3.97E-08	11974.32	16252.77	10569.33	10277.01	9804.488	9550.071	15767.9	16847.05	15435.13	12648.44	10090.54	10795.65	11952.65	11974.32	10352.54	17131.85	18070.71	17254.51	15847.39	16452.07	16760.98	12043.33	13906.34	12991.27	14794.99	13737.98	13154.56

Probe ID	ID	p-value	Period	Phase	EBI ID	Annotation	GO category	Go term	E-value	0	0	0	3	3	3	6	6	6	9	9	9	12	12	12	15	15	15	18	18	18	21	21	21	24	24	24
CUST_27921_PI429010886	N56946	8.87836E-05	24	3						389.053	93.21779	241.4743	304.7866	695.3717	508.3099	86.53109	190.8547	105.242	133.6627	544.9477	590.8215	76.44155	207.4056	240.1565	12.76114	6.092867	13.05352	37.58637	142.2314	50.21608	187.9422	234.4659	171.2731	48.90035	382.3723	144.3231
CUST_43079_PI429010886	M62019	8.96237E-05	21	6						10.30511	9.724209	12.71343	118.9614	22.01591	56.93121	111.9958	65.3511	94.45356	37.05226	69.6376	24.1058	106.3835	112.683	19.83531	31.57047	41.48089	39.03115	28.41463	21.52069	20.35695	8.826303	9.673796	18.49152	NA	NA	6.111488
CUST_37600_PI429010886	M19735	8.99219E-05	24	15			Protein metabolism	Proteolysis		1745.528	1047.687	767.3697	342.6821	123.4933	327.4664	4134.923	1702.984	3467.123	2930.842	1524.311	430.5713	1741.604	1692.044	605.9768	10605.81	3546.981	8631.841	2179.188	2538.621	3324.822	1743.671	68.35388	1890.428	2208.268	575.6253	1672.68
CUST_22529_PI429010886	N47191	9.06289E-05	24	21	321455420	hypothetical protein DAPPUDRA	Metabolic process	Carbohydrate metabolic process	1.44E-12	739.0418	622.9027	704.142	783.6564	521.6397	542.9066	250.5828	480.5983	384.9395	582.785	461.4101	339.8451	589.338	349.8278	501.3701	489.2868	687.9587	174.2207	1404.214	628.502	855.0422	1105.917	1138.567	736.2732	1619.49	937.9336	1135.893
CUST_37996_PI429010886	M22680	9.10601E-05	24	15						1782.659	987.1103	871.592	379.2367	194.7103	330.9231	4055.849	1731.406	3154.32	2855.927	1297.493	443.4999	1739.873	1615.517	974.6663	10746.95	4101.099	8025.596	1902.67	2679.375	5184.879	2201.915	85.64416	1818.148	2379.92	607.3706	1506.647
CUST_39035_PI429010886	M33770	9.10601E-05	24	15						256.573	119.9092	154.0154	46.70567	16.3304	52.45299	800.3618	337.6329	740.5837	593.6835	366.6368	156.8946	168.6428	455.1948	105.3328	3145.521	1512.535	2030.062	756.8703	566.888	1187.041	920.4596	7.142339	723.3991	590.1632	113.7894	407.0205
CUST_7679_PI429010886	N21906	9.10601E-05	24	15						359.3488	608.2737	400.146	352.5182	331.1886	324.5457	687.6817	567.5384	563.8477	630.7177	403.8629	450.6028	664.4758	424.0822	453.9545	860.0989	838.3409	871.1645	775.9607	571.5137	787.5865	575.9481	435.8605	523.8156	773.0893	592.8013	657.1039
CUST_15001_PI429010886	N33841	9.10601E-05	24	9						159.497	128.8445	142.5791	166.9595	212.1849	167.0102	226.7781	133.8434	130.8147	267.6739	232.0442	175.151	216.0939	149.786	218.2926	110.571	223.1202	147.7301	168.7741	158.3229	186.3924	124.7151	92.64515	108.1961	113.6995	169.2478	126.6958
CUST_8567_PI429010886	N23230	9.19914E-05	18	9	242022067	RAC protein kinase DRAC-PK85,	Signal transduction	Intracellular signal transduction	9.29E-132	23.23426	22.84451	25.62847	18.8838	11.4865	23.22374	44.9365	14.97433	28.73586	27.72879	26.84779	32.1177	27.19181	28.5784	24.4823	31.39708	25.81956	36.88652	27.15149	25.02711	25.21673	22.25604	19.67938	22.95957	24.53712	28.36418	27.46604
CUST_51605_PI429010886	M94205	9.25967E-05	24	21						7.444772	19.66736	15.84702	6.687789	18.02157	12.96341	NA	12.47654	12.21582	NA	6.270949	8.739672	9.494876	3.836599	11.63897	11.76147	8.032833	11.10613	10.12509	13.50493	19.51476	52.39688	32.8057	19.66385	11.55881	21.22876	25.2621
CUST_38746_PI429010886	M29929	9.32346E-05	18	15						41610.17	38228.71	36195.31	29520.35	25555.67	30065.36	46193.24	38996.88	33749.71	43090.8	25888.13	40595.56	38719.75	31108.81	36724.45	43955.79	42014.5	41258.32	36043.3	34757.71	41419.19	30821.46	30065.36	30821.46	34173.92	31967.8	34173.92
CUST_34032_PI429010886	M8558	9.32346E-05	18	15						186.6281	268.7276	224.6222	183.411	154.2231	134.1449	199.7474	208.0191	239.2059	223.053	218.3332	234.1695	229.7888	230.9439	226.0802	281.2628	295.3547	261.2478	267.1988	253.2927	218.1148	234.791	175.4589	185.9639	270.1339	237.439	212.0999
CUST_22133_PI429010886	N46491	9.39395E-05	12	0						10.54947	8.009934	NA	9.8644	5.588826	6.180183	5.658568	3.221456	3.064213	12.66493	6.645065	9.947363	12.41522	13.90922	5.972458	4.369107	4.72816	9.459821	4.715645	4.308443	4.19775	5.375793	5.977356	8.388209	7.085828	12.66963	6.909134
CUST_34207_PI429010886	M9058	9.44749E-05	24	15						100.1918	171.8869	132.5675	136.5823	113.7264	123.305	199.6427	163.8654	169.4819	188.7447	138.4795	195.7919	174.172	150.2055	166.8118	239.6808	245.5909	247.2845	211.5442	169.7494	207.1407	176.9877	175.63	220.3669	188.5193	168.5856	211.2708
CUST_2632_PI429010886	N12797	9.44749E-05	24	15			Nucleic acid metabolism	RNA metabolic process		743.0295	956.4678	774.5071	609.2645	491.802	562.4363	1043.605	813.2524	999.9612	1053.276	553.413	779.846	921.0975	565.424	819.3739	1373.039	1071.049	1314.736	1054.885	773.6805	1061.158	931.8674	764.0639	989.9825	1048.117	704.9081	979.9752
CUST_7485_PI429010886	N21614	9.53267E-05	24	15	321463650	hypothetical protein DAPPUDRA			5.37E-59	48.18114	26.03031	43.8213	36.93269	32.26704	33.77215	28.96764	53.28017	16.15024	22.38325	37.17924	34.3577	26.43013	29.90874	55.47729	60.28486	63.33433	55.57314	55.91239	60.88384	57.8427	35.53128	53.75479	53.68776	37.70404	32.22863	46.29703
CUST_8492_PI429010886	N23121	9.58985E-05	24	15	154416763	viral A-type inclusion protein [Tr			5.32E-11	44.42841	42.04861	26.87774	54.31927	59.73624	49.42933	48.02557	61.12576	66.79456	55.0054	50.43341	47.89413	74.06253	48.68665	47.7452	79.62962	74.39469	84.9077	53.14759	53.90894	63.08459	57.781	46.21146	58.9258	50.49566	40.48622	38.8005
CUST_26697_PI429010886	N54721	9.61716E-05	18	0						41.94361	27.06969	26.35385	16.84041	19.52695	9.698616	20.29507	8.709124	9.165105	14.66707	3.276039	NA	7.557804	5.939175	6.427241	16.99266	30.98678	41.04183	13.5245	25.55884	40.90141	23.88306	9.743639	13.51154	16.98896	6.936046	9.607864
CUST_19083_PI429010886	N40949	9.68979E-05	15	0	405959130	Elongator complex protein 1 [Cr			8.45E-14	21.83271	27.93535	23.14529	20.11563	18.41787	18.92222	8.970532	15.20809	18.03136	7.196175	12.08782	17.99711	21.1126	14.12155	19.7654	22.63447	30.81577	31.25004	15.17671	16.31227	24.80368	18.36487	13.3283	24.15387	11.1896	11.51019	24.7708
CUST_54814_PI429010886	M107791	9.6964E-05	18	15						26.08621	48.76461	34.88249	22.39421	17.04459	25.09267	57.16004	42.36924	46.15921	43.21465	33.01818	32.66605	44.38354	31.06867	25.10206	64.57877	66.48433	43.18567	41.8114	36.94243	42.6374	21.0497	22.97677	24.92323	43.80331	28.35837	24.20903
CUST_24348_PI429010886	N50460	9.88328E-05	12	6						72.77442	74.97441	71.93057	93.30975	92.60339	85.33414	229.8852	90.8123	114.0114	104.3299	96.03692	69.89861	70.72448	79.92025	62.73874	75.38714	82.66079	82.00982	132.6306	81.02819	96.30356	81.9979	80.24156	69.326	87.10926	60.39038	69.45852
CUST_34780_PI429010886	M10631	9.97264E-05	24	21						157.4759	247.0676	310.7267	189.9808	196.581	121.718	166.0779	208.5809	228.253	198.84	155.6724	202.7406	121.1808	149.4831	150.113	114.1092	163.6909	161.6606	206.2187	184.4435	193.3912	317.0103	335.3114	334.3239	191.5757	208.6394	351.8456
CUST_8670_PI429010886	N23390	0.000100693	18	3						51.30921	40.57567	52.73695	143.0166	106.0765	139.7737	39.00565	49.85973	52.29097	71.43667	82.42776	105.2156	71.03566	60.31817	38.27962	44.77327	32.1267	47.41757	42.9709	78.69416	26.54663	113.187	119.5301	125.8966	62.23993	82.79094	56.22801
CUST_24063_PI429010886	N49965	0.000101092	24	3	260785490	hypothetical protein BRAFLDRAF	Metabolic process	Other	1.12E-10	122.0207	59.40177	110.822	129.2072	121.0882	112.8635	71.78767	77.19972	65.59134	67.7048	92.35614	98.43645	85.1761	85.79452	109.1508	39.28264	76.89496	52.17564	28.21781	54.79166	48.07833	85.71168	54.88564	62.96095	106.3076	75.28314	130.0819
CUST_17029_PI429010886	N37258	0.000101268	15	0						266.8019	289.7731	NA	153.318	NA	119.4286	197.3334	132.6306	122.5572	16.8144	11.05247	NA	47.66934	178.7734	47.89413	367.876	195.2581	169.2197	143.902	39.84392	149.8586	NA	187.8413	NA	19.31587	37.34026	NA
CUST_24438_PI429010886	N50627	0.000101628	12	3						11.62281	13.85499	NA	46.67221	NA	39.57166	48.10521	14.37528	24.24843	14.14112	6.269189	NA	13.84416	13.85654	9.710211	150.1608	64.58573	60.81905	19.02577	5.202657	18.76293	NA	3.90246	NA	41.65544	27.00456	7.020474
CUST_55648_PI429010886	M111121	0.000102899	21	15	13898841	CUB-serine protease [Panulirus a	Protein metabolism	Proteolysis	5.66E-20	126.1572	343.5695	283.351	147.9373	186.6982	88.75	237.439	279.8938	270.3715	254.1458	242.3852	83.51378	215.6896	315.1053	262.7025	484.5238	338.0427	428.3837	242.0464	272.7898	348.3823	292.3452	109.892	205.5946	188.2411	143.7214	166.0466
CUST_23571_PI429010886	N49046	0.000102946	21	18						184.1725	188.4169	183.1326	160.0636	151.6954	180.1092	111.3836	149.1062	142.5791	136.6363	148.5751	186.1968	88.42474	127.9686	147.5974	204.6434	153.8272	212.5696	232.64	183.3606	232.7864	164.8664	169.1748	212.3717	183.8614	178.3834	189.6824
CUST_29585_PI429010886	N60149	0.000103805	12	9						54.80637	57.77356	81.64232	58.43105	30.45211	56.54714	41.55628	61.88614	30.97694	86.84479	75.24855	58.6281	69.53147	50.87138	23.75813	23.09193	26.06752	18.97931	68.99631	50.10507	79.32602	82.69673	94.33013	113.0702	56.61327	65.25923	128.6276
CUST_6711_PI429010886	N20494	0.000104126	24	21						21.76426	49.54311	50.20269	34.02124	36.38972	29.64248	20.40806	44.20101	44.41457	32.15717	34.08501	45.18702	17.87527	17.15225	24.12669	26.57189	22.4617	36.81564	28.00878	39.50981	34.50654	45.76721	60.29296	73.32545	48.06623	37.60099	55.42058
CUST_42534_PI429010886	M60524	0.000104289	21	15	321457450	hypothetical protein DAPPUDRA			1.45E-29	406.3531	622.7305	500.8894	377.6097	417.5037	361.1904	435.1623	528.1103	421.977	422.5065	548.4345	476.9732	514.7289	469.2787	399.8777	473.3422	698.508	669.7814	433.3852	459.3044	425.5802	420.1391	395.1137	450.1126	430.8354	362.195	380.6302
CUST_6208_PI429010886	N19771	0.00010568	21	15	260797582	hypothetical protein BRAFLDRAF			1.11E-74	1488.548	2297.179	1644.807	1205.686	1241.8	1175.829	1798.348	2091.62	1876.444	1658.691	1570.104	1731.406	2022.811	1444.952	1499.429	2198.578	2862.188	2455.967	1659.781	1795.363	2171.504	1466.746	1509.047	1731.406	1483.632	1232.079	1692.044
CUST_31271_PI429010886	M1112	0.000107021	24	15	529367583	ecdysteroid receptor E75 [Litope			1.39E-68	1102.848	2173.027	910.2628	751.2377	705.8467	705.624	1839.274	1536.792	1586.354	1187.041	999.9612	1100.835	1097.014	1740.73	1654.651	2434.185	2112.104	2233.186	1464.031	1355.75	1482.511	912.2418	1716.333	1004.862	1059.777	1412.941	1054.373
CUST_12118_PI429010886	N28958	0.000107039	24	6						45.3219	58.4887	57.30682	154.9682	63.85058	131.8934	115.6261	101.0257	204.6859	63.5234	180.4687	72.70716	145.6336	159.5977	85.6799	62.92312	96.17648	73.50137	40.44359	54.53262	33.74242	32.29617	45.746	44.3077	23.54776	17.44773	27.02166
CUST_57215_PI429010886	E2510	0.00010707	21	15	296317283	eukaryotic translation elongatio	Protein metabolism	Regulation of translational elongation	1.06E-24	35862.04	45502.32	37427.64	29725.44	32798.44	30721.41	46674.67	45964.24	43280.43	42224.23	33402.18	39713.59	36302.66	37427.64	38996.88	51269.31	48559.97	49329.8	40217.51	45257.85	43955.79	33176.01	37427.64	37427.64	36043.3	37932.98	28858.93
CUST_45510_PI429010886	M69333	0.000107693	15	12						NA	26.805	NA	4.947558	8.652491	29.48091	33.04298	41.97957	19.25364	37.57549	33.9482	14.39483	54.55547	57.12984	54.63268	18.19714	72.59454	35.81706	10.74202	25.00095	17.2236	26.9245	14.1199	16.72346	36.1335	30.72686	30.6146
CUST_6997_PI429010886	N20891	0.000108525	24	18						213.6621	255.9552	260.9827	189.9429	192.5776	161.6353	196.9337	219.6551	234.0578	266.6324	245.8656	239.348	200.3302	242.3379	242.5947	199.3048	302.0161	270.8095	278.1319	251.5087	263.4037	258.0601	311.4501	326.1422	232.0442	243.726	252.0561
CUST_49582_PI429010886	M86246	0.000109547	21	12						80988.9	85264.67	89785	66456.69	69586.63	94790.65	89785	99004.2	103630.6	94019.51	79085.89	98191.21	116387.2	100092.3	102933.3	84653.3	110385	102933.3	97373.16	87746.38	102393.8	96349.38	89298.33	95305.17	85264.67	79747.61	70445.35
CUST_35902_PI429010886	M13860	0.000109992	24	18						114.8083	152.2628	148.4641	110.5494	122.5392	99.58967	106.6009	152.5349	129.6764	95.83012	107.5789	81.38488	98.15697	104.4233	124.0673	168.9237	171.8869	185.8751	162.1415	176.6888	177.9078	145.3783	168.7131	182.794	165.1857	159.6186	217.7022
CUST_16641_PI429010886	N36615	0.000110012	24	18						25.13614	93.6437	54.26213	57.74381	43.94355	63.10394	75.6543	74.30536	59.37073	69.15316	73.4442	58.2972	68.36093	59.25391	70.60324	85.51486	113.8069	81.80549	119.971	85.95962	242.6247	93.22902	110.9763	82.40323	83.0128	80.8714	62.44379
CUST_38661_PI429010886	M28919	0.000110084	15	3						1210.563	744.7409	1379.279	2573.227	861.7915	1633.469	458.8651	1638.477	813.5332	754.9422	400.6266	460.1149	661.8815	860.0989	362.1172	456.4069	1180.964	1153.859	1238.715	1650.878	1690.112	1484.484	843.8571	477.8297	1119.38	277.6692	626.641
CUST_43228_PI429010886	M62469	0.000110635	12	9	512883568	PREDICTED: putative ferric-chela			3.05E-08	59.57649	69.24371	100.2259	56.65028	60.80606	37.60099	50.57582	61.46387	80.84339	70.42129	140.9438	61.3418	103.4513	51.75429	49.07635	33.93799	26.91606	30.63675	95.29319	73.73506	83.84766	108.4697	166.3542	157.386	55.19896	91.19065	162.3594
CUST_7287_PI429010886	N21321	0.000111567	24	15						191.5757	243.5905	222.0168	254.1458	261.5479	256.0953	166.3542	275.0049	248.4962	214.0769	273.4227	218.5686	303.3581	273.134	311.7361	313.6779	306.7294	300.0533	235.8737	273.6644	252.8102	218.889	258.6669	244.2151	227.4568	183.0375	228.163
CUST_84_PI429010886	N558	0.000112001	12	9						43701.56	9167.401	40217.51	44511.54	18715.71	21459.8	18910.88	24984.55	38719.75	60953.29	79747.61	39875.67	74264.89	25978.53	18715.71	2551.022	1054.885	3426.429	42496.4	31435.61	47826.44	62699.58	72257.2	82051.33	43955.79	66456.69	53773.55
CUST_44562_PI429010886	M66324	0.000112985	24	6						121.8531	81.55186	168.2637	166.158	170.0181	183.2355	146.2442	178.1189	220.2176	123.8539	257.8891	138.575	174.0123	210.4367	150.8283	122.0207	123.6152	127.0236	116.3471	88.67629	85.53397	91.32281	104.8028	108.1577	72.55053	79.76299	117.5696
CUST_461_PI429010886	N5429	0.000113366	12	9						5769.472	952.6113	4841.865	10464.54	2539.976	3633.442	1980.694	1127.159	3011.975	23162.84	32064.51	10408.52	25403.62	4577.512	12141.91	222.9863	567.7111	431.4309	5467.822	2732.247	5068.287	30965.56	38228.71	29647.7	11805.59	29396.88	33838.77
CUST_1976_PI429010886	N11212	0.00011375	15	9						324.3641	61.49211	299.0693	937.1039	438.2874	200.5807	246.3057	380.5457	600.2455	724.9498	2477.377	611.6853	1324.412	430.4406	495.5669	12.70892	15.82041	21.55177	873.5747	386.6278	836.9003	1457.953	3516.69	3763.397	1072.507	1453.134	2125.115
CUST_38087_PI429010886	M23281	0.000115154	15	9						2443.901	1041.894	3024.414	6936.974	2820.736	717.7206	3017.306	3307.225	6696.753	6696.753	10532.72	4229.758	10232.82	3193.902	3472.797	158.6683	81.32217	203.9603	7653.847	5365.583	7681.431	9516.402	16323.5	13685.2	9653.786	9444.83	17555.23
CUST_31181_PI429010886	M908	0.000115865	21	0						2402.724	2962.88	2440.627	157.5876	588.164	247.0114	435.6254	472.6199	633.9987	759.3274	1833.616	2278.847	50.30791	915.406	686.4707	3428.918	1185.011	1875.312	2022.811	1704.256	2592.232	2829.469	1843.608	3588.155	1302.487	1368.116	1041.115
CUST_32393_PI429010886	M3994	0.000116098	12	9	321465430	hypothetical protein DAPPUDRA			2.59E-32	48.22901	42.25942	65.60638	45.01993	63.59914	25.1528	46.59051	31.18062	45.90076	53.10397	117.2948	51.9988	71.25639	48.98205	20.22587	30.35985	17.63971	17.28976	46.3034	63.94916	64.2252	81.9335	78.86032	76.69145	76.22837	32.91719	57.08761
CUST_1271_PI429010886	N9090	0.000117445	24	6	307169370	Chorion peroxidase [Camponotu	Response to stress	Response to oxidative stress	2.53E-104	52.33176	155.5805	78.5278	131.6454	169.9743	115.7411	1137.925	502.5054	1522.629	115.5828	101.0154	45.25376	115.3698	90.05411	99.92196	115.0038	290.9304	339.5673	73.94525	37.14951	65.45785	57.20828	27.69715	50.87138	38.79677	40.27654	35.42699
CUST_24025_PI429010886	N49890	0.000117765	18	9						12.81539	12.58849	11.19796	16.73592	NA	24.33859	26.73995	31.73936	NA	46.81937	40.10788	28.86811	24.31278	NA	27.09196	32.78488	21.21931	33.08133	10.12825	5.268864	7.016143	45.07182	16.09693	32.10997	32.85632	25.74697	8.974877
CUST_1948_PI429010886	N11143	0.000118256	24	15						15.9111	22.16329	17.90327	10.98475	14.39646	15.99496	23.41063	17.50283	19.42414	36.69692	17.15876	24.37754	48.12738	18.03262	34.25718	43.84283	36.22402	24.6637	23.90768	12.21743	22.31416	22.20534	9.632394	23.5397	19.89427	16.34777	19.95957
CUST_40505_PI429010886	M55307	0.00011883	12	9						8886.515	294.3919	8818.425	8269.17	1670.335	5913.632	615.2672	370.7223	720.2377	17061.92	21113.92	11395.49	12344.85	2342.087	9444.83	92.99115	106.2889	160.2997	2333.596	1148.933	2575.646	11206.93	10277.01	12255.54	1922.582	3499.526	6041.324
CUST_2856_PI429010886	N13255	0.000118932	24	21						317.1978	363.297	320.552	361.1904	264.9162	294.9139	151.5059	251.4219	204.5091	357.3731	291.7534	186.728	401.2544	252.3	369.8268	435.8605	327.283	210.2297	562.0064	261.0327	296.3981	530.6367	497.4085	532.6369	678.5721	415.4114	604.3308
CUST_35080_PI429010886	M11510	0.000119522	12	0						114.7972	63.88253	154.9154	75.55461	53.06387	70.56233	50.66742	50.84751	39.96697	76.34956	52.65281	58.04431	221.8149	76.3265	144.2677	20.02186	34.37139	35.18743	41.03792	38.36719	33.6985	133.4406	54.50441	94.70581	75.51715	65.69754	114.7972
CUST_37445_PI429010886	M19056	0.000120006	12	6						1022.798	366.242	518.791	770.3832	165.9817	472.502	2323.856	2782.49	2181.465	1346.472	386.3361	417.734	919.6173	546.2084	253.1207	2744.675	1851.473	2149.415	1034.581	3598.269	5388.872	2261.704	814.3635	972.4265	418.2862	161.8178	222.0168
CUST_7661_PI429010886	N21884	0.000121514	24	12						9.22306	25.03508	20.72028	34.77477	59.71475	55.65083	12.34301	31.84126	22.94104	29.40702	30.28156	36.93887	48.28205	46.90598	67.21667	39.62294	29.55174	25.01301	40.80027	42.73834	37.46702	30.78396	21.35401	26.01383	15.95467	19.59461	17.46154
CUST_51762_PI429010886	M94928	0.000121635	24	21						87.83185	62.67742	95.39909	83.15856	57.97707	37.77999	72.92411	59.64058	94.27306	72.16617	46.14458	62.70293	87.92365	56.82231	81.43322	101.0057	21.70753	76.04559	192.4672	110.8393	101.5466	187.0768	142.9154	157.2786	139.5452	100.9118	169.1748
CUST_42011_PI429010886	M59144	0.000122091	18	9						11.97294	15.65876	25.37685	13.61629	14.17656	13.44807	35.25348	39.25338	25.56436	49.23192	47.88053	37.45982	49.09284	19.55073	31.80176	29.91879	23.36033	32.99109	43.62046	11.48186	16.84945	21.30099	18.67988	16.11644	15.96858	20.16722	21.73698
CUST_56383_PI429010886	M113643	0.000123092	24	0						65.71584	58.98036	55.63371	57.29374	44.7597	39.80553	56.57137	41.50445	38.70471	48.42501	36.82399	48.04838	33.75549	37.62021	32.40475	52.15678	43.36291	50.56209	54.45212	46.51413	45.05271	66.54171	47.04453	45.60384	69.51507	43.79306	58.65468
CUST_2021_PI429010886	N11345	0.000123296	24	15	241859217	purple acid phosphatase, putativ			1.09E-29	NA	11.5439	6.782652	15.18659	NA	4.500661	11.28594	5.634563	17.19685	8.844931	11.0285	NA	62.23993	48.45851	55.49194	28.45078	97.73797	83.55746	36.44264	21.48961	21.31707	9.61862	NA	16.25004	NA	15.56681	NA
CUST_49990_PI429010886	M88118	0.000123316	24	15						22.58254	11.10999	6.931329	5.270725	3.245721	10.85553	36.71405	11.89711	35.38735	73.10715	32.09525	NA	23.38714	18.57121	12.80959	125.9513	32.55747	77.40803	26.80709	24.58293	31.78112	21.46595	NA	15.83532	43.81166	10.58533	20.3727
CUST_48653_PI429010886	M81489	0.000123324	24	15						137.7837	197.1276	152.6002	165.8065	161.4729	171.436	180.9657	180.2649	151.9479	186.4449	139.2612	229.393	182.7293	190.613	184.6434	278.1702	283.5165	231.9279	247.7427	207.3047	257.7877	170.3073	177.1027	183.7151	185.4692	202.9634	198.7466
CUST_37844_PI429010886	M21834	0.000123861	18	3			Protein metabolism	Ribosome biogenesis		1605.911	171.3909	1699.905	2551.022	1292.456	1669.427	460.5885	623.6004	983.8429	2308.81	2191.923	1654.651	1160.436	1047.687	1150.764	42.48457	36.8828	47.59053	1324.412	1311.742	1179.062	3356.595	3316.279	3543.046	1194.342	2988.845	2871.837
CUST_39521_PI429010886	M40763	0.000123861	18	15	40887062	Cherax quadricarinatus beta acti			3.12E-108	212.7978	40.78067	169.6212	196.526	41.20908	161.8447	122.7362	219.1089	83.94977	209.9184	86.28411	164.7136	287.4579	488.3385	139.5903	393.358	566.6051	803.3036	228.7822	396.9028	524.0943	49.25099	16.15024	36.37756	439.2301	197.4586	185.2774
CUST_20895_PI429010886	N44271	0.000124501	15	3						16.33412	5.47375	24.80919	52.33755	NA	38.66936	7.99047	14.74793	9.206401	8.150003	3.819605	6.866061	10.01207	6.976782	NA	13.10871	8.394142	23.24382	11.57295	29.97653	14.01651	6.147251	NA	4.613513	7.166484	4.495694	4.736698
CUST_55608_PI429010886	M110944	0.000125354	21	15						47.92699	53.69322	49.58225	47.88053	35.17211	50.28629	26.68249	26.86103	27.67043	39.36134	33.59889	45.96012	53.39865	41.84178	57.30284	68.69203	61.25771	57.98766	37.43054	42.64535	53.13079	52.05076	39.85654	56.53387	56.60172	33.83846	45.9512
CUST_50058_PI429010886	M88452	0.000125659	12	3	521027703	CUB and sushi domain-containir			4.33E-10	82.39127	58.22453	83.11893	130.3258	75.66353	103.1504	98.57909	223.3777	114.6993	66.07592	41.60531	46.39504	29.37844	126.1572	48.11395	192.1667	151.6707	266.8703	100.8962	53.56063	102.3598	75.45664	46.64371	39.29429	130.0993	92.52233	85.64416
CUST_44563_PI429010886	M66327	0.000128284	15	6						57.29374	64.2158	46.54974	48.21771	33.53246	50.60801	87.75883	79.49714	73.42732	65.63872	27.12725	53.087	44.31933	44.83771	42.92276	57.1768	57.58975	55.63811	70.72448	65.27026	59.26363	53.13532	77.49407	74.94714	47.80328	50.22696	69.29751
CUST_38706_PI429010886	M29290	0.000128392	24	15						721.4643	347.2002	352.227	114.5184	79.07706	92.62501	1214.037	659.603	1066.406	900.553	487.7778	125.025	744.5255	705.4218	412.9087	3284.996	1543.558	2757.04	635.1199	1071.939	1628.405	848.0782	33.7495	640.9263	796.612	232.9121	467.458
CUST_6641_PI429010886	N20390	0.000128392	24	15						85.98865	117.6651	119.713	53.73825	83.40132	61.87915	80.42543	123.988	130.2699	150.6257	113.4582	32.52246	143.1622	121.3554	125.3097	139.6859	164.0887	168.7741	120.0145	105.7112	125.9947	123.4933	115.4443	140.549	135.2901	76.21108	114.6239
CUST_19724_PI429010886	N42071	0.000128432	15	9						37.64058	42.88477	33.28109	29.06179	22.80532	35.13452	21.06721	33.02453	39.45708	52.16829	53.54287	61.26619	43.45077	41.56038	47.9205	53.35547	34.43069	49.9905	27.87026	36.40731	36.25072	36.49381	23.48629	58.84073	104.2173	40.19911	102.1933
CUST_53718_PI429010886	M102905	0.000128739	21	0						326.855	359.6695	352.8279	263.4946	200.0776	226.3778	212.9726	196.6122	226.0802	345.0411	283.351	213.5563	258.7813	228.9149	252.3	246.3057	342.9765	231.0435	266.3762	311.1974	265.9831	349.6396	347.1137	316.4278	341.6702	324.3008	407.4228
CUST_37834_PI429010886	M21809	0.000130081	24	21						48.6216	63.01324	70.14984	37.73322	42.316	32.32834	46.28626	56.33633	62.08412	58.97313	39.93038	37.82991	51.38302	43.77016	54.31269	47.03106	40.69275	61.4292	61.45632	69.34953	78.48989	86.51281	72.79587	74.81691	59.43294	59.73624	69.06046
CUST_17096_PI429010886	N37354	0.000130097	21	15						4.952547	16.81757	12.14006	13.66542	13.07681	13.08993	10.21986	11.23239	7.565346	9.849381	14.80803	14.88353	16.10664	7.808788	16.99789	15.36855	17.83378	24.08402	18.7288	14.73812	20.65942	NA	14.67376	16.0845	11.56593	11.2226	10.86736
CUST_43840_PI429010886	M64131	0.000130392	24	9	242006829	conserved hypothetical protein	Transport	Transmembrane transport	0.00000099	93.00549	184.9936	116.8305	264.2234	206.5859	218.8175	238.167	229.0002	158.0565	215.0483	190.1223	249.4346	177.1027	206.0425	115.5105	96.94761	215.4758	225.5545	46.13913	89.68248	79.57438	68.2334	169.6715	178.0895	95.173	186.728	184.9278
CUST_56174_PI429010886	M113027	0.000131771	24	15						103.0843	47.50128	62.196	20.01045	27.74342	8.722159	97.07924	95.93997	84.36167	100.1289	70.19469	23.2019	82.31732	91.46995	53.87582	249.5605	188.9335	176.549	73.7088	129.8849	108.8896	93.74241	12.8517	51.07791	106.7719	31.70434	41.71225
CUST_5662_PI429010886	N19006	0.000131771	24	15						206.8179	132.7868	136.103	141.6972	57.07543	80.58239	129.5473	230.4127	137.0649	303.7038	147.1038	88.42474	196.875	156.4627	98.93161	507.3489	307.7409	338.0427	262.831	226.6091	333.2833	273.8856	65.25923	222.6501	296.1058	87.85118	203.7183
CUST_33826_PI429010886	M7965	0.000132131	24	0						21710.8	21590.74	18273.52	11780.77	12288.42	9905.822	3890.917	8984.262	7225.192	17254.51	5502.319	7090.073	22598.48	4217.01	15340.52	15585.38	21398.95	5144.706	22847.77	13879.42	18235.97	31206.9	28120.27	21459.8	42900.93	25611.3	38719.75
CUST_7987_PI429010886	N22373	0.000133112	21	15						124.7151	709.9527	355.3919	246.0227	215.748	202.5102	539.9257	491.5182	526.8439	349.5554	181.0227	257.4304	385.6128	651.9522	388.031	587.0902	1354.492	1113.188	386.528	336.4491	415.6373	180.5859	269.4621	211.3776	191.3515	185.1157	131.0082
CUST_24777_PI429010886	N51235	0.000133426	21	15						20.82118	33.34145	16.71139	26.43339	6.742613	24.37229	63.72422	56.8797	46.9424	42.06094	21.49428	19.46418	55.51551	64.46055	42.6001	71.77497	64.39267	93.78493	42.26958	36.64369	58.06923	12.4535	27.23544	23.61619	23.15721	31.2936	37.87395
CUST_38200_PI429010886	M24096	0.00013387	18	15						511.0453	314.9794	246.356	112.669	64.60254	79.67772	414.9089	354.7453	384.6227	363.3984	194.5781	90.01966	434.0618	321.4419	191.1071	1544.613	569.3405	1430.525	335.0962	573.6451	870.8828	260.0376	43.17122	214.9733	414.7402	121.8531	170.3943
CUST_3900_PI429010886	N15523	0.000134978	24	0	443721341	hypothetical protein CAPTEDRAI			1.06E-13	60.63273	57.99332	62.71091	44.76603	50.3146	51.38302	40.16189	33.55937	37.50453	46.67638	39.50225	34.7801	43.32608	30.62762	49.48565	42.48109	37.78857	43.27193	54.0322	41.86801	38.62045	68.76743	42.4997	48.72562	54.73663	54.61235	54.1005
CUST_38842_PI429010886	M31115	0.00013515	24	3	149675211	cuticle proprotein proCP5.2 [Cal			1.43E-55	48051.75	1094.513	41865.42	57775.61	52165.44	59861.19	1560.774	3774.541	2361.017	42496.4	46875.63	48559.97	27665.22	26740.38	43701.56	424.0822	734.7144	593.3017	3715.857	3886.966	3706.021	44511.54	21710.8	39713.59	4565.036	12523.21	7714.571
CUST_28524_PI429010886	N58112	0.000135217	21	15						22.82225	26.89679	38.00971	18.39442	29.43236	22.52975	20.3727	20.80721	14.40631	23.69096	33.88276	23.74852	33.29588	27.47705	32.63627	30.77017	44.57504	37.8531	38.14824	25.87427	29.66835	29.47583	20.4836	27.65161	22.67247	19.61789	32.3182
CUST_3800_PI429010886	N15301	0.000135568	24	21						9.586868	17.36534	12.1946	7.082133	12.79576	8.350162	3.80698	5.542133	8.407667	16.10164	10.49733	7.344785	11.33295	7.266309	7.094278	9.550476	15.85755	14.81156	13.38008	22.87902	18.6092	15.53301	19.53794	17.25784	6.467201	17.54	14.80397
CUST_4235_PI429010886	N16291	0.000136247	18	3						70.95708	27.67304	95.9971	116.5916	115.1318	125.4804	32.63321	35.66495	52.15113	32.05226	57.48416	43.59179	68.75665	68.75665	67.18486	30.92216	17.87345	35.0396	43.34	68.99631	59.01526	69.05459	128.5661	97.16144	44.39167	65.97229	76.61576
CUST_33758_PI429010886	M7812	0.000136839	24	15						300.549	129.1152	193.3026	75.22137	28.56603	59.5633	1219.279	389.1575	1021.074	667.6928	437.5336	180.7742	222.4664	563.1838	114.4742	4140.108	1873.347	2622.032	845.9441	671.2531	1452.249	932.1979	17.52199	762.1289	821.2893	125.025	595.2796
CUST_6805_PI429010886	N20621	0.000136839	24	15						321.8126	324.2431	338.7418	316.0187	251.655	260.557	404.7958	343.8057	382.8	364.2049	288.3047	322.8267	390.6122	313.2852	350.2239	405.9933	385.9551	438.9261	299.1209	360.5101	380.83	368.8823	348.473	383.132	342.9247	295.6886	304.4188
CUST_34144_PI429010886	M8882	0.000140826	21	0	339254310	1,4-alpha-glucan-branching enzy	Metabolic process	Generation of precursor metabolites ar	5.62E-18	54.6004	45.53592	57.28678	54.36935	51.32137	49.21384	35.93556	47.88053	43.68293	44.79025	44.98933	43.70979	46.73349	33.03392	33.91504	35.13173	38.83274	39.09234	39.29873	24.04549	43.51485	60.26506	57.38746	62.3082	53.10397	32.50967	67.97224
CUST_50104_PI429010886	M88638	0.000142229	15	0						130.3258	153.1648	42.80558	107.7085	79.96781	111.1646	78.35549	68.21622	59.797	23.42954	18.45165	41.10401	72.55053	153.409	153.3857	110.3077	145.73	112.6234	129.9905	60.05066	98.19279	49.9386	289.0575	34.84867	31.93885	43.89958	13.46872
CUST_1994_PI429010886	N11264	0.000142631	24	15						193.7855	320.1071	243.3699	173.5608	156.2876	181.9587	218.5351	248.0205	271.3104	211.695	209.6968	183.5316	234.6515	220.1523	221.5335	231.3331	319.3967	299.9228	248.439	225.6261	271.4195	194.5781	223.1465	206.7386	196.2571	189.7104	222.5957
CUST_3083_PI429010886	N13755	0.000142798	24	18	321459356	hypothetical protein DAPPUDRA	Metabolic process	Carbohydrate metabolic process	3.24E-107	122.8067	291.3655	170.1187	95.75319	136.5009	98.65475	178.0114	167.1289	188.2604	199.7474	171.9376	99.2507	138.9709	184.5623	146.3562	235.9311	334.3239	312.4862	210.955	296.5728	363.2101	160.5879	223.2399	206.8179	200.7824	250.2264	205.143
CUST_53277_PI429010886	M101066	0.000143046	21	15						59.13513	34.6494	29.13534	24.91503	21.3198	19.49003	42.316	21.70891	24.98919	47.60041	30.04497	34.96168	39.4611	31.03777	34.57091	50.77134	35.78309	46.22189	41.18704	23.27568	40.25595	35.30149	24.22714	27.77843	29.01519	20.31011	26.99058
CUST_35273_PI429010886	M12112	0.000143933	12	0						35.95603	15.54734	27.97979	18.82259	6.940213	12.45733	18.0705	12.56182	14.59784	25.69361	18.78876	9.904747	44.90369	23.02558	27.10462	11.88037	8.931679	11.78126	17.39841	14.23813	13.62301	28.72644	21.85461	26.89377	18.09671	16.36409	38.18648
CUST_32023_PI429010886	M3029	0.000145032	21	15	13898841	CUB-serine protease [Panulirus a			0.000000605	1125.978	1415.173	1202.808	824.4785	657.9421	628.2632	1754.468	2239.116	2478.84	1240.711	1302.487	763.8013	2076.904	1620.506	1092.583	2103.097	2105.784	2294.993	1509.889	1238.715	1575.523	1122.574	863.8645	1289.967	1321.627	720.2377	900.3004
CUST_22445_PI429010886	N47040	0.000145032	21	9						65.19605	49.71921	50.60267	120.5417	125.8592	94.59748	105.3966	63.87134	61.89891	108.3596	102.2726	130.8473	94.51495	100.9673	105.946	88.74114	69.79048	89.30494	75.81657	76.49084	77.31152	80.61166	67.34563	76.19255	90.08744	84.75661	70.15908
CUST_36923_PI429010886	M17235	0.000145565	24	3						21.3436	20.45768	23.09017	40.63005	24.77785	23.63019	26.46648	20.13002	37.46348	6.063885	17.96345	12.31255	7.503112	14.32082	9.519989	9.506985	8.604047	16.44171	21.58133	26.0166	18.14784	14.39043	16.06359	20.81138	20.17982	19.43519	37.20056
CUST_2063_PI429010886	N11469	0.000145565	24	21						25.06929	22.47011	30.09371	35.32719	28.05953	27.54823	23.84404	21.9811	20.86102	43.13077	22.21596	17.56701	17.72698	17.07639	24.40659	22.91081	9.578088	24.58511	54.29543	41.42074	29.24902	84.28713	33.49837	72.81177	68.74828	28.98683	57.51884
CUST_12630_PI429010886	N29775	0.000145607	12	0						101.5579	107.4885	191.1727	67.31002	105.4292	104.437	69.79983	86.18133	126.3605	111.6419	123.0482	124.8106	132.6815	175.768	193.7076	81.56078	82.137	91.63916	100.4521	108.2394	118.928	137.044	172.4491	171.6937	134.1449	153.9033	167.7764
CUST_7994_PI429010886	N22389	0.00014962	15	0						3272.412	5131.503	2618.303	1136.139	1706.299	1938.89	860.0989	1012.36	1632.718	2928.861	56.70114	1979.658	4125.439	2732.247	3598.269	10209.43	5350.891	7653.847	2129.384	2787.074	3835.495	1213.608	617.0017	1819.509	11052.65	4114.144	5073.866
CUST_22260_PI429010886	N46708	0.000149917	15	9						1182.042	412.5149	1458.832	3135.16	1352.876	419.2614	1098.155	2320.751	3295.67	2816.959	6333.445	1580.709	4976.553	1337.377	1538.316	61.86512	66.2384	78.10699	3321.698	1923.884	4767.094	3460.858	10260.07	7900.843	4326.077	5826.27	10311.23
CUST_19369_PI429010886	N41464	0.000150432	18	6						9.280922	15.49957	15.41819	10.69163	14.41191	11.71679	32.28853	17.91133	17.4076	10.7159	12.47813	15.59668	6.001367	21.93249	NA	11.37944	6.306457	4.237098	9.295776	11.91014	18.50385	15.40578	18.27355	11.92197	23.59507	24.63804	19.84259
CUST_53330_PI429010886	M101298	0.000152557	21	0						25.2621	38.12534	21.77362	30.6586	20.97645	21.04316	15.82272	30.84622	18.36487	17.46742	19.51476	21.0238	18.96877	16.79507	14.97194	20.68379	26.10261	21.44313	14.93971	19.86079	18.34559	26.56674	32.51285	27.96875	21.1126	19.57461	25.22202
CUST_45216_PI429010886	M68365	0.000154511	21	18						11.30453	19.72473	22.75502	10.96971	10.13279	5.339243	6.298914	15.72841	15.82272	10.30948	10.42743	11.1896	13.91913	15.63819	16.68416	12.77646	31.83006	22.5758	26.22059	23.92527	20.11384	8.687501	21.45593	22.45014	10.21488	6.921979	16.12259
CUST_4043_PI429010886	N15869	0.000154621	24	3						23.5588	15.19323	23.67523	21.21298	317.2644	183.166	8.005861	14.06254	16.71925	13.95493	123.0345	101.334	NA	56.36738	76.36789	7.168799	14.16584	5.956757	5.865277	8.525673	4.962987	7.865315	11.48082	11.91165	9.981463	18.60288	8.984476
CUST_38971_PI429010886	M32801	0.000155683	15	9						3561.33	1441.224	3975.62	10311.23	4499.92	1084.752	4852.406	5284.133	9791.81	9226.663	14828.55	5137.74	15553.93	4772.224	5807.4	234.6515	143.1622	307.9283	10987.48	7769.948	14256.4	13790.46	23016.75	16553.17	13812.43	15585.38	25888.13
CUST_47865_PI429010886	M77925	0.000156066	21	15						15.35881	9.203065	9.71776	34.85212	12.7792	48.27587	31.02035	22.57786	27.96022	23.09515	34.58873	13.26584	40.83466	33.45999	35.38735	88.98877	60.71646	28.03915	44.83298	11.97624	37.38952	11.33713	22.9969	10.60299	13.72622	17.79852	10.16395
CUST_24466_PI429010886	N50674	0.000156112	24	0	29134779	projectin [Procambarus clarkii]			2.85E-54	37.44599	40.68416	42.42056	27.1479	38.48209	37.09003	19.96477	26.29331	19.93458	22.10476	18.48914	30.36386	35.32216	33.3478	31.11011	33.75905	32.83178	31.1591	35.32719	30.92873	47.14137	26.97869	42.70736	48.38982	43.69757	36.41438	48.91741
CUST_25562_PI429010886	N52649	0.000156114	24	9						39.52957	21.88339	19.04623	28.14237	21.22177	33.08424	15.88417	28.81419	20.18794	35.93556	26.32889	29.72388	22.00575	31.98798	16.0336	14.32405	20.9364	13.74955	8.651473	5.859138	8.035027	15.41819	10.68195	16.70829	29.82341	18.79971	13.31624
CUST_51515_PI429010886	M93809	0.000156254	12	3	345488143	PREDICTED: carbohydrate sulfot	Metabolic process	Carbohydrate metabolic process	1.66E-09	15.72693	43.00281	NA	35.29764	NA	37.9908	32.71622	36.29464	17.25616	23.12555	19.94147	9.9869	28.71759	51.53986	29.40702	80.12221	92.95196	66.96376	36.02118	5.75072	29.79347	7.107604	27.83932	12.87353	44.70872	39.56727	NA
CUST_29910_PI429010886	N60802	0.000157111	24	6						11.82291	14.83214	9.815468	6.68956	9.187163	15.78015	17.9285	12.00032	13.23504	12.63505	16.19225	11.52408	8.473586	NA	5.368642	8.433282	5.210289	5.441289	NA	4.979025	4.976733	NA	7.340202	7.713675	9.032116	7.608242	7.336788
CUST_34641_PI429010886	M10217	0.000157566	24	21						2670.787	2674.816	3017.306	2933.397	1790.499	1598.489	1228.932	1472.69	1146.806	3095.903	1537.641	1698.955	2708.146	804.0438	2017.363	2276.393	2210.04	771.7617	2811.043	2194.43	2765.552	5125.495	3212.539	4058.337	8846.332	3777.656	6854.464

Probe ID	ID	p-value	Period	Phase	EBI ID	Annotation	GO category	Go term	E-value	0	0	0	3	3	3	6	6	6	9	9	9	12	12	12	15	15	15	18	18	18	21	21	21	24	24	24
CUST_23739_PI429010886	N49374	0.000158311	24	15						12.48707	12.12405	10.95928	17.23263	17.26844	13.31417	16.98717	19.82019	10.44732	23.78991	16.99096	9.815468	23.93863	20.99116	16.60231	28.55984	19.1261	27.74851	24.07838	22.52125	23.4544	16.11271	15.7218	19.04623	15.55385	13.14544	16.75338
CUST_39309_PI429010886	M37533	0.000158625	24	0						4319.299	3960.355	2940.045	2519.472	1584.461	1592.484	1356.93	1754.468	2075.256	4545.107	2159.534	1929.095	2355.337	1292.456	1580.709	1733.335	2539.976	1144.272	4000.901	2645.058	2571.491	5166.879	3702.472	4217.01	7993.785	5558.806	5833.315
CUST_6558_PI429010886	N20268	0.000158637	24	3	345479564	PREDICTED: wee1-like protein ki	Protein metabolism	Cellular protein modification process	6.34E-95	15.19175	11.13644	14.38486	26.21426	15.82424	16.81027	10.5238	9.419511	NA	8.809458	11.3488	12.60408	14.47877	13.27388	7.840494	7.698781	4.502353	5.446073	3.8005	8.649765	3.855481	14.64829	16.81592	6.695947	10.11713	10.62708	9.411311
CUST_8814_PI429010886	N23632	0.000159657	12	9						98.46402	19.06607	80.91667	184.1143	76.3423	80.96563	41.85107	70.332	54.31927	259.0732	674.8039	241.8418	211.9389	128.8445	131.1212	18.95217	19.0327	19.73488	102.6964	76.6076	75.55461	340.1047	530.8049	455.9995	129.3882	391.3071	386.6278
CUST_53749_PI429010886	M103047	0.000160333	24	21						71.75645	76.53128	62.34647	42.88477	61.95078	44.03643	32.2766	132.5156	81.98831	24.76487	33.21716	48.68211	33.13557	27.18421	21.23607	49.22608	87.03898	45.73174	39.05213	35.91186	35.59906	104.3412	91.85221	93.57741	85.25564	50.84751	98.94639
CUST_292_PI429010886	N3553	0.000161604	12	9						9684.529	4889.847	10464.54	11974.32	7021.693	6515.192	8644.801	8334.379	13360.37	12496.19	17254.51	11780.77	19960.25	9684.529	11052.65	720.7726	527.3184	772.0259	17345.96	14764.65	17453.24	19052.86	29725.44	27832.29	11206.93	16070.74	25247.34
CUST_41927_PI429010886	M58897	0.000161943	24	18						103.4513	69.37968	82.00982	63.43943	53.19546	52.8068	98.17006	65.63171	76.36789	115.9671	67.69664	113.3707	113.23	111.5206	165.6517	154.4269	87.36259	182.9511	227.7173	105.9124	140.2224	147.3274	95.22353	100.9118	120.7668	108.3124	121.0405
CUST_2428_PI429010886	N12327	0.000163272	21	3						110.0054	58.55963	162.1415	192.1066	95.21224	172.8416	72.63288	86.83154	136.2005	91.47922	139.4798	96.58233	168.4308	47.64701	103.4374	41.84178	40.60853	45.33911	83.79749	85.90563	124.7762	168.511	147.1247	191.3796	137.7644	174.1094	251.655
CUST_40024_PI429010886	M48768	0.000163325	21	9						26.00555	19.21128	37.44318	29.77371	56.01896	5.256252	90.50145	78.62482	59.00011	72.55053	166.0161	33.31109	27.08528	135.2901	62.40045	11.62724	61.51056	12.72035	16.73229	14.82588	16.57521	24.32624	29.0511	15.14057	NA	34.03486	29.6926
CUST_52187_PI429010886	M96683	0.0001651	15	0						36.90197	43.80331	NA	18.44379	NA	32.70202	38.58803	9.269929	31.74988	14.05555	7.264645	NA	25.3148	7.523467	27.09821	69.66595	43.37645	21.9474	42.2303	NA	33.23134	NA	30.14806	NA	8.58477	9.903662	2.88368
CUST_38112_PI429010886	M23418	0.000165498	12	9						12211.42	1330.649	12191.65	24040.66	5284.133	10352.54	4499.92	4388.629	8269.17	20762.38	19635.13	20059.08	29520.35	8451.427	15673.03	300.3594	109.3748	476.0531	13635.69	8227.781	11780.77	30721.41	35022.2	32969.41	16252.77	16106.84	28943.19
CUST_11960_PI429010886	N28693	0.000165528	24	3						1328.27	15.67952	1257.339	1764.635	1001.688	1321.627	9.066919	65.7755	61.77408	363.5741	305.2389	333.7063	229.1648	57.27204	226.205	4.9725	29.02458	12.85346	11.19367	35.07134	41.95139	542.3764	145.0185	319.6543	172.9496	NA	209.0675
CUST_18306_PI429010886	N39544	0.000165725	24	0						17.22926	25.19867	14.67792	10.83531	8.177364	NA	6.908019	17.18912	14.16364	19.36587	9.13994	12.04777	5.059873	NA	11.83302	4.606356	6.256607	4.416366	8.829167	5.944674	4.39103	NA	5.727894	9.05635	14.64996	16.42269	21.37846
CUST_48078_PI429010886	M78789	0.00016632	24	3	241716804	RB13-6 antigen, putative [Ixodes	Metabolic process	Other	0.0000006	197.796	11.30326	187.5751	274.5666	128.5506	244.4841	20.96269	12.48201	11.35899	35.87747	108.298	144.058	34.48395	83.97595	135.2901	15.9111	11.76708	11.18301	8.822371	9.05285	9.848403	52.05686	17.97466	35.40359	45.95593	18.94246	29.67188
CUST_26083_PI429010886	N53628	0.00016632	24	21						21.89409	28.39888	23.75813	20.62453	27.90262	19.5987	18.54665	10.81711	18.60288	23.25613	12.84632	17.90327	21.82795	13.48819	24.96583	23.76471	30.31074	39.84152	21.49936	24.56549	20.45768	36.34979	27.27615	36.63747	25.10206	25.63724	32.55747
CUST_11810_PI429010886	N28439	0.000166662	24	15	321473475	hypothetical protein DAPPUDRA			1.85E-18	44.31933	43.99925	65.93698	54.71691	73.12505	61.60595	41.72651	43.25692	36.56104	66.59115	37.4753	72.30147	43.0146	66.53367	56.87317	95.95298	80.64549	115.0551	66.06005	78.21211	62.49633	72.48391	75.34564	84.45954	79.72468	55.86654	69.41702
CUST_37890_PI429010886	M22076	0.000168005	24	6	498785	PAPI I [Pacifastacus leniusculus]			6.34E-33	227.7726	331.9294	223.7905	550.2129	492.5086	546.6865	1553.379	980.6718	866.8631	1050.367	351.2785	1054.373	537.5716	808.5577	297.188	209.031	595.1579	717.9895	166.5631	360.6967	207.6942	165.4536	477.3178	424.6765	97.52903	424.3124	403.4693
CUST_38058_PI429010886	M23122	0.000168384	21	12	196000326	hypothetical protein TRIADDRAF			4.53E-21	21.37846	27.79405	18.57121	268.7276	149.73	96.97291	180.5334	82.60618	223.5617	48.5966	294.9139	178.576	247.1999	76.36789	203.8839	28.30304	98.14527	107.2586	146.8225	93.35268	240.6532	8.596616	24.11615	15.3929	26.69535	62.16088	60.18712
CUST_37949_PI429010886	M22419	0.000168508	24	3						773.6805	569.3405	578.5106	1006.869	1139.062	1250.489	727.7655	900.3004	591.3029	878.8076	810.6462	942.7053	751.8458	549.679	1008.089	472.8686	433.3852	458.4811	303.4454	658.7898	262.9461	745.3715	534.1168	798.0084	417.9126	813.5332	562.7989
CUST_12986_PI429010886	N30396	0.000168508	24	21						44.56728	21.34009	28.49131	22.89373	22.02084	31.53769	29.33369	16.72797	19.78464	31.67008	27.2791	22.98107	43.22841	33.36729	34.48922	38.61632	33.53619	43.42121	65.12931	30.48738	33.95508	78.04556	36.99237	43.26708	44.61738	25.81487	35.28538
CUST_38749_PI429010886	M29975	0.000169393	12	9						8378.475	727.4839	8683.686	15967.93	3702.472	7337.918	2704.306	2583.285	5119.273	16726.21	13098.74	11719.99	22092.06	5966.983	11179.97	181.8309	73.53729	266.9539	10156.68	5726.275	8698.04	20662.81	23820.74	23324.95	10277.01	11179.97	20099.82
CUST_87_PI429010886	N699	0.000169393	12	9	426237987	PREDICTED: keratin-associated p			0.000000513	11588.48	1134.386	10311.23	21177.49	4934.022	8121.327	3193.902	3426.429	6791.114	21710.8	13906.34	15731.96	24040.66	6481.59	11588.48	246.0227	101.6403	333.2833	12765.38	7147.632	9629.338	22658.35	27210.5	24721.57	12043.33	10727.23	18758.28
CUST_143_PI429010886	N1541	0.00017134	12	9						13360.37	6956.592	17771.79	15871.24	7546.585	9653.786	10090.54	11052.65	17287.24	17210.94	19791.01	14307.54	22658.35	9285.648	17453.24	2719.085	3664.856	3992.329	17692.35	13737.98	17771.79	21922.49	23658.24	23231.4	11719.99	14982.33	22279.42
CUST_55423_PI429010886	M110026	0.000172431	18	3	353228410	Schistosoma mansoni strain Pue	Cellular Process	Cell death	1.69E-61	372.1298	249.0225	907.3817	1450.664	243.1406	1046.706	58.89558	230.4612	96.60811	61.76701	38.77522	38.87535	75.49296	639.8078	167.5116	80.36141	245.4997	92.53323	169.3049	161.9294	42.34791	641.1736	244.6301	193.6659	181.9587	198.0524	80.76921
CUST_54318_PI429010886	M105551	0.000172885	24	18						7643.683	9056.645	6421.864	5578.907	2640.776	2820.736	10448.35	6265.89	8284.715	6179.344	3387.12	3452.681	11588.48	5751.95	4672.963	12699.11	9923.978	12141.91	16288.46	12605.8	15012.16	7721.817	10550.02	7558.705	13685.2	12699.11	10464.54
CUST_51355_PI429010886	M93237	0.000172885	24	21						162.7381	192.1066	198.9127	102.1108	151.5412	85.785	50.59749	85.07992	97.47634	170.9641	163.6128	126.2375	213.4477	144.5793	189.1569	145.5507	188.5193	110.0991	278.9665	185.3668	134.27	402.436	202.9634	194.9048	404.8745	128.179	244.6301
CUST_2624_PI429010886	N12783	0.000172885	24	18						92.31393	164.5654	166.7877	125.2728	119.4286	104.258	158.0791	153.6189	169.9145	160.217	142.0375	130.3988	168.0943	144.7781	150.9231	159.1789	197.088	209.8119	173.1388	181.2583	221.3966	142.0036	141.4085	171.8413	154.6661	138.5034	179.0245
CUST_720_PI429010886	N6913	0.000172885	24	18						5197.224	7138.174	5856.992	4863.888	2466.158	2598.143	8818.425	5407.94	8945.499	4616.026	2858.309	2904.283	10294.69	5110.752	3827.862	10727.23	7861.824	11475.48	14100.35	10277.01	11836.96	7303.286	8296.544	6533.018	10178.51	9791.81	9684.529
CUST_8651_PI429010886	N23359	0.00017296	21	0	110763495	PREDICTED: MORN repeat-conta			9.85E-63	823.7754	977.1077	896.981	825.0827	612.5918	538.4885	499.0568	582.1367	543.6971	675.6901	720.7726	523.1285	808.8122	587.5636	830.4195	577.5373	939.9518	641.6007	728.0598	661.1838	626.641	1004.431	905.0625	950.7112	867.6626	727.0088	1065.144
CUST_35881_PI429010886	M13798	0.000173132	15	6						845.9441	473.3422	1415.173	709.9527	450.1126	243.8189	2646.596	1310.327	3843.133	1035.146	1875.312	750.0607	2201.915	783.6564	1071.049	178.6979	185.0545	222.0531	1855.874	1694.196	2114.8	2110.599	3495.947	2738.485	1818.148	2061.689	4349.21
CUST_21171_PI429010886	N44762	0.000173287	12	9						41.8114	39.6267	71.66506	37.44318	33.67973	31.36919	49.12781	26.23532	47.94598	64.66012	69.11926	53.22588	51.56297	51.48244	51.05459	43.15923	37.21408	52.06198	62.34647	44.24302	60.50917	67.48124	66.26258	108.7426	56.28495	39.55139	90.74217
CUST_53821_PI429010886	M103323	0.000173478	18	0	229892279	uncharacterized protein LOC100	Transport	Transmembrane transport	2.51E-10	42.20483	34.44551	40.76682	37.40246	16.50439	33.39499	22.45692	32.50381	32.49411	18.08639	19.26106	15.65876	19.95957	12.97286	8.440387	18.55929	25.1	32.42666	23.34012	50.93881	49.65645	15.73263	38.09775	25.45483	15.43474	38.03345	25.76854
CUST_23503_PI429010886	N48942	0.00017395	15	12						29.36452	39.86153	23.8882	15.62519	20.96992	9.232864	21.2559	31.14654	31.23137	27.0245	34.11681	52.41365	37.01424	33.54781	34.60522	35.89334	43.84283	47.16128	22.49747	18.44774	26.90764	23.99454	30.03343	29.95843	41.38164	24.34505	44.10093
CUST_35615_PI429010886	M13024	0.000175457	21	15	383858515	PREDICTED: protein fat-free hon			1.42E-46	78.12052	149.786	84.48811	91.6033	84.29772	53.14759	44.58927	58.79368	20.10181	124.2158	83.75475	71.89808	69.10515	109.6621	117.3251	116.0279	143.144	139.5639	109.6359	100.4234	82.9019	119.6392	64.13867	91.6033	50.96545	71.78767	107.567
CUST_4428_PI429010886	N16735	0.000175686	21	15	321476145	myosin heavy chain isoform 3 [D			2.07E-44	174.0123	136.424	146.5694	188.0176	170.9048	163.3644	161.4729	143.8649	137.9345	173.1143	109.5487	123.263	178.5273	143.9614	94.85229	221.8149	237.835	229.5682	214.2511	190.5205	290.029	224.1099	192.9931	190.7291	177.9078	132.0358	142.4297
CUST_3251_PI429010886	N14108	0.000175785	15	0						33.4748	31.28202	NA	52.96326	6.937084	58.75919	26.6468	7.484013	20.52844	5.088426	9.880999	4.790585	18.37444	71.06688	22.74972	69.84655	35.48951	33.90697	29.27204	9.83045	26.30298	12.80959	31.53193	12.55197	7.479546	6.993373	NA
CUST_19659_PI429010886	N41961	0.000176127	24	21						10.77609	9.342102	9.75683	10.45586	NA	9.05285	7.444772	8.565689	8.072209	5.243557	6.273022	8.188093	8.734334	6.652007	13.02868	17.75966	7.319642	9.614822	6.871633	6.432193	12.83426	24.4823	11.87453	19.99907	12.06381	8.334969	18.59753
CUST_5455_PI429010886	N18727	0.000177262	24	3	321472375	hypothetical protein DAPPUDRA	Signal transduction	Cell-cell signaling	6.11E-19	76.16835	50.32489	61.15056	105.3673	79.37286	81.75003	67.78785	57.9604	77.59604	70.38705	46.69101	56.18757	78.8865	56.15909	89.13599	51.36995	41.08386	37.22016	49.87102	37.71176	33.1947	71.28516	36.1335	53.73347	40.51538	49.23962	39.35783
CUST_7673_PI429010886	N21899	0.000177262	24	3	321455993	hypothetical protein DAPPUDRA	Metabolic process	Other	7.62E-60	44.3077	30.31506	42.9547	52.33176	61.65559	50.36291	37.7689	45.1102	43.98563	31.58885	39.79966	36.6589	40.92146	44.43314	34.90937	39.96323	29.29243	39.22978	19.04208	32.75774	28.28196	44.35011	41.50754	43.35728	29.66274	33.25783	44.9498
CUST_12314_PI429010886	N29276	0.000177262	24	3	380029118	PREDICTED: uncharacterized prc			1.3E-30	53.29185	38.62045	58.84948	66.83189	85.72132	100.4643	45.72512	54.39842	49.37308	64.37978	44.92669	69.66595	71.97753	44.39167	65.69754	37.77333	37.09773	31.96059	30.93515	41.75149	28.93482	53.8516	51.18755	61.70924	37.03485	51.7695	54.87487
CUST_53053_PI429010886	M99919	0.000177411	12	0						128.3075	94.36986	99.56289	62.88268	64.91949	47.66075	41.81524	45.40562	59.21121	54.90594	93.14583	72.67801	78.15502	68.88761	96.47869	100.264	75.84873	78.49976	49.18092	64.46055	56.75304	107.5789	85.27841	92.16269	89.91214	90.35029	119.7806
CUST_37467_PI429010886	M19131	0.000178775	24	21	321455420	hypothetical protein DAPPUDRA	Metabolic process	Carbohydrate metabolic process	9.82E-92	1722.827	1818.148	1964.046	1721.8	1302.487	1206.945	917.5193	1489.353	1155.769	1604.901	1147.873	1042.748	1843.608	746.7514	1338.68	1451.369	1792.472	534.4784	3232.565	1640.994	2420.859	2519.472	2895.969	1950.295	4048.748	2466.158	3783.527
CUST_33442_PI429010886	M6896	0.000178775	24	18						13.62679	11.10263	14.04923	9.994225	10.38364	8.203427	3.742775	9.03778	6.485846	14.38127	12.32463	22.77868	22.66351	16.46161	12.14425	11.04536	16.38009	16.98896	17.53785	18.87745	17.2724	14.95707	8.982204	20.41963	14.98689	11.09687	13.12953
CUST_36404_PI429010886	M15810	0.000179293	15	12						757.1366	857.1902	1019.08	663.1483	527.3184	594.6179	570.8807	702.7679	609.2645	677.9076	756.356	656.7134	903.1163	845.9441	802.1705	601.1087	854.1319	648.9592	580.6289	601.5188	558.2968	691.181	582.3778	645.8971	592.215	886.7077	805.476
CUST_31817_PI429010886	M2483	0.000179928	18	3	193676271	PREDICTED: kelch domain-conta			5.58E-32	35.22067	56.70114	69.71083	63.07317	38.85319	75.76283	43.18058	57.98315	59.15199	32.86579	38.82052	29.0511	33.3824	29.71551	48.16684	41.20908	62.5781	58.18757	43.30339	72.96939	61.295	82.50222	68.55444	73.39156	61.60595	73.10715	16.71379
CUST_50120_PI429010886	M88705	0.000179928	18	15						24.78354	14.49982	23.32746	12.87704	19.35085	9.141868	12.23088	19.90569	23.51553	38.00456	28.8005	20.9364	12.97492	25.84066	27.95476	50.75534	37.84952	34.62846	19.59198	22.9881	27.32212	12.63354	12.43008	28.0193	19.46418	20.77024	24.26872
CUST_8968_PI429010886	N23867	0.000179942	24	12						79.14448	88.85544	111.4946	94.1914	83.9852	74.18947	139.755	85.19452	136.3336	144.5615	126.521	150.3467	176.9444	138.5034	147.2789	177.9861	133.196	165.2368	159.9018	94.95617	121.9486	128.797	101.572	113.823	134.7975	87.63468	153.0208
CUST_48239_PI429010886	M79483	0.000181258	24	21						52.98141	92.32672	52.53638	40.28522	30.01509	21.71446	42.78996	46.64691	48.27158	37.09003	29.01782	39.05992	102.5557	37.8045	32.78884	64.65354	75.53689	64.02245	89.47762	77.96514	48.54356	93.29743	79.81483	81.4582	41.50754	53.21446	53.33381
CUST_38783_PI429010886	M30336	0.000182706	12	9						28.67152	15.88853	36.56941	532.6369	422.8235	33.4688	375.5874	267.0346	313.6779	1488.548	3175.147	1041.894	241.4743	249.9933	151.7575	16.1942	47.61057	17.67823	7.370654	378.9491	633.4883	1631.254	420.3226	1294.853	12.26172	12.93513	19.71264
CUST_13413_PI429010886	N31133	0.000186224	24	3	512950785	PREDICTED: plectin isoform X6 [			1.05E-11	25.5836	32.70202	25.5566	42.82003	56.72705	65.50385	31.58885	28.8864	28.47101	35.99566	30.96768	53.76691	20.568	33.99416	35.83737	23.98146	15.62701	25.0378	25.28008	25.2621	19.10119	30.99477	25.9824	32.11277	24.79879	31.11011	28.9184
CUST_30054_PI429010886	N61090	0.000186394	12	0						37.82991	32.09525	53.04738	20.66998	22.96931	17.69464	16.80584	23.50843	22.01795	12.78825	33.2543	25.28249	36.45739	30.08709	33.80478	22.06613	28.1014	19.92192	14.57376	22.0686	21.61569	31.07591	40.80836	25.97276	27.89685	29.4873	98.70542
CUST_12916_PI429010886	N30263	0.000186465	24	21						38.49859	31.91281	48.20502	30.32915	38.93757	33.87813	28.54797	28.67393	52.56738	45.69732	43.58292	40.97531	30.35985	38.39235	44.85722	59.797	46.62193	57.01672	65.30254	78.80045	68.27965	79.02299	58.33297	68.08811	82.34733	28.18616	56.32908
CUST_36492_PI429010886	M16052	0.000186641	21	15	501415066	C-type lectin domain-containing			1.27E-17	226.6524	209.1018	242.8747	284.5651	242.833	232.8383	282.8745	293.2877	274.8211	369.4099	300.0533	373.3349	308.7489	303.4901	259.3203	435.731	317.784	464.5257	241.9195	284.4024	324.6618	263.6527	185.2204	290.7989	320.2208	189.0814	291.5804
CUST_42411_PI429010886	M60209	0.000187425	18	3						90.52573	99.34395	87.74995	120.9827	88.27501	111.3972	109.561	111.4113	98.07401	71.45149	69.45139	72.08616	98.32351	78.66288	65.97229	108.8028	94.87741	87.98812	110.5494	73.08823	84.12527	113.1748	124.4425	99.93518	83.12776	87.33991	104.6457
CUST_23541_PI429010886	N49004	0.000187425	18	9						17.83143	15.19667	21.10822	16.57982	46.45178	25.17932	25.60467	18.13805	19.79478	32.03079	49.35536	54.37537	29.33369	26.88183	27.9125	19.64055	28.25712	23.40815	24.44847	17.9492	16.36771	25.71638	23.5397	19.8984	24.37455	28.29802	24.28968
CUST_53798_PI429010886	M103232	0.000187641	18	12						38.17853	36.37398	34.4378	17.33337	20.01976	13.68381	25.89606	25.35952	30.83657	28.81419	28.35562	28.16647	40.3254	35.97078	29.64248	47.62949	36.42929	24.91804	32.14243	21.21298	13.21612	16.41161	20.30456	16.42417	39.63021	25.33441	38.27451
CUST_50549_PI429010886	M90237	0.000187654	18	3						24.71696	50.1322	34.63788	71.93885	68.06818	81.4132	40.96379	53.71014	42.23497	47.42663	43.31704	50.1877	49.364	34.17741	33.92477	38.27962	51.52222	61.04906	67.66335	64.75498	56.33633	73.23385	69.63156	48.9712	51.03976	66.31026	58.36343
CUST_1632_PI429010886	N10288	0.000190373	24	18	270014040	hypothetical protein TcasGA2_T	Metabolic process	Carbohydrate metabolic process	9.86E-107	2511.558	2247.929	2066.858	1754.468	829.47	968.7419	2367.057	1944.792	1927.794	3060.056	2197.153	2006.118	2020.363	1510.508	1883.877	3808.458	3250.257	2853.1	3373.758	3175.147	3193.902	1769.667	1882.752	2359.281	3145.521	1638.477	2480.784
CUST_44455_PI429010886	M66038	0.00019119	24	3						227.8584	22.94579	160.139	419.4465	472.8686	515.2465	24.65528	40.35274	13.71845	287.9808	388.382	468.8585	110.3861	193.2061	289.3915	5.278973	6.774828	6.766809	8.203427	45.86429	12.24986	231.374	78.74455	233.7667	15.14334	102.1002	92.88966
CUST_8068_PI429010886	N22493	0.00019119	24	21						45.90992	48.2963	57.59663	37.43476	25.23371	18.2969	41.38631	42.34405	67.01762	22.49527	27.37466	23.70162	65.72115	32.80252	30.58943	19.85649	31.84882	43.27654	79.25889	56.52239	63.41449	67.15403	83.53855	82.03251	31.61388	40.16939	80.52797
CUST_24297_PI429010886	N50377	0.000193673	24	3						79.93916	46.28626	58.18021	109.2373	99.99679	100.2134	88.75	41.81524	49.94405	108.374	68.35388	81.72936	65.29504	69.03939	72.00199	39.66978	49.23192	45.93656	55.58555	53.45027	59.37073	72.03186	68.60832	55.60957	55.55609	86.70327	71.64925
CUST_1207_PI429010886	N8833	0.000193673	24	3						2184.345	1765.847	2356.919	2547.238	3140.215	3706.021	1577.474	2419.664	2452.653	1605.911	2744.675	2439.09	1597.44	2489.305	2472.65	1065.144	1050.844	941.3897	1127.686	1719.004	1016.824	1420.017	1635.354	1449.959	912.8746	2033.334	1255.327
CUST_16337_PI429010886	N36065	0.000194952	15	0						52.22249	70.14984	56.32908	46.03411	62.03291	36.26885	26.21182	45.21941	28.09462	42.6001	60.04487	53.94311	51.47751	44.6746	56.24698	54.54964	73.50942	73.79944	37.81203	51.8805	51.14752	42.54185	51.03976	49.08691	44.91446	42.75542	50.8191
CUST_12973_PI429010886	N30373	0.000195589	21	0			Nucleic acid metabolism	RNA metabolic process		11.39786	20.47681	17.15642	10.27866	7.638129	12.05475	14.90046	13.92996	10.72533	6.198566	3.966345	3.989228	7.938175	6.984847	5.37232	4.878495	13.73044	17.24504	14.12155	9.336607	8.32998	15.85451	13.34535	23.70418	12.05333	16.98352	13.28376
CUST_55309_PI429010886	M109516	0.000196156	24	15						27.97586	23.69946	36.41874	36.58087	33.17225	29.44247	60.00388	41.58423	36.56623	37.53076	23.84965	33.746	37.77999	36.63747	39.36134	46.83625	48.80597	44.10584	43.86651	42.6775	42.70736	43.56248	38.74886	37.95648	35.7142	35.53128	40.38904
CUST_41471_PI429010886	M57812	0.000197389	15	6	321458428	hypothetical protein DAPPUDRA	Metabolic process	Lipid metabolic process	4.45E-59	22.52125	17.50958	16.77811	31.47125	24.90228	30.68536	26.65221	24.05806	35.14671	25.90137	22.39995	25.81236	26.56161	28.11335	23.86873	20.23215	15.37628	16.09693	27.46007	23.02109	24.09442	27.48203	27.1944	40.97531	28.17748	17.08471	26.63581
CUST_11207_PI429010886	N27444	0.000197421	18	6						971.9693	232.9982	1191.479	780.4581	488.5919	239.2973	3525.214	1149.369	4334.63	792.599	1338.68	694.9037	1680.655	865.0124	643.379	452.3252	123.305	490.8866	1544.613	1181.431	1605.911	1492.942	2330.806	2090.105	2696.535	1889.404	5336.579
CUST_37346_PI429010886	M18730	0.000198639	24	15						14956.1	22946.38	13635.69	11455.94	10550.02	11112.95	19960.25	18146.2	19855.57	17424.76	13532.16	12648.44	17424.76	18198.77	16520.01	19522.5	29294.9	25888.13	19434.65	20708.31	21177.49	15673.03	19679.49	16954.37	19150.48	14885.24	18522.28
CUST_4345_PI429010886	N16556	0.000199728	15	6						1397.328	482.8348	1927.794	4173.107	1792.472	621.9301	1575.523	3940.028	5973.032	3664.856	9937.479	2302.573	5879.268	1976.903	1927.794	60.45821	55.94559	71.70119	4388.629	3696.265	6922.365	5502.319	13685.2	12605.8	5317.598	7769.948	12743.08
CUST_10433_PI429010886	N26222	0.000199973	21	0						603.0231	289.9679	601.9971	327.8924	372.7272	340.2357	194.8463	280.0406	246.1645	294.6856	310.9804	304.9349	309.9338	263.4524	309.878	201.0489	279.1951	205.8558	266.9539	307.8719	326.9964	457.5988	375.1194	520.7346	268.6876	322.75	404.5068
CUST_27597_PI429010886	N56345	0.000200752	18	12						9.069476	6.180949	5.916949	8.884176	74.21946	17.40265	11.31857	NA	6.656681	14.71976	61.81014	76.88599	10.78499	113.187	100.8962	9.470236	6.62957	6.567996	5.060452	6.204836	6.946877	10.06884	11.56722	14.78165	9.824108	17.59575	11.87749
CUST_54735_PI429010886	M107376	0.000201122	24	15	308512650	ubiquitin b [Eriocheir sinensis] >			2.04E-20	2322.285	4185.629	2837.693	1965.787	2419.664	2201.915	3460.858	4150.213	2568.829	2740.835	2524.402	2554.596	2615.936	2702.508	2736.432	4045.654	5091.533	4705.762	2862.188	3463.912	3672.992	2375.903	3098.696	2846.594	2757.04	2945.018	3036.167
CUST_35659_PI429010886	M13165	0.000201122	24	15						142.2521	211.625	151.1899	139.5198	128.0875	113.7264	228.4713	189.8339	210.9122	161.9294	107.7215	153.1144	180.8125	164.9977	186.2275	223.5106	235.738	236.3422	170.0456	178.1474	233.0433	193.0271	164.8928	184.9936	173.7132	156.7919	171.8137
CUST_37769_PI429010886	M21476	0.000201122	24	15	225382096	protein disulfide isomerase [Lito	Metabolic process	Oxidation-reduction process		13658.74	30447.23	14982.33	13761.91	8911.047	13481.03	28943.19	27918.92	16014.56	17931.09	14363.02	15141.22	17729.56	17088.5	15585.38	34065.02	39540.76	32798.44	25065.69	21834.29	25403.62	14189.5	14982.33	15705.46	24040.66	20708.31	15367.74
CUST_47225_PI429010886	M75276	0.000201122	24	15						265.6845	90.44348	140.0779	33.25783	19.14248	26.22317	917.9436	299.4177	722.3326	508.9078	311.2489	156.7437	151.9479	402.0757	77.711	2870.034	1731.406	1643.622	595.0044	525.5146	1060.229	717.7206	16.51171	511.4855	620.2559	119.6713	357.1234
CUST_47657_PI429010886	M77066	0.000201122	24	3	54042615	arthrodial cuticle protein AMP6.			1.82E-08	10352.54	943.8069	7316.568	17388.9	16520.01	17555.23	1529.136	1322.312	1112.702	13906.34	14548.28	14285.49	10664.62	9257.227	13222.77	656.7134	559.5956	687.2962	772.7965	2116.149	792.4227	11138.2	5311.077	10764.25	1129.142	3781.313	3777.656
CUST_2217_PI429010886	N11893	0.000201122	24	15			Transport	Protein transport		2599.331	3825.101	2615.936	2785.011	2534.505	2484.399	3709.96	3220.778	2803.748	2968.824	2809.488	2584.904	3067.239	2803.748	2833.623	3739.191	3801.741	4105	3164.281	3426.429	3513.064	2811.043	2926.153	3105.612	2998.296	2960.558	3360.114
CUST_52289_PI429010886	M96963	0.00020173	15	0	321468138	hypothetical protein DAPPUDRA	Transport	Transmembrane transport	3.36E-16	37.01072	64.99766	74.20038	40.30598	30.88085	18.94794	15.48837	29.4575	18.95217	17.91933	32.62695	10.35047	45.93656	23.55652	30.59991	46.84134	42.5644	70.57647	44.85722	32.20247	37.67421	27.24405	15.75922	29.07923	24.28968	24.91503	48.56063
CUST_5031_PI429010886	N18176	0.000202714	21	18	405973238	RRP5-like protein [Crassostrea g	Nucleic acid metabolism	RNA metabolic process	2.61E-71	289.56	65.27494	65.45785	40.50202	45.43624	30.73939	37.71641	59.28006	50.88852	45.05649	49.61593	44.87713	21.94131	28.37326	23.2664	49.59683	85.32321	64.51751	68.20088	57.01067	71.22472	53.68776	63.45821	70.78121	50.52247	58.75919	66.2054
CUST_39485_PI429010886	M40156	0.000203605	24	3	149675211	cuticle proprotein proCP5.2 [Cal			2.89E-51	49649.01	955.5815	44285.99	59443.36	51269.31	62699.58	1570.104	3499.526	2549.09	42900.93	49113.82	45257.85	27832.29	27210.5	42900.93	391.4124	667.9516	525.7914	3024.414	3555.208	3253.191	41419.19	20616.2	36302.66	3230.906	11413.74	8146.369
CUST_47774_PI429010886	M77560	0.000203605	24	15						125.0519	116.7758	80.25802	47.77802	32.15355	35.16517	640.7195	221.6521	475.8315	364.8648	210.8033	91.97617	109.762	276.8158	77.67929	1231.406	855.307	905.9928	293.835	251.9987	472.502	446.2058	22.52125	294.0126	253.8613	75.49296	240.84
CUST_48054_PI429010886	M78709	0.000203605	24	3						597.9185	139.1141	531.9945	819.3739	1987.037	1569.464	122.9044	278.8807	116.3062	401.9784	1377.595	999.3353	327.5322	767.6438	359.587	49.97298	78.79009	45.7369	99.74451	317.9491	71.62515	347.3018	321.2079	381.5238	77.02123	613.3285	114.4474
CUST_305_PI429010886	N3718	0.000204918	18	3			Metabolic process	Carbohydrate metabolic process		521.7908	159.5774	523.8156	781.2177	793.2617	792.9194	224.7103	224.2885	219.538	515.2465	676.1529	677.6753	668.9797	413.108	675.5044	150.1836	126.4187	111.9372	200.5474	271.1875	211.4099	1063.832	694.4759	849.9671	215.9444	408.5695	397.1925
CUST_370_PI429010886	N4626	0.00020602	24	3						392.9019	131.752	279.7784	1093.204	557.6299	1071.939	124.3957	436.8129	86.9167	90.87055	354.3285	463.1658	218.6001	259.2692	435.1623	42.14237	26.81873	56.92501	30.37407	114.1614	20.29057	334.719	168.3104	441.7626	51.24511	213.4477	92.33626
CUST_39803_PI429010886	M44949	0.000206088	24	3	170069696	conserved hypothetical protein			1.77E-15	22526.09	252.989	24984.55	28561.19	24199.32	29122.17	1198.741	775.0084	1580.709	20871.55	20871.55	26056	19791.01	13301.65	23273.41	88.0075	69.14386	240.6532	1090.482	1890.428	1528.323	19522.5	4598.208	18117.06	2547.238	3751.984	6826.356
CUST_1197_PI429010886	N8808	0.000209909	24	18						558.8184	2528.059	2900.703	282.4469	410.2255	482.5812	3419.816	2381.656	1544.613	199.4867	282.5034	34.9506	4913.686	1477.508	3164.281	1695.11	4527.493	5856.992	7937.877	13658.74	8378.475	798.2619	1162.935	837.2458	1192.533	892.0968	771.0455
CUST_41897_PI429010886	M58829	0.000210836	24	15						32.60314	37.61646	27.88813	56.51566	33.79728	42.39624	59.65828	51.33067	41.51987	38.72594	37.58094	32.39325	54.65618	50.75534	61.04906	79.46471	54.48769	63.99137	53.53275	32.95195	47.41757	33.71063	28.57519	50.21608	30.13995	31.66341	36.92118
CUST_37768_PI429010886	M21474	0.000211144	24	6						225.9834	649.4957	248.3542	2192.968	739.9176	1769.667	1340.842	2924.097	4270.698	1086.78	2075.256	783.1683	486.4482	1856.844	2213.256	620.0365	1290.575	796.9187	103.5395	401.2544	78.91149	389.053	257.4878	309.5645	123.2141	16.91699	139.7737
CUST_44210_PI429010886	M65268	0.000211279	15	12						76.6349	74.25833	63.49903	52.40229	64.96779	67.44859	86.6454	48.09966	53.57855	102.4622	132.8564	108.5015	103.0566	92.47181	121.9377	79.76299	107.5502	71.3958	60.65875	41.13599	69.39785	87.56469	75.2089	90.27688	90.8123	63.83347	54.41132
CUST_9826_PI429010886	N25259	0.000212284	24	18						19.18647	46.90198	38.61632	10.37676	13.58386	13.68542	28.97384	31.23137	42.38836	7.505551	15.91453	5.690758	20.11384	10.59474	22.8581	41.33969	23.44192	26.57459	106.2889	58.21158	102.0268	44.10093	23.42467	21.44037	56.60765	12.28362	32.05226
CUST_3317_PI429010886	N14255	0.000212995	24	9						49.87102	38.15601	34.15266	49.57184	44.74502	51.08158	33.45999	53.50459	49.22048	81.23215	51.49011	55.04966	50.73158	53.67167	47.88053	40.7495	49.49937	44.48574	38.30361	53.84528	44.66421	30.6146	42.65681	38.27451	39.44209	49.37308	39.70866
CUST_1504_PI429010886	N9849	0.000213233	12	9						17.91133	12.19014	29.10637	24.76741	16.91902	24.17927	16.8613	73.61793	80.50976	104.7064	94.87741	104.3593	NA	77.78826	NA	15.90721	17.07298	8.042805	51.82191	5.480213	36.73639	69.92903	612.3531	194.5138	22.13345	43.42121	9.133693
CUST_46598_PI429010886	M73113	0.000214043	21	0						18.55929	20.09331	18.89628	9.857053	10.19704	14.38836	15.99496	16.13855	7.519886	8.34915	8.086218	10.482	8.737286	11.94299	13.80235	9.683226	24.7491	16.69306	14.19705	15.82642	13.17346	16.57137	21.73277	14.87819	18.18639	14.45239	15.18237
CUST_7530_PI429010886	N21683	0.000216598	24	18	241171158	oxidase/peroxidase, putative [Ix	Response to stress	Response to oxidative stress	3.43E-18	94.86558	122.5392	116.0897	98.56741	84.07229	115.9671	67.05704	167.2311	103.5796	49.1314	101.7744	56.55991	71.44611	101.4138	63.81233	204.2081	121.8217	175.0487	166.242	124.8558	185.9927	188.9335	135.0204	147.7951	136.2737	110.298	132.952
CUST_2232_PI429010886	N11929	0.000216859	21	0			Protein metabolism	Protein folding		2645.058	3321.698	3130.289	2166.525	1718.114	1701.993	1761.545	2140.692	2400.564	2132.096	1462.033	1808.449	3046.537	1504.447	1609.728	1862.343	2179.188	2277.337	2454.398	2292.947	1929.095	3247.587	3031.683	3135.16	3033.527	1851.473	3532.198
CUST_37647_PI429010886	M20226	0.000219138	21	0			Nucleic acid metabolism	Regulation of gene expression		767.3697	1043.236	669.1662	560.1392	772.5597	434.7194	590.8215	335.4857	405.5854	588.9309	446.0553	621.441	657.9421	369.4962	756.356	774.5071	655.6894	860.9694	885.5204	925.9054	495.3901	1108.83	807.6213	868.9348	679.224	365.1669	258.3703
CUST_18012_PI429010886	N38995	0.000219676	21	15						25.36487	28.8005	9.69295	14.69726	11.37814	13.75793	17.88521	19.49414	17.72848	23.87227	23.5695	17.0552	23.80751	16.82245	11.15315	37.36858	30.3574	34.88898	21.82795	18.80957	17.12368	29.53009	19.53602	24.90332	8.577815	12.59046	14.18163
CUST_30978_PI429010886	M364	0.000220003	24	0						262.1946	312.8082	303.8109	243.1902	174.9627	180.1324	86.84479	153.502	147.0639	227.1085	165.742	137.2489	244.1747	107.0064	228.9149	215.2207	301.8062	119.807	352.5932	211.4988	322.8914	331.3594	293.835	295.7504	531.0942	325.1203	468.1951
CUST_32951_PI429010886	M5549	0.000221972	21	0						281.2628	339.5673	307.3354	257.8376	171.5325	182.2404	239.2427	156.8946	168.8433	346.2958	187.1331	213.0837	169.6456	127.4883	132.5859	143.647	293.0253	186.4716	239.5846	168.8433	293.1739	339.6934	399.8777	296.2664	327.216	144.6793	272.7395
CUST_491_PI429010886	N5633	0.000222254	15	0	321476026	hypothetical protein DAPPUDRA			3.43E-43	310.9804	93.78493	80.07305	119.4286	168.6714	96.12752	186.3541	62.71091	75.31313	68.59824	22.71736	43.69757	258.3703	39.86943	244.0506	266.3181	367.6429	637.2271	64.18044	51.15383	34.52717	103.3596	36.4809	76.83706	34.49352	37.45982	33.83846
CUST_37013_PI429010886	M17539	0.000222359	12	0			Protein metabolism	Proteolysis		47.96277	36.43616	53.14113	68.21622	34.3049	55.61954	30.12869	35.44329	18.06134	55.2321	50.88852	35.98384	53.57855	63.86465	59.23509	22.1135	28.21177	24.22177	17.60727	23.35729	21.68924	57.40907	60.84718	32.21584	68.38347	60.76642	61.27138
CUST_38650_PI429010886	M28840	0.000222614	24	12						131.0644	146.449	152.0064	125.0424	478.7694	270.4208	187.4758	150.5356	139.9139	155.3899	313.3524	238.0667	166.2848	302.9304	319.5317	156.7919	161.2407	155.8444	162.0267	131.4508	123.3207	113.5376	107.935	112.5544	148.7099	166.7877	107.7409
CUST_16307_PI429010886	N36016	0.000222614	24	18						77.67929	78.11286	84.65768	64.9627	61.09188	68.91312	66.87814	84.70621	73.46998	69.52362	47.77802	63.62492	90.26753	85.40071	69.98305	71.63426	102.3686	83.4637	86.39437	104.9838	86.16832	102.8535	75.17439	96.62082	79.23954	81.49613	87.63468
CUST_44817_PI429010886	M67085	0.0002227	24	15						14.0177	17.63804	14.69726	8.437268	8.132185	11.89711	19.50236	17.59285	13.63697	20.84897	15.54537	15.79305	23.03897	10.62708	18.99164	28.14643	23.03273	29.43019	31.89668	NA	19.80764	14.34967	12.49835	16.02629	15.23987	22.56021	19.92947
CUST_31074_PI429010886	M619	0.000225308	21	15	383860799	PREDICTED: cyclin-dependent ki	Protein metabolism	Cellular protein modification process	3.25E-124	54.33188	82.87384	76.96435	57.05091	43.98563	30.56946	63.77866	89.48954	84.67585	75.61315	51.31552	57.53339	75.44765	50.15161	66.63719	67.27409	114.9073	89.95321	83.02117	71.22472	85.06849	64.94907	83.52767	63.28348	42.55235	51.97249	50.98858
CUST_57109_PI429010886	E1074	0.000226687	24	3	152949487	calcification associated soluble r			8.64E-11	3408.763	3284.996	2711.745	4787.419	6132.817	4672.963	3636.712	2958.733	2098.966	3568.199	5251.996	5119.273	3098.696	4829.887	5533.093	1047.687	1680.655	1796.427	458.7704	1414.36	660.1893	2310.632	2713.39	2940.045	1066.406	2608.106	3796.503
CUST_12012_PI429010886	N28773	0.000227587	15	3	163868840	autotransporter [Bartonella trib			0.000000612	79.60935	20.52147	63.62492	517.3443	856.9537	471.7045	366.556	363.3984	321.0799	NA	15.67594	5.367764	5.790172	907.1782	9.781107	310.4859	124.1127	97.77174	383.478	738.2631	313.2852	39.09638	53.86859	12.48313	NA	NA	NA
CUST_40002_PI429010886	M48221	0.000228426	12	9						14703.33	3717.963	12807.83	23016.75	6734.459	12211.42	15071.82	12097.54	22469.11	23435.22	29949.84	17982.7	26491.1	9376.495	13559.72	783.6564	1684.207	2133.67	25247.34	21010.2	25247.34	25888.13	38996.88	31659.39	17131.85	27073.29	36724.45
CUST_21563_PI429010886	N45439	0.000228714	21	0						9.316038	14.21176	24.10826	8.750703	27.03618	21.24736	NA	4.916376	7.140165	7.643306	12.89468	3.481467	5.066072	7.87171	3.840437	5.129966	7.610237	11.81411	7.626272	10.11839	15.12403	NA	25.97013	24.08979	13.74764	12.42147	9.03053
CUST_55849_PI429010886	M111881	0.000228975	18	12						22.31213	15.01335	12.55746	18.87745	12.22382	12.34025	10.04216	8.537397	7.394511	24.92804	15.28035	16.74981	26.6468	29.68907	24.1706	15.37441	42.03616	11.61031	32.86579	15.66801	29.04471	14.77271	11.84234	17.11031	14.66893	6.193801	13.0149
CUST_57144_PI429010886	E1501	0.000229169	24	9						20.65415	25.97276	15.13397	17.32703	36.80764	26.00555	27.66435	26.96496	19.65297	28.21781	37.66871	79.30248	26.09525	55.60414	27.02166	23.90768	29.32605	28.74405	17.92495	24.54621	23.51819	19.43519	32.93904	23.65361	11.78676	21.65776	20.51331
CUST_40907_PI429010886	M56361	0.000231841	21	18	443696893	hypothetical protein CAPTEDRAI			1.1E-09	38.66936	97.83763	144.7602	25.26967	56.3623	31.21069	51.35849	40.64205	65.27494	59.14641	38.85987	32.8057	69.17898	16.87383	50.15592	55.28196	52.33176	60.78548	170.1187	108.3275	216.6807	65.27494	93.47893	45.53592	37.81203	21.67269	23.97222
CUST_18226_PI429010886	N39387	0.000231841	21	18						248.6058	85.40783	246.7787	53.95129	19.73758	7.878601	89.1801	84.92778	50.04164	90.20095	56.37975	24.29464	174.0791	43.18567	113.8465	139.8503	58.73466	160.8884	54.11401	641.1736	925.9054	58.87877	18.0302	59.5633	49.33322	14.81846	32.19938
CUST_48842_PI429010886	M82441	0.00023189	15	0						19.89427	20.20385	19.85896	19.47927	10.04856	11.07717	8.663589	9.801925	10.3668	16.81757	14.00343	16.24433	17.63683	14.38127	13.24351	19.30163	14.64685	18.28716	18.97114	13.0479	14.72662	13.0253	20.12578	6.92838	9.840114	11.45884	13.91758

Probe ID	ID	p-value	Period	Phase	EBI ID	Annotation	GO category	Go term	E-value	0	0	0	3	3	3	6	6	6	9	9	9	12	12	12	15	15	15	18	18	18	21	21	21	24	24	24
CUST_38500_PI429010886	M27409	0.000232341	24	12	156349174	hypothetical protein NEMVEDRA			2.37E-08	303.1963	558.8184	431.4309	1620.506	630.0078	1194.342	1493.774	2297.179	3135.16	233.7126	3679.2	646.3558	1862.343	2434.185	846.219	990.2906	1331.208	1356.93	444.7023	785.5802	940.7158	254.1863	824.7782	905.9928	114.5807	388.9715	447.4189
CUST_2903_PI429010886	N13357	0.000232977	24	21	321455420	hypothetical protein DAPPUDRA	Metabolic process	Carbohydrate metabolic process	4.73E-96	1715.071	1552.466	1867.24	1109.766	1176.913	885.1856	489.8482	839.3829	764.2764	1342.206	991.1291	757.3112	1898.75	403.3152	1423.667	1365.984	1574.714	335.0962	3415.242	1400.583	1938.127	2335.81	2920.075	1715.071	4140.108	2230.426	4004.603
CUST_12255_PI429010886	N29173	0.000232977	24	21						214.2511	294.7488	282.2716	270.4208	284.8134	220.0563	227.0272	228.0728	252.0561	227.5418	234.1695	262.4813	206.4029	263.1307	236.43	253.9896	285.6824	300.8806	268.4876	280.3649	284.9168	287.3232	296.2197	315.2731	273.7185	280.2395	360.9619
CUST_29102_PI429010886	N59206	0.000232977	24	21						258.5192	268.6423	284.9168	262.7505	216.7919	200.3693	161.5503	217.8031	198.0827	237.5901	180.676	209.295	469.784	245.6701	365.1669	297.1434	354.4742	242.3852	402.3519	359.6695	287.1316	465.5812	319.7298	388.031	464.7851	197.3334	404.0425
CUST_52536_PI429010886	M97849	0.000233353	15	6						23.56774	37.90412	41.43387	40.32229	29.60122	21.31707	72.5147	43.27193	46.87435	35.32216	19.38793	29.40141	29.72829	15.87884	21.52625	28.1833	36.53396	37.07514	47.82733	35.60356	47.83813	41.55628	49.5263	48.66684	31.52277	29.4575	63.88807
CUST_2259_PI429010886	N11977	0.000233353	15	6	291241875	PREDICTED: protein phosphatas	Protein metabolism	Cellular protein modification process	4.04E-127	11.75186	20.43156	15.80426	16.59163	23.17996	22.61608	24.40409	17.46553	22.75207	16.70652	16.83626	16.74788	21.14686	13.84533	11.12989	21.19789	13.03491	18.2576	21.2307	20.55179	21.3828	21.75638	25.73037	22.32831	21.06899	17.56163	21.70341
CUST_54359_PI429010886	M105717	0.000233785	24	3						62.77844	13.34035	67.96481	206.1814	81.91952	128.645	26.30298	236.2202	11.19922	10.3668	3.046465	NA	12.37254	57.99332	8.929849	4.430571	10.24111	3.267022	NA	13.3556	6.592192	24.06305	NA	8.846413	NA	NA	NA
CUST_40627_PI429010886	M55683	0.000233961	21	15						13.77125	22.63957	11.4759	13.7086	12.78545	NA	9.868311	10.78499	7.529095	5.05262	10.96209	10.2931	13.70444	NA	7.348066	NA	23.98408	17.96765	15.09107	16.00437	12.34692	17.72848	14.06607	17.9492	13.05352	11.3477	15.64595
CUST_43450_PI429010886	M63093	0.000235287	12	9						162.2343	227.7726	235.1283	226.2788	210.991	186.4449	197.088	249.2986	237.5415	264.7345	239.2973	233.0433	230.3457	210.3744	219.2338	162.4812	205.1165	218.0311	231.134	224.1479	247.9707	284.0207	260.1908	280.7417	264.8694	257.0037	305.7009
CUST_18019_PI429010886	N39007	0.000235349	24	3						52.68847	NA	30.63675	43.95645	75.87682	67.39104	19.03842	NA	NA	19.64801	37.4892	53.43212	23.48308	33.66656	47.47767	15.60667	5.881307	10.8747	8.518628	10.00814	14.0598	21.50122	6.233337	16.22323	16.08645	8.83019	18.10455
CUST_38389_PI429010886	M26166	0.00023548	24	12			Symbiosis, encompassin	Other		3770.678	14015.31	7439.177	23231.4	8984.262	18198.77	25792.96	38719.75	52165.44	4650.166	62699.58	8484.924	29647.7	41865.42	19150.48	13419.52	17210.94	17886.89	5726.275	10569.33	11881.91	4490.88	11228.98	11094.64	1478.028	5564.697	7048.129
CUST_39996_PI429010886	M48168	0.00023548	24	12			Symbiosis, encompassin	Other		4247.1	13391.14	7653.847	25065.69	8962.903	16913.15	28414.17	36195.31	46875.63	4988.039	66134.01	8886.515	31967.8	42900.93	20320.15	15045.33	19485.9	17345.96	6506.137	9937.479	11738.35	4680.36	10430.71	11738.35	1507.382	6018.305	7240.469
CUST_32022_PI429010886	M3021	0.000237345	24	6						9.734639	14.2083	22.81991	30.81966	23.33593	15.65667	20.58296	53.081	44.80668	12.67448	68.36093	5.661376	15.18237	38.27962	24.96813	22.80251	23.98146	15.73083	3.880845	3.749863	7.451763	5.481109	12.00512	8.467723	7.457449	NA	6.226721
CUST_6932_PI429010886	N20798	0.00023752	18	15						6.685275	16.72797	NA	9.025697	3.373033	14.49774	24.78354	14.55272	11.3488	25.43981	18.77676	7.214528	18.11297	37.85657	23.51724	36.58776	58.49428	28.31962	12.10629	4.061438	15.9251	10.26259	15.65515	4.281841	23.10726	18.06394	NA
CUST_7796_PI429010886	N22088	0.000238125	18	15						31.79073	39.64997	25.51207	38.57077	NA	19.77651	10.43499	11.45318	13.65032	152.0267	NA	69.19342	131.7371	145.5507	72.20641	23.89085	295.6289	182.5585	216.6807	144.1758	141.5029	23.22612	220.4736	22.248	17.61898	25.61401	5.201318
CUST_35200_PI429010886	M11883	0.00023936	24	18	524869040	PREDICTED: acyl-CoA synthetase	Metabolic process	Other	4.68E-48	157.3439	253.4735	207.8484	140.9588	145.843	146.2607	162.6193	179.241	181.8118	150.0295	155.6009	177.6455	179.3306	167.3834	191.0479	186.3924	225.3383	262.2441	229.1648	209.4822	238.4834	186.4449	226.9376	236.1021	209.6366	142.5791	285.8938
CUST_46119_PI429010886	M71441	0.00023936	24	18						44.00986	47.24216	55.78477	3.39905	12.18449	9.804365	11.44051	18.89336	13.99198	53.04738	20.83583	19.06321	37.82047	7.308662	31.51184	22.29844	44.635	69.64615	63.98495	108.9908	61.11985	89.44264	25.01801	70.5683	209.2563	53.04169	188.0574
CUST_17764_PI429010886	N38551	0.00023936	24	18	325303532	TPA_inf: hypothetical secreted p			1.22E-12	13.59581	15.74607	25.61949	13.48307	15.47714	13.13409	21.08201	16.31963	17.47661	12.03178	18.92593	19.37891	18.29235	18.32237	20.90369	30.15056	20.26341	21.46399	21.94131	26.85225	21.7758	14.72888	14.05324	20.3727	18.07496	15.35763	19.30987
CUST_51751_PI429010886	M94865	0.000240296	15	9						20.45768	12.65578	26.18151	16.0336	13.47249	15.70113	15.36685	16.13355	19.90073	29.29782	26.77083	29.7311	28.84319	15.60788	32.8057	16.94155	9.040984	13.7873	19.43327	11.7976	13.31819	22.21977	21.27506	20.1119	22.51895	21.08968	27.22159
CUST_52473_PI429010886	M97567	0.000242187	12	9						858.7677	275.2462	928.5423	764.2764	289.8261	313.739	429.1442	496.3451	772.7965	839.7101	1384.734	611.1595	1914.352	475.9252	859.0835	119.1916	196.8426	204.9422	884.7471	490.2242	825.4048	1261.275	1744.803	1368.726	889.3882	569.6083	1438.372
CUST_32542_PI429010886	M4380	0.000243734	18	12						10.83965	7.531208	9.79002	75.48453	58.81576	33.83846	33.35975	306.2147	379.6086	19.23032	1528.323	48.10521	1526.163	1218.143	186.3368	38.3812	75.9364	97.84796	9.375499	23.59034	28.66457	NA	224.5032	212.8318	NA	102.326	120.6137
CUST_13145_PI429010886	N30655	0.000243874	12	3						23.59807	25.10509	16.38009	NA	44.6225	28.55675	22.36702	41.20512	15.97495	18.64717	15.89978	10.01392	26.01047	19.4293	18.21898	44.89831	37.8045	29.2298	28.82648	70.97468	30.30161	15.87086	9.930505	21.1126	30.52605	50.60267	31.54655
CUST_48992_PI429010886	M83441	0.000244474	24	3						299.7411	163.9403	199.4568	264.3686	252.6173	385.9551	131.6028	335.7533	123.018	103.4024	95.57738	74.92905	77.73937	101.5579	92.67361	71.46735	75.6543	55.70355	55.74024	225.0911	48.69222	138.3092	101.4958	116.2734	43.57254	206.98	30.84327
CUST_18359_PI429010886	N39646	0.0002449	24	0	118403706	NRDE-2, necessary for RNA inter			0.000000988	37.51496	43.90368	46.39504	32.3135	36.63348	34.17398	22.58052	21.33758	30.26428	35.54384	27.85573	38.39235	21.03678	26.64465	23.5934	29.96518	36.58388	35.79767	35.45282	26.23786	36.34385	25.13355	49.35016	47.71964	37.09003	48.30656	40.29806
CUST_1155_PI429010886	N8676	0.0002449	24	12						111.4394	242.474	178.3834	818.7919	354.9347	651.369	441.5527	995.9721	1235.811	114.9362	2133.67	260.798	568.0849	1113.622	500.3221	288.4809	514.9905	460.3468	176.8599	413.2217	457.8873	117.7279	398.088	405.7918	31.86686	142.5641	177.9078
CUST_9855_PI429010886	N25306	0.000245742	24	21						55.49194	60.18712	64.97632	34.02915	37.77333	42.96134	44.95536	54.58501	50.47099	48.2662	52.35235	59.37073	56.39523	45.83845	63.41449	70.94231	56.99251	54.85916	60.61437	57.15552	76.96435	80.31138	63.2206	80.50976	67.3848	63.26218	100.783
CUST_20017_PI429010886	N42655	0.000245742	24	21						5947.47	9804.488	6929.595	5365.583	4940.836	4809.493	2767.762	5467.822	5233.042	7402.627	4572.831	4650.166	9643.272	4450.275	6906.237	9041.082	11030.98	6567.976	9684.529	9565.255	8107.941	12084.47	8400.194	10053.8	14703.33	6999.724	11612.18
CUST_28347_PI429010886	N57772	0.000245742	24	18	380005623	fatty acid binding protein 10 [M			1.91E-10	719.258	673.5376	606.3479	472.1134	394.0157	416.4698	519.4495	522.0737	623.4767	569.1918	606.3479	670.1687	555.2007	524.8326	598.7661	649.314	533.7924	701.5555	633.691	839.3829	996.7263	789.6051	547.8118	947.0352	748.709	503.6798	901.5762
CUST_3735_PI429010886	N15159	0.000247691	12	3						19.92759	28.16647	27.87333	34.76036	45.69732	36.68361	34.49352	29.14227	27.17222	15.75178	25.34024	6.313999	40.02352	24.37455	34.2198	29.45413	24.39353	27.88568	24.83403	28.50902	26.02721	17.01954	14.12309	15.84358	26.64196	21.00978	40.61754
CUST_13294_PI429010886	N30924	0.000248502	21	3						423.0504	335.0962	251.6934	310.5741	480.3553	366.3486	278.1319	262.0968	361.3178	253.3528	242.7903	148.4289	288.5968	263.7057	296.5728	204.7184	315.3455	279.6802	356.4497	282.4469	369.0567	291.3249	338.1914	215.3959	382.2543	434.2782	421.7429
CUST_35543_PI429010886	M12833	0.000248934	24	21	242019466	serine/threonine-protein kinase	Protein metabolism	Cellular protein modification process	2.78E-11	86.41587	36.34596	94.71637	76.55656	69.55765	71.74885	58.05667	40.57158	57.1768	63.31668	52.55424	62.35301	75.81657	58.72904	72.66211	89.81747	56.11745	62.68994	106.4901	65.62334	88.69555	125.4485	96.99292	94.14191	118.7327	49.11012	132.9992
CUST_52004_PI429010886	M96029	0.000248934	24	3						135.8928	85.00598	90.62398	114.7628	154.6439	161.9124	89.60857	89.7803	67.53847	88.90086	112.8276	157.3672	87.21789	80.66827	147.9838	82.40323	54.10933	59.38892	59.13513	71.52252	44.95996	105.7762	69.41702	108.5389	66.89231	90.26753	91.33505
CUST_31854_PI429010886	M2580	0.00024912	15	6	380016613	PREDICTED: protein FRG1-like [A			1.91E-28	32.52808	59.04856	70.13338	56.4602	56.98746	54.04744	68.92058	69.2197	82.55332	48.03485	38.37685	51.86305	40.0019	35.99333	46.05498	52.49766	76.37498	70.76996	63.00026	66.55746	66.11978	70.25686	89.0262	93.46078	50.33808	44.47158	62.94384
CUST_23049_PI429010886	N48168	0.00024975	24	0						23.73807	19.97953	16.80584	29.38566	14.65162	16.5534	23.2872	19.08766	15.23818	19.12392	NA	13.69119	14.18475	8.78406	8.272162	14.16065	10.11839	16.17146	15.95307	15.36351	9.684334	18.16224	17.21886	17.75016	22.87315	18.43914	17.6031
CUST_26254_PI429010886	N53928	0.000252125	24	21						34.34753	17.18912	41.62674	27.11011	27.63333	31.18737	10.63037	19.31939	18.40737	34.88249	17.52792	5.723799	35.84121	38.23951	44.42841	44.15404	26.10539	28.3301	92.45168	39.6633	37.51953	67.63991	35.0975	55.1065	54.36391	38.25885	46.77426
CUST_19637_PI429010886	N41916	0.00025306	24	21						24.41183	21.32203	21.10164	30.84622	19.60994	19.44173	13.57805	16.47278	16.1959	11.67372	11.39498	15.67594	17.25784	13.3975	21.11041	31.66341	23.34465	25.77932	15.68645	28.84319	16.38009	27.43793	24.13409	24.84254	29.03664	19.45847	38.15236
CUST_36230_PI429010886	M15101	0.000254385	24	15						35.15002	28.8322	26.38647	25.46958	36.1335	33.63302	40.65621	26.11949	35.13853	47.10201	37.40246	52.27137	39.20049	47.2578	35.97416	52.61688	41.58882	58.35105	55.06272	36.97883	53.09948	53.07683	44.92669	43.86995	42.5735	29.74732	41.65139
CUST_46341_PI429010886	M72300	0.000254385	24	21						73.06306	88.31999	76.47464	71.47305	78.02817	67.81869	58.67081	62.52685	56.72705	66.05158	57.81074	58.31116	62.03291	74.48675	86.20512	78.64253	73.56286	74.66025	75.31313	60.47985	63.68621	82.14842	76.76723	90.76223	86.56139	78.08717	86.11117
CUST_7537_PI429010886	N21696	0.000255317	24	18	291237650	PREDICTED: predicted protein-li			2.8E-44	85.25564	161.8917	135.9051	77.34627	65.14299	71.67272	109.9913	121.5461	118.3083	97.61964	96.81529	92.9385	129.5648	105.5425	98.71772	112.8468	158.8716	143.92	144.3231	148.0265	136.9113	88.70868	135.056	106.8499	100.4391	133.1266	136.1879
CUST_27242_PI429010886	N55701	0.000256019	18	9						NA	5.300759	NA	9.369673	7.038408	4.562771	11.4263	NA	7.745918	10.35736	10.00684	13.72622	5.897801	7.930223	7.153945	6.606839	21.1277	6.220036	5.833172	4.175973	3.630829	NA	8.582162	NA	8.013913	NA	NA
CUST_2019_PI429010886	N11341	0.000256202	21	15						546.2084	848.3549	382.6455	1322.312	5.879006	962.741	857.4356	748.1962	465.972	1890.428	198.2813	734.4362	1761.545	1050.367	1142	3629.795	4326.077	4740.787	2679.375	1379.279	2393.197	369.0567	1810.6	377.789	490.7221	740.2235	129.6335
CUST_3365_PI429010886	N14367	0.000256675	24	9						206.1397	251.7959	192.6063	187.8413	575.8046	401.876	196.0115	257.0734	279.0664	190.7291	435.2817	429.8212	234.2298	333.3795	372.6214	262.806	283.7827	184.1477	141.4085	191.8512	140.8152	120.1066	184.4145	127.2771	164.741	194.8463	148.1939
CUST_23648_PI429010886	N49200	0.000257321	24	21						468.4112	448.8304	496.4508	603.3505	446.5017	385.4238	275.1168	377.0504	276.0746	364.6437	301.3368	219.0705	384.2558	172.9954	351.8456	221.7013	383.7931	94.7749	1053.829	379.0965	844.8862	667.2339	799.0901	525.7914	1000.752	593.171	902.4975
CUST_30705_PI429010886	N62437	0.000258095	15	0						59.21803	42.98811	43.02903	25.82506	28.10987	25.28008	27.46378	10.93377	25.1445	26.45917	19.71328	28.49433	37.90056	16.07386	40.4113	48.85342	29.64248	33.89397	26.48825	25.91577	23.85156	30.98678	23.22612	18.03823	31.8265	32.96543	35.90614
CUST_39556_PI429010886	M41199	0.000259497	24	3			Metabolic process	Carbohydrate metabolic process		271.6574	165.5194	80.86273	139.3207	934.7113	577.8423	33.33002	59.36408	25.49446	33.07256	167.261	196.4999	54.26213	147.2789	119.5301	19.13355	8.611312	7.657844	20.02992	75.2089	27.73284	45.96577	62.51652	33.32109	NA	382.8	11.74077
CUST_39253_PI429010886	M36728	0.000260291	24	15						2093.838	3297.935	2459.181	2063.235	1471.771	1717.387	2219.678	2348.232	2659.616	2320.751	1716.333	1722.827	2573.227	2839.956	2184.345	3197.119	3405.379	3555.208	2012.206	2886.102	2459.181	2661.468	2369.531	2855.927	2283.014	1967.079	2396.659
CUST_48765_PI429010886	M82087	0.0002617	24	18						2886.102	4168.103	3210.455	3101.358	1522.629	1035.146	5402.577	2654.193	4173.107	2681.804	1766.713	1699.905	5744.161	3356.595	2626.057	5048.826	3506.645	5705.148	7983.519	6770.352	7928.164	3699.413	4870.528	4041.944	6285.074	5420.715	4928.217
CUST_50654_PI429010886	M90559	0.000262436	12	9						45.17249	4.469209	52.40731	72.30895	23.47009	49.75986	NA	14.6763	19.09461	110.3707	716.6417	106.5406	255.7014	42.16901	133.9994	4.281841	5.233224	4.086937	30.34849	41.37426	50.03259	326.1422	333.1336	309.7415	50.51216	234.8735	256.0953
CUST_43398_PI429010886	M62936	0.000262451	21	12						15.58431	13.42904	10.64424	16.64948	15.67395	23.74852	33.29327	17.09586	39.54727	12.36534	40.49223	NA	49.91114	47.23231	26.85513	12.91477	14.58842	15.8055	7.078115	8.013913	12.62446	7.496542	8.604047	17.43476	NA	4.209816	NA
CUST_33961_PI429010886	M8367	0.000262708	18	3						50.69876	62.60527	60.73958	71.14732	73.02048	57.29374	76.73936	40.56201	32.32436	50.23652	33.82765	15.84904	49.25099	46.86994	17.07151	8.763023	63.47982	26.77351	133.3487	47.45891	71.99448	187.6112	112.9762	77.2068	53.69851	60.72297	21.34628
CUST_49321_PI429010886	M85184	0.000263906	24	15	546675121	hypothetical protein D910_0377			1.26E-09	132.6978	92.98043	175.1243	126.0731	71.48244	108.568	146.8225	133.239	169.4561	187.0768	97.05434	171.912	194.7103	202.7406	148.2715	243.2347	175.8219	263.4946	213.2675	111.4113	220.2176	155.3531	120.3757	157.4759	176.549	118.4588	175.3842
CUST_7143_PI429010886	N21102	0.000263906	24	21						54.31269	93.19639	87.40333	79.73452	84.39985	77.08119	70.81536	70.46471	73.21671	62.94384	65.63171	79.46471	69.03939	70.98403	78.48038	76.7295	110.9878	92.06463	90.35029	67.61972	87.40333	89.29338	104.661	93.53318	82.47763	87.38427	90.78766
CUST_20121_PI429010886	N42848	0.000266971	24	21						300.3594	453.0125	420.8457	295.1045	253.8613	239.8291	268.0293	217.6621	220.5454	295.4891	234.5996	348.8785	356.7674	254.459	344.3047	425.7716	372.4691	447.1876	299.2328	282.1054	367.8176	328.3409	432.5544	412.9087	317.7216	291.1427	370.9049
CUST_37011_PI429010886	M17535	0.000267521	24	21						531.7033	958.49	773.969	704.142	823.2974	555.5824	651.369	757.3112	790.1557	570.427	664.6847	572.7695	743.0295	623.4767	607.3706	610.9907	728.6567	685.653	794.9446	815.2223	929.1604	879.1441	873.0203	1036.158	833.5137	696.3331	927.0038
CUST_39409_PI429010886	M39006	0.000267521	24	15	32527554	Squilla empusa 28S ribosomal RI			5.91E-105	56377.06	65558.65	69178.17	38719.75	47826.44	64638.29	55705.47	67856.09	73328.57	64273.41	58747.16	79747.61	78492.44	75071.87	76090.53	93147.81	80425.69	71692.31	79085.89	61365.17	70445.35	70445.35	61768.02	69931.06	79747.61	60953.29	66134.01
CUST_50792_PI429010886	M91026	0.000267951	12	6						31.96656	346.0708	365.4954	428.2506	339.077	131.5172	439.8767	1355.75	1786.238	88.20477	232.4886	61.0126	125.3435	144.1943	73.45313	NA	392.7267	381.9172	987.5397	753.9335	2060.105	174.857	4140.108	4118.017	76.55656	26.97175	92.18916
CUST_17274_PI429010886	N37690	0.000268684	12	0						113.5235	98.89641	106.2034	118.8436	91.63916	83.83975	91.80105	80.05281	74.20038	94.98744	103.8143	95.04674	105.8148	86.68025	130.3114	101.4138	103.6284	99.80727	88.26602	89.80371	91.92491	85.40071	110.4792	93.37419	116.3995	116.2567	144.474
CUST_14078_PI429010886	N32291	0.000271136	24	15						370.1622	211.4099	442.9918	193.8172	71.21793	90.37428	385.3052	213.0104	328.6973	805.2136	165.6517	352.0796	574.4065	359.3488	231.0141	675.2885	596.9264	801.3203	405.1127	214.4299	427.0381	243.7665	357.3731	331.3594	392.8052	172.8416	379.6086
CUST_38453_PI429010886	M26822	0.000271421	18	15						448.4659	273.3536	213.3048	109.8814	66.52473	77.9272	390.7107	435.6254	412.8294	315.8899	222.3991	98.28823	380.6302	338.8265	211.9007	1372.283	579.9447	1340.037	291.0948	480.4617	736.9876	262.4014	39.95086	212.7978	423.2638	152.4151	190.7935
CUST_19442_PI429010886	N41590	0.000272108	21	18						209.7702	317.6549	277.2002	185.717	201.6917	192.1066	177.7913	248.8552	241.7074	200.6093	180.1092	154.7361	118.3504	185.7829	95.01092	342.9765	358.6508	329.8991	401.757	286.9821	325.6483	392.3777	250.7612	241.4097	331.4329	300.3068	251.7468
CUST_7367_PI429010886	N21439	0.000273376	21	15						248.4962	386.6278	82.67536	131.274	239.2059	215.1114	142.0207	247.4344	76.67528	189.4468	232.9522	263.8276	297.6356	367.2044	346.1134	715.6987	494.3291	390.5287	173.3624	335.4857	206.5859	351.7434	242.0929	284.8134	174.0123	244.4841	60.66586
CUST_20989_PI429010886	N44426	0.000273712	21	0						34.89501	26.69535	30.91451	22.92862	18.89628	17.00862	21.98453	16.36771	13.46097	16.51006	26.87774	22.15452	19.60329	10.99972	23.30722	17.02795	14.39849	13.99541	18.34365	14.69094	14.76753	33.2866	22.96488	27.10462	24.44473	24.8172	24.11115
CUST_49532_PI429010886	M85988	0.000274751	24	18						28.77495	29.6152	37.78456	11.52123	21.51658	30.98189	6.110374	20.03497	18.55713	10.61215	32.47195	33.59889	20.18416	18.44173	21.30099	15.23242	21.56373	21.54067	46.23699	43.28585	48.3568	27.6757	39.07837	34.27395	34.44933	35.92153	39.97068
CUST_39102_PI429010886	M34586	0.00027713	24	15						17.60139	11.10613	7.465917	11.71874	NA	6.12072	35.78309	26.13302	16.86908	54.37537	36.62239	NA	15.25101	20.66314	15.62048	102.411	39.24334	61.68316	16.26492	31.90671	33.2866	32.19938	NA	15.78726	30.02336	6.267202	11.20316
CUST_46162_PI429010886	M71612	0.000277338	21	15	13898841	CUB-serine protease [Panulirus a	Protein metabolism	Proteolysis	1.31E-17	235.1738	599.3024	478.9906	223.3777	304.0274	145.2758	287.2425	380.915	365.3294	289.1658	287.8854	141.2321	299.8648	465.6755	413.2862	607.6204	418.5156	569.1918	376.0943	442.6514	475.4976	400.0672	224.4256	294.6856	251.1902	249.928	253.4735
CUST_45251_PI429010886	M68484	0.000277949	12	0						176.6314	215.0483	280.7417	165.5949	184.7904	137.2351	114.3193	132.5291	177.9861	197.7072	362.8242	170.1715	281.3641	173.6995	270.7536	183.9367	180.2441	189.5124	158.7682	175.5738	141.6537	225.3038	210.6226	186.9296	336.1378	241.4097	317.0778
CUST_35458_PI429010886	M12622	0.000278978	12	9						47.93427	28.9184	42.58958	14.32273	24.78664	15.19477	NA	75.31313	NA	71.76549	158.9628	153.8597	7.413131	60.39038	28.39612	NA	NA	4.107116	15.32206	4.102172	11.02011	NA	133.5489	157.6143	22.23772	32.86579	17.53011
CUST_29882_PI429010886	N60745	0.000280584	18	3						73.08027	71.3958	56.63612	72.94835	74.99403	68.10466	70.01129	67.51408	59.14128	61.08411	62.50267	63.64767	53.36088	43.63653	57.47217	52.6814	72.36965	55.22014	60.78548	75.71093	54.76127	76.31853	74.12935	67.15403	53.17104	65.63872	53.48617
CUST_40388_PI429010886	M54962	0.0002813	21	15						369.1521	483.7942	295.9223	447.0887	168.1532	262.6219	590.6124	531.3775	381.6653	1064.673	468.8585	139.2427	606.9823	540.2756	366.0882	1908.561	732.0587	1551.452	906.8146	823.5364	1171.1	1326.72	144.5963	794.9446	424.3124	124.0485	338.993
CUST_50861_PI429010886	M91260	0.000281667	12	3						30.40311	33.5653	18.26774	71.3181	19.49003	85.38787	63.14866	51.78206	48.46283	23.6825	21.64165	17.89355	61.39667	83.09656	65.309	185.3397	74.42622	94.47843	57.08761	20.23623	58.42497	7.99563	49.07635	18.13167	32.62695	63.59136	10.83697
CUST_29617_PI429010886	N60223	0.000286444	15	9						24.98354	27.09506	44.62591	50.37855	42.45213	43.65579	38.11477	49.72483	24.25128	56.93927	54.16578	72.42903	42.77417	42.69941	43.99536	43.55661	22.17612	39.36946	35.77181	36.04308	32.22863	57.56952	49.62	59.33452	52.68847	50.36694	63.84124
CUST_40772_PI429010886	M56050	0.000286601	21	0						15.88147	19.49649	23.64075	9.991081	14.39043	13.39103	7.234145	13.47557	10.82105	9.6637	14.83387	8.798487	6.218813	10.9779	12.67619	21.46399	12.67448	15.26868	17.36938	13.59114	11.50709	28.68485	12.93627	22.20311	19.55278	16.68416	16.31601
CUST_51234_PI429010886	M92742	0.000287227	18	3	121484199	cuticle protein CUT8 [Portunus p			1.73E-18	11395.49	377.2326	13360.37	18146.2	22221.29	19341.82	613.1951	1940.134	2057.214	8631.841	18198.77	12236.09	8430.296	13222.77	11881.91	169.022	327.3332	146.7486	3114.68	2867.707	3098.696	13419.52	17210.94	14161.49	3349.165	9999.789	9301.136
CUST_43553_PI429010886	M63341	0.000287607	21	12	241105590	conserved hypothetical protein			6.12E-40	80.46117	96.07003	116.9474	50.0937	53.47447	55.42689	112.4069	62.65711	120.4478	120.8427	56.26015	134.0408	177.6455	103.9393	124.8368	155.0383	58.78081	143.902	148.0023	114.5807	41.84178	113.9699	73.99941	106.6811	56.03078	63.96849	54.19934
CUST_45520_PI429010886	M69365	0.000289212	24	3	220172369	cuticle protein [Rimicaris exocul			1.62E-09	12191.65	185.653	11112.95	16491.53	12921.27	16760.98	304.096	800.3618	430.5713	8321.498	16622.66	11475.48	6584.064	7872.926	11138.2	101.0257	100.6344	120.9342	531.7033	1026.487	708.4537	9759.347	6068.533	10017.83	890.9879	2744.675	2670.787
CUST_19237_PI429010886	N41228	0.000289212	24	18						144.5615	157.847	138.4795	144.495	72.59454	126.1267	127.9686	68.95833	124.0046	178.447	133.5662	114.6993	76.01297	140.1608	151.8566	229.9275	131.0463	186.5368	157.4566	213.4477	231.626	180.729	138.4144	183.7819	218.1822	159.3992	172.6851
CUST_39992_PI429010886	M48025	0.000292827	24	3	220172369	cuticle protein [Rimicaris exocul			1.29E-22	29219.06	482.1823	25703.26	36302.66	31659.39	25888.13	762.4429	1631.254	909.1828	23324.95	35157.95	25065.69	14363.02	18613.15	21590.74	149.0864	267.3212	243.2347	1164.205	1935.604	1069.345	24289.54	9629.338	17623.06	1834.729	4646.072	4616.026
CUST_7594_PI429010886	N21780	0.000292827	24	15						210.4014	432.1282	347.0422	272.0128	75.23071	198.0058	639.2868	512.7881	357.296	547.3976	226.9579	243.5905	555.5824	288.1795	381.849	727.0088	665.59	724.6231	530.4682	207.6552	578.6613	279.6157	379.158	362.8786	336.212	336.5251	387.1258
CUST_47693_PI429010886	M77197	0.000293158	18	12	321464079	hypothetical protein DAPPUDRA			1.29E-65	4.421565	NA	NA	NA	NA	6.770096	14.63859	17.19904	5.844628	27.6893	19.5652	7.414538	18.10243	21.81818	22.38961	14.73247	13.3975	15.8318	10.05625	NA	4.056541	7.328935	NA	10.78644	18.56115	13.61097	NA
CUST_33070_PI429010886	M5896	0.000293501	24	21	260816654	hypothetical protein BRAFLDRAF			2.43E-134	26.81298	47.88053	45.75395	30.61698	43.88916	32.09223	25.16449	40.53975	30.06311	30.05953	31.50642	25.76125	25.01036	26.87469	29.59163	40.2126	56.08051	46.59051	44.60802	53.55568	41.57926	41.65139	56.97506	49.4765	42.68213	38.35003	43.00281
CUST_2170_PI429010886	N11768	0.000293501	24	15						49.55941	85.09153	64.75498	61.5382	53.45604	54.68333	18.7792	63.54768	42.33888	80.6925	79.25889	74.00845	106.1184	90.98859	106.5634	105.2562	117.5696	130.1383	60.70253	84.40775	57.18737	122.1883	72.24298	106.2214	103.5272	73.60215	78.49976
CUST_47352_PI429010886	M75744	0.000296442	24	15	328899629	ribosomal protein L36 [Procamb	Protein metabolism	Ribosome biogenesis	1.7E-14	980.3183	1505.771	978.9259	996.7263	1133.812	895.1277	1676.984	1281.397	1171.1	1512.535	996.3906	1513.287	1141.401	1164.205	1228.382	1812.631	1842.758	2080.932	1543.558	1288.693	1562.544	1215.073	1449.959	1581.87	1356.93	1202.808	1509.047
CUST_38691_PI429010886	M29132	0.000297755	24	12	238630920	rhoptry protein [Plasmodium fal			3.8E-14	3124.619	3731.866	3043.672	6845.752	1749.36	4427.539	12743.08	5336.579	11455.94	3237.866	13503.69	3050.493	13078.51	16221.54	8417.477	2037.287	11475.48	9194.226	877.7497	3222.8	1517.515	1022.285	2717.569	2809.488	681.5295	1124.051	1661.512
CUST_20224_PI429010886	N43050	0.00029817	18	9	242009647	conserved hypothetical protein			8.28E-46	24.6974	34.5207	38.99311	26.28128	32.62382	24.13994	38.02572	29.666	39.69061	39.62294	83.4637	41.57553	44.22224	31.80823	67.5936	52.6597	42.18222	38.01289	39.88097	30.44146	38.89512	30.77017	33.6128	31.98798	29.33369	34.82911	33.97232
CUST_9489_PI429010886	N24724	0.000299112	24	21						28.51774	43.87393	43.32213	27.62298	40.31371	31.83006	22.22355	36.99679	32.46193	27.7582	33.2143	21.55742	35.38735	32.4427	26.44115	28.3901	35.58267	37.42766	27.65161	33.49057	36.71405	45.43624	48.31614	42.11956	32.58867	38.21258	37.86955
CUST_2077_PI429010886	N11512	0.000300057	24	3						345.7435	182.2609	272.2877	379.7764	468.9491	444.244	293.835	217.9435	297.0848	201.6917	263.893	298.6917	266.1014	333.1336	287.1316	117.5273	85.77379	61.0054	239.0862	220.2503	220.2503	312.8962	327.1238	283.4149	181.6944	244.537	261.3125
CUST_55216_PI429010886	M109148	0.000302008	24	6						42.74631	75.6543	62.50267	67.56678	126.2375	75.00197	135.209	65.67004	118.9614	49.69782	99.79674	46.63196	33.56909	77.01155	46.36381	17.2551	36.31991	32.38584	45.81835	67.54737	33.13557	55.53662	94.50451	28.18616	80.41731	58.14687	38.89189
CUST_37803_PI429010886	M21665	0.000302073	21	15						712.6985	3611.195	1587.909	802.4687	217.8383	563.9832	4685.458	4080.702	4058.337	1401.131	439.4254	633.4883	2194.43	1645.987	1401.718	3371.075	5467.822	5209.792	2053.392	2065.743	2127.944	1009.169	1994.973	999.3353	1285.646	1148.933	772.308
CUST_24820_PI429010886	N51300	0.000302226	21	0	383856504	PREDICTED: actin-related protei	Cellular component orga	Cytoskeleton organization	4.57E-52	17.69186	14.80209	13.43787	4.7072	NA	8.550634	11.10613	8.447449	9.252611	7.800199	9.048157	8.376444	NA	11.12634	9.11873	13.33647	5.490942	14.17824	9.710211	8.38931	9.328141	10.39886	14.33538	15.89766	13.40089	12.23889	13.16499
CUST_13016_PI429010886	N30443	0.000303526	15	9						479.1083	297.492	360.8837	187.8136	150.9231	112.7172	183.411	158.2397	129.1152	163.1045	531.8423	416.778	192.4289	395.6891	361.5772	248.9012	312.7556	416.3476	90.63409	155.6261	127.7162	151.1207	263.7057	354.8596	210.0927	610.6881	847.8182
CUST_19275_PI429010886	N41294	0.000304407	21	6						76.5633	82.91936	62.04596	79.40591	198.1201	127.8904	108.1859	105.946	88.95701	88.27501	111.371	96.79055	82.67536	94.54537	119.3462	96.60811	104.6784	79.45422	66.28735	86.76212	75.28314	54.5198	72.14124	60.36476	105.0177	114.8354	97.18737
CUST_12899_PI429010886	N30230	0.000304543	24	0						42.4997	112.3846	48.68665	NA	48.68665	40.90141	34.18834	59.93426	40.82537	35.01636	34.63148	38.36719	62.1238	53.04169	33.94092	23.17242	17.00074	5.698886	43.68742	4.585303	28.63092	43.21465	50.97745	3.369289	94.62002	99.37198	109.6887
CUST_26136_PI429010886	N53730	0.000307287	24	15						105.4159	119.8471	108.663	96.79055	84.88526	85.16727	150.2498	88.43455	136.8369	169.9145	110.2586	104.173	137.4424	86.956	97.60846	158.2931	160.9133	167.1289	158.3928	124.8106	148.2557	161.4921	117.4173	117.3106	153.7917	110.8779	147.5021
CUST_19862_PI429010886	N42337	0.000308244	18	15	321461251	hypothetical protein DAPPUDRA	Transport	Protein transport	4.23E-18	448.34	576.0861	404.2408	375.7494	279.563	364.2801	511.6359	448.0132	436.108	559.2886	348.2785	402.5585	496.3451	345.4356	386.8846	540.61	555.2007	607.8959	412.4307	436.3894	444.5929	317.0778	380.5457	344.6427	388.9715	402.1648	463.7632
CUST_14005_PI429010886	N32161	0.000308819	21	3						101.508	110.7598	112.073	129.6173	130.7693	136.0268	123.5107	109.0197	130.174	123.5457	128.8978	98.5911	148.4879	100.3265	127.9454	105.9898	122.6496	89.08902	88.4112	102.8535	114.8511	98.61391	124.1913	111.5617	137.6019	131.3388	145.1606
CUST_32827_PI429010886	M5202	0.000310516	24	15						29.55431	20.73675	43.21882	28.65291	23.93602	29.56665	27.03345	30.47418	18.01013	31.72419	27.56589	22.60966	13.53119	47.75189	55.02143	48.07833	68.64219	65.79182	39.99568	35.16853	43.98997	32.03079	30.70425	37.53998	54.71169	41.22261	31.96656
CUST_5957_PI429010886	N19425	0.000310743	15	9			Nucleic acid metabolism	Regulation of gene expression		96.13685	99.56289	105.8028	73.14068	114.6993	83.58359	80.48196	92.31393	97.3611	104.9226	119.1547	117.2948	96.19976	86.73529	89.3199	95.45574	76.82742	86.02033	91.22685	89.53408	72.55053	105.746	98.40391	102.2726	119.9092	118.7939	134.5963
CUST_32686_PI429010886	M4811	0.000316399	12	6						812.4851	1168.291	798.0084	1303.295	1074.917	1027.321	1077.531	1512.535	1278.88	1121.501	889.0282	1191.902	1038.012	997.2176	1070.493	1513.832	1441.945	1564.909	1371.826	1807.527	1802.44	1202.808	1546.88	1455.41	1042.748	1047.139	982.3643
CUST_43451_PI429010886	M63096	0.000316987	24	18						12.28481	9.082829	11.75627	6.9169	3.671989	3.207123	6.531481	26.23786	22.74972	33.13557	NA	12.20801	98.79304	94.51495	NA	7.56114	16.90983	17.25943	52.87075	217.909	210.7377	22.38325	NA	32.39325	78.18007	18.70945	56.9001
CUST_34485_PI429010886	M9806	0.000319023	24	15						346.1837	494.8296	429.8212	365.3294	340.7195	315.0449	302.504	356.9618	247.7427	493.5638	363.1656	375.7494	386.0289	346.5231	329.3762	485.1077	520.31	530.4682	441.7626	462.9596	528.895	435.0369	387.4512	467.5708	329.6536	457.0998	539.7737
CUST_7369_PI429010886	N21441	0.000319023	24	18						109.1836	183.9971	155.2643	134.5716	139.0478	141.7833	137.8428	154.9682	150.5856	141.1702	136.7201	119.1208	146.1694	158.5618	138.9364	187.286	171.5864	185.4321	186.3924	193.2061	162.3844	176.5842	161.0739	198.0058	183.9744	151.6381	190.2455
CUST_2276_PI429010886	N12010	0.000319697	18	6						17.09822	20.6404	21.09878	39.27561	28.27048	4.113049	60.87753	229.5682	74.78378	28.80453	15.13397	12.32661	54.91706	55.75291	110.0054	15.97652	4.002598	8.300487	10.43842	NA	6.333731	25.02499	NA	29.11747	NA	407.2747	NA
CUST_39673_PI429010886	M42689	0.0003198	24	12						4279.69	13193.16	6864.217	22092.06	8713.974	16323.5	24825	36802.85	50283.97	4378.72	55705.47	7912.634	28743.72	39540.76	16801.9	12765.38	19287.83	16411.2	6079.326	8750.959	11138.2	4548.63	9980.233	9345.369	1282.19	5068.287	6622.666
CUST_282_PI429010886	N3400	0.000320252	12	9	82795517	arthrodial cuticle protein AMP1			0.00000042	8984.262	6079.326	8786.531	7937.877	9056.645	6592.219	6885.506	3804.856	7704.294	11228.98	13988.25	10852.86	13790.46	6523.544	10090.54	2659.616	2482.212	5197.224	6249.252	7597.158	7290.969	15367.74	16106.84	15553.93	6500.649	11822.99	14642.96
CUST_29532_PI429010886	N60051	0.000320568	24	15	240989783	adenylsulfate kinase, putative [I	Metabolic process	Nucleobase-containing compound meta	0.000000347	72.22265	68.02537	76.22837	84.45954	85.45046	96.68164	96.19976	105.1207	112.6997	87.71525	127.8904	18.94041	100.4643	106.2889	76.19975	159.497	147.7951	107.9954	128.2877	105.0886	144.5963	116.0037	81.07096	89.19083	99.72851	59.93426	82.85507
CUST_14380_PI429010886	N32785	0.000323277	24	18						210.6647	322.5903	253.5194	208.1829	226.4631	203.0154	187.8778	302.9304	252.9258	188.7199	245.4129	198.334	259.5745	306.1538	170.7219	287.938	366.6368	326.4068	300.0533	386.4267	478.8721	257.1925	367.876	309.2002	339.5673	319.1468	348.473
CUST_35716_PI429010886	M13289	0.000323305	18	6	157134262	mmr1/hsr1 GTP binding protein			2.07E-63	35.55192	67.17319	47.16128	40.83819	45.74256	57.83575	73.93804	97.21065	91.29948	94.67757	71.12138	63.67462	58.72904	66.65338	69.76252	58.60167	92.2966	68.67272	52.05686	57.52657	61.45632	57.53339	84.6671	70.26755	70.36116	88.57697	79.62962
CUST_9602_PI429010886	N24904	0.000323369	15	9						81.1468	83.40132	85.43893	61.93359	117.9157	100.3625	74.73696	84.47977	82.67536	133.5489	143.0166	186.6982	59.26831	113.7563	116.6823	79.30248	48.8281	54.29543	59.74362	61.11356	67.58778	122.2649	85.26437	169.34	86.55254	130.0053	168.902
CUST_83_PI429010886	N536	0.000324111	12	9						45731.6	11503.97	44511.54	64273.41	24984.55	33076.81	25888.13	32798.44	53773.55	70445.35	112823.1	49649.01	100092.3	33402.18	77887.6	3862.287	1620.506	4415.56	54162.9	43280.43	69178.17	79085.89	111377.9	115488.3	58747.16	82776.25	120946.6
CUST_56163_PI429010886	M112998	0.000324627	24	3						41.29379	17.4695	10.45873	24.53712	68.35388	55.87434	32.33466	30.69521	20.37081	NA	22.71218	48.1462	10.10725	21.70891	26.66045	20.56418	4.535494	5.243557	8.362495	16.45406	5.210982	NA	15.16581	3.896876	NA	28.88374	NA
CUST_6575_PI429010886	N20291	0.000326929	21	6						52.29097	42.42877	50.40096	62.96095	54.48188	44.83298	66.24674	307.6702	71.46735	70.50377	52.03532	44.69425	52.79948	29.00584	46.2068	34.37139	42.57027	58.04431	39.80982	38.55172	49.96129	42.75542	38.25388	47.39234	57.47217	74.35572	96.69796
CUST_4822_PI429010886	N17554	0.00032738	15	6						296.1058	734.4362	1159.214	654.5389	430.8354	66.54171	1543.558	2524.402	4602.898	272.9604	338.7418	80.11297	284.7236	169.1505	129.5473	215.8727	999.5752	826.9656	2654.193	1008.859	3327.482	696.7992	5872.99	6559.55	978.9259	308.1912	946.146
CUST_10085_PI429010886	N25668	0.000327734	18	0	348576308	PREDICTED: molybdenum cofact	Metabolic process	Other	1.07E-30	19.13763	17.08055	20.55824	10.77882	16.79753	8.241594	10.36053	12.01856	6.533775	13.98312	17.14498	8.573258	18.08416	9.483977	17.46376	16.56014	14.31736	15.90885	19.48308	18.14415	19.77172	16.45936	8.249733	10.30655	8.217462	10.48651	17.38217
CUST_38016_PI429010886	M22785	0.000329004	24	15						31.8936	133.4611	88.17603	391.9617	198.9444	319.3342	265.541	376.8731	426.1948	52.33755	584.9829	254.6677	221.8856	434.1663	130.586	525.6391	343.8057	530.0108	189.054	94.29714	223.5106	115.9671	71.98615	122.51	28.90914	49.57699	47.42168

Probe ID	ID	p-value	Period	Phase	EBI ID	Annotation	GO category	Go term	E-value	0	0	0	3	3	3	6	6	6	9	9	9	12	12	12	15	15	15	18	18	18	21	21	21	24	24	24
CUST_15416_PI429010886	N34537	0.000331419	18	6	383849023	PREDICTED: mitochondrial folat			8.61E-43	87.38427	142.8249	96.26661	104.1889	130.6615	110.9284	109.8243	119.9092	113.9812	113.4873	135.8804	41.36945	112.5544	76.6076	109.9654	81.08936	81.00976	95.57738	91.52744	95.53167	100.5377	103.7748	113.5495	110.7898	122.1097	120.6989	138.2294
CUST_1862_PI429010886	N10901	0.000331827	12	9	260793808	hypothetical protein BRAFLDRA			1.05E-13	7695.811	251.9129	6358.535	8098.961	2141.9	3712.526	1981.815	549.8031	1769.667	11932.74	11881.91	7950.355	12473.12	3281.159	8269.17	185.8751	322.8914	284.3354	3786.16	1929.095	4503.776	10532.72	13446.51	16106.84	4368.987	6592.219	11378.62
CUST_52661_PI429010886	M98363	0.000332943	24	0						505.369	442.1242	433.9196	632.4923	362.3548	421.977	247.8796	298.8719	383.0448	442.023	265.6462	409.6221	326.9292	203.7183	234.5996	315.18	452.6806	257.3902	320.381	230.859	234.791	376.7239	468.6225	403.3938	489.7282	424.2083	442.8695
CUST_16877_PI429010886	N37011	0.000334486	12	0						100.1798	98.84501	95.38758	66.1472	58.29201	53.21446	52.50511	72.64707	94.16687	85.1761	86.31943	83.3741	81.36078	87.68846	95.39909	61.83411	87.53029	70.89953	54.64003	76.40541	63.4481	84.51406	69.73149	70.80726	119.4473	80.91667	111.9958
CUST_11102_PI429010886	N27266	0.00033522	24	3	443731537	hypothetical protein CAPTEDRA			2.1E-25	8.174815	6.804117	8.737286	12.99802	39.72002	26.96013	5.58216	12.55527	6.956141	8.494969	17.54652	12.46202	NA	5.897189	6.422559	7.187709	4.372438	5.178823	NA	NA	5.195666	6.004064	5.22459	10.21893	6.070219	12.99937	5.890677
CUST_47157_PI429010886	M74994	0.000335686	12	3						20.83583	25.60678	19.28481	30.52273	27.22159	32.42334	15.06042	21.4181	28.13189	11.09687	11.00087	19.94626	24.92985	22.41889	17.93358	23.53464	28.7875	28.15447	21.87746	15.78015	22.38325	20.41963	14.70241	22.87902	34.76036	16.52067	18.80373
CUST_30117_PI429010886	N61207	0.000336292	18	3						21.76706	15.59063	25.2729	40.59088	23.38479	33.94389	11.98023	15.19323	36.53104	23.61619	24.4152	25.16166	14.68427	11.52497	17.20227	12.6478	12.10978	11.45624	15.37323	23.94374	23.18509	23.58742	27.13005	33.22428	13.97597	24.32431	31.65828
CUST_15762_PI429010886	N35097	0.000336696	24	3						50.97745	27.75337	33.33002	41.55628	94.815	53.081	37.18674	50.98258	31.66129	44.05907	47.28223	57.35611	36.73983	52.2967	38.87958	43.55336	36.67874	31.22862	29.87124	30.90184	22.64435	32.88915	32.3135	23.19056	47.11505	46.63617	32.23579
CUST_41920_PI429010886	M58884	0.000338292	12	9						93.89302	52.76213	101.2406	144.389	82.49497	71.61601	56.18757	110.6946	123.3207	130.3816	324.6618	103.7569	179.7028	96.91497	80.63078	44.04067	50.93881	49.00524	106.2214	135.6848	77.62481	176.1711	327.0634	248.4015	118.6245	213.853	188.3904
CUST_31453_PI429010886	M1557	0.000339544	12	3						183.9744	218.3991	178.5273	256.3549	254.3715	271.3104	267.555	257.5532	237.2821	235.4202	219.5016	234.1016	243.453	218.5351	251.9129	341.177	308.2378	296.3981	287.3062	245.8656	279.8449	201.258	217.1972	239.7136	263.3104	247.5032	241.2716
CUST_6118_PI429010886	N19653	0.000340603	18	6						24.15844	28.64244	34.39364	28.28196	18.14784	25.34302	43.48958	34.42224	55.22014	33.42535	28.75373	36.28429	35.43842	38.42576	15.74083	27.23388	29.16024	28.75654	23.98666	17.77119	13.55428	24.12361	30.8343	19.32331	29.36117	59.5633	81.10019
CUST_45180_PI429010886	M68247	0.000341166	18	9						4.750576	13.31134	14.66204	11.29632	7.164507	13.87017	13.00824	12.1946	16.55114	31.86686	14.81156	22.19592	19.30987	17.41463	10.87896	15.52955	9.528739	14.69726	13.41897	9.875893	10.21185	10.10592	5.704366	11.57746	16.38858	14.60927	16.28863
CUST_35529_PI429010886	M12799	0.000341309	24	21	321472565	hypothetical protein DAPPUDRA			5.04E-32	80.85368	61.57198	96.45691	85.00598	60.45051	60.0766	40.08729	43.67914	51.02509	83.6658	54.46319	55.25246	96.22172	46.07508	80.1827	59.21803	63.2206	44.90369	134.3827	63.14073	72.14124	156.0675	89.37424	120.4647	195.8396	77.08119	174.1094
CUST_48032_PI429010886	M78614	0.000341309	24	21						100.1152	113.0037	107.6547	75.88424	108.298	88.15439	65.31526	80.24156	84.14449	80.12221	83.24757	111.0363	67.06332	82.05798	107.3799	108.3981	102.437	98.01972	104.8945	97.97101	122.7362	118.8608	110.3412	117.8598	110.0862	116.8305	136.7059
CUST_20952_PI429010886	N44362	0.000341704	24	12						7.255904	39.26844	15.58431	48.69222	46.6169	31.62985	66.79456	45.24721	58.72446	60.11411	52.06198	42.75542	74.10459	68.32149	37.15987	25.84968	52.9338	41.81524	50.30283	69.95424	68.67272	21.27506	37.28248	7.817615	10.84443	19.51186	18.25369
CUST_3961_PI429010886	N15668	0.00034171	12	0						92.55588	71.3181	88.91387	69.22757	53.38027	67.05704	29.95632	52.65281	58.78776	69.4807	76.11372	64.26444	63.66743	84.69767	103.3338	76.78462	63.54017	85.66787	70.10685	69.04786	59.64761	114.2058	68.68072	84.37355	114.0013	86.54102	123.0785
CUST_22591_PI429010886	N47290	0.00034324	24	0						NA	18.7418	NA	8.746049	NA	11.63737	8.366851	10.23503	11.98023	11.84993	14.5111	NA	10.0894	7.016143	NA	4.296884	6.25226	4.325219	12.06492	10.3668	13.42624	11.34074	7.230351	16.09389	15.60788	14.60252	NA
CUST_764_PI429010886	N7213	0.000343375	21	15			Metabolic process	Carbohydrate metabolic process		20.62453	1099.849	22.07673	1147.873	46.27549	1087.147	2153.531	1758.225	80.73093	658.1324	588.9309	64.12746	2214.163	752.4222	1799.736	3022.554	2368.349	2177.847	963.404	39.05628	889.0282	37.9908	114.8223	48.89327	514.7289	379.6985	13.75076
CUST_41681_PI429010886	M58300	0.000343403	12	9						5184.879	233.3746	4392.913	5451.324	1234.088	3801.741	389.8933	368.0554	537.0249	10815.88	11455.94	7327.614	9345.369	1585.44	7111.995	46.49868	58.58735	74.27281	1717.387	772.308	2168.846	7673.313	6201.489	8619.889	1279.64	1965.01	3851.521
CUST_1137_PI429010886	N8611	0.000344545	24	9						25.97276	66.94807	17.79195	50.58432	58.48301	51.01468	19.60994	35.96649	35.02468	65.06618	63.64767	36.9244	38.12534	45.93656	23.39255	18.77257	51.79467	31.53769	12.3285	4.676114	11.33181	21.03874	19.35309	13.15166	10.40975	25.46688	19.75521
CUST_14908_PI429010886	N33673	0.000345077	15	12	321470439	hypothetical protein DAPPUDRA			6.89E-11	36.80294	29.58887	41.40683	38.65654	29.68598	30.92873	24.57559	27.85041	31.84584	33.39499	32.28459	35.01298	42.6416	32.87709	37.07884	32.48749	33.9424	34.8722	28.23949	30.80078	28.12879	25.68152	21.3729	31.4973	45.62734	31.62493	45.44669
CUST_12529_PI429010886	N29607	0.00034604	18	15						17.5783	105.9997	24.55645	50.41173	10.06809	53.92132	80.23561	85.83911	36.9244	92.90072	58.82712	27.90958	70.13338	88.15439	80.97133	149.6649	165.3421	159.4235	106.9946	20.37574	63.76387	31.04302	19.79906	35.02468	79.90962	59.96603	19.27054
CUST_39951_PI429010886	M47399	0.000346085	24	12						339.1735	410.2255	272.2328	1275.515	595.2796	819.0741	2317.529	793.2617	2611.542	437.4032	3105.612	219.923	3899.124	3062.683	725.3859	773.969	2188.815	971.9693	697.9099	420.3226	739.59	206.0776	1041.894	1166.709	198.0058	153.595	291.4994
CUST_16937_PI429010886	N37107	0.000346293	21	15						13.30176	52.73695	6.411708	7.26329	NA	NA	19.5898	17.06087	3.7666	54.344	NA	38.25388	50.69492	60.90419	44.67899	8.949447	87.92365	103.2872	76.59026	42.92652	58.13362	34.33515	228.7039	NA	4.72816	3.817178	4.248297
CUST_39599_PI429010886	M41629	0.000347603	24	21	326781695	crustacyanin subunit A [Fennero	Transport	Other	2.6E-30	319.3967	84.33008	333.6191	496.9611	123.675	291.8765	23.40329	77.78826	117.4006	176.0973	235.738	128.2119	331.2753	NA	254.7629	29.3403	256.2294	25.43349	440.5339	132.9821	334.8644	1191.068	332.7289	941.7984	515.109	142.9154	294.0637
CUST_38638_PI429010886	M28729	0.000347829	21	12						444.7023	243.6336	535.2394	450.4965	98.05836	332.8862	347.4772	624.2116	554.6469	455.6054	585.434	195.0513	621.614	579.6417	626.2886	136.4064	347.9094	115.6552	73.27988	137.3189	36.56104	163.4271	162.7602	265.9831	279.4339	238.025	355.7553
CUST_22349_PI429010886	N46870	0.000350533	24	3						64.57231	92.2966	62.50961	196.2948	181.7506	163.4271	80.68221	104.2473	88.96871	80.41026	212.8318	156.0305	99.00226	106.8499	141.6537	58.18021	68.20885	62.86957	48.21771	53.91461	46.11508	115.5828	114.5979	139.3908	57.08761	94.54537	99.22393
CUST_26597_PI429010886	N54510	0.000350533	24	21						126.7896	212.5696	162.9694	140.5662	105.6309	140.8152	109.3748	109.4096	120.2076	134.0408	146.6816	134.1449	127.8904	111.2293	90.07438	175.0487	246.5526	196.0115	170.9936	172.0707	210.4367	144.035	244.4333	258.7813	180.5627	229.393	211.2323
CUST_16490_PI429010886	N36326	0.000351479	15	6						6.077489	5.847915	8.542873	11.708	11.72346	11.45197	15.30443	13.77603	12.78096	9.442403	NA	7.409255	8.158467	6.323496	7.446593	7.414538	9.585902	5.063453	11.85264	6.491698	18.21466	14.43829	17.32917	10.73437	16.8636	9.197706	17.29812
CUST_8265_PI429010886	N22795	0.00035223	12	6						1088.703	672.9412	606.7201	865.141	112.0856	702.325	3029.351	3379.597	2516.066	1497.856	362.9291	775.0084	1014.541	948.7571	353.2411	5736.233	2383.479	3401.586	1540.275	4150.213	6183.806	2268.13	812.4851	1001.191	436.5051	198.4091	218.889
CUST_35451_PI429010886	M12607	0.000353047	21	15	295639972	chitinase 2 [Penaeus monodon]	Metabolic process	Carbohydrate metabolic process	5.36E-143	26.77594	58.03893	17.85602	48.56063	20.32715	36.63747	75.95975	46.26072	55.26296	259.2692	108.4858	31.07888	59.07254	63.12641	36.47269	333.4754	182.9006	260.3413	153.5706	29.01236	147.5021	34.29441	33.65745	30.12442	58.18757	39.98414	19.91041
CUST_37800_PI429010886	M21652	0.000355102	21	3						528.6354	533.5339	89.7242	262.5725	8321.498	7653.847	658.2775	2432.608	466.3373	31.673	4234.05	4727.546	22.83375	1608.753	1880.339	4.120124	17.8915	10.28202	37.61646	4253.847	28.0761	57.39735	722.545	53.89829	NA	4090.127	NA
CUST_12995_PI429010886	N30409	0.000355145	24	21						182.2908	175.63	255.7585	178.7424	122.5288	201.983	144.0796	132.4344	153.47	194.6096	149.1772	167.4695	142.8448	174.3818	159.9962	227.0582	198.4987	217.6136	257.3491	185.2539	224.2516	265.3922	220.5454	198.5315	270.3263	292.1626	240.3468
CUST_39480_PI429010886	M39996	0.000355207	12	9						3331.869	1235.266	4190.519	10017.83	4517.506	1314.02	3724.515	3918.531	8098.961	9516.402	15585.38	6171.545	13503.69	3715.857	6461.658	173.8666	132.0176	236.9175	11395.49	6226.747	14858.64	16183.31	30821.46	29949.84	14189.5	14548.28	25343.01
CUST_691_PI429010886	N6778	0.000356851	24	15			Cellular Process	Cell differentiation		194.821	238.6215	243.0247	227.4234	187.0484	196.7753	188.4673	225.7463	237.8029	227.3167	189.6097	212.0999	239.5431	182.547	200.6783	265.6462	282.172	271.3703	243.9147	280.5879	258.4714	231.4591	253.4358	234.9137	241.0614	201.8328	250.0252
CUST_31916_PI429010886	M2729	0.000359758	24	18	187608367	uncharacterized protein LOC100			3.88E-31	23.0693	49.76639	35.01298	21.68208	33.77513	28.39612	46.47246	49.45288	49.77832	41.59303	39.43512	39.67383	39.44669	45.87769	41.32287	38.64548	62.34647	47.20876	65.0271	76.47464	58.37796	49.46595	46.21776	48.62526	39.87751	40.27208	43.17122
CUST_2191_PI429010886	N11830	0.000359758	24	21			Protein metabolism	Cellular protein modification process		818.3565	647.0623	715.878	649.4957	377.6097	447.6336	324.0369	323.9561	367.4169	763.8013	518.2326	555.0536	954.4926	274.0938	818.7919	735.5518	587.0902	399.0134	1380.017	479.1083	794.0736	1512.535	795.5533	949.6232	1879.179	660.4277	1403.719
CUST_32119_PI429010886	M3277	0.000362736	21	18	321476145	myosin heavy chain isoform 3 [D	Cellular Process	Cell adhesion		80.13048	99.79674	88.81146	120.8147	109.762	87.61045	112.5697	91.62496	92.56838	81.78148	72.46811	82.1262	78.5366	75.054	55.09433	83.59991	145.4106	146.5222	101.1701	137.2489	212.7978	110.3861	128.5824	124.991	95.74045	96.18658	97.47634
CUST_50165_PI429010886	M88895	0.000363538	24	12						48.43357	34.28012	14.30044	53.13079	192.8968	163.1533	93.19639	72.52587	89.97113	34.32863	100.6045	120.5852	34.28288	205.9122	184.7904	105.9997	49.26677	60.53935	51.41072	42.92948	52.07351	26.63581	12.99612	16.54048	18.11193	40.40244	22.80766
CUST_15320_PI429010886	N34395	0.00036437	24	21	478259909	hypothetical protein YQE_0376	Developmental process	Nervous system development	6.96E-69	140.5297	114.4742	152.1914	103.6054	126.7896	120.6989	96.07979	104.2696	107.332	138.7083	85.22534	129.0291	106.2659	129.4603	153.1826	159.7027	131.3179	178.2736	118.4588	141.1475	128.9165	176.3724	148.4879	195.9604	187.9849	135.8001	171.7821
CUST_22379_PI429010886	N46925	0.00036437	24	21						182.5355	223.4669	234.5996	194.8463	127.4702	129.7338	99.69334	188.4673	163.488	127.4883	218.7863	163.6909	243.1406	189.1569	250.6739	146.2442	279.7784	242.5947	224.2026	205.8558	198.257	249.8271	271.3703	282.172	170.1187	220.2176	246.8683
CUST_33706_PI429010886	M7662	0.000365099	24	6						132.2497	130.3258	166.838	152.096	121.8531	172.3438	203.6502	175.2065	319.3967	157.1577	211.8418	140.1608	208.5384	184.5044	119.8471	204.9422	171.2114	158.8716	105.4571	82.50222	76.89496	106.5634	90.05411	143.3502	125.0086	78.70208	126.521
CUST_7018_PI429010886	N20921	0.000366029	24	21						1823.25	3010.08	2393.197	1536.792	1779.656	1491.134	922.5025	1780.678	1789.218	1931.709	1818.148	1840.408	2954.482	1734.409	2642.585	2879.516	3675.29	2727.371	3751.984	2685.938	2447.506	3627.136	2736.432	2771.365	4539.587	2056.189	2835.943
CUST_40472_PI429010886	M55225	0.000367855	15	9	242008949	protein disulfide-isomerase TXN	Protein metabolism	Protein folding	6.44E-35	5.283967	5.486086	6.800335	7.915424	8.248627	9.466716	11.84049	12.80959	11.72519	22.37256	15.32656	8.81061	NA	7.495356	4.693356	4.897954	12.3901	8.601925	9.905888	8.394142	5.551574	14.2562	NA	7.423378	16.89173	13.28096	12.7792
CUST_44923_PI429010886	M67426	0.000368982	24	21						25.8625	35.95046	38.07503	36.48523	50.36694	38.89512	27.80262	33.15311	35.01636	37.63739	34.05764	9.398999	35.55503	30.52779	39.02298	41.19655	50.72013	44.03097	34.62127	35.11692	43.07048	54.27691	51.35849	58.16145	44.06952	42.52944	45.36304
CUST_8872_PI429010886	N23731	0.000368982	24	18						111.2105	113.1524	120.7979	109.2995	97.07924	97.64083	117.7627	118.0753	131.4868	115.984	135.8001	110.298	136.5823	124.0147	125.025	113.8465	146.5474	142.0601	122.9511	133.7211	163.4636	129.6975	130.2225	150.7041	128.9772	114.7628	149.2547
CUST_34711_PI429010886	M10429	0.000369902	24	0						6.029956	13.42334	18.15913	NA	5.78951	12.54744	NA	8.641728	15.36202	13.03008	5.396608	10.15678	5.446073	5.755702	3.683163	7.439076	4.636857	5.607023	17.54875	6.827125	8.243095	7.300631	10.51838	9.350524	25.41015	14.35144	14.75102
CUST_10494_PI429010886	N26323	0.000370409	18	3						3039.361	839.3829	3085.208	4217.01	5947.47	6255.734	1200.665	2915.434	2787.074	3428.918	6018.305	5601.899	6333.445	2608.106	4164.365	922.1615	624.2116	842.6773	2320.751	3744.093	2837.693	7270.382	9194.226	8962.903	3552.655	6298.27	8334.379
CUST_15665_PI429010886	N34948	0.000370409	18	3						11.28749	22.24587	18.85869	27.48203	48.33387	34.78692	27.29078	27.69944	34.72341	15.03281	34.86879	28.00878	29.54223	11.26701	34.48071	16.38187	16.66197	14.75816	30.03343	21.87085	33.11578	35.78636	37.29691	42.1572	33.54341	20.74174	34.4378
CUST_50754_PI429010886	M90892	0.000372136	12	0						266.8703	575.4805	547.0285	736.0921	278.4293	283.224	132.6606	381.849	178.576	460.5885	163.0633	535.4815	1212.026	305.7738	616.7748	129.7798	193.0271	159.0392	54.23896	117.6781	7.354982	35.45592	44.74502	21.46595	842.2205	570.1346	1205.686
CUST_48375_PI429010886	M80053	0.000373595	24	18						79.19058	32.84255	102.7749	54.03718	14.09547	22.9969	225.5545	307.9283	232.9121	93.88506	26.42112	53.47447	483.0822	242.4377	175.5738	254.4938	235.5705	407.9106	369.9026	1321.627	1426.897	228.6642	32.44868	156.3694	711.071	170.2707	412.5149
CUST_39547_PI429010886	M41111	0.000374801	24	21						20.41652	29.23556	34.1983	11.11405	20.81559	18.06394	17.15077	17.79852	13.85499	15.73263	11.55337	10.42463	14.41795	20.32505	15.01335	32.41159	30.77878	35.80909	24.22714	15.89391	17.5656	34.39723	19.72473	25.93001	30.4229	19.07007	25.57888
CUST_2033_PI429010886	N11371	0.000375283	18	3						1330.649	134.3589	1116.506	1268.009	960.5506	1190.571	173.8872	260.6535	244.5822	554.6469	851.1928	1053.829	593.6835	429.9294	514.8454	52.17564	33.33002	37.10235	325.1203	371.8649	204.7788	1573.048	887.2093	1375.234	287.3062	560.4998	549.1826
CUST_14222_PI429010886	N32511	0.000375283	18	15						43.65579	67.71277	44.32847	33.39115	39.62294	38.70471	60.50212	75.80655	38.7383	65.36387	34.51373	50.34979	56.734	52.52277	46.0486	78.33025	67.96036	65.41169	52.92831	47.15551	46.91122	44.6542	41.04183	40.32964	49.02346	56.84633	41.02997
CUST_48242_PI429010886	M79487	0.00037553	24	9	260795348	hypothetical protein BRAFLDRA	Transport	Transmembrane transport	4.84E-22	53.43823	25.54219	31.47125	45.93201	202.3915	113.0557	57.11305	41.55059	50.51216	41.3582	113.0557	179.4316	37.93535	106.9946	111.653	43.79783	33.72382	32.43638	46.66635	28.29802	38.4343	24.36566	20.19245	27.59053	32.08859	37.97184	57.8894
CUST_51757_PI429010886	M94903	0.000379678	12	0						732.552	1282.861	1087.147	901.1852	730.0615	609.4415	527.3184	798.2619	684.8522	757.3112	1135.35	1219.279	821.2893	1074.104	1370.424	605.6304	817.7537	848.0782	566.888	591.4732	602.8578	941.0274	943.8069	976.1525	940.3723	867.6626	1404.214
CUST_9168_PI429010886	N24169	0.000380695	24	18						534.593	541.6936	430.3398	463.096	527.7959	426.5559	406.254	521.4036	520.31	496.2739	396.2592	457.8873	502.3264	394.8687	457.7423	535.2394	591.6487	611.6853	587.8441	592.0137	654.5389	585.434	641.1736	607.8959	708.4537	514.8454	679.224
CUST_41209_PI429010886	M57134	0.000380746	24	3						101.3884	92.16269	57.8894	67.13611	325.4289	286.8701	92.31393	206.2187	76.81667	28.87134	151.5648	140.5662	51.57473	34.12139	42.14237	17.24674	21.79685	15.25955	21.89409	221.9252	18.35318	80.47316	125.4804	39.17612	27.2264	364.4673	109.2995
CUST_13890_PI429010886	N31948	0.000380746	24	15						43.86651	98.41457	49.81991	37.34026	48.29273	45.34342	68.64759	51.24138	52.82485	45.59864	57.8427	45.76721	68.79187	77.19972	47.13519	69.96485	90.3848	71.27374	59.94044	69.326	59.00011	47.6343	67.40377	58.06923	60.39565	47.72449	46.43858
CUST_31300_PI429010886	M1166	0.00038119	24	9						3.078953	4.626825	NA	8.143822	14.20626	5.734979	5.989665	5.326271	3.679546	7.938175	14.70241	18.67988	4.17009	7.360727	12.38102	6.207673	3.717075	4.667487	NA	NA	4.135823	NA	4.0751	NA	3.587457	3.677248	4.516364
CUST_15019_PI429010886	N33874	0.000382819	24	18						172.8084	193.1768	183.1939	179.7279	174.5649	166.5631	192.2095	203.5811	214.5554	158.418	154.9422	192.4672	176.0219	247.3301	258.8836	211.9007	207.4443	239.0006	273.0279	266.8703	329.6536	227.1397	192.8532	245.5335	242.9432	212.4427	282.9651
CUST_12809_PI429010886	N30084	0.000382859	21	3	13898841	CUB-serine protease [Panulirus	Protein metabolism	Proteolysis	1.85E-20	184.7058	299.3012	253.1207	180.3409	416.1276	337.6971	171.7541	179.7279	222.3734	179.4936	165.3421	173.9043	166.2655	169.2749	98.63138	204.7458	248.8552	192.7408	125.5101	93.8595	87.09988	190.0383	229.8267	232.0442	245.059	316.2435	232.9522
CUST_39829_PI429010886	M45317	0.000382951	24	3						1450.664	148.7099	855.0422	1648.551	7058.228	6116.36	164.071	1019.654	124.8855	179.9884	2795.798	2706.184	111.3048	2105.784	2584.904	39.8934	58.38713	45.48556	37.43476	1436.914	29.07492	376.5416	235.4574	329.7179	NA	1475.969	NA
CUST_41116_PI429010886	M56909	0.000385962	24	21	546685137	hypothetical protein D910_1193			4.37E-83	356.7674	379.0965	424.9756	345.514	323.2204	334.4355	327.5322	328.6973	359.5095	344.2242	321.4975	317.8651	346.5231	301.3784	339.077	514.4981	416.251	562.7989	411.5622	379.6086	410.9137	462.0354	389.8933	487.7778	410.7073	428.2506	540.61
CUST_6664_PI429010886	N20427	0.000387431	24	21	546686493	hypothetical protein D910_0015			1.85E-67	78.76138	98.29871	101.9483	73.80869	105.2262	95.98627	68.63358	77.87097	79.06068	91.82394	81.02819	96.27921	85.31441	83.08821	90.79873	87.75883	98.73078	113.0301	87.66036	88.85544	92.82706	106.3489	110.8928	126.4468	84.13391	86.07541	118.1075
CUST_31590_PI429010886	M1915	0.000391177	24	15						800.6803	1340.037	855.8718	902.1594	745.3715	654.1448	1145.735	1144.859	1129.142	961.4773	782.9344	792.599	1168.291	819.0741	830.1589	1274.879	1410.73	1583.518	1214.037	1265.639	1396.045	969.8589	1117.04	1150.764	1106.912	1013.387	1306.636
CUST_873_PI429010886	N7648	0.000391177	24	3			Metabolic process	Lipid metabolic process		24.49701	19.44404	18.63724	26.99962	33.75196	35.33768	25.5566	22.58254	10.08687	23.51724	21.63837	23.27322	16.31963	21.66219	14.61885	16.87762	17.68949	13.93814	17.44435	21.06721	4.200134	19.71996	21.05828	17.84159	18.69379	24.67085	17.01004
CUST_53911_PI429010886	M103676	0.000392611	15	6						909.9142	349.6396	1341.551	779.5767	463.1658	279.9265	3237.866	1519.636	4577.512	910.719	1984.393	762.8618	2285.617	799.5929	1063.832	350.0691	183.7151	384.0543	1770.862	1260.62	1988.355	1791.446	2968.824	2409.846	2221.308	1972.842	4863.888
CUST_19703_PI429010886	N42040	0.000393418	24	21						146.4225	158.0057	160.6804	133.2817	106.9946	108.8194	143.7853	142.4472	140.612	168.9237	118.6698	115.5725	127.1435	113.9812	102.3834	152.5742	134.4487	120.8608	126.7896	147.2139	135.9631	184.9936	172.748	167.0808	159.5977	136.8604	153.5202
CUST_31308_PI429010886	M1186	0.000395356	21	18	124626535	Litopenaeus vannamei actin T2				91.54439	139.1591	121.0004	110.0862	135.0936	89.88767	79.82778	95.44831	78.97649	102.8535	71.36454	79.1181	76.31853	75.2089	79.56634	106.1034	149.3159	132.5675	154.1589	106.1034	188.2922	93.60566	146.0289	137.7249	148.6649	91.56473	142.7301
CUST_19904_PI429010886	N42420	0.000399652	18	15						14.90046	32.86579	24.37229	17.89974	12.91017	13.4773	47.33949	28.01303	28.67614	47.1467	14.25789	26.30298	25.90356	19.66385	22.00011	51.72314	33.67688	37.04481	20.2341	17.96765	24.93768	11.16309	8.842249	19.28057	25.59017	25.24183	23.49891
CUST_30058_PI429010886	N61102	0.000400263	15	12						5.140584	13.0692	7.713675	7.484013	11.08963	3.664081	7.676016	10.32176	8.710796	13.66542	13.35744	18.22518	19.35085	18.92222	16.18532	3.883878	4.68663	9.382464	10.05333	4.252369	7.217315	NA	7.491826	4.459243	8.638059	13.21801	11.67212
CUST_39834_PI429010886	M45358	0.000400497	21	0	170574983	T-cell receptor beta chain ANA 1			4.06E-08	20.6563	9.043827	16.91699	16.23247	9.51357	8.015981	8.568381	NA	3.936804	8.739672	4.867806	6.359637	10.914	7.153945	8.259511	12.35137	10.21893	8.79359	9.24446	9.117714	7.792211	14.83032	10.06809	27.77493	10.26423	9.613655	7.113497
CUST_5731_PI429010886	N19116	0.000401609	24	15						96.36643	102.2726	107.424	77.72928	73.71889	64.45499	88.72914	140.662	125.0613	102.1002	96.9591	91.92491	122.247	106.0382	49.35016	190.289	145.5033	171.097	176.8903	119.971	136.7882	83.4815	103.8862	94.71637	161.6353	91.18387	115.7246
CUST_6493_PI429010886	N20174	0.000401609	24	15			Nucleic acid metabolism	Regulation of gene expression		1101.918	2283.014	1579.509	1306.13	1356.303	1200.665	1224.109	1813.66	1816.946	1373.039	1396.628	1164.632	1356.303	2007.702	1715.071	2422.76	2893.96	3058.086	1379.279	2317.529	2424.1	1373.039	1800.506	2419.664	1659.781	1796.427	2213.256
CUST_24237_PI429010886	N50279	0.000401609	24	3	91076860	PREDICTED: similar to zinc finge			1.02E-11	307.3354	18.51954	248.9453	383.67	356.871	380.35	18.00685	34.84492	35.91688	291.0502	341.732	383.5875	275.1168	193.3377	331.2753	15.75024	15.68854	4.747329	14.0887	28.24828	18.02378	269.2665	115.0679	252.4627	36.77637	54.44021	71.29445
CUST_26462_PI429010886	N54282	0.000401609	24	21	158300505	AGAP012123-PA [Anopheles gar			6.31E-26	13.80235	10.54698	13.81116	21.97251	15.18659	9.826732	12.72466	10.51731	9.189042	8.499991	5.554011	6.437589	12.08433	16.71139	4.571298	11.59806	20.60634	14.60762	7.854749	13.50832	13.37213	17.64252	16.33804	25.37982	14.13494	16.50853	13.28237
CUST_22019_PI429010886	N46278	0.000402173	24	9	321458259	hypothetical protein DAPPUDRA			6.49E-22	45.37131	32.03897	23.30722	20.59213	85.79452	86.85325	32.2735	26.54663	34.16687	NA	57.73811	88.75	6.76308	46.36802	31.34548	23.61457	23.11901	26.92179	5.902123	13.05606	12.41089	NA	17.42256	5.031459	10.22939	21.85804	9.71776
CUST_91_PI429010886	N732	0.000404211	15	0						21010.2	12021.42	18365.76	23739.57	7279.608	15102.78	10532.72	10077.64	9629.338	6864.217	9226.663	7450.782	8996.679	14703.33	13419.52	15630.09	15673.03	12648.44	9167.401	8911.047	10464.54	7714.571	14743.32	8899.45	6622.666	9643.272	8713.974
CUST_17339_PI429010886	N37797	0.000406475	24	21	270012294	hypothetical protein TcasGA2_T			2.36E-08	43.16883	43.37645	29.37054	39.09638	28.1833	30.81577	24.32178	33.75196	33.51959	23.90042	24.11397	24.30986	30.54671	30.09026	29.68598	43.30726	39.16842	33.53984	31.12503	27.10746	38.67341	49.07635	32.78884	41.12558	40.71158	30.06574	33.64987
CUST_11269_PI429010886	N27540	0.000406824	24	15						17.8915	22.84762	15.16316	17.23127	8.977003	8.358144	20.03269	18.00112	18.10043	19.96477	9.839028	9.939018	21.89139	13.95642	36.54404	26.36566	55.88006	46.03907	22.8831	18.61738	20.5703	16.21895	19.65914	29.90874	23.95576	15.9018	23.48308
CUST_18788_PI429010886	N40407	0.000406824	24	15						37.34538	54.52711	38.82932	22.74717	22.81449	16.7995	34.69476	27.25852	40.18248	37.77609	33.15745	46.59051	33.05084	44.15404	42.20935	47.69479	51.02011	61.37944	25.82769	42.2303	45.40562	24.96379	61.03917	42.24367	39.71151	36.70398	48.48882
CUST_42261_PI429010886	M59827	0.000409141	21	15						82.52242	129.4034	100.4391	73.09797	120.749	112.585	113.9542	109.425	129.9006	117.3396	119.3879	137.8214	97.3763	123.2894	139.6676	140.6785	139.755	136.9442	109.1677	107.3485	120.2238	96.85513	96.73368	107.4552	112.7612	95.83012	115.186
CUST_22714_PI429010886	N47520	0.000409394	21	9						49.0091	72.35325	46.42563	54.42945	115.7715	71.89808	116.0279	93.65434	164.5342	50.41623	183.4751	201.983	73.39156	131.6748	152.5572	126.1085	112.5697	152.7137	105.9898	105.242	106.2214	40.79604	56.86184	65.86088	97.95973	187.8778	56.86184
CUST_51499_PI429010886	M93736	0.000410265	21	0						125.3559	173.9585	128.2119	136.0891	98.10696	146.7486	89.68248	103.801	83.28215	107.332	29.22669	109.2822	74.71995	65.86788	48.79532	118.5947	86.62987	199.8339	78.64253	94.815	45.52154	201.5487	124.3957	267.7885	110.3707	83.70817	52.0293
CUST_36075_PI429010886	M14547	0.000410718	24	0						152.9126	257.4304	116.4725	48.28814	206.1814	75.47204	192.4289	152.5137	112.2698	44.82733	81.70379	23.93602	67.66335	111.894	88.86561	50.55672	94.10473	45.82718	172.4755	205.453	196.4115	184.9052	112.8927	168.4308	329.3072	156.0492	230.9439
CUST_49234_PI429010886	M84840	0.000410718	24	0						9500.977	7007.731	7415.157	6025.479	3801.741	3371.075	3914.879	4134.923	3755.671	9345.369	4889.847	1877.836	6343.146	2470.84	4667.622	5907.39	5244.429	2356.919	8073.399	5601.899	6461.658	9226.663	10871.96	6956.592	16288.46	9500.977	13812.43
CUST_57182_PI429010886	E2172	0.00041204	24	15	258406678	QM protein [Penaeus monodon]			1.23E-12	23231.4	34620.42	29037.53	24721.57	21177.49	21224.39	35862.04	35445.2	32064.51	20913.02	22847.77	29037.53	25611.3	25703.26	26299.14	38719.75	40595.56	44913.68	32454.44	29294.9	33508.11	27459.44	29647.7	27918.92	27459.44	27347.94	28210.59
CUST_55591_PI429010886	M110875	0.00041204	24	3	220172369	cuticle protein [Rimicaris exocul			6.83E-09	14982.33	333.3795	15394.16	24426.06	22526.09	24590.28	571.6616	1640.282	1133.285	12473.12	28492.44	17931.09	7439.177	12424.28	16655.53	134.5963	260.9505	166.7408	845.2107	1397.328	1179.914	12921.27	8061.991	12921.27	1185.011	5002.917	4211.995
CUST_35688_PI429010886	M13238	0.00041204	24	3	270002194	hypothetical protein TcasGA2_T	Metabolic process	Carbohydrate metabolic process	1.06E-84	161.6606	83.9852	135.1327	142.3304	225.6261	271.8944	62.94384	88.6659	77.67929	116.8167	146.1939	208.1054	113.0037	100.7211	157.5876	37.28898	32.19938	40.5192	40.78815	61.01777	27.30031	154.3725	64.11411	131.2222	68.20885	87.18835	63.09832
CUST_6071_PI429010886	N19588	0.00041204	24	15	156537747	PREDICTED: adenylosuccinate ly	Metabolic process	Nucleobase-containing compound met		46.73964	99.32904	83.32737	54.25064	58.77493	61.52728	78.23111	69.48765	85.30314	108.3124	47.46968	78.40447	86.24643	60.16557	64.26444	100.6934	120.035	116.804	74.20038	87.00868	88.86561	62.80714	66.58185	71.33011	86.06068	61.67659	65.87993
CUST_6154_PI429010886	N19697	0.00041204	24	15	528469909	PREDICTED: poly(A)-specific ribo	Nucleic acid metabolism	RNA metabolic process	1.79E-68	10.58439	25.52576	22.35906	13.22308	22.02956	12.82614	36.41114	25.31848	29.06579	46.06991	20.35695	23.37085	36.42637	35.45915	26.49338	41.64718	35.55192	44.61738	25.57669	47.52511	40.91608	26.46648	22.26852	25.46423	31.50316	24.79569	28.69791
CUST_37494_PI429010886	M19227	0.000417256	24	15	242017286	Pediculus humanus corporis Hsp	Protein metabolism	Protein folding	1.15E-103	3151.384	7389.276	3767.349	2965.89	2350.317	2797.556	5179.003	5939.375	5394.696	4403.488	3020.109	3220.778	3864.779	3774.541	2990.827	5992.677	7459.987	8846.332	4685.458	4998.113	6255.734	3064.959	4470.918	4253.847	3960.355	3902.533	4539.587
CUST_31918_PI429010886	M2731	0.000417256	24	15						2006.118	2611.542	2201.915	1813.66	1543.849	1570.104	2422.76	2050.749	2404.745	2299.057	1573.941	1864.874	2395.468	1630.398	2028.877	2928.861	2956.694	2807.452	2107.433	2204.749	2584.904	2195.531	2498.654	2348.232	1825.053	2093.838	2354.096
CUST_48638_PI429010886	M81386	0.000417256	24	15	213493458	nascent polypeptide-associated			2.47E-13	919.3983	1271.688	1048.117	992.2362	888.236	832.328	1114.294	988.5868	1036.158	1132.475	841.994	1149.369	1158.095	1036.158	1026.869	1314.736	1692.044	1635.354	1025.404	1100.835	1322.806	877.3403	1185.011	1307.951	1118.881	976.1525	1257.339
CUST_51633_PI429010886	M94342	0.000418629	12	9	524974426	PREDICTED: zinc finger protein 8			3.82E-29	39.38283	21.84014	31.23137	20.36911	13.33647	14.53482	13.98648	21.18377	17.22926	28.00878	19.4293	23.62689	26.09025	22.55063	10.24506	12.45971	11.03537	15.71789	24.0298	26.74791	21.81628	36.8404	37.12911	30.40674	21.52992	16.67353	21.58672
CUST_987_PI429010886	N8132	0.000418629	12	3						25.24448	31.88585	24.28357	34.08743	32.28165	37.14951	15.20646	25.81236	30.37143	21.43699	18.24533	27.32212	26.46184	25.9662	24.12932	40.86304	33.02712	30.31787	29.26951	30.81263	41.77836	19.5898	29.42662	24.62171	23.85663	28.41121	30.04801
CUST_5464_PI429010886	N18740	0.000418886	24	18	291222709	PREDICTED: deoxyhypusine hyd			3.62E-96	881.8202	868.9348	1193.688	1114.745	952.2766	649.4957	774.5071	837.2458	729.7963	834.3772	619.5951	642.7419	850.2576	560.3033	532.2614	804.3138	1670.335	1322.312	1261.961	1087.769	1251.245	899.2992	1235.811	1099.075	1064.673	971.1067	1637.38
CUST_31161_PI429010886	M856	0.000420459	21	3	19921888	CG8026, isoform B [Drosophila	Transport	Mitochondrial transport	1.84E-29	34.96168	54.02691	62.44379	49.47094	67.55887	63.15515	35.32216	54.25668	48.41784	41.7466	60.83425	31.95397	53.6019	45.26359	41.14564	27.86499	45.5838	29.64873	46.87885	34.24084	44.44058	47.57931	64.39747	43.89532	49.66595	67.64673	55.95718
CUST_34156_PI429010886	M8906	0.000420459	21	15						911.7924	779.0482	818.7919	717.3191	538.3095	843.8571	816.314	819.3739	723.6856	791.0912	758.1822	616.0609	794.9446	814.3635	1085.159	1453.134	1445.709	1275.515	771.2654	432.2687	927.9648	851.8736	597.7182	818.7919	554.8747	351.2785	570.2732
CUST_32151_PI429010886	M3372	0.000421012	21	3						823.2974	645.4936	328.9434	714.712	5179.003	4616.026	295.7504	844.4833	247.9707	NA	3321.698	3137.141	34.47208	1500.263	1644.807	46.65753	32.05226	20.31267	33.07741	1826.319	23.72167	115.9325	556.4241	103.2365	NA	3598.269	NA
CUST_38475_PI429010886	M27162	0.000422471	24	3	149675211	cuticle proprotein proCP5.2 [Cal			1.7E-55	11339.63	192.6657	11339.63	17424.76	14307.54	15731.96	298.7578	691.4224	343.8057	7376.103	15071.82	10178.51	5712.375	7558.705	10294.69	84.07229	92.49079	111.0926	489.7282	998.2787	702.325	9980.233	6116.36	9425.197	851.4962	2809.488	3031.683
CUST_7263_PI429010886	N21283	0.000422471	24	15						73.00128	166.0779	140.4269	92.61293	71.60883	60.36476	129.7968	142.1557	141.539	170.4605	98.15697	33.14322	136.3915	116.8754	99.35613	209.4822	193.3701	215.7105	194.635	139.0478	173.4147	151.6707	117.7735	159.5479	190.613	114.0013	155.1073
CUST_15749_PI429010886	N35079	0.000423547	24	18						21.88339	29.12719	27.06162	27.2823	14.48348	20.87993	25.92125	12.16729	29.47583	13.03847	24.83403	18.43758	27.22807	17.6212	19.47577	36.16345	28.28196	38.66006	62.28036	31.27903	45.98151	25.69634	27.61985	34.98417	24.73755	27.40728	40.85968
CUST_38160_PI429010886	M23772	0.000427687	24	15	24646605	squid, isoform C [Drosophila me	Reproduction	Reproduction	2.15E-09	63.04493	98.68778	77.6897	53.59606	41.36333	54.41132	88.80408	88.15439	80.57219	83.62172	54.44704	95.33452	58.05667	70.1867	67.60668	101.3081	128.6276	109.1371	91.97617	82.70695	91.96128	69.76252	80.00199	67.3848	95.42344	79.60935	74.05592
CUST_31446_PI429010886	M1545	0.000428072	24	0						730.3491	775.4008	746.9967	554.0705	491.5182	399.0134	588.0127	675.5044	559.5956	536.6512	447.759	578.5106	416.9152	472.1134	484.4076	443.9249	416.9152	509.6459	352.8279	425.5802	302.504	323.446	694.9037	539.0498	722.1173	657.3507	673.8771
CUST_462_PI429010886	N5434	0.000429363	12	9						427.8043	61.87143	456.4069	905.3835	211.8745	330.4034	155.3804	95.95298	208.9983	1300.047	4211.995	880.5962	1684.207	382.3723	709.4562	19.11764	59.53613	35.98384	341.965	185.7829	362.9994	1849.733	4118.017	4045.654	659.0035	3441.376	5571.385
CUST_19899_PI429010886	N42414	0.000429512	24	18						72.19272	82.95322	76.00489	58.08676	56.60172	45.47301	71.36454	66.77855	58.06369	52.05076	49.9386	39.52112	59.51158	48.51397	65.61458	81.23215	65.91522	77.57132	76.86672	76.97367	95.24948	72.06957	81.9432	69.6885	87.44315	63.96236	99.72851
CUST_10300_PI429010886	N26026	0.000429859	21	15	322791130	hypothetical protein SINV_0637	Metabolic process	Oxidation-reduction process	2.33E-28	32.73765	22.54151	23.11231	11.67524	7.402375	12.36934	NA	48.78475	34.3611	31.09316	25.31216	NA	21.7829	20.37574	18.57947	78.3065	35.33768	66.41804	40.06809	44.97817	62.92312	37.35922	7.228642	24.65307	27.17222	10.13279	18.30441
CUST_33732_PI429010886	M7737	0.000431523	21	15	506968903	eukaryotic initiation factor 1 [Co	Protein metabolism	Regulation of translational initiation	1.71E-31	513.8347	549.989	588.164	547.3976	382.4457	377.789	562.954	667.6928	525.9937	589.7452	488.48	512.6794	495.7052	467.458	409.3888	668.9797	753.373	762.4429	533.0556	603.191	668.297	501.6566	612.7708	600.2455	475.2146	441.6606	439.5135
CUST_1191_PI429010886	N8789	0.000431523	21	15	187610687	small nuclear ribonucleoprotein			3.39E-20	26.99962	46.09707	65.11364	4.096565	5.784206	4.728836	30.54026	18.2303	22.13988	42.5644	13.73333	30.84927	28.85219	45.46717	15.4862	16.85312	97.64083	34.80421	27.09821	17.95536	27.77493	26.24406	32.98843	17.12548	13.05124	10.50374	13.67378
CUST_48016_PI429010886	M78537	0.000431987	21	0	156374930	predicted protein [Nematostella	Response to stress	DNA repair	1.04E-21	73.54748	84.11819	98.10696	66.6173	45.51721	44.61277	37.36406	54.28141	43.27193	62.82209	60.50212	57.31946	56.18757	61.70126	48.68211	60.97733	85.03859	55.17016	48.77487	64.60254	63.88253	75.0185	60.71091	65.66275	72.62444	60.83425	55.03418
CUST_35813_PI429010886	M13580	0.000435464	24	21						652.5211	956.0225	896.6099	629.4202	630.7177	497.7865	485.4687	735.8335	683.6777	457.8873	683.4131	564.8143	588.6767	511.2321	622.1839	826.4146	826.9656	647.9305	553.2936	682.1048	728.0598	962.741	783.1683	996.3906	999.9612	782.9344	942.2704
CUST_31857_PI429010886	M2584	0.000439106	21	15						265.5849	440.7516	265.5849	331.7259	360.9619	369.0567	452.4209	524.6943	354.7453	336.8186	379.2367	346.1837	365.0376	524.2711	508.513	386.8846	527.0091	544.0973	383.132	399.2455	385.0272	260.2914	355.1717	291.6767	308.2886	347.8293	342.5433

Probe ID	ID	p-value	Period	Phase	EBI ID	Annotation	GO category	Go term	E-value	0	0	0	3	3	3	6	6	6	9	9	9	12	12	12	15	15	15	18	18	18	21	21	21	24	24	24
CUST_31073_PI429010886	M617	0.000441194	24	21						495.3901	543.9588	593.0437	455.4195	482.5812	381.5238	442.9918	438.3717	534.7331	477.3178	428.6927	411.1376	651.2334	451.5651	580.31	554.6469	585.6903	573.4698	698.717	541.6936	679.6938	534.593	738.8137	690.3228	649.314	542.5585	815.2223
CUST_45404_PI429010886	M69006	0.000441194	24	12						39.59705	33.22138	40.4062	108.0057	31.48667	83.28215	110.0556	33.41357	78.74455	38.68202	118.7499	71.58388	138.4795	102.4735	112.4069	23.76085	81.17631	82.30731	22.43407	42.84015	43.15443	10.60847	26.05879	43.28585	7.405722	15.1605	30.49064
CUST_53940_PI429010886	M103791	0.000442939	21	0						64.42993	88.10909	NA	37.10697	19.89174	55.24742	42.1373	88.96871	31.11011	12.64646	18.86717	10.19338	10.43842	68.04046	15.87243	69.31429	38.78785	28.12273	39.87751	52.92294	56.86184	NA	105.5804	NA	NA	64.88783	NA
CUST_16803_PI429010886	N36896	0.000445426	24	0						263.6152	267.884	240.2645	189.5433	202.2706	219.7679	205.7884	213.89	204.2969	180.3689	264.9858	246.9107	184.8821	212.4024	263.9734	186.2275	163.4636	197.8282	215.2625	231.5378	219.7344	207.8484	227.1783	266.8019	285.8344	286.4692	381.9172
CUST_37253_PI429010886	M18368	0.000446311	24	12						NA	5.256818	NA	6.35162	6.162699	6.86757	5.944674	10.30243	18.39792	19.64262	7.405722	7.884574	15.44623	NA	8.707003	10.25273	8.560595	24.12109	5.241251	NA	NA	5.643166	6.444484	4.800498	NA	3.344721	3.487187
CUST_51950_PI429010886	M95795	0.000448398	24	0						25.02228	24.04549	73.89284	15.08312	16.46966	11.97294	10.70872	9.164016	15.97652	20.07484	15.08312	16.52701	14.60927	16.31227	6.014616	5.084507	10.39762	18.0705	9.053925	19.70046	13.03156	18.00497	20.55179	19.22058	18.47174	22.5078	23.73245
CUST_7844_PI429010886	N22167	0.00044855	24	15	291237036	PREDICTED: Saccoglossus kowal	Cellular Process	Cell cycle	1.49E-73	314.367	438.2151	344.705	343.2592	297.575	272.8455	552.7385	432.5544	490.4622	443.4999	436.8129	439.4254	510.9141	360.8077	483.9464	596.5924	560.6594	688.4549	434.9251	398.5907	575.3242	440.6377	451.4694	486.0305	531.2109	429.2293	591.1008
CUST_32553_PI429010886	M4415	0.000449253	12	3	242010289	salivary plasminogen activator b	Protein metabolism	Proteolysis	3.22E-13	22.14763	24.94507	16.34183	45.75798	NA	37.74193	40.76292	22.28353	15.85285	30.08206	21.45062	NA	29.7549	27.54823	23.8882	103.9519	54.41132	51.58002	32.38584	7.745918	33.29327	NA	20.95911	5.120189	24.80919	30.02799	NA
CUST_41076_PI429010886	M56823	0.000449604	21	18						20.65147	50.98258	50.20946	44.69809	36.30672	42.46657	34.49684	34.06404	35.06077	38.30361	34.74447	9.970616	53.05853	36.69203	34.63434	39.94687	45.78026	41.91606	77.99973	44.83771	43.32213	45.68256	44.74977	45.40562	43.63653	36.08229	43.75819
CUST_10828_PI429010886	N26852	0.000450831	12	9						391.9617	146.093	512.1954	881.258	208.9983	457.5988	134.3827	131.9106	191.2458	1212.026	1519.636	764.7593	1203.381	199.0615	681.2288	28.67393	46.73349	33.73205	222.3734	186.0781	269.6374	935.0962	1365.984	1624.639	371.2053	431.1432	922.5025
CUST_29869_PI429010886	N60720	0.000450831	12	9	37780426	hemocyanin 2 [Pacifastacus leni	Transport	Other	1.07E-12	2787.074	535.89	2806.331	5048.826	1135.35	1802.44	915.0007	1618.404	2645.058	4181.398	6649.946	2799.9	7100.104	1653.487	3122.414	89.91214	60.43161	110.8072	2727.371	2114.8	3000.392	6333.445	10987.48	10109.9	3670.22	6132.817	9096.626
CUST_38216_PI429010886	M24213	0.000456198	12	9						9466.456	1363.035	7531.236	10569.33	3819.978	5550.987	4783.413	4055.849	8135.669	14218.13	14526.85	8565.196	17692.35	5009.272	10193.49	605.9768	652.1093	791.3056	9923.978	6143.541	8669.063	15900.37	20059.08	16183.31	9364.597	11305.05	17487.6
CUST_39686_PI429010886	M42915	0.000456198	12	9						6385.128	641.3942	6826.356	7376.103	5388.872	5251.996	1711.883	1571.574	2631.802	9330.025	9532.564	7861.824	10193.49	5420.715	5955.414	683.9311	477.5471	617.1935	6292.839	3336.354	4027.931	14411.75	10641.47	10987.48	6601.274	5021.117	9598.965
CUST_22748_PI429010886	N47601	0.000459497	15	12						46.51413	51.3028	61.05496	24.53204	52.94098	26.94942	24.30298	49.02346	43.01953	53.70551	51.20424	60.40971	58.5677	85.1599	82.06727	88.42474	102.9729	68.73418	37.04769	49.91114	41.69944	45.54712	55.88006	30.13664	64.49711	49.81307	93.19639
CUST_28596_PI429010886	N58260	0.000461565	12	3	157125192	chondroitin 4-sulfotransferase [	Metabolic process	Carbohydrate metabolic process	5.23E-08	53.48046	37.65544	51.82191	68.30294	73.1662	61.37268	46.83625	45.13797	32.50066	31.31906	48.83893	42.87131	52.38529	93.15792	91.37677	50.29492	57.99775	54.38582	54.07873	24.4823	47.21493	32.02499	28.75907	35.76914	51.52222	43.78128	36.50656
CUST_6351_PI429010886	N19988	0.000462409	21	18	321479073	hypothetical protein DAPPUDRA	Metabolic process	Other	1.11E-60	69.06046	145.1992	121.2999	67.67367	107.935	83.30459	84.69767	144.3062	119.1547	126.521	141.598	74.8864	146.9401	147.4173	127.9261	124.4898	174.3634	100.3755	139.5903	176.429	154.9682	130.3816	170.7496	120.9166	105.9605	81.11075	76.40541
CUST_11577_PI429010886	N28061	0.000462781	24	0	321454600	hypothetical protein DAPPUDRA	Protein metabolism	Cellular protein modification process	1.42E-105	47.46968	40.99664	50.82457	21.3828	39.16223	31.42433	24.99187	34.67259	29.76443	34.20583	33.83378	31.20114	37.97588	28.52077	34.30861	34.91434	39.72867	30.18014	34.2198	34.47208	35.82514	38.61107	29.19296	35.27117	45.52672	51.11212	45.9767
CUST_11647_PI429010886	N28175	0.000464596	18	15	241358643	flavonol reductase/cinnamoyl-C	Metabolic process	Oxidation-reduction process	7.21E-54	42.82494	44.98293	28.10987	42.50868	27.55104	34.06922	44.49025	47.69479	34.48395	47.83813	44.04067	45.40562	28.82109	56.49038	40.36492	73.15055	56.31298	53.96936	45.64087	33.77513	43.64157	20.71687	37.98103	28.96499	36.29093	51.43678	41.57926
CUST_33938_PI429010886	M8298	0.000466932	12	9						3342.747	40.08729	3672.992	2067.912	690.0542	1702.984	87.81083	160.1108	233.8809	2561.777	4061.362	4288.608	2261.704	1871.7	3963.836	44.32333	22.14517	44.37502	406.3531	267.0729	370.7223	4919.057	4121.132	7122.178	766.5607	130.828	725.6388
CUST_38841_PI429010886	M31102	0.000467383	24	12						2016.054	5015.024	3011.975	9759.347	3825.101	6649.946	11052.65	14703.33	20871.55	2061.689	20172.23	3975.62	12165.66	15900.37	7090.073	5593.889	7248.769	7531.236	2375.903	3475.106	4229.758	1555.177	4041.944	4334.63	558.9714	1956.496	2451.472
CUST_19502_PI429010886	N41681	0.000467383	24	0						25.16714	28.39338	28.14	22.90061	22.56843	20.45768	12.56759	24.83115	20.19023	32.4427	26.82917	10.06884	22.24082	19.09461	16.70829	20.91851	18.87745	27.33153	20.82118	20.60839	24.94781	25.49661	29.44247	33.63302	38.37232	36.62645	55.37116
CUST_23037_PI429010886	N48148	0.000469126	24	18						132.8026	76.11372	126.8236	44.38734	43.44652	44.81289	39.63441	68.70029	62.57277	68.72832	95.90246	72.9865	69.71651	57.16004	90.55203	122.2305	156.1362	139.6859	148.7726	78.35549	104.2957	163.8654	46.90198	192.6977	140.0958	74.29925	97.66082
CUST_15995_PI429010886	N35476	0.00047127	24	21						123.1603	136.103	143.8476	132.304	118.653	101.5332	133.171	136.2532	122.604	110.8928	63.88807	115.2516	110.6946	96.35679	95.44831	180.5859	110.6039	157.2999	143.92	81.78148	161.3159	139.7737	155.3994	187.2522	143.92	125.8771	151.7904
CUST_23256_PI429010886	N48519	0.000471329	18	6						36.10172	24.09912	41.85499	41.37426	39.41457	22.72575	56.96365	47.97707	44.39167	42.88477	50.92071	32.6167	44.2489	25.92708	27.18132	19.80398	28.34234	29.92819	36.28088	30.36962	33.67688	31.34548	61.11985	57.01672	38.43	49.82997	65.84152
CUST_57153_PI429010886	E1745	0.000471385	24	3	387878122	hypothetical protein W7S_2375			4.17E-09	63.27624	30.5648	42.16077	33.3276	75.84873	65.92209	18.35653	31.99205	26.35385	24.28357	27.05785	49.54311	18.39792	12.14919	25.71381	27.61361	10.17714	11.40929	11.5282	NA	7.147875	9.974531	18.04144	10.66221	6.81845	122.5288	11.68057
CUST_55631_PI429010886	M111036	0.000475733	24	21						238.3932	246.6961	216.7919	178.6434	182.3885	195.7919	155.6261	172.783	175.2702	205.453	164.741	180.0489	239.1254	179.1574	114.9073	279.2412	195.5458	246.356	208.6833	213.6621	236.9699	319.2736	242.2901	203.1887	318.7834	234.533	294.796
CUST_37370_PI429010886	M18811	0.000477666	12	9	189242051	PREDICTED: similar to DNA repli	Response to stress	Defence responce		173.4439	133.0187	199.2769	182.5355	128.1317	133.8983	154.8931	148.7488	173.7538	233.8101	218.0736	237.2505	230.8967	142.7301	147.3568	99.72851	142.5641	150.2981	178.9277	158.0565	198.9717	183.5316	189.7707	204.9134	126.2717	160.389	234.9137
CUST_8444_PI429010886	N23046	0.000477973	21	0	260798388	hypothetical protein BRAFLDRA	Nucleic acid metabolism	RNA metabolic process	2.11E-15	76.97367	83.13651	99.23862	84.70621	53.71014	67.67367	42.72194	73.58526	64.91207	65.56898	59.31427	67.88505	72.70716	47.84164	39.31755	50.41623	69.13756	76.49084	80.60289	86.34178	84.25166	74.08077	84.70621	86.06068	81.66229	71.89101	93.631
CUST_5584_PI429010886	N18906	0.000480311	21	15	321473690	hypothetical protein DAPPUDRA			2.33E-33	1707.189	2379.92	2064.359	1746.391	1595.825	1626.549	1772.069	1991.351	1909.699	1675.406	1687.279	1579.509	1610.862	1938.89	1527.348	2511.558	3052.051	3786.16	2231.56	2805.21	3654.032	1647.856	2143.564	1964.046	2031.77	1689.176	2108.887
CUST_51367_PI429010886	M93290	0.000480709	24	18						8.879476	12.9801	NA	8.727182	8.789171	9.429304	11.88037	7.945183	6.196488	14.00894	NA	NA	14.74663	NA	9.712808	15.09289	16.40108	12.17199	24.39861	12.10509	14.97844	12.15207	16.98717	14.01914	13.0678	14.42132	11.91165
CUST_32116_PI429010886	M3269	0.000480915	12	0						497.4085	369.9777	455.6054	487.1319	335.2209	361.2613	303.4901	244.3968	302.0161	431.8791	334.6481	371.0059	546.8689	324.0982	511.0453	470.9814	438.8282	451.4694	327.1238	318.8359	316.9378	621.1319	382.8713	517.9425	609.4415	390.5287	624.4152
CUST_4258_PI429010886	N16339	0.000481346	15	6						4.7202	10.87896	16.49157	20.42404	16.29946	12.6806	18.72709	42.44693	22.55063	22.36969	15.64595	18.97777	14.48202	21.68208	12.05742	10.24111	20.98524	18.11564	19.43702	35.89734	14.29901	32.4397	30.30161	33.97881	24.38317	19.02985	26.99645
CUST_38418_PI429010886	M26391	0.000483033	12	9						10464.54	267.9719	10430.71	10077.64	2269.737	6727.299	808.5577	499.6833	801.5781	19855.57	20968.09	14161.49	21881.51	2304.738	13988.25	76.07871	113.622	152.3742	3774.541	1348.208	4036.235	13635.69	12344.85	14743.32	2913.515	4825.163	8631.841
CUST_42173_PI429010886	M59601	0.000483033	12	3						20.02415	14.11179	15.15361	20.01482	22.81773	19.70573	17.13272	10.56095	105.4679	8.569437	14.82873	11.31144	11.33713	22.29626	18.22518	21.44677	37.04177	16.87615	10.96478	16.73426	7.564006	6.279977	13.6553	5.200057	21.07339	17.9492	11.78676
CUST_24553_PI429010886	N50830	0.00048378	24	21						12.7417	16.06184	10.52106	13.99736	13.62435	5.35265	6.455238	8.083025	5.33569	NA	5.912371	12.50576	9.648862	10.23432	10.78353	11.80456	8.930779	14.96491	15.65667	13.78304	8.1939	20.76141	13.16217	16.77585	12.42262	8.455242	14.59197
CUST_2862_PI429010886	N13267	0.000485517	24	0	321465151	hypothetical protein DAPPUDRA	Protein metabolism	Ribosome biogenesis	2.76E-74	34.55816	51.21191	64.57877	42.73341	50.91261	34.05393	39.92572	35.5246	34.28012	40.55055	22.03549	47.57381	15.3494	5.281798	17.21562	22.74972	26.0838	41.17764	21.18591	44.79451	47.39234	53.50459	47.9205	32.07806	46.8318	34.36788	48.47854
CUST_38120_PI429010886	M23440	0.000485593	24	12						272.3474	388.566	262.04	1187.041	518.6591	702.7679	1921.473	671.0624	2271.439	399.0879	2904.283	202.9059	3349.165	3127.267	560.3033	655.1561	1901.421	869.5599	523.9454	400.5287	672.9412	198.9444	1048.117	1115.136	200.3302	161.5503	256.539
CUST_39273_PI429010886	M37072	0.000485593	24	12						140.1401	165.8549	134.7253	684.8522	218.1148	304.7866	1251.736	301.0722	1125.5	201.4995	1742.558	98.68778	1712.689	1601.274	302.7771	229.3211	975.7238	413.2217	208.404	200.4453	243.9147	98.83232	505.6819	505.369	113.187	90.04305	131.6618
CUST_3946_PI429010886	N15629	0.000486849	24	21						63.40282	61.99234	53.83595	49.75454	43.42575	39.8332	35.89734	48.23305	33.4664	51.77472	34.33958	35.73797	45.59864	54.23194	41.51987	48.38472	30.58395	38.64876	78.26455	40.28969	65.23465	54.33723	72.17918	65.3616	58.73466	43.08599	56.39523
CUST_41426_PI429010886	M57702	0.000490466	18	0						25.37685	93.77335	86.28411	45.49999	103.3596	70.71767	4.832163	NA	10.98917	6.872635	70.56233	23.09193	14.1426	49.30833	47.42663	51.11657	58.78776	46.91122	NA	117.8448	81.07922	25.69105	71.73966	59.01526	43.90368	6.552316	39.93436
CUST_4813_PI429010886	N17531	0.000492365	12	0						57.7126	68.46482	44.1311	55.29943	63.69892	54.2724	23.71956	35.33768	24.45128	57.06084	87.33081	66.6173	69.89861	44.26795	86.30982	54.17106	56.9001	29.67957	51.92761	47.33564	44.16317	59.35684	56.00071	54.54964	125.9947	77.6465	103.2365
CUST_3919_PI429010886	N15567	0.00049254	15	0						92.36671	98.7449	56.38348	135.7428	25.14815	98.00625	16.37588	34.2605	31.65828	52.40731	61.33441	31.69335	47.6242	41.79513	42.28696	79.84725	51.18195	58.78776	45.57229	48.32075	47.23775	29.07923	12.47186	21.81393	19.57916	62.66376	63.56244
CUST_17818_PI429010886	N38644	0.00049254	15	6	321469866	hypothetical protein DAPPUDRA			0.000000265	770.3832	909.1828	1135.35	2698.998	1552.466	198.9444	4976.553	2962.88	6710.162	827.5626	2371.181	434.3661	4173.107	2012.206	709.0094	185.1417	608.2737	263.9286	6430.973	3755.671	2846.594	2956.694	4710.772	3222.8	3024.414	977.1077	3349.165
CUST_26240_PI429010886	N53900	0.00049254	15	9						40.66228	42.66175	48.60251	27.90588	38.59195	29.96081	38.15601	41.3426	25.81956	57.33715	49.75986	43.48958	50.86361	35.07936	49.92214	38.94229	37.66871	43.75819	43.27654	29.60122	37.05862	35.92153	47.46533	56.11745	61.78991	44.3395	54.20497
CUST_31867_PI429010886	M2616	0.000493767	12	9						132.0703	107.509	139.1998	87.01734	127.6164	119.5167	100.8435	111.1388	170.5897	179.2719	119.3614	159.5875	158.518	123.9057	161.9124	101.7015	122.9221	92.83981	149.4086	122.3002	126.9762	175.3405	196.4999	223.0844	145.9321	94.93084	154.4146
CUST_44267_PI429010886	M65450	0.000495808	21	0						27.11917	25.92708	24.8172	13.88039	17.55051	13.24014	16.79206	8.590245	14.49179	16.3934	11.27021	30.865	15.84702	12.44774	17.50958	14.83032	15.70113	15.48991	21.57883	22.16839	31.05988	40.90141	21.04763	32.97277	22.44464	20.28215	23.53208
CUST_39511_PI429010886	M40667	0.000497264	24	15						212.6978	112.5697	122.771	55.63371	30.52953	57.25934	311.8576	93.22902	305.1178	481.0599	347.2002	67.22535	119.329	344.4073	54.05215	947.0352	626.9839	505.6819	276.8728	356.3573	616.5627	160.2766	9.794238	103.6896	508.9078	72.02191	376.7778
CUST_23656_PI429010886	N49213	0.000497708	21	18						53.23808	53.52577	59.77969	50.43341	44.06952	29.01519	37.08249	46.23157	45.50498	38.82052	46.80566	49.39632	36.36056	44.9365	42.69638	53.52577	44.98933	49.62618	63.81233	62.73163	72.73375	74.18037	61.19316	38.30361	51.94695	38.48209	53.40427
CUST_31718_PI429010886	M2239	0.00049809	12	6	291239931	PREDICTED: GK11735-like [Sacc			1.65E-13	216.5705	452.142	338.5986	347.5429	312.1009	360.4299	411.2361	471.8286	483.6608	516.2255	284.7236	465.4767	256.573	267.4193	291.6226	342.6821	507.231	437.9976	489.2868	405.8942	489.2868	349.8278	344.4073	302.7771	248.7029	192.1306	404.3968
CUST_53916_PI429010886	M103700	0.000498262	18	0						107.0354	80.41026	84.16166	84.35052	54.90137	58.70057	80.24156	61.43496	78.16561	49.54311	39.14896	40.33676	72.4473	70.17999	59.38272	58.24276	67.44192	63.9762	82.47763	83.29367	64.73297	80.74224	56.37975	79.5268	61.13851	79.05137	73.15055
CUST_57101_PI429010886	E874	0.000499003	24	21	110456474	Ac1147-like protein, partial [Dia			1.82E-16	194869.8	190951.6	182841.9	185304.1	189760.3	181822.5	190951.6	178249	169275.7	172120.5	167901	161853.1	142420.7	175227.8	146987	203126.6	161853.1	196812	189760.3	180368.9	182841.9	189760.3	185304.1	208728	193307.4	180368.9	191967.5
CUST_14271_PI429010886	N32608	0.000499003	21	15						14.94152	19.20499	19.37143	7.431224	11.60747	32.82904	15.86706	17.27868	13.81559	12.32971	12.83603	30.45492	24.01242	24.53712	31.06592	25.12027	33.40515	24.66557	11.61798	23.58164	23.44477	21.21056	15.88417	21.47115	7.86169	13.49255	12.83829
CUST_54794_PI429010886	M107706	0.000502163	24	3						2200.686	1422.675	1130.969	2228.732	1500.89	2021.719	1663.655	1871.7	1696.829	1647.856	749.3538	1965.787	1504.447	1210.563	1315.424	1142.458	434.8192	1095.073	869.9307	1244.137	502.5054	1339.398	449.8325	1313.269	855.5436	1691.116	535.4815
CUST_41563_PI429010886	M58041	0.000502163	24	21	260833120	hypothetical protein BRAFLDRA			2.86E-24	26.18151	30.18014	39.03604	17.68234	28.24507	14.23378	19.40349	22.34874	20.73429	14.59502	31.23427	28.22616	26.35385	22.80532	27.39507	24.73755	30.63129	38.81639	30.5034	29.65959	37.25229	36.83272	43.89958	36.53804	26.50208	27.96875	45.21325
CUST_18545_PI429010886	N39987	0.000502163	24	21						30.29561	36.69203	30.28156	20.59845	29.02823	25.02499	19.49414	30.2752	31.4461	8.037102	39.36134	27.67843	22.32831	31.92535	33.17225	34.66403	33.51959	40.95208	35.55192	35.65021	46.35195	35.45915	35.78636	46.57555	42.6519	34.25718	44.52224
CUST_36767_PI429010886	M16796	0.000503348	15	9						26.34323	28.88222	NA	78.20138	232.6083	4.893177	67.07909	18.99164	56.56581	NA	631.8697	347.6198	25.71961	452.4209	554.0705	16.14862	12.47033	14.50203	37.9908	14.21628	38.59753	186.1647	266.8703	89.2783	117.6349	219.7679	267.7311
CUST_21173_PI429010886	N44764	0.000503681	21	0						42.62368	54.41132	44.00986	43.56248	43.96327	30.15331	33.84542	40.80027	37.14266	33.79296	47.71964	37.45153	43.65204	32.32834	37.84952	23.80897	24.88406	35.45592	38.11869	43.18058	39.38571	73.9144	47.61903	49.29631	46.39912	35.8443	37.47838
CUST_32302_PI429010886	M3745	0.000503803	24	0						14015.31	15297.04	12886.71	15102.78	8016.887	7704.294	12097.54	10966.45	11780.77	11738.35	5258.496	11503.97	8362.147	5467.822	6439.853	9425.197	13135.1	11228.98	10641.47	8899.45	8430.296	9392.481	14071.14	9444.83	11588.48	14256.4	13635.69
CUST_14408_PI429010886	N32834	0.000503815	12	0						28.67614	13.67194	21.00454	24.89149	18.27355	11.48082	16.78485	7.257747	7.273639	18.43758	17.27642	19.37652	25.18191	14.35144	21.43051	21.06519	16.0253	19.90073	11.93224	15.83716	10.46956	13.83903	13.1424	19.55672	25.49661	18.56346	26.34323
CUST_13571_PI429010886	N31397	0.000505207	24	15						104.4781	236.6326	157.1785	144.1169	159.0794	108.8028	116.4725	169.9945	185.3668	215.8727	253.1919	180.5627	226.5718	214.693	220.9943	243.543	275.5775	192.2662	155.3531	228.1062	225.4359	146.4666	204.6434	205.7884	151.7575	164.741	181.4589
CUST_44115_PI429010886	M64986	0.00050664	24	18						NA	7.462458	NA	4.621629	3.641476	3.148261	5.019429	5.642491	8.602712	8.817048	8.602712	NA	13.43624	NA	7.793073	8.580119	9.558194	6.621148	24.60171	8.934291	25.64786	8.748029	10.72798	4.682234	23.11514	6.206844	17.66478
CUST_48282_PI429010886	M79652	0.00050877	24	21	242009950	hypothetical protein Phum_PHU			5.15E-31	66.39483	73.60215	85.38787	50.34979	72.20099	78.34836	47.65574	45.24265	68.02537	62.338	65.15059	64.64642	96.05666	68.34584	61.57198	66.87814	97.85715	65.04377	84.09371	67.10107	73.69276	89.7803	77.57976	91.85221	74.03602	81.4582	83.64481
CUST_6830_PI429010886	N20654	0.00050877	24	18						21.16225	23.33045	31.39494	18.14187	10.45084	9.973024	23.71399	19.85412	28.49433	23.46744	15.0452	19.57916	34.09348	16.43173	18.86919	28.80996	19.96887	23.86386	33.48437	30.01509	36.41114	31.27903	32.01377	24.42377	30.64441	24.85202	30.67349
CUST_10941_PI429010886	N27026	0.00050877	24	18						23.61884	41.75149	37.07514	27.6893	33.35662	24.68095	62.00499	34.83345	48.54356	36.45739	41.26235	35.31786	57.76704	33.32412	45.49065	50.0937	58.17376	45.60384	53.55568	50.44651	63.18725	35.63404	43.61117	46.37409	42.50386	27.09821	46.74583
CUST_31816_PI429010886	M2478	0.000511606	21	3						115.5105	92.82706	81.61482	173.6159	127.2771	139.866	57.27829	71.88037	56.55385	79.30248	94.62002	46.78844	83.08821	33.82765	53.42717	82.53807	95.2367	36.06722	283.4621	78.84424	190.613	104.7345	127.1629	121.56	191.0167	148.298	178.5273
CUST_45737_PI429010886	M70099	0.000514642	24	15						125.2132	258.2061	192.5776	146.6816	92.80135	50.45617	211.2323	98.097	188.8287	187.773	183.2355	96.51035	220.2833	141.3689	125.9134	252.989	234.5732	331.8463	240.84	198.5315	244.0026	235.1738	191.6851	242.2514	188.4419	135.0936	233.0433
CUST_30986_PI429010886	M383	0.000515378	24	21	91078850	PREDICTED: similar to tan CG12	Metabolic process	Other	1.14E-13	474.0818	555.7599	582.785	480.2531	445.1432	394.1154	304.0274	379.037	438.485	482.9421	589.8797	279.2412	480.3553	446.5017	494.8296	545.1995	667.0187	602.4086	519.1671	516.7278	461.4101	672.7456	535.89	674.5328	746.9967	625.4995	881.8202
CUST_32612_PI429010886	M4573	0.000515378	24	21	321455420	hypothetical protein DAPPUDRA	Metabolic process	Carbohydrate metabolic process	1.42E-86	1365.442	1321.627	1507.382	1148.933	718.7132	767.3697	752.6445	814.3635	657.3507	1328.27	887.7791	962.3475	1940.134	791.3056	1508.513	1384.734	2100.692	1221.709	1844.624	1170.327	1346.991	2097.341	1406.649	1612.928	2793.635	1212.026	2294.993
CUST_12921_PI429010886	N30276	0.000515378	24	18						41.99548	57.26697	44.07411	25.08941	19.30617	19.67938	34.79855	54.81901	39.34641	44.73116	28.14985	21.82795	52.96757	31.90012	32.45355	49.75454	36.56941	38.92916	47.37075	72.66933	68.99631	46.44642	61.08411	42.29849	36.23977	44.65936	57.3502
CUST_33321_PI429010886	M6590	0.000517254	18	3	156550289	PREDICTED: ileal sodium/bile ac	Transport	Ion transport	9.59E-33	30.89131	31.34171	32.21229	40.82537	58.7415	35.82514	38.93757	26.84286	31.08176	17.90502	46.41316	52.62861	13.90922	24.88953	30.95428	37.67803	29.09733	29.96081	39.35783	32.96923	35.66495	48.95167	42.62886	48.56063	45.31246	33.6128	41.19213
CUST_36821_PI429010886	M16951	0.000518131	15	0						13.38505	NA	NA	6.619984	14.23378	14.96903	13.6668	5.985076	NA	4.716104	5.385232	4.320866	7.414538	NA	9.75683	27.95071	22.08254	13.57539	9.843601	11.78536	8.177364	4.062742	9.982867	NA	7.632214	9.298932	4.202379
CUST_53093_PI429010886	M100122	0.000518915	21	0	390345459	PREDICTED: zinc finger protein 8			9.09E-29	30.75945	32.36608	35.3123	25.53483	24.00156	25.00095	27.45637	26.0838	34.37539	21.59473	18.11193	17.68505	24.20903	15.47146	3.689386	17.78857	25.44757	23.89085	26.41027	35.00954	17.29812	37.61646	28.52646	25.1173	35.4467	24.09713	22.9969
CUST_53503_PI429010886	M102015	0.000519318	12	0	152949487	calcification associated soluble r			9.59E-10	50.87686	28.09462	56.71975	36.30353	21.11455	28.08693	16.03181	17.24019	20.17168	33.71063	36.11568	29.88246	36.10701	32.03169	33.87813	10.86736	15.86334	11.7375	23.10443	31.32237	20.16039	50.06206	52.0131	26.13567	48.64263	40.86304	48.39409
CUST_31636_PI429010886	M2029	0.000519318	12	9	321463221	putative glycogen synthase [Dap	Metabolic process	Generation of precursor metabolites ar	2.54E-80	125.4485	141.9587	175.9736	175.4084	123.4753	167.8222	113.878	146.2889	149.3159	173.9043	190.3661	218.2236	136.0065	78.21211	102.2621	109.4096	137.7031	151.8112	178.033	181.7225	208.5809	237.5415	197.6371	245.2781	100.8009	147.5789	164.2936
CUST_57261_PI429010886	E3119	0.000519532	21	0	242015079	RAB6-interacting protein, putati			3.66E-17	50.55672	44.17652	48.99934	33.5727	24.69162	30.07738	31.55692	34.28288	33.12003	37.96647	18.8882	26.61351	51.20424	17.8457	29.03394	43.76293	34.10624	38.04316	63.54017	43.79783	42.75542	57.11305	36.33638	46.68118	46.50411	35.66159	41.80345
CUST_42756_PI429010886	M61169	0.000519594	12	3						18.48914	15.44846	NA	160.4236	1232.079	1363.035	20.07184	57.40907	42.05841	12.13203	11.79177	6.632628	46.68118	21.79685	50.6873	236.4795	237.0529	26.61351	16.06042	4.26876	17.93943	7.181893	122.754	NA	96.58233	9.435046	86.45703
CUST_24424_PI429010886	N50598	0.000521985	24	18						13.96378	24.9499	23.86386	4.240642	12.5832	8.979767	19.99489	18.77458	22.30972	10.374	13.07084	14.73953	25.32145	17.47501	18.46268	16.87953	19.54026	31.85963	31.50642	27.55963	32.48271	15.02551	26.3515	18.82499	31.07275	18.69988	27.1479
CUST_51577_PI429010886	M94057	0.000523292	24	0						88.78315	112.2837	108.7083	104.8028	105.8805	96.54391	71.64925	76.40541	89.50979	77.6465	132.6815	82.28293	82.137	86.46711	93.76286	82.71729	93.15792	91.16754	103.7192	96.87922	88.0075	149.047	99.82243	100.2134	174.2606	141.8167	140.8381
CUST_56202_PI429010886	M113092	0.000525195	18	6						6.281077	10.85018	7.513141	12.69375	10.30824	13.97597	11.5008	16.63618	14.18007	5.619603	15.83532	11.87593	16.63419	9.045745	13.95828	3.747707	10.79559	6.542048	11.22739	10.02667	11.10457	13.93814	16.23086	15.68303	20.41963	15.16581	13.77957
CUST_1544_PI429010886	N10005	0.000525195	18	3	121484199	cuticle protein CUT8 [Portunus p			3.01E-38	3053.693	104.5613	4061.362	5407.94	6818.249	6101.131	182.3885	753.373	484.5238	2173.027	9759.347	2498.654	2646.596	4539.587	3929.52	65.76047	95.74045	56.79852	957.7854	1083.848	1193.156	4733.525	6592.219	6132.817	1240.074	4450.275	3224.848
CUST_52593_PI429010886	M98110	0.000528592	24	3						26.39512	31.673	35.12339	40.39391	40.25595	38.61632	28.12879	27.19756	25.19327	22.9969	23.76937	24.46439	23.12555	37.82047	22.02956	31.36138	26.03303	20.66717	26.51031	21.88581	20.37769	25.15651	22.61447	18.73801	24.10212	26.41027	30.20493
CUST_1235_PI429010886	N8949	0.000534799	21	6						584.6183	573.9026	492.5086	511.8036	709.6774	690.7831	643.379	737.8151	832.6018	357.2181	362.0388	321.2079	387.6202	346.8165	334.5246	452.5351	385.6128	449.8325	439.7541	819.3739	586.0404	534.9189	367.2975	360.4299	602.7354	600.0613	732.552
CUST_36376_PI429010886	M15752	0.0005352	24	21						132.0358	168.2637	194.2319	154.5458	183.2355	129.7798	135.3048	142.749	141.8167	159.7027	171.8137	160.6506	161.4921	148.6897	144.1303	152.6411	184.2288	162.3101	202.1456	158.6164	190.3933	171.7541	201.8882	203.1325	190.0902	169.022	227.9494
CUST_31863_PI429010886	M2602	0.0005352	24	18						59.72832	42.38319	83.77516	56.29065	14.57595	22.81234	127.9261	217.7022	180.1324	62.82646	32.17634	47.10201	542.5585	252.884	215.2207	159.8296	130.1961	231.1683	370.0497	975.2848	1245.448	197.3334	16.18229	149.3381	646.8706	132.7509	359.174
CUST_9954_PI429010886	N25463	0.000535473	21	0						272.7395	206.8979	290.511	205.95	198.1577	188.8287	153.2663	160.9374	170.7219	196.6443	235.0418	235.738	257.0403	216.4424	239.7853	162.7885	228.8193	190.0165	229.6141	204.9134	221.8149	265.9831	269.707	304.7058	222.3734	191.9057	269.1635
CUST_44690_PI429010886	M66681	0.000544477	24	15						74.66025	91.02301	55.99408	63.0922	60.30614	63.57042	69.65321	70.25686	63.53279	104.3062	62.41935	63.10394	113.9393	80.52797	91.76448	120.6508	92.61293	113.4137	115.1005	71.1642	109.1085	89.39537	78.65231	96.99292	98.64233	79.82778	90.48608
CUST_18896_PI429010886	N40609	0.000544537	21	15	478252715	hypothetical protein YQE_10251	Protein metabolism	Regulation of translational initiation	9.87E-39	73.29648	138.8001	101.0861	57.99775	31.18062	89.3199	69.28139	78.81902	84.58847	93.56602	91.54439	61.52728	104.1579	84.02947	106.3489	119.6854	101.3206	105.8028	94.94344	74.89382	88.09968	111.6232	72.22265	102.7497	79.66914	62.02618	57.73811
CUST_36956_PI429010886	M17313	0.0005454	12	3						11.00269	10.89115	NA	28.21525	NA	20.43703	28.43678	30.7975	18.49709	11.55337	12.07314	4.792589	19.84259	51.55397	15.4017	61.72867	38.4508	37.03185	36.6656	10.15066	24.47097	5.94666	16.58591	14.31897	19.22794	24.02389	NA
CUST_5899_PI429010886	N19348	0.000548415	24	18			Cellular Process	Cell death		400.2135	1405.334	760.24	575.4805	508.7574	328.9434	676.869	836.1158	1426.211	1178.539	660.1893	834.7743	1271.126	896.2629	801.9356	1282.861	1510.508	1373.039	1804.854	1212.445	1772.069	1024.829	1353.691	1149.936	1893.112	1197.753	1760.199
CUST_38159_PI429010886	M23769	0.000551316	24	18						488.2191	2343.924	2424.1	254.3715	443.0968	541.4917	3321.698	2489.305	1923.884	250.0724	331.2753	39.28264	5420.715	1443.341	3552.655	1979.658	4077.152	5227.74	8945.499	12084.47	8857.18	704.9081	1040.528	687.4827	1288.168	821.966	761.2797
CUST_23533_PI429010886	N48992	0.000551316	24	18						397.5976	2348.232	2237.142	242.9871	420.9575	419.5373	2558.16	2350.317	1609.728	227.8105	338.3225	42.30272	5166.879	1276.929	3222.8	2041.036	4031.99	4767.094	8334.379	11413.74	8189.692	638.4715	975.2848	797.1924	1180.964	814.573	697.5829
CUST_44268_PI429010886	M65454	0.000551935	24	15						16.59434	12.37254	17.33723	7.991764	16.0188	13.44284	18.5449	40.44814	16.78653	19.5015	15.16769	17.78857	21.96464	26.62997	21.47745	33.2543	27.69944	38.34001	17.43685	10.5374	19.73758	21.26556	14.53482	19.39937	16.67805	13.53805	26.14108
CUST_48920_PI429010886	M82928	0.000551935	24	15						18.82499	11.06998	16.84945	10.97116	10.2893	7.590267	14.50367	13.89586	17.01954	20.93117	10.38364	22.83159	26.90034	13.67002	18.33509	22.2941	22.68184	23.90042	16.2053	17.01732	26.4274	12.75342	20.60634	15.2486	6.834174	17.26262	18.68212
CUST_52460_PI429010886	M97511	0.000551935	24	21						54.44704	109.8412	86.19309	105.0177	61.03293	39.6267	86.42647	102.411	175.7896	52.5487	91.7922	23.64777	122.5728	52.27625	80.96563	120.5967	110.5322	100.2009	323.6649	119.4473	192.3094	284.3354	257.8891	257.1925	297.575	102.4622	215.4758
CUST_7966_PI429010886	N22345	0.000551935	24	18	410901921	PREDICTED: RPA-interacting pro			7.19E-28	83.82183	144.495	139.9464	101.8399	127.579	96.13685	85.18385	112.096	103.6461	122.51	124.4273	91.14348	120.5552	120.4953	136.2532	113.878	167.5398	136.4616	130.2882	154.8276	155.1864	120.8275	147.8425	141.1475	130.7958	119.3614	136.9272
CUST_9016_PI429010886	N23938	0.000551935	24	9						24.65528	14.48424	18.08089	16.21895	26.39512	24.08402	28.60128	15.23242	24.15387	28.52375	27.28807	49.48225	24.07128	23.84965	24.95132	24.5682	18.37247	26.63911	20.11731	7.99563	19.5625	17.73313	3.85052	23.31389	16.0527	21.01206	23.87984
CUST_741_PI429010886	N7085	0.000551935	24	21	219815997	crustacyanin-A2 precursor [Pan	Transport	Other	2.75E-10	88.96871	102.2469	160.766	159.1067	148.7099	13.63697	82.1818	91.17437	132.6428	58.72446	157.1785	41.04967	204.4319	21.28674	191.5119	29.11025	304.1662	19.70573	882.2277	345.4356	305.5103	985.4686	553.926	721.4643	348.8785	116.5916	232.64
CUST_47167_PI429010886	M75021	0.00055209	24	15						116.804	207.8778	239.7853	105.7619	92.32672	93.14583	187.3336	167.6498	199.2156	162.8199	105.242	88.08839	180.0167	179.5567	98.86808	209.5934	414.9089	252.9258	262.2441	180.9657	216.3545	213.8259	204.5441	171.3909	175.4908	190.8547	160.2766
CUST_40609_PI429010886	M55622	0.000557176	21	12						72.20641	94.60841	101.5332	83.3741	67.20028	71.29445	88.13366	111.2006	107.1698	86.61603	109.6887	85.4597	113.1524	162.4126	132.3608	63.63996	142.5195	117.6965	59.15199	67.35191	54.15073	54.99013	77.19972	84.61891	68.4557	72.6054	72.58638
CUST_29558_PI429010886	N60101	0.000557498	24	21	344297999	PREDICTED: zinc finger protein 2			4.5E-15	59.34282	83.11893	97.93073	59.6688	52.57215	42.75542	71.87251	64.7406	82.82852	50.89474	78.9339	38.13279	63.26798	47.2724	68.28737	64.7701	48.80246	70.91565	96.71212	84.9077	111.1891	102.1108	86.18133	89.48954	85.22534	85.785	94.34203
CUST_55277_PI429010886	M109414	0.000559394	24	6						47.61497	46.34771	58.35105	41.33969	49.75986	40.23682	68.19401	51.95311	55.67726	39.74893	61.48782	75.12194	52.63501	42.59404	38.293	40.92496	40.04635	54.81291	32.07806	36.10701	34.17398	38.293	35.93556	37.13937	30.90807	52.3424	48.39798
CUST_1743_PI429010886	N10586	0.000559394	24	18						256.4943	241.54	375.5874	168.0227	202.5815	223.6663	167.4488	162.8199	149.3774	165.5949	238.4366	226.7528	221.3576	166.6038	235.9311	138.6664	164.1609	191.4824	450.2424	354.4742	345.2692	274.4544	298.0705	248.3542	334.0615	240.5209	338.8265
CUST_44472_PI429010886	M66082	0.000561629	24	21						112.3135	133.7558	137.8214	139.1591	140.9228	131.1212	116.8467	111.6419	110.3077	157.9625	145.3783	163.3644	176.8339	169.7003	170.8814	159.2709	178.447	154.2424	149.8147	157.3439	171.1773	208.0191	189.0814	225.6648	228.1333	145.8127	208.4776
CUST_1554_PI429010886	N10049	0.000561629	24	21						1095.073	848.0782	1386.294	1091.584	698.508	828.8502	684.4289	668.1223	1189.941	1849.733	878.1427	1142.458	1510.508	702.7679	1001.191	1035.674	811.3473	1497.856	1791.446	1188.558	1699.905	2374.203	1667.986	2361.017	1361.853	1199.116	2369.531
CUST_24032_PI429010886	N49905	0.000565217	18	15						172.0707	209.031	134.2942	203.8465	122.3346	148.1737	395.1137	214.9733	231.4591	314.1834	143.7853	291.6226	270.3263	162.9278	189.1836	405.5854	399.9663	365.8262	192.6657	168.511	250.9574	143.7613	135.3048	157.1328	144.0984	139.1998	210.4634
CUST_10022_PI429010886	N25575	0.00056535	24	0						42.78996	37.70404	53.72874	37.59004	21.31454	25.90137	45.69732	36.07467	43.43371	41.79513	34.12139	42.07726	32.37762	24.20903	23.61619	34.33958	28.46437	34.48395	38.87958	34.36788	28.84319	61.21971	60.65875	38.40048	50.46073	45.5523	50.99975
CUST_14533_PI429010886	N33033	0.000565595	18	15						39.71647	61.55774	27.27615	40.63802	18.53553	32.54688	70.80726	63.96236	35.27964	66.97909	41.28281	35.12021	39.78094	69.73149	60.39565	155.4522	105.9997	104.6237	49.78973	37.4401	45.142	35.00954	47.54634	31.50316	61.85717	55.53179	36.09076
CUST_47165_PI429010886	M75017	0.000566852	24	15						116.9098	139.9842	140.1401	74.31745	82.1985	60.79074	77.79664	131.1766	104.3299	106.2528	108.9605	115.0343	115.0551	116.2357	152.3312	159.203	264.6763	186.848	152.6002	121.4525	115.908	207.1791	124.4898	165.8977	180.1324	130.6083	176.4771
CUST_29200_PI429010886	N59381	0.000569453	24	3						25.1528	21.59198	12.71903	24.74687	53.80944	42.73834	21.63236	39.75608	28.80996	NA	30.43834	24.68878	16.06184	27.65783	18.75881	23.8882	16.56735	10.37766	7.930223	21.5284	3.884984	NA	22.20311	3.372963	4.831368	6.911194	NA
CUST_15967_PI429010886	N35426	0.0005727	24	12						751.8458	129.3882	221.6228	1149.369	243.8189	979.3611	211.1406	129.0645	199.588	253.9457	256.2719	638.3206	256.46	937.1039	844.4833	127.1629	174.2606	134.0991	71.37579	167.3313	116.2567	76.28125	67.13611	117.0265	145.0599	130.8473	102.2878
CUST_31505_PI429010886	M1685	0.000573202	24	21	443725219	hypothetical protein CAPTEDRAI			9E-39	26.1001	32.80252	38.48962	29.76725	24.51729	32.1177	26.56161	31.09684	31.19127	24.49039	29.74537	25.70782	27.92224	25.0443	25.13614	33.59889	41.66058	49.16853	47.66434	29.0216	33.762	49.8658	31.17381	39.95843	33.95508	34.86478	33.56909
CUST_11340_PI429010886	N27650	0.000574844	24	18						543.5625	1168.934	1020.798	795.25	554.0705	248.8552	947.6406	1992.843	2602.477	1528.323	717.3191	620.2559	1985.342	913.8824	1103.344	1390.116	1669.427	1794.235	2844.645	2491.674	4153.924	1947.583	1608.753	2285.617	1988.355	1189.292	3101.358
CUST_31189_PI429010886	M931	0.000579062	18	15	60652230	protease [Homarus americanus]	Protein metabolism	Proteolysis	1.11E-24	1085.159	1058.187	972.4265	781.7261	753.6723	617.7634	974.9689	1096.072	1033.091	792.9194	1158.095	1432.11	633.4883	1230.097	853.7719	1131.397	1374.54	1266.211	951.856	1143.807	677.0488	623.6004	845.2107	866.5613	961.8532	959.7319	868.2894
CUST_43126_PI429010886	M62125	0.000579339	18	6	240979808	phosphorylase kinase gamma su	Protein metabolism	Cellular protein modification process	5.49E-72	5.026423	14.30523	4.473118	NA	NA	6.813064	9.472484	9.105368	7.935999	5.52012	5.624991	7.441018	NA	5.57486	4.93815	3.888277	6.072592	7.215408	4.050994	7.398978	7.879556	NA	8.013913	8.340014	8.744802	13.8182	9.027928

Probe ID	ID	p-value	Period	Phase	EBI ID	Annotation	GO category	Go term	E-value	0	0	0	3	3	3	6	6	6	9	9	9	12	12	12	15	15	15	18	18	18	21	21	21	24	24	24
CUST_41906_PI429010886	M58852	0.000581	24	18						75.81657	122.6496	171.2731	81.56078	72.36145	23.45708	127.0615	105.3002	210.6647	66.70374	25.22785	61.74127	109.9078	59.51158	47.2578	78.15836	70.28135	96.65486	210.4014	343.5695	287.3401	154.4146	181.5978	122.4755	83.15856	114.0296	161.2674
CUST_46803_PI429010886	M73771	0.000581777	24	15						100.4907	109.0197	91.73279	72.15825	62.04596	54.6004	140.6454	104.8945	115.7057	124.3455	94.70581	99.31589	142.1426	105.0583	137.0649	155.8635	111.3418	170.8146	124.1301	84.62659	138.1387	114.0114	87.29007	130.6255	129.5211	89.62874	105.5565
CUST_14195_PI429010886	N32467	0.000581777	24	15						270.0299	109.0861	219.2811	59.45846	37.54916	31.80555	157.6398	330.1913	95.37708	760.7688	490.3414	85.90563	145.6782	892.4292	25.55069	1113.954	638.835	1743.671	305.2389	363.1656	495.5669	489.8482	25.75751	374.7006	627.1961	70.7612	416.0009
CUST_33398_PI429010886	M6789	0.000582884	21	15	383858866	PREDICTED: LOW QUALITY PROT			2.24E-27	22.74972	28.81777	17.20227	12.32463	16.65157	12.13039	19.96887	20.11563	16.70652	21.47745	26.58505	17.39282	19.01591	17.38353	15.86113	26.12451	72.19272	25.58133	21.53519	19.22058	21.27915	20.24228	15.66009	30.35985	15.07768	18.00851	15.9251
CUST_39982_PI429010886	M47801	0.000585289	12	9						15.20248	46.57945	26.94025	13.15696	18.96294	15.93269	21.29702	42.07458	39.44669	49.36855	25.86911	56.48171	15.2772	23.30899	10.52106	8.097597	14.23642	29.91504	6.147251	23.5426	16.71139	50.83559	34.02915	60.55423	22.44752	32.62087	17.42012
CUST_7191_PI429010886	N21174	0.00058579	18	0	225711953	Lepeophtheirus salmonis Pacific			1E-105	20.75814	28.08693	23.42467	16.23086	16.94573	11.38131	8.210092	14.55087	10.72225	19.48764	8.120127	NA	9.226214	13.05124	4.005676	11.55982	26.86667	19.43519	12.60565	28.14237	19.6314	17.78243	16.28228	4.357137	10.55091	5.48672	8.026216
CUST_54366_PI429010886	M105738	0.000587317	24	18						6.084151	9.562348	6.170982	4.836209	4.922133	5.995784	10.06086	14.75102	5.603167	5.290133	7.98476	7.095312	8.020864	6.472863	6.943759	8.595293	17.04641	10.68893	12.72466	8.578942	10.19561	11.44329	12.28481	16.67961	5.855488	7.673574	7.295483
CUST_2719_PI429010886	N12975	0.000587317	21	15						237.1976	385.0272	266.3181	195.2227	156.9719	166.3995	305.1744	274.6135	277.3623	408.3007	262.1593	271.8944	263.0087	208.2391	242.9432	348.1933	395.6891	378.5271	317.7216	284.9823	313.1767	256.6162	205.536	274.7035	265.6845	250.621	217.9731
CUST_37809_PI429010886	M21707	0.000589228	24	3	149675211	cuticle proprotein proCP5.2 [Cal			1.51E-39	14764.65	239.4831	16580.62	17487.6	21459.8	23231.4	341.5157	1335.17	894.0902	10727.23	26191.89	16655.53	5666.924	13419.52	9565.255	93.29743	308.6292	139.3563	526.2718	1154.406	641.9649	10949.85	6470.198	9791.81	666.6396	3879.479	2618.303
CUST_45656_PI429010886	M69832	0.000589228	24	15	170033026	conserved hypothetical protein	Transport	Ion transport	5.87E-09	1363.035	1447.238	1171.1	1196.962	1293.42	1155.769	1348.208	1651.623	1412.179	1474.205	1155.769	1245.448	1489.353	1265.639	1490.226	2179.188	2201.915	2297.179	1388.047	1541.174	1798.348	800.6803	1570.104	1324.412	1302.008	1036.601	1499.429
CUST_4476_PI429010886	N16849	0.000589228	24	15						43.50595	43.38791	63.28914	36.46097	24.52363	40.34223	40.99664	46.72845	50.01635	52.06823	28.62359	44.52658	34.46101	45.81369	43.65204	68.60832	88.0075	71.50913	69.37968	53.56818	74.79444	43.73128	57.14912	66.13826	66.82276	64.23608	62.88893
CUST_23957_PI429010886	N49767	0.00059003	18	15						32.79575	34.39364	NA	24.52594	6.756203	24.4665	51.97249	41.4895	16.16198	34.20262	30.58943	11.27508	51.91074	34.03869	40.10788	97.1413	62.62916	51.7501	32.79575	NA	45.5838	12.28923	21.9708	4.123145	53.60597	39.64997	NA
CUST_38633_PI429010886	M28704	0.000592528	18	15	152949487	calcification associated soluble r			1.8E-19	29725.44	51616.89	23575.84	23084.2	12344.85	14885.24	28120.27	27210.5	22037.75	28492.44	24289.54	25555.67	26299.14	21758.66	19679.49	45964.24	40757.76	40024.21	25611.3	26891.88	18146.2	20563.31	20822.29	17254.51	32295.88	21881.51	24136.69
CUST_42394_PI429010886	M60173	0.000592528	18	15						122.0546	137.7837	184.1725	116.0759	104.2173	114.9878	150.6257	136.3513	158.224	170.4812	115.8791	123.5457	148.131	143.4863	110.7454	192.0736	205.7884	171.3126	159.1067	120.0493	138.0213	133.6073	120.8147	107.7706	158.7682	139.0644	138.7083
CUST_11019_PI429010886	N27135	0.000593146	21	0	299782457	kinesin 2B [Nasonia vitripennis]	Cellular component orga	Cytoskeleton organization	4.87E-30	29.55798	16.25789	15.72693	NA	7.57781	12.47302	NA	NA	NA	13.6951	NA	9.036413	8.176241	3.342467	4.034525	3.306786	6.001367	5.013117	20.95678	5.773049	7.039214	14.48348	15.63819	NA	18.50842	7.519886	NA
CUST_44967_PI429010886	M67556	0.000593418	12	9						23.63327	24.1706	29.17375	7.461548	17.79852	8.593041	9.24062	20.58996	38.25885	41.28987	31.97992	40.75473	NA	14.9705	NA	NA	NA	NA	15.28613	NA	16.58793	25.7544	51.27142	64.45499	34.97876	50.12052	14.73247
CUST_32346_PI429010886	M3890	0.000594751	24	3	126571555	hexokinase [Litopenaeus vannar	Metabolic process	Carbohydrate metabolic process	1.05E-46	37.00674	24.64742	33.4937	57.53971	79.35979	68.30294	27.0245	35.92598	25.0378	15.11243	38.47726	29.05473	26.6719	26.49945	30.7666	21.87534	24.05036	24.64222	17.17797	27.76408	15.27294	31.90974	25.23036	25.38551	17.11834	45.01222	24.4203
CUST_28633_PI429010886	N58326	0.000596687	24	15						115.6114	168.5358	150.0449	98.7449	136.7882	124.4425	141.0774	224.2516	203.0356	186.0544	154.4454	167.0939	139.5452	156.9197	127.8904	279.1333	257.5954	279.3607	154.6882	202.5463	194.6096	137.7837	160.4874	166.7408	152.6181	110.4792	183.9085
CUST_34980_PI429010886	M11229	0.000598271	24	18	302887532	hypothetical protein NECHADRA			2.52E-09	4.285677	10.39641	8.58477	6.029956	3.657752	NA	NA	8.213544	6.313999	NA	NA	4.843001	NA	10.07424	14.1486	5.344742	9.166589	12.39193	17.94579	14.0733	27.99655	NA	12.2009	14.54604	11.38277	6.663215	15.12028
CUST_2848_PI429010886	N13237	0.000599261	18	3						519.1671	371.8649	745.3715	1517.515	1671.759	1690.112	431.6201	544.8319	653.1145	329.0788	1316.811	981.1472	1117.04	644.7162	964.6221	243.9595	152.4015	180.5627	454.1788	921.4706	919.9655	864.8838	1159.214	1040.528	341.3363	623.6004	687.0247
CUST_39650_PI429010886	M42210	0.000601426	24	12						1368.726	3747.441	2177.847	7714.571	3064.959	5380.838	9376.495	10485.81	17487.6	1393.878	19572.59	2599.331	9565.255	11643.13	4783.413	5840.985	6094.281	6559.55	1851.473	3197.119	3561.33	1254.672	3983.53	3755.671	440.7516	1785.094	1849.733
CUST_44272_PI429010886	M65458	0.000602186	24	15						185.7539	224.2516	205.5946	201.7698	193.7855	175.768	211.7618	172.0985	198.4987	232.4494	164.4418	223.8638	251.0374	200.7515	257.4304	286.2998	294.8466	319.3967	250.7116	197.7606	251.7468	240.479	280.0915	286.9821	260.6027	220.8506	331.4329
CUST_8823_PI429010886	N23650	0.000603206	18	9						49.82997	71.24846	56.71352	56.49038	33.96811	56.57137	105.2701	77.57132	43.55661	77.00089	72.92411	82.65009	74.14723	71.35943	88.84654	118.8608	69.39085	112.201	58.82275	52.18229	73.0273	38.16633	70.87494	57.83575	67.41125	68.13325	67.7048
CUST_55300_PI429010886	M109487	0.00060415	24	15	258406678	QM protein [Penaeus monodon]	Protein metabolism	Ribosome biogenesis	6.04E-44	8238.918	11763.32	8911.047	6358.535	6109.222	6826.356	8522.605	12584.9	11612.18	8430.296	7337.918	8051.463	5380.838	8996.679	7122.178	12191.65	16014.56	15367.74	10448.35	11952.65	11932.74	8025.596	11112.95	9056.645	7872.926	10626.1	10374.24
CUST_38256_PI429010886	M24747	0.00060415	24	15						1043.605	945.6613	689.5858	244.9453	229.2847	236.5827	2436.323	1202.808	2103.097	2095.253	1041.894	560.9756	572.9401	1330.649	535.748	7177.873	3617.55	4667.622	1532.345	1209.897	2189.919	1715.071	212.5696	1663.655	1735.82	493.5638	1146.806
CUST_39250_PI429010886	M36712	0.000604145	24	3	149675211	cuticle proprotein proCP5.2 [Cal			8.92E-46	2584.904	48.85342	2909.148	3620.969	4539.587	4061.362	47.03106	265.3922	144.345	1720.155	5336.579	2853.1	841.3097	2559.917	2461.211	13.5456	42.28696	14.66099	94.14191	201.6125	97.63248	1736.886	1316.811	1614.735	139.7918	724.4063	434.1663
CUST_23111_PI429010886	N48277	0.00060655	21	6						43.10891	63.92981	81.48573	86.67082	117.6651	111.894	195.8784	121.9822	187.5023	130.0446	171.5325	107.0354	158.0791	82.59378	94.90821	135.6611	185.1417	194.9048	132.1106	79.90962	79.23954	111.3972	48.69222	80.37006	96.03692	67.78785	101.6124
CUST_46599_PI429010886	M73115	0.000607002	24	15						17.05701	14.73062	16.11644	13.73223	22.74199	23.42467	19.23998	22.62431	20.8897	41.38164	23.00637	24.09713	31.98442	17.57039	24.68337	33.65745	32.69926	27.11011	20.81357	15.31518	28.56603	34.23416	16.44507	38.6867	10.71017	9.697101	22.67074
CUST_13672_PI429010886	N31568	0.000608272	21	0						209.4303	36.16824	139.4559	124.6688	35.89334	124.3819	17.63035	22.30617	17.51582	22.57092	63.74918	42.5735	34.97876	30.75559	44.7597	44.52658	34.86879	38.78289	38.36376	53.52013	35.96302	66.15369	64.54817	61.47323	52.59937	60.25895	56.68053
CUST_39702_PI429010886	M43114	0.000609779	24	12						1422.675	3992.329	2408.139	7807.75	3292.245	5482.841	10178.51	11138.2	17886.89	1505.771	18673.37	2891.912	10337.3	11667.94	5769.472	6612.476	6837.265	7844.789	2126.752	3076.623	3786.16	1298.646	3767.349	4482.635	540.7576	1552.466	2159.534
CUST_44380_PI429010886	M65762	0.000611604	24	3						19010.63	611.5217	13419.52	33508.11	23575.84	33637.86	505.5201	1647.162	403.2127	19434.65	22221.29	24659.23	16655.53	23016.75	22526.09	65.2491	184.0897	209.9538	459.6187	1854.805	305.7738	17837.79	7071.199	17023.68	981.9828	5840.985	4168.103
CUST_958_PI429010886	N7987	0.000611604	24	15						3590.769	7695.811	2879.516	3590.769	1180.964	2060.105	14032.06	9804.488	6999.724	6515.192	2575.646	2650.032	5415.358	4598.208	3215.844	12021.42	15798.02	11395.49	8451.427	8061.991	7832.882	3921.719	8503.163	3636.712	4880.699	7484.678	4311.402
CUST_50152_PI429010886	M88841	0.00061347	21	6	512816354	PREDICTED: protocadherin Fat 1	Cellular Process	Cell adhesion	6.87E-16	17.18131	16.91535	9.337932	22.52125	17.82488	10.7519	22.28065	21.23798	20.83109	23.11901	18.37247	15.7815	14.36072	15.42242	18.77257	6.202056	15.16316	9.228702	6.046138	11.77966	12.4535	15.8318	13.67194	23.07404	17.429	17.14498	11.27666
CUST_7145_PI429010886	N21105	0.000613558	21	15						903.1163	1701.993	1078.02	1226.655	1271.126	1316.811	1546.88	1543.849	1249.87	1564.909	1310.327	1227.139	1338.071	1273.67	1359.053	1795.363	1727.221	1628.405	1485.178	1380.017	1566.672	1222.23	1173.789	1142.458	1303.86	1271.688	1371.826
CUST_28431_PI429010886	N57935	0.000614885	24	0						481.8774	437.0635	423.2638	395.3057	283.5676	281.8133	393.1863	263.7574	304.3391	341.3363	268.9804	283.2866	93.00549	302.274	291.8765	356.9618	408.1953	308.8707	370.8194	329.7868	320.381	439.5135	363.084	303.5583	483.2043	344.5549	343.6496
CUST_54197_PI429010886	M105035	0.000616604	21	0						47.36163	56.08051	51.47126	27.81464	34.87919	36.34085	32.22003	35.59906	24.83681	47.07501	42.94276	31.61388	40.0648	35.27117	47.85839	43.59179	43.43371	38.36376	41.53886	38.98386	42.97957	63.78974	47.97707	45.93656	53.21955	42.77967	53.30891
CUST_716_PI429010886	N6899	0.000618132	24	0						73.95531	80.03641	118.0906	82.89284	59.76447	64.00415	106.0541	74.36638	89.10635	52.86067	55.65872	91.84111	11.95115	41.88081	42.73341	53.30891	30.2752	62.39412	149.8334	83.9013	122.0941	53.96936	112.599	89.35207	128.3674	125.2342	125.2132
CUST_6302_PI429010886	N19923	0.000619062	24	3	321470470	hypothetical protein DAPPUDRA			4.18E-55	231.2512	131.4002	211.1114	212.598	373.0445	357.3731	88.12342	202.5463	107.4395	103.8305	279.563	206.4922	119.7806	150.6494	203.5144	56.28495	39.90611	51.38302	81.43322	168.944	73.75221	190.553	136.6874	174.6876	51.58532	149.1062	81.22064
CUST_57285_PI429010886	E4142	0.000622332	12	9						6897.446	2736.432	9532.564	9684.529	5649.913	4834.858	5166.879	3043.672	7807.75	9629.338	11763.32	8929.115	16252.77	6249.252	9500.977	601.5188	394.0157	626.8237	11164.38	11179.67	11179.97	14071.14	19738.31	18715.71	8387.726	13559.72	18365.76
CUST_9110_PI429010886	N24077	0.000622768	24	0						586.5026	607.6204	633.2997	509.6459	457.5988	447.1876	544.3805	449.8325	555.2007	499.5403	481.4474	436.108	579.318	397.7869	526.2718	523.4696	455.7266	605.9768	506.2756	451.4694	584.9829	586.0404	654.3402	581.3213	621.7856	548.9123	711.4323
CUST_33314_PI429010886	M6573	0.000625841	15	9						13.66845	5.585409	16.83626	11.66013	4.828222	10.95623	33.43295	22.06613	32.60933	29.66274	44.10093	22.28576	24.37754	11.24821	9.812664	16.15211	10.31356	15.69552	19.31757	11.83721	15.22111	20.61232	20.61453	10.60847	30.27186	32.34328	87.60092
CUST_14774_PI429010886	N33450	0.000625916	21	0						512.6794	167.261	670.4033	260.752	88.84654	254.2305	157.4759	171.4564	189.3839	240.3468	136.5677	182.794	206.3618	143.0339	96.9591	117.1964	88.31999	98.19966	320.9643	309.0132	295.973	367.4169	283.1093	243.4049	161.754	245.9078	274.2134
CUST_36313_PI429010886	M15550	0.000626521	24	15						14.03865	20.10181	28.8005	11.43195	13.6951	13.00824	25.0865	24.03743	24.2121	39.40243	23.33315	33.22829	27.29484	16.38536	19.69265	38.30668	40.91608	43.76612	28.14643	20.36911	20.56418	36.53636	13.99541	34.12419	34.02124	15.95307	31.58885
CUST_37499_PI429010886	M19247	0.000626521	24	15	357619639	hypothetical protein KGM_1093	Protein metabolism	Proteolysis	1.32E-10	11836.96	18802.8	13716.26	8135.669	11780.77	8786.531	18522.28	16411.2	16847.05	13532.16	14956.1	11822.99	12191.65	11859.41	12424.28	25888.13	23739.57	25792.96	17524.49	17589.58	18320.9	15492.49	14470.28	14956.1	16801.9	15673.03	17131.85
CUST_39366_PI429010886	M38425	0.000626521	24	3	149675211	cuticle proprotein proCP5.2 [Cal			8.69E-19	15942.89	271.3104	15705.46	21881.51	17023.68	20871.55	317.9491	897.3252	423.936	11503.97	21224.39	12402.11	8204.372	11763.32	12743.08	86.61603	132.1793	153.1826	714.4899	1350.405	879.1441	13616.29	8768.122	11503.97	1023.762	4004.603	3284.996
CUST_18051_PI429010886	N39058	0.000626521	24	3						19522.5	65.66275	17131.85	23503.18	18408.63	24040.66	133.8983	335.9787	171.6359	18198.77	20222.93	18146.2	13301.65	10897.12	18802.8	20.9266	16.32398	130.1528	169.2197	711.8674	229.5267	15435.13	4857.819	13761.91	948.7571	3145.321	3230.906
CUST_27024_PI429010886	N55314	0.000628167	24	21						54.1566	51.29815	68.40054	45.73174	39.39814	52.92294	47.20253	43.92043	46.48232	50.47099	55.0756	57.8755	59.79025	50.30791	50.8307	80.70329	60.94991	78.34836	71.88037	49.89252	59.69202	82.40323	54.78117	75.3636	78.49976	52.6814	72.69874
CUST_47739_PI429010886	M77390	0.000628686	12	3						64.06114	23.51724	36.97883	41.83378	86.23628	85.66787	NA	5.590518	NA	NA	5.32791	15.27616	23.5588	26.62131	9.563744	39.03115	23.61457	28.79058	36.13796	20.10354	4.897248	NA	6.100675	5.903147	22.29988	9.970616	11.85566
CUST_47479_PI429010886	M76286	0.000629131	12	6						NA	7.85968	5.730283	19.88951	NA	13.41897	187.4109	NA	132.9353	22.15192	19.67166	NA	8.078793	9.632394	NA	19.02154	NA	31.83006	225.5195	309.4924	530.8049	27.9424	8.90453	31.97992	45.68256	9.884796	28.26579
CUST_37559_PI429010886	M19499	0.000629787	24	15						63.0922	25.15651	11.09357	4.674714	NA	6.562196	74.58857	31.94579	79.86465	112.6108	48.75454	6.124786	55.96387	40.01172	19.7933	318.482	93.51275	199.3483	49.3434	50.3146	90.39488	56.68053	NA	35.63867	76.5464	15.19175	21.91645
CUST_19001_PI429010886	N40809	0.000633088	12	0						56.51566	47.36534	51.261	54.19504	37.89734	40.11539	38.24515	28.35837	35.32216	49.62	44.20101	41.98856	37.89734	45.64087	44.98293	38.73465	30.89406	43.42575	40.62628	28.0761	36.53396	48.37004	37.31661	41.26887	43.70538	46.54974	51.18195
CUST_6174_PI429010886	N19727	0.00063715	12	9						4548.63	142.7301	4173.107	6051.213	675.0754	3423.157	216.3545	395.0243	600.8289	6999.724	5921.175	5617	7721.817	819.7622	5137.74	27.44734	82.02164	69.39785	1280.159	733.9767	1314.02	9211.498	6430.973	8768.122	880.92	1286.883	2345.461
CUST_8016_PI429010886	N22424	0.00063715	21	9						615.4627	52.6597	701.2976	449.3664	84.78861	372.7272	10.02667	67.58103	94.01958	1204.7	3886.966	668.9797	2275.002	272.2328	1128.19	28.31242	42.68213	44.95536	199.1806	155.3531	192.2662	1565.636	1356.93	1751.317	149.2223	1076.368	1357.702
CUST_11254_PI429010886	N27518	0.000637415	18	9						23.77227	25.22785	45.36684	29.38788	48.26184	32.85935	10.06668	55.57314	46.05207	10.21743	42.51804	19.8869	22.71122	27.26144	20.24228	18.73195	22.40935	16.25246	23.33803	57.24758	27.38699	81.529	70.5683	54.91386	46.31434	55.47729	42.92652
CUST_31068_PI429010886	M605	0.000637443	21	15	488560926	PREDICTED: putative uncharacte			1.45E-10	23.07724	20.2757	NA	28.4735	NA	21.43699	43.46338	25.77691	15.37323	38.55172	10.60534	NA	44.00493	32.78182	32.48749	82.91071	46.35836	48.0027	46.84848	11.68418	28.71759	NA	15.07604	4.733337	27.79405	29.00237	8.977003
CUST_7357_PI429010886	N21424	0.000639123	21	15						30.98678	59.93426	37.1161	26.23262	28.73586	32.4427	33.99416	47.41757	35.43842	29.04471	59.15199	35.15355	34.96815	44.73509	42.82494	57.51884	61.10664	74.39469	28.6788	56.20706	42.12383	35.40128	32.23135	30.15331	45.72512	28.90914	27.39507
CUST_22805_PI429010886	N47703	0.000639238	12	0	4894766	phospholipid phospholipase C b	Signal transduction	Intracellular signal transduction	3.2E-21	143.0944	185.2774	159.9694	154.3027	145.4106	114.5979	110.3412	146.4225	90.89393	156.0011	186.8705	184.0897	174.4367	209.7386	200.9666	144.5227	231.9647	179.9541	156.7437	120.964	104.0803	184.6434	163.8342	179.7937	280.2395	207.5504	247.1564
CUST_50045_PI429010886	M88373	0.000640809	12	0	13624645	arrestin [Libelloides macaronius	Signal transduction	Other	4.55E-23	47.34599	42.5561	44.39683	45.5101	34.0139	33.91249	23.11901	31.72419	23.5588	35.56743	36.12041	23.39833	51.24511	25.32605	37.72215	49.82997	32.17634	38.17039	9.225171	35.40841	23.82015	34.18125	38.79203	44.20516	37.11143	39.14498	65.18818
CUST_9375_PI429010886	N24509	0.000640809	12	0	71159516	C-type lectin [Penaeus monodor			3.26E-11	2940.045	3614.192	3029.351	2195.531	1488.548	1579.509	1836.765	1733.335	1736.886	1850.533	2222.683	1728.138	3154.32	1691.116	2721.255	2216.187	2150.693	2365.258	1340.037	1843.608	1664.278	2842.045	3197.119	2755.522	2790.956	2589.848	3112.057
CUST_44790_PI429010886	M66990	0.000641438	24	3						1393.878	61.59148	1555.177	2203.327	2239.116	2809.488	71.93057	178.447	51.2209	619.5951	1891.416	2028.877	319.0787	1456.541	1796.427	29.77656	27.50147	30.16221	68.32149	244.0506	66.74201	672.5292	155.3804	575.6253	43.09131	288.1795	78.3726
CUST_44867_PI429010886	M67257	0.000644558	12	9	220172363	putative articulin p60 [Rimicaris			8.12E-12	16622.66	1807.527	15731.96	31289.71	8042.108	13962.41	6999.724	6976.622	12836.63	35022.2	30721.41	24199.32	47826.44	12402.11	25555.67	475.3372	195.1701	570.5532	22658.35	13559.72	19522.5	35607.66	45731.6	47123.31	20501.42	21224.39	42900.93
CUST_44391_PI429010886	M65811	0.000647005	15	6						NA	38.66936	45.42128	68.65785	38.73021	NA	76.57491	188.0574	230.3457	NA	28.04985	NA	8.079781	7.958428	NA	NA	33.82382	31.98798	154.3283	57.8755	179.4316	11.33953	503.1242	560.9756	5.115776	11.30735	5.76381
CUST_25122_PI429010886	N51819	0.000651967	12	9						19.75023	6.544441	41.16872	56.16991	43.34	7.397642	8.770103	21.58992	38.35932	56.49639	93.94191	58.0985	74.44615	19.77651	65.28656	7.174372	6.511029	13.57661	63.89963	40.80027	51.01468	164.5889	134.4639	118.5066	69.92903	41.82506	120.0673
CUST_15414_PI429010886	N34534	0.000656558	21	15	91086587	PREDICTED: similar to NADH:ubi			3.99E-48	1015.318	1480.822	1154.406	867.6626	955.5815	931.8674	1068.554	1183.911	1104.992	883.0931	933.5904	1003.961	807.6213	987.1103	958.49	1478.028	1636.385	1716.333	1245.954	1235.811	1402.437	1072.507	1244.709	1208.723	1202.808	1044.931	1260.081
CUST_10652_PI429010886	N26572	0.000659353	18	6						197.1967	205.3407	152.5572	274.0938	173.9842	149.1062	342.1248	286.0534	257.678	252.3498	254.3715	249.0225	238.8661	204.0717	192.5048	276.3303	202.9968	205.0181	226.022	156.9414	210.4014	290.1517	182.7533	303.3581	316.4278	270.4772	464.1815
CUST_39133_PI429010886	M35017	0.000659376	12	3	239799185	ACYPI005467 [Acyrthosiphon pis			1.68E-28	143.8296	360.8837	197.796	215.9822	208.8218	222.505	249.4346	292.7159	223.277	170.3275	140.0779	79.84725	175.9736	211.0809	211.8745	343.9474	258.1145	256.8109	213.3048	166.9271	232.8674	156.6283	201.8328	162.8755	246.0227	205.2018	172.0135
CUST_46502_PI429010886	M72842	0.000659376	12	9	307208663	ER degradation-enhancing alpha			7.4E-15	24.5939	25.15875	28.5452	33.18845	16.28863	19.86462	16.37294	35.90062	21.66729	42.41232	50.88233	40.88884	48.2963	19.04014	28.99912	33.53246	27.30031	20.23623	32.32436	34.02535	34.17027	44.83771	37.52227	59.55202	29.58887	29.85892	31.84584
CUST_43057_PI429010886	M61960	0.000663971	12	0						75.25623	58.18757	87.39589	69.19342	70.51234	73.14068	46.35836	47.96277	46.43071	59.25874	62.08412	56.66829	76.93465	63.54017	84.01764	50.61162	75.0097	52.37742	61.67659	51.55397	49.85348	92.41119	61.79558	87.70118	76.6349	60.96191	100.2259
CUST_40731_PI429010886	M55957	0.000666785	12	9						113.1214	129.5035	121.236	92.50275	81.96005	79.76299	106.8903	113.5034	110.1773	119.8471	111.0547	129.9905	150.0105	93.42816	115.7982	106.9403	62.50961	112.2102	101.9985	116.9474	100.783	113.823	142.6928	137.1887	107.935	110.0054	138.9709
CUST_33527_PI429010886	M7166	0.000669933	18	15						11.02183	14.22535	10.66102	NA	5.223414	6.457152	5.912371	6.743875	4.218831	NA	NA	9.168643	12.65073	NA	6.799426	NA	13.10078	12.85037	8.463106	6.654189	8.372078	8.407667	9.832908	9.225171	6.111488	5.3907	8.169501
CUST_46349_PI429010886	M72321	0.000670067	24	15						95.50727	92.77365	92.22131	115.9671	126.1425	126.2557	121.7107	96.34162	104.6784	144.4328	66.96376	101.5332	144.2213	130.586	108.9908	170.1396	150.3698	195.2227	101.6403	95.65727	124.4273	148.55	96.79055	136.5009	124.9059	93.91412	87.15453
CUST_34330_PI429010886	M9343	0.000673182	24	15						NA	NA	5.570449	5.303946	NA	3.661945	5.122505	5.272315	5.3662	6.950855	6.436561	5.526054	9.476005	6.66152	6.775818	9.120228	9.651486	7.024734	5.910876	NA	6.727557	9.810166	3.763199	10.76987	5.389175	4.80327	8.588027
CUST_52088_PI429010886	M96376	0.000674193	12	9						24.93768	21.0526	24.5729	10.10592	16.48308	15.33609	25.52339	23.04915	26.73698	23.40329	19.49414	29.15439	18.67325	24.89861	21.21527	13.47047	19.71996	13.92807	17.41092	24.65035	15.99496	33.40515	23.90768	33.12802	14.84278	15.60281	29.6361
CUST_34345_PI429010886	M9389	0.000674366	18	9						107.0618	136.6874	125.3758	95.14144	112.1849	124.8368	143.938	143.4692	108.4189	148.0023	133.6627	134.2321	127.4016	132.3168	121.9201	142.9612	126.4865	146.1694	87.56469	103.8566	129.4725	83.18277	97.72445	98.10696	105.1035	98.10696	132.6815
CUST_55009_PI429010886	M108391	0.000674406	21	0						97.77174	85.53397	75.95345	35.96302	72.02191	13.18326	69.39785	61.07789	49.07635	74.01825	48.37508	85.61207	40.60404	79.56634	42.45213	78.41566	75.81657	69.88293	77.18489	89.91214	92.78173	90.8123	93.34171	98.7449	65.36387	52.7243	63.43943
CUST_19688_PI429010886	N42013	0.00067545	24	21						361.0483	501.9234	387.6202	201.7307	334.5246	208.2391	264.9162	304.0274	221.6521	414.9926	303.88	248.4015	377.7197	198.1815	364.3837	430.2082	478.0895	359.4486	318.6428	397.2907	459.1993	484.7365	484.0256	593.5138	240.4351	216.7613	325.1203
CUST_52730_PI429010886	M98691	0.000676585	15	9						422.3982	232.8383	715.4355	1191.902	523.3495	199.4867	375.8148	913.5277	1487.991	803.027	2767.762	625.6951	1578.545	668.7382	609.6218	25.88702	31.50316	36.62239	1418.598	1333.239	2054.56	1497.206	4672.963	4055.849	1734.409	2615.936	4710.772
CUST_45280_PI429010886	M68569	0.000679805	18	6	478262481	hypothetical protein YQE_0251	Protein metabolism	Proteolysis	9.55E-13	71.20264	195.9604	218.1432	203.1887	381.6653	NA	NA	368.9684	420.7391	747.3198	140.4108	438.2874	4.433971	NA	NA	NA	31.40589	NA	105.8493	79.23954	NA	544.9477	86.74668	371.0946	1232.801	566.3117	721.1898
CUST_43062_PI429010886	M61966	0.000681889	24	15						5009.272	5191.892	3783.527	4302.905	1326.216	2402.724	9923.978	6864.217	8929.115	12765.38	5298.376	1813.66	7632.364	5137.74	4121.132	26299.14	9013.443	16035.79	13837.12	9643.272	18365.76	11030.98	1861.323	10260.07	6491.663	1158.095	5125.495
CUST_4471_PI429010886	N16835	0.000683604	18	3						2507.724	2261.704	1486.662	1890.428	2650.032	3575.021	1566.672	4093.741	2108.887	1113.954	1561.658	1316.811	2482.212	1392.832	1626.549	234.1695	295.8069	353.7833	544.9477	5021.117	442.6514	2681.804	3179.907	2459.181	573.9026	9153.451	1856.844
CUST_27265_PI429010886	N55734	0.00068513	24	3						57.9604	25.57409	25.62254	39.11911	33.41645	30.37143	29.15011	22.36152	28.3901	39.41926	25.51207	25.86911	53.95129	17.66478	40.32964	25.34302	20.30731	20.52844	17.48074	14.57035	16.49157	27.4271	25.38551	20.05681	24.67313	33.13955	36.35313
CUST_2272_PI429010886	N12003	0.000686114	24	15	443697440	hypothetical protein CAPTEDRAI	Transport	Protein transport	1.75E-23	36.615	26.39512	19.26727	12.38845	18.60754	22.99952	19.25567	20.41652	22.51326	25.28249	27.9284	32.07484	32.02255	28.23406	26.37866	37.84952	32.79916	30.27748	23.1769	25.25638	26.05591	33.54341	25.63215	23.0873	25.20375	18.30441	31.9639
CUST_35309_PI429010886	M12199	0.000694516	24	3						39.25338	29.77371	56.95109	81.40497	113.3845	73.22433	48.2227	73.17158	46.9602	32.3516	65.71042	49.42933	46.04292	78.38177	62.46312	45.63539	52.41843	24.30986	39.90993	33.39115	34.00168	38.70891	35.73305	32.22003	42.59404	49.53863	40.09585
CUST_44242_PI429010886	M65366	0.000694516	24	21	321469964	hypothetical protein DAPPUDRA			2.15E-33	41.84178	53.80944	58.53483	32.97743	30.80426	36.76462	29.21241	34.02535	44.12566	46.00851	29.22381	41.62674	42.29849	32.3737	37.9964	45.49544	40.56201	49.17415	56.21355	45.98151	55.35616	47.16802	46.7009	61.51933	60.31108	43.7275	49.8658
CUST_907_PI429010886	N7776	0.000695916	21	3						981.1472	1087.147	511.4855	554.5386	2637.871	2605.771	1261.275	2194.43	1238.715	271.0264	808.5577	1133.812	512.9523	428.8199	490.8866	217.1239	354.0991	358.5953	349.5554	2918.383	273.0815	735.8335	977.5298	606.3479	357.8637	3855.701	1300.047
CUST_3291_PI429010886	N14199	0.000702121	24	15	225713504	Probable rRNA-processing prote			6.74E-59	165.0194	257.2967	222.4375	143.144	130.7209	142.2962	184.6852	194.3971	211.803	192.5776	174.3193	181.8309	198.2813	173.2871	171.5592	225.1246	279.6802	266.1986	223.2399	234.0578	270.4208	200.0776	191.0479	214.2039	192.9642	191.0479	237.6854
CUST_47420_PI429010886	M76036	0.000702818	15	12						14.21628	12.16729	8.640415	6.795326	7.534185	10.26699	13.65032	9.079938	9.37834	27.39004	14.99779	9.701216	24.19026	19.8408	20.80953	9.992623	10.16286	10.9779	16.60231	8.246623	5.334914	6.209536	7.202814	12.6791	15.68492	11.29273	9.896366
CUST_35797_PI429010886	M13537	0.000703886	12	3						93.38599	498.5642	38.08216	358.97	NA	321.0799	742.4077	441.1218	270.0743	141.0554	49.58225	47.71104	181.0227	423.0504	172.561	514.7289	633.4883	487.3173	237.0529	53.96355	194.3126	87.19961	145.8855	85.04871	214.8953	217.3138	22.5758
CUST_31624_PI429010886	M1994	0.000703901	24	21	380026333	PREDICTED: uncharacterized prc			1.75E-35	32.25164	37.96647	57.11305	33.18448	22.11134	71.73966	34.3577	26.56674	34.59782	38.2274	36.55792	19.82888	36.36383	37.58094	25.99138	38.69008	52.09654	41.64305	60.77123	39.35132	54.74825	38.0933	73.54748	61.49211	53.5836	48.90741	72.16617
CUST_32494_PI429010886	M4268	0.000703901	24	21						1098.155	1212.445	922.8654	802.1705	694.1978	466.4341	376.9694	678.3225	518.3699	1109.283	675.2885	606.7201	1147.873	509.2951	807.6213	824.4785	1074.53	480.5983	1560.774	900.3004	1093.204	1678.527	1348.208	1034.116	2187.303	981.6108	1407.191
CUST_37681_PI429010886	M20572	0.000711048	21	3	371591210	Euphausia superba voucher 980			1.45E-177	16.73229	13.48654	NA	18.66401	18.41119	23.0638	20.19825	17.54	4.347979	17.95668	6.24949	NA	6.981927	15.29719	NA	4.381137	4.724092	5.252307	10.53078	9.007628	NA	25.06028	NA	7.2899	13.81116	20.55348	NA
CUST_37831_PI429010886	M21802	0.000711318	18	15						560.6594	1082.664	593.171	607.6204	532.1418	525.1123	1048.592	895.1277	733.0037	736.2732	578.6613	631.4625	784.4035	629.8536	646.3558	1007.214	1239.555	1221.044	659.7683	617.0017	786.3486	449.0619	644.0162	537.4657	670.6338	640.2875	527.455
CUST_41834_PI429010886	M58656	0.000711318	18	15						102.0887	488.48	331.6434	27.68187	40.06052	10.97548	411.6604	257.2967	280.7963	37.51496	40.3971	35.8443	240.6532	60.64674	199.3242	653.5411	429.3727	394.9633	408.0121	162.3844	384.8278	36.29792	27.30898	51.39588	150.073	141.598	341.5971
CUST_8647_PI429010886	N23354	0.000711433	24	18	478259671	hypothetical protein YQE_0397			0.00000009	19.03842	NA	23.70418	8.853913	NA	23.10247	28.27682	44.05427	28.89871	41.13599	11.46286	18.29038	115.3572	73.1662	19.9833	92.7489	56.96848	88.61521	170.8363	309.7415	259.4587	42.26958	9.741497	24.92085	132.131	65.50892	65.82678
CUST_31943_PI429010886	M2790	0.000711951	24	18	339247611	putative myosin head [Trichinell	Metabolic process	Nucleobase-containing compound meta	3.06E-141	234.9525	325.1877	375.1194	299.7411	348.2785	308.9438	335.9787	310.8187	334.8644	261.7871	338.3895	285.1454	466.8411	351.8456	327.8085	466.3373	509.999	531.7033	458.147	516.2255	830.1589	305.5103	362.6643	377.3111	381.9964	376.3542	420.2294
CUST_53417_PI429010886	M101615	0.000713259	18	0	62126070	C-type lectin protein [Fennerope			2.84E-09	1207.663	1343.626	1138.567	633.4883	407.2747	444.4782	399.0879	685.4537	559.4339	1077.531	65.49568	665.873	291.198	188.0944	302.0161	1880.339	708.4537	2573.227	1291.739	1801.332	3670.22	1074.53	351.422	726.7698	1087.769	473.9767	369.1521
CUST_42721_PI429010886	M61067	0.000713286	24	3						142.4928	66.65338	149.73	190.6927	265.1693	270.4772	144.3228	88.04091	101.7595	181.4589	151.7139	243.331	162.0684	87.12904	218.889	62.80714	102.6378	108.3124	44.635	68.11052	82.20662	160.4657	82.99391	146.3771	166.9271	134.7418	106.9297
CUST_3246_PI429010886	N14096	0.000713286	24	21						6559.55	8073.399	6506.137	5374.176	3546.981	4217.01	3759.237	4247.1	4181.398	6710.162	4311.402	3350.998	6310.155	2589.848	4889.847	6452.703	8644.801	3224.848	11246.8	6533.018	8135.669	9669	11094.64	6696.753	12886.71	7316.568	11030.98
CUST_1829_PI429010886	N10823	0.000715519	21	0			Nucleic acid metabolism	DNA metabolic process		99.53837	126.5797	109.3497	73.05642	44.69425	59.53613	79.10899	95.173	67.83335	86.54102	55.76605	84.84924	69.00406	57.34418	61.11356	76.82742	66.34289	93.24932	78.70208	102.4622	95.62259	93.88506	120.5704	110.8559	107.4112	85.59262	56.08545
CUST_41631_PI429010886	M58187	0.000715868	24	9	321463719	hypothetical protein DAPPUDRA	Transport	Protein transport	4.13E-98	43.7275	25.74697	43.33093	38.48559	41.72651	33.12802	48.7032	49.41907	34.79498	49.06918	53.76136	44.09481	28.20414	45.54022	42.50868	37.46702	30.00507	38.7574	35.76583	25.75751	23.80052	36.04308	37.19414	24.64222	30.58395	33.35975	46.1952
CUST_31471_PI429010886	M1612	0.000720598	24	18	321474328	hypothetical protein DAPPUDRA			5.28E-34	13.16313	18.24533	17.51139	13.96239	9.394115	13.11625	13.65748	27.94793	30.5034	8.870244	16.15024	11.86355	12.7945	14.85832	18.05597	17.57258	16.53385	25.47189	23.76317	32.77954	33.43972	19.60994	23.43945	19.12194	19.25825	17.78324	31.55692
CUST_17244_PI429010886	N37642	0.000722672	24	21						28.44753	26.34871	33.5847	21.03035	18.67988	25.74697	14.13121	19.64801	14.94547	17.57258	32.16695	21.8116	29.84354	15.86706	39.34641	27.47239	26.65836	27.85388	31.83006	25.0865	23.22144	44.2137	29.82705	33.92168	36.30353	25.35423	32.29945
CUST_29188_PI429010886	N59357	0.000722672	24	21						103.3894	86.57172	102.2094	106.9803	92.34598	81.91112	89.26977	83.28215	93.8595	101.9343	72.00199	104.6122	107.509	94.22856	100.9249	82.46867	102.8659	103.8983	144.2876	104.7064	134.4639	152.0785	113.7264	137.1639	113.9812	82.26609	123.1603
CUST_48872_PI429010886	M82628	0.000727329	15	0						157.2786	178.9851	122.3002	138.1611	137.1425	125.0424	137.0649	183.2834	102.2878	125.9339	81.95267	92.15089	81.1468	170.8814	128.3867	244.8878	173.9043	175.4908	144.9793	119.5848	144.4561	101.0057	127.4016	105.4796	130.9866	98.78131	115.3698
CUST_12300_PI429010886	N29254	0.000727329	15	0						26.82167	28.56322	27.19181	28.44753	18.72709	28.84875	25.39051	25.75751	21.98717	19.87183	19.38387	9.257058	24.13409	25.60929	22.30308	27.79405	30.445	29.53381	23.72726	24.21438	21.91441	27.29712	26.98744	19.71101	18.88946	27.42138	18.39269
CUST_55273_PI429010886	M109408	0.000727583	18	0						16.5468	NA	20.87673	7.931706	17.68949	9.518164	NA	11.35345	6.287167	3.790529	NA	NA	12.31491	21.87009	16.11449	6.998897	16.79206	10.23432	NA	21.23798	24.66091	11.85089	14.55467	18.31223	7.555163	9.764026	6.233337
CUST_4391_PI429010886	N16657	0.000728914	18	15	321467999	hypothetical protein DAPPUDRA	Metabolic process	Carbohydrate metabolic process	1.76E-11	116.7583	170.8635	106.0881	142.2138	70.11515	140.717	310.1796	203.2614	202.8396	206.5228	182.2404	93.42816	258.9723	195.2973	229.9746	377.9818	324.9732	341.84	225.7047	85.87925	225.1246	127.4388	153.502	148.2361	220.9943	247.0114	158.2397
CUST_29645_PI429010886	N60281	0.000729794	18	15	195393330	GJ18862 [Drosophila virilis] >gi|			7.58E-34	78.20138	106.786	126.7723	71.33011	49.04219	69.6885	77.02123	90.28649	99.04563	95.80871	50.44651	83.03055	76.2209	103.1963	77.38763	155.2643	111.1532	118.8772	96.66958	72.30147	73.02048	68.68072	75.97892	63.1189	113.7264	96.73368	55.57314
CUST_11707_PI429010886	N28274	0.000732057	24	21						201.3668	441.5527	313.0268	245.7379	268.869	259.7345	150.7445	238.4366	231.588	266.1484	284.1966	236.8007	400.6266	256.0012	433.2807	418.5156	510.3128	373.2381	349.1843	385.0272	264.176	636.7343	346.9643	465.2004	618.3282	312.008	483.3359
CUST_29119_PI429010886	N59234	0.000733638	24	0						50.42215	74.67002	74.4544	47.26148	54.23194	69.99598	49.98481	47.30405	56.86607	52.44815	48.71388	46.24282	37.38294	37.33345	42.96134	73.82805	69.12973	56.97506	84.16166	66.72605	74.42622	55.91239	70.7612	49.48225	68.52496	79.59885	77.08874
CUST_50011_PI429010886	M88236	0.000735714	24	15						71.9695	44.97817	18.17381	17.29971	19.80764	17.66478	280.9343	161.6606	311.1315	251.655	177.7281	NA	23.06634	72.10955	17.29188	548.9123	384.7267	409.117	87.33991	196.1489	265.541	202.0727	8.771483	185.7829	144.3231	37.32223	60.20069
CUST_40118_PI429010886	M50563	0.000737241	24	3						59.55202	40.30598	42.62886	76.16835	39.0142	30.07238	48.36443	23.10443	27.93535	34.36428	21.68674	27.39507	19.9436	16.15468	7.762013	21.63236	39.21711	32.2704	15.89978	17.32058	21.36249	22.68661	23.09515	36.18124	25.87214	25.21148	NA
CUST_10623_PI429010886	N26527	0.000739075	24	15	260832812	hypothetical protein BRAFLDRAF			2.4E-59	13.21287	15.483	20.0391	14.46435	14.66551	15.84904	22.6604	15.01335	15.7786	19.34308	10.74484	28.5396	19.02154	19.34679	25.70122	33.76876	28.76456	34.13318	38.84447	15.40578	33.78234	31.19127	27.01424	33.50165	29.76143	16.06768	32.1177
CUST_5774_PI429010886	N19176	0.000741442	24	3	330371797	lactate dehydrogenase [Litopen	Metabolic process	Cellular amino acid metabolic process	2.91E-141	262.8953	270.6292	247.0676	220.7835	149.5552	227.3555	138.9709	134.7253	154.0925	160.5687	122.0709	174.1094	146.9973	98.94639	106.8903	109.6621	104.7861	97.97101	69.54417	125.0613	64.57231	135.7196	139.8503	106.1445	118.5066	134.5506	73.60215

Probe ID	ID	p-value	Period	Phase	EBI ID	Annotation	GO category	Go term	E-value	0	0	0	3	3	3	6	6	6	9	9	9	12	12	12	15	15	15	18	18	18	21	21	21	24	24	24
CUST_6307_PI429010886	N19928	0.000741442	24	21						37.67421	45.32861	48.75822	37.20732	38.04561	43.38791	35.0064	37.34896	42.74631	44.23722	42.98811	37.43476	58.59505	65.31526	38.62391	46.29157	43.07048	57.90877	38.92149	50.67391	37.25639	72.29255	61.33441	68.40753	43.76612	43.71813	49.70798
CUST_28079_PI429010886	N57251	0.000741442	24	18						16.29003	16.4966	21.95217	13.62435	13.85654	10.46343	12.52433	16.0527	16.98352	13.93501	13.81	22.35516	23.70635	15.929	23.66496	26.6898	22.8101	28.65621	29.48451	23.02305	21.14216	32.93541	17.64762	35.67153	27.21287	18.1064	37.31972
CUST_36003_PI429010886	M14261	0.000741506	12	3	321454610	hypothetical protein DAPPUDRA			1.13E-10	49.93016	71.03566	NA	56.20706	NA	54.57944	97.3611	51.61367	70.84605	13.22574	11.93633	NA	42.52944	149.8147	39.41926	144.345	69.6714	107.332	81.14024	40.80836	88.58571	6.788846	26.13038	8.236488	20.11384	39.37754	10.64305
CUST_50719_PI429010886	M90772	0.000744367	15	0						36.97106	46.96617	NA	14.42489	9.226214	20.33677	29.40141	14.28982	29.41761	7.511244	15.67736	3.202569	14.73062	20.02577	13.30754	63.62492	25.00095	51.25301	20.7112	17.2724	17.91767	5.625819	33.17225	10.66953	11.78821	10.70872	NA
CUST_38689_PI429010886	M29125	0.000746562	24	21			Symbiosis, encompassin	Other		1967.079	3161.765	2315.807	1979.658	1394.635	1306.636	780.1728	1551.452	1341.551	1879.179	1514.569	1368.726	2408.139	1421.92	2183.114	2049.491	2517.49	1971.443	3401.586	2388.437	2656.978	3145.521	1501.763	2666.243	4482.635	2477.377	3672.992
CUST_49980_PI429010886	M88081	0.000746996	21	0						28.57269	28.91213	27.79864	18.48559	17.8457	16.08274	23.34465	19.25567	24.33043	22.56644	24.70901	18.22098	13.65748	10.71329	19.20294	20.53592	14.5601	19.25364	27.20363	28.6464	24.3283	39.59398	23.27322	31.98442	27.15652	18.29498	33.44269
CUST_56336_PI429010886	M113533	0.00074733	24	9	91090940	PREDICTED: similar to tenebrin [			1.8E-26	106.7181	55.99408	89.7803	122.0363	75.94639	105.7619	110.1325	63.2086	72.67801	146.093	135.2901	136.0268	135.6453	128.5506	134.9488	160.1108	91.89616	126.4187	126.7723	58.24276	124.3244	124.0306	88.81146	107.8755	96.60811	85.99964	102.1812
CUST_51690_PI429010886	M94592	0.000750425	24	21	262305015	acetylglucosaminyl-transferase [			8.97E-55	32.75387	28.87134	36.04017	26.56161	14.96193	25.7879	22.38961	19.99907	20.92153	26.21426	25.84326	29.07923	21.21931	25.25638	28.49433	29.74537	26.73097	34.29815	30.86168	28.00501	30.28912	51.62622	26.32889	29.57468	38.61632	28.3766	31.07591
CUST_13544_PI429010886	N31361	0.000750425	12	0						27.07259	25.0763	30.31787	44.73509	25.91063	27.99322	24.42528	14.28195	20.70448	31.90012	32.63017	30.87082	28.19237	33.3337	26.73304	19.68623	22.35516	23.26849	20.20024	16.90269	17.60727	21.34009	28.99912	27.87026	39.22978	36.56941	35.07936
CUST_51304_PI429010886	M93001	0.000750828	24	21						16.32616	17.64448	24.57064	11.23132	11.41659	19.47348	15.43474	16.65693	12.52146	20.00187	24.81983	11.48082	18.08853	26.36566	26.73097	20.32505	19.77738	19.0444	27.60403	20.51742	20.90369	28.64244	22.91081	27.31402	26.805	18.77458	24.25128
CUST_4371_PI429010886	N16615	0.000753856	24	21	291236607	PREDICTED: zinc finger protein 1			6.21E-50	8.956638	NA	14.61747	8.419629	9.27828	6.455238	NA	8.684295	5.8709	NA	8.681841	6.145604	6.378654	6.612027	7.262291	11.05247	12.62197	14.28982	8.409122	10.16523	8.823825	13.56135	13.41211	10.62147	8.573258	9.93804	10.51323
CUST_9711_PI429010886	N25080	0.000755239	21	0	357619676	hypothetical protein KGM_2193			5.3E-16	15.81757	9.499412	17.65737	9.665024	NA	8.009934	7.201851	10.60449	12.84908	12.64908	12.32167	11.51457	8.219759	10.28394	7.565346	5.085084	7.904365	5.21464	8.289866	6.06107	10.56586	12.77798	17.54223	14.41307	9.946155	15.80426	11.64898
CUST_39324_PI429010886	M37811	0.000757508	18	3	121484199	cuticle protein CUT8 [Portunus p			1.13E-54	6727.299	158.6683	6010.065	10550.02	9669	8387.726	314.9794	883.7364	783.6564	6041.324	9653.786	6897.446	6559.55	5157.305	6791.114	130.4451	143.6223	101.0861	1735.82	1236.613	1671.759	8269.17	8786.531	9241.731	1915.575	4349.21	4906.082
CUST_5059_PI429010886	N18205	0.000757508	18	15	321472021	hypothetical protein DAPPUDRA	Metabolic process	Cellular amino acid metabolic process		149.0265	244.4333	167.708	142.126	140.6285	144.2213	251.6934	226.205	216.2603	188.7958	169.8826	144.389	174.4941	215.5592	165.5194	263.251	190.1223	305.3804	179.3019	186.9296	213.5938	130.702	164.8179	148.5751	214.5153	187.0996	163.5388
CUST_37191_PI429010886	M18140	0.000758468	21	15						62.97498	83.41575	80.6925	62.02618	56.2716	54.17106	61.31201	49.67166	36.21321	68.31562	66.47509	65.31526	80.99203	67.09453	58.58735	70.3428	86.29714	86.11117	50.62088	78.40447	72.79587	62.47784	93.27554	84.72348	59.51724	60.3351	69.53147
CUST_23637_PI429010886	N49182	0.000758648	21	15						16.45776	13.40595	10.15324	14.18326	14.3566	16.43671	18.26175	17.17977	9.659899	22.71992	15.47982	17.55426	19.33949	16.28228	18.49355	26.97701	23.14702	21.80903	17.00672	11.68939	12.9801	11.01416	8.086218	13.7624	10.33927	25.52576	8.18229
CUST_29755_PI429010886	N60484	0.000758858	12	9						9.497226	8.156982	14.16364	3.556285	4.790015	8.348262	9.802866	7.404706	14.53618	17.02991	NA	13.56792	10.63333	NA	9.145931	8.627581	12.43497	9.472484	11.24042	6.583562	12.00378	17.53011	12.78681	19.83069	16.85312	11.80456	14.60077
CUST_36284_PI429010886	M15418	0.000760213	24	6						854.1319	949.6232	52.09654	330.3379	2279.977	1212.026	1467.602	2051.901	1021.91	76.06374	803.738	906.8146	113.2744	1219.279	826.1267	1199.116	702.937	585.1407	551.8317	721.1898	419.0196	41.47035	417.2642	34.17741	144.389	488.0472	12.83208
CUST_37133_PI429010886	M17951	0.000760213	24	21	345484846	PREDICTED: glycogen debranchi	Metabolic process	Generation of precursor metabolites ar	2.19E-65	10.11558	11.48313	22.78054	14.51805	9.949274	15.38937	10.26259	7.142339	11.15951	11.68247	5.879724	12.54489	20.97185	6.631805	15.57359	20.51989	11.53064	12.40192	22.53181	16.34183	11.38924	25.37072	21.2559	24.7525	26.10539	12.35338	19.04623
CUST_3452_PI429010886	N14533	0.000760213	24	6	390338472	PREDICTED: pentatricopeptide r			0.000000793	1085.793	1288.168	71.07642	416.6449	2924.097	1564.909	2140.692	3286.945	1460.387	99.99679	1211.071	1230.097	50.83861	1823.25	1338.071	1998.783	866.3088	829.47	826.745	979.7526	653.3386	31.61388	427.9123	34.46463	122.2649	550.4921	11.88441
CUST_10441_PI429010886	N26239	0.000760213	24	21	332376843	unknown [Dendroctonus ponde			3.67E-34	550.9285	540.0952	647.9305	525.9937	401.5328	450.4965	365.6513	364.7211	502.9461	507.073	570.5532	563.3118	501.9234	406.4569	574.9115	556.4241	1016.824	867.2487	641.1736	635.9298	597.9185	708.7213	566.7623	739.9176	906.8146	776.4904	834.3772
CUST_27291_PI429010886	N55793	0.000760213	24	18			Cellular component org	Chromosome organization		199.3483	241.4743	246.7327	172.9739	207.2734	145.5507	254.1458	188.2081	315.6063	250.9574	129.0812	236.6326	221.5001	232.7425	238.9099	236.2759	278.1702	317.4998	393.8421	309.3501	449.1793	293.095	254.0305	267.6739	308.5071	151.1899	309.9865
CUST_20310_PI429010886	N43212	0.000761389	24	15	23505740	cAMP generating peptide precu			6.23E-09	1280.159	1494.458	1109.283	984.5856	825.7252	850.8759	948.1481	1255.327	996.3906	1113.188	935.873	935.4596	1059.777	1069.345	1001.688	1519.636	2194.43	2092.723	835.3759	1455.41	1554.179	968.0088	1591.635	1340.037	1198.216	1179.062	1179.914
CUST_1975_PI429010886	N11210	0.000764962	12	9						207.6167	9.346935	137.2826	178.5544	50.95848	45.24265	33.70651	106.7298	70.70842	1444.129	203.8066	1130.969	563.6439	283.4621	107.0354	NA	NA	5.451604	45.61863	83.62172	84.40775	536.0532	2337.634	1680.655	30.63415	541.4917	766.804
CUST_38499_PI429010886	M27402	0.000766745	18	3	220172369	cuticle protein [Rimicaris exocul			2.04E-23	4602.898	155.7485	4048.748	10506.53	7769.948	9056.645	421.3144	1415.173	1523.69	5317.598	14218.13	5693.503	6913.964	6101.131	7518.482	106.3359	154.2023	99.78003	2086.93	1649.323	2152.198	7497.741	11138.2	9954.878	1987.037	6665.157	7157.757
CUST_13680_PI429010886	N31579	0.000766745	18	15						37.55337	30.68002	38.15236	25.7341	27.97979	16.96032	39.76397	37.91239	33.21573	38.87958	40.3323	27.29484	34.07334	34.47718	34.88898	38.4343	69.88293	45.60889	31.54655	36.88652	50.17642	29.99869	29.53381	32.52808	35.44968	28.43101	43.78502
CUST_306_PI429010886	N3728	0.000766745	18	3			Metabolic process	Carbohydrate metabolic process		482.8348	107.6658	502.2054	596.9264	664.4758	640.2875	165.3421	175.63	164.3433	383.8668	569.7522	490.3414	542.0349	353.1568	544.8319	125.9134	108.8478	74.47864	185.1157	217.7355	151.261	703.6419	575.9481	567.5384	190.3204	369.9026	284.2929
CUST_32821_PI429010886	M5190	0.000768035	21	9	33468738	calcification-associated peptide-			1.48E-16	12.67448	12.83208	18.52205	14.7567	76.25504	46.3034	13.02032	21.39542	15.7695	28.34528	86.57172	98.63138	25.59303	34.14439	34.39051	19.10801	10.22653	13.54908	5.858162	11.97009	16.95871	14.39646	22.23772	7.794152	20.44981	16.75714	25.44962
CUST_25604_PI429010886	N52724	0.00076863	12	0						NA	13.99541	7.40828	5.362097	9.662411	6.077489	6.678572	3.777473	5.823197	6.365294	8.110275	5.873873	NA	NA	11.80456	6.603742	10.64209	5.880569	4.883912	9.844862	4.020996	11.22739	7.159232	6.89341	12.41522	10.74743	7.322474
CUST_37616_PI429010886	M19862	0.000768682	24	15						80.82353	172.1367	139.9317	501.7792	218.5351	302.504	436.0075	313.4133	494.2109	84.07229	970.2746	274.6613	907.8057	798.2619	130.9524	976.531	857.4356	777.3446	88.84654	181.7766	163.4271	37.18271	193.3026	119.8767	62.33316	63.22855	98.24098
CUST_56331_PI429010886	M113518	0.000769598	24	6	546682942	hypothetical protein D910_1012			6.83E-08	329.7868	222.9863	105.242	119.3101	1063.435	471.601	657.5452	781.7261	400.5287	105.9898	423.0504	295.7504	54.15073	525.1123	223.5106	176.0973	41.67619	74.42622	118.7939	370.4445	53.76136	58.07534	53.7784	50.41623	37.25639	196.3457	9.501041
CUST_38606_PI429010886	M28491	0.000769598	24	3	13898841	CUB-serine protease [Panulirus a	Protein metabolism	Proteolysis	2.34E-55	34.48071	20.34099	29.73407	26.7573	52.2967	27.49657	36.53396	39.93436	14.9705	35.72049	39.86153	17.76942	26.51031	25.58795	16.2936	20.1058	26.56409	14.51648	13.50148	18.9538	7.193219	20.03497	18.9538	9.166589	21.54974	21.27915	11.56292
CUST_3647_PI429010886	N14955	0.000769598	24	21						70.64915	61.88614	67.43392	76.09471	54.41681	50.77623	56.46685	56.00544	54.47023	81.45042	56.734	59.28822	74.81691	61.46387	52.32559	59.92793	62.92982	59.88108	74.36638	65.69754	62.50961	98.73078	70.66441	89.49919	78.0963	67.63991	92.69475
CUST_10922_PI429010886	N26999	0.000769598	24	3						284.7236	338.993	347.2002	551.429	554.5386	545.1995	253.7732	578.8472	393.5689	426.7646	467.6907	257.6419	314.9328	273.3536	542.3764	170.4812	419.8124	234.791	207.0521	124.5521	190.289	282.551	554.3874	321.1394	171.3909	409.117	197.9185
CUST_11752_PI429010886	N28345	0.000769598	24	21						822.9225	1025.404	775.6832	586.5026	546.0695	542.1922	571.5137	669.1662	716.6417	921.0975	710.8823	846.219	1200.665	672.5292	1168.291	896.6099	1191.479	1062.767	998.2787	916.8554	639.2868	1414.36	1218.815	1414.36	1220.331	925.9054	1104.345
CUST_31739_PI429010886	M2282	0.000769971	21	15						157.4566	1730.472	66.27041	2191.923	220.7089	1659.781	2915.434	2327.901	264.3686	1074.917	1115.579	595.0044	3185.221	1355.196	2581.822	4971.086	3177.941	2965.89	2645.058	106.1914	1857.76	250.3956	311.7361	289.3393	1615.517	978.4034	88.93364
CUST_37824_PI429010886	M21772	0.000770984	24	18						544.5236	2427.484	2443.901	252.1635	428.6927	518.9718	3401.586	2371.181	1935.604	220.9724	326.4068	35.45282	6304.559	1480.103	3307.225	1765.847	3875.533	5110.752	9041.082	14161.49	8348.895	689.0797	1092.583	715.878	1251.245	963.1582	757.5703
CUST_39701_PI429010886	M43090	0.000771964	24	15						5157.305	7653.847	5336.579	4719.533	2962.88	3247.587	4012.41	6333.445	6116.36	4388.629	5131.503	3879.479	5625.087	3324.822	4261.241	7039.176	7731.941	8362.147	5947.47	5496.275	5482.841	5489.861	4798.856	6226.747	4646.072	4482.635	4548.63
CUST_38678_PI429010886	M29081	0.000774817	21	15						1961.653	6818.249	3027.036	1157.507	374.1555	684.1238	7663.711	6988.188	7731.941	2285.617	766.5607	867.2487	3843.133	2551.022	2330.806	5244.429	8911.047	8051.463	3272.412	3360.114	3712.526	1038.012	2598.143	1130.969	2499.817	1979.658	1992.843
CUST_15079_PI429010886	N33972	0.000777668	21	15						7.87171	30.23399	22.12314	16.6131	11.09087	19.94017	23.19651	37.88972	26.69789	42.24367	8.267617	16.06359	44.37921	26.93742	35.91186	92.27655	64.64642	45.59558	29.41761	19.17932	15.4299	24.54259	37.94596	25.5482	18.80121	23.10946	10.84565
CUST_34938_PI429010886	M11122	0.000778984	24	18						17.42676	65.20578	64.52421	21.3257	21.56906	17.23652	26.72462	76.19975	70.14984	21.42365	45.84356	19.91453	29.24032	27.46909	41.24111	39.89679	58.47152	46.46187	122.1883	108.4697	155.1073	62.94384	33.66656	40.4228	45.05271	22.95726	49.78973
CUST_33392_PI429010886	M6768	0.000781294	21	15	330371797	lactate dehydrogenase [Litopena	Metabolic process	Cellular amino acid metabolic process	6.38E-60	104.6237	173.4784	133.7558	167.564	159.3669	166.8948	142.9786	209.1753	205.4207	172.0396	230.3006	56.25386	140.9846	251.4577	173.332	393.5689	290.9943	273.5066	257.1241	269.0492	291.198	131.6454	111.766	143.8043	182.4139	130.3441	139.3908
CUST_49052_PI429010886	M83815	0.000782035	21	0						290.5678	310.4343	332.0817	253.6738	238.5827	230.2055	248.439	242.7465	264.0732	304.9349	268.7721	233.412	265.3922	247.9236	244.4333	322.315	285.0355	274.4093	270.9698	265.4524	277.5468	286.1136	289.7731	298.5019	265.6462	261.3125	263.4524
CUST_22719_PI429010886	N47528	0.000782539	24	21						258.2414	364.6437	333.1336	297.8825	253.4735	302.1401	277.3623	321.0799	293.5137	283.9592	232.64	279.9809	279.6802	235.3802	301.5476	428.2506	323.747	420.7391	371.4802	280.4189	392.057	507.231	316.8051	489.8482	361.4106	330.6328	406.9392
CUST_4538_PI429010886	N16987	0.000787137	12	3						29.53009	27.53743	19.73488	40.3323	36.56623	34.68278	33.54781	31.31571	28.19779	40.38567	17.67253	30.81577	23.12326	22.45692	22.20069	39.81401	35.62951	32.92024	29.25153	28.05953	24.78012	23.14702	27.88813	25.13144	23.98146	26.76018	31.0164
CUST_46548_PI429010886	M72956	0.000788369	24	18						5.65772	6.429816	15.96647	5.752268	4.670269	9.055283	11.34206	7.24584	19.35535	12.16843	4.596206	15.05262	17.22544	12.46897	12.00512	18.67654	20.40806	16.19743	24.73755	20.36712	15.55749	32.5431	18.04733	20.2236	24.22714	17.97466	20.13304
CUST_52710_PI429010886	M98586	0.000788369	24	3						28.24507	21.70164	14.03865	16.48308	22.33262	30.05065	21.01206	10.99972	15.07768	16.45591	13.25797	9.349393	16.65548	18.1699	16.74205	18.87533	8.410091	12.07805	5.906938	5.361132	6.95828	14.22358	10.85699	10.96209	15.0561	14.80561	8.867841
CUST_13268_PI429010886	N30885	0.000788369	24	18						105.1035	166.9271	150.2258	102.1199	80.00199	82.66079	103.4513	176.7718	165.7618	110.45	118.7615	103.9866	152.096	101.3719	144.4561	121.1808	139.5639	135.209	209.2118	199.3048	243.7665	195.2973	126.3851	163.0243	233.7126	134.8921	166.1333
CUST_15503_PI429010886	N34687	0.000791834	21	0	321473177	hypothetical protein DAPPUDRA			9.52E-20	260.5042	568.2556	460.1149	309.3501	394.4626	383.7931	203.057	310.4859	302.7771	189.6492	264.3686	177.2739	172.7187	338.1196	370.6325	232.4494	287.3062	312.3683	224.7103	456.7205	312.6928	420.9575	418.9116	390.8971	354.9347	439.1006	279.7318
CUST_45511_PI429010886	M69334	0.000791861	15	6	321473002	hypothetical protein DAPPUDRA			2.84E-14	7.114823	11.4247	8.796309	10.86311	12.50469	11.37088	21.42365	16.74981	20.61709	18.36173	11.87175	21.93034	16.98223	33.15745	13.5155	7.64439	12.58686	13.50832	14.0796	16.07009	15.20809	17.61537	16.40534	22.91081	13.51353	7.9866	13.61982
CUST_51775_PI429010886	M95016	0.000793114	24	15	321475716	hypothetical protein DAPPUDRA	Metabolic process	Carbohydrate metabolic process	2.52E-29	56.48171	36.42929	48.7032	29.15694	30.12442	20.16039	60.58102	67.63991	38.8005	55.22014	25.91063	30.02506	58.71184	44.50543	37.57177	82.60618	60.62225	94.3568	54.04744	67.80459	73.76003	33.41357	37.24906	39.06825	53.081	49.67166	53.7784
CUST_2371_PI429010886	N12205	0.000793114	24	15	321473526	hypothetical protein DAPPUDRA	Metabolic process	Carbohydrate metabolic process	1.07E-17	1642.989	4606.27	2437.73	1758.225	850.8759	995.0788	1653.487	1827.788	1930.481	2593.566	1066.406	1444.129	2837.693	1793.169	2276.393	5678.164	5251.996	5336.579	3091.943	2514.613	2909.148	3393.901	1464.031	2900.703	5162.169	1041.115	3940.028
CUST_42206_PI429010886	M59688	0.000794227	12	0						324.1912	354.4054	313.4133	244.7472	259.9818	225.0517	174.3193	255.571	238.0667	268.3647	301.3784	314.4452	359.587	253.4735	331.6434	240.9301	346.9643	288.117	258.4714	291.2685	278.8807	308.9438	355.3919	349.6396	428.3837	349.7302	437.5336
CUST_29636_PI429010886	N60271	0.000794345	15	0						32.17634	34.64088	31.36138	21.26556	28.28966	32.22585	20.66314	25.46688	25.48697	27.69481	15.19667	22.0234	20.07037	23.93602	21.60514	42.67386	34.70595	31.03564	31.92217	25.58573	22.82508	27.30572	26.76257	24.80109	22.78318	25.32605	23.86079
CUST_16536_PI429010886	N36420	0.000795763	18	0						105.5064	239.7136	348.0379	108.8321	76.46737	4.542234	11.55069	10.18329	5.882831	28.90326	30.05065	26.0166	39.40243	15.02551	16.7377	NA	62.04596	48.77487	25.17016	43.36291	50.98858	53.56818	39.24909	27.13523	36.5095	4.021257	25.70439
CUST_13622_PI429010886	N31489	0.000798406	24	18						45.73174	73.09797	56.51566	40.93434	41.57553	41.25004	49.1413	65.53387	70.3428	55.11106	54.6004	45.90076	63.61175	44.17172	47.13	73.54748	65.03673	69.25445	71.47305	66.60304	94.25033	78.34836	87.34939	92.54225	88.76092	69.16443	76.38149
CUST_37582_PI429010886	M19596	0.000800488	24	12						28.0791	20.36405	7.489289	22.2434	4694.435	1430.525	30.5922	63.32579	23.09515	NA	2498.654	2261.704	NA	4190.519	4499.92	29.76143	15.84059	14.50725	22.27865	62.05756	18.09453	9.201788	21.66219	8.790565	NA	21.84014	4.950558
CUST_29077_PI429010886	N59152	0.000800711	24	0						43.67283	54.13743	40.40244	36.73983	36.66337	24.87517	39.70565	36.98876	36.03288	44.81684	36.1335	22.44233	36.29093	34.36788	25.57409	18.1573	33.09557	28.76131	32.10702	32.63627	29.9217	33.63302	39.44669	25.13355	76.69145	34.23102	41.60067
CUST_11139_PI429010886	N27329	0.00080146	24	15	270008578	hypothetical protein TcasGA2_T	Nucleic acid metabolism	RNA metabolic process	3.26E-31	81.95267	66.31026	67.48124	68.60832	65.82127	67.87655	100.9526	77.63531	75.09386	83.59122	68.41738	115.1151	92.92674	83.42667	91.13599	126.2905	86.41587	108.623	82.95322	69.45139	84.35052	78.86032	70.90586	84.89672	87.47672	64.24425	76.8564
CUST_21887_PI429010886	N46043	0.000801902	12	3						20.11731	21.62003	NA	20.57512	42.03961	35.6426	21.26556	15.66009	9.545474	15.81139	6.817435	13.63037	16.77811	16.23247	9.167675	27.27378	16.84041	24.33371	9.459821	13.0267	16.36134	NA	14.21628	8.954353	6.934132	28.26373	NA
CUST_52303_PI429010886	M97004	0.00080284	24	15						6.374074	6.339415	9.477425	9.14315	7.740604	4.060355	3.318441	4.171723	7.665823	NA	NA	8.018435	10.98054	NA	11.18027	15.41819	NA	10.33423	14.13121	NA	11.99083	15.90721	9.331963	9.320269	9.386871	6.563887	6.121824
CUST_39863_PI429010886	M45893	0.000803689	24	21	371591236	Euphausia superba voucher 760			5.22E-160	101686.5	98191.21	104498.1	100092.3	102393.8	98191.21	103630.6	95305.17	94019.51	99678.14	94790.65	89298.33	101035.5	99004.2	111377.9	109451.2	95305.17	118638.4	103630.6	102393.8	118638.4	105237	104498.1	122649.5	102933.3	102933.3	115488.3
CUST_6374_PI429010886	N20018	0.000803689	24	9	308512298	Caenorhabditis remanei CRE-ME	Cellular component org	Cytoskeleton organization	6.89E-158	95.79812	153.0619	92.23687	131.468	208.2743	181.1454	175.4589	162.7885	113.6067	139.0644	183.8614	177.05	97.6745	184.2288	103.0964	94.87741	180.3196	106.3076	73.88293	130.496	86.41587	50.47099	95.37708	71.07642	82.57504	162.0267	62.48982
CUST_1361_PI429010886	N9404	0.000803689	24	15	512913175	PREDICTED: proteasome matura			2.58E-08	338.1914	523.3495	340.4211	249.0801	267.3602	263.4524	280.879	412.604	384.1573	346.4474	287.2425	354.5733	273.1848	438.3717	317.784	427.5284	511.6359	525.1123	309.2795	425.7716	450.8164	263.893	398.2963	361.4833	337.4051	325.2782	374.1555
CUST_12501_PI429010886	N29563	0.00080394	21	15						102.222	95.9971	28.82648	74.75403	185.4321	213.4477	150.8283	87.80111	86.06068	126.0731	113.2432	131.7896	93.57741	148.8378	128.9375	316.7134	190.4302	162.4391	143.5433	53.72874	120.4803	45.44328	76.16048	39.18322	85.09153	81.68207	31.81746
CUST_1673_PI429010886	N10408	0.000804703	21	18	321474958	hypothetical protein DAPPUDRA			1.51E-84	59.08609	121.0252	97.77174	28.73196	60.58102	41.67991	45.42128	92.27655	79.88277	44.37921	115.286	49.63135	63.69892	46.12726	102.9045	60.32347	59.9228	72.80134	114.9651	121.5027	128.8978	97.25517	63.88253	82.34733	49.75065	45.26784	56.34222
CUST_8101_PI429010886	N22537	0.000806262	21	9						19.58386	24.19322	22.24082	16.84413	18.47386	14.41931	34.19427	31.50642	24.44847	39.61156	16.96414	37.6918	26.99424	30.41718	28.81777	33.15311	36.66952	29.62066	29.64248	27.18917	28.17748	15.35106	29.95378	20.465	19.7864	27.49657	20.73851
CUST_55851_PI429010886	M111885	0.000806755	21	0						132.3608	53.04738	184.2613	60.68646	34.89501	83.99404	40.7495	34.07747	40.4113	73.67616	39.72368	62.35954	35.24577	36.42265	16.2921	121.3825	46.98969	103.7292	41.31439	35.01636	51.32137	208.6394	112.6548	63.74337	116.4848	62.58458	76.2209
CUST_6331_PI429010886	N19960	0.00080714	24	21						935.4596	1091.098	1011.981	812.2115	543.5625	616.5627	451.4694	635.3318	803.3036	1025.772	988.9686	923.7499	1037.623	781.2177	999.9612	1185.011	1049.887	1109.283	972.8403	969.4842	790.1557	1110.326	1068.824	1288.693	1105.917	905.3835	1533.458
CUST_12475_PI429010886	N29524	0.00080714	24	21						1119.38	246.8683	1289.967	1545.477	1005.566	382.7119	532.2614	207.8484	440.6377	863.8645	1981.815	630.5448	2738.485	1217.388	884.1094	944.1713	707.6592	1084.272	2192.968	2061.689	875.0518	2629.319	2430.729	1718.114	1241.315	446.2058	1548.254
CUST_16949_PI429010886	N37128	0.000810868	24	3						NA	19.01388	16.14215	25.74697	28.3766	43.24154	18.64133	28.8807	11.99083	NA	22.76835	10.03041	10.28202	8.04727	11.71113	12.17645	18.85869	11.57988	14.80803	19.78846	19.43327	14.85493	23.29768	14.43282	18.80957	21.42827	11.0716
CUST_8548_PI429010886	N23206	0.000811015	12	3						13.33462	10.33423	3.283408	21.62449	25.18191	28.93158	9.381399	12.6478	13.69771	6.427241	9.75094	7.610237	13.27288	23.96408	14.75483	28.8322	22.36969	15.87551	14.24675	7.782145	17.22926	NA	14.5199	13.55744	NA	13.87165	NA
CUST_19486_PI429010886	N41660	0.000811387	24	0						31.18313	25.20375	31.45292	24.5024	17.44275	14.24384	29.20612	15.68303	16.69734	22.22979	14.48646	18.35004	22.33547	12.87704	18.59104	19.47577	10.17169	13.18326	14.27002	15.92395	20.72028	31.30624	16.04092	25.74022	39.17239	22.46441	27.37887
CUST_38872_PI429010886	M31412	0.000812149	12	9	195447950	GK25804 [Drosophila willistoni]			0.000000501	580.6289	34.12419	539.9257	1416.762	121.6909	712.4529	66.19633	47.73425	93.70997	1805.629	3724.515	946.5528	1729.252	314.9794	1127.159	7.702073	23.00207	20.30258	210.6647	100.1016	164.9813	1877.836	1964.046	2702.508	393.4684	1649.323	2317.529
CUST_11318_PI429010886	N27616	0.000812949	21	0	414151636	prophenoloxidase activating enz	Protein metabolism	Proteolysis	2.15E-40	1160.436	1083.233	1143.279	1113.622	459.3044	692.1008	837.7142	1104.992	1074.104	729.6122	736.7743	594.2073	830.1589	927.3328	786.1643	521.7908	1041.115	662.3233	1005.566	874.6609	1040.528	968.3563	1397.328	1118.482	789.3467	951.856	1185.739
CUST_57211_PI429010886	E2487	0.000814263	24	15	151579928	40S ribosomal protein S2 [Arten	Protein metabolism	Ribosome biogenesis	1.83E-29	14502.16	18365.76	13503.69	12631.63	9684.529	12344.85	18613.15	18910.88	19434.65	15435.13	11780.77	12378.85	14389.61	12699.11	13154.56	20501.42	19522.5	22847.77	18758.28	19635.13	20271.85	12402.11	14580.94	14411.75	16070.74	14032.06	17088.5
CUST_32808_PI429010886	M5156	0.000815263	21	9						20.55029	39.63021	12.93513	8.440387	3.800251	12.40192	39.70866	24.62932	34.59443	43.74801	31.51924	42.38836	28.67614	16.32616	29.50684	20.55619	16.04092	21.98953	31.60415	24.83937	18.01822	30.37998	13.88603	19.89174	15.80013	18.91131	17.41782
CUST_7965_PI429010886	N22344	0.000815263	21	15	76158140	Taeniopygia guttata clone 0063F			4.28E-54	15.76545	35.84121	19.99907	20.95393	27.31402	32.97277	30.31994	33.07741	18.05597	37.33345	36.32694	29.38566	30.14473	21.07179	36.71092	37.87395	37.71641	46.64371	24.47097	32.56117	34.22563	25.46041	18.29498	23.87086	28.30304	24.58293	20.20229
CUST_52312_PI429010886	M97027	0.000816715	21	0						952.6113	616.0609	1350.405	162.2114	118.7214	18.08853	282.172	558.6463	373.6591	118.8608	187.773	90.13423	103.4374	48.80246	48.88764	169.6212	351.92	236.3014	230.6071	195.3229	194.7406	426.4459	174.0791	414.226	128.1168	232.0765	377.9818
CUST_12066_PI429010886	N28871	0.000819144	24	21						621.285	829.47	698.2425	695.8214	627.1961	553.2936	365.0376	525.2596	625.4995	815.2223	548.75	595.1579	1116.065	647.0623	855.0422	712.9201	1043.236	631.2553	817.4569	917.9436	837.9532	1099.423	826.745	833.7381	1333.239	695.1268	1057.154
CUST_16671_PI429010886	N36662	0.00081977	24	6						4.87786	13.31417	10.42633	3.216882	7.383186	10.69163	17.34464	30.81263	17.76418	11.08814	NA	NA	NA	13.24014	11.02607	14.68136	8.376444	13.46545	10.42743	6.856268	13.63196	5.79893	3.127206	5.316215	9.383476	5.908768	13.3485
CUST_20867_PI429010886	N44214	0.000822063	24	9						31.50058	16.74205	17.78243	42.76535	37.39816	37.17059	25.22536	21.69652	22.37256	33.47158	54.77517	35.13173	37.40246	30.31074	26.65508	19.99907	31.66604	21.68449	18.40546	21.39999	13.64203	18.34559	16.8338	24.64466	17.46553	19.87929	14.6763
CUST_36322_PI429010886	M15588	0.000822301	12	9						28.47679	29.81738	23.57211	16.0253	13.06456	13.45946	15.33068	21.16897	23.8982	26.32557	22.03386	30.30695	39.76763	18.04733	41.85499	23.90042	17.44594	16.28066	46.68552	19.97115	36.34085	68.64759	53.67622	51.72801	52.82485	26.14375	35.36547
CUST_43407_PI429010886	M62971	0.000824838	24	3						498.4242	100.0827	537.5716	433.4692	1091.098	561.3058	222.8685	184.6434	260.557	514.7289	557.4698	586.0404	256.0394	332.5739	518.3699	93.96854	85.58451	138.2385	200.863	185.1715	225.8314	384.1573	268.6876	445.0179	263.0994	372.4691	433.4692
CUST_34068_PI429010886	M8653	0.000824838	24	15						926.6453	889.3882	891.3462	922.5025	655.1561	828.3699	901.877	843.2459	976.1525	1024.289	548.5685	803.027	1017.256	941.3897	630.2951	1764.635	1324.412	1890.428	1265.098	1189.941	1591.635	1026.487	686.4707	1164.205	1196.459	837.7142	1078.02
CUST_1244_PI429010886	N9002	0.000824838	24	15						5678.164	7861.824	6584.064	5489.861	4101.099	4130.841	4998.113	6193.269	6837.265	6885.506	4253.847	5166.879	6744.57	4606.27	5578.907	9096.626	8334.379	8400.194	7900.843	7090.073	7769.948	7950.355	7111.995	7303.286	8016.887	6304.559	8189.692
CUST_34314_PI429010886	M9303	0.000825351	21	3						350.5162	226.4631	414.9089	464.677	419.1486	380.5457	239.0006	284.5232	287.4074	289.8261	163.8916	193.0974	493.728	281.628	249.888	180.4687	195.3229	150.3174	335.8993	331.9294	388.7734	313.739	453.1048	344.3047	426.7646	298.3765	446.0553
CUST_49856_PI429010886	M87457	0.000827908	15	9						124.5835	38.23223	87.91078	189.1569	157.4103	133.239	56.35449	60.75955	42.42877	167.7409	301.9582	203.8465	168.7741	120.8608	149.4369	55.8305	32.01377	27.46378	52.35235	56.03663	45.84807	193.3912	221.9709	202.6223	58.16145	196.9638	219.9666
CUST_25344_PI429010886	N52236	0.000829424	15	12						20.38623	18.2969	14.23378	14.29468	13.88603	13.35388	7.555163	15.80426	11.29793	22.06175	8.599165	20.42168	19.41383	24.75929	23.64329	18.86536	77.57976	21.2032	32.44868	15.10467	8.512367	11.76421	11.67855	12.81384	16.16637	7.335668	20.90135
CUST_1566_PI429010886	N10096	0.00083116	21	0						7.091187	10.97116	10.38364	6.391026	9.595961	6.518117	9.106589	5.142937	7.196175	NA	5.985076	6.5874	7.870945	8.958741	6.405558	4.422432	5.668943	4.900801	5.639861	3.484263	9.752082	11.79624	10.76826	8.640415	9.647611	8.822371	9.274547
CUST_30938_PI429010886	M266	0.000832663	24	0						867.6626	969.0998	1004.431	869.3099	710.633	583.0039	601.6893	571.0817	664.9053	704.9081	783.939	509.2951	923.7499	673.8771	666.8579	617.7634	782.0893	859.0835	796.2861	806.2686	605.287	1176.913	890.6088	834.3772	1306.13	844.8862	943.4447
CUST_7536_PI429010886	N21695	0.00083353	24	6						39.27561	36.96718	8.374367	7.512221	108.9127	12.95419	96.40725	126.4021	112.7746	NA	51.03976	46.29157	NA	49.66108	33.4748	78.44375	45.04591	27.36851	13.28567	21.68674	11.21915	NA	NA	NA	NA	4.484845	NA
CUST_7880_PI429010886	N22212	0.000835413	24	15	153791718	probable global transcription ac	Developmental process	Embryo development	1.88E-57	17.43071	25.50246	29.29243	17.14735	18.14584	16.34992	24.87849	24.15387	29.13251	23.67748	17.99307	25.00095	25.10509	33.15012	19.95957	42.28326	26.83157	39.55957	32.13276	23.79553	27.53522	23.29214	31.87401	33.59136	27.55409	18.5449	30.54385
CUST_14958_PI429010886	N33760	0.000835413	24	15						50.70492	70.5281	60.58781	46.67638	43.20988	33.61683	61.60004	62.49633	61.97446	50.73158	45.54022	61.91527	62.28713	50.92486	68.62585	72.14124	82.11629	78.33025	70.45005	68.9382	72.5147	67.97224	57.20828	70.45005	71.40475	69.35987	105.0583
CUST_18097_PI429010886	N39135	0.000835413	24	15	121512010	S10e ribosomal protein [Xenops			2.74E-25	5558.806	8189.692	5900.066	5350.891	4875.223	4610.9	7861.824	7415.157	6885.506	6430.973	4740.787	5533.093	5736.233	5197.224	5686.353	8683.686	8984.262	10464.54	6752.376	7122.178	8605.765	5073.866	6696.753	6265.89	6171.545	5792.477	7506.653
CUST_52888_PI429010886	M99339	0.000835941	21	3						26.71367	24.02389	22.1912	29.6361	22.33779	30.09026	18.25369	22.12314	17.56328	21.60932	23.87086	25.53705	12.8639	20.55029	13.35744	25.77932	24.19784	24.24247	24.46253	22.10476	22.46441	34.24084	24.99752	29.5453	25.0443	28.82404	29.9255
CUST_38518_PI429010886	M27613	0.00083655	24	21						1494.458	1383.977	1600.243	1707.189	1344.214	1427.593	1697.826	1426.211	1497.856	1653.487	1221.709	1373.039	1204.169	1285.646	1020.798	1954.479	2314.026	1885.037	1789.218	1574.714	1638.477	2092.723	1913.068	2343.924	2322.285	1462.785	2001.707
CUST_3113_PI429010886	N13825	0.000836814	21	3						21.92764	15.40374	17.08884	22.09313	27.2558	30.26815	19.79114	25.32953	27.24094	18.28716	33.09951	17.31771	30.45211	14.27175	18.89628	11.86035	12.50576	14.43282	35.20206	20.80025	25.13805	37.60099	22.37256	26.6049	40.93434	29.72829	31.50316
CUST_55374_PI429010886	M109818	0.000842605	12	9						2035.999	199.5249	1971.443	3633.442	1361.235	1816.059	451.2333	793.634	1401.131	3098.696	5571.385	3376.686	4045.654	1579.509	2889.672	45.9872	27.03986	58.12746	1847.559	1340.842	2097.341	3801.741	5659.622	6143.541	1941.073	3811.795	6317.157
CUST_47779_PI429010886	M77588	0.000842605	12	9						16452.07	1061.158	14922.71	19195.08	6491.663	10294.69	4253.847	2332.204	5686.353	21974.84	24721.57	17131.85	27073.29	7484.678	15045.33	419.1486	580.6289	585.6903	8827.334	5826.27	10127.21	21459.8	26491.1	25792.96	11305.05	14015.31	24040.66
CUST_6517_PI429010886	N20203	0.000842605	12	9						15.07177	18.60754	12.30413	9.928002	10.5335	10.5238	14.17193	18.42634	13.00488	21.4181	13.02177	12.17645	15.36995	11.60477	6.031062	8.128515	10.95928	9.373767	17.29448	11.95362	14.64996	12.72466	20.23623	22.01795	16.07832	12.12705	19.01591
CUST_38939_PI429010886	M32275	0.000843415	24	9						111377.9	135762.5	133343.7	142420.7	143808.2	139320.7	135762.5	140605.6	146308.3	151266.3	148414.4	184129.5	146987	142420.7	142420.7	146987	154561.7	135762.5	154561.7	145543.1	104498.1	136545.1	137201.5	135762.5	136545.1	141877.5	130632.6
CUST_38562_PI429010886	M28010	0.00084346	18	15	157111095	myosin heavy chain, nonmuscle			8.56E-09	1075.462	731.2874	1239.086	1219.83	734.7144	902.4975	927.9648	1312.243	905.7144	956.8208	661.1838	673.8771	1466.161	1251.736	995.0788	2414.182	2060.105	2201.915	1955.527	1974.35	3205.37	668.9797	395.3057	594.6179	2139.451	754.9422	1135.35
CUST_45500_PI429010886	M69310	0.00084346	18	0						50.75197	35.5871	28.03594	19.65735	19.2221	11.37814	8.747232	18.94794	13.34178	20.50908	5.02057	6.229277	45.29856	47.1467	20.14753	26.79915	9.177369	28.98683	42.42432	62.45522	96.58233	18.91649	8.730033	24.9499	41.45634	11.46624	11.29
CUST_2017_PI429010886	N11332	0.000845988	24	15	222160395	Sox14 protein [Scylla paramamo			4.31E-20	26.51323	44.9498	28.2373	21.17165	30.11811	25.14223	28.53195	33.06934	36.69203	35.68728	23.51724	32.11277	35.99005	42.16378	44.05006	65.73258	58.06369	56.44652	23.93259	33.47158	40.46451	33.48088	37.63739	33.44947	34.7801	39.92572	35.95603
CUST_88_PI429010886	N710	0.000845988	24	3						11179.97	164.9432	9791.81	14982.33	14137.09	19485.9	269.1036	409.8716	244.1329	7577.055	9791.81	15340.52	3255.97	12378.85	18235.97	63.74337	79.55681	102.6229	203.4357	604.6962	282.8745	4177.108	1472.69	4513.062	778.7232	1556.87	1594.029
CUST_49633_PI429010886	M86465	0.000847437	15	0	291235783	PREDICTED: galactose-3-O-sulfo	Metabolic process	Lipid metabolic process	8.07E-13	57.36964	73.65204	NA	53.04738	3.619597	58.32536	59.22327	54.36935	46.76121	27.27615	15.45147	7.913226	19.54299	27.18917	20.64275	111.8445	80.37006	76.78462	63.16716	20.81559	59.25874	8.290919	52.75072	9.715358	49.9052	44.80331	3.377397
CUST_16754_PI429010886	N36812	0.000848009	24	21						189.2095	259.0205	213.3383	167.4695	196.5522	169.3865	150.6011	183.6827	208.5115	211.4988	206.4029	184.6852	157.5397	195.3229	132.2008	202.4277	168.7741	167.3834	211.7465	193.3377	145.9095	285.8344	270.2277	231.9279	214.8629	238.1099	237.6384
CUST_57336_PI429010886	E6444	0.000848493	12	0	374095290	voltage-dependent non-L-type c	Transport	Ion transport	5.32E-64	46.17368	43.01095	56.59797	42.29117	37.48759	24.27221	18.97529	34.01769	31.6335	34.46463	37.33345	48.98753	32.29221	39.2895	59.82583	40.7919	33.92168	39.45708	41.28281	33.53619	33.42535	43.77016	44.49549	43.05532	52.86649	44.96721	65.78378
CUST_164_PI429010886	N1947	0.000852757	12	9						64995.27	4080.702	57453.95	66134.01	33076.81	52429.1	14015.31	15394.16	26891.88	74264.89	92404.68	69931.06	85264.67	40595.56	80988.9	848.3549	1105.498	1841.638	32540.66	27832.29	40217.51	76090.53	95305.17	94790.65	46674.67	65558.65	88732.25
CUST_53670_PI429010886	M102717	0.000852789	15	12						36.02994	31.09316	36.56623	31.28585	32.33214	29.75777	31.36138	35.07936	36.03727	36.12859	39.64997	48.37508	49.66108	34.25309	43.27654	52.37142	32.72443	41.39951	28.96499	25.5482	37.9908	39.33451	31.64075	41.24525	42.29117	33.92845	40.3175
CUST_26045_PI429010886	N53558	0.00085518	21	15	242009301	conserved hypothetical protein			5.21E-08	1640.994	2231.56	1610.862	1190.571	1412.179	1432.11	1881.294	1681.402	1910.884	1676.984	1267.497	1649.981	1770.862	1465.124	1529.961	2256.232	2221.308	3122.414	1762.47	1669.427	1925.502	1470.023	1888.631	2031.77	1330.649	1339.398	1681.402
CUST_5890_PI429010886	N19338	0.000856457	12	6	321463770	putative triacylglycerol lipase [D	Metabolic process	Carbohydrate metabolic process	1.92E-126	NA	5.044846	NA	30.24945	54.66763	55.47226	214.2039	43.03704	164.6878	28.42265	53.63563	29.22381	4.788815	20.26341	11.26818	47.7452	9.275544	41.76233	195.3229	680.5705	988.9686	40.4228	NA	41.4895	122.6496	27.4271	78.01928
CUST_55145_PI429010886	M108868	0.000856563	24	15						707.1593	1065.144	1009.468	768.79	713.4537	738.5083	872.0312	826.1267	1096.577	867.6626	839.7101	781.2177	913.1879	736.9876	895.1277	1389.498	1179.476	1307.951	1098.155	831.9483	1120.742	1037.623	981.1472	1102.848	1089.998	845.5682	1195.535

Probe ID	ID	p-value	Period	Phase	EBI ID	Annotation	GO category	Go term	E-value	0	0	0	3	3	3	6	6	6	9	9	9	12	12	12	15	15	15	18	18	18	21	21	21	24	24	24
CUST_55844_PI429010886	M111855	0.000856563	24	3						20968.09	457.4761	22092.06	31801.71	31801.71	35022.2	833.5137	2384.985	1940.134	15798.02	38861.2	22279.42	10178.51	17729.56	22037.75	219.7344	410.7073	251.3401	1535.771	2167.915	2018.868	18198.77	11356.17	16491.53	1743.671	7270.382	6592.219
CUST_39239_PI429010886	M36617	0.000856563	24	3						562.2603	75.51715	450.394	807.6213	816.5697	686.6456	57.65509	66.54894	43.45889	241.3201	649.9373	746.9967	207.9512	707.3888	756.8703	36.44264	46.97626	48.68211	50.35761	86.92889	50.4892	280.0406	94.93084	218.8175	50.75534	129.3679	97.14951
CUST_50725_PI429010886	M90798	0.000856563	24	3						10532.72	2033.334	9712.243	19094.08	17555.23	20662.81	1743.671	2713.39	1852.945	14161.49	17287.24	18522.28	10035.81	11179.97	16372.71	1003.255	1229.495	1453.877	1837.894	4031.99	2141.9	14439.79	8644.801	14703.33	2010.612	7232.62	7138.174
CUST_2317_PI429010886	N12095	0.000856563	24	15	225711970	7-dehydrocholesterol reductase	Metabolic process	Lipid metabolic process	6.6E-94	24.04549	100.7491	40.45937	37.87395	30.72686	50.51811	81.68207	105.3673	61.3302	78.87059	51.94695	54.64003	91.08467	94.15594	67.97894	88.30662	126.5609	165.3213	76.33415	77.61728	120.2404	64.02245	45.78026	72.46811	108.6123	80.06182	50.57582
CUST_10418_PI429010886	N26199	0.000856563	24	3						1582.625	543.3224	2299.057	2809.488	4641.846	3485.281	677.2359	716.3366	1113.188	1103.734	3043.672	3478.142	1373.854	3460.858	4284.075	304.7866	323.5934	446.6878	893.2381	941.0274	1026.487	1335.17	1303.295	1852.945	943.4447	1561.658	1967.079
CUST_1010_PI429010886	N8210	0.000856563	24	3						1660.744	49.4079	1717.387	2839.956	3336.354	3825.101	95.18929	213.0483	106.1339	1109.766	2254.423	2482.212	711.4323	1651.623	2102.006	21.85461	4.230836	16.62453	37.29216	209.1018	31.88942	2080.932	225.0517	1681.402	35.02108	203.2256	76.19975
CUST_19484_PI429010886	N41658	0.000859469	24	18	260836230	hypothetical protein BRAFLDRAI	Transport	Protein transport	5.5E-34	15.0561	10.76358	14.09206	11.66134	11.25219	6.683325	10.19338	18.26175	18.84481	10.03041	6.307711	5.44689	10.27251	6.37788	14.23996	19.15475	9.553409	17.40595	42.96134	26.85004	37.05226	16.09693	13.67551	17.17004	27.36342	6.205904	18.91474
CUST_21856_PI429010886	N45981	0.000859469	24	9						135.4737	100.8605	123.8841	155.3061	254.6677	191.1071	156.3694	143.0166	103.1619	160.139	186.2473	280.5224	111.1784	170.7874	174.3634	123.5107	100.4112	103.9647	114.0573	105.2701	82.3809	112.5075	99.58967	116.0591	128.78	93.44995	100.59
CUST_28759_PI429010886	N58565	0.000862315	24	3						21.94131	11.40119	26.77594	28.38277	59.98742	65.9928	13.7086	14.84138	12.99612	31.50944	26.27112	34.41579	27.29712	19.86967	27.12484	12.93352	12.62763	15.52955	17.98946	12.6791	5.578787	25.9357	18.50635	22.05458	19.27844	62.87718	26.99424
CUST_40898_PI429010886	M56332	0.000862889	15	3						8.320354	8.023716	4.892286	9.200402	10.01527	NA	7.046728	6.460713	9.203065	NA	3.784383	4.800498	NA	5.393032	NA	6.394884	9.916433	8.3196	NA	12.93352	17.20767	NA	NA	7.587167	8.165142	5.938065	7.370654
CUST_26266_PI429010886	N53950	0.000862909	12	9						144.9158	78.73755	256.7541	187.0768	72.74057	91.89616	99.23862	114.6686	172.9954	203.7183	166.8948	161.691	134.9672	133.4287	168.944	112.6234	65.82678	104.8815	160.0873	128.0247	201.4162	480.9139	194.0105	433.3852	175.0487	152.6639	293.8829
CUST_9352_PI429010886	N24480	0.000864471	15	3			Transport	Protein transport		106.1034	122.7625	90.45329	112.6548	131.6748	93.3967	65.20578	99.74451	96.79055	96.7742	88.43455	108.298	58.19693	69.58412	63.5234	91.34986	193.2061	87.54238	156.2876	155.1864	143.1244	98.91443	128.1168	92.02415	106.5192	104.4233	93.03552
CUST_30858_PI429010886	M21	0.000865738	21	15			Metabolic process	Cellular amino acid metabolic process		530.8049	642.7419	450.2424	281.7476	311.2489	381.2204	709.2377	631.6446	594.8478	571.5137	419.4465	639.5264	404.7001	476.8665	363.5741	707.6592	762.8618	789.8715	454.5758	444.7023	584.6183	321.3471	458.6774	515.8767	402.436	428.6927	348.964
CUST_31349_PI429010886	M1320	0.000867138	24	15	390350564	PREDICTED: ribosome-binding p			7.88E-40	3475.106	6826.356	2631.802	2663.717	2216.187	2622.032	6165.981	5777.198	4284.075	4819.372	2715.678	2727.371	3085.208	2924.097	2676.834	8269.17	7138.174	6945.783	5380.838	4616.026	5496.275	2736.432	3699.413	2871.837	5292.711	3952.736	3449.27
CUST_32352_PI429010886	M3906	0.000867138	24	15						219.538	452.0404	289.3393	201.8328	222.9863	211.4988	296.8699	367.6429	306.7699	265.8169	234.6515	238.1099	282.2255	269.9329	247.116	379.4005	503.6798	423.1563	338.993	299.5787	346.8977	219.2811	332.0817	283.8964	290.3829	308.8707	283.9592
CUST_39439_PI429010886	M39522	0.000867138	24	15	196008589	expressed hypothetical protein [	Protein metabolism	Proteolysis	5.57E-22	253.6231	154.7361	126.5797	89.26114	16.24433	63.53279	1249.87	344.6427	908.7057	861.2738	507.073	27.58489	168.1851	322.9826	82.77125	2362.508	612.5918	1965.787	492.5086	616.2414	1150.764	509.5667	50.03259	472.8686	362.8242	66.58185	269.8306
CUST_5270_PI429010886	N18474	0.000867138	24	15	345498130	PREDICTED: E3 ubiquitin-proteir	Protein metabolism	Cellular protein modification process		637.4326	957.7854	779.344	627.9112	621.285	568.455	951.856	868.6022	965.4976	926.2026	676.3457	789.6051	960.8612	663.1483	748.709	1117.567	926.6453	1145.735	888.236	859.4584	1063.832	666.0922	806.9344	864.5859	772.5597	741.6851	1022.285
CUST_32647_PI429010886	M4685	0.000871879	21	3						60.30356	50.55672	77.08874	80.21511	82.88351	94.74514	32.29945	55.6648	40.66863	36.41874	46.11508	45.95593	43.49402	62.49633	60.05636	49.9905	46.281	59.65828	58.05667	44.13653	46.91713	59.07967	51.8805	56.45276	60.68646	57.70575	89.26977
CUST_13867_PI429010886	N31911	0.000872059	12	6	155966352	putative cuticle protein [Lepeop			9.86E-23	40.62184	31.07591	6.486846	121.0252	203.8465	193.9616	296.8179	422.2995	230.7682	NA	4.433475	NA	NA	315.7654	NA	148.7876	70.16535	47.33949	178.7734	626.0846	151.0262	NA	29.04471	NA	5.460737	53.60597	NA
CUST_52510_PI429010886	M97708	0.00087266	15	9						548.9123	80.82353	435.509	1029.68	413.2217	501.6566	156.7032	131.6454	202.8062	1439.606	4140.108	1241.315	1710.003	480.4617	806.9344	37.73828	56.11275	56.71352	386.3361	218.1148	314.367	2013.288	2900.703	3303.981	608.2737	3289.37	4602.898
CUST_40917_PI429010886	M56387	0.00087306	12	3						11.19641	25.06692	26.14707	22.5078	41.93463	31.13164	16.89173	16.7995	20.96269	13.24894	19.97632	16.38688	26.86667	20.01045	23.67991	26.48825	31.77537	24.68337	21.78716	11.12989	24.68878	18.9186	21.82795	14.94729	19.08766	21.76828	19.16283
CUST_32861_PI429010886	M5293	0.000876296	21	15						630.5448	2064.359	1247.204	488.8743	190.7291	252.2559	2824.276	1853.898	1925.502	2060.105	642.342	921.4706	1441.224	992.6472	722.7897	1271.688	2755.522	2485.974	1529.136	1453.134	1177.962	979.7526	493.1272	1189.292	886.9505	611.5217	736.0921
CUST_34507_PI429010886	M9874	0.000877713	24	3						129.5648	33.19846	105.1207	170.9936	155.3531	203.0356	23.29768	65.15776	39.321	28.51273	70.43485	79.63863	45.08608	122.6389	128.8131	20.99538	27.07259	24.26356	17.8915	48.54356	33.73205	32.65194	37.75646	46.39504	28.06923	82.91936	49.51848
CUST_16486_PI429010886	N36317	0.000877713	24	3						50.97241	21.43051	55.29402	108.2549	91.38505	128.4222	23.92952	21.7849	29.99869	27.44415	56.23453	56.06577	34.75367	52.90159	74.17237	10.89769	7.373784	10.7714	16.90805	21.52625	13.47047	100.3493	23.19392	69.69671	39.55957	14.99779	41.42853
CUST_22370_PI429010886	N46909	0.000877713	24	3						3532.198	13.90121	3755.671	3921.719	4334.63	5191.892	69.326	68.69203	99.94989	773.6805	3549.354	3509.087	1520.832	4153.924	3253.191	30.80078	9.655344	37.03485	520.8578	394.6878	687.6817	2517.49	984.2262	2010.612	28.89871	3011.975	3899.124
CUST_23555_PI429010886	N49025	0.000877713	24	3						2648.467	117.5411	1916.926	2962.88	2177.847	2793.635	161.0998	207.5504	199.8689	2276.393	2291.48	2442.216	1488.548	1930.481	2228.732	93.61951	78.23111	105.1748	254.9324	376.7778	366.0882	1720.155	1158.095	1504.447	338.1196	686.6456	498.1009
CUST_36777_PI429010886	M16834	0.000878928	21	0						1042.748	1090.482	1152.623	1013.822	800.6803	814.3635	1006.869	814.9423	1002.608	1001.191	898.3377	867.9312	1040.093	818.7919	960.5506	1024.829	1062.332	1061.955	1028.813	961.4773	1109.283	1091.584	1044.09	1350.405	1085.159	916.8554	1420.017
CUST_44419_PI429010886	M65902	0.000881227	18	15						27.86183	30.8222	44.29702	45.93656	28.13497	36.74386	23.78446	31.05581	34.04952	53.33823	20.52618	37.91239	31.32937	27.57162	44.43314	55.09433	54.50978	52.0293	49.66595	37.93177	35.20206	39.09234	31.50316	39.59705	29.30711	18.86307	21.2559
CUST_39531_PI429010886	M40855	0.000882388	24	9						20.04202	21.19056	17.93943	21.36623	66.66302	41.98487	12.02986	25.30919	17.83143	24.39028	61.8234	52.58831	16.99603	60.0855	60.60875	16.98514	38.01559	15.13188	6.079186	15.97824	8.342949	13.76082	17.36739	14.12309	8.992091	14.59784	10.57189
CUST_17924_PI429010886	N38843	0.000884417	15	0						22.93309	22.09313	21.16473	20.47402	39.42252	29.83769	17.35447	14.56346	14.19869	30.25225	17.63804	27.9284	20.15147	12.91333	20.49098	22.77605	50.64865	22.77027	15.64744	20.02764	18.41364	15.74083	13.33769	14.95707	15.12219	16.09247	15.23818
CUST_41453_PI429010886	M57768	0.000888287	24	3						5125.495	127.048	5137.74	8284.715	7794.971	11094.64	95.68309	667.2339	109.5722	487.5393	4945.667	5550.987	315.1053	4863.888	5697.98	10.10725	9.220034	31.52277	35.04281	798.2619	29.12719	1438.372	130.0819	1197.753	16.35396	1570.104	16.93597
CUST_14109_PI429010886	N32341	0.000888287	24	15						563.8477	1707.189	1170.327	618.3282	549.8031	499.3569	1343.626	1002.608	1299.503	1094.513	634.2073	631.0985	991.8349	763.5241	813.0168	1723.472	2063.235	1902.67	1251.245	1212.026	1596.49	759.9978	1163.513	1405.334	1311.742	890.9879	1382.183
CUST_32656_PI429010886	M4715	0.000889883	12	0						307.6702	250.9574	231.4591	162.4604	250.7612	86.61603	94.41489	179.0245	153.1388	292.95	125.0917	218.1148	231.134	104.9484	189.4468	179.3019	236.7186	184.9648	88.38967	181.8309	21.81393	247.2845	176.0973	187.1331	447.1876	339.3438	361.3178
CUST_38878_PI429010886	M31485	0.000890685	24	12						163.4636	246.7327	75.29284	371.0059	290.9943	344.0704	633.2997	289.9679	905.0625	121.5027	1776.485	89.47762	1209.897	1508.513	350.791	306.6661	1034.581	264.2234	416.4698	205.6562	451.8198	63.88807	608.2737	390.7107	57.26697	66.74201	78.87059
CUST_31592_PI429010886	M1920	0.000893364	12	9						203.9603	201.5828	220.9078	190.9659	219.9666	169.2749	222.4375	214.693	240.0325	270.5842	185.4692	265.6845	273.6644	192.0736	246.7327	166.4486	186.9009	181.1959	250.3956	224.3337	243.494	233.6818	259.4124	276.6101	239.7136	229.2847	333.0274
CUST_30297_PI429010886	N61574	0.000893364	12	9						19.39036	23.72726	34.79855	23.95909	17.60727	15.93445	22.29844	19.80965	22.62698	28.44038	24.71432	24.33371	42.53392	16.52701	30.31787	15.77285	21.80903	15.31705	18.73195	20.7057	19.09012	37.8045	35.80909	33.3478	30.46011	17.10849	24.57834
CUST_47126_PI429010886	M74882	0.000893848	24	18	366984573	putative ribosomal protein S25 [			1.59E-12	60.71091	77.86312	94.45356	70.1867	63.31428	64.36702	74.37918	65.12931	91.63916	50.47976	62.62916	56.32908	69.80795	87.30779	74.83312	99.54889	119.1208	107.6017	128.4802	69.84655	123.1444	106.7719	84.9728	97.244	113.666	74.81691	138.9534
CUST_21335_PI429010886	N45034	0.00089413	18	12						54.85564	20.84466	50.56209	8.980966	16.35946	11.57897	16.3304	31.70434	40.28089	32.17224	31.76944	19.77435	40.98681	22.62431	27.9576	24.61634	29.64873	23.42954	28.08693	24.20903	18.6455	12.72606	10.83233	19.6201	21.49158	21.09878	15.65279
CUST_22587_PI429010886	N47282	0.000895036	15	9						23.61457	5.177049	19.94017	52.86067	40.12721	26.9108	6.000495	25.19608	11.41917	86.29714	340.9357	57.70575	70.7612	51.79467	32.76978	4.829023	5.443763	4.291282	13.40297	21.00723	8.606721	93.21779	106.0382	52.61041	13.22168	115.7982	47.48815
CUST_39107_PI429010886	M34661	0.000895183	12	0						15.31518	11.25219	16.4194	7.113497	8.799574	NA	6.774081	6.488336	NA	19.48546	10.90094	14.70059	11.988	NA	12.8443	13.13774	11.68791	4.037653	7.888139	5.81402	6.73927	14.71778	13.73333	10.98917	28.49433	13.9779	15.70678
CUST_31552_PI429010886	M1815	0.000898862	24	15	321475814	hypothetical protein DAPPUDRA			5.72E-42	181.6282	337.4051	164.3905	164.794	82.7532	154.4146	346.1837	257.3902	286.2354	348.1933	179.5826	174.3818	281.0852	198.2176	197.1631	492.831	421.0625	446.5017	220.8506	174.5649	295.8069	211.625	190.4954	297.492	280.5224	207.3653	224.3833
CUST_40635_PI429010886	M55709	0.000898862	24	15						433.4692	599.8602	489.0279	466.0849	312.3683	288.5336	871.592	590.8215	701.8416	812.7394	436.9283	474.7066	782.0893	450.8164	563.1838	944.8425	808.8122	977.5298	773.6805	611.8901	946.5528	670.1687	483.5315	644.5321	905.3835	587.3135	708.1548
CUST_5404_PI429010886	N18655	0.000898862	24	21			Metabolic process	Oxidation-reduction process		85.6799	782.4302	569.8831	362.6643	168.9237	216.5044	1186.54	447.0887	806.9344	183.3606	700.5788	30.23708	965.1042	745.8583	162.165	1089.998	1106.912	1464.031	552.4266	1622.787	2108.887	2043.437	2528.059	2073.458	1865.951	1629.282	3168.826
CUST_17972_PI429010886	N38927	0.000902104	24	0						33.66061	42.58958	32.8145	22.21765	23.02305	23.96712	28.81777	23.05329	39.32943	10.73071	33.42941	18.86919	23.8475	17.55561	17.75609	21.03678	21.46595	26.23532	44.70348	30.26124	40.31371	26.22964	31.62493	23.47597	43.35728	30.48373	42.13235
CUST_34717_PI429010886	M10447	0.000903516	12	3						206.781	370.7223	170.1396	264.176	523.2386	456.7205	328.3409	325.4289	295.4303	215.3674	270.8095	224.3833	244.1329	312.9583	241.8418	417.9126	366.4491	331.1317	369.1521	262.6219	325.3527	180.0167	282.551	204.1061	277.5468	301.2002	207.0521
CUST_49547_PI429010886	M86062	0.000904809	21	3						33.34498	29.40507	33.65745	45.77544	40.11539	41.3582	36.76462	12.67619	32.69926	41.82506	25.74471	34.36428	22.74435	21.82087	24.95325	24.2121	21.24956	31.57973	51.29815	30.46605	29.94695	66.6173	26.16413	36.53396	34.11363	35.55503	39.94687
CUST_56458_PI429010886	M113843	0.000906115	12	3						36.11568	50.1974	46.04292	59.00011	87.92365	66.97219	61.1324	61.16253	58.8025	59.11715	46.1196	66.62309	43.34	51.36995	58.94126	66.74201	52.41365	55.03418	58.50483	51.14752	62.44813	54.44704	39.02642	41.98856	40.80027	35.64607	38.07838
CUST_41772_PI429010886	M58521	0.000906224	15	9						832.328	325.2782	632.6818	1384.734	1130.969	1307.951	373.0445	546.6865	303.7038	1537.641	3667.762	1643.622	1368.116	1304.861	1201.304	321.2836	282.9282	260.798	377.789	547.9685	369.3009	1733.335	1903.836	1573.941	452.4209	1888.631	1734.409
CUST_45153_PI429010886	M68149	0.000913213	18	0						17.59285	19.02985	20.81138	12.36934	11.57897	6.136811	15.06825	7.123709	10.01645	12.52789	9.165105	7.847769	NA	15.55519	11.1169	NA	15.63953	7.491826	19.17932	19.64434	12.99098	12.04588	11.08814	8.049997	9.203065	7.54952	9.416468
CUST_36599_PI429010886	M16328	0.000915268	15	6	443682399	hypothetical protein CAPTEDRAI	Metabolic process	Other	5.31E-24	32.73463	35.59076	37.96647	51.92761	70.22094	65.52411	81.40497	83.92194	98.478	70.71767	43.67283	64.77885	70.75071	60.76642	64.47775	65.32192	70.09576	75.49296	68.44391	56.17586	80.1827	88.27501	135.397	155.2845	48.85342	65.54992	103.0843
CUST_11748_PI429010886	N28341	0.000915268	15	9	374095290	voltage-dependent non-L-type c	Transport	Ion transport	1.74E-76	48.53255	38.5262	39.88527	30.63675	26.10261	30.02336	24.16626	22.58797	30.43559	49.67735	40.19489	44.73509	36.70742	38.70471	45.0895	44.48574	23.62454	37.37325	38.52068	29.26951	27.48203	40.2126	41.80345	42.91273	58.42497	42.36924	65.86088
CUST_50571_PI429010886	M90304	0.000922333	21	0						21.47745	23.29768	25.7844	24.32624	17.54652	19.26727	7.177997	12.04956	14.20021	19.28668	14.84138	23.07724	15.63819	10.61538	18.86717	23.34913	19.46213	23.12968	23.49626	19.87183	18.3141	28.39338	21.96936	23.08008	26.02167	10.13408	24.54259
CUST_2429_PI429010886	N12328	0.000922461	18	3						70.36116	29.20882	72.23349	78.46823	49.67735	61.55047	29.76143	36.48523	60.60875	49.19138	67.01094	49.0091	77.5233	27.49298	62.05097	15.81139	30.30695	23.45708	49.62	52.63501	46.57555	98.46402	93.50035	124.3819	66.73428	65.26399	87.33991
CUST_17161_PI429010886	N37462	0.000928226	24	18						83.08821	86.04303	106.9297	92.36671	72.72426	57.95506	89.13599	105.4679	101.8124	56.64171	71.1558	63.74918	103.8566	93.33147	73.01092	78.36365	92.99115	85.1761	143.0944	151.5144	176.9714	100.1686	114.9651	100.9354	126.2905	66.09647	144.9379
CUST_20583_PI429010886	N43700	0.00092852	24	15	307187382	Ribonuclease P/MRP protein sul	Nucleic acid metabolism	RNA metabolic process	2.79E-17	16.09247	24.15844	28.81419	10.12825	12.92804	10.4666	16.14215	43.83834	29.87687	19.71754	26.44115	25.51773	18.87393	21.23798	20.49572	31.26771	74.95343	30.905	24.83681	18.34193	28.42265	16.48308	18.9186	23.19651	22.03729	16.77987	24.67313
CUST_43766_PI429010886	M63916	0.000931273	12	0	291241819	PREDICTED: p90 ribosomal S6 ki	Protein metabolism	Cellular protein modification process	7.84E-10	76.58195	61.82712	66.02974	65.03673	57.34418	81.5061	29.08789	53.49503	49.10462	60.81905	60.96816	63.2086	63.2086	101.3475	135.7682	61.94047	46.26381	43.94355	60.66586	47.47767	43.85961	77.67075	48.19092	77.60656	62.01214	61.06467	81.04337
CUST_5770_PI429010886	N19172	0.000931273	12	0						126.2182	73.68465	113.1524	139.866	63.59136	61.02485	75.9364	39.86943	50.51811	112.6234	73.37425	74.97441	123.9728	74.82596	94.86558	90.57569	54.11854	61.05496	50.31996	55.93014	53.98068	113.2744	54.08431	69.53147	92.20119	70.35289	112.7612
CUST_43984_PI429010886	M64555	0.000933845	21	15						11.59928	27.06704	19.87479	16.80772	19.22794	21.48468	19.95043	22.20069	13.64348	27.5259	25.46688	33.49057	30.58701	23.73807	26.41027	30.37998	30.96219	26.0084	39.60134	21.20837	21.64409	24.16626	19.7933	31.11334	20.13887	17.47317	16.7995
CUST_18343_PI429010886	N39619	0.000938977	18	0	390352602	PREDICTED: zinc finger protein 8			7.47E-15	31.99537	33.92477	45.68766	35.79767	33.39499	17.13499	27.41576	21.48216	31.07275	25.66233	19.59742	17.311	19.95709	24.13994	23.45708	24.82555	41.93907	32.8731	39.63818	31.12893	31.73936	34.26345	43.26708	30.04497	31.48667	18.61955	15.66009
CUST_4016_PI429010886	N15791	0.000939763	24	12						8.203427	6.882653	3.016986	7.444772	NA	NA	8.416104	25.79299	13.07493	9.94845	7.971271	5.829325	23.43945	16.58967	14.25928	13.82262	NA	7.404706	18.43607	19.44615	22.97146	10.04386	9.471206	12.34559	3.432355	5.272947	7.694205
CUST_50864_PI429010886	M91274	0.000941271	24	0						38.29909	52.47367	41.36333	NA	21.07756	5.049709	25.23036	37.40246	20.93117	12.89468	21.84605	10.98475	18.11998	17.19316	23.15112	29.55174	24.5682	35.66159	35.28538	43.58292	25.30132	19.96659	36.41114	16.50625	53.36513	23.2872	43.92043
CUST_2801_PI429010886	N13137	0.000942668	24	15	260836807	hypothetical protein BRAFLDRAI	Metabolic process	Other	5.74E-70	41.93463	88.14296	57.51072	35.00954	55.41495	33.84921	48.53921	80.97133	64.11411	25.48421	49.22048	24.73755	66.97219	55.01648	69.2197	87.43473	125.8771	80.19562	57.48416	78.48989	99.52529	72.42124	67.16357	64.85257	54.49956	46.49241	71.77497
CUST_33183_PI429010886	M6211	0.000944924	24	18	506969159	DNA-directed RNA polymerase [	Metabolic process	Nucleobase-containing compound meta	2.36E-24	14.2026	17.98498	16.42417	13.75076	13.52973	4.78806	10.55727	11.80584	9.708019	10.78499	10.99842	11.14467	22.49107	18.84709	10.06366	19.9833	22.42596	17.37448	28.03915	21.66483	29.93334	11.81288	13.56135	43.8251	32.85244	8.105145	18.7439
CUST_43942_PI429010886	M64411	0.000947915	18	12	308220078	LIM class homeobox transcriptic			1.63E-19	18.69191	6.46829	13.65748	18.2488	3.489258	13.10704	19.70046	17.30627	18.23431	28.25136	13.74572	21.90402	36.00369	38.98881	46.48232	21.1933	18.61345	16.03181	21.06899	8.447449	17.93558	21.84014	5.256252	13.36606	16.90111	18.33955	16.33991
CUST_12407_PI429010886	N29413	0.000947953	24	21						46.39912	55.65477	47.87148	39.26035	50.07055	48.0027	45.91877	44.82733	42.80558	51.8805	40.13911	12.6262	44.7597	39.95086	46.64063	61.81635	71.89101	51.74535	60.65305	46.983	55.59367	80.24156	50.97241	79.15396	86.92889	56.87317	71.33856
CUST_42778_PI429010886	M61230	0.000950976	15	9						17.47661	14.12309	22.78318	18.3768	20.7112	11.68791	28.72062	19.42744	28.71068	39.34641	64.80138	19.2221	34.88249	18.00685	23.36828	16.63618	12.08782	20.68602	22.41168	19.95277	25.8625	34.2712	29.85271	29.42662	35.80613	18.34559	49.65203
CUST_2720_PI429010886	N12976	0.000951737	24	15	332376786	Dendroctonus ponderosae clone			3.05E-162	328.2183	506.2756	382.9427	297.6356	250.0724	232.1097	466.9715	458.4811	422.6098	483.0822	342.9247	361.2613	422.0714	309.878	365.989	505.8465	677.0488	548.5685	461.522	428.8199	398.5907	394.5922	358.5953	393.8421	495.3083	373.8469	431.5244
CUST_1069_PI429010886	N8399	0.000951737	24	15			Nucleic acid metabolism	Regulation of gene expression		2650.032	4880.699	3108.034	2369.531	2061.689	2216.187	4295.699	3667.762	3672.992	3808.458	2393.197	2469.347	3284.996	2648.467	2491.674	4602.898	5342.932	5265.552	2895.969	3445.555	3543.046	2666.243	3336.354	3342.747	3269.912	3220.778	3770.678
CUST_57312_PI429010886	E5180	0.000956054	18	0			Metabolic process	Carbohydrate metabolic process		63.62492	55.95026	117.0136	70.46471	15.18461	57.16004	15.03718	44.81289	50.1322	13.92104	6.239298	14.81286	11.624	36.22942	NA	15.22691	98.19279	51.55397	48.66684	79.03209	32.8145	61.87915	30.01855	30.02506	35.4183	9.223983	34.33515
CUST_28861_PI429010886	N58744	0.000956761	18	15	443715571	hypothetical protein CAPTEDRAI			9.05E-08	26.4274	57.80626	28.72317	53.43212	22.6604	34.04952	50.20946	37.56673	40.95208	56.12288	69.24371	43.28077	41.46642	47.83813	33.34498	85.24573	117.4173	77.808	33.14692	54.6495	69.5761	29.56665	22.75769	20.37081	58.4887	44.84883	47.56782
CUST_36157_PI429010886	M14833	0.000957927	15	3	321463201	hypothetical protein DAPPUDRA			1.16E-15	20.80721	20.14368	11.77605	21.53884	26.38195	21.74375	28.16376	16.36929	18.75881	21.29385	11.7375	10.86736	15.29719	14.20021	11.36638	33.08731	19.67391	13.42624	29.35503	27.29078	28.28966	15.78875	16.35184	16.17305	25.0865	13.45766	21.62696
CUST_5708_PI429010886	N19078	0.00095943	24	21						200.7515	152.6002	185.937	134.826	176.076	143.1622	108.5947	135.3432	116.1695	151.7315	144.9581	115.9325	151.5867	148.6436	152.4355	195.511	200.5182	144.7602	205.536	164.0034	116.2155	240.802	176.6888	155.7231	208.6833	203.7183	253.7732
CUST_44777_PI429010886	M66956	0.000965427	21	0						229.203	1080.452	1325.644	355.0829	334.9433	310.0635	316.8751	274.7682	705.624	99.26059	306.2147	174.2396	242.133	201.0938	207.6167	256.539	56.39523	321.03	508.6367	207.4056	361.1904	406.6714	401.876	588.5037	349.1843	184.1477	448.34
CUST_39058_PI429010886	M34034	0.000969349	18	3						130.5163	50.30791	141.2631	208.6833	117.0919	191.1727	26.59448	79.68621	59.19847	25.90356	115.1501	68.25591	173.502	139.0252	187.7437	15.20646	26.99058	32.41501	76.3423	56.67374	71.70119	266.1986	188.2411	252.6661	119.2957	117.3668	161.754
CUST_45420_PI429010886	M69045	0.000971172	24	6						28.05953	39.09638	49.3138	22.7622	46.44642	37.28516	41.24111	56.93121	41.19213	36.26515	31.49032	39.14896	35.32719	25.1528	27.53522	31.58885	27.66084	38.4343	29.57468	25.24183	21.12553	29.09733	25.52576	36.07467	39.70565	45.02349	36.69203
CUST_13253_PI429010886	N30854	0.000972402	24	21	291239959	PREDICTED: peroxisomal membr			1.25E-26	13.53611	17.10224	15.37323	8.405629	10.90468	3.927512	15.32337	19.64434	10.51731	5.787811	7.876547	8.835842	9.412668	6.960251	7.197173	NA	16.85775	10.22133	13.34035	15.92717	9.109484	14.79074	18.26962	21.08968	19.73758	13.16694	20.78179
CUST_39828_PI429010886	M45292	0.000973821	24	18	197940771	Euphausia superba mitochondri			1.08E-162	554.2471	863.266	831.3637	621.9301	555.5824	590.9592	326.6163	704.3483	720.5302	381.6653	543.9588	383.9729	461.1854	724.6231	417.6111	643.379	414.6538	555.7599	733.9767	736.2732	946.146	697.9099	742.7432	654.3402	731.2874	638.835	691.8963
CUST_7801_PI429010886	N22101	0.000981033	24	21						2570.156	2988.845	2419.664	2167.915	1667.986	1503.022	1674.551	1718.114	1796.427	2203.327	1655.603	1028.452	3336.354	1234.088	2412.569	3292.245	2952.044	2586.608	3292.245	2378.51	3313.258	3561.33	2952.044	3224.848	4750.447	2327.901	4517.506
CUST_36897_PI429010886	M17175	0.000981938	18	15						103.4374	168.4308	97.30769	97.77174	64.91207	92.91315	192.3394	152.4772	120.9506	155.7011	82.35709	90.8123	129.1825	105.6058	113.6067	195.082	223.5106	184.8821	140.4269	131.2392	137.7031	96.30356	93.83228	111.4113	101.1475	111.6045	111.1784
CUST_34326_PI429010886	M9334	0.000984149	24	18	62126070	C-type lectin protein [Fennerope			0.000000271	NA	23.36033	NA	9.425176	4.019051	7.136768	NA	NA	NA	NA	3.13128	NA	25.90662	196.8082	26.0759	98.82385	55.4869	52.62255	300.4274	16.46661	237.366	308.0511	25.3952	238.7217	9.538918	10.68359	11.2206
CUST_8552_PI429010886	N23212	0.000991616	24	18	332023058	Protein lap4 [Acromyrmex echin			8.57E-68	31.90012	36.1335	45.17758	35.59474	22.58496	28.49131	32.66605	30.6586	29.89602	39.73627	23.74587	39.55139	49.51361	38.12957	39.02642	50.36694	36.05055	42.42056	51.87516	40.85573	42.26354	48.87241	29.9505	40.6742	44.48574	27.02166	38.15601
CUST_28125_PI429010886	N57342	0.000991616	24	21						42.59404	46.09063	58.96212	35.92153	23.33045	30.31994	42.24729	36.89171	27.33338	48.7205	46.51413	38.9642	40.32229	23.49112	36.37036	43.13077	16.22893	53.62536	72.02191	43.5104	60.65875	105.7899	71.26688	97.80698	121.7034	49.74586	86.9167
CUST_39842_PI429010886	M45566	0.000994191	15	3						573.9026	339.636	463.096	1156.716	336.9544	858.1052	155.0166	797.7145	372.7272	205.9794	204.5441	180.676	298.3249	426.3157	174.4367	186.13	443.2849	442.8695	689.5858	584.4771	604.6962	633.4883	365.6513	193.7593	443.355	123.9057	220.5131
CUST_39674_PI429010886	M42712	0.000994527	18	15						2534.505	3182.56	1695.11	1260.081	559.7322	667.6928	2536.537	2263.041	1965.787	2646.596	655.1561	1799.736	2210.04	1061.955	1388.047	3650.983	4288.608	4841.865	1870.445	2276.393	1882.752	1117.567	1691.116	1774.191	1806.683	1837.894	1818.148
CUST_5138_PI429010886	N18306	0.000994527	18	15			Nucleic acid metabolism	RNA metabolic process		677.0488	1149.936	915.7404	697.732	611.8901	705.4218	1074.104	895.8876	939.4725	1069.78	711.5947	793.634	977.9831	762.6542	886.9505	1188.024	1380.586	1294.853	985.8154	861.2738	1170.327	688.6527	773.0893	848.9964	1005.258	669.7814	1034.116
CUST_2669_PI429010886	N12873	0.000996194	24	9			Metabolic process	Nucleobase-containing compound meta		11.87453	6.49677	5.688322	NA	8.042805	6.564831	NA	15.31063	11.24141	NA	35.4224	11.6712	NA	17.37611	NA	21.37846	10.55458	6.834174	NA	9.532396	6.450227	6.029155	4.72696	6.809331	9.800749	5.138058	NA
CUST_43979_PI429010886	M64545	0.000999194	21	6						19.17715	18.84878	13.76817	33.02453	10.44922	26.1748	40.44359	30.3832	51.58532	21.72565	30.17742	32.51285	35.0064	25.97804	13.94762	39.60134	26.52653	26.22964	21.2559	12.47654	15.06042	14.33104	6.64674	18.00324	17.92495	36.64741	14.1279
CUST_44451_PI429010886	M66032	0.00100206	12	9						21.67269	25.69361	34.88562	30.43559	NA	8.188858	35.31786	10.07137	34.54667	59.19241	25.6591	43.55336	101.4958	42.38319	6.110374	22.11983	20.01232	19.92575	16.25246	30.47418	39.33451	260.9147	58.87393	214.2802	33.48088	38.52068	35.71025
CUST_32831_PI429010886	M5211	0.001004837	24	18						61.68316	149.6649	97.16144	17.44275	21.80093	25.02902	44.05006	74.5042	97.51431	28.00501	40.64205	25.69105	40.93434	53.69851	39.42252	81.43322	60.74813	95.52209	153.502	96.71212	184.7058	90.64448	40.34223	74.23643	136.1879	54.97558	117.6349
CUST_17465_PI429010886	N38018	0.001004837	24	18	478253811	hypothetical protein YQE_0907	Developmental process	Multicellular organismal development	1.29E-43	56.4057	42.60881	50.15592	45.63101	32.95886	30.905	66.46575	45.92624	49.80766	42.52944	39.53461	40.74313	72.82177	45.01683	61.1324	82.32889	55.03418	79.43494	89.98471	46.02907	91.10731	87.11871	52.91229	73.62565	88.24057	46.39063	72.20099
CUST_7798_PI429010886	N22092	0.001005102	24	15	443727911	hypothetical protein CAPTEDRAI			7.68E-08	53.80406	57.0448	47.38682	45.16295	35.01298	43.62652	105.9997	117.4173	117.9968	139.9842	88.92336	59.00011	107.4986	89.21288	56.10448	200.9398	105.6309	175.9965	108.8028	91.10731	138.824	103.9866	35.23848	62.05756	105.3837	25.40416	77.79664
CUST_14674_PI429010886	N33272	0.001007116	18	15						343.1775	306.0384	306.5038	318.5699	286.6983	319.3967	257.4878	179.6067	246.7787	413.5169	257.3491	320.1464	301.3368	256.6162	307.6081	417.734	377.6097	515.4837	251.0873	258.5627	316.4278	348.5318	287.0864	452.0404	273.2424	198.6089	238.025
CUST_49109_PI429010886	M84132	0.001008604	24	9						12.70063	6.681327	11.16309	20.47903	NA	7.886951	13.64348	11.68057	5.979128	17.425	18.11022	14.48348	12.46071	10.4115	6.81204	6.778794	9.666191	4.387226	7.991764	4.504529	5.785239	6.330656	7.916441	4.716104	14.11031	6.854397	10.45708
CUST_27051_PI429010886	N55357	0.001009355	21	0						181.1959	162.4391	150.0295	162.5329	164.0459	155.9036	113.1623	132.6428	135.5555	163.8654	118.3236	116.7758	84.34054	76.14147	99.65569	111.1293	204.1061	125.1074	160.1694	103.9647	126.1425	168.4808	151.5867	131.568	181.3504	127.1629	139.9842
CUST_18543_PI429010886	N39985	0.001010747	21	3						26.62694	21.55177	25.12907	26.73995	23.16492	29.6926	24.88691	24.4152	28.7398	26.91606	15.53825	15.14518	27.86183	29.17859	18.5866	11.86651	18.88946	15.15512	14.48646	18.21898	7.429163	14.10253	22.07673	30.8222	27.60039	30.43834	32.41159
CUST_641_PI429010886	N6502	0.001011325	21	18						300.7552	8.588027	393.924	NA	NA	NA	89.63749	18.10243	76.49879	2463.526	14.85124	1767.886	4971.086	6622.666	3654.032	70.17999	6897.446	6298.27	8713.974	5184.879	6481.59	NA	9013.443	NA	64.91949	15.14996	34.86224
CUST_49609_PI429010886	M86385	0.001015903	15	0						36.17071	48.05999	33.6985	32.29221	21.65945	26.08621	28.85513	37.92736	24.5682	26.68521	18.69594	22.1135	32.15923	30.09371	41.58882	42.92276	40.11852	40.18962	41.13599	16.23247	39.47125	30.48044	34.00502	40.3254	26.37524	19.65735	11.10041
CUST_4449_PI429010886	N16781	0.001017134	24	15						8.589131	8.922458	6.451204	11.20316	8.771483	4.64216	11.12735	11.5439	17.20923	15.99305	7.567646	NA	15.02117	13.16859	7.555163	22.75502	18.86307	17.54223	12.31866	13.1174	15.61685	11.3488	10.36312	15.33232	15.32543	5.941028	15.60667
CUST_51721_PI429010886	M94725	0.00101792	21	0						134.8607	168.564	248.2232	58.60789	35.33485	12.42147	259.1614	28.19779	234.322	25.9662	59.71475	18.77257	49.27254	48.74993	8.311471	71.90542	90.62398	163.2328	357.9365	124.1913	245.7379	186.8705	264.2762	172.3736	121.6645	30.87082	123.5591
CUST_52279_PI429010886	M96944	0.001018059	24	18	328790393	PREDICTED: argininosuccinate ly	Metabolic process	Cellular amino acid metabolic process	8.36E-46	38.69748	20.81726	53.15878	25.43515	23.82619	20.55029	31.32237	27.75069	36.72861	34.82552	38.07503	32.4427	48.81917	43.76293	41.50754	52.32559	38.32469	46.54974	62.49633	34.03869	48.15075	78.50931	38.12224	53.52577	53.39865	50.28629	64.74689
CUST_6906_PI429010886	N20761	0.001018059	24	18	321470670	hypothetical protein DAPPUDRA	Metabolic process	Cellular amino acid metabolic process	7.76E-97	326.0295	419.8124	426.9219	260.798	204.4688	239.9783	367.6429	344.0033	384.3395	354.6555	281.3108	280.8283	363.6624	258.7813	256.6606	418.6884	386.8846	471.4786	503.5534	440.6377	643.6241	406.8432	317.1285	372.6214	546.0695	285.0355	440.7516
CUST_7694_PI429010886	N21932	0.001018059	24	18						213.2675	362.6643	316.9378	212.2981	216.1701	183.1939	261.9111	315.7654	280.9343	311.5041	314.6496	238.3045	250.8083	219.4592	274.4544	348.0379	328.3409	361.1904	371.9503	310.4343	361.7315	358.4079	331.7259	412.4307	330.1913	296.1605	360.6127
CUST_8660_PI429010886	N23374	0.001018059	24	21	260793621	hypothetical protein BRAFLDRAI	Metabolic process	Carbohydrate metabolic process	5.94E-66	137.9345	145.6152	177.2393	112.4891	129.2072	105.1035	45.07182	52.43555	99.98511	152.2628	105.6633	115.6552	183.9971	122.8503	146.1482	110.519	158.6448	101.2137	175.317	122.7895	113.9542	219.8774	167.5116	149.3774	293.568	106.7181	180.2873
CUST_11711_PI429010886	N28283	0.001018059	24	18						22.52707	32.92749	37.21408	28.57519	16.24785	17.78589	24.46253	28.79283	28.99688	24.60722	30.29264	27.43264	31.79073	29.35503	22.68453	26.51854	38.13279	47.36534	38.02572	43.15923	35.32216	36.66952	35.02108	42.71727	30.08709	35.13452	50.62088
CUST_30473_PI429010886	N61970	0.001018059	24	18						157.3672	211.185	203.7183	170.8635	126.5408	129.4034	211.5442	198.84	203.6782	217.2347	129.1403	144.8971	217.1972	136.5457	167.9403	184.6434	195.9604	211.8745	236.0521	222.9426	235.284	222.5585	223.1202	221.4635	204.3745	177.9861	257.1925
CUST_35730_PI429010886	M13333	0.001019705	18	15						947.0352	1493.774	1090.482	623.3324	649.4957	627.4387	1245.954	1585.44	1018.651	1289.967	1256.036	999.9612	917.1646	1177.962	584.9829	1572.421	1612.928	1247.204	1073.164	1151.814	1195.535	956.0225	852.5352	702.7679	1128.705	946.5528	955.5815
CUST_4050_PI429010886	N15882	0.001019705	18	15						37.48759	63.43943	47.59455	33.11195	7.198567	8.274559	43.51785	40.94392	23.30496	61.98746	20.64485	36.18627	37.60724	19.38387	23.70418	56.79387	75.77051	84.16808	64.27657	50.58432	40.52376	8.807925	23.69696	20.19611	40.62184	41.31086	36.63038
CUST_21886_PI429010886	N46041	0.001019758	24	15	260808223	hypothetical protein BRAFLDRAI			2.96E-11	66.60981	39.12873	47.75189	59.17675	28.35837	43.52338	50.36694	47.16802	41.60531	78.06756	45.872	69.82715	64.74689	59.79025	51.37499	90.55203	63.27624	100.9118	96.35679	54.0024	71.14732	64.61049	69.5671	96.02016	59.77062	44.73509	74.56881
CUST_34317_PI429010886	M9309	0.001024088	24	9	154417199	ankyrin repeat protein [Trichom			5.08E-09	31.4461	52.43098	41.97418	57.47217	40.4281	63.45821	52.85457	74.55481	94.66861	60.45821	152.7399	43.60777	49.04219	93.24932	53.33823	62.5913	132.9149	124.0832	22.81991	46.52866	35.45282	29.16024	34.28997	26.77786	22.03984	32.25543	26.84779
CUST_3431_PI429010886	N14485	0.001026235	24	18						10.765	9.351659	14.00174	11.51019	8.708353	11.79955	3.737478	NA	6.758311	6.96624	6.200844	6.203165	20.80721	8.616008	7.663806	8.81061	NA	11.00412	NA	83.34871	88.33336	NA	12.07465	5.304563	79.45422	9.324284	69.55765
CUST_6028_PI429010886	N19528	0.001029649	12	9	50539914	alpha- and gamma-adaptin-bind			1.87E-26	497.5258	208.8218	506.2756	769.5468	355.841	459.6187	233.5329	270.5362	362.8786	895.4864	1787.383	704.9081	854.7576	435.0369	640.2875	250.1769	216.0628	262.8559	424.7669	351.2785	428.0259	1145.735	1416.762	1450.664	522.6994	1154.885	1649.323
CUST_15134_PI429010886	N34059	0.001030816	18	9						13.43266	22.04447	10.40534	15.53301	16.31049	13.34178	16.81027	20.95393	22.63685	26.88006	20.52844	16.91351	17.68505	17.31531	NA	18.89336	14.29468	23.21157	9.612207	15.36528	12.03178	11.87026	15.71789	11.58566	19.75521	17.12042	16.52245
CUST_10139_PI429010886	N25758	0.00103128	24	21	321471060	hypothetical protein DAPPUDRA			1.39E-15	77.56319	64.33318	64.03624	77.10772	67.10463	75.51715	40.33676	52.50511	64.0447	69.19342	61.4469	28.94147	68.39377	60.5484	77.97296	86.26011	73.80869	73.89284	74.63974	61.89058	75.73146	75.61315	78.76138	95.33452	101.7333	75.17439	88.16698
CUST_10486_PI429010886	N26310	0.00103128	24	3						86.40352	54.32526	66.84176	80.70329	103.9519	93.69945	74.40311	76.96435	43.06564	71.26688	89.85516	103.0566	69.37968	80.16932	90.23368	58.445	63.17902	68.71816	58.24898	57.89687	47.56782	62.01214	61.68316	67.12887	64.6674	65.53387	51.75429

Probe ID	ID	p-value	Period	Phase	EBI ID	Annotation	GO category	Go term	E-value	0	0	0	3	3	3	6	6	6	9	9	9	12	12	12	15	15	15	18	18	18	21	21	21	24	24	24
CUST_26392_PI429010886	N54165	0.00103128	24	21						39.28587	31.83324	45.0895	42.97957	34.72664	43.74801	31.04302	34.13318	52.49766	39.56268	40.63005	41.47035	45.83287	47.85099	30.21215	49.4765	54.94337	40.50707	52.66618	48.33387	50.82457	58.15501	57.06084	54.93619	40.48248	47.14137	46.6272
CUST_6720_PI429010886	N20510	0.001032968	21	3						651.0737	593.5138	NA	20.90369	1216.51	1158.756	523.8156	1787.383	490.8866	NA	223.2399	277.8871	NA	211.3027	20.38177	30.76277	8.540806	10.99089	16.18229	2406.654	20.60087	NA	919.0444	NA	NA	3568.199	NA
CUST_5552_PI429010886	N18853	0.001034729	24	15	321460932	hypothetical protein DAPPUDRA			2.5E-99	23.86386	23.01193	23.58164	29.04471	10.75635	31.48421	25.65567	22.5078	30.92492	34.80724	33.94092	30.43314	34.47718	30.44744	24.40118	53.83595	34.13078	46.47735	27.43264	21.43051	31.15433	20.78179	20.56563	35.85354	22.142	14.31582	24.40118
CUST_11141_PI429010886	N27332	0.001037452	24	15						NA	11.9832	9.304777	7.517792	3.566301	4.901556	17.58836	20.78429	11.4247	13.91913	NA	7.31445	10.35412	8.409122	14.04735	NA	24.12932	30.96531	7.61802	22.25011	23.23151	12.93095	NA	11.63109	11.45478	NA	3.860932
CUST_46258_PI429010886	M71970	0.001038886	18	0	298204347	endonuclease-reverse transcript			3.69E-09	86.30982	66.51767	77.74804	79.99233	60.4258	71.33856	60.65305	51.64343	57.52657	65.96155	49.97848	63.22855	75.58044	49.71921	52.14659	76.71191	50.00771	72.24298	87.74995	76.42243	78.50931	62.84262	61.80424	62.93557	57.03545	57.48416	71.60883
CUST_53377_PI429010886	M101458	0.001044502	24	18						21.80093	16.22893	10.73543	19.77825	13.76817	15.99154	27.37201	10.21986	18.41787	23.69946	13.94885	23.36033	20.08349	11.3139	29.15439	27.50901	32.53094	25.61401	39.1588	25.59575	29.00837	31.33292	26.30097	36.54404	24.24527	18.66636	24.91804
CUST_9133_PI429010886	N24117	0.001044502	24	3	332017603	PTB domain-containing adapter			2.16E-29	27.14205	21.15973	25.9824	29.25153	35.63404	26.6468	15.35298	26.78263	15.40011	25.84968	17.68714	34.42594	25.14815	21.7119	35.30558	21.08201	20.27243	16.92486	9.657459	17.13499	15.63819	21.18377	15.78726	18.28904	23.81554	23.38956	19.30372
CUST_13293_PI429010886	N30923	0.001044502	24	21						94.30839	88.8899	117.3396	82.95322	87.96067	86.84479	56.65739	77.14782	78.56039	78.81049	59.3284	95.08224	58.12065	90.88256	72.02191	128.9772	83.69522	96.5246	120.5967	71.79771	93.35268	126.327	91.47922	121.6645	128.5506	98.04747	117.2793
CUST_53351_PI429010886	M101378	0.001044883	18	3	121484195	cuticle protein CUT5 [Portunus p			6.31E-14	4809.493	164.8407	6060.248	8146.369	10815.88	8723.793	250.3956	853.7719	773.3668	3658.191	12939.25	5462.129	3827.862	5578.907	6255.734	81.17631	143.938	77.36861	1226.655	1178.539	1202.203	6818.249	7558.705	7348.003	1455.41	4266.005	4101.099
CUST_36837_PI429010886	M16994	0.001044883	18	3						242.9432	58.52453	193.2061	571.8635	273.8856	382.8713	107.3799	197.0569	140.549	421.3837	960.1156	389.1575	580.4723	196.9337	221.9252	3.227448	3.98717	4.304099	122.201	347.7046	99.44651	746.1539	1296.153	1039.149	223.4669	644.9039	506.3774
CUST_39630_PI429010886	M42011	0.001044883	18	3	121484199	cuticle protein CUT8 [Portunus p			1.92E-34	11413.74	446.7799	14256.4	19341.82	24426.06	21881.51	718.7132	2422.76	2463.526	9313.798	33076.81	12765.38	9613.776	15071.82	13098.74	220.4736	309.4924	191.823	3247.587	3428.918	4000.901	16372.71	18365.76	18476.28	3712.526	12725.99	10764.25
CUST_44110_PI429010886	M64974	0.001044883	18	15						60.15379	72.02191	72.83024	54.80156	42.28696	45.91877	46.93369	62.73163	63.03855	47.01945	45.52154	62.3082	64.24425	54.591	49.57699	77.32899	72.99439	72.30895	49.014	45.45322	60.78187	41.02313	42.32017	53.21446	59.50711	64.74689	60.95576
CUST_33747_PI429010886	M7781	0.001047918	24	21						6.004064	NA	10.73905	4.267308	NA	11.35653	7.780482	5.665078	NA	5.680908	7.99563	NA	NA	NA	NA	13.78137	9.949274	19.99783	11.09687	12.42147	9.137298	24.71432	31.673	29.9255	8.558176	11.76598	7.515502
CUST_38924_PI429010886	M32005	0.00105283	24	9						38.21258	46.21146	47.25275	197.7606	48.72562	140.038	120.4647	97.39106	177.9352	87.38427	215.0811	64.82247	230.2055	150.8908	65.40116	82.9019	46.24985	62.96898	75.02912	51.84236	76.45795	82.03251	39.8332	67.01094	28.66457	44.09481	52.26773
CUST_16218_PI429010886	N35866	0.001054722	18	3						27.58739	38.86739	30.88085	36.07006	51.32543	62.80714	36.66952	53.20844	32.29945	31.31216	26.85225	46.21146	27.79642	26.21426	28.04788	32.32834	42.1572	29.4638	28.12044	36.32916	31.90974	40.80443	43.31181	50.98858	26.61853	50.21608	30.98378
CUST_39355_PI429010886	M38223	0.001054762	12	9						6226.747	919.6173	6559.55	9980.233	2844.645	3190.966	2294.993	2244.174	4266.005	12317.37	15170.38	7122.178	15394.16	3882.503	6854.464	197.2216	127.8764	285.0355	7459.987	4655.696	7653.847	12725.99	15258.82	15394.16	9129.704	12807.83	18146.2
CUST_56325_PI429010886	M113499	0.001057723	24	21						250.35	251.2413	271.0733	235.6895	210.5427	215.2938	183.2355	234.0079	249.5605	284.5651	209.1753	245.3228	315.4118	202.3368	249.6908	226.6524	265.4961	220.1875	251.7468	263.7057	323.2204	329.7868	300.9971	374.6189	264.5418	222.4375	339.1735
CUST_6619_PI429010886	N20359	0.001057723	24	18						121.4525	191.4096	188.4673	149.6649	147.7951	119.3462	141.6695	169.4561	203.5459	175.317	165.1857	174.7665	211.4099	147.2411	172.6851	207.0168	254.1458	239.9273	221.1582	198.4091	260.0953	202.4277	183.9085	244.3561	214.6479	190.3204	299.1753
CUST_9156_PI429010886	N24150	0.001057723	24	18						49.85973	58.62146	61.75273	37.57549	44.82232	40.57567	50.97745	50.89474	62.41935	47.6343	61.145	57.88315	49.71376	47.95196	65.2491	51.24138	87.24349	63.72422	76.42243	62.76751	80.68221	76.24747	81.60481	104.9484	77.3983	63.07768	93.60566
CUST_13115_PI429010886	N30598	0.001060879	24	3	545193871	PREDICTED: heterogeneous nucl			3.76E-18	116.7899	89.19083	142.9612	111.1784	179.2149	140.8575	153.9744	102.5315	112.2837	146.8442	88.4931	110.2867	107.8221	178.033	114.3854	64.35228	89.64766	60.94356	78.01174	90.18372	46.78844	91.50373	90.32894	51.5089	95.01092	107.1012	77.17435
CUST_12265_PI429010886	N29190	0.001067883	24	21						12.3024	41.42487	38.70891	21.75638	10.46073	16.58967	30.52273	20.28589	44.17172	32.20511	18.2969	14.95849	8.551917	7.469059	9.132496	20.15147	19.96229	25.31848	66.05158	24.65812	47.08587	45.82718	28.2373	48.71388	33.63302	24.50769	37.25639
CUST_8946_PI429010886	N23835	0.001069932	21	18	321479379	hypothetical protein DAPPUDRA			2.8E-17	13.44969	10.02353	NA	NA	3.501373	8.724815	NA	6.940213	8.158467	5.286558	9.676757	8.525673	7.402375	NA	4.031451	15.65279	8.724815	24.71116	15.11126	25.17932	16.45591	9.133693	9.801925	14.86509	14.0591	3.21043	9.824108
CUST_1558_PI429010886	N10064	0.001070945	24	21						49.39254	58.77493	66.76294	51.0346	60.5484	48.82419	41.87629	55.97133	55.05576	49.82997	67.11217	59.9023	50.1877	57.01067	58.07534	53.98617	61.57198	67.66335	65.67573	56.99251	63.69892	73.60215	68.80951	90.44348	51.53362	60.19335	70.3428
CUST_24512_PI429010886	N50750	0.001075078	12	3						NA	12.50022	17.88097	8.782333	13.95493	15.65279	NA	10.28394	12.6121	9.798222	4.808312	8.1002	11.16576	19.7864	10.14305	29.2298	20.78429	25.21391	41.45022	16.43359	12.53384	8.233633	9.695023	9.563744	9.607864	20.04802	12.86584
CUST_42852_PI429010886	M61426	0.001075343	24	15						13.06086	32.39325	NA	49.5489	26.0166	48.36443	129.7163	41.13217	90.09793	66.00136	18.68614	33.45999	73.56286	47.19052	46.09063	96.1174	154.9154	127.3889	68.72832	27.46378	51.25301	54.40508	35.34705	58.78081	40.18962	31.28202	8.120127
CUST_47262_PI429010886	M75374	0.00107561	12	0						29.42662	24.43918	29.24902	15.32656	31.96971	23.11733	6.040983	24.34505	18.76293	23.86079	30.4229	42.0865	19.41864	36.55366	47.22395	33.33002	28.15447	28.34861	16.71688	13.67002	17.08471	35.69023	26.57189	32.86579	38.08917	40.59088	43.13077
CUST_30128_PI429010886	N61220	0.001076135	24	12						88.36719	74.23643	99.83532	79.97607	53.43212	67.01094	76.84692	80.63831	96.99292	133.4611	45.31246	108.0539	141.1475	102.1812	108.3596	104.173	134.2942	95.71962	115.5552	108.583	88.8899	116.2009	110.2733	85.45508	103.616	81.72083	87.28041
CUST_36739_PI429010886	M16712	0.001084167	24	21						29.90874	27.99655	35.02468	27.21619	31.51924	25.45483	14.78891	16.69734	19.25825	28.1014	38.27077	32.37762	22.34874	20.41652	39.08397	26.22964	30.84927	24.94781	28.73586	27.36342	27.04259	39.80982	41.36333	34.9059	27.50901	36.48955	47.58659
CUST_14821_PI429010886	N33528	0.001084167	24	21						306.3603	391.4124	421.8557	415.0852	340.6386	370.6325	355.0829	370.0497	405.1127	377.4146	383.8668	338.507	441.3596	380.5457	444.1379	392.253	463.096	445.6007	391.1906	406.8432	432.4641	504.3232	458.2399	559.5956	452.8102	376.0142	525.6391
CUST_42599_PI429010886	M60683	0.00108532	12	3	321459460	hypothetical protein DAPPUDRA			1.74E-19	NA	16.61149	10.87896	NA	31.31216	18.83308	15.76131	20.57512	16.93224	15.26493	16.91699	14.74345	6.413865	17.19904	9.228702	23.01448	42.05307	24.13994	19.05161	15.87551	15.56681	6.824523	10.41882	9.678103	16.2921	8.440387	11.32723
CUST_616_PI429010886	N6335	0.001085421	24	21	321455420	hypothetical protein DAPPUDRA	Metabolic process	Carbohydrate metabolic process	5.01E-93	2676.834	2719.085	2719.085	2867.707	1707.189	1863.699	1412.941	1797.322	1702.984	2188.815	1458.832	859.4584	2945.018	621.1319	1969.061	1991.351	2646.596	493.0213	5125.495	1931.709	4109.069	3783.527	5002.917	3390.028	6032.614	3899.124	5215.78
CUST_5622_PI429010886	N18959	0.001087383	21	18	351542215	exonuclease 3'-5' domain contai	Metabolic process	Nucleobase-containing compound meta	7.41E-77	6.010955	17.67253	12.67284	7.941808	10.45586	7.869127	5.698034	9.095729	7.509062	9.279573	7.562913	4.479614	6.550174	NA	10.01073	9.989717	13.77957	9.551996	NA	17.08055	13.99873	13.82584	16.06582	9.781107	10.32743	5.415947	12.17199
CUST_1733_PI429010886	N10564	0.001088628	21	15	15425681	I-connectin [Procambarus clarkii			5.61E-27	91.58124	78.74455	74.26533	87.06038	80.87979	93.50035	82.66079	68.44391	71.74885	80.43757	61.76701	30.93515	66.02134	81.28557	71.24846	120.0673	108.4697	119.2442	89.99586	73.39823	102.3977	76.80903	95.29319	98.40391	81.11075	72.30895	89.91214
CUST_32198_PI429010886	M3501	0.001090483	24	9			Signal transduction	Cell-cell signaling		137.5078	124.2158	85.69239	106.8499	163.338	156.7242	228.9535	158.0057	137.0969	149.8334	168.9955	173.5314	137.6616	151.922	146.2607	236.5379	128.8295	170.0181	190.9332	124.0673	154.4454	77.91051	118.1166	93.38599	101.8893	105.6512	106.7994
CUST_15428_PI429010886	N34567	0.001090773	21	3						14.03403	14.05324	9.485255	16.20857	14.9705	18.51168	NA	14.28509	13.08598	8.109042	16.44507	14.22003	7.10526	17.24304	8.634832	NA	9.619909	14.01651	10.89953	12.98896	11.9073	13.16859	NA	15.80769	17.52474	19.74485	15.48837
CUST_26016_PI429010886	N53499	0.001092654	24	12						13.68542	11.14748	13.59757	20.15147	32.04577	19.58744	38.06969	6.238498	16.12259	NA	14.26068	15.21701	159.7501	23.18254	21.45343	15.51385	13.82436	12.24304	5.343407	6.994855	41.77121	3.989872	7.931706	11.32007	11.43195	10.63475	13.09363
CUST_29259_PI429010886	N59499	0.001097125	21	3						37.47176	27.70466	35.97078	45.00629	42.71727	43.39176	32.77297	33.98225	31.57973	39.89679	32.42666	34.12419	47.53117	37.34538	41.25004	33.14322	25.09267	28.75373	30.98678	30.89131	29.63864	49.41341	36.40347	36.5095	50.87138	40.89712	40.04223
CUST_55538_PI429010886	M110577	0.001097388	24	18						32.91719	25.86911	56.05212	24.46439	10.55248	24.80109	134.3589	135.4737	103.0566	42.88959	17.44773	16.39186	83.1934	39.25338	32.53452	141.352	232.2336	374.5443	153.318	640.002	640.7195	120.7183	16.74062	72.52843	447.0887	110.6819	227.3167
CUST_34986_PI429010886	M11248	0.001097388	24	18						195.511	239.9783	296.2664	203.1024	198.7466	154.5211	249.888	251.9547	305.3804	252.7366	219.187	229.8267	262.3549	222.8685	241.9944	303.5583	303.3581	243.4049	264.2762	310.6389	324.1912	265.2779	257.7274	323.2204	242.2901	251.4577	328.4824
CUST_5210_PI429010886	N18403	0.001097388	24	3	332023022	Ornithine aminotransferase, mit			4.84E-176	165.9265	115.9507	115.1679	150.6494	203.6782	191.5119	112.6387	80.41026	80.65861	89.7803	157.6143	236.2759	72.88356	128.0075	142.2741	94.04538	85.91672	76.05422	77.38763	86.43772	68.2334	82.70695	80.50976	75.23071	83.74447	100.7337	98.52549
CUST_6540_PI429010886	N20237	0.001097388	24	18	506968263	p23-like protein [Coptotermes fo			1.07E-27	21.52069	28.7956	30.96768	20.94297	17.22127	10.73905	25.01036	32.64901	25.04161	32.33684	25.44757	12.70744	34.09591	22.45228	22.21134	22.35516	25.47879	25.65359	30.81577	34.60522	40.4281	31.80176	40.86304	33.84162	24.92085	27.63333	32.32834
CUST_57314_PI429010886	E5221	0.00110029	24	15	151579928	40S ribosomal protein S2 [Artem	Protein metabolism	Ribosome biogenesis	7.05E-29	16580.62	20271.85	15967.93	13935.1	12021.42	14015.31	19387.17	20871.55	22526.09	19052.86	12084.47	15367.74	16760.98	15871.24	13616.29	23739.57	23961.37	23961.37	21710.8	21645.19	22777.74	15045.33	15630.09	15435.13	17658.04	14743.32	16913.15
CUST_32768_PI429010886	M5066	0.00110029	24	15	321468780	hypothetical protein DAPPUDRA	Metabolic process	Oxidation-reduction process	1.72E-41	13.63522	7.669956	7.821834	5.486086	4.421238	6.228711	15.99964	13.38306	9.709038	19.79478	12.2785	24.86359	14.81416	15.04296	18.7867	37.15316	12.21743	26.79339	22.326	13.11926	16.09566	12.40482	11.97624	30.37143	18.73595	13.80235	12.15684
CUST_9018_PI429010886	N23940	0.00110029	24	3						185.0283	114.4851	112.7999	120.5852	270.4208	213.4873	164.4172	177.2128	132.0703	101.2279	149.7452	210.4014	113.6801	128.9165	149.2223	59.36408	77.61728	50.72554	94.51495	183.8806	93.8595	132.4149	158.8243	127.6164	80.81117	209.6968	133.196
CUST_19564_PI429010886	N41785	0.001100686	21	3						45.3971	36.72861	43.07453	54.07225	33.42535	44.52658	57.69269	31.90974	53.56063	30.18887	29.34349	30.89721	32.35917	31.47936	31.23137	53.01138	28.20584	32.85935	33.01818	27.29712	31.07888	61.28823	52.98897	55.63811	63.00637	34.5207	78.72715
CUST_7580_PI429010886	N21757	0.001101058	15	9						18.51954	24.63328	16.07832	11.7024	23.76684	12.08928	NA	6.372855	5.397248	29.00837	23.99912	22.80532	18.69191	19.21337	28.46788	21.7713	15.73759	23.16492	9.078909	15.4541	14.15899	25.24448	19.15475	26.12151	24.45965	32.57078	49.79667
CUST_39228_PI429010886	M36410	0.001104832	21	15						6744.57	6592.219	4633.616	4945.667	1658.691	2900.703	14100.35	9837.56	11246.8	15630.09	6799.146	1936.909	9392.481	6018.305	4847.527	27665.22	10464.54	19791.01	14189.5	11822.99	21113.92	13281.09	2374.203	10394.61	8818.425	1358.461	5564.697
CUST_36896_PI429010886	M17174	0.001106585	24	9						301.2626	424.2083	274.8753	305.0003	720.7726	467.5708	372.9744	377.2326	383.2384	400.7915	545.361	597.9185	341.965	418.2862	538.4885	394.1154	410.9137	412.7396	322.1133	405.1127	366.7915	319.7298	348.6137	353.1568	335.6252	343.406	340.5595
CUST_5397_PI429010886	N18645	0.001106585	24	0	301607176	PREDICTED: threonine synthase-	Metabolic process	Cellular amino acid metabolic process	2.38E-180	100.5778	116.2357	153.0619	115.5828	47.35759	79.43494	84.11819	91.16754	112.9762	50.84163	79.58331	36.00369	90.19219	41.11802	50.28629	81.70379	55.89295	70.23956	91.36194	88.25185	113.3845	82.67536	76.29019	86.02033	57.7126	117.0413	185.6848
CUST_52542_PI429010886	M97868	0.001107828	18	3						344.8862	89.16759	379.6086	415.6373	494.8296	423.6	124.0485	143.6223	119.0449	263.3579	253.3528	291.4248	470.1035	240.9716	399.0134	101.852	90.91573	64.83629	148.4438	164.8407	147.2139	512.3561	482.0377	419.8124	118.0753	331.5173	218.2926
CUST_142_PI429010886	N1521	0.001107828	18	15	460838092	strongly chitin-binding protein-1			4.83E-14	29396.88	36302.66	20020.97	17771.79	9211.498	12359.97	21590.74	19855.57	20020.97	23379.75	16690.76	17623.06	17837.79	16760.98	16348.66	35738.11	37427.64	30447.23	23016.75	23016.75	12765.38	15942.89	11998.69	12963.97	27347.94	16655.53	18579.76
CUST_20240_PI429010886	N43078	0.001107828	18	3						548.5685	269.3499	737.8151	1284.327	1265.639	1387.245	369.9026	516.6055	565.6114	266.255	1143.279	858.51	840.0505	623.7953	697.0808	146.6342	108.9271	142.6928	303.7619	725.6388	635.9298	708.1548	887.5066	768.539	259.0732	530.1492	521.6397
CUST_46608_PI429010886	M73149	0.00111061	24	21	321458613	hypothetical protein DAPPUDRA			2.09E-12	42.28326	48.17616	53.1876	44.05427	39.47125	40.78067	27.60747	27.38152	31.55061	70.28922	42.04861	48.43013	42.86161	41.23754	50.46073	54.98542	47.90004	56.59797	50.78807	37.88233	49.25099	79.83656	45.35771	84.11819	61.32398	50.26083	65.43257
CUST_14054_PI429010886	N32259	0.00111061	24	21						50.28036	43.23211	58.9258	62.367	60.12159	46.17368	45.40562	45.28285	51.14197	67.06332	50.24652	57.66836	64.02245	49.70798	63.38213	67.83335	58.19234	55.09974	69.13756	64.52421	95.86932	93.42816	74.95343	78.43006	52.83788	42.10224	84.78861
CUST_36022_PI429010886	M14313	0.001111455	21	9						42.14237	44.95996	40.3323	56.68053	325.1877	239.7853	63.77213	76.51051	68.55444	66.93928	350.2239	252.6661	49.50343	303.7619	221.7509	92.69475	60.29296	61.30321	53.02213	98.2265	69.42506	68.8017	39.76763	51.44117	64.80829	150.4308	66.49647
CUST_24774_PI429010886	N51232	0.001114352	15	12						36.03288	45.27328	27.79096	39.58266	26.61351	37.90056	33.3478	49.73252	40.23235	62.1238	45.80812	45.49065	45.96012	53.31643	58.3384	46.97174	43.90368	59.12156	30.20183	43.74801	32.83861	47.57381	36.55366	49.82997	41.21274	53.05853	41.25004
CUST_56306_PI429010886	M113460	0.001114996	24	12						214.3618	273.802	179.2719	456.5305	100.2539	340.9357	746.7514	224.7721	730.3491	263.2054	2049.491	386.1445	1074.917	1218.815	447.0887	61.17453	1121.501	523.3495	67.71277	159.1592	112.669	44.0823	187.0121	164.5342	42.80111	60.95576	63.61175
CUST_41876_PI429010886	M58765	0.001114996	24	12						121.4398	114.2058	91.6852	160.9576	199.4568	113.0037	127.2375	107.424	85.1761	147.2139	119.4647	237.6854	132.6086	162.8755	189.9429	106.9179	174.3818	136.8929	99.32904	103.8983	63.21401	90.7511	90.28649	83.7199	128.2695	104.3593	92.64515
CUST_29684_PI429010886	N60343	0.001114996	24	21						44.58927	68.21622	65.28031	68.73418	67.3285	60.64152	59.04856	67.25975	65.80074	57.57578	63.21401	64.06114	41.59303	54.26863	49.67735	78.7116	58.14687	53.21446	71.24846	62.20434	65.12931	87.34939	72.36965	68.64219	93.8595	56.31897	81.68207
CUST_6784_PI429010886	N20595	0.001115159	24	21						1857.76	2167.915	2116.149	1848.594	1683.28	1457.953	1367.344	2002.791	1943.332	1584.461	1666.041	1423.667	2167.915	1440.472	1674.551	2108.887	2494.519	1998.783	2201.915	1960.346	2023.772	2228.732	2333.596	2048.35	3017.306	1916.926	2954.482
CUST_25271_PI429010886	N52096	0.001116861	24	3	543335593	innexin 2 [Penaeus monodon]			2.82E-09	6.36732	7.195349	9.603299	12.13203	14.63983	12.91646	13.74025	9.970616	8.851485	6.607812	9.865694	10.5335	7.611325	6.685275	11.88037	4.238252	17.55763	4.328821	7.436551	4.474535	3.862761	9.904747	5.476566	5.362967	NA	10.80654	5.967574
CUST_4712_PI429010886	N17321	0.001118465	15	9						106.5773	99.18844	130.7209	97.50175	103.5272	92.30682	65.9928	97.47634	96.36643	113.0557	125.9134	132.7723	105.0092	76.59026	130.6615	95.18929	98.2265	86.60823	90.93696	61.07006	88.36719	120.2238	121.9756	123.8539	131.5859	119.4286	150.0295
CUST_45515_PI429010886	M69346	0.00111918	21	15						804.9022	676.1529	581.8311	598.5974	229.4919	339.5673	1398.823	941.3897	1397.328	2133.67	1114.745	234.7388	1188.024	819.3739	537.7378	3048.498	1260.081	2447.506	1444.129	1104.345	2075.256	1945.845	227.3921	1365.984	914.5652	186.3924	644.0162
CUST_49001_PI429010886	M83522	0.001124128	24	9						31.61388	27.92534	23.10726	32.75387	53.33823	49.08263	33.3824	39.26427	32.15717	61.70924	50.15161	35.57898	45.69147	37.95648	31.56676	24.68095	37.88972	40.23682	47.46968	45.04591	29.84906	28.16113	19.95709	22.65356	29.666	43.74346	21.0408
CUST_23341_PI429010886	N48667	0.001124976	15	9						15.29086	7.079941	NA	12.67619	6.211681	11.55732	9.683226	9.734639	NA	23.3113	31.91889	20.945	17.69677	NA	20.47128	6.662217	7.830046	NA	5.597349	5.329949	4.083237	23.16703	16.03888	29.82341	11.21915	11.87453	23.37894
CUST_49615_PI429010886	M86406	0.001129145	18	0						146.658	1340.842	1329.124	31.50642	NA	17.88097	12.51777	65.25923	65.15059	78.97649	98.84501	67.4639	175.0487	10.57446	121.1272	5.585409	165.9615	213.2675	274.8211	39.8332	285.1454	46.42563	32.04295	17.65126	4.325219	4.465464	NA
CUST_31822_PI429010886	M2492	0.001130027	24	15						527.9796	1593.454	994.565	500.4612	460.4529	487.1319	736.5204	719.4386	842.2205	791.8334	783.6564	282.8745	960.1156	602.1887	751.2377	1074.53	2107.433	1826.319	927.7273	492.3638	1149.936	577.2434	821.966	747.3198	694.7413	592.4023	805.2136
CUST_38298_PI429010886	M25115	0.001130027	24	9						35.72473	37.73828	11.0388	40.14624	69.98305	53.42134	17.06953	45.18209	31.20114	64.63944	56.75304	43.15014	40.48622	43.12031	16.97814	30.40072	58.9878	29.81738	16.35946	5.730283	30.07738	22.1072	16.77585	12.64082	15.12403	13.08598	22.16019
CUST_32019_PI429010886	M3013	0.001130027	24	21						1034.116	1445.709	1462.033	1132.862	957.3975	852.5352	990.2906	1088.256	1315.424	1108.83	944.508	836.1158	1366.714	1096.577	1149.936	1010.428	1466.746	1268.009	1212.026	1274.133	1476.878	1328.27	1566.672	1666.955	1107.545	1080.452	1638.477
CUST_3015_PI429010886	N13605	0.001130027	24	3	478251671	hypothetical protein YQE_11184	Metabolic process	Carbohydrate metabolic process	1.48E-49	2129.384	940.7158	1292.456	2536.537	5073.866	4036.235	1764.635	1196.459	1169.783	1835.563	3122.414	3948.157	2494.519	2663.717	3670.22	588.3344	515.8767	547.5307	1087.769	1493.774	932.6252	1500.89	1847.559	1502.443	1024.829	1989.721	1695.833
CUST_478_PI429010886	N5571	0.001130027	24	15						9364.597	16801.9	9578.799	7166.836	4378.72	4548.63	8121.327	10464.54	9179.508	9889.351	5966.983	7632.364	11612.18	7731.941	9142.021	20708.31	19387.17	19485.9	13962.41	12496.19	12743.08	11552.67	8857.18	8061.991	22658.35	8802.072	14502.16
CUST_33286_PI429010886	M6501	0.001130271	15	6						18.17381	59.26831	26.24865	25.81487	24.09912	17.28062	50.53473	74.48675	115.3698	23.1537	21.17352	25.65567	30.36386	16.19958	9.672539	13.84795	25.72152	17.4695	78.79009	29.84906	26.54115	39.73245	101.4865	53.39865	62.86145	19.75521	66.22157
CUST_5668_PI429010886	N19015	0.001130434	21	15	405966738	Eukaryotic translation initiation			3.87E-14	54.08928	52.05076	62.47784	69.04786	67.42662	75.12894	95.69298	80.14476	73.55381	108.9127	56.01896	66.13826	91.14348	63.29655	77.08874	93.8166	95.32122	102.2878	81.24878	51.78825	69.77877	66.77855	58.41256	56.3623	66.37527	64.59327	71.19421
CUST_55962_PI429010886	M112335	0.0011307	24	12						38.93757	39.47955	53.45604	126.7723	31.69335	90.55203	114.4277	44.62591	95.173	43.00754	118.5461	74.39469	130.0221	145.3375	109.4096	31.96059	90.83407	92.99115	32.40475	38.00971	36.25597	10.02094	34.74447	51.26614	4.762326	26.79132	29.9505
CUST_55483_PI429010886	M110321	0.001132273	15	9						602.7354	753.1726	762.4429	569.4689	598.4778	533.1871	397.5976	557.4698	619.5951	832.894	1065.144	710.1661	740.8082	657.1039	717.3191	548.4345	790.776	519.4495	527.6644	666.0922	436.0075	652.3046	731.069	586.6628	793.898	673.1503	1002.608
CUST_36262_PI429010886	M15211	0.001132273	15	6						25.63724	37.18271	33.14322	40.18248	37.70788	24.15233	54.44704	49.7839	49.28164	40.49642	52.52723	38.56693	62.22572	24.38607	28.08439	14.10469	28.51774	11.44051	48.22901	64.39267	41.87629	47.88919	48.73482	39.88527	48.88152	41.64305	47.12456
CUST_42269_PI429010886	M59838	0.001132273	15	6						69.66595	40.10788	56.62992	87.48976	72.24298	56.78277	70.50377	71.7184	85.06849	51.93257	206.3618	68.91312	118.7939	65.06618	71.89808	35.2239	28.83489	32.88915	66.93928	85.49462	83.14687	106.2889	207.9512	169.1953	68.11052	160.9133	183.9367
CUST_14344_PI429010886	N32731	0.001132547	12	3						341.84	439.9905	218.9179	332.484	536.489	617.3702	499.9903	357.9365	358.0959	287.5765	350.791	438.9261	276.9413	354.9347	464.7851	914.2819	548.9123	577.9786	460.2218	277.2489	444.046	235.338	275.0049	260.8728	457.0998	353.6905	156.0675
CUST_13450_PI429010886	N31211	0.001133148	24	3						34.13078	15.28035	12.03632	25.83025	41.73123	28.86593	22.80532	15.14334	16.46966	25.80839	25.76307	19.6075	25.19608	17.26651	20.26699	5.144631	7.715831	13.89134	12.71903	16.51333	7.925431	11.35653	7.417941	13.90357	NA	14.13349	13.24592
CUST_28171_PI429010886	N57415	0.001134303	24	21						6.218813	11.26399	14.4099	7.16048	5.765353	10.61794	6.203165	5.984179	10.63933	7.339187	NA	7.119344	6.415726	13.35908	8.24012	11.80135	7.276912	9.121309	12.15876	7.991764	9.116536	17.79195	15.0561	11.02183	12.36665	10.96116	10.9534
CUST_36261_PI429010886	M15210	0.001134742	24	18	344299267	PREDICTED: zinc finger protein 8			7.14E-16	13.764	12.86759	20.51331	11.28257	9.967046	8.302503	NA	12.3345	NA	17.83143	12.90521	19.90276	10.70188	18.48189	28.77495	19.0327	42.065	23.34012	20.80218	20.01232	32.3135	10.77882	26.33431	19.44853	19.62692	7.10668	25.17263
CUST_26997_PI429010886	N55252	0.00113538	24	21						NA	NA	7.639644	8.383304	7.802942	NA	NA	3.855136	4.777208	4.865404	6.092867	5.748942	6.015676	NA	NA	10.30948	NA	7.872788	12.02678	6.606839	9.950651	9.615742	11.15025	20.71518	30.82949	5.895298	34.57091
CUST_31620_PI429010886	M1983	0.001143152	21	3						56.12288	60.0766	122.8067	133.8844	102.1002	NA	28.1014	87.51637	80.21511	7.592519	12.39952	9.295776	93.89302	56.62992	13.16313	13.67378	15.84205	24.92804	154.5458	92.96907	85.66787	30.75559	81.86695	87.78171	165.4536	46.983	218.1432
CUST_51776_PI429010886	M95019	0.001144896	24	15						285.1454	370.4445	312.8082	268.2532	280.5224	245.5909	328.0523	296.3981	265.8169	350.8666	274.4544	324.7451	355.9245	296.4601	261.5986	412.3443	407.5243	498.6872	340.1047	304.6294	427.5284	317.784	350.8666	445.4238	315.8899	299.5787	454.4018
CUST_43691_PI429010886	M63704	0.001145308	21	15						109.3497	146.8442	159.5246	150.1836	137.2208	134.4124	220.6244	159.9962	209.4822	230.1142	125.3559	194.2665	187.3112	173.7269	169.9743	234.5732	204.1402	246.1645	156.6771	157.6143	257.5532	132.8203	124.362	160.766	164.8928	122.0363	151.6954
CUST_20311_PI429010886	N43214	0.001146359	12	9						35.72049	22.20534	29.4483	34.25309	28.098	24.69407	36.52323	27.11917	36.31158	32.63627	38.93069	47.54035	34.51373	28.79058	25.45723	15.48134	27.2264	13.62301	37.15987	19.87663	20.96118	34.63434	57.86776	70.24722	17.68949	32.42334	37.18674
CUST_10502_PI429010886	N26334	0.001146404	24	21						15.92281	28.61053	22.54151	16.26097	21.13533	10.06884	28.46437	19.7414	20.32505	19.79906	16.27462	11.33295	13.7591	9.89934	11.66134	14.98342	28.12044	21.42827	22.86026	22.50019	31.37822	22.81449	23.85867	29.3468	20.19245	21.0554	28.93158
CUST_23780_PI429010886	N49450	0.001146644	24	3						62.25184	11.29211	50.70492	28.76849	106.1914	52.71315	17.40595	28.00501	14.93713	13.9939	60.31108	70.99538	14.67985	102.3466	111.7284	9.400412	7.865315	8.124053	8.135191	5.252307	4.450194	14.17824	5.336459	19.08041	NA	19.87663	8.056452
CUST_3179_PI429010886	N13961	0.001147877	24	18						37.17059	48.20014	31.57579	18.65702	12.79123	7.395633	124.6061	22.65356	110.6677	135.6611	64.97632	73.49385	120.0493	85.86734	37.8457	105.8493	60.69474	150.7041	116.0279	76.3265	156.5402	103.1619	65.97229	78.49976	68.42492	62.88893	92.01146
CUST_37999_PI429010886	M22705	0.001147969	24	3	300123208	unnamed protein product [Blast			2.43E-13	11.53968	7.54952	12.52789	15.09289	13.73882	12.89253	8.089365	11.88722	15.12564	8.639252	NA	12.57883	7.890721	7.964923	10.51731	10.32895	8.843446	9.836576	7.156045	6.067823	5.961032	6.011975	5.114487	12.89468	9.179179	4.399978	12.66963
CUST_31050_PI429010886	M559	0.001149699	24	0	405959297	hypothetical protein CGI_10022	Metabolic process	Carbohydrate metabolic process		782.4302	586.0404	702.1022	636.9918	435.1623	425.8369	439.4254	335.2209	378.2634	741.4284	438.2151	420.9575	646.1155	352.8279	373.2381	419.8124	487.5393	330.4891	467.0581	333.6191	282.0632	564.2041	624.767	611.8901	659.1588	658.1324	816.5697
CUST_23861_PI429010886	N49607	0.001149699	24	0	156361850	predicted protein [Nematostella			8.02E-11	42.08196	69.35987	58.89558	32.71622	24.34343	20.80953	32.17885	38.01913	41.67991	30.98678	28.89871	20.59574	41.0185	34.93951	18.16767	30.64441	33.93523	44.19365	33.93523	43.19513	33.36729	24.23762	36.51861	44.47768	37.23075	47.10689	48.8586
CUST_39608_PI429010886	M41736	0.001150274	24	3						2204.749	1556.093	1403.719	2050.749	2320.751	2732.247	1733.335	1816.946	974.36	1281.397	1471.771	1901.421	961.4773	1605.911	1836.765	1108.146	1423.667	1272.378	555.7599	1304.861	443.0968	1085.793	1553.379	1011.215	405.5854	1602.248	247.7035
CUST_7075_PI429010886	N21003	0.001150274	24	21						88.92336	96.9319	125.9947	78.94493	103.4975	66.43371	53.98617	70.91565	58.91249	128.7298	102.9161	107.5871	120.4309	95.33452	113.8967	120.8275	111.539	75.74761	122.1528	115.7411	104.8945	160.217	119.272	124.2633	203.2256	97.60846	137.4877
CUST_34096_PI429010886	M8760	0.001152976	12	6						41.47035	12.47654	22.92037	542.7794	23.72726	452.8102	147.0744	NA	166.6038	57.14064	173.3126	227.2502	64.49711	NA	60.83425	105.7112	79.92025	31.17381	559.1602	341.3363	147.043	NA	NA	NA	11.23899	10.57564	27.81736
CUST_12262_PI429010886	N29184	0.001154515	24	18						383.5875	438.1085	414.0319	527.5856	561.5074	506.9058	363.4898	506.2756	412.2409	473.2258	435.731	380.915	516.0491	528.6354	364.0288	503.7654	676.1529	610.9907	706.8768	923.7499	762.8618	495.9709	635.3318	497.2634	466.3373	670.1687	561.6697
CUST_17655_PI429010886	N38355	0.00116017	12	9						703.1577	86.8615	714.4899	1368.116	410.9137	426.4459	314.5854	540.0952	764.7593	1030.655	2602.477	687.4827	1717.387	550.3617	604.6962	37.21408	55.06272	36.21321	895.4864	794.0736	1106.912	1873.347	3423.157	3371.075	937.5478	2110.599	3475.106
CUST_30966_PI429010886	M335	0.001162108	24	18	242019954	DEAD box ATP-dependent RNA h			2.64E-19	68.82621	113.636	113.878	99.28639	36.37756	38.05697	91.4929	84.06095	100.9354	94.69266	55.28754	52.541	94.63322	49.69173	39.28587	101.8004	124.0485	128.0075	120.2404	130.6442	145.7916	104.9484	102.5417	120.7259	128.3075	98.12003	121.7517
CUST_52574_PI429010886	M98003	0.001162108	24	12	241647006	Grb2 associated binder (Gab), pu			1.45E-29	22.9911	25.16714	38.05697	19.73488	37.16411	32.76978	39.52957	29.56947	42.11529	36.19278	27.7211	42.50386	56.69444	54.37537	54.81901	60.49503	48.4073	40.1235	31.43412	40.11539	51.5089	36.66337	35.39516	48.94113	34.4378	32.45355	50.6873
CUST_45800_PI429010886	M70333	0.00116389	21	0						246.4965	265.8681	235.738	223.4669	177.5137	206.0093	160.6506	199.1442	187.6435	221.6521	152.2217	228.6642	217.8657	233.3271	190.2455	196.5522	188.9592	232.8674	232.9982	222.6501	197.796	253.4016	216.8711	246.356	249.8271	187.773	222.6813
CUST_40716_PI429010886	M55925	0.001166539	21	15	260835820	hypothetical protein BRAFLDRAF			1.6E-55	6.583562	13.3485	11.5043	4.52375	6.621148	4.78576	12.08433	11.40638	9.689688	8.378958	9.550476	9.184105	12.24624	6.92309	15.0452	15.78015	19.99086	11.03688	7.918292	10.69359	14.71359	8.368746	12.15359	NA	9.01805	9.856067	5.292582
CUST_38654_PI429010886	M28880	0.001172287	24	0						4767.094	4650.166	NA	822.3417	NA	614.3598	3811.795	457.222	2521.203	322.3894	NA	224.6812	129.6511	221.0978	361.8298	249.4346	653.3386	235.7954	NA	2711.745	NA	172.5296	462.531	350.9469	1367.344	1273.044	915.0007
CUST_39631_PI429010886	M42021	0.001173982	12	9						9999.789	64.61049	8802.072	9211.498	7260.089	9578.799	502.2054	513.9521	886.3549	11528.01	10949.85	12473.12	11822.99	4685.458	11378.62	183.3606	42.27337	258.7439	1677.845	2108.887	2948.008	12991.27	13301.65	15731.96	3640.441	11164.38	12344.85
CUST_11952_PI429010886	N28682	0.001173982	12	9	321469341	hypothetical protein DAPPUDRA			2.3E-09	60.0855	69.91553	87.43473	42.20935	49.35016	48.73882	44.99422	60.0628	51.39126	53.47447	126.327	77.19972	43.03288	58.41256	49.70331	56.2194	47.7452	48.41784	37.91672	59.45846	71.6009	60.60875	120.2076	132.6606	69.98305	56.08545	99.57709
CUST_38774_PI429010886	M30255	0.001174634	24	15	117414788	Euphausia mutica 18S small sub	Protein metabolism	Regulation of translational elongation	2.59E-91	13078.51	20059.08	14502.16	10408.52	11076.77	10142.18	20172.23	19434.65	15847.39	15340.52	14389.61	13360.37	14764.65	10871.96	13135.1	20434.9	24426.06	22092.06	14548.28	20099.82	19094.08	12523.21	17524.49	14389.61	14411.75	15967.93	12836.63
CUST_5879_PI429010886	N19326	0.001174634	24	15	62126070	C-type lectin protein [Fennerope			1.29E-13	598.0964	279.8449	588.5037	288.0498	386.528	226.022	99.65569	1293.784	828.8502	425.4449	307.6081	183.8046	830.4195	250.3956	463.5485	1439.002	1273.67	1717.387	990.2906	1286.883	2461.211	787.5865	29.90874	741.1426	1183.911	451.5651	620.9302
CUST_472_PI429010886	N5524	0.001174634	24	21	171189511	glutathione peroxidase [Metape	Response to stress	Response to oxidative stress	8.59E-48	13737.98	11643.13	12921.27	14071.14	10835.48	10626.1	14548.28	12236.09	15102.78	11264.76	9613.776	10035.81	16622.66	10109.9	12836.63	13360.37	11822.99	12991.27	14307.54	11881.91	15553.93	16221.54	17345.96	17315.8	14526.85	11475.48	14071.14
CUST_31284_PI429010886	M1137	0.001177812	24	18						49.1413	112.0615	84.2744	55.52101	66.46575	35.81706	64.62434	64.29327	63.46562	42.86666	56.75829	55.38305	66.43371	87.83185	44.45113	65.93698	112.3711	127.633	59.14128	115.7715	123.2322	85.3597	114.7841	99.79674	69.31429	93.31746	95.92856
CUST_32293_PI429010886	M3711	0.001177812	24	12	291222411	PREDICTED: adaptor-related pro	Transport	Protein transport		27.18421	36.26082	39.98058	31.98442	21.17352	31.62493	40.5799	36.53804	42.31229	56.86607	35.90614	15.51385	56.59797	59.00719	57.92587	61.13851	40.64686	41.61011	48.48882	36.03288	48.43013	54.22723	24.92085	38.60711	46.70567	33.43626	44.3395
CUST_40149_PI429010886	M51049	0.001177812	24	18						125.2461	134.8921	133.5862	116.0037	111.9654	131.6618	105.7112	113.23	120.5852	123.0947	102.6861	101.4459	142.7074	118.9614	68.9382	169.7003	133.8434	132.2497	157.5632	136.2198	142.4472	188.767	133.7038	119.6392	157.6143	137.8885	197.3639
CUST_46983_PI429010886	M74391	0.001177812	24	18	149622942	PREDICTED: N-alpha-acetyltrans			6.27E-09	35.25711	56.36738	53.80406	42.02831	42.33335	35.22718	45.67933	58.3384	46.6272	36.37756	41.00325	12.94826	46.11055	46.32151	39.52957	52.46839	69.63156	76.71918	65.309	52.98141	61.78111	59.49271	59.11715	54.75432	60.34528	45.99276	43.01095
CUST_52394_PI429010886	M97288	0.001177812	24	18						28.42785	57.08138	50.65841	40.49223	42.6519	28.84319	36.58388	45.63539	46.99801	33.32412	41.1126	23.15112	38.09775	31.99902	39.11401	41.56856	51.80751	51.33067	65.13726	52.06823	56.34222	50.69492	38.43	52.97194	60.55423	45.3971	49.18503
CUST_12980_PI429010886	N30385	0.001177858	15	9						73.60907	26.0759	71.33856	131.375	108.4697	85.93897	38.66936	42.73834	33.71363	149.047	488.5919	154.566	157.4103	82.2265	128.4972	30.08206	31.43843	25.7844	39.86943	43.89532	39.11401	165.4279	194.1709	195.3485	63.37029	164.6109	212.4427
CUST_1308_PI429010886	N9228	0.001177858	15	0						10683.23	14670.24	114.0013	2027.732	386.8846	1582.625	3164.281	1544.613	1651.623	274.2134	231.5378	254.5783	1013.822	551.5575	294.0126	13017.21	6559.55	2164.77	5609.633	3879.479	5157.305	132.4508	695.8214	643.379	395.0243	1534.389	615.2672

Probe ID	ID	p-value	Period	Phase	EBI ID	Annotation	GO category	Go term	E-value	0	0	0	3	3	3	6	6	6	9	9	9	12	12	12	15	15	15	18	18	18	21	21	21	24	24	24
CUST_2227_PI429010886	N11915	0.001181253	18	15	321455574	hypothetical protein DAPPUDRA	Metabolic process	Other	1.06E-148	216.4424	316.2435	177.0792	198.4987	216.7269	201.86	319.1468	314.8641	242.4377	254.5783	259.5151	199.3242	285.3818	263.3579	260.798	459.5171	467.3293	422.1689	266.255	234.6515	352.3291	165.5194	280.7417	204.1402	268.869	256.1905	183.9971
CUST_2465_PI429010886	N12435	0.001183627	21	15	506968903	eukaryotic initiation factor 1 [Co	Protein metabolism	Regulation of translational initiation	1.57E-37	1041.894	1166.709	969.4842	859.0835	695.1268	593.5138	1088.703	975.2848	817.1951	991.1291	772.5597	957.7854	910.719	668.1223	837.9532	1157.507	1259.516	1203.381	934.3693	1036.158	1098.155	806.5559	988.0134	1082.092	882.5044	631.8697	829.1725
CUST_50116_PI429010886	M88685	0.001187793	12	9						23.93863	24.06897	20.84466	16.32195	17.18571	17.49943	22.56219	17.94579	23.53743	15.17119	27.1944	29.61117	23.91323	25.53977	17.88814	15.72841	16.65336	21.34152	12.36934	27.28807	20.64644	32.28853	33.01146	38.49334	21.16633	28.65875	37.69822
CUST_38527_PI429010886	M27681	0.001189502	24	3	149675211	cuticle proprotein proCP5.2 [Cal			2.13E-40	5884.771	93.37419	4207.659	7785.133	6864.217	6317.157	161.8917	364.9578	251.4577	6132.817	8296.544	5374.176	4508.46	3179.907	4852.406	51.34712	69.73149	39.37754	937.1039	476.1651	543.3224	6837.265	4622.321	5525.521	1260.62	1269.337	2068.831
CUST_3652_PI429010886	N14966	0.001189502	24	15	321478257	hypothetical protein DAPPUDRA			1.24E-59	54.344	98.81307	70.28135	53.04169	62.88268	60.63273	73.22433	86.36335	92.43032	104.4062	79.43494	60.86142	109.7513	61.19974	57.12065	110.7898	104.3181	104.9484	96.57151	80.26815	96.09055	84.10925	59.84299	92.13598	85.2914	74.25833	77.27754
CUST_90_PI429010886	N718	0.001189502	24	3						11164.38	148.956	9313.798	14470.28	12631.63	16035.79	273.1848	353.0555	284.3354	8227.781	9013.443	15102.78	3135.16	12921.27	15871.24	54.09509	89.83016	126.7723	166.6574	628.6837	254.1863	4453.231	1366.714	4150.213	699.7985	1813.66	1836.765
CUST_42176_PI429010886	M59607	0.001189939	24	18						21.0554	31.50944	31.9639	30.64789	25.36192	19.69014	34.41229	32.80252	35.60799	33.86491	42.61451	33.40515	39.86153	32.20247	34.74862	42.73341	45.89596	43.96941	50.53965	36.50276	41.95985	59.41357	31.65179	41.04183	49.50343	40.37732	59.80532
CUST_33723_PI429010886	M7708	0.001193517	24	15						92.71133	100.7337	66.87814	87.57759	73.66383	69.79983	53.6019	59.62083	73.76756	108.8896	65.309	80.77747	79.1181	99.31589	83.05204	110.7268	117.7735	102.1346	100.4907	74.67002	103.9282	59.58718	116.6401	81.62587	84.31911	96.13685	93.67836
CUST_40928_PI429010886	M56407	0.001193948	15	6						73.9144	100.6934	54.23896	92.31393	63.80586	91.72224	93.27554	104.7861	102.9594	92.03434	67.56678	71.9236	70.99538	64.60254	81.36078	77.09727	105.0177	55.14025	98.55805	112.5544	111.6045	193.5997	129.1955	122.771	76.14902	105.5941	96.81529
CUST_6611_PI429010886	N20349	0.00119518	15	6	260819927	hypothetical protein BRAFLDRAI			1.36E-09	8.000536	43.52816	46.68118	NA	55.51071	42.43675	105.1338	120.4106	98.70542	25.8029	20.41652	11.1169	18.07496	11.95545	24.17927	33.16894	23.09778	30.49064	53.20844	24.62171	84.80932	31.55061	34.17027	38.89189	29.99869	31.45923	36.31158
CUST_1896_PI429010886	N11002	0.001197226	18	6	532759728	antimicrobial peptide type 2 pre			7.86E-34	76.21108	217.7355	108.7869	191.9733	223.3238	140.0039	403.4693	340.1047	393.8421	196.6122	141.2055	222.5585	248.554	196.3832	102.411	157.5632	322.75	241.7973	131.8579	222.1744	111.8609	149.3774	295.8069	246.3057	171.0499	398.6975	404.2408
CUST_6564_PI429010886	N20275	0.001198061	21	15						604.6962	3667.762	1245.448	956.0225	297.1434	656.3228	2965.89	2100.692	1682.203	2257.369	1075.892	869.5599	2048.35	1178.539	1377.595	2152.198	5420.715	3354.131	1542.881	678.7605	1721.8	1319.093	964.2235	1324.865	1340.037	917.5193	325.2782
CUST_2034_PI429010886	N11373	0.001198977	18	3						22.69882	NA	15.85928	33.82382	20.50908	13.61765	6.398645	NA	NA	24.27221	10.9364	7.914201	12.6121	4.651424	3.97152	NA	NA	4.090324	3.249116	6.717766	6.206844	25.45723	39.32943	18.81483	16.39583	14.44887	26.86103
CUST_48479_PI429010886	M80552	0.001201605	12	9						53.21446	37.42032	65.3293	55.69808	50.67391	42.39291	53.66499	43.44652	60.20069	59.51724	56.29733	80.54573	54.64003	42.66175	45.99276	34.63148	42.4997	27.32212	63.61864	51.86305	56.38348	81.00976	46.84134	88.31999	52.27625	44.13653	55.09974
CUST_11939_PI429010886	N28661	0.001201605	12	3						45.63539	59.46532	44.03643	61.05496	86.65164	64.42993	56.30361	71.26688	49.81307	40.89205	45.63101	35.95938	37.35559	69.91553	42.52366	55.70902	62.57277	55.45831	37.82047	40.96049	41.2262	38.06969	32.44579	43.21465	37.38685	55.38965	37.70788
CUST_53462_PI429010886	M101813	0.001202044	15	6						4.312968	4.247024	NA	11.98927	9.057903	6.719445	8.009934	6.561084	14.95172	9.550476	NA	11.783	17.41463	5.643166	NA	4.825812	4.766502	4.602944	12.11997	12.09389	11.43891	13.37213	24.67768	18.99164	13.60764	7.677339	17.44773
CUST_53434_PI429010886	M101688	0.001204371	24	3						141.9587	33.59136	127.2771	118.6245	95.46305	157.8871	29.22381	37.86516	16.61523	47.5648	84.39985	85.21665	65.47283	37.73322	57.02244	15.42607	17.39282	19.47927	23.82872	60.79882	19.24962	87.40333	52.98141	70.91565	32.8057	66.56707	31.78702
CUST_49359_PI429010886	M85357	0.001204371	24	3						4048.748	76.8564	4655.696	6922.365	6999.724	7769.948	86.92889	461.6561	266.6798	3091.943	8802.072	4069.14	2230.426	4965.926	3647.267	28.77495	59.69831	43.01953	445.4238	580.9599	358.4079	4450.275	3633.442	3715.857	521.5155	2091.62	1377.009
CUST_7185_PI429010886	N21167	0.001204371	24	15						309.7415	464.9207	231.0141	276.0746	228.5093	246.4061	322.6893	472.1134	254.714	273.8482	278.9665	271.2533	290.4456	317.0778	276.1893	493.4583	423.4844	418.6884	366.3486	423.7011	384.0543	246.3057	336.4491	307.6702	330.4034	356.871	319.8754
CUST_24138_PI429010886	N50099	0.001204371	24	15						146.6816	175.5187	163.4024	147.7951	153.9033	142.7074	171.436	167.7409	150.3928	149.9126	176.6544	178.0895	153.0882	160.713	182.8746	202.9059	218.4783	262.1946	170.3943	173.05	191.9733	146.0664	174.8361	193.6334	149.7452	176.8599	194.1257
CUST_38425_PI429010886	M26487	0.001204737	12	6	210062957	C-type lectin 5 [Fenneropenaeus			3.24E-11	9.207701	10.65615	8.869172	15.32048	NA	14.47219	88.65754	NA	69.36872	15.865	10.35412	NA	NA	NA	8.335988	20.14949	NA	23.79152	72.59454	124.7322	265.0552	22.54848	NA	18.45644	45.60384	7.157752	25.77383
CUST_41640_PI429010886	M58204	0.001207184	24	0						16.33991	21.86932	24.53204	15.69552	13.73044	20.33857	20.47681	22.5581	15.51127	9.269929	10.7159	17.34089	13.13946	17.91593	12.66337	26.28423	20.49572	18.32878	18.56908	20.53256	15.82642	23.27971	12.61395	18.68765	28.0155	23.5588	36.14198
CUST_42507_PI429010886	M60457	0.001207305	15	0						289.5149	124.0944	192.6657	57.43595	153.7672	126.1993	393.0833	296.0318	326.9964	34.53159	42.88477	24.88691	90.11127	141.9587	108.0846	596.1129	282.7416	247.1999	185.7829	72.75115	183.0069	159.0392	36.91722	144.5793	32.28165	57.9604	4.990083
CUST_49189_PI429010886	M84589	0.00120833	18	6						179.5567	143.9979	85.90563	157.0068	144.3231	156.338	242.222	181.1959	238.9649	129.6975	93.54583	139.9139	146.953	124.7322	124.517	134.9328	82.48416	122.3631	130.0993	175.545	135.6848	159.7974	256.2719	136.1215	145.8127	165.4991	142.2521
CUST_43518_PI429010886	M63258	0.001210072	18	0						220.9505	125.3435	265.7303	193.0974	53.78431	59.19241	82.42094	180.8767	89.43021	50.69876	10.1984	28.38277	108.1577	47.9205	40.63802	30.4229	112.201	93.75176	201.0489	364.9578	214.4735	245.4997	97.44091	58.82275	317.2644	9.355486	201.4635
CUST_18362_PI429010886	N39653	0.001214622	18	6						8.420719	10.27251	7.87171	4.820294	11.04699	10.67194	NA	8.452888	28.75373	12.04588	8.416104	NA	NA	13.07815	14.63182	10.29813	9.450084	10.33813	5.228764	4.71102	4.508663	16.21072	8.348262	5.457353	18.98812	13.7624	12.36934
CUST_144_PI429010886	N1544	0.001215417	12	9						8107.941	6642.147	17167.45	14189.5	5666.924	11138.2	7430.648	9096.626	14526.85	16014.56	10337.3	12743.08	19738.31	10764.25	9613.776	2478.84	4798.856	3598.269	11588.48	9629.338	9905.822	20059.08	23379.75	21270.53	8451.427	8073.399	13988.25
CUST_38121_PI429010886	M23445	0.00121924	24	3	121484199	cuticle protein CUT8 [Portunus p			1.7E-39	40415.13	1053.276	21645.19	49329.8	40217.51	42496.4	3104.628	5442.505	7248.769	34877.59	40217.51	36802.85	31543.85	23892.85	37083.68	735.2877	747.5407	687.6817	10277.01	8503.163	11429.91	38861.2	33838.77	38996.88	10699.09	21010.2	28561.19
CUST_39815_PI429010886	M45092	0.00121924	24	3	220172369	cuticle protein [Rimicaris exocul			1.21E-13	31967.8	753.9335	39540.76	40415.13	27758.19	46875.63	1146.245	3333.312	2424.1	24721.57	48252.93	27758.19	12141.91	25065.69	23575.84	201.2812	699.1348	346.0708	1545.477	2715.678	1731.406	27210.5	14828.55	19855.57	2119.199	9653.786	6018.305
CUST_7465_PI429010886	N21586	0.001224639	21	6						352.0102	463.4514	89.01154	932.6252	125.5382	657.7333	1592.484	1769.667	1985.342	1715.071	725.9659	498.7889	1470.023	432.3981	791.5844	1296.827	1582.625	3114.68	67.3007	130.24	22.39421	314.7154	167.4488	38.66006	1800.506	55.8366	1538.316
CUST_49365_PI429010886	M85395	0.001225236	24	0						141.7369	174.2921	190.3661	142.1733	136.4064	126.859	110.8559	133.335	165.5194	158.0791	140.7362	128.7522	138.6295	112.6108	123.1774	151.5867	148.4879	157.8268	110.9878	114.1769	128.5165	149.7452	154.6661	167.708	198.4091	174.5649	249.7708
CUST_33671_PI429010886	M7561	0.001225787	15	3						382.191	189.5806	455.4195	501.3701	303.7619	499.6833	132.5859	460.5885	365.989	210.8033	219.2811	179.2719	129.3882	221.1265	61.63911	159.5246	413.108	296.1058	361.5772	531.5525	418.5156	468.5187	233.8101	137.6198	451.6859	91.73279	193.2061
CUST_12763_PI429010886	N30000	0.001227116	18	15						26.26876	22.05134	35.15693	18.62882	17.39987	24.96379	26.75452	13.64203	21.95424	37.43476	23.52466	21.7802	24.21438	21.26818	27.97586	27.66435	36.48955	34.43322	30.99121	23.16492	28.04788	18.04144	18.96527	10.37881	23.67078	25.78185	14.57376
CUST_44112_PI429010886	M64979	0.001228931	15	9						53.15363	61.9265	51.87516	34.03869	53.20844	30.8995	41.00325	55.56014	41.59688	52.37142	61.43496	77.14782	35.76914	61.295	53.88747	38.88446	63.03163	52.0131	45.32861	38.03753	43.03704	69.23652	45.75395	61.04906	59.15801	69.93972	70.59518
CUST_24767_PI429010886	N51221	0.001229228	12	9						90.42094	38.40373	185.653	153.9962	160.737	61.15056	67.68769	65.90991	146.1482	227.1397	237.2505	210.2297	446.2058	136.4791	309.797	45.90556	17.14108	36.66952	178.9536	144.7386	156.0305	256.3549	252.9258	249.3839	199.588	119.0641	389.6444
CUST_35146_PI429010886	M11721	0.001234109	24	15	241733201	myosin-IX, putative [Ixodes scap	Signal transduction	Intracellular signal transduction	9.91E-18	335.0962	525.2596	441.6606	357.6352	406.3531	299.9819	479.2755	423.4844	487.7778	458.5926	381.4494	474.9425	482.8348	420.3226	499.8092	559.4339	528.772	623.3324	459.3044	435.2817	608.1132	467.458	518.0684	549.069	442.1242	442.7601	705.624
CUST_37842_PI429010886	M21831	0.001234109	24	3						3519.517	237.0207	3382.058	5921.175	9550.071	9153.451	242.4377	448.9533	249.2196	2826.773	4334.63	4637.293	1693.271	3067.239	4125.439	72.63826	53.54287	25.24448	109.0546	797.7145	66.44259	3992.329	656.7134	3000.392	95.10568	662.9752	105.1207
CUST_38785_PI429010886	M30344	0.001234109	24	15	13898841	CUB-serine protease [Panulirus a	Protein metabolism	Proteolysis	5.72E-41	6461.658	7402.627	5021.117	2900.703	4275.766	2975.418	8768.122	8899.45	7194.306	8484.924	4224.791	3929.52	6506.137	4834.858	4560.074	11305.05	7714.571	9392.481	8309.047	7484.678	8736.825	6298.27	5073.866	5502.319	7177.873	5467.822	7497.741
CUST_2336_PI429010886	N12130	0.001234109	24	3						102.3111	59.14128	105.3222	121.7034	578.1548	467.458	45.81369	91.85221	51.67361	73.79109	374.6189	323.0951	69.99598	124.2158	157.8028	38.48559	42.316	46.70567	66.53367	126.2375	49.80766	100.821	85.20409	84.09371	67.83335	140.2946	91.56473
CUST_15037_PI429010886	N33902	0.001234109	24	15	321454587	hypothetical protein DAPPUDRA	Transport	Other	7.64E-31	146.2607	195.6118	158.9628	159.2248	145.6616	142.5791	175.768	166.158	201.3668	221.1981	154.1589	188.1844	181.9919	152.4946	186.2715	285.9469	221.596	240.5209	202.9968	191.6851	314.7154	182.1852	163.0857	203.5811	230.8967	161.2674	280.5224
CUST_996_PI429010886	N8156	0.001234109	24	15						107.2961	225.3038	153.2663	102.9449	105.2701	121.1967	156.4112	197.5438	162.9278	112.3846	129.0975	107.8039	115.186	148.5106	144.7781	247.3301	307.9283	255.3925	164.3905	176.3993	230.1554	133.6243	144.7602	159.9018	186.728	148.0804	176.0523
CUST_52439_PI429010886	M97449	0.001235364	21	15						10.46073	16.62059	13.75275	23.40815	29.32605	21.58992	14.81156	23.48894	29.22014	23.32746	21.39542	21.70164	29.11025	28.46788	30.24252	41.35381	33.24527	41.77836	29.64523	17.34255	32.81904	22.73887	16.89763	24.91228	25.68422	14.95707	30.63675
CUST_95_PI429010886	N760	0.001236751	15	0	255653874	crustin 4 [Panulirus japonicus]			5.1E-27	20271.85	10999.91	17210.94	18966.14	6897.446	13098.74	10035.81	8605.765	9041.082	6310.155	8135.669	6132.817	8016.887	14100.35	10485.81	12496.19	14956.1	10852.86	7844.789	8605.765	8146.369	6292.839	14526.85	7090.073	5907.39	10127.21	7007.731
CUST_27297_PI429010886	N55801	0.001240629	24	15						24.39028	6.735712	23.12968	25.89856	17.87709	19.01967	8.306588	15.37784	14.36256	16.70465	8.611312	30.2752	17.425	12.15059	16.89173	28.5396	32.75774	41.42853	33.5653	9.871812	30.19163	27.87602	26.30298	34.93184	16.96577	17.49602	27.72605
CUST_38541_PI429010886	M27801	0.001240669	12	0						17.24504	22.97677	21.04316	14.83948	17.35867	17.26262	4.281841	11.09687	16.6676	12.38845	10.3228	11.7546	15.0452	19.55278	22.49107	15.66225	14.50725	18.3082	5.809954	7.060112	7.061549	21.18195	5.348053	14.24966	24.57064	10.80862	16.65693
CUST_2732_PI429010886	N13000	0.001241262	21	15	321473292	hypothetical protein DAPPUDRA	Metabolic process	Carbohydrate metabolic process	3.37E-103	8.052062	17.75202	15.48134	5.256252	6.968697	11.91165	17.89355	15.87405	12.45866	16.1892	20.34099	10.11286	10.62708	14.71023	15.40578	16.95396	31.4973	25.35697	12.86873	16.9559	17.74047	12.86873	7.95024	13.92645	9.817879	15.23242	11.1702
CUST_9560_PI429010886	N24839	0.001241706	15	12						54.16578	29.90099	52.2967	37.96647	29.73756	33.31109	25.19327	29.6741	28.52375	54.27691	53.30473	43.89958	57.89687	41.77836	57.37441	34.90937	38.66676	40.7919	41.05749	33.50527	34.29815	35.65021	40.27654	38.96726	44.54779	31.60141	40.50202
CUST_50934_PI429010886	M91502	0.001246396	24	9						18.31223	8.58477	14.60927	24.26356	44.37502	25.55884	22.6315	25.65567	11.12735	14.74196	32.73039	27.24917	14.99779	17.85411	19.88951	15.96179	6.706445	11.23607	14.60467	13.42904	4.648358	7.071377	NA	9.099343	7.785469	19.11039	11.48755
CUST_44193_PI429010886	M65226	0.001248978	24	15			Transport	Protein transport		465.024	951.1847	554.3874	493.2216	476.5395	524.517	632.1075	826.9656	649.314	611.6853	649.4957	529.0583	534.9189	682.3134	530.8049	858.7677	1023.762	849.7305	671.5725	723.9274	695.3717	510.3128	792.067	611.5217	665.873	686.4707	651.2334
CUST_5050_PI429010886	N18195	0.001248978	24	15	388548526	ubiquitin-activating enzyme E1 [	Protein metabolism	Cellular protein modification process		2356.919	4291.944	2539.976	2526.145	1998.783	2018.868	4456.862	4008.648	2747.205	3120.055	2208.268	2194.43	3261.337	2149.415	2684.097	4177.108	5644.907	5847.84	3717.963	3069.207	4012.41	2700.891	2811.043	3376.686	3202.946	2795.798	3522.391
CUST_6776_PI429010886	N20586	0.001248978	24	3						2076.904	104.953	2251.39	3036.167	3516.69	4221.228	106.9297	280.3649	108.5148	952.9844	2797.556	3007.18	641.1736	2112.104	3151.384	39.72867	32.7919	45.57229	84.53983	351.8456	103.7569	921.4706	184.1934	857.1902	77.78042	415.3144	88.27501
CUST_394_PI429010886	N4808	0.001248978	24	3						14642.96	16.67154	13098.74	14764.65	13193.16	14764.65	142.1072	87.31684	115.8934	12548.11	11572.84	18613.15	9804.488	6799.146	13812.43	3.490872	12.16729	86.28411	104.2696	177.9078	167.2859	10746.95	3786.16	8886.515	741.1426	1318.185	2402.724
CUST_27527_PI429010886	N56217	0.001248978	24	3						58.62146	53.49041	58.19693	68.64759	90.14554	79.00471	49.78973	52.37742	50.99975	62.90417	70.83005	59.3284	61.81014	53.27399	67.09453	36.89757	34.73799	37.21408	49.39632	51.93257	39.93436	66.1472	55.95026	46.1952	56.52874	28.72062	50.14527
CUST_49999_PI429010886	M88160	0.001251474	15	9						15.25605	11.27021	24.17321	21.98453	14.90623	20.56044	16.23526	30.09026	23.41063	39.92572	33.24527	26.38647	33.55937	12.45205	25.85498	28.67152	18.00851	22.5248	28.8322	26.52653	19.9379	26.6049	22.82225	27.38152	42.6416	34.16048	43.46921
CUST_34305_PI429010886	M9285	0.001252286	18	15						216.6056	228.7039	152.2217	258.3202	154.8085	243.453	233.1818	132.7723	160.1694	378.1731	239.051	247.5845	187.8413	277.4932	251.7959	467.5708	215.2625	370.5351	360.4299	276.8158	360.3638	251.0374	136.6185	223.1202	171.8137	134.4304	132.8402
CUST_9047_PI429010886	N23983	0.001254274	21	18						29.55174	18.93048	21.3055	18.32623	19.45847	29.5773	18.10243	13.23813	10.76257	14.91234	9.531461	26.0759	15.60918	18.15913	13.68381	17.89974	18.51735	20.90369	26.77351	24.25971	42.22249	24.17698	19.33949	23.97898	24.13218	27.73866	18.05145
CUST_34045_PI429010886	M8583	0.001256333	24	21						473.9767	599.0901	487.0065	255.5294	513.5436	441.2406	377.3111	535.0429	474.8239	417.2642	470.7608	413.9053	397.4943	458.147	381.4494	595.447	692.1008	506.8228	456.9596	453.1048	397.6869	641.6007	647.785	626.8237	485.4687	617.7634	618.9801
CUST_9703_PI429010886	N25070	0.001256333	24	15						22.58052	13.76632	15.88417	15.77596	11.24321	17.63035	12.80411	7.180584	6.694797	35.1142	26.14707	28.98683	18.85869	18.20133	17.78589	37.62021	44.03643	18.87393	21.61108	9.27828	22.43161	27.64775	18.73426	29.10319	18.28134	11.25743	17.72698
CUST_51908_PI429010886	M95638	0.001258495	21	0	328724047	PREDICTED: hypothetical protei			0.000000448	13.83245	24.34343	NA	12.87838	14.95849	18.34365	4.351926	10.34767	12.39952	9.036413	11.51457	9.040984	11.35215	5.645713	15.18833	9.007628	17.36938	21.87306	11.7024	5.171462	11.07393	32.72443	15.47146	29.94695	11.81016	11.57491	13.73223
CUST_38871_PI429010886	M31408	0.001261202	18	3						3492.822	192.2397	3154.32	4934.022	2474.336	3197.119	898.3377	957.7854	1922.582	3274.995	4470.918	3172.092	4392.913	1798.348	3182.56	45.48556	31.69795	83.84766	2170.443	1727.221	2354.096	5712.375	5442.505	5080.07	2457.62	4610.9	6809.882
CUST_56016_PI429010886	M112540	0.001263846	24	3	341650466	Spz1 [Litopenaeus vannamei]			4E-40	704.142	33.9635	830.4195	744.5255	840.9882	973.3791	28.4962	93.37419	84.16166	437.6531	943.4447	694.1978	438.2874	656.3228	737.8151	21.55398	21.95937	21.60709	114.8511	204.2969	136.8369	561.6697	507.6273	652.9219	179.8173	274.5666	324.6065
CUST_31914_PI429010886	M2723	0.001263846	24	3						435.509	54.88564	532.2614	600.8289	1098.604	927.3328	29.86767	86.37483	32.94207	336.6729	842.6773	423.6	235.1738	543.6971	872.0312	14.44743	29.00837	19.98159	29.21491	120.9827	27.81464	389.3624	216.5705	363.8565	42.5482	152.6411	34.55282
CUST_7939_PI429010886	N22300	0.001270663	12	9	195436302	GK22112 [Drosophila willistoni]	Transport	Transmembrane transport	2.97E-48	36.41438	56.12288	66.11318	49.99479	44.52224	53.74306	43.31181	36.17395	53.8298	59.79025	77.6897	63.55637	61.58528	29.4638	54.78117	23.18831	46.2068	34.21691	45.63539	50.33808	68.48192	75.25623	61.49909	91.13599	49.1413	25.77169	60.25895
CUST_29093_PI429010886	N59192	0.001270663	12	3						177.8505	274.2134	247.4344	237.0529	335.8993	239.0006	247.2393	275.9447	295.3036	170.8363	203.9965	232.5186	217.7635	216.7919	238.3932	278.592	334.1482	331.5173	188.2081	214.8056	244.7472	142.2962	214.0769	211.7618	192.3094	250.7116	355.686
CUST_34863_PI429010886	M10932	0.001271029	24	9						62.45522	60.59689	9.621436	23.23151	109.097	108.623	NA	74.21946	NA	16.62453	220.7835	245.9747	6.844388	105.9717	45.1102	43.49681	30.9462	13.4565	6.16087	9.666191	3.988211	NA	38.9748	4.953316	NA	73.01092	5.025678
CUST_51351_PI429010886	M93219	0.001271264	21	15						26.38647	39.22582	28.68485	20.68379	23.89085	19.55846	27.81087	23.64329	21.90135	26.78263	19.71754	13.50148	34.58217	25.95165	29.50027	37.61238	42.46222	44.98293	30.31787	34.55816	33.37054	24.65307	26.81873	30.72426	42.00836	29.60455	48.66684
CUST_52694_PI429010886	M98521	0.001272037	24	21						135.0024	139.6676	126.3605	120.4309	132.1609	127.8764	111.539	129.6027	124.4425	142.749	114.6993	150.2258	106.4901	97.2956	117.7903	141.2055	132.3168	120.6508	149.7056	125.4319	117.6781	155.8635	147.1038	153.6655	178.5273	131.4171	159.7027
CUST_4824_PI429010886	N17564	0.001272037	24	21						26.12451	34.56666	33.07741	21.16225	15.56129	19.56944	28.90562	21.62003	25.87214	35.46629	19.10616	31.27222	18.77458	18.03645	18.68449	30.44146	25.91063	28.13497	29.57251	22.55486	31.27544	35.52825	34.1106	39.39293	29.91279	27.7842	69.43522
CUST_42386_PI429010886	M60158	0.001274822	24	12	241622413	transmembrane protein 41A, pu			9.8E-15	22.83159	10.83046	11.24947	26.0713	11.708	23.88306	17.34089	14.87819	16.45406	18.9075	24.78354	17.53011	35.48599	19.45602	22.11624	18.54288	20.81726	16.07009	18.75264	19.3675	15.52247	14.42489	13.02868	15.64744	14.42653	16.39583	10.86311
CUST_52507_PI429010886	M97699	0.001275672	15	9						8.612579	NA	9.057903	6.221535	7.64439	4.002427	NA	3.228466	7.451763	10.20667	11.38568	NA	10.77609	12.81264	4.067669	4.649542	4.393119	4.973238	4.258288	NA	3.036368	8.131518	NA	5.572484	12.62994	11.10881	13.37563
CUST_23981_PI429010886	N49813	0.001278246	18	3						30.56946	11.94299	26.29331	29.76995	44.76603	29.11195	20.89693	28.70362	38.21258	34.04952	22.77868	25.0378	43.26321	19.93458	28.75045	14.98175	20.79753	17.9492	38.36719	30.72933	34.25309	47.08587	51.6582	47.40679	40.01543	27.34971	55.35616
CUST_31044_PI429010886	M545	0.001278715	24	18	321463706	hypothetical protein DAPPUDRA	Metabolic process	Cellular amino acid metabolic process	1.07E-53	197.3862	300.4795	322.75	309.797	284.4662	134.3827	195.7159	234.6915	213.3048	377.5103	285.598	258.4078	380.4577	222.5957	315.0449	255.2927	312.8082	296.9431	488.0472	319.2736	482.8348	306.6661	411.0555	441.3596	331.7854	393.0106	606.3479
CUST_17039_PI429010886	N37271	0.001278715	24	3						725.9659	30.27186	874.1945	963.1582	2123.927	1658.691	22.97455	86.92889	35.44329	54.23194	1242.354	769.5468	61.72095	1281.883	1195.535	9.985418	20.84897	11.57491	31.34548	96.62834	28.04521	94.80138	50.47099	67.90691	33.24527	172.3736	37.90412
CUST_38919_PI429010886	M31957	0.001289665	15	9						2881.653	1163.513	3851.521	8547.585	4015.947	1147.873	3434.818	3790.113	7950.355	8591.806	4319.299	5638.594	13591.98	3235.804	5558.806	175.1243	96.16362	204.1061	10506.53	7303.286	12084.47	13658.74	27273.84	25188.43	12548.11	14502.16	21177.49
CUST_43345_PI429010886	M62794	0.001291121	12	0						281.1928	216.9998	379.5535	282.1054	176.9714	231.4277	139.1812	194.0511	218.8639	274.8753	256.3549	254.1458	583.3052	331.6434	526.6092	132.7723	98.80176	172.5012	139.2203	122.771	147.5241	585.1407	306.279	433.1774	292.0008	237.8029	528.895
CUST_48915_PI429010886	M82890	0.001292405	24	18						7.818651	NA	13.56629	7.584815	4.419065	7.640953	5.379769	3.66908	6.932272	3.505965	NA	6.220734	12.02986	6.142774	9.451468	8.879476	16.42848	10.06809	15.5158	6.793494	13.83903	11.55626	10.98177	10.80862	9.925179	11.23899	20.66527
CUST_7343_PI429010886	N21406	0.001295289	18	15	339896293	cytochrome P450 [Bemisia taba	Metabolic process	Oxidation-reduction process	2.79E-48	404.7001	632.4923	717.5574	157.2999	331.2753	191.5757	217.1972	570.7447	418.6023	414.4128	377.1487	246.8683	355.686	443.7289	325.2782	600.2455	535.3308	680.9887	486.8888	898.791	1025.772	190.829	313.2232	197.9185	343.406	393.7655	496.1354
CUST_36133_PI429010886	M14744	0.001296183	15	9	91090308	PREDICTED: similar to mandelat	Metabolic process	Other	4.31E-34	NA	10.42463	9.325616	4.899473	5.898552	4.854712	7.702073	7.937099	NA	NA	15.0891	NA	5.623142	11.03119	6.481391	12.06806	9.11075	5.718914	5.963982	8.97599	3.925432	NA	9.734639	8.694186	13.21441	15.00684	NA
CUST_55420_PI429010886	M110018	0.001306867	15	0	2499378	RecName: Full=Compound eye c	Signal transduction	Other	1.41E-13	262.8559	892.7134	968.7419	102.6741	113.0827	99.60174	57.01672	111.1646	44.03643	247.1999	277.1553	240.6532	254.6677	183.411	241.1372	147.1665	396.05	294.5533	98.56741	299.1209	126.2182	125.3435	90.47296	138.3923	268.4876	321.9997	302.7771
CUST_42494_PI429010886	M60414	0.001307695	24	0						93.01673	92.71133	68.27308	79.03209	77.56319	86.13364	51.64343	90.87055	62.44813	34.76331	54.91706	43.43013	44.51618	74.57856	41.41215	36.44629	46.76121	27.96022	53.24219	92.87822	48.73108	60.09389	76.46737	51.09652	58.09261	93.13119	48.11986
CUST_35687_PI429010886	M13233	0.001311858	24	6						829.8307	994.565	861.5088	1331.208	817.1951	973.9492	1695.833	1462.033	2001.707	1168.291	1311.742	832.328	1375.234	1133.812	924.3063	1360.696	1501.763	1538.316	688.6527	819.7622	744.1912	653.1145	736.2732	930.4398	755.4761	569.4689	948.1481
CUST_46304_PI429010886	M72137	0.001311858	24	21						4650.166	7518.482	5002.917	3895.544	2965.89	2692.671	3786.16	3932.655	4409.168	4329.518	4275.766	4000.901	5015.024	4114.144	4705.762	5068.287	5783.496	5322.868	4589.607	4598.208	4221.228	5973.032	5110.752	5826.27	5277.675	4015.947	6999.724
CUST_42491_PI429010886	M60404	0.001312094	18	0	9186884	BCS-1 [Amphibalanus amphitrite			0.000000011	242.7012	397.7869	351.2785	210.991	126.1085	45.54022	181.5802	129.6173	218.5351	135.4351	173.1143	81.19478	275.2462	134.9672	168.5358	140.182	182.5875	229.2847	502.8105	227.5059	251.2891	200.7515	328.7563	305.863	208.2068	105.1748	305.5795
CUST_13136_PI429010886	N30643	0.001312332	18	3	7008007	DD9B [Marsupenaeus japonicus			2.07E-08	2524.402	2140.692	2225.697	3324.822	3220.778	3658.191	1030.655	1776.485	1032.593	1968.185	3117.642	3014.898	1897.754	2922.057	3555.208	447.0887	423.6	502.9461	669.5828	1390.116	1859.635	3339.651	2767.762	4023.648	624.767	2571.491	3538.483
CUST_7968_PI429010886	N22347	0.001322133	24	9	328776125	PREDICTED: hypothetical protei			1.16E-94	195.1701	152.096	176.8599	195.5794	413.2862	303.2777	213.6241	241.7074	183.5316	161.8447	293.7564	341.177	157.6922	231.9647	273.4227	228.9149	204.3998	182.6495	161.5143	97.84796	190.4954	132.6606	139.5903	170.4812	155.7673	186.3541	175.4908
CUST_39059_PI429010886	M34047	0.001323322	24	3						2297.179	61.5382	2065.743	2629.319	2877.124	2694.146	100.264	153.3375	159.8736	557.4698	2577.27	1887.557	751.5322	1957.624	2568.829	31.95741	49.25556	44.96264	241.2716	263.6527	157.1328	703.1577	487.4115	699.1348	150.2258	409.6221	405.3815
CUST_16276_PI429010886	N35956	0.001323322	24	3						8786.531	138.9364	8400.194	13616.29	13335.43	15258.82	63.29655	879.8906	99.47239	2965.89	5973.032	7138.174	2411.386	7240.469	10178.51	4.332102	27.00709	46.26072	36.54177	1207.663	127.0236	3774.541	454.9515	3043.672	100.5671	1420.628	345.7435
CUST_10437_PI429010886	N26226	0.00132677	15	3	253314416	peroxisomal biogenesis factor 1(	Cellular component org	Other	8.99E-17	38.55552	48.88764	53.73825	52.24236	66.18945	71.6009	59.92793	38.90574	41.05749	41.71225	55.61954	33.78234	77.48265	31.34971	41.07927	102.7974	35.60799	58.86906	74.52512	75.38714	131.1212	67.49593	40.17351	66.21459	35.59906	26.54115	36.41438
CUST_7532_PI429010886	N21688	0.001329825	21	0	291237105	PREDICTED: CG9119-like [Saccog			2.34E-91	490.2242	943.0143	709.6774	281.4085	322.8914	287.7668	318.7834	351.8456	513.8347	346.3619	394.3642	392.3777	384.5219	274.8753	418.6884	441.8514	370.8194	496.8458	427.9123	608.2737	555.7599	500.8894	567.7111	519.2716	458.3577	464.2927	530.8049
CUST_1219_PI429010886	N8897	0.001330186	24	9	321469994	hypothetical protein DAPPUDRA			2.09E-20	36.8117	21.24956	7.996777	29.80661	1529.136	505.1911	74.91993	82.0771	47.38035	NA	633.0621	880.92	NA	798.0084	868.6022	31.32591	11.21079	23.49112	16.35547	59.21803	13.38724	NA	13.09661	6.85944	NA	160.7833	NA
CUST_46651_PI429010886	M73306	0.001333387	18	9						75.73146	69.87542	88.28749	94.20496	77.36861	67.3007	90.98859	80.1827	90.97666	113.92	87.00868	98.33769	90.90449	67.54737	76.49084	75.92637	66.58185	103.1963	59.85233	75.67783	56.53387	80.77747	79.47605	81.60481	88.57697	77.44274	122.2183
CUST_3629_PI429010886	N14920	0.001336012	21	9						21.91441	13.74367	16.225	21.48468	20.81138	12.40062	24.09442	12.56899	17.41612	22.3189	26.37524	20.12395	18.05145	21.38085	18.01657	13.42476	16.10883	15.67181	11.63381	4.652688	6.298914	20.07037	14.35294	17.53224	14.07002	21.69176	17.88666
CUST_14974_PI429010886	N33786	0.001338993	12	0	4725963	EG:103B4.4 [Drosophila melano			2.79E-14	15.67736	15.47714	20.77492	13.62679	12.50865	8.120127	5.545745	7.831289	4.356715	10.48413	NA	7.651711	9.220034	19.9833	10.73437	9.404259	20.53256	15.42011	6.270949	11.40479	8.671876	21.22392	17.42676	10.72798	16.18229	18.1573	28.24507
CUST_12116_PI429010886	N28954	0.001345806	21	15	405956121	Carbohydrate sulfotransferase 1	Metabolic process	Carbohydrate metabolic process	5.77E-16	27.34045	35.78636	26.25902	33.746	21.73515	16.83166	36.94988	45.34892	37.24162	63.99691	22.43161	18.7288	42.91838	45.70717	35.71776	64.58573	34.25309	59.67529	41.1635	60.77123	61.09188	33.97232	12.85647	18.56908	27.77843	7.598096	19.08041
CUST_29357_PI429010886	N59703	0.001347556	24	15						58.95577	68.03257	77.73937	69.28931	65.28031	57.05091	80.09123	73.05642	79.66104	87.20964	66.50532	77.07091	77.34627	72.34444	88.10909	99.51051	80.77747	84.47977	78.87059	70.98403	89.49919	75.3401	52.67704	81.9335	66.76294	67.12887	78.33954
CUST_8530_PI429010886	N23180	0.001348811	24	21						62.1238	48.28205	64.36702	32.4427	46.39063	40.36492	29.41053	36.06722	40.50202	30.0974	46.13169	37.40599	54.41132	40.2126	53.66499	50.53965	60.48555	55.32159	49.84168	36.21321	40.80027	63.66743	52.38529	64.02943	63.2206	52.21427	71.46735
CUST_32648_PI429010886	M4687	0.001352402	24	18						8.622045	13.37051	15.84358	6.285156	9.69295	6.734835	5.120189	13.90777	9.415553	9.477425	5.795835	5.937279	5.451604	NA	6.803316	16.54899	16.55665	14.06051	15.96403	15.74083	17.90502	22.92262	20.01976	9.064402	11.32305	16.17305	14.64529
CUST_3004_PI429010886	N13580	0.00135387	21	6						17.84997	36.70742	26.25684	19.93458	28.51774	29.74732	37.37642	38.70891	27.96875	29.0511	24.64466	31.13895	17.66478	24.52363	19.72255	29.07492	28.6389	28.60396	15.06042	22.56219	7.693398	17.82881	25.30391	18.38558	30.48373	38.13647	33.51959
CUST_17817_PI429010886	N38643	0.001359577	24	9						42.84965	251.1346	186.2275	123.7768	142.6225	100.8605	471.0794	284.1966	574.4065	341.965	267.2693	327.9636	221.4294	289.7381	99.00226	142.749	177.2962	351.2056	24.28735	65.93698	93.16926	26.83656	213.9341	240.5209	33.57662	116.8939	134.3961
CUST_6841_PI429010886	N20669	0.001360639	21	15						35.74914	63.31668	6.698312	71.59289	14.89846	74.46237	100.2259	60.57425	39.65854	68.49163	39.60134	21.14493	79.39688	115.4959	71.71217	186.8218	134.6492	104.9085	92.27655	24.45965	85.91672	13.30176	57.16525	21.36108	76.16048	73.03741	10.07008
CUST_11372_PI429010886	N27709	0.001360789	24	21						208.4776	177.4557	218.8639	173.7269	154.4146	172.1631	80.78521	99.93518	140.8575	189.7104	146.3771	184.2949	257.4878	153.8597	292.4122	329.3072	216.3545	221.2615	327.216	197.3334	198.7013	437.6531	168.902	255.6336	427.9123	138.8443	342.4681
CUST_38933_PI429010886	M32210	0.001365287	24	15						839.3829	518.4861	449.8325	461.0647	179.0994	267.1275	443.4999	1717.387	849.2679	1623.703	1045.59	83.70817	650.5235	675.0754	300.3068	2846.594	1390.646	2661.468	737.8151	1965.01	2519.472	1352.876	64.6835	1100.835	772.0259	271.2533	435.391
CUST_24881_PI429010886	N51416	0.001366328	18	12						179.1861	215.748	199.0877	181.1959	171.3724	206.98	551.2795	274.4544	232.7425	217.8657	219.702	216.0939	234.4388	354.6555	406.254	379.0965	308.1102	419.6327	117.0413	112.7327	103.7054	221.3966	186.3541	228.0375	242.6247	145.5634	225.1246
CUST_32690_PI429010886	M4820	0.001377492	21	18	321472416	hypothetical protein DAPPUDRA			9.99E-25	30.445	26.70669	86.05106	21.40753	31.61116	39.12428	23.21157	25.50686	44.60802	31.55061	36.615	25.40663	16.6676	37.43054	49.55479	58.12746	33.6303	53.087	55.89971	114.9233	118.5947	35.27964	24.85437	33.00277	20.31011	30.02948	20.31011
CUST_55033_PI429010886	M108448	0.001380505	18	3	242013209	hypothetical protein Phum_PHU			1.09E-23	92.96907	33.07256	70.39513	89.47762	76.79264	87.30779	52.66618	41.403	35.69673	33.67346	65.59134	66.33824	63.45821	38.32178	37.01072	54.19934	27.96533	32.48271	42.79442	36.34596	34.26775	96.94761	48.32944	100.9815	47.03106	53.28421	68.92908
CUST_48162_PI429010886	M79168	0.001380505	18	3						118.6245	65.44741	88.15439	94.25033	123.988	137.0649	88.45765	77.9006	64.1954	50.51811	81.36078	75.40109	73.00128	72.38862	84.2314	69.05459	64.34403	55.34035	47.11911	80.63831	43.90368	101.7482	90.00835	89.3199	50.58009	87.9342	52.541
CUST_2223_PI429010886	N11906	0.001382052	15	0						512.0349	666.3032	420.2294	382.8713	36.53396	301.6158	252.0561	232.8674	149.0265	293.095	588.3344	529.579	488.0472	300.6771	216.222	1248.523	392.057	1068.26	357.0377	777.9259	761.5417	284.9823	75.70089	185.3999	683.9311	230.0139	338.3225
CUST_38957_PI429010886	M32578	0.0013833	12	9	321459463	hypothetical protein DAPPUDRA			0.000000273	2611.542	8.235338	2571.491	1562.544	165.0873	1043.236	34.18577	66.25687	107.8221	5630.843	5799.539	3561.33	4740.787	114.8223	3804.856	29.62856	NA	22.24587	251.6934	36.52323	275.1168	7832.882	3319.225	5678.164	306.8476	926.2026	1745.528
CUST_42857_PI429010886	M61441	0.001387449	24	9						18.93239	47.90004	43.57254	30.84927	17.4518	29.5607	67.64673	45.57662	77.12608	74.10459	47.56782	92.22131	48.15989	40.10788	14.47877	18.80558	32.73765	34.39723	8.81061	14.4099	17.49943	NA	39.63441	38.29632	4.42489	33.39499	9.926343
CUST_42620_PI429010886	M60766	0.001388184	24	18	390352602	PREDICTED: zinc finger protein 8			8.14E-33	12.40631	44.10584	41.99235	22.85306	23.5268	13.09533	34.94683	36.514	47.91548	15.51127	5.015515	15.13759	25.49204	20.39956	16.22893	26.6468	51.37499	32.97743	54.18142	46.39504	57.75543	39.19706	32.07806	39.19188	49.53225	18.35004	26.69299
CUST_46659_PI429010886	M73322	0.001389752	18	6						3.992031	NA	6.44666	4.252607	7.789818	6.758311	10.08205	8.395174	NA	NA	6.548842	5.669669	3.946695	5.151983	NA	6.387046	5.913114	6.033137	7.364433	4.069793	NA	NA	7.02166	6.218813	NA	9.482594	12.94217
CUST_12085_PI429010886	N28903	0.001391316	24	21						10.90833	13.905	18.28716	NA	NA	NA	NA	NA	4.211861	7.84449	7.137762	NA	NA	5.251169	4.865404	23.30899	19.01388	21.18826	12.56491	4.988076	11.98927	27.89978	22.2434	23.43945	17.75202	15.47714	16.09928
CUST_52481_PI429010886	M97597	0.001393595	18	0						70.85676	47.79385	85.00598	87.09988	43.34384	58.88834	37.74598	48.21063	46.88996	76.16835	24.40844	28.59585	29.51504	24.30298	16.33412	41.82506	69.76252	41.10799	27.12725	111.355	112.073	86.60823	59.26363	46.22711	21.06356	18.64133	32.20511
CUST_44577_PI429010886	M66374	0.001394099	21	9						265.3922	160.623	218.1822	292.8798	484.3203	181.8521	219.9666	229.3211	357.7827	279.8938	265.1126	585.2891	247.5032	266.1484	257.4878	248.9453	227.2167	235.1738	157.9287	196.3246	200.9087	229.3211	179.5567	229.9746	190.5205	263.0994	226.205

Probe ID	ID	p-value	Period	Phase	EBI ID	Annotation	GO category	Go term	E-value	0	0	0	3	3	3	6	6	6	9	9	9	12	12	12	15	15	15	18	18	18	21	21	21	24	24	24
CUST_40491_PI429010886	M55274	0.001397549	18	3	121484199	cuticle protein CUT8 [Portunus p			0.000000213	6018.305	175.9965	7568.31	9923.978	11805.59	10764.25	316.0187	1050.844	1010	4783.413	16874.05	7058.228	5039.165	6988.188	6612.476	95.9971	177.2739	84.16166	1567.333	1334.416	1531.626	8189.692	8484.924	8348.895	1579.509	5277.675	4650.166
CUST_14614_PI429010886	N33174	0.00140048	15	0	321471327	hypothetical protein DAPPUDRA			3.66E-47	172.2349	181.7225	163.0243	140.4625	151.8995	138.0005	119.4159	113.8607	154.2424	122.5728	131.1766	103.1963	171.3724	113.6995	149.3381	134.9488	167.0939	200.1232	175.7896	164.1609	167.0102	82.9737	63.94916	86.71039	145.0834	157.519	217.0412
CUST_37683_PI429010886	M20594	0.001400749	24	15	32527554	Squilla empusa 28S ribosomal RI			1.27E-67	1760.199	1809.494	1266.853	1585.44	537.0249	1060.229	4383.744	2622.032	4130.841	6439.853	2634.199	723.6856	3224.848	1722.827	1475.969	9872.039	3264.887	7546.585	5427.368	3292.245	6430.973	4545.107	652.9219	3675.29	2583.285	421.4753	1910.884
CUST_53318_PI429010886	M101251	0.001403849	24	9						11.81411	42.20483	42.97957	3.454726	47.68605	50.17119	16.90621	84.55241	74.37918	61.145	115.7411	86.97617	46.47735	75.7215	65.63171	11.88574	54.36935	40.31371	17.44594	78.71874	46.6272	33.10297	56.91288	42.30272	23.89085	30.11519	6.653114
CUST_50248_PI429010886	M89158	0.001403849	24	0						20.79753	16.70282	18.46268	12.49082	20.24952	14.98175	16.04875	9.503205	8.097597	12.45733	9.913032	15.81757	14.39152	8.689291	19.40157	13.58986	11.23454	15.95845	18.87895	15.37323	23.50114	24.34343	12.82511	23.84404	30.49064	25.123	21.6346
CUST_6322_PI429010886	N19946	0.001404929	21	6	548531516	PREDICTED: E3 ubiquitin-proteir			2.72E-124	82.53807	79.25889	67.78785	67.9004	63.29655	71.93057	138.4394	140.2011	184.7436	110.7598	133.4766	81.9432	120.9166	94.41489	100.3493	112.7612	131.0265	81.24878	81.6931	102.015	83.29367	51.24511	108.9605	66.55746	107.0748	81.9335	78.91149
CUST_10067_PI429010886	N25641	0.001412116	18	6						20.92431	19.42414	8.94045	24.78664	25.19327	13.15166	24.49986	20.12782	20.50908	23.51188	17.78589	13.7873	18.94041	8.23062	12.45733	16.71688	19.23789	18.7792	21.92026	19.59461	14.74793	27.18663	24.07838	21.19518	27.79864	21.3198	21.72789
CUST_37689_PI429010886	M20672	0.001414592	18	3						1292.915	31.94938	1203.381	3475.106	1883.877	2389.711	134.7613	254.6677	231.6588	2171.504	2685.938	2245.768	1546.88	1550.781	1856.844	23.10726	7.046728	26.39512	495.3901	428.6927	409.3888	2411.386	2176.241	2288.957	527.3184	1242.354	1424.355
CUST_48459_PI429010886	M80436	0.001415581	21	3						81.81979	71.1558	94.80138	108.2084	70.03526	103.8305	77.61728	63.77213	57.56952	85.66787	57.32544	92.23687	74.59679	55.65083	64.45499	72.00199	70.96595	61.56482	77.66076	75.28314	62.61945	88.92336	65.74218	77.76407	87.19961	100.315	86.40352
CUST_33144_PI429010886	M6079	0.001422719	24	18	321479170	hypothetical protein DAPPUDRA			2.28E-71	51.19385	55.40345	53.31643	38.57471	38.51685	31.06867	67.88505	53.90894	36.97883	46.94606	59.97246	29.87124	66.01029	55.59842	49.32564	63.20291	65.14299	64.26444	76.45795	54.76807	79.98506	42.9709	54.26863	60.31817	70.7916	46.62193	53.41031
CUST_45215_PI429010886	M68354	0.001422719	24	21						207.8099	228.5899	247.6563	203.9965	166.1819	176.1711	105.3837	162.6721	150.4929	216.222	215.6896	193.9353	256.6606	182.1021	231.7623	207.9921	193.3377	198.8083	188.9592	221.0978	186.1968	296.7459	240.3937	239.2059	271.4702	207.1201	247.8796
CUST_15213_PI429010886	N34194	0.00142308	24	15						16.1693	8.697442	8.355785	11.74246	12.51608	14.81846	16.43018	17.50097	11.75315	15.47323	10.58169	25.49661	17.71533	17.18362	20.56563	18.84339	22.4289	19.9833	11.43741	12.50303	16.39884	17.1638	21.81393	10.14958	10.36406	4.129818	18.40907
CUST_33471_PI429010886	M6971	0.001431168	18	15						401.3498	518.3699	346.0708	512.9523	318.1116	343.406	212.598	186.2275	260.1465	173.9289	364.8028	159.3169	375.8148	689.88	208.2068	785.8536	958.9028	1281.883	178.8343	786.1643	868.9348	236.7562	176.6026	218.7135	396.7957	173.9842	448.5869
CUST_20967_PI429010886	N44387	0.001431295	24	9						NA	8.432245	NA	8.381188	11.65098	8.223081	4.839088	8.794886	NA	11.55435	9.690677	18.07496	4.821255	10.51838	13.37894	NA	NA	5.766617	8.126376	5.329428	NA	NA	3.708703	4.533493	4.81366	6.410283	6.68462
CUST_43015_PI429010886	M61864	0.001431475	24	12						22.32375	65.32192	52.70059	33.09951	29.89281	26.48147	33.89756	38.32178	60.77123	41.81524	95.34191	6.982881	61.15056	92.53323	69.10515	61.47323	50.91261	48.72562	14.99036	43.98563	32.92494	20.1	24.83403	30.49532	25.38297	23.73245	25.91869
CUST_35618_PI429010886	M13027	0.00143839	21	0						111.4237	67.66335	94.53661	114.8652	41.10799	77.72005	51.90036	35.47671	63.83347	53.74902	29.37844	52.65281	73.26943	35.95046	56.3623	28.78071	14.50907	32.51998	55.47729	43.78128	35.86995	71.04436	71.08646	57.10009	40.06052	40.48248	55.62648
CUST_25151_PI429010886	N51876	0.00143839	21	3						58.97313	50.92071	46.09063	46.36381	64.2158	52.55424	30.70816	44.88116	38.5634	42.32505	42.60881	37.71176	44.22724	34.93669	44.32847	36.05612	48.14157	35.02108	49.82313	29.24359	43.96941	44.66421	46.22711	54.81291	46.67638	47.58263	57.30682
CUST_43551_PI429010886	M63338	0.001440368	21	12						7.342734	7.60439	9.551996	11.33713	NA	10.65068	5.029315	11.47734	14.32082	9.398999	NA	11.36956	16.78177	17.06708	11.77966	5.93112	8.141288	8.00908	NA	4.078932	3.841109	NA	6.257426	8.505122	3.764987	10.68598	7.21871
CUST_36515_PI429010886	M16111	0.001441196	24	18						612.5918	486.1663	616.5627	361.1116	317.1978	338.8265	520.31	424.7669	449.9406	635.7483	435.8605	491.5182	457.222	483.6608	375.0062	737.5198	541.2118	658.1324	629.0343	739.59	597.4035	533.7924	329.0788	510.3128	691.6444	421.8557	539.0498
CUST_34525_PI429010886	M9914	0.001441196	24	3						125.5101	155.8635	132.2346	104.6784	176.1473	147.2992	109.6621	148.3156	125.123	90.95134	108.0539	97.74585	85.38009	145.9501	95.80871	112.3401	128.9945	118.1715	73.1869	73.34259	85.0123	94.25033	83.26396	83.32737	103.801	141.1162	124.1127
CUST_30101_PI429010886	N61180	0.00144231	24	0	345483347	PREDICTED: hypothetical protei	Protein metabolism	Cellular protein modification process	4.48E-09	69.48765	44.23722	70.87494	52.6416	51.49597	59.45846	49.91114	52.61688	54.62717	48.96189	49.75454	44.96264	48.84433	56.16991	56.56581	59.7098	56.21355	57.36964	59.93426	56.22476	57.23466	78.5366	59.99359	46.3034	64.20079	57.64367	66.63719
CUST_43143_PI429010886	M62188	0.001442389	12	0						206.1814	249.0801	206.6211	221.2615	201.258	191.4824	162.9694	173.3624	171.5033	181.8521	220.4205	220.4205	233.2689	182.9809	234.2298	184.4707	239.4831	188.3253	190.7683	216.6807	171.9853	234.9137	223.9415	240.7581	284.3354	251.7468	295.7504
CUST_24178_PI429010886	N50175	0.001443038	21	3						11.58271	7.026862	23.50114	12.28744	15.87243	17.6345	21.618	11.85264	23.85663	8.451625	11.3477	11.60186	8.698736	10.914	9.832908	4.412786	23.82872	5.964848	6.150357	6.697207	7.284615	8.285959	19.18647	3.191416	NA	13.5245	15.94747
CUST_15191_PI429010886	N34164	0.001445532	12	9						29.98295	24.26872	33.73873	20.61917	19.32331	19.11039	23.22612	27.75337	37.8457	27.49657	35.62359	36.07467	56.03663	51.04378	54.08431	30.98378	18.43607	26.65836	75.6711	60.06954	48.02557	102.8924	92.82706	96.71212	57.46642	29.78433	81.05496
CUST_30227_PI429010886	N61440	0.001445773	15	9						47.67532	40.14297	36.1598	46.84848	44.73509	52.37742	23.35468	57.68508	41.71225	68.21622	55.06272	53.43823	46.56066	49.39254	42.14752	33.45208	29.66274	36.86325	32.2766	58.01234	35.15002	72.21507	65.92976	58.47581	62.1826	66.40286	50.90051
CUST_5183_PI429010886	N18369	0.001446898	21	15						191.21	266.5309	228.9149	251.2891	263.2054	264.3178	245.4129	232.9522	252.2559	191.4096	242.7012	96.9319	231.9984	233.4998	232.7425	299.3538	275.2113	282.2716	297.3019	271.2533	262.4813	256.3164	269.0492	253.4735	239.5431	273.2919	217.3996
CUST_42806_PI429010886	M61308	0.001448678	18	15	32527554	Squilla empusa 28S ribosomal RI			1.96E-100	20913.02	24507.87	20059.08	17023.68	16372.71	16622.66	22711.46	25555.67	23273.41	18673.37	18476.28	19287.83	19195.08	17771.79	19387.17	27210.5	27347.94	27210.5	24507.87	22711.46	24040.66	16288.46	17982.7	20172.23	18435.82	20762.38	22037.75
CUST_2176_PI429010886	N11794	0.001450642	21	9						122.3466	104.3412	104.6237	169.0749	123.6928	130.6255	128.7173	119.4647	126.5057	142.3304	136.8772	138.8443	117.3803	140.1401	130.828	155.6724	129.4603	122.622	118.7615	98.83232	131.568	104.8322	100.1289	120.6665	131.4868	131.4985	135.056
CUST_33023_PI429010886	M5757	0.001451878	21	0						49.84168	59.38892	76.36789	26.67982	41.35381	36.80294	36.67403	43.08271	45.56407	30.72076	42.66588	34.02915	28.8807	42.32017	26.29331	30.03767	72.06957	40.66863	43.10438	53.29185	44.87713	40.69852	70.36116	50.88233	42.97957	46.83625	63.77866
CUST_51778_PI429010886	M95035	0.001451878	21	0						2793.635	3538.483	3264.887	2696.535	2698.998	2747.205	2104.842	3368.791	2639.099	2322.285	2526.145	2684.097	1842.758	1979.658	1848.594	2418.009	2958.733	3020.109	1521.677	3390.028	2320.751	2971.539	2512.997	3093.621	2396.659	3250.257	2713.39
CUST_57271_PI429010886	E3460	0.001455532	12	3						191.7974	339.8451	309.7415	293.6993	475.3372	402.7185	175.8219	264.3178	218.9179	167.4144	326.4698	287.938	201.4162	588.9309	300.8806	368.8823	679.9519	694.9037	317.3631	388.875	510.3128	107.2586	283.9592	112.8135	210.8765	337.9166	292.8798
CUST_34684_PI429010886	M10337	0.001459673	24	21						3082.237	3405.379	2851.067	2319.232	1839.274	1777.355	1311.742	1989.721	1799.736	3470.741	1705.593	2003.765	4217.01	1708.901	2911.669	3790.113	3960.355	2694.146	3561.33	2797.556	3043.672	4372.597	3210.455	3647.267	6000.152	2870.034	4910.228
CUST_55630_PI429010886	M111030	0.001459673	24	6	321456220	hypothetical protein DAPPUDRA			6.77E-12	586.6628	435.731	445.6007	644.9039	544.2292	500.7179	780.7545	738.0615	917.1646	681.5295	612.5918	379.037	907.1782	435.6254	661.1838	693.9081	657.3507	431.5244	595.6417	469.2787	572.1963	434.4459	451.8198	390.5287	556.2705	443.9249	379.2367
CUST_36613_PI429010886	M16352	0.001460647	12	0						110.1907	125.3559	129.9006	112.0316	84.13391	97.84796	67.63991	86.21825	82.72734	125.2132	110.45	91.62496	136.0268	73.97753	103.4142	111.8116	88.27501	69.5671	81.1468	87.99932	65.1084	130.7693	118.5815	118.7615	135.1327	111.0547	142.8249
CUST_54026_PI429010886	M104151	0.001461541	24	15	321458008	hypothetical protein DAPPUDRA	Transport	Transmembrane transport	1.34E-46	25.39051	21.51186	22.45014	42.51804	17.69464	31.07591	26.23262	32.68105	32.22297	52.13985	25.97013	68.09581	37.42032	39.02298	42.06849	42.07458	52.7448	52.6597	24.31278	16.25968	13.51981	48.61045	28.61425	41.84717	26.14707	19.76134	15.7947
CUST_53827_PI429010886	M103348	0.001465045	21	9						3.592956	10.34409	NA	7.86169	25.73695	28.51273	6.233337	15.76766	NA	16.96814	23.44924	29.6926	NA	4.932654	10.96478	13.25197	34.02124	14.27475	8.716574	4.482599	6.586668	4.401339	3.664265	5.494001	12.85037	11.01879	NA
CUST_25603_PI429010886	N52721	0.001468308	18	6						NA	12.03789	7.282372	18.25109	7.836931	16.26976	28.59028	15.57359	20.26341	23.69696	6.362179	19.38111	26.76519	14.58537	21.03259	17.29188	12.70197	18.89832	14.27946	8.219759	11.95545	19.88951	25.32953	18.80373	22.56644	24.93768	23.82872
CUST_52801_PI429010886	M98971	0.001475866	24	21						67.79476	85.37067	112.473	64.14599	64.03624	65.37786	60.86727	63.01997	83.59122	58.58735	76.8564	64.27657	61.43496	81.97833	55.1847	81.68207	78.57224	95.79812	67.35191	76.98432	82.85507	97.77174	88.20477	87.91078	105.2701	81.47133	89.45518
CUST_39731_PI429010886	M43606	0.001476166	12	6	260801594	Branchiostoma floridae hypothe	Protein metabolism	Proteolysis	7.28E-90	8.723632	NA	14.83387	297.7548	NA	225.0911	3650.983	NA	2749.137	633.0621	420.9575	NA	160.2439	293.8829	103.5395	772.308	NA	713.1241	5420.715	10852.86	17287.24	885.1856	NA	786.3486	2771.365	412.0092	2021.719
CUST_48322_PI429010886	M79828	0.001476166	12	6						6.881395	NA	6.374074	26.16413	NA	26.17262	523.2386	NA	451.4694	79.87529	54.25668	NA	26.05879	45.35253	15.80013	120.4953	NA	115.5552	677.4857	1017.712	2206.592	206.6211	NA	169.9945	338.3895	50.1974	311.055
CUST_49914_PI429010886	M87714	0.001477801	24	18						106.2889	236.013	269.707	117.1085	167.1507	227.5418	100.0635	196.6443	258.2061	103.3439	98.51271	218.0311	121.5461	215.2207	176.6314	347.1137	268.6876	306.4312	394.8687	219.0312	322.1133	316.2435	255.9023	308.4293	383.0448	228.8193	370.2353
CUST_12144_PI429010886	N28998	0.001477801	24	18						370.3281	506.9058	442.023	368.5924	362.8242	418.6884	362.7316	401.2544	416.3476	368.1539	416.1276	180.4396	362.1172	365.0376	482.9421	454.5758	503.262	452.9256	454.0605	472.1134	505.1911	464.1815	476.8665	474.7066	482.8348	433.7137	598.5974
CUST_42299_PI429010886	M59946	0.001478149	24	3						23.76317	5.209393	17.25943	49.49937	55.2321	35.52825	9.848403	6.335605	8.314533	27.66729	24.67768	35.97882	16.51333	27.69481	22.12314	7.664784	5.688322	9.361001	6.112777	6.279198	5.463559	17.2551	19.36009	7.142339	5.780077	9.251212	13.40671
CUST_45329_PI429010886	M68748	0.001478149	24	21	405973852	Protein Daple [Crassostrea gigas			0.000000475	315.18	280.3032	386.1445	345.1684	255.4927	290.6243	210.5427	211.695	295.5482	303.9295	241.364	282.2716	356.6056	256.2294	291.1427	383.3073	358.247	296.1605	273.5066	294.5533	331.9294	488.7695	323.747	391.1173	385.3052	338.9144	323.3549
CUST_31132_PI429010886	M785	0.001478149	24	21	383864755	PREDICTED: glycogen debranchi	Metabolic process	Generation of precursor metabolites ar	4.7E-156	435.2817	577.1013	628.1097	626.0846	651.2334	505.6819	336.9544	460.4529	503.9395	532.3756	497.6488	505.0907	664.9053	462.9596	570.8807	562.1389	833.2103	513.0768	753.6723	662.7709	541.2118	712.1062	702.7679	653.7367	952.9844	886.9505	745.1009
CUST_3434_PI429010886	N14492	0.001478149	24	3						484.4076	291.4248	479.3697	407.6903	531.7033	671.9924	256.3549	311.1974	265.2295	388.6637	327.283	564.5167	384.4369	262.9461	439.7541	212.7978	183.1939	193.9037	171.8869	251.1902	124.6245	501.3701	215.2207	461.4101	235.8737	285.3215	223.277
CUST_13054_PI429010886	N30503	0.001478655	18	3						112.0182	151.7904	190.613	170.0181	241.4097	237.366	130.3988	191.7176	128.645	178.6585	227.5418	218.7863	85.26437	128.3867	114.952	162.0267	112.5075	168.9624	124.0673	170.5634	147.3924	265.4961	196.3832	233.1508	73.27988	235.9714	143.5284
CUST_13560_PI429010886	N31380	0.001478905	12	0						46.43451	38.34001	44.56728	30.46326	31.39279	34.43322	28.10459	29.01236	28.4735	30.43559	25.50997	24.28357	39.34209	32.73765	30.77878	35.48221	28.81419	26.87469	26.11298	28.51548	24.37229	35.08331	28.87134	28.5396	38.88446	33.90314	25.9662
CUST_39113_PI429010886	M34698	0.001480772	24	15						77.93692	86.38253	70.15908	137.9765	37.58637	97.53962	111.0263	132.8996	148.5965	272.4392	163.2927	28.2205	100.3986	164.5127	75.73146	407.9106	199.5583	296.8179	157.2999	201.4635	225.2142	241.0614	46.14868	139.5903	111.2894	28.6389	79.58985
CUST_304_PI429010886	N3717	0.001482765	18	3			Metabolic process	Carbohydrate metabolic process		540.7576	133.0371	557.162	818.3565	804.9022	816.8898	222.4664	239.348	183.381	531.5525	690.0542	656.0648	799.0901	431.346	761.8025	167.261	157.6609	114.413	272.1212	268.0924	214.2802	1124.051	790.1557	835.3759	223.1202	447.1876	334.795
CUST_22465_PI429010886	N47078	0.001483688	24	0						11.85264	69.33519	25.1506	4.007214	10.54698	NA	8.078793	NA	13.01309	6.397769	6.05695	10.8747	12.22514	11.59198	7.033009	6.456174	4.274871	7.286501	8.673027	12.24754	12.49454	12.11716	14.21449	13.38505	12.99098	NA	19.74485
CUST_44536_PI429010886	M66263	0.001486149	24	12						637.2271	485.5854	655.1561	1254.672	441.4649	711.4323	1216.51	549.069	1143.279	953.6942	2057.214	665.1463	1262.927	1676.387	961.8532	687.9587	1401.131	1124.497	500.8894	632.6818	569.4689	599.0901	534.9189	719.258	452.5351	412.1414	643.0042
CUST_31811_PI429010886	M2471	0.001486374	21	0						1059.201	474.3448	1112.302	1041.115	654.9488	1006.869	469.9074	596.2588	543.3224	772.308	712.9201	753.1726	857.7889	417.3805	705.624	380.4577	421.7429	364.3837	488.8743	591.3029	486.0305	1322.806	788.7271	1218.815	581.8311	668.9797	836.3643
CUST_24919_PI429010886	N51482	0.00148643	24	0						51.83065	50.55101	50.36291	36.22402	29.86767	29.72388	29.92819	28.56322	16.68416	25.20922	15.93995	33.82054	26.90354	30.78396	35.0975	44.18768	35.17583	37.12091	54.45775	22.33262	33.84542	38.413	23.34913	48.00737	33.62389	43.63653	41.91606
CUST_21460_PI429010886	N45262	0.001489829	24	21						6.126911	11.18155	NA	5.391687	7.640953	3.596764	5.016947	7.463335	10.21986	3.355805	6.009874	8.056452	5.551574	9.261783	3.744973	4.60408	10.97548	5.921908	13.49255	9.307362	4.09305	15.19014	18.61345	12.77084	10.77882	12.18599	9.902198
CUST_1841_PI429010886	N10861	0.001495571	24	18	321466121	hypothetical protein DAPPUDRA			9.61E-09	365.0376	431.0524	840.6574	454.1788	262.7025	113.6995	533.1871	353.4279	935.0962	276.3303	139.9317	192.8297	510.5943	281.3108	138.9184	320.1071	248.6058	341.5157	816.8898	1135.893	1255.327	514.4981	672.5292	606.9823	408.6556	455.3201	702.937
CUST_42727_PI429010886	M61083	0.001496325	15	0	4585366	extracellular superoxide dismuta	Metabolic process	Oxidation-reduction process	9.71E-44	55.76605	111.2462	90.71076	71.26688	34.57091	35.55837	95.46305	46.85381	71.99448	17.86679	14.3798	22.53586	118.0597	32.69926	79.92025	84.3918	48.4991	84.84924	NA	53.39229	42.60412	44.86243	84.13391	43.15923	29.4515	15.70448	35.5206
CUST_35743_PI429010886	M13381	0.001496626	24	18						276.0746	991.5499	995.9721	753.1726	383.478	83.81296	1140.807	750.3368	1135.35	1069.345	527.1701	816.8898	1144.859	490.077	390.0801	1114.745	1063.832	1984.393	1215.754	1999.848	2666.243	928.2282	999.3353	1302.487	816.0394	370.8194	1339.398
CUST_6436_PI429010886	N20098	0.001496626	24	18						275.5152	394.1154	352.1552	364.2801	279.6802	279.6802	351.2785	378.9491	289.6241	360.8837	377.5103	350.1584	384.7267	294.2691	417.2642	345.1684	527.5856	425.0628	432.4641	385.6874	430.4406	376.0142	400.0672	437.2723	386.2259	367.8176	412.7396
CUST_19109_PI429010886	N40993	0.001496626	24	21						181.0227	164.1331	223.0844	202.7113	112.9448	137.8428	141.898	140.444	170.5634	207.2734	127.7854	149.6248	218.3669	137.5443	184.3858	144.1497	180.5627	189.1836	229.9746	166.6574	211.8418	224.9241	228.0375	256.9518	203.5459	190.9064	279.9265
CUST_24247_PI429010886	N50294	0.001496626	24	18						26.87774	43.20078	42.68735	20.38818	16.04505	15.87729	34.32123	38.35932	43.86283	39.6633	41.53886	30.3404	49.35984	12.67148	32.50381	100.9815	25.24715	77.44274	55.20546	65.82678	49.38843	45.38696	27.99655	66.1068	42.13235	21.99703	50.36694
CUST_24718_PI429010886	N51143	0.001496626	24	3						11.72699	9.575352	9.753338	11.18434	73.35252	69.60024	11.29	11.48486	8.078793	4.417002	10.55248	20.49843	16.74981	16.68416	30.3574	7.569757	8.00437	8.846413	6.66539	5.765353	4.158939	10.03947	7.027807	8.040102	9.016507	9.271842	5.3907
CUST_25854_PI429010886	N53174	0.001496626	24	18						95.93997	149.1509	109.9913	82.03251	77.9206	88.70868	94.99849	94.76517	90.03155	100.3755	98.38918	142.1948	93.78493	64.13419	112.8635	123.9057	108.4858	141.2321	121.7034	129.3077	176.1711	109.7081	96.49913	166.6346	146.3562	104.3062	173.9289
CUST_29006_PI429010886	N59015	0.001496816	18	9						53.90529	44.6542	40.71158	63.47982	37.73828	57.85506	44.40388	52.76213	43.38791	94.83653	59.72832	65.0271	115.0193	64.88783	54.07225	56.64171	43.67283	52.70059	40.13104	37.9908	24.55645	59.97246	32.82904	55.06924	63.92981	45.30575	55.95026
CUST_10116_PI429010886	N25724	0.001497163	12	0	321464005	hypothetical protein DAPPUDRA	Transport	Ion transport	2.17E-77	277.6138	194.635	282.8745	195.9057	179.5327	156.6771	150.0907	152.1662	155.0651	256.3549	186.2275	231.1683	273.4227	184.1934	206.3209	274.2662	164.3213	207.5504	166.3995	165.6814	171.8137	290.3235	234.4037	285.7364	371.5772	222.6199	362.5984
CUST_12897_PI429010886	N30228	0.001497307	24	3			Metabolic process	Carbohydrate metabolic process		204.2476	27.34045	198.7781	251.3401	2038.719	1565.636	25.67433	188.656	19.10616	33.9183	792.599	703.6419	17.22771	589.338	475.4976	3.508635	12.01555	8.219759	4.094973	273.9926	NA	49.20954	44.42034	34.10624	NA	345.1684	NA
CUST_36422_PI429010886	M15864	0.001497505	24	21						98.29871	121.5027	143.4269	116.2567	102.3362	96.5246	79.33651	97.25517	85.09153	99.16406	132.9821	90.25881	116.6925	81.61482	132.8996	98.17006	119.713	80.66827	179.0567	139.4559	146.9178	162.1178	125.9747	133.7211	176.9877	147.8169	172.4229
CUST_10093_PI429010886	N25681	0.001497505	24	18	54042615	arthrodial cuticle protein AMP6.			4.12E-15	1183.356	699.1348	1416.053	1802.44	1301.148	591.1008	1722.827	1069.345	1708.901	846.219	569.8831	354.4742	1920.18	679.6938	925.0688	1232.801	1639.362	1331.208	1629.282	2362.508	3751.984	1536.792	2659.616	2160.965	1500.263	2369.531	3525.214
CUST_15155_PI429010886	N34094	0.001500003	24	0	332024397	Phosphofurin acidic cluster sorti			3.83E-14	40.46925	43.45889	50.86361	40.58521	34.88249	39.87272	28.38772	39.61156	36.5903	38.61632	35.78924	19.50992	39.27561	35.20827	40.61255	40.80443	36.69692	33.56251	33.26748	36.12473	35.95938	50.64865	38.69748	45.5838	51.26614	39.31755	54.53262
CUST_1028_PI429010886	N8267	0.001500849	21	0						2389.711	2755.522	2598.143	1568.389	1253.748	1091.584	837.9532	1289.967	1039.627	1377.595	1119.38	1071.049	1587.909	1183.356	1163.513	816.314	1385.605	1260.62	1375.747	2235.693	1066.707	1996.997	1816.946	1255.327	1162.935	856.5689	1082.092
CUST_43401_PI429010886	M62945	0.0015122	18	6						58.28361	62.1238	83.15856	63.10394	67.95059	40.85573	100.0492	78.25384	86.15946	60.93815	110.8559	87.61045	60.20446	49.31951	81.66229	61.32398	62.51652	50.70492	67.54737	39.09998	61.55774	108.7869	79.95529	94.88668	115.1501	87.44315	98.60183
CUST_44544_PI429010886	M66286	0.001513861	24	6						20.47903	21.91441	27.08528	27.48873	29.84142	30.87082	31.8936	36.12041	32.05523	27.69715	35.99913	22.71992	32.69608	19.52436	16.88307	27.982	28.14985	33.51137	16.58591	13.03685	12.21743	23.0693	11.46286	33.05798	26.24091	10.31105	25.96348
CUST_7302_PI429010886	N21342	0.001515103	24	21						118.9614	114.9651	148.728	105.1035	92.84934	78.99466	108.1208	69.96485	122.201	138.575	89.99586	109.2676	124.8368	109.097	121.0004	167.0102	155.2093	116.4978	151.8566	91.32281	127.8764	165.9265	241.5804	270.0299	170.9936	87.87185	161.0998
CUST_15825_PI429010886	N35209	0.001515919	24	0						92.39943	77.17435	87.47672	66.33824	59.87629	60.15379	70.66441	67.17319	84.6671	72.82177	53.92132	59.65828	53.88185	53.8516	75.86824	74.31745	67.71277	68.9382	79.67772	68.82621	78.39296	89.90152	62.08412	78.96469	80.11297	71.1642	92.7216
CUST_19103_PI429010886	N40985	0.001516046	24	15						66.27962	104.1325	89.97113	85.40071	77.84495	66.11318	137.8885	122.0709	126.2717	164.4172	90.88256	106.6811	148.0804	90.50145	98.60183	130.9866	177.2393	140.182	159.0574	128.5165	150.4102	137.1269	139.8319	120.513	137.8428	118.3083	145.0834
CUST_5695_PI429010886	N19061	0.001516851	18	15	383854650	PREDICTED: aspartate--tRNA liga	Developmental process	Nervous system development		128.1107	191.1727	184.2288	132.4508	100.1918	118.4747	177.9861	179.4021	178.033	171.3126	141.2631	128.3485	172.8084	151.5229	146.0664	205.9794	261.3674	247.0676	147.7552	152.4015	171.1487	143.0807	151.261	117.7118	175.4084	165.7618	163.8102
CUST_20446_PI429010886	N43458	0.001520811	24	15	443732865	hypothetical protein CAPTEDRAI	Metabolic process	Oxidation-reduction process	1.13E-42	4.475565	NA	NA	5.437296	NA	5.828206	NA	3.755811	4.928978	11.6258	NA	7.354982	8.208913	5.934466	4.710525	14.69279	14.89051	12.85763	NA	12.27129	8.368746	6.018964	NA	4.386394	5.975334	6.27007	11.17863
CUST_5264_PI429010886	N18467	0.001521612	21	3						NA	24.44216	12.53267	23.48308	17.12368	26.90354	12.23254	9.952015	21.69176	5.99174	20.9364	12.71213	15.13981	11.45758	31.52277	14.68427	8.474912	12.18717	7.415808	9.482594	5.409442	NA	9.838142	10.80202	20.51536	20.73144	48.07163
CUST_17620_PI429010886	N38289	0.001527825	12	0	241555876	TATA element modulatory facto			5.61E-18	5.121	13.41897	11.46286	7.869127	3.363808	7.755188	4.693356	7.903245	4.484845	6.685275	8.326096	6.050143	19.82019	9.058939	14.13842	9.865694	6.62579	4.844365	4.772325	6.108532	7.267337	10.21893	NA	4.256486	11.12735	12.66652	9.425176
CUST_33558_PI429010886	M7272	0.001528413	21	15	270010437	hypothetical protein TcasGA2_T			3.55E-11	21.58422	32.40814	21.0554	25.55069	27.42419	19.13568	25.95548	22.81234	19.43519	22.61447	17.18571	9.619909	29.79027	21.23607	32.56737	28.61425	30.58943	35.34111	26.47878	26.51323	30.11519	32.82904	26.37524	27.75337	23.80543	27.2558	26.49338
CUST_18535_PI429010886	N39975	0.001529636	24	21						7.496542	18.66636	13.92807	11.82291	12.8517	19.27844	4.634489	16.57693	13.19768	13.08105	11.10457	11.3284	7.628654	14.29658	10.54698	13.97991	16.25968	18.46557	15.80769	5.866363	15.02898	26.92179	24.6758	24.79	23.15112	17.30739	14.73062
CUST_45462_PI429010886	M69182	0.00153358	24	21						414.8181	448.2125	364.2801	411.3393	465.5812	336.1378	274.1568	385.1879	375.6666	414.136	359.7575	392.7267	482.9421	368.0554	307.3952	437.9976	537.9838	498.4242	428.9483	360.0833	425.7716	571.8635	499.0568	531.9945	535.2394	459.5171	466.0849
CUST_23038_PI429010886	N48149	0.00153358	24	21						113.2581	110.7326	173.1388	110.5907	113.1088	118.6368	103.6054	97.244	123.4547	131.8269	91.51354	146.658	142.2962	112.9084	169.1748	146.798	113.3227	149.6459	171.0708	115.2322	155.6261	195.3485	137.4674	198.7013	191.8796	115.5105	183.166
CUST_51678_PI429010886	M94546	0.001534599	24	15	332029065	Transcription elongation factor S			5.48E-16	24.77298	18.71237	33.80478	17.46376	25.60118	20.34644	19.69265	26.9815	21.82087	23.50326	27.53522	26.12451	48.76461	19.0327	31.48667	43.21882	52.62861	34.57879	31.92535	26.01047	27.85041	26.38902	18.34767	35.05743	23.40815	25.42047	32.8145
CUST_41344_PI429010886	M57509	0.001536913	24	18						36.54404	33.2361	32.72443	19.57461	50.20269	18.1802	33.36729	43.07453	27.43793	30.37998	32.19227	24.11615	46.16308	37.47838	53.36513	32.42334	37.19414	28.61945	57.781	53.02947	87.59003	86.40352	65.79182	52.32559	56.47386	41.89699	60.05636
CUST_10512_PI429010886	N26353	0.001536913	21	15	260820477	hypothetical protein BRAFLDRAI	Metabolic process	Other	8.24E-55	64.9627	97.17225	98.86808	80.36141	72.30147	57.37441	84.06095	96.13685	84.70621	63.33988	77.61728	62.5913	92.34598	84.99467	72.18458	122.3466	119.5848	121.7366	102.8149	96.7742	140.6785	73.10715	104.5338	85.60411	78.57224	94.22856	103.9763
CUST_38299_PI429010886	M25120	0.001541968	21	18						16.1297	19.59461	23.18509	7.299732	8.012359	16.51006	19.75896	17.1309	20.4836	22.45014	13.83564	12.01555	22.03386	21.00723	20.54013	21.85917	18.89336	22.85306	18.82754	25.65567	27.21619	18.97317	14.95172	23.07724	10.36519	17.93755	19.3323
CUST_36637_PI429010886	M16430	0.001550432	21	0	91081481	PREDICTED: similar to cyclin d [T			1.49E-26	6.597671	10.4451	14.55272	6.415726	6.912398	4.963645	NA	13.11926	10.36406	5.833172	5.833172	5.075019	4.89273	5.699934	NA	4.928978	3.720792	4.589395	5.648752	4.365639	4.282737	NA	NA	9.259524	6.440377	4.967934	7.446593
CUST_31859_PI429010886	M2590	0.001552057	24	21						592.5764	567.7111	690.5452	551.8317	437.9976	459.3044	441.3596	464.1815	560.022	520.31	489.0279	494.3291	623.4767	546.5288	697.2436	510.3128	599.566	565.0242	506.1561	549.8031	596.5924	746.7514	638.835	765.1767	620.7278	539.7737	808.5577
CUST_40854_PI429010886	M56236	0.001552057	24	18						101.8399	201.8882	157.8028	121.1043	94.21867	92.83981	150.2055	152.3049	164.7136	172.7187	137.9056	130.5722	164.6109	132.304	131.7896	155.6488	182.6192	174.5369	198.3111	174.5369	222.4664	181.4338	135.3635	176.6026	176.6314	143.4863	231.8452
CUST_47128_PI429010886	M74889	0.001552057	24	21						103.1963	61.55047	85.51486	86.54102	72.38107	84.55241	69.94875	59.11715	72.79587	97.53962	49.22608	88.13366	82.42094	63.1617	81.04337	109.6043	76.91277	94.86558	93.69945	76.25504	92.36671	111.371	105.1748	145.3183	93.91412	62.2112	105.034
CUST_13076_PI429010886	N30533	0.001552057	24	18						40.87182	58.37071	56.16512	42.75885	39.54209	27.9424	43.44652	44.24302	56.63612	44.87713	51.44911	71.6009	47.90611	49.18503	46.52866	67.68103	57.8755	73.88293	67.44192	59.14128	62.367	74.12935	53.66499	71.59289	69.99598	57.00268	94.10473
CUST_38254_PI429010886	M24695	0.001555337	24	15						549.5192	1574.714	942.7053	593.9206	332.8061	328.1119	583.3052	620.9302	740.2235	817.1951	373.5884	561.5074	844.6913	695.5915	704.9081	1785.094	2258.648	1967.079	1038.339	813.2524	798.5585	1210.563	580.31	928.5423	1640.282	423.2638	1203.381
CUST_25454_PI429010886	N52447	0.001555337	24	21						126.8773	115.921	144.5963	123.0024	115.5105	81.9335	115.4585	95.61224	113.0037	108.6739	72.26194	83.62172	129.4034	78.44375	118.3083	100.2539	130.4451	128.8774	142.9154	109.1979	124.7322	168.7131	128.7652	116.9614	162.3844	110.2215	138.3923
CUST_57284_PI429010886	E4140	0.00155548	24	0	332376232	unknown [Dendroctonus ponde	Metabolic process	Other	4.77E-09	37.23663	66.02134	88.70868	39.74479	4.581292	37.78857	29.79027	35.84121	24.57064	9.093643	24.37455	NA	38.35003	12.55197	64.10142	5.936222	27.99322	4.696463	8.999366	18.74013	5.262435	11.48313	21.67269	13.34178	51.23064	51.34712	38.11089
CUST_33385_PI429010886	M6755	0.001558996	18	3	281337645	hypothetical protein PANDA_00			0.000000426	105.6309	99.03286	134.0408	125.6495	163.782	145.0599	81.76793	81.9335	99.116	107.7085	110.721	137.3189	114.4623	93.16926	84.61068	72.01042	93.83228	87.81083	126.1572	150.7445	128.645	131.9455	153.1826	137.3382	132.1477	120.1554	181.173
CUST_41554_PI429010886	M58016	0.001566142	18	12	242019410	Diacylglycerol O-acyltransferase	Metabolic process	Other	2.56E-84	9.339408	10.47852	NA	7.878601	9.395362	7.133224	7.176989	11.42758	NA	NA	40.44359	7.849927	10.14417	19.6251	28.84319	19.0444	12.71903	9.496049	NA	13.46097	9.581479	5.729424	4.368233	8.704745	13.7624	29.23276	NA
CUST_38119_PI429010886	M23439	0.001566994	21	6						4.660637	4.70019	9.346935	5.182985	14.01914	NA	9.561117	21.18591	13.61629	9.44382	8.601925	8.421879	5.420396	9.559713	7.062362	6.557583	10.60449	9.381399	6.118751	5.239057	6.984847	NA	4.050732	7.259084	7.924882	9.540789	5.217523
CUST_33660_PI429010886	M7538	0.001569876	24	0						70.47134	60.87753	47.37075	58.40669	45.31246	40.80836	54.06274	60.94356	50.61747	53.39229	34.60956	45.63539	50.57112	40.47822	26.32359	30.68789	40.90642	52.24236	43.48425	49.94958	32.05523	55.76605	51.95311	42.39624	66.15369	53.53717	56.01078
CUST_45814_PI429010886	M70381	0.001570195	12	0						53.02947	69.60024	52.09068	51.73381	66.09101	59.84299	13.72472	41.27805	33.32109	52.81201	93.97841	56.89319	70.62321	58.5677	75.70089	30.54385	63.74918	28.37326	44.47768	54.56863	52.26773	68.70749	57.74953	53.30473	108.7311	80.09649	122.4178
CUST_45320_PI429010886	M68725	0.001576075	24	3						125.9513	10.23574	87.37285	206.2187	138.6295	172.3736	11.68791	4.314637	22.97455	64.02245	177.9078	175.5738	50.6629	93.69945	136.0437	3.849147	6.827125	3.651602	12.76017	12.34025	3.436245	65.90991	36.28429	67.3285	14.65289	17.63804	361.2613
CUST_11031_PI429010886	N27156	0.001576075	24	15						9.382464	26.95753	15.20453	10.34673	16.44171	21.49158	21.0408	21.12553	23.80751	21.1516	20.61453	12.89633	25.70122	18.6435	29.72829	35.17211	34.76331	30.45754	27.30572	17.83378	31.69013	21.2032	23.93863	29.08241	27.01424	24.23607	23.51913
CUST_27681_PI429010886	N56507	0.001576075	24	15						11.4263	11.04829	15.4017	11.46799	6.801504	5.765353	17.1543	7.671252	15.40578	14.69477	16.38187	10.38036	17.58375	10.90468	14.88235	31.27222	12.44465	23.69096	22.03729	13.39577	21.59473	14.9436	13.65748	20.00439	16.45776	10.11056	10.85858
CUST_15018_PI429010886	N33870	0.001579942	24	9						6.649922	8.624273	6.523317	9.10076	15.33433	12.03475	6.387046	9.658463	4.893793	NA	16.03181	15.61374	11.76947	12.04588	NA	9.604194	7.782145	6.845572	7.709748	8.445011	9.239178	4.229226	7.642085	4.462111	7.99927	12.21233	6.206844
CUST_11271_PI429010886	N27542	0.001581032	24	18						18.02618	28.70897	23.1769	22.70484	17.93755	14.68872	11.20074	19.37652	22.96931	16.94573	14.46435	18.63267	17.30627	17.51375	24.45655	21.85018	29.01782	22.35262	38.96726	24.10826	39.00145	22.44464	27.13523	19.23397	34.33958	19.35535	28.71526
CUST_20817_PI429010886	N44116	0.001581032	24	9						119.0641	125.5382	95.15335	94.66861	138.6464	124.3244	152.8269	139.5198	110.2215	144.2677	153.9484	141.598	96.46623	121.1643	134.125	138.3312	115.2516	115.4585	85.79452	107.5871	99.47239	86.08655	155.4749	86.96518	123.1089	103.657	107.6658
CUST_40121_PI429010886	M50578	0.001586157	12	9	222476500	hemocyanin [Fenneropenaeus c			4.92E-14	2622.032	544.6702	2622.032	5317.598	1046.706	1890.428	1084.752	1622.787	2767.762	4322.847	7058.228	1875.312	7937.877	1626.549	3284.996	85.18385	61.47323	109.1677	2690.376	2239.116	3460.858	6385.128	11246.8	10485.81	3706.021	6358.535	9699.55
CUST_43222_PI429010886	M62454	0.001586157	12	9						2833.623	246.2584	2860.025	4329.518	1533.458	2075.256	835.0606	675.2885	1247.204	3601.999	4229.758	3071.483	4761.143	1659.781	3022.554	93.50035	144.2876	111.2293	1517.515	1429.783	2538.621	4719.533	6809.882	7785.133	2047.134	2950.533	6079.326
CUST_359_PI429010886	N4421	0.00158715	15	3	220172369	cuticle protein [Rimicaris exocul			2.35E-29	65.87261	60.73958	66.07592	74.09264	479.6967	69.13756	121.6759	155.1393	150.2498	64.6835	3060.056	55.52101	60.59689	54.20975	47.7609	65.82127	5872.99	55.31238	5227.74	4015.947	79.49714	4383.744	3434.818	62.5403	62.98013	58.29201	95.34191
CUST_7136_PI429010886	N21090	0.001587525	24	15	308500844	hypothetical protein CRE_31104			1.26E-08	20.26911	18.02157	12.74304	9.546386	13.15696	7.870945	9.518164	10.96116	11.86035	25.66507	10.76649	13.59227	11.59662	13.52139	7.816284	23.0873	14.5966	26.85225	14.95298	18.85311	16.44171	18.56115	18.17588	19.31939	15.12866	13.69771	12.47033
CUST_30969_PI429010886	M338	0.001587865	21	15	524881459	PREDICTED: pre-rRNA-processin	Nucleic acid metabolism	RNA metabolic process	1.62E-16	160.139	190.2229	222.9426	201.5487	112.1553	103.106	252.7743	194.3126	309.6367	207.6942	197.796	187.3112	202.3368	216.014	172.7187	281.8768	291.5804	311.4501	171.664	151.7315	211.9713	190.5906	150.0449	187.7437	173.1388	145.0599	134.3368
CUST_3363_PI429010886	N14363	0.001587865	21	15	321477410	hypothetical protein DAPPUDRA			6.57E-76	73.35252	161.9628	62.65711	75.054	19.43172	67.72081	185.2204	189.5806	120.1066	132.2682	79.08754	77.27754	111.7284	120.2238	74.92905	173.3126	227.8105	174.857	159.3169	92.05562	158.518	100.315	104.2797	113.9393	105.7248	73.88293	62.85549
CUST_44010_PI429010886	M64639	0.001588453	12	0						19.94017	20.3194	21.20564	12.62197	12.92228	15.80013	6.949768	8.93938	13.91119	16.4789	15.63819	19.94147	17.32703	18.43066	25.00741	18.64717	11.53299	22.81773	7.187709	11.02011	20.02186	21.9474	16.33604	26.38383	19.11956	22.5581	31.92217
CUST_13086_PI429010886	N30551	0.001588453	12	0						15.59925	23.6675	16.7995	15.62347	18.36321	16.66	8.91225	12.69375	14.18835	11.98023	25.67959	23.75813	19.79478	20.7793	25.35027	9.24446	22.60699	12.78321	15.83716	10.27659	8.105145	24.87248	21.70753	16.48722	26.86909	22.87902	28.70137

Probe ID	ID	p-value	Period	Phase	EBI ID	Annotation	GO category	Go term	E-value	0	0	0	3	3	3	6	6	6	9	9	9	12	12	12	15	15	15	18	18	18	21	21	21	24	24	24
CUST_17478_PI429010886	N38038	0.001589309	24	18						9.946155	19.80398	14.93075	9.628869	13.41756	10.38657	13.93661	5.201984	16.44171	18.23129	5.258405	8.346975	19.74619	7.976904	3.795435	20.9985	21.0238	17.44104	37.08249	19.49003	21.34152	15.23659	32.85244	17.12239	16.72545	12.70744	19.53602
CUST_42466_PI429010886	M60348	0.001592782	24	18						682.5361	237.366	313.9334	18.91649	NA	8.880632	151.0701	230.7682	24.55379	32.39325	16.10883	NA	61.16761	348.7073	NA	1434.548	45.8224	1246.549	1188.558	659.9573	1088.703	450.4965	NA	488.2191	979.9752	98.28823	949.6232
CUST_42785_PI429010886	M61253	0.001596025	24	3						1261.961	1302.008	1049.045	2757.04	3193.902	3349.165	1375.747	2269.737	2086.93	1132.862	2001.707	1849.733	1142.458	2174.954	1778.328	697.732	841.3097	868.2894	1007.681	1832.717	816.0394	509.6459	1275.515	795.9001	609.0112	2100.692	766.0458
CUST_32450_PI429010886	M4146	0.001596813	24	15						391.6595	451.1176	359.174	350.9469	406.6714	350.3187	414.226	405.1127	388.382	404.3238	358.4079	414.325	433.097	355.324	426.6472	469.5128	464.4221	461.6561	412.8294	449.9406	429.3727	387.048	410.7073	407.0205	443.9249	400.7915	507.6273
CUST_55863_PI429010886	M111912	0.001606711	12	0						87.50644	91.18387	91.97617	82.56422	80.72426	83.92194	56.03663	64.88783	56.48171	85.10841	67.1207	91.18387	74.39469	83.52767	90.35029	81.9335	59.38892	81.02819	76.25504	69.31429	69.80795	70.63954	73.55381	83.06309	74.90044	84.52278	80.01131
CUST_12836_PI429010886	N30129	0.001606711	12	0			Metabolic process	Other		48.78916	111.2293	159.1592	26.45629	24.73755	18.98812	18.22769	22.61608	27.62832	39.88903	23.87506	38.52068	23.95198	38.12224	34.67932	24.80368	32.81904	45.74936	16.54419	30.70093	16.1275	34.46864	36.61091	29.16024	31.85157	31.84882	56.42712
CUST_33216_PI429010886	M6309	0.001607468	21	3						62.80714	45.7721	59.92793	76.19255	114.9878	95.39909	27.96875	50.55101	44.47768	45.98151	112.6234	74.04749	58.40669	78.34836	75.92637	37.34026	22.70713	47.18539	70.1867	61.72867	41.01272	70.32352	60.12159	56.99251	73.72575	78.71874	83.19883
CUST_7804_PI429010886	N22108	0.001607468	21	15	321476509	hypothetical protein DAPPUDRA	Metabolic process	Other	1.65E-62	67.9004	392.3777	234.2807	130.9003	98.60787	40.76292	302.274	278.3094	248.8065	302.7771	172.9954	176.8903	226.2405	130.2225	179.7937	335.8993	361.1116	307.5442	175.3842	285.8344	386.0289	232.2882	328.9434	89.79089	133.0187	70.24722	140.7536
CUST_27420_PI429010886	N56030	0.001615142	24	15	242002324	sugar transporter, putative [Ixoc	Transport	Carbohydrate transport	2.1E-20	7.310973	13.22308	NA	6.784619	4.214527	11.48896	11.93827	13.84533	10.12695	19.22503	NA	4.539514	12.93095	NA	13.51154	22.16839	18.46729	22.73253	13.47047	8.640415	32.21584	NA	13.19589	5.527945	12.38261	8.885052	12.19826
CUST_18696_PI429010886	N40246	0.001615724	21	15						NA	13.87312	NA	12.77084	11.08682	12.60992	13.13244	9.991081	9.141868	6.346484	NA	9.508309	25.18751	17.94579	21.72565	21.74621	28.88967	16.8613	12.53864	14.90046	10.36863	14.25789	17.22544	13.61444	12.90391	7.477063	NA
CUST_34101_PI429010886	M8775	0.001615729	21	15						30.18612	59.75785	41.76233	24.19322	29.17859	28.98015	38.9748	48.69222	53.37496	38.57077	45.79664	45.9512	38.85987	45.66053	28.33805	55.84882	64.63944	54.84307	32.40814	51.08158	51.62622	51.44911	53.80944	51.9164	38.61107	34.46463	38.43848
CUST_3040_PI429010886	N13661	0.00161605	15	6						1345.742	2381.656	1392.216	1256.746	1833.616	1189.292	1994.973	2041.036	2499.817	1253.748	1291.096	1238.715	906.4938	928.5423	860.5197	1000.752	1885.037	1497.206	914.2819	794.3963	853.4119	1962.886	1940.134	2509.266	1511.222	1005.566	1883.877
CUST_9120_PI429010886	N24094	0.001617374	24	21						198.9127	163.7578	223.0141	295.6289	165.2368	187.5258	105.7248	150.3174	84.83411	120.1066	135.258	75.43056	183.8352	67.44192	175.7896	104.0183	149.1062	55.41495	446.0553	165.5396	337.7577	256.1444	319.6543	213.6241	373.9602	244.6301	346.1837
CUST_34794_PI429010886	M10694	0.00161755	24	15	321463376	hypothetical protein DAPPUDRA	Transport	Ion transport	2.28E-17	15.28234	20.50908	19.25825	5.692618	12.01165	11.76947	35.85354	32.56414	51.68947	28.14461	28.73196	32.74097	29.06853	18.2576	21.94323	51.34157	48.69222	65.27494	32.46562	16.38536	43.36808	18.80558	26.88006	19.56045	33.42941	12.06245	20.07484
CUST_43561_PI429010886	M63354	0.00161755	24	21						93.04693	89.33073	76.82742	79.86465	60.15379	73.4442	99.4113	70.23956	67.63991	88.93364	50.6629	68.71816	82.95322	78.46823	81.5061	97.75803	83.62172	100.3016	86.40352	81.96955	107.365	91.26396	112.4501	107.9954	95.56729	75.40109	117.1888
CUST_7520_PI429010886	N21668	0.00161755	24	21	225711306	Mitochondrial 39S ribosomal pr			9.28E-88	135.5781	203.2256	200.5182	120.5967	143.2998	109.2539	127.5275	178.1474	191.1727	111.0263	134.0192	113.0301	141.6537	147.7301	149.0864	107.0461	159.3669	161.3354	90.84788	179.1861	176.3183	258.7439	177.0208	239.1254	154.2758	167.2859	168.4308
CUST_19182_PI429010886	N41125	0.00161755	24	15						50.27602	113.666	66.64484	62.16915	67.65721	48.8328	72.89302	101.1175	73.34259	88.58571	77.83598	83.29367	123.0345	78.97649	89.44264	85.47045	96.27921	99.49612	68.33012	85.785	94.1914	91.01249	92.47181	83.40132	75.79833	67.45544	86.29714
CUST_19383_PI429010886	N41485	0.00161755	24	15	91085205	PREDICTED: similar to AGAP003	Response to stress	Defence responce	7.18E-50	64.15258	94.98744	81.44134	69.45852	76.42243	62.7253	80.99203	78.87943	95.58893	80.25802	82.91071	38.64548	66.36737	93.57741	78.03831	108.7707	114.6133	107.567	87.17685	89.00153	104.9377	80.85368	103.1963	89.55714	92.79509	74.04749	92.77365
CUST_39644_PI429010886	M42144	0.001623478	12	3	371591236	Euphausia superba voucher 760	Metabolic process	Generation of precursor metabolites ar	5.89E-103	27273.84	44511.54	34620.42	36724.45	36195.31	32969.41	33508.11	39213.8	33838.77	26056	31967.8	23961.37	22711.46	35607.66	24289.54	41419.19	49329.8	50560.8	29520.35	35607.66	35862.04	26056	32969.41	29843.45	33304.41	40415.13	38996.88
CUST_1861_PI429010886	N10900	0.001623478	12	9						3706.021	93.13119	3185.221	4439.592	1416.053	2016.054	596.8192	455.7266	891.7043	7585.802	5048.826	4598.208	9179.508	1521.677	5884.771	201.1205	203.6502	244.5822	2913.515	935.873	3064.959	6219.297	6439.853	9272.221	1918.312	4499.92	8086.369
CUST_38832_PI429010886	M31027	0.001623762	24	15						1844.624	3519.517	1572.421	2025.285	1223.646	1492.153	1065.973	2361.017	813.2524	1563.253	1602.248	1596.49	1492.942	2152.198	1287.523	7731.941	5222.203	3721.413	3050.493	3699.413	2381.656	2478.84	3354.131	2197.153	5137.74	3373.758	3426.429
CUST_42610_PI429010886	M60723	0.001625183	24	0						13.96585	14.87284	8.026216	9.279573	12.42262	12.26439	10.4597	13.52139	11.29335	10.33927	5.481109	10.92283	8.797471	5.337184	5.3575	6.397769	10.92283	4.901556	5.262435	23.03724	15.62519	15.88417	13.51353	7.732666	11.84866	30.27748	25.83296
CUST_10604_PI429010886	N26497	0.001627216	24	0						116.3209	103.4258	176.9444	74.66025	108.3596	36.07006	62.76751	96.7742	72.20641	78.69416	30.3404	45.1102	63.57872	51.77472	61.68316	61.33441	68.06818	48.34401	69.51507	79.29416	38.24975	74.63974	108.5947	92.87822	133.3046	75.53689	50.17119
CUST_41326_PI429010886	M57453	0.001637013	21	18						209.5934	98.478	300.9971	112.6997	111.0926	81.9979	101.2406	59.63292	116.4561	113.23	69.64615	94.40167	115.2014	85.09153	109.9768	222.1744	88.6659	192.9259	256.4131	119.4647	318.6428	126.7369	116.8305	112.096	122.3466	108.9908	105.4159
CUST_38595_PI429010886	M28404	0.001637597	15	12						382.5381	425.8369	215.6896	97.20086	156.0675	103.0843	106.7994	442.9918	245.1417	363.9263	271.3703	169.4171	197.2544	280.5224	285.6824	110.3412	270.4772	259.2192	49.93448	39.45301	53.13532	112.669	122.9221	80.50166	94.71637	170.8635	205.453
CUST_41717_PI429010886	M58382	0.001638288	24	15						10.60141	14.64332	12.94453	6.348391	7.756367	4.475565	5.338332	13.32317	9.245564	18.34365	14.77972	11.10999	7.422436	6.162699	9.6637	25.06692	22.44752	13.44675	15.85618	16.66935	14.60077	9.770244	10.16286	17.39841	8.45839	14.74196	7.718356
CUST_2078_PI429010886	N11513	0.001638288	24	3	238480901	Bifunctional inhibitor/lipid-trans			1.64E-08	3975.62	2509.266	3120.055	3699.413	10035.81	8857.18	3172.092	6481.59	3250.257	2090.105	5197.224	5467.822	2751.324	4036.235	4919.057	1692.044	1124.051	1058.746	1665.146	6906.237	2244.174	3240.436	3819.978	3014.898	1680.655	7714.571	2637.871
CUST_41991_PI429010886	M59102	0.001640035	15	9						254.8611	335.3114	293.0253	275.5775	262.0968	220.4205	199.4867	278.9665	259.7345	306.9062	294.1356	308.4293	278.7033	277.2002	315.4675	289.3915	277.3007	289.2307	241.4097	266.4734	275.3403	406.766	317.8651	317.3105	336.6729	301.7541	426.4459
CUST_21360_PI429010886	N45078	0.001640035	15	9						28.23103	16.71925	40.082	17.85791	13.54422	15.48134	26.17884	23.87506	15.13397	78.45326	22.35516	57.44168	18.12496	16.15024	10.15208	18.55369	67.28242	27.74551	17.48796	11.69612	14.13668	21.2447	22.83576	18.92052	21.24736	79.64838	90.40607
CUST_45000_PI429010886	M67675	0.001642139	12	3						402.6495	389.8933	359.943	292.0008	408.1953	367.5711	337.4051	344.5549	372.1298	261.0327	283.224	275.7417	270.1829	376.9694	358.6508	385.121	445.4238	404.9858	262.525	208.3716	282.6016	284.9168	280.7963	318.4002	315.8311	323.2204	342.3913
CUST_32805_PI429010886	M5150	0.001643227	12	0						87.59003	70.09576	68.50963	44.09004	60.18029	41.38164	41.77489	46.2714	35.67153	64.06806	107.8221	61.84336	94.47843	51.25725	90.66543	59.45846	74.78378	51.98033	33.13955	78.24597	55.97582	73.4071	78.42276	69.92347	107.8333	88.69555	112.3711
CUST_20425_PI429010886	N43413	0.001643694	21	0						413.5169	477.4346	NA	137.7644	NA	197.4942	440.9544	798.8065	402.0757	15.02898	13.62868	4.475565	43.07828	397.9279	49.7839	404.3238	242.4377	113.9699	338.7418	780.7545	266.6798	NA	629.6411	NA	35.37225	534.9189	NA
CUST_33403_PI429010886	M6802	0.001645128	24	18						488.7695	312.4862	652.3046	421.7429	283.1538	148.3156	324.2431	215.7897	417.5037	320.8974	240.0325	173.1737	606.5345	349.9023	125.6161	319.3342	150.2981	274.2134	820.9036	750.0607	563.1838	495.3083	521.9217	477.4346	541.2118	328.7563	791.3056
CUST_6883_PI429010886	N20729	0.001645128	24	18	2660868	fast tropomyosin isoform [Homa			8.9E-109	75.43056	41.63464	71.78136	81.9432	74.59679	65.44741	73.36846	62.87718	64.82967	68.50963	61.89891	60.0766	72.63288	60.00388	64.56632	100.0738	93.90326	85.63473	156.338	118.4277	150.9904	89.09696	52.42496	72.42903	133.4287	94.34203	88.76092
CUST_32314_PI429010886	M3782	0.001645265	18	6	155966352	putative cuticle protein [Lepeop			1.17E-18	341.2447	348.2785	NA	48.03034	1393.878	1553.379	797.1924	2570.156	696.5086	NA	318.8359	316.9378	NA	212.7294	120.2238	67.19251	43.41687	13.16499	103.106	2149.415	74.55481	NA	931.8674	NA	NA	1545.477	NA
CUST_44234_PI429010886	M65341	0.001646943	24	6						6.101511	8.490098	NA	NA	7.500782	10.10472	NA	19.20962	13.73044	NA	17.8915	6.777795	17.86876	11.12081	16.27254	6.385639	14.44307	7.944417	9.943813	4.281007	5.947537	NA	6.518824	4.864276	5.345768	6.618689	10.99576
CUST_26842_PI429010886	N54973	0.001650702	18	9						58.36343	75.3401	71.5317	51.55397	85.37067	62.25886	90.14554	85.64416	88.30662	101.1175	69.45852	124.7762	86.74668	88.87721	86.43772	96.33044	70.73344	81.98831	63.92344	54.31927	69.34161	96.91497	82.0771	76.08583	82.66079	67.16357	93.88506
CUST_38180_PI429010886	M23948	0.001653603	24	12						12.78825	37.96148	20.59213	281.8768	32.40149	157.3269	191.7176	237.5415	229.393	28.56322	123.7963	19.30617	183.1082	300.6157	56.23453	107.0876	122.9378	226.0802	45.43179	57.29886	61.68952	13.36606	70.59518	95.02561	9.355486	9.858804	10.38475
CUST_51342_PI429010886	M93152	0.001653754	21	0						25.43349	30.69787	30.84044	27.16394	24.83681	18.09243	28.47101	16.75338	28.551	23.40815	17.20227	15.31897	20.92863	18.18231	13.4622	20.03269	28.48764	20.51331	34.72664	25.57119	19.20294	23.10946	38.13279	30.08709	22.8273	18.49152	29.98996
CUST_7394_PI429010886	N21477	0.00165782	15	0						92.87822	99.83532	110.2483	112.9762	80.9995	61.97033	105.9124	95.98627	100.0492	71.20264	65.34281	78.94493	93.34171	94.80138	88.17603	108.5389	107.7528	127.2771	85.53397	80.1827	105.3536	80.05281	94.3568	107.3228	73.39156	69.22757	88.72914
CUST_1324_PI429010886	N9290	0.001658463	15	6	321455804	hypothetical protein DAPPUDRA	Metabolic process	Nucleobase-containing compound meta	8.14E-60	55.8305	105.6867	97.11689	81.24878	122.6496	88.07773	119.807	141.0311	134.125	85.73084	79.35979	90.65467	117.4337	87.29007	116.9474	77.79664	86.04303	88.59515	101.2701	116.5476	107.4986	115.7715	122.1215	124.0832	99.22393	89.05929	98.93161
CUST_37921_PI429010886	M22249	0.001659026	24	15			Protein metabolism	Ribosome biogenesis		349.1007	715.2101	437.1787	367.6429	388.382	343.906	678.0803	646.1155	469.5128	492.5086	356.871	431.1432	483.6608	507.073	480.2531	568.2556	616.7748	786.6307	451.2333	715.4355	716.6417	411.6604	545.1995	532.8364	416.251	489.2868	524.517
CUST_244_PI429010886	N2985	0.001659026	24	15						713.6664	1054.885	704.6212	893.2381	554.8747	730.5469	905.0625	934.3693	784.1859	1083.233	635.7483	744.5255	1049.395	948.407	636.1587	1181.431	1452.249	1212.98	1361.853	948.7571	793.634	919.9655	969.0998	823.5364	898.3377	774.2411	873.2461
CUST_28585_PI429010886	N58241	0.001659026	24	3						126.712	14.89459	112.1403	95.44831	134.9488	128.1975	38.36376	27.3201	39.26035	80.64549	111.007	106.2768	78.64253	67.71277	106.5192	30.81966	15.20248	29.25965	38.32469	32.43638	43.33538	65.53387	75.82447	80.87979	57.67493	45.63539	88.36719
CUST_12117_PI429010886	N28956	0.0016608	12	9						71.5317	54.55547	46.52866	58.41936	49.54311	54.21756	50.41173	58.6281	66.0686	79.02299	102.5315	110.2215	62.94384	49.82997	92.7356	53.78431	52.13265	60.41841	81.58851	50.98258	67.41761	83.61292	61.73575	85.98865	77.33923	55.56698	77.96514
CUST_38006_PI429010886	M22744	0.001662934	24	21	157420220	Penaeus monodon Ubc protein			3.47E-114	3371.075	4490.88	4177.108	3431.358	2818.904	3356.595	2911.669	4173.107	2973.957	2779.473	2676.834	3393.901	3079.233	3929.52	3426.429	3731.866	5265.552	4761.143	3286.945	4880.699	4339.098	3437.158	4378.72	4733.525	3995.862	3660.946	4432.869
CUST_45365_PI429010886	M68857	0.00166331	24	15						77.24207	57.11305	67.01094	74.6809	34.19123	40.36067	48.66684	149.769	84.61068	109.1508	76.02418	13.53357	93.29743	49.25556	62.25184	264.9858	96.76253	216.3545	112.0615	148.107	223.2399	105.3095	NA	61.46387	97.72445	18.56115	52.71315
CUST_13466_PI429010886	N31238	0.001666278	21	15						146.3771	157.8028	111.3237	88.24057	78.39296	91.93441	135.3294	105.5804	127.8235	123.4012	105.0583	172.3438	100.1686	113.4137	115.984	136.4438	144.5227	160.5173	124.0485	123.9222	123.354	129.29	145.1769	135.9752	99.44651	93.68896	106.7562
CUST_1404_PI429010886	N9534	0.001667518	15	6	91082977	PREDICTED: similar to sugar trar	Transport	Transmembrane transport	8.26E-12	105.5425	233.6818	308.6874	157.2166	146.129	198.2176	140.6285	216.6807	321.03	130.7958	265.8681	101.6014	98.33769	74.81691	97.83763	123.7628	133.2817	136.2198	103.8305	171.6067	151.9667	207.5176	243.543	183.6198	222.505	133.4611	201.0489
CUST_54301_PI429010886	M105482	0.001671257	12	0						15.49793	33.19205	20.76789	4.822966	NA	11.85089	13.35744	8.218417	12.43008	NA	38.66936	14.36072	NA	30.54385	11.13904	6.770096	8.730033	4.527432	6.398645	10.14167	5.278973	8.669674	36.10172	23.96408	16.00106	28.90075	32.06918
CUST_4580_PI429010886	N17074	0.001673617	24	15						10.28025	8.168708	8.106954	15.97824	12.301	14.4297	19.71996	9.457347	6.455238	19.90788	7.940901	NA	13.24351	12.0284	12.31377	25.57409	18.76458	20.43485	14.05708	15.29443	11.78126	18.56513	12.43497	12.20395	11.39786	10.05212	11.14899
CUST_32126_PI429010886	M3294	0.001673629	18	9	321471368	hypothetical protein DAPPUDRA	Protein metabolism	Proteolysis	1.08E-28	151.8995	193.0588	157.985	274.3383	233.0433	292.8189	281.1928	174.5908	191.0479	276.9819	323.865	283.4149	302.345	244.266	234.8735	222.8209	259.7699	240.2302	192.1066	187.7082	109.2676	268.4876	286.8701	210.4367	194.9048	233.0946	195.9869
CUST_13011_PI429010886	N30435	0.001673629	18	15						55.28754	104.0463	75.63115	67.73599	73.60215	72.02191	79.68621	81.5975	72.73375	70.88457	99.10109	84.51406	75.58915	85.3597	78.67787	92.54225	108.9478	96.04821	77.03004	80.09649	77.17435	50.95275	70.42129	79.16248	66.97219	85.90563	70.23956
CUST_36841_PI429010886	M17005	0.00167946	12	9						31.78394	25.69634	35.70711	70.68343	12.70197	30.62439	46.16801	25.07913	34.52717	37.86516	101.8646	31.40066	95.88853	26.70103	55.53662	18.22518	18.47851	18.96294	31.6335	53.10397	62.57277	78.80045	306.7294	269.4056	32.22585	34.45662	63.50827
CUST_53459_PI429010886	M101794	0.001679764	24	3	54042615	arthrodial cuticle protein AMP6.			1.57E-12	10815.88	4517.506	9937.479	14032.06	16981.94	15045.33	3798.572	3832.521	3187.955	13419.52	14885.24	13098.74	13716.26	8086.369	11503.97	2116.149	2978.418	3052.051	2891.912	5966.983	4118.017	14956.1	8984.262	12021.42	4536.055	8669.063	8846.332
CUST_55709_PI429010886	M111387	0.001679764	24	15						5671.62	7327.614	6393.352	5807.4	3688.899	4461.089	6343.146	6500.649	7937.877	8107.941	4190.519	5233.042	6906.237	4740.787	5435.742	10448.35	8204.372	8736.825	8658.641	6988.188	8565.196	6752.376	6551.409	7007.731	8269.17	6710.162	8644.801
CUST_36243_PI429010886	M15143	0.001679764	24	15						79.90962	74.46237	69.67842	49.07329	27.51681	44.11057	93.3634	79.5268	87.45577	106.8423	40.03196	58.82712	81.75003	50.08183	55.89295	152.3312	99.23862	134.4124	96.58233	61.96517	89.91214	63.77213	42.69638	87.24349	85.1599	55.41495	77.67929
CUST_8958_PI429010886	N23854	0.001679764	24	15	291411894	PREDICTED: Oryctolagus cunicul			6.15E-52	548.4345	579.5063	502.9461	435.1623	385.3052	446.7799	581.9588	593.3017	512.9523	522.4996	404.2408	700.5788	475.8315	518.9718	504.3232	691.4224	734.4362	628.1097	548.5685	517.7643	598.7661	444.5929	570.1346	503.1242	479.4997	528.4781	534.9189
CUST_12327_PI429010886	N29291	0.001679764	24	15						513.9521	985.8154	707.937	540.0952	617.1935	574.9115	659.7683	766.0458	779.846	635.3318	732.552	610.6881	659.9573	626.8237	685.082	881.258	1118.139	1193.156	707.6592	770.1221	798.2619	527.455	782.9344	716.6417	617.6234	684.4289	710.1661
CUST_29880_PI429010886	N60743	0.001679764	24	15						90.62398	142.126	96.25473	130.7958	106.6278	112.3953	142.6458	173.4784	159.645	125.4627	142.2741	91.27426	132.7314	132.7723	135.4737	203.5459	186.5755	162.844	151.0875	128.0875	158.0297	146.8681	146.1149	132.0176	124.4273	144.345	123.0947
CUST_21919_PI429010886	N46096	0.001680692	15	3						20.89958	26.62387	22.11624	27.28807	37.49445	23.71399	8.991143	28.7875	20.74399	14.88982	23.09515	25.47189	17.87188	17.82881	17.66056	22.97146	22.35906	15.68096	33.45613	33.63623	25.23652	17.48581	28.23406	25.85237	16.9478	23.3163	22.23439
CUST_38786_PI429010886	M30347	0.001681364	24	18						575.4805	655.6894	326.6921	386.7231	304.2261	288.4188	454.9515	315.6961	351.6631	350.7074	260.0376	289.3393	360.5101	306.5038	247.6161	3724.515	3222.8	351.7434	507.6273	1398.823	400.7915	380.6302	568.8248	583.1603	486.4482	523.4696	368.2385
CUST_39926_PI429010886	M46877	0.001685996	18	6						75.77051	257.3491	92.46133	348.6137	186.13	286.6404	174.3634	458.2399	317.2644	244.7879	270.0299	225.5195	50.72013	167.4144	84.80017	148.0265	278.6422	125.7971	306.6661	210.1903	165.7121	132.304	209.5934	134.6275	350.3187	320.4829	388.382
CUST_5041_PI429010886	N18186	0.001687858	24	18			Cellular Process	Cell cycle		374.9091	563.1838	459.4079	323.1624	432.7554	359.7575	389.2749	557.8175	610.1834	346.8977	337.1412	310.357	514.9905	398.2963	385.121	419.6327	537.4657	474.9425	516.0491	693.5896	659.0035	392.057	603.3505	490.077	372.873	412.3443	631.8697
CUST_28613_PI429010886	N58293	0.001689987	15	0						147.3924	131.3179	135.6132	178.863	120.5852	118.4588	90.44348	97.22863	109.5906	139.0252	93.83228	108.6527	166.6346	88.91387	110.2215	208.7171	182.2404	183.1326	126.3449	134.6107	100.9966	97.39106	85.73084	144.0796	134.9139	79.13617	111.371
CUST_33073_PI429010886	M5901	0.00169161	15	6	256033192	triacylglycerol lipase [Litopenaeu			1.27E-90	8.1002	7.629488	7.994051	19.53602	38.5634	10.38947	51.93946	NA	54.02691	14.21176	9.542112	8.924839	4.903455	16.86908	13.96239	12.26439	4.810747	9.289439	116.3995	281.628	463.8737	37.28248	NA	30.86801	73.32545	16.99422	47.64318
CUST_40331_PI429010886	M53439	0.001694545	24	3						761.2797	232.3243	576.6349	1250.867	1856.844	1497.856	159.8296	496.4508	168.7741	375.1194	856.5689	1086.19	249.4703	917.9436	968.7419	53.71769	87.17685	104.7593	60.57425	458.7704	43.58292	541.3579	299.4177	424.5467	53.92132	556.8156	66.84952
CUST_13432_PI429010886	N31179	0.001696374	15	0						59.62625	109.6493	124.3455	53.612	35.83339	45.7721	91.86363	38.35367	69.55079	20.57977	25.32953	22.53853	36.55032	12.08661	42.26354	77.78826	78.91149	76.19975	43.19513	25.49446	25.49661	18.99164	29.91279	25.49446	47.72449	29.54223	29.19743
CUST_18368_PI429010886	N39663	0.001697992	21	15						7.752733	15.38937	6.080857	5.449648	6.597671	9.155794	4.203167	NA	4.870551	8.11425	NA	10.08072	14.61747	4.954518	5.132329	22.62431	15.93445	8.326096	7.973645	11.37944	NA	NA	12.41811	15.7745	5.399766	9.243151	5.37508
CUST_48987_PI429010886	M83413	0.001699581	24	12						19.53602	13.98478	7.848782	NA	12.87704	NA	13.38505	6.539229	NA	NA	19.78464	35.5206	18.57337	33.39499	38.86739	18.51735	13.28096	6.948808	9.628869	23.71956	NA	3.461713	8.506507	4.971185	8.269575	4.837682	11.45318
CUST_39519_PI429010886	M40749	0.001700502	24	3						10260.07	171.3126	9819.956	13685.2	12743.08	14670.24	263.4524	368.1539	226.9173	7474.416	8309.047	13591.98	2706.184	13658.74	16183.31	38.42178	85.85149	120.5852	187.2522	602.8578	267.9719	4291.944	1513.287	4221.228	617.3702	1666.041	1803.597
CUST_39808_PI429010886	M44980	0.001700502	24	15	13898841	CUB-serine protease [Panulirus a	Protein metabolism	Proteolysis	1.58E-54	511.6359	287.1316	297.7147	328.1119	69.14386	253.9457	1617.033	803.027	1483.086	1617.033	908.0084	96.36643	616.0609	502.5054	429.9294	4302.905	1357.702	3588.155	990.5961	1613.784	2170.443	1130.351	149.5552	979.3611	1033.621	258.4714	690.0542
CUST_20327_PI429010886	N43243	0.001700502	24	3						1002.608	123.2011	374.7006	1177.475	1062.767	826.1267	109.7929	159.6647	144.5793	1361.853	605.287	1064.289	996.3906	525.5146	738.2631	78.70208	105.1495	90.09793	114.5608	148.2557	117.6651	598.5974	112.9897	506.6858	124.9582	272.9604	210.0683
CUST_20972_PI429010886	N44400	0.001700502	24	15						63.16716	63.84719	44.37502	51.58002	40.50202	56.75829	64.59327	124.2633	61.04906	64.36702	49.31951	60.51578	67.25975	74.30536	52.18733	116.9965	80.68221	74.5042	79.04147	63.78974	69.10515	44.32333	69.2197	63.84124	62.98884	56.65739	73.52981
CUST_21982_PI429010886	N46206	0.001700502	24	15	395539349	PREDICTED: RNA-binding protei	Nucleic acid metabolism	RNA metabolic process	0.000000199	86.06068	84.80932	98.36388	75.34564	69.07482	72.29255	119.6713	77.35718	104.0183	111.5617	72.4082	115.4443	120.8608	70.44138	93.27554	140.2618	122.8685	153.9744	94.45356	84.46858	116.3209	96.59602	76.55656	116.4561	116.2734	78.5451	120.6396
CUST_33281_PI429010886	M6482	0.001701004	21	3						130.2565	163.5142	162.7145	236.2759	213.1128	233.5329	90.01966	132.5501	221.4294	75.12194	195.6436	100.0492	43.12031	157.3269	75.48453	59.31427	119.7663	64.00415	271.577	80.50166	165.8756	151.6381	105.1495	181.5127	245.3228	109.0861	173.3624
CUST_1586_PI429010886	N10158	0.001704396	21	9						474.5724	350.791	536.6512	1049.395	859.0835	825.7252	147.5021	1294.363	917.5193	1074.53	2123.927	811.0498	1065.617	633.2997	1307.951	510.0998	1122.961	926.6453	329.6536	581.1787	254.7629	100.3625	696.0096	615.2672	571.5137	815.7902	448.4659
CUST_1644_PI429010886	N10324	0.001710348	24	15	291399278	PREDICTED: UDP-galactose-4-ep	Metabolic process	Nucleobase-containing compound meta	3.51E-122	140.0219	166.3221	111.9372	98.08719	92.18916	109.2676	187.5258	166.2848	128.8774	170.6411	114.6554	123.1267	133.3824	89.36499	136.3915	194.5781	234.322	219.2338	149.3939	148.7099	176.6314	131.771	123.354	109.9526	168.8172	143.2644	149.3159
CUST_6863_PI429010886	N20701	0.001710348	24	15	345497387	PREDICTED: LOW QUALITY PROT	Transport	Protein transport	3.23E-66	205.536	388.156	198.0827	175.63	167.6498	162.9278	362.6643	317.6549	263.6152	287.0134	182.4721	235.8737	240.802	199.944	215.0483	322.75	411.6604	395.2192	247.6563	261.8722	245.2392	163.563	218.2236	210.2297	202.4277	241.0614	270.1829
CUST_8471_PI429010886	N23092	0.001715344	21	15						38.25388	26.67745	28.00501	25.03965	14.44307	7.376965	25.30132	50.40096	72.50711	48.94113	33.14692	3.990586	21.45062	29.13534	6.301132	147.9113	61.95078	125.4804	38.64548	75.12894	103.4258	20.96118	NA	14.21628	8.628754	NA	9.838142
CUST_151_PI429010886	N1727	0.001716782	12	9						33.3824	26.58505	31.4617	13.84038	19.57119	15.40374	31.73936	20.91626	16.7377	46.80045	27.87602	26.90764	27.4271	15.5042	23.91323	20.17508	14.58537	21.20564	34.53159	16.75714	36.79006	28.27048	24.99187	30.3574	23.12134	21.55928	23.26132
CUST_41264_PI429010886	M57279	0.001718706	24	18	543364768	PREDICTED: dehydrogenase/red	Metabolic process	Oxidation-reduction process	8.68E-55	261.3125	210.0683	194.3971	88.45765	110.3551	80.08063	140.8152	379.6985	269.4621	122.9659	166.9271	149.3578	329.6536	167.3552	179.4936	221.596	266.1986	206.561	219.538	307.7409	426.8467	149.0864	113.6067	156.1618	241.54	147.5021	151.1486
CUST_31206_PI429010886	M972	0.001719481	18	15	410509308	myosin heavy chain type 2 [Pena				1363.959	774.2411	1439.002	1024.829	659.9573	861.5088	783.1683	1556.093	904.627	930.4398	622.5028	855.5436	1161.162	1704.256	730.7886	2242.949	2304.738	3033.527	2159.534	2777.197	3801.741	592.8013	485.8608	499.3569	2073.458	1115.136	1196.459
CUST_33344_PI429010886	M6657	0.001719937	24	18						142.9786	143.5029	115.7715	126.6732	126.2905	115.6407	107.8755	133.3487	118.6869	148.2557	126.1425	132.3168	157.9287	109.7186	179.7408	168.2637	190.8765	136.4791	170.3275	146.0289	165.742	142.5791	168.7955	189.5124	164.8664	137.396	170.5634
CUST_41314_PI429010886	M57436	0.001721239	24	15						230.6808	362.5008	264.0266	226.4976	244.5822	237.5415	301.5476	318.5699	311.2489	316.1661	309.878	250.35	295.4303	251.7468	314.1834	372.9744	447.0887	431.8791	255.7585	443.6171	323.5302	283.1093	284.7236	304.875	287.5765	288.3603	329.8464
CUST_43272_PI429010886	M62598	0.001721239	24	15						69.46672	110.0735	69.84655	54.23194	69.6376	53.7784	103.1229	78.22197	80.08063	100.6344	74.4166	66.1472	90.00835	95.52209	73.21671	147.2992	121.0882	166.6574	98.81307	96.23561	107.8221	77.89321	65.33664	89.5211	162.5035	86.58046	102.8306
CUST_3114_PI429010886	N13827	0.001721239	24	3						3467.123	659.1588	3480.931	3744.093	7138.174	6276.978	673.1503	1580.709	649.1337	2166.525	5021.117	2824.276	1398.823	3142.5	4258.14	477.7276	442.9918	263.3104	452.0404	1701.286	449.9406	1864.874	1497.206	1967.079	377.2326	3423.157	713.8123
CUST_18661_PI429010886	N40187	0.001721239	24	15						47.51371	48.78475	31.61116	31.36138	26.09525	22.78318	54.93024	48.95167	51.86305	57.07543	33.97881	46.84134	52.03532	31.17639	67.68769	102.1728	78.12052	67.22535	53.27399	26.32359	64.61677	39.34209	29.81035	55.3772	61.62446	48.85342	54.78529
CUST_21107_PI429010886	N44643	0.001721239	24	3						446.2058	20.64919	412.0092	861.5088	806.2686	813.5332	10.01527	35.82991	27.90262	173.259	491.802	493.5638	61.25239	846.219	674.2308	4.51184	7.542922	10.1544	10.04386	32.40149	8.526821	78.66288	69.94875	76.5464	19.92947	70.1867	57.44168
CUST_29990_PI429010886	N60958	0.001721239	24	3						2782.49	228.9535	3598.269	4432.869	1601.274	3090.222	129.29	187.4404	238.9099	111.5065	444.8216	441.5527	220.0563	3543.046	3899.124	64.24425	93.75176	69.81806	122.0941	125.2132	100.3265	1074.917	123.4012	878.4972	81.75003	164.1331	137.2652
CUST_30816_PI429010886	N62802	0.001721239	24	15						100.3265	122.5572	86.04303	77.79664	62.45522	67.7048	165.4776	120.1882	153.8104	128.1317	89.84164	130.6442	85.53397	113.9393	129.5849	166.6574	160.5687	203.4357	138.5034	94.66861	148.956	105.9124	85.07992	130.6615	105.0092	124.8106	165.8977
CUST_30617_PI429010886	N62242	0.001725986	24	3	443730657	hypothetical protein CAPTEDRAI			1.65E-14	4.429018	8.210092	NA	28.26579	NA	10.96971	NA	5.67171	5.592145	NA	NA	9.825217	8.086218	NA	NA	7.237005	4.730812	6.479989	3.451831	3.455026	3.849491	6.662217	6.5874	6.573571	7.688774	4.344418	5.604207
CUST_32347_PI429010886	M3894	0.001730589	24	9						1377.009	1108.146	1232.801	2374.203	1588.642	2174.954	835.3759	1428.28	1219.83	1803.597	2275.002	1923.884	1360.186	1405.334	1035.146	1241.315	2228.732	1647.162	914.5652	394.7866	814.3635	887.2093	1343.626	704.9081	963.404	1154.406	1606.909
CUST_51900_PI429010886	M95585	0.001731727	21	0						1112.302	1074.104	NA	267.8251	16.55114	238.775	633.0621	711.4323	451.9245	12.14919	4.714407	NA	53.69322	480.1172	29.49099	702.1022	430.0583	158.1747	468.0876	1500.263	432.7554	NA	780.4581	NA	39.70188	635.7483	NA
CUST_32070_PI429010886	M3153	0.001741796	21	0	13898841	CUB-serine protease [Panulirus a	Protein metabolism	Proteolysis	9.53E-43	249.0801	620.9302	516.7278	165.4776	75.45664	65.32192	83.87393	NA	69.81606	318.5699	139.003	212.4427	64.33747	180.3409	117.8103	21.7849	21.93896	4.303683	NA	41.02997	NA	380.1903	692.1008	527.3184	59.5223	157.7362	168.6714
CUST_36572_PI429010886	M16259	0.001741977	24	3						192.1306	82.16147	156.8658	234.9137	1024.829	845.2107	75.8587	136.5823	60.79882	99.96271	533.1871	583.5463	99.49612	222.1744	278.592	55.31238	65.80773	62.99459	93.1813	191.21	67.36564	130.5722	130.9346	145.6946	88.92336	261.4074	127.7313
CUST_31715_PI429010886	M2230	0.001741977	24	15	291239561	PREDICTED: metalloproteinase S	Protein metabolism	Proteolysis	3.26E-46	907.1782	481.4474	467.6907	142.4074	27.39004	87.12904	1151.33	717.7206	1117.567	1880.339	1177.962	93.59078	505.369	279.9265	324.8189	5350.891	1534.973	4020.487	1187.041	365.8262	1194.342	1418.063	38.40048	1243.512	1357.702	239.0862	1043.236
CUST_39399_PI429010886	M38859	0.001741977	24	15						140.7536	288.8335	188.7447	151.7139	107.1994	132.5859	253.8231	238.7217	195.1701	218.2236	128.2695	141.4255	172.259	153.7124	163.4636	280.0406	372.7272	295.0388	166.2848	172.1367	229.0726	174.2606	187.3814	213.853	168.7955	156.2636	164.7136
CUST_50502_PI429010886	M90037	0.001741977	24	3						66.88415	28.63092	63.28348	96.99292	83.87393	109.8412	16.11449	28.81777	18.48559	59.14128	32.14643	42.4928	26.37524	81.85533	19.06607	16.48073	22.2434	16.89615	15.23394	27.12484	16.91133	26.2902	29.95843	27.74851	25.71638	26.60721	18.24171
CUST_1722_PI429010886	N10539	0.001741977	24	15			Nucleic acid metabolism	RNA metabolic process		115.1151	205.0181	207.6942	162.3594	144.2876	159.0392	234.4037	225.1246	221.3966	228.6642	176.2933	166.7642	205.0181	173.8666	156.3126	276.7645	321.4419	286.8043	218.5686	223.3238	257.3902	192.7408	174.5137	185.0283	212.0999	179.6067	229.4464
CUST_5475_PI429010886	N18754	0.001741977	24	15	195153137	Drosophila persimilis GL21499 (I	Transport	Protein transport	8.81E-104	508.513	824.4785	564.8143	406.4569	493.9591	455.5301	687.2962	686.4707	748.449	507.9246	556.047	469.2787	558.2968	560.9756	529.2519	695.3717	783.4167	873.9122	606.9823	731.2874	745.5832	496.5813	665.1463	550.9285	634.8724	599.6889	595.447
CUST_8326_PI429010886	N22875	0.001741977	24	15						590.6124	540.9421	548.4345	422.3982	434.5642	352.0796	645.7283	483.6608	659.337	647.785	373.8469	437.5336	685.2688	414.9926	400.7915	968.3563	919.9655	1007.214	570.427	599.4277	780.1728	655.1561	488.48	681.8481	682.1048	541.0757	711.071
CUST_8532_PI429010886	N23182	0.001741977	24	15	242022061	eukaryotic translation initiation	Protein metabolism	Regulation of translational initiation	1.01E-78	119.077	152.7876	118.0123	113.878	117.7118	102.5048	115.8648	168.6428	132.5675	143.2998	115.4443	136.2737	140.8978	126.6202	140.9228	147.8633	178.9851	175.2384	119.0641	136.6363	148.7488	135.9051	165.7618	147.4173	138.1611	134.4124	167.0102
CUST_9665_PI429010886	N25016	0.001741977	24	15	11869966	engrailed-b homeobox protein [	Developmental process	Multicellular organismal development	3.31E-26	50.20269	42.77417	31.4218	39.17239	21.14493	19.38594	54.05823	47.39716	45.09415	56.68583	32.76569	37.88233	61.23583	25.49854	30.44744	65.53387	70.13338	69.41702	42.39291	60.03992	61.145	47.04453	38.49334	47.83193	44.57504	49.70331	52.98141
CUST_10414_PI429010886	N26193	0.001741977	24	3						136.983	29.2298	212.4427	168.2226	152.7399	174.1094	19.04014	50.29492	31.16674	45.872	167.2859	97.44091	54.31927	149.769	126.2905	18.67083	21.11731	20.46072	41.65544	36.75514	25.77691	97.87129	47.97144	80.75468	36.98315	45.04591	57.26697
CUST_25700_PI429010886	N52908	0.001742408	18	15						36.55792	35.26837	26.41027	25.35027	37.8457	31.64405	24.6865	29.4638	30.61698	29.53809	26.74265	33.762	34.84492	32.29221	34.23416	47.7665	46.88337	51.0346	30.36962	27.99056	30.16583	38.95176	30.64789	40.08729	27.47705	21.7225	28.36166
CUST_47118_PI429010886	M74855	0.001744502	24	18	74828242	RecName: Full=Techylectin-5A; I	Signal transduction	Other	4.47E-27	168.564	215.9178	336.6041	266.1014	68.20885	155.1864	185.4321	242.133	144.5227	131.9825	100.3265	52.33755	216.3545	175.0235	112.6997	277.1553	340.8616	168.9831	319.5317	267.3602	317.1285	378.5271	308.2378	179.2149	438.5777	200.9087	106.2034
CUST_48241_PI429010886	M79485	0.001745334	15	12						21.03035	16.69306	20.80953	17.94352	12.28362	9.599842	21.33315	18.71717	17.48074	23.28183	16.57982	8.844931	22.93584	20.55348	27.13005	17.99307	20.39286	11.32723	5.474648	7.635827	12.68845	10.28796	12.4892	19.47164	36.97883	12.99098	17.94579
CUST_55822_PI429010886	M111814	0.001749775	21	3						12.84776	11.33953	6.110374	16.90269	12.26303	7.190374	12.91333	NA	11.53968	6.92838	11.39973	3.650996	6.163656	10.70762	5.639861	NA	NA	6.838349	5.891833	4.549946	3.829182	8.794886	NA	NA	13.22574	9.914778	NA
CUST_15117_PI429010886	N34035	0.001757856	24	9	1764107	pacifastin heavy chain precursor	Metabolic process	Oxidation-reduction process	4.83E-17	38.2274	54.97558	54.2431	59.8383	37.35922	34.92175	73.31512	50.72554	73.60907	78.77099	43.93928	62.51652	66.25687	48.5966	39.39293	56.97506	53.1876	78.18007	47.59053	23.26849	50.6306	28.95548	27.53743	30.90807	36.43276	35.57898	40.66544
CUST_35749_PI429010886	M13402	0.00175876	12	6						48.63014	49.21384	83.79749	90.73151	100.658	70.24722	116.6092	101.7856	118.4103	74.71995	50.58009	52.81957	69.29751	115.8122	72.81177	100.9966	161.5503	132.7314	83.38146	164.3905	159.3374	122.1735	51.14752	71.93885	60.38394	48.84814	49.49501
CUST_32663_PI429010886	M4745	0.001761052	24	0						257.0037	259.675	254.5783	200.0418	256.6606	226.4187	186.4162	253.6738	222.6501	225.0911	202.7719	198.9717	211.3776	196.4999	173.6159	258.3202	259.5745	259.4124	237.2821	255.4282	261.0851	236.2759	249.7255	244.0506	256.6162	260.798	262.4014
CUST_31879_PI429010886	M2649	0.001761705	21	3	294860896	ubiquitin conjugating enzyme-4			4.21E-90	7.2855	22.23772	14.74939	10.2223	15.74083	16.15902	11.40638	15.97033	8.215865	5.559073	16.84413	8.925992	14.28509	13.21612	8.282821	3.86771	3.004471	5.470296	12.47435	8.996542	13.53119	7.298709	NA	7.502046	35.16074	NA	21.69652
CUST_6718_PI429010886	N20506	0.001763536	24	18	321471179	hypothetical protein DAPPUDRA			2.14E-68	108.2549	198.1815	239.4831	172.6519	213.853	154.5458	57.73811	116.3062	185.1715	119.5167	81.91112	85.09948	169.8371	104.1208	151.2161	96.16362	169.6212	149.8871	336.9544	692.8899	458.6774	106.4901	200.9087	175.4908	220.7835	161.8663	335.6891

Probe ID	ID	p-value	Period	Phase	EBI ID	Annotation	GO category	Go term	E-value	0	0	0	3	3	3	6	6	6	9	9	9	12	12	12	15	15	15	18	18	18	21	21	21	24	24	24
CUST_12491_PI429010886	N29552	0.001765334	18	3						38.12224	30.12082	34.76331	38.38502	38.15601	35.98686	31.20826	34.37139	33.40167	29.71551	22.27865	26.88508	42.5735	36.11568	32.23946	31.43843	28.25337	21.88142	21.68674	25.89322	35.06662	49.84575	38.12957	37.92065	44.39683	33.77215	36.67874
CUST_17333_PI429010886	N37791	0.001769103	21	15	242011892	conserved hypothetical protein	Cellular Process	Cell adhesion	4.14E-45	13.03491	23.32054	18.87533	20.84673	26.74553	40.41914	34.74153	18.2969	20.55824	38.79677	35.17993	22.78318	36.20611	31.04302	35.76914	52.67255	40.59839	39.41457	23.1743	11.34074	27.77264	22.4723	20.26569	19.56731	23.03013	35.92906	12.93352
CUST_33191_PI429010886	M6229	0.001773249	15	3						355.7553	1335.991	689.3323	890.9879	1155.365	855.307	679.9519	679.9519	856.5689	633.8464	565.0242	537.5716	678.5721	350.791	691.8963	1228.932	1068.554	982.8111	1021.527	959.3506	1463.189	1028.452	811.3473	1139.817	641.3942	438.6999	845.9441
CUST_8955_PI429010886	N23850	0.00177332	24	15	291234007	PREDICTED: H1 histone family, n	Cellular component org	Chromosome organization	6.56E-21	863.5664	973.9492	921.0975	844.8862	614.3598	609.2645	620.2559	802.1705	663.8187	1082.092	752.0705	1011.588	796.2861	568.6219	799.3476	1059.777	1224.109	1225.455	1080.06	1044.09	1034.116	966.0248	1041.115	1141.401	1412.179	925.0688	1507.382
CUST_48977_PI429010886	M83306	0.001775108	21	12						12.77277	10.17169	6.952262	8.831604	NA	10.68598	13.22574	10.22494	4.924109	15.5351	15.70678	10.98177	15.4529	NA	13.76968	14.58324	4.314333	9.231247	7.203709	5.086765	6.444484	7.322474	3.90781	6.302453	9.99914	11.27323	10.7519
CUST_23285_PI429010886	N48567	0.001776834	24	15						43.38217	44.79025	47.92699	41.1635	32.88066	40.24112	37.10697	55.11106	38.42576	39.93436	38.7574	48.87241	43.78502	47.28223	56.4602	71.61601	56.57986	58.75296	48.78475	46.00851	41.50033	75.16291	50.86361	67.78024	58.19693	42.45638	58.0985
CUST_12455_PI429010886	N29486	0.001783453	24	15	321469647	hypothetical protein DAPPUDRA			1.81E-13	39.17239	90.14554	91.27426	45.96577	39.17612	30.5034	127.4702	90.00835	126.0374	126.1425	41.79921	87.48976	106.9545	51.39126	46.18377	173.259	152.4151	182.702	127.8074	80.89811	135.258	95.16242	62.00499	107.6466	141.898	99.46029	126.4021
CUST_45455_PI429010886	M69167	0.001784247	15	0	444708250	Canalicular multispecific organic	Transport	Other	5.84E-23	15.59668	16.80772	18.44595	13.1174	12.69014	9.869484	4.770671	10.17265	10.22494	6.78575	19.23397	6.974516	22.8581	11.00412	17.02795	15.78373	19.68126	16.70465	18.40393	12.34813	19.04208	8.027278	5.310051	12.54285	14.9436	NA	15.6646
CUST_46051_PI429010886	M71199	0.001788261	18	15						27.2697	23.47009	28.62655	26.6049	23.19056	17.96345	31.03564	22.64182	27.60747	25.98049	28.16647	36.80764	30.45754	28.71526	24.58018	36.16824	33.17685	31.68069	36.48955	17.20391	32.67736	13.2682	19.72255	21.83479	35.16853	14.40631	30.22406
CUST_3829_PI429010886	N15360	0.001788429	15	3						255.866	33.97881	379.0965	564.2041	553.5806	275.386	53.33381	386.2259	363.9263	210.1175	115.1318	171.3397	55.19896	201.8328	22.32831	31.13164	225.1246	189.5806	284.3354	245.6327	459.9674	297.492	128.2877	74.86678	104.6784	10.88798	81.33153
CUST_7274_PI429010886	N21303	0.001794376	21	15						17.04641	21.87979	11.1169	20.2236	15.36995	23.89577	21.82087	33.19846	20.47903	25.48121	27.10746	20.74174	24.45655	26.97533	33.79023	40.1121	31.30301	34.36788	20.44056	8.474912	18.3768	26.0759	12.3846	20.12578	24.6974	28.51548	18.54162
CUST_34893_PI429010886	M11006	0.001804191	24	15	225719214	Longitudinals lacking protein-lik			6.52E-15	78.81902	98.08719	57.98766	72.5147	57.81074	78.06756	111.2714	88.51224	96.13685	123.9057	59.66359	102.0603	107.7085	77.99973	86.23628	142.2314	111.6045	137.6386	136.6003	74.28032	94.98744	109.6493	83.87393	110.0556	124.0944	83.34871	94.50451
CUST_37394_PI429010886	M18892	0.001804191	24	15			Developmental process	Multicellular organismal development		1694.196	2873.993	1758.225	1470.868	1657.457	1665.146	2485.974	2284.471	2195.531	2030.062	1551.452	1684.207	1751.317	1827.788	1742.558	2775.294	2708.146	2978.418	2180.402	2211.381	2427.484	1603.304	2208.268	1993.835	2070.818	2050.749	2058.438
CUST_32090_PI429010886	M3208	0.001804191	24	15	193575575	PREDICTED: heterogeneous nucl			6.49E-36	1323.872	2818.904	1483.632	1252.458	879.8906	978.9259	2157.924	1886.448	1758.225	2035.999	1116.506	784.8506	1573.941	1180.964	1393.878	2696.535	2711.745	3281.159	1808.449	1695.833	2409.846	1296.827	1524.311	1570.104	1763.47	1419.346	1659.781
CUST_39818_PI429010886	M45131	0.001804191	24	6						654.9488	599.6889	667.2339	507.3489	661.6594	588.9309	553.5806	869.9307	1154.406	410.4537	433.8311	390.3118	344.9779	391.8616	297.4214	341.4287	801.9356	387.249	155.4749	415.6373	254.9746	234.2298	421.5919	188.9335	207.2734	354.2621	275.9079
CUST_45716_PI429010886	M70031	0.001804191	24	3	9186884	BCS-1 [Amphibalanus amphitrite			5.1E-19	433.2807	102.4924	420.3226	528.895	2971.539	2404.745	111.4813	227.7173	150.4473	123.988	1224.109	1398.059	205.6925	1335.17	1492.942	61.78991	52.10354	42.88959	196.6122	322.8914	130.7693	148.1939	264.0732	113.098	161.1455	384.3395	220.9943
CUST_56362_PI429010886	M113599	0.001806607	18	3						14.96631	10.05113	25.79038	31.64405	20.04802	33.26748	13.78443	15.60667	10.04481	30.49532	34.04257	16.06582	18.29498	7.23313	9.442403	15.26493	NA	13.24735	18.55514	14.45802	17.68077	42.76898	32.29945	51.93257	24.40659	16.07632	34.17741
CUST_25765_PI429010886	N53017	0.00181605	24	9						379.037	59.87629	319.3342	370.7223	343.0574	410.9137	89.16012	75.39478	75.28314	396.5838	502.9461	613.7905	326.9964	204.8848	342.4681	93.11317	87.79232	85.22534	47.61057	56.11745	71.88037	270.1339	127.4216	296.3076	53.62536	75.264	113.1748
CUST_40650_PI429010886	M55743	0.001816521	24	0	195436302	GK22112 [Drosophila willistoni]	Transport	Carbohydrate transport	2.76E-15	505.8465	780.4581	812.2115	500.5747	520.31	543.8441	507.7884	625.6951	832.894	522.0737	435.391	496.9611	447.6336	314.5854	489.5895	383.67	173.3126	377.0504	516.3556	491.9043	784.4035	512.9523	561.6697	639.2868	844.6913	324.3641	675.2885
CUST_2020_PI429010886	N11342	0.001816521	24	15						477.1875	1026.123	535.748	2340.704	4.81828	1886.448	839.0592	751.8458	636.1587	1685.048	407.0205	536.9014	1793.169	967.7143	1074.53	7937.877	5777.198	5271.495	2310.632	1428.28	2487.704	610.6881	1479.335	686.1628	497.1127	1331.886	204.8843
CUST_43479_PI429010886	M63155	0.001820298	12	9						5.526054	NA	8.870244	5.451604	NA	5.06766	15.56129	20.57977	6.617801	29.89832	29.43236	27.16164	13.6668	NA	9.745235	13.03847	3.648485	14.44158	15.71789	8.702496	16.06042	22.79876	NA	13.21612	14.6246	5.135432	28.63477
CUST_33739_PI429010886	M7755	0.001820534	21	0						376.0943	335.8125	NA	119.4775	6.747508	109.3613	298.5479	270.7536	244.266	7.562913	6.381994	NA	16.51673	232.4494	14.46435	176.7718	112.0316	91.34986	229.393	258.7813	163.2927	NA	335.8993	NA	11.59098	206.4922	NA
CUST_54148_PI429010886	M104807	0.001824929	24	3	321472482	hypothetical protein DAPPUDRA	Response to stress	Response to oxidative stress	4.97E-43	318.1116	25.62254	138.3312	402.0757	357.4621	367.9854	30.42641	32.71218	25.92125	240.1565	221.4635	216.4739	173.7818	292.0008	244.6301	10.59744	18.6092	5.379272	36.66952	57.33149	30.49064	191.21	81.19478	120.4953	33.55937	62.38008	65.92209
CUST_51962_PI429010886	M95840	0.001824929	24	15						6500.649	13481.03	9285.648	6885.506	6226.747	6125.57	10987.48	13419.52	11695.66	8698.04	7950.355	6461.658	8698.04	7632.364	6929.595	14828.55	17287.24	14580.94	10142.18	10626.1	10532.72	7653.847	10178.51	7886.969	8984.262	10485.81	9410.039
CUST_54778_PI429010886	M107637	0.001830097	24	15	343183153	myosin heavy chain type a [Mar			3.94E-49	14.44307	3.911615	13.41756	9.965241	7.17884	23.01448	17.7035	13.32474	21.43051	32.16999	21.02758	9.239178	23.76684	21.64409	10.63475	44.88978	27.02687	25.59017	24.47696	41.80345	25.66233	32.51998	19.60329	20.23015	39.75997	12.51777	17.75202
CUST_29085_PI429010886	N59171	0.001830097	24	15	321462004	hypothetical protein DAPPUDRA			1.1E-13	55.47226	74.54603	64.0447	42.05841	18.42634	26.76257	77.21485	63.27624	61.65559	59.83108	21.06519	35.54628	105.4902	66.31625	48.38102	69.92347	120.1351	112.6387	112.5245	83.82183	97.84796	59.16434	52.78692	45.09415	75.23071	79.35979	93.65434
CUST_45132_PI429010886	M68092	0.001831523	15	12						37.11143	47.88919	63.6314	16.44342	26.85004	9.559713	41.23754	50.08183	63.54768	32.49678	40.50707	53.28017	99.57709	62.53388	57.67493	23.70635	59.21121	41.06867	23.2872	47.22395	28.79283	20.72897	59.58718	36.23977	27.09506	76.02418	67.35825
CUST_3730_PI429010886	N15147	0.001835462	24	0						41.13599	31.44937	38.74481	32.49411	31.22074	33.72382	27.02166	30.70816	30.73939	31.55396	29.51805	26.39253	18.39609	28.77495	21.83061	26.18926	23.97557	28.10987	45.90556	30.64441	25.41015	37.10235	20.51167	29.88935	26.0733	32.26704	35.78012
CUST_5847_PI429010886	N19287	0.001838399	24	18	260791035	hypothetical protein BRAFLDRAF	Metabolic process	Cellular amino acid metabolic process	8.2E-125	112.9084	162.1178	150.2055	127.1242	111.5887	86.89375	135.9051	152.5742	146.3133	127.0615	104.7711	113.7405	132.3168	121.5849	110.4898	150.073	200.7173	192.4063	165.4279	170.9048	193.8538	164.5342	145.8669	135.9219	166.0466	142.1948	157.4324
CUST_53116_PI429010886	M100266	0.001845666	24	3						5756.98	123.018	5420.715	6448.141	9643.272	9490.363	141.2055	711.8674	107.696	677.2359	6506.137	8348.895	584.3107	8605.765	5284.133	92.12733	110.2026	90.32894	192.8532	781.2177	157.1577	776.4904	317.4305	559.9215	192.6657	1024.829	264.6223
CUST_20216_PI429010886	N43035	0.001858781	24	18						81.61482	25.36487	101.0339	147.2139	92.95971	20.91286	106.529	86.69353	171.0708	38.29909	71.79771	33.36729	394.8687	109.5906	223.6663	82.54419	34.19123	86.87274	262.04	815.7902	959.7319	486.8888	400.4361	563.6439	274.2662	258.5192	583.1603
CUST_48038_PI429010886	M78623	0.001860019	15	9						35.62951	NA	34.32863	28.95548	NA	5.612247	55.88006	25.34518	55.04149	54.05823	64.39267	52.21052	21.51378	7.335668	18.81696	16.46484	14.24832	14.0796	44.38354	14.80397	37.3089	42.2303	17.37771	35.85739	47.53117	39.02642	93.57741
CUST_37829_PI429010886	M21791	0.001863896	12	6						4.571766	NA	NA	18.17381	NA	16.28228	301.3368	NA	239.7136	51.59532	39.56727	NA	11.89453	6.833148	11.49825	63.76387	NA	57.39735	480.3553	957.7854	2188.815	111.0263	NA	89.97113	185.5629	27.78158	181.8118
CUST_15180_PI429010886	N34141	0.001864252	18	9						243.494	56.78277	254.0305	325.7976	323.3549	201.9179	1420.628	857.1902	1037.05	1904.856	1129.142	488.5919	1111.944	769.5468	232.9522	369.9026	1361.853	899.9958	193.0974	439.5135	208.6051	487.1319	538.9124	523.3495	432.7554	40.97117	424.6765
CUST_11942_PI429010886	N28667	0.001865458	24	12						17.89735	16.84207	19.20294	65.12931	21.94563	NA	27.78158	7.786617	11.20074	37.02719	17.70936	24.54259	52.78141	33.27509	34.96815	11.40929	31.4461	30.81577	22.39225	21.55398	9.921498	10.88798	15.18659	8.525673	21.9811	17.07151	15.67395
CUST_43478_PI429010886	M63153	0.00186638	24	3						100.9815	71.89101	129.7428	268.6876	419.6327	462.531	33.06158	134.0991	86.51281	137.6019	445.0179	369.756	51.0501	239.2427	255.6336	6.190304	59.51158	14.73247	17.93113	137.7481	68.37633	130.5494	162.4391	180.3689	31.01416	404.9858	267.7885
CUST_35682_PI429010886	M13225	0.001866404	24	3						624.4152	170.4605	744.1912	530.9393	995.515	925.4144	137.3189	204.1816	186.4449	184.3599	722.545	474.3448	216.7919	645.2626	768.2904	105.746	116.9474	112.8276	154.3971	266.1014	162.5035	338.6752	194.2665	333.1336	167.5116	245.1417	221.8856
CUST_30873_PI429010886	M70	0.001866404	24	15						247.8796	302.9304	244.2151	211.3776	205.1648	183.3329	281.4085	253.0362	272.7395	280.0915	252.3498	211.9713	228.8193	210.7737	232.3662	316.081	359.0835	370.0497	276.0279	249.0225	268.8247	255.6336	251.3401	264.7345	272.9604	250.5828	262.806
CUST_15103_PI429010886	N34016	0.001870527	21	3						284.6794	244.9453	602.8578	716.3366	641.6007	NA	221.3068	180.9657	467.8312	24.05806	82.02164	16.76557	598.4778	363.084	42.0926	48.46739	79.43494	87.11871	882.5044	617.3702	466.9715	237.3958	503.9395	480.3553	566.6051	433.6142	948.7571
CUST_13685_PI429010886	N31590	0.001875528	21	12						8.62661	10.37881	9.725094	14.19023	11.28749	14.04039	5.29504	14.49503	NA	NA	9.228702	16.32398	58.90806	14.23509	30.08709	9.303503	6.557583	4.476541	NA	5.713125	4.092338	NA	NA	9.856067	NA	13.24735	NA
CUST_4143_PI429010886	N16102	0.001878257	24	3						36.79436	21.49936	43.98997	71.83398	126.9272	144.3231	25.61949	45.31246	29.38788	30.40311	122.1364	110.8779	39.95456	50.06206	67.93128	7.625156	15.72542	16.84945	11.90435	29.00837	17.92495	22.12314	34.00804	24.6637	13.42167	47.8851	15.18461
CUST_21916_PI429010886	N46093	0.001881604	15	12						11.68939	19.85199	15.96858	6.839885	12.51386	8.330941	8.843446	8.917284	9.279573	25.01801	12.61565	20.06249	23.37894	15.48991	26.27801	19.51476	4.789351	12.53267	16.76975	10.79815	4.714407	25.67685	17.24019	13.02032	18.90266	18.99164	34.48071
CUST_12013_PI429010886	N28776	0.001902745	12	0						167.9984	215.7105	249.7255	158.418	174.3818	150.8682	171.2911	176.6544	180.5627	193.6659	198.4672	173.9585	211.695	177.9861	176.6026	185.9639	182.4721	165.4408	177.7613	150.7719	170.8363	186.2275	193.4888	190.289	199.1806	199.421	213.5563
CUST_1678_PI429010886	N10417	0.001902893	18	15			Transport	Protein transport		190.0624	286.745	210.1567	191.2458	183.4751	211.9713	231.4946	226.7781	207.0168	231.3331	236.8696	200.83	204.9812	273.7185	259.2192	301.0722	332.8862	335.2209	181.5802	185.9023	237.8915	162.6721	222.2239	209.1753	254.9746	242.7465	209.5586
CUST_13994_PI429010886	N32142	0.001909343	24	18	290462133	tRNA 2-thiocytidine biosynthesis	Metabolic process	Other	1.81E-26	31.51584	49.87102	59.3284	38.36376	28.30304	36.44629	29.64873	43.53777	41.12558	40.53493	41.77489	44.69809	43.86651	34.00804	45.5523	46.43451	55.90731	56.98746	41.88562	49.4079	57.39735	31.36138	50.33808	47.82733	45.01222	30.80841	41.70713
CUST_30985_PI429010886	M382	0.001909447	15	0						36.11092	114.7628	84.53983	54.77517	68.4765	68.87137	70.15908	65.27026	56.3623	64.53182	63.93775	58.7415	60.95576	55.22716	61.71621	98.33769	97.11689	101.572	90.64448	98.03098	81.24878	50.41173	70.41437	59.46532	53.18236	52.35235	48.02557
CUST_20264_PI429010886	N43124	0.001910496	21	0	390362924	PREDICTED: zinc finger protein 8			4.25E-35	138.0005	147.9838	158.1573	128.6628	120.0145	97.12878	127.1242	112.7999	81.22064	147.7301	88.81146	109.8412	135.0204	116.4848	78.75209	112.7891	149.3939	126.0081	146.8442	144.7014	119.3614	170.4291	154.5211	129.9594	168.8433	119.8645	146.3365
CUST_12424_PI429010886	N29439	0.001911922	12	3						20.44483	8.267617	6.16087	14.18835	28.5989	12.08147	3.687656	9.133693	NA	NA	10.50491	3.351724	9.568695	11.03688	4.037804	34.44933	20.24396	11.24321	15.76131	10.782	10.57446	6.441192	7.019216	NA	NA	8.293234	5.438256
CUST_52361_PI429010886	M97196	0.001914997	15	3						20.31479	17.00074	22.95059	28.61945	32.3057	27.2823	21.48468	26.47689	25.76565	15.62171	11.14343	11.11102	18.6455	16.95396	9.572414	18.82499	32.2766	19.02154	22.0686	38.46934	26.79339	26.25684	26.15603	11.91014	30.57991	16.67597	34.48922
CUST_6780_PI429010886	N20590	0.001919969	21	0	405970761	Phosphotriesterase-related prot	Metabolic process	Other	6.43E-102	287.8256	471.8286	501.1967	204.4688	278.9665	240.9301	181.8521	243.2848	198.6494	221.0978	245.5909	210.2979	184.7904	228.7039	379.158	258.5627	275.2462	337.1412	233.9614	265.8169	333.9088	244.9453	414.7402	263.1307	348.5318	267.6236	261.4619
CUST_18308_PI429010886	N39547	0.001920741	21	0						224.6222	236.7186	312.2924	204.0717	196.9337	253.0362	119.272	195.9057	198.257	123.1603	256.9518	126.4571	176.3724	139.755	202.7406	153.5706	157.0354	148.4438	114.5807	197.0196	184.8821	251.0873	244.6993	287.0864	184.7904	162.3844	232.5186
CUST_34300_PI429010886	M9275	0.001921527	24	21						126.2717	65.49056	141.352	93.61951	72.86661	84.2744	63.50827	61.55774	84.98418	98.28823	94.63322	90.08744	152.3548	61.86512	109.4578	54.99013	163.8654	88.71852	115.0832	109.8522	145.4106	148.7876	137.7644	120.3127	104.8681	121.1808	106.4281
CUST_54248_PI429010886	M105283	0.001923292	15	9						7.12908	14.59005	12.64397	6.331516	NA	7.490317	8.428222	6.869765	7.807697	15.72841	16.28066	23.02109	7.073354	15.97824	14.09744	11.78126	14.33104	12.61861	4.300968	13.10546	10.38036	17.40415	12.98636	12.86584	16.6387	21.19056	11.86355
CUST_19037_PI429010886	N40874	0.001923599	12	0						60.77123	42.81475	56.39944	37.48759	205.0181	99.4113	16.76758	29.72388	28.33805	38.00971	121.8684	152.8269	46.23699	155.885	198.9717	31.98442	39.35132	25.85738	14.47219	27.39273	24.43421	28.36418	32.48061	41.19655	25.88702	43.83834	49.43562
CUST_38892_PI429010886	M31700	0.001925819	18	3	121484199	cuticle protein CUT8 [Portunus p			4.89E-21	32064.51	1013.387	24426.06	61365.17	44511.54	41865.42	3004.305	6292.839	7122.178	29725.44	69931.06	34286.52	36724.45	28943.19	39389.41	694.9037	758.1822	613.1951	8683.686	9041.082	12424.28	39875.67	40595.56	44511.54	12836.63	26491.1	29520.35
CUST_47897_PI429010886	M78084	0.001925819	18	15						340.4211	512.7881	431.346	274.6613	372.873	332.484	505.6819	474.5724	429.5829	455.7266	293.3331	356.7674	361.6608	420.6276	422.7276	713.4537	638.3206	647.0623	407.1156	394.5922	433.0002	394.4626	338.3895	346.1837	548.1274	378.4533	417.5037
CUST_14136_PI429010886	N32379	0.001925819	18	9						10.03476	17.1543	20.63272	13.45294	22.24082	19.77172	20.84466	15.33068	21.40258	35.40359	32.2766	29.03664	18.51735	18.39269	18.3141	19.83069	20.67803	16.61873	14.34289	17.57258	9.841307	34.80724	20.97645	19.51852	23.85156	29.24359	16.03034
CUST_25600_PI429010886	N52718	0.001925819	18	3						7832.882	140.8769	8378.475	14218.13	13988.25	13248.98	881.5382	1548.998	2564.444	10987.48	21922.49	9067.463	15297.04	9013.443	10641.47	104.1325	58.30071	187.5258	2942.677	3636.712	3859.53	13532.16	15141.22	15102.78	2820.736	10966.45	12165.66
CUST_36622_PI429010886	M16375	0.001927502	21	3	383862409	PREDICTED: uncharacterized pro			1.8E-16	8.705867	7.792211	9.629964	14.68671	26.40704	15.77596	5.127027	8.262222	4.348615	3.669692	10.29995	10.25099	8.102905	10.74336	9.917631	5.743745	5.154544	6.891795	6.040983	8.76542	9.550476	NA	9.934505	11.56152	10.42021	10.14167	NA
CUST_29065_PI429010886	N59137	0.001928306	21	9						37.31972	4.510613	26.0242	55.76126	204.8848	103.8983	24.40844	62.54833	53.70551	NA	191.1071	221.2615	18.04496	285.6824	358.8543	3.570664	4.280652	13.81248	10.05496	11.48896	8.567205	23.90534	15.62519	27.7211	4.885026	22.39225	11.01286
CUST_20370_PI429010886	N43324	0.001928861	21	18						168.1289	31.08176	106.8649	74.58857	23.64075	53.00589	15.35763	19.57461	5.150194	41.08386	43.03704	36.05612	163.5388	17.59796	82.30731	43.53457	5.778815	32.18818	138.5964	138.3677	78.29555	48.96189	94.96672	179.3019	14.11506	40.46451	38.54735
CUST_27001_PI429010886	N55260	0.001930343	18	12						19.04623	7.595815	14.24675	23.34465	36.44966	21.55177	15.63496	6.709355	5.425939	13.61765	13.42773	9.685449	28.8864	45.0895	20.00187	25.83296	20.94045	26.57189	7.257747	12.6121	12.03366	10.00814	18.81329	6.841078	4.010793	9.096904	7.161558
CUST_1505_PI429010886	N9851	0.001934704	12	9						26.18388	15.28431	45.33911	59.09232	NA	33.11578	25.6877	154.1096	200.6093	273.2919	161.1455	231.134	19.37891	154.1589	17.9285	25.89856	13.35388	9.928002	105.285	7.403761	92.2128	151.5229	344.0033	471.0794	3.921944	11.60929	NA
CUST_7158_PI429010886	N21123	0.001935419	24	21	345484364	PREDICTED: DNA-directed RNA p	Nucleic acid metabolism	Transcription, DNA-dependent	5.43E-20	56.18757	62.82646	58.27052	58.02727	48.28205	40.35274	49.08263	52.96326	67.82594	56.36738	40.88884	49.68059	55.31238	37.93177	65.64875	65.57565	61.69489	67.49593	67.7438	63.05176	73.06306	75.3636	59.797	79.02299	64.11411	54.53262	72.22265
CUST_39038_PI429010886	M33821	0.001936341	21	15	528314838	ubiquitin [Schizosaccharomyces	Metabolic process	Oxidation-reduction process	1.77E-22	52.35235	32.00302	59.29331	72.93884	63.05696	52.09654	29.8647	49.28164	48.96667	49.03029	48.23305	54.2724	52.35798	56.11275	62.86145	83.23692	63.01324	76.77599	54.49956	78.86032	62.71091	58.72446	51.44911	49.75454	58.06923	46.67221	55.69808
CUST_43119_PI429010886	M62112	0.001937645	24	3	402470219	hypothetical protein EDEG_0121			0.000000475	44.50237	NA	36.44264	51.60135	29.33753	25.28496	13.3283	7.401064	NA	18.17588	16.00633	26.9514	13.41897	16.19041	14.66707	5.803119	4.399522	6.239298	NA	3.944038	9.076199	16.21562	8.392924	18.44938	4.241364	6.180949	18.80121
CUST_45465_PI429010886	M69188	0.001938444	21	15						1574.714	2388.437	1818.148	1812.631	1725.737	1477.508	1496.297	2230.426	1857.76	1463.189	1585.44	1593.454	1525.217	1701.286	1406.649	2343.924	3220.778	3215.844	1994.973	2300.958	2559.917	1640.282	1996.997	1999.848	1887.557	1876.444	1938.127
CUST_53702_PI429010886	M102844	0.001944459	18	6						6.401717	11.84049	9.458406	17.59422	7.428342	12.38845	20.11563	17.59422	25.70617	19.32091	9.860254	7.660563	9.499412	10.11056	13.97433	12.96173	14.51258	16.37112	6.883456	13.02868	4.98459	18.0705	13.81248	9.389205	17.0552	18.34193	13.35017
CUST_1415_PI429010886	N9562	0.00194596	24	3						334.9433	274.6613	79.85553	198.1577	4598.208	4589.607	220.4205	1297.493	174.7665	13.57384	1741.604	1483.086	5.897189	1033.091	1041.115	NA	3.547606	6.117612	23.41335	1638.477	16.59434	55.06924	226.1282	50.29015	NA	1553.379	NA
CUST_37225_PI429010886	M18271	0.001947072	15	12	395507594	PREDICTED: DNA-directed RNA p	Response to stress	Defence responce	1.54E-24	12.94826	18.79354	16.10329	15.48134	15.19175	22.90896	13.04163	11.2094	9.982867	16.01569	28.23406	16.4789	26.78121	25.54534	25.92708	15.25101	15.1083	12.74437	16.64399	18.34365	13.82436	10.32743	NA	9.854974	9.326879	12.55344	13.82584
CUST_34413_PI429010886	M9601	0.001947148	24	21						102.326	140.387	149.2223	106.5406	100.2386	79.17186	98.43645	104.0936	99.43494	99.51051	108.5389	106.9803	104.8456	89.86465	114.8354	138.2385	98.67888	145.5847	109.1836	133.0726	126.4187	126.2905	140.5297	145.0834	104.4062	121.6309	145.7916
CUST_52897_PI429010886	M99361	0.001953749	24	3						12.86873	8.247584	4.542644	14.39152	NA	12.65731	8.878239	9.329172	10.96784	17.14735	NA	6.025114	8.06529	6.220036	12.60871	5.699934	9.893288	7.930223	3.726877	5.429962	4.578326	15.60281	5.790172	4.788815	8.078793	11.34206	9.461435
CUST_6222_PI429010886	N19797	0.001956016	12	0						165.1445	159.9313	185.4692	127.8074	109.2539	130.2882	88.56702	118.3395	137.5851	134.4487	179.0245	125.8093	182.0698	157.2999	145.843	97.64083	180.4963	116.6401	136.5677	158.5618	98.36388	218.6641	145.9208	159.4491	236.5827	176.2933	191.8796
CUST_13097_PI429010886	N30573	0.001957304	21	0						198.334	187.0484	217.3699	183.9085	126.9698	170.1187	165.3867	144.495	117.6965	208.1829	107.8333	109.5722	151.2854	130.1154	157.1984	138.5964	202.8062	200.9666	162.844	148.7726	135.2233	240.3468	204.1816	191.082	173.5608	124.8855	134.6492
CUST_14561_PI429010886	N33078	0.001958844	15	0	459178862	PREDICTED: annexin A7-like [Cio			7.96E-16	28.62359	81.1468	60.79882	58.445	50.20946	51.92761	47.10999	75.68836	69.35987	38.86739	26.36041	48.73482	41.76233	58.37796	21.0756	86.27418	106.4806	87.26779	59.87629	56.01896	61.05496	42.57027	47.22812	37.2113	49.71921	37.5978	36.63747
CUST_26092_PI429010886	N53648	0.001958914	24	6						16.72046	20.86335	NA	11.0388	24.46253	31.00311	36.97455	59.8383	26.27328	NA	22.44233	17.19316	NA	17.04105	25.88702	20.01139	NA	4.241019	11.32723	25.48923	17.20227	7.797479	13.83116	8.284739	NA	39.28587	18.53335
CUST_27140_PI429010886	N55515	0.001960611	24	21						39.14498	52.34706	42.32912	43.58688	36.24394	35.75247	20.63759	20.228	29.08789	40.56201	27.37673	37.39308	35.07549	43.4735	29.41053	21.33758	11.08511	34.09891	47.09024	46.15443	49.13607	44.18092	38.27451	52.45861	46.13169	48.20502	37.06569
CUST_5819_PI429010886	N19242	0.00196088	24	15	260828211	hypothetical protein BRAFLDRAF			3.39E-118	35.36937	41.50754	40.76995	32.43254	24.36005	21.46191	60.75955	51.22586	52.77429	56.76432	35.02108	41.17764	46.8318	29.15694	46.48748	60.14273	67.3285	54.74265	38.65223	38.58331	56.81546	48.10991	29.38152	44.3395	37.41195	34.14439	41.30394
CUST_45540_PI429010886	M69423	0.001963234	18	3						79.69545	72.06957	71.43667	94.50451	80.71612	101.7015	88.54675	81.04337	50.92486	66.09101	72.86661	61.61447	70.39513	79.49714	84.78014	58.60789	57.64367	83.40132	83.88489	83.00392	75.2001	92.84934	85.13676	88.81146	86.98659	78.18007	107.3799
CUST_48250_PI429010886	M79549	0.001968474	12	0						85.14751	87.85118	98.88646	47.83193	52.42496	45.21941	54.07873	67.76221	57.05634	75.47204	68.11052	109.0861	66.34289	108.0539	106.7424	56.72705	35.53128	31.46705	27.36657	65.37178	50.55101	45.86771	113.098	102.5048	50.62618	68.07757	73.17158
CUST_27192_PI429010886	N55607	0.001968593	21	6						8.688519	5.31236	9.259524	22.23218	9.356835	9.506221	14.36256	11.84993	24.11615	20.452	7.301642	6.41682	5.90416	7.168799	5.975334	12.88736	9.492246	16.60777	7.516744	5.588826	4.709967	7.302349	6.799426	10.93144	13.54726	9.361001	33.71063
CUST_54727_PI429010886	M107342	0.001970029	24	0			Protein metabolism	Ribosome biogenesis		19.60994	28.94147	23.44192	17.94579	7.975034	7.430249	15.96403	10.30948	15.23394	14.08381	13.50964	9.205084	18.3141	20.28829	20.93117	15.74607	22.71992	20.58643	11.85412	20.21086	23.75071	16.69937	25.23652	24.70381	23.7799	22.79012	25.41015
CUST_34230_PI429010886	M9116	0.001970029	24	0						91.7443	117.2201	98.14527	85.34679	110.822	84.04962	98.83232	103.0349	90.48608	82.92495	75.34564	95.64524	97.91705	89.14903	105.4159	91.39547	102.0444	91.15714	93.96854	121.1808	94.50451	107.3799	112.0316	110.1325	112.6108	118.8089	123.7628
CUST_39137_PI429010886	M35145	0.001971324	24	3						85.13676	78.8865	83.83043	80.41026	78.32421	80.86273	53.77161	62.74684	90.18372	74.18037	76.34956	53.74306	77.57976	34.06673	9.432312	53.89331	42.94276	50.69876	76.99156	48.86558	50.42732	52.42496	68.04046	46.95394	92.52233	60.60875	62.43555
CUST_21177_PI429010886	N44770	0.001972462	21	15						336.1378	864.8838	281.1928	657.3507	5.118547	643.2149	466.0849	984.2262	108.623	485.7316	79.62962	264.8694	968.0088	1349.624	573.048	1841.638	2915.434	1607.994	1260.62	968.0088	1099.849	315.0449	1814.798	278.1319	424.8811	512.4944	34.66403
CUST_5359_PI429010886	N18589	0.001972768	24	21	405968415	Gem-associated protein 5 [Crass			9.2E-58	476.8665	403.2127	624.9341	476.244	357.575	495.3083	373.2381	357.7827	423.4844	475.2146	500.4612	401.9784	577.7104	433.1774	397.1925	374.9091	407.9106	420.2294	468.6225	450.9209	446.5017	805.476	645.4936	706.2599	496.692	471.2164	661.1838
CUST_27926_PI429010886	N56955	0.001972768	24	21						123.5457	125.7784	149.4602	89.24382	107.2144	121.9377	102.2726	105.3328	108.3596	110.5024	110.3861	125.2728	96.58233	98.85767	139.7386	157.4103	103.9393	173.6159	120.9827	110.1773	134.4996	180.3689	136.9653	173.1143	141.8724	116.1695	146.8442
CUST_21444_PI429010886	N45236	0.001995614	24	0						85.81977	226.5718	170.9936	63.72422	39.70188	32.01377	93.99213	128.4802	148.892	25.39283	38.00456	26.14707	85.43893	43.126	36.8828	142.8894	51.32543	118.3837	163.4271	48.71388	151.6547	114.1224	110.2215	72.65503	199.421	48.56519	162.4812
CUST_25114_PI429010886	N51808	0.001995614	24	0	291410057	PREDICTED: zinc finger protein 7			1.82E-37	31.30624	18.72412	27.24405	23.24382	7.179669	15.49423	20.15802	9.081076	17.91767	15.70448	13.07357	10.49595	17.94168	13.36916	8.658203	16.59861	24.11862	15.45782	16.80349	18.52205	16.54419	20.2995	20.77024	18.10455	22.36152	12.46634	36.40013
CUST_56697_PI429010886	M114441	0.001998388	24	21						68.56321	72.95425	99.92196	62.01214	61.98061	73.05642	53.42717	61.67047	67.44192	72.70716	53.66051	71.66506	56.20706	71.18779	72.42124	71.49082	77.00089	84.2314	75.91716	62.5403	81.22064	77.00089	89.59602	73.92515	85.55473	75.98892	91.28627
CUST_37383_PI429010886	M18847	0.001998388	24	21	321455420	hypothetical protein DAPPUDRA	Metabolic process	Carbohydrate metabolic process	5.49E-92	7585.802	12097.54	8547.585	6430.973	5507.513	5322.868	5856.992	7248.769	6752.376	8984.262	5666.924	5697.98	11763.32	5502.319	8962.903	10394.61	13685.2	9026.68	12084.47	11030.98	10337.3	13988.25	11429.91	10897.12	17287.24	8362.147	12584.9
CUST_34027_PI429010886	M8547	0.001998388	24	21						277.2002	385.5175	324.3008	271.2533	355.6308	276.4387	267.4596	309.0132	318.6428	292.4745	324.7451	270.6292	300.4274	326.6163	303.2777	312.1666	347.9647	363.1656	310.7267	303.3581	399.8074	355.324	432.7554	453.6305	327.3954	286.745	406.5634
CUST_53047_PI429010886	M99895	0.001998388	24	21						9759.347	16491.53	12860.58	9804.488	7203.647	7663.711	6922.365	11112.95	10699.09	11859.41	9345.369	3886.966	15705.46	8503.163	12043.33	18117.06	17982.7	13419.52	14411.75	14982.33	14100.35	18758.28	15967.93	13503.69	23379.75	13503.69	15585.38
CUST_2729_PI429010886	N12995	0.001998388	24	21	321455420	hypothetical protein DAPPUDRA	Metabolic process	Carbohydrate metabolic process	4.97E-96	1443.341	2184.345	1536.792	1175.329	1018.651	929.9574	712.9201	1053.276	1026.487	1642.989	1154.885	1013.387	2294.993	1026.123	1938.127	1951.028	2276.393	1369.19	1848.594	1634.309	1670.335	2137.853	2086.93	1956.496	2524.402	1263.423	2277.337
CUST_50142_PI429010886	M88803	0.00199982	21	3						5.009787	12.55867	7.791167	NA	19.32091	13.26377	NA	11.57897	4.913749	NA	16.72545	12.25196	8.960965	8.111553	4.808869	4.387604	3.527703	NA	NA	18.53335	4.149591	NA	NA	4.691936	9.168643	21.28456	13.09533
CUST_46461_PI429010886	M72721	0.002003449	12	9						219.6103	56.55991	208.1054	482.9421	202.0095	427.5284	39.81401	83.64481	67.40377	523.8156	1173.346	470.6338	380.83	347.4772	238.8236	22.5248	32.49411	22.9023	81.87749	119.7313	63.73076	845.5682	706.2599	720.7726	129.5849	660.1893	376.2092
CUST_14710_PI429010886	N33334	0.002014327	21	6	321473992	hypothetical protein DAPPUDRA			0.000000632	12.69375	50.47099	47.33949	33.54781	47.60041	42.92948	52.82485	58.91866	78.08717	19.15475	50.46073	49.49501	27.68187	48.84433	50.37855	19.6075	40.26358	36.514	11.10613	39.22978	39.4828	13.55744	41.92879	33.5806	20.4836	45.84807	58.5677
CUST_31034_PI429010886	M523	0.002017525	18	3	341925955	Dugesia japonica Djtuba mRNA f	Cellular component org	Cytoskeleton organization		352.9709	362.195	530.4682	639.8078	566.3117	659.337	344.8027	384.0543	319.7298	387.4512	458.5926	552.4266	490.8866	307.6702	482.5812	225.5195	441.4649	239.6808	447.1876	457.3588	412.3443	632.6818	474.4729	617.7634	284.1348	412.9894	581.4931
CUST_8619_PI429010886	N23313	0.002017525	18	15						137.7249	245.3228	184.4145	119.807	94.38205	150.6011	230.1554	209.7386	221.8495	212.0595	159.7718	108.4189	210.955	145.4834	153.502	223.4224	298.135	237.3259	165.8756	218.6641	166.9821	74.89382	165.1648	108.2549	186.9296	166.8658	150.1329
CUST_21420_PI429010886	N45185	0.002021199	24	0						51.00867	47.32106	49.78682	42.53768	51.11212	20.29507	41.30748	49.25556	32.93541	25.57119	37.45982	28.28966	35.72862	48.04838	46.54514	31.55396	77.67929	33.47158	49.59199	56.02531	37.53998	41.50754	60.25264	49.15533	62.40045	49.87102	63.80586
CUST_44041_PI429010886	M64738	0.002022739	24	9						406.8432	323.0408	380.1903	376.3542	363.297	382.8	374.6189	303.8109	361.4106	376.9694	400.3546	415.0852	343.1775	343.9474	343.5695	370.0497	307.9833	324.4202	283.5676	293.095	307.5442	369.4962	322.5153	359.2536	343.1184	319.0038	367.8176
CUST_19345_PI429010886	N41423	0.002030152	21	18	321478973	hypothetical protein DAPPUDRA			1.15E-23	22.69623	32.59654	27.21287	12.66176	17.00672	17.84342	22.30308	21.66729	21.1126	16.09389	11.78676	18.38558	12.09251	14.77271	11.84049	27.36008	17.58375	27.37466	30.81966	25.53705	23.81296	25.13996	13.37051	25.31848	25.9662	17.87709	23.1769
CUST_30188_PI429010886	N61350	0.002036689	24	21	395532342	PREDICTED: alanyl-tRNA editing	Nucleic acid metabolism	RNA metabolic process	1.51E-23	15.69371	16.96963	16.75338	12.99937	8.839932	8.718132	11.98172	13.6545	16.21072	8.550634	8.346975	NA	78.23111	15.71978	12.37092	18.04496	21.04513	16.79507	12.84199	15.09289	18.44774	17.86679	24.33617	19.38793	12.47654	12.79576	14.40144
CUST_5943_PI429010886	N19407	0.002042654	21	15	118136260	Penaeus monodon beta-actin m				1489.353	2795.798	1776.485	1329.875	1316.811	1104.345	1640.994	1424.355	1471.771	1348.794	1127.686	1185.011	1588.642	1613.784	891.3462	1635.354	2692.671	2922.057	1812.631	2020.363	2278.847	1390.646	2389.711	1936.909	1326.216	1751.317	2037.287
CUST_36077_PI429010886	M14559	0.002046747	18	12						24.7708	22.44464	18.54037	23.76085	17.43071	22.95726	37.53076	29.78433	28.27048	40.27654	14.13668	26.76519	34.58559	31.07888	38.02217	35.32719	29.59753	35.56153	23.24382	26.66393	32.86278	23.41549	9.840114	26.23786	30.03767	20.51331	34.51642
CUST_38396_PI429010886	M26199	0.002046784	24	0						2824.276	9629.338	8503.163	1913.068	1287.523	16.72346	7750.118	6171.545	12359.97	1142.815	451.8198	897.658	1608.753	933.0577	202.183	1796.427	2455.967	2790.956	5137.74	4565.036	4733.525	1293.42	1916.926	2279.977	6109.222	2685.938	7597.158
CUST_54868_PI429010886	M108037	0.002046819	12	9						53.52577	53.97594	69.85306	58.65468	47.10201	35.88107	54.25064	52.7448	60.0766	65.50892	72.48391	64.47775	90.18372	51.49011	69.53723	54.44704	54.87487	56.79387	78.5451	61.78991	88.80408	112.2698	92.22131	96.07003	89.20331	65.27026	76.09471
CUST_10444_PI429010886	N26245	0.002049264	24	15						16.4194	30.64441	21.50122	25.87214	20.84897	11.81016	16.77811	21.07339	17.05046	20.24017	24.97096	18.67083	26.58261	24.28512	21.04763	34.76331	30.73256	34.89809	25.93001	31.53465	21.03259	19.08041	23.64554	22.57092	23.46744	25.8664	25.5566
CUST_37344_PI429010886	M18725	0.002049629	24	21						20708.31	27832.29	23084.2	15798.02	15871.24	12473.12	11475.48	17886.89	16801.9	21398.95	16372.71	13790.46	29725.44	14548.28	22221.29	30965.56	28025.82	21710.8	27665.22	23961.37	25555.67	37632.98	25247.34	26567.15	45502.32	25555.67	36043.3
CUST_3705_PI429010886	N15089	0.002049629	24	18						48.38982	81.04337	84.19786	60.86142	66.51767	51.93946	60.17344	74.67002	72.4082	60.49503	67.41125	76.19255	62.73163	67.60011	66.71306	59.67529	93.60566	79.83656	146.0508	91.62496	105.7007	85.88791	95.34191	85.21665	89.36499	67.76221	116.7048
CUST_22356_PI429010886	N46883	0.002049629	24	21						152.7673	159.4657	167.7764	150.2498	164.8664	104.8815	110.9429	145.5507	138.4394	181.173	133.4406	149.3159	149.2626	136.5823	131.568	202.9634	162.6466	175.7896	152.2838	150.7849	174.2606	233.8101	161.7701	202.0095	208.4776	154.0154	173.4147
CUST_53840_PI429010886	M103385	0.002049856	21	12						48.1462	46.5849	35.77451	41.73602	30.49064	28.6788	49.16277	39.56268	33.82382	51.09282	43.18567	64.63225	57.36369	53.02947	45.25376	49.06918	47.12456	36.48523	33.33002	40.44359	37.07109	47.79939	42.28326	51.33067	32.76569	38.87535	27.89685
CUST_430_PI429010886	N5155	0.002050077	18	0						27.89086	20.60839	24.15844	21.33315	13.0678	20.84897	17.68714	10.30349	11.98927	11.03119	12.09577	21.28456	22.83896	6.970533	11.12212	18.3904	21.92764	26.61853	15.49957	30.53244	31.48667	18.47	17.34089	27.01619	11.62165	15.87243	16.34366
CUST_38430_PI429010886	M26541	0.002055247	21	12	321478880	hypothetical protein DAPPUDRA	Metabolic process	Carbohydrate metabolic process	1.86E-27	44.21653	8.26064	27.36851	40.98319	166.4486	71.47305	16.73896	44.67899	12.11716	NA	56.56581	58.02072	NA	121.5207	107.9218	12.49181	11.93992	8.591323	6.404646	11.39401	4.37985	21.56373	5.516194	8.956638	NA	NA	NA

Probe ID	ID	p-value	Period	Phase	EBI ID	Annotation	GO category	Go term	E-value	0	0	0	3	3	3	6	6	6	9	9	9	12	12	12	15	15	15	18	18	18	21	21	21	24	24	24
CUST_13649_PI429010886	N31528	0.002057139	15	12						217.909	193.8172	215.4758	175.0235	171.8413	173.9585	107.4986	189.4144	177.5447	214.2039	243.5905	229.5682	218.5686	238.5314	309.5645	245.6701	241.9944	247.3301	157.7724	233.9614	171.722	233.2281	190.9992	241.1815	231.3331	221.5686	282.8745
CUST_177_PI429010886	N2124	0.002057974	12	9	321466528	hypothetical protein DAPPUDRA			6.39E-22	35.00954	7.491826	44.70872	26.46408	25.53977	27.16746	NA	7.940901	13.13097	44.47158	50.53965	50.47099	37.47176	25.85237	35.00954	8.851485	4.62804	NA	14.70241	5.134295	16.74788	77.06177	55.14025	101.6655	21.21056	10.75288	33.86827
CUST_5008_PI429010886	N18125	0.002059298	15	9						52.11743	23.28393	59.06511	42.23986	27.97412	21.90793	77.67929	46.11055	90.32894	79.05137	103.2627	61.80424	48.11395	33.26748	39.72867	35.72473	44.26452	34.03486	66.27041	37.40246	64.33747	51.07791	44.55836	48.68665	80.86273	65.54266	107.3485
CUST_35296_PI429010886	M12167	0.002066179	24	21						208.941	341.0815	298.135	215.748	221.8149	204.4688	178.3071	289.1171	337.8262	154.6661	256.9019	217.7635	248.2232	266.9539	212.598	214.693	287.3401	233.7667	294.9654	328.8423	293.6993	298.6917	306.3603	369.9026	313.6779	289.9679	468.5187
CUST_47446_PI429010886	M76166	0.002068042	21	15	91080041	PREDICTED: similar to AGAP005!	Transport	Protein transport	1.41E-25	281.0852	253.9896	325.1877	237.1976	209.4822	238.9099	319.4595	231.626	327.9636	300.9971	262.9461	329.3762	410.3258	204.8124	390.7977	381.5915	327.5322	434.8192	310.2931	294.9139	381.5238	252.7366	239.6808	383.8668	300.0533	199.0025	325.0373
CUST_11228_PI429010886	N27478	0.002068042	24	6						15.95963	22.79379	17.86363	24.63541	20.05184	30.19163	24.24247	28.32731	40.89712	30.54026	26.63281	8.838822	18.35004	20.36911	16.12609	20.76565	17.46742	16.79051	19.6075	13.84795	17.77119	16.23895	17.93113	14.36577	18.44938	24.33371	17.09586
CUST_2161_PI429010886	N11726	0.002073848	12	9						17.85602	30.96219	9.802866	33.44592	40.18962	35.0975	52.11743	30.7358	41.35381	52.35798	37.20732	47.59053	24.05036	20.40329	18.08416	26.05879	90.68839	54.79166	32.63627	20.52844	40.93434	30.83657	52.58831	43.78952	23.75571	8.371114	22.0686
CUST_43424_PI429010886	M63012	0.002075249	24	21						14.47477	61.79558	32.28459	33.72001	32.96215	35.40841	18.50063	6.755262	22.70303	40.15753	31.22074	23.03273	10.74484	29.4237	14.90046	37.632	33.91504	35.4732	15.97495	36.90512	43.20988	44.51618	51.10469	52.10827	39.60406	33.62051	22.29187
CUST_1931_PI429010886	N11103	0.002075249	24	18	321468336	hypothetical protein DAPPUDRA			1.79E-09	34.45309	36.40013	40.1541	28.49433	33.59136	28.34861	29.18122	62.08412	56.10448	32.3182	27.4271	41.63922	44.99721	45.17758	40.1121	43.98997	49.88072	35.8443	50.87138	62.41935	83.83043	37.81634	63.05696	66.77244	53.7784	45.42128	61.27966
CUST_39282_PI429010886	M37187	0.002077882	15	6						492.3638	362.4397	377.9818	730.5469	348.964	215.4411	1125.025	2108.887	2789.551	711.5947	825.4048	476.0531	1945.845	672.9412	712.4529	766.804	865.141	600.669	1568.389	1335.17	1918.312	1852.945	2906.899	3470.741	1075.462	2140.692	3220.778
CUST_28442_PI429010886	N57952	0.002078127	24	15						26.11634	26.61562	25.83296	19.87479	18.21466	13.85499	21.43051	13.17136	9.370822	21.51658	24.2121	22.06613	29.08241	10.83965	22.03984	26.78263	31.02823	28.13189	26.60721	21.84014	27.97586	16.69734	28.44753	21.82795	31.79774	26.96277	21.82087
CUST_189_PI429010886	N2220	0.002078579	12	9						6906.237	552.6048	5271.495	8503.163	2700.891	3871.525	998.7674	1603.304	2711.745	9712.243	9364.597	7248.769	13616.29	3546.981	7832.882	65.30254	53.37496	111.793	2584.904	3721.413	5166.879	11911.49	12699.11	12317.37	4508.46	7844.789	14218.13
CUST_4186_PI429010886	N16189	0.002097703	18	12						47.22395	83.18277	44.10093	18.80121	69.16443	36.69203	45.21941	48.92732	50.62088	52.96326	72.66211	67.40377	73.3591	80.96563	60.81352	17.17977	175.4908	173.8408	55.67726	56.49038	44.73509	30.03767	43.38791	26.21182	38.05697	90.08744	60.93307
CUST_41816_PI429010886	M58616	0.002099404	12	0						130.1961	120.8608	136.3718	107.9823	107.5363	95.10568	87.96067	94.63322	97.97985	104.1064	132.5675	98.56741	116.4848	111.4394	120.6508	94.40167	109.8092	104.6904	97.11689	121.9822	114.0573	111.4813	123.2141	135.3294	116.2567	143.902	168.2866
CUST_41993_PI429010886	M59110	0.00210087	24	21						124.991	160.0873	145.3581	99.54889	118.606	99.116	60.60875	115.3868	97.75803	92.53323	136.1879	135.9219	112.3135	137.2059	165.4991	129.3077	141.598	153.9484	111.6232	134.703	116.8939	166.108	154.3283	190.0902	203.5144	142.0207	213.1791
CUST_34197_PI429010886	M9027	0.00210087	24	21						114.4851	116.0138	66.43371	119.971	68.73418	86.51281	45.05271	57.97707	39.30986	64.51751	80.70329	27.7842	96.14621	89.06904	83.75475	54.87487	76.76723	62.4694	71.77497	89.0262	60.04487	151.4662	133.4406	231.588	103.0218	176.9444	66.93928
CUST_37914_PI429010886	M22219	0.002103622	12	9						20762.38	1848.594	20762.38	22711.46	15230	18408.63	4777.94	4453.231	7585.802	21224.39	27665.22	25611.3	31801.71	14580.94	24426.06	1806.683	1027.619	1613.784	11643.13	10921.28	12043.33	33402.18	30352.12	37083.68	12288.42	14794.99	24341.41
CUST_25941_PI429010886	N53342	0.002103622	12	9	528522579	PREDICTED: gastrula zinc finger				4.98E-27	88.17603	45.33378	83.26396	71.28516	72.70716	60.35593	77.49407	62.10736	78.94493	101.5332	85.06849	79.23954	93.99213	62.23304	37.98103	51.58532	55.26895	62.70293	83.76355	72.74057	69.00406	83.87393	85.49462	121.6162	96.13685	61.17453	85.02933
CUST_36026_PI429010886	M14323	0.00210699	24	18						18.18791	28.59291	41.90191	17.82226	13.44428	13.552	9.640926	25.64515	19.26369	10.17872	15.29086	11.29	21.52069	31.78394	27.00266	15.00294	26.51323	21.02758	39.0719	40.97531	63.02678	18.18231	19.7654	22.04447	45.85217	25.98049	30.68789
CUST_34471_PI429010886	M9770	0.002115503	24	0						30.25225	33.02712	43.36291	NA	15.48837	12.80959	24.97563	25.49661	24.07838	26.30298	9.133693	20.465	12.23088	19.51186	3.945831	34.19123	14.50529	31.91659	15.30251	35.48951	30.98378	18.07496	28.98374	8.248627	52.43098	56.12288	47.6242
CUST_48220_PI429010886	M79390	0.002115959	12	6	197940771	Euphausia superba mitochondri			2.12E-159	95843.19	95843.19	98191.21	107696.8	83454.29	101686.5	98191.21	105237	104498.1	102393.8	96349.38	102933.3	98191.21	80988.9	7021.693	107696.8	112823.1	100092.3	107696.8	110385	101686.5	101035.5	105804.6	99678.14	100092.3	87131.39	94790.65
CUST_28563_PI429010886	N58191	0.002116005	21	9						14.6635	23.38479	14.22535	15.00133	33.51959	33.25164	20.71518	26.12451	12.55746	24.51729	42.32505	44.27544	23.2664	22.28941	30.8769	36.29464	28.17546	28.49433	21.23607	18.60288	28.01116	14.46435	8.476423	17.01732	35.53128	21.73011	26.53236
CUST_42577_PI429010886	M60630	0.002118174	18	15						73.04616	94.76517	79.34748	50.98258	101.9053	76.24747	81.61482	84.80017	70.66441	71.36454	82.47763	76.27282	84.29772	95.92856	100.3986	98.10696	91.57245	94.93084	73.67616	81.34169	67.91644	62.98884	49.20954	60.30614	90.3848	80.29411	80.13048
CUST_27253_PI429010886	N55715	0.00211906	24	15						74.82596	80.05281	97.84796	79.44437	72.73375	55.36247	111.6675	100.3755	111.6865	103.8143	75.04432	105.7007	112.7474	89.64766	100.0738	100.3986	120.6137	133.5862	129.0812	105.074	110.822	101.7856	98.64233	142.2741	97.70277	91.14348	165.4536
CUST_38889_PI429010886	M31662	0.002121757	24	21						9923.978	11805.59	9954.878	9345.369	4803.622	6710.162	5718.956	7762.077	6988.188	10077.64	6165.981	5847.84	12424.28	3851.521	6818.249	9923.978	12141.91	5191.892	15435.13	9613.776	10664.62	14502.16	15526.29	11264.76	18673.37	11881.91	14015.31
CUST_41374_PI429010886	M57581	0.002123573	24	18						72.31873	68.52496	74.90044	51.7695	67.63991	55.96387	42.17431	38.33215	60.37267	57.41489	57.34418	85.82929	37.86114	66.77244	76.16048	76.59647	83.67777	60.84053	95.45574	92.03434	66.87814	75.0839	66.68933	71.33856	83.32737	58.98036	77.18489
CUST_43005_PI429010886	M61831	0.002123573	24	21	307169645	Protein suppressor of forked [Ca	Nucleic acid metabolism	RNA metabolic process	1.02E-19	38.06125	49.9052	29.74161	42.30746	39.66978	25.16166	45.37131	36.46097	36.78636	36.11568	26.96277	37.4753	39.6633	39.60406	25.06319	41.42487	58.21158	45.2017	56.75304	38.67341	45.88546	52.70059	45.50498	50.15161	41.403	42.75885	50.25143
CUST_47475_PI429010886	M76272	0.002124323	21	18						73.65204	79.88277	80.37006	57.83575	88.71852	52.35798	53.91461	92.7356	67.51408	78.9339	62.98013	69.84655	48.35178	36.98315	53.21446	55.47729	96.74886	73.23385	122.3191	96.45691	113.1623	90.1637	60.03374	86.18133	68.83252	55.34035	81.64232
CUST_54846_PI429010886	M107937	0.00212649	24	3						1912.144	1238.023	1173.346	1859.635	4470.918	2795.798	1418.598	1233.546	831.6922	1193.688	3313.258	3210.455	1170.841	3095.903	1183.911	1118.881	984.5856	1234.088	1104.345	985.0577	866.5613	1117.04	1205.686	1173.789	952.2766	1449.243	1073.581
CUST_35500_PI429010886	M12728	0.00212649	24	18	260813390	hypothetical protein BRAFLDRAI			1.78E-38	96.09055	149.3939	115.6407	94.51495	101.1304	95.36659	110.6555	138.5964	105.7691	114.5807	123.8699	106.0881	157.0815	109.9768	131.5859	110.1596	150.0449	130.6255	132.1609	132.2204	147.2789	118.5815	169.9145	134.4304	103.6807	140.2426	131.4002
CUST_10622_PI429010886	N26526	0.00212649	24	18	118369130	hypothetical protein TTHERM_0			6.45E-08	184.5623	146.3133	142.6928	55.71616	62.21897	27.7582	169.1253	207.9921	354.0991	79.1181	62.33316	77.91051	113.2581	89.85516	104.7345	162.3844	156.5799	221.9709	494.3291	205.1648	430.0583	209.4303	57.19268	152.0267	379.0965	103.5646	224.1099
CUST_21384_PI429010886	N45122	0.00212649	24	3	321475473	hypothetical protein DAPPUDRA			6.71E-56	165.1205	176.5278	89.5211	122.2183	183.0375	192.8297	117.5696	171.7821	156.8401	135.1633	126.9826	112.0316	79.67772	114.952	116.8623	135.6453	111.4237	94.42757	101.3719	92.77365	93.59078	138.7083	118.6869	96.87922	123.9057	98.4933	76.88599
CUST_21427_PI429010886	N45199	0.00212649	24	21	541906785	period [Eurydice pulchra]	Metabolic process	Lipid metabolic process	1.54E-47	36.28732	34.87559	32.51568	26.35742	29.88536	33.17685	23.02558	26.45217	35.8443	42.34405	29.66274	35.35463	35.47011	30.18014	37.16734	51.80267	42.03292	44.79451	35.99566	30.65511	38.86739	55.84882	37.53998	51.43184	51.3517	34.18577	52.48812
CUST_43465_PI429010886	M63128	0.002128091	21	15						50.64118	58.15501	67.76221	52.92294	57.39387	52.61041	47.6242	42.68213	46.03411	57.20137	45.87769	42.46657	53.60597	43.22401	48.81917	69.06598	62.17466	64.74689	68.46482	57.35611	59.38892	56.20706	44.43314	56.57499	71.1038	56.39944	55.89971
CUST_36426_PI429010886	M15871	0.002128665	12	3						101.9985	88.29653	81.42279	125.4804	113.9542	103.5544	136.5218	100.0919	105.8148	74.06253	98.91443	103.0964	100.821	87.03898	84.33008	120.8427	90.52573	106.4281	109.2822	94.69266	93.63735	76.6076	75.10319	77.29301	100.3986	87.66036	88.5599
CUST_23551_PI429010886	N49021	0.002128665	12	9						205.6244	231.809	234.5732	185.1417	215.0811	162.9468	174.7345	163.8102	164.6109	222.9863	293.5137	258.5192	189.351	192.5776	179.4021	185.3051	185.9639	194.2319	225.9507	187.8413	192.4672	240.84	262.2441	218.889	209.666	225.3038	221.7509
CUST_31192_PI429010886	M941	0.002131864	24	12	261289819	hypothetical protein BRAFLDRAI	Metabolic process	Other	2.73E-136	38.7574	44.47158	58.9013	46.18377	29.26681	49.27254	56.44269	82.63781	74.33815	51.18195	56.34856	53.05853	72.19272	60.68039	88.90086	46.16308	28.2205	48.08352	53.07048	67.40377	86.07541	30.36386	29.00584	31.30624	52.62255	34.48922	51.21624
CUST_41272_PI429010886	M57321	0.002140004	12	0						199.0318	212.2981	240.8917	204.3745	152.1914	182.1611	149.3578	154.7131	175.6627	194.1916	217.4288	246.2015	239.348	200.181	225.1246	160.6506	205.6925	171.4846	169.8611	184.9648	156.1818	242.6247	263.5563	183.1082	207.2425	237.9791	267.9409
CUST_18973_PI429010886	N40751	0.002140004	12	0	270016018	hypothetical protein TcasGA2_T	Transport	Ion transport	2.42E-21	143.3201	101.2809	126.2375	87.79232	88.51224	88.90086	57.05091	67.52243	92.69475	110.7326	92.37878	119.8471	107.9064	100.1424	121.8684	111.1646	83.22682	100.5377	114.8959	77.30188	66.03803	135.5781	100.3986	106.5903	139.5013	94.30839	167.2859
CUST_38384_PI429010886	M26098	0.002142455	21	15	262401203	mitochondrial ubiquinol-cytochr	Transport	Ion transport	1.69E-19	783.1683	1328.27	679.5306	793.2617	667.6928	619.843	932.1979	812.7394	759.9978	857.1902	814.0506	771.2654	905.0625	831.3637	667.2339	932.6252	1128.705	1024.829	638.3206	851.4962	751.2377	771.7617	927.0038	796.2861	403.8629	785.5802	531.8423
CUST_39934_PI429010886	M46994	0.002143425	24	9	268607710	cuticular protein precursor [Trib			5.59E-10	16.94155	6.515712	24.68878	14.67792	87.28041	62.68994	22.00575	30.18887	15.3561	81.56078	123.4349	120.5704	51.64343	76.01297	109.0351	NA	21.10356	22.30617	11.89578	10.6926	12.2372	39.2895	32.99838	47.36534	4.409339	22.16019	15.11126
CUST_8437_PI429010886	N23037	0.002144735	18	15						3.069095	6.3587	8.420719	6.550174	6.459779	3.567253	8.152582	6.78752	8.590245	5.816259	9.271842	NA	9.730417	9.932014	7.350928	9.248817	11.41336	11.06753	NA	6.881395	4.422432	5.917822	5.513093	7.148694	5.931811	9.322519	6.765229
CUST_1605_PI429010886	N10216	0.002145391	18	15	343183153	myosin heavy chain type a [Mar:				692.8899	517.0399	934.3693	700.8468	481.6057	603.766	417.5037	1317.605	481.3104	640.9263	601.1087	542.9066	818.0478	1371.826	521.3103	1431.337	1748.513	2189.919	2171.504	1721.8	2312.635	399.2455	303.1234	202.183	1406.649	915.0007	682.1048
CUST_6870_PI429010886	N20710	0.002150737	21	0						244.6553	275.0563	NA	74.18947	182.4362	273.8856	206.3618	843.8571	158.3685	8.505122	30.9435	23.30722	37.16734	207.9512	34.33515	310.2309	98.82385	130.479	170.064	520.8578	174.9928	NA	439.1006	NA	21.7225	1091.098	NA
CUST_7659_PI429010886	N21882	0.002151219	15	6						159.645	133.3192	162.5329	169.1505	151.1486	79.42387	225.0911	408.0121	579.8505	118.3236	292.6099	128.0875	432.7554	256.4943	207.5504	206.6211	113.4137	133.196	404.5068	290.2762	439.1006	475.8315	630.9023	392.8052	339.9532	274.4093	532.8364
CUST_40675_PI429010886	M55813	0.002151941	21	15						43.65204	28.71759	30.47714	40.13911	28.21177	39.38283	26.9677	32.54957	29.3403	29.00237	33.83846	35.99005	43.40182	31.50944	25.02711	43.63653	62.11363	43.04131	35.05094	51.70196	49.60673	34.40862	34.84184	46.5095	11.88179	31.00311	33.49837
CUST_5659_PI429010886	N19002	0.00215211	24	18	268370207	integrator complex subunit 3 isc			1.72E-155	414.226	534.2591	441.1218	402.3519	377.0504	409.0189	482.9421	529.0583	449.1793	456.0887	493.728	550.6893	553.926	408.6556	476.5395	544.8319	520.7346	665.1463	584.3107	484.6312	595.447	567.3781	475.0917	577.1013	589.8797	465.2004	646.5974
CUST_11953_PI429010886	N28683	0.00215211	24	21	390333216	PREDICTED: RNA-directed DNA			3.24E-11	179.4316	237.5901	254.4938	199.0318	201.229	195.6436	166.3995	169.3865	217.5757	212.6978	340.7195	216.54	221.0978	192.8085	225.9834	257.1925	242.133	262.4374	258.3202	203.5459	260.752	314.9794	250.6739	301.1497	295.1045	224.9241	338.4518
CUST_30937_PI429010886	M264	0.00215227	24	21						69.45852	145.1105	117.1549	79.31123	128.179	88.82328	78.45326	110.5806	110.4383	53.30891	102.2094	87.84235	78.15502	96.57151	100.821	109.9215	136.7882	135.2233	105.1748	136.5823	118.4277	119.6854	136.9272	152.6002	124.3455	96.42996	156.5986
CUST_23251_PI429010886	N48510	0.00215227	24	15	262401367	putative ribosomal protein S23e	Protein metabolism	Ribosome biogenesis	1.73E-27	62.3254	40.02841	51.84875	25.25032	22.49107	31.77263	49.40203	46.12726	47.08587	66.55746	37.38294	32.57446	41.51987	61.77408	34.12419	68.91312	55.59367	63.65381	63.17461	51.63077	56.79387	45.93656	39.88903	41.99895	64.74689	46.55494	35.96302
CUST_38991_PI429010886	M33089	0.002153708	12	9						9080.902	4952.328	12991.27	13301.65	7643.683	6631.875	8857.18	8006.37	15553.93	14307.54	18235.97	4767.094	21834.29	9041.082	12378.85	698.508	421.5919	823.2974	16553.17	15492.49	15871.24	19635.13	30257.2	25555.67	11612.18	16151.95	21270.53
CUST_190_PI429010886	N2228	0.002153708	12	9	407408426	hypothetical protein MOQ_0042			0.000000993	29520.35	3460.858	28210.59	42662.49	17886.89	22469.11	6799.146	9330.025	14642.96	36802.85	56377.06	29520.35	42496.4	20708.31	31108.81	1106.912	1157.507	1395.332	19094.08	18320.9	23324.95	42014.5	66946.06	67450.12	21922.49	41095.52	56054.28
CUST_1145_PI429010886	N8631	0.002155541	21	15	321476544	hypothetical protein DAPPUDRA	Metabolic process	Carbohydrate metabolic process	8.16E-09	245.1933	486.6807	168.9831	384.2558	99.58967	292.3452	795.25	481.7121	310.9804	568.455	198.84	223.5617	634.4332	366.7062	414.325	1324.865	845.9441	1089.089	624.9341	142.4928	523.3495	412.604	251.9129	452.2327	359.5095	275.4561	163.3155
CUST_23427_PI429010886	N48826	0.002158898	24	21						60.3351	97.26952	84.91891	66.48433	83.09656	78.13239	35.97416	70.49708	50.16621	77.6897	76.80903	76.94539	53.22588	64.41139	43.78952	45.0895	90.58585	62.77181	59.96133	86.53109	62.21897	88.0075	93.13119	101.5579	81.26825	77.40803	109.8092
CUST_19296_PI429010886	N41332	0.002160618	21	3						7.300631	12.4892	6.825911	NA	23.60614	10.66221	9.309902	14.42653	8.360391	11.75064	12.68845	7.587167	5.330713	7.069413	6.842059	5.442056	9.838142	6.618689	6.503833	7.417941	11.44626	NA	12.41089	6.236495	NA	15.13188	12.66493
CUST_16496_PI429010886	N36333	0.002164049	12	0	380028136	PREDICTED: long-chain-fatty-aci	Developmental process	Multicellular organismal development	8.3E-33	420.8457	503.5534	544.2292	444.8216	376.3542	360.2285	237.6854	396.7957	394.0157	431.4309	507.5036	430.2082	509.5667	393.358	492.831	375.415	609.0112	424.9756	356.1688	358.247	335.3952	529.579	477.4346	493.2216	519.7601	465.3594	590.6124
CUST_32757_PI429010886	M5048	0.002167784	24	0						38.78785	NA	11.09601	8.090541	20.20385	25.49854	13.98478	13.79706	NA	14.04576	11.27831	18.1699	7.423378	9.065482	9.726899	5.997609	11.52249	8.822371	10.33674	12.25196	NA	16.37294	6.921979	13.56445	18.23869	26.12451	14.39043
CUST_6940_PI429010886	N20808	0.002175322	24	15	15425681	I-connectin [Procambarus clarkii				52.63501	56.3623	54.52711	47.57381	60.23194	52.84329	39.42252	54.73663	53.69851	57.94759	47.09644	34.84867	46.91713	45.39182	44.70348	76.3955	63.03855	74.86678	61.19316	56.54714	72.74057	63.59136	58.58735	72.55053	61.908	66.87814	55.7467
CUST_13448_PI429010886	N31208	0.002175473	18	15						137.7031	248.554	143.5029	152.0064	68.01679	100.0492	250.4566	210.7377	262.04	150.3928	103.1504	131.5353	193.3701	183.4751	135.0936	276.9413	305.1178	287.4579	181.7506	205.8252	239.9783	106.1914	162.4604	130.496	175.2065	171.7821	161.8447
CUST_48053_PI429010886	M78707	0.002177731	24	21						74.36638	74.44615	91.29948	63.81233	60.75399	61.06467	60.34528	58.14687	70.69946	116.4848	87.60092	94.7296	105.7387	68.83252	92.2966	95.33452	67.37607	84.35052	119.2573	72.10171	93.51275	137.4234	95.03516	126.2905	127.8904	72.89302	129.4725
CUST_3553_PI429010886	N14738	0.002177731	24	3						9613.776	34.6239	6285.074	6584.064	4185.629	5847.84	178.1189	163.6405	175.0979	322.1133	3011.975	3801.741	336.0374	5357.194	4981.65	80.9995	46.96617	112.1293	18.74013	48.27158	34.05393	2454.398	70.80726	2345.461	109.9078	78.07876	173.7269
CUST_15455_PI429010886	N34608	0.002177731	24	21						35.92153	62.94384	42.2771	46.02219	36.73639	43.08599	32.85013	42.29499	37.15316	49.09284	44.72843	49.58749	62.76751	41.00325	43.70538	49.74586	54.07225	57.31291	47.9867	55.36247	45.13797	57.42791	51.1228	75.35645	55.6648	51.77472	50.25586
CUST_43381_PI429010886	M62888	0.002178751	12	9						623.3324	274.3383	985.8154	1331.886	637.4326	817.7537	376.7239	294.3919	441.8514	1609.728	1236.613	1162.935	1535.771	737.8151	1227.139	197.4586	321.5516	270.7536	862.533	575.9481	557.4698	1819.509	1731.406	1742.558	854.7576	690.3228	1274.133
CUST_16828_PI429010886	N36926	0.002178751	12	3						21.87085	24.87248	26.17884	22.81652	30.85244	27.5259	13.24159	19.26879	26.56161	10.78644	15.84205	16.36929	17.60895	24.25128	23.44477	24.84514	21.82549	34.09348	26.98424	15.55385	22.62211	18.47386	20.37998	14.15162	27.36657	10.85018	21.97709
CUST_25868_PI429010886	N53201	0.002180398	18	0						369.9777	783.4167	720.7726	369.8268	662.5571	607.3706	467.8312	550.9285	550.3617	414.325	490.2242	337.1412	352.227	405.1127	497.1127	436.5051	554.8747	499.8092	651.2334	793.634	721.6825	524.6943	611.8901	476.1651	664.4758	525.1123	545.9351
CUST_48435_PI429010886	M80310	0.002180967	24	15						18273.52	23273.41	16981.94	16954.37	15847.39	15461.72	20382.6	23162.84	23016.75	17345.96	15630.09	13658.74	16580.62	17692.35	19195.08	26678.14	26678.14	27918.92	20382.6	21974.84	25130.01	15630.09	18758.28	18579.76	20913.02	16252.77	20320.15
CUST_17412_PI429010886	N37921	0.002181191	21	0						35.14671	4.561895	48.76461	29.90099	NA	25.42657	14.6763	7.685875	17.6701	NA	14.10253	13.80069	16.39186	6.860532	23.68893	13.50346	11.54561	8.549067	15.21327	4.643571	19.53187	42.69638	25.9357	45.34342	28.80735	NA	26.06752
CUST_45685_PI429010886	M69908	0.002184715	18	3						801.5781	67.68769	797.5472	698.717	557.162	577.7104	95.14144	123.9222	120.3932	284.6216	462.8312	408.3889	340.4948	195.3762	313.9334	32.18818	21.81818	30.42641	191.741	235.6895	113.6067	939.0464	451.3395	728.6567	165.6234	277.5468	208.3375
CUST_93_PI429010886	N737	0.002184864	15	0	532759730	antimicrobial peptide type 2 pre			1.31E-26	9937.479	6631.875	6171.545	5783.496	2860.025	3929.52	6592.219	5550.987	4727.546	3549.354	4023.648	3046.537	4628.184	6358.535	3978.701	6132.817	6155.993	5892.313	4705.762	3232.565	6051.213	3988.54	7048.129	3004.305	2684.097	3393.901	1840.408
CUST_52872_PI429010886	M99276	0.002189714	18	12						87.54238	95.80871	102.8397	136.3336	96.33044	84.61891	91.13599	89.08902	94.38205	101.0612	105.5685	77.9272	147.6657	99.6269	150.7041	116.0138	143.8832	95.98627	115.6711	71.70119	102.3834	93.06282	75.9364	77.09727	66.55746	74.22801	84.56114
CUST_5752_PI429010886	N19148	0.002189836	24	15						4090.127	6310.155	5277.675	3832.521	3604.261	3346.038	4740.787	4740.787	5336.579	3843.133	3686.257	4008.648	4101.099	4045.654	4487.184	6358.535	6210.057	6999.724	4554.072	6155.993	6219.297	4041.944	5009.272	5913.632	5929.402	5073.866	5693.503
CUST_39971_PI429010886	M47663	0.002193578	24	9	152949487	calcification associated soluble r			3.31E-22	442.3593	159.4235	148.7876	487.0065	857.1902	540.61	440.4343	155.0383	104.1208	555.8829	771.7617	828.8502	387.5344	554.2471	569.1918	244.537	93.94191	292.5506	119.4647	147.1902	134.125	149.2467	140.2011	180.4687	114.0896	233.4638	142.4928
CUST_7295_PI429010886	N21335	0.002196733	24	21						448.0872	530.6367	618.3282	362.5984	321.9997	355.3919	281.4753	293.5137	368.4034	400.4361	353.3256	447.3237	420.9575	380.35	494.9456	456.5305	450.4965	550.3617	417.9126	360.8077	418.1662	616.4216	446.9571	585.8731	605.6304	528.895	789.6051
CUST_49761_PI429010886	M87028	0.002200111	15	9						144.7781	134.826	142.2962	125.0755	109.9215	99.97142	123.2894	126.9272	130.6083	163.488	110.7454	162.844	124.1767	127.9102	143.9979	143.2463	123.3867	142.6733	138.9184	125.4627	117.7425	148.1501	138.824	156.2107	151.7315	153.502	180.6504
CUST_49967_PI429010886	M87978	0.002203794	12	9						45.33378	14.46087	45.04182	47.65574	15.99305	32.34672	27.79405	10.02353	13.28567	61.16253	86.59225	42.86666	48.27587	28.24507	25.13996	14.53951	8.766471	24.03275	33.26372	24.38607	26.98744	52.52723	85.79452	69.94875	41.85879	22.43161	62.5403
CUST_20333_PI429010886	N43252	0.002203794	12	9						105.87	31.71788	134.27	169.9145	83.9013	61.73575	62.78718	99.22393	214.3972	114.413	845.5682	102.1346	235.0418	125.0917	165.9817	9.465451	12.61861	8.980966	170.7874	171.3126	115.921	221.7872	325.0373	340.7195	164.7136	172.5012	133.9146
CUST_18075_PI429010886	N39093	0.002206688	18	9						80.94269	64.24425	67.24604	65.66275	51.22586	62.93557	82.51294	77.99973	56.11275	100.646	91.86363	82.51294	73.99941	93.22902	79.02299	133.2506	85.45046	73.15055	77.13441	66.15962	73.79109	75.15569	77.37879	76.99156	77.62481	68.38347	78.26455
CUST_20592_PI429010886	N43715	0.002206688	18	3						8.668203	5.27516	9.45577	22.0572	19.1981	12.67619	12.53109	12.07805	12.5832	21.31454	9.900546	11.40479	10.89769	7.409255	12.79576	4.745385	6.686795	10.81013	10.63933	10.77956	11.62281	13.40089	11.36795	14.9267	9.528739	6.990911	12.68388
CUST_53528_PI429010886	M102121	0.002207329	15	9						15.56376	12.12132	22.20311	13.70168	11.15484	12.39357	9.528739	11.29793	12.682	21.55177	20.76789	17.82881	15.93269	NA	15.23394	17.17582	19.65735	18.34193	9.267762	11.0388	12.64537	21.75367	17.12756	12.66493	45.9872	13.46714	21.85461
CUST_12344_PI429010886	N29321	0.002209664	24	15						12.69014	16.56978	14.01914	7.647383	7.356172	8.622045	11.13904	14.47089	11.2094	17.66478	12.80236	9.603299	14.87141	11.96398	22.53853	18.56513	15.4529	16.27834	19.67697	12.86584	15.36018	16.07632	6.987091	19.40949	13.05606	10.93877	15.94166
CUST_20752_PI429010886	N43998	0.002209664	24	3						82.137	118.7214	77.38763	130.3816	159.5774	186.728	100.8115	134.9856	165.3213	82.62828	127.9862	99.71725	117.5411	100.5529	125.1571	46.50411	82.23633	52.59937	111.0547	89.57069	105.034	79.43494	95.14144	102.7615	63.14866	110.7898	164.8407
CUST_39467_PI429010886	M39889	0.00221801	21	15						11738.35	16372.71	12523.21	10035.81	5712.375	4988.039	9179.508	11455.94	9743.786	11667.94	7130.037	9111.496	14285.49	9923.978	8911.047	22711.46	20968.09	23231.4	16520.01	15435.13	13017.21	12939.25	10337.3	9313.798	24199.32	11667.94	17210.94
CUST_22622_PI429010886	N47345	0.002221678	21	0						55.43214	51.25725	57.85035	53.53717	40.0019	43.8296	43.63223	35.62662	37.03485	51.02509	49.23192	49.04743	52.2471	52.03532	52.27625	39.70565	34.77477	43.13538	46.65167	44.44554	52.39131	60.58102	58.71759	54.37948	50.97241	39.91801	61.96517
CUST_45631_PI429010886	M69725	0.002224323	18	15	209418466	serpin 8 [Branchiostoma lanceol			5.77E-31	1465.124	2454.398	1855.874	1189.941	1425.394	1201.71	1901.421	2152.198	1891.416	1854.805	1710.003	1704.256	1857.76	1511.222	1587.909	2240.494	2913.515	2844.645	1724.425	2057.214	2045.264	1644.807	1265.639	1752.658	1716.333	1373.854	1863.699
CUST_37529_PI429010886	M19379	0.002228837	12	9	321466528	hypothetical protein DAPPUDRA			1.5E-21	10077.64	275.7417	9889.351	10921.28	2378.51	6818.249	772.7965	423.2638	732.0587	19387.17	18522.28	13737.98	22946.38	2257.369	16411.2	82.61597	102.1812	171.2412	3838.854	1524.311	4940.836	14161.49	12789.24	14828.55	3379.597	4548.63	9080.902
CUST_4514_PI429010886	N16941	0.002229188	24	3						190.463	35.5246	166.1333	278.2449	220.6244	360.8837	12.37796	37.71176	18.5449	22.03729	143.5988	145.6782	58.75919	51.261	126.7723	28.05593	10.07278	43.55661	18.52811	32.10035	16.77987	126.9491	23.57971	101.4459	35.61538	66.78529	60.84053
CUST_6563_PI429010886	N20273	0.002237336	18	15	189031345	trehalose-6-phosphate synthase	Metabolic process	Carbohydrate metabolic process		16.69306	18.32878	19.91267	16.02196	18.61475	8.781043	19.10315	21.72789	18.43253	23.14702	11.34631	13.0149	19.14803	19.37652	21.31454	24.39353	24.93525	18.67988	20.44736	7.9866	20.34644	12.8517	11.60045	9.45412	15.75736	16.79346	16.06582
CUST_24185_PI429010886	N50191	0.002237336	18	9						16.20192	6.044071	24.37029	12.46071	11.99886	9.728753	18.56513	11.05843	15.93995	15.62347	17.6322	20.09102	15.75563	13.99873	17.41092	10.54024	15.11126	17.98274	5.842145	14.84471	13.50346	8.559475	8.213544	9.428061	13.0253	10.35153	14.51258
CUST_53795_PI429010886	M103219	0.002238361	24	15						46.17834	59.797	32.08466	23.85395	15.69552	40.15753	49.91567	75.30381	56.93121	59.20501	35.3444	21.1516	32.60595	51.44117	47.06434	138.0571	68.62585	106.0103	71.4584	59.93426	50.06722	38.4343	52.22249	35.05094	33.03392	68.28737	23.80543
CUST_36689_PI429010886	M16549	0.002238361	24	21						151.7139	196.2133	172.3736	176.549	193.3701	147.8169	141.3689	171.4846	173.2168	141.5281	169.1953	126.8065	160.9374	147.0639	176.0219	164.8179	197.2544	196.4414	162.188	178.576	194.6568	189.1287	191.8796	230.649	190.6544	198.7013	248.9012
CUST_5310_PI429010886	N18526	0.002238361	24	15	242000104	ribosome biogenesis protein - N			4.89E-172	480.6993	1106.912	892.0968	489.5895	473.1245	407.0205	771.0455	887.5066	1055.851	666.8579	534.4784	459.8781	801.0558	589.7452	617.6234	1044.527	1163.513	1160.436	980.6718	969.8589	1333.943	825.4048	777.3446	836.1158	890.2317	716.8261	1022.798
CUST_11258_PI429010886	N27522	0.002238361	24	21						26.05879	38.27451	44.46024	26.93742	34.24084	63.00026	29.7085	41.90666	44.45113	26.54115	33.06459	33.79023	30.36962	38.90861	35.78636	45.95593	50.02722	37.02396	61.80424	48.84433	43.77016	96.9319	54.35675	44.0823	48.79532	51.06045	46.1895
CUST_16059_PI429010886	N35580	0.002238361	24	15						95.68309	47.25275	73.76003	44.70872	7.705814	23.81296	97.14951	105.6058	89.63749	60.22483	17.89547	43.0146	95.16242	102.4313	29.38152	224.0178	163.211	139.3356	144.345	118.9736	156.493	81.19478	17.28062	62.04596	206.0093	58.04431	96.62834
CUST_7649_PI429010886	N21870	0.002246037	24	0						428.5726	465.972	440.2791	359.3488	462.9596	329.3762	309.9865	394.8687	385.1879	326.6921	397.0876	386.7231	286.0534	378.6955	321.7248	396.4646	439.3042	424.4332	320.6536	428.5726	433.2807	358.247	497.6488	462.9596	415.7252	492.2464	480.3553
CUST_54667_PI429010886	M107092	0.00225388	12	3						151.5412	313.6779	209.4303	269.4056	286.4183	281.9984	234.7388	248.9453	159.203	127.4555	181.3844	247.7871	197.0196	290.2155	298.3765	280.3649	289.0575	343.906	194.2665	222.4664	212.5212	196.9638	236.2759	178.033	253.1919	246.3057	255.7585
CUST_18351_PI429010886	N39633	0.00225388	12	9						14.03213	11.36795	23.27767	20.80218	12.73763	17.29812	13.74764	10.38947	9.706075	21.10356	25.80839	21.50122	16.9478	22.905	23.53743	4.58897	14.44521	12.08433	12.96468	19.53187	19.01772	23.4616	28.34528	25.17263	21.08201	22.25295	21.98717
CUST_24567_PI429010886	N50856	0.002254592	24	3	307176630	DOMON domain-containing pro			1.64E-09	46.9424	13.75552	84.20844	90.76223	128.6059	111.7404	14.26389	23.8324	20.60839	16.90428	100.1424	47.21493	28.71238	104.9377	87.39589	19.52955	21.2307	12.5772	13.07815	9.41785	8.367548	23.75379	34.97876	29.99314	12.55344	21.13752	15.41819
CUST_13035_PI429010886	N30473	0.002267058	24	15						22.72983	39.60406	31.52857	21.31272	10.49199	19.47024	51.63077	38.73465	27.68187	36.21685	20.83366	21.45886	37.97184	31.74988	32.66921	55.59367	42.2509	50.15592	26.23262	24.19322	24.95132	25.97013	25.35697	31.59758	22.08254	37.24162	23.44477
CUST_16531_PI429010886	N36404	0.002267058	24	15						470.9814	671.9924	464.677	420.5189	463.5485	455.4195	617.0017	553.413	614.0454	468.9491	444.9419	471.0794	487.4115	508.513	463.1658	703.4578	574.7506	643.379	611.1595	670.4033	559.4339	508.1759	573.048	542.9984	508.7574	495.0948	588.164
CUST_41032_PI429010886	M56688	0.002274596	21	3						6.041845	15.76131	8.04855	12.59794	12.44897	18.20572	NA	8.959857	NA	7.10526	NA	10.36519	NA	4.631993	NA	8.028381	NA	6.831045	6.334584	11.9269	3.891686	9.330978	6.742613	NA	15.79305	11.6476	12.23254
CUST_38578_PI429010886	M28205	0.002277745	24	3						138.4394	34.37139	124.3957	102.411	135.1633	117.4793	26.14707	58.34712	27.06409	40.48248	116.0897	106.0765	37.01072	32.29617	32.3057	NA	4.115522	12.1946	18.1529	29.06579	9.88254	107.0064	46.82329	92.22131	15.02898	90.83407	36.37036
CUST_23397_PI429010886	N48766	0.002277956	24	0						167.261	211.0451	186.1038	163.5388	162.9694	125.025	148.892	160.9133	160.139	160.6506	162.7145	161.8447	170.8814	154.7361	94.55136	158.0057	189.0201	184.7058	153.2144	166.3995	182.8221	177.8505	205.3015	195.7159	208.3055	176.3993	257.956
CUST_214_PI429010886	N2612	0.00228019	24	3						2249.486	30.11519	2051.901	2853.1	2871.837	3342.747	16.73426	124.2797	18.16224	117.5967	1958.772	2696.535	87.85118	2642.585	2110.599	18.06836	7.237005	19.12392	8.920491	110.6677	7.42127	108.0685	75.0185	76.12107	NA	265.1126	8.143822
CUST_5729_PI429010886	N19113	0.002282903	18	15	260790067	hypothetical protein BRAFLDRAI			4.05E-61	156.0492	357.7827	171.7821	84.08221	160.3187	112.5405	231.4591	268.3054	256.0012	192.6063	203.2256	170.6675	196.9837	239.8775	225.5545	281.5792	355.7553	290.511	192.3671	194.7103	236.8007	149.2791	235.4202	164.4678	236.2202	234.322	181.0227
CUST_36555_PI429010886	M16221	0.002284019	24	15	242011547	conserved hypothetical protein	Metabolic process	Carbohydrate metabolic process	9.17E-17	118.4103	270.0299	77.15711	190.6544	64.20079	134.0991	402.0757	295.6886	189.7429	234.8735	93.16926	116.7899	338.993	281.6983	207.3346	534.7331	497.1127	406.0638	332.3383	58.5159	167.7409	224.6812	154.7807	196.6698	161.8663	144.9581	86.92889
CUST_46101_PI429010886	M71369	0.002288929	15	6	332026908	hypothetical protein G5I_04441			0.000000579	10.0277	16.78485	21.35916	17.26114	9.887656	19.68126	14.85124	29.29497	27.17008	26.52119	35.28538	12.1659	10.85699	10.6734	5.653024	11.02357	23.21635	11.29903	25.41267	16.76354	18.89832	27.85388	34.38713	33.85619	34.46864	19.55846	26.11949
CUST_42948_PI429010886	M61693	0.002295755	24	18						27.47971	44.16317	37.455	42.2265	47.47428	29.63864	38.04859	33.85619	36.70398	29.84906	47.40679	37.9908	66.2384	37.71641	47.60041	40.16574	58.02072	55.58555	49.4765	61.70126	65.53387	44.73509	56.72705	46.00851	38.45768	40.06052	71.28516
CUST_32276_PI429010886	M3685	0.002298633	18	15	345498356	PREDICTED: F-actin-capping pro	Cellular component org	Cytoskeleton organization	1.05E-138	151.8566	140.0219	133.8983	119.0143	90.06479	123.9057	147.9573	125.3097	119.0641	162.9942	113.5495	125.6606	144.8055	96.00931	156.436	229.203	173.8666	181.9587	150.3467	99.38318	177.2739	128.0468	97.33698	123.2011	141.753	121.236	115.1679
CUST_51217_PI429010886	M92656	0.002298633	18	3	196000927	hypothetical protein TRIADDRAI	Metabolic process	Oxidation-reduction process	5.49E-08	33.15745	29.3303	39.41032	50.06206	158.4947	94.18113	30.59991	49.53225	28.4851	30.41718	21.93896	30.92492	34.90937	28.94999	20.10978	35.3123	28.05304	42.61939	32.05523	34.33958	30.8343	43.06184	38.01289	32.23135	31.14654	26.73097	23.30899
CUST_11377_PI429010886	N27715	0.002298633	18	15						43.84283	19.89427	8.956638	7.633196	5.776661	16.99096	17.30435	13.60565	12.34692	15.96403	14.51805	6.204057	27.9125	15.94747	18.1656	27.30031	26.17884	19.8984	25.30391	9.365338	18.08256	13.31624	10.17872	10.16018	10.92142	16.98717	9.889204
CUST_44737_PI429010886	M66836	0.002301186	21	3						37.83797	44.67899	60.00388	47.2578	50.4405	44.63889	38.19091	47.59053	37.43054	48.21063	32.88915	30.20183	29.4935	38.99311	45.28285	37.38294	39.18322	28.14237	39.77797	57.02244	26.58261	39.90127	32.12942	43.78128	48.27158	64.91949	55.60957
CUST_2487_PI429010886	N12487	0.002301499	24	15						141.1811	336.8186	222.3734	697.3828	313.3524	474.4729	123.7135	178.6979	161.4729	3717.963	1201.304	888.6241	2150.693	794.0736	1586.354	1082.092	1982.92	2906.899	6439.853	997.9064	4531.71	34.47208	476.1651	323.5302	6.476119	1849.733	6.906183
CUST_13707_PI429010886	N31630	0.002302188	24	0						127.1242	120.1554	87.92365	110.4624	72.91519	96.09055	125.5852	59.14641	105.5272	115.2014	69.75403	95.15335	79.18277	55.98105	65.69029	114.8354	84.88526	91.17437	100.3755	72.90397	98.67888	95.98627	100.5213	108.4697	132.6978	97.00725	435.0369
CUST_26779_PI429010886	N54860	0.002302188	24	0						37.29216	38.38927	30.25516	23.54538	12.8639	6.718737	26.206	24.17321	30.36386	19.30372	14.14112	24.21058	16.21895	7.595815	20.06847	20.84897	17.19904	25.99676	20.41652	13.75552	20.60839	26.51031	16.33412	23.49323	41.93463	22.49107	43.8213

Probe ID	ID	p-value	Period	Phase	EBI ID	Annotation	GO category	Go term	E-value	0	0	0	3	3	3	6	6	6	9	9	9	12	12	12	15	15	15	18	18	18	21	21	21	24	24	24
CUST_34123_PI429010886	M8826	0.002303593	24	15						318.9156	415.3144	360.2879	335.8993	333.3795	311.4501	376.2092	295.6289	404.3238	352.6665	308.6292	308.5661	426.6472	327.9636	375.5874	439.2301	455.5301	472.7524	434.3661	373.7341	430.6456	379.158	401.757	447.0887	427.8043	358.0959	477.1875
CUST_53411_PI429010886	M101591	0.002310659	21	0						30.30413	10.17265	41.38164	17.04105	9.164016	36.93887	8.267617	NA	8.740614	16.15211	17.14735	21.13979	16.84596	NA	8.753354	4.322519	6.92099	3.045822	7.99927	15.42768	11.29793	36.58388	25.91869	25.60929	12.57044	5.261269	18.08089
CUST_33623_PI429010886	M7451	0.002312852	24	15						3056.068	5162.169	2499.817	2566.656	2197.153	2434.185	4363.515	4295.699	2593.566	3382.058	2600.653	2960.558	2992.811	2911.669	2826.773	5277.675	5110.752	4945.667	3647.267	3535.101	3036.167	2558.16	3331.869	2787.074	3948.157	3571.414	2659.616
CUST_33927_PI429010886	M8267	0.002312852	18	9						1858.682	2618.303	2302.573	1420.017	1912.144	1531.626	2432.608	2763.96	1785.094	2849.078	2696.535	2820.736	1623.703	1685.048	1943.332	3647.267	2790.956	2814.474	3289.37	2449.312	2137.853	1473.47	1369.757	1047.687	3792.758	2323.856	2881.653
CUST_45803_PI429010886	M70340	0.002320621	21	18						23.64329	59.15801	58.06923	10.69455	19.61588	15.53825	19.16781	40.71656	27.47971	3.903545	27.11917	NA	12.11716	6.752779	16.32911	29.88536	30.62124	33.11195	53.53717	95.28229	98.24098	32.64246	22.21765	22.62003	55.70902	15.67395	47.54634
CUST_41531_PI429010886	M57974	0.002321502	21	0						49.89765	25.76307	47.21843	35.78309	21.77362	35.68286	31.66806	24.3283	32.84675	28.18616	22.62211	27.68667	41.403	22.79631	19.28269	21.08718	28.39338	23.87506	15.41819	20.26699	26.29555	50.56209	50.39653	45.33378	39.43097	31.61116	47.28223
CUST_44333_PI429010886	M65652	0.002323855	24	18						382.6455	254.6677	471.2164	139.0478	123.5107	138.1387	398.6975	333.6191	343.5695	152.4772	186.9296	131.274	213.152	240.6532	190.9332	414.4974	341.2447	352.8944	247.4668	535.2394	882.5044	220.8506	236.5827	304.58	295.9223	168.2637	230.5014
CUST_35800_PI429010886	M13545	0.002324452	24	15						368.4918	376.4343	276.6685	350.1584	286.2354	257.4878	482.0377	302.6664	374.5443	391.5717	256.6162	347.3018	407.5835	287.632	367.876	451.2333	420.9575	558.4737	421.4753	300.549	444.1379	391.3071	417.1463	469.1525	392.7267	199.1806	380.4577
CUST_18533_PI429010886	N39968	0.002324452	24	15						2654.193	5374.176	3759.237	3289.37	2948.008	3073.818	4403.488	4633.616	4450.275	3972.222	3154.32	3924.629	3384.461	3890.917	3324.822	5467.822	5630.843	6010.065	4211.995	5055.201	5265.552	3079.233	4363.515	3948.157	3364.257	3688.899	4545.107
CUST_39990_PI429010886	M48006	0.002325952	24	18						NA	NA	NA	11.42944	4.732358	10.72798	1310.327	5.085084	1277.638	44.96264	34.33222	4.247945	NA	11.62846	NA	72.5147	NA	69.61335	1594.801	2045.264	3538.483	262.9461	NA	233.0946	262.4374	40.4228	241.364
CUST_35875_PI429010886	M13789	0.002329282	18	15	307176866	Cubilin [Camponotus floridanus]			5.95E-15	21.93249	21.7987	18.68765	17.69816	18.71959	16.74981	26.2902	27.16394	30.74897	27.17008	18.78483	21.41005	24.73091	21.40753	26.77083	40.59456	26.9245	28.48764	27.63973	17.46154	20.58397	22.40935	16.23247	12.99454	21.5031	13.75076	18.27116
CUST_13893_PI429010886	N31952	0.002337903	24	18						153.9962	171.3397	171.8649	148.7099	144.1943	153.2899	129.7636	127.4016	148.131	156.6283	161.3598	147.9573	129.7968	149.9126	148.9789	214.2511	166.8118	193.9616	201.3401	152.0267	180.8125	185.653	147.6657	194.6568	209.9842	158.6448	204.0717
CUST_8334_PI429010886	N22893	0.002339745	24	15	321479105	hypothetical protein DAPPUDRA	Protein metabolism	Protein folding	9.9E-61	2139.451	7632.364	4031.99	2422.76	2256.232	2108.887	5783.496	5558.806	5251.996	4342.518	2971.539	3041.467	3281.159	3286.945	2962.88	6966.12	10448.35	9410.039	4746.115	4261.241	4825.163	2552.554	2681.804	3243.823	4727.546	3975.62	3670.22
CUST_8558_PI429010886	N23221	0.00234645	15	9						6.486846	8.576647	12.3285	9.858804	8.407667	5.158329	NA	14.9436	7.445689	NA	12.59667	NA	11.76598	13.37563	105.8953	11.30735	10.54947	13.72622	9.528739	7.220518	11.87323	16.02388	14.48348	12.99289	16.40534	14.17028	14.97655
CUST_55661_PI429010886	M111183	0.002353149	24	3	121484195	cuticle protein CUT5 [Portunus p			2.53E-15	1034.581	22.16839	1492.153	1649.981	2512.997	2256.232	20.31011	126.0542	58.23269	562.0064	2710.483	1398.823	243.331	1403.011	1129.795	6.933026	22.68453	8.600876	49.06379	109.3921	45.96577	1068.554	529.2519	786.1643	69.45852	387.9483	185.5629
CUST_55841_PI429010886	M111850	0.002353149	24	3						5091.533	132.1609	4275.766	8439.929	6125.57	7138.174	168.9624	757.5703	256.2719	5533.093	5222.203	7785.133	3675.29	4093.741	6837.265	44.30249	49.85348	86.13364	284.6216	1110.746	549.1826	4319.299	2981.297	5099.888	315.4118	2288.957	1583.518
CUST_38821_PI429010886	M30948	0.002353149	24	3	7008007	DD9B [Marsupenaeus japonicus			4.12E-15	15492.49	476.9732	14470.28	24909.79	22092.06	24984.55	330.7777	1110.326	282.2255	17487.6	22658.35	23231.4	11503.97	19960.25	26891.88	53.23808	63.5234	131.0644	254.1863	1284.327	172.9739	15847.39	7353.952	13193.16	551.429	4456.862	3847.793
CUST_33421_PI429010886	M6843	0.002353149	24	15			Protein metabolism	Proteolysis		99.20976	88.60558	24.21718	84.6858	13.55607	35.07549	938.592	282.5034	748.1962	542.9984	312.008	67.31002	123.2141	233.2689	56.22476	1988.355	868.9348	811.9435	320.1071	411.2361	831.6922	376.6399	5.331625	415.8002	247.1999	36.56623	107.365
CUST_2748_PI429010886	N13031	0.002353149	24	15	328698812	PREDICTED: Acyrthosiphon pisu				2849.078	5322.868	3319.225	2893.96	2734.019	2457.62	5864.557	5197.224	5625.087	4077.152	2844.645	3230.906	3952.736	3145.521	3069.207	5545.402	6809.882	6976.622	4177.108	4368.987	5322.868	2962.88	3798.572	3739.191	4031.99	3717.963	4513.062
CUST_9910_PI429010886	N25392	0.002353149	24	15						54.60623	106.8499	87.29007	49.83616	22.36969	13.09781	143.5988	64.82967	79.4857	94.33013	24.19535	62.19095	108.7185	45.57662	58.69436	147.2789	171.1268	201.4162	141.1702	113.0702	124.3819	67.75299	49.70798	79.94645	162.0504	97.83763	138.2736
CUST_258_PI429010886	N3133	0.002353149	24	15	345494337	PREDICTED: S-adenosylmethioni	Metabolic process	Generation of precursor metabolites ar	2.45E-162	1941.073	4177.108	2592.232	2329.582	1705.593	1761.545	4388.629	3855.701	4150.213	3112.057	2598.143	1913.068	3124.619	2634.199	2291.48	4319.299	4910.228	4329.518	3441.376	3715.857	4159.327	2152.198	2679.375	2685.938	2867.707	3124.619	3264.887
CUST_53469_PI429010886	M101847	0.00235993	18	3						9285.648	120.8147	9905.822	11572.84	11998.69	13301.65	362.4397	1149.936	1378.754	11339.63	16452.07	12548.11	9999.789	8146.369	9855.129	103.801	60.23194	136.8369	1015.827	2214.163	2224.156	10077.64	11076.77	13301.65	1931.709	9226.663	11395.49
CUST_50285_PI429010886	M89321	0.00235993	18	15						51.82749	76.49879	24.23762	27.47705	13.81373	37.66142	89.99586	83.15856	62.82209	68.82621	28.57269	18.01657	84.45149	75.58915	63.73076	78.73755	95.22353	96.40725	43.86995	18.33066	35.60799	21.47276	45.00629	20.80953	40.83819	24.74687	25.69634
CUST_11411_PI429010886	N27763	0.00235993	18	15	91081549	PREDICTED: similar to muscle pr			2.64E-56	171.8649	141.3865	165.368	90.20095	110.2733	94.30839	91.44099	192.8968	147.7301	159.0077	151.4448	175.9736	179.4936	202.0727	72.22265	200.7515	269.1635	271.8414	182.5021	148.7876	228.7822	138.6664	129.7968	159.2971	150.7849	242.5407	317.7216
CUST_26147_PI429010886	N53750	0.00235993	18	15						351.6631	627.6794	312.1666	275.386	349.2496	333.4754	494.7611	494.606	404.7958	444.046	462.9596	385.121	437.8715	328.6362	365.9284	534.593	465.6755	506.1561	449.3664	412.8294	502.3264	275.9873	357.296	316.6599	380.2708	423.4844	298.928
CUST_31064_PI429010886	M600	0.002367136	18	15	91079214	PREDICTED: similar to histidine t	Nucleic acid metabolism	RNA metabolic process	6.34E-40	10.90671	17.58836	11.9073	8.297607	8.438338	11.57491	10.45264	14.11179	13.84677	16.5317	15.28613	14.82044	18.53553	12.05888	15.17119	10.14305	21.53675	23.60614	11.33295	11.29793	17.89974	3.446905	10.38947	13.17136	20.00439	13.32644	15.30631
CUST_3576_PI429010886	N14797	0.002367136	24	15						253.4358	103.106	192.8085	81.75855	44.18092	62.97498	222.2997	260.5042	246.5526	207.2425	131.2891	94.74514	104.0463	106.7424	63.74337	671.0624	313.739	560.8143	260.9147	304.282	558.6463	109.7929	30.54671	124.0485	187.5559	90.1637	132.1477
CUST_34257_PI429010886	M9182	0.002371094	18	15						58.33297	115.7824	95.87944	70.89953	99.42287	79.25889	44.51618	94.52718	89.21288	50.36291	91.02301	58.9258	59.46532	92.61293	81.4582	113.3227	148.0804	109.762	100.7211	101.9053	96.49913	63.13263	94.63322	51.73911	89.98471	92.23687	60.43161
CUST_37732_PI429010886	M21285	0.002377521	24	15	6831612	RecName: Full=60S ribosomal pr	Protein metabolism	Ribosome biogenesis	8.7E-82	3071.483	4554.072	1576.537	2568.829	1926.392	2466.158	3053.693	2386.8	3654.032	3048.498	2474.336	3215.844	5131.503	4499.92	4368.987	3975.62	5769.472	3838.854	2451.472	5502.319	6612.476	2960.558	3267.789	2992.811	4427.539	1806.683	2710.483
CUST_57057_PI429010886	E302	0.002377883	24	9	429327037	putative cysteine-rich protein 1			4.48E-31	329.3762	444.7023	115.4443	389.1575	1868.336	1290.575	464.5257	551.2795	197.5273	443.355	1191.902	1303.295	224.4256	920.7381	986.164	509.4445	594.8478	653.3386	276.1256	564.3622	332.8862	196.9337	231.374	213.1791	293.6993	477.1875	133.6243
CUST_47028_PI429010886	M74571	0.002377883	24	21						246.3057	233.1508	195.4747	195.4372	191.0167	189.7104	208.4398	182.794	200.181	184.1143	172.4022	114.8959	195.2227	157.7362	184.5933	260.557	216.6807	226.6959	208.8952	162.8755	207.6552	222.1435	204.7184	237.5901	236.0521	192.4063	236.6326
CUST_31425_PI429010886	M1496	0.002380124	18	0	345495075	PREDICTED: uncharacterized pr			2.67E-56	38.55172	40.18962	92.05562	39.53772	43.26054	30.67349	28.3794	39.79062	37.34538	30.00507	37.37642	34.15718	32.18818	31.08176	25.50997	35.20547	43.28942	38.51685	56.51566	43.64769	47.33022	32.09525	34.53535	31.84584	24.33043	35.10272	41.29379
CUST_51207_PI429010886	M92595	0.002381288	18	15						49.16277	45.86771	38.23559	34.86879	62.55614	38.52068	36.29093	29.20612	31.89668	41.21871	53.82263	31.72419	42.96134	40.65621	42.40871	52.27625	75.29284	44.66421	49.07635	40.91214	46.03907	41.83854	34.9059	40.9293	37.14951	33.75905	51.81869
CUST_37242_PI429010886	M18347	0.002381845	24	15	505845202	PREDICTED: THUMP domain-cor			1.13E-56	28.42265	60.13153	38.07838	31.03777	31.23427	23.97898	49.33322	46.56066	56.58591	57.81843	33.75549	28.52077	65.01496	40.26043	45.18702	66.63152	62.21897	60.93307	51.88798	37.90412	68.24011	55.34035	48.75822	60.52841	52.95714	36.14885	64.37351
CUST_39835_PI429010886	M45382	0.002381845	24	3						30965.56	160.766	30141.56	23575.84	21834.29	26299.14	228.253	555.3803	282.7416	16954.37	24199.32	20099.82	19287.83	16252.77	14502.16	82.80797	106.5406	178.596	570.2732	937.1039	797.7145	16411.2	8042.108	16221.54	1102.848	2306.791	2656.978
CUST_2208_PI429010886	N11874	0.002381845	24	15	321454284	hypothetical protein DAPPUDRA	Protein metabolism	Regulation of translational initiation		946.146	1913.068	1196.459	1082.664	1009.169	1038.012	1538.316	1663.655	1574.714	1421.92	1146.806	984.2262	1177.475	1324.412	1119.882	1803.597	2350.317	2188.815	1325.644	1505.065	1567.333	1069.78	1262.927	1233.546	1228.932	1281.397	1235.811
CUST_3763_PI429010886	N15223	0.002381845	24	15						2978.418	2887.827	2516.066	2271.439	1915.575	2363.552	3528.773	2369.531	3112.057	3327.482	2192.968	2598.143	3620.969	2252.908	2761.678	4224.791	3122.414	3492.822	3522.391	2416.236	3255.97	3120.055	2469.347	2983.608	3272.412	2566.656	2367.057
CUST_259_PI429010886	N3151	0.002381845	24	15	156554747	PREDICTED: S-adenosylmethioni	Metabolic process	Generation of precursor metabolites ar	4.11E-166	1253.748	2881.653	1975.778	1600.243	1349.624	1217.388	3060.056	2930.842	3327.482	2103.097	1667.986	1625.527	2082.19	1765.847	1794.235	3580.711	3516.69	3390.028	2249.486	2281.476	2782.49	1470.868	1801.332	1854.805	1829.191	2154.965	2463.526
CUST_41801_PI429010886	M58587	0.002384623	12	3	242003910	conserved hypothetical protein			3.28E-35	14.61747	36.93269	20.67803	26.90354	140.9846	68.78391	7.901772	6.24358	9.149353	3.072009	10.9183	NA	NA	45.5523	57.9144	25.62847	34.23772	26.4274	7.614422	12.24496	19.46213	NA	3.647433	NA	3.268042	29.22381	10.97416
CUST_35197_PI429010886	M11868	0.002388572	12	3	321478381	hypothetical protein DAPPUDRA			1.97E-17	42.96548	68.51645	55.92368	68.95833	58.05667	114.8083	98.38918	89.26114	94.7296	67.89364	75.39478	84.42785	78.57224	77.59604	44.6542	101.522	101.2406	121.3713	83.00392	56.87317	65.60638	76.19255	50.48397	70.69946	61.34753	47.27657	51.55894
CUST_16066_PI429010886	N35591	0.002391757	15	6						21.33315	29.64873	26.76018	30.69787	29.61912	23.23426	119.4647	39.58685	40.23682	44.6225	32.64246	41.36333	29.8647	39.12873	34.2465	33.81651	40.71656	30.66752	30.9435	27.50147	41.80733	44.16317	53.36513	49.364	34.07334	50.0221	62.03291
CUST_53474_PI429010886	M101861	0.002395619	24	21	242023499	conserved hypothetical protein			1.18E-23	77.38763	79.85553	50.45617	65.17942	79.82778	61.40643	70.74232	72.3378	74.4544	70.54441	55.42058	76.70125	52.46241	61.23583	55.72212	84.45954	56.24698	77.26866	61.85717	55.53179	68.26223	94.27306	89.35207	81.56927	77.03004	81.21161	66.67393
CUST_3254_PI429010886	N14114	0.002397878	21	15						389.3624	357.3731	392.9019	302.6134	324.8189	259.4124	369.0567	406.1674	411.0555	397.5976	427.5284	318.8359	340.8616	377.2326	287.3062	577.2434	457.8873	563.4896	351.8456	425.1755	473.7195	388.382	305.6416	343.3314	397.867	298.6917	355.0829
CUST_35715_PI429010886	M13288	0.002410542	24	15						283.6079	634.2073	377.4146	325.4289	346.1837	341.4287	586.3741	534.4784	555.7599	486.7832	486.8888	392.253	433.3852	382.4457	375.7494	651.2334	813.2524	672.9412	464.677	447.3237	499.0568	419.6327	388.875	447.5258	424.9756	389.1575	464.5257
CUST_30998_PI429010886	M416	0.002410542	24	15						1811.687	3393.901	2416.236	2051.901	2031.77	2034.424	2491.674	2978.418	2805.21	2245.768	2149.415	1468.759	2335.81	2278.847	2517.49	3577.841	3441.376	3699.413	2237.142	2799.9	3137.141	2206.592	2634.199	2887.827	2491.674	2634.199	2853.1
CUST_2180_PI429010886	N11803	0.002410542	24	15	524908000	PREDICTED: 60S ribosomal prote	Protein metabolism	Ribosome biogenesis	2.13E-33	21645.19	27347.94	26740.38	21113.92	16847.05	19572.59	28306.31	26987.8	28306.31	22658.35	19341.82	22154.05	22401.51	22154.05	20871.55	28743.72	30721.41	31435.61	27918.92	28210.59	31206.9	22279.42	23739.57	22279.42	28492.44	23084.2	22598.48
CUST_2598_PI429010886	N12729	0.002410542	24	15	307198170	Putative protein tag-53 [Harpegi				284.5651	335.0262	240.5209	254.4938	251.2891	225.4711	460.1149	306.0384	361.5772	335.8993	253.9457	301.1497	388.9715	243.5905	339.9532	497.6488	404.3238	449.9406	397.4943	271.1207	351.92	309.2002	273.8482	294.4308	381.0871	261.4619	360.5101
CUST_18472_PI429010886	N39862	0.002410542	24	3						148.4641	165.9817	381.849	182.8511	411.2361	436.5051	100.5529	136.4438	223.6033	76.61576	372.3645	344.4852	119.6589	352.5932	296.3981	30.58943	46.86356	40.26358	207.5504	115.908	104.437	160.5413	166.5631	160.8211	132.6428	162.5329	152.4772
CUST_10185_PI429010886	N25834	0.00241449	24	0						135.8928	84.28713	141.7833	51.5265	98.78131	115.8934	91.82394	71.72818	157.8268	66.84176	85.97141	81.97833	59.03583	26.69535	69.41702	181.6282	88.29653	112.3401	81.80549	82.72734	108.9478	83.4747	182.35	64.82247	259.1614	116.4561	251.0374
CUST_18654_PI429010886	N40175	0.002415482	21	6						65.01967	39.2334	68.62585	214.8629	182.136	65.85526	225.0517	147.6402	131.0265	141.4438	122.754	167.6224	78.40447	81.19478	79.46471	52.39131	171.4108	116.3642	71.94693	60.0628	50.0221	94.62002	53.77161	73.15055	66.19633	79.17186	58.86475
CUST_20408_PI429010886	N43385	0.00241662	24	21						46.71131	123.9057	95.78807	58.3569	69.45852	63.75599	44.44554	67.62577	75.47204	45.45721	69.86885	77.63531	38.12957	68.74221	44.12566	84.5309	96.91497	88.80408	94.67757	85.73084	97.45374	82.79094	109.5487	107.6151	102.2878	118.8436	96.54391
CUST_38267_PI429010886	M24833	0.002421227	18	15						64.29833	70.23956	57.45443	51.92132	47.79385	52.05686	66.16604	80.38647	59.59396	60.5679	37.64058	67.71277	55.32819	59.58718	59.62625	95.58893	80.62092	93.73127	68.70749	49.19586	65.04883	52.43555	56.09226	51.75972	53.74902	61.95862	45.55903
CUST_34173_PI429010886	M8947	0.002421227	18	15	242000802	AP-3 complex subunit delta-1, p	Transport	Protein transport	6.68E-34	47.53635	79.27327	72.29255	36.72221	64.56632	58.6831	65.72695	83.18277	49.99888	56.57986	72.91519	76.04559	63.17461	59.42381	67.15403	104.5179	79.88277	79.54469	45.66053	57.44712	64.95625	45.142	58.08676	50.89474	56.20706	60.52841	62.40045
CUST_38012_PI429010886	M22771	0.002424481	24	18						224.2885	98.93161	260.3413	329.6536	222.3298	41.47761	363.297	203.057	498.4242	161.7929	210.955	146.7041	945.2143	286.1798	487.5393	250.5426	52.79948	271.4195	714.0141	2277.337	2971.539	1014.541	1073.164	1606.909	589.7452	322.6893	1042.748
CUST_33937_PI429010886	M8293	0.002433798	15	6						136.3336	207.9512	222.1435	182.7293	275.7847	175.4084	278.9665	229.9746	299.7411	211.2323	249.1748	269.5152	216.6056	201.4635	179.9541	170.6985	338.6752	217.0918	221.9252	253.5695	249.8271	261.6417	359.5095	323.747	289.4524	230.3006	217.0918
CUST_17684_PI429010886	N38408	0.002433798	15	12	193705824	PREDICTED: ankyrin repeat dom			1.25E-19	14.89459	22.26642	12.57527	4.697614	6.773043	6.700321	15.67594	15.82957	12.40824	16.13855	15.80202	13.27761	35.04281	9.782412	28.3901	14.08999	20.11384	18.92052	14.86987	9.671582	7.946301	15.92281	14.68671	12.90391	17.96997	12.62329	13.36916
CUST_47308_PI429010886	M75554	0.002439239	24	3						15394.16	470.2656	13043.65	17692.35	26056	32454.44	59.70409	1359.053	105.3966	7695.811	13812.43	16221.54	9194.226	14526.85	23739.57	12.53109	27.20363	87.41282	47.19838	840.0505	122.3002	5085.201	998.7674	4928.217	69.55765	1244.709	354.1831
CUST_2984_PI429010886	N13543	0.002439239	24	3	194910179	GG11231 [Drosophila erecta] >g			1.81E-41	651.9522	176.549	862.8898	991.8349	1068.26	935.0962	115.5105	177.0208	175.63	152.4355	765.466	559.9215	215.3375	968.7419	808.8122	125.3559	162.3101	123.7963	167.6224	162.0504	150.9231	306.7699	352.1552	344.2242	163.8342	180.2441	186.6982
CUST_19259_PI429010886	N41266	0.002439239	24	15						140.7023	341.3363	212.2192	158.0988	83.1934	115.6711	323.5934	248.2232	238.5827	305.7009	146.7486	171.6359	188.2081	336.3654	151.5059	408.1119	475.4976	450.1126	233.7126	175.8219	238.2083	190.0165	237.439	175.6041	242.0464	234.7388	181.0435
CUST_20012_PI429010886	N42645	0.002439239	24	21	242023449	conserved hypothetical protein			0.000000199	64.96779	104.9676	76.16048	42.93819	21.0164	49.18092	42.45638	216.222	130.8809	7.925431	35.67533	12.56829	115.7562	59.797	104.7235	110.6039	97.09091	140.5088	107.3228	119.272	111.9491	104.4062	166.7131	150.5856	189.2095	81.91112	138.7794
CUST_1159_PI429010886	N8695	0.002439239	24	15	115382607	Litopenaeus vannamei clone c0(	Protein metabolism	Ribosome biogenesis	1.91E-95	26740.38	33838.77	22332.92	23658.24	20563.31	22092.06	27273.84	31543.85	30577.23	29037.53	24136.69	25403.62	26403.54	24040.66	22332.92	36043.3	31967.8	36195.31	31108.81	30821.46	31967.8	27347.94	25343.01	29037.53	32540.66	24040.66	25703.26
CUST_1440_PI429010886	N9638	0.002439239	24	3						17623.06	931.5266	24341.41	43280.43	36302.66	38384.01	2710.483	4358.067	4234.05	30065.36	34486.72	38719.75	26129.84	23324.95	31206.9	683.1382	712.1062	596.2588	11138.2	7232.62	9889.351	38228.71	21224.39	28743.72	10156.68	20320.15	19903.59
CUST_55735_PI429010886	M111521	0.002449892	15	3						19.12194	7.889571	23.67748	20.83109	24.79569	14.74196	13.31417	8.670444	20.29709	8.85643	19.21337	11.56862	NA	NA	5.848596	7.315528	3.167357	7.164507	18.20758	51.55894	51.75429	10.36053	10.3429	13.36241	10.22494	10.09623	15.87405
CUST_32990_PI429010886	M5644	0.002453591	12	3	28465341	c-type lysozyme [Marsupenaeus			1.16E-35	510.3128	1588.642	39.39293	741.4284	NA	768.2904	1389.498	1151.33	456.2678	613.5208	335.4857	178.3834	1025.772	1442.661	640.5041	3060.056	2355.337	2384.985	702.325	347.0422	602.1887	251.6089	626.641	270.5362	1369.757	1002.076	12.00687
CUST_44500_PI429010886	M66151	0.00245628	12	6						20.95678	14.21628	17.99135	24.1562	19.66532	19.74485	32.66921	20.86335	27.1944	16.85925	12.00687	15.85755	31.03777	20.41366	10.23574	19.99659	24.95817	15.63254	24.03275	34.66846	33.08424	22.19592	21.52336	25.96075	7.854749	12.4284	13.44127
CUST_40596_PI429010886	M55564	0.00245902	21	3						96.1174	172.3736	282.2255	371.6485	309.0132	NA	133.335	168.9237	298.3249	6.623584	47.7609	7.199986	239.0006	196.0361	11.88441	45.75395	NA	49.99479	418.3708	167.0939	314.5854	174.172	307.8719	240.802	564.5167	78.41566	598.2839
CUST_30678_PI429010886	N62372	0.002459362	21	15						23.82015	14.70588	28.27902	13.27288	10.59156	13.56135	42.25498	15.4862	18.39609	43.10891	19.48546	19.41759	42.54185	17.64762	16.10664	43.8169	23.4592	28.75907	28.98015	17.86526	25.13355	20.92431	11.75186	23.81296	15.94932	14.20021	25.01301
CUST_13995_PI429010886	N32143	0.002460231	21	15						667.4336	621.285	840.9882	707.937	568.2556	580.6289	482.5812	627.4387	614.3598	558.8184	805.2136	591.786	992.6472	571.0817	854.1319	861.7915	826.4146	812.7394	663.421	752.0705	782.4302	715.878	690.7831	578.3316	656.7134	574.4065	740.8082
CUST_29139_PI429010886	N59269	0.002461886	15	9						77.25936	57.69989	87.75883	77.32154	62.68994	81.24878	71.00454	56.97506	37.38685	93.15792	83.14687	93.02834	101.1701	89.45518	94.95617	86.38253	89.39537	78.59166	70.89193	73.29648	79.74416	103.9282	77.91051	87.18835	97.55312	89.65818	111.91
CUST_50789_PI429010886	N91018	0.002462772	15	12						13.49972	9.006528	17.21755	8.316005	9.153547	9.615742	13.76968	10.69163	9.048157	13.6932	10.27866	18.97114	18.79764	15.14334	19.8627	14.25286	5.709589	19.56045	17.14108	8.580119	8.880632	11.0716	9.161427	17.78405	18.97529	11.85747	15.87551
CUST_42943_PI429010886	M61684	0.002467808	12	9						32.64246	NA	48.14157	80.25802	24.75929	45.81835	10.69163	31.88585	55.56698	110.1596	166.7131	92.7216	123.6581	37.18271	91.6033	NA	NA	NA	51.80751	48.6277	77.14782	180.7742	207.6552	246.1645	63.40756	81.36078	190.4954
CUST_2579_PI429010886	N12689	0.002467936	24	15	449493118	PREDICTED: Taeniopygia guttata	Nucleic acid metabolism	DNA metabolic process	3.24E-77	727.0088	1396.628	1031.478	723.6856	594.442	537.4657	1280.159	941.7984	977.1077	1208.338	721.1898	793.2617	896.2629	680.2076	723.9274	1398.823	1546.88	1573.941	1118.482	944.508	1089.998	871.1645	881.5382	1010.854	1084.272	823.5364	1037.623
CUST_5617_PI429010886	N18953	0.002467936	24	15	383860221	PREDICTED: eukaryotic translati	Nucleic acid metabolism	Regulation of translational initiation		1515.59	2532.427	1490.226	1480.822	1413.759	1421.92	2026.561	2092.723	1980.694	1761.545	1554.179	1420.628	1774.191	1596.49	1365.984	2514.613	2918.383	3217.866	1981.815	1984.393	2228.732	1596.49	1890.428	1616.307	2123.927	1816.946	1854.805
CUST_10420_PI429010886	N26201	0.002467936	24	3						8204.372	385.6128	4700.854	6864.217	8348.895	7785.133	364.8028	745.1009	1475.969	3767.349	6348.681	5342.932	4388.629	3267.789	2816.959	536.489	181.2834	472.502	3316.279	1766.713	1242.354	5763.929	2285.617	2973.957	2384.985	2965.89	2577.27
CUST_2921_PI429010886	N13393	0.002481934	21	0						372.0362	260.9505	324.6618	301.6948	187.0768	214.0023	143.5591	148.5106	163.6628	266.4734	225.8691	254.0874	258.8256	208.7594	252.8102	294.481	220.4736	297.7548	286.2354	240.1155	209.5237	428.6927	229.6548	357.7047	336.3654	258.1145	362.8242
CUST_11344_PI429010886	N27654	0.002482524	18	15	347967235	AGAP002135-PA [Anopheles gar			3.35E-17	62.196	46.81135	47.10689	35.21621	34.91725	16.75714	46.0811	45.29434	46.59051	37.48759	50.51811	40.72568	36.49885	52.46839	31.74988	63.14073	41.09779	53.56818	30.32287	41.05749	31.54655	26.47281	27.37201	29.39105	40.55409	22.86438	43.93928
CUST_1076_PI429010886	N8416	0.002482524	18	15			Transport	Protein transport		698.508	1091.584	762.8618	634.2073	589.8797	637.6043	797.7145	907.3817	931.5266	720.2377	704.142	710.8823	667.0187	776.2782	689.88	911.7924	1180.485	1099.423	865.141	748.449	823.2974	561.3058	804.3138	601.887	605.4928	901.1852	648.4382
CUST_35503_PI429010886	M12738	0.002483896	24	0						140.387	109.9215	161.0998	97.12878	92.98043	84.80932	96.55775	137.6386	100.1424	99.2507	84.62659	76.25504	75.12194	168.9831	57.8261	62.27316	84.6671	94.60841	113.23	92.80135	100.3016	96.19976	87.33081	94.43966	109.0351	105.0475	127.048
CUST_36952_PI429010886	M17302	0.002484988	21	15						6.970533	134.1449	19.11579	107.424	10.51838	116.4978	240.2302	183.2542	44.88978	220.6244	134.0782	45.80079	288.8335	90.09793	238.7217	372.2898	442.1242	241.9195	75.0839	6.98399	62.78718	66.52473	27.21869	79.58985	183.4751	131.5353	17.0913
CUST_47951_PI429010886	M78268	0.002489658	18	6						NA	6.098024	NA	10.83233	7.982621	NA	12.30677	11.22435	9.753338	5.276347	16.88729	5.129966	12.60227	6.949768	11.32146	10.1544	10.33674	NA	9.331963	3.286271	4.632417	9.925179	9.509852	10.43238	13.5167	19.43327	18.80558
CUST_37601_PI429010886	M19739	0.002491746	15	6	13898841	CUB-serine protease [Panulirus	Protein metabolism	Proteolysis	1.55E-48	2811.043	3899.124	3052.051	3356.595	3932.655	2519.472	6132.817	6079.326	4628.184	4398.359	3862.287	3264.887	2292.947	2968.824	2348.232	3452.681	5482.841	3039.361	3658.191	3506.645	2443.901	3565.017	3715.857	3862.287	2807.452	3532.198	2842.045
CUST_37498_PI429010886	M19245	0.002496633	24	15						10178.51	19341.82	10921.28	9041.082	6826.356	7348.003	17023.68	15942.89	14502.16	12605.8	8713.974	9613.776	11455.94	12255.54	7415.157	17287.24	24040.66	18966.14	13281.09	14828.55	14743.32	10109.9	14161.49	11076.77	11289.6	13281.09	11998.69
CUST_31754_PI429010886	M2315	0.002496633	24	15						177.1691	226.4187	186.9529	173.9289	176.3183	160.3612	197.9185	202.4552	218.2535	211.0451	190.6927	166.9821	221.0635	168.9624	173.9585	240.3058	235.338	260.8728	202.5815	198.7013	234.4388	184.7904	180.1675	214.4735	196.8082	192.4289	249.888
CUST_39033_PI429010886	M33726	0.002496633	24	3						6304.559	117.2468	5726.275	5884.771	12548.11	11289.6	83.62172	889.3882	113.8344	1031.076	10683.23	10835.48	690.0542	9804.488	11859.41	51.46661	53.64587	97.44091	139.1364	785.8536	106.5192	1017.712	376.2092	821.5501	113.636	1020.798	173.1143
CUST_1721_PI429010886	N10536	0.002496633	24	15	307352167	voltage-dependent anion-select	Transport	Ion transport	1.27E-147	4787.419	10532.72	6240.674	5166.879	5131.503	4495.71	7937.877	9272.221	7721.817	6292.839	5244.429	5525.521	5601.899	5907.39	4754.165	9532.564	11179.97	11763.32	7048.129	9111.496	8911.047	5265.552	7100.104	6470.198	7844.789	6956.592	5913.632
CUST_5259_PI429010886	N18460	0.002496633	24	15	321470538	hypothetical protein DAPPUDRA	Metabolic process	Carbohydrate metabolic process	6.2E-149	324.4202	1065.973	551.8317	434.9251	355.7553	393.254	769.305	802.754	769.5468	775.4008	538.3095	459.0911	558.4737	529.0583	473.8372	995.0788	1231.406	1192.533	635.3318	583.7299	763.8013	472.4073	533.5339	576.2705	701.0526	524.4136	593.0437
CUST_941_PI429010886	N7888	0.002496633	24	15	91088233	PREDICTED: similar to Elongatio	Protein metabolism	Regulation of translational elongation	3.78E-25	5666.924	9330.025	5807.4	5131.503	5039.165	5068.287	8086.369	8461.114	7663.711	6922.365	5317.598	6068.533	5502.319	5638.594	5144.706	9226.663	9179.508	10156.68	7316.568	8146.369	8362.147	5847.84	6193.269	6210.057	7653.847	7081.435	6481.59
CUST_11717_PI429010886	N28291	0.002499756	12	9	530415838	PREDICTED: zinc finger protein 6	Nucleic acid metabolism	Regulation of gene expression	1.51E-10	5.419661	8.750703	9.027928	11.90584	4.945161	6.016685	7.091187	7.621112	11.45624	14.41667	9.504797	NA	15.95467	9.317073	NA	4.373352	4.578326	5.210289	12.3024	11.80135	5.247423	16.6895	17.88521	8.958741	8.748029	4.62804	NA
CUST_53380_PI429010886	M101474	0.002507457	21	0						14.78505	16.59861	15.14134	14.28873	11.07918	14.71235	8.829167	13.85925	12.10978	20.13887	13.21287	5.362097	12.53864	8.913392	8.04506	11.7976	NA	7.344785	11.69458	5.072802	4.898624	21.23798	13.06456	19.47777	13.2682	12.54368	13.13409
CUST_46036_PI429010886	M71146	0.002512025	21	15						22.28353	27.45637	19.71754	16.75127	16.03888	9.383476	26.32889	20.465	17.17357	13.30754	16.87953	14.66099	15.77596	19.22794	24.21942	40.16939	35.65289	40.86771	26.9514	22.20311	32.17224	17.69677	21.58672	28.23949	40.57158	21.64165	30.21832
CUST_30948_PI429010886	M288	0.002513172	18	15	321463870	hypothetical protein DAPPUDRA	Metabolic process	Cellular amino acid metabolic process	1.07E-161	66.22878	96.81529	42.80977	46.1895	38.77869	42.83599	80.5628	75.29284	67.34563	92.05562	60.2163	62.367	70.55138	60.77638	61.42131	69.50462	101.4339	96.22172	69.72385	51.09282	60.35053	41.1126	47.7452	53.06387	91.37677	69.72385	68.17964
CUST_47881_PI429010886	M77998	0.002514591	24	18	170819724	reverse transcriptase [Daphnia p			3.93E-11	17.38217	24.54621	20.92153	14.37747	17.95536	13.06086	16.28686	10.75824	16.10664	16.1297	19.55672	30.78396	17.35195	16.06184	19.55672	23.29768	23.05545	18.23431	23.58453	26.64196	23.80751	16.09389	23.14702	31.52497	24.42377	24.08402	20.21086
CUST_2188_PI429010886	N11826	0.002514591	21	0	459937334	putative TRPL channel protein [F	Transport	Ion transport		1282.861	1425.394	1321.031	1206.945	858.51	839.7101	773.0893	844.6913	950.0092	1231.406	898.0277	860.9694	1331.886	926.2026	1100.835	1064.673	1113.622	1307.316	1346.472	1125.5	921.74	1720.907	1121.925	1238.715	1765.847	1221.709	1671.759
CUST_56667_PI429010886	M114381	0.002517544	24	6						399.7084	320.7176	519.2716	251.7959	331.0671	304.4972	713.6664	939.0464	843.2459	450.3327	433.6142	341.5157	226.4187	408.0121	403.2127	367.8176	1616.307	202.7406	295.7504	254.3008	219.0705	337.2109	293.4022	297.7548	361.4833	396.7957	313.2232
CUST_12037_PI429010886	N28818	0.002522064	21	0						202.3915	127.7162	152.6639	120.7183	140.8978	128.9375	115.2129	123.5107	101.1701	136.1215	109.4578	112.7891	147.3274	110.2733	107.9954	117.494	89.01154	125.8093	190.0165	107.6466	152.8709	188.7199	146.9806	151.7904	229.6548	143.0339	139.8319
CUST_16170_PI429010886	N35785	0.002523504	24	18						7.733714	8.084291	9.269929	10.8487	6.040983	5.320114	5.219516	5.567914	5.508782	6.516558	NA	6.843272	7.153945	NA	27.2697	NA	3.61091	5.534335	14.00531	12.67619	8.529923	NA	12.6791	12.16037	NA	7.934974	7.713675
CUST_27710_PI429010886	N56558	0.00252533	24	15						246.7787	397.3902	274.1568	259.634	198.257	230.4127	406.766	353.6905	380.7227	362.3548	248.9012	231.9279	320.381	239.0006	253.9896	501.6566	508.513	521.206	507.231	316.4278	438.1085	292.8189	366.9286	325.1877	386.3361	332.2617	333.7805
CUST_34188_PI429010886	M9000	0.002528571	24	21						209.2118	252.5592	213.9341	195.082	198.8809	166.2655	195.9869	209.7702	260.9827	235.8737	164.6109	178.1474	196.7017	177.0208	194.5455	195.2973	262.7025	241.3201	189.8339	266.6324	260.1465	267.1988	253.5695	271.9604	239.6808	196.3832	223.3777
CUST_14737_PI429010886	N33379	0.002539893	15	12	189239442	PREDICTED: similar to Nedd4 CG	Protein metabolism	Cellular protein modification process	9.96E-53	14.68671	19.06805	23.1348	15.37441	15.01335	20.55348	20.28589	21.9474	17.14735	21.60709	26.35385	18.99955	21.65776	30.82949	23.66239	17.07151	16.9401	20.78179	10.70343	12.28032	17.927	13.54908	21.44037	19.97369	20.20024	16.12609	19.83069
CUST_50990_PI429010886	M91709	0.00254176	12	6	47078721	trypsin [Marsupenaeus japonicu	Protein metabolism	Proteolysis	1.13E-10	5.354125	5.066072	6.393169	135.5353	NA	123.018	1026.869	NA	707.6592	232.0765	147.5974	NA	35.4467	130.8809	41.03485	313.4731	NA	259.9818	1708.051	4928.217	8439.929	305.1178	NA	286.6404	1185.739	177.0792	1050.367
CUST_10106_PI429010886	N25709	0.002543624	18	3						27.82474	15.5366	17.74472	24.31278	23.13824	20.7057	42.24367	25.66507	22.83375	15.68492	20.61088	9.404259	20.26109	13.85925	19.66181	13.77125	11.51019	17.01558	21.45343	18.73426	20.6613	27.6893	18.8838	29.24902	21.12176	11.07276	20.141
CUST_13621_PI429010886	N31488	0.002543624	18	6						139.4798	180.3689	148.6211	95.28229	151.4852	167.708	171.6067	227.9064	279.4339	126.6578	116.9965	170.3275	143.9614	146.2442	213.853	169.9743	123.4188	87.87185	224.5862	160.5413	97.42977	154.2231	236.8356	171.0275	181.8118	263.4037	265.8169
CUST_33597_PI429010886	M7378	0.002544066	24	21						308.3526	426.5559	339.3438	295.0388	152.9638	238.167	334.4355	286.1798	264.4742	302.6134	194.1916	162.7381	231.0141	236.3014	165.9615	570.2732	284.6216	466.8411	462.8312	202.2179	419.5373	607.8959	358.6508	407.5835	353.1568	252.1994	302.8616
CUST_1992_PI429010886	N11255	0.00254572	21	6						67.52243	79.03209	57.27204	142.5641	110.8779	169.515	295.1045	160.623	130.0053	118.9736	76.59647	144.6203	122.3466	115.4156	93.71999	153.0882	148.4641	107.509	97.42977	107.8435	101.5332	141.2484	85.09948	94.64399	122.8685	114.3521	115.9671
CUST_1393_PI429010886	N9500	0.002546143	12	3	270014040	hypothetical protein TcasGA2_T	Metabolic process	Carbohydrate metabolic process	6.85E-151	174.1457	271.4195	111.355	509.1116	62.5781	415.6373	560.3033	283.7827	366.0882	429.0423	162.0267	245.6327	159.9962	298.3249	58.00342	1038.012	464.7851	672.5292	249.2196	137.9056	229.5682	251.2891	171.7821	198.1577	58.34712	57.94141	57.77356
CUST_5883_PI429010886	N19330	0.002546174	21	21						10.28626	51.19908	30.75559	19.83269	42.31229	31.92217	27.59053	31.19486	29.00837	39.6417	40.87561	44.83771	42.76898	61.39667	51.32137	44.54779	54.03718	51.96584	23.19392	30.76277	44.93169	37.96647	36.29792	43.24498	42.76535	27.66729	30.46326
CUST_17179_PI429010886	N37505	0.002550752	12	0	383847601	PREDICTED: microtubule-associa			1.83E-08	44.56209	39.90993	49.30286	41.3785	38.34449	45.80079	28.47997	28.23103	33.34498	28.55402	34.76743	41.38957	37.25229	26.31789	50.45124	33.78234	31.25385	35.10272	35.02108	25.1445	14.5233	35.80613	36.86782	29.99634	46.78844	29.71923	46.13913
CUST_30860_PI429010886	M32	0.002554027	24	15						523.1285	1073.581	762.1289	539.0498	439.4254	498.9218	870.8828	811.9435	906.8146	752.6445	578.3316	595.8749	702.1022	579.5063	601.1087	859.0835	1179.914	1149.369	709.2377	783.4167	913.1879	563.6439	681.8481	694.1978	655.1561	669.5828	746.9967
CUST_19976_PI429010886	N42546	0.002555064	24	15						5.33569	8.409122	NA	NA	9.858804	8.91225	5.464473	6.78016	6.814859	NA	10.05212	9.053925	7.133224	20.84466	NA	10.35947	30.07738	13.02177	14.46618	13.74572	11.81562	8.984476	6.338566	9.14743	4.509462	11.4117	5.022765
CUST_490_PI429010886	N5629	0.002564163	21	15						1519.636	2239.116	1837.894	1765.847	1686.38	1462.785	1440.472	2120.795	1766.713	1339.398	1586.354	1431.337	1445.709	1696.829	1269.839	2104.842	2855.927	2831.186	1802.44	2249.486	2526.145	1447.238	1958.772	1758.225	1661.512	2105.784	1656.675
CUST_40738_PI429010886	M55970	0.002574469	18	3	371591223	Euphausia superba voucher 702	Metabolic process	Generation of precursor metabolites ar	4.23E-127	1377.595	43.59878	2085.588	2611.542	3664.856	2965.89	68.70749	309.878	265.0552	1023.228	5407.94	1787.383	1138.567	2315.807	2418.009	32.95886	47.79385	20.91286	434.5642	482.1823	482.9421	2251.39	3046.537	2437.73	408.4889	1687.279	1448.453
CUST_15641_PI429010886	N34907	0.002575082	12	0						173.4147	100.9354	177.5447	84.4188	70.75071	89.53408	58.87877	70.19469	87.42396	74.90044	93.8595	84.55241	151.1486	58.87877	106.506	91.33505	78.72715	88.35558	82.60618	89.95321	82.68763	172.8084	99.83532	131.8851	116.3326	105.2562	146.7041
CUST_52040_PI429010886	M96176	0.002579905	24	15			Metabolic process	Carbohydrate metabolic process		28.09462	32.1629	30.78396	19.16061	36.75862	42.92276	17.25943	55.64654	44.9365	33.62389	30.905	38.71639	41.14081	50.91261	45.31745	131.7371	61.65559	126.0731	80.38647	74.70021	80.30375	43.0146	28.34861	53.14113	81.87749	58.05667	44.53304
CUST_6524_PI429010886	N20214	0.00258041	24	18						44.73116	29.94695	46.21146	50.18213	42.35304	43.39655	28.99688	45.89143	35.38316	35.26049	35.26837	33.98225	41.20908	35.77451	41.66708	48.27587	40.92496	46.35836	54.00515	52.16829	54.5198	67.84273	31.9432	59.64761	60.50917	37.00354	60.12159

Probe ID	ID	p-value	Period	Phase	EBI ID	Annotation	GO category	Go term	E-value	0	0	0	3	3	3	6	6	6	9	9	9	12	12	12	15	15	15	18	18	18	21	21	21	24	24	24
CUST_12913_PI429010886	N30255	0.002582087	21	3						19.91267	17.88948	42.24729	52.541	57.31291	36.72586	34.53973	40.7495	28.47997	49.75454	37.72807	42.14237	19.32331	26.34051	25.68422	14.40443	40.2472	25.02499	23.53464	22.01591	27.2558	46.24985	26.71617	30.80841	26.85809	58.86475	54.79166
CUST_13078_PI429010886	N30536	0.002582243	12	0						209.8119	226.8625	227.2167	198.1201	201.6125	161.6606	157.2569	149.0714	170.9048	211.6596	196.6443	230.9439	173.9043	160.4657	203.4662	185.653	194.2319	198.8809	152.1194	191.7176	164.6109	213.6621	216.5044	237.0207	237.0529	250.4996	288.9345
CUST_8586_PI429010886	N23262	0.002582724	24	3						608.2737	282.5034	641.6007	669.9449	608.1132	694.1978	276.9819	428.0259	380.35	330.1913	499.3569	532.1418	435.0369	490.2242	703.6419	179.0994	281.5287	224.2885	288.7179	404.7958	346.6392	447.1876	453.8518	436.3894	289.8261	396.9983	448.2125
CUST_14953_PI429010886	N33750	0.00260093	24	15						130.496	113.4282	123.0785	86.16832	83.99404	71.31082	97.51431	88.75	97.44091	106.2889	71.69067	95.35371	108.7311	104.8028	127.4883	114.2304	160.8732	125.6606	109.9215	113.7264	97.2956	90.96558	101.8646	124.3455	114.3521	93.11317	110.5322
CUST_57151_PI429010886	E1735	0.002605118	18	15	242007134	elongation factor 1-beta', putati	Protein metabolism	Regulation of translational elongation	2.31E-13	5311.077	9837.56	6410.91	5244.429	4560.074	5015.024	9013.443	8451.427	7111.995	7316.568	4919.057	5769.472	6385.128	5442.505	5311.077	9819.956	10699.09	10109.9	7731.941	7353.952	8160.518	5110.752	4700.854	5593.889	6913.964	7558.705	6676.906
CUST_37752_PI429010886	M21387	0.002605118	18	3	149675211	cuticle proprotein proCP5.2 [Cal			2.53E-77	25403.62	814.0506	24659.23	34877.59	35738.11	31206.9	2168.846	4258.14	4398.359	26678.14	40595.56	24721.57	28210.59	16874.05	30352.12	721.1898	679.6938	561.2356	9179.508	5744.161	10260.07	37213.84	28943.19	38060.46	9392.481	15731.96	20871.55
CUST_18091_PI429010886	N39123	0.002605118	18	15						61.03293	78.76138	31.41902	32.68105	27.83033	33.97586	69.06598	52.51146	53.41659	48.23892	37.14951	37.22016	51.44911	52.11743	38.00971	85.95962	52.0293	68.57845	52.17564	71.14732	53.21955	24.40409	33.95966	32.5401	36.26082	58.16712	29.39693
CUST_46372_PI429010886	M72422	0.002606052	12	0						15.08312	20.71687	22.95726	11.53299	15.50088	12.36534	16.07832	12.51777	14.83387	10.67194	10.8257	9.048157	14.21806	14.84278	16.35547	25.13355	15.64425	22.96223	5.758808	14.67376	5.831039	8.457185	8.891795	13.88944	38.12534	14.57595	27.42998
CUST_37376_PI429010886	M18825	0.00260611	24	0	256539086	fatty acids binding protein [Fenr	Symbiosis, encompassin	Viral process	1.79E-31	2777.197	4109.069	3069.207	2721.255	1401.718	1767.886	3336.354	1910.884	1926.392	2706.184	2204.749	2130.512	3301.049	1363.959	2047.134	1514.569	1971.443	1125.978	2775.294	2477.377	2906.899	3346.038	3052.051	3356.595	5329.024	2816.959	5756.98
CUST_30853_PI429010886	M5	0.002612562	24	18						5119.273	10294.69	6845.752	5311.077	5578.907	4569.485	2981.297	5394.696	9013.443	10641.47	5402.577	7886.969	8736.825	2915.434	4928.217	10966.45	10311.23	10209.43	18320.9	12317.37	10550.02	12236.09	10193.49	10727.23	22777.74	5456.239	14137.09
CUST_9166_PI429010886	N24167	0.002614191	21	0						243.4049	261.9111	157.4954	172.2349	136.5218	129.6173	196.526	107.2724	120.4647	223.1865	135.1327	201.9179	125.679	111.0263	109.2995	134.6275	219.3902	143.3201	127.1117	132.1793	184.1934	241.668	207.9921	204.3998	236.43	129.3215	209.6968
CUST_15100_PI429010886	N34009	0.002614468	18	15	322789712	hypothetical protein SINV_0326			1.11E-13	50.69492	63.67982	46.1196	47.28223	53.5836	46.52035	61.16761	79.84725	61.83411	52.46839	60.48555	51.68671	54.6004	56.9001	155.3994	64.97632	132.8203	96.80429	40.7495	63.61175	67.88505	33.08424	52.01796	47.91548	58.28361	52.29097	45.23829
CUST_36602_PI429010886	M16332	0.002615777	21	15						47.88919	63.77213	16.66622	74.98208	12.11179	72.47632	141.9587	68.71816	62.09142	93.91412	88.84654	38	122.0941	76.98432	70.06906	261.0851	296.5339	153.1388	115.6931	16.82245	91.15714	33.26372	51.33814	38.41822	118.1166	96.44195	14.31736
CUST_40227_PI429010886	M51899	0.002624188	21	9						57.20137	27.39273	13.82768	46.85381	35.1903	56.76432	64.54113	44.80668	50.03686	99.57709	57.51072	62.44379	101.6403	39.53772	48.7032	63.51585	41.12558	53.01786	41.56038	34.1983	44.99422	42.60881	31.45923	43.86651	46.57555	33.99771	43.74346
CUST_53660_PI429010886	M102676	0.002635766	18	15	284027780	trypsin 2 [Panulirus argus]	Protein metabolism	Proteolysis	1.17E-11	18966.14	20616.2	16288.46	10699.09	11503.97	10897.12	22332.92	28414.17	23379.75	21758.66	16913.15	16981.94	17623.06	16553.17	13837.12	26803.66	21645.19	23739.57	16622.66	18235.97	20099.82	16014.56	12359.97	16151.95	19522.5	14764.65	20172.23
CUST_5350_PI429010886	N18577	0.002644266	24	18	260796465	hypothetical protein BRAFLDRAI			3E-14	52.9338	107.0876	102.5191	56.31298	62.19095	46.74583	77.53014	89.39537	85.99964	75.12894	68.74221	62.03768	95.65727	78.92328	68.33852	88.71852	107.8039	103.7748	80.26815	106.7719	129.1152	81.36078	113.187	117.4173	81.42279	97.22863	95.70549
CUST_9172_PI429010886	N24178	0.002644266	24	3						31.97992	40.92496	28.48764	44.38354	59.55786	52.19714	47.32106	39.73245	31.60141	29.46145	59.51724	38.18197	37.03848	42.90506	33.43972	25.87711	42.71727	30.6656	29.79615	26.11949	33.81651	37.94596	30.6774	36.02118	26.32889	42.50386	42.69941
CUST_55561_PI429010886	M110723	0.002645219	24	18						1958.772	3584.754	2588.32	1698.955	1969.061	1628.405	1158.095	2171.504	2185.802	2239.116	2114.8	1712.689	3331.869	1999.848	2915.434	3272.412	4383.744	2952.044	3706.021	3073.818	2734.019	3862.287	3085.208	3090.222	5233.042	2113.55	3608.266
CUST_23630_PI429010886	N49167	0.002648474	21	15						33.24054	60.53935	41.65139	60.37856	49.89765	68.30948	51.21191	57.24758	52.07351	61.2068	56.76972	8.822371	73.07238	57.95506	60.79074	136.6363	87.43473	92.66549	45.97159	41.65544	58.31899	41.62236	36.30672	48.04838	60.74813	72.5233	43.7275
CUST_36997_PI429010886	M17495	0.002648869	24	15	13898841	CUB-serine protease [Panulirus a	Protein metabolism	Proteolysis	1.28E-67	102.8659	72.85755	68.99631	156.9654	24.44847	146.1482	316.7134	233.8101	273.8856	501.1967	285.4767	46.14458	277.8871	200.5182	173.3886	709.6774	283.351	751.2377	252.5592	552.9475	649.1337	254.3008	44.89359	168.564	403.3152	173.3886	225.5927
CUST_43195_PI429010886	M62375	0.002653097	24	18						48.49422	75.24855	80.47316	37.23401	35.18743	12.89756	61.78991	42.69638	71.17214	56.38348	47.39716	33.44592	65.48356	43.57748	43.6037	83.4637	67.97894	97.14951	84.02947	72.4917	67.50363	62.65711	60.98236	74.37918	61.74773	37.68424	84.47977
CUST_45481_PI429010886	M69244	0.002654822	21	18						20.59845	26.25684	28.6389	9.547929	21.95424	4.797128	11.78676	14.42283	22.99386	14.06396	25.83296	27.65161	21.74375	14.98899	20.75814	22.52975	25.08449	24.62618	27.23388	23.99679	31.87047	9.095729	25.46423	22.60509	4.151518	18.76293	21.74621
CUST_38063_PI429010886	M23139	0.002665961	12	0						1230.895	662.7709	1143.807	1298.045	570.8807	565.424	488.3385	467.6907	869.9307	938.2916	923.7499	711.5947	2787.074	1227.139	2204.749	270.6947	605.6304	540.2756	707.937	659.7683	801.0558	1718.114	1187.586	1033.091	1007.681	581.4931	1670.335
CUST_23709_PI429010886	N49331	0.002666414	18	15			Protein metabolism	Ribosome biogenesis		33508.11	45257.85	40415.13	26299.14	28025.82	27665.22	37765.06	34757.71	36571.4	36724.45	28210.59	31801.71	30257.2	34173.92	29122.17	40024.21	40217.51	43955.79	33838.77	36571.4	44112.52	24136.69	30577.23	30577.23	30821.46	28561.19	35607.66
CUST_9151_PI429010886	N24141	0.002667861	21	15						125.5382	141.8013	111.6675	117.9281	107.567	92.13972	129.7428	107.3799	102.4481	138.9837	103.5272	130.2225	110.145	243.9595	274.6135	166.5631	141.8724	135.9219	155.9816	109.3497	110.4109	117.7425	94.00187	125.7133	121.9377	100.8009	103.8712
CUST_5812_PI429010886	N19230	0.002672023	24	18	321463625	hypothetical protein DAPPUDRA	Metabolic process	Nucleobase-containing compound meta	8.36E-133	260.9147	563.4896	340.6386	247.0114	419.8124	338.3225	338.9144	385.6128	479.9831	269.4056	314.7808	318.037	332.0817	298.3765	337.5198	347.9647	869.3099	526.2718	442.7601	679.6938	937.1039	441.3596	466.4341	570.5532	340.3327	313.2232	445.1432
CUST_33304_PI429010886	M6546	0.002673801	24	15						23.97222	37.50107	24.40118	46.81135	29.43929	41.03485	47.7452	43.85961	35.42699	53.20126	31.45292	43.07048	57.8427	41.96551	37.31263	54.47023	86.7231	47.30405	37.95004	36.15228	37.20732	28.42265	44.6746	44.43625	42.92276	36.16345	24.38824
CUST_42887_PI429010886	M61517	0.002679523	24	21						12.48201	12.99289	15.78726	6.805347	6.233337	13.89687	7.733714	9.468977	12.14749	15.62048	11.78126	10.5385	22.69623	16.35396	11.9832	17.94579	21.43699	11.33953	15.1083	13.93184	15.17465	18.57337	17.26844	20.57756	15.0661	10.60676	16.24245
CUST_21846_PI429010886	N45963	0.002679523	24	21						30.81966	24.73949	30.8769	25.82769	23.96123	25.69634	18.68212	18.9132	25.30391	28.30304	19.99659	22.08061	25.94465	26.06422	30.74499	31.75441	23.56774	31.94579	28.70137	18.7439	28.16926	32.31005	31.50944	31.92217	25.05534	23.8982	37.55747
CUST_5412_PI429010886	N18666	0.002682483	12	3						315.6063	335.6891	376.9694	370.8194	360.6967	298.928	294.7488	272.9604	286.1136	287.4579	309.3501	240.3468	299.3012	370.6325	233.6058	569.7522	605.4928	708.4537	395.9506	528.2931	684.1238	225.3383	230.9439	272.5621	632.8123	546.5288	441.3596
CUST_56455_PI429010886	M113840	0.002687267	12	9						242.0929	28.11739	406.8432	284.1348	172.0707	188.656	41.83378	141.1162	242.8747	369.5842	275.293	291.198	490.7221	268.3054	446.9571	15.48991	NA	14.70794	177.7913	301.6948	333.4754	884.1094	991.5499	1010	210.3322	299.5341	614.2194
CUST_3014_PI429010886	N13604	0.002689111	12	9	241999376	ubiquitin protein ligase [Ixodes s	Protein metabolism	Cellular protein modification process	6.53E-34	153.8272	134.5506	165.6234	222.1435	144.474	165.8233	119.5461	115.6711	107.2961	250.621	575.8046	213.2149	264.7345	169.4561	221.2277	73.22433	71.13619	64.64642	121.2679	94.96672	102.9729	265.4524	291.7534	315.5362	132.3978	288.7764	348.0379
CUST_45766_PI429010886	M70222	0.002697063	18	15	345493892	PREDICTED: protein spinster-like	Transport	Transmembrane transport	1.28E-18	33.55937	31.43117	23.37452	17.51582	24.25396	26.93456	34.59167	28.3093	30.46011	41.53886	19.93149	33.06459	36.08683	25.06692	24.89861	51.43184	33.2143	38.02922	25.49204	24.4152	26.5296	22.87607	21.73011	26.41747	30.77357	30.21832	27.97412
CUST_39368_PI429010886	M38452	0.002702914	24	3						578.1548	9.759969	569.7522	1179.476	724.6231	1201.304	NA	6.692466	NA	205.0491	436.0075	365.7666	300.9971	375.5874	587.3135	4.255823	NA	6.324357	NA	20.15584	7.350212	388.4776	379.3272	533.6749	10.53196	196.1132	265.6845
CUST_3349_PI429010886	N14331	0.00270563	21	0						399.6202	590.9592	NA	129.4034	NA	156.1141	248.6699	166.4205	229.7477	5.544575	6.065608	NA	29.80233	382.6455	25.35952	498.4242	255.3925	149.9126	507.9246	37.8045	399.4473	NA	676.6048	NA	14.94968	47.84693	NA
CUST_36706_PI429010886	M16598	0.002706212	24	21						201.7698	200.9398	229.393	201.155	189.1836	198.4395	185.8563	187.8136	178.6434	183.7151	201.258	197.0569	236.8696	184.7436	224.9241	189.0814	245.7379	199.1806	180.5334	191.2458	187.0996	264.1326	248.9012	231.2123	246.6369	214.8056	308.8707
CUST_51834_PI429010886	M95322	0.002707973	24	9						5002.917	3911.515	4819.372	4727.546	5068.287	4114.144	4772.224	4445.119	4680.36	5191.892	4193.691	5162.169	6470.198	3614.192	3327.482	2371.181	3835.495	3098.696	2264.823	4207.659	2926.153	4279.69	2858.309	3120.055	3717.963	4077.152	4326.077
CUST_19547_PI429010886	N41759	0.002708206	12	0						21.70891	23.26403	31.17381	30.00811	26.0838	21.82274	14.65727	19.95043	21.3859	20.76565	21.60709	22.67946	27.38458	21.0526	34.15718	21.06899	31.85963	28.94147	26.72462	18.16224	20.63528	28.92548	22.07129	29.53009	33.46312	33.72798	56.07467
CUST_15292_PI429010886	N34343	0.002708297	15	9						139.5639	265.0552	237.835	159.4235	213.3048	209.4303	204.1061	228.7481	244.6553	271.2533	308.9438	326.5385	238.0667	256.2719	264.3686	338.8265	337.4051	303.4901	252.989	249.0801	182.2908	321.1394	278.7033	326.5385	365.989	303.9295	385.121
CUST_3454_PI429010886	N14537	0.00271478	24	21	326781695	crustacyanin subunit A [Fennero	Transport	Other	2.29E-30	2097.341	336.6729	2122.683	2988.845	917.5193	1323.872	203.3258	563.3118	822.6543	945.6613	1019.08	706.4981	2037.287	65.08113	1512.062	343.7299	1509.047	204.2969	4238.105	1111.297	2844.645	9285.648	1909.699	7518.482	4453.231	993.1872	2494.519
CUST_25524_PI429010886	N52570	0.00271478	24	21						886.7077	735.2877	706.8768	662.0947	730.7886	636.7343	680.4062	547.6961	654.9488	885.5204	547.6961	769.0192	916.1745	565.6114	595.0044	821.2893	575.6253	665.873	822.9225	667.0187	781.7261	1032.593	994.081	942.2704	909.1828	645.2626	1075.892
CUST_16414_PI429010886	N36198	0.002719114	21	15						79.28417	65.17942	NA	48.5088	9.257058	52.8767	72.43858	38.30361	48.02051	22.48894	27.32212	3.696889	49.49501	205.2018	42.3598	237.2821	127.579	71.31082	151.3538	4.906349	90.53932	NA	108.7551	NA	25.56436	38.2274	NA
CUST_20849_PI429010886	N44184	0.002719322	21	15						138.8656	277.8871	50.51216	202.8718	80.53824	167.3834	357.575	263.9734	153.7917	281.2628	197.6734	85.41835	205.1165	263.251	211.4988	444.244	395.5893	362.5984	282.9282	53.33823	229.0326	106.0881	175.8857	83.65467	207.1791	185.4321	40.20336
CUST_48395_PI429010886	M80127	0.002727711	18	15	371591236	Euphausia superba voucher 760			5.72E-101	26.42112	22.01591	23.34681	8.4248	14.2083	11.50838	14.71023	30.19163	22.9911	35.69023	25.56702	9.994225	17.19685	14.27815	15.06825	26.74791	26.83914	27.68187	19.1981	19.63566	17.85602	14.51258	8.38931	14.79429	27.87602	17.43861	19.98159
CUST_3523_PI429010886	N14681	0.002731697	24	15	390331875	PREDICTED: early endosome ant			3.99E-27	30.35094	35.82162	33.22428	23.8475	34.79855	34.58873	34.35183	46.25621	47.70295	46.43451	49.70798	45.17758	32.70202	49.22608	43.57254	59.55786	42.29499	50.26433	39.75608	37.24532	52.70656	48.00737	36.08683	47.32579	25.92125	33.48088	45.56749
CUST_10858_PI429010886	N26906	0.00273359	24	15						166.3542	240.2645	195.7159	187.3336	179.6371	199.908	197.4942	213.9775	190.289	199.0025	177.4557	219.7957	200.0099	180.676	160.2997	257.4304	257.3902	253.8231	215.0483	235.8737	245.6327	204.7458	227.4568	221.0978	204.3998	199.6163	239.5431
CUST_20147_PI429010886	N42888	0.002736176	24	0						13.71572	64.08368	63.9762	15.82642	9.810166	NA	9.633908	20.40329	35.86186	23.62454	13.95642	14.30221	10.30511	9.464633	6.662217	8.395174	15.33232	10.45084	15.69192	40.51232	24.64222	5.877513	18.66636	16.69734	50.8191	17.34255	43.32608
CUST_39257_PI429010886	M36788	0.002737937	12	9						4012.41	45.70271	4536.055	7993.785	3640.441	4981.65	663.421	689.88	1306.636	8996.679	10966.45	5258.496	11998.69	4302.905	8400.194	16.62679	23.16272	48.31065	2168.846	1816.946	2797.556	10569.33	10017.83	13154.56	3731.866	5427.368	8016.887
CUST_43606_PI429010886	M63489	0.002737937	12	9						36571.4	15394.16	40915.77	51269.31	15942.89	25555.67	26491.1	28210.59	30721.41	44722.61	50283.97	30257.2	42662.49	25130.01	26740.38	4545.107	8098.961	7506.653	41258.32	36302.66	35157.95	45964.24	49895.87	38228.71	31289.71	46458.58	32454.44
CUST_2058_PI429010886	N11448	0.002739696	12	6						7821.557	9410.039	7270.382	11289.6	6515.192	6854.464	14439.79	11719.99	12765.38	10248.36	4247.1	4885.181	10260.07	8631.841	6233.119	10532.72	8929.115	9855.129	13222.77	12068.94	11503.97	10641.47	6109.222	10337.3	9466.456	5202.819	8238.918
CUST_41746_PI429010886	M58445	0.002741492	24	15						32.54957	29.4237	34.09055	38.82932	55.97133	21.17352	41.57926	43.73952	40.48622	44.14843	49.91567	38.19516	36.48955	47.69014	40.7495	62.96898	54.96965	80.54573	31.12893	55.35616	55.15568	47.92699	44.76312	64.26444	32.5401	30.75945	45.74936
CUST_43004_PI429010886	M61827	0.002741492	24	0						174.4633	122.1528	127.8402	87.64548	111.105	103.4258	122.9878	62.3082	121.1493	97.40632	88.01649	60.16049	132.4149	90.28649	106.8102	59.77062	105.9309	49.96679	83.86117	101.7744	90.14554	113.222	126.8065	89.76139	181.5475	104.2473	178.896
CUST_50520_PI429010886	M90126	0.002741492	24	9						29.24032	27.77493	47.45455	41.51635	54.02691	49.63135	49.39254	29.5453	41.86801	51.27875	51.62156	67.01762	44.19365	63.28348	60.97733	50.0221	36.69203	50.64118	26.18926	20.19611	32.57078	46.34194	39.60825	44.29702	28.26373	19.98159	43.99536
CUST_35597_PI429010886	M12985	0.002747114	18	12						395.8718	387.8304	395.4956	198.5315	318.037	217.9731	320.3348	478.8721	354.2621	380.4577	378.6955	406.254	379.9317	454.3052	444.5929	330.6996	426.5559	423.7011	342.3913	373.2381	319.3342	327.1238	374.1555	360.3638	298.135	298.3249	472.1134
CUST_24423_PI429010886	N50597	0.002748479	15	6						10.72911	8.58312	10.98328	18.27547	16.71379	9.237704	12.55746	17.17582	12.11646	9.526147	9.321353	12.34692	5.38307	7.140165	6.13309	8.664483	4.127575	17.12756	9.959247	9.221685	10.59156	16.65836	13.38008	9.695023	4.415227	12.97694	7.575524
CUST_56353_PI429010886	M113575	0.002750037	24	3	32527554	Squilla empusa 28S ribosomal RI			6.18E-116	389.5477	226.4976	352.9709	430.2082	633.9987	787.5865	193.4185	511.8036	156.7032	134.7853	240.9716	238.3483	122.8067	239.9273	77.01155	91.22685	169.34	115.921	49.13607	615.1114	51.49011	124.2158	289.3915	110.8928	68.30948	618.1517	73.54748
CUST_32327_PI429010886	M3809	0.002750037	24	21						919.6173	1510.508	1260.62	891.7043	778.2044	868.9348	431.346	841.994	1043.605	1179.476	1048.592	538.9124	1058.187	821.2893	1348.208	1584.461	1152.159	1218.815	883.7364	1279.64	957.3975	1573.941	1328.27	1582.625	1971.443	1155.769	1758.225
CUST_11116_PI429010886	N27292	0.002750037	24	21	66556185	PREDICTED: exocyst complex co	Transport	Other	1.97E-63	38.03345	48.92732	45.93656	44.03643	33.75549	44.72435	31.40589	44.29702	35.92906	42.2771	34.79855	39.34641	44.32333	44.49549	38.86376	59.35684	45.53057	49.65645	48.99281	32.36331	49.15533	51.22586	47.06434	52.14659	59.01526	44.03643	55.01217
CUST_22522_PI429010886	N47183	0.002750037	24	21	158285793	AGAP007367-PA [Anopheles gar	Transport	Neurotransmitter transport	6.24E-17	137.9543	143.2463	184.4707	103.7292	109.2373	140.9846	100.4112	120.4803	132.329	173.2168	93.52245	144.4115	140.2946	101.4001	144.5963	184.1477	109.6621	178.1474	187.3503	151.8112	146.093	197.4226	155.8206	149.769	183.9971	168.4596	163.5142
CUST_3036_PI429010886	N13652	0.002754827	21	0						636.7343	890.6088	731.5275	562.0064	541.0757	512.0349	488.48	663.421	646.5974	580.9599	623.7953	536.0532	585.1407	410.1393	426.5559	578.8472	805.8552	696.0096	674.8039	608.6613	746.9967	687.4827	739.9176	673.8771	645.7283	617.4977	655.1561
CUST_39963_PI429010886	M47548	0.002758063	12	9	157129412	hypothetical protein AaeL_AAEL			2.19E-17	1731.406	174.5649	1661.512	3050.493	785.0219	1395.332	450.4965	726.2724	1127.159	2355.337	4101.099	1666.955	3182.56	891.7043	1555.177	72.46811	105.3222	112.7999	1083.233	958.9028	1875.312	2855.927	3243.823	3426.429	1393.878	3699.413	6240.674
CUST_30203_PI429010886	N61380	0.002766872	12	3						31.62493	35.55192	NA	34.92495	29.67188	33.12003	30.02336	35.83339	20.84244	17.51375	23.33045	4.974069	33.95212	32.51285	14.64332	39.57166	16.04505	35.95603	25.68152	28.13497	29.65224	7.266309	17.87527	5.689801	19.95043	33.4664	17.11411
CUST_11182_PI429010886	N27399	0.002769135	18	9						10.16523	13.93184	17.91933	29.4515	29.51504	21.2594	29.66835	25.91577	21.54067	29.12453	23.60789	31.64075	20.57756	19.95957	24.4203	24.29655	12.08147	22.53586	15.71978	16.73229	18.24693	37.03485	22.5248	23.01448	23.30032	14.71575	29.0511
CUST_6727_PI429010886	N20520	0.002772862	24	3						54.21756	28.27682	24.2956	27.87953	55.8305	72.0781	18.83308	NA	17.15077	NA	6.710675	15.61685	16.88502	23.35468	60.27161	19.14033	5.085926	5.952255	NA	11.49713	NA	23.08008	8.922458	26.32557	NA	47.00541	NA
CUST_12797_PI429010886	N30067	0.002774124	21	18			Metabolic process	Carbohydrate metabolic process		387.249	162.844	197.3862	252.2559	81.67118	121.8217	455.7266	770.1221	642.5938	479.3697	244.266	80.09123	624.9341	374.6189	180.8767	1094.513	757.8952	920.7381	1288.168	682.7328	1341.551	250.2264	24.75929	172.9954	402.7185	138.8656	142.8894
CUST_28887_PI429010886	N58790	0.002776964	15	9						1965.01	152.4015	1999.848	2911.669	1896.629	2037.287	297.4214	301.7541	308.4293	3036.167	4383.744	4145.789	4097.003	1455.905	2867.707	144.474	123.6404	108.3364	423.8193	367.2975	454.7075	2698.998	2996.567	3393.901	680.9887	2710.483	4093.741
CUST_13399_PI429010886	N31113	0.002785294	24	3						160.389	93.26197	63.24067	257.5954	860.5197	706.2599	163.4271	198.5715	123.6581	36.63747	458.4811	794.9446	45.36684	466.4341	438.9261	34.99565	31.33292	24.22486	7.017134	105.3966	16.17475	79.31123	85.90563	68.26223	10.0854	272.3474	26.20838
CUST_892_PI429010886	N7721	0.002785294	24	18	383864352	PREDICTED: RNA-binding protei	Nucleic acid metabolism	RNA metabolic process	3.13E-135	237.6384	307.9283	288.0498	280.4572	198.7013	196.1132	227.9494	264.4742	268.6876	337.6971	231.7623	229.5267	387.4512	226.0802	295.2364	284.1348	388.382	290.6243	379.2367	301.1497	343.906	350.791	305.9094	331.9294	364.3837	319.4595	295.973
CUST_49782_PI429010886	M87090	0.002788198	24	3	343082698	thioesterase superfamily memb			6.27E-09	62.05097	64.06806	71.58388	75.45664	68.51645	66.50532	39.05992	64.05318	31.74591	45.44328	53.09289	37.77333	44.53859	37.87395	37.17059	42.4761	49.5489	60.79882	57.7126	54.49956	41.15865	41.66708	37.60365	51.68947	46.85381	52.09654	73.77771
CUST_38066_PI429010886	M23150	0.002800292	21	3						258.2061	394.8687	319.0038	567.0251	468.0876	440.1079	386.8087	508.513	458.2399	383.7931	286.4183	310.9083	429.0423	439.0112	309.3501	344.6427	542.7794	285.8344	305.6416	284.8685	412.5149	412.3443	388.4776	417.734	619.5951	501.7792	424.4332
CUST_931_PI429010886	N7861	0.002800292	21	0						76.73936	114.4623	73.06306	9.45577	20.59574	23.21157	18.09243	26.0733	15.34413	25.48923	63.57042	23.7799	36.1964	37.19414	26.23262	24.75763	54.67791	24.75929	46.48232	41.82858	20.19245	64.70441	31.3559	42.69273	71.03566	26.63911	30.92492
CUST_1553_PI429010886	N10045	0.002802678	12	6						86.53109	94.50451	95.34191	77.07091	63.88807	67.9925	104.6904	103.6807	95.63382	100.4907	83.21526	101.3081	71.76549	69.39785	64.84578	85.56504	86.36335	87.70118	117.9775	96.76253	101.4611	78.08717	89.06904	91.51354	48.73882	78.33025	84.80017
CUST_54006_PI429010886	M104054	0.002804716	12	9						22154.05	2504.226	21459.8	24507.87	20320.15	19960.25	4814.394	6310.155	10017.83	22154.05	32969.41	27832.29	31435.61	16981.94	32639.19	2463.526	1345.001	2304.738	15967.93	11667.94	16106.84	38719.75	33402.18	43701.56	16014.56	17555.23	31206.9
CUST_38114_PI429010886	M23423	0.002804716	12	9						25703.26	1267.497	29725.44	33637.86	21066.26	29037.53	4242.57	6601.274	9669	38228.71	36967.46	38060.46	45502.32	16491.53	35445.2	356.5119	195.7481	620.7278	14670.24	11015.18	14703.33	51269.31	38540.49	50283.97	15435.13	26299.14	39875.67
CUST_49323_PI429010886	M85198	0.002804716	12	9	544735066	FI21229p1 [Drosophila melanog	Nucleic acid metabolism	DNA replication	2.99E-11	183.411	11.56292	135.9631	268.9316	41.47342	70.17999	21.83271	18.3904	19.4293	259.8283	105.1495	151.7139	326.0295	80.43757	64.23608	7.156722	6.146523	5.296825	59.20501	46.05207	42.04361	337.9981	287.2425	274.9461	103.5895	122.5728	200.3302
CUST_10883_PI429010886	N26943	0.002804716	12	3						142.1426	135.7008	11.65578	167.6224	8.734334	171.1487	264.3686	141.2484	146.5474	99.6269	82.67536	12.56829	133.4909	156.0011	121.8066	616.4216	220.2503	268.7721	244.3561	25.97804	208.9613	7.697503	104.1579	23.90534	157.8028	106.3835	13.81948
CUST_50021_PI429010886	M88286	0.002806504	21	0						16.49344	36.01668	28.75045	8.767695	11.05673	23.99266	NA	21.3518	17.25337	11.63004	17.79195	5.583754	6.376979	18.12971	NA	16.57521	19.54566	16.21373	17.54652	23.14702	11.72201	23.5426	31.02823	31.02823	20.91626	26.18151	17.30194
CUST_25584_PI429010886	N52689	0.002807075	21	15						NA	283.224	271.1207	73.16089	81.49613	5.998488	28.93158	194.635	218.2926	51.22586	148.1501	45.9872	163.338	150.4692	36.12473	315.96	374.7855	385.1879	116.0037	108.3596	130.6083	120.5552	7.179669	108.6308	66.04384	21.1126	99.42287
CUST_17351_PI429010886	N37819	0.002808504	24	18						385.6874	409.0189	536.7837	674.2308	465.8295	412.9087	358.0309	324.5457	405.6944	500.4612	556.4241	515.3493	927.7273	611.5217	466.588	689.0797	444.3539	571.0817	848.7243	686.1628	588.5037	820.9036	628.2632	666.3032	737.8151	400.146	634.6359
CUST_49918_PI429010886	M87736	0.002808732	12	0						NA	10.11839	19.69443	5.494001	11.07276	7.639644	5.774867	5.337184	5.697081	7.518583	4.871944	NA	13.02868	6.505387	12.91646	8.265196	4.878987	4.773427	NA	6.328784	5.401098	7.932927	10.99326	6.180949	13.34709	8.03382	9.184105
CUST_6610_PI429010886	N20346	0.002809183	24	0	374842188	opsin protein [Charybdis japonic	Signal transduction	Other	1.93E-120	4945.667	8713.974	7232.62	4685.458	4247.1	3137.141	3281.159	5697.98	4847.527	3408.763	4940.836	3390.028	5202.819	4439.592	4857.819	4616.026	7695.811	5317.598	4319.299	5712.375	4134.923	5609.633	6304.559	5137.74	6481.59	7058.228	8135.669
CUST_34122_PI429010886	M8825	0.002820551	24	6			Developmental process	Other		302.2155	484.1868	348.964	291.198	316.8751	326.6921	483.9464	467.458	484.9644	419.8124	314.1834	326.9964	326.0747	292.3452	323.0408	400.0672	362.9291	288.1795	305.0003	210.8765	335.8125	271.3703	306.4312	253.1207	415.4114	309.4924	372.3645
CUST_6276_PI429010886	N19886	0.002820551	24	18	345488494	PREDICTED: hypothetical protei			3.24E-63	77.54349	147.7301	112.669	89.45518	106.5634	98.89641	115.7246	139.2795	132.7164	116.4725	117.4173	133.7038	118.0906	114.9073	124.9756	120.749	189.8002	156.5402	142.5195	138.9837	167.708	103.5646	110.2586	136.6874	128.8774	121.8684	122.6496
CUST_9231_PI429010886	N24272	0.002820551	24	21						127.3332	152.7399	255.2927	111.7284	150.073	109.8522	125.2902	110.2586	132.8026	141.1162	123.5457	200.5474	185.5286	84.11819	169.729	130.5722	138.0734	182.2041	204.1061	167.7409	187.4758	263.7574	181.5475	299.9228	269.9763	194.2665	294.3919
CUST_14453_PI429010886	N32910	0.002820551	24	6						103.9763	129.3679	120.4953	106.0765	123.4188	125.6969	143.144	129.2736	115.0832	109.8522	122.604	133.0536	91.4929	110.2026	90.27688	102.9161	120.1223	108.6527	118.5947	118.8271	74.93498	84.01764	73.06306	90.69879	116.6823	117.3803	113.4282
CUST_29236_PI429010886	N59450	0.002820551	24	21						67.51408	70.06906	76.75947	57.74953	48.0027	61.11985	61.42589	47.56782	68.67272	68.94498	55.35616	55.77263	77.07091	62.00499	74.21946	88.92336	68.74828	75.09386	95.02561	58.75919	109.9654	84.43872	80.72426	91.6714	132.8564	51.87516	122.9659
CUST_30643_PI429010886	N62302	0.002820551	24	3						3295.67	1261.961	2245.768	3565.017	5233.042	5144.706	1481.716	2777.197	925.9054	2867.707	4349.21	4880.699	1429.079	3879.479	4798.856	692.8899	736.0921	796.2861	511.0453	1747.584	501.0897	2719.085	1522.629	2266.512	627.6794	3010.08	754.2071
CUST_991_PI429010886	N8143	0.002820551	24	18			Protein metabolism	Protein repair		366.3486	529.7211	469.4191	334.9433	344.6427	303.6159	444.4782	430.2082	326.3053	403.3938	350.5935	457.5988	453.8518	354.4742	419.7244	434.5642	571.5137	596.4198	441.4649	458.3577	603.0231	406.0638	445.0179	545.361	477.9381	358.97	601.3269
CUST_44953_PI429010886	M67516	0.002821917	24	21						532.6369	527.0091	667.9516	484.1868	429.8212	331.7854	615.9354	425.2574	388.6637	529.0583	421.7429	441.4649	270.5362	360.2879	305.3804	467.458	583.3052	527.455	618.1517	392.9019	660.6448	594.2073	537.3205	505.8465	783.6564	479.8831	487.671
CUST_22627_PI429010886	N47359	0.002821917	24	3						109.8814	169.9945	174.9928	225.8314	179.7537	232.7013	108.2394	183.6198	157.1328	125.5101	167.1507	92.2128	107.2144	149.1062	96.00931	141.4438	203.8066	134.1808	122.6496	152.5572	109.762	141.0554	132.3442	135.5176	171.1773	182.136	155.8444
CUST_12628_PI429010886	N29770	0.00282252	21	9						23.84404	30.36962	25.07412	26.26876	164.8407	118.1075	36.44264	39.68181	6.875647	55.82058	156.0492	151.8566	23.49323	44.53859	56.54714	18.59104	26.86103	15.47982	11.84234	34.56666	12.52949	23.32264	29.14856	31.19486	44.72435	77.96514	53.33823
CUST_7678_PI429010886	N21905	0.002826923	24	15	242021693	TFIIH basal transcription factor c	Response to stress	DNA repair	1.02E-80	23.52466	35.62359	27.94531	21.64409	31.03967	28.90914	14.73953	25.70782	24.23762	29.66835	22.08061	19.62896	30.99121	34.68278	32.69018	49.27688	35.91186	58.40669	30.60362	29.79615	45.01683	21.91441	26.9108	28.42566	35.40359	33.30353	36.1964
CUST_40797_PI429010886	M56102	0.002827146	24	3						186.0781	NA	201.4635	147.6402	1165.385	677.9076	NA	19.15316	7.800199	133.6073	719.0646	835.0606	59.27468	659.337	871.1645	4.409339	10.37676	12.08661	14.07625	16.74788	10.39496	86.05106	23.19651	72.21507	14.0225	35.04281	28.16926
CUST_2380_PI429010886	N12227	0.002830711	12	3						1158.756	1113.622	203.5144	1268.665	1548.254	1447.238	557.6299	342.5433	66.41804	425.7716	170.0181	460.2218	5.886646	960.8612	17.37448	2779.473	NA	2570.156	953.6942	643.379	NA	44.98933	102.015	89.26977	236.013	34.30861	196.7413
CUST_874_PI429010886	N7649	0.002835422	21	18	255983833	glutathione S-transferase [Erioc			2.23E-55	75.30381	151.8566	64.51751	35.44329	35.19371	28.66752	30.22132	31.37614	23.1537	45.8224	87.48976	66.87814	21.2559	36.96261	45.52672	44.44058	50.03259	55.80638	57.07543	98.21374	177.2962	33.79728	NA	39.6947	59.26363	35.77181	60.58102
CUST_22604_PI429010886	N47318	0.002836786	21	12						103.1504	103.3038	108.0057	129.1403	201.9478	142.8448	129.0291	107.1382	116.6823	124.5835	153.2663	186.3924	127.3506	195.2581	160.9993	107.1873	119.1916	83.59122	103.0566	113.8344	110.1907	104.5613	118.7775	91.6852	115.1679	114.5488	133.4909
CUST_46097_PI429010886	M71354	0.00283696	21	15						44.89831	52.68847	39.30986	34.35444	40.38567	40.88884	61.06467	61.55047	70.06906	62.71091	43.26708	62.61945	54.66763	51.30921	34.11363	72.31873	64.10142	67.72081	52.36478	43.87779	59.28822	44.80668	57.31946	53.58837	54.11401	37.51953	37.86114
CUST_1770_PI429010886	N10658	0.00283696	21	3						538.4885	303.1234	769.305	652.3046	1834.729	1282.861	282.3174	396.5838	406.8432	328.2183	1143.807	1046.212	539.0498	813.2524	1071.939	141.4616	149.3002	173.3126	345.8925	717.7206	363.6624	744.1912	590.6124	695.3717	500.7179	1261.275	959.3506
CUST_49008_PI429010886	M83580	0.002837378	24	15	32527554	Squilla empusa 28S ribosomal RI			2.39E-72	54162.9	64273.41	70857.23	37765.06	49113.82	66456.69	52165.44	64638.29	79747.61	56938.34	48051.75	57453.95	72257.2	79747.61	71692.31	88223.14	82051.33	73328.57	71289.88	60953.29	93147.81	67856.09	61365.17	66134.01	42224.23	53773.55	80988.9
CUST_37087_PI429010886	M17810	0.002841161	24	18						255.2927	188.9036	398.7982	279.563	124.1301	220.2503	176.2281	191.5757	304.2261	304.1662	289.1658	222.2656	451.2333	143.647	357.4621	334.6481	138.3312	291.8297	983.3131	515.2465	592.5764	539.585	399.7084	337.294	533.7924	306.9528	646.8706
CUST_50392_PI429010886	M89675	0.002843236	15	6						29.98633	140.8152	125.2902	18.93239	142.345	133.684	310.7267	360.6967	283.9592	88.35558	52.77429	50.07687	59.80532	42.73341	67.69664	106.1766	96.47869	102.5926	159.5479	96.27921	233.3746	95.88853	111.7011	111.0779	79.92781	70.7916	91.6714
CUST_45876_PI429010886	M70591	0.002844688	24	0	270005635	hypothetical protein TcasGA2_T			8.59E-19	24.68337	23.64554	31.06592	21.99164	24.43634	17.82488	33.59136	20.39056	22.72787	19.65914	13.82118	22.21134	19.80181	10.53196	15.81562	21.14216	43.38217	23.21885	25.56181	23.27971	20.7271	18.82754	30.49064	25.60118	27.14447	23.6675	30.71702
CUST_8126_PI429010886	N22570	0.002848546	24	0	345497849	PREDICTED: prominin-1-like [Na			1.73E-31	121.5342	137.4775	290.3235	132.8026	120.2076	133.1101	115.9325	74.16527	88.02817	75.87682	106.9179	84.01764	192.1066	80.79724	247.4668	90.06479	122.4622	37.74193	100.1424	125.1377	96.40725	130.0819	34.84492	123.4188	223.1865	194.9465	286.5115
CUST_54212_PI429010886	M105107	0.002848847	18	9						7.966091	4.830349	7.664784	5.336459	7.985663	13.05124	12.64397	NA	5.939175	15.31063	13.7861	19.8984	15.12219	7.985663	18.02157	9.027928	14.56346	10.47216	NA	3.997012	6.337569	35.31786	NA	8.969422	7.991764	6.640038	16.65548
CUST_57270_PI429010886	E3439	0.002855361	18	3	224613508	Xanthine dehydrogenase/oxidas	Metabolic process	Oxidation-reduction process	5.6E-18	4.542644	5.047195	14.9705	10.79175	6.969562	41.3785	11.64284	NA	8.628754	10.02234	6.41682	13.26937	7.608242	3.457537	8.818102	6.483685	7.697503	6.316445	11.15315	5.957935	10.05113	15.07942	16.38009	16.44852	11.63897	10.51838	13.2617
CUST_33542_PI429010886	M7219	0.002855808	24	21	242021988	cAMP-dependent protein kinase	Signal transduction	Intracellular signal transduction	2.78E-22	39.59398	42.87131	35.85739	39.31755	33.84921	32.62382	14.78309	23.70922	27.17795	38.39581	42.3598	34.34338	43.18567	32.64017	35.95046	34.04952	33.16894	36.47671	45.89596	29.43929	25.76307	61.81014	47.57931	52.10354	64.94456	50.64118	52.6814
CUST_50655_PI429010886	M90566	0.002855808	24	3						29.67957	28.79058	36.5095	30.59991	62.91277	62.92982	23.8324	33.50951	21.43518	22.75207	45.93656	48.05348	20.85648	30.94126	18.30245	15.02898	17.63683	21.89139	9.868311	23.79152	17.14363	25.89606	24.68878	35.23848	8.768661	20.51167	15.4541
CUST_15151_PI429010886	N34088	0.002855808	24	3						148.6649	94.87741	120.0673	159.3992	176.6026	167.2859	133.196	100.8009	83.08821	88.6659	124.6493	176.1204	140.275	151.6381	151.0875	88.27501	92.88966	72.36145	97.22863	71.56582	72.42124	59.33452	113.23	94.24227	77.23272	126.9272	79.75332
CUST_23459_PI429010886	N48867	0.002864612	15	12						58.94126	43.37269	41.83378	33.14322	26.72167	34.80724	31.82327	33.68871	22.29626	40.06809	39.01005	62.20434	66.65338	53.72417	59.03583	51.78206	29.5607	44.64909	34.50993	35.52825	38.55172	46.13557	39.64543	57.0448	37.82506	32.93904	46.73349
CUST_1366_PI429010886	N9420	0.002864612	15	12	321470487	hypothetical protein DAPPUDRA			5.71E-108	275.6746	241.0942	337.6971	226.6959	251.1902	200.1232	164.9616	195.8396	259.2192	403.4693	229.6548	446.2058	478.2442	450.1126	470.3803	353.0555	284.1966	271.4702	353.5003	243.7665	326.6163	374.1555	249.5189	350.8666	365.9284	340.3327	361.0483
CUST_23949_PI429010886	N49752	0.002865648	24	15						13.16067	14.21449	12.34978	13.85771	6.526253	8.930779	10.35947	11.69612	8.454103	8.652491	8.112296	26.19566	12.82511	6.111488	17.44435	19.29579	38.12224	28.58437	12.38845	22.28576	11.99886	15.50564	14.94547	19.79114	14.67236	7.368945	16.44032
CUST_50566_PI429010886	M90295	0.002869505	15	9						239.5431	289.3393	226.9579	196.3246	160.0414	167.3552	165.6517	218.4783	186.5169	264.3686	270.4208	240.4351	274.4093	181.5475	227.0272	229.9746	243.1902	248.2232	201.7698	214.3188	156.768	290.7575	223.5617	283.9592	264.176	231.626	331.9974
CUST_6591_PI429010886	N20316	0.002872422	21	15	260811568	hypothetical protein BRAFLDRAI	Nucleic acid metabolism	RNA metabolic process	6.27E-95	38.34001	62.46312	36.49381	27.96533	33.98645	21.42114	43.07048	51.80751	61.15056	50.70492	34.60122	31.30624	33.07479	34.73082	31.60141	56.62456	83.07439	75.66353	31.97992	49.35984	58.57419	42.78489	59.16434	49.30286	38.06125	31.37822	31.13895
CUST_9442_PI429010886	N24630	0.002872422	21	0						22.73253	35.71025	31.76584	27.64345	24.21718	13.905	15.929	26.05349	22.09536	15.95467	17.66478	22.38105	23.26132	20.1058	21.56157	28.69416	28.40206	26.32034	17.51782	17.96345	19.40949	37.78456	32.92024	28.45606	22.93584	22.56219	31.37286

Probe ID	ID	p-value	Period	Phase	EBI ID	Annotation	GO category	Go term	E-value	0	0	0	3	3	3	6	6	6	9	9	9	12	12	12	15	15	15	18	18	18	21	21	21	24	24	24
CUST_38801_PI429010886	M30619	0.002875299	12	3	391333258	PREDICTED: uncharacterized prc	Response to stress	Response to oxidative stress	1.56E-25	588.5037	342.9247	736.5204	697.2436	437.4032	621.1319	733.0037	567.7111	313.4731	330.8653	173.4784	62.88893	1193.156	1410.73	803.3036	2469.347	785.3422	2007.702	1214.453	1132.862	2904.283	182.8221	111.371	148.3567	891.7043	239.8291	517.9425
CUST_37696_PI429010886	M20783	0.002876874	24	9						28.3766	14.34832	20.68602	49.06379	26.71901	16.81913	37.50107	18.12289	8.324734	62.35301	29.3468	31.09684	28.84875	19.68364	24.09713	26.12743	15.87729	34.70197	19.78002	16.0845	24.03275	14.68671	17.67979	22.36702	8.293234	10.77751	14.19869
CUST_20334_PI429010886	M43256	0.002881965	24	21	321468336	hypothetical protein DAPPUDRA	Cellular Process	Cell adhesion	1.74E-27	5.344742	21.46191	20.60087	20.46072	23.81811	25.74022	13.30593	25.45723	17.97858	8.26064	16.8271	12.72209	10.49914	18.35653	12.49542	20.29057	23.92281	11.5282	7.660563	25.79038	16.73896	29.18456	31.03967	24.0921	25.74022	26.37524	22.46641
CUST_26856_PI429010886	N54993	0.002882774	12	0						146.4834	222.2656	274.6135	97.97985	344.0033	261.3125	26.80709	75.77051	33.80913	125.3559	343.4733	165.1445	110.45	273.8482	271.6574	109.9399	143.902	77.56319	55.63371	100.7083	56.72705	56.08545	81.29518	88.06844	50.90626	244.2151	290.2762
CUST_27183_PI429010886	N55593	0.00288495	24	18						70.01827	114.5608	104.8456	85.54538	74.56212	61.78991	53.37075	80.02644	87.61045	104.2957	94.17512	108.8028	85.49462	102.9317	87.55235	101.5466	127.7453	137.7249	122.9659	104.0575	110.519	102.015	100.7618	95.27189	98.01972	104.0463	129.6975
CUST_37205_PI429010886	M18179	0.002891064	24	21						20.81559	14.86874	41.14081	17.47911	20.42799	21.37846	12.36397	11.92532	15.1083	16.8613	20.21086	20.32505	16.96414	18.1992	21.98953	30.69521	9.113167	30.96768	24.23607	20.945	18.12496	36.53396	25.83559	26.75452	46.43071	30.445	42.72425
CUST_26518_PI429010886	N54381	0.002891086	15	12	242025134	conserved hypothetical protein			3.63E-17	NA	12.50469	9.468977	3.307438	10.70051	8.438338	10.52106	12.55999	10.17394	11.00269	6.479989	10.54437	70.63176	NA	12.33889	9.194171	5.767359	10.70872	5.349722	5.415947	4.861279	5.339243	5.656717	13.66542	9.724209	16.79206	14.87994
CUST_3611_PI429010886	N14881	0.002900737	12	6	212283656	gamma-interferon-inducible lysc			2.76E-79	NA	NA	4.152717	38.85319	NA	46.91122	743.9121	NA	561.5074	95.65727	58.49962	NA	21.66219	25.0763	34.54981	121.7787	NA	105.242	577.9786	1534.389	3142.5	159.3169	NA	183.6827	473.1245	84.61068	481.3104
CUST_20529_PI429010886	N43614	0.002901747	15	9						30.54671	25.30919	35.06077	21.51186	18.22098	30.54385	24.26872	13.6668	29.21491	47.61057	90.60669	33.80053	30.99993	22.45487	30.71702	26.43885	24.45965	29.24359	33.9482	22.65356	27.43571	38.31063	26.23262	32.3737	35.99333	37.34896	36.42929
CUST_56691_PI429010886	M114428	0.002903323	15	6						537.3205	624.019	508.0836	746.1539	564.2041	484.0256	630.5448	844.8862	962.3475	490.8866	492.3638	473.8372	558.9714	386.7231	394.9633	385.5175	614.0454	623.1184	791.8334	555.8829	443.355	782.6486	615.2672	617.1935	740.5837	515.6628	511.8036
CUST_54188_PI429010886	M105004	0.002904373	24	21						323.446	326.855	329.3762	314.8641	354.1831	309.6955	302.7218	288.0498	342.7829	300.8131	285.8344	330.9231	300.1105	302.7771	252.7366	365.2368	348.11	384.9395	338.6752	292.8798	427.1681	343.8057	368.8823	390.0801	318.8359	394.7866	409.7628
CUST_53520_PI429010886	M102096	0.002904736	21	15	321466278	notch 2 [Daphnia pulex]			1.17E-41	132.2682	102.8763	79.66914	142.8894	96.86942	176.076	227.7173	114.2058	124.3819	289.3393	135.6611	183.8614	202.8062	96.48781	209.9538	397.2907	242.6247	241.4743	180.4189	55.73019	174.3193	129.1955	87.47672	119.6854	198.84	141.753	77.91051
CUST_45136_PI429010886	M68098	0.002904884	12	9						145.1606	25.93791	142.9786	168.511	72.92411	119.4045	42.2265	35.86186	45.68766	381.3569	154.3027	338.6752	110.1325	66.58185	103.1735	11.93224	27.56835	14.7781	47.38682	35.4467	31.92535	277.5468	277.3007	326.855	48.84433	51.63077	93.51275
CUST_46530_PI429010886	M72909	0.002904905	24	15						7.27562	9.033804	9.20883	12.80411	9.796996	22.10929	17.23263	28.93796	13.11503	19.15894	17.59796	17.54	9.779895	12.29048	NA	29.01782	19.2072	20.84897	17.25943	13.52139	13.21801	10.95623	23.99266	7.795202	7.709748	18.53553	6.253609
CUST_25695_PI429010886	N52895	0.002905117	24	9						7.660563	7.606464	NA	12.18092	352.9709	183.5637	7.118638	24.22177	5.873873	NA	152.8076	236.6856	NA	152.9126	221.0978	15.50088	NA	NA	15.59239	7.293154	9.551996	3.854367	4.81366	5.608764	NA	19.52695	NA
CUST_48836_PI429010886	M82401	0.002906501	24	15	428186095	hypothetical protein GUITHDRAI	Protein metabolism	Protein folding	0.000000506	10.06809	10.55325	18.03458	9.445121	9.492246	4.328821	NA	10.64561	7.186563	12.939	12.02014	11.94845	17.34255	NA	12.86215	23.61225	15.74936	11.99226	8.641728	43.4735	16.44032	18.21466	10.99207	11.36956	14.80803	11.92197	9.578088
CUST_22442_PI429010886	N47037	0.002906539	24	3						20.03497	9.180205	13.40089	11.42758	26.16413	25.29001	12.31866	14.92039	15.63819	10.16286	17.89355	20.80218	3.83344	21.01433	15.15512	14.11696	14.91234	8.987086	8.484214	4.452263	8.713271	NA	9.730417	8.544323	11.16104	10.28796	8.176241
CUST_31081_PI429010886	M653	0.002907504	24	18						41.73602	70.70842	58.62146	40.01947	47.18539	46.64063	46.33211	55.8305	58.42497	54.41132	52.07927	54.81901	58.52453	54.8814	54.68833	56.93121	89.5211	82.36909	64.96779	55.99408	86.37483	48.65167	58.38713	64.81491	59.33452	56.37375	69.22757
CUST_10613_PI429010886	N26507	0.002912807	15	0						NA	26.06752	10.97548	7.008745	10.08281	9.659899	9.533566	9.45412	6.443218	8.025098	6.805347	NA	NA	10.56907	9.350524	10.17169	27.74851	14.15522	13.59928	7.482905	27.75587	6.68956	9.203866	8.235338	10.08072	11.57988	9.180205
CUST_38129_PI429010886	M23511	0.002916194	24	15	498935050	PREDICTED: neuroglian-like isofc			3.29E-08	132.2204	572.5541	291.8765	207.4443	103.7748	102.7615	372.1298	177.388	485.8608	300.9346	95.93997	162.2343	351.2785	53.05853	263.0087	623.7953	1269.337	1183.356	399.2455	663.5871	588.164	218.7535	194.3553	289.1658	295.8529	136.314	307.2712
CUST_46859_PI429010886	M73967	0.002916194	24	9						26.60721	33.57662	31.16363	38.086	68.27308	47.71104	28.00501	46.14868	30.13995	45.17249	59.15801	48.96189	43.5104	52.2471	38.76999	36.23648	38.53699	23.79553	34.18125	32.19586	13.84795	14.31392	40.02352	21.32425	28.25712	40.53493	30.26124
CUST_3272_PI429010886	N14157	0.002916194	24	21	260828941	hypothetical protein BRAFLDRAI			1.18E-88	17.38353	39.2895	36.91722	32.66921	34.20262	29.64873	27.2264	36.56941	30.53772	23.71643	27.45155	25.87214	32.26395	31.88159	20.41652	23.4616	51.63636	29.8472	33.86491	34.27669	36.34979	41.54368	37.03185	36.27638	31.68332	32.00302	53.14759
CUST_48366_PI429010886	M80008	0.002923819	24	3						278.0108	75.60649	112.6387	503.6798	1783.882	1239.815	77.78826	243.0739	65.90164	18.79764	954.4926	1695.11	24.53447	959.7319	1296.827	51.74535	18.59557	20.4011	14.90815	259.0205	9.261783	119.9522	74.51372	100.8009	NA	431.0524	NA
CUST_38728_PI429010886	M29691	0.002926321	24	6	238625719	SpAN-like protein [Rimicaris exo			5.94E-19	5061.439	9129.704	7474.416	3485.281	3924.629	3629.795	11206.93	9980.233	10506.53	4900.819	5751.95	6201.489	4934.022	3688.899	5482.841	4461.089	6906.237	5947.47	4450.275	4545.107	3408.763	3104.628	4295.699	3731.866	7577.055	4427.539	4610.9
CUST_42526_PI429010886	M60501	0.002926321	24	21						232.5608	319.7298	283.2866	261.1366	274.0938	219.8774	208.4776	270.2277	229.3211	274.6613	244.1329	274.1568	255.4927	239.5431	317.4998	343.906	337.0445	369.6716	299.4177	241.7527	278.9665	377.1487	279.9809	386.4267	366.7062	268.9804	408.0121
CUST_46033_PI429010886	M71137	0.002926321	24	3	21357745	Glutamine:fructose-6-phosphate	Metabolic process	Carbohydrate metabolic process	1.12E-30	941.0274	501.7792	540.0952	681.2288	760.24	1162.935	418.1662	1062.332	372.5479	264.9858	253.6738	342.0443	297.7147	294.2148	397.9279	219.5016	217.9435	147.6215	119.8645	809.2125	99.74451	269.0492	340.7195	212.598	95.06795	999.9612	63.27624
CUST_9869_PI429010886	N25327	0.002926321	24	9						40.91214	57.39735	49.51848	42.52944	40.99989	35.31786	34.37978	36.95293	45.87769	49.74586	51.68671	54.79166	43.77424	56.734	54.48769	46.90198	60.95576	48.01317	53.12256	52.15113	51.92132	64.69656	54.66218	67.01762	70.48984	52.26773	76.3423
CUST_14651_PI429010886	N33235	0.002926321	24	3						89.57069	123.2141	72.27956	173.7538	164.2684	121.6759	94.20496	96.31678	99.22393	115.7057	123.1444	156.0011	85.07992	94.87741	79.37901	75.50155	128.4972	90.3848	80.16932	41.57553	60.44338	94.94344	112.0615	77.58626	79.06068	73.95531	75.83949
CUST_19751_PI429010886	N42127	0.002926321	24	18						217.8383	268.2532	322.9826	233.5329	224.2885	221.0978	265.1126	268.5317	317.9491	279.305	265.6845	319.3342	308.3526	249.1748	281.9984	334.9433	297.8139	458.9701	325.8846	290.3829	374.7006	271.7825	309.5645	354.9347	351.5196	252.1635	400.7915
CUST_25003_PI429010886	N51618	0.002926321	24	6						107.6017	97.6745	60.98236	114.3312	96.97291	101.8124	102.222	98.40391	103.106	61.51056	91.08467	84.69767	68.70029	97.51431	71.1642	99.47239	75.22137	62.52685	67.82594	78.07876	63.2086	57.14912	74.65174	47.51371	68.31562	87.30779	63.38213
CUST_1499_PI429010886	N9825	0.002926321	24	21						166.4755	232.2882	209.9842	172.9496	161.8178	211.2323	185.4966	152.096	290.7575	185.1715	218.6368	235.6895	210.7377	191.823	230.2547	315.3455	197.5273	220.7429	388.4776	214.2511	245.2781	552.9475	460.1149	378.3539	340.6386	259.9408	199.3145
CUST_7645_PI429010886	N21865	0.002932026	18	15	28465341	c-type lysozyme [Marsupenaeus			4.15E-37	214.8953	740.8082	25.25032	177.4828	3.421294	190.4302	464.4221	474.3448	195.2227	250.2647	138.3312	54.99013	391.3071	872.2903	299.6699	1672.68	1383.977	1249.272	319.2736	279.1951	236.6856	127.3889	337.2109	84.74637	553.926	584.6183	5.144631
CUST_17365_PI429010886	N37835	0.002933405	24	15						19.02985	9.42167	17.49199	9.249852	9.547929	4.882539	14.98899	5.212339	4.276156	35.6426	3.520724	33.65745	39.24334	31.50316	36.14605	14.24201	36.33342	48.27158	46.80566	20.90896	36.03727	14.08189	45.19064	8.110275	23.51913	9.237704	21.66219
CUST_3468_PI429010886	N14563	0.002943346	21	15						395.1137	530.0108	513.5436	295.8529	369.756	352.9709	587.5636	565.179	590.1632	547.8118	461.6561	408.8076	531.5525	496.1354	462.6929	501.5231	655.9099	685.9099	538.6242	380.5457	468.6225	425.6386	521.4036	443.6171	478.9906	392.4757	371.3812
CUST_6005_PI429010886	N19493	0.002945843	24	18						117.7279	108.6739	127.3153	83.68571	74.46936	40.03773	114.8223	78.63104	79.17186	97.21065	73.75221	109.6043	116.9614	86.47683	78.49976	111.766	140.8152	95.74045	95.34191	198.334	128.0875	138.0384	164.6589	146.3365	51.37499	77.43333	110.5907
CUST_7219_PI429010886	N21209	0.002951579	21	18	390331900	PREDICTED: protein SpAN-like [S			1.07E-20	351.1109	320.1464	189.5124	220.2833	361.7315	291.4248	150.6494	401.876	125.4627	412.8294	280.6399	334.1482	370.8194	252.7366	284.6794	392.9019	444.4782	360.6127	453.3141	508.7574	365.8262	422.5065	236.013	359.8294	202.7406	92.12733	204.0335
CUST_2511_PI429010886	N12535	0.002954607	15	0						9.530142	13.14676	NA	9.883782	12.939	8.879476	10.83803	9.01805	5.414126	NA	6.743875	7.812621	10.09352	15.00826	12.01275	13.40595	13.68243	11.27154	10.63475	9.625637	12.19654	6.830046	15.03281	7.295483	8.66264	4.378175	8.43105
CUST_7446_PI429010886	N21564	0.002955602	24	21	291243192	PREDICTED: hypothetical proteir			2.11E-79	77.62481	134.1618	114.3193	71.73966	65.42578	39.93436	99.96271	95.84452	131.8269	75.06278	81.84916	42.39291	110.519	57.56952	59.37073	101.0477	102.6964	88.79346	107.6336	132.1609	224.7434	132.9661	129.2573	166.1819	82.88351	87.97629	156.4112
CUST_18778_PI429010886	N40391	0.002957182	18	3	321476640	hypothetical protein DAPPUDRA	Transport	Transmembrane transport	2.3E-19	29.89602	5.853456	20.50908	37.39308	31.42758	38.39235	15.29086	17.37168	32.42666	12.32049	12.37092	29.07103	21.2447	13.15906	20.02764	15.88853	10.47216	20.74174	27.34627	21.41461	9.877137	32.49678	29.07492	22.54559	39.11911	17.34867	31.7949
CUST_36530_PI429010886	M16159	0.002961578	24	21						233.5705	253.9457	255.3925	252.6661	230.8178	210.4634	192.8085	244.5822	217.5404	248.0205	257.4878	267.1275	248.4015	240.2302	248.1114	247.2845	266.0382	268.2532	295.2364	246.9107	225.4711	301.4388	288.3047	310.6389	324.8189	270.3263	357.0377
CUST_44758_PI429010886	M66898	0.002961578	24	18						69.60581	114.2468	91.43014	67.75299	78.10699	73.9144	73.75221	77.2068	72.16617	99.7036	92.75983	74.05592	81.72083	72.86661	87.75883	85.03859	92.90072	102.4622	120.513	116.6823	109.5188	54.54304	85.76428	83.92194	106.9946	109.1371	103.7292
CUST_2298_PI429010886	N12047	0.002961578	24	18	443688979	hypothetical protein CAPTEDRAI			2.33E-13	43.02903	51.7501	52.60565	44.23244	36.98315	35.99333	40.70251	57.66836	43.21465	52.71315	45.25376	47.22395	54.26863	39.89679	43.86651	50.36291	55.53179	64.78842	59.3504	60.52369	70.39513	40.83819	48.83893	49.18503	53.95129	50.71375	64.92793
CUST_11597_PI429010886	N28097	0.002961578	24	18						88.71852	105.2562	82.63781	74.5042	88.39235	79.60935	120.3932	68.05647	97.12878	124.4898	71.95656	108.7707	91.11591	53.01138	88.75	141.5173	94.14191	120.4309	128.3674	126.8958	133.3192	101.9053	65.6817	117.5411	106.0382	81.43322	127.1117
CUST_14585_PI429010886	N33118	0.002961578	24	21						179.9258	174.8016	109.0351	57.70575	154.9893	63.33433	100.783	196.0361	91.58124	137.6772	183.0069	230.2055	165.5673	207.3346	256.4131	157.0532	258.8256	165.9265	172.5012	190.5906	247.8341	287.8854	266.9539	447.1876	235.6895	140.9846	180.9942
CUST_2934_PI429010886	N13421	0.002971663	12	9						321.5516	97.53962	291.8297	273.556	57.9689	175.7096	167.7764	129.9006	165.2118	404.7001	302.0716	360.8837	588.9309	181.1092	425.957	148.6211	142.3304	135.3048	322.3894	123.8699	316.6599	505.1911	323.0951	450.394	260.752	195.511	450.6028
CUST_34632_PI429010886	M10191	0.002975042	21	9						449.7223	217.3138	436.9283	462.9596	381.5238	618.5099	442.7601	757.8952	480.1172	1355.75	760.24	824.0838	316.5315	583.5463	NA	329.0788	777.0993	545.1995	530.6367	677.4857	186.9009	404.0425	299.8024	398.3951	888.236	455.8434	1285.646
CUST_39595_PI429010886	M41570	0.002977931	15	12						NA	NA	NA	NA	3.711245	5.177049	3.552913	3.055923	3.650634	9.021406	9.910461	NA	9.164016	7.807697	6.208575	5.329949	6.773043	5.758808	3.595434	9.298932	3.170868	6.008078	3.1054	3.032779	6.946877	9.117714	5.843012
CUST_17644_PI429010886	N38330	0.002978808	21	15						194.3126	171.2412	154.1589	86.97617	135.7682	96.36643	206.4029	182.6495	237.7241	181.2265	159.5977	177.5137	157.866	181.7506	123.9222	226.022	187.8778	199.8063	152.0785	143.0807	161.1223	138.8001	144.1169	157.3439	135.3782	132.3442	151.9479
CUST_55627_PI429010886	M111021	0.002980203	24	15	13898841	CUB-serine protease [Panulirus ;	Protein metabolism	Proteolysis	3.25E-23	188.6256	143.8476	128.1605	349.3947	49.42284	264.8027	569.1918	367.0194	596.2588	722.545	380.1903	47.31029	437.6531	316.0187	387.5344	1710.003	603.766	1448.453	1020.042	853.7719	1447.815	1198.741	86.8615	1126.574	487.3173	116.3209	336.6041
CUST_4297_PI429010886	N16433	0.002983213	24	15						25.70617	18.21058	18.24171	20.38307	21.55549	24.12932	18.04324	27.08947	20.25105	25.59906	21.62961	25.93791	19.92947	24.93768	27.05785	25.23036	47.2578	36.92118	21.99164	32.85632	25.25032	20.89958	17.35683	22.52975	19.07677	11.50709	20.82813
CUST_22021_PI429010886	N46283	0.002994954	21	9						9.533566	5.797914	37.03485	17.54	41.82858	24.1058	NA	NA	NA	16.54899	77.69968	59.62083	17.12548	14.74793	35.42699	3.860527	4.320563	NA	NA	6.103744	4.47227	21.65776	22.34076	21.81818	NA	27.89407	14.58115
CUST_5644_PI429010886	N18984	0.00299501	24	15	242022215	Chromaffin granule amine trans	Transport	Transmembrane transport	4.71E-43	532.3756	1341.551	923.1653	662.3233	395.0243	463.1658	1118.881	912.5849	1219.279	1116.065	528.1103	668.1223	929.4814	645.4936	553.1321	1443.341	1268.665	1529.136	1112.702	1271.688	1294.853	816.0394	723.3991	818.0478	1095.073	737.5198	911.7924
CUST_30674_PI429010886	N62362	0.00299501	24	21						9.841307	16.7995	13.0479	16.22323	6.917829	8.886295	7.339187	10.82822	14.61386	7.078115	4.76961	9.879786	11.29903	3.542058	8.74922	11.66418	10.29658	9.878387	10.90094	13.5943	10.09062	13.95828	21.80903	12.65302	8.922458	13.47437	20.60839
CUST_32308_PI429010886	M3767	0.002996835	24	6						118.6368	64.65354	51.00442	32.02499	42.91273	37.92065	85.75696	154.0616	153.2351	48.80597	62.96095	53.23278	48.88152	47.92699	47.23231	50.07687	57.69269	22.35516	29.23556	65.78378	20.52618	29.84906	21.55928	23.16272	46.55494	22.47797	58.82275
CUST_15681_PI429010886	N34970	0.002996835	24	3						12.98896	13.12827	18.07903	11.38407	5.758808	9.35283	10.53196	35.02813	29.65224	21.04763	21.78937	12.27349	22.38961	18.46268	18.43607	9.426819	21.69176	19.84259	26.03821	44.42841	47.62949	25.05534	18.39978	20.74399	19.88527	9.610362	28.098
CUST_31630_PI429010886	M2015	0.003000795	21	18	260782199	hypothetical protein BRAFLDRAI			1.45E-35	51.73381	113.4582	120.6665	31.97696	25.41267	34.06404	45.68256	82.31732	79.01425	30.55564	49.01904	18.37247	41.06867	NA	27.982	57.85506	38.31686	81.38488	129.3499	220.1523	196.8082	61.145	43.99925	50.10131	127.0236	30.9318	45.90556
CUST_18752_PI429010886	N40352	0.003005053	12	9						57.58975	62.04596	57.17098	59.06511	35.7142	39.99568	64.13419	59.6688	62.48283	73.52254	54.28141	76.30972	73.02048	47.90004	75.32925	58.73466	44.49025	60.71646	86.1454	52.10827	70.06906	89.91214	78.67205	105.6867	66.85645	39.06825	80.86273
CUST_29364_PI429010886	N59716	0.003005053	12	3						145.6336	241.7074	68.20088	179.5327	260.6535	288.3047	259.8283	232.7425	139.1591	107.424	134.125	168.9237	141.539	205.8558	167.0808	223.7905	262.1593	212.0595	121.1043	163.6909	119.2442	78.31361	163.3644	67.19251	131.8067	218.9572	26.75452
CUST_55211_PI429010886	M109130	0.003013471	18	15	530723035	cardiac muscle actin [Portunus t			3.88E-19	2807.452	3127.267	3243.823	2646.596	1719.004	2159.534	2225.697	3264.887	1947.583	2477.377	2088.583	1863.699	2650.032	3164.281	1471.771	4513.062	6132.817	6210.057	4027.931	5558.806	7232.62	1329.124	1819.509	1223.105	4181.398	3838.854	3048.498
CUST_18901_PI429010886	N40616	0.003013471	18	3	307715368	arginine kinase 1 [Macrobrachiu			0.000000342	10.1984	13.60764	9.524903	18.73028	11.69775	13.34535	15.39651	15.7122	11.36156	14.96903	10.96336	14.56607	9.389205	12.29213	6.347447	5.659483	8.93938	8.585661	11.76708	10.03167	9.835386	12.14327	15.47982	17.89355	6.545559	7.858193	6.232148
CUST_2219_PI429010886	N11897	0.003016535	18	15			Transport	Protein transport		1071.48	2135.546	1180.485	1075.892	869.9307	1055.306	1897.754	1747.584	1590.527	1557.736	1055.306	1136.139	1194.794	1234.557	1141.401	2291.48	2727.371	2269.737	1392.832	1342.206	1611.643	982.8111	1204.169	1148.933	1601.274	1177.475	1348.794
CUST_3162_PI429010886	N13933	0.003021321	12	0	321462849	hypothetical protein DAPPUDRA			4.03E-14	13.91274	13.73706	43.26708	7.724427	6.088117	7.176989	38.75298	NA	6.257426	11.07393	5.255483	9.550476	19.81642	18.67654	29.77656	6.152029	5.512222	10.93505	11.58271	8.428222	NA	7.37464	8.509638	14.70059	27.08528	11.07918	10.16181
CUST_4834_PI429010886	N17583	0.00302603	15	18						15.74461	33.762	25.52339	NA	9.494876	6.245436	10.22339	21.15973	18.81483	NA	13.51813	23.23426	83.79749	69.54417	86.94104	27.2264	4.42489	25.01036	13.97991	15.3929	NA	19.30796	7.458633	45.07182	NA	11.95545	NA
CUST_43593_PI429010886	M63447	0.003027553	21	9						8.091775	12.62329	11.82408	17.58836	13.6932	8.555797	15.5828	15.50564	21.60215	22.85306	NA	19.84675	7.783571	17.60895	9.904747	14.56607	16.88729	15.97824	8.173581	10.49199	15.60164	12.9826	8.91045	8.888649	15.33898	12.49082	18.58143
CUST_40168_PI429010886	M51312	0.003031839	24	0						154.0154	191.8512	176.6544	136.8929	136.1215	139.5452	102.3977	138.0005	111.3972	113.622	171.0275	135.0024	103.9956	129.5473	130.4587	136.8093	165.2945	183.6198	87.44315	137.7031	172.3438	128.1449	143.3201	200.0099	147.0201	199.6427	244.4333
CUST_1793_PI429010886	N10719	0.003031839	24	0						176.2041	192.4289	199.6427	162.7602	162.8199	160.8514	189.2321	170.2213	181.2265	176.3429	159.0574	162.7885	158.0565	152.1456	122.8685	190.463	194.4561	170.8814	205.9122	157.866	159.6186	201.1205	161.2407	188.2604	201.155	184.5933	199.6837
CUST_45767_PI429010886	M70225	0.003032092	24	3						348.3823	56.32436	332.8862	449.1793	401.4434	381.3569	198.4395	52.95714	24.65307	78.60579	184.7904	202.2706	79.96781	248.0205	264.3178	47.93427	29.98295	23.37085	33.89153	52.47367	17.38916	57.64367	43.35728	56.31897	15.13981	120.9506	18.18791
CUST_26899_PI429010886	N55088	0.003032092	24	3						18.16423	15.64744	9.654225	22.07129	21.58422	22.77225	10.95477	6.664444	7.336788	14.92455	22.46441	18.8606	20.39286	10.40166	15.45147	10.13108	10.67466	8.776321	10.02667	5.808702	3.888398	7.640953	13.99004	8.841399	7.439076	12.15592	8.651473
CUST_26914_PI429010886	N55117	0.003032092	24	21						26.84779	15.14518	27.41265	23.93602	32.67736	27.12484	12.99937	10.4115	18.81696	32.55747	15.29246	33.72798	28.30062	10.52106	24.79273	27.11011	23.79553	21.51875	37.48234	33.77215	46.57945	38.43	43.77016	29.3303	70.03526	26.43885	76.16048
CUST_31526_PI429010886	M1720	0.0030338	24	15	195493694	GE21870 [Drosophila yakuba] >g	Metabolic process	Carbohydrate metabolic process	0.000000175	979.3611	481.8774	484.9644	534.9189	96.58233	345.3559	753.9335	1067.709	903.938	1426.897	630.7177	318.1938	908.7057	611.8901	408.6556	3532.198	1101.918	2761.678	1825.053	1444.129	2528.059	684.5912	31.98442	589.5468	1328.27	295.1045	800.6803
CUST_7562_PI429010886	N21736	0.003034418	24	6	260803087	hypothetical protein BRAFLDRAI	Protein metabolism	Proteolysis	1.12E-69	70.95104	153.1975	77.35718	41.30394	125.1571	42.03616	80.84339	107.52	122.2649	72.42903	117.5817	92.99115	41.82104	128.6956	97.00725	36.08229	71.25639	61.87915	31.28202	34.78692	40.43868	36.79887	51.76488	39.49765	26.83914	45.22389	49.7839
CUST_11871_PI429010886	N28544	0.003034418	24	18						27.30572	39.8934	36.14198	42.98408	33.28409	28.75045	23.91812	19.54299	26.05879	43.76293	24.74446	32.81904	48.69844	26.99962	21.24278	54.55547	64.76383	27.18917	47.69479	48.43357	55.38965	60.78187	38.4343	26.71901	42.96134	38.5634	49.31951
CUST_47829_PI429010886	M77765	0.003038442	12	3						21.32425	32.15717	20.94891	33.6699	21.9474	55.70902	43.00754	28.1721	38.39235	28.55984	20.67357	25.05787	20.23623	20.4836	26.2515	52.31981	33.12003	48.53921	29.37844	21.52992	29.66274	17.213	28.05953	11.82584	30.90184	31.0164	20.53256
CUST_32437_PI429010886	M4120	0.003054193	18	15	321469659	hypothetical protein DAPPUDRA	Metabolic process	Cellular amino acid metabolic process	6.44E-56	84.75661	216.8296	162.5822	101.0477	104.7345	102.2726	160.8211	230.3006	171.6937	119.2267	148.0265	99.30177	99.74451	131.5353	112.1553	190.3933	344.705	291.8765	135.0731	112.2407	131.4508	89.37424	80.13048	98.45312	128.8445	107.935	80.50166
CUST_12325_PI429010886	N29289	0.003054193	21	15						29.92819	19.7274	29.09987	27.94793	19.29579	17.37448	33.25164	26.14108	19.07253	44.97359	29.25965	26.92179	32.57817	26.54663	28.25136	39.68636	32.44868	38.293	31.18313	18.3768	34.67932	17.51375	20.7112	29.91879	21.2559	24.03743	38.12224
CUST_34833_PI429010886	M10836	0.003059273	24	15						38.63208	40.48248	48.21063	53.9262	38.14116	43.01095	28.9184	32.41885	39.76397	54.83706	36.79436	41.76233	44.29702	41.15865	40.76682	64.71994	67.45544	51.94695	65.92976	43.22401	41.24111	50.16621	36.07852	59.47135	50.0131	46.44259	39.08885
CUST_6076_PI429010886	N19597	0.003059273	24	18	391336523	PREDICTED: uncharacterized prc	Metabolic process	Lipid metabolic process	1.55E-106	84.56114	105.3536	123.5992	99.61307	93.3967	91.03605	105.9898	87.40333	112.1553	97.97101	68.10466	88.71852	103.657	116.2897	84.48811	107.2361	94.97719	113.622	126.0246	154.0925	219.3545	94.28531	120.4647	139.8503	133.684	90.09793	135.5937
CUST_25483_PI429010886	N52497	0.003063929	18	6						14.00343	10.70762	NA	17.02795	23.23737	24.00968	34.04608	38.60191	38.35003	NA	9.937052	4.543679	5.764556	8.007057	NA	15.02762	12.64397	5.782025	7.417941	58.99377	9.923975	NA	49.13607	NA	NA	76.45795	NA
CUST_51538_PI429010886	M93902	0.003067349	24	3	391326909	PREDICTED: UDP-N-acetylhexos	Metabolic process	Other	2.98E-11	90.23368	15.7786	90.09793	101.508	129.29	170.064	24.22332	29.42092	16.37588	14.65575	92.60339	110.6946	12.82717	117.7425	122.3466	22.45014	13.11503	25.68152	7.786617	15.26143	10.45873	21.96936	17.96765	20.68983	7.828751	28.3093	12.7329
CUST_7396_PI429010886	N21479	0.003072265	24	9						4.606791	7.705814	9.914778	9.451468	8.560595	14.39646	6.511029	5.397248	11.25938	21.54205	17.94749	27.70466	12.5848	27.63973	23.0693	17.34666	13.56629	14.50907	12.87353	12.97623	11.00269	13.11625	10.79559	12.69158	15.55124	12.78321	8.631384
CUST_44694_PI429010886	M66696	0.003073826	24	15						105.0177	137.1269	130.479	108.1961	107.7528	100.9249	145.9095	104.661	111.7011	157.386	140.6454	149.1062	145.73	110.571	165.8233	144.6793	166.8948	168.5358	111.793	136.2348	160.217	216.5044	127.5138	143.5591	138.8443	135.3782	166.3542
CUST_17138_PI429010886	N37416	0.003073826	24	21						71.1558	64.70441	54.40508	48.8586	60.51578	61.27138	53.8516	70.92326	71.5149	43.52816	48.18525	47.18539	79.37901	54.12459	49.4478	76.94539	54.56309	53.31643	50.44651	84.9728	77.33923	77.2068	57.34418	88.83368	75.88424	53.03534	76.99156
CUST_18173_PI429010886	N39287	0.003073826	24	15	241151381	protein C10, putative [Ixodes sca			5.56E-18	21.38839	17.04459	12.78545	16.8811	9.975583	8.958066	17.6031	30.24945	30.95428	39.4752	19.20962	24.62171	19.39208	22.96223	27.24405	38.03345	41.23754	36.05055	20.05426	9.706075	24.83937	19.99489	26.61351	22.60023	21.7849	23.40563	28.58081
CUST_25491_PI429010886	N52512	0.00307472	21	18						34.54334	23.30899	3.842954	15.72693	8.716574	11.88574	23.8982	6.600616	22.10047	6.74823	NA	5.70609	18.35318	21.65776	18.90899	32.81019	36.20611	21.76196	35.7142	33.80913	31.91889	14.35464	34.74153	25.44962	NA	5.756344	NA
CUST_28926_PI429010886	N58856	0.00308235	24	3	83628250	arthrodial cuticle protein AMP1(			2.28E-11	158.224	112.2997	99.72851	267.0729	370.4445	264.5418	78.41566	74.10459	69.49719	236.013	433.0002	215.0811	184.4435	141.2799	176.1473	26.78121	45.25971	51.92132	NA	45.23287	24.51492	156.0931	105.6776	145.9501	26.30647	97.91705	96.49913
CUST_3119_PI429010886	N13835	0.003090959	24	6	195335314	GM19962 [Drosophila sechellia]	Signal transduction	Cell-cell signaling	6.4E-36	NA	15.67594	11.86483	25.96075	18.59104	24.49326	18.11998	21.17352	25.63724	NA	47.28718	10.47216	15.98963	22.73887	14.36072	NA	20.02186	14.16908	4.059031	4.446727	8.249733	10.16395	14.62836	13.00242	5.774867	4.295357	7.773057
CUST_44718_PI429010886	M66769	0.003095568	21	15						73.79109	47.71104	42.41232	50.72013	40.28089	49.94405	97.93073	64.50416	94.28531	118.653	59.91376	64.32511	127.0776	81.5975	75.89652	144.5615	66.96376	96.26661	77.28495	50.6629	88.51224	75.64874	37.53076	72.88356	78.76138	42.43675	64.1954
CUST_25438_PI429010886	N52419	0.003096878	15	12						29.84906	19.84536	24.52363	22.38744	23.37724	25.63724	14.59197	18.7867	24.95555	30.15056	29.87687	19.83531	30.31787	32.68105	26.06089	27.24094	19.86462	26.16413	26.59145	20.11563	11.5008	23.47308	17.4076	16.58793	29.62856	20.36405	20.0391
CUST_34954_PI429010886	M11156	0.003113234	24	3						252.5113	49.35536	211.7312	493.9591	3558.043	2147.917	67.78024	227.5418	59.81196	32.09223	2580.428	3197.119	37.02719	1515.59	1807.527	30.84927	30.18325	33.9905	31.47125	325.7976	27.18917	49.81307	46.35836	50.75534	36.50276	549.069	32.0098
CUST_37726_PI429010886	M21269	0.003113234	24	15	238683720	Palaemonetes varians ribosoma	Protein metabolism	Ribosome biogenesis	8.69E-59	22598.48	25611.3	22946.38	19855.57	15435.13	17345.96	26740.38	21758.66	20822.29	23820.74	15798.02	20563.31	21758.66	21066.26	17345.96	27347.94	31289.71	28561.19	23273.41	26403.54	27577.78	17061.92	22946.38	21177.49	21974.84	24136.69	23435.22
CUST_40623_PI429010886	M55674	0.003113234	24	21						51.39126	83.4637	81.02819	84.10925	46.26072	55.92368	64.02943	68.4765	73.19641	68.40054	48.03034	61.24535	72.48391	54.14388	65.87993	79.79406	63.62492	96.51035	82.99391	74.98208	87.72633	86.35128	87.14131	93.50035	102.3686	71.91459	133.0187
CUST_4860_PI429010886	N17629	0.003113234	24	15			Protein metabolism	Ribosome biogenesis		40024.21	51875.49	43090.8	38228.71	33838.77	37213.84	38228.71	54598.59	42014.5	45731.6	36802.85	42014.5	39713.59	41258.32	40024.21	58090.3	54925.27	54162.9	48051.75	47342.04	57775.61	38060.46	34757.71	43955.79	44511.54	42014.5	44722.61
CUST_9028_PI429010886	N23960	0.003113234	24	15	546684179	hypothetical protein D910_1117	Protein metabolism	Proteolysis	1.03E-26	24.17321	27.90588	12.08661	9.884796	22.1273	12.99289	26.20838	16.83843	18.64717	9.11873	11.36284	10.59607	28.65291	22.67515	13.7861	18.48727	37.00025	16.86789	22.16839	29.98633	21.13533	33.26748	38.23559	20.13887	18.53553	25.21925	19.31757
CUST_10092_PI429010886	N25678	0.003113234	24	9						222.0168	52.40229	149.3381	221.1265	233.2689	331.4329	111.105	126.859	61.13851	185.4321	138.5532	312.7556	120.1554	68.2334	148.7876	73.07238	27.69715	53.49503	59.26831	107.509	46.03411	183.2834	90.92644	190.829	87.37285	100.783	96.41916
CUST_43972_PI429010886	M64502	0.003113301	18	0						102.6964	75.04432	75.50995	50.04692	51.61367	47.27657	55.90731	34.81137	47.58659	68.32149	64.2158	73.51603	97.99445	44.38354	70.49708	81.53976	43.67914	68.11779	90.78766	58.96212	98.91443	74.56881	48.54356	63.80586	59.40177	34.55816	61.74127
CUST_15006_PI429010886	N33848	0.00311679	24	0						46.13913	47.22395	49.4478	46.46695	40.59088	39.41032	32.21229	46.00245	40.84161	46.15921	48.45428	43.40869	40.76995	41.04967	44.50543	42.6332	32.64017	34.57091	39.04354	44.99721	30.52953	56.29065	42.83599	50.60801	62.66935	52.88884	57.76119
CUST_34098_PI429010886	M8764	0.003125268	24	6						28.13189	76.8759	79.62962	91.21622	61.84336	95.65727	325.1203	52.31438	202.9315	33.31109	383.132	19.33464	104.9377	6.064642	81.53976	8.057556	370.1622	192.5048	22.79379	NA	16.22155	6.582672	5.390022	9.94845	22.67074	11.4924	6.603742
CUST_646_PI429010886	N6533	0.003125567	24	3			Response to stress	Defence response		3739.191	1103.734	3258.199	4253.847	6906.237	5763.929	939.0464	2634.199	880.92	1739.059	3832.521	4761.143	1485.178	3777.656	4715.833	385.0272	636.3867	535.89	395.2192	2183.114	397.3902	2740.835	1429.079	2375.903	355.9245	2666.243	514.2205
CUST_55219_PI429010886	M109166	0.003135589	15	6						95.11745	113.1748	118.9614	95.11745	176.4771	159.2473	162.1178	193.3912	216.0628	173.9842	178.033	102.4622	101.7482	124.8855	75.16291	116.8939	126.9272	155.2093	95.12721	117.7735	112.3401	160.0414	149.9991	219.6103	144.2876	136.8369	112.1553
CUST_45636_PI429010886	M69752	0.003135589	15	12						43.03704	47.55964	50.30791	46.81937	54.12459	40.71158	38.29632	36.64741	34.17741	38.31063	43.8076	48.56063	39.54727	54.81291	68.21622	48.98753	53.96355	35.20547	33.66061	24.28126	43.38217	27.49657	28.80996	40.03773	33.27799	40.32964	47.2724
CUST_8235_PI429010886	N22753	0.003135639	18	15						483.6608	854.7576	366.7062	488.5919	434.8192	441.0424	851.4962	789.0657	504.4324	485.8608	469.6287	482.4747	507.6273	586.5026	527.455	986.649	880.3029	890.6088	553.926	506.6858	599.0901	313.0268	549.679	355.841	503.9395	650.2714	285.6824
CUST_36793_PI429010886	M16874	0.003136723	24	15	429508137	troponin T isoform A [Spodopter			2.07E-41	20.90135	12.13203	19.83531	12.41357	25.41015	16.81592	11.17615	14.50725	7.928135	9.567571	17.04949	22.4839	20.48939	12.1946	11.51601	39.41926	30.70816	26.70669	26.10539	19.81642	27.27378	28.4851	5.971284	21.45886	26.0838	20.93117	27.77009
CUST_3411_PI429010886	N14451	0.003137863	24	18	386376737	hematopoietic prostaglandin D s	Metabolic process	Cellular amino acid metabolic process	1.12E-46	662.9752	540.438	837.7142	558.8184	478.8721	400.146	573.9026	1422.675	1428.28	1017.256	796.2861	282.6016	789.3467	589.1435	574.0759	659.603	964.6221	850.4847	1149.936	1659.781	2240.494	813.5332	678.5721	859.0835	971.1067	578.5106	1083.848
CUST_14071_PI429010886	N32282	0.003144709	24	0	443708401	hypothetical protein CAPTEDRAI	Protein metabolism	Proteolysis	3.99E-66	43.8169	60.18029	75.59966	58.27052	84.98418	46.15921	35.40128	60.89212	57.16004	49.66108	52.15678	53.53275	54.83706	38.11477	52.65281	40.83819	56.50326	66.73428	54.23194	61.85109	79.46471	65.82678	74.55481	77.28495	128.797	85.06849	107.4752
CUST_41854_PI429010886	M58702	0.003149291	21	18						165.0873	523.9454	367.9854	224.5463	237.2505	226.7528	248.7719	203.919	198.1577	209.9538	277.7202	243.2347	134.5963	115.4585	161.175	375.7494	444.046	356.0841	396.7957	312.2172	489.9777	178.2736	341.0171	303.7038	280.5879	265.2295	306.9528
CUST_5139_PI429010886	N18307	0.003149291	21	15						514.2205	780.7545	700.5788	509.8873	419.3654	490.077	491.3879	542.3764	486.4482	548.4345	459.3044	466.0849	492.2464	487.1319	477.5471	662.9752	780.4581	795.5533	552.7385	671.0624	674.2308	560.6594	659.0035	623.6004	576.0861	578.1548	798.2619
CUST_6495_PI429010886	N20176	0.003152642	24	15						679.5306	858.51	852.2653	736.7743	613.0157	640.5041	822.9225	745.5832	949.6232	874.1945	724.4063	663.5871	1051.789	677.6753	729.1294	1029.68	1058.187	1093.204	935.873	878.8076	1124.497	798.0084	966.3163	947.376	969.8589	746.4733	1093.204
CUST_89_PI429010886	N717	0.003152642	24	15						11206.93	157.519	9872.039	14338.96	11112.95	17131.85	245.3228	393.0106	298.7578	8348.895	9855.129	14743.32	3292.245	13737.98	19434.65	51.19385	96.99292	114.2673	176.6026	694.9037	301.9582	4164.365	1510.508	4270.698	670.1687	1720.155	1841.638
CUST_50435_PI429010886	M89803	0.003161316	24	15						19.15475	17.17004	11.32628	25.76854	29.25965	44.23244	28.72644	14.29782	17.54	55.05576	31.57047	41.46154	24.33043	108.9753	42.178	44.18092	49.30286	38.80533	40.88884	39.97592	47.36534	44.04608	14.28982	25.30391	30.61698	24.90578	16.19225
CUST_39632_PI429010886	M42026	0.00316191	18	15						2871.837	8736.825	4185.629	2975.418	2150.693	2696.535	7032.355	7203.647	6068.533	5601.899	3364.257	3031.683	3688.899	3683.886	3014.898	8547.585	9727.143	9954.878	5048.826	5374.176	6060.248	2924.097	3135.16	3274.995	5826.27	4238.105	3721.413
CUST_2918_PI429010886	N13387	0.003162386	15	6	291230052	PREDICTED: predicted protein-li			1.93E-27	63.4939	60.26506	73.70166	92.26014	64.58573	53.15878	614.9534	50.91261	80.77747	60.35053	53.34992	63.42167	58.11342	40.78067	35.88107	44.69425	51.49011	52.78692	74.54603	70.60324	69.49719	68.36775	69.92903	80.65861	67.09453	66.60981	92.2473
CUST_52654_PI429010886	M98332	0.003174235	15	0						351.2056	NA	253.4735	176.4986	170.5061	227.8584	134.9328	105.9309	204.8443	79.54469	119.4159	99.52529	234.9525	14.63519	286.926	117.8894	183.2834	NA	183.5082	201.8328	140.612	155.5805	NA	137.8056	73.50137	140.5662	56.53387
CUST_12524_PI429010886	N29600	0.003175448	24	21	391328467	PREDICTED: SCAN domain-conta			1.48E-36	151.8112	145.3783	188.1589	172.5896	131.5353	173.4439	169.7494	150.4692	138.8888	140.7362	152.3312	111.5466	123.5457	101.4459	136.983	218.6368	159.9962	225.2142	189.5806	114.7172	131.4315	221.3576	168.7741	223.4224	166.6906	157.1085	200.6093
CUST_56119_PI429010886	M112844	0.003176361	18	15	157117627	thrombospondin [Aedes aegypti	Cellular Process	Cell adhesion	7.96E-22	113.0702	124.4898	158.5618	79.81483	115.1679	52.18229	15.15187	179.6371	81.71011	252.6173	150.5856	33.48437	110.3551	100.9354	71.97753	537.4657	224.1099	428.4643	103.9282	136.2737	110.775	184.5044	65.10135	75.13872	110.145	43.31704	32.02499

Probe ID	ID	p-value	Period	Phase	EBI ID	Annotation	GO category	Go term	E-value	0	0	0	3	3	3	6	6	6	9	9	9	12	12	12	15	15	15	18	18	18	21	21	21	24	24	24
CUST_20215_PI429010886	N43033	0.003176361	18	15	321464482	hypothetical protein DAPPUDRA	Protein metabolism	Protein folding	4.83E-42	22.25295	28.7468	24.34231	16.43671	19.47577	16.62267	18.24533	29.19296	26.22059	25.86911	17.83143	22.18214	20.37769	22.71736	22.93309	28.45348	23.7984	27.33627	21.8966	22.58254	21.67518	19.86967	22.28065	18.87895	21.27506	21.23798	20.02764
CUST_22060_PI429010886	N46350	0.003176361	18	15						34.74862	40.74313	25.79299	25.12907	27.25185	33.06699	17.30627	42.47062	36.42265	34.7801	33.3478	44.28109	34.20583	47.44751	29.51504	60.5679	51.2209	42.91838	31.8144	22.09002	33.30723	28.36166	26.01383	26.00178	30.32915	37.7689	48.96667
CUST_10670_PI429010886	N26600	0.003181799	21	9						17.53011	19.33464	25.58573	15.56798	36.67874	27.04259	45.40234	24.71696	31.95397	77.69968	38.98881	49.72483	15.50088	24.87248	18.02618	19.49861	30.3404	20.9383	13.93951	28.76456	14.5111	23.96712	31.95397	24.5682	24.72495	41.23754	22.32173
CUST_27667_PI429010886	N56476	0.00319133	21	15						30.72076	47.41757	41.50033	28.55984	29.81035	38.01913	61.63075	39.39293	68.34584	24.36566	54.61715	33.9635	38.61107	62.4694	40.67888	48.95729	41.14081	62.80714	51.11657	44.85722	65.79182	45.87769	39.64997	32.10035	37.47838	23.10033	33.70156
CUST_51453_PI429010886	M93600	0.00319205	24	15			Transport	Protein transport		88.4446	204.5091	99.99679	112.8635	154.5921	143.144	160.6804	182.4139	167.0939	152.2838	159.0077	132.6815	160.766	169.2478	168.3482	222.5585	347.6198	198.4987	185.0283	164.9977	216.54	158.0297	159.6864	227.9064	175.317	168.9955	202.2179
CUST_5056_PI429010886	N18202	0.00319205	24	15	321465952	hypothetical protein DAPPUDRA	Transport	Protein transport		756.8703	1684.207	1062.332	901.877	852.2653	766.3188	1249.272	1201.304	1433.817	1071.049	822.3417	839.3829	934.3693	958.1681	945.2143	1377.595	1702.984	1710.003	1269.839	1200.665	1429.079	944.8425	1091.584	1265.098	1168.934	936.2266	1219.83
CUST_5143_PI429010886	N18311	0.00319205	24	15	321475505	hypothetical protein DAPPUDRA	Transport	Protein transport		693.3608	1204.7	805.476	707.6592	673.7135	600.4266	955.1294	961.8532	883.0931	892.4292	737.8151	628.2632	915.0007	785.5802	868.9348	1140.288	1447.238	1082.664	941.7984	915.7404	1026.869	606.1742	964.6221	769.5468	844.8862	953.6942	897.3252
CUST_694_PI429010886	N6790	0.00319205	24	15	356651208	chaperonin [Macrobrachium ros	Protein metabolism	Protein folding		1802.44	4461.089	2579.047	1577.474	1582.625	1420.628	3168.826	3739.191	3354.131	2736.432	1835.563	1946.675	2225.697	1923.884	1993.835	3071.483	5571.385	5558.806	2992.811	3046.537	3767.349	1562.544	2988.845	2482.212	2554.596	2499.817	2886.102
CUST_49248_PI429010886	M84909	0.003193474	24	15						92.58214	109.9654	95.57738	87.36259	88.09968	89.73784	109.7774	121.8066	109.2676	105.3095	108.8788	95.73042	110.5494	87.74995	99.07571	119.2089	144.849	133.9615	110.7898	98.71772	116.4262	91.09751	121.3713	81.96005	109.2676	95.9971	92.03434
CUST_37110_PI429010886	M17873	0.003193647	21	18			Cellular component orga	Chromosome organization		164.8928	208.794	234.9939	148.1501	156.0931	130.9346	162.5583	183.411	209.3429	148.3283	143.9807	142.8894	138.3092	118.5289	130.0446	200.2792	220.2176	227.9934	255.2927	216.014	203.392	226.8625	177.388	184.8516	263.4946	167.5116	259.5151
CUST_48913_PI429010886	M82878	0.003193846	18	3						17.47911	6.128343	14.23813	17.61709	15.03854	16.04969	8.656479	10.25273	4.291129	NA	4.724847	15.59925	7.016143	9.700016	NA	6.937084	4.226334	6.5566	13.85151	6.191209	12.54489	20.78429	12.89756	15.73083	18.82944	4.669189	11.36156
CUST_25060_PI429010886	N51716	0.00319942	21	15	291244435	PREDICTED: angiopoietin-like 2-l	Signal transduction	Other	8.16E-33	58.80984	57.78766	89.88767	117.3251	69.97581	25.41267	143.4863	84.09371	195.4747	211.9007	106.9946	95.95298	165.1205	118.606	91.94456	187.0768	135.209	196.4115	126.8388	117.6651	105.242	43.22401	120.9506	51.36995	141.4255	101.852	128.8295
CUST_16706_PI429010886	N36726	0.00320059	15	6						7.335668	NA	NA	102.4255	8.128515	102.7497	33.31109	700.8468	792.599	590.6124	42.68735	481.4474	42.1373	26.85004	52.37742	84.57655	15.09656	92.27655	118.8271	NA	105.0475	1029.68	159.5246	1187.041	41.50033	181.5475	301.8993
CUST_822_PI429010886	N7420	0.003203126	24	21						6010.065	4145.789	4055.849	3956.525	2491.674	2330.806	1701.993	2765.552	2886.102	4998.113	2455.967	2451.472	4487.184	1482.511	3565.017	4020.487	4342.518	1105.917	8565.196	3956.525	5564.697	6612.476	8750.959	4622.321	9425.197	5966.983	9056.645
CUST_13755_PI429010886	N31716	0.003205841	24	0	291235351	PREDICTED: zinc finger protein 3			2.25E-22	39.90127	50.59319	39.68181	24.66091	35.74914	32.60933	29.85271	23.69096	29.98633	24.61357	29.96518	17.12548	36.97455	26.32034	29.03961	42.316	44.1972	54.00791	32.41159	45.66053	40.73959	23.30296	49.46595	38.86376	44.3395	39.42679	56.55385
CUST_43179_PI429010886	M62299	0.00321089	21	15	296033922	caspase [Fenneropenaeus chine	Protein metabolism	Proteolysis	3.43E-19	4.671352	6.388858	3.308888	4.078932	15.07177	8.512367	6.902664	8.112296	11.12081	7.139105	NA	7.234885	15.25955	6.691551	11.65578	NA	19.7654	15.69192	6.246654	4.273535	11.82584	NA	3.643104	4.622854	7.979072	3.367532	3.838746
CUST_56628_PI429010886	M114250	0.003215774	24	6						11.68247	8.056452	11.23132	6.226721	7.934974	34.88562	16.29695	17.02151	19.75521	14.50529	14.41307	13.27388	12.79576	11.54932	9.207701	10.80074	14.74345	5.415947	NA	5.133564	11.71553	8.248627	4.906349	9.465451	19.50817	17.23127	8.487659
CUST_46704_PI429010886	M73458	0.00322189	24	9	321473261	hypothetical protein DAPPUDRA			1.26E-10	18.67988	10.09352	8.885052	14.19525	NA	15.54734	8.531174	10.13898	3.899392	19.10119	19.90569	24.12515	10.69641	20.18225	9.879786	15.84702	9.613655	9.892309	10.16181	5.869146	4.446727	7.31445	4.68783	7.429163	9.38001	4.622854	13.31819
CUST_29312_PI429010886	N59616	0.003228637	21	12	20331094	Bodo saltans 18S ribosomal RNA			1.23E-64	7.112539	10.3429	NA	8.025098	107.5789	93.79597	40.6742	18.03262	26.3515	27.94531	176.3429	214.0023	NA	130.3816	159.6864	114.9651	65.72695	10.22653	32.74097	39.26844	37.61646	11.36418	45.76721	17.9949	22.35703	63.04493	12.3901
CUST_57183_PI429010886	E2187	0.003231458	24	15	119134	RecName: Full=Elongation facto	Protein metabolism	Regulation of translational elongation	1.7E-22	29843.45	37213.84	31659.39	27918.92	23231.4	30257.2	40915.77	42662.49	37083.68	35157.95	29219.06	39389.41	33402.18	30965.56	35305.99	47826.44	41610.17	41419.19	35022.2	38228.71	41610.17	26891.88	34486.72	32540.66	33838.77	33637.86	36571.4
CUST_38417_PI429010886	M26390	0.003231458	24	15	62126070	C-type lectin protein [Fennerope			5.9E-25	684.5912	445.6007	608.2737	576.2705	374.7006	423.3824	2496.293	1405.334	2532.427	3250.257	1782.659	466.8411	710.633	657.7333	480.3553	6945.783	1902.67	6060.248	1019.654	1940.134	2636.073	1165.385	350.9469	790.4703	1471.771	324.6065	909.1828
CUST_31087_PI429010886	M665	0.003231458	24	3						570.0085	130.1154	482.4747	597.5894	445.9579	639.0445	164.3644	162.4812	124.5255	302.345	359.174	479.1083	364.0288	363.7634	543.5625	63.69387	58.70606	36.02118	98.37749	122.4622	78.16561	462.9596	339.4175	359.7575	79.29416	195.1701	128.8978
CUST_49119_PI429010886	M84191	0.003231458	24	3						38.35932	20.00439	27.37887	46.00851	38.24515	38.11869	21.9474	20.20229	25.45723	35.83737	18.96294	45.11511	31.06592	29.43019	46.31434	16.17764	8.106159	20.85338	20.26341	15.25605	23.37894	26.60187	16.51861	21.60709	29.51262	21.41005	29.60122
CUST_6660_PI429010886	N20421	0.003231458	24	15						514.8454	788.0337	519.1671	535.89	475.9252	517.6361	776.9293	599.8602	645.2626	574.2528	509.6459	339.9532	648.6912	536.3161	577.9786	959.3506	882.2277	819.3739	656.4851	539.9257	698.2425	549.679	634.4332	571.0817	690.9865	583.0039	557.4698
CUST_1073_PI429010886	N8411	0.003231458	24	15	270013832	hypothetical protein TcasGA2_T				4211.995	7430.648	3460.858	3286.945	2702.508	3327.482	7459.987	7090.073	6025.479	4560.074	3222.8	2860.025	4302.905	4134.923	3532.198	10035.81	7632.364	7993.785	5162.169	5879.268	5913.632	3014.898	5032.372	3835.495	4545.107	5678.164	4207.659
CUST_1962_PI429010886	N11174	0.003236599	18	15						265.6462	299.1209	306.0769	292.95	229.3564	293.568	259.2692	181.2583	256.539	395.5893	235.4202	264.5903	335.5615	263.0994	319.9838	350.791	316.1661	333.0825	347.7046	220.3669	325.7046	274.3383	290.2155	284.2929	305.4328	219.0312	268.1336
CUST_22647_PI429010886	N47395	0.00323844	12	9						47.26618	41.85499	63.92981	50.93881	66.06005	53.72417	50.01635	51.171	70.90586	50.77134	81.60481	119.4045	105.4292	84.21664	98.45312	19.1841	25.14223	28.8322	47.43208	56.31897	43.98563	108.8194	154.5458	199.0025	104.1579	45.64468	139.698
CUST_45738_PI429010886	M70102	0.003251036	21	0	194905042	GG11794 [Drosophila erecta] >g	Metabolic process	Cellular amino acid metabolic process	2.73E-10	12.92087	16.95871	10.37552	6.239298	NA	11.16455	9.007628	11.23899	11.27154	8.903229	12.1699	6.136811	10.26947	NA	8.529923	6.854397	5.272315	7.869127	11.52944	12.97758	4.451761	15.25271	13.69996	17.52474	6.643179	NA	11.48186
CUST_3355_PI429010886	N14349	0.003258212	21	15						2604.346	3940.028	2409.846	2420.859	2287.533	2039.749	2950.533	3215.844	2913.515	2063.235	1802.44	2211.381	2551.022	2777.197	2846.594	3522.391	3792.758	3929.52	3022.554	3022.554	3588.155	2895.969	3706.021	3289.37	2983.608	2549.09	3577.841
CUST_32889_PI429010886	M5387	0.003260267	24	15	37196918	thrombospondin [Marsupenaeu	Metabolic process	Carbohydrate metabolic process	5.2E-53	16.43875	12.69799	26.85225	31.6869	19.95957	22.55486	71.4584	68.79187	54.344	45.80079	29.47872	31.22862	103.5395	41.45634	50.07687	145.5262	53.84032	125.1706	71.48244	30.22406	50.36694	127.0776	37.57549	96.16362	43.33093	43.51785	32.38272
CUST_31740_PI429010886	M2284	0.003270867	24	3						37.12091	20.82309	39.96697	74.92905	118.7775	113.1381	20.99538	37.10697	27.91891	17.1309	96.20921	33.32109	45.49065	66.69697	38.11477	8.23062	14.0887	12.73763	21.73892	52.31438	26.82547	38.04316	65.60638	31.51184	25.03218	63.94916	31.31571
CUST_32627_PI429010886	M4627	0.003270867	24	15	195438179	Drosophila willistoni GK24252 (D			1.03E-74	77.76407	80.91667	82.41127	93.03552	66.31625	54.13743	108.1961	104.6904	119.4473	103.8712	71.64167	86.13364	115.5968	76.81667	92.15089	137.2967	136.6363	125.2342	108.7185	109.7081	155.4118	94.92219	94.99849	105.3837	116.4725	95.93997	144.0796
CUST_32715_PI429010886	M4905	0.003270867	24	15	224459352	adhesion regulating molecule 1			7.77E-70	557.162	1176.428	706.4981	591.786	736.9876	647.2976	967.3181	1027.619	954.137	561.6697	772.7965	652.5211	847.4628	853.4119	941.0274	1195.535	1253.748	1278.279	814.0506	834.0907	932.1979	700.359	900.3004	921.4706	813.7492	712.9201	877.3403
CUST_40489_PI429010886	M55271	0.003270867	24	3						9669	376.7778	9999.789	8750.959	8238.918	10448.35	413.5619	587.3135	749.0313	5271.495	8818.425	7021.693	6873.81	5284.133	8321.498	74.46237	129.8849	152.1914	573.4698	617.6234	651.2334	6448.141	6094.281	7337.918	1221.044	2300.958	3313.258
CUST_47845_PI429010886	M77822	0.003270867	24	3						23820.74	1501.763	17658.04	25703.26	23658.24	21710.8	943.8069	1480.103	1294.363	22332.92	22332.92	18408.63	19679.49	12523.21	12963.97	547.9685	571.0817	673.1503	2389.711	2198.578	2670.787	26678.14	12569.14	20434.9	3525.214	6717.703	4164.365
CUST_11464_PI429010886	N27855	0.003270867	24	15	156552229	PREDICTED: mediator of RNA po	Nucleic acid metabolism	Regulation of gene expression	3.01E-52	309.6367	353.5003	299.8024	259.8835	293.6993	291.3655	324.6065	451.5651	359.943	284.2467	256.539	300.549	294.4308	309.5645	281.628	380.0182	381.0871	513.5436	317.4998	388.4776	375.2193	350.649	377.0504	358.4079	349.5554	357.1234	365.4091
CUST_534_PI429010886	N5871	0.003270867	24	3	291242887	PREDICTED: hypothetical protein			0.000000108	35157.95	781.7261	32295.88	40595.56	40595.56	47342.04	149.786	2627.758	216.7269	18613.15	23084.2	37083.68	11881.91	31206.9	43090.8	53.72874	45.47301	200.2294	137.6019	2079.426	217.8657	18273.52	1483.086	15900.37	139.9842	3914.879	518.9718
CUST_3412_PI429010886	N14454	0.003271082	18	3						22.53853	150.3467	161.4921	154.3725	651.0737	518.791	72.97666	6.187929	99.32904	21.48061	324.921	210.0927	83.54731	12.7417	33.61683	111.6045	385.6874	294.7488	449.5658	145.7707	291.0948	245.3707	1194.794	1204.169	118.9453	290.7067	92.66549
CUST_31647_PI429010886	M2050	0.003273418	12	3	443734191	hypothetical protein CAPTEDRAI			3.95E-70	180.0776	241.3201	128.1317	145.7707	188.5963	203.6782	202.2179	224.6222	157.1085	160.8514	157.9625	121.1043	239.642	217.4779	223.8255	301.3368	313.1767	316.4278	202.6489	167.4144	213.3383	116.3642	172.8989	121.1043	181.5978	225.9834	166.8118
CUST_20097_PI429010886	N42805	0.003277209	21	6						43.94355	75.94639	53.55076	107.1698	82.61597	94.14191	129.7798	99.57709	56.55991	79.5268	54.40508	77.46257	39.60825	61.68316	32.81019	25.64786	55.42689	66.40286	53.99104	65.08113	32.98496	43.66107	105.4292	82.25472	50.10507	89.07659	81.60481
CUST_33654_PI429010886	M7523	0.00327771	15	9						40.80027	19.25567	24.86911	52.84329	47.49734	27.79405	19.6201	21.88142	24.28735	52.61688	166.4755	55.02895	68.89904	58.40039	34.35183	10.76358	24.74194	18.88946	25.70617	31.49411	29.13899	106.1766	115.7057	98.33769	40.11852	113.1524	74.18947
CUST_42588_PI429010886	M60658	0.003281553	12	6						68.54812	90.05411	69.23652	91.62496	81.529	74.52512	85.79452	105.619	104.5953	100.6757	92.80135	69.85306	126.4468	84.72348	78.91149	95.02561	97.44091	105.4902	106.6543	112.0615	107.5789	91.21622	96.20921	100.386	83.30459	79.70599	91.00224
CUST_13216_PI429010886	N30794	0.003281553	12	0						100.0919	139.1364	137.9543	103.8983	102.4735	93.91412	106.7719	88.5599	92.79509	93.47893	83.34871	101.4339	107.9954	122.1883	107.8039	127.3153	135.8804	118.1364	120.5552	80.49207	94.67757	114.5608	98.35456	113.0557	151.421	137.4424	153.5706
CUST_36430_PI429010886	M15876	0.003282056	21	18	321478944	hypothetical protein DAPPUDRA			1.4E-16	40.65621	97.07924	69.79048	46.43858	77.32899	63.59136	47.27657	73.90571	57.02931	48.50466	55.53179	43.58688	70.78121	47.53635	68.48192	71.43072	74.63073	77.97296	70.60324	83.67777	98.25227	65.26399	69.6885	78.15836	66.45173	55.55019	76.07226
CUST_1842_PI429010886	N10862	0.003282056	21	18						393.4684	445.4238	793.634	440.6377	306.0769	100.386	636.3867	277.7202	912.8746	415.0852	130.4587	265.7303	537.9838	295.6289	216.3545	400.3546	265.3286	612.3531	890.6088	1112.702	1265.639	614.3598	651.9522	511.369	292.2917	332.0817	775.6832
CUST_51576_PI429010886	M94055	0.00328236	21	15						27.47239	39.2136	37.24906	20.92431	30.47018	23.05545	23.50565	30.99758	29.34996	28.40807	27.71884	28.20584	36.50656	27.68667	27.43264	39.19706	55.35616	39.96323	36.16824	39.55957	33.68871	32.10443	31.40589	29.64873	36.79436	30.49532	36.95845
CUST_39791_PI429010886	M44735	0.003292128	18	3						10.88552	9.03053	11.88179	34.08118	14.80006	10.69455	NA	3.211354	8.630404	5.751246	7.455208	8.200935	15.33898	5.217523	15.07177	5.300759	5.939175	6.86757	9.271842	9.24174	6.119695	19.27271	16.16065	15.7695	5.200057	6.806327	9.595961
CUST_17563_PI429010886	N38193	0.00329523	12	0						14.47089	15.36202	18.35487	14.44887	NA	NA	11.18684	NA	5.05334	17.77566	12.96341	13.39577	18.85511	NA	11.92894	13.31624	7.456173	11.63004	13.47437	6.477013	5.758808	14.32656	14.04167	8.835842	27.1944	9.817879	19.5625
CUST_35956_PI429010886	M14106	0.003298383	24	15						290.2762	469.2787	494.2109	308.5661	319.9319	319.0038	335.6891	397.9279	483.3359	330.1216	394.1154	385.4238	368.4034	365.6513	409.117	474.7066	491.802	536.3161	458.9701	424.7669	548.1274	429.0423	461.522	473.6093	383.2384	412.2409	496.4508
CUST_37712_PI429010886	M21135	0.003298383	24	18						18.42053	15.23242	18.20758	11.63897	10.60847	13.87165	9.586868	9.942624	11.11796	17.87188	11.46799	16.59434	17.08055	13.25323	12.22964	20.74858	16.43018	15.9967	23.7799	19.39459	12.82342	27.36657	13.0692	16.28426	25.57669	15.47982	17.82084
CUST_25333_PI429010886	N52213	0.003298383	24	21						41.17764	51.87516	69.63156	46.57945	53.7784	45.91529	37.46702	41.97418	61.85109	43.55336	43.56248	41.29379	46.69609	44.22724	44.35011	42.06094	80.3181	68.41738	54.14388	63.99691	60.20069	82.53807	48.64263	77.25059	50.44651	63.07317	59.27468
CUST_39429_PI429010886	M39249	0.003298529	18	15						1342.206	5365.583	2575.646	851.8736	338.3225	531.0942	4140.108	4798.856	4177.108	1534.389	513.2014	542.0349	2609.919	1821.979	1675.406	3506.645	6401.205	4554.072	2543.39	2078.188	2239.116	788.7271	1777.355	826.9656	1725.737	1528.323	1262.424
CUST_6429_PI429010886	N20090	0.00330165	24	15						8827.334	11356.17	11974.32	9500.977	7459.987	6171.545	12765.38	11356.17	14285.49	10699.09	8121.327	7750.118	10815.88	7157.757	7138.174	11228.98	10871.96	12496.19	12921.27	14032.06	16252.77	12789.24	13716.26	14580.94	13616.29	11246.8	16452.07
CUST_36652_PI429010886	M16457	0.003304008	24	9						13.9939	24.75929	17.97858	7.202814	9.63872	13.63853	16.50059	21.54205	19.12988	20.84244	9.963988	19.85199	24.08979	20.17168	18.14584	14.61224	18.12496	16.20857	14.06396	14.54128	19.48121	23.82334	20.93117	18.7507	24.16626	22.21344	23.94642
CUST_57177_PI429010886	E2054	0.003310275	24	15	509391552	ribosomal protein L37 [Acartia p	Protein metabolism	Ribosome biogenesis	4.25E-34	7007.731	6799.146	6696.753	5227.74	4319.299	4275.766	8378.475	6826.356	6758.872	6799.146	4565.036	5292.711	7376.103	4560.074	6727.299	10569.33	9364.597	10448.35	7389.276	6025.479	9578.799	4467.089	7021.693	6010.065	6826.356	6461.658	8869.924
CUST_2224_PI429010886	N11908	0.003310275	24	15	308052945	endoplasmic reticulum protein S	Metabolic process	Carbohydrate metabolic process	3.67E-172	2228.732	7081.435	3675.29	2177.847	1492.942	2114.8	5892.313	5571.385	5265.552	4159.327	2790.956	2367.057	3647.267	2867.707	2575.646	7337.918	10294.69	8461.114	3770.678	3281.159	4517.506	2521.203	3499.526	2837.693	4685.458	3485.281	3159.077
CUST_5240_PI429010886	N18439	0.003310275	24	15	242002944	translational activator GCN1, pu				327.6087	620.5792	384.5219	354.7453	301.8993	304.3391	574.7506	500.3221	471.3761	416.6449	358.5953	381.9172	414.4974	349.9633	355.3919	548.75	716.3366	737.2275	516.2255	448.0132	552.4224	407.2747	433.6142	436.7155	510.5943	394.8687	454.1788
CUST_6365_PI429010886	N20006	0.003310275	24	15	260832213	hypothetical protein BRAFLDRAI			6.02E-67	250.4996	345.3559	242.133	205.4884	204.8124	242.9432	368.9684	293.1739	318.8359	328.2775	267.3602	321.3471	305.3135	214.7712	286.2998	410.0384	397.9279	440.7516	340.2357	216.5705	286.9821	311.1974	249.6908	306.4312	286.5775	259.5151	291.7534
CUST_6432_PI429010886	N20094	0.003310275	24	15						169.34	309.132	218.3669	183.5637	138.4615	152.7137	277.4932	306.0769	238.1099	238.025	165.5673	190.9659	240.2645	194.7406	212.0999	324.0982	328.4033	300.2438	221.596	220.8177	250.5426	208.1829	231.9647	187.9849	225.8691	180.5859	252.2559
CUST_10550_PI429010886	N26411	0.003310275	24	15	321461928	hypothetical protein DAPPUDRA			1.9E-29	106.9545	187.8136	112.2407	106.0541	87.25531	84.92778	196.2948	261.6417	146.4834	165.9265	164.071	133.171	125.4319	192.8532	128.2695	227.4234	282.8745	222.505	138.3923	119.6392	167.0939	119.329	364.5679	183.4751	159.203	124.5255	131.2558
CUST_39596_PI429010886	M41608	0.003313229	15	9						4378.72	2575.646	5558.806	6642.147	3130.289	1532.345	6010.065	4863.888	9532.564	6631.875	7039.176	4503.776	10835.48	3431.358	4288.608	438.5777	660.6448	761.5417	10727.23	8736.825	13812.43	10127.21	14922.71	12097.54	13043.65	12605.8	24507.87
CUST_18590_PI429010886	N40068	0.0033155	24	9						5.726423	11.76421	3.196574	13.70583	11.35345	5.900002	NA	19.25364	9.804365	26.76257	7.714788	34.35183	17.15642	8.285959	NA	16.64023	14.42774	6.103744	12.74304	12.54744	14.1297	NA	NA	4.500151	3.074213	6.451204	6.259465
CUST_14017_PI429010886	N32181	0.003316662	18	12	242015762	conserved hypothetical protein			1.07E-58	137.1269	180.9657	166.158	172.3438	169.9145	185.4692	138.9364	165.1648	136.2348	158.6164	214.8629	184.6136	191.5757	212.8617	241.3201	249.7255	172.8766	239.348	196.2133	162.6193	183.6827	207.8778	148.6211	205.4207	156.2342	168.3995	164.188
CUST_23194_PI429010886	N48423	0.003322573	12	0						34.98417	43.5104	43.75295	30.55564	31.51184	28.90075	13.88764	23.00207	25.123	34.41579	41.47342	30.83657	46.06605	26.86667	28.72062	34.313	36.32694	35.43842	22.86904	31.15433	32.96215	31.2986	37.40246	38.16299	41.81524	34.94683	47.45891
CUST_34848_PI429010886	M10883	0.003322781	18	9						6.414885	7.662672	20.45768	7.873998	7.062362	10.24931	15.1605	16.71688	16.18715	18.93459	19.63812	28.25337	12.35025	18.11564	17.25784	13.80069	17.46154	17.39841	10.16942	13.1553	8.802939	25.34518	15.22851	16.62865	13.28376	10.94476	21.12976
CUST_1403_PI429010886	N9533	0.003327667	21	0	291461591	sugar transporter 16 [Nilaparvat	Transport	Transmembrane transport	1.76E-21	65.20578	66.2054	123.1089	47.59455	41.97418	58.27052	41.3582	74.20038	71.76549	62.4694	63.0922	53.48046	38.47726	34.70595	38.22258	48.28205	38.51685	54.63268	50.69876	61.67659	46.61175	73.79944	46.66302	53.087	84.06095	45.50498	40.25595
CUST_51659_PI429010886	M94458	0.003332592	24	3						29.27764	4.860603	24.43634	75.15569	51.03976	70.5281	NA	3.527703	4.417312	26.74791	42.3639	52.01796	11.56464	33.79023	39.38571	NA	NA	5.673079	3.811284	5.196761	4.838499	20.79498	5.786969	18.0729	8.5322	11.34493	6.3219
CUST_11799_PI429010886	N28420	0.003332991	24	15						NA	5.264041	10.23134	15.4541	10.50051	9.201788	9.768932	6.969562	13.80416	22.45935	13.01309	14.42132	10.06086	12.45971	8.016857	20.83583	16.16198	19.39663	9.050756	9.731693	10.94976	10.03828	NA	9.836576	6.914242	8.755998	6.005973
CUST_5994_PI429010886	N19479	0.003339252	18	15						79.56634	115.6552	104.0674	86.28411	75.48453	70.29674	102.6964	79.43494	82.89284	87.87185	89.43021	94.82698	90.79873	99.28639	93.31746	102.1603	122.622	101.7333	63.88807	103.0349	100.9815	75.31986	90.20095	86.60823	83.58359	93.02834	112.599
CUST_53456_PI429010886	M101781	0.003349683	24	15						6240.674	105.1903	5298.376	8713.974	4900.819	6897.446	303.8109	517.6361	443.2849	3790.113	6913.964	4857.819	3774.541	4207.659	4906.082	62.33316	80.00199	65.17942	910.719	707.3888	956.8208	5202.819	3172.092	5227.74	912.2418	2400.564	2858.309
CUST_54983_PI429010886	M108325	0.003349683	24	15						590.4595	557.9393	547.285	423.936	489.7282	446.6878	852.5352	709.6774	932.6252	863.5664	701.0526	252.8102	749.5653	563.9832	508.6367	1200.042	831.6922	893.7709	832.328	513.7138	688.6527	881.258	603.0231	613.1951	724.186	444.1379	448.6833
CUST_37303_PI429010886	M18547	0.003349683	24	15	66774601	Penaeus monodon 14-3-3-like p				2696.535	5913.632	3513.064	3393.901	2822.582	2773.888	4349.21	4326.077	4164.365	4101.099	3205.37	3717.963	4453.231	3441.376	3535.101	5744.161	5271.495	5879.268	4064.827	4234.05	4436.509	3715.857	4247.1	4008.648	5119.273	3292.245	4125.439
CUST_38127_PI429010886	M23503	0.003349683	24	3						4436.509	268.8247	4517.506	3654.032	4284.075	5558.806	186.8218	313.2232	285.7894	2179.188	4109.069	3346.038	2661.468	3253.191	4622.321	52.77429	47.47767	81.10019	266.6561	418.6023	259.2192	3029.351	2889.672	3924.629	512.0349	1597.44	2251.39
CUST_51219_PI429010886	M92662	0.003349683	24	21						48.24507	42.83388	59.96133	43.72166	35.21261	53.97594	52.9338	40.9293	65.93698	36.56736	36.29464	33.34498	60.85412	43.9077	48.64708	38.78014	48.12354	48.24962	91.73279	42.90092	73.7433	99.2742	56.55385	68.89904	107.5363	50.30791	81.4132
CUST_5590_PI429010886	N18912	0.003349683	24	15	345495736	PREDICTED: signal recognition p	Transport	Protein transport	1.98E-133	345.8925	499.9903	367.4917	298.135	336.3654	361.4106	528.2931	515.8767	428.6927	362.5984	365.8262	359.8294	353.8605	390.7107	388.382	527.9796	667.4336	600.976	442.2313	488.2191	509.1116	285.4272	496.2739	382.8713	390.4253	389.8933	452.6806
CUST_20874_PI429010886	M44224	0.003363151	24	18						NA	10.70343	12.01275	4.514414	7.940901	8.522432	3.537824	7.301642	11.92532	4.635169	10.14816	6.216366	9.358571	3.991284	6.707537	8.805786	12.03475	10.12124	11.03537	12.6478	10.73336	12.26439	10.40166	9.266709	11.98172	9.273221	9.130244
CUST_13736_PI429010886	N31686	0.003386855	15	12						508.6367	425.365	557.3127	420.9575	671.2531	560.8143	223.5106	356.6938	324.6065	460.1149	527.3184	627.9112	432.2687	756.8703	1010.854	468.6225	540.61	456.7205	353.5795	373.0445	453.3141	372.6214	489.5895	423.8193	455.7266	437.5336	536.3161
CUST_20157_PI429010886	N42915	0.003396073	24	15	524893067	PREDICTED: TOM1-like protein 2			2.7E-12	188.2411	185.1715	201.2812	183.6827	150.3928	179.0245	160.713	166.2022	158.7144	216.9264	177.9607	252.7743	188.656	179.1302	174.7665	221.5686	254.9525	232.1097	203.3258	159.4052	218.8175	201.9478	191.9474	234.9939	233.3746	180.1675	237.4902
CUST_47861_PI429010886	M77892	0.003396073	21	3	242332534	uncharacterized protein LOC100			5.91E-13	9.590111	13.24351	12.27699	19.0017	17.16197	9.93804	12.75698	9.15859	9.555938	18.04327	13.46872	6.285156	4.969275	14.18007	9.675629	13.66341	9.755769	11.76147	17.23574	10.20394	15.10953	18.01822	16.87953	16.03517	6.303517	10.30981	14.16983
CUST_2185_PI429010886	N11816	0.00337033	21	6						25.6877	38.37685	41.66708	55.05576	35.30917	37.87771	36.49885	32.46193	24.26356	40.26043	42.91838	52.56738	28.18989	23.86873	29.20036	39.36946	29.20882	29.78824	29.81738	37.17438	31.88585	52.69375	31.20826	38.53227	51.80267	45.25971	46.13557
CUST_4329_PI429010886	N16524	0.00337033	21	12						57.9689	57.8261	54.07225	32.21229	53.3873	35.47671	60.0187	90.42094	53.96355	49.17415	65.86788	31.02823	70.8379	79.24842	70.36913	52.18229	107.9218	57.3802	29.06853	74.79444	58.70606	43.5017	52.3424	40.57567	38.02922	34.94317	50.20269
CUST_35016_PI429010886	M11343	0.003379974	18	15						393.0106	618.3282	442.6514	372.2898	466.2067	384.0543	447.6336	474.227	436.9283	487.4115	438.8282	489.7282	482.7166	452.6806	524.6943	483.9464	675.5044	532.8364	439.0112	499.8092	441.0424	429.8212	445.6007	457.8873	498.7889	482.0377	463.5485
CUST_417_PI429010886	N5097	0.003379974	24	15	332271601	protein disulfide isomerase 2 [Fe	Metabolic process	Oxidation-reduction process		10448.35	16106.84	13078.51	7966.321	6470.198	7821.557	19485.9	17837.79	18408.63	12288.42	8669.063	6032.614	13193.16	9272.221	11356.17	22469.11	31659.39	17658.04	16726.21	10987.48	18117.06	7983.519	9598.965	8073.399	17487.6	12442.6	11643.13
CUST_16090_PI429010886	N35637	0.00338634	24	9						45.98151	23.66239	30.1739	72.84435	28.12879	65.87993	33.74242	32.54688	13.82436	69.12973	40.65621	40.45135	49.12192	27.45155	46.27549	18.75881	32.51998	21.74943	9.477425	14.43107	9.814559	43.57254	15.60788	23.28963	34.14439	30.09026	10.74067
CUST_17440_PI429010886	N37966	0.00338634	21	0						179.8173	185.3668	188.9036	166.7131	138.3923	148.5106	138.0213	131.9825	144.9581	212.2981	142.3135	165.2368	164.794	141.6972	141.2484	171.8413	161.3598	155.4522	229.7005	145.2758	164.8407	207.6167	163.782	197.961	235.1738	152.1194	163.6909
CUST_40127_PI429010886	M50675	0.00338909	21	15						6533.018	13135.1	7900.843	6854.464	5227.74	5609.633	12236.09	12359.97	10921.28	8025.596	5558.806	6734.459	6368.381	7337.918	5593.889	13532.16	15297.04	15141.22	10035.81	10871.96	10035.81	7147.632	8321.498	7912.634	8669.063	9067.463	7430.648
CUST_3457_PI429010886	N14543	0.00338909	24	15						4363.515	23.26849	5043.311	5678.164	4577.512	5947.47	15.21169	44.9498	47.09644	4415.56	4965.926	5110.752	2432.608	2948.008	5388.872	3.884331	23.37085	21.49158	28.87134	35.51709	19.07007	4130.841	1407.95	2586.608	103.2627	51.24511	113.7264
CUST_49812_PI429010886	M87266	0.003394791	18	12						7.002504	5.230371	8.679248	8.208913	NA	5.864027	14.61747	7.546327	5.573122	11.18155	NA	46.75132	16.5534	15.4017	NA	8.648652	6.212857	15.90721	5.394829	4.111851	5.94666	NA	NA	7.095312	4.359608	8.978286	14.5445
CUST_26129_PI429010886	N53720	0.003395902	21	0						57.93583	60.0628	45.95593	53.53275	40.5799	36.67403	27.74851	39.44209	34.85461	49.13607	52.41365	46.99801	35.83737	32.39325	30.3832	49.13607	37.1161	41.99235	51.66124	39.91458	44.19365	53.21446	49.67166	48.76855	45.9767	30.20183	45.35771
CUST_15771_PI429010886	N35119	0.003397388	24	15						4.784925	6.815755	8.096327	8.069641	3.526847	3.992176	7.198567	10.07565	5.908768	9.586868	NA	NA	NA	4.816293	9.346935	10.8747	9.365338	14.72662	NA	9.005083	11.60345	NA	6.269189	7.596603	6.921979	6.108532	7.548659
CUST_22815_PI429010886	N47726	0.003403719	18	12						25.26967	22.04202	22.62698	33.72382	29.282	42.69638	4.051233	17.26651	19.30372	42.49657	24.6865	48.7032	31.48421	34.38418	60.0855	35.17993	26.91338	35.19853	20.66998	19.0327	21.35681	35.41491	19.09012	22.67515	18.75264	16.73896	17.49425
CUST_10572_PI429010886	N26446	0.003406124	12	3						229.7888	330.7777	40.72568	388.6637	74.8864	372.2167	399.7084	206.5228	159.4235	223.7463	161.4921	127.8904	357.3731	351.1109	305.9094	585.2891	453.0125	364.8648	342.4681	59.49271	269.4056	50.80105	198.334	63.62492	256.539	214.8358	32.99109
CUST_18260_PI429010886	N39451	0.003409908	21	0	169931080	cuticular protein [Artemia franci	Metabolic process	Carbohydrate metabolic process	8.43E-14	63.66074	109.9526	167.5116	97.244	44.1442	32.98843	30.4977	51.02509	31.18313	70.51234	61.04906	24.48777	48.2662	58.3384	52.16829	21.48685	30.26815	61.17453	30.58943	40.7919	37.20362	78.67205	129.6335	141.539	48.99934	93.19639	110.3551
CUST_8901_PI429010886	N23769	0.003416935	24	15						45.37684	35.74167	37.58094	53.31643	49.04743	45.54712	54.85916	36.24723	40.84998	52.79948	22.06613	30.69521	41.33036	26.346	33.6128	39.34641	26.40704	33.64233	34.20583	38.57897	31.94938	40.52376	33.9635	33.09557	46.51413	43.75819	34.53535
CUST_27230_PI429010886	N55676	0.003416935	21	9						28.45078	21.24736	20.24017	24.96813	25.48121	21.01951	26.99962	32.86579	22.4723	38.16299	34.88562	35.02108	16.46322	26.1001	17.26262	18.86717	34.89175	14.5445	19.32331	19.50992	10.02667	27.0245	17.83143	30.06574	19.74297	28.58437	21.49936
CUST_36926_PI429010886	M17243	0.00342187	18	3	156390869	predicted protein [Nematostella			5.71E-34	16.64224	6.029155	15.29086	27.90958	26.25902	27.60747	10.21185	25.95809	22.0234	26.86103	89.65818	16.58358	27.69715	24.68095	23.37724	4.253486	12.51148	4.929922	31.56412	19.7654	12.2416	35.3123	88.70868	61.36611	18.53166	59.28006	68.51645
CUST_37296_PI429010886	M18536	0.003428499	24	15	546673984	hypothetical protein D910_022			5.89E-17	11998.69	21524.84	12473.12	11112.95	6326.89	9142.021	20662.81	19635.13	16655.53	15461.72	9142.021	11076.77	13043.65	13391.14	9699.55	21334.13	24199.32	24040.66	16801.9	16690.76	16726.21	11395.49	13812.43	11164.38	13419.52	13906.34	12725.99
CUST_49949_PI429010886	M87835	0.003428499	24	15	262401305	ribosomal protein L19e [Scylla p	Protein metabolism	Ribosome biogenesis	4.37E-21	7769.948	12344.85	8296.544	7900.843	6060.248	7130.037	10727.23	11836.96	10966.45	8669.063	7643.683	8160.518	8321.498	8683.686	4988.039	13737.98	12725.99	13737.98	9500.977	11719.99	12496.19	7240.469	10077.64	9096.626	9444.83	9345.369	10949.85
CUST_9401_PI429010886	N24556	0.003428499	24	15						143.5809	312.2924	212.1398	173.1737	143.9979	139.7918	237.1374	215.2207	238.3483	201.5828	185.5286	180.5334	221.0264	229.3211	174.0123	239.9273	288.9896	331.0671	212.9394	225.7047	271.3703	179.7279	219.4592	238.1099	234.2807	218.0311	230.2547
CUST_10249_PI429010886	N25944	0.003428499	24	15						42.16901	85.59262	71.70119	50.80319	55.06272	46.38563	84.35052	78.35549	73.1869	88.92336	53.43212	52.91761	69.31429	59.73624	80.13048	100.3625	108.6527	111.105	66.45173	65.50385	101.7015	64.39747	81.529	100.1686	81.71011	74.46237	90.90449
CUST_222_PI429010886	N2830	0.003428499	24	15						4772.224	11179.97	5693.503	4577.512	2891.912	3039.361	8321.498	8996.679	7430.648	5202.819	3988.54	4093.741	4475.935	5311.077	3747.441	10156.68	10587.95	11503.97	7597.158	7081.435	7364.792	4229.758	6999.724	5251.996	5979.853	6265.89	5388.872
CUST_26746_PI429010886	N54806	0.003428499	24	15						44.30249	66.1068	55.91239	35.63867	44.95536	56.15909	59.03583	62.5781	46.41625	59.40177	64.06114	54.63268	48.45851	48.0027	58.11342	66.33824	94.17514	74.10459	59.51724	66.22878	60.4258	58.47581	68.65785	65.44143	58.4567	64.42288	73.7088
CUST_5807_PI429010886	N19223	0.003431983	24	15						203.4357	277.3623	285.4272	200.3043	236.386	195.4372	181.5978	222.8209	249.5189	243.0739	242.5947	251.4219	254.618	234.493	248.7029	234.8306	298.1868	293.095	259.0205	263.4037	306.5038	293.934	242.5407	314.6496	262.5725	229.1648	372.2167
CUST_18391_PI429010886	N39710	0.003431983	24	15						249.2581	198.7466	246.8231	245.4565	206.8179	205.5946	219.3902	223.7905	235.5705	260.2442	193.7362	244.7472	219.4592	203.2614	275.4561	330.4891	267.6236	288.5336	298.1868	252.1189	298.8719	318.3284	270.5362	289.3915	273.134	215.601	312.3683
CUST_94_PI429010886	N744	0.003444455	15	0	255653874	crustin 4 [Panulirus japonicus]			3.95E-27	19434.65	11822.99	19195.08	20871.55	7090.073	13419.52	11643.13	9013.443	10248.36	6461.658	8857.18	6421.864	8309.047	15526.29	6385.128	14307.54	14363.02	12963.97	8786.531	9129.704	10077.64	6966.12	15900.37	8547.585	6430.973	10464.54	7643.683
CUST_3714_PI429010886	N15115	0.003446321	12	6						127.048	190.829	25.09267	138.8888	214.8358	133.6449	434.7194	1337.377	217.4288	63.87749	223.1202	234.533	54.08928	130.5329	8.539795	52.48031	309.9338	106.9946	151.7904	127.6698	147.7749	7.702876	188.3253	121.5849	3.745693	5.314769	3.09224
CUST_161_PI429010886	N1840	0.003446381	24	3	242018020	chitin binding peritrophin-A, put	Metabolic process	Carbohydrate metabolic process	2.52E-51	1072.507	204.1402	1050.367	1249.272	5336.579	5110.752	188.5963	962.741	164.741	157.9135	2629.319	2522.496	83.24757	2097.341	2090.105	34.05764	45.18209	30.41985	40.73959	1392.832	37.94299	356.1688	326.7696	259.2192	NA	1338.071	4.187337
CUST_12543_PI429010886	N29630	0.003450319	18	12	241818211	BRO1 domain-containing protei			1.21E-25	18.30245	6.209536	10.54562	10.7195	7.807697	7.17081	15.50904	15.3561	20.12782	13.71185	17.5521	12.83426	19.41654	19.90073	12.15592	8.534644	16.99422	8.246623	11.44514	18.07705	12.55197	7.966091	10.782	8.060296	11.0324	10.94203	12.87838
CUST_13813_PI429010886	N31819	0.003450359	12	3						336.6041	452.5351	129.9776	755.266	50.47976	604.0998	638.6943	252.8102	514.7289	220.1523	124.2323	60.98236	459.1993	452.5351	296.2664	1436.159	632.4923	835.3759	671.9924	340.7848	673.8771	84.6023	534.593	93.31746	431.346	270.1339	203.2877
CUST_12443_PI429010886	N29469	0.003455654	21	3						62.09609	40.29385	44.57171	56.56581	48.14157	57.16525	48.68211	41.15339	50.03259	41.47761	28.75045	54.73135	29.4575	31.3526	35.99333	45.27328	42.14237	58.26212	45.35253	54.05823	31.98442	44.08647	41.87629	58.5159	54.48188	62.82209	55.1065
CUST_51873_PI429010886	M95497	0.003456229	24	21						19.95957	18.89628	11.21622	NA	9.239178	9.684334	10.32176	5.168998	11.44626	10.63779	16.45776	12.36205	11.42315	11.67524	18.79083	15.87551	17.49722	15.91453	25.76307	9.745235	16.25246	36.36056	15.99305	26.28725	24.33617	23.85395	31.04885
CUST_37788_PI429010886	M21589	0.003459785	21	15						1448.453	2396.659	1949.314	1675.406	1626.549	1513.287	1257.339	2030.062	1824.284	1362.378	1530.86	1465.124	1361.853	1711.15	1321.031	2247.929	3027.036	3230.906	1895.668	2147.917	2470.84	1559.979	2063.235	1996.997	1781.913	2009.095	1906.005
CUST_55382_PI429010886	M109848	0.003461419	18	15						82.3809	106.2889	67.85229	84.09371	55.17588	82.17196	106.3489	99.2507	80.24156	104.8169	50.78318	65.04377	85.785	86.85325	74.5359	160.5879	136.2005	140.2946	97.88781	76.12107	108.298	63.45821	61.38551	59.37073	82.67536	89.95321	86.48763
CUST_37495_PI429010886	M19231	0.003461419	18	15	507950122	PREDICTED: transmembrane pro			2.21E-30	1189.941	2117.814	1465.124	1152.159	576.2705	753.1726	1898.75	1449.959	1466.746	1813.66	858.1052	1014.938	1638.477	922.5025	1005.944	1787.383	2627.758	2507.724	1657.457	1253.748	1738.077	887.5066	1080.452	1178.539	1631.254	1264.606	1589.718

Probe ID	ID	p-value	Period	Phase	EBI ID	Annotation	GO category	Go term	E-value	0	0	0	3	3	3	6	6	6	9	9	9	12	12	12	15	15	15	18	18	18	21	21	21	24	24	24
CUST_38738_PI429010886	M29811	0.003461419	18	3						3735.581	842.4709	4027.931	5777.198	5587.034	5833.315	1060.229	1311.001	1624.639	5502.319	6060.248	6533.018	4602.898	3611.195	2168.846	421.2021	608.6613	718.207	2105.784	2031.77	2112.104	5718.956	4536.055	5992.677	1179.062	2829.469	4015.947
CUST_26181_PI429010886	N53807	0.003461853	12	9	339234267	ETS factor [Trichinella spiralis] >	Nucleic acid metabolism	Regulation of gene expression	2.58E-15	14.54604	28.75907	26.72777	17.15642	20.29507	NA	21.31707	28.0155	37.52227	61.8234	52.51146	54.73663	620.2559	23.60789	51.30921	28.08693	30.54671	34.78378	56.68053	34.25718	50.63563	87.79232	47.6242	83.0128	46.26072	35.46315	66.77244
CUST_20791_PI429010886	N44060	0.00346329	24	3						2512.997	40.39391	2173.027	3272.412	2983.608	3851.521	19.7274	155.9036	12.46469	112.7474	2091.62	2102.006	81.26825	2820.736	2981.297	23.03897	5.196761	17.93358	11.50587	126.4865	4.629932	130.7209	77.05387	79.57438	8.332244	251.3401	NA
CUST_35861_PI429010886	M13731	0.003467225	15	3						56.27761	79.14448	38.0933	93.01673	13.17618	72.3378	128.4629	17.93943	59.42381	13.61629	11.68418	7.869717	68.98955	51.98569	55.85351	51.02011	60.32929	13.73223	192.8297	62.35301	174.8981	18.30046	56.24032	51.59074	45.5523	45.01683	38.61107
CUST_39852_PI429010886	M45764	0.003467907	24	3						14363.02	55.25246	10248.36	9727.143	14285.49	13685.2	85.99964	235.3802	162.6466	8387.726	10193.49	13248.98	4895.993	7643.683	12211.42	22.95059	16.0336	74.8864	264.9858	837.2458	341.732	7966.321	2012.206	7290.969	381.9172	1794.235	1949.314
CUST_48762_PI429010886	M82075	0.003467907	24	15	160552257	putative 60S ribosomal protein I	Protein metabolism	Ribosome biogenesis	1.59E-28	28492.44	37083.68	33963.44	25130.01	17623.06	23379.75	36967.46	36406.05	33508.11	35305.99	25978.53	18320.9	32064.51	29122.17	26491.1	40757.76	39713.59	38540.49	31801.71	35445.2	33402.18	27577.78	34286.52	30352.12	32798.44	35022.2	29647.7
CUST_7252_PI429010886	N21266	0.003467907	24	3						12860.58	472.6199	12165.66	13335.43	14794.99	17424.76	651.5453	1225.977	1213.608	4427.539	11952.65	7389.276	4783.413	13193.16	17658.04	372.7272	306.6661	463.5485	1512.535	2222.683	1475.969	4727.546	3382.058	4880.699	1535.771	2627.758	2837.693
CUST_9874_PI429010886	N25338	0.003467907	24	15						62.10736	134.7253	87.30779	62.09609	69.73149	58.09261	90.74217	111.7011	90.45329	85.62565	83.59122	107.1233	81.45042	70.22094	113.3335	103.1229	125.6495	141.753	94.16687	104.3412	116.6229	90.29464	83.45736	104.4489	95.2367	85.43893	99.20976
CUST_7134_PI429010886	N21087	0.003470838	24	21						2136.93	2775.294	1968.185	1653.487	1346.472	1098.604	1316.811	1426.897	1365.442	1827.788	1750.013	1433.817	2023.772	1587.121	2021.719	1797.322	2325.222	2268.13	1415.173	1526.163	1607.994	2507.724	2166.525	2429.15	2467.704	2076.904	2793.635
CUST_50204_PI429010886	M89031	0.003478085	21	15						38.7383	20.66527	33.84162	16.47491	30.84327	31.34171	26.71367	15.99964	25.22202	21.54473	16.35738	43.70538	43.01095	27.6893	37.19071	38.17426	36.49381	38.14824	43.84283	23.80329	34.8722	34.16687	31.83006	31.74591	29.6361	26.38195	26.48147
CUST_15484_PI429010886	N34654	0.003480673	21	12						9.144684	10.44258	9.902198	NA	6.740547	6.534983	14.33104	14.49041	12.86873	13.6668	9.486624	9.820162	10.48413	12.96776	20.05681	18.8308	8.874019	13.41897	5.797914	10.97416	10.8869	5.044846	6.768812	14.84138	3.899392	3.92559	7.451763
CUST_10254_PI429010886	N25953	0.003485995	24	3						1427.593	6.222395	1239.555	2016.054	1559.979	2180.402	7.932927	20.07958	14.27946	212.598	606.1742	496.1354	261.0851	1383.977	1949.314	4.372897	6.790621	4.72816	4.448757	28.26843	6.76308	522.4996	105.9997	496.9611	NA	54.5198	39.07837
CUST_50052_PI429010886	M88422	0.003491846	12	9						16.4966	16.33991	15.31705	9.411311	5.58165	12.08009	NA	157.6143	164.3905	49.97848	95.01092	20.20612	27.56258	73.3346	12.99289	3.628158	7.685875	5.961032	NA	NA	4.177755	24.485	64.71262	54.28814	7.24682	43.45077	13.43146
CUST_55229_PI429010886	M109204	0.003493048	15	0						2445.736	3499.526	3064.959	2258.648	2292.947	2566.656	2489.305	3145.521	3717.963	2043.437	1812.631	1914.352	1903.836	2579.047	1787.383	3804.856	3371.075	4690.093	3112.057	3217.866	3796.503	2350.317	2251.39	2159.534	2287.533	2552.554	2454.398
CUST_36452_PI429010886	M15933	0.003496171	18	15						117.5967	138.6868	119.2442	127.7313	93.87326	110.7598	132.1609	83.10688	132.0014	105.746	125.8093	107.6466	143.3201	113.2744	183.8806	142.9974	172.3438	142.8249	145.6152	152.5137	173.6159	101.0612	111.0684	94.00943	115.9671	103.3216	136.8369
CUST_9152_PI429010886	N24144	0.003502043	24	21						53.80944	56.66829	59.67529	60.53935	40.082	49.04743	33.83079	49.91114	46.46187	42.75074	45.91877	43.40182	59.48241	40.86304	67.3975	44.58117	56.13868	52.43555	51.20424	51.49597	58.2972	59.51724	65.34281	93.42816	44.32333	47.64701	75.24855
CUST_51413_PI429010886	M93425	0.003505639	21	3						96.00931	156.6283	193.4608	193.9616	121.236	163.7578	111.653	170.1972	218.0311	121.9377	197.8282	137.3382	93.88506	69.71651	95.73042	89.0262	123.2485	114.2182	121.5461	148.131	128.0633	163.0633	181.7766	133.8434	237.3259	97.40632	159.5479
CUST_56113_PI429010886	M112812	0.003507315	24	15						2536.537	2613.493	2771.365	1751.317	1809.494	1420.017	4727.546	2948.008	3940.028	3679.2	2279.977	3261.337	2629.319	2065.743	2332.204	5489.861	5144.706	3755.671	3709.96	2447.506	2719.085	3373.758	2391.051	3124.619	3692.065	2210.04	2719.085
CUST_39308_PI429010886	M37531	0.003507315	24	3						14100.35	61.89891	12725.99	10949.85	17453.24	17287.24	76.8564	255.866	149.5014	8869.924	12860.58	14614.52	5671.62	9241.731	13935.1	13.19225	12.83208	73.67616	266.9079	826.9656	487.7778	9466.456	3058.086	8321.498	474.9425	2112.104	2088.583
CUST_41828_PI429010886	M58647	0.003507315	24	3						13503.69	177.673	11738.35	14161.49	19010.63	19195.08	145.5847	805.476	229.4919	5644.907	14764.65	15394.16	4181.398	17487.6	16553.17	48.23305	47.00541	99.84349	231.8895	1277.638	377.8683	6368.381	2511.558	6481.59	301.2002	4105	1720.155
CUST_12890_PI429010886	N30215	0.003507315	24	15	328777305	PREDICTED: hypothetical protein			3.13E-52	565.0242	999.9612	570.2732	447.3237	409.6823	484.1868	616.0609	685.2688	572.5541	620.4556	496.692	624.4152	551.2795	513.3858	497.9545	823.2974	839.3829	1053.829	607.8959	576.0861	785.8536	504.201	637.7947	620.2559	600.2455	629.0343	629.8536
CUST_19194_PI429010886	N41142	0.003507315	24	15						226.0802	279.7784	248.439	206.2842	207.3653	194.7103	282.8168	221.8856	296.1058	285.6824	223.3777	256.2294	260.6535	200.6783	250.4996	313.5422	338.9144	334.0615	260.557	222.7248	290.511	238.3932	281.4085	268.1336	267.4596	248.3542	317.1978
CUST_1923_PI429010886	N11085	0.003508364	12	6	320090149	C-type lectin-1 [Litopenaeus van			1.84E-18	5.918723	NA	10.79953	20.61709	NA	12.28744	436.7155	NA	335.3952	57.60444	42.66175	10.14816	8.825422	26.12743	3.808698	75.8587	11.74876	78.62482	493.1272	752.6445	1636.385	65.3293	NA	46.9383	152.0785	27.57421	128.0633
CUST_1934_PI429010886	N11109	0.003518869	24	3						394.3642	173.332	252.6173	406.6714	2795.798	1831.816	207.1791	208.941	209.1018	82.53131	2402.724	2595.878	133.1266	1831.816	2135.546	164.0459	123.1444	88.74114	124.6988	59.25391	92.69475	84.11819	91.88292	51.43678	94.18113	152.3312	14.31215
CUST_16518_PI429010886	N36375	0.003522698	15	12						357.6352	687.0247	530.3219	290.6243	272.1212	225.3383	331.8463	406.4569	470.6338	369.0567	341.3363	315.6063	379.037	497.9545	464.9207	372.3645	386.8087	465.4767	331.7259	327.6087	160.342	344.705	433.9196	462.283	416.1276	381.9964	437.7611
CUST_56396_PI429010886	M113674	0.00352767	12	6						8284.715	8846.332	8439.929	12424.28	5482.841	6393.352	13391.14	11179.97	12886.71	11030.98	4097.003	6348.681	10532.72	6393.352	5061.439	11323.45	9743.786	9684.529	13532.16	13154.56	10746.95	10999.91	7653.847	10550.02	9699.55	5736.233	7872.926
CUST_48502_PI429010886	M80692	0.00352767	12	0						49.10462	46.75132	33.06459	29.96518	43.70538	38.14116	26.51031	31.54041	28.61945	32.63017	31.09684	41.49384	44.96721	31.87401	36.98315	29.84354	24.8172	39.12873	31.11334	33.54341	17.35447	25.02074	25.1	32.79916	36.49885	70.80726	46.34194
CUST_13213_PI429010886	N30789	0.003531607	21	12	345489120	PREDICTED: hypothetical protein			0.000000484	3.358927	3.155733	3.351795	4.131407	14.46986	NA	NA	6.863677	5.964848	3.76011	13.82436	23.43251	NA	10.97252	14.25102	9.15859	5.253352	6.375942	NA	6.279977	4.075478	NA	3.362915	8.66264	5.817195	5.061979	10.95081
CUST_11894_PI429010886	N28587	0.003531801	18	15						35.84878	35.33768	NA	50.69492	NA	41.77489	38.07503	9.458406	28.0791	10.47216	6.232148	25.71638	19.00748	80.82353	27.13842	153.9962	67.7294	79.10899	79.23144	3.796933	74.76519	NA	39.70866	NA	10.9325	12.46346	NA
CUST_7610_PI429010886	N21805	0.003542864	18	15	321477670	lectin, mannose-binding, 1 [Dap			1.32E-123	24.91228	19.42744	7.935999	8.422869	8.606721	18.55514	17.0629	22.261	12.54744	18.80121	17.50097	14.39282	9.598517	16.11271	19.3323	37.94299	24.23412	23.99912	15.97652	12.90656	13.59757	11.84866	9.799617	10.12124	19.35309	14.68136	11.12989
CUST_8712_PI429010886	N23461	0.003544822	21	15						73.62565	123.4012	21.55928	48.05999	NA	31.87855	329.015	215.3375	120.3757	403.5639	44.26452	241.6209	48.07833	244.5822	26.24091	258.9303	445.1432	410.2255	99.88608	87.88247	115.5416	55.96387	14.93559	56.29733	75.28314	55.7467	32.91165
CUST_37794_PI429010886	M21622	0.003546723	24	15	242007350	15 kDa selenoprotein precursor,			4.84E-43	2595.878	4164.365	2600.653	2365.258	1778.328	2066.858	3924.629	3914.879	4023.648	3478.142	2332.204	2291.48	3120.055	2264.823	2541.875	5137.74	4415.56	4965.926	3333.312	3098.696	3584.754	2637.871	2661.468	3022.554	3767.349	2789.551	3212.539
CUST_41072_PI429010886	M56817	0.003546723	24	3						70.70842	33.39856	82.91936	78.72715	116.1509	104.9676	24.76219	20.97815	30.71104	50.43341	60.86142	77.35718	48.95729	73.24973	102.1812	22.88115	19.89427	25.1445	33.66656	33.71363	24.9948	68.15639	30.29561	50.43341	29.84906	37.38952	40.29385
CUST_45905_PI429010886	M70684	0.003546723	24	15	321461384	hypothetical protein DAPPUDRA	Metabolic process	Generation of precursor metabolites an	7.92E-31	19.34486	26.99645	13.62435	12.3846	8.124053	16.28066	37.9964	16.15211	22.46641	33.95966	18.15913	12.86759	21.83271	9.840114	27.99927	57.61594	32.22297	45.67933	33.22829	15.84358	32.36608	29.6222	24.49326	28.47101	33.75905	21.23607	29.26681
CUST_34437_PI429010886	M9678	0.003551814	24	3	242017921	conserved hypothetical protein	Protein metabolism	Proteolysis	1.15E-57	121.1272	91.94456	77.47208	78.83688	147.3274	145.6946	88.10909	112.473	104.9484	71.40475	98.00625	133.335	103.2365	65.94538	106.4444	51.21624	38.64131	37.03848	87.9342	87.18835	61.46387	76.64241	103.1735	77.59604	57.07543	82.48416	84.12527
CUST_30234_PI429010886	N61450	0.003551814	24	21						174.3407	198.7013	222.7248	220.1875	112.683	140.444	137.6386	183.4518	216.3545	188.1844	117.9775	142.0207	199.3483	119.8645	73.88293	319.1468	182.8221	233.7126	218.3332	176.3183	222.9426	353.5795	223.6033	250.4566	342.3913	186.13	272.2328
CUST_46364_PI429010886	M72392	0.003553526	24	3	121484201	cuticle protein CUT9 [Portunus p			4.12E-10	1720.907	26.24625	1883.877	2600.653	2704.306	2436.323	31.46705	134.8435	58.24276	960.1156	2228.732	1383.294	393.8421	1584.461	1043.236	NA	23.50843	11.8917	60.92733	169.8826	44.63889	1308.545	733.4921	876.6144	70.55138	496.2739	183.5918
CUST_13287_PI429010886	N30915	0.003559186	12	9						NA	24.39861	13.95828	5.161139	9.044852	3.506178	6.527016	21.73892	30.64789	21.1277	40.26358	20.18794	15.82424	23.44924	10.75515	3.310609	9.426819	3.97447	7.870331	61.21121	6.146523	9.651486	36.37756	24.30578	13.27761	25.41498	33.76496
CUST_4406_PI429010886	N16692	0.003562046	24	0						124.3455	121.0405	73.56286	22.81234	62.90417	35.90062	121.4525	65.37786	86.16832	40.3475	65.42578	8.150003	51.63077	54.66763	105.1035	80.96563	23.73991	54.8814	100.875	94.31617	121.6759	97.48939	66.63152	83.70817	184.9052	96.19976	133.3669
CUST_32743_PI429010886	M4996	0.003562253	24	15			Metabolic process	Carbohydrate metabolic process		367.5711	180.4396	195.9869	184.5342	42.13235	122.436	336.8186	724.9498	551.2795	868.2894	395.2192	218.3991	478.4126	525.4149	239.9273	1399.933	286.9821	1468.759	736.2732	685.4537	1132.862	545.4629	51.21191	432.0096	1005.566	211.7618	648.9592
CUST_44663_PI429010886	M66602	0.003563873	24	18						206.4029	496.8458	572.9401	178.9851	109.5487	122.3346	247.6563	360.8837	462.9596	186.1968	208.3716	143.1622	192.3394	141.4616	257.0403	340.1047	208.3055	335.0262	787.5865	612.0937	1128.19	387.6202	265.6845	236.8696	412.5149	151.6078	398.088
CUST_24933_PI429010886	N51504	0.003564853	24	6	321477434	hypothetical protein DAPPUDRA			2.78E-25	51.80751	56.89319	63.72422	103.9282	67.7438	94.04538	108.0539	70.73344	99.84349	74.15901	96.23561	90.76223	47.09644	46.8318	48.02557	74.72923	125.3249	109.8644	89.5211	49.81991	59.27468	42.48778	48.26184	38.20339	66.11318	90.13423	67.53158
CUST_32156_PI429010886	M3385	0.003579318	21	15	291241353	PREDICTED: hypothetical protein	Metabolic process	Carbohydrate metabolic process	3.68E-38	68.39377	75.49296	50.24098	37.59336	94.22856	60.52841	64.90539	100.4391	84.00818	69.77207	114.1426	82.73518	65.81456	111.8609	102.9977	77.6465	122.7013	87.22486	87.18835	86.81003	107.6401	35.63867	61.45632	74.4166	62.73874	77.46257	60.86727
CUST_30495_PI429010886	N62014	0.003582933	24	0						43.85321	47.73425	54.17655	37.7689	27.34326	33.53984	26.68521	27.33338	24.76487	20.7271	36.89171	26.6719	20.2155	63.94264	26.56674	27.2697	24.00968	27.75069	25.41794	21.02168	20.94297	27.39507	57.49967	46.768	53.61862	59.13513	29.95632
CUST_6232_PI429010886	N19820	0.003583065	12	9	443719661	hypothetical protein CAPTEDRA	Metabolic process	Lipid metabolic process	3.26E-114	169.6212	374.7006	345.8925	194.635	221.1981	187.1971	262.2441	258.3703	273.0815	276.7278	339.5673	328.2775	224.8574	151.8271	207.9512	219.2338	175.182	244.8381	277.1553	321.9997	343.1184	330.3379	318.5699	355.5754	347.8293	248.0559	285.7894
CUST_8857_PI429010886	N23710	0.003583587	18	15	157126301	latent nuclear antigen, putative			0.000000111	111.8445	151.0071	144.9158	114.1426	95.44831	99.80727	180.3196	157.847	152.1456	180.4963	107.6658	118.4747	164.1331	112.963	131.2891	243.3699	190.463	229.4919	154.6439	122.3191	163.0243	145.2758	100.3265	138.5257	168.5856	157.7159	167.7409
CUST_20403_PI429010886	N43379	0.003583587	18	15	196014851	hypothetical protein TRIADDRA	Metabolic process	Carbohydrate metabolic process	3.65E-09	14.86033	25.74022	29.16979	10.22653	8.640415	4.260292	41.49384	34.6494	27.56835	44.04067	22.39651	17.2724	17.52792	3.749863	14.18007	35.18743	54.30068	48.38472	33.64233	26.11634	31.22074	14.41191	5.563331	15.15512	16.70829	31.68069	38.55552
CUST_46595_PI429010886	M73104	0.003591009	21	15	238683723	60S ribosomal protein RPL23 [Pa	Protein metabolism	Ribosome biogenesis	5.63E-14	55.87434	26.98744	36.11092	59.57649	17.39841	41.72041	52.19714	81.71011	70.04249	52.99968	62.60527	13.98648	55.62648	62.3254	24.87517	69.45139	107.4395	94.815	112.2997	73.92515	66.65338	61.58	20.37574	46.27549	42.14752	23.39604	23.25083
CUST_55798_PI429010886	M111738	0.003591343	24	6	542173512	PREDICTED: nuclear RNA export	Transport	Other	2.07E-57	8.837166	6.547691	11.25075	10.47216	7.339187	13.78304	16.96246	11.15315	15.25766	9.104193	NA	12.13497	10.66525	13.61629	7.562132	8.898826	9.707188	9.317073	NA	14.42653	5.443763	6.569065	NA	5.181037	NA	NA	7.024734
CUST_11542_PI429010886	N27994	0.003594074	15	9						178.3834	318.7134	189.4144	132.8203	212.5212	177.5731	168.902	213.0837	288.7179	217.7355	354.4054	231.8452	289.5149	315.8899	276.0279	193.4888	350.2239	305.1178	165.368	246.5526	148.3156	241.7973	294.6856	310.5741	326.0295	293.2244	356.7674
CUST_39657_PI429010886	M42308	0.003596816	18	12						37.07514	30.30413	28.46437	27.24634	16.01417	20.41052	33.17685	19.33037	20.53051	34.96505	31.02823	18.45917	44.81684	38.00971	22.37256	32.40814	36.16824	35.48951	21.85917	27.982	31.19486	15.16316	20.86861	28.42785	46.20239	27.37466	25.09771
CUST_2186_PI429010886	N11822	0.003606232	24	15			Protein metabolism	Cellular protein modification process		1206.191	1833.616	1273.044	1211.071	1079.588	1002.076	1491.134	1340.842	1521.677	1466.746	1064.289	1583.518	1359.653	1154.406	1193.688	1660.744	1836.765	1788.478	1757.509	1450.664	1941.997	1356.93	1373.039	1619.49	1565.636	1196.459	1662.168
CUST_6327_PI429010886	N19951	0.003606232	24	15	260818051	hypothetical protein BRAFLDRA			7.68E-56	343.9474	547.9685	507.7884	360.0833	348.0379	326.4698	526.4775	598.7661	634.6359	474.3448	385.0272	367.5711	468.8585	418.1662	415.5107	535.6201	667.2339	707.3888	585.434	577.2434	898.3377	463.1658	442.023	462.6929	484.9644	432.9222	656.3228
CUST_6903_PI429010886	N20755	0.003606572	24	0	328779109	PREDICTED: Apis mellifera vincu	Cellular Process	Cell adhesion	1.43E-84	36.01668	27.13842	28.27048	24.96583	12.94453	23.24571	30.20493	26.17673	21.7802	19.53602	13.40748	13.14087	19.15769	16.01569	17.10849	17.40265	27.20683	20.61709	29.33753	20.74399	24.83115	20.9266	19.56731	19.82888	27.06704	17.10366	32.68841
CUST_47648_PI429010886	M77027	0.003609709	12	0						45.35771	31.31571	49.11594	56.11745	29.95843	46.82329	27.69246	23.94642	27.24634	55.16243	28.7875	39.77797	40.61754	34.61745	42.62886	35.40841	30.51443	33.84162	19.55478	25.90137	28.21781	42.17431	26.22609	31.0164	41.25004	30.14806	38.06618
CUST_16647_PI429010886	N36625	0.003612034	24	21						109.9399	151.2383	132.7314	115.2516	103.5143	100.6344	78.33954	104.8565	101.6524	128.6276	120.513	128.3867	76.80903	124.1583	96.58233	136.6675	93.37419	131.9106	125.7244	116.3062	152.0785	150.8113	134.0586	181.663	153.9033	139.4089	190.9064
CUST_52780_PI429010886	M98878	0.003612478	15	0						93.51275	275.6274	315.3455	163.5866	258.3202	151.5059	71.44611	89.76967	26.79915	56.76972	100.1289	45.5523	90.41336	130.479	118.8271	141.6972	145.1769	104.1434	18.58892	84.58847	85.03859	37.93869	141.6695	137.3537	23.98408	42.29117	42.42056
CUST_7564_PI429010886	N21738	0.00362431	18	15	241652778	galactosyltransferase, putative [			1.4E-64	67.41761	82.66079	89.76139	61.32398	63.17902	48.87241	77.45378	66.65338	73.60215	71.09432	50.13895	79.14448	71.63426	66.8086	53.1876	70.0874	104.0803	95.02561	63.06627	69.58412	63.03163	45.72512	61.67047	63.66743	70.56233	61.81014	68.30948
CUST_11659_PI429010886	N28192	0.00362431	18	15	110671430	putative ribosomal protein L34 [	Protein metabolism	Ribosome biogenesis	5.42E-30	36195.31	38060.46	31967.8	28943.19	24289.54	28414.17	34877.59	36967.46	35305.99	35445.2	26678.14	35862.04	33637.86	30141.56	33304.41	44913.68	45731.6	40915.77	30065.36	33304.41	30821.46	27832.29	33637.86	33076.81	33176.01	31206.9	33508.11
CUST_27132_PI429010886	N55501	0.003630162	21	15						135.8408	113.4005	203.392	61.89058	58.30569	36.10172	42.87617	99.7036	41.63464	42.08196	25.7107	27.01035	203.6502	46.03907	276.2644	73.52254	179.3632	147.0201	186.728	77.69968	147.9838	77.17435	65.81456	48.07163	113.4582	99.97142	134.6275
CUST_39744_PI429010886	M43810	0.003630951	12	9						31.91659	5.602333	34.5207	198.6089	86.32974	39.30626	13.82584	451.4694	460.2218	804.3138	351.1109	575.3242	983.8429	250.1228	444.9419	NA	NA	NA	346.3619	29.52107	289.1658	1087.769	1403.719	1926.392	22.9023	221.4294	215.8263
CUST_34516_PI429010886	M9897	0.003632015	21	3						55.22014	111.9372	86.66046	95.61224	101.0861	82.42776	50.91261	83.19883	102.0776	64.1954	37.3522	54.45212	66.95538	56.64171	61.17453	67.73599	103.8143	92.95971	77.98875	70.62321	92.33626	89.41928	95.78807	82.32889	85.70234	109.2206	97.21065
CUST_24623_PI429010886	N50963	0.003634881	15	0	321459829	hypothetical protein DAPPUDRA	Developmental process	Nervous system development	1.43E-13	45.79664	60.71646	NA	41.57252	20.76565	53.45604	58.86906	48.67088	48.77948	20.7793	21.04763	5.534335	34.6494	31.34548	36.22402	92.36671	50.90051	49.78973	71.80743	22.01795	45.17249	5.675951	41.85499	54.29543	21.23607	39.63818	4.888737
CUST_34844_PI429010886	M10870	0.003636358	15	9	321474388	CG13830-PA-like protein [Daphr	Signal transduction	Other	2.66E-12	66.32665	60.22483	74.42622	66.67393	59.16434	49.92591	46.88337	68.50135	59.38272	80.99203	74.92905	60.58781	49.28723	59.00011	56.05872	76.08583	76.64241	60.83425	45.07182	46.21146	52.20454	77.21485	54.17655	72.31873	80.03641	90.62398	96.62082
CUST_7915_PI429010886	N22267	0.003643234	24	0	443691851	hypothetical protein CAPTEDRA	Reproduction	Reproduction	1.42E-41	26.78121	25.42047	19.05953	23.01448	18.66832	21.39999	NA	18.65321	20.67803	14.1084	8.256752	35.19371	9.941387	10.79953	17.08255	22.60277	9.33567	17.95932	19.23624	21.35401	17.34867	21.78716	17.06435	15.88272	39.28264	25.13355	20.41963
CUST_32412_PI429010886	M4050	0.003645079	24	9			Metabolic process	Carbohydrate metabolic process		60.69474	29.07923	28.85219	71.25639	873.0203	510.5943	79.28417	101.4772	43.84283	55.3772	780.7545	695.5915	NA	631.6446	588.3344	72.88356	32.74766	32.96923	19.16548	95.16242	23.29214	29.97653	24.19784	20.4836	6.569065	198.1815	NA
CUST_45096_PI429010886	M67989	0.00364768	18	6						23.352	5.536298	29.96998	NA	16.55821	11.20316	26.38195	27.65783	22.35703	35.40841	14.85493	25.53977	6.876832	8.419629	3.039739	26.56947	14.37528	20.40806	10.17265	21.40492	24.71432	22.14763	26.35385	41.43789	27.2558	37.33345	71.48244
CUST_34831_PI429010886	M10820	0.00365021	24	15						242.222	340.0191	311.1315	262.4813	308.4293	248.6699	319.0038	332.4145	329.8991	349.2496	264.176	361.9725	352.7205	272.1212	320.552	399.8074	393.5689	467.3293	351.343	357.4621	445.9579	341.177	389.7239	455.8434	447.3237	381.5238	493.4583
CUST_50903_PI429010886	M91408	0.003650728	12	0						36.31991	40.03773	52.3424	29.43929	43.19513	33.97881	15.87405	29.90874	30.00163	23.5545	35.80613	30.07238	41.02997	29.79915	48.04838	40.84998	31.75895	43.86283	29.95632	38.21742	22.91325	47.44242	30.89721	25.43349	61.40643	57.20137	39.03604
CUST_4275_PI429010886	N16381	0.003659423	24	15						184.0518	230.7269	231.7623	176.3993	210.6988	201.7307	177.5447	215.1466	217.0412	197.6734	169.1253	196.4414	228.7822	227.1397	253.2436	271.7261	302.4553	259.3203	197.8624	223.982	274.6613	231.588	257.1925	245.783	249.9933	194.3971	275.6746
CUST_2450_PI429010886	N12377	0.003660194	24	21						70.5683	89.36499	88.46676	100.8867	81.91112	100.6934	73.92515	80.71612	76.78462	97.32812	60.23886	105.0475	80.76921	71.89101	85.19452	128.5165	95.39909	109.0704	125.4006	86.89375	107.5502	128.6628	96.71212	122.201	121.56	109.9526	101.361
CUST_47338_PI429010886	M75679	0.003667607	21	6						15.62926	5.820987	6.494807	9.807589	15.36528	14.80962	13.68243	16.80772	7.205732	12.80236	11.9307	36.57741	13.74367	10.44732	7.065157	7.571002	NA	8.252165	8.076758	4.362541	5.632345	7.855592	12.20542	8.814223	14.97655	12.47302	12.7897
CUST_51570_PI429010886	M94034	0.003668539	24	21						39.75608	43.126	43.22841	41.28574	36.53104	29.53809	50.34979	31.08965	34.97876	45.80468	36.39662	43.36291	26.0242	15.46584	26.70669	35.86607	31.26513	39.68636	44.21087	41.88081	38.04561	44.71461	46.44259	43.63223	54.33723	41.3785	32.64901
CUST_43245_PI429010886	M62518	0.003670925	24	12						286.926	516.7278	367.1233	1282.861	379.5535	884.7471	2100.692	1125.978	2920.075	313.2232	1505.065	179.3019	1537.641	1553.379	922.1615	919.6173	1306.13	1900.457	177.1027	577.3756	262.525	134.3589	684.5912	551.2795	124.5835	152.7673	278.2449
CUST_20627_PI429010886	N43778	0.003674366	18	3						56.64171	90.32894	75.96972	82.36909	130.7958	107.509	68.26223	60.79074	105.4571	70.99538	100.8115	16.2053	43.8076	37.16734	50.70492	80.41731	116.6562	100.8867	74.25196	71.26688	45.872	89.98471	117.4337	101.7015	44.83298	83.42667	69.87542
CUST_34776_PI429010886	M10621	0.003678641	15	6	545854183	PREDICTED: zinc finger protein 7	Nucleic acid metabolism	Regulation of gene expression	2.24E-50	14.85124	17.29613	18.43914	28.86593	22.34334	15.4862	20.50908	20.84673	23.56042	20.8665	19.56045	25.39952	20.95393	15.63074	24.29464	15.95845	14.41667	19.6697	31.79774	18.32237	20.25356	40.30891	22.58052	36.56104	18.69793	24.28735	22.22355
CUST_50332_PI429010886	M89483	0.003678781	18	15						16.40816	8.949447	NA	5.104435	15.483	11.59928	18.28516	32.70202	15.02898	NA	10.22339	19.9436	NA	13.51154	25.83809	32.68105	34.88898	27.54533	27.40728	13.96051	21.5031	NA	5.045715	NA	12.50167	20.68187	3.321803
CUST_26528_PI429010886	N54401	0.003680822	12	9						NA	NA	11.15025	6.885217	10.30511	3.988917	NA	NA	10.17714	13.01662	21.42827	11.09087	17.11834	NA	8.116522	4.723529	NA	5.50597	5.979128	5.762763	NA	16.24051	11.48896	17.64448	4.110361	7.022671	6.330656
CUST_52728_PI429010886	M98678	0.003685509	21	3	328776957	PREDICTED: putative vacuolar pr			4.45E-33	4.514414	14.62836	19.43702	20.53404	109.7081	70.80726	16.83166	15.6646	20.5047	7.444772	58.78081	49.62618	20.41366	20.35695	24.31278	4.772786	4.172602	9.404259	20.16039	32.18121	19.65508	14.65727	16.19958	21.92764	26.78523	22.1804	34.85943
CUST_46875_PI429010886	M74033	0.003686693	24	18	321468336	hypothetical protein DAPPUDRA			3.33E-10	36.94988	41.85107	60.90419	30.377	29.59501	21.94131	39.86153	48.15989	47.24767	25.16166	30.56155	27.63659	53.27399	45.89143	43.58292	44.90369	41.72651	46.6272	47.10689	64.86228	94.17512	43.72166	39.38869	88.52477	56.19317	39.73627	73.26943
CUST_1917_PI429010886	N11056	0.003686693	24	3						1647.856	1462.785	693.3608	2835.943	2119.199	2113.55	792.599	783.939	716.3366	1447.815	3310.069	2849.078	1176.913	787.5865	1218.815	768.79	449.5658	835.0606	287.938	328.6362	356.3573	1155.365	1167.815	1701.993	217.4779	518.6591	789.3467
CUST_48946_PI429010886	M83095	0.003689043	21	15						83.83043	132.3768	33.86491	104.7454	29.52349	113.5034	273.2919	192.2662	95.09356	207.4443	142.5641	67.62577	203.4357	98.50388	182.7293	330.3379	297.9395	275.1575	153.9962	67.4639	127.6534	54.02066	103.7909	69.71651	195.6831	193.2448	31.74183
CUST_42584_PI429010886	M60646	0.003691747	12	0	383853984	PREDICTED: uncharacterized pro			0.000000047	95.80871	32.95505	100.0635	71.22472	66.3578	37.93535	24.19322	30.5648	48.84433	84.96211	49.96679	38.04561	442.023	60.10219	104.2173	56.12959	36.74804	60.49503	51.86305	45.61423	32.55747	138.1161	78.59166	91.86363	106.4444	51.41632	137.3537
CUST_33408_PI429010886	M6813	0.003691747	12	0						196.6122	201.2	209.9184	223.8968	156.0492	168.3482	159.4657	142.8249	158.6683	232.9522	185.7539	195.1701	214.0358	162.2114	183.4751	204.8124	206.0425	175.8219	187.5258	166.6906	154.7131	202.3368	191.9733	203.3258	244.4841	251.655	261.6417
CUST_39820_PI429010886	M45152	0.003691897	15	9	532759728	antimicrobial peptide type 2 pre			1.52E-30	8.064044	15.12028	6.57946	14.09922	NA	5.076455	NA	8.253616	4.574832	19.46213	NA	14.74793	11.87749	10.05496	NA	NA	12.44307	5.807914	8.884176	4.092338	9.917631	10.00684	14.23378	21.63837	29.71551	24.57559	72.38107
CUST_32503_PI429010886	M4292	0.003695623	24	15	242011463	conserved hypothetical protein	Transport	Protein transport	1.08E-79	118.5289	143.3711	116.1695	123.6152	151.7315	120.7872	161.2169	152.1194	167.7764	128.4025	141.2484	121.8066	126.6423	146.7486	131.2222	204.8848	243.0247	275.2462	166.158	135.0024	153.9248	167.3103	156.1818	128.179	123.1774	143.5809	123.2141
CUST_27584_PI429010886	N56317	0.003695623	24	0						102.6861	86.42647	79.92025	59.11174	71.94693	58.78776	52.0131	75.12194	85.51486	33.88742	71.33856	39.95456	81.73809	71.33856	60.3351	25.49854	27.69481	58.57419	69.88293	79.16248	3.692657	64.13867	83.74447	39.39814	92.48145	83.19883	59.2979
CUST_7383_PI429010886	N21463	0.003708354	24	18						272.2328	623.4767	402.1648	309.7415	349.9633	313.6779	370.4445	499.0568	361.1904	309.797	366.242	320.8212	390.4253	378.0804	328.5727	425.365	562.7989	465.2004	537.3205	522.6994	520.4605	403.3938	468.7122	394.193	570.0085	379.9317	568.6219
CUST_22270_PI429010886	N46722	0.003708354	24	21						80.65861	58.39443	76.03311	76.71918	43.33538	49.5263	21.59473	44.25351	31.78394	68.18657	31.11715	47.77307	99.34395	30.46011	126.0081	52.3424	59.83108	33.29327	103.8143	64.30529	78.21211	127.1242	60.93307	96.84289	141.3689	51.0346	125.5852
CUST_22403_PI429010886	N46974	0.003708354	24	21						174.8799	282.9282	247.1999	208.8218	223.8255	198.6494	173.2871	198.9444	225.7754	222.0531	217.3699	207.3047	194.821	221.2277	219.187	359.0835	273.556	312.2924	318.4002	239.4431	284.7236	351.2056	241.9195	295.6886	469.6287	269.8306	391.4746
CUST_40949_PI429010886	M56460	0.003715772	12	9						37.6281	40.60853	49.83616	39.9983	38.08917	29.282	21.48061	25.22536	33.33788	54.22249	42.53768	85.57553	42.36924	36.29792	38.33215	25.91341	27.39004	32.75387	37.93869	36.04692	39.74086	55.93014	37.77609	48.97575	53.42717	42.06094	50.19242
CUST_4633_PI429010886	N17176	0.003715772	12	9						319.9838	11.08066	312.8962	1080.452	48.25642	620.0365	35.79767	22.64904	41.57926	1322.312	2777.197	817.7537	1031.076	151.3085	621.9301	4.782171	11.90435	15.11427	98.51271	40.45487	63.31668	947.0352	1031.076	1193.688	122.436	626.8237	1028.452
CUST_337_PI429010886	N4053	0.003715772	12	9						1730.472	3875.533	3781.313	4545.107	3202.946	2104.842	4622.321	3324.822	6491.663	4539.587	6523.544	4048.748	7762.077	5540.01	3274.995	1555.177	3643.551	3660.946	4055.849	3692.065	4998.113	6285.074	9226.663	8996.679	3811.795	6515.192	9516.402
CUST_28842_PI429010886	N58710	0.003719175	18	12						22.99386	39.24909	27.5259	16.2921	6.843272	24.28968	42.178	31.79774	43.02903	18.42193	25.93287	13.18497	39.61493	44.07915	35.45915	29.98295	25.71961	28.17748	16.79346	52.51146	37.42403	10.74484	19.43702	7.512221	24.21058	31.52857	23.36033
CUST_49774_PI429010886	M87065	0.003722108	21	15	260781241	hypothetical protein BRAFLDRA			0.000000154	28.82109	42.42056	35.53811	36.34596	21.34628	35.86607	38.69332	47.96813	43.51485	49.93016	32.94858	42.48109	47.57931	39.18322	39.44209	59.27468	45.01683	50.16621	32.68105	31.36919	32.61311	22.74435	35.47671	34.65363	30.10077	35.56743	42.6001
CUST_31554_PI429010886	M1821	0.003732241	18	6	383859284	PREDICTED: uncharacterized pro			4.31E-48	4.413116	8.694186	9.354156	10.11713	8.109042	5.501586	10.41528	9.44382	11.41659	NA	8.846413	12.08928	5.899397	11.64284	7.635827	10.24931	5.729424	6.393169	5.554011	8.977003	4.705386	8.502559	8.862207	6.4695	12.3595	12.32971	NA
CUST_19104_PI429010886	N40988	0.003732562	24	3						14.55272	9.879786	8.109042	15.36995	NA	12.33765	5.319445	11.61456	8.77273	6.963509	NA	3.938542	6.571137	NA	3.738027	NA	6.36996	5.644653	5.689801	3.860932	NA	7.111158	9.411311	9.617132	6.418865	6.077489	18.55182
CUST_16491_PI429010886	N36327	0.003735156	15	9						660.4277	374.3365	753.6723	951.1847	775.0084	763.8013	402.7185	474.0818	676.3457	825.4048	1445.709	848.9964	998.2787	700.5788	753.1726	197.796	230.3457	241.4097	587.5636	684.1238	758.1822	844.4833	1535.771	1393.298	672.7456	1095.073	1340.842
CUST_23970_PI429010886	N49799	0.003738128	15	12						593.9206	529.8591	514.6065	429.4693	288.5336	339.9532	596.1129	452.5351	455.4195	550.4921	366.7062	578.6613	493.3336	541.3579	440.9544	551.0918	464.1815	596.8192	355.841	358.97	335.0962	397.867	363.2101	364.3837	559.1602	472.6199	595.0044
CUST_36317_PI429010886	M15560	0.003738167	24	18			Protein metabolism	Proteolysis		589.1435	1010.854	915.406	720.7726	519.7601	265.1126	904.3497	974.36	1722.827	774.5071	697.732	546.0695	1318.185	814.0506	620.9302	915.406	759.9978	749.5653	1809.494	1439.606	1960.346	1154.885	1648.551	1359.653	1262.424	1180.485	1984.393
CUST_42933_PI429010886	M61659	0.003740148	24	15	478255816	hypothetical protein YQE_0740	Nucleic acid metabolism	DNA metabolic process	1.08E-08	34.71797	57.8755	54.06732	56.22801	44.58593	48.90035	62.98013	79.62038	69.76252	67.69664	43.16345	61.67047	64.41139	81.26034	64.29833	79.51886	96.71855	80.31138	40.98681	93.49221	78.40447	54.91386	80.78521	79.30248	57.8755	48.61521	62.86145
CUST_9680_PI429010886	N25036	0.003741967	24	18						24.60722	24.1562	13.24159	12.90391	9.283589	9.54303	14.19185	21.83061	18.87745	12.55999	10.73336	11.56593	10.36863	16.96577	13.20115	14.61522	17.311	23.0873	17.28788	24.40659	23.98408	22.59724	22.57092	16.76758	14.81846	17.55051	20.69452
CUST_33467_PI429010886	M6964	0.003750477	24	3						87.43473	63.98495	30.81263	81.10019	554.6469	354.0092	21.62227	30.51072	31.13895	NA	271.577	228.7481	6.795326	112.4404	127.0615	6.170982	NA	4.090763	9.933103	114.0755	NA	40.56783	19.10315	20.86861	NA	186.4449	NA
CUST_43780_PI429010886	M63944	0.003756515	24	21						111.6675	121.9822	107.5871	102.0776	83.31874	86.04303	81.97833	84.40775	79.1181	129.3679	91.0714	103.9763	100.3755	89.96257	96.9319	89.3399	77.84495	128.7298	96.41916	111.8116	124.9247	129.4725	109.5906	131.4508	154.8931	93.57741	129.5648
CUST_8432_PI429010886	N23030	0.003756515	24	18	405960555	Fanconi anemia group J protein	Response to stress	DNA repair	1.35E-21	30.85244	22.96223	27.85852	13.39289	18.6092	17.86526	105.8493	18.62596	22.06407	24.004	11.7546	24.10826	24.75478	20.38818	18.93459	27.7616	21.32425	25.64786	24.82357	31.50944	36.46888	45.33378	20.97185	24.77298	32.60314	23.12968	24.19322
CUST_10764_PI429010886	N26750	0.003756515	24	18						26.37129	17.99135	29.74537	22.03984	21.12341	17.02151	23.10033	14.59197	33.71063	26.28423	16.62267	36.90512	28.83977	26.00178	9.040984	28.0761	18.6455	26.46648	30.13995	31.69013	38.03753	30.34849	28.68225	29.94161	26.2515	23.87984	25.85738
CUST_55691_PI429010886	M111304	0.003760007	12	9						3801.741	258.4714	4295.699	5697.98	3798.572	3786.16	910.2628	789.3467	1861.323	5992.677	6873.81	4913.686	7821.557	3303.981	5456.239	238.2083	211.4477	238.167	3437.158	1994.973	2445.736	10193.49	5666.924	8160.518	2881.653	2529.949	4633.616
CUST_18074_PI429010886	N39092	0.003760007	12	9						208.404	374.5443	392.6361	204.1061	245.9078	196.6443	116.7048	275.386	383.3865	324.5457	511.0453	296.8179	292.4745	204.3745	259.4587	185.0545	149.5014	162.3594	203.0154	313.2852	256.539	304.3391	366.0882	338.8265	517.7643	201.7698	590.1632
CUST_905_PI429010886	N7771	0.003760007	12	9	445958583	hypothetical protein [Escherichi			0.000000212	1719.004	199.1806	2013.288	1283.49	1235.266	1525.217	52.8068	668.297	183.3329	1773.012	3525.214	2171.504	3907.106	3135.16	4242.57	12.22382	36.56623	57.1768	114.9362	346.1837	163.2328	5792.477	4125.439	5025.735	320.4829	617.1935	1193.688
CUST_31519_PI429010886	M1709	0.003760076	24	21	321460800	hypothetical protein DAPPUDRA	Transport	Ion transport	1.18E-30	1930.481	2451.472	1606.909	1584.461	1449.243	1315.424	2228.732	2755.522	2477.377	1438.372	1369.757	1346.991	1848.594	1278.279	1470.868	1539.37	1505.771	1462.785	1674.551	2568.829	1754.468	1930.481	3114.68	2710.483	1604.901	2378.51	2467.704
CUST_53910_PI429010886	M103670	0.003760188	24	18	260830643	hypothetical protein BRAFLDRA	Metabolic process	Other	1.68E-21	18.08256	13.6553	7.542058	5.640641	7.187709	12.46071	12.85647	15.85755	11.66289	7.393617	18.21662	17.82488	15.38937	13.20115	17.91362	22.13507	13.0479	17.60535	12.16729	21.1933	19.39459	22.0572	16.12455	14.84915	14.43641	11.42315	12.47654
CUST_42258_PI429010886	M59819	0.003760188	24	18						23.78991	19.83817	22.68453	14.78505	19.71392	21.59674	23.38956	21.14493	17.66478	24.54621	23.4647	22.42596	20.63272	25.55884	16.66408	34.72341	23.28183	39.88097	28.10459	23.5588	28.15267	26.33135	29.89602	27.37887	25.36192	17.12239	30.31074
CUST_45753_PI429010886	M70170	0.003760795	24	6						14.87141	7.768671	9.745235	14.1735	13.7861	15.94333	22.03386	16.72046	11.39292	13.95828	11.29903	12.03632	13.95366	12.03366	10.15557	9.753338	15.08312	11.45478	10.29065	3.931438	8.348262	8.529923	9.372363	11.52944	14.5404	7.004515	NA
CUST_7174_PI429010886	N21145	0.003763207	15	9	321479037	hypothetical protein DAPPUDRA			0.000000307	54.98064	56.94521	60.37267	34.96505	47.10689	49.05136	53.68198	45.08105	69.17285	89.01154	42.20935	107.3485	68.14816	60.11411	61.908	61.2068	53.4446	58.54288	61.2068	48.51397	58.76496	76.3265	63.03163	71.20823	139.4798	60.41841	106.2768

Probe ID	ID	p-value	Period	Phase	EBI ID	Annotation	GO category	Go term	E-value	0	0	0	3	3	3	6	6	6	9	9	9	12	12	12	15	15	15	18	18	18	21	21	21	24	24	24
CUST_30331_PI429010886	N61647	0.003763207	15	6						54.56309	25.63539	40.73056	42.44693	24.23948	29.08789	60.84053	174.9928	240.1963	44.36058	148.107	39.579	58.9878	25.18427	45.08608	27.42138	17.15642	30.88222	44.11057	88.30662	92.26014	74.70953	137.3189	105.0258	24.16363	36.01433	30.36962
CUST_32445_PI429010886	M4137	0.00376336	24	9						47.42663	33.47158	40.41914	43.93243	32.94207	39.95843	52.88225	35.27964	61.62446	74.39469	52.6814	48.18525	48.81332	42.45213	33.40941	65.65546	74.18037	68.38347	39.36544	37.60365	30.05953	29.61117	39.41457	23.04458	46.94606	32.28459	43.93243
CUST_38563_PI429010886	M28021	0.003765107	18	15	157127583	troponin i [Aedes aegypti] >gi|1			4.06E-41	105.8493	91.4929	111.3972	119.4045	91.11591	74.89382	64.50416	135.6294	84.93839	79.66104	118.5815	113.2938	138.575	149.0067	66.72007	123.2754	165.8065	163.6628	109.6769	153.1826	141.7704	79.10279	78.73755	72.29255	108.7869	130.415	83.96708
CUST_37093_PI429010886	M17830	0.003767615	24	15						17.13689	19.21808	17.54223	4.79296	17.97466	11.12634	8.310351	10.56444	14.86234	11.04699	17.81491	17.82226	26.4046	18.83693	23.58164	22.57092	23.92527	25.75942	32.67436	14.74345	19.56045	12.18092	26.46648	24.14234	14.43107	23.64329	31.91659
CUST_11500_PI429010886	N27915	0.003772087	24	15						596.2588	773.3668	591.3029	555.0536	517.3443	467.6907	326.5385	461.2704	414.9926	645.8971	413.8083	401.2544	879.1441	370.5351	731.2874	1022.285	850.8759	706.8768	878.4972	630.7177	659.603	1116.065	611.3316	735.5518	1262.927	405.6944	935.0962
CUST_51728_PI429010886	M94747	0.00377636	18	9						9.850773	12.25846	14.98689	4.795354	16.74205	10.56207	20.55348	NA	18.53796	23.422	49.51361	21.75638	12.00163	14.12665	11.94299	13.93501	12.92228	15.04296	11.67372	11.61625	18.84481	14.06051	7.769994	14.48646	19.10315	16.11449	18.88058
CUST_29668_PI429010886	N60323	0.00377829	12	9						NA	5.307449	6.144785	7.865315	5.37508	11.50267	9.003802	NA	8.318366	10.07945	11.04053	11.24141	5.929819	3.720792	7.569054	9.562348	NA	10.78499	5.366961	NA	5.592145	13.23504	11.21493	11.51954	4.57399	9.829222	4.372897
CUST_37475_PI429010886	M19163	0.003779258	24	0						16106.84	13248.98	14285.49	7844.789	5726.275	5666.924	6936.974	8227.781	6897.446	15170.38	7704.294	7166.836	10746.95	4329.518	7248.769	10506.53	9516.402	4834.858	14956.1	8786.531	9613.776	15012.16	19237.66	13635.69	29843.45	18910.88	26803.66
CUST_49581_PI429010886	M86244	0.003780918	21	3						190.8547	177.8178	NA	63.1189	2078.188	2082.19	198.8809	758.1822	171.9853	NA	390.7977	398.4892	NA	158.6925	111.793	38.14824	15.30443	9.396737	8.851485	1164.632	14.21299	NA	271.0264	NA	NA	2264.823	NA
CUST_45008_PI429010886	M67698	0.003781269	24	15	321466590	hypothetical protein DAPPUDRA	Metabolic process	Carbohydrate metabolic process	3.97E-32	41.39421	60.14723	52.89528	31.91889	29.38566	34.66846	57.92587	61.87143	62.38786	54.18142	36.04017	24.83115	52.75072	37.31661	46.03907	82.02164	82.69673	77.83598	53.92132	47.05022	64.85257	45.25376	37.50453	45.81835	65.67004	44.02111	48.32075
CUST_50872_PI429010886	M91294	0.003787252	15	12						6.203165	10.22133	8.231507	4.414781	NA	6.865089	NA	4.448272	9.946155	NA	19.52016	9.942624	12.55593	15.44438	13.51981	8.284739	7.27001	10.21326	4.73725	11.4865	6.692466	10.26423	10.61353	6.471559	11.43085	8.937861	13.14544
CUST_39428_PI429010886	M39237	0.003789157	21	12	47211322	unnamed protein product [Tetra			0.00000034	32.65563	39.6417	28.65621	60.10219	68.40054	50.98858	45.03269	40.2126	50.4892	65.15059	49.00524	62.60527	100.8962	50.42732	50.68042	34.70595	61.36611	50.96545	70.76996	27.40129	43.73547	33.73518	41.58882	35.61538	32.14998	41.17764	42.10969
CUST_51082_PI429010886	M92093	0.0037938	21	6						11.34354	14.73636	10.3228	23.30899	16.99898	7.885832	18.68973	29.22381	27.06162	21.07922	9.206401	10.63475	9.11221	17.6345	7.830046	6.515712	20.86335	18.85113	6.89548	8.259511	8.173581	15.09289	26.72167	6.891795	19.17715	24.82357	13.58051
CUST_31415_PI429010886	M1476	0.00379531	12	6	270005507	hypothetical protein TcasGA2_T			1.72E-53	NA	10.04386	NA	NA	22.61447	8.923377	NA	36.21321	34.38418	12.85464	5.107275	14.35464	3.82594	NA	14.08381	10.78353	5.879724	7.904365	17.6212	36.20611	40.56201	6.81204	6.356289	9.821487	NA	4.681705	NA
CUST_12158_PI429010886	N29022	0.003796649	18	12						21.3257	19.35309	21.90629	27.84184	25.51207	22.35063	21.7802	33.64987	43.8296	22.86026	161.8447	25.28782	116.0279	102.8763	28.79283	23.26132	24.63804	27.6757	22.56843	14.01076	24.11615	19.04623	61.77408	32.52246	22.248	26.69299	35.02108
CUST_16613_PI429010886	N36568	0.003804242	12	9	54042615	arthrodial cuticle protein AMP6.			1.05E-08	2314.026	202.4552	1584.461	3022.554	2467.704	1819.509	97.60846	115.5552	149.6459	4064.827	3636.712	3611.195	3319.225	1319.093	2672.714	13.31134	18.56115	40.07582	314.254	196.9837	260.696	3654.032	2558.16	3478.142	534.4784	1099.075	1844.624
CUST_47195_PI429010886	M75150	0.003804675	24	18						695.5915	883.7364	941.0274	638.4715	573.6451	660.6448	680.2076	733.0037	895.8876	716.3366	655.3101	752.85	702.325	607.8959	773.0893	1283.49	833.7381	1005.944	1541.174	735.8335	983.8429	1273.67	734.9802	980.6718	1252.458	654.1448	1110.326
CUST_46850_PI429010886	M73935	0.00380549	15	9	246372819	voltage-gated sodium channel [(	Transport	Ion transport	1.29E-98	26.52653	24.09442	16.1275	9.534814	21.53313	26.30298	22.83159	17.43071	21.86479	30.31074	27.75069	33.75196	13.19901	22.6466	27.77843	27.95476	23.69096	25.59906	22.88864	17.425	19.78002	32.21229	24.43421	26.49621	31.08176	25.41015	51.07097
CUST_16922_PI429010886	N37078	0.00380549	15	9						67.07909	75.30381	55.84882	63.87749	63.5234	47.13519	70.51234	68.15639	56.12288	90.3848	71.89101	68.27308	62.61027	55.45245	66.58185	89.45518	92.50275	60.89951	57.50494	54.69951	62.85549	71.65566	82.95322	66.47509	87.59003	82.08894	113.9079
CUST_44348_PI429010886	M65681	0.003809022	24	18						33.25783	53.98617	65.95736	58.70057	42.96134	44.73509	38.93222	58.23269	59.7098	53.8169	46.15921	30.5034	38.46213	59.80532	45.99785	66.9011	81.30602	62.05097	64.56632	69.41702	60.26506	66.8709	61.06467	70.25686	78.85311	59.25391	62.5781
CUST_6469_PI429010886	N20139	0.003809022	24	15	242005831	WD repeat domain phosphoinos	Metabolic process	Autofagy	7.93E-117	97.55312	73.21671	90.39488	86.62987	67.11217	83.70817	93.60566	54.12459	72.97666	99.93518	78.3065	106.7181	132.6428	81.2035	99.49612	133.0536	90.41336	126.6578	114.5333	88.02817	115.8122	123.3372	82.19156	110.7454	105.3437	62.89605	103.5796
CUST_29420_PI429010886	N59830	0.003809022	24	18						337.0445	469.6287	418.1662	309.6955	266.8703	266.6798	449.5658	443.6171	460.4529	454.5758	254.533	251.7959	503.6798	277.8246	332.484	484.4076	501.1967	502.8105	494.2109	444.1379	590.9592	507.5036	431.4309	571.8635	473.6093	379.0965	494.454
CUST_3854_PI429010886	N15411	0.003818894	18	15	72094941	PREDICTED: protein SpAN-like [S	Protein metabolism	Proteolysis	0.00000032	39.20662	65.24075	38.57897	39.04694	78.06756	59.09838	30.19875	56.46685	24.79879	47.41217	56.30361	44.38354	45.95593	57.19268	40.6742	61.68952	78.23924	52.53638	76.5464	74.85952	56.85229	70.39513	48.20014	43.03704	36.62239	13.44675	35.83339
CUST_15031_PI429010886	N33891	0.003818894	18	3						27.4271	14.53951	18.12496	26.29555	28.65875	32.72443	66.54171	22.88663	22.9795	10.55859	18.28904	17.48259	21.65257	23.47814	20.0975	19.14248	23.55652	14.44521	23.24733	29.05473	24.49701	21.48961	28.9184	30.71702	18.46557	22.82225	32.28165
CUST_3443_PI429010886	N14509	0.003819226	24	21						144.6342	149.6459	122.8211	128.8295	99.18844	107.5363	119.1723	119.807	146.4666	126.0374	99.38318	108.3275	111.7787	96.89897	98.46402	165.742	94.12797	147.1665	175.6627	127.4216	132.8564	199.1806	130.479	159.9495	174.9627	111.8445	206.3209
CUST_52599_PI429010886	M98124	0.003819746	24	0						61.2068	191.9057	222.6813	15.88417	27.702	17.50283	77.08119	45.01683	102.9449	19.46755	49.92591	57.81843	28.8322	13.28735	24.80109	14.42774	39.87272	35.14363	207.9716	85.27841	160.5687	128.1605	50.7989	87.43473	198.1201	60.93815	159.2971
CUST_12389_PI429010886	N29386	0.003821361	24	18						507.073	513.0768	598.5974	499.2313	412.7396	432.5544	437.6531	463.6603	530.6367	405.9933	422.5065	533.5339	588.164	487.4115	595.447	574.2528	480.3553	496.692	616.0609	573.048	642.342	581.3213	560.3033	646.8706	644.0162	512.9523	771.7617
CUST_34667_PI429010886	M10290	0.003827382	24	15	31338868	glyceraldehyde-3-phosphate del	Metabolic process	Generation of precursor metabolites ar	9.81E-124	9598.965	20020.97	14642.96	8786.531	7762.077	6358.535	16847.05	13658.74	18146.2	12378.85	7621.268	7364.792	15340.52	12765.38	8846.332	16070.74	22279.42	20020.97	12442.6	15553.93	14764.65	9194.226	11881.91	9643.272	14389.61	11643.13	11305.05
CUST_20287_PI429010886	N43173	0.003827382	24	15						481.7121	992.2362	592.5764	526.1263	482.4747	535.0429	1038.012	787.5865	799.983	981.1472	664.4758	657.9421	814.9423	659.603	408.1119	1153.859	1083.848	1161.622	865.6479	625.077	814.573	603.0231	618.1517	681.5295	854.3715	649.7567	696.0096
CUST_40043_PI429010886	M49195	0.003848478	12	9	155966159	hypothetical protein [Lepeophth			2.43E-12	6734.459	159.3669	5545.402	6791.114	3390.028	5726.275	623.4767	603.9154	1077.231	9129.704	7203.647	8996.679	10352.54	5558.806	8522.605	54.70587	4.176443	91.50373	1780.678	1798.348	1329.124	10587.95	6929.595	10035.81	3079.233	3342.747	3995.862
CUST_26948_PI429010886	N55169	0.0038498	24	0						30.19535	39.06825	35.22067	17.50536	27.70822	24.44216	15.87086	21.68208	17.21141	23.89372	27.55711	16.32616	7.932927	18.47386	32.59982	45.80468	22.94332	25.81706	28.70626	26.64465	27.66729	21.68674	26.60187	26.56161	30.94993	32.69926	54.69951
CUST_35533_PI429010886	M12815	0.003852836	24	21						400.6266	455.0731	365.7666	313.2852	352.0796	278.6422	191.2871	308.3526	313.0268	370.9049	365.2368	348.1933	332.6579	274.6613	297.3675	470.5066	379.6985	380.83	456.2678	360.8837	309.2002	567.1816	375.8148	435.731	610.1834	422.0714	605.287
CUST_41117_PI429010886	M56910	0.003852836	24	21						15.42242	15.68303	25.82506	28.0635	23.775	19.95709	15.03411	12.91146	11.83721	16.34183	14.36256	22.21977	17.29188	14.4625	18.32029	22.94579	18.76673	32.26395	20.53404	16.51171	19.67547	18.53005	32.76569	40.84998	14.87284	20.81559	26.2902
CUST_47849_PI429010886	M77840	0.003852836	24	21						80.15283	88.75	86.02033	71.79771	50.75197	51.90562	72.55053	72.17918	66.05158	99.65569	64.8708	76.30002	87.61045	63.81233	73.12505	85.83911	86.37483	102.9594	77.5538	67.94466	86.04303	103.6807	90.07438	102.6229	83.91189	74.40311	89.55714
CUST_11864_PI429010886	N28533	0.003852836	24	21						77.49407	105.7248	101.2545	98.57909	89.26114	74.46936	69.13756	93.26197	77.2068	86.06068	115.5416	100.264	84.78014	99.116	102.5557	82.72734	99.67801	103.4374	94.98744	127.0776	90.79873	125.9513	112.2837	127.4388	116.2357	103.9282	128.1975
CUST_6689_PI429010886	N20465	0.003866024	21	3	157134143	glucosyl/glucuronosyl transferas	Metabolic process	Other	1.12E-69	160.9133	10.15772	10.62276	10.57021	21.76828	9.969471	14.07796	7.85968	4.819687	12.70386	11.08375	10.87768	3.763751	6.191209	3.66961	NA	6.571137	7.886951	8.593041	4.237452	8.468746	11.54243	10.88798	NA	7.087249	14.60762	17.5783
CUST_15430_PI429010886	N34569	0.003867507	24	21	505844696	PREDICTED: tyrosine-protein kin	Protein metabolism	Cellular protein modification process	1.55E-27	49.9052	70.26755	85.47045	84.9077	65.1725	43.13538	61.18015	69.71651	89.61931	63.10394	62.14833	58.39443	67.45544	50.16118	66.79456	65.69754	61.27966	83.18277	73.84495	93.83228	94.57461	76.27282	97.2956	113.9699	86.36335	73.72575	146.6123
CUST_53620_PI429010886	M102512	0.003869353	24	12	443716906	hypothetical protein CAPTEDRAI	Transport	Protein transport	3.34E-35	199.588	258.1565	273.4227	1464.031	593.3017	581.9588	2666.243	781.2177	2514.613	439.8767	3354.131	196.6443	4109.069	3058.086	509.999	341.6702	1992.843	910.2628	135.8804	345.7435	312.008	210.955	1165.385	1227.139	200.5182	209.1419	320.381
CUST_46788_PI429010886	M73726	0.003870837	24	21						1765.847	1767.886	1435.616	1686.38	1353.691	1369.757	1625.527	1352.876	1634.309	1620.506	1172.08	1469.262	1447.815	1170.841	1408.778	2132.096	1604.901	1669.427	1886.448	1470.023	1614.735	1904.856	1624.639	1768.674	1776.485	1559.979	1708.901
CUST_10133_PI429010886	N25745	0.003872682	18	15						55.35053	74.63073	72.6168	69.43522	39.78094	69.64615	104.2797	71.33856	53.31643	78.81902	61.81635	67.5936	89.01896	66.17125	85.62565	112.1849	90.18372	106.7298	80.51893	62.80159	95.92856	64.71994	42.51318	72.43858	80.12221	56.34856	69.70354
CUST_32599_PI429010886	M4539	0.003900996	24	18						305.1744	378.2634	386.8846	299.5341	275.3403	268.9804	281.8133	380.83	329.7868	316.3525	315.6961	338.3895	332.3383	289.2838	323.8056	384.9395	315.8899	412.0092	437.2723	399.4473	433.9196	366.6368	344.0704	420.7391	506.5099	425.365	527.3184
CUST_5227_PI429010886	N18423	0.003900996	24	18	345481436	PREDICTED: putative rRNA meth	Nucleic acid metabolism	RNA metabolic process	4.01E-74	37.15316	44.5991	51.39588	45.89143	32.43638	24.39861	41.95985	53.88747	47.85839	40.4228	35.53472	34.86224	55.36247	62.48283	41.76233	34.23416	70.54441	48.76461	61.01777	63.23529	66.22878	50.35761	47.77802	46.7009	51.5089	44.52224	49.96679
CUST_9329_PI429010886	N24451	0.003900996	24	18						451.8198	445.9579	571.9784	372.5479	350.4372	284.4024	353.6905	557.036	608.8174	654.1448	347.0422	610.5014	505.1911	384.5219	420.0475	495.7052	620.5792	586.8713	701.8416	510.5943	680.4062	584.4771	558.6463	777.3446	429.9294	440.1079	770.1221
CUST_22343_PI429010886	N46858	0.003900996	24	21						41.99895	28.70626	56.54124	53.09948	40.56783	37.69532	31.65179	28.21525	38.99638	41.31086	32.70763	50.86361	40.74313	32.64246	52.01796	71.6009	35.27117	46.91713	67.41125	43.20988	48.51397	58.14687	45.17758	58.25453	55.78477	39.65854	56.00544
CUST_18817_PI429010886	N40470	0.003908927	12	0						22.5248	16.33222	18.24693	16.22685	10.60003	17.13864	NA	6.834174	6.660555	3.037331	6.598734	5.850805	12.00378	NA	3.747178	8.67534	10.99842	9.856067	9.251212	4.426892	10.21326	3.202569	9.800749	5.233224	12.53109	10.38947	6.490355
CUST_48938_PI429010886	M83040	0.003910185	15	9						468.9491	431.8791	438.485	399.5386	319.8754	271.0733	237.8029	343.406	286.6404	489.0279	478.8721	393.7655	395.794	311.2489	420.2294	386.2259	400.7119	453.1048	341.5157	373.3349	336.7218	390.4253	353.4279	449.1793	452.0404	485.5854	673.3593
CUST_48378_PI429010886	M80075	0.003915773	12	0	21358761	ionotropic glutamate receptor s	Signal transduction	Cell-cell signaling	1.64E-78	12.08433	8.354153	10.8257	14.45239	5.592145	11.11546	NA	3.874608	3.671748	6.532525	5.948304	13.64203	10.45264	NA	NA	5.251169	5.561634	6.455238	NA	3.991072	5.718914	9.237704	NA	5.710174	NA	8.413648	13.84795
CUST_41592_PI429010886	M58100	0.003919601	18	15	321458587	hypothetical protein DAPPUDRA	Metabolic process	Oxidation-reduction process	9.45E-65	52.41843	67.13611	64.65354	64.20079	61.40643	65.58263	66.33824	75.89652	62.35301	68.81679	59.28822	49.71376	76.14147	82.46867	70.42129	91.15714	71.64925	88.04091	64.52421	77.45378	95.85439	37.20732	65.1725	60.41841	66.296	58.13362	63.10394
CUST_55388_PI429010886	M109869	0.003922449	24	15						330.1913	331.5173	353.0555	377.5103	205.5639	281.4085	341.177	295.9223	243.7665	421.8557	217.2732	274.2134	330.4891	307.8719	226.3355	559.5956	581.9588	532.6369	455.4195	246.4965	414.4974	405.2976	358.97	486.4482	331.0671	248.8065	412.2409
CUST_16595_PI429010886	N36525	0.003922449	24	12						57.76704	51.42014	29.04471	52.08619	84.95059	66.56707	43.92787	35.53811	50.52247	44.16632	71.73966	80.47316	60.35053	116.0897	115.8934	67.60011	47.81362	58.18021	35.57898	47.72449	32.65194	46.1196	42.70334	26.40151	40.91214	40.0082	25.89856
CUST_38761_PI429010886	M30131	0.003926469	18	15	71726281	Euphausia eximia voucher KCeu			3.49E-121	396.9028	144.058	376.0943	407.4228	176.1711	294.796	290.2155	301.8062	293.095	416.251	213.89	260.9505	441.8514	400.5287	304.3391	934.7113	472.1134	796.612	844.6913	392.6361	888.6241	262.525	108.3488	205.7216	733.7832	290.4456	367.876
CUST_43717_PI429010886	M63783	0.003926469	18	9						110.8779	209.5934	146.4666	128.1449	99.94989	79.46471	151.9479	169.8127	138.3467	153.7917	159.3992	178.4173	93.12448	240.603	160.4657	87.25531	143.5433	156.4627	123.5591	144.389	133.0187	92.2128	123.018	109.3218	79.86465	157.0815	152.8487
CUST_50583_PI429010886	M90352	0.003926469	18	15						268.4249	169.4819	225.5927	211.4477	159.9694	191.9733	213.0837	143.1244	188.2922	262.1946	160.8732	199.5583	385.8737	191.5119	283.351	935.0962	692.3061	330.3379	400.7915	156.7032	326.7696	210.9122	132.6086	196.7017	277.7663	175.2702	242.9432
CUST_4835_PI429010886	N17585	0.003929059	21	9						67.21667	26.58916	40.69852	72.86661	508.6367	137.4234	30.67349	62.1017	44.43314	NA	224.2516	186.9529	24.469	289.5149	333.7805	13.68542	4.069548	3.639443	9.701216	28.26373	8.951537	37.51315	32.75067	46.55494	NA	23.69696	13.35908
CUST_51716_PI429010886	M94702	0.003936948	12	9						413.9686	417.0366	638.1559	334.8644	77.99973	189.8628	336.8769	111.7147	378.9491	357.7827	773.0893	278.9328	828.1272	422.3982	124.7151	163.9175	219.538	183.8806	172.3438	354.0991	379.6985	621.7856	470.9814	1048.592	255.866	337.2109	560.6594
CUST_53035_PI429010886	M99823	0.003936948	12	9						96.73368	139.8891	91.04847	58.60167	107.4552	90.00835	111.0547	117.2468	143.4442	153.0358	124.2797	147.1038	131.2392	86.41587	156.768	142.2138	83.94977	122.0207	150.5356	118.7775	106.9803	165.4776	170.8146	177.7913	139.3356	158.9852	219.1432
CUST_8243_PI429010886	N22763	0.003944483	24	0						49.57184	76.24747	65.95736	59.23509	52.39131	59.64761	69.46672	59.45185	53.39229	42.77967	55.86654	14.20998	51.04378	46.02219	53.49041	48.84814	65.90991	49.18503	58.2972	62.21897	70.61194	68.24011	62.87718	63.10394	58.9013	63.2206	80.20598
CUST_34724_PI429010886	M10465	0.003949157	24	21						75.46497	86.62987	86.09864	83.11893	74.16214	64.08368	55.59367	73.22433	70.0011	84.93839	68.04046	75.46497	86.51281	77.00089	82.27528	78.49976	82.14842	67.12887	72.15056	74.61536	75.76283	98.81307	87.00868	100.875	87.40333	82.7532	89.70467
CUST_42710_PI429010886	M61034	0.003949157	24	3	545188040	PREDICTED: E3 ubiquitin-protein			1.02E-14	53.60597	23.03724	63.64767	55.50618	43.29935	45.80468	20.23015	28.52973	20.73144	34.22791	29.4575	44.79025	21.34152	17.29971	24.22486	28.06923	17.10019	29.40702	20.9828	14.60927	17.39101	43.09613	18.25575	38.9748	32.04295	22.42596	37.62021
CUST_19075_PI429010886	N40938	0.003949157	24	3	157127444	amino acid transporter [Aedes a			5.23E-40	74.93498	65.01967	58.82275	52.35798	62.1017	54.08928	50.00771	51.49597	46.01299	50.22	25.47189	50.48187	48.43927	40.49223	41.86279	58.85992	26.78523	44.06379	37.86114	36.27638	33.16469	49.7367	43.78502	44.17172	54.0024	30.84622	45.96012
CUST_56817_PI429010886	M114706	0.003955522	24	15						1333.943	2715.678	2314.026	2039.749	1555.177	1785.094	2300.958	1914.352	1710.003	2807.452	1792.472	1269.337	2436.323	1830.285	1934.597	2900.703	2820.736	2681.804	2652.405	2285.617	2558.16	2467.704	2173.027	2478.84	3159.077	2214.163	2398.339
CUST_21278_PI429010886	N44939	0.003955522	18	15						112.7891	135.2743	80.97133	80.93024	82.45499	76.79264	135.4532	96.24348	99.07571	124.6061	97.00725	121.6645	103.8712	110.5024	125.7244	130.8473	136.2198	114.3676	114.8354	85.18385	124.5085	102.3834	104.7235	96.9319	131.468	92.50959	107.1873
CUST_39589_PI429010886	M41504	0.003965721	24	15						882.793	2021.719	1050.844	672.5292	306.6661	388.382	1377.009	1604.901	1406.008	1265.639	484.9644	824.7782	1055.306	612.5918	581.1787	2188.815	2777.197	3056.068	1617.033	1579.509	1407.191	641.3942	747.5407	909.1828	875.8259	1054.885	1052.857
CUST_53994_PI429010886	M104013	0.003967821	12	0	321466304	adenylyl cyclase [Daphnia pulex]			8.99E-16	26.68521	27.03986	31.90974	26.55272	26.64465	22.8101	21.90402	24.67313	31.12893	26.99058	37.36406	19.49861	27.54823	29.20612	34.66072	17.02991	24.32624	15.79305	26.79132	28.52973	31.40901	37.51315	39.63818	24.7069	46.21776	38.87958	44.79025
CUST_50431_PI429010886	M89794	0.003968568	24	0	383860842	PREDICTED: metal transporter C			5.71E-44	36.69203	36.15228	41.99895	31.13507	31.74591	21.17165	31.74183	34.64088	34.29441	32.61311	23.62162	35.63404	28.23103	23.07724	27.47971	41.09379	28.0231	34.32507	52.95714	33.87486	38.90574	34.13318	33.20541	33.28109	48.15989	38.23223	56.38972
CUST_11962_PI429010886	N28698	0.003968568	24	0						39.76397	39.44924	40.03196	29.46145	24.4823	21.01433	30.04801	33.04583	45.29856	36.17758	34.09591	28.42064	23.58164	21.54473	19.18145	20.28829	35.25014	30.59991	38.51685	33.06699	39.47955	35.99913	49.41341	34.54667	43.36808	40.10315	31.66341
CUST_13610_PI429010886	N31465	0.003969572	24	15			Metabolic process	Carbohydrate metabolic process		1819.509	950.7112	1211.537	1230.097	468.6225	526.4775	1879.179	3197.119	2369.531	2831.186	1293.784	576.0861	1890.428	1275.515	876.3228	6575.891	2489.305	5644.907	3940.028	2773.888	6018.305	1498.652	192.5048	984.5856	1960.346	747.8771	1092.583
CUST_4520_PI429010886	N16956	0.003970307	21	18						60.48555	117.9028	85.21665	35.67153	16.77811	6.536266	105.6776	48.73882	49.30833	37.27103	40.48622	22.33262	34.93669	12.26816	15.20248	168.4308	62.1017	184.9052	122.8853	52.60565	131.468	60.23194	75.30381	73.98792	37.35922	29.59163	19.55846
CUST_46368_PI429010886	M72411	0.00397406	24	15						126.9105	124.2797	142.749	121.3554	137.044	121.8365	106.2416	98.42724	107.1382	190.0165	146.3365	137.5078	216.9998	103.1842	220.5454	270.4772	147.6402	197.6371	214.2039	150.2498	192.1667	229.5267	124.9059	219.8774	230.1554	91.77441	206.0425
CUST_36456_PI429010886	M15939	0.003974806	18	15						312.5578	231.9279	165.6814	255.1811	176.6888	281.5287	327.7409	231.809	223.3777	292.6648	220.1011	313.4133	313.9334	313.5422	323.9561	526.1263	402.9178	374.2544	306.0384	131.468	307.1973	201.0489	273.4227	229.8267	352.0102	236.8356	177.1027
CUST_2140_PI429010886	N11668	0.003980256	18	9						49.71376	34.45662	56.24032	50.84163	59.92793	62.1826	91.94456	60.91215	99.6269	61.4469	114.7172	82.36909	70.19469	52.48812	55.57314	32.82904	119.5461	76.53128	48.58376	34.84492	60.56117	68.68508	76.19255	43.06564	56.10448	107.567	85.69239
CUST_35164_PI429010886	M11780	0.003981184	12	9						68.50135	66.70374	72.5147	72.42903	61.91527	52.7448	64.93737	71.08646	73.90571	77.56319	85.66787	76.55656	80.33191	60.40403	73.4442	72.23349	75.88424	59.9228	61.71621	70.95708	74.62293	83.51378	86.43772	75.6543	69.10515	75.30381	96.99292
CUST_33524_PI429010886	M7156	0.003981184	12	9						24.12669	14.44743	18.19202	12.03178	10.58439	11.81992	23.12759	23.08008	15.40884	40.84998	44.64909	23.31389	13.79706	11.4924	17.14498	19.16781	16.1275	18.0495	12.37599	14.86987	24.04006	23.72974	15.483	25.19327	24.15387	22.44233	22.83159
CUST_2143_PI429010886	N11680	0.003989715	18	15						70.43485	107.0748	54.65618	60.57425	65.95317	65.42578	95.70549	103.9956	99.67801	58.64151	80.08063	71.23962	104.2317	85.785	92.36671	104.1579	99.75898	102.4735	91.43014	85.31441	94.58629	64.41139	87.50644	53.99735	67.68769	81.98831	59.86525
CUST_53491_PI429010886	M101952	0.003997317	24	21	383857835	PREDICTED: long-chain-fatty-aci	Metabolic process	Other	0.000000766	72.5147	72.4473	90.64448	74.63073	60.75955	67.06332	67.02911	44.26452	57.08138	76.46737	69.41702	54.5743	82.54419	56.30846	83.86117	60.84718	86.85325	68.35388	73.42732	57.45443	63.96236	104.5741	77.31152	89.69199	93.80651	76.5464	78.23111
CUST_33593_PI429010886	M7368	0.003997317	24	21	7160789	guanine nucleotide-binding prot	Response to stress	Defence responce	1.43E-108	280.3032	289.4524	394.1154	221.3576	257.8891	283.2866	149.1509	215.4758	260.0953	230.5014	328.0523	317.3631	276.0279	252.1189	342.9765	338.4518	305.1744	281.2628	304.58	271.0733	252.5592	429.1442	336.6041	406.0638	478.7694	315.1053	474.3448
CUST_6286_PI429010886	N19898	0.003997317	24	3						815.2223	161.0268	565.424	1102.526	878.4972	1025.772	143.5284	175.4908	151.0875	985.4686	1060.655	1337.377	628.502	558.1235	936.5204	148.4641	169.0749	140.8152	139.6429	135.9752	159.7027	673.7135	305.5795	628.8213	198.5315	220.1523	317.784
CUST_11137_PI429010886	N27327	0.003997317	24	21						51.66124	50.53965	39.27561	35.47671	34.84184	51.78825	16.99096	29.91879	24.15844	52.70656	49.67166	35.59474	76.08583	28.27682	72.15056	38.19894	52.0048	17.65558	67.18486	39.61981	46.69609	113.3132	54.54304	68.99631	87.12904	32.10997	61.64797
CUST_24317_PI429010886	N50405	0.003997317	24	3						171.3126	384.7267	515.2465	210.6988	164.2936	121.0882	64.32511	122.9878	72.63826	21.00124	180.6504	33.6303	113.8465	68.85376	116.7446	107.4395	67.86728	110.775	25.37403	35.69311	28.67614	32.52808	48.95729	32.69018	54.60623	54.81291	36.05963
CUST_44101_PI429010886	M64957	0.004007302	24	0						892.0968	1124.051	1041.894	763.2886	525.7914	514.8454	475.0917	963.1582	827.5626	852.2653	800.3618	645.7283	673.5376	770.1221	341.3363	367.2044	711.071	498.5642	963.73	855.307	729.4004	615.6816	612.0937	531.5525	801.3203	1116.506	1267.497
CUST_47462_PI429010886	M76220	0.00401141	24	21						121.6909	97.48939	158.0791	85.26437	26.79132	69.24371	46.14868	49.3434	38.09775	84.46858	48.68665	23.8982	363.4898	35.64607	226.6091	352.0796	33.60313	307.0639	606.3479	83.42667	513.9521	721.6825	32.2766	668.9797	201.86	97.94351	122.4907
CUST_50200_PI429010886	M89007	0.004025419	12	9						38.85553	16.01033	34.97876	43.78502	15.07942	14.28982	16.74788	17.48796	20.29057	42.05307	56.18215	32.71622	54.11854	23.66496	25.53483	29.64523	23.49891	19.71996	29.00237	19.6314	26.21182	54.41132	65.54992	65.82127	30.51443	40.57567	42.9547
CUST_3377_PI429010886	N14390	0.004025419	12	9						37.69532	40.67888	55.51071	34.31749	33.37375	48.48882	24.04754	42.87617	37.77609	50.29492	44.12566	51.7172	55.55609	49.41907	53.98068	24.22177	25.05534	36.83272	22.39651	65.12931	51.38302	89.5211	58.9258	82.39127	37.72504	33.24054	53.22588
CUST_19900_PI429010886	N42415	0.004026319	21	6						93.24932	111.3972	95.62259	183.2834	150.7719	168.9955	208.5384	180.4963	239.051	325.3527	163.9723	138.9184	267.1275	174.0478	163.5142	94.07474	190.289	123.2322	198.5715	227.4234	116.8623	131.1766	116.2155	83.09656	113.9699	212.4024	90.73151
CUST_5006_PI429010886	N18119	0.004028653	24	15						52.07351	45.80468	55.06924	51.03976	45.61423	32.10035	72.56894	40.96049	44.62591	74.47864	52.29097	68.98107	73.01092	56.75304	64.57877	88.79346	66.54171	75.82447	60.81905	47.42663	51.83708	62.95202	65.9928	53.74306	73.20517	48.33387	61.4814
CUST_40825_PI429010886	M56181	0.004029135	18	3	307175251	Cystinosin-like protein [Campon			1.71E-48	4.531688	9.626714	6.015676	NA	23.6675	17.77566	18.11022	14.0591	15.78373	8.029464	13.60347	3.089094	NA	7.865315	11.51199	4.344875	3.820553	4.38328	47.45455	8.994279	33.02712	7.970211	16.70064	31.01416	5.29504	21.65484	6.836029
CUST_11899_PI429010886	N28596	0.004029651	21	12	152949487	calcification associated soluble r			3.74E-28	34.2712	10.14048	9.084966	47.34599	290.7989	168.1851	51.08768	42.64535	32.74766	19.08178	173.05	203.9965	52.28327	467.5708	613.7905	72.43858	78.06123	32.96215	22.7226	56.77661	10.67743	NA	40.87561	6.098854	19.8869	72.73375	NA
CUST_20871_PI429010886	N44218	0.004037419	21	0						21.16473	23.8882	23.80751	27.07551	22.6315	20.2155	13.43787	20.81357	23.422	10.91277	18.18791	22.31416	19.3644	18.82754	13.01662	21.91441	20.91286	20.41235	10.2951	30.86801	23.19965	28.92859	22.27391	23.36545	21.14216	19.83269	33.27799
CUST_41294_PI429010886	M57374	0.004045478	24	21						224.7434	323.8056	269.4621	256.8674	322.0602	278.0706	211.9389	246.4965	252.6661	226.7528	288.117	270.9307	255.1811	291.9252	241.2245	259.9408	296.3076	348.0379	219.187	257.7274	294.6196	297.6356	385.9551	407.6903	257.2967	268.3647	339.4771
CUST_44616_PI429010886	M66480	0.004045478	24	21	543747099	PREDICTED: peroxisomal memb	Signal transduction	Other	4.27E-19	27.45155	32.15355	41.33036	15.47146	33.01818	19.35718	13.56629	22.66607	18.28516	27.67043	22.70713	30.40311	24.57559	31.6335	19.25145	24.59592	37.40599	33.02712	30.83657	13.8625	23.28393	35.38035	38.89512	33.69474	29.03104	30.59603	41.55628
CUST_49635_PI429010886	M86478	0.004045478	24	21						54.00791	62.61027	62.3082	51.6516	49.97848	70.95104	59.37537	59.88108	54.45212	77.60656	56.33633	59.20501	72.63826	64.58573	57.78766	63.17461	69.73801	71.84187	60.89212	76.77599	69.99017	81.62587	73.21671	89.12634	79.37286	81.56078	84.6671
CUST_13586_PI429010886	N31424	0.004045478	24	21						9.082829	12.45866	11.98667	13.60764	5.142338	7.325387	5.343407	11.36638	10.12975	14.34474	6.633834	6.020478	19.28057	5.354125	14.33538	11.0324	8.864412	11.79624	20.28365	12.84776	14.62238	36.97455	27.84184	14.26253	16.61873	10.46956	16.3145
CUST_21686_PI429010886	N45665	0.004045478	24	18	225710648	Charged multivesicular body prc	Transport	Protein transport	1.66E-30	41.08386	51.03976	55.08838	42.92652	38.92511	50.97745	40.91608	57.56013	56.50958	62.55614	43.53457	56.36738	50.06722	62.87718	47.55964	61.27138	62.68299	58.48301	70.38705	51.67361	71.56025	60.93815	49.03029	64.88027	55.91239	57.39387	64.31661
CUST_53279_PI429010886	M101073	0.004060956	15	6						10.5335	12.07005	12.75519	8.521029	NA	6.010955	21.10822	19.49861	12.301	7.256866	8.421879	6.112777	6.547691	NA	7.341589	16.45776	5.377304	12.18858	17.73565	8.058837	15.40374	19.31939	NA	21.3518	18.85713	9.093643	10.43842
CUST_14740_PI429010886	N33385	0.004061177	18	6						948.407	1131.397	1896.629	5091.533	2656.978	140.7362	4531.71	2710.483	6551.409	2189.919	4004.603	1600.243	5644.907	2013.288	1734.409	166.5929	56.3623	202.9315	7762.077	4852.406	7643.683	3230.906	6051.213	5157.305	5507.513	5913.632	10260.07
CUST_23288_PI429010886	N48573	0.0040629	24	0						64.10757	79.84725	90.63409	78.5366	79.21267	59.72208	46.29703	82.50222	84.46858	62.76751	77.23272	48.66253	75.0839	67.91644	73.56286	55.29943	68.84252	61.47323	76.3955	77.8161	88.5599	76.77599	112.2407	101.6524	89.09696	95.28229	142.6225
CUST_7956_PI429010886	N22325	0.004067782	24	0						69.51507	95.96208	92.28753	55.52583	82.7815	42.11956	70.69299	83.95829	75.78646	67.11217	59.47811	66.97219	48.39409	57.09472	41.00325	69.74823	115.4302	91.02301	65.74218	65.98466	78.02817	111.4113	116.1322	58.29201	233.9171	194.1709	80.98106
CUST_3642_PI429010886	N14949	0.004069655	12	3						487.1319	454.0605	371.6485	555.8829	394.7866	506.2756	510.7551	423.936	406.0638	453.9545	283.749	351.7434	485.4687	394.7866	459.1993	729.7963	512.3561	579.8505	541.8648	299.3012	429.5829	490.5865	257.8376	384.2558	521.6397	459.9674	387.6202
CUST_7041_PI429010886	N20953	0.004069655	12	3						59.00719	125.8966	72.48391	95.55779	98.5911	101.0477	97.73797	135.0204	79.23954	61.97033	81.07922	60.67275	52.07351	99.71725	86.7231	105.7899	163.6628	121.7034	81.63425	90.13423	90.96558	38.72594	101.1701	32.85244	109.762	86.78193	74.79444
CUST_39998_PI429010886	M48184	0.004073746	21	15	195117704	GI22815 [Drosophila mojavensis			0.000000554	216.7919	209.4822	145.4304	220.5798	130.6442	157.0532	409.6823	255.1321	194.7711	322.6893	186.762	244.1747	275.0049	161.9628	262.0968	335.8125	459.4079	260.752	302.345	129.29	235.6895	285.1454	205.2819	201.3668	200.0418	154.8735	132.3608
CUST_51966_PI429010886	M95851	0.004073746	24	0						397.3902	395.5893	346.8977	378.6955	309.4164	336.3654	342.2376	318.3284	288.5336	394.269	228.6642	278.1702	302.274	316.4801	211.1114	378.2634	421.8557	381.9964	282.8745	306.7699	325.1877	397.3902	360.2285	347.4772	437.8715	376.7778	434.4459
CUST_34606_PI429010886	M10133	0.00407404	24	15						194.5138	253.7263	251.6089	185.6007	202.4552	142.3304	191.0167	240.4351	234.8306	194.8762	196.1778	175.0487	197.5603	223.0141	187.8413	244.999	301.3784	344.4073	191.5119	256.6162	296.7459	231.134	283.8964	252.5113	237.0207	182.4139	207.1791
CUST_26582_PI429010886	N54486	0.004076023	15	9						8.404592	6.141714	NA	4.570658	4.481776	5.887576	NA	3.947669	5.173108	7.903245	19.06109	11.988	9.078909	NA	8.380003	12.01856	NA	7.864064	10.59156	7.817615	NA	11.36284	NA	11.9269	15.51771	12.36665	14.87284
CUST_28081_PI429010886	N57256	0.004076147	24	0						60.25895	21.19789	81.85533	10.84443	NA	8.734334	13.9939	27.35507	5.386403	5.597349	9.057903	10.41882	19.39663	21.65484	7.57444	24.51979	NA	22.95059	12.40953	13.31624	5.272947	15.82957	NA	21.70164	84.40775	23.22374	55.20546
CUST_32442_PI429010886	M4131	0.004082223	15	12	344291315	PREDICTED: hepatocyte growth	Transport	Protein transport	2.87E-66	11.37517	3.423805	13.79706	NA	9.946155	11.7387	4.037393	5.847915	3.47844	13.90121	11.08963	5.228764	15.94932	19.83817	8.980966	7.350928	6.704581	8.734334	NA	3.698093	4.333589	NA	4.777208	4.330302	7.070367	7.430249	5.190574
CUST_31238_PI429010886	M1047	0.004082368	24	15						469.5128	729.7963	555.5824	400.7915	412.8294	342.4681	453.9545	527.3184	476.0531	517.7643	505.5201	440.8715	591.786	359.174	634.8724	740.8082	772.0259	744.1912	520.31	464.4221	719.7217	557.9393	581.9588	751.2377	708.1548	546.0695	842.6773
CUST_4278_PI429010886	N16391	0.00408863	12	3						222.7682	260.4457	27.47449	99.04563	145.3783	92.22131	98.32351	400.2135	86.98659	55.52583	15.23659	26.79339	41.89699	186.4716	18.69191	154.9682	613.3285	545.361	323.5302	311.3849	280.3649	13.85312	13.34397	NA	124.9247	53.33823	65.15776
CUST_35610_PI429010886	M13016	0.004093638	24	18						156.0931	207.8099	233.4998	139.3563	134.6822	114.7628	164.7688	205.3015	214.8056	230.7269	172.3736	173.6723	238.4366	132.5291	197.3639	130.479	197.1631	182.0698	261.9111	270.3715	283.351	204.8443	240.7581	242.0929	225.5927	177.05	315.8899
CUST_44375_PI429010886	M65747	0.004093638	24	3	270006792	hypothetical protein TcasGA2_T			1.8E-24	49.33322	36.27201	45.65444	48.73482	46.06104	49.9905	34.59782	35.26049	47.37075	38.08917	45.9512	55.05576	37.19071	42.81475	40.72145	42.61451	41.82506	38.53699	28.56603	28.17748	34.44933	44.635	37.96647	42.80977	60.59689	40.22045	39.75608
CUST_10672_PI429010886	N26602	0.004093638	24	3	242022154	conserved hypothetical protein			4.21E-17	13.17346	8.560595	14.92315	24.13637	17.87188	21.0554	15.29607	9.084966	12.76907	4.791983	10.56765	19.38793	15.59063	12.8289	10.30824	16.44852	11.68618	10.1905	8.364605	8.505122	8.655185	30.49532	11.08375	10.42867	17.458	12.10084	13.33647
CUST_37973_PI429010886	M22529	0.004097941	24	3						3747.441	12.94453	3419.816	2198.578	953.2644	1806.683	NA	158.3229	NA	269.0492	493.1272	648.9592	115.5309	334.5246	441.3596	5.362097	7.403761	9.365338	NA	18.79511	5.709589	1543.558	209.7386	1443.341	5.827121	58.64981	48.75822
CUST_51469_PI429010886	M93647	0.004102023	21	15						667.2339	2489.305	1074.917	492.5086	119.8471	322.3894	1740.73	2098.966	1897.754	666.6396	217.7022	263.7057	1005.566	956.0225	613.5208	1314.02	2386.8	2529.949	957.0763	1129.795	1183.356	393.0833	1105.917	399.8777	719.258	865.141	391.1173
CUST_17854_PI429010886	N38722	0.004102023	21	15						611.1595	2326.394	1003.961	392.1412	104.7861	278.9665	1927.794	1971.443	2010.612	735.5518	229.4464	284.4662	923.4428	849.9671	550.3617	1526.163	3709.96	2482.212	966.6426	969.0998	1172.651	393.924	1076.368	356.4497	735.2877	761.5417	510.4694
CUST_11579_PI429010886	N28064	0.004115602	24	21						657.5452	912.2418	878.1427	632.3124	681.8481	636.3867	683.9311	794.3963	771.2654	728.8717	697.3828	692.8899	717.5574	627.6794	651.369	863.8645	840.0505	914.5652	794.3963	767.8994	890.9879	934.3693	1053.276	1089.998	798.2619	698.9223	1098.604

Probe ID	ID	p-value	Period	Phase	EBI ID	Annotation	GO category	Go term	E-value	0	0	0	3	3	3	6	6	6	9	9	9	12	12	12	15	15	15	18	18	18	21	21	21	24	24	24
CUST_30469_PI429010886	N61957	0.004117389	24	12	307199398	Placental protein 11 [Harpegnat			1.2E-12	17.75966	12.48451	25.82213	30.34519	36.87514	21.01206	23.39604	13.58701	36.55366	41.84178	22.56843	34.92175	38.48962	33.24806	67.78785	24.97563	36.09076	19.88951	33.73518	38.14116	40.27654	17.79852	32.14998	31.17639	28.99089	18.17588	19.19457
CUST_2313_PI429010886	N12085	0.004123265	24	0						334.3239	391.3071	NA	51.94695	208.0191	267.8251	358.5953	1010	410.2255	5.358996	10.03167	13.91758	NA	69.55765	NA	123.5992	57.49102	34.26345	105.1035	716.9976	122.9659	NA	329.8991	NA	NA	778.454	NA
CUST_33791_PI429010886	M7892	0.004127477	18	3						61.37944	33.62051	71.00454	50.20269	78.31361	65.83412	23.86617	26.86328	42.82832	30.3832	48.56908	28.70897	69.15316	40.01947	48.51961	31.94579	29.63277	37.51496	37.50844	38.97889	37.77333	114.6414	92.80135	101.1475	85.23471	45.29856	105.2026
CUST_48165_PI429010886	M79191	0.004128893	24	9						242.133	146.6342	186.792	215.3375	214.3188	226.1282	179.5826	140.4803	162.5329	227.9494	215.8263	260.0376	219.5016	160.4874	212.3284	192.5776	155.3061	142.3895	150.3174	125.6161	109.3613	195.6436	161.6064	146.8442	196.6122	151.2854	124.8682
CUST_51253_PI429010886	M92816	0.004129194	24	18						12.96468	42.17431	31.56412	4.293487	5.40299	NA	13.48654	15.7815	17.86363	32.43638	16.6041	16.5534	18.30245	9.496049	7.111158	17.18571	34.09891	18.79083	34.2465	23.80052	28.3093	22.95506	11.68618	28.25712	18.82259	10.01645	20.02992
CUST_53363_PI429010886	M101413	0.004131556	15	0						19.20962	17.61537	18.28134	18.33509	12.72035	15.89766	14.12665	11.37944	13.17479	10.79175	11.80135	13.64514	13.04927	14.2733	14.00699	19.49649	15.82272	20.5047	143.3711	16.89173	75.64874	15.2772	10.1544	10.12695	19.36238	17.52199	16.38858
CUST_46180_PI429010886	M71687	0.004133749	18	9						23.18254	5.800929	17.97466	46.40767	33.74242	31.81746	45.90076	33.06699	48.45851	79.55681	53.49898	84.75661	23.77756	29.5773	12.14006	NA	3.162548	NA	45.10508	18.77059	28.88967	48.98205	6.08524	44.76603	5.021175	31.78112	48.88152
CUST_19031_PI429010886	N40863	0.004135254	12	0						19.69443	9.519076	11.4263	5.775662	5.552409	10.48651	3.53189	3.59378	5.836101	13.15906	5.527945	6.049144	7.95024	NA	10.80654	13.28237	6.246654	9.064402	8.036199	5.679021	5.458067	17.92495	9.709038	15.95467	10.66656	11.57021	11.61188
CUST_37085_PI429010886	M17806	0.004136084	24	15						54.0322	83.97595	70.54441	56.16512	54.67791	38.69748	63.07317	58.10634	72.53637	64.9627	57.3802	50.51811	59.45846	66.36737	52.0131	74.56881	100.4774	75.56156	72.93006	63.48698	73.93804	79.58331	73.39156	75.91716	59.88723	64.31661	72.31873
CUST_41711_PI429010886	M58365	0.004136084	24	21						23.12555	23.04198	24.04006	16.54048	18.87134	21.64409	8.316005	17.85411	21.49662	18.52205	13.68729	20.58195	21.41224	18.86536	21.48061	25.65087	28.99912	17.29448	24.45965	27.38458	15.98138	23.36545	24.93951	26.62997	23.92779	32.41501	31.64857
CUST_42899_PI429010886	M61546	0.004136084	24	21	470294077	homogentisate 1,2-dioxygenase	Metabolic process	Cellular amino acid metabolic process	2.24E-60	67.61402	93.99213	74.96434	66.44259	80.82353	37.9964	86.60076	78.44375	112.1712	61.79558	60.75955	51.75972	85.16727	64.24425	53.70551	93.55501	66.83189	69.53147	92.63177	77.49407	105.3437	105.5272	104.4489	95.16242	102.722	58.37071	107.9667
CUST_44292_PI429010886	M65529	0.004136084	24	15	324497825	putative serine palmitoyltransfe	Metabolic process	Other	4.64E-25	24.07838	18.52811	25.57888	23.25881	18.45165	26.10756	36.02118	16.19225	27.97586	30.13664	21.61108	27.10146	29.96998	25.40839	25.90662	45.42698	29.49099	39.34641	34.16048	29.59753	34.03486	32.32436	27.03345	18.93816	27.94531	22.1273	27.2558
CUST_7257_PI429010886	N21273	0.004136084	24	21						660.1893	870.8828	781.2177	651.7291	620.0365	600.8289	648.2686	726.7698	750.0607	687.2962	581.9588	620.7278	789.0657	568.2556	673.8771	845.9441	1103.344	931.1977	708.1548	766.0458	941.0274	790.776	886.7077	954.7966	895.4864	747.5407	990.5961
CUST_35002_PI429010886	M11305	0.004136308	24	15						556.047	621.614	46.70567	825.7252	62.73874	734.7144	1008.499	596.5924	499.5403	339.8451	193.3701	81.32217	691.181	1150.764	843.8571	2650.032	1408.778	1306.636	1099.075	197.4226	1027.321	104.9226	689.5858	79.58331	394.1154	523.3495	60.50212
CUST_42260_PI429010886	M59826	0.004136308	24	15						65.85526	140.2011	20.04588	237.4902	13.54205	245.1933	200.181	125.1377	33.66656	165.1039	179.9884	24.62618	209.9184	150.2981	196.6443	513.8347	247.0676	311.7988	129.7968	81.88951	101.9686	27.50901	128.2527	28.72644	68.40753	69.25445	18.48559
CUST_45538_PI429010886	M69417	0.004136308	24	15						201.8328	141.9148	163.4271	223.4224	180.8767	201.258	198.9444	173.8666	216.0939	161.6353	313.6779	150.2981	215.4758	297.575	195.8784	296.2197	338.1196	257.2967	269.4056	222.3298	237.4902	204.8848	154.7361	191.135	218.4319	129.007	143.2259
CUST_3034_PI429010886	N13645	0.004140121	21	12	195499483	GE24761 [Drosophila yakuba] >g			6.62E-31	58.90806	174.857	70.28922	273.7566	126.4865	106.786	185.4692	267.7311	298.6917	170.0456	133.2506	204.2969	155.7485	336.6729	208.6051	74.80515	226.4631	201.5487	74.51372	96.9842	72.87552	101.98	101.8893	127.4702	153.4511	69.10515	93.35268
CUST_45374_PI429010886	M68900	0.004141618	18	3						52.52277	55.42689	51.78206	66.68058	60.81905	53.34499	31.4973	36.14605	30.77357	50.36694	44.85324	61.40643	41.47342	55.38965	47.17279	39.54727	29.15694	41.76732	54.20975	49.92214	49.16853	64.7701	52.58375	64.9627	40.26358	52.15113	51.62622
CUST_11369_PI429010886	N27703	0.004141618	18	15						2042.326	3709.96	917.5193	926.6453	15.60788	579.318	2648.467	1891.416	942.2704	1961.653	610.864	425.6386	2566.656	1049.887	1458.832	3217.866	5593.889	3371.075	1089.56	305.0003	1007.214	1001.191	986.649	899.2992	1760.199	1698.955	366.4491
CUST_56395_PI429010886	M113673	0.004141798	24	18						8.333645	15.95845	12.31866	16.26708	9.252611	13.42476	81.53976	10.91561	85.00598	16.95871	8.949447	29.19743	18.20572	24.90125	17.09822	19.73213	17.5656	23.51724	112.9324	106.2768	238.5827	43.04426	16.14034	39.28587	33.06934	13.16499	22.2941
CUST_44717_PI429010886	M66766	0.004141798	24	18						194.9048	324.4873	211.7618	235.5705	241.6209	200.5474	250.4996	241.4097	335.8125	256.4943	235.284	250.4996	269.707	240.6918	303.1963	268.3054	311.5041	287.6916	331.3594	301.2002	340.9357	296.3076	304.7866	331.1317	287.4074	230.1554	366.6368
CUST_13568_PI429010886	N31394	0.004141798	24	6	262401023	cytochrome c oxidase subunit VI	Metabolic process	Generation of precursor metabolites ar	0.000000462	406.4569	877.3403	510.0998	506.8228	299.3538	450.8164	571.0817	947.376	667.0187	445.9579	722.1173	770.3832	354.1831	303.1963	349.3947	434.9251	851.4962	553.413	304.7058	335.7533	382.2543	330.8653	333.9088	278.7672	449.4542	482.3026	278.1319
CUST_28543_PI429010886	N58146	0.004141798	24	21						55.42058	47.08587	65.6817	43.45077	63.54768	36.15228	41.02313	34.87559	33.23134	54.45775	62.46312	72.77442	50.23264	70.04249	70.43485	69.86685	49.18092	57.20828	53.67622	76.10344	42.44693	92.08367	72.15825	76.07871	89.68248	58.40039	89.16012
CUST_29457_PI429010886	N59901	0.004141798	24	21						97.10574	104.203	112.585	107.332	107.7409	68.54812	92.23687	81.47133	91.19065	111.1154	109.9215	95.53167	105.5272	87.57759	115.5725	105.2701	105.285	117.3251	111.1154	103.9282	133.5862	152.1194	109.762	151.4852	123.354	100.4907	196.2133
CUST_34700_PI429010886	M10388	0.004150857	24	18						331.9294	429.9294	392.8052	297.7147	290.511	288.7764	341.732	404.9858	491.3879	354.0092	321.4419	348.11	393.1863	351.7434	333.4754	415.2055	408.9306	484.7365	464.1815	387.5344	464.2927	411.5622	404.2408	416.9152	477.4346	341.5971	406.3531
CUST_38550_PI429010886	M27872	0.004150857	24	3						51.11657	17.14498	36.48955	77.76407	52.67704	52.96757	28.36733	13.75552	6.199384	25.38297	41.62674	30.11046	40.14297	22.33547	31.48421	15.93269	13.45294	17.63035	17.95668	10.49733	11.65578	39.37754	18.71959	20.20024	16.46484	18.20758	19.39459
CUST_45894_PI429010886	M70652	0.004150857	18	15	256079279	procollagen-lysine2-oxoglutarat	Protein metabolism	Other	2.36E-12	49.04743	68.40753	46.9424	24.75763	9.629964	26.80709	124.4149	48.7205	67.2671	92.39145	31.82327	48.63014	57.60444	39.55139	24.5939	182.1852	119.1014	135.5555	75.58915	84.17542	99.61307	46.0811	45.72137	34.01063	127.8074	56.18757	76.73936
CUST_34488_PI429010886	M9814	0.004150857	24	21						51.39588	48.78916	49.80766	48.75454	43.15923	33.71712	27.36851	52.62861	41.23205	48.36443	48.94611	34.38418	64.03624	32.08859	43.67914	32.94858	50.45124	58.23269	48.89327	66.74201	39.72867	69.49719	80.12221	80.15283	42.48778	65.56898	74.35572
CUST_145_PI429010886	N1558	0.004150857	18	3	83628250	arthrodial cuticle protein AMP1			6.04E-16	9257.227	106.3359	8321.498	12097.54	9226.663	12939.25	130.9169	374.7006	119.713	7389.276	10294.69	9056.645	9889.351	8175.098	7353.952	42.16077	80.05281	65.0067	302.4553	907.1782	259.7699	12743.08	5693.503	10430.71	339.8451	2598.143	2203.327
CUST_19535_PI429010886	N41737	0.004150857	24	3						62.35954	44.01625	61.295	86.78193	104.1325	68.50963	34.06673	29.09105	23.35729	47.71104	94.56207	78.32421	47.55101	77.93692	85.09948	32.61311	17.62376	30.3261	21.51875	31.02035	27.67304	48.93341	39.55568	60.68646	29.3005	54.74265	39.37754
CUST_977_PI429010886	N8057	0.004150857	18	15	260801622	Branchiostoma floridae hypothe	Protein metabolism	Ribosome biogenesis	2.86E-145	13935.1	22221.29	14363.02	11164.38	10999.91	13761.91	17131.85	22847.77	18320.9	14828.55	10641.47	14389.61	15673.03	15394.16	12765.38	23324.95	23273.41	23503.18	17131.85	20913.02	19341.82	11413.74	14189.5	13737.98	17771.79	16288.46	15871.24
CUST_18744_PI429010886	N40337	0.004151461	24	9	321466665	hypothetical protein DAPPUDRA			1.8E-26	311.2489	421.0625	156.493	433.9196	1970.101	1313.269	487.7778	586.8713	270.3263	425.8369	1025.404	1531.626	222.7248	932.1979	1192.533	451.6859	523.2386	617.3702	272.0128	549.679	347.4772	202.9059	302.9304	205.2408	262.7505	549.8031	131.8934
CUST_25625_PI429010886	N52765	0.004160543	15	0						35.62359	54.85916	NA	62.7534	NA	44.39683	47.82733	44.57504	42.61451	17.10366	10.928	NA	59.47811	161.586	34.58559	145.1992	65.20578	73.9144	50.74506	40.26358	47.28223	NA	126.859	NA	13.17949	17.21032	5.282495
CUST_12729_PI429010886	N29945	0.004176315	18	6						8.130511	6.02664	6.228711	11.5008	15.84508	25.46423	12.89112	18.67811	25.99138	15.18033	15.9251	7.075663	8.396446	10.88367	6.554748	4.788815	22.00011	19.06321	8.549067	13.01662	8.298928	22.27113	NA	12.89112	35.82162	19.4916	29.72829
CUST_54978_PI429010886	M108312	0.004180504	24	15						57.35611	36.29792	46.10518	51.01468	45.68766	58.55433	45.85631	21.76828	41.20033	50.0875	30.47018	51.89343	53.02213	56.2194	38.95566	138.4795	75.34564	101.9592	91.55686	43.93243	82.14842	63.75599	22.84451	60.60875	110.5494	61.98746	73.39156
CUST_15919_PI429010886	N35352	0.004187269	18	6						167.6498	159.2473	336.212	294.8466	213.0837	NA	330.8653	375.2193	1086.78	168.7398	215.1466	86.78193	459.4079	258.0601	47.35759	63.47982	30.1739	48.51397	1057.612	686.8238	1017.712	324.4873	1018.186	864.8838	685.9099	601.5188	1430.525
CUST_29305_PI429010886	N59599	0.004188629	15	3						15.09289	15.579	20.01045	206.561	119.4775	NA	253.0838	39.03986	267.1988	NA	47.64318	NA	13.13409	11.47222	NA	23.02109	NA	9.405457	92.9385	666.3032	572.7695	81.58851	42.43263	14.61885	65.41169	75.7215	159.3669
CUST_783_PI429010886	N7300	0.004189799	24	15	506968851	chitin binding peritrophin-A don	Metabolic process	Carbohydrate metabolic process	2.85E-08	301.8062	126.4468	106.5406	190.8765	32.21229	111.2293	355.2635	403.6726	379.158	656.4851	297.8139	144.474	298.5983	254.7629	119.8645	876.9516	427.5284	832.328	429.2293	601.9971	728.6567	369.1521	46.42563	192.3671	495.9709	130.9524	228.5093
CUST_50451_PI429010886	M89861	0.004189959	24	21						102.8763	80.84339	134.9488	100.59	127.9454	59.81196	34.18125	50.87138	45.59558	102.1199	152.0514	81.18389	96.82856	87.63468	114.1426	88.51224	114.2673	84.31911	140.7771	86.27418	92.7356	229.4919	97.69073	196.4999	237.2821	146.4666	175.9436
CUST_38826_PI429010886	M30982	0.004190441	24	15						10987.48	19195.08	14580.94	8461.114	6094.281	4261.241	9669	12084.47	10485.81	11455.94	6559.55	7039.176	9444.83	9111.496	9041.082	23575.84	24825	21974.84	17287.24	14470.28	12725.99	16655.53	9712.243	11030.98	22332.92	9727.143	14922.71
CUST_41955_PI429010886	M58972	0.004194597	18	6						19.46755	26.09025	29.45413	28.8296	37.45153	35.30149	44.17652	35.6426	119.4159	36.71863	24.97351	15.24489	45.46717	16.58591	21.52069	20.50045	23.85663	26.0166	58.58735	21.86932	26.14707	55.28754	33.48738	21.10532	65.39166	37.22647	33.76876
CUST_35561_PI429010886	M12885	0.004195405	18	9						40.30891	44.22724	36.55032	35.47011	26.27536	37.05226	391.9617	39.81879	35.64607	36.32916	58.47152	58.62146	45.60889	36.23977	47.6242	50.64865	49.12192	47.68118	47.84164	28.19441	35.89734	35.67884	31.54041	37.03485	55.06924	50.4892	38.28366
CUST_12215_PI429010886	N29115	0.004195405	18	15	321459849	troponin C [Daphnia pulex]			8.62E-46	339.636	166.5929	323.5302	275.3403	158.7682	222.7248	322.5903	316.081	180.5334	297.9948	182.2041	243.8189	297.8825	424.6765	223.8638	828.8502	653.8973	1122.574	924.3063	788.3617	1414.36	221.3068	90.53932	172.5296	715.2101	428.5726	245.9747
CUST_27505_PI429010886	N56179	0.004195405	18	3						399.8074	40.00526	331.4329	490.077	333.6191	299.0693	109.9526	75.51715	235.7954	227.3921	794.0736	162.8199	563.3118	246.5526	197.4942	27.85852	22.39225	26.2182	255.6336	287.7668	226.3355	516.2255	763.8013	416.6449	136.983	389.2749	415.7252
CUST_28082_PI429010886	N57258	0.004195405	18	3						17.56163	11.21079	41.89159	26.67745	24.42859	23.04683	20.0084	19.50236	19.63427	19.69857	14.03865	9.649837	26.97175	19.32331	37.27103	11.7948	14.1735	14.1735	21.50571	17.87527	14.07625	55.12067	46.53342	37.66415	31.30301	14.36734	36.53804
CUST_36504_PI429010886	M16088	0.004197209	15	0						36.34979	42.6416	34.53535	27.40435	31.68069	29.96518	25.77691	16.44342	32.35564	24.49701	27.69246	21.39254	27.03618	13.08598	21.64165	40.82014	36.91722	43.51785	21.86479	28.5452	37.58094	30.43559	35.69023	31.09316	21.09303	35.99566	23.82015
CUST_40044_PI429010886	M49197	0.00419969	24	21						60.74813	97.22863	69.74823	41.06421	56.83684	53.54287	62.1826	67.53847	64.93737	52.7243	68.96449	52.15113	55.06924	60.78187	49.41907	61.11356	75.45664	73.01092	48.67088	59.69202	70.68343	82.96235	82.91071	78.36365	67.69664	69.48765	72.83024
CUST_55465_PI429010886	M110254	0.00420344	21	18						47.55587	57.06949	53.6019	53.85584	39.64997	45.81369	42.07458	45.44328	52.06198	41.26887	41.55059	68.81679	41.91066	40.45937	45.01993	67.20028	51.29155	65.50892	84.65768	57.0448	68.80951	74.79444	54.39234	66.66302	67.52243	39.80553	46.69101
CUST_37872_PI429010886	M21968	0.00420344	21	3						153.6189	180.8767	84.38135	137.4877	493.5638	476.244	137.0267	241.9944	114.0896	17.83592	231.9279	219.538	59.15801	40.20755	36.13796	49.63757	43.35233	49.09284	37.53429	144.5227	33.6128	151.8772	173.05	102.222	31.4617	659.603	187.2195
CUST_25923_PI429010886	N53311	0.00420344	24	3						17.38739	20.84673	14.67985	23.27322	28.83977	25.08941	29.41761	15.41425	16.73229	36.21321	24.20066	22.4679	16.31601	26.38647	22.01115	8.531174	19.64634	12.17947	17.76942	9.381399	14.56816	16.39186	25.01301	17.07418	21.64165	21.44482	17.6701
CUST_31623_PI429010886	M1992	0.004208046	24	15						73.02048	76.14147	66.97219	108.9605	142.9974	169.8127	77.09727	330.7777	105.9997	125.0917	115.7246	52.56129	338.507	312.4862	376.3542	366.867	715.6987	329.5816	295.8529	322.6893	112.7945	227.2807	66.63719	226.4631	174.2207	173.8121	115.5828
CUST_35864_PI429010886	M13747	0.004214678	15	6						82.32889	97.31511	78.85311	86.9167	82.08894	62.99459	111.8276	113.4873	115.286	75.53689	75.54551	70.05016	85.69239	58.07534	54.36391	59.57649	95.62259	93.27554	122.0207	79.34748	95.03516	98.97699	100.0233	85.58451	111.3237	80.79724	78.20138
CUST_10556_PI429010886	N26424	0.004214678	15	12	321463419	hypothetical protein DAPPUDRA	Metabolic process	Other	2.7E-85	9.935802	9.858804	13.178	8.042805	3.508961	5.078835	16.98896	7.877193	15.28733	10.93759	9.24446	3.639661	13.78137	12.85346	19.15056	15.91774	11.2094	13.93951	3.237617	4.429653	8.690166	9.38001	9.099343	3.979632	16.16198	5.000935	9.320269
CUST_23610_PI429010886	N49120	0.004216603	24	0						1061.537	1342.206	1064.289	1047.687	922.1615	935.4596	757.3112	966.3163	954.7966	941.0274	1018.186	662.0947	762.1289	872.6161	765.466	1051.228	1455.905	1204.7	934.0598	855.0422	935.873	725.6388	990.2906	952.6113	1088.256	1316.174	1199.116
CUST_15075_PI429010886	N33966	0.004225217	18	0						20.69452	23.52466	NA	NA	NA	NA	3.975621	5.077304	7.146978	12.10978	7.425904	NA	11.67524	NA	NA	13.15166	13.7144	5.653997	14.81286	9.733163	8.806809	12.64646	5.923896	6.861282	10.63333	4.031607	5.436199
CUST_35101_PI429010886	M11577	0.004235025	21	0						56.00071	93.66451	90.27688	65.38557	58.75296	64.54817	55.73395	52.52277	52.53638	67.63991	54.87487	65.49056	68.65785	66.55746	66.64484	67.04993	50.70492	78.32421	78.80045	64.80829	72.27008	63.67462	88.93364	84.99467	66.60304	56.86607	82.29898
CUST_48865_PI429010886	M82600	0.004243515	24	15						1021.527	1528.323	1363.959	961.4773	800.3618	926.6453	1345.001	1073.164	1451.369	1404.695	836.1158	1155.769	1112.702	850.8759	1251.245	2122.683	1653.487	2263.041	2072.212	1108.146	2078.188	1627.485	1221.044	1716.333	1950.295	1064.673	2144.939
CUST_2576_PI429010886	N12683	0.004243515	24	15	410509308	myosin heavy chain type 2 [Pena				3825.101	8238.918	4772.224	4322.847	4513.062	3390.028	6717.703	8269.17	5558.806	3506.645	4261.241	4432.869	4900.819	4672.963	3972.222	6543.051	8135.669	9272.221	6155.993	6481.59	6452.703	4910.228	6032.614	5233.042	4633.616	6125.57	5284.133
CUST_37783_PI429010886	M21562	0.004248524	18	15	13898841	CUB-serine protease [Panulirus a	Protein metabolism	Proteolysis	7.54E-60	9153.451	11612.18	7721.817	5879.268	4814.394	4439.592	13879.42	9257.227	12165.66	9855.129	7568.31	6780.642	8484.924	6304.559	5960.592	16726.21	14922.71	14256.4	12021.42	8802.072	10949.85	7337.918	7240.469	7983.519	11076.77	8051.463	9727.143
CUST_3455_PI429010886	N14540	0.004248524	18	15	238683660	Palaemonetes varians ribosoma	Protein metabolism	Ribosome biogenesis	2.39E-130	26299.14	29396.88	28414.17	24199.32	21710.8	20616.2	35738.11	28561.19	29949.84	34286.52	21010.2	24909.79	25463.11	22777.74	26129.84	37765.06	30065.36	38384.01	30257.2	29725.44	30352.12	22401.51	24659.23	25065.69	27577.78	28414.17	29843.45
CUST_56423_PI429010886	M113763	0.004249192	18	15						21.61569	69.25445	46.88337	19.43327	29.73407	23.59807	40.30598	46.97626	41.85499	37.6281	41.33391	29.25551	30.9318	26.99645	37.48759	52.06198	47.70295	56.82231	48.67665	54.42434	44.07915	32.33466	20.47402	24.4665	36.27201	35.42699	36.17071
CUST_3846_PI429010886	N15387	0.004249192	18	15	290908989	myosin light chain [Marsupenae			3.12E-46	244.7879	106.365	219.5016	168.4596	48.77948	129.3499	136.9442	256.9019	68.89904	133.3487	67.2671	120.2076	161.2869	295.1045	77.23272	233.3271	292.6099	377.5103	168.8682	262.4014	341.0171	67.39104	42.59404	45.89596	270.3715	177.388	114.1769
CUST_12792_PI429010886	N30052	0.004249192	18	3						143.7017	137.0649	173.1143	150.2258	336.8186	229.8267	154.4681	145.7532	177.4828	148.6436	152.4151	195.2973	158.3685	145.2258	112.5245	76.02418	70.39513	97.58001	161.4106	247.5032	143.5284	191.823	209.6366	143.1878	192.7777	126.6423	143.0807
CUST_37558_PI429010886	M19495	0.004254096	24	18	518355741	hypothetical protein [Streptomy			0.000000056	1888.631	1157.507	2048.35	2140.692	840.6574	1148.486	1065.617	2404.745	1712.689	1345.742	666.8579	651.7291	1614.735	1118.139	820.9036	1103.734	2275.002	2018.868	3043.672	3480.931	4814.394	3168.826	1723.472	1114.745	3011.975	199.1442	2312.635
CUST_12401_PI429010886	N29406	0.004259291	21	18	390334252	PREDICTED: DNA mismatch repa	Response to stress	DNA repair	7.58E-15	31.33584	70.06067	57.99332	32.91482	40.85573	40.36855	52.25815	46.32151	53.14759	41.52352	61.0054	41.73123	60.98236	47.07893	32.71218	50.80319	56.06577	61.67659	58.08676	61.49909	49.77832	52.23206	42.40053	47.18014	38.93757	33.72001	46.02219
CUST_45045_PI429010886	M67833	0.004261408	15	9						22.16637	27.72605	36.92692	45.50498	50.39653	15.48991	46.86356	34.74153	53.68198	55.90731	63.38745	37.71641	43.94355	22.66607	20.43895	33.05472	40.73056	35.65021	33.60676	34.63148	44.38354	58.11342	57.93583	32.82226	46.07508	59.72832	68.29523
CUST_50385_PI429010886	M89655	0.004263944	18	3						39.05628	17.4518	83.05204	33.10821	46.17834	NA	24.44847	32.7919	44.32333	29.30404	45.33378	17.01131	52.35235	19.55478	30.24945	NA	11.32723	7.161558	23.27971	35.59474	24.85202	54.45212	83.9013	70.82447	38.23223	97.82425	110.2867
CUST_14311_PI429010886	N32670	0.00426523	24	18						29.58249	30.69119	24.59083	14.47659	19.24962	16.1942	12.04777	14.53482	18.26373	21.28456	24.83115	7.602024	21.7802	19.31397	33.75196	35.80613	20.52147	26.58916	19.98159	34.26345	31.28202	24.64222	22.07863	28.6464	27.93535	24.30986	31.57579
CUST_38139_PI429010886	M23612	0.004265914	21	12						1226.655	1569.464	1447.815	2958.733	1632.718	2002.791	3495.947	2219.678	4221.228	789.8715	3240.436	1401.718	4772.224	5947.47	3875.533	1322.312	4913.686	2924.097	856.2286	977.1077	761.2797	549.5192	2160.965	2119.199	677.9076	1758.225	1943.332
CUST_24208_PI429010886	N50229	0.004284897	21	15						1242.744	467.1523	331.5852	271.1875	258.2061	251.2891	317.1285	375.9296	323.9561	255.9023	357.575	204.3998	325.2782	315.8311	267.1988	422.6098	560.4998	383.67	355.5754	314.367	389.8933	303.2777	332.484	370.6325	321.6405	269.1635	273.7566
CUST_41052_PI429010886	M56765	0.00428628	24	3						384.2558	70.71767	288.1795	411.4386	774.7662	631.2553	53.79961	63.51585	48.58376	222.3734	419.5373	579.8505	168.4808	385.6128	570.7447	68.06818	60.52841	52.16829	46.44642	49.51848	37.18271	249.7255	64.91949	208.3375	47.66434	84.38135	29.46925
CUST_52244_PI429010886	M96835	0.00428628	24	3	125710716	glycosyl-phosphatidylinositol-lin			8.26E-14	57.56952	23.04458	75.25623	72.63288	51.78206	61.55774	32.85632	31.40066	20.17341	17.16197	34.16048	32.84255	24.3283	32.75387	37.34538	16.71139	45.91877	14.03518	11.52249	18.19714	11.85566	29.46646	26.77594	27.49657	14.93075	30.64789	8.820637
CUST_34979_PI429010886	M11225	0.004286297	24	12	383864139	PREDICTED: protein ECT2-like [N	Cellular Process	Cell cycle	1.24E-21	15.21701	9.075187	NA	53.54287	9.079938	40.1235	40.85573	24.76219	NA	31.19842	25.8029	NA	237.9355	NA	218.4783	54.35675	NA	59.47811	48.45851	14.39043	39.49333	49.04743	61.70924	7.804205	9.133693	10.08816	NA
CUST_54078_PI429010886	M104430	0.00429723	24	3						4981.65	65.27026	4680.36	7138.174	3792.758	5571.385	101.588	109.8092	60.16557	4432.869	3148.451	6333.445	1204.7	4809.493	5966.983	26.34323	28.89254	46.45729	74.94714	181.0227	90.15514	1871.7	590.8215	1610.862	275.5152	472.8686	504.0705
CUST_31635_PI429010886	M2028	0.00429723	24	15			Metabolic process	Carbohydrate metabolic process		479.9831	2983.608	755.266	869.9307	56.11275	785.5802	2362.508	1864.874	709.9527	1151.814	741.8916	441.2406	1288.693	2162.694	880.92	4767.094	8591.806	4976.553	1418.063	431.2488	1074.917	1173.346	1261.961	995.515	1247.935	935.0962	152.1914
CUST_40063_PI429010886	M49607	0.00429723	24	3	83628250	arthrodial cuticle protein AMP1			5.51E-16	4754.165	247.3301	3247.587	8387.726	5929.402	8135.669	128.3075	629.4202	87.0776	7203.647	8387.726	9550.071	2149.415	2690.376	5872.99	25.9824	27.83542	67.65721	53.98617	520.6055	58.94726	4834.858	1420.017	3859.53	140.4108	1008.859	476.8665
CUST_42543_PI429010886	M60547	0.00429723	24	15						212.6443	529.3696	382.3723	356.0841	141.6357	207.6552	517.5058	476.1651	552.0297	315.0449	241.1372	106.3835	549.5192	359.2536	300.1105	610.5014	551.2795	631.8697	449.4542	395.1137	579.5063	289.9679	371.3022	406.6714	490.4622	266.3762	436.108
CUST_5271_PI429010886	N18475	0.00429723	24	15	241780604	E3 ubiquitin ligase, putative [Ixo			2.9E-42	697.5829	1053.829	840.0505	733.9767	561.6697	543.1972	827.5626	787.2783	913.5277	843.5336	668.7382	690.7831	957.3975	632.1075	777.3446	1046.212	1037.05	1110.746	944.508	775.4008	1108.146	880.3029	861.7915	935.873	925.0688	656.7134	1053.829
CUST_20106_PI429010886	N42821	0.00429723	24	15	242023524	Heterogeneous nuclear ribonucl			6.17E-25	57.31291	112.0856	98.98816	64.2086	68.39377	66.79456	82.47763	81.21161	100.2134	117.2468	63.28914	76.28125	83.58359	91.55686	104.437	143.5433	113.0037	111.5206	95.63382	94.46419	114.4006	82.52242	94.83653	115.5828	101.9186	90.8123	102.3598
CUST_36507_PI429010886	M16091	0.00430298	18	9						15.9967	21.74375	14.5147	10.3455	12.88005	12.21582	17.70936	15.2696	20.2757	31.49411	19.57119	21.20564	16.63236	16.34571	14.65575	23.27568	23.34913	26.2515	13.95043	13.28567	15.24733	22.04646	7.18431	15.06155	24.27662	12.24304	20.55619
CUST_31604_PI429010886	M1948	0.00430298	18	15	339236599	coiled-coil domain-containing pr			9.44E-11	176.7718	347.8293	273.2919	183.5918	171.2412	200.9666	287.5765	227.3167	224.5862	219.7957	256.8109	164.1609	258.5192	168.6714	273.2919	363.1656	266.7409	283.351	262.7025	215.9178	251.8474	163.4636	211.9007	199.6837	206.9345	201.258	335.2209
CUST_32452_PI429010886	M4158	0.00430298	18	15	114053033	Ef1alpha-like factor isoform 1 [B			1.22E-08	177.9861	175.1243	142.2314	177.1325	111.6865	141.1345	287.9808	214.8358	188.1589	217.909	145.5033	177.9861	198.84	151.8772	152.0514	252.2559	250.3014	254.5783	125.3559	148.5291	247.7035	132.8996	140.2426	143.2644	184.0897	178.0606	131.4508
CUST_2731_PI429010886	N12999	0.00430298	18	15	321473292	hypothetical protein DAPPUDRA	Metabolic process	Carbohydrate metabolic process	4.47E-53	4.93131	18.99164	14.52179	7.212797	3.06341	4.230496	10.83531	15.27129	9.161427	5.454383	11.97009	6.839885	7.446593	9.774001	13.06599	15.34065	22.8581	11.66876	9.666191	8.265196	9.37834	3.104553	9.187163	8.503753	3.429856	12.81141	9.725094
CUST_22780_PI429010886	N47660	0.00430298	18	15						7.74189	33.92845	26.73097	9.319096	25.64005	19.15316	34.12419	35.02108	27.71884	33.2361	26.41198	29.16024	24.54963	29.64248	30.69521	40.44814	52.70059	34.75367	36.17395	33.9635	29.90099	23.10033	10.38657	28.51774	30.70425	17.32058	40.23682
CUST_10427_PI429010886	N26211	0.004305108	12	0	380019209	PREDICTED: uncharacterized prc			2.92E-15	53.69851	27.57725	48.03034	34.3577	47.9867	43.44652	22.11134	21.28456	19.13165	24.52901	26.92758	44.15404	23.57416	34.86224	47.36534	15.92717	23.75379	24.02389	25.02711	15.41819	20.14753	33.762	26.64918	36.53636	26.89103	27.45637	43.30726
CUST_6900_PI429010886	N20751	0.004310016	24	0						NA	37.80064	39.18322	13.18135	NA	7.864064	10.18759	6.81204	16.87615	15.88417	6.583562	13.67711	9.811153	8.599165	10.85363	6.64183	14.29143	19.19097	9.301907	14.72302	11.39401	16.92091	10.12825	15.58609	16.19041	18.1699	19.14803
CUST_54638_PI429010886	M106987	0.004311082	24	0						2143.564	2990.827	2472.65	1996.997	1437.647	1355.196	947.0352	1537.641	1515.59	2098.966	1500.263	1306.636	2740.835	1512.535	2244.174	1903.836	2681.804	2027.732	2920.075	2472.65	2609.919	2968.824	1947.583	2494.519	4470.918	2713.39	3709.96
CUST_20210_PI429010886	N43026	0.004311189	21	15						47.34933	36.61868	20.81357	24.69162	45.7369	35.45915	39.72368	40.5799	41.49384	47.7452	48.96189	39.92196	35.65289	64.10142	70.26755	56.4602	40.6742	88.25185	57.22003	25.01036	38.64131	30.39689	28.4962	28.2373	25.58795	41.13599	26.5557
CUST_31610_PI429010886	M1958	0.00431535	15	9	321473202	hypothetical protein DAPPUDRA	Metabolic process	Oxidation-reduction process	1.97E-107	784.8506	244.1747	727.0088	1066.406	1333.239	1089.089	290.029	426.1127	283.4149	1244.137	2973.957	1386.294	1167.815	950.4596	1282.19	294.2691	285.7894	288.8335	443.8257	487.4115	345.0411	1248.523	1664.278	1257.339	359.174	1678.527	1764.635
CUST_40038_PI429010886	M49068	0.004317891	24	15			Protein metabolism	Ribosome biogenesis		6.362179	8.34915	NA	15.70678	NA	15.73574	19.91267	40.38119	17.2236	17.02795	11.75064	7.811501	13.18326	26.09836	NA	31.33584	42.73834	37.88664	28.5396	35.72049	21.45886	12.80411	NA	13.79063	28.23406	7.832139	4.99561
CUST_21431_PI429010886	N45206	0.004319802	24	3	260793489	hypothetical protein BRAFLDRAF	Transport	Transmembrane transport	1.9E-18	23.07404	5.420396	22.56021	31.60721	55.56698	33.77513	NA	9.411311	9.075187	4.327295	35.34111	26.55873	NA	22.26642	27.03986	8.526821	NA	8.051031	NA	4.717564	4.885026	12.09389	15.2887	10.07424	10.05333	13.36765	7.811501
CUST_30546_PI429010886	N62101	0.004333057	18	12						16.57521	46.38563	15.09656	16.02076	26.28128	16.52918	29.15439	36.81564	19.83531	18.01233	8.643882	3.443598	62.34647	65.01967	48.45428	46.29703	53.74902	50.1322	38.56693	31.08617	41.67167	NA	20.05184	3.820656	39.2895	36.3002	7.655695
CUST_8664_PI429010886	N23381	0.00433444	24	18						115.9671	142.5641	146.726	136.1461	106.7181	106.1184	142.8045	151.2161	160.389	174.2396	120.4803	121.3126	142.5641	122.1364	123.7768	167.1713	153.47	162.5583	181.2265	155.2845	181.9587	166.8948	162.8755	142.9974	188.0574	146.4045	180.7102
CUST_19151_PI429010886	N41063	0.004350261	12	0	152013711	putative cuticle protein [Artemia			8.45E-14	51.81869	89.90152	97.90232	32.09223	78.90154	47.46968	49.17415	55.59367	46.74583	13.9779	33.39856	37.72215	97.85715	151.4662	175.9436	50.42732	44.5991	51.41072	40.07856	61.91527	32.78884	39.6947	32.63321	78.68427	45.64916	47.56782	73.3346
CUST_56814_PI429010886	M114701	0.004350645	21	9						70.35289	83.69522	65.18818	73.43473	78.43693	73.10715	89.83016	75.03704	68.60832	90.59636	86.07541	93.51275	79.00471	67.64673	105.746	93.21779	65.08782	76.43278	56.82896	45.00194	74.00845	68.06818	62.37324	78.40447	89.58853	55.30643	80.87979
CUST_56990_PI429010886	M115065	0.004350945	24	21						506.6858	483.5315	523.9454	498.5642	403.6726	427.8043	494.8296	400.9911	495.5669	477.5471	393.6626	463.4514	479.3697	400.9911	460.4529	572.9401	492.0502	574.4065	614.0454	396.9983	598.2839	626.0846	533.0556	544.3805	782.6486	425.8369	678.0803
CUST_35796_PI429010886	M13536	0.004350945	24	3						200.2792	111.8276	154.5211	216.9264	168.9831	242.1802	112.3846	77.41498	138.2964	140.5297	159.2248	231.0435	121.0405	106.5519	150.1836	91.38505	41.16872	87.66036	117.3396	108.5015	82.86519	196.3457	73.69276	182.9006	133.1101	94.39327	96.85513
CUST_37171_PI429010886	M18084	0.004350945	24	15	307180204	Alkylated DNA repair protein alk	Metabolic process	Oxidation-reduction process	3.52E-20	55.17588	71.14732	66.88415	54.30068	55.15568	46.54074	62.58458	84.76892	82.69673	57.09472	65.45292	33.83079	61.16253	57.68508	57.10009	77.99973	94.07474	85.52485	75.06278	69.70354	70.62321	68.79187	75.31986	88.85544	71.36454	62.82646	78.46823
CUST_37407_PI429010886	M18923	0.004350945	24	15						2809.488	6116.36	4439.592	3755.671	2736.432	3124.619	6298.27	5451.324	5032.372	4495.71	3172.092	2547.238	4266.005	3405.379	3339.651	6885.506	6430.973	7937.877	4798.856	5907.39	5960.592	3804.856	5751.95	4329.518	3588.155	4814.394	5671.62
CUST_37926_PI429010886	M22266	0.004350945	24	3	220172369	cuticle protein [Rimicaris exocul			9.81E-27	27459.44	664.2531	27832.29	36043.3	32969.41	38060.46	2092.723	1927.794	2315.807	24289.54	44913.68	32798.44	28492.44	27347.94	47826.44	128.2119	74.3865	214.4299	1394.635	2200.686	2706.184	22154.05	18070.71	27758.19	4238.105	5233.042	9565.255
CUST_334_PI429010886	N4022	0.004350945	24	15						3319.225	6368.381	3808.458	3879.479	2849.078	2833.623	5966.983	5525.521	5166.879	4012.41	3177.941	2920.075	3851.521	3346.038	3237.866	6988.188	6051.213	7225.192	5258.496	5233.042	5587.034	3528.773	5055.201	4565.036	4740.787	4319.299	5380.838
CUST_50833_PI429010886	M91179	0.004352559	21	0	321464068	hypothetical protein DAPPUDRA	Metabolic process	Lipid metabolic process	6.03E-12	60.23194	88.74114	99.85694	49.4478	61.68952	74.05592	41.65544	73.50137	62.06796	29.81999	90.8123	40.34223	51.7695	48.10521	47.29117	30.17742	62.88893	48.52454	37.455	58.84948	54.591	64.72689	80.96563	78.59166	49.22608	77.01155	58.16712
CUST_290_PI429010886	N3536	0.004352664	24	18						7450.782	12084.47	8619.889	5055.201	6922.365	5329.024	8565.196	6018.305	6358.535	7058.228	4415.56	6249.252	7111.995	7750.118	5402.577	6515.192	11052.65	8945.499	10852.86	8713.974	10352.54	5649.913	10852.86	9211.498	8911.047	8591.806	10017.83
CUST_17311_PI429010886	N37755	0.004354052	24	12						53.98068	59.2979	67.95059	44.12566	46.41625	49.78973	57.19268	46.5992	65.2491	53.08995	64.38614	74.11009	75.47204	61.15056	74.3865	73.7088	87.21789	58.59505	70.02512	53.76136	71.66506	69.03939	41.79513	62.55614	46.04292	60.36476	98.85767
CUST_18636_PI429010886	N40146	0.004355305	12	0						21.31707	14.37528	15.95307	11.56152	14.29468	14.15703	6.361012	10.07945	11.19099	17.04802	21.60932	11.47734	17.01732	18.29038	17.93943	7.621112	NA	7.634361	4.378175	6.273022	6.027376	12.23558	10.02094	11.03403	8.00437	9.24062	12.39819
CUST_12430_PI429010886	N29450	0.004358023	21	18						23.14702	63.96849	61.59148	18.02618	12.80411	11.11102	20.71285	23.96123	21.77362	26.14998	15.61374	27.23388	11.02357	10.11927	15.75024	21.94131	30.52273	20.68187	37.79767	31.88159	30.82525	29.4515	16.37588	26.61351	27.35733	29.62856	27.74342
CUST_6702_PI429010886	N20482	0.004358257	12	0						46.1196	71.68028	85.33414	75.1848	87.50644	70.55138	69.02489	47.46533	60.77638	73.56286	71.64925	76.92175	77.29301	84.95059	90.64448	67.00003	74.70953	57.12984	55.45831	55.73395	58.98036	72.61063	66.52473	64.52421	73.35252	79.84725	69.88293
CUST_38284_PI429010886	M25016	0.004367121	24	9	2414483	Crithidia fasciculata ribosomal D				67.60011	169.2197	33.91249	173.502	1125.025	1080.06	315.0449	245.8656	343.6496	306.7699	1562.544	2494.519	98.2265	1338.071	1701.993	1352.876	747.8771	205.7884	520.6055	367.4169	544.2292	168.6428	571.6616	208.2743	224.1099	605.6304	116.3995
CUST_37248_PI429010886	M18360	0.004367539	24	18	543735448	PREDICTED: LOW QUALITY PROT			7.54E-15	27.96022	55.7467	75.68836	38.46589	26.00555	35.41196	24.45128	38.95566	48.61045	33.02712	26.86103	41.14081	37.73828	34.53535	37.66871	38.16299	57.54767	40.9293	49.71376	63.12641	66.26258	49.93016	51.29155	47.97707	53.86055	44.63889	61.908
CUST_27529_PI429010886	N56221	0.00436934	24	9						25.95809	20.86861	33.13254	29.32221	12.39501	42.53392	53.33823	32.51568	32.83861	40.25187	71.1126	61.85109	48.39798	21.67269	42.53392	64.29833	10.40166	35.84878	46.57025	5.967574	20.96508	24.97826	30.94126	27.69944	18.38558	12.34692	8.105145
CUST_23916_PI429010886	N49697	0.004369431	21	15						21.75932	17.76942	25.94197	19.05953	31.95741	15.83716	31.52277	44.98293	34.67614	14.56816	49.94958	26.67431	27.33627	60.83425	56.46685	57.74381	78.3065	92.67361	13.23058	27.34326	28.40807	16.8613	18.95876	29.7085	16.64745	32.98496	43.76612
CUST_37151_PI429010886	M18014	0.004374165	21	0						71.18104	44.61277	96.85513	44.99721	86.12428	67.68769	19.61789	39.12873	28.67393	54.30655	32.84255	45.06652	19.94871	25.20136	40.78485	29.73407	52.6814	81.60481	49.71921	39.56268	77.74804	56.61327	70.27366	71.13619	40.84629	26.0733	41.83378
CUST_55769_PI429010886	M111639	0.004386949	24	18						1587.909	1525.217	1369.757	1293.42	818.3565	488.0472	2214.163	881.258	1568.389	1128.19	656.7134	625.2657	2297.179	1331.886	469.5128	1085.159	1837.894	1764.635	3274.995	2228.732	2698.998	1527.348	2122.683	1122.961	2252.908	2216.187	1929.095
CUST_18703_PI429010886	N40254	0.004386949	24	15	156386840	predicted protein [Nematostella	Nucleic acid metabolism	DNA replication	6.13E-14	53.77161	56.4602	41.84717	56.14643	38.99311	57.74953	47.57381	54.65618	48.8281	55.29943	59.58172	51.63077	71.13619	52.10354	56.78277	123.3372	69.01316	76.5464	75.76283	45.60889	61.70924	80.41731	59.9228	65.69029	73.51603	75.63115	67.48124

Probe ID	ID	p-value	Period	Phase	EBI ID	Annotation	GO category	Go term	E-value	0	0	0	3	3	3	6	6	6	9	9	9	12	12	12	15	15	15	18	18	18	21	21	21	24	24	24
CUST_19331_PI429010886	N41398	0.00439859	24	3						8.600876	19.13165	NA	15.60164	44.03097	48.2963	NA	NA	6.720337	NA	24.58511	26.32359	23.08008	47.6343	34.84492	12.92804	14.68296	15.63953	4.088808	11.14206	5.305303	NA	NA	7.307485	26.83424	20.43703	12.21233
CUST_3752_PI429010886	N15193	0.004403078	24	6	298162780	early cuticle protein 6 [Callinect			0.000000249	12.7329	5.596537	10.14958	5.489948	16.99266	16.25789	49.42933	84.56114	57.36369	14.44743	NA	NA	7.690049	7.919117	46.90198	9.44382	13.62144	4.160011	9.642309	33.5727	7.903245	NA	5.996867	4.083445	NA	43.44035	3.545954
CUST_38322_PI429010886	M25410	0.004404661	24	3	290462391	Cuticle protein 6 [Lepeophtheiru			5.98E-10	25130.01	1086.19	21881.51	20662.81	23273.41	26403.54	598.2839	656.0648	718.4732	21590.74	18910.88	26491.1	17658.04	27832.29	12288.42	150.0105	186.0781	383.3073	869.5599	1228.932	706.4981	18910.88	4705.762	16801.9	1204.7	1868.336	3310.069
CUST_32044_PI429010886	M3088	0.004404661	24	15						70.67656	107.4986	82.7532	75.38714	65.54266	76.73936	78.01174	101.6124	89.08902	106.0382	58.58735	77.21485	88.01649	76.3423	85.1761	97.42977	133.6073	125.1917	88.04091	87.28041	101.9985	78.92328	90.40607	86.60076	76.06374	80.20598	109.8644
CUST_40751_PI429010886	M55999	0.004404661	24	15						273.9926	290.2155	329.8464	281.1468	187.3814	260.798	317.5843	274.5666	310.357	459.3044	249.6908	345.3559	344.8862	235.738	312.0575	447.1876	349.8278	468.9491	427.391	342.9247	452.142	398.6975	238.4366	363.7634	445.3005	294.6196	396.1202
CUST_44645_PI429010886	M66561	0.004404661	24	15	213408433	40S ribosomal protein S6 [Schizc	Protein metabolism	Ribosome biogenesis	2.93E-11	11572.84	19287.83	13248.98	12402.11	10156.68	11378.62	16372.71	18435.82	10209.43	13135.1	11503.97	11030.98	12836.63	14015.31	11612.18	20099.82	18758.28	22895.57	13503.69	16801.9	18673.37	11455.94	14439.79	14307.54	15847.39	14956.1	15394.16
CUST_47551_PI429010886	M76564	0.004404661	24	6						89.58853	59.95532	47.73985	17.67253	39.72867	50.95848	85.21665	57.76704	60.23194	72.73375	59.07967	38.57471	40.08729	65.3511	48.47854	41.69944	54.04214	15.61171	37.90412	3.96145	15.30631	44.61738	43.13077	13.49776	33.21927	56.17586	14.12155
CUST_6027_PI429010886	N19526	0.004404661	24	15	321475563	hypothetical protein DAPPUDRA	Protein metabolism	Proteolysis	3.85E-52	383.9729	710.633	507.5036	347.7046	471.7045	389.053	461.0647	601.6893	525.5146	474.5724	584.7373	409.5077	420.5189	497.5258	424.7669	570.5532	682.1048	706.4981	574.7506	566.1586	513.7138	488.2191	535.3308	540.0952	568.455	486.1663	474.7066
CUST_11812_PI429010886	N28441	0.004404661	24	15						67.31813	141.1811	114.5979	74.82596	62.57277	61.908	107.2724	107.6466	163.5388	112.963	86.08655	76.02418	124.0673	119.3462	90.53932	124.3819	186.2275	174.1457	146.9973	97.25517	126.8065	123.1089	110.7326	130.8809	139.9317	119.9522	167.9137
CUST_16194_PI429010886	N35823	0.004404661	24	21						122.9878	142.8045	156.5799	123.5992	120.2562	107.332	106.0251	128.179	131.9825	121.3291	92.46133	109.6769	110.4251	104.8322	129.2736	125.5101	138.9364	166.8658	105.2026	112.9762	145.1606	124.9411	167.6498	167.3552	135.1101	137.1269	187.5023
CUST_24222_PI429010886	N50254	0.004404661	24	9						41.86801	45.8224	43.84747	51.12885	65.70417	54.79166	57.18737	35.44968	27.3201	81.84916	65.76047	64.29327	63.02678	46.69101	37.69532	25.80026	80.6925	30.53244	42.57027	43.79306	18.17826	45.54022	25.39744	22.69361	20.06847	46.91713	20.32291
CUST_28080_PI429010886	N57252	0.004404661	24	15						1692.044	2252.908	1625.527	1788.478	1409.395	1444.952	1888.631	1662.168	2039.749	2135.546	1453.134	1916.926	1999.848	1384.734	1844.624	2954.482	2299.057	2442.216	2826.773	1831.816	2452.653	2400.564	1742.558	2276.393	2895.969	1847.559	2180.402
CUST_1379_PI429010886	N9447	0.004404661	24	15	350420678	PREDICTED: Bombus impatiens g	Metabolic process	Oxidation-reduction process	6.94E-100	4329.518	9153.451	5166.879	3516.69	3843.133	3798.572	5907.39	7316.568	6041.324	4705.762	3963.836	3688.899	4190.519	4495.71	4221.228	7415.157	7337.918	7032.355	5482.841	6642.147	7389.276	4398.359	6249.252	5162.169	4981.65	5777.198	5507.513
CUST_37152_PI429010886	M18015	0.004410554	18	0						549.679	756.6003	528.2931	601.887	560.022	451.3395	541.3579	531.7033	467.5708	420.1391	432.0096	370.7223	451.6859	571.5137	364.2801	437.7611	707.3888	655.9099	693.3608	692.1008	735.8335	430.4406	520.6055	463.8737	620.0365	452.3252	601.1087
CUST_43365_PI429010886	M62854	0.004410554	18	15						151.5648	110.8634	196.7017	85.75696	59.50711	43.83834	171.1268	118.4437	205.3015	153.318	64.1954	72.62444	141.3865	165.3867	50.87138	157.847	175.6041	194.1489	115.6407	127.9261	168.1851	72.5233	85.63473	66.49052	114.8652	109.3052	99.26059
CUST_2252_PI429010886	N11966	0.004410554	18	15	225714106	Cytosolic non-specific dipeptida:	Protein metabolism	Proteolysis	5.28E-177	49.08263	91.39547	49.15533	44.1311	55.49194	49.89252	63.53279	94.33013	54.73663	55.49194	72.97666	79.53675	67.91644	58.19234	70.50377	71.41406	95.11745	83.07439	59.70409	61.19974	89.38934	61.22809	50.1974	56.28495	62.51652	59.02352	63.00637
CUST_9906_PI429010886	N25384	0.004410554	18	15			Metabolic process	Carbohydrate metabolic process		112.7612	264.0266	117.6716	127.1629	24.38607	119.5667	371.7268	242.0929	230.4612	290.4456	101.6014	142.3895	208.1428	162.2564	170.1972	501.0897	415.7252	392.1412	247.116	79.87529	194.0917	140.2618	101.9592	140.5662	187.1657	148.4641	73.24134
CUST_21466_PI429010886	N45269	0.004410593	24	0						973.9492	845.2107	533.7924	560.9756	808.5577	713.8123	598.5974	506.6858	651.0737	308.6292	412.7396	523.4696	605.6304	514.354	552.4266	523.3495	456.0887	415.0852	433.8311	674.5328	399.6202	502.0555	701.0526	657.7333	517.9425	713.1241	985.0577
CUST_7735_PI429010886	N21994	0.004411406	12	0						431.5244	420.2294	387.7178	436.7155	319.3342	268.9316	244.5822	333.0825	250.7116	319.1468	307.4677	308.5071	407.4228	327.4664	333.0825	328.0523	403.1106	229.3564	324.7451	253.3528	285.5358	400.9911	328.6973	361.3178	367.5711	353.9275	380.1903
CUST_41508_PI429010886	M57902	0.004411484	21	18						35.86186	50.47426	90.65467	26.96496	39.96323	43.29935	51.42591	66.64484	44.26795	49.98481	51.9164	31.77803	40.90642	64.99766	43.70979	49.4478	169.3865	50.02722	74.15901	61.68952	55.32159	49.44159	49.81991	51.47751	27.72605	49.29631	47.2578
CUST_15322_PI429010886	N34399	0.004421036	24	9						34.74447	37.76079	32.82562	41.29379	49.6421	38.11477	27.61361	42.32017	35.41491	57.66836	50.1322	46.13557	38.78785	33.37375	38.54735	37.17059	36.40013	42.90506	36.63038	34.75367	46.98969	34.84867	27.54823	32.80252	51.47126	26.60979	33.29327
CUST_5454_PI429010886	N18726	0.004423233	15	0						405.9933	498.6872	236.2202	405.7918	303.5583	212.0999	160.0636	125.8417	69.23652	116.0591	70.64915	37.03485	328.6973	60.56117	368.8823	799.5929	351.0025	508.9078	100.646	84.91891	155.1663	171.9553	432.0096	74.74645	314.4452	267.7311	71.26688
CUST_22984_PI429010886	N48054	0.004427598	24	15						14.39152	10.43238	12.74848	12.2567	21.0408	21.24956	14.93971	17.14498	19.22058	10.30349	13.96855	19.16548	15.72542	23.34913	16.61149	28.75907	22.79379	29.40141	15.63074	19.98537	21.45062	14.95172	15.89766	25.81487	8.956638	11.9085	20.01045
CUST_7515_PI429010886	N21662	0.004430384	24	9						40.24112	NA	22.20069	21.35401	43.79306	33.3337	16.06768	10.45873	NA	21.62961	42.62368	58.28361	14.75816	44.29702	44.21087	NA	14.49352	7.372894	NA	10.41738	5.713125	11.87323	7.35203	10.04481	11.41494	15.23987	10.4666
CUST_53203_PI429010886	M100710	0.004458376	24	15						6032.614	13360.37	6041.324	6559.55	3452.681	6401.205	12378.85	14032.06	8750.959	8911.047	5482.841	6439.853	7071.199	6592.219	6210.057	17424.76	17424.76	15585.38	10949.85	8996.679	10605.81	6523.544	7531.236	6770.352	9889.351	7872.926	6649.946
CUST_31176_PI429010886	M895	0.004458376	24	15						1670.997	2424.1	1984.393	1760.199	1396.045	1489.353	2256.232	1922.582	2168.846	2177.847	1461.32	1690.112	2035.999	1695.833	1781.913	2573.227	2837.693	2948.008	2037.287	1786.238	2251.39	2275.002	2076.904	2575.646	1954.479	1677.845	2570.156
CUST_2596_PI429010886	N12725	0.004458376	24	15	242005887	eukaryotic translation initiation	Nucleic acid metabolism	RNA metabolic process	3.59E-108	2170.443	3053.693	2222.683	2014.653	1594.801	1874.483	2851.067	2411.386	2384.985	2504.226	1786.238	2228.732	2785.011	1721.8	2378.51	3142.5	2985.873	3601.999	2371.181	2299.057	2751.324	2108.887	2322.285	2595.878	2636.073	2058.438	2790.956
CUST_5153_PI429010886	N18326	0.004458376	24	15						779.5767	1028.452	893.2381	845.5682	612.0937	681.2288	1218.815	956.4678	1001.688	1106.369	654.3402	859.7804	1002.076	721.9277	866.0744	1481.716	1454.668	1509.889	1224.109	802.1705	1144.272	827.8854	857.7889	884.1094	998.2787	803.027	1096.072
CUST_6217_PI429010886	N19790	0.004458376	24	3	119117126	Cryptocercus punctulatus alpha-	Cellular component org	Cytoskeleton organization		1354.492	1666.498	1329.875	1335.991	2696.535	2026.561	1284.327	1278.88	1432.11	761.5417	1637.38	1471.771	1253.748	1390.116	1593.454	872.0312	965.4976	680.2076	800.8587	1540.275	1137.925	1444.129	1144.272	1118.881	884.1094	2149.415	1602.248
CUST_8317_PI429010886	N22863	0.004458376	24	15						125.6969	186.9529	159.203	125.6969	102.222	111.6865	171.664	148.131	149.9126	185.6007	101.3475	143.5591	169.1505	119.4159	128.4423	215.3674	227.2807	213.3383	166.9821	141.0554	178.6434	143.92	129.3882	158.2397	161.8917	136.2532	174.8361
CUST_13267_PI429010886	N30884	0.004458376	24	15						196.5522	278.7033	228.0728	182.0165	192.6063	186.658	282.7416	271.3104	275.0049	230.649	213.853	260.1908	196.7753	215.1868	238.6788	296.8179	314.3155	332.1692	198.7013	256.8674	281.2628	173.3126	250.0724	269.8306	209.8119	231.374	255.866
CUST_22497_PI429010886	N47138	0.004458376	24	21	242010833	conserved hypothetical protein	Nucleic acid metabolism	Regulation of gene expression	1.7E-22	49.15533	65.86088	75.24855	62.10736	48.02051	59.90743	35.47927	65.30254	68.22331	43.06564	63.43943	35.82514	60.00388	76.35805	67.43392	59.58718	46.14868	72.20099	59.64761	76.03311	81.96955	97.01999	68.02537	87.00868	93.37419	75.46497	87.59003
CUST_27172_PI429010886	N55577	0.004458376	24	3						206.0776	27.97979	147.1247	322.1133	135.0204	176.6314	51.84875	51.14752	45.83845	140.5949	70.01129	120.1554	110.8634	61.92068	92.61293	16.12259	13.31268	12.80801	36.39325	37.93869	15.68645	178.1819	46.67638	123.1089	25.42345	53.55076	34.51373
CUST_38782_PI429010886	M30321	0.004464341	18	3	149675211	cuticle proprotein proCP5.2 [Cal			0.000000398	17061.92	536.6512	15553.93	31108.81	22777.74	27832.29	1782.659	4829.887	5751.95	17315.8	39713.59	18235.97	23658.24	18365.76	24590.28	486.8888	507.7884	378.627	8591.806	6240.674	9129.704	23084.2	29122.17	28414.17	8215.523	21270.53	23503.18
CUST_3901_PI429010886	N15525	0.004464556	12	0						254.714	288.0498	170.246	395.1137	182.0409	324.3008	34.11681	39.84392	20.85648	264.1326	436.7155	428.0259	350.4372	267.884	58.21158	41.66058	873.2461	93.40586	30.73939	29.03104	30.29848	52.48031	103.106	44.36058	149.578	516.8815	422.0714
CUST_4400_PI429010886	N16679	0.004466813	15	9	546685236	hypothetical protein D910_1203			2E-15	52.01796	54.04744	46.91713	35.21261	44.05907	56.10448	47.81362	34.88562	43.66107	76.74926	57.71976	72.96939	70.30569	55.26895	54.0024	78.76138	72.78292	60.03374	54.51407	35.17583	41.14081	54.74265	37.5978	61.0985	71.1642	64.54113	70.17999
CUST_16441_PI429010886	N36245	0.00446823	24	18	405963507	Calcium-transporting ATPase typ	Transport	Ion transport	4.76E-25	14.59005	12.52949	25.41794	15.03411	8.493194	9.523868	8.355785	9.978491	12.89468	21.14901	7.120472	13.53119	19.76134	6.520876	15.36202	22.87088	16.15024	20.67143	22.20918	23.9201	14.93397	15.64262	17.03783	15.34413	17.78405	12.70197	16.26097
CUST_41839_PI429010886	M58675	0.004468627	21	9						269.5843	312.9583	181.0435	337.2109	704.9081	598.4778	403.0078	394.0157	286.8043	290.9943	508.3099	453.6305	356.0841	453.0125	453.1048	632.4923	438.5777	447.4189	340.0191	276.8728	348.0379	185.6261	359.174	219.923	382.191	348.5318	194.2665
CUST_28518_PI429010886	N58100	0.004475716	21	3						NA	8.217462	5.308855	17.33539	21.62961	6.071056	7.244787	13.39289	11.08066	11.62165	9.872817	8.804391	5.595606	14.44887	5.785986	7.611325	7.151367	3.021506	8.212284	7.595815	4.198663	16.13855	11.38718	7.715831	12.22964	16.41382	NA
CUST_44900_PI429010886	M67356	0.004477175	15	0						39.86943	66.50532	52.24236	42.15192	40.20336	45.48556	34.88562	47.90004	40.38119	31.59217	42.19985	28.05953	43.26708	47.68118	44.43625	43.67283	65.70417	54.50441	41.23205	30.75945	46.60711	38.09775	56.47386	42.10224	36.59345	39.26844	34.34753
CUST_12721_PI429010886	N29935	0.004480982	24	15						366.7062	473.8372	377.2326	366.6368	510.9141	413.5169	344.8862	385.121	293.6993	321.4975	503.7654	494.9456	355.6308	384.2558	538.3095	629.0343	531.7033	641.9649	407.2747	471.4786	544.9477	391.1906	407.9106	631.8697	460.1149	317.0103	494.606
CUST_36312_PI429010886	M15539	0.004485855	24	0						42.00294	33.5564	38.17039	38.16041	13.49972	19.05161	35.71025	21.15511	37.12565	24.91804	23.67523	17.72698	45.05271	23.4592	38.19894	32.8057	25.94024	22.07863	30.19535	29.96518	24.62618	38.37685	77.12608	41.90191	48.54356	43.87779	54.08928
CUST_46043_PI429010886	M71171	0.004486021	21	0						40.01543	25.24183	19.07869	21.44313	23.50326	22.13507	26.20109	20.57756	18.35988	21.16897	17.16673	26.4908	12.80801	12.82342	13.79706	22.89571	21.39542	27.61361	22.1912	26.34051	16.30898	30.03343	24.43634	25.24715	20.62924	21.26556	27.59053
CUST_18424_PI429010886	N39774	0.004486021	21	12						60.94356	49.42284	65.19605	45.25971	48.5496	51.60691	36.49381	67.93128	67.61402	52.83788	58.83501	56.37375	71.49956	63.49903	79.34748	74.58857	58.8025	61.33441	33.64987	55.8366	63.67982	67.56678	21.72565	70.3428	47.26618	31.55994	48.37508
CUST_24783_PI429010886	N51245	0.004486021	21	3						283.4149	293.4576	339.8451	370.1622	322.4558	347.9094	283.1093	257.3491	307.6081	327.283	199.982	305.5795	243.5905	274.9461	287.6916	366.7062	206.8179	292.3452	325.3527	266.8019	182.5585	324.8189	277.6692	306.5038	376.5416	310.5741	345.514
CUST_52889_PI429010886	M99340	0.004488592	24	0						22.51636	42.96134	34.60956	28.97134	20.41052	19.40349	30.70425	35.12339	38.23951	22.6585	16.06582	13.96378	12.33142	16.43018	21.89919	12.10203	21.0526	20.61917	24.2121	31.31906	28.3766	23.83774	35.41196	22.27865	22.66607	30.7975	39.88097
CUST_16438_PI429010886	N36236	0.004490822	15	9						173.8872	146.798	145.3375	161.0998	133.4611	155.0803	178.5544	127.1629	187.286	169.5998	173.8872	209.7702	180.7102	160.0873	163.211	182.1611	166.5631	178.2173	120.6989	137.0045	158.9852	145.5262	134.4815	145.3783	269.5152	196.4999	163.4636
CUST_56616_PI429010886	M114224	0.004496559	15	0						25.12027	13.70744	21.25376	13.04927	9.580454	10.67874	13.04021	10.75635	8.437268	10.92533	NA	7.157752	10.31745	8.18229	14.29658	14.29283	12.36294	12.75176	NA	10.47591	11.15823	16.06359	7.463335	8.460507	7.794152	5.866363	11.7375
CUST_17565_PI429010886	N38195	0.004498223	21	9						NA	8.623114	8.241594	10.55615	11.94454	16.85775	9.821487	14.19525	10.46195	11.5765	13.55428	NA	NA	12.36665	12.6262	NA	8.25537	7.729186	NA	6.429065	3.677678	5.534335	10.80202	5.061979	NA	9.800749	12.88005
CUST_13747_PI429010886	N31702	0.004502221	15	6						11.31857	27.34627	20.67603	25.63539	17.25616	26.66658	47.80328	20.75137	51.00867	14.98689	21.97462	22.5248	20.31267	25.72369	14.39152	19.80764	34.1415	19.05575	31.14331	10.14816	34.34753	79.67772	42.93819	49.91567	40.65182	45.97159	39.19188
CUST_39377_PI429010886	M38581	0.004503013	24	21	326781695	crustacyanin subunit A [Fennero			4.5E-25	201.258	39.99078	204.7184	336.9544	103.616	172.9496	21.39039	57.99332	72.61063	95.14144	160.9576	73.26943	255.1321	NA	229.7888	33.3337	169.515	27.57421	449.8325	139.6859	291.0502	889.3882	170.4291	718.4732	334.4355	114.6686	247.3301
CUST_22543_PI429010886	N47214	0.004503782	12	0						25.02499	8.788031	16.36929	11.16741	10.59607	8.128515	8.817048	4.006086	9.678103	22.66786	19.45417	12.44307	20.35931	11.26073	12.90825	12.84776	NA	6.692466	8.718132	8.112296	5.495792	16.10329	8.00437	12.83052	11.72346	14.1199	23.36828
CUST_31240_PI429010886	M1053	0.004512091	24	15						322.3894	572.7695	478.2442	361.7315	280.9343	274.5195	478.7694	412.4307	521.086	421.977	342.1248	407.5835	412.9087	313.7912	373.5884	515.6628	617.3702	586.0404	484.0256	430.8354	584.1152	371.2053	490.2242	500.1661	461.4101	407.5243	487.7778
CUST_35011_PI429010886	M11327	0.004512091	24	15	242004275	cAMP-dependent protein kinase	Signal transduction	Other	5.89E-28	107.1012	153.2663	143.647	127.0776	126.6578	112.473	132.329	128.7173	142.0601	85.93897	149.7056	144.7386	139.9139	152.8947	149.8871	171.8649	160.713	169.8127	119.3879	155.9594	150.9062	140.6454	157.5397	175.1243	139.2612	163.8916	180.5627
CUST_2405_PI429010886	N12283	0.004512091	24	15	443683291	hypothetical protein CAPTEDRAI			1.12E-45	2559.917	3555.208	2521.203	3056.068	2065.743	2487.704	3159.077	3495.947	3297.935	4109.069	2887.827	2661.468	3731.866	2312.635	2958.733	4885.181	4145.789	5043.311	2865.238	3767.349	3811.795	2785.011	2541.875	3277.985	3679.2	2700.891	3988.54
CUST_2658_PI429010886	N12849	0.004512091	24	15	321466865	hypothetical protein DAPPUDRA	Nucleic acid metabolism	RNA metabolic process	2.37E-134	1364.845	2436.323	1530.86	1375.234	1157.025	1163.513	1974.35	1950.295	1829.191	1754.468	1244.709	1309.684	1627.485	1463.189	1362.378	2064.359	2457.62	2583.285	1684.207	1521.677	1720.155	1377.009	1610.862	1409.957	1676.387	1435.616	1548.998
CUST_5164_PI429010886	N18344	0.004512091	24	15	383853115	PREDICTED: Megachile rotundat	Protein metabolism	Regulation of translational termination	5.39E-147	325.0373	535.4815	411.6604	280.1957	321.03	286.0534	517.1871	522.858	552.6048	413.2217	335.0962	369.4962	311.3849	380.83	322.5903	541.6936	641.3942	532.1418	524.8326	424.4332	476.7459	272.1942	410.0384	470.5066	406.0638	369.4962	405.4699
CUST_6480_PI429010886	N20156	0.004512091	24	21	321461563	hypothetical protein DAPPUDRA	Protein metabolism	Ribosome biogenesis	1.31E-95	57.06084	90.67658	76.58195	61.55047	76.23852	52.0131	50.1322	98.28823	78.70208	40.96049	46.24985	56.8797	54.62254	64.35228	72.06126	77.58626	75.28314	92.52233	80.30375	65.73258	42.07726	79.78242	109.3921	82.69673	90.82582	75.54551	63.15515
CUST_8475_PI429010886	N23096	0.004512091	24	15						342.0443	536.3161	470.5066	360.8077	373.9602	354.5733	377.9818	421.3144	420.4184	469.5128	347.9094	433.1774	412.2409	373.2381	436.601	570.0085	678.3225	667.0187	458.6774	417.6111	528.2931	477.9381	527.0091	488.5919	518.6591	441.2406	563.8477
CUST_16960_PI429010886	N37145	0.004512091	24	15						42.13235	45.22907	55.77263	49.60146	34.80421	27.65783	72.46811	57.94141	70.69299	66.28735	33.97586	12.876	57.08138	41.52352	49.09951	85.97811	66.62309	93.96854	70.56233	51.05459	63.4939	63.31187	49.88072	56.85229	56.70679	43.15443	68.50963
CUST_712_PI429010886	N6888	0.004512091	24	3	210062962	Fenneropenaeus chinensis C-typ			6.17E-78	12141.91	5625.087	13222.77	15731.96	19341.82	16580.62	6744.57	7610.57	9923.978	7762.077	14285.49	15435.13	9937.479	19855.57	19903.59	3815.958	3489.361	3455.897	9194.226	8522.605	10323.84	11094.64	13360.37	11356.17	6945.783	11805.59	13616.29
CUST_41849_PI429010886	M58689	0.004516095	18	0						10.49284	16.90428	13.08993	17.61537	9.213352	3.026834	NA	4.051944	7.852776	4.151802	3.671989	6.292668	7.174372	8.208913	5.110851	6.306457	4.988076	4.722347	21.29948	NA	9.726899	NA	NA	4.446727	10.23574	8.342949	8.829167
CUST_48990_PI429010886	M83418	0.004517705	12	0						38.48962	38.3287	40.73959	33.47807	38.29632	31.23655	18.02378	38.38927	27.43571	36.45493	35.0396	43.58292	21.80903	33.52669	35.80198	32.76221	37.51953	30.39689	19.85649	28.82109	26.11949	33.3276	36.91238	39.61981	45.67933	45.01993	54.56863
CUST_34451_PI429010886	M9706	0.004517705	12	0						90.68839	106.9035	122.436	81.21161	67.83335	74.76519	53.46841	80.77747	85.01861	90.62398	93.47058	69.5761	108.583	76.34956	102.5417	77.72928	114.1912	106.7719	66.13826	80.47316	90.52573	109.0071	91.39547	109.5188	119.7663	115.5309	149.1772
CUST_2645_PI429010886	N12823	0.004517705	12	0	242024982	hypothetical protein Phum_PHU			1.12E-158	63.95679	80.99203	79.42387	62.23304	37.81634	50.13895	39.99568	55.55019	50.61162	62.50961	45.39182	48.06623	67.96481	63.63996	78.10699	60.11411	50.52783	74.6809	64.75498	57.99775	51.19385	70.88457	58.19693	72.84435	79.18277	61.37944	81.5061
CUST_13810_PI429010886	N31814	0.004521959	15	9						161.1223	164.9172	182.9809	133.6627	151.5059	153.0208	179.4021	156.768	206.0776	196.581	225.5195	212.5212	221.9709	144.4115	187.922	189.9176	190.0383	197.5273	161.8447	181.6944	187.1331	195.5458	133.6627	222.3991	244.7472	175.0698	252.6173
CUST_43941_PI429010886	M64408	0.004523069	15	6						16.38359	28.32731	36.94988	20.4836	16.12455	18.97114	40.19489	41.43387	36.09484	27.86499	28.35837	16.56573	15.51771	29.18456	6.155042	30.27186	29.8647	31.29616	16.81913	29.19743	21.79685	35.0005	48.1462	38.87958	18.96527	24.52363	29.78059
CUST_72_PI429010886	N395	0.004527022	24	21						70.44138	63.5234	80.28093	56.07467	58.10634	65.67004	71.9695	69.93972	75.58915	59.21803	56.8039	47.35759	72.43858	69.6376	61.145	107.5789	84.28713	72.11693	82.52242	71.27374	83.68571	107.2724	80.6925	74.30536	90.03155	65.62334	91.86363
CUST_8618_PI429010886	N23312	0.004528866	24	15	340905485	hypothetical protein CTHT_0072			0.000000263	848.3549	1388.047	861.2738	686.4707	533.1871	635.9298	1105.917	1111.944	1137.334	897.3252	776.7493	785.0219	834.0907	703.1577	646.5974	1203.381	1271.126	1317.605	1383.977	928.898	1081.498	974.6663	673.5376	1063.435	908.3858	682.9458	718.207
CUST_8563_PI429010886	N23226	0.004535482	21	18	307184380	Carbohydrate sulfotransferase 1	Metabolic process	Carbohydrate metabolic process	1.37E-19	273.7185	291.8765	343.2592	282.9651	272.7898	234.2807	218.1822	326.1422	204.9134	241.364	280.3032	253.1631	157.2166	153.1648	231.5378	281.9984	407.1156	291.7534	309.9865	330.4034	340.1047	283.5676	289.6241	318.6428	385.1879	293.095	355.2635
CUST_53699_PI429010886	M102832	0.004537703	24	15						156.6513	189.3839	133.7949	154.7807	138.1387	142.4928	112.8927	138.2078	133.6627	197.3334	143.9614	190.0383	163.1238	129.9594	188.2411	192.3671	197.4942	215.4411	162.5329	150.0105	196.6443	174.6876	133.0726	205.95	185.1417	136.0891	178.5544
CUST_9170_PI429010886	N24172	0.004538617	21	12	321475395	hypothetical protein DAPPUDRA	Metabolic process	Oxidation-reduction process	8.99E-47	4.757189	13.88764	14.83032	14.76753	67.86728	34.10624	NA	42.66175	21.47276	NA	36.14885	29.51504	9.86298	71.89808	49.50741	17.06087	37.87771	17.92297	5.381324	25.33737	19.05761	9.771765	8.312327	13.0253	5.847915	23.92952	5.026958
CUST_8749_PI429010886	N23520	0.00454222	18	3						25.12586	73.47778	NA	70.40519	NA	58.445	24.40659	16.00633	53.68776	24.23053	22.7355	20.1	19.83531	3.875919	22.93848	65.59808	17.68028	40.58521	9.435046	30.49064	11.2206	27.12725	222.4375	37.87395	NA	25.3148	25.10747
CUST_1210_PI429010886	N8848	0.004545773	12	6	374719840	trypsinogen 1 [Litopenaeus vanr	Protein metabolism	Proteolysis	5.43E-50	6.771066	NA	NA	63.33988	NA	61.08411	949.2292	NA	655.6894	136.6003	108.3596	NA	26.83424	101.4772	26.93456	140.1401	NA	136.1664	1251.736	1561.658	3744.093	187.5258	NA	155.6724	461.1854	91.53685	353.6905
CUST_25503_PI429010886	N52535	0.00454734	24	3						40.23682	16.16198	29.30996	41.12558	50.58863	59.40866	14.86033	5.396608	5.447639	27.60039	24.99752	38.16299	12.31866	26.34871	38.70148	13.92807	13.64203	4.737879	NA	4.68663	6.613138	20.60366	11.45478	24.42859	29.15439	19.6201	5.574105
CUST_42532_PI429010886	M60511	0.004549294	24	3						3449.27	221.9709	3717.963	5435.742	4971.086	5671.62	518.6591	1258.787	1078.02	4863.888	7071.199	6430.973	4048.748	4470.918	4383.744	48.88152	38.54735	68.07757	919.3983	1742.558	1033.091	4641.846	4203.047	5514.378	1003.255	3932.655	4322.847
CUST_36698_PI429010886	M16576	0.004550078	24	3						NA	7.242699	6.657442	NA	10.65068	10.52851	NA	8.196534	6.822802	NA	NA	6.618689	NA	9.129149	5.436199	7.362979	5.061086	6.124786	6.251438	3.965637	4.360057	6.883456	5.632949	9.251212	4.829619	10.36406	5.602333
CUST_9237_PI429010886	N24281	0.004551811	21	0						963.1582	911.7924	931.8674	1123.543	349.1843	773.6805	857.7889	831.0254	935.4596	739.3679	575.6253	538.6242	906.8146	436.2064	652.1093	459.6187	331.5173	381.7573	625.2657	879.5119	627.4387	754.5011	1037.623	842.2205	529.0583	797.5472	975.7238
CUST_34948_PI429010886	M11141	0.004565807	24	21						167.7764	187.2522	130.6083	89.86465	43.08599	68.86246	105.285	291.0948	206.8179	45.76238	62.80714	37.59336	217.9435	114.1426	165.4096	203.8839	171.6067	260.3883	200.6093	180.1092	258.7813	194.0511	279.4339	254.1863	352.5932	107.9218	267.6739
CUST_3115_PI429010886	N13828	0.004565807	24	3						3777.656	566.4722	3715.857	3709.96	7376.103	6696.753	755.8283	1347.499	616.5627	2085.588	4554.072	3620.969	1388.826	3467.123	3808.458	412.9087	379.7764	229.9275	395.6891	1862.343	382.8	1895.668	1673.566	1793.169	287.5406	3277.985	718.4732
CUST_34127_PI429010886	M8838	0.004570855	12	0						204.7458	207.209	263.8276	218.6368	200.6783	216.1701	123.1089	174.6626	185.7829	151.1698	222.8685	179.5327	228.5093	208.4776	247.7427	231.134	181.8309	176.6314	205.2018	199.6163	161.6353	240.0694	199.2156	241.4743	216.5044	230.3457	269.5561
CUST_30609_PI429010886	N62219	0.004570855	12	0						527.7959	353.7833	445.4238	329.3762	266.3762	262.806	388.382	239.4012	265.3286	419.6327	297.9395	351.6631	401.757	355.6308	300.0533	465.3594	317.4305	436.108	253.2436	326.0295	241.2245	415.4114	334.5944	328.8423	568.0849	395.4956	389.053
CUST_53921_PI429010886	M103714	0.004571916	18	3	371591210	Euphausia superba voucher 980			2.57E-68	42.87131	19.35535	44.25351	68.03257	61.89891	63.92344	25.21673	19.50817	27.90588	53.45027	91.61189	55.42058	56.92017	48.2963	63.65381	20.18794	11.68247	21.23274	28.61744	33.04298	34.76036	67.68103	57.66836	75.22137	22.4723	35.0005	57.50494
CUST_37451_PI429010886	M19072	0.004571916	18	3	149675211	cuticle proprotein proCP5.2 [Cal			1.49E-72	35445.2	1124.497	33637.86	54598.59	40415.13	43456.54	3792.758	6226.747	8698.04	35607.66	50560.8	42224.23	42014.5	29037.53	40595.56	820.0431	945.2143	821.5501	13419.52	10260.07	13043.65	40024.21	44511.54	46193.24	13935.1	28743.72	30352.12
CUST_44425_PI429010886	M65932	0.004571916	18	3	220172369	cuticle protein [Rimicaris exocul			1.46E-17	42014.5	1181.431	34173.92	58456.96	48051.75	51616.89	3535.101	6612.476	9129.704	41610.17	67856.09	41258.32	43280.43	36043.3	46193.24	865.141	1077.531	712.1062	12344.85	10127.21	13281.09	43955.79	47342.04	45502.32	11015.18	32064.51	32064.51
CUST_1372_PI429010886	N9435	0.004571916	18	15						172.6192	369.6716	257.5954	245.2392	129.007	231.809	448.5869	326.6163	358.4079	403.9297	170.8363	178.4979	334.795	252.1635	265.541	588.6767	520.8578	521.9217	322.2486	224.2885	426.9219	196.1778	221.8856	229.4919	373.4093	287.3062	261.0327
CUST_45940_PI429010886	M70816	0.004574305	24	18	405970469	Monocarboxylate transporter 9			0.000000259	29.94695	27.97586	12.5832	7.572085	11.60633	15.61908	7.513141	13.92645	17.66664	18.05597	15.5193	20.45768	7.068342	11.98023	18.73426	26.89103	24.01703	25.44274	20.26699	50.7989	34.50993	20.66998	12.15684	12.7502	19.6075	11.83721	24.44473
CUST_8247_PI429010886	N22768	0.004574305	21	6			Signal transduction	Other		152.5137	144.8704	180.8125	156.0492	189.6097	147.5974	167.9137	184.4707	194.4274	153.7124	164.9813	262.8559	133.5115	160.2766	178.1819	99.69334	165.1857	132.7509	120.7668	147.5505	137.6198	171.8137	170.6411	156.4627	156.0305	165.6814	169.2749
CUST_45760_PI429010886	M70202	0.004575125	24	21						76.34956	124.3819	87.42396	62.89605	70.78121	63.79933	78.21211	72.57836	62.73874	73.39823	80.78521	102.7889	64.91207	41.82104	53.51076	94.90029	78.8865	85.43893	84.61068	77.2068	75.2089	111.793	92.9385	110.5322	121.4044	92.78173	116.3818
CUST_28087_PI429010886	N57273	0.004580706	21	18						127.7453	188.5193	166.2848	176.4512	112.7746	113.2744	155.3994	108.7311	133.9377	142.7777	122.8067	125.4319	107.1994	121.3825	132.1609	218.5686	145.6946	244.6993	195.3485	159.6864	207.9512	206.1048	158.0565	178.3071	176.0973	133.239	172.7187
CUST_33893_PI429010886	M8166	0.004584959	24	3						22.20069	22.36969	24.34505	31.91659	25.20667	33.33002	25.50686	40.99664	10.75951	25.35423	20.96508	29.82705	23.80329	16.96963	10.96336	20.62243	22.05134	20.88553	15.25101	21.49428	17.8915	33.14322	19.60559	24.87849	21.56373	30.07738	22.60966
CUST_39117_PI429010886	M34798	0.004587943	24	21						192.2397	291.3249	232.8674	252.8481	270.2762	212.5696	201.0489	237.6854	253.1919	207.9118	244.8381	229.6548	217.1534	218.3991	268.4249	264.5903	285.7364	270.3263	245.4997	240.0694	289.5149	268.7276	320.1071	327.6721	305.5103	276.6685	409.8716
CUST_52810_PI429010886	M99011	0.004601196	12	9						10921.28	63.93775	9067.463	10587.95	7327.614	9669	486.1663	484.7365	865.6479	15102.78	9872.039	12359.97	12743.08	4803.622	8061.991	173.1969	29.46145	228.4713	1647.162	2335.81	2790.956	13391.14	14764.65	16070.74	3321.698	11719.99	14764.65
CUST_24167_PI429010886	N50151	0.004602105	18	3						10.26132	5.939175	13.31417	18.47174	20.03269	13.45294	NA	6.963509	7.045737	12.30813	17.63035	16.07386	7.925431	NA	11.26538	4.299782	9.369673	4.436424	8.132185	9.03053	4.504529	18.75704	12.31491	12.70386	31.03777	NA	6.476119
CUST_10868_PI429010886	N26918	0.004602371	24	0						38.51685	48.87698	44.37921	28.51273	48.67088	31.02469	22.43161	40.33676	50.23264	27.96875	45.61863	23.83774	27.46378	33.52305	45.7171	42.05307	50.88852	40.98681	42.68735	51.73381	47.04453	50.93191	47.7889	49.16277	73.36846	64.67753	63.73816
CUST_39291_PI429010886	M37330	0.004610577	24	3	152949487	calcification associated soluble r			1.58E-12	488.8743	130.6083	460.3468	954.7966	1929.095	1398.059	185.937	169.34	206.1048	1176.913	1474.205	1175.829	434.5642	764.7593	1283.49	47.7889	49.3138	26.06422	93.13119	377.5103	103.5272	942.7053	821.2893	1195.903	111.371	613.1951	709.2377
CUST_37850_PI429010886	M21866	0.004619522	24	3	291242887	PREDICTED: hypothetical proteir			9.36E-09	6410.91	76.81667	6183.806	6125.57	4910.228	6285.074	44.85324	209.9538	53.73347	2710.483	3918.531	3755.671	2009.095	4852.406	4527.493	14.12473	25.7341	20.32505	97.75803	293.3331	89.01154	2571.491	1142.458	2615.936	115.1318	725.9659	383.7931
CUST_30991_PI429010886	M394	0.004619522	24	15						1513.287	1926.392	1540.275	1382.183	1095.073	1182.042	2010.612	1485.843	1738.077	1971.443	1333.943	1653.487	1980.694	1148.486	1489.353	2063.235	2097.341	2652.405	1946.675	1683.28	2362.508	1921.473	1652.642	2332.204	1929.095	1965.787	2583.285
CUST_32654_PI429010886	M4706	0.004619522	24	15	240995606	conserved hypothetical protein			4.89E-76	367.1233	688.6527	509.7706	344.5549	425.5802	371.7268	598.0964	626.0846	675.9355	602.7354	532.4935	536.1823	645.4936	446.7799	514.354	657.5452	726.5143	1076.743	496.692	667.9516	847.4628	441.5527	607.6204	662.7709	533.1871	530.6367	644.7162
CUST_3183_PI429010886	N13967	0.004619522	24	9			Protein metabolism	Protein folding		35.70122	28.40807	35.36547	29.282	34.4378	25.75202	38.38502	29.43649	25.13144	42.82494	32.93138	42.19985	36.40347	30.22686	28.66457	27.62537	33.43626	31.4973	17.87188	32.31005	33.79296	24.01703	22.34605	24.02389	24.08402	21.85804	22.19592
CUST_19145_PI429010886	N41049	0.004619522	24	3						1309.684	29.91504	523.1285	2184.345	950.4596	1291.739	56.24698	53.57855	72.86661	1398.059	1206.945	1201.71	1099.423	925.0688	1091.098	13.68381	27.34971	20.3622	130.5163	159.3992	207.7685	816.5697	642.342	779.344	188.2922	334.2355	462.3981
CUST_44124_PI429010886	M65005	0.004625703	18	3						67.35191	34.47208	82.79094	95.21224	83.0128	86.07541	41.11802	41.31439	67.9004	69.08897	111.5206	84.65768	104.173	64.63944	99.84349	8.874019	32.51568	22.51636	36.55792	51.171	48.4073	172.5012	116.804	173.332	43.8251	52.15678	65.46439
CUST_50524_PI429010886	M90136	0.004625703	18	15						25.23036	63.31187	55.08315	24.90332	16.72346	6.709355	77.54349	53.3873	72.35325	98.68778	22.96931	18.19202	117.5696	39.0193	38.52366	86.16832	114.2468	109.7186	57.8755	38.38502	55.59842	57.39387	15.17316	53.95129	83.31337	64.42993	66.3493
CUST_18217_PI429010886	N39369	0.004625703	18	15	270011121	slalom [Tribolium castaneum]	Transport	Transmembrane transport	1.52E-31	84.10925	129.8158	120.3588	85.20409	56.41359	78.67205	166.2093	107.8039	118.8271	184.8821	134.4124	96.66958	131.2558	73.17975	90.22636	221.7509	108.3124	173.5314	98.88646	113.9079	143.2785	92.59408	67.01762	79.06068	155.9036	94.06019	110.1907
CUST_23862_PI429010886	N49608	0.004625703	18	15						18.24052	24.11397	28.05953	22.15002	16.18229	16.60777	28.6389	23.21157	29.84906	31.32937	13.16067	22.22815	28.05304	23.07404	29.27204	33.19205	32.25543	32.95195	24.59083	15.12403	22.35516	25.62847	18.68449	21.65776	30.48044	25.64515	27.3523
CUST_51879_PI429010886	M95512	0.004636392	12	9	156356316	predicted protein [Nematostella			4E-12	499.2313	NA	516.0491	316.1661	373.1569	241.9195	13.52663	55.56698	69.17898	715.6987	779.344	760.7688	470.5066	210.6988	508.3099	NA	NA	10.75951	52.06198	73.97753	99.4113	942.2704	1552.466	2187.303	62.74684	201.0489	327.1238
CUST_8994_PI429010886	N23905	0.004637298	24	3						59.3284	72.57836	50.41623	71.99448	103.1619	90.68839	73.19641	64.36702	72.85755	61.58528	64.7701	61.85717	51.8805	48.08963	41.25814	50.99345	78.18007	46.22711	59.24085	45.95593	56.94521	44.87342	69.8604	52.67704	39.5247	69.47326	74.58857
CUST_322_PI429010886	N3887	0.004639149	21	0						2261.704	962.741	1877.836	2156.524	273.802	1354.492	906.4938	818.7919	727.0088	1304.861	1321.627	825.7252	1098.604	761.5417	907.8057	357.1234	441.5527	280.5879	819.3739	912.5849	894.7312	1651.623	2135.546	1754.468	543.1972	810.1648	982.8111
CUST_50398_PI429010886	M89685	0.004654016	18	3	148841064	relish [Litopenaeus vannamei]			7.62E-09	30.95428	28.64933	23.64554	29.99634	42.80977	28.67614	13.16859	23.25083	31.07888	11.93633	25.93001	34.80724	24.30986	21.69939	24.42859	25.28249	23.66496	23.86873	26.71367	31.64857	30.16221	37.95648	29.84354	39.67383	27.72879	19.46038	35.61115
CUST_1659_PI429010886	N10363	0.004660629	24	9	157108236	hypothetical protein AaeL_AAEL			4.64E-08	96.40725	69.85306	80.89811	101.8747	108.5015	102.0603	70.86476	79.58985	95.16242	93.3967	107.8884	137.044	92.23687	100.783	129.8158	109.3218	79.84725	91.84111	75.23071	47.93963	86.71039	90.79873	77.32899	91.09751	70.69946	64.68944	74.16527
CUST_13998_PI429010886	N32147	0.004660629	24	0	395540285	PREDICTED: peptide-N(4)-(N-ace	Metabolic process	Nitrogen compound metabolic process	1.33E-12	50.05856	57.81843	80.35316	71.85783	61.67047	49.16277	38.30361	60.60253	55.73019	42.5735	57.86776	48.56908	52.71315	67.50363	47.10689	47.66075	67.20028	59.6688	50.12052	66.79456	68.83252	54.85564	70.57647	69.62329	76.05422	71.07642	76.92811
CUST_11012_PI429010886	N27127	0.004667605	24	9						5.044846	3.042775	7.873998	9.294827	36.58776	30.7666	6.409298	17.213	10.86877	14.40728	42.24367	26.06422	NA	14.11506	19.6201	NA	4.532239	5.434281	NA	5.350473	4.650934	8.503753	3.048366	7.171959	3.490315	6.941367	NA
CUST_40746_PI429010886	M55988	0.00466962	18	0	149579996	PREDICTED: VPS33B-interacting			4.79E-11	40.10315	48.24507	29.29782	26.69789	35.53472	36.29464	23.00944	26.60721	40.32964	32.98496	42.40452	36.08229	31.6335	27.22363	38.40373	39.30254	28.89871	39.19188	35.88575	44.45113	42.71301	13.44807	29.59753	28.85937	23.47009	22.04646	21.3436
CUST_18517_PI429010886	N39939	0.004670455	15	0						106.1445	207.5504	173.05	111.8609	204.8443	108.3883	106.6126	157.3672	121.9377	104.7345	147.4493	118.1075	156.8658	144.0147	176.5842	179.3306	213.8259	222.4375	143.0807	136.3915	155.0166	102.4924	128.6276	118.1715	136.6555	140.6285	154.4681

Probe ID	ID	p-value	Period	Phase	EBI ID	Annotation	GO category	Go term	E-value	0	0	0	3	3	3	6	6	6	9	9	9	12	12	12	15	15	15	18	18	18	21	21	21	24	24	24
CUST_56028_PI429010886	M112581	0.004673238	24	3	312072580	hypothetical protein LOAG_035			2.99E-19	272.6276	167.3103	267.884	230.5374	305.9701	312.0575	139.5639	181.8872	160.5879	82.7532	261.5986	252.2559	90.08744	267.4596	369.5842	119.7806	91.45416	95.75319	97.28384	179.7028	127.1843	121.6468	139.7162	143.7214	126.5057	171.7541	147.5241
CUST_31456_PI429010886	M1564	0.004673238	24	15						47.51044	68.39377	46.66635	22.39225	42.1373	34.70595	35.23063	55.80136	50.65434	51.82749	55.97582	48.64708	37.42403	47.79385	45.58935	56.52239	72.52843	58.30569	43.34384	61.43496	52.27625	48.13748	54.96965	59.12751	56.22476	51.28488	43.30339
CUST_12046_PI429010886	N28831	0.004673238	24	15						38.40373	42.58344	36.04308	30.0529	28.00501	31.46346	60.96191	39.03604	44.58927	55.73395	33.26372	33.33788	49.38313	35.33485	38.7574	88.69555	56.35449	56.38348	53.33823	36.00722	58.01467	32.75774	45.05271	53.13532	47.72966	54.99763	59.11715
CUST_1298_PI429010886	N9172	0.004673238	24	15	348505893	PREDICTED: Oreochromis nilotic	Protein metabolism	Ribosome biogenesis	6.11E-69	21177.49	26491.1	20968.09	17931.09	14764.65	16183.31	27665.22	25403.62	27073.29	20708.31	17388.9	19195.08	22221.29	19195.08	15967.93	29396.88	27758.19	29122.17	23575.84	27577.78	28025.82	16580.62	22037.75	19485.9	20968.09	25188.43	22154.05
CUST_2258_PI429010886	N11974	0.004677265	15	3	443686771	hypothetical protein CAPTEDRAI	Metabolic process	Oxidation-reduction process	2.85E-155	22.16839	16.16791	19.88289	35.92598	37.61646	25.23371	31.5845	19.19666	21.10822	22.23439	22.15692	31.46705	20.36712	17.02151	12.1659	15.17831	25.20375	30.6774	29.25551	42.00294	40.43404	22.9157	11.27154	21.67269	25.94024	19.03842	35.66495
CUST_46313_PI429010886	M72168	0.00467949	18	3						87.62517	58.24898	104.9377	113.8607	129.5035	114.0755	70.43485	68.73418	83.26396	68.90523	101.1475	79.37286	97.69073	73.50137	108.8321	52.26773	49.35984	52.10827	78.94493	66.89231	79.38788	126.4187	74.57856	125.8771	78.63104	48.87698	77.35718
CUST_9864_PI429010886	N25318	0.00467949	18	15						22.2762	22.99386	21.62003	10.06086	13.86062	17.67823	24.24527	23.1769	25.18427	24.06072	19.91817	8.883273	20.43156	19.04208	15.02551	27.96239	20.72028	31.02823	23.96712	18.21177	26.27328	13.33647	17.20227	8.109042	18.18461	18.28134	22.52125
CUST_10835_PI429010886	N26867	0.00467949	18	15						107.9218	183.166	207.5504	126.3152	67.97894	140.387	275.9079	170.625	138.6295	232.6717	132.5859	133.2817	164.071	87.61045	146.9806	359.2536	203.6782	289.56	178.2425	89.20331	138.4394	98.56741	113.1623	84.28713	208.6394	130.5329	125.3559
CUST_12048_PI429010886	N28837	0.00467949	18	15						252.9258	336.5251	290.6243	220.6649	259.0205	218.2236	321.6405	266.4292	347.9094	253.5194	294.4308	265.1693	282.7416	269.3499	298.1868	302.6134	385.0272	330.9231	313.5422	284.0207	354.5733	240.3937	249.9933	280.9977	311.4501	262.8953	344.5549
CUST_24937_PI429010886	N51512	0.00467949	18	15						15.93823	21.37642	17.10849	8.508175	5.235046	12.12405	14.79074	13.54422	11.63109	16.56735	20.05184	15.82642	7.45426	4.911674	12.57356	27.47971	26.65508	18.65093	9.473667	16.36409	9.947363	13.10871	9.231247	7.763694	10.80517	11.16309	14.79505
CUST_2957_PI429010886	N13475	0.00468003	24	18	118136260	Penaeus monodon beta-actin m				1526.163	3190.966	2162.694	1486.662	1772.069	1433.817	1696.829	1972.842	2233.186	1385.605	1676.387	1285.646	1559.979	1904.856	1322.312	1537.641	2073.458	4754.165	1865.951	3120.055	3346.038	1848.594	3460.858	2652.405	1717.387	2837.693	1821.979
CUST_18595_PI429010886	N40075	0.004691825	24	21						38.71226	41.61011	40.20755	16.99422	27.98737	31.30624	19.16983	25.66849	25.92708	33.44592	32.25543	22.69087	28.74405	38.91258	39.17612	34.81487	38.82932	43.15923	44.77327	39.32943	32.32834	37.62021	37.78857	41.69222	42.90901	41.41654	47.42663
CUST_4127_PI429010886	N16077	0.004692442	24	9						25.82213	20.01232	13.74955	11.38924	2442.216	946.5528	47.9302	30.51884	52.75604	NA	1694.196	1543.849	12.61565	1988.355	1708.901	18.6455	5.264041	10.31893	3.979131	21.70891	6.841078	NA	7.234885	NA	NA	14.09206	NA
CUST_50495_PI429010886	M90010	0.004713683	18	12	426236068	PREDICTED: Ovis aries ATPase fa			1.83E-55	6.669026	10.71329	14.36577	10.19704	9.005083	11.85412	26.33135	12.47435	11.61456	13.72622	4.968715	15.0891	19.11579	17.20923	18.47	8.551917	14.36256	16.28426	10.75824	15.9018	13.70744	7.587167	9.356835	12.97888	11.49713	13.79482	13.8625
CUST_33972_PI429010886	M8387	0.004722527	24	9						61.69489	97.71482	45.26784	105.8953	143.7853	206.7386	42.19574	182.5875	112.7999	112.1712	222.0168	206.8979	77.47208	138.3312	77.77436	72.34444	106.6278	86.31462	79.72468	8.434585	86.19309	55.0054	100.2539	35.72862	65.0271	60.94991	113.878
CUST_57103_PI429010886	E903	0.004726953	24	15	48142173	PREDICTED: proteasome subuni	Protein metabolism	Proteolysis	2.29E-11	1922.582	3356.595	1918.312	1870.445	1542.881	1508.513	2975.418	2799.9	2487.704	2359.281	1564.909	1803.597	1946.675	1725.737	1729.252	3329.804	3660.946	3907.106	2266.512	2230.426	2849.078	1807.527	2054.56	2184.345	2512.997	1988.355	2149.415
CUST_36650_PI429010886	M16453	0.004726953	24	15	17221659	ribosome like protein [Marsupe	Protein metabolism	Ribosome biogenesis	3.12E-32	19903.59	26403.54	19010.63	18476.28	15258.82	16553.17	26403.54	23961.37	25403.62	22777.74	16348.66	10871.96	21066.26	18146.2	19572.59	28561.19	28561.19	31108.81	21758.66	25188.43	27665.22	17287.24	20662.81	21066.26	21710.8	21066.26	23892.85
CUST_31967_PI429010886	M2859	0.004726953	24	15	291223821	PREDICTED: early endosome ant			2.32E-10	820.6216	1875.312	1093.204	826.745	619.3687	773.0893	1382.183	1563.997	1545.477	1554.179	1084.752	728.8717	1301.148	787.8266	977.1077	1968.185	2140.692	1849.733	1201.304	1023.762	1433.817	1046.212	1101.531	1369.757	1378.281	968.3563	1079.588
CUST_26_PI429010886	N109	0.004726953	24	15	188572483	Phoronis muelleri putative 40S r	Protein metabolism	Ribosome biogenesis	1.22E-61	23162.84	26129.84	21177.49	17837.79	15297.04	16690.76	29520.35	23658.24	24825	24590.28	17088.5	20871.55	22847.77	20099.82	19237.66	30447.23	26403.54	32454.44	23379.75	25888.13	28414.17	19855.57	21645.19	22221.29	24136.69	23016.75	25130.01
CUST_6710_PI429010886	N20493	0.004726953	24	15						821.5501	1672.68	1049.887	932.1979	744.5255	785.0219	1383.977	1273.67	1285.646	1443.341	920.7381	478.5299	1247.204	817.7537	962.741	1738.077	1846.233	2075.256	1126.574	1068.554	1453.877	1137.334	1046.706	1476.878	1334.416	1020.042	1365.984
CUST_50625_PI429010886	M90466	0.004730303	12	0						119.6589	141.0115	150.2498	130.9169	124.0485	134.4639	85.64416	118.5947	104.5846	119.6589	159.1592	147.6402	133.0536	135.6132	141.5897	139.7162	134.2508	129.2198	117.4793	128.9542	109.0351	144.9379	142.5991	161.4506	159.0794	142.7074	216.7613
CUST_37598_PI429010886	M19725	0.004733278	18	15	478503880	PREDICTED: kinectin isoform 5 [	Cellular component orga	Cytoskeleton organization	4.65E-08	74.57856	78.25384	57.00268	52.09068	42.72194	55.77263	92.62501	83.78528	96.54391	109.3748	47.26148	64.96779	88.4112	54.58501	85.22534	121.0882	86.71039	111.3048	86.59225	48.60251	77.54349	69.44476	47.18014	74.42622	104.7711	77.00089	76.90215
CUST_38565_PI429010886	M28032	0.004733278	18	15						769.5468	2851.067	982.3643	1011.588	176.2933	745.5832	3069.207	1352.068	1652.642	2097.341	804.9022	917.5193	1462.033	1314.736	792.9194	4427.539	4234.05	6000.152	1704.256	588.3344	1686.38	786.6307	507.5036	889.3882	2679.375	1516.421	670.4033
CUST_4110_PI429010886	N16039	0.00473338	21	15	290908989	myosin light chain [Marsupenae			3.4E-41	535.89	530.1492	562.7989	571.275	565.823	495.8587	482.1823	564.8143	458.6774	398.2132	535.3308	475.7174	465.024	481.7121	425.7716	692.5483	864.5859	1011.588	711.4323	725.9659	1035.146	423.2638	539.585	583.7299	555.2007	694.7413	459.4079
CUST_6696_PI429010886	N20476	0.004734217	24	9						124.2323	101.1475	122.8853	138.9364	116.6562	121.7517	183.5918	112.0316	146.7827	228.0375	100.9673	153.1975	169.7003	143.2463	112.2698	166.8118	148.1939	157.1984	95.2367	122.7362	89.35207	100.264	100.0093	101.3206	106.4806	113.7707	103.9866
CUST_13765_PI429010886	N31732	0.004756989	21	0						123.8699	122.9044	100.6344	98.55805	74.72923	80.03641	87.66036	106.6009	105.746	94.75478	85.40071	104.7593	89.62874	109.5302	98.04747	100.875	102.6741	115.1005	130.9003	113.4582	107.6401	114.8223	118.6368	115.9268	85.63473	117.7627	110.145
CUST_39563_PI429010886	M41234	0.004764403	24	3	341650466	Spz1 [Litopenaeus vannamei]			8.36E-88	211.7618	26.88183	115.2322	159.3669	166.9821	182.9006	52.28327	62.20434	21.50871	86.53109	114.4623	228.1333	76.3265	63.01997	40.9561	9.610362	22.36702	22.71026	6.580479	82.29248	4.979025	98.03098	37.51134	60.49503	10.37266	91.92491	34.58217
CUST_5336_PI429010886	N18559	0.004764403	24	15	91078808	PREDICTED: similar to Protein SI	Transport	Other	1.63E-165	102.1933	141.898	103.6693	101.4138	94.31617	91.40987	104.258	146.3365	148.8152	99.75898	95.80871	110.1907	107.4552	130.0221	93.21779	126.2557	206.4029	166.2848	139.4798	140.0039	163.782	136.8604	112.4501	149.7056	135.6132	110.3077	156.2107
CUST_13817_PI429010886	N31827	0.004764403	24	15	321479469	hypothetical protein DAPPUDRA			2.27E-19	291.4994	258.9723	284.1966	260.2914	186.5368	234.533	323.0408	247.0676	304.7058	305.4328	227.7173	373.9602	342.0443	249.0801	359.2536	487.7778	296.8179	409.0189	480.4617	261.7871	444.9419	338.9144	268.7721	375.6666	440.1863	244.1747	421.3837
CUST_39473_PI429010886	M39930	0.004771531	24	15						4415.56	5763.929	4217.01	3258.199	2005.153	1909.699	2829.469	3519.517	3944.006	2881.653	3956.525	3020.109	4329.518	2363.552	2445.736	4467.089	5913.632	5462.129	3390.028	3617.55	4058.337	3835.495	3561.33	4637.293	3324.822	2937.545	3122.414
CUST_42673_PI429010886	M60918	0.0047718	24	0						295.9223	201.3091	237.1976	387.9483	222.8685	221.3576	162.8755	174.935	208.9613	291.3249	112.3135	285.8938	170.8146	101.6014	144.058	35.07936	381.5238	94.66861	177.6012	139.1998	94.75478	171.1773	237.0529	166.5055	234.791	250.2647	241.668
CUST_53697_PI429010886	M102830	0.00477335	18	12						85.37067	75.82447	91.26396	76.92811	71.72818	53.48046	82.31732	69.14386	104.437	95.74045	62.26618	95.75319	91.71339	98.43645	109.5906	89.93986	81.61482	93.631	91.30891	75.9364	99.48565	70.96595	65.42578	85.47045	79.2021	78.06756	121.1808
CUST_28280_PI429010886	N57660	0.004778689	24	21						38.15601	73.0273	63.24693	48.52901	37.54467	23.00207	42.37353	29.74161	40.22947	39.10716	21.52336	36.79436	33.20989	22.25864	31.08965	68.98107	51.83065	62.48283	51.86945	32.26395	43.22841	69.55765	42.97495	49.71376	66.2384	43.07828	91.87245
CUST_38108_PI429010886	M23386	0.004780668	24	3						12743.08	68.76743	11094.64	9080.902	18476.28	15798.02	97.55312	287.0864	196.3832	8238.918	12442.6	14642.96	5467.822	9855.129	15141.22	15.67395	16.33804	67.60668	351.5907	790.776	498.6872	8886.515	3093.621	8098.961	463.3449	2156.524	2208.268
CUST_34240_PI429010886	M9145	0.004780668	24	15	498943826	PREDICTED: transmembrane prc	Protein metabolism	Proteolysis	8.61E-34	104.0674	22.68661	80.8714	19.94626	9.654225	5.955482	295.3547	94.10473	281.1928	313.6779	188.9036	5.684912	65.84893	54.48769	22.52975	816.5697	325.7046	782.9344	78.73755	56.80856	134.6107	411.0555	29.40141	311.3298	236.5379	11.27021	222.4375
CUST_5063_PI429010886	N18212	0.004780668	24	15	212656629	cellular apoptosis susceptibility	Transport	Protein transport		37.12565	59.07967	59.37537	29.96804	28.03915	29.81035	38.65654	58.13362	68.82621	57.47217	40.93762	47.69479	49.72483	35.74167	33.5847	72.82177	67.39104	73.56286	56.05872	55.35053	63.79933	34.81487	46.39504	52.13265	55.05576	31.42758	78.85311
CUST_5130_PI429010886	N18297	0.004780668	24	15	340725023	PREDICTED: hypothetical protei			1.08E-70	2956.694	5879.268	4072.42	2945.018	2294.993	2499.817	5311.077	4976.553	5091.533	4392.913	2879.516	2889.672	3918.531	3105.612	3046.537	5979.853	6966.12	7303.286	4740.787	4436.509	5233.042	3555.208	4193.691	3832.521	4694.435	4919.057	5025.735
CUST_11069_PI429010886	N27217	0.004780668	24	15						138.0734	239.5431	198.0524	134.5227	127.9686	105.2262	187.1331	218.3332	232.8674	251.0873	154.0925	136.4616	196.1132	127.4555	168.9955	266.8703	261.3125	280.9343	182.1611	177.4828	273.6644	183.1939	141.3156	220.2833	217.0918	154.4146	207.4056
CUST_13198_PI429010886	N30770	0.004780668	24	15						371.3812	980.6718	461.7785	340.1047	293.4022	357.0377	740.8082	688.887	600.0613	733.2499	422.3982	408.5695	535.2394	461.0647	403.5639	545.7936	895.8876	952.2766	629.6411	553.926	779.0482	464.5257	420.7391	500.4612	595.1579	469.0501	376.0142
CUST_23127_PI429010886	N48300	0.004780668	24	6						559.4339	614.5401	615.2672	738.0615	394.9633	723.9274	747.5407	954.4926	578.5106	641.3942	463.1658	348.6137	531.2109	908.3858	551.2795	645.1212	454.5758	929.4814	339.5673	376.9694	277.4142	535.6201	339.7941	520.0326	501.5231	481.8774	440.1079
CUST_38064_PI429010886	M23144	0.004783453	12	6	229577134	glycine N-methyltransferase-like	Metabolic process	Cellular amino acid metabolic process	8.58E-18	24.62618	40.89712	7.589324	20.69224	36.22402	37.81634	43.84283	36.53104	46.45729	25.0378	27.31402	28.2426	14.58842	28.84875	23.49626	36.10172	21.55742	28.73196	37.28516	50.23264	41.85879	12.10203	27.6893	37.63739	22.02084	31.59503	23.49323
CUST_42813_PI429010886	M61329	0.004783453	12	0	15718457	peroxinectin [Penaeus monodon	Response to stress	Response to oxidative stress	1.15E-11	91.61189	68.30294	84.07229	58.79368	74.5359	55.55609	70.93399	59.12156	33.57662	46.71775	94.34203	63.94916	96.20921	55.74024	80.77747	71.9236	82.30731	81.16582	22.62211	35.74554	21.74119	67.72081	63.69892	69.21158	115.2129	123.8841	54.70587
CUST_51459_PI429010886	M93616	0.00479021	15	6						1657.457	330.6328	1804.854	3455.897	2237.142	904.3497	1103.539	2049.491	3379.597	1993.835	2631.802	1552.466	4319.299	1512.062	1080.452	72.20641	195.511	80.98106	3269.912	3151.384	3692.065	2536.537	7844.789	4740.787	3177.941	4650.166	2839.956
CUST_48437_PI429010886	M80320	0.004800826	15	0						22.79631	39.17612	33.93179	23.7984	37.05862	42.61451	35.80198	45.95593	23.25881	22.58797	21.76828	18.84709	24.28126	38.84447	30.35424	30.3574	46.39063	29.38788	25.48421	28.68856	22.30972	22.00011	42.5482	19.64801	21.49428	18.41787	19.03842
CUST_19814_PI429010886	N42243	0.004805421	21	3						28.87489	34.54334	48.13265	54.84307	53.73347	63.87134	40.74313	26.20354	35.15693	47.15185	23.70635	63.63996	43.63223	20.60087	28.91213	35.51402	20.04588	32.92494	67.9004	36.10172	42.98811	69.34953	36.19278	36.12041	57.63804	43.50595	79.68621
CUST_1401_PI429010886	N9525	0.004805852	24	21						211.9389	260.9827	294.9654	296.2197	221.7509	252.6173	244.2151	189.9808	284.1348	197.4226	222.2239	266.583	233.6058	247.6563	233.9614	294.2148	280.2395	288.7179	438.6999	258.9723	342.2376	429.2293	260.6027	361.2613	434.7194	257.4304	293.4576
CUST_16400_PI429010886	N36162	0.004820913	12	9						37.47838	52.14659	56.12288	42.07726	19.77651	22.14517	36.59345	31.38536	45.39182	48.73482	38.90861	26.6468	58.13362	39.76763	39.06481	16.00633	23.25083	23.10726	25.90356	33.4192	48.86558	49.57184	62.367	59.93426	34.22791	38.26324	58.23791
CUST_40713_PI429010886	M55919	0.004820985	24	18	321475764	hypothetical protein DAPPUDRA	Signal transduction	Other	5.42E-10	45.54712	90.12112	125.6161	34.50418	51.75972	33.87201	48.2963	149.6818	132.4508	61.35249	37.23532	45.85631	63.03163	42.67386	78.70208	61.65559	47.26148	58.51048	347.5429	178.896	196.9036	166.9595	74.26533	142.0036	193.9353	121.1967	120.9166
CUST_25666_PI429010886	N52837	0.004821935	24	0						29.74537	33.76496	38.35932	23.61457	21.13144	20.39754	13.21612	15.73415	9.213352	18.74814	19.09908	16.84768	22.07863	17.54	14.80397	15.50904	12.89253	19.38594	30.68002	22.38961	15.56129	25.0865	9.63872	20.42799	33.00869	17.75966	25.47189
CUST_31366_PI429010886	M1357	0.004823223	24	15						91.32281	88.4446	104.4886	68.32149	88.06844	71.21793	96.33044	97.45374	93.41761	98.71772	78.23111	94.54537	86.89375	108.2549	79.90962	115.7715	87.85118	126.0246	108.298	104.8456	91.47922	95.04674	107.7085	103.4513	73.79109	91.30891	102.4481
CUST_36813_PI429010886	M16931	0.004823223	12	0						65.13726	68.95176	64.13867	43.24498	66.27962	32.92494	35.74167	50.70492	35.74914	62.28713	62.82646	51.49597	89.54662	54.04214	68.04046	51.60691	47.85099	40.67888	41.84717	71.00454	53.68198	93.12448	78.92328	92.13972	82.39127	57.94759	95.24948
CUST_9080_PI429010886	N24031	0.004824637	21	18						NA	NA	NA	13.95233	11.56464	8.3196	14.66893	13.24351	5.807099	7.856768	6.801504	NA	14.56607	27.2264	11.61625	17.10019	19.43519	15.14334	17.44275	28.22301	29.92819	22.57786	3.901574	14.32824	15.714	5.742729	8.11425
CUST_17177_PI429010886	N37502	0.004829446	15	12						20.14949	41.99235	NA	17.35867	6.603742	17.9949	8.928675	NA	10.19179	44.48574	23.4616	NA	20.85079	20.20024	27.65783	29.01519	18.80558	12.09251	25.58133	6.939412	15.40578	11.6476	10.13279	10.10062	19.01388	13.61982	NA
CUST_42927_PI429010886	M61651	0.00483324	24	21						207.6552	185.1157	233.0433	212.8318	148.0804	177.1027	173.7538	140.612	161.2407	189.8002	142.6225	173.7818	198.1815	174.8361	184.1477	253.0362	166.0161	209.9184	227.6714	145.7916	212.7294	255.3925	182.1021	229.7005	265.9313	176.6026	229.9275
CUST_15818_PI429010886	N35198	0.00483324	24	21						125.4319	98.31087	106.0765	107.7947	85.10841	99.2507	69.86685	70.2307	73.14068	122.5392	85.7431	69.04786	154.2424	76.79923	105.2562	160.1938	136.7438	114.1224	175.4084	105.6512	102.6378	211.803	92.39145	149.3002	190.3661	83.96708	125.0424
CUST_6133_PI429010886	N19672	0.004834168	12	3	242016587	hypothetical protein Phum_PHU			5.63E-13	179.1302	234.1352	120.5417	172.561	280.2395	322.4558	244.266	206.6211	182.9511	141.4085	205.7616	202.7719	155.0166	203.0154	208.5809	440.9544	256.9518	294.2148	246.2584	116.8754	232.0765	128.4629	160.5413	119.4647	193.0588	150.8283	79.30248
CUST_57324_PI429010886	E5966	0.004834384	24	15	91083583	PREDICTED: similar to adenosylh			1.13E-26	3992.329	7225.192	4976.553	3702.472	2858.309	3056.068	7643.683	7232.62	6559.55	5215.78	3936.583	3862.287	4415.56	4193.691	4319.299	6956.592	7260.089	8713.974	5025.735	6717.703	6500.649	3721.413	4450.275	5061.439	5131.503	4685.458	5644.907
CUST_17005_PI429010886	N37215	0.004834384	24	3						30.50653	17.49199	19.19318	24.56289	33.5847	38.74481	12.77084	17.35683	11.89304	6.452099	21.05828	29.24609	11.60045	42.93819	30.97404	8.865478	5.539656	11.93633	11.81992	25.21673	9.733163	12.38102	14.87474	12.60041	15.01514	15.31282	10.99723
CUST_12410_PI429010886	N29420	0.004835403	24	0						581.8311	787.2783	483.0822	491.2558	524.517	441.5527	346.6392	542.9066	494.0593	432.1282	528.2931	376.3542	448.5869	479.3697	437.0635	323.0951	658.7898	590.3145	433.4692	530.0108	553.7643	496.3451	591.4732	536.7837	595.0044	712.1062	718.7132
CUST_55569_PI429010886	M110772	0.004836835	21	15						353.8605	676.6048	453.0125	374.7006	201.3401	304.0274	663.1483	628.502	606.3479	669.9449	507.7884	320.552	621.9301	388.382	365.7068	661.1838	856.2286	648.2686	536.7837	410.5544	441.2406	370.3281	420.1391	406.3531	463.1658	380.83	371.2053
CUST_39423_PI429010886	M39190	0.004841701	18	3	255653868	crustin 1 [Panulirus japonicus]			0.000000014	41.90666	63.76387	51.45353	154.5211	35.71025	73.3346	43.99925	53.9367	75.2001	86.20512	43.23211	81.79336	67.83335	40.43868	34.97429	34.95791	61.42131	22.45935	55.72212	39.44209	48.8281	100.2009	86.99822	103.2872	143.902	65.82678	143.6704
CUST_162_PI429010886	N1910	0.004841739	15	6	518355741	hypothetical protein [Streptomy			4.45E-08	11.07529	7.23313	10.26259	18.22769	NA	10.87185	NA	88.87721	96.04821	10.6362	NA	10.1802	10.80654	14.27002	3.926108	3.92479	10.69017	18.81483	10.8487	35.81401	25.80026	43.83834	13.4622	25.45303	24.88953	NA	29.85598
CUST_36078_PI429010886	M14561	0.004844753	12	3	91079482	PREDICTED: similar to AGAP002			2.72E-20	16.79753	24.56549	NA	48.94611	7.695174	63.01324	17.11222	14.89285	19.64055	NA	6.441192	3.637552	10.07839	39.77797	18.64884	102.3598	43.24154	58.50483	58.23791	4.464401	59.81752	NA	14.49179	NA	21.57697	19.92002	NA
CUST_21099_PI429010886	N44628	0.004846156	21	9						5.935469	4.947558	NA	6.721195	12.18283	9.008672	NA	7.469059	4.412405	13.05918	11.21185	8.278081	NA	NA	8.115316	4.364494	4.190132	4.32996	NA	3.857484	3.172222	NA	NA	8.727182	6.544441	4.374986	9.288536
CUST_39529_PI429010886	M40852	0.004852019	18	6						339.4771	419.9233	448.4659	393.0106	328.6973	395.3057	406.0638	399.2455	465.8295	375.7494	348.1933	355.6308	360.4299	264.2234	260.6535	323.1624	349.5554	287.2425	335.7533	407.1156	369.756	441.4649	362.1172	332.7289	495.8587	464.5257	340.1047
CUST_21796_PI429010886	N45869	0.004852834	15	0						597.4035	532.4935	602.1887	515.109	452.9256	446.2058	368.8823	363.7634	509.4445	563.1838	456.1764	536.7837	472.502	328.5727	334.6481	603.9154	603.0231	556.4241	522.1886	514.6065	477.8297	475.9252	439.8767	503.5534	525.1123	508.3099	576.9572
CUST_17097_PI429010886	N37355	0.004854768	15	0						191.7677	68.80951	168.944	118.4103	58.41936	94.15594	129.7338	108.0539	79.63863	11.36638	82.68763	22.17034	103.8983	169.3609	43.22401	235.6412	162.8755	267.6739	61.08411	28.39612	33.41357	52.541	69.67842	40.73438	49.05504	63.77866	30.34849
CUST_22832_PI429010886	N47764	0.004858971	24	3						19.38111	21.57697	20.72247	16.40975	176.8026	51.24138	14.45802	NA	32.64521	12.7417	92.0968	214.2802	15.95963	75.53689	68.5694	4.698974	10.95746	13.10078	12.62763	14.23509	9.665024	19.46213	13.65032	6.364152	13.05918	18.10043	16.13549
CUST_10046_PI429010886	N25611	0.00487359	18	3	321458259	hypothetical protein DAPPUDRA	Metabolic process	Nucleobase-containing compound meta	7.11E-39	35.63404	67.82594	53.09948	73.78491	42.5644	124.9582	21.42599	97.90232	95.85439	74.89382	44.93169	41.38164	39.25729	23.76085	33.72001	28.70362	33.32109	32.04577	19.63566	42.72823	31.19127	56.63612	79.24842	58.35105	37.54467	67.87655	34.01769
CUST_11831_PI429010886	N28482	0.00487359	18	3						122.1215	118.0753	191.4096	163.8916	145.2758	152.3742	103.2872	145.2758	141.9148	112.7891	149.3578	140.2946	156.3694	134.0782	131.7124	101.2545	138.6868	104.8681	158.2397	164.2345	166.2093	164.6109	176.8903	217.9435	141.352	149.6035	215.0091
CUST_48569_PI429010886	M81078	0.004880273	15	12	321466112	hypothetical protein DAPPUDRA			1.06E-21	19.12837	NA	14.69279	12.10978	10.35412	17.47661	19.22503	14.63303	8.545551	14.24557	13.25495	20.70771	19.81371	21.06721	20.67603	11.73655	12.10629	14.79074	10.8869	10.01392	11.00412	13.96855	13.30593	11.43354	15.07053	9.120228	16.2952
CUST_39659_PI429010886	M42343	0.004892411	12	9						17167.45	118.8089	13812.43	17210.94	15045.33	20172.23	1129.142	1200.665	1976.903	25463.11	21524.84	24289.54	20762.38	9791.81	21066.26	451.1176	74.89382	523.631	2593.566	3633.442	5009.272	21066.26	17487.6	23892.85	6179.344	14161.49	20382.6
CUST_40557_PI429010886	M55453	0.00490798	15	9	443687501	hypothetical protein CAPTEDRAI	Signal transduction	Other	2.48E-21	129.5211	255.1811	201.9179	57.8755	173.6159	97.11689	185.0545	212.598	220.6649	153.0208	381.7573	374.2544	149.6818	233.9614	152.6909	154.757	211.8745	262.0968	57.12065	141.3865	107.332	162.7885	309.4164	339.5673	134.3827	303.4901	347.0422
CUST_13181_PI429010886	N30729	0.004913632	12	6			Metabolic process	Carbohydrate metabolic process		4.035824	17.87527	9.58783	6.627872	NA	15.18033	132.6815	NA	96.68164	30.08483	82.2265	5.101568	5.65772	15.32206	NA	23.80329	NA	28.18616	279.305	337.4051	769.0192	37.84952	NA	33.44592	90.57569	4.893177	66.55746
CUST_55539_PI429010886	M110578	0.004920462	24	9	321478442	hypothetical protein DAPPUDRA			4.18E-28	65.50892	78.518	83.51378	37.455	92.42138	49.39254	82.21745	75.53689	89.0262	84.48811	82.29248	92.11335	74.13678	87.62517	72.58638	70.30569	76.3955	54.12459	54.61235	54.29543	46.84134	72.13266	76.19975	61.08411	76.98432	65.09533	69.18571
CUST_43022_PI429010886	M61879	0.004922602	12	3						25.8664	36.58388	49.45942	66.78529	46.45729	57.14912	82.96235	76.25504	86.53109	77.19174	23.52214	61.39667	54.81291	28.35209	41.18226	60.68039	68.8804	91.21622	45.66597	49.37835	48.11986	33.76496	52.20454	42.42877	22.61447	24.66091	16.47491
CUST_43369_PI429010886	M62861	0.004925888	18	0	386376737	hematopoietic prostaglandin D s			9.58E-20	69.64615	138.824	140.1608	49.66595	82.72734	59.42381	45.50498	99.52529	115.2993	49.21384	43.16883	45.56749	47.41757	44.73914	45.47674	38.58803	44.62591	54.88564	71.49956	141.9587	88.97825	57.70575	54.02691	42.96134	76.57491	72.75115	49.27254
CUST_50383_PI429010886	M89648	0.004935769	15	0						62.21897	71.97753	62.35954	45.90992	56.97991	41.50033	34.10236	41.49384	37.88972	59.62083	51.55397	48.66684	41.02313	41.51987	55.8305	56.88517	64.90539	52.01796	48.98205	69.05459	48.69844	45.49544	58.95577	44.38734	52.31438	59.48775	43.32213
CUST_47621_PI429010886	M76930	0.004940862	24	3	117414788	Euphausia mutica 18S small sub			7.2E-117	5420.715	330.2787	5233.042	6592.219	17982.7	12424.28	251.4219	562.2603	275.9873	1529.136	9550.071	11413.74	1028.106	11339.63	11719.99	192.4063	183.381	213.6621	190.7291	521.086	170.9641	2383.479	416.1276	1770.862	254.4938	783.1683	293.934
CUST_2698_PI429010886	N12929	0.004941924	24	15	241997760	meprin A metalloprotease, puta	Protein metabolism	Proteolysis	9.02E-54	74.59679	NA	72.43858	7.763694	NA	8.026216	98.63138	NA	66.98556	68.11779	38.76155	7.713675	18.85311	102.5315	23.97557	627.6794	77.96514	519.9072	97.69073	101.6524	214.693	137.8214	NA	133.4052	136.2198	44.37034	75.35645
CUST_34763_PI429010886	M10584	0.004950654	12	9						447.5258	416.778	357.296	307.3952	288.9896	364.8028	287.632	397.7869	434.8192	430.4406	380.9979	482.5812	382.3723	321.1394	515.6628	221.8495	259.5151	316.2435	396.9983	369.3009	280.8283	509.8873	378.1731	541.6936	572.3479	338.5986	825.0827
CUST_33601_PI429010886	M7397	0.004950654	12	3						134.9672	278.8215	68.9724	237.5415	198.84	252.5113	278.7672	177.6012	121.6645	191.4474	166.6906	181.0435	170.3606	200.1541	244.2151	421.4753	274.2134	307.3354	220.0171	102.8306	199.982	64.6674	147.7301	109.3921	219.5016	203.7634	71.87251
CUST_24474_PI429010886	N50687	0.004950737	24	21						97.18737	63.87749	88.50381	138.7489	96.71212	88.78315	41.77121	77.10772	36.3002	56.4057	57.81843	50.63563	87.68846	44.46024	89.08902	55.08315	86.9167	37.02719	195.2227	61.69489	162.099	135.1633	136.983	114.5488	168.2637	106.365	155.3804
CUST_14995_PI429010886	N33821	0.004952482	24	12	291226842	PREDICTED: TTC3 protein-like, p			1.07E-08	48.30191	56.18757	53.9367	50.21608	44.3077	51.68111	62.80159	55.14747	61.58528	67.48124	47.01945	65.1725	70.14984	105.6309	61.81635	73.69276	60.0766	78.20138	62.76069	49.5263	63.01997	55.77263	57.16004	70.27366	63.65381	59.81752	71.25639
CUST_21320_PI429010886	N45009	0.004961744	21	0						70.41437	71.62515	52.84329	21.73698	14.62992	16.25968	5.06501	26.12151	8.47965	59.85927	12.56491	38.6365	54.78117	28.99089	54.74825	47.01008	10.72088	41.65139	30.72076	21.86479	19.7654	123.2011	49.24543	58.49962	19.48121	35.97882	33.79296
CUST_22081_PI429010886	N46390	0.004969498	24	3						27.34627	19.31126	25.90662	19.74619	25.62254	29.09105	23.78251	26.94942	22.78614	27.08528	23.16492	29.46145	21.62449	15.78015	12.37796	15.85618	18.00112	18.75482	21.62449	18.08746	17.6322	23.53464	22.8831	21.68208	25.40663	16.55665	25.06028
CUST_13341_PI429010886	N31024	0.004988802	24	3						37.4753	31.55692	38.58803	41.84717	35.58267	37.49081	22.46441	41.0185	25.80839	20.53051	34.77477	7.873998	23.61619	21.66483	23.69696	11.69094	35.46629	21.92026	31.3526	30.81966	17.33156	33.746	28.62655	27.32212	36.10701	26.88183	24.9948
CUST_1351_PI429010886	N9373	0.004990245	24	0	345484812	PREDICTED: carbohydrate sulfot			1.24E-09	80.51893	89.93986	81.2035	55.19896	86.96518	74.25196	43.54271	68.91312	36.40013	53.86055	80.61166	64.9627	44.77796	37.70788	34.28012	57.39735	101.361	63.88807	78.13239	50.84163	52.87075	58.55963	101.3475	64.44827	58.81576	90.90449	71.89101
CUST_45372_PI429010886	M68883	0.004993365	18	0						33.10821	45.5838	32.54957	31.40901	43.30339	30.06892	22.74199	32.64521	26.67982	23.81043	26.58017	32.38938	21.58992	26.33431	21.95678	26.81873	30.10077	31.43412	29.30711	41.42853	40.7495	34.06404	23.15721	27.93535	21.75638	40.45487	40.90642
CUST_33632_PI429010886	M7471	0.004999682	12	9						342.4681	35.25348	601.1087	376.0943	203.4662	222.5957	59.53613	154.3451	226.4631	342.9765	360.6967	365.9284	708.7213	366.157	551.5575	27.04586	5.192651	18.52564	157.4759	385.6128	463.096	1127.159	1241.315	1142.815	281.3108	435.1623	783.6564
CUST_48592_PI429010886	M81169	0.005001919	12	9						8.232598	5.001528	NA	20.819	4.612947	NA	14.11179	10.14305	4.650934	NA	NA	NA	17.6536	14.40825	8.586447	4.671965	6.57946	3.556917	30.12082	25.54534	16.92091	22.97455	27.37466	29.5607	6.220734	17.95536	22.6466
CUST_56126_PI429010886	M112877	0.005008897	12	9						44285.99	5415.358	46875.63	56938.34	29219.06	35738.11	11503.97	13635.69	22154.05	49895.87	101686.5	44511.54	57775.61	32064.51	51875.49	2113.55	2485.974	2595.878	26987.8	25703.26	38228.71	54598.59	89785	105804.6	26056	52815.17	65558.65
CUST_38987_PI429010886	M33019	0.005008897	12	9						414.9926	74.56212	416.1276	716.8261	180.0489	333.3795	128.645	158.0791	241.9195	584.4771	733.9767	349.8278	713.4537	362.0388	290.029	82.82852	148.3567	85.38787	202.0095	229.0326	240.4351	818.3565	709.9527	968.3563	275.9873	477.9381	517.7643
CUST_46979_PI429010886	M74378	0.005008897	12	9						2930.842	16.11271	2661.468	2722.979	30.15984	1826.319	43.30726	15.11819	21.9811	4834.858	3397.779	3519.517	1861.323	24.90332	1233.546	7.435217	13.22168	23.94163	45.28285	32.12359	9.102869	4027.931	492.2464	3503.626	51.2209	281.2628	354.4054
CUST_30886_PI429010886	M114	0.005017646	21	6						7568.31	16183.31	9598.965	10999.91	8006.37	7832.882	15394.16	13193.16	16035.79	14071.14	11094.64	11305.05	11395.49	7130.037	6575.891	11805.59	11998.69	10394.61	7531.236	7900.843	5617	9111.496	9392.481	9699.55	9759.347	10323.84	8996.679
CUST_1833_PI429010886	N10841	0.005017646	21	0						197.088	196.8426	240.6532	209.9842	155.9594	168.6714	153.9033	162.3594	204.3745	206.4922	145.7707	149.0714	186.3541	182.6192	171.8413	207.1201	207.7328	164.5342	209.3816	189.054	165.1648	219.4299	235.0418	193.1329	216.3122	173.9585	206.2484
CUST_42547_PI429010886	M60558	0.005018569	24	0						374.2544	513.8347	549.1826	423.2638	514.354	318.7834	496.8458	586.6628	580.117	407.1156	397.7869	310.5741	426.5559	360.3638	227.6714	432.5544	462.0354	465.5812	524.4136	631.8697	534.593	464.055	655.1561	478.0895	578.3316	564.5167	663.8187
CUST_38905_PI429010886	M31812	0.005018691	24	6						136.6874	107.7706	111.5617	308.6874	858.1052	883.7364	2254.423	263.6527	1607.994	232.4886	122.5288	287.0864	44.3077	84.87009	95.26274	83.49206	108.8788	203.9965	110.5907	202.4277	91.41914	27.75587	120.2717	83.80666	6.076625	6936.974	487.1319
CUST_48943_PI429010886	M83059	0.005019457	15	12	326925459	PREDICTED: cytochrome P450 2.	Metabolic process	Oxidation-reduction process	3.28E-12	7.422436	11.16309	NA	5.056249	6.566709	3.896217	5.925916	7.975034	8.286877	NA	6.255758	9.869484	11.24042	NA	20.61088	7.605705	19.70573	12.52789	6.164371	7.695174	5.771961	8.869172	6.714878	15.73574	34.59782	13.51154	5.201984
CUST_13452_PI429010886	N31218	0.005019742	15	9						79.86465	129.1607	94.815	69.23652	106.3722	98.51271	174.9928	127.4388	134.5716	103.0089	223.7463	143.6704	100.4907	115.7562	105.1748	84.03656	129.8635	166.9821	31.15433	86.84479	75.80655	103.4024	157.1785	163.3644	85.33414	216.9684	245.6327
CUST_50139_PI429010886	M88792	0.005021619	18	6	296245397	inhibitor of apoptosis protein [Li			8.8E-24	13.22017	13.06086	6.998897	15.19323	21.2032	14.11179	15.67181	21.93034	15.17119	14.74939	15.84508	16.89173	11.70489	14.21176	13.81116	19.56944	13.73333	14.45383	12.84908	11.34493	13.91913	14.22159	15.49209	12.28362	21.03435	16.82471	11.16741
CUST_37957_PI429010886	M22464	0.005029182	24	18	241121691	RNA polymerase I/III 16Kd polyp	Metabolic process	Nucleobase-containing compound meta	1.97E-15	59.65352	80.71612	62.51652	49.8852	48.64263	45.5523	48.41784	81.56078	68.69203	61.58	55.08315	64.34403	61.36043	53.09289	49.23192	70.93399	86.96518	64.28341	68.39377	81.56927	91.90805	55.98105	104.0337	105.0886	64.00415	70.41437	67.41125
CUST_2520_PI429010886	N12552	0.005029182	24	3						30.77357	33.13557	54.78117	74.14723	117.9606	88.31999	49.70798	78.41566	53.19546	44.53859	70.14108	49.53225	39.59705	83.11893	71.77497	45.41788	50.1322	38.05228	40.5192	36.44629	39.88527	56.26015	42.20935	31.90671	56.87317	55.44525	33.65745
CUST_37866_PI429010886	M21946	0.005042666	12	3						177.2739	43.21465	203.8465	1514.2	683.1382	1010.854	581.654	1197.753	1743.671	441.4649	309.2002	293.1739	103.1619	708.4537	618.9801	1504.447	2377.165	2564.444	15.9111	931.5266	197.6371	50.76093	567.0251	302.1401	38.21258	361.6608	410.1393
CUST_34204_PI429010886	M9052	0.005056484	18	6	307180351	Retinol dehydrogenase 11 [Cam	Metabolic process	Oxidation-reduction process	4.82E-66	5.943856	20.32715	21.58672	NA	7.23313	14.25789	NA	16.97405	16.4966	10.41636	NA	10.23043	16.68577	13.24592	NA	7.119344	8.3196	4.165547	NA	NA	4.768426	14.72302	15.23394	NA	17.23263	28.34861	23.4544
CUST_40901_PI429010886	M56340	0.00506714	12	9						79.22393	40.17794	153.7917	76.30002	34.12139	48.61045	52.55424	62.38786	98.97699	98.56741	75.66353	71.5317	98.87854	78.56039	75.66353	72.63288	29.25965	52.9338	85.09153	67.58778	84.24007	222.1018	178.3834	171.0708	115.2993	83.9852	167.1998
CUST_51131_PI429010886	M92307	0.00506714	12	9						9330.025	251.6934	7032.355	12084.47	9129.704	10699.09	1195.535	955.1294	1708.051	18146.2	16981.94	17589.58	15767.9	8334.379	13503.69	128.4629	88.4112	202.3368	3702.472	3240.436	4577.512	15340.52	10746.95	13591.98	3715.857	5514.378	9804.488
CUST_12400_PI429010886	N29401	0.005068646	21	3						178.596	182.2041	185.6007	202.4277	275.1168	336.9544	204.6095	191.21	174.4941	129.3077	235.4891	250.8664	211.5442	161.754	254.6677	166.7131	132.9992	127.0776	160.3612	278.1702	171.7821	236.6326	194.1916	182.1021	221.0978	234.8306	221.6228
CUST_32365_PI429010886	M3938	0.005076076	15	6						844.4833	730.0615	885.8693	878.1427	930.8409	684.8522	955.5815	1051.228	1063.435	936.2266	768.79	1199.116	736.7743	804.3138	744.5255	890.9879	857.7889	1120.742	1061.158	1097.014	1218.143	909.4866	1344.214	1123.543	895.1277	1057.154	889.8299
CUST_16728_PI429010886	N36763	0.00508281	24	15	374079276	GalNAc/Gal-specific lectin [Cren	Cellular Process	Cell death	1.37E-18	268.4876	223.2399	289.1171	296.5339	251.2413	243.1406	233.3271	266.5309	288.6545	365.989	411.5622	291.8765	292.6648	346.0708	250.9574	449.7223	453.6305	422.7276	248.8065	338.6752	288.9345	308.3526	315.8899	471.3761	307.7409	240.7581	321.8126
CUST_31539_PI429010886	M1780	0.005085485	18	15	321473526	hypothetical protein DAPPUDRA			1.84E-15	172.4755	1562.544	231.9279	786.9042	103.7192	655.1561	2659.616	1307.951	284.7236	1787.383	826.9656	652.6836	2188.815	730.7886	2173.027	3205.37	2844.645	3434.818	860.0989	74.28907	915.406	858.51	213.1128	817.1951	1701.286	1081.498	170.1396
CUST_51611_PI429010886	M94226	0.005085485	18	0						27.45352	40.45487	39.76763	21.71688	24.41183	30.18612	22.25011	36.50276	19.54026	26.0838	17.47911	16.44342	23.54776	30.70425	26.94621	25.53705	34.73082	42.65681	30.00163	33.56909	34.98417	25.29614	34.83345	23.10946	18.95217	30.98678	26.17
CUST_49817_PI429010886	M87287	0.005089598	24	15						54.05823	231.3331	139.5639	123.3207	29.76725	54.02691	255.4927	170.246	153.0882	204.8848	96.13685	108.0194	218.9572	114.0573	85.51486	267.8251	344.3047	324.4873	189.1039	129.5035	236.8356	125.4154	83.23692	143.4022	244.0506	143.144	163.4024
CUST_35133_PI429010886	M11681	0.005094496	24	18						155.1393	142.9786	172.748	169.3609	117.7279	138.8656	208.7171	140.8978	202.2179	162.0504	105.6776	134.5227	262.5725	169.729	232.3243	188.6256	234.5996	219.7679	213.4123	248.8552	345.993	161.2869	168.5358	216.6056	219.5694	186.1647	238.8236

Probe ID	ID	p-value	Period	Phase	EBI ID	Annotation	GO category	Go term	E-value	0	0	0	3	3	3	6	6	6	9	9	9	12	12	12	15	15	15	18	18	18	21	21	21	24	24	24
CUST_7727_PI429010886	N21979	0.005094496	24	18	322799594	hypothetical protein SINV_1070			2.01E-66	21.55398	25.45723	41.18226	19.01388	11.22607	17.49602	28.43101	16.43359	21.95678	18.25369	22.57786	21.53519	26.84779	25.17677	21.14493	28.41796	47.54634	52.45299	36.27201	29.47583	29.72388	26.346	19.84259	28.95548	44.20516	33.74242	51.31552
CUST_10411_PI429010886	N26189	0.005094496	24	18	321473526	hypothetical protein DAPPUDRA	Metabolic process	Carbohydrate metabolic process	1.9E-19	178.6979	199.2769	287.0134	156.493	95.83012	51.75429	91.56473	178.3071	188.0176	226.4187	77.02123	234.791	212.0165	106.529	200.7173	243.1406	204.4688	437.0635	266.1484	295.5482	282.7416	311.055	130.8147	157.9135	290.7989	36.95293	300.7552
CUST_11586_PI429010886	N28074	0.005094496	24	18						145.3375	416.1276	331.7259	253.6231	140.7023	90.48608	280.6926	305.3804	530.8049	229.0326	240.479	171.1268	353.5003	246.4546	157.1085	340.7848	239.0862	371.0059	599.3024	385.9551	610.6881	398.3951	504.4324	382.6455	405.1127	139.6676	381.4494
CUST_48320_PI429010886	M79821	0.005111264	24	3						485.7316	NA	649.4957	1020.042	1266.211	902.1594	NA	4.825812	NA	83.73063	182.6192	250.7116	102.4255	117.7903	198.4987	NA	NA	NA	4.394228	66.40286	3.948789	109.9078	16.44032	82.86519	4.429018	28.85937	9.37907
CUST_32401_PI429010886	M4012	0.005115403	24	3						14.33334	7.611325	10.32033	12.11309	10.13898	13.58834	9.171096	33.07256	4.922133	NA	434.3661	4.271228	8.91225	NA	6.169063	5.753193	4.204185	5.232424	NA	4.340526	NA	9.499412	6.078184	7.352636	6.293716	14.28052	10.68598
CUST_39381_PI429010886	M38633	0.005117322	24	3	83628250	arthrodial cuticle protein AMP1			1.75E-15	1286.883	297.1434	1156.716	1330.649	1521.677	1457.293	261.1366	350.4372	301.0722	1307.316	1237.412	1387.245	1001.191	1020.798	1240.711	208.0709	214.7361	265.4524	253.2927	359.7575	179.4641	1442.661	957.7854	1002.076	177.5731	508.4006	419.3654
CUST_4606_PI429010886	N17117	0.005117322	12	9						616.5627	331.8463	918.7311	786.1643	473.8372	161.3598	923.7499	311.8576	983.3131	1906.005	1383.977	1338.071	1653.487	522.1886	626.5423	561.5074	311.1315	739.3679	3721.413	2461.211	3475.106	1119.882	2184.345	2247.929	1203.381	1037.623	2335.81
CUST_8810_PI429010886	N23626	0.005125383	12	9	241333881	zyxin/trip6, putative [Ixodes sca			2.43E-87	51.18195	37.69532	42.6416	26.76519	30.19535	26.81873	29.34996	29.82341	30.40072	48.92732	34.38418	53.80406	39.70565	40.04223	44.87713	29.68598	35.65778	37.6918	25.07913	36.01433	39.90127	46.96617	43.19513	55.94559	45.74256	27.14205	56.93121
CUST_9840_PI429010886	N25282	0.005131222	21	0						197.5603	163.338	182.2908	148.2138	101.2008	156.5604	113.823	130.6083	151.5412	126.4021	127.4388	148.3838	131.8067	152.3742	150.0449	143.1244	143.6704	116.2009	140.6785	111.9372	130.0819	185.717	162.9694	193.9353	149.7056	132.8754	179.5826
CUST_12879_PI429010886	N30197	0.005138378	12	0						37.94596	38.69748	48.75454	30.865	43.01953	45.78637	31.83324	40.97531	29.66835	35.15355	42.32912	40.64686	43.28942	40.61754	43.16345	38.5634	37.10235	36.03288	21.42599	29.65588	32.51285	77.31152	49.99888	49.25099	51.57473	49.30286	51.78825
CUST_42850_PI429010886	M61419	0.005147975	24	15						39.19706	44.32847	40.28522	50.54447	56.06983	48.96667	35.82162	70.30569	56.23453	48.3568	61.4814	34.81137	62.26618	89.90152	67.62577	69.21158	79.10279	92.85843	61.58	64.7701	69.16443	47.50671	90.51162	74.92905	63.21401	40.20755	61.77408
CUST_6358_PI429010886	N19997	0.005147975	24	21						283.6844	341.8735	411.5622	282.2716	230.1554	255.7585	323.446	297.8825	369.9026	339.2657	270.0743	296.3981	321.1394	267.2693	268.2532	400.5287	382.2543	362.195	409.0189	282.682	326.9292	423.0504	407.5243	447.8863	395.1137	322.0602	359.8294
CUST_44692_PI429010886	M66691	0.005153572	24	6						NA	4.727516	7.146978	30.04204	6.163656	18.52406	15.4677	17.08672	11.34354	8.917284	27.33627	8.529923	11.97918	15.33068	8.718132	NA	8.418641	9.670124	6.573571	3.912739	4.283307	10.56095	NA	7.24682	4.560078	NA	NA
CUST_54934_PI429010886	M108203	0.005156117	18	15						52.51782	64.98274	46.82329	47.24767	46.04292	42.33335	63.36224	45.14878	58.30071	52.12177	58.60167	46.72845	69.03139	49.37308	49.13607	64.75498	66.86503	74.22801	32.03897	30.24252	31.73534	25.52339	36.21981	46.51413	34.82138	56.53387	55.75291
CUST_31925_PI429010886	M2743	0.00515981	24	18						390.8971	544.2292	560.9756	401.9784	367.2975	367.0194	414.7402	388.875	472.1134	418.5156	494.2109	392.057	494.0593	378.4533	446.0553	592.4023	534.7331	596.1129	480.9139	551.0918	613.5208	433.9196	508.9078	585.1407	601.9971	492.831	690.3228
CUST_33029_PI429010886	M5776	0.00515981	24	18						32.50967	44.43314	44.64909	34.98836	35.00954	30.30413	25.59303	36.02528	47.77802	43.15923	34.80112	35.47927	45.49544	28.00501	41.20512	90.3848	49.37308	50.47099	52.56738	54.12459	48.48353	56.59797	47.49734	49.69782	64.78842	30.76277	65.17942
CUST_31191_PI429010886	M940	0.00515981	24	21						1996.997	3140.215	2706.184	1854.805	1666.041	1448.453	1516.421	2306.791	2365.258	2010.612	2480.784	1626.549	2464.909	2141.9	2627.758	2711.745	3090.222	2855.927	2315.807	2457.62	2053.392	3457.999	2860.025	3284.996	3411.4	2797.556	4193.691
CUST_8091_PI429010886	N22526	0.00515981	24	3	242020334	conserved hypothetical protein	Cellular component org	Cytoskeleton organization	3E-34	38.06969	28.4962	39.98414	55.84882	271.4702	214.3188	42.77417	52.29097	35.48599	19.39208	143.5284	141.8433	28.0193	140.8769	144.1077	41.03792	39.22128	27.63973	21.96708	46.2068	22.35063	23.25613	22.39651	25.36192	33.18448	65.86088	27.45352
CUST_48716_PI429010886	M81810	0.005169974	21	18						44.96264	30.71104	64.66012	39.67832	53.03534	34.53973	28.91213	27.06969	17.39682	30.15331	55.32159	24.52363	45.64916	22.96931	26.46988	29.38788	25.6877	39.56727	79.62038	64.23608	79.18277	37.17059	32.3516	40.18248	48.18525	30.43314	54.59539
CUST_42023_PI429010886	M59183	0.005171367	15	9						12.41522	12.49835	32.99109	16.5389	15.91592	16.24611	197.7325	16.05617	29.62518	24.08726	58.77493	21.66483	35.38316	21.38839	24.50935	19.87663	15.00684	15.53132	22.41344	18.17826	17.94579	21.47745	18.42193	20.01679	35.01298	19.64434	29.18456
CUST_34741_PI429010886	M10523	0.005183626	12	3	344267827	PREDICTED: LOW QUALITY PROT			3.29E-26	112.9448	153.7124	97.52903	116.724	169.3609	137.9056	173.259	132.8564	132.6815	85.54538	158.6925	90.47296	120.6396	174.4633	118.3592	141.7369	177.4189	162.9278	96.81529	128.3674	127.9686	89.64766	97.97101	90.93696	122.6677	168.1851	136.3915
CUST_45094_PI429010886	M67983	0.005183626	12	3						19.41383	35.79767	14.06396	19.12392	24.27221	30.54671	20.31267	20.33173	25.47879	18.72412	15.49793	15.7122	13.34035	20.00591	20.1119	39.2784	35.07936	35.36547	29.70306	19.20294	23.77756	18.18461	19.23998	22.55486	28.67152	23.36545	21.41461
CUST_8933_PI429010886	N23816	0.005191341	21	0						171.9853	170.1396	184.9648	176.3429	173.5895	160.5879	102.7615	141.7174	162.2564	150.0907	162.3594	68.29523	166.6906	151.7575	168.564	122.4178	161.9945	156.7919	106.9674	216.1701	155.6488	178.9277	196.9837	211.2708	149.2013	155.4242	194.3971
CUST_12717_PI429010886	N29931	0.005193821	24	15						80.79724	91.52744	111.8276	66.95538	70.28135	89.04608	58.55433	89.2783	73.48645	85.41835	87.19961	96.94761	65.12931	88.22605	74.84749	148.5965	96.86942	116.5916	111.1293	104.8815	95.67012	119.971	78.48038	110.4109	91.69645	105.9898	82.81729
CUST_51732_PI429010886	M94766	0.005200273	18	15						6.852265	9.289439	6.679536	4.666875	NA	3.67551	21.00124	NA	13.6782	10.45447	7.048882	3.925762	15.18833	NA	13.25637	18.41119	24.59083	30.3404	5.627759	NA	4.181523	NA	NA	6.323496	11.88441	NA	10.18759
CUST_39440_PI429010886	M39535	0.005212972	21	18						734.7144	991.1291	216.5044	647.785	180.3196	461.522	904.627	1923.884	1158.756	1102.526	611.6853	403.3938	1652.642	1882.752	981.9828	805.8552	442.2313	825.7252	1941.073	2185.802	3073.818	1089.089	362.8242	662.0947	545.4629	279.6157	368.0554
CUST_52160_PI429010886	M96592	0.005212972	21	0						21.37086	24.62932	23.04198	11.93827	12.60227	12.33889	13.22308	17.56163	13.54908	18.49709	17.79422	14.44521	15.89253	22.20069	17.46154	9.247073	21.3286	18.11402	11.15823	19.6327	16.15468	22.5078	23.4647	22.00011	14.47089	18.50842	19.61183
CUST_36177_PI429010886	M14897	0.005213098	12	0	241595281	NudC domain-containing protein			6.12E-30	76.38149	114.6414	99.74451	52.67704	64.77885	54.14388	40.49642	88.6413	41.43789	106.2659	89.29338	98.73078	94.21867	95.57738	97.88781	109.9913	89.53408	85.49462	57.18227	40.12721	43.54722	124.7562	92.99115	126.6732	87.38427	126.2182	120.6665
CUST_35271_PI429010886	M12108	0.005213655	24	0						110.8634	162.2856	308.1912	158.1747	131.2222	142.1948	58.37071	118.702	122.8503	118.1364	102.4481	84.45954	128.7173	86.44935	94.38205	94.60841	163.7162	111.7284	141.4616	121.1272	95.61224	136.8093	126.8958	104.6237	176.1711	108.6386	168.1851
CUST_41325_PI429010886	M57452	0.005222761	12	3						75.06882	96.51035	21.41224	130.8809	708.1548	548.4345	201.5487	436.9283	235.1738	158.0297	126.1993	103.4638	75.31313	220.4205	41.38957	223.4669	192.6977	269.4056	136.8093	308.2378	177.5447	77.59604	42.21331	92.32672	210.7737	188.7199	129.6173
CUST_6630_PI429010886	N20371	0.005222761	12	9						198.4395	198.0827	249.2581	240.603	247.1564	235.9714	252.6661	254.9324	255.4927	261.3125	263.3104	258.7813	188.9036	234.4037	279.563	224.4256	258.2414	295.5482	139.3356	243.5905	289.3915	252.9258	267.1275	314.5854	211.185	192.3094	195.1305
CUST_967_PI429010886	N8015	0.005222761	12	0						1112.702	660.9643	1097.014	999.5752	408.0121	418.8351	315.5362	475.2146	708.4537	749.8532	563.8477	624.9341	1968.185	944.8425	1543.558	301.8993	547.5307	563.1838	614.7112	628.8213	673.5376	1870.445	1067.709	1031.076	740.8082	373.3349	1109.766
CUST_10290_PI429010886	N26010	0.005224436	21	3	195329440	GM24062 [Drosophila sechellia]			1.02E-12	39.48899	19.54174	38.72594	39.68181	24.71432	41.52789	30.63129	40.80836	53.17104	13.97597	27.59657	NA	40.7495	21.47115	37.50844	10.34053	17.03584	27.16746	17.23263	14.45239	29.41321	16.15211	11.17145	14.5311	73.96504	33.26996	76.14147
CUST_31261_PI429010886	M1098	0.005225124	24	18						311.1315	452.8102	394.9633	284.8134	342.3913	299.1753	338.3225	442.7601	476.7459	350.1584	343.6496	375.9296	352.5182	370.3281	344.9779	363.8565	521.9217	376.4343	405.9933	452.9256	462.1954	382.8	505.1911	453.8518	351.1109	406.1674	470.7608
CUST_15351_PI429010886	N34443	0.005227496	24	0						44.7999	53.14759	41.32674	16.30105	29.97378	10.74336	30.13347	29.37054	21.83479	11.19367	29.18122	10.374	22.98107	15.14134	28.68856	15.69371	48.91526	30.85244	31.70122	25.64005	15.07353	21.09878	13.32146	12.95701	96.5246	60.14723	79.37286
CUST_20123_PI429010886	N42850	0.005229047	24	3						NA	22.0572	8.323564	21.28132	22.09706	19.42557	14.3393	12.55197	14.09206	NA	28.30062	12.66822	18.68765	9.791478	8.381188	15.23987	14.03865	13.70168	5.029898	11.43891	9.273221	10.0986	11.26916	14.87626	12.90521	20.82118	17.77119
CUST_10863_PI429010886	N26911	0.005229255	24	0						68.04801	77.75578	79.46471	83.42667	54.42945	60.48555	65.309	57.12984	60.30614	76.99156	53.68776	63.57042	70.21276	57.01672	49.75454	72.83024	65.01496	67.31813	65.87993	69.48765	71.43072	85.38009	60.50212	70.60324	85.51486	73.41723	90.88256
CUST_45005_PI429010886	M67688	0.005235243	18	9						4.424304	7.014131	7.018124	9.785757	15.19667	15.32206	21.18591	12.80557	13.73223	14.08558	15.4847	13.10406	12.34178	25.29384	12.9512	26.14108	8.826303	18.96038	4.72137	7.101655	4.605694	12.2337	7.649773	12.51386	6.41682	16.50059	3.603436
CUST_38103_PI429010886	M23360	0.005241869	12	3	371591223	Euphausia superba voucher 702				42224.23	58747.16	54162.9	52429.1	46193.24	46458.58	44112.52	56377.06	51269.31	39540.76	43090.8	37932.98	38060.46	50951.28	36302.66	52165.44	58090.3	54925.27	42662.49	52815.17	50951.28	35445.2	40415.13	41095.52	43701.56	59443.36	44511.54
CUST_6052_PI429010886	N19566	0.005242169	24	9						4.420558	13.23504	5.700788	6.136811	77.12608	60.3351	NA	10.01645	7.587167	NA	82.00982	72.42124	8.523259	57.63804	54.89599	7.306229	5.178823	5.551574	NA	35.60799	3.879505	NA	NA	4.093308	NA	21.55177	9.465451
CUST_36024_PI429010886	M14316	0.005262718	21	6	262303234	Libinia emarginata isolate 11_12	Metabolic process	Cellular amino acid metabolic process	4.15E-68	59.52846	52.99968	44.56209	43.78128	60.78548	58.98036	64.03624	63.58392	70.64915	62.64991	62.367	66.02134	59.23509	61.63075	68.72832	79.88277	51.42014	65.53387	63.50827	57.65509	61.96517	51.68947	52.96326	54.41681	57.9604	52.81201	66.15962
CUST_11211_PI429010886	N27450	0.005272027	24	3						9.392936	10.01935	8.24518	10.05113	15.15187	6.72839	8.212284	9.036413	4.407448	NA	8.373343	NA	5.203228	4.322242	3.406318	5.839233	8.806809	3.409742	6.285156	11.53833	5.180466	7.340202	8.401043	9.003802	NA	14.25457	6.343539
CUST_53421_PI429010886	M101631	0.005275401	12	6			Metabolic process	Nucleobase-containing compound met		11.859	33.11578	33.13557	40.6742	31.81746	32.66605	43.78128	76.93465	43.53457	49.41907	49.27254	43.03704	15.56129	104.3299	13.15696	62.66935	29.56947	56.60172	68.16127	37.58094	39.85168	20.29057	35.4467	36.33638	23.20911	11.57188	25.46423
CUST_46661_PI429010886	M73332	0.005278303	24	21						362.8242	435.0369	384.3395	427.8043	320.3348	305.863	360.2879	282.3692	369.5842	415.7252	316.8051	147.2543	326.3053	263.4946	409.2932	403.5639	375.5874	420.3226	503.262	376.5416	379.8743	500.9726	411.2361	425.8369	543.6971	383.3865	491.3879
CUST_30878_PI429010886	M98	0.005278303	24	18	326579691	JHE-like carboxylesterase 1 [Pan			1.98E-83	80.86273	106.0382	137.2351	174.4941	99.20976	151.9875	115.4959	125.5586	158.8716	156.3694	104.8322	95.57738	235.5705	143.2044	137.4424	180.7516	153.4511	202.6784	251.5671	267.7885	376.6399	172.748	130.9524	235.7954	191.8512	118.6245	240.9301
CUST_5042_PI429010886	N18187	0.005278303	24	18	343183153	myosin heavy chain type a [Mar				489.2868	321.8126	419.1486	350.7074	313.5422	377.5103	685.2688	386.0289	494.454	499.3569	326.6163	447.8863	498.6872	411.8854	358.0309	1041.462	421.4753	773.969	614.2194	663.421	803.738	498.7889	381.9964	522.1886	654.5389	312.2924	599.3024
CUST_5196_PI429010886	N18387	0.005278303	24	21	383860504	PREDICTED: 7SK snRNA methylp			3.18E-82	133.8434	143.144	133.7779	125.0086	108.5274	101.522	111.7147	126.8958	138.2078	138.7489	105.6058	118.3592	159.7027	113.3335	121.9689	128.797	144.2677	155.9594	119.713	153.3624	164.0459	150.7041	156.988	173.1969	140.1138	127.1435	210.9122
CUST_12303_PI429010886	N29258	0.005278303	24	21						19.98159	29.80661	17.43071	21.73011	29.44247	28.91544	18.46729	23.60134	17.88666	19.88527	18.93048	12.69158	23.83774	21.07756	27.57725	23.68606	26.18151	37.51953	25.33737	30.17742	24.83403	28.05304	25.82506	33.84921	34.41857	29.21491	31.93549
CUST_18486_PI429010886	N39885	0.005278303	24	15						165.8977	142.0375	95.73042	206.3618	118.2766	154.8735	296.2197	197.3334	258.7066	291.4994	169.0416	158.4455	263.6152	124.7912	229.6141	420.4184	272.1942	321.2079	232.6083	168.6714	178.6979	228.7481	208.8662	250.8664	221.8149	149.6459	184.828
CUST_4192_PI429010886	N16197	0.005279919	24	0	54042615	arthrodial cuticle protein AMP6.			1.18E-16	323.747	115.2322	332.5739	392.3777	561.1654	307.2712	14.26605	175.8857	76.02418	9.896366	54.73663	6.433509	623.3324	266.583	349.3011	35.45282	41.4895	45.142	92.12733	1142.458	1343.626	516.3556	99.66557	317.0103	677.4857	562.7989	1223.646
CUST_29661_PI429010886	N60311	0.005282793	12	0						1346.991	1835.563	1224.109	1094.513	656.4851	712.1062	568.455	767.3697	865.141	1211.071	948.1481	1005.566	1296.153	796.9187	958.1681	908.0084	769.5468	919.3983	1331.886	960.8612	980.6718	1119.38	689.88	1022.798	1425.394	1280.159	1503.641
CUST_15029_PI429010886	N33888	0.005290438	24	18						36.83655	51.53986	36.53104	47.25275	22.86904	21.3436	47.55964	40.98319	47.22395	45.90992	28.2373	35.84121	55.92368	34.72341	49.23962	47.25275	62.55614	65.47283	66.33824	55.96387	62.1238	42.0926	51.25725	32.63627	44.47158	39.41032	63.49903
CUST_22738_PI429010886	N47579	0.005290438	24	21						21.23274	27.32212	22.10476	20.7057	20.28829	26.82547	28.75373	20.06847	22.06613	25.15651	24.97826	23.4544	30.18612	28.63477	25.38297	23.76317	31.91281	34.19427	22.96723	30.68536	23.69696	42.80111	49.59199	34.22235	34.50418	23.12326	31.60141
CUST_48161_PI429010886	M79158	0.005290738	15	12	359270180	TIMELESS [Thermobia domestica			4.09E-13	24.09713	12.94058	46.85381	6.88957	16.14473	4.022042	9.92008	8.025098	9.412668	12.1921	36.34979	25.83296	17.85411	18.19202	18.37247	21.16897	17.01131	16.85534	17.08255	11.71113	12.10349	14.80209	11.84393	14.68671	25.3952	15.90348	14.01288
CUST_49069_PI429010886	M83897	0.005290738	15	12	291230402	PREDICTED: predicted protein-li	Transport	Lipid transport	1.66E-22	50.87138	52.6416	61.63075	50.02722	50.10131	57.11305	47.71964	38.47726	47.80868	38.28366	49.65645	54.09509	64.31661	64.64642	70.38705	61.67047	62.46312	54.2431	66.65338	51.90036	62.35954	47.24216	50.0937	64.42288	67.98511	53.50459	86.37483
CUST_39775_PI429010886	M44332	0.005293433	15	3						1801.332	1255.327	2031.77	3159.077	1186.54	1853.898	728.8717	1985.342	1180.485	1480.822	599.4277	997.2176	1096.072	1109.766	606.5345	775.4008	2085.588	1853.898	1488.548	2636.073	2958.733	2093.838	1225.455	735.2877	1696.829	369.1521	1060.229
CUST_31414_PI429010886	M1474	0.005294621	24	0	524906190	PREDICTED: zinc phosphodiester			3.41E-35	32.03079	69.98305	70.21276	26.86909	26.31789	25.36783	27.4271	53.83595	49.81991	36.49885	31.96971	21.44482	28.3301	19.82627	20.79498	27.84463	37.38294	42.26354	39.46646	50.72554	67.35825	37.54467	33.07741	36.65461	67.58778	48.49422	64.36702
CUST_26625_PI429010886	N54558	0.005296861	24	21						93.4363	86.71039	80.54573	94.78884	77.31152	74.20038	81.10019	68.52496	52.51146	70.80726	62.40732	78.12052	86.956	55.27672	64.37351	93.14583	71.84826	89.5211	80.98106	75.91716	77.3983	126.1267	81.87749	90.32894	101.7154	75.43954	101.105
CUST_22986_PI429010886	N48057	0.005300112	12	3						133.1101	139.1998	57.56952	141.3156	154.7131	181.3504	173.2392	101.8004	132.5224	118.2313	125.8242	147.5974	139.7386	110.2586	151.1486	265.3286	167.4488	152.8709	167.261	81.66229	153.5202	67.00003	114.7343	90.8123	142.3635	132.0014	74.17237
CUST_45621_PI429010886	M69699	0.005300936	24	0	242021006	conserved hypothetical protein			1.11E-25	11.3477	15.432	13.59581	12.88293	12.31491	NA	5.267179	12.92228	11.46447	10.56444	NA	11.39194	7.893062	6.016685	10.57189	11.31534	4.059622	10.09352	7.125638	9.791478	14.83547	8.148792	8.668203	10.02234	13.92104	12.65578	12.2567
CUST_8813_PI429010886	N23631	0.005306194	24	21						496.5813	743.6336	853.7719	694.7413	391.6595	603.3505	470.7608	682.5361	1003.255	648.4382	477.1875	335.3952	576.2705	445.7269	523.0011	1193.688	789.3467	811.6064	1101.531	664.6847	922.5025	1160.436	718.4732	931.1977	1264.606	691.8963	890.9879
CUST_742_PI429010886	N7090	0.005306194	24	21						132.4344	113.9393	245.783	299.1753	252.884	10.04079	82.0771	87.84235	134.6492	59.11174	91.11591	56.42712	238.2083	59.81752	175.2702	34.93184	154.036	20.53404	728.8717	208.1829	461.0647	1041.462	706.8768	641.3942	233.8101	54.9218	183.6447
CUST_39518_PI429010886	M40736	0.005309164	18	3						11.48186	12.79123	22.00575	39.30254	23.03013	NA	4.913749	12.60992	7.547517	6.796162	41.39951	4.634489	43.86283	23.12326	11.54243	4.96729	4.610862	10.13108	10.96971	15.19323	27.30898	30.93515	117.2793	111.9491	11.22607	59.22932	76.08583
CUST_10408_PI429010886	N26186	0.005329109	24	3						8387.726	603.0231	7459.987	10683.23	11588.48	11805.59	339.5673	543.5625	480.3553	9257.227	7316.568	7558.705	8768.122	6265.89	10795.65	235.4202	272.5621	230.5014	1058.187	999.3353	745.8583	16760.98	6956.592	9111.496	1329.875	2645.058	3258.199
CUST_13986_PI429010886	N32129	0.005330501	24	18						176.3724	370.3281	389.053	271.1207	261.3125	137.2652	286.8701	264.176	391.6595	254.7629	203.057	214.0358	346.6392	228.8193	217.5404	258.2813	277.9454	296.8699	447.6336	428.1289	429.6749	312.8082	325.8846	400.3546	478.5299	259.8835	688.887
CUST_28288_PI429010886	N57673	0.005330501	24	12						28.83489	42.178	29.75114	36.30672	28.70626	38.3287	51.53986	34.43322	53.32942	47.26148	43.09613	36.83655	46.91122	60.75955	72.62444	70.53623	42.93819	51.85541	42.93819	46.92175	49.54311	39.36946	55.85351	73.82805	28.76131	51.54626	38.12534
CUST_5555_PI429010886	N18859	0.005332888	18	6						13.00116	17.44104	17.95932	22.01115	29.74732	23.09017	18.87895	24.50769	23.67991	8.219759	12.52305	27.5289	24.26626	18.85113	20.92431	12.77798	18.99016	14.56169	15.24733	21.19789	18.57773	18.85311	19.1261	19.79906	19.1752	27.47239	28.01116
CUST_54928_PI429010886	M108192	0.005338876	24	21						NA	11.65231	13.96855	9.937052	7.979072	15.13981	10.58723	8.461781	8.452888	10.34183	6.516558	7.833379	7.934974	4.738336	14.92315	10.74067	16.44342	10.63151	NA	12.2567	14.22003	18.06394	16.87762	10.10871	NA	10.41528	14.98029
CUST_24177_PI429010886	N50173	0.005343134	15	0	219809644	Kazal-type serine proteinase inh	Protein metabolism	Proteolysis	1.33E-11	73.50942	82.9019	NA	95.67012	28.71526	114.7172	65.08782	38.12224	62.97498	24.1706	18.66832	9.916433	51.68671	117.4337	74.01825	272.2328	117.2201	109.0197	111.6865	4.925341	88.25185	NA	93.14583	7.217315	37.64453	41.93907	NA
CUST_46187_PI429010886	M71708	0.005343467	18	3	152949487	calcification associated soluble r			1.57E-12	23961.37	1180.485	21834.29	26056	22946.38	24136.69	635.1199	1217.388	1410.73	19791.01	20822.29	20172.23	17487.6	14161.49	25888.13	437.2723	522.3491	502.2054	2063.235	2003.765	2457.62	27918.92	11305.05	25611.3	2581.822	6193.269	10746.95
CUST_22592_PI429010886	N47292	0.005343467	24	3	226374370	peroxinectin [Fenneropenaeus c	Response to stress	Response to oxidative stress	1.89E-21	214.693	143.0166	101.98	115.186	624.9341	554.5386	110.5806	105.1748	108.583	19.11378	253.5695	294.2691	44.98293	142.0823	211.9389	31.2986	25.39744	28.2373	20.80025	131.4171	11.86651	46.53342	61.9265	43.02401	36.07852	247.0676	12.18717
CUST_50881_PI429010886	M91327	0.005350139	24	3	189031345	trehalose-6-phosphate synthase	Metabolic process	Carbohydrate metabolic process	1.13E-26	6.040325	7.832139	9.138593	14.44743	12.5816	9.481245	7.772157	9.767511	8.82821	6.814859	6.842059	7.006651	4.904795	9.223983	4.264028	10.17265	8.11425	7.971271	7.002504	4.177755	5.343407	5.873873	NA	7.787574	13.24735	NA	7.503112
CUST_45445_PI429010886	M69132	0.005355752	24	3						41.61011	66.32665	50.06722	64.41139	76.71918	65.81456	51.07097	67.9004	48.74364	34.72873	55.34035	49.28164	57.16004	40.70251	9.61862	25.29001	80.02644	33.63302	37.2113	45.13269	18.88946	47.82733	47.01508	37.64058	20.35228	62.29516	48.32509
CUST_45866_PI429010886	M70560	0.005355752	24	18						840.4028	1987.037	1335.17	1020.366	809.8174	542.3764	1580.709	1242.744	2237.142	1218.815	1078.593	826.745	1801.332	1019.08	1097.014	2284.471	2273.452	2480.784	2501.925	1511.222	2757.04	1374.54	1499.429	1961.653	2181.465	1706.299	3166.856
CUST_49325_PI429010886	M85202	0.005355752	24	21						12.61101	16.96414	32.53094	12.78096	25.02499	14.6763	6.886703	12.66963	17.75609	21.27281	16.3934	16.48308	21.24736	10.91964	13.69996	16.29003	19.82888	17.59796	21.3055	27.83033	12.24052	34.48922	26.4908	38.04316	33.44269	30.33619	32.15355
CUST_50896_PI429010886	M91372	0.005355752	24	3						20.51331	13.48307	21.76706	29.13899	32.64246	37.38294	23.36033	15.579	9.129149	30.02799	20.63528	38.70891	19.59198	11.16104	27.55409	18.17826	6.180949	21.30308	18.60134	6.594004	6.16087	30.01509	10.49914	15.40578	19.92575	25.32145	12.1461
CUST_5332_PI429010886	N18552	0.005355752	24	18	321478820	hypothetical protein DAPPUDRA			9.46E-72	31.94579	56.66348	54.31927	37.65544	33.25783	27.96239	39.80553	50.66742	54.04214	47.33022	41.64718	47.32579	41.89159	30.66224	45.53592	56.00544	53.10397	57.20137	69.94875	51.261	59.07254	66.88415	39.59398	55.4869	64.93737	48.21063	64.48863
CUST_24199_PI429010886	N50214	0.005355752	24	21						1286.33	1397.328	1423.667	1280.738	1294.853	1143.279	1174.279	922.5025	1002.076	1429.079	998.7674	1125.025	1493.774	993.6309	1247.935	1527.348	1240.711	1361.853	1588.642	1028.452	1112.702	1909.699	1504.447	1384.734	1542.337	1205.686	1749.36
CUST_49619_PI429010886	M86416	0.005358568	15	12						31.1712	26.48825	20.58643	31.28202	23.20404	22.11134	18.96877	40.7919	16.83626	17.5997	18.36667	29.40702	27.9424	39.8332	40.16189	34.89175	25.27733	35.35463	22.33262	26.6049	25.2729	12.56182	11.3741	21.31707	31.66129	22.261	18.73426
CUST_14653_PI429010886	N33237	0.005363299	24	15	395507903	PREDICTED: tRNA (adenine(58)-I	Protein metabolism	Regulation of translational initiation	3.03E-09	74.35572	90.06479	91.7443	69.08897	57.21256	64.07605	87.44315	86.9167	93.13119	92.7356	65.25923	75.13872	104.0936	69.16443	79.60935	84.70621	120.9342	98.55805	104.9226	80.34247	103.9647	75.2001	71.64167	97.11689	87.53029	59.21121	87.00868
CUST_39277_PI429010886	M37136	0.005364205	21	18						21334.13	22777.74	22526.09	11179.97	4041.944	3437.158	13248.98	21645.19	23503.18	8051.463	3825.101	3297.935	21645.19	9194.226	5649.913	20968.09	13193.16	21590.74	30352.12	25463.11	25463.11	13761.91	13391.14	11429.91	31543.85	15197.96	17623.06
CUST_45166_PI429010886	M68183	0.005368012	18	3						45.1102	52.62861	56.56581	53.56818	58.89558	62.79403	57.0448	60.94991	47.7889	51.69582	66.68058	56.72705	47.88053	41.28281	59.67529	51.1228	55.27672	44.91446	53.19546	49.4765	52.30892	63.28914	95.09356	71.62515	49.73252	61.63911	59.88108
CUST_44337_PI429010886	M65660	0.005368192	12	9						62.07591	41.77489	53.35547	157.1328	94.80138	15.29246	145.3783	91.63916	101.7856	146.4045	153.0882	54.95988	79.24842	NA	87.89749	10.41738	97.56774	99.58967	124.4582	106.8649	122.9511	150.4102	143.2463	133.4287	32.15717	116.4725	84.62659
CUST_1425_PI429010886	N9594	0.005376268	24	18						12.72606	10.0986	17.10603	16.06184	7.989246	11.29211	7.009565	10.20538	4.983426	11.48896	7.852776	5.923896	8.013913	NA	12.6806	4.437704	6.292668	NA	44.89359	13.2108	27.83932	11.93992	27.31187	20.30731	38.00971	17.47131	27.97133
CUST_43471_PI429010886	M63137	0.005386211	24	6						27.63973	35.21261	8.919527	32.66215	172.6851	111.9225	47.77307	36.14198	40.02841	15.7218	103.801	77.17435	20.13002	107.9543	77.62481	38.43	25.58573	31.9432	30.69787	25.44962	35.29322	7.457449	21.43518	4.167976	NA	27.19756	7.87171
CUST_46111_PI429010886	M71411	0.005398841	24	15						6.205904	10.10871	19.37283	11.7093	6.537448	5.917822	6.9169	5.442961	10.16286	9.844862	9.174534	14.65727	8.683022	12.46756	12.10629	27.96533	13.08598	20.70915	14.01651	10.2223	12.74046	12.13039	14.64332	13.74025	15.60918	13.55428	11.70657
CUST_25048_PI429010886	N51694	0.005398841	24	18						19.21337	23.21885	30.5034	10.93877	19.3675	16.38536	23.81043	19.22794	21.66219	19.42557	11.38718	14.49179	17.55561	4.000891	25.08941	24.00968	3.583429	19.61588	26.96145	26.76018	50.3744	31.89668	24.26356	25.91869	37.4401	18.43607	24.84769
CUST_49393_PI429010886	M85487	0.005399493	12	9						34.11681	10.42995	34.72341	37.92736	17.13689	18.76458	NA	16.41767	14.34832	45.34892	82.79094	28.99688	40.82537	14.96903	20.65147	9.603299	NA	7.699921	12.1659	21.86715	12.52146	68.56321	63.68621	52.81957	12.95701	62.91855	24.07412
CUST_19510_PI429010886	N41693	0.005402856	12	0	443709437	hypothetical protein CAPTEDRAI			5.33E-08	27.34326	31.74591	27.96022	16.19743	24.58018	22.07451	24.07128	22.07129	20.75814	18.51328	33.22428	31.39708	27.56835	21.91263	39.19188	17.92495	27.32212	12.26439	71.71217	26.92758	16.72046	24.62618	26.0733	27.53522	44.00493	42.42877	52.69375
CUST_51408_PI429010886	M93415	0.005409339	18	0						8.91225	5.708684	3.918853	4.020773	5.820987	5.267874	NA	4.001642	NA	6.12072	NA	5.280386	8.591323	NA	5.407145	NA	7.826429	4.343239	7.38545	9.058939	7.091187	NA	5.379272	3.679982	3.662558	3.978545	3.485221
CUST_54669_PI429010886	M107095	0.005410533	21	3						36.04017	14.42132	29.18871	30.77017	21.55177	22.9795	30.12082	16.40816	21.26556	24.78354	17.94352	18.02767	21.43518	18.18791	17.40415	15.04687	15.75178	15.79824	28.54797	7.338359	12.1461	29.09987	16.44171	8.158467	35.85739	21.11994	25.19867
CUST_9554_PI429010886	N24828	0.00541858	18	12	307214894	Protein preli-like [Harpegnathos			2.01E-50	46.96617	55.67726	67.75299	59.61189	56.29733	61.85717	46.5992	55.01217	40.04223	55.28196	57.50494	59.71475	56.31298	77.711	78.14029	53.41659	70.14108	68.68508	34.43069	44.511	28.72062	41.31439	43.28077	48.46739	40.47381	36.71092	43.42121
CUST_52720_PI429010886	M98646	0.0054186	12	3	242019712	tripsin, putative [Pediculus hum	Protein metabolism	Proteolysis	2.31E-11	10.78353	27.38458	8.348262	29.16979	NA	45.16295	27.69481	72.74057	8.652491	15.49584	13.71572	7.862835	40.86771	59.34282	44.90369	97.06787	112.4213	69.87542	28.66457	23.84171	19.33037	13.81248	58.23791	13.36058	24.65307	37.54916	NA
CUST_53709_PI429010886	M102862	0.005421067	24	21						414.4128	403.8629	457.3588	394.4626	368.7646	361.3178	299.2328	339.636	369.2112	445.8569	396.4646	348.2785	453.2213	325.0373	433.8311	353.8605	480.8108	481.7121	394.193	377.9818	381.2824	486.6807	392.6361	503.9395	472.1134	395.5893	467.6907
CUST_49212_PI429010886	M84701	0.005421067	24	21						37.50844	33.50527	36.5903	49.56614	27.92534	29.4873	22.93584	40.10788	24.73549	30.97694	37.60724	42.47062	57.6297	35.83737	20.78849	26.45217	48.5731	44.1584	41.16872	47.44242	49.9905	54.68833	43.36291	66.22878	31.23655	36.11092	52.42496
CUST_7266_PI429010886	N21288	0.005421067	24	3	405965651	Mitochondrial sodium/hydroger			1.57E-17	578.6613	504.4324	544.6702	1086.19	3627.136	2672.714	329.9676	778.7232	285.2599	396.6952	2383.479	2676.834	604.3308	1008.499	1387.245	294.3919	364.6437	372.8037	186.848	503.262	177.2128	508.0836	351.1109	513.3858	180.1675	1055.851	210.8385
CUST_19742_PI429010886	N42106	0.005421067	24	18						44.49549	121.6309	86.49398	47.93963	27.93918	20.35931	103.0218	43.76612	86.21825	108.2394	61.58528	66.2384	93.02834	48.34401	25.5566	101.3475	124.8682	132.8875	154.9552	103.9956	132.6306	74.92905	58.24898	85.54538	151.0496	99.48565	125.9134
CUST_21952_PI429010886	N46149	0.005423944	21	12						55.01217	42.44272	43.18058	28.80453	37.34026	50.73158	44.32847	26.90764	39.96323	45.06652	38.21742	61.5382	66.66302	76.90215	63.12641	24.49986	49.05917	52.27137	62.20434	34.85943	65.67004	43.30339	40.69852	48.28205	37.53429	38.33655	39.77797
CUST_12682_PI429010886	N29864	0.005426398	15	6						46.40767	34.27669	39.63818	39.4828	46.25621	36.73639	42.04861	55.31584	49.5263	40.64686	48.44449	52.62255	38.31063	37.67803	33.72382	60.75399	41.67991	48.90035	46.31434	59.64761	53.15878	60.79882	60.37856	58.54288	43.06184	52.40229	51.78206
CUST_273_PI429010886	N3247	0.005429539	21	6						768.539	2485.974	1119.38	929.4814	1334.416	1546.88	1349.624	1650.878	1488.548	1358.461	1286.883	450.2424	994.081	1038.012	1464.031	615.2672	914.5652	787.5865	677.9076	487.5393	867.2487	603.9154	1749.36	891.3462	1444.952	1620.506	944.8425
CUST_30391_PI429010886	N61781	0.005435342	21	0	157129622	hypothetical protein AaeL_AAEL			1.12E-18	18.77458	10.93505	19.2072	10.44922	13.07357	19.67697	9.429304	13.24014	13.86405	15.01034	10.04079	14.62238	14.18679	10.39886	12.94453	9.807589	9.412668	8.807925	17.10603	6.850695	14.63666	17.93943	15.15361	16.15468	10.57698	17.4092	17.37611
CUST_34918_PI429010886	M11078	0.00545157	24	15	156555475	PREDICTED: protein FAM192A-li			1.23E-26	23.6825	23.00444	27.66729	17.67622	11.10881	13.26729	14.72189	21.16633	17.6134	32.64901	18.57121	17.86526	18.58143	18.73595	22.23439	32.05943	25.62527	25.26406	15.41425	10.72088	24.62932	25.53189	22.15452	29.64523	17.7621	21.3518	20.46701
CUST_44231_PI429010886	M65332	0.00545157	24	21						249.6908	261.9707	330.0358	314.5002	191.5757	254.9324	270.5362	212.8941	213.0483	248.9012	177.9352	265.4524	294.3919	192.5284	243.2848	333.1336	200.3302	305.1744	351.92	257.0403	269.5152	364.8648	281.2628	331.8463	348.0379	272.8455	308.9438
CUST_49042_PI429010886	M83757	0.00545157	24	21						396.9983	418.1662	397.2907	396.1202	471.4786	375.6666	365.4091	340.0191	408.9306	353.5003	408.1119	360.1634	294.1356	380.0182	440.8715	351.5907	473.2258	384.8278	393.924	411.5622	473.8372	432.6388	448.0132	506.3774	435.6254	451.9245	406.8432
CUST_20962_PI429010886	N44382	0.005463272	24	6						26.93079	23.48894	40.23235	36.47269	39.95086	35.78309	38.21742	40.54423	96.9591	44.34431	50.33808	38.91258	35.86186	32.90484	43.26054	17.75609	37.98103	36.12041	30.06574	28.62655	38.10649	39.81879	23.45171	28.19441	24.17321	33.23134	41.62674
CUST_32005_PI429010886	M2976	0.00546718	24	15	307715367	Macrobrachium rosenbergii argi				148.6897	154.2023	186.4449	126.0081	134.4815	142.126	107.3228	151.1207	107.509	93.4363	164.3433	154.2231	81.56078	144.3062	126.4865	262.0968	223.5617	262.4813	181.8118	234.493	229.6141	93.40586	164.1609	126.6202	218.9179	185.1417	169.4819
CUST_6996_PI429010886	N20890	0.005473796	18	15	383853263	PREDICTED: mediator of RNA po			3E-69	102.6378	166.3679	128.3075	83.88489	103.1504	92.36671	140.444	162.5035	174.0791	146.953	135.3635	107.8221	132.329	128.8774	138.3092	167.7963	224.7434	184.4707	125.7784	141.352	173.0186	91.75455	122.4907	140.8978	137.6198	131.1766	165.8233
CUST_52470_PI429010886	M97546	0.005480883	12	9						6.097172	3.354259	NA	NA	9.068193	4.819136	4.107769	7.155078	10.84058	18.57573	33.17225	11.49063	3.42519	16.51333	17.66267	6.473882	5.748942	7.181893	6.656681	7.517792	14.55087	13.14847	19.78846	17.38353	13.72334	9.212546	13.3975
CUST_54427_PI429010886	M105964	0.005482146	24	15	348572387	PREDICTED: glycogen phosphory	Metabolic process	Carbohydrate metabolic process	4.36E-10	13.11503	18.69594	18.95021	10.96784	19.87183	20.03497	17.10849	8.284739	13.05352	14.48048	20.945	13.35017	18.70945	17.425	16.62679	30.46326	23.9493	22.78614	21.85691	15.16581	24.04754	12.65976	18.68765	19.27271	18.7867	14.13494	22.03386
CUST_42913_PI429010886	M61583	0.005486381	24	21						81.36078	73.26943	82.88351	73.52981	58.21158	68.98107	62.71779	56.26482	71.97753	93.38599	59.57649	85.13676	76.35805	67.45544	88.04091	89.62874	65.37178	60.79074	103.4142	65.15776	107.0748	118.5659	78.94493	107.9667	112.3537	74.6809	115.5552
CUST_31136_PI429010886	M798	0.005486381	24	18						98.67888	107.9954	132.5859	83.81296	63.78974	78.01928	92.35614	113.5376	131.8747	149.2467	76.90215	99.12783	131.3179	101.522	79.95529	118.8271	139.3356	112.8927	131.6939	121.4663	131.9106	143.5433	126.4673	128.1317	120.6989	108.8788	123.1267
CUST_14645_PI429010886	N33223	0.005486381	24	3						162.188	55.61954	128.9542	187.4109	300.549	294.2148	48.94113	58.07534	31.17381	122.771	194.9048	219.3545	102.3362	127.8764	149.9126	44.05427	40.53157	28.59028	22.17869	88.08839	12.39193	109.8243	73.60907	87.33991	26.26607	88.27501	19.407
CUST_15489_PI429010886	N34661	0.005486381	24	21						196.4999	361.4106	350.4372	250.1228	233.9171	218.9179	272.2877	280.2395	342.9765	276.9413	306.6661	286.4692	323.1624	265.2295	279.8449	284.3354	309.6367	358.247	300.9346	344.0704	362.5008	393.8421	413.5169	456.5305	373.8469	305.3804	477.3178
CUST_51501_PI429010886	M93755	0.005488337	18	3						75.47204	70.07704	102.3466	78.44375	83.00392	103.0349	25.58573	60.26506	50.26083	51.14752	84.80017	34.41857	59.71475	69.92347	79.5082	39.61493	50.99975	42.25498	43.02401	48.04838	58.00342	101.9985	74.46936	107.7528	54.81291	65.1725	52.35798

Probe ID	ID	p-value	Period	Phase	EBI ID	Annotation	GO category	Go term	E-value	0	0	0	3	3	3	6	6	6	9	9	9	12	12	12	15	15	15	18	18	18	21	21	21	24	24	24
CUST_22331_PI429010886	N46836	0.005494228	15	12						78.44375	72.64707	93.00549	60.79882	68.86246	74.23643	74.21946	71.23962	68.33012	82.29248	55.82058	83.61292	167.2859	72.3378	175.5187	130.3988	123.354	87.25531	54.07225	74.11009	40.85968	73.07238	80.82353	86.89375	107.696	69.59216	93.44995
CUST_31517_PI429010886	M1704	0.005500819	21	18	380023928	PREDICTED: protein RCC2 homo			4.96E-125	44.31359	34.06404	67.03577	31.83762	30.6407	18.99955	76.95384	22.78614	70.87494	75.37573	42.83599	59.38272	51.43678	44.39683	29.22381	60.17344	51.68671	67.83335	73.51603	54.75432	96.87922	50.80105	32.78182	50.8307	43.98563	27.64775	43.63223
CUST_10651_PI429010886	N26571	0.00551452	24	6						NA	14.58685	3.905043	14.82588	7.878601	10.95081	9.492246	10.13408	19.48546	21.74119	6.860532	13.00372	8.485387	NA	NA	17.72698	NA	12.31866	3.87502	3.41858	4.986564	5.521695	5.830281	NA	7.130952	11.36795	7.654544
CUST_16611_PI429010886	N36566	0.005514639	21	9						13.35908	21.04763	9.794238	23.61225	14.49041	13.43787	17.01732	27.65463	19.20294	NA	71.43667	16.19041	23.1696	31.06592	12.54629	6.720337	11.68057	8.893955	5.441289	11.58452	17.91133	NA	9.753338	16.8271	NA	12.95302	16.99096
CUST_24073_PI429010886	N49983	0.005516125	15	0						10127.21	14580.94	10178.51	9376.495	5292.711	5979.853	9613.776	9855.129	11164.38	8547.585	7632.364	5644.907	7177.873	6109.222	6665.157	13154.56	13154.56	14389.61	10127.21	10053.8	9425.197	8631.841	7130.037	9905.822	8439.929	7260.089	8269.17
CUST_32569_PI429010886	M4455	0.005517041	24	18						793.898	559.2886	1066.707	1098.155	616.5627	265.6845	907.8057	493.9591	1292.456	2918.383	1582.625	2047.134	1938.89	712.1062	889.3882	672.9412	530.1492	1291.096	4875.223	3640.441	4238.105	1753.571	2773.888	3522.391	1636.385	1486.662	3292.245
CUST_21729_PI429010886	N45746	0.005522209	12	3						15.61685	15.865	10.13898	17.39841	NA	21.92026	13.13552	NA	14.57595	15.53957	5.945578	16.93035	19.01144	12.64908	13.39443	18.76907	16.87615	18.69379	14.79581	6.932272	14.13494	12.97492	9.304777	11.35065	15.10046	16.3775	15.98963
CUST_48002_PI429010886	M78473	0.005524257	24	15						1240.711	2773.888	1732.526	889.8299	1160.436	901.0046	1235.266	1746.391	1316.174	1068.824	1241.8	937.5478	1019.654	1373.039	1089.56	1844.624	2920.075	2217.875	2203.327	1789.218	1670.997	1152.159	1678.527	1134.386	1609.728	1308.545	1383.294
CUST_24413_PI429010886	N50580	0.005524257	24	6						106.2214	228.7822	197.4586	208.2743	225.5545	196.6698	207.2734	227.2167	241.5804	198.2176	204.3287	207.0168	169.4171	215.601	234.9525	201.3091	153.7672	190.6927	145.2125	169.5339	150.2816	146.8225	232.1097	190.1479	190.7291	117.6349	203.36
CUST_56867_PI429010886	M114795	0.00553552	24	21						323.0951	317.0103	649.9373	238.2083	182.35	175.8857	192.4063	222.6199	430.9236	357.4621	172.3129	285.6824	189.3285	196.1132	148.3156	341.0815	257.2967	347.5429	684.4289	255.6336	319.8754	582.785	366.7062	421.4753	657.7333	284.7236	390.7107
CUST_22336_PI429010886	N46846	0.005538111	21	18						49.09284	68.79187	28.14643	23.50114	16.51508	12.60763	28.65291	42.80977	8.467723	35.62359	21.0238	29.55798	39.29873	15.07768	28.87134	38.32178	50.95848	53.7918	51.41632	38.09775	52.61688	17.61898	26.22059	21.34628	40.44359	15.31518	26.03631
CUST_32208_PI429010886	M3527	0.00553896	18	15						88.12342	172.8084	164.7136	112.0451	109.2995	92.46133	133.7558	161.0498	157.8871	129.5648	138.9837	156.5187	149.578	131.1604	134.2321	170.4605	223.1865	206.8979	145.0834	147.2789	153.2351	138.8888	122.1097	141.4085	126.7723	151.7315	175.8857
CUST_5744_PI429010886	N19137	0.00554378	21	6						7.443803	77.16447	56.44269	61.46387	134.2508	165.3213	14.84278	166.5929	174.3407	7.697503	104.9484	24.5729	6.766809	29.60752	18.58892	12.9826	43.56758	38.88914	7.645491	33.4664	23.0873	7.327637	58.32536	48.20502	NA	73.36846	84.52278
CUST_54194_PI429010886	M105024	0.005549448	12	0						44.6746	41.47342	44.94568	33.90314	32.59982	26.37524	33.67346	26.67745	32.51285	28.5989	35.17583	16.12259	48.36443	23.80751	45.66597	40.37344	40.83819	33.75196	33.3626	29.04223	33.57662	49.08691	42.11956	40.76292	59.25874	39.38283	67.72081
CUST_863_PI429010886	N7610	0.005549448	12	0			Cellular component org	Cytoskeleton organization		826.745	493.0213	798.8065	713.1241	745.8583	722.7897	318.2644	390.0801	353.4279	351.7434	692.1008	463.3449	401.9784	783.4167	579.5063	353.6905	467.458	403.5639	362.2694	363.9263	358.8543	365.1669	491.1439	404.5068	421.7429	449.4542	443.8257
CUST_43235_PI429010886	M62485	0.005551695	24	18						17.12042	12.67448	19.11039	15.88592	14.42132	8.022388	14.30855	19.21808	16.90269	10.03167	16.98352	13.86535	24.04006	16.46484	18.03262	18.42891	18.11193	18.66401	23.44192	20.38307	19.44615	20.14753	16.4194	19.63812	32.19227	11.29273	28.46437
CUST_49367_PI429010886	M85404	0.005551695	24	21						25.14815	19.2763	18.38125	25.57669	16.18088	16.8613	9.275544	9.907467	7.222375	11.87886	18.51735	17.23263	19.5987	14.77073	21.28833	12.30813	22.62431	17.08471	12.36397	26.39512	21.1277	36.16345	25.3148	14.29658	31.53193	19.26727	21.58133
CUST_5540_PI429010886	N18835	0.005551695	24	18						321.8933	540.2756	420.0475	336.1378	320.0483	337.9981	433.8311	478.9906	528.1103	398.7982	385.9551	399.8074	433.7137	368.4918	398.2963	406.766	541.6936	524.2711	568.0849	510.9141	646.5974	448.8304	476.5395	449.0619	519.4495	384.2558	594.8478
CUST_9171_PI429010886	N24177	0.005551695	24	21						47.66075	59.01526	51.35849	48.7205	64.42288	62.03768	34.94317	47.43208	40.98319	43.07048	50.69492	54.13071	49.30286	53.39229	49.69173	44.99422	65.3293	55.71616	49.05136	47.81362	53.65203	60.71646	66.38702	64.98274	60.0628	55.88596	58.49962
CUST_5863_PI429010886	N19308	0.005554216	21	18	189240220	PREDICTED: similar to Cytochror	Response to stress	Defence responce	1.76E-63	25.1445	19.64634	28.31962	7.144159	13.07958	11.75627	23.90216	11.43354	11.18155	21.35916	12.69375	19.57461	13.51353	7.777922	22.92037	19.15316	13.60098	33.55276	38.12957	19.71101	34.23416	26.3515	13.72622	11.59198	16.36409	7.653718	14.3798
CUST_6950_PI429010886	N20821	0.00556437	21	3						44.81289	43.61117	44.18092	53.83595	52.22742	35.30558	50.60267	43.32608	41.3426	53.28421	50.55101	45.69732	46.43858	35.59474	29.6976	41.56038	44.80331	42.93819	33.44269	36.92118	34.16687	53.36513	48.66684	52.82485	53.19546	54.11854	48.68665
CUST_20762_PI429010886	N44011	0.005566744	18	0						50.73868	14.98175	67.73599	12.33889	6.241144	9.897949	8.477265	18.47674	10.20394	11.72201	26.26607	6.214729	8.373343	8.696601	14.20448	22.8581	40.77521	14.85832	15.5366	16.96577	15.74847	15.50721	12.38102	8.097597	10.05997	4.418678	8.649765
CUST_42686_PI429010886	M60970	0.005579907	18	15						38.24515	48.56908	55.47226	27.79864	38.62045	38.29909	36.51861	46.65753	49.03029	43.20078	36.38972	40.66544	47.65044	44.45113	44.53859	58.77493	45.02349	51.45353	60.03374	47.47428	35.61538	38.76155	16.30105	33.71063	51.79467	31.40589	43.34823
CUST_7526_PI429010886	N21676	0.005582567	21	0						173.6723	115.5105	196.5522	98.2692	92.08367	96.53451	97.66082	70.75071	70.67656	80.96563	89.44264	109.4907	92.9385	94.25033	118.4103	126.6732	99.01871	125.0755	96.69796	117.7425	131.7896	132.4344	80.43757	103.4024	109.5487	119.3879	113.5235
CUST_22946_PI429010886	N47984	0.005593545	15	3						6.031062	11.21493	8.937861	NA	11.23607	NA	3.97025	NA	NA	3.790068	NA	5.301599	5.197416	9.268739	7.682187	5.552409	13.79883	11.02468	NA	14.72497	6.89341	7.45936	11.64284	7.413131	NA	3.735034	5.165098
CUST_53953_PI429010886	M103850	0.005596945	24	9						478.2442	220.0171	384.0543	338.3895	456.7205	542.5585	208.3055	371.7268	250.0724	223.982	409.0189	414.226	208.794	404.1681	550.4921	285.2064	208.0191	237.0207	205.8252	263.251	175.6627	171.4846	160.1694	169.6715	187.8413	269.7799	177.362
CUST_55475_PI429010886	M110288	0.005596945	24	21						240.6918	274.1568	277.6138	233.1508	195.1036	156.4627	186.4449	206.5859	185.9639	158.6448	156.338	144.6342	250.3014	275.4561	297.7147	168.3104	396.5838	341.2447	277.4142	155.4242	222.5957	279.7318	260.3883	266.2866	195.2581	252.884	372.2898
CUST_49795_PI429010886	M87141	0.005601345	21	12						25.33441	10.79815	15.82642	11.60477	11.46447	NA	15.98731	13.6932	10.14417	15.41425	19.11764	26.15317	55.39639	30.36386	21.9708	14.78675	16.07832	26.96496	5.966459	38.06969	42.9937	28.49433	7.504118	13.74367	7.299732	12.69474	14.45802
CUST_56926_PI429010886	M114916	0.00560386	24	15						302.6134	336.0374	362.8786	329.7179	254.8611	333.7063	328.2775	255.3385	354.3285	396.4646	286.926	331.3594	331.7259	275.9079	302.7218	451.5651	403.4693	374.0444	390.7107	339.6934	384.1573	377.3111	358.0959	386.528	470.1035	363.084	354.2621
CUST_33252_PI429010886	M6401	0.005613566	24	21	157115557	mitochondrial uncoupling protei			0.000000579	41.28281	33.49837	36.58776	21.53675	26.60979	26.13856	22.45014	31.2401	26.95753	18.27547	22.98354	18.74591	24.24247	30.11046	36.61091	42.23986	17.70936	44.09481	25.04161	17.86363	27.17222	37.3305	32.6846	38.74481	33.09195	33.29588	40.36492
CUST_37841_PI429010886	M21826	0.005617009	24	18						1271.126	1200.042	1701.993	476.0531	312.7556	59.33452	2143.564	1236.613	2738.485	599.6889	550.9285	489.9777	1881.294	934.7113	380.83	699.7985	1534.389	2517.49	2620.241	2613.493	4513.062	726.5143	1364.845	1871.7	1487.355	2127.944	4436.509
CUST_43436_PI429010886	M63042	0.005617009	24	18	255729756	S-adenosylmethionine syntheta	Metabolic process	Generation of precursor metabolites ar	6.47E-26	158.132	182.0165	167.1998	159.4491	135.3432	110.3983	195.2581	185.2539	183.2355	219.3222	177.5137	187.2195	194.4561	196.7413	164.9616	222.4664	166.4205	209.6366	220.1523	179.3019	226.205	227.1783	189.1039	192.7777	215.2207	148.2715	205.6925
CUST_8219_PI429010886	N22727	0.005617009	24	21						8.77847	17.6134	13.10871	11.09202	17.74472	16.6895	8.355006	12.90067	11.60633	19.28815	11.25219	13.11926	7.669956	16.66408	17.13864	20.54391	39.2713	26.48147	15.80953	16.14034	15.90721	25.1445	23.71643	27.5238	19.83069	20.84244	28.58712
CUST_26115_PI429010886	N53693	0.005617009	24	21	242004748	hypothetical protein Phum_PHU			3.97E-24	24.42528	13.85151	23.32054	24.2063	15.58835	9.329172	9.13994	16.56978	12.23088	17.79852	9.952015	16.42269	24.02712	18.68765	21.11994	26.43013	13.114	15.88592	21.9826	18.47851	16.97215	26.92758	18.68973	26.67982	19.1841	24.68337	30.54026
CUST_14189_PI429010886	N32458	0.005621398	24	3	405975393	Allene oxide synthase-lipoxygen	Metabolic process	Oxidation-reduction process	1.19E-14	14.28509	14.84471	11.60186	20.80953	10.41528	16.40534	NA	NA	12.96002	5.599884	19.55278	NA	25.54219	7.847769	9.595961	13.16499	12.81539	4.828222	7.383186	3.502449	3.651969	10.65808	6.842059	6.264574	11.95174	6.532525	12.13635
CUST_15495_PI429010886	N34674	0.005621449	24	0	156552355	PREDICTED: hypothetical protei	Metabolic process	Other	3.69E-09	25.39283	44.75431	30.72426	24.39353	26.25392	24.6758	11.45478	26.93742	25.89322	16.58358	23.22612	13.43959	11.27508	16.17764	10.27106	16.63419	32.76569	21.13144	16.13183	21.3859	19.64262	7.536259	21.60215	17.311	12.83829	27.13842	33.95508
CUST_18882_PI429010886	N40575	0.005621449	24	12	334326753	PREDICTED: LOW QUALITY PROT	Protein metabolism	Proteolysis	9.26E-13	34.81137	29.13534	28.38277	59.66359	49.76639	66.39483	37.28516	34.67614	37.72215	37.50107	51.28488	40.3323	68.83252	84.00818	67.61402	38.03753	26.00178	30.68226	37.77333	44.43314	23.41335	30.10418	29.97653	46.281	29.97997	27.93535	36.12041
CUST_15406_PI429010886	N34523	0.005632129	21	9	489303282	agmatinase [Pseudomonas] >gi|	Metabolic process	Nitrogen compound metabolic process	3.91E-29	32.15923	35.86995	38.23951	62.60527	62.42709	75.97892	31.88585	36.71092	46.14458	84.14449	74.46237	72.0781	44.48574	60.99085	70.83005	26.70669	36.03288	35.12713	39.23752	32.36969	22.57324	43.32213	49.63757	34.2465	41.96937	34.78692	39.11911
CUST_20814_PI429010886	N44110	0.00563239	12	6						NA	NA	NA	14.81636	NA	15.06342	1201.304	NA	1014.541	41.28574	28.76456	NA	6.706445	15.5366	3.548747	63.2086	5.323745	63.66074	1546.88	1565.636	3555.208	223.0141	NA	217.3996	227.3921	25.57119	183.7151
CUST_19417_PI429010886	N41544	0.005635213	18	3						25.5566	21.35401	35.68728	38.69008	51.55397	73.83526	18.93653	20.86861	31.98442	25.89606	11.55982	23.00444	11.15025	21.44902	34.08118	33.18845	27.16394	29.04223	22.61783	12.97286	11.46286	77.44274	31.08617	51.27875	27.07259	21.29948	29.11607
CUST_4752_PI429010886	N17406	0.005639559	24	0						428.6927	408.8076	377.0504	387.5344	339.636	270.5842	241.7074	433.8311	346.1837	412.0092	336.9544	362.195	347.7046	257.3902	374.9091	236.7186	362.1172	328.0523	411.776	412.7396	362.6643	494.0593	346.1134	467.6907	503.3681	519.2716	671.9924
CUST_1555_PI429010886	N10051	0.00564071	12	9	7008007	DD9B [Marsupenaeus japonicus			0.000000165	1797.322	537.8527	1863.699	1251.736	1003.544	975.7238	858.1052	720.0128	1487.355	1247.935	1483.632	1491.134	1718.114	1033.621	1145.735	669.7814	612.7708	746.1539	1793.169	1296.153	1861.323	2277.337	2066.858	2522.496	1622.787	1205.159	2477.377
CUST_48530_PI429010886	M80826	0.005649031	15	12						562.6231	475.5631	533.0556	395.4956	300.9971	346.5231	573.4698	434.2782	454.7075	472.6199	551.5575	583.7299	346.3619	592.8013	491.5182	508.513	573.9026	595.0044	400.146	328.6973	338.507	305.7738	521.3103	332.8061	400.9039	580.6289	656.7134
CUST_21611_PI429010886	N45524	0.005650539	18	9						6.563263	6.899584	4.369637	7.525743	11.00713	7.040601	9.902198	8.147248	11.77451	8.834386	18.43253	20.78179	9.83045	6.982881	11.4263	9.506221	16.91902	8.645163	8.928675	3.205031	5.571313	18.81696	16.13355	13.56135	9.942624	6.828268	15.83889
CUST_52237_PI429010886	M96821	0.005651098	15	9						23.4544	57.58975	43.00754	32.18818	55.32159	36.32916	74.27281	81.78148	64.2158	81.05496	80.42543	59.47135	64.71262	55.80638	74.14723	42.2509	90.32894	74.70953	58.60789	62.90417	57.98315	55.43214	45.79664	60.84053	95.91396	86.21825	81.95267
CUST_50110_PI429010886	M88657	0.005655068	18	3						285.8344	405.1127	509.999	447.1876	469.0501	481.1553	231.7623	420.5189	364.3837	234.6915	469.784	347.7046	208.1054	304.6294	243.0739	237.4902	373.9602	232.2336	219.8774	372.0362	251.9129	430.7389	474.9425	363.4898	285.598	458.5926	261.5031
CUST_37487_PI429010886	M19208	0.005663395	12	6	321479210	hypothetical protein DAPPUDRA			7.64E-18	5.143723	NA	NA	18.93459	NA	16.11449	172.4022	NA	152.6411	36.02118	25.41015	NA	NA	12.67448	7.333794	47.6242	NA	37.52618	339.3438	540.9421	916.8554	29.67693	10.81194	12.83426	94.1914	12.45014	63.92981
CUST_6326_PI429010886	N19950	0.005669632	24	21						124.2797	141.4085	192.7777	131.7896	103.7054	99.78003	120.4803	132.1793	144.5615	123.4349	104.2696	127.6821	154.0154	120.035	137.2351	129.3882	117.6125	151.421	142.2962	166.158	187.7437	168.902	173.6159	225.3038	161.4729	108.2549	226.6524
CUST_11903_PI429010886	N28600	0.005669632	24	21						26.2182	23.422	27.23544	28.77829	24.09442	27.54194	24.78664	23.59507	19.23236	18.95217	20.25957	18.74013	34.92175	23.81296	15.33773	27.55409	16.51171	28.19779	23.88306	26.04805	25.36192	34.84492	27.03986	41.78309	28.0791	23.18509	40.07582
CUST_21254_PI429010886	N44895	0.005669632	24	18	261331295	T. brucei spp.-specific protein [T			1.27E-09	149.73	172.8766	138.5532	156.7242	153.6889	156.5799	129.3077	130.496	146.1694	220.4205	164.1331	174.3193	178.6979	118.1871	172.1631	355.324	134.8769	355.841	185.0545	202.3368	315.0449	165.2368	137.8885	168.7955	186.13	165.3421	157.6922
CUST_47918_PI429010886	M78152	0.005678284	12	9						9211.498	656.9404	8827.334	16760.98	5476.032	9313.798	1449.243	2687.973	4740.787	13193.16	25611.3	11339.63	15967.93	6717.703	12084.47	126.2717	245.6327	230.7269	6240.674	4922.978	7844.789	16323.5	21177.49	22332.92	9080.902	14703.33	22469.11
CUST_47298_PI429010886	M75519	0.005680662	21	15			Metabolic process	Carbohydrate metabolic process		6.846349	5.444614	3.783482	11.70368	NA	10.76077	5.781108	30.90807	8.958741	24.78664	19.9436	NA	10.64561	14.36388	8.936436	66.53367	35.35794	38.17853	51.70196	74.66025	89.86465	9.832908	NA	8.334969	22.38744	10.41636	7.340973
CUST_27923_PI429010886	N56949	0.005686471	12	6	410509308	myosin heavy chain type 2 [Pena			9.41E-21	28.72644	16.50439	28.04521	24.40118	24.5024	23.78446	38.79677	27.92224	40.68971	34.60522	10.32476	19.16283	31.73175	23.00444	10.12509	30.47418	36.6656	32.70464	45.58935	32.64521	43.02401	31.92217	32.15923	27.33338	28.71238	23.70922	24.83681
CUST_13128_PI429010886	N30630	0.005689282	21	3						71.37579	80.1827	67.83335	74.06253	100.1686	85.73084	67.23484	83.56745	81.4582	80.16932	96.02016	74.61536	60.68646	73.34259	70.71767	43.28585	86.51281	48.36443	59.69831	76.69145	42.73341	65.27026	90.79873	62.61027	72.26194	96.76253	83.45736
CUST_26495_PI429010886	N54345	0.005690511	18	6						10.66656	35.82991	35.48599	39.08885	39.58685	37.11143	69.37968	37.72504	47.75189	67.48873	37.23532	51.16396	54.6495	50.23264	49.9905	86.69353	56.60765	73.63397	41.89159	16.51673	32.26704	57.53971	35.69673	34.96168	125.1571	170.0929	62.09142
CUST_54987_PI429010886	M108336	0.005714221	18	0	242020610	protein kinase C and casein kina	Metabolic process	Other	2.69E-23	NA	36.75514	33.35975	13.78137	25.82506	21.79422	5.323176	22.04447	20.2799	16.21373	17.311	20.46917	10.09062	32.47615	27.23544	15.1271	47.18014	23.15721	10.97252	29.7549	28.18616	13.58986	26.12151	26.29331	4.580134	7.609031	13.3386
CUST_11904_PI429010886	N28601	0.005715064	18	15	260792312	hypothetical protein BRAFLDRAI			6.04E-26	NA	13.47047	13.31268	19.24654	5.465099	10.34183	20.30258	7.883393	16.52067	38.83274	7.872788	20.13002	105.3328	80.38647	9.815468	55.22716	74.44615	28.43101	115.8934	44.95996	45.61863	6.200844	7.488137	9.024323	98.56741	59.91376	22.34334
CUST_28678_PI429010886	N58414	0.00571713	21	0						16.36134	12.24754	14.35294	14.67236	10.99842	9.050756	8.640415	8.169501	6.627872	4.616179	7.791167	11.40248	3.989228	6.498165	9.324284	12.41233	9.889204	11.67212	4.400307	7.068342	8.550634	16.17629	6.855412	16.4789	10.44258	5.23145	10.28796
CUST_49332_PI429010886	M85239	0.005721171	18	15						27.98453	38.98881	37.38685	24.94781	33.27509	14.2562	26.81517	32.87709	48.98205	36.34085	35.14671	23.64075	28.42566	39.37754	34.63148	57.29374	50.35296	43.96327	30.24945	28.7398	49.24543	16.51006	14.92893	36.63348	29.48091	38.44506	46.14868
CUST_31547_PI429010886	M1802	0.005722369	15	6						3428.918	3274.995	4672.963	6109.222	3434.818	44.65936	11015.18	4875.223	15230	2913.515	2674.816	1707.189	8945.499	5514.378	567.0251	704.6212	449.0619	963.1582	17589.58	10746.95	12317.37	5705.148	14885.24	12359.97	10949.85	9855.129	18117.06
CUST_47718_PI429010886	M77299	0.005732901	21	12						131.5859	94.53661	109.7081	154.854	182.1611	134.7613	95.12721	98.79304	69.71083	138.0928	142.126	174.857	1462.785	207.2734	149.786	136.0065	101.105	127.8553	63.47256	81.35177	84.75661	113.2008	97.16144	122.0363	89.50979	105.7762	118.5947
CUST_29359_PI429010886	N59707	0.005734453	24	21						101.8893	105.2262	166.6346	191.2871	51.92132	110.4898	79.03209	55.98105	42.98811	194.1709	233.0946	113.2432	636.3867	43.64769	470.0131	94.51495	153.6889	88.81146	243.6336	324.1912	210.2639	531.9945	227.5795	468.9491	131.9274	498.7889	96.47869
CUST_54362_PI429010886	M105728	0.00574232	24	9						84.16166	38.78289	91.16754	74.10459	156.6771	99.83532	50.8191	64.37978	61.09188	77.06177	91.82394	129.9594	49.33812	144.7602	102.7615	88.4112	45.59558	83.30459	68.57845	46.06605	74.12141	41.95534	32.45769	44.31933	64.01323	52.98897	67.98511
CUST_38843_PI429010886	M31132	0.00574232	24	6	532759728	antimicrobial peptide type 2 pre			2.51E-30	1433.817	3815.958	2035.999	4690.093	2799.9	2332.204	8683.686	3284.996	5374.176	2787.074	1755.972	2924.097	4857.819	2646.596	2117.814	1732.526	4900.819	5357.194	1401.131	1842.758	1764.635	591.786	2609.919	2568.829	2166.525	1836.765	2048.35
CUST_51204_PI429010886	M92583	0.00574232	24	15						42.86666	98.097	53.19546	32.58867	30.42641	89.65818	91.40987	70.60324	90.77498	48.06623	71.98615	149.2626	107.9218	89.24382	119.8767	98.60183	126.1572	125.6025	114.0013	87.05088	111.1388	115.7246	83.02117	116.6562	39.4752	81.78148	130.3988
CUST_5777_PI429010886	N19183	0.00574232	24	15						136.7882	396.2592	281.4085	201.6917	160.623	182.5875	339.8451	337.5198	417.0366	301.3784	201.155	55.28754	260.0376	278.3094	232.4494	438.9261	594.6179	470.5066	385.9551	304.58	406.1674	239.2973	248.6699	237.439	366.7915	277.9454	300.549
CUST_9327_PI429010886	N24444	0.00574232	24	15						345.2692	284.0823	343.9474	443.2849	196.9036	262.8953	449.1793	317.784	345.674	474.8239	241.4743	354.8596	564.5167	425.5802	340.4948	542.0349	506.3774	549.069	508.1759	310.8187	676.869	436.601	299.9228	442.9918	392.057	305.1178	444.7023
CUST_8155_PI429010886	N22611	0.005743657	24	3						298.1868	168.3685	108.0846	304.7058	1996.997	1640.994	61.97033	201.5487	57.88315	NA	859.0835	1032.593	21.95424	437.9976	573.2384	9.621436	NA	NA	14.88235	540.2756	10.31498	44.66993	99.09115	38.13279	NA	693.9081	NA
CUST_52645_PI429010886	M98302	0.005743925	21	15	380005810	cyclin K [Artemia parthenogenet	Cellular Process	Cell cycle	5.53E-50	6.411708	5.49503	9.793016	6.683325	NA	NA	9.801925	NA	6.733117	NA	7.010551	10.07008	16.90983	14.45151	6.955349	15.56129	10.78353	8.874019	10.89368	7.808788	10.61538	9.910461	4.829619	4.661531	5.8709	12.42421	6.78016
CUST_33666_PI429010886	M7550	0.005747637	24	18						62.40045	63.12641	74.14723	66.3493	41.81524	50.39653	66.85645	59.19241	64.57231	73.73506	45.17758	60.15379	80.51893	61.50475	70.75071	77.74804	60.09389	61.21121	102.3362	89.30494	87.94793	53.49041	63.21401	80.34247	75.49296	54.67195	66.2384
CUST_3436_PI429010886	N14495	0.005747637	24	3						17729.56	66.59115	15012.16	21270.53	18198.77	25463.11	61.0985	419.0196	60.43767	4284.075	11305.05	14137.09	2911.669	14470.28	11974.32	24.58293	15.13981	56.78944	12.66963	522.858	14.38127	8846.332	397.3902	7248.769	10.97548	973.3791	71.95656
CUST_56664_PI429010886	M114372	0.005749389	24	6						33.97232	84.37355	85.85149	141.6695	84.31911	39.17612	254.6677	115.5725	52.96757	42.47062	29.98295	32.63321	46.38563	50.25143	16.14215	41.51635	77.03919	30.40311	23.5771	24.60432	19.78273	58.05667	18.29878	57.66115	48.87698	95.09356	34.95791
CUST_53901_PI429010886	M103627	0.005758992	12	0	443685111	hypothetical protein CAPTEDRAI	Protein metabolism	Cellular protein modification process	7.25E-53	23.38714	32.49093	32.29221	17.19904	22.86698	27.5238	20.72504	24.32624	24.6637	19.52187	29.5773	33.85933	27.97133	31.91659	37.40246	21.11731	19.39937	27.11349	19.19457	23.76937	16.04875	33.29588	29.39693	30.00811	34.27669	22.3639	38.18197
CUST_16472_PI429010886	N36294	0.005762333	24	0						130.7332	175.2922	186.5169	120.513	142.9612	108.1711	113.6995	126.5408	121.0252	91.13599	96.47869	90.07438	109.7332	101.5466	118.9453	126.6578	157.5876	120.9	143.0339	114.7343	169.6715	68.29523	103.4258	127.048	182.3239	86.71039	190.1878
CUST_13939_PI429010886	N32050	0.00576237	24	9						9.991081	14.76338	8.223081	18.50063	16.42848	6.615596	16.27655	13.44127	38.02217	19.5625	18.27698	23.5588	15.21169	13.07084	15.76545	11.84537	14.34967	12.88005	16.40534	13.02177	10.40034	20.84673	7.181893	9.415553	9.68199	14.73458	9.939018
CUST_27770_PI429010886	N56663	0.005765548	15	12						23.99912	25.21391	23.95198	33.03392	23.11733	21.95678	21.87306	23.92527	23.09193	33.49837	23.49891	28.84569	31.57579	32.94464	24.78012	19.68364	25.99676	24.35366	14.38486	20.90369	20.21346	22.75991	23.26132	43.10891	26.86103	24.25128	27.2791
CUST_53156_PI429010886	M100436	0.005779941	24	21						40.59839	8.834386	15.63254	18.47851	6.569065	15.61374	18.29235	11.75777	6.586668	13.89283	5.507602	7.784678	15.89978	14.49352	7.045737	23.55114	NA	21.78716	12.59667	6.353992	7.985663	25.25868	26.51323	22.18889	32.16695	18.58143	18.81483
CUST_2610_PI429010886	N12755	0.0057853	24	0	91082923	PREDICTED: similar to transgluta	Protein metabolism	Cellular protein modification process		530.6367	1271.126	925.0688	775.0084	901.5762	724.186	676.3457	850.2576	807.3597	697.732	975.2848	636.7343	634.6359	452.3252	579.065	510.9141	976.531	603.3505	618.7694	668.7382	510.4694	675.9355	939.4725	701.0526	961.2009	844.1613	964.2235
CUST_31134_PI429010886	M793	0.005791803	18	3						1925.502	1389.498	1320.156	1452.249	2975.418	3156.873	956.0225	3633.442	1296.827	952.6113	1390.116	1429.079	1795.363	1389.498	2206.592	128.9165	166.108	223.277	433.7137	5202.819	400.146	1944.792	2573.227	2002.791	351.92	7568.31	1675.406
CUST_204_PI429010886	N2465	0.005791803	18	15	270010491	hypothetical protein TcasGA2_T	Metabolic process	Carbohydrate metabolic process	5.76E-15	5015.024	8577.765	5840.985	3792.758	1786.238	1089.998	4606.27	4217.01	5462.129	5558.806	1409.957	3164.281	4880.699	2240.494	1900.457	7111.995	9345.369	9241.731	6116.36	5407.94	4450.275	4097.003	1505.065	2924.097	6976.622	2299.057	5298.376
CUST_13422_PI429010886	N31160	0.005791803	18	15						30.07238	41.1635	41.77836	31.07888	19.41383	18.43758	60.75399	38.55552	54.68833	56.25386	33.34145	45.36684	39.0193	30.69119	35.48951	58.16712	55.51551	49.53863	47.55101	28.65875	33.28109	34.85943	24.32624	29.16267	49.75454	28.89871	40.66863
CUST_56399_PI429010886	M113678	0.005793161	12	3						1641.783	1071.049	1055.851	2222.683	486.6807	1116.065	673.3593	2541.875	1781.913	839.3829	431.0524	138.7794	1233.546	1082.664	510.4694	2674.816	1508.513	2711.745	3350.998	2088.583	4105	611.8901	236.5379	295.2364	2013.288	754.678	1028.813
CUST_15388_PI429010886	N34502	0.00579487	15	9						60.4258	35.05094	51.34157	42.60881	36.31991	33.18448	51.60691	43.00281	54.15073	53.62536	44.53304	49.4765	57.01672	44.80331	47.11505	39.44209	42.52944	44.6542	26.51577	42.5735	32.68841	54.74825	41.78309	48.39409	73.19641	47.47767	44.53859
CUST_44470_PI429010886	M66075	0.00580354	24	6						142.2962	207.2425	136.1215	273.7185	98.097	136.9272	610.6881	283.1538	395.6891	120.0673	589.1435	162.1415	819.3739	820.0431	86.79002	7.055723	324.8189	173.332	33.50951	69.02489	35.99566	33.98645	53.58837	86.78193	16.22685	25.48421	30.07238
CUST_30146_PI429010886	N61257	0.005812951	24	21	499003904	PREDICTED: protein retinal dege	Transport	Other	5.76E-34	104.4886	95.71962	118.2766	92.03434	121.1493	124.6061	93.93436	86.28411	92.63177	96.41916	91.28627	141.1475	104.8169	103.106	123.4753	149.047	105.6512	108.4189	106.8102	90.84788	110.5907	164.5654	109.4738	166.6574	144.9379	101.6806	142.3895
CUST_54646_PI429010886	M107015	0.005815008	24	15						156.394	211.1406	163.7162	179.8716	156.2636	143.6223	174.6876	206.9345	205.143	227.4234	152.2431	135.7428	213.0837	159.4491	175.4398	282.9651	232.9982	236.6326	225.8691	180.5627	287.3062	199.8689	182.9511	231.374	225.1699	192.1066	237.835
CUST_40009_PI429010886	M48360	0.005815008	24	15						1679.651	6255.734	2790.956	1256.036	318.7134	784.8506	6897.446	5763.929	6343.146	1859.635	660.4277	749.8532	2898.397	2054.56	1591.635	4253.847	8713.974	7058.228	2822.582	2990.827	4045.654	992.2362	2708.146	1020.798	2110.599	2180.402	1886.448
CUST_48413_PI429010886	M80204	0.005815008	24	15	238683657	ribosomal protein S10 [Palaemo			2.78E-17	6125.57	9285.648	6210.057	5705.148	4783.413	5021.117	8591.806	9056.645	7983.519	7048.129	5697.98	5365.583	6929.595	5659.622	6481.59	10311.23	8750.959	10626.1	6976.622	7821.557	9855.129	5002.917	6727.299	6491.663	6543.051	6791.114	7695.811
CUST_11520_PI429010886	N27959	0.005815008	24	15						233.9614	149.2791	216.6807	189.6097	148.9789	156.3126	207.8778	179.4316	185.2204	237.835	130.8641	216.6056	274.6613	138.575	213.3383	305.1744	235.2069	287.1877	275.85	172.9739	271.7825	240.1963	146.4225	254.6677	239.8291	190.0624	250.2264
CUST_12198_PI429010886	N29090	0.005815008	24	3	169931080	cuticular protein [Artemia franci	Metabolic process	Carbohydrate metabolic process	4.77E-28	1501.763	266.1484	1432.11	2427.484	3415.242	3267.789	337.2109	554.6469	329.8464	2100.692	2719.085	3914.879	1602.248	1998.783	3202.946	210.1175	314.5854	196.875	234.1695	593.9206	435.1623	1548.998	808.8122	1662.168	288.3603	872.6161	852.8973
CUST_23747_PI429010886	N49392	0.005815008	24	15						135.6611	219.6551	195.9322	154.3283	134.703	117.2948	191.5119	210.8033	216.8711	166.2655	167.4883	168.3482	171.8137	170.8635	166.2655	189.4144	237.366	249.4346	220.3669	185.1157	220.1875	188.0944	187.8136	190.4302	160.4874	182.4721	205.6562
CUST_1471_PI429010886	N9733	0.005815008	24	15	242024401	sorting and assembly machinery			5.29E-106	465.2004	648.9592	512.9523	491.3879	512.0349	445.0179	530.8049	532.3756	504.3232	553.926	443.6171	613.3285	600.976	461.522	549.1826	660.1893	629.6411	744.5255	523.1285	518.2326	593.171	511.0453	543.9588	635.1199	552.9475	456.2678	632.3124
CUST_34859_PI429010886	M10918	0.005823495	12	0	321475426	hypothetical protein DAPPUDRA			1.25E-31	429.1442	300.1105	403.4693	354.6555	228.3322	222.2239	223.9415	221.5335	221.8149	394.8687	285.6412	322.0602	487.5393	250.1769	400.9039	416.3476	281.9365	342.9247	381.5238	285.6824	273.134	491.2558	306.9062	383.3865	579.065	367.9854	536.0532
CUST_35480_PI429010886	M12684	0.005824075	24	15						658.2775	597.7182	672.5292	551.2795	598.0964	585.8731	595.2796	645.7283	685.082	801.9356	762.8618	637.6043	556.6392	602.8578	636.9918	757.3112	878.8076	817.7537	694.7413	581.4931	675.5044	619.2009	639.2868	713.4537	601.3269	531.2109	703.6419
CUST_52198_PI429010886	M96711	0.005829474	24	21	358442122	Broad-complex protein isoform			8.1E-09	17.06953	9.508309	10.07565	12.84299	NA	10.49914	14.03865	6.163656	20.03269	7.276912	12.49326	8.591323	11.13493	7.537524	10.68195	12.5072	11.16741	10.26259	22.08254	21.10356	13.40748	18.7219	13.4515	27.06704	19.8984	18.32029	18.17381
CUST_47809_PI429010886	M77687	0.005833605	21	0						81.529	54.61715	65.13726	60.73126	51.85541	52.49326	55.93542	51.92132	58.06923	76.27282	43.64769	43.04131	70.57647	33.47807	51.10469	50.384	37.3305	50.28629	58.55963	58.66126	70.54441	86.50032	64.14599	54.65618	63.14073	59.45846	78.02817
CUST_12413_PI429010886	N29424	0.005837163	21	6						14.76151	21.58133	23.96123	20.63528	24.45381	23.30899	26.84779	33.39115	27.22363	31.18737	24.58293	NA	22.73253	19.88098	24.63541	20.70212	24.26356	25.33441	23.48629	18.5845	22.41344	20.30258	18.32237	26.33708	19.87663	26.52431	23.50843
CUST_6971_PI429010886	N20852	0.005838153	24	0	291222534	PREDICTED: hypothetical protei			2.24E-25	96.14621	149.2223	176.9714	82.94083	42.6374	56.96848	68.16127	181.9919	181.663	62.98013	68.27308	55.58555	101.7333	62.90417	53.9262	105.5565	41.25004	101.6124	161.8178	116.8305	251.1902	115.9507	112.5405	122.0045	269.2665	137.7837	204.3745
CUST_34214_PI429010886	M9073	0.005838261	21	15	61369380	ribosomal protein S13 [synthetic	Protein metabolism	Ribosome biogenesis	2.41E-69	25611.3	27153.68	24289.54	21524.84	12191.65	15630.09	28743.72	23575.84	23435.22	20172.23	13391.14	17729.56	21177.49	20913.02	16690.76	30257.2	27665.22	29520.35	27073.29	28414.17	27758.19	20271.85	19960.25	18613.15	24909.79	24590.28	17345.96
CUST_50844_PI429010886	M91215	0.005853938	18	3						25.56932	5.151983	8.814223	70.07704	53.59606	20.40638	24.63804	19.73213	47.01008	14.83214	23.09778	34.25718	31.25815	33.38645	40.20755	3.322905	NA	3.53297	25.91869	20.11934	17.03783	32.74766	69.52362	48.39798	16.30898	23.87506	48.2662
CUST_347_PI429010886	N4202	0.005855077	24	15	342356378	Heliconius melpomene cythera r	Protein metabolism	Ribosome biogenesis	8.8E-69	29647.7	35022.2	31543.85	26191.89	22279.42	23658.24	34065.02	31108.81	28492.44	29122.17	21834.29	27073.29	28414.17	24984.55	27665.22	37083.68	34620.42	40217.51	30721.41	34486.72	32798.44	22777.74	29949.84	29520.35	29520.35	29294.9	31967.8
CUST_53849_PI429010886	M103409	0.005862434	18	15						21.70516	41.89699	21.53675	20.26109	28.00193	15.38078	23.7984	37.1161	23.14891	19.78002	35.82991	16.59163	36.11092	23.37452	31.47125	44.1584	55.69128	48.46283	46.41625	25.83296	26.04551	15.03094	21.98953	29.15166	34.26345	31.10668	32.89506
CUST_53398_PI429010886	M101541	0.005865696	15	0						104.7345	101.1175	126.8958	126.327	111.4113	118.3395	63.84719	66.90744	79.1244	93.67836	89.26114	97.28384	114.8223	107.2961	134.8921	224.7103	259.2692	168.7955	90.3392	85.50328	99.10109	131.8851	84.87009	106.2528	141.1702	113.8465	119.272
CUST_24726_PI429010886	N51155	0.005867264	21	9						13.95493	20.87445	11.22943	40.13474	34.34338	52.03911	35.80909	43.65579	28.79283	39.17612	32.96215	44.00986	27.01619	19.33949	22.39833	56.57137	56.38348	58.29201	29.98633	14.41931	23.44192	9.822779	20.56246	12.84088	37.44318	40.3971	NA
CUST_8171_PI429010886	N22641	0.005868023	18	15			Protein metabolism	Cellular protein modification process		15.54537	9.003802	13.07357	8.696601	6.5566	5.725273	19.34308	8.148792	7.576581	16.65836	19.82627	3.957912	10.05496	12.37463	9.783945	19.57289	18.08089	20.13731	13.53357	16.54419	5.092765	NA	6.617801	9.284798	14.55686	15.99154	14.26253
CUST_38666_PI429010886	M28956	0.005869387	12	9						7303.286	407.5835	7353.952	8296.544	5783.496	6799.146	1357.702	2343.924	3686.257	9578.799	7861.824	8189.692	10795.65	4490.88	7232.62	69.44476	92.7216	140.9438	3801.741	3921.719	4672.963	14526.85	13335.43	13222.77	5792.477	9774.949	14670.24
CUST_26042_PI429010886	N53552	0.005869387	12	9						55.49774	59.84299	53.38027	47.20253	47.45455	44.64386	63.55637	52.03532	47.21843	53.46273	60.37267	50.81159	61.84336	41.73123	44.04608	35.08331	52.22742	40.2252	55.69808	47.26618	51.85541	67.76221	56.78944	65.08113	60.61437	53.14759	67.3975
CUST_45869_PI429010886	M70576	0.005877028	15	9						354.5733	223.982	263.0087	371.2053	294.3919	292.7676	214.0769	269.0492	332.2617	319.218	590.1632	276.9413	370.4445	269.8812	273.802	266.1986	142.1557	212.1398	309.5645	258.2414	262.5725	361.9725	415.3144	443.9249	288.6545	373.2381	431.7294
CUST_40699_PI429010886	M55893	0.005878265	24	18						307.5442	354.3285	352.8944	288.4809	189.9429	199.4568	390.8971	338.2602	391.4124	432.9222	304.58	368.0554	527.9796	262.525	367.9854	393.924	362.8786	471.7045	525.9937	440.1863	516.7278	579.8505	237.3259	360.0208	493.3336	321.7248	633.2997
CUST_19025_PI429010886	N40854	0.005884327	24	21						177.8178	96.87922	171.2263	106.9674	86.22741	76.02418	117.2468	112.5697	107.567	96.64158	129.3679	85.42964	51.35849	89.41928	150.7445	111.5617	112.4891	106.5406	216.4424	104.6237	153.2663	200.3302	204.4319	224.3337	270.2762	141.2484	241.7973
CUST_28866_PI429010886	N58754	0.005884327	24	15						31.24275	23.76085	26.75452	16.63419	28.47101	27.3523	24.9867	32.9944	39.45301	27.59657	14.67376	38.52366	45.36304	42.98408	47.51371	48.56063	29.80233	56.58591	44.53859	27.91891	37.31972	41.18226	30.71702	49.03029	40.04635	32.83861	34.06072
CUST_10908_PI429010886	N26975	0.005886905	18	9	291229341	PREDICTED: N-methylpurine-DN	Response to stress	DNA repair	4.34E-42	5.111708	7.186563	6.275153	8.839932	NA	8.480906	7.153945	11.75315	9.896366	13.178	14.71023	19.7654	8.958741	5.57575	20.90638	7.308662	12.65456	11.38407	6.375942	8.891795	12.29213	13.40748	5.442961	12.39656	14.16065	11.52408	18.06394
CUST_37860_PI429010886	M21904	0.005887695	24	3	82795517	arthrodial cuticle protein AMP13			2.06E-16	1281.397	37.93535	1057.154	1505.065	3643.551	2698.998	71.98615	173.4147	77.42501	1422.675	3132.033	2865.238	1041.462	2085.588	2870.034	19.69655	15.81139	20.09522	31.75895	264.9858	37.58637	1417.404	413.9053	1311.742	77.9206	479.9831	243.1902
CUST_38596_PI429010886	M28428	0.005887695	24	15	546673984	hypothetical protein D910_029C			1.71E-18	16655.53	37765.06	23503.18	20382.6	11836.96	14828.55	30352.12	33304.41	29725.44	23575.84	16070.74	5840.985	21010.2	24136.69	16288.46	36302.66	38540.49	35022.2	24984.55	28025.82	27918.92	17388.9	24825	18026.32	24341.41	27210.5	20616.2

Probe ID	ID	p-value	Period	Phase	EBI ID	Annotation	GO category	Go term	E-value	0	0	0	3	3	3	6	6	6	9	9	9	12	12	12	15	15	15	18	18	18	21	21	21	24	24	24
CUST_1436_PI429010886	N9625	0.005887695	24	6	238476875	crustin type I [Macrobrachium r			2.87E-10	148.5291	463.6603	96.51035	434.7194	414.7402	208.6051	845.2107	318.1938	702.1022	340.6386	44.50237	240.9716	171.2911	413.5169	344.4852	58.57991	158.132	128.0247	81.44134	81.33153	78.31361	41.17764	168.0725	217.8383	7.94894	117.5411	57.94759
CUST_44387_PI429010886	M65791	0.005888167	21	3						151.6381	139.2928	NA	33.4192	392.9019	374.7855	75.63115	310.0635	84.95059	NA	122.9378	121.7787	NA	111.793	48.84814	50.73158	18.32878	11.49366	26.47689	286.5115	32.08859	NA	178.033	NA	NA	352.5932	NA
CUST_29444_PI429010886	N59871	0.005893671	21	15						288.7179	80.13048	159.7501	132.6306	NA	127.1117	110.9878	184.1143	114.8652	224.0655	40.78815	152.3312	155.0803	125.3758	47.69823	844.6913	468.7122	892.4292	205.7884	384.1573	594.8478	248.2232	6.773043	221.3068	227.0272	62.14225	142.9612
CUST_37071_PI429010886	M17773	0.005896959	24	0						8.142557	18.49557	10.69163	3.310167	NA	NA	13.03303	10.15772	9.576311	3.458466	11.00713	6.12072	6.301132	3.273688	NA	7.393617	7.249218	6.666981	8.596616	10.83359	3.868331	6.381994	12.41357	9.169725	13.01662	12.89253	10.9035
CUST_11988_PI429010886	N28735	0.005901208	15	3						154.6882	204.3287	219.0011	209.3429	178.0114	167.6498	153.7124	172.8766	198.84	149.0067	178.5273	172.6192	119.1399	103.6054	162.1415	170.4291	136.296	150.0295	199.8689	237.7581	200.2294	112.1293	187.3336	107.8039	123.9495	151.5648	127.4883
CUST_54332_PI429010886	M105621	0.005912438	24	3						155.3337	18.07705	145.1606	206.6626	191.6851	235.284	17.4518	46.27549	26.85809	54.80637	54.11401	66.3578	9.11551	149.3939	108.0194	6.342459	15.52247	15.38781	14.15358	24.71997	10.37109	32.17634	12.76907	22.73253	4.514985	26.05591	NA
CUST_45262_PI429010886	M68512	0.005922385	24	15						45.56407	92.11335	48.18525	45.9767	51.57473	61.51056	58.62146	56.47386	51.93257	55.01217	55.1065	7.820884	53.64587	63.81813	69.91553	70.66441	96.73368	81.87749	76.46737	59.88108	72.13266	46.88337	59.47811	69.17285	59.37073	31.68332	56.64171
CUST_5989_PI429010886	N19473	0.005933066	18	15	215541377	putative hemomucin [Schistocer	Metabolic process	Other	5.08E-60	57.97707	91.40987	70.17999	42.30272	47.03106	47.41757	62.10736	77.8809	94.48817	90.43411	44.16317	59.3504	56.63612	40.21667	49.82313	113.3335	96.25473	137.5443	89.20331	51.45353	78.42276	52.33755	30.55564	45.06652	68.92058	46.01666	80.22358
CUST_18358_PI429010886	N39644	0.005937229	15	6						28.64933	20.50662	19.88098	49.23192	26.12451	37.42766	47.53635	36.57306	32.23946	28.2092	22.10476	37.07884	32.26017	30.52273	37.46348	21.12976	22.75991	24.90332	31.04885	15.48837	26.39253	55.4869	36.96718	43.26054	32.25543	20.20612	24.73549
CUST_9218_PI429010886	N24253	0.005937496	24	15						98.68778	119.5301	105.3437	89.14903	78.20138	72.2509	68.79187	90.24484	87.12904	94.26006	87.75883	78.28413	97.40632	79.17186	97.40632	111.4755	124.5085	103.2488	100.3265	89.5211	113.9393	96.16362	111.894	92.79509	107.2361	86.61603	124.0046
CUST_41271_PI429010886	M57320	0.005938878	15	0						155.5805	333.5586	NA	132.5675	3.185503	144.5227	170.2707	186.9296	71.93885	155.3061	93.68896	23.42954	127.5646	259.5151	106.2659	324.6065	274.8211	322.2486	198.334	44.12566	189.1703	51.08768	278.4811	63.18725	23.68606	112.201	4.875945
CUST_43307_PI429010886	M62699	0.00594213	18	15						39.50981	31.52277	39.16842	42.68213	367.4917	34.27395	39.34209	27.20363	29.06853	33.24054	30.00507	32.74766	41.34776	37.57177	35.9428	52.07927	42.6416	54.49956	45.86771	36.59345	42.91838	28.98015	29.33369	49.13607	33.89153	32.32188	31.78112
CUST_34845_PI429010886	M10873	0.005945613	12	3	321468185	hypothetical protein DAPPUDRA			3.83E-11	105.7007	167.872	33.08133	178.033	109.2676	191.4474	196.7413	68.89904	79.39688	130.5163	104.9943	111.8116	121.7034	154.2758	150.0907	258.2061	233.2281	213.152	179.7279	81.19478	187.1657	54.42434	97.82425	72.78292	171.9376	158.132	34.63434
CUST_30995_PI429010886	M409	0.005960383	24	15						809.2125	1105.917	962.741	927.7273	701.8416	821.5501	883.0931	957.0763	969.8589	964.6221	842.9082	886.9505	1002.608	758.5678	1016.824	1146.806	1143.807	1043.605	944.1713	830.4195	1061.955	995.515	937.1039	1143.807	888.6241	727.4839	1039.627
CUST_44630_PI429010886	M66522	0.005960383	24	15						60.86142	164.188	98.01972	75.23071	119.1547	95.173	124.4898	159.203	147.7126	167.6498	120.3757	143.902	144.849	104.7064	111.0779	148.728	300.1843	236.8007	114.7628	158.8716	182.3885	154.6882	137.6386	176.0219	106.6811	138.6664	123.2322
CUST_44856_PI429010886	M67221	0.005960383	24	15	321473486	hypothetical protein DAPPUDRA	Nucleic acid metabolism	Transcription, DNA-dependent	4.82E-81	77.28495	69.79983	63.99137	52.37142	47.61497	58.12065	64.99766	103.7192	53.33823	70.62321	72.59454	90.92644	71.75645	70.11515	38.65223	126.6423	122.6677	85.13676	81.18389	49.28164	64.33318	84.85915	70.3428	65.98466	63.80586	75.63986	52.62861
CUST_2165_PI429010886	N11749	0.005960383	24	15	328899797	ribosomal protein S6 [Procamba	Protein metabolism	Ribosome biogenesis	9.86E-83	15258.82	20913.02	16655.53	14285.49	11429.91	12605.8	16655.53	19387.17	16690.76	15230	12743.08	11552.67	16874.05	13685.2	14389.61	21459.8	22946.38	23162.84	18408.63	19150.48	21459.8	11356.17	17453.24	14858.64	18235.97	18673.37	18673.37
CUST_5151_PI429010886	N18322	0.005960383	24	15	326579691	JHE-like carboxylesterase 1 [Pan	Metabolic process	Other	2.45E-85	6.974516	19.78846	15.15187	12.75176	14.71235	16.32616	15.12028	66.31625	44.74977	27.17503	34.71797	11.84707	18.94604	21.33527	37.42403	40.38567	28.98015	27.35507	39.71647	27.83542	27.09196	26.45629	17.71771	38.26638	26.79915	21.8116	16.60926
CUST_5463_PI429010886	N18739	0.005960383	24	15	321476030	hypothetical protein DAPPUDRA			1.72E-130	48.07833	85.6799	71.75645	46.0486	43.86651	46.21776	59.6688	63.55637	89.7803	55.25246	54.83219	47.45455	61.60595	77.08874	59.83108	55.84366	102.3977	91.45416	78.33954	60.2163	86.03269	48.33387	69.89215	58.97313	60.96816	65.87261	48.38472
CUST_8072_PI429010886	N22499	0.005960383	24	15						32.88452	105.285	86.53109	55.14747	51.76488	40.1121	76.92175	82.49497	99.2507	66.71306	47.38682	51.28488	56.37975	56.41359	58.71759	93.89302	113.5495	115.6407	91.40987	87.68846	95.90246	67.9925	67.3848	70.06067	84.01764	62.85549	80.03641
CUST_8924_PI429010886	N23802	0.005960383	24	15						49.75986	100.1686	58.72904	70.0874	60.50212	65.54992	59.05616	85.21665	67.84273	70.24722	80.65861	70.56233	62.93557	77.40803	82.45499	87.47672	96.81529	96.69796	84.47977	72.02191	92.05562	56.08051	71.23962	85.12597	70.78121	76.3955	82.29248
CUST_15118_PI429010886	N34038	0.005960383	24	15						159.4235	243.0247	190.9659	148.8378	143.8476	138.2964	178.9277	170.4812	166.3995	187.8136	180.0167	167.4144	181.2583	152.8076	204.6434	212.5212	232.0442	249.8271	209.7386	201.8882	161.7147	213.4123	178.2425	215.9822	222.5957	184.5044	250.5828
CUST_15743_PI429010886	N35069	0.005960383	24	15						45.30575	69.70354	62.73874	56.57137	39.3253	39.14896	49.85348	61.0985	58.96212	71.35943	47.51371	40.82537	49.8658	53.71769	37.88233	81.80549	79.51886	89.90152	67.84273	73.39156	77.95933	53.1876	72.96184	53.48046	68.8804	69.04786	77.51144
CUST_21884_PI429010886	N46038	0.005960383	24	15						2870.034	8522.605	2911.669	2713.39	805.2136	1528.323	6612.476	9613.776	5879.268	3798.572	2700.891	1589.718	3867.347	2928.861	1747.584	5407.94	11859.41	6292.839	4655.696	5630.843	5191.892	3274.995	8238.918	4319.299	3237.866	4655.696	2971.539
CUST_27871_PI429010886	N56849	0.005962731	15	9						7.098506	4.712103	10.03307	7.43445	9.58783	NA	9.775246	8.439304	11.33181	14.13349	NA	11.82291	11.83302	7.264645	5.629485	7.017134	NA	9.398999	6.841078	11.55069	5.787811	NA	NA	10.21185	13.14087	9.749549	13.03229
CUST_35067_PI429010886	M11471	0.005969038	15	6						13.13946	20.07184	13.72472	20.9828	18.04324	12.87353	22.02084	31.83006	48.51961	17.54223	15.70113	21.65257	14.41491	16.21373	12.43008	16.69789	39.77797	26.80971	20.44259	10.91277	20.47903	34.66846	21.67982	27.36851	30.94126	19.29948	32.14243
CUST_2530_PI429010886	N12568	0.005989793	24	0						23.27767	50.6873	19.98797	24.92085	15.23394	25.39952	6.687789	11.69289	9.586868	12.13803	8.665928	18.45165	23.96123	8.519876	18.28516	19.61413	11.16104	24.62442	13.97125	16.4194	11.51329	21.70753	9.280922	20.31011	35.51059	36.03727	27.68187
CUST_14329_PI429010886	N32704	0.005989793	24	15						17.67403	16.65693	15.33773	19.84536	17.30194	8.988284	17.54652	19.52016	22.30617	17.87345	23.81043	26.99058	23.32054	29.43649	22.261	25.46423	21.00454	22.37256	17.16197	34.1106	30.31994	31.03967	24.97563	13.10871	16.41382	15.97275	20.44483
CUST_1110_PI429010886	N8525	0.005989793	24	0						147.7951	129.6764	125.8242	122.7221	121.56	125.5382	120.4478	121.4525	123.2141	102.9977	109.6043	94.93084	146.093	97.45374	104.0803	129.7428	144.2441	115.4156	109.4907	115.1501	57.8755	126.4673	112.6108	109.7081	126.1085	140.0577	123.2485
CUST_38641_PI429010886	M28764	0.006003698	18	3	121484199	cuticle protein CUT8 [Portunus p	Signal transduction	Other	2.27E-32	33838.77	951.856	33304.41	48051.75	35445.2	40915.77	2855.927	5110.752	5777.198	29520.35	51875.49	37427.64	34286.52	26129.84	44511.54	798.0084	735.2877	760.7688	10764.25	7621.268	12939.25	40415.13	37213.84	50560.8	11138.2	21398.95	30447.23
CUST_33014_PI429010886	M5727	0.006005077	24	18	157130417	serine protease [Aedes aegypti]	Protein metabolism	Proteolysis	7.07E-27	9.015243	14.41667	12.07005	4.09305	NA	4.203167	13.68928	9.089798	18.02378	NA	9.781107	NA	16.40534	15.53957	9.654225	28.53702	12.09389	13.75793	63.13263	14.81286	14.98175	11.91866	26.63139	17.38916	8.913392	15.07768	10.66525
CUST_36762_PI429010886	M16777	0.006008762	15	6						22.44233	19.23624	17.41278	21.83061	18.13556	9.622741	25.39744	46.6272	43.90368	42.80558	36.05612	25.54219	19.38387	34.50101	26.24865	21.13533	22.58797	24.25396	27.79642	24.53994	37.62384	29.70619	36.85694	34.84184	20.66998	31.94938	45.27328
CUST_41668_PI429010886	M58269	0.006013525	15	3						22.34605	20.66314	14.26253	69.77207	30.36663	31.71101	25.77169	8.355006	7.295483	6.919048	11.41802	5.220991	16.2936	NA	4.936239	7.254794	NA	NA	92.36671	31.53193	49.99479	7.02166	17.0028	28.87134	30.11519	31.22451	33.19846
CUST_46024_PI429010886	M71100	0.006021245	21	0						636.9918	696.7992	530.1492	493.0213	390.0801	320.4829	638.835	297.4214	421.3144	481.3104	300.4795	454.3052	474.3448	256.3549	354.0991	413.6069	472.9954	488.3385	513.2014	318.5699	608.6613	622.7305	575.1095	580.117	611.6853	279.305	706.8768
CUST_54395_PI429010886	M105851	0.00602903	12	6						16760.98	21758.66	16622.66	23324.95	17487.6	15340.52	27153.68	25611.3	28025.82	21922.49	11246.8	14794.99	21334.13	17287.24	17388.9	23379.75	20913.02	23435.22	31435.61	26056	28210.59	26491.1	17061.92	22946.38	25343.01	14071.14	22946.38
CUST_33653_PI429010886	M7515	0.00602903	12	6						160.3612	149.4831	136.983	242.2514	279.9809	234.322	368.5924	247.1999	284.6216	145.9095	193.7855	152.2628	188.2604	224.5032	198.9127	278.6422	174.7505	250.6739	283.4149	251.7468	338.8265	200.6783	280.6926	184.5342	262.04	211.1114	223.6033
CUST_35105_PI429010886	M11596	0.00603307	24	15						124.2158	203.6222	182.8746	162.4812	150.2816	145.3183	184.3599	249.1319	195.0025	187.8778	152.3049	134.3589	196.0115	185.2204	185.6007	229.8852	300.4274	251.3778	165.7121	219.7679	211.3776	135.0936	221.7013	166.3221	213.0483	193.7362	179.4021
CUST_32247_PI429010886	M3624	0.00603307	24	15	498532503	C-type lectin [Procambarus clark			2.06E-18	77.08874	100.8307	82.56422	88.12342	80.62092	73.7433	92.84934	83.43947	97.14951	126.6022	88.95701	113.5235	102.2726	96.65486	87.26779	170.2213	167.3552	145.0969	141.0115	71.04436	103.616	96.35679	76.68447	101.4138	116.1062	92.03434	151.4662
CUST_48134_PI429010886	M79039	0.00603307	24	15	380023724	PREDICTED: Apis florea polyubiq	Cellular Process	Cell cycle	3.2E-88	592.4023	1188.558	789.3467	640.2875	844.8862	612.3531	889.8299	947.6406	850.2576	1048.117	606.9823	760.24	624.019	999.9612	677.2359	1201.71	1314.02	1412.941	979.3611	1038.87	939.9518	900.553	864.8838	775.9607	906.4938	1013.822	625.9001
CUST_51115_PI429010886	M92261	0.00603307	24	15						287.4579	614.9534	450.1126	328.3409	347.5429	331.2753	410.2255	518.2326	463.1658	385.3052	443.191	391.5717	357.2181	423.4844	403.7519	553.5806	615.2672	696.7992	471.9849	471.3761	645.7283	308.6292	447.8863	483.6608	423.3824	376.0943	501.5231
CUST_2346_PI429010886	N12153	0.00603307	24	3	270161184	i-type lysozyme-like protein 2 [P	Metabolic process	Carbohydrate metabolic process	1.06E-43	9392.481	5587.034	8061.991	10156.68	9257.227	11030.98	5769.472	8683.686	6240.674	6255.734	6421.864	6642.147	6649.946	8284.715	8818.425	5209.792	4503.776	4234.05	5317.598	7632.364	5144.706	8051.463	7484.678	7704.294	3683.886	6780.642	5718.956
CUST_6214_PI429010886	N19783	0.00603307	24	3						1753.571	36.73639	1791.446	1862.343	1505.771	1825.053	58.9878	55.71616	40.86304	198.7013	948.407	870.6321	289.3915	1506.647	1493.774	39.12428	31.31216	38.52874	43.35728	49.08263	34.08501	442.3593	126.1993	408.5695	50.22	89.08902	119.4286
CUST_8735_PI429010886	N23498	0.00603307	24	15						18.97114	56.15422	32.49678	20.00439	18.45917	18.09857	45.74936	42.57027	42.92652	45.54022	37.40599	34.93669	45.84356	21.57883	27.51681	40.06052	53.9367	59.74362	36.514	40.46925	49.60146	31.30932	28.85513	39.61156	37.19071	33.11195	39.579
CUST_20110_PI429010886	N42829	0.00603307	24	21						49.7367	19.21337	59.43938	43.86995	29.41321	23.53208	27.75069	42.62368	40.27208	23.15983	35.0005	17.78243	82.51294	32.19586	26.92758	26.41747	33.05472	32.98843	52.53638	35.85546	25.14815	74.4166	64.51751	54.91706	39.20662	41.02997	40.70597
CUST_26296_PI429010886	N54008	0.00603307	24	15						237.8029	244.6993	153.1648	151.3538	101.0257	145.9321	313.4133	247.374	356.9618	332.5739	123.0024	185.084	274.0413	185.6261	157.0068	344.4073	288.8335	302.8616	263.4946	221.0264	286.2998	190.2455	210.955	162.6193	224.2516	210.0927	225.9127
CUST_7042_PI429010886	N20954	0.006034669	24	15	443695700	hypothetical protein CAPTEDRAI	Signal transduction	Other	1.26E-74	522.3491	549.069	607.3706	743.0295	769.8174	618.3282	670.6338	540.438	653.5411	618.7694	516.3556	621.9301	703.9211	586.1434	632.1075	764.7593	707.1593	1010.854	518.9718	537.8527	635.1199	503.6798	661.1838	665.59	415.8814	441.0424	466.8411
CUST_13202_PI429010886	N30774	0.006037167	24	15						17.07418	20.19245	20.85079	16.24433	15.81904	9.408973	23.09778	15.33433	4.464401	43.43371	19.72473	25.98049	18.7507	9.890547	17.17227	31.17639	21.67982	30.00507	23.63019	24.97351	28.89254	24.67085	16.96246	27.68431	29.57468	14.46804	22.71992
CUST_34515_PI429010886	M9896	0.006065614	24	0						45.93656	64.47129	53.4446	37.64453	30.72933	37.17438	36.42929	25.06028	45.36304	23.06154	36.33342	24.55645	40.5192	31.99205	50.85543	39.86153	50.26083	51.1596	39.56727	32.44579	36.61868	50.26083	38.82583	39.10373	57.47701	39.67832	43.11409
CUST_3080_PI429010886	N13751	0.00607433	18	15	195997716	Trichoplax adhaerens conserved	Protein metabolism	Other	1.83E-144	218.2535	304.2261	223.8255	198.2176	225.2142	190.829	292.1626	317.1978	227.2502	253.1919	240.7581	242.5947	256.2294	198.4091	120.1066	260.2914	363.6624	334.5246	202.1125	244.8381	232.2882	127.7621	204.2969	225.9127	229.3211	237.0906	181.6282
CUST_5262_PI429010886	N18465	0.00607433	18	3	526195870	hypothetical protein H072_8992			3.91E-10	1469.262	466.0849	1563.253	3175.147	2589.848	2327.901	374.4283	435.8605	387.048	2799.9	2156.524	2640.776	2645.058	1346.991	2430.729	309.4924	355.686	251.2891	575.8046	678.5721	678.0803	3095.903	2388.437	3611.195	538.8011	1187.586	1604.089
CUST_1559_PI429010886	N10073	0.006087548	24	21						17.18571	8.956638	31.8633	16.23526	NA	8.707003	NA	3.545145	5.793138	10.42743	8.445011	NA	17.34666	8.744802	4.160011	14.28325	11.56593	23.20911	5.096629	6.162699	5.795835	41.45634	23.86617	33.52669	16.64399	7.693398	18.48189
CUST_21181_PI429010886	N44774	0.006089082	18	12						6.423762	6.049144	NA	NA	8.594232	3.532397	3.861538	6.860532	13.30035	14.09547	9.395362	6.674088	12.52146	8.985774	10.441	9.104193	5.252307	11.97777	4.886621	7.542058	4.524122	5.906938	NA	3.812894	8.351717	7.14512	7.156045
CUST_30920_PI429010886	M218	0.00609006	24	15	91076896	PREDICTED: similar to upheld CC			3.32E-67	453.0125	775.0084	631.0985	474.5724	695.8214	446.3323	471.8286	629.8536	567.5384	472.8686	496.2739	549.989	520.4605	754.2071	505.9666	765.466	1063.435	1399.933	554.6469	706.0661	892.4292	484.1868	860.5197	877.3403	526.4775	597.5894	523.8156
CUST_53845_PI429010886	M103400	0.006105758	24	15						83.91692	130.5163	91.80105	93.04693	67.03577	78.94493	126.7896	125.6161	130.1383	132.0014	99.57709	69.35987	108.4697	89.80371	73.84495	153.8823	116.6092	157.4566	110.4251	105.5804	112.9324	112.2221	93.31746	96.39493	107.6017	96.14621	99.31589
CUST_39662_PI429010886	M42387	0.006105758	24	3	68486571	questionable orf [Candida albica			2.47E-10	5172.899	49.58749	4224.791	6543.051	3847.793	5304.997	95.56729	126.3605	85.19452	4027.931	1765.847	5357.194	1208.723	4719.533	5833.315	16.26708	30.91451	42.90092	83.55746	186.1038	81.5975	1649.981	569.6083	1516.421	231.8452	455.7266	443.0968
CUST_45518_PI429010886	M69362	0.006105758	24	15						24.25396	43.53777	41.41215	29.61912	21.93034	32.64017	34.1415	32.60314	51.81269	38.46589	37.88233	40.9561	37.04481	32.57817	28.18989	50.98858	61.49211	56.32436	42.5786	43.84747	48.08963	40.35645	38.14824	54.53868	47.96813	38.18648	54.25064
CUST_34191_PI429010886	M9012	0.006105758	24	15						449.9406	466.9715	475.9252	427.5284	337.6329	391.7638	505.9666	422.5065	367.4917	537.7378	422.7276	465.3594	503.1242	356.6938	500.1661	602.7354	594.442	576.0861	590.6124	391.4124	531.8423	594.6179	510.9141	549.5192	573.4698	484.1868	636.7343
CUST_4403_PI429010886	N16686	0.006105758	24	15						22.18889	28.88967	20.59574	15.37929	21.49662	22.34874	29.82341	20.46917	27.89685	31.11011	22.0686	24.03468	29.22669	25.76307	24.71116	30.99121	24.55123	34.65363	23.95576	30.3832	30.13995	31.41185	26.96013	23.83774	28.84319	23.02558	29.40702
CUST_5569_PI429010886	N18886	0.006105758	24	15						97.64083	149.578	122.3466	91.04847	70.94231	76.96435	101.1304	117.7279	117.1403	142.0207	89.05929	117.3396	120.7872	93.92415	111.6045	136.314	172.2349	128.1605	128.4025	105.4159	124.2797	100.4112	128.0247	107.6017	120.3588	117.0136	159.2709
CUST_8990_PI429010886	N23897	0.006105758	24	15	405950156	Exosome complex exonuclease F			8.27E-48	187.6789	239.9273	180.9657	164.2936	150.8283	137.7481	228.8663	233.5329	270.6292	206.1814	187.1971	246.6369	178.863	222.7248	182.5355	237.366	310.0635	353.1568	215.748	247.1564	214.3188	161.5503	192.639	206.2484	204.6434	207.1407	238.6215
CUST_9228_PI429010886	N24269	0.006105758	24	21						17.63035	31.54041	28.4048	20.44056	20.67603	13.26937	18.9132	24.46439	24.65812	21.26818	17.61709	19.94017	29.1953	20.465	24.6637	18.67811	22.142	33.83079	24.55645	28.23103	23.45708	29.52349	31.47125	30.99993	25.56181	28.32731	32.65194
CUST_29250_PI429010886	N59468	0.006105758	24	15	194270637	nuclear receptor E75 protein [Fe	Signal transduction	Other	1.22E-20	117.3396	146.953	74.6809	97.64083	60.14723	73.06306	183.4518	132.6978	128.1975	112.1293	102.3362	100.3986	94.7749	138.8443	100.2259	259.1236	167.5904	180.5334	160.2439	120.7872	111.4113	94.76517	123.9222	76.5633	120.3932	113.015	105.2262
CUST_1423_PI429010886	N9588	0.006105758	24	15	222875570	selenium-dependent glutathione	Response to stress	Response to oxidative stress	3.14E-78	683.6777	1898.75	1165.385	727.7655	696.7992	642.5938	1643.622	1403.719	1557.736	714.9939	935.0962	765.7762	1079.142	742.4077	911.4252	1473.47	1941.997	2240.494	1799.736	1160.436	1584.461	1039.627	1231.406	1012.891	1225.977	967.7143	1284.957
CUST_36612_PI429010886	M16351	0.006110738	24	21						NA	21.74621	12.10084	9.723255	9.933103	8.928675	7.480571	10.41528	5.891833	10.16018	9.886318	10.57446	14.43423	6.508862	12.88584	7.482905	23.37894	37.41529	19.55073	12.13039	14.21449	14.89626	18.03645	13.89421	4.661173	13.73044	18.81173
CUST_34203_PI429010886	M9051	0.006113587	24	18	321466121	hypothetical protein DAPPUDRA			1.96E-10	165.0428	264.1326	396.6952	136.3718	112.2837	6.413865	182.7293	197.1967	357.2181	193.9353	72.88356	134.0192	253.0838	128.1605	50.35761	186.2473	238.025	302.274	462.531	387.1258	681.5295	171.5864	226.4631	282.7416	244.6993	242.6247	654.7479
CUST_6960_PI429010886	N20837	0.006119091	24	0	241174483	conserved hypothetical protein			0.000000733	29.29243	21.12553	5.712015	11.10164	116.3995	75.83949	36.54707	60.47985	15.69192	NA	36.06378	32.68841	10.25642	29.52588	22.20311	5.19356	5.367764	5.025678	16.63236	40.43868	NA	6.836029	34.08118	NA	5.528891	75.1848	NA
CUST_43913_PI429010886	M64314	0.006119943	24	6						12.46469	10.4115	9.633908	12.68546	14.01446	NA	34.60122	12.94453	13.08105	17.62807	6.235237	5.019429	12.71343	5.877513	9.254546	17.63804	9.685449	5.045715	8.618077	6.449511	5.398149	8.459548	5.721366	4.871076	8.574366	13.25033	6.997156
CUST_24678_PI429010886	N51068	0.00612225	21	18						17.0552	12.49454	6.550174	13.32474	14.00343	9.880999	3.55928	16.27834	18.29038	8.092996	11.85264	6.734835	19.60559	7.64439	20.81357	26.0759	36.01433	16.17475	22.79876	27.23892	21.84297	9.533566	19.04999	10.64699	NA	3.803636	9.42167
CUST_11996_PI429010886	N28751	0.006141434	24	0						30.41985	39.88903	41.54672	36.8828	28.60128	26.92758	12.74437	31.50316	35.90062	19.73036	28.44439	18.79083	33.39499	31.23427	24.17495	37.14266	46.11055	34.94317	24.7223	33.23134	25.03218	33.81651	35.98384	46.09707	50.88233	31.57579	51.90562
CUST_52530_PI429010886	M97831	0.006144173	24	0						22.07673	26.46184	24.06537	15.54937	18.63942	12.70605	22.56843	17.14363	22.82925	11.08511	20.25567	15.53825	21.93034	13.02105	18.50385	22.40935	21.8116	24.96583	21.82274	16.92486	23.11514	21.41461	25.23371	28.26843	26.04224	18.26373	39.06481
CUST_39993_PI429010886	M48035	0.006144961	18	15			Transport	Ion transport		1978.294	4685.458	2076.904	1873.347	1855.874	1667.986	3036.167	2992.811	2558.16	2345.461	2184.345	2083.733	2228.732	2005.153	1770.862	3835.495	3114.68	4342.518	2937.545	2445.736	3709.96	1503.022	1581.87	1930.481	2589.848	2263.041	2430.729
CUST_33342_PI429010886	M6654	0.006144961	18	15						304.7866	428.5726	438.6999	244.0026	330.4891	302.7218	504.902	350.3187	512.7881	422.7276	364.6437	420.0475	405.8942	348.6137	362.195	499.6833	546.5288	455.8434	363.6624	359.5095	391.4746	366.867	322.2486	355.6308	418.3708	355.4873	420.5189
CUST_44721_PI429010886	M66780	0.006151772	24	15			Protein metabolism	Protein folding		14.53618	20.63528	6.607812	NA	7.40723	3.852828	8.409122	4.451761	11.13292	NA	13.41122	6.473882	NA	9.930505	NA	22.51109	16.02629	25.00095	15.58835	12.59794	26.92758	14.72189	5.731637	11.12081	19.07007	8.776321	5.499811
CUST_21035_PI429010886	N44510	0.006157604	12	0						101.9686	91.03605	100.658	97.47634	97.244	66.89231	55.31238	79.33651	49.92591	53.56818	115.0832	64.61049	99.80727	93.30975	121.7787	40.99664	40.53975	39.77797	68.80951	84.80932	86.38253	96.13685	106.4281	99.74451	94.07474	98.08719	108.568
CUST_52608_PI429010886	M98151	0.006166292	24	0						193.6659	128.8774	142.7301	124.6061	140.4803	98.1824	103.8438	151.3949	159.2473	140.1608	127.4702	100.038	113.8344	112.599	88.13366	151.1486	77.82741	137.3715	68.48192	98.1824	112.7612	141.6158	155.3337	151.5412	155.2845	154.0616	210.7737
CUST_20420_PI429010886	N43407	0.006172762	12	6						14.52682	4.944352	5.136576	8.558176	NA	11.75388	13.70168	98.57909	14.55686	NA	NA	5.329428	8.198822	9.203065	NA	20.38623	10.02667	12.41357	34.40062	27.79405	26.86909	NA	13.08263	3.832296	4.583941	13.88764	7.276912
CUST_6165_PI429010886	N19712	0.006173404	24	15						161.3598	191.8796	200.7515	170.246	124.8106	123.8841	176.4986	184.2949	159.3669	179.7279	130.24	184.5044	229.1263	159.3169	174.5908	207.3653	200.7824	255.0769	210.039	162.9942	238.9099	208.9983	167.0371	217.8657	198.4672	142.2138	234.5996
CUST_10559_PI429010886	N26427	0.006173404	24	9						41.72651	29.51017	41.39421	74.15901	69.03939	56.38348	32.93904	26.07855	20.84897	58.9258	66.48433	117.4006	40.57158	42.62368	59.21121	23.12968	34.52377	28.01116	17.79422	27.19756	16.82065	43.35728	30.45492	34.05764	14.82226	32.54957	32.43254
CUST_29594_PI429010886	N60164	0.006173404	24	18	242023295	guanine nucleotide exchange fac			0.000000386	210.039	160.2997	270.3263	80.46117	111.9491	116.3209	193.0271	129.4603	179.6766	197.0196	221.8149	131.4315	197.4226	174.1934	130.1961	192.3094	194.7103	196.7413	180.0489	199.6427	202.6223	252.1189	281.628	363.297	175.2702	129.0975	186.5368
CUST_57232_PI429010886	E2689	0.006178446	24	15						15871.24	20172.23	16151.95	14100.35	13281.09	13879.42	19195.08	22221.29	21974.84	16847.05	14470.28	11572.84	15630.09	16580.62	15367.74	22895.57	22154.05	21645.19	17771.79	19960.25	20871.55	14703.33	16913.15	17167.45	17931.09	19387.17	18715.71
CUST_36462_PI429010886	M15958	0.006178446	24	15	225713352	Sideroflexin-1 [Lepeophtheirus s	Transport	Ion transport	8.4E-21	10.04079	28.1721	20.36911	5.027801	19.77172	17.92297	24.04549	23.88306	19.66736	16.85775	29.30996	20.50277	26.28725	17.76942	29.24609	29.28939	34.95499	38.84896	23.22906	31.88942	9.179179	37.50107	27.14447	33.41645	21.53313	18.53796	31.31216
CUST_39426_PI429010886	M39211	0.006178446	24	3	341650466	Spz1 [Litopenaeus vannamei]			3.53E-86	3489.361	766.3188	2749.137	4086.34	21270.53	16014.56	1019.08	3277.985	892.4292	1637.38	15012.16	13301.65	1789.218	12991.27	12991.27	462.283	485.2701	419.5373	492.0502	4036.235	551.8317	2144.939	1926.392	1801.332	412.604	4531.71	1097.014
CUST_2325_PI429010886	N12108	0.006178446	24	3						2454.398	878.4972	2130.512	2690.376	3565.017	3264.887	783.6564	989.4983	847.8182	948.407	1918.312	2408.139	909.1828	3292.245	4680.36	784.4035	584.1152	578.5106	840.0505	664.2531	784.8506	1297.493	862.533	1361.235	604.8923	687.4827	891.3462
CUST_2936_PI429010886	N13425	0.006178446	24	3						1485.178	149.6035	1501.763	1641.783	2558.16	2803.748	79.00471	345.7435	127.2771	828.8502	1623.703	2213.256	1048.592	822.3417	1753.571	81.65394	88.57697	73.3346	182.6769	454.8526	167.1507	2072.212	813.2524	2171.504	241.2716	966.3163	480.8108
CUST_2983_PI429010886	N13539	0.006178446	24	3						3604.261	107.8221	3499.526	3932.655	8899.45	7966.321	70.47134	436.2064	96.62082	580.31	5992.677	6143.541	566.7623	6734.459	7450.782	59.59396	76.28125	85.51486	101.8893	390.0801	64.54817	1005.258	450.6028	784.8506	134.0782	784.4035	220.3669
CUST_5750_PI429010886	N19144	0.006178446	24	15			Protein metabolism	Protein folding		726.2724	1712.689	600.8289	588.0127	329.8991	466.2067	1736.886	1320.156	899.9958	950.7112	538.4885	651.0737	733.9767	804.9022	677.9076	1209.443	2050.749	1386.294	1072.507	882.793	915.7404	469.5128	1005.258	605.287	858.1052	956.8208	484.9644
CUST_7803_PI429010886	N22107	0.006178446	24	15	242020722	acetylcholinesterase, putative [F			5.34E-26	71.43667	81.529	67.66335	59.9228	23.14239	28.90562	174.3818	63.63996	83.81296	180.8452	67.60668	81.34169	126.5609	49.02346	72.19272	137.2826	186.3924	134.2942	141.8013	82.42776	147.5974	113.1088	48.94113	101.7333	183.0375	132.4959	102.0268
CUST_8079_PI429010886	N22511	0.006178446	24	15						371.9503	481.6057	359.2536	374.9091	370.5351	333.0825	464.677	511.369	479.2755	418.2862	401.757	406.1674	446.9571	397.5976	437.9976	515.8767	662.3233	497.9545	408.6556	440.4343	545.5749	390.5287	474.7066	466.3373	437.0635	354.4054	508.4006
CUST_10933_PI429010886	N27016	0.006178446	24	15						74.14723	182.4362	100.1798	83.32737	90.32894	97.87129	149.1772	142.3635	128.4423	145.6782	98.64233	105.9124	116.8305	116.9474	141.898	165.6234	246.6369	170.9358	144.474	101.2008	149.4369	97.16144	127.3678	124.184	133.6627	132.5156	148.2715
CUST_11437_PI429010886	N27804	0.006178446	24	15	262401203	mitochondrial ubiquinol-cytochr	Metabolic process	Generation of precursor metabolites ar	8.45E-21	1139.817	1535.771	1010.854	1078.593	1072.507	886.3549	1281.397	1361.235	1166.063	1288.693	1132.862	940.3723	1083.848	1143.279	976.531	1652.642	1656.675	1766.713	1205.686	1321.031	1266.853	1179.476	1352.068	1130.351	741.6851	1371.161	1115.136
CUST_22086_PI429010886	N46399	0.006178446	24	6						33.62389	46.64371	42.03616	40.5799	50.56675	41.63922	55.47226	42.19574	36.35313	64.92793	41.3582	77.77436	41.78309	47.82733	35.98686	47.40679	60.03992	32.65852	40.34223	35.51059	44.00493	28.23406	27.75587	28.90326	48.50466	24.469	50.42732
CUST_28048_PI429010886	N57172	0.006178446	24	15						32798.44	35157.95	29294.9	33838.77	25888.13	29520.35	43280.43	42496.4	38540.49	37765.06	27758.19	39540.76	33963.44	32184.55	32064.51	43280.43	39389.41	41095.52	40024.21	40024.21	40595.56	31543.85	35445.2	36967.46	35305.99	35305.99	36406.05
CUST_1106_PI429010886	N8515	0.006178446	24	3	342326199	protein spaetzle [Cherax quadric			1.92E-14	541.0757	354.1831	849.7305	830.1589	666.6396	641.1736	156.1362	266.9539	376.5416	326.4068	593.9206	685.082	328.4824	617.6234	682.7328	132.1793	189.4144	176.3429	180.8452	317.8651	320.2208	453.9545	373.7341	555.5824	224.2026	309.2002	464.2927
CUST_440_PI429010886	N5188	0.006186345	21	15	498985671	PREDICTED: thioredoxin domain			1.07E-68	241.1815	447.8863	296.3076	252.401	238.6788	264.9162	372.6214	380.1186	368.4918	289.1171	286.9821	243.2848	300.8131	340.0191	253.2927	380.915	587.8441	464.1815	302.2155	301.4388	341.3363	206.5228	340.6386	244.3968	240.0325	317.3631	251.6934
CUST_23597_PI429010886	N49090	0.006189087	15	3						42.48778	15.66635	40.82537	104.3712	20.11934	45.8224	14.12155	72.10955	65.76047	NA	11.36956	NA	13.79213	31.31906	5.699934	33.56251	29.30996	55.7467	77.56319	60.86727	77.02123	25.74022	17.27868	7.46045	57.781	16.68199	19.54174
CUST_6924_PI429010886	N20785	0.006202065	21	15	443694909	hypothetical protein CAPTEDRAI			2.6E-44	34.04952	38.34449	32.19586	34.47208	39.83652	36.45739	44.88521	42.88959	41.68317	36.58388	33.08731	51.92761	46.76121	50.27602	43.85961	63.94916	46.57945	55.60414	34.46864	31.11334	39.44669	44.9498	30.28489	33.53619	53.37075	33.51137	42.93375
CUST_22580_PI429010886	N47272	0.006214853	21	15						3.933996	9.599842	8.701063	27.2791	6.086343	9.932014	34.0139	20.51742	16.225	35.24577	14.29901	14.98689	32.64521	9.311282	23.59807	35.92598	34.86478	28.42566	28.86297	NA	19.16983	7.240266	9.917631	15.61171	25.35697	19.68364	30.43314
CUST_39542_PI429010886	M40978	0.006217254	24	0						60621.99	38996.88	61365.17	21758.66	11015.18	5807.4	38719.75	36571.4	50951.28	15012.16	8215.523	11695.66	31108.81	13446.51	10664.62	44511.54	29122.17	41865.42	57169.5	44511.54	34286.52	25555.67	24341.41	26129.84	62699.58	29647.7	51616.89
CUST_49764_PI429010886	M87033	0.006217254	24	15						67.29151	189.8869	214.7712	32.60595	22.13345	29.98996	57.7947	183.0682	187.1331	42.0865	52.58375	21.86932	44.3343	31.81746	47.64318	88.07773	31.04592	86.69353	310.0635	167.1289	328.6973	231.809	104.2473	151.2161	434.5642	138.9837	300.4274
CUST_9064_PI429010886	N24006	0.006218879	21	0						72.4917	95.50727	83.92194	77.91051	98.38918	85.37067	72.20641	71.98615	78.67787	78.57224	82.03251	71.69067	64.57877	78.18007	57.60969	87.17685	73.39823	84.83411	77.25936	83.27222	74.86678	85.34679	87.33991	81.15666	89.21288	83.03055	83.69522
CUST_35196_PI429010886	M11862	0.006223361	15	9						450.9209	556.047	517.1871	378.2634	288.117	280.879	384.2558	547.0285	458.147	498.6872	540.2756	505.8465	398.2132	458.4811	385.0272	397.1925	455.4195	378.8803	473.9767	430.4406	447.759	439.1006	354.2621	458.6774	533.9662	497.2634	598.4778
CUST_36743_PI429010886	M16723	0.006223361	15	9						293.2877	373.4093	297.8139	170.1715	233.6384	170.0929	241.4743	352.1552	365.8262	260.2914	331.1317	267.7885	215.1868	408.3007	401.1245	490.7221	234.0578	316.6599	292.2414	235.284	181.1959	160.9374	316.4278	246.7787	471.2164	532.4935	822.3417
CUST_18422_PI429010886	N39772	0.006223361	24	6						83.7199	106.8903	11.05843	77.73937	17.72282	49.85348	15.59925	20.84897	8.068353	63.92981	16.03512	25.53483	4.573473	12.12705	5.618815	36.54707	62.40732	55.21231	85.33414	59.34282	51.03976	43.89532	20.24751	16.80584	NA	37.56673	9.984306
CUST_27701_PI429010886	N56540	0.006234525	21	12						6.932272	9.659899	6.792509	8.610034	NA	NA	6.16087	6.740547	5.217523	7.24682	NA	4.594536	40.89712	7.725643	73.4071	19.18875	13.09661	14.81846	NA	7.505551	12.26439	5.826272	7.796517	8.438338	11.26241	8.224117	6.325259
CUST_9132_PI429010886	N24115	0.006234565	12	0	443719184	hypothetical protein CAPTEDRAI			4.64E-08	99.3998	68.64219	116.981	90.87055	77.76407	83.0128	52.15113	57.37441	79.54469	83.7199	69.96485	78.39296	88.36719	94.52718	91.97617	96.86942	71.07642	71.80743	88.97825	68.39377	44.72002	102.6229	65.90164	84.36167	139.2795	72.90397	107.509
CUST_32811_PI429010886	M5164	0.006242702	24	21						17.41092	5.554011	20.65942	7.007853	11.66876	11.5008	15.10953	7.202814	16.22893	7.985663	NA	10.61794	11.29	14.61747	7.216326	19.29052	11.7375	10.78069	20.20229	15.03411	16.39186	16.09002	42.2771	17.78857	16.38688	16.0548	14.53734
CUST_19544_PI429010886	N41755	0.006245092	24	15						33.17685	63.53279	53.32445	27.74342	21.70753	32.94207	48.32944	69.6714	49.364	57.20828	24.67085	44.23722	36.74804	37.44318	28.3093	53.96355	74.35572	69.16443	45.25376	52.01796	57.66115	33.59889	40.30891	37.29691	32.94858	41.86279	41.64718
CUST_11715_PI429010886	N28287	0.006248231	24	9						16.04092	14.68671	17.21141	36.86027	108.0685	83.24757	33.01146	57.28678	34.06404	31.26158	64.59327	73.73506	15.60281	31.18313	52.25815	32.8731	20.61232	15.44101	8.652491	49.39632	NA	18.2303	8.372078	20.05184	10.98054	27.20683	7.031281
CUST_15620_PI429010886	N34874	0.006250516	12	9						47.90004	44.69809	49.94405	50.3146	40.82014	64.12039	55.54321	81.85533	54.41132	62.51652	78.38177	63.33433	112.6234	86.36335	49.75986	35.64607	45.03269	36.73202	61.34753	41.72041	45.9872	108.9605	58.91866	103.6054	32.38002	52.5487	57.70575
CUST_41959_PI429010886	M58997	0.006251133	24	3						166.1333	10.73207	172.4491	245.1008	133.0898	251.1346	8.00908	25.93791	21.23274	19.80764	74.21946	78.783	21.75638	132.8996	168.5856	7.567646	10.3228	7.194555	20.27243	26.0759	18.05364	38.89794	26.51854	40.45937	16.74205	22.23772	25.34024
CUST_42425_PI429010886	M60249	0.006251133	24	15	307202216	PAX-interacting protein 1 [Harpe			6.85E-24	149.3774	180.9438	194.6568	162.165	152.7137	121.2158	211.8418	186.9009	181.0435	214.2802	138.8888	186.5169	188.4898	141.539	157.8268	231.097	229.3564	264.0266	214.3618	187.5258	220.2833	192.7777	159.3992	180.3196	209.1419	174.7074	273.0815
CUST_33380_PI429010886	M6747	0.006251133	24	15						1458.832	2393.197	1746.391	1434.548	1372.283	1346.472	1826.319	1852.945	1954.479	1583.518	1538.316	1645.987	1757.509	1406.008	1712.689	2363.552	2659.616	2577.27	2021.719	1626.549	2198.578	1534.973	2006.118	2297.179	1840.408	1947.583	2541.875
CUST_33868_PI429010886	M8091	0.006251133	24	3						7886.969	152.4151	7643.683	6656.173	10683.23	11429.91	132.8402	554.0705	102.4924	1847.559	6143.541	7402.627	1531.626	9026.68	9819.956	49.00524	42.2303	59.50711	73.08027	819.0741	170.8635	2393.197	619.3687	2268.13	52.05076	2558.16	403.8629
CUST_4103_PI429010886	N16013	0.006251133	24	15						29.06179	26.0838	25.30919	26.49621	14.27175	24.21942	26.52119	25.15875	31.93549	27.42998	26.54409	25.57888	43.04426	21.94323	33.05798	49.92591	37.03485	57.17098	35.70711	25.07913	46.01299	41.09779	25.07412	39.22582	41.73123	34.13826	43.18058

Probe ID	ID	p-value	Period	Phase	EBI ID	Annotation	GO category	Go term	E-value	0	0	0	3	3	3	6	6	6	9	9	9	12	12	12	15	15	15	18	18	18	21	21	21	24	24	24
CUST_5088_PI429010886	N18243	0.006251133	24	15	321469650	hypothetical protein DAPPUDRA	Metabolic process	Cellular amino acid metabolic process		88.60558	107.1382	90.3848	63.54768	79.97607	81.28557	110.8072	102.5417	107.1233	101.2809	85.27841	109.4424	112.3401	50.59319	75.19367	155.2093	188.3607	130.7693	121.9689	80.24873	118.5289	97.18737	117.3251	69.112	120.513	93.37419	78.40447
CUST_5868_PI429010886	N19313	0.006251133	24	15	157115862	leucine-rich transmembrane prc			5.39E-20	429.0423	682.5361	499.8092	446.2058	364.3837	413.2217	697.9099	597.4035	557.8175	710.1661	439.7541	565.6114	665.1463	521.206	453.8518	748.709	620.7278	678.0803	546.8689	660.6448	615.9354	551.0918	530.6367	505.0907	670.9198	524.2711	583.9375
CUST_915_PI429010886	N7816	0.006251133	24	15	183397235	innexin 2 [Penaeus monodon]			9.07E-18	3907.106	8547.585	5304.997	5144.706	2183.114	2539.976	9804.488	6368.381	8061.991	7966.321	3284.996	4988.039	7794.971	3522.391	3721.413	8786.531	8146.369	9712.243	8577.765	5792.477	8723.793	5317.598	5678.164	6734.459	6710.162	5402.577	8362.147
CUST_33360_PI429010886	M6683	0.00625127	21	3						294.7488	256.0394	54.77517	115.3398	1864.874	1753.571	127.7854	360.5101	107.3799	NA	1468.759	1452.249	NA	592.0137	482.4747	24.14912	16.65693	7.869717	10.42021	544.0973	9.331963	16.74981	142.1072	35.33106	NA	1767.886	NA
CUST_21644_PI429010886	N45576	0.006255711	18	9	332016241	Lin-9-like protein [Acromyrmex			6.02E-30	NA	4.728836	6.525596	3.354922	5.785239	8.324734	13.38177	4.748557	NA	11.84866	8.252165	10.7714	6.566709	7.439921	NA	6.220036	6.006861	12.01431	5.001528	4.351926	6.947668	9.50239	3.799167	8.086218	8.848966	10.44258	10.60534
CUST_32943_PI429010886	M5531	0.006263675	15	0						37.23401	38.413	26.92758	26.08093	25.37982	21.45062	22.24587	36.36056	25.02074	25.75202	30.74237	24.19322	30.51884	20.0391	30.59603	33.15012	32.13276	38.6867	25.95165	32.06918	26.88893	19.13763	24.33371	30.90184	13.50493	29.84354	20.08859
CUST_51441_PI429010886	M93544	0.006266427	24	15						15.63697	7.05762	12.65731	7.095312	12.88736	9.913032	11.62724	10.72225	11.04387	42.92948	9.92008	19.75521	15.74279	12.79751	22.56644	29.46646	13.25797	22.52707	16.3304	14.99981	15.9967	22.9969	16.15698	21.59961	21.38839	13.17618	15.68854
CUST_40871_PI429010886	M56268	0.006271372	18	3	242007036	class B secretin-like G-protein cc	Signal transduction	Other	6.78E-59	139.7162	118.702	107.3485	150.1329	139.9317	132.4508	110.0417	130.415	141.1811	132.9353	183.0682	100.59	146.4225	107.2361	92.88966	85.2914	125.3758	75.264	88.59515	100.59	61.15687	150.0907	180.729	132.7314	117.1403	145.139	131.8408
CUST_52712_PI429010886	M98593	0.006276668	15	0	339233402	conserved hypothetical protein	Cellular Process	Cell cycle	7.37E-42	9.939018	9.697101	11.52497	7.956098	7.093434	5.350473	NA	5.21464	9.029102	7.821834	3.085787	NA	NA	6.327914	6.660555	8.135191	12.9826	11.47222	4.756073	5.708684	9.164016	3.366669	3.75717	6.112777	11.59198	10.18923	7.228642
CUST_30892_PI429010886	M124	0.006286225	18	15	332374616	unknown [Dendroctonus ponde			8.46E-58	816.314	1616.307	823.7754	709.2377	583.9375	587.5636	1234.557	964.6221	926.6453	1000.348	625.6951	823.2974	839.3829	666.0922	814.573	1601.274	1682.203	2042.326	1151.33	808.5577	1167.815	720.5302	814.573	787.5865	1171.1	882.2277	1077.231
CUST_39264_PI429010886	M36900	0.006286225	18	6	225712016	Cuticle protein 6 [Lepeophtheiru			2.62E-19	16348.66	14743.32	24825	20271.85	11695.66	3532.198	29949.84	25463.11	43701.56	16411.2	27153.68	14338.96	36195.31	14764.65	15942.89	3988.54	4847.527	7316.568	32295.88	29520.35	48252.93	28943.19	34065.02	26987.8	40415.13	30821.46	62699.58
CUST_46912_PI429010886	M74147	0.006286225	18	15						383.2384	385.6874	382.4457	362.0388	352.1552	303.3581	465.972	334.1482	489.8482	441.7626	345.8925	473.4786	413.108	348.473	393.7655	558.2968	494.0593	604.8923	390.6122	322.1601	368.7646	323.6649	346.2958	379.3272	483.7942	352.8944	448.0132
CUST_41226_PI429010886	M57179	0.006294894	15	9						36.20921	41.17309	36.06722	42.31229	47.79939	43.93243	27.39507	34.35444	33.26748	52.01796	76.45795	64.59327	54.0024	44.10093	55.7791	55.40345	34.08743	48.56908	36.98315	41.03792	35.55503	53.95129	49.48225	45.91529	53.04738	65.37786	57.74953
CUST_2425_PI429010886	N12319	0.006294894	15	6						40.82956	45.42128	30.30161	88.75	60.25895	66.74201	55.23799	53.21446	78.46823	63.0922	37.1161	58.19693	66.04384	45.45322	56.57499	55.39639	52.92831	39.54209	87.20964	52.52723	49.96679	95.27189	90.41336	88.6659	48.65167	70.36913	40.27208
CUST_52087_PI429010886	M96372	0.006304574	12	3	307176782	Probable E3 ubiquitin-protein lig			1.19E-83	16.62267	6.930237	10.85699	15.91258	10.98917	25.15651	10.15066	10.79953	9.45412	10.73905	9.260771	7.052579	7.740604	5.786969	10.22339	13.87312	13.16987	10.97416	19.60068	NA	14.31582	8.064044	9.061533	4.758462	17.51582	9.973024	12.15359
CUST_38506_PI429010886	M27519	0.00630516	24	6						87.74995	310.4859	184.5623	175.317	44.96721	75.88424	1978.294	213.5938	1242.354	299.2328	114.5333	214.3618	1478.028	885.8693	97.58001	60.90419	169.8611	151.6707	13.64057	59.87629	63.72422	35.37225	108.0685	84.1875	47.18014	16.60777	48.19549
CUST_1908_PI429010886	N11038	0.00630516	24	18	345329480	PREDICTED: RRP15-like protein-l			5.87E-16	99.26059	159.8296	104.9085	111.7011	82.42776	50.47426	147.5974	108.8896	92.23687	112.7172	71.6009	104.0674	136.9442	94.95617	98.57909	109.0861	143.4022	161.1965	138.0928	152.0064	129.6764	115.1318	135.5176	122.4178	82.46867	102.1346	117.4669
CUST_49118_PI429010886	M84190	0.006306574	15	9						54.84874	6.913289	64.50416	14.17028	32.91719	39.47125	67.81869	69.16015	57.28678	80.37006	78.81902	NA	146.8225	66.04384	72.95425	47.54634	18.5449	30.06311	99.1481	17.95668	38.89794	80.96563	75.53689	7.287837	172.7187	55.59367	135.9631
CUST_43_PI429010886	N174	0.006319738	21	15	226731904	C-type lectin 13 [Perca flavescer			4E-15	102.722	102.8659	111.1646	148.1737	123.354	119.1547	410.8005	405.6944	491.2558	569.3405	367.0194	74.82596	135.8226	132.6428	95.30674	1212.026	413.2217	851.8736	267.9409	451.0149	414.325	169.2197	69.6714	97.32812	268.3054	50.93191	116.0138
CUST_14057_PI429010886	N32263	0.006321723	24	15						9.190116	21.85917	20.54806	7.353952	NA	8.545551	17.52792	15.64904	15.54937	24.45381	10.4597	14.44158	35.26461	14.69878	17.50753	25.81706	17.89974	32.22297	15.68492	17.33539	23.25083	26.29812	15.32337	28.53195	14.29901	11.18862	21.17165
CUST_57129_PI429010886	E1279	0.006323821	24	3	7008007	DD9B [Marsupenaeus japonicus			3.86E-18	8846.332	344.0704	7007.731	13591.98	7714.571	11763.32	314.1834	650.5235	255.9552	10966.45	10506.53	10683.23	5693.503	9301.136	10260.07	94.54537	138.3923	216.4068	466.0849	840.6574	478.9906	9041.082	4101.099	7203.647	337.8262	2650.032	924.7313
CUST_37727_PI429010886	M21270	0.006323821	24	15	506965393	40S ribosomal protein S10 [Copt			7.26E-48	10641.47	16874.05	11932.74	10966.45	9410.039	10053.8	15340.52	16014.56	15526.29	12141.91	10156.68	9684.529	11052.65	11998.69	10408.52	17167.45	16070.74	18715.71	12359.97	15871.24	15258.82	9013.443	13559.72	11998.69	11822.99	13135.1	13193.16
CUST_32724_PI429010886	M4937	0.006323821	24	15	54400676	ORM1-like protein 3 [Danio reric	Protein metabolism	Regulation of translational elongation	3.53E-52	120.0673	126.6958	100.4907	107.6151	87.71525	113.1748	124.5521	140.0779	120.4309	111.7284	93.01673	126.327	125.4154	83.84766	137.1639	205.1165	169.2478	184.6434	133.8226	117.5696	142.9419	129.2736	115.5552	158.6448	144.7781	116.1322	194.4274
CUST_40277_PI429010886	M52382	0.006323821	24	3						2803.748	37.93869	1535.771	4069.14	882.793	3033.527	23.23737	89.54662	54.1566	2575.646	2098.966	1906.005	1508.513	1429.079	1590.527	8.742604	19.24274	10.33053	108.9605	116.6925	80.47316	2038.003	1028.813	1152.159	116.1822	440.5339	268.3054
CUST_40826_PI429010886	M56182	0.006323821	24	15						857.1902	1751.317	886.9505	1024.289	802.4687	914.5652	1666.041	1396.628	1289.967	1179.914	940.3723	1031.076	1299.503	1062.767	1069.78	1807.527	1945.845	2041.036	1294.853	1040.528	1345.742	950.7112	1055.851	1189.941	1447.815	1164.205	1221.709
CUST_4639_PI429010886	N17187	0.006323821	24	3						81.70379	61.16761	70.22094	66.79456	239.9273	139.7386	82.20662	49.96679	50.55101	50.0131	197.4226	142.5641	81.42279	93.29743	111.3237	45.76238	45.85631	39.88527	64.06806	56.60172	53.14113	67.08754	74.39469	44.34431	94.38205	66.21459	53.49041
CUST_5735_PI429010886	N19123	0.006323821	24	15						157.8871	274.7682	211.1406	153.8823	126.6022	107.7528	219.3222	192.0736	211.5442	237.3259	161.3159	176.7718	197.2974	147.5021	192.0309	267.9409	276.9413	293.568	224.888	200.4116	263.5563	171.5325	171.9376	233.6058	222.4375	179.7279	310.0635
CUST_5787_PI429010886	N19197	0.006323821	24	15			Protein metabolism	Cellular protein modification process		675.5044	1059.201	744.7409	505.6819	560.8143	486.8888	577.8423	791.0912	738.5083	555.2007	617.6234	931.5266	563.8477	735.2877	655.4746	844.4833	1140.807	1094.513	773.969	822.6543	749.8532	602.5729	761.0743	852.5352	789.0657	792.067	850.8759
CUST_14814_PI429010886	N33513	0.006323821	24	3	9186884	BCS-1 [Amphibalanus amphitrite			1.47E-16	632.4923	138.4795	667.6928	874.1945	1574.714	1711.883	98.478	243.2347	75.94639	242.833	931.8674	854.7576	126.8065	830.7266	949.6232	84.45149	73.4442	49.95516	59.12156	431.7294	74.25196	175.8219	252.989	215.2625	35.20827	557.036	127.3065
CUST_15514_PI429010886	N34704	0.006323821	24	15						231.2123	283.5165	247.6161	206.4541	171.4108	161.2674	323.2809	263.5563	302.7218	279.9809	192.6977	210.5038	287.0864	206.2842	206.0093	334.3239	368.4918	404.8745	301.4906	267.6236	345.674	217.4779	253.9087	268.9316	726.2724	226.2788	329.015
CUST_23260_PI429010886	N48527	0.006323821	24	15						19.11039	23.69696	41.1635	19.17715	19.67391	23.02109	25.01557	30.10418	26.6719	35.36195	25.29384	33.37664	26.68521	24.78664	26.346	37.12091	38.04316	42.35625	30.15331	20.05017	37.632	42.29499	31.61624	28.75654	34.25718	21.79685	36.19278
CUST_28573_PI429010886	N58209	0.006326239	18	3						9.370822	12.56491	12.43497	10.64424	NA	NA	19.43702	11.09925	17.44773	23.05545	11.69458	9.755769	22.43976	NA	7.596603	4.949676	5.182324	4.417312	14.86691	12.33142	21.65484	15.21906	25.02902	26.47281	28.08693	14.14112	16.95258
CUST_30142_PI429010886	N61250	0.006329692	21	0						59.8943	55.28754	64.39747	42.45638	43.45077	40.98047	28.82648	35.00336	31.22862	57.98315	47.43208	51.27142	45.2017	41.68317	42.64535	47.38682	43.42575	44.79025	45.19064	46.62193	35.02108	67.16357	48.88152	64.65354	67.37607	46.46695	73.39823
CUST_467_PI429010886	N5465	0.006341004	24	15	84043336	Fenneropenaeus chinensis calre	Protein metabolism	Protein folding		7439.177	14470.28	8417.477	7058.228	4229.758	4976.553	15526.29	12765.38	13812.43	10017.83	6575.891	7303.286	9013.443	7147.632	6567.976	15367.74	17771.79	16580.62	12211.42	10337.3	13078.51	6343.146	7794.971	7695.811	11413.74	8547.585	9129.704
CUST_39124_PI429010886	M34919	0.006356233	21	12						481.6057	413.8083	440.4343	1298.646	853.7719	786.3486	1684.207	1075.462	2133.67	257.956	2064.359	485.5854	2180.402	2104.842	1988.355	429.8212	2088.583	1020.366	388.382	460.5885	339.2657	238.3483	957.3975	832.894	314.5002	572.1963	722.1173
CUST_40559_PI429010886	M55457	0.006356857	18	3						7577.055	815.4763	6249.252	7518.482	8605.765	9980.233	593.0437	1559.979	1762.47	6543.051	8683.686	10156.68	5073.866	7937.877	8160.518	429.4693	195.7159	502.0555	5451.324	3033.527	4616.026	8135.669	10897.12	13078.51	2541.875	2185.802	3731.866
CUST_45038_PI429010886	M67802	0.006359647	18	0	123316129	Portunus pelagicus actin mRNA,			3.53E-56	47.80868	50.1877	39.75997	51.74535	49.57184	32.58198	46.48748	36.58388	35.83339	36.65135	36.3567	31.53193	26.80971	49.14925	30.37143	34.00502	47.79939	33.99771	56.28495	52.83788	52.86067	39.41926	41.82104	34.21691	53.79961	37.75646	31.44182
CUST_41148_PI429010886	M56989	0.006362421	12	0						14032.06	NA	10587.95	2711.745	1291.096	1625.527	30.91451	15.94507	17.44435	303.1963	723.3991	609.0112	2893.96	1471.771	3397.779	29.94161	26.66045	8.218417	13.20883	12.56182	13.38008	7415.157	75.45664	5840.985	6.453374	NA	NA
CUST_22536_PI429010886	N47201	0.006376772	21	15	345481600	PREDICTED: hypothetical protei			2.33E-56	223.8255	201.229	167.7409	258.3703	180.729	298.7578	441.7626	225.8314	214.2511	362.1172	208.8662	313.6779	329.3072	187.8136	317.9491	708.4537	284.9168	541.3579	273.1848	111.0363	231.9279	215.5592	101.1475	210.2639	281.4085	231.9279	150.9904
CUST_42847_PI429010886	M61406	0.006382882	12	3						121.8684	113.5928	131.8067	173.0814	155.7485	152.2217	141.8433	131.4985	146.2169	140.9438	100.3493	83.87393	147.89	125.7647	133.8983	162.9942	207.9921	170.0456	125.6969	136.5009	166.6038	138.0571	156.2876	170.5634	136.7201	104.5953	125.5691
CUST_24834_PI429010886	N51328	0.006395593	18	3						27.05424	28.2608	34.82138	48.09966	27.7842	24.50769	31.99902	22.12571	24.17495	38.51205	13.20883	41.92055	20.71687	21.57697	23.04915	32.86278	23.08008	23.85663	26.51323	23.65361	27.24917	47.15551	28.46788	49.10462	28.95792	22.6585	27.54194
CUST_34528_PI429010886	M9921	0.006396508	24	15						233.2281	294.0637	255.4282	243.494	224.0655	213.2149	316.5315	268.3647	316.1661	297.3675	240.3468	293.0253	318.2644	225.4359	248.439	346.5231	368.2385	340.3327	314.4452	236.013	331.2753	294.0637	236.9699	318.7134	329.2075	267.8251	341.84
CUST_5556_PI429010886	N18864	0.006396508	24	15						521.6397	1214.453	820.9036	608.8174	519.1671	580.7924	814.0506	884.3862	713.6664	1021.527	725.1557	737.8151	755.266	704.3483	603.3505	998.7674	1365.984	1295.587	740.2235	778.2044	851.8736	605.6304	825.7252	733.0037	714.0141	817.4569	815.7902
CUST_27532_PI429010886	N56226	0.006396508	24	21						11.81016	14.96006	15.08699	12.44585	11.24141	10.22133	10.81386	9.738777	32.76221	10.19442	4.791983	5.896106	16.36594	17.2236	14.41667	16.16366	7.404706	24.53447	7.705814	10.94976	17.80604	14.82588	15.75335	19.74297	13.12827	14.04923	13.79883
CUST_1382_PI429010886	N9459	0.006396508	24	15	314122335	Mus musculus DEAD (Asp-Glu-Al			1.57E-120	1553.379	3452.681	1931.709	1534.389	1534.389	1411.448	2586.608	3046.537	2846.594	2389.711	1873.347	2475.896	1818.148	2216.187	2037.287	3161.765	5197.224	4072.42	2281.476	2467.704	2842.045	1541.174	2332.204	2143.564	2338.958	2269.737	2816.959
CUST_1506_PI429010886	N9853	0.006396508	24	15	219644788	Rimicaris exoculata 70 kDa heat				12886.71	26678.14	17453.24	15526.29	14703.33	15071.82	21834.29	26803.66	26403.54	18966.14	17658.04	13078.51	18673.37	18673.37	15847.39	32798.44	32184.55	32639.19	22895.57	24590.28	27832.29	15731.96	17388.9	18966.14	22895.57	22037.75	23820.74
CUST_8163_PI429010886	N22630	0.006400761	24	15						12.17947	14.96774	23.45171	17.43071	26.2182	18.70643	16.59434	27.53743	18.49355	29.94161	26.86328	35.26837	34.13078	21.46595	29.21241	39.54209	31.74988	35.44329	37.57549	20.02577	33.24806	39.84392	28.16376	45.31745	29.6948	21.33315	24.18434
CUST_1532_PI429010886	N9957	0.006400761	24	0						61.86512	61.0985	58.79928	63.68621	42.98408	55.7467	39.42679	56.42125	47.03106	57.8427	53.89331	41.57926	56.39944	42.41648	47.54634	54.33188	46.66198	48.38102	72.14124	51.25725	56.38972	59.81752	53.39865	54.99763	61.10664	56.21355	65.12931
CUST_39150_PI429010886	M35302	0.006402968	12	9	493789484	hypothetical protein, partial [La			9.09E-08	6276.978	4403.488	6612.476	6710.162	3972.222	2928.861	7303.286	4705.762	7966.321	6116.36	9129.704	4388.629	9167.401	6298.27	3303.981	1926.392	3972.222	4058.337	4803.622	5271.495	6448.141	9167.401	10605.81	11339.63	4761.143	8086.369	11179.97
CUST_16868_PI429010886	N36994	0.006402968	12	9						19052.86	844.8862	19960.25	20501.42	14256.4	19522.5	2211.381	3122.414	4439.592	26129.84	24909.79	21974.84	35022.2	10430.71	26987.8	178.5273	109.4578	325.1203	9516.402	6837.265	9194.226	36302.66	21834.29	33304.41	11738.35	15900.37	19094.08
CUST_51670_PI429010886	M94518	0.006405039	15	0	328899961	Procambarus clarkii clone pc-97	Protein metabolism	Ribosome biogenesis	1.86E-73	5777.198	6845.752	4715.833	5080.07	3667.762	4363.515	5507.513	6060.248	6913.964	4690.093	2765.552	3624.383	4719.533	4583.265	4185.629	7058.228	5671.62	8996.679	5233.042	6430.973	6837.265	5329.024	4513.062	5456.239	4825.163	4291.944	2942.677
CUST_11164_PI429010886	N27371	0.006417013	21	6						1505.065	3650.983	134.0586	611.6853	22.7226	538.6242	3882.503	2998.296	1971.443	2795.798	1868.336	NA	NA	NA	NA	3827.862	1213.608	2066.858	60.16049	52.10354	143.7613	273.556	9.978491	271.5356	2474.336	1890.428	294.6856
CUST_1592_PI429010886	N10173	0.006418333	24	3						2260.134	49.02346	2224.156	3692.065	2064.359	3426.429	69.74823	429.9294	298.997	509.7706	392.253	394.6878	189.5124	312.3683	278.9665	75.76283	98.60183	138.3677	285.7364	364.4673	608.8174	1128.19	294.9139	771.7617	244.3091	9.44382	241.0144
CUST_27857_PI429010886	N56824	0.006418333	21	18						133.4406	294.9654	298.3249	22.00575	35.68286	10.07945	85.40071	255.4927	136.9442	41.95985	7.784678	20.80428	102.9045	91.6714	101.1544	41.45022	35.89334	34.74447	419.1486	307.6702	539.585	87.86087	100.7337	68.46482	151.0875	95.97338	111.8609
CUST_7154_PI429010886	N21118	0.006420852	24	18	156549734	PREDICTED: FAM203 family prot			8.25E-60	166.0779	212.7978	225.5545	147.3731	147.9113	124.2958	194.3971	179.7537	192.9642	158.2397	156.0931	172.6519	186.9781	162.099	170.7496	203.3258	205.7216	222.6813	203.5144	191.7677	208.2743	226.9579	157.2283	216.9264	197.8872	183.2355	177.2962
CUST_14856_PI429010886	N33581	0.006425972	12	9						1585.44	11.40638	1762.47	2001.707	213.0483	1269.839	53.30473	112.0182	170.7874	4193.691	12317.37	2487.704	4910.228	208.1428	3269.912	5.742729	NA	20.66527	496.5813	400.146	504.0705	4288.608	3360.114	3899.124	517.1871	2975.418	4242.57
CUST_11544_PI429010886	N27996	0.00642724	24	18						15.9251	37.7496	51.42591	23.24571	22.91325	13.76082	23.0638	30.00507	41.30394	32.19938	20.93451	NA	37.69822	28.04521	31.91659	33.04583	39.2334	35.17583	40.78485	37.51496	42.30272	31.25004	40.14947	33.80053	35.52825	31.33958	41.40683
CUST_34254_PI429010886	M9176	0.006434724	21	15						4.846566	7.907113	NA	5.100826	3.576199	NA	NA	7.904365	6.523317	3.270319	7.180584	NA	5.016308	6.079186	4.319008	9.73754	9.15859	8.83019	8.568381	7.212205	15.95845	9.104193	3.494935	7.717206	5.176369	4.520414	3.169854
CUST_38343_PI429010886	M25653	0.006437307	18	3						3208.57	313.739	3601.999	6999.724	2361.017	3565.017	522.4996	832.6018	1377.009	4750.447	8006.37	3161.765	6999.724	2184.345	3763.397	58.30071	34.59443	76.45034	2589.848	1871.7	2952.044	5992.677	9937.479	9041.082	4004.603	5872.99	8984.262
CUST_50687_PI429010886	M90648	0.006464125	21	15	291223589	PREDICTED: carbohydrate (N-ac			2.34E-08	63.96849	119.7663	29.30404	124.9411	28.42785	89.39537	152.0785	113.9812	51.82749	132.8026	89.76967	63.18725	147.7749	155.0803	99.12783	251.4219	219.5694	184.3285	98.97699	36.4809	84.13391	42.66588	101.7595	41.15339	98.46402	120.6508	28.17546
CUST_36769_PI429010886	M16802	0.006469196	24	3						111.1532	29.98295	73.17158	86.74668	202.2179	157.2999	50.19242	37.56185	19.29052	64.78842	70.90586	133.684	56.28495	61.36043	89.35207	20.16972	9.531461	14.99367	19.75896	71.54776	11.92894	82.85507	45.33911	107.5502	33.0209	49.28164	41.13599
CUST_3324_PI429010886	N14265	0.006469196	24	15	443734659	hypothetical protein CAPTEDRAI	Protein metabolism	Regulation of translational elongation	6.51E-38	10550.02	14439.79	9211.498	9613.776	9345.369	8945.499	15045.33	14670.24	12043.33	11112.95	9211.498	9980.233	10852.86	10448.35	8484.924	15705.46	16580.62	16491.53	11455.94	13790.46	13790.46	10156.68	11264.76	11413.74	11881.91	11206.93	12860.58
CUST_11853_PI429010886	N28515	0.006469196	24	15						83.9852	105.6776	97.07924	65.70417	66.95538	65.09533	71.70119	102.7889	102.1108	102.9161	101.0612	84.02947	93.30975	81.32217	78.33954	115.9507	124.1913	151.9667	103.9866	74.95343	73.66383	103.0566	80.47316	117.8448	99.61307	96.45691	96.37995
CUST_21075_PI429010886	N44580	0.006469196	24	15						68.10466	79.81483	117.5547	87.38427	77.61728	70.50377	108.374	99.4113	124.9756	103.4513	111.9654	89.26977	128.1168	91.05781	106.2416	130.4316	149.9991	130.8147	76.96435	121.3126	136.296	110.8559	121.7034	136.103	103.1963	122.8067	159.9313
CUST_1164_PI429010886	N8712	0.006469196	24	3						15767.9	70.89953	14389.61	13906.34	19287.83	19010.63	109.2373	303.7619	192.6977	9179.508	15900.37	19791.01	6410.91	10127.21	16491.53	15.81757	13.70744	80.00199	346.1134	923.4428	574.2528	10835.48	3390.028	10835.48	520.6055	2595.878	2440.627
CUST_55312_PI429010886	M109528	0.006479193	12	9						418.1662	63.03163	396.9028	145.4304	27.31187	136.983	59.11715	151.8995	81.31408	177.5731	249.6361	137.6019	217.4779	101.361	128.3485	56.45276	49.35536	47.07055	21.58422	73.49385	67.04993	360.8077	191.823	328.2775	77.63531	153.3375	209.295
CUST_38188_PI429010886	M24010	0.006479193	12	9	91086861	PREDICTED: similar to cuticular p			9.03E-17	3588.155	27.40435	3952.736	6523.544	2826.773	3686.257	547.3976	441.8514	876.0896	6201.489	8577.765	4490.88	9743.786	2822.582	5184.879	8.544323	13.84533	23.72726	1576.537	1158.756	1844.624	8204.372	7506.653	8296.544	3020.109	3411.4	6165.981
CUST_2072_PI429010886	N11500	0.006479193	12	9						4456.862	396.9983	3731.866	4427.539	3568.199	3774.541	355.324	1257.339	1335.991	3735.581	4825.163	4710.772	4207.659	3104.628	5900.066	319.8754	167.8421	295.6289	1631.254	1225.455	1920.18	6348.681	4690.093	6401.205	1480.103	1843.608	4048.748
CUST_165_PI429010886	N1995	0.006479193	12	9						66946.06	3983.53	62215.85	68735.71	39540.76	52165.44	14614.52	15585.38	25978.53	75512.6	95305.17	62699.58	95843.19	42224.23	88732.25	958.1681	1449.959	1689.176	38228.71	29843.45	46193.24	80425.69	102933.3	83454.29	46875.63	71289.88	95305.17
CUST_39532_PI429010886	M40884	0.006508809	24	18						836.1158	736.9876	887.5066	452.142	314.7808	312.9583	297.492	615.9354	527.7959	452.3252	677.9076	716.1458	1426.897	1132.05	897.658	1270.268	603.562	1201.304	1248.523	1272.378	786.9042	389.5477	1284.957	1301.148	870.6321	1727.221	2436.323
CUST_11676_PI429010886	N28222	0.006508809	24	21						752.85	771.7617	841.3097	751.8458	571.6616	694.7413	610.334	553.2936	642.342	771.0455	636.7343	745.8583	854.3715	597.4035	818.3565	817.1951	852.5352	824.0838	773.0893	720.9725	770.1221	886.3549	764.2764	1026.487	914.2819	668.7382	1083.233
CUST_36724_PI429010886	M16662	0.006509492	15	6						191.5454	195.4372	168.7955	202.3368	194.6568	258.2813	194.4274	258.1145	288.7764	233.0433	221.9709	202.9634	275.7417	178.4726	228.163	221.8856	221.6521	184.1934	253.9087	247.2393	204.9812	256.46	275.6746	257.241	231.0435	224.8574	193.5997
CUST_44659_PI429010886	M66592	0.006512745	21	0						40.17351	128.6059	138.9709	61.06467	41.67991	8.818102	47.93427	60.25895	89.44264	49.12192	63.43243	23.99266	62.99459	14.62238	22.3639	22.79379	52.27137	29.72388	114.6414	100.1798	151.3747	102.7497	153.8597	174.4941	51.74535	34.59167	93.40586
CUST_16500_PI429010886	N36341	0.006524352	24	0						47.71964	24.17927	32.50066	38.79677	27.34971	27.37201	36.21981	26.09525	29.87124	26.38383	28.21525	27.28454	40.78815	22.92037	9.515144	15.50564	26.58505	19.69014	21.17352	20.6613	16.30527	31.13164	30.02053	30.77878	39.57166	36.04308	27.03345
CUST_26952_PI429010886	N55176	0.006526666	24	12						24.64466	9.547929	20.77024	15.12219	143.9807	35.95046	NA	17.63035	3.934999	NA	67.76221	64.8708	19.37143	175.182	173.7538	9.941387	15.55385	4.506631	14.80962	5.356052	10.82253	3.870107	11.32723	5.466472	9.231247	6.735712	10.7971
CUST_27818_PI429010886	N56749	0.006533068	12	0						50.42732	54.99763	57.46642	17.58375	43.72166	32.33684	41.89159	25.21148	25.41498	62.91855	27.39507	63.59914	77.33923	36.1335	58.17376	18.43253	19.91267	6.231156	23.29521	21.25166	37.8457	48.15075	23.81296	63.08459	50.25586	9.506985	76.42243
CUST_51210_PI429010886	M92603	0.006537214	18	9						22.04646	8.063144	3.659972	3.880736	NA	14.10469	14.48824	22.94332	20.43703	24.67313	18.05945	12.42007	NA	NA	4.7817	5.322281	8.539795	8.273433	10.01073	6.570223	7.571002	12.2009	11.16309	12.45971	15.35298	8.593041	18.75881
CUST_37374_PI429010886	M18821	0.006541884	24	15	219842400	mitochondrial ATP synthase cou	Metabolic process	Generation of precursor metabolites ar	4.01E-24	4517.506	7821.557	4727.546	3593.994	3611.195	3577.841	7337.918	6448.141	6183.806	4889.847	3992.329	4020.487	4090.127	4432.869	3859.53	7558.705	8006.37	9837.56	4981.65	6780.642	6744.57	3535.101	5826.27	4798.856	5227.74	5659.622	5879.268
CUST_40763_PI429010886	M56033	0.006541884	24	15						215.6541	361.3178	259.2192	240.9716	182.8221	179.8439	364.3837	296.2664	318.037	273.1848	201.7307	259.1236	291.4994	205.6562	226.1282	345.674	444.8216	365.7068	307.1973	265.8681	357.2181	269.8306	349.3011	333.2833	253.8231	302.274	326.9964
CUST_12197_PI429010886	N29089	0.006541884	24	15	321462004	hypothetical protein DAPPUDRA			5.66E-13	533.1871	1412.179	910.9934	544.6702	238.3483	261.0851	1814.798	1026.869	1333.239	1441.945	523.631	684.8522	1100.835	499.5403	664.6847	1909.699	2461.211	2516.066	1321.627	893.7709	1523.69	837.9532	610.864	960.5506	1656.675	839.0592	1419.346
CUST_22726_PI429010886	N47544	0.006542708	24	9						15.89978	28.36166	17.63683	23.27971	27.81736	30.01855	55.33608	39.87751	53.17689	67.0724	22.81449	53.99104	17.02151	26.66393	24.43634	27.24405	81.60481	78.48989	22.95726	46.5095	43.80331	5.834481	23.35729	9.8644	17.58836	13.43959	11.04247
CUST_43721_PI429010886	M63793	0.006551478	21	15	507681436	PREDICTED: galactoside 2-alpha-	Metabolic process	Carbohydrate metabolic process	7.95E-24	18.85311	132.2497	10.86172	45.99785	23.4616	51.16396	158.0297	142.7301	23.85395	162.6193	110.6819	62.69677	164.5889	70.60324	152.7137	157.2569	304.0274	226.9579	38.95566	38.24975	55.42058	59.9023	23.30496	68.43368	164.0224	131.0463	5.877513
CUST_7728_PI429010886	N21981	0.006551478	21	0	307197312	Sodium-dependent neutral amir	Transport	Neurotransmitter transport	9.9E-47	125.1074	204.0335	315.0449	194.1709	172.187	88.05908	127.6821	132.2204	185.8563	79.5082	156.5187	88.80408	158.0297	84.3918	107.9543	80.07305	114.3676	88.6659	196.4115	78.97649	166.5055	186.5169	240.2302	170.6675	138.0734	94.66861	209.1753
CUST_46176_PI429010886	M71681	0.006555278	21	18	321468589	hypothetical protein DAPPUDRA	Signal transduction	Intracellular signal transduction	5.66E-62	10.77956	4.044259	13.61444	3.429579	6.267202	8.459548	NA	NA	5.318595	3.302643	NA	3.833007	7.190374	16.04505	NA	7.423378	8.524647	4.557982	15.51127	12.49687	9.320269	8.91225	3.808097	12.14006	4.035399	NA	7.633196
CUST_9093_PI429010886	N24055	0.006562805	21	9	194452590	Cruzella marina small subunit rit				40.75864	73.85955	18.50635	71.3852	649.314	544.2292	159.3374	127.2978	173.05	169.2749	909.1828	487.7778	44.9217	803.3036	781.034	592.0137	399.0134	75.07482	187.0121	164.0459	195.1701	68.14816	263.8276	72.61063	95.21224	314.367	53.82263
CUST_55122_PI429010886	M108800	0.006564276	24	18						3166.856	5222.203	3175.147	2895.969	2414.182	2514.613	3120.055	3972.222	3313.258	2906.899	2605.771	3056.068	2920.075	2738.485	2692.671	4798.856	4118.017	4998.113	4203.047	3948.157	4857.819	3062.683	3240.436	3568.199	3561.33	3232.565	4203.047
CUST_36673_PI429010886	M16505	0.006564276	24	21						575.9481	337.9981	641.7824	513.9521	247.8341	318.6428	280.7963	467.9385	508.6367	538.9124	323.8056	328.6973	521.086	272.7395	449.8325	446.7799	333.2203	478.9906	666.6396	519.6084	682.7328	744.9105	568.6219	630.7177	531.7033	509.999	763.8013
CUST_39297_PI429010886	M37403	0.006570376	12	9						28.9184	11.57746	42.26958	55.72212	20.9383	21.91645	25.23371	20.26699	33.76496	53.59606	72.4917	55.91798	55.50618	40.80443	44.6225	NA	6.533775	5.204831	20.68785	35.22718	49.48225	37.19414	78.67787	80.47316	16.63236	39.53461	83.59122
CUST_5410_PI429010886	N18663	0.006572992	24	15	443726020	hypothetical protein CAPTEDRAI	Metabolic process	Cellular amino acid metabolic process		218.8639	437.4032	239.0862	241.54	184.7058	247.1999	436.601	462.531	396.4646	367.9854	263.251	192.5776	365.6513	296.9431	281.5287	478.2442	594.2073	445.3005	336.3654	360.3638	393.0106	236.8007	336.1378	285.0355	338.7418	360.5101	259.2692
CUST_1387_PI429010886	N9476	0.006572992	24	15	112420518	Gasterosteus aculeatus clone CF	Metabolic process	Carbohydrate metabolic process	1.41E-98	185.9927	439.3042	168.564	178.3394	117.1085	183.7819	360.6127	343.5695	274.5666	269.5843	234.4037	259.8283	245.5909	205.1165	190.3204	375.4894	452.3252	362.7316	287.6916	247.1999	319.5833	190.3933	189.476	226.5718	382.7119	215.0811	194.5455
CUST_11638_PI429010886	N28162	0.006575745	12	3						50.77134	9.875893	11.87886	NA	NA	NA	41.82858	5.470296	10.80517	3.723982	7.462458	4.954518	12.3846	NA	14.60077	NA	12.61565	8.710796	9.133693	5.768746	11.02468	5.486086	4.872616	5.257454	6.387046	7.313381	8.895556
CUST_1307_PI429010886	N9226	0.006580519	15	0						18910.88	26987.8	82.14842	5476.032	459.8781	4746.115	3608.266	4467.089	1173.789	1082.664	807.865	875.8259	3478.142	1208.338	1806.683	18851.7	11429.91	4284.075	7785.133	5125.495	4710.772	624.767	4234.05	2085.588	1396.628	4750.447	528.6354
CUST_21675_PI429010886	N45635	0.006581025	15	9						13.48819	27.00709	21.95009	13.21801	20.68602	14.97844	18.52564	16.96414	21.79422	22.23218	20.37574	30.21215	18.02157	20.62243	17.63683	29.75777	23.9201	24.97096	14.13842	12.73103	21.28833	23.91545	21.91645	17.50536	32.2704	26.54663	28.99089
CUST_31966_PI429010886	M2853	0.006584803	15	6	321479500	hypothetical protein DAPPUDRA			6.6E-28	373.6591	626.9839	742.1526	571.0817	438.1085	281.5792	541.8648	683.6777	928.898	530.0108	499.2313	478.6398	423.8193	360.4299	221.6521	415.7252	379.9317	438.3717	910.9934	842.9082	1007.681	630.9023	947.0352	902.8628	447.4189	1103.734	1488.548
CUST_25798_PI429010886	N53079	0.006584803	15	12						8.785038	16.03696	7.032137	4.193462	15.75024	11.7948	7.660563	8.886295	10.03828	19.15316	15.44623	17.90158	20.70771	16.88729	36.11092	14.61747	11.00269	11.11975	9.934505	6.379749	9.9869	13.76968	9.045745	17.33337	21.54473	9.122345	11.54756
CUST_5159_PI429010886	N18335	0.006585065	21	18						NA	5.849741	4.278961	NA	NA	5.448429	6.670759	4.982299	6.081959	9.032116	11.93992	NA	5.53049	7.992817	NA	5.937279	9.394115	NA	7.91734	15.00684	28.75654	7.639644	21.06519	5.75072	4.144296	3.858606	6.633834
CUST_14510_PI429010886	N32999	0.006606889	18	12	444709098	Heparan sulfate glucosamine 3-	Metabolic process	Carbohydrate metabolic process	9.9E-16	NA	5.134732	29.28939	12.17645	8.47965	10.34888	7.840494	4.956673	NA	NA	12.62197	6.990911	24.82357	17.2724	13.74572	14.26605	14.23051	12.76017	11.7546	7.982621	21.91017	10.15066	3.776889	13.66341	4.99561	8.25537	NA
CUST_26168_PI429010886	N53790	0.00660816	12	0						56.38972	17.00862	69.07482	25.52339	19.11039	26.39799	34.1983	34.2198	26.06752	35.25014	21.58133	23.73807	97.22863	29.57468	153.0619	20.9383	18.88565	20.76789	25.48923	26.4485	33.31402	27.26773	30.91821	27.32487	38.0933	30.28912	33.82765
CUST_42013_PI429010886	M59149	0.006610729	12	6	190610833	trypsin [Marsupenaeus japonicu	Protein metabolism	Proteolysis	4.79E-32	4.00586	NA	NA	369.3009	NA	262.4813	4339.098	NA	2692.671	636.9918	384.4369	NA	171.0499	316.3525	132.3978	795.5533	NA	745.5832	5277.675	11264.76	20822.29	816.314	NA	778.7232	3441.376	486.0305	2545.232
CUST_11886_PI429010886	N28574	0.00661995	18	6	46240812	crustin-like peptide type 2 [Mars			1.52E-08	322.1601	458.2399	444.5929	868.2894	420.7391	432.6388	603.9154	454.7075	782.6486	730.5469	335.8125	566.6051	813.2524	459.6187	253.1919	235.0418	551.0918	405.2976	305.3135	231.4591	321.0799	523.631	828.1272	979.7526	716.9976	737.8151	1369.757
CUST_20613_PI429010886	N43753	0.006629775	12	9						210.955	NA	190.0902	156.6513	NA	139.3563	8.890942	NA	10.65615	434.9251	1645.987	357.3731	498.5642	15.84358	279.305	NA	NA	3.949314	35.76914	45.84356	58.31116	625.077	328.0523	405.5854	76.71918	369.5842	333.1336
CUST_18803_PI429010886	N40440	0.006629957	18	15	3907620	actin 1 [Penaeus monodon]			2.06E-28	1399.933	1137.334	1377.009	1549.756	686.4707	1327.159	1090.482	1355.196	903.1163	1251.736	881.8202	931.1977	1797.322	1647.856	382.8713	2279.977	2656.978	3017.306	1759.396	1988.355	2803.748	704.142	571.8635	562.1389	2185.802	1572.421	1132.475
CUST_39329_PI429010886	M37907	0.006631645	12	9	220172363	putative articulin p60 [Rimicaris			3.83E-26	26567.15	1101.918	25343.01	26567.15	18026.32	25611.3	3672.992	5840.985	8996.679	32184.55	33508.11	35445.2	39213.8	14363.02	38540.49	312.8962	198.9717	528.772	13193.16	10017.83	13716.26	42496.4	36724.45	45257.85	14885.24	22711.46	36302.66
CUST_25405_PI429010886	N52356	0.006638831	21	3						103.2488	94.54537	84.31911	91.96611	109.4578	88.29653	80.39714	75.77805	61.55774	97.6745	57.18737	75.58044	105.2156	74.63974	84.2744	91.6852	74.93498	86.34178	83.73063	80.43757	63.71474	104.4489	96.25473	105.7248	105.074	112.8927	125.7784
CUST_1020_PI429010886	N8241	0.006650321	24	3						372.873	72.06126	355.324	573.4698	822.3417	835.7807	147.1665	230.6071	210.991	420.9575	834.0907	594.8478	370.5351	441.1218	578.8472	58.86475	29.07923	32.34672	235.4202	292.2917	221.1265	382.5381	505.8465	516.2255	75.67783	655.6894	240.603
CUST_51534_PI429010886	M93883	0.006650648	24	3	444299464	dual oxidase [Marsupenaeus jap			1.27E-22	199.6427	93.70997	113.0417	97.61964	100.264	135.0936	98.01972	75.37573	90.96558	110.4251	75.97892	139.2612	85.37067	66.09101	98.27861	82.63781	72.32818	65.20578	84.96211	54.2431	72.21507	94.82698	67.23484	82.16147	111.9766	95.68309	69.82715
CUST_23403_PI429010886	N48778	0.006650648	21	0						15.10046	19.56045	20.84673	4.248874	9.991081	16.90983	15.62701	11.26701	13.48819	7.658856	14.62836	8.740614	8.376444	14.15358	17.94168	15.61539	14.28509	20.27243	19.19097	8.870244	19.1752	23.38714	12.44156	19.25145	15.69763	19.19457	17.39841
CUST_42969_PI429010886	M61751	0.006662628	18	12						10.55458	17.4076	17.25784	6.734835	11.08188	6.823514	15.57359	8.306588	14.86691	NA	23.08008	12.19342	21.25376	17.65126	17.48796	14.64829	18.47386	17.19316	20.38818	16.83166	15.5945	12.12405	11.33295	17.19146	15.47982	8.949447	23.21157
CUST_57171_PI429010886	E1939	0.006672991	24	15	225443760	PREDICTED: 60S ribosomal prote	Protein metabolism	Ribosome biogenesis	2.22E-14	19572.59	28210.59	21398.95	17524.49	16070.74	15767.9	22946.38	24341.41	14256.4	19150.48	16288.46	16252.77	15798.02	16726.21	17254.51	28306.31	25611.3	29396.88	24659.23	24289.54	29725.44	18522.28	20708.31	21834.29	24659.23	21459.8	23231.4
CUST_26738_PI429010886	N54792	0.006675432	12	3						12.59667	14.22159	12.27496	10.26132	11.21622	14.18007	13.0479	15.85928	13.82118	7.423378	8.43105	10.08816	13.40297	8.852359	13.44969	15.29607	13.69996	18.01552	10.26132	NA	12.26816	8.750703	10.21029	11.05247	12.939	10.70051	17.14108
CUST_30957_PI429010886	M305	0.006684722	24	18						36.07467	52.55424	57.0448	33.51959	38.293	20.32715	39.31361	44.85722	47.42168	36.17395	42.53392	49.16853	40.76292	33.71712	43.70175	44.55279	59.84299	63.13263	56.4057	47.84693	71.21793	49.43562	52.97194	56.35449	53.1876	38.98881	56.67374
CUST_21998_PI429010886	N46236	0.006684722	24	21	260836805	hypothetical protein BRAFLDRAI	Metabolic process	Oxidation-reduction process	5.45E-28	297.1434	306.3603	314.4452	270.8095	233.5705	228.29	179.7937	237.4902	240.8917	318.037	260.2442	102.6964	345.1684	259.0205	326.4068	368.3282	290.4456	347.9094	311.1315	283.1093	244.7879	424.2083	275.9447	395.5893	423.6	257.3491	417.3805
CUST_28038_PI429010886	N57158	0.006684722	24	21						87.9342	63.75599	88.12342	97.71482	66.08255	78.67787	80.52797	55.40345	57.11305	81.35177	62.05756	74.83312	84.1875	62.16088	74.56881	95.21224	67.61402	73.50942	121.0114	80.41026	94.06019	117.5273	82.21745	110.2733	126.5797	103.2365	78.3065
CUST_32953_PI429010886	M5551	0.006685744	15	3			Metabolic process	Generation of precursor metabolites ar		180.2649	414.6538	225.4359	190.3661	284.0207	214.0358	209.7702	385.9551	222.5585	178.576	205.4207	106.1914	116.0445	235.5705	136.3513	245.4997	369.3009	371.2053	245.6327	372.9744	447.1876	128.9165	224.0178	200.6783	181.0227	166.1819	182.5355

Probe ID	ID	p-value	Period	Phase	EBI ID	Annotation	GO category	Go term	E-value	0	0	0	3	3	3	6	6	6	9	9	9	12	12	12	15	15	15	18	18	18	21	21	21	24	24	24
CUST_27566_PI429010886	N56285	0.006686572	12	6						4.903455	8.542873	6.420652	12.08298	12.84299	7.141342	4.26876	14.64332	12.1921	11.98443	2.954576	10.36406	5.460737	NA	5.140584	12.12627	10.20394	8.041645	18.33289	NA	14.2562	11.13493	7.717206	9.398999	9.313657	4.38328	9.568695
CUST_47810_PI429010886	M77692	0.00668783	24	0						33.51959	35.43409	25.41498	34.92878	17.62607	23.90042	59.69831	27.702	29.99314	22.37507	16.73896	19.33949	15.82642	19.30987	10.18329	21.03874	25.27502	22.97146	16.16065	14.91026	17.32449	18.70388	28.53464	17.4076	25.13996	25.85498	24.51492
CUST_57286_PI429010886	E4164	0.006690377	12	6	461857	RecName: Full=Chymotrypsin BI	Protein metabolism	Proteolysis	1.07E-45	NA	NA	4.756073	71.18779	NA	46.57945	599.566	NA	412.5149	115.2129	64.01323	NA	19.26106	48.22901	20.37769	87.29938	NA	87.61045	435.8605	1711.15	2696.535	144.8287	NA	117.7735	486.0305	69.62329	341.0171
CUST_52731_PI429010886	M98696	0.006703027	21	9						7.545184	5.627759	7.035061	13.01662	10.88552	7.702073	14.76151	14.01651	14.27002	14.84471	12.14425	15.62926	15.13397	6.588475	12.9826	12.54285	14.81286	9.295776	10.53196	10.83233	10.71849	17.05355	9.628869	7.511244	10.49595	7.552026	18.15454
CUST_46947_PI429010886	M74264	0.006703412	18	12						34.75696	33.56251	39.26035	50.10507	34.65363	42.0926	48.12354	38.07838	41.33036	53.14759	39.21711	38.33655	57.10009	40.2252	47.03106	46.68552	43.16345	55.49194	33.0209	23.49323	27.56589	47.10689	36.74386	32.96215	50.47099	40.50202	42.78489
CUST_9937_PI429010886	N25439	0.006704696	24	15						12.99289	11.33539	NA	5.342246	8.137599	6.666981	5.2497	12.51148	6.848153	77.9206	18.75482	13.29816	19.92667	13.40595	18.86536	23.02558	22.20755	11.988	14.45063	6.721195	11.73655	26.00178	11.03403	15.47146	NA	8.49758	15.24733
CUST_54533_PI429010886	M106412	0.006707516	18	9	260810893	hypothetical protein BRAFLDRA			0.000000299	18.89336	12.96468	7.234885	5.272947	NA	4.249699	NA	9.710211	NA	NA	15.97652	14.24201	9.874092	12.95701	NA	7.343689	5.491771	10.16018	NA	9.342102	10.14167	5.489262	4.861279	NA	5.35265	10.46773	11.0285
CUST_29009_PI429010886	N59020	0.006716831	24	3						609.6218	44.26795	501.7792	963.73	1181.431	1143.807	37.86516	158.5618	34.69476	318.6428	1559.979	1276.272	195.8396	899.2992	806.2686	7.391099	NA	14.14657	26.54115	180.0776	22.75502	332.1692	191.082	266.8703	26.58017	311.9509	42.36924
CUST_1998_PI429010886	N11270	0.006719499	18	9						11.9671	16.20707	17.91133	21.03435	25.43349	21.21931	25.24183	17.93358	18.63267	27.00456	21.44902	28.83489	11.32969	19.01144	12.91017	18.51168	13.85499	18.02767	NA	10.83531	9.720534	21.62227	23.03273	6.217149	25.51773	8.687501	24.32178
CUST_1942_PI429010886	N11131	0.006720414	12	0	321475659	hypothetical protein DAPPUDRA			3.42E-15	222.7248	215.1114	243.0739	246.356	158.5985	174.4941	125.4154	136.7201	147.9113	262.0968	161.586	57.8261	310.0635	173.332	267.2693	315.8899	211.9713	193.7362	170.6985	168.9237	152.3312	419.5373	177.0792	394.6878	340.5595	217.5068	354.4742
CUST_8849_PI429010886	N23693	0.006720414	24	15						678.5721	898.791	806.5559	684.4289	571.9784	465.6755	991.5499	799.0901	1068.554	981.9828	579.9447	847.8182	1039.627	651.369	719.4386	952.6113	1377.009	1039.627	1086.19	881.8202	1190.571	879.8906	848.3549	905.7144	956.8208	797.7145	1099.075
CUST_47303_PI429010886	M75536	0.006720471	18	0						1818.148	2891.912	2883.204	2057.214	2188.815	2308.81	1900.457	2795.798	2757.04	1687.279	1701.993	1697.826	1369.757	1814.798	1573.048	3177.941	2447.506	3796.503	2906.899	2570.156	2805.21	1826.319	2075.256	1902.67	2422.76	2041.036	2422.76
CUST_38020_PI429010886	M22805	0.006726184	21	0						944.1713	355.841	1167.815	1093.904	163.9723	820.6216	236.4795	433.4692	296.8179	321.0799	766.804	296.0318	367.9854	464.4221	449.7223	132.3768	225.4359	157.9135	292.6648	348.6137	338.5986	629.8536	908.7057	782.4302	174.3818	665.3418	660.4277
CUST_37064_PI429010886	M17750	0.006732503	18	3						10.98054	15.4541	18.97777	15.58431	25.52339	22.56387	205.5946	9.236157	14.33334	18.53553	12.66963	24.96583	20.36911	10.38947	15.36855	12.8517	12.27699	8.725887	13.6023	16.23526	11.29632	23.13824	19.35535	25.12586	22.49527	13.67378	38.02572
CUST_4185_PI429010886	N16187	0.00673702	24	21						119.1547	101.6806	133.3046	117.5696	71.75645	93.76286	111.7284	64.2086	97.28384	117.0413	98.19279	69.09764	111.5466	80.57219	82.84289	121.5461	91.46995	117.123	141.753	99.03286	152.1914	151.9667	130.0637	121.1643	159.8296	82.85507	175.0698
CUST_12227_PI429010886	N29136	0.00673702	24	18						6.064642	23.19965	26.88183	16.17305	16.46833	9.562348	16.91535	16.48073	29.7549	25.04161	7.810134	22.45228	25.99892	11.48755	30.865	34.27395	34.4378	30.14227	36.43974	18.08746	35.47671	18.84709	19.00338	24.58795	28.64933	11.95232	35.27117
CUST_20424_PI429010886	N43412	0.00673702	21	9						11.51019	20.14533	20.43895	24.50769	28.75045	21.43518	19.25145	26.53236	23.9895	17.41782	26.72777	34.79145	27.18663	25.21673	11.72201	23.36033	28.94999	21.04513	12.32348	22.99386	19.93149	9.466716	11.66554	16.40975	21.45886	27.24917	17.01732
CUST_46288_PI429010886	M72080	0.006737174	21	6						44.54327	47.29636	47.01945	109.7929	91.94456	71.35943	120.0493	133.7779	94.17512	101.8124	61.85717	92.65597	112.1553	66.02134	126.7896	92.75983	101.1544	141.9148	81.62587	45.01222	62.16088	97.58001	49.3138	76.30002	98.08719	64.17487	95.29319
CUST_33135_PI429010886	M6056	0.006746019	12	0	521028669	Zinc finger protein 2 like protein			1.78E-09	21.41005	13.51154	15.69371	14.07152	20.82813	14.08999	12.59667	NA	13.46381	12.07145	12.3345	17.75202	24.34766	19.22503	21.54473	13.04163	20.41235	12.2337	16.34777	18.1573	11.66289	12.04777	16.55821	10.73646	21.77362	15.90885	20.96118
CUST_36319_PI429010886	M15574	0.006755351	21	15						230.1554	425.957	305.9094	229.2847	253.1919	247.8796	323.9561	357.7047	315.4118	330.2787	237.7581	265.8169	283.0383	248.4015	281.5792	349.3011	353.9275	399.4473	294.0126	276.0746	297.0848	261.5986	293.0253	263.893	252.401	246.0227	285.6412
CUST_42799_PI429010886	M61285	0.006758878	21	6						12.02167	47.24767	26.14108	28.34234	58.8025	39.39293	43.93243	63.69892	27.32212	20.30731	53.39229	38.29909	20.95678	43.28585	36.36056	31.01077	35.49229	19.89174	28.93482	12.60871	16.16198	28.46151	27.40129	20.81559	34.07747	59.47135	38.32469
CUST_35315_PI429010886	M12227	0.006763167	24	21	390334981	PREDICTED: cytochrome c-type	Metabolic process	Other	0.000000875	21.87746	34.25309	65.15059	19.07007	35.36937	33.00869	19.09012	33.48437	34.32863	20.03497	26.08093	30.85737	27.18421	30.55784	24.00156	28.00193	270.3715	38.19894	38.86376	40.73438	41.75149	46.33666	34.85943	46.94606	19.82888	44.85324	38.48962
CUST_31120_PI429010886	M739	0.006763167	12	0	291231880	PREDICTED: predicted protein-li			1.48E-41	189.9176	143.0807	171.8869	132.2204	104.0048	116.2897	103.07	120.1351	123.5107	114.5333	155.7673	135.8001	153.8823	197.088	229.8852	158.7443	131.8067	126.8236	183.3606	166.2655	180.9438	168.4596	135.8001	222.3298	170.3943	171.722	295.3036
CUST_12461_PI429010886	N29497	0.006772679	24	18						298.439	732.7914	650.5235	357.9365	268.5828	259.8283	521.3103	471.7045	743.6336	537.8527	356.3573	452.9256	538.4885	403.4693	319.1468	552.4266	613.0157	642.342	559.5956	713.6664	729.7963	633.2997	517.3443	799.983	552.2424	535.89	645.1212
CUST_26827_PI429010886	N54951	0.006772679	24	6						124.4425	108.623	42.67003	67.22535	110.2026	96.84289	144.1943	73.47778	103.4024	54.99013	89.74912	99.03286	76.7295	146.5049	197.1967	81.79336	60.0126	47.93427	76.53128	50.34979	53.86055	45.60384	53.85584	21.42827	41.10799	70.44138	32.85935
CUST_3150_PI429010886	N13901	0.006784097	12	3			Protein metabolism	Proteolysis		82.79094	79.82778	61.45632	57.66836	99.13779	70.14108	41.69494	68.01679	56.68583	35.48951	67.3848	76.3955	50.60801	38.56693	19.37143	114.4474	80.9995	92.77365	66.74201	91.08467	57.16525	23.19651	21.44902	22.38325	62.14225	82.70695	51.59532
CUST_323_PI429010886	N3890	0.006784097	12	9						3182.56	1280.738	2570.156	3071.483	512.3561	2031.77	812.2115	882.5044	806.2686	2595.878	1713.82	2140.692	1622.787	1441.224	1852.945	628.502	829.1725	374.7006	1938.127	1393.298	1654.651	2626.057	3558.043	3467.123	1025.772	652.3046	1328.27
CUST_38196_PI429010886	M24045	0.006787744	24	15	514765630	PREDICTED: astacin-like metallo			0.000000171	83.10688	35.48599	31.9432	33.05798	3.638334	34.83345	241.9195	46.42038	224.1479	208.2068	113.6801	25.30919	89.69199	58.64981	44.26795	514.8454	138.6089	353.8605	103.0482	55.19896	136.8772	193.6659	NA	163.9723	144.849	34.62127	95.16242
CUST_16275_PI429010886	N35954	0.006789992	18	15						126.6732	169.8611	180.0776	184.7904	129.9594	165.2368	187.5751	174.8981	186.9781	192.3394	152.9126	173.3886	205.3015	160.7833	193.1329	212.2553	225.6648	230.649	192.8968	134.8435	190.7291	152.8076	171.6359	167.2859	209.1753	177.4557	196.3832
CUST_43037_PI429010886	M61923	0.006795853	24	3						2702.508	23.80052	2559.917	3862.287	3525.214	4027.931	82.29248	107.2144	117.6125	3522.391	4242.57	3640.441	3122.414	2168.846	3604.261	NA	8.790565	18.38125	59.43294	125.8592	132.2851	2018.868	2383.479	2491.674	296.0318	280.4572	520.4605
CUST_14084_PI429010886	N32303	0.006803223	24	0						68.62585	180.4963	172.783	68.54812	55.43882	93.03552	27.00266	278.374	276.1256	43.92787	28.67614	28.47679	29.7085	20.71687	48.69844	17.82084	68.77617	31.48421	148.5106	287.9808	396.9028	132.1106	61.67659	76.8759	469.2787	57.12065	414.6538
CUST_45048_PI429010886	M67846	0.006804102	24	3						NA	NA	20.87837	22.54343	136.6003	68.19401	9.03778	11.23132	NA	5.925916	64.71994	62.14833	9.34334	93.12448	60.64674	4.324178	3.919198	5.486086	9.960257	10.28202	8.969422	7.822887	7.665823	21.01951	NA	10.54562	6.140611
CUST_23817_PI429010886	N49523	0.006805872	24	0						162.5583	124.5835	179.7937	153.3857	116.981	132.4791	130.6442	99.09115	137.8056	158.9424	128.9945	118.3083	124.9582	104.6457	102.1603	127.8235	122.3346	101.6524	170.8635	103.1504	129.7428	180.3409	123.7474	127.1629	176.2041	148.9789	163.8654
CUST_17284_PI429010886	N37708	0.006809627	12	0						NA	10.15886	11.69458	3.425929	12.32661	10.75824	6.100675	7.371989	10.46343	8.604047	10.12252	11.81841	12.90521	16.16366	8.306588	9.214634	11.15823	7.822887	7.180584	7.511244	6.89548	9.042493	6.883456	9.585902	11.07529	9.522507	13.28567
CUST_422_PI429010886	N5133	0.006819053	21	18	220172371	putative elastin a [Rimicaris exo			7.65E-13	15900.37	32639.19	36571.4	16070.74	11612.18	596.5924	18435.82	33963.44	48559.97	10605.81	11356.17	7484.678	16726.21	8098.961	4159.327	12921.27	14858.64	14858.64	40915.77	39213.8	51875.49	20172.23	31659.39	31435.61	31801.71	26803.66	51269.31
CUST_5194_PI429010886	N18385	0.006823392	24	18			Metabolic process	Oxidation-reduction process		159.8538	154.4681	143.2044	140.038	102.722	117.3668	203.1024	108.5274	117.7279	166.4755	124.4898	152.4355	190.5906	123.988	190.6927	219.8774	128.1605	225.7754	247.4344	146.1482	189.7104	164.4172	130.6442	195.5794	141.2321	119.8257	202.7406
CUST_243_PI429010886	N2980	0.006823392	24	15						2948.008	3310.069	2668.728	2952.044	1708.901	2211.381	2893.96	2757.04	2710.483	3349.165	1708.051	2721.255	3145.521	2412.569	2284.471	4450.275	3929.52	3767.349	3956.525	2785.011	3463.912	3039.361	3056.068	2862.188	3401.586	2891.912	3011.975
CUST_20377_PI429010886	N43336	0.006823392	21	18						19.29052	14.82383	29.7085	27.75337	11.48486	18.52205	10.07278	15.47714	14.47477	24.29464	12.3901	20.77024	30.92873	17.68234	15.88272	30.89131	17.90158	19.6327	28.35837	27.88249	34.93951	19.92002	7.565346	32.60595	23.14702	13.84795	26.16165
CUST_22172_PI429010886	N46564	0.006823775	12	9	260831342	hypothetical protein BRAFLDRA	Metabolic process	Oxidation-reduction process	1.92E-34	7.684798	7.088641	12.62197	8.654198	10.89235	5.213865	9.820162	8.737286	5.858162	10.9534	9.877137	12.95419	13.51154	7.657844	10.49414	4.446328	4.378175	8.147248	13.13097	NA	4.550378	20.69888	12.18599	15.86706	9.787245	5.01999	8.282821
CUST_54063_PI429010886	M104333	0.006827445	21	0						30.73939	NA	37.95004	47.45455	16.02076	34.14921	3.308738	7.792211	19.40949	23.62689	15.69371	24.14234	33.73205	10.01392	32.66605	3.102376	NA	4.193462	17.10366	8.581038	14.27815	73.62565	40.68416	57.02931	22.62211	11.97009	27.73866
CUST_3656_PI429010886	N14974	0.006827714	24	9	532759720	antimicrobial peptide type 1 pre			1.16E-18	51.49597	139.095	108.8788	144.8704	60.43767	98.2265	249.928	93.24932	284.5651	333.4754	151.3342	280.3649	155.7969	160.139	48.52901	46.13557	100.2386	97.70277	35.56153	52.79302	46.92175	18.1802	188.4673	157.6398	7.518583	124.1767	57.00268
CUST_9941_PI429010886	N25444	0.006828983	24	21						644.0162	586.5026	656.0648	522.858	403.9297	401.9784	412.3443	429.4693	443.0968	606.5345	521.7908	596.1129	586.8713	506.8228	625.2657	603.562	562.954	539.7737	631.6446	563.6439	487.5393	712.9201	565.179	703.9211	594.8478	508.0836	792.599
CUST_36754_PI429010886	M16756	0.006833439	12	3						35.51402	45.29856	57.70575	71.56582	89.01154	75.66353	39.82654	77.18489	58.80984	46.65753	56.36738	58.71759	31.84584	57.37441	51.23629	67.41125	98.36388	77.62481	57.7947	53.35547	46.13169	68.62585	51.79467	63.20291	55.6719	62.41935	53.13079
CUST_9704_PI429010886	N25071	0.006833869	24	6	260792635	hypothetical protein BRAFLDRA			1.52E-31	401.9784	1249.272	1080.452	353.7833	376.7239	330.8653	532.4935	842.9082	928.2282	405.1953	557.8175	512.4944	149.6649	172.0135	177.05	123.935	228.0375	136.1879	305.5103	421.5919	401.757	332.3383	276.6685	317.4998	258.5192	218.6641	345.4356
CUST_37049_PI429010886	M17683	0.006841839	18	9	157107416	n-acetylgalactosaminyltransfera	Metabolic process	Carbohydrate metabolic process	4.13E-48	44.58117	69.03139	49.46595	59.27468	70.5683	58.68809	85.13676	74.42622	111.5887	108.0685	67.37607	68.95176	75.40109	58.4887	53.97594	85.12597	121.9689	106.506	53.58837	34.37539	44.86788	69.55079	71.79771	49.23192	76.00489	62.7253	62.7253
CUST_47937_PI429010886	M78231	0.006841839	24	6						33.18047	24.66557	26.01047	24.32178	16.89615	22.21344	68.20885	54.56863	39.04354	62.68994	21.10532	26.24091	24.28357	19.05953	12.81539	26.0084	97.65071	19.22794	14.75102	15.11599	10.02506	34.32507	19.19457	21.71688	21.58992	34.57525	17.13272
CUST_40861_PI429010886	M56245	0.006860322	12	3						73.57016	88.6659	93.21779	81.08936	116.4561	102.2878	75.42049	114.8835	100.8115	82.87384	82.41127	80.98106	113.469	84.80932	90.3392	124.4273	112.6997	114.1426	91.45416	107.4885	107.424	80.95294	85.23471	95.90246	101.0722	97.66082	95.83012
CUST_8908_PI429010886	N23779	0.006860322	12	3	345482651	PREDICTED: LOW QUALITY PROT			6.16E-27	59.25874	63.4481	50.97745	92.83981	74.14723	90.15514	62.42709	60.27161	39.84392	64.33747	49.4765	52.22249	47.85099	57.66115	62.5403	93.46078	92.17501	67.40377	54.62717	30.25225	49.38313	54.90137	40.78067	40.46925	42.3639	81.53976	24.54963
CUST_39692_PI429010886	M42973	0.006860636	24	3	7008005	DD9A [Marsupenaeus japonicus			8.15E-09	5251.996	4513.062	4650.166	4445.119	4829.887	4522.071	3843.133	4470.918	3963.836	5039.165	4130.841	4569.485	4517.506	3886.966	4809.493	3101.358	3076.623	3702.472	2412.569	4109.069	2710.483	4339.098	3480.931	3382.058	3452.681	4253.847	4527.493
CUST_51400_PI429010886	M93394	0.006860636	24	21						183.7151	151.3342	189.1965	158.2614	127.9261	148.2715	129.3339	136.3915	159.6186	182.6769	151.0875	175.2702	144.3231	142.4472	152.3742	194.2665	173.9842	201.86	214.7361	155.2643	197.7325	187.773	184.5623	215.7105	235.5705	139.4798	242.2514
CUST_2676_PI429010886	N12887	0.006860636	24	21						525.5146	967.3181	684.4289	544.2292	487.1319	515.4837	444.046	629.6411	620.0365	579.318	667.0187	604.3308	589.5468	608.4831	613.1951	653.3386	834.7743	778.454	615.4627	709.2377	677.4857	730.5469	733.7832	893.2381	755.8283	620.2559	932.6252
CUST_10902_PI429010886	N26966	0.006860636	24	21						53.95129	63.06627	43.66107	47.78312	60.13944	50.97241	58.6281	42.45638	46.47246	63.76387	54.42434	48.62526	65.87993	56.12959	67.44859	67.88505	58.72446	71.98615	70.99538	48.77948	57.8894	72.96184	59.08609	71.56582	51.83708	67.60668	56.84633
CUST_17318_PI429010886	N37766	0.006860636	24	21						48.92244	59.05616	35.72862	21.57697	30.06892	26.13567	23.31843	28.551	28.49131	38.24975	41.92055	40.56201	38.17039	27.17222	34.88562	39.93436	62.50267	40.85573	40.99989	40.14624	31.80176	42.83599	47.10201	53.70551	56.04246	39.02298	60.68646
CUST_35966_PI429010886	M14137	0.006871379	24	15	241999578	thrombospondin, putative [Ixod	Cellular Process	Cell adhesion	1.81E-66	96.22172	153.1826	156.6513	111.894	226.5718	148.4289	79.22393	254.5783	275.85	204.9422	310.6389	171.2412	111.5731	313.0953	386.6278	252.1189	348.7073	422.9545	80.94269	253.8231	141.1702	149.533	108.1711	83.95829	62.21897	130.5163	91.88292
CUST_13525_PI429010886	N31326	0.006874874	24	3						519.2716	66.91624	576.7675	759.5695	1732.526	1393.298	58.12065	128.0075	53.53717	303.1234	981.1472	809.2125	170.8635	552.2424	569.4689	13.24735	7.094278	NA	7.155078	129.2198	14.26774	422.9545	95.97338	336.1378	15.71	101.5466	29.36452
CUST_3501_PI429010886	N14631	0.006875294	18	0	504163658	PREDICTED: D-dopachrome dec			1.07E-13	138.0571	127.3506	204.3287	97.66082	123.5754	117.5817	138.2078	167.4695	228.163	77.93692	130.9169	115.4156	93.96854	61.67047	69.64615	75.14729	123.5457	97.25517	162.3101	211.1406	308.5071	113.5495	153.2663	84.19786	118.6368	92.68642	136.1215
CUST_14724_PI429010886	N33356	0.006875294	18	6	443689529	hypothetical protein CAPTEDRA			1.44E-35	50.52783	85.38009	58.16145	73.99941	72.4004	59.07254	72.10171	97.05434	81.33153	79.35979	62.92312	62.81204	56.46685	56.01078	52.07927	77.29301	85.07992	72.70716	73.73506	70.63954	79.33651	61.83411	89.36499	100.6344	133.3487	90.28649	157.9287
CUST_48170_PI429010886	M79210	0.006876703	24	3						14580.94	64.37978	11138.2	18026.32	16183.31	18613.15	55.28196	348.3823	50.8307	3528.773	8886.515	10090.54	2096.347	11805.59	12699.11	12.12132	6.364152	35.04644	11.66711	394.8687	10.56444	7750.118	335.3952	6461.658	9.733163	869.9307	53.02947
CUST_40895_PI429010886	M56317	0.006882184	18	15	321479210	hypothetical protein DAPPUDRA			6.41E-12	NA	16.95396	9.196346	6.352714	6.664444	4.029299	NA	8.189998	6.520876	NA	8.26064	11.99886	NA	9.370822	9.462955	19.25364	5.79988	12.70605	6.908019	9.633908	9.843601	7.877193	NA	5.49503	10.66953	7.230351	NA
CUST_54985_PI429010886	M108332	0.006891621	24	0						2466.158	2652.405	NA	517.1871	241.7527	550.4921	4928.217	8348.895	3499.526	7.350928	34.15266	30.23961	31.26158	1175.329	32.04908	1345.742	638.6943	356.871	842.2205	7459.987	837.2458	NA	3217.866	NA	NA	6226.747	NA
CUST_53034_PI429010886	M99822	0.006896418	24	0						116.1822	106.0541	109.4907	123.1444	100.3377	95.57738	113.8069	84.34054	104.5741	95.86932	93.89302	88.24057	104.7064	68.9724	83.18277	100.4112	72.94835	94.64399	79.28417	102.1483	68.78391	104.1325	102.3362	83.4815	109.8814	110.5024	91.09751
CUST_56750_PI429010886	M114574	0.006902155	24	0						152.9408	67.49593	93.07316	96.53451	78.956	82.45499	105.0886	64.27006	77.8809	118.5815	72.81177	45.10104	67.88505	70.29674	78.04556	61.295	54.28814	66.53367	72.0781	85.7431	62.85008	107.7788	73.64351	78.02817	107.2724	71.13619	107.9218
CUST_4695_PI429010886	N17291	0.006903075	18	3	444726426	Zinc finger protein 84 [Tupaia ch	Nucleic acid metabolism	Regulation of gene expression	4.58E-12	15.73083	24.88093	21.1337	NA	25.99892	21.15337	16.67154	30.22132	18.79511	25.29614	10.49414	17.17357	17.14108	15.96403	18.97529	19.12392	13.89283	15.29086	29.53009	16.42131	17.01395	18.8838	32.63017	33.6843	24.01242	21.51378	26.31588
CUST_42825_PI429010886	M61358	0.006908531	12	0						59.03042	54.1566	36.50656	50.50655	47.21493	48.56908	54.2724	61.57198	48.18114	18.17826	25.71381	30.59603	49.45288	107.0748	53.57855	52.43098	45.25376	22.86182	48.24507	50.15161	38.01559	30.47714	46.26381	24.17927	59.38272	70.81536	48.33387
CUST_56190_PI429010886	M113060	0.006909764	24	15						110.3077	120.7183	101.9686	184.1477	48.47854	110.8634	291.6767	189.3285	280.4189	513.8347	230.2055	60.76642	212.7624	190.553	114.4277	680.9887	249.1748	491.5182	269.7799	264.2234	356.6056	361.3178	74.89382	261.4074	239.0862	45.7369	155.8635
CUST_39464_PI429010886	M39822	0.006909764	24	3	13898841	CUB-serine protease [Panulirus	Protein metabolism	Proteolysis	3.01E-18	94.59748	312.4297	117.3668	137.0649	320.7176	318.5699	138.7794	285.6824	288.4188	91.75455	388.566	131.3179	105.1207	203.5459	95.12721	43.59878	391.4746	171.8413	89.50979	88.50381	78.07876	93.56602	180.676	110.2359	79.88277	279.1333	206.6626
CUST_42031_PI429010886	M59201	0.006909764	24	0						211.8418	315.18	368.2385	181.3844	263.4946	262.8559	187.773	250.9068	362.5984	208.3375	255.4282	218.9179	233.0433	276.4994	264.4742	347.5429	288.3603	288.4188	367.2975	226.4976	308.5661	390.6122	272.2877	319.8028	451.0149	298.1868	440.9544
CUST_45797_PI429010886	M70323	0.006910551	21	3						14.17513	12.55015	17.89355	43.6037	37.47176	69.49719	16.41382	27.37887	47.47767	35.64607	33.4688	34.62846	27.1592	16.38984	25.67959	9.388241	19.81371	15.17119	40.97117	20.64275	NA	26.77351	58.6281	15.82642	27.31739	32.88452	NA
CUST_34546_PI429010886	M9982	0.006912527	24	15	321469961	hypothetical protein DAPPUDRA	Metabolic process	Cellular amino acid metabolic process	5.26E-54	43.69757	103.1504	63.23529	39.64997	45.29856	38.9642	57.781	56.18757	54.6004	58.66126	42.50386	52.19714	74.57856	61.87143	76.2209	59.78407	65.36387	76.01297	57.25175	49.76639	77.69968	53.73347	60.61437	65.56898	56.71352	59.92793	58.78081
CUST_8138_PI429010886	N22590	0.006916534	21	0	321462691	hypothetical protein DAPPUDRA	Metabolic process	Lipid metabolic process	1.44E-86	82.51294	76.54043	102.3977	67.51408	68.03257	82.95322	37.23401	78.518	60.53935	73.77771	62.43555	52.17564	88.94466	53.47447	66.70374	92.49079	71.04436	73.39823	73.07238	70.05016	70.79864	89.36499	74.87652	88.93364	79.1244	49.68059	75.62098
CUST_9646_PI429010886	N24981	0.006916955	18	15						55.20546	118.0123	78.74455	19.68364	36.12473	28.3093	40.91214	88.92336	79.37901	29.93607	65.84893	27.39273	89.81747	64.7701	32.57817	63.41449	140.2426	186.9529	118.4437	120.4309	117.4524	21.86212	35.63867	32.16999	92.30682	44.26795	67.2518
CUST_45506_PI429010886	M69323	0.006923788	24	9						25.98862	20.67357	24.80622	74.78378	29.6976	40.68416	41.46642	18.67654	25.76854	52.6416	40.2126	31.5432	27.10746	42.80977	32.38938	14.79252	19.29579	25.7107	12.51522	17.90791	23.03897	20.47903	31.85157	19.53794	15.13397	16.84207	22.57092
CUST_49084_PI429010886	M84003	0.006923788	24	12						118.7327	54.89599	140.038	98.96283	41.31086	44.49549	189.1039	44.26795	68.98955	110.3861	104.6237	165.5949	136.4064	174.0791	192.1306	133.7339	131.2891	65.16557	185.1715	105.1748	139.4089	170.6411	128.6059	131.8934	133.3824	107.8755	99.04563
CUST_8140_PI429010886	N22593	0.006936087	15	12	260798408	hypothetical protein BRAFLDRA			7.02E-41	99.47239	142.1948	131.4985	78.67787	85.01861	81.57806	105.9605	102.1603	107.5363	166.9271	118.4103	105.619	144.3651	92.18916	118.7327	143.1878	104.8945	131.8269	98.19279	59.39503	94.27306	112.7999	74.75403	87.96067	131.8408	72.74057	105.7387
CUST_42360_PI429010886	M60095	0.006936548	12	3	260811974	hypothetical protein BRAFLDRA			3.95E-17	121.0405	86.22741	102.2993	98.81307	139.5903	121.8844	125.4991	92.28753	115.6114	91.04847	72.30147	84.93839	74.94714	113.2581	100.315	143.5029	101.0612	131.6618	92.52233	90.78766	68.46482	90.15514	120.513	82.7985	119.5667	87.71525	93.34171
CUST_22452_PI429010886	N47057	0.006937148	12	0						335.4857	NA	239.0006	136.1879	193.6659	98.32351	11.65752	NA	18.82754	116.4413	234.2807	94.90029	234.9137	64.82247	248.6699	NA	22.39833	NA	23.26403	18.39792	30.88831	142.9612	233.0433	62.62916	37.38294	68.21622	168.3685
CUST_35205_PI429010886	M11895	0.006942501	21	6						41.71225	160.1694	102.0887	111.4813	155.2093	124.3072	106.1339	194.2665	188.8608	114.3521	145.8127	96.40725	82.23633	78.23111	57.7126	61.08411	131.568	86.55254	71.42098	35.35085	37.40246	160.5173	161.5143	108.2818	137.1639	79.93916	136.4791
CUST_38698_PI429010886	M29190	0.006943518	24	12			Symbiosis, encompassin	Other		65.25923	156.5986	130.4587	321.5516	153.8823	273.7185	297.9948	366.7915	509.1116	83.14687	1278.279	126.7896	254.533	683.1382	301.9582	80.09123	600.2455	261.7118	69.50462	250.8083	120.4647	41.18704	169.5621	122.7895	24.75929	100.9354	78.16561
CUST_17772_PI429010886	N38565	0.006943518	24	18						98.478	92.05562	138.8888	93.20756	87.39589	53.52013	86.90266	64.65354	126.8065	82.77125	83.38925	96.62082	119.0449	75.0097	85.20409	101.4339	93.33147	104.203	165.6517	124.3455	244.9453	114.0896	126.6578	170.9358	186.6982	74.32824	236.3014
CUST_53431_PI429010886	M101679	0.006944671	18	3			Response to stress	Defence responce		10.5103	14.80397	17.65126	28.92251	24.58511	7.786617	NA	20.60839	12.72353	5.216636	50.48397	3.874399	19.17715	11.21915	9.349393	7.824872	16.72797	4.101043	43.78502	24.17495	21.51378	105.8028	53.61862	84.08221	34.64509	12.37092	27.7842
CUST_35824_PI429010886	M13609	0.006948593	24	18						34.13318	62.27316	96.97291	34.50654	46.7009	35.22067	87.14131	63.74337	68.81679	34.82138	46.13557	22.26355	48.21063	100.9526	40.68971	87.46732	75.46497	100.2134	83.23692	173.8121	195.0513	49.95516	78.06756	46.57945	74.89382	49.9386	80.71612
CUST_38625_PI429010886	M28638	0.006948593	24	21						1536.792	1500.89	1469.262	1426.897	903.1163	1083.233	720.2377	1005.258	863.8645	1526.163	1171.488	828.1272	1956.496	566.4722	1245.954	1018.186	1970.101	501.6566	2478.84	1249.272	1480.103	2058.438	2275.002	1383.294	2981.297	1499.429	2424.1
CUST_4120_PI429010886	N16059	0.006948593	24	21						151.5229	119.9872	148.2138	102.1002	89.5211	86.83154	126.0542	92.55588	118.606	148.9308	79.47605	132.4791	155.7231	86.97617	131.4171	152.4015	107.2586	147.1038	163.211	115.4443	170.3073	151.7904	170.6985	174.3407	170.7874	102.9594	199.4568
CUST_9973_PI429010886	N25491	0.006948593	24	18	321461754	ABC transporter [Daphnia pulex]			3.53E-62	28.06923	24.69162	34.85461	21.10532	14.46618	8.293234	15.28234	18.85713	30.50653	18.05945	21.32425	12.10978	33.66656	17.20227	27.34971	58.13362	25.13996	21.22638	43.87779	42.83599	36.514	37.34896	14.91234	21.47745	49.03029	23.06154	37.94596
CUST_24505_PI429010886	N50736	0.006948593	24	6						87.17685	84.9077	66.86503	92.75983	137.3537	142.0375	133.5662	112.1133	101.7482	79.28417	85.34679	111.5617	72.12484	80.13048	97.45374	111.3048	65.98466	87.96067	98.03098	81.51736	87.91078	72.16617	60.40971	75.12194	64.12039	90.14554	113.2581
CUST_9905_PI429010886	N25383	0.006950679	24	3						62.55614	5.532456	36.47671	77.36861	131.6242	105.619	NA	12.57527	NA	7.853693	65.67004	82.1081	21.0408	50.51216	49.85973	7.812621	4.470838	NA	8.390628	12.00512	15.78984	21.32425	10.16181	21.57697	9.835386	20.16972	7.475842
CUST_45923_PI429010886	M70765	0.006955463	21	3						54.59539	72.96184	58.98036	62.30179	96.00931	88.79346	53.18236	62.60527	58.08057	46.44642	75.63115	60.91215	52.39688	62.92982	66.1068	58.24898	52.24236	57.25934	48.8328	45.5838	60.93307	50.87686	65.23465	68.55444	61.22809	69.29751	80.95294
CUST_9367_PI429010886	N24499	0.006955463	21	0	358339569	hypothetical protein CLF_10057			0.000000838	46.85381	62.58458	415.7252	45.43179	44.6746	42.06094	18.09671	46.05498	21.46766	30.78094	44.68965	43.91443	59.24756	44.24302	51.90036	43.86995	74.28907	25.50997	31.0164	38.89189	42.49657	78.57224	79.80578	77.53014	29.40702	43.5104	28.81419
CUST_46989_PI429010886	M74416	0.006962673	24	15	256002943	ribosomal protein L10a [Fenner	Protein metabolism	Ribosome biogenesis	6.55E-35	9345.369	13935.1	9550.071	7750.118	5209.792	5939.375	11695.66	11076.77	6727.299	7415.157	6310.155	7681.431	8215.523	7681.431	6421.864	12255.54	13222.77	13193.16	11378.62	11339.63	10248.36	7643.683	9345.369	8121.327	10852.86	8605.765	9285.648
CUST_28671_PI429010886	N58401	0.006970476	24	18						90.88256	11.19507	84.84924	34.35183	18.22518	29.35282	8.578942	78.3065	75.63986	34.55282	NA	37.59004	163.6405	72.92411	74.00845	50.97241	28.04985	65.85526	103.8862	124.9059	151.4852	99.1481	NA	84.78861	81.36078	30.17026	45.90076
CUST_55120_PI429010886	M108792	0.006989612	24	0						77.32154	63.2086	76.92811	75.28314	49.58749	55.67726	56.59098	41.47035	52.43098	57.39387	40.04635	62.33316	60.79074	41.26235	53.41031	59.28822	45.80812	55.04966	64.67753	59.94044	66.08255	61.39194	46.96617	67.29151	80.59242	53.79961	66.41015
CUST_51380_PI429010886	M93328	0.006992972	24	21			Metabolic process	Cellular amino acid metabolic process		39.82958	28.00501	45.28285	31.6335	31.70122	26.87774	30.69119	22.35063	36.17758	36.89757	22.33547	24.6188	24.15387	31.89668	34.09891	28.47679	34.24988	26.66658	40.23682	24.66829	37.15316	43.97914	39.26035	40.59088	35.68286	28.03915	46.10158
CUST_37567_PI429010886	M19523	0.006996136	18	15	321468738	hypothetical protein DAPPUDRA			7.58E-14	21.09303	27.02166	21.75932	19.79114	18.99558	13.61629	16.92486	26.87774	20.91626	30.04497	38.93069	21.08718	21.70516	15.1484	19.08041	29.89602	39.26035	36.12473	39.34209	27.40728	31.14654	8.00437	26.0166	18.02618	21.41461	22.38744	33.72798
CUST_3003_PI429010886	N13579	0.006996136	24	21						240.4351	176.8599	243.494	228.8193	206.5228	188.4673	206.2187	155.7485	200.4453	228.1062	175.5187	205.7884	268.0924	205.4207	244.6993	256.7541	211.1406	368.7015	237.7581	243.4049	327.3954	282.3174	226.7781	322.2486	312.1009	201.229	378.9491
CUST_30542_PI429010886	N62090	0.006996136	21	18						74.24613	81.96955	73.57745	79.53675	79.03209	98.55805	55.88596	60.32347	73.13284	74.4166	78.82605	76.22837	57.64995	89.81747	69.112	83.73063	72.87552	85.76428	92.47181	95.08224	92.43955	58.69436	89.70467	100.6757	74.48675	89.2783	70.64915
CUST_14604_PI429010886	N33158	0.007001641	21	18	338815387	copper chaperone Atox1 [Crasso	Transport	Ion transport	4.57E-16	13.24735	15.56681	10.01527	6.043075	6.417883	5.873873	5.954504	12.25196	7.776877	NA	9.20883	6.697207	NA	16.8271	10.30948	9.672539	16.33222	9.287131	15.29443	11.09357	7.562913	NA	9.292415	10.89769	9.276918	8.787188	7.356928
CUST_54415_PI429010886	M105915	0.007002498	21	15						142.2314	149.8586	183.9367	98.65475	84.21664	97.70277	163.7162	118.9453	169.1253	212.9394	122.201	152.3049	140.1608	123.2322	113.8607	179.4641	199.944	165.0873	142.4297	132.6428	121.8066	143.9979	133.0898	133.1475	131.6748	125.1074	119.4981
CUST_42909_PI429010886	M61577	0.007004085	24	21	242006996	conserved hypothetical protein			1.45E-18	38.08216	35.24577	47.16802	37.8045	19.49861	28.95229	23.23426	18.43066	26.29331	32.12942	26.21615	31.90671	34.77078	30.95428	38.69332	43.26708	28.29271	39.81401	35.4732	29.54223	24.00968	67.85905	30.8995	47.46533	30.89131	40.60404	48.45851
CUST_2352_PI429010886	N12165	0.007004563	24	6						NA	2552.554	91.02301	306.279	20.77492	270.7536	6966.12	2198.578	3240.436	3423.157	2496.293	NA	6.418865	NA	NA	1205.159	1116.506	657.9421	18.36487	17.32917	35.41196	251.3401	NA	207.9118	19.64634	118.702	5.394829
CUST_48171_PI429010886	M79221	0.007012774	12	3						109.8644	171.1773	199.421	176.0523	169.6212	196.0115	147.4173	173.6995	170.246	179.8439	119.1014	121.2532	126.6958	157.1785	108.9271	198.0058	204.5441	225.1699	116.3326	143.5433	137.0045	128.5824	132.5156	116.5476	174.4633	128.5323	156.0305
CUST_360_PI429010886	N4424	0.007014759	15	6	220172369	cuticle protein [Rimicaris exocul			4.64E-29	7032.355	3087.565	9111.496	14502.16	8786.531	5137.74	7762.077	12605.8	16913.15	8042.108	12473.12	4900.819	25792.96	10966.45	9257.227	709.4562	2378.51	794.0736	21334.13	16183.31	20382.6	15071.82	31289.71	29725.44	21010.2	27665.22	37932.98
CUST_40082_PI429010886	M49881	0.007020395	24	21						NA	11.41917	14.18835	10.15772	8.574366	7.805069	3.24149	4.919698	6.622118	13.52663	12.67448	4.477295	12.1699	NA	11.99886	8.610034	86.66046	5.836101	12.4892	9.311282	7.424438	28.21177	24.37229	12.24304	24.53447	9.34077	9.321353
CUST_51934_PI429010886	M95736	0.007023176	15	6						NA	6.776767	4.249325	5.481109	NA	7.183028	NA	NA	NA	4.615229	7.020474	6.9169	4.822966	4.657406	3.320798	3.538348	5.206785	3.181666	NA	7.124762	4.71102	NA	11.46624	9.096904	5.793138	8.417136	3.534662
CUST_15218_PI429010886	N34205	0.007030256	15	12						16.99603	20.7793	16.5815	12.85647	13.19901	12.29576	7.883393	13.10078	9.01805	12.60227	17.24304	5.734979	16.26097	17.69677	NA	12.62015	15.69192	14.65289	8.947221	16.39705	5.217523	8.63283	9.723255	4.348218	5.187353	12.9375	5.58216
CUST_50956_PI429010886	M91568	0.00703204	24	15						26.76803	42.89742	26.70669	34.62846	13.71845	25.57409	27.73284	26.31789	28.38277	35.86186	43.52816	42.01939	58.31116	33.20239	57.39387	46.64691	50.51216	44.1972	44.53304	37.27834	38.93222	66.47509	33.0209	42.41232	58.48301	26.87774	77.25936
CUST_5267_PI429010886	N18470	0.007033693	24	21	113931406	ubiquitin specific peptidase 25 [	Protein metabolism	Proteolysis	1.01E-38	63.14866	90.57569	104.2317	69.67842	76.06374	71.81502	73.97753	88.27501	87.26779	74.59679	93.04693	68.9382	82.56422	78.83688	82.51294	77.72005	101.7482	86.70327	86.80106	95.45574	96.25473	92.0968	106.6278	113.3845	88.69555	77.57976	91.0714
CUST_35024_PI429010886	M11367	0.00703655	24	21						208.7594	290.6243	241.6209	258.1565	231.289	209.8556	221.8856	217.1239	251.6934	225.1699	238.3483	236.7186	233.6818	253.7263	232.4149	235.738	333.7805	267.555	260.0953	235.4574	295.3036	254.7629	377.4146	333.4754	241.1815	307.8005	352.6665
CUST_44941_PI429010886	M67470	0.00703655	24	18						197.961	434.8192	699.1348	106.2034	162.6193	225.5927	175.9101	321.8126	748.709	265.4524	261.3674	397.9972	252.401	295.4891	329.0788	444.7023	661.4243	375.5874	836.9003	367.1233	479.2755	729.7963	596.2588	555.2007	1075.892	202.6784	473.7195
CUST_2599_PI429010886	N12731	0.00703655	24	21	345483293	PREDICTED: LOW QUALITY PROT				1554.179	1669.427	1691.116	1371.161	1048.117	1058.746	1094.513	1159.706	1406.649	1694.196	1363.035	1300.047	1894.407	1161.162	1642.989	1964.046	1991.351	1795.363	1494.458	1328.27	1379.279	1964.046	1808.449	2003.765	2586.608	1648.551	2554.596
CUST_12442_PI429010886	N29468	0.00703655	24	18						312.4862	217.5757	474.227	214.2039	186.0544	102.3362	201.7307	267.6739	360.4299	340.7195	147.8425	168.3685	409.3888	265.4524	373.4093	275.1168	402.7185	378.9491	410.0384	373.5014	426.7646	572.5541	237.2821	365.7068	653.1145	153.7401	700.359
CUST_34904_PI429010886	M11039	0.007053751	24	18						6.874984	30.6407	22.17869	18.45644	13.12379	15.4594	12.62197	27.17008	20.83994	14.64996	14.83032	20.15802	21.2307	17.67253	18.87134	20.141	33.62051	34.73799	22.79631	28.19237	31.55061	21.48216	13.77769	24.13409	18.34559	17.22127	17.96574
CUST_36153_PI429010886	M14819	0.007060406	18	15						1070.493	1481.716	1226.655	908.7057	971.1067	953.6942	1288.168	1503.022	1622.787	1077.231	2017.363	839.0592	1702.984	1557.736	1158.095	1445.709	2110.599	1757.509	1039.627	1120.742	910.2628	913.5277	1130.969	1104.345	1175.329	906.8146	1017.712
CUST_16855_PI429010886	N36978	0.00706203	24	15						15.58609	26.63911	NA	66.64484	NA	47.28223	38.85987	NA	19.1752	229.7005	348.6137	69.96485	99.44651	150.564	78.22197	214.0358	151.4448	243.1406	26.99058	5.594771	24.7491	192.0011	99.47239	195.8396	37.8081	38.76999	37.40246
CUST_22211_PI429010886	N46633	0.007082508	24	21						105.2701	77.67929	108.8321	93.91412	41.17764	59.09232	67.61402	70.17999	82.1262	107.0461	54.28814	75.23071	150.2816	50.00334	89.70467	71.36454	49.47372	80.03641	109.561	79.07706	96.02016	177.2128	117.0564	154.2231	110.6946	88.62537	128.4629

Probe ID	ID	p-value	Period	Phase	EBI ID	Annotation	GO category	Go term	E-value	0	0	0	3	3	3	6	6	6	9	9	9	12	12	12	15	15	15	18	18	18	21	21	21	24	24	24
CUST_7511_PI429010886	N21654	0.00708379	24	15						643.2149	966.3163	399.4473	538.8011	391.3071	555.8829	1071.049	827.1853	734.7144	786.1643	557.9393	440.6377	652.1093	508.1759	543.9588	1362.378	1014.938	1038.012	750.3368	677.6753	903.938	441.8514	617.9354	557.8175	613.1951	579.318	537.1828
CUST_11436_PI429010886	N27802	0.007094654	18	3						33.65261	11.19796	34.56666	45.68766	NA	43.25692	35.68286	25.39283	33.08731	17.00074	20.68983	42.76535	32.53452	11.06478	32.25543	22.29187	26.78779	21.73515	23.5934	35.93896	36.49381	40.82956	34.86224	36.65135	15.29841	22.855	10.75824
CUST_51122_PI429010886	M92275	0.007099026	24	9						64.68944	58.55433	25.13996	104.0936	19.97115	75.64874	155.3531	132.7868	209.0675	190.4954	74.94714	95.93997	192.2662	60.62225	68.22331	104.3412	216.2603	298.135	30.37143	21.21527	27.69715	51.39126	46.40366	22.58254	25.99138	26.40151	19.65095
CUST_44191_PI429010886	M65213	0.007100671	18	9						11.04053	8.399033	12.42421	16.81757	11.43741	14.0307	10.33927	23.75813	18.40393	72.56894	48.41454	19.52187	14.82873	NA	11.88893	33.22829	16.55821	22.99952	12.21743	10.35153	13.52973	10.89769	20.73144	12.59237	19.46755	9.445121	23.24733
CUST_8264_PI429010886	N22794	0.007101407	18	15	241742424	geranylgeranyl pyrophosphate s	Metabolic process	Lipid metabolic process	2.87E-41	93.8595	414.226	92.0742	164.6109	73.56286	121.6162	297.3019	289.7731	163.2608	343.8057	249.0225	155.7231	187.286	215.8727	163.9403	358.6508	580.4723	447.759	186.2473	111.259	185.9639	120.5852	142.2521	146.9806	289.9679	243.1902	87.91078
CUST_12637_PI429010886	N29786	0.007101407	18	3						910.9934	233.9171	796.9187	1241.315	757.1366	670.4033	424.6765	387.5344	578.6613	830.1589	963.73	766.3188	1368.726	656.7134	739.3679	166.4755	216.9264	191.9474	685.4537	654.5389	598.5974	1232.801	1676.984	1483.632	728.3477	1047.687	1389.498
CUST_22300_PI429010886	N46769	0.007101407	18	15						1256.746	1760.199	964.2235	1021.91	399.2455	528.6354	1922.582	1491.134	1396.628	1459.585	505.0907	948.7571	1337.377	718.7132	734.7144	1941.997	2330.806	3031.683	1673.566	1203.381	1306.13	635.7483	881.258	950.0092	902.4975	1003.544	1105.917
CUST_17343_PI429010886	N37801	0.007103627	24	21						73.85285	88.70868	96.54391	81.79336	64.99079	70.76996	57.23466	87.67177	82.92495	81.24064	68.99631	82.63781	78.7116	60.25895	74.56212	61.60595	93.74241	85.69239	84.45149	78.08717	111.007	108.1577	92.42138	127.9862	96.86942	81.16582	109.9768
CUST_20476_PI429010886	N43522	0.007107552	21	9						NA	3.528285	6.281077	4.722896	7.3032	11.66289	10.07727	6.878064	7.259084	16.18394	7.920298	NA	NA	6.13309	11.81841	7.595815	5.656717	8.963971	7.117232	9.808744	4.187833	6.245436	NA	3.390682	4.979931	5.610497	6.793494
CUST_15318_PI429010886	N34392	0.007108015	12	0	1703416	RecName: Full=Arrestin, lateral (	Signal transduction	Other	1.99E-39	26.6049	20.05879	23.18254	13.56287	19.99659	12.36397	12.08009	14.7781	11.85264	12.27349	37.3274	19.15894	22.53368	21.80903	24.14234	15.53957	33.26038	20.12179	NA	22.75502	14.10098	17.75786	22.56387	16.74788	23.41549	29.70306	21.77362
CUST_523_PI429010886	N5820	0.007110028	21	15						2085.588	3124.619	3368.791	1681.402	2570.156	1936.909	2083.733	2356.919	2629.319	1889.404	1707.189	1853.898	2924.097	2235.693	3142.5	3914.879	2722.979	3528.773	2496.293	3654.032	3289.37	2933.397	2538.621	2670.787	3428.918	2152.198	4159.327
CUST_3127_PI429010886	N13849	0.00711218	21	12						6.574572	7.319642	10.23947	10.52214	7.759578	6.324357	11.59544	9.354156	3.679982	8.947221	10.68359	10.09352	9.228702	10.93377	12.94612	6.670759	7.712272	4.14568	4.439237	4.034525	5.213865	NA	7.070367	5.167289	NA	6.943759	4.71358
CUST_26987_PI429010886	N55229	0.007117415	12	0						77.58626	60.60875	69.326	77.61728	69.30661	56.82231	67.08754	57.34418	35.35794	85.37067	73.21671	77.3983	77.77436	74.80515	75.37573	53.02213	65.61458	58.91249	64.12039	49.95516	52.99594	76.07871	61.19974	56.68053	89.69199	70.8379	73.64351
CUST_41348_PI429010886	M57522	0.007124507	24	3						47.63863	12.48035	58.08676	78.80045	98.15697	76.3955	13.20553	37.46702	28.86297	46.39063	110.519	92.31393	55.15568	48.47319	63.2086	21.70516	15.57566	19.32541	12.44897	23.64777	15.7786	39.49333	36.828	45.97159	16.76354	48.84433	24.87248
CUST_13429_PI429010886	N31174	0.007124507	24	18						502.6586	2269.737	897.3252	828.3699	322.5903	579.8505	837.2458	474.9425	870.1856	1219.83	464.9207	844.6913	1251.245	37.81634	1203.381	2025.285	896.981	1311.742	2057.214	1965.787	2095.253	1568.389	501.5231	1295.587	3142.5	1189.941	3428.918
CUST_18992_PI429010886	N40792	0.007124507	24	3						47.38682	69.17285	54.07873	60.69474	93.12448	74.44615	60.37267	64.74689	53.64076	68.19401	49.45288	60.84053	32.6846	60.23886	71.46735	52.18733	46.39504	54.57944	48.38982	53.68198	39.30626	48.98753	45.13797	43.08599	58.47581	51.81269	38.83587
CUST_32645_PI429010886	M4677	0.007125625	18	6	321462910	hypothetical protein DAPPUDRA	Protein metabolism	Proteolysis	6.83E-62	10.10472	214.0358	14.39043	324.6618	140.5662	232.3662	575.6253	746.7514	641.1736	16.76758	20.06471	6.805347	276.1893	192.9642	478.5299	NA	274.3383	6.532525	137.6386	16.05617	157.2786	161.7929	349.4617	86.36335	22.71026	596.5924	349.9023
CUST_16689_PI429010886	N36697	0.007128953	24	3	530655347	PREDICTED: dual specificity testi	Protein metabolism	Cellular protein modification process	1.18E-39	11.69775	NA	NA	13.74225	73.26943	66.37527	5.396608	7.000055	4.222474	NA	11.17738	4.007761	4.899473	5.504548	NA	NA	5.247423	6.320355	10.10592	3.643187	4.175395	8.355006	5.191314	5.65063	6.116784	10.19338	7.177997
CUST_11176_PI429010886	N27390	0.007129707	24	21	44890292	LOC100045488 protein [Mus mu			1.16E-34	50.83559	91.15714	60.05066	66.63152	77.63531	45.75395	59.64058	75.0097	84.34054	61.25771	74.66025	47.27657	67.90691	59.62083	73.35252	53.70551	68.43368	78.11286	101.9343	89.74912	80.85368	88.53491	104.3299	85.57553	88.54675	89.12634	80.49207
CUST_38318_PI429010886	M25374	0.00713038	12	6	3006086	trypsin [Litopenaeus vannamei]	Protein metabolism	Proteolysis	1.3E-66	14.19023	NA	8.174815	461.7785	NA	313.5422	4803.622	NA	3360.114	661.1838	484.8492	NA	164.8179	346.4474	115.2993	972.4265	NA	688.6527	6265.89	12236.09	23379.75	856.9537	7.352636	854.1319	4349.21	413.2217	2584.904
CUST_54781_PI429010886	M107652	0.007141679	24	12						4.659382	11.44781	7.878601	17.73565	7.609031	13.30754	6.42494	7.760758	11.03403	12.15684	NA	23.34681	16.89173	19.28815	19.63812	19.04623	18.81827	15.5158	NA	5.679722	3.899392	NA	12.10816	11.57491	5.266343	8.300487	9.388241
CUST_36722_PI429010886	M16660	0.007146505	21	12	85677399	pigment-dispersing hormone 3 [	Signal transduction	Other	0.000000345	142.0207	269.8306	208.404	262.1593	311.1974	243.331	341.6702	311.6703	240.479	261.5986	438.6999	295.1733	233.412	671.9924	522.1886	299.0693	459.9674	367.9854	131.8269	390.8971	209.1419	175.317	219.8411	120.0145	284.5232	475.4976	457.8873
CUST_45838_PI429010886	M70455	0.007157699	21	18						149.5014	192.6063	181.8309	133.3192	151.5648	180.4189	113.5034	158.6164	176.9444	166.5631	134.4996	182.1852	117.5967	150.9904	135.1633	173.2168	205.2408	156.1141	261.6417	171.2114	254.4159	190.2229	201.86	188.4419	174.8799	175.3842	208.2743
CUST_46441_PI429010886	M72659	0.007165791	18	6	498966874	PREDICTED: rho guanine nucleot			1.43E-42	12.90521	8.982204	9.301907	7.10668	13.62679	NA	NA	12.301	11.41494	10.11839	14.68427	11.38924	9.271842	11.69775	15.75736	4.72816	5.643166	4.516364	7.120472	5.266343	12.83603	6.720337	NA	11.69775	9.704976	16.32616	24.33617
CUST_57330_PI429010886	E6184	0.00716888	18	15	397611322	hypothetical protein THAOC_18	Protein metabolism	Proteolysis	3.72E-13	10053.8	11455.94	8073.399	6439.853	6612.476	5298.376	13135.1	16288.46	13988.25	12569.14	8962.903	9516.402	9855.129	9425.197	7762.077	17315.8	12236.09	16553.17	10626.1	10550.02	10727.23	9410.039	7673.313	9872.039	10550.02	8025.596	11695.66
CUST_37309_PI429010886	M18573	0.00716888	18	15						884.7471	2170.443	1301.148	1056.763	961.4773	922.1615	2210.04	2067.912	2113.55	1998.783	917.9436	1513.832	1475.969	1522.629	966.6426	2529.949	3899.124	3185.221	1622.787	1341.551	1829.191	991.8349	1058.746	1310.327	1390.116	1566.672	1301.148
CUST_39169_PI429010886	M35554	0.00716888	24	3						178.7203	60.02726	108.7707	196.6122	157.847	185.9023	80.74224	89.43021	65.1725	164.206	149.9126	111.894	219.702	118.7327	116.8305	51.56856	66.6173	30.32287	64.02245	53.55076	44.96264	178.619	136.314	114.3003	67.91644	139.9842	98.54648
CUST_31154_PI429010886	M844	0.00716888	18	15						1943.332	4746.115	1972.842	1531.626	454.3052	840.9882	4560.074	2694.146	2883.204	3269.912	1015.318	1214.037	2452.653	1142	1294.853	3886.966	4772.224	5578.907	3156.873	2066.858	2924.097	1091.098	2231.56	1741.604	2231.56	2067.912	2393.197
CUST_13931_PI429010886	N32033	0.007174608	18	3						28.27902	31.73936	42.51318	36.66337	35.87747	34.20961	19.407	22.53586	25.73695	19.46418	13.53241	18.28904	36.72221	25.32953	28.05304	25.70617	25.12027	13.87626	14.57803	29.03664	16.81913	24.82357	37.89317	26.94621	23.17242	23.53464	24.33207
CUST_14165_PI429010886	N32421	0.007191159	15	0	242002276	topoisomerase TOP1-interacting			2.44E-12	46.23157	56.71352	48.44449	43.68293	36.61868	34.9506	39.24909	48.99281	41.42853	35.82162	28.47285	31.41902	45.72512	40.94392	41.60531	52.59937	71.27374	54.33188	37.69822	48.21366	52.5487	21.95009	40.61754	40.82014	35.36937	46.65167	56.2716
CUST_23883_PI429010886	N49641	0.007193522	15	9						18.86919	22.82508	17.79044	23.11733	22.8831	19.52016	21.0526	21.80903	24.28126	25.56702	27.5238	44.55836	23.99266	28.80735	27.17222	26.08093	16.40332	26.18643	23.85867	18.68765	19.1261	31.45292	49.33322	36.28088	23.61457	29.73407	33.67973
CUST_53087_PI429010886	M100098	0.007193546	24	18						303.3581	254.4159	207.4823	187.3112	266.9079	157.0068	859.0835	262.2441	527.5856	193.3912	168.2226	131.5172	173.8666	259.1614	234.7388	148.892	276.3758	337.46	995.0788	1327.159	2402.724	324.7451	258.3703	340.5595	343.6496	252.4627	352.7205
CUST_53098_PI429010886	M100133	0.007193633	18	3						40.27654	64.27006	33.87201	59.36408	58.66639	40.05085	68.4557	78.61195	77.8161	33.57662	21.18195	33.17685	47.71104	35.4183	37.7496	40.83466	21.44313	29.36117	39.11041	28.45348	39.55139	93.3967	44.1442	95.93997	37.49842	22.905	36.81564
CUST_2432_PI429010886	N12332	0.007193633	18	3						42.02407	20.26569	53.9262	53.23808	36.92118	40.84998	28.61425	22.35703	29.79347	29.90874	21.82549	36.35313	31.05988	18.65471	27.00456	25.38779	16.97648	20.25356	31.12088	27.51459	25.60929	51.17522	24.30578	40.75864	27.2791	20.67143	40.07856
CUST_10442_PI429010886	N26241	0.007194701	15	9						521.206	289.2838	456.7205	846.219	924.3063	773.3668	338.5986	435.0369	280.6399	873.2461	2070.818	961.4773	932.1979	684.4289	835.7807	337.9166	253.3528	284.1966	344.8027	383.2384	291.1427	927.3328	1124.497	1180.964	355.841	899.9958	1063.435
CUST_44390_PI429010886	M65804	0.007201064	21	15						5.036752	9.970616	9.858804	NA	5.800929	14.12665	10.51976	14.73062	14.62025	11.84707	13.0692	13.85312	16.98514	13.81248	9.323328	18.27116	16.63236	16.75338	18.05364	12.23558	14.6763	13.36386	4.733337	12.3024	15.68854	11.25075	6.808227
CUST_53196_PI429010886	M100666	0.007207689	15	3						467.6907	478.8721	365.2368	460.1149	549.1826	540.9421	484.0256	526.4775	679.5306	352.1552	443.4999	285.0355	379.3272	363.2101	247.374	374.3365	540.2756	400.6266	628.8213	383.3073	509.8873	378.1731	498.4242	275.3403	501.3701	435.509	365.1669
CUST_48858_PI429010886	M82527	0.007207689	15	6						19.46418	18.5866	9.429304	11.6258	11.79955	16.83843	22.21596	22.74199	20.57977	11.23239	10.20667	12.05475	8.818102	5.745179	12.44307	22.04447	13.97125	12.1699	13.5943	12.59237	17.69186	24.43634	16.84945	17.28062	22.35262	15.22111	20.5703
CUST_52727_PI429010886	M98669	0.007207689	15	12						32.06294	22.39651	23.45708	13.09661	33.82765	41.31439	15.60447	7.096311	14.32824	12.51777	19.68859	33.77888	35.88985	23.72167	40.05085	37.6281	13.55744	23.50565	12.99937	24.98047	18.57947	12.5848	8.068353	18.40393	14.74345	14.53307	12.42007
CUST_25579_PI429010886	N52679	0.007212374	24	18	443711993	hypothetical protein CAPTEDRAI			1.04E-08	39.0719	59.88108	75.2001	47.60041	64.12746	54.61715	49.96129	43.95645	43.17122	36.97106	68.33012	25.00404	42.37791	43.00281	75.59966	29.57251	81.15666	22.10476	120.7334	91.71339	137.6772	48.43927	165.4776	104.5021	55.03418	35.86607	77.36861
CUST_53092_PI429010886	M100121	0.007212464	24	3						46.7009	19.1752	29.89281	41.74139	131.9825	103.6461	20.02186	46.22711	17.48796	17.87709	70.98403	49.93016	11.51954	45.9767	44.97359	15.5193	14.15703	10.81194	6.205904	40.10788	10.07008	28.89871	27.66084	26.33431	7.569054	59.39503	7.646455
CUST_49075_PI429010886	M83935	0.007212464	24	21	408474813	calcium-activated potassium cha			4.65E-41	60.43767	40.38904	49.35984	40.83466	28.7398	36.86027	28.52646	39.27561	27.52155	38.70148	40.14947	56.71975	42.90092	36.12473	44.73116	43.96941	45.56749	48.84433	40.81486	34.27395	40.38119	53.42717	56.18215	46.15443	56.13868	45.49544	52.03911
CUST_5388_PI429010886	N18630	0.007212464	24	18	405973122	hypothetical protein CGI_10019			1.36E-49	78.3065	130.3816	118.7615	100.821	88.13366	70.15908	100.821	114.1092	121.6468	103.4638	83.13651	80.48196	135.0399	98.08719	92.34598	110.2359	141.6537	146.658	143.5591	133.0536	176.8903	120.4106	100.771	124.2158	145.5033	102.5557	134.1449
CUST_9446_PI429010886	N24639	0.007212464	24	21						240.3468	287.938	371.3812	270.5362	245.3228	207.2734	200.7173	253.4735	242.3852	228.163	251.3778	251.6089	307.7409	233.5705	271.1207	218.6001	369.5842	278.592	303.1963	264.6223	256.6606	323.8056	352.7205	317.3631	362.5008	322.5903	414.226
CUST_48736_PI429010886	M81922	0.007214044	24	6						62.38786	71.33856	89.39537	77.22486	79.4857	75.44765	73.49385	70.20407	92.77365	73.20517	85.40783	71.70119	85.54538	65.11364	77.03004	49.32564	61.145	58.22453	58.01001	53.10853	70.87494	50.45124	82.04507	64.50416	63.1617	69.61335	65.1084
CUST_2556_PI429010886	N12615	0.007216897	15	6						20.91626	NA	9.64326	16.79051	NA	11.02965	60.72297	60.32929	53.66051	56.96848	27.88249	25.61174	114.7628	63.99137	7.256866	18.12971	21.64409	8.891795	10.11056	52.37742	35.84121	64.80829	119.9264	110.3412	72.15825	15.29443	33.60676
CUST_11173_PI429010886	N27383	0.007222993	18	3						4253.847	1732.526	4487.184	6317.157	3360.114	3815.958	501.9234	1587.909	1572.421	3200.557	1329.124	2570.156	1767.886	473.1245	1143.279	676.6048	1774.191	2152.198	1542.337	4041.944	6864.217	6143.541	2162.694	3327.482	3053.693	46.24282	2940.045
CUST_53344_PI429010886	M101344	0.007223318	24	18						11.26818	9.902198	NA	12.38585	NA	8.487659	16.34183	12.46756	16.33604	13.33462	7.646455	8.020864	17.83378	18.08853	NA	23.98408	10.84292	27.17008	21.0164	20.69694	17.5198	15.69192	17.93943	17.71533	12.54903	17.89735	14.64332
CUST_16055_PI429010886	N35575	0.007227374	24	15						47.93963	41.1126	54.44704	33.40941	47.30405	31.50316	56.05872	40.16574	45.21941	54.90137	55.84366	50.23264	59.64058	43.96327	64.76383	53.64587	57.97707	65.78378	42.3639	53.43212	57.27829	50.90051	48.45428	54.19934	46.5992	55.31584	56.4602
CUST_21773_PI429010886	N45827	0.007232579	21	3						32.48749	25.25292	9.411311	11.07276	85.02933	63.31187	19.35535	47.19838	18.90503	20.95393	41.17764	56.71352	7.534185	17.55051	12.79947	15.14134	4.11703	9.711241	6.799426	48.19092	9.258479	NA	36.20611	7.389162	NA	72.69874	NA
CUST_33725_PI429010886	M7713	0.007255253	24	6						28.12879	56.9001	47.68605	44.02723	41.33969	48.76658	49.49083	60.3351	54.38582	32.62087	23.63569	25.35423	50.34388	55.69128	43.10438	34.42594	34.90142	32.33466	19.75767	38.9642	31.7949	22.38961	34.5207	22.86438	41.51987	33.59889	31.20454
CUST_9786_PI429010886	N25204	0.007255253	21	15						29.31643	80.6925	65.58263	46.41625	39.2784	32.91482	41.79921	50.1322	59.33452	53.17689	66.11978	53.8169	46.45178	51.02011	42.94799	74.8864	62.77181	65.86088	58.61403	47.61497	57.33715	47.3524	55.30643	62.73874	52.44815	33.53246	50.15161
CUST_23538_PI429010886	N48998	0.007255921	24	3	345486561	PREDICTED: disintegrin and met	Signal transduction	Other	5.19E-16	12.9801	6.854397	8.338749	13.09363	13.37422	16.25246	NA	6.324357	9.045745	7.95738	10.35502	13.0685	9.974531	12.91477	18.30046	9.406434	7.789818	10.70343	4.750576	4.736071	8.143822	7.485899	5.865277	13.2526	12.03366	11.41659	11.7339
CUST_25714_PI429010886	N52929	0.007266738	24	6			Protein metabolism	Proteolysis		85.62565	112.5544	114.1092	116.4413	188.3253	165.0676	124.2797	134.4304	173.7269	133.3046	130.8809	113.5495	128.0875	89.13599	113.878	125.6161	181.3504	100.8009	120.0673	108.298	105.7619	105.3536	94.78884	82.98239	65.48356	139.6859	165.1648
CUST_45089_PI429010886	M67974	0.007269197	24	9						149.533	114.9233	124.2958	120.3588	192.4672	149.7452	194.9703	106.696	190.8547	202.0095	132.4959	298.1868	192.3094	165.7121	202.0727	197.3862	152.8709	184.2613	162.5974	121.6162	171.3397	160.2439	154.2424	161.4921	123.5252	137.5078	187.5258
CUST_52934_PI429010886	M99514	0.00727965	24	12	443734178	hypothetical protein CAPTEDRAI	Protein metabolism	Cellular protein modification process	5.04E-29	5.754541	20.51167	10.0854	11.08814	18.28324	16.97405	364.2049	13.63348	246.6961	89.29338	128.3075	221.2277	583.9375	167.7764	306.0769	11.32628	17.52199	28.82648	288.6545	183.1939	7.69611	568.8248	232.4149	206.0425	6.64183	14.66893	11.78126
CUST_44827_PI429010886	M67120	0.007282441	15	9						255.571	153.3624	378.3539	139.3356	313.1767	257.5532	531.0942	400.3546	414.4128	611.8901	328.4824	460.5885	682.3134	364.1244	437.7611	368.2385	234.1352	103.3439	319.1468	251.7959	365.6513	878.8076	219.1089	733.4921	675.6901	417.0366	479.1083
CUST_45131_PI429010886	M68089	0.007284363	18	0						2552.554	601.9971	2611.542	360.4299	533.6749	393.358	253.2927	289.3915	403.4693	535.3308	268.6876	246.083	451.4694	333.0825	546.0695	2437.73	323.0408	2103.097	810.1648	383.8668	917.5193	422.0714	1600.243	377.0504	669.3491	367.6429	598.0964
CUST_37916_PI429010886	M22224	0.007285859	18	9	242007821	Pediculus humanus corporis mit	Cellular Process	Cell cycle	8.63E-84	84.3918	105.7387	95.36659	68.40753	69.6714	65.98466	147.3924	105.4796	105.5425	106.4281	123.6581	94.76517	103.8438	73.73506	93.94191	98.94639	136.2532	125.4006	64.28341	90.04305	100.658	79.03209	86.52356	75.9364	130.702	88.77171	122.771
CUST_21442_PI429010886	N45229	0.007285859	18	15						55.40345	52.8068	64.08368	37.83797	32.94858	26.79339	36.1598	39.24334	47.84693	41.53336	44.35011	86.8615	37.66871	57.39387	59.79025	76.57491	48.09966	61.38551	61.26619	35.77181	33.00277	35.2239	50.73868	35.19853	70.332	45.76238	50.45124
CUST_45692_PI429010886	M69944	0.007291658	15	9						17.07151	7.225798	8.917284	10.1984	3.41144	5.063083	6.136811	6.435594	3.991284	8.103931	9.575352	12.41357	4.882539	7.873998	5.11654	6.415726	6.063885	4.549127	6.340508	3.82208	3.681468	13.6782	10.4031	8.741642	25.97276	9.953391	10.61964
CUST_36556_PI429010886	M16224	0.007293154	12	0						105.0258	114.4006	94.20496	100.0635	81.64232	70.8379	79.92025	54.78117	68.71816	71.4584	98.87854	98.89641	96.02016	107.8039	113.1748	105.4679	79.13617	96.09055	86.04303	88.27501	81.9979	125.2342	86.65164	116.8939	117.6515	95.02561	114.6414
CUST_20513_PI429010886	N43585	0.007296673	15	12	321472706	hypothetical protein DAPPUDRA			8.22E-09	2294.993	1541.174	1436.914	3073.818	2642.585	2439.09	952.6113	1102.848	912.5849	1460.387	2306.791	3010.08	2126.752	2965.89	4427.539	3667.762	2056.189	2449.312	1044.09	1687.279	1243.512	1563.997	1592.484	1863.699	2078.188	2372.615	1677.845
CUST_42452_PI429010886	M60317	0.00730042	24	6	321455342	hypothetical protein DAPPUDRA			5.53E-51	144.9581	200.7173	205.453	98.52549	119.4159	129.7163	235.4202	217.8657	243.543	192.8085	188.5629	161.754	199.944	136.296	203.392	182.136	191.1727	176.429	152.6411	185.3051	127.2978	127.633	114.8083	154.3027	185.6007	117.8103	187.1657
CUST_6444_PI429010886	N20108	0.00730042	24	18						200.9398	268.9316	351.1109	244.7879	240.0325	179.241	235.2069	253.1207	319.4595	272.7898	257.678	312.0575	309.0132	205.9794	254.618	281.4753	262.9461	365.1669	322.4558	336.9544	388.6637	319.9319	283.224	436.5051	338.993	255.4927	503.262
CUST_14335_PI429010886	N32714	0.00730042	24	18						956.8208	1049.395	1223.646	537.3205	375.3353	290.7575	965.1042	921.74	1196.459	1003.544	389.7239	660.9643	1063.832	501.3701	430.7389	886.9505	1190.571	1198.216	1225.455	1359.053	1927.794	529.2519	488.0472	770.3832	988.0134	650.8946	1323.872
CUST_12076_PI429010886	N28890	0.007306358	18	9						36.56941	50.05302	42.2265	37.61238	46.65167	31.39279	51.42014	62.11363	54.78117	50.0221	55.36247	65.27494	61.52728	59.88108	47.63863	67.41761	54.62254	58.87393	50.384	53.5736	45.06212	43.48425	50.24098	52.03911	62.71091	55.91239	60.03374
CUST_39311_PI429010886	M37562	0.007308499	18	3			Metabolic process	Oxidation-reduction process		21.47499	45.24721	36.8949	51.1228	33.89756	44.79025	17.6134	16.78894	15.88592	16.90805	16.91351	27.13842	33.68871	26.28423	26.76018	26.76018	33.48088	36.33342	27.24094	54.9218	30.23399	26.97175	39.90127	41.29379	31.32237	50.50655	29.21779
CUST_54916_PI429010886	M108155	0.007325041	24	9	157120354	hypothetical protein AaeL_AAEL	Cellular component org	Cytoskeleton organization	3.07E-33	24.21438	18.70247	11.58971	23.14239	263.0994	254.4938	29.90427	30.30161	23.55652	30.12869	203.1024	203.392	19.05953	200.3302	216.2603	8.464303	17.70184	17.23263	20.24017	37.73828	5.259486	11.35215	21.02168	9.423248	16.05064	60.78187	14.18007
CUST_35479_PI429010886	M12683	0.007340597	21	15						84.09371	124.1767	115.3273	72.27008	89.25205	95.11745	81.73809	93.54583	93.42816	92.50275	102.8659	92.42138	120.2717	109.6359	98.83232	95.79812	152.2217	145.3581	108.8657	113.0417	81.75855	103.3439	134.5716	112.9084	74.93498	105.4902	81.96955
CUST_41569_PI429010886	M58051	0.007341625	21	15	2501170	RecName: Full=Protein 60A; Alt	Cellular Process	Cell proliferation	2.87E-29	122.5288	217.5404	225.6261	160.5687	161.2169	129.5648	205.6244	218.4319	198.3747	189.5433	157.6398	186.792	191.741	191.741	193.8796	230.2055	241.1815	301.3368	168.5856	178.5273	215.2938	178.7734	186.1647	216.54	173.9289	147.2992	199.3483
CUST_20816_PI429010886	N44114	0.007362392	24	12	390333473	PREDICTED: probable ubiquitin c	Protein metabolism	Proteolysis	4.63E-57	13.29403	14.50203	17.12908	12.4383	17.64923	18.31541	11.45197	19.60068	23.91003	12.97623	18.26175	21.68208	18.89628	28.13712	35.83339	26.30298	23.53208	17.00672	21.46399	18.98812	23.21157	15.79824	21.00124	24.36005	15.17465	13.37051	19.25825
CUST_53029_PI429010886	M99805	0.007363888	21	9						12.90391	15.9111	9.63137	14.22894	15.08105	20.17341	20.51536	17.48259	19.75023	21.48685	10.31356	26.58916	19.4293	17.46154	28.37038	19.45088	19.28481	16.09566	18.27547	16.90983	14.18007	13.82118	11.26701	18.66832	19.75023	25.13144	12.46897
CUST_2399_PI429010886	N12265	0.007370181	15	9						24.01937	6.434401	25.41015	30.22988	19.42744	32.25543	8.035027	3.504057	15.10046	33.84921	53.25991	52.84948	25.91869	18.81696	29.37844	12.52305	14.17513	10.38475	11.18546	16.35946	14.61747	41.34776	26.26607	42.3639	24.17698	29.7085	51.67361
CUST_2251_PI429010886	N11963	0.007373385	24	18			Metabolic process	Generation of precursor metabolites ar		84.72348	52.86067	94.01958	47.01945	46.76121	65.86788	65.41928	65.17942	62.04596	40.82537	67.83335	16.70282	38.38502	84.73544	109.5188	64.50416	45.54022	73.00128	126.1085	87.47672	130.3988	60.0855	108.6386	69.42506	93.09894	62.14833	94.80138
CUST_22702_PI429010886	N47501	0.007373774	18	9						9.381399	8.984476	11.20316	25.67685	24.53447	16.39705	8.295728	18.48018	8.548335	22.80766	88.78315	33.86827	25.36192	13.13774	56.06983	9.336607	13.34178	NA	8.248627	4.598398	10.62708	14.90046	29.39937	42.69941	11.62846	31.71402	33.68871
CUST_9551_PI429010886	N24823	0.007378085	18	15						249.3839	374.6189	397.9279	299.8024	254.4938	282.172	322.4558	222.5585	303.9295	344.5549	327.0634	337.8262	369.756	293.7564	359.6695	415.8002	409.2932	430.5713	278.6422	288.4188	360.0833	296.2664	281.5287	372.9744	329.7868	311.2489	480.6993
CUST_34972_PI429010886	M11207	0.007388377	24	3	225712430	C10orf58 homolog precursor [Le			2.31E-19	44.48574	28.57269	21.51378	75.63986	37.11143	39.27561	30.62762	33.06459	19.82019	41.45022	29.02823	41.00859	28.21781	29.07923	30.62762	42.91273	36.56104	27.39752	19.82888	16.56168	23.67342	22.84192	28.22616	22.40935	25.39744	30.57247	28.59028
CUST_37076_PI429010886	M17781	0.007388377	24	18						106.3228	193.0974	146.0508	139.9317	145.4834	98.01972	155.1073	158.0297	151.3949	155.8444	126.6423	145.4304	167.9984	132.4149	140.4269	181.9919	159.2709	189.1287	206.5859	178.619	201.2812	175.6882	146.8681	188.1589	221.2615	137.0267	232.1999
CUST_32660_PI429010886	M4728	0.007406279	21	15	270009250	hypothetical protein TcasGA2_T	Response to stress	Response to oxidative stress	4.45E-83	2918.383	2965.89	2330.806	2613.493	1100.835	1711.15	2765.552	4392.913	2359.281	1536.792	1444.129	1267.497	2312.635	1776.485	1594.801	7597.158	3179.907	6922.365	4012.41	2338.958	5955.414	4121.132	2744.675	3172.092	3558.043	2319.232	3735.581
CUST_1538_PI429010886	N9985	0.007406279	24	21						239.6808	210.5853	287.0864	234.791	225.7754	232.64	237.9791	199.6837	278.0108	221.9709	194.1219	216.0628	224.7434	185.2539	252.0561	332.6579	264.4099	286.4692	306.279	209.031	307.8005	299.8024	284.6216	260.0376	272.3474	251.5671	328.3409
CUST_41832_PI429010886	M58654	0.007419233	24	18						5.05016	8.178473	8.524647	8.095073	5.505311	7.947466	5.548779	5.23145	5.014884	17.2236	5.370224	3.716787	7.621112	7.121664	NA	9.006528	5.928689	14.41667	12.17844	6.73927	9.857053	NA	9.667776	9.943813	NA	5.969391	6.921979
CUST_7800_PI429010886	N22098	0.007422709	24	21						92.17501	147.2411	120.3127	110.7898	69.91553	61.93359	126.3449	128.2695	89.41928	109.8412	77.808	91.44099	149.73	79.80578	102.3466	133.7779	124.4425	142.345	119.8767	127.7313	148.4879	148.9789	133.4611	168.6123	113.015	123.1089	146.4045
CUST_39714_PI429010886	M43359	0.007423374	24	21						14.81846	NA	17.45601	47.30043	9.472484	27.11011	7.461548	7.094278	21.24037	14.68671	37.97588	15.20248	41.67991	14.96631	13.76082	NA	NA	NA	16.00633	22.71736	19.19097	46.5849	53.087	47.25275	10.1544	44.25351	42.42877
CUST_46342_PI429010886	M72303	0.007427997	24	21						92.69475	79.44437	122.0045	88.58571	56.59797	75.25623	76.49879	53.4446	89.06904	104.2173	58.13362	56.28495	82.7815	78.67787	79.55681	89.69199	69.17285	97.94351	98.64233	76.06374	74.72923	101.8646	104.5021	157.7362	114.2468	80.12221	98.1824
CUST_703_PI429010886	N6835	0.007427997	24	18						868.2894	546.0695	1097.521	1176.913	639.5264	225.1246	1100.31	527.0091	1155.365	2829.469	1729.252	1888.631	2133.67	733.2499	939.9518	956.0225	491.2558	1544.613	3670.22	3624.383	4284.075	1681.402	2950.533	3699.413	1627.485	1385.605	3620.969
CUST_43532_PI429010886	M63290	0.00743319	24	18						11.29793	13.95828	18.68212	9.75683	8.032833	6.129524	4.175017	18.96294	20.28829	NA	11.84537	NA	16.21246	10.74484	17.06435	12.17483	17.34867	20.31717	26.27801	22.8273	16.29003	19.63812	8.17078	16.75714	6.540338	14.08701	20.01679
CUST_45281_PI429010886	M68570	0.007435034	24	21	393910677	hypothetical protein LOAG_033(	Transport	Ion transport	1.05E-08	192.6063	83.38925	166.7642	131.0265	75.8587	101.2137	97.94351	101.6228	87.86087	95.33452	77.86312	102.6741	112.3953	98.87854	124.0944	94.55136	78.81902	113.5034	108.9271	125.7435	140.2426	152.1456	142.3304	216.65	154.8276	84.5309	222.7682
CUST_54448_PI429010886	M106062	0.007438295	12	3						204.1402	194.3553	17.93755	292.0796	16.21373	276.0746	431.2488	263.0994	202.9968	193.9616	94.82698	39.99078	228.1062	253.9087	241.668	873.0203	353.8605	432.4641	353.8605	49.60146	316.8751	32.59982	163.5866	39.74479	245.2392	197.7072	22.99386
CUST_15947_PI429010886	N35391	0.007446178	12	0						21.26236	NA	NA	11.33839	6.647624	7.411034	8.015981	NA	5.796902	10.21893	10.18923	7.153945	24.35366	10.98177	10.74067	4.219133	12.22382	5.16664	10.35301	7.806436	7.606464	11.79315	9.957432	NA	13.60565	14.66099	14.45617
CUST_40706_PI429010886	M55902	0.007447061	24	15						2271.439	1748.513	1785.094	1779.656	1407.191	1377.595	2147.917	1557.736	1800.506	2315.807	1317.605	1920.18	1916.926	1400.583	1551.452	2432.608	2129.384	2230.426	2119.199	1740.73	1790.499	1854.805	1877.836	1867.24	1695.833	1718.114	1670.997
CUST_31155_PI429010886	M848	0.007447061	24	15	340376494	PREDICTED: molybdopterin synt	Metabolic process	Other	0.000000854	136.7059	169.5621	137.2652	126.9491	94.74514	110.9429	166.1333	163.5142	185.4321	178.1474	124.8855	124.685	149.6035	112.5697	105.242	177.2128	214.5153	176.3183	159.5246	154.5458	162.8755	125.0755	147.9373	131.2008	141.7833	115.7715	143.2463
CUST_44097_PI429010886	M64945	0.007460354	24	0						19.86678	60.45051	45.86429	29.56947	24.50546	22.45692	29.25965	33.87201	48.37508	12.62446	31.9639	23.5771	26.4908	31.16674	18.47174	26.28128	23.48051	30.75233	51.00867	40.36067	38.93757	44.60387	40.71656	40.44814	65.43257	45.2017	39.61981
CUST_7681_PI429010886	N21911	0.007460354	24	0	321474141	salivary alkaline phosphatase [D	Metabolic process	Lipid metabolic process	2.53E-77	506.8228	547.5307	445.3005	469.0501	389.7239	459.4079	514.6065	658.2775	469.9074	321.7248	308.0511	246.2584	360.8837	362.4397	390.2201	450.4965	211.3027	359.8294	426.8467	608.4831	401.3498	378.3539	506.5099	337.1412	527.1701	621.7856	427.0381
CUST_11917_PI429010886	N28624	0.007460354	24	0						971.4356	1663.655	1343.626	1099.423	921.0975	838.3409	745.3715	1008.499	1099.075	844.6913	1075.462	961.8532	951.5195	849.7305	1007.214	1062.332	1399.933	1166.063	1189.292	1128.19	1031.076	1367.344	1038.87	1132.475	1475.028	1122.574	1463.189
CUST_16682_PI429010886	N36684	0.007461015	21	9						32.08466	27.2823	23.22144	35.78636	15.26143	33.79023	34.45662	20.56563	23.5397	47.71567	33.92477	41.12558	38.92916	43.7275	27.48203	25.79299	14.57035	27.30898	25.36192	21.11731	7.367001	38.17039	19.66181	22.76482	26.37129	39.24909	27.40129
CUST_35623_PI429010886	M13041	0.007463304	24	15	383848137	PREDICTED: pumilio homolog 2-			3.6E-91	38.14824	34.46101	50.29492	46.98969	31.21069	39.94084	43.2948	34.39051	30.05595	57.45443	35.9428	43.07828	60.69474	33.60972	49.44159	81.71011	42.3598	57.05634	56.60765	41.98856	60.96191	53.58837	52.60565	51.24511	64.47129	47.7665	48.40188
CUST_9753_PI429010886	N25145	0.007468892	12	0						69.84655	73.99941	77.84495	72.6168	91.87245	66.07592	56.97991	67.97894	76.27282	73.96504	68.91312	94.96672	75.95345	69.51507	76.91277	73.39156	76.88599	71.99448	57.77356	49.06379	55.74024	79.5082	62.69677	74.61536	101.334	86.34178	101.1544
CUST_38182_PI429010886	M23963	0.007470311	18	15	302668071	Trichophyton verrucosum HKI 0	Protein metabolism	Ribosome biogenesis	7.96E-70	28414.17	40217.51	37083.68	24341.41	25978.53	29294.9	42496.4	44285.99	40217.51	32969.41	27073.29	28858.93	30821.46	32454.44	32184.55	40415.13	44722.61	42224.23	31289.71	38719.75	44913.68	27073.29	28414.17	29122.17	31206.9	38996.88	26567.15
CUST_44245_PI429010886	M65373	0.007470311	18	15						24.45655	36.31991	23.86617	25.20922	25.12586	15.98012	18.79083	27.47239	44.69425	31.02823	25.68152	12.70892	33.66355	40.30891	24.19784	38.55949	42.48109	28.2092	29.15166	32.10702	33.48088	10.82822	18.77059	18.10836	25.8029	41.96551	25.84643
CUST_3163_PI429010886	N13935	0.007470311	18	3						620.5792	758.5678	754.678	859.7804	1080.903	624.2116	633.691	739.9176	667.4336	684.4289	850.2576	936.5204	712.1062	611.3316	700.8468	566.888	651.9522	650.5235	828.8502	816.8898	693.3608	983.3131	1075.892	1091.584	844.1613	626.2886	1014.938
CUST_16581_PI429010886	N36501	0.007471095	15	12			Metabolic process	Lipid metabolic process		30.3574	22.90896	17.52792	12.09746	6.256607	15.5158	10.59156	32.95886	20.4296	42.04861	10.51838	28.89871	27.34971	26.57189	33.08424	12.83977	32.05943	26.9677	9.187163	8.720871	10.14417	21.58992	32.66921	17.01954	NA	13.72176	9.952015
CUST_12965_PI429010886	N30362	0.007474641	15	12						33.69474	191.2871	71.5149	24.4795	38.70148	58.63553	84.6858	91.58124	88.67629	113.4005	68.24816	41.53336	141.4085	88.84654	126.9698	80.75468	456.2678	185.0283	52.13985	24.05563	44.12566	56.05212	114.6239	46.75132	125.6161	94.27306	26.09836
CUST_37847_PI429010886	M21852	0.007476334	24	3						4834.858	160.4433	4253.847	5502.319	4655.696	5184.879	339.9532	292.1626	114.6133	656.7134	3604.261	3832.521	489.1563	4641.846	4863.888	254.7629	93.40586	220.7089	89.08902	276.2644	114.413	311.3298	237.6854	294.8466	95.02561	517.1871	132.3768
CUST_6182_PI429010886	N19737	0.007476334	24	21						21.54756	18.28716	40.54423	23.25083	16.4504	16.46661	25.91577	20.18416	18.47386	30.5922	20.1058	26.50208	29.94695	19.5987	27.83542	29.96998	34.06673	32.93541	34.70965	23.86873	36.53636	34.36428	33.14692	37.57177	42.4997	38.97889	36.03727
CUST_2890_PI429010886	N13325	0.007485917	12	0	242025486	conserved hypothetical protein			1.68E-166	52.58375	52.08844	42.4928	32.59654	15.28431	30.77017	25.42047	30.37143	38.63208	54.67791	42.1572	41.83378	66.39483	33.3626	65.04883	44.53304	35.02813	42.45213	51.72314	53.53275	45.24721	48.8281	34.4378	37.76461	63.95679	57.30682	64.71994
CUST_53149_PI429010886	M100409	0.007486021	15	0						141.539	127.8235	NA	44.46588	15.64145	33.53007	94.90821	34.27669	81.118	20.51742	24.96126	10.15208	17.34867	59.57649	18.01822	141.7833	58.85992	58.64151	114.7172	55.23799	104.8681	NA	222.9863	4.140032	12.85464	23.47308	6.423762
CUST_52075_PI429010886	M96328	0.007497841	12	9						1140.807	193.8796	1802.44	3827.862	770.3832	1791.446	383.132	885.1856	1632.022	2237.142	4077.152	1666.041	3437.158	996.3906	1610.862	27.25185	25.49661	33.51493	1644.807	1331.886	1758.225	2440.627	5048.826	4353.655	1195.903	2805.21	4368.987
CUST_11742_PI429010886	N28333	0.007504979	21	0						130.24	136.8929	NA	56.76972	NA	47.64701	140.662	93.3634	113.9699	5.461689	3.452627	NA	16.39884	112.5405	9.361001	103.4513	71.02716	30.6407	126.5057	55.95026	88.62537	NA	116.6562	NA	NA	52.73066	NA
CUST_30110_PI429010886	N61193	0.007505267	24	18	443707500	hypothetical protein CAPTEDRAI			0.000000231	NA	NA	6.363307	5.59915	NA	4.453212	7.267337	7.976904	4.576981	NA	NA		10.3668	7.277984	5.585409	5.730283	6.833148	7.982621	11.988	12.67284	13.47249	7.465917	6.717766	5.882831	10.56207	NA	10.28394
CUST_26272_PI429010886	N53960	0.007513493	12	9						7.544076	NA	12.8639	4.873307	3.453033	6.948808	NA	5.297656	6.325259	13.25495	3.245659	18.92414	7.308662	NA	8.558176	5.246901	5.794673	4.42489	6.472863	4.330868	10.08687	15.32206	NA	10.39641	10.98917	7.564006	8.494969
CUST_12098_PI429010886	N28920	0.007514816	24	0						8.747232	6.601408	8.386993	5.837765	NA	7.253451	NA	NA	7.457449	5.768746	NA	NA	8.506507	5.916014	4.128588	7.20082	7.860942	5.770813	6.459779	5.990546	7.361901	13.26584	12.73565	9.519989	10.35153	14.84471	17.43476
CUST_33496_PI429010886	M7056	0.007520376	24	15	321467207	hypothetical protein DAPPUDRA	Protein metabolism	Proteolysis	2.36E-34	206.2842	325.5716	257.0734	152.5742	173.0814	189.054	196.2571	200.5807	212.2981	234.1352	192.2662	240.84	196.4999	207.4823	220.9078	220.5454	301.4906	327.8085	200.3043	209.0675	269.8306	239.5431	195.6118	224.5032	208.8218	183.8614	258.1565
CUST_5096_PI429010886	N18251	0.007520376	24	21						1931.709	3017.306	2687.973	1941.997	1803.597	1571.574	2356.919	2434.185	2831.186	2538.621	1879.179	2010.612	2829.469	1744.803	1849.733	2038.719	2877.124	3064.959	2648.467	2960.558	3101.358	3182.56	2870.034	3516.69	2811.043	2862.188	3798.572

Probe ID	ID	p-value	Period	Phase	EBI ID	Annotation	GO category	Go term	E-value	0	0	0	3	3	3	6	6	6	9	9	9	12	12	12	15	15	15	18	18	18	21	21	21	24	24	24
CUST_1072_PI429010886	N8409	0.007520376	24	15						547.9685	951.5195	689.88	569.3405	369.3009	443.2849	773.3668	651.7291	788.7271	524.517	508.9078	561.1654	700.359	558.8184	611.3316	753.9335	957.7854	985.4686	729.6122	758.5678	969.0998	651.5453	753.9335	896.6099	680.5705	506.9058	869.9307
CUST_1556_PI429010886	N10057	0.007526748	24	3						16.98033	11.66289	24.54963	17.53011	14.70588	14.57803	19.63427	12.75342	10.31356	5.942887	8.704745	10.68195	19.68364	7.95738	12.75519	5.783029	11.01416	NA	13.79706	9.570901	6.939412	11.37517	12.90656	8.71517	17.0629	14.31897	17.52622
CUST_15395_PI429010886	N34511	0.007527652	21	0						2509.266	2359.281	2374.203	1407.95	1638.477	1955.527	1045.59	994.081	1349.624	1916.926	869.9307	1410.73	1550.781	543.3224	1053.276	1719.004	669.1662	1282.19	2484.399	1091.584	949.2292	2935.737	1338.071	2584.904	1496.297	700.1848	1156.223
CUST_20826_PI429010886	N44136	0.007538992	18	15	328721059	PREDICTED: hypothetical protei			0.000000843	127.6164	229.2847	47.6242	192.9259	51.9988	218.3332	247.1999	247.5439	141.6537	197.5933	148.9308	109.8243	349.1843	319.3967	348.2785	619.2009	492.5086	410.9137	307.0061	117.8103	222.9112	71.75645	314.3155	68.38347	421.8557	406.254	40.00526
CUST_14542_PI429010886	N33053	0.007549622	18	3	321464484	hypothetical protein DAPPUDRA			1.14E-71	11.35215	13.24735	17.12042	12.55746	11.45478	22.18677	17.26262	13.3975	9.265375	6.331516	4.541722	13.47249	10.42867	6.606839	8.452888	13.32146	6.473882	10.66525	12.11179	10.65432	9.985418	15.68492	18.96294	12.15359	14.485	11.66554	12.53713
CUST_28339_PI429010886	N57757	0.007550293	15	12						36.29093	17.01954	29.55431	28.51548	18.55929	10.86736	29.38566	21.31272	20.19611	34.86224	32.98843	44.22224	37.1161	21.53519	38.6365	27.08528	7.406463	30.48044	25.30627	19.99659	17.27642	28.4048	15.56798	24.62932	30.06311	28.13497	27.87333
CUST_31430_PI429010886	M1507	0.007556762	12	0	443727635	hypothetical protein CAPTEDRAI			1.86E-28	55.56698	50.56209	45.50498	47.52511	31.36919	41.06421	39.18663	29.79615	35.32719	46.28626	30.99993	34.06404	56.50958	40.06052	37.54467	37.82506	34.09591	25.97804	21.3859	36.72586	22.55249	51.80267	43.37269	44.84883	42.46657	37.12565	47.72966
CUST_7874_PI429010886	N22203	0.007556762	12	0						78.70208	42.5644	57.69989	42.6374	28.61744	23.91545	27.87026	20.31011	20.90369	43.22841	34.16334	21.11455	37.64453	23.43505	32.62087	35.59076	30.38583	33.63811	28.3766	25.23371	39.50225	28.14643	29.86767	28.61945	35.91186	38.85987	53.91461
CUST_28251_PI429010886	N57597	0.007556762	12	6						86.81904	69.00406	78.71874	117.0413	87.88247	95.02561	264.6223	153.3624	306.0384	108.623	89.70467	159.6864	129.8456	113.7894	89.4662	235.7954	101.2925	163.1238	174.2606	144.3062	240.3058	126.4468	111.3972	136.4438	181.9215	143.6223	108.4858
CUST_38853_PI429010886	M31235	0.0075585	24	9						795.5533	439.1006	562.954	1738.077	501.1967	997.2176	748.709	1013.822	1197.753	583.1603	4015.947	958.49	1047.139	1459.585	472.4073	848.9964	2047.134	1446.549	253.5695	705.8467	434.2782	359.6695	505.369	710.4288	79.07706	179.4316	160.8732
CUST_43304_PI429010886	M65561	0.007558517	24	0						37.39308	28.78434	37.93535	9.713824	22.72575	16.15211	32.73463	35.76279	65.69029	14.42489	25.43515	22.68661	18.17826	19.95709	17.54443	10.84565	19.71754	9.659899	10.28025	50.56675	30.44744	35.82514	28.551	23.80329	44.52224	26.4274	40.991
CUST_51083_PI429010886	M92094	0.007558517	24	0						71.85783	66.3493	77.2068	63.62492	53.76691	64.37351	45.57662	41.31713	50.19242	71.44611	48.96667	41.27805	80.65861	76.65834	43.24498	58.51048	65.62334	46.10518	89.44264	68.03257	59.67994	106.9674	77.01155	44.70348	92.88966	94.90029	83.31337
CUST_7672_PI429010886	N21898	0.007558517	24	0						274.2134	310.9804	416.251	324.9732	270.7536	390.2201	218.889	307.9833	377.4146	227.5059	292.5506	278.2449	297.2551	219.923	231.4277	215.1868	213.4123	241.8418	284.2467	281.5287	336.6729	232.1097	276.0279	310.2309	318.2644	279.6802	456.5305
CUST_32849_PI429010886	M5265	0.007562537	18	15						250.9068	438.2874	126.4468	159.5479	32.80252	84.98418	316.3525	203.5459	161.2674	225.8691	135.1101	9.394115	385.6874	256.8674	231.134	373.7341	677.2359	225.3038	327.6721	85.26437	256.6162	108.2084	51.83708	171.0499	448.4659	243.4049	125.4154
CUST_48740_PI429010886	M81953	0.007562537	18	15	332023809	Vigilin [Acromyrmex echinatior]			9.52E-32	833.5137	716.1458	525.9937	615.1114	381.849	525.6391	1147.195	649.314	789.8715	885.8693	383.2384	560.4998	724.6231	534.2591	594.6179	1337.377	862.1694	1040.093	904.627	490.7221	707.3888	539.4622	433.0002	490.5865	687.0247	597.9185	519.6084
CUST_717_PI429010886	N6903	0.007563625	15	12	345482582	PREDICTED: aminopeptidase N-l	Protein metabolism	Proteolysis	3.93E-51	475.8315	931.8674	285.7364	424.0822	124.6245	325.7046	911.7924	677.9076	341.84	962.3475	624.4152	235.4891	1009.169	661.8815	628.1097	2177.847	1342.206	2034.424	172.2067	140.6285	129.1607	441.2406	506.3774	324.921	1400.583	876.9516	281.6983
CUST_39252_PI429010886	M36727	0.007564291	24	3						2088.583	22.89373	1375.234	3851.521	3660.946	3208.57	93.13119	72.81177	36.43276	39.09998	2150.693	2447.506	67.62577	8644.801	6897.446	16.00252	47.33022	54.26863	6.429065	24.25396	7.457449	215.9178	94.76517	292.2414	16.0188	48.99281	20.72897
CUST_5127_PI429010886	N18293	0.007566318	24	0	260801994	PREDICTED: hypothetical protein BRAFLDRAF	Metabolic process	Carbohydrate metabolic process		777.6219	577.9786	773.0893	536.9014	408.9306	431.8791	415.3144	361.3178	432.6388	822.9225	429.5829	558.6463	720.9725	267.6739	353.9275	404.7001	466.8411	287.1316	383.8668	343.8057	218.4783	467.8312	600.976	500.7179	523.1285	662.3233	777.0993
CUST_37265_PI429010886	M18434	0.007568556	15	9	13898841	CUB-serine protease [Panulirus a	Protein metabolism	Proteolysis	8.86E-31	174.4941	210.991	320.8212	108.568	243.6336	37.19071	150.7849	139.1998	236.2202	262.806	357.296	299.2328	201.7307	224.7721	356.9618	181.3177	264.8027	144.2677	158.9852	197.4942	102.8306	203.4662	44.79451	198.7781	115.5828	425.7716	589.8797
CUST_14990_PI429010886	N33813	0.007569279	15	9						49.78973	22.30972	107.8039	165.2661	82.27528	141.2799	58.08676	61.75273	107.0876	407.7964	418.6023	366.7915	80.25802	50.23652	43.9077	NA	10.31745	NA	184.1143	63.68621	171.0275	182.6495	197.7072	155.0651	126.6423	108.6739	197.2216
CUST_19263_PI429010886	N41271	0.007584467	18	6						10.06086	22.44883	NA	204.6859	59.6688	165.7618	93.04693	424.8811	412.9087	53.37075	59.80532	8.681841	41.13599	87.20964	86.07541	43.27654	80.16932	72.22265	101.361	47.21843	57.64367	53.17689	40.48248	87.42396	131.0463	145.9895	234.4037
CUST_18999_PI429010886	N40807	0.007598202	21	6	321478953	hypothetical protein DAPPUDRA	Transport	Protein transport	6.56E-67	58.69436	41.65544	37.51134	52.34706	52.44267	32.41159	59.47811	49.48565	44.69809	41.50754	39.22128	69.06046	51.73381	38.94229	42.27337	38.81388	22.60023	42.62886	42.11956	14.5199	28.72917	46.82802	43.8169	39.89679	43.34	47.13	80.77747
CUST_55179_PI429010886	M109018	0.007600801	24	3						1039.627	NA	1150.764	1378.281	1260.081	1380.017	NA	20.81138	9.003802	450.9209	1798.348	366.4491	677.6753	1193.156	1532.345	NA	4.403942	4.222134	8.914356	28.41121	13.56629	828.1272	569.3405	805.476	47.87148	137.8056	126.4673
CUST_44698_PI429010886	M66704	0.007606884	21	9						72.5607	56.65028	52.6814	66.49647	109.8092	66.22157	79.30248	66.63719	72.23349	85.98865	77.87097	95.69298	76.69145	80.01788	127.8764	76.31853	41.47035	75.98892	84.57655	56.44652	92.68642	66.05158	65.51653	64.18993	72.75115	83.91189	107.7215
CUST_34474_PI429010886	M9776	0.007615493	18	15	291236799	PREDICTED: MGC80816 protein-	Metabolic process	Cellular amino acid metabolic process	1.62E-91	18.50635	24.61357	12.1921	13.76817	13.45946	20.0084	19.06805	33.5806	17.90327	25.54534	35.35085	18.71717	16.10329	NA	23.52214	26.87222	38.23559	28.51548	14.37116	14.0177	18.87134	20.62724	11.13493	12.08009	26.86328	17.75786	23.31843
CUST_13854_PI429010886	N31892	0.007616956	15	6						9.721747	13.36765	10.87054	8.360391	12.78321	9.078909	10.7714	18.01233	13.24663	7.920298	11.21773	5.396608	NA	10.35834	5.873873	7.248302	6.534983	10.70603	11.68939	13.09533	11.87886	16.78894	29.58629	12.16843	18.94246	15.29246	8.460507
CUST_5030_PI429010886	N18173	0.007618043	24	21	345497365	PREDICTED: semaphorin-2A-like				235.4891	379.5535	338.0427	275.7847	211.3422	227.3167	299.9819	319.9319	323.2204	303.7619	215.1868	294.6196	350.2239	254.0305	273.5066	305.3135	379.6086	334.9433	393.358	337.0445	340.7848	367.1233	346.5231	398.6975	353.5003	295.7504	393.8421
CUST_44028_PI429010886	M46698	0.007626422	24	18						NA	7.156722	9.928002	10.17714	6.378654	5.510486	182.9511	6.537448	134.3368	17.08055	12.48615	NA	28.46437	11.22943	NA	15.41425	5.140584	20.25781	258.2813	401.3498	913.5277	46.69609	53.86055	30.98378	86.62987	11.62724	71.12138
CUST_7889_PI429010886	N22228	0.007632387	24	18	321477199	hypothetical protein DAPPUDRA	Cellular Process	Cell growth	1.52E-73	77.34627	118.1364	96.91497	75.77051	77.8809	66.2384	62.95202	90.68839	91.87245	90.35029	72.49896	87.53029	82.35709	74.49329	78.80045	81.42279	137.9765	105.1495	91.75455	112.5697	125.3758	96.9591	104.8945	104.0183	99.49612	82.19156	104.1208
CUST_22043_PI429010886	N46318	0.007632387	24	3	15718457	peroxinectin [Penaeus monodor	Response to stress	Response to oxidative stress	3.99E-32	33.75196	21.30308	18.74814	43.56758	93.55501	68.33012	26.36566	31.80176	15.58835	22.53853	43.40182	44.28707	35.23063	36.04692	45.8224	20.84673	15.96403	17.92151	21.43699	27.87602	12.94982	21.20837	10.42743	18.02157	21.77765	37.2113	23.23151
CUST_92_PI429010886	N735	0.007633402	15	0						25978.53	18758.28	20563.31	33176.01	9629.338	20708.31	15900.37	14256.4	13481.03	8827.334	13685.2	9889.351	12496.19	23231.4	19791.01	16553.17	19010.63	17837.79	13737.98	13098.74	14642.96	10626.1	22092.06	12648.44	9376.495	13988.25	11952.65
CUST_37496_PI429010886	M19243	0.007644631	12	6	290908989	myosin light chain [Marsupenae			1.34E-40	825.7252	711.4323	851.1928	973.3791	753.1726	833.7381	1462.033	909.9142	1069.345	889.3882	816.5697	789.0657	848.3549	717.7206	442.5726	1631.254	1183.911	1393.298	1537.641	1166.063	1888.631	906.8146	828.8502	937.5478	1132.475	764.2764	1102.526
CUST_43785_PI429010886	M63977	0.007644631	12	0	157134727	hypothetical protein AaeL_AAEL			1.64E-18	40.28089	41.86801	52.68847	42.92948	37.31972	35.10272	33.22428	33.57662	35.62951	42.18222	38.21258	27.89407	60.45051	31.67678	45.55903	34.09891	34.76743	26.15886	26.06089	20.8587	27.51459	49.42933	23.88306	50.84751	41.89699	36.84812	47.57931
CUST_50801_PI429010886	M91042	0.007648959	21	15						138.6464	332.0817	25.01557	279.7318	43.78128	320.8212	509.7706	391.6595	182.702	378.8803	279.8938	118.8608	537.8527	325.5716	488.2191	836.1158	564.8143	664.2531	414.4128	98.37749	352.0796	82.08894	312.1666	100.0827	771.0455	617.6234	23.78991
CUST_38781_PI429010886	M30311	0.007652248	24	3						69.93972	27.40129	72.71537	104.9484	401.3498	189.5806	33.89153	70.14984	18.86919	25.25032	364.5679	293.934	17.98274	205.6925	185.8751	26.30097	28.87489	11.53597	15.79093	24.56009	12.02318	38.07838	38.65223	30.84622	5.472738	56.00071	13.08598
CUST_7715_PI429010886	N21964	0.007652248	24	15	169931080	cuticular protein [Artemia franci	Metabolic process	Carbohydrate metabolic process	4.97E-27	564.5167	454.5758	398.4892	593.3017	1453.134	1177.475	356.4497	616.0609	273.8482	415.6373	1049.045	1015.318	267.8251	685.2688	722.3326	479.2755	385.4238	332.944	260.1465	399.6202	238.775	370.5351	371.2053	328.6973	306.0384	675.5044	360.2285
CUST_9068_PI429010886	N24013	0.007652608	15	12	291238891	PREDICTED: RAS protein activato	Signal transduction	Other	4.4E-14	18.95217	30.3574	23.92527	38.45768	28.89254	24.16822	12.48615	17.07418	16.05064	28.99089	25.55323	47.20876	22.51636	30.90807	51.83708	28.13189	23.95909	26.33135	21.89919	25.18751	29.28939	15.68303	14.21299	22.67946	34.37978	25.7341	32.98204
CUST_25095_PI429010886	N51774	0.007654763	18	15						29.02458	30.44744	22.41168	19.01967	17.75609	23.5771	44.22224	42.93819	36.98876	34.18834	33.33788	27.92224	25.17932	22.74717	20.61232	42.94276	37.68818	46.7009	30.3832	32.69318	25.02902	26.0713	21.38839	19.56944	29.36748	22.1072	29.666
CUST_1373_PI429010886	N9437	0.007654763	18	15						178.3394	368.9684	249.5189	192.3394	115.5416	202.183	438.1085	331.8463	289.7381	389.6444	191.2871	191.082	365.3294	213.1128	216.5705	552.0297	476.9732	522.858	345.8264	197.1967	410.1393	175.1243	224.8574	220.4205	394.193	277.4142	243.543
CUST_38027_PI429010886	M22886	0.007656679	24	0						8430.296	17658.04	20616.2	12211.42	3859.53	3968.622	10897.12	10871.96	13559.72	5394.696	4913.686	3085.208	11289.6	6452.703	4090.127	13281.09	8321.498	13098.74	15630.09	14580.94	16690.76	7610.57	10142.18	10294.69	14764.65	10605.81	16070.74
CUST_47018_PI429010886	M74523	0.007660856	15	9						45.24721	33.16469	49.63757	42.24729	30.69119	33.48088	44.97817	29.67693	33.746	54.56863	34.94317	59.9228	50.94583	37.17438	42.89355	39.16223	32.73463	28.69416	31.90012	28.24507	31.08617	47.97144	36.06722	47.71104	58.9878	37.31263	39.23752
CUST_12473_PI429010886	N29519	0.007685118	18	3						8.15959	20.44981	13.6668	12.02678	30.72076	21.81818	13.905	12.40953	23.69946	11.27508	15.49423	8.950493	9.101709	9.548881	9.326879	8.428222	21.38085	15.41579	13.1424	25.09267	23.80329	13.20553	44.7999	35.07134	9.569825	19.32802	17.32917
CUST_55603_PI429010886	M110921	0.00770253	21	0			Transport	Other		17.82688	10.5374	21.61569	14.54604	9.708019	17.05355	27.71884	14.20626	5.561634	12.08009	8.378958	13.71712	6.968697	9.640177	7.21144	15.37929	23.9895	11.61625	12.61861	14.42132	14.97194	28.23949	26.0713	13.48116	12.2567	15.0561	9.843601
CUST_44357_PI429010886	M65706	0.00770253	24	21						18.49557	14.91599	18.8308	17.39682	15.18833	17.61709	14.96774	16.96246	11.15725	6.467201	16.59434	18.10243	11.81841	7.578741	18.99016	19.47164	12.39656	13.66174	15.93995	14.80006	18.19202	22.629	17.6345	23.85395	22.81234	23.40815	17.54223
CUST_35496_PI429010886	M12718	0.007707865	24	0	72160341	PREDICTED: WD repeat domain-	Metabolic process	Other	8.4E-86	56.3623	59.30659	68.41738	41.52352	34.39051	43.05092	36.96718	44.03097	47.47428	47.28223	69.55079	44.42034	30.73256	33.94092	42.90901	51.93257	50.16621	52.56129	49.32564	50.84751	40.88884	37.71176	54.71169	49.19586	55.34035	52.84948	53.65203
CUST_27308_PI429010886	N55821	0.007707865	24	0						43.56248	31.45292	43.5104	19.87929	35.02468	29.7549	32.76569	28.37326	29.35282	11.21622	40.43868	36.72221	29.52349	25.58133	17.02151	35.93556	25.53483	31.64857	38.38502	24.41916	25.0763	33.67688	28.66131	29.18456	37.45982	40.97531	47.83193
CUST_57048_PI429010886	E154	0.00771571	24	21	399936392	voltage-gated calcium channel b	Transport	Ion transport	1.63E-45	396.05	507.7884	441.5527	331.6434	435.8605	374.9091	333.8469	435.1623	445.9579	422.8235	406.5634	284.9168	472.7524	371.2053	459.6187	506.9058	498.1009	562.4363	402.5585	412.5149	538.9124	480.6993	538.8011	522.0737	488.48	462.0354	617.3702
CUST_43559_PI429010886	M63351	0.00771571	24	21						168.8172	261.3125	207.4443	197.5933	231.2123	156.768	156.8206	257.4878	199.2769	178.2425	203.0154	176.1711	168.7955	181.4338	208.0709	178.619	277.0946	206.5859	195.8784	231.0141	263.8276	235.6412	308.6874	302.6664	231.9647	241.0942	324.1912
CUST_15386_PI429010886	N34500	0.00771571	24	3						508.1759	308.1102	343.6496	409.9614	405.5854	500.1661	242.8747	302.6134	183.6447	198.1815	292.7159	295.6289	157.1785	270.4208	264.9542	230.1554	156.3694	183.6827	229.3211	215.5592	184.5342	380.5457	218.9179	322.6893	161.5143	213.3759	193.0588
CUST_20197_PI429010886	N43005	0.00771571	24	15	321468304	dishevelled-like protein [Daphni	Developmental process	Multicellular organismal development	5.05E-43	18.1699	17.91593	18.58892	18.77059	9.160132	17.81281	19.10616	15.13566	18.69594	15.52247	16.36771	17.84997	22.12571	11.3602	21.65945	23.17996	26.4274	24.71432	20.76264	15.39457	21.2032	23.5397	20.78849	26.20109	20.96508	14.62836	19.78273
CUST_21921_PI429010886	N46098	0.00771571	24	15	443717346	hypothetical protein CAPTEDRAI			4.4E-31	34.37539	46.24985	17.84342	24.19026	17.82226	26.83914	30.26815	46.43451	23.90216	52.21427	33.63302	28.12273	35.97882	38.81388	28.5396	41.77489	52.43098	55.24742	29.83399	39.43512	27.59657	45.93201	31.00644	24.54963	22.41344	47.46533	30.84622
CUST_988_PI429010886	N8133	0.00771571	24	15						124.9247	179.9884	125.4804	111.6045	101.0339	113.015	139.8891	128.0875	129.4603	150.7719	89.73784	115.4585	110.2733	148.2361	109.7513	159.9694	176.6026	197.8624	136.9021	163.782	133.3046	131.9274	153.4348	142.4928	141.5029	150.7849	137.7481
CUST_43311_PI429010886	M62710	0.007721142	15	0						22.86698	25.70782	12.96173	10.33674	14.29283	21.73515	20.53592	23.65361	12.76402	6.110374	11.87593	9.082829	11.91014	26.4485	19.6251	21.86715	22.48649	23.38714	14.57956	18.46268	10.60676	15.07053	45.08105	19.33678	8.559475	8.96678	11.51019
CUST_44649_PI429010886	M66572	0.007733062	24	12						73.93804	35.78924	67.52243	85.62565	97.52903	105.1602	50.0221	43.05532	67.14461	63.88807	79.82778	97.21065	83.83043	111.4813	194.1916	62.76751	46.72845	53.48046	67.10463	50.47426	74.60421	49.11012	39.4828	43.70175	60.04487	46.69609	65.42578
CUST_47756_PI429010886	M77488	0.007738053	24	21						25.61659	20.21086	24.73091	32.75067	34.13078	14.58324	9.11075	14.67236	14.77631	17.5656	13.15696	18.80957	37.28248	10.31608	36.10172	11.34354	13.72761	26.09025	28.19237	38.07503	37.86955	29.97997	35.03557	45.5101	16.61149	19.24274	46.32151
CUST_16503_PI429010886	N36346	0.007738879	21	15	321473698	hypothetical protein DAPPUDRA			2.89E-22	59.59396	109.5906	73.3346	86.31943	77.83598	64.71262	85.16727	78.18913	76.00489	63.14073	63.81813	54.16069	80.72426	76.68447	72.84435	116.3062	107.8221	125.6495	73.46044	90.05411	92.85843	77.808	78.5366	89.70467	105.619	66.94807	81.07922
CUST_53245_PI429010886	M100965	0.007740205	24	18						20.77726	53.41659	38.1445	20.3194	21.99164	22.9157	42.5644	41.18226	37.31972	34.93951	36.32296	35.06662	27.72385	33.5806	32.45769	42.80111	40.80443	43.72166	52.15113	57.62248	62.73874	29.85271	32.56414	46.2714	48.86558	33.86827	61.2068
CUST_53969_PI429010886	M103921	0.007740205	24	12						75.78646	54.46319	69.47326	65.52411	70.72448	57.58975	74.42622	59.07254	63.14866	78.39296	68.02537	105.4571	73.52254	41.94644	71.49082	78.18913	74.22801	79.2021	82.1818	69.93972	79.56634	109.3613	87.45577	91.27426	88.34301	83.64481	101.572
CUST_32704_PI429010886	M4879	0.007740205	24	18	242019841	Flotillin-1, putative [Pediculus hu			4.75E-77	72.50711	97.25517	88.59515	75.95975	84.83411	77.98875	75.43954	79.08754	85.98865	99.58967	82.54419	105.87	80.1827	85.71168	108.4858	92.02415	95.08224	115.186	99.19887	102.4924	133.2629	73.05642	101.508	120.3932	99.66557	93.27554	152.4015
CUST_33673_PI429010886	M7566	0.007740205	24	18						197.2216	125.4154	147.4173	120.6837	64.49711	84.21664	148.4289	188.3607	185.198	193.3026	138.9709	144.4561	155.8206	143.2998	114.7343	362.5008	121.8844	284.4662	537.7378	215.6896	441.4649	188.1296	108.1041	147.3568	394.7866	147.0849	285.4767
CUST_6970_PI429010886	N20851	0.007740205	24	3						503.6798	562.7989	656.9404	609.6218	714.2812	714.4899	333.7063	465.6755	481.8774	389.8933	673.8771	516.6055	430.8354	505.5201	579.318	386.8087	495.5669	496.3451	340.9357	436.7155	419.9233	454.5758	570.5532	486.1663	484.1868	443.4999	546.6865
CUST_15849_PI429010886	N35242	0.007740205	24	21	321473690	hypothetical protein DAPPUDRA			4.85E-17	29.97378	40.98047	51.17522	41.24111	42.70736	37.36406	33.18448	19.86462	25.61949	44.72843	26.66045	54.27691	55.14747	33.2361	48.47319	70.06906	32.77297	52.66618	40.87561	40.83466	45.84356	62.88893	47.70751	74.20038	72.18458	39.33451	61.33441
CUST_1524_PI429010886	N9934	0.007740205	24	3						57.47217	51.15383	32.65563	75.43954	36.41114	69.65321	53.11562	43.78128	38.02572	53.65203	44.28707	56.86607	41.79921	28.5396	39.82958	35.97416	54.22723	36.8404	22.35262	19.63812	19.62318	47.97707	34.6494	44.87713	68.16127	16.9478	38.3812
CUST_1184_PI429010886	N8771	0.007741592	15	12	124495004	putative cuticle protein [Artemia			6.45E-15	2547.238	1504.447	1520.832	2881.653	3232.565	2604.346	870.1856	1082.664	623.3324	1222.23	2058.438	2561.777	1641.783	3401.586	3851.521	3679.2	2030.062	2698.998	689.0797	1655.603	1063.435	1307.316	1443.341	1462.785	1292.915	2904.283	1180.485
CUST_47870_PI429010886	M77959	0.0077419	24	9	383850494	PREDICTED: stromal membrane-	Signal transduction	Other	6.56E-43	108.7426	52.11425	83.65467	60.5679	70.64915	50.88233	77.00089	54.93024	74.59679	85.63473	156.5799	85.38787	99.38318	66.66302	119.3101	76.83706	33.19846	57.63804	54.61715	49.0355	48.6073	64.27006	69.00406	69.48765	55.28196	52.2967	69.8604
CUST_31451_PI429010886	M1553	0.007746989	18	15						217.1972	223.3777	230.7682	163.0633	141.9808	129.9202	229.1263	206.3209	193.0974	185.1157	129.2573	251.6934	190.4954	178.8078	193.2061	311.6703	279.5075	289.3393	159.6647	203.4357	214.2511	180.2873	178.0114	199.5583	256.7087	222.5585	240.2302
CUST_26506_PI429010886	N54362	0.007747069	15	0						156.0675	174.2396	195.511	123.5754	197.2974	108.7707	79.25889	194.1489	107.4885	87.43473	155.1393	120.513	127.2055	145.6336	148.6436	174.935	248.554	168.8172	172.0985	163.8654	124.5521	140.717	158.518	130.9169	147.1495	90.15514	134.6822
CUST_25463_PI429010886	N52462	0.007779433	18	0	296245397	inhibitor of apoptosis protein [Li			5.03E-27	77.9006	96.26661	86.74668	83.21526	63.00637	84.35052	35.68728	115.3116	155.6724	50.1974	56.64171	45.64916	62.05097	56.26482	51.99386	69.91553	52.67704	51.59074	69.17898	96.37995	100.7211	82.44777	76.47464	74.00845	62.66376	84.65768	59.3203
CUST_50648_PI429010886	M90544	0.007785025	21	15	342326340	sphingomyelin phosphodiestera	Metabolic process	Lipid metabolic process	4.65E-20	128.0633	250.2647	82.9737	166.0161	47.86566	140.5949	268.869	205.9122	177.1325	198.6494	107.6017	94.41489	165.5194	217.8383	152.1662	477.058	366.157	323.1624	251.3778	100.8867	206.4922	84.25166	173.2392	102.7974	157.3269	151.7904	52.78692
CUST_39298_PI429010886	M37413	0.007793399	18	9						11.51735	11.71271	NA	14.93971	7.945183	16.01239	24.82867	18.34559	16.58967	55.15568	NA	18.3082	25.6591	18.14584	14.78309	13.86535	45.27328	5.125327	8.129627	13.25323	NA	22.30308	NA	15.87729	8.996542	22.91325	NA
CUST_41204_PI429010886	M57121	0.00780128	21	9	225581173	GA11316 [Drosophila miranda]			1.29E-48	15.5042	23.35468	12.92976	19.82476	10.79559	12.05333	22.27113	23.93863	15.27512	67.61972	16.1693	27.15396	22.06175	6.578516	13.5456	19.20294	13.95233	22.69882	13.11189	11.20517	10.54024	10.54947	14.20021	16.0336	18.61475	16.98896	14.67062
CUST_24826_PI429010886	N51308	0.00780128	24	21						109.4578	69.76252	93.35268	84.53983	62.49633	52.7182	63.61864	70.63954	57.8894	81.75855	74.43638	91.08467	121.0882	104.6784	74.18037	87.54238	133.6627	148.728	79.27327	94.80138	56.83684	115.6931	106.3228	109.4578	108.1961	81.45042	94.78884
CUST_44589_PI429010886	M66406	0.00780265	18	15						255.7014	226.9173	NA	135.9752	NA	144.1758	359.4486	75.63115	314.1063	36.72861	27.02166	4.434508	59.94713	129.1152	67.63991	597.5894	246.2584	278.4293	380.5457	27.96239	291.4248	NA	153.8104	NA	90.84788	87.75883	NA
CUST_17893_PI429010886	N38786	0.007807538	24	6						17.40595	NA	10.36977	35.5246	10.24111	17.88814	19.98797	NA	13.36765	13.88039	37.69822	9.167675	10.72353	10.07008	NA	11.27831	15.87243	12.79123	5.125327	NA	9.840114	7.21144	6.621148	4.329502	10.98177	6.870771	19.15475
CUST_27789_PI429010886	N56698	0.007809735	24	0						294.2691	250.3014	254.459	253.9457	292.7676	244.6553	231.2123	257.4304	195.9057	293.934	229.3211	368.8823	250.1228	189.7707	195.5458	202.1125	257.1241	220.2833	264.5903	208.0191	215.0811	281.0341	252.2559	264.5903	277.6138	290.029	308.4293
CUST_36343_PI429010886	M15669	0.007813377	24	15						349.1843	527.5856	505.1911	375.8148	343.3314	299.9228	478.9906	373.9602	395.1137	473.4786	477.3178	613.0157	507.5036	428.4643	436.8129	515.2465	563.1838	566.7623	363.9263	425.2574	545.7936	442.8695	424.7669	518.9718	439.7541	408.8076	680.9887
CUST_39786_PI429010886	M44700	0.007813377	24	15						7506.653	5456.239	4311.402	5025.735	1375.747	3052.051	9819.956	13248.98	12860.58	11572.84	4048.748	2933.397	9500.977	6310.155	3558.043	34620.42	11289.6	23379.75	22279.42	11305.05	17487.6	9056.645	1961.653	5726.275	16411.2	4041.944	6612.476
CUST_31060_PI429010886	M586	0.007813377	24	15	405970980	Cytosolic Fe-S cluster assembly f	Metabolic process	Other	1.73E-122	82.72734	115.4959	88.26602	81.17631	68.54812	63.43243	114.1912	126.1993	92.53323	155.5483	119.8471	128.9375	105.619	83.75475	79.72468	111.6675	123.8198	143.9807	101.4865	130.0221	126.5057	123.2485	105.3437	118.0906	81.01838	87.56469	126.4187
CUST_12727_PI429010886	N29942	0.007813377	24	15						343.5695	470.3803	354.4054	268.5317	285.1148	362.5984	340.8616	414.9926	386.7231	390.7977	400.7915	266.0382	354.0092	307.7409	268.9804	493.9591	588.5037	458.5926	420.3226	426.3157	390.2201	408.3007	449.5658	406.4569	441.8514	396.6952	378.6955
CUST_16734_PI429010886	N36773	0.007813377	24	15						38.15236	90.82582	123.263	76.54043	41.66058	52.67704	125.1706	148.892	235.9311	87.31684	18.1064	96.71212	97.74585	103.9647	47.68605	201.4635	224.1479	245.783	161.0739	193.1768	247.1999	85.14751	124.2158	77.48265	180.6504	41.85499	168.1289
CUST_43064_PI429010886	M61973	0.007813512	21	0						79.63863	109.6359	105.0177	76.30972	86.6454	77.13441	61.295	84.9077	80.86273	79.31808	73.42732	92.08367	71.80743	70.50377	72.06957	65.7755	90.08744	91.28627	59.06511	100.5671	96.69796	79.79406	112.0077	94.26006	91.52744	96.57692	75.9364
CUST_8868_PI429010886	N23724	0.007816001	21	12	157117495	golgi reassembly stacking protei			3.01E-13	39.31755	50.95848	45.60889	51.41632	173.05	94.10473	51.24138	60.78548	55.87434	59.82583	117.3251	60.12159	56.62992	93.74241	104.6237	81.10019	95.27189	83.92194	35.22067	39.03115	47.55964	35.92906	84.37355	45.55903	45.49065	75.09386	48.67088
CUST_39645_PI429010886	M42153	0.00782037	12	6						14389.61	17589.58	14703.33	23435.22	13879.42	16070.74	22526.09	23231.4	28858.93	18476.28	8547.585	13616.29	19855.57	17131.85	12921.27	24721.57	18802.8	18365.76	24341.41	23162.84	23084.2	21710.8	15340.52	21113.92	21524.84	11932.74	17424.76
CUST_48568_PI429010886	M81077	0.00782037	12	0	383859776	PREDICTED: transient receptor p	Transport	Ion transport	0.000000441	32.76978	36.78636	36.21321	21.35916	24.71116	37.7496	17.98748	21.75367	17.31771	42.62368	27.48203	43.51485	42.0926	34.74447	44.05907	38.48962	27.60403	31.61116	40.03196	30.68789	17.08255	47.54035	26.10756	35.25348	49.87572	42.27337	43.57254
CUST_6295_PI429010886	N19913	0.00782037	12	0						105.0092	54.86877	121.4525	106.4281	95.37708	72.75115	38.94629	48.90741	45.40562	92.31393	89.54662	126.1085	129.9202	97.18737	143.92	46.38013	62.99459	70.01129	53.49898	55.15568	49.7367	170.9048	121.8066	134.1808	113.575	57.58254	158.0057
CUST_12576_PI429010886	N29685	0.00782037	12	0						93.90326	79.00471	86.76212	98.91443	66.68058	74.4544	72.84435	57.93583	77.78042	99.87189	70.97468	85.11492	102.0603	61.88614	88.74114	83.7199	84.02947	81.4132	80.72426	72.85755	64.10142	88.22605	76.5464	75.82447	104.6237	85.50328	114.662
CUST_45816_PI429010886	M70390	0.007827124	18	9						13.18497	15.29719	14.18475	14.54879	32.79575	46.281	30.92492	15.90508	22.02956	25.35027	25.29891	36.06378	20.84673	23.90768	16.50853	19.04014	13.413	22.12571	14.62238	16.20857	13.59581	20.10978	5.718078	22.72787	13.00372	21.85018	29.47872
CUST_4970_PI429010886	N17953	0.007828162	24	18	226316411	retinoid X receptor isoform 2 [Ca	Signal transduction	Other	1.45E-42	43.37269	39.51395	43.96327	40.16189	49.44159	34.21333	55.95718	36.8117	60.90419	39.78508	45.03724	51.2209	49.48225	49.46595	73.24973	77.03919	40.34223	51.75429	67.47234	56.39944	77.63531	51.55894	47.14137	54.88564	53.56818	48.56908	71.80743
CUST_38240_PI429010886	M24541	0.007831009	21	15						124.1767	186.5368	150.4929	131.4002	160.5173	149.7056	154.6882	160.8211	160.5173	126.0542	149.533	148.2715	156.1618	179.4936	134.9328	191.3144	183.2355	187.922	184.1725	169.9145	161.4506	169.1505	176.6544	140.717	168.6428	171.5864	168.511
CUST_36214_PI429010886	M15031	0.007834779	12	9						7.301642	8.91045	13.58701	9.090753	10.63333	8.494969	14.67985	14.65418	11.06604	25.45303	8.668203	21.7713	15.10648	7.798673	9.187163	16.81278	15.05878	17.99711	13.81373	17.1166	17.07418	19.35535	16.34992	12.65731	8.429466	11.07815	8.847627
CUST_49599_PI429010886	M86355	0.007835004	12	9						233.5329	185.6848	283.8964	265.4961	169.4819	205.143	183.0682	187.0484	231.097	240.603	219.7957	278.6422	268.869	179.9884	217.1972	152.5349	182.7293	195.7481	297.1434	196.475	287.6916	309.7415	287.3401	375.3353	217.7355	188.0574	349.3947
CUST_40604_PI429010886	M55603	0.007836584	24	12						70.46471	50.64865	26.98424	34.99565	2930.842	1037.05	51.33814	49.1413	41.04596	9.412668	1664.278	1949.314	22.13988	2466.158	2457.62	33.94389	14.42489	19.23624	17.21141	25.89088	15.97652	5.754114	14.95707	8.093745	10.26947	32.23135	3.791233
CUST_19867_PI429010886	M42350	0.007837114	18	6						26.71617	22.26355	16.38858	20.40329	29.20036	27.57421	27.29484	57.08138	33.15745	11.88179	17.29448	18.59394	9.416468	21.11041	14.4297	24.65528	9.206401	13.87312	34.35183	32.48271	23.8882	19.35085	26.36289	15.39125	28.00501	26.7573	25.49446
CUST_2168_PI429010886	N11755	0.007839215	18	15						450.3327	1109.283	731.069	613.3285	494.7611	459.1993	1180.485	1162.325	997.2176	786.6307	581.654	590.1632	737.8151	649.1337	618.7694	860.9694	1453.877	1241.8	768.539	740.2235	1040.093	515.3493	666.8579	517.3443	703.9211	684.1238	697.732
CUST_48681_PI429010886	M81606	0.007845455	15	6						663.421	796.2861	872.0312	1159.706	1026.869	498.3024	2455.967	776.4904	2312.635	1598.489	2039.749	1284.327	2389.711	1810.6	867.9312	255.4927	1131.397	840.4028	969.4842	818.3565	1269.839	1504.447	2742.804	3350.998	721.9277	1933.198	2501.925
CUST_50674_PI429010886	M90612	0.007849369	18	6						8.601925	5.934466	13.51353	8.135191	10.74202	14.83547	14.15162	15.14706	23.42712	15.57359	14.42774	15.77123	12.38845	11.86035	18.72559	17.43071	7.515502	19.52955	9.536214	12.61395	8.804391	15.29086	13.19768	22.96223	23.9493	15.96179	16.12104
CUST_702_PI429010886	N6827	0.007852865	18	0						843.5336	3914.879	4719.533	1447.238	939.4725	3.738396	1601.274	2913.515	4554.072	294.2148	613.5208	185.9927	558.1235	481.6057	75.38714	1500.89	798.5585	1767.886	3108.034	2551.022	2889.672	842.9082	2083.733	2057.214	2045.264	1085.793	2924.097
CUST_27214_PI429010886	N55645	0.007853004	24	15						700.1848	759.3274	459.1993	711.5947	820.3087	604.6962	716.6417	700.5788	717.7206	872.0312	246.956	798.5585	704.142	853.7719	611.5217	862.533	1054.373	1196.962	660.1893	622.1839	843.5336	661.1838	817.4569	931.5266	634.6359	592.215	602.4086
CUST_28259_PI429010886	N57617	0.007861995	15	3						161.5143	123.8699	285.2599	552.4266	260.2442	167.872	134.9856	301.2626	267.8251	217.8383	48.39798	120.4309	225.1699	67.9925	96.44195	350.8666	725.3859	951.856	422.1689	1039.149	1594.801	262.2441	151.421	79.14448	254.459	15.15512	280.9977
CUST_22306_PI429010886	N46785	0.007876368	12	0						10.82105	NA	8.81061	13.68243	14.87994	6.7642	36.34385	40.3254	21.21779	20.79498	10.6734	8.449479	11.60633	17.32449	19.5652	15.68096	15.52429	12.18092	31.38536	39.579	18.11723	14.45063	NA	10.38364	7.833379	NA	10.5335
CUST_44764_PI429010886	M66909	0.00787737	18	15						155.2643	183.5637	177.9861	156.1818	186.6281	167.8421	146.3562	177.1325	134.2508	136.3915	122.8503	153.6189	131.6028	134.2073	153.4511	291.1427	185.1157	194.821	194.7103	155.8444	163.8102	155.4242	159.0077	173.4784	178.596	167.7409	157.1328
CUST_32710_PI429010886	M4889	0.007884178	15	9						147.0639	65.54992	218.3991	271.7825	289.1658	82.73518	158.0988	197.088	343.9474	201.6125	415.0852	141.4787	631.2553	201.7698	296.5339	36.47269	77.34627	40.68971	357.6352	334.4355	378.5271	771.0455	728.0598	552.7385	488.0472	395.4036	978.9259
CUST_29999_PI429010886	N60975	0.007887481	15	6						144.2677	77.85193	128.2877	125.1571	121.3422	105.3095	135.0936	84.16808	47.32106	153.9962	91.7443	119.9872	158.418	89.2783	134.6822	125.3249	153.1388	131.8067	116.2734	108.7185	129.4913	105.6776	116.2567	103.8862	102.0887	103.9866	43.08271
CUST_43142_PI429010886	M62184	0.007892824	18	6						36.72221	37.42766	32.28165	26.57189	36.8445	21.21056	61.5382	22.50572	47.60538	43.64769	29.85271	41.3426	32.96923	49.29631	49.62	55.24242	29.0216	42.62368	41.65139	37.45713	58.33297	22.89867	26.22317	25.74471	39.63818	30.26815	34.09591
CUST_51874_PI429010886	M95499	0.007908239	12	0						57.07543	39.67832	68.66348	43.57748	49.23192	37.64453	35.8443	31.01077	38.12534	52.61041	43.0146	60.43161	41.94361	54.83706	47.95196	56.55385	33.93179	39.08885	39.73627	34.74447	36.22402	36.56104	42.1373	49.81991	52.25224	43.17122	47.36534
CUST_25159_PI429010886	N51888	0.007908239	12	6						22.05957	12.71074	25.12027	14.03403	18.73426	13.01309	13.58834	11.64005	7.713675	17.50283	12.16843	24.43421	16.11449	16.22893	27.47705	22.71736	14.90324	12.06381	11.1702	11.6476	13.25948	23.0352	16.42634	13.84038	28.84569	12.96468	18.16767
CUST_54510_PI429010886	M106293	0.007911045	24	15						377.0504	135.4737	220.7089	165.742	11.38568	137.2208	312.008	783.6564	739.0418	680.4062	470.9814	65.34281	289.56	304.096	120.0673	1339.398	686.6456	1116.065	800.3618	699.596	759.3274	356.2511	28.44038	233.7126	664.0464	290.3829	322.2486
CUST_56302_PI429010886	M113439	0.007911045	24	3						25247.34	804.0438	49113.82	72257.2	66456.69	75071.87	4710.772	8768.122	12699.11	58747.16	91877.59	66946.06	70445.35	48051.75	54162.9	937.1039	345.993	1379.279	9376.495	17837.79	17315.8	52429.1	52815.17	66946.06	18070.71	54162.9	52429.1
CUST_49810_PI429010886	M87260	0.007911045	24	15						22037.75	31967.8	22037.75	22598.48	19485.9	20222.93	27918.92	30447.23	25555.67	23231.4	18966.14	23084.2	25188.43	22598.48	24984.55	33508.11	29520.35	36802.85	25555.67	27665.22	29219.06	20708.31	24289.54	23575.84	28414.17	23503.18	27347.94
CUST_2686_PI429010886	N12905	0.007911045	24	15			Protein metabolism	Other		418.0505	757.8952	670.9198	463.4514	503.262	473.3422	622.1839	756.6003	777.3446	551.5575	561.8923	414.4974	522.1886	490.3414	482.8348	840.0505	986.649	903.1163	682.7328	693.3608	840.4028	538.9124	579.318	530.9393	604.3308	504.7574	664.0464
CUST_39952_PI429010886	M47406	0.007913282	12	9						16.40332	19.32331	23.81811	23.15112	13.23504	4.740256	NA	10.17169	16.38536	9.084966	41.86801	16.93807	29.28939	13.1174	14.69477	4.932654	5.025274	4.757963	17.16545	16.08057	12.91815	25.00741	82.9737	76.11372	6.686074	40.61255	44.18768
CUST_38296_PI429010886	M25105	0.007926119	18	6						28.86297	21.71446	29.38788	49.53225	22.95294	32.23135	20.21346	16.78356	14.99561	14.29901	17.85791	17.83592	32.18121	27.00456	28.88967	10.74906	11.97294	23.75071	18.74814	21.48061	17.33337	51.46015	42.48109	36.81898	17.8457	29.83087	52.76723
CUST_43928_PI429010886	M64361	0.007931441	18	3						23658.24	813.2524	22221.29	32295.88	22711.46	30352.12	1877.836	3890.917	4114.144	22946.38	33749.71	32969.41	25065.69	17167.45	27459.44	781.7261	639.0445	511.369	8669.063	6101.131	9712.243	34486.72	26803.66	36406.05	8006.37	15102.78	17287.24
CUST_13624_PI429010886	N31492	0.007931441	18	15						943.8069	1627.485	653.3386	208.8662	131.7896	61.15687	2266.512	2076.904	1288.168	1110.746	175.9436	609.8498	1228.932	541.8648	602.5729	1289.967	2343.924	1273.67	850.4847	2086.93	673.3593	186.0544	551.0918	254.8611	1027.321	1238.023	654.5389

Probe ID	ID	p-value	Period	Phase	EBI ID	Annotation	GO category	Go term	E-value	0	0	0	3	3	3	6	6	6	9	9	9	12	12	12	15	15	15	18	18	18	21	21	21	24	24	24
CUST_16794_PI429010886	N36883	0.007931441	18	15						426.1948	719.7217	470.6338	386.2259	263.6152	315.8311	665.1463	511.4855	419.0196	495.8587	278.5346	318.6428	446.5017	338.5986	369.2112	507.9246	898.791	860.0989	417.1463	493.0213	485.2701	330.1216	304.875	372.0362	538.3095	423.6	368.7015
CUST_20806_PI429010886	N44092	0.007931441	18	9						11.83438	17.24019	14.1297	26.41027	17.1309	16.51861	18.61955	16.69103	23.97222	45.10508	29.21779	29.58249	27.23128	14.01651	27.41265	25.88343	22.78614	26.94621	16.78485	19.80567	20.77278	44.07915	18.85565	47.42663	29.46145	21.98453	43.04131
CUST_30534_PI429010886	N62075	0.007931441	18	15	321465392	hypothetical protein DAPPUDRA	Transport	Vesicle-mediated transport	9.84E-31	51.03976	40.75864	34.39051	21.0164	19.19666	24.06305	31.07591	47.45891	43.31704	35.44968	17.95932	34.39723	76.25504	35.63867	19.47348	48.76855	51.33814	40.82956	36.72586	15.73759	39.30986	25.89322	27.34045	19.97115	26.70103	34.9506	45.7721
CUST_37113_PI429010886	M17882	0.007935781	21	9	321472705	hypothetical protein DAPPUDRA			5.81E-31	1123.543	1534.973	196.475	1215.754	3914.879	3647.267	3620.969	2300.958	1904.856	1154.885	4841.865	5826.27	1121.501	2751.324	2484.399	3675.29	2271.439	3166.856	1207.663	1743.671	1522.629	236.5379	1769.667	288.5968	748.449	3240.436	116.3326
CUST_14704_PI429010886	N33326	0.00794697	18	15						32.90484	50.69492	45.18209	31.2401	34.39364	26.20354	52.58831	67.72081	30.22132	51.99386	30.35985	30.10754	46.80045	42.2303	42.66588	51.76488	88.12342	61.63075	44.50543	48.98205	45.44328	27.2264	43.22401	44.9498	36.32694	48.39798	40.11852
CUST_14284_PI429010886	N32632	0.007947816	24	18						42.57027	52.49326	39.49765	28.43382	20.85079	30.27186	19.52955	38.8005	45.74936	36.00369	28.2205	33.59475	32.47195	36.52621	20.19825	30.33619	49.83616	38.46934	42.40452	47.30405	50.29015	36.73202	61.97033	43.72166	46.49241	38.52874	44.56209
CUST_38696_PI429010886	M29152	0.007951167	24	0						67.33902	72.97666	78.43006	73.85955	73.65204	65.61458	67.68103	69.71083	52.45299	48.58376	61.57198	72.32818	67.3285	64.83629	74.58857	74.4166	58.30569	76.58195	50.58885	74.63073	66.51767	97.46284	72.26194	78.3726	90.37428	79.5268	84.2744
CUST_14466_PI429010886	N32934	0.007951167	24	0						267.1275	422.3982	397.7869	247.374	356.4497	254.459	305.0003	361.1116	398.8954	307.0639	276.1893	367.1233	292.7159	295.973	336.212	339.8451	372.6214	399.349	355.1717	373.8469	407.4228	388.9715	399.8074	457.0998	450.6028	410.5544	549.8031
CUST_9492_PI429010886	N24728	0.007962266	15	6						50.47426	53.92132	52.81957	30.79372	76.43278	58.73466	52.35235	78.87059	77.01155	40.08729	34.40062	52.70059	42.80977	54.49956	53.8169	58.70606	40.53493	47.44242	60.11411	83.10688	65.35932	73.58526	100.8435	73.7088	52.73695	63.88807	67.0724
CUST_18262_PI429010886	N39457	0.007968609	15	0						NA	89.4662	NA	41.50445	31.04885	37.89734	79.66914	77.47208	5.259486	53.7784	73.86844	19.64634	87.59003	59.35684	69.6885	87.56469	166.7877	105.285	47.18539	14.24966	34.04952	12.98636	12.60408	14.53482	104.661	99.53837	NA
CUST_14197_PI429010886	N32471	0.007997101	24	6						9.273221	8.512367	NA	8.015023	16.46322	13.39577	13.95493	NA	NA	8.460507	NA	9.700016	12.57883	7.630778	20.39754	8.388209	NA	8.979767	3.651166	NA	3.790811	10.28202	5.239057	4.065752	5.579461	5.035991	NA
CUST_47953_PI429010886	M78273	0.007998781	24	21	328751381	carbonic anhydrase I [Litopenae			1.33E-18	49.66108	45.11511	60.66586	87.15453	101.9186	31.45923	40.5192	41.19213	56.30846	31.51184	44.38734	37.76079	47.99288	54.70587	68.82621	51.171	53.15363	58.06369	53.41659	97.52903	106.8768	62.73163	80.28093	58.42497	92.71133	57.46071	111.6232
CUST_25543_PI429010886	N52606	0.007998781	24	21	213512304	Inosine triphosphate pyrophosp	Metabolic process	Nucleobase-containing compound meta	1.88E-28	39.55568	38.95176	30.39026	50.78318	33.44592	36.40731	37.80064	32.32188	29.26288	41.78715	21.9474	31.36138	38.2274	33.06699	25.81487	32.57817	42.07458	37.42403	40.23235	22.16329	33.80478	51.46661	46.8318	45.65444	44.97817	40.04223	40.02352
CUST_4563_PI429010886	N17035	0.008004075	24	18						41.27805	34.01063	26.79132	42.64535	5.241251	33.6985	89.69199	100.875	120.9827	156.988	73.99941	21.61569	151.0875	25.41267	133.5115	111.6865	58.68809	165.8977	213.3383	219.1089	401.1245	117.7735	23.05084	58.68809	270.8095	16.23895	205.3407
CUST_12859_PI429010886	N30165	0.008007661	24	3						NA	16.13712	10.68459	NA	22.51636	23.21362	11.48896	NA	12.25846	NA	8.550634	6.955349	6.410283	14.19705	5.421258	11.80858	8.991143	10.6926	NA	9.392936	7.237005	11.05532	8.448438	12.92804	12.84299	17.50536	10.69641
CUST_46734_PI429010886	M73555	0.008007764	12	0						113.8465	617.0017	159.8296	76.45795	28.90914	49.87572	28.19441	NA	25.97013	28.76849	24.03275	42.70334	327.6721	4.516364	254.714	61.4292	242.9432	190.2229	7.171959	7.041787	7.622177	31.50642	21.7713	35.29322	20.04588	111.8276	158.8243
CUST_20933_PI429010886	N44330	0.008008544	15	12						16.74062	17.33337	16.31227	7.485899	7.58043	9.748353	11.39082	24.5682	8.503753	21.57456	15.88853	4.864944	18.52564	23.27767	16.80349	10.66953	9.313657	11.10999	15.52603	6.327133	4.586777	20.94698	21.64409	9.712808	26.19258	11.82162	11.48486
CUST_33636_PI429010886	M7482	0.008008712	24	21						1547.502	2300.958	2420.859	1608.753	725.9659	615.9354	2400.564	1541.174	2702.508	1708.051	1128.705	1056.414	3611.195	1825.053	1278.279	1512.062	1200.665	1938.89	2245.768	2060.105	2263.041	4105	2990.827	3151.384	2950.533	698.717	3205.37
CUST_52948_PI429010886	M99579	0.008008712	24	15	459353537	ribosomal protein L18 [Litopena	Protein metabolism	Ribosome biogenesis	1.09E-21	8362.147	12523.21	8736.825	8227.781	6439.853	7248.769	10815.88	12165.66	11475.48	9425.197	7402.627	8025.596	7983.519	7832.882	7316.568	15012.16	12648.44	15045.33	9889.351	11094.64	14285.49	7807.75	10485.81	9759.347	8417.477	9550.071	12043.33
CUST_5357_PI429010886	N18586	0.008008712	24	15						371.8649	759.7943	551.71	508.513	336.5251	383.2384	744.9105	741.1426	849.7305	628.1097	376.8731	422.5065	594.8478	444.244	431.7294	773.3668	948.7571	952.9844	668.1223	554.2471	767.0668	480.3553	521.086	526.4775	589.5468	457.7423	610.1834
CUST_10944_PI429010886	N27030	0.008008712	24	21						17.86008	23.18254	40.59839	17.91133	35.27538	23.42954	23.01802	25.38551	19.87929	17.81491	14.67985	24.07412	24.89861	18.60134	20.83811	23.41694	30.37998	29.96804	27.34971	21.56373	20.24228	45.04182	26.21182	37.85657	33.56909	33.77513	37.42032
CUST_21408_PI429010886	N45160	0.008008712	24	15						509.4445	897.658	508.7574	578.6613	447.5258	443.191	795.9001	698.2425	745.8583	703.6419	436.108	505.5201	639.2868	527.9796	505.1911	1050.367	900.553	904.3497	723.9274	606.3479	792.9194	477.7276	649.1337	659.9573	606.1742	570.8807	693.3608
CUST_21794_PI429010886	N45867	0.008008712	24	18						41.62674	37.98103	55.23799	43.62046	30.73939	39.86612	33.5564	30.99993	24.61357	139.698	28.31962	127.7313	39.41926	30.65511	31.52857	45.84356	83.83975	71.43667	319.3967	73.01092	225.0187	61.50475	29.30404	43.98997	132.3608	27.54533	89.86465
CUST_29124_PI429010886	N59246	0.008008712	24	15						308.8108	308.5071	268.6876	256.7541	248.0205	273.3536	303.7038	257.1241	337.9166	339.3438	217.5757	316.7134	318.1116	261.9111	337.7577	413.2862	340.9357	352.0102	397.6869	269.1036	334.5246	365.0376	295.4891	341.4287	362.6643	273.556	387.9483
CUST_24707_PI429010886	N51124	0.008012585	21	0						108.1041	80.3791	51.9988	53.8516	43.09613	50.85543	43.38791	60.05636	46.17834	45.08105	54.80637	39.6536	42.35625	36.29093	53.90529	43.01095	53.88747	29.95378	46.77922	45.83845	31.8265	74.36638	51.15383	68.88761	52.6416	72.38862	44.47768
CUST_37881_PI429010886	M22033	0.00801815	24	3	152949487	calcification associated soluble r			1.1E-23	144.5431	65.83412	95.30674	364.5679	1495.315	1063.435	364.9578	269.2064	87.84235	35.18384	743.9121	902.1594	45.49999	1122.961	1337.377	117.6349	87.01734	106.4017	31.72419	57.23466	36.5095	17.80399	72.78292	39.70565	30.37998	141.9587	51.83065
CUST_53141_PI429010886	M100385	0.008030054	15	0						32.87709	31.50316	39.63441	29.51805	28.12044	36.32296	18.99799	19.71392	32.84255	40.22045	21.34009	27.35507	35.93244	25.44962	28.0728	39.6536	31.20454	42.37353	22.44464	32.33214	31.60721	27.51459	24.05563	26.97533	36.78126	31.2401	27.97979
CUST_43664_PI429010886	M63625	0.008030054	15	0						39.95843	38.58331	29.79027	26.61853	16.02629	26.15603	7.96838	22.91081	25.50686	21.29142	26.0838	32.7919	26.0759	27.14447	14.67236	25.21148	63.14866	67.85229	21.87979	15.31282	27.50901	24.85202	18.5845	23.48051	31.84126	25.14223	17.2236
CUST_33009_PI429010886	M5713	0.008033843	24	18	291220823	PREDICTED: Saccoglossus kowal				39.11041	95.52209	71.86601	88.16698	62.53388	50.78318	50.69876	48.20014	56.80856	33.51959	63.06627	55.7467	56.9001	65.75115	71.5149	55.72212	63.02678	64.42288	77.54349	89.05929	87.34939	71.7184	61.03293	79.45422	70.12343	66.99064	95.98627
CUST_10255_PI429010886	N25954	0.008046307	21	15	512919574	PREDICTED: zinc finger protein 5			8.18E-13	23.79553	21.52625	27.09196	25.01557	27.61361	25.17677	26.06752	26.71367	24.34343	34.38418	27.99322	35.53128	33.63623	23.23737	26.32889	31.1712	38.21258	37.68818	36.51861	22.74717	31.2936	20.55179	21.56373	25.7844	32.10997	33.26372	29.04788
CUST_47958_PI429010886	M78292	0.008047361	12	0						55.05576	39.98058	59.66359	36.97883	41.92055	50.99975	40.78815	34.42594	39.40714	34.85943	50.77623	54.06274	80.02644	40.48622	71.56025	39.77946	28.3901	39.33847	50.85543	45.01993	42.10601	56.45276	48.09451	67.96481	61.98746	46.38013	79.92781
CUST_18666_PI429010886	N40192	0.008061058	18	0	133754273	inhibitor of apoptosis protein [P			5.25E-16	346.4474	401.6396	384.6227	429.8212	276.3758	342.3203	218.7535	445.4238	451.0149	222.4664	269.1036	175.1243	241.668	279.0664	243.3699	304.3391	272.2877	251.4219	404.8745	371.9503	435.509	395.794	295.8529	333.0274	389.053	369.3009	297.188
CUST_23416_PI429010886	N48803	0.008069864	24	15						38.01913	76.66571	61.9265	67.34563	87.87185	75.13872	59.19241	75.50155	55.88006	65.83412	57.9032	60.75399	75.96972	68.51645	68.06818	102.0887	86.83154	69.83801	58.60167	67.29151	69.30661	71.66506	55.06924	64.78842	54.94337	54.37537	47.59053
CUST_55002_PI429010886	M108370	0.008076792	12	6	260787569	hypothetical protein BRAFLDRAI	Metabolic process	Other	2.57E-16	4.396195	6.179101	NA	4.476541	NA	NA	20.4296	NA	18.76673	11.65887	NA	4.79788	NA	NA	4.023157	21.62227	7.514308	15.68854	17.87345	90.15514	138.9534	16.01417	4.989346	13.35908	18.25369	4.549127	11.98927
CUST_24219_PI429010886	N50248	0.008083714	24	15			Metabolic process	Other		7.103758	9.11551	51.39126	5.922716	3.32888	7.934974	3.874399	3.703598	8.64742	6.633834	5.983227	NA	14.45929	7.830046	7.338359	12.62994	26.56674	13.50493	9.317073	NA	10.70762	10.36312	6.627872	8.817048	18.41119	13.64514	7.309623
CUST_33220_PI429010886	M6320	0.008092032	24	18						15.60281	20.08558	18.69988	13.71972	13.89788	17.39101	63.47256	14.60762	50.4405	31.08965	19.67547	38.42576	22.45935	34.95499	21.14216	23.19651	26.70669	22.02084	87.81083	157.2166	251.6089	39.60134	10.8487	47.26148	42.69941	29.74161	59.61189
CUST_33858_PI429010886	M8062	0.008097531	24	21	210062959	C-type lectin 6 [Fenneropenaeus			6.29E-28	782.9344	756.8703	694.7413	782.9344	626.641	620.4556	605.8123	677.4857	659.7683	838.6312	638.3206	655.1561	867.9312	550.4921	637.7947	834.7743	704.6212	625.4995	876.0896	637.2271	776.1493	1012.36	1083.233	915.7404	881.5382	642.7419	873.9122
CUST_5386_PI429010886	N18625	0.008097531	24	18						270.5842	470.8549	466.9715	193.8538	178.1189	157.4954	231.626	488.7695	538.9811	212.9726	263.4037	131.1766	291.3655	149.0265	326.2417	458.3577	257.7877	396.5838	632.6818	512.9523	720.9725	341.6702	265.6462	320.4829	616.0609	279.7318	429.0423
CUST_9946_PI429010886	N25450	0.008103576	18	0						142.9419	159.645	164.9432	106.6432	121.9035	148.9148	115.3572	179.2149	147.7552	114.6554	115.5105	98.13418	122.9659	96.68164	121.4525	107.5363	161.4729	117.4173	141.0969	144.1943	186.3368	147.5241	148.4289	132.8026	140.8381	114.4277	130.415
CUST_8322_PI429010886	N22871	0.008105103	24	15						256.4131	277.4142	304.4972	231.9647	215.2938	217.5757	248.0559	208.8662	236.8356	334.6481	272.1212	298.928	261.5479	234.533	220.7429	292.1626	288.7764	356.4497	276.0746	261.5031	294.2691	262.04	244.2151	289.9011	248.9012	224.7721	343.9474
CUST_55657_PI429010886	M111165	0.008106379	24	3						707.3888	117.4669	687.6817	706.0661	3101.358	3098.696	103.9763	868.9348	75.6543	176.2669	1723.472	1374.54	98.85767	1289.967	462.8312	32.04908	32.71622	23.98666	56.4602	684.8522	35.26049	310.2931	304.2261	194.1709	7.097241	1072.507	7.076886
CUST_38304_PI429010886	M25156	0.008106379	24	15						4419.639	5251.996	3342.747	2978.418	2389.711	2314.026	9226.663	6744.57	5666.924	5960.592	4398.359	3968.622	4819.372	3031.683	3145.521	5814.883	10323.84	5514.378	5292.711	6094.281	5298.376	3911.515	4531.71	3480.931	5350.891	4061.362	4150.213
CUST_4144_PI429010886	N16104	0.008106379	24	15						62.5403	81.86695	87.70118	82.53131	55.54321	71.37579	89.37424	119.2957	108.9908	75.07482	52.41843	65.15776	90.77498	101.1304	67.25975	119.3462	141.7704	155.7011	87.16322	105.9605	119.5461	57.81843	93.53318	94.57461	84.63533	101.7482	108.2696
CUST_7699_PI429010886	N21943	0.008106379	24	15						41.57926	45.69732	49.06379	53.98617	45.2886	32.53452	53.30891	50.41173	53.5836	79.37901	40.55409	55.39639	81.14024	38.82932	56.78944	80.24873	60.47985	75.79833	72.68883	47.61903	63.78974	71.24846	46.01299	58.04431	53.4446	51.51478	64.83629
CUST_10458_PI429010886	N26270	0.008106379	24	15						287.6916	316.4278	286.5115	238.1099	178.033	206.4029	408.1953	321.9997	336.4491	368.4918	236.8007	222.0168	391.4124	222.9426	307.6702	533.3565	463.3449	380.0182	486.2941	280.5224	398.2963	268.8247	303.88	258.1565	456.5305	266.3181	367.1233
CUST_13437_PI429010886	N31188	0.008106379	24	15						92.82706	119.4775	101.2137	94.33013	90.69879	83.45736	96.20921	108.1041	113.1214	97.77174	90.28649	95.06795	339.8451	86.51281	118.928	122.7221	117.9028	141.1702	111.8116	118.0906	135.2412	102.1603	114.1092	133.7779	108.5947	102.9161	148.728
CUST_47023_PI429010886	M74559	0.00811401	24	3						162.1178	150.5856	152.4355	204.1816	427.2889	522.6994	147.3568	161.4506	158.8243	221.8495	166.6038	119.3879	157.9135	137.0649	127.7013	199.2769	136.7059	151.4448	159.0794	146.4045	142.9154	205.143	145.2758	165.8065	217.7635	147.5241	161.2169
CUST_5372_PI429010886	N18608	0.008115894	18	15	344288174	PREDICTED: Loxodonta africana	Metabolic process	Carbohydrate metabolic process	9.6E-104	273.556	354.9347	351.8456	284.4662	196.4115	249.8271	418.6884	301.8993	428.2506	533.7924	253.7732	312.4862	454.9515	244.999	308.3526	584.6183	540.438	558.9714	522.3491	231.809	335.4857	325.8846	287.632	258.7066	512.6794	279.5075	387.7178
CUST_15275_PI429010886	N34308	0.008122353	15	9						38.85987	48.51397	47.5648	30.15056	29.19296	37.3089	22.4679	40.22045	36.48955	36.18124	44.68483	54.05823	51.88798	43.83432	46.7009	36.05308	54.5198	36.90512	24.9094	31.95397	28.22804	63.89423	54.73663	57.46071	48.91311	57.12516	55.15568
CUST_33626_PI429010886	M7461	0.008124167	18	3						47.45455	6.671924	63.21401	65.9928	46.90598	47.25275	NA	14.30855	13.69996	59.99813	289.3393	51.69582	57.29886	17.66839	31.46346	5.583754	4.777689	4.200561	39.29429	29.25965	38.97889	47.64318	80.38279	68.82621	30.68536	37.8081	64.95625
CUST_50846_PI429010886	M91224	0.008140935	12	0						23.25373	27.88568	23.72726	8.04855	10.5238	10.40534	15.70448	17.12368	14.29782	11.80584	13.56287	12.94453	19.72473	9.212546	16.45406	23.22612	20.62243	17.41463	19.33949	8.15128	12.15359	8.953032	7.45015	5.437296	54.37948	13.93323	66.05158
CUST_37987_PI429010886	M22615	0.008147016	21	6	225680773	predicted protein [Paracoccidioi			0.000000848	3.97901	29.57251	16.06768	28.94386	11.13203	19.41383	49.29631	15.07604	56.49639	12.31491	5.393985	11.16576	7.613359	32.98204	NA	NA	9.973024	6.558697	NA	4.165547	3.529865	17.99711	17.6701	10.13279	4.170472	20.0391	26.32359
CUST_48939_PI429010886	M83044	0.008147492	24	0						164.0887	219.4592	216.5705	187.0484	176.2669	193.9353	162.7145	196.0115	176.429	147.5974	148.55	171.8137	174.5137	194.9703	194.0917	200.48	221.4635	215.601	171.1773	183.166	186.848	179.9021	204.1061	210.8765	200.0776	237.835	242.9871
CUST_54624_PI429010886	M106907	0.008147988	21	15						51.5265	NA	52.99594	44.57171	20.24751	31.18313	14.81286	16.01239	18.43607	65.1084	18.69379	33.81651	46.03411	23.0638	41.72651	3.718814	NA	4.295696	51.18755	27.72605	30.65156	56.16512	50.59749	36.95293	48.48882	34.54334	34.22791
CUST_38463_PI429010886	M27015	0.008148089	24	15						6696.753	8256.789	6906.237	6770.352	4211.995	4531.71	8430.296	6791.114	7794.971	7364.792	4803.622	6304.559	6376.466	5021.117	5644.907	11612.18	7147.632	10017.83	9872.039	7316.568	8644.801	8006.37	7138.174	8430.296	8400.194	7071.199	7762.077
CUST_1022_PI429010886	N8244	0.008148089	24	21	449482914	PREDICTED: gem-associated pro			9.06E-08	119.9872	193.2061	146.7486	131.8394	127.1435	108.5581	133.5115	163.9175	148.4289	183.8352	148.131	157.3269	169.5998	114.4623	143.7853	125.8417	202.183	166.5631	184.0263	168.2866	187.2195	200.6419	204.3998	213.4477	154.854	164.7688	185.937
CUST_40852_PI429010886	M56233	0.008152309	18	3						14.00699	20.93117	NA	41.82104	NA	30.03343	31.60415	27.73284	28.3301	7.304174	5.683307	NA	12.76732	17.11834	8.11338	24.24527	34.9506	6.203165	43.47993	7.37464	37.75646	NA	111.2714	NA	NA	33.27799	NA
CUST_52377_PI429010886	M97234	0.00818139	24	3	321477583	hypothetical protein DAPPUDRA	Metabolic process	Cellular amino acid metabolic process	4.58E-20	44.09004	25.71961	34.00168	35.13853	53.22588	34.40524	NA	31.77803	8.995322	6.758311	33.91504	32.9944	15.79609	35.57898	38.40964	21.64707	26.32557	25.9479	13.14676	16.0427	15.97275	10.9364	18.01822	17.23806	26.12743	17.49425	29.59163
CUST_39370_PI429010886	M38470	0.008188631	24	12						28.39338	29.18456	30.36663	77.93692	11.96077	56.44652	75.23071	20.99331	59.43938	28.73586	80.53824	57.16525	73.65204	70.81536	68.01679	15.76131	50.15161	48.51961	19.50992	26.78779	22.7622	7.652903	20.13304	30.86168	10.648	23.07724	20.95678
CUST_37412_PI429010886	M18942	0.008204046	24	15	260829625	hypothetical protein BRAFLDRAI	Metabolic process	Cellular amino acid metabolic process	1.93E-160	10294.69	20662.81	9272.221	12807.83	2219.678	7673.313	28858.93	20563.31	19150.48	14642.96	7430.648	4976.553	13481.03	9056.645	7032.355	22092.06	28492.44	24659.23	17658.04	14614.52	16411.2	10374.24	14363.02	12473.12	12165.66	13098.74	9613.776
CUST_39015_PI429010886	M33378	0.008204046	24	15						9194.226	23324.95	6734.459	9056.645	2386.8	5456.239	22221.29	22279.42	13193.16	10408.52	6226.747	5099.888	9111.496	7260.089	5277.675	21113.92	26129.84	22279.42	13790.46	12211.42	14015.31	9241.731	9410.039	9889.351	9490.363	13248.98	6906.237
CUST_46514_PI429010886	M72873	0.008204046	24	15						27.32487	42.41648	27.97133	18.9004	21.16633	29.666	33.08424	36.39662	35.75247	38.58331	25.59017	28.46151	32.29945	31.64075	34.14439	33.48088	44.80668	45.83845	33.15745	44.81289	38.07503	30.33321	35.76279	40.44359	38.96726	31.05581	33.31402
CUST_11295_PI429010886	N27577	0.008204046	24	21	321466080	hypothetical protein DAPPUDRA			5.22E-52	19.92192	7.718356	22.08755	16.60777	14.81846	14.67792	14.38836	13.0547	15.7161	9.642309	11.04829	14.99561	25.33166	26.48644	13.03847	15.17316	13.42476	20.46288	21.11731	12.09251	18.79971	25.76565	20.53592	53.17104	18.73801	14.41191	35.67884
CUST_16730_PI429010886	N36766	0.008204046	24	21						28.72917	24.23053	22.94579	26.09309	16.85925	21.31454	17.79556	21.98953	25.69105	13.14087	18.42634	20.81559	23.28393	23.26403	20.45505	29.06179	19.35718	25.05787	15.9512	23.48308	27.09821	38.52366	25.26967	25.02074	83.54731	24.57834	27.57162
CUST_22296_PI429010886	N46764	0.008204046	24	15						19.00538	23.23426	20.09331	9.935802	23.01193	16.14215	30.03343	25.20922	32.70763	23.72974	25.18427	19.54299	30.63129	12.16262	29.65959	29.27764	35.00954	27.53522	21.08479	25.23036	28.82109	21.6346	28.45078	26.87774	19.77825	25.97276	20.98727
CUST_40879_PI429010886	M56287	0.008204383	18	12	321468927	hypothetical protein DAPPUDRA	Metabolic process	Nucleobase-containing compound meta	6.56E-69	8.322606	15.43901	11.61625	6.963509	15.43656	7.673574	12.63858	14.2562	17.17797	8.901364	19.75226	9.709038	28.51273	23.8982	23.03724	15.7393	13.6951	25.5836	19.34679	12.3024	14.19023	16.58591	8.519876	10.54823	25.56702	15.03854	20.79498
CUST_32337_PI429010886	M3860	0.00820812	18	15						24.43106	42.12383	41.10401	21.44902	20.39494	17.60535	41.94644	44.36058	41.13217	35.0005	43.01095	30.62762	18.27547	31.02469	41.25469	67.53158	60.66586	48.21771	19.47164	32.41885	50.90626	18.19202	34.80421	25.13614	27.01915	43.39655	48.70868
CUST_32474_PI429010886	M4203	0.00820812	18	15	56968003	Anaspides tasmaniae internal tra				2257.369	2858.309	2227.296	1008.499	1519.636	1117.04	3161.765	3098.696	1485.178	2054.56	1877.836	1483.632	1501.763	1561.658	1221.044	3168.826	3522.391	3145.521	1676.387	2589.848	703.1577	1459.585	456.9596	1636.385	2319.232	1190.571	1559.979
CUST_18145_PI429010886	N39237	0.008228468	18	15						27.06969	23.72424	29.88246	30.23708	18.8838	23.50843	12.01555	21.92026	19.10801	20.51989	32.60595	24.84514	27.80576	24.77785	24.91503	34.05393	22.43407	24.19026	25.41015	28.86811	23.05329	29.24902	20.72897	21.97251	17.17582	16.28426	23.13661
CUST_236_PI429010886	N2899	0.008233373	18	15						1985.342	7415.157	4012.41	1670.335	439.2301	913.5277	6410.91	7130.037	7327.614	2383.479	684.4289	824.4785	3608.266	2727.371	1637.58	5587.034	10966.45	7389.276	3781.313	3258.199	3509.087	1174.279	2782.49	1283.49	2496.293	2291.48	1717.387
CUST_6159_PI429010886	N19705	0.008234509	12	3						167.3103	300.6771	228.163	239.051	656.7134	424.3124	293.2244	405.5854	484.4076	242.6675	334.719	194.9465	297.7548	283.224	247.8341	407.0205	830.4195	628.2632	305.863	644.0162	375.4894	255.5294	423.4844	350.1584	247.2845	342.0443	343.8057
CUST_19875_PI429010886	N42361	0.008241969	15	9						NA	5.102347	6.912398	3.1086	6.701529	NA	7.847769	4.128588	4.121459	12.05475	11.44051	12.16729	9.824108	4.476859	6.327133	4.981512	4.757189	8.83279	4.340032	5.542893	4.92688	NA	3.404059	7.402375	7.041787	15.26314	7.214528
CUST_1548_PI429010886	N10027	0.008251229	24	9						42.5482	48.68665	47.20876	58.50483	57.11305	37.14598	77.91051	61.36043	54.65618	79.77056	55.29943	57.42791	60.82571	38.97094	30.98678	27.57162	61.44094	49.91114	47.21493	49.99888	60.75399	24.29996	45.03269	44.99422	31.72143	45.79664	48.21771
CUST_16817_PI429010886	N36913	0.008253542	24	12						13.74764	7.376965	8.476423	7.488137	33.30723	15.91774	10.3014	7.282372	10.95203	10.27251	17.86876	19.84259	16.4155	24.63804	21.46191	5.290922	8.139109	7.994626	NA	8.664483	9.532396	8.005861	6.943759	4.396759	6.532525	11.94845	5.8709
CUST_16643_PI429010886	N36617	0.008258956	21	9						7.027807	6.879187	NA	5.187353	8.499991	6.092068	5.237244	NA	NA	11.79041	NA	11.924	10.27866	14.83214	4.116502	5.545745	4.616639	8.116522	5.196761	3.39433	3.901717	8.284739	5.450845	4.181523	13.30593	9.5666	NA
CUST_18030_PI429010886	N39024	0.00826082	24	6						14.07152	10.89769	10.41738	12.66493	13.0253	14.55841	14.36936	15.96647	47.23231	12.26816	22.91325	15.48991	12.45205	15.93823	12.92228	7.74189	15.3561	13.95043	14.59906	12.04424	10.92283	11.92894	9.428061	13.89788	15.7122	11.4038	8.498492
CUST_5469_PI429010886	N18746	0.008295032	24	15						566.6051	873.0203	684.1238	614.3598	554.2471	609.6218	742.7432	484.5238	702.7679	754.5011	515.4837	562.2603	873.0203	488.2191	647.2976	1045.59	940.3723	1164.205	760.601	566.7623	799.5929	901.1852	685.082	899.9958	827.5626	672.9412	887.2093
CUST_54485_PI429010886	M106211	0.008301714	24	15	260875744	peroxiredoxin [Litopenaeus vani			4.49E-15	1710.003	4072.42	2725.344	1834.729	1949.314	1839.274	2395.468	2973.957	2862.188	2047.134	1857.76	2185.802	2367.057	2337.634	2549.09	3052.051	3948.157	3890.917	2294.993	2663.717	2604.346	2022.811	2853.1	2820.736	2482.212	2451.472	2645.058
CUST_31420_PI429010886	M1484	0.008301714	24	15	242024143	translation machinery-associate			5.66E-76	255.0769	436.7155	319.9319	295.5482	233.2281	228.6197	402.8177	415.8002	386.0289	385.4238	236.3014	86.52356	327.8924	311.9509	312.1009	398.8954	647.5505	507.9246	293.7564	336.6041	397.7869	292.7676	376.5416	330.1913	328.4033	328.2183	275.2462
CUST_41048_PI429010886	M56756	0.008301714	24	15						491.1439	679.5306	580.4723	523.1285	438.9261	499.8092	569.6083	571.6616	547.8118	558.9714	481.8774	445.4238	618.1517	474.5724	553.2936	730.7886	860.0989	797.7145	546.2084	562.4363	600.2455	512.6794	789.0657	714.9939	596.5924	542.9984	610.334
CUST_52650_PI429010886	M98316	0.008301714	24	15						10.79318	8.208913	6.613138	4.486081	6.841078	8.542873	10.43842	16.58967	20.37998	14.07796	18.94794	8.806809	16.98717	17.47131	12.76017	16.06042	20.86102	16.55114	18.08853	9.758637	20.10978	16.25968	30.36663	19.00985	10.73071	13.83737	14.80006
CUST_2703_PI429010886	N12943	0.008301714	24	15						324.9732	483.2043	452.0404	314.5854	312.2924	296.3981	367.5711	458.8651	580.31	429.6749	314.5854	306.3603	441.6606	330.1913	395.0243	466.2067	554.3874	551.5575	452.4209	448.9533	586.5026	344.4073	430.6456	479.4997	438.2151	344.9779	513.3858
CUST_239_PI429010886	N2946	0.008301714	24	15						7177.873	19855.57	7731.941	8996.679	3179.907	4388.629	23575.84	19522.5	16981.94	6551.409	3696.265	2297.179	10949.85	9819.956	5892.313	28025.82	34877.59	36302.66	16981.94	14285.49	17555.23	6452.703	12674.82	7270.382	9142.021	10897.12	15297.04
CUST_285_PI429010886	N3483	0.008301714	24	3						7762.077	998.2787	5736.233	6799.146	9999.789	9774.949	1821.979	2886.102	2023.772	3696.265	8042.108	8962.903	3855.701	8430.296	9376.495	1280.159	547.3976	772.308	1653.487	3048.498	1352.876	2883.204	3376.686	3513.064	1065.973	6317.157	2950.533
CUST_23075_PI429010886	N48215	0.008301714	24	21						50.50174	41.60531	42.93819	54.28141	36.18124	38.58331	44.96264	34.57525	50.0221	42.51318	35.06409	25.21673	56.65028	39.82654	54.53262	49.07329	34.53973	50.65841	39.50565	40.64205	52.7182	70.26755	53.26755	58.4887	62.22572	50.45124	60.99956
CUST_32910_PI429010886	M5448	0.008302362	21	6	532759728	antimicrobial peptide type 2 pre			8.99E-30	99.78003	167.5904	47.67532	28.45078	71.40475	52.03532	141.0311	163.1045	139.3753	102.2469	14.83785	74.52512	40.84629	28.44439	5.72244	7.952277	112.473	122.9221	28.29271	24.38035	5.336459	17.55426	21.54067	45.46233	117.9606	158.8716	164.4678
CUST_16672_PI429010886	N36664	0.008302921	21	15						46.29157	66.93928	58.60789	46.6169	21.0497	31.49032	52.12833	45.05649	39.31361	62.70293	44.67899	77.87097	63.32579	41.05749	50.47426	70.46471	54.00791	91.32281	51.261	49.16277	54.17106	76.96435	46.281	64.38614	54.31269	37.98691	39.58685
CUST_49179_PI429010886	M84543	0.008305175	21	18	51104825	Euphausia superba small subuni			2.28E-168	74264.89	102393.8	99004.2	78492.44	88732.25	70445.35	63245.57	59861.19	72257.2	72257.2	53496.25	77410.19	92404.68	94019.51	75512.6	64995.27	103630.6	90599.39	90599.39	92404.68	85264.67	86457.06	101035.5	84138.56	92404.68	77410.19	78492.44
CUST_55451_PI429010886	M110185	0.008306952	15	9						105.5565	95.98627	125.1706	92.28753	89.23195	84.37355	58.90806	100.3986	89.48954	95.73042	124.3957	111.9491	94.74514	105.9898	111.1154	114.4851	121.6759	125.8592	84.21664	89.1801	82.53807	123.6404	116.7899	131.1419	122.771	122.201	189.476
CUST_47613_PI429010886	M76877	0.008306952	15	3						26.7042	15.96858	27.67043	24.80368	27.67843	29.61912	22.33058	16.21562	16.32195	24.85014	16.52918	15.47714	21.35916	25.22785	22.61153	19.48546	23.58742	23.16272	19.37461	24.31913	22.86026	20.15802	23.73245	20.49572	11.4924	14.92455	14.51648
CUST_46155_PI429010886	M71585	0.008316284	15	9	530649818	PREDICTED: double-stranded RN			7.44E-10	16.14673	10.52564	7.669956	10.33279	9.480246	6.206844	9.947363	6.936046	15.89063	10.53608	17.62607	17.8703	9.854974	7.773057	22.83576	9.025697	13.84795	15.29841	6.078184	NA	11.29211	12.00687	10.28202	14.22003	25.35423	12.25846	16.70829
CUST_54886_PI429010886	M108081	0.008330891	12	9						6649.946	39.75608	5496.275	6481.59	4727.546	5900.066	352.9709	263.893	538.1212	8786.531	6249.252	8846.332	8631.841	3364.257	6592.219	134.703	15.84508	160.1938	901.877	1103.344	1680.655	7912.634	7663.711	9392.481	1979.658	5350.891	9241.731
CUST_38515_PI429010886	M27598	0.008330891	12	0						15170.38	1420.628	15197.96	20762.38	13532.16	15141.22	3664.856	4159.327	6622.666	19572.59	20708.31	19738.31	24426.06	10178.51	23084.2	1407.95	814.0506	1348.794	9153.451	7832.882	9872.039	31108.81	27459.44	34486.72	11695.66	11356.17	21974.84
CUST_1802_PI429010886	N10742	0.008330891	12	3	405950185	Cubilin [Crassostrea gigas]			0.00000066	312.8082	196.4999	310.4859	543.5625	146.1694	293.7564	69.61335	629.0343	387.8304	153.1648	91.58124	11.84049	206.3209	280.1957	117.8719	457.0998	376.5416	484.1868	716.1458	438.1085	809.8174	117.5817	67.31813	38.9642	569.3405	187.1971	247.4344
CUST_44074_PI429010886	M64858	0.008334428	24	15						104.9484	72.76543	137.5851	53.67167	92.13598	51.39126	63.49903	65.23465	72.43858	106.1184	116.4848	112.8635	122.3631	116.7583	122.8067	116.6823	117.6515	126.6423	126.6423	89.54662	94.40167	135.3048	87.38427	151.4448	116.4413	63.56244	135.7428
CUST_39647_PI429010886	M42173	0.008337836	12	6						NA	NA	NA	58.47581	NA	47.87148	971.1067	NA	694.1978	141.5029	104.9576	NA	28.70362	45.35771	27.85388	178.596	3.621299	158.5985	1081.498	1920.18	4234.05	202.6223	NA	207.4056	596.9264	101.2137	523.1285
CUST_30890_PI429010886	M121	0.008342066	21	12	345487802	PREDICTED: hypothetical protein	Nucleic acid metabolism	Regulation of gene expression, epigene	4.84E-99	19.01869	11.90136	29.53381	25.18751	16.74788	14.65162	21.16473	17.34089	10.32033	17.83951	22.55063	30.01855	30.48044	30.95789	23.8324	22.8513	24.47097	28.30304	20.88553	26.57189	17.48796	31.77803	19.04623	24.6758	14.37528	18.53335	17.28062
CUST_33117_PI429010886	M6009	0.008343817	24	9						126.1425	38.36376	133.196	72.06957	1029.68	880.92	204.9812	204.1402	368.1539	278.592	759.3274	560.3033	595.8749	245.6327	303.1234	36.49885	38.18468	7.893062	469.784	409.0189	392.1412	29.22669	198.1577	9.808744	5.912371	15.43901	7.050764
CUST_21684_PI429010886	N45660	0.008354369	15	9						53.31643	53.90894	64.58573	40.68971	39.34641	47.9302	30.61037	29.42662	37.57177	71.81502	75.82447	72.63288	58.74713	77.32899	77.66076	66.68933	62.367	50.51216	46.36802	61.47862	49.16277	94.11531	88.40099	70.61194	86.48763	59.18726	66.18945
CUST_36644_PI429010886	M16445	0.008363571	18	15						66.25687	167.6498	43.62652	158.9852	39.49765	121.0252	160.1694	154.8276	64.36702	128.8978	78.10699	52.60565	236.43	218.9179	187.3112	387.1258	294.5533	289.6843	96.71855	39.28264	84.84924	58.4887	118.2092	63.22855	183.7477	169.2197	33.45208
CUST_14278_PI429010886	N32621	0.008372453	18	6	193878325	crustin-1 [Eriocheir sinensis] >gi			2.93E-08	4.724092	12.53963	6.96624	17.09383	22.52125	17.4695	14.57956	29.14856	15.44262	12.42724	16.31776	11.20074	15.84205	13.84038	10.09062	4.193932	25.20667	10.32367	9.283589	7.609031	8.852359	13.95043	24.39623	17.97858	11.48082	17.22926	23.2395
CUST_27164_PI429010886	N55561	0.008385655	21	6						14.65904	12.42724	13.93951	12.23558	19.14803	18.65093	41.85499	23.05795	34.77477	10.59016	30.68226	20.75137	17.42256	17.32917	25.98862	24.05563	22.95059	24.4823	18.24171	19.50236	15.14518	16.1297	16.81913	23.33315	22.02956	19.37652	26.62997
CUST_50915_PI429010886	M91445	0.008391022	12	6	291245169	PREDICTED: bone morphogeneti			1.77E-11	22.46441	NA	15.01185	371.4802	21.00723	335.2209	100.3986	NA	80.38647	8.94045	120.0493	121.5342	47.45455	NA	35.05094	60.66586	47.57931	17.44773	415.6373	220.2176	99.1481	NA	NA	NA	12.4535	3.817897	5.669669
CUST_5625_PI429010886	N18962	0.008392334	21	15	164685128	pyruvate kinase 2 [Litopenaeus \	Metabolic process	Carbohydrate metabolic process		320.3348	635.9298	491.1439	335.6252	634.6359	576.6349	189.6824	567.871	493.9591	222.5957	509.5667	185.2774	245.1008	227.9064	246.083	293.3331	654.3402	265.7303	358.5218	421.977	505.6819	366.556	584.7373	304.4188	501.9234	577.3756	524.0943
CUST_53571_PI429010886	M102302	0.008393783	24	6	444516848	Tankyrase-1 [Tupaia chinensis]			2.59E-14	23.67748	38.04561	36.8828	22.61783	26.66045	25.95548	34.06072	39.65854	34.19427	23.74286	37.34538	18.2488	14.76753	30.79372	20.86102	14.95486	25.83559	19.26106	17.21886	18.28904	22.11134	24.5939	22.60023	21.62696	17.94168	20.12578	20.21875
CUST_56004_PI429010886	M112505	0.008399381	24	15						6060.248	8160.518	5814.883	6101.131	4349.21	4508.46	7673.313	7663.711	8503.163	6656.173	4467.089	5960.592	6109.222	4889.847	4922.978	10683.23	9578.799	9872.039	9096.626	7450.782	8503.163	6734.459	6752.376	6913.964	9301.136	7100.104	8461.114
CUST_39871_PI429010886	M46031	0.008399381	24	15						10949.85	28306.31	10746.95	11264.76	4121.132	6410.91	30257.2	27347.94	26299.14	9699.55	5032.372	3601.999	16372.71	14885.24	6913.964	36571.4	42496.4	46674.67	21066.26	18579.76	24825	9067.463	19791.01	10795.65	13301.65	18522.28	19052.86
CUST_39885_PI429010886	M46208	0.008399381	24	3	152013699	putative cuticle protein [Artemi			1.46E-18	1074.53	46.91713	746.1539	787.5865	1716.333	1608.753	72.86661	95.69298	37.18674	190.1479	1338.071	1322.806	128.9165	1221.709	1179.914	44.47158	35.4183	22.17612	43.04426	162.9694	33.85619	250.35	147.6402	195.7919	16.44507	267.5084	47.84693
CUST_45121_PI429010886	M68059	0.008399381	24	15						77.83598	116.8623	61.92068	59.94044	73.62565	75.50155	100.7337	129.1607	78.49976	111.1784	70.37823	98.07401	84.58847	86.74668	87.01734	167.0371	106.3228	142.8672	125.0424	85.00598	117.2039	88.14296	90.19219	89.38331	128.5824	102.6632	72.79587
CUST_2076_PI429010886	N11511	0.008399381	24	15						4203.047	3237.866	3633.442	4315.085	11138.2	10835.48	3033.527	7794.971	4415.56	2734.019	6430.973	3148.451	3346.038	5227.74	6183.806	2200.686	1487.991	1301.148	1931.709	7966.321	2668.728	3696.265	4577.512	3960.355	1903.836	9080.902	3535.101
CUST_8632_PI429010886	N23331	0.008399381	24	15	321456752	hypothetical protein DAPPUDRA			5.76E-31	95.28229	113.0301	111.766	89.05929	93.22902	85.97141	116.5916	102.0776	105.1035	128.8295	92.95196	135.0731	133.684	95.05524	141.4085	195.2227	112.9897	177.1691	126.0081	112.9204	141.1345	146.5474	113.1381	174.5137	171.6937	114.1614	220.0171
CUST_11200_PI429010886	N27430	0.008399381	24	15						16.57845	10.66953	21.17352	26.62694	32.30205	32.66921	13.52973	22.21134	14.81286	22.02956	7.682187	23.15721	20.88371	12.68845	23.71154	7.648589	20.43895	9.713824	14.87474	12.76017	13.03491	15.36018	12.62015	16.84413	15.27294	17.84342	12.84199
CUST_26709_PI429010886	N54747	0.008399381	24	15						340.0191	348.3823	327.7409	316.8051	280.7963	251.0027	393.358	269.9763	376.0142	398.088	250.6739	330.8653	406.0638	234.1695	368.5924	491.802	419.6327	492.5086	406.8432	325.0373	449.3664	370.8194	344.8027	417.5037	433.1774	316.6599	539.9257
CUST_47825_PI429010886	M77739	0.008416093	24	12	498956561	PREDICTED: centrosome-associa			1.58E-09	22.37256	13.1553	27.17795	28.11335	21.46399	20.18794	22.08755	17.94352	22.92037	41.16872	24.28512	40.94392	28.20584	24.11862	44.53304	26.84286	18.51328	29.05473	22.25295	12.58686	21.70164	20.56044	20.819	17.60895	21.27028	14.45802	26.66658

Probe ID	ID	p-value	Period	Phase	EBI ID	Annotation	GO category	Go term	E-value	0	0	0	3	3	3	6	6	6	9	9	9	12	12	12	15	15	15	18	18	18	21	21	21	24	24	24
CUST_26104_PI429010886	N53674	0.008421657	12	0	117670124	notch protein [Parhyale hawaier			2.74E-41	11.86355	12.682	16.23086	9.044852	16.6949	12.44156	5.6115	10.67743	11.78821	11.72848	8.588027	5.388376	14.60077	10.75288	17.38739	10.68031	8.340014	11.4759	7.830046	5.862152	10.59744	11.44514	6.068633	7.177997	11.17299	13.71712	10.44732
CUST_22866_PI429010886	N47822	0.008426217	24	9						NA	NA	NA	4.791328	7.581791	13.764	23.7799	NA	NA	26.33135	22.80532	22.58052	15.67181	NA	11.44176	15.5828	29.70619	6.669026	11.63897	15.44262	16.18229	10.26541	7.506514	8.378958	9.154669	3.824652	NA
CUST_10731_PI429010886	N26698	0.008430068	12	9	270009810	hypothetical protein TcasGA2_T			7.51E-21	511.369	368.2385	644.2122	566.4722	507.231	436.8129	391.1906	363.9263	568.455	631.6446	496.8458	581.4931	754.5011	338.2602	486.0305	274.7035	327.1238	362.4397	574.0759	463.096	550.3617	926.2026	636.1587	671.2531	504.6417	461.2704	856.2286
CUST_57297_PI429010886	E4568	0.008430747	24	3	322800055	hypothetical protein SINV_0516			2.5E-18	890.6088	793.898	172.4022	348.8785	8929.115	10311.23	766.5607	2877.124	633.691	34.30861	4719.533	4403.488	18.26962	2320.751	2333.596	10.60847	7.307485	18.87745	38.48559	5402.577	37.02719	75.84873	952.6113	73.35252	NA	4934.022	5.640641
CUST_33101_PI429010886	M5971	0.008443656	12	9	10280992	DD5 [Marsupenaeus japonicus]			6E-49	1955.527	61.48782	2170.443	3506.645	2261.704	2112.104	191.6575	438.9261	644.9039	2895.969	3835.495	2478.84	3660.946	1375.747	2928.861	8.785038	8.271083	18.70247	733.7832	452.5351	616.4216	3712.526	3627.136	4145.789	1315.424	1768.674	3222.8
CUST_12771_PI429010886	N30016	0.008456218	15	9	195376227	GJ13140 [Drosophila virilis] >gi			1.92E-19	4.483644	NA	8.378958	11.42158	NA	3.472149	5.647748	6.040325	7.324518	8.413648	19.96887	12.07145	6.978065	NA	5.271402	5.710982	8.128515	9.947363	4.94817	4.280007	3.531459	9.848403	7.619983	10.17486	14.02546	5.532456	11.63897
CUST_31443_PI429010886	M1538	0.00845751	24	15	317107865	nucleotide excision repair protei	Protein metabolism	Proteolysis	2.1E-68	868.9348	2288.957	1426.211	1101.531	1348.794	1092.1	1316.174	1679.651	1581.87	1318.185	1351.154	1212.445	1278.279	1227.762	1481.716	1667.986	2080.932	2135.546	1602.248	1782.659	1874.483	1429.079	1628.405	1677.845	1474.205	1525.217	1743.671
CUST_22603_PI429010886	N47315	0.00845751	24	21						34.21333	31.78394	32.94464	35.3759	29.10319	32.87709	28.0155	32.37762	24.03743	32.49411	26.89377	25.36783	41.42487	37.86955	33.64233	30.67349	32.04577	33.37664	46.17368	26.22964	40.29385	54.19504	34.31749	35.30149	51.42591	42.39291	42.14752
CUST_50888_PI429010886	M91350	0.008466848	12	9						7.994626	26.71617	20.7793	36.04308	9.476005	5.891833	6.90056	19.15056	20.89958	60.24608	41.30748	34.79498	52.541	21.1337	5.627759	NA	19.69655	5.428328	12.36822	6.691551	10.65972	46.14458	35.94651	41.48089	14.78505	29.65959	50.68042
CUST_42274_PI429010886	M59849	0.008481183	21	3						10.86877	9.451468	NA	22.21344	11.5869	10.60534	3.173836	10.98328	7.729186	9.772857	NA	14.67236	3.018249	5.614947	9.872817	NA	NA	5.619603	6.645886	NA	4.922612	11.78821	NA	6.051391	12.19342	10.34183	11.95232
CUST_17061_PI429010886	N37298	0.008484086	21	15						35.62662	57.44168	21.55549	28.20584	23.86873	20.98524	30.17026	30.3261	39.68636	24.58293	48.15989	41.60531	32.20247	42.75074	32.14243	25.68152	62.67742	36.828	35.66495	35.03557	51.90036	32.88066	22.01795	46.60311	24.47696	22.01409	32.09525
CUST_3053_PI429010886	N13694	0.008484798	18	15	156553829	PREDICTED: coatomer subunit z	Transport	Other	1.03E-70	99.49612	139.7386	96.40725	81.34169	84.16808	88.4798	114.7972	139.9842	92.68642	120.7872	84.5309	99.16406	80.00199	105.442	97.41787	136.8369	145.5847	165.0428	92.02415	130.586	140.1608	74.44615	98.97699	84.63533	100.7491	136.5457	123.935
CUST_47791_PI429010886	M77631	0.008491551	15	9						53.18236	50.31996	57.90877	30.05595	41.41654	48.94113	53.16566	49.63135	58.62146	48.81332	75.45664	94.86558	40.13911	49.25994	47.59053	110.0556	124.0673	56.67374	48.02051	38.18648	43.17591	55.59367	81.9979	67.3007	128.8978	54.6004	132.7868
CUST_57225_PI429010886	E2609	0.008497048	24	15	260794300	hypothetical protein BRAFLDRAF	Protein metabolism	Ribosome biogenesis	6.55E-27	8522.605	12424.28	9056.645	7872.926	6688.584	7032.355	12043.33	10699.09	11413.74	9013.443	6401.205	7807.75	7928.164	8269.17	7621.268	13098.74	12765.38	14642.96	9226.663	11228.98	10506.53	7621.268	8334.379	9067.463	9153.451	8857.18	9330.025
CUST_56033_PI429010886	M112594	0.008497048	24	15						49.08691	72.34444	83.29367	64.11411	62.51652	50.93191	59.81196	83.59122	84.91891	82.40323	65.48356	66.92448	57.9144	68.84252	79.10279	86.71039	92.13972	88.53491	77.75578	65.74218	93.13119	89.68248	79.45422	80.83391	77.61728	47.97707	77.78042
CUST_49723_PI429010886	M86856	0.008497048	24	15						167.6224	207.9118	185.2539	152.3742	172.4491	162.0267	177.6996	197.5438	201.9179	203.5811	145.5847	195.4747	147.3924	172.8416	170.3606	255.3385	263.6527	249.0225	216.1701	200.0776	202.8062	168.5856	182.5875	191.7974	208.794	196.9837	195.0513
CUST_34266_PI429010886	M9196	0.008497048	24	3			Protein metabolism	Proteolysis		258.4078	144.1943	273.556	257.241	2281.476	1856.844	102.6488	338.0427	163.0857	163.7578	1400.583	838.6312	125.4627	1012.891	850.4847	90.71076	79.76299	80.70329	105.1495	520.31	92.47181	171.0499	153.9744	121.0882	114.4623	789.3467	80.89811
CUST_5057_PI429010886	N18203	0.008497048	24	15						841.3097	1436.914	817.4569	873.2461	694.9037	782.6486	1086.19	1359.653	1224.714	1136.139	900.553	851.1928	944.508	816.5697	610.6881	1307.951	1436.159	1260.081	1200.042	930.4398	1235.266	891.3462	1089.56	1000.752	983.3131	896.981	875.0518
CUST_6040_PI429010886	N19549	0.008497048	24	15						209.1753	416.5526	276.7278	215.3674	214.117	212.598	324.6618	380.2708	357.575	263.6527	239.5846	265.541	302.2155	289.6241	239.348	366.7915	433.1774	414.7402	273.802	313.739	337.8262	241.54	341.732	350.5162	270.0299	278.8807	325.8846
CUST_314_PI429010886	N3820	0.008497048	24	3	321473209	hypothetical protein DAPPUDRA			6.62E-123	509.8873	281.0341	758.5678	813.0168	1514.569	1176.428	284.0207	390.8971	397.9279	478.6398	1135.893	835.3759	561.1654	817.4569	901.5762	183.2542	336.8186	252.884	371.3022	441.3596	383.0448	749.5653	669.1662	724.9498	419.2614	520.7346	593.3017
CUST_27262_PI429010886	N55729	0.008497048	24	3						48847.04	241.1372	48051.75	37632.98	36724.45	40415.13	379.158	729.7963	503.262	27832.29	33304.41	33176.01	29843.45	25888.13	51269.31	185.5629	221.7872	361.7315	1046.212	1125.025	1535.771	29037.53	11932.74	27210.5	1991.351	3686.257	5686.353
CUST_15857_PI429010886	N35254	0.008501669	21	15						57.64995	62.40732	11.09473	101.105	16.26288	117.7735	120.2717	78.85311	36.05055	89.35207	57.14064	31.18313	92.01146	124.991	73.7433	246.956	137.3715	137.1425	110.2215	16.81161	89.84164	33.49837	34.03869	30.11519	119.8892	102.8306	11.46366
CUST_18247_PI429010886	N39425	0.008501669	21	15						30.03767	67.97224	6.632628	62.70293	7.399935	56.3623	81.15666	90.22636	37.87395	98.46402	47.36534	37.28248	72.34444	87.94793	74.70953	156.7437	131.4315	133.4909	63.38213	14.52179	53.62536	21.00723	50.24652	28.28966	97.42977	130.7693	8.581038
CUST_49688_PI429010886	M86711	0.008502623	18	3						96.57151	107.3485	75.0097	99.92196	85.93897	99.12783	84.3918	80.10622	37.67803	61.36043	53.84032	38.83587	89.10635	67.82594	73.43473	88.21411	55.43882	66.17125	80.58239	73.94525	60.67275	109.762	59.21121	89.91214	95.64524	63.61864	59.62083
CUST_31499_PI429010886	M1672	0.008529245	12	9	321469546	hypothetical protein DAPPUDRA	Metabolic process	Other	4.02E-173	283.224	428.1289	371.3022	289.4524	332.0817	224.961	379.8743	399.349	488.2191	402.3519	404.6157	378.4533	481.3104	268.8247	400.4361	313.9334	374.2544	380.915	427.5284	429.6749	458.8651	514.9905	514.354	575.4805	438.6999	382.5381	569.4689
CUST_1726_PI429010886	N10548	0.008529245	12	9						1396.045	58.16145	1604.089	1220.331	1247.935	1184.477	86.36335	162.6466	232.1097	1413.759	1592.484	1490.226	1584.461	887.5066	1895.668	14.7656	11.80456	13.22755	297.3675	408.5695	365.5842	1673.566	1534.389	1593.454	408.5695	709.4562	1361.853
CUST_51685_PI429010886	M94571	0.008529824	24	21						139.6157	163.4271	173.7538	146.9973	131.274	150.7849	130.4451	126.521	158.2614	176.3993	134.7418	69.58412	170.3275	135.1327	155.2093	176.3183	153.1975	154.5921	326.0747	147.3731	186.2473	239.2059	164.741	182.4362	213.5938	159.1322	114.2673
CUST_55060_PI429010886	M108575	0.008530525	15	9						26.43885	20.90896	18.99164	24.00632	17.50645	21.3859	26.89679	22.06407	21.37086	24.57064	26.13302	45.70271	27.58739	23.02752	26.32034	23.81554	17.82688	25.81487	16.4194	20.95138	22.27391	18.11193	17.21562	25.41498	28.54278	26.89103	23.46744
CUST_28854_PI429010886	N58730	0.008532152	24	0						46.5095	37.44599	45.41169	50.53965	25.28782	43.68742	32.87709	36.86782	25.7544	47.80328	25.54219	25.05161	32.45769	27.01424	22.29626	28.96499	32.06918	22.89373	23.94163	21.57697	27.89407	39.39293	24.90578	31.25385	43.44035	32.78182	45.32861
CUST_56431_PI429010886	M113779	0.008533923	21	6						17.32058	18.89832	16.0548	22.31697	119.807	78.5278	29.39693	33.44269	32.13989	22.91325	63.99137	48.88152	11.68791	56.03078	31.57973	23.74852	26.02167	23.03013	20.51989	28.36166	18.42634	12.37254	33.06158	17.19685	20.95138	42.94799	22.44464
CUST_27439_PI429010886	N56060	0.008538462	18	15						49.35984	12.22964	13.92645	16.93597	13.2617	NA	16.87762	15.94932	6.578516	32.83861	23.61619	23.5397	117.0564	16.36929	101.8893	166.7877	105.3328	29.77371	3.208247	18.05945	12.73904	12.24754	30.06311	4.477295	20.63759	6.588475	18.21662
CUST_49645_PI429010886	M86529	0.008542993	24	3						94019.51	1518.512	90599.39	112114.8	115488.3	127330.9	6523.544	15967.93	21758.66	88732.25	146308.3	107696.8	104498.1	77410.19	117120.5	2449.312	762.6542	2913.515	23084.2	30352.12	34757.71	84653.3	108703	119519.5	39540.76	83454.29	108703
CUST_15499_PI429010886	N34679	0.008550038	18	6						10.13728	NA	18.14187	4.011251	NA	5.05016	17.72529	12.99454	20.30731	23.00207	22.58797	8.267617	4.9656	7.914201	4.356715	9.000381	8.015023	8.77273	15.01034	5.025274	11.76845	11.97918	9.558194	12.07005	17.89355	25.7107	20.11934
CUST_37172_PI429010886	M18089	0.00856065	24	15						799.0901	1931.709	1506.647	480.6993	331.6434	89.30494	1647.856	1303.295	2396.659	950.4596	706.4981	565.823	1422.675	685.9099	585.434	1518.512	2252.908	2235.693	1489.353	1555.177	2775.294	1162.325	1325.644	1749.36	1230.895	1128.705	2197.153
CUST_8161_PI429010886	N22626	0.00856065	24	15	242011695	Cyclic AMP-dependent transcrip	Nucleic acid metabolism	Regulation of gene expression	1.11E-11	358.1637	352.1552	373.3349	252.5113	224.2516	222.1744	342.85	260.798	335.4857	450.394	264.4099	326.0295	353.4279	260.6535	265.8169	362.4397	399.1589	399.8777	309.9338	375.7494	405.7918	370.2353	301.7541	369.4099	363.5741	324.0369	438.3717
CUST_46130_PI429010886	M71487	0.008562065	24	3						27.94531	29.3468	NA	32.53452	48.94113	43.48958	26.47689	26.48347	27.29712	18.41119	45.28285	38.78785	14.87994	44.21087	26.37129	17.17227	13.96585	14.6246	16.97215	12.47302	16.50853	18.33712	9.816681	9.648862	5.165709	15.06155	3.936211
CUST_1674_PI429010886	N10413	0.008563063	12	9	91086861	PREDICTED: similar to cuticular p			9.61E-17	1865.951	20.91286	2629.319	3093.621	1563.253	2291.48	237.9355	393.0833	712.6985	3558.043	4295.699	3000.392	4153.924	1689.176	3549.354	3.429196	NA	13.00649	1143.807	801.0558	1219.83	3215.844	5292.711	5209.792	1837.894	2126.752	3546.981
CUST_3256_PI429010886	N14119	0.008569217	21	0	9186886	BCS-2 [Amphibalanus amphitrite			1.79E-28	1416.053	1907.271	2188.815	1293.784	597.5894	788.7271	1483.086	1436.159	1676.984	1157.507	1268.009	975.7238	1289.967	827.1853	686.1628	1371.161	1360.696	1739.059	1595.825	1449.243	1490.226	2340.704	1243.512	1777.355	1416.762	1269.839	1371.161
CUST_54244_PI429010886	M105270	0.008588369	15	12						49.6421	41.57252	34.62846	41.34776	50.77134	46.27549	30.07738	34.38713	33.93179	49.47094	53.02947	51.06045	48.41454	53.51076	47.8851	44.63889	43.96941	39.53772	32.86278	33.746	32.60595	38.12224	41.95534	33.43972	37.09385	37.65544	41.47761
CUST_18596_PI429010886	N40076	0.008590015	15	6						NA	15.89978	8.866716	5.202657	12.1414	9.11221	6.981132	27.20683	17.77566	6.664444	3.277462	NA	2.963111	NA	NA	4.342023	3.800894	5.141064	NA	9.363648	4.105363	NA	11.04829	10.21488	4.412786	7.234145	3.028026
CUST_28426_PI429010886	N57927	0.008591283	24	6						137.0769	96.85513	14.01651	102.2094	79.67772	105.5425	130.1154	136.8369	113.469	57.75543	48.56519	66.41015	69.85306	76.79264	88.54675	64.7406	38.75298	43.24154	46.13169	60.40403	46.71131	61.73575	71.54776	43.93243	35.35463	68.24011	39.26844
CUST_36356_PI429010886	M15699	0.008594715	24	3						115.2516	35.50803	56.96365	172.5296	112.963	126.6202	68.80951	33.05472	39.75997	54.53868	84.56114	99.65569	52.92831	107.365	96.91497	10.88207	40.73959	13.69119	49.76639	45.24721	14.4297	91.43014	30.57632	18.08089	68.91312	52.07351	8.550634
CUST_37420_PI429010886	M18967	0.008594715	24	3						12344.85	438.9261	11289.6	16014.56	16760.98	21177.49	642.342	1446.549	867.2487	12674.82	17837.79	12699.11	10109.9	10408.52	19341.82	289.3393	353.4279	406.6714	1436.159	1597.44	1991.351	15461.72	5992.677	14470.28	450.394	3112.057	2937.545
CUST_5447_PI429010886	N18716	0.008594715	24	15						379.8743	456.4069	346.1134	327.3332	343.5695	311.1974	475.3372	388.9715	369.3009	437.9976	286.6983	396.2592	397.1925	303.4454	397.9972	483.2043	496.9611	587.3135	382.3723	383.478	379.0965	381.3569	437.7611	456.0887	415.3144	430.8354	443.355
CUST_14236_PI429010886	N32547	0.008594715	24	15						32.05523	85.03859	69.81806	33.92845	11.51457	27.00709	81.26034	47.39234	53.05277	100.0093	34.34338	49.94405	64.99079	26.47878	40.07582	151.7904	141.3689	135.9468	89.3399	44.37034	69.44476	66.2384	11.77451	62.19095	123.6928	62.27316	53.45604
CUST_21817_PI429010886	N45907	0.008594715	24	6						60.52841	95.37708	98.097	140.0039	96.40725	116.3642	209.1419	84.01764	174.935	40.62628	125.7435	44.34431	120.8275	65.87993	99.28639	58.30569	137.2826	134.0408	21.19789	49.61113	16.21373	45.45322	88.06844	104.0463	60.86142	69.28139	55.80136
CUST_1267_PI429010886	N9078	0.008594715	24	15						1793.169	2831.186	1991.351	1497.206	1305.464	1240.711	2146.15	2342.087	2221.308	1888.631	1511.222	1956.496	1656.675	2035.999	1503.022	2826.773	3580.711	3960.355	1968.185	2420.859	2166.525	1493.774	2167.915	2379.92	2271.439	1935.604	2184.345
CUST_52119_PI429010886	M96462	0.008619795	18	12						118.5461	98.97699	88.76092	44.97817	65.60638	62.40732	70.3428	104.9377	88.27501	70.69946	67.66335	126.1993	110.1907	97.78469	104.5613	92.95196	103.106	91.71339	84.84135	101.572	92.20119	67.63278	69.13756	94.28531	116.5476	72.49896	126.6022
CUST_6418_PI429010886	N20073	0.008624077	15	9	154416763	viral A-type inclusion protein [Tr	Cellular Process	Cell cycle	4.93E-13	4.360057	9.12812	3.289897	12.32348	5.569014	NA	8.303928	14.96631	13.12953	NA	15.71789	10.61353	13.86062	NA	9.776816	7.602024	8.445011	8.323564	NA	24.92804	10.89368	15.40884	17.21755	10.60003	10.07727	18.31223	24.40409
CUST_3064_PI429010886	N13722	0.008626412	21	15	380853794	proteasome alpha 3 [Penaeus m	Protein metabolism	Proteolysis	2.61E-114	367.4917	1059.777	584.3107	527.1701	513.0768	428.2506	560.9756	781.034	695.5915	631.8697	504.7574	519.7601	564.8143	596.1129	575.2243	608.2737	788.3617	1081.498	615.1114	760.7688	547.9685	509.999	740.2235	559.1602	472.2533	563.8477	536.6512
CUST_35187_PI429010886	M11836	0.008628423	12	9	189241537	PREDICTED: similar to GA13696-			3.58E-70	40.74313	8.731098	47.96277	31.44937	12.09577	15.37158	11.66711	11.66554	7.894226	47.7665	62.5403	24.45655	86.60823	21.52336	25.41498	18.97114	7.037282	7.909773	28.65291	15.83379	17.25616	34.55816	59.09232	56.91288	22.68661	46.21776	48.81917
CUST_33414_PI429010886	M6828	0.008628423	12	3						786.9042	1167.232	820.3087	851.4962	1386.294	1130.351	1212.445	907.8057	1209.897	662.3233	865.141	762.6542	649.314	1021.074	753.6723	1655.603	1165.385	1223.646	1341.551	901.0046	1441.945	546.3173	1089.998	677.4857	1045.59	780.7545	916.8554
CUST_47769_PI429010886	M77541	0.008628423	12	3	291241680	PREDICTED: mannose receptor (			5.59E-18	6.457842	10.88552	7.843567	26.18151	19.72473	25.44274	8.635855	22.03138	11.85747	13.47047	7.410322	6.286258	28.8296	26.32359	16.57982	26.10539	13.72176	28.0728	8.326096	14.5601	25.93287	7.634361	8.404592	14.4297	19.38387	14.51805	4.470838
CUST_8974_PI429010886	N23878	0.008628423	12	9						78.85311	81.87749	122.5728	75.77805	56.03663	52.45861	76.97367	72.03186	73.09797	76.01297	94.95617	87.23514	98.17006	60.48555	79.26449	61.53216	85.64416	63.56244	69.71651	53.75479	78.65231	124.1301	119.1547	113.5495	91.72224	75.24855	137.7837
CUST_13748_PI429010886	N31703	0.008628423	12	9	46575823	calcification-associated peptide-			9.75E-10	2400.564	22.4617	2014.653	1555.177	815.7902	1031.076	86.35128	123.0024	180.7102	4266.005	4952.328	3437.158	1875.312	663.8187	1739.873	25.99138	9.152091	32.72443	223.5617	109.0197	229.2492	2598.143	1269.839	1505.771	110.2733	516.0491	997.6033
CUST_4261_PI429010886	N16349	0.008634864	12	0						188.4673	181.6282	177.6012	167.8421	154.3971	136.2532	104.3593	117.7425	134.826	169.022	135.0204	150.0105	184.5933	95.68309	190.3933	198.1577	147.9573	154.4454	170.7219	118.0401	139.6157	215.9444	135.988	208.5384	269.4621	168.1532	252.8481
CUST_49989_PI429010886	M88117	0.008638488	15	9						61.31201	51.29155	54.22249	50.29015	47.15551	50.20269	49.31951	53.94311	46.06605	58.5677	57.06949	72.73375	67.01094	59.30659	60.40971	65.04377	45.76238	58.96212	53.33381	43.14425	55.20546	70.19469	53.29749	78.97649	78.86032	54.98542	77.24207
CUST_41947_PI429010886	M58949	0.00864233	12	9						195.3762	59.8383	248.1643	351.422	177.4557	326.3053	96.05666	31.39708	102.2094	209.1419	570.2732	637.2271	203.2877	108.3124	247.7035	20.56044	9.548881	17.52474	186.1968	84.10925	160.4657	420.8457	300.6771	785.0219	49.18092	41.4895	77.98181
CUST_24246_PI429010886	N50293	0.008643555	24	21						46.09063	99.84349	42.72194	56.19317	44.02723	50.15592	41.86801	59.21803	45.2886	43.54722	44.88978	30.72686	36.07852	31.27903	45.49544	79.45422	58.85484	90.92644	41.71225	49.39254	52.02384	58.67768	59.93426	61.2068	51.84236	49.18092	57.97707
CUST_9683_PI429010886	N25039	0.008643659	21	15	184198734	Lit v 3 allergen myosin light chai			2.1E-36	1416.762	1489.353	1923.884	1652.642	1269.337	1133.812	1865.951	1903.836	1214.037	1383.977	1079.142	1239.555	1418.598	1695.11	1157.025	2670.787	3148.451	3715.857	2149.415	2180.402	3495.947	1219.279	1390.646	1373.854	2144.939	1565.636	1600.243
CUST_15548_PI429010886	N34767	0.008653018	24	3						23.4647	16.89173	25.57409	45.7369	44.17172	49.23192	5.055557	54.50978	13.43624	42.73341	20.24017	43.31181	NA	10.41882	13.52973	18.68765	35.73797	8.281737	NA	10.42633	14.56346	26.47878	17.24881	15.7218	NA	23.52466	24.07636
CUST_35637_PI429010886	M13079	0.008663791	24	3	477513443	hypothetical protein FOC1_g10C			0.000000685	42.58958	17.74883	51.40281	64.2252	66.67393	65.22345	30.43834	32.22297	27.0512	48.64263	57.30682	35.78636	47.69479	25.62254	27.63973	20.85079	22.88663	28.1575	36.04308	27.34045	16.59163	42.89355	24.35086	26.94322	32.70464	31.75895	15.73415
CUST_53774_PI429010886	M103128	0.008664156	24	15						10.56305	4.695111	NA	6.712305	NA	7.140165	12.70386	16.90621	22.92862	25.36783	18.55369	4.93079	8.083025	21.43699	5.867408	56.99733	23.64777	34.99565	9.057903	18.90899	38.27451	22.00305	25.94024	18.29235	11.98927	NA	10.14305
CUST_39461_PI429010886	M39772	0.00867299	24	0						102.5048	106.3076	117.4669	97.41787	63.96236	65.12931	81.38488	56.24032	59.07254	72.43858	69.60024	52.63501	87.62517	93.76286	23.08542	NA	134.9328	76.59647	100.4391	102.3466	121.0574	146.1482	55.37116	39.04694	136.3336	127.3889	115.8263
CUST_40652_PI429010886	M55754	0.008686414	24	6						110.6677	94.59748	28.99304	37.55337	192.0736	196.2571	137.4424	109.2373	98.75554	NA	67.54737	70.10106	72.24298	97.85715	55.71616	78.18007	32.86972	83.52767	20.37081	133.7558	30.42951	13.114	54.21756	19.87479	43.75819	129.9594	20.74858
CUST_55675_PI429010886	M111222	0.008692382	24	3						8451.427	126.0542	7402.627	6165.981	8417.477	9804.488	146.7658	540.7576	94.14191	2141.9	6491.663	7597.158	1572.421	7081.435	8802.072	41.09	26.62997	56.95761	78.69416	671.8022	176.2933	2900.703	695.1268	2469.347	82.26609	1950.295	434.5642
CUST_39474_PI429010886	M39938	0.008692382	24	3	7008007	DD9B [Marsupenaeus japonicus]			2.62E-17	5979.853	270.4208	5025.735	9516.402	14338.96	11572.84	180.8452	607.8959	198.4395	5593.889	9301.136	9301.136	3449.27	8846.332	14218.13	14.07002	9.221685	58.71759	111.7284	890.2317	110.2733	9532.564	1587.121	8522.605	31.21307	1580.709	272.2877
CUST_39974_PI429010886	M47690	0.008692382	24	15	160552257	putative 60S ribosomal protein I	Protein metabolism	Ribosome biogenesis	4.36E-47	36043.3	47826.44	34486.72	32184.55	26803.66	25065.69	44511.54	44511.54	34286.52	38861.2	30141.56	32454.44	37083.68	33838.77	34286.52	46458.58	46193.24	48847.04	39540.76	36406.05	39540.76	31967.8	39540.76	40024.21	38996.88	39213.8	40024.21
CUST_43623_PI429010886	M63524	0.008692382	24	3						69.19342	25.56436	56.10448	75.07482	121.9201	115.0193	34.87559	32.81019	26.42527	55.19896	79.01425	63.27624	58.70057	73.31512	91.72224	13.69996	21.73892	23.59507	31.76275	44.10093	24.7753	69.07482	64.26444	57.44712	28.53464	44.20101	28.70626
CUST_33530_PI429010886	M7171	0.008692382	24	15						96.71855	158.3229	124.7322	74.86678	110.7898	79.35979	99.97142	139.9464	129.9419	104.5613	114.0296	102.2993	129.5035	115.8648	119.2089	134.7253	164.5127	162.1178	93.50035	123.9728	131.375	129.7798	179.2719	121.3422	116.4561	137.9765	125.7971
CUST_3908_PI429010886	N15541	0.008692382	24	3	270002154	hypothetical protein TcasGA2_T			1.44E-15	461.906	45.5523	544.3805	602.1887	1365.442	1328.27	38.6365	73.80869	58.22453	170.8814	695.5915	603.766	180.676	837.9532	889.8299	28.43382	24.59944	20.465	50.23652	131.2008	44.98293	296.5728	115.3572	309.0756	38.53227	137.3715	75.07482
CUST_4203_PI429010886	N16225	0.008692382	24	15	242004807	60S ribosomal protein L37, puta	Protein metabolism	Ribosome biogenesis	3.74E-36	5131.503	6929.595	5502.319	4910.228	3811.795	3972.222	6688.584	5693.503	5807.4	5545.402	3921.719	5678.164	5336.579	4610.9	4606.27	8669.063	7663.711	10077.64	5678.164	5545.402	7714.571	4415.56	5467.822	5744.161	5374.176	5166.879	6897.446
CUST_5929_PI429010886	N19391	0.008692382	24	15	537240071	diphthine synthase-like protein	Protein metabolism	Cellular protein modification process	6.75E-84	166.7525	266.583	214.4735	136.0437	122.9044	150.9666	247.5439	252.3	251.3778	240.7581	185.1157	174.5369	215.3959	176.6314	194.8762	228.7039	295.9223	256.3549	230.9748	244.3968	242.5407	182.2908	226.3355	215.0091	197.088	214.4735	246.4546
CUST_8183_PI429010886	N22664	0.008692382	24	15						100.821	116.9271	126.2905	81.64232	34.09591	73.07238	172.8416	113.5495	162.8755	182.2908	77.36861	117.2039	129.3215	70.46471	102.437	236.1021	129.6027	203.0356	226.9173	95.173	166.3679	135.3782	108.0539	133.171	189.5806	97.41787	126.0081
CUST_9951_PI429010886	N25457	0.008692382	24	3	195485926	GE13571 [Drosophila yakuba] >g	Developmental process	Multicellular organismal development	3.01E-61	47.06434	26.80971	33.03819	31.18062	72.42903	59.04201	21.08718	26.4908	19.19097	20.88172	52.7182	64.58573	3.971108	56.93121	95.10568	14.70588	9.688124	13.23504	15.79824	27.15652	14.90815	22.93584	14.82044	24.13637	18.5449	29.77371	10.05212
CUST_533_PI429010886	N5870	0.008692382	24	3	291242887	PREDICTED: hypothetical protein			4.53E-09	33176.01	823.5364	33508.11	38384.01	41610.17	53191.72	149.4369	2742.804	196.1778	18365.76	27832.29	39213.8	11015.18	33508.11	41419.19	59.94713	57.27829	196.3832	163.338	2021.719	186.0781	17729.56	1408.778	16372.71	121.5849	3590.769	520.31
CUST_33306_PI429010886	M6552	0.00870129	24	18						2217.875	2871.837	3590.769	1879.179	2002.791	2063.235	2319.232	2200.686	2755.522	2558.16	1781.913	1742.558	2447.506	2014.653	2167.915	3124.619	3117.642	3091.943	2962.88	3327.482	3095.903	3175.147	2402.724	2842.045	3611.195	2599.331	3627.136
CUST_51229_PI429010886	M92702	0.008705268	21	6						10.66525	25.56181	12.29417	8.109042	7.929151	21.81393	19.49861	30.27186	18.83693	20.65147	24.0921	28.76456	7.444772	6.89852	4.619721	6.586668	14.47659	7.509062	6.62957	4.696988	6.677458	21.61318	17.2345	20.56044	10.60847	22.54559	14.78675
CUST_1790_PI429010886	N10712	0.008705329	24	3						688.4549	1114.294	665.1463	1433.817	14032.06	6734.459	1326.216	1433.817	821.966	713.8123	5514.378	4777.94	523.0011	14614.52	11738.35	228.8193	437.8715	478.7694	182.8221	1396.045	198.2176	267.7885	688.2575	639.5264	170.9048	1940.134	867.9312
CUST_32766_PI429010886	M5064	0.008722492	21	15						307.1364	444.3539	250.5426	360.2879	435.391	589.8797	620.7278	582.599	357.7047	533.1871	400.146	418.5156	449.1793	367.9854	489.8482	879.8906	843.2459	660.1893	491.2558	258.0601	399.0134	300.8806	372.873	298.0705	454.7075	486.6807	218.4783
CUST_50791_PI429010886	M91020	0.008723611	24	21						29.60122	45.74936	64.98274	27.0512	28.88967	6.558697	44.17172	24.31682	67.91644	22.59279	19.52436	24.65035	21.91017	16.42634	14.40144	40.48248	52.90722	52.46839	31.73534	29.12719	34.40862	43.50595	38.23223	52.44815	33.27509	47.29117	106.3359
CUST_55476_PI429010886	M110291	0.0087276	12	9						5191.892	321.03	2971.539	6676.906	4847.527	4482.635	1324.412	1098.604	2451.472	7353.952	6219.297	6317.157	9056.645	2281.476	7430.648	229.5682	225.9834	276.0279	3843.133	1788.478	3731.866	10178.51	10090.54	10569.33	3735.581	3036.167	6936.974
CUST_50253_PI429010886	M89184	0.0087276	12	3						439.0112	745.8583	373.9602	522.0737	524.4136	567.0251	628.2632	750.0607	493.3336	387.9483	417.3805	425.5802	359.7575	555.3803	420.6276	646.8706	620.2559	630.2951	459.5171	534.2591	527.1701	337.6971	517.6361	361.1904	408.8076	521.3103	351.6631
CUST_218_PI429010886	N2768	0.008730086	21	15						692.1008	581.8311	492.0502	1208.338	625.9001	343.7299	342.7829	1007.214	225.8691	604.8923	231.3331	194.3126	221.3068	501.1967	228.7822	2409.846	1361.235	2445.736	666.4362	981.1472	1487.355	1257.339	513.8347	409.5077	1266.853	333.2833	776.2782
CUST_929_PI429010886	N7856	0.008734242	21	15						416.251	562.2603	700.1848	265.7303	547.0285	265.2295	352.5932	548.9123	325.1877	417.0366	626.9839	673.3593	406.8432	310.8187	321.5516	1119.38	991.5499	1185.011	218.7535	399.1589	475.0917	311.5873	358.0309	373.7341	273.802	321.2836	338.5986
CUST_9612_PI429010886	N24921	0.008736467	24	0						49.06379	62.73874	65.63872	38.02922	29.46145	13.96051	38.67879	40.13474	32.3182	13.37213	42.19221	18.11022	24.00968	34.45309	47.34599	25.47532	52.53211	46.73964	27.39752	36.37756	17.41278	34.67932	33.77513	24.34505	53.15363	64.66012	68.68072
CUST_41646_PI429010886	M58225	0.008742134	18	0	321479162	hypothetical protein DAPPUDRA	Metabolic process	Lipid metabolic process	6.49E-43	19.14033	11.47734	13.76968	11.7948	12.54903	9.315034	9.414522	8.233633	9.273221	6.494807	4.415227	11.21321	12.99289	10.97548	13.5943	12.93627	8.493194	15.84508	13.88386	7.985663	14.28195	10.93505	11.02607	12.20801	12.45205	6.723319	10.77609
CUST_19745_PI429010886	N42115	0.008747005	12	0						29.24359	27.95476	27.57162	28.99304	25.7544	26.65836	23.5397	13.56629	16.35738	20.42799	21.90402	27.7582	21.3729	18.38358	26.9514	17.429	25.17932	26.04224	20.89693	15.29246	16.94155	30.70093	18.11564	28.42265	34.40524	23.29521	37.05226
CUST_1967_PI429010886	N11186	0.008757286	24	21	241695651	secreted protein, putative [Ixode	Cellular Process	Cell growth	0.000000153	411.0555	163.3852	435.1623	142.749	139.5452	102.8924	62.54833	96.17648	105.6776	988.9686	122.1215	683.6777	143.1244	112.096	118.3923	155.5483	362.3548	175.5187	471.7045	112.2837	439.0112	119.9872	563.3118	522.3491	156.5986	172.0707	155.2643
CUST_18432_PI429010886	N39787	0.008775081	24	0						169.1748	169.8127	89.69199	8.785038	65.37178	58.91866	110.9564	302.5618	102.7974	NA	56.01078	62.04596	NA	61.95862	25.25292	20.2155	10.56444	4.819687	8.983138	270.1829	13.5155	NA	96.16362	NA	NA	305.9701	NA
CUST_15136_PI429010886	N34062	0.008781196	15	0						38.29632	44.83298	30.20183	28.02939	19.82888	26.97533	43.27193	45.55903	30.47018	17.74699	18.49915	12.41089	24.7753	35.5246	21.0164	58.78081	44.57171	41.88081	32.69926	26.63911	39.94084	22.19382	39.74893	27.67304	27.59053	32.26017	21.81818
CUST_18403_PI429010886	N39737	0.008785336	24	0						30.73256	27.12725	29.20311	40.23682	25.08941	28.28966	14.9705	24.6865	23.76937	42.07458	27.89685	27.22363	27.66435	14.08558	25.93001	19.26557	22.40411	26.99962	21.1516	16.32911	23.05084	26.03031	22.55249	26.04805	39.35132	27.18132	43.85321
CUST_37322_PI429010886	M18620	0.00879005	24	15	53830708	putative fructose 1,6-bisphospha	Metabolic process	Carbohydrate metabolic process	4.43E-154	4539.587	10374.24	4746.115	4229.758	4140.108	3202.946	7821.557	9653.786	8387.726	6233.119	4971.086	3154.32	5884.771	5151.779	4885.181	8886.515	11738.35	10897.12	5571.385	8215.523	7327.614	4981.65	6132.817	6826.356	8309.047	6439.853	6376.466
CUST_5936_PI429010886	N19399	0.00879005	24	3						2252.908	294.0126	2194.43	2256.232	2096.347	2342.087	312.5578	531.8423	505.6819	1061.955	1491.134	1896.629	876.9516	1638.477	2561.777	273.802	309.5645	215.3375	412.9087	622.7305	471.3761	1143.279	859.4584	1391.531	466.0849	545.361	769.0192
CUST_6755_PI429010886	N20559	0.00879005	24	3						669.5828	334.9433	534.593	772.5597	1080.06	1023.762	322.315	439.8767	364.2049	267.884	732.7914	971.4356	591.1008	1011.215	1160.436	308.4293	243.4049	244.8878	344.4852	423.6	392.253	450.6028	476.6448	535.3308	357.3731	389.9629	465.6755
CUST_9308_PI429010886	N24413	0.00879005	24	15						359.943	623.1184	497.1127	328.0523	325.2782	311.2489	511.9251	518.791	611.3316	522.858	419.1486	421.5919	408.4889	425.2574	353.8605	604.0998	718.4732	738.0615	512.9523	436.108	583.5463	425.7716	449.7223	494.9456	455.3201	434.3661	602.8578
CUST_11041_PI429010886	N27172	0.00879005	24	15	156537005	PREDICTED: U3 small nucleolar r			2.02E-68	207.2425	246.7787	225.8691	172.187	140.1608	152.7673	234.2298	241.5804	242.7465	241.7527	176.8339	210.6988	236.6326	181.0813	177.6996	275.6274	268.7276	303.9295	204.9134	222.3991	301.8062	202.1456	220.9505	258.0178	203.0154	225.4359	275.9447
CUST_27666_PI429010886	N56475	0.00879005	24	3						110.8559	40.84629	93.26197	131.2392	142.3135	176.3993	53.20844	42.54185	58.99377	48.4991	116.1695	140.2618	65.44143	142.5367	158.4947	50.92486	22.9969	42.16378	45.78026	59.58172	52.08619	66.22157	69.79048	72.73375	53.55076	53.01786	78.39296
CUST_1268_PI429010886	N9079	0.00879005	24	15						1253.031	2883.204	1711.883	1378.754	1455.41	1152.623	2003.765	2039.749	2107.433	1745.528	1495.315	1657.457	1713.82	1612.928	1522.629	2201.915	3161.765	2975.418	1962.886	1836.765	2100.692	1472.69	2073.458	2176.241	2001.707	1676.984	2112.104
CUST_14584_PI429010886	N33117	0.008790749	12	9	390335036	PREDICTED: uncharacterized pro			0.000000661	5.191314	NA	16.00252	10.26541	5.226998	3.192087	NA	NA	4.744251	13.09781	21.07179	16.35946	20.3622	8.341692	15.21029	NA	NA	NA	8.465429	12.35025	12.34813	16.78485	21.91017	25.33166	4.537992	13.82436	24.81147
CUST_56448_PI429010886	M113828	0.008798042	21	6						28.2009	41.72041	32.36969	37.79201	37.24532	45.28285	56.55385	50.88233	60.75955	41.48089	49.0091	29.51805	45.18702	36.64369	34.92878	60.20069	68.96449	60.51578	35.1903	40.49223	43.75295	33.35088	35.20206	26.13302	40.32964	59.12156	36.07467
CUST_2160_PI429010886	N11724	0.008809453	18	0						27.77493	18.72559	21.27734	24.43918	16.6676	21.9826	18.65093	9.438548	17.1309	14.28195	7.074597	15.90508	31.87047	16.98223	16.56329	24.74446	19.4293	15.81139	22.51109	22.37256	17.38549	22.75502	10.36053	16.71139	15.59239	9.35283	20.10181
CUST_33261_PI429010886	M6428	0.008818875	24	18						30.59603	24.49701	36.32694	31.4218	26.18388	27.33627	25.34024	31.5432	35.39516	26.81517	11.59198	20.20024	39.53461	19.72255	24.21718	30.1739	22.81652	30.78094	64.94907	32.92024	58.9013	31.44937	30.07472	35.54628	56.50326	43.28942	38.63208
CUST_15942_PI429010886	N35384	0.008818984	24	3						129.8849	139.5639	108.3488	125.4627	148.6649	157.6398	124.1913	127.048	102.3686	113.7405	100.6344	98.4933	128.3674	110.4624	120.1351	117.5411	111.6675	100.5213	108.8896	102.4255	89.62874	131.4002	99.94989	108.8028	127.2978	122.51	124.9059
CUST_15396_PI429010886	N34512	0.008820487	15	12	242019615	conserved hypothetical protein			6.17E-57	26.28725	19.10315	35.57524	28.56603	42.20483	30.43314	21.28833	19.4115	19.55073	24.32431	43.6037	42.64535	32.19586	41.72651	58.98036	30.23708	26.22059	25.29001	12.73904	23.0873	25.59906	16.93597	19.56045	29.9505	29.00584	19.99489	29.60455
CUST_44819_PI429010886	M67087	0.008822968	24	21						101.2925	56.18215	77.25936	94.22856	77.10772	83.80666	72.73375	40.55409	57.10693	79.23144	62.03291	88.22605	114.1092	71.07642	65.90991	69.64615	49.6421	65.03673	69.86685	85.24573	98.32351	108.1961	103.1842	123.1603	101.105	80.21511	138.8443
CUST_47366_PI429010886	M75794	0.008826777	12	3						37.06569	50.86361	36.26082	38.46934	49.75065	40.22045	22.77225	40.98681	48.81332	31.60415	28.87751	25.34518	36.50276	24.39353	48.39798	61.37944	58.88834	35.68286	50.70492	35.41491	52.57215	20.3622	35.21621	25.19608	43.94763	27.16746	56.17586
CUST_47934_PI429010886	M78219	0.008829461	15	9						16.08645	7.475842	14.57595	10.41283	NA	6.825911	13.95366	11.10613	8.843446	19.17715	NA	19.60559	12.50576	16.72797	11.39973	9.50239	12.65302	15.68645	11.01286	6.282956	12.26651	15.16953	7.444772	7.02166	11.10263	14.97194	19.97953
CUST_29427_PI429010886	N59841	0.008842072	21	0						3185.221	3552.655	2626.057	2467.704	1701.286	1465.124	2452.653	10795.65	3140.215	1655.603	980.6718	1003.544	2320.751	5507.513	898.0277	1748.513	3387.12	2031.77	2228.732	3148.451	1789.218	3408.763	4637.293	3175.147	2437.73	3085.208	2219.678
CUST_54258_PI429010886	M105316	0.008851205	21	9						4.612947	13.73044	15.9565	16.25439	11.63737	6.190304	44.46024	15.45147	13.31134	16.36929	24.73755	10.55248	13.43146	10.81386	14.79074	9.05635	5.736085	4.665682	9.93804	14.68296	9.24174	12.60565	9.704976	8.294501	10.1905	14.34289	22.59481
CUST_27999_PI429010886	N57077	0.008861069	24	3						27.05785	NA	25.77383	28.93158	32.29221	30.97112	5.672619	15.86706	11.65231	17.28062	29.76995	18.66832	4.45798	21.80093	18.76458	3.943255	NA	5.458067	6.967623	21.92273	4.230109	17.08255	7.238058	16.96414	5.338332	NA	12.03178
CUST_53447_PI429010886	M101740	0.008861889	18	3						84.83411	42.36924	93.93436	114.5038	50.62088	97.79679	28.17546	47.13519	40.4062	32.14243	38.64131	50.59749	42.42877	43.67283	54.04744	37.53429	69.17898	42.09646	45.34342	41.23205	36.1335	98.00625	55.11106	91.54439	73.57016	50.57582	58.37796
CUST_6909_PI429010886	N20765	0.008861889	18	3	440211664	Wormaldia moesta voucher Wm	Metabolic process	Cellular amino acid metabolic process	1.46E-69	65.14299	85.75696	76.28125	97.93073	137.396	155.1393	81.24878	99.75898	88.01649	56.52874	112.1712	71.59289	95.55779	77.65458	96.41916	58.72446	168.1532	53.47447	129.3215	92.37878	48.61045	210.6647	205.1165	129.4034	136.296	211.3776	198.4987
CUST_39394_PI429010886	M38819	0.008865771	24	18	528766854	hypothetical protein CB1_0003C	Response to stress	Defence responce	6.11E-33	12378.85	19903.59	15492.49	12236.09	10485.81	12442.6	16035.79	17131.85	16553.17	13335.43	9684.529	9598.965	13559.72	14032.06	10852.86	18320.9	20382.6	16655.53	17210.94	18365.76	16874.05	14743.32	17167.45	15526.29	17982.7	15630.09	15435.13

Probe ID	ID	p-value	Period	Phase	EBI ID	Annotation	GO category	Go term	E-value	0	0	0	3	3	3	6	6	6	9	9	9	12	12	12	15	15	15	18	18	18	21	21	21	24	24	24
CUST_31728_PI429010886	M2255	0.008870022	18	3			Metabolic process	Carbohydrate metabolic process		550.4921	192.8297	483.2043	625.077	666.4362	665.873	218.6001	220.8506	213.7437	563.4896	707.6592	719.4386	684.5912	412.5149	758.5678	174.0123	135.1101	117.8448	220.9943	217.4779	223.7463	958.1681	741.8916	806.5559	268.9804	475.5631	432.4641
CUST_51736_PI429010886	M94786	0.008871017	24	21						61.23583	73.50942	94.18113	67.31813	68.30294	50.47099	46.23699	58.26212	75.22137	56.35449	64.92793	65.122	57.54767	72.47632	66.57534	68.14166	75.17439	91.6714	74.98208	68.35388	61.15056	79.60935	83.93495	83.29367	83.21526	51.02849	94.76517
CUST_35219_PI429010886	M11943	0.008875311	24	3						113.0827	43.42575	142.5641	150.8682	364.9578	301.7541	24.98354	72.93006	18.46268	87.08887	243.453	199.982	79.94645	145.1105	214.2039	16.39884	41.93463	76.98432	8.565689	43.50595	12.96173	91.51354	41.38631	73.50137	14.81286	36.73983	NA
CUST_41564_PI429010886	M58042	0.008905627	21	0						21.2559	10.782	15.04022	10.12252	20.05879	17.60895	6.5566	8.724815	3.850281	8.016857	10.89368	9.548881	13.45766	16.32398	10.54823	8.94306	10.25273	12.43497	17.24674	11.19922	11.94189	13.82262	17.42012	10.29813	7.31445	13.66174	12.16262
CUST_32433_PI429010886	M4105	0.008923836	24	0						3862.287	7194.306	8256.789	3039.361	1985.342	NA	5550.987	7531.236	11094.64	1599.332	1022.285	1091.584	2978.418	1551.452	263.3579	2801.6	2003.765	3686.257	5864.557	6818.249	7993.785	1528.323	3543.046	3709.96	6734.459	2983.608	9257.227
CUST_3235_PI429010886	N14075	0.008925954	12	3						1442.661	1455.905	72.30147	186.8705	1843.608	212.7294	729.7963	11974.32	73.68465	20.24952	26.64465	9.884796	3.795435	6171.545	25.91341	143.2998	178.596	81.76793	211.1114	44.60387	116.6925	7.126641	11.37814	4.704254	29.89602	58.31116	6.885217
CUST_20759_PI429010886	N44007	0.008925954	12	3						75.77805	80.33191	20.57512	53.25991	67.7294	105.3328	77.99973	86.30982	63.11334	29.39937	36.40731	24.89149	36.18124	87.06917	36.40347	130.3441	98.50388	79.64838	50.7989	53.13079	39.11041	19.67166	90.58585	19.21337	29.59501	63.03163	6.95948
CUST_1316_PI429010886	N9266	0.008925954	12	3	406817026	selenoprotein M [Penaeus monc			1.69E-40	2605.771	5678.164	1900.457	2105.784	2883.204	2865.238	4311.402	4368.987	2713.39	2371.181	2330.806	2354.096	2179.188	2496.293	2740.835	5131.503	4606.27	4940.836	2704.306	2870.034	2631.802	1509.889	2577.27	1690.112	3390.028	3786.16	1783.882
CUST_45781_PI429010886	M70273	0.008930617	24	6						118.8089	120.1733	130.3988	120.8608	237.439	198.1201	185.2539	110.4109	185.9023	130.415	160.1938	148.2361	118.2092	124.2633	132.1106	156.3126	121.3554	135.9051	137.4234	118.1715	130.7958	89.12634	93.65434	71.40475	124.2469	159.2971	155.2845
CUST_53695_PI429010886	M102826	0.00893065	24	15	283854611	DEAD(Asp-Glu-Ala-Asp) box poly			2.92E-47	399.9663	537.9838	426.7646	362.3548	243.7665	302.6134	547.9685	459.8781	542.7794	487.9361	311.5041	309.6955	492.5086	330.9867	358.0959	509.999	884.3862	605.287	579.8505	400.9911	537.4657	412.2409	387.7178	352.0796	473.3422	411.2361	355.6308
CUST_41867_PI429010886	M58740	0.00893065	18	15	410509306	myosin heavy chain type 1 [Pena			1.21E-09	1843.608	2524.402	2018.868	1775.368	1695.11	1916.926	1988.355	2442.216	2288.957	1494.458	1190.571	1376.514	1538.316	1710.003	1487.355	2299.057	2244.174	3104.628	2807.452	2013.288	2960.558	1799.736	1863.699	1480.103	2093.838	1688.167	1810.6
CUST_10745_PI429010886	N26719	0.008930706	12	6						NA	6.885217	7.367873	14.08999	12.70063	20.8587	27.47971	NA	12.96601	15.07942	9.703667	16.23086	8.937861	6.132264	9.743639	NA	6.508862	18.25369	15.74083	21.00454	49.8852	22.59481	12.9826	5.371414	12.29417	9.025697	15.10195
CUST_12016_PI429010886	N28781	0.00893646	24	21						67.23484	79.31808	76.01297	75.95345	50.22	54.97558	62.06796	61.58528	69.07482	67.27409	65.88877	64.88783	61.91527	54.53868	49.09284	105.87	73.92515	87.72633	89.60857	63.59914	80.59242	85.4597	71.64925	95.86932	103.4755	66.24674	95.08224
CUST_14918_PI429010886	N33688	0.00893646	24	21						401.6396	505.1911	476.244	430.0583	383.9729	405.8942	413.108	372.6214	354.5733	457.7423	337.46	433.7137	370.0497	345.674	293.2877	551.71	354.1831	482.7166	494.9456	373.6591	447.4189	544.8319	378.2634	525.9937	491.9043	401.2544	521.5155
CUST_1880_PI429010886	N10942	0.008936546	15	12						20.55824	15.68096	14.10253	11.16309	11.53833	7.067325	20.5422	12.24304	11.3139	17.31771	11.55069	12.77277	29.55431	13.76632	16.07632	12.09079	14.15899	15.81757	11.61327	8.213544	4.668232	16.32911	13.22017	5.401098	12.44585	10.43678	10.80517
CUST_43194_PI429010886	M62372	0.008940608	24	3	459172798	PREDICTED: endothelin-converti	Protein metabolism	Proteolysis	1.24E-16	51.85541	59.69831	65.47772	74.62293	98.46402	134.3961	61.15687	72.83024	67.86728	47.60538	41.29379	43.56758	37.60099	46.33666	52.61041	36.01668	53.86055	57.66115	33.05084	74.80515	37.81203	40.60853	44.85324	46.43451	33.6128	60.77123	38.37232
CUST_50040_PI429010886	M88361	0.008940608	24	3						148.2361	57.93583	133.2506	113.622	178.2173	123.7963	73.85955	68.54812	57.70575	68.01088	177.6455	125.2902	89.58853	175.9965	204.8848	53.5736	59.67529	44.67899	59.40866	47.32106	41.03485	65.81456	85.06849	54.33723	74.12141	105.242	57.66836
CUST_11000_PI429010886	N27109	0.008940608	24	21						159.4491	166.8948	190.0624	158.9424	126.712	131.8408	132.6306	131.4171	136.5009	171.2114	142.9974	71.86601	163.5142	139.5013	124.0485	203.7634	164.794	193.3912	166.7408	144.9793	183.2542	206.3618	149.533	180.8767	220.4736	149.3578	189.351
CUST_23163_PI429010886	N48362	0.008940608	24	18			Transport	Ion transport		4875.223	6343.146	5365.583	4403.488	4403.488	4125.439	5476.032	4809.493	4705.762	5435.742	4522.071	5222.203	4841.865	3755.671	4694.435	5507.513	5099.888	6116.36	5144.706	5736.233	6758.872	4772.224	6025.479	6452.703	4499.92	4981.65	6506.137
CUST_32582_PI429010886	M4478	0.008943706	24	15						8.391548	12.60041	6.093975	NA	12.36205	8.493194	8.614696	24.83681	16.38009	17.35447	8.680288	18.1064	13.413	14.31033	13.92483	17.425	16.96963	18.68449	18.22327	4.488887	10.44372	7.206892	22.21596	12.05097	6.783466	7.015162	10.34183
CUST_17152_PI429010886	N37446	0.008946457	24	21						156.9414	152.8269	160.2997	157.1785	126.0246	127.0776	131.0883	137.2351	144.8287	153.47	132.952	130.24	139.7918	125.025	128.6956	160.4433	147.89	138.1161	152.9969	119.2267	146.2442	170.7496	151.2161	163.1533	177.5137	140.275	188.5629
CUST_566_PI429010886	N6078	0.008951581	24	18	380853796	protaglandin reductase 1 [Penae	Metabolic process	Oxidation-reduction process	6.05E-104	30.02053	29.81385	39.08885	34.90142	52.3424	28.55984	28.87489	26.00178	28.2205	29.76143	54.59539	33.70156	26.71617	36.71092	21.10822	47.97707	39.03986	36.65461	48.56063	72.48391	75.48453	31.14654	27.46604	50.69492	33.6985	32.64246	40.26043
CUST_7746_PI429010886	N22012	0.00896228	21	0	528507108	PREDICTED: zinc finger protein 7			6.85E-70	14.32273	25.42657	13.93814	6.635748	13.30593	10.98475	11.25743	15.35462	13.71972	11.72699	7.054812	7.397642	7.645491	6.376979	6.3941	14.3566	11.1896	10.27542	11.12081	18.67654	12.59237	9.675629	14.45383	19.76869	5.729424	15.73263	21.34884
CUST_32518_PI429010886	M4332	0.008969647	24	0	91094771	PREDICTED: similar to AGAP000			9.81E-16	1129.142	3543.046	2787.074	815.2223	569.0422	329.6536	2261.704	191.6575	1747.584	967.7143	1160.436	656.3228	929.9574	327.5322	588.0127	1151.814	4680.36	1832.717	2599.331	1298.646	1538.316	2116.149	2016.054	2971.539	2237.142	2157.924	4445.119
CUST_24922_PI429010886	N51488	0.008971287	12	0						46.78844	34.73082	36.26885	33.97881	25.75202	11.61798	22.4165	8.819479	18.0495	29.27204	39.87751	26.11634	21.55928	23.60361	44.1972	17.83378	36.18124	28.05953	29.36452	25.99676	23.30032	49.53225	33.10821	35.76583	57.32544	61.58	42.05307
CUST_5611_PI429010886	N18945	0.008973212	24	18						173.05	159.1592	150.4473	211.588	135.9468	162.3101	159.4235	174.4941	171.5592	231.5378	179.9541	211.625	288.6545	176.7166	177.1325	202.8062	210.7737	185.084	241.668	214.5153	227.2502	255.7014	213.4477	234.4388	252.8481	186.0544	241.5804
CUST_41428_PI429010886	M57706	0.008975617	15	9						187.5559	89.50979	114.9771	216.8711	134.6275	130.8147	102.1199	114.7343	77.75578	262.525	555.5824	254.3008	320.7735	111.8276	209.3816	92.86727	93.27554	93.49221	131.4868	102.7497	101.4865	257.2967	273.556	294.481	123.5992	198.2813	278.9328
CUST_13561_PI429010886	N31381	0.008977622	15	9	321468833	hypothetical protein DAPPUDRA			1.84E-48	5.795835	11.19238	9.908964	5.219516	3.548825	4.95154	NA	4.902008	5.110851	10.5117	12.50576	NA	5.486086	6.512115	11.96077	11.37254	8.218417	6.322751	7.010551	7.052579	4.020773	10.17714	5.267179	13.22574	13.56287	NA	11.1284
CUST_48024_PI429010886	M78575	0.008984748	12	0						3046.537	3688.899	4482.635	3177.941	1654.651	2583.285	2418.009	2889.672	3067.239	3156.873	2337.634	2362.508	4499.92	2722.979	3890.917	2219.678	2411.386	3085.208	2996.567	3346.038	3629.795	6018.305	3827.862	3449.27	5921.175	3316.279	6101.131
CUST_10220_PI429010886	N25894	0.008984748	21	9						61.06467	72.56894	68.15639	79.39688	128.9165	88.14296	69.98305	64.10142	61.35249	84.78861	91.6852	93.28654	72.03186	94.40167	85.0591	74.67002	81.63425	78.518	71.70119	51.79467	64.6835	67.69664	62.30179	63.88253	98.75554	66.26258	73.31512
CUST_42512_PI429010886	M60471	0.008985384	24	15	242019722	transmembrane serine protease	Protein metabolism	Proteolysis	3.27E-30	58.60789	41.32287	31.77263	19.52695	17.17227	14.4099	59.5223	36.28088	34.72664	42.19574	32.62382	32.50066	44.42841	26.87222	34.85461	57.42791	53.21955	52.2365	40.72145	34.02915	30.12442	47.31561	46.00245	41.55059	41.16872	29.10319	36.86176
CUST_12027_PI429010886	N28801	0.008990235	21	6						13.10546	17.71771	21.59473	15.12028	22.83896	11.83609	33.46312	24.05325	22.49527	25.33441	17.10019	20.37998	8.681841	NA	14.95172	14.33765	14.33538	11.15823	15.22691	16.70652	16.81027	17.51139	15.23987	15.24489	19.60068	14.47477	15.38781
CUST_21609_PI429010886	N45522	0.008995779	18	0	2499378	RecName: Full=Compound eye c	Signal transduction	Other	1.82E-45	NA	24.02712	26.32034	14.59502	23.32264	10.35153	NA	22.01115	12.71074	NA	11.19796	NA	4.530774	12.50022	5.935469	4.30741	22.74199	21.86212	26.19258	13.92807	28.52077	NA	NA	4.077341	8.080815	12.52146	NA
CUST_29902_PI429010886	N60791	0.009001535	18	9						7.338359	12.70605	18.94794	9.192907	15.78875	19.1841	17.8371	21.98717	15.71	26.43013	22.4839	23.86873	15.33232	16.6131	22.11134	22.00814	10.08403	21.62961	10.07137	13.48307	25.32605	11.21622	15.60447	20.4296	19.00748	16.6387	20.10978
CUST_7011_PI429010886	N20912	0.009004443	15	0						59.81196	65.04883	61.87143	59.48241	53.54287	54.25668	51.95311	48.96189	48.73482	53.14113	59.06511	48.20014	45.91877	46.94606	49.78973	66.34289	73.96504	64.93737	49.06918	53.12256	56.82896	56.30361	43.21465	53.56818	54.5743	67.3007	53.84032
CUST_34770_PI429010886	M10610	0.009011646	21	3						54.2724	46.64691	50.53965	77.35718	56.31897	71.66506	52.05076	38.71226	45.96577	65.72695	65.84152	60.79074	45.9512	41.27319	46.39504	55.88006	42.76898	57.29374	47.97707	27.50428	35.85739	68.51645	34.23416	48.61521	57.56454	50.62618	77.808
CUST_50998_PI429010886	M91728	0.009025132	12	9						575.3242	943.4447	657.3507	650.2714	501.9234	530.3219	652.6836	534.7331	739.3679	704.3483	764.2764	697.5829	837.2458	688.6527	736.9876	553.1321	476.5395	601.887	582.3778	912.8746	1103.344	674.5328	792.599	789.8715	658.7898	662.7709	908.3858
CUST_6582_PI429010886	N20302	0.009025132	12	3	321468529	hypothetical protein DAPPUDRA			2.03E-93	195.7919	333.2203	206.98	220.8506	309.9338	223.7463	258.7066	348.0379	279.7784	180.0167	207.8778	184.3858	225.1246	224.3833	197.4226	255.0769	435.509	382.9427	187.3112	263.0521	270.3263	180.8767	251.5671	169.3049	243.9147	247.2845	199.2769
CUST_42794_PI429010886	M61268	0.009058247	24	21						84.67585	94.1914	106.1625	91.82394	78.66288	60.43161	82.57504	78.67787	78.7116	97.44091	77.44274	84.45149	105.7899	60.94356	72.96184	80.5549	107.6737	97.10574	89.57069	104.0463	112.2837	106.2214	113.6471	95.68309	86.99822	98.7449	139.0644
CUST_6193_PI429010886	N19753	0.009058247	24	18						347.1137	572.9401	574.0759	440.9984	337.1412	300.7552	513.5436	487.4115	535.2394	529.3696	405.5854	352.7205	489.4418	372.1298	376.8731	555.3803	665.3418	653.5411	686.4707	531.8423	743.3385	541.4917	523.3495	566.6051	652.1093	515.2465	694.4759
CUST_7792_PI429010886	N22080	0.009058899	24	9	321473113	hypothetical protein DAPPUDRA	Transport	Vesicle-mediated transport	1.01E-24	71.12138	65.88877	60.27161	76.25504	68.14816	84.25166	88.84654	76.02418	79.98506	76.89496	77.21485	92.03434	63.04493	73.35252	73.72575	117.2793	69.96485	91.08467	86.16832	49.97848	58.69436	75.31313	62.76069	61.48782	70.67656	67.36564	57.65509
CUST_51758_PI429010886	M94914	0.009060992	12	0	442769035	glycosyl-phosphatidylinositol-lin	Metabolic process	Nitrogen compound metabolic process	3.23E-13	206.2187	73.42732	323.3549	159.9694	130.496	60.90419	125.8093	103.5544	227.0055	109.762	203.7183	114.4474	257.7877	156.5986	211.2323	34.13826	61.59148	57.73118	155.5805	104.5613	165.5396	284.1966	395.794	396.2592	188.4169	286.0534	530.9393
CUST_18430_PI429010886	N39784	0.009070068	21	3	321473037	hypothetical protein DAPPUDRA	Metabolic process	Oxidation-reduction process	1.11E-34	103.3338	94.27306	NA	29.98633	210.8033	216.014	78.70208	184.0518	63.29655	NA	55.62648	48.81917	24.80368	97.17225	58.41936	43.73952	27.7582	25.46423	20.30731	187.9422	24.39861	38.92916	122.3191	42.29499	NA	356.4497	NA
CUST_1085_PI429010886	N8443	0.009071823	18	0						542.7794	776.9293	613.0157	500.9726	620.4556	540.0952	445.0179	610.1834	448.5869	535.748	604.6962	573.4698	494.606	535.4815	494.454	527.7959	791.8334	606.9823	574.4065	655.6894	635.7483	362.4397	643.0042	656.7134	423.0504	499.3569	469.2787
CUST_8820_PI429010886	N23647	0.009073678	15	12						23.82334	27.44415	14.7532	6.863677	6.624695	27.80262	10.88981	14.1084	7.920298	8.884176	NA	12.14327	23.4544	36.26885	23.98666	14.51648	21.99437	28.96132	19.26369	27.93243	13.71712	12.50303	10.91685	12.31866	20.78429	17.77361	13.39577
CUST_15862_PI429010886	N35261	0.009074053	21	15						74.18037	81.80549	15.76545	71.02716	11.09601	64.02245	66.296	39.74893	45.5101	68.36775	31.89668	26.87469	51.3517	67.41125	36.39325	123.8198	137.6772	123.0482	56.49639	13.1424	49.39254	28.55984	89.97113	21.8116	13.42624	21.13533	12.80063
CUST_48580_PI429010886	M81126	0.009083219	24	21						149.7056	93.3634	151.2383	104.3299	57.02244	71.1038	118.4277	89.84164	113.4582	124.4149	65.90164	75.11158	105.6776	72.69874	81.84916	116.4978	102.411	149.7452	137.7644	121.6759	174.0791	119.5848	140.2946	147.2992	132.3168	136.8093	183.5316
CUST_26548_PI429010886	N54432	0.00908946	24	18						12.66337	5.356052	NA	7.050764	NA	4.618704	3.563833	NA	5.200057	5.920801	6.293716	6.405558	12.28624	6.013724	5.879724	8.869172	4.230109	8.184195	27.29484	9.913032	11.3602	9.989717	9.585902	10.44922	14.13668	9.893288	3.264445
CUST_36989_PI429010886	M17470	0.009092791	21	0	321465867	hypothetical protein DAPPUDRA			2.07E-12	16.97405	14.00699	15.74607	9.37834	8.396446	12.48451	6.317629	9.735966	11.94299	8.47849	11.74876	6.899584	6.609031	6.49677	12.59237	6.192212	5.270725	9.218794	7.964923	17.49722	5.103026	14.6635	5.709589	15.36351	13.87626	10.15772	9.656421
CUST_964_PI429010886	N8007	0.00909708	21	9						5.797914	13.45455	NA	7.092464	53.42717	33.4192	16.72046	12.40482	8.243972	NA	31.13507	48.03034	15.57705	8.015981	NA	5.523343	7.209009	10.4031	4.426892	8.348262	5.668943	6.447745	13.96735	7.56114	11.16455	8.797471	NA
CUST_55705_PI429010886	M111378	0.009098479	18	15						91.38505	70.63954	46.05207	65.7688	56.26482	37.00025	109.2822	102.3111	34.43069	167.0595	95.78807	53.91461	80.09649	106.7181	49.20954	265.8681	108.2246	224.6812	94.7296	138.6664	155.8635	84.73544	27.62832	54.06274	124.0673	66.0686	35.35085
CUST_9544_PI429010886	N24811	0.009098479	24	3	321460073	eIF2B-alpha protein [Daphnia pu	Protein metabolism	Regulation of translational initiation	2.13E-15	85.0591	30.01166	24.88093	112.3276	37.10235	34.56311	91.00224	28.60711	46.38013	108.7185	32.5401	33.92168	37.14598	43.23631	12.19654	27.37201	35.75247	27.79405	14.92455	20.95678	25.53977	14.32943	36.34596	18.48189	15.81562	59.15801	22.62211
CUST_28450_PI429010886	N57965	0.009098479	24	6						19.60329	41.67619	30.99758	53.087	32.36969	20.945	27.20014	54.04744	58.48301	32.1267	37.13496	27.1479	49.6421	5.797914	37.44318	22.54343	34.22563	26.92758	23.80751	18.68449	24.11862	17.15876	12.06091	32.14243	35.75916	19.32091	32.3057
CUST_42758_PI429010886	M61171	0.009124309	12	9	321471759	hypothetical protein DAPPUDRA			1.36E-08	105.3673	63.88807	103.1963	94.45356	55.19896	71.78136	75.16291	73.13284	87.31684	148.131	72.93884	105.6776	131.1419	78.4622	83.70817	65.04883	51.59074	79.90962	128.8131	70.42723	90.03155	177.6996	93.07316	141.7833	104.3593	69.53723	145.7532
CUST_7406_PI429010886	N21493	0.009126325	18	9	307175084	Nardilysin [Camponotus floridan			4.82E-63	27.90588	25.70617	36.40347	18.98605	22.53586	18.67083	92.7356	30.58701	47.79939	43.126	26.31272	42.26958	33.26038	43.07453	42.12804	60.76642	28.85219	47.15185	36.615	22.86182	38.22258	32.15923	20.9364	36.31991	42.26354	30.11811	37.77999
CUST_6043_PI429010886	N19552	0.009130712	21	3						74.10459	57.3502	54.62254	73.75221	88.07773	95.68309	55.49194	54.87487	51.29155	52.60565	28.34861	68.66348	50.7989	45.89596	82.86519	65.94538	33.63302	52.86649	74.21045	63.66074	72.75115	61.74127	35.82991	63.49903	83.68571	79.54469	72.48391
CUST_19079_PI429010886	N40945	0.009135833	24	18	260801100	hypothetical protein BRAFLDRAI	Transport	Protein transport	5.27E-32	35.40128	27.9152	41.86279	33.44947	23.44192	18.59269	41.31439	17.8371	15.54537	27.20683	21.43294	16.99789	20.68983	22.87315	28.93158	40.3175	22.4839	24.78012	62.89605	34.6239	46.90598	44.1311	20.77278	33.62763	42.94276	15.8055	56.56581
CUST_29652_PI429010886	N60298	0.009140646	24	15	241705172	ubiquitin specific protease, puta	Protein metabolism	Proteolysis	7.78E-23	8.30504	6.559869	NA	9.150568	6.017589	3.93697	10.00971	8.421879	9.090753	NA	5.868192	NA	9.300553	6.265386	12.67284	13.58834	12.71578	14.24675	NA	9.33567	9.00236	19.33464	NA	6.987091	7.416957	10.68459	8.092996
CUST_27285_PI429010886	N55781	0.00915025	24	0	524880969	PREDICTED: exostosin-2-like [Ap	Developmental process	Other	6.45E-30	22.13004	23.95198	20.16722	17.57495	16.92315	16.39884	20.04347	18.60577	24.86359	15.32048	16.66197	16.34183	13.90121	18.46557	16.28228	14.89846	22.82508	22.49527	13.84198	20.2995	7.333794	24.42528	18.25109	18.67988	26.6049	26.65836	20.17508
CUST_44364_PI429010886	M65721	0.009156696	24	6						211.3027	178.3834	178.7424	223.3609	156.1818	174.857	373.5014	295.8069	424.7669	197.5603	569.6083	124.3455	292.95	354.7453	214.5942	320.552	384.9395	530.8049	120.6137	145.1769	139.3061	130.2565	128.4025	151.6547	98.53709	141.0969	141.9587
CUST_3700_PI429010886	N15078	0.009162775	15	0						317.4305	449.9406	NA	173.8121	NA	172.1367	286.5115	118.1532	288.117	8.595293	14.11364	12.08433	72.71537	325.6483	72.64707	382.5381	201.3091	143.144	242.133	158.6683	189.476	NA	347.9647	5.057633	49.28164	92.71133	NA
CUST_16571_PI429010886	N36484	0.009168665	15	12						22.93848	24.22177	24.4152	12.65976	22.14763	17.24019	14.24832	12.67148	12.95302	7.356172	12.4284	24.53994	19.96477	27.77843	28.4851	24.85014	18.67083	15.3457	19.51476	17.57039	14.79429	15.9565	13.84795	21.87306	24.469	6.81204	16.34992
CUST_11650_PI429010886	N28179	0.009175887	24	18						62.03291	56.45276	119.6854	47.46533	49.40203	43.97459	59.3203	48.8328	54.03718	62.64213	41.25814	62.56704	64.54113	48.51961	59.99359	64.47129	76.59647	65.42578	92.33626	53.69851	80.74224	66.49052	50.74506	70.57647	91.86363	61.34753	78.25384
CUST_5969_PI429010886	N19442	0.009179495	21	9						83.30459	94.55136	110.9878	104.3843	95.80871	78.43006	97.97101	119.077	134.9856	138.7794	123.2894	150.9062	104.7454	116.0445	107.2361	84.91891	141.3011	101.7482	108.3364	115.3398	116.4413	85.92835	123.0345	122.1215	92.00118	96.9591	97.46284
CUST_1272_PI429010886	N9093	0.009179684	24	15	13898841	CUB-serine protease [Panulirus a	Protein metabolism	Proteolysis	3.55E-58	2256.232	1594.801	1480.822	1578.545	767.8994	1203.381	2350.317	4064.827	3509.087	3258.199	1191.479	1033.621	2933.397	2561.777	1125.5	6559.55	3331.869	6929.595	3688.899	4981.65	5427.368	2363.552	435.2817	1446.549	3620.969	793.2617	1964.046
CUST_37441_PI429010886	M19040	0.009184485	15	6						30.83149	4.038133	12.00032	22.74199	29.6926	28.61425	338.6752	NA	303.5583	28.26843	15.01185	24.76487	9.370822	24.9948	22.59279	53.92132	7.485899	19.71101	367.2044	367.9854	835.0606	65.44143	NA	74.49329	86.50032	23.57416	98.12003
CUST_37288_PI429010886	M18519	0.009194861	21	6	321479310	hypothetical protein DAPPUDRA	Protein metabolism	Ribosome biogenesis	2.53E-102	5.081378	22.0686	15.09107	8.537397	9.605488	12.15059	26.66658	26.7042	NA	NA	4.269174	NA	3.798423	NA	9.488162	7.622871	5.084507	4.175724	NA	8.00437	4.212213	NA	7.024734	7.214528	9.346935	19.31126	31.21665
CUST_20155_PI429010886	N42911	0.009200272	15	0						61.07789	144.849	187.3814	113.8344	121.7107	32.51285	25.17016	37.47838	51.32543	7.572085	58.78776	92.2473	297.9948	12.38261	273.9417	363.5741	74.7748	211.1114	78.40447	63.37029	87.33991	54.76807	24.87517	NA	NA	102.7497	101.0722
CUST_50211_PI429010886	M89053	0.00921221	24	6						24.90578	36.09821	28.82404	33.60972	26.9815	22.93848	40.35645	28.65875	34.87919	35.16074	29.97378	31.37614	34.61745	14.65575	39.32943	28.23406	30.02948	23.42712	26.2515	23.6825	30.77878	11.12735	24.71116	22.65596	24.40409	30.72076	25.23036
CUST_6812_PI429010886	N20632	0.00921221	24	21	291243678	PREDICTED: La autoantigen-like			1.2E-12	74.49329	107.8755	110.0417	65.82127	75.02912	53.612	47.2724	66.34289	75.34564	45.67933	125.5382	62.73163	118.3592	60.69474	125.3249	63.89423	109.2822	68.72832	68.91312	82.20662	93.01673	135.056	128.9542	125.9747	166.4755	81.62587	146.5474
CUST_2628_PI429010886	N12791	0.009213102	21	3						19.11378	18.39609	18.20572	23.86617	17.61537	21.21527	10.90567	13.88386	10.34409	8.753354	12.93095	18.11193	11.98023	10.43678	9.565216	16.77338	18.64884	7.584815	68.70029	12.63683	13.04163	13.05606	16.87615	19.3323	9.580454	19.98797	20.23623
CUST_21097_PI429010886	N44626	0.00921334	24	21	189234454	PREDICTED: similar to AGAP003	Metabolic process	Oxidation-reduction process	1.72E-22	19.09713	20.141	25.39952	27.99927	32.61311	20.44259	18.19714	32.57446	32.44868	15.49209	26.27536	21.38839	21.14901	24.55645	20.96508	22.01409	21.68674	25.5482	30.3574	28.72644	29.48451	32.63017	33.97232	35.22718	34.80724	17.97858	33.78234
CUST_7892_PI429010886	N22233	0.009215735	24	15	342326382	hnRNP protein [Cherax quadrica			4.56E-54	441.8514	558.2968	511.8036	377.2326	273.0815	431.5244	399.9663	473.6093	516.495	563.6439	302.8616	123.3207	443.7289	401.3498	319.7298	929.9574	740.5837	755.266	622.5028	437.1787	600.976	631.2553	472.6199	531.0942	688.6527	532.1418	553.5806
CUST_38201_PI429010886	M24122	0.009230062	24	0						1430.525	1903.836	1640.994	1305.464	1272.378	1044.527	1055.851	1379.279	1368.116	1057.154	1304.861	1118.139	1092.583	1155.365	1251.736	1540.275	1505.065	2338.958	1298.646	1266.211	1864.874	1153.203	1449.243	1824.284	1604.089	1804.854	2327.901
CUST_20556_PI429010886	N43655	0.009230062	24	15						33.4664	29.54782	33.48738	36.36523	18.73595	36.8404	71.62515	57.08761	37.24906	57.19268	39.34209	36.5095	38.4343	29.26951	52.16251	67.61402	49.75065	57.27829	37.07109	27.47705	42.36924	35.57898	26.53793	47.36163	32.99109	32.00663	36.74386
CUST_26770_PI429010886	N54844	0.009241194	21	0						45.78637	59.25874	60.4658	57.69269	53.63563	45.23287	41.97418	41.47761	53.72417	52.26773	41.25004	49.12192	41.3426	45.5101	46.10518	55.0756	46.61175	39.97592	57.94141	42.07726	58.20456	71.89808	58.67768	50.6306	62.84262	42.20935	51.22586
CUST_11975_PI429010886	N28717	0.009241402	15	9	242013098	protein groucho, putative [Pedic	Nucleic acid metabolism	Regulation of gene expression	1.27E-41	11.25563	16.77158	18.38558	7.852776	14.82044	9.683226	14.61052	16.40534	19.34308	28.71526	18.14584	18.24533	23.62162	16.11868	21.20564	19.90569	25.16714	22.59481	17.98274	14.46435	17.80998	16.86582	19.11039	21.40258	32.22863	17.74047	25.85237
CUST_51579_PI429010886	M94076	0.009252813	21	6						94.54537	65.33664	82.1818	81.1468	99.89645	100.9526	130.0637	84.03656	114.0755	85.1599	104.6784	102.0776	79.41371	90.95134	133.0536	81.00976	51.34157	77.22486	77.42501	76.30972	91.98987	85.90653	87.44315	101.5332	112.1849	67.98511	94.83653
CUST_44530_PI429010886	M66240	0.009253444	21	15	546676006	hypothetical protein D910_045			1.39E-08	168.7398	222.5585	177.388	153.4511	132.0014	98.70542	239.6808	202.6784	228.4713	165.8756	161.1223	102.2878	178.576	173.8872	99.53837	254.1863	289.2307	285.9469	188.1844	252.5592	186.762	191.7974	105.3222	144.5227	221.4294	150.7445	129.2573
CUST_45141_PI429010886	M68114	0.009260798	18	0	91090522	PREDICTED: similar to mitochon			6.79E-10	284.3354	465.6755	378.2634	338.993	287.4074	297.575	253.7263	403.5639	302.6664	243.1406	290.1517	285.8344	230.649	265.1126	204.7788	283.5676	373.5884	253.7263	328.6973	352.8279	357.6352	205.4884	321.8126	283.2866	295.4303	327.0634	189.2095
CUST_5261_PI429010886	N18464	0.009274548	21	15	242017789	conserved hypothetical protein	Transport	Protein transport	1.5E-110	89.44264	142.4472	104.7235	115.9325	168.0498	130.8641	173.3126	171.5592	189.1569	116.5745	132.9661	104.9377	120.6665	176.1473	118.702	177.1691	215.0811	187.0121	120.7979	136.4438	105.746	99.64105	131.274	80.64549	120.035	131.9455	106.4017
CUST_51561_PI429010886	M93990	0.009274955	24	9	410509312	myosin heavy chain type 2 [Litop			1.47E-42	6.887831	16.73592	13.92483	13.87626	12.62197	16.92486	14.11853	27.87953	7.386536	17.88948	28.14237	21.7225	11.42944	21.71688	NA	13.52297	8.327384	14.69878	10.53608	14.30523	9.897949	18.86536	11.90033	13.22308	7.020474	4.922612	7.037282
CUST_31800_PI429010886	M2443	0.009284724	15	12						814.3635	805.8552	880.3029	847.8182	937.5478	800.3618	631.8697	620.5792	708.1548	641.7824	876.3228	883.0931	815.2223	986.649	1137.925	1022.798	815.7902	989.9825	1150.764	659.0035	1146.245	647.0623	679.5306	756.8703	774.5071	671.2531	1091.098
CUST_23326_PI429010886	N48638	0.009286224	21	6						34.88898	35.26049	24.49039	50.76484	35.05743	26.0084	34.17027	42.55235	39.67832	74.55481	31.79774	33.64987	14.85278	22.62211	21.49936	36.53804	24.9499	30.41385	31.88585	24.14912	32.26017	44.58927	31.2401	32.99838	35.98686	29.4483	35.56153
CUST_4268_PI429010886	N16368	0.009287962	21	0						4.669189	7.509062	11.5869	8.889617	4.549457	6.429065	4.007214	3.640727	5.304563	4.156092	3.586107	NA	4.73273	NA	3.179343	3.298832	NA	NA	NA	3.685308	3.963874	5.398885	6.420652	9.00236	5.180466	18.05945	3.435667
CUST_56785_PI429010886	M114649	0.009289215	24	21						736.7743	904.627	795.5533	805.476	640.2875	687.9587	747.3198	741.4284	714.9939	705.624	670.6338	830.7266	486.7832	665.3418	697.5829	857.1902	932.6252	934.3693	967.3181	870.8828	439.5135	936.5204	833.2103	903.1163	1029.336	855.5436	1087.769
CUST_14530_PI429010886	N33028	0.009289215	24	15						37.3522	40.32229	40.05574	32.2735	25.3952	35.3759	27.90262	36.42265	34.13318	53.15878	20.71687	33.01146	43.46338	27.75069	38.40048	59.15801	52.73066	48.56519	48.84433	35.8443	41.14564	46.79452	53.34499	46.14458	54.40508	38.22258	55.56698
CUST_38056_PI429010886	M23116	0.009293527	24	18	77743875	Euphausia superba isolate DGGE				731.5275	1313.269	923.4428	690.7831	643.6241	499.5403	909.4866	751.2377	902.1594	883.4797	689.3323	772.0259	942.2704	643.0042	831.0254	1092.1	1409.395	981.9828	912.8746	1066.707	1354.492	833.2103	939.9518	1085.159	1115.579	836.1158	1375.747
CUST_42708_PI429010886	M61025	0.009293527	24	3						224.961	168.3995	190.0165	187.5258	267.884	242.833	171.4108	179.1574	159.9495	185.2204	250.1769	213.6241	188.3904	226.9173	240.3702	163.9723	195.8784	133.8983	134.7418	155.0651	121.7366	179.3019	168.1289	146.4225	141.1811	189.476	172.3736
CUST_49427_PI429010886	M85605	0.009293527	24	21						115.984	135.7196	139.2795	102.8535	97.60846	92.85843	78.97649	98.43645	118.928	120.749	115.7562	136.6555	139.2203	113.6067	147.0201	160.5173	122.5897	138.3092	165.5949	106.6543	122.5897	195.1305	122.436	154.4681	165.4279	120.4953	157.8871
CUST_23657_PI429010886	N49216	0.009293527	24	18						22.09706	23.49891	24.42377	17.18571	18.55369	20.38177	20.15584	28.29271	20.33677	27.63659	21.85249	10.58439	23.01193	19.39036	18.24171	40.04635	23.02109	27.73485	36.33638	30.13347	31.30624	37.56673	16.25968	30.02799	32.25164	23.19965	30.31787
CUST_441_PI429010886	N5191	0.009293527	24	18						8818.425	14338.96	10323.84	6087.174	4685.458	4086.34	11339.63	11264.76	13135.1	9999.789	5960.592	6665.157	9872.039	6727.299	6201.489	9313.798	14411.75	12886.71	12674.82	12991.27	12021.42	10852.86	12548.11	12836.63	12496.19	8984.262	10727.23
CUST_42339_PI429010886	M60047	0.009302126	24	18						20.17508	16.03034	11.32442	10.89368	9.71776	8.329107	19.19666	11.16909	16.13549	22.74199	7.457449	13.34035	23.90042	11.79315	23.77227	12.94058	19.35085	12.76402	30.14227	14.7567	25.48923	14.59906	NA	15.38078	12.08147	9.774001	20.39956
CUST_25507_PI429010886	N52544	0.009320771	24	21						3240.436	2440.627	2143.564	2056.189	1306.13	1380.586	1306.636	1965.01	1504.447	4234.05	2188.815	1528.323	2014.653	1297.493	1758.225	2206.592	2035.999	727.7655	3434.818	1759.396	2430.729	3598.269	3739.191	2722.979	5151.779	3692.065	3819.978
CUST_37408_PI429010886	M18933	0.009325941	24	3	242018020	chitin binding peritrophin-A, put	Metabolic process	Carbohydrate metabolic process	7.81E-52	983.8429	548.75	404.1681	988.0134	3636.712	3331.869	536.7837	1496.297	317.6549	391.6595	1728.138	2170.443	284.4024	1156.716	1214.037	249.3411	263.6152	267.2693	116.5333	1841.638	99.67801	374.0444	711.4323	379.7764	97.58001	2260.134	147.3731
CUST_23063_PI429010886	N48192	0.009325941	24	18						68.27308	84.25166	81.5061	61.87915	43.06564	47.71567	61.86512	49.71376	56.08545	81.40497	56.25386	78.06756	77.60656	60.28486	87.30779	79.68621	55.04149	89.37424	103.5544	64.53182	91.63916	78.5278	67.3975	93.65434	90.60669	65.51653	108.2394
CUST_30559_PI429010886	N62122	0.009325941	21	15						19.73488	21.7713	9.013796	17.67823	3.612717	17.48796	27.41834	18.70643	13.4515	22.34076	18.9186	14.24832	22.60023	14.37116	13.77444	59.5443	35.78636	23.82619	30.22988	12.06806	24.53994	17.73766	19.8029	15.19667	21.00252	13.84198	10.77373
CUST_20279_PI429010886	N43151	0.009335943	21	6						2034.424	2459.181	948.407	2886.102	2968.824	2702.508	4266.005	2789.551	2484.399	3193.902	2637.871	2116.149	2729.922	3230.906	2586.608	3091.943	2302.573	2883.204	2251.39	2609.919	2085.588	2130.512	2227.296	1833.616	1782.659	3079.233	1746.391
CUST_48310_PI429010886	M79757	0.00934091	18	3						32.47851	22.16019	17.82226	29.00237	32.17224	16.2172	29.6976	18.48357	12.07145	38.52366	21.54473	9.779895	36.03727	24.68337	15.50271	12.6423	9.515144	13.79482	27.9424	20.819	21.89409	41.78309	36.52621	37.07514	28.68856	26.46184	22.36152
CUST_12871_PI429010886	N30180	0.009341131	18	15	321457051	hypothetical protein DAPPUDRA			2.42E-36	72.0781	97.46284	27.39004	82.29898	13.85499	80.60289	197.3639	113.0827	74.25196	200.6783	89.41928	61.64797	122.9878	68.14166	109.4424	388.6637	173.6445	221.5335	164.9977	21.48685	125.3097	59.45185	46.78844	63.69892	204.4319	111.7284	38.71226
CUST_4587_PI429010886	N17084	0.009352244	21	3						25.33166	28.50564	28.0231	30.80078	46.01666	27.85852	33.93179	23.01802	19.40349	26.48825	37.9964	27.72605	24.23948	22.78318	27.21869	25.02499	21.96464	23.38956	34.67259	31.00311	33.3337	27.01619	27.20683	41.1126	34.69476	37.9908	40.60853
CUST_30376_PI429010886	N61745	0.009352244	24	15						76.3955	44.63889	39.6947	77.9006	22.41168	45.41169	124.7762	66.52473	133.6243	263.0994	110.3077	35.58267	74.95343	85.04871	42.11529	226.9173	100.2134	142.2521	122.2183	95.55779	129.0467	143.7425	19.8408	97.63248	72.02191	8.468746	39.96323
CUST_28185_PI429010886	N57452	0.009354915	21	0						33.83378	45.04591	37.19414	26.02721	22.54151	27.51681	23.41335	26.66393	21.22638	34.8722	25.9357	18.61738	32.39325	14.49503	25.16714	42.01431	35.66495	51.43184	33.13254	30.95789	31.11715	29.00837	40.55768	37.91672	32.79575	31.36481	50.03259
CUST_16479_PI429010886	N36305	0.00936486	21	3	211922663	opsin [Triops granarius]			5.3E-21	36.63747	60.04487	NA	96.22172	NA	84.09371	NA	19.0327	18.43758	17.89974	43.35233	32.58484	31.26771	NA	30.08206	64.96779	31.94938	34.87919	41.4895	68.02537	33.84162	57.24189	5.434281	61.95078	180.8767	NA	195.4747
CUST_35627_PI429010886	M13058	0.009372346	24	18						14.26774	22.14763	18.37444	16.58793	10.83531	10.06086	24.58293	10.9779	17.43071	14.9184	7.824872	NA	14.17028	13.34709	11.49555	22.68453	20.48939	22.50572	32.3516	19.02577	24.39623	18.51484	18.36834	26.34871	23.53208	11.68791	26.60187
CUST_13688_PI429010886	N31597	0.009388843	21	18						399.0134	514.9905	462.9596	458.5926	421.4753	363.9263	428.8199	383.7931	392.253	392.6361	322.6893	415.3144	419.4465	404.7001	359.0835	427.8043	490.2242	402.9178	465.972	486.2941	477.058	329.6536	466.3373	441.0424	448.0872	476.6448	413.8083
CUST_12088_PI429010886	N28906	0.00939587	21	18						15.51385	39.96697	40.9561	27.38458	30.97112	37.27834	20.6404	23.80751	22.69087	24.60171	29.70619	45.41169	26.26876	18.2303	26.85004	23.33803	39.55568	29.55431	32.7202	53.40427	71.84187	31.25815	38.92916	38.92149	23.25881	23.1406	46.40767
CUST_23558_PI429010886	N49029	0.009397511	21	12						6.89341	16.36134	13.13409	15.28234	12.48798	15.84358	67.04107	14.57376	24.36005	18.95021	12.50865	12.21076	20.24017	14.93264	43.84283	27.04805	19.70882	14.07152	12.7792	12.3846	18.22769	8.777341	11.40929	12.301	12.65456	19.0444	10.38791
CUST_39534_PI429010886	M40909	0.009398567	21	3						506.9058	647.785	425.8369	333.4754	1795.363	1999.848	776.7493	1727.221	680.5705	221.2615	679.9519	707.937	391.5717	383.8668	365.4954	146.953	277.6138	262.8953	243.331	2877.124	162.0504	556.2705	884.3862	399.4473	234.6915	2759.556	738.5083
CUST_32454_PI429010886	M4163	0.009400783	15	12	345482582	PREDICTED: aminopeptidase N-l	Protein metabolism	Proteolysis		303.0592	585.2891	170.3606	231.626	68.5694	226.6524	570.5532	512.3561	203.8839	615.4627	415.6373	138.1161	746.7514	463.9651	548.5685	1155.769	733.2499	1073.164	177.8816	68.79187	104.9676	234.322	310.2931	203.0356	878.4972	571.8635	136.6675
CUST_51709_PI429010886	M94664	0.009400783	15	12						321.1394	286.5115	326.7696	266.9539	213.6621	263.893	305.5795	272.5621	266.1014	426.1127	249.7255	292.8798	376.7239	284.5651	303.3581	349.0153	300.3594	270.0299	243.6749	276.0279	258.5627	265.3286	210.5853	291.4248	277.9454	265.6462	375.7494
CUST_54876_PI429010886	M108067	0.0094068	24	9						624.2116	228.29	722.3326	649.314	404.3968	557.4698	142.749	1124.497	1049.045	890.9879	1603.304	666.8579	537.4657	220.2176	657.7333	323.446	1048.592	1281.397	201.5487	283.749	95.06795	81.96005	460.8197	450.6028	370.3281	742.4077	299.4177
CUST_37027_PI429010886	M17603	0.0094068	24	18						74.58857	96.46623	85.42964	67.25975	84.70621	68.39377	69.80795	82.05798	83.09656	59.47135	79.17186	78.67787	81.66229	76.2639	104.3593	78.04556	65.47772	66.34289	98.85767	106.3722	116.981	88.68681	92.00118	84.65768	95.27189	74.4544	99.54889
CUST_47575_PI429010886	M76668	0.009411166	24	18						1011.981	2365.258	1302.008	764.7593	897.3252	812.7394	1005.258	1458.832	1414.36	1038.339	1080.06	940.7158	723.134	1247.935	875.3699	1402.437	1728.138	1522.629	1846.233	1652.642	1427.593	951.1847	1309.684	905.3835	1373.039	1138.567	1055.306

Probe ID	ID	p-value	Period	Phase	EBI ID	Annotation	GO category	Go term	E-value	0	0	0	3	3	3	6	6	6	9	9	9	12	12	12	15	15	15	18	18	18	21	21	21	24	24	24
CUST_2559_PI429010886	N12624	0.009411166	24	21						284.8685	340.7195	292.4122	302.7218	302.9304	274.7035	169.5998	260.9505	196.875	286.745	250.7116	272.3474	305.6416	266.1014	314.254	308.8108	273.6157	334.719	344.3047	256.1905	207.3653	394.1154	294.481	314.0471	382.5381	283.5165	328.7563
CUST_4183_PI429010886	N16185	0.009411166	24	18						631.2553	678.0803	709.0094	565.424	490.7221	497.1127	677.6753	539.585	522.858	690.7831	518.9718	702.1022	689.88	532.2614	629.0343	905.7144	690.9865	769.5468	889.3882	576.7675	836.1158	827.5626	618.3282	798.5585	872.2903	614.0454	896.6099
CUST_7459_PI429010886	N21579	0.009411166	24	18	383853357	PREDICTED: cell growth-regulati			1.39E-40	74.04749	156.4627	119.5848	78.3726	72.13266	68.31562	103.0089	123.0345	93.80651	75.71093	90.69879	88.0075	99.94989	82.76148	87.71525	101.7482	142.6733	121.0574	132.5291	119.9522	155.7969	88.94466	89.21288	99.46029	118.5659	111.4946	119.329
CUST_13474_PI429010886	N31252	0.009411166	24	21						156.2876	233.0433	236.1021	165.5673	143.92	165.4991	144.7014	165.4991	164.8179	186.4716	166.8658	156.0931	234.7388	150.073	215.3959	186.5755	203.0154	215.1114	240.1963	190.3204	200.1232	232.7013	182.2041	257.5954	216.9998	209.7386	250.621
CUST_26825_PI429010886	N54947	0.009424619	15	0						54.55547	125.8093	90.41336	53.61862	68.71816	40.38119	20.38623	43.46921	42.06094	47.59455	36.27638	29.5607	187.0484	34.80724	195.2227	201.0938	31.53193	152.8269	117.5547	83.9852	96.74886	47.66934	35.46629	17.35683	18.61738	57.10009	53.9262
CUST_26213_PI429010886	N53857	0.009439672	21	0						37.84952	36.53104	35.34111	34.74447	36.83655	25.80525	26.92758	22.75502	40.72145	19.28481	14.30371	28.05304	25.31848	29.65588	44.45593	39.25729	14.62836	31.22074	45.7721	31.22451	26.55873	46.77922	29.67957	55.87434	28.52646	22.27865	49.85348
CUST_22066_PI429010886	N46359	0.009448122	15	9						197.7072	130.0637	113.3335	177.4557	129.4913	157.2166	193.6659	140.9438	180.4963	243.8189	157.7724	189.8869	152.2431	151.9875	163.9175	134.6492	150.4308	163.2927	189.4144	154.854	148.298	213.1791	150.6011	175.63	241.1372	164.9432	226.5718
CUST_8564_PI429010886	N23227	0.009449749	18	15	157131921	mitochondrial ribosomal proteir	Protein metabolism	Ribosome biogenesis	2.37E-88	289.6241	462.531	292.0796	235.9714	452.5351	304.9349	326.9964	473.2258	379.9317	279.9265	344.1561	366.9286	332.1692	348.1933	378.7986	480.9139	492.2464	529.7211	291.8297	381.5915	370.3281	246.6369	319.5317	313.5422	323.2809	323.5934	372.7272
CUST_35227_PI429010886	M11964	0.009454684	24	9	339787896	calmin-like protein [Tigriopus ca			8.24E-14	4.179167	14.46618	11.1896	12.17385	22.40935	19.13763	20.38818	40.96757	28.82404	NA	38.06618	18.07705	16.66408	51.32543	19.35718	16.02388	22.18677	26.51031	11.9085	30.09026	19.86967	17.41612	22.16839	7.442251	7.625156	20.25957	6.065608
CUST_52916_PI429010886	M99417	0.009460665	18	15						94.10473	146.9401	93.03552	71.03566	70.44138	66.0686	178.7203	108.8028	106.506	69.92347	70.0011	67.81145	115.2516	96.16362	87.06917	107.4885	115.6931	111.2714	75.10319	75.87682	53.88185	66.1068	60.30614	71.86601	74.05592	61.39194	83.6658
CUST_16105_PI429010886	N35660	0.009460665	18	15	356651196	heat shock protein 21 [Macrobr			5.03E-13	655.6894	547.1222	586.1434	633.8464	513.2014	511.0453	563.8477	490.4622	512.3561	622.9027	476.9732	680.9887	546.0695	521.086	543.6971	664.9053	720.7726	722.7897	589.5468	506.8228	709.4562	546.6865	576.7675	655.3101	465.972	488.2191	659.603
CUST_48238_PI429010886	M79478	0.009484085	18	0						12.37965	26.86667	16.04875	18.49355	9.902198	21.87746	15.15361	NA	16.98223	8.198822	9.62424	NA	8.771483	8.102905	NA	21.93249	20.7057	19.66532	26.42112	19.51325	19.25567	17.65737	12.45866	19.92947	16.36929	13.98648	12.4892
CUST_4899_PI429010886	N17737	0.009487271	18	15	390345455	PREDICTED: zinc finger protein 1			4.45E-27	14.24675	13.34709	11.7024	10.25273	9.273221	NA	9.149353	11.4038	8.297607	13.42904	NA	10.63933	12.52949	10.39641	14.23642	11.52944	23.08542	13.905	6.926192	9.555384	15.21531	11.18155	8.790565	8.807925	7.737208	8.410091	10.89368
CUST_39181_PI429010886	M35737	0.009490981	24	3						7316.568	19.88289	5514.378	7568.31	6500.649	8215.523	33.78234	94.60841	46.64691	6304.559	6752.376	8768.122	5080.07	3972.222	6125.57	12.63962	NA	34.23102	53.51539	221.7013	47.63863	5080.07	1457.293	4522.071	258.1145	869.3099	909.4866
CUST_35174_PI429010886	M11806	0.009498697	21	12						41.59303	22.3639	40.29385	250.3014	144.6793	122.4622	729.4004	119.1399	754.678	127.5443	886.9505	24.74446	1201.71	901.5762	98.36388	33.92168	316.4278	66.70374	10.38475	49.59199	12.21582	21.97251	264.7345	161.9628	71.80743	44.98293	37.5978
CUST_38951_PI429010886	M32423	0.009512087	24	12						29.79615	31.2986	33.38645	68.24011	43.17122	44.56209	71.30275	75.50995	112.4597	24.79879	83.08821	29.32605	52.25815	142.8894	85.785	44.64909	141.8167	99.57709	21.55928	38.11477	29.84354	8.30152	43.55336	61.17453	15.09523	33.67346	43.60777
CUST_21039_PI429010886	N44516	0.009513978	24	21						28.00501	34.04257	57.9144	15.24489	9.000381	13.73882	12.4892	48.94611	55.1847	13.69771	22.37507	12.07805	33.5806	11.82584	25.23652	12.59509	21.91017	12.68701	56.74562	46.24985	74.58857	70.84605	58.4567	36.78126	87.97629	24.95325	83.62172
CUST_968_PI429010886	N8016	0.009525659	12	0						893.2381	493.5638	1092.583	707.3888	340.4948	297.7548	304.2261	324.7451	471.9849	468.1951	349.3011	427.0381	1348.208	854.1319	1399.933	165.2661	200.5807	302.6134	600.976	561.1654	595.1579	1318.185	877.7497	804.0438	517.5058	219.538	675.0754
CUST_33400_PI429010886	M6795	0.009528806	24	21						158.6448	133.4766	194.0105	154.4454	165.3213	171.9553	110.8779	139.5639	153.9744	160.7833	151.922	146.4225	149.5552	175.4398	157.0354	172.0396	190.6927	187.3112	194.2319	160.139	148.7876	179.4641	179.7937	187.6435	215.0091	194.7406	202.9059
CUST_49208_PI429010886	M84686	0.009528806	24	21						227.5418	337.5799	421.977	263.4037	303.88	316.6599	249.6908	315.1053	204.4688	261.5479	338.507	311.5041	254.9324	286.2998	293.0253	365.1669	412.604	343.5695	381.849	299.4747	288.5968	449.3664	334.8644	394.5922	568.8248	340.9357	307.6081
CUST_31640_PI429010886	M2035	0.009531993	12	0	524893554	PREDICTED: uncharacterized prc	Metabolic process	Cellular amino acid metabolic process	5.26E-44	124.8682	234.8735	211.4099	158.0057	180.3948	149.2467	115.8263	160.0191	147.1247	182.2609	171.436	186.658	152.4151	197.5933	193.3377	170.5897	42.49657	167.0808	154.8931	140.9228	120.6508	191.8512	255.0769	249.8271	211.9713	230.0694	222.3991
CUST_22631_PI429010886	N47366	0.009532095	24	21	321478800	hypothetical protein DAPPUDRA			3.53E-28	12.60763	15.93445	23.93602	4.250002	29.19743	15.68492	4.733913	19.55073	11.56292	8.880632	11.94454	5.990546	11.0155	12.92087	8.62544	7.534185	14.66099	13.52973	8.178473	21.42827	20.71285	16.42269	24.80919	23.72167	14.93559	18.12289	26.79915
CUST_30308_PI429010886	N61596	0.009548438	18	15						18.53796	19.15475	NA	12.09389	13.99736	19.1752	6.258419	27.81087	14.52479	24.09713	17.49722	14.3393	29.1452	10.59882	18.40393	19.00338	20.77278	35.49916	15.7218	14.85649	14.72189	11.7024	NA	31.05988	3.847413	4.266835	3.994186
CUST_49838_PI429010886	M87376	0.009549535	18	0						17.79556	14.18007	NA	12.52949	NA	12.46469	9.942624	7.07223	6.669932	15.80426	8.567205	13.03303	5.461689	15.49957	39.46646	13.50832	7.309623	11.73235	46.10158	11.52651	11.77605	11.4759	10.80862	8.010707	6.564831	4.648358	10.41528
CUST_38571_PI429010886	M28126	0.009553403	24	3	33468738	calcification-associated peptide-			4.54E-12	968.0088	197.3862	857.1902	1057.154	2769.46	1981.815	199.6837	358.1637	162.5974	489.8482	1359.653	1426.897	283.4621	1632.022	2061.689	89.1801	82.9737	74.84749	65.71584	467.0581	48.05348	517.7643	422.0714	440.2791	82.49497	624.9341	157.3439
CUST_34593_PI429010886	M10106	0.009554	21	15						207.1407	360.1634	191.3144	309.0132	436.2924	412.4307	341.2447	273.8482	186.1038	320.7176	534.1168	543.0846	251.3401	461.2704	523.2386	431.5244	601.3269	491.0486	231.2512	156.338	220.9078	171.3397	230.5014	264.5418	214.7361	318.6428	214.5942
CUST_9264_PI429010886	N24334	0.009557334	21	15	380011359	PREDICTED: E3 ubiquitin-proteir			1.55E-40	68.58944	92.48145	94.7296	68.22331	77.79664	86.24643	69.64615	84.9728	88.34301	83.70817	83.89283	61.96517	85.63473	92.0742	76.5633	104.2797	115.0193	110.519	76.09471	92.95196	88.78315	90.07438	90.56235	81.47133	89.65818	74.60421	92.28753
CUST_21190_PI429010886	N44791	0.009559277	15	0						1649.981	2629.319	441.8514	624.767	1415.173	500.3221	702.937	13988.25	447.8863	218.889	181.663	155.3531	91.17437	6688.584	126.4468	443.8257	851.1928	449.1793	382.8713	402.436	374.3365	320.2208	349.6396	196.7413	331.6434	325.0373	145.5507
CUST_52581_PI429010886	M98048	0.009582641	18	3						744.9105	764.2764	904.3497	909.4866	1317.605	1522.629	821.5501	699.1348	876.6144	811.9435	548.9123	802.1705	890.6088	581.4931	555.0536	1107.545	814.9423	828.3699	1000.348	712.9201	829.1725	1030.175	800.6803	973.9492	921.74	724.9498	923.7499
CUST_41499_PI429010886	M57884	0.00958605	24	15						142.6225	98.98816	128.4222	107.935	67.44192	90.66543	185.1157	95.43553	142.1072	183.5082	93.61951	144.5431	175.7096	95.47293	125.8771	186.792	137.5078	169.8371	150.1329	92.39943	147.7951	131.2558	109.9399	135.8226	126.8388	91.95645	139.095
CUST_3079_PI429010886	N13749	0.00958605	24	15	242017839	hypothetical protein Phum_PHU			5.02E-45	93.56602	153.6889	128.78	83.23692	99.64105	91.84111	101.4772	140.4269	122.9659	82.05798	100.0827	124.1583	103.9647	129.9776	115.0193	128.2527	185.937	157.985	100.6757	121.5207	165.7838	88.34301	138.7489	131.8408	88.45765	121.6162	116.804
CUST_5592_PI429010886	N18918	0.00958605	24	15	148226898	phenylalanine--tRNA ligase alph	Metabolic process	Cellular amino acid metabolic process	4.33E-173	23.41335	63.17902	36.33342	21.618	33.55276	26.31272	38.89512	57.05634	43.35233	31.3587	38.89794	26.80276	38.18197	36.74804	29.59501	48.17461	63.71474	52.78141	39.61156	35.33106	42.74631	28.87751	33.35662	42.12804	34.91434	38.89189	24.11397
CUST_41355_PI429010886	M57537	0.009593672	21	9						4.002972	13.80069	5.404267	12.36112	12.76575	12.83977	6.415726	14.91026	15.68492	21.35681	16.00106	NA	11.40479	15.72841	12.00032	9.718797	19.58546	12.1414	6.72839	10.31745	11.35653	10.46073	12.78545	7.652903	15.28234	12.32049	6.058085
CUST_41128_PI429010886	M56936	0.009606049	15	0						48.48353	78.68427	61.37944	63.08459	69.54417	53.04738	41.67991	43.77016	40.17351	42.09646	35.04281	47.2578	52.56129	45.1573	48.30656	53.9367	64.82967	45.96012	41.40683	40.9561	49.4765	45.66053	43.02401	50.07055	41.86801	43.61505	48.07833
CUST_57029_PI429010886	M115152	0.00962368	24	15						511.2321	470.9814	574.7506	509.999	422.6098	440.4343	463.4514	444.1379	500.4612	542.5585	414.4128	365.8262	474.5724	464.5257	476.0531	697.5829	635.4957	567.5384	496.2739	503.1242	549.679	567.0251	501.3701	576.7675	536.489	506.2756	600.976
CUST_57301_PI429010886	E4652	0.009624225	24	21	345316531	PREDICTED: beta-1,4-mannosyl-	Metabolic process	Carbohydrate metabolic process	2.61E-15	29.58887	16.6131	28.08905	26.05349	17.0552	14.90496	8.883273	27.69715	20.72028	13.71572	20.1	23.08542	12.71713	15.01335	25.56702	18.99558	18.28134	23.78251	21.28132	24.9948	26.42112	29.51504	27.15396	28.71238	31.84584	16.4789	32.67436
CUST_2483_PI429010886	N12478	0.00962606	18	15						45.65444	61.37944	54.62717	41.84178	62.367	53.45027	22.79379	37.09003	33.60313	23.73245	35.5206	53.06387	41.82858	82.24574	72.3378	57.67493	55.19896	77.48265	31.44937	54.11401	63.55637	34.96815	66.96376	66.73428	38.81639	51.18755	28.57269
CUST_24559_PI429010886	N50840	0.009636845	18	3						23.44667	31.75441	30.4977	32.41501	35.56743	31.33584	18.82944	27.9284	21.82795	26.95753	31.11334	22.80766	30.99758	31.12503	24.58293	17.90502	25.12907	23.422	17.94749	20.4836	17.90949	31.43843	33.71363	31.64405	29.56314	23.4544	35.38316
CUST_50706_PI429010886	M90719	0.009644134	12	0						40.45487	32.50381	33.74242	29.35282	64.95625	42.3598	23.70635	29.5607	27.89407	31.43843	37.07514	40.56783	35.05094	38.07838	43.73952	30.36386	25.85738	32.68841	30.51072	26.4046	31.26771	34.88562	31.07591	21.38839	33.60676	36.80764	41.12174
CUST_32335_PI429010886	M3851	0.009644307	24	15	345495410	PREDICTED: hypothetical proteir	Cellular Process	Cell death	3.08E-128	59.09232	58.64151	59.97246	51.44911	55.08838	62.01214	68.13325	57.48416	55.94132	80.90741	57.60444	86.01043	58.80984	75.12894	62.12865	74.48675	73.06306	80.60289	74.8864	62.06796	59.8943	69.02489	59.22932	65.22345	74.49329	55.32819	73.88293
CUST_13772_PI429010886	N31740	0.009644307	24	15						620.9302	501.0897	489.9777	538.6242	399.349	345.674	615.4627	494.3291	563.9832	581.654	413.108	330.9867	699.9941	416.778	433.7137	717.5574	619.2009	712.9201	722.545	493.5638	701.5555	620.2559	484.5238	625.2657	531.8423	422.7276	542.7794
CUST_26225_PI429010886	N53877	0.009644307	24	21						26.80276	23.73807	21.47499	24.25636	16.2921	19.05953	20.97185	20.80025	14.85493	11.83609	20.03269	10.03167	25.46041	17.80167	20.97645	27.9125	34.63148	21.72789	34.94683	17.16673	23.10033	32.34328	25.24715	20.62924	47.71567	22.08254	32.10443
CUST_54608_PI429010886	M106834	0.009646445	24	6						8.396446	11.86651	8.815762	14.72662	18.85869	10.39079	11.48186	13.64514	11.75933	10.75635	8.168708	8.561749	8.243972	9.217287	9.184105	11.18301	8.944558	8.133753	9.068193	9.389205	12.64397	7.530036	6.87399	6.070219	9.808744	7.401064	10.99842
CUST_52986_PI429010886	M99693	0.009646445	24	21						19.50063	21.05828	21.39039	17.33723	12.16037	4.017884	9.215946	10.15208	11.23899	30.06892	11.44329	18.53166	21.75367	13.02105	18.49915	24.09442	15.10046	16.98033	21.63837	19.30617	11.63737	34.82138	23.43732	17.21886	23.0873	21.87085	34.53159
CUST_10991_PI429010886	N27098	0.009646445	24	18						27.55963	80.63078	70.02512	46.22189	43.92787	38.28366	50.18213	45.49065	57.79948	67.09453	45.76238	54.12459	63.55637	41.89699	64.97632	57.86776	55.47729	82.91071	62.58458	99.06526	70.44138	64.01323	62.33316	75.39478	59.81196	71.5317	67.81869
CUST_22053_PI429010886	N46338	0.009646445	24	21						25.69634	30.94993	36.97106	18.30651	35.0064	28.65291	24.54259	31.52277	32.24502	32.13276	24.71696	31.72823	25.13355	38.16633	29.24032	38.83274	46.9424	33.5564	45.67001	24.69407	34.1983	50.48397	40.83819	39.74086	43.70175	32.05943	36.7
CUST_10729_PI429010886	N26694	0.009647783	21	9						45.39182	46.14868	44.66421	45.96012	61.86512	45.17758	62.66376	58.48301	62.14225	78.80045	63.50827	68.11052	50.51811	42.03292	53.39229	62.196	59.797	64.23608	73.39823	58.30071	53.40427	58.79368	56.95109	56.15422	61.53216	59.77969	58.72904
CUST_36079_PI429010886	M14565	0.00965919	18	15	328796226	mitochondrial ATP synthase gan	Metabolic process	Generation of precursor metabolites ar	4.83E-98	1091.098	1664.278	1296.153	1049.887	1370.424	1407.191	1806.683	1384.734	1034.116	1415.173	1744.803	1139.062	491.3879	1392.216	544.0973	2543.39	1934.597	1834.729	1649.981	1087.147	1657.457	916.8554	1060.229	882.793	676.6048	844.6913	811.3473
CUST_42690_PI429010886	M60979	0.009667134	24	18						2030.062	3643.551	2174.954	2058.438	1485.178	1643.622	3538.483	3258.199	3350.998	2514.613	2277.337	2127.944	2549.09	2177.847	2886.102	2889.672	3588.155	3568.199	2419.664	3724.515	4527.493	1749.36	2335.81	2487.704	2842.045	2098.966	2740.835
CUST_49987_PI429010886	M88107	0.009667134	24	3						1169.783	162.6721	971.9693	1171.488	4895.993	3478.142	177.7613	550.3617	154.3451	457.0998	3161.765	3124.619	403.3938	2945.018	3601.999	107.6547	132.9821	114.1769	106.4149	799.0901	54.10933	573.6451	430.9236	467.9385	91.8129	1057.612	275.386
CUST_263_PI429010886	N3192	0.009667134	24	3						4069.14	216.2603	4229.758	4965.926	11094.64	9444.83	157.7724	415.3144	179.4936	1139.817	6897.446	7376.103	824.4785	8309.047	7484.678	138.0734	141.9148	171.4108	119.329	542.3764	106.8262	1747.584	300.8806	1322.312	186.3541	589.8797	212.7294
CUST_13927_PI429010886	N32029	0.009667134	24	15						32.37762	51.85541	50.12052	62.14225	44.79025	35.87313	39.78508	48.12738	23.3163	75.64874	61.75951	73.98792	50.12052	58.59505	94.26006	67.0724	64.15258	77.78042	64.91949	72.14124	76.53128	56.36738	53.09948	85.52485	89.25205	55.88006	66.52473
CUST_307_PI429010886	N3740	0.009667134	18	3			Metabolic process	Carbohydrate metabolic process		116.0759	46.82802	103.2209	154.9154	121.2532	144.9793	77.11712	66.11978	54.67195	126.1702	151.3085	112.473	133.5862	113.5681	102.3977	49.17415	36.36523	31.53193	71.62515	64.1954	34.47208	166.2093	142.0375	92.65597	70.41437	114.9651	81.15666
CUST_29436_PI429010886	N59859	0.009675667	24	21						4.564041	6.208575	8.700256	4.920386	NA	5.038532	NA	4.056541	6.652007	NA	NA	7.634361	NA	6.413865	6.112777	4.202898	6.259465	8.831604	NA	13.6796	7.929151	13.2072	12.22228	9.799617	8.109042	NA	8.202317
CUST_52562_PI429010886	M97957	0.009685069	24	15						42.98408	NA	25.21391	20.33677	24.47097	49.28723	12.30813	20.61453	19.33037	6.339415	38.92511	NA	32.59654	16.25789	40.36855	59.09838	93.08615	59.15801	42.19221	20.53404	50.01635	31.96059	NA	44.72002	47.1467	18.99016	60.30614
CUST_22765_PI429010886	N47631	0.009697423	24	21						38.52068	NA	71.1642	22.01409	10.81853	6.168158	13.68381	47.52511	7.975034	NA	29.11467	NA	3.550267	NA	NA	32.54688	127.5443	26.60187	270.7536	22.98601	187.7082	NA	153.9248	432.3981	532.4935	52.82485	424.0822
CUST_50953_PI429010886	M91549	0.009700176	18	15						42.96134	45.64468	42.38319	29.84354	26.29555	29.41321	52.56738	44.68965	33.63623	62.09609	10.83531	44.53859	57.48416	30.5648	26.49945	66.64484	49.94958	69.01316	59.41831	46.0486	29.82341	45.83287	23.38714	27.83033	49.9905	39.57166	30.02336
CUST_1221_PI429010886	N8903	0.009702953	24	15	197210409	60S ribosomal protein L30 [Hapl	Protein metabolism	Ribosome biogenesis	1.29E-43	14526.85	19522.5	11667.94	13837.12	10127.21	11475.48	17658.04	17210.94	16221.54	15705.46	9629.338	13812.43	14256.4	11112.95	13248.98	20020.97	19791.01	21224.39	15553.93	15705.46	18435.82	13335.43	14307.54	14794.99	15630.09	14363.02	10871.96
CUST_26425_PI429010886	N54220	0.009705792	12	0						32.42666	63.26218	46.75132	30.1739	28.68856	24.26626	17.93755	30.27748	15.41819	11.1169	48.32075	42.316	58.27906	18.3141	65.57565	24.16121	26.76018	25.58573	10.20268	12.30677	11.02719	40.30598	44.39167	30.82525	31.43843	14.92893	NA
CUST_3527_PI429010886	N14691	0.009709165	24	3						6864.217	125.8592	5678.164	12473.12	7912.634	11339.63	48.66253	597.9185	42.70736	3890.917	4705.762	5043.311	2698.998	6515.192	6999.724	NA	16.31776	47.61903	25.40663	700.1848	57.78766	4181.398	797.7145	3010.08	75.97892	857.4356	246.356
CUST_2319_PI429010886	N12099	0.009712045	24	21						35.22718	73.59425	69.02489	49.97848	46.11055	66.02974	48.5496	51.36995	46.66635	50.02722	51.81269	53.90894	46.85827	56.72705	47.4031	52.8767	53.68776	58.36343	54.25668	52.06198	60.5679	60.44338	59.28006	75.80655	55.33608	45.58935	67.42662
CUST_56402_PI429010886	M113689	0.00971532	15	9						15.98731	26.42527	29.87493	4.234096	20.17748	7.439921	21.52069	25.70122	23.56505	44.58927	38.69008	22.42126	20.50662	13.70168	14.85832	24.01703	23.88306	17.02151	18.02157	19.82326	14.16364	22.21134	17.84752	17.03584	32.74766	37.39308	19.60329
CUST_32560_PI429010886	M4435	0.009735501	18	12	443717635	hypothetical protein CAPTEDRAI	Nucleic acid metabolism	RNA metabolic process	2.15E-50	16.11644	22.68184	8.804391	10.67743	13.82584	13.52297	24.92323	14.35881	23.59034	15.46584	24.7069	16.37588	23.23426	NA	17.56163	14.20626	22.78318	22.48649	15.27294	17.59796	20.53404	12.45442	15.67181	13.33462	10.89646	16.36594	17.95536
CUST_25829_PI429010886	N53129	0.009742306	15	0						19.0538	13.57005	8.417136	11.13904	12.85647	NA	5.129351	3.769753	13.86062	15.25449	NA	NA	15.9967	11.33295	12.50576	15.18237	18.16224	24.50935	6.385639	5.479271	6.618689	3.590037	14.04576	5.186782	9.651486	4.786641	10.52851
CUST_34025_PI429010886	M8540	0.009746101	18	0						916.1745	1011.588	NA	47.31029	NA	53.58837	928.2282	37.45982	802.754	8.361378	13.28096	8.611312	26.85809	1127.686	23.27075	79.37286	33.05084	27.90588	98.71772	195.9057	92.08367	NA	435.6254	2.990286	28.10987	55.08838	NA
CUST_37413_PI429010886	M18944	0.009754607	12	9						7225.192	3264.887	11052.65	10626.1	6622.666	6744.57	7558.705	7270.382	13419.52	10127.21	8417.477	8522.605	19341.82	7821.557	9669	873.9122	301.2626	1003.961	18070.71	14956.1	19635.13	19485.9	26567.15	25703.26	13248.98	20616.2	27758.19
CUST_45138_PI429010886	M68103	0.009757317	12	9						11.05673	14.30044	3.755811	4.54022	10.01207	NA	9.404259	NA	6.068633	14.95486	53.10853	8.485387	13.76968	15.20248	12.77277	NA	NA	8.419629	NA	17.8371	6.92099	24.27393	32.50381	30.43314	11.41802	10.96613	11.88722
CUST_9116_PI429010886	N24087	0.00975735	24	18						69.8604	65.61458	58.99377	67.41125	49.0355	41.84178	47.01945	50.51216	48.4991	69.69671	45.2017	97.50175	61.07006	55.50618	67.36564	65.17942	76.31853	75.32925	88.8899	63.58392	87.31684	60.37856	70.75071	56.76972	77.72928	52.31438	72.89302
CUST_9342_PI429010886	N24466	0.009757992	12	3						34.42594	37.3274	39.579	43.77016	42.29499	58.22453	47.1467	42.94276	39.13314	47.66434	40.52376	47.28223	36.97106	26.01047	38.11089	38.16633	43.15443	56.87317	34.09055	36.38725	39.8934	26.57189	42.30746	39.97068	28.87751	18.28716	18.97317
CUST_25555_PI429010886	N52635	0.009757992	12	0	443731507	hypothetical protein CAPTEDRAI			7.17E-21	10.44732	10.62828	11.51457	8.321502	6.736601	5.715989	8.326096	7.991764	6.164371	3.614024	3.964254	5.360268	7.545184	8.923377	10.94861	12.05475	8.638059	10.90671	6.029956	5.974507	13.14087	6.828268	4.258288	6.756203	9.394115	7.309623	7.062362
CUST_30241_PI429010886	N61469	0.009759555	24	15						194.7103	228.7481	303.4454	244.4333	204.9422	253.2927	189.1569	219.3545	279.563	235.7954	229.1648	286.1798	213.853	222.1435	257.5954	756.8703	540.7576	310.2309	350.649	279.7318	330.3379	394.269	252.884	330.9231	379.497	264.6763	348.11
CUST_56014_PI429010886	M112538	0.009764085	24	3						50.43341	28.95229	31.37822	52.41843	102.1812	78.36365	32.16999	27.10746	31.90671	61.92068	91.77441	64.50416	34.04952	49.53863	32.7919	24.73949	44.61738	49.12781	22.43722	21.68208	20.2995	63.60621	49.96679	44.74502	45.26359	31.5845	34.46101
CUST_45387_PI429010886	M68938	0.009764085	24	3	321465965	hypothetical protein DAPPUDRA	Metabolic process	Autofagy	4.84E-22	28.13497	22.06613	32.06918	25.98862	54.43474	37.37325	17.56328	18.39269	16.20857	29.60752	35.72862	29.11467	20.84897	26.18926	27.2558	15.75178	21.59473	29.07923	15.62701	18.06394	10.74743	22.62431	10.05997	19.09216	16.93807	22.11134	27.85041
CUST_6102_PI429010886	N19633	0.009764085	24	18						297.2551	510.4694	434.3661	334.1482	354.5733	346.8165	354.3285	432.2687	486.2941	426.6472	377.9818	351.2785	482.1823	365.4091	346.5231	459.8781	598.2839	518.9718	549.069	462.6929	612.5918	554.2471	530.0108	473.7195	577.1013	441.8514	564.6439
CUST_264_PI429010886	N3197	0.009764085	24	3	91077530	PREDICTED: similar to cuticular			0.000000998	6143.541	260.0376	5197.224	7147.632	12584.9	11881.91	294.4308	472.4073	212.2192	1692.044	7681.431	10664.62	1155.365	9345.369	10017.83	196.581	190.7935	231.4946	185.1417	550.2129	136.2005	2299.057	406.766	1655.603	189.5124	770.1221	282.4469
CUST_15874_PI429010886	N35282	0.009764085	24	3						81.80549	47.36163	23.87767	56.96365	130.9346	124.6245	53.13532	43.11409	34.24988	16.21246	64.47775	84.76892	11.11257	56.39944	68.58944	37.03848	15.67395	8.243095	3.783114	38.62045	13.55041	11.60633	34.21333	14.99367	3.345143	52.0048	6.973469
CUST_18748_PI429010886	N40344	0.009764085	24	18						202.8396	289.1658	256.4943	236.8007	235.2412	188.2922	260.752	260.3413	328.0523	283.0383	208.9613	243.6749	288.7179	229.2492	209.5934	286.5775	401.6396	336.0374	355.4873	289.1658	347.1137	288.4188	253.7732	265.2779	322.315	279.1951	290.9943
CUST_20644_PI429010886	N43805	0.009764085	24	18	2500500	RecName: Full=40S ribosomal pr	Protein metabolism	Ribosome biogenesis	4.29E-42	20501.42	27210.5	18966.14	16035.79	16726.21	16221.54	25703.26	24040.66	25247.34	20616.2	16183.31	16411.2	18476.28	20662.81	16622.66	23273.41	22037.75	27665.22	25888.13	27073.29	28658.52	18579.76	21398.95	19635.13	24590.28	19903.59	23016.75
CUST_1194_PI429010886	N8797	0.009764085	24	18						3137.141	4140.108	3441.376	3232.565	1142.815	1401.131	6326.89	6873.81	6575.891	4981.65	3593.994	2290.485	6642.147	2534.505	3611.195	4913.686	6854.464	5960.592	4976.553	7673.313	8619.889	6265.89	9954.878	10209.43	4787.419	4772.224	7531.236
CUST_1797_PI429010886	N10730	0.009769472	21	15	291245006	PREDICTED: Saccoglossus kowal	Metabolic process	Oxidation-reduction process	7.48E-67	21.3518	39.43934	37.55337	32.44579	42.76535	44.99721	42.74261	37.4401	31.59503	23.1348	23.81811	7.908515	22.89097	42.78996	35.27964	43.80331	53.43212	49.8658	43.22841	42.02831	56.45276	39.36544	74.4544	41.21871	53.16566	38.47726	21.40492
CUST_1417_PI429010886	N9568	0.009774232	18	0						349.0153	NA	278.592	502.9461	824.4785	517.1871	NA	5.975334	3.613491	61.03917	161.0998	89.26114	75.10319	83.33786	150.4929	NA	NA	NA	NA	40.95208	NA	64.11411	14.71575	40.28522	9.290836	18.25369	5.504548
CUST_3565_PI429010886	N14765	0.009774635	24	21						64.99766	32.98204	92.43955	212.7294	129.3339	28.13189	25.69105	89.3199	104.0575	48.10521	66.60304	42.82494	141.6357	56.05872	52.85457	32.83178	97.01999	25.79528	375.6666	213.3759	100.4643	468.4112	198.3747	433.097	174.8361	70.5683	175.6627
CUST_16108_PI429010886	N35669	0.009774635	24	15						67.86728	45.40234	79.73452	51.30921	42.82003	40.53493	52.97194	49.39254	64.34403	96.30356	102.6378	86.24643	100.0919	53.85584	82.98239	175.8219	67.50363	172.1367	91.01249	75.43056	62.41331	77.25059	88.4931	100.6045	92.68642	67.81869	117.8719
CUST_39032_PI429010886	M33722	0.009774728	24	15						340.6386	295.9223	422.1689	415.4114	225.0911	281.0341	360.9619	1040.093	637.4326	651.9522	344.4073	198.8083	467.9385	694.1978	196.9837	1201.304	943.4447	1060.229	918.7311	887.2093	889.8299	458.2399	118.0123	250.0252	797.1924	249.8271	349.7302
CUST_37409_PI429010886	M18934	0.009780865	18	15	321470906	hypothetical protein DAPPUDRA				2269.737	3759.237	2489.305	2210.04	2170.443	2086.93	3274.995	3071.483	2536.537	2549.09	2153.531	2315.807	2343.924	2584.904	2113.55	3770.678	4295.699	4055.849	2498.654	2676.834	2867.707	2057.214	2355.337	2519.472	2945.018	2799.9	2491.674
CUST_41484_PI429010886	M57844	0.009780865	18	15	195493694	GE21870 [Drosophila yakuba] >g	Metabolic process	Carbohydrate metabolic process	2.91E-08	8962.903	4503.776	6818.249	1912.144	725.6388	753.6723	6988.188	9410.039	2842.045	15258.82	8086.369	3568.199	3117.642	9643.272	208.2391	19791.01	8296.544	22401.51	8605.765	10795.65	16372.71	5322.868	967.7143	3546.981	18026.32	4372.597	13248.98
CUST_2594_PI429010886	N12719	0.009780865	24	18	91082911	PREDICTED: similar to dre4 CG1	Cellular Process	Other		197.2544	319.218	253.9457	227.2502	167.5116	168.2226	197.6734	232.7864	296.3076	296.1605	223.5617	281.7476	285.9469	214.3972	245.1417	291.9252	317.1978	342.6821	307.1364	255.1321	383.2384	213.7108	261.1993	324.9732	244.5822	271.1875	385.5175
CUST_37763_PI429010886	M21466	0.009781586	15	0						27758.19	13017.21	23324.95	33076.81	8802.072	14922.71	15258.82	11588.48	10464.54	8309.047	9330.025	8256.789	13879.42	15435.13	18070.71	16411.2	15900.37	14922.71	11030.98	11206.93	13532.16	8713.974	18715.71	9153.451	7673.313	15492.49	10795.65
CUST_34241_PI429010886	M9146	0.009784325	21	0	345484330	PREDICTED: hypothetical proteir			8.02E-15	114.4474	55.52101	155.8206	33.37375	22.83159	52.11743	35.34111	52.70656	78.3726	49.44159	55.3772	66.06005	43.6037	47.32106	52.48812	79.10899	55.55019	58.23791	123.7307	51.02509	89.43021	81.84916	99.13779	84.85915	91.05781	62.52243	85.60411
CUST_51029_PI429010886	M91852	0.009792711	12	6						NA	14.98899	21.18591	31.04302	NA	41.65544	331.9294	NA	299.9819	36.53396	31.59503	NA	7.623786	49.05504	10.05333	46.73964	NA	42.00294	316.0187	1139.062	1609.728	57.58254	25.08449	77.54349	211.1406	32.76978	161.9124
CUST_23262_PI429010886	N48534	0.009802462	18	15						368.7015	426.8467	241.7074	332.4145	60.3351	348.8785	42.42056	85.38787	85.11492	203.2614	353.8605	411.6604	333.2833	204.3287	318.2644	1308.545	293.6993	954.4926	166.0161	332.8061	291.6226	450.9209	123.935	258.6669	385.4238	165.8065	153.9248
CUST_48543_PI429010886	M80903	0.009815686	21	3						80.16082	87.19961	84.98418	64.63225	93.91412	99.79674	57.67493	77.65458	76.09471	74.76519	71.12883	82.7815	44.79025	89.55714	61.52728	80.58239	93.69945	76.00489	71.56582	75.83149	78.06123	87.08887	96.9842	87.36259	90.07438	94.88668	107.2961
CUST_49341_PI429010886	M85262	0.009818257	24	3						319.8028	3.721395	322.8267	332.3383	329.2075	384.2558	10.03604	16.3604	9.997663	136.1879	271.3104	361.0483	102.2347	207.5176	271.1875	3.899103	NA	4.06827	36.80294	28.86593	18.81827	150.9231	52.06823	96.73368	32.63627	40.77521	41.69494
CUST_32307_PI429010886	M3764	0.009819856	12	6	91094127	PREDICTED: similar to GA20276-	Protein metabolism	Proteolysis	5.84E-146	161.3354	218.1148	150.6767	156.1362	152.4355	117.0698	229.5267	202.6489	270.2762	174.3634	152.4772	193.9616	126.6732	171.1773	138.2078	179.8439	357.7047	283.749	198.7781	185.7539	222.4375	141.0311	193.0588	115.3698	130.2882	137.4674	125.9747
CUST_30999_PI429010886	M418	0.009819856	18	15	157108878	mitochondrial ribosomal proteir	Protein metabolism	Ribosome biogenesis	2.85E-44	56.94521	99.09115	84.73544	55.34035	56.04689	49.20176	68.84252	101.6904	96.81529	56.4602	55.89295	65.17942	83.78528	83.25572	63.10394	72.17918	121.8066	103.1229	66.52473	73.42732	70.28922	53.51539	80.93024	58.89558	51.59074	63.90528	76.3265
CUST_5854_PI429010886	N19297	0.009819856	21	12						76.99156	92.85843	82.34733	71.56025	72.89302	60.03374	78.90154	91.44099	99.71725	80.50166	84.57655	78.07876	93.71999	88.43455	88.4446	76.89496	94.22856	90.1637	81.75855	96.58233	84.47977	73.21671	84.74637	94.18113	85.80718	71.0111	69.36872
CUST_53449_PI429010886	M101753	0.009819931	18	15	321474003	hypothetical protein DAPPUDRA			1.25E-29	9.486624	21.73277	14.80397	9.372363	10.14417	15.93995	32.59222	18.98361	22.71026	24.25396	15.06825	16.99096	27.21287	6.914242	13.86851	26.23262	35.92153	25.83559	16.53385	11.76421	21.57883	13.61629	3.359434	12.9826	24.01937	11.33713	15.62171
CUST_36248_PI429010886	M15168	0.009819931	18	15	195383978	GJ20063 [Drosophila virilis] >gi|	Transport	Protein transport	3.27E-18	50.10131	68.89904	57.88315	65.63872	35.22718	51.45353	91.55686	66.32665	68.16127	85.92835	49.4478	79.99233	90.65467	58.57991	67.31813	98.83232	92.30682	83.78528	83.44953	53.24793	112.6997	60.20069	38.1445	60.51578	88.93364	53.18236	72.21507
CUST_8350_PI429010886	N22914	0.009819931	18	15	225718238	CREG1 precursor [Caligus clemei	Metabolic process	Oxidation-reduction process	6.83E-40	37.63739	42.83599	39.78508	31.34971	66.01029	45.38696	20.75814	18.30947	22.67723	18.61143	39.41926	52.95129	26.24625	45.37131	43.10028	40.75473	39.66978	50.24652	41.79513	35.59076	30.41006	34.23102	35.49229	46.92838	27.5331	23.17996	30.39026
CUST_13151_PI429010886	N30666	0.009819931	18	6	307181945	RNA exonuclease 1-like protein			1.15E-27	272.1212	247.5439	292.5506	145.4586	353.5795	272.0128	306.9528	483.5315	394.3642	175.768	209.295	160.4657	223.0844	292.4122	355.2635	153.1144	218.7535	189.7707	200.181	474.0818	210.8385	251.0027	275.0563	232.4494	315.5362	454.0605	288.8335
CUST_13746_PI429010886	N31700	0.009819931	18	3						105.1602	62.14225	83.82183	100.2539	75.75538	106.9297	88.74114	63.81233	75.02912	80.89811	50.98258	68.01679	66.83189	79.16248	98.89641	56.10448	52.37142	61.295	65.09533	49.82313	67.07909	107.6151	75.12194	94.21867	75.98892	57.61594	75.28314
CUST_11900_PI429010886	N28597	0.009852916	24	3			Metabolic process	Carbohydrate metabolic process		31.10352	17.52622	21.10356	15.07604	50.36291	46.40366	16.00106	10.57446	7.427327	NA	23.03013	26.22609	NA	33.48437	19.407	6.783466	6.53016	4.941591	7.455208	11.85264	8.740614	15.58609	5.350473	8.972832	5.580551	21.87534	NA
CUST_18494_PI429010886	N39894	0.009862258	24	0						362.195	231.1683	293.3331	310.9083	260.6027	125.3097	480.4617	268.6423	283.1538	190.0383	185.6848	162.5974	287.7668	168.3482	135.5353	195.8784	321.7248	175.6041	349.9633	215.1868	207.9921	262.7025	348.5318	199.4568	489.4418	235.284	357.8637
CUST_13828_PI429010886	N31849	0.009865113	21	15	157813780	putative transmembrane 9 supe			1.94E-84	322.1133	533.6749	363.297	377.9818	397.1925	410.7073	554.6469	621.285	513.7138	488.2191	469.5128	384.6227	430.6456	565.179	425.8369	710.633	614.3598	594.442	484.3203	334.795	405.5854	383.67	368.5924	442.8695	455.9995	380.7227	529.7211
CUST_35306_PI429010886	M12193	0.009868416	12	0						595.1579	588.6767	541.8648	532.4935	733.2499	623.4767	377.4146	452.3252	402.6495	504.201	611.8901	701.8416	477.1875	698.2425	857.7889	516.495	623.3324	476.7459	460.1149	456.0887	399.5386	534.4784	489.8482	527.5856	561.1654	465.024	547.1222
CUST_14853_PI429010886	N33578	0.009868416	12	6						11.06998	18.63267	12.88915	12.59237	18.10243	20.56418	28.75907	18.83308	28.32731	18.39609	7.007853	19.92192	12.9375	15.73415	22.55249	18.63267	6.592192	23.26132	24.51979	21.44482	34.92175	21.22876	16.15468	22.629	15.38634	19.09216	8.297607
CUST_52918_PI429010886	M99429	0.00987189	12	0	168824627	Euphausia superba isolate I241 (			5.71E-93	304.58	1265.098	958.49	22.4679	166.3679	43.14425	60.20446	59.84299	51.42591	327.5322	80.93024	236.4795	301.8062	1134.773	732.3207	13.07815	1420.628	42.23497	527.3184	97.05434	302.4553	491.3879	1411.448	865.6479	68.26223	729.7963	581.8311
CUST_44549_PI429010886	M66295	0.009873645	24	18						105.7387	122.6389	134.5227	109.7774	110.6946	79.62038	123.0785	127.2209	125.3249	112.0856	94.66861	101.2406	122.4907	92.7356	78.76138	141.6357	121.1043	146.9973	213.6241	127.7621	154.3725	159.2971	127.6164	151.5229	180.5334	107.0876	178.9277
CUST_44993_PI429010886	M67659	0.009873645	24	18	321473762	hypothetical protein DAPPUDRA			5.54E-10	43.96327	33.99416	43.76293	36.88652	32.81904	31.06306	39.88903	27.36008	39.88903	33.01451	29.64873	43.52338	47.89413	32.36331	33.90314	60.13153	39.58266	38.74481	51.44117	38.15601	47.65574	51.73911	41.00325	46.03411	40.68416	46.28626	42.07458
CUST_55723_PI429010886	M111475	0.009881724	24	21						100.7083	89.47762	111.9958	91.2559	85.03859	85.56504	82.7815	74.8864	87.99932	124.0673	101.6228	89.12634	127.2375	83.59991	100.8605	127.5138	86.97617	99.30177	138.2964	88.45765	120.2717	153.5452	87.39589	129.8849	129.3339	121.8844	128.2695
CUST_45850_PI429010886	M70496	0.009881724	24	3						55.54321	40.71158	34.79145	47.79939	71.87251	64.33747	40.78067	36.32296	35.21621	32.30205	57.75543	44.46024	16.85118	43.51785	51.66717	35.53472	47.41217	30.24615	23.13661	36.87514	32.34672	26.27536	33.6843	27.48515	37.84156	46.60711	30.56946
CUST_45946_PI429010886	M70838	0.009881724	24	3						115.5968	41.88081	40.87182	165.0873	54.42434	66.59115	99.38318	40.82014	30.96768	144.9158	67.18486	49.4079	84.72348	36.55792	40.18962	24.90125	41.42853	62.98884	39.47955	30.27186	16.31601	36.5903	41.21274	34.91725	30.69119	53.45027	33.27509
CUST_9977_PI429010886	N25498	0.009881724	24	21						140.9846	158.3476	154.6661	153.0619	164.6109	130.6615	86.02033	138.9709	134.8108	135.4114	165.2661	146.5474	132.2497	133.2218	175.6882	136.7059	183.5316	167.7409	152.4523	147.2411	124.0147	194.1489	176.7421	187.6112	202.6223	176.3183	245.8278
CUST_13291_PI429010886	N30919	0.009881724	24	21						241.4743	270.6947	341.965	284.3354	226.6959	219.4592	264.4099	216.4424	280.9977	312.4862	228.4093	230.4127	301.7541	206.781	279.4339	297.492	286.745	336.8769	310.6389	227.9064	303.4901	343.906	314.1063	385.3052	326.0747	271.4195	423.8193

Probe ID	ID	p-value	Period	Phase	EBI ID	Annotation	GO category	Go term	E-value	0	0	0	3	3	3	6	6	6	9	9	9	12	12	12	15	15	15	18	18	18	21	21	21	24	24	24
CUST_19868_PI429010886	N42351	0.009881724	24	21						375.2193	365.989	418.2862	352.7205	303.6159	287.5765	299.5341	270.9307	320.6536	491.1439	323.0951	309.6367	363.8565	247.5032	322.75	330.9231	319.4595	312.4297	379.4005	278.6422	369.6716	419.2614	421.8557	378.627	432.4641	309.132	403.3152
CUST_27133_PI429010886	N55506	0.009881724	24	18	157132998	4-aminobutyrate aminotransfer	Metabolic process	Cellular amino acid metabolic process	2.06E-36	119.3101	141.753	131.5172	83.13651	103.3038	104.3593	114.6133	126.3851	115.7982	154.0925	119.7969	163.9723	99.47239	129.4391	112.9084	127.1117	137.5443	151.9875	195.5458	136.7882	164.7688	95.92856	119.9872	118.1871	116.724	125.0424	167.6224
CUST_12033_PI429010886	N28813	0.009886038	24	9						6.198566	8.593041	8.203427	7.657844	18.43476	4.709967	NA	14.72662	9.055283	6.882653	20.2236	23.13174	NA	NA	5.059102	NA	5.090935	4.816293	4.77556	NA	4.344074	8.655185	4.819136	4.100789	4.824842	NA	5.031459
CUST_41560_PI429010886	M58036	0.009888364	24	15						21.90135	20.71285	34.10624	32.73039	23.76471	23.20404	30.37998	18.51954	26.5557	30.76277	11.93224	40.61255	30.20935	27.88813	27.60403	48.78475	30.79372	48.89327	45.49999	36.91722	35.96649	38.60711	28.1575	27.39004	27.39273	29.37527	30.35985
CUST_21599_PI429010886	N45501	0.009894596	18	3						42.6416	17.88521	48.33941	84.38135	98.73078	65.11364	30.31506	14.09394	14.56346	41.36333	73.56286	57.94141	56.05212	63.51585	69.5671	12.37965	7.096311	12.74046	27.30898	29.19296	32.69318	57.42169	51.93257	68.49163	20.452	33.58881	45.67001
CUST_23626_PI429010886	N49161	0.009894596	24	18						63.77866	57.6297	56.88517	40.98047	42.94799	43.19018	59.16985	62.81204	77.9272	91.95645	56.97991	62.80714	72.08616	44.7597	61.58528	86.9167	86.48763	86.71039	93.59078	57.9144	91.87245	67.64673	54.72488	68.16905	100.0093	48.48882	103.616
CUST_5145_PI429010886	N18313	0.009894931	18	15			Nucleic acid metabolism	RNA metabolic process		53.46273	117.494	95.69298	65.92976	80.31138	74.03602	82.14842	75.0185	82.91936	66.66302	75.25623	65.92209	78.61195	98.38918	73.02048	82.62828	130.9003	103.8143	88.98877	110.2733	89.01896	75.04432	81.73809	84.01764	74.09264	83.15856	61.01777
CUST_5043_PI429010886	N18188	0.009896348	21	15						14.58842	27.17503	18.42193	16.15468	22.53853	16.07632	16.55821	14.34474	25.62847	17.05701	7.691112	14.18163	20.452	13.7591	17.83143	40.97117	22.63447	24.66829	25.32953	20.36405	22.6604	23.27322	19.38111	24.2919	18.13981	17.54875	31.26513
CUST_13562_PI429010886	N31382	0.009902069	24	15						33.22829	38.32178	25.35423	25.62527	24.19026	23.31389	26.60187	14.26389	20.24228	26.97175	8.386993	20.11384	31.53465	22.8581	29.0216	38.46589	29.4515	35.76279	19.72255	27.51681	31.71101	25.31216	34.2198	26.03303	26.40704	26.34323	26.67982
CUST_31122_PI429010886	M748	0.009904964	24	3	443699454	hypothetical protein CAPTEDRA	Cellular Process	Cell cycle	3.05E-10	57.85035	77.91051	74.55481	76.27282	95.28229	71.63426	56.26015	81.81979	53.081	80.3181	86.6454	71.67272	66.17651	69.46672	55.25246	56.24698	62.5913	53.07048	58.20456	66.02134	57.51884	86.18133	70.50377	67.48873	63.46562	81.65394	69.12973
CUST_1501_PI429010886	N9836	0.009904964	24	21						3426.429	3571.414	3855.701	3528.773	2519.472	2575.646	3376.686	2740.835	4193.691	3437.158	2009.095	2620.241	3455.897	2670.787	2466.158	4392.913	2940.045	4583.265	4329.518	3091.943	3992.329	4705.762	3650.983	4847.527	4097.003	2102.006	4403.488
CUST_3467_PI429010886	N14561	0.009909633	24	9						5.807099	23.87984	18.11564	6.345561	11.08375	NA	11.5282	15.38937	17.98748	17.20063	19.90276	16.06184	18.67988	14.28195	8.34915	4.369955	14.64529	7.455208	5.279543	5.698886	10.66383	NA	3.680503	10.20667	8.540806	6.265386	13.26584
CUST_253_PI429010886	N3026	0.009924011	24	21						894.7312	1582.625	1357.702	1136.704	1196.459	1108.83	1022.798	1719.004	1675.406	958.9028	1055.851	892.4292	916.8554	1054.373	864.3189	1080.452	1503.022	1293.784	1095.503	1516.421	1360.186	1291.739	1766.713	1370.424	1340.842	1455.905	1529.136
CUST_56031_PI429010886	M112588	0.009930502	21	15						57.49967	80.76921	65.7688	59.65828	39.03604	52.82485	39.86612	69.55765	57.29886	47.54634	69.17285	20.47128	41.70713	78.8865	54.04214	112.2514	139.7162	149.4086	41.90666	77.42501	88.65754	51.9988	100.3493	121.0707	59.98742	78.94493	58.55963
CUST_12439_PI429010886	N29465	0.009954522	15	9						9.807589	9.615742	16.27834	15.74847	12.75519	18.02618	17.20923	13.83903	4.048817	21.39999	8.124053	22.71026	22.01409	6.699326	11.3602	9.320269	6.949768	9.057903	13.30754	12.27496	11.93992	20.9383	18.75264	33.72001	18.74814	17.8915	22.96931
CUST_22459_PI429010886	N47068	0.009980557	12	0						234.6915	68.48192	176.2281	84.01764	97.63666	88.95701	58.97313	57.46071	64.18044	90.07438	80.07305	86.38253	77.44274	72.81177	91.22685	97.66082	57.6297	80.59242	59.5223	56.61772	63.27624	85.79452	65.04377	84.45149	86.96518	98.07401	74.94714
CUST_56044_PI429010886	M112613	0.009999364	24	21						70.79864	62.38786	63.59136	53.99104	59.18726	82.35709	58.49962	50.28629	58.31899	67.10463	66.71306	52.53638	59.88723	49.4478	64.96779	86.97617	72.30895	67.48873	61.65559	64.65354	62.91277	103.7054	66.74815	76.70125	76.61576	73.0273	61.74773
CUST_33845_PI429010886	M8011	0.009999364	24	21						274.1568	265.6462	301.9582	236.8356	287.632	224.3337	147.9373	225.6648	225.0187	249.6361	267.1275	224.1099	239.8291	223.3238	290.7575	283.5165	263.7057	255.4927	249.3411	234.8306	224.4732	319.8754	326.188	293.6993	344.0033	240.3937	397.9972
CUST_49873_PI429010886	M87535	0.009999364	24	6						148.1501	120.964	61.85717	84.02947	101.7015	120.4106	199.2769	105.1903	164.1609	106.5519	75.40109	92.91315	109.2206	75.46497	94.59748	104.0048	127.7621	111.5887	83.87393	79.54469	78.43693	45.80468	78.87059	54.80637	91.58124	89.70467	76.12999
CUST_5199_PI429010886	N18390	0.009999364	24	6	260807245	hypothetical protein BRAFLDRA	Cellular Process	Cell cycle	1.32E-112	26.46988	20.62724	14.59502	11.45884	7.525743	9.419511	33.07479	22.60966	24.64742	17.22544	13.94414	24.30578	19.11764	13.11926	8.264292	7.335668	10.72225	10.40534	16.92091	7.872788	10.65808	7.26874	12.8605	8.908946	17.80788	6.425488	9.897949
CUST_15895_PI429010886	N35311	0.009999364	24	21						349.6396	346.4474	364.9578	349.2496	225.4359	312.1009	315.2731	317.1285	326.4698	441.5527	297.7147	366.556	395.0243	294.4308	329.6536	465.5812	357.0377	392.7267	476.9732	297.8139	396.6952	499.0568	410.8005	437.5336	507.9246	316.4278	410.3258
CUST_20135_PI429010886	N42869	0.009999364	24	18						152.096	160.9576	198.7466	141.5813	99.84349	68.48192	180.2873	166.9821	168.9955	196.7753	128.7298	161.5143	172.6519	106.0881	162.0267	165.5194	185.0283	216.5044	233.1818	174.2921	251.5671	174.1457	121.5207	184.6434	185.6261	136.7882	204.7788
CUST_29351_PI429010886	N59693	0.009999364	24	18						26.14707	40.97531	13.58834	20.77492	18.79971	17.04459	21.11731	33.6843	15.41425	31.99205	29.08472	31.25385	25.6877	26.27536	33.82765	57.44168	24.61634	37.29216	48.79532	26.37866	50.69492	25.72152	27.22807	27.41576	42.75542	25.51207	37.3089
CUST_29700_PI429010886	N60366	0.010003894	15	9	307203419	hypothetical protein EAI_00199	Metabolic process	Other	2.93E-14	30.22686	38.37232	37.94299	53.37496	53.35547	24.80368	48.21771	36.42637	39.74893	70.83005	48.7205	63.77213	79.37286	36.19278	76.68447	37.29216	37.27834	47.53117	55.71616	42.6332	54.39234	72.42903	39.85168	76.77599	85.91672	56.66348	108.4526
CUST_1209_PI429010886	N8842	0.010008327	18	15	478415058	eukaryotic initiation factor 2 sub	Protein metabolism	Regulation of translational initiation	1.76E-146	866.5613	1507.382	1038.339	766.5607	750.3368	744.7409	1304.861	1342.206	1232.079	1165.385	806.5559	820.0431	1118.881	902.1594	873.5747	1152.623	2113.55	1774.191	1129.795	1164.205	1289.52	802.754	951.856	992.2362	1051.228	1184.477	1081.498
CUST_6697_PI429010886	N20477	0.010021651	24	18	527273256	PREDICTED: protein AATF [Melo			2.01E-45	75.0097	133.196	111.1388	87.82302	89.47762	71.20823	98.70542	104.6237	108.0397	78.21211	71.05641	91.46995	86.81003	105.3673	86.88214	92.7356	159.1067	142.4928	110.1907	129.5335	154.3283	95.73042	98.37749	120.1066	118.4901	95.34191	119.7663
CUST_12862_PI429010886	N30168	0.010031631	21	3						6.692466	12.55593	15.91979	10.09352	19.31757	18.31793	NA	NA	5.706917	6.443218	27.63973	15.94507	4.939673	6.510028	14.68296	5.476566	6.345561	9.480246	NA	6.038255	3.924589	12.09577	17.31771	13.58565	NA	19.75767	13.95366
CUST_15798_PI429010886	N35167	0.010037138	18	6	358442120	Broad-complex protein isoform			6.82E-32	12.8639	17.50536	18.09857	14.16203	14.28325	14.30855	15.14996	17.96131	32.20511	17.15225	14.10098	18.9571	16.43359	9.060475	12.13203	12.83208	10.43122	17.458	17.59048	8.335988	20.36911	13.79063	18.33509	22.75502	22.01591	17.94579	28.10459
CUST_38864_PI429010886	M31337	0.010048332	18	3						601.887	565.3173	648.4382	699.596	554.3874	613.1951	495.0948	694.1978	535.4815	373.5884	633.2997	545.9351	320.552	454.1788	332.0817	362.8786	644.5321	450.3327	387.3552	727.7655	547.6961	651.369	602.8578	600.8289	397.4943	815.4763	549.3721
CUST_38719_PI429010886	M29577	0.010050697	12	6	259166841	collagenolytic serine protease [F	Protein metabolism	Proteolysis	1.88E-13	4.433037	NA	NA	58.9878	NA	33.71063	754.2071	NA	549.1826	138.0005	87.62517	NA	36.72861	67.20028	25.63724	114.7841	NA	106.3722	1171.1	2234.498	2583.285	193.1768	NA	138.4615	535.6201	99.00226	334.9433
CUST_25965_PI429010886	N53400	0.010052849	24	9						19.28057	10.57979	13.46545	12.58686	13.67551	12.96002	13.29816	11.0716	9.483977	17.75786	7.419066	17.1166	31.02469	16.75504	12.64397	7.866638	11.45007	10.91685	3.884984	7.307485	4.055566	9.801925	3.655347	6.189073	8.490098	5.84003	NA
CUST_57207_PI429010886	E2456	0.010059442	18	15						19485.9	19791.01	15435.13	10248.36	11179.97	10337.3	23892.85	29843.45	25703.26	21010.2	17487.6	19150.48	16801.9	16520.01	14137.09	27832.29	23162.84	22598.48	17424.76	17524.49	19855.57	15798.02	13616.29	16580.62	18758.28	14439.79	19150.48
CUST_33124_PI429010886	M6029	0.010059442	18	15						150.7719	288.7764	211.8745	181.8521	114.8835	142.1426	275.1575	217.5068	220.5131	316.081	155.0651	212.3284	268.1336	122.9221	180.9942	277.4932	324.3008	307.9833	246.5526	156.8206	271.0733	202.2179	137.8653	197.5603	219.7344	153.318	201.3401
CUST_8583_PI429010886	N23257	0.010059442	18	3	443689912	hypothetical protein CAPTEDRA			1.93E-58	23.71154	29.93334	27.6757	53.16566	64.59327	57.00268	20.60634	36.6885	31.59758	24.33371	59.53613	45.9767	38.71226	36.11092	41.4895	25.75942	36.08229	18.08639	24.12109	30.43559	19.6201	47.42663	50.3744	43.07828	20.08349	47.1467	39.08397
CUST_10910_PI429010886	N26977	0.010059442	18	15	321467474	hypothetical protein DAPPUDRA			1.82E-40	87.15453	130.3441	145.9411	91.18387	67.02911	75.56999	126.4187	114.4742	122.8853	92.16269	92.82706	85.14751	100.9966	114.3312	128.645	114.1224	149.0864	141.352	111.4237	116.9185	139.8319	75.50155	109.7513	94.29714	114.1769	83.83975	129.9006
CUST_15926_PI429010886	N35360	0.010059442	18	3						108.1208	28.77495	121.0574	105.7899	153.1648	117.0265	50.30283	59.12751	85.73084	109.8814	113.23	148.4879	115.0038	93.28654	102.1933	26.64196	14.05555	32.36969	86.79002	73.87601	93.11317	111.1784	179.4021	139.8095	51.5265	182.5355	94.01958
CUST_33227_PI429010886	M6336	0.010070758	15	3						480.8108	444.8216	362.5984	500.3221	442.4459	574.7506	430.2082	461.7785	447.6336	346.5231	402.5585	402.1648	389.053	545.5749	422.5065	513.9521	464.677	434.1663	501.7792	464.5257	459.8781	433.0002	493.728	420.6276	298.3765	487.671	392.6361
CUST_38062_PI429010886	M23133	0.010074843	18	6						145.7916	234.5732	78.12052	286.3491	NA	291.7534	536.1823	152.2838	622.1839	104.0674	83.80666	NA	NA	NA	NA	2051.901	1027.321	1500.89	11.7948	11.44899	8.446033	243.5905	NA	110.2586	4415.56	3117.642	511.4855
CUST_31129_PI429010886	M775	0.010080049	18	6	321464420	hypothetical protein DAPPUDRA	Metabolic process	Lipid metabolic process	1.81E-72	NA	11.783	9.531461	10.17394	8.311471	10.54437	17.43861	29.62518	10.90252	9.284798	24.12669	16.7377	8.983138	13.3485	24.5024	8.527799	6.699326	11.29632	10.81711	8.178473	11.9832	10.80344	5.165709	18.21058	20.91286	29.76995	39.74893
CUST_7669_PI429010886	N21893	0.01008837	21	6						12.99802	10.14167	6.813903	11.07815	13.75552	18.44938	18.19202	15.45607	16.49157	19.39663	8.63283	7.626272	25.68422	15.87551	NA	4.868818	6.722248	6.956141	12.28032	14.45617	12.13803	11.57188	4.727516	6.599501	15.00826	15.5193	18.05945
CUST_6101_PI429010886	N19632	0.010097477	24	21	358442122	Broad-complex protein isoform			7.46E-37	9.738777	8.037102	5.807914	NA	6.432193	4.975896	NA	6.557583	3.381503	NA	7.230351	NA	7.287837	5.698034	3.878432	4.340526	4.487743	6.848153	NA	7.461548	10.95746	7.939364	13.3975	10.41636	10.10725	5.036752	9.019056
CUST_7088_PI429010886	N21023	0.010109454	21	18	518897355	hypothetical protein [alpha prot	Metabolic process	Other	1.23E-27	74.62293	134.0586	98.35456	66.53367	92.20119	89.80371	71.77497	157.3269	174.9928	97.39106	66.18945	77.72928	125.1706	97.31511	116.3209	85.1761	121.6645	107.9823	180.729	162.7381	215.601	70.36116	90.84788	94.62002	81.15666	68.42492	89.41928
CUST_11016_PI429010886	N27132	0.010115136	24	0						82.80797	85.49462	89.20331	82.05798	86.19309	76.94539	83.3741	67.31813	67.2518	96.25473	83.04063	91.37677	79.06068	58.60789	80.73093	73.83526	74.96434	69.66595	80.24873	57.20828	70.47134	81.01838	64.34403	81.17631	98.51271	81.81979	97.12878
CUST_54515_PI429010886	M106316	0.010117004	24	21						160.3187	105.3002	140.0577	144.9984	78.9339	114.3676	52.42496	61.52728	103.7292	136.2348	104.9085	96.23561	191.8796	105.8953	139.1364	166.4205	107.9543	125.9134	150.3698	105.4679	102.326	230.8178	118.7499	180.1999	276.2644	97.48939	173.6995
CUST_35189_PI429010886	M11845	0.010117004	24	21	242022916	phosphatase and actin regulator			4.46E-69	78.48038	64.10142	80.31138	80.78521	93.47893	65.82127	51.85541	39.38571	52.02384	63.80586	60.70253	67.12887	70.66441	65.61458	70.19469	81.21161	52.6471	80.78521	72.58638	58.74713	56.91288	90.87055	71.47305	98.24098	98.42724	61.41445	82.43712
CUST_32842_PI429010886	M5251	0.010117004	24	3						213.4873	47.10201	165.8977	196.4999	1331.208	998.7674	49.45288	83.70817	21.57456	38.45768	1062.767	1085.793	43.35728	1106.369	1105.498	28.27048	22.33547	17.31531	19.65297	20.60634	24.15844	60.64674	28.55984	36.43616	20.62453	62.11363	4.924109
CUST_33260_PI429010886	M6427	0.010117004	24	18	170585770	RNA recognition motif containin	Protein metabolism	Other	0.000000289	83.31337	96.9591	98.91443	74.90044	80.91667	70.51234	118.3504	100.386	94.33013	93.3634	96.9319	77.30188	102.4735	99.65569	58.79928	94.16687	145.4586	149.3939	112.7474	124.4149	157.5397	104.2797	82.60618	98.89641	127.8402	102.2347	130.2225
CUST_26490_PI429010886	N54335	0.010117004	24	18						19.23397	13.11625	14.93713	17.85189	13.78897	12.93513	18.43253	11.87749	16.38359	22.16637	19.11956	18.25979	20.24751	9.771765	20.83109	22.06175	24.5729	16.58967	23.76471	18.69191	24.99752	18.13167	27.50147	20.59574	29.35503	20.02415	17.96765
CUST_38687_PI429010886	M29121	0.010135959	12	9						64273.41	32295.88	81434.38	91877.59	36043.3	59131.8	50560.8	49895.87	71289.88	69931.06	110385	52165.44	76090.53	49113.82	63245.57	11738.35	18910.88	16452.07	67450.12	71289.88	66456.69	80988.9	121965.4	98191.21	56054.28	84653.3	87131.39
CUST_45505_PI429010886	M69321	0.010143003	21	15			Protein metabolism	Regulation of translational initiation		34.32507	57.63804	46.67221	44.76312	55.91798	49.87102	47.55101	55.19052	59.97997	70.63954	89.53408	47.70295	56.16512	55.52583	47.62949	63.49903	66.93125	74.89382	43.08271	52.40229	47.02455	27.47971	52.15678	46.39912	51.66124	50.64865	46.65167
CUST_3916_PI429010886	N15559	0.0101501	21	0						274.5666	319.9319	269.2064	244.0894	240.0694	140.6454	293.4022	247.1564	254.5783	258.7439	199.116	209.666	183.7151	187.286	246.1645	247.2393	249.2196	270.8525	282.551	218.8639	301.2626	286.6404	269.1036	298.8084	278.374	206.7386	332.6579
CUST_18325_PI429010886	N39580	0.0101501	21	12						27.92534	39.79966	26.58505	24.91804	25.99138	39.0142	26.15317	53.74902	31.62493	49.61593	28.27902	59.88723	57.86143	68.61527	51.29815	38.48209	38.37232	47.7609	50.23264	26.10539	36.48955	51.09652	35.02468	45.7171	73.4442	24.94507	24.38317
CUST_7836_PI429010886	N22155	0.010155561	24	3	210062959	C-type lectin 6 [Fenneropenaeus			1.66E-35	2147.917	974.6663	1862.343	2624.319	3480.931	4164.365	789.3467	3310.069	765.1767	1330.649	3267.789	2790.956	2234.498	1532.345	2211.381	148.8622	145.4834	66.46575	369.0567	4863.888	451.6859	2327.901	2294.993	1949.314	327.6721	4409.168	756.0941
CUST_31291_PI429010886	M1153	0.010165621	24	21	548364570	PREDICTED: partner of Y14 and			1.79E-20	68.61527	110.4898	86.37483	76.17615	91.33505	55.1353	77.77436	99.71725	106.2889	74.25196	87.16322	46.06991	70.3428	76.2209	86.66046	82.29898	135.0731	97.97985	87.54238	109.6178	103.6054	89.74912	112.1849	117.7425	96.55775	108.1394	110.4624
CUST_37761_PI429010886	M21443	0.010165621	24	15	321460819	hypothetical protein DAPPUDRA	Transport	Other	1.53E-28	82.42094	145.393	85.91672	87.71525	91.58124	78.5451	113.0301	142.1426	98.44478	98.5911	83.69522	82.7532	91.80105	103.3216	81.32217	121.1493	164.8928	146.2889	113.3707	123.675	115.6261	86.03269	112.2102	98.67888	70.97468	110.0862	99.82243
CUST_1768_PI429010886	N10655	0.010165621	24	15						2114.8	2654.193	1783.882	1338.071	1476.878	1168.291	1528.323	1854.805	2092.723	1907.271	1769.667	2023.772	2079.426	1916.926	2222.683	2097.341	3360.114	3431.358	1856.844	2396.659	1710.003	2862.188	2734.019	2230.426	4247.1	2443.901	2704.306
CUST_19431_PI429010886	N41570	0.010173784	21	12						12.53713	21.45593	12.58849	15.8055	430.2082	219.4299	28.25337	67.81145	36.84812	NA	216.4068	128.7298	NA	239.348	221.596	17.1309	28.44439	9.485255	10.17714	68.16905	8.735819	NA	12.04588	NA	NA	107.7215	NA
CUST_4491_PI429010886	N16890	0.010178233	18	3	386376749	thromboxane A synthase [Pena	Metabolic process	Oxidation-reduction process	1.37E-08	26.67982	10.62276	22.29844	31.49032	20.61917	28.2608	31.27222	NA	17.84752	9.470236	9.497226	12.39819	25.8664	20.6563	19.80181	10.73769	10.75824	16.99603	13.81948	24.15387	23.55114	42.35304	32.59222	22.70303	30.54385	8.433282	28.95548
CUST_42066_PI429010886	M59307	0.010179197	18	3						48.21366	27.5331	40.36067	51.24138	49.49937	48.59053	34.72664	18.11564	32.5431	43.36808	63.85692	38.95566	38.40048	34.24084	33.21716	36.23977	26.29331	24.6188	44.84231	28.6389	36.86782	94.43966	39.37754	58.61403	33.9635	43.77424	23.15721
CUST_10831_PI429010886	N26857	0.01018098	18	3						123.6404	164.8928	162.844	137.9345	215.9822	158.224	94.42757	154.8931	168.6428	102.8763	90.48608	129.3882	80.28093	88.0075	117.7279	147.5505	182.7533	237.5901	95.79812	125.9339	142.3895	148.8152	219.923	214.7361	83.9013	145.9321	135.0024
CUST_21565_PI429010886	N45443	0.01018098	21	6						44.37502	45.49544	54.78529	45.47301	70.85676	24.77298	34.92175	58.46402	47.36163	48.88764	60.0628	51.07791	27.27615	21.33315	18.87393	22.58496	38.00456	21.17745	30.84927	40.32229	43.70175	23.37452	23.04198	34.61745	32.89506	58.72446	64.88027
CUST_25213_PI429010886	N51985	0.010196246	12	6						8.689291	13.15346	NA	8.559475	20.22133	9.617132	36.99679	24.21718	36.16345	NA	8.101583	6.438537	8.766471	23.53743	7.307485	32.58484	25.64234	35.20206	72.77442	52.88884	22.08755	42.51804	17.20767	26.37129	6.694797	9.675629	24.15387
CUST_36699_PI429010886	M16577	0.010197153	24	18						11.96493	NA	24.99187	37.33345	61.52728	NA	20.48628	16.87953	49.56614	15.7786	23.88544	8.447449	63.48698	45.49999	18.2969	NA	NA	4.35423	94.62002	68.95833	101.8893	43.07453	69.37968	45.69732	54.93619	36.19278	96.71212
CUST_3021_PI429010886	N13617	0.010204839	12	6						602.4086	811.6064	446.3323	633.9987	559.2886	688.887	915.406	1097.014	921.0975	667.0187	642.1289	578.3316	440.4343	578.1548	480.9139	1044.09	1021.91	781.034	759.5695	805.2136	799.983	413.9053	762.8618	426.9219	553.413	733.7832	537.4657
CUST_39984_PI429010886	M47883	0.01022858	24	0						5792.477	4224.791	4398.359	3321.698	2275.002	1956.496	2187.303	1856.844	2020.363	5939.375	3007.18	1938.127	3739.191	1786.238	2811.043	2710.483	2842.045	1212.445	2687.973	2659.616	2191.923	5921.175	6559.55	4981.65	10569.33	6210.057	6500.649
CUST_28808_PI429010886	N58659	0.01022858	24	0						39.26427	22.03729	42.96134	31.73175	13.58051	27.76408	24.73755	24.37455	21.39782	36.1964	21.20837	23.22144	17.76942	16.66	32.47195	23.64554	21.81393	22.07673	14.69094	20.88172	27.23128	27.70466	28.99304	24.72495	42.40053	35.06409	29.50311
CUST_23480_PI429010886	N48905	0.010229895	24	9						73.55381	152.4355	80.23561	104.6457	54.36391	109.762	103.1842	21.28674	59.8943	124.362	113.2938	117.7425	77.76407	75.2384	11.60345	19.66736	239.2973	47.34599	21.85018	20.52286	19.81148	19.66181	31.08965	17.88521	54.12459	101.572	63.20291
CUST_562_PI429010886	N6056	0.010230251	15	0	521020557	Titin [Myotis brandtii]			7.94E-08	28.39612	9.77844	17.55426	12.20234	8.421879	6.945676	7.593608	6.735712	10.27659	8.437268	8.505122	3.746379	10.57851	5.37232	18.16033	17.77566	16.25246	16.10883	12.14749	12.44585	8.748029	17.30194	8.189998	5.837765	11.91333	7.290889	10.90833
CUST_16343_PI429010886	N36076	0.010234643	24	3						17.18912	10.21029	9.553409	17.00862	22.75207	20.34099	14.80676	13.88241	11.90136	20.42404	7.732666	6.861282	18.24171	16.37294	15.49209	20.04202	9.766099	14.30676	7.298709	6.200844	6.981132	12.03475	8.125179	11.18301	10.20667	11.39082	10.68409
CUST_19441_PI429010886	N41587	0.010234643	24	18						32.35917	29.63864	35.71025	11.90435	14.04576	12.50022	18.52406	59.15801	56.37375	20.90135	22.88663	22.72983	35.06077	22.02956	26.805	43.85321	33.52669	48.59053	48.21366	33.45613	49.75065	42.65681	41.45022	45.1573	75.68836	21.28833	50.99345
CUST_45776_PI429010886	M70255	0.010241575	24	0						70.01129	83.09656	101.8276	55.56698	57.03545	46.37409	47.3524	64.23608	64.33747	41.76233	59.16434	48.02557	53.46273	80.81117	64.59327	42.70334	63.88807	59.97997	36.18918	85.93897	71.3958	50.33133	131.8747	115.0832	98.55805	75.59966	112.0182
CUST_41776_PI429010886	M58535	0.010246384	21	15						94.33013	144.4115	108.8657	98.10696	135.6294	102.0887	95.21224	114.6993	121.2532	78.5366	113.469	99.6269	106.4585	117.8103	91.92491	152.4523	160.342	136.7882	125.6025	126.712	158.418	102.8306	128.8445	138.3677	111.0779	125.4006	109.1677
CUST_17409_PI429010886	N37917	0.010257457	24	15						79.51886	118.6698	54.75432	120.0145	72.63826	102.1346	127.4216	129.0467	81.83086	136.4064	104.661	120.7183	118.8271	140.9438	123.0345	212.4024	207.6167	130.3441	109.5188	108.4858	74.65174	79.47605	146.9973	86.7292	113.3845	133.9778	60.5679
CUST_26288_PI429010886	N53988	0.010264968	15	0						43.13077	62.3082	21.46595	13.87165	23.51553	21.35401	20.16486	48.1462	47.13	NA	6.528927	4.797128	8.846413	22.42351	6.734835	24.51729	60.45051	44.42034	NA	38.45427	23.06154	6.485846	28.73586	9.349393	5.06766	26.71059	10.10062
CUST_22618_PI429010886	N47338	0.010276977	21	0						45.41788	41.00325	52.76213	36.58776	56.04246	50.04692	31.78112	34.00168	40.86771	48.37508	57.26697	38.73021	28.50564	23.15112	33.13557	37.77609	47.19838	37.74598	43.38217	51.5089	35.33106	37.81203	96.59602	62.39412	44.69425	37.22016	71.09432
CUST_31222_PI429010886	M1008	0.010280013	24	18						220.2176	281.5792	297.188	227.1783	216.5044	194.3553	205.0491	219.5016	271.7825	241.0144	213.3383	232.6717	259.4124	220.3669	256.0953	251.3778	286.3491	307.1364	247.8796	267.9719	310.1796	260.557	253.6231	354.1831	275.6274	223.3777	346.0708
CUST_37174_PI429010886	M18094	0.010295949	24	15						1055.306	1283.49	590.4595	1173.346	251.4577	716.8261	1460.387	933.0577	1251.245	877.3403	521.5155	560.1392	1221.709	1240.711	760.7688	2532.427	1857.76	1659.781	1429.783	962.3475	1627.485	830.1589	1257.339	876.3228	1407.95	866.5613	1164.632
CUST_37791_PI429010886	M21604	0.010295949	24	15	260798008	hypothetical protein BRAFLDRA			4.19E-30	839.7101	794.0736	737.5198	866.5613	520.4605	646.1155	2528.059	2412.569	3286.945	3503.626	1848.594	599.6889	1113.954	671.2531	789.6051	8439.929	2234.498	7290.969	1720.907	2026.561	3611.195	1465.124	455.9995	1004.431	1891.416	363.9263	1221.044
CUST_31100_PI429010886	M697	0.010295949	24	21						91.4929	112.473	128.2402	93.97841	75.92637	91.20065	114.5333	132.5859	120.0145	67.68769	96.80429	59.87629	73.85955	112.8135	79.89483	99.32904	97.72445	70.67017	103.1619	109.1085	122.8381	123.2894	127.2375	118.702	100.159	120.7334	96.12752
CUST_33951_PI429010886	M8345	0.010295949	24	15						988.0134	1119.882	1160.436	1053.276	769.5468	850.4847	1124.051	848.9964	1159.706	1249.272	914.5652	873.0203	1021.527	791.8334	917.1646	1408.778	1236.613	1429.079	1333.943	908.7057	1276.272	1362.378	1144.859	1214.037	982.3643	949.2292	1304.861
CUST_5624_PI429010886	N18961	0.010295949	24	15						22.20918	47.22812	26.67745	33.35088	21.06045	23.21635	24.96813	39.99568	24.21058	30.41006	35.40359	21.66219	34.39051	25.71638	28.85219	42.26958	40.65182	36.40013	37.21408	37.41529	31.83006	33.53619	35.44329	30.55784	26.63281	28.88967	39.68636
CUST_24868_PI429010886	N51387	0.010295949	24	21						184.0263	215.1466	178.9277	175.5738	132.5291	168.0725	181.3177	185.717	199.5583	172.6192	153.9962	139.2203	194.635	188.9036	131.3023	208.9983	226.3355	235.1283	187.0996	150.4308	216.65	202.7113	242.0464	210.1567	199.3483	207.0521	216.54
CUST_27427_PI429010886	N56041	0.010295949	24	15						126.3851	108.5148	128.2695	111.1646	81.63425	91.45416	142.8672	95.33452	113.0417	133.3669	86.67082	106.9179	112.5697	65.84152	132.3168	138.2736	147.6215	129.7636	131.6242	114.8652	130.1528	109.9526	123.7628	131.375	122.8211	93.41761	126.8065
CUST_27755_PI429010886	N56643	0.010295949	24	21	307188872	mRNA-capping enzyme [Campo	Metabolic process	Cellular amino acid metabolic process	2.15E-17	21.51378	30.24252	21.21527	10.82728	20.57512	20.13731	22.4617	25.19098	25.27733	20.95678	14.2026	6.483685	27.44734	15.26143	19.68859	28.70137	27.89086	23.73505	30.39342	20.12395	27.84184	26.16165	32.1629	25.32432	39.42252	21.37086	25.64005
CUST_52763_PI429010886	M98825	0.010298952	18	3	220172369	cuticle protein [Rimicaris exocul			2.76E-18	28658.52	964.2235	26299.14	40024.21	24507.87	37427.64	3368.791	5892.313	7531.236	31801.71	43456.54	28658.52	40024.21	23162.84	23435.22	678.0803	812.2115	647.2976	11667.94	8160.518	11094.64	34620.42	41419.19	33402.18	10897.12	23273.41	24590.28
CUST_24300_PI429010886	N50383	0.01031698	12	0						124.2958	106.7424	133.3824	125.5691	97.75803	95.16242	94.80138	70.89193	81.49613	118.7214	93.02834	153.7917	143.2044	66.72007	139.3908	125.3758	82.49497	101.8276	91.44099	65.67004	79.19058	161.2674	87.77262	144.0984	163.9403	107.4552	151.0701
CUST_6245_PI429010886	N19844	0.010329255	15	12	321478487	cyclin G-like protein [Daphnia pu			9.29E-58	84.63533	75.28314	90.67658	91.44099	110.2359	102.8535	68.99631	58.60789	69.93972	76.93465	97.42977	86.30982	78.3065	119.4473	114.5184	111.5206	76.14147	67.11217	79.66914	72.04345	68.53151	67.40377	74.4166	78.41566	88.13366	75.58915	74.59679
CUST_17680_PI429010886	N38401	0.010336352	18	6						7.626272	7.16974	17.98748	20.6404	14.70059	14.98489	19.46755	9.799617	20.75594	13.48522	4.154581	24.37229	13.63697	NA	10.08281	11.59098	4.394743	6.616767	9.703667	NA	12.5072	19.69655	15.57359	22.42351	27.23544	14.32943	20.43703
CUST_11682_PI429010886	N28232	0.010342868	21	3						32.18121	10.6102	19.07253	41.86801	15.14996	39.89679	16.57137	11.51457	11.90255	13.40297	33.72001	10.29403	30.91821	9.814559	18.9132	22.70303	32.34036	29.38152	39.06825	30.50653	11.41659	79.74416	79.87529	33.59889	37.04177	84.71691	107.8619
CUST_32040_PI429010886	M3079	0.010352283	24	18	91084671	PREDICTED: similar to CG9914 C	Metabolic process	Lipid metabolic process	2.93E-79	182.4362	288.4188	180.676	185.9639	170.8814	176.5278	228.5455	258.0601	203.1024	236.6326	168.5856	199.3483	227.5795	177.9352	215.1868	228.3322	284.1348	247.7427	270.9307	271.6574	409.2932	188.2922	194.0917	176.1473	259.4587	228.5093	263.3579
CUST_24036_PI429010886	N49918	0.010382682	21	3						23.05545	18.28324	14.59784	18.37962	20.12578	23.4647	7.714788	17.47317	26.50821	20.36712	22.24082	13.13946	23.48629	18.17164	17.48074	16.47491	13.10238	16.53525	16.39583	7.392041	13.3386	20.26569	13.25948	18.69191	22.905	27.29712	19.15056
CUST_4044_PI429010886	N15871	0.010384908	21	18						108.2696	90.15514	88.01649	109.1371	56.50958	79.87529	68.59824	62.3254	70.75071	92.0742	89.2783	103.106	117.0919	57.33149	91.50373	119.9092	66.28735	90.69879	123.6666	119.7443	114.5488	101.4235	63.9762	75.46497	92.87822	67.00003	77.31152
CUST_39802_PI429010886	M44947	0.010392566	12	9	54042615	arthrodial cuticle protein AMP6.			3.04E-08	121.6468	30.68789	90.21633	119.7313	105.9898	69.22757	58.31899	36.55366	66.1068	143.8476	178.6979	132.5291	136.6003	82.70695	130.415	14.83547	34.53535	29.85271	34.57091	38.35003	68.77617	91.58124	132.4344	106.6696	60.13944	75.22137	101.1839
CUST_23901_PI429010886	N49675	0.010392761	21	15						31.4218	24.77785	48.11986	59.34282	15.3561	66.3578	18.62127	57.06084	68.65785	19.69655	24.85808	25.03508	41.43387	9.838142	25.52907	108.7185	101.2545	68.8804	95.24948	69.19342	88.74114	76.55656	19.62896	86.59225	24.28512	26.78263	65.37178
CUST_13780_PI429010886	N31756	0.010402739	15	9						25.17016	25.71381	25.1528	NA	22.45014	11.81163	15.91258	38.57077	28.14643	7.352636	29.68907	34.42224	21.58422	29.60455	28.16113	33.09951	24.88093	29.03104	10.20394	17.43476	16.47719	24.0804	19.52436	33.86827	33.62763	30.36386	32.2766
CUST_12352_PI429010886	N29335	0.010404372	24	15	530610369	PREDICTED: COBW domain-cont			3.99E-40	95.52209	225.256	201.229	94.54537	88.68681	107.1698	168.6123	161.8663	217.1239	160.9993	192.7777	137.2967	148.0265	138.0928	148.55	189.6824	241.887	228.1062	151.4852	164.3433	211.803	134.9328	167.0939	166.242	140.3412	115.0343	177.673
CUST_31375_PI429010886	M1392	0.010415277	21	0						2189.919	2648.467	2189.919	2021.719	1461.32	1509.889	1673.566	1609.728	1532.345	2143.564	1596.49	2038.719	2139.451	1634.309	2078.188	2234.498	2395.468	2200.686	1916.926	1944.792	1886.448	2447.506	2056.701	2539.976	2509.266	1897.754	2595.878
CUST_5981_PI429010886	N19464	0.010418707	18	15	405959643	Thimet oligopeptidase [Crassost	Protein metabolism	Proteolysis	5.88E-149	60.23886	130.8147	89.95321	81.83792	61.31201	57.06084	126.1702	118.1871	86.71039	103.657	70.45005	82.79094	84.71691	89.01896	74.36638	123.2011	139.3563	141.7369	103.9956	65.57565	98.45312	64.03624	85.89725	77.83598	98.65475	81.00976	80.02644
CUST_14652_PI429010886	N33236	0.010426197	15	9						22.8273	14.03403	12.89756	15.25861	3.446413	10.27866	19.50478	18.45414	14.69726	39.33847	14.58115	19.73758	18.21898	16.23526	16.70064	29.03664	23.74286	24.31913	17.33337	8.755998	13.07681	18.47674	7.356172	20.31717	29.3403	21.58992	23.37724
CUST_36054_PI429010886	M14468	0.010426278	24	15	443715877	hypothetical protein CAPTEDRA			6.87E-36	31.50642	37.30482	42.13235	18.40546	24.65528	23.51724	31.65485	36.1335	30.19163	31.80555	26.07855	33.67346	44.13653	31.4973	33.17685	37.49842	46.22189	41.77121	37.94299	26.30955	28.05304	35.65289	30.3832	34.50654	38.413	30.04204	32.32436
CUST_31777_PI429010886	M2380	0.010426278	24	3			Metabolic process	Nitrogen compound metabolic process		442.1242	290.511	821.5501	1157.025	820.0431	860.9694	241.0614	474.8239	349.3011	824.0838	860.0989	865.141	796.612	479.9831	440.7516	322.9826	197.961	302.1401	215.3959	324.4873	359.2536	859.7804	669.9449	716.9976	325.3527	438.2874	359.6695
CUST_45484_PI429010886	M69255	0.010426278	24	15	358443193	Heliconius erato control protein	Protein metabolism	Ribosome biogenesis	3.82E-51	27577.78	29949.84	32969.41	24590.28	22598.48	23820.74	34173.92	34286.52	27347.94	29219.06	23162.84	21758.66	32969.41	24426.06	29219.06	37932.98	37632.98	35607.66	33749.71	30447.23	38060.46	20616.2	31435.61	26491.1	30447.23	30257.2	34877.59
CUST_10457_PI429010886	N26267	0.010426278	24	15						52.05076	98.05836	76.8564	48.45428	49.93448	68.21976	54.0151	63.76387	64.95625	72.20641	63.5234	68.82621	73.79944	66.59706	70.54441	69.70354	104.8028	91.6852	54.91706	90.95134	80.26815	50.75197	98.2692	68.95833	63.90528	73.69276	81.02819
CUST_19533_PI429010886	N41735	0.010426278	24	15	321459826	hypothetical protein DAPPUDRA	Metabolic process	Carbohydrate metabolic process	3.12E-16	565.823	461.1854	543.3224	477.9381	476.8665	452.3252	555.7599	476.244	471.7045	580.117	420.6276	1004.431	575.4805	458.022	629.6411	647.0623	654.7479	614.3598	624.767	436.5051	609.4415	597.4035	517.0399	601.1087	611.5217	417.8265	576.6349
CUST_24491_PI429010886	N50710	0.010426278	24	15						73.7433	71.05641	62.05097	88.53491	34.57525	53.17104	96.69796	80.54573	62.33316	107.8221	46.85381	61.41445	143.1878	80.58239	88.14296	150.3928	103.1345	144.345	123.6152	73.20517	111.4237	105.2156	70.24722	74.5359	127.6698	85.85149	82.33668
CUST_34552_PI429010886	M9997	0.010447738	24	21						604.5265	591.786	542.9984	583.0039	439.3042	485.1077	644.5321	488.5919	511.9251	746.4733	389.3624	578.8472	260.1465	397.0876	425.2574	518.791	577.2434	551.0918	513.8347	462.0354	584.7373	779.846	552.0297	701.2976	725.9659	521.086	637.9796
CUST_15490_PI429010886	N34663	0.010465065	24	18	328793009	PREDICTED: structural maintena	Response to stress	DNA repair	6.67E-19	18.25575	16.94426	21.1758	23.45708	19.45088	22.36969	20.52618	21.12341	27.32487	12.52529	16.98717	15.25766	25.59017	18.27116	22.28065	27.46909	21.36108	24.9499	26.78779	23.86617	36.00722	28.5396	24.84254	20.56246	37.87395	23.84171	25.94024
CUST_54337_PI429010886	M105632	0.010480959	21	3						28.79811	21.21056	26.44115	26.95458	28.7956	30.9318	36.64369	21.0526	25.74471	50.95275	18.49557	21.12341	24.92323	26.50821	24.32624	22.39225	15.9111	19.3675	23.88544	22.82225	22.47011	33.13254	27.56258	24.25128	39.54727	22.04447	28.38277
CUST_7697_PI429010886	N21939	0.010482462	24	18	383849910	PREDICTED: cullin-3-A-like [Meg	Protein metabolism	Proteolysis	2.02E-99	154.6661	127.7313	159.497	150.4102	120.6665	132.6815	143.8649	100.5213	119.4647	170.2213	107.3228	127.3332	142.8672	113.1214	135.6848	183.4518	147.0639	165.0676	192.3094	141.5173	186.0269	172.9739	69.91553	152.6639	163.211	133.6627	138.7489
CUST_40550_PI429010886	M55437	0.010490851	12	0						247.1564	78.80045	270.2277	95.64524	54.19934	67.25975	31.69013	33.43972	58.97313	71.20823	125.0755	44.64386	168.902	12.65302	135.5555	23.32264	21.53519	30.13995	38.20771	68.17964	77.96514	155.6261	90.36229	117.354	20.47681	321.8126	430.0583
CUST_3666_PI429010886	N14995	0.010490851	24	21						41.03792	40.45135	47.07501	36.55792	37.455	32.50656	20.17508	38.62717	32.19938	26.81298	28.32278	50.92486	38.79203	30.00811	42.35625	48.80246	36.82399	32.0098	44.51618	34.50418	38.8005	44.05907	45.5838	45.18209	50.32489	44.43314	46.60711
CUST_35849_PI429010886	M13695	0.010494454	24	15						48.32075	66.63719	46.49868	143.8476	10.76649	106.4585	109.0351	75.14729	98.5911	115.8934	38.90574	28.5784	100.9354	118.0401	69.19342	171.8137	168.9237	160.713	68.11052	66.18945	54.71169	57.44168	71.89101	49.11012	57.16004	70.03526	27.60403
CUST_2064_PI429010886	N11470	0.010507461	21	0						58.77493	61.93359	99.42287	85.95962	66.25687	53.25197	66.64484	57.07543	80.26815	70.13338	42.6416	61.82712	78.38177	34.36428	62.82209	62.06796	31.51184	54.15073	55.02143	47.2724	57.20137	176.1473	79.66914	172.6192	145.9662	99.116	99.3998
CUST_45807_PI429010886	M70352	0.010511568	12	0						13.7624	17.39841	13.94414	13.03303	10.33053	7.463335	9.173417	8.508175	8.470203	6.025811	25.90137	5.059102	9.504797	27.06969	14.61747	8.273433	7.708304	9.397916	NA	8.284739	3.595563	3.141406	9.24446	3.178737	12.07805	13.43787	6.457842

Probe ID	ID	p-value	Period	Phase	EBI ID	Annotation	GO category	Go term	E-value	0	0	0	3	3	3	6	6	6	9	9	9	12	12	12	15	15	15	18	18	18	21	21	21	24	24	24
CUST_52900_PI429010886	M99366	0.010513579	24	18						NA	NA	NA	28.61053	NA	23.47009	387.1258	NA	317.7216	46.40366	27.07259	5.638853	14.96631	59.98742	6.634618	64.8708	NA	66.02134	332.484	726.5143	1501.763	94.01958	NA	74.44615	178.619	28.53195	153.5452
CUST_17449_PI429010886	N37979	0.010514235	24	0	307188510	hypothetical protein EAG_1140			5.62E-10	16.81913	15.05439	12.62841	4.543125	9.779895	8.563015	NA	7.779225	8.196534	3.333763	NA	NA	NA	5.768746	14.03865	6.968697	NA	7.078115	NA	6.609031	3.939668	13.28567	3.366443	7.002504	12.24304	13.65032	11.53833
CUST_52887_PI429010886	M99331	0.010520869	12	9						192.7408	29.43236	232.7864	277.9454	286.3491	243.3699	30.96219	49.3138	73.85285	237.7581	460.3468	339.3438	276.2644	262.04	349.7302	35.20827	22.92149	15.95963	182.4721	138.0005	130.828	426.9219	448.4659	463.3449	179.3632	297.8139	334.719
CUST_14059_PI429010886	N32265	0.010521397	24	18	371929005	ecdysteroid regulated-like prote			4.7E-30	5.571313	20.80218	24.91228	9.570901	10.12016	23.86873	100.8009	NA	85.97811	26.63281	13.11503	NA	NA	5.389175	NA	36.65135	NA	30.45492	225.4359	317.6549	759.9978	34.42224	NA	37.28248	66.8709	23.32264	64.76383
CUST_37777_PI429010886	M21530	0.010536395	24	15	383859002	PREDICTED: Megachile rotundat	Protein metabolism	Ribosome biogenesis	1.82E-58	22221.29	31206.9	20822.29	24136.69	19150.48	20822.29	32454.44	31659.39	29647.7	28743.72	19434.65	9194.226	29219.06	24659.23	25188.43	33838.77	35305.99	34620.42	26056	28743.72	29949.84	22037.75	26056	23739.57	27153.68	27273.84	24659.23
CUST_53846_PI429010886	M103404	0.010537926	21	0						14.53307	19.4056	20.60634	22.38961	14.03213	16.84945	9.838142	10.27106	20.56044	10.59607	14.41795	13.64057	3.559221	17.42676	10.00971	4.910986	18.06134	16.86908	13.37051	22.0263	6.466135	21.40258	33.89397	23.96992	14.52879	8.677561	10.52474
CUST_55642_PI429010886	M111084	0.010538463	18	15	197940771	Euphausia superba mitochondri	Metabolic process	Oxidation-reduction process	1.08E-125	112.2698	217.5068	150.4102	130.9714	91.17437	123.2141	181.4867	163.1238	201.86	127.3678	136.5457	154.6661	159.5977	108.8896	139.755	222.4375	261.5479	167.3313	169.9145	141.3689	185.7539	118.0906	135.7682	111.8276	193.5276	125.5586	133.8226
CUST_38293_PI429010886	M25096	0.010538463	18	3						2545.232	545.0797	1981.815	2789.551	2853.1	2593.566	407.7964	516.3556	420.5189	2759.556	2365.258	2725.344	2067.912	1388.047	2755.522	390.6122	337.1412	460.1149	563.6439	771.2654	602.7354	3808.458	2306.791	3633.442	254.1458	972.8403	1130.969
CUST_42971_PI429010886	M61757	0.010538463	18	3	321454186	hypothetical protein DAPPUDRA	Metabolic process	Cellular amino acid metabolic process	2.72E-31	30.42641	25.79817	54.67791	57.27829	52.52723	32.48749	28.96499	27.85388	44.49549	26.87469	40.55055	39.38869	54.17106	33.35088	39.50981	14.05102	7.808788	9.331963	45.49065	29.58629	43.4735	64.74689	46.2714	43.59179	25.34819	36.94243	68.06179
CUST_1680_PI429010886	N10420	0.010538463	18	15	195425743	GK10316 [Drosophila willistoni]			9.74E-16	33.85933	79.13617	71.9236	35.45592	27.99927	23.77756	37.03848	48.8281	50.58863	43.88384	40.44814	47.84693	29.38152	34.40862	39.25729	58.02727	59.15801	65.2491	52.25815	46.71775	43.21465	41.59303	24.77298	31.77803	32.64017	51.36995	37.77609
CUST_14028_PI429010886	N32205	0.010538463	18	15	242012463	lachesin precursor, putative [Pe			2.49E-11	61.30321	123.3372	73.03741	47.16128	11.43891	55.28754	165.2368	89.53408	102.3111	144.849	58.4567	87.45577	76.22837	78.36365	63.76387	268.6876	211.4988	205.5946	122.0546	42.31229	65.59808	90.03155	22.89571	61.91527	167.2859	85.58451	50.26983
CUST_997_PI429010886	N8157	0.010538463	18	15	6760654	neuroglian [Drosophila melanog	Reproduction	Reproduction	7.23E-56	1370.424	6219.297	2498.654	1421.92	451.2333	1142.458	6506.137	2871.837	2915.434	4548.63	1380.017	1801.332	2377.165	1656.675	1595.825	7832.882	10142.18	9153.451	3675.29	1915.575	3717.963	2194.43	1227.139	2086.93	5427.368	2584.904	2194.43
CUST_44951_PI429010886	M67511	0.010544676	15	9						41.51987	31.2936	48.81332	36.85694	31.77537	40.50202	43.25692	36.76966	54.69951	54.62254	216.0628	46.05207	33.27509	45.16295	46.9383	47.90611	25.96348	52.73695	44.85324	31.59758	44.635	46.34771	25.33166	51.47126	61.37944	48.74364	67.63991
CUST_32962_PI429010886	M5575	0.01055054	24	18	62858575	proteasome 26S non-ATPase sul	Metabolic process	Lipid metabolic process	7.33E-48	150.4929	274.8753	211.4988	181.1454	227.1085	171.5325	165.7838	239.6808	200.4116	227.2167	203.5459	189.3285	217.5068	357.1234	203.2877	240.1565	244.9453	290.9943	229.0726	246.0227	286.5115	263.7057	261.3125	346.2958	189.0201	198.4987	305.049
CUST_30687_PI429010886	N62401	0.01055054	21	0						98.66873	95.74045	107.52	102.9729	63.36224	95.35371	73.87601	68.83252	72.42124	84.84924	80.47316	106.8649	85.80718	88.31999	74.55481	130.1961	82.7532	92.50275	154.5211	70.74232	96.20921	142.9154	83.18277	112.7612	138.9837	75.73146	90.50145
CUST_53036_PI429010886	M99825	0.010552581	18	0						52.97194	132.9353	185.5286	54.73663	46.63196	NA	NA	NA	NA	19.68364	39.26427	15.38213	21.92026	13.30917	NA	NA	27.88249	5.714052	6.445408	43.76612	46.9424	42.4997	32.02822	31.18062	16.89933	NA	27.11011
CUST_25030_PI429010886	N51663	0.010554507	12	0						42.40452	27.18917	37.2113	24.11615	20.56246	27.40728	13.53241	23.17996	11.08511	23.16703	29.28594	24.85808	24.44847	12.17199	34.3611	24.80622	15.07942	21.47745	16.26856	36.71405	18.43758	46.06605	27.94531	31.78394	48.69222	32.46898	39.94084
CUST_18034_PI429010886	N39028	0.010555357	21	12						8.15128	18.70945	17.89974	14.99561	9.066919	10.41528	14.1279	14.25457	12.36665	21.56373	10.21488	17.08255	24.97826	19.07869	20.68785	8.109042	17.82084	16.35547	11.37088	16.37588	23.7984	NA	9.675629	16.44507	10.83803	6.768812	9.743639
CUST_35741_PI429010886	M13375	0.010556606	24	15						50.80319	65.15059	58.42497	63.20291	43.89532	43.74346	62.338	62.96095	63.84719	78.92328	52.27137	60.81905	74.52512	54.57944	75.87682	77.53014	79.41371	92.59408	65.40116	46.16801	73.70166	90.60669	47.62949	72.5147	74.71995	55.57314	72.42124
CUST_31599_PI429010886	M1939	0.010556606	24	3						551.71	440.5339	464.055	653.5411	830.4195	933.0577	473.2258	533.9662	417.2642	459.9674	642.5938	690.9865	312.2172	457.3588	404.2408	418.0505	407.7964	338.1914	370.3281	619.3687	344.8027	468.7122	521.206	440.9544	225.3861	653.1145	274.2662
CUST_38605_PI429010886	M28484	0.010556606	24	15						305.0003	481.0599	433.097	261.8722	275.2462	211.803	410.9137	450.7184	549.679	331.5173	312.1666	305.3135	346.8165	328.4033	288.6545	398.2132	523.4696	505.9666	367.9854	394.269	472.7524	350.7074	372.2898	358.7305	345.0411	327.3954	436.9283
CUST_39180_PI429010886	M35723	0.010556606	24	15	9367086	Euphausia superba large subunit				1500.263	2722.979	1614.735	999.3353	1175.329	2333.596	1632.022	2608.106	2654.193	2793.635	1490.226	3232.565	3819.978	3073.818	1629.282	5184.879	3224.848	2469.347	3217.866	2227.296	1984.393	3924.629	1779.656	2663.717	2940.045	1738.077	2136.93
CUST_39525_PI429010886	M40815	0.010556606	24	9						36.41874	86.99822	74.3865	37.97588	46.80566	45.08105	71.42098	64.44827	76.21108	98.53709	48.19549	82.71729	37.06569	99.09115	23.80329	51.25301	73.19641	78.33954	58.19693	24.92085	61.4814	32.83487	33.73873	21.76828	62.87718	45.38207	36.21685
CUST_9842_PI429010886	N25285	0.010556606	24	3						234.4388	227.5418	90.91573	333.6191	379.7764	299.5341	157.3672	221.3576	222.9863	225.5195	280.1334	458.8651	226.7781	256.573	199.6163	96.18658	63.61864	62.51652	84.52278	229.5267	119.6589	124.2469	371.7268	324.1912	105.3222	200.3043	136.424
CUST_17340_PI429010886	N37798	0.010556606	24	15	391328842	PREDICTED: transient receptor p	Transport	Ion transport	3.63E-09	4589.607	7950.355	6018.305	4531.71	3744.093	3747.441	3907.106	5322.868	5678.164	5304.997	4663.073	4436.509	5630.843	4895.993	4646.072	8189.692	8269.17	8016.887	7337.918	5489.861	5601.899	7872.926	4641.846	6116.36	9727.143	5879.268	7886.969
CUST_29026_PI429010886	N59059	0.010556606	24	15						47.27657	51.82749	39.6536	34.88898	26.45217	25.07913	36.57306	52.09068	34.08743	33.4748	47.19052	37.82047	43.45515	39.2334	36.27201	43.35233	59.75785	63.74337	32.77297	52.53638	37.92348	43.24154	54.2724	54.74825	41.25469	47.4031	42.60881
CUST_9438_PI429010886	N24620	0.010569772	21	3						6.584517	10.23432	20.26699	7.338359	10.39886	12.44585	13.44284	9.591858	16.63236	3.00467	6.96245	5.366961	8.076758	8.561749	8.757129	5.327092	7.210206	10.20538	5.103645	9.169725	6.614583	12.41522	9.961233	15.28613	13.26377	14.6059	10.9364
CUST_12036_PI429010886	N28816	0.010573483	24	15	37595925	signal transducer and activator c	Signal transduction	Other	1.14E-85	23.8982	25.75007	70.57647	18.67325	15.02117	19.23624	11.16104	22.80532	25.03218	18.00112	26.24091	26.59797	22.71122	39.26035	23.69946	32.73039	36.95845	34.72664	25.77932	24.1706	46.78449	40.02352	22.70303	32.45355	27.75069	24.8137	35.05094
CUST_26797_PI429010886	N54898	0.010591565	18	0	242006863	fgf receptor activating protein, p			2.53E-26	NA	14.77433	8.871249	NA	9.799617	9.551996	NA	4.945161	8.613517	3.657413	4.438558	2.93509	21.54205	6.404646	5.593157	5.987089	10.15066	NA	15.26143	14.41491	5.71978	NA	5.015515	3.755392	4.598867	7.643306	13.96735
CUST_24853_PI429010886	N51365	0.010593722	24	0						24.19535	43.46338	35.59906	15.5945	42.47062	22.04646	12.81892	26.29555	30.07738	18.08416	31.03188	18.83943	7.365868	8.66264	9.622741	18.61345	35.15002	18.28134	37.3305	18.1699	27.87026	12.87838	49.15533	45.67405	7.866638	74.12935	57.56013
CUST_6350_PI429010886	N19986	0.010596569	21	12	321467972	hypothetical protein DAPPUDRA	Metabolic process	Lipid metabolic process	1.06E-48	17.20923	25.01557	15.79609	17.72529	30.81577	30.75945	17.76942	52.39131	46.02907	18.06696	22.88864	9.979978	49.99479	38.89512	51.00442	21.39254	41.13599	26.21426	35.99333	32.1853	38.51685	16.71139	23.92281	10.86017	31.9639	28.97384	32.34328
CUST_5382_PI429010886	N18621	0.010609259	21	15	91076488	PREDICTED: similar to Bm44 [Tri	Response to stress	Other	1.07E-93	66.56707	93.65434	88.5599	44.84231	73.26051	63.62492	74.89382	86.26011	72.4917	60.39038	63.15515	62.66376	77.99973	75.19367	58.13362	88.36719	125.3559	109.5487	111.3048	77.56319	83.99404	58.71184	88.45765	71.02716	78.87059	73.52981	69.51507
CUST_8389_PI429010886	N22972	0.010609259	15	0	91093000	PREDICTED: similar to g1/s-spec	Cellular Process	Cell cycle	3.54E-111	113.1524	169.9462	138.4144	147.1038	129.4391	115.6114	126.8065	110.7898	147.7951	100.2009	126.1702	100.4643	125.9747	169.9145	137.9056	160.2766	224.5463	194.2665	101.4958	193.3912	188.3607	83.17096	174.1094	116.1062	113.4282	105.285	132.3442
CUST_3374_PI429010886	N14386	0.010611505	15	9	241998746	conserved hypothetical protein			1.58E-12	7.913226	10.73336	9.03778	17.04802	14.31736	5.503681	17.93113	9.926343	12.91477	18.47851	NA	12.93513	12.49181	13.06086	10.42867	6.676397	10.4451	9.160132	17.91767	10.61538	13.66341	14.91234	9.725094	15.54337	19.91817	11.53429	16.42634
CUST_16240_PI429010886	N35900	0.010612313	15	3						47.3524	58.29201	42.75885	64.94907	54.55547	50.25586	35.90614	37.00354	39.79966	14.15358	37.79767	33.31402	43.21882	30.88358	31.93086	41.86801	31.11011	47.26148	41.80345	59.77062	48.93341	35.46315	51.90036	32.8145	23.39255	42.6001	60.20446
CUST_39254_PI429010886	M36746	0.01061961	24	12						492.5086	272.1212	423.1033	845.2107	160.0873	514.0806	1042.748	324.4873	772.308	413.6069	1210.563	286.8701	1380.017	1616.307	417.734	209.3429	660.1893	522.3491	101.8646	186.5169	126.2717	190.9064	206.7075	299.7411	75.63986	88.28749	118.9736
CUST_39042_PI429010886	M33880	0.010620893	24	15	233957366	ribosomal protein L3 [Penaeus n	Protein metabolism	Ribosome biogenesis	4.05E-54	75.34564	93.41761	94.11531	86.46711	106.0103	101.8747	93.01673	113.7405	125.2461	108.7311	106.8903	96.81529	100.8115	126.0081	103.3596	125.8592	116.0897	118.8089	110.6819	89.7242	105.4159	111.5466	91.95645	110.2867	102.2094	72.36145	108.8657
CUST_50714_PI429010886	M90760	0.010620893	24	0						434.1663	610.9907	536.9014	379.037	389.8933	329.0788	236.386	409.6221	385.6874	445.4238	476.6448	116.2567	461.6561	375.1194	451.0149	464.7851	556.8156	477.9381	384.8278	460.2218	353.3256	605.287	498.3024	490.7221	627.4387	541.6936	654.3402
CUST_36719_PI429010886	M16650	0.010621987	24	12						47.68605	35.4467	39.61981	47.9302	66.39483	45.53057	39.17612	54.68833	43.2535	33.35088	56.22801	53.02213	54.98064	73.85285	50.56675	51.68111	58.84948	58.02072	41.20512	47.89413	45.41169	56.24698	50.03686	48.40188	37.34026	49.33322	37.72504
CUST_49438_PI429010886	M85643	0.010639498	21	3						7.469869	8.30152	NA	25.123	28.64244	18.22327	NA	6.698312	4.703837	2.982746	NA	11.43195	3.434408	3.452283	12.56491	4.748557	10.04736	4.628432	NA	4.658677	8.81143	NA	5.615391	7.436551	23.78446	NA	8.869172
CUST_32674_PI429010886	M4771	0.01064079	24	9						8.131518	23.5934	21.28132	32.52808	102.9161	79.07026	23.87227	28.54278	14.67062	25.83559	79.23954	38.10178	18.7418	61.55774	39.29429	10.7267	31.70729	12.90825	20.40806	14.72189	4.142754	14.3798	26.36566	11.77966	18.17164	23.41549	NA
CUST_38993_PI429010886	M33137	0.010649172	12	9						24289.54	1182.698	25247.34	28858.93	20708.31	24909.79	3584.754	6326.89	8256.789	28561.19	33963.44	35157.95	37632.98	14743.32	36406.05	299.9228	172.6192	559.4339	12991.27	9980.233	14470.28	49649.01	33508.11	47553.77	15170.38	23324.95	38384.01
CUST_24186_PI429010886	N50193	0.010655911	21	6						8.071281	9.569825	12.66963	21.18195	13.14676	10.83046	30.6656	22.17359	19.06607	20.87837	8.723632	14.16065	21.76828	14.11364	13.33001	36.94988	16.95396	4.953959	10.78353	9.728753	10.42995	8.214848	19.18145	5.27516	11.86035	15.65515	16.13355
CUST_43023_PI429010886	M61883	0.010658218	18	3						13906.34	850.8759	12068.94	22332.92	13837.12	22526.09	365.9284	832.894	669.7814	15731.96	13591.98	17088.5	12963.97	10260.07	19522.5	333.5586	247.0114	313.8573	1473.47	1573.941	1946.675	24507.87	8911.047	24136.69	1470.023	4461.089	6665.157
CUST_49269_PI429010886	M84968	0.010658218	18	15	343183155	myosin heavy chain type b [Mar	Protein metabolism	Protein folding	4.93E-24	556.4241	1563.253	982.8111	620.7278	571.5137	630.5448	1007.214	1139.817	940.3723	850.2576	799.0901	717.9895	655.4746	889.8299	641.1736	1358.461	2170.443	1777.355	949.2292	968.3563	1044.09	579.065	759.7943	685.2688	1073.164	906.4938	780.4581
CUST_1374_PI429010886	N9438	0.010658218	18	15						254.533	411.6604	362.1172	218.3991	269.8306	298.1868	313.739	431.8791	340.4211	322.2486	404.0425	320.6536	261.7871	385.3052	318.8359	336.4491	509.5667	436.9283	299.4747	397.867	314.1063	313.8573	257.7877	261.9111	273.5066	412.8294	364.2801
CUST_50776_PI429010886	M90965	0.010663339	21	15						126.0542	214.5942	64.2086	120.4478	13.72472	101.7154	296.2664	209.0675	163.8916	197.3639	72.36145	76.24747	161.0498	175.4084	162.9694	346.3619	252.4627	263.9734	134.7975	82.60618	111.355	80.11297	123.0785	81.96955	113.0827	117.4669	40.80836
CUST_5761_PI429010886	N19162	0.010685803	24	15	260800271	hypothetical protein BRAFLDRAF			4.48E-30	85.04871	137.2208	133.3669	83.49206	67.47234	62.33316	115.2014	135.8408	141.2631	98.96283	73.1869	99.32904	111.8116	97.41787	90.09793	114.0755	159.497	146.4045	108.3981	145.1105	156.5799	109.0351	115.3273	118.2313	100.9815	95.29319	121.3126
CUST_23710_PI429010886	N49332	0.010685803	24	6						13.77957	15.78015	13.27566	17.79195	28.46788	19.41654	24.39028	22.35906	26.36041	8.654198	38.4508	11.91707	12.59794	10.95623	21.09601	11.30587	13.75076	8.526821	23.02109	5.053978	16.16791	10.08281	11.77966	10.82728	11.31707	36.44966	12.05742
CUST_1066_PI429010886	N8388	0.010685803	24	9	321455267	hypothetical protein DAPPUDRA			1.76E-45	47.09644	12.80236	13.67551	46.87435	321.4975	159.5246	22.49527	27.22159	6.86903	28.97134	166.7408	223.0844	16.21373	105.3437	111.4946	23.73807	10.66765	9.470236	6.100675	20.07958	4.063236	10.02353	23.47009	14.10658	3.735584	46.02219	10.25993
CUST_1974_PI429010886	N11207	0.010686935	24	15	238477343	ribosomal protein S11 [Rimicaris	Protein metabolism	Ribosome biogenesis	9.1E-64	17524.49	25247.34	20222.93	18613.15	14502.16	17167.45	23503.18	23892.85	22598.48	19485.9	13481.03	12939.25	17771.79	17453.24	17837.79	28414.17	26740.38	30721.41	21974.84	25130.01	26678.14	18715.71	21334.13	21590.74	21590.74	22469.11	23575.84
CUST_2178_PI429010886	N11801	0.010686935	24	21						10.89953	23.50843	17.38353	16.37112	14.27475	17.14825	21.9826	18.65957	26.73304	16.72545	18.44379	12.26303	17.43476	18.62338	18.70643	30.6774	18.94794	19.08041	30.8222	25.35423	19.60329	31.64075	19.50478	33.36729	29.49731	15.78373	32.83178
CUST_5375_PI429010886	N18611	0.010686935	24	15	321457584	hypothetical protein DAPPUDRA				135.5937	250.7612	200.1232	136.5457	168.8682	181.5802	191.7677	213.9341	183.0682	171.8869	167.5904	168.2637	200.7173	209.9538	183.6827	217.7355	254.8129	233.9614	240.0325	187.0768	209.3816	165.6234	217.3996	196.9638	203.057	185.3999	217.5404
CUST_12444_PI429010886	N29470	0.010686935	24	15	91087591	PREDICTED: similar to GA18111-	Protein metabolism	Ribosome biogenesis	9.5E-28	207.3653	419.3654	291.6767	226.8253	197.4226	204.0717	231.2512	313.8573	247.1999	267.4193	221.3068	228.5093	233.9614	235.9714	260.3883	328.4033	371.8649	412.9894	230.1554	290.3235	375.7494	243.726	248.4962	323.1624	256.6606	274.1568	339.077
CUST_26549_PI429010886	N54435	0.010686935	24	21						283.9592	500.1661	473.9767	280.879	375.1194	323.8056	383.8668	485.2701	496.4508	403.7519	311.7988	287.0134	419.8124	299.1753	386.8087	501.9234	527.7959	459.7509	457.0998	479.4997	470.0131	537.4657	537.1828	470.0131	493.5638	448.9533	536.489
CUST_35790_PI429010886	M13516	0.010687493	24	18						23.71643	25.61949	21.96464	21.48061	14.49179	14.88235	27.05785	11.36156	14.18163	20.62453	18.38558	13.27566	16.93597	17.1166	22.78318	24.04006	23.67078	25.30391	24.62932	24.71696	30.47714	22.1804	19.77651	20.16722	26.81298	15.10648	33.53984
CUST_39374_PI429010886	M38510	0.010689872	21	18	148841019	hemocyanin subunit L [Marsupe			2.16E-50	6.694797	30.28156	24.62932	5.128731	14.17028	9.073899	6.11387	10.52564	16.4155	NA	9.480246	9.991081	14.86987	7.092464	20.1058	13.19395	8.888649	14.11031	24.50935	33.6843	24.28126	13.93501	13.88764	18.56513	12.3901	13.76817	9.569825
CUST_3409_PI429010886	N14449	0.010698784	21	15						75.74761	631.0985	65.54266	1127.686	236.0521	909.1828	2333.596	1492.942	109.762	1235.811	800.8587	267.884	1402.437	645.7283	1038.339	3212.539	2224.156	2851.067	791.5844	110.6304	638.4715	299.3538	291.8297	351.7434	1746.391	1291.739	16.97648
CUST_11771_PI429010886	N28373	0.010702498	24	21						5.117121	11.71874	11.35065	12.55197	9.459821	9.633908	8.958066	NA	9.63644	10.81984	8.160789	7.669956	6.863677	5.102347	8.426205	12.71213	NA	16.88956	19.06607	6.226721	9.01805	17.20923	13.54205	14.85278	12.58686	6.138191	10.20093
CUST_6904_PI429010886	N20758	0.010704307	24	21						775.0084	949.2292	707.3888	789.3467	628.6837	649.7567	746.1539	687.2962	785.0219	844.4833	768.2904	912.8746	885.5204	630.0078	827.8854	887.2093	952.6113	984.5856	803.738	861.7915	936.5204	927.0038	901.5762	1083.848	902.1594	789.0657	1114.294
CUST_53260_PI429010886	M101024	0.010705201	24	21						34.20262	46.13913	48.78475	52.22742	55.90731	50.59749	24.56009	47.58659	42.48778	59.25391	46.16308	46.06605	55.25246	36.83655	53.78431	49.99479	58.94726	52.99968	50.05302	53.50459	63.253	72.24298	66.3578	58.10634	61.25239	42.87617	76.51861
CUST_27042_PI429010886	N55342	0.010736346	15	0	260814085	hypothetical protein BRAFLDRAF			3.53E-12	89.64766	73.97753	NA	58.0985	47.92699	89.57931	70.54441	46.67638	55.51551	10.1544	8.102905	10.43238	18.35487	58.3569	15.63496	72.4004	73.08027	21.10164	40.21667	32.38272	19.38387	13.92807	78.7116	4.155742	14.72662	69.54417	7.747958
CUST_52784_PI429010886	M98907	0.010744147	21	12						12.21076	8.001639	7.605705	NA	3.570062	NA	29.38152	37.6281	20.05879	14.46804	17.4695	8.407667	30.41985	22.37825	19.62318	14.0733	29.10478	47.7665	21.04763	16.50059	8.293234	NA	6.926192	NA	14.5601	14.62671	19.36238
CUST_20161_PI429010886	N42921	0.010750715	15	0						13.28376	18.11564	19.43327	16.50059	11.06478	13.2072	10.79318	18.85113	16.42131	4.072095	12.41644	8.087041	7.40723	10.04615	17.09586	20.25781	24.85202	16.1105	11.46624	14.43423	21.8966	11.94663	15.52429	14.27475	12.59667	12.19342	13.29617
CUST_32462_PI429010886	M4184	0.010751282	21	15	321473526	hypothetical protein DAPPUDRA	Metabolic process	Carbohydrate metabolic process	1.57E-21	68.68072	167.1998	36.34385	299.5787	40.10315	282.7416	290.8608	201.983	37.87771	243.331	167.1713	39.77499	374.0444	68.5694	256.1905	484.3203	257.8376	320.2208	112.8135	35.78012	100.8867	39.64997	34.93184	28.80453	232.9121	170.2213	32.58198
CUST_24212_PI429010886	N50235	0.010758827	24	15						37.59336	54.91386	16.76758	40.92146	NA	33.66656	126.4468	91.6714	77.11712	64.88783	24.92085	15.69941	117.3396	44.37921	111.3972	189.5433	156.3126	149.2223	131.6618	22.2941	107.567	32.69608	95.49781	34.18125	59.59948	89.01154	14.98689
CUST_7433_PI429010886	N21544	0.010769653	24	18	291222345	PREDICTED: MEGF7-like [Saccog			1.7E-12	37.22016	20.75594	25.90356	14.01446	22.42351	22.09313	19.17216	18.3141	12.89633	24.40844	27.04586	19.07677	29.08789	28.13189	16.99898	33.76496	19.47348	29.37527	39.82319	26.24406	26.76018	28.41463	11.80717	29.58249	21.00454	22.88663	30.83657
CUST_27895_PI429010886	N56897	0.010776866	24	15						12.46756	12.66337	17.51582	11.02011	9.952015	6.408089	19.04623	9.394115	10.22653	17.35683	7.576581	14.15899	21.1277	10.37552	11.27021	22.27391	11.28859	22.27391	18.32623	17.75202	17.12908	17.425	13.86535	8.92176	21.18195	9.930505	7.773983
CUST_39514_PI429010886	M40686	0.010777476	12	9						27665.22	1198.741	24909.79	30721.41	19791.01	24721.57	3509.087	4461.089	6656.173	30577.23	32540.66	28306.31	41258.32	14338.96	37932.98	252.884	168.6428	479.2755	12963.97	10178.51	11974.32	45257.85	32639.19	44112.52	14580.94	23961.37	35445.2
CUST_43686_PI429010886	M63686	0.010777476	12	9						89.87221	19.48308	73.19641	89.01154	34.47718	51.59532	18.15042	18.51328	24.63804	83.4747	100.7491	57.31291	102.2469	45.55903	48.27158	3.339719	7.164507	4.330532	20.47903	42.13235	45.30575	70.21276	136.8772	148.5106	32.69018	64.76383	100.821
CUST_17463_PI429010886	N38013	0.010777476	12	3	321476315	hypothetical protein DAPPUDRA			8.71E-11	81.19478	171.2911	27.40435	155.0166	48.21771	200.6419	140.9588	123.6581	84.2744	46.86994	60.5484	34.08118	126.4865	248.8065	90.76223	205.3407	167.708	113.666	110.9564	31.51924	104.3593	25.85738	110.5806	20.84673	95.97338	117.1549	15.2772
CUST_38988_PI429010886	M33024	0.010777973	18	15	478257781	hypothetical protein YQE_05601			3.09E-19	2140.692	5601.899	4168.103	2330.806	1862.343	1478.028	3774.541	3172.092	4490.88	3000.392	2816.959	1667.986	2835.943	2571.491	2107.433	3774.541	6642.147	6533.018	2824.276	3243.823	2702.508	2247.929	1987.037	2125.115	3838.854	2494.519	1518.512
CUST_40449_PI429010886	M55140	0.010777973	18	15	13898841	CUB-serine protease [Panulirus a	Protein metabolism	Proteolysis	3E-17	1672.68	2928.861	1787.383	1133.285	1360.696	1053.276	1968.185	3763.397	2858.309	2386.8	2310.632	1263.923	1556.093	2061.689	1191.068	3286.945	3056.068	2753.439	1685.048	2160.965	2001.707	1564.909	1618.404	1518.512	2575.646	1488.548	2030.062
CUST_49263_PI429010886	M84945	0.010777973	18	15	321479105	hypothetical protein DAPPUDRA	Protein metabolism	Protein folding	6.64E-13	1201.71	3978.701	2039.749	1422.675	1166.709	1279.64	2889.672	2818.904	2552.554	2310.632	1604.089	1678.527	1704.256	1851.473	1412.941	3056.068	3552.655	4242.57	2033.334	2414.182	2083.733	1273.044	1679.651	1661.512	2251.39	2120.795	1971.443
CUST_5098_PI429010886	N18253	0.010777973	18	15	527246877	PREDICTED: tyrosine-protein kin			1.95E-75	16.56573	21.0756	18.13167	12.24902	8.116522	8.700256	20.24017	9.785757	25.94024	20.79105	12.91146	22.17612	13.19145	10.33053	14.16203	23.11231	23.97222	23.80751	19.97632	10.57698	19.41654	13.94762	10.64424	17.34867	16.87383	17.62807	19.69265
CUST_16358_PI429010886	N36102	0.010777973	18	15	1888451	pigment dispersing hormone pr	Signal transduction	Other	6.23E-17	11528.01	15798.02	11395.49	9129.704	6240.674	6481.59	13906.34	15012.16	12288.42	11413.74	10485.81	11588.48	11323.45	9999.789	12097.54	14764.65	15394.16	15942.89	9727.143	13988.25	12674.82	8175.098	9041.082	11378.62	14285.49	12211.42	19434.65
CUST_50771_PI429010886	M90945	0.010790813	15	0	321455583	hypothetical protein DAPPUDRA			1.52E-15	19.71754	28.1833	15.7745	17.89547	97.73797	14.43997	5.984179	6.174251	18.92789	7.168799	7.004515	7.905688	25.9479	15.7695	10.21185	14.50367	68.14816	31.83324	24.55123	26.24625	19.63427	12.44307	10.9183	4.884572	5.668943	18.13981	33.01451
CUST_34501_PI429010886	M9862	0.010790813	18	0	242025642	conserved hypothetical protein			5.47E-12	25.22202	24.3283	28.34528	21.49158	21.7225	27.08219	23.43505	17.65737	12.84199	13.32146	15.01335	22.26642	29.31643	22.19826	20.82813	22.39421	32.33214	20.92863	16.2921	32.62087	41.74139	22.67723	15.0661	16.41767	15.52429	14.44743	23.8882
CUST_33884_PI429010886	M8129	0.010817263	24	15	81230851	Cyamus scammoni hemocyanin			2.85E-72	33.5653	48.21063	30.74237	34.52377	18.65471	25.9357	34.28012	35.50803	42.20483	48.17616	25.55884	41.33036	40.93762	33.6985	39.64997	53.8516	44.73509	56.90618	38.89512	34.14921	38.4508	36.02118	40.9293	34.00804	40.19911	33.3478	47.0382
CUST_52903_PI429010886	M99381	0.010817263	24	15	321463451	hypothetical protein DAPPUDRA	Metabolic process	Oxidation-reduction process	1.82E-32	24.79569	45.48093	26.53512	26.72462	16.34183	23.422	51.3517	33.95508	38.36376	45.49544	21.08718	57.1768	46.83625	27.38152	35.3759	57.57578	58.02727	65.72695	40.634	24.41183	40.30169	53.39229	25.28249	42.79442	65.38557	29.88246	32.82562
CUST_2670_PI429010886	N12875	0.010817263	24	15	321451794	hypothetical protein DAPPUDRA	Metabolic process	Cellular amino acid metabolic process	1.4E-88	989.4983	1404.695	1106.369	1209.443	983.0621	915.7404	1324.865	1214.037	1182.698	1271.126	1061.537	1212.98	1443.341	1112.702	1401.131	1392.832	1853.898	1642.989	1402.437	1023.228	1438.372	1123.543	1210.563	1340.842	1550.781	967.3181	1503.022
CUST_7901_PI429010886	N22247	0.010817263	24	15						112.5245	146.4225	120.4803	115.7982	127.9102	101.1839	110.9284	174.6876	169.3049	130.4587	149.2626	150.073	148.0504	139.698	158.9424	136.8604	215.4411	169.3609	150.0449	137.2652	196.5522	164.3213	125.3249	165.4776	149.1062	133.8226	135.5555
CUST_9204_PI429010886	N24228	0.010817263	24	15	524898940	PREDICTED: elongator complex p	Nucleic acid metabolism	Regulation of gene expression	1.79E-44	89.11547	108.2549	91.69645	90.29464	89.7803	84.11819	106.7181	136.0737	136.0437	67.43392	96.71212	81.75003	91.18387	93.34171	95.02561	119.7663	136.3424	136.6003	107.1698	130.9346	145.2258	123.0785	101.4001	104.2473	112.0615	106.8423	117.8448
CUST_7788_PI429010886	N22076	0.010821067	24	15	321469671	hypothetical protein DAPPUDRA			3.09E-98	256.46	461.906	354.8596	230.9439	229.9746	230.1554	298.3249	324.921	407.9106	341.732	247.374	309.4924	281.6983	222.9863	276.1256	358.247	525.6391	509.4445	405.4699	374.7855	457.5988	328.4033	352.8944	354.5733	361.5772	299.1753	414.8181
CUST_17961_PI429010886	N38910	0.010824475	12	6	291235508	PREDICTED: carbohydrate (N-ac	Metabolic process	Carbohydrate metabolic process	3.87E-11	19.71264	10.92685	14.27475	19.06321	11.55626	20.10181	27.21287	21.30878	17.36739	18.34559	16.53724	18.87134	11.82584	11.02965	13.76486	31.11715	46.99361	36.34979	20.58996	37.36858	38.293	33.77215	18.17164	28.89871	19.77172	10.89488	19.91267
CUST_44309_PI429010886	M65590	0.010827619	15	0						6.978065	10.43499	10.24819	6.740547	10.55091	10.34409	4.136368	8.341692	6.193801	8.30152	12.13203	NA	10.18184	12.46346	6.823514	16.56735	13.08105	15.57359	NA	10.64938	5.854267	NA	5.548779	8.968044	11.79955	9.073899	9.481245
CUST_3806_PI429010886	N15313	0.010831892	21	18	255985514	Kazal-type proteinase inhibitor [			1.02E-24	61.0126	85.41835	78.9339	82.99391	57.12984	49.47094	39.24334	108.9478	78.92328	74.28032	90.15514	48.35178	40.0727	48.46283	16.8338	81.39312	148.7488	164.9616	95.173	178.7734	211.185	110.7326	57.98315	119.713	141.3865	63.54768	116.8623
CUST_33199_PI429010886	M6256	0.010831918	24	21						128.2244	6.548842	202.0727	183.0375	29.68907	144.4561	17.18732	20.64644	21.40492	326.2417	1956.496	306.8476	349.3947	66.54894	311.5041	NA	NA	NA	35.73305	161.175	154.566	673.8771	911.4252	929.1604	139.1591	449.3664	645.7283
CUST_25890_PI429010886	N53246	0.01083684	15	6						10.648	6.004064	16.94573	20.37769	18.84481	14.08558	43.77016	13.6782	28.19441	14.34832	13.01662	7.335668	7.664784	22.61153	18.62127	12.64537	10.29403	10.42633	31.97376	21.24956	32.67436	21.71446	NA	20.39956	22.83576	42.58344	23.37452
CUST_5775_PI429010886	N19179	0.01085	21	3	359475044	PREDICTED: uncharacterized pro			2.25E-32	941.7984	1005.944	3.776889	52.07351	4777.94	5462.129	903.4294	2110.599	804.3138	NA	736.2732	813.5332	NA	176.0973	46.5095	82.35709	42.30272	19.67938	45.68766	2522.496	39.88097	NA	455.3201	4.817638	NA	3864.779	NA
CUST_32563_PI429010886	M4439	0.010855176	18	6						38.08917	50.8191	97.91705	33.40167	52.66618	NA	45.66597	60.95576	116.2897	NA	43.61505	NA	46.7009	29.65224	16.94155	13.905	17.37069	5.866363	97.16144	95.15335	93.73127	58.01001	101.6014	51.35849	109.0546	70.19469	114.5608
CUST_34308_PI429010886	M9292	0.010897728	18	3						495.5669	129.3339	626.9839	775.9607	760.7688	718.4732	265.8169	233.6384	314.9794	419.4465	743.3385	633.691	706.4981	614.2194	923.4428	67.47234	101.7595	86.43772	337.8262	414.0319	527.3184	704.6212	658.1324	819.0741	266.3181	433.9196	595.6417
CUST_246_PI429010886	N2999	0.010897728	18	15						35.33768	82.69673	39.31755	30.4977	13.53611	15.26143	56.38348	50.1087	44.31359	59.49858	7.244787	42.3598	38.53699	28.21525	22.38538	61.34753	73.86844	90.84788	45.33378	46.95061	40.88497	26.43013	20.66998	40.082	40.37732	43.51485	45.33378
CUST_1325_PI429010886	N9292	0.010897728	18	15	390343335	PREDICTED: Strongylocentrotus	Protein metabolism	Ribosome biogenesis	4.58E-67	53191.72	56377.06	44722.61	45964.24	41095.52	36802.85	58747.16	58090.3	56938.34	49329.8	35305.99	51875.49	44511.54	47123.31	48559.97	57169.5	59443.36	59131.8	50283.97	50283.97	52165.44	38996.88	38060.46	47826.44	49649.01	49113.82	49649.01
CUST_39590_PI429010886	M41523	0.010905779	12	9						28306.31	982.8111	28025.82	33749.71	29843.45	33749.71	4517.506	4275.766	8086.369	39713.59	41095.52	44112.52	41610.17	26803.66	49113.82	451.3395	89.81747	717.5574	10209.43	11164.38	15526.29	43701.56	36195.31	49113.82	15197.96	18613.15	33963.44
CUST_1827_PI429010886	N10820	0.010905779	12	9						157.1577	475.0917	477.1875	537.0249	290.5678	122.1364	538.6242	396.2592	697.5829	594.8478	695.3717	403.9297	607.6204	319.8754	166.9595	320.3348	266.4734	394.193	409.5077	314.4452	238.7217	601.887	871.1645	739.3679	345.4356	453.9545	711.8674
CUST_47045_PI429010886	M74636	0.010906626	24	12						28.61425	29.65224	32.91719	32.05523	57.3502	55.62648	45.26784	43.86995	32.92024	25.56436	52.23206	44.41457	30.69521	61.41445	51.5265	31.8936	48.77487	71.29445	23.38184	26.00178	19.74793	18.87134	26.21615	30.37998	20.44259	29.94161	22.89097
CUST_33546_PI429010886	M7241	0.010916756	24	21	345315538	PREDICTED: zinc finger protein 3	Nucleic acid metabolism	Regulation of gene expression	0.000000154	NA	5.433499	NA	11.05984	7.474455	6.973469	NA	NA	5.104435	4.443343	NA	4.045737	4.323172	5.764556	7.085828	12.23558	9.355486	13.39103	7.146148	NA	10.27388	10.25993	NA	13.63522	17.38217	5.84695	15.49584
CUST_32020_PI429010886	M3015	0.010933794	21	9	443692673	hypothetical protein CAPTEDRAF			0.000000254	75.09386	89.88767	94.27306	878.8076	192.8532	735.2877	107.8333	224.2026	323.3549	173.2392	2436.323	390.6122	361.3178	292.8189	NA	56.19317	45.00629	8.372078	49.37308	154.7131	195.1305	52.92831	268.9804	395.794	NA	126.5408	105.8148
CUST_16421_PI429010886	N36208	0.0109436	12	6						11.54107	5.954504	8.176241	23.43505	16.52701	11.53968	40.4281	8.06529	26.55272	12.96173	14.79799	22.9023	10.12016	14.69094	11.74246	18.64133	7.717206	8.428222	25.74022	24.0921	55.9876	18.55514	16.57693	19.38387	12.57527	16.89173	22.14517
CUST_54872_PI429010886	M108062	0.010947591	24	3						12836.63	188.4419	11805.59	14439.79	12473.12	14032.06	463.9651	1329.875	1379.279	12211.42	17131.85	14828.55	13446.51	7993.785	13962.41	224.3337	166.9821	272.8455	1364.845	2345.461	2440.627	11228.98	11836.96	13935.1	2911.669	10430.71	12021.42
CUST_39392_PI429010886	M38800	0.010947591	24	3	225712016	Cuticle protein 6 [Lepeophtheiru			1.35E-17	19237.66	747.3198	15141.22	17287.24	17388.9	18235.97	384.3395	393.7655	446.5017	13790.46	14982.33	17487.6	11805.59	21974.84	29843.45	63.99691	91.28627	160.9133	603.191	784.1859	479.1083	12699.11	2937.545	12068.94	799.3476	1102.526	1802.44
CUST_50144_PI429010886	M88807	0.010947591	24	15						129.6511	149.6818	104.8028	146.953	86.62987	131.568	187.3503	147.3568	144.7781	186.3368	140.549	132.7314	198.5315	143.1878	116.4978	227.4568	221.2277	189.7429	163.0857	111.007	163.9403	148.107	168.4596	151.1899	176.1473	139.7737	141.9808
CUST_5720_PI429010886	N19097	0.010947591	24	15	229366981	Anoplopoma fimbria clone afim-	Metabolic process	Oxidation-reduction process	1.3E-81	368.7646	669.7814	476.5395	357.296	344.5549	354.4054	522.3491	524.2711	590.8215	444.8216	452.4209	489.8482	525.7914	454.8526	457.222	495.3083	807.3597	640.002	475.9252	671.5725	610.334	314.8641	620.0365	548.2784	492.5086	463.6603	476.6448
CUST_6270_PI429010886	N19877	0.010947591	24	15						1317.605	1695.833	1497.206	1325.644	1047.139	1042.748	1749.36	1208.338	1587.909	1595.825	1023.228	1454.668	1439.002	1104.345	1357.702	1985.342	1870.445	2406.654	1707.189	1481.716	2030.062	1393.298	1474.205	1743.671	1768.674	1363.959	2164.77
CUST_16467_PI429010886	N36280	0.010947591	24	15						44.61738	70.80726	60.62225	40.56783	40.07856	39.00145	79.78242	61.76701	78.86032	74.6809	40.97117	74.37918	54.68333	53.64895	58.33297	72.24298	94.46419	77.31152	67.34563	64.72689	69.75403	52.17564	49.08263	68.60832	76.11372	47.89413	65.01496
CUST_27416_PI429010886	N56021	0.010947591	24	15	321458298	hypothetical protein DAPPUDRA	Cellular Process	Cell adhesion	1.6E-14	52.17564	66.2384	57.94141	66.77244	32.07144	45.18702	76.48161	67.85905	57.63804	82.09637	51.15383	53.71769	72.04345	58.18757	55.73019	98.03098	88.45765	107.7947	106.8768	55.85351	96.29109	62.60527	63.83347	79.37901	91.08467	59.68511	92.55588
CUST_8840_PI429010886	N23678	0.010971152	18	15						36.67874	96.76253	25.32145	7.975849	NA	11.55982	82.63781	62.67742	49.20176	12.27496	9.602206	NA	40.35274	17.38916	33.4664	80.85368	165.8977	119.077	15.58431	4.173492	20.72504	16.35396	8.122754	12.13497	33.64233	34.23772	5.385232
CUST_3138_PI429010886	N13881	0.01097245	12	9						75.3636	47.23231	69.34161	114.4742	81.42279	42.92652	36.08683	40.27654	49.78973	110.2586	327.216	103.736	126.4021	63.43943	72.09362	7.415808	58.31116	34.44149	49.74586	48.46739	40.01947	159.645	270.2762	188.2922	66.33276	234.1695	248.4015
CUST_41467_PI429010886	M57799	0.010976675	21	0						324.8189	293.2877	374.6189	350.5935	242.6675	285.0355	306.8476	237.0529	299.9228	325.1877	221.5001	296.3076	284.8134	268.1907	274.5666	215.6896	228.1062	281.9365	282.9651	313.3524	364.2801	407.9106	304.9349	455.7266	269.5561	221.9709	338.7418
CUST_10384_PI429010886	N26153	0.010989909	24	0						108.2394	121.7787	140.8575	115.8263	137.2489	124.6988	110.7082	112.2698	126.4021	110.4792	101.361	119.5301	104.5953	101.0861	87.74995	126.6202	100.1424	145.0599	137.7249	121.4836	130.752	152.7673	89.7803	131.0265	133.239	130.8809	159.7718
CUST_657_PI429010886	N6595	0.010999604	18	12	516327276	hypothetical protein [Oscillatori			1.5E-11	368.3282	390.3118	395.2192	380.6302	356.6938	396.1202	241.5804	323.5302	359.174	317.6549	439.1006	333.9851	445.6007	404.5068	449.5658	300.9971	597.4035	318.482	298.5019	342.6036	283.9592	325.0373	300.3068	316.5959	275.9079	227.2502	234.2807
CUST_6912_PI429010886	N20768	0.01100021	24	15						29.1953	24.86911	30.75233	39.73627	27.55963	32.93904	48.23305	29.35503	34.71439	34.24988	29.67957	33.09557	43.70175	38.61107	43.28585	45.19709	36.22942	44.50543	43.54722	22.95506	36.36523	42.42877	34.90937	36.1335	38.72594	26.20109	34.3577
CUST_46688_PI429010886	M73418	0.011004456	24	3						72.52843	54.25668	57.66115	50.52247	75.34564	64.95625	52.07927	69.58412	42.178	34.48922	36.43276	59.78407	33.99771	35.83339	40.57567	43.93243	32.83487	46.25621	31.23655	67.16357	45.41788	57.8427	42.29499	51.63077	34.02535	94.62002	47.71567

Probe ID	ID	p-value	Period	Phase	EBI ID	Annotation	GO category	Go term	E-value	0	0	0	3	3	3	6	6	6	9	9	9	12	12	12	15	15	15	18	18	18	21	21	21	24	24	24
CUST_3493_PI429010886	N14615	0.011004456	24	18	170039990	conserved hypothetical protein			1.33E-14	92.2966	91.7043	94.31617	96.62834	109.3108	87.33991	66.74201	137.7644	96.14621	46.16801	73.80869	40.84998	73.4071	98.81307	66.28735	74.28907	74.51372	79.23144	118.0906	153.7917	150.6011	93.8595	111.6045	70.0011	125.7244	118.8772	102.1199
CUST_6151_PI429010886	N19694	0.011004456	24	15	499003452	PREDICTED: splicing factor 3B su			5.99E-134	80.09123	73.97121	71.91459	65.81456	45.00629	71.62515	56.00071	55.50618	64.80829	86.15946	53.72874	81.45042	88.13366	83.53855	76.65834	97.63248	80.43757	84.62659	89.13599	73.9144	72.0781	109.425	69.112	90.45329	129.6027	70.69946	87.96067
CUST_52711_PI429010886	M98592	0.011006165	24	21			Signal transduction	Other		36.74804	39.23752	43.69757	NA	24.63804	8.355785	10.85858	11.73655	10.06884	27.71884	35.71776	22.16637	25.72369	22.51326	9.870583	16.74549	24.54963	25.45723	21.48216	20.51167	6.285156	43.7275	43.79306	49.49501	47.79385	41.2262	29.15694
CUST_22318_PI429010886	N46804	0.011010896	24	12						28.58081	21.84879	31.80555	16.36594	26.31588	28.81419	39.55139	19.18145	24.06897	28.74405	19.96229	21.89919	55.91798	31.84584	52.75072	41.79513	42.24367	33.43626	36.03288	29.56665	27.97979	45.13797	85.24573	34.22791	25.54534	28.14237	30.62439
CUST_46231_PI429010886	M71876	0.011022089	21	0						NA	7.231758	9.473667	8.096327	4.681705	6.636812	8.04727	5.121843	3.660981	4.026677	3.212059	NA	4.090966	NA	NA	6.795326	7.139105	9.491244	NA	4.978112	4.7993	8.758815	NA	7.916441	7.766169	3.716442	6.180949
CUST_32281_PI429010886	M3693	0.011025321	12	3	241745122	WD-repeat protein, putative [Ixo	Metabolic process	Other	3.17E-66	11.25075	20.64485	12.01431	14.88678	21.91645	9.519076	14.39849	12.71343	13.49586	12.11716	13.38505	14.03518	5.074363	4.495694	NA	18.00685	13.43266	23.48894	11.41917	19.44173	16.5317	11.99361	11.80456	11.22739	9.767511	10.99723	11.52497
CUST_37163_PI429010886	M18058	0.011025424	21	3						137.7481	78.48038	138.4394	175.1243	162.3101	152.8947	147.7749	111.6675	108.4697	175.9736	73.43473	157.2569	101.0154	141.3011	142.0823	121.3291	55.02895	112.0077	140.5088	129.1403	36.46491	157.5876	138.0734	140.7362	148.3156	154.4681	128.8601
CUST_32776_PI429010886	M5089	0.011034082	12	3						1089.998	2045.264	1181.431	1789.218	1573.048	1676.984	2251.39	4598.208	1793.169	1706.299	723.9274	327.8085	1129.795	1377.009	482.3026	2320.751	6744.57	5955.414	2312.635	5671.62	4372.597	1079.588	1202.808	684.1238	3073.818	1125.025	1289.52
CUST_40386_PI429010886	M54959	0.011034082	12	9						21459.8	220.6649	16348.66	23961.37	22847.77	22401.51	1656.675	2685.938	4153.924	20320.15	36406.05	32540.66	30721.41	14137.09	26191.89	336.6729	85.61207	389.3624	6688.584	6448.141	7260.089	32454.44	31206.9	36724.45	10795.65	17210.94	25611.3
CUST_44584_PI429010886	M66385	0.011038088	24	15	260814680	hypothetical protein BRAFLDRAF			4.99E-11	5.06766	11.11975	NA	9.929168	NA	7.881914	10.21029	5.129351	7.810134	9.211369	8.371114	8.694186	10.86736	11.57897	3.938144	11.89711	13.94762	13.21441	NA	5.68169	9.090753	5.228125	5.061979	8.850254	4.381432	7.433712	NA
CUST_32684_PI429010886	M4807	0.011042464	15	3	321477586	carbonyl reductase [Daphnia pu	Metabolic process	Oxidation-reduction process	3.58E-85	113.23	191.1071	164.9813	172.4491	196.8082	193.8796	169.4171	232.2882	154.8085	156.2107	159.2971	146.3771	104.1889	158.9424	115.7411	189.9808	206.781	193.8796	190.1878	202.183	171.436	156.5799	190.7683	154.7361	156.8946	176.5842	164.9432
CUST_47690_PI429010886	M77183	0.011044173	24	15						66.17125	136.2348	90.92644	82.00982	51.69582	78.65231	164.3213	131.7896	104.3181	172.5296	73.36846	118.5815	104.9085	84.61891	81.55186	250.7116	191.9733	223.1865	112.6108	80.16082	150.113	94.48817	64.50416	114.2468	163.2608	104.6237	89.74912
CUST_7998_PI429010886	N22397	0.011044173	15	6						40.28522	45.2017	58.66639	57.9032	108.4356	74.98208	55.93014	83.07439	69.66595	27.59053	77.29301	51.44117	65.64875	61.15687	64.83629	42.92652	44.83298	36.43276	53.96355	72.63288	46.29703	61.64797	82.51294	63.46562	47.99288	77.16447	60.60875
CUST_34940_PI429010886	M11130	0.011050776	24	15			Protein metabolism	Proteolysis		412.4307	206.1048	208.1428	107.567	NA	97.72445	223.6663	668.9797	411.3393	346.1837	182.9809	97.82425	180.8767	236.8356	40.22947	1225.977	603.3505	952.6113	554.3874	731.5275	958.9028	310.1796	29.17375	249.4703	464.9207	149.4602	220.9505
CUST_20802_PI429010886	N44078	0.011059289	24	18						56.20706	26.28128	35.47671	18.7507	NA	11.9085	75.51715	52.63501	60.03374	62.196	9.511013	25.20136	35.45915	38.15601	18.0705	100.8867	70.01129	76.41348	77.72005	67.22535	102.5417	40.13911	NA	29.90427	75.12194	32.48271	41.10401
CUST_3230_PI429010886	N14067	0.011067256	18	3						2608.106	360.0833	1796.427	2139.451	1470.023	1139.817	507.6273	892.0968	896.6099	1640.994	1106.912	1021.074	2031.77	1173.346	940.3723	170.625	225.9507	191.7974	266.5309	1011.215	439.2301	2470.84	1875.312	1702.984	582.1367	1042.247	1271.126
CUST_57316_PI429010886	E5308	0.01107792	24	3						3952.736	242.6247	4363.515	4819.372	4456.862	5507.513	250.8664	956.8208	413.5169	1509.889	5451.324	3871.525	1644.807	4900.819	4733.525	113.6471	325.0373	209.5934	202.8396	847.0248	327.3332	3052.051	798.8065	2092.723	335.3952	1376.514	458.147
CUST_38022_PI429010886	M22822	0.01107792	24	3						26403.54	20.452	23231.4	35445.2	6348.681	22332.92	41.70713	186.4449	94.82698	5329.024	10699.09	7100.104	5099.888	13154.56	11552.67	5.961032	10.45264	37.90412	88.52477	166.242	91.52744	6210.057	2556.357	4761.143	276.55	562.1389	802.754
CUST_34005_PI429010886	M8485	0.01107792	24	15						643.8008	1426.897	878.8076	685.653	222.9112	444.7023	1351.154	1391.531	1060.229	692.8899	393.4684	270.0743	1293.42	820.9036	888.236	1557.736	2264.823	2637.871	1066.406	687.6817	1199.116	528.1103	2722.979	528.1103	519.1671	950.4596	1183.356
CUST_49410_PI429010886	M85542	0.01107792	24	3						1580.709	10.37552	1378.754	1835.563	2198.578	1907.271	48.81332	143.7017	183.1939	1412.941	2568.829	2173.027	1401.131	1091.584	2054.56	9.459821	18.0729	15.54537	115.5416	257.241	261.8722	1253.031	1440.472	1875.312	244.9453	990.2906	1287.523
CUST_5442_PI429010886	N18709	0.01107792	24	15	321469939	hypothetical protein DAPPUDRA	Transport	Protein transport	5.42E-31	143.8832	170.3073	187.0121	160.713	126.0081	118.2092	188.695	184.9052	168.5856	161.0498	143.5809	168.8433	158.3476	155.8206	168.7741	191.7176	239.0006	218.8175	135.1633	216.8296	169.9462	174.9928	211.1114	181.4063	168.4808	156.3126	190.9992
CUST_8769_PI429010886	N23559	0.01107792	24	15						145.3581	307.4677	231.588	131.0082	121.2063	100.783	237.3958	204.7788	208.5384	258.3703	164.206	176.8339	280.7963	170.625	179.1861	223.9415	318.037	297.4214	265.7303	204.5441	237.7241	187.1657	219.0705	197.7325	274.5666	169.3049	268.1907
CUST_14728_PI429010886	N33360	0.01107792	24	21	512882510	PREDICTED: zinc finger protein v			6.85E-13	34.12139	43.06564	25.59575	32.45769	26.4485	27.30898	40.83466	37.39308	18.6092	15.36855	18.97114	23.4647	32.36608	43.36291	30.90807	42.97957	34.51373	28.24828	28.37038	33.14322	39.90993	38.88446	44.09481	42.33335	22.39421	31.53465	38.85553
CUST_19489_PI429010886	N41663	0.01107792	24	15						104.173	116.3062	103.8143	94.96672	96.47869	99.90955	105.6309	139.3061	108.6123	114.0114	115.4959	134.6492	133.4287	108.5015	119.1399	151.5412	106.4281	144.474	134.7253	119.077	118.653	121.3825	133.2629	149.2013	129.2072	103.4374	157.2166
CUST_20810_PI429010886	N44100	0.01107792	24	3						74.96434	34.41579	74.13678	103.8438	331.9294	358.0309	25.97276	57.73118	58.82712	64.42288	216.3545	166.7408	33.47158	139.6676	192.6063	12.48615	12.29756	12.90521	20.68379	86.90266	27.61361	37.22647	101.9686	105.8953	11.82408	124.1127	77.10772
CUST_31117_PI429010886	M735	0.01109332	24	18	494952537	oxidoreductase [alpha proteoba	Metabolic process	Lipid metabolic process	7.05E-67	6.396737	NA	21.56373	NA	9.366695	4.791328	13.1101	NA	16.42848	4.841768	4.059378	NA	16.86908	16.93391	NA	5.736085	5.220446	19.50817	30.0529	48.21063	91.21622	9.078909	NA	11.68939	18.76106	NA	15.58609
CUST_35063_PI429010886	M11462	0.011095096	21	12	123507472	ankyrin repeat protein [Trichom			1.42E-12	293.568	570.0085	403.7519	330.6996	71.20264	311.3298	548.1274	216.0628	597.9185	404.9858	616.5627	206.1814	331.6434	732.3207	813.7492	677.9076	178.3394	624.767	145.7157	218.0736	279.2412	62.63588	227.7726	249.2581	123.0345	48.31614	96.07003
CUST_30417_PI429010886	N61848	0.011095508	21	0						51.46661	NA	52.30892	68.74221	13.17346	46.09063	6.381994	21.91645	15.04022	48.1462	20.82118	35.30917	37.13496	8.874019	35.0064	3.491618	NA	10.87185	23.05545	9.889204	21.0526	81.44134	34.49352	51.23629	22.51109	24.92804	39.53461
CUST_15747_PI429010886	N35075	0.011106274	12	0						286.2354	260.3883	237.0529	190.1878	218.8639	184.5933	176.1473	207.7328	195.3229	193.4888	236.013	189.6097	225.5927	179.9258	234.5732	219.0312	278.4811	221.1265	204.4319	216.2603	207.8099	220.2503	232.7864	237.8915	226.2788	236.8696	260.0376
CUST_11527_PI429010886	N27972	0.011108001	21	6						14.58115	14.85124	14.15899	16.66408	6.62957	20.28365	14.95172	18.86536	15.2887	20.87255	15.06042	13.73882	12.38731	15.82272	10.01392	11.87323	10.05625	12.36665	14.39849	7.961062	12.89112	16.46661	10.83359	7.773983	18.52205	14.84915	17.01558
CUST_10425_PI429010886	N26208	0.01110933	12	0	270005256	hypothetical protein TcasGA2_T			1.73E-52	190.0902	133.0898	149.0265	120.1066	155.0803	148.2361	133.7339	100.59	132.4959	142.1948	134.0586	25.46688	199.6427	102.2013	203.1325	131.1039	85.95962	91.12829	134.8108	74.55481	94.59748	173.5895	131.1039	149.2467	200.181	153.6189	202.9968
CUST_21731_PI429010886	N45749	0.01111562	21	0						44.04608	21.7225	38.44506	37.29216	27.09506	30.95428	20.91044	10.91277	17.78015	26.35742	11.66991	29.71228	43.76293	27.72385	38.83587	10.1235	13.6023	8.767695	22.75769	16.6676	11.54561	69.62329	41.64305	41.71225	31.17639	19.16548	41.76732
CUST_37449_PI429010886	M19065	0.011135421	18	3			Cellular component orga	Cytoskeleton organization		44.93169	42.21839	72.72426	67.7048	45.83287	56.734	35.45282	52.46839	33.20541	26.346	98.45312	39.321	44.70348	75.10319	29.47872	24.26626	43.5017	14.3393	32.23946	18.0495	27.29712	76.42243	81.48573	104.5846	68.40054	28.79058	66.17651
CUST_27949_PI429010886	N56997	0.011156926	24	12						50.45617	10.27771	8.385883	51.11657	14.90324	22.22585	44.1442	12.85037	13.76486	46.57025	27.98737	15.75024	56.83684	21.95217	23.11901	17.13272	7.95738	18.99955	15.07604	16.29003	4.14739	10.35047	9.712808	10.45873	15.2696	18.65471	16.51006
CUST_56681_PI429010886	M114413	0.011159692	24	21						63.23529	61.8234	76.99156	58.58735	29.30404	68.01679	67.84273	45.5523	77.06177	55.31238	58.30071	42.68213	62.50267	46.83625	39.40714	71.58388	52.58375	72.6168	108.8788	55.24742	80.98106	99.56289	72.82177	71.99448	112.2102	58.11342	92.65597
CUST_13863_PI429010886	N31907	0.011175506	12	9						2452.653	740.5837	2679.375	2249.486	1752.658	1876.444	317.0103	1225.455	927.0038	2698.998	2642.585	2414.182	4064.827	2795.798	4275.766	310.1796	217.3409	260.2442	907.3817	980.6718	464.7851	4503.776	4027.931	3608.266	1518.512	1507.382	3696.265
CUST_53879_PI429010886	M103525	0.011189807	24	15	328899962	ubiquitin [Procambarus clarkii]	Protein metabolism	Ribosome biogenesis	3.95E-19	19195.08	21834.29	17023.68	16801.9	12886.71	14071.14	21922.49	19572.59	20172.23	16622.66	12344.85	12584.9	15258.82	15297.04	14858.64	21758.66	23435.22	24507.87	19485.9	20382.6	21270.53	14389.61	17729.56	17589.58	17210.94	17345.96	17771.79
CUST_40897_PI429010886	M56331	0.011193643	15	12	156552029	PREDICTED: hypothetical protein			4.19E-42	44.22724	44.02111	36.86325	40.25187	80.26815	60.14723	44.72002	41.1126	29.50027	45.5101	44.73116	49.81991	49.61113	57.76704	57.57578	66.92448	55.84366	52.27625	31.17639	38.70471	33.92168	35.74167	44.53304	33.20541	49.63135	57.05634	45.01222
CUST_5822_PI429010886	N19248	0.011200439	24	18	328705463	PREDICTED: hypothetical protein			7.6E-62	185.937	208.4398	252.1189	187.6435	126.0374	103.6807	106.4683	92.0968	124.0944	130.8147	168.511	159.5875	281.0341	162.5822	113.7707	120.3127	180.1675	128.5824	241.7973	232.5608	223.1465	151.0262	130.9169	167.261	213.3383	197.6371	316.4278
CUST_57148_PI429010886	E1597	0.011208248	24	15	119134	RecName: Full=Elongation facto	Protein metabolism	Regulation of translational elongation	4.28E-50	30447.23	42662.49	29122.17	26403.54	29949.84	32540.66	41095.52	48051.75	37765.06	34757.71	33176.01	31289.71	36043.3	30352.12	34877.59	47123.31	38060.46	43280.43	37932.98	38861.2	43456.54	30447.23	34620.42	33838.77	36195.31	33304.41	37083.68
CUST_34968_PI429010886	M11197	0.011208248	24	15						269.3499	618.7694	296.4601	276.6685	200.3693	244.0026	457.3588	462.0354	426.4459	459.1993	327.3954	314.5854	423.4844	275.5775	236.3014	555.5824	686.4707	600.0613	367.4917	357.9365	435.731	336.6041	314.1834	362.7316	454.4018	337.6329	367.0194
CUST_56554_PI429010886	M114084	0.011208248	24	15			Protein metabolism	Ribosome biogenesis		27.69944	34.24988	36.40013	30.12442	23.26132	38.02572	30.28489	32.26017	54.97558	61.03293	34.13318	35.44968	40.49642	36.70398	33.56909	71.85783	56.22801	55.91239	49.58225	35.15693	38.62717	46.02907	32.07144	45.78637	67.51408	50.23652	36.71405
CUST_31910_PI429010886	M2719	0.011208248	24	3	345491954	PREDICTED: hypothetical protein			2.74E-56	162.6466	46.68552	151.0262	164.0887	171.664	169.729	70.58682	47.90984	69.45139	98.42724	104.8169	177.7913	128.4802	112.0182	167.9984	56.63612	46.0486	51.55894	75.94639	51.11212	62.66376	127.0936	79.23144	128.3075	81.57806	67.3285	90.04305
CUST_39415_PI429010886	M39101	0.011208248	24	15	347966902	Anopheles gambiae str. PEST AG	Cellular Process	Cell death	1.14E-94	2381.656	4761.143	2975.418	3202.946	2839.956	2891.912	4545.107	3419.816	3899.124	3992.329	3079.233	2985.873	3875.533	3217.866	2654.193	5659.622	5502.319	5162.169	3952.736	4238.105	4242.57	3289.37	3751.984	3565.017	4041.944	4487.184	3808.458
CUST_46715_PI429010886	M73496	0.011208248	24	15						89.85516	124.4425	108.9478	123.0345	92.71133	87.72633	117.7903	128.4972	97.1413	127.9454	114.2182	80.95294	123.3723	124.4898	113.7563	134.8108	167.6224	130.3816	124.5521	127.1435	101.2279	116.4413	124.2323	125.8417	173.8666	121.9377	115.2656
CUST_8175_PI429010886	N22649	0.011208248	24	3	321460082	hypothetical protein DAPPUDRA	Metabolic process	Other	2.1E-42	322.75	125.1571	459.5171	651.0737	876.9516	980.3183	120.5552	160.737	132.952	215.2625	633.8464	470.5066	257.4304	935.873	593.6835	91.7443	118.3752	122.7895	96.24348	215.9444	118.7327	339.8451	191.1727	281.9365	101.0154	318.482	145.7157
CUST_11384_PI429010886	N27726	0.011208248	24	15	260793968	hypothetical protein BRAFLDRAF	Metabolic process	Cellular amino acid metabolic process	1.38E-47	326.7696	485.2701	371.2053	321.4975	324.0982	344.1561	450.7184	456.9596	499.6833	469.2787	310.1796	246.6961	418.3708	325.1203	310.9804	621.614	531.5525	513.3858	588.5037	362.7316	525.6391	437.0635	400.146	391.9617	509.5667	365.8262	392.8052
CUST_11449_PI429010886	N27826	0.011208248	24	3						5826.27	34.02535	5659.622	8309.047	7983.519	10277.01	105.5685	92.0742	47.82263	967.3181	5659.622	6401.205	795.25	5726.275	6864.217	45.01222	18.40249	90.74217	22.9969	165.4536	48.66253	2528.059	99.10109	2167.915	47.66075	201.6588	64.10757
CUST_14357_PI429010886	N32750	0.011208248	24	3						36.33342	25.8625	41.09779	45.37684	96.12752	83.08821	31.91889	50.0221	28.01116	33.01146	61.66326	55.40921	28.66131	37.92736	52.92831	19.99489	25.97013	26.34871	33.59136	44.46588	37.69822	29.04223	33.762	27.04586	26.89679	38.01913	36.26515
CUST_1435_PI429010886	N9622	0.011208248	24	15	546673984	hypothetical protein D910_029C			2.03E-19	7872.926	15942.89	9410.039	6966.12	4637.293	6559.55	12141.91	9872.039	10987.48	11429.91	5091.533	5402.577	7872.926	8713.974	5693.503	12584.9	16491.53	17088.5	11112.95	13078.51	10374.24	8378.475	10408.52	8016.887	11228.98	11780.77	5209.792
CUST_47025_PI429010886	M74563	0.011226857	12	9						43.19018	39.86612	44.7999	34.91434	32.49093	23.9493	28.42566	25.76854	30.78396	52.57814	29.03394	42.83292	38.46589	33.29588	47.30043	33.55937	12.91646	30.60362	36.14198	29.97997	41.42074	62.68299	46.47735	72.36145	51.02509	29.23556	64.01323
CUST_2406_PI429010886	N12285	0.011226857	24	18	386376737	hematopoietic prostaglandin D s			4.25E-61	432.3981	483.9464	466.4341	424.2083	177.6012	212.4893	454.1788	826.4146	726.7698	296.2197	125.0424	177.2393	883.0931	456.0887	370.8194	493.3336	624.4152	712.4529	717.3191	700.359	1129.142	591.6487	332.3383	507.9246	801.0558	413.7039	561.5074
CUST_30537_PI429010886	N62082	0.011226857	24	3						217.1239	84.21664	173.8121	288.3603	367.8176	262.1946	137.396	105.1602	137.2826	169.9945	180.6161	268.5828	145.5033	205.95	234.3588	102.3362	95.88853	83.26396	145.2125	109.5302	73.34259	165.368	127.048	103.2732	74.06253	152.0064	104.2173
CUST_55035_PI429010886	M108453	0.011229719	21	15	225712480	60S ribosomal protein L11 [Lepe	Protein metabolism	Ribosome biogenesis	2.43E-30	25343.01	32454.44	30965.56	22154.05	19635.13	19635.13	28561.19	28943.19	28414.17	25188.43	18435.82	18851.7	23503.18	24341.41	22895.57	32969.41	33176.01	34757.71	27758.19	30721.41	33076.81	23324.95	24507.87	26403.54	27073.29	28210.59	29122.17
CUST_1463_PI429010886	N9717	0.011260073	24	3						1006.38	1257.92	356.9618	437.2723	2942.677	2571.491	979.3611	2526.145	1062.767	294.1356	922.8654	918.7311	302.0716	663.5871	599.566	356.6056	328.9434	226.2788	314.3155	1950.295	247.5439	263.0087	915.0007	325.7046	170.246	2137.853	308.8108
CUST_22192_PI429010886	N46598	0.011271772	15	0	405964657	Putative exonuclease mut-7-like			9.92E-15	16.96963	13.16694	15.11819	NA	NA	9.858804	6.247573	5.40299	8.143822	10.62276	6.691551	14.1426	13.85654	11.29903	8.352666	4.994211	18.98812	14.98899	NA	8.239061	7.760758	7.027807	9.708019	5.588826	9.945045	NA	4.648358
CUST_52403_PI429010886	M97333	0.011288708	15	6	321461236	hypothetical protein DAPPUDRA			2.12E-09	459.3044	625.4995	1005.566	1240.074	757.3112	22.10476	1080.903	876.9516	1975.778	103.0843	44.86243	90.28649	598.2839	23.0352	476.7459	35.46629	22.09536	33.90314	1403.011	2245.768	4772.224	629.2438	2600.653	2873.993	809.8174	4.001878	859.4584
CUST_31036_PI429010886	M530	0.011290689	12	9						332.7289	298.2569	340.8616	370.6325	329.4879	321.4975	271.2533	332.8061	301.8062	450.6028	346.0708	503.6798	340.1047	621.7856	811.0498	212.8617	294.3326	323.2204	293.568	393.7655	372.2898	379.6985	455.6054	490.4622	388.4776	333.1336	458.9701
CUST_9597_PI429010886	N24896	0.011291024	21	3						16.47278	10.94976	16.06582	28.96132	18.13805	16.34777	14.17824	7.776877	14.30855	11.68939	4.997977	14.34832	9.221685	11.7024	9.089798	12.4284	6.240405	14.30371	24.92323	3.761052	10.74906	17.04802	9.03053	18.63493	17.6031	14.28873	16.93224
CUST_33418_PI429010886	M6832	0.011299718	24	6	326579691	JHE-like carboxylesterase 1 [Pan			1.91E-22	44.10093	47.49734	48.08352	52.19714	59.24756	33.6843	65.71042	39.52957	59.35684	35.99333	66.93928	55.70355	23.44667	22.69882	29.20882	33.71363	13.05352	26.73995	21.03259	26.10261	12.67757	34.88898	77.29301	81.79336	41.64305	20.46288	40.81486
CUST_21311_PI429010886	N44994	0.011337151	24	18			Transport	Transmembrane transport		30.60362	26.13038	41.55628	22.21596	23.82872	23.35729	24.93525	32.47851	20.71518	25.34302	17.40265	17.81281	31.82327	30.24945	32.1267	38.38502	31.34171	31.8633	29.8647	30.31994	39.38283	33.50165	28.8322	38.11869	38.11477	26.19566	41.52789
CUST_57073_PI429010886	E547	0.011338577	24	15	478252315	hypothetical protein YQE_1054(			4.28E-08	323.865	521.206	503.7654	356.1688	344.4073	356.6056	533.0556	504.4324	529.2519	436.5051	394.0157	326.6163	458.4811	421.3837	408.3007	573.4698	668.5027	684.8522	477.7276	462.1954	587.8441	388.875	501.1967	424.2083	476.6448	446.9571	498.4242
CUST_39490_PI429010886	M40285	0.011338577	24	3						40217.51	185.8751	35305.99	27210.5	30257.2	37765.06	302.0161	745.8583	429.0423	21834.29	31206.9	24426.06	22526.09	22332.92	35862.04	134.2073	138.4394	251.0873	867.6626	1020.042	1049.887	20871.55	8827.334	20020.97	1582.625	2636.073	4284.075
CUST_32680_PI429010886	M4795	0.011338577	24	15	321453648	hypothetical protein DAPPUDRA			4.68E-21	26.29812	26.44427	25.34518	30.83657	19.65095	22.6604	44.5991	26.62131	31.10668	31.43117	22.30617	34.82552	32.3182	27.29712	29.97653	40.91214	33.80478	38.96134	38.23223	25.10206	38.61632	33.51493	26.38647	29.81999	31.76944	29.37054	28.6389
CUST_31209_PI429010886	M979	0.011338577	24	3						669.3491	72.90397	811.6064	796.612	749.3538	745.3715	63.87749	70.3428	79.51886	111.0547	577.8423	577.9786	176.6314	582.785	720.2377	53.5836	67.7048	61.16253	61.3302	71.1038	74.08077	250.621	104.8565	204.2969	59.63292	91.15714	101.2008
CUST_2839_PI429010886	N13217	0.011338577	24	15	321456034	hypothetical protein DAPPUDRA	Nucleic acid metabolism	RNA metabolic process	1.08E-16	477.058	723.6856	682.3134	496.5813	614.5401	551.429	563.1838	979.3611	827.1853	560.6594	724.9498	530.3219	621.1319	702.5663	710.633	733.7832	1097.014	955.1294	763.2886	801.5781	881.8202	661.4243	785.3422	704.142	811.9435	700.8468	861.7915
CUST_3496_PI429010886	N14623	0.011338577	24	3						16035.79	37.78456	12939.25	11695.66	11932.74	16348.66	70.42723	190.9064	79.58985	4358.067	11836.96	8945.499	2958.733	9490.363	12317.37	16.70829	10.76257	43.15014	121.9486	551.429	108.4526	9272.221	3598.269	7861.824	112.8135	1261.961	1148.933
CUST_18072_PI429010886	N39090	0.011338577	24	15	465954970	Beta-1,4-galactosyltransferase 3	Metabolic process	Carbohydrate metabolic process	3.48E-20	51.87516	111.0363	46.71131	64.71262	24.89861	61.01777	83.30459	119.713	96.85513	62.78718	64.70441	44.82232	86.23628	92.12733	66.8709	126.327	119.272	96.05666	95.49781	79.1244	87.03157	56.62456	71.1558	84.07229	114.6414	79.47605	70.44138
CUST_381_PI429010886	N4737	0.011338577	24	15	307195421	hypothetical protein EAI_14436			2.2E-09	4746.115	7071.199	5540.01	4767.094	3532.198	3434.818	6656.173	6179.344	7021.693	5137.74	3838.854	3327.482	5564.697	4783.413	3664.856	6717.703	8768.122	8698.04	5350.891	6018.305	6791.114	5157.305	5777.198	5380.838	4834.858	5799.539	5489.861
CUST_21262_PI429010886	N44908	0.011338951	18	9						8.439304	15.29443	7.971271	11.85566	10.95477	9.03053	9.805457	12.97087	14.47877	16.38187	12.52529	12.55344	10.00971	13.54422	12.11309	7.678598	NA	8.931679	12.74046	8.62661	7.195349	10.6362	NA	9.066919	11.08188	13.37894	10.72533
CUST_43102_PI429010886	M62070	0.011348692	12	9						19.73758	15.31063	23.65923	5.93112	15.87086	19.7414	5.325106	24.35366	9.806325	19.68859	22.35063	31.61624	15.22541	13.88764	23.07404	3.668475	12.93513	4.412786	NA	17.49602	11.66991	21.35401	23.13824	22.15192	8.806809	16.62453	NA
CUST_28324_PI429010886	N57725	0.011349477	15	0						129.0467	133.2629	153.2144	132.5501	105.3837	110.8559	112.585	90.23368	112.096	122.2838	93.74241	130.479	135.0731	121.7517	115.0832	164.188	130.0637	136.6675	114.3312	106.2034	117.5817	123.305	110.0735	116.7899	138.6868	122.1735	157.1984
CUST_40891_PI429010886	M56311	0.011369987	12	3						85.34679	116.8939	NA	114.3193	NA	118.5461	143.5433	59.06511	53.8169	50.55101	36.95845	11.76598	61.26619	87.71525	42.32912	203.1619	74.48675	78.94493	78.01928	17.83143	68.95176	NA	113.5034	6.671924	72.15056	44.18092	NA
CUST_12220_PI429010886	N29126	0.01137558	18	0						225.4359	375.5874	320.9643	246.3057	317.3631	255.3925	262.8953	268.2532	304.0274	231.7623	234.5732	203.5144	220.9505	257.5954	262.806	269.7799	260.3883	269.5843	325.7046	226.9173	322.6893	352.9709	299.5341	279.7784	222.8209	248.6699	223.4669
CUST_10878_PI429010886	N26933	0.011391948	18	3						9.963988	NA	NA	NA	48.88152	NA	8.550634	3.63284	110.6946	7.769994	4.146307	4.971185	8.645163	NA	3.955482	7.773057	7.292117	15.25605	18.67811	NA	14.1279	15.55385	51.82749	7.792211	8.440387	6.842059	9.840114
CUST_20180_PI429010886	N42963	0.011401956	18	9						35.20206	39.38283	42.48778	55.91798	41.96937	47.10689	62.84262	51.25301	32.0326	74.86678	45.59558	66.28735	49.62	47.35759	45.38207	34.48071	38.54386	36.80764	44.1442	44.40388	25.04742	78.5451	39.60134	47.99749	43.43013	47.90984	43.63653
CUST_53796_PI429010886	M103226	0.011417399	21	15	307190527	F-box/LRR-repeat protein 7 [Can			1.2E-12	17.15077	17.57039	12.34429	15.05878	20.92863	12.20542	8.539795	18.38558	13.80631	11.65578	13.28237	14.20021	14.83547	17.75202	8.486725	22.22171	22.07129	25.47879	20.53592	22.63957	17.43237	20.3194	19.14658	11.88722	16.81592	22.41889	17.18571
CUST_54330_PI429010886	M105618	0.011418992	12	3						30.98378	34.88562	21.82087	35.51059	25.06929	28.64933	25.60929	38.01559	31.43843	26.85225	21.55742	13.25323	18.14187	28.15267	17.1543	47.32106	49.57699	38.59195	12.4535	24.12361	16.19041	20.23015	17.95932	13.69996	6.364152	27.72385	11.33539
CUST_47272_PI429010886	M75421	0.011418992	12	3	321476689	hypothetical protein DAPPUDRA			2.8E-11	25.52339	32.8731	35.69311	42.96548	41.14942	56.63612	14.32273	37.77609	41.55628	21.02758	34.26775	32.32188	34.12419	29.61117	39.24909	37.86516	130.0993	62.3082	33.79023	20.63759	14.66204	25.83296	35.65778	36.61091	45.88844	36.05055	39.72867
CUST_6984_PI429010886	N20869	0.011418992	12	9						124.0944	116.6092	118.4901	137.1425	93.97841	115.1501	95.39909	123.6152	139.8319	195.1305	143.0944	142.126	115.6931	101.0339	119.8257	102.3977	74.28032	97.64083	96.26661	102.222	114.7492	150.3174	106.9803	144.6606	113.9393	61.74127	95.44831
CUST_16094_PI429010886	N35645	0.011418992	12	3						111.9225	220.8506	198.8809	134.0586	168.9955	158.4947	72.87552	134.6107	135.9219	63.21401	126.7539	71.3958	80.31138	205.0491	223.2399	122.6936	280.8283	140.6285	76.49084	146.8953	106.786	49.35536	85.40783	69.28931	60.03992	147.1038	130.828
CUST_990_PI429010886	N8142	0.011418992	12	9			Protein metabolism	Protein repair		554.5386	551.0918	630.5448	657.7333	516.0491	547.285	497.9545	556.8156	677.6753	663.5871	1013.387	643.6241	759.3274	552.0297	523.1285	685.9099	468.8585	561.8923	857.4356	566.3117	627.9112	944.508	1066.707	827.5626	824.4785	814.9423	895.1277
CUST_36814_PI429010886	M16933	0.011420903	24	9						22.56387	23.71956	15.21531	17.44104	94.83653	53.49041	32.58867	37.72807	36.23287	28.99688	72.56894	36.05055	3.073896	59.8383	51.24511	14.89626	26.14108	16.21246	8.329107	18.23869	15.71978	18.01233	34.44551	15.11126	25.28249	31.50058	13.33769
CUST_17377_PI429010886	N37865	0.011423722	24	0						49.5263	54.96474	76.09471	45.42698	64.25198	48.40188	44.79025	49.25994	52.48031	69.42506	41.98487	42.32505	31.00644	34.23772	18.97114	47.66434	38.63208	67.51408	27.99927	49.40203	28.70897	49.77832	38.88446	44.23244	47.25275	59.99813	47.15185
CUST_18150_PI429010886	N39247	0.011423722	24	0						169.5339	223.053	183.8046	127.4702	214.2039	165.8065	111.894	181.7506	140.8381	155.0651	178.0606	177.2128	122.1215	159.9694	149.8147	155.4242	209.1419	145.8855	137.8428	214.117	137.044	181.4589	221.1981	213.7108	186.3368	235.4574	236.2759
CUST_45356_PI429010886	M68835	0.011429034	15	0						107.8333	49.06918	43.41187	11.7354	37.55747	22.43722	15.93445	29.90427	17.00672	30.53244	5.209393	33.73518	6.222395	39.31755	36.59345	42.87131	50.26983	41.38957	23.13174	33.15745	41.69222	14.54311	50.53965	13.06456	46.72264	39.04694	51.64343
CUST_41253_PI429010886	M57249	0.011437322	24	12						25.63215	9.055283	15.64904	26.46648	51.70731	64.33318	8.595293	19.14803	13.65748	NA	31.50944	39.00565	NA	33.71363	42.47062	11.47222	5.506966	8.511016	NA	19.33678	7.071377	9.842297	3.752206	4.377759	3.283128	12.04424	5.809954
CUST_40966_PI429010886	M56505	0.011439224	15	6						158.9852	151.5412	178.447	223.5617	73.78491	199.0877	407.6903	185.2774	157.8028	159.2971	61.18015	127.2055	98.01972	58.87393	137.4877	116.7899	219.0011	157.519	299.3012	188.1296	97.85715	404.8745	192.5284	314.8641	232.2882	87.89749	346.2958
CUST_32120_PI429010886	M3279	0.011455708	18	15	410509307	Penaeus monodon MYH2 mRNA			2E-129	87.48976	31.6869	111.2006	80.53824	42.93819	71.42098	60.35593	68.19401	53.30891	76.54043	38.47393	42.96134	86.90266	125.2132	62.84262	147.3568	119.7443	121.6025	139.7386	88.34301	161.691	71.99448	33.13955	34.85212	115.7824	76.44155	55.38305
CUST_3855_PI429010886	N15415	0.011459207	21	9						33.61683	40.65182	31.25815	62.1017	38.94629	51.42014	44.81289	44.36561	42.91838	68.34584	44.40388	52.43098	45.20901	49.35984	22.85306	31.79774	42.92948	37.76079	32.87709	31.5432	33.73873	60.41841	42.05841	44.46588	44.36561	34.97429	33.5653
CUST_11999_PI429010886	N28755	0.011462975	24	18						601.5188	642.342	734.4362	605.287	603.9154	553.1321	608.2737	595.0044	554.8747	574.4065	564.6439	568.6219	589.1435	526.6092	680.2076	640.002	768.79	662.9752	680.8236	609.6218	715.6987	597.1413	707.1593	651.7291	699.1348	648.2686	944.508
CUST_38115_PI429010886	M23424	0.011468905	24	3						2992.811	155.9243	3179.907	4450.275	3654.032	4203.047	221.7872	581.1787	362.195	1763.47	3528.773	2551.022	1812.631	2234.498	3227.68	53.081	228.7481	184.3599	167.8421	550.4921	299.3012	2561.777	613.3285	1898.75	124.3244	969.8589	290.511
CUST_44757_PI429010886	M66893	0.011468905	24	3						242.1802	110.2733	260.752	452.8102	369.6716	391.1173	87.77262	642.342	86.60823	58.91866	149.8147	98.29871	138.1161	348.964	114.3521	63.74918	82.33668	42.19985	96.05666	151.8271	65.05732	230.4612	131.5529	100.9249	74.16527	77.6897	57.22713
CUST_1614_PI429010886	N10240	0.011468905	24	15	321479460	hypothetical protein DAPPUDRA				386.528	1139.817	808.8122	443.6171	469.5128	456.0887	706.4981	859.0835	957.0763	735.8335	659.7683	475.4976	649.4957	550.9285	554.6469	1026.487	1219.83	1143.279	726.2724	718.7132	883.4797	665.3418	717.9895	869.5599	754.2071	702.937	851.1928
CUST_5394_PI429010886	N18637	0.011468905	24	15						266.9539	561.3058	506.9058	307.4677	265.5849	266.6324	573.2384	549.5192	622.9027	523.3495	325.7046	358.3361	485.5854	301.4388	331.8463	573.6451	677.6753	640.2875	603.562	512.0349	665.3418	381.2824	371.5772	417.6111	584.6183	404.3968	535.3308
CUST_6049_PI429010886	N19561	0.011468905	24	15						1776.485	2308.81	1858.682	1880.339	1523.69	1515.59	2159.534	2227.296	2126.752	2271.439	1473.47	1827.788	2222.683	1583.518	1763.47	2777.197	2865.238	2659.616	2509.266	1790.499	2363.552	2113.55	1880.339	2499.817	2558.16	1798.348	2469.347
CUST_8115_PI429010886	N22554	0.011468905	24	15						119.3727	173.05	133.8226	93.75176	115.0343	110.145	128.1046	135.5555	143.8296	167.7764	132.6606	144.9984	127.9862	109.4424	134.7253	157.9135	176.1473	152.4151	137.0267	137.5628	173.2871	133.5304	144.5615	180.3948	140.7771	131.7124	165.4096
CUST_12684_PI429010886	N29868	0.011468905	24	3	411125289	alkaline phosphatase, partial [M	Metabolic process	Other	9.96E-30	93.14583	100.3265	157.847	129.1825	365.5842	289.7381	57.47217	94.30839	101.0477	78.63104	231.289	227.4568	105.2562	211.4477	271.3703	43.49402	89.04608	55.40345	60.20446	82.33668	106.696	143.0944	91.24191	136.3718	86.1454	100.264	150.4929
CUST_16473_PI429010886	N36295	0.011468905	24	15						94.63322	131.8747	83.06309	60.20069	50.30283	70.92326	129.1955	122.0363	111.8609	139.755	64.75498	89.98471	108.374	79.62962	106.6009	135.0024	121.5461	156.768	151.2383	98.68778	129.6511	93.35268	109.2676	103.9393	137.2208	91.59236	124.2958
CUST_19026_PI429010886	N40855	0.011468905	24	15						1102.526	1629.282	1020.042	863.8645	782.9344	753.9335	1344.214	1155.365	1331.886	1107.545	781.7261	748.709	1119.38	863.5664	948.7571	1471.771	1641.783	1708.051	1139.817	1109.766	1302.487	977.9831	1109.283	1128.705	1208.723	1029.68	1375.234
CUST_17840_PI429010886	N38694	0.011475596	21	3						204.3287	127.1843	150.9904	207.209	183.4518	229.4919	263.8276	153.47	145.249	136.8093	176.9877	153.595	152.8947	155.8444	301.6158	162.1178	94.25033	155.8635	151.3342	79.68621	119.3879	207.6942	118.606	161.3598	433.6142	155.3531	391.6595
CUST_33338_PI429010886	M6644	0.011476065	15	0						2064.359	2706.184	NA	2068.831	NA	1824.284	4522.071	1632.022	2283.014	855.5436	608.8174	3.546828	1151.33	2001.707	579.9447	7147.632	2795.798	2684.097	3093.621	240.1565	2681.804	NA	2114.8	4.412405	1965.787	1632.718	NA
CUST_6752_PI429010886	N20556	0.011497383	24	3	443707651	hypothetical protein CAPTEDRAF			3.17E-98	497.2634	264.176	428.0259	442.023	444.046	407.5835	258.8836	325.5716	250.2647	381.9172	668.1223	433.0002	353.1568	330.1216	405.5854	207.7685	219.6103	222.0168	188.0574	281.3641	244.0894	342.4681	353.5795	303.7619	206.4541	339.4771	227.4234
CUST_25971_PI429010886	N53411	0.011505165	24	0						30.21523	38.5634	35.30558	41.20512	33.15311	29.67957	23.67342	26.46408	25.9357	14.17513	21.96708	23.76937	25.31216	19.23397	30.8343	36.12041	30.56946	34.63434	20.79352	29.02458	37.86516	18.65471	43.2948	27.22807	27.16746	36.17758	44.23722
CUST_23120_PI429010886	N48289	0.011511909	21	9						8.847627	6.931329	3.790918	13.82768	13.83564	8.825422	NA	14.34832	10.31745	24.55123	NA	8.568381	10.06209	14.67062	10.12975	8.28383	11.16576	16.56978	14.22653	11.59098	9.17267	7.991764	6.378654	7.896654	4.915537	18.10243	14.09206
CUST_4165_PI429010886	N16145	0.011521188	15	12						19.35718	12.44156	13.33253	6.059208	7.653718	8.263401	6.266017	15.78875	13.16499	17.35867	NA	11.36156	23.75379	8.97599	NA	6.088117	14.28701	19.01388	5.489948	11.35215	4.197074	9.467901	7.695174	5.554504	13.4622	8.48334	8.85643
CUST_29876_PI429010886	N60735	0.011525259	18	15						260.752	249.3839	228.253	243.8189	195.3229	199.5249	230.3457	200.3043	230.5806	280.9343	192.8085	247.5439	254.714	192.3671	175.4908	258.5192	279.2412	282.551	276.6101	210.3744	284.3354	239.051	146.449	232.4149	208.3716	177.6012	186.2473
CUST_7352_PI429010886	N21419	0.011532456	21	0						207.3346	274.6135	411.8854	133.684	328.4824	91.21622	142.5367	139.1812	134.3827	97.03233	321.6405	153.8823	151.7315	34.40062	137.1269	18.38358	171.1773	83.09656	140.0958	56.96365	133.9377	196.526	374.6189	327.283	204.7184	99.10109	233.6384
CUST_53720_PI429010886	M102914	0.011538556	15	0	157813616	putative myosin VIIa [Nebalia he			1.74E-35	14.1297	14.60762	8.295728	7.784678	7.224793	7.428342	9.86298	3.762799	7.308662	13.2682	8.607441	10.02506	4.767082	8.192393	12.77084	18.5449	15.5193	9.905888	15.2887	4.209816	12.28032	5.439271	NA	6.993373	12.44897	5.970314	9.498033
CUST_41709_PI429010886	M58360	0.011547295	12	9						15.7393	52.95129	54.1005	36.68361	16.2952	20.33173	20.53404	34.73082	32.47615	84.76892	66.15369	61.95078	64.27657	19.31757	47.31029	18.85869	38.33655	36.76966	25.13144	21.08718	26.38902	61.88614	67.97224	86.01043	10.08403	16.15024	31.6869
CUST_34935_PI429010886	M11116	0.011565364	24	6	189236353	PREDICTED: similar to signal rec	Transport	Protein transport	1.19E-30	30.35985	29.93607	20.4836	17.52792	27.36342	33.51137	33.55937	36.5903	29.45413	32.13644	30.37143	23.09515	36.26885	32.11277	23.84965	41.04596	24.51215	33.19205	26.73304	21.31272	27.32756	18.39792	18.89832	20.58397	25.1	24.3283	23.73505
CUST_20685_PI429010886	N43876	0.011568326	24	0						16.07187	19.13355	24.16121	14.0171	12.72781	12.84199	6.828268	10.96784	6.890806	16.46484	14.39646	14.49503	14.64332	12.78825	12.12132	16.88502	12.91017	13.81948	14.65727	11.80858	16.38187	20.96269	14.24832	14.38342	26.03303	17.09822	42.21839
CUST_49198_PI429010886	M84648	0.011576025	24	15						4.542234	5.059873	NA	NA	6.598734	5.005302	NA	7.638129	6.13309	6.989686	NA	10.24225	3.199826	NA	10.4654	14.485	11.34354	12.42147	11.6258	NA	8.445011	14.98689	7.08329	14.90324	5.708684	8.092996	7.117232
CUST_55766_PI429010886	M111634	0.011577773	24	15						22.75502	13.93501	22.38744	16.82471	16.7995	19.16983	12.48451	13.34035	14.30044	18.52564	9.288536	NA	19.88951	39.12428	16.89763	38.13279	22.58496	21.39039	26.4046	17.00074	33.30353	18.2576	16.09247	27.00266	28.99912	14.87474	26.98744

Probe ID	ID	p-value	Period	Phase	EBI ID	Annotation	GO category	Go term	E-value	0	0	0	3	3	3	6	6	6	9	9	9	12	12	12	15	15	15	18	18	18	21	21	21	24	24	24
CUST_54738_PI429010886	M107402	0.011589741	15	12						68.50963	60.48555	80.33191	59.797	80.11297	78.72715	52.67255	43.59878	45.93656	167.2311	90.51162	102.7615	194.0105	62.22572	191.6851	105.3328	54.09509	103.2365	77.94963	71.01745	69.45852	77.45378	56.82231	74.31745	106.1445	55.45245	85.40783
CUST_44788_PI429010886	M66985	0.011589741	15	12	52630412	guanylate cyclase PcGC-M2 prec	Protein metabolism	Cellular protein modification process	1.02E-55	27.9152	26.11298	38.14116	26.20838	43.86283	42.4928	29.3303	23.63327	30.11046	47.46968	33.93799	40.04223	42.2265	46.9602	49.80184	60.38394	38.53227	32.3516	37.14951	17.66839	34.37978	22.03729	20.49098	35.48221	54.25064	28.61425	39.5247
CUST_34738_PI429010886	M10514	0.011591712	15	3						200.863	128.3075	324.1912	299.2328	178.9536	257.956	75.46497	292.0008	157.0815	106.1034	86.29714	75.38714	49.55479	172.8989	86.69353	68.42492	239.7136	126.3851	198.2813	253.9896	262.1593	280.9343	121.6645	51.56297	215.3674	116.0037	78.29555
CUST_54345_PI429010886	M105653	0.011599234	24	15						6551.409	11138.2	7225.192	6276.978	4531.71	4650.166	10142.18	9699.55	8857.18	7704.294	5587.034	6255.734	6799.146	5847.84	6333.445	11911.49	11763.32	12860.58	8484.924	8591.806	9550.071	6051.213	7993.785	7872.926	8713.974	8334.379	9759.347
CUST_37838_PI429010886	M21822	0.011599234	24	3						13716.26	312.5578	12141.91	15767.9	14411.75	19237.66	45.0273	923.1653	43.37269	8644.801	10815.88	15798.02	3091.943	12631.63	14703.33	5.348053	8.165142	87.34939	38.99638	955.5815	50.0875	10248.36	376.0943	8658.641	16.25968	1716.333	86.50032
CUST_30859_PI429010886	M25	0.011599234	24	15	260833030	hypothetical protein BRAFLDRAI	Metabolic process	Cellular amino acid metabolic process		222.9863	609.8498	343.7299	197.6371	202.9315	280.8283	235.6105	412.9894	420.3226	584.1152	339.9532	476.7459	162.9468	444.3539	424.9756	584.4771	721.6825	907.8057	392.9019	333.9851	284.9823	361.5772	592.8013	441.6606	462.0354	144.2677	451.9245
CUST_17557_PI429010886	N38179	0.011599234	24	3	195452816	GK14160 [Drosophila willistoni]			0.000000587	628.502	35.02468	644.9039	955.1294	2517.49	1888.631	20.45768	101.0612	25.7879	152.9126	1808.449	1345.742	78.87059	1780.678	1553.379	13.35744	16.02196	23.30899	29.44247	95.73042	29.22669	198.7466	48.23305	143.0807	16.61697	123.7768	15.53301
CUST_25157_PI429010886	N51885	0.011599234	24	15						69.90555	104.3299	82.55332	67.48124	73.81857	75.09386	80.47316	91.72224	97.48939	87.25531	79.03209	83.97595	76.49084	76.5633	81.21161	102.2878	125.9747	131.6939	82.68763	80.46117	89.98471	71.89101	112.963	106.5406	77.12608	79.08754	96.65486
CUST_1433_PI429010886	N9612	0.011599234	24	3	519662799	hypothetical protein [Geobacillu			0.000000222	12097.54	351.0025	9301.136	16411.2	21524.84	28025.82	45.62418	1494.458	74.30536	6155.993	10764.25	15045.33	6922.365	12496.19	18117.06	20.87837	18.18231	87.71525	39.95843	691.6444	86.94104	5311.077	683.9311	5467.822	32.88066	685.653	209.7702
CUST_46089_PI429010886	M71335	0.01160608	21	15	238683705	NADH dehydrogenase [Palaemo	Transport	Ion transport	4.29E-17	1159.214	2039.749	1307.951	1221.709	1108.83	1026.487	1721.8	1469.262	1458.832	1296.827	892.7134	1044.931	1199.116	1303.86	1124.497	1802.44	2291.48	2292.947	1480.103	1755.972	1916.926	1146.245	1724.425	1423.667	1424.355	1818.148	1513.287
CUST_42718_PI429010886	M61052	0.011619361	24	12						47.77307	145.4834	20.95138	169.2197	18.64133	160.9576	242.133	67.96481	200.7515	175.151	171.912	16.21246	210.5427	141.0774	257.1241	462.0354	102.0887	386.3361	40.2252	36.99679	29.5773	33.21716	30.11519	37.71641	47.68605	29.6152	19.05575
CUST_392_PI429010886	N4801	0.011631533	18	6	321466528	hypothetical protein DAPPUDRA			2.58E-19	2759.556	2906.899	4787.419	5571.385	1711.883	1154.406	3549.354	5884.771	7714.571	3166.856	6000.152	1895.037	6631.875	2475.896	2002.791	594.0785	844.4833	769.305	6945.783	7032.355	10921.28	4767.094	10727.23	8605.765	7568.31	8439.929	12569.14
CUST_47456_PI429010886	M76206	0.011632647	12	3						48.56519	71.88037	64.99079	149.0265	53.66051	76.11372	79.07706	35.96302	52.27137	45.2017	43.41187	30.26428	97.60846	35.05094	61.37944	43.45889	140.1401	85.54538	26.39253	26.22317	10.25382	38.26324	40.66863	26.12743	44.56728	84.19786	68.50135
CUST_32685_PI429010886	M4810	0.011651356	15	6	149675211	cuticle proprotein proCP5.2 [Cal			5.32E-71	6758.872	3261.337	9500.977	16348.66	9067.463	5693.503	8061.991	12631.63	18435.82	7993.785	13248.98	5380.838	24984.55	10464.54	11528.01	772.0259	2811.043	881.8202	20616.2	16913.15	22332.92	14670.24	29396.88	30721.41	20320.15	29843.45	35305.99
CUST_43934_PI429010886	M64378	0.011666149	12	9						42.28696	44.66993	52.92294	45.34342	51.56297	39.51778	33.42156	49.21384	27.79864	50.39653	46.44259	52.67255	39.84739	36.27201	37.22647	17.9949	35.04281	34.88249	35.33106	42.91273	63.05696	62.02003	64.54113	60.37856	48.66253	31.40901	36.45493
CUST_56425_PI429010886	M113765	0.011667419	24	21						87.29938	83.26396	73.47778	84.51406	69.5671	65.47772	71.58388	58.55963	79.73452	88.05908	69.50462	71.54027	80.66827	56.4057	76.45795	78.38177	79.10899	78.44375	85.98865	79.36702	47.15185	88.78315	91.36194	109.3497	78.40447	71.88037	86.70327
CUST_38974_PI429010886	M32870	0.011671348	24	9	152013699	putative cuticle protein [Artemia			1.06E-17	28.03213	16.61523	13.30458	36.99237	43.75295	25.16449	17.98498	19.27271	13.1553	21.82274	51.52222	67.65721	14.7532	91.7043	59.19847	26.0733	24.7525	21.01433	20.08859	17.68077	14.36936	20.02415	11.85089	13.07815	11.87453	24.28512	19.46213
CUST_37105_PI429010886	M17858	0.011672026	24	18						116.1322	249.1319	203.919	278.1319	189.0201	100.038	194.4561	286.1136	353.6905	241.8418	247.4668	189.0814	369.5842	207.8484	253.9457	249.9933	270.6292	281.0852	376.2092	316.5315	423.3824	312.008	407.5835	508.7574	304.4188	265.541	541.2118
CUST_30928_PI429010886	M238	0.011674938	24	15	72049918	PREDICTED: condensin-2 comple			2.72E-31	63.92344	104.3593	75.63986	59.5223	74.12935	65.71584	70.78121	84.39985	74.82596	88.37671	67.64673	62.77181	81.32217	79.82778	62.11363	103.1345	91.75455	116.0897	93.40586	74.78378	107.0876	73.17975	78.33954	69.59216	61.72867	68.03257	98.7449
CUST_46884_PI429010886	M74064	0.011674938	21	0						29.5453	40.6742	46.18377	16.46966	17.6345	12.47813	34.95791	26.11029	26.01383	27.11635	21.96464	11.56292	15.76131	20.4296	19.34867	23.44192	36.78126	39.70565	26.70669	21.73011	31.2986	25.53705	44.25991	41.38164	22.67515	26.87222	30.69787
CUST_245_PI429010886	N2986	0.011674938	18	15						10.61538	26.91338	15.9111	4.650562	3.7268	10.69888	24.72767	4.690007	13.68243	36.35313	6.716683	27.30294	11.63269	15.86113	16.26288	22.13004	27.80576	18.1573	27.96875	8.801336	16.42131	9.63644	14.49774	9.688124	9.416468	8.922458	17.73313
CUST_4213_PI429010886	N16247	0.011679399	18	15	345482651	PREDICTED: LOW QUALITY PROT			1.97E-09	35.0318	30.10754	22.62211	27.85041	20.84244	37.16734	25.85738	25.62254	19.16781	44.25351	28.4048	45.20901	37.65544	33.49837	38.15236	47.45891	45.44328	42.70334	34.32863	22.2879	25.41794	27.11917	25.62847	31.8265	32.23946	29.30404	20.57977
CUST_5161_PI429010886	N18339	0.011693643	24	15	156544470	PREDICTED: hsp90 co-chaperone			7.19E-106	285.6824	433.8311	352.0102	249.3411	243.2848	262.04	244.1747	286.5115	278.8807	286.5115	339.8451	224.7103	299.1753	316.8051	252.5113	305.5795	452.142	315.18	288.7764	331.7854	330.0358	292.4122	372.1298	288.1795	289.1171	308.4293	301.4906
CUST_13473_PI429010886	N31248	0.011695376	15	3						35.98686	63.19478	58.78776	46.48232	139.1998	99.19887	88.5599	65.44741	75.41256	34.11681	28.93482	46.21776	47.09024	33.77888	60.02726	38.11477	45.42128	44.70348	52.21427	60.15379	80.05281	42.37353	64.86228	79.63863	50.81159	25.9357	44.86788
CUST_44748_PI429010886	M66867	0.011700291	24	9	443735013	hypothetical protein CAPTEDRAI	Metabolic process	Cellular amino acid metabolic process	1.81E-57	12.20542	18.64038	8.364605	26.84779	NA	24.79569	10.96116	26.52431	13.73539	7.843567	95.26274	48.80246	41.19213	27.2558	20.34912	10.93759	25.51207	20.04588	7.64439	18.11022	NA	14.55841	19.8869	17.25129	7.26329	16.56168	9.052014
CUST_23008_PI429010886	N48102	0.011700377	21	18						18.13167	17.6322	15.58609	NA	NA	NA	6.777795	11.90584	3.673243	17.53224	6.145604	19.36587	13.66542	NA	12.67448	11.63109	25.19098	14.45866	19.57119	16.69734	18.63267	10.36406	15.51127	20.17748	4.794421	13.0547	NA
CUST_2303_PI429010886	N12061	0.011715528	12	6	241997760	meprin A metalloprotease, puta	Protein metabolism	Proteolysis	9.04E-57	10.97416	11.11796	6.150357	16.92091	NA	26.74265	146.5694	9.762817	124.4273	27.74551	20.58643	8.203427	9.852481	14.86874	4.483644	34.87919	17.89355	48.61521	187.8778	386.1445	675.0754	57.16525	18.63267	57.9032	112.6387	24.24843	88.96871
CUST_17053_PI429010886	N37287	0.011717022	24	0						102.3686	96.71212	97.01999	43.42575	56.76432	67.56678	73.70166	90.48608	61.95078	93.42816	56.08051	107.7085	70.26755	72.10171	55.23799	86.28411	63.77866	72.62444	92.54225	69.69671	87.46732	90.74217	100.5778	106.2659	107.2833	74.62293	99.84349
CUST_34598_PI429010886	M10120	0.011718354	24	12						47.30405	112.7999	52.35235	51.25725	88.26602	95.75319	75.0839	91.11591	68.59824	67.88505	77.48265	78.87059	64.27006	92.41119	79.06068	72.86661	113.9079	77.16447	62.05756	73.89284	70.8379	38.52068	56.75304	33.1607	59.17675	61.84336	52.21052
CUST_52156_PI429010886	M96582	0.011718354	24	9						54.48188	47.70295	54.83706	55.06272	112.2997	141.8433	81.80549	58.53483	44.05427	84.09371	82.7985	65.91522	84.03656	54.74265	53.13532	63.06627	56.52874	47.29636	62.4694	41.76233	32.7919	57.54767	40.16574	45.26784	71.48244	53.74306	83.61292
CUST_37855_PI429010886	M21884	0.011721652	24	21						3014.898	2277.337	1944.792	2960.558	645.8971	1908.561	1874.483	1653.487	1827.788	2805.21	1587.121	1663.655	2983.608	2237.142	2189.919	2814.474	1453.134	2566.656	2747.205	2132.096	3349.165	2352.61	4825.163	3724.515	1263.923	1962.886	2757.04
CUST_39882_PI429010886	M46134	0.011721652	24	18						439.635	797.7145	602.4086	537.7378	415.2055	325.4289	525.5146	690.9865	660.6448	418.6884	510.7551	580.117	522.4996	496.692	497.6488	592.5764	716.8261	617.7634	809.8174	683.6777	788.3617	627.9112	539.3693	695.1268	658.5111	526.1263	784.4035
CUST_45910_PI429010886	M70726	0.011721652	24	6	71159516	C-type lectin [Penaeus monodor			2.2E-14	40.07856	29.16979	48.32075	37.95648	32.77639	33.09951	49.04743	40.25187	41.73123	55.40345	8.24518	35.56153	56.73963	27.80576	26.00178	42.82494	36.63038	31.66129	18.42193	23.74852	28.63477	24.53204	15.9251	20.36712	19.20499	16.90428	17.91593
CUST_36753_PI429010886	M16754	0.011729562	24	15	91089713	PREDICTED: similar to ubiquitin	Protein metabolism	Proteolysis	2.22E-68	584.9829	1034.116	680.8236	631.4625	584.6183	588.3344	785.5802	928.898	982.3643	818.0478	679.5306	653.8973	715.6987	591.4732	707.3888	1096.072	997.9064	1137.334	850.2576	740.8082	952.9844	910.9934	756.356	840.6574	955.1294	693.3608	988.0134
CUST_39955_PI429010886	M47421	0.011729562	24	3						17931.09	14.63182	18320.9	22037.75	25792.96	28943.19	10.23726	218.5686	13.14847	3227.68	12725.99	13532.16	2181.465	21922.49	22847.77	11.16909	4.939673	50.52783	14.55686	375.5874	23.69946	4863.888	570.8807	4616.026	23.43505	691.181	94.00187
CUST_49842_PI429010886	M87391	0.011729562	24	15	528746119	C-type lectin 4 [Fenneropenaeus			3.99E-08	44.63889	98.17006	98.63138	41.08386	13.14847	27.49895	110.519	78.26455	97.50175	103.6054	31.39708	78.29555	82.34733	31.25385	85.99964	201.8882	156.493	223.3609	100.8605	50.63563	87.06038	95.01092	25.77932	109.1979	120.2974	49.73252	103.914
CUST_7008_PI429010886	N20908	0.011729562	24	3						1989.721	37.2113	1547.502	1816.059	1606.909	1989.721	115.2993	98.38918	127.7013	497.6488	1195.903	2393.197	455.0731	1416.762	1621.358	33.95966	24.04754	43.91443	163.563	205.2018	147.2543	579.9447	257.3491	551.0918	133.2817	244.0506	229.9746
CUST_8453_PI429010886	N23060	0.011729562	24	15	383863595	PREDICTED: uncharacterized prc			3.62E-23	107.8435	227.0055	183.5082	128.5506	149.3381	126.8388	186.9529	202.9059	182.2908	148.8622	190.3933	163.1533	167.3834	163.8916	186.9009	204.2476	314.4452	287.3062	177.388	176.5278	256.2294	145.1992	171.0499	219.4299	183.9367	166.7408	221.9709
CUST_13715_PI429010886	N31644	0.011738313	24	9	345496695	PREDICTED: hypothetical protein	Developmental process	Multicellular organismal development	2.7E-33	31.38536	19.44853	24.13218	33.75905	121.0707	59.02352	30.15628	30.68002	21.1337	34.16048	45.41788	73.90571	29.11467	52.51146	58.78776	21.28132	25.0763	20.65147	25.19098	24.34766	21.66219	27.57162	15.9018	25.87711	17.00074	27.60039	33.26038
CUST_9338_PI429010886	N24461	0.011740791	15	9						25.58795	NA	9.704976	11.64898	4.861279	3.746474	25.64786	11.51199	NA	21.7987	NA	12.74848	18.68212	6.537448	14.36577	26.57459	8.202317	5.854267	20.39494	4.174325	8.43105	16.75127	11.57188	7.410322	36.8445	27.79642	31.70122
CUST_23526_PI429010886	N48980	0.011754056	18	9						37.96647	19.34106	16.63618	23.51724	25.67433	29.55431	42.68213	37.12565	35.26837	82.7985	56.20706	70.54441	41.46154	31.69795	31.89668	61.67659	52.38135	58.66126	29.5453	30.91821	43.62652	61.62446	39.51778	58.57991	48.8586	33.10297	45.95593
CUST_24808_PI429010886	N51279	0.011754056	18	9						14.00894	5.085926	4.977375	8.378958	22.95506	11.16576	3.723073	6.371533	8.90453	17.26262	14.6059	15.929	12.00855	18.69191	8.631384	7.875391	7.619983	9.840114	8.286877	5.438256	6.724289	8.036199	9.771765	8.394142	12.09079	11.00341	12.87704
CUST_39369_PI429010886	M38460	0.011754168	18	0	321472284	hypothetical protein DAPPUDRA	Response to stress	DNA repair	1.25E-46	20.93117	87.46732	50.79258	44.511	68.0002	32.68105	32.70763	43.63653	26.83656	32.85632	39.6267	27.57725	44.24302	45.33378	43.36291	36.40347	76.96435	53.09289	56.59797	62.02618	69.33519	42.43263	40.30169	32.0326	34.52717	41.0185	33.72382
CUST_31831_PI429010886	M2517	0.011754876	24	18	321477497	hypothetical protein DAPPUDRA	Nucleic acid metabolism	RNA metabolic process	2.08E-89	74.33815	155.0803	137.0045	89.54662	75.25623	82.1985	89.0262	133.1475	99.51051	124.8855	103.9866	99.93518	116.8467	99.16406	98.15697	104.6784	170.8635	151.2161	129.5648	130.6255	110.3861	125.2728	109.1979	115.7057	105.9997	97.51431	127.1629
CUST_44434_PI429010886	M65978	0.011754876	24	9						335.6891	214.5554	195.6436	221.2277	460.5885	411.3393	274.9461	260.1908	234.5732	310.5741	282.9651	431.8791	231.8895	273.802	354.1831	287.7668	199.421	216.7613	238.6215	264.6763	173.6995	232.3662	197.2216	235.0418	202.6784	212.1398	215.2938
CUST_28154_PI429010886	N57386	0.01177928	18	9						74.73696	18.35318	51.15383	8.250802	NA	11.9307	42.50386	33.64233	36.48523	248.6058	172.6851	NA	23.05545	138.2203	11.38277	87.89749	108.6739	56.09749	69.85306	101.1701	130.3816	25.87214	NA	23.43251	61.01777	9.195054	25.95165
CUST_51592_PI429010886	M94152	0.011782362	24	15						NA	11.7387	9.567571	5.167289	15.00826	10.54122	11.64898	14.44887	14.70588	7.794152	7.653718	11.98667	10.27771	17.90158	15.05439	24.5939	18.7792	17.95536	15.56376	18.68212	16.28426	11.02719	13.65957	6.176204	17.21886	16.43671	11.46624
CUST_52201_PI429010886	M96716	0.011784823	24	18						5.303114	10.58533	16.32616	4.08496	8.859308	5.449648	6.810212	7.843567	8.771483	11.08375	10.84292	NA	12.59391	6.552316	11.56593	20.45505	5.128731	14.54604	38.07838	12.26023	11.10715	18.75264	7.627631	18.59394	16.2936	6.864225	14.485
CUST_48873_PI429010886	M82629	0.011802672	15	9						20.19825	4.62478	16.0427	14.8883	13.97125	9.544263	18.28716	9.24062	4.671352	26.61562	15.36685	18.48018	18.23869	14.39043	13.95366	11.40479	11.81562	14.5147	15.14518	9.536214	11.97474	20.45505	15.95845	23.42467	26.0713	20.33173	22.29626
CUST_46757_PI429010886	M73623	0.011803902	12	9						77.05387	33.47807	67.68103	86.96518	38.34001	49.54311	59.57009	51.68111	82.74445	54.00791	113.3845	64.6674	108.4526	70.85676	89.16012	38.95176	50.88233	36.76462	67.92426	59.62083	89.22264	120.6396	134.9139	126.7539	62.38008	34.36428	72.46811
CUST_11919_PI429010886	N28628	0.011803902	12	9	226374370	peroxinectin [Fenneropenaeus c	Response to stress	Response to oxidative stress	4.73E-23	186.792	104.437	204.0335	168.6428	67.64673	112.3135	120.9	134.1618	132.2346	242.1802	150.7445	170.9358	212.3717	122.1097	163.8102	135.7682	126.0899	108.5581	127.3332	126.9826	180.0489	352.0796	174.4633	321.3471	96.81529	120.1223	180.1675
CUST_22523_PI429010886	N47184	0.011803902	12	9						39.71151	46.54074	40.49223	24.93525	23.40563	32.77297	21.00978	55.70902	36.49381	46.38013	53.20844	48.33941	42.88477	29.00837	37.73322	32.40475	46.08569	46.98969	45.24721	32.01836	19.24274	54.33188	56.10448	51.3632	40.81486	38.50846	47.07893
CUST_8328_PI429010886	N22878	0.011813404	21	15	66499523	PREDICTED: peroxisomal memb	Transport	Mitochondrial transport	5.73E-85	33.3276	30.74499	46.80566	37.60099	26.52431	31.02823	27.98737	14.31215	20.18225	22.38961	25.10747	33.50951	38.89512	31.62985	32.49093	37.89317	36.85694	40.80027	38.06969	31.52857	35.18384	27.9284	35.14363	54.22723	37.39816	25.34819	42.96548
CUST_5406_PI429010886	N18659	0.011814982	21	18	240978029	tetratricopeptide repeat protein	Metabolic process	Other	7.92E-50	29.29782	105.0092	66.83189	41.13217	75.76283	55.99408	45.3219	62.23304	40.53493	39.44669	51.82749	41.16872	41.01272	63.13263	61.78111	59.66359	82.19156	74.82596	57.64367	89.99586	84.57655	47.20876	70.65416	58.88834	56.78277	45.94658	62.98884
CUST_39717_PI429010886	M43395	0.011815444	12	6	321479210	hypothetical protein DAPPUDRA			4.41E-08	NA	NA	7.499635	137.6386	NA	116.9614	1396.045	NA	1082.092	178.0114	138.2203	NA	52.21427	129.9202	45.41169	275.7847	4.067669	259.3611	1853.898	2672.714	5533.093	396.5838	NA	334.6481	601.5188	146.6816	393.358
CUST_10611_PI429010886	N26505	0.011821466	12	0						76.76723	76.19975	94.48817	88.93364	70.04249	68.16905	68.12566	43.38217	49.98481	91.41914	72.14124	55.17016	102.6488	65.37786	63.08459	74.90044	73.7088	72.53637	66.69697	73.62565	55.93542	78.8865	76.89496	71.75645	82.16147	82.55332	82.70695
CUST_13932_PI429010886	N32034	0.011824786	21	0						40.02841	68.27965	22.69087	26.47878	15.04687	12.94826	8.141288	17.74254	26.15603	36.71405	9.032116	24.72767	11.00269	15.5722	7.322474	21.13144	15.78015	41.38631	32.75774	38.35367	24.29996	58.84073	21.88581	38.78289	29.13251	18.68973	7.442251
CUST_24326_PI429010886	N50422	0.011824876	24	3						630.9023	11.77271	687.2962	372.3645	490.2242	462.8312	12.88584	38.51205	14.03646	63.85058	376.5416	167.6498	46.02907	356.9618	372.3645	6.051391	11.33713	4.28871	16.20707	38.91666	23.91323	47.06434	38.35367	26.61853	14.25102	44.21087	NA
CUST_16714_PI429010886	N36735	0.011829254	24	0						17.91593	25.89322	22.39651	27.08947	13.44127	18.33066	20.81357	9.161427	19.46213	22.2434	19.42183	12.57356	16.46161	17.10849	16.69306	15.36018	15.2486	13.91642	17.42256	12.49082	21.2594	19.21592	21.26236	23.23151	58.6831	17.61898	22.53368
CUST_36071_PI429010886	M14527	0.01183345	21	12						38.47726	41.33391	42.49657	37.48234	37.79767	44.1584	37.08608	33.37054	36.60063	37.41529	34.68278	48.65167	55.75291	49.60673	47.29636	62.52685	38.34001	49.0091	47.87148	30.63415	48.00737	42.2265	42.6374	46.13913	43.21882	34.64088	49.87572
CUST_25076_PI429010886	N51746	0.011839006	24	0						48.4073	45.00629	49.12192	25.85738	55.24242	47.61903	45.17249	44.22724	46.81135	27.20014	29.21241	32.46562	21.33527	45.47301	31.36138	24.58018	26.58261	22.79876	45.51721	50.28629	70.88457	29.65224	60.39565	43.37645	47.24767	52.6416	41.89699
CUST_35031_PI429010886	M11389	0.011842122	24	3	221056120	hypothetical protein, conserved			0.00000041	133.2218	36.75099	105.0475	139.1998	126.5797	145.9662	51.74535	47.80328	50.46073	107.8619	122.4018	50.38885	161.586	84.16808	112.0451	42.83599	39.44924	19.95277	35.15002	43.45515	30.29561	184.1725	117.4793	110.3983	46.32725	71.44611	48.48353
CUST_39123_PI429010886	M34915	0.011854977	24	3						2681.804	37.7689	2904.283	3267.789	3629.795	6025.479	23.60614	371.2053	27.10462	230.3006	3660.946	3033.527	175.317	2988.845	3513.064	NA	8.62544	9.027928	NA	463.9651	19.49861	602.8578	81.08936	537.1828	NA	992.6472	6.565637
CUST_10282_PI429010886	N25998	0.011857534	24	12						7.824872	8.350162	11.04999	11.84866	12.99802	14.34832	13.43624	16.9284	5.866363	16.97814	12.66176	13.413	14.57595	15.73759	17.33888	9.179179	8.452888	10.11558	11.82895	11.93827	22.40231	13.40443	12.70063	12.46469	11.13644	10.98177	6.277745
CUST_38718_PI429010886	M29563	0.011859891	24	15						2066.858	1867.24	2179.188	1348.794	1380.586	1111.297	3295.67	2217.875	2867.707	2924.097	1566.672	2835.943	2050.749	1599.332	1873.347	4234.05	3593.994	2809.488	3140.215	1545.477	2028.877	2777.197	1745.528	2221.308	2898.397	1491.134	2104.842
CUST_45378_PI429010886	M68912	0.011859891	24	15						29.86767	38.89189	43.94763	21.59198	22.78614	19.66181	36.37036	27.3523	36.34385	29.46925	25.42047	33.06699	28.05953	29.89832	37.28898	49.1314	45.70271	56.06983	35.07134	19.55672	32.38002	42.29117	26.46988	34.15266	42.29849	23.28963	41.7563
CUST_4908_PI429010886	N17760	0.011859891	24	3						295.6886	468.6225	807.865	597.1413	1509.047	1346.991	292.7159	397.0876	562.7989	249.6908	1109.283	921.74	402.9178	1042.247	801.5781	139.5903	126.4468	174.9627	616.7748	619.2009	428.2506	455.8434	488.2191	557.6299	466.2067	722.545	586.1434
CUST_8047_PI429010886	N22466	0.011859891	24	15						1903.836	2865.238	2028.877	2078.188	1645.987	1600.243	2935.737	2216.187	2494.519	2529.949	1634.309	1782.659	2965.89	1848.594	1931.709	2988.845	3050.493	3050.493	2242.949	1925.502	2414.182	1802.44	2356.919	2626.057	2184.345	2085.588	2744.675
CUST_8188_PI429010886	N22672	0.011859891	24	15						95.21224	193.1329	124.3072	117.4337	99.85694	81.32217	160.389	144.9581	132.1106	153.2144	115.6711	131.9455	132.0703	120.8427	122.6496	181.5475	239.1584	214.5554	126.6958	121.8066	159.4235	134.6492	117.6349	141.4438	149.9518	108.2084	149.3578
CUST_5422_PI429010886	N18680	0.011860203	24	15						359.7575	367.1233	355.7553	351.2785	375.6666	340.5595	250.7612	294.0126	294.4308	276.55	402.8177	330.6996	430.3398	364.2049	493.2216	407.4228	427.2889	421.8557	360.2879	365.4091	315.4675	437.2723	392.8052	397.3902	491.802	392.1412	549.989
CUST_45695_PI429010886	M69952	0.011860882	24	18						7.037282	14.09394	13.45766	12.35025	5.210289	5.156021	8.411227	15.26868	16.89933	12.09389	6.077489	6.845572	10.97416	NA	11.97624	25.25638	14.86691	13.21287	17.10849	20.55029	14.66893	16.78356	17.39841	14.36936	13.20883	19.47348	25.05534
CUST_34644_PI429010886	M10222	0.011877941	24	18						108.9127	138.9709	114.4474	104.6237	77.00089	77.57132	82.02164	132.1477	110.7898	98.31087	58.03893	99.26059	102.437	113.7264	83.41575	127.048	138.5034	174.2606	132.2682	146.0081	165.2118	78.27256	108.7869	89.83016	137.8056	124.7762	129.7163
CUST_7827_PI429010886	N22140	0.011877941	24	18						287.8854	454.7075	380.2708	306.9528	246.2584	235.338	344.9779	366.3486	335.6252	328.3409	255.8139	322.4558	406.6714	275.6746	299.7411	361.6608	462.1954	385.3052	385.8737	378.7986	466.4341	342.3203	396.9028	421.5919	380.6302	341.177	454.8526
CUST_24013_PI429010886	N49863	0.011879851	18	9						18.23869	21.92764	11.41917	16.50625	17.23806	19.26106	31.4461	23.20911	39.43934	20.06249	21.23798	43.46921	30.43559	18.60577	19.94626	19.75521	37.54916	34.93184	10.24506	13.63037	23.69096	26.79132	14.36388	13.91274	27.19181	23.73807	28.3901
CUST_31061_PI429010886	M593	0.011901453	21	12						454.3052	590.3145	474.3448	587.8441	697.3828	673.8771	619.3687	568.0849	536.6512	511.8036	728.0598	677.9076	557.4698	641.1736	783.4167	724.6231	568.2556	635.4957	590.4595	472.4073	724.186	451.1176	527.455	504.902	584.1152	518.2326	567.0251
CUST_34392_PI429010886	M9529	0.011903671	21	0	170576227	unspecific monooxygenase [Bru			0.000000758	33.72001	51.50149	56.78944	35.66495	37.6281	30.58701	33.11195	42.67003	50.05302	33.04583	27.63116	39.96323	38.96134	28.03915	40.38904	29.42662	56.56581	49.10462	39.91458	47.1467	45.56407	51.44117	51.19908	54.73135	30.61037	44.1584	55.43214
CUST_31416_PI429010886	M1478	0.011906811	18	0						386.0289	511.2321	420.5189	312.8082	380.4577	360.0833	443.355	548.75	458.5926	358.247	316.5315	350.3187	326.6163	342.3203	339.4175	364.4673	371.6485	363.6624	514.8454	412.1414	473.2258	349.9633	469.784	414.4128	403.9297	380.6302	474.227
CUST_11601_PI429010886	N28105	0.011911936	18	3						316.4801	224.0178	163.9175	405.6944	262.4014	295.7504	214.8953	166.0779	233.7667	393.6626	297.2551	394.4626	302.7218	278.6422	240.0325	208.794	206.1397	167.9137	279.9809	246.6961	237.0906	366.7915	402.436	426.1948	307.6702	333.8469	288.9896
CUST_8252_PI429010886	N22774	0.011923572	24	3						147.5974	7.066092	153.0208	453.0125	862.1694	544.5236	10.0184	13.94283	5.053978	NA	442.4459	454.1788	7.113497	413.9053	508.1759	NA	4.644471	6.934132	NA	15.45782	4.189168	10.57851	4.408932	6.980145	6.457152	25.20136	5.764556
CUST_10866_PI429010886	N26915	0.011935312	12	9	260821810	hypothetical protein BRAFLDRAI	Cellular component org	Cytoskeleton organization	1.58E-57	5.673888	7.679886	8.259511	4.932	7.805069	8.04727	11.89304	8.596616	9.567571	24.0921	NA	9.171096	14.0225	NA	3.794398	5.964848	4.997291	5.862879	11.75627	4.208597	11.25938	12.2416	8.23062	15.74607	8.369875	NA	9.941387
CUST_17634_PI429010886	N38316	0.011939219	18	3						42.1572	41.95139	NA	13.2886	71.28516	47.35759	41.64718	117.5547	45.38207	7.916441	30.70816	8.937861	6.070219	31.66604	7.737208	31.41595	17.93558	9.372363	26.69535	134.9672	22.10047	NA	78.66288	NA	9.215946	139.1591	NA
CUST_56142_PI429010886	M112935	0.011943399	18	3						69.40758	57.85506	66.3578	99.07571	72.93006	79.22393	75.54551	61.28823	49.58749	70.70842	43.83834	44.88116	59.62083	47.88053	41.17309	73.7433	48.68665	49.7839	85.24573	46.66198	46.0811	103.3216	60.79074	56.78277	65.122	56.50958	47.23231
CUST_32403_PI429010886	M4015	0.011946667	21	15			Metabolic process	Cellular amino acid metabolic process		107.2361	212.9726	130.4316	86.27418	138.0384	114.2058	102.8397	101.6014	102.9977	84.87009	169.4561	74.74645	129.9419	150.3928	127.2055	222.6501	235.7954	226.205	112.2514	85.19452	135.0731	139.2612	156.0011	234.1695	100.0492	116.0279	89.65818
CUST_32063_PI429010886	M3142	0.011969853	21	6						200.4453	200.1232	204.2969	235.1738	314.0471	336.0374	216.896	221.3966	282.3692	218.4319	257.6419	273.2919	247.374	220.0171	276.4387	253.9457	202.5102	185.7829	201.4162	211.3776	207.1201	164.8407	231.2512	190.613	165.7121	238.5314	246.5526
CUST_7377_PI429010886	N21450	0.011973044	24	18						1421.441	2228.732	1831.816	1493.774	1273.67	1120.742	1842.758	1682.203	1907.271	1567.333	1259.516	1457.953	1796.427	1251.245	1248.523	2088.583	2180.402	2429.15	1816.059	2177.847	2264.823	1676.984	2191.923	2275.002	2038.719	1946.675	2170.443
CUST_28233_PI429010886	N57557	0.011990219	24	15	383848450	PREDICTED: vesicle-trafficking p	Transport	Other	5.33E-29	90.25881	110.3707	99.05353	86.24643	71.14732	96.04821	101.3719	134.67	134.4487	106.4585	94.06019	107.2586	103.0482	96.94761	113.4137	139.4798	127.4555	154.9422	106.9297	99.89645	126.521	113.1524	103.9866	114.9362	121.3825	120.7979	141.598
CUST_15875_PI429010886	N35284	0.011991298	24	9	321475343	hypothetical protein DAPPUDRA			7.27E-26	22.70394	20.79498	32.57078	32.22863	31.85157	19.39459	34.16048	19.14658	41.65139	43.28942	62.5781	32.23135	41.79088	22.59279	31.81746	44.57504	34.78378	29.44247	26.58916	34.06072	16.51333	26.30298	26.10539	31.30932	16.44032	23.05545	29.96804
CUST_30017_PI429010886	N61011	0.012002138	24	12						7.816284	17.70184	13.8182	12.60227	17.79044	24.33617	32.0326	10.94861	14.35294	15.17316	17.24118	18.9186	23.70162	18.98361	31.12503	23.13824	15.26493	19.15316	15.05878	18.51484	20.34099	19.39208	14.35144	18.26373	7.904365	13.04021	19.34867
CUST_34869_PI429010886	M10941	0.012006626	24	21						928.5423	778.7232	1032.593	705.8467	617.9354	602.7354	457.7423	583.0039	624.5971	822.6543	752.6445	633.0621	907.3817	616.2414	764.5562	960.5506	703.4578	682.3134	985.0577	693.9081	563.8477	1089.56	792.4227	767.8994	1468.759	726.5143	1100.835
CUST_15658_PI429010886	N34932	0.012014281	21	15						26.11949	52.24236	NA	81.75003	NA	81.6931	103.9647	95.15335	28.26579	103.2732	67.7048	NA	142.4928	98.76623	108.8478	215.2938	152.9638	125.9947	82.33668	5.033666	73.67616	NA	27.10146	NA	165.4776	116.8467	NA
CUST_9418_PI429010886	N24594	0.012026029	21	3						250.7116	187.3503	336.8186	391.4124	223.4224	153.8823	299.1209	288.117	188.991	267.3602	165.368	217.8031	271.1207	144.495	227.4568	65.49056	166.2848	154.566	152.0267	133.7779	140.8978	289.7381	247.116	327.3332	218.6641	270.0743	394.9633
CUST_3131_PI429010886	N13861	0.012029125	18	12	242008705	conserved hypothetical protein	Nucleic acid metabolism	RNA metabolic process	5.33E-137	NA	NA	4.666875	NA	NA	6.089114	8.417136	NA	14.65418	11.04536	7.656774	11.55231	11.30046	11.49825	26.62997	8.746049	7.828751	6.760669	NA	4.107116	5.098512	10.76826	NA	8.511016	17.30901	4.09358	6.404646
CUST_44138_PI429010886	M65046	0.01203423	24	21	312986079	gamma-interferon-inducible lys			1.93E-41	150.5856	40.90642	169.8611	251.655	157.866	169.3609	76.92811	59.22327	130.5163	78.03831	276.7645	186.302	304.4188	168.3995	197.3334	11.94454	11.89711	13.7591	256.1444	202.9968	278.4293	366.3486	467.458	558.8184	260.5042	324.7451	580.4723
CUST_30507_PI429010886	N62029	0.012052467	21	15	321462910	hypothetical protein DAPPUDRA	Protein metabolism	Proteolysis	1.05E-24	285.7894	379.037	367.0194	209.5586	94.78884	84.51406	425.6386	237.0906	297.7548	235.284	86.66046	112.6548	330.1913	291.8297	153.2663	431.1432	405.5854	418.1662	478.7694	260.1908	327.216	267.4193	324.4873	257.1241	322.4558	280.0915	350.2239
CUST_55007_PI429010886	M108388	0.01205759	15	0						29.53381	30.63129	25.74697	44.72435	18.16224	23.10946	18.08089	38.90187	11.52497	15.07053	6.131248	29.84354	18.78127	18.38792	21.08718	21.26556	29.93607	35.65289	21.80414	28.69791	23.21362	16.22155	19.44173	11.61327	28.08439	12.60565	12.38585
CUST_34441_PI429010886	M9688	0.01206152	15	0						18.83308	20.03269	20.12578	8.963971	13.90121	9.884796	7.867933	8.846413	22.23218	16.43018	13.34397	25.01036	17.35683	15.70264	21.38085	19.59198	20.70212	15.08105	9.785757	18.71505	19.81642	5.34003	14.66099	10.06884	17.28976	18.63267	15.52795
CUST_3853_PI429010886	N15407	0.012074334	24	21						254.9746	440.1079	376.7239	299.4177	352.5182	272.5092	273.0815	236.1602	363.2101	402.8177	325.5716	269.5843	246.4965	314.7154	172.8416	294.5533	320.8974	336.8186	377.3111	267.7311	277.2002	456.5305	425.6386	367.7282	329.7179	421.3144	457.7423
CUST_49719_PI429010886	M86847	0.0120747	21	0						135.7196	117.7118	187.5023	99.32904	197.796	120.513	99.79674	106.7994	148.8378	58.55963	66.74201	88.91387	90.50145	105.9124	122.1097	44.14843	100.6934	54.14388	88.54675	95.75319	90.36229	122.9878	129.3679	119.1208	65.50892	90.66543	362.195
CUST_45598_PI429010886	M69628	0.012095192	12	9						301.3368	207.1201	248.6058	208.941	314.1834	284.9823	318.6428	176.2933	349.0153	321.2079	352.3291	388.156	273.8856	178.596	372.873	291.8297	186.848	278.1319	269.707	210.3322	323.6649	428.3837	374.3365	398.2963	250.5426	230.8967	288.7179
CUST_12381_PI429010886	N29378	0.012104623	24	15						8.03382	7.892182	7.352636	6.664444	7.033822	5.872215	11.67855	5.461689	12.05528	6.770096	9.917631	9.957432	9.330978	8.871249	13.87446	15.9967	8.934291	13.5167	13.24894	8.217462	8.204835	8.742604	NA	12.03789	11.28749	10.20667	12.47302
CUST_32436_PI429010886	M4118	0.012105305	15	0						245.7379	358.6508	293.2244	183.7819	279.8449	234.0079	203.36	272.0606	243.1902	269.8306	261.8228	291.3655	240.479	281.9984	209.5586	287.3401	344.6427	320.3348	237.6854	278.592	226.022	219.7344	265.2295	208.3055	209.8556	225.3383	273.1848
CUST_3116_PI429010886	N13829	0.012106321	12	6						2167.915	2627.758	2640.776	2648.467	2381.656	2168.846	3384.461	2408.139	3744.093	3069.207	1573.048	2112.104	2960.558	2010.612	2162.694	3039.361	2332.204	3060.056	3202.946	2831.186	3405.379	2645.058	2221.308	2742.804	2371.181	1555.177	2517.49
CUST_19321_PI429010886	N41373	0.01213302	24	0						24.6865	15.38634	29.52349	19.14803	12.67757	17.41782	14.80962	12.01165	11.50709	14.99981	8.930779	13.56445	16.6131	9.279573	10.92971	11.04387	14.35464	10.6102	8.554104	15.90721	11.27508	16.01239	12.48451	15.18659	19.09461	13.77603	12.29918
CUST_46880_PI429010886	M74048	0.012133823	21	6						167.6755	399.0879	153.318	643.2149	440.2791	173.7818	578.1548	638.4715	640.002	625.9001	83.3606	404.7958	549.989	364.2801	176.4512	158.9219	352.1552	285.5358	284.4662	180.5859	215.9178	279.8449	457.4761	464.677	314.5854	356.7674	387.3552
CUST_2261_PI429010886	N11981	0.012133823	21	9						196.2948	265.1693	263.0994	191.0479	275.2113	284.7236	237.5901	290.3829	334.4355	286.1798	291.0502	284.8685	232.9121	246.1645	285.3215	247.4344	231.2512	263.3579	207.4056	225.2142	215.7105	245.7379	255.571	308.0511	209.295	236.4795	288.0498
CUST_27749_PI429010886	N56633	0.012146742	24	0						95.35371	134.125	117.2948	90.42094	103.7292	102.1812	91.6714	90.14554	102.437	111.5206	90.96558	93.77335	80.21511	81.71011	93.44995	133.6449	108.1961	114.6686	131.7124	85.47045	95.39909	141.8013	98.24098	104.0936	138.1892	104.5613	142.0207
CUST_5329_PI429010886	N18549	0.012148713	15	6	156553799	PREDICTED: lipoyltransferase 1,	Protein metabolism	Cellular protein modification process	2.11E-79	54.13071	87.11871	104.1325	90.21633	110.3707	45.40562	62.93557	106.0881	122.3191	74.37918	144.2441	73.79944	89.16012	57.22003	61.23583	63.85058	74.67002	56.35449	119.4045	124.9582	131.6748	132.7314	161.1965	157.0532	87.9342	125.4319	147.482
CUST_17163_PI429010886	N37471	0.01215033	24	0						58.43105	107.4552	118.8436	34.20961	25.29384	22.54848	32.429	176.1711	180.0167	19.86678	20.49843	6.524433	11.0114	28.56931	13.22308	46.14868	68.42492	68.27965	135.7196	141.9381	261.7871	36.02994	53.76136	4.041904	255.1811	41.94644	234.6915
CUST_14102_PI429010886	N32330	0.012164997	24	21						103.5895	96.25473	91.12829	101.0057	82.48416	83.10688	69.01316	100.8867	89.47762	93.51275	88.4798	97.90232	105.3222	66.51317	82.69673	79.78242	99.61307	88.14296	108.663	94.14191	101.3719	123.3723	101.8276	122.3191	92.75983	103.1504	125.5101
CUST_38356_PI429010886	M25815	0.012190518	24	3						729.7963	384.3395	498.4242	761.5417	1122.961	927.7273	497.2634	603.191	565.9653	534.4784	857.1902	712.1062	593.3017	809.8174	745.1009	327.3332	379.037	231.6588	304.282	549.069	260.2914	320.552	511.2321	300.6771	213.8259	686.1628	236.4795
CUST_2736_PI429010886	N13007	0.012190518	24	18	512127157	checkpoint kinase 1 [Daphnia pu	Protein metabolism	Cellular protein modification process	1.3E-142	229.8852	392.1412	488.2191	263.1307	214.4735	212.8617	335.3114	361.4833	473.1245	315.96	221.1981	307.0061	365.7068	260.0953	272.1212	343.4733	513.3858	426.4459	416.5526	443.355	504.7574	371.8649	358.3361	427.2889	437.4032	336.3654	429.6749
CUST_6560_PI429010886	N20270	0.012190518	24	21	268573893	Caenorhabditis briggsae Hypoth	Metabolic process	Nucleobase-containing compound meta	5.76E-69	29.56314	24.83937	31.12893	20.72028	17.84752	19.05575	21.3436	20.05879	26.30955	24.62171	23.68606	29.6976	25.39283	25.19098	18.49557	28.92251	22.07673	26.17	29.18456	24.51979	35.97416	36.64741	29.11467	32.23946	33.67973	22.2762	33.80053
CUST_8623_PI429010886	N23320	0.012190518	24	21						31.53465	38.27962	34.93669	21.67518	25.30627	26.14998	23.32054	30.98189	18.59394	24.04323	38.70891	26.93742	26.10539	25.40839	21.2032	63.57042	30.03767	42.24367	38.29632	36.14198	29.91879	39.35132	35.74167	39.44924	56.57499	40.44814	45.1536
CUST_17294_PI429010886	N37728	0.012190518	24	3	189237914	PREDICTED: similar to CG4733 C			0.000000449	41.14564	33.77888	42.6374	51.34712	102.3834	69.00406	41.18226	42.39291	38.83987	43.35233	70.04249	79.90962	31.69795	68.30948	62.23304	43.42121	42.10969	36.08683	25.43712	34.20961	36.6885	43.99536	45.88546	37.8081	28.38772	47.90611	45.96577

Probe ID	ID	p-value	Period	Phase	EBI ID	Annotation	GO category	Go term	E-value	0	0	0	3	3	3	6	6	6	9	9	9	12	12	12	15	15	15	18	18	18	21	21	21	24	24	24
CUST_41016_PI429010886	M56636	0.012195921	24	3						28.41463	15.03718	20.32056	31.50944	42.0865	38.90187	17.78015	22.43976	24.29464	27.68431	21.85691	15.53301	26.6049	19.7414	NA	22.34334	20.15802	19.34867	15.5351	17.75609	21.3518	18.09671	18.37444	15.4109	27.3201	20.05017	16.01802
CUST_33051_PI429010886	M5843	0.012201263	24	18						4105	5467.822	4415.56	4105	3509.087	3796.503	5336.579	3875.533	6010.065	5055.201	3675.29	4378.72	5394.696	3060.056	4392.913	7695.811	5462.129	5921.175	6791.114	4589.607	6649.946	4847.527	4490.88	5197.224	6727.299	4064.827	5979.853
CUST_20775_PI429010886	N44037	0.012201263	24	21						136.0268	116.0897	126.4865	114.8083	123.3723	91.58124	125.8592	108.8478	131.5172	132.3768	107.7528	191.1727	144.7781	123.0024	145.1769	197.6734	140.2618	175.9736	161.2169	125.5586	157.9625	198.5715	170.3943	219.7957	137.5851	107.8333	245.1933
CUST_32644_PI429010886	M4676	0.012218032	18	9	241846314	SEL-1, putative [Ixodes scapulari	Metabolic process	Other	1.07E-122	42.3598	53.10853	63.78974	45.60384	21.41224	27.32212	96.9842	64.88027	57.42169	71.90542	52.39131	67.17926	35.11692	49.56614	42.5482	92.79509	60.97733	84.42785	40.68971	37.08608	51.98569	39.61481	41.24111	40.94392	70.38705	47.70751	54.93619
CUST_43641_PI429010886	M63564	0.012218032	18	15						33.28109	36.52621	26.36566	41.69944	20.37081	25.84326	40.23682	36.28732	30.25516	48.33941	29.65959	6.478825	37.20362	26.82198	30.32915	60.74813	48.11395	51.08768	20.02415	28.95792	29.63277	22.88864	21.65257	23.59507	22.6315	23.65361	27.21287
CUST_52256_PI429010886	M96867	0.012218032	18	15						27.1592	48.12354	41.51635	33.62051	29.01236	27.85573	27.1592	32.06294	36.83272	43.13936	34.72664	24.28735	55.88596	31.82049	53.04169	47.33022	54.20975	61.15056	19.32802	31.4218	38.14824	24.73549	34.98836	29.36452	42.91273	39.20662	48.04899
CUST_16529_PI429010886	N36400	0.012230674	21	0	195162205	GL14252 [Drosophila persimilis]	Cellular Process	Cell death	5.61E-10	59.38892	65.42578	79.57438	72.66933	63.68621	63.91561	58.2972	56.95109	44.93169	56.84633	70.65416	68.21622	46.39912	54.53262	53.5836	54.39842	71.18104	53.65203	68.18657	43.94763	56.49038	72.15056	68.73418	68.03257	61.95862	85.10841	67.93128
CUST_37491_PI429010886	M19220	0.012235696	24	18	383857871	PREDICTED: zinc finger protein 2	Protein metabolism	Ribosome biogenesis	4.95E-44	154.7688	268.6876	249.9933	172.4755	149.4369	115.2656	187.6435	186.9529	206.8689	169.3049	127.5994	118.0753	184.1477	132.1793	123.6152	154.5725	260.4014	197.5603	218.9572	231.9647	296.5728	162.4812	227.9494	174.8465	190.289	194.1257	180.8452
CUST_48424_PI429010886	M80256	0.012235696	24	3						54.69276	68.67272	66.63719	107.3485	246.1269	220.6244	78.20138	170.0929	98.85767	84.84135	191.7176	144.7602	84.9077	121.1967	79.31808	37.72215	77.77436	43.73128	54.33723	113.23	35.49916	119.6201	111.5065	94.33013	64.99766	156.1362	69.73801
CUST_39888_PI429010886	M46266	0.012237514	15	9						16553.17	109.3354	14743.32	17555.23	13098.74	17623.06	1153.859	993.6309	1889.404	24825	19509.04	25343.01	23084.2	8857.18	19094.08	376.4343	65.16557	417.1463	2554.596	3783.527	4819.372	18408.63	16252.77	19791.07	5814.883	15340.52	19791.01
CUST_53040_PI429010886	M99859	0.012266193	18	3						20.32291	19.71754	32.10035	26.16822	29.00237	24.87849	25.5482	23.14239	16.14473	13.17346	16.91133	23.70418	20.46917	16.42848	10.8747	20.64919	24.64033	18.80957	28.3301	18.14584	17.70572	25.70782	19.29948	26.2902	24.06897	15.98548	23.64554
CUST_2664_PI429010886	N12862	0.012266193	21	15	224486244	glutamine-oxaloacetic transamir	Metabolic process	Cellular amino acid metabolic process	2.99E-153	135.3635	290.8608	197.1631	196.1489	191.1071	136.1461	125.7133	244.8878	148.728	208.7171	188.4898	223.4669	189.6492	227.1085	174.857	216.8711	283.841	309.132	173.9842	275.386	261.7118	144.2441	239.642	210.039	142.8249	205.4884	127.2055
CUST_17027_PI429010886	N37253	0.012266193	15	0						12.66176	22.52975	15.16953	7.537524	17.04802	9.346935	8.197876	11.29632	10.62408	10.59744	14.62238	19.83269	16.51861	17.40595	16.8636	22.16019	18.41601	18.51484	19.86967	12.44307	10.17265	14.64996	6.046147	12.76732	14.3393	17.46376	22.10047
CUST_14469_PI429010886	N32938	0.012273652	24	15						6.945676	26.54409	3.873863	12.56182	3.896595	14.88508	7.956098	12.77798	10.15678	13.43146	15.23086	13.95493	14.18007	11.90033	13.70168	45.18702	20.54013	15.9111	12.13203	4.932	15.57566	18.81827	15.3089	23.24733	6.051391	10.79066	10.94364
CUST_883_PI429010886	N7689	0.012273652	24	15						1787.383	1284.957	643.0042	937.9336	472.7524	659.0035	2269.737	3384.461	2935.737	1717.387	797.7145	460.9197	2078.188	1526.163	584.3107	4628.184	2618.303	5304.997	3039.361	1720.155	1863.699	2005.153	550.2129	1180.485	1898.75	665.873	616.7748
CUST_3579_PI429010886	N14811	0.012275493	24	3	326781697	crustacyanin subunit C [Fennero	Transport	Other	8.31E-42	61.19316	16.63618	25.51539	95.36659	116.9614	97.45374	NA	NA	NA	12.54368	32.36608	45.65444	21.15337	69.326	81.96955	10.94364	NA	6.346484	42.10601	21.70753	46.54974	26.88183	NA	27.18132	65.01496	48.03485	9.157379
CUST_54973_PI429010886	M108296	0.012290404	24	21	334328835	PREDICTED: zinc finger protein 3			6.73E-60	40.0082	46.84134	49.49501	48.80246	50.73868	29.22669	42.80977	46.90598	41.55059	35.13853	40.06809	34.53159	40.53493	41.47761	29.24359	42.16901	49.3434	40.16189	47.23231	38.85319	39.2136	51.92132	51.71141	41.85107	43.01953	42.87131	51.60135
CUST_28417_PI429010886	N57909	0.012291346	24	12	242004632	zinc finger protein, putative [Pec			8.07E-09	11.12735	12.96601	7.168799	24.46253	11.49825	7.764959	10.56907	25.03508	7.219437	13.55744	12.87017	16.50625	16.84207	17.43685	26.60187	11.31707	11.3477	15.03411	17.25784	12.48035	16.5029	15.91592	19.14248	21.43518	6.228711	8.881447	6.831045
CUST_20579_PI429010886	N43696	0.012309815	24	21						8.888649	NA	NA	4.87786	12.47813	10.07424	5.448429	7.103758	7.635827	6.957271	NA	NA	6.631805	NA		6.520876	7.886951	9.218794	10.79559	4.518426	10.79066	15.75563	14.92039	NA	17.37069	13.16499	NA	7.132043
CUST_4663_PI429010886	N17235	0.012316108	24	3						9.24062	NA	11.14899	40.07856	14.41795	16.66622	NA	NA	3.900363	11.16576	18.10243	19.35085	9.372363	NA		10.70343	6.938245	4.571766	4.21594	7.836931	10.39496	7.912113	12.54285	6.406844	7.653718	5.260365	7.162363	12.60041
CUST_28049_PI429010886	N57174	0.012322266	21	15						355.5754	329.8464	469.5128	355.324	328.2775	370.3281	310.4343	298.6917	351.5196	381.4494	220.9943	347.9094	413.9053	308.1912	294.6196	568.8248	367.876	517.0399	535.748	280.9343	451.2333	511.2321	433.1774	422.1689	351.343	293.7564	389.7239
CUST_6478_PI429010886	N20154	0.012343302	24	21	242017806	purine biosynthesis protein 6, pu	Metabolic process	Nucleobase-containing compound meta	8.46E-151	146.3365	521.6397	502.8105	306.0769	253.1207	97.80698	278.6422	226.6091	405.7918	229.3564	522.858	154.0154	500.9726	214.9733	322.9826	185.8563	414.8181	265.5849	593.3017	616.7748	796.2861	493.728	1106.912	1131.397	511.8036	272.0128	672.7456
CUST_31969_PI429010886	M2866	0.012346807	24	18						76.5464	130.8641	108.2696	80.30375	83.42667	73.16089	67.39104	95.64524	91.69645	82.9737	86.35128	91.96611	89.60857	84.61068	96.79055	84.6858	137.2351	128.9945	97.58001	101.9686	138.7489	82.19156	94.53661	97.99445	94.66861	78.783	119.2573
CUST_11762_PI429010886	N28358	0.012350059	24	3	82795527	arthrodial cuticle protein AMP1(			1.09E-09	505.5201	225.7047	375.0062	629.6411	3177.941	2442.216	158.7144	997.2176	177.388	188.4169	1208.338	1255.327	163.2328	604.6962	988.9686	157.1984	49.55479	95.63382	18.40249	990.2906	41.38631	250.5828	405.1127	266.2866	13.88764	2197.153	112.683
CUST_1094_PI429010886	N8475	0.012350059	24	3						93.02834	154.3027	131.8579	134.7853	179.0567	163.4024	80.03641	124.2323	124.991	94.25033	119.713	109.6493	93.16926	100.8435	102.6229	53.19546	171.8649	95.27189	61.49211	114.9771	101.7744	80.3791	119.7443	104.8565	66.54171	123.0024	128.5824
CUST_42958_PI429010886	M61728	0.012366127	21	15	546680978	hypothetical protein D910_0849			1.94E-48	274.0413	329.6536	212.9726	363.297	264.3686	423.1563	560.4998	253.3528	483.7942	336.1378	354.9347	392.9019	317.2644	411.8854	901.0046	560.9756	763.5241	395.3057	186.2715	415.3144	286.3491	223.3777	332.2617	442.6514	419.0196	203.3258
CUST_26325_PI429010886	N54055	0.01237269	18	9						52.77429	52.9338	56.60172	70.17999	78.65231	69.70354	70.82447	54.60623	67.04993	81.63425	73.11579	93.74241	84.74637	50.30283	38.51205	70.72448	64.61049	61.49909	69.75403	46.95394	54.41681	78.81049	30.9435	53.5836	84.51406	71.49082	70.7612
CUST_36217_PI429010886	M15039	0.012380013	24	15	241829153	Ixodes scapularis traf2 and nck i	Protein metabolism	Cellular protein modification process	2.64E-86	60.91215	77.31152	51.41632	79.51886	67.2518	52.6471	53.82263	64.35228	45.67405	70.89953	43.79783	70.26755	83.74447	79.07026	60.86727	68.19401	90.44348	96.20921	64.50416	67.7048	81.35177	65.41169	76.02418	89.61931	67.85905	64.72689	65.01967
CUST_19147_PI429010886	N41053	0.012380013	24	21						133.9146	104.8681	118.702	82.73518	83.69522	144.7781	91.72224	87.29938	148.2459	107.0195	113.9079	116.1062	90.7511	89.93986	66.97219	182.8511	101.1475	163.1045	200.83	102.411	110.9564	218.2926	137.8214	160.9374	203.4357	119.9264	183.2834
CUST_48511_PI429010886	M80731	0.012391176	12	0						57.8894	55.80136	45.61863	56.44652	34.87559	35.62359	44.58117	27.47971	28.66752	40.67888	30.84927	33.0209	51.42014	48.06623	54.86291	38.37232	32.72715	50.29015	37.27834	47.24767	36.15638	44.13653	49.64751	31.87401	41.59303	44.78302	51.61367
CUST_50020_PI429010886	M88284	0.012391176	12	0						104.2173	86.13364	87.06038	68.52496	46.57945	42.19985	49.42284	45.17758	48.60251	65.97767	46.52866	44.62591	86.85325	77.23272	87.51637	93.47893	49.39632	69.06046	43.79306	37.59336	50.74506	137.7481	67.54737	104.4062	65.95317	60.77638	80.5628
CUST_32610_PI429010886	M4568	0.012392929	18	9						51.23064	67.75299	42.82003	47.23775	32.70464	40.43868	75.34564	61.13851	49.11012	49.42933	66.11318	71.49082	69.88293	53.5836	53.20126	46.43858	60.30614	55.6719	34.27395	45.39182	52.0293	30.53594	53.56818	56.20706	51.0346	55.31238	60.64674
CUST_17978_PI429010886	N38938	0.012422863	24	3						187.8136	206.98	289.7381	254.618	318.8796	225.256	52.05686	181.4063	119.3727	149.578	79.94465	88.43455	116.7446	144.4328	102.8659	133.196	27.47449	109.9215	86.956	153.6189	106.5773	185.3668	131.9455	86.44935	214.0023	109.9768	119.8892
CUST_21813_PI429010886	N45899	0.012437626	24	9						227.2807	25.91869	55.09974	258.7813	48.94611	211.185	226.5403	76.23852	71.59289	508.513	155.3804	73.15055	435.509	83.74447	102.2993	213.152	34.54334	136.2005	19.53794	50.13895	37.31263	16.50439	60.94991	52.58831	8.066527	70.332	38.73021
CUST_2616_PI429010886	N12768	0.012454824	21	9	393827419	innexin 1 [Cancer borealis]	Signal transduction	Cell-cell signaling	1.7E-174	230.9748	256.1905	227.1085	195.2581	365.7666	309.7415	368.7015	307.6081	313.3524	343.7299	326.0747	364.4673	280.8283	277.4142	402.436	297.8139	193.0974	191.3515	369.9777	219.8774	239.0862	244.1329	217.7635	244.8381	359.7575	256.2719	329.7868
CUST_26907_PI429010886	N55102	0.012461199	24	12	395858607	PREDICTED: uncharacterized pro			2.33E-24	5.965489	NA	NA	7.040601	9.350524	7.172971	NA	NA	8.030713	10.70051	3.070047	NA	9.528739	9.234299	10.00814	3.667091	5.868192	4.177755	NA	4.248874	5.06501	3.639661	4.307915	5.513814	5.524222	4.843669	7.410322
CUST_46984_PI429010886	M74401	0.01246397	12	9						8.38931	5.230931	NA	5.860466	6.27007	NA	9.389205	9.798222	10.32476	14.54879	10.41528	12.70605	8.440387	NA		3.405836	15.84904	6.683325	12.13039	5.917822	NA	10.05788	NA	11.88037	22.37507	5.971284	NA	8.620448
CUST_31828_PI429010886	M2510	0.012482613	24	15	48097764	PREDICTED: 26S proteasome no			2.26E-20	46.11508	36.74804	45.99276	35.48951	36.60452	35.85354	41.26235	32.71622	40.82014	45.78026	34.85461	57.55394	47.45891	35.43409	49.80766	55.76605	45.08105	63.15515	51.37499	40.75473	58.45199	54.73663	37.71641	48.53921	55.47226	36.68361	62.60527
CUST_49061_PI429010886	M83857	0.012482613	24	21						97.87129	165.368	121.3126	99.74451	130.2882	127.8235	83.28215	147.043	121.9486	98.66873	144.1169	165.0676	107.7409	141.2055	161.4291	147.2411	142.9419	173.6995	178.8078	142.8448	174.0123	159.3992	160.4657	218.1148	157.1085	140.8381	197.0196
CUST_25355_PI429010886	N52253	0.012484425	15	12						23.26403	38.2274	22.47797	17.57039	23.30296	19.02985	15.77285	10.21185	13.97433	25.3148	26.96013	25.04161	24.92561	19.70573	24.50769	20.48185	25.06319	19.97369	27.74851	19.0444	13.81	20.24017	17.81281	17.04105	27.05785	21.88915	32.38938
CUST_41799_PI429010886	M58579	0.012492837	15	3						34.23772	33.87813	29.40141	80.09649	32.31005	38.98881	31.18062	36.6589	28.42566	28.82404	32.59982	28.79283	20.3727	32.00302	21.03678	27.29078	26.53512	36.14198	24.0804	37.42032	37.43476	36.34596	30.02506	27.90262	25.16714	22.43407	31.3587
CUST_31301_PI429010886	M1168	0.012498855	21	15	321477760	hypothetical protein DAPPUDRA	Metabolic process	Cellular amino acid metabolic process		117.2637	210.6226	147.8633	101.3884	75.42049	120.2076	203.919	291.2685	247.8796	188.2922	117.7425	168.511	118.653	123.1089	111.6232	230.2547	338.2602	280.9977	167.564	184.7058	221.0264	114.6686	238.9099	110.5907	101.572	158.5985	136.4616
CUST_75_PI429010886	N459	0.012498855	24	3						1114.294	34.3611	1227.762	1195.903	2873.993	1715.071	69.83801	281.8133	83.18277	248.4015	1593.454	1338.68	215.0091	1778.328	1363.035	23.27322	81.97833	55.38965	39.05628	213.0483	46.38013	322.5153	139.5452	185.2774	71.05641	436.2064	129.8158
CUST_4614_PI429010886	N17137	0.012499552	24	15						1904.856	1338.68	306.279	1704.256	110.9089	1235.266	4031.99	6906.237	4875.223	4945.667	1087.147	1788.478	2498.654	2330.806	715.6987	9804.488	3921.719	8929.115	5322.868	4329.518	6041.324	2054.56	225.1699	1669.427	2620.241	968.7419	1514.569
CUST_39906_PI429010886	M46634	0.012500386	12	0						222.5585	226.1282	314.6496	74.28032	44.94128	21.81628	109.3497	167.7963	172.4491	145.0362	161.754	74.14723	341.6702	54.43474	229.3564	83.93495	103.2732	124.6061	104.3181	124.3819	187.0484	252.7743	228.6642	207.1407	181.0813	49.89765	216.4739
CUST_53228_PI429010886	M100831	0.012503096	24	21						253.8613	208.2068	246.5526	160.8732	208.404	183.6447	162.5329	174.6338	213.8259	196.3246	173.9842	239.2059	242.6247	181.8521	150.073	299.8648	215.9822	232.4149	324.4873	233.7667	239.642	337.6329	238.6215	234.2298	310.1796	257.956	337.6971
CUST_45412_PI429010886	M69022	0.012503096	24	21						242.5407	375.415	317.1978	410.4537	326.5385	323.0951	373.7341	312.7556	297.188	359.0835	239.2427	412.9894	443.355	331.7259	100.2134	506.1561	467.9385	448.8304	510.5943	289.5149	510.7551	523.1285	459.5171	515.2465	565.179	303.6159	506.2756
CUST_52563_PI429010886	M97963	0.012503096	24	21						238.4834	260.1465	371.5772	215.1868	233.4638	347.2002	200.7515	235.2412	273.6644	241.668	245.1933	282.172	223.053	260.3413	280.6399	479.3697	293.568	296.7459	499.3569	243.1406	313.9334	451.3395	277.4142	343.6496	493.4583	296.4601	286.2354
CUST_1968_PI429010886	N11187	0.012503096	24	21						237.4902	170.9358	217.4288	153.6189	155.1663	96.62834	76.41348	114.0434	89.04608	380.83	113.622	312.1666	159.9495	118.3752	144.6203	112.3135	292.0796	130.752	409.7628	96.64158	357.4621	123.5992	430.3398	501.3701	121.0574	160.1108	156.2636
CUST_3171_PI429010886	N13949	0.012503096	24	18	68349616	Marsupenaeus japonicus Ras-rel	Transport	Protein transport	2.96E-135	445.9579	827.5626	678.0803	537.4657	412.1414	405.5854	550.8269	574.7506	627.9112	608.6613	537.1828	501.7792	636.1587	512.3561	455.0731	678.3225	1061.158	807.6213	650.7176	689.0797	840.6574	457.0998	782.4302	709.4562	638.6943	582.1367	873.5747
CUST_22265_PI429010886	N46715	0.012508432	24	9						36.14885	25.00404	35.93896	35.27538	30.48373	44.70348	35.57091	38.46589	29.55431	29.6741	41.42487	28.1833	31.8144	25.89606	23.4647	27.83542	23.8882	25.11323	26.6898	27.21869	29.1953	41.60531	10.0026	40.77521	28.56931	30.0974	44.02111
CUST_3105_PI429010886	N13803	0.01252658	24	15	392348507	PREDICTED: zinc finger protein 7			4.79E-66	9.476005	18.68973	10.45447	4.456932	10.67194	6.028187	8.838822	10.30655	14.40303	7.032137	7.925431	NA		12.0429	11.86794	9.471206	12.82069	19.59742	12.82069	10.99438	14.50203	11.76598	NA	16.09389	6.970533	3.534991	10.70603	10.92142
CUST_22001_PI429010886	N46239	0.012526892	18	3						220.5131	275.9079	361.6608	350.0691	296.6356	236.5379	151.0496	252.1994	154.177	257.0037	234.2298	293.095	217.8011	247.4344	290.2762	43.75819	212.0165	190.3661	164.741	280.3032	132.8026	350.9469	562.2603	588.9309	125.3559	523.0011	584.7373
CUST_20536_PI429010886	N43624	0.012531423	21	18						31.93086	36.46097	42.52366	27.48515	23.06634	21.08201	26.70669	21.96464	26.96645	34.32123	35.34705	23.46416	31.93086	18.20572	28.40807	44.00493	32.74097	35.24152	29.52588	60.98236	27.48515	23.51188	30.03767	23.1537	25.91577	36.90923
CUST_47355_PI429010886	M75749	0.012533603	12	0						108.7311	87.31684	92.06463	62.53388	88.72914	54.16069	55.63371	68.82621	54.87487	85.85149	56.45276	108.2246	71.05641	59.10442	74.15901	57.16525	89.16759	56.70114	55.70902	48.81917	61.72867	86.13364	48.88152	91.98987	83.0128	88.92336
CUST_10947_PI429010886	N27033	0.012533603	12	0						112.963	60.89951	142.2138	80.99203	67.60011	25.63539	50.69492	32.62695	9.144684	68.41738	38.293	62.09609	91.32281	69.67842	122.754	13.7086	59.19241	38.12534	57.58975	58.78776	44.43314	117.6515	130.5329	75.99695	110.8559	31.25815	141.5813
CUST_11302_PI429010886	N27589	0.012535046	15	9						19.67166	14.18326	23.59807	99.98511	20.95393	NA	8.825422	25.53483	37.32223	38.74481	21.00978	25.80525	60.97733	26.08621	21.7829	16.67961	19.12392	9.345975	37.51007	22.59279	33.14322	44.31359	55.68441	50.87686	52.48031	47.51827	86.01538
CUST_53574_PI429010886	M102315	0.012559182	18	0						24.62171	12.40192	13.28376	10.6734	9.92008	15.66225	10.68195	5.591367	14.63182	6.911194	11.77271	NA	NA	9.45577	8.078793	6.86903	NA	7.18431	16.7995	37.34538	39.36946	5.889572	18.01938	14.78505	12.56325	9.728753	14.48202
CUST_43526_PI429010886	M63277	0.012559254	15	0						84.04962	26.79132	77.99973	38.57897	38.89512	34.86224	29.9505	23.95576	24.36566	37.97184	31.69013	29.67188	34.40524	43.54271	32.94464	43.21465	35.99913	47.61057	55.26895	30.00811	21.67775	35.33768	25.05534	31.68069	30.88831	52.25815	34.76743
CUST_35340_PI429010886	M12319	0.01257301	12	6						106.4444	129.4391	114.5807	113.5034	96.53451	109.207	162.2856	183.166	165.0194	155.2845	108.8478	131.3023	134.2321	114.5979	102.4481	158.5385	207.8778	154.2023	139.8319	122.8853	161.4291	90.45329	136.0891	128.3867	127.5275	107.0618	95.47293
CUST_35738_PI429010886	M13355	0.012593999	24	6						12.20234	10.61964	NA	7.823808	5.455303	107.2833	10.43678	41.61011	45.20901	NA	8.402176	15.2887	10.78069	10.75402	NA	6.531481	12.52305	6.438537	NA	4.920386	5.644653	6.101511	NA	8.169501	10.35834	18.1529	9.575352
CUST_38847_PI429010886	M31172	0.012598727	18	15	321473526	hypothetical protein DAPPUDRA	Metabolic process	Carbohydrate metabolic process	2.14E-20	2166.525	3069.207	2549.09	1624.639	763.8013	546.0695	1762.47	1141.401	2119.199	1614.735	448.2125	761.8025	1950.295	842.2205	1098.155	2983.608	3431.358	3516.69	3130.289	2014.653	2136.93	1779.656	545.4629	1184.477	3992.329	851.8736	2849.078
CUST_49694_PI429010886	M86738	0.012611565	15	9						19.97115	15.14996	23.65361	20.88172	83.05204	42.84582	23.98408	21.06356	28.78071	30.7975	39.05992	67.7438	13.764	38.18197	26.05879	20.50662	15.90885	19.03459	21.0526	19.66181	23.02305	40.57158	30.98189	23.86079	17.52792	34.33958	38.7383
CUST_19041_PI429010886	N40884	0.012620972	24	18	321456174	hypothetical protein DAPPUDRA	Signal transduction	Other	1.4E-16	21.97462	25.02711	15.94333	18.65093	22.50019	14.64996	26.82917	15.26121	29.75114	17.59048	12.79751	12.33606	14.12309	16.47719	16.90269	36.12859	28.10303	32.29221	39.94084	37.76079	38.13279	30.8995	21.96464	19.77172	35.4224	17.42042	25.48121
CUST_19705_PI429010886	N42042	0.012630523	21	15						83.45736	76.7295	82.40323	47.18539	32.83487	26.92179	74.34865	100.2134	120.6989	96.55775	68.26223	72.36965	89.23195	55.22014	64.00415	80.40126	110.1161	162.6959	67.3975	47.46533	59.09838	114.4006	69.86685	88.71852	43.41187	43.98563	58.39443
CUST_40416_PI429010886	M55020	0.012636028	21	9						20.61709	40.991	23.91545	43.53457	32.55747	39.99078	47.85839	28.60128	46.1952	53.19546	30.36962	40.99664	48.6216	31.59758	42.40452	52.61041	42.43675	42.27337	27.48873	22.38538	43.19513	26.01047	30.84327	24.51979	46.22711	27.59053	43.31704
CUST_19189_PI429010886	N41136	0.012641589	12	3						44.06379	27.36657	44.08647	112.8927	39.27561	46.49868	22.68661	12.19826	15.69941	34.2605	21.27734	29.67957	54.14388	32.95195	21.87534	48.20014	83.64481	88.37671	56.57499	40.02352	24.61634	34.30109	22.9911	39.07837	35.51402	28.81419	56.43411
CUST_53646_PI429010886	M102627	0.012643961	24	15						129.2573	81.17631	131.2008	102.4735	37.67803	104.2317	111.9225	61.05475	87.33991	136.7679	73.92515	117.9968	175.151	65.04377	156.6283	223.8968	154.6164	231.5378	120.2238	79.96781	103.0566	194.1916	132.7164	200.5807	97.11689	61.97446	158.469
CUST_27241_PI429010886	N55700	0.012643961	21	15						182.8511	151.2161	264.3686	179.7537	227.7173	157.1984	138.6295	129.5035	154.7131	208.941	184.5933	170.4812	236.8356	160.217	207.3653	266.5309	204.0335	311.7361	212.0165	212.5212	190.9992	266.6798	143.1878	233.9171	226.4976	156.988	221.8149
CUST_34661_PI429010886	M10279	0.012647651	18	3						1539.37	173.2392	1441.244	1495.315	1307.951	1716.333	130.702	165.742	173.1143	1185.011	1170.841	1549.756	357.7827	1068.824	1349.624	76.24747	83.09656	71.61601	74.5042	219.3902	118.3923	2026.561	413.6069	1571.574	123.4753	262.0968	402.2649
CUST_31440_PI429010886	M1533	0.012659384	24	21						2112.104	2790.956	1814.798	1982.92	1701.993	1428.28	1278.279	1760.199	2050.749	1974.35	1898.75	1670.997	2310.632	1681.402	2197.153	2425.524	3604.261	2593.566	2157.924	2137.853	1604.901	2922.057	2284.471	2753.439	2393.197	2337.634	3472.797
CUST_54_PI429010886	N237	0.012659384	24	21						255.8139	359.174	493.1272	406.8432	341.965	384.5219	348.473	322.5903	434.0618	382.4457	294.9654	428.1289	365.4954	400.7915	465.6755	432.9222	419.7244	511.6359	439.9905	385.3052	532.6369	524.0943	444.244	665.1463	322.75	467.0581	486.4482
CUST_13698_PI429010886	N31614	0.012659384	24	21						63.67982	27.37887	57.57578	26.76803	38.82583	31.05988	26.346	26.3515	21.65257	34.28012	38.61107	31.23655	41.80345	30.5034	33.9635	39.39814	36.02528	21.3729	58.63553	24.97563	32.54688	46.08569	72.75695	55.0054	83.80666	60.79882	91.40987
CUST_21693_PI429010886	N45675	0.012659955	18	12						6.235237	42.44693	NA	65.07547	27.60039	31.84126	6.768812	16.73229	5.975334	6.487467	NA		84.16808	158.0988	176.9714	17.05355	58.37071	37.24162	15.03495	96.20921	128.9542	7.114823	36.77637	20.55179	7.029612	NA		9.442403
CUST_52450_PI429010886	M97479	0.012667599	24	9						9.519076	6.607812	NA	9.471206	12.56759	7.419066	6.976782	NA	NA	16.715	15.63254	13.14355	NA	NA		12.78321	12.10084	13.81116	8.944558	9.438548	9.068193	5.587905	9.556905	10.52564	5.062707	8.823825	11.14599
CUST_52414_PI429010886	M97363	0.01267603	12	0						139.4307	73.85285	146.4225	113.1453	86.7231	82.49497	61.37944	75.35645	104.3593	134.6107	105.3328	96.62834	126.2717	79.22393	109.5548	84.25166	96.49913	70.36116	79.19058	67.42662	64.78842	121.0252	96.81529	99.88608	171.3397	96.40725	120.4647
CUST_7887_PI429010886	N22220	0.01267603	12	0						127.1435	83.6658	95.14144	92.33626	68.20088	70.21276	77.33923	56.05872	63.77213	112.2837	86.27418	91.00224	107.1873	69.37968	118.8436	113.5034	81.21161	90.8123	86.85325	68.61527	70.2307	139.4307	66.36737	120.9506	151.8112	95.05524	128.6789
CUST_49982_PI429010886	M88099	0.012682008	18	3						17.27059	21.47115	26.28423	26.89377	21.37846	23.71354	20.14949	16.06042	21.13752	12.8605	27.65164	17.6701	16.66935	21.2594	21.49082	17.67823	26.23786	24.66091	29.64523	29.84354	19.9833	20.42404	39.24909
CUST_35527_PI429010886	M12796	0.012684649	21	12	321477216	hypothetical protein DAPPUDRA	Transport	Other	8.12E-17	210.1175	76.57491	95.48478	211.9007	71.3976	99.64095	227.1783	77.73937	61.26616	264.8027	133.1501	100.8755	247.2393	115.0193	142.1027	137.8214	120.6508	141.2448	63.42167	71.3958	73.74883	80.01788	82.17196	77.57976	78.79491	107.1549
CUST_44299_PI429010886	M65541	0.012685693	24	21						136.424	146.6123	160.1108	130.174	128.7298	118.1715	140.4803	110.8779	145.139	174.3193	142.5367	123.1089	126.7896	124.0944	118.7939	164.3644	143.2404	153.3624	155.2093	135.4737	131.6618	154.9154	173.8408	176.2281	152.3742	133.171	168.6123
CUST_15778_PI429010886	N35135	0.012687586	24	0	260798324	hypothetical protein BRAFLDRAI			5.87E-15	9.287131	8.449479	9.363648	6.92309	3.907151	6.453374	5.102347	NA	10.52214	6.484978	3.912608	5.941854	4.386394	8.273433	8.295728	10.62276	9.843601	6.170982	8.835842	5.78951	8.168708	7.011775	7.130952	12.27349	10.81194	12.64646	16.91133
CUST_38239_PI429010886	M24536	0.012703086	21	18						288.2433	283.9592	319.218	210.7377	288.4809	224.5463	218.8639	250.2264	274.6613	248.1114	204.4688	274.4093	264.9858	244.6993	182.2404	311.1974	211.2323	401.757	320.552	260.4457	346.5231	303.88	309.0756	284.0207	248.554	251.4219	437.0635
CUST_46239_PI429010886	M71904	0.012709555	12	3						77.84495	88.12342	9.10076	150.4692	11.86483	155.7969	150.3467	87.9342	69.28931	60.06954	47.30405	13.47437	102.5315	147.0201	169.1953	381.6653	197.0196	231.8895	242.3852	20.5703	199.8063	23.08289	72.50711	16.53385	62.49633	73.60907	7.329988
CUST_28789_PI429010886	N58622	0.01271057	15	6						14.29658	8.394142	6.85944	12.18283	8.432245	9.870583	28.68225	13.44675	19.10315	23.05795	13.72622	15.34065	26.70103	10.04481	6.921979	13.63522	6.170099	19.30163	10.70762	4.60841	13.88386	38.86739	27.03026	44.45593	20.75357	17.25784	14.89285
CUST_14281_PI429010886	N32627	0.012718684	24	15						152.4015	231.9984	206.2842	186.1968	178.7734	170.625	221.596	189.476	233.8458	227.7726	184.9052	215.601	253.7732	207.1791	228.8193	216.7613	308.8707	245.9747	181.173	238.9099	227.3555	186.9529	195.4062	248.9012	193.2741	200.5182	227.3921
CUST_44725_PI429010886	M66795	0.012750385	24	3						31.76944	33.51959	11.93224	15.23086	39.95843	37.1161	12.91815	48.98205	23.16492	10.65068	21.31047	17.39987	12.38585	26.61351	16.80772	11.55881	11.48082	6.25226	10.95923	22.905	12.08298	23.74286	45.90556	14.93713	9.055283	24.84254	14.35294
CUST_17269_PI429010886	N37681	0.012750385	21	15						107.8755	148.8152	151.3468	88.35558	82.85507	122.604	117.1549	158.2614	113.8069	117.7199	121.1967	98.46402	109.207	130.5494	111.6865	191.6851	187.3814	171.9553	118.6245	113.1623	112.5405	95.95298	138.3923	95.44831	89.06904	141.6537	85.54538
CUST_29937_PI429010886	N60846	0.012755718	12	3						73.39156	84.42785	8.360391	54.20497	NA		56.28495	141.4616	63.9762	99.99679	55.36247	38.73021	5.073594	64.88783	63.88253	70.36116	282.3174	120.5704	111.9225	164.9432	NA	112.0077	7.567646	47.42663	3.952416	70.90586	60.99956	5.694875
CUST_48145_PI429010886	M79088	0.012772526	24	18						12.09948	10.12509	12.76732	9.528739	9.586868	7.725643	28.22616	NA	31.91281	19.61588	NA	7.514308	23.27075	9.466716	NA	18.67988	9.545474	6.046138	42.4761	40.67888	93.27554	15.93995	17.74047	18.79971	18.79083	8.24518	11.19922
CUST_37362_PI429010886	M18791	0.012783064	24	15						1186.54	1930.481	1356.93	1156.223	1086.78	1028.106	1471.771	1306.636	1350.405	1409.957	1139.062	1087.769	1355.196	1005.258	1261.275	1563.253	1821.979	1666.955	1302.008	1461.32	1635.354	1219.83	1717.387	1506.647	1347.499	1136.139	1484.484
CUST_14620_PI429010886	N33184	0.012783064	24	18	332372624	unknown [Dendroctonus ponde	Cellular component orga	Chromosome organization	2.91E-22	23.28393	33.37664	21.3518	24.54259	15.33232	9.647611	17.91933	16.76036	13.48522	31.76584	15.89766	12.01856	16.31277	16.82471	20.91851	24.68878	20.19611	31.41185	32.0098	31.69335	26.60979	19.7414	23.93602	19.02389	25.6877	29.82705	26.68755
CUST_21397_PI429010886	N45146	0.012783064	24	3	260813040	hypothetical protein BRAFLDRAI	Metabolic process	Nitrogen compound metabolic process	0.000000235	12.4284	5.921908	13.31624	15.35106	69.99017	87.80111	12.65976	18.59394	7.951293	10.44922	93.60566	65.71584	15.88272	61.80424	55.16243	3.727461	6.584517	4.590859	7.156722	16.65548	10.05333	9.568695	13.17136	14.05555	16.18394	14.19525	13.08993
CUST_9983_PI429010886	N25511	0.012783356	18	6						56.36738	112.7612	76.3265	67.64673	59.67994	64.17487	91.18387	92.11335	89.57931	108.7707	83.83975	90.89393	44.88816	23.16272	55.08838	40.26778	122.9659	27.23388	63.40282	52.84329	89.57069	70.17999	73.22433	109.6043	117.9968	99.84349	82.54419
CUST_57276_PI429010886	E3651	0.012787067	12	9	4585366	extracellular superoxide dismut	Metabolic process	Oxidation-reduction process	2.4E-21	78.57224	37.65275	108.4858	150.9904	121.3713	52.09068	44.82733	106.7562	157.1577	110.0991	158.9852	122.201	320.8974	200.5807	132.5675	26.55272	34.35444	31.62493	208.5115	268.4249	336.3654	274.5666	452.3252	470.2656	173.7818	217.7022	408.3889
CUST_35892_PI429010886	M13825	0.012787752	12	15	157118739	oligopeptide transporter [Aedes	Transport	Other	1.82E-37	41.59688	41.97418	33.92168	56.65739	77.95933	92.63339	54.48188	97.30769	71.79771	38.56693	75.96439	62.16915	54.70587	84.31048	47.82733	69.6376	89.16012	66.71306	67.51408	64.25198	63.47982	62.52777	75.7215	66.15369	54.8814	54.77517	41.33036
CUST_24487_PI429010886	N50703	0.012793869	15	6	390353410	PREDICTED: crystallin J1A-like [S			8.61E-11	32.58484	27.37201	38.43	31.45703	27.1479	38.90574	44.77327	33.87813	53.22588	34.26345	34.32507	33.97881	44.43314	26.71617	25.14815	33.06699	40.86304	33.85619	30.865	38.59753	41.21871	51.51478	49.77832	44.52658	43.64769	39.78508	34.5207
CUST_53365_PI429010886	M101416	0.012796447	18	3	220172369	cuticle protein [Rimicaris exocul			1.44E-17	21974.84	592.5764	19387.17	36406.05	28414.17	28743.72	2598.143	4900.819	6051.213	23961.37	34877.59	23435.22	29294.9	19237.66	24136.69	559.9215	500.9726	557.3127	9026.68	6439.853	9313.798	34877.59	21590.74	32064.51	9257.227	18802.8	17524.49
CUST_35126_PI429010886	M11657	0.012796447	24	18						1316.811	1752.658	1578.545	1691.116	1224.714	975.2848	1713.82	1957.624	2130.512	1900.457	1347.499	1200.042	2216.187	1607.994	1131.397	3235.804	1824.284	2983.608	2491.674	2042.326	3053.693	1998.783	1322.312	1712.689	2281.476	1323.872	2416.236
CUST_35290_PI429010886	M12157	0.012796447	24	18						65.84152	90.77498	86.68025	67.02911	65.88877	62.61027	70.22094	94.54361	105.3095	73.60907	82.42094	60.60253	89.37424	75.70089	73.17975	96.42996	104.2696	97.3611	76.58195	100.3755	108.623	94.87741	96.40725	97.01999	98.2692	94.21867	121.6162
CUST_40077_PI429010886	M49819	0.012796447	18	15						47.9302	66.68933	63.07768	33.4748	17.99307	3.577182	448.4659	59.16985	280.8283	277.4932	39.88527	159.9313	70.89953	36.04308	32.60933	193.2741	345.8925	187.4758	152.5572	65.24075	116.3062	46.97174	49.82997	54.32526	87.54238	72.53637	103.6054
CUST_38620_PI429010886	M28605	0.012803661	18	15						8750.959	17524.49	12648.44	8321.498	3670.22	1976.903	11289.6	8387.726	12631.63	10587.95	2684.097	2144.939	10394.61	4694.435	5625.087	16106.84	20434.9	18320.9	13559.72	11998.69	13635.69	8309.047	4020.487	5697.98	15900.37	4940.836	12648.44
CUST_34309_PI429010886	M9294	0.012806063	24	21						163.2927	280.3649	233.412	186.0544	197.8282	219.5694	191.3144	263.6152	233.1508	162.2343	188.1296	178.863	192.9642	187.2522	207.1407	254.8129	263.251	223.7206	250.6739	244.2151	241.7973	260.3413	232.1589	240.6918	258.6669	235.338	265.7303
CUST_52002_PI429010886	M96025	0.012815673	24	21	383851597	PREDICTED: synapsin-like [Mega	Signal transduction	Cell-cell signaling	1.42E-44	59.05616	49.85973	60.65305	51.19908	44.46588	38.38927	38.48962	37.89734	35.74554	60.14723	39.78508	61.99865	53.98617	42.00294	56.31298	52.65281	38.79203	69.06598	58.52453	33.48437	50.36291	60.68646	61.8234	71.08446	78.08717	51.60691	87.89749
CUST_4734_PI429010886	N17371	0.012815673	24	21						1136.704	474.5724	1042.247	308.1102	278.1319	179.2719	217.3409	371.0946	287.0864	512.4944	655.9099	708.4537	2368.349	994.081	939.4725	966.6426	281.5792	1071.48	830.1589	1274.879	1406.649	245.3228	1520.832	1630.398	1081.498	2329.582	4291.944
CUST_13415_PI429010886	N31139	0.012815673	24	18						415.3144	565.424	478.0895	456.4069	339.5673	352.3291	383.7931	329.5816	484.6312	444.5929	563.6439	514.2205	481.4474	486.8888	503.9395	518.3699	386.6278	520.7346	435.731	637.7947	863.5664	483.3359	518.2326	667.4336	419.0196	465.4767	764.2764
CUST_5428_PI429010886	N18688	0.012818458	12	0						57.26697	48.20502	59.57649	52.2471	196.4999	123.675	17.77119	31.61116	25.09771	39.12428	69.29751	13.01831	70.23956	162.5329	206.9345	33.1947	24.45128	41.92055	23.53208	33.67973	27.5331	43.70538	35.61909	66.84176	38.17853	50.07055	32.1267
CUST_761_PI429010886	N7195	0.012818458	12	6						78.04556	75.2384	35.06409	49.99888	49.9052	72.16617	220.2503	359.0835	170.6675	44.80668	45.68766	53.46841	30.42641	57.76119	44.07411	49.04219	43.70175	48.32509	86.51281	273.2919	76.92175	39.06825	211.9713	41.06421	28.08202	78.73755	32.94464
CUST_28973_PI429010886	N58940	0.012833912	21	6						26.13038	41.77121	32.14243	31.87855	33.43972	22.8273	40.32964	29.26951	36.40731	33.48738	31.02469	33.1607	24.21058	27.37201	18.69793	8.605501	28.3766	29.93109	13.98821	9.165105	15.62048	30.06311	32.33684	34.62846	26.10261	32.33684	39.76397
CUST_24909_PI429010886	N51465	0.012838532	24	18						82.23633	157.9135	161.586	132.0358	49.476	88.62537	69.79048	168.7955	183.1326	128.8131	64.95625	65.90164	316.6599	106.3359	129.5648	78.4622	157.1785	148.0265	202.7719	229.2847	311.4501	261.5031	84.42785	269.4621	211.0148	104.9377	133.8642
CUST_52329_PI429010886	M97076	0.012838611	21	0	334326889	PREDICTED: zinc finger protein 2			2.97E-26	44.3343	79.93916	65.85526	61.77408	42.10969	34.86478	59.81752	53.6019	43.26708	44.77796	43.93243	38.89189	38.53951	32.67316	28.88374	38.49334	55.59842	40.76995	49.19586	45.37684	33.07479	46.07508	48.21063	45.99785	43.73128	43.49621	46.43071
CUST_1443_PI429010886	N9654	0.012839392	24	3	121484216	cuticle protein CB6 [Portunus pe			1.3E-09	331.5173	132.6978	182.5585	852.2653	3295.67	2123.927	130.5329	357.575	113.8967	149.8459	2002.791	2388.437	19.46038	2103.097	2356.919	97.51431	39.74479	35.74167	16.05617	416.1276	13.66845	206.5859	66.83189	166.3995	NA	621.1319	NA
CUST_609_PI429010886	N6315	0.012851129	21	15			Protein metabolism	Proteolysis		1373.039	944.508	414.4974	864.5859	108.0194	608.8174	1680.655	2761.678	2306.791	1945.845	361.5772	1194.342	1173.346	1272.378	314.5854	3463.912	2026.561	4224.791	2922.057	2201.915	2247.929	1064.289	107.4112	823.2974	1460.387	538.8011	810.1648
CUST_13912_PI429010886	N31994	0.012861886	15	9						62.80159	55.69808	72.26194	40.30891	53.01138	58.0985	66.51767	65.33664	63.45821	67.7438	59.48775	70.7916	51.84236	38.43848	70.2307	65.22345	62.02003	62.03768	46.79452	50.74506	52.79948	60.4658	53.45027	54.77517	82.41127	54.13743	68.69203
CUST_6746_PI429010886	N20549	0.012868806	21	18	256574617	Marsupenaeus japonicus MjGo r	Signal transduction	Other		40.99989	38.16041	35.97416	38.00456	35.59906	32.04577	19.43519	24.28735	37.01424	30.17026	23.56774	43.75295	31.93549	36.97455	27.42138	35.55192	31.16363	32.90163	39.50981	42.61451	39.41926	47.70295	34.27152	35.99913	40.98681	38.99638	31.07888

Probe ID	ID	p-value	Period	Phase	EBI ID	Annotation	GO category	Go term	E-value	0	0	0	3	3	3	6	6	6	9	9	9	12	12	12	15	15	15	18	18	18	21	21	21	24	24	24
CUST_25056_PI429010886	N51706	0.012872228	18	15						26.30955	48.54356	44.9217	41.07927	25.39051	30.02053	33.18845	48.59053	43.12031	28.4735	34.03486	22.23439	34.15718	41.99895	28.17546	57.30682	59.39503	61.31764	39.90127	42.04361	37.93869	30.26815	38.39581	36.8828	32.33684	35.96302	36.64741
CUST_2709_PI429010886	N12956	0.012874749	24	18	321466698	hypothetical protein DAPPUDRA			1.35E-41	6.232148	22.9969	16.51333	5.156021	8.878239	3.70239	8.649765	14.12473	10.83697	4.033349	10.74743	6.045196	4.284906	15.36528	10.31236	10.9325	17.00862	20.63115	9.838142	23.2664	28.5784	15.58431	10.60141	16.80142	14.43997	17.62376	16.26708
CUST_28357_PI429010886	N57795	0.012877635	18	0	290792704	nitric oxide synthase [Litopenae	Metabolic process	Other	7.49E-23	27.53743	11.3139	25.64005	15.00826	7.907113	6.706445	6.071811	12.8517	7.782145	NA	6.385639	13.49776	12.88445	11.25075	8.887381	11.1375	14.1426	15.9565	14.02697	29.38788	39.80982	24.92085	3.966136	5.741944	11.58143	4.42489	13.69119
CUST_28674_PI429010886	N58406	0.012911782	24	15						75.95975	72.30895	60.69474	64.67753	66.76294	49.1413	76.53128	70.90586	64.69656	94.60841	55.2321	92.12733	90.57569	44.89359	88.37671	94.38205	92.75983	108.9127	96.17648	53.66051	86.11117	67.25975	52.33755	89.67162	83.05204	66.72007	77.32154
CUST_13021_PI429010886	N30453	0.01293503	18	15						134.5227	216.9684	243.9595	158.5618	176.5278	143.3201	138.5257	200.1541	169.8826	168.511	104.4886	160.1938	193.5276	124.9059	142.7074	179.7279	316.2435	271.9604	175.4589	237.4902	233.1818	147.2139	228.9535	169.0416	204.8443	131.3179	132.4149
CUST_49343_PI429010886	M85273	0.012942632	21	0						19.9833	17.50753	18.1573	16.79206	11.10457	9.149353	8.084291	6.576754	15.42768	12.30677	NA	8.648652	14.42489	9.647611	3.385856	17.58246	18.77257	13.19225	14.73062	20.46917	13.13946	11.81016	17.57495	15.67014	21.7119	12.83052	14.52682
CUST_828_PI429010886	N7437	0.012945243	18	15	321459544	hypothetical protein DAPPUDRA			1.24E-49	1408.778	5476.032	1842.758	1745.528	350.8666	1056.414	5015.024	2564.444	3064.959	4291.944	1719.004	1620.506	2742.804	2225.697	1543.849	6854.464	8417.477	7785.133	3179.907	1227.762	2968.824	2181.465	1444.952	2005.153	4086.34	2593.566	1639.362
CUST_56201_PI429010886	M113088	0.012960885	12	6						56938.34	55277.47	35157.95	72794.71	53496.25	55277.47	67856.09	66456.69	82051.33	62699.58	40915.77	37632.98	63245.57	58747.16	33963.44	73758.94	57775.61	62215.85	72794.71	69178.17	71692.31	61768.02	34877.59	62215.85	60302.68	43701.56	56938.34
CUST_17938_PI429010886	N38867	0.012963566	24	0	321456733	hypothetical protein DAPPUDRA			9.81E-29	28.16647	34.04952	37.74193	35.7936	20.47681	21.93896	24.70036	33.17685	32.33684	16.66408	21.48061	13.94414	23.73245	23.01193	20.21346	36.6656	42.70334	17.94352	30.10418	24.73755	35.02813	26.37524	33.05472	30.32287	36.61091	34.22791	24.52594
CUST_32037_PI429010886	M3069	0.012971962	24	21	546679138	hypothetical protein D910_070C			1.01E-148	22.66351	22.38325	56.48171	19.8408	28.54797	23.6825	17.47317	22.53181	22.38744	24.68878	23.85395	27.76693	19.69655	15.54182	27.20944	33.43295	31.66129	35.47011	35.72049	22.30617	23.8475	45.00194	31.09966	33.88276	38.48559	29.97997	36.97455
CUST_39135_PI429010886	M35032	0.012971962	24	18						2722.979	6791.114	4093.741	2125.115	1908.561	1650.878	4270.698	3584.754	4548.63	3277.985	2095.253	2477.377	3269.912	2602.477	2201.915	3310.069	5304.997	5769.472	4392.913	4427.539	6533.018	2605.771	3286.945	3215.844	4334.63	2708.146	4145.789
CUST_6534_PI429010886	N20226	0.012971962	24	18	270007790	hypothetical protein TcasGA2_T			8.43E-79	98.79304	162.9942	124.0306	103.4755	77.47208	81.70379	105.4796	132.9661	139.0252	119.4159	100.315	134.7613	129.2573	106.365	103.8143	119.1399	156.2107	172.0135	128.7652	148.3838	173.1388	134.8435	141.8433	167.7963	144.2213	128.1449	170.9358
CUST_8768_PI429010886	N23558	0.012971962	24	3						56.8039	44.40911	58.67768	46.9602	124.2958	83.78528	42.9709	49.08691	34.8722	23.89577	85.95962	102.8149	38.16041	69.99598	63.92344	38.73021	37.34896	34.67259	27.70822	43.66648	23.32566	42.98811	29.09105	45.23287	32.56737	53.09948	35.49229
CUST_22652_PI429010886	N47403	0.012971962	24	18						25.71638	43.13077	34.99184	28.24828	30.36962	23.40563	30.32915	42.49657	28.87751	29.22014	43.51485	30.05953	34.76331	30.63675	33.29588	54.42945	33.3337	36.47269	51.22586	36.63348	35.38735	33.91504	34.37978	38.52068	48.78916	32.98204	53.66499
CUST_56061_PI429010886	M112656	0.012986547	24	0	321478408	hypothetical protein DAPPUDRA	Transport	Ion transport	2.78E-88	306.8476	391.8616	321.3471	279.5075	291.198	273.0279	228.1333	272.7395	304.4972	297.1434	343.5695	285.0842	275.7847	270.8095	308.8707	272.9604	363.297	316.2911	272.0606	306.3603	277.7202	333.2833	381.849	393.1863	365.0376	406.9392	563.4896
CUST_42105_PI429010886	M59408	0.012991325	18	15						84.87009	101.3719	96.59602	109.6178	71.80743	67.94466	95.57738	75.27273	82.54419	100.1918	88.97825	78.69416	110.4898	69.29751	83.67777	105.442	105.3437	117.7425	88.53491	89.86465	96.89897	75.59966	74.72923	75.58044	96.73368	81.4132	113.9812
CUST_44125_PI429010886	M65006	0.013013203	15	0			Transport	Transmembrane transport		15.77285	11.55337	20.2155	25.39952	11.04247	63.23529	10.98592	14.61522	7.333794	19.78273	12.6579	12.28481	22.54151	3.916503	17.68234	28.31587	25.26406	20.80025	12.41811	12.42618	15.58166	11.87749	6.817435	8.684295	9.140908	12.69014	19.31397
CUST_310_PI429010886	N3767	0.013017894	18	12	3907619	Penaeus monodon actin 1 (act1)				64.94907	13.50832	30.81577	23.29521	13.31994	22.45935	13.28096	24.04754	8.645163	NA	9.217287	5.202657	89.3399	108.1577	NA	30.3404	78.07876	60.30099	50.58863	85.3597	99.30177	NA	3.575449	NA	NA	NA	NA
CUST_6301_PI429010886	N19921	0.013020759	15	0			Metabolic process	Cellular amino acid metabolic process		21.74943	58.80984	51.00867	40.20755	56.64171	37.12911	37.88233	43.33538	35.73797	37.60365	27.83033	27.68431	27.69944	23.21635	31.83324	42.75074	57.14912	47.90004	38.67879	50.22	42.38319	32.77954	44.50237	40.11852	21.35401	39.17612	36.1598
CUST_35485_PI429010886	M12690	0.013022477	15	6						2580.428	1910.884	2948.008	3154.32	1071.939	1680.655	3426.429	3179.907	6799.146	3847.793	2954.482	2922.057	5456.239	2228.732	3835.495	1326.216	855.5436	2379.92	709.9527	1688.167	2455.967	2978.418	5329.024	5032.372	3082.237	2224.156	4885.181
CUST_14044_PI429010886	N32234	0.013025337	18	9						3.574738	5.74117	5.762763	21.65484	7.958428	7.189279	112.5544	5.874713	92.60339	30.16583	10.30511	25.34302	44.77327	8.962349	61.06467	11.49555	NA	8.959857	8.009934	4.552592	11.09797	26.19566	10.99723	11.98667	13.35744	5.333703	15.4109
CUST_4337_PI429010886	N16535	0.013034266	21	15						7.537524	NA	8.11425	4.706457	NA	4.904795	9.844862	NA	10.12016	NA	8.060296	9.534814	14.76924	NA	9.625637	8.506507	22.54343	10.9534	12.06381	5.665078	9.645129	18.53166	10.94861	NA	7.662672	8.295728	5.605713
CUST_5414_PI429010886	N18669	0.013044997	24	15	260790497	hypothetical protein BRAFLDRAI	Metabolic process	Nucleobase-containing compound meta		104.0463	149.3002	146.2607	75.61315	82.25472	89.86465	144.8055	116.7583	133.7558	117.1717	101.3884	97.3611	106.7424	87.70118	115.4302	135.6848	188.7958	199.2487	152.8709	119.2442	170.1396	138.5257	107.5789	154.3725	129.4391	81.55186	184.2288
CUST_51614_PI429010886	M94232	0.013045708	15	0						40.634	31.37822	NA	23.70418	NA	24.08726	28.27902	12.60565	16.41767	NA	NA	NA	8.084291	23.85867	12.50303	63.0922	25.48121	53.72417	44.99422	4.12101	29.39363	7.085828	7.187709	NA	13.4773	10.18184	NA
CUST_26966_PI429010886	M55196	0.013071224	18	12						8.246623	9.010934	3.930033	4.44137	NA	4.074046	5.758808	6.794495	11.73235	9.810166	8.28049	10.81711	12.79123	9.155794	16.88729	4.279633	NA	12.34025	3.534327	13.54908	15.04687	NA	5.270725	NA	6.570223	4.749956	9.524903
CUST_24184_PI429010886	N50189	0.01307798	24	3						51.22586	33.10297	NA	32.22297	123.305	79.74416	45.94141	13.96585	53.90529	13.31009	42.90092	47.44242	7.514308	43.27193	30.01509	44.90938	39.79966	5.111708	7.831289	26.40151	12.16843	12.85905	12.84199	7.030628	NA	42.30272	NA
CUST_25538_PI429010886	N52593	0.013092101	15	12						17.30194	9.405457	17.01558	11.79624	23.74852	12.1699	12.60565	15.15744	14.91599	11.77966	25.48121	16.16065	24.62171	20.32715	22.43976	16.49344	23.4616	14.9436	10.9183	10.33559	11.97918	4.188537	11.06316	14.96903	12.8105	14.72662	15.87729
CUST_1716_PI429010886	N10515	0.013092238	18	12	270015067	hypothetical protein TcasGA2_T	Metabolic process	Oxidation-reduction process	4.39E-80	34.75367	49.50343	43.24994	48.43927	32.13989	28.11335	59.79025	50.04692	59.01526	52.19714	50.57582	35.10272	72.06126	56.21355	49.63757	41.95534	34.99565	49.37308	41.76732	53.09948	75.82447	28.53195	41.67619	38.48209	36.92118	38.48962	44.46588
CUST_1872_PI429010886	N10927	0.013094039	24	3						1971.443	345.1684	1913.068	2020.363	4450.275	2952.044	335.8993	1370.424	357.0377	312.1009	2395.468	3289.37	197.1967	2125.115	2171.504	258.8256	142.8045	81.49613	135.5781	690.7831	121.6909	669.7814	162.9278	621.9301	33.50527	663.1483	30.54671
CUST_47560_PI429010886	M76608	0.013103312	12	0						312.8962	33.74242	252.8481	300.4274	73.69276	211.4099	31.01416	15.75178	32.43254	147.9373	65.69029	117.8103	314.6496	110.45	257.7274	50.53473	32.65194	53.73825	27.63116	23.76085	20.1058	412.0092	141.9381	302.0716	60.4658	58.72904	91.46995
CUST_15073_PI429010886	N33964	0.013103312	12	0						22.20311	18.28134	36.61868	25.34518	21.48216	18.85511	13.14676	13.91642	11.97624	19.35535	18.06696	22.2879	19.00985	20.17748	24.24843	19.91817	15.21327	13.86535	16.80772	15.61685	16.4789	13.75076	22.52975	17.33156	20.57756	17.78324	18.74591
CUST_41350_PI429010886	M57524	0.013103661	24	21	356651196	heat shock protein 21 [Macrobra			1.35E-14	81.5975	90.41336	103.5895	67.00003	71.24846	90.05411	55.2321	79.82778	70.5683	74.63974	69.66595	103.2365	64.54817	78.26455	70.91565	108.1394	92.47181	111.4813	84.73544	62.09142	76.67528	99.98511	92.50275	124.8368	88.52477	78.84424	80.8714
CUST_42692_PI429010886	M60985	0.01312561	24	15						16.61523	30.63415	28.35837	35.64607	24.56549	40.23235	38.25388	33.07479	33.06699	45.90076	30.33619	43.64157	35.84878	31.09316	32.03897	39.63021	48.91526	46.47246	19.09012	24.84514	34.81487	42.25498	26.2902	34.82552	19.73975	32.45769	25.67959
CUST_45595_PI429010886	M69613	0.01312561	24	15						42.78489	27.11349	27.87026	37.51953	41.85107	34.60522	69.71083	35.18743	54.96965	38.46213	38.56693	34.19123	38.04561	57.9144	36.43974	78.57224	45.67933	41.92879	86.8615	25.9357	59.29331	25.95809	60.68646	44.83771	18.27698	22.4679	18.20374
CUST_37125_PI429010886	M17932	0.01312825	24	18						109.892	295.6289	229.6141	78.84424	87.99932	68.36093	122.8685	237.2821	242.1802	118.1715	119.6713	97.12878	191.823	199.718	170.6675	159.4491	196.6698	175.317	252.6661	224.3833	309.9338	122.9659	157.3672	150.073	243.4049	173.8872	251.9129
CUST_10121_PI429010886	N25730	0.01312825	24	21						56.77661	42.64535	68.07757	67.21667	57.24189	37.84156	19.11201	22.39225	36.04017	45.16793	25.08941	59.77062	56.70114	43.78502	62.38786	62.28036	53.90894	57.11305	63.80586	41.1635	62.95202	73.09797	30.41006	68.4765	48.02051	38.12224	79.2021
CUST_10996_PI429010886	N27103	0.01312825	24	21						62.29516	53.22588	56.87317	55.11762	50.41173	52.17564	52.03911	41.54672	47.40679	70.14108	48.95167	48.80597	59.11174	40.19489	59.17675	65.64875	61.2068	61.24535	48.8281	44.74502	51.57473	67.53847	68.37633	66.88415	70.15908	57.24189	74.95343
CUST_25910_PI429010886	N53284	0.01312825	24	3	546675596	hypothetical protein D910_041E			1.98E-15	25.30132	17.47501	13.08835	35.4224	40.96379	32.16999	21.79222	7.240266	8.692858	24.59592	23.94374	23.21362	24.09713	27.18132	16.55821	18.73028	30.89131	11.26538	12.47033	9.605488	15.65667	18.78483	16.18532	17.10849	19.55672	22.36969	27.30572
CUST_30015_PI429010886	N61005	0.01312825	24	18						261.6417	342.1248	345.3559	309.9865	244.3561	247.9236	347.9094	300.7552	333.5586	327.3332	268.2532	318.2644	362.0388	256.0953	294.3326	336.8769	375.3353	395.794	350.5162	355.4873	424.3124	320.1071	338.4518	404.6157	388.6637	346.1134	524.9759
CUST_53128_PI429010886	M100321	0.013128552	24	9						27.7582	18.06134	17.50536	28.84569	20.03497	14.31897	24.83681	20.42168	18.38358	21.44482	21.48961	25.56181	29.87961	19.16983	18.47386	14.03865	27.25852	17.73086	19.18875	11.85566	15.65079	14.47877	14.30221	17.72282	9.715358	15.44438	16.26288
CUST_38883_PI429010886	M31592	0.013128552	24	15						2393.197	3441.376	2844.645	2747.205	1819.509	1754.468	3728.752	2811.043	2761.678	2652.405	2053.392	2666.243	2816.959	2554.596	2480.784	3415.242	3683.886	3467.123	3546.981	3470.741	2532.427	3336.354	2765.552	3331.869	2650.032	2174.954	3033.527
CUST_25911_PI429010886	N53285	0.013128552	24	15	242022418	heat containing protein, putative			1.01E-11	86.09864	90.62398	84.88526	54.93619	48.74364	55.84882	66.70374	70.16535	79.82778	93.8595	61.82712	80.93024	88.96871	72.5147	85.14751	116.8305	90.36229	94.34203	114.7972	59.19847	77.51144	118.702	64.89736	84.69767	124.1583	60.02726	98.98816
CUST_55942_PI429010886	M112226	0.013128852	18	12						66.17651	48.2227	66.54894	54.67195	72.21507	56.52874	50.4056	61.01777	53.53275	51.7172	119.5461	114.4851	77.08874	91.72224	123.988	82.21745	56.26482	64.70441	51.83065	43.69757	99.53837	61.4292	42.92276	61.07789	46.2714	44.19365	50.33133
CUST_36559_PI429010886	M16233	0.013130267	24	9						435.8605	284.4024	470.2656	344.2242	300.1105	382.9427	361.4106	226.5718	383.7931	637.6043	229.7477	546.8689	381.0871	338.1914	376.2092	285.6412	237.5415	284.9168	319.8754	202.1456	220.3217	233.7667	172.6519	223.3238	281.2628	343.5695	232.3662
CUST_43467_PI429010886	M63131	0.0131266	21	0						147.2992	67.77134	128.5506	50.46073	47.11911	50.40096	51.94695	43.89958	43.84747	53.88747	46.0486	65.35932	64.40257	42.84582	56.61772	71.12138	49.50741	50.0131	87.98812	56.89319	63.12641	94.33013	44.71461	66.95538	80.89811	47.39234	71.91459
CUST_31677_PI429010886	M2148	0.013144212	24	18						721.6825	708.4537	765.1767	591.4732	691.8963	537.7378	725.1557	721.9277	996.7263	430.8354	544.0973	522.1886	765.7762	848.7243	671.5725	599.8602	609.6218	763.8013	1016.824	972.8403	1223.646	719.7217	597.4035	813.7492	782.4302	547.5307	1049.045
CUST_34544_PI429010886	M9977	0.013144212	24	18						751.2377	698.717	925.9054	660.1893	782.6486	609.8498	826.1267	747.5407	1020.798	512.7881	679.224	737.5198	997.6033	834.7743	692.3061	583.1603	820.3087	826.1267	1096.577	999.5752	1220.331	918.4461	814.0506	896.2629	866.3088	624.5971	1043.605
CUST_54994_PI429010886	M108354	0.013164519	15	0	391346464	PREDICTED: myosin heavy chain			3.25E-20	28.80996	30.6586	35.23063	30.89721	39.7958	15.98138	13.70444	38.55172	17.5656	11.91866	22.81652	25.0443	19.70046	27.77009	31.77803	33.55276	53.63076	33.79728	15.82041	19.9436	51.86305	22.78318	18.05364	4.077908	24.46253	20.54806	31.12893
CUST_16813_PI429010886	N36909	0.013164519	15	9						248.2232	329.3072	379.4005	253.3528	292.8189	235.3802	195.082	317.9491	272.901	293.6993	372.4691	324.9732	317.3631	311.8576	335.8993	258.4078	357.3731	286.2998	284.6794	297.3675	273.5066	306.279	254.3715	281.1928	374.7855	356.6938	436.0075
CUST_21376_PI429010886	N45105	0.013164519	15	9						124.9756	127.633	179.3306	127.2209	153.1388	123.1089	103.2365	133.8844	142.6458	130.3114	174.6626	157.7159	130.1528	137.4424	177.4189	128.0468	146.7827	131.274	107.9667	143.4863	117.8323	134.5227	151.0262	138.1892	162.7885	197.2974	191.9474
CUST_38402_PI429010886	M26281	0.013174156	18	15	9367086	Euphausia superba large subunit			2.98E-175	117.9775	7.964923	72.53637	73.81857	82.51294	69.69671	NA	4.830349	5.60961	43.31181	48.5966	94.12797	27.2558	56.52239	68.59824	NA	NA	NA	NA	NA	NA	27.79864	31.48667	44.40911	6.772005	24.14564	27.3201
CUST_19894_PI429010886	N42406	0.013183217	21	18						258.2813	301.4388	330.8653	242.3379	222.4375	230.8967	264.9858	282.6016	241.7527	254.4938	196.1132	254.714	266.8703	191.9474	177.4557	399.2455	207.8099	283.1093	379.9317	293.0253	290.5678	298.3249	270.6292	305.7738	364.9578	247.6563	248.554
CUST_7810_PI429010886	N22117	0.013185415	12	3	459175521	PREDICTED: beta-hexosaminidas	Metabolic process	Carbohydrate metabolic process	2.53E-99	15.2887	41.79921	28.77495	57.49102	40.16189	55.60957	37.18271	40.99989	25.6877	21.2447	202.4277	41.32674	9.015243	28.28966	13.26377	59.25874	148.9148	153.6425	60.30614	106.1184	159.8736	14.23996	40.99989	18.96527	29.27204	23.50114	23.27971
CUST_34588_PI429010886	M10089	0.013191121	18	15	321457051	hypothetical protein DAPPUDRA			2.92E-43	16.52701	17.28788	NA	17.62807	3.191967	21.87085	20.2926	31.38832	6.95828	31.77537	9.207701	8.183217	15.4594	14.0887	14.82044	46.9602	41.44302	33.42941	16.44032	7.122688	17.18362	5.742729	3.662016	6.53016	29.28594	23.81043	8.378958
CUST_3637_PI429010886	N14939	0.013193826	15	6						906.4938	365.7666	1209.443	1265.098	833.2103	72.17918	748.1962	1249.87	2068.831	533.5339	882.793	362.2694	1381.205	601.9971	205.0181	121.6909	86.19309	182.9809	988.0134	802.754	742.7432	1974.35	1897.754	944.508	1127.159	247.8341	1458.832
CUST_5493_PI429010886	N18774	0.013221281	21	15	57525634	nuclear pore complex protein Nu	Transport	Protein transport	9.58E-126	57.41489	131.1039	94.28531	71.09432	79.43494	55.46697	91.6033	75.98892	94.1914	84.00818	102.222	75.76283	111.9372	80.74224	66.30335	76.30002	109.1085	96.27921	95.39909	114.3003	105.5272	89.86465	101.2809	78.85311	53.8169	83.79749	85.75696
CUST_18095_PI429010886	N39131	0.013225151	12	0	91078850	PREDICTED: similar to tan CG12			2.48E-26	12.01781	8.616008	12.74437	6.861282	9.00236	13.15346	3.960862	5.14946	NA	11.06604	NA	11.55626	10.33053	10.07727	19.08766	13.91758	10.10725	11.07815	8.232598	8.317068	4.289475	17.43476	4.893177	13.7591	23.30496	12.3169	24.6974
CUST_37380_PI429010886	M18841	0.013227453	24	18	478503308	PREDICTED: Ceratotherium simu				340.5595	696.3331	602.5729	403.4693	459.1993	309.9865	358.5218	563.6439	477.9381	432.6388	445.7269	370.5351	504.201	475.8315	394.5922	419.7244	611.3316	514.7289	531.8423	617.7634	621.441	537.7378	452.0404	574.0759	369.9777	525.9937	407.1156
CUST_31452_PI429010886	M1554	0.013233887	18	12	388523609	glutathione s-transferase d1 [Cr			1.88E-40	61.19974	13.58386	18.9004	4.93131	19.6697	8.876488	9.969471	NA	9.590111	NA	30.38583	21.65945	NA	34.74153	33.82382	NA	44.594	9.600991	20.66527	28.10987	18.98812	6.3219	17.26114	13.12827	7.852776	14.5111	18.52811
CUST_19878_PI429010886	N42370	0.013245418	15	12						40.48248	77.33923	27.3523	22.02084	19.61413	28.70626	28.55984	36.46888	36.8117	47.70295	24.22486	41.09779	43.8296	18.16767	43.126	40.3254	43.00754	77.69968	34.75696	7.933892	18.83693	29.37527	19.60068	37.7689	37.69532	47.80868	42.66588
CUST_5874_PI429010886	N19320	0.013259402	21	15	91085789	PREDICTED: similar to CG11418			8.31E-74	16.23895	37.3305	31.6335	23.43732	19.97953	21.0756	29.37844	42.27337	14.38486	34.39364	26.68521	25.3148	36.21981	41.18704	21.42365	32.81904	35.99005	57.42169	36.54707	8.93938	31.65828	29.83087	18.23431	29.95632	25.65087	16.29695	9.294827
CUST_36579_PI429010886	M16283	0.013284539	24	3						61.15687	55.40921	41.29878	57.30682	93.51275	64.91949	54.79166	45.26359	58.05044	44.42841	56.48171	76.53128	66.73428	68.08811	48.99934	44.83771	42.53392	50.80319	37.74193	45.76721	37.56673	55.0756	45.38696	56.97506	43.11409	53.02213	43.88384
CUST_50260_PI429010886	M89214	0.013284539	24	3						70.93399	56.38972	50.59319	79.83656	212.6443	165.3867	57.39387	58.69436	37.20362	30.28156	138.8443	97.69073	28.42265	174.857	152.3548	64.30529	51.12885	36.71863	22.87315	33.51959	29.09105	27.57725	46.1196	23.20404	30.43314	45.64087	14.44521
CUST_2622_PI429010886	N12779	0.013284539	24	21	15425681	I-connectin [Procambarus clarkii			2.17E-113	82.95322	83.14687	88.77171	83.59122	91.6714	92.78173	72.27956	75.55461	71.65566	73.86844	67.10107	105.4159	81.44134	95.48478	95.11745	101.3206	88.65754	150.073	107.509	69.4807	101.7482	101.3719	97.14951	113.92	91.11591	85.60411	102.0776
CUST_14076_PI429010886	N32387	0.013284539	24	18						118.0597	137.6772	206.5859	45.22389	18.33289	14.01914	96.99292	251.3401	236.5379	155.0166	243.0247	157.9287	70.94231	33.87813	60.03992	164.9813	89.54662	244.0026	208.2068	309.878	456.7205	167.0371	211.3027	260.798	250.1769	27.5259	296.2664
CUST_25031_PI429010886	N51664	0.013284539	24	18						142.5991	264.9542	255.6336	128.797	164.5889	158.4455	223.4224	265.9313	235.4891	296.6356	185.3999	256.4943	188.6256	139.3356	171.6359	340.4211	239.5846	332.3383	378.3539	309.2795	355.4873	381.5915	239.2973	330.3379	414.4128	273.9926	400.4361
CUST_35061_PI429010886	M11457	0.013300642	18	15						100.2745	169.0416	169.515	137.4424	141.0554	111.5065	182.4362	186.6058	143.5591	141.9381	146.9401	134.4304	168.9955	132.6606	161.3784	174.7074	278.8215	193.6334	142.3895	97.39106	157.4954	105.7387	141.0115	129.2198	156.9654	169.5998	158.4947
CUST_35224_PI429010886	M11957	0.013300642	18	9						5.558159	19.87929	23.26132	12.07314	17.29188	16.62059	19.09216	17.11834	18.28904	28.2959	21.74375	20.29057	19.70573	25.12907	15.65876	18.8606	24.67313	19.16983	13.81248	17.58246	19.30987	18.32878	15.58609	18.54037	13.55744	20.6404	26.49945
CUST_32316_PI429010886	M3785	0.013300642	18	15						55.58555	78.32421	24.95132	20.40806	27.6893	19.75767	76.2209	92.71133	54.54304	46.17834	50.36291	42.92276	40.63005	56.61772	48.69222	79.06068	73.37425	78.8865	40.48622	63.33988	59.77062	29.87493	67.19251	25.82506	44.34431	69.23652	41.53886
CUST_34184_PI429010886	M8985	0.013300642	18	15	383865231	PREDICTED: polypeptide N-acety			9.07E-16	83.77516	97.61964	55.04149	54.18142	71.02716	75.32925	100.1424	86.42647	65.71042	90.05411	66.1068	106.9545	81.39312	66.96376	87.68846	111.1388	113.1524	92.45168	86.92889	62.51652	56.37975	75.30381	55.1847	67.24604	72.24298	77.75578	57.34418
CUST_5390_PI429010886	N18632	0.013300642	18	15			Metabolic process	Oxidation-reduction process		20.02186	64.03624	47.49302	26.04805	21.27281	24.7753	35.35794	46.88337	31.22074	48.94611	35.85354	23.88544	41.02697	25.89322	28.56931	57.46071	53.43823	64.80138	27.55963	26.51854	21.69939	22.39651	33.09557	26.50821	31.96059	23.48308	30.40072
CUST_302_PI429010886	N3623	0.013300642	15	15	70909487	ribosomal protein S4e [Mycetop	Protein metabolism	Ribosome biogenesis	4.7E-96	28120.27	36043.3	33076.81	31659.39	24040.66	28492.44	35022.2	32295.88	36406.05	35738.11	27210.5	26987.8	30141.56	30065.36	24825	41865.42	41419.19	43701.56	30577.23	35738.11	38719.75	23658.24	25611.3	27665.22	32184.55	33176.01	31543.85
CUST_31912_PI429010886	M2721	0.013301296	24	15	242006797	smile protein, putative [Pediculu			1.09E-53	193.9616	292.95	245.9747	197.5273	237.5901	179.3019	192.4672	268.8247	224.1099	210.1567	229.9275	205.1648	277.3007	233.3746	237.6854	252.401	335.5615	315.2731	249.7255	275.1168	239.7136	200.2294	295.5482	262.806	228.7481	269.2064	300.1105
CUST_45229_PI429010886	M68404	0.013301296	24	21						219.2811	195.9057	201.5487	174.6626	172.2349	157.1577	183.2834	185.5286	208.941	207.5504	141.2321	171.2731	250.6739	170.7496	194.3126	210.6647	203.4662	228.8193	199.6163	199.5583	232.9522	245.9078	219.702	248.9453	197.1967	193.3026	265.3922
CUST_2759_PI429010886	N13047	0.013301296	24	21						234.6515	409.117	342.85	298.2569	308.6292	280.5879	295.5482	315.96	317.3105	257.5954	298.1868	259.7699	270.0743	295.6886	275.85	287.4074	418.6884	365.989	279.5075	326.2417	331.6434	330.7777	403.4693	392.3777	290.2155	360.6967	331.9294
CUST_45239_PI429010886	M68428	0.013328365	21	0						309.9338	NA	358.7305	310.4859	278.4811	328.3409	NA	7.526841	6.369185	203.0356	287.938	309.9338	135.4737	168.5856	230.9748	NA	8.058837	4.738336	8.622045	34.93184	30.48044	461.0647	256.2294	497.1127	19.64055	68.53151	41.43789
CUST_48635_PI429010886	M81373	0.013331504	18	3						108.8321	72.38107	101.6124	145.2258	135.0399	129.4913	82.04507	71.45149	68.91312	122.201	171.8413	127.9862	111.5617	88.51224	118.5947	88.95701	40.55768	85.20409	85.70234	56.09226	91.30891	121.1043	126.7723	121.5207	109.0071	105.5425	118.7775
CUST_1769_PI429010886	N10656	0.01333668	24	21	321473724	hypothetical protein DAPPUDRA			2.77E-38	4536.055	7111.995	5601.899	3428.918	3011.975	3140.215	2971.539	4097.003	4565.036	4118.017	4173.107	4329.518	4363.515	3503.626	4432.869	5251.996	6612.476	6025.479	3759.237	5777.198	5442.505	4475.935	5900.066	6125.57	5756.98	5322.868	7327.614
CUST_50280_PI429010886	M89343	0.01335761	18	15						2028.877	3000.392	2320.751	2393.197	2181.465	2315.807	2742.804	2826.773	3640.441	2180.402	1679.651	1854.805	2604.346	1861.323	1509.889	3437.158	3329.804	3851.521	2971.539	2329.582	3307.225	2414.182	2474.336	2240.494	2173.027	2245.768	2147.917
CUST_22512_PI429010886	M47166	0.013365174	21	0						80.5628	70.36913	110.2867	86.1454	64.15258	64.2158	52.0048	52.83064	33.88276	55.77263	41.94361	93.16926	48.73482	62.48982	65.49568	44.92669	57.75543	55.69808	65.34281	81.07922	70.32352	93.68896	55.80136	55.92368	44.9217	76.19975	58.31116
CUST_53173_PI429010886	M100542	0.013369791	12	3						186.8705	153.318	219.7957	197.1967	197.2216	184.0518	150.9904	169.2749	178.5273	142.4683	267.0027	123.5992	268.9804	301.6158	178.7424	204.3745	371.3022	187.0768	176.8026	160.6804	162.5822	157.8028	173.3624	155.8444	159.7718	172.6851	164.5889
CUST_2074_PI429010886	N11504	0.013395934	15	3	306530847	fatty acid synthase [Litopenaeus	Metabolic process	Other		30.7666	26.4908	15.55124	40.10315	22.35262	25.21148	26.90034	18.73426	15.74279	21.15511	22.58254	32.83178	12.74046	13.28376	14.92175	19.8869	22.55063	23.1537	19.52436	39.2895	37.84156	10.57979	20.66527	16.55502	16.28147	16.30898	19.1841
CUST_31516_PI429010886	M1703	0.013395974	24	15						58.72446	123.7963	106.365	78.55333	80.47316	81.48573	70.40519	98.56741	101.2137	105.3536	105.285	46.45729	104.7593	81.96955	92.0742	117.7735	146.4225	142.9786	93.80651	81.07096	114.7172	93.34171	93.42816	97.20086	104.7064	94.45356	90.3848
CUST_12905_PI429010886	N30244	0.013397565	24	18	391325031	PREDICTED: zinc finger protein 4			2.3E-11	25.59906	20.56044	34.06404	22.94332	19.2763	19.31126	19.71754	10.33559	19.92192	36.23648	14.60927	21.13144	23.0352	10.57021	24.08979	20.85648	25.37685	26.52119	25.30132	25.48697	29.03664	24.72767	23.92527	28.08202	29.19296	23.91323	30.15331
CUST_10536_PI429010886	N26386	0.013426689	15	9						12.71074	12.80959	17.22544	13.73539	13.28971	21.09601	21.29385	15.28613	28.18989	25.19098	26.346	17.63035	18.81827	14.60762	15.31897	28.98683	14.94729	16.4194	28.82109	9.657459	15.3929	28.17748	47.5648	24.04549	58.86906	36.62239	38.10178
CUST_54109_PI429010886	M104610	0.013424753	18	3						12.39819	17.99711	22.59724	29.88536	19.71875	31.75895	7.843567	9.531461	10.04856	13.97433	18.99955	27.0512	13.67378	21.91645	26.16413	NA	8.499991	8.250802	14.0225	9.207701	12.22382	38.19091	23.48308	36.57741	17.74254	12.1995	20.13887
CUST_31832_PI429010886	M2518	0.013440828	24	18	321474529	hypothetical protein DAPPUDRA	Protein metabolism	Ribosome biogenesis	7.48E-90	25.68152	54.09509	44.12566	32.20511	27.41834	26.6719	32.58484	47.98232	39.53772	35.35463	28.18026	35.67884	37.83455	40.64205	33.99771	35.45915	49.72483	44.94568	45.94658	47.26148	44.70872	33.93799	46.5849	35.57091	38.086	35.12021	46.68118
CUST_41402_PI429010886	M57656	0.013440828	24	18						170.064	162.0684	136.0268	198.0827	200.3302	188.9036	150.0105	149.0265	126.1825	146.449	160.1108	171.436	179.2149	159.8538	216.9264	148.0804	154.4876	147.9838	136.2348	101.7482	135.3635	141.4438	153.595	161.4291	118.653	148.8152	143.7214
CUST_48619_PI429010886	M81297	0.013440828	24	21						47.48815	52.90722	56.13868	49.33322	36.8117	39.87751	34.12139	35.87747	32.58198	50.63563	31.14654	56.56581	42.4761	31.3587	49.05917	52.44815	36.44264	49.33322	53.71769	36.35313	43.28942	73.1662	46.54514	58.71184	68.05647	43.60777	83.08821
CUST_7478_PI429010886	N21605	0.013440828	24	21			Protein metabolism	Proteolysis		33.91249	34.16048	44.1972	23.72424	35.55192	29.68598	14.37747	23.91003	25.70122	38.61107	33.99771	37.38952	38.26638	33.16894	38.06618	35.24152	24.87517	39.60825	35.21261	39.17961	33.71063	38.61632	36.31158	42.03292	55.45245	37.54467	35.77181
CUST_9613_PI429010886	N24924	0.013440828	24	15						2162.694	3117.642	3148.451	2549.09	1587.909	1028.813	3220.778	2383.479	3445.555	3706.021	2108.887	2204.749	4234.05	1982.92	2203.327	2753.439	2816.959	3549.354	4952.328	3525.214	4965.926	4069.14	4181.398	4415.56	4885.181	2846.594	6430.973
CUST_9912_PI429010886	N25394	0.013440828	24	18	3925803	pro-phenoloxidase activating en	Protein metabolism	Proteolysis	1.23E-14	25.18751	28.72644	47.86566	25.93791	20.80025	11.35899	51.8805	25.87427	47.70751	53.89829	23.00444	30.52273	59.36408	27.56589	50.16118	62.40732	24.86103	66.78529	74.44615	30.12869	55.40345	53.31643	28.14237	39.52112	49.24543	25.63215	55.65083
CUST_13120_PI429010886	N30609	0.013440828	24	18						106.6811	112.2698	82.96235	118.3504	83.78528	63.73816	94.29714	89.68248	78.9872	96.51035	94.43966	96.59602	140.2224	80.36141	111.2894	103.106	92.60339	136.3513	130.479	129.4391	89.1801	136.3336	136.0599	140.0958	106.7424	115.5725	165.7121
CUST_25756_PI429010886	N53005	0.013440828	24	18	119487704	betaine homocysteine methyl tr	Metabolic process	Cellular amino acid metabolic process	5.75E-15	451.6859	688.2575	655.3101	485.1077	535.2394	375.415	686.8238	533.7924	801.3203	632.6818	570.1346	423.7011	692.1008	491.1439	403.8629	757.8952	815.4763	672.7456	926.6453	811.9435	1004.862	617.3702	605.6304	601.3269	935.873	473.4786	920.4596
CUST_12294_PI429010886	N29246	0.013447078	15	9						10.80074	NA	10.67743	26.9245	12.69799	22.79631	NA	NA	NA	15.10467	33.92168	34.16334	20.99538	6.097172	24.7708	8.77972	NA	NA	NA	14.46087	12.71903	30.25516	31.52857	43.20383	6.936046	47.95196	50.38885
CUST_52039_PI429010886	M96174	0.0134497	24	0	345486208	PREDICTED: neither inactivation			6.94E-08	14.1735	30.19705	29.76143	26.45917	27.72605	20.54391	7.036384	17.39282	19.78846	18.44379	21.0554	15.43474	27.27378	18.9004	13.21287	19.78273	32.09223	27.1479	23.59807	21.04316	18.03992	31.06592	21.73515	27.1592	35.0396	39.39814	30.94993
CUST_18958_PI429010886	N40724	0.013449891	24	15						23.21885	51.70196	50.51811	33.83846	62.48283	21.47499	13.13097	44.92669	12.76017	53.93137	54.33188	54.78117	47.24767	55.56014	28.87751	65.43257	117.3106	105.0886	42.18222	29.95843	56.00071	34.97429	97.00725	65.26399	43.13936	101.8893	45.7171
CUST_22952_PI429010886	M47996	0.013449891	24	18						45.40234	71.67272	104.9226	40.16939	38.11089	26.69946	63.28914	121.6309	102.4255	33.80913	35.12021	24.4795	56.87317	53.77161	52.06198	60.31108	47.94598	60.55423	102.6861	124.5255	141.5813	48.96667	67.24604	46.41625	156.4627	54.18142	130.0993
CUST_7201_PI429010886	N21184	0.013455301	18	3						309.6955	347.3018	333.7063	354.2621	364.2801	352.6665	222.7682	300.3594	280.0915	278.1319	361.9035	394.5922	286.1798	319.1468	365.0376	191.6218	288.5968	239.1254	250.1769	315.8311	313.739	342.0443	399.349	438.6999	246.8231	295.9223	343.0574
CUST_15187_PI429010886	N34155	0.013455301	18	3						147.0201	92.46133	116.3995	299.9819	65.59134	208.9983	123.5992	102.015	99.52529	63.53279	31.10668	48.50466	165.3213	153.5452	78.74455	26.99962	101.8646	41.73602	108.4697	60.91215	83.9013	148.5291	224.1099	126.5408	66.15962	91.57245	26.85004
CUST_21808_PI429010886	N45889	0.013467151	15	0						39.76763	58.6831	57.7947	48.55532	43.87779	36.61868	59.5443	48.18525	51.72314	64.13867	82.40323	62.11363	57.38746	48.14157	43.13538	45.78637	49.8852	59.17675	61.94047	52.52277	43.97459	50.57112	70.47134	51.98569	71.01745	54.44704	70.86476
CUST_28516_PI429010886	N58093	0.013467151	15	9						83.03055	73.81857	40.46451	52.45861	104.0936	92.7356	44.88978	50.28036	54.31269	62.86145	60.61437	52.95714	74.63073	82.14842	70.81536	81.19478	75.95975	74.15901	57.98315	45.85631	59.17675	48.21771	50.4892	62.196	59.21121	73.89284	71.07642
CUST_46981_PI429010886	M74386	0.013468216	24	21						8.140119	20.90369	13.56287	6.465123	16.16637	11.69289	6.349458	9.292415	5.362097	15.27866	10.88981	11.86794	7.109967	7.728021	7.61802	9.946155	10.92685	8.673027	12.04588	9.113167	11.83032	16.13549	10.70343	21.45062	9.532396	13.58565	17.84752
CUST_4560_PI429010886	N17029	0.013468216	24	15						86.43772	85.2914	94.94344	73.17158	61.4814	61.95862	60.51578	64.94907	76.30972	83.3741	66.1472	91.03605	67.65721	62.99459	56.08545	100.2889	59.37073	85.40783	110.6304	72.06126	79.5268	135.6132	79.89483	96.60811	102.9045	80.10622	83.97595
CUST_31903_PI429010886	M2709	0.01347404	24	21			Signal transduction	Other		84.1875	64.44827	100.159	103.4024	73.00128	79.73452	80.41731	73.1662	82.50222	76.86672	77.03919	82.03251	86.70327	78.33025	60.91215	70.88457	98.78131	81.83086	92.7489	85.56504	82.98239	132.9353	99.30177	97.79679	89.16759	86.59225	105.9898
CUST_40415_PI429010886	M55018	0.01347404	24	15						499.6833	532.3756	453.4842	395.0243	294.0126	373.6591	513.0768	433.0002	507.5036	573.048	307.3952	397.7869	424.7669	347.9094	347.7046	701.0526	528.895	590.0254	588.0127	438.8282	534.1168	588.5037	412.0092	526.6092	562.4363	420.7391	374.5443
CUST_25011_PI429010886	N51628	0.01347404	24	18						345.1684	310.2931	291.2685	351.8456	230.6808	235.7954	299.8648	359.174	300.2438	396.9983	247.5032	299.6699	312.7556	267.0346	269.2064	556.2705	395.794	486.5784	426.9219	330.1913	511.8036	385.748	191.5454	367.4917	344.1561	248.9453	321.8933
CUST_51773_PI429010886	M95008	0.013484932	21	0						78.83688	18.87895	77.69968	20.50277	16.67154	22.15002	7.821834	42.75542	29.36117	9.132496	4.902653	10.21029	40.05574	3.867026	43.75819	NA	12.03789	6.301132	7.19184	27.33338	29.60122	27.1479	41.99895	59.41831	20.945	12.18283	15.5351
CUST_202_PI429010886	N2380	0.013498239	12	6	395539984	PREDICTED: macrophage mannc			0.000000135	3.42333	6.571137	NA	29.22269	4.874615	15.21169	471.3761	NA	349.3947	79.25889	60.73958	NA	9.619909	26.95753	14.90324	52.68847	11.87453	61.61447	729.4004	606.7201	1268.009	86.41587	5.577723	73.84495	187.4404	25.74471	146.9401
CUST_48297_PI429010886	M79714	0.01351375	24	18						41.19655	46.01666	55.43882	56.18757	39.56268	31.96059	26.56409	43.03288	31.78112	37.45713	42.17431	23.78251	46.26072	52.35798	31.3526	44.1972	48.08963	50.74506	50.20269	48.83893	45.1536	42.84965	46.92175	51.62622	49.65203	42.52366	45.86429
CUST_56348_PI429010886	M113559	0.013534851	12	3						2455.967	1820.572	1573.941	3200.557	815.2223	1818.148	983.3131	4719.533	2891.912	1347.499	672.5292	156.0492	1816.059	1567.333	846.6405	4880.699	2466.158	5329.024	5442.505	3568.199	6696.753	1071.48	375.9296	495.8587	3835.495	1307.951	1958.772
CUST_9825_PI429010886	N25258	0.013535884	15	12						11.17145	3.327403	6.731178	6.669026	NA	4.238625	8.396446	5.141647	4.144296	12.03994	8.788031	9.905888	11.02468	23.99454	NA	12.09746	4.456667	7.524476	NA	4.791983	5.922716	NA	7.973645	13.43959	7.013107	15.15187	11.81992
CUST_33625_PI429010886	M7460	0.013540426	18	15						573.4698	1220.331	819.3739	631.2553	487.4115	460.9197	1049.395	814.573	990.2906	1056.414	675.0754	583.3052	782.4302	646.5974	626.8237	1212.445	1556.87	1400.583	955.1294	507.9246	843.8571	654.7479	681.5295	626.641	948.1481	620.7278	685.9099
CUST_47206_PI429010886	M75186	0.013541593	24	18						2769.46	2230.426	2889.672	2719.085	1688.167	1895.668	2906.899	2261.704	2962.88	2499.817	1522.629	1314.736	2652.405	1667.986	2177.847	3431.358	2757.04	3156.873	4045.654	2501.925	3200.557	3549.354	2805.21	3145.521	4253.847	1719.004	2406.654

Probe ID	ID	p-value	Period	Phase	EBI ID	Annotation	GO category	Go term	E-value	0	0	0	3	3	3	6	6	6	9	9	9	12	12	12	15	15	15	18	18	18	21	21	21	24	24	24
CUST_5185_PI429010886	N18371	0.013557372	24	15	345494023	PREDICTED: Nasonia vitripennis	Transport	Protein transport	3.33E-104	130.0637	193.0271	149.533	119.1547	97.82425	131.4508	145.8669	154.9154	145.3183	149.1062	114.9651	131.2008	170.246	150.1608	141.0554	207.9118	203.8839	188.7958	153.3375	121.9035	151.8271	126.1993	149.3939	152.5572	167.261	152.2628	158.224
CUST_5431_PI429010886	N18692	0.013558889	21	18	241601186	aldehyde dehydrogenase, putati	Metabolic process	Cellular amino acid metabolic process		378.6955	633.9987	497.9545	449.8865	397.7869	385.6874	493.2216	545.5749	470.2656	389.053	383.67	393.6626	364.8648	430.2082	356.1688	504.3232	659.0035	630.7177	517.6361	594.442	698.9223	456.7205	565.0242	608.6613	396.2592	509.7706	675.5044
CUST_5709_PI429010886	N19079	0.013573849	24	9			Metabolic process	Carbohydrate metabolic process		39.3738	25.7341	22.93309	43.52338	33.14322	35.72473	25.90137	34.30109	30.12082	58.445	43.12031	27.61709	31.12893	50.47099	25.44962	27.39507	27.50901	18.93459	22.6604	23.42467	13.5245	19.48546	32.35917	21.31047	13.72761	42.54185	12.15207
CUST_30865_PI429010886	M48	0.013579984	21	15						72.26194	99.30177	85.34679	74.85952	107.1698	107.7788	90.88256	96.89897	96.71855	76.80903	131.9455	101.0722	106.9403	118.8271	119.9092	143.4863	127.9862	121.4836	88.70868	97.16144	97.82425	68.22331	101.8747	82.3809	132.7314	109.5906	103.07
CUST_20796_PI429010886	N44067	0.01358334	24	12						92.37878	47.48311	35.0396	113.23	67.00003	65.28656	92.30682	74.37918	62.25184	153.7672	59.43294	66.39483	106.1034	73.64351	66.76294	66.31625	74.58857	89.00153	42.76535	69.06046	70.0874	48.58376	59.27468	51.57473	55.63371	61.13851	54.33188
CUST_54723_PI429010886	M107323	0.013584952	24	6						113.7264	135.0024	66.52473	125.7435	184.5044	201.1205	183.7819	134.1808	134.6822	132.3608	139.9464	158.7144	128.9945	145.1769	115.2322	194.1489	124.5255	178.0606	91.4929	107.0748	99.83532	102.411	101.6124	85.43893	115.0343	186.2715	68.21622
CUST_16424_PI429010886	N36215	0.013591743	21	15						8.041645	8.179712	8.963971	7.02166	NA	6.251438	12.18092	3.85351	4.582875	4.76901	5.6288	5.769843	NA	3.198189	NA	11.4924	10.1847	10.55458	10.48413	NA	6.62579	7.282372	NA	8.276959	8.1002	6.487467	NA
CUST_9061_PI429010886	N24003	0.013597117	24	18						433.1774	581.4931	583.7299	592.215	388.156	502.0555	595.447	431.6201	687.0247	545.361	527.7959	481.8774	714.9939	505.6819	573.6451	608.1132	580.31	685.082	723.3991	651.0737	832.328	628.502	637.9796	750.0607	879.5119	520.1918	774.5071
CUST_16538_PI429010886	N36427	0.013597117	24	18						49.364	90.00835	72.96939	69.17285	25.67187	23.69696	106.1034	66.22157	96.44195	82.84289	48.32509	57.74381	86.80106	43.42121	40.082	107.2724	128.3485	121.0252	129.5849	112.6108	165.4776	67.20874	67.63278	119.6201	131.2008	92.69475	157.2999
CUST_29553_PI429010886	N60094	0.013597117	24	21	549834	RecName: Full=Zinc finger Y-chr	Nucleic acid metabolism	Regulation of gene expression	2.16E-09	17.13499	10.91685	13.60934	22.80766	8.312327	10.45873	17.72698	7.827649	13.12104	15.27129	11.91333	28.51273	8.544323	19.06321	15.62701	19.61789	16.29695	19.78002	19.03648	18.35004	22.905	28.86811	19.69443	21.28674	25.44274	14.61522	27.10146
CUST_29849_PI429010886	N60685	0.013598206	15	6						11.10164	11.04387	11.68057	17.5656	5.616552	9.504797	NA	17.20391	17.95932	17.58375	10.67743	24.83403	17.79556	6.503133	14.96491	7.377947	4.405558	10.4666	24.51492	16.68577	33.17685	28.5784	17.59422	28.93158	43.65579	12.50469	30.00507
CUST_53829_PI429010886	M103353	0.013602731	24	15						1940.134	1916.926	1850.533	2017.363	1586.354	1692.044	2027.732	1579.509	1490.226	2384.985	1509.889	1859.635	2487.704	1513.287	1269.337	2853.1	2338.958	2631.802	2184.345	1901.421	2367.057	2179.188	1666.955	2136.93	2361.017	2130.512	2447.506
CUST_41384_PI429010886	M57598	0.013609959	18	15						135.0731	194.1916	167.5398	137.5078	125.679	141.7704	198.0524	179.8173	168.7955	181.1959	165.0194	146.9973	181.9587	136.0737	188.1589	195.3485	242.7465	216.4424	158.3229	152.1662	187.6789	164.7136	170.1972	158.6164	188.6256	180.7516	187.4404
CUST_11381_PI429010886	N27722	0.013616573	24	18						261.1993	359.943	245.3707	311.055	245.1008	291.0948	404.1681	267.7885	296.6356	302.5618	266.7409	271.1207	444.3539	252.0561	279.9265	364.2049	328.6362	394.7866	463.3449	397.0876	483.5315	348.473	467.5708	344.0704	376.9694	324.0982	511.2321
CUST_13559_PI429010886	N31379	0.013616573	24	15						17.49425	28.19237	25.91869	21.25376	13.26584	9.066919	8.739672	16.20353	29.24902	32.90888	14.38342	37.83455	20.24396	21.3518	22.43161	38.78289	36.01668	31.08617	33.6303	23.19392	20.2757	34.60522	19.07869	29.59163	38.54386	17.90158	30.01166
CUST_16298_PI429010886	N35997	0.013618468	15	9						4.246772	16.9505	16.49866	7.119344	6.790621	7.399935	13.21801	5.521695	12.91333	9.886318	29.56665	33.16469	9.491244	10.40818	22.51109	13.82584	34.67259	29.11025	6.566709	16.95871	8.452888	21.66483	21.39782	18.5449	18.00685	35.90614	34.56666
CUST_10762_PI429010886	N26748	0.013622673	21	9						11.99886	23.09515	20.1078	17.07298	131.1212	109.0704	37.57549	42.24367	30.75233	26.63911	90.90449	79.57438	24.31682	33.9635	68.50135	19.2221	59.21803	30.09026	13.09533	47.24216	25.68152	19.47577	21.59473	14.35881	15.55999	52.60565	54.39234
CUST_9186_PI429010886	N24201	0.013624904	18	9	242017398	conserved hypothetical protein			3.41E-35	55.16243	84.50226	58.84073	87.45577	73.0273	46.71775	61.12576	66.98556	82.32889	98.33769	106.0765	99.94989	67.80459	81.40497	76.21108	33.90314	67.53847	70.90586	67.78024	60.86142	73.54748	50.06722	78.956	62.77844	87.70118	66.296	87.01734
CUST_42545_PI429010886	M60556	0.013635347	12	3	321463530	hypothetical protein DAPPUDRA	Metabolic process	Carbohydrate metabolic process	1.35E-16	10.94203	16.21562	6.862158	60.11411	39.50981	33.28409	9.834143	5.025678	11.76147	7.99927	18.54665	9.612207	49.30833	28.65621	10.44732	15.4529	55.96387	21.77765	10.29658	15.49957	NA	15.27129	8.155855	7.273639	4.56093	10.25642	19.63812
CUST_39585_PI429010886	M41434	0.013646784	24	15						1579.509	4791.878	2157.924	3197.119	1076.368	1358.461	5884.771	5061.439	4569.485	3914.879	2584.904	2167.915	2820.736	1816.946	1720.907	5043.311	7621.268	6240.674	4650.166	6410.91	5525.521	3759.237	6452.703	5872.99	4211.995	3379.597	4487.184
CUST_18092_PI429010886	N39125	0.013646784	24	21						158.3229	264.9162	227.1397	174.1094	172.6192	124.0046	198.5715	209.031	201.0027	164.8407	162.8199	150.3928	206.6211	190.9332	169.34	189.7568	349.1843	230.8178	206.0425	217.1972	212.2981	197.4942	278.0108	236.5379	205.9122	192.2095	258.6163
CUST_49470_PI429010886	M85780	0.013648621	24	15						1950.295	1533.458	1676.984	1509.889	1251.245	1189.941	1929.095	1281.883	1865.951	1594.029	903.938	1242.354	2547.238	1387.245	1006.869	3423.157	2367.057	2799.9	2213.256	1547.502	1679.651	1965.787	1812.631	2443.901	2235.693	1217.388	2103.097
CUST_4218_PI429010886	N16258	0.013649547	15	6						5.051862	15.56566	6.937084	12.06492	14.08701	NA	6.285156	20.12578	27.18421	12.61702	NA	13.12104	5.05016	6.074391	NA	9.098207	4.084094	13.48654	4.699544	15.64904	22.30617	19.14803	17.47501	14.18163	5.05016	8.486725	8.204835
CUST_39450_PI429010886	M39617	0.013662521	24	3						155.6488	NA	121.8531	166.5055	147.2411	174.7074	NA	NA	11.26701	156.0931	133.0187	156.6513	98.04747	96.9591	162.3101	4.217699	6.227359	NA	NA	5.504548	NA	123.8841	37.56673	120.2238	6.422559	24.0921	22.87902
CUST_23274_PI429010886	N48550	0.013696966	15	6						25.43515	18.93239	14.2083	24.30578	9.617132	14.69094	40.1121	27.04586	39.07435	20.6404	10.02506	14.85832	21.9811	15.68096	13.96378	19.65095	18.9132	26.83914	21.31707	26.16822	20.74174	22.31697	26.68521	22.59481	19.91453	20.41963	21.43699
CUST_1107_PI429010886	N8518	0.013696966	15	3	342326199	protein spaetzle [Cherax quadric			4E-17	204.5091	217.1534	219.8411	219.702	280.6926	240.3468	198.257	298.439	265.1693	135.4532	239.2059	199.8201	134.7853	248.3542	140.9846	199.7755	217.7635	208.6051	266.7409	277.7202	264.8694	145.5033	241.6209	180.0776	209.5237	226.8253	199.7755
CUST_19905_PI429010886	N42423	0.01369991	12	9						182.5585	73.75221	152.0267	113.5495	94.75478	134.826	131.4508	102.9977	173.8872	201.7698	107.9064	158.6683	104.6457	97.34674	127.9686	131.568	87.11871	83.08821	132.0886	188.7199	168.1289	227.8584	193.9616	311.7988	180.7102	88.35558	247.5439
CUST_2923_PI429010886	N13397	0.013707871	21	15	498987305	PREDICTED: platelet binding pro			5.56E-15	291.6226	590.4595	91.58124	446.6878	144.1497	543.5625	722.1173	605.0439	421.5919	489.5895	372.7272	208.404	889.3882	677.4857	748.1962	1683.28	1092.583	1006.38	777.3446	296.8179	671.5725	193.6334	714.2812	186.4449	1124.497	1063.832	86.04303
CUST_1724_PI429010886	N10544	0.013710412	24	18						9.916433	5.053978	NA	6.655389	NA	8.645163	70.1726	NA	58.7415	24.17495	14.93264	NA	9.225171	12.6791	NA	36.615	NA	42.67003	316.7134	542.1922	752.4222	30.26124	NA	45.27328	213.6621	19.14033	174.5908
CUST_31878_PI429010886	M2648	0.013718769	24	15						443.355	776.7493	446.9571	441.7626	407.9106	410.1393	687.0247	618.9801	558.1235	431.346	462.283	442.023	462.1954	546.8689	475.3372	762.1289	726.7698	690.5452	546.3173	509.999	536.489	380.7227	581.3213	434.0618	574.5775	489.0279	533.3565
CUST_14155_PI429010886	N32406	0.013718769	21	9	321471310	hypothetical protein DAPPUDRA	Signal transduction	Other	2.04E-56	10.96209	9.301907	11.77856	5.322281	15.4541	12.6262	9.62794	7.758333	8.266276	14.15162	17.08884	20.42404	12.04162	13.50346	12.45014	19.86967	10.44732	13.81	12.25196	10.98749	12.2567	8.806809	4.643571	10.42633	24.97563	11.27666	19.92002
CUST_11724_PI429010886	N28300	0.013751515	15	9	242017012	conserved hypothetical protein	Signal transduction	Other	1.12E-11	NA	12.63354	NA	6.758311	12.09251	7.675103	13.53611	19.19318	16.45591	22.74972	13.66542	NA	NA	24.30578	5.695825	4.56093	11.43891	8.562382	8.239061	14.82383	11.02965	9.908964	35.54077	23.27971	16.45406	37.23401	22.55063
CUST_51759_PI429010886	M94916	0.013753405	24	18						77.06177	95.70549	102.1603	103.3038	81.24064	55.1065	132.4344	59.49271	109.5188	121.7366	71.22472	93.46078	168.7398	117.6651	83.96708	62.50961	125.9513	99.53837	176.4771	195.082	240.5773	127.7162	216.896	191.1913	100.3493	127.9454	240.5209
CUST_49826_PI429010886	M87336	0.013754086	21	9						109.9913	91.92491	97.47634	96.45691	81.95267	82.7985	102.3466	92.68642	93.52245	126.3605	104.7454	170.8635	106.3359	79.55681	101.8646	118.8951	94.95617	87.51637	88.82328	95.95298	108.0539	101.7015	71.34612	92.56838	89.87221	65.29504	84.83411
CUST_48754_PI429010886	M82029	0.013755423	24	21						161.754	9.255882	157.3439	449.4542	145.2258	102.8763	63.20291	122.1215	102.3977	260.6535	726.7698	156.493	614.3598	187.3814	237.0529	15.36995	NA	11.04053	292.6099	170.4812	304.282	743.0295	949.2292	924.3063	334.9433	654.7479	972.8403
CUST_55364_PI429010886	M109778	0.013764618	18	15	13898841	CUB-serine protease [Panulirus	Protein metabolism	Proteolysis	3.17E-16	6041.324	6897.446	5992.677	3679.2	3284.996	3379.597	8160.518	9791.81	7506.653	6559.55	5807.4	5197.224	5085.201	4998.113	4064.827	8818.425	7518.482	7928.164	4928.217	6333.445	6116.36	4527.493	4875.223	5322.868	5482.841	5726.275	6285.074
CUST_31637_PI429010886	M2032	0.013764618	18	15						318.7134	478.0895	327.1238	334.795	255.8139	278.592	389.7916	395.4036	371.5772	304.0274	330.1216	331.0671	344.4073	392.057	368.9684	371.0946	420.1391	453.4842	304.6294	335.0962	2659.616	271.2533	310.6389	335.0962	353.0555	337.6971	363.1656
CUST_31709_PI429010886	M2216	0.013764618	18	15	342326446	GrpE protein [Cherax quadricari	Protein metabolism	Protein folding	2.95E-45	50.0875	116.724	69.49719	49.20954	46.15921	44.1311	73.24973	93.33147	49.22608	48.6216	39.36544	76.74926	62.28036	64.88027	65.81456	92.78173	112.683	91.61189	71.09432	60.71091	73.04616	59.77969	48.15075	57.3802	92.84934	67.97224	67.05704
CUST_32730_PI429010886	M4946	0.013769784	15	3						40.16574	25.72369	25.0443	58.69436	66.74201	46.10158	39.30626	29.71923	33.52305	43.10438	31.97696	12.65073	63.20291	34.78692	24.33859	28.22616	55.32819	19.57584	102.7615	47.57381	45.42128	84.26462	19.29052	55.86654	31.97696	17.2551	22.4068
CUST_44532_PI429010886	M66243	0.013773789	24	15						10.23432	21.68208	14.50203	20.30731	23.12326	20.82813	27.03026	24.12669	25.20922	10.88207	16.42269	22.90061	19.78846	20.01482	24.62932	37.46348	28.11739	29.54223	18.00112	16.21373	28.56322	23.56505	14.64529	18.8606	18.99164	18.83509	13.37213
CUST_335_PI429010886	N4034	0.013782298	15	6	170040829	predicted protein [Culex quinqu			9.59E-10	366.0882	525.7914	505.5201	680.4062	855.5436	359.6695	1466.746	470.3803	1421.92	949.6232	2449.312	1081.498	2025.285	1280.159	928.5423	208.4776	714.712	703.9211	433.6142	356.2511	582.599	1394.635	2721.255	2906.899	404.6157	1392.832	1775.368
CUST_11123_PI429010886	N27303	0.013790872	15	0						55.89971	96.20921	63.26218	78.39296	68.05647	80.05281	75.61315	81.38488	73.65204	53.88185	82.24574	48.34401	65.88877	74.76519	111.5466	88.93364	117.5148	97.55312	75.3401	92.67361	81.30602	43.98997	66.51317	73.46998	61.93359	55.21231	55.72212
CUST_54970_PI429010886	M108291	0.013800873	24	3	220172369	cuticle protein [Rimicaris exocul			6.83E-13	205.6562	NA	613.3285	1097.521	707.3888	892.7134	39.95456	28.43678	23.81043	468.6225	859.7804	417.3805	627.6794	350.1584	231.626	NA	NA	6.917829	11.55337	30.23399	20.33393	404.5068	332.1692	554.3874	43.98997	84.06095	108.1577
CUST_54542_PI429010886	M106457	0.013819529	24	21						5.066072	19.19097	16.64224	16.35396	9.15859	9.590111	18.39792	29.53809	27.95476	9.62794	5.305984	5.327092	23.72726	13.33462	7.814775	12.53384	15.21169	9.126089	35.06409	21.86212	19.42557	20.7112	35.40128	34.29815	20.77024	21.35916	39.57166
CUST_46906_PI429010886	M74132	0.013819529	24	15	328899662	ribosomal protein L44 [Procamb	Protein metabolism	Ribosome biogenesis	2.2E-33	23324.95	29294.9	23658.24	19485.9	17524.49	18910.88	26191.89	27273.84	23961.37	23016.75	18117.06	19572.59	19522.5	20172.23	18146.2	29725.44	28858.93	32295.88	29396.88	27459.44	29037.53	23379.75	22221.29	21398.95	25188.43	26191.89	25188.43
CUST_48539_PI429010886	M80892	0.013819529	24	3						51.63636	25.21148	42.55235	86.32974	54.98542	78.12052	40.082	34.8722	31.13507	38.69332	135.4989	176.076	30.35094	75.9364	79.10899	24.96126	25.01301	27.64345	22.08497	44.79025	26.23262	39.84739	34.54334	46.72845	26.6898	44.76603	37.74193
CUST_3781_PI429010886	N15253	0.013819529	24	3						110.3983	82.09637	59.72832	88.60558	8025.596	4791.878	67.94466	584.6183	60.52841	12.50576	5578.907	5184.879	19.13763	3577.841	2790.956	4.284666	5.966459	31.74591	6.842059	1187.041	9.120228	7.649773	100.8115	11.11546	8.419629	2279.977	11.79624
CUST_20965_PI429010886	N44385	0.013819529	24	15	542207028	PREDICTED: zinc finger protein 2			0.000000155	15.7947	20.08349	20.6613	8.796309	7.276912	8.683022	13.33099	15.91979	17.96997	10.56586	9.562348	22.12571	13.46097	10.00971	11.2795	22.53181	21.07922	26.76803	21.27506	16.94573	18.51168	14.3566	22.58254	13.67002	18.43607	14.55686	28.34861
CUST_33510_PI429010886	M7110	0.013823603	18	9			Transport	Other		4041.944	514.6065	3145.521	5666.924	6298.27	6622.666	260.3413	974.6663	449.4542	8846.332	11179.97	11228.98	4258.14	5402.577	8619.889	138.2964	67.82594	137.4877	564.8143	1404.695	597.5894	5659.622	4207.659	7632.364	510.3128	4560.074	4857.819
CUST_28806_PI429010886	N58652	0.013823603	18	6						28.88967	48.94611	22.83576	41.64305	41.27805	44.43625	54.43474	45.72512	61.51933	34.40524	42.52944	29.27764	18.62882	36.40013	24.00968	46.983	39.37754	39.15453	36.26885	38.04859	35.21621	39.24909	24.97826	31.8144	45.23287	49.28723	38.86739
CUST_3936_PI429010886	N15614	0.013839243	21	12						5.242802	13.81373	9.959247	3.439072	8.851485	12.65302	10.9325	NA	9.084966	9.917631	23.94163	7.349073	13.83903	17.88666	9.62424	6.597671	7.217315	7.433712	3.058048	5.117121	3.699519	13.15166	4.978112	6.89852	5.733242	6.609031	NA
CUST_38133_PI429010886	M23564	0.013861471	24	3						2575.646	45.65444	2159.534	3316.279	2291.48	3295.67	NA	17.15225	NA	2722.979	1587.909	3604.261	1852.945	1426.211	2873.993	NA	13.07357	16.69103	15.24489	73.88293	18.84878	1903.836	506.9058	1293.784	3.494399	376.2092	313.2852
CUST_37538_PI429010886	M19413	0.013862307	24	3						664.0464	7.734794	624.767	907.3817	623.4767	661.8815	NA	14.86033	NA	190.3933	525.4149	573.048	132.0176	499.3569	757.5703	3.969898	6.894282	13.13244	15.267	11.00713	4.091824	331.7259	22.37825	268.0924	3.791995	14.18163	10.6362
CUST_46805_PI429010886	M73777	0.013863118	18	15						95.83012	218.6641	80.14476	62.68299	NA	51.44117	101.3206	114.6414	63.59136	102.3111	63.60621	37.03185	122.3191	49.48225	118.8608	185.4321	300.8131	158.7682	74.34865	43.16345	61.42589	59.78407	57.42169	59.77062	142.8672	146.5049	32.40475
CUST_25406_PI429010886	N52357	0.013879095	21	9						82.28293	65.87993	69.44476	64.29833	75.95975	80.01131	92.2128	72.91519	90.11127	87.79232	87.12904	78.92328	86.68025	50.41623	66.27041	74.09264	54.58501	69.03939	72.91519	62.196	66.09647	81.60481	68.40054	66.3493	76.83706	69.43522	76.36789
CUST_18488_PI429010886	N39887	0.013890428	15	0						47.56782	39.93038	NA	43.27193	6.574572	33.88276	63.75599	21.65257	31.79774	18.75264	12.12405	6.238498	34.28702	41.41654	32.6846	74.99403	31.84126	48.29273	39.89679	8.397801	37.59004	24.58018	33.18845	10.50904	35.56743	22.52707	8.639252
CUST_55862_PI429010886	M111911	0.013904955	24	9						39.02298	NA	23.23426	62.98884	10.40534	5.974507	40.79604	16.19743	NA	45.66053	NA	26.04551	32.2704	19.56731	7.702876	5.410427	11.94958	19.51186	7.548659	8.088527	4.145953	8.326096	NA	4.477878	7.273639	17.52474	NA
CUST_5656_PI429010886	N18999	0.013907198	18	0						955.1294	473.1245	1249.87	1017.712	761.0743	914.2819	284.9823	406.3531	429.1442	326.0747	465.5812	361.1904	390.7977	772.5597	1038.012	345.5951	384.1573	372.873	474.227	581.8311	529.579	517.5058	471.3761	524.9759	426.3157	468.6225	596.8192
CUST_37641_PI429010886	M20124	0.013919277	18	15						8658.641	11206.93	8107.941	5329.024	6368.381	4602.898	12402.11	13446.51	9804.488	10323.84	8996.679	8461.114	7007.731	8321.498	6179.344	14015.31	12789.24	11429.91	8929.115	11528.01	7861.824	7900.843	7518.482	8051.463	9999.789	7912.634	9669
CUST_45912_PI429010886	M70733	0.013919277	18	15						6845.752	6906.237	6523.544	2636.073	3552.655	2615.936	7983.519	8869.924	8899.45	6770.352	4754.165	3411.4	4490.88	3864.779	3168.826	9653.786	10430.71	7704.294	5783.496	5451.324	5227.74	3205.37	4093.741	3571.414	7203.647	6584.064	5258.496
CUST_3271_PI429010886	N14156	0.013919277	18	15						1563.997	6873.81	2761.678	834.7743	247.5845	500.9726	4910.228	2079.426	2777.197	2637.871	746.1539	1321.031	1495.315	994.565	1030.175	3319.225	5601.899	5671.62	3575.021	2213.256	2851.067	1264.606	505.9666	1050.844	4012.41	2014.653	2690.376
CUST_56796_PI429010886	M114665	0.013926416	24	15						518.3699	637.2271	680.9887	654.9488	493.4583	533.5339	546.0695	495.8587	646.1155	650.2714	515.2465	635.9298	587.8441	510.4694	571.0817	830.4195	713.8123	736.2732	776.9293	578.6613	703.9211	750.6839	675.9355	769.8174	653.7367	620.4556	957.7854
CUST_2877_PI429010886	N13297	0.013966999	24	21	321473740	hypothetical protein DAPPUDRA	Metabolic process	Carbohydrate metabolic process		421.8557	583.9375	NA	234.9525	NA	236.8007	644.0162	1132.05	642.1289	17.71771	27.24405	10.69359	48.92244	445.4238	68.40753	755.8283	315.7654	319.4595	547.285	432.5544	481.1553	NA	710.633	NA	68.33012	685.9099	NA
CUST_10522_PI429010886	N26368	0.013979225	24	0						203.1619	256.7087	336.1378	247.116	249.0801	229.1648	181.9587	226.0802	234.1695	225.5927	263.4946	269.0492	225.7463	190.0902	225.6648	198.2176	303.9295	214.2802	194.5455	259.0732	235.1283	250.1769	315.6961	319.3342	282.9282	327.283	361.8298
CUST_51665_PI429010886	M94496	0.013984516	24	21						138.0213	235.0418	191.135	135.1633	201.8019	173.6159	127.3506	171.5033	115.3868	122.9044	151.3747	124.7562	119.2267	170.6985	125.5852	155.885	226.9579	242.6675	150.9904	201.2	195.082	143.1622	246.8231	212.8941	125.0613	198.0827	220.7835
CUST_57228_PI429010886	E2641	0.013992273	24	15	328899662	ribosomal protein L44 [Procamb	Protein metabolism	Ribosome biogenesis	0.00000044	4971.086	7474.416	5085.201	4829.887	3503.626	4012.41	5209.792	6439.853	5227.74	5298.376	3855.701	4719.533	4633.616	4315.085	3463.912	7681.431	7032.355	7807.75	5872.99	6592.219	7610.57	4554.072	5215.78	5718.956	5244.429	5705.148	6348.681
CUST_35704_PI429010886	M13265	0.013992273	24	3						295.6289	293.0253	242.5407	1479.335	6285.074	2536.537	229.2492	502.8105	141.5813	175.4398	2447.506	2906.899	102.9729	13098.74	15461.72	24.485	38.46934	68.49163	55.76605	864.8838	60.87753	56.25386	138.2964	108.3883	18.53796	559.9215	191.7677
CUST_31593_PI429010886	M1921	0.013992273	24	15						213.0104	334.1482	242.5947	180.2649	180.1999	191.3144	232.1999	237.835	266.255	307.8719	182.794	210.1903	310.6389	181.2834	222.2656	248.1114	348.7951	296.3076	286.6404	267.0027	248.1643	245.8656	284.4662	270.6947	247.0676	236.013	320.7176
CUST_30862_PI429010886	M38	0.013992273	24	15	242041648	Sorghum bicolor hypothetical pr			3.48E-94	765.466	1036.601	957.0763	790.4703	856.2286	815.7902	1122.574	1226.655	1188.558	951.1847	876.9516	755.8283	985.8154	903.4294	947.376	1215.754	1319.617	1373.854	939.9518	1082.092	1135.35	732.0587	1019.08	934.0598	767.8994	966.6426	1013.387
CUST_50849_PI429010886	M91234	0.013992273	24	18						49.51361	220.5798	211.185	65.88877	85.00598	39.56268	55.32819	42.40053	72.93006	39.58685	327.5322	49.70798	274.8211	176.3183	62.5913	81.91952	396.4646	67.96481	626.0846	237.3958	203.0356	401.876	196.875	187.8778	289.6241	230.4127	294.2148
CUST_12282_PI429010886	N29228	0.013992273	24	21						191.9474	410.3258	264.4099	236.9699	289.9679	278.1319	248.2992	323.3549	301.4906	193.7076	199.1806	65.63171	250.7612	273.0279	232.1999	377.789	245.8656	291.198	256.9518	279.9265	296.1605	295.6289	312.7556	313.739	308.8108	317.1978	243.9595
CUST_24139_PI429010886	N50100	0.013992273	24	3	321478908	hypothetical protein DAPPUDRA	Protein metabolism	Proteolysis	4.72E-25	39.70866	19.25364	19.02154	27.5259	35.67153	43.32608	20.80953	20.19611	17.21755	10.03737	20.74399	34.3049	14.28701	12.61702	26.41747	21.18826	6.348391	4.00805	19.21808	24.88093	18.82754	17.0629	25.50686	16.98896	13.07493	28.56603	24.1058
CUST_24201_PI429010886	N50219	0.013992273	24	15						31.43843	22.05957	26.35742	22.9881	15.8055	17.67979	19.19457	19.11579	24.32178	26.76018	26.0166	28.82109	23.99679	16.37588	28.21177	28.8296	33.11195	32.55747	29.01782	21.27028	26.48825	28.31242	17.95343	26.10261	26.85225	16.0253	37.74598
CUST_25019_PI429010886	N51640	0.013992273	24	15						17.56328	34.1415	28.96132	12.88736	16.05064	14.39849	16.5534	24.8137	28.31962	32.41501	18.28324	22.34605	22.7226	24.91503	31.54041	29.39105	27.39752	33.61683	13.99736	26.72167	26.05879	23.50843	26.03031	26.38902	27.06162	21.13144	26.15886
CUST_6749_PI429010886	N20552	0.014009948	24	15						33.52669	42.2265	36.31683	32.49093	29.27528	28.71238	43.05532	33.5727	35.82514	26.17673	30.7975	40.36492	24.8962	22.54151	31.96971	63.88807	53.93137	63.04493	39.81401	42.16378	38.95566	49.6421	43.11409	39.62294	27.34627	29.16626	38.04561
CUST_55005_PI429010886	M108380	0.014030028	12	9						21.1758	18.96527	12.29576	11.87453	15.03094	11.00974	22.50019	15.72693	19.52187	18.66832	22.38744	25.82506	14.5147	14.38836	16.77585	10.86311	4.712418	11.42758	17.89974	17.29812	20.30731	12.28362	13.01831	20.18416	9.563744	12.11179	13.91119
CUST_51649_PI429010886	M94401	0.014030028	12	9						25555.67	1135.893	25888.13	34620.42	20099.82	27577.78	3555.208	4791.878	6676.906	30821.46	36043.3	28025.82	43090.8	14922.71	37632.98	293.934	152.3548	488.5919	12631.63	9774.949	13360.37	47826.44	35305.99	45731.6	14439.79	22598.48	33749.71
CUST_51807_PI429010886	M95179	0.014030028	12	9						26.62997	62.88268	53.24793	40.44814	59.67529	23.10726	31.52497	73.59425	34.36428	42.11529	295.8069	189.5433	62.66376	203.1887	123.3867	14.0307	28.0193	26.13567	36.64113	34.12714	24.47696	52.45299	54.27691	63.66074	30.67349	48.61045	51.35849
CUST_30585_PI429010886	N62173	0.014030028	12	9						106.5519	45.30575	44.13896	80.44707	74.56881	61.82712	21.3859	50.0937	36.28732	81.67118	155.7011	95.24948	67.78785	82.72734	62.90417	21.31707	15.93653	16.38688	43.00754	40.34223	43.45515	79.66914	83.0128	104.5338	32.90888	59.90743	99.06526
CUST_32221_PI429010886	M3560	0.014043049	12	0						15.27294	9.05635	10.33158	15.22691	16.50625	10.32668	6.88957	8.983138	6.698312	9.127031	8.906998	9.609273	8.973853	19.88951	13.11189	8.701063	7.975034	11.66711	9.808744	5.097995	3.800894	12.51986	16.00252	11.97918	15.96647	11.05126	12.31377
CUST_10190_PI429010886	N25847	0.014065983	24	21						291.8297	250.7116	317.0778	331.2753	253.8231	239.1584	220.9078	210.1903	208.8218	345.514	276.6685	328.5727	365.5842	255.0769	324.4202	458.9701	269.9329	478.8721	375.1194	207.8778	323.0951	489.1563	324.1912	478.5299	402.0757	241.1815	508.7574
CUST_23953_PI429010886	N49759	0.014065983	24	6	118403526	sialin [Xenopus (Silurana) tropica	Transport	Carbohydrate transport	1.5E-23	133.4766	183.5082	235.0418	149.4369	106.5406	147.7749	201.8882	168.511	190.4954	146.6123	95.63382	154.2758	146.6342	145.6946	136.7438	176.7166	130.6255	222.6199	128.7298	125.4991	132.8402	84.09371	128.8978	104.7064	107.3952	83.59991	80.05281
CUST_40478_PI429010886	M55239	0.014073935	18	15	345496857	PREDICTED: Nasonia vitripennis	Protein metabolism	Ribosome biogenesis	8.27E-95	6584.064	9425.197	6809.882	6780.642	4754.165	5202.819	9167.401	8484.924	8658.641	7681.431	5525.521	5879.268	7663.711	5840.985	5587.034	10142.18	10815.88	10532.72	6179.344	9167.401	9041.082	5913.632	6368.381	5617	7007.731	7157.757	7474.416
CUST_18061_PI429010886	N39076	0.014079245	21	15						257.3491	318.1938	336.9544	274.7035	278.374	306.1538	332.1692	339.6934	307.3952	353.9275	313.739	265.2295	334.1482	228.163	312.5578	360.2285	428.9483	389.9629	295.9223	230.7682	297.7548	338.4518	277.8246	346.9643	261.3674	211.185	274.6135
CUST_34977_PI429010886	M11219	0.014080565	18	15						30.84622	30.5034	43.34823	32.57446	20.29057	40.64205	13.47557	22.925	35.02813	28.19237	26.30955	40.01172	49.42933	30.46605	42.19574	36.7727	41.03485	43.30726	23.90042	36.87898	44.18768	35.07936	42.02407	32.2735	25.76307	31.54655	26.61562
CUST_41139_PI429010886	M56966	0.01408297	21	0						15.89391	27.79864	17.39987	18.07903	9.758637	7.713675	13.28971	NA	5.330713	10.59474	NA	11.58452	18.62338	6.040325	15.63697	12.40631	16.46484	18.99331	28.51548	14.04103	13.10406	23.5545	14.05555	26.12451	20.2757	17.67979	10.97924
CUST_16460_PI429010886	N36270	0.014088042	21	18						24.30298	20.63272	30.60362	24.53447	15.35881	25.98049	13.93501	8.523259	20.69452	22.93584	8.995322	17.62376	19.80567	22.44752	16.25968	24.46253	13.50148	24.40409	27.33627	23.10946	26.14108	28.94839	14.70455	22.53586	19.04999	25.01036	20.42404
CUST_20626_PI429010886	N43776	0.0141027	24	18						86.67082	53.19546	66.2384	69.73149	77.96514	62.28036	62.40732	66.2384	62.64213	82.76148	52.87075	71.85783	77.08119	63.10394	95.16242	81.33153	63.15515	78.71874	88.0075	79.72468	104.7454	110.4383	77.40803	90.87656	94.41489	45.61423	74.84749
CUST_5231_PI429010886	N18427	0.014108573	21	15						135.3432	253.8613	184.5933	162.4126	159.9313	132.9661	158.418	195.4747	184.9648	156.5799	172.8989	146.9401	152.2838	182.3239	156.988	240.1155	284.9823	224.1099	193.9616	209.1753	213.8259	138.3677	203.392	215.1868	195.9057	174.3634	208.1829
CUST_52734_PI429010886	M98717	0.014114433	24	21	390333701	PREDICTED: zinc finger protein 5			2.02E-15	NA	5.872215	12.87838	4.548186	5.731637	4.218831	NA	7.55889	5.117121	5.267874	6.514898	4.904093	NA	4.822457	8.243972	NA	5.304563	6.290529	NA	8.288676	5.567914	9.580454	54.53262	12.09948	7.231758	9.356835	14.57595
CUST_55929_PI429010886	M112176	0.0141212	24	21						99.7036	52.27625	111.3418	45.82718	32.36608	23.8882	30.09371	41.42487	37.01072	55.97133	25.30627	55.23799	78.76138	35.72862	54.73135	40.50202	27.80868	58.67768	70.20407	58.77493	45.79273	153.9248	70.36913	78.15168	178.0895	16.78177	148.4065
CUST_36315_PI429010886	M15552	0.014121947	24	21	357130274	PREDICTED: uncharacterized RN			2.71E-08	69.59216	69.326	59.40866	25.38551	26.99058	22.44752	45.52672	57.06949	30.4977	43.26321	40.05085	41.33391	66.33276	65.49568	37.62384	36.20921	73.62565	64.15258	64.33747	62.71091	44.61277	69.92347	65.69754	75.6711	57.64367	96.05666	54.02691
CUST_38846_PI429010886	M31163	0.014165017	24	3						15197.96	13.178	13532.16	14580.94	12569.14	16847.05	79.01425	87.20964	107.7528	13716.26	12584.9	20271.85	9699.55	8107.941	14580.94	10.14578	17.28062	95.78807	110.519	174.4633	169.2197	11378.62	4000.901	9285.648	743.9121	1406.008	2556.357
CUST_40169_PI429010886	M51317	0.014165017	24	15						199.3048	444.1379	274.4093	170.4291	222.0168	195.2581	216.9264	376.6399	267.7311	228.5455	254.2305	165.9615	209.7386	283.1538	240.3058	294.796	433.097	423.3824	208.8218	324.2431	368.3282	199.5583	319.0038	250.35	223.6663	243.6749	274.8211
CUST_44683_PI429010886	M66668	0.014165017	24	15	516024011	hypothetical protein, partial [He	Protein metabolism	Regulation of translational elongation	3.07E-08	6448.141	8786.531	6988.188	5992.677	4445.119	5061.439	8547.585	7643.683	7166.836	6809.882	4655.696	5792.477	6087.174	5415.358	5507.513	10109.9	8802.072	9759.347	7861.824	7430.648	8591.806	5840.985	6018.305	6688.584	6885.506	6631.875	7832.882
CUST_50775_PI429010886	M90961	0.014165017	24	15						28.5784	44.61738	76.12107	36.53104	19.25825	30.20493	50.72554	63.59914	87.96067	70.26755	42.5482	35.46315	58.87393	51.75972	18.00851	101.2137	105.7007	87.91078	96.23561	48.91311	65.41928	42.56107	60.57425	25.39283	80.14476	23.25613	64.08368
CUST_4986_PI429010886	N18079	0.014165017	24	15	383864701	PREDICTED: transcription elonga	Nucleic acid metabolism	Regulation of gene expression	1.53E-61	625.6951	1071.48	780.7545	639.0445	489.4418	549.8031	937.9336	966.0248	985.8154	856.9537	651.7291	500.4612	831.0254	661.4243	651.7291	917.9436	1308.545	1044.527	1003.961	858.1052	960.8612	802.4687	808.2743	735.8335	958.49	840.9882	527.9796
CUST_8773_PI429010886	N23565	0.014165017	24	21	260827732	hypothetical protein BRAFLDRAI			1.86E-20	61.59148	50.62618	65.92209	40.43404	41.53886	47.12456	49.66595	61.145	62.1017	55.99408	43.59179	48.92732	47.21843	48.60251	56.13868	67.44192	59.43938	60.70253	73.38208	50.17119	54.36935	73.64351	62.09142	62.11789	61.50475	52.53211	65.11364
CUST_20463_PI429010886	N43492	0.014165017	24	15						58.65468	73.69276	38.90574	36.615	45.04591	36.02994	76.10344	52.36478	65.66275	52.39688	25.20667	38.54386	50.3744	43.79783	47.55587	110.6946	67.60011	85.83911	71.97753	54.19934	72.9865	40.98681	56.03078	46.45729	55.51071	46.64371	52.84948
CUST_12258_PI429010886	N29176	0.014171003	15	12						24.89149	25.48421	20.65147	20.35228	20.96269	38.07503	20.30731	20.37998	21.17352	23.30032	27.51162	32.57078	31.71402	36.33342	41.72041	43.23631	23.01802	33.93179	28.39612	31.85963	23.82619	18.50063	28.49131	24.43918	36.31683	27.50901	20.84466
CUST_55293_PI429010886	M109469	0.014183272	21	0						85.33414	73.1869	87.01734	89.07659	67.57316	101.4235	51.46661	60.29296	51.44117	72.12484	74.04749	78.90154	89.30494	78.32421	76.88599	85.93897	71.49956	99.74451	72.3378	82.36909	56.57137	106.1339	83.17096	120.0673	65.12931	54.25668	60.09389
CUST_2832_PI429010886	N13201	0.014189998	24	18						NA	24.79879	33.51959	12.48798	8.600876	7.770794	29.08241	6.811238	16.60231	15.50271	8.376444	11.33839	NA	35.35085	NA	13.13552	9.856067	23.98408	27.14205	28.77495	35.54384	13.19064	27.90262	28.48764	21.26556	12.99612	18.65471
CUST_53074_PI429010886	M100017	0.014192855	21	15	321478571	hypothetical protein DAPPUDRA			2.96E-11	4.018228	9.231247	NA	12.42421	5.809954	15.34805	20.64275	9.003802	4.203313	9.542112	9.764026	NA	9.832908	14.05708	14.42489	31.64075	22.26852	13.09533	7.608242	7.273639	13.12827	NA	9.565216	4.879601	8.041645	10.07008	5.450845
CUST_32594_PI429010886	M4522	0.014195087	12	3	241611809	peroxinectin, putative [Ixodes sc	Response to stress	Response to oxidative stress	1.38E-99	90.7511	18.43758	55.0054	255.2315	59.12156	202.1456	486.5784	203.6502	597.4035	15.58835	9.603299	10.50374	45.1573	46.05498	33.93799	715.4355	310.9804	408.1119	54.02066	536.1823	620.7278	153.502	12.87838	115.0551	150.2055	48.41454	113.622
CUST_33772_PI429010886	M7852	0.014205252	24	15						11.66991	15.72693	20.47903	13.88764	16.59163	25.91341	14.53618	44.5991	10.73437	45.07182	25.37982	10.43122	52.97639	55.28196	40.23682	41.64305	164.206	77.75578	36.37398	65.61458	16.0548	37.12091	13.85925	23.68893	43.46338	40.26043	12.17645
CUST_41805_PI429010886	M58594	0.014220253	21	15						21.73698	16.37294	102.4255	12.02986	17.18131	12.54058	7.185538	7.646455	11.89834	18.42634	5.89243	18.27698	10.90094	13.80235	20.34309	23.98666	24.56549	24.44473	8.575367	4.842515	13.92483	18.03458	23.04458	20.34644	13.2617	16.56978	18.55514
CUST_46414_PI429010886	M72608	0.014221916	18	9	405959130	Elongator complex protein 1 [Cr			1.77E-16	38.38502	25.7844	17.08055	16.15211	15.15744	13.57384	21.98953	18.3082	20.13002	29.6222	73.4071	15.69763	32.04295	13.38505	22.34605	21.17352	19.78846	27.38458	21.40258	16.99266	19.52955	14.71023	13.10406	20.07958	20.7112	14.88982	25.39051
CUST_29040_PI429010886	N59084	0.014245023	24	0						21.08968	11.708	NA	3.97653	21.08201	17.15225	14.23208	15.97275	16.23526	7.359305	8.798487	9.245564	3.58776	10.96033	5.343407	NA	4.619721	5.478147	7.213648	37.80064	3.199333	NA	5.329949	4.907005	NA	27.87333	NA
CUST_53890_PI429010886	M103587	0.014259922	24	15						110.2026	138.0571	111.539	111.5887	130.4587	161.1455	180.676	176.9444	183.8352	187.1657	115.1151	117.1403	151.9875	135.3048	135.8804	222.1435	196.875	203.8465	139.7918	121.6645	134.2321	138.3923	148.1939	129.3882	155.8444	117.3251	124.8855
CUST_38514_PI429010886	M27586	0.014259922	24	9						21.28833	20.96508	18.20871	20.19394	22.07451	30.62124	20.69694	24.5024	21.91017	32.38272	24.86103	23.98666	29.68598	20.92153	19.76134	28.482	19.43327	25.34819	24.28126	22.37825	19.56731	27.50428	21.49936	18.91131	17.46742	21.3729	14.96491
CUST_41687_PI429010886	M58310	0.014259922	24	0						138.2385	163.8654	164.6381	151.9667	119.1208	109.1228	119.3462	145.0834	145.1992	138.3312	131.752	104.6784	128.4629	132.9149	136.1879	161.175	154.2424	128.0875	109.6493	145.0185	136.2532	153.595	132.6306	165.6814	152.4355	159.7252	207.4823
CUST_49200_PI429010886	M84652	0.014264427	24	18	77743875	Euphausia superba isolate DGGE	Protein metabolism	Proteolysis		13.18135	NA	7.478329	210.4367	7.076886	170.6985	1964.046	NA	1382.183	379.5535	211.9389	NA	69.6885	233.8101	68.27308	415.5107	NA	409.8716	2217.875	7750.118	12548.11	466.0849	NA	453.4842	1751.317	250.0724	1558.626
CUST_3522_PI429010886	N14679	0.014276129	24	15	321473526	hypothetical protein DAPPUDRA	Metabolic process	Carbohydrate metabolic process	3.49E-23	2333.596	8086.369	2091.62	2666.243	1628.405	1870.445	2144.939	2461.211	1836.765	3247.587	1174.851	2139.451	2793.635	2620.241	2043.437	5847.84	7130.037	6410.91	4602.898	3419.816	3310.069	2171.504	2478.84	1944.792	6193.269	2430.729	4372.597
CUST_12369_PI429010886	N29360	0.014277095	12	0						20.99538	20.51536	20.19611	17.38549	12.91146	15.7786	3.717725	12.64082	12.57883	17.64923	19.37283	29.45413	18.80815	20.25105	24.12361	16.09566	12.74437	12.06245	13.89586	16.06042	19.49003	22.74717	10.78644	23.32566	27.30294	14.71235	20.16722

Probe ID	ID	p-value	Period	Phase	EBI ID	Annotation	GO category	Go term	E-value	0	0	0	3	3	3	6	6	6	9	9	9	12	12	12	15	15	15	18	18	18	21	21	21	24	24	24
CUST_25004_PI429010886	N51619	0.014277095	12	6						12.05333	13.37563	11.37944	18.15042	15.40374	23.5545	15.91774	16.10452	17.53638	32.12055	15.33068	28.45606	17.58118	12.04956	14.34967	12.99454	14.75984	18.12289	30.80078	24.62618	22.54343	13.46714	25.70782	17.23263	22.26642	12.50576	14.91234
CUST_45992_PI429010886	M71020	0.014278984	21	0						22.11624	17.82881	16.47278	16.39186	15.5722	11.01286	9.213352	14.79074	9.228702	18.96527	9.398999	21.86212	9.799617	10.70188	15.56566	13.65957	16.7995	19.201	18.16224	15.20453	17.04949	25.67187	17.30435	18.7418	22.23218	6.57946	7.345945
CUST_11872_PI429010886	N28545	0.014292753	24	12	512996801	PREDICTED: COMM domain-con			6.85E-40	23.68893	24.46253	27.90262	38.12957	45.22389	26.27801	26.54949	30.38583	28.00878	18.87745	20.76565	37.83797	38.43848	41.23205	43.05092	26.7042	42.86666	38.20771	20.18416	24.33371	24.32178	19.19097	18.86919	25.5836	18.2303	32.36608	29.282
CUST_39017_PI429010886	M33394	0.01429984	24	0						323.6649	263.0994	333.0274	274.1568	253.9457	196.5522	149.4086	260.9827	246.8231	316.8051	361.4106	249.3839	291.8765	227.2167	251.655	212.6443	231.588	208.9613	238.4366	219.4592	210.1175	255.2315	226.5718	207.8778	359.587	268.3054	304.9349
CUST_30341_PI429010886	N61669	0.014303602	24	15						6.968697	4.658677	5.460737	3.434184	NA	4.119385	NA	3.318033	3.594724	4.647584	NA	7.712272	6.449511	NA	5.198229	8.131518	5.584553	12.39501	5.659483	4.127227	5.042233	7.598096	5.043586	NA	3.831778	NA	7.611325
CUST_5209_PI429010886	N18402	0.014315015	21	3	7414466	phosphoenolpyruvate carboxyki	Metabolic process	Generation of precursor metabolites ar		402.7185	482.5812	708.4537	711.071	1799.736	1603.304	434.5642	523.8156	605.0439	644.2122	1647.162	1425.394	685.653	972.4265	1113.954	300.9346	673.1503	392.3777	472.1134	360.6127	414.0319	1033.621	844.8862	1416.762	484.6312	1061.158	1432.914
CUST_20109_PI429010886	N42827	0.01433139	24	21						15.32206	16.73229	8.996542	8.799574	NA	6.504644	7.05159	8.133753	3.537562	9.214634	7.223776	8.426205	9.850773	5.39578	5.934466	8.243095	9.300553	6.010955	18.84709	5.48672	13.77603	18.46729	10.90567	14.39646	17.31903	7.130952	18.19714
CUST_36450_PI429010886	M15926	0.014337761	24	15						10.57564	11.69289	18.37247	8.652491	16.70829	9.531461	14.77073	9.512184	21.48216	23.47597	10.31893	25.79038	34.50101	12.70605	33.08133	36.48523	24.02389	30.73256	24.49326	9.642309	25.18191	24.97096	38.52366	28.84569	26.38647	16.04653	25.69105
CUST_37525_PI429010886	M19370	0.014337761	24	15	321477568	hypothetical protein DAPPUDRA			1.48E-33	27.93243	63.61864	50.4405	28.85937	34.06404	36.14198	37.93177	47.37518	33.64987	48.43013	46.55494	30.58395	44.21653	48.8281	43.28942	46.82329	59.36408	58.70057	40.71158	40.41488	51.75972	44.74977	44.3395	49.50741	43.30726	38.40373	46.41625
CUST_32538_PI429010886	M4371	0.014337761	24	15	332025010	Copper chaperone for superoxid			1.73E-12	83.43947	133.4287	136.2348	104.8945	108.1711	100.1016	105.4292	171.9376	165.2945	119.1399	101.2137	79.15396	112.7891	108.4356	90.18372	152.0785	189.476	153.2351	115.5309	166.1333	150.6257	108.5947	149.8147	139.2427	132.6815	130.6255	138.4795
CUST_52657_PI429010886	M98353	0.014337761	24	15						17.36938	30.445	31.00311	27.5331	8.385883	17.07816	30.84927	19.83269	26.99058	43.75295	16.20353	21.20837	88.29653	33.16469	30.47418	35.87313	41.70283	42.68735	32.6167	24.1058	30.99993	36.95293	20.89693	35.94651	29.1953	33.39115	42.316
CUST_8969_PI429010886	N23868	0.014337761	24	15						157.6398	180.2649	223.8968	139.3753	131.9106	112.8764	207.8099	173.5895	215.3674	173.8121	160.7996	94.47843	228.253	162.3371	131.0644	232.6083	239.642	241.0614	179.2149	179.3306	234.6915	222.9863	180.4396	177.2128	260.696	142.8894	230.6808
CUST_20861_PI429010886	N44204	0.014337761	24	18						88.65754	120.3127	117.4006	122.5392	101.2809	84.17542	93.80651	105.3437	103.2627	119.8645	98.75554	103.9647	135.0936	94.16687	106.8649	97.00725	131.5859	128.8131	134.8921	130.3607	130.479	124.4425	125.8771	125.9339	142.1948	104.5846	146.0508
CUST_38989_PI429010886	M33025	0.014349877	15	0						470.0131	1275.515	735.5518	531.9945	387.048	510.3128	344.6427	746.9967	918.7311	270.2277	673.3593	188.656	424.6765	720.0128	994.081	794.9446	1104.345	1485.843	805.2136	771.5383	594.0785	597.7182	494.606	327.1238	541.0757	688.4549	303.2777
CUST_7317_PI429010886	N21371	0.014354216	24	9	321458061	hypothetical protein DAPPUDRA	Transport	Protein transport	3.14E-50	5.768746	4.606356	3.796093	NA	14.07152	4.877189	NA	10.60847	7.02166	12.46897	11.12358	11.83609	4.280426	6.19314	16.56978	7.781119	7.453005	7.939364	4.726313	6.080857	6.62957	5.973561	NA	12.21743	6.724289	6.254674	4.527432
CUST_34712_PI429010886	M10430	0.014360146	12	9						174.2207	37.74193	122.0207	219.5016	158.0791	109.892	63.38213	28.88374	47.72449	259.3203	169.7886	315.5362	201.3668	132.7164	80.14476	20.06249	24.34094	28.36418	36.80764	91.56473	40.1121	258.4078	137.524	224.0655	60.96191	86.88214	57.66115
CUST_34435_PI429010886	M9671	0.014360146	12	9						160.8732	122.8853	181.2265	191.741	154.036	132.304	107.5871	126.4673	119.7663	252.5113	605.0439	231.4277	292.0008	163.1533	238.3483	108.9127	126.8773	133.2629	126.3605	141.7369	137.8428	303.7038	400.5287	370.7223	135.258	341.8735	422.8235
CUST_32084_PI429010886	M3197	0.014364358	24	15	156549535	PREDICTED: developmentally-re			7.17E-147	473.1245	836.9003	600.669	413.7039	417.2642	399.8074	697.2436	752.6445	651.7291	666.0922	492.658	510.7551	569.1918	484.8492	442.8695	739.59	792.067	900.553	588.3344	730.0615	719.4386	470.0131	594.442	502.5054	623.6004	583.5463	642.1289
CUST_6914_PI429010886	N20771	0.01437856	24	21						99.05353	101.8893	118.2919	135.0024	88.35558	113.4582	97.09091	81.118	95.52209	122.3191	85.37067	104.203	113.3707	103.4638	122.5728	119.4159	86.34178	119.3879	111.5206	81.86695	118.606	189.8869	133.8844	133.4406	142.4297	116.9098	128.9945
CUST_34215_PI429010886	M9077	0.014383825	15	6						150.6767	196.4115	212.7294	157.3672	153.1975	174.2396	221.8149	206.1397	173.5314	108.9271	130.5494	120.6396	131.4508	144.389	122.0941	104.7593	181.5475	190.9064	173.4439	213.5938	218.0311	180.1675	195.4372	218.7535	157.5876	129.5648	214.1578
CUST_40769_PI429010886	M56047	0.014384294	24	15						18.42634	26.79339	3.261653	23.65361	13.94586	26.91338	39.88527	53.00589	45.81369	12.25513	23.51188	20.74399	76.53128	40.78485	71.41406	92.92674	61.71621	48.73482	13.77125	22.08061	13.389	NA	165.0873	158.2614	9.865694	34.86879	9.189042
CUST_32717_PI429010886	M4912	0.014402673	18	15						29.16267	18.61475	NA	12.05333	7.207883	8.416104	16.64745	14.71235	16.90805	24.11615	12.20921	26.12151	17.73086	23.17242	11.75933	18.35318	20.55348	18.39978	25.79817	19.31397	14.7656	16.1942	13.89586	10.97673	19.14555	12.00032	8.567205
CUST_43852_PI429010886	M64155	0.014410522	24	0	383847096	PREDICTED: acyl-protein thioest			1.54E-31	186.1038	315.5362	215.1868	154.4269	169.8371	149.578	142.1426	219.8411	195.3485	153.4348	232.3243	160.9133	139.3753	213.3759	162.5035	151.5648	265.541	172.4491	187.1657	206.98	179.9541	208.8807	200.863	223.5617	247.2393	237.2505	294.8466
CUST_43949_PI429010886	M64437	0.014417556	24	15	260802151	hypothetical protein BRAFLDRAI	Metabolic process	Lipid metabolic process	1.97E-30	22.20534	32.36331	35.60356	27.57421	17.90158	31.20826	19.42557	17.01395	29.95378	30.68789	40.14624	28.94999	46.05207	19.55672	43.69757	46.34194	35.55837	38.04859	41.99895	21.23274	29.7311	38.62045	30.59991	36.89171	36.87514	25.80839	27.63333
CUST_4374_PI429010886	N16622	0.01442015	24	21						144.0796	143.8832	162.6466	123.7474	112.6234	111.91	125.0613	114.0755	151.8112	144.9379	101.6524	107.0618	126.4338	106.3722	115.2656	165.4279	169.7003	128.7173	182.2404	103.7428	127.633	191.9474	148.131	149.533	192.6063	142.9419	190.0902
CUST_41490_PI429010886	M57859	0.014452441	24	15						184.9648	443.6171	NA	NA	10.12695	4.874615	7.57781	142.126	42.37791	35.28538	58.11342	NA	16.30277	NA	20.44056	701.5555	109.9078	869.9307	611.3316	32.18818	491.3879	NA	112.1712	NA	81.08936	79.07706	11.69458
CUST_14312_PI429010886	N32672	0.014454114	18	9						11.19238	5.396608	7.471039	14.1199	6.759425	18.35487	18.30245	7.770794	23.40329	14.40443	20.48939	17.12368	12.8517	15.43474	8.45839	12.28923	14.06051	12.37254	10.16395	NA	4.05425	23.30496	5.013948	8.965368	12.31866	4.324511	13.09923
CUST_2307_PI429010886	N12070	0.014455267	12	0						34.83692	32.8057	23.82015	24.7491	21.55742	25.22536	15.90508	27.06704	15.16581	25.40416	18.00324	21.82274	19.07677	22.36969	19.11764	18.01822	24.07128	16.33991	15.65876	14.49179	14.2733	17.00074	20.58996	18.32237	41.60067	17.03584	25.14223
CUST_53194_PI429010886	M100662	0.014456298	12	6						38.47393	10.78499	18.13366	54.53262	75.98892	63.03163	191.9733	11.25219	136.9272	49.29631	59.9228	44.68483	30.85244	53.15363	27.53743	35.05094	11.11102	26.33708	191.7974	209.9538	338.9144	54.31269	13.19225	49.87102	40.09585	27.68431	58.57991
CUST_54721_PI429010886	M107312	0.014459064	15	12						11.624	27.3523	19.44615	5.163494	10.76987	8.066527	7.876547	16.79346	9.666191	15.66801	NA	13.87743	22.33058	13.88603	21.30878	10.19442	15.18461	16.1297	6.806327	15.54937	5.479271	8.211347	28.97725	22.20755	18.06134	14.71359	10.92283
CUST_34988_PI429010886	M11251	0.01447503	21	18						156.6283	485.7316	340.7848	226.5403	248.3542	229.6548	236.3014	232.3662	198.0058	193.9037	276.55	243.7665	130.702	121.0004	175.2384	357.8637	456.4069	320.6536	342.2376	262.7505	513.2014	183.6198	316.2911	259.7345	262.0968	280.4189	291.1427
CUST_18382_PI429010886	N39683	0.014488785	18	6	321471081	hypothetical protein DAPPUDRA	Protein metabolism	Cellular protein modification process	3.77E-43	13.96735	19.47024	16.10452	12.75519	23.66239	17.66267	17.21755	17.41278	24.59944	11.14899	14.79429	14.37116	14.06805	17.13499	15.14134	9.010934	12.60227	13.48307	16.3145	28.91213	14.65575	18.9075	13.62679	17.78243	17.23729	18.62882	21.56906
CUST_51931_PI429010886	M95730	0.014496252	24	15						10.9112	10.52474	NA	9.645129	10.7943	12.60408	NA	8.442843	8.752118	14.65289	13.59581	10.209	11.35795	10.90833	18.79971	18.69594	22.34076	18.87533	7.069413	13.34178	10.20667	12.92976	NA	11.31857	4.423444	15.89253	12.7945
CUST_32224_PI429010886	M3574	0.014510505	24	15	332023027	Pre-mRNA-splicing factor SLU7 [			3.91E-87	106.5903	177.388	119.1208	108.1394	108.2394	91.02301	133.6243	137.2967	160.342	132.2204	128.8131	67.93128	153.1144	83.31874	105.6633	171.912	181.5978	177.0792	159.9694	145.73	166.7642	134.67	135.7428	153.4511	159.5479	134.826	160.0191
CUST_39750_PI429010886	M43866	0.014510505	24	3						3321.698	121.9377	2871.837	3786.16	5884.771	5840.985	144.8971	271.4702	92.41119	944.1713	4203.047	4952.328	609.6218	5002.917	5712.375	81.16582	79.44437	117.0564	83.97595	281.3108	69.31429	1206.191	210.0683	950.4596	104.9943	418.6884	121.1043
CUST_15836_PI429010886	N35222	0.014510505	24	15			Protein metabolism	Ribosome biogenesis		4445.119	5826.27	4064.827	3532.198	3467.123	2992.811	6304.559	4880.699	5258.496	4456.862	3376.686	4004.603	4548.63	4159.327	4508.46	5686.353	5744.161	7157.757	4787.419	5068.287	7090.073	3790.113	4086.34	4988.039	5002.917	4145.789	5947.47
CUST_22123_PI429010886	N46473	0.014510505	24	15	13898841	CUB-serine protease [Panulirus a	Protein metabolism	Proteolysis	1.79E-16	57.51072	31.05581	36.84812	51.53986	31.06306	27.29712	94.58629	69.55079	109.1836	224.5463	128.797	24.93525	57.18737	49.71376	23.19392	196.1489	164.8407	259.5151	109.0861	157.5632	198.1201	85.21665	25.93001	51.171	142.0036	42.93819	65.74218
CUST_28006_PI429010886	N57093	0.014510505	24	21						91.34986	124.5085	103.7748	128.9945	82.68763	89.85516	132.2346	90.15514	66.69697	83.4815	90.19219	90.12112	138.824	94.71637	94.78884	108.0539	112.7172	116.3471	105.3837	116.5745	149.2626	123.5457	120.1882	148.9148	125.6344	129.9776	159.3169
CUST_43098_PI429010886	M62062	0.014518737	21	3						97.11689	103.8566	NA	27.03986	1224.109	1219.83	107.52	444.3539	111.766	NA	213.1791	204.3745	NA	75.43056	40.64205	18.55182	9.946155	NA	3.933121	590.3145	5.19429	NA	156.436	11.04247	NA	1482.511	NA
CUST_45680_PI429010886	M69891	0.014525205	12	3	321474894	hypothetical protein DAPPUDRA			1.24E-17	361.1904	705.1169	406.5634	486.0305	474.8239	541.2118	647.5505	632.1075	608.2737	498.7889	399.6202	128.2244	535.4815	531.7033	506.8228	791.3056	1263.423	939.4725	597.5894	738.5083	668.7382	321.0799	528.6354	364.2049	665.1463	652.5211	432.6388
CUST_9480_PI429010886	N24704	0.014525205	12	9						8269.17	1992.843	6922.365	9013.443	6523.544	9855.129	2922.057	2646.596	3624.383	8577.765	13658.74	10248.36	11164.38	9142.021	16726.21	1247.204	1485.178	1391.531	4140.108	4710.772	4928.217	11763.32	10795.65	13017.21	2634.199	5900.066	8522.605
CUST_10357_PI429010886	N26112	0.014525205	12	9	156549330	PREDICTED: protein kinase C, br	Signal transduction	Intracellular signal transduction	5.02E-78	20.34099	9.592986	27.60039	16.90111	25.66849	18.91474	20.37081	5.943856	19.407	35.80909	22.56387	33.14692	38.69748	31.1712	34.22791	15.58431	12.70063	11.56152	43.96941	34.04257	22.90061	37.82991	42.29117	26.77351	17.02991	27.44415	34.25718
CUST_37854_PI429010886	M21878	0.014530852	21	15						588.9309	783.939	605.287	602.4086	629.8536	570.2732	651.9522	644.9039	733.2499	745.1009	651.9522	839.7101	792.4227	597.7182	617.1935	660.6448	851.8736	766.3188	663.1483	611.1595	717.5574	658.1324	646.5974	725.9659	524.8326	588.9309	676.869
CUST_40189_PI429010886	M51523	0.014530852	21	15						7993.785	11475.48	8430.296	7832.882	4045.654	4928.217	7653.847	9905.822	9727.143	7439.177	7364.792	5496.275	8591.806	5048.826	6448.141	10550.02	11339.63	12378.85	8238.918	8886.515	9013.443	7327.614	6864.217	9301.136	6929.595	6752.376	7673.313
CUST_49201_PI429010886	M84656	0.014538394	24	0						47.10999	49.11594	51.57473	61.58	38.06969	43.75819	51.58532	41.85107	40.78815	49.38843	38.94229	41.50445	39.13627	35.51059	35.66495	58.07534	42.2265	35.88575	43.31704	45.12599	40.82014	54.37537	43.30726	43.53457	64.67753	38.28366	52.30441
CUST_51621_PI429010886	M94269	0.014538394	24	0						83.41575	114.2871	113.7563	88.59515	89.95321	100.4391	81.01838	111.3418	121.7366	95.57738	96.35679	117.9412	75.82447	86.35128	66.83189	117.123	95.45574	94.78884	89.4662	86.52356	92.0742	140.387	101.0057	104.5953	124.0147	126.4673	121.9035
CUST_15651_PI429010886	N34921	0.014538601	12	6						4.179896	NA	NA	17.29188	NA	17.89974	127.3889	NA	112.5075	37.9964	19.52695	NA	8.454103	9.820162	6.059208	37.12911	NA	37.82991	170.0181	402.7185	857.1902	36.92118	NA	39.05213	156.3126	22.32831	151.3538
CUST_40417_PI429010886	M55023	0.014573653	24	9						15.73574	14.85832	NA	16.10329	61.95862	23.60614	25.49446	11.61188	19.6075	5.973561	112.6108	101.9343	21.65945	18.0495	19.2072	28.1014	9.131262	6.494807	18.66159	16.39884	4.422108	5.429962	12.39193	4.156837	20.13552	15.41579	10.25642
CUST_42993_PI429010886	M61807	0.014583166	12	0						293.5137	325.8846	329.7179	207.3653	226.7781	175.2702	166.8118	234.5996	220.3669	242.9871	289.5149	278.3094	266.1986	256.8109	421.977	257.7877	406.766	364.4673	279.2412	236.1602	233.7667	440.2791	260.9505	356.7674	431.6201	291.6767	489.2868
CUST_4596_PI429010886	N17101	0.014585763	21	15						22.84762	44.53859	56.00071	24.88691	23.32566	33.84542	24.71116	18.88946	23.21635	27.56589	17.74254	25.61949	31.8265	28.6464	30.01855	34.48922	33.45999	31.73936	43.55336	27.04586	26.09309	36.25072	25.76854	27.87333	37.58637	26.65508	29.15166
CUST_54033_PI429010886	M104178	0.014593197	24	0						17.53785	17.33723	23.11733	18.13556	13.78137	18.44774	NA	10.12252	9.62794	NA	6.142774	6.515712	16.52409	NA	3.849031	9.893288	29.91279	21.01951	4.37898	11.51735	13.11625	12.20801	7.05159	11.21493	24.82555	18.41119	21.74119
CUST_32631_PI429010886	M4634	0.014615607	15	0						738.8137	2225.697	1839.274	1012.36	868.6022	829.1725	457.5988	936.2266	291.198	548.2784	486.6807	363.5741	673.7135	368.5924	285.598	1658.691	1065.144	1412.179	529.7211	337.2109	447.3237	267.9409	835.7807	937.9336	565.3173	322.9826	628.1097
CUST_7534_PI429010886	N21690	0.014667462	15	6	354491334	PREDICTED: CD209 antigen-like			0.000000122	8.380003	NA	14.6246	25.1173	NA	4.868818	17.51582	810.1648	966.6426	68.09581	135.1633	92.49079	18.16423	116.8305	10.39496	18.51954	7.254794	11.91333	12.28923	6.911194	16.98223	28.16376	314.367	338.2602	17.1309	6.484978	34.58559
CUST_48075_PI429010886	M78772	0.014679427	24	15						813.0168	530.9393	623.6004	754.9422	420.6276	611.3316	2375.903	1988.355	2734.019	3342.747	1640.994	494.0593	1003.544	593.0437	706.2599	6922.365	1848.594	5929.402	1553.379	1840.408	3166.856	1149.936	436.2924	753.6723	1570.104	341.732	1095.073
CUST_53285_PI429010886	M101096	0.014683249	24	3						220.7429	113.9699	225.0187	266.9079	139.9464	265.9313	109.5722	99.01871	77.26866	149.4369	144.035	169.8611	142.3304	136.3336	157.9135	87.59003	103.7292	88.45765	100.038	93.25565	102.0776	169.1953	121.1493	145.5033	111.8276	102.0887	112.1712
CUST_37178_PI429010886	M18104	0.014683249	24	15						57.8755	110.4251	100.7618	69.58412	75.40109	60.37267	78.24597	106.3228	95.15335	93.73127	76.40541	80.82353	100.4391	88.77171	84.67585	95.12721	118.4588	126.327	77.40803	98.96283	103.4755	89.97113	90.46312	91.57245	92.22131	76.28125	109.7513
CUST_37382_PI429010886	M18846	0.014683249	24	15	195438859	Drosophila willistoni GK16371 (D	Protein metabolism	Proteolysis		640.2875	1469.262	979.7526	688.6527	896.6099	704.3483	934.7113	1262.927	1025.404	996.7263	912.5849	848.3549	895.8876	978.4034	978.9259	1204.169	1542.881	1291.739	923.1653	1010	1261.961	813.7492	1098.604	1044.527	822.6543	968.0088	1061.158
CUST_43002_PI429010886	M61823	0.014683249	24	15	242023915	Importin-7, putative [Pediculus h	Transport	Protein transport	4.53E-19	112.8135	175.4398	158.2614	141.0774	128.0468	139.4559	195.9322	169.5621	144.495	191.823	129.007	138.7489	159.3374	172.6519	83.13651	214.117	273.134	179.7028	205.7216	139.003	170.0929	172.0396	162.5974	147.5789	164.7688	147.3568	145.3012
CUST_45845_PI429010886	M70480	0.014683249	24	3	341650466	Spz1 [Litopenaeus vannamei]			7.35E-50	1568.389	471.601	1100.835	1689.176	9490.363	6601.274	526.6092	1371.161	427.6634	469.784	5826.27	6470.198	508.9078	6051.213	4616.026	361.3178	246.1269	203.36	272.5621	2017.363	281.7476	600.669	830.7266	504.4324	123.4012	2506.266	284.1348
CUST_46925_PI429010886	M74190	0.014683249	24	15	188572522	putative 60S ribosomal protein L	Protein metabolism	Ribosome biogenesis	6.42E-22	6523.544	10464.54	7100.104	7353.952	6032.614	6000.152	9111.496	9364.597	8736.825	7497.741	6155.993	4253.847	7731.941	6000.152	7506.653	12725.99	10683.23	12473.12	7832.882	8577.765	11164.38	6656.173	7402.627	7439.177	8296.544	7531.236	9226.663
CUST_33852_PI429010886	M8037	0.014683249	24	3						268.0293	35.96649	296.6874	388.382	359.587	449.9406	34.1106	38.45427	26.13038	207.209	423.7011	315.7654	164.188	224.5862	427.1681	21.93453	30.18014	8.25537	18.84141	58.9878	23.29521	181.2583	102.411	154.036	15.69941	66.15369	49.56614
CUST_157_PI429010886	N1750	0.014683249	24	15						18715.71	18966.14	16221.54	16622.66	10374.24	12496.19	21398.95	16874.05	18273.52	19341.82	7290.969	17210.94	18365.76	12084.47	13591.98	27459.44	19855.57	23084.2	22037.75	17658.04	21224.39	18613.15	16520.01	19237.66	21758.66	18579.76	19522.5
CUST_7951_PI429010886	N22314	0.014683249	24	15						1807.527	3145.521	2219.678	1869.564	1242.744	1369.19	3472.797	2581.822	2759.556	2014.653	1722.827	1511.222	2299.057	1782.659	1647.856	3441.376	3364.257	4238.105	2404.745	2290.485	2881.653	1587.121	1935.604	2056.189	2624.319	2228.732	2047.134
CUST_9103_PI429010886	N24068	0.014683249	24	15						222.1435	317.3631	199.8063	177.6455	212.1398	189.8869	298.5983	335.0962	314.5002	273.0279	226.7528	233.4638	225.9127	213.9341	254.9746	333.6191	316.3525	295.4891	243.2347	260.798	271.7261	220.7835	271.3104	276.8158	254.3008	211.0809	234.1695
CUST_238_PI429010886	N2929	0.014683249	24	15						2640.776	8899.45	1951.028	4334.63	1713.82	1701.286	17623.06	13391.14	9490.363	1752.658	1790.499	467.1523	8042.108	9937.479	3090.222	13716.26	25130.01	22332.92	8160.518	4803.622	6179.344	4628.184	14389.61	2687.973	6717.703	8577.765	7653.847
CUST_15748_PI429010886	N35076	0.014683249	24	15	345478826	PREDICTED: hypothetical protei	Nucleic acid metabolism	Regulation of gene expression	1.08E-13	63.07768	54.03718	63.07317	55.54321	37.64453	44.594	75.14729	43.15014	49.23192	89.4662	45.79273	55.73019	83.53855	41.50754	58.9013	89.16759	71.43667	91.36194	88.83368	49.80184	71.47305	66.59115	64.57231	70.94231	84.28713	55.09433	105.8493
CUST_16452_PI429010886	N36259	0.014683249	24	15	390362922	PREDICTED: zinc finger protein X			1.77E-10	8.405629	13.42624	11.4038	8.739672	9.167675	10.08205	12.32463	11.7375	12.80236	15.70113	13.7624	14.60467	19.48546	7.915424	12.16427	23.38479	15.89565	20.2155	15.00133	12.66652	7.359305	17.43237	13.45766	20.28829	11.81163	14.04735	24.60432
CUST_21997_PI429010886	N46235	0.014683249	24	15	242010311	UGA suppressor tRNA-associate			7.78E-36	10.91685	17.73086	10.99842	13.51154	3.726948	10.22653	17.34666	12.45971	19.55672	23.56505	16.33604	12.49326	6.108532	11.73235	8.117736	26.76257	22.19592	23.05329	14.11031	20.24228	30.70093	14.29468	16.87953	16.16065	10.18635	16.8271	12.99802
CUST_22495_PI429010886	N47135	0.014683249	24	21						15.43656	11.80274	14.33538	10.51838	5.365616	15.7393	8.061769	15.7085	15.73263	7.371989	11.78536	9.621436	10.29813	8.052062	14.99779	18.32237	11.88179	17.49021	14.64332	12.34301	11.68418	24.85622	15.0452	17.82688	22.78614	14.19705	21.69176
CUST_27587_PI429010886	N56323	0.014683249	24	3						133.6449	107.8619	158.8243	124.3072	1412.941	1275.515	105.6633	195.2973	158.3476	82.59378	1200.665	720.5302	86.92889	981.9828	800.6803	46.02907	52.29097	51.99386	129.6173	292.3452	107.1382	117.7627	104.8169	115.6711	97.90232	480.8108	149.8147
CUST_28252_PI429010886	N57598	0.014683249	24	3						1149.369	51.24138	1211.071	1315.424	565.6114	986.164	58.94726	48.86558	61.4814	265.8681	549.8031	567.5384	392.1412	863.8645	1126.574	31.25385	24.73091	33.12802	29.35853	45.0273	66.2054	544.2292	88.24057	465.6755	38.80533	71.1558	102.9045
CUST_1469_PI429010886	N9729	0.014683249	24	15	334349652	PREDICTED: Monodelphis dome	Cellular component orga	Cytoskeleton organization		112.5697	274.7035	194.7103	166.8118	143.6704	133.6073	297.8825	312.5578	230.8967	285.4767	246.6059	55.27672	231.4946	193.9037	235.3802	276.1893	445.9579	391.0137	257.956	196.0361	291.5804	199.6427	201.9478	231.4591	218.9572	252.989	247.6161
CUST_7550_PI429010886	N21714	0.014683585	18	6						214.8056	834.3772	289.2838	502.0555	345.993	124.5085	1012.891	858.51	1060.655	329.4331	183.7819	246.4965	430.7389	372.9744	190.1223	321.3471	754.678	613.7905	563.9832	548.75	324.4873	574.0759	799.3476	802.754	768.2904	897.3252	1239.086
CUST_56726_PI429010886	M114523	0.014690265	12	9						34.19427	14.81156	34.80421	25.30627	20.17168	17.23127	30.44744	32.06918	38.19091	24.29996	41.82858	25.58573	43.83432	23.41694	38.56693	19.84536	17.91133	16.90983	36.17071	43.34384	24.37455	40.78067	61.26619	37.88972	48.73108	33.78234	40.50707
CUST_30905_PI429010886	M181	0.014690265	12	9	328699090	PREDICTED: polyserase-2-like [A	Protein metabolism	Proteolysis	5.51E-86	254.8129	702.937	653.7367	198.8083	437.0635	235.6895	434.3661	679.224	571.6616	440.6377	756.6003	459.9674	432.0096	280.1334	393.358	277.9454	358.7305	206.98	359.8294	488.0472	404.7001	302.6134	574.5775	510.5943	378.3539	444.7023	331.3594
CUST_178_PI429010886	N2127	0.014690265	12	9	321459497	hypothetical protein DAPPUDRA			9.74E-21	109.097	14.52179	67.3975	118.7615	51.81269	76.78462	35.27538	19.64262	34.38713	105.6633	143.4863	96.7742	90.12112	57.51884	107.8435	30.86168	27.91891	18.21058	53.76691	47.09644	47.47428	112.8468	111.4697	143.9979	59.20501	50.00771	95.09356
CUST_1140_PI429010886	N8622	0.014690265	12	9						53.73347	24.78664	67.23484	59.51158	26.78523	15.59668	51.7172	10.77751	59.07967	59.24085	95.35371	45.746	131.8269	85.19452	30.74237	35.88107	35.15355	28.86297	45.2886	81.24064	81.23215	136.7201	70.40519	135.8408	39.12428	59.34282	120.964
CUST_37827_PI429010886	M21788	0.014695252	24	3	390517064	hemocyanin alpha subunit 2 [Ca			4.26E-32	19.21128	12.76402	NA	22.63957	29.91879	27.30031	18.6435	28.35209	9.275544	NA	16.59861	11.73655	8.962349	3.401349	NA	5.845515	8.374367	7.487104	3.348615	43.77016	6.645065	15.40723	16.60566	5.838572	5.092156	49.05136	NA
CUST_6542_PI429010886	N20239	0.014701895	18	0						201.0489	236.4795	207.8099	192.8532	178.6979	167.6224	106.8768	193.8538	95.86932	137.8653	47.47428	149.6035	167.5904	191.4824	203.6222	152.0064	96.5246	171.4564	192.2397	106.9946	234.9137	191.0167	84.40775	208.7171	147.9113	168.6123	182.0698
CUST_20128_PI429010886	N42856	0.014702301	24	18						341.8735	182.8746	307.6081	300.9971	159.6647	218.889	279.1951	166.8658	211.8745	349.3011	238.3045	232.0765	698.2425	314.3155	542.5585	322.5153	364.8648	318.4002	687.4827	267.2693	617.9354	356.9618	191.0167	336.8186	400.9911	245.7379	470.5066
CUST_26614_PI429010886	N54541	0.014702301	24	15	471417319	PREDICTED: unconventional myo			8.28E-38	83.21526	87.73981	98.60183	80.54573	60.5679	82.24574	87.55235	78.64253	55.40345	125.0086	95.58893	93.50035	137.2489	73.3591	124.8682	159.8538	103.07	145.0969	88.22605	73.36846	97.30769	107.7706	82.16147	111.5466	133.7038	82.45499	116.4262
CUST_42217_PI429010886	M59709	0.014718618	24	0						35.0064	62.37324	47.17279	29.45413	26.90034	23.42712	36.40013	51.7695	46.89534	36.31683	25.21673	26.24625	21.87306	38.24975	22.71026	37.68818	23.99912	24.72767	18.99955	40.77521	26.88006	41.57553	38.64131	44.42034	39.84739	46.79452	36.61868
CUST_12062_PI429010886	N28864	0.014721858	12	0	242004297	leucine-rich transmembrane pro	Protein metabolism	Cellular protein modification process	1.01E-26	27.56258	35.08725	21.85018	12.43346	54.10933	47.80328	8.311471	10.39189	9.308623	10.54024	30.92216	48.46739	NA	31.62493	58.5159	11.32305	7.113497	7.907113	6.933026	24.61634	4.047703	14.39849	6.258419	14.13121	14.68872	28.87134	NA
CUST_495_PI429010886	N5675	0.014757353	21	0						462.3981	622.3504	318.3284	222.5585	576.6349	620.9302	435.509	1680.655	536.489	174.1094	236.1021	181.4589	287.5406	340.7195	285.0842	75.56999	115.6261	90.55203	132.4791	2356.919	136.0891	384.6227	876.3228	301.5476	108.2246	2025.285	421.3144
CUST_1385_PI429010886	N9470	0.014759747	21	15	1168684	RecName: Full=Blastula proteas	Protein metabolism	Proteolysis	1.15E-38	11.27831	19.57289	16.73592	66.296	217.2347	84.78014	175.2702	404.5068	73.30672	169.6715	55.95718	113.0417	200.9087	912.5849	21.77471	175.5738	276.1893	429.8212	386.0289	522.4996	733.2499	40.7919	6.074391	41.02997	220.2503	NA	187.3112
CUST_56988_PI429010886	M115061	0.014807835	24	15						15526.29	17023.68	15367.74	14137.09	8945.499	10966.45	19010.63	15767.9	17658.04	16760.98	10127.21	15230	17315.8	11076.77	14032.06	25343.01	19341.82	21334.13	22598.48	15847.39	21524.84	18026.32	13837.12	17131.85	24289.54	15141.22	21881.51
CUST_23603_PI429010886	N49104	0.014807835	24	21						45.34892	70.02512	70.49708	50.98858	61.92068	41.78715	47.5648	47.46968	65.80773	58.40039	73.15055	43.88384	48.50466	51.3028	52.33176	46.84848	61.26619	52.33755	63.4481	55.34491	48.85342	70.10106	79.25889	82.04507	68.07757	67.96481	79.22393
CUST_5426_PI429010886	N18686	0.014808068	21	3			Metabolic process	Other		6.478825	11.30212	15.59925	21.70341	24.96126	11.81717	17.92151	12.46202	13.83737	10.96971	14.30855	10.75031	7.19184	9.127031	10.18635	11.81163	16.64948	10.648	13.89788	11.52123	9.491244	17.04105	5.137454	15.929	18.12496	11.31144	15.67952
CUST_40733_PI429010886	M55962	0.014812557	24	18						66.46575	160.9374	214.8953	163.4636	106.3228	237.9355	132.0536	251.6934	281.3641	107.4552	137.2351	147.6657	95.36659	177.6455	108.3883	403.8629	267.1988	298.1868	430.0583	263.1307	411.0555	482.7166	219.3222	284.3354	400.5287	165.0428	334.2355
CUST_7439_PI429010886	N21553	0.014827313	18	3						14.2733	17.20063	20.42799	30.45754	20.08558	17.73565	8.865478	16.39186	17.4518	17.74254	13.05918	12.10816	10.53476	13.96378	10.68733	20.43485	17.17977	20.13304	8.76542	8.117736	15.49793	15.9018	20.63115	21.95678	20.05426	14.49041	15.7161
CUST_33003_PI429010886	M5689	0.014836197	21	12						667.9516	748.709	182.136	566.3117	137.8653	374.6189	953.6942	1764.635	1035.146	785.8536	527.5856	268.0293	1401.718	1621.358	709.6774	722.3326	354.2621	690.7831	1405.334	1475.028	2511.558	692.3061	254.714	376.7239	404.3968	176.6888	268.8247
CUST_6213_PI429010886	N19781	0.014855324	12	3	225713778	Transmembrane emp24 domain	Transport	Other	1.89E-74	478.8721	844.4833	414.7402	541.0757	589.338	669.5828	624.9341	898.0277	570.0085	536.489	450.6028	213.3048	448.6833	567.1816	404.3968	700.359	759.3274	781.7261	491.0486	645.7283	625.4995	393.358	609.6218	389.8933	502.6586	630.1325	376.5416
CUST_17830_PI429010886	N38662	0.014855324	12	9						79.72468	77.02123	60.92036	67.05704	87.20964	72.74057	61.49211	64.73297	67.3975	81.96005	84.21664	82.9737	61.19974	77.62481	91.41914	62.1826	60.2163	61.85717	65.65546	58.43593	75.24855	93.76286	79.05137	105.619	67.81145	73.00128	76.84692
CUST_35751_PI429010886	M13413	0.014855993	24	15						548.75	826.4146	705.1169	517.6361	571.0817	484.8492	655.9099	643.379	741.1426	709.0094	511.9251	571.6616	670.1687	501.7792	564.8143	871.1645	927.9648	956.8208	651.7291	712.1062	787.8266	624.2116	740.5837	744.5255	742.4077	634.4332	792.4227
CUST_39703_PI429010886	M43162	0.014855993	24	3						7597.158	346.1134	6744.57	11228.98	6799.146	9194.226	1402.437	1791.446	2356.919	9056.645	10323.84	9241.731	8911.047	6601.274	8474.042	101.1701	69.90555	131.2558	2846.594	2409.846	1876.444	7762.077	6276.978	8006.37	1958.772	5032.372	7203.647
CUST_39953_PI429010886	M47414	0.014855993	24	15						275.85	173.0186	157.4324	107.7788	25.17016	92.63177	677.9076	86.35128	583.9375	817.7537	510.3128	86.94104	50.04164	401.1245	34.89175	1673.566	951.856	804.3138	482.1823	570.1346	930.8409	223.3238	5.356052	160.5879	597.9185	95.74045	470.9814
CUST_46270_PI429010886	M72021	0.014855993	24	3	77743875	Euphausia superba isolate DGGE				13616.29	75.98892	11429.91	14956.1	6567.976	11246.8	92.52233	117.7735	82.98239	2123.927	8348.895	8121.327	2187.303	8962.903	5467.822	74.74645	87.94793	87.38427	92.39943	124.2958	112.3711	2566.656	654.7479	1894.407	148.4289	173.6723	193.9037
CUST_52041_PI429010886	M96180	0.014855993	24	6	524912976	PREDICTED: papilin-like [Aplysia	Protein metabolism	Other	1.82E-08	264.8694	735.8335	589.1435	459.8781	691.4224	780.1728	538.9124	790.1557	894.3502	309.5645	913.5277	359.5095	481.0599	673.1503	423.4844	379.5535	598.944	468.5187	268.5317	441.7626	253.0838	269.7799	655.4746	396.5838	354.0092	437.2723	387.048
CUST_52449_PI429010886	M97478	0.014855993	24	21	242008812	conserved hypothetical protein			1.32E-11	20.25567	11.18684	13.6951	17.32266	14.57035	18.28134	13.4565	13.14087	12.8443	10.1116	9.776816	12.28744	21.83479	39.97068	16.18394	19.03842	20.39754	21.17952	29.39693	10.75951	8.136232	26.206	90.14554	15.23659	19.51476	26.33708	19.4056
CUST_5253_PI429010886	N18454	0.014855993	24	15						871.592	1920.18	1041.115	910.2628	1049.395	1037.623	1445.709	1429.079	1311.001	1055.306	1157.025	879.1441	1063.435	1006.869	1051.789	1639.362	1941.073	1588.642	1386.294	1206.945	1332.694	1082.664	1278.279	1132.05	1276.929	1240.711	1149.369
CUST_5817_PI429010886	N19240	0.014855993	24	15	345486496	PREDICTED: hypothetical protei	Cellular Process	Cell cycle	4.58E-32	152.9638	246.5228	218.9572	161.4506	187.9849	133.3824	208.6833	247.0114	246.083	210.4367	170.5061	152.1914	221.2277	175.2922	188.8287	245.059	344.0033	279.7784	215.9822	255.1811	221.4294	194.1257	232.9522	215.9178	243.1902	212.0595	239.7523
CUST_7371_PI429010886	N21443	0.014855993	24	15	226510662	Penaeus monodon eukaryotic tr			1.04E-55	3269.912	6060.248	3558.043	2753.439	2543.39	2425.524	5807.4	6068.533	5073.866	3956.525	2881.653	2913.515	3801.741	3827.862	3112.057	5238.942	4945.667	7071.199	3924.629	4606.27	4976.553	2609.919	4322.847	3269.912	3798.572	3549.354	4572.831
CUST_7851_PI429010886	N22175	0.014855993	24	15						59.27468	113.3707	47.10999	59.37073	42.34405	59.14128	79.66104	77.42501	57.9144	60.04487	66.73428	62.48283	70.36116	75.38714	63.253	87.16322	94.34203	87.75883	61.0054	65.90164	75.27273	46.57555	73.50942	70.71767	70.14108	62.86957	206.1814
CUST_7434_PI429010886	N21545	0.014872865	21	3	405958337	Nucleolar protein 66 [Crassostre	Nucleic acid metabolism	Regulation of gene expression, epigene	4.13E-62	104.0803	71.23242	67.20028	118.7939	157.0815	165.0194	64.29327	83.31874	60.47985	122.4622	249.2196	144.3062	110.7454	123.7474	106.2214	53.24219	74.57856	55.77263	69.00406	81.48573	52.78692	117.4669	143.2785	119.1723	73.10715	145.843	116.0897
CUST_51941_PI429010886	M95754	0.014874466	24	6						51.19908	38.64876	71.40475	57.20828	43.83432	15.4529	22.66351	92.13972	85.70234	28.31587	NA	22.16839	35.82162	6.963509	15.07768	3.808482	91.71339	NA	4.376836	6.286258	13.9779	43.88384	10.92685	12.53864	NA	NA	NA
CUST_40779_PI429010886	M56060	0.014886017	21	15	126282077	PREDICTED: golgin subfamily A r	Transport	Vesicle-mediated transport	6.04E-17	30.14227	27.01424	18.21466	33.33788	30.07472	75.43056	31.44182	18.55514	30.36663	22.905	20.00187	31.81107	40.40244	33.5564	43.63223	33.92845	38.19894	36.60452	29.72606	18.84878	34.52377	24.64033	24.4823	25.93791	30.445	14.05708	30.05953
CUST_33952_PI429010886	M8346	0.014895753	21	0						331.9974	398.2132	333.8469	289.1171	302.6134	268.3054	302.9977	295.4303	295.8529	303.0592	248.4962	262.8953	355.841	226.6524	336.6729	283.749	335.6891	292.1626	406.254	303.5583	320.7176	371.3022	350.791	324.8189	367.4917	267.555	326.188
CUST_50615_PI429010886	M90438	0.014895753	21	3						98.2692	138.0384	124.4149	97.63248	211.625	158.3476	123.4188	134.6822	121.6025	91.36194	109.3921	117.4173	104.5741	107.0461	122.1735	87.08887	79.4857	111.1293	113.622	114.8835	133.5662	124.2323	105.7831	115.908	145.5634	102.4255	134.9139
CUST_5117_PI429010886	N18281	0.014895753	24	18	170037075	Culex quinquefasciatus glycogen	Metabolic process	Carbohydrate metabolic process		735.8335	1711.15	1103.734	866.8631	1069.345	965.1042	650.8946	1148.486	1108.83	699.7985	1039.627	789.3467	872.0312	858.51	903.4294	1064.289	1253.031	896.6099	1212.445	1146.806	1375.747	1005.566	1475.028	1203.381	1005.944	1136.704	1327.748
CUST_14103_PI429010886	N32331	0.014895753	21	9						41.22261	70.36116	56.52239	63.49903	72.34444	80.1827	55.70902	76.11372	78.77099	74.73696	70.60324	97.3763	60.10787	59.09838	66.42663	81.76793	95.38758	90.03155	52.52277	57.85506	57.62248	40.61255	70.38705	51.11212	62.48283	49.05504	76.19255
CUST_23265_PI429010886	N48538	0.014895753	24	0						48.38102	68.70029	68.4765	50.70889	56.25386	44.44554	47.48815	60.77123	59.47135	47.39716	37.22016	32.94858	55.28196	44.04608	60.84718	48.28814	61.30321	55.42058	56.01896	53.36088	67.24604	116.3209	51.48244	53.41031	50.85543	70.28135	71.00454
CUST_38741_PI429010886	M29865	0.014911173	24	12						42.64856	49.55479	54.55547	262.4014	60.88384	140.8769	269.2064	339.4175	431.4309	51.23629	781.4453	41.1635	542.7794	637.9796	194.4885	176.8903	119.4775	240.1565	16.10664	138.9534	21.7849	31.67008	340.4211	341.2447	15.35763	47.5648	49.08691
CUST_17183_PI429010886	N37518	0.014955998	24	6						55.19896	42.72823	6.89852	5.430904	110.0991	57.08761	59.99359	143.059	57.47217	NA	44.43314	47.50128	6.063885	44.97359	29.53009	11.98023	16.70064	8.71204	6.464013	182.702	4.000016	5.771961	32.14643	10.8869	NA	226.3355	11.09202
CUST_46310_PI429010886	M72160	0.015017793	18	12						6.246654	6.692466	8.061769	14.25286	NA	12.93352	16.31601	12.32348	12.97623	24.89383	8.62544	17.77566	26.45386	10.43499	22.08061	19.11039	8.441492	21.0164	8.865478	7.079125	8.618077	26.95458	11.31707	14.93264	15.05045	12.86584	5.961953

Probe ID	ID	p-value	Period	Phase	EBI ID	Annotation	GO category	Go term	E-value	0	0	0	3	3	3	6	6	6	9	9	9	12	12	12	15	15	15	18	18	18	21	21	21	24	24	24
CUST_36478_PI429010886	M16020	0.015020383	12	9						86.88214	126.5609	120.3426	101.3081	89.45518	92.56838	108.2696	125.2728	95.38758	112.6387	130.6083	97.87129	120.7979	100.3755	121.7034	107.2144	84.40775	102.1199	143.2998	115.8648	143.5809	128.3867	184.7904	166.8658	117.0564	92.28753	150.7041
CUST_31595_PI429010886	M1924	0.015020383	12	9	270007800	hypothetical protein TcasGA2_T			0.000000511	512.9523	592.0137	588.3344	878.4972	326.2417	417.9126	774.7662	549.3721	1027.619	1175.829	684.8522	790.1557	1671.759	670.9198	856.5689	629.8536	611.8901	581.8311	1039.149	756.6003	1126.574	1246.549	1162.325	1175.829	953.6942	745.5832	1422.675
CUST_20270_PI429010886	N43136	0.015020383	12	9	242011966	predicted protein [Pediculus hur			4.92E-14	47.7609	5.801895	41.71675	30.73256	16.15902	29.84354	18.03992	12.02678	14.64529	78.43693	22.01795	70.0011	65.59134	24.33371	51.34712	18.92222	6.097172	16.59634	35.52825	21.64707	36.92118	68.90523	32.73463	77.38763	25.28496	24.6758	51.25301
CUST_163_PI429010886	N1912	0.015022963	24	18						1138.567	939.4725	1654.651	1701.993	731.069	756.6003	848.0782	1830.285	1367.344	797.5472	579.318	536.489	801.3203	1001.688	472.6199	968.7419	2216.187	2299.057	2283.014	3000.392	3792.758	1814.798	1054.885	565.6114	2157.924	206.5859	1685.048
CUST_30950_PI429010886	M291	0.015028737	24	15						238.4366	472.502	316.5315	245.8656	250.9068	243.5905	370.1622	442.5726	436.2924	350.791	330.7287	288.0498	331.1317	264.4742	319.0038	406.3531	577.3756	467.8312	351.422	336.3009	435.8605	295.7504	356.7674	329.6536	354.0991	317.8651	342.2376
CUST_39126_PI429010886	M34936	0.015028737	24	3	341650466	Spz1 [Litopenaeus vannamei]			2.02E-31	77.37879	18.76106	61.0985	59.82583	111.8855	96.17648	26.19863	36.11092	12.53963	26.11634	130.4316	103.5395	31.20454	94.70581	107.1698	6.822802	7.425904	4.309811	14.16203	48.30191	9.181284	38.96726	31.15138	22.17612	5.68169	60.82571	18.9004
CUST_49701_PI429010886	M86781	0.015028737	24	3						33304.41	1363.959	29396.88	50283.97	32184.55	53773.55	1261.961	3882.503	982.8111	42662.49	34757.71	52429.1	34065.02	34620.42	47342.04	561.3058	558.1235	690.3228	1309.131	7531.236	1038.012	41258.32	16874.05	35445.2	1945.845	11455.94	2956.694
CUST_51978_PI429010886	M95900	0.015028737	24	15	294860856	ubiquitin/ribosomal S30 fusion p			1.21E-11	17692.35	19485.9	16954.37	14703.33	10699.09	12191.65	20968.09	21113.92	17345.96	17623.06	11822.99	12807.83	15900.37	12442.6	12631.63	22526.09	20762.38	24199.32	19387.17	19855.57	19791.01	13879.42	15705.46	15630.09	15967.93	16183.31	18910.88
CUST_5620_PI429010886	N18956	0.015028737	24	15						293.7564	494.7611	469.1525	274.7682	283.351	259.0732	357.0377	464.7851	416.4698	381.0871	296.8179	330.7777	339.4771	371.6485	347.0422	404.6157	551.5575	547.0285	351.2056	440.9544	443.9249	354.0991	396.05	450.3327	358.6508	361.3178	392.5562
CUST_5661_PI429010886	N19004	0.015028737	24	15	91080451	PREDICTED: similar to AGAP009	Nucleic acid metabolism	Regulation of gene expression	4.37E-164	100.8867	157.6143	120.6137	100.0233	102.326	114.0573	125.2342	132.2497	109.3354	122.0045	135.8408	114.952	114.9651	135.7196	108.9478	136.7882	170.6675	187.2522	112.0856	154.0616	149.6248	118.9114	160.139	137.0267	131.5353	139.2612	155.3061
CUST_5719_PI429010886	N19096	0.015028737	24	3	321476311	hypothetical protein DAPPUDRA	Protein metabolism	Proteolysis	2.48E-140	226.6959	145.8669	275.1575	234.533	819.0741	542.9984	136.7059	275.4561	174.6626	131.5859	563.3118	297.3019	206.8689	371.8649	376.6399	116.7048	109.6178	127.7854	164.0459	291.8765	143.8296	214.2802	235.9311	193.8538	138.2964	416.3476	211.625
CUST_9998_PI429010886	N25539	0.015028737	24	15						253.2927	261.5031	348.7951	259.0732	176.3993	196.2948	275.2462	265.1693	274.0413	331.4329	240.8917	288.3603	268.2532	231.374	231.374	310.9083	370.6325	390.7107	318.9156	290.029	300.4795	293.3331	248.0205	348.1933	298.2569	235.0418	317.6549
CUST_11072_PI429010886	N27222	0.015028737	24	15						182.5875	292.8798	218.4319	204.0335	130.0446	164.1331	244.6301	331.7854	332.8061	216.4739	217.1534	195.4062	191.3796	236.8696	227.3921	253.1631	338.993	290.1517	244.0506	311.1315	280.4189	219.3902	214.6479	225.9834	238.0667	220.4736	272.4392
CUST_19521_PI429010886	N41709	0.015028737	24	21						574.5775	658.5111	733.9767	704.3483	618.1517	557.8175	668.1223	661.8815	814.9423	671.9924	490.7221	570.427	790.1557	590.8215	469.0501	795.9001	888.236	937.9336	757.3112	519.9072	869.3099	849.9671	857.4356	1001.688	759.3274	795.5533	830.7266
CUST_26574_PI429010886	N54476	0.015028737	24	15						22.34334	15.54537	13.85151	8.509638	12.20395	4.226021	18.95876	14.54727	15.37784	18.23129	6.709355	19.83817	14.71778	9.753338	13.79355	22.88864	26.31588	26.72462	22.96223	8.744802	19.71754	17.88341	10.43842	18.85791	18.89628	7.127819	25.17016
CUST_2203_PI429010886	N11858	0.015058382	24	18	478260277	hypothetical protein YQE_0350	Nucleic acid metabolism	DNA replication		221.6228	259.2692	300.549	225.1246	240.3058	177.7613	257.3902	279.3607	317.8651	217.9435	218.3991	226.1282	253.1919	194.8762	221.5686	287.1316	278.5346	309.0756	394.6878	378.8803	310.1119	273.1848	217.2347	262.2969	281.9984	258.6669	227.5418
CUST_32057_PI429010886	M3130	0.015058493	24	18	405960295	Fumarylacetoacetase [Crassostr	Metabolic process	Cellular amino acid metabolic process	7.91E-177	672.9412	1702.984	1096.072	894.0902	496.4508	481.6057	927.3328	1021.074	1022.285	631.0985	657.9421	626.9839	1179.476	733.4921	699.596	975.2848	1093.904	959.7319	1368.116	1049.045	1449.243	687.0247	755.4761	759.3274	947.6406	647.2976	951.856
CUST_49021_PI429010886	M83649	0.015075759	21	15						27.9125	80.41731	66.0686	27.82789	27.0245	14.7656	19.53602	54.35078	22.905	51.94695	63.91561	20.17982	35.5246	48.20014	23.54776	50.98258	60.20069	61.64797	21.03874	36.0804	13.00649	33.14692	40.1235	32.72443	16.66935	19.28815	14.07446
CUST_38837_PI429010886	M31074	0.015089204	24	6	156938905	profilin [Penaeus monodon]	Cellular component orga	Cytoskeleton organization	2.42E-30	108.9271	107.9823	113.9699	104.258	122.8211	121.1643	104.2957	125.7647	119.8892	113.0037	105.1748	124.2158	102.1933	119.5986	108.6739	154.2758	130.1383	108.568	96.9591	118.0123	94.14191	74.98208	100.783	74.97441	103.0964	118.5289	95.04674
CUST_47127_PI429010886	M74886	0.015089204	24	0						28.00193	36.36056	34.67614	19.65297	31.52497	17.30901	24.92804	21.30099	29.68907	22.33779	22.94332	21.23607	29.30711	23.61225	32.75067	26.83424	34.21333	37.13496	26.57459	31.12503	26.83424	35.15002	28.67614	35.28906	39.22128	26.26192	39.63021
CUST_12063_PI429010886	N28865	0.015089204	24	12						42.10969	31.02035	28.63477	40.99664	430.7389	231.9647	80.60289	61.15056	34.04257	42.46657	66.05158	335.6252	44.41457	268.7276	341.0815	38.40964	25.19867	36.29464	30.905	63.30474	24.65812	34.74447	36.31683	24.63804	24.43634	67.18486	32.00302
CUST_12362_PI429010886	N29348	0.015089204	24	18						31.96059	44.53304	44.69809	42.24367	30.04204	32.86972	35.15002	56.74562	45.24265	46.9383	44.76312	54.71691	46.983	48.37508	40.26778	38.86739	63.07317	52.09654	44.67899	62.5781	55.7467	41.97418	51.64343	48.87241	48.17305	42.01431	42.85396
CUST_17137_PI429010886	N37415	0.015089204	24	21						28.18616	39.03604	27.78668	29.38152	31.41595	37.77333	30.8769	28.14	25.45483	22.95957	27.83932	32.22585	32.40149	26.26607	26.9245	36.53104	39.579	34.58217	33.22138	31.45923	31.44182	45.69732	30.99758	48.55532	61.51933	39.93038	44.80668
CUST_32608_PI429010886	M4562	0.015099243	18	15	443728134	hypothetical protein CAPTEDRAI			8.25E-97	53.70551	31.5432	37.53076	36.83655	30.13995	26.87469	31.51924	30.85244	26.56947	35.36547	29.48451	44.86788	28.53195	38.79677	44.71461	49.63135	36.20921	46.01666	37.91239	37.41195	23.98146	28.74405	31.27222	34.22563	32.50381	28.41463	30.3832
CUST_14128_PI429010886	N32370	0.015099243	18	15						272.0128	242.7903	17.16197	236.1602	28.93158	212.7624	400.0672	232.64	206.2187	201.0027	120.8275	62.1362	291.3249	222.6813	242.0464	666.5379	291.0502	324.6618	268.9804	95.68309	224.3337	50.25586	169.9743	62.64213	179.1302	193.5276	20.28589
CUST_49062_PI429010886	M83862	0.01514162	15	12	11494216	Limulus polyphemus internal tra			1.5E-150	70445.35	80425.69	78492.44	60621.99	58090.3	83454.29	74264.89	82051.33	69586.63	80425.69	71692.31	69178.17	89785	85913.71	87746.38	101686.5	84653.3	76615.52	71692.31	65558.65	71289.88	72794.71	67450.12	78492.44	78492.44	68214.13	89785
CUST_48059_PI429010886	M78726	0.015143512	24	6	197940771	Euphausia superba mitochondri			1.59E-149	56054.28	50951.28	46193.24	56377.06	53191.72	59443.36	71289.88	60621.99	66456.69	30447.23	56938.34	44722.61	40217.51	56377.06	28561.19	60621.99	57453.95	75512.6	40415.13	54925.27	45502.32	36043.3	29294.9	32184.55	64273.41	61365.17	43090.8
CUST_42314_PI429010886	M59982	0.015143882	15	9						21.48685	20.72028	34.74862	29.33369	34.16687	34.89809	25.61174	37.96647	23.5934	25.96348	38.99638	73.43473	50.10507	21.24956	38.89189	27.97133	27.2697	24.10212	32.96543	27.11011	27.25185	48.67088	34.62846	28.49131	57.44712	38.86376	72.65503
CUST_36377_PI429010886	M15753	0.015160402	21	0						31.77537	41.33036	33.76496	26.4274	32.99838	31.68332	29.68102	17.29613	21.14493	18.72559	35.3759	36.22942	24.01452	22.35063	39.6536	31.06306	27.90262	26.93456	33.05798	36.67403	22.86438	34.31749	38.77522	39.08397	21.10356	30.37998	51.10469
CUST_36755_PI429010886	M16759	0.015160402	21	0	321466417	hypothetical protein DAPPUDRA	Metabolic process	Lipid metabolic process	1.87E-65	52.13265	62.80714	66.67393	43.5017	47.42168	41.27319	27.35733	47.68605	34.21691	49.22608	45.66053	38.11089	35.85739	45.36304	47.47428	44.78302	45.90076	72.63826	44.61277	41.43789	51.21624	72.11693	66.71306	58.91249	69.34161	38.63208	69.2197
CUST_8501_PI429010886	N23134	0.015160402	24	3	390342290	PREDICTED: ectonucleotide pyro			7.16E-24	189.7104	23.3163	179.1574	249.888	163.4271	227.9934	18.84141	13.83383	11.53429	33.09557	132.8402	176.0219	24.88691	70.28135	136.1461	20.13552	14.75102	18.22769	21.38839	10.66221	17.91362	67.04107	23.69096	49.50343	17.41092	24.04895	26.97175
CUST_7421_PI429010886	N21519	0.015167279	18	3						112.1133	89.23195	148.4289	124.8855	118.3504	95.86932	81.05496	104.173	102.1933	58.05667	95.48478	70.57647	61.67047	72.01042	97.97101	40.1541	83.17096	63.80586	69.33519	91.33505	66.32665	93.27554	106.8768	113.4137	55.31238	51.86305	49.71376
CUST_31855_PI429010886	M2582	0.015185442	12	3						274.7682	349.2496	232.3662	347.0422	338.8265	302.5618	285.7894	297.0192	324.7451	289.5149	174.0478	242.4377	232.3662	278.2449	295.6886	347.0422	368.1539	315.4118	286.6983	270.0743	345.8264	234.7388	322.8914	253.4358	345.1067	251.0873	390.6122
CUST_30990_PI429010886	M393	0.015185442	12	3						167.1998	317.9491	165.9817	247.2393	358.0959	318.9156	253.9457	354.0092	185.4692	188.1296	124.0673	201.3401	193.7593	243.543	198.2813	243.4049	275.6274	316.8751	206.0093	262.806	174.5369	178.3071	254.533	176.076	226.205	308.2886	223.8968
CUST_6500_PI429010886	N20182	0.015185442	12	9						41.12174	39.08397	44.01625	32.93138	26.79608	28.04788	32.71218	34.74447	43.41187	41.67167	35.82162	44.3077	43.7275	19.37283	39.09998	36.33342	26.11298	34.42594	38.76155	37.29216	46.59051	51.61367	32.96543	58.46402	38.70471	36.83655	53.27399
CUST_16578_PI429010886	N36494	0.015185442	12	3						125.1377	159.0483	88.71852	201.9179	106.2528	160.6506	122.2305	209.2118	131.8579	99.42287	96.40725	87.06917	128.6789	180.0776	92.2473	161.4506	145.3783	187.2195	147.8169	200.9398	73.85285	100.2889	91.44099	78.22197	161.691	125.5852	84.57655
CUST_23269_PI429010886	N48543	0.015185442	12	3						38.20339	35.33106	25.75751	36.14885	34.35444	40.55409	36.35313	32.60595	38.02922	35.55837	23.75379	32.98204	45.69732	33.09557	31.03188	48.39798	36.80764	39.41032	35.6426	31.87401	40.13474	25.54219	29.43649	29.84906	58.22453	39.07435	48.35178
CUST_28908_PI429010886	N58824	0.015185442	12	9						27.74851	9.209853	30.88358	18.67988	7.030628	13.82436	22.54151	17.7667	26.82167	21.81393	21.35032	27.04805	35.42699	23.43945	20.26341	11.75186	13.83564	9.013796	21.33066	35.99333	30.45492	27.29484	21.70164	33.19205	12.30677	21.46191	17.12548
CUST_23918_PI429010886	N49699	0.015190552	24	21						NA	NA	46.46187	19.92759	4.546146	3.703509	NA	3.350525	18.4638	18.98361	12.79263	4.982299	21.40492	17.82688	16.3304	3.695184	17.33156	NA	10.55458	12.56491	28.14	49.37308	62.83512	50.96545	25.77383	7.930223	30.37143
CUST_14101_PI429010886	N32329	0.015191465	15	6						29.87687	36.05612	26.9108	39.09998	40.61754	29.70619	40.88884	48.97575	35.65289	34.47208	20.89443	40.5799	37.54916	28.0635	40.82537	29.49099	28.60396	26.46408	52.25224	58.22453	41.44758	43.8169	40.39391	44.77796	38.02572	42.78489	57.76704
CUST_38091_PI429010886	M23287	0.015201481	24	15	190702313	cytochrome c oxidase-like prote	Metabolic process	Generation of precursor metabolites ar	6E-12	2475.896	3675.29	2568.829	2096.347	2050.749	2085.588	3289.37	3590.769	3190.966	2200.686	1712.689	2524.402	2622.032	2447.506	2402.724	3393.901	3478.142	3747.441	2440.627	3114.68	3277.985	2167.915	3142.5	3132.033	2975.418	3217.866	2268.13
CUST_32541_PI429010886	M4378	0.015201481	24	3	242005182	conserved hypothetical protein			1.03E-25	154.2231	76.09471	295.6886	222.1744	342.9247	287.2425	81.72083	65.96155	123.6152	141.2055	213.7796	171.8869	199.2156	186.3541	224.7103	60.64152	94.15594	62.23993	120.5417	86.77435	91.51354	199.1442	157.3269	164.188	134.6492	134.2942	172.8989
CUST_44016_PI429010886	M64656	0.015201481	24	15	17221659	ribosome like protein [Marsupe	Protein metabolism	Ribosome biogenesis	4.18E-26	16288.46	22847.77	16252.77	15942.89	12765.38	14307.54	22154.05	21066.26	20968.09	18851.7	13335.43	15585.38	16035.79	15967.93	16580.62	23084.2	20099.82	26740.38	19522.5	20968.09	24909.79	14363.02	16151.95	18851.7	18851.7	17315.8	18966.14
CUST_46242_PI429010886	M71923	0.015201481	24	15						4906.082	6348.681	5184.879	4906.082	3721.413	3463.912	7531.236	5144.706	6317.157	5979.853	3647.267	5238.942	5726.275	3759.237	4322.847	8522.605	6791.114	9743.786	6285.074	5666.924	6385.128	6171.545	6601.274	6897.446	6770.352	5435.742	7983.519
CUST_5545_PI429010886	N18842	0.015201481	24	15	242018509	Splicing factor 3B subunit, putat			1.46E-131	46.28626	80.61166	72.4004	59.07254	42.52366	38.1445	69.77877	79.25889	83.3741	59.51158	67.27409	71.06688	77.53014	60.74813	62.02003	73.96504	107.6507	86.92889	63.72422	62.61945	87.56469	54.04214	72.63826	73.01092	68.13325	67.95059	69.35987
CUST_5859_PI429010886	N19304	0.015201481	24	15	321469736	hypothetical protein DAPPUDRA	Protein metabolism	Proteolysis	1.84E-60	1491.134	2814.474	2269.737	1459.585	863.8645	881.5382	2469.347	2103.097	2367.057	1953.31	1198.741	1126.574	2171.504	1447.238	1291.739	2541.875	2891.912	2820.736	2363.552	2056.189	2620.241	1426.211	2091.62	1842.758	2501.925	1598.489	2522.496
CUST_13101_PI429010886	N30579	0.015201481	24	15						202.6223	266.255	299.9228	183.2355	200.863	194.5781	199.2156	238.6215	240.5209	261.8722	237.366	281.3108	222.6501	205.6244	216.54	341.84	308.2886	298.8719	299.3538	221.3576	294.0126	277.7202	271.8414	326.188	302.5618	246.6369	353.1568
CUST_14062_PI429010886	N32269	0.015201481	24	15						496.9611	575.2243	461.522	362.9291	432.4641	406.0638	558.8184	523.4696	499.2313	484.8492	451.0149	419.6327	529.7211	423.8193	535.6201	525.2596	691.4224	588.9309	467.5708	535.6201	518.0684	470.2656	501.0897	471.2164	511.9251	488.7695	552.2424
CUST_16717_PI429010886	N36746	0.015201481	24	15						36.07852	48.30191	26.69789	36.28429	41.12558	28.21781	47.16802	38.4508	29.1296	47.58263	37.67803	39.07837	37.00674	40.28089	41.38164	54.48769	49.7839	51.22586	45.41169	39.72867	44.47768	41.16872	42.41232	49.67166	36.36523	44.6542	59.75033
CUST_19344_PI429010886	N41422	0.015201481	24	15	321464697	hypothetical protein DAPPUDRA			3.78E-59	239.9783	263.7574	306.1538	251.4219	194.9465	195.8396	264.2234	231.9279	263.3104	329.6536	217.9435	262.525	285.4767	208.6833	282.0632	382.4457	305.7738	346.9643	315.7654	196.9638	274.3383	304.7866	226.1685	319.3967	285.2599	269.2665	342.0443
CUST_1398_PI429010886	N9515	0.015201481	24	15	321459536	hypothetical protein DAPPUDRA			4.55E-37	225.3038	268.9804	247.7871	200.4453	161.3159	187.4404	244.537	173.9842	201.4635	271.4702	234.791	246.3057	292.1626	171.8413	223.7206	312.5578	281.1468	331.7854	302.274	183.7151	228.5093	264.9542	174.2207	272.9604	293.2244	193.7076	292.2414
CUST_26424_PI429010886	N54219	0.015205035	21	9						6.03591	5.983227	NA	7.740604	8.620448	9.41785	4.655424	NA	9.969471	12.62763	8.589131	8.023716	4.031607	12.89633	7.205732	4.429018	12.71074	4.815635	NA	3.979414	4.294508	5.932716	7.482905	5.368642	9.893288	9.41028	11.85264
CUST_35744_PI429010886	M13383	0.015205368	24	15	443700354	hypothetical protein CAPTEDRAI			2.84E-14	14.88235	17.95932	12.24304	7.21871	14.14112	16.28426	12.34178	14.81156	11.32305	17.4076	19.31939	5.40299	10.6102	9.372363	6.045196	42.06849	26.41747	34.40062	43.44652	24.76741	55.44525	14.5966	7.307485	18.84481	18.83509	5.181037	24.88953
CUST_20565_PI429010886	N43666	0.015205752	12	9						53.02213	258.6669	187.286	95.46305	19.51629	61.67047	115.1679	103.8566	180.3948	122.604	109.4738	101.588	92.68642	61.48782	85.81977	38.47726	112.1293	57.27204	95.41451	74.46936	99.96679	165.2945	199.718	245.8278	117.5148	154.757	285.9995
CUST_36386_PI429010886	M15772	0.015206492	21	15						31.80823	42.2771	30.95428	33.62389	41.32674	38.02217	24.74446	50.26433	32.70464	38.20771	44.1311	47.47767	49.4478	35.17583	44.42034	48.65167	49.99479	50.93191	21.13533	34.90142	36.61091	30.40674	34.89175	35.80198	43.9077	23.68606	47.22812
CUST_21462_PI429010886	N45264	0.015207559	24	21						206.4922	334.6481	285.5358	231.7623	194.3971	184.5623	251.9547	259.2192	248.4202	228.8663	264.9162	182.7533	202.0727	206.3618	181.0227	222.6813	590.8215	328.2183	366.9286	247.0114	274.2134	376.4343	297.6356	255.8139	261.7871	325.7976	252.9258
CUST_7782_PI429010886	N22068	0.015233885	21	0						80.3181	67.10107	75.54551	64.34403	76.53128	48.31065	64.79523	32.33214	49.7367	68.74828	61.27966	58.24276	86.96518	57.00268	74.78378	52.01796	69.95424	73.76003	66.34289	53.8169	70.13338	94.50451	82.76148	74.09844	58.36343	42.60412	55.71616
CUST_12234_PI429010886	N29145	0.015233885	21	0						51.89343	47.33949	45.57662	47.13	48.24962	42.53768	34.41857	27.2823	25.39744	57.40298	35.18384	38.14116	39.11401	56.66348	46.36802	37.69532	39.94084	34.51373	41.48089	44.95536	34.27669	47.94598	51.61367	42.97957	32.93138	36.46491	39.28264
CUST_12645_PI429010886	N29796	0.015242293	21	15						24.20903	53.27399	38.62391	23.74587	35.38735	33.05798	23.48629	33.10297	32.94858	19.08766	29.05473	29.63277	18.30947	47.09644	24.04323	42.60412	56.36738	43.13077	44.10093	39.07837	43.20383	43.62046	22.98354	42.91838	47.09644	36.21321	39.76763
CUST_22763_PI429010886	N47629	0.015251709	21	12						21.49936	13.1221	16.1105	37.1975	8.702496	18.93816	27.13842	24.01452	27.44734	26.72777	23.73991	26.04224	46.57025	32.70464	23.64075	17.32058	6.2741	16.52245	18.8308	13.57539	17.41463	17.38353	19.1981	15.52429	21.62227	14.34116	15.03281
CUST_55416_PI429010886	M110005	0.015257394	21	15						31.91281	43.20383	12.15876	13.04564	11.58143	28.39338	59.44629	36.72861	54.8814	45.61423	26.5557	23.03013	27.98453	26.91606	7.113497	34.33222	63.38213	61.02485	38.22258	40.07856	37.10235	27.22363	31.26771	20.1	18.51328	23.56042	23.72726
CUST_21573_PI429010886	N45454	0.015257394	21	15	86609863	DnaJ domain-containing protein	Response to stress	Other	1.91E-10	71.76549	87.23514	72.06957	46.29157	84.64677	55.35053	115.6114	82.7532	82.29898	82.16147	48.00737	65.93698	75.71093	76.59647	62.64991	119.4473	117.0919	127.6698	98.10696	68.30294	56.61327	68.06179	79.2021	85.2914	57.42169	61.70924	59.09232
[truncated: 1,453,594 more chars]
