# Supplementary material for: Analysis of the circadian transcriptome of the Antarctic krill Euphausia superba
Source: Sci Rep. 2019 Sep 25;9:13894. doi: 10.1038/s41598-019-50282-1 (PMC6761102; doi:10.1038/s41598-019-50282-1)
Supplement: Supplementary file 3 — Supplementary Table 2 [file 41598_2019_50282_MOESM3_ESM.pdf]

# Analysis of the circadian transcriptome of the Antarctic krill *Euphausia superba*

Alberto Biscontin[1,2,*], Paolo Martini[1], Rodolfo Costa[1], Achim Kramer[2], Bettina Meyer[3,4,5], So Kawaguchi[6], Mathias Teschke[3], Cristiano De Pittà[1,*]

[1]Dipartimento di Biologia, Università degli Studi di Padova, Padova, Italy

[2]Laboratory of Chronobiology, Charité Universitätsmedizin Berlin, Berlin, Germany

[3]Section Polar Biological Oceanography, Alfred Wegener Institute Helmholtz Centre for Polar and Marine Research, Bremerhaven, Germany

[4]Institute for Chemistry and Biology of the Marine Environment, Carl von Ossietzky University of Oldenburg, Oldenburg, Germany

[5]Helmholtz Institute for Functional Marine Biodiversity (HIFMB) at the University of Oldenburg, 26111 Oldenburg, Germany

[6]Department of Environment and Heritage, Australian Antarctic Division, Kingston, Tasmania, Australia

[*]Corresponding authors:

Cristiano De Pittà, Dipartimento di Biologia, Università degli Studi di Padova, via U. Bassi 58/B 35131 Padova, Italy; Phone: +39-049-8276210; Fax: +39-049-8276209; e-mail address: cristiano.depitta@unipd.it

Alberto Biscontin, Dipartimento di Biologia, Università degli Studi di Padova, via U. Bassi 58/B 35131 Padova, Italy; Phone: +39-049-8276228; Fax: +39-049-8276209; e-mail address: alberto.biscontin@unipd.it

**Supplementary Table 2. List of 6,561 genes with a sinusoidal expression pattern in DD.**

Expression levels of genes, identified by RAIN (adjusted $p$-value < 0.05) throughout the 24-hour cycle in DD regime, are shown. [a] Probe ID: ID of probe sequence in "Krill 2.0" Agilent microarray platform; [b]ID: accession number of krill master transcriptome [23];
[c]$p$-value: RAIN Benjamini-Hochberg adjusted $p$-value; [d]Period: period of oscillation; [e]Phase: maximum peak of expression throughout the 24 hours cycle; [f]EBI ID: accession number of EBI; [g]Annotation = description of the gene; [h]GO term: gene ontology biological process;
[i]GO child term: most fitting gene ontology child term to further refine the functional annotation; [j]e-value: score of GO functional annotation with BLAST2GO; [k-ak]mRNA expression levels.

| Probe ID | ID | p-value | Period | Phase | EBI ID | Annotation | GO category | Go term | E-value | 0 | 0 | 0 | 3 | 3 | 3 | 6 | 6 | 6 | 9 | 9 | 9 | 12 | 12 | 12 | 15 | 15 | 15 | 18 | 18 | 18 | 21 | 21 | 21 | 24 | 24 | 24 |
|---|---|---|---|---|---|---|---|---|---|---|---|---|---|---|---|---|---|---|---|---|---|---|---|---|---|---|---|---|---|---|---|---|---|---|---|---|
| CUST_37166_PI429010886 | M18062 | 7.35E-07 | 24 | 21 | 348565785 | PREDICTED: cell division cycle protein 123 homolog | Cellular Process | Cell cycle | 4.31E-74 | 37.27042 | 45.83957 | 29.51147 | 36.14912 | 29.08151 | 28.24369 | 19.33574 | 17.14965 | 23.04991 | 42.30947 | 26.49443 | 30.14577 | 26.73374 | 33.83173 | 26.6361 | 37.21299 | 32.6735 | 33.80016 | 29.06792 | 36.85933 | 36.19062 | 52.94516 | 43.65882 | 60.99798 | 39.43891 | 28.71965 | 41.33633 |
| CUST_9350_PI429010886 | N24477 | 1.02E-06 | 18 | 3 | 13095576 | cellulase [Coptotermes acinaciformis] | Metabolic process | Carbohydrate metabolic process | 2.54E-72 | NA | 55.75917 | 67.35597 | 956.7714 | 243.6339 | 669.4372 | 157.1495 | 130.1016 | 328.5456 | 228.3459 | 29.97309 | 32.41028 | 10.28696 | 21.70738 | 9.352687 | 96.70093 | 58.36102 | 177.5884 | 55.5381 | 180.4643 | 99.71807 | NA | 392.4795 | 223.5364 | 77.12763 | NA | 86.4072 |
| CUST_42369_PI429010886 | M60124 | 1.11E-06 | 15 | 0 | | | | | | 190.7563 | 245.7365 | 212.4051 | 178.7689 | 162.6286 | 167.982 | 126.2208 | 110.3002 | 102.4945 | 172.9946 | 170.6145 | 172.2644 | 135.9425 | 170.4477 | 174.1781 | 215.2456 | 217.2265 | 187.746 | 227.7485 | 131.1966 | 166.186 | 169.4032 | 137.5108 | 112.2867 | 179.2012 | 140.228 | 143.3418 |
| CUST_10872_PI429010886 | N26923 | 1.16E-06 | 15 | 0 | | | | | | 25.67033 | 24.41159 | 18.98793 | 18.7049 | 18.70053 | 18.98962 | 7.446083 | 11.61773 | 14.91349 | 20.06593 | 13.67972 | 9.226004 | 17.9331 | 18.19413 | 16.0016 | 25.76223 | 23.53335 | 19.95854 | 13.66405 | 18.38014 | 16.4888 | 12.52117 | 7.560847 | 8.721751 | 18.08701 | 26.03157 | 8.733788 |
| CUST_2556_PI429010886 | N12615 | 1.41E-06 | 21 | 9 | | | | | | 36.58662 | 9.844983 | NA | 33.84232 | 157.6076 | NA | 62.78892 | 69.56934 | 80.26839 | 227.5328 | 191.3655 | 146.902 | 151.0368 | 146.4231 | 63.37317 | 12.24951 | 143.3418 | 87.34049 | 8.082183 | NA | 6.451538 | 33.75702 | NA | 22.6805 | 91.87634 | NA | 40.92745 |
| CUST_11942_PI429010886 | N28667 | 1.42E-06 | 24 | 12 | | | | | | 5.997689 | 25.08483 | 13.60767 | 28.90009 | 25.4154 | 29.91175 | 39.63814 | 42.2531 | 15.19101 | 31.79359 | 84.42877 | 78.93556 | 101.1596 | 107.1241 | 43.46213 | 46.04195 | 29.71896 | 51.06646 | 37.33468 | 57.81384 | 64.95763 | 26.92776 | 32.60782 | 26.29806 | 25.30394 | 13.12705 | 39.02375 |
| CUST_37732_PI429010886 | M21285 | 1.62E-06 | 24 | 0 | 6831612 | RecName: Full=60S ribosomal protein L15 >gi\|4530 | Protein metabolism | Ribosome biogenesis | 8.7E-82 | 5882.858 | 7197.042 | 5396.825 | 7060.087 | 5528.982 | 5492.691 | 3100.565 | 3067.321 | 4257.291 | 4532.248 | 2659.902 | 3266.951 | 2024.906 | 2788.353 | 2599.94 | 271.2785 | 3624.616 | 2447.295 | 4267.625 | 4575.004 | 3991.112 | 3653.581 | 6049.937 | 2130.831 | 5299.221 | 5888.291 | 7212.951 |
| CUST_52918_PI429010886 | M99429 | 1.98E-06 | 15 | 9 | 168824627 | Euphausia superba isolate I241 cytochrome c oxida | | | 5.71E-93 | 206.8341 | 275.9866 | 226.1214 | 215.9695 | 284.093 | 1042.025 | 1280.61 | 1351.148 | 191.7997 | 1767.465 | 2727.828 | 1194.442 | 245.6875 | 1077.802 | 606.0546 | 232.3859 | 154.7492 | 114.9603 | 91.88705 | 901.5294 | 253.0671 | 1991.454 | 1061.893 | 2045.457 | 1421.77 | 2226.703 | 1331.007 |
| CUST_45109_PI429010886 | M68024 | 2.04E-06 | 24 | 18 | 308476721 | hypothetical protein CRE_19860 [Caenorhabditis re | Nucleic acid metabolism | DNA replication | 1.28E-14 | 81.27628 | 75.02406 | 90.08902 | 68.24733 | 75.35464 | 72.2954 | 59.98068 | 74.29802 | 95.88672 | 120.6113 | 93.71752 | 104.1211 | 83.63981 | 108.3037 | 115.1438 | 126.7035 | 104.6405 | 144.4075 | 147.0578 | 111.8634 | 131.0829 | 148.7626 | 110.0025 | 82.23746 | 103.5957 | 77.08541 | 85.4443 |
| CUST_52111_PI429010886 | M96435 | 2.26E-06 | 18 | 15 | | | | | | 59.3369 | 56.46834 | 86.46205 | 53.51251 | 68.88215 | 52.74471 | 36.73446 | 38.44815 | 60.6077 | 92.29442 | 61.5 | 64.14066 | 71.62646 | 63.07552 | 50.46214 | 120.4628 | 95.61724 | 88.60515 | 84.63515 | 57.16741 | 80.48768 | 49.21104 | 30.46627 | 37.71676 | 49.96135 | 28.93334 | 41.0817 |
| CUST_29585_PI429010886 | N60149 | 3.26E-06 | 15 | 12 | | | | | | 68.9769 | 45.71121 | 122.5181 | 42.16099 | 23.1151 | 37.08698 | 68.25442 | 84.43869 | 79.68658 | 76.03863 | 36.48665 | 78.53416 | 111.2973 | 164.8084 | 118.0563 | 50.61028 | 41.84077 | 32.6735 | 25.40127 | 29.9146 | 28.7982 | 82.02402 | 71.88591 | 45.81525 | 93.95638 | 88.6992 | 63.48536 |
| CUST_23265_PI429010886 | N48538 | 3.63E-06 | 24 | 0 | | | | | | 79.43105 | 81.18707 | 81.35601 | 64.01814 | 59.18336 | 64.07997 | 62.5758 | 69.72451 | 50.90018 | 56.19981 | 67.77286 | 58.52324 | 55.16431 | 53.80851 | 43.88387 | 66.86178 | 54.49444 | 49.45564 | 72.3636 | 72.74687 | 61.02204 | 81.95659 | 61.24287 | 56.93106 | 88.30586 | 64.62304 | 172.9097 |
| CUST_37254_PI429010886 | M18369 | 3.64E-06 | 15 | 9 | | | | | | 108.0902 | 140.2821 | 183.9787 | 81.25016 | 69.76028 | 64.51419 | 70.56572 | 185.8187 | 163.9462 | 212.0939 | 146.7819 | 178.0431 | 148.08 | 236.4903 | 144.9791 | 139.5841 | 135.6803 | 120.8183 | 118.6343 | 119.9589 | 103.2145 | 116.321 | 123.3865 | 104.976 | 205.5616 | 170.531 | 180.6083 |
| CUST_39473_PI429010886 | M39930 | 5.06E-06 | 18 | 12 | | | | | | 2465.06 | 4424.066 | 4110.832 | 1260.686 | 2411.696 | 2247.598 | 2741.645 | 2768.781 | 3120.564 | 5712.144 | 2697.082 | 4651.575 | 4528.171 | 5732.178 | 5133.687 | 3661.505 | 3187.857 | 4179.242 | 3016.912 | 3792.107 | 3225.426 | 3720.06 | 2203.176 | 2435.819 | 3227.523 | 4135.156 | 4028.762 |
| CUST_45308_PI429010886 | M68658 | 5.17E-06 | 12 | 0 | 358442122 | Broad-complex protein isoform 4 [Penaeus monodc | | | 1.04E-49 | 20.21835 | 24.75742 | 32.10055 | 18.14003 | 15.04869 | 9.419858 | 15.33921 | 14.4753 | 9.510155 | 24.30007 | 10.74056 | 19.39216 | 23.62815 | 44.53615 | 27.36061 | 14.37169 | 11.50377 | 15.75594 | 7.489011 | 8.359315 | 9.329547 | 10.73592 | 9.301993 | 24.65264 | 22.21414 | 21.8835 | 23.22371 |
| CUST_51049_PI429010886 | M91954 | 5.3E-06 | 21 | 0 | | | | | | 314.8877 | 385.4652 | 410.06 | 162.6527 | 300.3777 | 271.7176 | 174.0729 | 137.2513 | 187.2939 | 213.0864 | 185.4691 | 186.6799 | 189.9733 | 260.373 | 137.3935 | 196.7037 | 214.8824 | 139.3321 | 231.648 | 222.0327 | 239.9876 | 401.8543 | 227.704 | 279.387 | 263.7999 | 215.0865 | 251.0141 |
| CUST_46414_PI429010886 | M72608 | 5.34E-06 | 24 | 18 | 405959130 | Elongator complex protein 1 [Crassostrea gigas] | | | 1.77E-16 | 18.78752 | 26.14633 | 28.80255 | 19.31282 | 16.93182 | 17.39038 | 19.09041 | 22.78742 | 21.43971 | 23.36338 | 13.23457 | 26.70421 | 24.7547 | 32.53672 | 19.3955 | 36.96758 | 24.41741 | 36.33605 | 58.58633 | 34.12708 | 36.01731 | 26.6361 | 34.58379 | 24.77058 | 28.43336 | 21.50683 | 28.67873 |
| CUST_16275_PI429010886 | N35954 | 5.41E-06 | 24 | 21 | | | | | | 222.578 | 221.425 | 213.51 | 189.2062 | 182.2902 | 177.4848 | 153.5041 | 142.7507 | 151.386 | 299.389 | 178.3288 | 173.2007 | 162.3309 | 216 | 207.0153 | 260.7005 | 210.6402 | 215.4042 | 216.1856 | 230.9992 | 226.9563 | 272.7999 | 242.5645 | 218.2149 | 275.8443 | 192.7007 | 202.5771 |
| CUST_50106_PI429010886 | M88646 | 5.77E-06 | 18 | 15 | | | | | | 35.38892 | 56.61232 | 35.78945 | 33.39813 | 32.06361 | 35.28945 | 39.52535 | 37.01331 | 37.00901 | 38.28633 | 37.54361 | 39.15253 | 37.31224 | 43.35936 | 45.19753 | 52.56534 | 38.19149 | 47.01793 | 36.91363 | 48.28142 | 34.79371 | 33.68076 | 34.36554 | 27.23134 | 42.85678 | 35.18059 | 39.54096 |
| CUST_4336_PI429010886 | N16534 | 5.94E-06 | 12 | 0 | | | | | | 77.02903 | 63.3033 | 73.81964 | 45.26792 | 26.47171 | 22.16055 | 29.56366 | 46.51079 | 51.71837 | 63.45518 | 52.84586 | 80.10123 | 72.2954 | 128.4226 | 69.0257 | 37.27402 | 26.59751 | 17.39272 | 69.89082 | 60.40681 | 51.13662 | 80.34778 | 27.64159 | 38.3841 | 85.93471 | 69.9145 | 81.73916 |
| CUST_9535_PI429010886 | N24794 | 6.03E-06 | 24 | 18 | 443733871 | hypothetical protein CAPTEDRAFT_197081 [Capitell | | | 2.07E-07 | 35.27881 | 47.58792 | 46.09323 | 20.91887 | 28.40026 | 26.62182 | 29.04798 | 21.14283 | 24.0422 | 42.2531 | 27.19369 | 25.13331 | 23.17992 | 37.77885 | 34.79371 | 47.37598 | 41.23292 | 39.51821 | 48.15554 | 50.2407 | 52.77021 | 45.60489 | 55.35407 | 30.58011 | 46.6 | 38.37639 | 38.7854 |
| CUST_29351_PI429010886 | N59693 | 6.41E-06 | 18 | 0 | | | | | | 51.35337 | 35.97087 | 27.71495 | 23.20447 | 25.56884 | 28.05565 | 20.09831 | 16.82346 | 17.36105 | 25.70664 | 18.11108 | 25.63451 | 24.97464 | 71.88591 | 27.25419 | 27.74158 | 25.66207 | 44.39447 | 30.42832 | 35.53394 | 28.94552 | 60.97776 | 30.82801 | 25.68589 | 20.1154 | 18.10067 | 24.92176 |
| CUST_17054_PI429010886 | N37288 | 6.7E-06 | 15 | 6 | 478859730 | glutathione S-transferases [Macrobrachium nippon | Metabolic process | Cellular amino acid metabolic process | 1.01E-43 | 44.52812 | 40.81934 | 3.640382 | 57.256 | 49.59839 | 53.21879 | 65.68694 | 47.93244 | 64.41721 | 33.70252 | 40.1699 | 53.47338 | 30.89566 | 14.4635 | 21.62429 | 36.47986 | 24.70497 | 28.71965 | 37.78907 | 42.42919 | 53.51251 | 61.80458 | 49.22548 | 47.34914 | 60.99125 | 35.8336 | 36.52603 |
| CUST_19903_PI429010886 | N42418 | 7.27E-06 | 21 | 15 | 405974301 | hypothetical protein CGI_10026724 [Crassostrea gi | | | 4.89E-18 | 163.4951 | 214.5095 | 152.0834 | 110.3267 | 134.7752 | 160.2379 | 107.2229 | 134.1962 | 122.7515 | 160.1083 | 129.5236 | 140.8068 | 146.6794 | 162.1995 | 175.8023 | 192.7007 | 203.482 | 179.4229 | 203.5264 | 176.2225 | 142.6905 | 178.5202 | 184.4849 | 173.0247 | 158.2183 | 131.9438 | 149.3496 |
| CUST_1657_PI429010886 | N10361 | 7.28E-06 | 15 | 0 | | | | | | 217.1953 | 184.654 | 231.4586 | 215.2456 | 174.5672 | 229.1012 | 270.8602 | 164.5164 | 154.7492 | 134.8756 | 138.2955 | 136.6832 | 128.3451 | 172.3811 | 189.0068 | 179.9528 | 238.5792 | 229.8746 | 209.0603 | 168.4747 | 161.0222 | 178.7372 | 174.6614 | 166.4452 | 157.5 | 127.7418 | 114.1326 |
| CUST_18442_PI429010886 | N39806 | 7.93E-06 | 18 | 3 | | | | | | 129.8259 | 184.4849 | 182.965 | 369.4169 | 177.2677 | 284.7376 | 171.3591 | 126.3438 | 133.2626 | 140.8976 | 91.46281 | 148.4609 | 74.17182 | 92.47211 | 48.54759 | 116.8757 | 74.87405 | 133.8101 | 81.7755 | 90.15151 | 246.4698 | 176.2225 | 283.4361 | 172.2644 | 213.51 | 141.6171 | 115.7238 |
| CUST_51321_PI429010886 | M93068 | 8.56E-06 | 15 | 3 | 156539569 | PREDICTED: SET and MYND domain-containing prot | | | 2.08E-09 | 93.5311 | 114.4714 | 61.97713 | 120.7015 | 129.2719 | 101.6948 | 66.75063 | 63.50632 | 53.07285 | 41.09065 | 55.70082 | 31.79024 | 49.96135 | 50.6408 | 60.60207 | 105.3539 | 62.506 | 59.04082 | 88.6992 | 69.28034 | 54.01314 | 55.39951 | 80.66968 | 104.5737 | 46.17776 | 41.40356 | 36.08462 |
| CUST_52983_PI429010886 | M99678 | 8.59E-06 | 24 | 18 | | | | | | 28.29582 | 35.91866 | 41.43272 | 22.72662 | 25.43742 | 22.09691 | 27.00493 | 34.59277 | 30.59469 | 30.37057 | 28.90658 | 35.6143 | 30.24809 | 37.36668 | 38.01489 | 31.85967 | 43.19279 | 36.99055 | 43.47138 | 37.81392 | 39.10739 | 39.62195 | 41.92727 | 32.31352 | 37.96113 | 27.10385 | 37.70458 |
| CUST_11709_PI429010886 | N28278 | 9.38E-06 | 15 | 9 | | | | | | 9552.668 | 11673.13 | 23759.64 | 1946.546 | 2925.159 | 2478.692 | 15199.11 | 25196.95 | 33985.8 | 31396.1 | 23998.85 | 35686.35 | 20597.24 | 38394.55 | 28610.41 | 17031.04 | 14355.49 | 13044.56 | 11926.23 | 28610.41 | 10777.69 | 29696.39 | 29142.09 | 4549.28 | 52713.61 | 29210.68 | 44080.52 |
| CUST_39293_PI429010886 | M37357 | 9.61E-06 | 21 | 18 | | | | | | 149.0574 | 177.1792 | 169.3396 | 30.41187 | 84.54992 | 49.05985 | 37.86509 | 64.01814 | 52.37245 | 86.82244 | 25.52881 | 36.98139 | 169.3396 | 140.7918 | 72.72106 | 99.05161 | 156.9601 | 184.3916 | 185.2951 | 170.4767 | 196.6789 | 64.3345 | 106.4056 | 60.05019 | 47.51073 | 50.96015 | 71.13139 |
| CUST_11518_PI429010886 | N27952 | 1.03E-05 | 15 | 0 | | | | | | 40.62219 | 38.67688 | 47.2535 | 33.14786 | 39.68514 | 44.40267 | 27.97056 | 24.66785 | 33.59506 | 30.95206 | 25.56884 | 28.94785 | 34.55194 | 34.88082 | 30.74148 | 47.78664 | 43.363 | 52.52687 | 28.99306 | 21.71104 | 34.20754 | 30.45779 | 32.20521 | 27.04395 | 26.86002 | 25.34926 | 28.65923 |
| CUST_25111_PI429010886 | N51804 | 1.09E-05 | 18 | 9 | | | Protein metabolism | Proteolysis | | 5.990217 | 10.2754 | 14.65658 | 4.643844 | 6.831546 | NA | 4.803208 | 14.06725 | 14.96077 | 16.48255 | 37.30837 | 33.71073 | 25.73345 | 22.72441 | 29.29243 | 11.56577 | 9.824879 | 14.39787 | 14.73015 | 14.46988 | 15.03778 | 4.956681 | 12.61456 | 4.3423 | 17.61017 | 7.259344 | 13.03814 |
| CUST_7088_PI429010886 | N21023 | 1.15E-05 | 18 | 12 | 518897355 | hypothetical protein [alpha proteobacterium SCGC | Metabolic process | Other | 1.23E-27 | 99.81229 | 261.6417 | 135.3575 | 127.4262 | 123.669 | 76.05543 | 108.7837 | 176.601 | 155.7184 | 197.6332 | 180.4354 | 192.8746 | 214.2001 | 209.1953 | 175.8509 | 201.2707 | 124.5707 | 160.0546 | 133.0452 | 287.5478 | 112.064 | 106.3847 | 102.8187 | 75.05721 | 125.4314 | 164.0153 | 168.7266 |
| CUST_52957_PI429010886 | M99596 | 1.17E-05 | 12 | 0 | | | | | | 10.95668 | 15.79548 | 11.19977 | 7.160939 | 8.802891 | 16.91321 | 4.837943 | 5.688713 | 6.268183 | 11.99992 | 8.338462 | 7.360126 | 10.71325 | 16.90499 | 10.16134 | 7.953838 | 11.16911 | 12.59689 | 5.97138 | 6.436681 | 7.719905 | 8.780794 | 7.707868 | NA | 10.84809 | 14.33237 | 17.28408 |
| CUST_56538_PI429010886 | M114042 | 1.22E-05 | 21 | 0 | | | | | | 174.4788 | 146.0101 | 154.5247 | 136.3426 | 140.7358 | 134.6768 | 120.6877 | 111.5879 | 102.3344 | 153.2734 | 104.964 | 112.5301 | 110.9084 | 128.4766 | 117.1089 | 137.4615 | 118.0698 | 117.9497 | 129.9706 | 137.196 | 135.3397 | 138.0091 | 148.9131 | 148.08 | 158.2045 | 124.4949 | 130.2217 |
| CUST_13222_PI429010886 | N30807 | 1.23E-05 | 12 | 3 | | | | | | 237.2477 | 237.8356 | 230.0814 | 269.2314 | 261.511 | 390.2131 | 253.7689 | 257.8674 | 215.9695 | 256.281 | 259.3866 | 219.3834 | 205.1968 | 219.061 | 168.3796 | 257.9765 | 318.4396 | 301.4214 | 257.3277 | 251.4349 | 268.82 | 254.7271 | 272.638 | 239.146 | 217.2265 | 239.9876 | 178.3086 |
| CUST_8553_PI429010886 | N23215 | 1.39E-05 | 15 | 9 | 241326643 | programmed cell death-involved protein, putative [ | Nucleic acid metabolism | RNA metabolic process | 1.73E-38 | 198.7167 | 231.235 | 267.8998 | 192.6772 | 196.129 | 191.8566 | 249.3844 | 219.3834 | 201.8776 | 294.9448 | 216.741 | 293.9351 | 213.7215 | 276.7086 | 253.025 | 226.8032 | 173.6414 | 196.5633 | 171.6462 | 243.595 | 180.8415 | 230.7168 | 257.5143 | 202.4642 | 268.9771 | 261.0789 | 318.9508 |
| CUST_10318_PI429010886 | N26050 | 1.42E-05 | 18 | 6 | | | | | | 5.034012 | 4.555208 | 7.407239 | 7.785373 | 5.835764 | 14.25304 | 29.75085 | 8.502113 | 17.06111 | 8.167815 | NA | 6.734253 | 8.161195 | 7.624707 | 10.39893 | NA | 13.295 | NA | 4.987261 | 4.414933 | 5.065174 | 10.39055 | 18.37346 | 9.739447 | 13.31154 | 13.18932 | 19.72128 |
| CUST_6158_PI429010886 | N19703 | 1.44E-05 | 21 | 0 | 242010409 | conserved hypothetical protein [Pediculus humanus | | | 2.75E-34 | 818.9073 | 656.1679 | 734.477 | 660.5155 | 587.6341 | 674.2322 | 598.6499 | 627.4273 | 624.8531 | 631.2945 | 572.4248 | 583.066 | 634.4094 | 602.4628 | 648.0827 | 719.37 | 706.0046 | 586.2213 | 677.3854 | 687.0726 | 611.5405 | 722.955 | 692.0661 | 742.9123 | 619.9697 | 629.802 | 637.4283 |
| CUST_28377_PI429010886 | N57835 | 1.46E-05 | 21 | 0 | | | | | | 302.2555 | 209.6917 | 379.0179 | 196.157 | 96.30815 | 192.1174 | 97.33778 | 159.0362 | 117.1213 | 160.778 | 87.46258 | 87.8914 | 90.57431 | 156.6119 | 159.1475 | 245.2785 | 130.6215 | 160.259 | 109.3542 | 123.9921 | 268.1467 | 213.7802 | 257.0697 | 234.443 | 181.9242 | 161.827 | 168.6167 |
| CUST_28608_PI429010886 | N58283 | 1.49E-05 | 24 | 15 | | | | | | 360.8661 | 351.2576 | 327.9176 | 357.5465 | 345.7017 | 330.1179 | 348.4628 | 319.5751 | 304.942 | 377.8098 | 370.9949 | 321.2041 | 360.8661 | 404.6132 | 380.5555 | 452.1056 | 499.4614 | 383.4448 | 405.5199 | 418.2809 | 373.5295 | 433.0579 | 354.9858 | 375.4995 | 411.6609 | 296.5712 | 353.785 |
| CUST_45492_PI429010886 | M69280 | 1.64E-05 | 18 | 3 | | | | | | NA | 7.529742 | 13.08017 | 8.466065 | 12.24194 | 14.61247 | 10.14742 | 8.381146 | 8.689343 | 6.212927 | 7.338708 | 8.14453 | NA | 4.544747 | NA | 5.673212 | 6.28041 | NA | 11.37699 | NA | NA | 10.35813 | 16.82346 | 13.22412 | 6.776347 | 10.31326 | 8.876178 |
| CUST_25344_PI429010886 | N52236 | 1.69E-05 | 12 | 0 | | | | | | 23.65401 | 14.85609 | 21.02813 | 16.50813 | 8.084402 | 12.92626 | 10.34165 | 10.4969 | 7.162712 | 5.792463 | 15.88012 | 14.85271 | 15.26731 | 25.9425 | 18.29194 | 17.04303 | 11.80651 | 21.14283 | 10.65431 | 7.527497 | 6.127478 | 8.995799 | 10.27406 | 10.3662 | 28.26127 | 18.44505 | 19.30622 |
| CUST_36586_PI429010886 | M16295 | 1.73E-05 | 18 | 12 | | | | | | 179.7342 | 221.6212 | 200.21 | 144.6167 | 184.5383 | 164.7204 | 225.6051 | 262.5059 | 238.2441 | 287.4102 | 329.473 | 237.3703 | 257.4087 | 327.7237 | 310.0283 | 316.0527 | 194.6296 | 236.1903 | 277.6024 | 323.2711 | 219.2135 | 217.159 | 161.861 | 108.9026 | 243.4729 | 162.0241 | 286.423 |
| CUST_13190_PI429010886 | N30752 | 1.86E-05 | 15 | 12 | | | | | | 148.544 | 280.7168 | 207.128 | 87.65825 | 123.0707 | 84.49247 | 250.3612 | 170.3395 | 301.9881 | 332.1138 | 155.649 | 275.2295 | 459.2637 | 358.8712 | 289.3731 | 354.4714 | 196.032 | 183.9501 | 116.1332 | 192.442 | 135.6184 | 347.3822 | 279.1889 | 152.2072 | 1058.251 | 226.2861 | 704.9925 |
| CUST_38717_PI429010886 | M29543 | 1.89E-05 | 18 | 15 | | | | | | 521.8521 | 372.2873 | 537.3115 | 140.2989 | 473.8216 | 309.7028 | 517.6852 | 423.4903 | 385.5507 | 806.3497 | 330.8112 | 513.2283 | 823.8136 | 643.0031 | 451.6758 | 572.2834 | 622.7518 | 829.599 | 519.8548 | 513.6722 | 441.9667 | 356.4277 | 208.7483 | 309.2864 | 204.9855 | 393.1602 | 599.2901 |
| CUST_5203_PI429010886 | N18394 | 1.9E-05 | 24 | 21 | | | Signal transduction | Other | | 2754.789 | 5650.794 | 3062.827 | 3027.574 | 1877.805 | 2211.182 | 1353.303 | 1685.531 | 1841.426 | 2623.017 | 1914.117 | 3624.616 | 1293.201 | 4306.703 | 3886.072 | 3578.372 | 5144.095 | 2219.95 | 5650.794 | 7729.059 | 6273.384 | 9358.113 | 4186.218 | 4690.442 | 8448.526 | 5256.29 | 8106.278 |
| CUST_44237_PI429010886 | M65353 | 1.96E-05 | 18 | 3 | 388855385 | related to FPR2-FK506/rapamycin-binding protein c | Protein metabolism | Protein folding | 4.63E-15 | 293.3227 | 311.3229 | NA | 618.5189 | 345.5168 | 310.2077 | 230.4843 | 49.40423 | 195.2252 | 4.127327 | 5.673834 | 7.72233 | 196.129 | 135.9627 | 250.6384 | 416.4515 | 11.9014 | 297.9644 | NA | 148.9974 | 15.66502 | 581.5974 | NA | 348.906 | 116.7026 | 56.16844 | 4.516394 |
| CUST_17638_PI429010886 | N38321 | 1.98E-05 | 18 | 6 | | | | | | 21.36125 | 17.7744 | 14.05481 | 32.13072 | 17.8956 | 27.19369 | 40.6037 | 38.88089 | 45.53479 | 33.1843 | 37.80277 | 33.0225 | 27.00762 | 31.64126 | 30.93077 | 31.15965 | 29.75783 | 29.25886 | 32.67642 | 27.9072 | 15.70076 | 32.11685 | 17.71709 | 36.14573 | 30.90181 | 32.96805 | 43.55575 |
| CUST_30504_PI429010886 | N62026 | 1.99E-05 | 15 | 9 | | | | | | 28.14633 | 33.89729 | 28.65923 | 8.587225 | 27.75224 | 18.50615 | 36.31145 | 35.72924 | 27.79102 | 42.23347 | 28.77687 | 42.72999 | 28.59435 | 20.53586 | 29.36188 | 36.80914 | 20.29145 | 26.92502 | 21.25854 | 23.34144 | 17.70313 | 27.42777 | 31.03981 | 23.2348 | 29.02633 | 61.29672 | 40.73672 |
| CUST_47259_PI429010886 | M75364 | 2.01E-05 | 18 | 3 | | | | | | 23.59336 | 26.38687 | 20.08306 | 48.43308 | 42.34172 | 54.08762 | 30.01154 | 12.7435 | 24.38855 | 4.769711 | 12.80802 | 17.67943 | 11.12902 | 25.42315 | 21.04421 | 46.60859 | 11.87443 | 40.13494 | 12.95945 | 29.72938 | 21.12385 | 37.92303 | 30.62818 | 54.05301 | 20.84115 | 18.30186 | 17.91014 |
| CUST_30527_PI429010886 | N62061 | 2.01E-05 | 18 | 9 | 32565682 | Protein ABU-13 [Caenorhabditis elegans] >gi\|3510 | | | 9.87E-07 | 5.82746 | 17.75499 | 15.02374 | 4.713461 | 9.602601 | 7.065415 | 11.52917 | 8.025541 | 12.21189 | 44.63765 | 16.24662 | 23.23227 | 15.86747 | 15.21068 | 25.40127 | 18.72259 | 9.858236 | 9.489751 | 8.542736 | 16.43274 | 10.95916 | 5.075645 | 5.147446 | 9.409299 | 19.75734 | 6.870124 | 17.86554 |
| CUST_3697_PI429010886 | N15073 | 2.15E-05 | 24 | 21 | | | | | | 97.67416 | 111.6628 | 119.6154 | 85.17215 | 93.04202 | 94.91653 | 80.76562 | 91.17869 | 84.95079 | 100.8686 | 105.242 | 109.3742 | 96.07604 | 101.8998 | 107.4857 | 100.2191 | 103.397 | 105.005 | 95.20545 | 121.0641 | 117.2199 | 117.9048 | 130.4682 | 150.4806 | 138.7624 | 115.3266 | 111.4592 |
| CUST_18665_PI429010886 | N40191 | 2.16E-05 | 24 | 18 | | | | | | 30.21389 | 14.92301 | 19.35495 | 5.98865 | 17.68458 | 10.70475 | 17.07166 | 9.588389 | 11.17232 | 16.88136 | 27.73533 | 21.4174 | 24.78858 | 18.50772 | NA | 28.53867 | 54.15621 | 19.30381 | 33.05506 | 40.98703 | 41.31249 | 31.43244 | NA | 6.146383 | 28.67208 | NA | 25.96255 |
| CUST_47258_PI429010886 | M75363 | 2.55E-05 | 24 | 18 | 321460748 | hypothetical protein DAPPUDRAFT_326816 [Daphn | Metabolic process | Other | 1.68E-21 | 41.70336 | 67.61178 | 64.74408 | 42.37752 | 58.03044 | 49.18419 | 36.17076 | 34.36888 | 41.03316 | 69.29923 | 69.39648 | 84.48505 | 44.8896 | 46.37649 | 81.82955 | 81.22317 | 90.84494 | 56.16844 | 79.0243 | 88.98374 | 69.56167 | 69.53748 | 76.01313 | 66.51353 | 60.53815 | 64.28764 | 76.89998 |
| CUST_3828_PI429010886 | N15359 | 2.62E-05 | 21 | 12 | | | | | | 46.63569 | 5.47471 | 14.84035 | NA | 63.94718 | 309.517 | 492.1806 | 33.49626 | 327.9867 | 127.5671 | NA | NA | 939.4252 | 832.0351 | 528.3775 | 68.2813 | 113.2498 | 24.66303 | 783.5804 | 15.7022 | 695.332 | 3.482817 | 29.45836 | 12.14051 | NA | 410.7156 | 62.92532 |
| CUST_51305_PI429010886 | M93004 | 2.71E-05 | 18 | 3 | 242025030 | organic cation transporter, putative [Pediculus hum | Transport | Transmembrane transport | 7.7E-09 | 503.6617 | 1298.051 | 880.2112 | 1695.261 | 813.1389 | 1359.544 | 556.7295 | 664.7021 | 678.9878 | 645.4311 | 384.4603 | 446.0696 | 382.3424 | 258.2663 | 364.9554 | 612.4623 | 340.3246 | 731.6805 | 329.2355 | 305.3469 | 898.9921 | 746.1542 | 1127.801 | 741.9924 | 788.3593 | 1123.432 | 552.3175 |
| CUST_19308_PI429010886 | N41358 | 2.85E-05 | 12 | 0 | | | | | | 23.30384 | 24.86204 | 50.56808 | 13.63311 | 18.64355 | 18.84309 | 22.93439 | 39.17807 | 26.07154 | 32.73413 | 19.70553 | 25.94889 | 48.77148 | 60.61424 | 29.00151 | 19.14986 | 19.89384 | 15.07523 | 14.19691 | 15.98525 | 21.70738 | 23.81072 | 39.00381 | 21.61111 | 64.39273 | 33.45456 | 42.36861 |
| CUST_35762_PI429010886 | M13443 | 2.86E-05 | 18 | 12 | | | | | | 87.82151 | 130.7466 | 102.8709 | 100.1573 | 80.9345 | 59.8939 | 127.5003 | 109.5048 | 115.7833 | 102.8545 | 163.8724 | 135.0089 | 216.741 | 208.138 | 178.862 | 146.0474 | 100.566 | 81.46986 | 111.151 | 217.2477 | 103.9891 | 93.36286 | 77.1205 | 61.35068 | 134.79 | 199.9828 | 196.032 |
| CUST_39397_PI429010886 | M38840 | 2.88E-05 | 15 | 12 | | | | | | 687.5678 | 1378.873 | 889.7966 | 329.4092 | 590.6242 | 280.165 | 794.4801 | 673.3658 | 1315.839 | 1596.656 | 606.6133 | 1422.337 | 1831.59 | 1796.352 | 1299.658 | 1637.238 | 864.4857 | 771.3694 | 518.9265 | 1034.145 | 573.39 | 1276.133 | 1298.051 | 668.4719 | 3893.391 | 937.3911 | 3787.685 |
| CUST_43268_PI429010886 | M62578 | 2.88E-05 | 15 | 12 | | | | | | 27.80368 | 31.05167 | 31.39871 | 25.7467 | 24.37063 | 30.06738 | 23.49374 | 28.08233 | 39.32188 | 35.53394 | 28.42343 | 32.62765 | 50.49286 | 38.16452 | 35.23219 | 38.03336 | 32.63905 | 30.04146 | 21.77287 | 25.64332 | 27.42267 | 27.89184 | 34.14111 | 32.99848 | 36.06105 | 29.87357 | 40.84477 |
| CUST_47773_PI429010886 | M77559 | 3.08E-05 | 15 | 9 | | | | | | 14744.71 | 18377.26 | 36785.94 | 3597.224 | 4778.44 | 4639.445 | 25830.1 | 28610.41 | 50702.21 | 47937.32 | 33666.48 | 60028.43 | 34070.11 | 54708.78 | 35264.41 | 23998.85 | 25748.12 | 22723.68 | 21514.91 | 39179.95 | 18023.86 | 58209.54 | 44409.28 | 9207.498 | 67520.86 | 40354.05 | 78574.02 |
| CUST_10994_PI429010886 | N27101 | 3.11E-05 | 15 | 9 | | | | | | 45.17236 | 46.40223 | 40.62506 | 29.24887 | 35.51693 | 16.68253 | 33.62817 | 63.09759 | 78.93037 | 97.63395 | 56.16292 | 65.56207 | 58.77783 | 69.4087 | 50.39093 | 58.76571 | 21.30938 | 29.69394 | 43.60346 | 40.15414 | 45.42072 | 85.2824 | 67.64754 | 18.83394 | 104.4054 | 93.20644 | 85.34367 |
| CUST_4822_PI429010886 | N17554 | 3.15E-05 | 15 | 9 | | | | | | 251.1828 | 684.8725 | 365.1209 | 116.3712 | 182.5915 | 86.87439 | 821.7869 | 614.3849 | 1147.081 | 1285.231 | 1241.135 | 3295.06 | 963.9292 | 673.8089 | 1119.759 | 6454.955 | 145.8124 | 2206.497 | 408.5798 | 648.9401 | 381.1947 | 3805.814 | 383.351 | 1099.991 | 3243.287 | 2934.424 | 5217.309 |
| CUST_5466_PI429010886 | N18742 | 3.17E-05 | 12 | 3 | 63003719 | Ascidia sydneiensis samea 6-PGDH mRNA for 6-pho | Metabolic process | Carbohydrate metabolic process | 1.22E-57 | 1245.533 | 1012.109 | 822.3869 | 1942.982 | 2115.987 | 2040.559 | 1918.897 | 1234.248 | 1082.445 | 829.599 | 1946.546 | 1103.397 | 910.8643 | 732.691 | 815.0535 | 1216.535 | 2149.927 | 1574.342 | 2065.573 | 1213.834 | 1204.562 | 1255.497 | 1143.569 | 1618.557 | 726.9909 | 1035.008 | 685.1065 |
| CUST_11747_PI429010886 | N28340 | 3.2E-05 | 21 | 12 | | | | | | 37.06438 | 59.41199 | 30.05351 | 21.60669 | 32.75767 | 20.49313 | 34.92634 | 22.33694 | 22.77196 | 44.96572 | 24.929 | 37.62291 | 52.44678 | 49.55005 | 42.0436 | 45.7637 | 50.60456 | 41.18311 | 24.53437 | 40.59586 | 27.93572 | 35.33485 | 28.60828 | 37.87276 | 28.69117 | 20.45832 | 22.01177 |
| CUST_16581_PI429010886 | N36501 | 3.36E-05 | 21 | 15 | | | Metabolic process | Lipid metabolic process | | 34.03924 | 30.63709 | 34.23978 | 10.29533 | 17.24085 | 19.61118 | 14.79157 | 21.74647 | 17.38798 | 18.29602 | 16.21736 | NA | 14.98214 | 21.75394 | 36.58662 | 62.45053 | 30.42483 | 69.53748 | 29.76345 | 61.6776 | 63.3033 | 38.46101 | 24.26769 | 26.57271 | 22.84227 | 12.45327 | 19.34576 |
| CUST_43305_PI429010886 | M62695 | 3.4E-05 | 21 | 0 | 91079060 | PREDICTED: similar to Polycomb protein Suz12 (Sup | | | 1.55E-53 | 172.957 | 180.1028 | 153.4178 | 135.1036 | 147.4281 | 155.9986 | 130.8664 | 93.11072 | 105.5539 | 144.1866 | 123.056 | 138.2955 | 147.7682 | 145.726 | 140.194 | 192.9519 | 145.6295 | 155.7397 | 157.9551 | 165.0063 | 145.308 | 189.9402 | 156.0552 | 146.5479 | 169.9162 | 160.4822 | 152.2072 |
| CUST_21638_PI429010886 | N45566 | 3.42E-05 | 18 | 3 | | | | | | 31.44965 | 31.46943 | 28.98446 | 52.34171 | 28.32496 | 45.95551 | 17.74139 | 29.35151 | 10.45509 | 23.51614 | 16.57372 | 28.59095 | 20.06979 | 24.34006 | 19.91373 | 27.75224 | 9.098614 | 21.82435 | 24.5556 | 25.73345 | 28.888 | 52.89696 | 33.01533 | 31.46943 | 30.52691 | 38.68605 | 29.95175 |
| CUST_51428_PI429010886 | M93507 | 3.45E-05 | 21 | 9 | 260810430 | hypothetical protein BRAFLDRAFT_120622 [Branchi | | | 8.91E-12 | 7.168304 | 14.55387 | 13.10037 | 4.398168 | 18.88981 | 19.55335 | 31.18733 | 33.09246 | 33.25306 | 54.44415 | 39.61818 | 51.40578 | 54.3134 | 23.35652 | 32.60782 | 16.83181 | 10.85593 | 14.29954 | 20.34468 | 64.58903 | 42.91318 | 18.01669 | 8.588693 | 3.464036 | 14.79157 | 24.44489 | 37.80681 |
| CUST_1230_PI429010886 | N8938 | 3.52E-05 | 21 | 9 | | | | | | 77.53281 | 83.51601 | 94.53569 | 109.8741 | 117.4608 | 132.2443 | 115.4652 | 135.78 | 119.9123 | 237.2944 | 179.6526 | 146.3743 | 192.0915 | 167.8576 | 173.0953 | 110.8588 | 125.8931 | 133.3745 | 143.7672 | 133.5378 | 94.04891 | 133.1101 | 95.7753 | 108.6304 | 263.1362 | 142.7087 | 153.9132 |
| CUST_9884_PI429010886 | N25353 | 3.55E-05 | 21 | 18 | 48132665 | PREDICTED: ubiquitin-conjugating enzyme E2 J2-like | | | 1.66E-59 | 246.1025 | 211.9082 | 224.5142 | 180.1444 | 197.1531 | 166.3529 | 192.0915 | 203.8966 | 208.4275 | 219.9975 | 209.0873 | 262.6197 | 239.9082 | 303.4405 | 242.7679 | 210.2883 | 175.0831 | 228.6651 | 279.9837 | 271.0795 | 255.0348 | 245.6875 | 220.2228 | 225.2988 | 197.2228 | 158.9585 | 218.0605 |
| CUST_44569_PI429010886 | M66345 | 3.65E-05 | 24 | 18 | | | | | | 1024.808 | 978.1392 | 789.4136 | 516.8489 | 776.1766 | 487.9783 | 923.2313 | 736.5339 | 1057.689 | 969.5225 | 824.3794 | 1268.323 | 833.259 | 826.1357 | 937.1457 | 1265.003 | 1112.064 | 851.0651 | 1487.266 | 1600.359 | 1284.605 | 930.8848 | 1400.624 | 867.4252 | 1171.568 | 920.3504 | 1067.136 |
| CUST_47533_PI429010886 | M76503 | 3.83E-05 | 21 | 0 | | | | | | 44.72593 | 27.23432 | 32.86388 | 30.91745 | 37.78907 | 30.76819 | 26.99397 | 22.11463 | 25.64065 | 35.77964 | 29.43926 | 27.21328 | 16.17384 | 25.05489 | 16.5954 | 17.84023 | 15.50513 | 16.04975 | 20.45683 | 29.67419 | 44.48912 | 40.55619 | 61.12299 | 28.28294 | 32.10376 | 40.84477 | 38.15596 |
| CUST_7506_PI429010886 | N21644 | 4.01E-05 | 15 | 9 | 321463584 | carboxylase:pyruvate/acetyl-coa/propionyl-CoA [Da | Metabolic process | Cellular amino acid metabolic process | 8.15E-60 | 22.24779 | 30.31136 | 26.37567 | 19.62458 | 22.87323 | 15.7841 | 35.85651 | 36.58662 | 42.98911 | 44.54185 | 53.23845 | 47.00702 | 27.75224 | 37.71676 | 27.32084 | 38.27387 | 34.86128 | 22.23059 | 22.9227 | 33.6052 | 21.82911 | 47.35331 | 41.34285 | 15.2174 | 82.71117 | 32.97999 | 53.16758 |
| CUST_10188_PI429010886 | N25842 | 4.07E-05 | 18 | 15 | | | | | | 91.25978 | 102.9429 | 114.3038 | 92.1489 | 80.68886 | 94.44207 | 103.5071 | 90.38098 | 75.37057 | 105.1778 | 111.3513 | 112.6306 | 115.0528 | 115.202 | 123.0174 | 144.6868 | 119.2791 | 117.863 | 101.92 | 107.9913 | 115.5725 | 98.86383 | 110.4732 | 92.10717 | 133.5047 | 109.3629 | 103.3023 |
| CUST_30765_PI429010886 | N62607 | 4.13E-05 | 18 | 6 | | | | | | 416.8412 | 272.3366 | 383.4448 | 492.7259 | 294.7498 | 402.9446 | 673.8089 | 473.2302 | 641.17 | 390.0674 | 251.7054 | 277.3932 | 749.2477 | 454.3834 | 463.0515 | 226.8346 | 375.4169 | 250.2477 | 249.1725 | 265.2639 | 271.9546 | 423.5928 | 488.7915 | 418.9595 | 474.912 | 491.2272 | 460.8905 |
| CUST_49401_PI429010886 | M85512 | 4.17E-05 | 15 | 9 | | | | | | 15.55244 | 21.24533 | 14.19445 | 14.18613 | 17.79403 | 17.40574 | 17.99221 | 15.24223 | 18.39819 | 26.72979 | 22.17526 | 21.71687 | 20.42948 | 15.60189 | 21.9738 | 19.61118 | 13.87201 | 17.07599 | 11.63703 | 14.77486 | 7.363331 | 18.99317 | 19.60024 | 16.56737 | 19.04367 | 23.42758 | 18.03617 |
| CUST_28070_PI429010886 | N57225 | 4.19E-05 | 18 | 3 | 443694150 | hypothetical protein CAPTEDRAFT_110364 [Capitell | Metabolic process | Other | 6.13E-09 | 537.3115 | 1415.482 | 956.1852 | 2123.129 | 960.3576 | 1348.92 | 660.7381 | 704.7831 | 602.6317 | 572.8694 | 362.1101 | 442.2873 | 572.0401 | 301.1198 | 356.4277 | 607.9318 | 262.0585 | 218.2149 | 362.4934 | 154.6176 | 981.4865 | 969.1321 | 1221.17 | 657.8288 | 1037.274 | 563.6523 | 550.2491 |
| CUST_36245_PI429010886 | M15160 | 4.25E-05 | 24 | 18 | | | | | | 9.289553 | 11.57662 | 10.98742 | 6.906185 | 4.298534 | 10.52396 | 14.59227 | 11.70566 | NA | 14.74948 | 17.72667 | 7.208745 | 11.18584 | 14.09377 | 7.421979 | 17.76635 | 15.69846 | 8.630999 | 28.35783 | 16.23539 | 19.00858 | 16.68781 | 17.42826 | 18.50615 | 15.51946 | 11.27249 | 13.16084 |
| CUST_14923_PI429010886 | N33695 | 4.27E-05 | 21 | 0 | | | | | | 182.1781 | 153.6882 | 225.7607 | 92.88396 | 163.8459 | 199.344 | 176.2795 | 153.2103 | 144.9176 | 118.2591 | 131.4929 | 95.41159 | 156.4871 | 153.961 | 119.8071 | 128.3615 | 166.6372 | 111.9276 | 168.032 | 160.3955 | 170.596 | 193.0602 | 223.6419 | 205.1968 | 162.9606 | 214.5527 | 152.9208 |
| CUST_876_PI429010886 | N7655 | 4.28E-05 | 15 | 9 | | | | | | 81.42898 | 75.41714 | 84.96672 | 73.75273 | 78.49411 | 86.2674 | 74.17182 | 73.96327 | 84.7131 | 89.98836 | 146.1555 | 100.0727 | 85.82609 | 110.9171 | 98.55351 | 77.60106 | 87.85163 | 84.83474 | 78.69319 | 68.33006 | 68.44956 | 113.6011 | 115.5867 | 57.56812 | 138.094 | 120.6382 | 108.829 |
| CUST_17326_PI429010886 | N37780 | 4.29E-05 | 18 | 12 | | | | | | 18.39397 | 16.73161 | 11.67109 | 13.86905 | 8.616285 | 11.09239 | 26.35211 | 25.10875 | 17.85771 | 33.62524 | 19.94541 | 20.71812 | 26.52223 | 18.97043 | 27.03609 | 24.11276 | 21.39305 | 17.37759 | 20.71416 | 17.75695 | 22.91218 | 9.758601 | 17.67943 | 12.14778 | 13.25777 | 12.06262 | 22.3138 |
| CUST_46376_PI429010886 | M72436 | 4.49E-05 | 18 | 3 | | | | | | 137.5853 | 4.307455 | 144.4224 | 123.4738 | 116.8917 | 173.5211 | 16.40268 | 69.81278 | 68.34468 | 59.50102 | 17.40769 | 24.03945 | 22.98483 | 21.31694 | 65.55617 | 21.04144 | 19.2789 | 7.144042 | NA | 92.72381 | 61.23542 | 236.6472 | 103.2892 | 70.89744 | 61.80458 | 45.75802 | 50.03352 |
| CUST_39285_PI429010886 | M37210 | 4.67E-05 | 15 | 9 | 532759728 | antimicrobial peptide type 2 precursor IIb [Pandalo | | | 2.7E-30 | 687.964 | 560.8883 | 307.6262 | 747.806 | 932.818 | 157.9551 | 1254.757 | 497.9965 | 1741.833 | 1369.558 | 723.6629 | 2070.325 | 1210.833 | 1346.091 | 807.1828 | 658.6556 | 1217.47 | 750.3627 | 315.6417 | 319.745 | 342.0536 | 879.0511 | 596.1618 | 1430.775 | 3378.169 | 1272.526 | 3018.553 |
| CUST_31818_PI429010886 | M2485 | 4.9E-05 | 24 | 18 | | | | | | 361.2861 | 342.0536 | 348.8261 | 289.7899 | 283.2143 | 343.3378 | 311.7466 | 349.4134 | 239.7427 | 446.3108 | 534.4051 | 472.8988 | 320.693 | 487.9783 | 477.9922 | 707.6225 | 770.7121 | 813.6581 | 581.2638 | 861.813 | 641.887 | 597.939 | 407.9548 | 609.4542 | 1013.924 | 586.9437 | 829.1909 |
| CUST_17207_PI429010886 | N37572 | 5.03E-05 | 24 | 3 | | | | | | 28.56717 | 28.36898 | 21.16222 | 42.07479 | 33.41607 | 35.71639 | 21.11131 | 21.53946 | 25.11695 | 29.04226 | 17.947 | 19.40757 | 11.93292 | 9.454588 | 17.08381 | 23.12912 | 15.91783 | 18.11337 | 21.79421 | 9.887988 | 34.8131 | 16.13381 | 29.38995 | 21.55583 | 23.29568 | 25.98413 | 23.90824 |
| CUST_48554_PI429010886 | M80964 | 5.05E-05 | 21 | 3 | 9367047 | Euphausia superba 28S large subunit ribosomal RN | | | 1.5E-150 | 169441.2 | 175370.5 | 169441.2 | 178679.8 | 180118 | 173562.9 | 176759.5 | 165803.3 | 165207 | 190551.5 | 166443.6 | 165207 | 165207 | 160411.4 | 157589.4 | 148780.5 | 164488.1 | 153607.2 | 166443.6 | 159657.9 | 182883 | 161030.7 | 169441.2 | 171853.4 | 186937.6 | 174460.2 | 172547 |
| CUST_1840_PI429010886 | N10852 | 5.55E-05 | 24 | 9 | | | | | | 152.3321 | 106.908 | NA | 175.2355 | 281.0282 | 328.2844 | 193.8995 | 308.1438 | 248.7921 | 474.3801 | 370.8487 | 538.4051 | 274.5343 | 287.0591 | 189.1194 | 9.313914 | 178.0976 | 32.71805 | 7.178303 | 712.9294 | 105.2954 | 88.7408 | 22.07122 | 77.38462 | 34.43912 | NA | 35.83833 |
| CUST_9282_PI429010886 | N24367 | 5.81E-05 | 15 | 9 | 532759728 | antimicrobial peptide type 2 precursor IIb [Pandalo | | | 8.33E-31 | 881.5993 | 1528.209 | 646.5567 | 1115.976 | 1065.113 | 230.6606 | 2205.198 | 1053.349 | 3390.638 | 2464.001 | 922.8399 | 3010.148 | 2195.833 | 1525.167 | 1133.519 | 1358.952 | 1771.34 | 1165.381 | 502.6158 | 586.5167 | 580.3063 | 1614.376 | 984.9879 | 2166.108 | 4114.35 | 1929.904 | 5144.095 |
| CUST_22632_PI429010886 | N47367 | 5.83E-05 | 24 | 21 | | | | | | 6.017128 | 11.62742 | 7.066118 | 4.611815 | 8.041483 | 5.98944 | 3.756362 | 5.828885 | 5.838228 | 6.480429 | 4.943 | NA | 4.379882 | NA | 5.319597 | 12.20563 | 7.308005 | NA | 9.112459 | 59.22654 | 9.334862 | 16.98325 | 13.80154 | NA | 8.356596 | NA | 9.117443 |
| CUST_6257_PI429010886 | N19859 | 6E-05 | 21 | 18 | | | | | | 1162.112 | 1242.051 | 999.4148 | 938.0188 | 1055.576 | 978.4784 | 1013.618 | 919.3319 | 900.1864 | 1037.274 | 1070.179 | 1080.128 | 888.7344 | 1017.392 | 1023.389 | 1199.213 | 1293.201 | 1097.654 | 1242.81 | 1187.094 | 1128.352 | 1218.367 | 1187.094 | 1110.941 | 1072.188 | 989.1564 | 1054.214 |
| CUST_50402_PI429010886 | M89702 | 6.15E-05 | 15 | 12 | | | | | | 47.23018 | 27.97946 | 48.84597 | 14.78359 | 21.14577 | 11.26192 | 39.766 | 36.64631 | 26.56221 | 25.22969 | 49.49896 | 65.15734 | 70.24276 | 78.64329 | 116.0087 | 33.94434 | 75.09478 | 32.35284 | 21.73089 | 30.86679 | 15.06859 | 25.37 | 138.3904 | 172.1642 | 81.61454 | 51.6678 | 168.2962 |
| CUST_20203_PI429010886 | N43014 | 6.23E-05 | 24 | 18 | 507628788 | PREDICTED: LOW QUALITY PROTEIN: importin-4 [Ec | | | 3.59E-33 | 96.21869 | 125.2228 | 98.57526 | 141.5625 | 121.3759 | 111.8492 | 91.11943 | 98.02439 | 94.16491 | 112.5793 | 126.1331 | 104.0193 | 88.78018 | 133.4278 | 118.8413 | 150.8165 | 151.8121 | 134.3671 | 137.604 | 163.3967 | 187.0261 | 144.9326 | 123.4117 | 136.6377 | 146.2811 | 141.8325 | 138.2955 |
| CUST_5191_PI429010886 | N18380 | 6.24E-05 | 18 | 15 | | | | | | 209.3167 | 206.6019 | 150.434 | 139.216 | 147.3674 | 127.5221 | 127.8042 | 143.7068 | 118.8413 | 173.1201 | 147.0435 | 209.5249 | 151.6596 | 144.1656 | 207.7149 | 235.2181 | 169.5063 | 174.1781 | 145.0813 | 272.8585 | 156.0552 | 258.1237 | 147.3848 | 167.982 | 136.0993 | 125.4203 | 87.34049 |
| CUST_16417_PI429010886 | N36201 | 6.28E-05 | 24 | 0 | | | | | | 27.18447 | 22.05551 | 36.5122 | 25.03495 | 19.32542 | 15.35258 | 21.82911 | 14.74259 | 26.66659 | 12.48145 | 13.27508 | 33.87876 | 13.26351 | 28.77061 | 6.38992 | 12.23171 | 10.13438 | 7.68792 | 18.59923 | 22.44547 | 28.96358 | 22.4606 | 25.88089 | 18.87895 | 27.94887 | 29.68097 | 36.99055 |
| CUST_32361_PI429010886 | M3933 | 6.34E-05 | 18 | 9 | 386118315 | 56kDa selenium binding protein [Acropora millepor | | | 7.11E-66 | 16.62053 | 10.27942 | 16.69384 | 12.87351 | 12.44655 | 17.48685 | 19.5882 | 20.87902 | 26.62182 | 33.98233 | 16.29756 | 32.86117 | 24.35681 | 31.66159 | 27.29125 | 24.36596 | 26.46898 | 17.73942 | 15.42005 | 11.32033 | 16.74268 | 23.92174 | 22.43724 | 16.02003 | 32.13434 | 24.51944 | 17.85063 |
| CUST_45038_PI429010886 | M67802 | 6.37E-05 | 18 | 0 | 123316129 | Portunus pelagicus actin mRNA, partial cds | | | 3.53E-56 | 85.55586 | 103.5194 | 94.64608 | 122.1037 | 78.27999 | 80.33892 | 74.26685 | 85.3343 | 73.75273 | 67.1799 | 76.84434 | 65.11259 | 77.76127 | 70.99743 | 36.4354 | 75.22711 | 74.19557 | 53.12725 | 89.4301 | 134.282 | 76.52417 | 88.47454 | 87.07504 | 83.53882 | 60.51107 | 75.71395 | 76.98892 |
| CUST_47527_PI429010886 | M76474 | 6.43E-05 | 15 | 0 | | | | | | 38.30957 | 47.66523 | 50.40874 | 32.99565 | 29.73851 | 41.52857 | 26.97315 | 23.84605 | 31.74511 | 33.76678 | 33.26161 | 40.04237 | 32.29627 | 51.58072 | 40.57245 | 42.32826 | 43.04145 | 43.28801 | 28.97255 | 22.34585 | 29.5457 | 39.63103 | 17.4849 | 27.5502 | 33.00505 | 36.44258 | 41.98854 |
| CUST_33298_PI429010886 | M6531 | 6.5E-05 | 15 | 9 | 307202832 | Annexin-B11 [Harpegnathos saltator] | | | 3.99E-38 | 42.89021 | 51.42712 | 48.24061 | 40.96307 | 39.82044 | 34.8364 | 67.48171 | 39.71396 | 50.54005 | 55.42699 | 61.29672 | 63.16675 | 51.29496 | 63.98783 | 72.16312 | 72.55972 | 36.09309 | 53.81515 | 37.32614 | 52.86776 | 37.80277 | 67.28086 | 49.67787 | 41.66498 | 74.03663 | 91.99262 | 80.00094 |
| CUST_1694_PI429010886 | N10456 | 6.77E-05 | 24 | 12 | 380017443 | PREDICTED: carboxypeptidase B-like [Apis florea] | Protein metabolism | Proteolysis | 7.36E-65 | 502.9268 | 387.779 | 472.8988 | 43.24186 | 597.7736 | 347.5866 | 790.5889 | 504.3528 | 492.0126 | 518.0939 | 501.1913 | 623.8166 | 524.0613 | 1119.759 | 522.6581 | 270.3602 | 444.4951 | 345.5168 | 138.2244 | 297.7533 | 128.673 | 21.18631 | 278.8959 | 200.8116 | 449.3849 | 85.07901 | 337.1609 |
| CUST_32403_PI429010886 | M4015 | 6.87E-05 | 24 | 15 | | | Metabolic process | Cellular amino acid metabolic process | | 178.1697 | 104.2639 | 138.323 | 69.18954 | 141.7648 | 99.2894 | 86.20359 | 74.6525 | 118.7738 | 93.46673 | 115.9402 | 186.7272 | 210.0878 | 212.3072 | 204.0536 | 250.3151 | 178.2181 | 269.0643 | 132.4207 | 224.1873 | 148.6082 | 181.8837 | 149.4702 | 161.2028 | 138.323 | 147.1696 | 184.5131 |
| CUST_14650_PI429010886 | N33230 | 6.95E-05 | 21 | 9 | | | | | | 6.177678 | 10.93186 | 9.296266 | 16.23717 | 14.94965 | 9.230112 | 27.07724 | 9.267153 | 19.50089 | 47.20161 | 17.45875 | 17.89801 | 12.31693 | 10.99426 | 12.88708 | 10.79019 | 13.58569 | 11.49086 | 20.52907 | 11.33598 | 9.262293 | 7.897325 | 8.013121 | 8.695689 | 12.85526 | 21.74923 | 30.0998 |
| CUST_53843_PI429010886 | M103389 | 7.35E-05 | 24 | 3 | | | | | | 1008.924 | 3435.959 | 1545.528 | 4440.858 | 1776.211 | 2352.559 | 1782.524 | 1514.446 | 1857.991 | 1739.21 | 694.9126 | 1973.742 | 1377.193 | 1387.967 | 810.6402 | 304.6076 | 396.481 | 907.7978 | 748.0078 | 1305.705 | 1798.294 | 1072.188 | 2013.592 | 1264.445 | 2163.813 | 2743.533 | 1160.295 |
| CUST_2484_PI429010886 | N12482 | 7.38E-05 | 15 | 0 | | | | | | 1848.861 | 1744.83 | 1848.861 | 949.0578 | 1535.786 | 1675.386 | 999.0406 | 867.8685 | 1082.038 | 1508.034 | 1446.386 | 1236.95 | 982.8541 | 1406.196 | 1374.989 | 2180.377 | 1835.374 | 2257.795 | 1680.099 | 1805.829 | 1569.952 | 1587.608 | 1210.418 | 1233.197 | 1666.923 | 1111.525 | 1576.033 |
| CUST_37152_PI429010886 | M18015 | 7.41E-05 | 21 | 0 | | | | | | 907.4672 | 966.7908 | 793.9994 | 403.4054 | 686.1452 | 787.7599 | 560.1437 | 635.813 | 683.1376 | 672.3398 | 504.1608 | 686.896 | 503.6617 | 650.0167 | 376.1818 | 444.9718 | 665.6609 | 664.8684 | 509.2578 | 815.5774 | 670.3311 | 697.5569 | 683.7716 | 853.3995 | 826.6639 | 619.5429 | 702.0078 |
| CUST_25120_PI429010886 | N51814 | 7.49E-05 | 24 | 15 | | | | | | 8.822611 | 7.063727 | 8.319795 | 6.212154 | 7.226406 | NA | NA | 7.099269 | 5.558018 | 20.26123 | 6.533554 | 12.51404 | 8.968773 | 17.03233 | NA | 24.93189 | NA | 12.99648 | 16.84886 | 17.08729 | 20.09831 | 12.07272 | 16.88939 | 9.08845 | 14.57533 | 10.53371 | 9.792471 |
| CUST_42373_PI429010886 | M60129 | 7.51E-05 | 24 | 9 | | | | | | 94.25565 | 87.93229 | NA | 114.2745 | 262.9561 | 280.2255 | 132.7894 | 237.9979 | 187.2169 | 414.1007 | 317.0842 | 383.017 | 203.5264 | 188.2993 | 178.4456 | NA | 121.8132 | 11.41581 | 6.847004 | 589.9381 | 102.205 | 63.00905 | 17.71088 | 59.83655 | 21.12887 | 6.746227 | 20.40858 |
| CUST_15607_PI429010886 | N34856 | 7.82E-05 | 15 | 9 | | | | | | 168.7755 | 27.70404 | 317.9044 | 37.87644 | 106.4186 | 122.6286 | 284.3166 | 433.4824 | 356.8337 | 300.8257 | 369.8403 | 443.9779 | 202.0834 | 317.1399 | 195.9585 | 311.0329 | 267.983 | 264.5866 | 94.13978 | 196.0648 | 52.73287 | 229.3656 | 165.2714 | 69.90395 | 324.1857 | 279.7284 | 615.9204 |
| CUST_38478_PI429010886 | M27180 | 7.91E-05 | 15 | 12 | 242009985 | zinc finger protein CCCH domain-containing protein | | | 3.07E-48 | 46.9717 | 43.55173 | 65.64906 | 40.82788 | 27.99312 | 30.21389 | 34.49254 | 62.46895 | 49.27786 | 70.68641 | 49.38456 | 61.48326 | 56.03701 | 77.1366 | 64.35394 | 53.66581 | 42.9717 | 44.71419 | 40.11789 | 52.18439 | 45.68644 | 44.94616 | 48.13849 | 34.64857 | 76.94138 | 63.91001 | 66.00564 |
| CUST_1219_PI429010886 | N8897 | 8.09E-05 | 12 | 3 | 321469994 | hypothetical protein DAPPUDRAFT_318119 [Daphn | | | 2.09E-20 | 132.2831 | 5.330779 | 106.2399 | 488.5796 | 759.2915 | 946.7835 | 617.5712 | 181.9242 | 138.7005 | 4.82625 | 375.4995 | 8.516835 | 31.36762 | 8.058299 | 8.447696 | NA | 2861.487 | 1883.709 | 447.7446 | NA | 591.2615 | NA | 289.2197 | 361.8439 | NA | 338.5891 | NA |
| CUST_19662_PI429010886 | N41968 | 8.17E-05 | 12 | 9 | | | | | | 1485.346 | 8106.278 | 1200.709 | 612.2893 | 640.4514 | 436.2219 | 1915.9 | 2377.614 | 1283.349 | 5875.497 | 1133.023 | 3292.379 | 1292.668 | 624.13 | 1481.812 | 977.4233 | 823.2501 | 732.314 | 2771.702 | 1795.627 | 1490.779 | 3147.989 | 1941.576 | 4396.276 | 713.4223 | 1745.549 | 3254.31 |
| CUST_38843_PI429010886 | M31132 | 8.25E-05 | 15 | 9 | 532759728 | antimicrobial peptide type 2 precursor IIb [Pandalo | | | 2.51E-30 | 2462.824 | 4077.008 | 1328.52 | 3656.575 | 2478.692 | 1414.097 | 5396.825 | 3001.59 | 6694.984 | 5794.422 | 2201.928 | 7364.493 | 6065.211 | 4107.091 | 5402.577 | 6495.601 | 5271.276 | 3893.391 | 2089.376 | 1870.503 | 1397.451 | 3890.087 | 1964.934 | 5197.227 | 8611.654 | 6136.091 | 7404.352 |
| CUST_538_PI429010886 | N5886 | 8.27E-05 | 15 | 3 | 405973827 | Cytochrome c [Crassostrea gigas] | | | 4.91E-30 | 1878.86 | 2191.024 | 1387.428 | 2004.816 | 2246.519 | 2377.614 | 2144.378 | 1673.62 | 1493.465 | 1215.949 | 1774.992 | 1280.61 | 1361.981 | 1222.467 | 1196.575 | 1901.328 | 1867.343 | 2036.617 | 2323.132 | 1419.795 | 1692.652 | 1758.855 | 1497.781 | 1964.934 | 1367.805 | 1483.935 | 1390.385 |
| CUST_27133_PI429010886 | N55506 | 8.36E-05 | 15 | 9 | 157132998 | 4-aminobutyrate aminotransferase [Aedes aegypti] | Metabolic process | Cellular amino acid metabolic process | 2.06E-36 | 144.7965 | 198.4542 | 193.2364 | 128.0141 | 126.4932 | 131.9942 | 149.507 | 181.5941 | 194.777 | 204.9855 | 183.2048 | 221.7247 | 205.0143 | 177.7855 | 189.5057 | 178.1983 | 115.284 | 146.1555 | 131.863 | 160.9334 | 159.7611 | 193.8296 | 143.8077 | 153.8888 | 201.8347 | 181.1566 | 255.4386 |
| CUST_43228_PI429010886 | M62469 | 8.38E-05 | 15 | 12 | 512883568 | PREDICTED: putative ferric-chelate reductase 1-like | | | 3.05E-08 | 47.5011 | 65.25641 | 155.5527 | 33.51452 | 41.31249 | 34.50513 | 17.59799 | 98.0649 | 97.82533 | 178.4123 | 94.47657 | 133.6264 | 199.4834 | 341.6507 | 134.9903 | 75.75327 | 70.65347 | 51.6678 | 39.26949 | 73.5908 | 58.49374 | 114.7609 | 130.2384 | 23.61053 | 219.4136 | 73.31065 | 197.2296 |

| Probe ID | ID | p-value | Period | Phase | EBI ID | Annotation | GO category | Go term | E-value | 0 | 0 | 0 | 3 | 3 | 3 | 6 | 6 | 6 | 9 | 9 | 9 | 12 | 12 | 12 | 15 | 15 | 15 | 18 | 18 | 18 | 21 | 21 | 21 | 24 | 24 | 24 |
|---|---|---|---|---|---|---|---|---|---|---|---|---|---|---|---|---|---|---|---|---|---|---|---|---|---|---|---|---|---|---|---|---|---|---|---|---|
| CUST_16648_PI429010886 | N36626 | 8.48E-05 | 18 | 3 | | | | | | 67.57577 | 70.98423 | 90.19499 | 104.8092 | 78.26326 | 96.28021 | 63.65227 | 54.27879 | 66.73255 | 53.21879 | 62.85005 | 54.45697 | 52.10062 | 47.70628 | 39.49607 | 71.10646 | 63.34141 | 75.68737 | 59.38984 | 39.39152 | 74.4613 | 71.9913 | 82.31536 | 64.64151 | 63.71417 | 65.04995 | 45.74288 |
| CUST_38712_PI429010886 | M29383 | 8.67E-05 | 15 | 9 | 371591223 | Euphausia superba voucher 702K mitochondrion, pa | | | | 10510.22 | 7462.719 | 8675.932 | 8904.357 | 8313.066 | 19004.47 | 22140.21 | 15922.04 | 10200.91 | 29696.39 | 45970.08 | 33813.8 | 7306.322 | 13202.87 | 18727.55 | 13124.49 | 8176.608 | 8986.011 | 7828.426 | 35818.08 | 10559.01 | 26783.35 | 24777.72 | 51007.02 | 26153.6 | 34070.11 | 30972.22 |
| CUST_8092_PI429010886 | N22527 | 8.72E-05 | 18 | 3 | 345490220 | PREDICTED: hypothetical protein LOC100121192 iso | | | 1.4E-28 | 94.16491 | 95.97714 | 100.8435 | 122.3884 | 111.7039 | 114.3333 | 111.3772 | 85.50355 | 93.90071 | 90.8645 | 92.55609 | 74.75705 | 82.04247 | 71.94962 | 80.53149 | 91.96613 | 100.4233 | 99.6429 | 97.73561 | 76.79471 | 105.0723 | 101.4497 | 100.2916 | 100.4962 | 136.0755 | 93.58403 | 77.2785 |
| CUST_45641_PI429010886 | M69763 | 8.82E-05 | 15 | 12 | 224587574 | Exportin-1 [Salmo salar] | | | 4.13E-53 | 20.72467 | 27.78747 | 18.46255 | 13.55063 | 14.73662 | 24.53193 | 19.30172 | 15.03778 | 38.94819 | 37.30131 | 18.12996 | 27.9952 | 39.53783 | 34.26633 | 40.87212 | 35.88091 | 26.22826 | 22.81578 | 20.17321 | 23.24144 | 16.51414 | 24.94331 | 22.14748 | 27.39302 | 29.06247 | 28.53623 | 29.98387 |
| CUST_9339_PI429010886 | N24462 | 8.82E-05 | 15 | 12 | | | | | | 682.0753 | 1302.095 | 890.6855 | 293.013 | 518.5036 | 240.1049 | 766.6399 | 691.8386 | 1267.407 | 1385.628 | 626.8612 | 1323.05 | 1700.08 | 1720.979 | 1228.741 | 1782.524 | 807.9372 | 708.9187 | 491.1625 | 1015.729 | 532.0502 | 1332.781 | 1300.946 | 607.9318 | 3742.075 | 1016.074 | 3676.785 |
| CUST_715_PI429010886 | N6896 | 8.9E-05 | 15 | 9 | | | | | | 20168.54 | 36281.72 | 46148.42 | 6018.766 | 8814.529 | 5039.929 | 29537.19 | 49052.57 | 63067.91 | 54053.67 | 46673.5 | 61898.59 | 39918.27 | 73854.47 | 65192.57 | 38256.92 | 26713.02 | 32330.94 | 28815.19 | 55642.21 | 28694.35 | 63412.19 | 45790.2 | 8995.888 | 70499.75 | 63973.47 | 75608.53 |
| CUST_53318_PI429010886 | M101251 | 8.9E-05 | 24 | 9 | | | | | | 87.13975 | 103.1223 | 97.17307 | 62.71467 | 146.585 | 131.7341 | 72.20186 | 91.48208 | 157.4606 | 314.8877 | 149.2467 | 446.5232 | 136.8476 | 206.3839 | 86.58356 | 20.84589 | 87.15435 | 71.15715 | 42.58462 | 8.337419 | 32.94363 | 23.39495 | 51.67859 | 51.88614 | 20.44558 | 207.9906 | 226.5511 |
| CUST_49570_PI429010886 | M86174 | 9.01E-05 | 15 | 9 | | | | | | 1493.465 | 5251.069 | 5503.933 | 615.9204 | 928.4158 | 356.1302 | 1344.532 | 6336.037 | 11089.86 | 11161.59 | 6018.766 | 11838.35 | 7017.655 | 13375.82 | 9061.383 | 5980.233 | 2945.87 | 2986.925 | 3503.673 | 8527.974 | 4120.1 | 11454.19 | 9992.798 | 799.1588 | 14744.71 | 9898.02 | 16062.45 |
| CUST_25716_PI429010886 | N52933 | 9.18E-05 | 21 | 15 | | | | | | 41.14049 | 40.15861 | 51.52202 | 39.04691 | 40.50555 | 72.21756 | 44.87442 | 41.96975 | 39.414 | 41.14494 | 40.09244 | 48.87456 | 45.98092 | 43.83106 | 42.12575 | 57.99026 | 76.64595 | 66.07282 | 62.33854 | 47.3028 | 56.7825 | 45.25329 | 46.90544 | 73.90307 | 33.01824 | 37.81058 | 36.00037 |
| CUST_713_PI429010886 | N6893 | 9.23E-05 | 15 | 9 | | | | | | 20719.17 | 27464.78 | 54053.67 | 4318.11 | 6614.331 | 5985.533 | 38079.24 | 58446.35 | 72295.32 | 52551.15 | 43935.4 | 66128 | 37651.14 | 72732.64 | 47369.29 | 31716.06 | 32869.85 | 27142.29 | 21372.62 | 54708.78 | 23759.64 | 63688.85 | 60957.53 | 10831.29 | 87164.76 | 61640.76 | 103589.1 |
| CUST_34086_PI429010886 | M8733 | 9.7E-05 | 18 | 3 | | | | | | 67.77286 | 52.40185 | 62.26361 | 86.80804 | 67.86664 | 82.49999 | 47.93942 | 36.12998 | 44.25502 | 36.53021 | 33.58065 | 12.75496 | 47.8728 | 64.11406 | 37.11196 | 51.00626 | 44.97088 | 66.33801 | 33.23976 | 32.75472 | 48.22578 | 58.20859 | 53.95436 | 79.80797 | 50.20569 | 43.18074 | 35.67442 |
| CUST_38273_PI429010886 | M24900 | 9.73E-05 | 21 | 3 | | | | | | 11.44906 | 12.81122 | 25.86296 | 19.24083 | 30.8467 | 24.4716 | 15.21915 | 12.93585 | 20.5721 | 19.36786 | 19.87757 | 17.61934 | 11.33598 | 11.33779 | 18.02091 | 12.06791 | 14.90361 | 9.784863 | 17.34784 | 15.24563 | 13.023 | 19.54896 | 29.09612 | 13.17199 | 38.723 | 27.73533 | 22.84227 |
| CUST_46623_PI429010886 | M73200 | 0.000101 | 24 | 12 | 443708502 | hypothetical protein CAPTEDRAFT_185644 [Capitell | | | 7.91E-08 | 26.58056 | 28.86885 | 30.21934 | 23.26869 | 27.18226 | 27.4471 | 34.58049 | 30.18791 | 26.47532 | 33.42247 | 30.36684 | 43.09498 | 35.74458 | 41.07674 | 47.02813 | 34.7029 | 42.98324 | 38.13489 | 33.73552 | 40.86821 | 31.09756 | 35.37802 | 31.12763 | 19.65247 | 43.90259 | 30.87561 | 28.93334 |
| CUST_50532_PI429010886 | M90163 | 0.000108 | 15 | 9 | | | | | | 115.9402 | 144.0774 | 207.7747 | 159.32 | 163.2051 | 153.8239 | 175.5769 | 166.9868 | 167.6977 | 179.4229 | 180.1262 | 216.7674 | 154.1139 | 200.5697 | 132.7287 | 131.39 | 150.8429 | 164.8776 | 160.8644 | 154.0879 | 165.5902 | 161.6849 | 214.2583 | 166.8454 | 195.1331 | 169.0881 | 181.8579 |
| CUST_20246_PI429010886 | N43095 | 0.000109 | 12 | 9 | | | | | | 32.24298 | 25.67234 | 16.97989 | 43.81829 | 42.33366 | 41.11091 | 44.18048 | 33.71856 | 35.97953 | 50.0808 | 50.04999 | 40.57649 | 29.74822 | 7.077971 | 23.33698 | 37.34183 | 50.61028 | 32.01033 | 67.85743 | 33.22111 | 37.03334 | 43.08722 | 40.78034 | 82.86721 | 32.92321 | 25.82466 | 23.61475 |
| CUST_44716_PI429010886 | M66762 | 0.000111 | 18 | 12 | | | | | | 16.14566 | 16.97048 | 10.36994 | 12.47348 | 8.653249 | NA | 4.715895 | 11.54084 | 8.548078 | 19.05357 | 10.27406 | 12.49592 | 23.72179 | 18.34756 | 26.14965 | 12.49458 | 14.65365 | NA | 4.798002 | 13.08345 | NA | 11.0059 | 10.41181 | 11.99536 | 9.318343 | 7.281858 | 8.682476 |
| CUST_8968_PI429010886 | N23867 | 0.000112 | 24 | 15 | | | | | | 68.96573 | 89.59424 | 76.76597 | 101.2666 | 83.67885 | 94.70207 | 85.01801 | 82.65719 | 95.18251 | 134.5271 | 116.1174 | 164.4877 | 102.9204 | 120.363 | 117.3341 | 155.0502 | 133.0251 | 143.4947 | 92.69475 | 140.6429 | 130.8343 | 126.5103 | 109.2001 | 99.2894 | 100.5206 | 93.41435 | 106.9663 |
| CUST_36610_PI429010886 | M16348 | 0.000114 | 24 | 15 | | | | | | 5.91164 | 5.428277 | NA | NA | 4.014612 | NA | 5.961141 | 5.070641 | 7.107009 | 9.370503 | 10.94377 | 8.506994 | 9.676508 | 12.59239 | 9.610816 | 9.791272 | NA | 14.85731 | 16.54136 | 7.003494 | 13.7763 | 6.099554 | NA | 14.53785 | 9.534612 | NA | 4.968738 |
| CUST_36544_PI429010886 | M16194 | 0.000117 | 15 | 12 | 321459531 | hypothetical protein DAPPUDRAFT_309375 [Daphn | | | 8.23E-25 | 147.1503 | 198.8279 | 181.4093 | 130.3093 | 115.5186 | 133.7601 | 133.006 | 136.3782 | 145.0486 | 190.2685 | 146.3743 | 194.1717 | 154.8286 | 206.1326 | 208.5959 | 240.3735 | 151.7333 | 197.384 | 128.195 | 157.7478 | 140.2062 | 187.387 | 165.9249 | 142.2307 | 195.6579 | 135.0274 | 183.4152 |
| CUST_13571_PI429010886 | N31397 | 0.000118 | 24 | 15 | | | | | | 160.0546 | 177.8416 | 220.2228 | 108.8762 | 120.7762 | 213.6671 | 167.1087 | 228.1106 | 201.085 | 252.7105 | 224.858 | 431.462 | 206.9847 | 376.5573 | 380.7018 | 415.4709 | 277.7527 | 335.1917 | 248.0682 | 269.0643 | 250.7705 | 285.8071 | 238.5428 | 173.9653 | 269.9974 | 205.3314 | 189.1715 |
| CUST_16373_PI429010886 | N36123 | 0.000118 | 24 | 3 | | | | | | 14.40922 | 34.91043 | 23.34144 | 21.61111 | 17.47454 | 34.41491 | 15.39972 | 28.11091 | 15.96047 | 14.90976 | 8.367475 | 26.94089 | 10.7438 | 13.38001 | 4.507727 | 8.194751 | 7.518909 | 9.706284 | 8.927589 | 10.40557 | 18.84163 | 10.93801 | 29.30762 | 15.72409 | 23.11258 | 30.515 | 12.57708 |
| CUST_21983_PI429010886 | N46209 | 0.000121 | 18 | 12 | | | | | | 33.46305 | 39.00975 | 31.73942 | 27.53501 | 24.4665 | 24.72926 | 22.88862 | 29.87699 | 35.04514 | 36.606 | 35.24411 | 37.02998 | 42.22198 | 37.74522 | 37.74031 | 37.52021 | 31.88231 | 23.59619 | 29.45107 | 34.87551 | 40.06518 | 29.54391 | 28.45711 | 23.80497 | 31.88231 | 19.78047 | 32.87464 |
| CUST_35296_PI429010886 | M12167 | 0.000122 | 15 | 9 | | | | | | 278.5705 | 353.6127 | 326.1708 | 223.1189 | 259.2819 | 254.7271 | 315.2378 | 331.1696 | 368.7924 | 448.0031 | 274.1119 | 420.5887 | 311.7466 | 450.8925 | 339.6011 | 305.2873 | 235.2484 | 296.0927 | 308.7717 | 266.851 | 270.707 | 391.2629 | 379.0729 | 248.2496 | 467.8704 | 364.8104 | 411.1682 |
| CUST_25453_PI429010886 | N52446 | 0.000122 | 18 | 3 | | | | | | 52.43379 | 68.98284 | 47.82652 | 72.4241 | 53.12725 | 70.54693 | 61.40781 | 51.50234 | 41.7785 | 47.25743 | 33.7142 | 42.24487 | 31.1045 | 17.5491 | 25.29581 | 50.37311 | 51.75281 | 48.57108 | 49.20588 | 41.0381 | 55.71823 | 40.92745 | 72.6524 | 59.59032 | 58.81878 | 55.74802 | 38.94819 |
| CUST_39873_PI429010886 | M46045 | 0.000123 | 24 | 3 | | | | | | 4635.589 | 14035.86 | 8597.6 | 23818.16 | 12762.11 | 11583.53 | 9087.177 | 11243.07 | 4348.32 | 4827.614 | 10130.67 | 8827.561 | 3154.98 | 6767.816 | 9739.086 | 3925.917 | 1647.631 | 2809.847 | 4729.674 | 2118.286 | 8974.042 | 9448.983 | 10190.76 | 7974.043 | 5875.497 | 5635.242 | 8378.604 |
| CUST_42802_PI429010886 | M61293 | 0.000124 | 24 | 15 | | | | | | 26.21698 | 24.95013 | 23.01368 | 16.20611 | 17.98995 | 18.32903 | 24.40411 | 25.00056 | 23.29073 | 20.75304 | 18.8495 | 25.36437 | 22.94743 | 31.87606 | 31.24487 | 38.32695 | 50.0808 | 33.16699 | 25.41068 | 24.54416 | 31.00851 | 27.24651 | 12.89178 | 20.82676 | 26.72979 | 26.62834 | 26.4818 |
| CUST_2240_PI429010886 | N11945 | 0.000125 | 18 | 6 | 321455300 | hypothetical protein DAPPUDRAFT_64687 [Daphnia | | | 1.81E-51 | NA | 79.80797 | 48.20166 | 184.3916 | 271.7719 | 136.3625 | 142.2559 | 969.5225 | 936.0363 | 128.2056 | 290.5996 | 427.9524 | 293.3716 | 68.72308 | 227.5328 | 5.089219 | 105.3783 | 5.617915 | 111.8286 | 435.9739 | 527.7394 | NA | 276.2901 | 242.6822 | NA | 538.667 | 903.546 |
| CUST_714_PI429010886 | N6894 | 0.000125 | 15 | 9 | | | | | | 20102.84 | 32708.63 | 45401.69 | 4461.585 | 7248.432 | 4706.053 | 32330.94 | 52978.01 | 64571.61 | 58715.07 | 43024.52 | 73076.54 | 39731.64 | 81423.55 | 63067.91 | 31148.55 | 26037.41 | 26406.52 | 27975.24 | 49052.57 | 26532.79 | 64571.61 | 55819.51 | 10200.91 | 76927.64 | 58446.35 | 81077.7 |
| CUST_32645_PI429010886 | M4677 | 0.000126 | 12 | 9 | 321462910 | hypothetical protein DAPPUDRAFT_307535 [Daphn | Protein metabolism | Proteolysis | 6.83E-62 | 472.1948 | 315.3784 | 17.08982 | 353.3873 | 18.61312 | 35.1468 | 14.58501 | 333.4458 | 261.8083 | 587.1049 | 404.8823 | 897.4259 | 17.10365 | 21.34103 | NA | 6.433191 | 7.509358 | 216.8689 | 813.4135 | 288.4954 | 307.2905 | 791.8244 | 530.9675 | 661.3119 | 278.7763 | 402.2126 | 484.2538 |
| CUST_19676_PI429010886 | N41996 | 0.000126 | 18 | 12 | | | | | | 94.77604 | 84.89465 | 106.0522 | 89.98836 | 81.21391 | 85.62903 | 67.79099 | 88.42631 | 96.9916 | 61.48326 | 104.7823 | 144.7304 | 114.4015 | 112.7975 | 118.3064 | 116.9508 | 107.6119 | 108.8006 | 140.9566 | 109.4262 | 88.72989 | 93.35337 | 61.07112 | 66.26094 | 101.3304 | 95.81155 | 99.31284 |
| CUST_15230_PI429010886 | N34232 | 0.000127 | 21 | 15 | | | | | | 476.4015 | 559.1604 | 539.1803 | 482.753 | 447.2563 | 504.7239 | 407.5311 | 425.6417 | 431.93 | 507.5628 | 437.8204 | 541.117 | 472.5516 | 558.5528 | 590.4824 | 661.1589 | 585.0822 | 509.7336 | 550.2491 | 506.9726 | 524.4761 | 568.0071 | 513.797 | 532.1598 | 557.4875 | 450.3936 | 469.0438 |
| CUST_39150_PI429010886 | M35302 | 0.000128 | 15 | 12 | 493789484 | hypothetical protein, partial [Lactobacillus iners] >g | | | 9.09E-08 | 6744.801 | 8228.765 | 9739.086 | 2287.157 | 4988.507 | 1989.387 | 13673.74 | 14035.86 | 22307.76 | 11413.33 | 7701.667 | 8378.604 | 40638.34 | 36398.63 | 25407.19 | 22631.71 | 13339.53 | 4743.467 | 7266.955 | 6744.801 | 6168.628 | 12313.28 | 9398.296 | 6189.048 | 35569.55 | 6600.16 | 46511.8 |
| CUST_10210_PI429010886 | N25881 | 0.00013 | 15 | 9 | | | | | | 23759.64 | 33418.81 | 58209.54 | 5650.794 | 8255.78 | 6122.745 | 33059.64 | 54708.78 | 76141.25 | 66663.04 | 48876.8 | 72295.32 | 43755.93 | 72295.32 | 54992.02 | 35818.08 | 31716.06 | 29992.45 | 27353.46 | 58446.35 | 32528.46 | 74289.93 | 61341.21 | 9992.798 | 93952.87 | 58936.46 | 99779.5 |
| CUST_25532_PI429010886 | N52581 | 0.000131 | 18 | 3 | | | | | | 9.896666 | 15.28811 | 15.59665 | 14.62912 | 12.85526 | 16.43063 | 9.315276 | 13.48931 | 11.45088 | 8.848997 | 10.82989 | 7.90501 | 10.13647 | NA | 5.202739 | 8.951802 | 13.51055 | 10.20858 | 6.967135 | 7.665956 | 13.87348 | 16.33892 | 16.51743 | 23.531 | NA | 12.35209 | 8.434609 |
| CUST_29212_PI429010886 | N59407 | 0.000131 | 15 | 12 | | | | | | 35.18679 | 39.39152 | 51.57463 | 27.76975 | 33.38822 | 30.24466 | 29.77206 | 36.53489 | 35.05495 | 38.34742 | 29.86056 | 151.1026 | 61.55497 | 46.75333 | 43.63321 | 36.19462 | 34.02862 | 36.73877 | 36.5186 | 33.38385 | 28.7253 | 33.80408 | 43.09035 | 26.0691 | 53.77942 | 43.29309 | 53.8551 |
| CUST_32095_PI429010886 | M3220 | 0.000131 | 15 | 9 | | | | | | 65.99023 | 86.97645 | 65.52616 | 57.30764 | 56.80303 | 51.87045 | 58.90324 | 58.08045 | 57.46153 | 91.39106 | 81.13979 | 80.68886 | 68.64401 | 83.48026 | 59.90035 | 63.11008 | 64.78587 | 59.00313 | 57.21451 | 74.40778 | 58.79065 | 77.70756 | 72.20186 | 58.22987 | 90.60771 | 66.75063 | 71.82501 |
| CUST_51270_PI429010886 | M92879 | 0.000131 | 15 | 6 | | | Symbiosis, encompassing mutualism through parasitism | Other | | 24.20666 | 19.84167 | 21.7344 | 37.30837 | 35.68147 | 60.95703 | 50.65259 | 122.2713 | 77.63254 | 103.3151 | 64.91729 | 70.27659 | 55.32113 | 28.60108 | 44.9603 | 31.18382 | 44.74185 | 23.50136 | 71.07701 | 94.33627 | 194.9822 | 82.21549 | 75.17352 | 64.94243 | 22.35102 | 55.6932 | 74.97848 |
| CUST_51673_PI429010886 | M94531 | 0.000137 | 24 | 18 | | | | | | 16.1391 | 9.787782 | NA | 4.474584 | 9.501422 | 7.916656 | 16.90946 | 7.379797 | 8.0157 | 10.69206 | 21.89979 | 13.69809 | 8.096609 | NA | 8.275583 | 32.26938 | 22.11226 | 31.14323 | 23.32 | 26.65768 | 24.57313 | 18.35021 | 38.27387 | 6.077986 | 17.7958 | 17.11186 | 13.43349 |
| CUST_52430_PI429010886 | M97422 | 0.000138 | 12 | 0 | | | | | | 164.0893 | 162.1587 | 187.1474 | 175.8509 | 142.7507 | 150.286 | 124.513 | 159.0925 | 132.1519 | 133.1717 | 146.1977 | 160.804 | 314.246 | 202.5331 | 121.3613 | 158.2183 | 146.0723 | 156.1462 | 121.2504 | 134.162 | 127.7879 | 151.9486 | 169.8261 | 120.3747 | 179.0372 | 191.9229 | 182.6137 |
| CUST_47432_PI429010886 | M76089 | 0.000141 | 21 | 18 | | | | | | 41.36078 | 41.39181 | 46.1565 | 35.71039 | 20.38809 | 34.09884 | 18.57026 | 18.52898 | 23.65987 | 21.83532 | 21.94699 | 94.23139 | 36.40505 | 62.69277 | 41.83506 | 33.02612 | 40.85343 | 38.8107 | 45.04433 | 65.85741 | 41.06525 | 41.79247 | 28.01868 | 39.23735 | 34.14111 | 37.67218 | 37.77885 |
| CUST_12748_PI429010886 | N29978 | 0.000143 | 21 | 3 | | | | | | 1534.021 | 1888.53 | 1940.549 | 2729.519 | 2212.175 | 1873.43 | 1663.063 | 1012.516 | 1714.256 | 2086.783 | 981.4865 | 1010.24 | 394.4542 | 1088.106 | 645.1219 | 1377.193 | 757.2157 | 922.8399 | 785.0368 | 415.7405 | 2441.116 | 1597.443 | 2518.59 | 962.8744 | 2606.005 | 2254.33 | 1406.196 |
| CUST_44277_PI429010886 | M65466 | 0.000146 | 12 | 3 | | | | | | 11.84191 | 13.26879 | 22.6017 | 19.48202 | 28.70795 | 34.55538 | 20.00172 | 13.73363 | 16.44746 | 15.40175 | 9.366783 | NA | NA | 10.04937 | 11.8505 | 18.12511 | 66.87846 | 47.85374 | 16.25197 | 14.9864 | NA | 6.221163 | 5.132251 | 14.17556 | 11.30787 | 10.26419 | 9.996492 |
| CUST_51477_PI429010886 | M93668 | 0.000156 | 24 | 3 | | | | | | 38.30389 | 44.28069 | 30.66153 | 39.65895 | 35.77567 | 116.1033 | 33.92714 | 29.77528 | 24.35681 | 32.10376 | 18.50615 | 15.80258 | 14.75975 | 25.47702 | 9.648135 | 38.1599 | 32.00329 | 15.06859 | 25.25393 | 25.74383 | 15.228 | 31.62724 | 26.10969 | 30.13575 | 35.75443 | 40.87212 | 21.65726 |
| CUST_21666_PI429010886 | N45620 | 0.000156 | 24 | 15 | | | | | | 64.47443 | 57.41489 | 44.25502 | 50.8348 | 54.5454 | 57.70169 | 37.20061 | 50.80203 | 38.76201 | 61.75222 | 66.938 | 54.89215 | 62.24459 | 57.62155 | 47.17722 | 86.38663 | 68.2813 | 66.15125 | 71.00324 | 64.10151 | 64.1319 | 75.62563 | 57.60056 | 55.54708 | 67.89063 | 45.62376 | 58.36968 |
| CUST_50682_PI429010886 | M90639 | 0.000157 | 21 | 9 | | | | | | 26.61723 | 30.16372 | 35.38892 | 38.09477 | 34.119 | 48.71321 | 47.03357 | 75.06598 | 46.12593 | 48.16535 | 74.58008 | 64.70488 | 52.5097 | 36.19875 | 39.39544 | 28.35396 | 53.59509 | 34.7772 | 38.73964 | 106.6808 | 47.47988 | 33.7306 | 29.32288 | 34.03186 | 45.51068 | 46.16276 | 64.77801 |
| CUST_32427_PI429010886 | M4094 | 0.000159 | 21 | 0 | | | | | | 1450.058 | 1127.217 | 1574.342 | 936.7815 | 764.7939 | 946.2082 | 907.4672 | 748.7335 | 748.9581 | 960.3576 | 826.4016 | 1071.809 | 733.3828 | 916.49 | 930.6107 | 1289.132 | 1059.917 | 942.6383 | 1328.909 | 891.3926 | 1010.922 | 1063.907 | 1013.272 | 1115.083 | 1031.749 | 783.5804 | 873.1071 |
| CUST_47034_PI429010886 | M74605 | 0.000159 | 21 | 3 | | | | | | 38.36859 | 28.73729 | 37.70021 | 47.35331 | 38.19149 | 43.29309 | 45.95892 | 22.35568 | 35.54095 | 45.9505 | 43.85239 | 24.4665 | 30.65659 | 20.49973 | 29.18222 | 42.20687 | 27.33369 | 25.80821 | 24.92567 | 16.01119 | 28.47434 | 40.99957 | 27.46314 | 25.93979 | 72.55294 | 47.12699 | 75.84454 |
| CUST_43587_PI429010886 | M63421 | 0.000162 | 12 | 9 | 54042615 | arthrodial cuticle protein AMP6.0 [Callinectes sapid | | | 3.27E-15 | 1246.965 | 1817.335 | 1529.572 | 876.7586 | 448.4129 | 549.794 | 1659.929 | 1771.34 | 2708.301 | 4297.995 | 1743.746 | 3024.762 | 2022.724 | 2800.799 | 1750.319 | 803.7954 | 1901.328 | 786.706 | 861.1597 | 1608.297 | 1065.852 | 3485.611 | 2343.419 | 1988.345 | 3177.58 | 852.9478 | 2658.303 |
| CUST_41879_PI429010886 | M58780 | 0.000163 | 24 | 12 | | | | | | 118.8194 | 99.24333 | 112.5941 | 126.5833 | 150.1848 | 172.854 | 118.4488 | 74.93674 | 122.3475 | 166.8454 | 156.8208 | 220.0411 | 259.2453 | 203.7035 | 192.5048 | 190.6989 | 167.5706 | 183.4917 | 179.0014 | 157.4172 | 226.6782 | 174.6614 | 190.8837 | 147.2113 | 164.9622 | 137.0338 | 86.24828 |
| CUST_12158_PI429010886 | N29022 | 0.000163 | 24 | 0 | | | | | | 55.50473 | 106.1871 | 83.70947 | 38.65653 | 69.68816 | 26.16897 | 37.56967 | 46.16276 | 11.99669 | 164.6926 | 25.51724 | 5.739453 | 36.606 | 3.89124 | 19.47657 | 12.79969 | 36.87977 | 10.59486 | 42.37752 | 26.96892 | 34.0757 | 52.59799 | 66.16504 | 47.5835 | 31.86956 | 104.8657 | 79.80104 |
| CUST_46399_PI429010886 | M72543 | 0.000163 | 21 | 12 | | | | | | 49.16013 | 56.43061 | 56.22273 | 36.50455 | 36.22709 | 39.7525 | 34.42762 | 41.15103 | 39.90155 | 47.76338 | 49.04149 | 44.75473 | 114.4136 | 50.19562 | 74.93674 | 80.37733 | 52.22902 | 65.51399 | 49.99255 | 61.11029 | 39.14518 | 46.95122 | 43.51237 | 49.92232 | 45.38512 | 29.02323 | 71.59535 |
| CUST_34786_PI429010886 | M10661 | 0.000165 | 24 | 21 | | | | | | 60.33388 | 82.86721 | 67.31728 | 41.9189 | 53.04998 | 50.00256 | 44.56286 | 45.88881 | 48.11074 | 55.76383 | 49.28963 | 58.482 | 48.49494 | 52.00681 | 47.56602 | 72.51875 | 52.56971 | 66.09773 | 55.36009 | 62.91706 | 71.16635 | 60.95084 | 75.32357 | 67.84362 | 76.668 | 51.23628 | 71.33949 |
| CUST_7413_PI429010886 | N21510 | 0.000165 | 12 | 9 | 242020240 | senecionine N-oxygenase, putative [Pediculus huma | Metabolic process | Oxidation-reduction process | 5.47E-59 | 218.636 | 113.0678 | 162.0012 | 123.1087 | 162.0241 | 99.69352 | 207.7427 | 259.9699 | 246.7296 | 233.3626 | 271.6624 | 294.9448 | 205.9317 | 172.6647 | 128.969 | 156.8398 | 216.807 | 162.0012 | 193.3576 | 219.7388 | 221.4718 | 237.1988 | 252.7105 | 216.6058 | 192.1174 | 216.6952 | 203.4225 |
| CUST_24845_PI429010886 | N51349 | 0.000166 | 24 | 21 | 405974301 | hypothetical protein CGI_10026724 [Crassostrea gig | | | 8.46E-07 | 82.21549 | 68.84477 | 80.75638 | 67.07557 | 55.52574 | 70.44162 | 41.05593 | 68.4981 | 57.664 | 57.61472 | 61.59565 | 69.74095 | 73.63983 | 64.60382 | 71.45222 | 80.25744 | 72.4241 | 81.41269 | 61.64915 | 117.487 | 64.34064 | 74.10963 | 93.70583 | 95.06463 | 75.97215 | 70.31729 | 81.45472 |
| CUST_2191_PI429010886 | N11830 | 0.000167 | 15 | 0 | | | Protein metabolism | Cellular protein modification process | | 609.6635 | 650.2354 | 671.7237 | 525.3464 | 563.5263 | 851.7359 | 213.7556 | 261.2742 | 224.6063 | 519.1962 | 588.5777 | 501.5691 | 179.9161 | 486.0261 | 329.6909 | 630.1031 | 602.272 | 610.6415 | 436.4909 | 857.3719 | 471.0971 | 427.5571 | 304.5092 | 348.0871 | 550.4344 | 417.0088 | 414.8654 |
| CUST_29492_PI429010886 | N59972 | 0.000167 | 15 | 3 | | | | | | 27.19088 | 23.40827 | 60.19028 | 45.00065 | 39.19383 | 45.18256 | 36.78203 | 33.38032 | 22.6653 | 18.35581 | 28.37808 | 3.441743 | 10.99151 | 14.07399 | 7.446083 | 17.08079 | 29.5457 | 16.50592 | 42.99343 | 21.99795 | 41.43739 | 12.04909 | 50.92195 | 22.498 | 15.8658 | 8.845266 | 6.567114 |
| CUST_7700_PI429010886 | N21945 | 0.000169 | 15 | 0 | | | | | | 815.0535 | 914.6851 | 720.0434 | 669.8776 | 763.2137 | 765.4435 | 617.1846 | 642.1649 | 666.6962 | 895.8021 | 675.7327 | 776.8406 | 678.2021 | 853.9995 | 705.5663 | 1147.081 | 778.415 | 914.3958 | 778.5698 | 682.7483 | 680.3748 | 798.8942 | 551.5652 | 557.1607 | 825.1657 | 635.9764 | 646.0018 |
| CUST_33584_PI429010886 | M7338 | 0.000172 | 24 | 0 | | | | | | 150.0554 | 149.0167 | 143.4291 | 113.0325 | 114.9603 | 135.6803 | 122.2713 | 125.027 | 122.9865 | 101.4215 | 91.16786 | 97.96017 | 87.63509 | 136.4264 | 120.5952 | 146.722 | 153.9132 | 137.0179 | 123.9272 | 105.1 | 117.6603 | 206.3446 | 117.1631 | 126.1607 | 215.4042 | 146.5997 | 200.1336 |
| CUST_48881_PI429010886 | M82686 | 0.000172 | 15 | 12 | | | | | | 38.0368 | 100.1156 | 69.16983 | 42.88135 | 47.96622 | 39.126 | 70.36778 | 90.0502 | 59.19392 | 101.0393 | 83.63285 | 52.93772 | 195.3201 | 97.91604 | 117.2582 | 77.84895 | 126.8628 | 47.63195 | 56.77783 | 57.77025 | 36.60913 | 62.17487 | 57.46153 | 73.09177 | 114.9498 | 49.93144 | 64.30394 |
| CUST_24973_PI429010886 | N51567 | 0.000173 | 24 | 18 | 512856749 | PREDICTED: uncharacterized protein K02A2.6-like, p | | | 7.9E-14 | 21.44476 | 11.81616 | 23.36677 | 19.86545 | 20.10077 | 16.73161 | 16.69153 | 20.38984 | 18.64626 | 26.61439 | 29.9579 | 24.72926 | 19.75359 | 17.55503 | 31.81303 | 31.92933 | 25.56371 | 21.09489 | 30.56193 | 29.07551 | 37.83723 | 39.47942 | 30.07342 | 29.26565 | 26.19371 | 21.35775 | 23.61236 |
| CUST_8873_PI429010886 | N23732 | 0.000173 | 15 | 9 | 260799069 | hypothetical protein BRAFLDRAFT_124991 [Branchi | | | 1.37E-45 | 19.04736 | 35.41295 | 31.46674 | 19.87086 | 17.32667 | 14.40255 | 28.8539 | 19.85963 | 25.45721 | 35.10761 | 26.67547 | 42.35648 | 21.96101 | 24.67398 | 35.18679 | 31.267 | 25.47915 | 25.24216 | 19.26616 | 15.43461 | 19.82309 | 25.749 | 21.44644 | 13.07005 | 30.10928 | 31.87279 | 29.05988 |
| CUST_6036_PI429010886 | N19540 | 0.000175 | 18 | 12 | | | Protein metabolism | Proteolysis | | 288.8593 | 374.4434 | 264.4703 | 265.9049 | 245.5656 | 218.037 | 234.9764 | 352.188 | 350.5877 | 403.4054 | 390.7936 | 512.9488 | 450.3936 | 506.7573 | 446.3108 | 433.8707 | 233.5227 | 308.5953 | 326.5696 | 626.4662 | 322.593 | 354.4714 | 252.5773 | 209.0287 | 437.8204 | 304.1627 | 358.9421 |
| CUST_38836_PI429010886 | M31066 | 0.000175 | 15 | 9 | | | | | | 22457.41 | 35818.08 | 48465.49 | 6717.481 | 8483.725 | 5181.094 | 31492.49 | 53822.38 | 70499.75 | 65192.57 | 52713.61 | 71527.46 | 43573.57 | 78081.9 | 70048.09 | 40254.56 | 31801.7 | 30443.79 | 35065.14 | 58936.46 | 34870.47 | 65192.57 | 57482.98 | 10366.97 | 100388.2 | 63688.85 | 95014.94 |
| CUST_13279_PI429010886 | N30902 | 0.000175 | 15 | 9 | | | | | | 48.53823 | 54.40089 | 61.62124 | 44.99497 | 45.7637 | 48.59467 | 41.23729 | 48.91209 | 56.39846 | 79.48728 | 64.45839 | 68.63903 | 50.90637 | 75.33028 | 48.41716 | 73.23407 | 51.93952 | 50.00256 | 52.93292 | 45.91808 | 47.82182 | 49.05985 | 50.00676 | 38.00715 | 88.6791 | 66.13621 | 89.54002 |
| CUST_54087_PI429010886 | M104483 | 0.000177 | 21 | 3 | | | | | | 29.27339 | NA | 17.47741 | 34.58049 | 30.46087 | 29.32561 | 15.87666 | 19.02231 | 24.26769 | 19.76503 | 10.0999 | 12.54065 | 12.91176 | NA | 19.24305 | 30.12812 | 16.64136 | 17.52653 | 13.94631 | 33.66812 | 20.51216 | 27.24907 | 49.95685 | 24.97091 | 36.79834 | 43.1896 | 42.11724 |
| CUST_25663_PI429010886 | N52831 | 0.000178 | 18 | 12 | 469647763 | ryanodine receptor [Ostrinia furnacalis] | | | 1.66E-46 | 161.6167 | 159.9075 | 166.4452 | 151.9691 | 157.9764 | 178.3785 | 123.2283 | 150.3004 | 138.1676 | 196.1002 | 185.7359 | 214.921 | 170.3395 | 179.8682 | 226.9159 | 214.7684 | 236.6906 | 171.0028 | 166.519 | 213.6671 | 147.8481 | 170.2922 | 180.6894 | 151.6596 | 133.7926 | 146.3743 | 160.778 |
| CUST_11321_PI429010886 | N27619 | 0.00018 | 15 | 9 | | | | | | 17645.1 | 30617.99 | 37972.43 | 6833.331 | 8404.925 | 3421.62 | 23030.7 | 35264.41 | 58936.46 | 50017.11 | 40846.53 | 66663.04 | 34379.32 | 64571.61 | 56816.44 | 40986.13 | 19571.84 | 26594.86 | 29816.7 | 54053.67 | 35065.14 | 65559.81 | 44666.31 | 7221.522 | 96143.7 | 77297.7 | 91927.09 |
| CUST_13246_PI429010886 | N30842 | 0.00018 | 12 | 0 | | | | | | 261.8083 | 229.9936 | 208.936 | 199.6816 | 197.0135 | 216.1505 | 160.5038 | 196.7037 | 188.3253 | 204.9184 | 178.5686 | 191.3918 | 206.3446 | 226.1214 | 227.5634 | 235.9573 | 211.6272 | 238.2441 | 197.6731 | 158.101 | 193.5271 | 255.0746 | 202.6087 | 203.453 | 279.924 | 229.0179 | 204.1851 |
| CUST_55227_PI429010886 | M109198 | 0.00018 | 18 | 15 | | | | | | 668.0541 | 400.8956 | 552.0613 | 134.7426 | 480.7772 | 402.7742 | 585.5822 | 347.1114 | 441.6462 | 860.1543 | 409.4886 | 545.8082 | 918.9933 | 851.7359 | 673.5866 | 594.2743 | 919.6285 | 962.2957 | 603.9714 | 456.0552 | 456.7992 | 571.7132 | 267.2122 | 318.2985 | 206.1326 | 453.79 | 658.8544 |
| CUST_38850_PI429010886 | M31218 | 0.000181 | 15 | 12 | 493789484 | hypothetical protein, partial [Lactobacillus iners] >g | | | 2.45E-11 | 144.5986 | 276.2073 | 215.8652 | 76.2979 | 74.25035 | 34.78955 | 703.6838 | 474.7169 | 1036.891 | 416.7544 | 169.5547 | 379.0179 | 1780.629 | 1651.385 | 1059.917 | 1020.365 | 701.6345 | 148.7085 | 268.3232 | 197.6933 | 163.162 | 350.5238 | 298.7079 | 302.0506 | 2289.879 | 215.6344 | 3477.696 |
| CUST_12082_PI429010886 | N28897 | 0.000182 | 24 | 21 | 307183663 | WD repeat-containing protein 7 [Camponotus florid | | | 1.75E-22 | 21.76618 | 24.37698 | 26.45407 | 25.59155 | 24.43609 | 26.11231 | 23.4957 | 19.63631 | 19.95159 | 29.0109 | 32.05062 | 35.63135 | 21.42649 | 30.96598 | 30.12445 | 30.31136 | 24.43609 | 32.41678 | 29.16623 | 32.8525 | 38.14533 | 36.37866 | 33.87876 | 35.01361 | 29.16341 | 30.45321 | 32.79731 |
| CUST_2450_PI429010886 | N12377 | 0.000183 | 15 | 12 | | | | | | 80.18725 | 92.09827 | 101.4497 | 98.26203 | 67.64754 | 74.52928 | 76.33468 | 93.33448 | 75.98951 | 111.0142 | 70.96812 | 114.9975 | 136.0526 | 139.6592 | 149.9119 | 99.49562 | 64.54153 | 71.37464 | 63.46235 | 69.97891 | 99.48375 | 99.05161 | 70.77093 | 63.88437 | 110.6199 | 118.1013 | 113.9625 |
| CUST_15436_PI429010886 | N34577 | 0.000184 | 15 | 9 | | | | | | 22.30707 | 25.78586 | 31.12392 | 15.95324 | 17.49801 | 16.82798 | 19.16951 | 17.91608 | 27.07492 | 33.55523 | 24.73831 | 34.10344 | 24.66303 | 36.01332 | 26.80106 | 26.53007 | 14.32589 | 23.64962 | 26.03903 | 19.5508 | 21.15997 | 31.42286 | 23.22371 | 20.27847 | 32.50043 | 32.69544 | 27.23432 |
| CUST_2993_PI429010886 | N13563 | 0.000184 | 12 | 6 | | | | | | 14.37169 | 25.58242 | 13.91822 | 26.58995 | 36.50455 | 42.37343 | 56.11071 | 28.80979 | 46.81158 | 28.84595 | 49.55874 | 74.80865 | 25.35894 | 21.8835 | 24.36596 | 15.18342 | 30.83339 | 40.14953 | 65.60373 | 30.35236 | 31.65427 | 17.34013 | 26.28688 | 28.79502 | 23.65401 | 27.8201 | 11.35281 |
| CUST_35305_PI429010886 | M12187 | 0.000186 | 12 | 9 | | | | | | 118.6904 | 41.50969 | 36.7129 | 16.75121 | 9.360222 | 20.32136 | 15.10731 | NA | 188.8504 | 692.3236 | 223.1512 | 1004.727 | NA | 120.8512 | 49.75477 | 36.28401 | NA | 29.49942 | 230.2231 | 208.0738 | 231.7343 | 198.9664 | 48.40257 | NA | 84.57304 | 46.09323 | 140.6429 |
| CUST_359_PI429010886 | N4421 | 0.000187 | 15 | 12 | 220172369 | cuticle protein [Rimicaris exoculata] | | | 2.35E-29 | 86.19466 | 4461.585 | 6407.392 | 3079.902 | 346.8405 | 347.8665 | 80.02167 | 75.64933 | 72.05819 | 5515.427 | 435.1229 | 161.355 | 7509.168 | 1060.287 | 6174.548 | 103.7315 | 89.88488 | 98.43882 | 94.51496 | 126.7767 | 67.35597 | 80.68026 | 61.32192 | 53.32755 | 103.457 | 8854.389 | 70.29878 |
| CUST_53629_PI429010886 | M102570 | 0.000187 | 18 | 15 | | | | | | 46.00478 | 54.74747 | 80.38938 | 45.73272 | 38.13489 | 57.03251 | 72.40334 | 71.81768 | 67.59725 | 74.60621 | 43.56595 | 102.4647 | 61.89778 | 121.6835 | 108.5891 | 150.3171 | 76.68397 | 83.26471 | 57.73367 | 50.36639 | 43.53998 | 46.89105 | 50.81627 | 36.04391 | 67.05664 | 43.67532 | 69.03435 |
| CUST_20990_PI429010886 | N44427 | 0.000189 | 24 | 6 | | | | | | 77.69906 | 51.39386 | 69.15357 | 74.01735 | 70.90857 | 100.6811 | 114.3333 | 73.5908 | 127.3882 | 76.01313 | 52.83011 | 46.49731 | 86.66049 | 66.15125 | 67.30873 | 58.67766 | 50.20045 | 61.2083 | 67.27473 | 31.23466 | 48.76067 | 49.86955 | 49.9806 | 51.67197 | 62.18234 | 75.93804 | 56.39304 |
| CUST_44240_PI429010886 | M65361 | 0.00019 | 18 | 12 | | | | | | 6.383068 | 6.94159 | 16.58905 | 11.39273 | 4.679737 | NA | 12.60293 | 10.58518 | NA | 11.38971 | 13.47203 | 27.80089 | 20.41951 | 32.44398 | 24.15872 | 13.023 | 14.94965 | NA | 11.00785 | 15.77161 | 12.10746 | 8.233831 | 6.386719 | 9.526791 | 26.35513 | 18.23052 | 18.78752 |
| CUST_641_PI429010886 | N6502 | 0.000194 | 24 | 6 | | | | | | 287.5857 | 6091.908 | 447.351 | 1458.732 | 294.1484 | 27.42777 | 6587.101 | 225.8926 | 5819.009 | 2580.486 | 187.9811 | NA | 2984.905 | 398.993 | 1418.381 | 310.518 | NA | 181.7953 | 38.53966 | 396.1095 | 19.70849 | 6.767462 | 11.8448 | 10.96329 | 26.84868 | 684.5685 | 13.60949 |
| CUST_35532_PI429010886 | M12809 | 0.000203 | 24 | 0 | | | | | | 48.53244 | 38.04684 | 30.88907 | 32.17296 | 32.58533 | 40.59586 | 37.5119 | 31.07494 | 28.48649 | 43.50197 | 16.00379 | 41.57079 | 17.01109 | 22.66789 | 21.04144 | 26.05696 | 16.02878 | 16.81213 | 33.35513 | 22.72441 | 31.55772 | 46.17281 | 34.55538 | 35.32273 | 48.53823 | 37.40747 | 44.95044 |
| CUST_27473_PI429010886 | N56119 | 0.000206 | 21 | 18 | | | | | | 20.56111 | 17.96537 | 16.68074 | 19.91752 | 14.9294 | 13.49109 | 15.72131 | 11.18163 | 12.83327 | 11.46217 | 8.617079 | 16.5954 | 16.9221 | 9.602601 | 24.56849 | 28.29582 | 11.53748 | 14.81806 | 19.84686 | 30.14577 | 18.30885 | 20.61498 | 22.18079 | 19.40236 | 13.50427 | 16.60676 | 15.65479 |
| CUST_50447_PI429010886 | M89856 | 0.000211 | 24 | 3 | | | | | | 46.36142 | 108.0902 | 126.5686 | 65.09833 | 102.4176 | 53.30382 | 23.13968 | 81.70156 | 88.09249 | 22.70524 | 21.56682 | 14.80676 | 18.53102 | 11.43263 | 9.190342 | 27.9072 | 33.68931 | 41.19265 | 39.78898 | 9.787782 | 14.70096 | 37.19805 | 22.67397 | 87.96508 | 29.96972 | 47.76338 | 38.41593 |
| CUST_39411_PI429010886 | M39014 | 0.000211 | 21 | 3 | 77743875 | Euphausia superba isolate DGGE band AntEuk13-5 | | | 2.2E-138 | 170126.9 | 155394.6 | 166443.6 | 185961.4 | 181047 | 175370.5 | 170974.8 | 168734.7 | 169441.2 | 212288.9 | 165207 | 164488.1 | 170974.8 | 163757.1 | 155394.6 | 156147.9 | 150939.2 | 156147.9 | 163136 | 146237.4 | 188114.2 | 134984.9 | 174460.2 | 173562.9 | 188114.2 | 180118 | 174460.2 |
| CUST_15628_PI429010886 | N34884 | 0.000212 | 24 | 18 | | | | | | NA | 11.95978 | 12.0315 | 12.84641 | 12.60126 | 8.148866 | 9.126873 | 7.357022 | 9.155769 | 4.552819 | 11.80651 | 14.90751 | 7.792392 | NA | 17.00562 | 18.15323 | 8.41226 | 12.77316 | 20.16852 | 21.87538 | 22.03473 | 13.49109 | NA | 19.1106 | 18.67218 | 21.16222 | 21.3486 |
| CUST_4329_PI429010886 | N16524 | 0.000214 | 21 | 15 | | | | | | 54.19041 | 53.83853 | 73.55011 | 21.30503 | 40.20059 | 46.55991 | 67.08317 | 47.29164 | 61.51764 | 43.86454 | 56.27804 | 64.52054 | 70.79063 | 76.13932 | 66.78979 | 71.91814 | 66.29488 | 95.67336 | 48.49058 | 43.31707 | 69.88371 | 93.69637 | 68.04642 | 57.59047 | 46.23463 | 35.27392 | 52.89103 |
| CUST_9288_PI429010886 | N24377 | 0.000217 | 24 | 0 | 332016384 | Putative ATP-dependent RNA helicase DDX49 [Acro | | | 1.87E-65 | 28.18417 | 29.45107 | 21.7968 | 17.37315 | 28.52476 | 18.93037 | 21.21967 | 18.49081 | 15.87666 | 25.9871 | 20.81295 | 15.59123 | 7.772166 | 20.99142 | 15.14528 | 14.17659 | 13.50427 | 12.83461 | 13.79851 | 19.59849 | 16.67878 | 21.13287 | 20.58696 | 24.22062 | 23.82095 | 15.48498 | 25.93179 |
| CUST_12039_PI429010886 | N28821 | 0.000218 | 21 | 3 | | | | | | 21721.24 | 39067.89 | 21011.69 | 46673.5 | 35686.35 | 29378.84 | 21864.7 | 21318.49 | 23420.74 | 21067.41 | 17031.04 | 27524.03 | 16156.64 | 19773.16 | 12762.11 | 25677.47 | 2264.822 | 18620.02 | 14205.89 | 20248.42 | 31231.03 | 25081.91 | 25407.19 | 16238.88 | 32800.86 | 36046.8 | 19960.08 |
| CUST_13156_PI429010886 | N30678 | 0.000222 | 15 | 12 | | | | | | 230.6845 | 221.3126 | 253.5864 | 142.5643 | 107.4484 | 102.0005 | 143.5589 | 227.3449 | 215.6784 | 245.0284 | 180.5686 | 215.4716 | 243.2097 | 298.1495 | 260.2651 | 248.8723 | 213.6947 | 144.0112 | 191.0327 | 219.4136 | 159.3003 | 217.2265 | 285.3112 | 145.8124 | 317.3299 | 221.9975 | 259.7703 |
| CUST_16111_PI429010886 | N35673 | 0.000223 | 18 | 3 | 328770135 | hypothetical protein BATDEDRAFT_35110 [Batrach | Protein metabolism | Protein folding | 1.35E-29 | 1016.851 | 829.8828 | NA | 2070.325 | 1455.397 | 1387.428 | 1109.205 | 154.2676 | 827.8275 | 8.037888 | 17.60822 | 9.289553 | 607.2425 | 641.7483 | 861.4839 | 1501.641 | 59.26817 | 1133.519 | 15.32478 | 477.5078 | 87.91683 | 1923.113 | NA | 1373.036 | 343.8588 | 152.4947 | 10.71609 |
| CUST_54325_PI429010886 | M105588 | 0.000225 | 21 | 6 | | | | | | 236.449 | 170.3395 | 273.5534 | 279.4425 | 202.8118 | 343.2786 | 558.9364 | 545.6531 | 383.5597 | 383.225 | 376.6382 | 398.132 | 295.9544 | 339.4134 | 249.5102 | 200.6692 | 488.7915 | 237.5773 | 384.5478 | 415.5762 | 269.2555 | 224.1286 | 224.5779 | 178.6287 | 228.6362 | 336.3354 | 353.4537 |
| CUST_52541_PI429010886 | M97867 | 0.000226 | 21 | 18 | | | | | | 378.3563 | 78.74635 | 68.04642 | 18.50437 | 18.96046 | 16.96167 | 19.44722 | 30.32196 | 22.82362 | 46.50141 | 20.39186 | 21.7761 | 37.83427 | 24.5556 | 22.5025 | 28.96949 | 32.72653 | 29.6615 | 25.59863 | 71.08447 | 77.05404 | 67.77787 | 40.41641 | 16.55113 | 22.86827 | 11.84611 | 15.5304 |
| CUST_26874_PI429010886 | N55028 | 0.000227 | 21 | 9 | | | | | | 138.7005 | 147.8113 | 74.88917 | 151.6596 | 169.9686 | 144.8798 | 281.8261 | 97.24592 | 247.9972 | 428.7852 | 174.0405 | 286.3951 | 238.941 | 297.4174 | 159.2127 | 278.9995 | 117.621 | 144.2346 | 139.7005 | 56.64713 | 129.2905 | 122.7034 | 140.8407 | 135.0409 | 116.1614 | 157.9764 | 158.1524 |
| CUST_3125_PI429010886 | N13847 | 0.000229 | 21 | 0 | | | | | | 97.93806 | 120.905 | 124.8573 | 106.4697 | 98.86383 | 112.6894 | 79.31367 | 82.49999 | 87.43516 | 85.67865 | 88.33792 | 115.0228 | 99.35803 | 96.10628 | 84.04395 | 110.192 | 117.4327 | 94.34866 | 97.32524 | 101.3567 | 107.4359 | 127.7418 | 108.8877 | 114.6834 | 97.78298 | 110.192 | 117.4728 |
| CUST_43136_PI429010886 | M62161 | 0.00023 | 21 | 3 | | | | | | 56.57261 | 38.48153 | 56.08637 | 61.74032 | 64.4873 | 65.93142 | 26.26013 | 40.50125 | 37.92303 | 55.38216 | 46.22113 | 62.89604 | 27.72017 | 43.29309 | 35.89324 | 52.25652 | 41.03316 | 58.38557 | 41.47521 | 37.00487 | 45.95551 | 42.70658 | 53.75298 | 42.56152 | 74.15763 | 86.43501 | 104.4054 |
| CUST_24735_PI429010886 | N51167 | 0.000233 | 21 | 15 | | | | | | 64.45055 | 55.11221 | 44.80736 | 38.63168 | 41.9046 | 58.75213 | 39.19727 | 36.00037 | 29.00747 | 53.07285 | 43.38432 | 49.39411 | 44.94077 | 46.85704 | 63.3033 | 67.02702 | 59.18336 | 54.73744 | 51.34688 | 63.27271 | 64.41721 | 36.8424 | 61.3315 | 47.3028 | 49.60877 | 40.95384 | 51.30486 |
| CUST_30480_PI429010886 | N61984 | 0.000238 | 21 | 0 | | | | | | 80.48768 | 63.01798 | 63.05147 | 47.2535 | 45.39483 | 75.44459 | 49.91574 | 46.87582 | 51.59241 | 59.99556 | 46.83381 | 59.84805 | 48.19061 | 62.6571 | 49.11669 | 61.71609 | 64.04369 | 64.77051 | 59.8939 | 55.87623 | 66.41721 | 66.81139 | 86.45155 | 66.47359 | 73.39754 | 55.70082 | 60.57719 |
| CUST_51003_PI429010886 | M91759 | 0.000239 | 24 | 21 | | | | | | 97.80592 | 107.5224 | 109.7874 | 77.24703 | 98.75692 | 93.58403 | 74.86726 | 82.08362 | 83.37454 | 105.4619 | 83.61604 | 90.18249 | 86.28978 | 92.18368 | 81.22317 | 105.6941 | 105.6711 | 99.36845 | 93.40468 | 99.22324 | 105.5208 | 97.04503 | 113.2243 | 112.3805 | 106.4489 | 114.5891 | 112.226 |
| CUST_39742_PI429010886 | M43763 | 0.000241 | 24 | 21 | 220172369 | cuticle protein [Rimicaris exoculata] | | | 6.19E-17 | 27.84917 | 35.26822 | 32.22977 | 26.09533 | 24.61213 | 42.11302 | 24.19014 | 20.29379 | 22.15622 | 30.35236 | 19.5882 | 19.30028 | 32.18039 | 24.0648 | 21.048 | 36.26174 | 37.83723 | 39.54096 | 63.51336 | 29.60636 | 31.28165 | 31.38813 | 43.69222 | 42.1732 | 44.29926 | 28.66914 | 39.27655 |
| CUST_15688_PI429010886 | N34977 | 0.000242 | 18 | 12 | | | | | | 110.5811 | 117.7023 | 125.2377 | 112.2136 | 105.6711 | 101.7455 | 93.02061 | 118.3632 | 120.2042 | 165.324 | 109.6802 | 150.7548 | 132.9126 | 173.2729 | 146.6794 | 131.6679 | 138.5572 | 127.8459 | 103.6413 | 144.5043 | 121.7904 | 96.52778 | 124.2716 | 105.0213 | 144.8143 | 104.6652 | 145.3659 |
| CUST_53513_PI429010886 | M102051 | 0.000243 | 15 | 12 | | | | | | 15.4798 | 18.2774 | 28.02151 | 12.32386 | 17.09735 | 20.99337 | 19.96619 | 21.07029 | 18.02091 | 26.97315 | 20.38379 | 26.41986 | 31.38593 | 38.63759 | 27.74359 | 24.04687 | 16.20103 | 19.85963 | 19.19726 | 17.76828 | 11.92804 | 25.0272 | 19.57585 | 21.49416 | 31.42286 | 16.64328 | 21.01141 |
| CUST_20617_PI429010886 | N43758 | 0.000243 | 15 | 9 | 321473766 | hypothetical protein DAPPUDRAFT_194337 [Daphn | Signal transduction | Other | 1.16E-31 | 13.79851 | 9.520915 | 44.76277 | 6.584593 | 10.35487 | 13.03254 | 31.8423 | 32.80956 | 39.58944 | 39.14912 | 13.91491 | 38.80417 | 21.3795 | 35.46388 | 28.10839 | 20.77886 | 20.59775 | 15.08066 | 13.01989 | 26.23144 | 13.75107 | 31.48445 | 48.19648 | 14.4448 | 49.20084 | 32.74092 | 52.66699 |
| CUST_13548_PI429010886 | N31367 | 0.000244 | 12 | 0 | | | | | | 65.39564 | 60.87936 | 60.49322 | 39.68514 | 37.37431 | 38.39579 | 30.10233 | 31.61999 | 30.6438 | 50.74126 | 38.2775 | 39.76167 | 42.28532 | 56.16844 | 57.85675 | 55.15882 | 41.80456 | 46.76098 | 31.52633 | 44.69546 | 38.96658 | 61.11029 | 35.87751 | 46.11982 | 58.34728 | 37.0175 | 54.5227 |
| CUST_5299_PI429010886 | N18513 | 0.000244 | 24 | 9 | 321475394 | hypothetical protein DAPPUDRAFT_308494 [Daphn | Metabolic process | Oxidation-reduction process | 4.3E-160 | 78.79452 | 96.66502 | 91.72554 | 122.5481 | 95.87365 | 88.69099 | 97.54257 | 158.635 | 103.9666 | 178.3288 | 120.3504 | 142.0799 | 105.4997 | 126.5833 | 119.1024 | 116.1911 | 85.141 | 72.85867 | 125.9794 | 194.747 | 104.5737 | 99.59438 | 140.3976 | 102.8187 | 67.83607 | 53.20866 | 76.94138 |
| CUST_29427_PI429010886 | N59841 | 0.000246 | 24 | 3 | | | | | | 3469.507 | 6646.048 | 5911.575 | 15086.66 | 7729.059 | 6767.816 | 6941.938 | 7568.915 | 2553.637 | 3630.71 | 6059.267 | 4345.199 | 1891.601 | 4348.32 | 4484.875 | 2218.572 | 1416.429 | 1470.792 | 3172.097 | 1942.982 | 6941.938 | 4041.003 | 7605.917 | 4959.867 | 3184.108 | 2842.143 | 4711.518 |
| CUST_3066_PI429010886 | N13725 | 0.000246 | 15 | 9 | | | | | | 123.8883 | 71.55597 | 278.8318 | 46.80174 | 63.64812 | 43.31707 | 159.2127 | 340.1906 | 547.9393 | 372.6528 | 527.4806 | 600.9036 | 210.4575 | 706.7803 | 276.9556 | 136.1246 | 181.6707 | 163.4951 | 121.3759 | 442.0725 | 172.213 | 946.4318 | 724.3304 | 168.7959 | 1069.225 | 292.6509 | 757.419 |
| CUST_57098_PI429010886 | E842 | 0.000248 | 24 | 0 | 402485602 | glyceraldehyde-3-phosphate dehydrogenase [Scylla | | | 7.54E-07 | 2856.456 | 4025.068 | 2840.45 | 2850.368 | 2879.829 | 3022.502 | 2257.795 | 2817.021 | 2524.383 | 2576.961 | 2539.516 | 4544.383 | 2066.68 | 2468.346 | 2332.981 | 2908.57 | 2607.508 | 2834.55 | 2476.731 | 4150.569 | 2770.165 | 2621.227 | 2786.84 | 2784.905 | 3053.471 | 4579.426 | 3246.287 |
| CUST_21697_PI429010886 | N45685 | 0.000249 | 24 | 21 | | | | | | 21.36429 | 27.73257 | 16.42915 | 12.43952 | 9.938622 | 20.44558 | 9.121241 | 15.2034 | 13.79507 | 14.38182 | 12.4643 | 30.2284 | 17.8888 | 13.76394 | 19.17264 | 35.4058 | 15.23856 | 23.94075 | 15.00335 | 25.8162 | 21.40056 | 30.6195 | 36.89165 | 23.63048 | 19.55556 | 21.03394 | 23.21318 |
| CUST_18431_PI429010886 | N39786 | 0.00025 | 21 | 3 | 256574616 | GTP binding protein alpha subunit Gi [Marsupenaeu | Signal transduction | Other | 8.01E-41 | 40.02231 | 40.22531 | 39.17072 | 41.56303 | 61.65538 | 59.25893 | 57.83187 | 36.76731 | 53.92569 | 41.77165 | 35.48457 | 41.66889 | 20.00906 | 39.80101 | 36.72937 | 48.75136 | 22.59879 | 28.13335 | 40.59153 | 22.50662 | 45.53479 | 56.39304 | 262.2997 | 53.67173 | 67.44354 | 67.58357 | 45.55959 |
| CUST_40753_PI429010886 | M56006 | 0.000252 | 24 | 21 | | | | | | 20.88392 | 27.36061 | 25.89264 | 23.26041 | 16.53651 | 17.61934 | 15.83738 | 8.749912 | 12.92194 | 25.26918 | 5.414583 | 28.08233 | 14.7136 | 21.08444 | 18.9048 | 44.45813 | 13.25918 | 30.99487 | 12.49757 | 25.76223 | 26.13969 | 37.11196 | 35.69319 | 42.06392 | 27.78206 | 19.26978 | 23.38781 |
| CUST_6457_PI429010886 | N20125 | 0.000252 | 24 | 21 | 405968363 | Hepatic leukemia factor [Crassostrea gigas] | Nucleic acid metabolism | Regulation of gene expression | 3.02E-31 | 86.09048 | 80.08977 | 94.49669 | 80.77462 | 71.27533 | 74.33243 | 80.29877 | 73.9797 | 78.51483 | 95.27106 | 78.90095 | 67.16912 | 82.59837 | 123.0876 | 85.50355 | 122.9865 | 91.6735 | 81.63335 | 90.33731 | 90.8776 | 88.12787 | 97.67416 | 131.771 | 111.625 | 90.0502 | 95.41159 | 106.064 |
| CUST_22527_PI429010886 | N47188 | 0.000252 | 15 | 6 | | | | | | 145.3839 | 96.97877 | 160.5038 | 186.7272 | 157.17 | 165.1069 | 243.3928 | 409.1687 | 402.5662 | 186.167 | 128.0586 | 145.1631 | 169.6296 | 215.167 | 245.9792 | 130.1677 | 127.8994 | 156.7533 | 401.3938 | 123.6853 | 237.5773 | 263.5218 | 172.9784 | 297.8606 | 228.9832 | 173.5962 | 276.4162 |
| CUST_51416_PI429010886 | M93434 | 0.000255 | 12 | 0 | | | | | | 196.8017 | 220.8734 | 230.254 | 135.3397 | 179.7697 | 162.6286 | 165.5224 | 155.7184 | 163.8946 | 223.7478 | 176.7991 | 182.8109 | 204.8186 | 230.4393 | 212.0119 | 195.1585 | 166.2291 | 173.7221 | 161.861 | 223.676 | 185.3847 | 198.0363 | 200.6444 | 170.3686 | 346.4727 | 215.8293 | 223.1512 |
| CUST_12704_PI429010886 | N29902 | 0.000255 | 15 | 12 | | | | | | 534.8364 | 1138.766 | 985.7913 | 237.9203 | 246.3705 | 118.2899 | 2574.868 | 1561.567 | 4388.254 | 1727.428 | 507.983 | 1650.111 | 9739.086 | 9249.246 | 5762.468 | 5744.456 | 3402.667 | 513.4234 | 1264.445 | 657.1327 | 739.8087 | 1021.64 | 1273.563 | 1609.757 | 8745.677 | 608.5272 | 13070.61 |
| CUST_337_PI429010886 | N4053 | 0.000255 | 15 | 12 | | | | | | 3713.677 | 7306.322 | 5985.533 | 1778.896 | 2309.39 | 1177.081 | 11243.07 | 10282.77 | 18298.8 | 8176.608 | 4309.606 | 9087.177 | 35468.64 | 32800.86 | 24425.67 | 22574.76 | 12422.81 | 3667.985 | 6329.544 | 5382.99 | 4494.887 | 10597.5 | 7163.89 | 5492.691 | 32330.94 | 5528.982 | 44543.72 |
| CUST_33685_PI429010886 | M7608 | 0.000259 | 12 | 0 | | | | | | 18.34756 | 25.67033 | 31.96946 | 19.27537 | 18.12511 | 25.70084 | 18.04709 | 20.03192 | 12.79969 | 20.52725 | 14.58166 | 37.68293 | 31.35346 | 40.84912 | 42.60531 | 28.1987 | 22.07292 | 27.37719 | 17.69248 | 20.2329 | 21.98126 | 19.37939 | 22.55627 | 23.22371 | 30.37057 | 29.26565 | 32.22977 |
| CUST_1880_PI429010886 | N10942 | 0.00026 | 24 | 21 | | | | | | 22.14395 | 16.97989 | 18.13433 | 23.00326 | 17.53688 | 11.10462 | 14.67473 | 14.19901 | 8.893352 | 9.040661 | 14.16669 | 13.41425 | 15.40382 | 22.27045 | 5.908883 | 14.90976 | 16.90108 | 22.59036 | 12.41217 | 14.91553 | 24.47403 | 24.53942 | 25.08483 | 24.71578 | 22.4434 | 12.82143 | 16.03532 |
| CUST_1730_PI429010886 | N10559 | 0.000265 | 15 | 12 | | | | | | 12916.14 | 20597.24 | 19626.64 | 5991.557 | 9119.184 | 5445.033 | 19004.47 | 21431.7 | 37461.84 | 25136.37 | 11619.8 | 21318.49 | 33985.8 | 34197.64 | 26902.5 | 25360.97 | 17786.81 | 15424 | 14113.22 | 16925.4 | 15060.57 | 29142.09 | 24647.46 | 11566.7 | 47564.51 | 20646.45 | 60957.53 |
| CUST_3048_PI429010886 | N13687 | 0.000267 | 18 | 3 | 543719592 | PREDICTED: E3 UFM1-protein ligase 1 [Columba livi | | | 6.3E-109 | 42.38165 | 27.07911 | 35.09944 | 45.78297 | 38.96253 | 43.03291 | 74.16318 | 47.36571 | 36.37157 | 31.70409 | 27.65968 | 15.35034 | 33.25306 | 29.40744 | 26.64032 | 33.30987 | 44.38619 | 33.42509 | 38.64078 | 50.66205 | 34.35609 | 41.8722 | 45.93669 | 47.49605 | 31.95513 | 13.24553 | 20.16852 |
| CUST_28008_PI429010886 | N57096 | 0.000268 | 24 | 15 | | | | | | 58.00667 | 78.17016 | 46.2898 | 38.06764 | 62.27229 | 39.00627 | 46.80174 | 56.91814 | 53.67173 | 83.63981 | 82.95644 | 96.15886 | 68.1165 | 71.80124 | 87.48439 | 101.7598 | 70.43457 | 161.6619 | 82.80093 | 114.1326 | 80.19532 | 82.3571 | 75.24399 | 54.49444 | 81.87168 | 48.70779 | 76.55655 |

| Probe ID | ID | p-value | Period | Phase | EBI ID | Annotation | GO category | Go term | E-value | 0 | 0 | 0 | 3 | 3 | 3 | 6 | 6 | 6 | 9 | 9 | 9 | 12 | 12 | 12 | 15 | 15 | 15 | 18 | 18 | 18 | 21 | 21 | 21 | 24 | 24 | 24 |
|---|---|---|---|---|---|---|---|---|---|---|---|---|---|---|---|---|---|---|---|---|---|---|---|---|---|---|---|---|---|---|---|---|---|---|---|---|
| CUST_36539_PI429010886 | M16174 | 0.00027 | 18 | 15 | 91076040 | PREDICTED: similar to CG1814 CG1814-PA [Triboliu | | | 1.34E-48 | 120.0446 | 174.8053 | 130.9315 | 91.52825 | 120.0646 | 100.336 | 101.0126 | 96.07604 | 76.84997 | 127.0345 | 115.4652 | 177.2148 | 72.21756 | 115.7889 | 107.2112 | 135.5775 | 160.3955 | 145.2433 | 121.3613 | 230.2846 | 124.0734 | 106.7139 | 88.45598 | 116.0676 | 106.3073 | 100.8047 | 107.0039 |
| CUST_25181_PI429010886 | N51926 | 0.00027 | 18 | 12 | | | | | | 63.65837 | 47.20161 | 37.3381 | 28.37808 | 24.73262 | 32.74781 | 39.37758 | 33.92714 | 36.3007 | 38.53966 | 44.64991 | 61.14077 | 50.54005 | 64.8492 | 45.13249 | 42.06993 | 36.83164 | 44.40996 | 60.41389 | 58.75806 | 48.81237 | 18.7895 | 37.92604 | 22.57964 | 44.94616 | 49.76383 | 42.38554 |
| CUST_28523_PI429010886 | N58110 | 0.00027 | 18 | 12 | | | | | | 27.49426 | 19.44722 | 15.64687 | 10.97059 | 16.72767 | 8.096609 | 12.84641 | 19.00858 | 20.44558 | 20.57784 | 24.98792 | 12.41093 | 20.22452 | 29.68582 | 28.94552 | 15.62884 | 24.62928 | 25.91041 | 19.6198 | 20.03192 | 19.51952 | 12.16044 | 17.77636 | 16.70757 | 24.76116 | 11.96936 | 18.88563 |
| CUST_30533_PI429010886 | N62074 | 0.00027 | 21 | 0 | 193591726 | PREDICTED: zinc finger protein 271-like [Acyrthosipl | | | 1.22E-19 | 202.7615 | 111.8858 | 135.1566 | 125.1637 | 112.9639 | 128.5885 | 132.1901 | 97.13391 | 79.59942 | 60.84139 | 97.74764 | 53.02384 | 94.07093 | 81.04828 | 46.14783 | 94.56836 | 84.61863 | 74.15763 | 106.9318 | 78.86687 | 104.7823 | 155.1933 | 186.006 | 95.70219 | 57.01626 | 138.6787 | 118.2591 |
| CUST_11650_PI429010886 | N28179 | 0.000272 | 12 | 0 | | | | | | 60.99125 | 71.45222 | 62.49854 | 56.74499 | 51.05482 | 41.53959 | 41.35575 | 32.44657 | 44.15212 | 69.57456 | 46.42413 | 56.60227 | 55.57729 | 59.52835 | 61.97713 | 75.02406 | 56.41396 | 54.01314 | 48.88625 | 53.56418 | 50.54005 | 54.89215 | 57.83187 | 44.00078 | 71.96189 | 52.17878 | 79.94364 |
| CUST_20529_PI429010886 | N43614 | 0.000275 | 24 | 0 | | | | | | 29.72221 | 20.26494 | 27.75482 | 19.08808 | 26.67812 | 37.06438 | 26.42193 | 23.2298 | 24.983 | 24.8597 | 22.43724 | 18.79959 | 12.75871 | 18.49894 | 19.20292 | 23.60773 | 19.6916 | 21.30741 | 29.96361 | 15.5279 | 23.37181 | 30.14891 | 25.54793 | 34.35609 | 35.31634 | 23.81358 | 52.645 |
| CUST_4402_PI429010886 | N16685 | 0.000278 | 18 | 0 | 321479230 | hypothetical protein DAPPUDRAFT_299977 [Daphn | Metabolic process | Other | 1.79E-27 | 9.573493 | 11.58371 | 12.24951 | NA | 8.589723 | NA | 8.472888 | 13.68168 | 11.33598 | 9.316937 | 7.222005 | 10.4644 | 4.758947 | 7.395008 | 6.629157 | 10.94103 | 8.858469 | 9.106984 | 13.82074 | 12.71524 | 11.40993 | 14.83018 | 11.15122 | NA | 8.886857 | 10.27942 | 7.794721 |
| CUST_12690_PI429010886 | N29875 | 0.00028 | 18 | 3 | 307202134 | Rhotekin [Harpegnathos saltator] | | | 8.47E-13 | 51.91237 | 60.77736 | 73.64901 | 158.2442 | 65.44799 | 97.65941 | 69.65899 | 41.17264 | 55.99321 | 67.94324 | 52.02242 | 69.03435 | 51.92939 | 53.41869 | 37.34484 | 46.83381 | 44.94616 | 23.36932 | 52.80587 | 40.66388 | 103.7718 | 69.84364 | 136.25 | 77.99438 | 70.45058 | 38.71068 | 44.66782 |
| CUST_1857_PI429010886 | N10895 | 0.000282 | 21 | 0 | | | | | | 112.1218 | 158.6525 | 116.8442 | 112.064 | 106.4697 | 114.5891 | 108.3189 | 78.66048 | 98.14502 | 108.0186 | 87.14788 | 77.56897 | 83.81066 | 68.78674 | 78.88657 | 83.39175 | 160.8449 | 91.47195 | 109.902 | 72.37554 | 112.5301 | 117.2337 | 109.6112 | 129.419 | 93.80773 | 103.7132 | 58.34728 |
| CUST_36636_PI429010886 | M16420 | 0.000283 | 15 | 0 | | | | | | 432.3349 | 41.71517 | NA | 35.57263 | 26.59511 | NA | 16.82979 | 6.340641 | NA | 6.199171 | 4.551336 | 14.99273 | 18.85808 | 6.351896 | 16.75298 | 270.2125 | NA | 288.1636 | 9.371383 | 184.8121 | 215.7523 | 5.119427 | 22.04537 | NA | 8.406805 | 15.60189 | 27.32583 |
| CUST_13776_PI429010886 | N31748 | 0.000285 | 21 | 3 | | | | | | 49.69905 | 68.78674 | 53.73015 | 56.72265 | 72.3404 | 61.16623 | 30.01523 | 44.4293 | 65.51399 | 42.78573 | 65.22254 | 61.61655 | 50.01795 | 39.49159 | 38.5218 | 42.57633 | 37.11863 | 43.03291 | 43.15798 | 57.67685 | 51.36601 | 47.95704 | 69.03435 | 28.9666 | 68.33792 | 78.60763 | 83.98715 |
| CUST_14545_PI429010886 | N33057 | 0.000285 | 21 | 9 | | | | | | 498.525 | 491.5609 | 337.6916 | 535.6554 | 519.9761 | 548.7959 | 549.5559 | 644.7638 | 526.943 | 761.273 | 619.6845 | 708.7623 | 549.794 | 504.7239 | 496.1582 | 624.8531 | 442.7732 | 494.2193 | 504.1608 | 621.0146 | 564.8923 | 552.5713 | 410.594 | 449.6075 | 526.8072 | 592.1986 | 509.3966 |
| CUST_46438_PI429010886 | M72656 | 0.000286 | 18 | 12 | | | | | | 76.51274 | 83.16839 | 72.40904 | 73.09177 | 52.35268 | 28.68237 | 54.89215 | 74.06411 | 54.89562 | 83.29887 | 61.03938 | 88.98374 | 104.2347 | 135.5608 | 106.1754 | 88.90431 | 86.00719 | 75.1394 | 151.8603 | 76.04812 | 99.36845 | 96.11914 | 52.5097 | 48.34765 | 85.94103 | 58.58074 | 68.86603 |
| CUST_51740_PI429010886 | M94802 | 0.000289 | 15 | 12 | | | | | | 84.35593 | 17.76828 | 45.50813 | NA | 5.889332 | 6.853489 | NA | 13.99028 | 19.39216 | 47.36571 | 6.418804 | 77.38027 | 80.07884 | 56.86246 | 39.62708 | 11.15425 | NA | NA | 8.56209 | 6.281409 | 15.23556 | 14.42559 | 34.15181 | 18.22767 | 18.21393 | 20.88813 | 31.8793 |
| CUST_47035_PI429010886 | M74612 | 0.000293 | 15 | 12 | | | | | | 37.12735 | 35.24075 | 35.30842 | 27.03844 | 30.42483 | 27.20765 | 35.20549 | 16.84704 | 30.9788 | 41.76704 | 36.3679 | 44.41428 | 36.23666 | 39.11677 | 43.05396 | 44.66782 | 29.75464 | 48.49058 | 27.8201 | 32.73752 | 35.82003 | 33.06846 | 31.25517 | 35.90257 | 43.45323 | 38.01489 | 43.68382 |
| CUST_47278_PI429010886 | M75443 | 0.000293 | 21 | 0 | | | | | | 37.86509 | 33.52987 | 41.26062 | 25.72523 | 16.98127 | 27.68313 | 26.51868 | 16.81213 | 23.47569 | 17.37163 | 21.8835 | 39.03429 | 13.53636 | 22.56723 | 19.04736 | 31.18147 | 22.61839 | 27.79102 | 18.80919 | 35.36241 | 28.68237 | 43.46708 | 33.79348 | 30.99832 | 25.82998 | 22.6805 | 34.45811 |
| CUST_21396_PI429010886 | N45144 | 0.000293 | 21 | 3 | | | | | | 22.7209 | 29.59116 | 22.16629 | 26.95285 | 30.32196 | 32.21966 | 19.5413 | 28.98746 | 19.40477 | 22.51909 | 22.93702 | 27.45566 | 18.63533 | 20.00677 | 18.54361 | 24.45097 | 23.75128 | 26.00775 | 16.67016 | 21.55161 | 20.05897 | 26.41986 | 28.88408 | 43.35404 | 28.62456 | 26.78447 | 38.79646 |
| CUST_15206_PI429010886 | N34185 | 0.000293 | 18 | 0 | 156537221 | PREDICTED: PKHD domain-containing transmembra | | | 6.52E-22 | 19.42289 | 36.73446 | 32.88145 | 25.05744 | 25.55609 | 16.71152 | 9.535711 | 9.560965 | 18.58096 | 26.73668 | 13.93436 | 24.23014 | 11.42142 | 3.884673 | 21.13597 | 27.0727 | NA | 22.66107 | 18.65671 | 50.02348 | 33.17041 | 26.91798 | 21.76203 | 16.62935 | 6.659608 | 9.278391 | 11.52078 |
| CUST_15495_PI429010886 | N34674 | 0.000296 | 18 | 15 | 156552355 | PREDICTED: hypothetical protein LOC100117960 [N | Metabolic process | Other | 3.69E-09 | 39.73253 | 29.65821 | 16.8101 | 27.18775 | 51.50234 | 38.37256 | 10.24818 | 16.25775 | 15.32584 | 24.26504 | 14.85935 | 21.95843 | 11.52078 | 31.56711 | 26.42807 | 44.62034 | 33.42509 | 48.45297 | 24.94079 | 26.30031 | 27.80573 | 24.39614 | 22.16629 | 31.29954 | 21.25027 | 15.89011 | 15.4125 |
| CUST_30609_PI429010886 | N62219 | 0.000297 | 15 | 9 | | | | | | 327.535 | 267.1348 | 395.3575 | 312.3654 | 390.7936 | 319.9685 | 253.6933 | 378.3563 | 424.8243 | 448.199 | 423.9576 | 411.751 | 368.6847 | 440.6954 | 311.6128 | 371.8288 | 295.6383 | 282.3655 | 316.7444 | 378.6086 | 413.7293 | 667.7123 | 424.4597 | 407.4423 | 552.6602 | 446.4087 | 704.3489 |
| CUST_3169_PI429010886 | N13946 | 0.000301 | 15 | 3 | 326781695 | crustacyanin subunit A [Fenneropenaeus merguiens | | | 4.41E-16 | 23.2714 | 12.58978 | 25.11695 | 26.02914 | 28.68237 | 35.72108 | 29.69737 | 22.4878 | 23.16574 | 13.66038 | 13.56559 | 16.87735 | 5.813584 | 9.982619 | 12.44781 | 10.25442 | 20.24475 | 28.888 | 24.06718 | 15.59665 | 29.15809 | 14.06725 | 19.2383 | 22.43908 | 18.1208 | 36.00421 | 17.34629 |
| CUST_36700_PI429010886 | M16578 | 0.000308 | 24 | 0 | | | | | | 29.59116 | 21.78224 | 27.03027 | 26.28334 | 21.21095 | 30.65915 | 24.63224 | 19.02839 | 15.78045 | 11.21554 | 12.98669 | 13.11969 | 17.00366 | 30.45027 | 12.84324 | 22.64343 | 15.55875 | 25.47915 | 37.45905 | 15.51619 | 21.10666 | 26.44706 | 33.20988 | 25.73345 | 33.52987 | 31.47929 | 25.82998 |
| CUST_37377_PI429010886 | M18827 | 0.000309 | 15 | 0 | 317383196 | myosin light chain [Penaeus monodon] >gi\|357640 | | | 1.35E-32 | 119.0396 | 105.4781 | 73.00251 | 92.05581 | 81.46986 | 82.9954 | 49.32644 | 40.68573 | 46.11029 | 64.97549 | 86.71532 | 62.506 | 64.01814 | 69.26327 | 44.70035 | 93.70583 | 81.49546 | 112.517 | 68.90417 | 260.7973 | 73.71983 | 48.32558 | 86.03759 | 56.78823 | 63.78913 | 59.67663 | 58.66467 |
| CUST_50488_PI429010886 | M89981 | 0.000319 | 24 | 21 | | | | | | 55.42699 | 86.65167 | 65.72077 | 49.88882 | 57.35641 | 45.75802 | 50.1618 | 31.12392 | 55.34146 | 67.65513 | 68.12178 | 69.47796 | 47.64282 | 40.34574 | 44.10538 | 50.15777 | 65.02052 | 43.16663 | 61.60941 | 79.97123 | 65.42078 | 94.67269 | 91.6735 | 82.13647 | 81.08583 | 199.0448 | 73.56763 |
| CUST_49615_PI429010886 | M86406 | 0.00032 | 15 | 9 | | | | | | NA | 463.3861 | 457.3191 | 10.79421 | 20.07209 | 41.43739 | 14.21164 | 138.4518 | 100.4445 | 238.7409 | 432.3349 | 380.2117 | 186.6214 | 385.8989 | 168.177 | 188.427 | 56.85058 | 62.61352 | 7.41743 | 133.4937 | 15.0164 | 39.83487 | 22.36193 | NA | 558.5528 | 142.3547 | 667.377 |
| CUST_43975_PI429010886 | M64509 | 0.000323 | 15 | 3 | | | | | | 165.6398 | 169.7593 | 112.7849 | 189.7138 | 205.078 | 274.49 | 213.51 | 127.9775 | 99.05161 | 106.011 | 125.5232 | 110.2618 | 113.7089 | 113.0169 | 99.45401 | 197.3275 | 191.1131 | 250.4057 | 340.4019 | 131.2743 | 182.6771 | 148.7285 | 131.1566 | 188.4563 | 109.4422 | 135.5237 | 90.15151 |
| CUST_15666_PI429010886 | N34949 | 0.000323 | 15 | 9 | 443697703 | hypothetical protein CAPTEDRAFT_197561 [Capitell | | | 3.45E-27 | 24.09915 | 27.9072 | 21.54483 | 25.24001 | 23.40583 | 23.29073 | 25.62143 | 24.96736 | 29.73209 | 35.18059 | 25.80044 | 35.00193 | 29.5179 | 33.23976 | 26.38687 | 38.50305 | 30.04394 | 34.35136 | 17.10625 | 25.31087 | 22.31737 | 32.4969 | 26.96538 | 28.2496 | 31.8356 | 33.16347 | 33.28531 |
| CUST_14342_PI429010886 | N32729 | 0.000327 | 15 | 9 | 321476397 | hypothetical protein DAPPUDRAFT_97134 [Daphnia | | | 9.36E-17 | 42.62361 | 48.30048 | 51.14708 | 43.09974 | 32.65296 | 33.90948 | 43.2321 | 31.67438 | 37.30507 | 72.09388 | 58.7202 | 58.7202 | 50.80674 | 57.59047 | 55.20849 | 63.09759 | 50.53472 | 38.93338 | 49.27153 | 54.26995 | 50.67859 | 50.9755 | 53.35103 | 43.93802 | 62.91706 | 64.22812 | 76.88221 |
| CUST_346_PI429010886 | N4191 | 0.000328 | 18 | 3 | 288869496 | cuticular protein analogous to peritrophins 3-A1 pr | Metabolic process | Carbohydrate metabolic process | 3.52E-51 | 316.971 | 169.5547 | 189.5968 | 705.7628 | 346.3948 | 367.27 | 217.5163 | 170.7891 | 77.61759 | 185.0712 | 193.773 | 57.90878 | 63.92583 | 217.0599 | 127.374 | 121.9392 | 161.3901 | 183.266 | 215.1259 | 436.2219 | 212.0939 | 764.3328 | 183.7339 | 424.5435 | 305.5566 | 314.6224 | 67.06219 |
| CUST_33235_PI429010886 | M6356 | 0.000332 | 12 | 3 | 321466175 | hypothetical protein DAPPUDRAFT_305917 [Daphn | Protein metabolism | Proteolysis | 1.9E-23 | 46.31285 | 30.45779 | 49.54555 | 50.74126 | 51.5653 | 67.85099 | 99.18362 | 23.17075 | 50.00676 | 44.27668 | 15.27919 | 29.10242 | 30.32568 | 55.13629 | 39.667 | 65.87593 | 80.79066 | 67.77286 | 44.90024 | 37.22562 | 45.60489 | 35.12261 | 36.39221 | 26.84667 | 26.70632 | 28.6962 | 29.68336 |
| CUST_45000_PI429010886 | M67675 | 0.000332 | 24 | 21 | | | | | | 344.9163 | 387.5117 | 409.8763 | 377.0163 | 302.8941 | 478.5158 | 345.7818 | 275.4605 | 332.6812 | 470.2 | 268.3232 | 320.0372 | 378.265 | 349.6083 | 330.527 | 396.9865 | 422.6396 | 513.9071 | 409.4886 | 408.7569 | 502.7361 | 743.1041 | 434.2638 | 582.9606 | 381.8035 | 437.4103 | 301.3697 |
| CUST_17691_PI429010886 | N38417 | 0.000333 | 18 | 6 | 238476875 | crustin type I [Macrobrachium rosenbergii] | | | 3.66E-09 | NA | 4.898854 | 11.15425 | 28.74973 | 9.960996 | NA | 91.04016 | 133.891 | 160.2794 | 34.88582 | 16.66456 | 45.91191 | 64.68215 | 55.55289 | 56.85058 | 52.73287 | 91.78258 | NA | 4.138608 | 10.20974 | NA | 20.85111 | 7.970984 | 59.40092 | 101.9719 | NA | 130.8343 |
| CUST_28716_PI429010886 | N58484 | 0.000334 | 12 | 0 | | | | | | 45.81043 | 53.89507 | 60.57138 | 18.16319 | 35.35407 | 29.21265 | 21.61291 | 41.12787 | 39.93998 | 19.05109 | 24.0077 | 38.68605 | 41.10188 | 41.57987 | 55.2283 | 45.81525 | 28.34529 | 38.75769 | 35.1893 | 33.76983 | 33.33046 | 31.49395 | 33.43942 | 28.1812 | 59.09902 | 44.47376 | 48.41716 |
| CUST_41751_PI429010886 | M58460 | 0.000339 | 24 | 21 | | | | | | 549.9307 | 331.6154 | 375.6267 | 646.0018 | 404.5388 | 379.2779 | 326.9766 | 321.696 | 306.9203 | 386.3188 | 331.1696 | 348.7613 | 417.2023 | 382.3424 | 389.3563 | 385.1644 | 614.3849 | 375.1955 | 546.5203 | 543.3625 | 472.3127 | 915.3775 | 449.6075 | 517.0533 | 384.7174 | 291.3327 | 348.9836 |
| CUST_17225_PI429010886 | N37602 | 0.000339 | 24 | 21 | | | | | | 165.4769 | 70.58805 | 177.7855 | 125.0791 | 138.2766 | 205.8446 | 161.3292 | 134.5392 | 147.2771 | 61.97713 | 117.1499 | 135.7237 | 96.46972 | 82.45182 | 108.8415 | 118.2899 | 158.496 | 60.75286 | 196.157 | 131.0636 | 117.1631 | 207.3573 | 238.9663 | 176.1772 | 179.1099 | 196.0648 | 126.3708 |
| CUST_38751_PI429010886 | M30047 | 0.000339 | 15 | 9 | | | | | | 443.1374 | 677.5621 | 522.6581 | 385.0916 | 425.5183 | 339.1595 | 471.4881 | 386.5802 | 328.8667 | 701.6345 | 652.2033 | 882.5331 | 309.1695 | 454.9277 | 449.6922 | 406.06 | 398.3325 | 397.525 | 340.2645 | 395.6626 | 233.124 | 765.9632 | 345.5168 | 439.4127 | 525.2083 | 461.7538 | 506.0078 |
| CUST_9228_PI429010886 | N24269 | 0.000342 | 12 | 0 | | | | | | 27.2102 | 34.30707 | 72.92262 | 21.71916 | 24.63851 | 24.5556 | 12.42474 | 16.23047 | 24.21798 | 29.86288 | 16.90946 | 24.93429 | 23.71308 | 27.82378 | 32.7674 | 22.48589 | 25.72523 | 28.17746 | 15.80258 | 24.40654 | 17.18917 | 32.28425 | 34.33884 | 27.44977 | 40.03126 | 24.7547 | 31.24843 |
| CUST_16622_PI429010886 | N36583 | 0.000344 | 15 | 15 | | | | | | 1805.829 | 1762.677 | 1720.101 | 1171.074 | 1338.953 | 1293.201 | 1331.49 | 1180.515 | 1536.448 | 1531.156 | 1866.472 | 1863.078 | 1160.295 | 1528.209 | 1751.759 | 2102.543 | 1871.332 | 1986.907 | 1773.273 | 1936.604 | 1557.483 | 1772.36 | 1188.466 | 1094.71 | 1391.685 | 1488.789 | 1583.802 |
| CUST_45018_PI429010886 | M67720 | 0.000351 | 12 | 3 | | | | | | 12.09063 | 37.12735 | 18.4152 | 34.50513 | 64.94243 | 83.61604 | 82.95644 | 42.4817 | 45.98092 | 18.96681 | 16.61891 | 18.14494 | 21.27912 | 28.6993 | 31.277 | 54.03548 | 79.26902 | 67.08317 | 62.95412 | 29.99408 | 42.14262 | 32.47936 | 10.26743 | 23.59336 | 114.0128 | 57.58322 | 49.29932 |
| CUST_44485_PI429010886 | M66117 | 0.000352 | 15 | 9 | 1764107 | pacifastin heavy chain precursor [Pacifastacus leniu | Transport | Ion transport | 6.07E-47 | 29.53988 | 29.62583 | 25.48959 | 26.43024 | 23.1013 | 5.83676 | 21.96744 | 29.89152 | 37.10843 | 43.1379 | 23.75855 | 54.66707 | 30.08888 | 45.1918 | 35.38465 | 53.75795 | 21.10435 | 30.91745 | 19.76282 | 29.20057 | 12.96601 | 68.38918 | 56.42615 | 26.92163 | 95.06463 | 31.35097 | 72.78887 |
| CUST_23937_PI429010886 | N49732 | 0.000353 | 21 | 0 | | | | | | 87.87096 | 98.27597 | 114.2341 | 75.65779 | 81.56419 | 75.84454 | 56.71651 | 61.07112 | 74.66823 | 80.95222 | 84.80692 | 81.59575 | 85.40569 | 89.419 | 55.784 | 85.53529 | 80.52163 | 81.55572 | 49.79836 | 100.8965 | 86.38663 | 108.7243 | 86.63308 | 110.8479 | 101.3443 | 87.59441 | 110.0194 |
| CUST_37786_PI429010886 | M21581 | 0.000354 | 15 | 12 | | | | | | 2959.951 | 6779.053 | 5738.426 | 1762.677 | 2041.736 | 1477.505 | 4955.555 | 5933.737 | 9320.998 | 7617.588 | 3509.529 | 6976.379 | 9965.559 | 9131.511 | 8066.308 | 9096.106 | 5407.054 | 4571.032 | 3929.651 | 5641.729 | 4083.732 | 8986.011 | 7590.588 | 3206.496 | 18410.6 | 5917.438 | 22281.96 |
| CUST_39436_PI429010886 | M39375 | 0.000354 | 15 | 12 | | | | | | 1210.833 | 2638.062 | 1981.218 | 768.5376 | 877.6784 | 380.8862 | 2604.753 | 2232.866 | 3551.684 | 3546.259 | 1573.105 | 2898.401 | 4827.614 | 4635.589 | 3330.039 | 4490.741 | 2487.623 | 1513.56 | 1672.079 | 2217.377 | 1501.641 | 3823.851 | 2221.45 | 1298.64 | 8574.409 | 1769.215 | 10478.08 |
| CUST_6318_PI429010886 | N19941 | 0.000355 | 12 | 3 | 345319740 | PREDICTED: riboflavin kinase-like [Ornithorhynchus | Metabolic process | Other | 1.37E-50 | 25.20056 | 23.05797 | 14.27753 | 42.80624 | 22.33151 | 44.95443 | 27.2017 | 28.82038 | 21.93936 | 21.93936 | 17.03078 | 21.0901 | 29.43393 | 22.77379 | 23.78915 | 37.6674 | 28.02833 | 25.8552 | 27.07911 | 17.39906 | 15.44955 | 15.95142 | 17.23726 | 21.00917 | 21.98421 | 26.03397 | 24.53193 |
| CUST_50450_PI429010886 | M89860 | 0.000356 | 15 | 9 | 56121719 | ribosomal protein L26 [Penaeus monodon] >gi\|124 | Protein metabolism | Ribosome biogenesis | 4.38E-29 | 34.78955 | 55.14628 | 48.09585 | 18.36555 | 31.83203 | 19.66703 | 36.57353 | 26.18825 | 41.5428 | 59.26817 | 39.66277 | 68.43089 | 37.52322 | 63.41464 | 44.85914 | 46.04576 | 20.23079 | 26.04992 | 31.37784 | 39.98555 | 40.55619 | 49.01378 | 40.59153 | 40.64999 | 58.31001 | 50.10243 | 61.91569 |
| CUST_43817_PI429010886 | M46059 | 0.000364 | 24 | 18 | | | | | | 162.3514 | 162.0477 | 183.7339 | 161.2704 | 144.2346 | 169.0651 | 164.6012 | 162.5022 | 174.126 | 179.678 | 152.9004 | 175.4567 | 153.2997 | 215.4319 | 205.2221 | 209.83 | 183.0597 | 185.7639 | 197.5444 | 194.6296 | 200.9848 | 199.8756 | 219.4603 | 183.574 | 179.4229 | 188.0537 | 191.3362 |
| CUST_30404_PI429010886 | N61822 | 0.000364 | 24 | 21 | | | | | | 31.45383 | 44.4356 | 16.06632 | 24.87004 | 30.50322 | 25.21391 | 12.56927 | 23.36338 | 13.84831 | 26.33364 | 18.45187 | 35.73252 | 17.80424 | 33.65009 | 25.91379 | 23.61053 | 20.07638 | 31.59448 | 56.63828 | 37.21299 | 37.63897 | 60.77736 | 39.06616 | 28.3838 | 31.99517 | 38.33878 | 45.71121 |
| CUST_11986_PI429010886 | N28731 | 0.000369 | 24 | 21 | | | Metabolic process | Nucleobase-containing compound metabolic process | | 23.86269 | 23.09604 | 30.72781 | 25.16709 | 28.23857 | 28.00895 | 21.27634 | 18.63301 | 19.66187 | 28.60828 | 26.42193 | 22.47267 | 22.59328 | 29.21516 | 32.60067 | 38.84613 | 37.23147 | 22.71194 | 33.37654 | 25.42619 | 32.49365 | 33.42828 | 33.98937 | 29.94885 | 27.48409 | 27.07911 | 31.88591 |
| CUST_33186_PI429010886 | M6224 | 0.00037 | 18 | 3 | | | | | | 228.4677 | 232.0593 | 217.0599 | 279.3325 | 192.6772 | 524.2988 | 200.8116 | 260.7973 | 215.4319 | 159.0362 | 170.2284 | 249.422 | 156.539 | 157.8626 | 76.06574 | 184.5783 | 218.8861 | 246.1869 | 222.5078 | 169.7593 | 114.443 | 258.1697 | 243.3133 | 295.9077 | 208.7767 | 345.639 | 221.7247 |
| CUST_36338_PI429010886 | M15644 | 0.00037 | 18 | 0 | | | | | | 119.7354 | 86.46205 | 65.28922 | 27.8177 | 56.24668 | 42.52046 | 47.14099 | 36.43958 | 32.79034 | 44.59831 | 26.76517 | 25.96531 | 49.08702 | 35.72108 | 48.98001 | 48.52333 | 45.61086 | 32.50993 | 54.77183 | 49.96713 | 60.86143 | 43.19279 | 35.85651 | 18.13841 | 33.94054 | 36.5122 | 30.62818 |
| CUST_17286_PI429010886 | N37713 | 0.00037 | 18 | 9 | | | | | | 100.64 | 369.6113 | 289.9742 | 68.43089 | 60.58837 | 5.787319 | 97.29292 | 220.5819 | 269.4403 | 816.6897 | 390.973 | 1130.431 | 411.8683 | 375.2702 | 401.7586 | 66.54333 | 134.486 | 70.82822 | 226.7097 | 657.4366 | 177.3713 | 110.3777 | 305.7949 | 15.1999 | 218.9157 | 364.1216 | 782.2398 |
| CUST_17960_PI429010886 | N38908 | 0.000371 | 12 | 0 | | | | | | 255.5791 | 343.0578 | 244.2054 | 244.2379 | 227.6752 | 245.6875 | 232.5216 | 232.1489 | 203.5636 | 241.4448 | 243.5502 | 276.1481 | 266.722 | 272.5517 | 262.2997 | 303.1495 | 221.6459 | 194.0645 | 193.9282 | 246.0125 | 221.5477 | 282.6926 | 233.0195 | 268.8761 | 304.8602 | 305.6076 | 328.0964 |
| CUST_41969_PI429010886 | M59026 | 0.000372 | 18 | 3 | | | | | | 87.73688 | 90.23885 | 87.2755 | 103.3023 | 106.5352 | 101.7962 | 95.63731 | 227.3812 | 74.03663 | 75.03476 | 84.92511 | 82.01377 | 70.31729 | 48.84271 | 84.4182 | 76.78516 | 89.40029 | 77.7529 | 91.09799 | 60.03661 | 83.06141 | 94.11857 | 91.90153 | 75.56946 | 92.13928 | 87.42512 | 88.82378 |
| CUST_51529_PI429010886 | M93860 | 0.000373 | 21 | 9 | | | | | | 178.3541 | 159.1708 | 186.6214 | 203.3538 | 180.1262 | 151.9116 | 240.3735 | 236.8954 | 221.5886 | 259.5794 | 183.7672 | 265.3147 | 182.1159 | 207.9906 | 198.0625 | 237.799 | 240.1758 | 213.2126 | 198.0922 | 168.3529 | 171.0777 | 155.3131 | 176.3863 | 140.0797 | 220.685 | 157.283 | 235.0953 |
| CUST_35540_PI429010886 | M12826 | 0.000376 | 24 | 15 | 47209244 | unnamed protein product [Tetraodon nigroviridis] | | | 2.97E-17 | 101.5112 | 100.4233 | 96.30815 | 63.51336 | 74.23824 | 71.38963 | 78.18464 | 73.64901 | 89.76549 | 114.7111 | 105.9691 | 149.5758 | 94.20676 | 96.1698 | 129.2382 | 144.9545 | 103.1477 | 119.3258 | 100.1156 | 117.9048 | 91.66231 | 116.3422 | 112.3919 | 82.76433 | 86.49079 | 107.4216 | 101.9359 |
| CUST_49586_PI429010886 | M86258 | 0.000376 | 15 | 12 | | | | | | 28.06449 | 38.03336 | 36.30688 | 16.89803 | 14.71926 | 17.40769 | 27.9952 | 24.03757 | 22.04311 | 24.43342 | 23.05797 | 28.3608 | 29.57425 | 29.65821 | 30.04146 | 25.54596 | 23.63855 | 22.77196 | 16.62548 | 28.9598 | 16.39064 | 23.61236 | 24.72926 | 8.514476 | 28.27144 | 24.90512 | 32.68431 |
| CUST_1398_PI429010886 | N9515 | 0.000376 | 15 | 12 | 321459536 | hypothetical protein DAPPUDRAFT_309340 [Daphn | | | 4.55E-37 | 179.1099 | 287.1125 | 268.1939 | 173.0247 | 120.0792 | 198.0625 | 137.759 | 198.0922 | 167.5706 | 296.4182 | 240.1049 | 261.0789 | 299.502 | 297.9644 | 340.4766 | 190.3154 | 217.7055 | 257.9077 | 202.0834 | 192.9519 | 189.8316 | 327.6399 | 125.8218 | 249.3844 | 333.5908 | 375.1955 | 247.2314 |
| CUST_52381_PI429010886 | M97240 | 0.0038 | 24 | 15 | | | | | | 9.580047 | 5.361029 | 9.070174 | 7.07379 | 8.274264 | 4.762923 | 11.04457 | 5.932286 | 4.425369 | 7.223501 | 6.782422 | 10.55308 | 4.797228 | 12.65934 | 10.27148 | 15.01453 | 16.74792 | 12.48018 | 11.68238 | 10.35487 | NA | 12.31693 | NA | NA | 5.908014 | 8.376213 | 8.788925 |
| CUST_38014_PI429010886 | M22776 | 0.00038 | 15 | 12 | | | | | | 453.4712 | 879.6665 | 572.5618 | 224.9395 | 407.6622 | 212.1377 | 664.3088 | 441.8735 | 932.818 | 1047.491 | 411.2669 | 916.49 | 1452.711 | 1307.703 | 780.1203 | 1278.963 | 668.4719 | 538.1956 | 323.8952 | 712.3784 | 426.1051 | 1003.971 | 958.7977 | 473.327 | 3108.301 | 639.5245 | 2634.615 |
| CUST_19587_PI429010886 | N41822 | 0.000381 | 21 | 18 | | | | | | 920.0256 | 706.7803 | 995.7921 | 595.4395 | 893.1457 | 770.7121 | 633.6093 | 557.0435 | 591.3951 | 665.3114 | 975.7147 | 816.3533 | 823.2501 | 910.1372 | 1165.381 | 982.4374 | 952.6524 | 691.8386 | 1057.219 | 975.7147 | 1002.084 | 671.3234 | 813.9324 | 915.6162 | 1409.58 | 628.02 | 901.1709 |
| CUST_19617_PI429010886 | N41879 | 0.000382 | 21 | 15 | | | | | | 33.90631 | 21.99145 | 30.3942 | 17.08381 | 13.06814 | 26.75604 | 19.09504 | 12.57535 | 15.99616 | 25.9573 | 29.01472 | 22.26808 | 18.67864 | 24.99806 | 28.43719 | 33.42247 | 34.64857 | 27.2017 | 34.13499 | 27.025 | 26.15331 | 28.25151 | 26.58331 | 22.21682 | 27.6707 | 19.7328 | 24.49005 |
| CUST_24366_PI429010886 | N50492 | 0.000383 | 21 | 18 | | | | | | 80.81357 | 87.90722 | 76.38127 | 78.58831 | 79.97877 | 80.1659 | 54.51541 | 59.82999 | 49.67248 | 65.11259 | 71.32452 | 57.70169 | 77.65762 | 74.32582 | 77.39037 | 96.09446 | 91.24943 | 67.78689 | 100.2191 | 118.9466 | 75.81603 | 85.42654 | 77.0714 | 61.7839 | 86.87439 | 53.18901 | 65.63314 |
| CUST_35026_PI429010886 | M11369 | 0.000388 | 24 | 18 | | | | | | 7.077971 | 8.246449 | 18.01173 | 5.204531 | 10.01001 | NA | 10.20235 | 11.06786 | 7.715488 | 10.02652 | 12.91494 | 14.27247 | 16.57742 | 17.98635 | 13.30229 | 13.92993 | 7.952703 | 14.86659 | 17.14295 | 23.38605 | 19.33417 | 41.53622 | 18.23695 | NA | 11.36808 | 4.000088 | 8.632122 |
| CUST_47404_PI429010886 | M75982 | 0.000389 | 12 | 3 | | | | | | 108.4222 | 56.87504 | 14.15156 | 124.4818 | 143.9014 | 208.027 | 50.45571 | 103.2892 | 43.12973 | 16.9221 | 41.47521 | 17.18399 | 84.98778 | 30.71209 | 61.76631 | 55.91435 | 105.1778 | 158.496 | 87.31709 | 20.92287 | 24.24219 | 46.91616 | 11.20301 | 73.3681 | 36.04391 | 84.03407 | NA |
| CUST_17498_PI429010886 | N38072 | 0.000396 | 24 | 15 | | | | | | 45.89316 | 43.48001 | 37.83723 | 32.42053 | 25.27468 | 31.68743 | 35.25245 | 48.069 | 50.72389 | 123.1229 | 48.09045 | 27.62424 | 52.95745 | 46.11558 | 55.98779 | 65.28922 | 60.49322 | 61.02204 | 51.91715 | 55.9701 | 32.22977 | 53.00119 | 45.55959 | 48.1448 | 66.26094 | 47.57087 | 48.72124 |
| CUST_52398_PI429010886 | M97296 | 0.000399 | 24 | 15 | | | | | | 125.5041 | 137.3935 | 104.2766 | 73.46154 | 138.4518 | 67.05664 | 52.98961 | 15.80258 | 142.124 | 198.8279 | 216.8379 | 282.5343 | 157.1495 | 91.43481 | 160.2794 | 320.693 | 56.69692 | 242.9037 | 340.1906 | 326.5696 | 371.6995 | 226.768 | 404.8094 | 130.1016 | 89.75292 | 304.942 | 164.7204 |
| CUST_592_PI429010886 | N6265 | 0.000404 | 21 | 3 | | | | | | 54.5396 | 99.79034 | 56.07774 | 140.1704 | 65.99023 | 98.7787 | 63.05147 | 77.92082 | 64.05654 | 49.14494 | 47.43502 | 91.7472 | 67.45213 | 60.19028 | 44.64991 | 27.58029 | 46.69563 | 44.29399 | 46.43129 | 104.1327 | 82.1182 | 56.11071 | 80.9845 | 70.23629 | 74.80865 | 100.0297 | 90.95085 |
| CUST_16986_PI429010886 | N37188 | 0.000404 | 15 | 9 | | | | | | 13.44435 | 19.15845 | 9.468387 | 33.47616 | 12.06652 | 16.5713 | 19.10174 | 11.33427 | 21.40881 | 25.60312 | 27.15358 | 47.14099 | 12.36728 | 20.70392 | 22.57524 | 17.0998 | 11.71536 | 8.529362 | 14.38818 | 25.47915 | 22.60935 | 30.56491 | 26.17206 | 16.1391 | 25.63785 | 30.86679 | 26.39674 |
| CUST_41485_PI429010886 | M57845 | 0.000405 | 18 | 15 | | | | | | 200.4301 | 195.1585 | 224.5779 | 134.2642 | 175.4362 | 161.4808 | 124.9376 | 116.3867 | 121.3307 | 226.0436 | 215.7523 | 233.5692 | 163.5913 | 211.4751 | 149.8536 | 314.5564 | 222.9038 | 223.9394 | 157.8794 | 168.8426 | 125.7869 | 192.7007 | 130.3328 | 175.9447 | 171.2587 | 114.0443 | 154.542 |
| CUST_17549_PI429010886 | N38157 | 0.000406 | 24 | 21 | | | | | | 76.924 | 50.70349 | 74.22065 | 56.48454 | 51.01157 | 73.20424 | 55.85206 | 37.10254 | 61.57414 | 45.63167 | 40.95384 | 46.95122 | 56.25359 | 58.50555 | 39.766 | 45.62788 | 58.15995 | 65.12567 | 55.77394 | 54.8287 | 69.60585 | 73.803 | 105.7587 | 78.41704 | 42.79462 | 58.75213 | 78.32339 |
| CUST_12904_PI429010886 | N30241 | 0.000413 | 21 | 3 | 1561985757 | Crustacean hyperglycemic hormone [Armadillidium | Signal transduction | Other | 3.00E-06 | 53.8203 | 49.91173 | 48.34221 | 52.18439 | 56.58363 | 62.70075 | 50.19147 | 50.66735 | 51.67197 | 48.13345 | 34.1704 | 21.25262 | 40.68573 | 41.20843 | 30.05351 | 51.97626 | 49.59376 | 35.82359 | 65.4438 | 28.35096 | 39.06616 | 84.97695 | 14.51263 | 43.75577 | 67.85743 | 81.82955 | 66.39266 |
| CUST_51658_PI429010886 | M94455 | 0.000416 | 15 | 12 | | | | | | 250.0775 | 588.293 | 260.0898 | 156.7272 | 252.3543 | 113.5681 | 379.0729 | 233.6069 | 567.3609 | 628.8832 | 262.5059 | 793.0472 | 838.3296 | 792.4242 | 505.1623 | 989.9215 | 286.4677 | 388.0401 | 197.7545 | 405.7694 | 257.2859 | 683.3484 | 469.7466 | 221.2763 | 1819.925 | 451.7787 | 1606.601 |
| CUST_49607_PI429010886 | M86378 | 0.000418 | 24 | 21 | | | | | | 52.85191 | 49.56436 | 58.25978 | 51.83879 | 57.38433 | 84.42877 | 39.29828 | 40.98995 | 42.19897 | 52.56534 | 40.80069 | 33.28766 | 45.42508 | 53.28731 | 66.04832 | 82.82132 | 53.01853 | 81.28485 | 52.74988 | 62.62111 | 50.67294 | 108.555 | 64.83984 | 78.94301 | 60.42655 | 67.37749 | 59.17622 |
| CUST_41888_PI429010886 | M58803 | 0.000428 | 12 | 3 | | | | | | 75.36334 | 79.0243 | 49.41638 | 240.2928 | 181.649 | 237.799 | 160.1366 | 82.45182 | 61.27293 | 34.16228 | 117.6303 | 24.08352 | 63.15525 | 99.06334 | 67.04072 | 145.794 | 245.5292 | 207.5948 | 151.2216 | 122.1728 | 92.38118 | 166.1287 | 72.23286 | 156.562 | 66.46786 | 78.00387 | 19.22446 |
| CUST_54476_PI429010886 | M106170 | 0.000437 | 24 | 0 | | | | | | 46.84888 | 46.04195 | 45.08069 | 46.40711 | 33.16347 | 45.41664 | 43.30223 | 44.33871 | 49.62512 | 43.32695 | 39.55733 | 38.67688 | 40.17935 | 34.73538 | 16.48255 | 36.19062 | 30.56491 | 50.36639 | 37.14316 | 31.88231 | 57.59651 | 51.17489 | 60.94428 | 44.30287 | 64.55134 | 57.60056 | 51.38396 |
| CUST_19957_PI429010886 | N42514 | 0.000442 | 15 | 9 | 321459258 | hypothetical protein DAPPUDRAFT_61435 [Daphnia | | | 2.18E-46 | 20.94079 | 29.9579 | 29.97309 | 19.93792 | 20.55297 | 19.28798 | 21.5971 | 30.62521 | 38.8107 | 34.41851 | 28.47434 | 40.90354 | 21.4565 | 41.77517 | 30.32568 | 36.16119 | 26.90259 | 32.10959 | 17.15146 | 27.00978 | 19.84931 | 24.41159 | 31.7268 | 21.10225 | 28.93334 | 33.14786 | 32.38272 |
| CUST_53068_PI429010886 | M99996 | 0.000445 | 21 | 3 | 514741530 | PREDICTED: multidrug resistance-associated protei | Transport | Transmembrane transport | 6.29E-22 | 13.94631 | 11.24123 | 8.003112 | 25.82153 | 15.14331 | 22.15622 | 8.442875 | 7.515463 | 5.941184 | 5.121625 | NA | 7.635215 | 4.870293 | 10.91384 | 5.288214 | 7.224308 | NA | NA | NA | 7.893242 | 11.0429 | 5.519043 | 13.06014 | 12.86528 | 8.006014 | 14.53622 | 10.06856 |
| CUST_47532_PI429010886 | M76496 | 0.00045 | 18 | 9 | | | | | | 24.81549 | 13.56226 | 75.14455 | 15.86933 | 22.89626 | 16.99938 | 20.35314 | 89.80969 | 60.78457 | 70.94036 | 74.95386 | 79.29506 | 44.01036 | 42.7734 | 7.434035 | NA | 40.01136 | NA | 23.6747 | 26.28003 | 9.954929 | 8.970081 | 8.474659 | 12.66068 | 22.498 | 36.55801 | 60.95084 |
| CUST_10931_PI429010886 | N27013 | 0.000452 | 18 | 12 | | | | | | 42.44034 | 42.82585 | 53.11468 | 52.2877 | 65.56207 | 47.57729 | 49.21104 | 54.0232 | 64.37974 | 67.63514 | 62.506 | 88.06388 | 78.08745 | 142.5887 | 82.53975 | 48.30896 | 69.04216 | 50.78152 | 56.72941 | 38.11258 | 41.87571 | 89.76549 | 63.65837 | 49.38887 | 86.90523 | 71.69416 | 85.09002 |
| CUST_33456_PI429010886 | M6935 | 0.000457 | 24 | 0 | 470380336 | ubiquitin thiolesterase [Acanthamoeba castellanii st | | | 1.79E-33 | 19.25031 | 19.58231 | 27.53905 | 17.89088 | 16.10864 | 15.95142 | 12.54217 | 13.71806 | 19.91122 | 13.69809 | 13.0306 | 12.42822 | 10.89944 | 23.56736 | 10.84659 | 15.33118 | 14.80982 | 20.71416 | 8.419112 | 14.32917 | 14.59396 | 13.96931 | 18.89448 | 17.12833 | 22.63247 | 28.70508 | 27.18447 |
| CUST_56970_PI429010886 | M115029 | 0.000457 | 24 | 0 | | | | | | 63.3033 | 67.46884 | 97.79572 | 52.12912 | 53.9049 | 57.90878 | 68.01317 | 45.61086 | 67.04779 | 56.70249 | 47.5061 | 60.93069 | 55.52574 | 74.69976 | 50.14002 | 52.10062 | 57.10423 | 65.19644 | 66.2145 | 69.4087 | 61.25018 | 61.91569 | 76.46353 | 81.55572 | 96.4587 | 74.80865 | 107.073 |
| CUST_21327_PI429010886 | N45019 | 0.000458 | 15 | 0 | | | | | | 151.2216 | 102.0005 | 115.0393 | 86.02859 | 100.2724 | 98.08436 | 93.75113 | 102.3532 | 71.89357 | 84.95079 | 68.50419 | 72.75459 | 95.72441 | 103.9053 | 82.17099 | 123.794 | 117.2072 | 109.0596 | 119.228 | 85.926 | 85.29111 | 140.7918 | 93.15439 | 87.38085 | 102.0461 | 64.3475 | 75.31286 |
| CUST_392_PI429010886 | N4801 | 0.000459 | 15 | 9 | 321466528 | hypothetical protein DAPPUDRAFT_106124 [Daphnia | | | 2.58E-19 | 1497.781 | 2618.901 | 7188.247 | 339.8212 | 326.7925 | 72.43161 | 1801.38 | 8024.987 | 9784.529 | 10233.27 | 3315.042 | 8285.528 | 7383.427 | 14375.97 | 7901.818 | 6474.137 | 3161.551 | 1637.238 | 3899.401 | 5751.466 | 4128.792 | 14295.94 | 7819.87 | 512.4566 | 18023.86 | 7794.866 | 17457.06 |
| CUST_37011_PI429010886 | M17535 | 0.000465 | 15 | 9 | | | | | | 892.3589 | 1078.296 | 916.49 | 838.6949 | 763.4512 | 720.9482 | 947.8741 | 931.7811 | 933.7706 | 1093.92 | 1083.765 | 1155.257 | 894.0864 | 1145.003 | 938.0188 | 1285.231 | 1012.109 | 942.3167 | 856.8878 | 1007.431 | 861.4839 | 1109.205 | 1027.698 | 739.0931 | 1382.607 | 958.7977 | 1058.786 |
| CUST_39757_PI429010886 | M44093 | 0.000465 | 15 | 9 | 220172371 | putative elastin a [Rimicaris exoculata] | | | 1.56E-07 | 428.7852 | 952.9126 | 2314.02 | 191.4276 | 244.0822 | 117.6603 | 460.3656 | 2166.108 | 3630.71 | 3438.186 | 1403.267 | 3414.171 | 1933.842 | 4575.004 | 2319.618 | 1173.656 | 717.957 | 844.642 | 912.7145 | 1424.308 | 1406.77 | 3704.973 | 3071.798 | 259.8087 | 4377.65 | 2673.681 | 6136.091 |
| CUST_47920_PI429010886 | M78162 | 0.000466 | 21 | 18 | | | | | | 99.68173 | 156.0949 | 129.8259 | 111.0963 | 76.41163 | 123.1229 | 74.4613 | 54.89215 | 67.37108 | 117.5843 | 98.74368 | 103.4972 | 86.97645 | 120.5115 | 129.9842 | 128.2673 | 102.6011 | 130.9315 | 93.97578 | 134.3489 | 162.98 | 107.3346 | 135.5426 | 119.3258 | 99.32217 | 118.7227 | 75.63407 |
| CUST_55110_PI429010886 | M108763 | 0.000467 | 12 | 3 | | | | | | 65.70437 | 36.2246 | 54.43748 | 42.01359 | 38.43703 | 48.11605 | 48.36598 | 36.54259 | 37.09496 | 23.69143 | 26.57271 | 22.96293 | 21.39067 | 28.45961 | 38.92558 | 36.62028 | 48.67882 | 38.95593 | 25.25069 | 36.92111 | 26.58817 | 19.75021 | 26.04444 | 35.38465 | 27.38056 | 28.34245 | 27.27727 |
| CUST_24238_PI429010886 | N50280 | 0.000468 | 15 | 12 | | | | | | 88.03425 | 91.62991 | 80.01037 | 60.85469 | 93.14117 | 54.55709 | 63.36658 | 74.97848 | 59.28015 | 97.282 | 80.61162 | 97.56553 | 92.80312 | 92.37125 | 141.8752 | 88.63339 | 91.51834 | 71.93323 | 71.75603 | 84.7131 | 59.52835 | 74.17182 | 78.01256 | 82.00472 | 92.01375 | 96.1698 | 90.6343 |
| CUST_28040_PI429010886 | N57161 | 0.00047 | 15 | 9 | | | | | | 23.33049 | 183.091 | 111.6027 | 67.50801 | 81.79478 | 52.96132 | 109.2001 | 160.974 | 264.6685 | 544.0717 | 158.91 | 272.2987 | 154.0002 | 424.3711 | 255.1463 | 119.9123 | 138.323 | 108.6177 | 117.2428 | 337.8584 | 153.5542 | 466.9714 | 322.2737 | 112.4193 | 584.5557 | 538.8855 | 510.2851 |
| CUST_18606_PI429010886 | N40088 | 0.00047 | 15 | 9 | 322801097 | hypothetical protein SINV_06464 [Solenopsis invict | | | 2.61E-11 | 270.8602 | 234.853 | 325.9737 | 238.5792 | 269.4403 | 292.9566 | 334.5634 | 282.2712 | 284.5979 | 372.3592 | 353.689 | 388.966 | 278.6632 | 370.3508 | 360.4943 | 336.7538 | 277.4454 | 327.7237 | 258.9692 | 412.0745 | 250.6384 | 392.9826 | 296.7454 | 314.4386 | 337.4667 | 439.1396 | 400.0206 |
| CUST_33691_PI429010886 | M7617 | 0.000472 | 12 | 3 | 321463229 | hypothetical protein DAPPUDRAFT_324499 [Daphni | | | 4.93E-14 | 26.31122 | 19.5508 | 23.82763 | 30.32196 | 23.56736 | 24.62928 | 32.09701 | 16.87055 | 19.68008 | 21.24533 | 15.2714 | 20.65925 | 22.49622 | 26.53793 | 21.08658 | 30.0998 | 35.87751 | 25.2475 | 30.08532 | 24.5797 | 18.64164 | 13.12545 | 15.12966 | 20.13319 | 22.06655 | 25.14408 | 29.97696 |
| CUST_49295_PI429010886 | M85062 | 0.000472 | 12 | 3 | | | | | | 46.58823 | 40.17935 | 23.4177 | 90.26625 | 77.69279 | 97.77159 | 61.45704 | 27.67861 | 47.92719 | 11.00948 | 21.89788 | 4.160102 | 79.13143 | 14.9248 | 42.72555 | 88.33792 | 46.02417 | 95.33691 | 62.74739 | 10.98836 | 15.91598 | 40.02682 | 15.18342 | 46.98145 | 30.82801 | 29.36188 | 14.42559 |
| CUST_34468_PI429010886 | M9758 | 0.000473 | 21 | 18 | 345480130 | PREDICTED: bifunctional polynucleotide phosphatas | | | 8.36E-75 | 94.10691 | 139.359 | 68.05219 | 98.29921 | 97.1202 | 94.3579 | 65.19644 | 65.69324 | 68.36198 | 114.5891 | 114.9498 | 105.2698 | 118.2899 | 90.92371 | 107.898 | 188.3253 | 86.81547 | 135.78 | 115.6759 | 154.0002 | 123.6853 | 121.9609 | 111.6142 | 89.21981 | 87.47537 | 102.8077 | 86.08273 |
| CUST_5919_PI429010886 | N11066 | 0.000475 | 21 | 12 | 344244211 | Zinc finger protein 252 [Cricetulus griseus] | | | 5.46E-13 | 14.22183 | 19.09041 | 15.4125 | 11.78768 | 13.2544 | 10.34523 | NA | 11.96936 | 8.396527 | 8.329617 | 9.343228 | 12.47112 | NA | NA | 10.4969 | 21.24533 | 9.021728 | 16.14981 | 7.96115 | 16.66793 | 16.20947 | 19.5508 | 19.40477 | 17.33525 | 20.8959 | 13.17448 | 15.04503 |
| CUST_6490_PI429010886 | N20170 | 0.00048 | 12 | 0 | 342326276 | dynein light chain roadblock-type 2 [Cherax quadric | | | 1.25E-45 | 194.9182 | 149.2244 | 112.4193 | 128.6369 | 68.68381 | 91.87634 | 84.54494 | 144.6688 | 120.496 | 73.78486 | 96.56866 | 63.69473 | 57.58322 | 72.58192 | 71.37464 | 165.9503 | 112.8118 | 111.8492 | 172.6882 | 61.64915 | 91.20024 | 113.2611 | 136.0004 | 125.6996 | 172.408 | 131.259 | 154.0476 |
| CUST_19858_PI429010886 | N42331 | 0.000481 | 15 | 9 | | | | | | 60.82114 | 56.99104 | 73.803 | 35.45118 | 52.38937 | 45.51332 | 33.35513 | 61.12874 | 53.43124 | 78.38473 | 62.62665 | 67.18762 | 55.55289 | 60.47407 | 64.73839 | 79.07032 | 64.38672 | 63.46235 | 55.36449 | 54.56432 | 55.64622 | 66.96201 | 63.03207 | 42.25718 | 94.68098 | 65.02562 | 89.97695 |
| CUST_22095_PI429010886 | N46416 | 0.000482 | 24 | 18 | 328671709 | WDR13 protein [Clarias gariepinus] | | | 2.9E-14 | 15.69467 | 28.21594 | 24.37063 | 14.6546 | 19.00448 | 18.24494 | 17.9021 | 18.79291 | 17.26373 | 21.3486 | 17.12411 | 24.67666 | 35.60511 | 19.93091 | 30.95206 | 39.94582 | 25.56608 | 20.95699 | 26.00383 | 62.28748 | 80.44402 | 30.43439 | 19.74282 | 13.44283 | 25.87358 | 21.71104 | 25.49499 |
| CUST_4181_PI429010886 | N16181 | 0.000487 | 15 | 12 | | | | | | 18.42118 | 16.9342 | 23.15281 | 13.69402 | 20.02647 | 12.71344 | 4.881326 | 16.08632 | 14.31728 | 21.87774 | 15.26556 | 18.78041 | 27.71021 | 34.95466 | 26.21698 | 24.06718 | 14.78359 | 25.56371 | 11.77935 | 8.421448 | 13.47949 | 24.17061 | 13.19079 | 16.07442 | 14.62058 | 12.29647 | 30.90472 |
| CUST_19260_PI429010886 | N41268 | 0.000487 | 18 | 3 | | | | | | 89.20945 | 100.4336 | 101.6948 | 109.3629 | 83.31796 | 161.2704 | 77.00574 | 50.05524 | 79.89836 | 93.24144 | 66.54991 | 39.90155 | 45.2026 | 95.7582 | 29.1329 | 90.68288 | 45.42508 | 108.7711 | 99.25819 | 91.51834 | 89.68808 | 105.57 | 112.4734 | 115.9016 | 104.3119 | 82.11078 | 54.64196 |
| CUST_45346_PI429010886 | M68809 | 0.0049 | 12 | 9 | | | | | | 20.47619 | 49.03725 | 83.97119 | 8.111614 | 18.10692 | 15.00845 | 31.19824 | 38.24749 | 26.30787 | 60.47407 | 46.67853 | 102.9064 | 76.53451 | 29.32849 | 45.00065 | 37.77068 | 12.56262 | 7.92158 | 20.73391 | 207.853 | 24.69594 | 29.66445 | 116.8917 | NA | 61.1802 | 23.82095 | 37.33083 |
| CUST_41697_PI429010886 | M58337 | 0.000493 | 15 | 12 | | | | | | 82.53975 | 120.363 | 91.91837 | 53.59509 | 32.48902 | 6.756825 | 439.5513 | 254.3377 | 719.8537 | 214.5304 | 77.31411 | 194.3859 | 1165.064 | 1105.691 | 595.8659 | 688.3695 | 478.8398 | 80.87419 | 154.1347 | 99.52485 | 75.03476 | 151.2719 | 164.8298 | 186.883 | 1383.78 | 101.9838 | 2256.115 |
| CUST_10079_PI429010886 | N25659 | 0.000494 | 12 | 9 | | | | | | 145.9801 | 207.885 | 186.9949 | 169.2493 | 75.87721 | 128.2524 | 265.3779 | 403.9415 | 252.7637 | 137.5279 | 265.1519 | 311.4551 | 230.3934 | 386.2115 | 254.0367 | 186.3153 | 95.14966 | 152.5866 | 236.5825 | 166.8948 | 266.6447 | 367.3426 | 336.682 | 418.2809 | 224.7094 | 178.6581 | 231.6114 |
| CUST_14540_PI429010886 | N33050 | 0.000496 | 15 | 12 | 242015281 | cell adhesion molecule, putative [Pediculus humanu | | | 7.64E-08 | 58.80718 | 40.20429 | 73.79476 | 42.74237 | 41.35575 | 46.84472 | 56.35545 | 50.64584 | 59.70379 | 63.1995 | 55.49391 | 60.25585 | 68.68381 | 77.76127 | 81.91685 | 67.34293 | 68.70977 | 65.26348 | 59.6278 | 59.18762 | 39.76167 | 64.4321 | 50.51081 | 40.99424 | 90.36196 | 53.36025 | 87.69732 |
| CUST_13659_PI429010886 | N31540 | 0.000498 | 18 | 6 | | | | | | 20.18978 | 22.33967 | 26.70117 | 21.54483 | 31.81303 | 23.98555 | 39.05183 | 27.30026 | 30.67698 | 22.01737 | 20.6434 | 14.33237 | 26.55213 | 42.12575 | 17.94517 | 19.52587 | 17.32922 | 20.06979 | 27.0727 | 22.8445 | 26.66065 | 24.42322 | 74.4613 | 24.16065 | 38.20891 | 26.95815 | 55.64028 |
| CUST_37774_PI429010886 | M21508 | 0.000499 | 15 | 12 | 170040829 | predicted protein [Culex quinquefasciatus] >gi\|167 | | | 2.72E-14 | 610.9693 | 1397.451 | 1016.851 | 330.1788 | 321.5473 | 141.0258 | 3446.227 | 2267.686 | 5098.883 | 1726.714 | 606.0546 | 2244.992 | 13400.98 | 11510.47 | 6992.837 | 6969.97 | 3917.614 | 540.3776 | 1531.156 | 749.9064 | 828.3608 | 1106.942 | 1193.941 | 1897.37 | 12130.73 | 1134.016 | 17535.03 |
| CUST_34151_PI429010886 | M104815 | 0.000503 | 18 | 12 | | | | | | 77.16796 | 77.70756 | 70.48615 | 64.61143 | 63.52108 | 70.08131 | 63.89005 | 63.24801 | 51.92326 | 97.23707 | 71.11204 | 55.52574 | 57.77749 | 66.12218 | 33.00843 | 112.9639 | 76.27537 | 67.1799 | 78.60763 | 89.08731 | 58.74569 | 105.6505 | 84.49247 | 80.37038 | 77.80891 | 94.65769 | 74.66117 |
| CUST_8892_PI429010886 | N23757 | 0.000505 | 24 | 18 | 321470773 | hypothetical protein DAPPUDRAFT_317395 [Daphn | Nucleic acid metabolism | Regulation of gene expression, epigenetic | 1.12E-57 | 26.51217 | 23.61053 | 17.61145 | 12.03276 | 16.35941 | 10.19943 | 13.42725 | 22.87829 | 8.688469 | 11.64631 | 13.38764 | 23.87529 | 14.61247 | 17.85496 | 24.35681 | 31.1463 | 11.23845 | 21.26332 | 43.93042 | 27.8177 | 24.59907 | 24.01545 | 23.06016 | 15.77573 | 18.72625 | 17.9566 | 17.31011 |
| CUST_25746_PI429010886 | N52980 | 0.000505 | 18 | 15 | | | | | | 52.063 | 53.1338 | 55.04289 | 36.50859 | 58.78466 | 42.71532 | 46.37939 | 61.77428 | 58.23835 | 66.45252 | 70.21778 | 81.8019 | 61.87735 | 71.81025 | 58.66467 | 58.91707 | 64.90243 | 59.17148 | 62.583 | 83.49596 | 58.92815 | 57.5238 | 44.58058 | 58.69441 | 66.63641 | 65.69324 | 83.6912 |
| CUST_27041_PI429010886 | N55340 | 0.000505 | 15 | 12 | | | | | | 402.6962 | 480.5601 | 434.1796 | 348.6809 | 360.6396 | 393.6847 | 364.0324 | 365.8372 | 392.3971 | 484.0144 | 463.6969 | 508.1895 | 426.3129 | 519.9761 | 536.7924 | 484.5005 | 388.6405 | 422.0003 | 372.8033 | 510.9063 | 364.475 | 480.2626 | 398.2452 | 403.4054 | 494.0702 | 437.1883 | 518.627 |
| CUST_19010_PI429010886 | N40824 | 0.000505 | 18 | 12 | | | | | | 17.68838 | 16.86014 | 14.24194 | NA | 56.64379 | NA | 14.58166 | 22.15217 | NA | 52.89103 | NA | 167.6977 | 434.2638 | 399.2623 | 267.2122 | 95.98918 | 599.7852 | 15.39132 | 10.04605 | 5.075645 | 45.37849 | 59.16475 | 8.454339 | 13.46425 | 57.02142 | 59.99556 | 176.4383 |
| CUST_28117_PI429010886 | N57329 | 0.000506 | 21 | 18 | | | | | | 74.83385 | 79.77146 | 68.33792 | 60.00971 | 98.46582 | 74.99621 | 56.52083 | 56.72941 | 58.17407 | 71.73214 | 56.48454 | 59.37489 | 60.31192 | 66.69348 | 76.26718 | 100.9304 | 68.21242 | 79.88802 | 88.33792 | 97.67416 | 78.84468 | 79.1048 | 94.36809 | 68.09627 | 87.00147 | 69.62631 | 72.53579 |
| CUST_25179_PI429010886 | N51922 | 0.000506 | 21 | 0 | | | | | | 42.37752 | 37.35358 | 42.39985 | 55.43705 | 33.57359 | 67.06743 | 41.11909 | 46.18431 | 44.63407 | 34.97152 | 33.20988 | 35.1842 | 32.69174 | 29.76585 | 31.93715 | 36.90242 | 58.67101 | 51.97626 | 32.03971 | 31.52633 | 24.27705 | 33.30259 | 43.36723 | 34.05805 | 53.9331 | 59.91437 | 44.97088 |
| CUST_17457_PI429010886 | N37994 | 0.000507 | 12 | 9 | | | | | | 4.972361 | 5.229452 | 14.37012 | 3.636142 | 6.98493 | 5.55948 | 10.05076 | 14.4735 | 10.7559 | NA | NA | 27.025 | 10.02837 | 8.091683 | 6.02768 | 3.469404 | 5.063215 | 6.234005 | 5.092364 | 5.307045 | 12.58347 | 16.97989 | 13.00912 | NA | 7.194544 | 18.56579 | 10.23894 |
| CUST_18615_PI429010886 | N40100 | 0.000508 | 18 | 15 | | | | | | 17.28828 | 16.53144 | 20.79184 | 12.7744 | 11.90796 | 6.630038 | 28.98096 | 17.24517 | 22.32278 | 18.95062 | 22.02142 | 25.43424 | 14.37012 | 34.52588 | 29.52168 | 35.23712 | NA | 31.44747 | 28.03123 | 13.51511 | 18.32903 | 21.06491 | 9.935826 | NA | 16.11406 | 11.47955 | 28.35396 |
| CUST_55673_PI429010886 | M111215 | 0.000509 | 15 | 9 | | | | | | 79.33173 | 53.90994 | 132.7602 | 84.20531 | 92.85168 | 101.4018 | 80.19532 | 160.1541 | 139.47 | 155.1509 | 146.405 | 182.9432 | 71.87199 | 165.716 | 109.8902 | 64.76438 | 123.7165 | 70.36056 | 120.3338 | 137.058 | 110.9326 | 128.8011 | 196.359 | 57.72307 | 204.1851 | 138.7005 | 228.1106 |
| CUST_53091_PI429010886 | M100115 | 0.00051 | 12 | 0 | 225875099 | Euphausia superba cry gene for cryptochrome, exor | | | 1.61E-64 | 24.59542 | 20.56111 | 34.37176 | 18.5156 | 23.22645 | 27.00004 | 17.69892 | 17.28408 | 17.13668 | 31.16892 | 18.8381 | 28.48649 | 25.82466 | 30.81693 | 33.11671 | 19.00858 | 15.10731 | 30.81978 | 17.85258 | 19.12864 | 21.71687 | 24.83327 | 21.24048 | 23.01868 | 30.50065 | 26.18577 | 37.52021 |
| CUST_42415_PI429010886 | M60224 | 0.000513 | 15 | 9 | | | | | | 165.9249 | 103.0135 | 129.3018 | 92.13028 | 75.04971 | 111.8739 | 70.02979 | 72.41676 | 61.05172 | 94.56836 | 95.0012 | 66.86178 | 98.67215 | 128.7649 | 86.95691 | 167.9037 | 142.963 | 127.8994 | 154.3834 | 85.15981 | 134.0969 | 64.65683 | 130.1875 | 88.6791 | 144.3428 | 75.88452 | 83.95292 |
| CUST_42919_PI429010886 | M61589 | 0.000515 | 24 | 18 | 345498442 | PREDICTED: glycerol-3-phosphate acyltransferase 1 | | | 4.42E-16 | 156.0301 | 117.0372 | 106.2054 | 65.25641 | 147.0578 | 108.5891 | 123.9921 | 103.2645 | 123.6053 | 130.7837 | 153.3324 | 156.7533 | 142.0014 | 145.4206 | 113.3366 | 164.7416 | 248.652 | 174.8316 | 172.1063 | 247.8273 | 291.6371 | 199.3801 | 126.2814 | 103.8326 | 192.5322 | 130.2691 | 167.2554 |
| CUST_22687_PI429010886 | N47460 | 0.000517 | 24 | 21 | 543171838 | CD209 antigen-like protein D [Oncorhynchus mykiss | | | 9.1E-08 | 716.9224 | 833.259 | 672.0887 | 478.5158 | 508.0799 | 776.8406 | 457.7503 | 442.852 | 347.1819 | 619.2505 | 573.2606 | 462.7955 | 308.3972 | 526.943 | 562.3412 | 959.6386 | 585.6944 | 772.1398 | 702.2549 | 653.9628 | 683.1376 | 600.2864 | 774.9868 | 923.7156 | 1148.187 | 785.705 | 1035.582 |
| CUST_46762_PI429010886 | M73653 | 0.00052 | 21 | 3 | | | | | | 89.419 | 139.8337 | 130.3926 | 330.6123 | 129.2002 | 146.882 | 108.3037 | 91.7472 | 103.5071 | 119.1637 | 49.8924 | 145.201 | 74.15113 | 94.70207 | 23.80171 | 64.69663 | 36.3679 | 74.29008 | 36.39953 | 76.31563 | 172.5457 | 95.689 | 208.8792 | 76.668 | 130.7837 | 214.6269 | 87.19276 |

| Probe ID | ID | p-value | Period | Phase | EBI ID | Annotation | GO category | Go term | E-value | 0 | 0 | 0 | 3 | 3 | 3 | 6 | 6 | 6 | 9 | 9 | 9 | 12 | 12 | 12 | 15 | 15 | 15 | 18 | 18 | 18 | 21 | 21 | 21 | 24 | 24 | 24 |
|---|---|---|---|---|---|---|---|---|---|---|---|---|---|---|---|---|---|---|---|---|---|---|---|---|---|---|---|---|---|---|---|---|---|---|---|---|
| CUST_6463_PI429010886 | N20132 | 0.00052 | 21 | 18 | 345491105 | PREDICTED: DOMON domain-containing protein CG | | | 3.52E-40 | 40.53465 | 30.86679 | 36.34355 | 27.59937 | 29.82743 | 28.92063 | 30.91453 | 28.48649 | 31.03692 | 40.33441 | 32.38272 | 37.47537 | 29.81837 | 43.12973 | 25.75936 | 34.53803 | 41.33159 | 50.1495 | 46.38212 | 50.8107 | 32.43067 | 34.68835 | 31.18382 | 27.57506 | 31.78686 | 23.95916 | 28.27339 |
| CUST_4168_PI429010886 | N16153 | 0.000521 | 24 | 15 | | | | | | 10.66241 | 7.465747 | 13.69592 | 7.681885 | 4.432447 | 9.362608 | 8.10145 | 12.00403 | 7.72233 | 18.71355 | 10.54492 | 12.15231 | 11.24362 | 17.67128 | 15.52381 | 36.68661 | 15.22088 | 23.08011 | 16.16875 | 8.382851 | 6.252271 | 19.89653 | 13.72673 | NA | 13.68318 | 9.462739 | 14.00504 |
| CUST_20282_PI429010886 | N43158 | 0.000521 | 15 | 0 | | | | | | 171.0319 | 118.3632 | 139.4033 | 141.4128 | 123.1229 | 132.3882 | 152.4947 | 98.69728 | 118.6617 | 95.81155 | 86.76715 | 91.28115 | 103.4319 | 105.798 | 108.8513 | 127.3036 | 143.7439 | 118.8883 | 139.359 | 78.88657 | 100.1858 | 126.4765 | 116.8442 | 109.2372 | 113.0567 | 73.73041 | 90.17193 |
| CUST_8096_PI429010886 | N22531 | 0.000524 | 15 | 12 | | | | | | 21.87979 | 37.18815 | 35.31167 | 22.3538 | 20.00906 | 42.19441 | 24.67666 | 30.0571 | 33.66515 | 25.45224 | 26.41719 | 34.84252 | 53.98541 | 62.09543 | 60.20751 | 43.95205 | 29.0776 | 32.47618 | 21.18631 | 20.56111 | 22.95261 | 38.80762 | 37.97967 | 19.93306 | 60.43419 | 34.41851 | 58.70421 |
| CUST_17805_PI429010886 | N38624 | 0.000524 | 21 | 3 | | | | | | 34.71088 | 49.69406 | 55.01225 | 106.064 | 61.00953 | 90.04123 | 43.76947 | 32.43067 | 32.27766 | 37.65143 | 19.24611 | 41.30947 | 21.10951 | 49.34559 | 15.56261 | 33.07929 | 18.25337 | 32.73103 | 28.29864 | 21.09489 | 72.78118 | 34.1704 | 74.70996 | 40.84069 | 75.68737 | 38.49034 | 42.79462 |
| CUST_9508_PI429010886 | N24758 | 0.000525 | 15 | 0 | 240972665 | chloride channel, putative [Ixodes scapularis] >gi\|2: | Transport | Ion transport | 1.06E-51 | 59.45837 | 51.20005 | 58.22987 | 37.91955 | 30.9788 | 47.2535 | 50.80203 | 35.6179 | 23.52324 | 40.01887 | 37.81058 | 49.13393 | 50.55173 | 44.31444 | 53.84859 | 54.71071 | 61.33812 | 56.68336 | 55.75375 | 69.04997 | 47.26675 | 48.78617 | 43.30223 | 32.48902 | 53.16758 | 33.86006 | 47.3396 |
| CUST_49339_PI429010886 | M85248 | 0.000526 | 18 | 3 | 321473453 | hypothetical protein DAPPUDRAFT_314716 [Daphn | Protein metabolism | Proteolysis | 2.05E-20 | 24.30007 | 71.73214 | 18.04283 | 22.75922 | 45.52612 | 34.27813 | 41.15368 | 21.60382 | 23.26285 | 26.51217 | 19.15845 | 14.61247 | 14.1623 | 10.33277 | 17.51958 | 35.32948 | 33.50669 | 23.71704 | 59.80641 | 25.42066 | 41.56303 | 43.87581 | 39.10739 | 44.6585 | 35.79389 | 19.71872 | 26.76116 |
| CUST_10669_PI429010886 | N26599 | 0.000529 | 24 | 21 | | | | | | 39.61818 | 47.54347 | 25.27468 | 27.5887 | 31.60035 | 22.8445 | 22.1833 | 24.30245 | 22.11953 | 42.93137 | 28.03356 | 34.29372 | 34.40003 | 34.81682 | 25.9871 | 36.36466 | 27.30432 | 38.14903 | 25.84268 | 40.31382 | 40.22531 | 32.30965 | 93.28305 | 98.4006 | 42.71853 | 52.98238 | 34.46849 |
| CUST_38401_PI429010886 | M26254 | 0.00053 | 15 | 12 | | | | | | 191.7997 | 150.914 | 131.4476 | 121.8439 | 214.7012 | 161.7305 | 98.18381 | 119.4271 | 69.05841 | 173.4736 | 177.0286 | 139.5098 | 171.6689 | 258.7017 | 180.8735 | 139.4033 | 156.7999 | 119.9711 | 139.7854 | 148.3091 | 128.7123 | 133.561 | 113.0059 | 95.72441 | 354.2035 | 92.95468 | 146.6794 |
| CUST_46225_PI429010886 | M71845 | 0.000531 | 18 | 12 | | | | | | 686.896 | 460.3656 | 1163.545 | 336.4536 | 374.3107 | 362.2892 | 980.1159 | 760.5296 | 611.2062 | 757.419 | 402.7742 | 599.656 | 685.9612 | 1114.646 | 757.6325 | 526.8072 | 496.7325 | 400.4224 | 561.2769 | 400.3298 | 445.4133 | 520.4614 | 399.7554 | 356.1302 | 624.8531 | 588.9896 | 680.741 |
| CUST_42573_PI429010886 | M60611 | 0.000534 | 18 | 3 | 126277740 | PREDICTED: dyslexia susceptibility 1 candidate gene | | | 1.53E-13 | 23.46033 | 38.36859 | 28.96949 | 57.57631 | 33.51452 | 67.12072 | 24.27461 | 34.59566 | 24.99108 | 20.2329 | 23.36117 | 72.39117 | 22.47267 | 12.95753 | 12.68111 | 25.36189 | 20.73682 | 27.14607 | 34.28772 | 15.10512 | 26.67812 | 24.32948 | 29.28006 | 43.94258 | 28.90009 | 60.98516 | 23.71505 |
| CUST_26688_PI429010886 | N54697 | 0.000535 | 24 | 18 | | | | | | 43.58778 | 49.23067 | 44.38974 | 39.91268 | 40.63191 | 21.05599 | 39.93503 | 30.5332 | 56.28989 | 41.24587 | 41.84554 | 46.29572 | 46.90121 | 53.41262 | 32.22652 | 51.07827 | 76.58426 | 43.11747 | 72.89846 | 57.71214 | 75.30403 | 65.84223 | 46.76455 | 30.28193 | 70.08131 | 41.03316 | 59.9279 |
| CUST_32246_PI429010886 | M3621 | 0.000539 | 18 | 15 | 380015144 | PREDICTED: uncharacterized FAM18-like protein CG | | | 1.26E-61 | 450.7936 | 455.5337 | 352.3859 | 372.4888 | 400.4224 | 340.6846 | 320.9097 | 270.0917 | 314.8877 | 419.4237 | 354.3972 | 388.3479 | 370.0362 | 339.1053 | 353.8817 | 493.9819 | 358.4672 | 399.0919 | 378.1899 | 386.5802 | 341.396 | 413.2538 | 361.6235 | 335.2532 | 351.6009 | 329.0813 | 337.9324 |
| CUST_18000_PI429010886 | N38975 | 0.000542 | 15 | 12 | 395535054 | PREDICTED: ralBP1-associated Eps domain-containi | Transport | Vesicle-mediated transport | 2.16E-09 | 25.19666 | 12.4022 | 23.85453 | 16.39936 | 10.24518 | 11.68718 | 15.1999 | 22.74587 | 18.27067 | 28.8656 | 12.86855 | 17.45713 | 22.17314 | 25.2086 | 24.2722 | 19.82681 | 13.8081 | 20.22682 | 6.65508 | 14.21497 | 11.49378 | 23.05482 | 22.67397 | 15.04717 | 22.5725 | 19.87525 | 18.40643 |
| CUST_35513_PI429010886 | M12761 | 0.000543 | 21 | 0 | | | | | | 226.1214 | 322.4251 | 293.8285 | 67.31728 | 73.39754 | 89.51747 | 81.63335 | 109.3542 | 133.135 | 78.27179 | 236.6906 | 25.4551 | 151.7898 | 58.92815 | 89.69655 | 381.0616 | 87.44346 | 188.9157 | 104.8092 | 172.9946 | 218.9512 | 534.2439 | 235.0953 | 431.462 | 203.0622 | 194.4273 | 355.6761 |
| CUST_27565_PI429010886 | N56284 | 0.000547 | 15 | 9 | 338224495 | hypothetical protein [Scylla paramamosain] | | | 4.32E-34 | 5.022376 | 9.238126 | 25.76654 | 8.003112 | 7.204193 | 11.16458 | 16.33291 | 28.54216 | 18.91214 | 33.80408 | 13.22555 | 36.46183 | 18.1208 | 25.52881 | 26.27382 | 15.84016 | 15.85326 | 11.09239 | 18.61046 | 12.35815 | NA | 27.58029 | 30.21934 | 12.80086 | 28.66257 | 24.32948 | 39.32573 |
| CUST_34812_PI429010886 | M10769 | 0.000548 | 21 | 0 | | | | | | 42.89937 | 45.87335 | 55.14182 | 33.7306 | 46.2898 | 32.60782 | 25.82998 | 26.04992 | 44.86385 | 51.2626 | 40.79309 | 42.16099 | 22.89976 | 22.79184 | 23.13337 | 31.88591 | 38.57181 | 32.39121 | 34.49254 | 19.79173 | 33.70683 | 67.52161 | 55.87064 | 56.72265 | 76.57062 | 35.92246 | 24.78858 |
| CUST_3791_PI429010886 | N15275 | 0.000548 | 24 | 21 | | | | | | 16.96853 | 32.87464 | 27.70733 | 23.91073 | 22.05258 | 18.64164 | 12.93141 | 13.83327 | 16.78198 | 27.12267 | 23.88015 | 19.38333 | 20.6935 | 31.87279 | 25.12826 | 29.6237 | 17.53908 | 27.78747 | 17.08222 | 30.76441 | 25.08483 | 34.55538 | 31.91824 | 27.54224 | 30.35236 | 31.62724 | 31.267 |
| CUST_23396_PI429010886 | N48765 | 0.000551 | 21 | 3 | 443717209 | hypothetical protein CAPTEDRAFT_227704 [Capitell | | | 3.69E-07 | 21.37183 | 42.10422 | 29.21058 | 71.15715 | 25.96255 | 39.99701 | 16.41585 | 21.07965 | 25.77304 | 26.92502 | 8.060022 | 41.9189 | 7.704662 | 20.92823 | 6.15687 | 16.85623 | 15.08227 | 10.22956 | NA | 15.36478 | 48.55278 | 18.9048 | 49.09421 | 17.64263 | 33.00184 | 53.03364 | 26.93068 |
| CUST_17391_PI429010886 | N37888 | 0.000552 | 24 | 18 | 307199491 | Chromodomain-helicase-DNA-binding protein Mi-2- | | | 2.34E-40 | 153.2997 | 188.7198 | 147.3674 | 133.2626 | 131.259 | 106.9501 | 110.8992 | 96.4587 | 104.4971 | 259.3271 | 158.5396 | 207.128 | 162.8265 | 149.1678 | 172.957 | 282.0775 | 153.8044 | 170.6936 | 215.028 | 241.5575 | 227.604 | 157.9028 | 156.5208 | 114.8668 | 201.6148 | 147.7426 | 244.775 |
| CUST_15257_PI429010886 | N34277 | 0.000553 | 24 | 21 | 242005598 | conserved hypothetical protein [Pediculus humanu | Metabolic process | Other | 1.54E-47 | 12.24341 | 8.364324 | 6.666438 | 17.83033 | 7.716948 | 13.22835 | 5.685852 | 7.529742 | 4.613954 | 6.007021 | 4.842579 | 8.33181 | 7.566637 | 10.58768 | 8.29527 | 9.530867 | 7.049754 | 22.33967 | 13.57505 | NA | 13.01679 | NA | 14.01909 | 62.05533 | 12.78901 | 16.92637 | 10.43918 |
| CUST_52756_PI429010886 | M98803 | 0.000555 | 21 | 9 | | | | | | 46.08794 | 95.78605 | 59.78571 | NA | 9.403927 | NA | 12.4672 | NA | 53.13993 | 197.384 | 195.6579 | 331.4379 | NA | 211.2718 | 88.75833 | 983.8663 | NA | 54.14583 | 129.8445 | 226.151 | 105.4619 | 50.69847 | 23.26869 | 7.874648 | 8.220442 | 26.64821 | 32.67128 |
| CUST_45351_PI429010886 | M68819 | 0.000555 | 24 | 18 | 260841562 | hypothetical protein BRAFLDRAFT_118457 [Branchi | | | 3.1E-08 | 371.7567 | 30.28884 | 885.3464 | 77.92082 | 138.3904 | 72.76448 | 285.1566 | 614.705 | 90.74799 | 177.5884 | 151.0563 | 150.4117 | 853.6849 | 1353.303 | 586.3578 | 161.0687 | 457.9897 | 207.853 | 1446.944 | 866.5099 | 1912.382 | 620.7934 | 415.0913 | 545.1015 | 1325.228 | 515.8865 | 766.6399 |
| CUST_11477_PI429010886 | N27878 | 0.000558 | 18 | 9 | 302821822 | hypothetical protein SELMODRAFT_186850 [Selagir | | | 1.03E-26 | 26.1797 | 22.41713 | 26.46898 | 24.44087 | 20.10727 | 24.38855 | 27.88984 | 30.42483 | 46.48282 | 38.01489 | 33.57359 | 35.8336 | 35.31167 | 44.56843 | 36.38283 | 32.38272 | 24.20666 | 18.72053 | 32.58533 | 33.88263 | 23.47811 | 16.65858 | 26.39223 | 22.18079 | 21.44644 | 32.74781 | 35.42916 |
| CUST_14284_PI429010886 | N32632 | 0.000558 | 21 | 15 | | | | | | 47.40881 | 65.84868 | 49.09421 | 23.56991 | 48.35331 | 32.7674 | 44.07419 | 26.7682 | 42.72999 | 58.27728 | 34.42762 | 51.25067 | 36.8424 | 34.80955 | 33.17839 | 79.21035 | 56.91814 | 53.30382 | 51.273 | 51.4 | 54.08762 | 49.46138 | 46.78356 | 35.43589 | 46.80174 | 32.62278 | 46.12938 |
| CUST_4993_PI429010886 | N18089 | 0.000561 | 24 | 18 | | | | | | 4.66 | 8.830666 | 10.64869 | 6.99897 | 4.02272 | NA | NA | 5.370411 | 10.00209 | 14.60389 | 11.80273 | 7.666723 | 6.24043 | NA | 12.85919 | 18.35292 | NA | NA | 25.24216 | 21.26849 | NA | 9.883505 | 19.01018 | 7.82103 | 7.862605 | 8.039625 | 8.033791 |
| CUST_26714_PI429010886 | N54754 | 0.000562 | 24 | 18 | | | | | | 91.77189 | 93.25437 | 106.4186 | 85.09002 | 91.02196 | 90.12961 | 145.6105 | 98.129 | 100.336 | 101.2088 | 91.11943 | 129.3708 | 108.8415 | 110.5556 | 126.0122 | 104.3306 | 115.6518 | 113.6765 | 109.2515 | 135.4539 | 133.7601 | 127.9641 | 119.7932 | 109.5988 | 130.8991 | 106.1639 | 112.9777 |
| CUST_20872_PI429010886 | N44219 | 0.000574 | 18 | 3 | | | | | | 27.16436 | 38.08235 | 16.39504 | 53.04998 | 25.66555 | 48.80089 | 21.40624 | 13.84485 | 15.14895 | 12.28249 | 4.7017 | 9.233721 | 17.00562 | 6.668324 | 32.94363 | 27.85465 | 8.546868 | 26.12256 | 15.65092 | 14.80809 | NA | 24.97756 | NA | 47.37022 | 17.78837 | 21.96895 | 15.54199 |
| CUST_29050_PI429010886 | N59109 | 0.000574 | 24 | 21 | | | | | | 47.74853 | 44.83381 | 42.19897 | 34.52588 | 37.3381 | 51.21188 | 55.04289 | 39.42673 | 43.5925 | 31.52203 | 35.18059 | 30.24099 | 40.18446 | 35.44816 | 31.60035 | 47.87924 | 68.55373 | 32.35642 | 45.08069 | 35.07318 | 50.68297 | 52.10062 | 72.03066 | 68.74728 | 49.52577 | 34.56933 | 45.2026 |
| CUST_29544_PI429010886 | N60080 | 0.000574 | 24 | 21 | | | | | | 41.46468 | 45.33809 | 61.05172 | 34.46151 | 36.79155 | 68.68381 | 63.37918 | 41.82009 | 35.53745 | 33.75052 | 37.81592 | 34.33884 | 35.55848 | 50.82916 | 43.86454 | 46.16699 | 67.95716 | 47.21974 | 54.24943 | 36.53489 | 49.01735 | 56.55039 | 74.316 | 66.78979 | 42.29018 | 49.29515 | 43.70451 |
| CUST_31250_PI429010886 | M1078 | 0.000576 | 12 | 0 | | | | | | 207.6805 | 254.095 | 269.8218 | 195.1585 | 165.7972 | 163.7139 | 139.9664 | 193.3073 | 181.1348 | 146.9814 | 151.5201 | 184.3061 | 219.1881 | 287.3657 | 212.606 | 245.4886 | 193.4964 | 220.9369 | 182.9909 | 193.4709 | 141.1886 | 244.8254 | 182.3936 | 165.6162 | 317.5778 | 197.2228 | 220.3236 |
| CUST_52376_PI429010886 | M97232 | 0.000578 | 18 | 0 | | | | | | 19.66479 | 14.06114 | 11.17076 | NA | 8.711267 | NA | 6.17458 | 5.605736 | NA | NA | 10.25442 | 5.666175 | 8.173644 | 3.552205 | 5.327653 | 17.19461 | 14.44838 | 9.010507 | 11.38156 | 21.72694 | NA | 7.463527 | 9.008803 | 16.65858 | 10.82989 | NA | 6.099554 |
| CUST_21887_PI429010886 | N46043 | 0.000581 | 18 | 3 | | | | | | 28.41001 | 36.97731 | NA | 32.97999 | 49.96713 | 32.13794 | 29.73851 | 27.53031 | 19.26616 | 19.44722 | 16.12785 | 11.40858 | 18.51985 | 24.42075 | 18.57528 | 42.15075 | 18.86937 | 23.94321 | 22.3138 | 19.81675 | 7.081402 | 34.76295 | NA | 36.7953 | 23.64437 | 20.00906 | NA |
| CUST_45518_PI429010886 | M69362 | 0.000583 | 21 | 15 | | | | | | 33.75052 | 30.38917 | 27.35753 | 38.49034 | 33.92714 | 22.85699 | 31.34763 | 31.98439 | 36.40882 | 38.99636 | 38.83801 | 51.37508 | 39.71872 | 41.10188 | 49.998 | 47.71977 | 43.62743 | 39.0714 | 32.22274 | 79.22919 | 33.17446 | 42.52046 | 30.0998 | 21.16716 | 38.81815 | 34.74405 | 38.19149 |
| CUST_42427_PI429010886 | M60253 | 0.000592 | 21 | 0 | | | | | | 148.2153 | 161.3901 | 121.4843 | 125.5232 | 106.2054 | 104.6964 | 96.92806 | 101.8776 | 101.2274 | 134.6919 | 100.7206 | 97.10121 | 79.78328 | 112.609 | 120.0446 | 163.3073 | 122.2019 | 119.4271 | 113.8304 | 111.3943 | 112.7335 | 176.1434 | 121.649 | 137.8813 | 113.5137 | 136.2923 | 110.6458 |
| CUST_40558_PI429010886 | M55455 | 0.000593 | 12 | 3 | | | | | | 150.5899 | 180.016 | 94.01656 | 699.6538 | 659.6239 | 1230.999 | 525.5752 | 515.7103 | 97.18779 | 128.7498 | 459.9274 | 120.496 | 183.4152 | 97.95028 | 120.3232 | 181.7533 | 1030.678 | 780.9267 | 894.9707 | 172.1318 | 240.8224 | 148.9974 | 207.3956 | 397.3578 | 111.4084 | 405.2638 | 112.6604 |
| CUST_12256_PI429010886 | N29174 | 0.000593 | 12 | 9 | | | | | | 89.60537 | 207.5948 | 242.8547 | 42.48986 | 73.03522 | 86.12374 | 79.4217 | 105.6561 | 34.68251 | 173.9653 | 155.0724 | 136.5503 | 86.67899 | 91.01032 | 112.1834 | 112.8928 | 36.92465 | 64.29604 | 165.0586 | 151.0368 | 150.0554 | 169.2493 | 203.482 | 147.341 | 150.8963 | 99.23417 | 115.6327 |
| CUST_22231_PI429010886 | N46660 | 0.000597 | 24 | 18 | | | | | | 169.7841 | 185.59 | 174.8053 | 165.2222 | 162.3872 | 152.6523 | 137.2699 | 147.1104 | 139.9854 | 217.37 | 164.8084 | 230.2231 | 150.1195 | 163.6107 | 148.544 | 189.4567 | 165.8485 | 197.86 | 225.2614 | 215.4042 | 192.7945 | 180.1444 | 180.162 | 153.7558 | 223.1811 | 191.1131 | 189.0849 |
| CUST_34162_PI429010886 | M8915 | 0.000604 | 15 | 12 | | | | | | 42.74724 | 51.5653 | 43.58328 | 30.54779 | 45.51822 | 33.21734 | 52.13588 | 45.40667 | 34.15826 | 56.46834 | 47.77531 | 51.50234 | 59.61663 | 63.75313 | 54.09438 | 66.27543 | 60.09613 | 49.23067 | 38.28234 | 48.2481 | 30.41187 | 50.74837 | 48.02359 | 42.38979 | 65.36011 | 34.23165 | 45.91191 |
| CUST_44360_PI429010886 | M65711 | 0.000605 | 18 | 12 | | | | | | 19.46511 | 14.97314 | 26.45984 | 11.49378 | 6.881575 | 7.542588 | 15.34644 | 20.53115 | 18.07887 | 22.77196 | 11.83225 | 20.27391 | 21.18827 | 25.21717 | 16.35722 | 23.84605 | 21.6649 | 15.58555 | 13.91822 | 16.39936 | 10.6528 | 15.70628 | 11.64355 | 8.799195 | 26.73153 | 15.51056 | 19.32948 |
| CUST_10476_PI429010886 | N26294 | 0.000614 | 24 | 15 | | | | | | 50.8107 | 53.99759 | 60.53815 | 54.04881 | 39.20036 | 63.52784 | 51.79974 | 55.01668 | 43.68024 | 62.05533 | 49.87667 | 45.83957 | 68.53497 | 75.65779 | 85.55586 | 66.4223 | 93.42325 | 82.82132 | 78.45985 | 95.42654 | 55.45229 | 77.69279 | 73.1075 | 62.3113 | 82.09306 | 56.27319 | 55.91435 |
| CUST_33208_PI429010886 | M6274 | 0.000616 | 21 | 18 | | | | | | 9.467102 | NA | NA | NA | 11.22153 | 7.044099 | 8.487802 | 6.235044 | 14.26332 | 6.779829 | 7.683919 | 5.839168 | 11.71876 | 9.570885 | 5.569488 | 12.98486 | 13.98479 | NA | 27.47445 | 15.59286 | 15.63054 | 16.66456 | NA | NA | 12.45979 | 7.253431 | 6.837856 |
| CUST_42476_PI429010886 | M60368 | 0.000616 | 24 | 12 | 443695271 | hypothetical protein CAPTEDRAFT_209723 [Capitell | | | 3.53E-13 | 9.740573 | 9.529604 | NA | 19.58405 | 11.19977 | 18.38957 | 11.05595 | 56.17738 | 12.82841 | 28.50232 | 4.682659 | 13.84136 | 23.04237 | 21.9058 | 33.63387 | 21.54677 | NA | 19.87757 | 18.6803 | 16.30067 | 10.67448 | 16.39064 | NA | 19.98178 | 9.585988 | 12.55145 | 7.434035 |
| CUST_42148_PI429010886 | M59539 | 0.000617 | 15 | 9 | | | | | | 141.6349 | 140.036 | 125.5654 | 149.1678 | 130.0885 | 118.8194 | 114.5891 | 124.7036 | 148.1764 | 167.7564 | 150.2035 | 163.0758 | 132.1335 | 157.6285 | 164.8776 | 149.4856 | 144.0112 | 123.2134 | 122.1911 | 156.1462 | 117.5244 | 158.91 | 140.1347 | 121.4442 | 214.921 | 160.0108 | 206.9847 |
| CUST_12613_PI429010886 | N29744 | 0.000617 | 21 | 0 | | | | | | 214.9913 | 253.7689 | 292.0563 | 187.2939 | 135.5426 | 233.6523 | 158.5157 | 175.2864 | 171.1636 | 188.7857 | 166.0384 | 187.5065 | 207.0153 | 237.6429 | 188.3473 | 211.0801 | 175.9719 | 194.9477 | 145.201 | 185.168 | 213.1195 | 228.0216 | 255.7893 | 197.1531 | 190.0645 | 179.9875 | 173.9653 |
| CUST_43015_PI429010886 | M61864 | 0.000628 | 18 | 9 | | | | | | 34.26311 | 23.93394 | 61.94492 | 13.93866 | 29.98761 | 24.66033 | 56.24014 | 44.71939 | 46.93128 | 85.87525 | 50.546 | 92.30386 | 42.59897 | 44.07091 | 89.06701 | 72.4241 | 30.87561 | 35.73798 | 53.46776 | 21.33598 | 27.89681 | 39.36991 | 27.34913 | 36.02402 | 27.99024 | 33.50292 | 50.91028 |
| CUST_48616_PI429010886 | M81280 | 0.000632 | 15 | 9 | 395509381 | PREDICTED: zinc finger protein 850-like, partial [Sar | | | 6.27E-27 | 18.35581 | 18.73634 | 15.10731 | 14.75975 | 12.39128 | 7.662248 | 14.27406 | 5.809357 | 19.44155 | 23.92396 | 25.59587 | 22.2321 | 18.33968 | 22.06407 | 17.33078 | 20.72901 | 18.35292 | 15.9338 | 12.22747 | 17.15304 | 12.80617 | 24.47717 | 19.14544 | 8.908142 | 23.17379 | 13.52517 | 22.38584 |
| CUST_13701_PI429010886 | N31619 | 0.000632 | 15 | 9 | | | | | | 929.5412 | 984.9879 | 3981.392 | 238.0845 | 208.138 | 71.26057 | 1129.308 | 4623.212 | 5980.233 | 6587.101 | 2139.684 | 5193.144 | 4484.875 | 9193.951 | 4139.452 | 3124.162 | 2172.159 | 894.2724 | 2027.433 | 3044.565 | 2756.174 | 8404.925 | 5108.798 | 293.3227 | 12697.77 | 3809.024 | 12822.96 |
| CUST_16827_PI429010886 | N36925 | 0.000632 | 15 | 0 | | | | | | 168.8237 | 214.5304 | 142.8727 | 155.4149 | 181.2186 | 147.2771 | 133.0251 | 119.4919 | 104.7731 | 135.5237 | 133.8101 | 154.3359 | 122.1728 | 130.2863 | 158.4788 | 199.4521 | 158.5396 | 159.5233 | 150.78 | 166.7311 | 128.7498 | 154.222 | 114.6697 | 109.6112 | 166.9868 | 148.3091 | 124.1092 |
| CUST_15925_PI429010886 | N35359 | 0.000633 | 12 | 3 | | | | | | 231.8541 | 176.9135 | NA | 395.8483 | 332.0008 | 388.7869 | 290.8954 | 223.676 | 169.3778 | 30.60425 | 113.2611 | 27.51707 | 235.5779 | 84.96672 | 142.2307 | 275.7295 | 382.5164 | 434.8523 | 301.1198 | 92.4425 | 87.64594 | 306.0346 | 8.361603 | 251.0141 | 153.5542 | 203.7326 | NA |
| CUST_26559_PI429010886 | N54447 | 0.000634 | 15 | 9 | | | | | | 97.99145 | 69.33625 | 78.22974 | 196.0648 | 168.7503 | 168.7755 | 125.6996 | 187.0582 | 194.9822 | 413.0515 | 376.9333 | 267.2122 | 317.5249 | 202.1751 | 271.918 | 70.66226 | 205.1195 | 84.87934 | 282.166 | 209.9313 | 147.6693 | 204.8478 | 83.85911 | 99.95963 | 422.8357 | 221.2065 | 249.3375 |
| CUST_50050_PI429010886 | M88402 | 0.000647 | 15 | 9 | | | | | | 344.24 | 408.9972 | 900.4832 | 126.6031 | 158.4788 | 94.46416 | 482.0705 | 725.1936 | 901.1709 | 774.5083 | 604.4329 | 961.8209 | 545.3681 | 1078.601 | 677.7517 | 655.7469 | 470.0153 | 479.0678 | 396.481 | 772.946 | 458.3652 | 815.3757 | 793.5884 | 260.055 | 976.3736 | 723.6629 | 965.2192 |
| CUST_47759_PI429010886 | M77507 | 0.000653 | 18 | 12 | 307178252 | NADPH--cytochrome P450 reductase [Camponotus | Metabolic process | Oxidation-reduction process | 1.23E-30 | 159.3655 | 227.312 | 192.6124 | 213.0864 | 197.8235 | 179.2925 | 226.6782 | 315.5114 | 203.453 | 270.707 | 256.7514 | 335.0674 | 264.4268 | 329.648 | 328.5456 | 257.2108 | 281.0282 | 213.8469 | 221.165 | 495.2283 | 185.5593 | 261.0269 | 187.4158 | 165.9503 | 220.6402 | 248.7474 | 269.1618 |
| CUST_12066_PI429010886 | N28871 | 0.000655 | 15 | 0 | | | | | | 794.7159 | 1137.917 | 853.3995 | 486.8632 | 859.0461 | 961.1254 | 316.4275 | 167.4539 | 391.7449 | 863.4186 | 792.7128 | 853.3995 | 358.6229 | 587.9777 | 522.5582 | 1371.865 | 948.7418 | 1022.691 | 712.1447 | 804.5642 | 784.6927 | 670.3311 | 589.2877 | 457.5237 | 1063.11 | 498.2267 | 773.8104 |
| CUST_12672_PI429010886 | N29848 | 0.000655 | 15 | 9 | | | | | | 178.7072 | 254.189 | 203.7634 | 122.1188 | 172.7927 | 114.8344 | 148.5237 | 218.8408 | 218.5128 | 295.6383 | 186.8543 | 209.6917 | 191.3918 | 241.638 | 227.9715 | 259.7236 | 152.2767 | 181.4398 | 150.6728 | 197.6332 | 165.0799 | 228.9161 | 205.5044 | 155.0724 | 237.0425 | 257.5442 | 265.3147 |
| CUST_17684_PI429010886 | N38408 | 0.000659 | 18 | 12 | 193705824 | PREDICTED: ankyrin repeat domain-containing prot | | | 1.25E-19 | 25.32849 | 12.47875 | 25.1894 | 15.77161 | 20.74695 | 13.97554 | 15.62557 | 21.15749 | 23.59336 | 176.5718 | 24.15101 | 14.4465 | 93.55006 | 134.486 | 31.46943 | 39.51034 | 29.11372 | 25.89483 | 27.54647 | 14.42276 | 14.03191 | 23.1883 | 22.88862 | 10.29246 | 27.07724 | 16.06632 | 22.8004 |
| CUST_27053_PI429010886 | N55360 | 0.000659 | 18 | 0 | | | | | | 212.4411 | 211.9851 | 79.84151 | 113.6358 | 138.5572 | 88.23558 | 69.34114 | 57.89544 | 79.86307 | 132.8611 | 78.38473 | 102.5272 | 111.151 | 175.1768 | 133.355 | 132.1519 | 39.29501 | 88.78018 | 179.2612 | 195.0743 | 135.7577 | 116.9944 | 52.14738 | 80.76562 | 76.14971 | 77.7747 | 83.81066 |
| CUST_32879_PI429010886 | M5354 | 0.000659 | 12 | 3 | | | | | | 829.1909 | 529.2101 | 20.97642 | 1146 | 1008.924 | 1665.324 | 1022.968 | 959.9717 | 777.0504 | 50.22974 | 153.5334 | 21.92896 | 1018.327 | 277.1919 | 530.5435 | 1035.582 | 1215.238 | 1496.572 | 1468.172 | 112.517 | 90.11072 | 716.9224 | 16.23047 | 989.1564 | 547.117 | 795.9721 | 13.82239 |
| CUST_33042_PI429010886 | M5815 | 0.000668 | 21 | 18 | 149716541 | PREDICTED: zinc finger protein ZPR1 [Equus caballu | | | 1.6E-99 | 183.8412 | 221.873 | 144.7965 | 168.9486 | 166.9868 | 148.886 | 130.6003 | 119.6624 | 125.8598 | 164.2928 | 158.2183 | 158.0318 | 159.9648 | 192.4758 | 200.6114 | 214.2348 | 166.5412 | 157.3097 | 182.747 | 225.9932 | 168.1988 | 212.4411 | 158.2913 | 166.1109 | 202.7615 | 127.2256 | 135.061 |
| CUST_38246_PI429010886 | M24610 | 0.00067 | 18 | 3 | | | | | | 14.28302 | 14.28677 | NA | 15.17932 | 19.25031 | 14.79848 | 8.589723 | 4.660908 | 8.309227 | 4.024049 | 8.393397 | 4.688591 | 7.931172 | 11.37559 | 14.73825 | 14.35579 | 12.61125 | 11.66976 | 14.88378 | 7.02756 | 11.40403 | 16.17931 | 10.31326 | 14.38028 | 14.32589 | 7.128415 | 6.393088 |
| CUST_41116_PI429010886 | M56909 | 0.00067 | 15 | 9 | 546685137 | hypothetical protein D910_11939, partial [Dendroc | | | 4.37E-83 | 429.0337 | 452.2817 | 589.1626 | 344.803 | 371.2619 | 390.8735 | 377.6826 | 403.3076 | 538.4051 | 598.4797 | 399.8374 | 651.6164 | 463.5935 | 560.7028 | 446.0696 | 491.9013 | 477.5078 | 385.4652 | 389.2749 | 405.2638 | 251.6265 | 416.647 | 516.9528 | 523.5388 | 482.6309 | 465.2249 | 607.9318 |
| CUST_1191_PI429010886 | N8789 | 0.00067 | 15 | 9 | 187610687 | small nuclear ribonucleoprotein polypeptide G [Pen | | | 3.39E-20 | 27.75482 | 46.73511 | 48.26458 | 22.64343 | 10.88063 | 15.75401 | 40.8947 | 17.97978 | 28.81252 | 182.1781 | 79.21035 | 94.79905 | 50.78152 | 18.54155 | 37.78907 | 41.55928 | 20.44316 | 23.34857 | 17.73774 | 26.36533 | 48.04331 | 74.51983 | 32.60067 | 35.13593 | 43.53998 | 34.54419 | 114.3038 |
| CUST_54595_PI429010886 | M106779 | 0.000671 | 21 | 15 | | | | | | 33.8524 | 24.29748 | 22.24779 | 22.28917 | 19.76503 | 18.82741 | 16.11003 | 16.56737 | 26.54031 | 33.3136 | 18.13841 | 28.99027 | 28.98746 | 28.53867 | 52.46279 | 30.04719 | 67.34873 | 33.1987 | 32.46691 | 28.97523 | 34.4458 | 36.98139 | 26.36033 | 15.42005 | 20.61255 | 26.22564 | 13.5817 |
| CUST_47600_PI429010886 | M76816 | 0.000671 | 24 | 0 | | | | | | 14.24335 | 15.50857 | 13.71161 | 11.32307 | 6.462365 | 8.221669 | 13.39222 | 6.049495 | 10.14463 | 9.495063 | 10.02652 | 7.332766 | 5.23284 | NA | 10.16697 | 7.988553 | 9.641424 | 12.82338 | 8.665868 | 5.361665 | 10.62972 | 10.80337 | 14.90554 | 14.68167 | 19.50517 | 16.56157 | 10.39612 |
| CUST_31592_PI429010886 | M1920 | 0.000673 | 15 | 12 | | | | | | 257.2108 | 357.5465 | 279.5586 | 219.4603 | 167.982 | 211.3962 | 204.5562 | 260.8562 | 240.1418 | 300.2764 | 222.3148 | 250.1572 | 361.9781 | 403.8464 | 311.0329 | 308.8946 | 269.8655 | 231.5437 | 265.8178 | 245.3745 | 215.4319 | 263.9741 | 311.394 | 188.6253 | 288.2204 | 228.0795 | 280.8839 |
| CUST_28850_PI429010886 | N58723 | 0.000674 | 21 | 3 | | | | | | 110.0025 | 108.9403 | 118.8194 | 111.2059 | 109.3629 | 113.0059 | 123.4029 | 104.7611 | 115.7448 | 83.8218 | 105.9793 | 88.89139 | 90.25676 | 90.75806 | 77.14278 | 106.5084 | 105.779 | 94.42153 | 104.0321 | 90.04123 | 94.42153 | 108.2334 | 124.1278 | 88.49089 | 118.246 | 109.6904 | 117.0097 |
| CUST_42428_PI429010886 | M60256 | 0.00068 | 24 | 18 | | | | | | 27.69389 | 17.33078 | 19.51465 | 15.61765 | 16.12222 | 14.37805 | 23.39301 | 12.40402 | 14.9648 | 20.17771 | 20.42397 | 11.33051 | 24.13249 | 31.59448 | 36.42394 | 26.69563 | 33.7538 | 31.87606 | 36.64988 | 26.95285 | 29.30417 | 22.8759 | 16.14788 | 18.34511 | 34.90742 | 14.15378 | 23.71308 |
| CUST_44555_PI429010886 | M66307 | 0.00068 | 24 | 21 | | | | | | 40.77403 | 53.18901 | 56.57948 | 26.84667 | 32.87464 | 36.93108 | 30.14229 | 23.46033 | 33.71073 | 52.42324 | 45.09026 | 82.90947 | 37.71676 | 41.47521 | 42.62942 | 51.24529 | 49.86955 | 54.8287 | 53.8551 | 47.3803 | 46.34675 | 60.6077 | 63.77021 | 46.37939 | 45.82436 | 37.31704 | 36.72537 |
| CUST_14191_PI429010886 | N32460 | 0.000683 | 21 | 18 | | | | | | NA | 11.8786 | 8.268709 | NA | 4.762239 | NA | 5.701937 | 5.651396 | 7.127057 | NA | 14.09963 | 4.212624 | 6.11892 | 5.257423 | 64.03011 | 7.681885 | 8.373525 | 7.884889 | 12.55922 | 14.10863 | 14.94458 | 5.574158 | 13.65247 | 7.860635 | 4.464293 | 7.249166 | 4.194887 |
| CUST_51716_PI429010886 | M94702 | 0.000685 | 12 | 0 | | | | | | 369.7785 | 708.7623 | 734.2085 | 118.0864 | 189.1194 | 140.4954 | 223.4595 | 464.7526 | 707.6225 | 536.8972 | 328.7379 | 657.8288 | 794.9156 | 1303.313 | 640.8065 | 244.3927 | 712.9294 | 204.5562 | 356.5583 | 597.7736 | 328.4337 | 637.9884 | 527.4806 | 139.7482 | 818.0738 | 489.338 | 911.24 |
| CUST_41374_PI429010886 | M57581 | 0.000688 | 24 | 9 | | | | | | 58.85113 | 67.51475 | 37.44113 | 53.01337 | 68.33792 | 72.65998 | 73.58286 | 92.66541 | 64.99075 | 94.08412 | 133.9111 | 148.9131 | 111.1358 | 54.15621 | 62.30633 | 86.45155 | 63.65227 | 66.20236 | 75.50768 | 95.94174 | 56.42615 | 62.18923 | 42.54782 | 64.77801 | 46.29572 | 68.03939 | 51.33619 |
| CUST_2472_PI429010886 | N12457 | 0.000689 | 21 | 9 | 75022291 | RecName: Full=Oplophorus-luciferin 2-monooxyger | | | 6.43E-13 | 14.84745 | 17.60068 | 6.369665 | 36.20273 | 35.6143 | 28.82637 | 111.9276 | 71.06446 | 52.24698 | 31.88591 | 94.87745 | 114.3208 | 53.11468 | 18.88381 | 24.45097 | 22.1165 | 19.52762 | 14.3364 | 19.59849 | 107.2382 | 52.76183 | 17.17879 | 21.31486 | 27.03844 | 10.78772 | 45.42072 | 31.40253 |
| CUST_335_PI429010886 | N4034 | 0.00069 | 15 | 12 | 170040829 | predicted protein [Culex quinquefasciatus] >gi\|167 | | | 9.59E-10 | 756.1137 | 1511.146 | 1034.677 | 354.9858 | 302.8326 | 135.0089 | 2886.71 | 2097.241 | 4851.309 | 1912.382 | 633.8371 | 1964.934 | 12334.51 | 10435.28 | 6600.16 | 6949.82 | 4008.447 | 545.8082 | 1491.426 | 759.5836 | 806.0846 | 1139.145 | 1318.564 | 1566.438 | 12822.96 | 843.1506 | 14805.59 |
| CUST_13208_PI429010886 | N30783 | 0.000691 | 18 | 15 | | | | | | 248.652 | 276.9079 | 255.1161 | 236.2907 | 253.9339 | 214.2001 | 192.0579 | 185.3252 | 180.7887 | 221.8177 | 201.8776 | 235.3127 | 226.8032 | 278.6632 | 225.8611 | 299.5774 | 261.9745 | 257.2108 | 215.8293 | 271.5235 | 210.6402 | 300.5825 | 208.8093 | 192.6124 | 278.469 | 190.588 | 201.1076 |
| CUST_41165_PI429010886 | M57032 | 0.000693 | 18 | 15 | 380022244 | PREDICTED: poly(A) polymerase gamma [Apis florea | Nucleic acid metabolism | RNA metabolic process | 2.87E-28 | 12.78761 | 11.76966 | 27.68104 | 8.073322 | 5.97138 | 4.952016 | 12.83327 | 5.109965 | 8.167031 | 13.66257 | 16.80068 | 18.56806 | 7.531717 | 15.37785 | 18.05975 | 19.49405 | 35.51693 | 16.41585 | 15.28371 | 11.11919 | 12.95606 | 12.1537 | 6.938783 | 15.32123 | 14.36708 | 6.113641 | 15.5539 |
| CUST_33382_PI429010886 | M6751 | 0.000698 | 18 | 3 | 301624502 | PREDICTED: flavin reductase (NADPH)-like [Xenopus | | | 1.96E-47 | 38.22525 | 34.47136 | 41.20481 | 47.99919 | 30.92026 | 42.96262 | 29.12856 | 28.3396 | 33.35026 | 57.79515 | 31.84731 | 23.78457 | 27.41533 | 25.12351 | 23.45219 | 31.74172 | 28.94552 | 30.69155 | 36.8424 | 16.09243 | 45.84492 | 38.64362 | 51.98858 | 42.22821 | 31.8918 | 43.43615 | 38.50578 |
| CUST_6595_PI429010886 | N20321 | 0.000698 | 15 | 12 | 307176635 | Hamartin [Camponotus floridanus] | | | 4.1E-54 | 69.97891 | 84.83474 | 94.5989 | 62.31885 | 61.75222 | 53.26328 | 61.911 | 90.13978 | 80.86611 | 89.0456 | 82.21549 | 103.6058 | 94.81726 | 102.8432 | 102.6264 | 86.30175 | 69.65017 | 63.1995 | 72.71237 | 86.3406 | 62.98504 | 78.58831 | 90.44569 | 80.36337 | 97.29292 | 61.25018 | 104.7823 |
| CUST_20331_PI429010886 | N43247 | 0.000702 | 21 | 0 | | | | | | 534.723 | 722.474 | 492.1806 | 385.3789 | 463.9991 | 389.0546 | 378.5438 | 358.2812 | 342.8578 | 638.2757 | 407.8836 | 420.2091 | 410.9035 | 435.2247 | 352.4518 | 608.6975 | 451.7787 | 430.1259 | 491.7148 | 547.8035 | 530.2702 | 565.6978 | 555.7341 | 493.0904 | 640.8065 | 333.5908 | 571.296 |
| CUST_34280_PI429010886 | M9232 | 0.000703 | 15 | 9 | | | | | | 12.09699 | 14.60212 | 9.554371 | 5.86895 | 7.82601 | 5.514158 | 13.24259 | 12.63783 | 4.537233 | 13.42229 | 12.90269 | 16.037 | 7.301575 | 12.90802 | 6.428186 | 6.208299 | 8.826373 | 9.184475 | 8.723307 | 6.039035 | NA | 7.145089 | 11.29004 | 9.269541 | 13.04046 | 9.700584 | 14.14994 |
| CUST_32473_PI429010886 | M4202 | 0.000707 | 15 | 12 | | | | | | 199.6316 | 158.5656 | 261.922 | 79.043 | 80.81357 | 98.05949 | 143.5925 | 219.9262 | 209.8084 | 254.6874 | 109.9175 | 168.5577 | 244.0376 | 344.0193 | 226.3675 | 176.2795 | 152.5473 | 92.05581 | 104.8539 | 185.3252 | 127.8042 | 218.242 | 167.9037 | 125.0614 | 207.5722 | 146.3399 | 196.7037 |
| CUST_390_PI429010886 | N4792 | 0.000707 | 15 | 12 | | | | | | 1692.652 | 3549.404 | 3105.643 | 1036.441 | 1337.58 | 1140.062 | 3934.354 | 3785.297 | 5917.438 | 4213.146 | 1761.883 | 2894.613 | 4854.251 | 4992.636 | 3120.564 | 4598.774 | 3197.843 | 2698.741 | 2026.043 | 2284.352 | 1835.374 | 2829.871 | 3809.024 | 2362.762 | 7662.198 | 2377.614 | 10851.4 |
| CUST_23025_PI429010886 | N48132 | 0.000708 | 18 | 3 | | | | | | 68.37525 | 48.56156 | NA | 38.85028 | 83.5291 | 73.59863 | 32.46954 | 20.04708 | 10.7321 | 22.78989 | 12.82841 | 21.15229 | 17.7534 | 7.529742 | 21.38839 | 24.5797 | 28.48104 | 27.33167 | 32.04305 | 7.179499 | 13.59444 | 40.16219 | NA | 58.04233 | 28.61737 | 30.39754 | 8.695689 |
| CUST_46972_PI429010886 | M74360 | 0.000709 | 18 | 15 | 157135044 | pom1 [Aedes aegypti] >gi\|108870311\|gb\|EAT3453 | | | 2.9E-18 | 14.87852 | NA | 12.60293 | 7.72466 | 9.03782 | 30.88587 | 4.527621 | 5.793257 | 5.678179 | 9.197112 | 6.500221 | 11.09239 | 11.93851 | NA | NA | 17.71088 | 10.99994 | NA | 8.499443 | 12.04602 | 7.30902 | 11.85953 | 11.08478 | 10.01939 | 4.585512 | 7.156953 | 5.957887 |
| CUST_29813_PI429010886 | N60616 | 0.00071 | 15 | 12 | | | | | | 17.02313 | 22.81875 | 29.58412 | 14.84417 | 13.09722 | 9.303154 | NA | 12.82706 | 7.588896 | 27.13842 | 17.42611 | 19.80974 | 26.57746 | 23.59085 | 31.76384 | 35.02024 | 21.42376 | 24.03757 | 17.95875 | 23.33967 | 14.63109 | 19.26616 | 16.53332 | 14.75394 | 23.4177 | 10.2062 | 24.30839 |
| CUST_25371_PI429010886 | N52292 | 0.00071 | 15 | 6 | | | | | | 8.711267 | 5.308585 | 14.27082 | 10.38575 | 9.921574 | 8.630999 | 17.37482 | 19.28994 | 12.04133 | 6.19695 | 8.802891 | NA | NA | 5.354146 | 6.497327 | 9.240522 | 5.660086 | 10.20734 | 15.49062 | 12.08287 | 8.463865 | NA | 20.72239 | NA | 6.929742 | 11.37412 | 10.13796 |
| CUST_10646_PI429010886 | N26564 | 0.000711 | 18 | 9 | 512925775 | PREDICTED: monocarboxylate transporter 5-like [Bc | | | 8.03E-07 | 32.02169 | 38.0368 | 47.53397 | 16.78585 | 29.99067 | 23.92874 | 24.56849 | 38.13783 | 44.01036 | 56.67806 | 40.43694 | 63.66464 | 28.58408 | 78.05922 | 57.33188 | 47.76946 | 28.5492 | 49.43277 | 39.54096 | 46.1038 | 37.35358 | 39.43891 | 29.53645 | 18.7912 | 51.97066 | 48.59055 | 76.47488 |
| CUST_34616_PI429010886 | M10154 | 0.000723 | 24 | 18 | | | | | | 33.91698 | 30.6513 | 35.23447 | 26.80742 | 24.60687 | 21.67436 | 41.51371 | 34.70757 | 51.87045 | 28.54216 | 42.69742 | 59.25893 | 45.13249 | 56.60227 | 43.16663 | 49.00501 | 41.99242 | 28.41001 | 58.38951 | 60.12109 | 56.23475 | 36.15353 | 39.64114 | 24.87256 | 31.59448 | 23.31281 | 55.6932 |
| CUST_1126_PI429010886 | N8583 | 0.000724 | 15 | 12 | | | | | | 248.2061 | 261.8822 | 296.6312 | 209.9951 | 193.1764 | 216.9753 | 223.2254 | 226.8346 | 224.668 | 260.0898 | 240.8565 | 338.6596 | 238.3762 | 296.6312 | 314.8276 | 313.1633 | 233.6842 | 223.9704 | 205.4682 | 285.2578 | 231.2637 | 274.0115 | 232.7383 | 218.4381 | 274.7936 | 237.9979 | 306.8772 |
| CUST_50356_PI429010886 | M89564 | 0.000724 | 15 | 0 | | | | | | 15.2714 | 12.86372 | 17.73774 | NA | 13.06343 | 11.6989 | 6.611992 | 9.604718 | 10.47246 | 10.2415 | 6.763654 | 15.24223 | 13.56421 | 13.24864 | 13.45283 | 15.87666 | 13.57843 | 12.6398 | 10.11317 | 13.32448 | 10.57226 | 7.411834 | 13.08683 | 9.166269 | 13.28314 | 8.259244 | 9.925474 |
| CUST_46196_PI429010886 | M71736 | 0.000726 | 18 | 12 | 443709823 | hypothetical protein CAPTEDRAFT_223901 [Capitell | | | 1.18E-14 | 14.6546 | 18.0708 | 14.78546 | 11.18429 | 13.64415 | 14.71224 | 8.197045 | 14.9648 | 15.64186 | 17.05262 | 21.47442 | 17.71924 | 21.27347 | 23.5068 | 22.40881 | 20.77394 | 20.50176 | 17.08982 | 15.01303 | 24.0422 | 11.58541 | 19.69856 | 15.88012 | 7.389291 | 13.53636 | 14.90976 | 21.57749 |
| CUST_15253_PI429010886 | N34272 | 0.000733 | 24 | 15 | 498999091 | PREDICTED: transmembrane protein 53-like [Cerati | | | 3.5E-12 | 9.171762 | 8.425113 | 17.28035 | 8.372351 | 10.82989 | 18.4152 | 14.00188 | 12.09198 | 18.85135 | 20.47876 | 14.18613 | 31.64376 | 20.92287 | 15.65817 | 19.09941 | 25.14633 | 17.85258 | 20.63988 | 4.991219 | 13.49109 | 15.37345 | 17.46532 | 16.41585 | 12.6532 | 6.463315 | 9.664283 | 13.85408 |
| CUST_39023_PI429010886 | M33444 | 0.000735 | 24 | 3 | | | | | | 138.7197 | 87.6188 | 132.3017 | 649.7314 | 211.1514 | 406.244 | 170.3963 | 223.1189 | 105.8252 | 165.3002 | 90.62378 | 128.5685 | 151.2719 | 154.8105 | 104.6828 | 113.6118 | 55.784 | 63.79444 | 32.40014 | 155.979 | 126.7933 | 131.1671 | 302.8941 | 85.43667 | 221.852 | 409.385 | 78.92088 |
| CUST_45401_PI429010886 | M68997 | 0.000737 | 18 | 12 | | | | | | 268.7796 | 295.2655 | 185.7909 | 169.7593 | 218.9157 | 179.1099 | 549.794 | 307.1797 | 424.5435 | 276.8504 | 266.2859 | 239.571 | 606.0546 | 524.2243 | 313.7524 | 310.8131 | 246.0582 | 221.6212 | 525.5752 | 197.86 | 230.8944 | 233.6842 | 194.777 | 232.1034 | 306.9757 | 235.9095 | 517.5225 |
| CUST_9963_PI429010886 | N25476 | 0.000739 | 18 | 9 | | | | | | 146.6632 | 149.7134 | 137.1423 | 146.8007 | 99.42759 | 113.9467 | 97.14315 | 92.95468 | 116.9387 | 180.9857 | 193.4196 | 222.4725 | 146.5479 | 189.5968 | 154.3172 | 173.0442 | 107.7492 | 126.8079 | 175.8911 | 150.2252 | 140.036 | 125.3899 | 73.27672 | 86.22581 | 120.4829 | 167.8576 | 125.5654 |
| CUST_36012_PI429010886 | M14290 | 0.000743 | 21 | 18 | 321458840 | hypothetical protein DAPPUDRAFT_228516 [Daphn | | | 8.74E-52 | 87.16389 | 86.49079 | 124.3334 | 73.93015 | 81.55572 | 71.94024 | 61.49124 | 62.04074 | 60.80817 | 91.12979 | 98.34685 | 107.7787 | 72.49991 | 100.4962 | 93.49845 | 100.6291 | 81.7755 | 70.2561 | 91.18913 | 138.7812 | 99.20203 | 108.7837 | 87.08384 | 82.58698 | 103.7436 | 81.7755 | 73.64901 |
| CUST_12475_PI429010886 | N29524 | 0.000744 | 12 | 0 | | | | | | 598.8088 | 2814.544 | 2090.413 | 68.30614 | 260.6408 | 117.7597 | 869.5437 | 2016.816 | 1666.923 | 1612.797 | 964.9461 | 1845.048 | 2178.033 | 2701.759 | 2192.843 | 342.4231 | 660.5155 | 654.914 | 1906.946 | 1768.416 | 1271.998 | 1331.49 | 1617.488 | 153.8434 | 1960.986 | 1478.287 | 2688.606 |
| CUST_9291_PI429010886 | N24384 | 0.000747 | 24 | 0 | | | | | | 40.21556 | 45.05944 | 37.82192 | 19.28994 | 33.52107 | 32.91621 | 21.24533 | 30.34969 | 22.63948 | 23.94925 | 23.06016 | 28.08839 | 29.51147 | 24.59022 | 23.95482 | 36.92762 | 37.40452 | 30.29871 | 29.70687 | 33.07929 | 21.16443 | 24.09176 | 33.50292 | 23.10525 | 49.70586 | 36.73446 | 38.19855 |
| CUST_4289_PI429010886 | N16411 | 0.000747 | 24 | 18 | | | | | | 11.15122 | 14.52536 | 10.29115 | 13.69809 | 9.159424 | 16.13721 | 9.301993 | 6.512863 | 10.02996 | 14.52373 | 12.89444 | 16.06632 | 4.930335 | 6.215347 | 8.663836 | 19.06154 | NA | 18.16713 | 19.18101 | 20.95699 | 19.19079 | 16.47211 | 19.03731 | NA | 17.28035 | 5.681294 | 10.99994 |
| CUST_39930_PI429010886 | M46911 | 0.000748 | 21 | 3 | 371591210 | Euphausia superba voucher 980349 mitochondrion, | | | | 159657.9 | 150939.2 | 154783 | 157589.4 | 153078.2 | 156827 | 154243.9 | 158283.5 | 153607.2 | 156827 | 146237.4 | 119417.1 | 126138.3 | 118691.9 | 113412.4 | 123526 | 133987.5 | 147644 | 152308.4 | 143243.9 | 159046.1 | 152308.4 | 161558.4 | 144434.8 | 160411.4 | 148206.7 | 155394.6 |
| CUST_45747_PI429010886 | M70152 | 0.000751 | 15 | 0 | | | | | | 157.4172 | 165.3446 | 140.5892 | 150.449 | 131.39 | 147.7228 | 104.8657 | 114.8668 | 111.4954 | 115.4469 | 130.9454 | 120.2195 | 119.2623 | 124.8931 | 139.419 | 214.1014 | 139.4961 | 133.355 | 129.1875 | 143.9734 | 121.1153 | 153.8729 | 119.1161 | 120.8512 | 156.4459 | 120.7928 | 122.1188 |
| CUST_36919_PI429010886 | M17230 | 0.000751 | 24 | 18 | | | | | | 95.03994 | 107.4719 | 90.95085 | 63.46235 | 66.92364 | 49.01378 | 62.21773 | 74.35805 | 79.46381 | 112.8928 | 77.97597 | 70.40792 | 103.5071 | 105.8816 | 86.83335 | 73.55823 | 89.77998 | 93.87602 | 138.8045 | 100.2406 | 141.5508 | 135.7406 | 102.6264 | 56.99104 | 91.31323 | 109.6567 | 118.3919 |
| CUST_40518_PI429010886 | M55354 | 0.000751 | 24 | 18 | | | | | | 178.3086 | 110.1779 | 129.8119 | 79.26193 | 92.57501 | 97.83625 | 103.0626 | 119.5708 | 107.8556 | 125.2377 | 101.4215 | 123.1997 | 119.3927 | 125.6357 | 114.0128 | 141.4486 | 130.9454 | 121.0512 | 130.2384 | 137.0908 | 134.282 | 123.6853 | 117.7859 | 106.9501 | 175.7782 | 123.3055 | 173.9115 |
| CUST_5664_PI429010886 | N19009 | 0.000751 | 24 | 3 | 443686980 | hypothetical protein CAPTEDRAFT_218731 [Capitell | | | 5.2E-36 | 210.1562 | 273.965 | 246.7296 | 391.6722 | 266.3801 | 349.4134 | 248.7921 | 257.5943 | 258.4896 | 253.1914 | 175.6726 | 342.1102 | 221.9563 | 223.1189 | 260.1739 | 207.9906 | 173.9653 | 317.7577 | 244.2054 | 130.4535 | 188.1392 | 226.3317 | 159.9839 | 224.1873 | 265.7722 | 247.9972 | 200.8936 |
| CUST_39161_PI429010886 | M35427 | 0.000757 | 18 | 0 | | | | | | 147.9625 | 172.8801 | 145.6295 | 135.78 | 122.2713 | 92.60851 | 125.4876 | 119.8572 | 100.1974 | 145.9205 | 97.80592 | 105.9305 | 82.13647 | 85.55586 | 107.0816 | 144.8631 | 131.9084 | 153.5041 | 137.0179 | 155.3371 | 134.0609 | 154.3834 | 94.83175 | 132.0735 | 107.6119 | 114.443 | 110.2442 |
| CUST_7240_PI429010886 | N21246 | 0.000757 | 21 | 3 | | | | | | 29.04567 | 23.76609 | 36.62478 | 39.83487 | 31.75695 | 37.18815 | 32.89748 | 20.77162 | 13.37205 | 35.10359 | 21.19713 | 26.48831 | 15.44211 | 29.00481 | 17.81187 | 18.85554 | 20.06405 | 18.96046 | 25.46747 | 17.07166 | 33.9856 | 33.2742 | 36.94632 | 30.5069 | 41.8887 | 39.57456 | 21.63352 |
| CUST_57322_PI429010886 | E5811 | 0.000758 | 21 | 3 | 270008607 | hypothetical protein TcasGA2_TC015150 [Tribolium | | | 2.27E-11 | 178.2839 | 191.452 | 239.2282 | 288.0337 | 179.3194 | 238.6573 | 146.6794 | 165.6398 | 179.0014 | 161.9804 | 114.0935 | 170.596 | 120.2795 | 166.3173 | 101.3667 | 163.6601 | 113.5137 | 166.4042 | 143.6702 | 119.6154 | 231.3422 | 162.3872 | 258.8229 | 162.5528 | 197.0618 | 183.4152 | 175.39 |
| CUST_35143_PI429010886 | M11717 | 0.00076 | 21 | 9 | | | | | | 52.55217 | 64.36368 | 80.42722 | 86.86206 | 73.64901 | 85.51349 | 89.5493 | 73.47109 | 92.77688 | 88.49666 | 97.3988 | 85.52397 | 94.00857 | 83.16029 | 80.01037 | 74.40209 | 78.57748 | 56.12021 | 136.51 | 66.18886 | 74.74764 | 73.07849 | 66.85557 | 60.5947 | 77.40739 | 69.39137 | 92.5954 |
| CUST_41965_PI429010886 | M59020 | 0.000761 | 15 | 0 | | | | | | 292.7227 | 223.1512 | 247.0492 | 165.0319 | 175.011 | 200.8116 | 139.6417 | 92.69475 | 167.2734 | 173.881 | 177.5136 | 185.8695 | 127.4262 | 199.7111 | 194.1717 | 361.0091 | 255.3452 | 303.7146 | 154.0879 | 187.276 | 174.8583 | 168.2847 | 213.1848 | 185.6524 | 242.2528 | 209.5249 | 269.5764 |
| CUST_4338_PI429010886 | N16539 | 0.000761 | 12 | 0 | | | | | | 49.94515 | 33.06846 | 54.6863 | 29.49276 | 32.37332 | 27.92893 | 28.25408 | 26.81554 | 28.81481 | 38.76201 | 31.65427 | 41.88371 | 31.93715 | 51.05906 | 44.86761 | 31.34763 | 25.83289 | 33.22506 | 32.80342 | 34.26633 | 32.97999 | 46.30865 | 33.58065 | 36.64631 | 36.20575 | 35.82359 | 37.77438 |
| CUST_53936_PI429010886 | M103776 | 0.000763 | 15 | 9 | | | | | | 22.34831 | 41.96451 | 30.57199 | NA | 11.84611 | 14.21497 | 15.39416 | 5.04092 | 32.40747 | 35.27881 | 20.84836 | 43.10871 | 28.40282 | 43.49759 | 31.32413 | 28.37529 | 11.93404 | 32.29255 | 19.12737 | 11.77935 | 30.50923 | 41.81668 | 36.46662 | 15.18055 | 44.82913 | 51.14708 | 48.30048 |
| CUST_5176_PI429010886 | N18359 | 0.00077 | 24 | 6 | 585342 | RecName: Full=Arginine kinase; Short=AK >gi\|3113! | Metabolic process | Cellular amino acid metabolic process | 1.6E-150 | 174.8815 | 128.3451 | 185.2639 | 191.6398 | 185.6828 | 129.9706 | 187.0999 | 249.4607 | 193.3576 | 168.2678 | 230.36 | 169.1739 | 212.8755 | 146.2811 | 159.4512 | 141.4293 | 155.6736 | 108.8415 | 159.0925 | 318.6212 | 162.6286 | 97.92701 | 154.9129 | 73.57494 | 109.6686 | 152.3559 | 179.7938 |
| CUST_39300_PI429010886 | M37428 | 0.000771 | 21 | 15 | 270016056 | hypothetical protein TcasGA2_TC012929 [Tribolium | | | 5.48E-11 | 111.7362 | 101.7342 | 91.35555 | 68.24289 | 69.99368 | 64.09512 | 80.63305 | 101.7598 | 90.54311 | 125.8442 | 114.7609 | 108.3885 | 114.4836 | 97.85969 | 89.24114 | 180.4919 | 72.10026 | 168.5282 | 158.8804 | 111.9364 | 102.8937 | 103.3291 | 106.1754 | 88.81258 | 105.711 | 63.87879 | 76.93173 |
| CUST_50784_PI429010886 | M90999 | 0.000788 | 24 | 21 | 443728528 | hypothetical protein CAPTEDRAFT_178633 [Capitell | | | 2.53E-11 | 34.61952 | 26.8214 | 28.05779 | 31.31098 | 10.07859 | 26.41115 | 20.93304 | 19.43749 | 20.12366 | 64.94243 | 26.74645 | 25.45224 | 28.85125 | 29.09314 | 36.07548 | 43.65248 | 28.09932 | 30.89255 | 22.31957 | 33.57067 | 30.8467 | 34.95466 | 50.65756 | 35.57263 | 31.44965 | 25.76451 | 31.6082 |
| CUST_27254_PI429010886 | N55716 | 0.000792 | 15 | 0 | | | | | | 378.7944 | 407.8386 | 366.4064 | 268.7796 | 318.5558 | 307.1797 | 254.189 | 252.0389 | 251.0973 | 355.116 | 266.0394 | 304.437 | 367.921 | 283.0254 | 290.5449 | 430.2513 | 324.5263 | 325.3686 | 290.2748 | 355.3996 | 255.7893 | 381.2578 | 261.6417 | 274.4437 | 399.7554 | 255.8665 | 269.9093 |
| CUST_20563_PI429010886 | N43664 | 0.000798 | 21 | 3 | | | | | | 1171.568 | 2626.728 | 1652.941 | 3988.418 | 1814.412 | 2016.816 | 1384.531 | 1022.013 | 1462.44 | 2027.433 | 791.8244 | 1608.297 | 910.1372 | 1449.375 | 682.9708 | 493.5731 | 538.4051 | 944.9878 | 632.6214 | 651.9918 | 2218.572 | 1396.98 | 2346.537 | 578.6895 | 2623.017 | 2195.833 | 1189.103 |
| CUST_3617_PI429010886 | N14893 | 0.000802 | 21 | 3 | 384402278 | predicted carotinoid oxygenase [Bombyx mori] | | | 4.93E-15 | 3618.732 | 7749.236 | 4797.809 | 12916.14 | 6448.481 | 5157.962 | 4041.003 | 3627.549 | 4107.091 | 5498.252 | 2298.025 | 5411.773 | 2777.472 | 4937.213 | 3483.315 | 1539.294 | 1848.861 | 3288.575 | 2201.928 | 2763.052 | 6461.792 | 5875.497 | 6234.905 | 2934.424 | 8086.505 | 5868.528 | 3634.672 |
| CUST_28703_PI429010886 | N58459 | 0.000804 | 24 | 15 | | | | | | 60.5059 | 42.94589 | 92.64233 | 47.5835 | 47.04186 | 60.89252 | 56.20414 | 67.90618 | 97.91604 | 104.7962 | 91.85305 | 101.7048 | 79.33173 | 92.53187 | 78.1117 | 109.1441 | 90.08157 | 107.1382 | 107.8715 | 98.71598 | 86.39769 | 68.1165 | 82.8076 | 67.15618 | 95.66411 | 70.17729 | 86.06426 |
| CUST_10764_PI429010886 | N26750 | 0.000809 | 21 | 9 | | | | | | 21.67436 | 13.08017 | 23.94659 | 20.24846 | 22.01177 | 36.87107 | 25.71165 | 16.88939 | 21.59129 | 27.03844 | 29.95513 | 28.48875 | 25.5267 | 16.00911 | 25.94889 | 24.6038 | 25.11853 | 22.46676 | 18.7049 | 26.36365 | 15.09945 | 14.37169 | 20.57784 | 10.70732 | 22.74008 | 24.61539 | 15.62744 |
| CUST_25941_PI429010886 | N53342 | 0.000819 | 15 | 9 | 528522579 | PREDICTED: gastrula zinc finger protein XlCGF57.1-l | | | 4.98E-27 | 96.07604 | 77.57474 | 87.08384 | 63.78913 | 52.15768 | 68.51154 | 88.10192 | 114.9603 | 96.40299 | 110.8479 | 53.47338 | 105.4884 | 82.00472 | 116.0676 | 98.65062 | 70.59503 | 77.34848 | 68.95198 | 56.42615 | 76.22636 | 67.34873 | 78.77563 | 106.4577 | 89.37758 | 103.109 | 79.26193 | 109.8291 |
| CUST_10717_PI429010886 | N26676 | 0.000821 | 12 | 9 | 306478627 | ficolin-like protein 2 [Pacifastacus leniusculus] | | | 1.31E-25 | 9.707875 | 7.774382 | 13.26743 | NA | 5.147446 | NA | 9.330885 | 19.689 | 12.57374 | NA | 46.84888 | 15.89893 | 9.659531 | 7.966321 | 8.835496 | 13.00011 | 6.628016 | NA | 9.960996 | 13.20272 | NA | 42.0037 | 13.7932 | 19.02231 | 11.09239 | NA | 9.604718 |

| Probe ID | ID | p-value | Period | Phase | EBI ID | Annotation | GO category | Go term | E-value | 0 | 0 | 0 | 3 | 3 | 3 | 6 | 6 | 6 | 9 | 9 | 9 | 12 | 12 | 12 | 15 | 15 | 15 | 18 | 18 | 18 | 21 | 21 | 21 | 24 | 24 | 24 |
|---|---|---|---|---|---|---|---|---|---|---|---|---|---|---|---|---|---|---|---|---|---|---|---|---|---|---|---|---|---|---|---|---|---|---|---|---|
| CUST_41575_PI429010886 | M58064 | 0.000821 | 18 | 12 | | | | | | 107.9121 | 107.2744 | 115.6902 | 117.1213 | 119.4271 | 120.8319 | 115.7071 | 149.0574 | 168.9693 | 141.3981 | 142.2559 | 123.6359 | 148.4195 | 213.366 | 180.1532 | 143.5925 | 127.5003 | 145.0486 | 121.0641 | 137.0179 | 95.83965 | 159.6585 | 143.9903 | 120.1261 | 193.773 | 186.0881 | 168.3529 |
| CUST_18790_PI429010886 | N40416 | 0.000822 | 12 | 6 | | | | | | 12.54832 | 4.060656 | 8.847678 | 24.73576 | 10.4869 | 22.39942 | 26.07451 | 33.87601 | 19.7715 | 14.05923 | 35.52135 | 43.57431 | 7.799519 | NA | 6.645675 | NA | 22.19563 | NA | 17.13473 | 32.55113 | 35.84962 | NA | 21.43971 | 24.24502 | 10.34912 | 19.40477 | 10.58518 |
| CUST_29099_PI429010886 | N59200 | 0.000823 | 15 | 6 | | | | | | 19.0691 | 11.85674 | 14.25137 | 23.70044 | 25.73345 | 47.79169 | 25.71973 | 39.91731 | 28.25834 | 27.64723 | 18.50437 | 26.09533 | 23.45771 | 20.41093 | 16.1997 | 11.76966 | 17.08729 | 24.4538 | 31.99517 | 16.92856 | 36.22069 | 24.0077 | 35.92679 | 31.03981 | 21.60941 | 25.92266 | 17.92835 |
| CUST_30923_PI429010886 | M229 | 0.000825 | 24 | 15 | | | Protein metabolism | Proteolysis | | 1710.741 | 985.3973 | 1324.078 | 1977.124 | 1187.991 | 1084.753 | 1867.343 | 2351.436 | 1143.569 | 2571.52 | 3246.287 | 1134.542 | 1649.441 | 1972.844 | 2070.325 | 3368.325 | 3795.26 | 3046.724 | 2881.575 | 2482.039 | 1458.041 | 2227.948 | 2644.158 | 1816.167 | 1601.013 | 1576.033 | 2236.34 |
| CUST_44201_PI429010886 | M65242 | 0.000826 | 21 | 15 | | | | | | 344.4277 | 267.2735 | 220.4437 | 326.3773 | 332.6812 | 321.0207 | 252.0041 | 254.5401 | 194.2325 | 215.9452 | 185.8695 | 240.0651 | 305.7949 | 286.7556 | 303.7719 | 395.3575 | 348.4628 | 380.124 | 271.7176 | 357.2936 | 288.1636 | 412.9301 | 270.4039 | 316.8471 | 315.9615 | 332.5951 | 258.602 |
| CUST_3929_PI429010886 | N15593 | 0.000828 | 21 | 6 | 442633892 | CG7632, isoform B [Drosophila melanogaster] >gi\|4 | | | 5.67E-17 | 12.00275 | 17.55722 | 9.09768 | 15.31913 | 11.5308 | 18.43075 | 25.95428 | 22.05258 | 22.88357 | 17.84023 | 16.83181 | 19.5882 | 21.54483 | 18.48864 | 17.35586 | 20.49048 | 4.905725 | 10.86396 | 9.238126 | 17.22069 | NA | 17.00167 | 5.721438 | 11.97085 | 17.83264 | 18.61312 | 14.17556 |
| CUST_12822_PI429010886 | N30108 | 0.000833 | 24 | 15 | 410509310 | myosin heavy chain type 1 [Litopenaeus vannamei] | Metabolic process | Oxidation-reduction process | 4.74E-56 | 56.48454 | 64.54153 | 88.10192 | 88.04251 | 76.21767 | 117.4608 | 84.10524 | 162.7985 | 103.3291 | 122.2898 | 90.96114 | 123.321 | 136.6832 | 126.9474 | 106.5084 | 142.1826 | 124.9549 | 135.8973 | 118.2591 | 107.4359 | 76.10603 | 144.4421 | 88.75833 | 112.4734 | 90.69277 | 81.13095 | 117.1213 |
| CUST_21788_PI429010886 | N45853 | 0.00084 | 18 | 3 | | | | | | 10.6257 | 21.4795 | 11.09833 | 34.37569 | 15.38273 | 16.94046 | 22.12202 | 30.28193 | 21.28517 | 7.262826 | 6.637612 | 6.850987 | 6.230319 | 8.197045 | 10.51492 | 12.81767 | 11.07843 | 12.31301 | 11.73054 | 6.858047 | 16.23324 | 13.26879 | 19.67357 | 13.89803 | 11.22985 | 4.882948 | 14.67638 |
| CUST_33654_PI429010886 | M7523 | 0.000842 | 18 | 6 | | | | | | 27.83815 | 21.34103 | 233.2127 | 24.38566 | 39.92228 | 45.32916 | 52.07596 | 259.0619 | 179.3477 | 168.4747 | 49.3195 | 48.49494 | 159.1708 | 449.2825 | 33.41929 | 25.86598 | 127.9469 | 40.58787 | 23.37682 | 30.44767 | 23.37424 | 46.61752 | 385.1644 | 50.6298 | 264.5866 | 59.0899 | 302.8326 |
| CUST_13455_PI429010886 | N31223 | 0.000842 | 18 | 0 | | | | | | 283.5032 | 374.3698 | 296.7865 | 275.8944 | 263.345 | 291.5581 | 170.2053 | 220.8169 | 230.9612 | 277.7994 | 233.7572 | 324.1237 | 226.8346 | 267.5678 | 259.9314 | 276.2557 | 269.7173 | 285.1566 | 282.5938 | 327.535 | 255.1996 | 314.9323 | 260.1385 | 259.9114 | 273.6903 | 221.9188 | 242.8547 |
| CUST_23749_PI429010886 | N49395 | 0.000843 | 18 | 15 | | | | | | 106.2858 | 65.21686 | 88.30586 | 84.30944 | 117.0501 | 100.0618 | 66.78979 | 70.90857 | 48.22578 | 80.08977 | 70.3852 | 62.69277 | 89.19702 | 78.37536 | 76.24971 | 94.72175 | 133.7601 | 97.87109 | 115.8883 | 67.08317 | 92.50739 | 77.7529 | 88.41726 | 89.32488 | 72.79951 | 62.92532 | 65.6996 |
| CUST_30192_PI429010886 | N61356 | 0.000852 | 15 | 12 | | | | | | 7.686663 | 4.959572 | 12.91624 | 6.694465 | 4.43107 | 5.320278 | 5.011506 | 6.181821 | 12.72627 | 7.743875 | 6.781376 | 12.6532 | 15.39132 | NA | 24.57313 | 12.64236 | 8.903536 | 15.12815 | NA | NA | NA | 6.221936 | 14.06926 | 7.119665 | 9.445353 | 10.44922 | 15.33921 |
| CUST_36391_PI429010886 | M15789 | 0.000854 | 15 | 9 | | | | | | 7737.591 | 20510.07 | 26037.41 | 2406.498 | 3172.097 | 1285.231 | 6225.291 | 34070.11 | 58209.54 | 43935.4 | 31492.49 | 44666.31 | 36174.65 | 63973.47 | 49654.41 | 30891.2 | 15389.68 | 12673.02 | 15353.74 | 35686.35 | 18694.18 | 49654.41 | 50235.79 | 3536.586 | 58209.54 | 39316.25 | 81423.55 |
| CUST_15640_PI429010886 | N34906 | 0.000854 | 15 | 3 | | | | | | 72.72106 | 49.70586 | 76.30835 | 53.62183 | 101.8998 | 100.64 | 106.9318 | 46.96643 | 27.81399 | 39.33067 | 38.55767 | 20.17559 | 42.44777 | 55.4319 | 35.45118 | 32.52687 | 74.85405 | 56.40861 | 84.10524 | 40.1303 | 71.11204 | 36.29351 | 55.5381 | 55.68518 | 26.95815 | 40.98319 | 24.31347 |
| CUST_393_PI429010886 | N4805 | 0.000854 | 15 | 9 | 225712016 | Cuticle protein 6 [Lepeophtheirus salmonis] | | | 1.35E-19 | 1623.132 | 2067.695 | 7764.128 | 381.2578 | 407.8386 | 101.2666 | 1958.473 | 8304.282 | 10777.69 | 9839.748 | 3756.632 | 9670.846 | 7273.892 | 16156.64 | 8686.323 | 6577.842 | 3352.836 | 1760.95 | 4025.068 | 6160.933 | 4690.442 | 15221.8 | 7920.23 | 502.6158 | 18962.11 | 7901.818 | 18865.02 |
| CUST_37224_PI429010886 | M18269 | 0.000856 | 24 | 12 | 242005220 | Calumenin precursor, putative [Pediculus humanus | | | 4.36E-62 | 22.59879 | 13.42877 | 11.80651 | 11.64631 | 27.75759 | 14.79313 | 15.7577 | 20.71154 | 24.42322 | 20.00906 | 14.08001 | 27.13598 | 25.8552 | 84.59116 | 22.51476 | 26.99397 | 15.79313 | 31.35806 | 18.21981 | 19.51092 | 18.63533 | 17.20395 | 10.28317 | 15.87123 | 9.315276 | 11.6754 | 13.64087 |
| CUST_34852_PI429010886 | M10889 | 0.000858 | 21 | 18 | | | | | | 36.07274 | 31.39141 | 12.69853 | 28.08839 | 52.81394 | 39.12009 | 11.33971 | 16.55564 | 21.02441 | 43.02039 | 26.0691 | 16.04975 | 24.03757 | 27.39302 | 22.06161 | 50.12589 | 29.78998 | 45.17801 | 46.94189 | 88.3275 | 33.1299 | 34.18999 | 35.42916 | NA | 29.68582 | 25.9871 | 25.07182 |
| CUST_38793_PI429010886 | M30512 | 0.000861 | 15 | 9 | | | | | | 15.80722 | 7.631181 | 10.12281 | 8.761286 | 4.83618 | 11.42664 | 9.029764 | 6.364227 | 7.64912 | 49.04708 | 13.38206 | 18.21981 | 13.06814 | 13.31366 | 13.54942 | 13.00717 | 6.130259 | NA | 11.63036 | 7.441066 | 8.692145 | 11.78251 | NA | NA | NA | 14.10338 | 24.26769 |
| CUST_39178_PI429010886 | M35693 | 0.000863 | 21 | 3 | | | | | | 9.834889 | 13.54646 | 9.140795 | 15.67634 | 14.98523 | 23.5201 | 8.12515 | 10.13796 | 13.6708 | 8.203406 | 6.791617 | 13.67211 | 6.692384 | 9.632488 | 10.82471 | 9.269541 | 8.832139 | 8.688469 | 7.882812 | 12.07791 | 16.24815 | 11.50536 | 19.13431 | 12.31888 | 23.14834 | 10.36906 | 23.08011 |
| CUST_51758_PI429010886 | M94914 | 0.000864 | 15 | 9 | 442769035 | glycosyl-phosphatidylinositol-linked carbonic anhyd | Metabolic process | Nitrogen compound metabolic process | 3.23E-13 | 100.2082 | 106.5844 | 346.5286 | 34.21339 | 65.36594 | 36.90877 | 202.6087 | 236.4903 | 162.1787 | 389.6265 | 247.3742 | 204.946 | 145.4206 | 347.2399 | 139.6803 | 48.02842 | 152.5175 | 70.93175 | 156.9838 | 109.1296 | 116.3712 | 185.4691 | 411.6609 | 34.87758 | 400.4224 | 164.6258 | 339.8212 |
| CUST_2729_PI429010886 | N12995 | 0.000864 | 15 | 0 | 321455420 | hypothetical protein DAPPUDRAFT_218978 [Daphn | Metabolic process | Carbohydrate metabolic process | 4.97E-96 | 1659.051 | 2128.599 | 2013.592 | 1122.467 | 1692.652 | 2001.122 | 778.8569 | 444.0863 | 860.5065 | 1321.214 | 1978.873 | 2120.568 | 483.2456 | 1435.814 | 1287.066 | 2390.205 | 1467.042 | 2232.866 | 1247.588 | 1989.387 | 1569.266 | 1515.268 | 1076.18 | 1113.282 | 1472.13 | 1281.141 | 1663.063 |
| CUST_8211_PI429010886 | N22717 | 0.000864 | 15 | 12 | 321455306 | hypothetical protein DAPPUDRAFT_332203 [Daphn | Metabolic process | Nucleobase-containing compound metabolic process | 4.27E-67 | 36.19062 | 50.18617 | 29.42815 | 20.81747 | 33.99233 | 27.9603 | 20.63276 | 38.92288 | 28.71654 | 44.25502 | 53.01853 | 48.68836 | 63.23408 | 60.56579 | 55.57729 | 61.04516 | 31.96745 | 31.71188 | 31.08214 | 59.46444 | 31.51891 | 38.19149 | 32.52286 | 34.26964 | 69.35049 | 37.54361 | 38.56828 |
| CUST_45794_PI429010886 | M70313 | 0.000865 | 18 | 3 | | | | | | 11.81158 | 11.55683 | 19.40477 | 19.86843 | 36.5984 | 34.16228 | 17.87274 | 8.904805 | 14.04228 | 12.65013 | 7.376677 | NA | 5.095792 | 11.89186 | 7.220984 | 22.0851 | 9.482226 | 20.07855 | 18.08249 | 5.223476 | 13.45433 | 32.44398 | NA | 17.60432 | 12.24194 | 9.675354 | 15.44955 |
| CUST_26542_PI429010886 | N54422 | 0.000871 | 24 | 15 | | | | | | 35.02024 | 27.19369 | 24.45097 | 23.59619 | 33.27151 | 22.0325 | 22.41298 | 22.80223 | 27.22941 | 31.35346 | 26.69563 | 33.79348 | 27.92065 | 46.57205 | 31.48445 | 39.19727 | 34.00967 | 47.51073 | 36.96378 | 23.50904 | 37.39122 | 35.92246 | 31.99053 | 37.02134 | 31.03981 | 26.0056 | 38.88089 |
| CUST_39532_PI429010886 | M40884 | 0.000871 | 18 | 9 | | | | | | 546.2052 | 497.3582 | 603.4589 | 408.0489 | 605.8766 | 500.2189 | 593.331 | 713.4223 | 1538.611 | 1301.471 | 741.9924 | 797.3133 | 544.5308 | 1335.109 | 780.6851 | 1669.785 | 862.6591 | 1038.465 | 641.3583 | 756.4835 | 875.295 | 448.5246 | 476.5945 | 411.957 | 3295.06 | 609.0182 | 2253.027 |
| CUST_35186_PI429010886 | M11829 | 0.000872 | 18 | 0 | | | | | | 440.5078 | 273.733 | 448.0873 | 387.779 | 345.3079 | 352.0546 | 314.3091 | 303.9241 | 256.7514 | 348.4628 | 261.7284 | 322.593 | 247.0492 | 296.7865 | 314.3652 | 846.4177 | 340.1906 | 357.4809 | 468.9522 | 402.9446 | 435.5217 | 428.5138 | 354.5586 | 348.2614 | 427.2547 | 305.3469 | 303.3749 |
| CUST_15084_PI429010886 | N33979 | 0.000883 | 15 | 9 | 242007206 | conserved hypothetical protein [Pediculus humanus | | | 3.16E-13 | 13.67691 | 7.497984 | 25.09429 | NA | 11.9014 | 5.87006 | NA | 8.045043 | 7.451482 | 15.9244 | 20.96353 | 15.25233 | 13.33763 | 10.49992 | 10.01513 | NA | 12.12113 | 5.173365 | 7.105175 | 6.062184 | 7.934263 | 9.931851 | 9.128153 | NA | 14.26904 | 15.43461 | 18.18793 |
| CUST_56713_PI429010886 | M114496 | 0.000884 | 15 | 9 | | | | | | 42.34172 | 59.93656 | 79.06168 | 37.73221 | 36.22069 | 41.55928 | 59.31402 | 53.44719 | 73.49284 | 63.25496 | 60.41993 | 66.79779 | 49.47977 | 65.82296 | 48.86699 | 60.13681 | 43.83694 | 48.15042 | 48.78129 | 56.07148 | 46.05816 | 70.82822 | 87.54624 | 44.75473 | 89.73481 | 56.64713 | 99.86709 |
| CUST_6014_PI429010886 | N19508 | 0.000886 | 18 | 0 | | | | | | 331.4917 | 393.3268 | 423.679 | 291.4361 | 247.7341 | 329.0332 | 200.5001 | 302.3102 | 252.8872 | 391.5964 | 272.9055 | 330.6772 | 227.225 | 381.498 | 280.8839 | 310.2726 | 338.0611 | 244.5361 | 375.6905 | 355.5348 | 407.2671 | 310.1413 | 343.4753 | 229.6568 | 326.3773 | 239.3095 | 227.4855 |
| CUST_17726_PI429010886 | N38485 | 0.000893 | 12 | 9 | 321455725 | enhancer of split mgamma protein-like protein [Dap | Nucleic acid metabolism | Regulation of gene expression | 3.28E-42 | 33.59506 | 54.6811 | 58.40039 | 29.93503 | 25.14839 | 18.67864 | 33.24975 | 49.62512 | 44.59476 | 78.17016 | 56.03701 | 75.7296 | 40.2482 | 45.33363 | 35.7636 | 33.6052 | 36.3262 | 29.48277 | 56.95101 | 71.10646 | 53.47899 | 69.45519 | 56.01567 | 32.30965 | 62.31885 | 75.2717 | 66.83042 |
| CUST_32384_PI429010886 | M3981 | 0.000894 | 21 | 9 | 91086121 | PREDICTED: similar to adenine phosphoribosyltrans | Metabolic process | Nucleobase-containing compound metabolic process | 5.42E-47 | 25.5315 | 65.08 | 20.06593 | 49.09733 | 58.49978 | 12.31007 | 50.47418 | 119.0689 | 98.38324 | 87.53485 | 80.95222 | 101.187 | 89.95558 | 44.27668 | 71.2117 | 44.6585 | 85.38257 | 62.583 | 49.18958 | 141.2095 | 69.72451 | 6.900525 | 32.19156 | 30.03204 | 52.34805 | 50.70831 | 59.50688 |
| CUST_28215_PI429010886 | N57529 | 0.000895 | 21 | 0 | | | | | | 23.3505 | 26.25702 | 27.91811 | 26.08148 | 18.38957 | 26.48449 | 9.300708 | 14.67882 | 12.30062 | 14.76794 | 15.48498 | 20.71004 | 9.497582 | 20.37942 | 14.09753 | 7.605624 | 45.88881 | 21.0027 | 19.78047 | 14.20169 | 20.8155 | 22.26173 | 34.06396 | 26.17436 | 14.86396 | 16.62231 | 13.66936 |
| CUST_47909_PI429010886 | M78127 | 0.000897 | 15 | 3 | | | | | | 48.2481 | NA | 46.4361 | 167.2554 | 65.99744 | 213.9895 | 80.53149 | 20.90912 | 7.208745 | 3.869762 | 116.4359 | 16.90108 | 14.40546 | 12.62537 | 12.1266 | 22.62556 | 179.678 | 65.42078 | 111.4592 | NA | 190.6724 | 11.9801 | 89.66356 | 115.7448 | NA | 12.16205 | 3.788515 |
| CUST_26891_PI429010886 | N55073 | 0.000899 | 24 | 0 | 332025325 | Neurobeachin [Acromyrmex echinatior] | | | 6.78E-48 | 10.36474 | 11.56285 | 9.268485 | 8.510732 | 13.72174 | 8.954946 | 15.86046 | NA | 9.914777 | 8.877949 | 6.987277 | 9.549172 | 5.901635 | NA | 5.899189 | 14.63359 | 7.232248 | 7.979138 | 8.982813 | 8.789967 | 8.108101 | 9.592481 | 15.95534 | 8.789967 | 16.89118 | 16.83941 | 9.268485 |
| CUST_41066_PI429010886 | M56797 | 0.000905 | 15 | 9 | | | | | | 57.48318 | 58.87201 | 66.03995 | 37.62657 | 43.15798 | 42.63354 | 44.70933 | 66.12938 | 78.58831 | 70.108 | 65.31258 | 79.19112 | 60.28718 | 71.78694 | 64.77801 | 62.60675 | 81.03057 | 51.79552 | 52.46279 | 62.74069 | 62.95412 | 84.14675 | 89.02227 | 49.93531 | 123.5891 | 69.28034 | 92.24128 |
| CUST_26722_PI429010886 | N54764 | 0.000911 | 12 | 0 | | | | | | 24.11784 | 24.17719 | 37.91131 | 7.255725 | 19.62917 | 17.06111 | 8.997185 | 14.04066 | 17.94517 | 14.46988 | 17.63842 | 20.21835 | 27.1975 | 16.19477 | 24.5797 | 19.31282 | 18.57693 | 16.90254 | 19.0657 | 7.917706 | 14.11638 | 15.59858 | 19.02231 | 17.31897 | 23.14292 | 21.70044 | 17.04861 |
| CUST_51566_PI429010886 | M94008 | 0.000911 | 12 | 0 | 546680489 | hypothetical protein D910_08102 [Dendroctonus p | Metabolic process | Lipid metabolic process | 2.62E-07 | 11.81299 | 11.48488 | 21.2067 | 5.523184 | 4.848232 | NA | 7.648203 | 5.318672 | 3.815707 | 19.62917 | 11.2915 | NA | 9.255767 | 11.49531 | 9.140795 | 9.165228 | 10.06452 | 16.22239 | 5.500018 | 5.654986 | 8.083245 | 11.05702 | 11.42007 | 9.005077 | 32.59218 | 9.416182 | 26.2903 |
| CUST_52378_PI429010886 | M97235 | 0.000915 | 15 | 9 | | | | | | 65.53578 | 74.88 | 65.03693 | 57.75281 | 62.83869 | 56.28307 | 56.70973 | 52.56971 | 70.46616 | 78.61543 | 68.61072 | 92.04256 | 60.31958 | 75.70522 | 80.94247 | 62.90962 | 60.66262 | 91.90962 | 59.72331 | 61.42421 | 61.28097 | 89.73481 | 62.76241 | 63.32035 | 91.45169 | 81.37626 | 71.30124 |
| CUST_24027_PI429010886 | N49895 | 0.000915 | 12 | 3 | | | | | | 11.70432 | 15.51946 | NA | 182.747 | 30.68445 | 154.6176 | 43.64196 | NA | 42.53656 | 3.444096 | 3.880834 | NA | 13.37036 | 3.46548 | 13.14309 | 17.33333 | 117.6403 | 103.8205 | 19.98856 | 32.91963 | 4.992877 | 7.838281 | NA | 17.52398 | 12.52222 | 17.07599 | 5.116273 |
| CUST_3122_PI429010886 | N13839 | 0.000916 | 21 | 18 | 291226599 | PREDICTED: thioredoxin 2-like isoform 1 [Saccoglos | Metabolic process | Oxidation-reduction process | 6.15E-30 | 349.0566 | 397.5988 | 306.6802 | 319.6424 | 320.1082 | 290.7849 | 300.0118 | 277.125 | 282.8614 | 414.6925 | 301.0265 | 339.3609 | 292.7654 | 313.6967 | 305.9118 | 472.997 | 317.0842 | 394.6245 | 369.7785 | 388.2685 | 335.0674 | 541.4466 | 328.0426 | 388.0401 | 308.7717 | 231.0649 | 324.5803 |
| CUST_13297_PI429010886 | N30935 | 0.000921 | 12 | 0 | | | | | | 19.97907 | 28.17746 | 22.82168 | 17.81187 | 25.24216 | 17.74547 | 17.35176 | 14.79313 | 8.943967 | 22.90261 | 15.99975 | 22.92529 | 22.46932 | 25.29876 | 23.06645 | 20.98763 | 18.4393 | 19.13174 | 31.39141 | 15.44039 | 10.25766 | 22.06161 | 18.10904 | 22.81381 | 25.76975 | 27.18447 | 21.58196 |
| CUST_13281_PI429010886 | N30906 | 0.000922 | 21 | 0 | 443693165 | hypothetical protein CAPTEDRAFT_178555 [Capitell | | | 4.16E-14 | 60.26917 | 43.44238 | 95.66411 | 45.41664 | 49.92642 | 47.37598 | 39.72839 | 56.95101 | 37.63001 | 55.5206 | 40.97837 | 39.63814 | 36.19462 | 33.41607 | 43.90905 | 46.6 | 35.74774 | 39.21347 | 39.4307 | 30.88587 | 59.6278 | 50.5231 | 62.88366 | 72.44795 | 40.63765 | 51.57463 | 69.216 |
| CUST_9279_PI429010886 | N24362 | 0.000923 | 15 | 12 | 260813382 | hypothetical protein BRAFLDRAFT_243962 [Branchi | | | 1.95E-19 | 27.53031 | 27.54647 | 20.47372 | 13.72842 | 14.34109 | 23.87529 | 15.05605 | 16.77607 | 24.5371 | 19.98178 | 21.7873 | 44.92099 | 28.46803 | 25.99299 | 30.41942 | 28.41001 | 22.22394 | 16.35532 | 5.747087 | 21.27634 | 18.7688 | 17.79725 | 20.208 | 14.2605 | 27.14607 | 20.02214 | 26.58817 |
| CUST_50889_PI429010886 | M91355 | 0.000924 | 18 | 9 | | | | | | 4.578992 | 7.661152 | 8.585014 | 8.883037 | 18.19204 | 15.32123 | 33.81235 | 55.78991 | 34.42762 | 61.18601 | 9.905555 | NA | 59.56411 | 50.46214 | 32.30965 | 5.17051 | 44.87166 | 11.04906 | 5.853359 | 9.733637 | NA | 38.53317 | 52.43379 | NA | 39.58144 | 19.89653 | 42.05465 |
| CUST_29821_PI429010886 | N60631 | 0.000925 | 24 | 0 | | | | | | 76.24971 | 73.12656 | 78.9119 | 66.67878 | 68.5761 | 64.52054 | 52.40185 | 71.26057 | 72.4241 | 56.25359 | 69.24723 | 76.32596 | 45.65065 | 50.60456 | 66.37222 | 70.30533 | 69.88371 | 78.17016 | 70.04597 | 64.69663 | 62.44095 | 87.67204 | 68.69041 | 79.27531 | 94.85349 | 73.61433 | 133.9495 |
| CUST_24419_PI429010886 | N50592 | 0.000928 | 24 | 21 | | | | | | 18.92827 | 26.77939 | 31.51141 | 16.95326 | 19.5413 | 30.85032 | 16.45281 | 17.5765 | 16.79903 | 21.5046 | 8.468531 | 23.76908 | 10.54091 | 15.17526 | 15.03227 | 35.84962 | 14.35201 | 32.49365 | 20.67135 | 18.28609 | 11.37559 | 39.84647 | 31.13099 | 30.94122 | 33.08815 | 20.65497 | 21.9738 |
| CUST_43479_PI429010886 | M63155 | 0.000933 | 24 | 12 | | | | | | 5.710989 | 5.710989 | 10.06452 | 10.2558 | 6.376695 | NA | 4.4993 | 6.98368 | 13.2704 | 9.126873 | 9.695604 | NA | 20.85646 | 26.31693 | 34.49254 | 17.14965 | 13.28601 | 16.87203 | 8.55086 | 11.98592 | NA | 15.40925 | 16.72767 | NA | 11.678 | NA | NA |
| CUST_6387_PI429010886 | N20036 | 0.000935 | 15 | 12 | 321460734 | hypothetical protein DAPPUDRAFT_308742 [Daphn | Protein metabolism | Cellular protein modification process | 5.94E-75 | 27.45566 | 40.42484 | 39.14912 | 19.3658 | 29.8311 | 17.13072 | 26.26527 | 41.31796 | 51.0829 | 46.21511 | 39.65895 | 51.15134 | 33.39553 | 54.48925 | 56.48952 | 40.1303 | 41.42564 | 43.27701 | 36.06929 | 37.90169 | 35.67442 | 43.99167 | 44.85517 | 32.96039 | 52.93292 | 38.723 | 45.53479 |
| CUST_8774_PI429010886 | N23566 | 0.000935 | 15 | 12 | | | | | | 22.63948 | 24.42322 | 24.35681 | 20.00677 | 15.99431 | 16.37668 | 14.8771 | 20.76696 | 27.78422 | 24.08352 | 20.71812 | 23.77956 | 31.47236 | 38.49034 | 35.11564 | 41.8722 | 18.47667 | 28.86203 | 23.27419 | 18.75687 | 15.37156 | 31.0493 | 28.34245 | 11.94121 | 33.33635 | 19.83086 | 28.45154 |
| CUST_30169_PI429010886 | N61314 | 0.000936 | 21 | 3 | | | | | | 15.67217 | 38.2551 | 14.29518 | 70.42582 | 27.14357 | 32.48228 | 11.23137 | 14.68799 | 9.925474 | 16.8666 | 6.548124 | 26.80106 | 9.512792 | 5.265199 | 4.308429 | 13.96684 | NA | 18.6374 | 7.109363 | 7.319054 | 37.54698 | 11.78894 | 37.65143 | 23.1151 | 27.4471 | 16.46028 | 20.37797 |
| CUST_18473_PI429010886 | N39863 | 0.000938 | 24 | 3 | | | | | | 26.9152 | 39.46953 | NA | 177.2148 | 82.17099 | 231.5437 | 53.97066 | 143.7864 | 16.80437 | NA | 33.29463 | NA | 29.25886 | 8.912458 | 21.78224 | 13.54322 | 37.84181 | 44.30287 | 3.811422 | 3.824513 | 22.30707 | 7.050922 | 14.76465 | 61.07112 | 4.024879 | 27.00493 | NA |
| CUST_14234_PI429010886 | N32543 | 0.000944 | 18 | 15 | | | | | | 63.747 | 61.53357 | 55.23983 | 43.25525 | 48.16109 | 36.31145 | 49.28963 | 45.97157 | 47.3396 | 59.13459 | 48.61666 | 49.998 | 54.64962 | 68.26569 | 59.3952 | 69.89082 | 45.58039 | 68.38203 | 65.22887 | 66.5854 | 55.17531 | 53.70396 | 41.61311 | 35.56458 | 69.99368 | 39.88324 | 58.63339 |
| CUST_29877_PI429010886 | N60738 | 0.000945 | 15 | 0 | | | | | | 18.87506 | 30.77501 | 11.71083 | 11.92505 | 13.91626 | 18.08701 | 11.40993 | 6.889529 | 14.11311 | 13.23813 | 8.871706 | 7.602225 | 7.664993 | 10.73752 | 13.04571 | 16.80263 | 19.24611 | 19.82458 | 20.13934 | 15.25738 | NA | 19.75734 | 10.22324 | 18.40021 | 10.26272 | 11.30787 | 6.814705 |
| CUST_1087_PI429010886 | N8450 | 0.000946 | 15 | 3 | 241151593 | calcineurin B, putative [Ixodes scapularis] >gi\|2154 | Metabolic process | Oxidation-reduction process | 6.03E-64 | 316.122 | 191.3918 | 180.8735 | 442.852 | 343.3378 | 645.2555 | 313.2163 | 322.1425 | 204.5562 | 196.7297 | 268.2743 | 207.5377 | 222.5361 | 166.7311 | 192.0579 | 205.0447 | 547.465 | 339.3078 | 285.9546 | 260.7005 | 354.6967 | 292.6509 | 251.4731 | 439.0329 | 210.9764 | 292.0038 | 221.6212 |
| CUST_54626_PI429010886 | M106917 | 0.000948 | 18 | 12 | | | | | | 248.0331 | 228.7951 | 283.5419 | 143.5925 | 202.4642 | 186.1374 | 172.7143 | 198.2503 | 217.9121 | 316.122 | 252.5773 | 295.6383 | 236.8954 | 306.479 | 319.4017 | 318.3844 | 221.2763 | 254.6874 | 235.1413 | 271.8631 | 191.2652 | 263.7999 | 202.9438 | 175.4567 | 240.1758 | 214.8393 | 245.0768 |
| CUST_51141_PI429010886 | M92344 | 0.000948 | 18 | 12 | | | | | | 25.29061 | 17.06824 | 31.54453 | 8.469456 | 23.86522 | 19.51691 | 31.99053 | 17.20395 | 28.16847 | 24.37441 | 24.29748 | 37.52322 | 28.94552 | 30.45321 | 31.42645 | 28.90658 | 29.76585 | 25.38172 | 44.29926 | 20.62307 | 21.87774 | 16.39729 | 22.53631 | 15.05158 | 25.5782 | 13.85312 | 23.5177 |
| CUST_14632_PI429010886 | N33205 | 0.000948 | 12 | 0 | | | | | | 326.9265 | 255.0348 | 437.2736 | 196.8321 | 163.1844 | 211.6792 | 233.2471 | 294.2267 | 410.8257 | 371.054 | 214.1723 | 312.4226 | 364.0324 | 632.2521 | 349.238 | 269.6735 | 250.2094 | 261.4696 | 248.2927 | 291.4361 | 264.7951 | 350.912 | 365.9198 | 256.333 | 447.6451 | 323.2711 | 459.1503 |
| CUST_14600_PI429010886 | N33152 | 0.000959 | 24 | 18 | 242006716 | conserved hypothetical protein [Pediculus humanus | Transport | Protein transport | 8.17E-49 | 29.71613 | 29.70687 | 32.02169 | 25.46966 | 21.4795 | 30.61606 | 29.60636 | 16.55113 | 30.55547 | 32.14525 | 35.72502 | 30.06057 | 30.31987 | 23.17075 | 39.59389 | 41.59799 | 36.03165 | 40.1147 | 73.95574 | 39.7396 | 62.99752 | 36.74882 | 24.49848 | 27.76975 | 44.44423 | 19.32948 | 30.85363 |
| CUST_40077_PI429010886 | M49819 | 0.000968 | 24 | 21 | | | | | | 82.1182 | 237.4703 | 196.7297 | 149.5758 | 56.46018 | 25.82998 | 32.02557 | 7.650264 | 25.88533 | 67.9344 | 87.48439 | 113.2396 | 488.3379 | 144.5473 | 198.3716 | 203.6364 | NA | 135.074 | 272.8585 | 100.336 | 252.8082 | 257.0386 | 493.5731 | 66.18886 | 453.5801 | 70.90194 | 329.6909 |
| CUST_15636_PI429010886 | N34896 | 0.000969 | 15 | 0 | | | | | | 12.53922 | 14.6922 | 18.09635 | 9.060793 | 20.02885 | NA | 15.17161 | 5.807692 | 10.7039 | 7.578825 | 4.581089 | 6.807158 | 14.1448 | NA | 10.33003 | 15.28371 | 15.54049 | 20.33623 | 7.503213 | 16.51414 | 11.19204 | 17.66078 | 13.36864 | NA | 5.102168 | 7.584705 | 9.893302 |
| CUST_8062_PI429010886 | N22483 | 0.000969 | 18 | 9 | | | | | | 134.9686 | 130.3926 | 145.0185 | 101.4497 | 136.1436 | 147.5412 | 155.8377 | 154.4419 | 185.1977 | 161.2244 | 159.4512 | 195.8687 | 116.3563 | 168.8426 | 140.5991 | 130.6959 | 164.9826 | 154.5009 | 131.0289 | 127.3882 | 147.0578 | 157.1911 | 149.8948 | 160.8926 | 164.3967 | 148.6082 | 189.7994 |
| CUST_7539_PI429010886 | N21698 | 0.00097 | 15 | 12 | | | | | | 18.66797 | 17.31011 | 22.10673 | 19.46833 | 21.87979 | 14.52959 | 12.09545 | 12.71188 | 6.798747 | 16.96557 | 10.29246 | 22.87323 | 24.78255 | 21.87083 | 29.36585 | 25.20311 | 20.92458 | 13.5981 | 24.91723 | 24.71814 | 8.07058 | 13.4766 | 12.24024 | 7.213856 | 23.12065 | 12.6487 | 26.60971 |
| CUST_1004_PI429010886 | N8185 | 0.000973 | 12 | 0 | 321475177 | hypothetical protein DAPPUDRAFT_97958 [Daphnia | | | 4.23E-20 | 184.8973 | 186.7544 | 225.0823 | 124.1661 | 155.2361 | 140.4751 | 184.0345 | 175.5504 | 168.2432 | 236.6472 | 206.6992 | 233.9751 | 176.885 | 268.2351 | 207.1617 | 178.3288 | 185.5593 | 163.329 | 184.6775 | 233.3626 | 182.0796 | 184.2307 | 212.9853 | 200.6114 | 246.4698 | 199.5128 | 277.4454 |
| CUST_31223_PI429010886 | M1011 | 0.000974 | 21 | 9 | 126635325 | luciferase [Photinus pyralis] | Metabolic process | Other | 7.33E-55 | 44.21249 | 67.48171 | 40.22531 | 60.26917 | 73.86349 | 49.89799 | 74.48926 | 195.1585 | 107.0545 | 102.1333 | 87.31709 | 109.6371 | 96.41487 | 66.78979 | 71.81768 | 45.51068 | 80.31713 | 42.05204 | 59.66164 | 250.8965 | 152.6964 | 40.57981 | 57.86313 | 49.68201 | 70.39937 | 70.84411 | 144.2774 |
| CUST_17872_PI429010886 | N38747 | 0.000981 | 15 | 12 | | | | | | 119.1955 | 135.5237 | 159.3655 | 67.59725 | 118.3632 | 71.83386 | 110.9726 | 112.7335 | 135.8973 | 177.5136 | 134.5558 | 127.6283 | 234.443 | 160.778 | 207.5377 | 148.08 | 100.0618 | 133.8452 | 97.56553 | 135.1969 | 151.3078 | 156.213 | 129.5087 | 128.1595 | 128.3042 | 137.759 | 168.4504 |
| CUST_6787_PI429010886 | N20602 | 0.000982 | 18 | 9 | | | | | | 290.2221 | 279.6215 | 341.3294 | 264.9674 | 364.475 | 245.0284 | 255.7416 | 724.3304 | 580.0174 | 369.6879 | 671.7237 | 675.3956 | 498.6758 | 325.619 | 415.6575 | 407.8386 | 456.9093 | 372.9677 | 295.5774 | 600.2864 | 434.4861 | 317.7577 | 286.3672 | 187.6525 | 330.527 | 298.5904 | 606.8516 |
| CUST_33636_PI429010886 | M7482 | 0.000984 | 12 | 0 | | | | | | 1963.68 | 5013.208 | 3911.313 | 199.3801 | 1325.963 | 145.4402 | 1187.094 | 2809.847 | 2545.683 | 4399.213 | 1756.971 | 3769.878 | 3821.032 | 4672.866 | 2970.275 | 3526.312 | 1281.141 | 1284.158 | 2271.87 | 3590.082 | 2675.265 | 2437.103 | 3676.785 | 299.3397 | 5176.163 | 3526.312 | 5774.702 |
| CUST_43192_PI429010886 | M62366 | 0.000985 | 21 | 12 | | | | | | 15.444 | 17.64462 | 9.103248 | 16.79285 | 15.71146 | 27.06658 | 49.52577 | 19.18101 | 28.94552 | 23.15839 | 25.91041 | 32.34301 | 20.93093 | 38.05463 | 39.94945 | 27.25936 | 22.48589 | 29.13814 | 23.80497 | 17.64462 | 29.26863 | 16.18253 | 14.3333 | 20.02647 | 22.46676 | 35.6576 | 35.15805 |
| CUST_12213_PI429010886 | N29112 | 0.000993 | 21 | 0 | 321459624 | hypothetical protein DAPPUDRAFT_60952 [Daphnia | Transport | Ion transport | 1.61E-29 | 10.97439 | 9.651747 | 10.82471 | 10.21233 | 7.507529 | 16.08632 | 11.22985 | 7.743875 | 8.100321 | 6.324533 | 6.915938 | 9.525782 | 3.734429 | 6.376695 | 6.788713 | 5.301637 | 5.087451 | 4.531506 | 4.206837 | 6.44346 | 9.145807 | 4.71937 | 9.871686 | 17.38288 | 6.668324 | NA | 9.001207 |
| CUST_18828_PI429010886 | N40488 | 0.000993 | 18 | 0 | | | | | | 452.6313 | 773.1832 | 470.0153 | 368.9642 | 500.3626 | 460.255 | 373.7568 | 307.2905 | 448.9486 | 499.2279 | 460.0363 | 512.0216 | 318.7622 | 361.7651 | 285.0949 | 619.3802 | 447.4667 | 574.6243 | 509.603 | 730.0267 | 581.5974 | 631.1117 | 498.6758 | 581.1144 | 303.9241 | 123.1087 | 204.9184 |
| CUST_21739_PI429010886 | N45762 | 0.000993 | 15 | 12 | | | | | | 282.9535 | 361.2261 | 575.8176 | 257.772 | 254.6455 | 247.961 | 238.2063 | 450.3936 | 508.3277 | 563.9632 | 278.2072 | 518.2235 | 392.0959 | 657.2757 | 456.2722 | 349.8347 | 315.1705 | 347.794 | 278.6632 | 347.8665 | 259.5794 | 404.5388 | 532.5435 | 272.9055 | 620.3013 | 325.7489 | 589.4448 |
| CUST_23658_PI429010886 | N49222 | 0.000995 | 21 | 3 | 189238830 | PREDICTED: similar to CG11293 CG11293-PA [Tribol | | | 1.41E-13 | 50.10243 | 88.30586 | 47.99919 | 160.8926 | 60.71887 | 101.0962 | 53.65679 | 56.62309 | 37.48716 | 39.78898 | 35.37524 | 47.09025 | 57.16741 | 43.92643 | 23.12065 | 45.77355 | 23.59336 | 56.91814 | 33.70683 | 36.94269 | 68.38918 | 65.16761 | 55.77862 | 75.12567 | 68.08199 | 80.5678 | 47.23018 |
| CUST_53520_PI429010886 | M102096 | 0.000995 | 18 | 15 | 321466278 | notch 2 [Daphnia pulex] | | | 1.17E-41 | 109.8291 | 104.4333 | 90.57431 | 129.8445 | 90.23885 | 155.4149 | 87.64594 | 85.59472 | 52.42987 | 75.4776 | 66.32011 | 76.12074 | 92.66541 | 131.4266 | 126.9474 | 161.4133 | 116.8757 | 144.6491 | 103.0385 | 156.3918 | 97.85969 | 155.1735 | 59.66164 | 148.7085 | 87.67204 | 78.41704 | 50.45103 |
| CUST_51441_PI429010886 | M93544 | 0.000996 | 21 | 0 | | | | | | 22.66107 | 19.74119 | 24.7458 | 14.61766 | 14.23448 | 13.04046 | 18.93949 | 10.59651 | 13.28156 | 16.25929 | 10.93489 | 9.005077 | 11.98592 | 19.84686 | 17.16312 | 22.63948 | 20.30647 | 20.25261 | 10.91573 | 16.44885 | 14.26904 | 22.43497 | 19.92242 | 23.4104 | 16.17213 | 15.16642 | 12.84068 |
| CUST_35794_PI429010886 | M13532 | 0.000997 | 24 | 18 | | | | | | 101.5014 | 63.94078 | 54.78436 | 64.29604 | 50.277 | 66.54333 | 49.18419 | 38.22142 | 49.05985 | 73.14043 | 53.35562 | 52.17339 | 44.1892 | 61.54664 | 59.6812 | 87.2868 | 64.41721 | 79.84151 | 77.36396 | 67.13373 | 92.56423 | 85.493 | 55.68518 | 55.61005 | 96.33377 | 52.12912 | 70.72361 |
| CUST_16213_PI429010886 | N35856 | 0.001 | 18 | 3 | | | | | | 19.36095 | 9.635022 | NA | 22.1165 | 40.46037 | 27.40469 | 7.858321 | 16.71521 | 6.71361 | 22.87323 | 13.44859 | 7.809814 | 9.319645 | 11.24362 | 4.725076 | 16.41397 | 9.740573 | 10.47564 | 17.14965 | 13.36577 | 24.49848 | 20.09233 | 23.24391 | 15.00483 | 19.47912 | 9.025647 | 16.96853 |
| CUST_18775_PI429010886 | N40387 | 0.001003 | 21 | 0 | | | | | | 25.35696 | 22.0504 | 14.36868 | 12.55922 | 15.52381 | 13.67523 | 11.1247 | 7.755082 | 7.216307 | 11.29768 | 9.676508 | 10.47429 | 17.34013 | 15.57429 | 6.105375 | 17.00755 | 14.1623 | NA | 15.31744 | 18.15598 | 9.352687 | 14.28677 | 18.36323 | 19.15208 | 20.32379 | 10.39161 | 14.59087 |
| CUST_47894_PI429010886 | M78070 | 0.001005 | 24 | 21 | | | | | | 147.5068 | 148.1205 | 184.1431 | 108.5374 | 133.1717 | 132.0609 | 120.9878 | 122.1911 | 121.6208 | 142.2307 | 132.9281 | 147.4899 | 138.5998 | 185.3847 | 130.1016 | 140.7918 | 141.1309 | 171.1862 | 135.6184 | 156.1682 | 175.0831 | 147.8481 | 206.2132 | 156.6764 | 171.2071 | 161.5676 | 160.0108 |
| CUST_28861_PI429010886 | N58744 | 0.001005 | 24 | 21 | 443715571 | hypothetical protein CAPTEDRAFT_158241 [Capitell | | | 9.05E-08 | 49.56436 | 35.74194 | 44.7891 | 43.38432 | 24.44087 | 37.47945 | 40.63765 | 22.98966 | 31.81303 | 55.40965 | 64.04953 | 78.37536 | 51.5698 | 80.8031 | 48.69326 | 53.64846 | 54.56998 | 62.44095 | 47.02203 | 121.8737 | 75.3452 | 80.63947 | 97.13391 | 107.7242 | 57.8814 | 32.84186 | 43.93042 |
| CUST_31765_PI429010886 | M2349 | 0.001011 | 21 | 0 | | | | | | 36.8424 | 48.08 | 44.64586 | 35.74774 | 30.3731 | 31.88591 | 24.25926 | 28.87134 | 18.42911 | 36.92111 | 13.63385 | 25.78299 | 6.978868 | 16.61483 | 11.42142 | 37.56417 | 22.2285 | 31.88231 | 16.58905 | 41.77517 | 29.09314 | 44.76277 | 26.62548 | 24.56057 | 25.05018 | 30.72781 | 33.68381 |
| CUST_36992_PI429010886 | M17480 | 0.001014 | 15 | 12 | | | | | | 227.312 | 265.3147 | 281.0282 | 199.4211 | 206.2132 | 240.0651 | 197.2228 | 242.6123 | 257.3603 | 278.3016 | 256.6496 | 343.1257 | 264.269 | 353.0802 | 280.7168 | 307.9665 | 252.1679 | 260.7973 | 180.8997 | 280.4106 | 236.2907 | 277.4972 | 284.6976 | 252.0389 | 360.037 | 277.5533 | 328.1634 |
| CUST_17978_PI429010886 | N38938 | 0.001015 | 18 | 3 | | | | | | 168.1071 | 199.7913 | 301.4214 | 203.8966 | 215.4319 | 248.7014 | 275.6931 | 135.3397 | 76.94138 | 80.75638 | 252.7637 | 157.8167 | 45.51822 | 60.94428 | 118.7356 | 137.8094 | 127.0219 | 100.2579 | 176.2225 | 82.10096 | 233.6069 | 136.2319 | 402.8509 | 424.7309 | 65.63314 | 183.8985 | 97.15481 |
| CUST_18666_PI429010886 | N40192 | 0.001025 | 24 | 0 | 133754273 | inhibitor of apoptosis protein [Penaeus monodon] | | | 5.25E-16 | 414.0246 | 415.4709 | 506.24 | 504.8164 | 352.3859 | 299.285 | 534.0061 | 315.7204 | 362.3529 | 419.3343 | 332.0008 | 391.5301 | 258.4027 | 294.3403 | 362.2892 | 353.689 | 244.2054 | 331.4379 | 354.3972 | 359.2285 | 430.3425 | 421.8908 | 344.7288 | 367.1896 | 437.7044 | 527.0813 | 424.6374 |
| CUST_1688_PI429010886 | N10444 | 0.001029 | 21 | 18 | | | | | | 220.2915 | 215.8652 | 153.961 | 200.5357 | 174.5925 | 166.585 | 120.1371 | 145.9425 | 152.8418 | 178.1983 | 169.5063 | 223.676 | 189.5057 | 190.645 | 165.1872 | 207.8011 | 126.2324 | 193.4709 | 202.3705 | 343.9276 | 199.9465 | 259.2052 | 215.0865 | 186.0881 | 187.2169 | 136.2681 | 161.3046 |
| CUST_38955_PI429010886 | M32543 | 0.00103 | 15 | 3 | 33468738 | calcification-associated peptide-1 [Procambarus cla | | | 3E-19 | 482.976 | 33.07929 | 303.9813 | 29010.89 | 39067.89 | 41577.33 | 21514.91 | 9839.748 | 79.51299 | 21.91785 | 22231.21 | 5.901635 | 1123.855 | 25.8552 | 688.7233 | 7.897325 | 27524.03 | 20510.07 | 32962.39 | NA | 29010.89 | 21.49674 | 9697.645 | 12445.58 | 12.2288 | 14684.57 | 7.681885 |
| CUST_11864_PI429010886 | N28533 | 0.001031 | 24 | 21 | | | | | | 94.34866 | 118.4488 | 122.84 | 71.00916 | 102.3344 | 121.284 | 69.06525 | 75.62563 | 79.92252 | 111.7487 | 83.80399 | 98.42661 | 73.803 | 107.7388 | 97.46537 | 129.7547 | 99.32217 | 101.187 | 99.2894 | 102.8784 | 116.5651 | 125.8931 | 122.949 | 107.8715 | 140.5892 | 102.1823 | 121.1456 |
| CUST_4351_PI429010886 | N16571 | 0.001032 | 18 | 3 | 152949487 | calcification associated soluble matrix protein 2 [Pr | | | 8.03E-17 | 307.6894 | 274.5343 | 339.3078 | 625.484 | 892.3589 | 1231.516 | 350.5238 | 197.989 | 187.9811 | 156.2777 | 103.0135 | 10.7321 | 425.8413 | 266.2431 | 277.0736 | 297.4174 | 272.1926 | 311.8796 | 367.8562 | 73.48596 | 82.72689 | 198.9264 | 641.887 | 645.5341 | 578.3985 | 237.1286 | 16.52754 |
| CUST_42909_PI429010886 | M61577 | 0.001036 | 15 | 0 | 242006996 | conserved hypothetical protein [Pediculus humanus | | | 1.45E-18 | 38.67242 | 31.75969 | 38.97496 | 15.31157 | 27.14824 | 38.62431 | 26.57549 | 20.95213 | 24.71814 | 38.05769 | 24.71374 | 30.58787 | 27.72618 | 31.721 | 29.63867 | 43.65882 | 48.62912 | 42.17788 | 35.95337 | 36.82429 | 29.13595 | 38.49034 | 25.88998 | 17.40769 | 40.85856 | 21.18145 | 37.6674 |
| CUST_2378_PI429010886 | N12221 | 0.001038 | 21 | 12 | | | | | | 17.31897 | 14.41938 | 18.71899 | 17.59799 | 29.3424 | 18.61046 | 12.40087 | 32.19497 | 29.9971 | 24.4665 | 22.1415 | 30.15316 | 26.14767 | 31.11066 | 23.60773 | 25.12574 | 30.86679 | 26.26013 | 25.05128 | 17.50362 | 23.01368 | 16.8263 | 19.22658 | 21.97651 | 21.99145 | 14.63359 | 22.23059 |
| CUST_36435_PI429010886 | M15885 | 0.001039 | 21 | 3 | | | | | | 227.6752 | 243.8028 | 314.3091 | 304.9967 | 287.1125 | 365.6206 | 266.3801 | 317.9044 | 267.5328 | 258.7295 | 294.0478 | 412.9301 | 271.4917 | 285.6495 | 286.2536 | 57.17501 | 275.3091 | 187.9002 | 247.6825 | 301.1632 | 208.2464 | 273.7876 | 269.8655 | 234.8913 | 359.9556 | 343.9276 | 343.3863 |
| CUST_43359_PI429010886 | M62828 | 0.001041 | 18 | 12 | | | | | | 47.12699 | 36.77765 | 43.46213 | 35.31845 | 40.44483 | 23.58866 | 33.13396 | 64.16127 | 45.08069 | 36.97227 | 40.9233 | 67.89962 | 65.22254 | 75.14455 | 47.04186 | 64.50028 | 39.46953 | 49.40423 | 78.22974 | 45.09974 | 52.17339 | 38.63168 | 37.83427 | 22.24949 | 55.44777 | 47.88942 | 44.13738 |
| CUST_17929_PI429010886 | N38851 | 0.001051 | 12 | 3 | | | | | | 51.78005 | 35.04514 | 37.61547 | 109.1135 | 55.20237 | 69.22629 | 61.14077 | 37.75935 | 34.67799 | 56.44254 | 42.33801 | 28.09705 | 39.11234 | 29.81183 | 50.71291 | 55.784 | 70.6776 | 54.08762 | 78.00387 | 53.48278 | 40.41641 | 37.55188 | 48.87835 | 31.0625 | 29.02946 | 26.0929 | 32.69544 |
| CUST_17339_PI429010886 | N37797 | 0.001058 | 24 | 21 | 270012294 | hypothetical protein TcasGA2_TC006417 [Tribolium | | | 2.36E-08 | 31.98439 | 31.38593 | 48.12746 | 25.88326 | 24.34006 | 32.58962 | 27.66265 | 26.89381 | 33.17839 | 30.78076 | 29.99067 | 29.5457 | 29.97696 | 43.70884 | 32.16411 | 44.40996 | 29.78164 | 35.32273 | 36.62775 | 33.38822 | 32.61875 | 48.17972 | 47.40597 | 39.16114 | 51.43692 | 33.51061 | 37.73674 |
| CUST_24871_PI429010886 | N51393 | 0.001058 | 24 | 21 | | | | | | 28.81481 | 29.28006 | 25.20056 | 21.89788 | 24.39107 | 22.52377 | 20.30155 | 19.11687 | 24.23314 | 43.44584 | 16.75535 | 20.46326 | 29.91175 | 28.09932 | 25.55814 | 30.52691 | 21.75697 | 24.45953 | 27.61589 | 22.16055 | 28.03356 | 37.21745 | 34.05249 | 49.37485 | 34.23165 | 20.72467 | 33.55523 |
| CUST_28798_PI429010886 | N58638 | 0.001058 | 24 | 21 | | | | | | 36.01332 | 24.6789 | 30.71209 | 4.142037 | 33.56673 | 25.52007 | 18.74405 | 16.44239 | 27.72317 | 30.23517 | 27.50907 | 36.45907 | 11.76966 | 41.52389 | 16.6126 | 31.16892 | 41.74772 | 27.12757 | 42.65591 | 27.09726 | 49.69905 | 49.18958 | 41.7589 | 46.2898 | 35.77964 | 43.41776 | 29.90756 |
| CUST_16803_PI429010886 | N36896 | 0.001063 | 24 | 0 | | | | | | 283.2818 | 318.5103 | 295.1449 | 203.9899 | 303.7146 | 260.7973 | 259.5794 | 245.6075 | 207.6805 | 219.0379 | 252.4954 | 243.9881 | 230.6259 | 287.9804 | 253.9783 | 312.5863 | 234.555 | 226.242 | 373.826 | 270.5782 | 315.5114 | 460.7596 | 251.4349 | 326.0904 | 427.7928 | 339.7564 | 412.3362 |
| CUST_22263_PI429010886 | N46711 | 0.001075 | 21 | 3 | | | | | | 62.90335 | 79.11601 | 69.96115 | 134.3348 | 99.79034 | 96.94108 | 63.06213 | 58.23835 | 66.33339 | 65.79499 | 63.75313 | 50.4975 | 55.00033 | 68.761 | 45.96465 | 53.27432 | 63.8461 | 78.69319 | 70.32547 | 46.23463 | 95.01446 | 57.664 | 121.0216 | 70.96221 | 83.41229 | 94.53569 | 69.4087 |
| CUST_16401_PI429010886 | N36163 | 0.001078 | 21 | 3 | | | | | | 33.8279 | 40.78034 | 27.58029 | 44.41428 | 19.80135 | 36.30688 | 33.03027 | 33.29815 | 30.79653 | 31.34117 | 26.54351 | 30.66462 | 23.55513 | 31.53112 | 18.35581 | 33.48853 | 17.25469 | 23.52815 | 26.77723 | 31.13642 | 34.02396 | 30.52691 | 43.97432 | 34.89935 | 38.41593 | 44.22632 | 40.56586 |
| CUST_40769_PI429010886 | M56047 | 0.001079 | 18 | 3 | | | | | | 39.93998 | 38.81815 | NA | 88.87538 | 41.42564 | 59.59632 | 87.22914 | 14.76465 | 62.76241 | 14.5219 | 9.104602 | 13.57374 | 18.88563 | 19.68008 | 36.96378 | 31.51484 | NA | 22.092 | 14.89445 | 15.60189 | 11.00334 | 78.37536 | 176.8527 | 72.49088 | 236.1561 | 44.84606 | 11.05263 |
| CUST_9892_PI429010886 | N25364 | 0.001086 | 18 | 3 | | | | | | 5.555758 | NA | NA | 34.14111 | 7.3208 | 11.68092 | 7.755082 | 5.105169 | 4.799209 | 3.710121 | 4.192601 | NA | 4.929948 | 7.258252 | NA | 6.431841 | 5.015188 | 4.859225 | 5.770518 | 4.5493 | 15.15241 | NA | 15.78045 | 25.88089 | 12.83593 | 12.11676 | NA |
| CUST_21668_PI429010886 | N45623 | 0.001088 | 18 | 3 | | | | | | 69.23488 | 162.98 | 67.79099 | 257.6478 | 99.6429 | 175.7224 | 81.11384 | 70.94036 | 62.56969 | 54.23711 | 37.94143 | 99.82512 | 58.90324 | 56.8218 | 51.80926 | 90.26625 | 31.53429 | 51.99458 | 23.72419 | 62.31885 | 93.6434 | 83.58732 | 103.1588 | 101.4752 | 94.00857 | 195.0326 | 42.42919 |
| CUST_8466_PI429010886 | N23082 | 0.00109 | 24 | 15 | | | | | | 174.9558 | 210.6402 | 151.0563 | 151.0154 | 148.3091 | 156.6593 | 141.0258 | 145.7727 | 131.1802 | 184.0607 | 166.4887 | 198.3716 | 139.419 | 166.7787 | 203.6001 | 225.7224 | 180.2584 | 210.5891 | 182.3936 | 213.6068 | 180.4919 | 206.9847 | 174.2228 | 193.2088 | 218.4011 | 180.9317 | 189.4251 |
| CUST_7075_PI429010886 | N21003 | 0.001091 | 15 | 0 | | | | | | 103.2289 | 248.5464 | 149.2244 | 108.2888 | 126.8537 | 212.2832 | 53.64297 | 46.19956 | 60.76524 | 102.3756 | 119.4425 | 112.9777 | 27.24907 | 79.07032 | 76.62236 | 118.1115 | 133.8595 | 179.9528 | 102.3423 | 120.4034 | 102.7139 | 101.1328 | 79.94364 | 77.10171 | 118.2899 | 102.9808 | 102.3344 |
| CUST_42404_PI429010886 | M60197 | 0.001092 | 24 | 0 | 371591197 | Euphausia superba voucher 841M mitochondrion, p | Transport | Ion transport | 2E-118 | 706.1971 | 1218.367 | 739.8087 | 624.8531 | 222.6599 | 693.5345 | 683.1376 | 974.6113 | 449.0628 | 810.9217 | 621.5625 | 306.3264 | 92.53187 | 868.0572 | 551.3995 | 11.80005 | 568.2036 | 159.741 | 368.494 | 845.457 | 364.2987 | 1012.109 | 322.9274 | 585.0822 | 1136.377 | 976.6512 | 950.2628 |
| CUST_38642_PI429010886 | M28779 | 0.001093 | 24 | 12 | | | | | | 47.38756 | 24.44489 | NA | 23.08457 | 26.58995 | 54.64962 | 38.43703 | 41.80808 | 58.02431 | 237.1286 | 76.07191 | 91.83515 | NA | 591.7087 | 256.3774 | 125.8442 | NA | 111.6027 | 61.49124 | 81.82955 | 134.3671 | 78.17016 | 81.07113 | 198.0922 | 123.3865 | 111.1358 | 160.2968 |
| CUST_14826_PI429010886 | N33533 | 0.001096 | 12 | 3 | | | | | | 27.52478 | 888.7344 | 21.73902 | 86.31209 | 4001.148 | 4001.148 | 1460.496 | NA | 1351.741 | 5.679797 | 6.698064 | NA | 234.0614 | 660.3398 | 341.5068 | 8138.075 | NA | 4432.801 | 12.83923 | NA | 10.59173 | 31.25182 | 12.87509 | 70.31729 | 302.3576 | 25.92862 | NA |
| CUST_46329_PI429010886 | M72238 | 0.001098 | 21 | 3 | | | | | | 8.255312 | 24.71578 | 13.53805 | 41.28609 | 13.97054 | 30.68719 | 13.7828 | 11.83103 | 14.41094 | 6.740179 | 3.888564 | 23.42758 | 6.55575 | 13.67211 | 3.916081 | 4.999323 | 5.398604 | NA | 3.682229 | 5.770518 | 26.61953 | 13.43544 | 41.52003 | 17.71088 | 13.91309 | 113.3918 | 15.14528 |
| CUST_1013_PI429010886 | N8220 | 0.001102 | 24 | 21 | | | | | | 12.45327 | 12.33822 | NA | 7.137459 | 7.699213 | 3.735274 | 3.748106 | 8.098026 | 4.223313 | 11.18709 | 7.804985 | 6.843848 | 13.52517 | 9.989923 | 9.95062 | 14.25924 | 10.66649 | 18.99531 | 7.662248 | 15.19655 | 6.703633 | 14.19691 | 15.08577 | 16.93182 | 15.36816 | 9.125404 | 10.76236 |
| CUST_39701_PI429010886 | M43090 | 0.001103 | 18 | 12 | | | | | | 3988.418 | 6654.81 | 5927.379 | 2379.147 | 4680.081 | 3785.297 | 4851.309 | 4208.899 | 5188.094 | 9950.402 | 4235.531 | 5948.096 | 5794.422 | 7450.764 | 7930.333 | 8313.066 | 4887.374 | 7605.917 | 4842.442 | 6587.101 | 5201.099 | 6941.938 | 3306.501 | 3929.651 | 7119.249 | 4769.116 | 6289.971 |
| CUST_36652_PI429010886 | M16457 | 0.001105 | 15 | 0 | | | | | | 24.26504 | 28.8964 | 23.43812 | 20.37597 | 14.59908 | 14.45246 | 20.10077 | 15.88827 | 10.6935 | 19.26616 | 21.93695 | 25.00056 | 17.33696 | 19.05757 | 20.42948 | 29.20693 | 25.27468 | 30.11515 | 25.9425 | 14.93407 | 23.51614 | 18.69291 | 18.99531 | 14.56343 | 26.43705 | 22.31737 | 22.03029 |
| CUST_2826_PI429010886 | N13190 | 0.001105 | 15 | 0 | | | | | | 1794.046 | 1474.289 | 1503.323 | 1261.41 | 1414.097 | 1751.759 | 645.4311 | 473.1073 | 635.0709 | 1172.181 | 1525.167 | 1321.79 | 307.8404 | 1165.381 | 795.552 | 1876.716 | 1476.818 | 1717.641 | 921.6949 | 2417.914 | 1194.442 | 1395.696 | 795.358 | 744.0064 | 1122.959 | 1073.06 | 1074.496 |
| CUST_4608_PI429010886 | N17124 | 0.001105 | 21 | 15 | | | | | | 1172.181 | 905.268 | 1025.618 | 754.5887 | 466.4129 | 730.4799 | 1569.952 | 747.4574 | 907.1459 | 2016.816 | 974.3808 | 973.9722 | 1461.256 | 1036.891 | 696.7816 | 3132.563 | 1998.595 | 1004.403 | 2032.79 | 1268.323 | 884.0735 | 1041.277 | 1155.257 | 881.8414 | 815.8741 | 698.4337 | 1071.809 |
| CUST_20135_PI429010886 | N42869 | 0.001108 | 18 | 9 | | | | | | 174.8316 | 209.5944 | 237.4703 | 125.2116 | 139.545 | 130.4232 | 159.9242 | 177.8158 | 181.2412 | 230.8944 | 209.7628 | 227.2732 | 127.5003 | 199.9465 | 198.1505 | 221.5477 | 149.0785 | 184.7219 | 149.0336 | 214.7012 | 156.7058 | 172.7709 | 143.8832 | 132.3396 | 204.2578 | 231.7343 | 190.2377 |
| CUST_45383_PI429010886 | M68930 | 0.001114 | 15 | 12 | | | | | | 48.77148 | 41.36452 | 56.89861 | 39.4307 | 49.94515 | 32.10055 | 49.998 | 51.82417 | 74.79904 | 75.30403 | 47.09025 | 60.13681 | 56.87504 | 77.15161 | 61.64915 | 56.45424 | 45.86806 | 60.00971 | 38.17289 | 50.61028 | 43.439 | 75.13415 | 47.24387 | 43.13359 | 58.55654 | 58.56187 | 60.46074 |
| CUST_11457_PI429010886 | N27842 | 0.001115 | 24 | 12 | 321470018 | hypothetical protein DAPPUDRAFT_303750 [Daphn | | | 4.05E-41 | 21.61291 | 15.34846 | 14.36708 | 11.17946 | 17.35586 | 10.71996 | 19.83251 | 23.77465 | 15.38675 | 23.98555 | 19.70849 | 25.04386 | 27.65747 | 29.27092 | 28.25408 | 27.22266 | 20.95946 | 15.45319 | 21.79167 | 21.38369 | 30.17589 | 26.93068 | 17.57463 | 25.52353 | 27.36205 | 20.33392 | 25.37243 |
| CUST_40969_PI429010886 | M56511 | 0.001118 | 15 | 9 | 321479500 | hypothetical protein DAPPUDRAFT_39582 [Daphnia | | | 1.99E-27 | 94.70207 | 275.8443 | 240.3331 | 146.405 | 104.4835 | 42.51309 | 93.55006 | 213.8469 | 278.6632 | 600.5562 | 263.6263 | 406.415 | 388.5645 | 634.1523 | 342.3707 | 247.5005 | 104.1514 | 95.56359 | 142.1088 | 212.773 | 120.2042 | 324.9748 | 467.9877 | 60.73689 | 809.6278 | 215.4716 | 749.7018 |
| CUST_47724_PI429010886 | M77328 | 0.001119 | 15 | 9 | | | | | | 72.3532 | 65.9084 | 104.4835 | 72.3404 | 76.20212 | 85.48477 | 71.47658 | 81.31771 | 87.96508 | 114.6944 | 89.47031 | 118.4299 | 91.33174 | 105.2191 | 79.03234 | 78.18464 | 73.42232 | 76.00655 | 72.53579 | 53.02384 | 85.10975 | 75.75327 | 82.95644 | 82.47663 | 103.0626 | 85.39372 | 112.064 |
| CUST_12196_PI429010886 | N29087 | 0.001119 | 21 | 0 | | | | | | 15.71416 | 13.43914 | 12.16464 | 14.72238 | 9.265801 | NA | 9.989923 | 10.84532 | 6.540321 | 11.62614 | 6.911676 | 14.21644 | 6.783959 | 3.988949 | 6.792388 | 9.341797 | NA | 11.58371 | 8.919059 | 15.35382 | 10.55308 | 16.23324 | 27.27727 | 9.656691 | 10.89133 | NA | 11.41469 |
| CUST_55390_PI429010886 | M109888 | 0.001122 | 21 | 0 | | | | | | 10.48086 | 21.97161 | 16.87557 | 12.91769 | 4.390237 | NA | 7.733411 | 9.901573 | 7.465747 | 9.085039 | 9.987338 | 7.431826 | 4.346071 | 9.96806 | 9.930283 | 12.22036 | 10.31326 | NA | 7.543719 | 10.26272 | 13.2544 | 12.49899 | NA | 10.93666 | 10.44922 | 10.44754 | 15.91598 |
| CUST_29139_PI429010886 | N59269 | 0.001126 | 24 | 18 | | | | | | 62.25774 | 48.1847 | 73.74514 | 63.26276 | 53.50545 | 72.41676 | 48.1847 | 61.60388 | 56.37885 | 61.58977 | 54.12753 | 56.92511 | 55.91435 | 57.80685 | 77.01708 | 83.06954 | 64.16127 | 62.12557 | 74.23824 | 92.57501 | 84.27965 | 72.31183 | 68.92299 | 69.04216 | 82.67004 | 77.32264 | 76.11306 |

| Probe ID | ID | p-value | Period | Phase | EBI ID | Annotation | GO category | Go term | E-value | 0 | 0 | 0 | 3 | 3 | 3 | 6 | 6 | 6 | 9 | 9 | 9 | 12 | 12 | 12 | 15 | 15 | 15 | 18 | 18 | 18 | 21 | 21 | 21 | 24 | 24 | 24 |
|---|---|---|---|---|---|---|---|---|---|---|---|---|---|---|---|---|---|---|---|---|---|---|---|---|---|---|---|---|---|---|---|---|---|---|---|---|
| CUST_9352_PI429010886 | N24480 | 0.001127 | 18 | 12 | | | Transport | Protein transport | | 83.19226 | 93.82886 | 89.60537 | 119.9589 | 146.722 | 118.3064 | 158.4788 | 110.5811 | 134.9479 | 126.5253 | 156.3748 | 120.5115 | 172.054 | 132.5328 | 130.0689 | 223.7779 | 123.9578 | 169.2493 | 100.5297 | 109.399 | 89.58206 | 122.8574 | 94.10691 | 134.577 | 178.9173 | 118.4488 | 225.2614 |
| CUST_9586_PI429010886 | N24878 | 0.001128 | 24 | 0 | 543729426 | PREDICTED: protein FAM86A [Columba livia] | | | 8.67E-13 | 35.992 | 69.68816 | 37.50197 | 62.89021 | 24.94581 | 58.544 | 31.31664 | 19.70026 | 28.58747 | 30.73213 | 19.42289 | 53.62643 | 24.05603 | 32.75095 | 10.6654 | 20.70161 | 9.887988 | 33.3239 | 16.71353 | 29.11133 | 36.37581 | 24.69594 | 39.11234 | 33.01824 | 39.47357 | 57.03251 | 37.80277 |
| CUST_13113_PI429010886 | N30596 | 0.00113 | 15 | 12 | | | | | | 45.7289 | 58.14864 | 57.76074 | 36.68252 | 34.50768 | 35.77964 | 38.04057 | 38.51713 | 42.46725 | 47.04186 | 44.04837 | 68.80103 | 61.39492 | 69.25418 | 64.41009 | 83.62457 | 47.2395 | 65.10535 | 43.13359 | 64.36368 | 32.15505 | 75.64933 | 40.50125 | 45.24787 | 64.20475 | 42.80624 | 66.5854 |
| CUST_28312_PI429010886 | N57708 | 0.001133 | 21 | 0 | 321469590 | hypothetical protein DAPPUDRAFT_51329 [Daphnia | Developmental process | Multicellular organismal development | 1.2E-20 | 21.44644 | 27.91811 | 26.4784 | 18.18555 | 11.2694 | 30.07342 | 14.15895 | 10.65864 | 13.50907 | 19.91752 | 17.86001 | 15.17526 | 8.426447 | 11.73596 | 21.44277 | 24.05603 | 14.40255 | 30.06426 | 14.02096 | 22.75684 | 16.24458 | 35.62064 | 25.39871 | 25.22969 | 16.64512 | 24.34524 | 13.64729 |
| CUST_26155_PI429010886 | N53764 | 0.001134 | 24 | 3 | | | | | | 79.11601 | 210.7138 | 131.8938 | 344.24 | 155.1093 | 191.8245 | 117.1089 | 91.37896 | 110.5811 | 124.8573 | 57.37114 | 133.3139 | 70.57795 | 82.8076 | 75.97215 | 97.30444 | 36.33335 | 61.84039 | 46.04195 | 86.17533 | 177.7238 | 120.1692 | 195.5222 | 112.3919 | 165.3002 | 199.7685 | 74.08523 |
| CUST_13927_PI429010886 | N32029 | 0.001137 | 24 | 21 | | | | | | 78.07189 | 71.84934 | 68.48249 | 65.03148 | 63.48536 | 84.65687 | 46.95662 | 63.6369 | 44.30287 | 48.34221 | 70.64745 | 91.86368 | 54.38941 | 59.00876 | 92.09827 | 80.79066 | 72.61909 | 90.6343 | 82.58698 | 60.89775 | 94.5785 | 78.75847 | 102.5545 | 97.84604 | 110.3138 | 115.7889 | 74.38643 |
| CUST_41906_PI429010886 | M58852 | 0.001139 | 15 | 9 | | | | | | 66.18886 | 107.0816 | 135.4539 | 9.999252 | 29.62583 | 13.89365 | 47.04186 | 101.6806 | 196.8724 | 329.7729 | 165.8485 | 189.4567 | 259.439 | 150.5251 | 142.1633 | 193.4964 | 56.01567 | 58.98538 | 95.83965 | 357.356 | 97.46537 | 225.2988 | 199.7685 | NA | 503.9702 | 221.9563 | 442.852 |
| CUST_30068_PI429010886 | N61125 | 0.001144 | 12 | 0 | | | | | | 136.597 | 136.8075 | 134.5271 | 122.2551 | 93.11072 | 136.9989 | 89.33478 | 92.20534 | 94.19343 | 113.6252 | 95.04998 | 133.2264 | 105.4104 | 136.1838 | 133.9706 | 136.5503 | 101.5961 | 141.3582 | 91.84392 | 133.6116 | 119.0071 | 138.1939 | 127.916 | 119.9416 | 186.3412 | 144.4224 | 173.9876 |
| CUST_40566_PI429010886 | M55477 | 0.001145 | 24 | 18 | | | | | | 64.80696 | 133.987 | 98.28699 | 90.02313 | 74.73352 | 72.98413 | 31.08214 | 44.25982 | 52.60928 | 89.48953 | 95.25723 | 124.1278 | 80.86611 | 81.9929 | 113.5137 | 149.6657 | 82.44815 | 113.9031 | 118.0864 | 112.1531 | 156.4282 | 119.0883 | 89.08731 | 76.13058 | 85.23443 | 92.41138 | 149.0992 |
| CUST_33904_PI429010886 | M8197 | 0.001146 | 15 | 9 | | | | | | 116.8603 | 71.97535 | 223.4939 | 34.09109 | 82.84904 | 55.78991 | 114.8668 | 206.3839 | 266.3801 | 235.6934 | 272.1926 | 439.2082 | 165.0319 | 625.484 | 330.3612 | 99.4422 | 275.5051 | 86.68692 | 182.2299 | 148.8262 | 128.1087 | 729.1026 | 222.7411 | 49.34012 | 509.8767 | 230.0814 | 516.0613 |
| CUST_34943_PI429010886 | M11135 | 0.001147 | 21 | 0 | | | | | | 12.0298 | 28.48875 | 18.12711 | NA | 11.56997 | 26.8901 | 8.14453 | 10.88684 | 7.31497 | 9.796108 | 8.158718 | 6.256328 | 8.132198 | 12.84883 | NA | NA | 12.4383 | 7.657897 | 16.94759 | 9.946499 | 12.394 | 17.33078 | 22.06655 | 16.04761 | 11.9176 | 4.320994 | NA |
| CUST_39028_PI429010886 | M33635 | 0.001155 | 21 | 3 | 307197955 | hypothetical protein EAI_12947 [Harpegnathos salt | | | 7.02E-17 | 41.39686 | 19.08147 | NA | 71.81768 | 113.3684 | 122.5481 | 84.43869 | 69.63319 | 21.56454 | NA | 47.79729 | NA | 52.09002 | 14.68311 | 18.57889 | 14.18973 | 64.45839 | 64.26853 | 36.52603 | 6.048524 | 14.70767 | 17.99854 | 5.952327 | 54.53458 | NA | 71.91814 | NA |
| CUST_12353_PI429010886 | N29336 | 0.001157 | 18 | 0 | | | | | | 145.4633 | 136.1838 | 169.4763 | 155.2787 | 123.2747 | 143.2115 | 126.8764 | 101.7455 | 136.7329 | 159.1135 | 71.83386 | 146.765 | 123.4738 | 170.8077 | 159.0362 | 144.3925 | 160.1366 | 150.6483 | 182.5915 | 151.9691 | 185.414 | 157.0892 | 126.405 | 134.2279 | 116.9236 | 102.8301 | 109.5513 |
| CUST_13618_PI429010886 | N31479 | 0.001159 | 18 | 12 | | | | | | 38.80762 | 48.92098 | 51.97066 | 29.81837 | 33.26161 | 28.51242 | 35.9063 | 34.78102 | 31.68444 | 54.08177 | 23.95482 | 47.98542 | 47.96198 | 55.6932 | 64.66893 | 51.5698 | 47.62717 | 53.15159 | 32.48902 | 37.21745 | 34.92205 | 35.06362 | 36.01332 | 26.84868 | 59.41826 | 30.53983 | 62.07681 |
| CUST_43907_PI429010886 | M64303 | 0.00116 | 15 | 9 | | | | | | 67.11047 | 147.0211 | 265.9049 | 26.87538 | 25.60131 | 4.898275 | 49.79836 | 340.5239 | 505.3693 | 280.165 | 210.3675 | 419.5293 | 322.9938 | 648.6302 | 408.5798 | 267.9382 | 134.1249 | 136.4108 | 111.0142 | 233.088 | 150.5899 | 502.1624 | 472.1948 | 27.9866 | 426.5023 | 420.1077 | 692.3236 |
| CUST_28280_PI429010886 | N57660 | 0.00116 | 15 | 9 | | | | | | 33.19093 | 41.25188 | 34.32332 | 33.59767 | 17.77043 | 23.68272 | 33.68931 | 30.00897 | 52.34805 | 56.32248 | 40.71531 | 54.40089 | 41.91362 | 38.44815 | 49.48526 | 51.09668 | 35.62474 | 35.4058 | 51.28805 | 35.52135 | 33.7142 | 55.24489 | 41.94819 | 29.88865 | 71.94024 | 58.5117 | 47.99065 |
| CUST_33205_PI429010886 | M6266 | 0.001162 | 21 | 3 | | | | | | 202.9774 | 220.0942 | 196.3252 | 221.425 | 299.9648 | 247.6825 | 223.377 | 187.3307 | 232.3859 | 272.4368 | 217.3065 | 252.9891 | 158.5869 | 212.7113 | 185.1977 | 197.0393 | 195.0054 | 184.6172 | 219.6868 | 184.1659 | 230.0814 | 209.9677 | 187.954 | 250.9292 | 239.7427 | 252.0389 | 245.1598 |
| CUST_9153_PI429010886 | N24146 | 0.001162 | 24 | 15 | | | | | | 370.4877 | 341.396 | 373.896 | 181.6229 | 256.1518 | 346.965 | 228.3928 | 169.847 | 184.2011 | 350.6566 | 365.6765 | 342.3066 | 182.9909 | 296.0085 | 323.8952 | 372.2873 | 426.3841 | 387.1393 | 326.0904 | 391.5964 | 397.525 | 267.983 | 278.0457 | 378.1407 | 368.2489 | 355.6761 | 383.7894 |
| CUST_31175_PI429010886 | M894 | 0.001162 | 15 | 3 | | | | | | 437.1883 | 446.7333 | 402.8509 | 440.8097 | 483.4448 | 650.0167 | 604.9689 | 335.8595 | 212.606 | 350.314 | 623.0865 | 346.8405 | 249.3051 | 221.4446 | 261.5579 | 347.3329 | 971.7833 | 499.3294 | 739.338 | 460.5023 | 799.3451 | 328.9267 | 536.3308 | 615.3731 | 290.0281 | 315.7204 | 323.8952 |
| CUST_24839_PI429010886 | N51339 | 0.001168 | 18 | 6 | | | Protein metabolism | Proteolysis | | 122.1911 | 346.965 | 194.5472 | 158.5396 | 113.0325 | 117.67 | 354.4714 | 158.0318 | 333.6383 | 152.6328 | 113.2721 | 183.4397 | 73.14043 | 135.1566 | 112.4452 | 131.7223 | 16.67878 | 61.72981 | 153.376 | 121.1298 | 149.7495 | 212.1159 | 82.14765 | 199.1974 | 266.5285 | 157.6076 | 391.7449 |
| CUST_42287_PI429010886 | M59909 | 0.001173 | 24 | 18 | | | | | | 10.20858 | 10.40723 | 14.74259 | 8.632122 | 6.282328 | 15.09945 | 6.093268 | NA | 7.968722 | 8.287779 | 7.917706 | 8.117917 | 4.473733 | 8.137781 | 10.45086 | 11.88288 | 8.337419 | 8.670788 | 36.98717 | 35.00866 | NA | 8.255312 | NA | 11.01284 | 24.38855 | 8.690781 | 10.53088 |
| CUST_50709_PI429010886 | M90724 | 0.001174 | 15 | 0 | | | | | | 45.84492 | 101.6948 | 80.89652 | 36.29774 | 80.83362 | 59.80108 | 45.23797 | 41.99643 | 32.55113 | 92.15955 | 55.18575 | 87.6112 | 43.83694 | 61.89778 | 71.88591 | 74.71666 | 78.37536 | 93.19487 | 39.3477 | 44.66782 | 56.18196 | 42.20687 | 35.91157 | 40.09922 | 90.85633 | 46.06229 | 54.73192 |
| CUST_12322_PI429010886 | N29286 | 0.001174 | 12 | 3 | | | | | | 83.45905 | 38.33525 | 58.77193 | 71.63476 | 119.1637 | 210.4017 | 101.1982 | 82.70078 | 60.01583 | 29.57087 | 35.5525 | 101.0263 | 44.6026 | 38.3841 | 58.26543 | 58.62811 | 173.7706 | 158.7737 | 74.90972 | 29.92416 | 47.27714 | 27.52478 | 39.19383 | 35.97087 | 77.46325 | 77.1122 | 86.20359 |
| CUST_46470_PI429010886 | M72743 | 0.001175 | 24 | 18 | | | | | | 73.88403 | 70.39937 | 87.86031 | 59.9058 | 68.02073 | 72.50841 | 53.84406 | 69.80469 | 62.11683 | 88.7408 | 70.96221 | 76.96996 | 68.63903 | 99.30005 | 86.49079 | 102.17 | 93.78702 | 69.85985 | 88.65038 | 99.33533 | 84.33492 | 93.19487 | 102.7959 | 72.00003 | 96.80182 | 74.86136 | 88.31622 |
| CUST_33058_PI429010886 | M5855 | 0.001178 | 24 | 0 | | | | | | 20.49729 | 26.71614 | 28.53148 | 18.82155 | 6.978868 | 10.39055 | 14.05481 | 5.945633 | 11.5275 | 26.98754 | 6.398941 | 13.00375 | 13.66936 | 15.3449 | 12.67937 | 18.39819 | 6.850987 | 15.67417 | 17.48039 | 15.05871 | 13.23043 | 25.41871 | 15.50857 | 18.24494 | 38.82258 | 19.69614 | 19.14111 |
| CUST_14998_PI429010886 | N33828 | 0.001179 | 21 | 12 | 291239605 | PREDICTED: kinesin family member 20A-like [Saccog | Cellular Process | Cell cycle | 1.82E-40 | NA | 9.434112 | 9.098614 | 14.11311 | 5.9437 | NA | 6.158206 | 6.134526 | 7.503213 | 11.11474 | 12.24951 | 11.11202 | 17.60432 | 16.52955 | 12.88285 | 12.00666 | 20.11281 | 12.51566 | 21.9058 | 9.073537 | 16.48057 | 9.659531 | 9.743013 | 8.710242 | 17.08381 | 8.928641 | 8.042965 |
| CUST_44928_PI429010886 | M67442 | 0.001195 | 21 | 3 | | | Signal transduction | Other | | 37.69724 | 32.76173 | 68.88215 | 57.90878 | 82.86721 | 77.38027 | 48.73587 | 37.90698 | 33.01533 | 57.07573 | 38.87395 | 34.63494 | 30.63369 | 48.26458 | 27.85465 | 107.409 | 51.38779 | 56.50947 | 19.17918 | 26.90259 | 16.91691 | 78.43466 | 44.80736 | 56.53393 | 80.42722 | 61.24287 | 92.84239 |
| CUST_23206_PI429010886 | N48438 | 0.001196 | 18 | 3 | | | | | | 13.43914 | 14.99431 | 13.85986 | NA | 16.44381 | 19.42496 | 13.19581 | 16.47211 | 10.70265 | 9.691153 | 17.42054 | 13.34705 | 6.99897 | 6.268183 | 8.33181 | 13.40566 | 23.73591 | 13.89803 | 40.17403 | 7.230194 | 11.678 | 16.56157 | 17.55969 | 22.51238 | 17.17252 | 13.42877 | 10.7921 |
| CUST_42221_PI429010886 | M59716 | 0.001198 | 21 | 3 | | | | | | 38.93621 | 40.70657 | 26.99024 | 56.28307 | 35.31167 | 44.19495 | 30.01858 | 27.5887 | 30.02625 | 39.38447 | 20.19663 | 39.27986 | 30.9722 | 36.45907 | 32.54126 | 31.51141 | 20.90912 | 23.92584 | 26.08148 | 21.84747 | 31.24239 | 46.87582 | 27.11255 | 29.14178 | 41.96064 | 41.25578 | 43.63321 |
| CUST_36017_PI429010886 | M14298 | 0.001201 | 15 | 9 | 321478769 | structural maintenance of chromosome protein 2 [D | Cellular Process | Cell cycle | 6.34E-58 | 49.59839 | 70.33128 | 38.69306 | 39.26132 | 47.62012 | 31.33759 | 53.36598 | 42.5721 | 61.39492 | 70.53195 | 65.14977 | 81.37626 | 56.56684 | 59.72927 | 49.23067 | 81.29483 | 48.28635 | 43.95205 | 47.3594 | 54.5276 | 56.27319 | 73.82839 | 48.69326 | 46.22951 | 86.48234 | 61.20199 | 76.80142 |
| CUST_44955_PI429010886 | M67522 | 0.001201 | 15 | 9 | | | | | | 550.4344 | 818.9073 | 1010.24 | 278.4332 | 269.7651 | 290.9496 | 881.8414 | 937.3911 | 1422.337 | 1185.368 | 769.2478 | 838.4693 | 1380.785 | 1392.18 | 669.8776 | 1059.331 | 1043.343 | 689.8687 | 538.1956 | 696.7816 | 622.3874 | 1205.12 | 2101.303 | 746.9886 | 3421.62 | 921.954 | 4357.297 |
| CUST_34046_PI429010886 | M8584 | 0.001201 | 15 | 9 | | | | | | 102.2907 | 104.976 | 134.1249 | 91.80446 | 95.9293 | 82.59837 | 95.20545 | 109.7974 | 108.5891 | 129.0306 | 120.7611 | 150.9399 | 99.55807 | 135.4402 | 95.84863 | 102.3871 | 82.19075 | 108.7243 | 90.15151 | 101.281 | 119.8572 | 128.5885 | 119.1637 | 97.00878 | 135.4402 | 125.8034 | 129.0811 |
| CUST_27090_PI429010886 | N55430 | 0.001202 | 12 | 3 | | | | | | 210.9393 | 164.9622 | 65.66431 | 227.225 | 196.5633 | 341.0906 | 161.711 | 106.3744 | 126.2208 | 52.29382 | 42.29018 | 37.04031 | 166.6372 | 54.17626 | 122.2019 | 152.424 | 130.0221 | 190.3637 | 113.7193 | 52.78112 | 53.22735 | 91.04966 | 36.33335 | 176.4808 | 68.78674 | 251.5651 | 46.58823 |
| CUST_41951_PI429010886 | M58961 | 0.001209 | 21 | 18 | 241678636 | conserved hypothetical protein [Ixodes scapularis] > | | | 4.15E-45 | 35.94612 | 48.16535 | 49.94515 | 39.33067 | 33.13396 | 34.49959 | 28.26309 | 26.22286 | 23.51614 | 30.79365 | 29.24265 | 49.99255 | 21.75964 | 44.03128 | 37.30507 | 33.06433 | 32.86388 | 43.58328 | 71.67319 | 37.87644 | 40.2115 | 32.70344 | 37.90169 | 41.24587 | 32.71054 | 31.75695 | 37.27402 |
| CUST_1327_PI429010886 | N9300 | 0.001215 | 15 | 9 | | | | | | 191.1131 | 177.8158 | 201.0518 | 171.4799 | 162.719 | 145.1427 | 189.684 | 154.8889 | 202.8541 | 218.3592 | 157.378 | 274.8462 | 215.9452 | 203.1014 | 221.425 | 260.5083 | 150.6325 | 217.4803 | 150.8706 | 213.636 | 190.8326 | 217.7055 | 207.8011 | 175.6552 | 271.9546 | 286.19 | 299.5774 |
| CUST_42723_PI429010886 | M61076 | 0.001215 | 12 | 3 | | | | | | 112.2867 | 107.2382 | 101.3443 | 133.5378 | 116.178 | 135.6517 | 110.6458 | 83.65751 | 94.04891 | 70.42582 | 91.24255 | 68.95801 | 86.4151 | 104.4054 | 128.2673 | 115.478 | 107.9913 | 118.5941 | 119.9589 | 98.69728 | 71.71135 | 110.9624 | 93.11072 | 74.32582 | 119.9711 | 78.51483 | 87.57209 |
| CUST_7595_PI429010886 | N21781 | 0.001215 | 12 | 9 | 335346117 | Macrobrachium rosenbergii tropomyosin mRNA, co | | | 2.66E-66 | 44.0999 | 46.1807 | 37.75935 | 40.66388 | 42.10422 | 38.85386 | 42.16582 | 47.98542 | 48.96463 | 79.09571 | 48.08 | 55.17531 | 46.38676 | 86.81547 | 11.7971 | 36.55477 | 46.71536 | 36.04391 | 39.36593 | 49.41092 | 49.57451 | 55.46608 | 53.92569 | 52.39551 | 45.65482 | 28.48875 | 37.86103 |
| CUST_45132_PI429010886 | M68092 | 0.001218 | 21 | 15 | | | | | | 51.09155 | 82.43374 | 41.94819 | 90.91352 | 60.16545 | 73.60851 | 51.84431 | 46.89592 | 33.89069 | 54.77183 | 43.51975 | 42.83336 | 56.76781 | 69.05841 | 61.14806 | 196.129 | 92.18368 | 144.1199 | 70.88996 | 91.50805 | 59.54039 | 107.9913 | 58.76571 | 72.03066 | 37.36976 | 79.59377 | 46.682 |
| CUST_18589_PI429010886 | N40064 | 0.001219 | 24 | 15 | | | | | | 28.25408 | 24.40411 | 24.8123 | 19.22446 | 24.95013 | 18.8495 | 19.68008 | 26.31122 | 27.71267 | 28.43719 | 50.25535 | 27.28673 | 35.05495 | 24.64992 | 22.35102 | 37.26633 | 110.1637 | 46.98145 | 26.57549 | 34.21609 | 52.645 | 45.66434 | 32.92321 | 24.30839 | 29.95513 | 42.53957 | 35.44546 |
| CUST_17802_PI429010886 | N38617 | 0.00122 | 24 | 21 | 224095776 | SET domain protein [Populus trichocarpa] >gi\|2228 | Developmental process | Multicellular organismal development | 3.11E-08 | 22.8445 | 18.9533 | 28.59732 | 26.36808 | 22.22168 | 41.37701 | 28.39426 | 18.85554 | 18.96681 | 16.20429 | 29.67419 | 21.20179 | 12.05708 | 20.96791 | 18.43402 | 21.22872 | 29.5483 | 24.20666 | 30.04952 | 13.23323 | 43.41406 | 30.10928 | 61.27293 | 46.39268 | 22.75922 | 28.01496 | 24.62111 |
| CUST_41904_PI429010886 | M58847 | 0.001224 | 12 | 0 | | | | | | 573.9804 | 443.6521 | 611.8341 | 324.7416 | 361.6235 | 392.3971 | 349.8347 | 397.0596 | 461.882 | 434.7605 | 375.6905 | 475.874 | 431.3053 | 478.2977 | 503.8676 | 436.4909 | 422.1729 | 428.5138 | 378.7944 | 496.1582 | 410.508 | 564.3927 | 463.2589 | 413.7293 | 501.8237 | 517.4057 | 512.9488 |
| CUST_4331_PI429010886 | N16526 | 0.001226 | 15 | 12 | | | | | | 152.1158 | 172.3811 | 276.1029 | 164.9098 | 100.5554 | 88.3593 | 184.1183 | 115.9863 | 214.7012 | 176.4808 | 95.7582 | 232.7081 | 214.4445 | 360.1952 | 206.1326 | 179.0874 | 141.5508 | 98.94163 | 115.9694 | 48.13849 | 146.405 | 133.0821 | 89.80969 | 106.1871 | 210.5151 | 168.4747 | 200.337 |
| CUST_47585_PI429010886 | M76734 | 0.001227 | 12 | 0 | | | | | | 1679.237 | 2024.906 | 1808.217 | 1037.639 | 1299.165 | 1009.6 | 724.0591 | 689.0956 | 649.5445 | 1451.1 | 1713.527 | 2086.783 | 1273.05 | 755.2577 | 1400.033 | 1297.484 | 1209.913 | 1273.05 | 1008.924 | 1007.032 | 944.3898 | 1390.385 | 1150.482 | 1009.6 | 1334.604 | 1473.383 | 1220.236 |
| CUST_14574_PI429010886 | N33098 | 0.001227 | 18 | 15 | | | | | | 67.46087 | 68.03939 | 58.4895 | 70.6776 | 53.28731 | 66.15125 | 58.46315 | 41.52857 | 53.00119 | 50.4975 | 54.30581 | 89.0565 | 59.9743 | 65.64906 | 80.23994 | 106.1871 | 80.55811 | 86.46205 | 74.48926 | 92.23086 | 54.38941 | 75.45183 | 59.76158 | 47.3594 | 76.85915 | 51.54875 | 36.86394 |
| CUST_57005_PI429010886 | M115105 | 0.001229 | 15 | 9 | | | | | | 642.8014 | 662.5447 | 698.0293 | 524.2988 | 520.9934 | 571.9057 | 582.6377 | 578.1088 | 597.3714 | 715.3982 | 663.1345 | 720.48 | 555.5605 | 543.0155 | 621.2292 | 766.6399 | 559.2711 | 610.0761 | 608.3741 | 603.4589 | 556.0784 | 618.5189 | 720.2695 | 644.4395 | 897.9401 | 646.7171 | 742.6323 |
| CUST_31990_PI429010886 | M2932 | 0.001229 | 15 | 9 | | | Protein metabolism | Cellular protein modification process | | 20.73391 | 20.0133 | 29.99408 | 16.94555 | 13.027 | 12.36728 | 18.52719 | 20.67573 | 26.76116 | 21.62904 | 28.03992 | 28.55509 | 13.31013 | 21.74146 | 33.92452 | 23.1605 | 17.08381 | 20.93627 | 14.03086 | 17.1086 | 21.52428 | 22.39686 | 35.72502 | 21.94249 | 29.6775 | 25.68325 | 23.5068 |
| CUST_12943_PI429010886 | N30319 | 0.001229 | 15 | 9 | 197361188 | signal transducer and activator of transcription [Fer | Signal transduction | Other | 1.72E-63 | 217.8082 | 278.3016 | 331.4917 | 212.606 | 213.8921 | 199.9465 | 202.301 | 251.8789 | 293.9979 | 303.2078 | 236.3355 | 411.8683 | 226.9563 | 300.2764 | 321.1502 | 335.4811 | 242.7155 | 272.1441 | 265.532 | 265.1519 | 220.9933 | 326.7477 | 312.7841 | 239.571 | 354.4714 | 345.9305 | 386.4471 |
| CUST_54995_PI429010886 | M108357 | 0.001233 | 15 | 3 | | | | | | 79.77146 | NA | 93.32222 | 161.7557 | 129.718 | 321.696 | 186.3412 | 50.25117 | 28.36898 | 16.73161 | 108.1041 | 4.494435 | 18.86937 | 15.63054 | 20.10327 | 6.153881 | 189.3905 | 121.7904 | 142.7507 | NA | 228.3459 | 10.21823 | 137.9867 | 210.3335 | NA | 82.20584 | 4.920856 |
| CUST_19992_PI429010886 | N42592 | 0.001235 | 24 | 15 | | | | | | 9.757187 | 7.781741 | NA | 8.036889 | 11.99091 | 13.97903 | 13.60949 | 22.57964 | 8.656419 | 14.29954 | 9.118641 | 19.92665 | 11.40559 | 22.77856 | 25.56371 | 24.64442 | NA | 14.4666 | 17.47454 | 17.11186 | 22.49442 | 6.771584 | 18.45614 | NA | 9.168882 | 7.857013 | 5.24251 |
| CUST_50536_PI429010886 | M90172 | 0.001237 | 18 | 15 | 91092388 | PREDICTED: similar to CG9395 CG9395-PA [Triboliu | | | 1.38E-24 | 7.621463 | 9.688636 | 7.420586 | 3.832194 | 4.883329 | NA | NA | 5.162518 | NA | 12.41093 | 7.187407 | 6.120324 | NA | 17.82405 | 7.624707 | 16.35182 | 9.155769 | 9.179429 | 6.333698 | 6.864974 | 9.714422 | 6.203683 | NA | 7.106027 | 5.216226 | 3.866189 | 7.790904 |
| CUST_40177_PI429010886 | M51372 | 0.001238 | 12 | 3 | 371591210 | Euphausia superba voucher 980349 mitochondrion, | | | 3.69E-55 | NA | 5.031644 | NA | NA | 9.479243 | 15.17932 | 5.130091 | 4.915853 | 4.998758 | NA | 4.112474 | 7.076703 | 5.807692 | 11.6401 | NA | 15.2618 | 14.72748 | 11.97367 | 4.642534 | 4.179205 | 8.356596 | 9.84998 | 9.394559 | 8.726312 | 7.391703 | 9.525782 | 8.689343 |
| CUST_55573_PI429010886 | M110788 | 0.00124 | 15 | 12 | | | | | | 9.55327 | 9.043293 | NA | NA | 7.677713 | 6.183728 | 10.88684 | 7.712934 | 6.875138 | 6.401811 | 14.87518 | NA | 16.80832 | NA | 8.816732 | 11.32963 | 14.69551 | 7.525118 | 6.763654 | 5.352926 | 5.028881 | 7.789768 | 6.104204 | 7.171909 | 11.54241 | 8.024819 | 9.273731 |
| CUST_41447_PI429010886 | M57752 | 0.001241 | 18 | 9 | 383766182 | hypothetical protein PSMK_11070 [Phycisphaera m | | | 2.16E-07 | 229.6885 | 201.5302 | 210.4862 | 184.654 | 280.0237 | 168.154 | 166.4887 | 351.4461 | 246.3941 | 331.6846 | 353.785 | 381.498 | 186.9659 | 244.3511 | 274.7936 | 252.4045 | 231.1582 | 254.5885 | 181.3561 | 231.1968 | 236.9573 | 229.8746 | 250.036 | 227.704 | 347.0333 | 271.4065 | 339.3078 |
| CUST_9497_PI429010886 | N24736 | 0.001242 | 15 | 12 | | | | | | 557.82 | 1013.618 | 894.9707 | 309.4606 | 244.2054 | 85.60322 | 2119.433 | 1653.841 | 4048.081 | 1535.786 | 617.9947 | 1494.92 | 8550.193 | 8582.669 | 5108.798 | 4759.605 | 3459.777 | 464.9946 | 1157.228 | 619.3802 | 685.3204 | 989.5639 | 1010.922 | 1114.056 | 8814.529 | 661.6789 | 11729.06 |
| CUST_4121_PI429010886 | N16060 | 0.001246 | 15 | 9 | | | | | | 110.4991 | 140.147 | 135.2315 | 84.15456 | 110.1333 | 94.17604 | 85.27513 | 105.2326 | 114.3617 | 180.1262 | 113.4637 | 202.2232 | 166.2981 | 117.7722 | 148.5237 | 153.44 | 127.601 | 133.0951 | 108.2633 | 125.4876 | 120.496 | 130.7638 | 135.4539 | 112.6431 | 155.6736 | 131.2903 | 150.0759 |
| CUST_41393_PI429010886 | M57628 | 0.001251 | 15 | 0 | | | | | | 352.5288 | 259.1091 | 301.5058 | 311.8189 | 266.6935 | 376.27 | 314.6817 | 237.9203 | 261.1858 | 230.6259 | 234.7048 | 214.5527 | 272.638 | 261.6861 | 290.7849 | 317.032 | 377.8878 | 327.3251 | 281.447 | 236.3355 | 305.8476 | 237.7359 | 295.3248 | 350.5238 | 286.19 | 250.4345 | 220.2915 |
| CUST_18096_PI429010886 | N39134 | 0.001253 | 12 | 3 | | | | | | 6.839858 | 16.83754 | 11.5308 | 18.14702 | 21.62904 | 20.58435 | 12.86528 | 17.67558 | 15.72277 | 16.15147 | 14.13095 | 14.11461 | 14.06114 | 8.97147 | 11.88893 | 18.02234 | 37.0569 | 25.82153 | 15.18055 | 13.08017 | 20.65721 | 22.56982 | 13.72174 | 24.19522 | 9.743013 | 10.86536 | 17.47867 |
| CUST_3672_PI429010886 | N15015 | 0.001254 | 15 | 12 | | | | | | 13.09162 | 115.7331 | 182.6137 | 25.1646 | 61.95712 | 20.56296 | 73.39168 | 79.92252 | 104.9178 | 98.68398 | 198.6322 | 163.6892 | 150.5702 | 288.4469 | 206.0055 | 213.51 | 99.00087 | 87.62612 | 114.3743 | 112.169 | 84.63515 | 153.8044 | 63.46985 | 33.15216 | 289.3731 | 129.5236 | 276.6094 |
| CUST_12694_PI429010886 | N29883 | 0.001257 | 12 | 9 | | | | | | 254.189 | 539.016 | 949.0578 | 33.04443 | 32.16133 | 7.372745 | 203.006 | 514.0171 | 588.9896 | 542.7169 | 475.5299 | 910.1372 | 296.8223 | 441.6462 | 318.1453 | 121.9609 | 199.4211 | 42.59502 | 207.885 | 616.6013 | 199.4834 | 604.9689 | 948.7418 | 22.23809 | 499.7486 | 197.122 | 818.4343 |
| CUST_38637_PI429010886 | M28728 | 0.001258 | 15 | 12 | | | | | | 3116.138 | 7145.704 | 4887.374 | 1586.719 | 2295.055 | 1013.272 | 4377.65 | 5336.786 | 9950.402 | 10510.22 | 3644.859 | 7930.333 | 11454.19 | 12070.48 | 9485.91 | 9950.402 | 5065.56 | 4313.91 | 4515.16 | 6687.729 | 4399.213 | 10348.94 | 6307.408 | 2420.027 | 17110.44 | 5342.716 | 25496.93 |
| CUST_45419_PI429010886 | M69044 | 0.001259 | 21 | 12 | 321478626 | hypothetical protein DAPPUDRAFT_303213 [Daphn | Cellular Process | Cell adhesion | 2E-11 | 61.62124 | 60.92331 | 66.36423 | 56.85058 | 80.75638 | 72.55972 | 96.74817 | 71.45222 | 79.07776 | 101.5278 | 95.88672 | 74.22931 | 88.08405 | 98.36103 | 87.24093 | 98.29921 | 76.26718 | 61.28097 | 76.73952 | 71.03479 | 69.98559 | 81.91685 | 57.96208 | 60.25585 | 70.59503 | 83.7299 | 99.26813 |
| CUST_51806_PI429010886 | M95178 | 0.001261 | 18 | 3 | | | | | | 22.63247 | 113.1942 | 45.37412 | 184.9825 | 49.55874 | 119.9288 | 62.80799 | 61.10331 | 52.80587 | 30.36684 | 31.65107 | 43.57816 | 50.8686 | 50.32404 | 46.90544 | 45.69586 | 22.0325 | 28.5608 | 18.03887 | 43.08353 | 46.19013 | 79.95313 | 70.31729 | 47.19112 | 52.82166 | 92.03214 | 41.57987 |
| CUST_4149_PI429010886 | N16110 | 0.001267 | 12 | 0 | | | | | | 124.3983 | 110.4288 | 308.8946 | 19.46313 | 46.30865 | 25.07182 | 120.0446 | 177.896 | 171.6462 | 185.5593 | 93.68609 | 217.6655 | 233.0458 | 382.2635 | 140.0012 | 80.83362 | 65.46937 | 62.5636 | 193.4435 | 119.9711 | 225.2614 | 89.86196 | 266.0871 | 95.82559 | 583.066 | 139.6239 | 556.7295 |
| CUST_18060_PI429010886 | N39075 | 0.001267 | 12 | 0 | | | | | | 33.08815 | 33.98937 | 33.58412 | 18.73831 | 25.89716 | 24.82512 | 24.48268 | 36.22069 | 15.63419 | 28.20406 | 24.44489 | 28.13108 | 31.07173 | 29.20384 | 35.16453 | 32.20238 | 19.85432 | 22.78539 | 18.94687 | 27.56983 | 29.35866 | 27.31066 | 30.15647 | 29.71896 | 29.71169 | 40.75297 | 40.13905 |
| CUST_1271_PI429010886 | N9090 | 0.001271 | 12 | 9 | 307169370 | Chorion peroxidase [Camponotus floridanus] | Response to stress | Response to oxidative stress | 2.5E-104 | 1256.084 | 156.3918 | 194.6975 | 81.96733 | 27.12757 | 60.6915 | 859.0461 | 115.3428 | 243.4729 | 3772.58 | 104.9922 | 1202.61 | 235.0098 | 100.7905 | 129.4353 | 48.22578 | 388.5645 | 90.72052 | 611.2062 | 69.2413 | 77.39915 | 2276.234 | 724.7977 | 978.1392 | 358.7125 | 18.31489 | 738.4538 |
| CUST_22348_PI429010886 | N46868 | 0.001271 | 21 | 18 | | | | | | 7.393906 | 8.170169 | 16.46215 | 5.921666 | 4.022505 | 3.650294 | 5.75088 | 3.74833 | NA | NA | 7.561772 | 3.286433 | 6.31321 | 7.401629 | 3.536602 | 7.800886 | 15.32584 | NA | 22.65189 | 7.599564 | NA | 7.72615 | 6.666438 | NA | 5.788646 | NA | 5.028291 |
| CUST_34525_PI429010886 | M9914 | 0.001271 | 21 | 18 | | | | | | 122.7676 | 124.5927 | 165.8727 | 146.882 | 151.0154 | 198.8064 | 127.6161 | 92.1688 | 95.63731 | 147.2543 | 102.8545 | 124.672 | 149.9331 | 120.4315 | 138.0748 | 145.2433 | 139.2832 | 159.2127 | 165.5224 | 171.0777 | 191.7619 | 254.5885 | 138.8045 | 175.2604 | 140.036 | 115.2533 | 126.7035 |
| CUST_57165_PI429010886 | E1811 | 0.00129 | 15 | 12 | 532759728 | antimicrobial peptide type 2 precursor IIb [Pandalo | | | 1.55E-28 | 3222.703 | 3949.649 | 1928.541 | 2441.116 | 3659.313 | 940.5709 | 7119.249 | 3599.759 | 8924.795 | 5780.138 | 2663.095 | 6273.384 | 7669.986 | 5602.191 | 6544.343 | 5726.098 | 6468.264 | 4034.014 | 3134.449 | 2182.975 | 2975.526 | 5627.009 | 2363.842 | 4743.467 | 12866.6 | 5539.858 | 12983.85 |
| CUST_48171_PI429010886 | M79221 | 0.001295 | 15 | 6 | | | | | | 151.1026 | 138.4789 | 122.6587 | 155.8377 | 174.6367 | 161.861 | 179.7938 | 216.3677 | 189.5352 | 215.2857 | 150.0759 | 241.4805 | 133.1932 | 168.7959 | 153.9132 | 137.1423 | 124.7587 | 119.7503 | 200.7582 | 128.5363 | 194.6975 | 137.9678 | 183.8709 | 171.0319 | 174.2228 | 182.4397 | 171.1038 |
| CUST_3792_PI429010886 | N15276 | 0.001295 | 24 | 12 | | | | | | 15.41663 | 25.80044 | 16.51414 | 7.488246 | 9.137413 | 10.01115 | 15.14895 | 23.47811 | 26.08978 | 41.8887 | 15.80722 | 27.13002 | 25.38784 | 41.8496 | 28.92395 | 57.73367 | 13.023 | 24.66785 | 24.51394 | 29.23458 | 26.79789 | 18.09414 | 28.16035 | 9.075144 | 24.03089 | NA | 17.13072 |
| CUST_40639_PI429010886 | M55716 | 0.001295 | 18 | 9 | 196000550 | hypothetical protein TRIADDRAFT_20924 [Trichopla | Response to stress | Other | 4.26E-15 | 63.8461 | 82.26332 | 67.85099 | 58.94211 | 51.49762 | 46.15285 | 34.93733 | 58.08697 | 59.76845 | 92.65529 | 82.03238 | 74.88917 | 55.87064 | 72.40904 | 63.87286 | 110.5384 | 68.24733 | 60.34766 | 67.14861 | 68.98284 | 68.1054 | 55.42181 | 58.20366 | 55.26178 | 138.8541 | 72.79951 | 61.64233 |
| CUST_21810_PI429010886 | N45894 | 0.001295 | 18 | 9 | | | | | | 12.99803 | 15.33784 | 13.87668 | 12.82499 | 16.30451 | 12.3071 | 19.03069 | 30.25566 | 20.22864 | 21.37656 | 20.56951 | 23.61845 | 14.86089 | 25.49244 | 19.86545 | 13.7828 | 18.96681 | 29.53988 | 23.70771 | 13.72475 | 14.55066 | 13.95948 | 8.641166 | 11.70041 | 24.17334 | 24.5556 | 31.83796 |
| CUST_4538_PI429010886 | N16987 | 0.001296 | 18 | 3 | | | | | | 32.51286 | 31.09756 | 19.83086 | 38.89725 | 31.57401 | 45.84492 | 28.85946 | 18.98962 | 26.30532 | 19.70026 | 26.02628 | 22.55376 | 17.29225 | 15.80873 | 24.51625 | 30.96598 | 62.44095 | 36.1117 | 26.73668 | 25.54793 | 37.29408 | 35.22807 | 46.22541 | 35.35825 | 24.88119 | 44.50661 | 21.60382 |
| CUST_461_PI429010886 | N5429 | 0.001305 | 15 | 12 | | | | | | 3355.686 | 871.62 | 30800.89 | 55.05355 | 103.6887 | 63.98224 | 6253.288 | 41887.92 | 32610.41 | 46511.8 | 1292.191 | 2567.686 | 37561.18 | 63688.85 | 32869.85 | 597.0282 | 25136.37 | 338.6596 | 1997.22 | 2923.398 | 935.7487 | 15389.68 | 34379.32 | 1260.157 | 50443.76 | 1940.549 | 46673.5 |
| CUST_41709_PI429010886 | M58360 | 0.001307 | 21 | 12 | | | | | | 49.98628 | 93.24144 | 86.81547 | 29.84652 | 53.53627 | 21.38676 | 43.9229 | 110.2442 | 69.96115 | 163.3073 | 54.73192 | 75.0125 | 139.1216 | 129.896 | 101.5112 | 76.40353 | 87.69732 | 53.20475 | 56.85652 | 49.79493 | 55.48383 | 57.028 | 84.14675 | 31.46674 | 67.16912 | 27.68808 | 53.03364 |
| CUST_22810_PI429010886 | N47713 | 0.001318 | 24 | 0 | | | | | | 233.6523 | 195.2959 | 231.7343 | 173.9115 | 229.6885 | 179.2308 | 226.9159 | 198.2869 | 124.2579 | 225.9175 | 224.0471 | 156.7272 | 114.4563 | 150.78 | 113.0059 | 169.6552 | 268.545 | 175.4136 | 207.8011 | 197.5737 | 180.8735 | 221.9563 | 102.7622 | 199.4521 | 355.5348 | 201.9745 | 235.2181 |
| CUST_4622_PI429010886 | N17155 | 0.00132 | 24 | 0 | | | | | | 54.40089 | 34.47823 | 47.21974 | 54.84373 | 23.5201 | 24.27705 | 24.61812 | 14.65167 | 26.95285 | 30.55547 | 15.15404 | 55.32634 | 15.55047 | 28.67208 | 17.03814 | 21.99572 | 19.83923 | 16.5818 | 22.44161 | 17.76083 | 30.83339 | 27.52267 | 21.93936 | 31.04579 | 36.16119 | 46.67338 | 54.88431 |
| CUST_29552_PI429010886 | N60093 | 0.001321 | 15 | 12 | | | | | | 29.2332 | 25.68173 | 36.12687 | 20.6419 | 18.88563 | 23.92174 | 24.01772 | 33.99233 | 19.44948 | 30.32872 | 27.52766 | 24.80851 | 30.06426 | 37.75561 | 31.37421 | 35.11191 | 27.91558 | 26.89586 | 25.07182 | 24.13249 | 19.68474 | 27.51937 | 29.2332 | 27.8177 | 41.09904 | 17.60068 | 34.15181 |
| CUST_15247_PI429010886 | N34264 | 0.001329 | 18 | 15 | | | | | | 379.2779 | 575.0804 | 467.9877 | 537.701 | 355.2567 | 195.5884 | 972.1119 | 410.9035 | 877.9874 | 855.218 | 629.9728 | 1039.842 | 703.4699 | 1067.963 | 748.4841 | 1309.194 | 1002.452 | 863.6121 | 1729.171 | 282.166 | 1113.282 | 547.465 | 527.7394 | 279.5586 | 712.9294 | 428.0243 | 803.5259 |
| CUST_313_PI429010886 | N3807 | 0.001333 | 15 | 3 | | | | | | 537.701 | 8.527754 | 596.0223 | 4034.014 | 14744.71 | 12651.8 | 15922.04 | 577.6505 | 70.06431 | 26.37273 | 18661.44 | NA | 14.93582 | 12.42241 | 8.772272 | 25.51149 | 22767.31 | 6108.092 | 26956.65 | NA | 22307.76 | NA | 18337.68 | 20248.42 | 18.57232 | 1055.909 | 5.017942 |
| CUST_662_PI429010886 | N6619 | 0.001337 | 15 | 12 | | | | | | 2984.905 | 4759.605 | 4068.874 | 1335.109 | 1771.34 | 879.6665 | 3398.882 | 4672.866 | 7415.798 | 6767.816 | 2780.239 | 6142.292 | 11437.58 | 11762.42 | 8845.346 | 10802.96 | 4080.667 | 3278.647 | 3561.268 | 5503.933 | 3124.162 | 9937.704 | 4480.043 | 1905.993 | 19317.04 | 3772.58 | 21431.7 |
| CUST_22525_PI429010886 | N47186 | 0.001345 | 15 | 0 | | | | | | 33.90948 | 26.8663 | 50.33907 | 30.16372 | 27.32583 | 28.22332 | 21.84275 | 26.83276 | 23.83176 | 31.06527 | 20.39615 | 24.83327 | 22.32946 | 28.229 | 29.89152 | 31.8918 | 32.44398 | 34.30707 | 35.58868 | 27.21982 | 32.43577 | 18.93228 | 27.41751 | 22.89148 | 32.88602 | 20.87462 | 28.01868 |
| CUST_35573_PI429010886 | M12920 | 0.001346 | 18 | 3 | 332028676 | E3 SUMO-protein ligase PIAS3 [Acromyrmex echina | | | 3.28E-35 | 24.42522 | 18.58833 | 24.52447 | 43.26504 | 33.42828 | 33.58065 | 23.1883 | 11.72586 | 25.96869 | 9.012961 | 13.03387 | 12.89985 | 14.21497 | 36.22709 | 6.406079 | 24.43879 | 12.29952 | 29.45562 | 22.41939 | 24.35681 | 24.38855 | 40.86821 | 15.01814 | 41.62253 | 16.08632 | 10.01513 | 14.046 |
| CUST_53709_PI429010886 | M102862 | 0.001352 | 24 | 15 | | | | | | 471.3393 | 397.9322 | 530.8519 | 360.8661 | 412.1707 | 470.2 | 356.8337 | 426.0225 | 356.3531 | 443.5662 | 400.3298 | 468.0946 | 377.1617 | 507.1661 | 459.6219 | 510.2851 | 536.0428 | 502.4781 | 486.6501 | 487.8771 | 432.7317 | 520.2836 | 440.9199 | 404.0517 | 504.5252 | 393.782 | 483.7826 |
| CUST_19745_PI429010886 | N42115 | 0.001352 | 18 | 15 | | | | | | 30.18791 | 19.99756 | 22.75922 | 18.51256 | 21.53946 | 29.25253 | 14.44838 | 11.12231 | 13.2165 | 28.27144 | 21.75179 | 22.70735 | 18.20618 | 23.33698 | 16.30451 | 27.31503 | 35.1893 | 24.0648 | 25.96531 | 21.92688 | 26.57271 | 36.49052 | 15.43278 | 28.21594 | 19.48765 | 15.8263 | 18.55113 |
| CUST_25279_PI429010886 | N52108 | 0.001353 | 15 | 12 | | | | | | 277.4454 | 196.0648 | 440.3254 | 200.7582 | 205.2221 | 203.1953 | 327.3834 | 212.773 | 309.2252 | 242.5645 | 218.037 | 277.4972 | 337.6916 | 437.9769 | 340.3246 | 280.0685 | 268.4354 | 260.0114 | 209.8989 | 212.5355 | 259.0619 | 345.9305 | 312.6532 | 189.0327 | 415.6575 | 292.4718 | 407.5311 |
| CUST_83_PI429010886 | N536 | 0.001353 | 15 | 12 | | | | | | 53822.38 | 5417.146 | 104856.2 | 1937.537 | 4604.537 | 3425.229 | 61898.59 | 74289.93 | 101693.2 | 87648.66 | 30360.4 | 28160 | 108311.1 | 135996.9 | 108311.1 | 14950.27 | 87648.66 | 5342.716 | 44409.28 | 46511.8 | 22281.96 | 93952.87 | 98448.52 | 19626.64 | 110974.5 | 23632.71 | 115564.1 |
| CUST_1332_PI429010886 | N9316 | 0.001354 | 15 | 12 | | | | | | 408.5798 | 419.7481 | 525.4442 | 414.7856 | 399.0919 | 488.7915 | 378.7944 | 477.1155 | 501.3373 | 626.4662 | 477.4078 | 681.1329 | 519.7383 | 683.7716 | 638.2757 | 548.6331 | 474.912 | 508.4411 | 476.8506 | 488.5796 | 487.0643 | 611.9605 | 536.1688 | 507.4019 | 638.9314 | 551.2455 | 574.4912 |
| CUST_55222_PI429010886 | M109174 | 0.001361 | 21 | 3 | | | | | | 379.6269 | 369.4169 | 356.6027 | 352.1132 | 402.1311 | 409.1687 | 408.4945 | 337.2385 | 350.5238 | 325.8124 | 371.7567 | 392.6279 | 280.971 | 356.1302 | 301.6337 | 355.3996 | 411.8683 | 344.4708 | 361.8439 | 378.265 | 346.6366 | 420.9194 | 372.6528 | 386.742 | 403.6614 | 440.428 | 392.9826 |
| CUST_16742_PI429010886 | N36787 | 0.001361 | 21 | 0 | | | | | | 251.9665 | 268.7143 | 304.942 | 196.5334 | 180.4919 | 196.2921 | 206.7399 | 174.9214 | 215.0865 | 222.3465 | 211.5909 | 193.6314 | 168.5577 | 208.8093 | 154.463 | 193.8995 | 188.2189 | 228.586 | 234.443 | 205.6287 | 239.146 | 223.8084 | 229.0179 | 240.2315 | 245.7815 | 176.0509 | 275.1873 |
| CUST_42792_PI429010886 | M61265 | 0.001363 | 18 | 15 | 242009439 | conserved hypothetical protein [Pediculus humanus | | | 7.71E-28 | 27.8344 | 28.33335 | 25.69538 | 36.40882 | 28.84855 | 21.89563 | 5.313502 | 17.1086 | 17.65481 | 28.41001 | 17.43225 | 24.76741 | 19.9835 | 43.77433 | 22.14618 | 31.73044 | 20.43695 | 37.02134 | 27.1975 | 24.94079 | 18.66977 | 37.87276 | 18.25747 | 17.95297 | 17.79403 | 15.4721 | 17.74325 |
| CUST_55416_PI429010886 | M110005 | 0.001367 | 24 | 18 | | | | | | 22.97943 | 75.73714 | 43.53241 | 14.46988 | 28.84595 | 13.42033 | 14.50223 | 22.13275 | 26.59238 | 62.88366 | 42.24487 | 49.21104 | 33.68381 | 43.49365 | 28.229 | 75.61377 | 34.47136 | 59.2457 | 45.91808 | 78.74635 | 66.15751 | 52.86329 | 14.1448 | 26.04444 | 15.99247 | 42.29494 | 51.01709 |
| CUST_43731_PI429010886 | M63815 | 0.001367 | 24 | 21 | | | | | | 124.2426 | 72.07584 | 72.55972 | 117.0641 | 68.80724 | 120.0037 | 18.60572 | 60.58205 | 45.35819 | 63.96858 | 66.84482 | 91.02196 | 74.57029 | 78.39028 | 82.03238 | 140.5657 | 73.76805 | 109.1441 | 71.88591 | 116.5433 | 122.3193 | 109.6802 | 104.5986 | 733.1697 | 114.8783 | 106.1871 | 93.49845 |
| CUST_26538_PI429010886 | N54417 | 0.001367 | 21 | 0 | | | | | | 59.2457 | 51.70242 | 82.55661 | 43.27701 | 34.27356 | 40.03759 | 43.12293 | 33.98233 | 52.49227 | 39.11677 | 37.46264 | 40.10273 | 50.43461 | 37.83723 | 56.40373 | 50.6408 | 54.40089 | 56.30291 | 55.84573 | 45.55401 | 79.64311 | 68.85144 | 62.32467 | 44.13738 | 55.25124 | 48.41716 | 42.61497 |
| CUST_33314_PI429010886 | M6573 | 0.001368 | 12 | 9 | | | | | | 37.20061 | 17.4398 | 30.2585 | 4.967335 | 6.206401 | 12.028 | 19.3376 | 8.257726 | 16.68444 | 20.58261 | 39.93142 | 41.70879 | 14.64036 | 35.76089 | 12.95283 | 6.767462 | NA | 16.46215 | 30.08888 | 38.31739 | 44.03128 | 36.50003 | 31.15588 | 18.14921 | 28.87669 | 30.89913 | 20.84836 |
| CUST_43827_PI429010886 | M64085 | 0.001369 | 15 | 12 | | | | | | 98.18381 | 98.71598 | 104.3742 | 89.29805 | 99.17539 | 93.13261 | 79.875 | 70.42582 | 97.62464 | 97.82533 | 73.17053 | 113.6765 | 107.409 | 127.3036 | 140.3134 | 96.6566 | 99.88549 | 86.19466 | 89.92723 | 87.26148 | 93.66147 | 113.3786 | 75.63407 | 73.33005 | 103.6058 | 100.5781 | 93.12227 |
| CUST_42232_PI429010886 | M59745 | 0.001373 | 21 | 18 | | | | | | 100.9764 | 160.1083 | 123.6359 | 81.13979 | 107.4857 | 101.1328 | 51.85569 | 78.64329 | 79.30391 | 127.7418 | 78.2069 | 152.4947 | 99.08673 | 105.1778 | 89.80969 | 155.3616 | 84.59116 | 127.4851 | 153.961 | 136.1666 | 154.0879 | 110.192 | 122.2181 | 75.43644 | 61.24287 | 129.2002 | 104.4202 |
| CUST_174_PI429010886 | N2108 | 0.001375 | 24 | 15 | | | | | | 51.32239 | 33.69201 | 36.99055 | 31.49395 | 44.06361 | 44.60711 | 39.88324 | 34.33559 | 46.46451 | 57.42327 | 59.05765 | 58.74569 | 57.00887 | 47.80199 | 52.42324 | 97.23707 | 70.54693 | 64.73185 | 58.20859 | 60.10166 | 71.69416 | 53.57478 | 54.59618 | 40.6037 | 56.20414 | 111.3415 | 110.2048 |
| CUST_9365_PI429010886 | N24496 | 0.00138 | 21 | 9 | | | | | | 22.86232 | 15.98734 | 23.91073 | 17.47076 | 12.99318 | 15.87862 | 28.29331 | 27.24907 | 23.75855 | 24.74877 | 31.08481 | 31.82901 | 22.64182 | 23.87788 | 31.13983 | 32.42053 | 22.05258 | 26.50391 | 25.27217 | 26.38499 | 15.31744 | 24.90221 | 16.13721 | 17.75499 | 21.11131 | 14.48944 | 24.64442 |
| CUST_47356_PI429010886 | M75752 | 0.001386 | 24 | 21 | | | | | | 38.41183 | 15.06711 | 23.82568 | 9.392481 | 11.64631 | 17.07004 | 14.57184 | 14.32267 | 9.512792 | 14.75649 | 21.18827 | 13.46088 | 21.47183 | 16.64978 | 23.87245 | 75.06598 | 13.93436 | 20.64798 | 68.20531 | 19.87757 | 15.23737 | 32.62765 | 38.82938 | 26.91205 | 20.87462 | 13.95459 | 17.2249 |
| CUST_42382_PI429010886 | M60146 | 0.001398 | 18 | 9 | | | | | | 44.1334 | 63.73904 | 39.44549 | 37.81058 | 35.73252 | 27.40752 | 32.28425 | 67.79099 | 67.68545 | 44.42212 | 73.5908 | 95.87365 | 61.20199 | 41.95242 | 66.79779 | 56.37263 | 47.47988 | 51.98858 | 58.29022 | 134.1419 | 58.36968 | 36.06457 | 33.92452 | 37.4709 | 49.87667 | 59.56411 | 51.34688 |
| CUST_43806_PI429010886 | M64029 | 0.001398 | 18 | 3 | | | | | | 596.3171 | 735.196 | 859.4575 | 1998.595 | 745.0756 | 1321.79 | 613.6489 | 714.0634 | 679.9331 | 839.8931 | 356.6027 | 1057.689 | 488.5796 | 722.955 | 552.0613 | 857.1846 | 292.3195 | 485.4409 | 392.8804 | 509.603 | 1125.414 | 966.1688 | 1277.111 | 927.5266 | 963.151 | 905.6425 | 603.7585 |
| CUST_2542_PI429010886 | N12584 | 0.001399 | 12 | 9 | | | | | | 9.260769 | 12.36888 | 8.877949 | 6.363361 | NA | 8.664964 | 9.853418 | 10.59893 | 10.07591 | 12.35487 | 18.60572 | 13.72475 | 6.648806 | 16.62399 | 11.37559 | 11.55545 | 8.881606 | 3.741952 | 10.71609 | 10.02441 | 17.9331 | 13.92172 | 16.70546 | 16.76094 | 18.03617 | 11.44906 | 17.62133 |
| CUST_57208_PI429010886 | E2466 | 0.001405 | 15 | 9 | 532759728 | antimicrobial peptide type 2 precursor IIb [Pandalo | | | 3.84E-27 | 3269.374 | 4365.095 | 1899.44 | 3120.564 | 3604.11 | 964.5529 | 6717.481 | 3756.632 | 9182.945 | 6078.985 | 2712.01 | 6587.101 | 7647.424 | 5139.37 | 6593.488 | 7404.352 | 6018.766 | 3972.597 | 3100.565 | 2091.598 | 2727.828 | 5757.178 | 2475.35 | 4915.943 | 13487.6 | 5620.472 | 13147.15 |
| CUST_3307_PI429010886 | N14227 | 0.001411 | 24 | 9 | | | | | | 117.4728 | 141.7029 | 146.6632 | 99.11857 | 13.15672 | NA | 134.9903 | 158.9804 | 140.2989 | 199.4834 | 141.6752 | 191.2652 | 118.275 | 36.98363 | 130.1875 | 53.25416 | 43.52397 | 57.18099 | 21.98126 | 86.39769 | 26.75249 | 9.624456 | 110.4435 | 32.36622 | NA | 24.46354 | 44.25018 |
| CUST_3876_PI429010886 | N15469 | 0.001415 | 21 | 9 | 270013661 | hypothetical protein TcasGA2_TC012288 [Tribolium | Metabolic process | Other | 4.54E-34 | 4.468785 | 10.62697 | 10.80445 | 8.662743 | 34.76129 | NA | 31.00505 | 125.183 | 61.66407 | NA | 106.3333 | 108.2334 | 103.4225 | 12.34338 | 24.31347 | NA | 22.08937 | NA | 38.37256 | 229.7242 | 73.47743 | NA | 9.680237 | 5.995731 | 6.311063 | 42.78573 | 44.4293 |
| CUST_27261_PI429010886 | N55728 | 0.001415 | 21 | 3 | 260782407 | hypothetical protein BRAFLDRAFT_132313 [Branchi | | | 7.44E-10 | 10.79758 | 17.56349 | 10.06298 | 23.69577 | 15.69846 | 9.343228 | 13.05601 | 10.00064 | 12.57535 | 16.21134 | 4.797228 | 6.805373 | 5.135864 | 7.062308 | 5.684217 | 6.316998 | NA | 8.268709 | 9.662859 | 7.553772 | 21.05599 | 5.682194 | 25.3441 | 10.72419 | 25.64937 | 14.58359 | 17.86315 |
| CUST_36673_PI429010886 | M16505 | 0.001419 | 15 | 9 | | | | | | 402.4861 | 418.5656 | 745.5335 | 309.517 | 214.8393 | 147.7426 | 491.0083 | 905.952 | 857.3719 | 913.4609 | 395.6626 | 571.9057 | 708.1055 | 821.7869 | 636.5467 | 571.5932 | 266.722 | 346.2493 | 338.5891 | 392.5464 | 340.6846 | 619.0377 | 785.2299 | 281.599 | 803.077 | 417.1018 | 1213.318 |
| CUST_51229_PI429010886 | M92702 | 0.001428 | 21 | 3 | | | | | | 8.117917 | 6.153881 | 14.57533 | 24.48509 | 10.68951 | 9.928249 | 16.93603 | 7.92158 | 7.338708 | 9.668476 | NA | 8.910931 | 11.01558 | 6.31804 | 5.034012 | 6.508712 | 12.88285 | 9.757187 | 4.620483 | NA | 5.153689 | 17.12071 | 7.088352 | NA | 28.06449 | 27.54224 | 27.97946 |
| CUST_9969_PI429010886 | N25485 | 0.001429 | 15 | 9 | | | Signal transduction | Other | | 57.90171 | 125.6673 | 214.6655 | 15.18055 | 91.96613 | NA | 21.95843 | 32.60782 | 122.3764 | 185.2951 | 148.572 | 213.8469 | 166.0856 | 87.15435 | 113.5368 | 102.0005 | 64.56992 | 88.19572 | 65.85741 | 54.63526 | 89.01214 | 267.8998 | 155.5901 | 40.05116 | 388.4984 | 166.7311 | 445.5129 |
| CUST_21960_PI429010886 | N46161 | 0.001435 | 24 | 18 | | | | | | 50.48761 | 47.40881 | 51.35337 | 43.8289 | 53.03364 | 44.69027 | 38.49435 | 30.34164 | 43.21123 | 35.54827 | 34.37569 | 35.15442 | 49.48526 | 46.52112 | 40.03501 | 40.97284 | 55.90028 | 40.4721 | 44.98472 | 52.33717 | 63.48998 | 42.12986 | 38.50305 | 56.03701 | 48.68295 | 48.67019 | 56.22954 |
| CUST_40865_PI429010886 | M56251 | 0.001439 | 21 | 3 | | | | | | 205.4682 | 239.4245 | 304.8063 | 233.3995 | 240.1758 | 310.8131 | 236.1246 | 273.7876 | 233.6842 | 226.5039 | 243.718 | 274.9043 | 82.33655 | 307.2905 | 221.4718 | 146.622 | 229.9564 | 265.3779 | 205.2221 | 130.0411 | 171.8712 | 232.021 | 268.82 | 266.6447 | 333.2881 | 348.6809 | 294.596 |
| CUST_3583_PI429010886 | N14819 | 0.001443 | 12 | 6 | | | | | | 4.484368 | 22.29671 | 5.534791 | 78.84971 | 64.07157 | 24.78255 | 60.57138 | 196.4719 | 116.6682 | 41.13272 | 168.5577 | 197.3275 | 9.787782 | 57.23868 | 23.78208 | NA | 113.8579 | NA | NA | 322.0841 | 114.2883 | NA | 52.30377 | 34.88082 | NA | 67.25956 | 47.67267 |
| CUST_36137_PI429010886 | M14770 | 0.001446 | 18 | 6 | | | | | | 30.70184 | 31.71188 | 33.01533 | 26.55213 | 43.91811 | 38.12809 | 40.17403 | 37.45529 | 38.0116 | 31.65107 | 36.33605 | 39.52248 | 29.71613 | 29.85387 | 22.77856 | 36.04028 | 16.62775 | 24.28324 | 29.10242 | 35.42916 | 39.53783 | 32.95281 | 32.03583 | 37.33468 | 32.77524 | 43.2321 | 55.77862 |
| CUST_44471_PI429010886 | M66078 | 0.001446 | 15 | 9 | 345492959 | PREDICTED: NFX1-type zinc finger-containing protei | | | 8.21E-41 | 13.14971 | 27.34621 | 13.65469 | 16.88691 | 33.89729 | 16.80263 | 18.93518 | 21.70445 | 25.35894 | 27.28431 | 51.7675 | 46.11982 | 21.02215 | 15.59858 | 14.37805 | 13.03814 | 14.13431 | 7.93896 | 16.73905 | 32.43067 | 21.248 | 19.49159 | 17.86315 | 18.47842 | 15.23556 | 28.90402 | 29.18503 |
| CUST_40977_PI429010886 | M56534 | 0.001452 | 24 | 18 | | | | | | 284.0133 | 240.0259 | 262.4225 | 236.6472 | 225.8273 | 199.0699 | 179.0604 | 192.7647 | 212.9538 | 256.4811 | 200.0082 | 218.7209 | 238.8599 | 295.4403 | 255.3857 | 297.361 | 222.3148 | 229.6175 | 261.2237 | 277.0736 | 289.7899 | 301.0265 | 243.2786 | 221.9563 | 315.1705 | 238.8193 | 281.9612 |
| CUST_33243_PI429010886 | M6380 | 0.001452 | 21 | 0 | 403182806 | AAEL006520-PB [Aedes aegypti] | | | 1.18E-11 | 46.71536 | 41.16431 | 44.40267 | 36.38283 | 39.42313 | 37.62657 | 38.08646 | 40.39881 | 45.31583 | 44.89448 | 34.05805 | 28.99859 | 30.08229 | 33.20236 | 32.46164 | 35.2603 | 36.69017 | 55.88861 | 40.28418 | 35.6179 | 40.90354 | 44.27177 | 57.34886 | 42.01729 | 46.85704 | 32.92321 | 53.01853 |
| CUST_10052_PI429010886 | N25618 | 0.001453 | 24 | 15 | | | | | | 610.6415 | 771.9192 | 623.9709 | 588.293 | 533.4173 | 578.3985 | 518.5036 | 581 | 555.136 | 529.6585 | 575.9559 | 600.1331 | 619.5429 | 689.8687 | 630.4701 | 850.4702 | 645.2555 | 658.6556 | 654.914 | 720.9482 | 578.8676 | 856.5552 | 628.8832 | 537.701 | 709.8502 | 588.5777 | 615.211 |
| CUST_6440_PI429010886 | N20102 | 0.001454 | 18 | 0 | 213624459 | phosphoglucomutase 2 [Xenopus (Silurana) tropical | Metabolic process | Carbohydrate metabolic process | 3.12E-96 | 18.59558 | 22.0796 | 20.58261 | 21.82017 | 17.99612 | 18.72449 | 10.82989 | 15.51227 | 13.04874 | 23.33499 | 10.05433 | 25.28525 | 16.56322 | 12.83593 | 15.70756 | 32.01858 | 17.36105 | 16.22818 | 23.01368 | 16.35532 | 27.2627 | 15.444 | 14.47174 | 12.6619 | 13.57654 | 11.11202 | 11.43831 |
| CUST_56513_PI429010886 | M113973 | 0.001456 | 15 | 9 | 321464775 | miniature spindles-like protein [Daphnia pulex] | | | 3.88E-57 | 53.32755 | 41.94819 | 42.31884 | 45.36871 | 51.98058 | 41.78277 | 46.47908 | 44.48344 | 48.92704 | 57.57631 | 54.04063 | 56.99751 | 51.18041 | 53.53627 | 50.69191 | 51.44191 | 46.19013 | 48.23121 | 43.04145 | 45.1372 | 45.56467 | 72.08475 | 37.77438 | 49.77895 | 62.85005 | 48.9358 | 54.73744 |

| Probe ID | ID | p-value | Period | Phase | EBI ID | Annotation | GO category | Go term | E-value | 0 | 0 | 0 | 3 | 3 | 3 | 6 | 6 | 6 | 9 | 9 | 9 | 12 | 12 | 12 | 15 | 15 | 15 | 18 | 18 | 18 | 21 | 21 | 21 | 24 | 24 | 24 |
|---|---|---|---|---|---|---|---|---|---|---|---|---|---|---|---|---|---|---|---|---|---|---|---|---|---|---|---|---|---|---|---|---|---|---|---|---|
| CUST_16674_PI429010886 | N36668 | 0.001456 | 15 | 9 | 321478072 | hypothetical protein DAPPUDRAFT_304700 [Daphn | Metabolic process | Nucleobase-containing compound metabolic process | 8.65E-46 | 138.8541 | 179.5583 | 175.8509 | 148.7855 | 131.2414 | 118.6493 | 193.0385 | 213.366 | 269.7651 | 220.9933 | 120.0446 | 201.9396 | 164.8084 | 202.7615 | 171.8979 | 194.8405 | 124.1278 | 199.3094 | 153.9392 | 159.5474 | 147.4496 | 160.3693 | 158.0318 | 211.7804 | 263.2372 | 214.1332 | 279.7821 |
| CUST_44776_PI429010886 | M66953 | 0.00146 | 18 | 0 | | | | | | 18.63533 | NA | 20.65238 | NA | 7.817365 | 21.45407 | 14.93945 | 11.06447 | 12.28125 | 5.6033 | 5.177834 | 4.40452 | 6.382168 | NA | NA | 3.975481 | 12.29647 | 9.523047 | 14.39423 | 15.91783 | 18.61514 | NA | 18.4246 | 26.01055 | 4.335161 | 3.773251 | 5.021225 |
| CUST_15002_PI429010886 | N33842 | 0.00146 | 18 | 3 | 241779172 | conserved hypothetical protein [Ixodes scapularis] > | | | 3.22E-21 | 9.257427 | 11.50698 | 7.239142 | 26.89938 | 13.73929 | 28.70508 | 13.82537 | 13.54122 | 5.263094 | 9.264427 | 10.84227 | 14.265 | 7.884889 | 8.1716 | 9.607772 | 16.16875 | 10.36736 | 8.858469 | 16.83754 | 11.91576 | 6.224633 | 22.49197 | 18.40839 | 13.23625 | 22.31737 | 9.939882 | 12.15878 |
| CUST_56465_PI429010886 | M113865 | 0.00147 | 21 | 3 | | | | | | 12.63126 | 17.73942 | 27.34621 | 131.7576 | 38.6196 | 34.3148 | 15.47412 | 9.149574 | 17.22674 | 18.57528 | 12.11785 | 16.07659 | 12.2919 | 14.0442 | 6.66209 | 12.8607 | 6.149357 | 22.09437 | 16.31327 | 9.040661 | 29.18503 | 8.66034 | 28.32194 | 12.86855 | 23.13337 | 27.64478 | 19.78599 |
| CUST_32477_PI429010886 | M4220 | 0.001471 | 15 | 9 | | | | | | 111.7908 | 155.6229 | 318.2985 | 65.21686 | 93.01036 | 68.1054 | 169.3616 | 339.8212 | 335.2532 | 317.2087 | 145.8455 | 352.327 | 250.4057 | 468.2572 | 301.0804 | 214.4445 | 100.2315 | 194.8105 | 163.3073 | 148.7855 | 162.6071 | 241.3477 | 287.4102 | 78.73486 | 306.8029 | 231.9868 | 388.966 |
| CUST_10429_PI429010886 | N26215 | 0.001472 | 21 | 3 | | | | | | 124.0734 | 148.3869 | 115.7448 | 155.1093 | 150.286 | 198.6322 | 115.8195 | 132.1519 | 111.5352 | 145.2206 | 121.7341 | 123.1377 | 83.90478 | 99.82512 | 114.1048 | 115.9538 | 142.9849 | 127.7733 | 131.1566 | 126.1607 | 135.0274 | 143.6875 | 87.93229 | 132.2831 | 130.1016 | 142.9167 | 133.0452 |
| CUST_22670_PI429010886 | N47428 | 0.001472 | 12 | 0 | | | | | | 15.27919 | 15.12127 | 10.57353 | 10.53799 | 8.262678 | NA | 4.220454 | 5.356366 | 8.883037 | 12.24661 | 7.396068 | 6.370435 | 14.77486 | 17.41767 | 23.2714 | 12.68722 | 4.727984 | 11.98592 | 8.087563 | 11.89186 | 9.379442 | 17.04092 | 6.269689 | NA | 15.78638 | NA | 13.49626 |
| CUST_8104_PI429010886 | N22542 | 0.001478 | 24 | 21 | | | | | | 108.6304 | 91.1558 | 85.41552 | 115.9863 | 137.1215 | 117.1213 | 86.04961 | 83.5291 | 87.57209 | 103.1342 | 86.22581 | 114.4714 | 107.6827 | 119.7777 | 142.8955 | 128.4384 | 90.6343 | 151.5411 | 769.5294 | 125.1102 | 119.6528 | 139.376 | 123.6999 | 156.1886 | 104.8909 | 146.0101 | 99.77775 |
| CUST_31619_PI429010886 | M1979 | 0.001478 | 24 | 21 | 505804162 | PREDICTED: TP53RK-binding protein isoform X1 [So | | | 8.7E-13 | 18.4928 | 20.77886 | 18.86593 | 19.90626 | 16.39936 | 22.13451 | 12.4383 | 13.30045 | 9.629706 | 18.87168 | 10.52513 | 28.93067 | 11.60477 | 15.73055 | 31.14945 | 30.3584 | 13.28314 | 10.19195 | 39.26587 | 25.58242 | 16.94759 | 36.22709 | 28.71019 | 46.71536 | 30.44396 | 28.25408 | 19.63157 |
| CUST_4980_PI429010886 | N18069 | 0.001478 | 24 | 21 | 29134779 | projectin [Procambarus clarkii] | | | 7.8E-130 | 45.00065 | 47.51659 | 48.48054 | 52.32895 | 44.98472 | 45.74744 | 28.62243 | 42.20313 | 38.42916 | 45.82436 | 47.0488 | 37.11863 | 48.45867 | 51.32807 | 42.22821 | 48.78617 | 49.05418 | 47.84739 | 47.01326 | 59.27376 | 38.68605 | 49.57895 | 61.05832 | 53.88401 | 67.237 | 41.19711 | 41.52389 |
| CUST_31846_PI429010886 | M2554 | 0.001479 | 12 | 0 | | | Response to stress | Response to oxidative stress | | 189.5968 | 196.157 | 236.1903 | 219.4848 | 199.7913 | 194.5182 | 150.3901 | 172.054 | 175.4567 | 173.3233 | 177.3442 | 204.2578 | 175.4567 | 313.8962 | 253.0671 | 191.9558 | 168.3529 | 163.3073 | 188.7857 | 175.0831 | 184.2548 | 216.7674 | 192.363 | 189.7138 | 234.1955 | 200.21 | 238.9663 |
| CUST_5530_PI429010886 | N18824 | 0.00148 | 21 | 0 | 241828659 | Ser/Thr protein phosphatase, putative [Ixodes scap | Protein metabolism | Cellular protein modification process | 8.6E-172 | 93.1664 | 66.04832 | 73.33005 | 50.49286 | 50.72999 | 66.99196 | 72.72106 | 41.91362 | 43.62391 | 55.39377 | 54.86684 | 55.784 | 60.78457 | 47.61652 | 51.46537 | 67.09564 | 56.31266 | 59.58353 | 68.80103 | 48.10487 | 65.60373 | 68.95198 | 66.98492 | 71.40808 | 58.71363 | 46.68828 | 40.26187 |
| CUST_18218_PI429010886 | N39372 | 0.001491 | 12 | 0 | | | | | | 29.32561 | 26.97522 | 32.15824 | 18.61514 | 23.12065 | 16.19313 | 19.8522 | 15.92987 | 21.74647 | 36.29122 | 28.6142 | 27.11255 | 31.24487 | 46.13321 | 32.14525 | 33.46044 | 22.96785 | 23.86861 | 27.25419 | 24.65512 | 20.01979 | 33.61445 | 39.97721 | 27.21982 | 46.92598 | 29.61528 | 40.34891 |
| CUST_2876_PI429010886 | N13293 | 0.001491 | 21 | 15 | | | | | | 148.196 | 180.4919 | 179.2925 | 175.8911 | 188.6896 | 160.5038 | 155.4719 | 142.1826 | 135.074 | 258.8229 | 168.2678 | 163.3498 | 172.1063 | 157.2097 | 210.0511 | 209.9951 | 277.9987 | 204.7153 | 209.6917 | 223.6052 | 186.7835 | 172.957 | 218.3592 | 172.082 | 226.4213 | 153.1027 | 174.7413 |
| CUST_9411_PI429010886 | N24576 | 0.001497 | 24 | 21 | | | | | | 52.07596 | 64.96528 | 35.78224 | 37.36325 | 45.77928 | 31.56077 | 28.03123 | 31.78038 | 35.9369 | 66.87846 | 52.5224 | 35.25245 | 40.76277 | 47.3028 | 28.53867 | 47.15656 | 40.19775 | 43.99672 | 41.66889 | 68.29796 | 61.91569 | 56.39846 | 70.61832 | 66.57113 | 81.58984 | 44.12842 | 60.19936 |
| CUST_40840_PI429010886 | M56206 | 0.001498 | 24 | 15 | | | | | | 45.42508 | 40.86821 | 26.82709 | 39.55733 | 55.27747 | 48.76067 | 27.5502 | 30.29871 | 33.28766 | 54.75352 | 45.01588 | 38.48636 | 37.54075 | 37.61167 | 37.13804 | 73.88403 | 49.46731 | 55.22248 | 49.63154 | 58.4208 | 53.61134 | 51.87547 | 44.16686 | 57.1457 | 45.42072 | 46.991 | 43.03291 |
| CUST_4742_PI429010886 | N17385 | 0.001498 | 24 | 15 | 321479211 | hypothetical protein DAPPUDRAFT_300177 [Daphn | | | 1.04E-13 | 27.13002 | 26.9152 | 19.28994 | 19.76039 | 22.73323 | 19.51465 | 25.12351 | 17.02082 | 19.59849 | 31.43817 | 25.9425 | 30.81346 | 12.63126 | 32.87841 | 30.83942 | 38.87769 | 31.99053 | 49.21104 | 23.60284 | 31.59448 | 23.18582 | 27.085 | 32.25357 | 27.42267 | 29.49638 | 21.75964 | 32.91963 |
| CUST_5569_PI429010886 | N18886 | 0.001498 | 21 | 18 | | | | | | 137.5442 | 189.4567 | 107.0174 | 117.4158 | 134.3489 | 92.68449 | 76.17525 | 91.33174 | 86.28978 | 152.6964 | 113.3366 | 125.183 | 133.8309 | 125.2677 | 163.8253 | 234.4127 | 78.18464 | 141.0765 | 135.9425 | 190.4443 | 157.4606 | 169.2967 | 128.5235 | 94.75367 | 153.3563 | 103.8205 | 141.8494 |
| CUST_38184_PI429010886 | M23974 | 0.001502 | 24 | 3 | | | | | | 360.6396 | 1242.81 | 1281.734 | 538.0354 | 1031.749 | 1131.289 | 853.9995 | 776.1766 | 740.4281 | 677.7517 | 754.7954 | 1001.62 | 544.2536 | 806.919 | 639.3223 | 583.2176 | 118.6343 | 180.5428 | 605.4467 | 419.5293 | 352.1132 | 681.3848 | 862.1393 | 268.1467 | 925.4363 | 773.8104 | 933.7706 |
| CUST_55173_PI429010886 | M108996 | 0.001515 | 24 | 21 | | | | | | 567.7179 | 667.8908 | 760.0455 | 547.8035 | 612.2893 | 462.4656 | 542.7169 | 567.0781 | 580.436 | 622.7518 | 491.7148 | 768.7795 | 631.4343 | 653.4911 | 623.3295 | 752.5982 | 665.511 | 528.3775 | 648.0827 | 669.0767 | 662.7679 | 976.3736 | 670.112 | 716.9224 | 796.7421 | 627.0644 | 831.0172 |
| CUST_7843_PI429010886 | N22164 | 0.001515 | 24 | 21 | | | | | | 79.47713 | 78.28594 | 80.29877 | 68.01317 | 100.1275 | 104.474 | 48.05852 | 57.01626 | 56.07774 | 63.50632 | 71.73214 | 82.97709 | 72.84145 | 78.07189 | 70.23023 | 73.33005 | 81.16843 | 93.73888 | 82.28242 | 57.06419 | 78.43466 | 89.26714 | 88.54248 | 90.79757 | 86.6967 | 70.14592 | 76.34394 |
| CUST_35934_PI429010886 | M14015 | 0.001516 | 21 | 3 | | | | | | 1166.811 | 1514.446 | 1627.802 | 5086.964 | 2408.505 | 2684.729 | 1467.042 | 1837.082 | 1466.368 | 2286.095 | 744.0064 | 431.3819 | 1341.448 | 1439.161 | 790.3406 | 2074.248 | 655.5465 | 1293.201 | 868.3571 | 1281.141 | 2410.357 | 2095.03 | 2326.832 | 1589.601 | 2504.679 | 3065.467 | 1347.753 |
| CUST_38152_PI429010886 | M23690 | 0.00153 | 15 | 12 | | | | | | 9.658216 | 20.23721 | 19.2217 | 14.82674 | 18.93518 | 23.85972 | 14.91181 | 10.87219 | 10.93666 | 26.13131 | 24.91723 | 26.79563 | 13.25918 | 29.48584 | 33.14087 | 27.52766 | 25.68021 | 30.37057 | 18.15993 | 15.68875 | 20.95495 | 11.63852 | 8.400369 | 16.66074 | 11.67245 | 13.88119 | 19.26978 |
| CUST_15942_PI429010886 | N35384 | 0.00153 | 12 | 3 | | | | | | 133.1932 | 134.2642 | 125.4528 | 158.91 | 130.7466 | 189.7386 | 135.0409 | 110.3777 | 97.71155 | 100.64 | 97.24592 | 100.4818 | 119.6384 | 115.0228 | 113.7623 | 132.2831 | 157.1911 | 121.8291 | 118.1469 | 102.1333 | 112.2549 | 149.7905 | 82.67802 | 108.6889 | 109.0449 | 142.0394 | 137.0769 |
| CUST_38284_PI429010886 | M25016 | 0.001532 | 12 | 3 | 2414483 | Crithidia fasciculata ribosomal DNA repeat (complet | | | | 235.4879 | 135.9831 | 40.42484 | 1179.987 | 628.4644 | 1725.627 | 535.2858 | 313.0231 | 132.5094 | 32.22977 | 497.0198 | 63.60606 | 147.341 | 135.0881 | 364.6302 | 388.7869 | 1813.091 | 2340.111 | 225.9932 | 251.1408 | 680.5523 | 524.0613 | 537.701 | 940.8909 | 15.13318 | 321.9594 | 19.85636 |
| CUST_37830_PI429010886 | M21795 | 0.001534 | 24 | 18 | | | | | | 387.589 | 23.60773 | 852.9478 | 89.24944 | 144.1009 | 107.5224 | 319.6424 | 825.1657 | 155.4485 | 207.9906 | 258.1697 | 209.0603 | 1579.682 | 2117.402 | 997.0196 | 241.6881 | 540.6649 | 194.5472 | 1294.143 | 1428.874 | 2007.972 | 1098.078 | 394.7994 | 851.0651 | 1958.473 | 715.1399 | 928.4158 |
| CUST_10105_PI429010886 | N25705 | 0.001534 | 24 | 21 | | | | | | 231.7766 | 210.1562 | 245.2785 | 209.8084 | 198.8522 | 214.1723 | 202.8118 | 162.0477 | 211.2718 | 287.3657 | 210.4274 | 244.6702 | 210.2047 | 228.2456 | 220.8169 | 255.4957 | 218.5128 | 229.4944 | 223.5686 | 242.4336 | 233.8998 | 253.3366 | 256.4303 | 239.9082 | 250.7705 | 236.0009 | 252.9368 |
| CUST_40731_PI429010886 | M55957 | 0.001534 | 12 | 0 | | | | | | 137.719 | 168.329 | 151.1592 | 120.2795 | 122.4782 | 139.9278 | 109.6246 | 106.1754 | 119.9288 | 142.7087 | 114.443 | 113.9294 | 113.9294 | 146.3045 | 161.7557 | 172.831 | 134.7426 | 165.6637 | 112.2867 | 117.4463 | 90.62378 | 143.7864 | 133.5939 | 127.2704 | 139.4507 | 141.9837 | 140.548 |
| CUST_56697_PI429010886 | M114441 | 0.001538 | 24 | 12 | | | | | | 64.01028 | 64.3589 | 85.20601 | 62.63151 | 64.10151 | 49.47977 | 64.66893 | 69.83541 | 70.59503 | 59.21305 | 70.23629 | 107.4983 | 79.61927 | 85.50355 | 85.61256 | 73.82839 | 70.63158 | 91.48208 | 67.1407 | 71.61132 | 70.23023 | 80.50367 | 66.60678 | 65.27575 | 58.4576 | 77.46325 | 74.54819 |
| CUST_45936_PI429010886 | M70806 | 0.001538 | 24 | 15 | | | | | | 95.71563 | 108.7487 | 121.1828 | 90.56267 | 79.78328 | 92.45305 | 89.65503 | 87.71808 | 94.25565 | 100.2406 | 86.36905 | 136.597 | 102.5272 | 147.073 | 137.4615 | 154.1562 | 105.57 | 125.8769 | 106.5981 | 120.0958 | 126.4932 | 119.9009 | 123.6359 | 91.28115 | 98.94163 | 109.0596 | 124.3147 |
| CUST_46341_PI429010886 | M72300 | 0.001538 | 24 | 12 | | | | | | 67.25349 | 54.08762 | 73.0191 | 63.82351 | 54.49444 | 57.14083 | 63.50632 | 73.33005 | 64.16127 | 90.54311 | 65.61755 | 76.93173 | 99.54848 | 75.55792 | 75.4087 | 77.39915 | 63.96858 | 88.28345 | 75.91487 | 72.52738 | 73.53135 | 74.92755 | 81.30299 | 74.86136 | 72.6348 | 63.32035 | 76.15742 |
| CUST_16362_PI429010886 | N36110 | 0.001547 | 21 | 15 | | | | | | 207.5012 | 223.5364 | 185.168 | 135.4209 | 156.4459 | 164.6258 | 124.3489 | 132.7049 | 154.1562 | 202.1175 | 197.8235 | 226.2023 | 175.8509 | 179.4229 | 171.1195 | 268.1153 | 187.4158 | 203.9546 | 220.9933 | 172.1829 | 194.4538 | 203.8319 | 161.9364 | 156.1197 | 194.9477 | 154.2483 | 179.5583 |
| CUST_39026_PI429010886 | M33579 | 0.001548 | 15 | 9 | | | | | | 10415.22 | 31148.55 | 35818.08 | 4174.241 | 5349.606 | 1784.695 | 7259.073 | 50235.79 | 65559.81 | 61640.76 | 44794.66 | 58446.35 | 56054.85 | 85436.14 | 61640.76 | 38496.63 | 21478.53 | 22307.76 | 20926.24 | 51255.28 | 24707.51 | 69125.19 | 69125.19 | 4965.834 | 77729.71 | 50235.79 | 99042.74 |
| CUST_10093_PI429010886 | N25681 | 0.001548 | 15 | 9 | 54042615 | arthrodial cuticle protein AMP6.0 [Callinectes sapid | | | 4.12E-15 | 1355.659 | 1860.994 | 1917.136 | 921.954 | 388.6405 | 477.7327 | 1786.373 | 1448.723 | 2321.278 | 3597.224 | 1677.906 | 3380.982 | 1621.924 | 2052.131 | 1710.741 | 825.7659 | 1409.133 | 616.7782 | 1022.968 | 2834.55 | 1281.141 | 3440.367 | 1294.143 | 1335.768 | 2257.795 | 1903.519 | 1931.541 |
| CUST_342_PI429010886 | N4167 | 0.001548 | 15 | 9 | | | | | | 361.0091 | 780.9267 | 1444.613 | 310.6611 | 210.8661 | 87.11006 | 288.7609 | 1504.155 | 2472.114 | 1830.724 | 1132.649 | 2319.618 | 1506.739 | 3008.795 | 2278.385 | 1146.628 | 606.6133 | 749.487 | 756.4835 | 1210.418 | 834.6224 | 2428.103 | 2461.297 | 213.1567 | 2948.044 | 1743.746 | 3500.164 |
| CUST_43252_PI429010886 | M62542 | 0.001551 | 15 | 3 | 341650470 | Spz3 [Litopenaeus vannamei] | | | 1.26E-74 | 62.70814 | 28.80255 | 14.25474 | 156.8208 | 71.19859 | 275.1873 | 113.5681 | 55.90718 | 31.88231 | 3.764097 | 53.22735 | NA | 45.20819 | 6.85925 | 31.76715 | 9.139595 | 153.6651 | 96.5915 | 84.376 | NA | 121.6348 | 16.15147 | 69.26327 | 153.1912 | 9.376909 | 51.33619 | NA |
| CUST_41369_PI429010886 | M57564 | 0.001554 | 18 | 0 | | | | | | 35.70676 | 19.46511 | 26.73374 | NA | 25.791 | 9.743013 | NA | 9.873369 | 12.24024 | 32.73103 | 12.06489 | 18.64626 | 12.09063 | 11.5308 | 16.68444 | 13.72673 | 19.14986 | 32.19898 | 22.81192 | 25.23241 | 25.77304 | 11.69128 | 24.55314 | 16.31327 | 8.149338 | 14.06926 | 7.084189 |
| CUST_51641_PI429010886 | M94373 | 0.001555 | 24 | 15 | | | | | | 52.65717 | 53.44251 | 50.51683 | 38.54575 | 44.12317 | 44.34868 | 72.00841 | 18.44972 | 38.9709 | 82.56662 | 58.08697 | 45.01151 | 49.75477 | 53.20866 | 64.3699 | 75.11707 | 72.96725 | 58.19173 | 40.88839 | 67.88326 | 67.82256 | 52.74988 | 52.63381 | 57.07573 | 59.07548 | 26.27382 | 47.11037 |
| CUST_15344_PI429010886 | N34434 | 0.001558 | 18 | 6 | | | | | | 382.2635 | 451.7787 | 475.4311 | 389.4334 | 405.6393 | 402.1311 | 399.475 | 708.4547 | 674.0173 | 444.1766 | 605.3029 | 657.2757 | 355.116 | 360.7942 | 330.4301 | 331.029 | 305.6076 | 318.4396 | 357.5465 | 728.235 | 629.6206 | 397.5988 | 497.9965 | 457.4348 | 464.6636 | 658.8544 | 614.705 |
| CUST_21376_PI429010886 | N45105 | 0.001566 | 15 | 0 | | | | | | 205.5955 | 218.9512 | 193.6603 | 198.2503 | 181.7752 | 162.3309 | 125.2532 | 173.2308 | 150.6325 | 196.7621 | 133.9267 | 169.2493 | 154.9827 | 165.2714 | 168.2678 | 255.5333 | 212.4692 | 238.2716 | 176.8268 | 181.5662 | 146.3743 | 194.9182 | 116.1174 | 148.1764 | 217.843 | 145.3839 | 193.7403 |
| CUST_28778_PI429010886 | N58603 | 0.001569 | 15 | 12 | | | | | | 10.40254 | 9.828551 | 19.47657 | 16.63365 | 13.61134 | 9.805454 | 5.654986 | 6.954585 | 13.47393 | 11.50923 | 11.11474 | 11.31528 | 20.93304 | 27.025 | 20.73834 | 17.29417 | 12.18439 | 18.44318 | 9.731006 | 8.120251 | 4.277447 | 11.26684 | 10.52513 | NA | 19.09941 | NA | 10.59651 |
| CUST_41501_PI429010886 | M57892 | 0.001572 | 18 | 0 | | | | | | 13.86795 | 10.91945 | 12.38947 | 7.794721 | 10.68692 | 6.51898 | 5.467898 | 4.737841 | 10.71996 | 5.394188 | 6.21618 | 7.636147 | NA | NA | 4.068968 | 7.884889 | 5.350654 | 12.4096 | 10.68521 | 13.30523 | 12.59239 | 11.62339 | NA | 8.388399 | 12.86683 | 5.191146 | 8.692145 |
| CUST_32844_PI429010886 | M5254 | 0.001573 | 15 | 12 | | | | | | 23.98555 | 33.81529 | 32.11685 | 21.45154 | 22.5025 | 19.04367 | 10.94377 | 19.40477 | 22.38395 | 31.37784 | 28.27339 | 32.54126 | 30.77501 | 37.42448 | 42.16582 | 39.59771 | 28.53359 | 25.19447 | 25.30759 | 41.89757 | 24.34275 | 34.63858 | 16.81213 | 24.08567 | 30.59469 | 25.75936 | 28.6271 |
| CUST_17893_PI429010886 | N38786 | 0.001575 | 21 | 12 | | | | | | 4.880377 | 11.54499 | 8.680488 | 111.4954 | 43.88387 | 132.88 | 61.2655 | 60.72981 | 62.71467 | 93.65476 | 10.40896 | 11.60477 | 134.4739 | 19.81178 | 77.85613 | 66.48727 | 17.11553 | NA | 24.42797 | NA | 29.11768 | 6.658652 | 5.765954 | 9.943996 | 10.36736 | 16.36632 | 8.864496 |
| CUST_15566_PI429010886 | N34796 | 0.001582 | 18 | 3 | 390343662 | PREDICTED: zinc finger protein 182-like [Strongyloc | | | 2.96E-13 | 7.380862 | 34.58756 | 14.27406 | 55.88861 | 22.89148 | 31.40253 | 23.44768 | 12.55145 | 14.41938 | 5.452395 | 4.464734 | 12.55757 | 11.56997 | 17.00366 | 6.126585 | 14.77827 | 4.725717 | 14.49586 | 20.47619 | 9.753321 | 23.47003 | 15.62557 | 26.96182 | 16.57948 | 11.97713 | 31.23112 | 17.78016 |
| CUST_34380_PI429010886 | M9490 | 0.001585 | 15 | 3 | | | | | | 295.6383 | 249.6087 | 276.2557 | 358.1231 | 306.6802 | 422.7364 | 467.4407 | 350.2692 | 279.1338 | 153.5542 | 192.557 | 122.4191 | 243.3928 | 160.4369 | 235.7212 | 260.9414 | 292.8344 | 288.2901 | 300.2764 | 157.283 | 167.2954 | 241.9617 | 232.1818 | 345.5828 | 161.6619 | 169.1571 | 88.5487 |
| CUST_6906_PI429010886 | N20761 | 0.001588 | 12 | 0 | 321470670 | hypothetical protein DAPPUDRAFT_211091 [Daphn | Metabolic process | Cellular amino acid metabolic process | 7.76E-97 | 290.5429 | 440.6015 | 390.2834 | 281.2623 | 278.397 | 308.1438 | 227.9039 | 309.9578 | 328.7379 | 337.0926 | 351.9054 | 615.211 | 357.356 | 451.6758 | 467.8704 | 333.2881 | 249.8117 | 277.7171 | 315.5114 | 439.4127 | 338.6596 | 385.7092 | 329.8367 | 294.4013 | 348.0871 | 405.164 | 377.56 |
| CUST_1436_PI429010886 | N9625 | 0.001589 | 18 | 6 | 238476875 | crustin type I [Macrobrachium rosenbergii] | | | 2.87E-10 | 115.202 | 154.0476 | 69.39648 | 389.6265 | 336.8185 | 256.9966 | 1293.201 | 423.8598 | 1555.864 | 512.5692 | 118.2001 | 1385.628 | 741.4101 | 419.0394 | 654.4842 | 1619.494 | 995.2843 | 230.5203 | 169.847 | 362.4143 | 141.6867 | 712.9294 | 151.2719 | 637.7954 | 1174.011 | 455.5337 | 1493.465 |
| CUST_9711_PI429010886 | N25080 | 0.00159 | 24 | 15 | 357619676 | hypothetical protein KGM_21935 [Danaus plexippu | | | 5.3E-16 | 9.50058 | 9.424573 | 10.27676 | 4.404875 | 5.033531 | 3.659475 | 7.334339 | 11.84611 | 3.506989 | 13.26521 | 12.69324 | 11.11331 | 16.37326 | 11.18163 | 9.091001 | 10.66241 | 17.95875 | 13.57654 | 14.5219 | 9.361552 | 13.60227 | 5.04345 | 13.72842 | NA | 11.39726 | 9.766658 | 5.973441 |
| CUST_37264_PI429010886 | M18426 | 0.00159 | 15 | 0 | | | | | | 80.54851 | 90.19499 | NA | 266.722 | 116.4584 | 79.48728 | 82.85967 | NA | 59.04082 | 11.14291 | 5.635016 | NA | 24.38855 | 18.03887 | 51.63118 | 134.2991 | NA | 107.4216 | 9.488473 | 8.451827 | NA | 74.88 | 10.6654 | 50.38211 | 50.09195 | 4.930846 | 8.430029 |
| CUST_54037_PI429010886 | M104199 | 0.001598 | 24 | 0 | | | | | | 19.7129 | 10.96784 | 27.23134 | 16.07034 | 9.564156 | 9.020299 | 9.02447 | 13.00717 | 7.306974 | 14.54874 | 8.53722 | 14.62285 | 8.081076 | 6.51898 | 15.43278 | 6.698064 | 3.410235 | NA | 14.04977 | 29.95175 | 7.213856 | NA | 12.51827 | 15.69846 | 22.23551 | 24.93429 | 27.75224 |
| CUST_17857_PI429010886 | N38727 | 0.001602 | 12 | 3 | | | | | | 9.318343 | 10.97938 | NA | 13.89365 | 11.27392 | 22.20774 | 5.682194 | 9.956467 | 6.076503 | 7.641633 | 7.460722 | 8.804214 | 8.050377 | NA | 13.04417 | 14.5269 | 16.18956 | 35.82003 | 8.92426 | 9.778317 | 12.12977 | 7.182647 | NA | 13.26174 | 10.44214 | 17.08381 | 10.51643 |
| CUST_3180_PI429010886 | N13962 | 0.001603 | 15 | 9 | | | | | | 1070.582 | 1731.857 | 3823.851 | 1309.784 | 1606.044 | 762.5883 | 829.8828 | 3991.112 | 4253.825 | 8196.001 | 3332.072 | 7083.117 | 3020.819 | 5193.144 | 3988.418 | 5396.825 | 2030.719 | 1508.69 | 2397.429 | 3243.287 | 3006.75 | 10866.21 | 6091.908 | 1257.529 | 6267.492 | 4813.402 | 12214.41 |
| CUST_28068_PI429010886 | N57222 | 0.001603 | 15 | 3 | | | | | | 8.585014 | 6.541514 | 8.335093 | 12.78607 | 11.28708 | 16.38019 | 9.635022 | 9.384386 | 8.92426 | 7.544684 | 7.48441 | 7.501961 | 12.32047 | 9.689869 | 8.113982 | 7.331706 | 9.654274 | 16.04554 | 13.44962 | 12.05492 | 6.28041 | 9.680237 | 11.56285 | 7.879494 | 6.808243 | 4.153912 | 5.185762 |
| CUST_36709_PI429010886 | M16612 | 0.001605 | 18 | 3 | | | | | | 17.92979 | 12.75871 | 23.64437 | 24.0077 | 27.64723 | 36.96758 | 25.89264 | 22.1415 | 18.03173 | 16.71353 | 20.50362 | 16.73527 | 14.75247 | 26.90876 | 15.66178 | 12.91897 | 21.49011 | 19.84167 | 20.35771 | 16.75121 | 22.07787 | 27.90958 | 28.27971 | 20.36949 | 23.3977 | 21.28517 | 31.92079 |
| CUST_38535_PI429010886 | M27753 | 0.001606 | 12 | 0 | | | | | | 72.5683 | 65.66431 | 86.61515 | 36.55477 | 46.83015 | 55.05996 | 37.00487 | 71.2192 | 59.93656 | 64.67521 | 48.59467 | 70.13143 | 58.81306 | 89.65503 | 47.71565 | 59.85492 | 56.88203 | 53.00119 | 57.22072 | 48.61666 | 56.9044 | 67.43306 | 59.69745 | 62.91706 | 93.32222 | 72.8742 | 84.73278 |
| CUST_2525_PI429010886 | N12561 | 0.001611 | 21 | 0 | | | | | | 27.03609 | 28.40654 | 18.08701 | 17.69248 | 18.27296 | NA | 18.59144 | 15.2618 | 13.19231 | 5.76082 | 5.876373 | 7.548864 | 12.51827 | 16.25929 | 10.48257 | 19.2789 | 10.6528 | 29.74822 | 29.87357 | 7.596526 | 18.18378 | 22.33967 | 20.94928 | 13.63311 | 20.98935 | 18.38014 | 15.94162 |
| CUST_13081_PI429010886 | N30545 | 0.001616 | 21 | 18 | | | | | | 118.1855 | 171.7601 | 139.7854 | 94.02768 | 119.0071 | 90.33731 | 97.25472 | 86.97645 | 100.5206 | 120.6234 | 110.1779 | 123.3055 | 98.23281 | 136.3233 | 122.3344 | 153.8888 | 81.71205 | 113.8053 | 139.9078 | 169.0201 | 137.4373 | 183.528 | 130.9315 | 129.9123 | 148.1409 | 110.5556 | 93.09068 |
| CUST_44967_PI429010886 | M67556 | 0.001616 | 18 | 0 | | | | | | 64.87589 | 101.3025 | 66.54333 | 32.81991 | 21.12654 | 28.56717 | 23.12065 | 11.81158 | 17.70313 | 16.69153 | 24.46901 | 15.81697 | 36.41652 | 36.55124 | 20.43695 | 235.3955 | 6.090696 | 196.2247 | 43.19614 | 66.81139 | 54.04881 | 63.70797 | 4.690669 | 48.84923 | 25.69538 | 39.70836 | NA |
| CUST_22661_PI429010886 | N47416 | 0.001622 | 15 | 0 | | | | | | 36.43958 | 55.31379 | 56.93106 | 24.16344 | 33.38032 | 20.50575 | 38.10393 | 16.10718 | 22.498 | 19.79375 | 21.74369 | 27.30762 | 32.64658 | 21.76863 | 20.87902 | 71.03904 | 44.20781 | 57.28119 | 71.3655 | 13.21509 | 20.54194 | 14.22529 | 12.11037 | 10.6154 | 58.85113 | 16.86851 | 46.80174 |
| CUST_30940_PI429010886 | M272 | 0.001623 | 12 | 3 | | | | | | 1555.864 | 1691.46 | 102.6107 | 2573.258 | 1898.136 | 3380.982 | 1550.226 | 1502.524 | 1592.642 | 266.851 | 670.112 | 226.3675 | 1883.709 | 713.1739 | 1410.296 | 3116.138 | 2341.487 | 3815.349 | 2143.344 | 540.3776 | 429.7605 | 1828.604 | 70.3928 | 2107.396 | 1246.008 | 1718.489 | 34.02396 |
| CUST_44486_PI429010886 | M66118 | 0.001623 | 12 | 3 | | | | | | 149.2677 | 117.7597 | 22.12824 | 289.3248 | 181.2698 | 326.6831 | 140.8976 | 131.259 | 133.0821 | 5.898499 | 64.46821 | 9.801459 | 142.727 | 63.43828 | 99.2894 | 165.1872 | 230.4843 | 345.8615 | 245.7815 | 42.98059 | 58.19889 | 138.5572 | 11.55275 | 242.6123 | 85.24568 | 91.76014 | 9.071169 |
| CUST_21630_PI429010886 | N45556 | 0.001624 | 24 | 21 | | | | | | 29.93088 | 39.92653 | 34.34243 | 27.84094 | 27.61834 | 40.04686 | 30.19994 | 24.69322 | 27.09726 | 19.24083 | 37.79341 | 33.26161 | 11.92505 | 21.74647 | 21.72694 | 51.14708 | 41.85446 | 38.13783 | 32.16644 | 31.47618 | 42.36861 | 42.37343 | 78.18464 | 33.24682 | 52.45258 | 33.68381 | 48.21613 |
| CUST_42589_PI429010886 | M60659 | 0.001625 | 18 | 12 | | | | | | 66.022 | 40.39881 | 68.74728 | 59.01989 | 40.66714 | 44.1334 | 70.14592 | 77.0714 | 83.27348 | 84.42877 | 67.48761 | 79.72112 | 60.61424 | 86.31209 | 81.95659 | 72.55294 | 98.32258 | 70.05382 | 66.07282 | 48.02842 | 37.74522 | 61.03108 | 66.1724 | 76.21767 | 81.19526 | 65.21686 | 66.64899 |
| CUST_37023_PI429010886 | M17592 | 0.001625 | 12 | 3 | | | | | | 108.4314 | 58.92815 | 12.10865 | 559.3835 | 383.7251 | 914.3958 | 442.4276 | 354.9858 | 116.6405 | 21.0901 | 209.0603 | 11.68992 | 165.2432 | 15.7789 | 91.52825 | 17.74547 | 1036.073 | 623.3295 | 563.6523 | 146.5997 | 236.2204 | 20.87254 | 235.838 | 511.0593 | 5.204531 | 326.4884 | 9.621618 |
| CUST_36450_PI429010886 | M15926 | 0.001625 | 12 | 0 | | | | | | 18.84012 | 15.59665 | 14.39924 | 11.69456 | 13.33083 | 12.95606 | 7.838281 | 10.47246 | 5.456718 | 20.90047 | 14.15378 | 22.1046 | 16.33689 | 17.74547 | 18.81645 | 16.84352 | 9.473492 | 14.5269 | 15.43638 | 11.58541 | 7.728763 | 16.97579 | 15.1983 | 12.32751 | 20.59244 | 16.15388 | 14.79665 |
| CUST_5454_PI429010886 | N18726 | 0.001625 | 12 | 0 | | | | | | 321.4904 | 435.3919 | 487.5504 | 147.8481 | 249.9317 | 391.5964 | 57.64388 | 210.3141 | 201.1997 | 370.8487 | 142.6158 | 249.0492 | 286.9075 | 279.924 | 430.0642 | 278.4332 | 97.84604 | 285.0949 | 274.5781 | 110.8479 | 131.259 | 340.6846 | 67.04779 | 66.97079 | 529.2101 | 179.0014 | 450.7936 |
| CUST_55756_PI429010886 | M111610 | 0.001628 | 18 | 15 | | | | | | 1335.768 | 796.5277 | 1166.811 | 361.1547 | 1342.932 | 646.5567 | 1569.266 | 926.3799 | 1016.492 | 2184.967 | 996.654 | 1074.839 | 1518.655 | 1470.792 | 1193.503 | 1360.127 | 1517.877 | 1754.186 | 1363.213 | 1335.768 | 1249.958 | 1228.27 | 716.6989 | 552.1675 | 630.6794 | 979.7027 | 1368.903 |
| CUST_49486_PI429010886 | M85836 | 0.001628 | 18 | 6 | | | | | | 10.13796 | 17.97064 | 15.45319 | 15.74925 | 23.01614 | 19.34225 | 32.69174 | 22.40634 | 18.17081 | 13.80899 | 16.71789 | 22.83945 | 17.67558 | 20.78133 | 4.179746 | 13.78635 | 15.17526 | 18.39819 | 3.969366 | 10.13526 | 32.06361 | 19.23624 | 23.53859 | 37.06438 | 76.62962 | 32.09117 | 38.11258 |
| CUST_21189_PI429010886 | N44790 | 0.001628 | 18 | 9 | 310743898 | glycogen synthase [Marsupenaeus japonicus] | Metabolic process | Carbohydrate metabolic process | 2.44E-45 | 36.33335 | 36.77053 | 77.98525 | 23.99602 | 39.97268 | 25.58065 | 43.5699 | 70.46616 | 44.87442 | 81.73916 | 69.35049 | 98.88783 | 53.80851 | 46.93642 | 57.71752 | 24.94778 | 51.28378 | 39.59389 | 29.00747 | 71.11204 | 34.14862 | 36.4525 | 50.61028 | 25.71973 | 40.84912 | 39.31816 | 45.04433 |
| CUST_30129_PI429010886 | N61221 | 0.001633 | 24 | 0 | | | | | | 573.864 | 502.1624 | 425.1252 | 360.4943 | 431.3053 | 466.6293 | 436.6018 | 375.4995 | 418.7921 | 467.665 | 488.0718 | 339.5005 | 297.0813 | 261.5579 | 321.6396 | 683.9239 | 422.0003 | 347.0333 | 256.5714 | 346.7902 | 352.3859 | 1151.226 | 376.3756 | 599.2901 | 949.0578 | 505.0665 | 614.0398 |
| CUST_20061_PI429010886 | N42735 | 0.001638 | 15 | 12 | | | | | | 28.09932 | 34.32725 | 30.88019 | 22.43297 | 17.13916 | 22.13925 | 19.7129 | 22.45323 | 20.32835 | 34.45395 | 12.84068 | 26.89381 | 31.22768 | 36.44258 | 122.2962 | 30.52136 | 40.44714 | 28.47434 | 14.04228 | 23.18582 | 24.83327 | 27.46314 | 36.05492 | 18.4677 | 24.66303 | 25.20311 | 32.2333 |
| CUST_34961_PI429010886 | M11186 | 0.00164 | 15 | 9 | | | | | | 158.635 | 259.2819 | 242.9037 | 172.3811 | 154.4215 | 101.8251 | 126.3127 | 275.0029 | 223.2254 | 373.9776 | 188.4915 | 274.4437 | 216.9753 | 235.3127 | 228.9832 | 231.6114 | 180.2796 | 185.168 | 190.8326 | 212.2832 | 161.7305 | 182.0796 | 189.5057 | 85.89616 | 295.7028 | 243.595 | 292.4718 |
| CUST_6803_PI429010886 | N20619 | 0.00164 | 15 | 9 | | | | | | 60.5193 | 57.7399 | 61.99224 | 47.56602 | 39.39544 | 31.2907 | 36.16451 | 48.41716 | 51.18041 | 79.1048 | 54.77183 | 65.22887 | 54.79286 | 50.21619 | 48.15042 | 66.63641 | 47.71565 | 49.24687 | 43.16246 | 65.91381 | 47.60452 | 67.73286 | 53.02941 | 31.12088 | 83.91514 | 70.47227 | 82.73488 |
| CUST_54189_PI429010886 | M105005 | 0.001643 | 24 | 3 | | | | | | 807.4639 | 2078.653 | 1044.56 | 2361.621 | 1150.815 | 2029.752 | 845.2412 | 880.6305 | 982.113 | 931.1392 | 556.543 | 1769.215 | 775.2325 | 835.1893 | 399.3695 | 1050.102 | 381.1947 | 796.983 | 486.1656 | 865.174 | 1228.741 | 897.4259 | 1484.716 | 1128.352 | 1361.981 | 1914.945 | 770.1948 |
| CUST_45415_PI429010886 | M69037 | 0.001643 | 12 | 0 | | | | | | 22.6805 | 28.87134 | 28.6142 | 21.34103 | 14.35579 | 6.738368 | 17.70878 | 15.29709 | 15.8511 | 22.36804 | 15.55047 | 25.00635 | 22.85699 | 25.27468 | 23.09298 | 31.40893 | 16.55564 | 13.13227 | 15.28613 | 23.28835 | 20.37597 | 21.68921 | 17.17022 | 35.81064 | 33.04443 | 33.28088 | 28.88138 |
| CUST_10321_PI429010886 | N26054 | 0.001651 | 18 | 15 | | | | | | 536.3308 | 558.7985 | 417.8136 | 448.5246 | 376.1818 | 429.0337 | 422.8357 | 436.0745 | 422.4541 | 451.4433 | 495.8952 | 573.2606 | 520.2836 | 491.1625 | 485.2041 | 537.8198 | 460.3656 | 541.4466 | 553.0837 | 593.6703 | 363.2975 | 450.9914 | 392.7033 | 386.9736 | 447.4667 | 448.4129 | 432.5053 |
| CUST_12687_PI429010886 | N29872 | 0.001651 | 18 | 15 | | | | | | 1184.785 | 1441.16 | 257.9388 | 1004.403 | 691.1905 | 345.9305 | 171.1195 | 461.5523 | 240.0259 | 689.0956 | 549.9307 | 459.7686 | 634.2891 | 876.1774 | 990.5143 | 1172.181 | 869.0196 | 1325.228 | 383.5597 | 918.9933 | 804.5642 | 1765.654 | 805.2979 | 2112.767 | 80.1769 | 308.9771 | 347.5866 |
| CUST_23293_PI429010886 | N48582 | 0.001651 | 18 | 15 | | | | | | 92.28356 | 74.07672 | 49.3486 | 26.05504 | 25.31087 | 44.08227 | 35.34739 | 45.14299 | 38.96253 | 49.49896 | 64.6187 | 40.20735 | 76.40353 | 54.48084 | 58.8975 | 73.51179 | 60.53435 | 46.06229 | 62.23219 | 39.01912 | 64.83329 | 55.37064 | 31.00211 | 31.82074 | 59.22278 | 31.90818 | 31.60336 |
| CUST_6718_PI429010886 | N20506 | 0.001654 | 18 | 9 | 321471179 | hypothetical protein DAPPUDRAFT_302543 [Daphn | | | 2.14E-68 | 84.81787 | 335.9236 | 265.9908 | 251.7868 | 247.961 | 108.4414 | 343.4753 | 1101.197 | 750.8373 | 671.3234 | 767.811 | 1061.463 | 984.6642 | 334.379 | 558.6606 | 478.074 | 427.4471 | 213.6068 | 168.4961 | 2108.426 | 509.3966 | 165.5033 | 444.0863 | 168.7755 | 187.869 | 391.7449 | 508.4411 |
| CUST_36842_PI429010886 | M17008 | 0.001655 | 18 | 15 | | | | | | 1539.294 | 2097.241 | 335.0674 | 1486.604 | 853.1958 | 441.0416 | 228.3459 | 610.0761 | 254.9115 | 843.5117 | 685.1065 | 469.6397 | 731.4443 | 1011.26 | 1143.075 | 1575.017 | 1045.476 | 1494.238 | 469.1551 | 1209.365 | 1175.49 | 2448.925 | 829.599 | 3134.449 | 113.9625 | 408.1397 | 532.1598 |
| CUST_8031_PI429010886 | N22445 | 0.001655 | 24 | 6 | 507936066 | PREDICTED: LOW QUALITY PROTEIN: type I iodothy | Metabolic process | Other | 1.41E-36 | 352.6729 | 367.7937 | 568.2975 | 410.4134 | 421.3888 | 195.9916 | 537.3115 | 557.9517 | 486.8632 | 337.3994 | 785.0368 | 761.7734 | 442.1751 | 305.3843 | 271.2785 | 388.0401 | 203.8319 | 298.6335 | 324.5263 | 732.314 | 294.8656 | 201.1997 | 252.9891 | 123.1377 | 191.7619 | 196.157 | 343.1257 |
| CUST_28774_PI429010886 | N58595 | 0.001655 | 24 | 18 | | | | | | 43.83694 | 38.75385 | 36.45907 | 26.54593 | 40.41132 | 41.28609 | 42.32826 | 29.35866 | 33.1071 | 48.44291 | 41.67823 | 36.44639 | 43.1527 | 38.50305 | 41.00395 | 43.68382 | 49.24687 | 43.54805 | 51.16457 | 52.34805 | 46.53779 | 64.72483 | 35.96862 | 45.99667 | 55.29319 | 49.39411 | 35.11191 |
| CUST_25996_PI429010886 | N53462 | 0.001659 | 24 | 15 | | | | | | 180.9317 | 218.1405 | 362.5859 | 84.376 | 120.1854 | 137.7783 | 275.0029 | 124.5927 | 189.6625 | 225.7224 | 162.6703 | 151.2877 | 171.0319 | 222.6303 | 159.6853 | 356.0402 | 284.2107 | 486.1656 | 236.2498 | 258.602 | 248.1135 | 254.1554 | 364.2987 | 137.1593 | 384.8393 | 153.9132 | 230.6845 |
| CUST_48681_PI429010886 | M81606 | 0.001659 | 15 | 12 | | | | | | 819.682 | 1959.594 | 1355.174 | 401.0158 | 459.9274 | 175.5219 | 2982.876 | 1841.426 | 5356.619 | 2204.613 | 836.4238 | 2970.275 | 9320.998 | 10610.68 | 8709.952 | 8904.357 | 4058.299 | 958.7977 | 1918.015 | 1632.477 | 1291.558 | 2576.961 | 1543.721 | 1777.79 | 12610.19 | 1350.099 | 18178.95 |
| CUST_1621_PI429010886 | N10255 | 0.001661 | 12 | 3 | 345484953 | PREDICTED: hypothetical protein LOC100121042 [N | | | 3.5E-25 | 4839.291 | 5888.291 | 1450.058 | 8745.677 | 5125.683 | 8117.934 | 5039.929 | 3219.948 | 4232.305 | 1435.814 | 2408.505 | 1627.802 | 4208.899 | 1849.813 | 4274.83 | 4440.858 | 4787.683 | 6100.209 | 4371.861 | 2013.592 | 2067.695 | 3886.072 | 1627.802 | 4887.374 | 2459.721 | 3905.483 | 1594.166 |
| CUST_10099_PI429010886 | N25691 | 0.001661 | 12 | 3 | 152013699 | putative cuticle protein [Artemia franciscana] | | | 1.19E-09 | 193.4709 | 85.17215 | 18.32903 | 863.1365 | 396.1095 | 1315.839 | 159.5837 | 339.9729 | 135.2525 | 6.396975 | 80.55811 | 17.58162 | 156.1682 | 57.96698 | 125.7351 | 334.0181 | 211.4751 | 482.5207 | 221.9975 | 18.58338 | 55.78991 | 124.8931 | 27.17883 | 426.3841 | 11.30649 | 449.7775 | 167.8299 |
| CUST_17510_PI429010886 | N38094 | 0.001661 | 12 | 3 | | | | | | 704.7831 | 755.8427 | 429.2473 | 879.939 | 645.5341 | 1038.465 | 660.0703 | 536.8972 | 558.6606 | 385.8062 | 438.3419 | 483.0972 | 625.1793 | 512.1412 | 729.1026 | 718.6829 | 763.8572 | 855.518 | 646.7171 | 634.1523 | 513.6722 | 635.813 | 423.5928 | 657.6448 | 431.93 | 768.7795 | 379.4393 |
| CUST_39916_PI429010886 | M46775 | 0.001674 | 18 | 9 | | | | | | 60.6077 | 56.11071 | 66.73255 | 48.33618 | 60.95703 | 50.41901 | 48.72124 | 62.583 | 55.49391 | 83.45905 | 78.86687 | 88.64024 | 79.49534 | 87.51279 | 66.60001 | 86.99215 | 71.16635 | 76.17525 | 63.65227 | 75.46943 | 69.89082 | 84.1835 | 65.88576 | 46.53779 | 82.58698 | 76.21064 | 106.4186 |
| CUST_4334_PI429010886 | N16530 | 0.001674 | 18 | 3 | | | | | | 74.97848 | 95.62622 | 137.2358 | 151.12 | 122.4667 | 115.4907 | 82.30578 | 93.9354 | 58.01161 | 94.02768 | 44.41825 | 75.25456 | 77.70756 | 83.88709 | 100.0727 | 59.78571 | 67.10317 | 47.92719 | 86.45155 | 78.30552 | 121.2697 | 95.44 | 125.1948 | 85.29111 | 81.72278 | 110.0025 | 91.27166 |
| CUST_45309_PI429010886 | M68663 | 0.001676 | 21 | 18 | | | | | | 45.61505 | 41.98854 | 31.67165 | 23.32541 | 21.70901 | 31.71188 | 30.34438 | 31.77739 | 35.03522 | 45.79823 | 35.54095 | 42.18155 | 23.55239 | 43.54805 | 41.99242 | 63.55528 | 30.94543 | 45.59444 | 41.28276 | 42.78573 | 46.37649 | 54.79286 | 37.86509 | 35.77567 | 30.10596 | 29.06792 | 38.49034 |
| CUST_52026_PI429010886 | M96128 | 0.001679 | 12 | 3 | | | | | | 13.54942 | 11.20539 | 45.62376 | 49.73229 | 25.89483 | 75.2717 | 30.48251 | 28.05565 | 13.80995 | 8.964575 | 10.57353 | 7.387333 | 17.53908 | 10.20122 | 12.22313 | 10.65167 | 52.16399 | 51.88614 | 46.22541 | 6.788713 | 9.202277 | 3.784276 | 7.277507 | 27.53905 | 9.525782 | 27.63541 | 5.979704 |
| CUST_43574_PI429010886 | M63378 | 0.001681 | 15 | 3 | 322786964 | hypothetical protein SINV_02452 [Solenopsis invict | Response to stress | Other | 3.27E-83 | 43.57816 | 29.09978 | 29.08411 | 63.78205 | 30.38651 | 89.02227 | 48.94359 | 27.85864 | 29.29818 | 39.40978 | 56.7825 | 33.51766 | 25.25877 | 25.56096 | 29.86056 | 46.74005 | 80.71386 | 52.58293 | 74.06105 | 51.84431 | 61.64233 | 52.05083 | 51.11982 | 51.69686 | 40.34891 | 45.2026 | 31.34117 |
| CUST_21217_PI429010886 | N44836 | 0.001681 | 15 | 12 | | | | | | 106.7633 | 113.8462 | 126.7035 | 68.35087 | 92.07382 | 107.9228 | 68.4981 | 85.84413 | 87.07504 | 132.5919 | 82.19809 | 171.784 | 108.1985 | 114.9975 | 156.2777 | 118.04 | 107.3414 | 123.3316 | 84.78816 | 108.5891 | 98.52867 | 105.2326 | 106.8828 | 68.56896 | 100.4962 | 108.2254 | 118.9466 |
| CUST_35714_PI429010886 | M13287 | 0.001688 | 24 | 15 | 170574609 | Zinc finger, C2H2 type family protein [Brugia malayi | | | 1.86E-32 | 12.14051 | 20.09233 | NA | 5.548767 | 5.498601 | 6.152945 | 13.68809 | 7.752552 | 18.53665 | 24.62111 | 12.30868 | 14.4735 | 14.30893 | 18.15993 | 14.63359 | 27.27727 | 15.47031 | 20.03408 | 19.38333 | 19.39216 | 7.520119 | 30.2585 | 9.322057 | 8.92426 | 13.86795 | 8.607023 | 14.20169 |
| CUST_14678_PI429010886 | N33280 | 0.001688 | 24 | 18 | | | | | | 17.25469 | 14.34788 | 13.68935 | 18.74405 | 4.094799 | 19.06154 | 12.20158 | 16.51009 | 13.97054 | 10.62972 | 8.461505 | 14.97661 | 9.719917 | 26.36808 | 5.088083 | 24.2001 | 15.7577 | 29.26863 | 23.13514 | 21.13597 | 22.38584 | 23.71505 | 19.96449 | 18.00776 | 16.21863 | 19.63631 | 19.57413 |
| CUST_44336_PI429010886 | M65656 | 0.001695 | 18 | 12 | | | | | | 15.42925 | 38.60384 | 35.85651 | 20.50362 | 14.48423 | 13.76394 | 13.74195 | 18.56074 | 21.93409 | 27.49771 | 34.86128 | 39.77522 | 30.93804 | 65.99023 | 26.50702 | 43.81006 | 16.94759 | 26.28688 | 34.02862 | 35.48044 | 23.80171 | 23.18582 | 13.07663 | 6.652893 | 19.31076 | 38.74854 | 28.87134 |
| CUST_56200_PI429010886 | M113087 | 0.001697 | 18 | 3 | 56968035 | Homarus americanus internal transcribed spacer 2 a | | | | 93952.87 | 102282.3 | 97928.01 | 107232.5 | 108910.4 | 92382.94 | 98448.52 | 106276.7 | 92382.94 | 118691.9 | 93952.87 | 105375.3 | 104856.2 | 92382.94 | 93467.7 | 83322.86 | 91927.09 | 67921.27 | 88276.1 | 82404.75 | 107761.9 | 107761.9 | 126138.3 | 111801.3 | 101693.2 | 108311.1 | 102282.3 |
| CUST_32079_PI429010886 | M3184 | 0.001697 | 18 | 15 | | | | | | 86.56723 | 95.689 | 73.29485 | 62.70814 | 67.67878 | 58.60996 | 62.6571 | 97.4551 | 80.02167 | 110.0025 | 76.94138 | 88.85357 | 83.57768 | 74.41568 | 87.5248 | 105.9444 | 83.97119 | 90.89001 | 77.1205 | 90.85633 | 77.18845 | 67.52947 | 75.11707 | 74.80865 | 77.92872 | 60.77736 | 86.54824 |
| CUST_55948_PI429010886 | M112247 | 0.001701 | 24 | 21 | | | | | | 45.47762 | 38.33878 | 37.34484 | 37.34183 | 42.84657 | 32.16644 | 41.46823 | 35.51693 | 45.39895 | 37.23147 | 29.24887 | 31.87279 | 33.67567 | 38.44451 | 42.65121 | 46.08794 | 37.68293 | 41.82918 | 48.53823 | 28.27971 | 41.67274 | 52.26081 | 42.58462 | 47.0949 | 42.1461 | 45.58985 | 43.46213 |
| CUST_35243_PI429010886 | M12030 | 0.001701 | 24 | 18 | | | | | | 641.5416 | 708.2954 | 674.4353 | 657.8288 | 628.6974 | 644.1161 | 515.4056 | 617.3843 | 611.8341 | 712.5461 | 597.0282 | 810.6402 | 582.1613 | 754.7954 | 687.7759 | 959.9717 | 649.8854 | 678.3407 | 781.7106 | 844.3643 | 808.7225 | 842.6525 | 781.7106 | 682.9708 | 898.9921 | 693.7505 | 857.1846 |
| CUST_37641_PI429010886 | M20124 | 0.001702 | 15 | 12 | | | | | | 13104.17 | 9207.498 | 9530.181 | 4253.825 | 6903.073 | 6794.71 | 7890.624 | 7060.087 | 7266.955 | 7864.497 | 7560.283 | 9726.619 | 9855.517 | 11222.08 | 9756.741 | 9541.284 | 6577.842 | 9530.181 | 5811.899 | 12584.81 | 7373.59 | 11864.38 | 7450.764 | 7338.425 | 9046.466 | 7479.639 | 9588.886 |
| CUST_22038_PI429010886 | N46311 | 0.00171 | 18 | 12 | | | | | | 13.57374 | 23.5585 | 20.28061 | 7.752552 | 6.625606 | 9.119784 | 8.534666 | 13.91147 | 17.11368 | 13.37557 | 12.85382 | 8.838077 | 15.94807 | 21.92529 | 23.03149 | 20.33392 | 14.60212 | 16.1997 | 8.726312 | 19.23258 | NA | 12.16991 | 9.55327 | 10.17288 | 12.32604 | 18.78041 | 14.21644 |
| CUST_41548_PI429010886 | M58006 | 0.00171 | 18 | 3 | | | | | | 15.9338 | 32.52687 | 11.81981 | 38.25121 | 19.37234 | 24.16344 | 14.80182 | 15.55047 | 20.07409 | 20.55898 | 11.55822 | 18.12711 | 15.72131 | 11.4768 | 14.2617 | 13.80154 | 15.1699 | 16.54136 | 18.77363 | 23.29812 | 24.77587 | 18.72449 | 30.72417 | 23.4957 | 26.95518 | 15.51227 | 21.59935 |
| CUST_14130_PI429010886 | N32372 | 0.001712 | 24 | 15 | | | | | | 404.8094 | 565.0414 | 421.3888 | 383.1748 | 375.8398 | 430.8818 | 342.1102 | 342.5053 | 286.5693 | 328.7379 | 469.28 | 466.1947 | 360.1121 | 362.1101 | 418.1195 | 741.9924 | 646.5567 | 802.795 | 582.4599 | 531.9567 | 494.0702 | 300.3777 | 266.1879 | 435.1229 | 800.6759 | 400.6975 | 611.8341 |
| CUST_35865_PI429010886 | M13753 | 0.001719 | 21 | 3 | 270004597 | hypothetical protein TcasGA2_TC003961 [Tribolium | | | 1.44E-08 | 72.59952 | 85.99449 | 77.25415 | 151.4326 | 105.5089 | 96.19258 | 80.68886 | 71.11942 | 81.27628 | 65.10535 | 48.91662 | 60.80817 | 51.73412 | 86.13317 | 33.76983 | 72.44795 | 44.69546 | 69.98559 | 438.5463 | 57.42327 | 117.3341 | 75.23604 | 122.3764 | 67.02702 | 114.8221 | 129.735 | 72.84145 |
| CUST_7496_PI429010886 | N21629 | 0.001719 | 15 | 0 | 383861539 | PREDICTED: myosin heavy chain, muscle-like isofor | | | 2.66E-18 | 260.9034 | 312.9412 | 266.9045 | 167.2954 | 256.6122 | 232.0593 | 202.4041 | 245.8233 | 217.9412 | 276.4162 | 187.8401 | 306.8772 | 243.3133 | 240.3331 | 231.6114 | 280.2255 | 344.3603 | 273.6399 | 290.4297 | 263.345 | 271.3099 | 222.2031 | 245.7365 | 204.7693 | 252.9368 | 228.1481 | 246.3468 |
| CUST_34743_PI429010886 | M10527 | 0.001719 | 18 | 9 | 242014362 | conserved hypothetical protein [Pediculus humanu | Protein metabolism | Proteolysis | 3.45E-72 | 202.005 | 197.1531 | 207.7427 | 145.4402 | 181.8416 | 160.778 | 138.2104 | 151.6818 | 197.4111 | 245.0768 | 246.2665 | 290.5429 | 200.6981 | 219.1881 | 243.9881 | 272.2987 | 210.2559 | 203.006 | 183.8985 | 345.3079 | 199.0448 | 241.0332 | 175.5504 | 184.9236 | 199.4834 | 194.7223 | 253.3046 |
| CUST_56550_PI429010886 | M114078 | 0.001719 | 18 | 3 | | | | | | 56816.44 | 73854.47 | 57772.94 | 87164.76 | 67520.86 | 81077.7 | 54053.67 | 59179.05 | 59745.46 | 64571.61 | 45577.47 | 71869.37 | 53582.06 | 58936.46 | 28752.77 | 51921.46 | 40491.1 | 47937.32 | 45970.08 | 64763.62 | 73854.47 | 62855.9 | 66128 | 62198.15 | 64277.46 | 79324.15 | 49052.57 |
| CUST_26522_PI429010886 | N54391 | 0.001719 | 18 | 3 | | | | | | 1113.282 | 1248.908 | 1798.294 | 4165.771 | 1918.897 | 3108.301 | 1374.989 | 1801.38 | 1261.41 | 1081.506 | 642.4467 | 2475.35 | 1209.365 | 1327.226 | 1056.312 | 275.6487 | 713.6188 | 1377.953 | 423.767 | 1761.883 | 1472.13 | 2304.824 | 2005.797 | 796.983 | 1447.664 | 1791.281 | 1362.62 |
| CUST_45524_PI429010886 | M69376 | 0.001721 | 24 | 21 | 471224014 | ubiquitin conjugating enzyme, putative [Ichthyopht | | | 1.8E-16 | 17.03814 | 19.2011 | 14.66496 | 15.59858 | 13.87668 | 18.32343 | 17.33525 | 18.77767 | 14.75825 | 10.80445 | 11.4747 | 6.626935 | 15.30361 | 14.32775 | 15.78045 | 35.1468 | 5.801755 | 21.52267 | 14.66262 | 4.424481 | 21.45407 | 28.49131 | 22.80223 | 42.0037 | 29.28006 | 16.82798 | 22.60454 |
| CUST_10197_PI429010886 | N25855 | 0.001724 | 15 | 12 | 431838964 | Lymphoid-specific helicase [Pteropus alecto] | | | 6.84E-07 | 53.76881 | 47.80852 | 63.22526 | 37.68617 | 31.37091 | 35.33485 | 43.71768 | 95.02448 | 91.6203 | 81.48802 | 43.62743 | 63.96858 | 66.59221 | 89.50659 | 72.69379 | 53.9799 | 68.19793 | 57.00393 | 56.63191 | 40.34891 | 40.67771 | 63.1995 | 68.34468 | 40.34341 | 83.64909 | 47.01793 | 81.36657 |
| CUST_23425_PI429010886 | N48819 | 0.001724 | 15 | 12 | | | | | | 32.86388 | 16.42533 | 33.80016 | 31.19161 | 23.33049 | 25.5782 | 22.16397 | 20.42712 | 13.60227 | 32.27766 | 20.21835 | 49.74933 | 23.41598 | 37.70765 | 45.11939 | 29.08151 | 34.63137 | 42.71532 | 13.29917 | 23.92174 | 38.8107 | 13.23813 | 18.83139 | 20.87033 | 27.65968 | 29.97696 | 41.73826 |
| CUST_29939_PI429010886 | N60852 | 0.001724 | 15 | 12 | | | | | | 50.65259 | 49.38887 | 50.52879 | 39.60553 | 29.60636 | 34.26964 | 46.22951 | 42.34172 | 56.69148 | 55.55289 | 44.20781 | 53.38747 | 51.5559 | 62.66235 | 61.19321 | 66.18886 | 54.86156 | 47.15148 | 51.93952 | 42.9717 | 43.0244 | 54.87756 | 45.93195 | 45.44604 | 60.74611 | 40.90818 | 47.19565 |
| CUST_51862_PI429010886 | M95432 | 0.001729 | 24 | 18 | | | | | | 12.0173 | 10.8979 | NA | NA | 11.56735 | 8.563205 | 7.846171 | 4.745595 | 4.989644 | 6.435319 | 11.63036 | 6.881575 | 6.740179 | 6.989719 | 5.56425 | 6.641946 | 10.10842 | 15.60693 | 10.64343 | 31.25182 | 17.74547 | 14.16669 | NA | NA | 7.882812 | NA | 14.27753 |
| CUST_43448_PI429010886 | M63091 | 0.001731 | 15 | 0 | | | | | | 358.225 | 195.1137 | 343.6779 | 252.4045 | 223.3365 | 292.4718 | 306.1523 | 217.0351 | 184.2548 | 155.1933 | 173.9653 | 113.9999 | 200.5357 | 260.055 | 200.0776 | 219.9262 | 325.7489 | 225.8926 | 346.5286 | 214.59 | 209.1269 | 254.0567 | 265.9908 | 326.7477 | 193.7403 | 152.424 | 123.1377 |
| CUST_14007_PI429010886 | N32164 | 0.001735 | 24 | 0 | | | | | | 23.2088 | 33.1071 | 30.44396 | 49.56436 | 25.8552 | 26.91205 | 32.42745 | 11.4747 | 8.670788 | 21.99795 | 14.31095 | 13.49842 | 12.01073 | 18.56806 | 11.13919 | 18.84163 | 16.86404 | 19.58019 | 26.1771 | 19.51952 | 26.25702 | 24.33679 | 28.74055 | 13.93436 | 37.12735 | 27.44183 | 17.82897 |
| CUST_54788_PI429010886 | M107682 | 0.001742 | 21 | 0 | | | | | | 20.08095 | 42.45161 | NA | 33.12696 | 16.50813 | 27.79833 | 15.06535 | 12.23764 | 4.589743 | 5.743146 | 11.28527 | NA | 21.49674 | 10.14742 | 22.29224 | 46.19013 | NA | 30.14229 | 16.37326 | NA | NA | 42.85091 | NA | 44.42212 | 14.83326 | 29.21913 | 6.706567 |
| CUST_9370_PI429010886 | N24503 | 0.00175 | 18 | 0 | | | | | | 106.2741 | 144.1452 | 104.2639 | 80.23994 | 81.54717 | 74.40778 | 78.13459 | 65.99744 | 80.48768 | 88.83369 | 77.18047 | 89.41173 | 94.65769 | 104.6405 | 82.57755 | 125.408 | 67.86664 | 98.85463 | 84.93968 | 103.1477 | 82.13647 | 111.9132 | 94.51496 | 89.60537 | 88.82378 | 61.06386 | 71.5379 |
| CUST_15552_PI429010886 | N34772 | 0.001752 | 24 | 15 | | | | | | 633.8371 | 862.1393 | 581.9863 | 639.8175 | 575.5153 | 576.5142 | 557.82 | 472.0832 | 550.6217 | 773.1832 | 617.029 | 726.329 | 569.2573 | 593.1266 | 663.1345 | 880.2112 | 770.1948 | 885.0858 | 728.0084 | 666.5369 | 646.4459 | 929.9665 | 516.0613 | 607.2425 | 858.518 | 618.6744 | 710.22 |

| Probe ID | ID | p-value | Period | Phase | EBI ID | Annotation | GO category | Go term | E-value | 0 | 0 | 0 | 3 | 3 | 3 | 6 | 6 | 6 | 9 | 9 | 9 | 12 | 12 | 12 | 15 | 15 | 15 | 18 | 18 | 18 | 21 | 21 | 21 | 24 | 24 | 24 |
|---|---|---|---|---|---|---|---|---|---|---|---|---|---|---|---|---|---|---|---|---|---|---|---|---|---|---|---|---|---|---|---|---|---|---|---|---|
| CUST_41187_PI429010886 | M57091 | 0.00176 | 15 | 9 | | | | | | 70.63158 | 91.48208 | 47.47988 | 26.16563 | 50.20569 | 50.68297 | 45.18835 | 60.47407 | 68.8749 | 89.28732 | 56.85652 | 92.38118 | 60.80176 | 45.6737 | 52.99425 | 64.77801 | 51.65313 | 45.60489 | 42.81164 | 54.64962 | 29.73496 | 49.2211 | 31.33759 | 38.28234 | 76.82831 | 75.72117 | 77.50869 |
| CUST_23438_PI429010886 | N48844 | 0.001765 | 18 | 3 | | | | | | 105.4104 | 185.6237 | 134.8627 | 512.8422 | 133.7749 | 227.3812 | 100.3042 | 86.78972 | 92.28356 | 94.06166 | 45.9505 | 182.9296 | 57.1457 | 129.6562 | 74.7266 | 51.89822 | 33.23976 | 116.3422 | 57.5238 | 76.39741 | 139.8135 | 123.794 | 195.1137 | 135.3773 | 147.098 | 132.8034 | 91.29321 |
| CUST_27356_PI429010886 | N55899 | 0.001767 | 15 | 12 | | | | | | 2088.06 | 1718.489 | 5580.063 | 616.6013 | 814.2205 | 916.8157 | 2380.879 | 9087.177 | 4297.995 | 4729.674 | 2107.396 | 4566.893 | 7701.667 | 7890.624 | 4368.248 | 1331.007 | 2645.933 | 1268.692 | 2147.931 | 2336.801 | 1744.83 | 5620.472 | 4404.238 | 2325.953 | 5372.754 | 3046.724 | 6827.005 |
| CUST_18964_PI429010886 | N40733 | 0.001772 | 21 | 3 | | | | | | 13.86351 | 13.06491 | 10.38108 | NA | 10.79421 | 15.84737 | 15.1027 | 6.673444 | 12.72931 | 12.75871 | 10.76694 | 6.101292 | 9.915982 | 10.46038 | 14.24812 | 9.928249 | 6.712633 | 9.907267 | 6.264624 | 13.24259 | 9.868667 | 15.27365 | 11.00948 | 9.993532 | 16.27641 | 13.50907 | 16.15388 |
| CUST_24296_PI429010886 | N50376 | 0.001773 | 18 | 3 | | | | | | 25.6683 | 33.42247 | 25.97694 | 39.53783 | 35.31845 | 35.83833 | 19.08808 | 26.67261 | 27.99024 | 26.83617 | 26.86002 | 17.94144 | 17.25129 | 18.13922 | 23.63048 | 38.25818 | 19.45151 | 52.08136 | 29.93088 | 31.48252 | 39.93142 | 50.33169 | 29.42815 | 33.40939 | 36.34774 | 25.97297 | 32.15263 |
| CUST_15834_PI429010886 | N35220 | 0.001773 | 24 | 21 | | | | | | 25.57234 | 11.06119 | 16.56157 | 15.24741 | 13.16084 | 6.752371 | 10.25912 | 13.27508 | 13.32777 | 21.68053 | 12.14502 | 7.832427 | 7.159766 | 7.17066 | 9.099665 | 17.93802 | 26.0521 | 14.32141 | 24.60687 | 8.176012 | 11.24362 | 32.38525 | 26.74645 | 13.57654 | 23.91945 | 22.01914 | 20.07209 |
| CUST_8477_PI429010886 | N23099 | 0.001785 | 18 | 3 | | | | | | 26.37273 | 22.84941 | 41.92727 | 41.77165 | 38.92288 | 41.41686 | 57.45413 | 12.40087 | 41.76704 | 30.19656 | 15.64186 | 30.29325 | 11.51911 | 20.49048 | 6.718366 | 27.23432 | 35.6284 | 11.71083 | 23.62598 | 32.8723 | 59.15067 | 33.56673 | 80.24894 | 42.61497 | 49.37485 | 49.81001 | 23.5201 |
| CUST_34960_PI429010886 | M11185 | 0.001788 | 18 | 15 | | | | | | 275.8944 | 355.9452 | 285.3755 | 247.0952 | 290.2221 | 296.0085 | 235.8718 | 287.2755 | 202.4642 | 327.031 | 261.6417 | 244.1579 | 249.7124 | 339.1595 | 356.8337 | 364.6302 | 356.9822 | 263.9041 | 312.8606 | 272.019 | 225.1487 | 318.8664 | 245.2785 | 219.0917 | 238.8599 | 211.5909 | 239.3501 |
| CUST_12721_PI429010886 | N29935 | 0.001788 | 18 | 9 | | | | | | 592.5758 | 495.7193 | 594.7631 | 285.8071 | 408.0489 | 428.7157 | 477.4078 | 404.3563 | 445.0547 | 686.3511 | 702.9226 | 671.3234 | 541.9679 | 516.8489 | 636.3168 | 681.8773 | 641.17 | 602.798 | 484.9716 | 495.8952 | 472.1948 | 480.1775 | 592.7227 | 559.8475 | 533.5457 | 699.0502 | 446.8256 |
| CUST_16477_PI429010886 | N36299 | 0.001788 | 18 | 15 | | | | | | 14.54005 | 17.25706 | 22.61376 | 10.81017 | 21.22623 | 16.94555 | 9.39688 | 10.91573 | 8.062208 | 30.515 | 19.92894 | 25.27977 | 17.28605 | 20.06809 | 20.00381 | 30.67432 | 21.92688 | 23.23746 | 17.86761 | 16.67878 | 11.36635 | 20.10727 | 8.830666 | 11.07003 | 17.02696 | 3.244273 | 18.20163 |
| CUST_50790_PI429010886 | M91019 | 0.001792 | 18 | 15 | 283475796 | scalloped protein, partial [Parhyale hawaiensis] | Nucleic acid metabolism | Regulation of gene expression | 1.71E-59 | 18.82343 | 19.06154 | 34.62871 | 3.928419 | 10.91945 | 18.32163 | 30.50322 | 18.69493 | 26.05952 | 17.76635 | 18.61312 | 23.31758 | 28.49131 | 25.18558 | 31.03981 | 26.93068 | 24.91029 | 34.05422 | 27.7492 | 29.09612 | 8.41226 | 15.63777 | 17.84645 | 15.35699 | 31.0325 | 23.46752 | 17.57463 |
| CUST_16591_PI429010886 | N36516 | 0.001798 | 24 | 21 | | | | | | 21.57256 | 19.25031 | 12.38626 | 23.80497 | 8.559956 | 9.09473 | 4.424908 | 4.971102 | 10.10287 | 18.90775 | 12.47217 | 15.13796 | 8.488945 | 9.676508 | 10.14742 | 24.40654 | 12.85234 | 17.27797 | NA | 19.25911 | 38.87769 | 21.07244 | 18.10067 | 19.4542 | 21.39305 | 17.50362 | 15.76954 |
| CUST_22665_PI429010886 | N47420 | 0.0018 | 24 | 0 | | | | | | 86.97645 | 61.48326 | 64.6187 | 56.37885 | 43.07866 | 57.84964 | 60.82114 | 44.18048 | 68.48249 | 60.26272 | 53.02941 | 58.99785 | 36.95565 | 53.65979 | 48.56555 | 59.48755 | 60.86834 | 58.76571 | 57.10423 | 64.3589 | 59.20737 | 84.63515 | 42.22821 | 54.4744 | 118.1621 | 62.08961 | 81.54717 |
| CUST_45180_PI429010886 | M68247 | 0.001806 | 24 | 12 | | | | | | 12.94753 | 7.72352 | 13.16261 | 7.807289 | 10.68521 | 13.94454 | 13.98352 | 16.17213 | 10.36906 | 12.88124 | 15.7975 | 17.51958 | 13.64729 | 23.69772 | 16.95326 | 10.98274 | NA | 24.08827 | 13.6708 | 12.84456 | 19.15374 | 9.457848 | 14.37562 | 10.16697 | 12.81928 | 5.223476 | 6.673444 |
| CUST_29401_PI429010886 | N59793 | 0.001806 | 21 | 15 | | | | | | 206.8694 | 192.8746 | 169.802 | 182.1441 | 183.2048 | 203.9546 | 180.1966 | 171.0565 | 160.7305 | 191.0009 | 178.9755 | 169.3227 | 194.0018 | 193.9282 | 161.5046 | 229.3254 | 235.3617 | 208.5959 | 219.5762 | 206.4448 | 175.5504 | 205.7682 | 199.3094 | 177.9616 | 212.3072 | 172.854 | 172.5457 |
| CUST_42676_PI429010886 | M60934 | 0.001809 | 15 | 9 | | | Transport | Other | | 45.86806 | 50.42448 | 32.73413 | 37.05276 | 33.52987 | 29.35151 | 51.76265 | 64.10673 | 68.0898 | 60.74611 | 52.31892 | 69.16983 | 46.93642 | 69.16983 | 69.216 | 49.45564 | 70.58805 | 45.3426 | 45.82004 | 62.01844 | 44.7891 | 55.85206 | 39.63103 | 50.79816 | 74.316 | 50.84587 | 71.77967 |
| CUST_10763_PI429010886 | N26749 | 0.001812 | 21 | 18 | 260824441 | hypothetical protein BRAFLDRAFT_118643 [Branchi | | | 2.82E-78 | 9.712939 | 7.643809 | NA | 17.15702 | 5.608378 | NA | 6.963361 | 5.756675 | 4.701277 | 9.135099 | 4.035562 | 4.297229 | 12.98781 | 13.40222 | 9.810562 | 11.23845 | 11.2915 | 13.22555 | 12.42241 | 108.1985 | NA | 6.256959 | 11.6989 | NA | 7.437557 | 7.88886 | 7.544684 |
| CUST_48126_PI429010886 | M79000 | 0.001817 | 18 | 0 | | | | | | 102.8187 | 63.42473 | 86.58356 | 18.3383 | 62.54934 | 28.6993 | 40.30994 | 44.25502 | 53.26961 | 58.55654 | 34.60204 | 49.16013 | 54.69269 | 45.2318 | 46.11982 | 87.57209 | 62.44513 | 73.29485 | 56.68336 | 64.08758 | 65.47772 | 59.0899 | 87.899 | 31.90127 | 42.12986 | 48.30048 | 29.02633 |
| CUST_11358_PI429010886 | N27684 | 0.001817 | 21 | 3 | | | | | | 12.23764 | 9.554371 | 14.02425 | 17.23326 | 24.72089 | 17.25337 | 13.28694 | 13.15128 | 12.65519 | 17.33078 | 9.896666 | 12.82338 | 15.1999 | 15.21234 | 12.78901 | 8.847678 | 6.477097 | 11.2694 | 12.81312 | 9.447649 | 12.04766 | 17.61145 | 23.3977 | 5.638886 | 14.04066 | 16.40766 | 14.70096 |
| CUST_56081_PI429010886 | M112708 | 0.001821 | 21 | 3 | | | | | | 958.279 | 1644.909 | 1251.807 | 2608.992 | 1402.469 | 1469.038 | 1073.06 | 854.4458 | 1045.476 | 1296.243 | 609.6635 | 1020.365 | 409.4886 | 884.6458 | 758.1826 | 1137.917 | 428.9575 | 908.1498 | 534.723 | 587.9777 | 1901.328 | 749.2477 | 1918.897 | 866.5099 | 1982.266 | 1610.651 | 921.275 |
| CUST_13225_PI429010886 | N30811 | 0.00183 | 21 | 18 | 67083823 | vitamin K epoxide reductase complex subunit 1 pre | | | 8.46E-39 | 143.8077 | 119.3258 | 160.804 | 99.2894 | 107.884 | 89.59424 | 109.4422 | 105.2191 | 113.1427 | 157.6076 | 125.9453 | 180.4354 | 129.8119 | 136.2923 | 140.8807 | 199.8275 | 95.7582 | 131.1566 | 138.133 | 151.9486 | 144.2774 | 166.1651 | 136.6153 | 127.6888 | 120.1521 | 94.79905 | 122.8849 |
| CUST_38289_PI429010886 | M25061 | 0.001832 | 15 | 12 | 7320913 | masquerade-like protein [Pacifastacus leniusculus] | Protein metabolism | Proteolysis | 1.99E-47 | 4506.751 | 4471.709 | 4955.555 | 2587.199 | 1713.527 | 803.3312 | 4873.025 | 4201.227 | 6011.008 | 6474.137 | 2020.188 | 4823.273 | 8188.092 | 6336.037 | 8024.987 | 9193.951 | 4553.274 | 2932.434 | 3020.819 | 4992.636 | 2679.021 | 5553.587 | 5534.91 | 2906.073 | 12422.81 | 3187.857 | 13649.38 |
| CUST_11282_PI429010886 | N27556 | 0.001834 | 18 | 12 | | | | | | 315.3064 | 315.7757 | 302.6146 | 267.5328 | 269.6262 | 266.316 | 349.4134 | 249.3051 | 353.785 | 366.9733 | 290.8954 | 358.4083 | 423.4903 | 537.0505 | 423.0023 | 438.9413 | 320.2376 | 323.3503 | 417.0088 | 268.588 | 371.903 | 403.5814 | 303.854 | 265.2111 | 429.6585 | 294.9448 | 511.0593 |
| CUST_36319_PI429010886 | M15574 | 0.001837 | 21 | 18 | | | | | | 423.1161 | 475.5299 | 392.5464 | 422.4541 | 334.3349 | 304.0369 | 344.0991 | 271.1281 | 285.0949 | 378.3563 | 294.7498 | 417.2023 | 370.13 | 446.2007 | 447.351 | 478.2055 | 309.2864 | 355.2567 | 448.5246 | 445.1412 | 380.9707 | 537.4529 | 382.5164 | 395.2528 | 333.6383 | 375.5434 | 348.178 |
| CUST_55894_PI429010886 | M112034 | 0.001845 | 21 | 3 | | | | | | 13.97697 | 10.82077 | 12.96922 | 12.36577 | 13.70684 | 20.75541 | 8.287779 | 13.14971 | 14.05746 | 17.46899 | 11.91263 | 13.10203 | 7.451482 | 11.59303 | 6.417851 | 17.08553 | 4.733482 | 13.55604 | 5.452395 | 13.29212 | 10.04101 | 11.6401 | 14.28145 | 19.10728 | 17.54669 | 22.85123 | 19.38333 |
| CUST_42208_PI429010886 | M59692 | 0.001851 | 15 | 0 | | | | | | 12.49899 | NA | 8.167031 | NA | 11.77644 | 14.03086 | NA | 7.159766 | 7.678477 | 5.654986 | 4.166725 | 4.430407 | 10.70475 | 10.99683 | 7.0824 | 6.102462 | 21.3197 | 12.40589 | 10.2375 | NA | 6.990585 | 7.842686 | 6.048524 | 17.23883 | 4.885718 | 7.556212 | 4.875781 |
| CUST_21593_PI429010886 | N45491 | 0.001851 | 12 | 9 | | | | | | 18.60572 | 11.68718 | 9.560965 | 25.00865 | 4.917976 | 8.880423 | 6.902769 | 41.48365 | 8.585981 | 45.73763 | 25.54204 | 40.98995 | 11.18034 | 10.74554 | 10.68521 | 12.89444 | 6.177678 | 3.422715 | 11.54499 | 9.418869 | 4.025715 | 34.35609 | 27.01225 | NA | 14.39787 | 18.37554 | 12.98669 |
| CUST_44049_PI429010886 | M64763 | 0.001853 | 15 | 12 | | | | | | 140.3479 | 134.1249 | 149.8707 | 116.7797 | 102.8077 | 93.66147 | 110.6582 | 110.3267 | 118.0698 | 148.9974 | 125.5654 | 146.3194 | 110.3957 | 164.3802 | 147.4899 | 140.6597 | 98.05949 | 150.7391 | 107.3899 | 116.3422 | 125.3729 | 136.8075 | 118.7113 | 110.4288 | 145.4981 | 138.5342 | 192.8746 |
| CUST_51322_PI429010886 | M93072 | 0.001853 | 15 | 12 | | | | | | 34.70757 | 25.69538 | 33.42828 | 11.27249 | 27.74158 | 11.95869 | 14.05335 | 32.87464 | 43.37513 | 36.71638 | 26.6839 | 26.52223 | 34.78955 | 58.23835 | 33.7538 | 32.64236 | 28.34801 | 18.15141 | 21.70044 | 24.03363 | 30.85363 | 32.01382 | 24.01772 | 20.96144 | 49.0094 | 32.41392 | 59.55765 |
| CUST_32608_PI429010886 | M4562 | 0.001854 | 24 | 15 | 443728134 | hypothetical protein CAPTEDRAFT_180561 [Capitell | | | 8.25E-97 | 55.6145 | 36.04391 | 53.62183 | 21.20179 | 35.53745 | 36.86394 | 42.31429 | 43.97432 | 35.89324 | 44.43928 | 59.6278 | 52.00681 | 39.18988 | 48.45867 | 43.56595 | 47.06225 | 86.7376 | 48.98425 | 62.36898 | 56.89076 | 38.89335 | 39.52535 | 59.22278 | 62.96106 | 38.25818 | 35.97087 | 48.13849 |
| CUST_8992_PI429010886 | N23900 | 0.001857 | 18 | 0 | | | | | | 106.5352 | 46.95122 | 133.4278 | 44.48912 | 45.45196 | 40.53465 | 59.48243 | 53.22735 | 33.94751 | 42.52496 | 25.75668 | 20.29379 | 40.32605 | 26.75249 | 36.12998 | 34.71503 | 79.13143 | 27.72618 | 86.91384 | 36.63834 | 67.46884 | 31.54852 | 73.95574 | 69.2413 | 15.11766 | 23.35223 | 27.87681 |
| CUST_36158_PI429010886 | M14836 | 0.001868 | 24 | 18 | 328719285 | PREDICTED: band 4.1-like protein 5-like [Acyrthosip | | | 1.08E-21 | NA | 7.994315 | 12.21859 | 7.184973 | 5.636736 | 5.914907 | NA | 11.72847 | NA | 6.820714 | 5.489718 | 6.684024 | NA | 8.897078 | 7.022738 | 43.33449 | 8.309227 | 12.73861 | NA | 13.13514 | NA | 8.297472 | 12.36577 | 15.18207 | 12.00666 | 7.228787 | 4.917573 |
| CUST_45529_PI429010886 | M69392 | 0.001869 | 21 | 3 | 528521826 | PREDICTED: CCR4-NOT transcription complex subur | Nucleic acid metabolism | Regulation of gene expression | 2.07E-19 | 24.32573 | 47.26675 | 25.88533 | 81.86063 | 34.47136 | 56.22954 | 26.49443 | 35.09192 | 27.95174 | 22.96785 | 13.51311 | 36.37157 | 13.09346 | 46.45397 | 12.89444 | 23.54635 | 16.11142 | 27.68808 | 16.03532 | 18.18164 | 48.22079 | 25.56608 | 47.10091 | 24.04991 | 51.80467 | 40.74077 | 29.64209 |
| CUST_26986_PI429010886 | N55227 | 0.001872 | 24 | 0 | 443694929 | hypothetical protein CAPTEDRAFT_92139 [Capitella | Signal transduction | Intracellular signal transduction | 6.24E-17 | 16.16098 | 16.4753 | 15.16191 | 11.0429 | 12.85919 | 6.62121 | 10.06298 | 18.42118 | 8.413487 | 12.27296 | 17.4379 | 7.538194 | NA | NA | 9.965323 | 8.161195 | 5.274791 | 14.39574 | 15.13796 | 8.972594 | 9.339132 | 17.3225 | 20.35771 | 13.92025 | 22.63022 | 22.64961 | 26.48831 |
| CUST_24772_PI429010886 | N51228 | 0.001878 | 21 | 0 | | | | | | 31.78356 | 26.58577 | 38.32695 | 25.44661 | 20.40858 | 28.47733 | 23.64694 | 21.68228 | 24.39614 | 21.28517 | 20.8433 | 14.62912 | 11.78251 | 22.64182 | 25.42895 | 31.76384 | 18.48661 | 74.4613 | 27.7391 | 29.10818 | 21.77287 | 28.45405 | 25.9871 | 29.28327 | 27.87322 | 23.32541 | 17.22674 |
| CUST_35576_PI429010886 | M12933 | 0.001882 | 15 | 9 | 383855296 | PREDICTED: vacuolar protein sorting-associated pro | | | 1.98E-30 | 93.17664 | 94.41213 | 146.2811 | 68.14294 | 77.1205 | 53.91424 | 93.49845 | 128.7498 | 77.72793 | 180.731 | 131.8938 | 135.3397 | 137.2513 | 154.6801 | 115.9016 | 129.896 | 111.4482 | 111.637 | 161.5934 | 110.6582 | 100.468 | 111.5107 | 138.6787 | 102.2413 | 150.242 | 117.4071 | 177.758 |
| CUST_46535_PI429010886 | M72929 | 0.001882 | 15 | 0 | | | | | | 50.47205 | 27.43923 | 35.00516 | 35.63872 | 31.82074 | 26.16206 | 27.40178 | 25.83743 | 25.19666 | 40.64326 | 34.32332 | 22.18561 | 34.39152 | 28.62456 | 28.01496 | 43.60712 | 34.58379 | 38.24021 | 35.44133 | 31.31344 | 39.79718 | 24.35068 | 29.68582 | 16.48468 | 33.44293 | 18.32343 | 31.81697 |
| CUST_14142_PI429010886 | N32388 | 0.001885 | 21 | 15 | | | | | | 198.4783 | 202.0834 | 168.6167 | 100.5206 | 162.9465 | 136.4108 | 137.4942 | 103.2145 | 114.2624 | 157.5503 | 157.0047 | 121.1298 | 111.1235 | 130.3328 | 139.8135 | 249.3844 | 175.6005 | 200.8366 | 155.7184 | 215.8293 | 181.2412 | 202.301 | 182.8475 | 136.2319 | 178.0976 | 148.9131 | 156.9838 |
| CUST_11525_PI429010886 | N27969 | 0.00189 | 12 | 6 | | | | | | 8.618402 | 6.699155 | 14.1603 | 10.01823 | 19.01018 | NA | 16.32704 | 16.4683 | 12.90641 | 13.15317 | 27.06658 | 11.54827 | NA | 4.702682 | 6.9339 | 11.28527 | 14.86089 | 11.75603 | 18.4372 | 14.73825 | 16.69153 | 18.65856 | 12.64728 | NA | 5.277704 | 9.712939 | NA |
| CUST_4942_PI429010886 | N17855 | 0.001893 | 24 | 0 | | | | | | 65.80017 | 61.11029 | 57.60743 | 58.8975 | 38.06764 | 53.38747 | 44.01861 | 40.57649 | 50.403 | 39.40002 | 30.64706 | 35.83044 | 28.36576 | 58.92238 | 51.02755 | 55.99321 | 36.96378 | 65.13281 | 48.08498 | 49.52577 | 48.48483 | 53.0654 | 58.36485 | 48.99962 | 69.07358 | 43.74674 | 61.53942 |
| CUST_51324_PI429010886 | M93077 | 0.001893 | 21 | 3 | | | | | | 7470.617 | 12563.99 | 11524.4 | 24478.23 | 15271.23 | 18337.68 | 6350.776 | 7154.842 | 11729.06 | 9715.562 | 6840.698 | 17436.39 | 7435.628 | 7212.951 | 5454.8 | 12313.28 | 4909.967 | 10819.14 | 4353.113 | 9839.748 | 14054.76 | 7901.818 | 13177.04 | 10729.62 | 15389.68 | 20138.4 | 10259.45 |
| CUST_26190_PI429010886 | N53824 | 0.001896 | 24 | 21 | | | | | | 15.84016 | 23.12649 | 17.78837 | 13.10821 | 15.88827 | 10.78323 | 9.54588 | 12.79498 | 10.93186 | 14.88541 | 11.89545 | 24.73576 | 17.51307 | 15.16804 | 13.85408 | 26.0056 | 12.08102 | 19.80692 | 15.9031 | 18.15993 | 20.25674 | 22.45323 | 17.33525 | 21.8835 | 26.47171 | 15.42925 | 14.61408 |
| CUST_52516_PI429010886 | M97743 | 0.001896 | 15 | 12 | | | | | | 402.5662 | 387.8661 | 481.1352 | 267.7292 | 261.8822 | 170.8077 | 298.3902 | 347.3822 | 420.2091 | 518.4033 | 322.9938 | 383.7894 | 391.4581 | 563.6523 | 368.2489 | 350.8381 | 334.4771 | 286.6201 | 383.8497 | 246.1531 | 286.4677 | 364.5694 | 383.225 | 253.3366 | 535.7875 | 298.0928 | 410.3497 |
| CUST_39940_PI429010886 | M47110 | 0.001904 | 21 | 9 | 18266071 | collagenolytic serine protease [Paralithodes camtsc | Protein metabolism | Proteolysis | 4.48E-29 | 39.126 | 34.15826 | 58.14864 | 50.90637 | 43.18546 | 65.95253 | 68.24733 | 81.16843 | 69.68123 | 67.68545 | 87.63509 | 79.49534 | 48.39289 | 61.95208 | 57.92486 | 46.39793 | 81.73916 | 55.47209 | 53.20016 | 84.85912 | 64.98263 | 29.45836 | 62.66235 | 47.80852 | 60.5193 | 72.05058 | 62.26361 |
| CUST_6232_PI429010886 | N19820 | 0.001909 | 18 | 12 | 443719661 | hypothetical protein CAPTEDRAFT_160702 [Capitell | Metabolic process | Lipid metabolic process | 3.3E-114 | 329.7729 | 329.0332 | 379.6269 | 247.6419 | 295.7576 | 202.438 | 291.5581 | 488.7111 | 437.8204 | 480.665 | 440.5078 | 409.0918 | 513.2283 | 497.8615 | 409.4886 | 366.089 | 402.6363 | 280.5674 | 495.8952 | 639.1213 | 399.5497 | 246.4302 | 403.6614 | 210.3675 | 310.0283 | 419.5293 | 527.7394 |
| CUST_54948_PI429010886 | M108233 | 0.001909 | 12 | 0 | | | | | | 263.2372 | 229.6568 | 309.9578 | 214.4744 | 235.7566 | 219.7388 | 211.0494 | 244.9337 | 224.4715 | 249.9317 | 248.7921 | 261.6019 | 234.7869 | 269.1187 | 107.7093 | 218.5128 | 222.578 | 207.9553 | 223.377 | 243.718 | 260.8562 | 300.9186 | 237.1286 | 263.0952 | 299.7277 | 265.6599 | 329.0332 |
| CUST_3884_PI429010886 | N15483 | 0.001912 | 18 | 3 | | | | | | 68.88215 | 213.5752 | 85.62172 | 364.2987 | 296.3588 | 271.0212 | 113.8579 | 444.9718 | 7.952703 | 15.28613 | 268.7143 | NA | 6.00037 | 14.44266 | 26.11716 | NA | 4.74894 | NA | 61.79791 | 9.816503 | 83.27348 | NA | 193.714 | 201.2454 | NA | 30.16973 | 8.394638 |
| CUST_54842_PI429010886 | M107927 | 0.001912 | 21 | 12 | | | | | | 25.71165 | 20.16852 | 17.7649 | 16.40453 | 14.05126 | 9.035702 | 11.74472 | 11.83564 | 12.92501 | 19.24305 | 21.68053 | 26.61439 | 24.35068 | 29.50649 | 31.40553 | 26.36808 | 20.04961 | 26.45722 | 26.62548 | 18.57367 | 20.11281 | 28.3396 | 24.9077 | 23.59869 | 43.79306 | 15.50269 | 19.3376 |
| CUST_43013_PI429010886 | M61855 | 0.001918 | 15 | 12 | 7320913 | masquerade-like protein [Pacifastacus leniusculus] | Protein metabolism | Proteolysis | 3.51E-19 | 2604.753 | 2807.026 | 3202.001 | 1483.13 | 1090.829 | 547.465 | 3432.751 | 2573.258 | 3448.497 | 3558.109 | 1277.616 | 2690.41 | 5411.773 | 5510.561 | 3667.985 | 4228.899 | 3044.565 | 1542.825 | 2229.117 | 2626.728 | 1789.616 | 4667.789 | 4080.667 | 1611.105 | 7890.624 | 1629.467 | 8117.934 |
| CUST_24271_PI429010886 | N50330 | 0.001922 | 24 | 21 | | | | | | 105.8718 | 137.4942 | 176.7754 | 146.6109 | 119.585 | 167.2734 | 86.86206 | 123.5093 | 117.3341 | 121.8439 | 105.8984 | 119.5328 | 110.4435 | 152.3775 | 147.4281 | 183.9501 | 126.0834 | 161.0763 | 159.8607 | 155.4149 | 164.5164 | 184.458 | 145.1303 | 164.3967 | 181.1566 | 163.791 | 157.7275 |
| CUST_14677_PI429010886 | N33278 | 0.001928 | 24 | 15 | | | | | | 8.756045 | 11.51323 | NA | 12.90472 | 11.6754 | 6.900525 | 10.21556 | 5.508579 | 7.881658 | 25.42895 | 14.63359 | 6.755699 | 10.37567 | 26.52223 | 14.80809 | 19.80692 | 26.13131 | 15.31913 | 17.61017 | 20.43695 | 9.444187 | 18.64838 | NA | NA | 15.31326 | 14.19523 | 10.08291 |
| CUST_37939_PI429010886 | M22365 | 0.001931 | 15 | 9 | 532759720 | antimicrobial peptide type 1 precursor Ic [Pandalop | | | 4.14E-19 | 15.51227 | 119.7645 | 15.1027 | NA | 45.03054 | 14.01418 | 758.1826 | 50.99575 | 756.7863 | 117.3497 | 86.46205 | 262.2092 | 555.7341 | 559.3835 | 325.3686 | 453.8761 | 71.55597 | 31.86956 | 70.55676 | 6.082494 | 73.85269 | 126.7933 | 72.58192 | 67.64754 | 1139.145 | 369.2549 | 807.7825 |
| CUST_5960_PI429010886 | N19428 | 0.001947 | 24 | 18 | | | Metabolic process | Generation of precursor metabolites and energy | | 807.7825 | 1175.839 | 886.7923 | 710.4011 | 805.0049 | 678.5082 | 724.7977 | 813.4135 | 754.5887 | 1031.135 | 671.0656 | 894.0864 | 839.8931 | 914.9994 | 968.7311 | 1071.809 | 851.7359 | 918.6617 | 939.4252 | 1060.588 | 913.4609 | 1003.294 | 898.9921 | 932.1915 | 879.939 | 707.1584 | 1003.971 |
| CUST_39906_PI429010886 | M46634 | 0.001957 | 12 | 9 | | | | | | 77.3067 | 476.4015 | 268.545 | 121.0793 | 18.03617 | NA | 61.95208 | 237.166 | 271.1281 | 294.8062 | 352.7559 | 493.215 | 127.2574 | 248.1135 | 168.3078 | 100.1156 | 47.8959 | 46.02004 | 224.9139 | 460.6363 | 239.9392 | 281.9612 | 315.1705 | 8.07676 | 177.9616 | 253.6933 | 103.3395 |
| CUST_50267_PI429010886 | M89235 | 0.001961 | 24 | 3 | | | | | | 25.38492 | 20.99142 | 55.08741 | 63.1832 | 42.40387 | 48.95474 | 36.8879 | 20.37065 | 38.06764 | 20.59519 | 10.47118 | 31.85611 | 11.22985 | 32.66252 | NA | 14.86823 | 17.42213 | 3.653594 | 3.853904 | 10.53669 | 87.97637 | 19.11687 | 85.45263 | 23.81072 | 35.25245 | 32.56779 | 29.69737 |
| CUST_53470_PI429010886 | M101848 | 0.001969 | 21 | 18 | | | | | | 40.34341 | 64.62304 | 41.8887 | 61.55497 | 55.30305 | 31.46479 | 24.49566 | 27.49426 | 31.70959 | 45.29251 | 35.77964 | 47.39274 | 44.83381 | 30.01858 | 35.20549 | 71.58792 | 30.37957 | 47.80852 | 63.83268 | 68.77778 | 70.77767 | 61.6776 | 32.90861 | 24.67398 | 43.18546 | 31.59756 | 36.13819 |
| CUST_9709_PI429010886 | N25078 | 0.00197 | 24 | 3 | | | | | | 231.3049 | 5075.28 | 270.2714 | 6787.522 | 3934.354 | 2884.034 | 2558.435 | 3775.991 | 22.93214 | 62.91706 | 2941.835 | 53.44251 | 115.0894 | 873.2696 | 2119.433 | 42.61497 | 37.75935 | 40.94647 | 265.9908 | 131.6288 | 697.0634 | NA | 897.9401 | 864.8993 | NA | NA | 41.67823 |
| CUST_12851_PI429010886 | N30154 | 0.001972 | 18 | 15 | | | | | | 253.3806 | 272.1441 | 317.2703 | 187.7096 | 262.997 | 346.8405 | 165.2714 | 201.2454 | 193.1525 | 264.6314 | 175.8715 | 354.1413 | 228.4344 | 254.0567 | 240.1418 | 330.0503 | 279.5586 | 319.745 | 221.1299 | 248.8723 | 173.881 | 250.9845 | 296.6828 | 248.652 | 181.8416 | 185.168 | 158.2669 |
| CUST_20650_PI429010886 | N43819 | 0.001983 | 18 | 12 | 380013584 | PREDICTED: vascular endothelial growth factor rece | Protein metabolism | Cellular protein modification process | 1.81E-16 | 49.34559 | 50.13498 | 46.29899 | 44.16686 | 34.58379 | 46.13321 | 68.85742 | 32.36622 | 49.39411 | 46.20496 | 37.90169 | 55.78991 | 65.63314 | 87.46258 | 73.03522 | 65.49916 | 48.2905 | 46.61273 | 84.30944 | 25.38492 | 46.25392 | 44.06771 | 31.6776 | 39.69657 | 61.19321 | 33.61732 | 81.22317 |
| CUST_21794_PI429010886 | N45867 | 0.001987 | 21 | 0 | | | | | | 55.18575 | 47.71565 | 64.68958 | 25.84583 | 29.17357 | 36.387 | 33.11671 | 49.63154 | 37.69372 | 30.0232 | 30.19994 | 27.01225 | 29.42815 | 26.86268 | 28.65923 | 24.56592 | 40.69465 | 82.01377 | 35.96518 | 66.45252 | 45.86308 | 32.35284 | 291.5581 | 110.4575 | 33.7538 | 55.36449 | 33.24975 |
| CUST_523_PI429010886 | N5820 | 0.001987 | 24 | 21 | | | | | | 3225.426 | 6621.858 | 3128.702 | 3090.22 | 3138.529 | 3074.469 | 2097.241 | 2408.505 | 1960.986 | 3965.108 | 2693.745 | 3809.024 | 2483.895 | 2132.178 | 774.9868 | 4925.566 | 3067.321 | 3443.242 | 3332.072 | 3869.78 | 2456.459 | 5367.717 | 4036.57 | 3347.664 | 5836.719 | 3269.374 | 4036.57 |
| CUST_49816_PI429010886 | M87284 | 0.00199 | 18 | 0 | | | | | | 19.88848 | 9.601564 | 22.56443 | 17.06586 | 14.78673 | 10.41462 | 9.020299 | 6.616037 | 7.743875 | 12.32604 | 16.23539 | 21.7799 | 9.31137 | 15.2558 | 13.2803 | 18.84012 | 13.9022 | 19.15845 | 18.88381 | 16.43668 | 17.39272 | 15.11963 | 14.30234 | 11.26503 | 18.10243 | 6.574274 | 5.194327 |
| CUST_9456_PI429010886 | N24657 | 0.001996 | 12 | 0 | 17529698 | endonuclease/reverse transcriptase [Branchiostom | Nucleic acid metabolism | DNA replication | 9.62E-16 | 216.8379 | 227.642 | 243.7531 | 212.4411 | 216.0426 | 216.4729 | 171.0028 | 198.7745 | 195.0054 | 224.0471 | 198.0625 | 208.9685 | 222.578 | 278.9452 | 193.0602 | 264.9674 | 204.4866 | 217.8082 | 192.0215 | 223.5364 | 175.4136 | 251.9218 | 229.9564 | 205.4682 | 281.8261 | 244.5361 | 232.6257 |
| CUST_6886_PI429010886 | N20733 | 0.001997 | 18 | 0 | | | | | | 71.91218 | 94.06166 | 72.6348 | 94.42153 | 54.26471 | 64.61143 | 38.35427 | 57.92964 | 52.20238 | 91.30112 | 62.99128 | 78.50617 | 41.0381 | 77.7747 | 67.67314 | 70.36778 | 74.08523 | 67.02702 | 90.03399 | 119.245 | 96.4587 | 63.32035 | 58.60431 | 59.69745 | 73.19849 | 44.42567 | 62.5636 |
| CUST_11676_PI429010886 | N28222 | 0.001997 | 21 | 0 | | | | | | 1017.693 | 950.8038 | 1123.855 | 698.2239 | 753.7243 | 891.3926 | 646.5567 | 645.5341 | 702.9226 | 722.2644 | 907.4672 | 789.4136 | 597.5638 | 798.0318 | 817.7915 | 928.4158 | 941.2341 | 959.6386 | 1078.601 | 772.1398 | 918.6617 | 862.6591 | 818.9073 | 889.7966 | 917.4556 | 686.5061 | 973.2028 |
| CUST_21575_PI429010886 | N45456 | 0.001998 | 24 | 18 | | | | | | 52.47732 | 53.80851 | 45.39895 | 29.97309 | 32.31627 | 34.01329 | 28.22332 | 36.94269 | 36.25859 | 41.56303 | 39.40978 | 46.84472 | 32.75472 | 52.52687 | 41.20481 | 41.14845 | 50.38632 | 50.95506 | 51.20753 | 67.40392 | 55.39664 | 26.4818 | 46.06229 | 39.27986 | 65.59001 | 33.16699 | 56.38454 |
| CUST_37052_PI429010886 | M17696 | 0.001999 | 15 | 3 | | | | | | 122.0703 | 123.4738 | 124.3644 | 112.8802 | 120.3338 | 157.1911 | 131.9258 | 97.96017 | 120.8733 | 104.2505 | 105.4781 | 123.8085 | 90.59403 | 82.77162 | 102.9064 | 137.2358 | 116.9236 | 118.5239 | 140.7563 | 138.7624 | 136.6153 | 132.7741 | 100.3487 | 165.1069 | 96.5915 | 103.6887 | 130.9315 |
| CUST_11151_PI429010886 | N27350 | 0.002002 | 18 | 3 | 453088456 | hypothetical protein SEPMUDRAFT_145731 [Sphae | | | 5.89E-07 | 31.39582 | 18.59683 | 26.77489 | 46.9717 | 42.96262 | 100.668 | 33.0225 | 27.97946 | 20.84115 | 18.24285 | 32.28425 | 25.81311 | 18.14003 | 12.44781 | 10.11773 | 31.34117 | 79.21992 | 67.55015 | 45.52189 | 25.41068 | 29.13011 | 26.83617 | 35.00193 | 53.9049 | 22.46932 | 46.61273 | 26.11497 |
| CUST_4335_PI429010886 | N16533 | 0.002003 | 12 | 0 | | | | | | 36.06457 | 43.11747 | 52.01848 | 21.08658 | 20.35771 | 15.02774 | 22.22394 | 27.89681 | 24.14576 | 51.74182 | 22.34365 | 62.92532 | 27.55378 | 56.25359 | 38.86489 | 15.55875 | 9.651747 | 18.35726 | 40.68573 | 37.53618 | 24.40654 | 45.33363 | 16.72612 | 8.3117 | 39.24656 | 46.79377 | 46.629 |
| CUST_48961_PI429010886 | M83216 | 0.002021 | 24 | 0 | | | | | | 85.05317 | 69.80469 | 85.73944 | 95.689 | 69.35049 | 77.91084 | 75.3452 | 61.29672 | 61.63016 | 54.34169 | 62.21773 | 47.44495 | 56.00982 | 66.022 | 44.4293 | 78.98243 | 68.41131 | 59.46444 | 91.60957 | 57.82155 | 72.4658 | 78.70239 | 71.73214 | 73.55823 | 84.96672 | 56.80732 | 98.60004 |
| CUST_16367_PI429010886 | N36116 | 0.002021 | 18 | 12 | | | | | | 9.55551 | 11.68238 | 9.814804 | 6.752371 | 5.950777 | 5.707653 | 10.80175 | NA | 12.95945 | 11.41324 | 11.75603 | 18.91365 | 8.388399 | 20.07209 | 18.94172 | 14.99955 | NA | 13.84485 | 10.22054 | 10.45943 | 12.86372 | 12.83173 | 6.180449 | 8.326131 | 15.20148 | 27.06117 | 12.98903 |
| CUST_8903_PI429010886 | N23773 | 0.002027 | 12 | 0 | 91090228 | PREDICTED: similar to GA20768-PA [Tribolium casta | | | 3.4E-23 | 65.64906 | 69.95364 | 69.93057 | 52.85191 | 42.98059 | 51.10924 | 40.32605 | 46.81158 | 50.33907 | 65.45683 | 53.41262 | 93.66147 | 194.1492 | 62.54347 | 64.94243 | 88.47454 | 38.93338 | 45.09375 | 56.48454 | 51.92326 | 54.41242 | 67.31728 | 61.911 | 63.13278 | 62.05533 | 62.4741 | 60.26917 |
| CUST_50437_PI429010886 | M89819 | 0.002028 | 12 | 9 | | | | | | 75.7764 | 139.545 | 86.97645 | 72.3461 | 113.1265 | 68.2359 | 88.86362 | 107.0931 | 74.87405 | 114.6834 | 111.1235 | 142.2307 | 93.72794 | 69.16053 | 88.71715 | 83.48026 | 54.37221 | 106.4846 | 119.6576 | 285.6495 | 111.2973 | 137.9515 | 161.2028 | 157.4775 | 130.6003 | 87.40211 | 123.1539 |
| CUST_39596_PI429010886 | M41608 | 0.002029 | 15 | 9 | | | | | | 2826.022 | 2199.321 | 8404.925 | 324.5803 | 358.6229 | 99.73067 | 2975.526 | 11926.23 | 9686 | 13673.74 | 3742.075 | 9305.385 | 11838.35 | 17234.2 | 10130.67 | 7002.188 | 6490.163 | 1277.616 | 3659.313 | 8504.473 | 2926.98 | 13415.53 | 12354.92 | 543.1668 | 22231.21 | 6536.631 | 21011.69 |
| CUST_9084_PI429010886 | N24040 | 0.002034 | 15 | 0 | | | | | | 58.70799 | 28.14633 | 41.44167 | 27.384 | 36.90242 | 55.37064 | 37.35358 | 31.01661 | 31.79359 | 34.20754 | 26.02914 | 25.86598 | 34.16739 | 23.33262 | 31.33759 | 43.49365 | 50.7592 | 40.26663 | 46.83015 | 26.82962 | 39.87971 | 23.23883 | 43.60712 | 35.7998 | 30.77101 | 18.27067 | 21.92688 |
| CUST_21157_PI429010886 | N44732 | 0.002045 | 21 | 0 | | | | | | 17.17661 | 7.357022 | NA | 9.96806 | 10.79319 | 9.722181 | 9.610816 | 6.538957 | 4.689232 | 6.598712 | NA | 4.458762 | 5.460269 | NA | 5.009581 | 7.538194 | 10.07706 | 10.86011 | 7.316619 | 13.62607 | 9.071169 | NA | 11.73596 | NA | 13.96315 | 7.040982 | 12.42241 |
| CUST_44372_PI429010886 | M65738 | 0.002047 | 18 | 6 | | | | | | 11.40559 | 5.247077 | 14.59759 | 13.27508 | 12.0863 | 5.429174 | 18.19818 | 33.48853 | 25.01541 | 14.66789 | 6.085618 | 9.904227 | 16.10051 | 21.03394 | 13.21125 | 13.28694 | 11.65616 | 8.991035 | 6.090696 | 4.886513 | 10.74985 | 15.49062 | 15.68172 | 17.83632 | 26.54593 | 8.896204 | 59.92145 |
| CUST_23663_PI429010886 | N49229 | 0.00205 | 24 | 21 | | | | | | 30.75954 | 34.03924 | 27.41533 | 14.59227 | 21.70738 | 25.41068 | 11.96936 | 17.58731 | 26.0521 | 25.19447 | 22.40379 | 27.32583 | 23.02991 | 31.48445 | 31.6343 | 34.13499 | 25.15397 | 23.78915 | 28.04755 | 30.13158 | 23.82095 | 33.42247 | 35.1842 | 28.229 | 29.15459 | 32.42452 | 32.18854 |
| CUST_8602_PI429010886 | N23289 | 0.00205 | 12 | 0 | | | | | | 46.16699 | 53.10218 | 41.16894 | 37.07575 | 29.12321 | 30.558 | 28.49131 | 35.91866 | 47.23493 | 36.5186 | 31.31664 | 58.57388 | 38.96658 | 47.54347 | 37.19463 | 35.64771 | 25.94889 | 30.74148 | 33.18761 | 40.1229 | 33.32019 | 41.61311 | 34.8398 | 44.18048 | 46.2898 | 39.00381 | 44.64586 |
| CUST_45739_PI429010886 | M70113 | 0.002058 | 15 | 3 | 157115719 | hypothetical protein AaeL_AAEL007311 [Aedes aeg | | | 1.61E-09 | 14.73825 | 26.5649 | NA | 24.3684 | 34.6052 | 15.89427 | 21.08222 | 16.24815 | 14.86659 | 25.791 | 11.65798 | 6.380402 | 8.431174 | 6.739431 | 6.550085 | 13.11481 | 28.68856 | 18.11915 | 31.68444 | 9.628728 | 19.61572 | 18.00026 | 14.57957 | 13.8828 | 11.49378 | 7.953838 | 9.970778 |
| CUST_11356_PI429010886 | N27682 | 0.002064 | 15 | 6 | | | | | | 17.13473 | 23.55239 | NA | 69.20807 | 51.94426 | 45.04433 | 117.2199 | 57.10862 | 87.63509 | 43.08722 | 38.87013 | 37.84899 | 52.23527 | 8.17741 | 34.86849 | 44.54185 | 22.54854 | 42.64305 | 41.73826 | 41.27495 | 40.16678 | 78.88657 | 41.52857 | 57.86841 | 41.909 | 30.50923 | 31.78356 |
| CUST_12025_PI429010886 | N28795 | 0.002068 | 12 | 0 | | | | | | 40.16678 | 33.61051 | 53.8551 | 49.73773 | 30.82234 | 41.84077 | 29.25886 | 29.33184 | 34.53064 | 30.07667 | 32.67128 | 52.33717 | 33.58412 | 58.17945 | 44.26787 | 32.38525 | 35.12625 | 37.25067 | 29.73851 | 36.40505 | 35.53394 | 39.70495 | 41.63072 | 44.03947 | 51.87045 | 53.10218 | 60.11602 |
| CUST_43040_PI429010886 | M61927 | 0.002068 | 24 | 15 | | | | | | 210.2559 | 224.0801 | 236.4243 | 216.9071 | 206.7246 | 207.5377 | 181.5941 | 214.7985 | 225.6208 | 274.9043 | 209.2891 | 255.7006 | 195.2252 | 263.2135 | 238.1723 | 282.2114 | 281.7845 | 282.5343 | 246.3068 | 243.166 | 207.885 | 317.4402 | 244.2379 | 219.9262 | 247.3742 | 231.8976 | 244.6362 |
| CUST_45093_PI429010886 | M67982 | 0.002074 | 12 | 3 | | | | | | 373.1186 | 401.8543 | 257.8206 | 486.5202 | 518.3277 | 455.745 | 385.3789 | 352.5288 | 265.0157 | 324.7416 | 425.3087 | 313.2163 | 280.4605 | 315.3064 | 345.8615 | 492.1806 | 617.8466 | 472.3127 | 406.8847 | 420.504 | 366.4854 | 427.6746 | 324.9748 | 315.3064 | 452.4018 | 310.0283 | 274.404 |
| CUST_52373_PI429010886 | M97229 | 0.002074 | 12 | 0 | | | | | | 37.0569 | 39.78497 | 33.51061 | 18.57528 | 36.98717 | 21.28123 | 28.84183 | 30.04952 | 36.13374 | 31.92079 | 36.43958 | 23.35652 | 46.37163 | 33.61051 | 40.11037 | 31.2144 | 27.96355 | 12.27649 | 41.26062 | 28.88138 | 32.86388 | 33.26462 | 28.46803 | 31.36122 | 50.77711 | 37.43755 | 35.85975 |
| CUST_20212_PI429010886 | N43029 | 0.002078 | 24 | 0 | | | | | | 121.1966 | 107.9821 | 38.44451 | 47.90107 | 45.98571 | 39.3558 | 41.36789 | 48.48054 | 39.53295 | 46.58823 | 39.96079 | 25.04907 | 27.71495 | 26.86002 | 26.64821 | 40.31691 | 37.34484 | 35.02438 | 31.03692 | 30.03779 | 44.5467 | 39.97268 | 58.12189 | 43.73764 | 43.43161 | 43.48424 | 50.69847 |
| CUST_12364_PI429010886 | N29353 | 0.002078 | 15 | 0 | | | | | | 114.1927 | 113.4862 | 140.2062 | 65.96017 | 130.9315 | 156.213 | 68.02073 | 74.58958 | 32.66252 | 114.0301 | 134.02 | 107.0416 | 58.36968 | 100.3587 | 97.03372 | 149.1678 | 133.7926 | 186.167 | 123.6533 | 124.9049 | 85.62172 | 106.8281 | 67.17456 | 70.86123 | 93.1869 | 109.4422 | 113.8196 |
| CUST_21345_PI429010886 | N45050 | 0.002078 | 24 | 15 | | | | | | 237.8356 | 198.0363 | 238.1723 | 145.749 | 168.5282 | 178.1191 | 150.1517 | 146.2811 | 136.9283 | 197.122 | 190.7772 | 206.0341 | 135.2716 | 237.4703 | 270.6659 | 256.52 | 238.0845 | 229.6568 | 221.3847 | 252.8872 | 204.946 | 254.8048 | 213.8921 | 197.2228 | 273.4298 | 210.1804 | 217.5886 |
| CUST_17772_PI429010886 | N38565 | 0.002082 | 15 | 9 | | | | | | 86.6967 | 137.33 | 134.659 | 59.62185 | 72.90791 | 58.77783 | 50.15308 | 134.5033 | 132.473 | 146.8304 | 134.5033 | 203.4225 | 148.3091 | 162.2752 | 134.7752 | 138.0091 | 103.3151 | 88.33792 | 89.49881 | 157.9269 | 111.3637 | 111.4954 | 132.6337 | 90.34874 | 131.3133 | 180.7887 | 199.5921 |
| CUST_47128_PI429010886 | M74889 | 0.002084 | 18 | 15 | | | | | | 76.62962 | 77.0714 | 74.15113 | 48.81237 | 77.91084 | 90.74799 | 66.45252 | 65.91381 | 61.21559 | 84.60749 | 69.11088 | 99.63032 | 70.82822 | 55.58682 | 82.85967 | 86.4072 | 99.48375 | 84.52872 | 85.72992 | 97.55406 | 66.64899 | 63.62137 | 62.1958 | 63.05647 | 68.37525 | 60.02795 | 51.26857 |
| CUST_2370_PI429010886 | N12203 | 0.002084 | 18 | 3 | 270007848 | hypothetical protein TcasGA2_TC014587 [Tribolium | Protein metabolism | Proteolysis | 1.43E-67 | 38.51713 | 22.02378 | 35.19889 | 43.58778 | 27.85193 | 39.84647 | 31.2144 | 29.65544 | 19.79646 | 29.85007 | 17.19113 | 9.213601 | 20.6419 | 14.82548 | 20.50772 | 31.277 | 29.5983 | 30.17815 | 32.29627 | 31.55772 | 30.49715 | 19.04191 | 34.45011 | 48.8316 | 33.72458 | 16.61483 | 19.46313 |
| CUST_20154_PI429010886 | N42909 | 0.002087 | 18 | 3 | | | | | | 367.0612 | 1521.376 | 558.4057 | 1710.741 | 656.7764 | 1164.105 | 449.6922 | 625.8341 | 453.79 | 472.3127 | 316.122 | 1171.074 | 515.5917 | 426.7007 | 310.0812 | 112.4311 | 210.7453 | 522.2489 | 262.1121 | 417.8136 | 688.3695 | 692.7803 | 679.1732 | 590.4824 | 741.7837 | 1158.222 | 392.5464 |
| CUST_42595_PI429010886 | M60671 | 0.002092 | 12 | 0 | | | | | | 7.438777 | 25.93979 | 21.44879 | 6.749952 | 7.174468 | 15.55047 | 8.854627 | 4.911529 | 6.245942 | 21.42376 | 17.4527 | 17.13668 | 15.4798 | 15.73939 | 15.67763 | 12.96775 | 10.68832 | 12.09063 | 5.185762 | 8.824107 | 13.96315 | 15.02774 | 26.46898 | 10.19943 | 27.35173 | 18.6803 | 15.66178 |
| CUST_19480_PI429010886 | N41653 | 0.002097 | 12 | 0 | | | | | | 153.8729 | 101.7152 | 262.0228 | 77.57474 | 86.4151 | 106.6963 | 98.73097 | 128.8206 | 123.444 | 155.1735 | 95.28234 | 132.8409 | 125.6357 | 157.6076 | 160.598 | 88.7086 | 112.6604 | 92.79213 | 107.1382 | 120.5371 | 123.1656 | 85.20601 | 134.577 | 104.8539 | 110.8364 | 135.78 | 162.1995 |
| CUST_5010_PI429010886 | N18130 | 0.002098 | 18 | 6 | | | | | | 235.8718 | 140.6816 | 245.3246 | 214.5304 | 231.4586 | 264.4268 | 404.6132 | 292.6086 | 434.8523 | 214.3213 | 260.373 | 259.7236 | 374.7559 | 445.2354 | 293.2752 | 213.7215 | 374.7559 | 239.0491 | 324.8235 | 139.562 | 196.8724 | 255.3452 | 281.4899 | 330.7539 | 349.238 | 330.7539 | 648.9401 |
| CUST_32098_PI429010886 | M3223 | 0.002102 | 15 | 9 | 328778301 | PREDICTED: calsyntenin-1-like [Apis mellifera] | Cellular Process | Cell adhesion | | 692.7803 | 571.094 | 999.0406 | 671.8789 | 670.866 | 636.1084 | 703.0939 | 650.0167 | 820.7972 | 849.5817 | 822.5821 | 905.6425 | 595.305 | 958.7977 | 756.4835 | 1088.645 | 694.3101 | 760.0455 | 664.8684 | 627.0644 | 708.7623 | 966.7908 | 813.6581 | 723.9047 | 1119.369 | 819.4278 | 1277.111 |
| CUST_17190_PI429010886 | N37531 | 0.002106 | 24 | 0 | | | | | | 209.2252 | 224.9139 | 210.9764 | 204.1086 | 181.957 | 186.3153 | 157.4606 | 164.2252 | 174.4543 | 216.3391 | 162.0779 | 186.7032 | 148.0995 | 170.6936 | 156.213 | 244.904 | 173.8312 | 192.7945 | 173.7221 | 206.9847 | 212.5355 | 248.1506 | 178.9755 | 170.531 | 238.941 | 197.0951 | 223.1189 |
| CUST_20010_PI429010886 | N42642 | 0.002106 | 18 | 9 | | | | | | 10.41462 | 6.131047 | 9.853418 | 22.9227 | 23.72419 | 19.90393 | 35.42374 | 25.01801 | 9.889085 | 57.19607 | 43.38005 | NA | 25.0475 | 23.96204 | 12.32751 | 21.69445 | 72.08475 | 24.22062 | 44.1761 | 8.84632 | 7.833526 | 10.59651 | 14.9648 | 49.55005 | 30.53018 | 26.4245 | 24.19734 |
| CUST_40017_PI429010886 | M48594 | 0.002118 | 18 | 3 | 240114287 | hemocyanin subunit 1 [Eurydice pulchra] | | | 1.8E-103 | 29.5483 | 38.85207 | 40.46782 | 80.85722 | 37.76259 | 67.98998 | 30.6709 | 30.48948 | 44.75473 | 44.32414 | 12.7088 | 35.94919 | 18.19015 | 40.6037 | 8.257726 | 27.75482 | 18.36165 | 25.29581 | 18.30638 | 12.06387 | 76.78516 | 36.606 | 87.6188 | 49.52106 | 45.03911 | 56.83575 | 28.04755 |
| CUST_5663_PI429010886 | N19007 | 0.002121 | 12 | 0 | | | | | | 142.6905 | 126.9103 | 150.8429 | 102.5401 | 111.685 | 108.1041 | 97.83625 | 122.4191 | 125.751 | 180.1444 | 112.6306 | 141.2095 | 128.969 | 139.9078 | 161.4808 | 166.5412 | 101.6485 | 107.4359 | 119.6576 | 124.3983 | 114.4136 | 144.9176 | 126.0646 | 106.1322 | 219.8055 | 130.5118 | 149.9119 |
| CUST_19582_PI429010886 | N41811 | 0.002125 | 18 | 3 | | | | | | 73.91159 | 98.41575 | 98.38324 | 206.8341 | 117.3497 | 176.0053 | 189.1194 | 57.34886 | 69.58233 | 60.75286 | 56.94451 | 61.36946 | 45.72037 | 66.2145 | 41.81244 | 44.50661 | 115.5316 | 101.4215 | 108.7837 | 37.8309 | 184.6775 | 64.75716 | 293.7629 | 259.6731 | 63.53447 | 137.9867 | 38.05463 |
| CUST_3459_PI429010886 | N14547 | 0.002126 | 24 | 21 | 321454615 | hypothetical protein DAPPUDRAFT_204241 [Daphn | Metabolic process | Cellular amino acid metabolic process | 7.13E-27 | 21.98642 | 35.08626 | 28.90976 | 36.74882 | 18.53904 | 32.22977 | 22.92991 | 24.38855 | 20.21559 | 25.84583 | 15.35382 | 25.46248 | 28.6303 | 32.61136 | 30.90472 | 33.48083 | 33.68076 | 28.31129 | 20.97642 | 30.03474 | 29.02946 | 43.84173 | 32.45453 | 41.6388 | 36.94632 | 31.33759 | 37.04031 |
| CUST_18853_PI429010886 | N40527 | 0.002127 | 15 | 9 | 301776372 | PREDICTED: LOW QUALITY PROTEIN: zinc finger pro | | | 5.84E-07 | 22.92529 | 26.4818 | 21.48663 | 8.625409 | 13.58792 | 13.50266 | 14.87518 | 10.9768 | 16.93603 | 33.5333 | 26.08978 | 24.7854 | 18.87506 | 16.31744 | 17.18186 | 33.80016 | 16.98682 | 22.12202 | 13.61134 | 20.32835 | 14.65167 | 27.59147 | 17.88327 | 14.84417 | 30.28193 | 22.55762 | 21.63831 |
| CUST_40612_PI429010886 | M55641 | 0.002127 | 12 | 0 | | | | | | 496.5233 | 514.5197 | 563.5263 | 461.2479 | 399.3695 | 447.2563 | 398.7713 | 425.3087 | 457.4348 | 506.8788 | 450.4975 | 588.293 | 464.4064 | 632.4528 | 589.8071 | 483.0972 | 448.6531 | 499.4614 | 477.3001 | 459.0733 | 490.0839 | 499.3294 | 524.0613 | 447.4667 | 605.8766 | 495.7993 | 608.8662 |
| CUST_47669_PI429010886 | M77110 | 0.00214 | 12 | 0 | | | | | | 1327.226 | 1827.911 | 1937.537 | 1469.038 | 1400.624 | 1569.952 | 1259.651 | 1468.172 | 1461.256 | 1539.294 | 1394.642 | 1977.124 | 1559.259 | 2326.832 | 1821.885 | 1942.982 | 1618.557 | 1611.105 | 1492.759 | 1331.49 | 1437.865 | 1784.695 | 1640.228 | 1517.009 | 1872.445 | 1673.62 | 2015.591 |
| CUST_41427_PI429010886 | M57704 | 0.002151 | 15 | 9 | 260826966 | hypothetical protein BRAFLDRAFT_96574 [Branchio | Transport | Ion transport | 7.74E-07 | 818.0738 | 2241.403 | 2335.152 | 521.7265 | 663.7859 | 246.5478 | 738.4538 | 2085.292 | 2797.965 | 3838.985 | 2303.489 | 3206.496 | 2194.366 | 4461.585 | 2110.744 | 2676.76 | 1067.136 | 1635.051 | 2188.013 | 3037.946 | 2030.719 | 4330.695 | 2851.724 | 536.3308 | 4791.679 | 2944.104 | 4797.809 |
| CUST_20044_PI429010886 | N42703 | 0.002151 | 15 | 0 | 542183510 | PREDICTED: dynein intermediate chain 1, axonemal | | | 3.87E-07 | 44.05203 | 37.95272 | 41.33633 | 26.95815 | 31.87606 | 59.93656 | 20.07638 | 28.01496 | 22.64343 | 37.44766 | 19.19079 | 30.91161 | 20.82459 | 39.68929 | 36.00905 | 47.39715 | 37.70458 | 38.82938 | 17.01911 | 30.17589 | 30.60937 | 32.13434 | 22.82685 | 19.92242 | 46.83826 | 32.23691 | 24.60142 |
| CUST_5008_PI429010886 | N18125 | 0.002154 | 12 | 9 | | | | | | 67.68545 | 48.31638 | 61.18601 | 17.46274 | 12.84883 | 20.27214 | 12.68973 | 33.47616 | 40.62219 | 69.08941 | 92.13928 | 111.2973 | 61.98456 | 91.31323 | 49.61919 | 46.14783 | 44.92099 | 46.25026 | 82.17099 | 68.27282 | 83.81066 | 78.90095 | 98.09433 | 31.69618 | 63.03207 | 80.24894 | 86.07302 |
| CUST_6192_PI429010886 | N19752 | 0.002159 | 21 | 9 | 460027843 | prostaglandin F synthase [Penaeus monodon] | Metabolic process | Oxidation-reduction process | 4.8E-110 | 8.486348 | 16.30792 | NA | 30.55547 | 43.35404 | 10.96784 | 42.29494 | 137.33 | 71.68684 | 34.872 | 123.8883 | 121.6959 | 93.90071 | 16.80068 | 37.49092 | 12.79677 | 53.51858 | 9.773611 | 20.92648 | 256.3774 | 35.08626 | 4.882555 | 7.479587 | 28.48104 | 10.27804 | 60.90428 | 97.40947 |
| CUST_14566_PI429010886 | N33086 | 0.002162 | 24 | 0 | | | | | | 81.15299 | 79.67757 | 86.17533 | 65.19644 | 62.74739 | 63.79444 | 66.97799 | 60.18184 | 90.18249 | 80.57644 | 55.55289 | 81.48802 | 59.8939 | 70.76377 | 56.32832 | 79.74735 | 60.0207 | 65.01299 | 77.76127 | 67.78689 | 78.04542 | 75.22711 | 101.2537 | 68.02073 | 100.7779 | 91.6409 | 86.5251 |
| CUST_5717_PI429010886 | N19094 | 0.002162 | 15 | 0 | | | | | | 24.84355 | 13.72842 | NA | 6.88044 | 13.37036 | 13.62943 | 11.82406 | 10.45244 | 12.88026 | 12.59383 | 12.03567 | 12.75753 | 7.93896 | 12.33539 | 12.4451 | 16.87557 | 18.24734 | 14.63109 | 9.927073 | 13.38001 | 11.19316 | 9.562437 | 5.068125 | 10.51996 | 13.63622 | NA | 8.678808 |
| CUST_50873_PI429010886 | M91295 | 0.002171 | 24 | 18 | | | | | | 8.767176 | 27.47752 | 17.0738 | 7.660234 | 12.66733 | 7.410795 | 16.7895 | NA | 6.417851 | 20.60423 | 26.81554 | 24.12979 | 14.19203 | 19.79375 | 20.03408 | 43.74674 | 11.03039 | NA | 28.05064 | 26.83996 | 28.33335 | 48.15042 | 19.3376 | 14.75825 | 26.71164 | 13.41075 | 14.94602 |
| CUST_42588_PI429010886 | M60658 | 0.002176 | 15 | 9 | | | | | | 87.40211 | 104.9253 | 104.46 | 65.84223 | 83.94314 | 75.4776 | 66.06376 | 86.60546 | 93.09068 | 144.4421 | 88.82378 | 135.6641 | 66.46046 | 133.5378 | 116.7432 | 111.5879 | 86.7972 | 87.6188 | 82.48421 | 88.23558 | 96.97877 | 113.2976 | 122.84 | 74.79904 | 119.9123 | 115.1327 | 120.7928 |
| CUST_1341_PI429010886 | N9339 | 0.002176 | 15 | 9 | | | | | | 10949.63 | 18727.55 | 15271.23 | 4080.667 | 8627.68 | 4719.422 | 7930.333 | 10366.97 | 18962.11 | 20926.24 | 12130.73 | 26532.79 | 16000.49 | 17900.98 | 15000.31 | 23580.44 | 6962.573 | 18298.8 | 10044.17 | 13202.87 | 12422.81 | 19317.04 | 11400.82 | 8924.795 | 21636.27 | 16607.59 | 25546.19 |
| CUST_38016_PI429010886 | M22785 | 0.002176 | 18 | 9 | | | | | | 848.6156 | 314.6817 | 333.708 | 254.095 | 174.126 | 55.20237 | 374.2323 | 865.6927 | 727.1859 | 1287.748 | 542.3878 | 996.2576 | 717.1047 | 561.2769 | 964.2217 | 733.1697 | 159.7825 | 210.9764 | 699.2311 | 258.4896 | 321.5473 | 429.7605 | 230.7517 | 186.1103 | 62.23219 | 389.4334 | 883.1215 |
| CUST_10755_PI429010886 | N26736 | 0.002183 | 21 | 15 | 524881293 | PREDICTED: vacuolar fusion protein CCZ1 homolog | | | 3.72E-28 | 59.79177 | 116.1332 | 59.73525 | 64.40125 | 59.41199 | 56.74499 | 56.47886 | 52.73715 | 36.07274 | 68.38918 | 70.5402 | 87.2868 | 57.35641 | 77.92082 | 83.75206 | 108.3734 | 73.00251 | 85.34367 | 80.07884 | 60.41389 | 55.75917 | 57.40224 | 65.14347 | 66.39266 | 71.41994 | 53.95436 | 62.69277 |
| CUST_36161_PI429010886 | M14844 | 0.002189 | 21 | 9 | | | | | | 21.40624 | 63.75313 | 67.32952 | 124.4616 | 178.4972 | 78.01974 | 94.94128 | 252.2638 | 220.3236 | 77.5423 | 453.7002 | 483.3249 | 280.3633 | 101.8407 | 199.9465 | NA | 88.11918 | 18.43584 | 50.28834 | 467.5419 | 332.3269 | 7.132581 | 72.05058 | 42.35334 | 20.70807 | 174.6884 | 145.2433 |
| CUST_132_PI429010886 | N1320 | 0.002195 | 21 | 0 | 321464825 | hypothetical protein DAPPUDRAFT_306600 [Daphn | Protein metabolism | Cellular protein modification process | 1.83E-97 | 483.8764 | 526.019 | 514.5197 | 433.8707 | 439.659 | 482.1854 | 427.5571 | 460.0363 | 384.0577 | 390.7246 | 417.8136 | 441.5046 | 444.8946 | 505.3693 | 448.0873 | 429.0337 | 480.8955 | 452.0017 | 455.0104 | 390.2131 | 402.4861 | 499.6354 | 564.5345 | 520.9934 | 518.2235 | 426.3841 | 371.903 |
| CUST_25566_PI429010886 | N52657 | 0.002196 | 24 | 21 | 48106220 | PREDICTED: CDK-activating kinase assembly factor | Cellular Process | Cell cycle | 8.69E-17 | 5.698946 | 7.591069 | NA | 4.876287 | 4.880377 | 26.14333 | 4.52367 | 4.288757 | 4.711545 | 5.226041 | 5.472076 | 11.62614 | 7.97673 | NA | 10.52648 | 13.68648 | 6.122347 | 10.50262 | NA | 11.31889 | 7.31283 | NA | 14.58166 | NA | 7.188579 | 5.76082 | 14.60389 |
| CUST_46090_PI429010886 | M71336 | 0.002203 | 24 | 18 | 255653868 | crustin 1 [Panulirus japonicus] | | | 1.38E-08 | 38.153 | 53.81515 | 82.38056 | 42.1461 | 55.48383 | 12.94311 | 38.66452 | 63.11008 | 78.52355 | 92.32556 | 102.1823 | 73.75273 | 107.4216 | 112.5446 | 79.48728 | 116.0676 | 165.8727 | 98.56528 | 86.08273 | 153.2734 | 111.2168 | 125.719 | 351.7659 | 80.53149 | 944.9878 | 120.3899 | 742.4096 |

| Probe ID | ID | p-value | Period | Phase | EBI ID | Annotation | GO category | Go term | E-value | 0 | 0 | 0 | 3 | 3 | 3 | 6 | 6 | 6 | 9 | 9 | 9 | 12 | 12 | 12 | 15 | 15 | 15 | 18 | 18 | 18 | 21 | 21 | 21 | 24 | 24 | 24 |
|---|---|---|---|---|---|---|---|---|---|---|---|---|---|---|---|---|---|---|---|---|---|---|---|---|---|---|---|---|---|---|---|---|---|---|---|---|
| CUST_29555_PI429010886 | N60097 | 0.002208 | 18 | 15 | | | | | | 25.25877 | 23.79915 | 26.0056 | 14.92301 | 12.51059 | 7.069154 | 17.98401 | 23.12912 | 18.38425 | 20.50575 | 17.06111 | 30.34969 | 23.89866 | 30.67432 | 20.60423 | 26.21698 | 23.3229 | 22.43908 | 22.04311 | 24.03557 | 16.79729 | 16.52086 | 21.18391 | 19.71614 | 23.38781 | 21.25262 | 22.74775 |
| CUST_33027_PI429010886 | M5771 | 0.00221 | 24 | 3 | | | | | | 256.1518 | 522.6581 | 395.7641 | 693.0321 | 288.8593 | 525.1056 | 284.0133 | 309.5934 | 281.447 | 288.1296 | 245.1937 | 438.3419 | 232.0593 | 352.6063 | 210.9062 | 242.3388 | 130.5118 | 221.9188 | 187.6525 | 166.7568 | 467.3451 | 188.7986 | 517.2906 | 148.3671 | 357.356 | 533.0782 | 242.4714 |
| CUST_2046_PI429010886 | N11405 | 0.002217 | 21 | 9 | | | | | | 99.67314 | 136.6153 | 58.53699 | NA | 22.09691 | 21.75697 | 5.380664 | NA | 86.28429 | 366.8204 | 300.9857 | 441.3844 | NA | 359.4272 | 130.357 | 844.3643 | 6.154713 | 68.89154 | 157.0634 | 163.1226 | 185.8187 | 56.27804 | 50.46214 | 13.61134 | 18.32343 | 44.28888 | 35.13017 |
| CUST_56023_PI429010886 | M112564 | 0.002225 | 15 | 0 | 260831912 | hypothetical protein BRAFLDRAFT_115340 [Branchi | | | 1.1E-126 | 79.92252 | 73.88403 | 69.49923 | 63.92583 | 48.54231 | 58.60431 | 52.57619 | 55.09927 | 50.277 | 62.46096 | 59.41826 | 63.71417 | 69.46903 | 66.18886 | 67.237 | 77.7747 | 62.08961 | 69.65017 | 64.1319 | 68.41131 | 42.82196 | 84.99622 | 45.85649 | 60.33388 | 67.22918 | 46.92598 | 57.9941 |
| CUST_43521_PI429010886 | M63262 | 0.002228 | 24 | 21 | | | | | | 76.55655 | 118.3919 | 79.95313 | 62.34386 | 77.7529 | 65.46937 | 71.87825 | 63.00378 | 66.26094 | 86.24828 | 84.62693 | 107.1617 | 59.776 | 79.80104 | 92.57501 | 90.37187 | 82.13647 | 78.53416 | 96.67753 | 121.8291 | 87.02844 | 106.3446 | 105.6711 | 91.35555 | 77.1205 | 68.04642 | 92.95468 |
| CUST_30275_PI429010886 | N61538 | 0.002229 | 24 | 21 | | | | | | 768.2695 | 839.8931 | 921.275 | 794.1984 | 709.6744 | 828.1362 | 641.17 | 717.2947 | 757.8647 | 856.1081 | 736.8107 | 928.6222 | 738.6637 | 938.9786 | 789.7368 | 789.9286 | 798.0318 | 937.1457 | 745.3969 | 807.9372 | 785.4766 | 945.5514 | 892.8593 | 938.0188 | 917.9837 | 863.9077 | 816.9007 |
| CUST_26293_PI429010886 | N54002 | 0.002236 | 12 | 0 | | | | | | 24.61812 | 107.512 | 102.7959 | 17.58889 | 4.030477 | 12.10865 | 12.42241 | 14.84745 | 11.81616 | 95.87365 | 19.53272 | 17.15146 | 34.73538 | 109.9704 | 56.52669 | 13.47559 | 66.3431 | 28.25151 | 37.30837 | 4.383111 | 6.685823 | 73.77338 | 32.19497 | 42.6374 | 60.97252 | 35.52135 | 29.28327 |
| CUST_1086_PI429010886 | N8447 | 0.002236 | 12 | 3 | 241151593 | calcineurin B, putative [Ixodes scapularis] >gi\|2154 | Metabolic process | Oxidation-reduction process | 3.63E-64 | 280.6713 | 213.9895 | 205.5044 | 476.0617 | 300.5238 | 678.2021 | 372.2181 | 330.6772 | 212.6792 | 190.2377 | 260.055 | 244.775 | 237.1988 | 165.8485 | 235.6197 | 217.7746 | 524.2988 | 377.56 | 352.1132 | 253.8057 | 320.8355 | 262.1655 | 247.0952 | 361.6235 | 244.2054 | 300.7234 | 207.6194 |
| CUST_45553_PI429010886 | M69472 | 0.002236 | 18 | 3 | | | | | | 11.89374 | 7.592295 | 13.89131 | NA | 14.91553 | 17.10625 | 23.99863 | 7.943105 | 5.94813 | 6.887126 | 4.068177 | NA | 9.184475 | 4.519335 | NA | 7.39706 | 5.55948 | 8.014383 | 13.66257 | 18.80919 | NA | NA | NA | 74.0547 | 6.788713 | 17.28228 | 22.50936 |
| CUST_20684_PI429010886 | N43875 | 0.002238 | 21 | 18 | | | | | | 72.54647 | 60.46074 | 55.71375 | 48.3768 | 49.21104 | 40.58787 | 41.26402 | 41.29432 | 41.77165 | 50.51683 | 27.66624 | 53.04998 | 34.44235 | 53.08483 | 47.80199 | 61.07112 | 55.92617 | 48.15554 | 63.16045 | 53.32755 | 50.21157 | 66.97079 | 45.59444 | 49.71501 | 63.12362 | 43.44584 | 44.31012 |
| CUST_46643_PI429010886 | M73262 | 0.002238 | 24 | 9 | | | | | | 26.75249 | 33.33046 | 30.88328 | 33.52618 | 41.98854 | 10.41028 | 30.3731 | 61.36946 | 56.76781 | 59.80108 | 77.48229 | 74.06718 | 42.62004 | 27.23432 | 28.6271 | 30.26197 | 24.00983 | 10.82328 | 22.64182 | 59.81399 | 27.17883 | 17.5216 | 29.1329 | 16.12785 | 25.27468 | 27.42267 | 68.63241 |
| CUST_3900_PI429010886 | N15523 | 0.002245 | 24 | 15 | 443721341 | hypothetical protein CAPTEDRAFT_191048 [Capitell | | | 1.06E-13 | 36.82802 | 40.04686 | 46.36775 | 39.8885 | 39.27655 | 30.46421 | 38.46752 | 42.93137 | 39.25313 | 58.12189 | 46.33994 | 42.11302 | 31.66434 | 46.89105 | 33.44743 | 168.1346 | 61.60941 | 142.124 | 40.2482 | 51.23152 | 50.94926 | 49.75939 | 49.01378 | 31.50694 | 63.8461 | 45.22744 | 44.51031 |
| CUST_6035_PI429010886 | N19538 | 0.002248 | 21 | 0 | | | | | | 54.44415 | 73.04458 | 47.31501 | 44.35344 | 43.97896 | 42.78573 | 40.18446 | 27.25419 | 33.15924 | 39.39152 | 39.48403 | 35.03294 | 45.76846 | 58.67101 | 53.14405 | 64.14878 | 31.17481 | 56.07148 | 41.11091 | 58.31834 | 45.31985 | 58.99785 | 53.22359 | 45.64241 | 50.29336 | 43.51237 | 60.45419 |
| CUST_15407_PI429010886 | N34524 | 0.002249 | 21 | 3 | | | | | | 93.38696 | 77.10171 | 62.59195 | 95.0012 | 91.58478 | 81.56419 | 64.40125 | 68.77778 | 79.38753 | 76.42108 | 80.88726 | 69.9145 | 48.29446 | 78.02809 | 77.97597 | 59.07909 | 86.55946 | 89.84183 | 54.28784 | 56.9379 | 70.6776 | 78.13459 | 78.98243 | 65.38596 | 124.5927 | 92.02079 | 114.7999 |
| CUST_31721_PI429010886 | M2244 | 0.002254 | 12 | 9 | 291229165 | PREDICTED: eukaryotic translation initiation factor | Protein metabolism | Regulation of translational initiation | 3.54E-68 | 179.1629 | 181.6229 | 303.7146 | 163.791 | 107.0314 | 125.2677 | 176.5007 | 199.6541 | 222.7049 | 239.2282 | 159.5233 | 250.6863 | 166.5412 | 320.3175 | 235.9095 | 178.0648 | 152.9711 | 174.8815 | 191.6146 | 153.132 | 211.7142 | 207.7149 | 247.7794 | 227.642 | 199.344 | 189.9152 | 230.8944 |
| CUST_27705_PI429010886 | N56544 | 0.00226 | 15 | 12 | | | | | | 601.3413 | 677.9305 | 696.5534 | 574.6243 | 590.3441 | 592.9907 | 550.2491 | 645.7634 | 636.5467 | 763.6837 | 611.9605 | 789.9286 | 721.8143 | 835.782 | 773.4201 | 795.1421 | 636.3168 | 663.7859 | 628.3128 | 653.6532 | 606.3721 | 773.8104 | 705.5663 | 638.1084 | 718.2136 | 571.4497 | 788.0347 |
| CUST_44115_PI429010886 | M64986 | 0.002261 | 24 | 21 | | | | | | 9.419858 | NA | 8.438285 | 6.902769 | 4.787624 | 8.957551 | 7.704662 | NA | NA | NA | 4.429971 | 3.407042 | 4.579912 | 11.54241 | 3.494751 | 6.037096 | 6.283437 | NA | 6.19695 | 7.492805 | NA | 9.673986 | 8.834126 | 13.5897 | 6.296703 | 25.26607 | 13.21926 |
| CUST_38251_PI429010886 | M24665 | 0.002262 | 18 | 15 | | | | | | 35.01361 | 46.63569 | 25.85184 | 26.48831 | 25.50881 | 26.18295 | 22.78102 | 28.40654 | 23.35652 | 14.93945 | 26.41485 | 28.15071 | 29.26565 | 24.69322 | 34.54086 | 43.48424 | 49.26562 | 30.36422 | 50.09589 | 35.05162 | 26.52817 | 35.6576 | 20.6694 | 31.75969 | 30.18478 | 18.56806 | 20.88392 |
| CUST_51098_PI429010886 | M92178 | 0.002267 | 24 | 15 | | | | | | 26.43705 | 22.93214 | 16.85623 | 22.0997 | 14.9864 | 11.59149 | 14.97861 | 22.13024 | 26.20596 | 34.63494 | 23.33049 | 31.92608 | 23.46287 | 26.28688 | 25.03856 | 35.48686 | 26.64257 | 31.48729 | 26.38687 | 30.68445 | 25.62143 | 24.3983 | 29.49276 | 23.09604 | 35.28275 | 24.95635 | 37.82706 |
| CUST_50489_PI429010886 | M89983 | 0.002269 | 24 | 12 | | | | | | 26.65472 | 30.59469 | 24.40654 | 28.26309 | 26.49443 | 35.81535 | 21.42376 | 24.63631 | 36.37866 | 30.91453 | 37.96113 | 37.91438 | 37.12271 | 42.82585 | 46.58823 | 42.29494 | 37.41038 | 36.42724 | 48.06558 | 31.77439 | 26.9442 | 33.78308 | 27.41533 | 28.20406 | 35.89648 | 33.0759 | 24.1098 |
| CUST_2154_PI429010886 | N11707 | 0.002272 | 24 | 0 | | | | | | 2448.925 | 1049.194 | 2945.87 | 547.9393 | 668.6149 | 717.957 | 360.6396 | 499.9431 | 4931.33 | 326.9265 | 566.4251 | 808.2235 | 270.3602 | 1030.293 | 588.5777 | 1226.144 | 199.5128 | 694.7611 | 239.4694 | 539.7579 | 592.8448 | 682.2742 | 739.8087 | 1068.926 | 2915.652 | 4561.712 | 5391.461 |
| CUST_859_PI429010886 | N7593 | 0.002278 | 15 | 0 | | | | | | 3197.843 | 6607.963 | NA | 5342.716 | 212.046 | 195.4506 | 494.6999 | 71.39836 | 413.5257 | 5.315305 | 10.19353 | 8.767176 | 3105.643 | 894.2724 | 3618.732 | 861.4839 | NA | 668.4719 | NA | 204.5915 | 38.00094 | 356.3531 | NA | 561.4598 | 46.65817 | 25.10548 | NA |
| CUST_43935_PI429010886 | M64384 | 0.002286 | 21 | 12 | | | | | | 28.4998 | 74.98811 | 24.6789 | 14.38493 | 19.14544 | 17.4379 | NA | NA | 27.42267 | 36.67483 | 20.55693 | NA | 34.17683 | 39.90543 | 42.73857 | 42.16099 | NA | 31.21998 | 8.8599 | 38.61391 | 36.87977 | 21.2067 | 14.73825 | 25.352 | 15.15984 | 15.42332 | 21.05599 |
| CUST_47159_PI429010886 | M74997 | 0.002289 | 15 | 12 | | | | | | 355.9452 | 457.8737 | 488.3379 | 336.5091 | 331.029 | 328.0964 | 339.3078 | 394.7994 | 339.8879 | 407.3532 | 349.1356 | 500.5021 | 430.9519 | 497.6528 | 433.0579 | 486.3734 | 351.9054 | 411.1038 | 381.6649 | 420.2091 | 291.3805 | 447.2563 | 363.5385 | 348.7613 | 412.5292 | 353.5438 | 393.782 |
| CUST_23939_PI429010886 | N49734 | 0.002289 | 15 | 12 | | | | | | 62.506 | 110.5233 | 110.2618 | 74.97194 | 72.08475 | 76.41163 | 94.7334 | 75.0125 | 110.1183 | 113.2835 | 77.57474 | 126.1331 | 96.21869 | 138.1473 | 128.7123 | 136.6153 | 89.6226 | 127.601 | 84.25035 | 90.62378 | 87.08384 | 132.033 | 107.2382 | 88.07214 | 107.9228 | 114.9376 | 106.2741 |
| CUST_26244_PI429010886 | N53907 | 0.002294 | 15 | 3 | | | | | | 6.694465 | 6.889529 | 26.12256 | 10.32671 | 10.49198 | 29.49638 | 4.584527 | 10.66902 | 6.332988 | 37.09113 | 4.752758 | 7.23801 | 3.844435 | 3.824122 | 5.852448 | 5.241968 | 6.618021 | 15.72851 | 21.33841 | 10.77469 | 10.00064 | 6.374853 | 18.12711 | 8.435831 | NA | 4.347664 | 4.618065 |
| CUST_51380_PI429010886 | M93328 | 0.002299 | 18 | 9 | | | Metabolic process | Cellular amino acid metabolic process | | 37.9853 | 34.49959 | 44.07915 | 20.24204 | 22.70056 | 23.12912 | 34.71929 | 46.83015 | 45.60489 | 54.79286 | 37.69054 | 62.39657 | 44.4356 | 53.07285 | 44.90621 | 42.5721 | 35.38107 | 29.76053 | 37.70458 | 54.35449 | 33.03817 | 39.14912 | 40.9306 | 21.15997 | 42.98911 | 38.07688 | 54.0716 |
| CUST_36306_PI429010886 | M15519 | 0.002304 | 12 | 9 | 443720297 | hypothetical protein CAPTEDRAFT_167536 [Capitell | Transport | Ion transport | 6.2E-26 | 38.37256 | 19.98856 | 39.68514 | 21.28517 | 19.02363 | 25.86961 | 22.64182 | 36.25025 | 28.1812 | 49.25815 | 36.9176 | 53.38382 | 24.05186 | 21.52056 | 32.31627 | 33.3136 | 16.34592 | 29.25253 | 27.03336 | 33.47045 | 27.77642 | 39.20447 | 33.70683 | 28.8539 | 31.69618 | 31.40253 | 31.14323 |
| CUST_48972_PI429010886 | M83257 | 0.002305 | 15 | 12 | | | | | | 246.9496 | 265.9049 | 342.6195 | 249.5597 | 212.4692 | 236.449 | 243.2786 | 278.7763 | 298.3253 | 328.3197 | 249.8933 | 338.9665 | 280.165 | 384.1514 | 287.9804 | 343.4753 | 275.1873 | 296.9872 | 257.455 | 283.788 | 246.7296 | 274.5343 | 277.5533 | 228.4344 | 356.4277 | 281.9612 | 388.2685 |
| CUST_44167_PI429010886 | M65146 | 0.002315 | 15 | 9 | 260808229 | hypothetical protein BRAFLDRAFT_122441 [Branchi | | | 5.97E-10 | 8.463865 | 6.66543 | 3.695895 | 6.321198 | 8.494473 | 5.274301 | NA | 8.352146 | NA | 10.00064 | 63.76139 | 9.071169 | 9.403927 | 34.86394 | 10.36736 | 7.011247 | 5.227424 | 7.045477 | 9.744311 | 8.853279 | NA | 8.299606 | 12.7088 | NA | 13.82942 | 8.431174 | 13.23043 |
| CUST_14120_PI429010886 | N32359 | 0.002315 | 18 | 3 | | | | | | 516.7166 | 1145.481 | 744.2633 | 1558.572 | 669.0767 | 1020.365 | 571.7132 | 498.9877 | 614.2182 | 654.3393 | 327.535 | 776.1766 | 395.8483 | 725.8016 | 290.1265 | 284.6753 | 239.7427 | 501.9627 | 333.7959 | 386.8238 | 1095.161 | 639.3223 | 1193.094 | 573.9804 | 913.8189 | 1010.922 | 499.9431 |
| CUST_23349_PI429010886 | N48677 | 0.002329 | 18 | 3 | | | | | | 29.80377 | 86.5251 | 28.85125 | 131.6679 | 45.1372 | 65.43441 | 43.37513 | 31.44747 | 29.69991 | 40.44714 | 10.92713 | 47.46581 | 19.2011 | 37.84181 | 28.90009 | 10.22956 | 7.53941 | 36.41652 | 19.03731 | 17.70878 | 77.91084 | 39.59389 | 84.12226 | 41.72002 | 48.92704 | 76.19351 | 22.77196 |
| CUST_18155_PI429010886 | N39254 | 0.002338 | 24 | 15 | | | | | | 102.3423 | 101.6741 | 94.48599 | 64.96286 | 79.57182 | 83.77617 | 78.25555 | 66.57113 | 58.97452 | 150.8165 | 112.8267 | 146.622 | 115.3125 | 96.06297 | 130.8664 | 175.1291 | 109.7501 | 137.6985 | 136.3426 | 189.4794 | 100.9909 | 189.4794 | 102.3344 | 111.3143 | 132.3882 | 138.8758 | 132.6337 |
| CUST_45388_PI429010886 | M68943 | 0.002347 | 15 | 12 | | | | | | 223.4939 | 189.7693 | 207.3573 | 160.5038 | 165.7787 | 172.5998 | 121.2986 | 129.9842 | 172.498 | 204.5562 | 146.2811 | 251.0141 | 173.0247 | 270.6659 | 235.4879 | 248.652 | 155.4878 | 230.1517 | 180.8117 | 197.2996 | 154.463 | 230.5922 | 118.1291 | 112.1834 | 227.5328 | 181.7752 | 212.773 |
| CUST_358_PI429010886 | N4420 | 0.002349 | 18 | 6 | 478432608 | beta-thymosin 5 [Pacifastacus leniusculus] | Cellular component organization | Cytoskeleton organization | 8.4E-46 | 59.79684 | 35.58868 | 45.24266 | 46.73511 | 93.33448 | 38.24021 | 79.14778 | 52.10631 | 164.5463 | 58.40039 | 38.153 | 36.31618 | 46.9109 | 31.07173 | 39.70836 | 37.58456 | 42.19441 | 29.98387 | 44.4293 | 33.91251 | 24.28 | 60.95703 | 44.27177 | 51.192 | 64.26189 | 54.26471 | 38.41931 |
| CUST_31677_PI429010886 | M2148 | 0.002353 | 24 | 0 | | | | | | 1214.782 | 1140.952 | 944.3898 | 1022.691 | 705.5663 | 1035.582 | 932.818 | 696.3149 | 865.9484 | 795.9721 | 849.3126 | 893.1457 | 639.8175 | 844.933 | 868.0572 | 1663.806 | 904.8603 | 709.528 | 1399.299 | 995.7921 | 1118.208 | 908.82 | 1060.287 | 918.6617 | 1317.659 | 1019.767 | 1248.552 |
| CUST_43143_PI429010886 | M62188 | 0.002354 | 15 | 0 | | | | | | 247.0492 | 233.3995 | 241.9617 | 139.9664 | 196.1002 | 200.337 | 173.9653 | 161.1553 | 155.1312 | 248.1506 | 187.1216 | 184.8426 | 120.0958 | 191.2063 | 189.7138 | 278.6013 | 203.1221 | 245.0284 | 199.281 | 205.4212 | 220.51 | 199.1974 | 177.9987 | 184.654 | 304.9967 | 193.8001 | 240.5734 |
| CUST_39903_PI429010886 | M46604 | 0.002355 | 24 | 15 | | | | | | 29.00747 | 51.05906 | 16.79285 | 28.21862 | 24.91029 | 34.46849 | 16.87735 | 6.890716 | 30.15316 | 41.87987 | 51.20005 | 89.32488 | 80.25744 | 69.80469 | 54.73192 | 141.8752 | NA | 143.406 | 43.12973 | 41.96064 | 67.56871 | 70.08131 | 64.91729 | 45.93669 | 68.2359 | 80.4611 | 112.9419 |
| CUST_336_PI429010886 | N4036 | 0.002376 | 15 | 12 | 189242004 | PREDICTED: hypothetical protein, partial [Tribolium | | | 1.98E-08 | 311.394 | 378.1899 | 423.4903 | 145.2889 | 129.772 | 61.23542 | 1164.641 | 971.7833 | 2253.027 | 743.4007 | 280.8226 | 639.3223 | 4114.35 | 5836.719 | 3084.768 | 2716.899 | 2035.189 | 240.8565 | 685.9612 | 375.0345 | 362.1101 | 448.2914 | 638.9314 | 762.5883 | 5674.79 | 288.3979 | 7985.61 |
| CUST_49598_PI429010886 | M86342 | 0.002377 | 24 | 18 | | | | | | 84.55489 | 66.62115 | 61.71609 | 68.9769 | 57.15725 | 70.75343 | 70.39937 | 77.24703 | 75.96227 | 83.94314 | 84.88556 | 91.31323 | 83.35473 | 94.97923 | 92.68449 | 118.3632 | 84.79763 | 71.33278 | 76.72395 | 126.2208 | 91.79308 | 97.84604 | 82.46049 | 69.56167 | 67.52161 | 67.19434 | 97.3486 |
| CUST_54391_PI429010886 | M105836 | 0.002377 | 21 | 12 | | | | | | 4.65346 | 10.8172 | 11.25284 | 3.422354 | 5.214068 | 13.63796 | 4.556789 | 6.195387 | NA | 7.625766 | 11.39999 | 13.8468 | 7.516724 | 18.10445 | 17.11883 | 14.92766 | NA | 11.51911 | 11.26061 | 15.73055 | 15.14732 | 16.42533 | 8.988276 | NA | 6.680971 | 7.506584 | 7.329225 |
| CUST_15384_PI429010886 | N34498 | 0.002392 | 15 | 12 | | | | | | 28.52767 | 36.34774 | 35.66432 | 30.55085 | 25.78835 | 30.68045 | 19.57848 | 20.56557 | 21.70738 | 22.38112 | 16.64978 | 33.21734 | 27.56341 | 40.25661 | 35.6179 | 32.15263 | 24.03089 | 29.92689 | 25.87167 | 27.22266 | 16.78445 | 30.0571 | 21.43684 | 17.59447 | 28.87134 | 36.39578 | 25.68325 |
| CUST_35726_PI429010886 | M13312 | 0.002405 | 15 | 12 | 260821736 | hypothetical protein BRAFLDRAFT_83989 [Branchio | | | 1.52E-13 | 70.90857 | 76.21767 | 98.97177 | 66.94583 | 72.04379 | 58.11529 | 56.69692 | 84.84663 | 73.76113 | 91.47195 | 69.76028 | 79.34273 | 91.51834 | 98.84179 | 97.5299 | 86.61515 | 71.57464 | 62.03099 | 61.2655 | 69.40293 | 56.46834 | 95.89707 | 82.75397 | 38.18452 | 85.17905 | 64.80696 | 69.72451 |
| CUST_10086_PI429010886 | N25669 | 0.002405 | 15 | 12 | | | | | | 23.40229 | 30.57199 | 28.64267 | 22.87829 | 13.13021 | 13.10657 | 13.22714 | 18.2155 | 25.6517 | 22.5025 | 21.58196 | 31.48729 | 18.44752 | 33.49626 | 38.56175 | 26.49155 | 26.34508 | 29.32849 | 19.6024 | 20.78959 | 18.39671 | 33.28531 | 25.29278 | 17.22867 | 27.60563 | 25.65833 | 29.18222 |
| CUST_460_PI429010886 | N5425 | 0.002405 | 15 | 12 | | | | | | 3218.113 | 815.8741 | 28610.41 | 49.64767 | 113.3258 | 46.94189 | 5751.466 | 36398.63 | 30617.99 | 45084.56 | 1346.091 | 2676.76 | 34870.47 | 61640.76 | 28226.57 | 597.939 | 24915.2 | 316.8071 | 1787.33 | 3158.604 | 874.9124 | 15271.23 | 44923.37 | 1308.281 | 47369.29 | 1904.883 | 45272.91 |
| CUST_10743_PI429010886 | N26716 | 0.002408 | 18 | 3 | | | | | | 182.4687 | 91.0847 | 28.25834 | 72.84986 | 157.0244 | 156.0949 | 79.81539 | 59.40092 | 57.68381 | 49.02131 | 44.82913 | 37.19186 | 70.47227 | 26.83276 | 43.8479 | 83.83973 | 15.46051 | 46.37649 | 45.33809 | 82.90011 | 30.39192 | 263.2877 | 46.46836 | 247.0952 | 118.1469 | 91.27166 | 70.09922 |
| CUST_16765_PI429010886 | N36832 | 0.002418 | 15 | 9 | 390369713 | PREDICTED: WD repeat-containing protein 76-like [ | | | 9.84E-07 | 6.981775 | 17.09174 | 35.88829 | NA | 5.352926 | NA | 16.37326 | 35.11564 | 43.63321 | 40.81934 | 28.77061 | 34.54419 | 18.53665 | 26.20316 | 29.46496 | 6.001769 | 15.66989 | 13.30348 | 18.02463 | 9.602601 | 21.18145 | 40.82323 | 19.676 | NA | 92.34903 | 22.52377 | 63.64323 |
| CUST_55099_PI429010886 | M108732 | 0.002426 | 15 | 12 | | | | | | 518.0939 | 698.0293 | 780.6851 | 546.3542 | 592.9907 | 639.1213 | 463.3861 | 650.3692 | 671.0656 | 711.6467 | 635.437 | 823.8136 | 714.0634 | 706.1971 | 771.0971 | 948.7418 | 654.7049 | 633.3531 | 565.2058 | 610.8058 | 546.8107 | 793.3041 | 710.22 | 614.3849 | 817.4781 | 667.377 | 849.0108 |
| CUST_52234_PI429010886 | M96801 | 0.002442 | 15 | 0 | | | | | | 149.9119 | 110.8588 | 146.9588 | 119.3795 | 94.76564 | 113.2396 | 105.9793 | 132.5751 | 111.9958 | 95.50344 | 92.28356 | 105.2005 | 90.3266 | 111.9132 | 97.37669 | 122.0703 | 112.6761 | 119.228 | 119.0396 | 109.7731 | 109.6686 | 120.2795 | 116.7026 | 107.3414 | 105.5539 | 109.5513 | 75.41714 |
| CUST_1384_PI429010886 | N9464 | 0.002442 | 18 | 3 | | | | | | 85.03701 | 47.03357 | 57.22832 | 116.5433 | 78.77563 | 126.7579 | 51.52202 | 229.2792 | 33.38032 | 81.72278 | 64.85632 | 51.90738 | 31.15965 | 44.86761 | 19.61572 | 51.34112 | 31.88591 | 33.21734 | 116.0676 | 119.0071 | 59.90035 | 65.76284 | 66.00564 | 93.46673 | 34.11202 | 79.79104 | 197.9029 |
| CUST_12396_PI429010886 | N29395 | 0.00245 | 21 | 0 | | | | | | 2999.441 | 3842.983 | 1891.601 | 2377.614 | 1849.813 | 2120.568 | 1818.602 | 1455.397 | 1248.552 | 2004.816 | 2029.752 | 2082.977 | 1672.079 | 1634.318 | 2536.388 | 2955.19 | 2464.001 | 1690.584 | 2348.331 | 2656.499 | 2354.203 | 2256.115 | 2236.34 | 3060.282 | 1877.805 | 2483.895 | 1516.22 |
| CUST_4455_PI429010886 | N16792 | 0.002451 | 24 | 0 | | | | | | 51.04482 | 37.99335 | 65.68694 | 33.29463 | 40.81311 | 37.31224 | 51.23152 | 40.63191 | 54.14583 | 39.70836 | 31.56943 | 29.28327 | 35.59331 | 42.36335 | 27.20523 | 29.37651 | 44.44423 | 37.27042 | 37.10254 | 30.65915 | 45.71627 | 54.1859 | 46.41914 | 52.05083 | 85.54854 | 47.90583 | 45.7637 |
| CUST_7786_PI429010886 | N22074 | 0.002452 | 18 | 9 | 157105197 | glycine dehydrogenase [Aedes aegypti] >gi\|108869 | Metabolic process | Cellular amino acid metabolic process | 5.6E-128 | 88.52351 | 99.70619 | 92.57501 | 84.92511 | 63.27271 | 54.55243 | 61.61655 | 110.9478 | 104.5737 | 126.727 | 108.5779 | 123.9272 | 121.4843 | 140.3314 | 99.30005 | 105.798 | 84.96006 | 61.81309 | 113.0468 | 153.6441 | 84.05405 | 81.13979 | 89.97695 | 72.5683 | 103.9053 | 88.18553 | 127.6382 |
| CUST_36365_PI429010886 | M15717 | 0.002453 | 15 | 9 | | | | | | 32.88145 | 32.54126 | 71.40808 | 12.30553 | 8.572306 | NA | 53.48774 | 38.92558 | 91.55434 | 70.21778 | 39.34212 | 43.33729 | 68.38203 | 131.1004 | 32.27198 | 47.80199 | 49.17946 | 33.28531 | 10.04937 | 27.62695 | 17.88327 | 104.9178 | 153.9817 | 12.09699 | 269.9093 | 48.80089 | 476.0617 |
| CUST_52559_PI429010886 | M97939 | 0.002453 | 18 | 15 | | | | | | 10.09872 | NA | NA | 6.678981 | 27.4692 | 19.76282 | 14.30414 | 14.08168 | 20.98222 | 6.053883 | 4.823868 | 6.697246 | 10.62697 | 8.711267 | 9.410572 | 34.36064 | 25.75936 | 38.66828 | 23.65678 | NA | 22.81578 | 29.85007 | 18.74405 | 16.57544 | 46.32451 | 8.494473 | 13.49483 |
| CUST_17951_PI429010886 | N38892 | 0.002472 | 18 | 3 | 148224993 | nucleoside diphosphate-linked moiety X motif 17 [X | | | 1.47E-18 | 46.14411 | 36.59373 | 49.35995 | 53.8998 | 34.88888 | 63.3148 | 32.25819 | 32.78199 | 38.60692 | 33.37295 | 32.58533 | 28.14021 | 42.16986 | 38.31256 | 27.31503 | 32.82252 | 41.93393 | 34.27356 | 39.55733 | 20.94079 | 36.56569 | 41.50677 | 87.68861 | 63.95336 | 43.86454 | 42.09086 | 32.46954 |
| CUST_9425_PI429010886 | N24604 | 0.002484 | 18 | 0 | | | | | | 176.3863 | 180.9607 | 161.6447 | 147.6293 | 144.2537 | 122.1911 | 116.6682 | 133.8309 | 130.9642 | 175.0456 | 147.8113 | 163.3967 | 135.2179 | 169.275 | 128.7368 | 193.8001 | 162.1184 | 137.5108 | 213.2126 | 186.3412 | 213.0626 | 160.1366 | 129.9123 | 107.0931 | 193.9691 | 122.5998 | 121.562 |
| CUST_40134_PI429010886 | M50848 | 0.002486 | 21 | 12 | 37780426 | hemocyanin 2 [Pacifastacus leniusculus] | Transport | Other | 1.62E-09 | 3.967645 | 97.44558 | 47.15656 | 255.9554 | 195.3201 | 42.2531 | 116.4359 | 1177.547 | 236.5291 | 123.0412 | 1088.645 | 1253.307 | 2761.396 | 55.72398 | 1305.146 | 5.699756 | 494.8206 | NA | 54.88431 | 20510.07 | 357.9328 | 7.526258 | 36.97731 | 18.36981 | 10.10137 | 458.0989 | 820.5141 |
| CUST_9208_PI429010886 | N24234 | 0.002492 | 15 | 12 | | | | | | 246.92 | 269.1187 | 320.2376 | 229.2792 | 233.9751 | 166.4452 | 230.8646 | 216.6058 | 276.4162 | 322.671 | 211.4751 | 271.1281 | 280.7715 | 323.9834 | 254.2781 | 369.0374 | 262.4649 | 252.7105 | 226.8738 | 196.2921 | 243.5153 | 279.6762 | 244.6702 | 216.1505 | 300.5825 | 238.0845 | 292.0563 |
| CUST_24512_PI429010886 | N50750 | 0.002492 | 15 | 12 | | | | | | 31.07173 | 19.48105 | 16.74068 | 12.71932 | 27.53501 | 15.27554 | 10.37992 | 9.816503 | 10.76087 | 21.13287 | 13.90672 | 20.77394 | 25.749 | 27.86117 | 23.95916 | 14.26332 | 10.35975 | 20.44923 | 20.70392 | 14.79157 | 19.12122 | 23.07547 | 6.716238 | 12.80802 | 23.9514 | 17.24517 | 18.50615 |
| CUST_26049_PI429010886 | N53566 | 0.002492 | 21 | 3 | | | | | | 28.02553 | 87.94324 | 43.73764 | 168.2678 | 63.65837 | 66.27543 | 54.94382 | 35.07318 | 58.10187 | 37.22874 | 23.80497 | 57.92964 | 25.37501 | 45.82004 | 34.76682 | 37.58201 | 12.27296 | 50.6408 | 29.62034 | 38.84241 | 75.89665 | 53.28731 | 98.86383 | 54.10071 | 73.73837 | 79.59942 | 42.81625 |
| CUST_31921_PI429010886 | M2738 | 0.002507 | 18 | 12 | 465995229 | Protein TSSC1, partial [Chelonia mydas] | | | 4.67E-89 | 281.4899 | 446.4087 | 351.1194 | 286.3036 | 238.8599 | 242.6123 | 225.3596 | 349.1356 | 323.0511 | 386.4471 | 332.5951 | 427.5571 | 351.2576 | 381.4199 | 344.1554 | 461.7538 | 255.6621 | 350.7295 | 306.8029 | 386.5159 | 267.0878 | 326.6284 | 240.3331 | 260.5944 | 326.8661 | 292.6086 | 303.7146 |
| CUST_24073_PI429010886 | N49983 | 0.002508 | 18 | 12 | | | | | | 7717.52 | 10200.91 | 8117.934 | 5065.56 | 7876.866 | 6665.624 | 6894.743 | 6005.835 | 7995.416 | 14583.52 | 7034.347 | 11143.54 | 10415.22 | 11413.33 | 11838.35 | 12563.99 | 6694.984 | 10247.75 | 7188.247 | 11437.58 | 7828.426 | 10575.43 | 5819.009 | 5830.309 | 11243.07 | 8421.341 | 10190.76 |
| CUST_22630_PI429010886 | N47363 | 0.002509 | 18 | 3 | | | | | | 11.82579 | 8.567471 | 11.74252 | 43.35936 | 53.52565 | 58.85113 | 11.31889 | 15.36262 | 39.08441 | 11.35551 | 8.711267 | 14.42119 | 12.25276 | 10.71325 | 9.126873 | 64.18045 | 4.067469 | 41.53296 | 14.74805 | 16.59282 | 17.11711 | 21.15466 | NA | 23.07266 | 16.0587 | 25.40852 | 20.46051 |
| CUST_11309_PI429010886 | N27599 | 0.002514 | 18 | 0 | | | | | | 30.19387 | 16.94555 | 20.01489 | 14.74805 | 17.83843 | 6.500221 | 4.624212 | NA | NA | 15.81096 | NA | 15.25972 | NA | 15.99082 | 11.21554 | 6.041641 | NA | 14.09963 | 22.07787 | 9.085039 | 20.14785 | 14.91553 | 14.63523 | 8.73506 | 8.336358 | 10.32397 | 8.720501 |
| CUST_613_PI429010886 | N6328 | 0.002515 | 15 | 9 | | | | | | 65.68089 | 56.56158 | 46.19529 | 33.94434 | 47.68335 | 35.17499 | 52.37245 | 69.216 | 55.47668 | 86.23591 | 49.5373 | 87.84073 | 61.53357 | 72.8194 | 50.32404 | 67.36225 | 45.46228 | 48.19648 | 43.91811 | 51.2626 | 48.94831 | 46.52112 | 57.60743 | 54.28784 | 77.39037 | 44.24444 | 82.53975 |
| CUST_10754_PI429010886 | N26735 | 0.002517 | 24 | 0 | | | | | | 64.52054 | 53.97066 | 43.27178 | 31.40253 | 36.01332 | 61.29672 | 44.09799 | 28.53867 | 31.28664 | 33.1299 | 41.27495 | 32.60462 | 25.72523 | 39.8966 | 39.19383 | 41.12787 | 30.34438 | 30.06057 | 45.34827 | 23.61475 | 35.28275 | 56.03237 | 38.05146 | 43.31294 | 45.31583 | 48.04331 | 65.38012 |
| CUST_34438_PI429010886 | M9680 | 0.002517 | 21 | 0 | | | | | | 19.75359 | 7.030976 | 25.749 | 17.29225 | 6.992992 | 10.08763 | 6.343801 | 6.170344 | NA | 5.002446 | 6.268939 | 11.04742 | 4.852014 | 10.9288 | 6.559123 | NA | 7.07379 | NA | 8.191948 | 9.059534 | 11.16007 | 15.06859 | 10.6327 | 13.19779 | 10.9847 | 10.58947 | 9.177025 |
| CUST_549_PI429010886 | N5938 | 0.002518 | 21 | 15 | | | | | | 2693.745 | 3651.445 | 1731.857 | 4031.594 | 6380.497 | 5004.678 | 4.427429 | 37.20514 | 789.7368 | 1251.807 | 956.4623 | 3855.839 | 2230.655 | 1588.949 | 2789.972 | 10884.33 | NA | 7060.087 | 2274.622 | 4803.756 | 4598.774 | 9770.164 | 3197.843 | 3355.686 | 3087.397 | 818.9073 | 1007.032 |
| CUST_9749_PI429010886 | N25139 | 0.00252 | 15 | 9 | | | | | | 33.26462 | 126.2631 | 93.98995 | 25.73345 | 40.75734 | NA | NA | 31.82074 | 117.7859 | 198.5364 | 118.8883 | 273.1122 | 68.95198 | 174.4788 | 130.4973 | 114.9498 | 31.72455 | 88.24844 | 73.67904 | 174.8316 | 90.36196 | 163.1844 | 125.9169 | NA | 189.9402 | 227.312 | 308.9771 |
| CUST_50322_PI429010886 | M89442 | 0.002521 | 12 | 0 | | | | | | 32.3288 | 26.94089 | 36.41327 | 26.40308 | 19.89057 | 20.8959 | 20.03907 | 21.30062 | 19.01018 | 22.15217 | 23.4177 | 16.56922 | 26.4083 | 48.75681 | 23.07266 | 20.57577 | 22.40881 | 17.57242 | 23.85138 | 27.73533 | 7.405027 | 35.70225 | 25.86961 | 23.17075 | 29.08151 | 26.17206 | 34.9503 |
| CUST_2791_PI429010886 | N13115 | 0.002529 | 21 | 6 | 242008167 | vacuolar amino acid transporter, putative [Pediculu | | | 6.5E-99 | 71.19859 | 68.06658 | 66.32627 | 76.75659 | 68.61799 | 95.86282 | 109.5173 | 84.27092 | 100.9197 | 71.38963 | 62.74739 | 64.9512 | 76.02137 | 43.57816 | 65.63314 | 66.06376 | 55.82952 | 65.87006 | 53.66581 | 66.48727 | 64.90973 | 42.02786 | 67.52947 | 99.34504 | 72.38234 | 80.82297 | 52.36466 |
| CUST_3806_PI429010886 | N15313 | 0.002536 | 15 | 12 | 255985514 | Kazal-type proteinase inhibitor [Pacifastacus leniusc | | | 1.02E-24 | 97.18779 | 244.1579 | 217.159 | 39.87225 | 29.42815 | 33.26462 | 175.6552 | 108.555 | 256.8944 | 238.7009 | 150.8429 | 98.50813 | 311.8796 | 160.0546 | 160.6473 | 117.67 | 179.1099 | 65.02562 | 76.2979 | 108.9026 | 92.73234 | 120.5115 | 80.05215 | 45.07578 | 156.5208 | 75.49979 | 212.8309 |
| CUST_46054_PI429010886 | M71204 | 0.002538 | 24 | 18 | | | | | | 19.49159 | 23.70771 | 14.18775 | 15.29798 | 29.2558 | 12.0262 | 19.88479 | 10.75433 | 15.84192 | 22.18561 | 19.63409 | 12.81928 | 17.14547 | 27.42777 | 25.41871 | 32.85525 | 33.14786 | 25.50317 | 30.75644 | 29.5179 | 35.19263 | 13.68168 | 29.83725 | 14.34646 | 29.55524 | 15.73405 | 9.073537 |
| CUST_23038_PI429010886 | N48149 | 0.002544 | 18 | 9 | | | | | | 100.0068 | 79.14043 | 155.1093 | 89.30785 | 94.30105 | 92.23086 | 97.82533 | 99.79838 | 98.43882 | 122.6119 | 127.0638 | 124.1861 | 85.48477 | 117.2199 | 111.0532 | 113.6641 | 116.7026 | 96.25237 | 101.0393 | 95.32771 | 95.80114 | 77.50869 | 145.9425 | 72.21046 | 115.5 | 120.8512 | 119.3486 |
| CUST_22072_PI429010886 | N46372 | 0.002544 | 21 | 18 | | | | | | 683.1376 | 626.2648 | 818.4343 | 586.5167 | 569.6586 | 661.1589 | 568.3832 | 553.514 | 578.2355 | 650.8924 | 619.5429 | 631.6256 | 641.5416 | 666.1474 | 712.3784 | 787.7599 | 655.7469 | 603.7585 | 646.4459 | 739.0931 | 696.1501 | 655.9864 | 634.7435 | 588.8612 | 696.5534 | 610.3506 | 692.5393 |
| CUST_35206_PI429010886 | M11898 | 0.002552 | 18 | 0 | | | | | | 20.74032 | 16.01119 | 19.34811 | 19.80135 | 15.2714 | 25.85777 | 6.66209 | 10.68037 | 7.700308 | 30.06057 | 8.183936 | 14.18613 | 16.83531 | 17.91994 | 16.23932 | 16.5954 | 13.23457 | 27.77642 | 18.77555 | 24.07736 | 27.88984 | 17.25706 | 27.89681 | 12.82841 | 13.93866 | 5.982092 | 16.17036 |
| CUST_54036_PI429010886 | M104196 | 0.002565 | 24 | 18 | | | | | | 51.59241 | 47.02203 | 60.7605 | 42.85091 | 51.54393 | 36.37157 | 51.47065 | 39.02375 | 40.66714 | 54.46899 | 77.73408 | 61.57414 | 39.85765 | 48.58552 | 47.0845 | 61.20199 | 98.15841 | 55.44212 | 72.72106 | 77.99438 | 63.39728 | 50.09195 | 65.22887 | 54.07518 | 60.08391 | 41.7785 | 75.48481 |
| CUST_36411_PI429010886 | M15834 | 0.002566 | 24 | 15 | | | | | | 706.0046 | 481.5308 | 595.8659 | 439.2082 | 281.3948 | 450.3936 | 936.7815 | 478.7395 | 601.1869 | 1468.172 | 569.1252 | 541.31 | 909.4624 | 566.9632 | 387.2348 | 1730.048 | 1042.392 | 578.8676 | 1094.71 | 757.8647 | 533.6685 | 565.434 | 658.1422 | 562.1725 | 513.797 | 411.751 | 654.3393 |
| CUST_47938_PI429010886 | M78235 | 0.002566 | 21 | 3 | | | | | | 21.9058 | 20.96791 | 19.96619 | 30.93394 | 27.28673 | 42.74237 | 17.93568 | 5.253549 | 20.08697 | 18.87506 | 17.14965 | 21.22623 | 15.96275 | 24.31121 | 21.56963 | 16.79729 | 6.56829 | 26.39959 | 17.66287 | 15.63968 | 16.29239 | 20.07638 | 38.96658 | 17.01511 | 26.78191 | 22.93702 | 25.09429 |
| CUST_42840_PI429010886 | M61391 | 0.002574 | 24 | 21 | 525021360 | PREDICTED: TELO2-interacting protein 2 [Ficedula a | | | 6.64E-07 | 31.43244 | 35.55553 | 43.8479 | 18.7912 | 26.31693 | 19.9426 | 25.64937 | 21.01736 | 18.61312 | 43.95633 | 22.88601 | 21.56178 | 27.13299 | 30.95933 | 36.75997 | 39.93998 | 25.08483 | 32.81302 | 31.23744 | 35.61182 | 32.05856 | 42.61497 | 46.15285 | 28.73136 | 38.98692 | 33.83579 | 31.14945 |
| CUST_52192_PI429010886 | M96695 | 0.002574 | 24 | 18 | | | | | | 33.98937 | 30.12077 | 33.45023 | 20.87254 | 18.97668 | 15.2636 | 19.37009 | 21.80739 | 19.48299 | 42.95635 | 20.71979 | 21.61291 | 20.78572 | 25.37 | 22.11463 | 29.88134 | 28.11972 | 27.78422 | 28.20146 | 41.8496 | 26.35743 | 39.55173 | 25.79616 | 19.39031 | 32.89748 | 11.32793 | 22.4434 |
| CUST_9319_PI429010886 | N24434 | 0.002574 | 18 | 15 | 242011856 | zinc metalloproteinase, putative [Pediculus humanu | | | 1.31E-20 | 858.2338 | 763.6837 | 872.8348 | 466.4129 | 886.1313 | 759.5836 | 581 | 421.6001 | 596.4585 | 1214.293 | 665.6609 | 964.9461 | 504.2482 | 656.1679 | 536.4898 | 1374.989 | 793.5884 | 1035.582 | 832.9715 | 595.5631 | 753.9359 | 923.7156 | 452.6313 | 304.1627 | 364.2987 | 421.7857 | 401.1607 |
| CUST_18292_PI429010886 | N39514 | 0.002578 | 18 | 3 | 321469843 | hypothetical protein DAPPUDRAFT_318231 [Daphn | Transport | Protein transport | 6.13E-41 | 49.32644 | 149.4128 | 52.08136 | 132.5094 | 52.56971 | 93.39666 | 53.01853 | 40.18883 | 50.39093 | 47.68335 | 23.50904 | 59.06999 | 35.48457 | 50.74126 | 17.17879 | 39.98555 | 25.79784 | 40.53465 | 26.14767 | 41.29432 | 77.6254 | 44.78041 | 101.5278 | 71.85634 | 57.41889 | 79.49534 | 38.30389 |
| CUST_48484_PI429010886 | M80570 | 0.002579 | 15 | 0 | | | | | | 1511.146 | 1316.85 | 1642.855 | 1236.256 | 1107.396 | 2095.03 | 492.8667 | 528.3775 | 615.3731 | 918.9933 | 1278.309 | 1512.011 | 376.4458 | 1433.182 | 1004.987 | 1554.539 | 1202.61 | 1847.968 | 997.4048 | 2077.773 | 1315.293 | 1212.885 | 796.7421 | 685.5063 | 1154.447 | 1392.643 | 1098.078 |
| CUST_3440_PI429010886 | N14503 | 0.002579 | 15 | 9 | 512850564 | PREDICTED: von Willebrand factor D and EGF doma | | | 8.72E-18 | 1416.05 | 2774.636 | 1433.821 | 804.8286 | 3576.101 | 204.5562 | 486.9644 | 978.4784 | 2009.101 | 6329.544 | 3051.185 | 6260.184 | 2876.173 | 2596.508 | 2736.774 | 4161.995 | 856.5552 | 2009.101 | 1441.828 | 885.2161 | 1261.41 | 3065.467 | 1885.749 | 537.1757 | 4706.053 | 1497.781 | 4309.606 |
| CUST_27643_PI429010886 | N56429 | 0.002589 | 21 | 15 | | | | | | 116.3563 | 106.9663 | 98.31044 | 80.82297 | 97.61331 | 63.17657 | 97.37669 | 89.61164 | 91.066 | 82.50766 | 101.6576 | 120.8888 | 95.76592 | 97.47845 | 97.96017 | 108.3989 | 157.6076 | 125.6357 | 119.9827 | 88.06388 | 103.3649 | 108.0902 | 93.81682 | 108.657 | 112.5941 | 78.19261 | 73.84429 |
| CUST_6111_PI429010886 | N19643 | 0.002596 | 21 | 9 | 260810737 | hypothetical protein BRAFLDRAFT_66615 [Branchio | | | 6.74E-22 | 4.983069 | 5.205738 | NA | 20.35072 | 36.23985 | 15.3428 | 21.70044 | 81.9929 | 62.85005 | NA | 91.07591 | 92.8298 | 74.09449 | 12.59689 | 32.07598 | 3.694473 | 37.10254 | 12.74489 | 4.578086 | 176.5188 | 71.62646 | NA | 11.85953 | 10.20858 | NA | 21.9738 | 28.32791 |
| CUST_47787_PI429010886 | M77618 | 0.002606 | 12 | 3 | 478256790 | hypothetical protein YQE_06536, partial [Dendroct | | | 2.54E-18 | 99.4135 | 59.57796 | 62.90335 | 140.8068 | 217.8082 | 177.1792 | 212.046 | 98.18381 | 105.2542 | 44.50009 | 165.2021 | 39.18988 | 88.4815 | 52.70775 | 77.65762 | 103.9891 | 228.0483 | 140.5892 | 114.9603 | 36.16451 | 128.969 | 94.02768 | 88.39516 | 165.2714 | 83.19226 | 74.67944 | 33.23109 |
| CUST_4770_PI429010886 | N17444 | 0.002606 | 12 | 0 | | | | | | 130.6431 | 121.9963 | 152.8803 | 107.6007 | 112.5446 | 100.3232 | 93.83775 | 123.1997 | 88.51347 | 136.3782 | 115.9131 | 137.8813 | 119.6624 | 181.1348 | 150.3004 | 122.7515 | 123.5719 | 116.516 | 112.8928 | 125.4528 | 121.3759 | 146.7445 | 121.2504 | 138.0216 | 145.4402 | 141.6752 | 137.9332 |
| CUST_11340_PI429010886 | N27650 | 0.002606 | 12 | 0 | | | | | | 1209.913 | 3942.272 | 2642.053 | 734.7434 | 261.5579 | 287.9804 | 564.2409 | 1697.824 | 1599.783 | 1977.124 | 2273.444 | 3419.141 | 1863.799 | 3549.404 | 2582.117 | 796.983 | 675.5498 | 1832.42 | 1335.109 | 6536.631 | 1177.547 | 839.2544 | 2452.337 | 402.8509 | 2079.573 | 4303.084 | 3281.126 |
| CUST_22923_PI429010886 | N47927 | 0.002608 | 15 | 12 | | | | | | 18.2155 | 22.21414 | 18.76417 | 19.01762 | 16.1321 | 22.55627 | 15.3592 | 14.75394 | 9.570885 | 17.23726 | 11.7224 | 22.79581 | 17.02832 | 22.90764 | 32.38272 | 19.02839 | 18.97898 | 17.62531 | 11.69586 | 18.47407 | 19.11241 | 14.33237 | 12.47875 | 11.71536 | 22.67277 | 27.80089 | 21.54483 |
| CUST_29472_PI429010886 | N59934 | 0.002609 | 24 | 21 | | | | | | 11.91576 | 20.60205 | 18.36165 | 15.49062 | 13.89365 | 16.85251 | 10.23894 | 11.63473 | 9.895282 | 12.6884 | 9.473492 | 14.00504 | 7.160939 | 16.16487 | 12.53064 | 19.05757 | 13.66601 | 22.57964 | 13.37205 | 16.6126 | 28.42343 | 22.0851 | 19.06722 | 14.40546 | 24.8301 | 14.50945 | 18.12277 |
| CUST_35101_PI429010886 | M11577 | 0.002612 | 15 | 6 | | | | | | 73.33005 | 60.44797 | 72.44248 | 66.92364 | 95.50344 | 73.32053 | 101.6676 | 123.8281 | 115.478 | 90.82199 | 90.15151 | 100.668 | 101.3798 | 81.03057 | 79.71189 | 67.51475 | 102.8432 | 57.75281 | 106.2171 | 84.32383 | 73.42232 | 115.284 | 92.86535 | 76.27537 | 114.4136 | 73.86349 | 118.6617 |
| CUST_15731_PI429010886 | N35044 | 0.002613 | 18 | 0 | | | | | | 60.12109 | 78.84971 | 71.68684 | 66.76607 | 67.94324 | 65.26948 | 41.66889 | 50.58305 | 48.23121 | 77.36396 | 53.80266 | 53.76242 | 55.37764 | 56.64379 | 63.92583 | 82.07629 | 58.40782 | 76.46353 | 62.04982 | 78.44959 | 64.53642 | 81.55572 | 51.28805 | 50.91495 | 54.33444 | 47.86881 | 64.68215 |
| CUST_31428_PI429010886 | M1500 | 0.002614 | 12 | 6 | 478304706 | aldehyde dehydrogenase 3-1 variant B [Aedes aegy | Metabolic process | Cellular amino acid metabolic process | 6.8E-101 | 18.03368 | 12.89788 | 7.468703 | 23.32806 | 19.13703 | 27.42544 | 14.97143 | 50.41901 | 37.2354 | 23.53335 | 54.32031 | 55.5381 | 14.27944 | 13.00375 | 14.4465 | 14.0442 | 23.86861 | 13.80995 | 21.43684 | 72.2558 | 42.74724 | 20.76211 | 17.82561 | 14.46778 | 11.11474 | 24.72623 | 16.59282 |
| CUST_34740_PI429010886 | M10522 | 0.002614 | 18 | 15 | | | | | | 82.16288 | 70.34764 | 62.3113 | 64.73839 | 63.97505 | 53.07285 | 54.12178 | 53.88876 | 66.57113 | 108.7034 | 66.98492 | 71.80124 | 68.16131 | 64.72483 | 73.62986 | 93.46673 | 84.73278 | 60.55923 | 69.87848 | 57.34357 | 67.06219 | 59.74927 | 54.06396 | 49.54106 | 78.1117 | 61.07679 | 66.51857 |
| CUST_23444_PI429010886 | N48851 | 0.002617 | 21 | 18 | | | | | | 25.82739 | 28.44308 | 29.79862 | 7.638107 | 27.22266 | 17.34013 | 11.7952 | 12.51566 | 20.22094 | 32.43294 | 19.63939 | 15.69662 | 18.91018 | 19.20292 | 28.22043 | 32.96039 | 20.47619 | 20.37942 | 25.32849 | 31.86956 | 34.03541 | 22.97006 | 29.41419 | 23.93544 | 35.60156 | 17.08222 | 21.99795 |
| CUST_745_PI429010886 | N7111 | 0.002639 | 21 | 9 | | | | | | 476.8506 | 437.0609 | 231.1968 | 410.8257 | 165.3955 | 158.2913 | 497.1091 | 146.5685 | 288.3979 | 1217.974 | 343.216 | 1550.902 | 809.6878 | 679.9331 | 400.8956 | 392.8804 | 483.2456 | 489.338 | 1042.897 | 128.5885 | 712.5461 | 338.892 | 357.6255 | 208.8792 | 101.6676 | 216.1856 | 315.9925 |
| CUST_49139_PI429010886 | M84314 | 0.002644 | 15 | 0 | | | | | | 100.944 | 307.7422 | 201.008 | 70.14592 | 135.0409 | 139.0616 | 53.13993 | 18.07682 | 68.9445 | 82.78386 | 136.0993 | 92.40099 | 33.37295 | 116.2372 | 66.33339 | 127.5818 | 103.9053 | 177.6428 | 97.95028 | 97.73561 | 105.8358 | 102.8077 | 58.04233 | 56.37263 | 106.908 | 52.55999 | 66.6859 |
| CUST_50385_PI429010886 | M89655 | 0.002645 | 12 | 9 | | | | | | 43.57431 | 27.07492 | 77.53281 | 4.364395 | NA | NA | 38.78083 | 58.82472 | 28.26309 | 101.3025 | 31.14945 | 55.29787 | 50.12098 | 72.07584 | 35.12625 | 4.84199 | 27.03027 | 10.23206 | 39.25762 | 15.39298 | 19.49659 | 60.93646 | 62.60675 | 56.32248 | 90.38098 | 50.43461 | 86.47473 |
| CUST_36477_PI429010886 | M16012 | 0.002654 | 24 | 0 | | | | | | 198.2869 | 181.2996 | 265.4463 | 185.9766 | 174.3026 | 167.5472 | 148.1409 | 159.8062 | 127.5355 | 170.5103 | 157.8626 | 120.1854 | 128.9181 | 146.1108 | 160.974 | 228.9161 | 194.7223 | 167.9263 | 179.8168 | 201.1076 | 133.6865 | 268.6688 | 124.1467 | 153.9392 | 235.1729 | 200.9492 | 195.3573 |
| CUST_51518_PI429010886 | M93819 | 0.002675 | 21 | 3 | 321478548 | hypothetical protein DAPPUDRAFT_303085 [Daphn | Metabolic process | Carbohydrate metabolic process | 1.18E-07 | 13.66784 | 20.58261 | 18.14003 | 22.13925 | 13.99239 | 19.98178 | 7.327597 | 14.42559 | NA | 8.013121 | 4.091735 | 9.023167 | 9.755316 | 12.85382 | 7.837273 | 9.129182 | 8.447696 | 9.268485 | 12.20868 | 11.92804 | 20.91226 | 10.07966 | 12.81312 | 9.631079 | 13.09004 | 13.295 | 10.75755 |
| CUST_4120_PI429010886 | N16059 | 0.002676 | 15 | 9 | | | | | | 112.7975 | 118.5776 | 123.2283 | 86.17533 | 109.1565 | 91.83515 | 100.1974 | 114.8562 | 114.0301 | 166.6809 | 103.6058 | 170.3686 | 120.8733 | 119.5328 | 170.9831 | 140.4504 | 127.8836 | 123.5719 | 91.82524 | 132.0957 | 108.3989 | 150.8165 | 116.2372 | 96.55629 | 144.3246 | 154.7047 | 147.073 |
| CUST_33537_PI429010886 | M7207 | 0.002678 | 15 | 3 | | | | | | 1384.531 | 26.00775 | 1442.354 | 1399.299 | 2461.297 | 1814.412 | 1395.016 | 402.9446 | 162.7472 | 60.36203 | 1592.642 | NA | 216.4071 | 80.92356 | 87.00147 | NA | 2766.721 | 1052.364 | 4537.099 | NA | 1482.523 | NA | 1947.429 | 2363.842 | 17.92979 | 386.2115 | 14.5958 |
| CUST_9840_PI429010886 | N25282 | 0.002679 | 21 | 0 | | | | | | 298.9754 | 207.1975 | 228.0216 | 142.7994 | 184.7219 | 135.403 | 226.6353 | 157.378 | 164.7626 | 189.6264 | 166.5598 | 124.5589 | 141.8965 | 133.6452 | 130.2006 | 205.8829 | 208.936 | 139.8135 | 196.9122 | 151.752 | 171.3364 | 205.5616 | 183.021 | 224.9679 | 223.2901 | 143.2947 | 180.2584 |
| CUST_5035_PI429010886 | N18180 | 0.002691 | 21 | 0 | | | | | | 1210.418 | 1156.728 | 1334.604 | 1019.044 | 1073.527 | 1142.155 | 973.9722 | 962.8744 | 879.939 | 984.2764 | 989.1564 | 1225.452 | 871.9238 | 720.0434 | 1135.012 | 1209.913 | 1277.616 | 1242.81 | 1248.106 | 969.5225 | 1023.389 | 1104.958 | 1247.588 | 1311.23 | 1001.156 | 1080.655 | 876.1774 |
| CUST_28123_PI429010886 | N57339 | 0.002698 | 21 | 0 | | | | | | 9.059534 | 16.83531 | 18.18793 | 8.291054 | 14.80809 | 6.868272 | 9.902997 | 8.052649 | 13.16535 | 8.851901 | 6.449652 | 7.708974 | 10.7807 | NA | 14.01249 | 18.76152 | 15.79894 | 14.27753 | NA | 14.02581 | 11.93292 | 16.51414 | 27.27234 | 20.44752 | 6.756825 | 14.23639 | 4.601952 |
| CUST_47529_PI429010886 | M76486 | 0.002705 | 12 | 3 | 330434836 | hypothetical protein [Macrobrachium nipponense] | | | 3.26E-10 | 104.46 | 214.1332 | 118.275 | 128.3451 | 368.0783 | 48.30896 | 94.38745 | 106.9212 | 136.1436 | 77.02903 | 51.89227 | 29.42444 | 86.12374 | 67.90618 | 62.1467 | 251.6265 | 158.7513 | 172.9267 | 126.4932 | 165.9977 | 187.3568 | 64.05654 | 74.76616 | 90.50136 | 150.6483 | 153.8729 | 81.73063 |
| CUST_25463_PI429010886 | N52462 | 0.002708 | 24 | 0 | 296245397 | inhibitor of apoptosis protein [Litopenaeus vannam | | | 5.03E-27 | 96.09446 | 102.4647 | 121.0374 | 152.8609 | 87.59441 | 69.216 | 116.6217 | 83.06954 | 87.6188 | 92.31619 | 86.28429 | 92.01375 | 65.02052 | 83.74259 | 86.85226 | 74.84295 | 60.37132 | 84.86918 | 82.29118 | 83.58732 | 113.5368 | 92.06564 | 79.1048 | 72.73041 | 109.8902 | 92.61954 | 94.7334 |
| CUST_52403_PI429010886 | M97333 | 0.002708 | 15 | 9 | 321461236 | hypothetical protein DAPPUDRAFT_254702 [Daphni | | | 2.12E-09 | 68.38918 | 27.54447 | 839.5092 | 14.56343 | 5.893349 | 8.186201 | 88.71715 | 256.333 | 2250.529 | 1144.662 | 1197.916 | 1903.519 | 428.7852 | 3949.649 | 2203.176 | 790.3406 | 287.6352 | 470.1075 | 363.9558 | 1469.899 | 383.9376 | 3551.684 | 3599.759 | 67.79099 | 4044.665 | 1548.599 | 5830.309 |
| CUST_1027_PI429010886 | N8264 | 0.002708 | 15 | 3 | 546673984 | hypothetical protein D910_02907 [Dendroctonus p | | | 8.97E-20 | 40.63191 | 163.329 | 56.76781 | 134.5271 | 174.3314 | 90.45788 | 66.09037 | 7.584705 | 56.9044 | 34.06396 | 40.07867 | 50.29826 | 55.59887 | 83.83314 | 59.40544 | 187.0999 | 54.63011 | 70.83788 | 31.88591 | 219.9571 | 64.60382 | 84.93221 | 60.43419 | 43.86105 | 37.38397 | 35.60511 | 21.7873 |
| CUST_28359_PI429010886 | N57800 | 0.002712 | 18 | 3 | | | | | | 32.83834 | 23.96204 | 34.64486 | 28.24369 | 76.61613 | 39.16114 | 23.78208 | 10.01285 | 24.71578 | 24.21798 | 26.89586 | 18.40021 | 16.23047 | 20.61883 | 18.84309 | 30.53983 | 20.97008 | 12.54832 | 37.32162 | 17.95452 | 16.44239 | 40.28418 | 29.88417 | 28.69415 | 41.72002 | 37.74785 | 19.98178 |
| CUST_28900_PI429010886 | N58815 | 0.002728 | 18 | 0 | | | | | | 41.97456 | 42.45744 | 42.34172 | 37.13409 | 37.75935 | 31.06856 | 28.6142 | 31.75969 | 32.99848 | 19.55335 | 39.42313 | 19.86545 | 24.19346 | 32.99565 | 32.53672 | 38.43363 | 38.56543 | 38.51022 | 51.6678 | 29.6615 | 41.23729 | 34.92634 | 37.72041 | 46.16276 | 49.63154 | 28.40026 | 24.8201 |
| CUST_4734_PI429010886 | N17371 | 0.002731 | 21 | 9 | | | | | | 479.5081 | 501.702 | 397.525 | 718.4589 | 637.2498 | 537.0505 | 805.2979 | 320.5083 | 1991.454 | 2049.694 | 742.4096 | 492.3863 | 671.0656 | 1864.705 | 702.4802 | 1667.846 | 946.7835 | 937.6859 | 552.8917 | 642.8014 | 577.0235 | 228.6362 | 380.8862 | 271.8631 | 3696.299 | 705.2005 | 3001.59 |
| CUST_8953_PI429010886 | N23847 | 0.002738 | 12 | 0 | 332380580 | syntenin [Penaeus monodon] | | | 4.65E-86 | 59.29234 | 52.63381 | 90.43345 | 40.49813 | 41.95242 | 31.55142 | 48.34765 | 80.35671 | 66.62115 | 75.75933 | 52.97579 | 67.48171 | 72.54647 | 81.79478 | 64.10673 | 47.22465 | 66.53186 | 40.3031 | 59.06999 | 64.82649 | 45.62376 | 51.79552 | 55.16975 | 42.34616 | 97.25472 | 59.07548 | 144.5244 |
| CUST_6412_PI429010886 | N20067 | 0.002741 | 15 | 9 | | | Protein metabolism | Cellular protein modification process | | 111.1643 | 197.5444 | 125.8598 | 31.66766 | 35.88829 | 13.75707 | 16.2854 | 37.75561 | 38.38764 | 279.3325 | 455.8444 | 694.099 | 64.62929 | 427.0325 | 288.3979 | 257.0386 | 39.35128 | 95.99909 | 224.6396 | 254.3377 | 249.5102 | 121.2041 | 286.423 | 9.707875 | 510.619 | 381.2578 | 744.0064 |
| CUST_55401_PI429010886 | M109932 | 0.002749 | 24 | 12 | | | | | | 671.0656 | 378.7197 | 836.4238 | 587.1049 | 637.0757 | 768.7795 | 741.589 | 467.3451 | 745.0756 | 836.7397 | 718.6829 | 892.8593 | 1056.762 | 1322.445 | 1136.737 | 923.7156 | 857.3719 | 947.2744 | 783.8018 | 976.6512 | 758.445 | 711.9048 | 1057.219 | 827.8275 | 618.5189 | 768.5376 | 1418.381 |
| CUST_16131_PI429010886 | N35710 | 0.002762 | 21 | 18 | 260782339 | hypothetical protein BRAFLDRAFT_288962 [Branchi | | | 4.37E-55 | 53.59509 | 69.89586 | 79.16747 | 79.70393 | 54.75352 | 66.87846 | 60.31958 | 53.05965 | 45.42508 | 56.85652 | 73.67904 | 71.09841 | 61.81309 | 71.6044 | 86.64513 | 70.63158 | 77.39915 | 78.72104 | 82.02402 | 69.78353 | 98.49703 | 61.99224 | 77.95158 | 97.70002 | 60.14737 | 63.1832 | 70.75343 |
| CUST_56605_PI429010886 | M114208 | 0.002764 | 18 | 15 | | | | | | 388.0401 | 247.0088 | 341.9433 | 110.8992 | 425.8413 | 317.3817 | 450.7936 | 318.3844 | 321.5473 | 550.9319 | 276.7912 | 363.2398 | 517.8895 | 464.7526 | 406.415 | 445.1412 | 468.0946 | 655.5465 | 335.4299 | 348.906 | 324.9748 | 366.1623 | 184.8121 | 160.1083 | 163.6892 | 301.7456 | 379.0179 |

| Probe ID | ID | p-value | Period | Phase | EBI ID | Annotation | GO category | Go term | E-value | 0 | 0 | 0 | 3 | 3 | 3 | 6 | 6 | 6 | 9 | 9 | 9 | 12 | 12 | 12 | 15 | 15 | 15 | 18 | 18 | 18 | 21 | 21 | 21 | 24 | 24 | 24 |
|---|---|---|---|---|---|---|---|---|---|---|---|---|---|---|---|---|---|---|---|---|---|---|---|---|---|---|---|---|---|---|---|---|---|---|---|---|
| CUST_45634_PI429010886 | M69744 | 0.002771 | 12 | 6 | 443709493 | hypothetical protein CAPTEDRAFT_17949 [Capitella | Metabolic process | Oxidation-reduction process | 6.55E-20 | 17.7146 | 10.48086 | 11.14291 | 28.40026 | 15.25038 | 22.81875 | 58.892 | 21.0027 | 22.39942 | 11.77644 | 21.63606 | 20.30807 | 19.95159 | 16.57948 | 15.1245 | 12.98669 | 23.48661 | 23.82095 | 19.07974 | 33.61445 | 22.28718 | 17.86001 | 16.91321 | 23.85453 | 10.27148 | 18.70291 | 13.25603 |
| CUST_51280_PI429010886 | M92920 | 0.002771 | 12 | 0 | | | | | | 89.0565 | 91.59828 | 107.4484 | 97.04503 | 85.32395 | 79.86307 | 69.87848 | 77.87542 | 82.90947 | 97.77159 | 66.04832 | 88.14739 | 105.1 | 124.2142 | 131.7044 | 109.5513 | 75.52934 | 106.9318 | 50.77711 | 66.39266 | 89.0456 | 111.6728 | 85.18737 | 99.01075 | 94.94128 | 89.26714 | 105.8476 |
| CUST_56338_PI429010886 | M113541 | 0.002773 | 15 | 9 | 291242287 | PREDICTED: ribosomal protein L23-like [Saccoglossu | Protein metabolism | Ribosome biogenesis | 1.19E-30 | 842.9384 | 944.0597 | 931.4066 | 714.7333 | 946.7835 | 963.151 | 818.0738 | 801.0442 | 953.9498 | 1145.003 | 950.8038 | 1256.084 | 891.0101 | 1118.208 | 1076.944 | 1023.389 | 879.3705 | 966.1688 | 875.926 | 882.5331 | 897.6332 | 1242.81 | 920.3504 | 947.8741 | 1328.909 | 1000.493 | 1133.519 |
| CUST_11380_PI429010886 | N27720 | 0.00278 | 18 | 3 | 432958375 | PREDICTED: oocyte zinc finger protein XlCOF6-like [ | | | 1.92E-25 | 14.54491 | 11.88414 | NA | 18.7895 | 14.1684 | 17.3028 | 16.92026 | 8.674882 | 11.51778 | 17.78837 | 15.87262 | 8.870531 | 4.072154 | NA | 5.692545 | 7.187407 | 13.18006 | 9.53977 | 13.58792 | 10.07966 | 20.12612 | 10.65706 | 23.33049 | 22.27554 | 11.28199 | 7.167214 | 5.900143 |
| CUST_46108_PI429010886 | M71402 | 0.002781 | 12 | 3 | 260176409 | Euphausia superba isolate Hap_405 cytochrome c o | Metabolic process | Generation of precursor metabolites and energy | 6.42E-85 | 29.09978 | 19.83698 | 32.13434 | 49.61919 | 37.62291 | 50.77029 | 48.01455 | 42.95145 | 37.68293 | 25.03208 | 29.12589 | 31.56432 | 29.5273 | 26.67261 | 25.70354 | 27.18775 | 61.86332 | 40.62855 | 45.31583 | 30.25566 | 35.32948 | 31.66434 | 47.15656 | 29.72539 | 33.41929 | 33.61051 | 25.47702 |
| CUST_11112_PI429010886 | N27285 | 0.002784 | 21 | 18 | | | | | | 68.2359 | 67.58357 | 76.53451 | 43.93802 | 51.93952 | 83.8218 | 61.8328 | 64.11406 | 48.78617 | 56.08637 | 47.3594 | 40.89955 | 50.53472 | 60.78952 | 63.21853 | 57.31513 | 83.96253 | 61.94492 | 93.01036 | 78.13459 | 58.58074 | 87.94324 | 54.86156 | 84.02515 | 57.58322 | 55.98343 | 63.65227 |
| CUST_32869_PI429010886 | M5322 | 0.002785 | 24 | 21 | | | | | | 9.139595 | 10.05433 | 20.44752 | NA | 4.130332 | NA | 5.332222 | NA | 4.935503 | 8.0157 | 3.936787 | 4.36971 | 5.116273 | 11.33971 | 7.049754 | 10.96515 | 11.01416 | 7.541361 | 7.734699 | 11.70432 | 6.367638 | 12.7088 | 11.56735 | NA | 15.81822 | 6.667395 | 8.044043 |
| CUST_44980_PI429010886 | M67606 | 0.002787 | 21 | 15 | 196016816 | hypothetical protein TRIADDRAFT_33849 [Trichopla | Metabolic process | Cellular amino acid metabolic process | 1.22E-29 | 8.162714 | 7.447234 | 13.24259 | 10.20858 | 9.174525 | 3.834178 | 8.858469 | 14.98047 | 9.017882 | 15.67634 | 16.65148 | 10.54361 | 18.28004 | 7.559884 | NA | 19.95634 | 12.57101 | NA | 7.822452 | 33.24682 | 11.33427 | NA | 6.812001 | 12.6973 | 5.809979 | 5.010923 | 6.674655 |
| CUST_27857_PI429010886 | N56824 | 0.002788 | 18 | 0 | | | | | | 163.0758 | 234.5938 | 181.9887 | 45.09375 | 157.2097 | 55.09927 | 8.790994 | 58.91203 | 30.39754 | 62.76739 | 108.7837 | 103.9198 | 11.678 | 74.74764 | 58.50555 | 164.0153 | 11.11331 | 29.39265 | 242.1982 | 208.9955 | 320.5709 | 63.42473 | 41.87987 | 60.01583 | 28.06124 | 203.7634 | 17.84852 |
| CUST_3865_PI429010886 | N15440 | 0.00279 | 15 | 12 | | | | | | 56.4484 | 60.12109 | 60.68591 | 46.08794 | 52.97579 | 43.93042 | 55.76383 | 47.58792 | 56.11071 | 65.22887 | 60.16545 | 61.12299 | 62.78892 | 65.03693 | 63.67102 | 427.8624 | 54.04881 | 99.2894 | 63.45518 | 57.57631 | 48.98425 | 58.34728 | 59.86271 | 43.41406 | 82.73488 | 67.22077 | 70.41805 |
| CUST_54761_PI429010886 | M107512 | 0.002805 | 15 | 9 | | | | | | 23.49823 | 30.71209 | 34.03541 | 21.83723 | 20.25021 | 23.51171 | 27.57791 | 25.99897 | 26.3191 | 27.5887 | 24.26504 | 38.57181 | 27.32084 | 31.94483 | 17.25337 | 45.29693 | 19.34025 | 34.17683 | 16.54329 | 25.8552 | 15.96955 | 23.64437 | 27.83166 | 19.79173 | 36.8879 | 45.38512 | 46.90121 |
| CUST_32425_PI429010886 | M4090 | 0.002805 | 15 | 9 | | | | | | 127.7038 | 151.9294 | 144.1009 | 109.4643 | 105.8816 | 102.6772 | 78.40746 | 107.4857 | 138.9958 | 184.4394 | 158.5869 | 224.5446 | 135.7577 | 193.267 | 183.3031 | 201.9745 | 112.2867 | 145.7727 | 127.5355 | 183.6281 | 118.9612 | 193.8995 | 165.6637 | 104.7461 | 226.0436 | 169.0651 | 197.5444 |
| CUST_41249_PI429010886 | M57239 | 0.002805 | 15 | 9 | | | | | | 1704.986 | 2299.417 | 2050.871 | 1416.429 | 1621.23 | 1498.845 | 1354.655 | 1634.318 | 1984.848 | 2197.498 | 1682.368 | 2831.641 | 1914.945 | 2168.863 | 2254.33 | 2516.877 | 1566.438 | 1863.078 | 1573.105 | 1802.411 | 1779.751 | 2119.433 | 1987.549 | 1428.149 | 2738.341 | 2091.598 | 2782.19 |
| CUST_49469_PI429010886 | M85776 | 0.002805 | 15 | 9 | 197361188 | signal transducer and activator of transcription [Fer | Signal transduction | Other | 6.46E-22 | 96.1698 | 129.6714 | 144.0441 | 90.29804 | 73.83558 | 78.21131 | 98.4006 | 107.8449 | 143.5804 | 195.689 | 91.09799 | 191.7619 | 108.3037 | 166.5412 | 154.1347 | 137.759 | 96.85587 | 105.3086 | 113.9999 | 112.1069 | 109.8291 | 160.8926 | 126.6474 | 95.73603 | 188.3767 | 140.4954 | 194.605 |
| CUST_29515_PI429010886 | N60021 | 0.002805 | 15 | 9 | | | | | | 408.4945 | 482.4063 | 501.3373 | 411.4634 | 399.93 | 422.1729 | 350.0802 | 429.3715 | 475.874 | 561.2769 | 450.0163 | 594.7631 | 443.3821 | 596.3171 | 426.2225 | 511.8133 | 424.2001 | 521.3278 | 409.7595 | 436.8338 | 485.2041 | 544.5308 | 482.287 | 335.333 | 724.7977 | 552.8917 | 714.0634 |
| CUST_25271_PI429010886 | N52096 | 0.002806 | 18 | 3 | 543335593 | innexin 2 [Penaeus monodon] | | | 2.82E-09 | 5.366751 | 9.099665 | 7.450142 | 11.7681 | 10.73339 | 10.97825 | 6.878181 | 8.992487 | 6.981775 | 4.852014 | 9.212485 | NA | 7.104442 | 7.158847 | NA | 8.553452 | 9.479243 | 10.34037 | 7.728763 | 9.648135 | NA | 9.881836 | 13.40566 | 15.46253 | 8.193123 | 12.47694 | 6.917097 |
| CUST_8377_PI429010886 | N22958 | 0.00281 | 18 | 9 | | | | | | 7.989468 | 9.286054 | 10.59051 | 9.857121 | 10.29115 | 9.845679 | 15.41448 | 13.55063 | 14.17989 | 29.25886 | 15.2174 | 56.47886 | 14.046 | 18.74405 | 22.00022 | 13.58003 | 16.00911 | 13.89423 | 5.427639 | 26.93871 | 8.59214 | 16.0166 | 10.69206 | 17.23726 | 27.49426 | 5.879149 | 11.88011 |
| CUST_13909_PI429010886 | N31986 | 0.002824 | 18 | 3 | | | | | | 35.4902 | 56.54516 | NA | 131.9942 | 94.08412 | 84.25035 | 55.64622 | 5.863493 | 46.95122 | 5.141434 | 4.488345 | 7.496731 | 20.70392 | 56.69692 | 58.99785 | 112.8118 | 6.566142 | 79.07032 | 8.427427 | 56.85652 | 24.02357 | 135.5608 | 8.8599 | 117.3985 | 41.57514 | 10.90695 | NA |
| CUST_47882_PI429010886 | M78000 | 0.002827 | 24 | 9 | | | | | | 75.40046 | 219.061 | 109.19 | 227.4855 | 150.5005 | 84.36786 | 174.0963 | 298.7079 | 159.878 | 223.0044 | 261.767 | 268.545 | 97.21485 | 141.2568 | 89.75292 | 135.0881 | 65.99023 | 59.31402 | 22.2285 | 144.7517 | 63.55528 | 20.97008 | 169.9973 | 149.0992 | 20.61883 | 165.2021 | 71.09841 |
| CUST_44050_PI429010886 | M64764 | 0.002837 | 15 | 9 | | | | | | 44.75093 | 70.69633 | 67.11047 | 48.32025 | 34.30707 | 36.62775 | 60.88717 | 37.24643 | 58.03638 | 57.8814 | 54.61808 | 122.7034 | 54.80919 | 60.47979 | 69.58233 | 59.43076 | 29.41046 | 47.75268 | 40.29023 | 52.54081 | 37.81058 | 53.15159 | 75.55792 | 50.22468 | 73.26779 | 57.39701 | 81.52938 |
| CUST_11913_PI429010886 | N28612 | 0.002837 | 15 | 3 | | | | | | 191.9558 | 120.1854 | 58.27728 | 268.4354 | 174.6614 | 236.2498 | 250.036 | 115.7448 | 134.8409 | 36.98363 | 75.71395 | 35.77567 | 135.0881 | 142.727 | 175.4362 | 153.9817 | 158.6127 | 178.255 | 115.9538 | 188.6253 | 92.21711 | 199.1618 | 72.10026 | 176.9549 | 45.87664 | 77.43118 | 23.72179 |
| CUST_19483_PI429010886 | N41657 | 0.002837 | 15 | 9 | 241587680 | conserved hypothetical protein [Ixodes scapularis] > | Nucleic acid metabolism | DNA metabolic process | 9.04E-45 | 20.13696 | 27.31503 | 32.00329 | 20.32136 | 14.84953 | 21.10435 | 15.08066 | 25.02094 | 24.99293 | 35.93002 | 33.88658 | 33.28088 | 22.87829 | 33.87601 | 25.84583 | 32.91621 | 10.9847 | 23.04237 | 25.8552 | 33.11671 | 15.54199 | 29.40744 | 31.58406 | 16.72273 | 35.55848 | 29.82479 | 47.1063 |
| CUST_22974_PI429010886 | N48031 | 0.002865 | 15 | 12 | | | | | | 38.97496 | 60.21705 | 41.65923 | 53.61134 | 41.14049 | 41.45965 | 29.96361 | 41.49369 | 39.26949 | 55.98779 | 50.55699 | 59.35924 | 48.34765 | 46.51079 | 58.6875 | 44.331 | 54.12178 | 44.58058 | 36.75997 | 50.57194 | 34.46849 | 38.41931 | 29.90107 | 27.60111 | 51.7675 | 32.95281 | 52.80587 |
| CUST_25911_PI429010886 | N53285 | 0.002865 | 15 | 12 | 242022418 | heat containing protein, putative [Pediculus human | | | 1.01E-11 | 48.50046 | 42.34172 | 62.87881 | 54.75352 | 55.64028 | 62.94659 | 38.85386 | 36.98717 | 57.95385 | 68.37525 | 58.56187 | 53.1957 | 73.71983 | 83.79573 | 85.82609 | 69.04997 | 67.78689 | 58.36102 | 74.45078 | 83.09489 | 58.0737 | 67.237 | 49.94515 | 36.82067 | 75.15426 | 61.36332 | 71.11942 |
| CUST_53997_PI429010886 | M104021 | 0.002866 | 21 | 3 | | | | | | 199.4521 | 464.4064 | 291.1029 | 768.0399 | 342.1102 | 389.9711 | 226.0436 | 257.0386 | 230.6259 | 275.9326 | 144.8631 | 294.6992 | 55.28753 | 273.6903 | 133.8595 | 181.6707 | 93.11072 | 214.3213 | 117.863 | 148.6917 | 342.6195 | 266.5934 | 486.3734 | 138.6419 | 357.6255 | 405.4301 | 155.6736 |
| CUST_1966_PI429010886 | N11185 | 0.00287 | 15 | 9 | | | | | | 989.1564 | 1165.381 | 1148.695 | 916.176 | 865.174 | 977.4233 | 739.338 | 1095.161 | 988.4432 | 1156.123 | 1036.891 | 1772.36 | 1003.547 | 1517.877 | 1003.294 | 1346.091 | 957.8471 | 1251.807 | 1054.702 | 934.2348 | 1050.577 | 1076.944 | 1246.008 | 1040.9 | 1479.619 | 1361.387 | 1336.322 |
| CUST_49830_PI429010886 | M87361 | 0.002871 | 12 | 9 | | | | | | 116.4744 | 91.6409 | 103.384 | 103.8326 | 114.9975 | 98.32258 | 112.1069 | 109.0449 | 115.4907 | 178.5939 | 135.0409 | 141.0765 | 125.4703 | 145.6462 | 128.8389 | 129.5236 | 105.4104 | 86.49959 | 137.5651 | 98.62916 | 101.0962 | 155.0724 | 146.939 | 121.0512 | 135.0409 | 114.4836 | 142.5243 |
| CUST_2755_PI429010886 | N13041 | 0.002872 | 21 | 15 | | | | | | 124.672 | 66.01381 | 121.9609 | 72.03066 | 77.1951 | 73.15736 | 116.2535 | 106.064 | 80.29008 | 98.96023 | 89.5312 | 82.1182 | 96.32091 | 108.6469 | 104.9253 | 115.7071 | 178.3541 | 106.1066 | 118.675 | 92.86535 | 102.3344 | 113.5868 | 98.98439 | 106.6963 | 103.2145 | 68.22048 | 77.97193 |
| CUST_4247_PI429010886 | N16318 | 0.002873 | 21 | 0 | | | | | | 41.9046 | 66.24577 | 57.21451 | 71.08447 | 31.9232 | 45.86308 | 24.63851 | 11.59303 | 12.11493 | 11.29639 | 18.21042 | NA | 27.4871 | 39.37435 | 34.46849 | 66.26607 | 27.68618 | 23.03433 | 19.94541 | 15.43278 | 64.27512 | 50.29336 | 40.89955 | 64.1188 | 48.02842 | 28.08233 | 37.19805 |
| CUST_18649_PI429010886 | N40167 | 0.002874 | 15 | 3 | | | | | | 7.72466 | 16.00379 | 13.27182 | 50.19147 | 10.17045 | 116.4869 | 9.628728 | 13.2528 | 10.50787 | 14.85935 | 6.820714 | 7.995482 | 4.534132 | 14.12813 | 10.56079 | 30.62818 | 6.664096 | 16.66074 | 20.85111 | 28.40282 | 23.52815 | 21.45934 | 19.0691 | 33.92982 | 8.52275 | 8.753886 | 13.20796 |
| CUST_55972_PI429010886 | M112383 | 0.002876 | 21 | 15 | | | | | | 1672.079 | 1079.252 | 1272.526 | 539.2831 | 1964.934 | 1145.003 | 1701.136 | 1219.823 | 1249.348 | 2984.905 | 1159.33 | 1448.723 | 2426.131 | 1531.812 | 1734.303 | 1862.262 | 1712.532 | 2626.728 | 1507.459 | 1927.058 | 1289.132 | 1680.099 | 716.2921 | 602.6317 | 671.7237 | 1099.991 | 1272.526 |
| CUST_39175_PI429010886 | M35621 | 0.00288 | 21 | 15 | | | | | | 1278.309 | 871.9238 | 1055.17 | 397.8439 | 1293.201 | 1075.686 | 1281.141 | 975.9966 | 864.2169 | 2437.103 | 957.2333 | 1341.448 | 2043.676 | 1500.831 | 1111.525 | 1522.93 | 1659.051 | 1983.417 | 1220.626 | 1532.545 | 1319.125 | 1320.618 | 740.4281 | 636.8404 | 606.1944 | 1171.568 | 1174.011 |
| CUST_33617_PI429010886 | M7437 | 0.002883 | 15 | 9 | | | | | | 1067.598 | 1782.524 | 2022.724 | 495.2283 | 679.5311 | 357.7936 | 1611.105 | 2410.357 | 2923.398 | 3266.951 | 1248.908 | 1798.294 | 3251.962 | 4630.476 | 1688.453 | 2752.569 | 1904.883 | 1189.103 | 1400.624 | 1510.415 | 1469.899 | 3453.79 | 2629.007 | 726.7504 | 7221.522 | 1804.906 | 9756.741 |
| CUST_38302_PI429010886 | M25136 | 0.002891 | 18 | 6 | 166952332 | Lineus viridis ribosomal protein rpl40 mRNA, compl | | | 6.29E-54 | 1152.374 | 1091.604 | 1140.432 | 945.2395 | 1492.759 | 1255.497 | 1587.608 | 1274.525 | 1282.355 | 953.5865 | 1410.296 | 2013.592 | 752.1706 | 1405.495 | 914.9994 | 1241.637 | 891.3926 | 941.2341 | 746.4019 | 1157.228 | 994.4459 | 1286.564 | 1478.956 | 1039.457 | 1270.234 | 1890.591 | 1704.986 |
| CUST_12019_PI429010886 | N28784 | 0.002891 | 18 | 9 | 242012607 | protein purity of essence, putative [Pediculus huma | Protein metabolism | Cellular protein modification process | 2.12E-34 | 100.4445 | 111.6142 | 104.7362 | 92.66541 | 101.92 | 102.5545 | 87.00147 | 99.93216 | 94.87745 | 157.1911 | 110.7887 | 127.0638 | 111.7487 | 111.6728 | 117.1499 | 123.9789 | 115.9538 | 112.8267 | 111.9633 | 108.6177 | 85.37214 | 104.6153 | 90.77809 | 93.03197 | 145.201 | 85.30129 | 115.5725 |
| CUST_46319_PI429010886 | M72196 | 0.002897 | 21 | 0 | 262401177 | RNA pseudouridylate synthase domain containing 3 | | | 2.12E-09 | 10.94511 | 12.58679 | NA | 6.007021 | 14.10671 | NA | 4.681127 | 8.222884 | 7.156953 | 9.15176 | 7.240049 | 4.335589 | NA | 7.506584 | 3.424992 | 9.032579 | 4.818127 | 8.060022 | 5.867579 | 9.662859 | NA | 12.35967 | NA | 11.73913 | 11.44141 | NA | 8.591341 |
| CUST_56573_PI429010886 | M114140 | 0.002902 | 15 | 9 | | | | | | 711.432 | 886.7923 | 965.2192 | 708.9187 | 732.5018 | 701.3851 | 654.4842 | 764.0731 | 815.8741 | 976.6512 | 799.1588 | 973.2028 | 760.2724 | 1055.17 | 955.0872 | 972.1119 | 835.4969 | 830.4425 | 817.4781 | 886.3986 | 820.0337 | 951.2956 | 974.3808 | 832.672 | 1087.534 | 991.907 | 1170.02 |
| CUST_41563_PI429010886 | M58041 | 0.002902 | 15 | 9 | 260833120 | hypothetical protein BRAFLDRAFT_63860 [Branchio | | | 2.86E-24 | 41.50205 | 42.71137 | 48.72124 | 21.02215 | 24.4716 | 27.21982 | 32.73103 | 31.70409 | 36.72024 | 58.32968 | 34.34564 | 38.71949 | 34.01329 | 41.36789 | 31.59756 | 47.23018 | 33.33046 | 41.05122 | 36.8462 | 27.31066 | 29.55233 | 42.94589 | 43.63321 | 21.24309 | 51.20393 | 43.85239 | 48.60566 |
| CUST_5708_PI429010886 | N19078 | 0.002904 | 21 | 15 | | | | | | 170.5103 | 182.5193 | 132.4515 | 133.064 | 167.2734 | 126.4395 | 136.3926 | 103.7132 | 114.0624 | 195.1585 | 173.8312 | 157.1911 | 114.0624 | 153.9132 | 132.1901 | 231.4164 | 229.3656 | 261.1858 | 172.1829 | 192.2628 | 144.8631 | 145.1427 | 120.9334 | 148.0995 | 194.4273 | 144.1009 | 151.3617 |
| CUST_1345_PI429010886 | N9349 | 0.002905 | 12 | 9 | | | | | | 436.6018 | 538.667 | 560.5893 | 456.2722 | 435.1229 | 405.2638 | 396.9018 | 425.0392 | 464.198 | 837.9656 | 522.6581 | 689.4854 | 469.6397 | 641.887 | 528.1283 | 537.1757 | 394.958 | 469.7466 | 471.0971 | 553.6437 | 488.3379 | 765.0242 | 561.029 | 576.5142 | 1167.349 | 503.7682 | 1000.816 |
| CUST_18267_PI429010886 | N39467 | 0.002906 | 15 | 9 | | | | | | 52.47261 | 68.1364 | 94.17604 | 64.79479 | 58.54952 | 107.0039 | 122.452 | 76.77824 | 127.2103 | 132.2265 | 73.18236 | 137.9867 | 74.64865 | 126.3708 | 104.8392 | 58.2166 | 74.93674 | 131.4929 | 40.06113 | 60.34766 | 72.62704 | 102.205 | 98.90127 | 162.6527 | 94.48599 | 151.0563 | 133.2798 |
| CUST_40390_PI429010886 | M54965 | 0.002927 | 18 | 6 | 56968003 | Anaspides tasmaniae internal transcribed spacer 2 a | | | | 101693.2 | 97928.01 | 108910.4 | 102282.3 | 113412.4 | 77297.7 | 122512.8 | 102912.5 | 113975.2 | 122512.8 | 121014.1 | 101693.2 | 115015.4 | 83322.86 | 102282.3 | 87164.76 | 89222.73 | 95014.94 | 115015.4 | 97307.86 | 96660.96 | 113412.4 | 109583.6 | 95621.16 | 121965.7 | 118691.9 | 112462.6 |
| CUST_14147_PI429010886 | N32394 | 0.002928 | 18 | 15 | | | | | | 26.48449 | 42.52496 | 33.04717 | 23.88015 | 23.66736 | 22.08177 | 30.96855 | 44.55872 | 45.58535 | 42.51309 | 35.69319 | 39.01912 | 34.9939 | 48.50046 | 35.44816 | 35.82359 | 53.9799 | 43.87581 | 38.90216 | 27.00493 | 29.15169 | 28.85125 | 21.67714 | 36.23985 | 24.87526 | 20.18328 | 39.00381 |
| CUST_40526_PI429010886 | M55368 | 0.002932 | 18 | 15 | 345490044 | PREDICTED: LOW QUALITY PROTEIN: hypothetical p | | | 9.31E-50 | 13.02142 | 9.848126 | 15.22088 | 8.147639 | 14.45048 | 12.75631 | 16.58665 | 10.02996 | 8.457009 | 20.53586 | 14.11461 | 10.16036 | 17.132 | 18.04108 | 18.38957 | 24.3983 | 41.93775 | 15.50711 | 12.29647 | 20.77886 | 14.30893 | 15.4684 | 6.548611 | 10.33535 | 13.04571 | 13.26174 | 17.72667 |
| CUST_36376_PI429010886 | M15752 | 0.002933 | 15 | 12 | | | | | | 206.6992 | 217.9412 | 205.9692 | 160.3693 | 175.9719 | 157.0244 | 195.487 | 206.8341 | 199.8275 | 248.4954 | 231.9868 | 228.6651 | 236.0839 | 247.0492 | 202.1175 | 298.1495 | 207.885 | 187.954 | 189.144 | 214.5304 | 157.9764 | 232.9174 | 244.0822 | 164.9622 | 302.4916 | 188.9032 | 218.2859 |
| CUST_43583_PI429010886 | M63406 | 0.002933 | 15 | 12 | | | | | | 420.025 | 596.3171 | 595.1496 | 265.6599 | 289.679 | 163.4482 | 643.2335 | 663.7859 | 873.2696 | 1069.858 | 457.8737 | 686.6887 | 1069.514 | 1393.263 | 749.7018 | 1009.897 | 582.4599 | 489.6976 | 568.2975 | 643.0031 | 538.667 | 1424.855 | 699.0502 | 321.9594 | 2439.013 | 593.5055 | 2365.487 |
| CUST_14881_PI429010886 | N33624 | 0.002934 | 24 | 21 | 321470641 | hypothetical protein DAPPUDRAFT_195959 [Daphn | | | 1.99E-34 | 20.46944 | 18.14309 | 12.76773 | 20.72901 | 13.69402 | 9.102009 | 11.30247 | 10.39893 | 8.235102 | 13.74381 | 15.11766 | 10.69206 | 16.16705 | 12.96349 | 12.98148 | 21.15229 | 17.42826 | 10.02222 | 14.85609 | 22.56443 | 13.59657 | 22.41298 | 19.14299 | NA | 15.59665 | 14.25474 | 20.05683 |
| CUST_31959_PI429010886 | M2829 | 0.002938 | 12 | 3 | | | | | | 371.8288 | 659.4054 | 23.47569 | 1332.224 | 761.0622 | 680.9808 | 533.6685 | 27.80368 | 454.8231 | 19.0691 | 14.70984 | 6.506768 | 184.5783 | 132.5751 | 530.042 | 1019.044 | 71.92462 | 730.0267 | 72.51875 | 35.07632 | 14.20045 | 632.4528 | 30.10596 | 365.5076 | 262.5059 | 31.16262 | 51.10924 |
| CUST_6810_PI429010886 | N20630 | 0.002938 | 12 | 3 | 527252945 | PREDICTED: inter-alpha-trypsin inhibitor heavy chai | Metabolic process | Carbohydrate metabolic process | 4.54E-07 | 1302.095 | 723.9047 | 423.9576 | 997.4048 | 1223.726 | 1462.44 | 786.706 | 772.4833 | 441.149 | 630.254 | 211.6792 | 209.3167 | 1021.127 | 620.6433 | 777.7644 | 1332.224 | 1052.364 | 1127.217 | 750.1475 | 671.8789 | 382.1801 | 385.8062 | 624.8531 | 1315.293 | 592.481 | 747.806 | 65.87593 |
| CUST_31031_PI429010886 | M517 | 0.002943 | 21 | 9 | 386376739 | glutathione-dependent prostaglandin D synthase [P | | | 4.95E-20 | 26.40308 | 33.92452 | 23.49374 | 27.51937 | 88.04251 | 44.49519 | 74.15113 | 79.51299 | 51.22479 | 40.59908 | 76.02137 | 90.49352 | 79.1048 | 38.17289 | 55.27747 | 57.62563 | 38.15596 | 27.65747 | 57.29447 | 157.8626 | 61.99224 | 24.65512 | 33.65802 | 20.26285 | 22.42981 | 40.14284 | 76.00655 |
| CUST_48872_PI429010886 | M82628 | 0.002946 | 21 | 0 | | | | | | 188.9157 | 206.7552 | 141.6752 | 248.1506 | 155.8611 | 189.3407 | 97.98092 | 81.15299 | 121.2398 | 110.3616 | 96.61745 | 145.4832 | 136.2923 | 133.6116 | 161.6619 | 124.9549 | 120.7487 | 190.6724 | 138.0216 | 126.5253 | 114.3485 | 218.804 | 144.9176 | 194.605 | 108.3734 | 128.3244 | 132.652 |
| CUST_53568_PI429010886 | M102283 | 0.002955 | 15 | 3 | | | | | | 18.00505 | 38.71497 | NA | 47.41918 | 51.02123 | 48.96463 | 47.12699 | NA | 38.41183 | 9.216332 | 4.279499 | 15.94807 | 15.66738 | 18.12996 | 23.02707 | 33.98233 | NA | 38.78083 | NA | 28.05779 | 15.51227 | 67.67878 | 7.25708 | 34.61952 | 5.125109 | 5.541563 | NA |
| CUST_17821_PI429010886 | N38648 | 0.002956 | 24 | 21 | | | | | | 116.1614 | 71.38156 | 122.3193 | 106.7139 | 99.49562 | 133.8101 | 95.17141 | 98.01409 | 75.40046 | 95.25723 | 93.11072 | 73.76113 | 87.19276 | 128.4938 | 113.5868 | 107.1532 | 161.0687 | 139.419 | 116.5651 | 86.06426 | 113.8702 | 119.6269 | 154.3982 | 179.3194 | 112.9777 | 98.21029 | 104.7461 |
| CUST_20018_PI429010886 | N42657 | 0.002958 | 15 | 12 | | | | | | 7.627544 | 10.08763 | 9.200881 | 5.861533 | 3.762808 | 8.912458 | 12.6285 | 4.122897 | 4.440993 | 8.736759 | 9.020299 | 10.90827 | 14.48944 | 16.77252 | 15.51227 | 15.07833 | 9.248628 | 9.514057 | 8.656419 | 4.92839 | NA | 20.93844 | 6.63545 | 18.13226 | 13.3326 | 11.65447 | 11.35808 |
| CUST_42301_PI429010886 | M59950 | 0.002969 | 15 | 12 | | | | | | 64.03635 | 73.84429 | 58.51806 | 56.43061 | 56.05475 | 55.71823 | 45.56467 | 37.15687 | 53.53068 | 63.33459 | 49.96713 | 71.10646 | 56.36899 | 77.78755 | 61.93065 | 77.33177 | 57.3007 | 68.45737 | 61.54664 | 60.5059 | 37.23914 | 62.29412 | 41.83506 | 51.28378 | 70.05382 | 59.50688 | 53.7412 |
| CUST_17818_PI429010886 | N38644 | 0.002969 | 15 | 9 | 321469866 | hypothetical protein DAPPUDRAFT_317966 [Daphn | | | 2.65E-07 | 124.9216 | 573.9804 | 1321.214 | 312.3161 | 239.0183 | 111.485 | 112.064 | 1588.949 | 6372.368 | 3572.519 | 3143.095 | 5641.729 | 1964.934 | 6932.514 | 3752.095 | 1534.856 | 497.3582 | 853.3995 | 1732.866 | 2083.983 | 2043.676 | 4847.521 | 3254.31 | 323.8952 | 6654.81 | 2978.783 | 11650.8 |
| CUST_40737_PI429010886 | M55969 | 0.002993 | 12 | 6 | | | | | | 9.248628 | 4.969785 | 5.420008 | 8.092867 | 18.59923 | 11.35064 | 4.702682 | 18.17736 | 14.67882 | 25.10548 | 16.71936 | 20.11905 | 8.665868 | NA | 7.665956 | 13.80995 | 13.55063 | 18.52898 | 13.86351 | 32.61498 | 19.67133 | 9.320692 | 14.56714 | 11.36093 | 6.709669 | 9.952171 | 8.074741 |
| CUST_23889_PI429010886 | N49649 | 0.002994 | 18 | 3 | | | | | | 102912.5 | 119417.1 | 113412.4 | 119417.1 | 113975.2 | 146237.4 | 123526 | 120106.4 | 119417.1 | 105375.3 | 99779.5 | 99779.5 | 69125.19 | 109583.6 | 40070.03 | 101136 | 62198.15 | 137174.3 | 110974.5 | 117180.4 | 130067.6 | 125432.7 | 116331.2 | 134984.9 | 99042.74 | 129062.7 | 107761.9 |
| CUST_11970_PI429010886 | N28712 | 0.002995 | 24 | 9 | | | | | | 64.42438 | 100.0408 | 152.5866 | 28.29864 | 106.2399 | 107.5586 | 74.00809 | 98.56528 | 69.76759 | 168.7959 | 119.9711 | 174.8053 | 85.95 | 88.99049 | 84.48505 | 87.69732 | 75.91487 | 58.53699 | 62.87324 | 94.54715 | 41.05859 | 69.03435 | 49.16013 | 34.39152 | 77.64933 | 73.19849 | 100.1275 |
| CUST_57140_PI429010886 | E1410 | 0.002997 | 24 | 21 | 507559876 | PREDICTED: zinc finger protein 665-like [Jaculus jacu | | | 9.46E-26 | 11.8227 | 4.505667 | 11.42367 | 9.830811 | 9.666802 | 11.01907 | 6.759208 | 4.084411 | 8.218065 | 7.072564 | 12.35209 | 17.06824 | 10.22575 | 6.571354 | 12.36224 | 13.71331 | 7.404114 | 12.79969 | 12.2919 | 12.98486 | 12.62537 | 11.45279 | 13.93866 | 17.66287 | 13.92624 | 13.0885 | 13.03254 |
| CUST_44850_PI429010886 | M67199 | 0.003005 | 12 | 3 | | | | | | 16.74068 | 15.10512 | 23.34607 | 43.66151 | 28.99306 | 32.2333 | 25.82153 | 20.71004 | 8.932629 | 24.69848 | 20.30399 | 13.47559 | 17.0365 | 24.16344 | 20.01979 | 20.35771 | 39.54839 | 30.61198 | 29.8328 | 26.01832 | 28.50866 | 14.20045 | 13.04696 | 20.37597 | 25.93179 | 13.27381 | 14.9294 |
| CUST_15933_PI429010886 | N35371 | 0.003017 | 21 | 3 | | | | | | 85.3343 | 90.20594 | 105.8476 | 78.20249 | 91.62991 | 112.8702 | 104.9922 | 45.4027 | 80.89652 | 30.06426 | 51.39386 | 46.85243 | 28.05064 | 70.92151 | 69.89586 | 104.976 | 46.74367 | 74.08523 | 82.75397 | 68.16131 | 28.90009 | 105.9129 | 82.64391 | 85.99449 | 84.51009 | 55.58682 | 119.7645 |
| CUST_18746_PI429010886 | N40341 | 0.003024 | 15 | 0 | | | | | | 65.09833 | 67.34873 | 74.29802 | 61.21559 | 66.03172 | 62.34833 | 64.3475 | 54.10775 | 40.94065 | 49.63764 | 51.23152 | 43.16663 | 30.21036 | 64.87589 | 54.27461 | 74.98811 | 75.07319 | 58.4576 | 78.84468 | 36.40882 | 67.3827 | 53.61134 | 48.80606 | 75.44459 | 52.10631 | 50.2407 | 40.81934 |
| CUST_28264_PI429010886 | N57625 | 0.003032 | 24 | 0 | | | | | | 40.75297 | 39.64527 | 43.61355 | 23.71505 | 31.16623 | 41.75113 | 27.64478 | 39.30599 | 31.17772 | 31.62413 | 36.27978 | 40.38558 | 33.92046 | 22.40135 | 24.26118 | 34.58049 | 28.77687 | 37.59475 | 26.3141 | 20.92107 | 31.96138 | 43.67532 | 29.22688 | 36.04752 | 42.87529 | 41.13272 | 47.37598 |
| CUST_24343_PI429010886 | N50451 | 0.003037 | 18 | 3 | | | | | | 2097.241 | 1877.805 | 2712.01 | 9382.703 | 3896.561 | 6394.985 | 5125.683 | 2567.686 | 689.8687 | 158.3361 | 4515.16 | 123.7772 | 197.9679 | 86.7376 | 1342.338 | 206.6326 | 147.5412 | 285.4569 | 1897.37 | 147.6124 | 2918.106 | 260.4155 | 3835.814 | 4925.566 | 44.36476 | 209.3591 | 74.28436 |
| CUST_14149_PI429010886 | N32398 | 0.003038 | 12 | 3 | 261289661 | hypothetical protein BRAFLDRAFT_119484 [Branchi | | | 2.11E-11 | 53.14405 | 37.63001 | 34.06396 | 46.26383 | 43.439 | 49.38887 | 42.34931 | 36.89793 | 44.69546 | 33.06846 | 34.63494 | 31.83796 | 43.64762 | 36.28805 | 49.92232 | 43.66792 | 53.67582 | 49.50652 | 33.96314 | 39.26949 | 38.65653 | 41.56303 | 29.66445 | 49.28537 | 44.38038 | 42.19897 | 42.25718 |
| CUST_15467_PI429010886 | N34626 | 0.00304 | 18 | 15 | | | | | | 50.40874 | 57.76074 | 66.12938 | 36.05134 | 53.77942 | 26.36033 | 41.41188 | 53.22359 | 37.11863 | 64.26189 | 49.90548 | 47.88942 | 54.35965 | 49.72048 | 44.48344 | 73.27672 | 62.4936 | 66.85557 | 34.41169 | 49.59376 | 41.5428 | 46.1038 | 41.21277 | 33.26796 | 67.47589 | 48.40257 | 56.41974 |
| CUST_15596_PI429010886 | N34844 | 0.003046 | 24 | 15 | | | | | | 10.13899 | 6.474157 | 12.24457 | 12.77756 | 6.847004 | 8.712785 | 16.53993 | NA | 7.126224 | 11.24259 | 14.00504 | 10.43208 | 13.44283 | 11.26061 | 5.97435 | 14.20655 | 27.43563 | 19.04191 | 12.6973 | 10.24818 | 12.58824 | 10.14162 | 14.42559 | 23.28108 | 9.006549 | 6.145307 | 6.646603 |
| CUST_37705_PI429010886 | M20988 | 0.003051 | 18 | 3 | | | | | | 1046.057 | 4743.467 | 3090.22 | 14011.56 | 11276.69 | 7239.058 | 3567.184 | 4455.764 | 3483.315 | 3908.116 | 2125.97 | 6148.012 | 3067.321 | 5376.972 | 914.3958 | 1305.705 | 1710.741 | 5009.146 | 2792.05 | 4041.003 | 5635.242 | 7364.493 | 5023.801 | 4034.014 | 6168.628 | 8470.008 | 3558.109 |
| CUST_47772_PI429010886 | M77555 | 0.003058 | 15 | 12 | | | | | | 20.12612 | 22.15408 | 29.39991 | 16.9925 | 12.54381 | 20.26748 | 13.16535 | 20.27847 | 10.13438 | 21.50201 | 22.19008 | 35.61182 | 28.05304 | 28.86203 | 24.33153 | 28.47129 | 19.74682 | 24.67398 | 19.32763 | 23.99379 | 26.51493 | 21.84275 | 13.46619 | 19.97907 | 26.37038 | 30.88019 | 20.77394 |
| CUST_7373_PI429010886 | N21445 | 0.00306 | 15 | 3 | 260831011 | hypothetical protein BRAFLDRAFT_124267 [Branchi | | | 4.45E-21 | 246.4698 | 189.1194 | 104.8909 | 317.5249 | 240.2928 | 263.5785 | 269.0643 | 171.1636 | 176.8527 | 158.4583 | 127.2423 | 89.38803 | 162.2159 | 144.4836 | 144.5473 | 265.8546 | 185.5201 | 231.3759 | 208.027 | 160.3251 | 197.4111 | 181.2186 | 160.598 | 212.773 | 167.5472 | 178.833 | 118.7113 |
| CUST_13536_PI429010886 | N31350 | 0.003064 | 21 | 18 | | | | | | 13.7763 | 12.27788 | 17.24085 | 10.96072 | 9.773611 | 8.3117 | 6.822524 | NA | 7.024562 | 9.435368 | 7.659037 | 9.073537 | 5.78592 | 13.20951 | 9.703156 | 13.98352 | 13.00185 | 11.49378 | 17.04092 | 11.09833 | 14.48423 | 11.71408 | 6.584593 | 12.67376 | 21.88907 | 4.482425 | 10.47982 |
| CUST_32366_PI429010886 | M3939 | 0.003072 | 12 | 3 | | | | | | 56.16844 | 66.45252 | 64.11406 | 70.06431 | 72.3636 | 79.24559 | 68.1054 | 51.90738 | 43.26125 | 81.07113 | 58.29649 | 50.89404 | 58.50555 | 57.40815 | 56.99751 | 74.17182 | 74.61343 | 80.73902 | 67.9514 | 50.1495 | 64.04369 | 52.44678 | 54.33444 | 38.73055 | 69.42462 | 81.19526 | 63.45518 |
| CUST_12707_PI429010886 | N29905 | 0.003072 | 12 | 3 | | | | | | 149.8707 | 94.04891 | 166.0195 | 172.8801 | 175.7472 | 282.9109 | 152.675 | 167.3634 | 110.7787 | 93.14117 | 128.8934 | 165.1069 | 193.6848 | 95.99909 | 138.7812 | 156.2332 | 288.9328 | 197.0951 | 111.5879 | 165.1872 | 127.7418 | 123.2747 | 123.2747 | 132.551 | 184.7859 | 184.3916 | 165.4521 |
| CUST_23568_PI429010886 | N49042 | 0.003072 | 21 | 15 | | | | | | 14.14626 | NA | NA | 13.64729 | 51.66322 | 64.19796 | 4.432775 | NA | 6.125711 | 26.01607 | 12.51969 | 16.05394 | 42.14262 | 65.80832 | 32.73413 | 159.5684 | 21.0027 | 158.101 | 47.84739 | 74.73352 | 40.37974 | 226.0436 | NA | 23.78208 | 8.051681 | NA | NA |
| CUST_19782_PI429010886 | N42183 | 0.003078 | 15 | 12 | | | | | | 33.22111 | 23.39071 | 23.59619 | 15.43461 | 15.03227 | 24.61812 | 18.20852 | 22.18811 | 25.00865 | 22.19942 | 14.65894 | 23.38375 | 30.52473 | 25.95428 | 32.58962 | 29.49942 | 23.92584 | 27.49118 | 28.47129 | 28.10965 | 32.83834 | 22.19649 | 14.27406 | 13.83521 | 26.62182 | 27.11607 | 25.12351 |
| CUST_23040_PI429010886 | N48151 | 0.003079 | 24 | 12 | | | | | | 14.83888 | 22.21682 | 24.45653 | 12.18876 | 7.61532 | 14.0382 | 19.57585 | 22.38395 | 33.01824 | 28.72316 | 17.54318 | 25.77304 | 24.11276 | 31.47236 | 35.03522 | 22.12202 | 26.9442 | 23.98815 | 30.85708 | 21.41219 | 32.97206 | 22.62281 | 22.52113 | 16.41397 | 19.89057 | 24.99293 | 30.45027 |
| CUST_41889_PI429010886 | M58806 | 0.003089 | 21 | 3 | | | | | | 11.80273 | 28.49553 | 6.915938 | NA | 13.8146 | 14.65167 | 13.13981 | 6.537253 | 12.25435 | 12.60946 | 6.455442 | 10.1142 | 4.670634 | 9.493787 | 9.482226 | 11.33312 | 5.51343 | 10.2343 | NA | 4.888509 | 11.26822 | 10.47118 | 7.460722 | 3.475347 | 13.54942 | 2.161979 | 12.71344 |
| CUST_54852_PI429010886 | M107957 | 0.003095 | 18 | 9 | | | | | | 69.77447 | 81.5806 | 108.7093 | 72.31183 | 82.72689 | 68.82889 | 76.674 | 96.10628 | 86.11085 | 140.3314 | 89.86196 | 115.3926 | 184.1183 | 104.7611 | 94.76564 | 92.26296 | 77.23965 | 114.165 | 96.10628 | 89.82702 | 86.47473 | 102.7028 | 79.23712 | 75.39347 | 119.4111 | 103.0771 | 110.3138 |
| CUST_50613_PI429010886 | M90433 | 0.003097 | 15 | 3 | | | | | | 42.06993 | 57.81384 | 25.3441 | 47.77531 | 46.54874 | 40.91182 | 31.60035 | 44.84989 | 32.33268 | 30.64068 | 21.11131 | 36.85539 | 15.43461 | 22.99283 | 20.78133 | 32.42745 | 29.08714 | 46.50141 | 39.74462 | 41.73826 | 32.05527 | 38.71949 | 26.83996 | 20.18733 | 41.2187 | 25.45947 | 29.05431 |
| CUST_19766_PI429010886 | N42153 | 0.003097 | 15 | 12 | | | | | | 35.50382 | 36.29351 | 26.79789 | 28.03701 | 27.01225 | 18.21981 | 29.72221 | 41.22377 | 54.74747 | 24.4716 | 29.3424 | 29.96361 | 63.96144 | 54.74214 | 58.25978 | 39.11234 | 31.53803 | 42.84203 | 30.43159 | 31.10751 | 20.28753 | 54.48925 | 27.70404 | 31.80323 | 47.78664 | 28.68856 | 56.24668 |
| CUST_29908_PI429010886 | N60798 | 0.003103 | 24 | 21 | | | | | | 84.19357 | 102.3423 | 88.07214 | 64.88975 | 93.99876 | 98.87521 | 63.00378 | 54.058 | 60.12109 | 80.14138 | 87.88231 | 102.7422 | 84.57831 | 106.7749 | 93.33448 | 107.3899 | 78.88657 | 123.6231 | 59.41199 | 84.58422 | 90.38098 | 126.8628 | 103.6762 | 113.5525 | 106.6375 | 77.90078 | 122.6882 |
| CUST_33665_PI429010886 | M7549 | 0.003105 | 21 | 15 | | | Cellular component organization | Cytoskeleton organization | | 31.07874 | 13.86466 | 27.24907 | 27.28431 | 22.34365 | 19.70026 | 17.96396 | 16.49783 | 20.33108 | 15.04174 | 18.66273 | 9.07643 | 28.85616 | 25.48959 | 23.85638 | 30.50923 | 24.57116 | 31.56432 | 31.32056 | 20.81295 | 24.03945 | 26.20596 | 18.51985 | 34.28424 | 22.3138 | 22.39942 | 19.51952 |
| CUST_17891_PI429010886 | N38779 | 0.003107 | 24 | 0 | 157126968 | hypothetical protein AaeL_AAEL010695 [Aedes aeg | | | 5.86E-15 | 58.05303 | 50.28834 | 48.08498 | 31.86259 | 54.5276 | 57.99026 | 38.90216 | 45.91191 | 40.57649 | 45.73272 | 44.01861 | 30.67698 | 32.6566 | 21.55583 | 24.94079 | 48.73236 | 53.84406 | 63.45518 | 50.72999 | 30.99832 | 45.06422 | 44.72593 | 32.98902 | 42.74724 | 66.59221 | 49.20588 | 64.8815 |
| CUST_55905_PI429010886 | M112069 | 0.003107 | 24 | 15 | | | | | | 87.8914 | 125.6016 | 89.98836 | 71.98272 | 79.94364 | 69.76028 | 58.83154 | 61.35068 | 81.91685 | 95.23078 | 77.85613 | 99.94463 | 68.89697 | 97.5983 | 105.19 | 137.6307 | 90.67241 | 99.65085 | 89.16253 | 124.1467 | 95.67336 | 114.6697 | 78.31562 | 85.88841 | 86.61515 | 91.02196 | 102.2534 |
| CUST_24638_PI429010886 | N50996 | 0.003126 | 21 | 18 | | | | | | 80.41737 | 134.8934 | 67.7616 | 69.45519 | 103.9537 | 113.9031 | 56.95742 | 37.97967 | 55.59388 | 61.34598 | 67.40392 | 88.91586 | 105.3086 | 85.75328 | 74.54819 | 59.34275 | 61.63578 | 105.6351 | 104.5131 | 146.1108 | 134.595 | 112.7975 | 73.60851 | 70.66973 | 98.15841 | 91.96613 | 81.81059 |
| CUST_13688_PI429010886 | N31597 | 0.003129 | 15 | 0 | | | | | | 689.0956 | 707.3962 | 568.6876 | 457.5237 | 523.5388 | 540.0536 | 419.5293 | 473.5692 | 412.6269 | 644.1161 | 541.7506 | 608.6975 | 527.8694 | 488.5796 | 508.6284 | 637.0757 | 718.917 | 605.6379 | 618.1584 | 792.7128 | 496.875 | 610.2139 | 485.0905 | 467.7939 | 657.8288 | 500.2189 | 552.8917 |
| CUST_27136_PI429010886 | N55510 | 0.003136 | 15 | 12 | | | | | | 24.80851 | 24.08352 | 22.13925 | 30.12812 | 14.68043 | 25.71973 | 18.13841 | 11.01084 | 9.577239 | 11.24522 | 20.65925 | 20.86543 | 24.37229 | 34.75205 | 26.60787 | 46.22951 | 16.84886 | 38.08646 | 20.42948 | 16.40268 | 18.56074 | 25.48959 | 8.858469 | 16.72612 | 19.87525 | 17.99464 | 24.33153 |
| CUST_34727_PI429010886 | M10477 | 0.003136 | 18 | 15 | | | | | | 175.0831 | 149.2677 | 180.162 | 114.743 | 156.8398 | 159.2768 | 163.8459 | 166.2726 | 159.7825 | 169.0411 | 159.5684 | 182.5193 | 174.9558 | 208.5959 | 182.0796 | 216.0765 | 196.9351 | 196.7621 | 199.9107 | 189.8565 | 132.9657 | 209.8084 | 143.002 | 136.9283 | 217.8082 | 148.8262 | 170.4197 |
| CUST_21770_PI429010886 | N45820 | 0.003136 | 18 | 6 | 528761633 | histone-lysine N-methyltransferase MLL4 [Camelus | Reproduction | Reproduction | 4.26E-10 | 39.40978 | 42.46136 | 46.9717 | 27.07911 | 45.66434 | 44.30287 | 55.82371 | 57.84964 | 47.44008 | 54.74747 | 44.39886 | 54.58961 | 32.39752 | 42.02191 | 42.38165 | 41.36078 | 40.64728 | 31.90512 | 52.09599 | 39.98991 | 53.30954 | 48.80606 | 51.99458 | 30.19101 | 46.9109 | 57.68381 | 62.70814 |
| CUST_51842_PI429010886 | M95356 | 0.003137 | 18 | 12 | 242009979 | conserved hypothetical protein [Pediculus humanus | | | 8.76E-12 | 20.03192 | 14.07729 | 21.92688 | 24.08063 | 19.16725 | 10.71453 | 9.207048 | 36.55477 | 19.69401 | 23.97713 | 20.06979 | 37.63897 | 23.96699 | 34.37569 | 38.54575 | 31.36762 | 26.73668 | 38.24304 | 15.94162 | 21.21095 | 26.01607 | 19.48765 | 19.95854 | 17.60614 | 35.59734 | 29.64495 | 20.45436 |
| CUST_1533_PI429010886 | N9961 | 0.003139 | 12 | 3 | | | | | | 186.7032 | 13.2768 | 56.15273 | 4598.774 | 12955.59 | 13937.5 | 8228.765 | 2511.522 | 59.12378 | 7.858321 | 11182.42 | NA | 344.0991 | 14.96682 | 142.727 | NA | 15353.74 | 7617.588 | 10575.43 | NA | 7686.447 | 12.85718 | 5585.575 | 6833.331 | 6.251086 | 3177.58 | 8.670788 |
| CUST_10663_PI429010886 | N26587 | 0.003144 | 21 | 3 | | | | | | 86.54824 | 105.7694 | 128.4384 | 205.3646 | 102.8709 | 234.141 | 111.6728 | 130.9315 | 145.4206 | 103.384 | 67.64754 | 107.3414 | 113.1518 | 113.2396 | 105.7372 | 84.55489 | 122.6286 | 107.9913 | 88.34755 | 91.5749 | 61.87735 | 62.16132 | 65.89913 | 101.6576 | 110.5649 | 162.0477 | 130.6003 |
| CUST_9986_PI429010886 | N25514 | 0.00315 | 15 | 0 | | | | | | 20.97642 | 20.43255 | 16.95126 | 77.28747 | 4.149795 | NA | 5.274301 | 6.422087 | 12.20868 | 14.58939 | 11.13159 | 9.08845 | 16.04761 | 16.30281 | 16.89803 | 27.08746 | 15.81194 | 11.17076 | 5.601268 | 19.73096 | NA | 8.017122 | 10.7039 | NA | 11.64471 | 11.28527 | 11.76966 |
| CUST_40567_PI429010886 | M55479 | 0.003153 | 24 | 15 | | | | | | 94.43038 | 111.9958 | 116.332 | 96.88082 | 86.62519 | 77.56897 | 88.89139 | 99.91859 | 100.3848 | 137.8443 | 151.2035 | 113.0678 | 108.1306 | 96.29479 | 104.4333 | 171.0777 | 118.2591 | 121.1828 | 100.0297 | 153.5797 | 122.9325 | 121.5738 | 122.7194 | 95.04998 | 115.1745 | 85.17215 | 150.3171 |
| CUST_47939_PI429010886 | M78237 | 0.003174 | 15 | 12 | | | | | | 1284.158 | 1291.558 | 2562.095 | 1376.551 | 1159.33 | 1239.798 | 1548.599 | 2761.396 | 2064.29 | 2454.105 | 1324.694 | 1710.023 | 2153.407 | 3546.259 | 2833.332 | 1550.226 | 1880.44 | 1349.576 | 1330.395 | 1624.747 | 1296.243 | 1953.939 | 2361.621 | 1446.386 | 2673.681 | 1359.544 | 2495.356 |
| CUST_4075_PI429010886 | N15953 | 0.003174 | 15 | 12 | 291241925 | PREDICTED: predicted protein-like [Saccoglossus ko | Metabolic process | Generation of precursor metabolites and energy | 1.29E-47 | 1739.21 | 5445.033 | 2968.006 | 600.4305 | 1367.153 | 783.9719 | 1515.268 | 1251.3 | 2780.239 | 3942.272 | 2090.413 | 6717.481 | 3483.315 | 3300.062 | 4471.709 | 5800.891 | 1396.98 | 3360.345 | 1817.335 | 2764.643 | 2539.516 | 3411.47 | 2454.105 | 1021.127 | 4135.156 | 2935.657 | 5206.134 |
| CUST_13718_PI429010886 | N31651 | 0.003174 | 15 | 12 | | | | | | 14.63523 | 18.35292 | 20.94928 | 11.48646 | 6.540321 | 16.26691 | 8.52275 | 19.51255 | 13.10516 | 27.35532 | 6.800737 | 32.98902 | 25.90485 | 24.8998 | 21.58663 | 22.6805 | 7.770497 | 17.63614 | 15.32584 | 17.90553 | 6.212154 | 18.69088 | 24.67089 | 18.02634 | 26.01055 | 13.50761 | 18.72537 |
| CUST_21824_PI429010886 | N45919 | 0.003174 | 15 | 12 | 459179322 | PREDICTED: tudor domain-containing protein 7 isof | | | 2E-07 | 18.33968 | 70.40792 | 66.46786 | 4.779135 | 12.13343 | 13.29917 | 24.16825 | 15.13462 | 45.19753 | 39.20447 | 26.35513 | 57.04658 | 63.68458 | 86.14614 | 72.03066 | 63.08907 | 18.25579 | 106.2741 | 33.90175 | 34.68251 | 33.43569 | 86.70732 | 44.78592 | 32.94683 | 74.25631 | 74.54008 | 121.5738 |
| CUST_30904_PI429010886 | M175 | 0.003175 | 24 | 12 | | | | | | 93.78702 | 177.5884 | 156.8398 | 132.7602 | 169.8261 | 129.9123 | 188.4915 | 184.2307 | 159.4135 | 167.8053 | 167.8299 | 255.0348 | 179.2012 | 220.9369 | 208.027 | 130.0689 | 128.195 | 117.4728 | 156.213 | 281.447 | 192.2628 | 131.3569 | 117.8039 | 109.4106 | 129.735 | 99.82512 | 135.1566 |
| CUST_19401_PI429010886 | N41518 | 0.003176 | 18 | 3 | | | | | | 347.794 | 770.9286 | 410.8257 | 1296.822 | 479.6761 | 707.6225 | 355.6761 | 276.1029 | 371.3509 | 385.0916 | 181.5941 | 598.8088 | 301.291 | 381.6649 | 248.3781 | 323.0511 | 161.8906 | 259.6247 | 178.7072 | 319.8933 | 571.9057 | 553.0837 | 716.9224 | 391.7449 | 528.8856 | 720.2695 | 296.7865 |
| CUST_23419_PI429010886 | N48807 | 0.003183 | 15 | 12 | | | | | | 23.78208 | 36.86394 | 27.25206 | 24.34759 | 21.28123 | 22.49622 | 18.58338 | 24.68523 | 12.60126 | 37.43361 | 28.88408 | 28.27971 | 19.23258 | 37.58456 | 30.14577 | 25.5315 | 22.65868 | 27.10054 | 11.52352 | 22.80672 | 16.7444 | 30.09768 | 25.91379 | 19.39824 | 37.6014 | 24.89184 | 26.32739 |
| CUST_46166_PI429010886 | M71619 | 0.003199 | 18 | 12 | | | | | | 176.0861 | 222.1754 | 131.0034 | 303.3224 | 115.0894 | 304.5092 | 291.2799 | 159.6292 | 263.1737 | 318.2985 | 282.4695 | 252.4954 | 344.9163 | 377.6304 | 303.1495 | 332.8163 | 276.6094 | 166.7311 | 173.4253 | 173.944 | 114.5044 | 322.7291 | 210.9062 | 195.8687 | 401.3938 | 229.4529 | 247.961 |
| CUST_52359_PI429010886 | M97187 | 0.003199 | 21 | 3 | | | | | | 44.14193 | 76.0951 | 42.48584 | 117.8175 | 52.65189 | 61.89778 | 29.89732 | 49.60877 | 29.95513 | 35.55553 | 31.8 | 54.74214 | 37.71174 | 39.70082 | 43.17605 | 42.95918 | 35.15805 | 53.42416 | 34.95466 | 40.54346 | 58.8975 | 52.77021 | 66.40412 | 66.22546 | 63.23408 | 80.61916 | 59.64714 |
| CUST_10372_PI429010886 | N26134 | 0.003201 | 18 | 6 | | | | | | 6.099554 | 7.481739 | 15.1414 | 8.17741 | 5.102784 | NA | NA | 32.95758 | 57.91335 | 24.65764 | 8.906902 | 17.44803 | NA | 6.946375 | 6.738368 | 10.21711 | 8.949545 | 13.01232 | NA | 12.39561 | 5.967946 | 16.59282 | 25.78586 | 8.352146 | 27.61589 | 18.4246 | 33.40939 |
| CUST_12202_PI429010886 | N29097 | 0.003202 | 21 | 9 | 307177147 | Acetyl-CoA carboxylase [Camponotus floridanus] | | | 6.5E-21 | 8.027473 | 8.506994 | NA | 8.131006 | 15.19655 | 19.81911 | 15.00987 | 30.83339 | 26.90513 | NA | 21.78224 | 31.5882 | 64.04369 | 12.10209 | 21.83723 | 9.323293 | 13.84831 | 15.35034 | NA | 65.13281 | 10.18458 | 13.07517 | 5.49631 | 14.89445 | 12.95283 | 10.05721 | 11.85953 |
| CUST_756_PI429010886 | N7176 | 0.003217 | 24 | 0 | | | | | | 24.8221 | 36.17076 | 26.32456 | 14.29674 | 15.4684 | 16.67559 | 18.14003 | 16.14158 | 20.10077 | 22.43724 | 13.18285 | 20.00172 | 14.24656 | 11.6794 | 16.71936 | 16.51743 | NA | 10.38437 | 6.262467 | 20.87254 | 25.26092 | 16.88307 | 20.58016 | 26.02132 | 21.35972 | 18.43075 | 35.63135 |
| CUST_37212_PI429010886 | M18220 | 0.003225 | 12 | 3 | | | | | | 5.291541 | 5.683749 | 8.981694 | 14.51263 | 13.34705 | 32.06361 | 8.788925 | 17.46708 | 14.88378 | 13.33083 | 15.14895 | 14.28145 | 9.959454 | 8.434609 | NA | 13.24762 | 19.01959 | 20.61498 | 12.98903 | 42.9046 | 10.81295 | 12.72278 | 9.465987 | 10.00467 | 5.133869 | 25.50611 | 5.604736 |
| CUST_7545_PI429010886 | N21705 | 0.003226 | 18 | 12 | 242018362 | myst histone acetyltransferase, putative [Pediculus | Metabolic process | Other | 4.74E-20 | 32.09701 | 19.49659 | 25.73774 | 24.21006 | 21.48663 | 16.11615 | 24.24219 | 24.43037 | 21.89117 | 29.6775 | 24.94778 | 33.57754 | 29.73209 | 33.29815 | 42.29841 | 30.67698 | 33.16347 | 18.5156 | 32.03583 | 31.5882 | 27.84371 | 26.13604 | 23.60773 | 12.6884 | 40.99957 | 21.33093 | 37.52322 |
| CUST_34605_PI429010886 | M10132 | 0.00323 | 18 | 3 | | | | | | 23.2348 | 16.24458 | 43.50197 | 28.18833 | 61.89112 | 42.38165 | 47.27714 | 40.22053 | 24.71119 | 31.65825 | 24.67398 | NA | 20.68784 | 14.99273 | 11.50923 | 17.69248 | 48.54759 | 24.53193 | 23.26285 | 10.56773 | 36.12998 | 40.97837 | 54.91632 | 35.05162 | 32.04305 | 16.85623 | 26.17436 |
| CUST_51055_PI429010886 | M91982 | 0.003231 | 18 | 0 | | | | | | 18.65856 | 14.4005 | 9.54588 | 7.254393 | 8.863586 | 8.999498 | 10.07859 | 9.39688 | 5.167898 | 3.816856 | 7.009314 | NA | 9.284755 | NA | 7.382145 | 10.90827 | 8.459432 | NA | 35.46061 | 8.930028 | 13.4766 | 14.89947 | 11.72847 | 7.598655 | 8.343945 | 9.573493 | 8.652112 |
| CUST_53235_PI429010886 | M100882 | 0.003232 | 21 | 3 | | | | | | 31.01112 | 20.3261 | 30.4923 | 43.13359 | 31.96544 | 45.88881 | 40.75297 | 28.7253 | 29.68892 | 27.24092 | 37.70021 | 26.96182 | 21.66748 | 29.56124 | 21.30938 | 21.57749 | 31.94483 | 45.81525 | 33.05858 | 11.84611 | 21.60172 | 26.38969 | 33.99233 | 29.8705 | 36.00421 | 40.48072 | 40.67008 |
| CUST_35918_PI429010886 | M13910 | 0.003233 | 15 | 9 | 196123668 | cathepsin D-like protein [Homarus americanus] | Protein metabolism | Proteolysis | 2.4E-139 | 4.670634 | 2270.606 | 598.9907 | 2788.353 | 4773.42 | 1051.549 | 1374.518 | 5445.033 | 3956.693 | 1855.858 | 5732.178 | 8228.765 | 4381.739 | 994.7785 | 2169.749 | NA | 1673.62 | 12.14326 | 884.4098 | 1899.44 | 5430.703 | NA | 1939.579 | 1560.159 | NA | 6225.291 | 4813.402 |
| CUST_37283_PI429010886 | M18502 | 0.003242 | 21 | 3 | | | | | | 1478.287 | 5396.825 | 3378.169 | 5681.469 | 4135.156 | 5959.328 | 1732.866 | 2201.928 | 2915.652 | 2236.34 | 1478.956 | 4589.406 | 1614.966 | 2027.433 | 1901.328 | 2779.017 | 1426.735 | 2881.575 | 1172.181 | 2624.92 | 4808.982 | 3192.934 | 4719.422 | 3124.162 | 4348.32 | 6621.858 | 2549.933 |
| CUST_47684_PI429010886 | M77169 | 0.003242 | 21 | 3 | | | | | | 10.88063 | 10.36994 | 14.42999 | 13.81689 | 13.99986 | 15.57429 | 13.46425 | 10.96329 | 10.90993 | 13.62098 | 12.9711 | 13.84485 | 10.71844 | 10.39055 | 9.284755 | 8.984223 | 13.18773 | 12.84883 | 21.52705 | 12.55445 | 16.60676 | 21.39067 | 11.45611 | 13.32908 | 20.18529 | 17.91608 | 45.65948 |
| CUST_42998_PI429010886 | M61814 | 0.003252 | 15 | 12 | | | | | | 408.6642 | 403.3076 | 595.4395 | 335.677 | 328.4337 | 382.1801 | 409.277 | 599.494 | 535.4947 | 649.1033 | 417.4014 | 438.8255 | 486.6501 | 825.7659 | 552.4266 | 506.0078 | 608.0744 | 442.7732 | 389.0546 | 462.6916 | 402.9446 | 584.3722 | 706.0046 | 435.9739 | 780.4444 | 394.7279 | 800.3484 |
| CUST_37976_PI429010886 | M22555 | 0.003256 | 15 | 9 | | | | | | 1490.779 | 1286.564 | 2826.022 | 726.9909 | 1451.1 | 826.9906 | 1309.194 | 1737.368 | 2604.753 | 4613.187 | 2104.851 | 6388.757 | 2953.556 | 3318.146 | 4306.703 | 5492.691 | 1421.77 | 3690.445 | 1795.627 | 2495.356 | 2461.297 | 5402.577 | 2596.508 | 1319.603 | 4729.674 | 3336.627 | 5289.144 |
| CUST_18389_PI429010886 | N39708 | 0.003256 | 15 | 3 | 157112290 | hypothetical protein AaeL_AAEL000946 [Aedes aeg | | | 5.77E-07 | 70.27178 | 64.21464 | 46.0364 | 89.44557 | 67.48171 | 78.89557 | 82.31536 | 44.00542 | 58.45028 | 42.91318 | 64.75716 | 35.76744 | 49.59839 | 50.131 | 37.61547 | 40.37133 | 89.14278 | 65.44799 | 51.84431 | 50.32801 | 86.4151 | 54.95061 | 60.97252 | 89.58206 | 34.49959 | 40.75734 | 37.15687 |
| CUST_48510_PI429010886 | M80728 | 0.003258 | 18 | 3 | | | | | | 175.6284 | 111.9132 | 313.6383 | 187.6821 | 107.2622 | 243.8028 | 186.1103 | 145.5578 | 205.5305 | 99.4135 | 62.45524 | 54.35449 | 159.4333 | 119.8071 | 145.3659 | 94.86726 | 221.3497 | 108.3189 | 259.3866 | 98.56528 | 151.4536 | 219.715 | 231.235 | 458.6579 | 130.2006 | 129.6562 | 91.76014 |
| CUST_21288_PI429010886 | N44953 | 0.003259 | 15 | 12 | 307200017 | Merlin [Harpegnathos saltator] | | | 2.65E-44 | 28.53623 | 43.53998 | 46.92136 | 37.10254 | 30.09768 | 34.36064 | 21.12385 | 48.5148 | 42.06993 | 44.02245 | 36.57353 | 68.38918 | 47.8959 | 50.5231 | 57.05842 | 46.07297 | 36.80914 | 36.39578 | 33.22506 | 37.10603 | 30.60425 | 40.11037 | 28.19099 | 29.36926 | 55.81794 | 48.46881 | 55.46608 |

| Probe ID | ID | p-value | Period | Phase | EBI ID | Annotation | GO category | Go term | E-value | 0 | 0 | 0 | 3 | 3 | 3 | 6 | 6 | 6 | 9 | 9 | 9 | 12 | 12 | 12 | 15 | 15 | 15 | 18 | 18 | 18 | 21 | 21 | 21 | 24 | 24 | 24 |
|---|---|---|---|---|---|---|---|---|---|---|---|---|---|---|---|---|---|---|---|---|---|---|---|---|---|---|---|---|---|---|---|---|---|---|---|---|
| CUST_37125_PI429010886 | M17932 | 0.003259 | 21 | 0 | | | | | | 252.5773 | 342.5053 | 216.7674 | 269.7173 | 230.8271 | 178.3086 | 176.3616 | 166.0384 | 129.3018 | 187.5374 | 188.9473 | 203.1953 | 192.4131 | 241.9211 | 231.2637 | 296.0927 | 128.3042 | 205.5616 | 223.2901 | 301.5058 | 212.9538 | 371.4417 | 261.7284 | 176.9973 | 229.9564 | 209.7628 | 235.838 |
| CUST_57069_PI429010886 | E450 | 0.003271 | 18 | 12 | | | | | | 14.25137 | 19.50921 | 19.36786 | 18.22302 | 14.01249 | 15.65986 | 13.11271 | 31.02643 | 23.15048 | 36.36466 | 16.71152 | 27.51414 | 27.25206 | 34.93063 | 25.45947 | 27.82792 | 21.33598 | 19.10728 | 25.91941 | 24.30839 | 23.11042 | 23.27419 | 15.97633 | 10.77921 | 24.1375 | 12.59689 | 24.9855 |
| CUST_39588_PI429010886 | M41502 | 0.003276 | 15 | 9 | | | | | | 56.29594 | 89.69655 | 82.13647 | 79.30391 | 89.82702 | 104.192 | 122.1911 | 88.64024 | 101.7342 | 107.884 | 95.89707 | 152.424 | 59.47675 | 145.899 | 62.16132 | 46.72053 | 75.4776 | 132.2661 | 53.35562 | 62.86517 | 84.78816 | 83.27348 | 125.7681 | 139.6803 | 155.2181 | 115.3125 | 163.569 |
| CUST_1232_PI429010886 | N8940 | 0.003279 | 24 | 3 | | | | | | 114.1326 | 265.4892 | 301.0804 | 122.2181 | 194.9182 | 285.9079 | 196.359 | 247.2726 | 122.1037 | 153.9132 | 145.749 | 171.7375 | 132.7469 | 213.1848 | 130.9135 | 117.7161 | 51.51637 | 52.97067 | 121.0216 | 46.13321 | 81.92913 | 172.213 | 210.6087 | 116.4203 | 231.3759 | 137.8245 | 209.0287 |
| CUST_9927_PI429010886 | N25424 | 0.003286 | 12 | 9 | | | | | | 47.66523 | 9.227326 | 56.99104 | 6.544569 | 11.68992 | 6.064337 | 18.79689 | 17.64462 | NA | 18.14494 | 16.5578 | 25.23488 | 15.41663 | 21.75964 | 12.03567 | 7.88778 | 16.36343 | 6.535693 | 15.7841 | NA | 7.444212 | 42.7734 | 45.05092 | 35.48044 | 9.175781 | NA | 7.744827 |
| CUST_56255_PI429010886 | M113284 | 0.00329 | 15 | 12 | | | | | | 66.76607 | 76.41163 | 99.1645 | 102.0104 | 74.85405 | 105.779 | 56.00276 | 75.67767 | 51.31578 | 90.38978 | 71.17303 | 106.1516 | 111.2168 | 128.5685 | 100.0408 | 97.89619 | 61.76631 | 87.72886 | 81.22317 | 87.96508 | 74.92071 | 81.60393 | 70.34764 | 63.13991 | 100.3042 | 89.84183 | 79.53245 |
| CUST_47341_PI429010886 | M75696 | 0.00329 | 15 | 12 | | | | | | 576.8367 | 1993.696 | 1722.719 | 338.4841 | 528.026 | 356.5583 | 712.3784 | 1834.08 | 2725.555 | 2522.174 | 900.857 | 2266.195 | 3934.354 | 3372.782 | 2718.679 | 3168.052 | 1847.968 | 1515.268 | 1346.091 | 2232.866 | 1566.438 | 2926.98 | 2692.301 | 748.9581 | 5917.438 | 1463.822 | 9448.983 |
| CUST_18046_PI429010886 | N39049 | 0.003299 | 18 | 9 | | | | | | 37.42448 | 36.4309 | 40.37514 | 22.8445 | 16.62053 | 22.68707 | 23.89866 | 26.50391 | 38.90805 | 47.49104 | 44.06361 | 38.09477 | 44.05675 | 39.46953 | 34.97912 | 43.78734 | 38.06259 | 28.96949 | 40.59586 | 40.97837 | 33.50292 | 35.37524 | 35.38892 | 29.97696 | 43.98243 | 27.4118 | 51.65858 |
| CUST_26773_PI429010886 | N54854 | 0.003302 | 24 | 18 | | | | | | 22.3581 | 25.4154 | 29.66445 | 18.49479 | 16.97579 | 24.0077 | 29.28851 | 16.47383 | 10.68382 | 27.69389 | 20.12366 | 27.56643 | 22.33422 | 39.3477 | 37.35904 | 28.06711 | 43.898 | 19.05109 | 31.07173 | 31.54852 | 35.87751 | 18.58338 | 45.99667 | 31.24487 | 22.05258 | 24.83589 | 20.85366 |
| CUST_2314_PI429010886 | N12087 | 0.003316 | 18 | 3 | | | | | | 84.59116 | 81.60393 | 74.7826 | 83.59685 | 86.57696 | 74.94569 | 57.59651 | 77.64933 | 75.7764 | 67.31728 | 61.60388 | 74.45078 | 61.14806 | 49.26562 | 43.66792 | 73.39754 | 83.16029 | 67.08962 | 66.71625 | 105.5375 | 65.10535 | 120.4034 | 89.71539 | 66.37927 | 119.9288 | 53.38382 | 72.39117 |
| CUST_6209_PI429010886 | N19772 | 0.00332 | 21 | 15 | | | Protein metabolism | Macromolecular complex assembly | | 33.23404 | 30.90791 | 39.68132 | 25.66207 | 29.15809 | 31.25971 | 22.8042 | 27.80089 | 27.20523 | 39.79718 | 21.31486 | 31.45667 | 36.59373 | 29.9579 | 20.1154 | 33.01533 | 39.80783 | 59.27376 | 30.70822 | 45.04433 | 42.37752 | 28.83874 | 33.78634 | 25.75422 | 27.57791 | 36.33335 | 24.94079 |
| CUST_41115_PI429010886 | M56908 | 0.003327 | 21 | 12 | | | | | | 4.976399 | 14.19691 | 17.21077 | 10.62424 | 13.53636 | 7.700308 | 7.276095 | 12.56761 | 4.786961 | 10.6257 | 15.33784 | 23.53335 | 18.7709 | 18.1208 | 25.74156 | 21.15466 | 16.12222 | 15.11263 | 11.11331 | 15.8658 | 13.48931 | 22.58452 | 22.21414 | 14.51263 | 10.95448 | 15.67763 | 12.6619 |
| CUST_4356_PI429010886 | N16585 | 0.003328 | 21 | 0 | | | | | | 76.62236 | 85.48477 | 54.87191 | 57.79515 | 72.39117 | 52.60928 | 47.03677 | 47.49104 | 29.76053 | 50.93999 | 52.54081 | 42.22821 | 26.04672 | 31.34507 | 34.36554 | 50.65756 | 53.14405 | 61.52513 | 60.52796 | 33.39553 | 30.29325 | 66.83042 | 44.89448 | 55.80731 | 64.64151 | 30.76819 | 55.72785 |
| CUST_53416_PI429010886 | M101606 | 0.003329 | 15 | 12 | | | | | | 21.03331 | 20.24204 | 35.74774 | 16.62775 | 11.97713 | 9.840112 | 19.87525 | 39.85359 | 22.60935 | 35.50744 | 14.98837 | 35.19742 | 42.83757 | 53.86751 | 38.73964 | 23.59085 | 30.05351 | 20.93304 | 17.44803 | 28.08233 | 27.60111 | 34.04504 | 40.78834 | 16.06834 | 37.20896 | 22.3138 | 30.40899 |
| CUST_50849_PI429010886 | M91234 | 0.003339 | 18 | 9 | | | | | | 184.7859 | 372.1299 | 455.9675 | 264.8881 | 114.5445 | 100.3128 | 181.4858 | 280.8226 | 90.92371 | 490.7702 | 719.6282 | 589.1626 | 873.2696 | 261.922 | 465.7878 | 349.5009 | 134.2642 | 80.37733 | 271.1821 | 709.8502 | 217.8838 | 66.23254 | 66.86178 | 88.51347 | 1051.932 | 1084.753 | 765.2983 |
| CUST_36322_PI429010886 | M15588 | 0.003347 | 24 | 18 | | | | | | 28.13722 | 18.69493 | 11.40858 | 8.063274 | 8.916592 | 19.11453 | 7.517908 | 8.884013 | 7.1973 | 29.92091 | 17.20887 | 18.1616 | 9.411481 | 17.01358 | 23.77729 | 41.57079 | 15.9498 | 51.77357 | 24.5371 | 30.57426 | 41.59799 | 16.7895 | 18.58096 | NA | 53.86751 | 23.00326 | 41.80456 |
| CUST_31296_PI429010886 | M1161 | 0.003354 | 12 | 0 | | | Transport | Protein transport | | 129.9323 | 95.46826 | 108.6177 | 67.25956 | 92.9186 | 85.9812 | 65.26948 | 164.4613 | 100.9909 | 111.0532 | 134.595 | 154.3834 | 108.9599 | 207.1022 | 141.5789 | 84.02515 | 62.63918 | 71.00324 | 105.5539 | 110.4128 | 86.25852 | 168.6167 | 98.81579 | 105.1 | 98.73097 | 134.6396 | 173.3717 |
| CUST_21549_PI429010886 | N45407 | 0.003361 | 18 | 12 | | | | | | 120.1854 | 129.0466 | 183.8412 | 126.9103 | 118.3356 | 117.7722 | 89.20945 | 145.3839 | 135.6517 | 165.5033 | 134.2642 | 148.7626 | 147.8113 | 168.99 | 159.0029 | 168.0579 | 140.8976 | 116.5788 | 140.8407 | 136.8075 | 132.3781 | 129.8445 | 145.6295 | 94.92777 | 146.0101 | 134.2279 | 143.7253 |
| CUST_38390_PI429010886 | M26180 | 0.003364 | 12 | 0 | | | | | | 31.23744 | 27.03027 | 24.29748 | 11.9615 | 9.434112 | 15.99975 | 3.992853 | 14.50945 | 10.57226 | 15.02374 | 16.81213 | 29.62583 | 9.197112 | 17.64462 | 18.49721 | 21.46189 | 17.41182 | 9.979521 | 6.221163 | 13.95091 | 12.89633 | 18.10243 | 19.9496 | 25.30759 | 21.14763 | 23.25228 | 21.88907 |
| CUST_55605_PI429010886 | M110927 | 0.003366 | 18 | 12 | | | | | | 645.4311 | 718.4589 | 937.6859 | 833.259 | 1018.693 | 899.5738 | 710.6638 | 1079.252 | 1410.909 | 963.9292 | 2212.175 | 2680.458 | 1594.166 | 1150.482 | 1160.888 | 856.8878 | 978.9007 | 793.3041 | 701.8561 | 1735.328 | 854.4458 | 857.5648 | 961.1254 | 844.642 | 942.9477 | 904.3304 | 1439.161 |
| CUST_7716_PI429010886 | N21965 | 0.003368 | 15 | 12 | 225712258 | Replication protein A 32 kDa subunit [Lepeophtheir | | | 5.3E-37 | 520.6248 | 661.0472 | 659.4054 | 426.0225 | 480.999 | 438.9413 | 471.3393 | 453.8761 | 595.4395 | 616.4707 | 501.9627 | 604.9689 | 550.2491 | 671.5411 | 580.0174 | 810.0074 | 536.8972 | 554.4782 | 477.4078 | 535.2858 | 411.2669 | 616.7782 | 566.7662 | 469.825 | 721.8143 | 555.454 | 760.5296 |
| CUST_6653_PI429010886 | N20408 | 0.003376 | 18 | 9 | 5020110 | beta 1,4-endoglucanase [Cherax quadricarinatus] | Metabolic process | Carbohydrate metabolic process | 2.89E-87 | NA | 13.49626 | NA | 20.78351 | 32.69174 | 16.77093 | 32.24593 | 47.51659 | 25.72813 | 24.96359 | 74.74125 | 67.02702 | 67.71736 | NA | 36.60913 | 6.678981 | 28.73729 | NA | NA | 18.17921 | 9.709104 | 6.768573 | 22.13925 | 27.40752 | NA | 90.94074 | 45.36871 |
| CUST_3116_PI429010886 | N13829 | 0.003381 | 12 | 0 | | | | | | 3010.148 | 2823.396 | 2845.968 | 2221.45 | 2088.06 | 1667.846 | 2224.226 | 2024.906 | 1957.029 | 2840.45 | 2604.753 | 2858.942 | 3296.897 | 3262.349 | 3432.751 | 3664.722 | 2591.27 | 2088.06 | 2308.436 | 3116.138 | 2415.728 | 4377.65 | 2811.816 | 2547.522 | 3451.374 | 2937.772 | 2809.847 |
| CUST_37898_PI429010886 | M22142 | 0.003383 | 21 | 9 | | | | | | 155.2181 | 29.19475 | 180.5172 | 19.43524 | 23.50136 | 37.08279 | 186.6438 | 515.307 | 587.9777 | 1384.531 | 377.6826 | NA | 479.7862 | 412.703 | 199.5128 | 145.1839 | NA | 7.329225 | 96.28021 | 4.599604 | 121.4237 | 142.7994 | 121.9257 | 32.95758 | 4.054862 | 78.52355 | 85.55586 |
| CUST_49406_PI429010886 | M85527 | 0.003389 | 24 | 6 | | | | | | 1875.725 | 1190.301 | 35.59331 | 2972.831 | 191.9229 | 281.599 | 2434.013 | 752.5982 | 2642.053 | 30.21934 | 21.42376 | 20.6809 | 1705.674 | 32.22977 | 908.82 | 117.8039 | 25.77821 | 106.7139 | 21.05098 | 12.96349 | 25.65833 | 63.67902 | 26.95815 | 770.419 | 30.57659 | 230.3269 | 27.96608 |
| CUST_19625_PI429010886 | N41895 | 0.003391 | 21 | 0 | | | | | | 10.87219 | 19.00199 | 16.28372 | 16.36491 | 10.27148 | 13.08017 | 14.86089 | 10.33138 | 11.64471 | 11.99091 | 4.019948 | 6.413493 | NA | 7.528845 | 7.061176 | 6.745411 | 5.075645 | 16.65372 | 8.958591 | 7.004476 | 12.50339 | 17.61017 | 13.37697 | 19.59849 | 5.697389 | 10.82851 | 9.354027 |
| CUST_44767_PI429010886 | M66918 | 0.003403 | 21 | 18 | 291228863 | PREDICTED: hypothetical protein [Saccoglossus kow | Protein metabolism | Proteolysis | 4.6E-12 | 17.91608 | 15.47775 | 18.09887 | 17.11883 | 17.11883 | 7.800886 | 13.56063 | 14.85609 | 21.56963 | 29.14874 | 15.15808 | 20.32835 | 7.361352 | 22.33694 | 20.53586 | 25.6517 | 13.7877 | 19.52364 | 27.21684 | 38.57556 | 25.76223 | 12.4831 | 17.52398 | 12.68722 | 13.83685 | 7.587626 | 11.95978 |
| CUST_50318_PI429010886 | M89432 | 0.003406 | 18 | 3 | | | | | | 39.27986 | 37.06882 | 16.71936 | 58.12189 | 54.24943 | 42.60531 | 64.97549 | 29.35511 | 39.78497 | 32.26619 | 30.74148 | 17.16312 | 16.44239 | 14.96828 | 11.85674 | 48.28635 | 37.27402 | 33.66515 | 21.22623 | 26.61953 | 12.22313 | 51.273 | 32.29255 | 30.75309 | 38.95593 | 19.14544 | 21.35581 |
| CUST_43551_PI429010886 | M63338 | 0.003413 | 21 | 9 | | | | | | 9.629706 | 13.52202 | 8.841942 | 7.987477 | 7.657897 | NA | 9.203374 | 9.152945 | 12.63007 | 17.14756 | 18.69874 | 15.10927 | 9.801459 | 12.00862 | 12.20158 | 26.57271 | 10.9956 | 13.69985 | 23.30384 | 9.091001 | 10.35813 | 10.20858 | 9.810562 | NA | 9.960996 | 7.493829 | 11.15284 |
| CUST_22040_PI429010886 | N46314 | 0.003417 | 24 | 15 | | | | | | 236.5825 | 179.6526 | 276.0613 | 252.3543 | 260.9414 | 255.9554 | 274.8462 | 247.1436 | 243.3133 | 267.177 | 219.5144 | 298.0242 | 277.7527 | 325.3231 | 267.619 | 350.5877 | 352.5288 | 363.1719 | 256.8548 | 300.5238 | 286.1419 | 329.5922 | 298.53 | 249.1725 | 273.8764 | 248.2927 | 362.7402 |
| CUST_3622_PI429010886 | N14903 | 0.003419 | 15 | 9 | | | | | | 10.20235 | 8.270829 | NA | 5.905287 | 6.650683 | NA | 8.930028 | 8.254327 | 5.830065 | 16.51414 | 7.446083 | 9.658216 | 6.025275 | 10.20974 | 9.843184 | 8.390549 | 8.137781 | 8.466065 | 5.280397 | 6.522752 | 4.890019 | 9.688636 | NA | 12.45838 | 9.922626 | 21.10951 | 9.384386 |
| CUST_43956_PI429010886 | M64457 | 0.003425 | 15 | 9 | | | | | | 98.46582 | 96.09446 | 112.8438 | 66.80323 | 80.07884 | 99.84559 | 71.78694 | 103.8205 | 101.1196 | 137.865 | 112.2136 | 163.8459 | 98.52867 | 126.3916 | 183.1526 | 146.0878 | 93.00061 | 109.5638 | 94.19343 | 112.9777 | 98.36103 | 134.0378 | 79.45781 | 97.10121 | 140.548 | 125.719 | 155.0724 |
| CUST_50951_PI429010886 | M91546 | 0.003425 | 15 | 9 | | | | | | 26.36365 | 15.16804 | 24.38855 | 10.35813 | 13.53347 | 10.40896 | 27.0727 | 16.50813 | 16.59282 | 39.87566 | 25.53642 | 63.93448 | 22.78329 | 28.31921 | 81.51675 | 58.27233 | 25.91676 | 41.31249 | 19.30381 | 37.72041 | 16.81632 | 38.69306 | 97.3988 | 26.77723 | 51.99458 | 42.31429 | 64.05654 |
| CUST_33374_PI429010886 | M6734 | 0.003446 | 12 | 0 | | | | | | 212.8755 | 198.6003 | 175.6005 | 160.2794 | 140.6181 | 183.3264 | 133.3745 | 140.147 | 171.5722 | 163.051 | 135.0274 | 195.2959 | 182.1441 | 229.178 | 204.7153 | 200.4301 | 159.5684 | 207.1022 | 152.3321 | 190.0374 | 145.4832 | 251.8524 | 184.9236 | 195.9437 | 193.3576 | 276.2073 | 219.7749 |
| CUST_4786_PI429010886 | N17475 | 0.003452 | 18 | 15 | | | | | | 187.0999 | 247.4184 | 185.6828 | 160.0314 | 169.2493 | 227.4855 | 162.0477 | 125.2116 | 141.4293 | 203.8319 | 246.0125 | 258.545 | 168.8237 | 195.9585 | 152.8184 | 273.1684 | 204.9855 | 190.4443 | 196.2247 | 181.9242 | 207.6455 | 163.3073 | 188.4915 | 162.9163 | 180.4643 | 171.2587 | 166.9427 |
| CUST_17816_PI429010886 | N38642 | 0.003454 | 18 | 15 | 241643601 | sidestep protein, putative [Ixodes scapularis] >gi\|21 | | | 2.03E-20 | 8.326131 | 9.315276 | 14.78884 | NA | 7.369719 | 10.13899 | 6.917097 | 15.19477 | 7.737261 | 15.68172 | 15.58555 | 15.54049 | 20.61883 | 24.87256 | 10.74664 | 16.64978 | 11.92305 | 26.53469 | 19.51691 | 15.81194 | 6.801891 | 7.645054 | NA | NA | 12.90472 | 5.98944 | 12.72782 |
| CUST_42097_PI429010886 | M59389 | 0.003462 | 21 | 0 | | | | | | 21.17943 | 21.36125 | 9.858236 | 9.297397 | 16.16705 | 8.017122 | 12.20042 | 12.80273 | 7.525118 | 11.24123 | 12.64236 | 10.58518 | 7.650264 | 9.529604 | 10.33277 | 20.29145 | 5.781367 | 12.01444 | 10.59324 | 19.61118 | 10.644 | 26.75604 | 44.59476 | 19.56685 | 17.29225 | 13.33386 | 8.545589 |
| CUST_29186_PI429010886 | N59354 | 0.003462 | 24 | 21 | | | | | | 104.1068 | 125.5232 | 103.8326 | 91.05924 | 110.8992 | 122.2181 | 88.97552 | 106.4577 | 89.85114 | 97.44558 | 93.48735 | 96.66502 | 93.2268 | 109.7631 | 111.7143 | 129.7027 | 128.9813 | 122.452 | 110.8588 | 111.4482 | 119.9288 | 116.9508 | 125.8931 | 113.0059 | 118.6904 | 108.0345 | 135.7103 |
| CUST_40033_PI429010886 | M48968 | 0.003474 | 21 | 15 | 338809146 | hemocyanin [Exopalaemon carinicauda] | | | 2.71E-09 | NA | 9.858236 | 10.90286 | 39.57145 | 120.6234 | 13.8859 | 105.4104 | 469.28 | 177.456 | 42.29494 | 341.9433 | 390.2834 | 1656.441 | 27.25206 | 685.806 | NA | 536.1688 | NA | 60.77185 | 17436.39 | 392.5464 | NA | 23.81072 | 13.58003 | NA | 90.57431 | 490.4258 |
| CUST_23146_PI429010886 | N48332 | 0.003474 | 24 | 18 | 198412012 | PREDICTED: uncharacterized protein LOC10018495 | | | 1.55E-07 | 31.99053 | 40.09922 | 36.39221 | 27.61208 | 28.2598 | 31.85271 | 25.05128 | 40.03501 | 22.78539 | 33.20236 | 27.94887 | 39.1747 | 32.8295 | 47.76338 | 38.31739 | 39.24212 | 29.35866 | 37.39567 | 43.87014 | 55.40965 | 44.47376 | 31.39141 | 44.6026 | 38.121 | 42.18909 | 35.02904 | 56.76781 |
| CUST_52902_PI429010886 | M99371 | 0.003487 | 18 | 12 | | | | | | 8.267442 | 8.972594 | 28.57032 | 7.869843 | 23.17801 | 24.09666 | 56.18729 | 53.43124 | 75.13415 | 77.59246 | 32.40747 | 31.64722 | 95.97714 | 133.298 | 41.27495 | 8.418027 | 13.03814 | 14.41938 | 38.20891 | 13.06014 | 40.68988 | 30.83662 | 29.43648 | 19.65043 | 70.96812 | 29.13814 | 33.67217 |
| CUST_13031_PI429010886 | N30466 | 0.003495 | 18 | 12 | | | | | | 70.92151 | 83.40091 | 99.00087 | 49.92232 | 59.80641 | 59.12871 | 46.57707 | 98.67215 | 61.64233 | 77.18845 | 74.93674 | 83.99654 | 84.50068 | 88.62563 | 88.50576 | 116.8603 | 75.15426 | 61.81926 | 81.05888 | 86.85226 | 74.42211 | 55.15882 | 72.88198 | 49.25815 | 88.69099 | 72.58192 | 101.2666 |
| CUST_20939_PI429010886 | N44338 | 0.003495 | 18 | 3 | 405966762 | UPF0582 protein C13orf37-like protein [Crassostrea | | | 9.73E-10 | 66.48237 | 123.3055 | 67.36225 | 188.9473 | 74.22065 | 147.3674 | 60.26272 | 72.01712 | 56.4484 | 57.70169 | 53.01337 | 85.72262 | 46.36142 | 66.72453 | 47.35331 | 72.08475 | 71.69416 | 89.81869 | 51.70242 | 55.62148 | 84.36786 | 92.13928 | 94.95262 | 98.86383 | 49.26562 | 110.9624 | 39.68929 |
| CUST_39790_PI429010886 | M44731 | 0.003506 | 21 | 12 | 321455300 | hypothetical protein DAPPUDRAFT_64687 [Daphnia | Metabolic process | Carbohydrate metabolic process | 2.31E-19 | 282.0094 | 382.1801 | 241.3085 | 303.4935 | 235.3955 | 203.482 | 276.2901 | 229.3656 | 247.2726 | 335.333 | 263.5785 | 402.2817 | 301.7927 | 413.3489 | 347.2399 | 329.8367 | 301.9881 | 280.165 | 345.9305 | 302.7176 | 276.3669 | 264.3268 | 315.0245 | 261.3191 | 247.5396 | 256.9966 | 279.387 |
| CUST_35345_PI429010886 | M12331 | 0.003508 | 18 | 0 | 541906825 | 6-4 photolyase [Eurydice pulchra] | Response to stress | DNA repair | 2.3E-124 | 62.12557 | 71.47658 | 65.25047 | 57.39701 | 44.30287 | 38.81815 | 33.68381 | 42.30429 | 41.78277 | 55.05996 | 40.53079 | 55.1911 | 50.22122 | 41.34285 | 41.78704 | 72.23286 | 37.19805 | 49.57451 | 72.91578 | 44.22632 | 63.54619 | 43.70884 | 31.88231 | 44.76277 | 38.2775 | 45.95892 | 35.61182 |
| CUST_53953_PI429010886 | M103850 | 0.003509 | 15 | 3 | | | | | | 517.2906 | 215.6344 | 345.7818 | 508.6284 | 493.9819 | 809.6878 | 505.7885 | 286.1419 | 211.7804 | 195.0054 | 344.1554 | 183.2048 | 275.8944 | 148.9131 | 245.6875 | 224.9679 | 452.1705 | 318.2985 | 516.0613 | 153.6651 | 552.5713 | 306.093 | 475.6507 | 560.5893 | 185.6524 | 400.4224 | 152.2451 |
| CUST_39797_PI429010886 | M44879 | 0.00351 | 15 | 9 | | | | | | 354.4714 | 564.1196 | 301.9881 | 243.6699 | 998.2355 | 55.00728 | 97.11051 | 150.78 | 461.2479 | 1789.616 | 798.0318 | 1241.637 | 682.2742 | 412.6269 | 420.025 | 961.5038 | 169.0881 | 436.4909 | 350.7295 | 198.6003 | 332.5951 | 716.0786 | 563.7899 | 112.169 | 1406.77 | 481.5308 | 1036.441 |
| CUST_12266_PI429010886 | N29192 | 0.003516 | 24 | 21 | 321479374 | hypothetical protein DAPPUDRAFT_300082 [Daphn | Metabolic process | Other | 5.41E-08 | 164.5463 | 179.0372 | 146.5035 | 122.4667 | 141.1498 | 118.1621 | 120.5371 | 118.6493 | 112.2413 | 193.773 | 117.487 | 203.7035 | 114.8562 | 148.544 | 143.124 | 178.5686 | 156.3082 | 149.7495 | 193.773 | 138.9365 | 180.1444 | 224.3549 | 144.6491 | 194.6574 | 165.9249 | 129.2244 | 139.8015 |
| CUST_24796_PI429010886 | N51262 | 0.003516 | 15 | 6 | | | | | | 21.74146 | 16.24253 | 22.63508 | 6.889529 | 27.21684 | 11.83225 | 27.53274 | 26.33096 | 13.67972 | 5.788646 | 11.41324 | 10.82851 | 11.06303 | 13.42609 | 4.471589 | 11.86943 | 22.87323 | 19.6916 | 22.82168 | 17.78837 | 19.20292 | 25.01541 | 29.03947 | 25.07677 | 20.48821 | 24.63631 | 9.588389 |
| CUST_25643_PI429010886 | N52797 | 0.00352 | 21 | 15 | | | | | | 9.211504 | 9.528362 | NA | 6.864974 | 8.3117 | 9.750393 | NA | 4.958353 | 5.409501 | 9.739447 | 12.38626 | 4.56075 | 6.865815 | NA | 6.811352 | 13.72321 | 12.49592 | 15.63271 | 14.1603 | 6.319033 | 7.923943 | 10.28696 | 12.46866 | NA | 8.892334 | NA | 6.295535 |
| CUST_30590_PI429010886 | N62182 | 0.003551 | 18 | 3 | | | | | | 292.4718 | 147.5412 | 174.8316 | 317.6987 | 264.3268 | 268.0379 | 149.974 | 116.9508 | 184.0345 | 157.9551 | 92.53187 | 137.759 | 119.6154 | 151.1369 | 87.72886 | 256.2441 | 45.45196 | 102.9429 | 120.7171 | 73.43455 | 300.7234 | 187.5572 | 283.1467 | 149.2835 | 146.7819 | 262.6696 | 139.7005 |
| CUST_51355_PI429010886 | M93237 | 0.003552 | 15 | 0 | | | | | | 117.8313 | 159.9648 | 190.8326 | 115.202 | 177.9616 | 164.2252 | 82.12587 | 50.57194 | 69.13041 | 114.5044 | 215.0865 | 127.2423 | 42.12286 | 130.4535 | 99.88549 | 186.1103 | 192.6463 | 174.7413 | 124.1467 | 170.5502 | 141.5103 | 116.2535 | 100.0857 | 116.7168 | 144.5655 | 123.669 | 133.2442 |
| CUST_28754_PI429010886 | N58556 | 0.003552 | 15 | 9 | | | | | | 51.20393 | 44.43928 | 74.02683 | 63.42473 | 46.07745 | 49.75939 | 55.74249 | 64.57467 | 64.8125 | 78.01974 | 82.15481 | 84.58422 | 66.82399 | 67.48171 | 80.90583 | 77.89081 | 63.92583 | 51.76265 | 85.05317 | 63.33459 | 59.13459 | 106.872 | 71.15715 | 41.79247 | 87.50342 | 77.97597 | 115.5444 |
| CUST_46746_PI429010886 | M73579 | 0.003552 | 21 | 15 | | | | | | 494.6999 | 435.7052 | 504.2482 | 349.1356 | 450.8925 | 492.2884 | 403.3076 | 318.4396 | 377.6826 | 701.3851 | 488.97 | 504.6329 | 237.9203 | 460.8905 | 435.3009 | 648.7533 | 559.0577 | 672.3398 | 508.4411 | 573.5264 | 537.0505 | 485.4409 | 424.3711 | 434.3733 | 625.1793 | 390.8735 | 574.8968 |
| CUST_33864_PI429010886 | M8080 | 0.003552 | 24 | 21 | | | | | | 126.8192 | 154.3172 | 183.2331 | 145.5578 | 148.7446 | 172.9267 | 147.3218 | 126.7579 | 142.0799 | 154.2025 | 159.0362 | 142.418 | 171.9761 | 209.9677 | 127.8994 | 202.0834 | 165.8216 | 180.8415 | 158.0318 | 197.6731 | 150.5251 | 190.4729 | 199.6541 | 174.3314 | 190.0374 | 151.6596 | 164.0893 |
| CUST_26257_PI429010886 | N53932 | 0.003552 | 21 | 15 | | | | | | 17.40133 | 22.36193 | 20.71979 | 14.51567 | 17.81842 | 14.65028 | 15.45319 | 12.46111 | 13.13227 | 27.44977 | 15.60189 | 19.82899 | 16.8197 | 13.13227 | 13.67691 | 38.81418 | 19.92665 | 29.46496 | 23.69356 | 19.05757 | 28.55239 | 28.13722 | 15.64687 | 11.41581 | 23.93544 | 14.06235 | 19.16281 |
| CUST_28227_PI429010886 | N57545 | 0.003552 | 24 | 0 | | | | | | 129.2544 | 144.7965 | 204.4226 | 121.4562 | 140.7156 | 166.8948 | 119.1299 | 125.9794 | 100.5098 | 133.6865 | 165.7787 | 146.7019 | 96.23175 | 106.7278 | 95.0012 | 114.4563 | 135.1423 | 182.2638 | 132.4059 | 115.8594 | 196.9122 | 173.2007 | 175.2604 | 142.5643 | 240.5734 | 147.9921 | 180.3864 |
| CUST_22417_PI429010886 | N46990 | 0.003562 | 24 | 0 | | | | | | 114.6834 | 77.51881 | 78.49411 | 75.71395 | 60.84139 | 54.61608 | 69.84364 | 61.34598 | 65.87006 | 48.92704 | 54.25425 | 75.05721 | 73.38298 | 67.53545 | 49.95685 | 74.92755 | 58.56755 | 67.84362 | 62.97836 | 65.20347 | 73.39754 | 66.48727 | 76.27537 | 73.91159 | 71.01876 | 72.84145 | 82.67004 |
| CUST_10873_PI429010886 | N26924 | 0.003563 | 21 | 18 | 84996369 | Spm1 homologue [Theileria annulata strain Ankara] | | | 1.07E-08 | NA | 8.892334 | 15.2034 | 6.723673 | 8.887892 | 6.655856 | 10.51996 | 4.69711 | 6.294694 | 15.69084 | 5.620229 | 6.002692 | 6.522752 | 10.3075 | 8.56209 | 15.23737 | 9.591065 | 16.63804 | 19.02839 | 19.04191 | 14.57386 | 8.454339 | 5.380664 | 17.64462 | 7.421979 | 6.088354 | 8.84632 |
| CUST_10446_PI429010886 | N26247 | 0.003564 | 24 | 21 | 209420822 | cyclin B [Fenneropenaeus penicillatus] | | | 1.68E-23 | 17.20661 | 20.29845 | 17.09174 | 8.459432 | 14.86823 | 11.83103 | 5.486804 | 9.418869 | 5.604736 | 13.87516 | 10.57226 | 18.0488 | 14.10863 | 14.04854 | 26.88741 | 22.51909 | 8.250428 | 20.12612 | 13.50427 | 16.63365 | 11.16458 | 23.49374 | 21.80535 | 16.87203 | 19.18311 | 13.69985 | 37.85605 |
| CUST_8572_PI429010886 | N23238 | 0.003569 | 12 | 6 | 405965499 | Solute carrier family 35 member F5 [Crassostrea gig | | | 1.28E-30 | 41.6388 | 35.77567 | 33.90948 | 34.78474 | 49.79493 | 52.85888 | 61.14806 | 40.38893 | 40.64999 | 39.36593 | 63.1832 | 43.86454 | 36.25388 | 29.83725 | 38.64362 | 41.18849 | 58.77783 | 52.42324 | 61.53942 | 62.51292 | 49.67787 | 40.96307 | 49.69406 | 29.29243 | 31.6343 | 37.89385 | 33.64032 |
| CUST_25971_PI429010886 | N53411 | 0.003569 | 12 | 0 | | | | | | 47.86055 | 43.70884 | 42.6726 | 37.19463 | 26.76517 | 43.9859 | 30.60937 | 26.45722 | 28.88138 | 45.44604 | 49.43277 | 30.92026 | 38.5218 | 39.8936 | 28.28611 | 38.59321 | 37.66305 | 43.67532 | 35.75443 | 30.83099 | 17.21502 | 39.50511 | 34.59277 | 33.77621 | 40.24548 | 51.44191 | 44.08735 |
| CUST_10058_PI429010886 | N25628 | 0.00357 | 15 | 0 | 321477834 | hypothetical protein DAPPUDRAFT_311166 [Daphn | Protein metabolism | Proteolysis | 2.59E-41 | 279.1889 | 232.766 | 158.9804 | 175.9447 | 254.955 | 218.6882 | 206.6326 | 92.75345 | 118.5239 | 194.0962 | 158.8804 | 162.3872 | 152.9488 | 146.882 | 105.6617 | 281.447 | 289.2842 | 227.3812 | 204.7885 | 118.04 | 143.4291 | 240.0259 | 99.71807 | 64.80137 | 84.1757 | 99.03782 | 69.31235 |
| CUST_19054_PI429010886 | N40908 | 0.003592 | 24 | 0 | | | | | | 17.86761 | 29.42444 | 41.9046 | 19.16281 | 20.69091 | 25.4983 | 22.41713 | 15.59997 | 20.8155 | 31.00851 | 22.17757 | 28.79209 | 20.70161 | 27.99024 | 20.36949 | 16.76285 | 19.04932 | 14.84417 | 19.88004 | 18.26372 | 22.14748 | 30.19101 | 24.86204 | 21.39067 | 31.85271 | 30.35236 | 40.4329 |
| CUST_56726_PI429010886 | M114523 | 0.003594 | 15 | 9 | | | | | | 30.28193 | 21.17508 | 51.00123 | 13.49974 | 18.97043 | 17.31462 | 38.67688 | 58.10187 | 70.18575 | 44.34401 | 46.42845 | 50.01173 | 31.70409 | 82.30578 | 14.93407 | 26.07952 | 42.12575 | 37.0569 | 30.705 | 31.55142 | 18.48046 | 60.43972 | 50.20045 | 17.95097 | 61.57414 | 56.63191 | 84.96672 |
| CUST_41379_PI429010886 | M57589 | 0.003594 | 15 | 9 | | | | | | 176.7462 | 738.4538 | 564.8923 | 195.6184 | 331.7667 | 100.1729 | 205.1968 | 431.0477 | 826.6639 | 1034.145 | 487.3364 | 1129.308 | 700.9759 | 1040.536 | 659.0457 | 826.6639 | 696.1501 | 570.9837 | 653.9628 | 538.0354 | 604.157 | 1432.157 | 1105.302 | 209.9677 | 1438.603 | 752.9771 | 2038.918 |
| CUST_13201_PI429010886 | N30773 | 0.003597 | 21 | 15 | | | | | | 92.52049 | 80.61162 | 64.99854 | 66.1724 | 66.12218 | 61.91569 | 63.37317 | 49.29932 | 59.86951 | 84.93968 | 65.1875 | 124.0734 | 72.57482 | 73.43455 | 61.25792 | 92.85168 | 81.02279 | 93.81682 | 75.0125 | 59.15695 | 82.50766 | 69.60585 | 74.38643 | 83.2292 | 81.5056 | 61.30991 | 66.938 |
| CUST_35958_PI429010886 | M14116 | 0.003597 | 15 | 0 | | | | | | 142.4711 | 150.3647 | 145.4402 | 128.1757 | 137.8094 | 137.2919 | 151.0563 | 114.2091 | 104.8539 | 115.5578 | 171.5473 | 142.6543 | 118.1469 | 119.8247 | 153.6882 | 170.2702 | 177.4241 | 175.7472 | 160.1366 | 134.5392 | 126.2814 | 134.7225 | 117.0236 | 117.7859 | 151.1369 | 128.4766 | 110.765 |
| CUST_42887_PI429010886 | M61517 | 0.003598 | 15 | 12 | | | | | | 17.80566 | 9.28355 | 34.82944 | 11.80273 | 15.89674 | 14.56343 | 18.90163 | 28.45405 | 18.79454 | 27.99312 | 23.97181 | 25.19237 | 19.99756 | 31.70016 | 25.67234 | 24.03757 | 18.3198 | 9.662859 | 13.31647 | 16.89803 | 7.948839 | 25.19237 | 14.52793 | 12.34868 | 29.62857 | 14.571 | 22.22168 |
| CUST_12107_PI429010886 | N28934 | 0.00361 | 12 | 0 | 242010735 | conserved hypothetical protein [Pediculus humanus | | | 3.13E-19 | 37.9894 | 41.0817 | 68.29075 | 36.25025 | 35.6576 | 29.64988 | 21.23199 | 25.9573 | 32.16644 | 49.88882 | 36.82067 | 38.86122 | 33.63068 | 45.38957 | 43.21123 | 43.27701 | 28.92063 | 39.33444 | 39.68514 | 33.15924 | 33.62817 | 50.52879 | 41.28276 | 38.75385 | 68.70977 | 41.17841 | 54.22335 |
| CUST_13030_PI429010886 | N30464 | 0.00361 | 12 | 0 | | | | | | 68.15284 | 68.52038 | 54.60219 | 42.35334 | 47.55714 | 43.8795 | 61.74634 | 40.84069 | 41.39686 | 67.41072 | 50.51683 | 51.94426 | 58.73387 | 56.67223 | 48.5802 | 64.46821 | 53.8998 | 55.47668 | 46.27883 | 44.14193 | 46.14783 | 66.83529 | 52.7291 | 43.99672 | 65.04228 | 56.22273 | 76.99966 |
| CUST_24627_PI429010886 | N50968 | 0.00361 | 12 | 6 | 546675597 | hypothetical protein D910_04166 [Dendroctonus p | | | 1.48E-34 | 31.64722 | 42.09528 | 18.45401 | 51.24529 | 45.65482 | 52.56534 | 61.71189 | 51.73412 | 53.9397 | 25.13331 | 39.70836 | 27.42544 | 28.86203 | 33.3136 | 25.3224 | 39.72839 | 56.99751 | 34.00581 | 88.04251 | 28.5608 | 47.26147 | 43.70451 | 23.60076 | 34.31858 | 36.58662 | 14.36868 | 28.18833 |
| CUST_5994_PI429010886 | N19479 | 0.003612 | 24 | 21 | | | | | | 113.8871 | 133.1553 | 102.3756 | 113.976 | 113.0567 | 131.2193 | 74.22065 | 77.89081 | 91.92897 | 98.47983 | 90.17193 | 111.9276 | 93.1869 | 104.5986 | 117.9903 | 159.6585 | 85.75328 | 146.5035 | 106.4186 | 155.0084 | 128.1244 | 141.3582 | 124.7036 | 121.5886 | 113.6358 | 97.31477 | 136.8231 |
| CUST_20571_PI429010886 | N43684 | 0.003613 | 24 | 12 | | | | | | 25.8352 | 20.9954 | 32.72017 | 21.62181 | 21.19536 | 10.91844 | 28.32496 | 22.20953 | 23.26041 | 32.12754 | 28.78903 | 40.27597 | 32.21099 | 33.47284 | 36.34025 | 31.27013 | 22.32503 | 34.08686 | 60.01583 | 25.44022 | 30.78794 | 31.67438 | 29.73209 | 28.88408 | 30.9788 | 32.26335 | 26.50017 |
| CUST_16390_PI429010886 | N36148 | 0.003621 | 12 | 9 | | | | | | 12.54217 | 16.62231 | 10.63417 | 7.570554 | 5.397572 | NA | 9.318343 | 6.730364 | 18.11337 | 26.60787 | 11.51911 | 20.56557 | 12.35967 | 20.88813 | 8.477051 | 10.5667 | 4.281876 | 11.28199 | 6.00037 | 11.98126 | 10.07371 | 20.38379 | 15.00845 | 13.79131 | 17.37482 | 5.573433 | 13.8468 |
| CUST_2707_PI429010886 | N12952 | 0.003626 | 21 | 0 | | | | | | 33.63678 | 27.63304 | 37.28683 | 38.66828 | 28.06711 | 33.64323 | 24.12427 | 34.05076 | 22.07122 | 14.71509 | 14.2983 | 26.45984 | 21.87774 | 24.51625 | 17.68291 | 29.68582 | 17.20064 | 21.91188 | 9.650569 | 11.4747 | 35.88829 | 25.02448 | 65.36594 | 37.25844 | 28.1626 | 35.77567 | 28.79502 |
| CUST_3249_PI429010886 | N14105 | 0.003627 | 12 | 9 | 255732843 | Candida tropicalis MYA-3404 NADH-ubiquinone oxi | Transport | Ion transport | 6.53E-62 | 991.907 | 1097.654 | 973.9722 | 723.6629 | 862.42 | 896.5197 | 1104.958 | 1064.349 | 1232.019 | 1364.868 | 1232.59 | 1620.39 | 1190.301 | 1124.348 | 1207.044 | 1650.747 | 982.113 | 981.0866 | 988.0968 | 1244.647 | 904.8603 | 1455.397 | 1183.341 | 1157.816 | 1053.785 | 942.6383 | 1544.651 |
| CUST_14070_PI429010886 | N32281 | 0.00363 | 18 | 3 | | | | | | 48.6393 | 71.29059 | 38.96253 | 74.61343 | 62.96106 | 64.3699 | 46.58164 | 40.62219 | 47.26675 | 34.11202 | 39.28519 | 57.37859 | 27.29319 | 27.65968 | 44.80164 | 60.26917 | 39.01912 | 51.31578 | 48.34221 | 38.49435 | 44.28888 | 50.43461 | 57.82155 | 56.05475 | 41.52003 | 30.78076 | 16.51187 |
| CUST_33120_PI429010886 | M6019 | 0.003636 | 15 | 9 | | | | | | 416.647 | 363.0918 | 518.8148 | 393.8505 | 367.4512 | 397.5988 | 353.3222 | 427.6746 | 466.0044 | 537.8198 | 423.1161 | 603.7585 | 414.6092 | 579.5177 | 510.1908 | 496.2657 | 433.1734 | 475.5299 | 385.6213 | 518.3277 | 412.0745 | 495.3471 | 502.9268 | 421.6972 | 623.0865 | 492.4839 | 636.6917 |
| CUST_13359_PI429010886 | N31052 | 0.003636 | 15 | 9 | | | | | | 66.03995 | 129.6421 | 84.27092 | 103.4687 | 52.40666 | 75.93804 | 112.0953 | 117.3157 | 79.33173 | 169.7841 | 77.45737 | 127.5485 | 114.2341 | 80.43582 | 119.2118 | 96.77635 | 82.25514 | 91.81369 | 83.44238 | 78.50617 | 57.61472 | 104.5244 | 119.9827 | 144.3614 | 143.5925 | 131.7044 | 111.7143 |
| CUST_50046_PI429010886 | M88375 | 0.003638 | 21 | 3 | | | | | | 383.652 | 619.6845 | 339.7564 | 457.038 | 631.8289 | 511.0593 | 289.131 | 370.5308 | 470.5429 | 495.7193 | 382.2635 | 507.709 | 392.8804 | 447.5499 | 202.438 | 327.8511 | 366.9733 | 379.882 | 293.6394 | 439.2849 | 372.6528 | 626.2648 | 234.017 | 378.7944 | 715.6525 | 620.6433 | 505.4528 |
| CUST_21393_PI429010886 | N45140 | 0.003642 | 21 | 15 | | | | | | 42.2806 | 42.78187 | 35.59734 | 29.35866 | 42.29494 | 36.53489 | 27.9072 | 25.30072 | 25.56371 | 38.65653 | 40.57649 | 29.65544 | 44.45342 | 19.37009 | 69.39648 | 53.20866 | 46.87582 | 63.20604 | 51.89227 | 33.93687 | 28.03701 | 33.83173 | 38.77187 | 44.65427 | 36.37157 | 46.17281 | 33.45023 |
| CUST_29834_PI429010886 | N60653 | 0.003654 | 15 | 9 | | | | | | 43.88802 | 38.44451 | 34.40827 | 31.46178 | 45.62788 | 46.68828 | 50.09195 | 40.58399 | 42.71137 | 51.37925 | 47.01793 | 65.75656 | 35.71039 | 64.4321 | 47.69004 | 44.21249 | 42.38979 | 42.34931 | 38.93621 | 42.43356 | 57.59047 | 42.43356 | 61.85594 | 48.65694 | 61.01573 | 52.22902 | 59.87756 |
| CUST_24903_PI429010886 | N51451 | 0.00366 | 24 | 21 | 145342477 | predicted protein [Ostreococcus lucimarinus CCE99 | Cellular component organization | Chromosome organization | 1.24E-27 | 35.69903 | 8.877949 | 16.44885 | 12.26155 | 10.7321 | 18.99953 | 6.578227 | 4.532016 | 8.059328 | 17.97064 | 8.180112 | 11.80005 | 12.54687 | 18.53904 | 5.197464 | 16.27254 | 11.05401 | 10.04937 | 27.27234 | 12.04026 | 20.2329 | 72.16312 | 17.31011 | 12.76027 | 17.65649 | 16.84352 | 21.21095 |
| CUST_24650_PI429010886 | N51016 | 0.003663 | 21 | 6 | | | | | | 34.20754 | 36.89793 | 49.43277 | 41.13711 | 47.96198 | 43.34938 | 45.9505 | 54.79286 | 47.93942 | 42.34616 | 34.79371 | 36.99297 | 32.68774 | 52.76535 | 48.48483 | 39.27214 | 31.44965 | 29.8705 | 23.64095 | 36.78596 | 22.65189 | 30.88328 | 55.91435 | 25.61197 | 79.57182 | 34.0788 | 43.45754 |
| CUST_6133_PI429010886 | N19672 | 0.003672 | 12 | 3 | 242016587 | hypothetical protein Phum_PHUM405430 [Pediculu | | | 5.63E-13 | 194.0018 | 171.8979 | 85.77208 | 314.6817 | 231.7343 | 343.6141 | 162.719 | 159.6853 | 115.5444 | 129.4744 | 123.2882 | 103.1223 | 137.1215 | 215.5021 | 202.8821 | 312.1179 | 206.8026 | 317.4402 | 233.088 | 209.6694 | 128.0667 | 241.7291 | 95.61724 | 173.4736 | 137.649 | 193.8296 | 83.27348 |
| CUST_47678_PI429010886 | M77149 | 0.003679 | 15 | 9 | | | | | | 44.64991 | 58.40039 | 52.95164 | 38.00094 | 40.23396 | 34.36554 | 49.86266 | 53.57981 | 53.58897 | 66.97799 | 39.69364 | 54.04063 | 50.19147 | 51.54875 | 38.04406 | 44.17135 | 51.52202 | 41.75433 | 49.58479 | 46.35772 | 45.90609 | 39.50189 | 56.9379 | 51.0829 | 58.03638 | 68.81509 | 67.18762 |
| CUST_845_PI429010886 | N7521 | 0.003679 | 15 | 9 | | | | | | 10662.05 | 20168.54 | 11472.39 | 14083.21 | 5980.233 | 12034.22 | 14529.33 | 16033.46 | 20010.45 | 18896.93 | 8904.357 | 17900.98 | 25081.91 | 19438.74 | 11747.34 | 21690.05 | 15845.68 | 15327.79 | 11089.86 | 12354.92 | 8924.795 | 24081.12 | 16000.49 | 14559.32 | 27975.24 | 25748.12 | 46346.16 |
| CUST_47784_PI429010886 | M77612 | 0.003686 | 21 | 3 | | | | | | 34.55995 | 58.95549 | 34.4458 | 107.884 | 50.59835 | 54.058 | 38.723 | 37.82706 | 38.58369 | 38.12468 | 24.78008 | 53.35103 | 32.08077 | 44.70035 | 54.79286 | 31.22335 | 30.29325 | 39.84647 | 23.98555 | 32.86388 | 48.02359 | 57.51102 | 40.66714 | 33.03027 | 57.98645 | 57.14083 | 48.98001 |
| CUST_49177_PI429010886 | M84527 | 0.003686 | 21 | 3 | 29134779 | projectin [Procambarus clarkii] | Protein metabolism | Cellular protein modification process | 1.44E-58 | 437.7044 | 702.0078 | 839.2544 | 1642.855 | 898.6441 | 1070.179 | 679.3339 | 518.0097 | 683.7716 | 696.5534 | 380.3886 | 803.3312 | 261.2237 | 783.9719 | 314.0114 | 534.2439 | 252.6775 | 560.7028 | 253.5447 | 311.9303 | 1033.021 | 710.0284 | 1125.765 | 460.0363 | 1340.303 | 1090.456 | 508.1895 |
| CUST_13122_PI429010886 | N30615 | 0.003689 | 18 | 15 | | | | | | 63.04603 | 62.18234 | 54.94382 | 40.55619 | 43.49759 | 44.74185 | 38.52899 | 59.37489 | 34.89935 | 65.39564 | 55.25615 | 69.85985 | 62.95412 | 37.54361 | 38.05769 | 67.41072 | 86.20359 | 42.68895 | 63.65837 | 69.52372 | 57.04658 | 55.52574 | 55.87623 | 59.21305 | 49.79836 | 35.22469 | 28.71325 |
| CUST_46522_PI429010886 | M72891 | 0.003689 | 21 | 9 | 77743875 | Euphausia superba isolate DGGE band AntEuk13-5 | | | | 263.5785 | 425.5183 | 397.9322 | 262.5661 | 646.5567 | 448.4129 | 375.3397 | 532.0502 | 613.1363 | 715.1399 | 603.2889 | 924.8591 | 600.2864 | 481.2318 | 468.7286 | 777.3766 | 594.7631 | 292.3676 | 534.9773 | 504.2482 | 420.5887 | 424.7309 | 441.2532 | 498.7637 | 532.5435 | 549.9307 | 679.3339 |
| CUST_32914_PI429010886 | M5455 | 0.003691 | 21 | 18 | | | | | | 38.13489 | 51.20753 | 31.6343 | 24.69594 | 38.82583 | 25.74383 | 32.05062 | 27.40752 | 38.52489 | 30.3471 | 42.02191 | 45.65065 | 37.16398 | 35.26418 | 34.61592 | 48.94359 | 37.13409 | 23.76908 | 47.27187 | 56.41396 | 37.70458 | 46.1807 | 30.39754 | 40.37974 | 25.73562 | 21.13287 | 21.57533 |
| CUST_10896_PI429010886 | N26960 | 0.003693 | 15 | 12 | | | | | | 33.58859 | 37.50442 | 45.88252 | 18.44318 | 32.48585 | 18.45819 | 16.81795 | 31.68997 | 29.00151 | 34.60204 | 30.25566 | 47.19112 | 38.83398 | 41.99643 | 43.84173 | 45.95551 | 29.41419 | 31.74172 | 27.32319 | 33.19093 | 30.56491 | 39.39544 | 28.34801 | 19.00199 | 45.72547 | 34.82944 | 37.83427 |
| CUST_38973_PI429010886 | M32846 | 0.003696 | 15 | 9 | | | | | | 293.4659 | 312.4689 | 373.6828 | 160.598 | 373.9776 | 368.8573 | 217.2477 | 383.225 | 351.6868 | 434.8523 | 388.966 | 449.6922 | 350.4435 | 511.1819 | 343.8588 | 347.4939 | 237.3209 | 381.5724 | 531.3865 | 233.6842 | 384.1514 | 429.9553 | 439.8846 | 392.7033 | 475.1468 | 539.6207 | 676.7937 |
| CUST_41720_PI429010886 | M58388 | 0.003701 | 21 | 15 | | | | | | 12.65934 | 15.62557 | NA | 6.991817 | 12.57374 | 5.156542 | 12.62711 | 10.57449 | 9.935826 | 10.76694 | 6.59049 | 4.137751 | 8.398107 | 14.74027 | 16.94406 | 15.78805 | 18.53665 | 19.2347 | 9.864571 | 12.51827 | 14.22354 | 16.79903 | 16.49958 | 17.65858 | 8.065131 | 14.07874 | 8.096609 |
| CUST_21289_PI429010886 | N44954 | 0.003707 | 21 | 3 | | | | | | 40.37974 | 58.02431 | 50.73538 | 84.2614 | 49.90548 | 73.47743 | 42.07479 | 23.24391 | 42.61497 | 31.85271 | 21.52705 | 52.79712 | 21.29581 | 47.8959 | 18.01314 | 38.24304 | 5.472076 | 24.2252 | 9.209552 | 18.47168 | 79.24559 | 34.36064 | 82.48421 | 38.83398 | 59.94958 | 59.19994 | 28.61737 |
| CUST_16953_PI429010886 | N37135 | 0.003707 | 24 | 0 | | | | | | 18.53904 | 10.01676 | 14.83018 | 6.416874 | 5.026041 | NA | NA | 3.949758 | 8.275583 | 5.832428 | 5.921666 | NA | 10.68692 | 10.83635 | 6.129322 | 8.41226 | NA | 9.636382 | 11.97367 | NA | 10.09872 | 11.54995 | 6.521689 | NA | 14.9248 | 14.63709 | 10.59893 |
| CUST_31448_PI429010886 | M1547 | 0.003711 | 21 | 18 | | | Protein metabolism | Cellular protein modification process | | 1268.692 | 903.546 | 1094.71 | 747.4574 | 808.7225 | 712.1447 | 927.5266 | 773.1832 | 582.2842 | 805.8232 | 1018.693 | 686.1452 | 765.9632 | 697.0634 | 774.5083 | 1150.482 | 1403.267 | 834.3363 | 1483.935 | 855.218 | 1051.932 | 1007.431 | 1073.921 | 1131.289 | 970.3266 | 588.293 | 825.7659 |
| CUST_47851_PI429010886 | M77857 | 0.003714 | 15 | 3 | | | | | | 7.769107 | 7.299608 | 14.30732 | NA | 11.95869 | 9.592481 | 12.21352 | 8.255312 | 6.572882 | 3.80972 | 6.643636 | 6.320653 | 6.052528 | 6.535693 | 13.94032 | 7.070433 | 12.75631 | 12.09699 | 11.56849 | 12.6973 | 12.49458 | 8.388399 | NA | 10.80337 | 8.036889 | 7.146366 | 4.374409 |
| CUST_6182_PI429010886 | N19737 | 0.003716 | 12 | 9 | | | | | | 27.10385 | 35.83833 | 21.98421 | 18.08454 | 14.30234 | 13.55604 | 12.91624 | 12.15878 | 27.26909 | 46.40711 | 27.53905 | 31.59119 | 22.70735 | 24.96359 | 21.73902 | 34.11542 | 17.63183 | 13.35309 | 31.55772 | 30.10928 | 26.68081 | 30.61606 | 33.90948 | 58.77193 | 40.28049 | 25.68743 | 35.29256 |
| CUST_16207_PI429010886 | N35841 | 0.003716 | 24 | 12 | | | | | | 38.31256 | 47.85374 | 38.99636 | 52.80847 | 39.44549 | 35.8336 | 43.49759 | 47.83781 | 49.3486 | 110.8588 | 56.48952 | 55.18575 | 102.3532 | 79.85552 | 113.1145 | 46.52112 | 63.81737 | 54.2928 | 122.8574 | 52.37245 | 72.72106 | 54.11265 | 86.54045 | 105.5539 | 73.32053 | 47.67821 | 51.48154 |
| CUST_36722_PI429010886 | M16660 | 0.003716 | 24 | 21 | 85677399 | pigment-dispersing hormone 3 [Marsupenaeus japc | Signal transduction | Other | 3.45E-07 | 358.55 | 506.8788 | 654.3393 | 649.8854 | 232.9884 | 369.4169 | 338.2007 | 249.1725 | 549.4003 | 469.0438 | 351.303 | 182.4169 | 240.4265 | 158.3621 | 213.9538 | 754.5887 | 296.3588 | 580.436 | 327.7802 | 409.385 | 286.997 | 858.518 | 315.7757 | 696.7816 | 586.9437 | 429.0337 | 649.5445 |
| CUST_38587_PI429010886 | M28318 | 0.003721 | 15 | 9 | | | | | | 4048.081 | 18524.86 | 9784.529 | 1709.2 | 3124.162 | 902.7962 | 2529.511 | 5070.698 | 17031.04 | 20248.42 | 12313.28 | 22978.37 | 12214.41 | 17837.74 | 15604.09 | 17165.71 | 4399.213 | 10044.17 | 10524.41 | 19317.04 | 11601.88 | 19269.36 | 14529.33 | 1090.829 | 18051.51 | 27018.31 | 27642.69 |
| CUST_12631_PI429010886 | N29777 | 0.003721 | 15 | 0 | | | | | | 8696.127 | 10415.22 | 4817.775 | 9072.043 | 7686.447 | 7654.795 | 6005.835 | 5293.128 | 5391.461 | 6122.745 | 5417.146 | 7259.073 | 7239.058 | 5693.449 | 7647.424 | 9249.246 | 6607.963 | 7784.736 | 8378.604 | 7011.167 | 5650.794 | 6116.034 | 4773.42 | 5113.292 | 7647.424 | 7383.427 | 6321.861 |
| CUST_2757_PI429010886 | N13043 | 0.003738 | 15 | 12 | 7320913 | masquerade-like protein [Pacifastacus leniusculus] | Protein metabolism | Proteolysis | 1.33E-75 | 1520.198 | 1197.916 | 2346.537 | 558.0588 | 793.5884 | 471.9637 | 1684.016 | 2362.762 | 1725.627 | 2472.114 | 1163.545 | 1951.832 | 2455.41 | 2580.486 | 1855.057 | 1612.177 | 1598.096 | 917.7643 | 1625.702 | 1655.791 | 1264.445 | 1880.44 | 1635.051 | 935.3105 | 2604.753 | 1557.99 | 2752.569 |
| CUST_44526_PI429010886 | M66217 | 0.003758 | 18 | 15 | 317419745 | Diphthamide biosynthesis protein 2 [Dicentrarchus | Protein metabolism | Cellular protein modification process | 4.23E-19 | 67.67878 | 53.50545 | 57.42962 | 44.59476 | 44.65427 | 29.82235 | 27.49771 | 31.62413 | 51.77357 | 117.621 | 47.40164 | 79.77146 | 46.29572 | 88.69099 | 52.59262 | 56.26704 | 58.28332 | 58.84482 | 47.93244 | 63.1832 | 58.04233 | 52.88081 | 48.75681 | 35.99615 | 42.61032 | 50.0808 | 46.4361 |
| CUST_39509_PI429010886 | M40651 | 0.003763 | 15 | 9 | | | | | | 565.6978 | 1951.832 | 1356.986 | 365.1209 | 924.0246 | 293.0543 | 297.209 | 719.6282 | 1342.338 | 2365.487 | 1033.403 | 2821.243 | 1061.463 | 2153.407 | 1856.885 | 2395.771 | 542.5507 | 1224.871 | 416.7544 | 1409.58 | 773.8104 | 2918.106 | 1078.601 | 523.6559 | 2706.735 | 1897.37 | 4128.792 |
| CUST_33160_PI429010886 | M6130 | 0.003763 | 15 | 9 | | | | | | 299.3397 | 284.3166 | 282.9109 | 171.2286 | 240.2315 | 196.8724 | 190.5242 | 154.9129 | 266.4816 | 341.6507 | 297.9644 | 375.1955 | 227.704 | 306.2716 | 287.8054 | 346.6366 | 225.3596 | 294.0954 | 206.2132 | 345.1704 | 247.1436 | 350.793 | 286.1419 | 223.7779 | 390.6066 | 318.2412 | 391.346 |
| CUST_3167_PI429010886 | N13941 | 0.003781 | 18 | 0 | | | Nucleic acid metabolism | DNA metabolic process | | 30.78534 | 23.25736 | 25.32554 | 20.47619 | 12.08776 | 35.1685 | 40.45279 | 14.57386 | 10.84532 | 7.968722 | 9.881836 | 8.158718 | 5.803366 | 11.79386 | 11.77184 | 19.82309 | 15.37508 | 21.46189 | 33.92982 | 11.44011 | 25.95428 | 11.48128 | 12.1856 | 16.22239 | 17.22867 | 22.94293 | 14.21847 |
| CUST_12745_PI429010886 | N29974 | 0.003782 | 21 | 18 | | | | | | 156.9005 | 169.847 | 113.6252 | 110.7887 | 115.4907 | 117.0501 | 97.42055 | 97.32524 | 85.95967 | 103.3395 | 110.3616 | 126.2631 | 73.53135 | 126.1032 | 102.205 | 195.5884 | 120.0167 | 126.7579 | 124.9049 | 133.987 | 124.8573 | 166.9686 | 124.2868 | 92.41138 | 122.9711 | 121.0374 | 83.63981 |
| CUST_35204_PI429010886 | M11892 | 0.003787 | 21 | 18 | | | | | | 350.912 | 381.1294 | 342.6916 | 235.3617 | 288.7076 | 300.6388 | 233.1687 | 249.8933 | 271.6132 | 294.2267 | 332.3269 | 352.1132 | 266.0871 | 347.3329 | 289.423 | 391.819 | 282.5938 | 351.1934 | 329.5353 | 394.2069 | 328.2492 | 363.0918 | 337.8584 | 313.4226 | 345.1018 | 312.2468 | 323.1264 |
| CUST_40582_PI429010886 | M55535 | 0.003789 | 18 | 6 | | | | | | 24.59022 | 13.63796 | 26.08713 | 11.03039 | 13.89206 | 27.23432 | 22.59036 | 28.02833 | 21.20862 | 9.41784 | 13.87516 | 17.97541 | 10.34781 | 20.42128 | 16.03304 | 12.75137 | 17.947 | 11.00785 | 4.278153 | NA | 12.81601 | 20.67362 | 20.26748 | 29.48914 | 31.71799 | 27.23134 | 36.606 |
| CUST_8303_PI429010886 | N22843 | 0.003805 | 15 | 9 | 170522956 | cAMP response element-binding protein [Polyrhach | Nucleic acid metabolism | Regulation of gene expression | 4.35E-24 | 139.2501 | 192.7273 | 195.7607 | 168.2432 | 135.7237 | 128.5525 | 150.5702 | 162.8265 | 193.714 | 217.9939 | 123.8729 | 199.9465 | 166.2545 | 210.6402 | 201.7309 | 209.4896 | 105.1 | 172.7709 | 103.0015 | 164.4877 | 144.9989 | 183.8709 | 186.4927 | 172.5457 | 242.8033 | 174.7134 | 241.3477 |
| CUST_14086_PI429010886 | N32305 | 0.003805 | 15 | 9 | | | | | | 68.4981 | 78.84468 | 62.89604 | 53.32755 | 56.95101 | 58.91203 | 61.3758 | 61.86332 | 61.55497 | 71.64903 | 69.43885 | 80.59405 | 63.64323 | 74.79904 | 57.38433 | 58.83776 | 60.99125 | 74.15113 | 58.11529 | 81.79478 | 56.85652 | 65.04995 | 69.88371 | 70.11707 | 72.23965 | 74.07672 | 80.1769 |
| CUST_33237_PI429010886 | M6367 | 0.003809 | 18 | 12 | | | | | | 422.9234 | 358.55 | 620.4611 | 196.9594 | 220.6402 | 111.7039 | 1169.079 | 815.0535 | 1334.604 | 579.3813 | 192.6463 | 450.3936 | 2306.592 | 1768.416 | 1212.885 | 796.2928 | 779.3521 | 278.0457 | 784.2356 | 314.7546 | 461.0025 | 399.1635 | 487.1736 | 326.1708 | 1220.626 | 331.6154 | 1984.848 |
| CUST_49287_PI429010886 | M85022 | 0.003815 | 15 | 3 | | | | | | 61.55497 | 44.32754 | 55.56489 | 67.7681 | 96.40299 | 107.4719 | 68.45737 | 48.74075 | 21.96556 | 41.80062 | 31.70016 | 25.79784 | 25.15661 | 45.06422 | 41.86303 | 78.22974 | 104.5244 | 113.7469 | 60.3963 | 48.65694 | 54.5089 | 102.3756 | 41.94118 | 83.04083 | 46.81158 | 39.02375 | 25.89483 |
| CUST_49715_PI429010886 | M86841 | 0.003817 | 18 | 3 | 242000706 | conserved hypothetical protein [Ixodes scapularis] | | | 6.18E-09 | 4.398494 | 10.95448 | 16.14788 | 29.74088 | 24.52679 | 17.83843 | 7.089313 | 7.457269 | 8.317554 | 6.505637 | 6.008155 | NA | 8.693497 | NA | 5.528263 | 14.00927 | 8.400369 | 16.52754 | 13.59657 | NA | NA | 10.49848 | NA | 14.04066 | 12.31301 | 10.29115 | 14.44266 |
| CUST_53842_PI429010886 | M103387 | 0.003818 | 15 | 12 | | | | | | 6.905105 | 8.167815 | 8.219245 | NA | 5.630584 | NA | NA | 7.937594 | 6.497327 | 9.988677 | 6.959325 | 5.255986 | 7.108049 | 20.01489 | 7.26531 | 8.29527 | 4.786562 | NA | 4.986436 | 4.741329 | 5.005331 | 6.276753 | 6.089115 | NA | 6.765406 | 8.563205 | 10.55447 |
| CUST_32723_PI429010886 | M4933 | 0.003821 | 24 | 18 | 328790902 | PREDICTED: islet cell autoantigen 1-like [Apis mellife | | | 2.82E-17 | 38.75769 | 35.9063 | 42.62942 | 26.07451 | 32.22274 | 31.8918 | 36.23123 | 28.88408 | 31.23466 | 51.71837 | 35.98798 | 42.1391 | 38.13783 | 41.04712 | 36.57812 | 49.1644 | 41.96064 | 36.46662 | 37.40452 | 49.27786 | 40.49183 | 41.14494 | 37.07575 | 40.95384 | 35.24411 | 36.19062 | 43.38432 |

| Probe ID | ID | p-value | Period | Phase | EBI ID | Annotation | GO category | Go term | E-value | 0 | 0 | 0 | 3 | 3 | 3 | 6 | 6 | 6 | 9 | 9 | 9 | 12 | 12 | 12 | 15 | 15 | 15 | 18 | 18 | 18 | 21 | 21 | 21 | 24 | 24 | 24 |
|---|---|---|---|---|---|---|---|---|---|---|---|---|---|---|---|---|---|---|---|---|---|---|---|---|---|---|---|---|---|---|---|---|---|---|---|---|
| CUST_34217_PI429010886 | M9087 | 0.003824 | 12 | 9 | 321478511 | hypothetical protein DAPPUDRAFT_40294 [Daphnia | | | 3.17E-20 | 25.13924 | 11.98928 | 10.36906 | 6.022117 | 6.189473 | 4.859225 | 10.85593 | 11.89729 | 11.25734 | 18.74861 | 14.60539 | 113.1518 | 11.91408 | 14.58939 | NA | 13.34357 | 22.27295 | 7.373723 | 15.12292 | 15.12683 | 15.07172 | 23.3229 | NA | 16.01839 | 28.37808 | 10.30907 | 14.59396 |
| CUST_312_PI429010886 | N3803 | 0.003827 | 12 | 9 | | | | | | 471.9637 | 4.795846 | 608.2249 | 3432.751 | 12884.64 | 12465.11 | 15537.42 | 607.2425 | 56.02742 | 49.67248 | 16969.9 | NA | 18.69874 | 8.214888 | 7.242883 | 7.637236 | 20719.17 | 7273.892 | 29378.84 | NA | 19541.87 | NA | 21011.69 | 26783.35 | 9.827509 | 1122.959 | 6.453334 |
| CUST_43971_PI429010886 | M64500 | 0.003829 | 24 | 15 | | | | | | 5.994911 | NA | 9.333826 | 5.089752 | 5.449338 | 8.334073 | 5.469892 | 10.18836 | 6.730364 | 8.949545 | 8.146528 | 16.10718 | NA | 19.18487 | 9.910844 | 22.88862 | 12.7372 | 16.29078 | 6.283437 | 9.569411 | 11.92189 | 16.68994 | NA | 24.23621 | 6.543554 | 13.49483 | 15.89674 |
| CUST_31805_PI429010886 | M2456 | 0.003829 | 18 | 0 | 322797366 | hypothetical protein SINV_09654 [Solenopsis invicta | | | 2.86E-17 | 14.21497 | 6.578227 | 13.39222 | 9.53739 | 11.58179 | NA | 11.50377 | 5.839168 | 6.285427 | 7.952703 | 11.31627 | 5.719759 | 11.12353 | 4.31549 | 5.384634 | 6.447966 | 9.760194 | NA | 13.7661 | 16.32704 | 12.5608 | 16.24253 | 9.160667 | 8.597478 | 12.91176 | 6.994251 | 5.919141 |
| CUST_43647_PI429010886 | M63576 | 0.003829 | 24 | 0 | | | | | | 139.1216 | 154.7047 | 176.0053 | 88.40608 | 144.6167 | 134.9686 | 129.3018 | 135.1969 | 136.2095 | 130.2384 | 114.9009 | 109.0596 | 121.6208 | 152.8002 | 141.7869 | 164.6529 | 143.9734 | 113.3559 | 120.4829 | 150.78 | 119.7777 | 135.9627 | 144.5473 | 135.2716 | 183.8709 | 154.6585 | 149.7694 |
| CUST_54228_PI429010886 | M105185 | 0.003835 | 21 | 0 | 527254475 | PREDICTED: E3 ubiquitin-protein ligase UBR4 [Melo | Protein metabolism | Cellular protein modification process | 1.63E-22 | 51.93952 | 59.48243 | 57.15078 | 66.47359 | 57.1457 | 46.86688 | 48.55604 | 37.90169 | 38.51373 | 47.62717 | 42.48986 | 46.32451 | 44.4641 | 51.50234 | 55.65323 | 53.26328 | 50.34321 | 56.26704 | 52.53463 | 56.26704 | 56.2163 | 59.46938 | 38.54256 | 79.5827 | 58.92815 | 98.00231 | 49.5373 |
| CUST_44801_PI429010886 | M67043 | 0.003847 | 15 | 9 | | | | | | 6.474974 | 8.057073 | 7.862605 | NA | 6.300421 | 3.582315 | 6.289922 | NA | 15.7734 | 6.246892 | NA | 10.45509 | 7.91389 | NA | 11.32793 | 9.712939 | NA | 7.751416 | 6.256959 | 4.493901 | 5.237344 | 17.64462 | 9.239082 | NA | 13.71667 | 19.13027 | 10.12652 |
| CUST_31136_PI429010886 | M798 | 0.003848 | 12 | 9 | | | | | | 91.23322 | 127.601 | 130.9859 | 83.49596 | 98.56528 | 107.1945 | 72.6348 | 110.3616 | 123.5719 | 150.1517 | 143.2735 | 189.144 | 85.56392 | 157.5293 | 131.5284 | 93.55006 | 98.75692 | 107.3035 | 98.82662 | 100.1156 | 135.2315 | 180.0766 | 136.51 | 93.69637 | 139.7632 | 129.357 | 137.8094 |
| CUST_30163_PI429010886 | N61303 | 0.003858 | 21 | 6 | 321475863 | hypothetical protein DAPPUDRAFT_307810 [Daphn | Protein metabolism | Proteolysis | 1.56E-20 | 43.21123 | 20.31071 | 45.27903 | 29.94487 | 35.30186 | 57.63741 | 64.90973 | 42.95535 | 52.48792 | 45.54024 | 23.11258 | 52.02853 | 40.93552 | 34.99003 | 41.23292 | 26.81319 | 38.46752 | 38.61391 | 31.44965 | 15.09234 | 19.64586 | 30.72417 | 43.05742 | 54.8287 | 40.48521 | 41.84554 | 33.89069 |
| CUST_21403_PI429010886 | N45154 | 0.003866 | 12 | 3 | 33468738 | calcification-associated peptide-1 [Procambarus cla | | | 8.53E-19 | 347.2399 | 17.66078 | 176.601 | 21119.13 | 34070.11 | 33611.5 | 21906.22 | 7415.798 | 63.94078 | 6.338807 | 16515.38 | NA | 784.6927 | 28.25151 | 418.03 | NA | 22231.21 | 18556.73 | 28160 | NA | 27088.59 | 6.144642 | 9448.983 | 9611.158 | 5.19915 | 11959.99 | NA |
| CUST_11351_PI429010886 | N27672 | 0.003867 | 21 | 15 | | | | | | 20.40111 | 18.28397 | 25.12574 | 23.1151 | 13.85759 | 27.56643 | 18.51801 | 13.57374 | 8.66034 | 12.37856 | 13.21125 | 18.94687 | 18.15323 | 21.63606 | 11.78251 | 24.60142 | 25.92862 | 24.34275 | 17.16312 | 24.15613 | 28.7672 | 22.61839 | 24.74877 | 31.34117 | 21.77287 | 13.75107 | 15.19655 |
| CUST_22061_PI429010886 | N46351 | 0.003868 | 24 | 3 | 391343387 | PREDICTED: ephrin-B1-like [Metaseiulus occidentali | | | 4.89E-22 | 17.95452 | 20.63276 | 20.73391 | 47.14504 | 14.24656 | 26.73153 | 21.87774 | 19.95854 | 13.87348 | 22.94537 | 4.211828 | 18.85554 | 4.542912 | 18.49479 | 7.986139 | 6.086228 | 7.706127 | 7.783894 | 7.668108 | 5.661008 | 19.42864 | 11.12791 | 29.04226 | 19.08147 | 19.93091 | 21.7761 | 11.28356 |
| CUST_49759_PI429010886 | M87020 | 0.00387 | 24 | 21 | | | | | | 19.39031 | 19.62917 | 19.26372 | 8.272098 | 11.38749 | 10.42321 | 9.965323 | 8.132198 | 12.17805 | 13.2803 | 10.73906 | 23.65146 | 16.61891 | 17.19113 | 26.04992 | 13.83521 | 10.0911 | 15.77573 | 12.89788 | 13.06127 | 20.26285 | 25.59725 | 24.2001 | 17.25129 | 12.65638 | 13.02142 | 11.81798 |
| CUST_43896_PI429010886 | M64280 | 0.00387 | 12 | 3 | 321479008 | hypothetical protein DAPPUDRAFT_309638 [Daphn | | | 4.18E-10 | 8.588693 | 36.08462 | 14.71509 | 87.67204 | 44.66322 | 36.53021 | 23.41598 | 94.46416 | 22.58452 | 5.756675 | 5.779406 | 14.92131 | 13.65696 | 24.42322 | 31.44965 | 28.42343 | 17.29005 | 23.89866 | 6.339538 | 12.13695 | 5.799012 | 22.62086 | 6.667395 | 9.25417 | 13.5521 | 13.56887 | 18.00026 |
| CUST_7477_PI429010886 | N21603 | 0.003871 | 24 | 12 | 345493506 | PREDICTED: striatin-3-like [Nasonia vitripennis] | | | 4.39E-69 | 123.349 | 99.08673 | 115.6759 | 107.8324 | 89.84183 | 103.8532 | 92.70294 | 107.4618 | 117.0501 | 121.4004 | 112.8438 | 142.6158 | 116.2656 | 154.5663 | 151.2216 | 125.5041 | 112.0103 | 151.6013 | 126.9103 | 128.0504 | 113.0567 | 163.051 | 110.1494 | 123.6853 | 109.002 | 95.01446 | 130.3469 |
| CUST_34488_PI429010886 | M9814 | 0.003872 | 12 | 0 | | | | | | 84.93221 | 86.58356 | 117.6032 | 45.83381 | 30.65462 | 15.52639 | 73.22698 | 137.2146 | 93.9354 | 117.9903 | 69.4087 | 67.22918 | 93.45456 | 109.5748 | 72.6524 | 58.24456 | 64.8492 | 39.02375 | 64.64151 | 57.90171 | 55.27747 | 84.1293 | 92.01375 | 31.09152 | 120.5952 | 77.33177 | 84.36786 |
| CUST_16603_PI429010886 | N36549 | 0.003873 | 18 | 9 | | | | | | 32.90555 | 34.9676 | 27.085 | 39.53295 | 41.98366 | 16.07923 | 48.12117 | 31.8356 | 50.03352 | 71.57464 | 43.05396 | 69.78353 | 44.07915 | 36.25388 | 66.39938 | 78.21131 | 32.94969 | 30.40899 | 37.70021 | 13.32601 | 26.49815 | 48.34221 | 19.46833 | 24.27461 | 37.95272 | 33.29463 | 60.10166 |
| CUST_37536_PI429010886 | M19411 | 0.003873 | 18 | 12 | 532057848 | PREDICTED: zinc finger protein 709-like [Microtus o | | | 8.11E-24 | 37.77885 | 48.15042 | 57.06419 | 21.30938 | 24.51394 | 28.87669 | 33.57359 | 30.15647 | 32.01382 | 50.68297 | 37.59821 | 45.82893 | 76.07766 | 41.14049 | 54.16396 | 39.24656 | 36.19062 | 35.03522 | 48.90211 | 42.03093 | 35.12261 | 39.65367 | 40.76795 | 26.42193 | 40.68218 | 33.8457 | 36.41953 |
| CUST_1509_PI429010886 | N9868 | 0.003873 | 18 | 9 | | | | | | 27.89422 | 41.63417 | 24.6038 | 13.06127 | 23.72867 | 11.94752 | 32.12364 | 25.9871 | 31.92933 | 44.03536 | 24.31817 | 40.44483 | 22.46339 | 29.2624 | 29.19814 | 37.59059 | 41.16431 | 28.29582 | 23.21071 | 24.50538 | 30.74654 | 21.22623 | 26.38687 | 18.44318 | 38.54575 | 21.21658 | 24.40138 |
| CUST_43392_PI429010886 | M62922 | 0.003875 | 21 | 9 | 498946448 | PREDICTED: calcium-transporting ATPase type 2C m | Transport | Ion transport | 5.67E-41 | 18.01173 | 18.6314 | 15.55875 | 21.29823 | 11.92505 | 20.37797 | 9.080702 | 22.0053 | 24.2722 | 23.50407 | 24.78858 | 34.16739 | 24.33679 | 19.51465 | 20.29379 | 16.83375 | 20.85111 | 22.16629 | 19.15208 | 27.52267 | 19.85636 | 17.60432 | 19.45845 | 9.99769 | 17.12833 | 18.50047 | 25.67741 |
| CUST_48226_PI429010886 | M79436 | 0.00388 | 24 | 15 | | | | | | 22.17526 | 14.01595 | 14.62058 | NA | 9.115042 | 6.106434 | 26.61439 | 25.75169 | 15.85477 | 22.47719 | 10.80725 | 19.12346 | 26.35211 | 13.06649 | 26.69312 | 42.73857 | NA | 23.17992 | 29.33184 | 16.70939 | 17.47076 | 22.49442 | NA | NA | 17.03915 | 8.635923 | 16.33068 |
| CUST_35475_PI429010886 | M12665 | 0.003883 | 24 | 0 | 358339569 | hypothetical protein CLF_100574 [Clonorchis sinens | | | 5.84E-07 | 19.79173 | 18.80396 | 18.07527 | 20.55297 | 17.93802 | 19.34025 | 11.46096 | 16.24662 | 16.1391 | 14.30234 | 17.45713 | 17.23137 | 15.46253 | 14.80507 | NA | 15.44778 | 13.94454 | 15.18687 | 11.74252 | 9.558027 | 24.79214 | 7.837273 | 25.92533 | 17.13072 | 20.10327 | 17.49801 | 29.91744 |
| CUST_54630_PI429010886 | M106924 | 0.00389 | 15 | 9 | | | | | | 39.64527 | 37.56417 | 38.74344 | 25.36189 | 36.94632 | 16.94233 | 21.70738 | 32.90861 | 46.84888 | 51.13662 | 30.56765 | 60.27464 | 48.2905 | 46.08794 | 37.00901 | 41.68037 | 39.81145 | 48.61666 | 36.03688 | 46.69563 | 32.06599 | 62.36898 | 40.38558 | 38.70169 | 60.37722 | 49.49896 | 61.32192 |
| CUST_48350_PI429010886 | M79956 | 0.003891 | 24 | 21 | | | | | | 18.15141 | 15.84016 | 17.01911 | 17.8869 | 7.468703 | NA | 7.573982 | 16.8197 | 13.7763 | 10.17638 | 11.24123 | 13.51693 | 9.751886 | 12.11329 | 5.13952 | 15.54357 | 20.92107 | 16.41178 | 17.05906 | 21.27912 | 12.92033 | 16.61483 | 20.52202 | 17.84395 | 22.56443 | 14.70096 | 21.30062 |
| CUST_7356_PI429010886 | N21423 | 0.003901 | 24 | 18 | | | | | | 99.82512 | 133.8452 | 120.4628 | 77.10171 | 87.11963 | 82.10096 | 111.5107 | 113.4221 | 104.5873 | 118.9193 | 108.6696 | 120.2964 | 101.1196 | 122.4667 | 149.731 | 147.9921 | 104.5737 | 119.7777 | 139.376 | 133.9495 | 123.7589 | 122.6587 | 112.4908 | 85.69951 | 110.7482 | 128.6574 | 152.675 |
| CUST_39950_PI429010886 | M47341 | 0.003906 | 18 | 3 | 121484216 | cuticle protein CB6 [Portunus pelagicus] | | | 2.69E-12 | 665.3114 | 30.32196 | 655.0692 | 1924.188 | 2464.001 | 4091.647 | 1827.911 | 258.7295 | 47.3594 | 23.69356 | 1982.266 | 14.31095 | 51.89227 | 12.0262 | 10.09227 | NA | 3048.703 | 2879.829 | 1733.695 | 24.25592 | 1769.215 | NA | 3216.436 | 2957.256 | 11.62197 | 577.6505 | 12.23171 |
| CUST_55063_PI429010886 | M108586 | 0.003906 | 15 | 12 | | | | | | 22.1046 | 28.00512 | 47.17722 | 28.21594 | 22.04079 | 25.27217 | 28.06711 | 24.983 | 21.9738 | 33.20597 | 26.97875 | 24.51625 | 30.99084 | 42.91838 | 28.8539 | 22.37068 | 21.82183 | 28.49366 | 26.03397 | 20.42948 | 24.19346 | 13.36364 | 15.7841 | 18.15141 | 22.62556 | 26.08978 | 31.79024 |
| CUST_23063_PI429010886 | N48192 | 0.003913 | 15 | 12 | | | | | | 65.42947 | 85.80553 | 79.08585 | 51.89227 | 49.14895 | 43.28403 | 59.72331 | 51.34112 | 57.50407 | 70.4935 | 47.60452 | 66.75063 | 67.21241 | 77.14278 | 96.40299 | 88.4462 | 71.94962 | 59.28688 | 53.56751 | 76.78516 | 65.37264 | 65.65578 | 59.38223 | 51.52932 | 78.84468 | 66.39266 | 77.39915 |
| CUST_4508_PI429010886 | N16927 | 0.003921 | 24 | 21 | | | | | | 23.16293 | 29.88865 | 23.46287 | 51.23628 | 17.88028 | 29.76585 | 14.70379 | 8.000521 | 14.00188 | 10.63106 | 7.871072 | 15.43638 | 9.365159 | 25.1894 | 28.1987 | 18.59144 | 4.789111 | 16.70546 | 25.50317 | 6.036326 | 34.96354 | 105.242 | 22.93439 | 59.62185 | 31.76715 | 34.94529 | 38.42267 |
| CUST_56566_PI429010886 | M114119 | 0.003946 | 18 | 9 | | | | | | 665.8633 | 738.6637 | 776.3963 | 604.157 | 626.6888 | 508.8245 | 540.0536 | 666.8781 | 714.7333 | 983.8663 | 698.8251 | 1011.687 | 755.2577 | 955.4895 | 753.7243 | 837.729 | 709.3475 | 799.1588 | 720.7013 | 875.295 | 655.3909 | 900.1864 | 652.9291 | 542.5507 | 831.7121 | 667.8908 | 943.689 |
| CUST_42254_PI429010886 | M59808 | 0.003946 | 24 | 12 | | | | | | 123.6053 | 144.4836 | 138.9124 | 92.9186 | 100.3711 | 90.9816 | 95.23078 | 116.5533 | 129.6421 | 141.6171 | 100.8047 | 171.5473 | 126.9651 | 187.0803 | 169.3396 | 158.5656 | 122.7034 | 142.8955 | 129.2544 | 117.0501 | 128.9813 | 151.6818 | 124.513 | 93.58403 | 141.2095 | 112.2867 | 119.7777 |
| CUST_41837_PI429010886 | M58666 | 0.003957 | 18 | 9 | 156545597 | PREDICTED: enoyl-CoA hydratase domain-containin | Metabolic process | Other | 4.64E-49 | 6.00986 | 8.863586 | 8.252454 | 8.80889 | 8.906902 | NA | 11.38637 | 11.93851 | 17.76311 | NA | 21.28336 | 34.14862 | 11.48128 | 11.30649 | 16.14788 | 13.20796 | 13.20437 | 16.25929 | 10.84227 | 27.085 | 8.789967 | 10.47246 | 15.37345 | NA | 20.36175 | NA | 13.14638 |
| CUST_55019_PI429010886 | M108416 | 0.003958 | 15 | 12 | | | | | | 31.62413 | 30.74148 | 32.43067 | 25.82739 | 26.44212 | 35.17156 | 25.28193 | 20.09831 | 23.93111 | 31.08214 | 30.58363 | 61.00953 | 37.38397 | 46.37163 | 38.70169 | 45.44604 | 23.10307 | 52.16399 | 29.86645 | 31.68997 | 35.20228 | 37.41548 | 26.6702 | 16.68444 | 35.97087 | 44.10538 | 36.99297 |
| CUST_34091_PI429010886 | M8748 | 0.003969 | 24 | 0 | | | | | | 173.5738 | 145.9616 | 163.4134 | 138.6217 | 148.0647 | 132.5751 | 123.0876 | 124.0489 | 152.5175 | 142.5512 | 101.435 | 137.5651 | 95.99909 | 120.5371 | 109.7501 | 123.349 | 174.0405 | 139.9664 | 136.5811 | 101.565 | 151.5629 | 170.7553 | 103.1342 | 177.0624 | 212.773 | 136.0755 | 163.4134 |
| CUST_41816_PI429010886 | M58616 | 0.003971 | 15 | 0 | | | | | | 158.3894 | 134.4739 | 143.5925 | 109.3881 | 124.4818 | 139.1216 | 98.68398 | 88.11918 | 91.95171 | 132.1901 | 99.94463 | 111.4482 | 78.68384 | 133.0251 | 110.8879 | 158.2913 | 133.0821 | 131.3745 | 125.1451 | 120.9878 | 127.5221 | 132.3781 | 92.96667 | 114.7719 | 156.8208 | 124.672 | 159.7611 |
| CUST_42396_PI429010886 | M60180 | 0.003976 | 24 | 12 | | | | | | 54.60219 | 21.87774 | 13.90998 | 40.13494 | 28.81481 | 31.7268 | 41.37211 | 36.8424 | 39.31465 | 28.73411 | 27.86117 | 93.62063 | 57.09018 | 65.46343 | 63.38531 | 96.029 | 15.30179 | 93.94852 | 31.74172 | 49.19582 | 43.05396 | 72.26362 | 28.1812 | 50.18155 | 18.88046 | 10.51643 | 18.28004 |
| CUST_37113_PI429010886 | M17882 | 0.003981 | 12 | 3 | 321472705 | hypothetical protein DAPPUDRAFT_239811 [Daphn | | | 5.81E-31 | 2194.366 | 3152.492 | 383.225 | 8336.973 | 4833.67 | 6787.522 | 4120.1 | 1228.27 | 3219.948 | 640.949 | 1731.857 | 980.4603 | 3320.318 | 1636.442 | 2289.879 | 3283.608 | 5585.575 | 7930.333 | 1584.97 | 1394.642 | 1378.434 | 2107.396 | 1035.582 | 5590.669 | 1140.952 | 2803.212 | 691.1905 |
| CUST_34235_PI429010886 | M9134 | 0.003997 | 24 | 21 | 242004474 | conserved hypothetical protein [Pediculus humanus | | | 1.1E-66 | 149.2835 | 160.4822 | 138.094 | 103.9198 | 123.1377 | 95.42654 | 72.37554 | 101.187 | 105.0313 | 148.8666 | 152.5175 | 152.3321 | 158.0759 | 125.9322 | 140.4954 | 178.0976 | 133.006 | 134.282 | 148.4195 | 156.8837 | 146.9588 | 221.7682 | 161.1265 | 146.4351 | 160.2794 | 177.6724 | 142.7507 |
| CUST_9309_PI429010886 | N24416 | 0.003997 | 24 | 21 | | | | | | 175.1526 | 182.3936 | 191.9229 | 200.7871 | 151.2035 | 163.569 | 147.2452 | 156.7533 | 136.3426 | 171.9533 | 165.7972 | 204.629 | 148.5237 | 213.0864 | 273.5534 | 225.9175 | 180.4919 | 201.1422 | 173.7958 | 215.0582 | 206.4165 | 242.4714 | 207.7427 | 194.9182 | 215.4319 | 209.9313 | 228.5019 |
| CUST_20182_PI429010886 | N42966 | 0.003998 | 15 | 12 | | | | | | 17.18917 | 24.58201 | 26.62834 | 13.43544 | 18.99317 | 13.532 | 21.79421 | 18.60109 | 23.85972 | 21.43971 | 14.35732 | 20.91693 | 182.747 | 33.14406 | 21.98819 | 27.68313 | 22.46676 | 23.93394 | 21.32116 | 12.42241 | 20.41093 | 20.34468 | 20.45436 | 15.6436 | 28.10273 | 12.04261 | 25.18232 |
| CUST_46130_PI429010886 | M71487 | 0.003999 | 15 | 3 | | | | | | 38.38764 | 19.97907 | 24.46151 | 48.02842 | 37.33083 | 89.76549 | 52.75651 | 26.71388 | 26.10969 | 7.894781 | 18.28397 | 10.01676 | 7.620705 | NA | 14.21644 | 7.991765 | 20.35314 | 3.669423 | 80.61916 | 12.11676 | 23.16735 | 15.99082 | 18.46255 | 26.14767 | 8.832139 | 36.50859 | 4.991219 |
| CUST_45943_PI429010886 | M70825 | 0.004009 | 15 | 12 | 383859690 | PREDICTED: protein numb-like isoform 1 [Megachile | | | 2.35E-52 | 11.34588 | 17.26689 | 32.38272 | 12.41521 | 15.33784 | 14.73565 | 17.64732 | 22.5259 | 16.87557 | 19.64187 | 20.65041 | 27.88621 | 22.0851 | 29.57766 | 31.01661 | 13.74799 | 35.88091 | 22.58247 | 18.6166 | 17.9566 | 16.26351 | 23.33049 | 19.01959 | 10.59173 | 21.25262 | 20.51687 | 32.10959 |
| CUST_7728_PI429010886 | N21981 | 0.004014 | 15 | 9 | 307197312 | Sodium-dependent neutral amino acid transporter | Transport | Neurotransmitter transport | 9.9E-47 | 84.40117 | 97.4551 | 123.3316 | 103.8441 | 101.8542 | 123.2747 | 48.90211 | 159.8607 | 218.3262 | 177.4848 | 121.9963 | 137.3696 | 144.3143 | 182.709 | 179.3955 | 165.9249 | 212.7445 | 145.6647 | 67.29642 | 127.2704 | 65.31793 | 124.1661 | 155.979 | 134.79 | 199.9465 | 306.479 | 336.1486 |
| CUST_33435_PI429010886 | M6880 | 0.004016 | 12 | 3 | | | | | | 67.44354 | 60.30436 | 59.22654 | 80.61162 | 81.67574 | 88.30586 | 90.55235 | 58.18474 | 61.71609 | 71.88591 | 82.32602 | 61.91569 | 54.42343 | 62.77579 | 65.18101 | 98.91679 | 99.31284 | 83.4507 | 118.5367 | 50.55699 | 75.75327 | 73.71157 | 76.64595 | 93.70583 | 52.41832 | 46.23463 | 51.97066 |
| CUST_13432_PI429010886 | N31179 | 0.004022 | 12 | 0 | | | | | | 72.28479 | 65.32582 | 65.72733 | 55.62148 | 44.90621 | 38.18452 | 78.83085 | 50.78152 | 68.09627 | 68.99159 | 51.62562 | 112.3805 | 53.65979 | 114.8221 | 80.59405 | 26.01607 | 35.30842 | 35.8336 | 51.95466 | 54.97901 | 42.11302 | 57.20099 | 35.63559 | 21.35775 | 76.924 | 74.45078 | 94.2195 |
| CUST_26289_PI429010886 | N53992 | 0.004022 | 24 | 0 | | | | | | 59.11379 | 117.9302 | 91.77189 | 63.6369 | 76.26718 | 75.92185 | 45.14299 | 40.90354 | 52.45258 | 52.76183 | 50.98064 | 39.30599 | 51.87045 | 108.5374 | 100.0297 | 106.4846 | 58.96932 | 65.96017 | 65.48233 | 50.78152 | 86.27625 | 92.79213 | 107.1617 | 67.25349 | 92.48444 | 103.9053 | 125.9322 |
| CUST_43285_PI429010886 | M62630 | 0.004028 | 15 | 9 | | | | | | 31.41586 | 42.24809 | 36.12998 | 29.08714 | 32.35941 | 28.20666 | 30.55308 | 31.96138 | 18.36981 | 45.31583 | 30.32196 | 42.48584 | 29.83449 | 54.40089 | 42.47769 | 38.30957 | 33.44293 | 41.91362 | 25.26918 | 34.96061 | 17.38074 | 38.72684 | 23.21071 | 24.91029 | 40.34341 | 47.06225 | 46.36142 |
| CUST_5959_PI429010886 | N19427 | 0.004029 | 24 | 15 | 226510844 | beta-amyloid precursor-like protein [Neohelice grar | | | 3.69E-42 | 158.496 | 142.1633 | 173.1369 | 137.687 | 146.405 | 142.4711 | 166.4452 | 149.9331 | 133.1932 | 174.0405 | 151.5005 | 173.5738 | 137.616 | 205.7316 | 184.5383 | 186.9659 | 202.8541 | 162.5301 | 184.4394 | 160.7485 | 151.2877 | 182.3936 | 148.7446 | 155.1093 | 164.0893 | 147.8755 | 183.7672 |
| CUST_41746_PI429010886 | M58445 | 0.004039 | 12 | 0 | | | | | | 55.45229 | 45.54848 | 32.30669 | 37.6674 | 36.70412 | 30.09221 | 37.27402 | 28.65923 | 33.05858 | 31.09756 | 30.96855 | 40.51578 | 65.59604 | 69.74095 | 61.11029 | 76.674 | 45.17801 | 41.87571 | 43.81006 | 32.6735 | 38.02365 | 59.80641 | 40.1147 | 44.93417 | 72.4241 | 79.36987 | 57.49879 |
| CUST_51035_PI429010886 | M91879 | 0.004043 | 21 | 6 | | | | | | 46.13849 | 42.18155 | 40.07222 | 96.74817 | 45.60489 | 130.5118 | 52.86776 | 117.0962 | 51.54875 | 47.0949 | 40.35311 | 49.12303 | 50.4763 | 71.61132 | 67.69277 | 59.94587 | 42.54336 | 109.4535 | 39.87225 | 37.55636 | 36.13819 | 42.56152 | 42.38554 | 52.74988 | 51.49206 | 57.80004 | 60.37132 |
| CUST_13997_PI429010886 | N32145 | 0.004045 | 24 | 21 | | | | | | 499.3294 | 573.39 | 677.3854 | 508.1895 | 592.5758 | 603.7585 | 496.2657 | 440.5078 | 540.5402 | 579.8544 | 552.0613 | 624.2921 | 565.5557 | 689.3131 | 571.7132 | 785.705 | 567.0781 | 686.5061 | 595.5631 | 648.6302 | 555.7341 | 746.9886 | 698.6187 | 600.4305 | 598.8088 | 543.3625 | 730.7862 |
| CUST_43226_PI429010886 | M62465 | 0.00405 | 24 | 3 | | | | | | 270.0917 | 7443.214 | 279.0433 | 8955.736 | 4102.292 | 3832.303 | 2567.686 | 4635.589 | 48.00546 | 101.637 | 3659.313 | 97.44558 | 118.5239 | 1051.549 | 2328.414 | 46.20996 | 60.42655 | 60.21705 | 338.8337 | 157.231 | 839.5092 | NA | 1133.023 | 1007.936 | NA | NA | 44.61606 |
| CUST_12361_PI429010886 | N29347 | 0.004053 | 24 | 18 | | | | | | 51.10313 | 41.7241 | 30.98188 | 17.4379 | 33.62105 | 38.56175 | 24.65264 | 34.49621 | 38.26462 | 34.51534 | 43.12662 | 97.72266 | 44.91149 | 50.66205 | 51.97626 | 94.7426 | 29.38047 | 73.83558 | 54.74214 | 58.34728 | 53.20475 | 52.49227 | 40.04686 | 82.36794 | 59.84805 | 38.44451 | 55.27747 |
| CUST_4681_PI429010886 | N17268 | 0.004081 | 15 | 3 | | | | | | 65.30643 | 64.6187 | 37.70765 | 81.31771 | 54.82199 | 110.3002 | 53.22735 | 42.50508 | 45.93195 | 48.93831 | 33.1987 | 40.87555 | 35.41996 | 52.15768 | 44.96572 | 74.99621 | 53.20016 | 102.4647 | 71.91218 | 44.0607 | 53.02384 | 61.3315 | 52.74988 | 81.74728 | 40.1303 | 50.67294 | 33.7538 |
| CUST_14922_PI429010886 | N33693 | 0.004082 | 24 | 18 | | | | | | 25.39336 | 38.59321 | 36.90619 | 20.20612 | 18.09247 | 21.21393 | 23.43097 | 19.14544 | 25.31297 | 26.67261 | 21.78927 | 47.96198 | 30.89913 | 39.05183 | 32.10055 | 43.94789 | 18.28609 | 28.07627 | 38.65653 | 33.68931 | 46.06884 | 37.78545 | 21.65521 | 24.73262 | 25.8162 | 16.80832 | 32.78743 |
| CUST_14061_PI429010886 | N32268 | 0.004086 | 15 | 0 | | | | | | 744.8667 | 885.0858 | 670.866 | 35.91866 | 575.9559 | 22.00833 | 354.3972 | 9.358965 | 18.84701 | 12.29489 | 70.62647 | 16.65858 | 7.703897 | 466.8917 | 199.1036 | 720.0434 | 785.4766 | 526.2091 | 21.54181 | 381.9407 | 273.6024 | NA | 274.49 | 37.86895 | 10.40723 | 500.7784 | 649.7314 |
| CUST_52557_PI429010886 | M97930 | 0.004086 | 18 | 9 | | | | | | 32.73413 | 35.76089 | 47.68335 | 32.09117 | 29.76053 | 31.44965 | 32.22274 | 42.84203 | 28.95199 | 53.64846 | 31.61999 | 37.21299 | 35.31634 | 46.73511 | 41.40356 | 34.89935 | 32.90178 | 36.81255 | 30.83339 | 36.24375 | 31.89828 | 33.19476 | 28.3805 | 29.89732 | 45.7289 | 44.62585 | 43.50197 |
| CUST_36029_PI429010886 | M14333 | 0.004094 | 24 | 18 | 123471670 | ankyrin repeat protein [Trichomonas vaginalis G3] > | | | 3.89E-30 | 12.91624 | 26.49443 | 16.27412 | 10.31132 | 11.54995 | 12.13161 | 11.67245 | 13.93127 | 17.53908 | 25.72813 | 17.24517 | 21.23199 | 14.41094 | 18.05758 | 16.01505 | 22.30272 | 22.70929 | 23.5201 | 14.32775 | 23.83766 | 28.32496 | 26.55213 | 27.66624 | 13.31814 | 18.21241 | 16.64978 | 28.41298 |
| CUST_41349_PI429010886 | M57523 | 0.004094 | 24 | 18 | | | | | | 69.49923 | 121.2697 | 93.42325 | 72.06797 | 73.46154 | 79.44029 | 64.86139 | 90.33731 | 92.13928 | 134.0969 | 105.2081 | 135.2315 | 106.2399 | 87.37332 | 77.04659 | 101.5527 | 143.1059 | 102.9204 | 112.4033 | 137.5108 | 122.1188 | 120.7015 | 136.7329 | 83.43282 | 115.284 | 88.49666 | 93.46673 |
| CUST_50382_PI429010886 | M89639 | 0.004098 | 24 | 3 | 330434836 | hypothetical protein [Macrobrachium nipponense] | | | 2.08E-12 | 21.95569 | 28.27702 | 18.01669 | 28.37201 | 21.27079 | 27.13002 | 20.58261 | 20.45832 | 17.12071 | 23.70771 | 19.73096 | 26.34872 | 22.08937 | 26.30031 | 17.23326 | 19.19334 | 14.53096 | 13.44726 | 14.27406 | 15.79172 | 25.92533 | 16.55113 | 22.07569 | 17.03463 | 27.39792 | 25.47702 | 20.86543 |
| CUST_27280_PI429010886 | N55764 | 0.004107 | 12 | 0 | | | | | | 41.62253 | 47.83293 | 45.49238 | 32.56779 | 33.75052 | 23.78457 | 27.19088 | 25.35894 | 40.54346 | 49.90548 | 35.71333 | 52.12333 | 34.41851 | 49.09421 | 52.71293 | 47.27714 | 29.39265 | 36.12242 | 38.121 | 40.22531 | 33.59506 | 42.88574 | 48.09045 | 36.8491 | 56.28989 | 39.569 | 58.77783 |
| CUST_52086_PI429010886 | M96369 | 0.004111 | 12 | 0 | | | | | | 798.3051 | 1386.924 | 522.5582 | 292.9566 | 104.1514 | 115.0393 | 288.0337 | 254.0209 | 243.2786 | 679.9331 | 231.8541 | 466.4832 | 337.8584 | 451.916 | 282.3228 | 282.9535 | 187.1216 | 160.6473 | 412.8295 | 731.6805 | 701.8561 | 714.0634 | 434.9301 | 243.8695 | 1322.445 | 478.7395 | 917.4556 |
| CUST_41098_PI429010886 | M56865 | 0.004112 | 15 | 12 | 66508366 | PREDICTED: inosine-5'-monophosphate dehydroger | Metabolic process | Nucleobase-containing compound metabolic process | 5.73E-93 | 90.02313 | 52.59799 | 36.606 | 94.63457 | 53.47338 | 39.766 | 36.75622 | 26.69312 | 42.85678 | 46.62146 | 55.16431 | 71.88591 | 77.42171 | 82.01377 | 49.23486 | 71.96791 | 52.8414 | 49.70586 | 48.35331 | 41.66498 | 44.48344 | 43.21123 | 36.99652 | 47.86055 | 50.92195 | 44.59831 | 78.31562 |
| CUST_17959_PI429010886 | N38904 | 0.004117 | 24 | 0 | | | | | | 28.44909 | 27.8177 | 29.83725 | 15.4125 | 22.76882 | 22.63022 | 12.63126 | 12.01254 | 12.72475 | 76.88221 | 28.39147 | 18.66977 | 11.68092 | 19.14986 | 12.78166 | 28.43719 | 19.95383 | 23.64694 | 14.12813 | 24.01772 | 22.55762 | 23.91343 | 19.42496 | 30.17169 | 27.4692 | 28.85125 | 32.65296 |
| CUST_25733_PI429010886 | N52955 | 0.004122 | 24 | 18 | | | | | | 18.689 | 11.89878 | 25.28525 | 10.28317 | 14.46988 | 13.83521 | 9.970778 | 12.05594 | 10.32397 | 10.5119 | 5.662691 | 17.60614 | 17.71709 | 23.26561 | 14.06382 | 17.51086 | 12.69552 | 20.03687 | 29.74822 | 12.38076 | 23.53335 | 15.16412 | 23.66736 | 17.41767 | 14.48109 | 22.5259 | 17.80745 |
| CUST_38123_PI429010886 | M23454 | 0.004136 | 15 | 9 | | | | | | 149.5538 | 266.4363 | 225.8926 | 277.5533 | 175.5504 | 151.386 | 302.4151 | 205.6121 | 362.1966 | 334.3349 | 223.6419 | 364.475 | 274.9598 | 393.933 | 218.1069 | 193.6314 | 211.9082 | 265.9437 | 217.9121 | 186.2582 | 222.5361 | 266.9045 | 248.8723 | 253.2546 | 267.3316 | 393.933 | 348.3321 |
| CUST_8676_PI429010886 | N23400 | 0.004136 | 15 | 0 | | | | | | 124.1999 | 136.0526 | 127.5818 | 109.399 | 98.07031 | 87.45287 | 85.74608 | 88.12787 | 89.82702 | 101.1596 | 96.76761 | 122.3193 | 93.11072 | 117.8039 | 90.21463 | 153.3563 | 124.3147 | 113.0821 | 98.10524 | 121.5117 | 84.1293 | 123.8447 | 104.205 | 92.55609 | 112.5022 | 90.12961 | 101.6948 |
| CUST_4127_PI429010886 | N16077 | 0.004138 | 15 | 3 | | | | | | 66.62755 | 25.4551 | 19.24611 | 1170.02 | 2786.84 | 2437.103 | 3118.308 | 204.885 | 32.31352 | NA | 2489.582 | NA | 25.34638 | 4.266623 | 14.50604 | 6.852264 | 1963.68 | 826.1357 | 1178.599 | NA | 1811.1 | 7.893242 | 1016.492 | 1069.225 | 4.671595 | 283.788 | NA |
| CUST_18863_PI429010886 | N40547 | 0.004142 | 18 | 9 | | | | | | 77.63254 | 123.1539 | 109.6904 | 79.52449 | 81.89181 | 55.88131 | 60.71325 | 174.6367 | 109.5693 | 134.5558 | 106.4056 | 148.2854 | 104.2505 | 122.0534 | 104.474 | 104.8214 | 87.81308 | 85.91746 | 100.1395 | 117.3497 | 71.3655 | 100.4062 | 93.42325 | 60.22941 | 108.4668 | 97.17307 | 127.723 |
| CUST_48103_PI429010886 | M78891 | 0.004142 | 24 | 21 | | | | | | 25.70664 | 17.9311 | 19.16725 | 16.3435 | 24.96011 | 18.55589 | 15.89893 | 13.19779 | 18.19413 | 24.15872 | 22.13275 | 18.32163 | 25.22098 | 25.62143 | 20.4401 | 26.95815 | 17.87274 | 27.07911 | 16.53144 | 28.02151 | 27.57506 | 24.93429 | 25.60312 | 30.18124 | 39.85765 | 19.87307 | 33.03437 |
| CUST_52995_PI429010886 | M99710 | 0.00415 | 21 | 3 | | | | | | 86.16637 | 112.3305 | 74.28436 | 104.4433 | 94.55772 | 95.80114 | 81.82955 | 69.00612 | 83.83314 | 92.20534 | 57.97853 | 68.37004 | 61.59565 | 64.56992 | 81.59575 | 102.3344 | 65.43441 | 78.77563 | 87.20925 | 75.51588 | 84.09478 | 100.9304 | 62.80799 | 90.73889 | 115.3428 | 80.43582 | 104.0454 |
| CUST_30694_PI429010886 | N62413 | 0.004151 | 21 | 18 | | | | | | 37.80681 | 22.02831 | 29.02633 | 14.4635 | 14.9648 | 21.16222 | 21.12158 | 23.62378 | 27.4871 | 24.51087 | 24.47717 | 30.78076 | 22.96293 | 17.54469 | 31.83796 | 27.51169 | 30.57426 | 28.71325 | 57.24358 | 36.25388 | 19.3658 | 19.17264 | 28.10538 | 22.77196 | 21.73902 | 10.06452 | 23.57465 |
| CUST_44829_PI429010886 | M67126 | 0.004174 | 12 | 3 | 121484216 | cuticle protein CB6 [Portunus pelagicus] | | | 5.63E-13 | 124.3334 | 9.534612 | 130.4407 | 37163.2 | 56447.03 | 57772.94 | 40846.53 | 5086.964 | 33.70683 | 15.80527 | 52133.5 | NA | 460.8905 | 11.18709 | 214.5304 | NA | 53582.06 | 43247.3 | 36665.15 | NA | 34197.64 | 3.53961 | 40755.59 | 51514.17 | 8.185415 | 22054.78 | 5.310263 |
| CUST_1196_PI429010886 | N8807 | 0.004175 | 18 | 6 | | | | | | 34.11542 | NA | 10.95668 | NA | 34.60204 | NA | 64.11406 | NA | 144.1009 | 76.05543 | 47.15656 | 17.3861 | 39.79718 | NA | 130.5635 | 60.48684 | NA | 14.92766 | 13.54942 | 3.592026 | 7.737261 | 257.2108 | 25.16199 | 175.9719 | 73.63983 | 64.26853 | 116.6845 |
| CUST_3707_PI429010886 | N15093 | 0.004178 | 24 | 18 | 443714729 | hypothetical protein CAPTEDRAFT_224969 [Capitell | | | 4.77E-09 | 72.61909 | 106.8104 | 72.48039 | 70.82822 | 60.26272 | 48.16535 | 68.71451 | 61.66407 | 63.16045 | 122.5004 | 82.929 | 83.35473 | 76.60117 | 50.48761 | 86.59518 | 123.9921 | 69.18954 | 118.4632 | 97.98092 | 103.0771 | 93.2268 | 93.09068 | 94.06166 | 74.08523 | 101.4644 | 79.03234 | 96.80182 |
| CUST_41953_PI429010886 | M58970 | 0.004185 | 24 | 9 | 219809648 | Kazal-type serine proteinase inhibitor 3 [Fennerope | | | 4.26E-20 | 82.13647 | 140.3818 | 77.76831 | 163.3073 | 125.1451 | 124.5707 | 115.4907 | 89.03619 | 132.4401 | 172.9097 | 151.1369 | 238.3762 | 125.3173 | 280.971 | 156.8837 | 167.321 | 134.7571 | 66.03172 | 154.7492 | 218.3262 | 188.3253 | 140.194 | 92.21711 | 76.74889 | 79.12414 | 58.94211 | 86.49959 |
| CUST_25004_PI429010886 | N51619 | 0.004191 | 15 | 0 | | | | | | 24.10675 | 22.34831 | 29.23595 | 10.46311 | 13.92315 | 27.9866 | 20.48326 | 13.40566 | 9.54588 | 14.37169 | 18.18378 | 26.39458 | 9.904227 | 19.0844 | 20.46326 | 21.86356 | 23.47569 | 24.96736 | 20.49973 | 21.91471 | 38.79996 | 9.035702 | 13.99028 | 15.55047 | 14.28453 | 18.70704 | 23.71704 |
| CUST_31563_PI429010886 | M1844 | 0.004195 | 18 | 15 | 242021017 | conserved hypothetical protein [Pediculus humanus | Nucleic acid metabolism | Regulation of gene expression | 1.74E-52 | 77.89081 | 74.35805 | 62.03623 | 46.85704 | 51.83037 | 42.31429 | 49.26562 | 43.61355 | 53.15159 | 77.16796 | 63.11741 | 96.40299 | 49.00501 | 68.14294 | 65.25641 | 80.15079 | 75.67767 | 70.46616 | 77.95844 | 56.85058 | 50.41387 | 51.13662 | 59.74927 | 43.88802 | 82.38056 | 65.52616 | 63.1995 |
| CUST_22212_PI429010886 | N46634 | 0.004195 | 18 | 3 | | | | | | 355.193 | 1450.058 | 648.287 | 1807.03 | 761.273 | 1153.924 | 527.8694 | 594.2743 | 603.0908 | 603.2889 | 260.7556 | 1246.44 | 609.6635 | 447.351 | 33.03027 | 253.1078 | 206.3839 | 333.1788 | 249.7604 | 250.8185 | 725.4134 | 664.3088 | 933.1038 | 620.4611 | 827.8275 | 245.4514 | 426.3129 |
| CUST_22838_PI429010886 | N47774 | 0.004195 | 18 | 9 | | | | | | 14.64247 | 10.29246 | 12.5052 | 12.61292 | 16.42197 | 18.29426 | 17.78225 | 24.18144 | 19.45845 | 19.37939 | 24.9855 | 23.92174 | 12.48479 | 23.20138 | 15.8511 | 15.39132 | 18.38698 | 13.14971 | 9.166269 | 11.58179 | 9.568409 | 21.51397 | 30.12445 | 24.2228 | 15.70408 | 7.3856 | 20.51687 |
| CUST_22385_PI429010886 | N46935 | 0.004206 | 21 | 3 | | | | | | 22.84941 | 34.35609 | 31.57619 | 44.40451 | 28.1226 | 40.06518 | 50.14442 | 24.14576 | 28.76281 | 29.11372 | 25.83289 | 33.90631 | 22.48304 | 24.75161 | 21.80952 | 27.24092 | 23.05482 | 25.56884 | 19.69401 | 27.89422 | 38.66828 | 22.01737 | 44.21249 | 28.35783 | 41.06137 | 35.00866 | 30.19387 |
| CUST_34530_PI429010886 | M9925 | 0.004207 | 12 | 3 | | | | | | 181.0152 | 150.0554 | 151.0154 | 196.1855 | 201.9745 | 241.4448 | 211.4751 | 210.2272 | 178.3785 | 152.565 | 159.0669 | 153.6022 | 167.3467 | 163.4134 | 176.2423 | 184.4143 | 221.425 | 255.6621 | 207.1022 | 185.4691 | 150.6483 | 205.3646 | 126.4569 | 196.2537 | 153.0166 | 160.5038 | 141.8965 |
| CUST_22668_PI429010886 | N47423 | 0.004207 | 12 | 0 | | | | | | 40.00171 | 43.36723 | 46.46836 | 21.56178 | 34.28772 | 35.89984 | 27.72017 | 28.83204 | 35.9416 | 40.90354 | 35.04798 | 44.50009 | 38.66042 | 44.48344 | 45.27452 | 47.57087 | 26.99397 | 34.21339 | 35.96862 | 32.79731 | 38.49435 | 54.25943 | 51.71837 | 34.35393 | 57.32094 | 36.8879 | 67.72476 |
| CUST_45089_PI429010886 | M67974 | 0.004209 | 21 | 12 | | | | | | 117.0962 | 183.6687 | 181.9242 | 171.9761 | 149.811 | 222.6303 | 146.1555 | 147.5412 | 157.6874 | 210.6087 | 157.4409 | 225.6591 | 223.6586 | 249.5597 | 286.8668 | 207.885 | 196.7037 | 187.2939 | 228.4344 | 253.2546 | 193.9691 | 199.5478 | 229.9104 | 200.2689 | 156.0301 | 167.3841 | 223.6419 |
| CUST_38028_PI429010886 | M22894 | 0.004215 | 15 | 12 | | | | | | 12193.93 | 1485.965 | 23368.13 | 602.4628 | 1267.999 | 869.5437 | 16000.49 | 30891.2 | 28919.9 | 39918.27 | 6920.477 | 9009.958 | 31396.1 | 41094.47 | 27464.78 | 3610.458 | 18524.86 | 1163.545 | 9739.086 | 11089.86 | 4012.642 | 23368.13 | 31231.03 | 5447.86 | 42869.03 | 4973.61 | 38680.42 |
| CUST_51751_PI429010886 | M94865 | 0.004215 | 15 | 12 | | | | | | 16.25929 | 12.20563 | 20.06809 | 16.54774 | 11.51496 | 14.22799 | 13.59282 | 22.3138 | 13.96065 | 18.26869 | 15.91395 | 27.09386 | 22.72441 | 29.17928 | 18.94868 | 16.17624 | 23.39945 | 25.33771 | 19.04546 | 9.409299 | 13.29348 | 21.21967 | 13.52947 | 16.44566 | 22.21172 | 28.26127 | 20.74695 |
| CUST_52440_PI429010886 | M97452 | 0.004217 | 24 | 0 | | | | | | 61.25792 | 60.73689 | 75.33028 | 56.19324 | 47.03677 | 50.08673 | 47.77531 | 42.85091 | 47.01793 | 57.028 | 55.95035 | 52.63886 | 34.34243 | 47.2535 | 55.91435 | 53.8998 | 48.98425 | 60.7605 | 53.39323 | 61.74032 | 49.88882 | 63.52784 | 51.66322 | 35.52357 | 83.94314 | 62.3009 | 66.23254 |
| CUST_10689_PI429010886 | N26633 | 0.004223 | 15 | 6 | | | | | | 12.34868 | 9.437303 | NA | 9.238126 | 11.50819 | 10.83296 | 12.3155 | 15.40175 | 17.07004 | 11.02964 | 10.6154 | 37.1484 | 8.73506 | NA | 8.434609 | 19.59849 | 6.00037 | 8.930028 | 11.17351 | 16.64328 | 15.27919 | 27.2271 | NA | 21.52056 | 10.63784 | 11.44313 | 6.772515 |
| CUST_4709_PI429010886 | N17317 | 0.004232 | 24 | 15 | | | | | | 97.83625 | 89.06701 | 89.09879 | 89.19702 | 82.10096 | 94.49669 | 88.33792 | 80.78349 | 83.06954 | 103.4972 | 77.68249 | 88.60515 | 109.7061 | 132.4401 | 84.28941 | 92.90826 | 118.9329 | 124.1661 | 99.55807 | 93.40468 | 114.9376 | 108.1985 | 99.05161 | 106.4989 | 105.2191 | 103.202 | 121.284 |
| CUST_9670_PI429010886 | N25023 | 0.004233 | 21 | 15 | | | | | | 20.95699 | 8.589723 | 13.30863 | 15.66989 | 14.8771 | 11.27096 | 6.86682 | 6.745411 | 8.864496 | 14.78997 | 14.68167 | 15.33408 | 18.61046 | 17.04861 | 19.37939 | 20.47188 | 16.3435 | 18.71748 | 14.28453 | 20.29574 | 18.98392 | 9.970778 | 19.18487 | 13.02142 | 19.69614 | 11.33427 | 14.33088 |
| CUST_27924_PI429010886 | N56952 | 0.004244 | 24 | 21 | | | | | | 50.87205 | 63.91001 | 58.29649 | 38.73964 | 36.92762 | 40.34341 | 50.41387 | 18.95813 | 66.11527 | 37.9742 | 33.61445 | 53.0654 | 45.58985 | 55.03461 | 50.69847 | 55.54708 | 43.76068 | 49.38217 | 47.91527 | 55.39951 | 70.85223 | 83.88709 | 55.96041 | 78.29209 | 85.81376 | 42.5506 | 58.76571 |
| CUST_41120_PI429010886 | M56914 | 0.004246 | 15 | 0 | | | | | | 30.8467 | 38.0269 | 28.02553 | 29.17612 | 16.80263 | 19.67774 | 20.43418 | 21.34584 | 16.5492 | 28.95462 | 20.32136 | 27.93572 | 19.54896 | 23.90354 | 34.09109 | 40.63456 | 30.19387 | 41.61311 | 26.91205 | 33.99968 | 27.74608 | 32.2333 | 13.71161 | 19.44948 | 36.65319 | 30.07667 | 28.69117 |
| CUST_23999_PI429010886 | N49841 | 0.004253 | 24 | 0 | | | | | | 15.84566 | 21.92246 | 17.31011 | 42.23347 | 14.85125 | 17.75695 | 8.092867 | 9.792471 | 9.76387 | 5.348518 | 10.24932 | 9.733637 | 7.838281 | 15.98933 | 19.05109 | 55.0673 | 14.95825 | 15.44039 | 14.94259 | 17.7534 | 19.89384 | 20.11768 | 22.69834 | 10.73106 | 20.93093 | 22.55376 | 23.56991 |
| CUST_23773_PI429010886 | N49441 | 0.004255 | 12 | 0 | 321461754 | ABC transporter [Daphnia pulex] | | | 2.43E-21 | 53.24454 | 44.47376 | 75.5762 | 51.02123 | 37.13804 | 29.76829 | 36.72937 | 51.74182 | 43.9229 | 58.83154 | 37.52322 | 57.21451 | 56.33375 | 66.90174 | 59.98068 | 47.60452 | 37.54075 | 36.23985 | 47.79729 | 79.19112 | 40.20429 | 48.9688 | 52.54728 | 42.99716 | 57.2685 | 59.67038 | 48.91662 |
| CUST_42911_PI429010886 | M61580 | 0.004258 | 15 | 3 | | | | | | 21.8729 | 20.79607 | 25.83289 | 29.02946 | 25.01145 | 36.89793 | 15.16191 | 18.85808 | 14.34646 | 24.54709 | 23.25736 | 20.06593 | 11.74368 | 11.00169 | 9.15176 | 26.26256 | 17.52059 | 23.25736 | 24.96359 | 21.55583 | 14.93582 | 21.22218 | 20.74032 | 27.93572 | 14.69878 | 14.9864 | 18.53904 |
| CUST_29999_PI429010886 | N60975 | 0.004259 | 21 | 12 | | | | | | 114.3208 | 120.9219 | 126.8079 | 105.8816 | 98.92872 | 120.7171 | 116.7797 | 132.2049 | 109.9175 | 128.1445 | 120.2795 | 133.2074 | 123.8531 | 141.3792 | 138.1141 | 124.7036 | 123.5093 | 168.6167 | 121.4562 | 98.09433 | 112.457 | 124.3983 | 161.9153 | 156.3748 | 93.43338 | 115.9863 | 104.5737 |
| CUST_26165_PI429010886 | N53784 | 0.004265 | 12 | 0 | | | | | | 17.75139 | 21.14025 | 63.8461 | 16.13721 | 14.88378 | 37.9894 | 20.68333 | 6.888224 | 18.47407 | 22.21682 | 36.40882 | 36.04391 | 17.81636 | 34.10344 | 32.69544 | 11.06447 | 14.38818 | 32.93932 | 3.605068 | 9.150748 | 12.75297 | 8.067068 | 16.63365 | 11.34133 | 42.12286 | 22.14395 | 46.87213 |
| CUST_38872_PI429010886 | M31412 | 0.004266 | 15 | 12 | 195447950 | GK25804 [Drosophila willistoni] >gi\|194167528\|gb | | | 5.01E-07 | 415.2193 | 25.01801 | 3375.684 | 7.251886 | 6.064884 | 14.99598 | 1127.801 | 5396.825 | 3040.688 | 2639.982 | 58.70044 | 108.1195 | 4955.555 | 7701.667 | 3168.052 | 41.59399 | 2390.205 | 18.87168 | 176.9698 | 86.4072 | 57.60743 | 690.6945 | 3687.08 | 73.07849 | 3876.188 | 73.42972 | 3607.364 |
| CUST_52473_PI429010886 | M97567 | 0.004266 | 15 | 12 | | | | | | 651.8188 | 292.8985 | 1208.126 | 25.81867 | 129.8119 | 18.16713 | 844.3643 | 1974.817 | 1081.105 | 1740.053 | 300.1314 | 606.7586 | 1609.757 | 2049.694 | 1214.782 | 360.5617 | 980.1159 | 217.4042 | 630.4701 | 779.3521 | 232.1034 | 1284.605 | 909.1015 | 31.2636 | 1458.732 | 465.1151 | 1621.23 |
| CUST_13219_PI429010886 | N30798 | 0.004266 | 15 | 3 | | | | | | 153.7764 | 170.596 | 154.6176 | 239.1179 | 204.946 | 148.8262 | 152.7576 | 54.21774 | 124.0734 | 186.0881 | 178.3086 | 149.0574 | 32.22652 | 109.1135 | 65.14347 | 212.5688 | 186.5659 | 153.2997 | 213.3434 | 284.093 | 193.2088 | 207.8011 | 120.0646 | 119.9009 | 152.7576 | 145.7071 | 123.2882 |
| CUST_45323_PI429010886 | M68735 | 0.004284 | 24 | 21 | | | | | | 38.55322 | 69.93057 | 61.42421 | 44.61206 | 37.39122 | 34.50298 | 38.75769 | 23.35894 | 29.89381 | 61.87735 | 45.97615 | 25.25877 | 23.49823 | 34.71088 | 22.61587 | 23.1151 | 82.29118 | 66.94583 | 58.47643 | 44.42212 | 36.7953 | 71.86244 | 56.86246 | 74.07672 | 93.04202 | 56.33375 | 92.23086 |
| CUST_10891_PI429010886 | N26953 | 0.004296 | 24 | 0 | 225717736 | ACN9 protein homolog, mitochondrial precursor [Ca | | | 3.99E-27 | 36.34355 | 57.84964 | 34.96354 | 43.12293 | 32.91963 | 26.30221 | 33.23109 | 26.27705 | 20.64798 | 27.88984 | 27.03027 | 18.70704 | 32.17021 | 18.45187 | 22.46676 | 32.13434 | 44.80736 | 26.85728 | 26.80106 | 29.56366 | 30.87561 | 41.45965 | 28.64876 | 32.60067 | 37.79822 | 36.82802 | 30.58363 |
| CUST_10482_PI429010886 | N26305 | 0.004297 | 24 | 3 | | | | | | 35.21227 | 59.35924 | 25.61779 | 70.33128 | 38.81418 | 101.6485 | 36.48332 | 60.84782 | 27.80573 | 34.26311 | 19.25911 | 36.89165 | 14.21316 | 41.2187 | 56.56684 | 25.39589 | 6.256328 | 24.89895 | 10.40388 | 15.89011 | 36.75622 | 45.50557 | 31.43244 | 22.86023 | 53.7412 | 82.41691 | 13.90998 |
| CUST_22402_PI429010886 | N46971 | 0.004297 | 24 | 15 | | | | | | 311.3229 | 617.6618 | 480.7772 | 380.124 | 429.8795 | 339.9729 | 410.239 | 387.2348 | 390.3686 | 672.5182 | 401.5677 | 513.9071 | 443.8496 | 594.4635 | 410.1622 | 1176.647 | 689.0956 | 552.0613 | 377.6826 | 507.0685 | 511.5434 | 523.4011 | 676.9355 | 458.3652 | 558.0588 | 461.0025 | 487.5504 |
| CUST_55535_PI429010886 | M110559 | 0.004301 | 15 | 9 | | | | | | 11.13919 | 5.633982 | 13.55467 | 4.145629 | 4.475634 | 10.86137 | 14.35036 | 12.69853 | 10.95578 | 15.3449 | 12.0793 | 12.58978 | 17.3028 | 7.393906 | 7.199687 | 7.208745 | 6.408788 | 24.43609 | 6.675568 | 6.567114 | 10.2558 | 9.617174 | 5.587654 | 12.19537 | 21.46638 | 12.54832 | 17.13916 |
| CUST_50380_PI429010886 | M89627 | 0.004302 | 12 | 3 | 45550185 | organic anion transporting polypeptide 33Ea, isofor | Transport | Other | 2.04E-20 | 24.62111 | 21.68681 | 24.0618 | 84.43869 | 89.81869 | 79.73386 | 105.0723 | 70.14592 | 36.37581 | 30.35559 | 50.19562 | 23.54424 | 29.5457 | 31.37091 | 19.43524 | 22.07292 | 61.32192 | 41.80808 | 115.7777 | 8.664964 | 38.42916 | 37.4516 | 28.6303 | 30.55547 | 19.80135 | 29.68892 | 15.54715 |
| CUST_35134_PI429010886 | M11682 | 0.004303 | 18 | 15 | 194863562 | GG10667 [Drosophila erecta] >gi\|190662368\|gb\|EI | | | 5.25E-19 | 21.15466 | 23.48014 | 9.370503 | 15.40711 | 12.31693 | 7.666723 | 8.237262 | 11.97367 | 11.59303 | 15.43638 | 12.1266 | 12.98903 | 13.3326 | 12.59071 | 12.97303 | 17.56349 | 12.94894 | 13.93127 | 10.57913 | 9.541795 | 13.73929 | 8.257726 | 8.663836 | 14.66602 | 7.015011 | 9.102009 | 6.745411 |
| CUST_27647_PI429010886 | N56437 | 0.004304 | 18 | 3 | 239505087 | hypothetical cuticle protein [Rimicaris exoculata] | | | 3.4E-07 | 181.3801 | 421.7857 | 275.0769 | 828.3608 | 266.851 | 438.8255 | 301.1632 | 234.7048 | 268.8761 | 162.5528 | 85.21423 | 251.7868 | 166.2053 | 263.9219 | 152.5175 | 155.5309 | 97.04503 | 201.9396 | 110.0483 | 154.9678 | 308.8474 | 195.3201 | 400.3298 | 318.6961 | 280.7715 | 503.6617 | 144.8962 |
| CUST_19089_PI429010886 | N40964 | 0.004309 | 21 | 9 | | | | | | 4.638613 | 11.30463 | 19.67133 | 17.57797 | 17.33078 | 8.426447 | 12.98903 | 38.44451 | 29.38047 | NA | 33.00843 | 36.90242 | 15.11963 | 9.880246 | 11.34711 | 9.031084 | 16.06458 | 13.71331 | 9.357193 | 60.73689 | 9.604718 | 9.751886 | 7.518909 | 6.591511 | 11.34226 | 21.57953 | 22.66107 |
| CUST_46865_PI429010886 | M73996 | 0.004317 | 15 | 12 | 478269759 | hypothetical protein YQE_00243, partial [Dendroctc | | | 9.82E-07 | 94.01656 | 82.06433 | 55.48383 | 44.8824 | 60.22941 | 12.8856 | 301.1198 | 90.12961 | 286.19 | 99.13646 | 57.78767 | 82.39034 | 318.8664 | 306.1523 | 232.7081 | 171.0028 | 58.45028 | 62.71939 | 85.47277 | 42.57633 | 70.36778 | 73.97012 | 71.83981 | 131.0289 | 188.591 | 45.91808 | 234.9764 |
| CUST_51578_PI429010886 | M94062 | 0.004317 | 15 | 12 | | | | | | 40.86391 | 27.13842 | 34.03924 | 24.7854 | 24.97756 | 29.28327 | 30.82234 | 26.30532 | 34.18144 | 40.58399 | 29.93503 | 41.0381 | 32.94683 | 44.64586 | 274.1119 | 38.10393 | 34.33169 | 38.09087 | 28.75932 | 29.10242 | 44.87442 | 29.44199 | 34.5638 | 27.94605 | 52.68897 | 42.38554 | 46.15285 |
| CUST_6278_PI429010886 | N19889 | 0.004317 | 15 | 3 | 321476826 | darkener of apricot-like protein [Daphnia pulex] | Protein metabolism | Cellular protein modification process | 2.3E-166 | 52.92115 | 48.89187 | 43.44238 | 55.75375 | 73.05511 | 94.20676 | 79.16747 | 45.54024 | 32.54126 | 36.03165 | 50.30627 | 33.19093 | 29.93929 | 34.89593 | 20.49048 | 32.99167 | 88.83369 | 37.19805 | 54.71071 | 26.09805 | 71.47658 | 52.96132 | 57.78767 | 36.23666 | 27.43011 | 26.30532 | 28.24069 |
| CUST_7784_PI429010886 | N22070 | 0.004317 | 15 | 12 | 196004710 | hypothetical protein TRIADDRAFT_15248 [Trichopla | | | 1.76E-61 | 149.4495 | 199.0047 | 172.436 | 147.523 | 146.3743 | 139.8628 | 136.8231 | 149.0992 | 195.1585 | 240.7867 | 168.58 | 243.3595 | 218.4011 | 308.7172 | 264.6314 | 255.0746 | 142.6905 | 225.4439 | 156.3242 | 196.5038 | 160.6917 | 248.4663 | 188.1392 | 120.0792 | 321.374 | 246.8318 | 275.0389 |
| CUST_22260_PI429010886 | N46708 | 0.004317 | 15 | 12 | | | | | | 452.8393 | 255.1996 | 4131.811 | 36.36466 | 31.50694 | 19.2217 | 1240.783 | 7605.917 | 6350.776 | 6042.693 | 1334.604 | 2299.417 | 5139.37 | 14182.86 | 8228.765 | 685.5063 | 3336.627 | 256.4303 | 654.3393 | 3474.89 | 281.3948 | 9371.785 | 8582.669 | 240.7867 | 10610.68 | 1862.262 | 11161.59 |
| CUST_36937_PI429010886 | M17265 | 0.004318 | 24 | 3 | | | | | | 52.18439 | 101.4644 | NA | 175.6969 | 84.03407 | 71.91814 | 58.38951 | 7.179499 | 57.97363 | NA | 6.090696 | NA | 23.65678 | 17.32922 | 43.34277 | 48.27575 | 12.4672 | 43.84173 | 13.40222 | 4.039519 | 6.804229 | 10.43073 | 13.77293 | 30.82801 | 19.02231 | 8.367475 | 9.805454 |
| CUST_14582_PI429010886 | N33115 | 0.004328 | 18 | 0 | 321477423 | hypothetical protein DAPPUDRAFT_234511 [Daphn | | | 4.49E-37 | 85.37214 | 51.98858 | 56.47886 | 58.71363 | 63.23998 | 34.05076 | 14.78001 | 14.51104 | 4.992877 | 33.25728 | 34.7228 | 35.41996 | 34.40352 | 69.64081 | 56.48454 | 63.91986 | 11.4747 | 53.9331 | 15.228 | 107.533 | 68.81509 | 158.3621 | 28.09147 | 51.78377 | 44.35344 | 4.793918 | 11.76447 |
| CUST_7130_PI429010886 | N21082 | 0.004334 | 21 | 0 | | | | | | 788.3593 | 809.3886 | 763.6837 | 640.6647 | 586.2213 | 548.2244 | 591.2615 | 481.9422 | 532.1598 | 687.2662 | 594.2743 | 722.796 | 567.3609 | 585.6944 | 598.6499 | 915.6162 | 719.1308 | 680.1392 | 658.4007 | 746.9886 | 610.6415 | 814.8201 | 612.2893 | 645.2555 | 674.891 | 494.3438 | 650.7381 |
| CUST_36367_PI429010886 | M15721 | 0.004335 | 18 | 12 | | | | | | 89.24944 | 93.92221 | 119.6777 | 95.37902 | 94.52571 | 107.2622 | 97.06276 | 110.9853 | 106.9663 | 126.3127 | 117.4608 | 118.8883 | 102.639 | 119.7503 | 125.8034 | 106.6375 | 124.6867 | 112.0103 | 79.89836 | 109.3157 | 82.22696 | 107.073 | 97.49057 | 86.15616 | 114.2228 | 92.77688 | 120.6113 |
| CUST_28331_PI429010886 | N57740 | 0.004337 | 21 | 18 | | | | | | 3.448007 | 9.907267 | 5.702763 | 5.27848 | 9.029764 | 9.502629 | 7.823839 | 7.881658 | 11.19204 | NA | 12.75137 | 10.42653 | 9.644421 | 11.28527 | 11.14656 | 15.31326 | 8.468531 | 12.22205 | 16.1997 | 29.05988 | NA | 10.34037 | NA | NA | 7.923943 | 6.534722 | 8.279758 |
| CUST_5494_PI429010886 | N18775 | 0.004341 | 24 | 18 | 395856304 | PREDICTED: Otolemur garnettii protein phosphatas | Signal transduction | Other | 8.4E-129 | 50.43461 | 55.19772 | 70.36056 | 47.80199 | 50.02348 | 45.97157 | 48.33618 | 48.59467 | 49.23486 | 61.96298 | 60.5059 | 56.49623 | 62.97836 | 55.07381 | 41.09904 | 65.45683 | 63.91986 | 49.28537 | 66.11527 | 71.74926 | 57.97853 | 64.57467 | 49.55874 | 49.99255 | 68.12178 | 52.62093 | 60.63321 |

| Probe ID | ID | p-value | Period | Phase | EBI ID | Annotation | GO category | Go term | E-value | 0 | 0 | 0 | 3 | 3 | 3 | 6 | 6 | 6 | 9 | 9 | 9 | 12 | 12 | 12 | 15 | 15 | 15 | 18 | 18 | 18 | 21 | 21 | 21 | 24 | 24 | 24 |
|---|---|---|---|---|---|---|---|---|---|---|---|---|---|---|---|---|---|---|---|---|---|---|---|---|---|---|---|---|---|---|---|---|---|---|---|---|
| CUST_28941_PI429010886 | N58888 | 0.00435 | 12 | 9 | | | | | | 12.39128 | 10.80337 | 13.84485 | 16.35941 | 13.71506 | 13.98479 | 11.68857 | 14.53622 | 18.3679 | 26.0929 | 25.4983 | 14.86823 | 8.314282 | 10.90106 | 6.301983 | 19.86296 | 35.5525 | 12.46866 | 13.03592 | 15.84934 | 24.3684 | 23.76908 | 28.28611 | 16.69775 | 33.65406 | 4.291679 | 10.86868 |
| CUST_50100_PI429010886 | M88618 | 0.004353 | 24 | 18 | 321469421 | hypothetical protein DAPPUDRAFT_318579 [Daphni | | | 2.41E-61 | 29.56761 | 45.17801 | 46.22541 | 33.36125 | 33.36548 | 34.46151 | 23.23227 | 27.4118 | 28.33335 | 31.00211 | 35.24786 | 34.86394 | 32.72653 | 44.68637 | 51.5653 | 48.01961 | 27.36801 | 37.5742 | 67.5589 | 39.58144 | 34.97152 | 58.4208 | 48.27575 | 27.6707 | 38.33099 | 43.42703 | 41.88371 |
| CUST_10520_PI429010886 | N26364 | 0.004357 | 15 | 0 | 13898841 | CUB-serine protease [Panulirus argus] | Protein metabolism | Proteolysis | 8.78E-33 | 4313.91 | 4306.703 | 2986.925 | 1537.216 | 4384.874 | 2777.472 | 3071.798 | 2206.497 | 2235.234 | 3459.777 | 2040.559 | 3100.565 | 3158.604 | 2608.992 | 2784.905 | 6468.264 | 3618.732 | 4420.361 | 2648.384 | 2840.45 | 2863.818 | 4228.899 | 1638.912 | 1526.305 | 2091.598 | 2041.736 | 2408.505 |
| CUST_51021_PI429010886 | M91828 | 0.004357 | 18 | 9 | | | | | | 10.34165 | 18.34346 | 12.67185 | 8.637107 | 15.81986 | 7.516724 | 11.85349 | 21.40056 | 12.41359 | 25.12109 | 26.85111 | 25.91379 | 22.27554 | 7.496731 | 16.80263 | 17.64263 | 16.77252 | 14.68971 | 18.25579 | 29.26863 | 13.34705 | 16.20103 | 8.959775 | 8.932629 | 22.75029 | 10.76549 | 9.124 |
| CUST_10939_PI429010886 | N27024 | 0.004369 | 15 | 12 | | | | | | 149.8301 | 158.4321 | 186.7032 | 129.9323 | 139.8015 | 156.2777 | 160.8449 | 159.3655 | 177.1267 | 173.3717 | 158.7513 | 183.0597 | 147.0578 | 218.5128 | 193.1525 | 188.0209 | 172.8801 | 186.7272 | 146.8141 | 162.3689 | 139.2323 | 165.3446 | 175.6726 | 148.8666 | 200.8635 | 150.5005 | 194.1492 |
| CUST_19421_PI429010886 | N41549 | 0.004369 | 15 | 12 | | | | | | 52.59262 | 40.22053 | 46.47367 | 34.42762 | 24.40888 | 50.10243 | 39.60553 | 58.74124 | 68.20531 | 53.69394 | 31.39582 | 38.98692 | 73.03522 | 81.92166 | 60.86834 | 48.25178 | 42.56152 | 48.5802 | 32.9302 | 42.79014 | 35.52135 | 50.19147 | 74.63865 | 48.68295 | 93.81682 | 53.37885 | 110.6986 |
| CUST_21356_PI429010886 | N45071 | 0.004369 | 24 | 0 | | | | | | 34.65808 | 30.41942 | 29.78998 | 23.96699 | 26.02132 | 32.73413 | 26.64032 | 23.81679 | 23.60284 | 19.78961 | 27.54224 | 21.79421 | 18.62473 | 38.24304 | 27.37419 | 20.68333 | 32.45773 | 23.29812 | 24.69322 | 20.2137 | 20.77886 | 40.81311 | 25.72813 | 40.86124 | 43.40065 | 33.46305 | 41.74272 |
| CUST_25680_PI429010886 | N52868 | 0.004371 | 15 | 0 | 345484053 | PREDICTED: integrator complex subunit 2-like isofor | | | 1.15E-23 | 17.78225 | 17.6292 | 19.3658 | 13.70132 | 15.38978 | 16.43851 | 11.91576 | 10.60153 | 5.294318 | 16.18695 | 9.293656 | 15.53405 | 13.13833 | 21.95116 | 14.66496 | 22.15622 | 16.08188 | 20.9473 | 19.43524 | 18.78533 | 15.28613 | 32.48902 | 10.45086 | 6.43253 | 19.24807 | 6.535693 | 13.91626 |
| CUST_14168_PI429010886 | N32427 | 0.004374 | 18 | 6 | | | | | | 44.97678 | 51.89227 | 80.4086 | 46.47908 | 64.66893 | 68.03939 | 60.86834 | 62.36898 | 64.90973 | 63.80913 | 55.32634 | 78.74635 | 35.06647 | 30.46087 | 63.06953 | 39.40451 | 39.31465 | 56.17296 | 53.36025 | 38.26462 | 73.45438 | 71.18045 | 71.2192 | 66.84482 | 90.6343 | 106.097 | 101.7734 |
| CUST_12877_PI429010886 | N30191 | 0.004376 | 24 | 18 | 390368710 | PREDICTED: RNA-directed DNA polymerase from mo | | | 3.97E-23 | 33.58065 | 36.64988 | 40.73202 | 33.10392 | 29.51436 | 33.83905 | 28.86203 | 34.61952 | 27.69147 | 42.46725 | 35.21227 | 51.49206 | 41.6172 | 39.56122 | 51.54875 | 57.20678 | 39.49159 | 31.02055 | 46.57707 | 49.9806 | 47.06225 | 47.9222 | 46.86207 | 49.87667 | 58.63339 | 49.51746 | 46.43129 |
| CUST_43361_PI429010886 | M62830 | 0.004396 | 24 | 18 | | | | | | 162.8499 | 214.59 | 260.5944 | 193.4709 | 232.1489 | 203.0375 | 136.7329 | 154.9299 | 180.3315 | 184.7219 | 210.9764 | 286.9075 | 196.032 | 200.9848 | 278.0457 | 255.6621 | 174.1781 | 294.4572 | 211.0494 | 278.6632 | 273.1122 | 314.4386 | 236.0508 | 229.6885 | 192.0915 | 223.8346 | 234.3391 |
| CUST_39563_PI429010886 | M41234 | 0.004405 | 24 | 6 | 341650466 | Spz1 [Litopenaeus vannamei] | | | 8.36E-88 | 134.0969 | 28.14021 | 213.4377 | 170.4767 | 147.0435 | 305.5566 | 236.7681 | 219.6484 | 162.98 | 102.5774 | 187.3307 | 39.67377 | 182.709 | 100.8965 | 6.879194 | 31.36464 | 127.9952 | 159.9075 | 90.78956 | 24.40411 | 107.6537 | 27.72618 | 109.0752 | 111.5529 | 79.18359 | 155.3897 | 62.5561 |
| CUST_43142_PI429010886 | M62184 | 0.004411 | 18 | 15 | | | | | | 33.86006 | 28.48104 | 38.56175 | 42.29494 | 43.42703 | 53.58478 | 25.39871 | 16.0073 | 20.46944 | 33.14406 | 23.94659 | 31.05167 | 47.79169 | 27.61589 | 34.63494 | 48.15042 | 37.20896 | 44.61606 | 42.56702 | 23.32541 | 26.47532 | 40.28049 | 26.72979 | 26.98034 | 28.73136 | 18.59923 | 23.5415 |
| CUST_34184_PI429010886 | M8985 | 0.004411 | 18 | 15 | 383865231 | PREDICTED: polypeptide N-acetylgalactosaminyltrai | | | 9.07E-16 | 79.32298 | 88.17592 | 71.22702 | 76.45771 | 71.77967 | 99.68173 | 71.5379 | 61.43801 | 61.05832 | 95.18251 | 83.43282 | 82.55661 | 86.28429 | 64.22812 | 88.87538 | 97.07573 | 75.49979 | 103.0505 | 75.2875 | 78.96798 | 95.21856 | 114.0787 | 66.90864 | 111.3637 | 63.6301 | 65.15734 | 58.59263 |
| CUST_40392_PI429010886 | M54968 | 0.004411 | 24 | 18 | | | | | | 140.3976 | 107.9913 | 132.6083 | 96.19258 | 62.95412 | 63.6301 | 123.9578 | 128.4384 | 110.1637 | 92.45305 | 146.4472 | 93.70583 | 133.3745 | 179.3955 | 145.7727 | 142.8727 | 148.7085 | 113.9832 | 179.5033 | 130.5819 | 124.1861 | 225.0368 | 153.4593 | 160.974 | 130.1875 | 152.2767 | 126.6211 |
| CUST_11681_PI429010886 | N28230 | 0.004412 | 15 | 0 | | | | | | 124.9049 | 131.7223 | 133.9706 | 103.5678 | 122.2373 | 149.4702 | 81.92166 | 115.478 | 97.4551 | 118.8413 | 100.8686 | 135.9242 | 113.3258 | 93.33448 | 124.3822 | 181.5403 | 119.0526 | 127.4456 | 143.5925 | 169.4763 | 112.5446 | 107.6969 | 102.1011 | 88.04251 | 124.4126 | 90.58155 | 118.3239 |
| CUST_5096_PI429010886 | N18251 | 0.004415 | 12 | 0 | | | | | | 3360.345 | 4340.493 | 3372.782 | 1960.986 | 2200.508 | 1863.799 | 2258.839 | 3118.308 | 3154.98 | 4038.67 | 2759.437 | 4303.084 | 3332.072 | 3775.991 | 3905.483 | 4146.864 | 2630.661 | 2710.041 | 2977.308 | 3599.759 | 2487.623 | 3713.677 | 2823.396 | 2456.459 | 4461.585 | 3062.827 | 4575.004 |
| CUST_15228_PI429010886 | N34230 | 0.00442 | 15 | 12 | 270016638 | hypothetical protein TcasGA2_TC011584 [Tribolium | | | 8.85E-08 | 42.95535 | 40.69827 | 48.1448 | 35.70225 | 37.19805 | 50.31204 | 38.56175 | 45.05944 | 57.48318 | 43.93042 | 36.03688 | 59.21866 | 54.73744 | 61.2655 | 64.48135 | 45.98571 | 44.48344 | 44.16686 | 29.71386 | 38.40419 | 41.70336 | 30.99084 | 68.93293 | 53.66581 | 56.44254 | 52.49852 | 53.6056 |
| CUST_20896_PI429010886 | N44273 | 0.004427 | 15 | 0 | 195588021 | GD13897 [Drosophila simulans] >gi\|194195768\|gb | Nucleic acid metabolism | Regulation of gene expression, epigenetic | 7.91E-30 | 125.8769 | 115.2365 | 120.1692 | 99.26813 | 117.7859 | 118.2591 | 96.63027 | 108.5045 | 70.53195 | 92.07382 | 94.25565 | 101.7734 | 92.06564 | 98.79644 | 111.7487 | 133.1553 | 178.6581 | 105.7694 | 126.1032 | 136.2095 | 74.15113 | 115.5578 | 101.2912 | 101.7342 | 104.0454 | 89.82702 | 105.2191 |
| CUST_28655_PI429010886 | N58367 | 0.00445 | 24 | 9 | 501294111 | aldo-keto reductase [Riptortus pedestris] | | | 2.37E-16 | NA | 10.02351 | NA | 11.44313 | 18.65486 | 8.806922 | 10.77175 | 18.30638 | 14.39423 | 12.92383 | 28.7253 | 23.95732 | 9.922626 | 16.1574 | 7.382963 | NA | 9.007525 | 9.649231 | 5.743146 | 10.28962 | 20.208 | 7.955028 | 8.11273 | NA | 6.263837 | 7.678477 | 5.775477 |
| CUST_19075_PI429010886 | N40938 | 0.004451 | 18 | 6 | 157127444 | amino acid transporter [Aedes aegypti] >gi\|108882 | | | 5.23E-40 | 31.34117 | 27.91206 | 39.25017 | 47.59295 | 37.79341 | 46.19529 | 70.76377 | 42.0037 | 50.59835 | 46.40223 | 38.07688 | 32.02557 | 29.61528 | 44.99497 | 33.63678 | 38.87395 | 30.47101 | 38.14082 | 41.87571 | 22.98483 | 35.95633 | 37.86509 | 29.65277 | 33.90175 | 42.21202 | 45.17236 | 56.56158 |
| CUST_15965_PI429010886 | N35424 | 0.004453 | 24 | 6 | | | | | | 4.768974 | 145.8314 | 18.89688 | 177.3713 | 50.33169 | 66.82399 | NA | 1049.194 | 715.6525 | 9.778317 | 158.2045 | 166.6809 | 250.3612 | 23.01368 | 112.9528 | 12.07145 | 111.1358 | 9.611855 | 5.758268 | 630.1031 | NA | NA | 7.571579 | 8.040463 | 5.636736 | 272.25 | 47.41387 |
| CUST_16479_PI429010886 | N36305 | 0.00446 | 24 | 18 | 211922663 | opsin [Triops granarius] | | | 5.3E-21 | NA | 58.88281 | 18.8381 | NA | 39.96079 | 5.594409 | 58.51806 | 26.02132 | 6.274269 | 16.2854 | 130.3093 | 70.32547 | NA | NA | NA | 86.32314 | 63.4424 | 73.44541 | 257.1155 | 127.8836 | 93.81682 | 61.21559 | 20.77162 | 82.11078 | 89.21981 | 22.51476 | 222.6599 |
| CUST_14181_PI429010886 | N32445 | 0.004464 | 18 | 3 | | | | | | 8.431174 | 33.60838 | 7.650264 | 35.67075 | 17.73598 | 36.78203 | 12.61647 | 6.547053 | 12.82143 | 18.99123 | 4.649026 | 19.30622 | 14.02096 | 10.97186 | 11.87443 | 13.53636 | 6.115825 | 3.529748 | 5.047605 | 5.995731 | 32.41028 | 40.36651 | 20.59775 | 14.01418 | 24.87256 | 26.93871 | 12.27788 |
| CUST_48113_PI429010886 | M78918 | 0.004468 | 15 | 9 | | | | | | 22.39033 | 25.33771 | 21.43485 | 17.50524 | 24.16612 | 23.70999 | 16.4683 | 18.57889 | 14.85125 | 26.92163 | 34.22484 | 34.88582 | 23.88653 | 43.439 | 31.95868 | 24.93429 | 25.86025 | 35.50382 | 18.57693 | 29.49638 | 20.59244 | 20.35314 | 25.44826 | 22.97006 | 38.15596 | 34.28069 | 47.79729 |
| CUST_32125_PI429010886 | M3293 | 0.004472 | 15 | 12 | 7320913 | masquerade-like protein [Pacifastacus leniusculus] | Protein metabolism | Proteolysis | 5.2E-101 | 7901.818 | 8667.659 | 10701.36 | 3235.532 | 4647.339 | 2595.092 | 8165.772 | 11030.7 | 9978.004 | 10401.6 | 5974.692 | 8643.087 | 10851.4 | 11143.54 | 8147.956 | 11385.83 | 10296.63 | 6571.189 | 7568.915 | 7175.165 | 4415.4 | 8327.109 | 9320.998 | 4051.321 | 12388.76 | 5167.16 | 11632.77 |
| CUST_34435_PI429010886 | M9671 | 0.004472 | 15 | 12 | | | | | | 209.7275 | 194.2907 | 544.2536 | 148.544 | 174.2228 | 155.4298 | 277.2896 | 684.8725 | 460.8905 | 733.7989 | 210.8661 | 192.324 | 577.33 | 971.3866 | 379.8142 | 197.384 | 525.5752 | 199.0047 | 189.4794 | 215.167 | 181.6996 | 357.2284 | 822.5821 | 168.8733 | 675.0461 | 186.0597 | 703.8812 |
| CUST_12568_PI429010886 | N29668 | 0.004472 | 15 | 12 | | | | | | 192.4131 | 267.0284 | 196.4719 | 154.4419 | 138.2381 | 108.657 | 175.1526 | 282.1142 | 262.5059 | 283.5999 | 155.0502 | 189.6264 | 254.0209 | 368.6847 | 238.6203 | 228.4677 | 241.9617 | 177.1792 | 203.8732 | 166.2291 | 114.0624 | 301.6859 | 187.1474 | 131.2903 | 536.0428 | 158.2913 | 335.1161 |
| CUST_45966_PI429010886 | M70932 | 0.004475 | 24 | 3 | 56684214 | SERCA [Panulirus argus] >gi\|71534700\|emb\|CAH1( | Transport | Ion transport | 1.29E-22 | 27.10885 | 22.17314 | 49.80346 | 50.97047 | 37.0175 | 57.60743 | 34.23978 | 41.37211 | 31.56943 | 33.53684 | 30.58011 | 49.72682 | 26.15676 | 44.69027 | 32.45453 | 30.60709 | 18.36555 | 18.689 | 49.92642 | 22.0504 | 39.42673 | 26.49155 | 24.28 | 21.63606 | 38.94325 | 34.93063 | 45.50557 |
| CUST_34544_PI429010886 | M9977 | 0.004481 | 21 | 18 | | | | | | 1294.143 | 1385.628 | 1189.103 | 1049.625 | 837.9656 | 1112.909 | 1030.678 | 803.077 | 1037.639 | 978.4784 | 868.721 | 1063.638 | 870.5556 | 949.0578 | 980.7968 | 1498.316 | 1052.869 | 821.7869 | 1669.785 | 1150.815 | 1384.531 | 1142.567 | 1107.396 | 914.3958 | 1530.336 | 1099.35 | 1412.634 |
| CUST_41814_PI429010886 | M58614 | 0.004481 | 18 | 6 | 321463034 | hypothetical protein DAPPUDRAFT_252370 [Daphni | | | 9.94E-20 | 32.69174 | 21.11131 | NA | 38.2775 | 39.63814 | NA | 59.35924 | 51.59735 | 41.9571 | 22.49442 | 9.972812 | 23.09604 | 38.08646 | 28.17477 | 21.60382 | 7.58227 | 47.79729 | 20.73391 | 15.67763 | 19.53485 | NA | 61.65538 | 6.342428 | 40.1071 | 97.05352 | 43.90259 | 46.29899 |
| CUST_40669_PI429010886 | M55800 | 0.00449 | 24 | 15 | | | | | | 324.9748 | 251.662 | 266.4816 | 313.4993 | 285.3112 | 255.5333 | 252.8082 | 220.8472 | 249.9907 | 396.7445 | 268.4354 | 340.5982 | 208.6342 | 273.8248 | 300.5825 | 330.9853 | 351.983 | 388.1247 | 319.3199 | 380.124 | 292.7654 | 334.2433 | 318.2412 | 257.772 | 308.0795 | 305.7396 | 382.1161 |
| CUST_25618_PI429010886 | N52753 | 0.004497 | 24 | 3 | | | | | | 78.25555 | 52.1682 | 91.41127 | 82.03238 | 78.31562 | 97.80592 | 76.36771 | 63.94718 | 72.3461 | 90.45788 | 63.47762 | 43.81481 | 51.58632 | 61.1536 | 38.18452 | 55.45229 | 58.67766 | 73.55823 | 74.09449 | 41.2187 | 69.38283 | 68.18607 | 103.7436 | 57.24358 | 74.76616 | 87.12746 | 59.68907 |
| CUST_18946_PI429010886 | N40700 | 0.004499 | 18 | 0 | | | | | | 42.58108 | 27.14607 | 52.71293 | 19.27159 | 46.64901 | 55.2283 | 20.55898 | 19.87525 | 14.01418 | 27.35173 | 12.37319 | 50.77711 | 26.61223 | 34.28069 | 26.40308 | 37.80681 | 27.81087 | 34.83322 | 22.94962 | 46.78872 | 32.52687 | 24.67398 | 45.72547 | 41.14049 | 7.57659 | 15.39416 | 6.599438 |
| CUST_23673_PI429010886 | N49258 | 0.004502 | 15 | 12 | 1764107 | pacifastin heavy chain precursor [Pacifastacus leniu | Transport | Ion transport | 1.01E-11 | 138.1141 | 222.3148 | 193.5749 | 149.974 | 141.8494 | 110.3421 | 222.1142 | 152.988 | 331.4917 | 164.9826 | 205.8829 | 339.8879 | 203.3901 | 243.8404 | 291.4998 | 351.1194 | 160.6473 | 251.4731 | 147.098 | 179.4697 | 122.1911 | 239.521 | 169.3227 | 143.8508 | 391.2629 | 149.5445 | 399.641 |
| CUST_1994_PI429010886 | N11264 | 0.004512 | 18 | 0 | | | | | | 378.0737 | 427.6746 | 365.0271 | 319.2639 | 241.0332 | 244.775 | 254.9978 | 236.1903 | 284.7376 | 327.9867 | 247.9972 | 347.4939 | 281.2623 | 341.3294 | 329.1684 | 402.2817 | 269.6262 | 250.3151 | 416.5528 | 385.5507 | 321.0768 | 428.7157 | 290.7302 | 297.0259 | 309.7028 | 251.662 | 288.5334 |
| CUST_13012_PI429010886 | N30436 | 0.004522 | 15 | 0 | | | | | | 388.3479 | 375.5434 | 366.7345 | 296.0927 | 326.8661 | 321.5473 | 275.3515 | 280.0685 | 241.5575 | 387.2348 | 302.566 | 306.3807 | 222.7049 | 305.8476 | 318.8664 | 440.6015 | 319.007 | 389.9711 | 355.9452 | 336.2744 | 286.0114 | 380.5555 | 223.2901 | 269.8655 | 479.0678 | 299.389 | 380.7018 |
| CUST_23949_PI429010886 | N49752 | 0.004522 | 21 | 0 | | | | | | 23.3229 | 40.23874 | 27.62424 | 10.15159 | 14.26904 | 5.981371 | 4.363301 | 5.045546 | 4.978402 | 9.865884 | 18.41069 | 6.446625 | 13.93254 | 3.611805 | 8.806922 | 26.68667 | 27.37719 | 20.92287 | 4.364625 | 32.25357 | 21.02638 | 14.38818 | 23.90824 | 11.42997 | 37.9742 | 16.68781 | 21.47745 |
| CUST_32402_PI429010886 | M4013 | 0.004523 | 15 | 12 | | | | | | 2151.64 | 2921.018 | 2182.975 | 1302.095 | 1797.174 | 1353.303 | 1749.353 | 2527.895 | 2757.444 | 2789.972 | 1595.711 | 2447.295 | 2701.759 | 2638.062 | 2890.576 | 3466.9 | 2338.281 | 2168.863 | 1979.892 | 1991.454 | 1516.22 | 4034.014 | 2400.961 | 2042.507 | 4186.218 | 1728.225 | 4365.095 |
| CUST_30722_PI429010886 | N62482 | 0.004526 | 15 | 12 | | | | | | 51.56072 | 45.51332 | 61.36332 | 47.01793 | 47.5061 | 53.73593 | 51.42712 | 41.63417 | 48.44291 | 52.0001 | 47.45909 | 54.5396 | 57.71214 | 61.20199 | 67.58357 | 53.16284 | 54.64962 | 62.59848 | 41.33159 | 52.27598 | 47.29164 | 58.27233 | 42.61032 | 47.0488 | 52.35268 | 64.87589 | 55.4319 |
| CUST_40619_PI429010886 | M55664 | 0.004538 | 24 | 15 | | | | | | 206.1326 | 223.1189 | 198.7745 | 194.1963 | 191.2652 | 190.0964 | 176.0053 | 180.3315 | 172.3811 | 253.9339 | 185.7109 | 192.4758 | 172.213 | 221.9563 | 242.041 | 284.3166 | 236.5291 | 256.1116 | 206.2668 | 195.0326 | 184.7859 | 235.2484 | 182.204 | 201.008 | 223.9394 | 176.5494 | 242.5125 |
| CUST_28639_PI429010886 | N58336 | 0.00454 | 21 | 9 | | | | | | 8.286303 | 4.18815 | 3.964672 | NA | 6.065653 | 8.558946 | 14.926 | 8.701981 | 8.958591 | 8.627885 | 16.94406 | 10.88063 | 6.744508 | NA | 9.031084 | 4.630231 | 8.29257 | 8.790994 | 8.246449 | 6.804229 | 6.718366 | 5.628723 | 4.463887 | 17.17252 | 8.569879 | 9.042021 | 5.127575 |
| CUST_34638_PI429010886 | M10209 | 0.004561 | 21 | 15 | 342326294 | Mg2+ and Co2+ transporter [Cherax quadricarinatu | | | 1.44E-25 | 58.77783 | 51.11982 | 36.3007 | 11.33312 | 28.39724 | 13.0288 | 33.78634 | 17.92595 | 38.3841 | 36.57035 | 43.7836 | 26.3141 | 35.88462 | 25.15397 | 48.68295 | 49.26562 | 47.55714 | 46.40223 | 34.40352 | 61.53357 | 29.29818 | 62.03099 | 40.05116 | 39.77522 | 23.99863 | 31.62413 | 33.80016 |
| CUST_35571_PI429010886 | M12910 | 0.004571 | 12 | 0 | | | | | | 20.28061 | 16.63804 | 15.79172 | 5.296822 | 14.1264 | 10.37712 | 13.25045 | 8.916592 | 14.41234 | 17.14295 | 14.07874 | 4.496477 | 16.67239 | 16.95126 | 14.75649 | 16.29644 | 14.71698 | 17.62325 | 12.14778 | 4.806509 | 5.274301 | 11.32143 | 14.75394 | 11.58179 | 17.38798 | 8.931253 | 19.40005 |
| CUST_49790_PI429010886 | M87120 | 0.004571 | 12 | 0 | | | | | | 853.6849 | 1120.205 | 1115.54 | 883.6545 | 851.7359 | 952.9126 | 744.8667 | 765.9632 | 813.6581 | 992.543 | 790.3406 | 1110.941 | 852.6077 | 1166.392 | 1015.729 | 1318.096 | 748.7335 | 885.7556 | 816.1069 | 902.1996 | 938.367 | 1112.064 | 1072.188 | 1002.084 | 1180.515 | 1234.796 | 1234.796 |
| CUST_144_PI429010886 | N1544 | 0.004574 | 15 | 12 | | | | | | 10282.77 | 15246.84 | 23934.78 | 1382.607 | 4340.493 | 2325.953 | 10610.68 | 25677.47 | 13555.43 | 22054.78 | 14011.56 | 9219.301 | 15424 | 27219.28 | 20401.48 | 7516.748 | 23241.72 | 6342.066 | 12933.77 | 10102.29 | 10217.96 | 13959.79 | 16184.83 | 5245.334 | 26101.79 | 8245.915 | 29896.62 |
| CUST_54757_PI429010886 | M107488 | 0.004577 | 15 | 9 | 195386778 | GJ23610 [Drosophila virilis] >gi\|194148538\|gb\|EDV | Behavior | Behavior | 1.67E-40 | 14.38818 | 32.45148 | 36.55801 | 18.07682 | 14.94259 | 13.54122 | 20.69763 | 20.06809 | 30.38651 | 31.16262 | 19.45151 | 28.22332 | 22.87112 | 21.68053 | 19.23025 | 23.67058 | 6.095505 | 19.48299 | 13.34178 | 16.01839 | 18.51403 | 13.54646 | 39.30599 | 18.83592 | 36.96378 | 23.71704 | 31.74877 |
| CUST_20981_PI429010886 | N44415 | 0.004577 | 15 | 9 | | | | | | 32.30965 | 34.35136 | 30.13575 | 34.9503 | 31.04302 | 40.38893 | 30.04146 | 19.74451 | 32.22652 | 41.37211 | 36.68661 | 46.98145 | 37.70021 | 41.00395 | 38.65189 | 39.87566 | 32.21099 | 27.03609 | 29.76053 | 25.53352 | 35.75835 | 44.19983 | 39.14518 | 38.153 | 42.59897 | 43.06591 | 45.58039 |
| CUST_795_PI429010886 | N7343 | 0.004577 | 15 | 9 | | | | | | 1181.87 | 3269.374 | 3659.313 | 1691.46 | 2226.703 | 794.9156 | 650.5246 | 6969.97 | 14274.58 | 14113.22 | 8562.999 | 17943.08 | 8995.888 | 23241.72 | 16209.81 | 12003.36 | 2282.54 | 5794.422 | 4672.866 | 10006.61 | 5585.575 | 26713.02 | 14113.22 | 1406.196 | 28815.19 | 13322.16 | 38079.24 |
| CUST_29495_PI429010886 | N59981 | 0.004584 | 21 | 0 | | | | | | 14.9321 | 15.0164 | 16.23717 | 11.92638 | 12.11785 | 13.67832 | 12.73246 | 7.194544 | 5.109231 | 13.37205 | 12.48479 | 8.495784 | 5.675402 | 3.780882 | 6.292306 | 12.17281 | 11.6754 | 7.996886 | 9.405002 | 10.93036 | 8.678808 | 12.89288 | 11.76966 | NA | 26.64821 | 4.362159 | 9.776853 |
| CUST_34371_PI429010886 | M9463 | 0.004586 | 21 | 3 | 260829705 | hypothetical protein BRAFLDRAFT_219336 [Branchi | | | 1.3E-30 | 1440.453 | 5376.972 | 2541.147 | 7347.807 | 2754.789 | 3578.372 | 1914.945 | 2203.176 | 2304.824 | 2587.199 | 1300.415 | 4357.297 | 2139.684 | 1824.738 | 445.5129 | 2500.44 | 580.436 | 2124.898 | 245.5292 | 2188.013 | 2497.013 | 2939.878 | 3068.87 | 2246.519 | 3272.814 | 4194.154 | 1764.536 |
| CUST_6929_PI429010886 | N20794 | 0.004586 | 21 | 3 | 345490035 | PREDICTED: RUN and FYVE domain-containing prote | | | 1.8E-101 | 876.4661 | 2338.281 | 1048.248 | 3627.549 | 1452.711 | 1572.497 | 983.8663 | 901.5294 | 890.6855 | 1165.381 | 475.874 | 1413.603 | 656.1679 | 1195.55 | 402.3792 | 1209.365 | 363.8764 | 695.7952 | 521.3278 | 837.1072 | 1430.089 | 1353.303 | 1569.266 | 783.1618 | 2109.682 | 1818.602 | 713.6188 |
| CUST_12349_PI429010886 | N29329 | 0.004594 | 18 | 3 | | | | | | 193.8001 | 243.4369 | 337.5537 | 401.5677 | 614.705 | 905.6425 | 12.37644 | 707.6225 | 81.76577 | 91.66231 | 309.3892 | 243.8028 | 120.9579 | 97.73561 | 70.69633 | 69.66945 | 122.6119 | 85.2824 | 167.5706 | 107.0816 | 398.6724 | 224.1873 | 299.1597 | 314.5564 | 99.19224 | 156.9601 | 49.73773 |
| CUST_3798_PI429010886 | N15293 | 0.004624 | 15 | 9 | | | | | | 43.86454 | 118.9612 | 212.1159 | NA | 22.1165 | NA | 45.80326 | 172.498 | 435.3919 | 269.5045 | 273.3216 | 399.1635 | 137.4373 | 507.2776 | 269.8218 | 176.7462 | 77.88301 | 98.29921 | 228.7951 | 313.8184 | 202.4642 | 345.1704 | 235.6934 | 39.25017 | 823.8136 | 371.054 | 849.9143 |
| CUST_26349_PI429010886 | N54094 | 0.004624 | 12 | 0 | | | | | | 52.24124 | 27.1196 | 43.77433 | 33.62524 | 23.74079 | 37.06882 | 22.88115 | 24.65512 | 21.84275 | 36.6668 | 24.40138 | 49.71501 | 22.58247 | 39.85359 | 52.84586 | 35.87751 | 27.75224 | 40.1071 | 43.20694 | 21.52267 | 22.5259 | 36.55801 | 43.51593 | 30.98507 | 42.07479 | 53.12725 | 47.73713 |
| CUST_1622_PI429010886 | N10258 | 0.004626 | 18 | 9 | | | Transport | Transmembrane transport | | 167.0802 | 182.318 | 210.2047 | 131.4476 | 135.7103 | 131.5965 | 163.6107 | 133.4752 | 160.6473 | 242.5125 | 215.0582 | 246.1025 | 173.7706 | 209.2569 | 213.0864 | 274.6972 | 122.1037 | 151.3368 | 211.85 | 141.9384 | 167.2309 | 211.85 | 154.1347 | 134.2151 | 159.9075 | 194.2907 | 233.124 |
| CUST_16066_PI429010886 | N35591 | 0.004626 | 15 | 12 | | | | | | 40.3031 | 86.19466 | 37.78218 | 29.20384 | 30.45321 | 38.65653 | 37.90698 | 44.07915 | 44.41428 | 50.09589 | 40.38558 | 35.64343 | 44.92995 | 46.16699 | 70.79063 | 50.85184 | 40.93552 | 46.07297 | 39.87971 | 41.50969 | 36.31618 | 44.85517 | 47.73713 | 52.11537 | 58.74569 | 45.52189 | 66.28856 |
| CUST_12997_PI429010886 | N30419 | 0.004627 | 21 | 18 | | | | | | 303.55 | 303.4935 | 206.3065 | 258.1237 | 290.9496 | 227.1802 | 260.1385 | 190.8098 | 200.0776 | 228.3928 | 287.1573 | 215.3368 | 201.4604 | 220.4437 | 227.8356 | 308.6668 | 383.093 | 243.8404 | 291.9341 | 327.0942 | 341.1708 | 270.707 | 283.6942 | 250.7333 | 235.6566 | 305.1009 | 216.8689 |
| CUST_706_PI429010886 | N6864 | 0.004631 | 21 | 18 | 429326693 | peroxiredoxin-like protein [Coptotermes formosanu | Cellular Process | Cell death | 5.78E-54 | 214.1014 | 240.1049 | 204.3252 | 143.002 | 121.9054 | 83.00511 | 89.00024 | 92.29442 | 107.2874 | 368.1562 | 130.0042 | 256.6496 | 174.1781 | 66.54991 | 117.8738 | 423.2024 | 157.9269 | 225.5174 | 177.1267 | 328.7379 | 210.3141 | 194.5783 | 177.2358 | 166.4042 | 271.8631 | 161.1033 | 157.2097 |
| CUST_54753_PI429010886 | M107465 | 0.004632 | 15 | 6 | 317383196 | myosin light chain [Penaeus monodon] >gi\|357640 | | | 2.12E-15 | 57.1153 | 38.02052 | 54.85007 | 56.66591 | 60.6558 | 38.51022 | 100.7779 | 84.91262 | 72.18474 | 86.99215 | 122.949 | 58.65437 | 118.1621 | 46.78356 | 27.9072 | 55.36449 | 39.45385 | 19.77641 | 69.51764 | 59.62185 | 86.19466 | 71.56769 | 104.5344 | 67.45213 | 67.62921 | 34.40827 | 81.71205 |
| CUST_34872_PI429010886 | M10953 | 0.004632 | 15 | 9 | | | | | | 126.5686 | 308.0149 | 418.4668 | 65.27575 | 170.2053 | 49.00501 | 87.15435 | 422.8357 | 558.9364 | 673.8089 | 298.4347 | 552.4266 | 419.0394 | 920.7129 | 463.1518 | 656.1679 | 516.8489 | 305.8476 | 237.1286 | 395.0536 | 345.3911 | 875.643 | 783.5804 | 120.5371 | 1801.38 | 462.9458 | 2048.598 |
| CUST_25040_PI429010886 | N51677 | 0.004632 | 15 | 0 | | | | | | 31.21185 | 24.44701 | 36.21024 | 30.43732 | 44.71939 | 26.05696 | 15.3449 | 22.36193 | 17.77043 | 22.42354 | 13.30229 | 30.37957 | 22.39033 | 38.49903 | 30.558 | 38.91585 | 34.24814 | 42.93137 | 31.87279 | 24.65264 | 28.92063 | 32.55631 | 17.28828 | 19.0657 | 25.39336 | 31.81061 | 33.54956 |
| CUST_5842_PI429010886 | N19282 | 0.004642 | 18 | 3 | | | | | | 98.6386 | 89.43652 | 63.43062 | 92.60851 | 82.42534 | 131.5799 | 102.8937 | 69.84364 | 58.482 | 75.53734 | 80.842 | 53.04479 | 43.12973 | 53.10218 | 34.52588 | 68.83612 | 109.902 | 71.63476 | 80.22855 | 62.08961 | 79.61927 | 91.91837 | 66.48237 | 137.7783 | 53.18901 | 51.98058 | 51.49206 |
| CUST_28339_PI429010886 | N57757 | 0.004648 | 24 | 18 | | | | | | 15.32478 | 25.97694 | 33.8524 | 15.39298 | 17.15304 | 16.43668 | 6.707531 | 15.98363 | 16.64512 | 15.01997 | 14.10202 | 16.82346 | 16.29894 | 35.22469 | 32.43294 | 47.91527 | 16.24994 | 35.15805 | 53.89507 | 18.05975 | 21.66748 | 21.15229 | 26.77939 | 29.44853 | 24.03557 | 25.4983 | 24.03363 |
| CUST_40274_PI429010886 | M52368 | 0.00465 | 24 | 12 | | | | | | 8.906902 | 5.463105 | NA | 5.627939 | 4.131773 | 15.6362 | 9.982619 | 4.865987 | 12.68111 | NA | 13.22245 | 17.04092 | 19.11453 | 24.35448 | 11.32653 | 18.92827 | 8.055935 | 13.072 | 12.91494 | 10.8378 | NA | 15.0972 | 8.401293 | NA | 10.38684 | 7.495131 | 3.864843 |
| CUST_18697_PI429010886 | N40247 | 0.004654 | 21 | 3 | 157122986 | activin receptor type I, putative [Aedes aegypti] >gi | Protein metabolism | Cellular protein modification process | 2.37E-17 | 32.73752 | 55.04289 | 46.41914 | 60.5193 | 53.51858 | 56.20414 | 43.22077 | 41.41188 | 42.24487 | 48.5148 | 42.48584 | 52.063 | 34.6609 | 42.05204 | 46.57205 | 42.41594 | 40.41132 | 60.04192 | 33.7538 | 44.08735 | 45.99048 | 52.68156 | 53.28087 | 46.23463 | 58.29022 | 52.59262 | 54.67468 |
| CUST_13853_PI429010886 | N31890 | 0.004656 | 18 | 12 | | | | | | 31.5882 | 40.1147 | 29.71613 | 27.84642 | 39.80783 | 42.01729 | 37.70458 | 40.71927 | 31.18382 | 47.92719 | 66.34998 | 47.36571 | 192.363 | 55.37064 | 130.0411 | 39.51525 | 37.87644 | 41.58444 | 34.76682 | 47.62012 | 27.9685 | 54.86684 | 30.59095 | 41.89757 | 53.71904 | 37.60789 | 39.93142 |
| CUST_56610_PI429010886 | M114214 | 0.004659 | 18 | 15 | | | | | | 178.3288 | 76.55655 | 129.1185 | 51.23152 | 172.7406 | 115.478 | 110.8044 | 113.9625 | 92.75345 | 286.5169 | 87.5248 | 128.969 | 180.0476 | 182.8475 | 128.8934 | 141.0947 | 163.9899 | 269.7173 | 109.2001 | 133.7601 | 104.6153 | 107.0816 | 59.21305 | 44.06361 | 56.50947 | 72.09388 | 119.0242 |
| CUST_49003_PI429010886 | M83540 | 0.00466 | 24 | 12 | 405967141 | Ubiquitin carboxyl-terminal hydrolase 32 [Crassostr | Protein metabolism | Proteolysis | 9.37E-13 | 20.98935 | 11.0392 | 18.19413 | 9.347971 | 13.95459 | 24.5371 | 25.40127 | 19.56685 | 21.26038 | 17.9021 | 18.93228 | 27.90293 | 22.91836 | 31.86571 | 31.87279 | 22.86827 | 24.0044 | 41.87987 | 19.60466 | 21.17293 | 18.45187 | 20.55693 | 14.04066 | 22.65536 | 17.17661 | 17.86554 | 25.00056 |
| CUST_36296_PI429010886 | M15481 | 0.00467 | 24 | 3 | 321462126 | hypothetical protein DAPPUDRAFT_227138 [Daphni | | | 4.57E-16 | 83.63285 | 146.4231 | 25.14633 | 742.4096 | 301.5634 | 730.7862 | 207.5722 | 717.4734 | 22.14395 | NA | 179.0372 | NA | 35.82003 | 33.26462 | 94.83175 | 19.72128 | 24.68523 | 26.95815 | 45.65065 | 7.931172 | 88.20739 | 3.864627 | 104.6964 | 282.4695 | NA | 17.77043 | NA |
| CUST_27118_PI429010886 | N55473 | 0.004677 | 12 | 0 | | | | | | 71.08447 | 118.7885 | 124.513 | 129.4038 | 82.09306 | 75.64933 | 75.11707 | 64.70822 | 80.47827 | 106.4697 | 76.87346 | 101.4018 | 91.24943 | 97.81711 | 112.0385 | 115.4184 | 74.58008 | 117.2732 | 73.77338 | 74.68625 | 65.87593 | 80.27864 | 80.32797 | 102.6107 | 107.1033 | 131.3133 | 102.8432 |
| CUST_1489_PI429010886 | N9788 | 0.004679 | 12 | 9 | 242332536 | zinc finger protein 934 isoform 1 [Mus musculus] | | | 5.47E-26 | 25.19447 | 23.06972 | 17.49952 | 13.82942 | 14.87518 | 15.9338 | 24.24769 | 21.84275 | 24.14877 | 20.59884 | 27.20765 | 20.51216 | 16.32267 | 29.99067 | 14.76986 | 10.39893 | 19.09941 | 13.18579 | 18.82343 | 13.92993 | 19.08808 | 27.97387 | 19.10174 | 30.86904 | 37.96902 | 22.16629 | 37.10843 |
| CUST_32947_PI429010886 | M5538 | 0.00468 | 12 | 3 | | | | | | 20.06405 | 18.6166 | 21.13287 | 24.03089 | 21.63831 | 13.49842 | 9.966503 | 14.2617 | 14.92131 | 13.83521 | 12.86234 | 7.226406 | 8.763789 | 23.63855 | 6.592569 | 31.50694 | 24.80851 | 31.68444 | 21.93409 | 21.03394 | 15.11263 | 6.664096 | NA | 12.59689 | 34.9503 | 19.75734 | 35.06362 |
| CUST_39264_PI429010886 | M36900 | 0.004687 | 15 | 9 | 225712016 | Cuticle protein 6 [Lepeophtheirus salmonis] | | | 2.62E-19 | 7154.842 | 14135.18 | 27577.88 | 1437.865 | 1943.851 | 262.3746 | 7773.881 | 25496.93 | 36912.34 | 32528.46 | 15537.42 | 28752.77 | 33529.19 | 46511.8 | 35686.35 | 29696.39 | 11472.39 | 9235.263 | 17506.5 | 29896.62 | 15353.74 | 49418.23 | 26218.61 | 1725.627 | 49654.41 | 35818.08 | 50702.21 |
| CUST_48438_PI429010886 | M80321 | 0.004687 | 15 | 0 | 197940771 | Euphausia superba mitochondrion, partial genome | | | 2.34E-57 | 557.3105 | 522.2489 | 591.5595 | 413.1358 | 582.6377 | 731.6805 | 290.8448 | 248.8286 | 300.2063 | 508.1895 | 661.0472 | 563.9632 | 243.1192 | 472.1948 | 395.9375 | 830.6821 | 594.4635 | 548.6331 | 422.2989 | 680.1392 | 501.4801 | 471.8606 | 312.1828 | 309.3403 | 527.7394 | 420.7782 | 422.2989 |
| CUST_53827_PI429010886 | M103348 | 0.004689 | 18 | 3 | | | | | | 21.08222 | 18.48864 | 6.987277 | 26.44212 | 15.88699 | 20.79607 | 14.38818 | 9.776853 | 6.169502 | 12.22313 | 13.08554 | NA | 10.20122 | 4.727333 | 5.027489 | 14.25474 | 34.49254 | 14.89135 | 15.73055 | 21.92246 | NA | 34.97152 | NA | NA | 8.680488 | NA | NA |
| CUST_41268_PI429010886 | M57293 | 0.004692 | 24 | 3 | | | | | | 35.7998 | 35.72502 | 22.79581 | 47.70026 | 46.51613 | 40.67008 | 27.11607 | 24.29286 | 31.2907 | 33.33371 | 24.63224 | 21.05341 | 21.20179 | 22.75236 | 25.81867 | 31.65427 | 22.88601 | 24.01545 | 13.70505 | 30.14229 | 20.16852 | 47.02813 | 20.99955 | 33.54956 | 32.58533 | 28.92063 | 30.65915 |
| CUST_20175_PI429010886 | N42956 | 0.004693 | 15 | 0 | | | | | | 151.2035 | 104.3119 | 148.6709 | 129.8119 | 67.85743 | 118.1855 | 147.9625 | 103.7009 | 66.80323 | 53.91951 | 74.12883 | 34.02862 | 108.9165 | 58.40039 | 61.66407 | 79.91242 | 135.2886 | 93.32222 | 132.2831 | 55.39377 | 67.63514 | 40.52511 | 87.03787 | 122.3609 | 65.64906 | 60.72981 | 52.08646 |
| CUST_52088_PI429010886 | M96376 | 0.004698 | 24 | 18 | | | | | | 40.39432 | 37.52322 | 24.94079 | 24.90221 | 34.9676 | 21.09976 | 34.50513 | 14.34274 | 22.06161 | 36.6789 | 30.19387 | 30.00667 | 27.16781 | 35.81535 | 24.18418 | 39.87225 | 47.5835 | 31.20492 | 49.77447 | 41.31249 | 43.79306 | 33.45023 | 25.7467 | 32.24298 | 48.00546 | 24.2514 | 47.45909 |
| CUST_56389_PI429010886 | M113658 | 0.004702 | 15 | 12 | 345493682 | PREDICTED: hypothetical protein LOC100120957 isc | Developmental process | Embryo development | 2.29E-26 | 113.8053 | 99.09557 | 113.8053 | 90.43345 | 106.2285 | 85.34367 | 101.867 | 122.0311 | 140.6597 | 153.0373 | 139.5229 | 123.4117 | 139.8628 | 200.8936 | 132.8409 | 120.5667 | 185.104 | 102.6264 | 135.6517 | 115.9131 | 103.8532 | 106.6963 | 131.1966 | 115.1886 | 181.0004 | 113.9031 | 194.9822 |
| CUST_30200_PI429010886 | N61373 | 0.004703 | 24 | 18 | | | | | | 134.6768 | 104.8657 | 142.194 | 134.7225 | 123.5719 | 149.6919 | 112.7145 | 115.6165 | 117.4158 | 137.8245 | 100.2082 | 120.0167 | 120.7015 | 151.2877 | 172.1829 | 165.6637 | 131.327 | 167.1292 | 159.7611 | 142.34 | 148.0447 | 144.4149 | 142.194 | 136.9989 | 133.4505 | 137.604 | 169.6552 |
| CUST_32673_PI429010886 | M4766 | 0.004706 | 15 | 12 | 321455465 | hypothetical protein DAPPUDRAFT_302493 [Daphni | | | 4.2E-113 | 6.201123 | 9.011589 | 10.02222 | 5.550178 | 4.346917 | 3.776579 | NA | 5.021796 | 14.87852 | 5.574158 | 4.962271 | 18.87895 | 9.149574 | 12.14502 | 27.09043 | 13.07843 | 7.388302 | NA | 8.743791 | 3.577418 | 5.013199 | 9.722947 | 12.82338 | 5.934873 | 9.015517 | NA | 14.0658 |
| CUST_5287_PI429010886 | N18495 | 0.00471 | 21 | 0 | 260792442 | hypothetical protein BRAFLDRAFT_116394 [Branchi | | | 4.3E-171 | 259.2052 | 303.3224 | 264.9674 | 234.2832 | 264.5866 | 255.3013 | 211.5135 | 213.1848 | 238.0845 | 259.5337 | 223.2254 | 327.8511 | 202.6837 | 187.746 | 140.6429 | 265.6599 | 218.0605 | 261.7284 | 169.2967 | 293.414 | 229.8746 | 299.1597 | 254.9115 | 260.1739 | 245.4886 | 254.8434 | 217.9121 |
| CUST_46952_PI429010886 | M74288 | 0.004721 | 21 | 15 | | | | | | 1537.216 | 1010.24 | 1273.05 | 503.4817 | 1766.553 | 1021.127 | 1604.147 | 1108.664 | 1184.216 | 2672.36 | 1045.15 | 1407.859 | 2053.321 | 1769.215 | 1516.22 | 1687.609 | 1646.767 | 2127.118 | 1544.651 | 1551.584 | 1341.448 | 1826.751 | 661.8313 | 602.4628 | 689.3131 | 1012.95 | 1379.405 |
| CUST_1041_PI429010886 | N8301 | 0.00473 | 12 | 0 | | | | | | 469.6397 | 458.6579 | 622.024 | 332.2188 | 455.745 | 523.8983 | 407.0568 | 443.8496 | 452.4978 | 554.0852 | 427.3564 | 710.22 | 474.912 | 652.7665 | 632.2521 | 592.1986 | 428.7157 | 533.2467 | 418.3911 | 518.8148 | 448.5246 | 546.6515 | 524.2988 | 463.2589 | 689.8687 | 596.0223 | 602.9273 |
| CUST_32554_PI429010886 | M4420 | 0.00473 | 15 | 12 | 321456387 | hypothetical protein DAPPUDRAFT_218652 [Daphn | Metabolic process | Nucleobase-containing compound metabolic process | 2.29E-61 | 37.21745 | 40.57245 | 38.91955 | 19.23025 | 28.21594 | 30.72417 | 28.71965 | 18.78752 | 23.90131 | 51.05482 | 42.24809 | 52.21965 | 23.69772 | 45.79191 | 56.16292 | 45.55959 | 37.76644 | 31.69618 | 43.43161 | 36.53812 | 29.31792 | 31.48252 | 36.72537 | 25.09128 | 42.62942 | 31.02312 | 44.74185 |
| CUST_12796_PI429010886 | N30065 | 0.004734 | 24 | 0 | | | | | | 12.3155 | 13.22714 | 13.97054 | 7.682987 | 9.198308 | 11.21303 | 6.187658 | 5.533212 | NA | 7.189683 | 8.813762 | 8.625409 | 12.17582 | 8.832139 | 9.03782 | 15.64433 | 8.14453 | 14.15536 | 7.84373 | 9.669901 | 10.50364 | 10.35656 | 11.13159 | NA | 16.87557 | NA | 13.18285 |
| CUST_40904_PI429010886 | M56350 | 0.004734 | 12 | 3 | | | | | | 520.2836 | 396.3895 | 10.6044 | 560.0158 | 393.3268 | 841.0533 | 503.3426 | 368.0783 | 358.7125 | 11.43623 | 34.2549 | 13.80899 | 367.27 | 145.7727 | 350.793 | 542.5507 | 618.3611 | 786.4352 | 678.2021 | 96.07604 | 54.1974 | 336.5695 | NA | 609.308 | 206.9547 | 376.4458 | 5.175611 |
| CUST_46128_PI429010886 | M71481 | 0.004739 | 15 | 12 | | | | | | 64.54153 | 67.13373 | 60.15341 | 56.85652 | 62.86517 | 49.83081 | 56.30291 | 54.62458 | 83.60593 | 101.0607 | 62.3113 | 100.2724 | 84.35593 | 155.1509 | 99.19224 | 114.1927 | 30.12812 | 65.03693 | 51.88614 | 81.21391 | 58.03638 | 87.48439 | 56.16292 | 43.34938 | 89.16253 | 108.6016 | 123.6053 |
| CUST_48179_PI429010886 | M79246 | 0.004747 | 15 | 3 | | | | | | 14.51392 | 16.48255 | NA | 30.84259 | 21.49202 | 20.4513 | 15.03377 | 6.524534 | 7.26746 | 3.837688 | 13.94032 | NA | 5.017204 | 4.176791 | NA | 13.54646 | 28.11334 | 9.320692 | 21.248 | 9.924045 | 9.819588 | 15.78805 | 7.434035 | 19.49159 | 5.074237 | NA | NA |
| CUST_25315_PI429010886 | N52172 | 0.004762 | 24 | 18 | 334327670 | PREDICTED: zinc finger protein 850-like [Monodelph | | | 5.86E-14 | 47.56097 | 38.71068 | 41.78704 | 35.34739 | 37.93755 | 38.68605 | 21.8588 | 28.99536 | 35.85352 | 54.14583 | 45.2318 | 34.27356 | 37.43755 | 46.48282 | 28.6993 | 54.63875 | 44.90621 | 46.66745 | 46.83826 | 43.33061 | 49.20588 | 68.71451 | 39.288 | 38.68077 | 58.86655 | 43.8795 | 47.28699 |
| CUST_40844_PI429010886 | M56211 | 0.004769 | 15 | 0 | | | | | | 17.89329 | 12.11941 | 12.88285 | 9.202277 | 10.21387 | 11.48488 | 7.011247 | NA | 7.273581 | 3.761305 | 10.99038 | 7.264223 | 8.390549 | NA | NA | 20.2329 | NA | 17.51574 | 5.437546 | 8.395664 | NA | 4.884386 | 5.902954 | 10.96784 | 17.9566 | 16.18956 | 7.200933 |
| CUST_6515_PI429010886 | N20201 | 0.004773 | 18 | 12 | 123438677 | viral A-type inclusion protein [Trichomonas vaginali | | | 4.88E-10 | 218.2859 | 157.7661 | 192.898 | 75.54672 | 133.006 | 121.6959 | 178.7689 | 187.5065 | 145.5994 | 171.1862 | 219.2135 | 161.2431 | 238.4211 | 235.3955 | 219.1432 | 267.177 | 172.9267 | 141.4293 | 168.4127 | 169.6125 | 241.8211 | 181.1666 | 179.4697 | 116.8091 | 139.562 | 178.5939 | 185.7109 |
| CUST_10982_PI429010886 | N27086 | 0.004777 | 24 | 0 | | | | | | 28.68856 | 36.45455 | 24.26769 | 24.18723 | 21.9738 | 42.9427 | 15.99975 | 19.41782 | 28.45405 | 23.02438 | 16.80437 | 19.81178 | 19.22831 | 27.28895 | 23.83451 | 32.92321 | 11.47955 | 10.93186 | 19.84167 | 27.2627 | 22.98483 | 26.92502 | 14.0442 | 24.02102 | 43.20136 | 39.78497 | 37.47537 |
| CUST_42891_PI429010886 | M61528 | 0.004779 | 24 | 18 | | | | | | 55.32113 | 49.86955 | 64.68215 | 47.71977 | 34.74686 | 42.68463 | 38.59321 | 46.64901 | 43.48424 | 53.24987 | 55.66127 | 60.41389 | 43.30223 | 49.59839 | 51.46072 | 46.78356 | 53.02384 | 50.12098 | 69.83541 | 63.77772 | 58.7202 | 69.9145 | 52.8351 | 41.38053 | 48.76067 | 45.92841 | 53.42416 |
| CUST_47284_PI429010886 | M75476 | 0.004781 | 18 | 0 | | | | | | 88.71715 | 71.77101 | 128.4554 | 89.90347 | 43.08353 | 28.39724 | 26.09805 | 126.045 | 49.37977 | 49.41092 | 37.29408 | 35.93002 | 76.13058 | 138.2955 | 63.13991 | 109.2001 | 52.68156 | 95.34993 | 112.304 | 92.56423 | 120.7611 | 8.200624 | 37.34484 | 31.66766 | 35.83833 | 33.67567 | 20.28225 |
| CUST_4134_PI429010886 | N16089 | 0.004783 | 12 | 0 | | | | | | 77.60106 | 92.84239 | 90.89001 | 62.21773 | 67.77286 | 63.72139 | 69.26327 | 64.54153 | 52.45794 | 76.40353 | 70.3766 | 80.5678 | 63.96858 | 80.39867 | 71.34779 | 70.24276 | 67.9638 | 75.4087 | 72.08475 | 53.80851 | 65.73281 | 75.48481 | 66.25397 | 72.06797 | 74.69976 | 73.66521 | 81.5806 |
| CUST_45891_PI429010886 | M70641 | 0.004793 | 24 | 0 | | | | | | 57.74713 | 47.39274 | 61.61655 | 50.31204 | 57.02142 | 53.30954 | 52.42987 | 45.26245 | 51.96426 | 50.90637 | 46.6 | 64.28764 | 48.36598 | 42.81625 | 47.51659 | 51.28378 | 47.13105 | 45.63767 | 44.4641 | 47.02203 | 53.14405 | 47.81705 | 50.48246 | 46.16699 | 86.11085 | 59.6416 | 53.84406 |
| CUST_44593_PI429010886 | M66422 | 0.004794 | 18 | 9 | 321473765 | hypothetical protein DAPPUDRAFT_314628 [Daphni | | | 1.51E-59 | 45.82893 | 33.87601 | 48.63366 | 48.59055 | 38.83398 | 30.75644 | 50.51081 | 57.10423 | 56.46018 | 61.53357 | 53.15159 | 57.78122 | 54.89215 | 58.32457 | 46.507 | 54.66133 | 54.5396 | 37.4709 | 75.14455 | 54.5396 | 44.30287 | 50.42986 | 43.76068 | 39.60553 | 38.85028 | 43.60712 | 56.03237 |
| CUST_38414_PI429010886 | M26371 | 0.004798 | 15 | 9 | | | | | | 2583.846 | 8461.907 | 8138.075 | 1477.505 | 2159.598 | 717.4734 | 2803.212 | 3278.647 | 13400.98 | 6665.624 | 7450.764 | 18377.26 | 7228.251 | 14559.32 | 9795.872 | 11202.95 | 2502.493 | 6976.379 | 5447.86 | 12762.11 | 6073.086 | 14970.57 | 8024.987 | 1733.695 | 14583.52 | 14950.27 | 22978.37 |
| CUST_33162_PI429010886 | M6140 | 0.004798 | 15 | 9 | | | | | | 333.5908 | 227.9427 | 337.9324 | 254.4916 | 241.3477 | 290.175 | 333.1788 | 355.3996 | 353.0021 | 353.0802 | 327.031 | 384.4603 | 304.1627 | 424.4597 | 360.6396 | 375.7452 | 377.0163 | 299.837 | 349.8347 | 314.3091 | 256.1116 | 419.1452 | 341.2566 | 466.7074 | 452.4978 | 371.8288 | 470.2 |
| CUST_2996_PI429010886 | N13567 | 0.004798 | 15 | 9 | 329565704 | ribosomal protein L24 [Azumapecten farreri] | Protein metabolism | Ribosome biogenesis | 4.27E-40 | 301.1632 | 350.9859 | 327.031 | 237.5448 | 229.1012 | 205.1195 | 232.021 | 238.3252 | 279.3325 | 461.0025 | 287.7468 | 492.0126 | 321.803 | 318.0852 | 343.6141 | 527.2284 | 238.7409 | 221.6459 | 261.922 | 372.0481 | 230.5203 | 342.5512 | 315.4574 | 276.4683 | 418.2809 | 329.648 | 381.459 |
| CUST_18752_PI429010886 | N40352 | 0.004798 | 15 | 9 | | | | | | 50.82151 | 438.3419 | 60.58205 | 36.60129 | 41.97456 | 39.51821 | 40.71927 | 49.49896 | 48.069 | 68.20531 | 59.3369 | 84.31618 | 43.17605 | 63.52784 | 67.34873 | 66.20577 | 60.51107 | 68.77778 | 41.47082 | 48.01961 | 63.35686 | 56.60803 | 69.751 | 50.32801 | 71.07061 | 53.47338 | 84.722 |
| CUST_46131_PI429010886 | M71492 | 0.004801 | 18 | 9 | 321455267 | hypothetical protein DAPPUDRAFT_229417 [Daphni | | | 5.34E-21 | 3.928378 | 7.493829 | 11.34588 | 6.090696 | 8.664964 | 3.94856 | 10.70475 | 43.68024 | 37.44113 | 15.22654 | 30.18478 | 45.58985 | 31.80677 | 11.97517 | 39.27655 | 5.425239 | 26.98034 | 10.09344 | 5.272598 | 44.73069 | 35.49911 | 7.795868 | 9.958012 | 9.155769 | 10.19739 | 19.79173 | 13.21125 |
| CUST_21004_PI429010886 | N44459 | 0.004812 | 21 | 18 | | | | | | 287.8054 | 193.2088 | 287.5857 | 195.0326 | 170.3686 | 220.2228 | 257.2108 | 125.9453 | 176.1138 | 154.7689 | 192.7273 | 169.847 | 131.7044 | 105.2326 | 193.714 | 235.4286 | 289.2197 | 192.0915 | 292.1904 | 188.427 | 297.5373 | 185.2303 | 305.0519 | 362.1101 | 216.8689 | 190.0374 | 189.0589 |
| CUST_34540_PI429010886 | M9958 | 0.004816 | 15 | 0 | 528486751 | PREDICTED: cytoplasmic tRNA 2-thiolation protein 1 | Nucleic acid metabolism | RNA metabolic process | 8.43E-68 | 88.77007 | 122.9325 | 76.07191 | 82.8076 | 100.3232 | 73.89571 | 58.63339 | 59.49267 | 65.27575 | 104.8753 | 65.68089 | 101.4752 | 86.31209 | 83.40091 | 99.91859 | 128.7649 | 90.48379 | 71.40808 | 67.06219 | 111.2474 | 95.61724 | 81.36657 | 58.4895 | 59.46444 | 84.62693 | 79.70393 | 85.17905 |
| CUST_9359_PI429010886 | N24487 | 0.00482 | 18 | 3 | | | | | | 37.28683 | 85.18737 | 21.35291 | 120.7762 | 51.58072 | 70.93175 | 43.55173 | 51.45306 | 29.33547 | 30.48036 | 17.51574 | 49.03345 | 23.78727 | 40.61218 | 32.13072 | 42.04788 | 4.702682 | 35.52696 | 21.04421 | 38.74344 | 58.92238 | 48.20555 | 65.28922 | 44.61606 | 59.30743 | 50.36639 | 25.61779 |
| CUST_56893_PI429010886 | M114850 | 0.004822 | 21 | 0 | | | | | | 89.77998 | 85.24568 | 102.3996 | 74.48926 | 83.10449 | 71.57902 | 79.9054 | 80.10966 | 92.38118 | 77.29645 | 71.98272 | 81.04828 | 72.62704 | 79.31367 | 74.75705 | 84.57831 | 83.48682 | 66.4223 | 65.86348 | 84.64574 | 78.05922 | 93.01036 | 98.55351 | 89.17313 | 98.23281 | 80.03068 | 72.8924 |
| CUST_52584_PI429010886 | M98061 | 0.004834 | 21 | 3 | | | | | | 44.34868 | 43.22574 | 43.60346 | 55.4319 | 47.03357 | 62.53849 | 44.22939 | 43.5437 | 40.31382 | 51.29496 | 48.26458 | 40.63191 | 35.66432 | 35.85352 | 49.87667 | 42.89451 | 43.28403 | 47.20692 | 38.33525 | 22.36804 | 55.42699 | 51.57463 | 54.14016 | 52.73287 | 51.5559 | 64.03011 | 52.49227 |
| CUST_48042_PI429010886 | M78640 | 0.004843 | 15 | 3 | | | Metabolic process | Carbohydrate metabolic process | | 24.05844 | 13.96779 | NA | 21.63606 | 20.04708 | 32.3787 | 25.8352 | 9.204642 | 20.16285 | 5.791904 | 4.876942 | NA | 12.13896 | 10.16036 | 16.98819 | 27.76975 | NA | 9.751886 | 10.01676 | NA | NA | 21.11131 | NA | 25.5267 | 7.621463 | 8.746322 | 4.204939 |
| CUST_10236_PI429010886 | N25925 | 0.004852 | 24 | 12 | | | | | | 185.9383 | 177.7855 | 193.0385 | 167.4333 | 186.4927 | 133.6264 | 137.9678 | 189.5687 | 140.7918 | 227.4444 | 224.5446 | 194.9477 | 195.7607 | 281.9234 | 220.9933 | 220.0411 | 228.274 | 155.6229 | 207.5948 | 215.5021 | 166.4251 | 206.9547 | 217.0054 | 212.5355 | 196.157 | 159.9075 | 196.5038 |
| CUST_37185_PI429010886 | M18127 | 0.004853 | 15 | 9 | | | | | | 83.25716 | 113.2498 | 118.7356 | 86.7788 | 55.81794 | 76.93173 | 79.80797 | 87.48439 | 91.07591 | 104.3742 | 89.4807 | 125.3014 | 88.37626 | 114.813 | 117.4608 | 94.17604 | 103.1763 | 112.7975 | 98.07031 | 81.20466 | 97.97139 | 111.4592 | 100.4062 | 93.34264 | 109.2001 | 126.1892 | 127.4645 |
| CUST_43526_PI429010886 | M63277 | 0.004853 | 15 | 0 | | | | | | 46.29899 | 48.12746 | 40.20429 | 27.98319 | 32.59686 | 43.72098 | 15.9498 | 14.31543 | 23.43812 | 38.24021 | 33.01533 | 32.8723 | 42.54782 | 21.86136 | 24.63224 | 51.37093 | 38.2775 | 33.96314 | 35.1468 | 42.61497 | 30.88019 | 28.44909 | 32.20795 | 7.555334 | 62.97162 | 44.14193 | 56.01567 |
| CUST_12173_PI429010886 | N29044 | 0.004853 | 15 | 9 | | | | | | 47.16593 | 50.29336 | 68.69041 | 54.23711 | 41.2187 | 40.46037 | 44.71939 | 46.37163 | 50.9289 | 70.04597 | 63.747 | 68.35087 | 42.30947 | 76.674 | 53.73015 | 55.73505 | 44.41825 | 54.08711 | 34.89935 | 49.56937 | 54.19041 | 56.63191 | 72.00841 | 53.1338 | 73.67107 | 64.62929 | 61.66407 |
| CUST_19152_PI429010886 | N41064 | 0.004853 | 15 | 0 | | | | | | 68.17959 | 53.61735 | 42.53014 | 42.29841 | 51.46537 | 50.8107 | 40.98995 | 32.89038 | 26.04145 | 48.35331 | 46.82282 | 32.3288 | 31.05901 | 36.32974 | 37.40747 | 54.38291 | 50.72389 | 55.23417 | 43.39175 | 35.94919 | 28.1163 | 35.58457 | 20.60205 | 39.61818 | 64.21464 | 51.90738 | 42.58462 |
| CUST_50870_PI429010886 | M91288 | 0.004856 | 18 | 3 | 328792941 | PREDICTED: hypothetical protein LOC410161 [Apis r | Signal transduction | Other | 1.15E-27 | 13.99622 | 13.45887 | 16.12222 | 51.13109 | 57.47681 | 52.37245 | 54.74747 | 43.83694 | 17.64263 | 11.57286 | 45.3118 | 19.53746 | 9.689869 | 6.649752 | 21.58422 | 10.62249 | 26.86002 | 8.024819 | 36.40199 | 10.6198 | 84.96006 | 17.52814 | 93.87602 | 33.92452 | 7.748787 | 28.04382 | 14.69551 |
| CUST_13764_PI429010886 | N31731 | 0.004863 | 12 | 3 | | | | | | 16.64136 | 10.33897 | 11.37935 | 15.60949 | 23.32541 | 47.51073 | 25.14633 | 16.86404 | 10.58768 | 3.849533 | 24.99534 | 13.46279 | 10.21711 | 14.6546 | 8.467075 | 18.00505 | 57.8814 | 29.5179 | 22.84941 | 23.1013 | 26.75604 | 14.23448 | 36.09682 | 39.59389 | 6.630788 | 12.83786 | 9.338175 |
| CUST_2677_PI429010886 | N12891 | 0.004865 | 24 | 18 | | | Cellular component organization | Cytoskeleton organization | | 260.7973 | 249.9907 | 265.1519 | 233.2471 | 292.3195 | 350.3639 | 262.9091 | 196.8321 | 209.83 | 217.2265 | 259.7703 | 233.088 | 169.4763 | 261.9745 | 275.5051 | 255.3452 | 355.7822 | 314.246 | 380.7018 | 260.6408 | 298.3902 | 304.1627 | 298.1939 | 294.2267 | 253.0671 | 273.6903 | 234.4818 |
| CUST_44261_PI429010886 | M65429 | 0.00487 | 21 | 15 | 240114285 | hemocyanin [Porcellio scaber] | | | 6.85E-09 | NA | 7.023683 | 10.87219 | 40.04237 | 111.4592 | 6.768573 | 88.09249 | 488.3379 | 161.2704 | 51.75281 | 331.4379 | 346.5286 | 1406.196 | 28.05779 | 561.9199 | NA | 501.0805 | NA | 51.15134 | 15199.11 | 469.1551 | NA | 26.01379 | 3.838773 | NA | 82.74334 | 453.4712 |
| CUST_40391_PI429010886 | M54967 | 0.004872 | 24 | 18 | | | | | | 199.5128 | 196.5989 | 218.8861 | 178.8016 | 180.3041 | 214.0415 | 238.8964 | 196.9122 | 225.1942 | 229.13 | 253.1583 | 284.4719 | 298.8597 | 335.4811 | 341.1708 | 367.921 | 207.9906 | 309.0322 | 325.9737 | 300.6766 | 234.5938 | 426.7007 | 257.8206 | 261.4696 | 323.2059 | 316.2685 | 351.6009 |

| Probe ID | ID | p-value | Period | Phase | EBI ID | Annotation | GO category | Go term | E-value | 0 | 0 | 0 | 3 | 3 | 3 | 6 | 6 | 6 | 9 | 9 | 9 | 12 | 12 | 12 | 15 | 15 | 15 | 18 | 18 | 18 | 21 | 21 | 21 | 24 | 24 | 24 |
|---|---|---|---|---|---|---|---|---|---|---|---|---|---|---|---|---|---|---|---|---|---|---|---|---|---|---|---|---|---|---|---|---|---|---|---|---|
| CUST_33465_PI429010886 | M6960 | 0.004874 | 15 | 9 | | | | | | 1577.693 | 655.3909 | 14629.47 | 56.88497 | 91.46281 | 8.226818 | 3285.957 | 19822.05 | 20168.54 | 21318.49 | 2963.617 | 5367.717 | 19666.57 | 37322.31 | 20288.17 | 2615.876 | 11161.59 | 664.3088 | 3664.722 | 8086.505 | 1481.812 | 21372.62 | 27142.29 | 816.6897 | 41887.92 | 4318.11 | 37040.41 |
| CUST_45894_PI429010886 | M70652 | 0.004884 | 24 | 15 | 256079279 | procollagen-lysine2-oxoglutarate 5-dioxygenase [Sc | Protein metabolism | Other | 2.36E-12 | 55.28288 | 142.5512 | 65.74131 | 29.16954 | 67.65513 | 32.61136 | 12.14661 | 12.9342 | 41.9189 | 137.5442 | 29.10818 | 110.605 | 56.65296 | 87.13975 | 76.53451 | 110.3777 | NA | 99.54848 | 73.27672 | 145.6462 | 100.566 | 163.3967 | 60.39123 | 62.17487 | 73.99757 | 77.61759 | 74.57029 |
| CUST_41715_PI429010886 | M58374 | 0.004887 | 21 | 6 | | | | | | 24.41741 | 46.20496 | 86.94867 | 21.35581 | 116.5788 | 136.51 | 80.92356 | 59.93656 | 104.5344 | 98.55351 | 23.49823 | 54.43209 | 75.04275 | 96.35645 | 38.80417 | 7.44504 | 30.50322 | 18.23321 | 28.75284 | 47.59957 | 46.81158 | 64.23456 | 59.35924 | 36.08065 | 56.79499 | 63.94718 | 87.43516 |
| CUST_13316_PI429010886 | N30979 | 0.004892 | 12 | 3 | | | | | | 305.2258 | 993.7035 | 146.3194 | 1047.078 | 536.0428 | 632.4528 | 464.198 | 116.5927 | 310.3428 | 53.9397 | 58.16774 | 60.18184 | 155.5901 | 127.2103 | 415.2193 | 619.8196 | 139.8446 | 545.3681 | 179.7342 | 35.1434 | 75.74491 | 387.4359 | 65.25047 | 348.4045 | 482.976 | 159.1972 | 379.2779 |
| CUST_31745_PI429010886 | M2295 | 0.004893 | 18 | 12 | 260819212 | hypothetical protein BRAFLDRAFT_77219 [Branchio | | | 3.12E-07 | 179.3194 | 186.9387 | 247.9972 | 139.4033 | 143.002 | 168.6965 | 200.184 | 215.4716 | 240.7867 | 139.5841 | 229.6568 | 291.2128 | 224.9679 | 262.2092 | 279.8719 | 307.7422 | 216.8379 | 249.8541 | 219.4848 | 269.6735 | 140.6181 | 231.1582 | 176.0509 | 137.0338 | 245.6075 | 153.6022 | 255.1161 |
| CUST_34401_PI429010886 | M9560 | 0.004893 | 18 | 3 | | | | | | 48.39849 | 50.71291 | 34.31018 | 56.30906 | 58.85973 | 57.00393 | 50.4975 | 38.4072 | 29.2332 | 50.277 | 43.66151 | 46.507 | 36.42394 | 31.97801 | 37.80277 | 45.73272 | 49.02131 | 45.92841 | 53.86751 | 36.76731 | 36.4309 | 41.12468 | 55.00728 | 51.92939 | 53.59509 | 42.83757 | 24.42322 |
| CUST_54084_PI429010886 | M104479 | 0.004895 | 15 | 9 | | | | | | 8.819513 | 6.205806 | 11.64355 | 12.10746 | 8.992487 | 3.843996 | 5.746337 | 12.62711 | 12.36888 | 17.8888 | 14.75649 | 15.46627 | 13.80323 | NA | 10.96904 | 12.19537 | 79.16747 | 8.324601 | 7.82601 | 11.71536 | 12.79677 | 12.88708 | 15.10095 | 8.693497 | 18.01173 | 19.17918 | 13.61134 |
| CUST_10183_PI429010886 | N25827 | 0.004899 | 21 | 18 | 321459393 | hypothetical protein DAPPUDRAFT_61331 [Daphnia | | | 1.23E-56 | 69.68816 | 73.47743 | 76.11306 | 57.66915 | 61.0979 | 45.92204 | 43.51975 | 53.7412 | 56.40861 | 76.62236 | 49.89799 | 87.68051 | 63.12362 | 76.53451 | 66.70229 | 87.18471 | 44.50661 | 64.3589 | 63.05647 | 83.14826 | 79.97123 | 71.46149 | 61.90324 | 62.98504 | 65.31793 | 55.98779 | 61.2083 |
| CUST_2171_PI429010886 | N11772 | 0.004908 | 15 | 9 | | | | | | 115.1886 | 122.3884 | 136.7554 | 93.71752 | 100.1395 | 129.0306 | 106.097 | 126.9841 | 132.4207 | 141.3308 | 145.6647 | 152.9488 | 130.8664 | 136.5699 | 129.8119 | 154.7047 | 123.9789 | 120.7327 | 99.65085 | 143.5925 | 135.7237 | 143.3418 | 145.0813 | 112.9419 | 168.4127 | 132.7894 | 192.9519 |
| CUST_7742_PI429010886 | N22003 | 0.004908 | 15 | 9 | | | | | | 123.6853 | 160.6252 | 132.6733 | 110.192 | 103.8938 | 120.5952 | 89.97695 | 131.4929 | 103.8205 | 144.7064 | 133.355 | 136.25 | 119.4919 | 115.5186 | 122.2373 | 121.8439 | 124.4126 | 138.7005 | 93.06352 | 120.5952 | 110.1183 | 132.2661 | 140.6181 | 125.719 | 153.8434 | 126.3708 | 132.9657 |
| CUST_7938_PI429010886 | N22299 | 0.004908 | 15 | 9 | | | | | | 131.4476 | 145.578 | 153.5334 | 120.2652 | 121.1966 | 121.5472 | 132.4401 | 117.1499 | 142.1431 | 176.5007 | 170.5103 | 204.3871 | 128.6369 | 171.8172 | 116.4203 | 148.9974 | 132.2049 | 155.7638 | 133.8309 | 119.3927 | 150.6133 | 190.1658 | 171.6462 | 149.6184 | 204.3871 | 144.77 | 191.4276 |
| CUST_13223_PI429010886 | N30808 | 0.004908 | 15 | 0 | | | | | | 371.1996 | 494.6999 | 449.0628 | 396.0145 | 406.1396 | 369.2549 | 288.1296 | 332.8858 | 316.5285 | 435.0294 | 365.0271 | 485.4409 | 297.14 | 360.3606 | 405.9331 | 418.3911 | 416.4515 | 430.9519 | 469.9116 | 319.007 | 377.9719 | 356.1302 | 371.7567 | 332.1642 | 369.9548 | 379.8142 | 411.6609 |
| CUST_18654_PI429010886 | N40175 | 0.004908 | 15 | 9 | | | | | | 167.9263 | 174.1781 | 168.5282 | 110.7787 | 63.52784 | 44.78041 | 105.0857 | 180.0476 | 119.3486 | 321.4467 | 65.78559 | 183.7339 | 255.0746 | 164.4415 | 170.6145 | 95.57811 | 186.8543 | 87.99607 | 171.8979 | 147.2113 | 75.79683 | 265.2639 | 136.4264 | 175.1768 | 550.0892 | 197.6933 | 204.5562 |
| CUST_23445_PI429010886 | N48852 | 0.004916 | 15 | 6 | 380016322 | PREDICTED: proclotting enzyme-like [Apis florea] | Protein metabolism | Proteolysis | 1.36E-16 | 5.690439 | 193.0385 | 34.35609 | 202.1751 | 201.6411 | 41.77517 | 157.2097 | 479.189 | 226.6353 | 27.64006 | 182.3659 | 218.9157 | 161.0222 | 121.7699 | 147.0578 | 9.548233 | 39.288 | NA | 14.20484 | 195.9916 | 40.34574 | NA | 313.6967 | 194.0962 | NA | 121.4442 | 34.73538 |
| CUST_19841_PI429010886 | N42300 | 0.004918 | 24 | 0 | | | | | | 14.09963 | 32.29997 | 11.47799 | 7.792392 | 13.45887 | 7.874648 | 8.6774 | 12.43735 | 12.67743 | NA | 13.24115 | 8.218065 | 6.081579 | NA | 9.477835 | 10.48943 | 7.736177 | 3.734567 | 4.885315 | 13.98209 | 5.877156 | 13.2528 | 13.55604 | 10.30348 | 14.046 | 17.03463 | 10.53669 |
| CUST_9016_PI429010886 | N23938 | 0.004929 | 21 | 18 | | | | | | 17.81187 | 26.69563 | 24.62356 | 11.57436 | 16.84517 | 29.93088 | 14.62618 | 6.008155 | 12.19407 | 12.7848 | 15.33921 | 12.6233 | 21.8123 | 21.0027 | 12.76211 | 23.06016 | 19.69401 | 31.73942 | 34.81682 | 26.71164 | 11.98928 | 22.45542 | 27.02172 | 16.34592 | 21.03086 | 17.22867 | 14.28863 |
| CUST_41186_PI429010886 | M57088 | 0.004937 | 15 | 3 | | | | | | 241.9617 | 188.1904 | 112.9234 | 295.8079 | 284.2107 | 399.7554 | 241.2571 | 178.8821 | 118.905 | 139.0764 | 186.2582 | 111.7143 | 151.752 | 125.5041 | 173.3233 | 184.7219 | 351.303 | 303.3224 | 276.2557 | 159.4135 | 196.9798 | 163.329 | 162.6703 | 258.3561 | 154.8889 | 168.9219 | 120.2042 |
| CUST_42864_PI429010886 | M61459 | 0.004939 | 24 | 6 | | | | | | 257.0386 | 204.6898 | 217.4459 | 204.658 | 214.4744 | 208.1099 | 227.8356 | 222.1445 | 233.5692 | 196.7037 | 213.6068 | 269.1618 | 221.3126 | 207.0153 | 196.157 | 200.0776 | 139.8337 | 168.7503 | 190.5242 | 251.8789 | 200.9243 | 165.8977 | 191.6146 | 181.5941 | 171.8504 | 193.3576 | 208.2838 |
| CUST_4085_PI429010886 | N15972 | 0.004945 | 24 | 18 | 332374588 | unknown [Dendroctonus ponderosae] >gi\|5466849 | Transport | Protein transport | 2.38E-20 | 164.3174 | 180.731 | 141.3582 | 149.4856 | 161.1265 | 125.0002 | 102.4535 | 121.4973 | 138.3764 | 200.9848 | 122.7515 | 234.3704 | 129.3867 | 163.5179 | 154.4419 | 230.8944 | 90.72052 | 220.0411 | 165.4521 | 228.2235 | 230.5542 | 203.7918 | 151.2035 | 150.5005 | 135.074 | 176.3863 | 165.0319 |
| CUST_41839_PI429010886 | M58675 | 0.004951 | 12 | 3 | | | | | | 464.4064 | 408.8064 | 271.5735 | 661.6789 | 576.8367 | 834.9559 | 558.4057 | 379.3496 | 271.5735 | 259.4845 | 321.4904 | 185.9383 | 337.5537 | 383.1748 | 352.0546 | 629.0535 | 570.2153 | 687.2662 | 583.9827 | 457.038 | 261.2742 | 744.5402 | 226.8346 | 529.9042 | 525.8609 | 388.3479 | 184.7219 |
| CUST_4501_PI429010886 | N16907 | 0.004951 | 12 | 3 | | | | | | 33.27763 | 31.98148 | 13.99779 | 37.9894 | 34.13499 | 68.63903 | 31.60525 | 24.99806 | 25.8552 | 26.80742 | 19.4114 | 17.1412 | 26.40308 | 30.54779 | 22.26367 | 42.70658 | 25.58065 | 33.78634 | 31.62413 | 26.46898 | 27.74158 | 26.97522 | 28.64267 | 23.46752 | 30.09768 | 24.51087 | 36.08065 |
| CUST_52501_PI429010886 | M97674 | 0.004952 | 15 | 12 | | | | | | 41.77165 | 35.5525 | 39.26949 | 36.57353 | 37.85261 | 39.03429 | 41.33159 | 37.56088 | 38.33099 | 37.30837 | 44.8896 | 54.83755 | 40.44714 | 53.04998 | 49.35995 | 38.02365 | 45.66434 | 31.91513 | 30.19994 | 35.96518 | 39.11234 | 32.96039 | 36.44639 | 38.71497 | 49.14494 | 43.76947 | 41.76249 |
| CUST_43929_PI429010886 | M64363 | 0.004952 | 24 | 0 | 76150346 | autophagy protein 5 [Callinectes sapidus] | Metabolic process | Autofagy | 2.43E-24 | 56.00982 | 72.05819 | 58.98538 | 42.99716 | 39.53295 | 46.81158 | 40.52511 | 43.80561 | 73.36115 | 46.54214 | 35.2603 | 40.66388 | 41.65038 | 66.80323 | 44.82913 | 57.38433 | 50.8107 | 46.60859 | 49.83081 | 43.75138 | 38.23666 | 46.72755 | 61.1739 | 47.14504 | 59.21866 | 62.28748 | 73.91931 |
| CUST_18660_PI429010886 | N40186 | 0.004952 | 18 | 3 | | | | | | 249.1317 | 121.4237 | 411.2669 | 362.2892 | 417.7156 | 368.3133 | 519.7383 | 255.4386 | 96.25237 | 49.20084 | 376.27 | 91.34253 | 61.38183 | 80.04179 | 132.4401 | 76.11306 | 185.5006 | 116.8757 | 296.0927 | 61.40781 | 356.8337 | 97.32524 | 501.9627 | 506.24 | 83.89746 | 91.92897 | 65.48233 |
| CUST_47517_PI429010886 | M76441 | 0.004956 | 18 | 15 | | | | | | 137.604 | 108.8415 | 176.4808 | 126.6371 | 119.2623 | 117.7304 | 133.1932 | 130.4973 | 123.2423 | 117.1913 | 143.9244 | 161.3292 | 156.8398 | 181.7276 | 184.3634 | 165.3955 | 181.3801 | 184.0345 | 143.4291 | 158.7326 | 114.9009 | 135.5237 | 130.8991 | 117.2072 | 179.2925 | 112.2136 | 171.8979 |
| CUST_8295_PI429010886 | N22831 | 0.004962 | 21 | 18 | | | | | | 67.34293 | 65.04228 | 59.88574 | 45.88252 | 39.61818 | 46.52715 | 43.19279 | 54.70385 | 56.55039 | 67.7616 | 38.53689 | 76.0951 | 60.12109 | 52.56534 | 57.03251 | 62.52529 | 43.57816 | 70.87083 | 74.25035 | 67.83607 | 86.15616 | 64.74408 | 35.77142 | 46.78872 | 50.6408 | 35.10359 | 63.06213 |
| CUST_41040_PI429010886 | M56721 | 0.004963 | 15 | 9 | 225718978 | L-aspartate dehydrogenase [Caligus clemensi] | Metabolic process | Cellular amino acid metabolic process | 2.14E-36 | 190.4186 | 167.2954 | 388.7082 | 59.776 | 75.15426 | 55.24489 | 120.3899 | 169.6552 | 250.4345 | 512.4566 | 116.9944 | 286.8104 | 291.9341 | 353.8817 | 213.4663 | 217.1953 | 159.878 | 155.0302 | 180.9607 | 220.7455 | 149.3496 | 352.327 | 426.1051 | 84.25035 | 400.0206 | 284.3688 | 549.6818 |
| CUST_13239_PI429010886 | N30831 | 0.004964 | 24 | 21 | 114145758 | zinc finger protein 1 [Ciona intestinalis] >gi\|930032 | | | 8.83E-22 | 37.00901 | 48.10026 | 39.52535 | 30.83339 | 42.12986 | 30.90181 | 24.42797 | 33.14087 | 31.55772 | 39.67377 | 31.39141 | 37.12271 | 33.45023 | 55.64028 | 42.6726 | 58.81878 | 40.55619 | 38.66042 | 43.41776 | 39.37435 | 37.58456 | 50.78152 | 37.70021 | 183.3264 | 34.15487 | 38.93338 | 50.90018 |
| CUST_16726_PI429010886 | N36759 | 0.00497 | 15 | 9 | | | | | | 22.9275 | 19.79375 | 30.14891 | 11.63331 | 13.17448 | 17.66078 | 19.22658 | 41.43739 | 20.36028 | 33.38385 | 25.95428 | 42.98059 | 26.98251 | 38.01489 | 22.74008 | 22.17526 | 22.90466 | 35.98369 | 34.93327 | 13.30348 | 22.07569 | 18.44132 | 43.11192 | 19.25465 | 25.1452 | 24.91328 | 40.67771 |
| CUST_38087_PI429010886 | M23281 | 0.004976 | 15 | 12 | | | | | | 905.268 | 481.7893 | 7662.198 | 32.75095 | 60.22346 | 17.85496 | 1949.421 | 12388.76 | 12651.8 | 10130.67 | 2745.288 | 3946.907 | 8493.602 | 21220.67 | 11908.36 | 1753.366 | 6011.008 | 498.525 | 1315.293 | 6394.985 | 586.5167 | 17786.81 | 16428.12 | 440.22 | 18178.95 | 2884.034 | 19224.7 |
| CUST_18811_PI429010886 | N40455 | 0.004993 | 21 | 12 | | | | | | 32.75472 | 35.84962 | 35.90257 | 27.73257 | 19.56884 | 36.30339 | 17.59108 | 26.64522 | 35.1842 | 40.55619 | 33.49626 | 76.48318 | 35.67075 | 56.91814 | 49.60877 | 40.01136 | 243.718 | 34.0469 | 33.57359 | 39.88324 | 42.59502 | 48.67472 | 63.26276 | 48.74075 | 23.71505 | 28.35396 | 28.27971 |
| CUST_25787_PI429010886 | N53065 | 0.004996 | 21 | 0 | | | | | | 12.59071 | 9.002381 | NA | 16.41585 | 6.942833 | NA | 8.396527 | 6.967135 | 8.451827 | NA | 7.220984 | 10.9225 | 5.54498 | 4.294809 | 4.737841 | 10.1858 | 7.936545 | 12.32915 | 5.961141 | 13.66038 | 8.154793 | 10.57913 | 14.61408 | 8.525365 | 11.27734 | NA | 11.5947 |
| CUST_45688_PI429010886 | M69923 | 0.005001 | 24 | 15 | | | | | | 105.9691 | 95.54961 | 114.917 | 113.3684 | 90.25676 | 92.90826 | 90.61609 | 79.55126 | 84.722 | 103.9666 | 96.50257 | 108.4668 | 88.53305 | 110.3002 | 96.71335 | 134.7225 | 120.2505 | 111.027 | 110.5556 | 113.2835 | 106.5844 | 94.46416 | 92.74338 | 100.1573 | 115.5444 | 125.4314 | 110.2159 |
| CUST_11905_PI429010886 | N28602 | 0.005006 | 15 | 3 | 321473083 | hypothetical protein DAPPUDRAFT_315157 [Daphn | | | 1.09E-33 | 38.52489 | 16.99403 | 34.59885 | 72.1095 | 22.6017 | 104.164 | 29.93503 | 47.25743 | 28.74973 | 12.31888 | 23.6747 | 8.992487 | 52.27598 | 21.7647 | 21.45407 | 33.95872 | 82.73488 | 68.33006 | 66.50645 | NA | 19.03542 | 29.82235 | 14.10863 | 29.73496 | 10.46311 | 26.15331 | 8.648968 |
| CUST_35592_PI429010886 | M12974 | 0.005009 | 12 | 3 | | | | | | 84.10524 | 59.27376 | 60.89252 | 93.05164 | 103.6762 | 150.0554 | 120.9334 | 88.10192 | 82.76433 | 77.97597 | 62.78204 | 88.57155 | 105.8358 | 134.3863 | 104.2917 | 121.2272 | 89.11931 | 94.07093 | 102.2907 | 89.5493 | 89.81869 | 82.6351 | 76.34394 | 56.30291 | 90.03399 | 99.38154 | 99.7668 |
| CUST_40911_PI429010886 | M56375 | 0.005009 | 12 | 9 | 91081799 | PREDICTED: similar to Transmembrane protein 62 [ | | | 3.26E-11 | 70.51648 | 70.30533 | 81.78469 | 52.034 | 50.4975 | 43.97896 | 72.05819 | 70.99058 | 89.41173 | 106.1322 | 68.5761 | 88.97552 | 73.67107 | 76.02137 | 80.04179 | 102.0693 | 44.98924 | 72.30449 | 78.85902 | 69.06525 | 87.19276 | 96.32091 | 107.8715 | 89.11931 | 66.39266 | 79.07776 | 68.2359 |
| CUST_8214_PI429010886 | N22720 | 0.005018 | 24 | 21 | | | | | | 435.9739 | 403.4755 | 564.2409 | 311.3229 | 381.498 | 356.9822 | 482.8729 | 353.0802 | 417.6101 | 409.6774 | 389.2749 | 397.1398 | 443.7417 | 513.4234 | 460.8905 | 599.2901 | 399.641 | 429.8795 | 467.5419 | 500.3626 | 398.993 | 514.5197 | 603.9714 | 610.8058 | 456.6758 | 403.1472 | 522.1156 |
| CUST_12417_PI429010886 | N29430 | 0.005018 | 15 | 0 | | | | | | 41.5428 | 35.86299 | 48.27575 | 25.12109 | 27.01753 | 46.87582 | 26.25702 | 20.93093 | 28.43068 | 51.61587 | 25.76975 | 36.47407 | 27.89422 | 35.96223 | 34.86128 | 37.20514 | 93.37534 | 31.6082 | 38.14082 | 30.94543 | 42.89451 | 20.73682 | 25.83743 | 21.65726 | 30.13914 | 40.68988 | 42.79014 |
| CUST_56199_PI429010886 | M113086 | 0.005019 | 15 | 12 | | | | | | 79.61927 | 117.0641 | 105.9444 | 88.46488 | 59.76845 | 46.9109 | 75.22006 | 57.18099 | 71.00324 | 79.71189 | 90.0502 | 107.2744 | 104.8392 | 98.21029 | 99.40267 | 82.05363 | 71.81768 | 75.85108 | 85.64499 | 144.9989 | 81.31167 | 71.93323 | 68.761 | 57.43992 | 62.44513 | 86.67899 | 91.50805 |
| CUST_2338_PI429010886 | N12134 | 0.005033 | 15 | 0 | 332022211 | Poly(U)-binding-splicing factor half pint [Acromyrme | | | 3.06E-34 | 48.20555 | 60.14737 | 53.23418 | 52.49227 | 48.75681 | 34.24386 | 44.67201 | 43.25525 | 34.26311 | 63.44792 | 47.14099 | 50.131 | 47.03677 | 50.37741 | 74.87405 | 67.30267 | 53.65979 | 53.23845 | 47.53397 | 57.24915 | 51.22479 | 56.69692 | 46.37163 | 43.898 | 49.55005 | 46.78872 | 53.33275 |
| CUST_14453_PI429010886 | N32910 | 0.005035 | 18 | 15 | | | | | | 123.1539 | 136.9283 | 129.065 | 118.3064 | 116.3867 | 194.0018 | 98.82662 | 86.4072 | 88.95458 | 124.0734 | 88.15874 | 146.882 | 91.11111 | 112.0385 | 105.5931 | 149.9119 | 133.4278 | 156.3748 | 110.4732 | 97.74764 | 108.6016 | 115.6902 | 102.8937 | 120.6113 | 99.91859 | 110.1637 | 111.3143 |
| CUST_41673_PI429010886 | M58284 | 0.005043 | 12 | 0 | | | | | | 592.3414 | 813.4135 | 1038.033 | 342.6916 | 317.2087 | 278.0457 | 394.1043 | 499.6354 | 409.9605 | 626.6888 | 384.8393 | 369.4961 | 689.6848 | 849.0108 | 544.0717 | 842.6525 | 469.28 | 415.7405 | 531.8244 | 555.8637 | 477.6391 | 907.1459 | 553.2164 | 445.9535 | 1972.844 | 475.7608 | 1807.03 |
| CUST_49255_PI429010886 | M84924 | 0.005049 | 15 | 0 | | | | | | 84.27092 | 227.8356 | 77.64933 | 66.22546 | 69.62631 | 76.29064 | 65.64051 | 67.85743 | 53.49548 | 66.83042 | 55.90718 | 110.765 | 72.30449 | 77.5823 | 113.2976 | 79.11601 | 80.95222 | 98.07031 | 52.32373 | 83.91514 | 92.5954 | 55.87064 | 66.87846 | 59.6055 | 89.35703 | 99.33533 | 83.39175 |
| CUST_6120_PI429010886 | N19655 | 0.005051 | 21 | 3 | 466017551 | PREDICTED: Orcinus orca structural maintenance of | Cellular Process | Cell cycle | 3.17E-52 | NA | 84.87934 | 45.1918 | 145.4206 | 56.62309 | 62.63151 | 39.98555 | 31.64126 | 35.7636 | 49.57451 | 22.8759 | 56.40861 | 20.74295 | 24.9855 | 20.41093 | 36.64631 | 4.935503 | 28.9598 | 19.16079 | 27.92065 | 89.73481 | 15.03992 | 76.14971 | 46.33014 | 65.56207 | 72.89846 | 34.99807 |
| CUST_56667_PI429010886 | M114381 | 0.005052 | 21 | 12 | | | | | | 517.5225 | 339.7564 | 433.4824 | 139.2667 | 563.1994 | 329.4092 | 529.3841 | 453.4712 | 303.0793 | 694.099 | 325.0806 | 392.2917 | 843.8131 | 553.9482 | 430.573 | 520.8337 | 665.3114 | 552.7878 | 463.2589 | 579.5177 | 375.5434 | 523.7505 | 248.7014 | 256.3774 | 163.2245 | 234.3391 | 422.4541 |
| CUST_11155_PI429010886 | N27358 | 0.005052 | 21 | 0 | 345493781 | PREDICTED: peptidyl-prolyl cis-trans isomerase-like | Protein metabolism | Protein folding | 1.69E-72 | 74.6525 | 87.26148 | 71.67319 | 76.27537 | 71.83386 | 65.9084 | 68.34468 | 51.1689 | 52.10062 | 70.39937 | 47.41387 | 55.04733 | 51.59735 | 57.05184 | 72.39117 | 69.95364 | 52.7291 | 63.1832 | 62.24459 | 68.1054 | 71.50258 | 87.47537 | 58.69441 | 82.70078 | 61.1739 | 46.29572 | 76.21064 |
| CUST_15212_PI429010886 | N34193 | 0.005067 | 12 | 3 | | | | | | 684.1373 | 2155.605 | 132.473 | 2444.229 | 1236.95 | 1315.293 | 1271.51 | 75.7868 | 1393.263 | 67.57577 | 59.04082 | 20.92458 | 349.238 | 187.7096 | 1205.12 | 3237.592 | 190.2377 | 2129.816 | 164.3174 | 38.68605 | 60.76524 | 529.4905 | 76.98892 | 488.1693 | 445.5129 | 171.0565 | 271.5735 |
| CUST_27430_PI429010886 | N56047 | 0.005067 | 12 | 3 | | | | | | 17.85063 | 8.190659 | 15.22654 | 22.17526 | 24.68274 | 25.0475 | 22.39942 | 17.5614 | 10.19943 | 13.41579 | 9.044875 | 6.951132 | 16.04069 | 13.14806 | 20.63001 | 22.47267 | 26.96182 | 21.97161 | 16.80068 | 11.58812 | 15.86933 | 13.83685 | 23.05797 | 18.97043 | 16.07923 | 17.13668 | 14.42119 |
| CUST_6466_PI429010886 | N20136 | 0.005073 | 15 | 9 | 307192890 | Neural proliferation differentiation and control prot | | | 1.14E-22 | 214.2881 | 240.0651 | 257.237 | 199.6316 | 215.6784 | 213.366 | 209.1953 | 204.0536 | 256.0146 | 271.3099 | 224.668 | 293.8787 | 179.7058 | 275.2295 | 245.6501 | 229.8288 | 230.9236 | 303.2741 | 200.2396 | 188.658 | 233.8649 | 241.7686 | 269.6735 | 225.3596 | 263.8863 | 268.588 | 271.9546 |
| CUST_8587_PI429010886 | N23265 | 0.005073 | 15 | 0 | 291230131 | PREDICTED: hypothetical protein [Saccoglossus kow | Metabolic process | Other | 2.81E-09 | 25.39589 | 20.01489 | 23.59869 | 21.17293 | 14.27082 | 28.31689 | 7.833526 | 7.592295 | 8.672182 | 20.32835 | 20.40111 | 20.28484 | 4.727333 | 16.70546 | 8.682476 | 23.23883 | 19.81911 | 24.4716 | 9.400307 | 14.91728 | 21.036 | 15.71146 | 7.789768 | 9.22307 | 24.71578 | 12.93967 | 16.48057 |
| CUST_55576_PI429010886 | M110799 | 0.005081 | 21 | 3 | | | | | | 12.66068 | 37.0175 | 12.85382 | 73.09859 | 25.72198 | 31.39141 | 19.39824 | 18.46372 | 22.61587 | 20.86179 | 5.941184 | 26.53469 | 7.176452 | 25.93979 | 28.40026 | 16.53332 | 4.734662 | 18.09887 | 10.8172 | 14.28863 | 27.69389 | 25.95233 | 25.4055 | 16.89352 | 38.51713 | 40.13494 | NA |
| CUST_5139_PI429010886 | N18307 | 0.005085 | 18 | 15 | | | | | | 635.2846 | 938.6625 | 753.7243 | 575.8176 | 617.8466 | 629.802 | 504.2482 | 527.3498 | 550.0892 | 708.9187 | 602.1279 | 972.1119 | 453.79 | 686.6887 | 717.957 | 1016.851 | 483.6862 | 746.9886 | 638.1084 | 745.6971 | 667.0295 | 663.5689 | 654.7049 | 594.4635 | 547.2331 | 620.4611 | 606.4913 |
| CUST_29363_PI429010886 | N59714 | 0.005087 | 15 | 12 | | | | | | 622.9374 | 586.6898 | 721.5575 | 472.8988 | 629.4397 | 646.0018 | 507.1661 | 564.3927 | 525.7241 | 659.8695 | 663.9332 | 828.3608 | 664.1086 | 703.6838 | 845.2412 | 906.5131 | 618.5189 | 715.1399 | 582.2842 | 653.1387 | 667.7123 | 580.161 | 673.1948 | 605.6379 | 681.1329 | 652.3862 | 801.7718 |
| CUST_4010_PI429010886 | N15779 | 0.005087 | 21 | 3 | | | | | | 92.79213 | 50.01173 | 100.2579 | 121.8737 | 82.08362 | 163.2552 | 75.74491 | 178.4123 | 151.2427 | 107.3035 | 77.43118 | 63.09759 | 113.2976 | 109.3157 | 65.26948 | 53.26961 | 120.6877 | 84.1293 | 83.59685 | 49.29932 | 59.87756 | 60.93069 | 90.0502 | 115.4344 | 122.7816 | 136.7329 | 160.5038 |
| CUST_16775_PI429010886 | N36850 | 0.005088 | 24 | 21 | | | | | | 84.09478 | 65.26948 | 72.9942 | 61.72981 | 65.81652 | 99.23417 | 57.00393 | 55.9772 | 59.21305 | 60.96666 | 41.55123 | 42.6726 | 44.09424 | 85.72992 | 66.53867 | 78.54463 | 65.85741 | 74.7826 | 74.3404 | 68.61072 | 56.93106 | 98.28699 | 67.79763 | 77.4993 | 76.55131 | 60.95084 | 78.16064 |
| CUST_10221_PI429010886 | N25896 | 0.005094 | 24 | 21 | | | | | | 122.0182 | 105.7491 | 111.1235 | 76.80142 | 93.41435 | 84.54494 | 100.4336 | 100.4445 | 84.64574 | 95.01446 | 103.8804 | 96.5167 | 97.87109 | 112.3805 | 103.5565 | 115.5 | 91.6512 | 113.3258 | 81.33169 | 105.9897 | 108.6304 | 137.351 | 128.5095 | 118.4789 | 119.9589 | 128.8708 | 107.4618 |
| CUST_18305_PI429010886 | N39543 | 0.005094 | 24 | 21 | | | | | | 189.1715 | 214.2881 | 250.6863 | 170.3963 | 168.6167 | 228.0216 | 145.9801 | 156.4118 | 177.896 | 215.7896 | 181.9887 | 193.0075 | 166.3529 | 198.3716 | 173.1201 | 219.8889 | 191.4774 | 238.6203 | 183.8412 | 211.8227 | 238.7009 | 211.7142 | 232.3526 | 231.1582 | 244.9337 | 198.308 | 235.1729 |
| CUST_24092_PI429010886 | N50011 | 0.005099 | 24 | 0 | | | | | | 105.6351 | 78.44959 | 109.7974 | 93.41435 | 91.82524 | 103.5822 | 80.86611 | 94.04891 | 82.33655 | 91.52825 | 68.34468 | 95.27106 | 76.69076 | 76.12074 | 85.94103 | 101.0493 | 78.92088 | 89.34718 | 81.76577 | 99.7668 | 86.09876 | 85.39372 | 81.79478 | 102.3183 | 103.1763 | 97.25472 | 106.4846 |
| CUST_6142_PI429010886 | N19683 | 0.005099 | 18 | 12 | 67625699 | TPA: endonuclease-reverse transcriptase [Schistoso | Nucleic acid metabolism | DNA replication | 8.73E-68 | 121.3307 | 85.76295 | 137.3083 | 86.7376 | 94.27998 | 119.6624 | 99.79034 | 89.14278 | 112.4193 | 120.2195 | 107.4857 | 142.5643 | 110.2326 | 157.8794 | 151.2877 | 143.7439 | 122.3884 | 141.6567 | 121.649 | 128.4226 | 115.5 | 119.7503 | 114.9376 | 83.32632 | 123.1656 | 101.7455 | 165.7972 |
| CUST_24247_PI429010886 | N50294 | 0.005099 | 18 | 12 | | | | | | 37.38397 | 38.57556 | 41.57079 | 34.65148 | 16.57372 | 56.15718 | 31.25517 | 22.48304 | 16.32704 | 15.00335 | 33.80016 | 42.79462 | 42.95145 | 71.06446 | 42.00009 | 41.99643 | 62.75575 | 56.38454 | 23.83766 | 36.18187 | 19.60024 | 45.16608 | 30.81346 | 22.30073 | 24.88909 | 34.91922 | 21.26849 |
| CUST_43530_PI429010886 | M63285 | 0.005103 | 12 | 0 | 260807227 | low-density lipoprotein receptor-related protein 2 [ | | | 6.98E-14 | 212.5355 | 210.2272 | 225.6591 | 163.8253 | 121.0793 | 116.332 | 152.3321 | 153.8729 | 192.442 | 250.9292 | 135.061 | 197.384 | 196.2537 | 285.9546 | 268.2743 | 246.92 | 151.2719 | 167.5217 | 138.3904 | 224.4715 | 202.3705 | 196.9122 | 176.7077 | 178.833 | 223.9704 | 202.1175 | 287.9369 |
| CUST_17784_PI429010886 | N38581 | 0.005103 | 12 | 0 | | | | | | 33.06846 | 24.02788 | 33.91698 | 23.27419 | 26.6702 | 28.45405 | 17.49801 | 24.15363 | 24.18144 | 14.79848 | 19.19726 | 21.33841 | 20.95699 | 28.04382 | 39.8966 | 33.83579 | 28.16847 | 26.76517 | 22.55992 | 22.8042 | 31.41951 | 25.0475 | 20.81747 | 14.67098 | 26.80742 | 23.06972 | 32.34301 |
| CUST_35021_PI429010886 | M11355 | 0.005122 | 18 | 15 | | | | | | 130.9315 | 124.9715 | 159.0925 | 128.9944 | 95.29429 | 99.66003 | 120.0167 | 120.2964 | 113.475 | 156.5024 | 111.4592 | 104.164 | 122.6882 | 188.3253 | 138.5342 | 159.6044 | 157.8347 | 135.4539 | 146.5131 | 115.6002 | 129.0306 | 145.4981 | 97.22671 | 104.8753 | 147.5789 | 87.51279 | 99.94463 |
| CUST_12434_PI429010886 | N29455 | 0.005125 | 24 | 21 | | | | | | 112.064 | 135.4539 | 117.6882 | 114.917 | 151.707 | 122.5004 | 106.0325 | 93.92221 | 111.8286 | 121.1828 | 105.4997 | 121.3613 | 114.9975 | 107.319 | 69.32875 | 128.1595 | 100.3711 | 126.3127 | 101.3914 | 93.35337 | 125.7681 | 162.0946 | 141.0765 | 154.1139 | 202.1175 | 134.3348 | 199.3801 |
| CUST_49030_PI429010886 | M83697 | 0.005128 | 21 | 0 | | | | | | 11.72586 | 5.32901 | 15.31326 | 10.35656 | 4.756913 | 17.06824 | 5.342076 | 8.450172 | NA | NA | 7.419678 | 7.693635 | 4.653863 | NA | 6.104204 | 4.114201 | 5.422154 | 4.515942 | 6.715293 | 4.538832 | 7.798194 | 6.733142 | 14.36708 | 10.31819 | 6.572882 | 8.545589 | 14.4735 |
| CUST_6383_PI429010886 | N20032 | 0.005129 | 24 | 12 | | | | | | 15.73231 | 13.83327 | 19.33205 | 10.37992 | 23.6747 | 15.94381 | 12.40589 | 23.37424 | 32.81991 | 19.54896 | 34.16228 | 42.98324 | 34.47823 | 30.40511 | 41.60845 | 22.89148 | 18.4326 | 27.86411 | 23.37181 | 37.38785 | 27.78206 | 21.55161 | 30.43439 | 15.53244 | 12.75631 | 16.66286 | 28.09147 |
| CUST_16810_PI429010886 | N36905 | 0.005129 | 24 | 21 | 321471614 | hypothetical protein DAPPUDRAFT_302347 [Daphn | Metabolic process | Cellular amino acid metabolic process | 2.06E-20 | 5.369808 | 12.56262 | 13.45546 | 10.38108 | 12.59383 | NA | 4.708261 | 5.013897 | 4.604286 | 7.52281 | 4.972361 | 8.844206 | 6.149357 | NA | 6.284506 | 12.52875 | NA | 31.57619 | NA | 10.93186 | 6.307552 | 18.00776 | NA | NA | 26.48449 | 11.0861 | 5.155084 |
| CUST_22598_PI429010886 | N47302 | 0.005132 | 15 | 0 | | | | | | 76.93173 | 68.88215 | 61.22888 | 71.68684 | 70.03699 | 68.78674 | 58.0658 | 44.66322 | 65.36594 | 64.1188 | 58.544 | 66.4223 | 64.90973 | 65.12567 | 67.72476 | 80.10123 | 67.13373 | 75.69546 | 75.62563 | 60.78457 | 73.25256 | 64.125 | 57.15078 | 63.37317 | 70.20962 | 75.03476 | 66.45252 |
| CUST_12971_PI429010886 | N30371 | 0.005139 | 24 | 12 | 195380950 | GJ21465 [Drosophila virilis] >gi\|194144016\|gb\|EDV | | | 3.13E-12 | 7.878584 | 12.27925 | 9.496558 | 7.319054 | 9.645798 | 6.832319 | 5.033531 | 6.839858 | 15.1027 | 29.45836 | 11.43555 | 12.61292 | 17.55722 | 16.32267 | 17.10186 | 5.942845 | 13.97697 | 12.58679 | 11.50211 | 16.02878 | 8.1716 | 14.60212 | NA | 9.82119 | 7.128415 | 5.550742 | 13.92172 |
| CUST_21760_PI429010886 | N45800 | 0.005154 | 15 | 6 | | | | | | 14.53622 | 17.7958 | 8.07676 | 24.51394 | 29.58077 | 9.04617 | 41.08623 | NA | 27.8201 | 23.38781 | 11.60331 | 10.682 | 9.202277 | 10.50509 | 18.0096 | 20.56296 | NA | 17.90553 | 21.23846 | NA | 18.85349 | 25.86296 | 21.43485 | 20.95213 | 24.35938 | 21.37656 | 6.15687 |
| CUST_14772_PI429010886 | N33444 | 0.005155 | 15 | 12 | | | | | | 500.9078 | 683.5585 | 704.3489 | 508.0799 | 491.5609 | 509.4935 | 545.6531 | 602.798 | 646.0018 | 792.1417 | 595.5631 | 870.5556 | 675.9235 | 890.3835 | 883.1215 | 831.0172 | 724.0591 | 598.8088 | 697.3779 | 800.0152 | 452.8393 | 732.691 | 636.3168 | 574.4912 | 714.5213 | 758.445 | 976.3736 |
| CUST_42518_PI429010886 | M60488 | 0.00516 | 24 | 18 | | | | | | 73.51884 | 71.04758 | 89.88488 | 52.80587 | 70.16242 | 60.03661 | 60.80817 | 73.19849 | 71.49639 | 94.23139 | 74.58958 | 83.81066 | 60.5059 | 107.2622 | 119.1299 | 91.11943 | 75.92185 | 85.35234 | 95.94174 | 102.6905 | 91.31323 | 96.77635 | 86.39769 | 65.85741 | 102.6772 | 92.06564 | 120.2505 |
| CUST_12835_PI429010886 | N30128 | 0.00516 | 24 | 21 | | | | | | 71.47658 | 77.42171 | 96.86826 | 61.22888 | 66.28856 | 62.04074 | 42.17788 | 54.80919 | 39.19727 | 116.9761 | 61.79243 | 86.02859 | 69.54952 | 77.08541 | 59.0899 | 87.14382 | 46.66745 | 64.75716 | 88.39516 | 71.32452 | 75.26431 | 84.2393 | 77.37108 | 77.65762 | 82.39034 | 76.03863 | 73.04458 |
| CUST_3023_PI429010886 | N13621 | 0.00516 | 18 | 9 | 241116683 | reductase, putative [Ixodes scapularis] >gi\|2154931 | Metabolic process | Oxidation-reduction process | 1.45E-65 | 50.65756 | 67.89063 | 46.89105 | 43.48893 | 48.88625 | 17.43083 | 39.84998 | 77.4993 | 65.66431 | 48.51868 | 79.77146 | 95.36046 | 72.1551 | 66.70898 | 63.80077 | 80.31713 | 57.28119 | 52.80587 | 62.74069 | 134.5271 | 74.83385 | 51.02755 | 48.57594 | 28.7018 | 52.44678 | 71.40808 | 65.43441 |
| CUST_9405_PI429010886 | N24563 | 0.005165 | 12 | 0 | | | | | | 52.08646 | 39.10739 | 78.84971 | 77.7747 | 43.65882 | 74.63865 | 37.70765 | 34.13499 | 34.1704 | 51.93952 | 41.82918 | 44.05675 | 64.94243 | 59.65312 | 58.04956 | 54.84373 | 60.20751 | 59.90035 | 36.65626 | 52.10062 | 48.13849 | 38.50578 | 49.8819 | 50.52879 | 70.07132 | 53.8998 | 72.84986 |
| CUST_15800_PI429010886 | N35170 | 0.005165 | 21 | 6 | | | | | | 84.63515 | 46.08794 | NA | 52.10631 | 52.36466 | 90.62378 | 127.2704 | 147.341 | 97.5299 | NA | 39.49607 | NA | 9.039304 | 11.36478 | 5.822589 | NA | 38.06764 | 31.16262 | 52.73287 | 26.58331 | 10.79562 | 3.391679 | 9.355583 | 108.8762 | NA | 74.69976 | NA |
| CUST_45191_PI429010886 | M68274 | 0.005173 | 24 | 18 | 514466636 | PREDICTED: beta,beta-carotene 9',10'-oxygenase [C | | | 1.46E-30 | NA | 9.679037 | NA | NA | 8.254327 | 8.330776 | 5.93818 | 6.633519 | 12.28913 | 3.43865 | 6.243139 | 6.440539 | 5.670388 | 15.34115 | 8.096609 | 5.29544 | 6.092564 | NA | 18.11337 | 16.89118 | 12.49275 | 7.892117 | 12.8856 | 14.35201 | 14.26332 | 29.75085 | 10.76813 |
| CUST_28557_PI429010886 | N58173 | 0.005176 | 21 | 18 | | | | | | 95.72441 | 89.89772 | 98.08436 | 119.8693 | 61.30366 | 55.33882 | 65.69324 | 86.03759 | 80.10966 | 74.03663 | 88.29574 | 69.58984 | 50.4975 | 75.26431 | 58.39472 | 107.9821 | 91.62991 | 82.91863 | 93.59388 | 97.04503 | 112.8438 | 102.5401 | 104.9253 | 93.59388 | 96.43909 | 88.10949 | 60.78457 |
| CUST_34626_PI429010886 | M10177 | 0.005184 | 12 | 3 | | | | | | 458.895 | 1148.695 | 80.94247 | 2632.403 | 1386.212 | 1336.322 | 1266.139 | 84.96672 | 1223.726 | 68.97204 | 53.26328 | 63.97505 | 261.0789 | 406.8847 | 1067.598 | 2014.561 | 114.6578 | 1317.338 | 101.9095 | 173.6625 | 73.49284 | 1421.77 | 52.48792 | 766.9049 | 675.5498 | 150.914 | 272.25 |
| CUST_46770_PI429010886 | M73678 | 0.005191 | 24 | 3 | | | | | | 8.621822 | 25.85777 | 14.66107 | NA | 8.920567 | 22.12374 | NA | 9.324813 | 13.54494 | 7.029716 | 9.166269 | 15.82858 | 6.809216 | NA | NA | 7.155832 | 8.52275 | 6.492705 | 7.212424 | 4.687434 | 8.309227 | 6.576173 | 8.2512 | NA | 9.152945 | 17.4103 | 9.202277 |
| CUST_27201_PI429010886 | N55619 | 0.005193 | 21 | 0 | | | | | | 32.14209 | 29.69161 | 33.24975 | 29.76345 | 29.14874 | 23.48014 | 16.68444 | 19.90164 | 15.76192 | 16.99042 | 24.69054 | 17.53198 | 13.44588 | 38.25121 | 18.38425 | 32.34622 | 18.88215 | 23.17379 | 21.74146 | 16.34764 | 27.86689 | 33.45456 | 26.65768 | 34.00967 | 38.99142 | 19.78441 | 27.10885 |
| CUST_7663_PI429010886 | N21886 | 0.005206 | 12 | 0 | | | | | | 21.06491 | NA | 30.27449 | 11.89031 | 3.978454 | NA | 9.106984 | 8.492959 | 12.86372 | 14.68043 | 5.008748 | NA | NA | 18.13433 | 17.75695 | 5.607582 | 9.413901 | NA | 8.430029 | 12.6487 | 5.265199 | 10.92621 | 10.79319 | 9.278391 | 17.77043 | 26.29349 | 22.65536 |
| CUST_2108_PI429010886 | N11597 | 0.005206 | 21 | 3 | 383863879 | PREDICTED: paxillin-like [Megachile rotundata] | | | 8.1E-101 | 899.8161 | 2750.47 | 1116.604 | 3172.097 | 1372.611 | 1939.579 | 1027.698 | 1281.734 | 1006.198 | 1107.396 | 772.4833 | 1561.567 | 1074.839 | 831.3875 | 962.2957 | 1403.267 | 738.859 | 627.0644 | 873.6327 | 1057.689 | 1352.582 | 1456.136 | 952.9126 | 1181.87 | 1576.839 | 1958.473 | 955.0872 |
| CUST_32470_PI429010886 | M4199 | 0.005213 | 15 | 12 | 358442122 | Broad-complex protein isoform 4 [Penaeus monodc | | | 2.32E-47 | 66.72453 | 108.077 | 96.80182 | 79.53245 | 38.21245 | 59.68907 | 72.59952 | 95.91036 | 62.45053 | 121.5192 | 77.23965 | 101.9838 | 81.91685 | 87.67204 | 124.2868 | 93.48735 | 74.70996 | 82.60943 | 74.4613 | 71.78694 | 79.82568 | 92.42083 | 97.5983 | 77.60864 | 96.75892 | 103.0385 | 83.99654 |
| CUST_218_PI429010886 | N2768 | 0.005213 | 24 | 6 | | | | | | 1213.318 | 1627.802 | 1416.429 | 1048.248 | 883.1215 | 591.3951 | 2104.851 | 2551.399 | 1716.583 | 1331.007 | 811.5226 | 1522.93 | 396.9865 | 557.1607 | 1179.987 | 1045.15 | 171.451 | 481.2318 | 117.1353 | 1106.942 | 542.1254 | 865.4579 | 1471.445 | 487.0643 | 83.77617 | 1667.846 | 862.1393 |
| CUST_2780_PI429010886 | N13087 | 0.005223 | 12 | 0 | | | | | | 42.32826 | 47.57729 | 35.46793 | 13.32908 | 32.24939 | 32.00727 | 21.92688 | 18.47407 | 27.06954 | 42.31429 | 33.48853 | 55.10664 | 22.53922 | 33.49263 | 35.43589 | 26.67812 | 17.49952 | 31.18382 | 27.1196 | 26.35513 | 37.58201 | 32.91963 | 23.17801 | 35.07318 | 76.34394 | 51.06646 | 39.70836 |
| CUST_23052_PI429010886 | N48174 | 0.005223 | 15 | 12 | | | | | | 17.76635 | 18.4393 | 41.98854 | 15.37345 | 18.89922 | 24.08352 | 38.14082 | 16.02878 | 23.68579 | 30.59095 | 28.25408 | 62.67964 | 18.84701 | 34.49621 | 43.38758 | 31.35097 | 31.87606 | 30.72063 | 18.66441 | 20.35314 | 18.40021 | 25.91041 | 20.25261 | 25.77304 | 28.96949 | 30.66462 | 37.32162 |
| CUST_3483_PI429010886 | N14593 | 0.005239 | 21 | 0 | | | | | | 28.93678 | 42.89937 | 26.17206 | 28.05304 | 29.6237 | 23.063 | 19.07791 | 22.47468 | 16.89803 | 19.51092 | 15.00181 | 20.36313 | 20.55693 | 17.60068 | 22.39477 | 18.61977 | 22.24561 | 15.76549 | 26.85728 | 34.78474 | 17.11553 | 34.24814 | 20.51216 | 34.16228 | 19.11453 | 27.19088 | 9.41784 |
| CUST_36426_PI429010886 | M15871 | 0.005242 | 12 | 9 | | | | | | 131.0289 | 189.0849 | 126.3312 | 117.7023 | 123.6853 | 114.7111 | 133.298 | 104.7461 | 119.7044 | 163.162 | 142.5512 | 165.7972 | 128.3042 | 137.2699 | 122.949 | 144.8962 | 117.0501 | 124.2426 | 133.1101 | 134.7073 | 137.058 | 158.6525 | 128.5885 | 197.0393 | 167.0802 | 153.8434 | 158.0759 |
| CUST_7294_PI429010886 | N21331 | 0.005242 | 12 | 3 | 345491565 | PREDICTED: NAD kinase-like isoform 3 [Nasonia vitr | Metabolic process | Other | 1.4E-139 | 169.847 | 169.3396 | 101.4116 | 229.8746 | 194.6296 | 261.767 | 204.7416 | 152.5175 | 217.37 | 82.06433 | 66.40981 | 105.4471 | 185.8187 | 143.3816 | 205.6642 | 225.3596 | 111.685 | 260.0898 | 127.2256 | 96.66502 | 85.12067 | 122.404 | 83.89746 | 238.3252 | 95.47811 | 171.5942 | 109.0752 |
| CUST_25743_PI429010886 | N52973 | 0.005267 | 18 | 6 | | | | | | 10.38437 | 9.407802 | 15.32584 | 18.95062 | 7.785373 | 10.74664 | 16.01505 | 16.66622 | 29.0776 | NA | 14.83888 | NA | 15.82289 | 16.5596 | 19.05569 | 6.588073 | 5.271736 | 9.920366 | 6.792388 | 9.325475 | 13.56226 | 9.339132 | 27.24651 | 12.00275 | 21.8123 | NA | NA |
| CUST_35564_PI429010886 | M12892 | 0.005269 | 18 | 9 | 521037349 | WD repeat-containing protein 92 [Myotis brandtii] | | | 2.93E-65 | 35.82359 | 43.75577 | 26.43705 | 24.33459 | 25.76223 | 18.13226 | 27.72962 | 34.91364 | 26.39959 | 43.98243 | 37.03334 | 37.96902 | 29.47649 | 31.38208 | 28.77383 | 44.46924 | 32.47618 | 40.5937 | 25.61415 | 44.51031 | 24.97464 | 32.77838 | 19.74119 | 25.29876 | 41.88371 | 29.07242 | 42.62942 |
| CUST_25561_PI429010886 | N52646 | 0.005272 | 12 | 0 | | | | | | 31.16892 | 42.36335 | 42.91318 | 17.1412 | 21.38676 | 15.25738 | 15.86933 | 24.02788 | 29.68336 | 43.80188 | 33.0225 | 40.46037 | 18.77767 | 36.92762 | 36.04391 | 41.37701 | 22.44547 | 28.09437 | 28.26641 | 30.73213 | 35.80323 | 29.39664 | 31.71188 | 23.59619 | 42.58941 | 40.89955 | 47.31501 |
| CUST_14184_PI429010886 | N32449 | 0.005273 | 24 | 0 | | | | | | 15.16191 | 25.67741 | 27.384 | 4.693553 | 8.536561 | NA | 10.33535 | 6.070064 | 16.66074 | 11.0861 | 11.86943 | 9.55219 | 6.62402 | 3.466827 | 7.627544 | 11.11919 | NA | 15.70076 | 9.625872 | 14.4465 | 10.10937 | 12.40735 | NA | NA | 11.54995 | 14.43375 | 25.05489 |
| CUST_29535_PI429010886 | N60057 | 0.005277 | 21 | 3 | | | | | | 93.60773 | 57.96208 | 57.67685 | 140.2437 | 52.60357 | 110.5098 | 64.76438 | 59.50102 | 83.65751 | 95.88672 | 45.67726 | 108.7487 | 43.55575 | 62.82655 | 24.82512 | 75.7296 | 33.62105 | 46.3344 | 40.28049 | 52.84586 | 124.8716 | 68.58333 | 95.52923 | 83.29202 | 118.8596 | 142.1088 | 72.2954 |
| CUST_2617_PI429010886 | N12769 | 0.005278 | 24 | 6 | 321477204 | hypothetical protein DAPPUDRAFT_311761 [Daphn | Cellular component organization | Cytoskeleton organization | | 136.0993 | 160.6473 | 91.12979 | 188.1623 | 179.8948 | 91.24255 | 162.1587 | 350.4435 | 218.8861 | 136.1666 | 314.5564 | 206.6019 | 300.7661 | 120.9219 | 145.9205 | 133.2074 | 119.1476 | 75.02406 | 150.914 | 581.4141 | 188.0879 | 101.2666 | 57.16741 | 62.81643 | 90.13978 | 166.6372 | 162.9163 |
| CUST_41856_PI429010886 | M58706 | 0.005279 | 21 | 12 | | | | | | 1945.509 | 1351.148 | 1583.802 | 656.1679 | 2424.452 | 1318.096 | 1978.873 | 1560.88 | 1488.789 | 3621.392 | 1383.212 | 1874.82 | 2796.121 | 1993.696 | 2260.474 | 2402.318 | 1913.282 | 2896.285 | 1704.986 | 2136.057 | 1770.146 | 2015.591 | 887.5191 | 740.0272 | 847.5292 | 1340.855 | 1896.247 |
| CUST_48012_PI429010886 | M78527 | 0.005286 | 18 | 3 | | | | | | 22.3538 | 31.75695 | 25.07677 | 41.96451 | 35.70676 | 46.33994 | 53.56418 | 25.55609 | 37.06438 | 25.82466 | 21.96294 | 31.78356 | 28.91721 | 22.56982 | 26.43705 | 32.02169 | 31.98774 | 38.19149 | 45.12848 | 29.29243 | 28.93983 | 38.28633 | 53.6538 | 61.50815 | 44.34868 | 39.93503 | 53.75795 |
| CUST_36690_PI429010886 | M16551 | 0.005291 | 15 | 12 | | | | | | 208.138 | 196.5334 | 216.2628 | 210.6402 | 202.8821 | 201.1997 | 173.6625 | 176.9973 | 160.1541 | 220.2915 | 187.387 | 176.4634 | 214.9497 | 251.312 | 255.7893 | 278.469 | 190.6724 | 217.1338 | 209.0287 | 244.2054 | 203.1495 | 249.8541 | 169.2316 | 172.2354 | 238.5792 | 213.1195 | 230.1517 |
| CUST_25255_PI429010886 | N52072 | 0.005314 | 21 | 0 | | | | | | 78.31562 | 78.12789 | 64.41721 | 60.72981 | 62.69277 | 71.16635 | 47.99919 | 47.90107 | 50.8348 | 54.30012 | 51.21188 | 60.35512 | 50.30627 | 78.35921 | 64.91729 | 63.25496 | 59.15067 | 83.44238 | 58.35369 | 88.05483 | 63.37918 | 96.38791 | 62.94659 | 60.74611 | 69.46903 | 70.01711 | 68.33792 |
| CUST_1090_PI429010886 | N8459 | 0.005316 | 21 | 12 | 321452529 | hypothetical protein DAPPUDRAFT_305931 [Daphn | Developmental process | Multicellular organismal development | 6.19E-77 | 253.5058 | 292.6086 | 279.8319 | 158.1305 | 184.1183 | 204.1086 | 150.7201 | 223.0851 | 271.2223 | 302.8326 | 178.6581 | 373.896 | 325.8124 | 225.5174 | 306.5332 | 355.9452 | 184.3061 | 239.0183 | 212.9853 | 376.1162 | 233.6523 | 229.4529 | 222.1754 | 229.7242 | 188.2724 | 174.9558 | 168.8237 |
| CUST_6860_PI429010886 | N20696 | 0.005325 | 12 | 9 | | | | | | 13.17788 | 9.06304 | 7.061176 | 25.10042 | 72.40904 | 29.93503 | 86.4248 | 29.38309 | 17.29225 | 5.687945 | 96.36676 | 29.68336 | 11.61773 | 7.379797 | 7.040982 | NA | 193.9691 | 14.29027 | 30.64068 | 69.0849 | 71.83981 | NA | 83.39175 | 128.195 | 4.387938 | 18.70053 | 13.38001 |
| CUST_47548_PI429010886 | M76555 | 0.005328 | 18 | 6 | | | | | | 80.82297 | 121.6056 | 101.7238 | 88.12787 | 84.65687 | 92.00286 | 104.7461 | 102.639 | 129.4911 | 95.08289 | 95.4903 | 118.275 | 110.0751 | 110.8588 | 86.04961 | 95.24605 | 81.78469 | 80.36337 | 93.43338 | 92.88396 | 92.00286 | 99.52485 | 107.7242 | 101.9359 | 117.2732 | 105.2542 | 136.8476 |
| CUST_8739_PI429010886 | N23502 | 0.005339 | 21 | 0 | | | | | | 1763.663 | 2273.444 | 2191.024 | 1745.549 | 1842.625 | 1855.858 | 1139.583 | 1424.855 | 1481.201 | 2054.295 | 970.0099 | 1488.789 | 914.1643 | 1929.904 | 1977.124 | 2986.925 | 1551.584 | 1598.096 | 1509.413 | 1746.637 | 1418.381 | 3509.529 | 2212.175 | 1874.82 | 1497.212 | 1951.832 | 1679.237 |
| CUST_36725_PI429010886 | M16664 | 0.005343 | 12 | 0 | | | | | | 375.4995 | 957.8471 | 679.3339 | 236.856 | 142.7087 | 119.5972 | 373.4486 | 622.024 | 982.4374 | 521.963 | 278.3016 | 882.8865 | 1010.24 | 1103.397 | 626.2648 | 247.6825 | 526.6917 | 154.1768 | 544.3999 | 649.1033 | 454.9277 | 382.9442 | 762.5883 | 196.5633 | 748.4841 | 607.7896 | 944.3898 |
| CUST_45781_PI429010886 | M70273 | 0.005343 | 12 | 3 | | | | | | 130.5455 | 146.4651 | 120.0167 | 198.5726 | 185.6237 | 252.0389 | 134.02 | 163.8724 | 163.6107 | 100.0068 | 136.023 | 157.8626 | 106.4846 | 143.0236 | 145.6647 | 176.2423 | 169.6125 | 242.8033 | 181.8837 | 140.8068 | 127.0483 | 153.4178 | 110.3267 | 182.0483 | 134.4739 | 166.9208 | 117.7722 |
| CUST_13398_PI429010886 | N31112 | 0.005343 | 12 | 3 | 345489519 | PREDICTED: long-chain-fatty-acid--CoA ligase 4-like | Metabolic process | Other | 3.14E-48 | 143.2115 | 120.0167 | 166.8689 | 168.4747 | 236.0508 | 407.9548 | 265.532 | 158.7326 | 109.7974 | 158.2913 | 197.7545 | 122.9711 | 111.7621 | 147.2771 | 133.2442 | 164.0384 | 303.854 | 243.2097 | 234.1059 | 113.5368 | 229.9564 | 182.0483 | 172.082 | 178.0648 | 161.7557 | 106.6656 | 145.8124 |
| CUST_27655_PI429010886 | N56454 | 0.005343 | 12 | 0 | | | | | | 78.41704 | 68.42403 | 82.72689 | 62.44095 | 66.08238 | 65.62571 | 80.91461 | 55.18575 | 69.69491 | 54.16396 | 52.66699 | 56.9818 | 102.1333 | 105.8718 | 108.2888 | 76.05543 | 59.6055 | 51.90291 | 116.4869 | 63.78913 | 68.25944 | 71.78694 | 34.83322 | 60.5193 | 82.20584 | 72.77354 | 93.6298 |
| CUST_26977_PI429010886 | N55211 | 0.005344 | 12 | 0 | 307190527 | F-box/LRR-repeat protein 7 [Camponotus floridanus | | | 1.21E-12 | 100.1395 | 108.8877 | 121.8737 | 82.9954 | 74.48084 | 78.22974 | 63.88437 | 65.20347 | 79.5827 | 96.24145 | 74.90972 | 102.1576 | 97.30444 | 110.0751 | 82.69106 | 141.7869 | 66.23254 | 96.80182 | 74.28436 | 93.03197 | 89.48953 | 97.78298 | 98.96023 | 83.8218 | 104.976 | 83.83314 | 98.26203 |
| CUST_22647_PI429010886 | N47395 | 0.005356 | 24 | 21 | | | | | | 33.28531 | 49.67248 | 68.17174 | 39.41947 | 32.19156 | 74.85405 | 49.73501 | 57.71214 | 28.22595 | 61.40092 | 27.00004 | 60.08391 | 45.28288 | 68.22048 | 60.80176 | 89.54002 | 158.1305 | 120.1521 | 160.531 | 35.30492 | 96.9668 | 110.8588 | 281.2963 | 83.85047 | 110.9853 | 116.9082 | 255.7893 |
| CUST_53887_PI429010886 | M103570 | 0.005358 | 12 | 9 | | | | | | 21.05098 | 18.29194 | 24.63631 | 12.96066 | 13.30659 | 18.47407 | 23.33698 | 18.3679 | 21.12158 | 26.99665 | 22.84941 | 19.21762 | 17.44175 | 21.68228 | 16.93182 | 20.87638 | 20.1154 | 17.05678 | 19.08628 | 18.19605 | 20.70543 | 29.25886 | 21.9738 | 24.81825 | 27.02172 | 17.4379 | 24.50817 |
| CUST_30852_PI429010886 | M3 | 0.005359 | 15 | 12 | 32450591 | Xenopus laevis similar to histone deacetylase 2, mR | Nucleic acid metabolism | Regulation of gene expression, epigenetic | | 439.659 | 566.0346 | 495.3471 | 334.2433 | 361.8439 | 307.7804 | 327.2675 | 385.0122 | 459.6219 | 531.6819 | 378.0737 | 544.2536 | 512.6993 | 597.1763 | 476.472 | 554.2309 | 402.5662 | 554.3622 | 368.0062 | 472.8988 | 445.5129 | 483.0972 | 460.6363 | 366.2536 | 613.0465 | 417.9411 | 623.5116 |
| CUST_49965_PI429010886 | M87971 | 0.005364 | 21 | 0 | | | | | | 55.13187 | 68.85144 | 50.98504 | 38.91955 | 56.52083 | 44.1892 | 30.92757 | 41.21277 | 32.43294 | 49.91173 | 54.34709 | 49.78435 | 39.52535 | 44.73069 | 23.75572 | 36.05134 | 40.56158 | 48.91209 | 44.70933 | 72.04379 | 67.03346 | 63.29578 | 52.24124 | 52.59799 | 51.83879 | 54.92635 | 57.78122 |
| CUST_50871_PI429010886 | M91289 | 0.005375 | 21 | 18 | | | | | | 150.5005 | 265.2639 | 202.9175 | 169.3396 | 133.7417 | 62.18923 | 14.02096 | 39.05895 | 36.30339 | 165.1538 | 107.4618 | 187.0999 | 117.0501 | 254.955 | 164.5164 | 190.9766 | 26.71388 | 75.45906 | 132.109 | 294.1484 | 316.6467 | 203.0622 | 77.98525 | 113.6252 | 71.33949 | 227.4855 | 94.13978 |
| CUST_18593_PI429010886 | N40072 | 0.005383 | 24 | 21 | | | | | | 28.49131 | 22.66333 | 27.37719 | 17.98635 | 27.27727 | 12.71046 | 6.679809 | 18.18793 | 17.29417 | 19.5463 | 19.10174 | 15.1109 | 29.56366 | 31.69618 | 11.95482 | 34.59277 | 20.75737 | 43.18074 | 33.14087 | 24.42075 | 24.62111 | 19.70849 | 29.1916 | 94.25565 | 33.5333 | 17.30092 | 29.20693 |
| CUST_13495_PI429010886 | N31285 | 0.00539 | 24 | 6 | | | | | | 9.132253 | 5.72681 | 7.323042 | 35.57881 | 17.3695 | 27.62121 | 201.294 | 10.40723 | 138.4518 | NA | 10.17199 | NA | 65.92191 | 6.398941 | 42.52496 | 21.30938 | 51.6678 | 52.96132 | 5.420637 | NA | 3.83684 | 3.62632 | NA | NA | 16.17384 | 79.31367 | 4.766047 |
| CUST_4740_PI429010886 | N17382 | 0.005403 | 21 | 3 | | | | | | 62.99128 | 107.638 | 71.28268 | 148.7285 | 74.3404 | 97.4551 | 70.89744 | 79.44802 | 80.77462 | 83.74259 | 43.46213 | 87.73688 | 54.30012 | 73.18236 | 60.88717 | 72.5683 | 47.66523 | 64.24619 | 56.36899 | 81.34562 | 89.88488 | 74.63865 | 86.94867 | 68.35087 | 111.8008 | 97.38686 | 73.76113 |
| CUST_34447_PI429010886 | M9701 | 0.005407 | 18 | 3 | | | | | | 13.66936 | 14.9512 | 8.113982 | 22.63247 | 19.88004 | 13.33386 | 16.61483 | 12.92974 | 9.754482 | 10.67318 | 7.125214 | NA | 4.263995 | 12.14778 | 9.174525 | 10.32397 | 8.051681 | NA | 12.40735 | 4.003908 | 7.370996 | 16.21551 | NA | 13.43544 | 9.289553 | NA | NA |
| CUST_39063_PI429010886 | M34090 | 0.005413 | 15 | 9 | | | | | | 686.3511 | 863.6121 | 1140.062 | 920.3504 | 1178.063 | 933.1038 | 901.1709 | 2563.253 | 1970.653 | 1269.774 | 2913.591 | 3384.084 | 2336.801 | 1445.235 | 1161.62 | 828.5874 | 1302.682 | 857.5648 | 860.82 | 2328.414 | 1040.9 | 1027.997 | 1109.944 | 1067.963 | 1181.488 | 1047.078 | 2155.605 |
| CUST_24820_PI429010886 | N51300 | 0.005417 | 21 | 6 | 383856504 | PREDICTED: actin-related protein 2-like isoform 1 [M | Cellular component organization | Cytoskeleton organization | 4.57E-52 | 14.97661 | 17.2249 | 18.19015 | 14.82365 | 13.68168 | 14.59227 | 20.16852 | 15.14331 | 18.29602 | 8.553452 | 10.33138 | 15.74925 | 11.48274 | 4.683701 | 6.990585 | 9.389836 | 8.674882 | 8.10145 | 14.99759 | 11.67368 | 5.791904 | 10.45833 | 10.86868 | 22.3788 | 14.29202 | 11.28356 | 20.67135 |

| Probe ID | ID | p-value | Period | Phase | EBI ID | Annotation | GO category | Go term | E-value | 0 | 0 | 0 | 3 | 3 | 3 | 6 | 6 | 6 | 9 | 9 | 9 | 12 | 12 | 12 | 15 | 15 | 15 | 18 | 18 | 18 | 21 | 21 | 21 | 24 | 24 | 24 |
|---|---|---|---|---|---|---|---|---|---|---|---|---|---|---|---|---|---|---|---|---|---|---|---|---|---|---|---|---|---|---|---|---|---|---|---|---|
| CUST_26285_PI429010886 | N53982 | 0.005421 | 24 | 21 | | | | | | 122.2551 | 143.2327 | 173.4253 | 121.2116 | 149.3076 | 126.2074 | 118.9612 | 117.3776 | 119.4919 | 133.4505 | 113.2243 | 167.4333 | 112.8438 | 204.3548 | 149.2677 | 136.3105 | 124.2291 | 127.916 | 164.2007 | 144.1866 | 148.6709 | 163.1226 | 149.3268 | 172.5998 | 170.7553 | 137.7352 | 152.9004 |
| CUST_22368_PI429010886 | N46907 | 0.005426 | 21 | 15 | | | | | | 25.50611 | 25.10548 | 25.2086 | 23.40412 | 30.03779 | 29.56124 | 24.02788 | 26.6361 | 16.57372 | 29.28327 | 25.77304 | 22.24073 | 17.99464 | 41.96064 | 28.37201 | 45.57502 | 38.74854 | 37.71174 | 37.59059 | 28.51585 | 33.23976 | 33.71856 | 25.89716 | 21.28517 | 25.06956 | 22.31012 | 36.29351 |
| CUST_37962_PI429010886 | M22486 | 0.005426 | 15 | 12 | | | | | | 40.81576 | 165.0799 | 267.6598 | 122.3609 | 63.45518 | 16.9342 | 467.9877 | 271.8242 | 783.9719 | 466.1947 | 156.3501 | 227.9715 | 2048.598 | 2343.419 | 1326.638 | 1099.991 | 860.1543 | 246.3068 | 221.4446 | 435.9034 | 202.4996 | 469.28 | 785.0368 | 109.3629 | 3835.814 | 224.6063 | 4651.575 |
| CUST_21360_PI429010886 | N45078 | 0.005436 | 15 | 9 | | | | | | 20.60205 | 24.04991 | 21.69445 | 11.43263 | 53.72529 | 14.90751 | 18.09887 | 37.22147 | 33.74722 | 22.91836 | 55.09927 | 16.80437 | 20.3649 | 33.24682 | 28.55766 | 30.25328 | 19.48105 | 22.18811 | 9.396038 | 21.41219 | 12.76972 | 19.14986 | 16.27254 | 15.93573 | 57.00393 | 76.27537 | 83.20205 |
| CUST_56638_PI429010886 | M114273 | 0.005439 | 18 | 15 | | | | | | 244.4592 | 117.5373 | 250.0775 | 50.64584 | 247.5396 | 172.0275 | 295.9544 | 221.165 | 184.3634 | 236.0839 | 174.6176 | 217.843 | 333.5081 | 317.0842 | 193.4964 | 246.8318 | 327.7237 | 339.0413 | 172.5998 | 232.8922 | 159.5474 | 231.8976 | 112.9777 | 92.05581 | 99.38154 | 176.7991 | 241.0332 |
| CUST_2798_PI429010886 | N13131 | 0.005439 | 18 | 9 | 443725752 | hypothetical protein CAPTEDRAFT_149382 [Capitell | Metabolic process | Carbohydrate metabolic process | 5.5E-179 | 429.9553 | 637.7954 | 690.0315 | 568.0071 | 449.7775 | 289.8985 | 347.794 | 811.5226 | 675.5498 | 768.7795 | 695.1115 | 1106.942 | 702.9226 | 793.9994 | 577.33 | 735.196 | 465.6558 | 534.8364 | 423.9576 | 1328.909 | 678.3407 | 574.7836 | 622.1973 | 414.2198 | 635.9764 | 606.9446 | 1001.62 |
| CUST_14840_PI429010886 | N33557 | 0.005439 | 18 | 9 | 321458928 | hypothetical protein DAPPUDRAFT_202549 [Daphn | | | 1.28E-47 | 32.26619 | 33.1843 | 53.46055 | 45.58985 | 40.05116 | 36.5984 | 36.87977 | 47.99065 | 83.85047 | 79.0521 | 73.63983 | 65.6996 | 32.09117 | 70.81749 | 64.41721 | 40.23032 | 40.28418 | 53.69723 | 48.8316 | 41.6388 | 37.13071 | 46.1565 | 49.26562 | 26.62548 | 63.91986 | 50.3553 | 54.83755 |
| CUST_25585_PI429010886 | N52690 | 0.005439 | 18 | 9 | | | | | | 23.53568 | 35.81064 | 25.74156 | 17.64263 | 19.64586 | 21.01435 | 25.9425 | 42.43356 | 37.52322 | 27.87322 | 49.62512 | 54.95061 | 41.54682 | 30.3611 | 39.00975 | 31.02312 | 20.34669 | 14.82853 | 24.17719 | 81.5369 | 33.22506 | 8.800802 | 25.13331 | 22.81875 | 12.75297 | 31.67438 | 43.94258 |
| CUST_20677_PI429010886 | N43865 | 0.005452 | 24 | 9 | 310743898 | glycogen synthase [Marsupenaeus japonicus] | Metabolic process | Carbohydrate metabolic process | 2.81E-50 | 44.06771 | 29.0365 | 39.50511 | 30.34438 | 35.19889 | 17.6292 | 36.2246 | 71.28268 | 39.45879 | 61.93065 | 56.88497 | 57.49879 | 41.34656 | 32.65296 | 28.24659 | 23.81072 | 37.07935 | 25.27468 | 22.96573 | 49.34559 | 29.44539 | 29.51147 | 26.10687 | 19.50921 | 42.20313 | 27.75482 | 28.07255 |
| CUST_13017_PI429010886 | N30444 | 0.005474 | 21 | 18 | | | | | | 64.35394 | 66.09773 | 46.16276 | 56.17738 | 70.57795 | 41.05122 | 42.03557 | 33.51452 | 50.51683 | 72.55972 | 46.8066 | 65.89913 | 52.30747 | 59.48243 | 65.11259 | 70.12587 | 40.06113 | 55.93797 | 73.13314 | 74.14418 | 66.62755 | 107.1033 | 57.26217 | 58.49978 | 70.27659 | 45.98571 | 55.76859 |
| CUST_45584_PI429010886 | M69580 | 0.005482 | 21 | 15 | | | | | | 15.25972 | 13.60583 | 19.69401 | 13.64087 | 13.54808 | 14.5578 | 21.57533 | 13.4573 | 5.314464 | 13.47393 | 19.12864 | 16.16705 | 13.03814 | 14.14304 | 19.26372 | 22.36394 | 22.45323 | 16.89973 | 16.32095 | 21.34103 | 15.87666 | 15.18342 | 15.47031 | 21.96895 | 17.12595 | 12.71188 | 6.493972 |
| CUST_43061_PI429010886 | M61965 | 0.005483 | 15 | 6 | | | | | | 12.00666 | 11.28527 | 20.03907 | 6.053093 | 40.00557 | 19.2347 | 47.82652 | 53.81515 | 31.09423 | 49.69905 | 13.2768 | 13.072 | 21.88139 | NA | 5.271099 | 12.35209 | 45.09974 | 17.10625 | 50.039 | 23.30725 | 19.07608 | 26.57549 | 62.72678 | 53.14405 | 30.79653 | NA | 14.04854 |
| CUST_43917_PI429010886 | M64322 | 0.005484 | 24 | 0 | | | | | | 8.992487 | 9.664283 | NA | NA | 7.967454 | NA | NA | 8.769916 | 5.231414 | 6.064884 | 4.127474 | 20.07855 | 5.140846 | 4.017798 | 5.661008 | 7.20194 | 9.329547 | 4.813825 | 11.17076 | 5.079117 | 5.946298 | 14.12974 | NA | 8.937423 | 15.26731 | 10.65706 | 10.29908 |
| CUST_49167_PI429010886 | M84484 | 0.005486 | 24 | 21 | | | | | | 62.33854 | 61.86952 | 96.67753 | 49.10837 | 70.47227 | 61.05172 | 55.1911 | 63.7264 | 51.04482 | 67.79099 | 64.75173 | 70.65347 | 62.76739 | 69.28034 | 65.49916 | 74.42211 | 67.41072 | 60.82114 | 52.05083 | 72.44795 | 67.39787 | 77.92872 | 76.72395 | 81.64235 | 73.98788 | 74.74764 | 77.89081 |
| CUST_810_PI429010886 | N7379 | 0.005486 | 24 | 18 | | | | | | 16.31911 | 11.22699 | 10.28317 | 6.235994 | 20.90297 | 13.58792 | 8.64523 | 32.84186 | 18.26586 | 32.47936 | 27.32872 | 17.01109 | 21.10225 | 14.89599 | 11.13027 | 31.92608 | 36.65626 | 17.75499 | 40.59908 | 28.13722 | 45.52189 | 21.12887 | 27.29633 | 20.59519 | 21.72859 | 19.14986 | 19.17708 |
| CUST_12345_PI429010886 | N29322 | 0.00549 | 18 | 12 | | | | | | 25.18232 | 38.41593 | 68.20531 | 34.03541 | 32.20238 | 35.82698 | 26.89938 | 61.8328 | 30.50923 | 43.82673 | 33.09246 | 50.91495 | 55.92013 | 66.54333 | 75.49979 | 62.62665 | 40.95384 | 64.14878 | 74.88917 | 74.08523 | 46.72383 | 24.77312 | 50.41901 | 21.60382 | 46.16699 | 39.36991 | 41.06525 |
| CUST_57290_PI429010886 | E4431 | 0.005493 | 12 | 9 | 493564239 | hypothetical protein [Leptolyngbya sp. PCC 7375] > | | | 1.35E-11 | 20.27043 | 16.91691 | 11.8505 | 6.045321 | 5.372564 | NA | 11.17351 | 9.360222 | 7.862605 | 15.8263 | NA | 27.03844 | 25.58851 | 16.39244 | 26.62548 | 6.759208 | 4.908172 | 6.906185 | 18.07527 | 18.2356 | 5.83676 | 28.34529 | NA | 10.68951 | NA | 16.77252 | 27.84094 |
| CUST_4030_PI429010886 | N15847 | 0.005494 | 18 | 15 | | | | | | 54.6573 | 68.49231 | 51.30066 | 24.62356 | 42.08627 | 41.7957 | 47.39715 | 46.41206 | 21.3985 | 51.51637 | 53.00673 | 50.277 | 59.28015 | 56.15273 | 62.45053 | 63.06213 | 52.20745 | 63.1929 | 48.9688 | 61.74634 | 49.86955 | 52.97579 | 41.44605 | 52.17878 | 83.25716 | 44.00542 | 72.71237 |
| CUST_30887_PI429010886 | M115 | 0.005494 | 15 | 12 | 321479130 | hypothetical protein DAPPUDRAFT_186952 [Daphn | | | 3.24E-86 | 57.57631 | 63.85255 | 45.79823 | 40.95098 | 50.45571 | 30.71736 | 37.83427 | 34.64486 | 36.71638 | 73.01018 | 53.21879 | 55.28753 | 45.91191 | 60.07749 | 67.82256 | 74.59866 | 55.11221 | 55.66641 | 38.58794 | 58.97911 | 41.46468 | 48.90708 | 53.84406 | 38.50578 | 68.5761 | 63.32755 | 66.18105 |
| CUST_55302_PI429010886 | M109493 | 0.005524 | 24 | 18 | | | | | | 790.3406 | 937.3911 | 1003.294 | 716.5268 | 771.6454 | 834.6224 | 699.0502 | 714.5213 | 833.5719 | 889.0555 | 817.1902 | 1153.433 | 820.2371 | 1045.476 | 1021.127 | 1089.985 | 878.2313 | 828.8539 | 914.3958 | 1088.645 | 1007.936 | 1033.021 | 983.8663 | 862.6591 | 921.954 | 850.2192 | 1102.275 |
| CUST_41321_PI429010886 | M57448 | 0.005543 | 21 | 3 | | | | | | 2404.795 | 4388.254 | 2829.871 | 6571.189 | 3597.224 | 3405.484 | 2538.071 | 1994.557 | 2007.972 | 3362.672 | 1726.714 | 3459.777 | 2009.101 | 2911.974 | 1307.203 | 2487.623 | 1495.765 | 2143.344 | 1495.765 | 2076.382 | 3034.646 | 2863.818 | 4051.321 | 1977.87 | 4361.48 | 3590.082 | 2610.308 |
| CUST_15067_PI429010886 | N33956 | 0.005552 | 24 | 21 | | | | | | 112.9528 | 98.43882 | 110.4732 | 106.1516 | 106.3744 | 108.5223 | 81.78469 | 95.78605 | 102.2996 | 135.4209 | 104.7962 | 127.2574 | 87.32671 | 132.7602 | 109.9853 | 136.8231 | 107.073 | 135.4209 | 105.6225 | 115.837 | 135.7103 | 149.6657 | 134.244 | 114.917 | 147.5412 | 122.8849 | 146.2438 |
| CUST_54060_PI429010886 | M104323 | 0.005556 | 24 | 0 | | | | | | 68.25944 | 77.64933 | 52.71843 | 21.55583 | 68.72963 | 48.57594 | 56.11599 | 25.59863 | 56.20414 | 50.92195 | 46.24589 | 53.55772 | 40.35674 | 51.00123 | 13.46781 | 50.04478 | 44.43928 | 59.6416 | 40.79309 | 40.72449 | 75.88452 | 41.75433 | 67.78689 | 62.48029 | 90.29804 | 58.12189 | 67.81299 |
| CUST_37912_PI429010886 | M22216 | 0.005562 | 15 | 12 | | | | | | 3921.02 | 963.6143 | 35686.35 | 62.29412 | 140.2062 | 79.21992 | 8147.956 | 65872.24 | 41577.33 | 53582.06 | 1326.638 | 2937.772 | 44666.31 | 71255.08 | 37163.2 | 791.8244 | 30290.41 | 352.6729 | 2304.824 | 3208.657 | 1046.312 | 19200.93 | 48876.8 | 1208.768 | 58715.07 | 2075.258 | 52133.5 |
| CUST_35407_PI429010886 | M12509 | 0.005568 | 18 | 12 | | | | | | 146.8141 | 148.08 | 157.5747 | 115.6518 | 137.1056 | 124.4818 | 134.577 | 116.8442 | 144.7064 | 140.6181 | 150.9847 | 173.7958 | 145.1035 | 170.6697 | 181.0372 | 227.8652 | 143.5925 | 163.6601 | 144.8143 | 158.8004 | 102.7622 | 178.9173 | 84.1293 | 108.3734 | 165.5902 | 123.0876 | 134.282 |
| CUST_7287_PI429010886 | N21321 | 0.005579 | 21 | 15 | | | | | | 311.8189 | 247.4599 | 364.8884 | 239.571 | 256.9966 | 281.4899 | 237.506 | 264.9246 | 200.4624 | 303.9241 | 235.8718 | 309.8242 | 223.377 | 242.302 | 283.788 | 322.7948 | 306.4375 | 323.8163 | 297.5859 | 273.8764 | 295.7576 | 268.588 | 291.5938 | 277.9328 | 244.5842 | 291.2799 | 319.6424 |
| CUST_37444_PI429010886 | M19054 | 0.00558 | 15 | 9 | 194207957 | PREDICTED: mRNA turnover protein 4 homolog [Eq | Protein metabolism | Ribosome biogenesis | 4.16E-74 | 248.5974 | 418.3911 | 270.5782 | 212.4692 | 256.0552 | 162.5301 | 239.6937 | 273.9149 | 295.4403 | 502.4781 | 278.397 | 498.2267 | 373.7568 | 406.244 | 332.1138 | 358.1231 | 267.3316 | 243.0834 | 251.0973 | 351.0419 | 266.0871 | 419.7481 | 285.2001 | 180.5686 | 333.3682 | 287.5857 | 446.3108 |
| CUST_47930_PI429010886 | M78209 | 0.00558 | 21 | 3 | | | | | | 1510.415 | 3254.31 | 1930.669 | 5447.86 | 2382.34 | 2323.132 | 1623.972 | 1433.821 | 1614.376 | 2469.728 | 924.2614 | 2208.121 | 1216.535 | 1305.146 | 229.245 | 1899.44 | 653.4911 | 1101.197 | 681.8773 | 1012.516 | 2608.992 | 1954.96 | 2539.516 | 1294.143 | 3051.185 | 2624.92 | 1069.858 |
| CUST_40830_PI429010886 | M56187 | 0.005585 | 15 | 9 | | | | | | 32.46164 | 28.82038 | 53.43689 | 22.49442 | 81.66432 | 38.11258 | 45.29693 | 88.47454 | 70.7847 | 141.5103 | 113.9625 | 74.90972 | 55.54708 | 125.9625 | 84.08227 | 35.69319 | 79.73386 | 42.54782 | 110.7081 | 60.38361 | 54.56998 | 135.7577 | 107.3414 | 21.11367 | 144.5986 | 61.6776 | 112.3515 |
| CUST_36693_PI429010886 | M16563 | 0.005593 | 24 | 12 | | | | | | 19.61727 | 18.07527 | 17.73598 | 15.75243 | 18.99531 | 13.68485 | 10.95916 | 10.78602 | 26.55213 | 18.20852 | 14.89445 | 23.71956 | 22.04079 | 30.03204 | 26.72979 | 25.76654 | 28.46316 | 21.64092 | 23.76609 | 34.13499 | 19.65423 | 19.40005 | 18.91531 | 15.29468 | 31.40553 | 20.48326 | 14.70218 |
| CUST_3017_PI429010886 | N13609 | 0.005616 | 18 | 3 | | | | | | 7.215021 | NA | 16.53993 | 11.44906 | 11.77858 | 15.83311 | 8.874168 | 7.45522 | 8.429067 | 5.626214 | 9.979521 | 6.450449 | 4.463363 | 9.400307 | 4.965226 | 4.801968 | 15.16412 | 6.528004 | 10.11181 | NA | 13.26521 | 9.184475 | 9.486543 | 17.31232 | 11.3111 | NA | 10.37417 |
| CUST_34033_PI429010886 | M8559 | 0.005617 | 24 | 21 | | | | | | 20.17321 | 8.29527 | 15.28107 | 19.99274 | 16.39504 | 19.88268 | 12.30417 | 6.976589 | 13.89131 | 16.7895 | 14.18775 | 18.24494 | 22.43724 | 16.17931 | 15.95746 | 29.64209 | 16.91528 | 39.81638 | 32.98635 | 20.12103 | 15.42332 | 19.72128 | 28.48875 | 29.41742 | 27.68104 | 28.61965 | 48.09585 |
| CUST_4941_PI429010886 | N17853 | 0.005617 | 24 | 3 | | | | | | 49.18958 | 65.43441 | 65.61755 | 67.34873 | 56.12021 | 70.7847 | 55.5381 | 54.26995 | 55.88861 | 65.96017 | 46.62471 | 55.63288 | 50.93524 | 51.11368 | 62.09543 | 70.7166 | 52.30377 | 43.42171 | 42.12286 | 45.54446 | 45.26245 | 65.06553 | 43.5925 | 58.91707 | 57.55495 | 83.92582 | 52.11537 |
| CUST_40002_PI429010886 | M48221 | 0.00563 | 15 | 12 | | | | | | 16734.53 | 7938.371 | 24841.44 | 651.5039 | 1744.83 | 818.6448 | 13400.98 | 28866.67 | 32231.42 | 38947.83 | 12715.79 | 10610.68 | 31148.55 | 39731.64 | 24244.07 | 6694.984 | 20510.07 | 3526.312 | 11454.19 | 19120.04 | 7137.019 | 30082.79 | 23706.31 | 3968.239 | 50862.95 | 9207.498 | 38496.63 |
| CUST_51281_PI429010886 | M92921 | 0.00563 | 15 | 3 | | | | | | 1513.56 | 1426.735 | 1017.693 | 1509.413 | 1774.043 | 1888.53 | 1588.949 | 1282.355 | 1063.907 | 831.7121 | 1504.846 | 954.6687 | 1100.556 | 951.2956 | 902.5128 | 1548.599 | 1567.155 | 1336.322 | 1803.696 | 1170.459 | 1167.349 | 1223.156 | 1085.118 | 1382.151 | 1113.695 | 1261.977 | 920.9532 |
| CUST_1344_PI429010886 | N9348 | 0.005645 | 15 | 9 | | | | | | 433.2734 | 536.8972 | 603.0908 | 321.9594 | 455.9675 | 420.9194 | 419.6576 | 402.5662 | 502.1624 | 920.0256 | 506.7573 | 684.8725 | 451.4433 | 625.9634 | 522.1156 | 567.3609 | 348.178 | 470.9015 | 464.9946 | 547.2331 | 492.7259 | 742.2219 | 571.094 | 600.1331 | 1070.988 | 546.2052 | 986.4774 |
| CUST_43798_PI429010886 | M64005 | 0.005649 | 21 | 12 | | | | | | 9.871686 | 15.99431 | 11.04144 | 10.22956 | 6.279736 | NA | 13.86613 | 7.475526 | 14.85609 | 13.63953 | 8.992487 | 8.850436 | 23.1075 | 16.50418 | 14.13803 | 12.59547 | 20.22864 | 11.83887 | 4.219514 | NA | NA | 13.35156 | 12.10865 | 11.0938 | 8.204634 | 8.031655 | 10.65431 |
| CUST_52819_PI429010886 | M99080 | 0.005656 | 24 | 0 | | | | | | 130.2217 | 155.6736 | 124.1092 | 102.3756 | 66.33339 | 84.88556 | 96.8323 | 98.82662 | 82.97709 | 95.45887 | 90.75806 | 142.9849 | 107.8082 | 61.72335 | 92.46177 | 105.2803 | 92.79213 | 116.8442 | 105.5819 | 113.7768 | 128.6574 | 125.2116 | 118.6493 | 107.0416 | 102.2299 | 152.988 | 140.6816 |
| CUST_35398_PI429010886 | M12477 | 0.005665 | 18 | 0 | | | | | | 43.20136 | 63.49666 | 63.03943 | 53.50545 | 25.79784 | 23.84339 | 29.41419 | 23.88883 | 26.60252 | 27.56643 | 37.14508 | 33.69477 | 35.84618 | 55.5381 | 34.08252 | 44.74185 | 27.32583 | 32.18557 | 60.49989 | 40.33441 | 57.55495 | 51.47065 | 22.42981 | 40.17403 | 47.36571 | 32.83588 | 24.73831 |
| CUST_21916_PI429010886 | N46093 | 0.005669 | 24 | 21 | | | | | | 19.10728 | 7.690783 | 12.43137 | NA | 7.542588 | 8.21709 | 9.720983 | 7.528845 | 14.20045 | 16.26104 | 10.32521 | 14.93407 | 9.339132 | 14.25604 | 11.22153 | 16.58665 | 9.605462 | 12.47217 | 73.00251 | 16.69153 | NA | 20.66168 | 21.03821 | 20.07209 | 25.28525 | 7.681885 | 13.67832 |
| CUST_15832_PI429010886 | N35217 | 0.005678 | 15 | 9 | | | | | | 23.91343 | 27.74608 | 28.05064 | 15.32478 | 29.09314 | 23.75346 | 21.3486 | 28.9565 | 33.36125 | 53.73015 | 36.54259 | 41.27857 | 68.63241 | 37.81392 | 29.58077 | 28.45961 | 17.92364 | 31.22335 | 16.98462 | 47.01326 | 32.47618 | 38.31739 | 34.38812 | 21.29581 | 33.37016 | 39.68929 | 56.64379 |
| CUST_11838_PI429010886 | N28496 | 0.005685 | 21 | 12 | | | | | | 198.7421 | 187.9811 | 199.9107 | 119.5328 | 142.5243 | 157.378 | 139.0764 | 144.1452 | 150.8429 | 161.1553 | 160.0867 | 193.7403 | 168.3796 | 296.3588 | 250.036 | 217.2265 | 168.1071 | 207.0153 | 165.1538 | 192.8746 | 180.162 | 204.1442 | 190.201 | 172.9267 | 205.1968 | 162.9163 | 190.4443 |
| CUST_29265_PI429010886 | N59510 | 0.005698 | 15 | 12 | | | | | | 119.6887 | 147.523 | 125.0002 | 83.00511 | 76.00655 | 119.585 | 79.38753 | 78.48723 | 93.04202 | 133.9706 | 115.202 | 155.0892 | 105.5931 | 132.8952 | 139.0616 | 128.4068 | 102.7268 | 128.9553 | 126.6371 | 110.4575 | 134.4594 | 110.3002 | 109.4779 | 71.73214 | 120.3338 | 139.545 | 126.9841 |
| CUST_42692_PI429010886 | M60985 | 0.005705 | 21 | 0 | | | | | | 44.42212 | 59.75541 | 40.9306 | 34.56663 | 42.6624 | 55.19772 | 32.858 | 28.97255 | 20.19927 | 24.91029 | 26.51217 | 48.6393 | 39.27986 | 40.82323 | 43.51237 | 50.30627 | 28.40654 | 54.4284 | 43.85239 | 51.76265 | 30.72417 | 53.02941 | 41.71124 | 55.81208 | 33.45023 | 47.84187 | 54.86156 |
| CUST_15005_PI429010886 | N33847 | 0.005721 | 18 | 3 | | | | | | 79.70393 | 54.24943 | 46.0983 | 54.66133 | 66.73255 | 66.91698 | 33.61051 | 22.07292 | 20.0846 | 14.98047 | 16.29403 | 16.08792 | 38.60384 | 37.53618 | 51.38396 | 49.75939 | 9.606473 | 65.37264 | 12.02167 | 39.09942 | 27.15737 | 72.23965 | 24.26504 | 50.41901 | 38.29585 | 16.80437 | 27.96355 |
| CUST_33155_PI429010886 | M6122 | 0.005723 | 15 | 9 | 54042615 | arthrodial cuticle protein AMP6.0 [Callinectes sapid | | | 1.9E-13 | 94.19343 | 280.0237 | 287.9804 | 230.7517 | 142.2748 | 279.7821 | 637.0757 | 531.5557 | 825.4395 | 1044.227 | 423.8598 | 630.254 | 194.6975 | 867.8685 | 404.8823 | 145.861 | 127.7733 | 162.8499 | 306.9757 | 1056.312 | 684.7245 | 195.1331 | 666.8781 | 133.4052 | 1025.99 | 422.8357 | 1563.475 |
| CUST_12015_PI429010886 | N28780 | 0.005728 | 21 | 0 | | | | | | 37.91955 | 46.0983 | 36.98139 | 17.7146 | 28.2598 | 29.45836 | 24.68523 | 17.20064 | 29.46496 | 22.79184 | 32.15505 | 34.50298 | 22.30272 | 31.07874 | 28.99536 | 44.42567 | 23.6747 | 43.70884 | 32.79034 | 37.09113 | 35.82359 | 34.37569 | 39.33849 | 35.48457 | 38.6481 | 15.15241 | 32.55113 |
| CUST_639_PI429010886 | N6489 | 0.005731 | 15 | 12 | | | | | | 384.1514 | 758.8197 | 454.9277 | 299.285 | 470.9015 | 127.0833 | 901.9536 | 592.9907 | 1777.79 | 1209.365 | 636.5467 | 1457.169 | 2328.414 | 2754.789 | 2027.433 | 2271.87 | 2328.414 | 768.7795 | 950.8038 | 814.2205 | 1096.765 | 1678.528 | 1265.474 | 1333.908 | 4882.08 | 1140.062 | 6750.846 |
| CUST_41057_PI429010886 | M56778 | 0.005739 | 18 | 6 | 345488671 | PREDICTED: proclotting enzyme [Nasonia vitripenni | Protein metabolism | Proteolysis | 4.82E-29 | 18.85349 | 20.12612 | 17.71709 | 38.0116 | 16.24458 | 15.15645 | 41.9189 | 27.94276 | 54.26995 | 46.14783 | 20.04961 | 24.8123 | 33.16347 | 52.62093 | 26.8663 | 27.55378 | 30.07667 | 23.09604 | 21.8835 | 22.6017 | 16.44381 | 37.15687 | 24.21006 | 24.2954 | 51.71264 | 20.81747 | 43.27178 |
| CUST_29357_PI429010886 | N59703 | 0.005739 | 18 | 0 | | | | | | 101.5527 | 106.2586 | 111.4482 | 101.5112 | 83.85911 | 86.58356 | 70.19365 | 71.01876 | 55.14182 | 82.79408 | 74.66823 | 85.86351 | 68.92299 | 108.6177 | 82.39034 | 103.7436 | 78.97339 | 85.0717 | 77.15161 | 84.62693 | 82.46901 | 84.53623 | 90.20594 | 82.31536 | 81.95659 | 68.09627 | 78.13459 |
| CUST_42831_PI429010886 | M61369 | 0.005748 | 24 | 18 | | | | | | 16.99562 | 15.38835 | 21.25262 | 14.4666 | 5.816875 | 11.98753 | 21.46638 | 11.4368 | 20.46326 | 15.24047 | 19.06154 | 21.51171 | 17.63614 | 19.1562 | 22.20953 | 20.62099 | 21.51773 | 34.21609 | 20.75228 | 48.34221 | 22.64731 | 20.90912 | 10.76813 | 35.75443 | 26.4083 | 39.01912 | 29.08714 |
| CUST_19138_PI429010886 | N41036 | 0.005755 | 12 | 0 | | | | | | 26.8901 | 35.27881 | 28.60108 | 26.43705 | 35.07318 | 35.29256 | 25.75422 | 25.76654 | 20.03408 | 32.43577 | 24.73262 | 34.00967 | 21.41546 | 33.62105 | 45.35228 | 25.34638 | 35.16453 | 29.19814 | 23.42758 | 20.17559 | 15.91395 | 36.34774 | 23.73784 | 30.83339 | 34.35136 | 41.95242 | 53.57478 |
| CUST_3251_PI429010886 | N14108 | 0.005757 | 15 | 0 | | | | | | 20.75541 | 15.4684 | 18.65246 | 38.69306 | 44.63407 | 55.9701 | 29.29243 | 13.60023 | 13.98209 | 9.508624 | 7.032309 | NA | 8.058299 | 11.36093 | 24.0618 | 56.22954 | 35.44133 | 72.49088 | 26.26779 | NA | 6.036326 | 29.42444 | 15.40711 | 35.28945 | 11.2994 | 4.322357 | NA |
| CUST_38971_PI429010886 | M32801 | 0.005766 | 15 | 12 | | | | | | 1293.201 | 774.9868 | 10760.71 | 35.09944 | 52.15419 | 15.90511 | 3001.59 | 16515.38 | 18377.26 | 16480.39 | 4216.888 | 6986.943 | 11313.76 | 33268.08 | 18524.86 | 2032.79 | 8627.68 | 615.066 | 1953.939 | 9855.517 | 665.511 | 25976.81 | 21067.41 | 511.4224 | 25976.81 | 3676.785 | 25871.73 |
| CUST_14848_PI429010886 | N33569 | 0.005766 | 15 | 12 | | | | | | 64.68215 | 89.27715 | 139.9854 | 58.5117 | 58.94211 | 63.16675 | 74.79904 | 140.8407 | 108.8877 | 93.78702 | 78.01974 | 112.5615 | 116.8209 | 151.9691 | 111.1116 | 104.6828 | 85.22504 | 79.46381 | 79.00627 | 80.42722 | 74.84295 | 108.8172 | 116.0529 | 50.90637 | 162.1687 | 63.45518 | 133.8722 |
| CUST_40655_PI429010886 | M55759 | 0.005769 | 18 | 0 | | | | | | 217.9939 | NA | 339.9729 | 25.73774 | 96.89497 | 209.8989 | 124.6243 | 97.93806 | 70.08934 | 32.45148 | 189.0589 | NA | 38.42916 | 47.93942 | 17.55275 | NA | 190.0645 | 96.85587 | 207.1617 | NA | 301.0265 | NA | 81.63335 | 108.0186 | 6.779829 | 40.74811 | 8.589723 |
| CUST_20809_PI429010886 | N44098 | 0.005771 | 18 | 9 | | | | | | 29.70907 | 37.92604 | 29.2332 | 27.72962 | 29.84325 | 22.03875 | 20.72467 | 42.98324 | 49.1739 | 44.48344 | 28.11091 | 44.4641 | 45.81043 | 41.26062 | 45.15073 | 42.21564 | 22.62281 | 22.26173 | 38.82258 | 29.99067 | 40.27597 | 20.76453 | 21.47442 | 13.06649 | 53.12725 | 22.93214 | 49.05418 |
| CUST_23312_PI429010886 | N48617 | 0.005788 | 21 | 15 | | | | | | 6.357899 | 5.58378 | 10.41462 | NA | 9.138723 | 7.750203 | 4.320994 | 5.381309 | 7.184973 | NA | 8.530807 | 6.875138 | 8.974986 | 11.05033 | 5.322036 | 9.890597 | 12.54687 | 10.68382 | 10.8708 | 11.93962 | 7.092466 | 8.464949 | NA | NA | 5.79109 | 7.34732 | 7.552507 |
| CUST_12732_PI429010886 | N29951 | 0.005793 | 12 | 6 | | | | | | NA | 11.56735 | NA | 25.92533 | 15.23856 | 20.44752 | 10.61682 | 29.32561 | 25.56884 | NA | 28.82389 | 15.22654 | 8.793342 | 11.76966 | 11.42268 | 12.83593 | 10.46311 | 13.44053 | 22.24073 | 35.62064 | 31.60035 | 15.22088 | 26.41986 | 21.41385 | 8.037888 | 15.39724 | 20.27391 |
| CUST_7580_PI429010886 | N21757 | 0.005809 | 24 | 21 | | | | | | 29.44853 | 31.82901 | 24.10675 | 24.04426 | 29.38309 | 20.98222 | 28.29582 | 14.38683 | 23.06645 | 27.13598 | 33.58859 | 23.74918 | 15.97144 | 20.92648 | 18.7688 | 35.73798 | 39.43891 | 31.54852 | 20.74032 | 23.63583 | 21.09489 | 38.153 | 34.31018 | 31.65107 | 35.09192 | 31.69267 | 45.98092 |
| CUST_51513_PI429010886 | M93802 | 0.005815 | 24 | 21 | | | | | | 77.42171 | 86.38663 | 79.043 | 92.85168 | 89.09879 | 91.5749 | 83.98715 | 76.674 | 75.01889 | 74.79904 | 66.54333 | 84.376 | 70.68641 | 98.85463 | 67.09564 | 83.90478 | 92.767 | 84.88556 | 62.48649 | 81.19526 | 79.80797 | 101.9474 | 98.53734 | 110.3957 | 94.63457 | 91.04016 | 93.62063 |
| CUST_3456_PI429010886 | N14542 | 0.005815 | 24 | 18 | | | | | | 37.67574 | 52.70775 | 35.16159 | 28.11334 | 26.1905 | 19.99534 | 11.51639 | 32.13072 | 21.13597 | 52.62699 | 26.65768 | 52.1905 | 27.03609 | 44.19983 | 37.04031 | 61.6776 | 27.24907 | 32.21099 | 46.23463 | 53.37885 | 48.86699 | 55.78991 | 22.96573 | 50.40874 | 38.84613 | 33.35026 | 46.98558 |
| CUST_4489_PI429010886 | N16885 | 0.005815 | 24 | 18 | | | | | | 2274.622 | 3416.788 | 2217.377 | 2680.458 | 2003.234 | 1846.987 | 1651.385 | 1358.952 | 1700.08 | 1898.136 | 2153.407 | 4194.154 | 2326.832 | 2226.703 | 3630.71 | 5271.276 | 1410.296 | 2727.828 | 2526.384 | 3759.753 | 2695.193 | 3756.632 | 1990.565 | 2371.653 | 2344.689 | 3303.552 | 2502.493 |
| CUST_33562_PI429010886 | M7282 | 0.005823 | 12 | 6 | | | | | | 142.4348 | 1114.056 | 507.5628 | 308.3369 | 706.7803 | 1022.449 | 566.4251 | 477.9922 | 832.3284 | 535.1571 | 321.2041 | 963.151 | 172.498 | 634.6129 | 286.423 | 635.0709 | 276.5696 | 792.7128 | 1242.051 | 1068.447 | 1023.799 | 822.9643 | 377.1617 | 424.6374 | 127.723 | 279.1338 | 76.71565 |
| CUST_50659_PI429010886 | M90575 | 0.005833 | 15 | 12 | | | | | | 14.23976 | 19.57173 | 23.531 | 19.20978 | 14.77643 | 18.44752 | 19.68628 | 14.11041 | 21.7849 | 18.11915 | 24.72623 | 21.83532 | 36.28805 | 28.41713 | 19.67357 | 23.14173 | 16.5818 | 24.9077 | 19.3883 | 12.38626 | 6.86682 | 22.07569 | 23.04508 | 12.98903 | 33.38032 | 13.13514 | 24.12066 |
| CUST_51717_PI429010886 | M94708 | 0.005833 | 15 | 12 | | | | | | 67.74837 | 33.30259 | 21.45934 | 42.07991 | 28.35396 | 10.91255 | 19.38333 | 16.21551 | 23.94659 | 41.84554 | 29.55843 | 32.27766 | 37.24189 | 44.44423 | 38.36474 | 52.63381 | 16.17036 | 27.25682 | 28.77383 | 22.45136 | 19.83086 | 24.50817 | 23.36338 | 5.132251 | 33.73933 | 32.3288 | 46.96643 |
| CUST_10315_PI429010886 | N26045 | 0.005833 | 15 | 3 | | | | | | 61.97713 | 55.09927 | 43.20136 | 55.39377 | 83.63285 | 85.15981 | 72.52738 | 50.14002 | 31.55452 | 36.78596 | 88.89139 | 34.75551 | 50.67859 | 28.65357 | 27.18447 | 35.83833 | 158.9804 | 55.25124 | 99.20203 | 45.11939 | 99.66003 | 49.37977 | 63.54619 | 60.41389 | 46.89592 | 37.87276 | 36.74157 |
| CUST_542_PI429010886 | N5908 | 0.005833 | 15 | 12 | | | | | | 1787.33 | 885.7556 | 11908.36 | 62.63918 | 75.72117 | 46.64901 | 3861.408 | 22978.37 | 22003.59 | 23241.72 | 4680.081 | 7920.23 | 15922.04 | 46673.5 | 27975.24 | 1772.36 | 11366.05 | 656.5643 | 2667.238 | 11882.91 | 992.924 | 34870.47 | 29010.89 | 675.3956 | 32012.64 | 4461.585 | 33529.19 |
| CUST_38741_PI429010886 | M29865 | 0.005843 | 18 | 12 | | | | | | 1002.89 | 233.9335 | 376.7168 | 321.6396 | 311.8189 | 73.49284 | 450.1209 | 399.475 | 541.31 | 3090.22 | 461.9687 | 332.54 | 687.964 | 650.3692 | 796.2928 | 411.5645 | 130.4682 | 277.1919 | 532.0502 | 179.2925 | 438.9413 | 331.7667 | 90.19499 | 184.2548 | 88.41726 | 486.2625 | 655.2712 |
| CUST_40225_PI429010886 | M51892 | 0.005844 | 18 | 6 | | | | | | 187.2169 | 27.45566 | 91.60957 | 115.4184 | 108.2745 | 124.5429 | 75.03476 | 133.7601 | 181.1767 | 221.3126 | 54.4284 | 130.0555 | 10.39312 | 57.23179 | 7.550196 | 69.37437 | 88.33792 | 35.38107 | 85.9812 | 27.40469 | 32.22274 | 101.637 | 100.3587 | 620.7934 | 186.5931 | 71.95612 | 143.3816 |
| CUST_29923_PI429010886 | N60825 | 0.005846 | 24 | 6 | | | | | | 2555.115 | 4.113524 | 11182.42 | NA | NA | NA | 7811.413 | 11400.82 | 8245.915 | 8237.502 | 23.74918 | 17.67128 | 6168.628 | 6812.048 | 2298.025 | 5.93716 | 3838.985 | NA | 1391.685 | 8.285257 | 11.38749 | 39.8966 | 1633.35 | 29.0899 | 1684.561 | NA | 1066.79 |
| CUST_40512_PI429010886 | M55339 | 0.005854 | 12 | 3 | | | | | | 30.44767 | 4.939008 | 3.353534 | 110.8364 | 72.55294 | 245.8985 | 51.78005 | 76.73952 | 20.20434 | 11.74757 | 41.59799 | 15.37508 | 37.36325 | 12.95945 | 15.87452 | NA | 228.1106 | 166.3173 | 161.0687 | 6.491213 | 18.88563 | NA | 7.051803 | 48.069 | NA | 98.46582 | 5.98944 |
| CUST_57252_PI429010886 | E2966 | 0.005856 | 15 | 12 | 56118690 | ribonucleotide reductase M2, gene 2 [Xenopus (Silu | Metabolic process | Nucleobase-containing compound metabolic process | 8.78E-19 | 43.24186 | 46.72755 | 44.99497 | 27.59392 | 31.32413 | 4.86353 | 55.21772 | 78.36561 | 63.06213 | 75.2095 | 48.74075 | 57.93571 | 83.43282 | 61.34598 | 61.87735 | 103.0243 | 44.71419 | 35.30842 | 57.94186 | 50.00256 | 29.62034 | 55.49391 | 38.4769 | 35.69319 | 115.3266 | 34.6687 | 65.79499 |
| CUST_22411_PI429010886 | N46982 | 0.005861 | 24 | 21 | | | | | | 3775.991 | 3037.946 | 3154.98 | 3618.732 | 3320.318 | 2661.313 | 1833.381 | 3821.032 | 2574.868 | 2404.795 | 2140.816 | 2238.739 | 958.279 | 3208.657 | 2195.833 | 3246.287 | 1986.907 | 2680.458 | 4062.216 | 3805.814 | 2601.307 | 8772.974 | 2784.905 | 4537.099 | 4171.713 | 1842.625 | 3175.174 |
| CUST_36685_PI429010886 | M16541 | 0.005866 | 15 | 0 | | | | | | 665.035 | 792.7128 | 588.751 | 402.4861 | 440.6954 | 485.9211 | 276.7604 | 304.8063 | 333.4458 | 685.9612 | 488.4404 | 565.2058 | 494.2193 | 421.2748 | 411.6609 | 706.4958 | 464.1138 | 583.7272 | 514.0171 | 601.5918 | 477.3001 | 519.8548 | 458.5323 | 397.7001 | 508.8245 | 631.1117 | 552.0613 |
| CUST_4050_PI429010886 | N15882 | 0.005866 | 15 | 9 | | | | | | 16.98462 | 44.85914 | 54.08762 | 12.6973 | 46.74774 | 30.17169 | 33.14786 | 29.47907 | 45.32916 | 82.73488 | 64.78587 | 96.30815 | 58.74569 | 94.87745 | 46.1038 | 54.27879 | 7.301575 | 50.16596 | 44.80164 | 32.49365 | 51.4111 | 101.6113 | 33.61051 | 31.85271 | 71.43726 | 49.98628 | 110.8992 |
| CUST_297_PI429010886 | N3582 | 0.005866 | 15 | 9 | | | | | | 51.46537 | 135.8973 | 119.841 | 102.7422 | 89.85114 | 113.2976 | 129.3867 | 85.91746 | 136.7775 | 162.9606 | 134.1249 | 193.0913 | 112.304 | 198.3971 | 143.6248 | 89.96525 | 88.30586 | 163.1486 | 93.48735 | 77.44837 | 115.0528 | 103.1763 | 235.7566 | 126.4395 | 182.3936 | 122.3764 | 200.0776 |
| CUST_37868_PI429010886 | M21949 | 0.005877 | 18 | 12 | | | | | | 3120.564 | 4154.657 | 1076.18 | 1298.64 | 1550.902 | 991.3315 | 4519.983 | 1469.038 | 6174.548 | 5396.825 | 1846.987 | 8995.888 | 5580.063 | 5503.933 | 4813.402 | 4778.44 | 1884.761 | 2303.489 | 6717.481 | 882.8865 | 3448.497 | 1270.943 | 2155.605 | 1654.803 | 3472.182 | 1186.311 | 7163.89 |
| CUST_1932_PI429010886 | N11106 | 0.005884 | 21 | 12 | | | | | | 60.67368 | 53.76881 | 56.48454 | 42.65121 | 48.12117 | 44.19983 | 56.77264 | 52.034 | 70.20247 | 54.00554 | 56.92511 | 64.80696 | 71.18669 | 55.25124 | 88.91586 | 79.59377 | 49.28963 | 65.08 | 74.18777 | 72.45575 | 46.65817 | 58.01161 | 57.63741 | 47.45553 | 52.37245 | 45.46228 | 66.33801 |
| CUST_35267_PI429010886 | M12096 | 0.005892 | 12 | 6 | | | | | | 9.479243 | 6.117837 | 9.207048 | 10.59324 | 8.763789 | 7.558567 | 13.73574 | 19.53485 | 13.75845 | 10.6654 | 13.33386 | 10.67013 | 13.37697 | 6.909158 | 10.67164 | 7.82506 | 8.134417 | 12.14502 | 9.992565 | 23.65678 | 15.23398 | 32.89329 | 10.93941 | 9.267153 | 4.231174 | 10.39491 | 6.603602 |
| CUST_38788_PI429010886 | M30395 | 0.005901 | 15 | 12 | | | | | | 1745.549 | 1300.946 | 2580.486 | 606.3721 | 980.4603 | 721.3883 | 872.488 | 1575.017 | 1939.579 | 2078.653 | 860.82 | 1879.633 | 2607.508 | 3489.908 | 2132.178 | 1574.342 | 1665.324 | 1253.921 | 929.0194 | 1132.32 | 1137.521 | 3405.484 | 3480.516 | 471.4881 | 2483.895 | 624.6679 | 3592.482 |
| CUST_49970_PI429010886 | M87987 | 0.005901 | 15 | 12 | 322791059 | hypothetical protein SINV_09638 [Solenopsis invict | | | 6.24E-09 | 72.21046 | 73.71983 | 133.7417 | 47.39715 | 37.81058 | 26.88741 | 64.59746 | 136.4264 | 73.00251 | 142.8582 | 51.99458 | 56.54516 | 103.1477 | 165.6162 | 79.80797 | 65.42947 | 83.45905 | 56.03701 | 73.36115 | 46.01155 | 46.70127 | 63.27271 | 97.30444 | 40.09244 | 94.53569 | 40.53465 | 91.83515 |
| CUST_15693_PI429010886 | N34985 | 0.005901 | 15 | 3 | | | | | | 78.95924 | 78.48723 | 53.35562 | 138.3413 | 148.7085 | 142.3547 | 120.9718 | 92.13928 | 61.88462 | 61.89778 | 133.5801 | 54.32031 | 104.6652 | 41.33922 | 48.95474 | 58.35369 | 186.4588 | 72.9595 | 162.6527 | 78.77563 | 79.37795 | 66.50645 | 94.33627 | 101.5396 | 57.60056 | 81.16843 | 55.98779 |
| CUST_17863_PI429010886 | N38734 | 0.005922 | 18 | 12 | | | | | | 52.05749 | 98.96023 | 128.8206 | 30.15647 | 41.67274 | 3.635183 | 182.747 | 103.4079 | 284.2642 | 137.4942 | 155.2537 | 202.262 | 294.6436 | 274.9043 | 196.1002 | 437.0609 | 209.9951 | 153.0166 | 102.6905 | 337.6916 | 143.7672 | 98.80707 | 128.1757 | 163.5179 | 1902.402 | 269.9093 | 1583.066 |
| CUST_49447_PI429010886 | M85663 | 0.005937 | 24 | 15 | | | | | | 10.92379 | 11.12846 | 7.183846 | NA | 8.607023 | 12.27121 | 14.05126 | 11.25926 | 10.19501 | 20.65721 | 15.06062 | 12.24779 | 6.396975 | 14.37562 | 12.59311 | 20.05201 | 23.50407 | 21.72859 | 19.73455 | 42.2764 | 12.12816 | 30.54779 | 16.33068 | 16.1997 | 19.60862 | 3.574702 | NA |
| CUST_43564_PI429010886 | M63358 | 0.005941 | 18 | 15 | | | | | | 803.7954 | 525.3464 | 661.9537 | 262.6197 | 809.6878 | 532.6708 | 819.1664 | 621.7238 | 591.157 | 1073.527 | 519.8548 | 590.4824 | 1073.921 | 819.4278 | 748.0078 | 784.4175 | 877.6784 | 881.8414 | 644.7638 | 685.1065 | 664.4926 | 742.6323 | 380.7812 | 312.052 | 318.6212 | 531.6819 | 709.6744 |
| CUST_38148_PI429010886 | M23676 | 0.005952 | 21 | 3 | 340728251 | PREDICTED: venom serine protease 34-like [Bombu | Protein metabolism | Proteolysis | 4.83E-16 | 35362.62 | 52978.01 | 31492.49 | 55819.51 | 46148.42 | 39434.25 | 28469.11 | 28160 | 31581.61 | 43151.77 | 27773.77 | 33351.13 | 28752.77 | 32092.43 | 20138.4 | 37322.31 | 16676.56 | 21372.62 | 20339.21 | 24132.63 | 45790.2 | 41094.47 | 36174.65 | 29896.62 | 42284.08 | 45577.47 | 31492.49 |
| CUST_40444_PI429010886 | M55114 | 0.005959 | 24 | 0 | 75022291 | RecName: Full=Oplophorus-luciferin 2-monooxyger | | | 5.95E-09 | 26.95285 | 38.39957 | 28.1812 | 31.21998 | 26.41485 | 44.40636 | 25.92862 | 21.43684 | 21.79167 | 13.35156 | 13.75362 | 41.17264 | 16.55564 | 26.98251 | 11.84337 | 19.8522 | 6.710606 | 19.1562 | 6.315643 | 17.81842 | 40.16219 | 23.24689 | 53.62643 | 23.29369 | 33.16347 | 45.93669 | 22.43297 |
| CUST_34307_PI429010886 | M9289 | 0.005961 | 12 | 0 | | | | | | 36.20273 | 57.19607 | 58.58074 | 39.38447 | 32.69544 | 69.99368 | 39.08441 | 19.88479 | 21.83957 | 52.60928 | 45.35228 | 38.84613 | 48.96007 | 54.07518 | 36.72537 | 31.35806 | 47.00108 | 19.97113 | 30.12077 | 25.54596 | 33.94751 | 67.85099 | 44.16197 | 48.01961 | 76.31563 | 53.80851 | 43.6379 |
| CUST_14565_PI429010886 | N33085 | 0.005961 | 12 | 0 | | | | | | 140.4217 | 167.2734 | 159.3888 | 138.2381 | 105.0213 | 109.3742 | 121.1153 | 101.92 | 121.562 | 141.2731 | 105.6827 | 136.9989 | 130.5299 | 151.4536 | 150.9664 | 174.6367 | 98.37238 | 144.6317 | 112.7145 | 133.9999 | 140.5313 | 123.6533 | 131.0159 | 140.1157 | 150.6728 | 156.7058 | 160.531 |
| CUST_16735_PI429010886 | N36774 | 0.005961 | 12 | 0 | 117670124 | notch protein [Parhyale hawaiensis] | Metabolic process | Lipid metabolic process | 2.41E-58 | 50.45571 | 48.93138 | 47.63767 | 53.98541 | 28.60372 | 32.83834 | 26.65472 | 36.60913 | 40.6037 | 50.19562 | 40.76795 | 43.72098 | 63.46235 | 43.78734 | 51.28378 | 58.44181 | 26.21698 | 41.43739 | 34.08252 | 43.9135 | 42.39985 | 36.41953 | 43.45323 | 32.02812 | 55.83606 | 85.62172 | 53.83853 |
| CUST_51687_PI429010886 | M94577 | 0.005967 | 21 | 15 | | | | | | 20.59775 | 12.67545 | 7.635215 | 12.11941 | 5.022376 | 6.610857 | 17.07599 | 10.5066 | 7.348282 | 6.991817 | 15.76779 | NA | 6.716238 | 18.60353 | 12.49458 | 14.38334 | NA | 55.95035 | 21.89788 | 10.97439 | 11.75719 | 18.4393 | 8.284158 | NA | 6.303067 | 4.843892 | 8.916592 |
| CUST_44977_PI429010886 | M67600 | 0.005972 | 24 | 15 | 546675447 | hypothetical protein D910_04042 [Dendroctonus p | Transport | Protein transport | 3.24E-66 | 13.2803 | 10.11317 | 24.69322 | 8.840456 | 11.17643 | 13.38957 | 26.1905 | 14.00927 | 13.70338 | 14.11808 | 19.79173 | 21.7849 | 18.36323 | 26.23528 | 27.18775 | 27.52478 | 23.51171 | 18.57232 | 17.0365 | 31.07874 | 15.06199 | 21.35072 | 12.79677 | 14.99759 | 28.30216 | 11.47799 | 21.52705 |
| CUST_50333_PI429010886 | M89487 | 0.005974 | 21 | 0 | | | | | | 720.0434 | 797.3133 | 995.2843 | 710.6638 | 719.37 | 724.0591 | 591.3951 | 653.8395 | 687.4211 | 671.0656 | 698.2239 | 737.0066 | 560.5893 | 799.1588 | 732.5018 | 782.2398 | 892.8593 | 859.9982 | 692.5393 | 735.5143 | 699.6538 | 786.706 | 841.7695 | 756.9801 | 799.1588 | 685.3204 | 710.9044 |
| CUST_2160_PI429010886 | N11724 | 0.005979 | 24 | 21 | | | | | | 14.94106 | 15.97144 | 14.40255 | 10.70994 | 11.67368 | 12.54217 | 6.685823 | 10.84659 | 10.27034 | 10.83296 | 14.62779 | 20.61073 | 6.837856 | 13.98077 | 12.30553 | 12.9382 | 7.737261 | 16.88939 | 16.80832 | 9.530867 | 16.51187 | 29.08151 | 22.80672 | NA | 27.95174 | 11.49378 | 25.99016 |
| CUST_56388_PI429010886 | M113656 | 0.00599 | 15 | 9 | 9367086 | Euphausia superba large subunit ribosomal RNA ger | | | 1.51E-65 | 97928.01 | 70816.79 | 88771.33 | 98448.52 | 95014.94 | 84866.33 | 102912.5 | 87648.66 | 93952.87 | 107761.9 | 108311.1 | 101136 | 93467.7 | 52978.01 | 88276.1 | 71527.46 | 106276.7 | 73854.47 | 92382.94 | 72732.64 | 81423.55 | 96660.96 | 92382.94 | 90253.54 | 109583.6 | 97928.01 | 102912.5 |
| CUST_27722_PI429010886 | N56580 | 0.005998 | 21 | 3 | | | | | | 28.17154 | 88.47454 | 50.01173 | 121.5472 | 45.92841 | 71.72709 | 29.44539 | 36.15353 | 40.27597 | 29.79322 | 14.61093 | 47.23018 | 27.16077 | 28.64267 | 32.99848 | 41.43739 | 11.84191 | 42.44777 | 18.08051 | 31.63804 | 65.76284 | 47.88942 | 53.78486 | 38.30389 | 60.17363 | 91.21234 | 31.16892 |
| CUST_50984_PI429010886 | M91683 | 0.006001 | 24 | 15 | | | | | | 7.115399 | 14.84618 | 16.65372 | 6.19041 | 11.7681 | 10.6044 | 6.022117 | 10.91708 | 4.585271 | 10.78323 | 7.512851 | 14.26692 | 10.62424 | 16.15147 | 9.490926 | 17.21502 | 35.27881 | 15.26556 | 13.63096 | 5.479138 | 14.09377 | 15.2174 | 16.98819 | 7.990456 | 10.5712 | 11.09741 | 12.81767 |
| CUST_29798_PI429010886 | N60574 | 0.006009 | 15 | 9 | | | | | | 241.3477 | 791.5217 | 756.9801 | 681.1329 | 721.5575 | 328.7379 | 119.0071 | 1318.096 | 2081.036 | 2599.94 | 1338.117 | 2915.652 | 1639.617 | 4627.233 | 3233.011 | 2133.627 | 456.5493 | 1202.61 | 1048.763 | 1974.817 | 942.6383 | 6614.331 | 2226.703 | 335.8009 | 5515.427 | 2442.431 | 7107.78 |
| CUST_8945_PI429010886 | N23832 | 0.006014 | 24 | 6 | 546685290 | hypothetical protein D910_12090 [Dendroctonus p | Metabolic process | Carbohydrate metabolic process | 5.19E-42 | 65.54862 | 41.63072 | NA | 82.1182 | 41.50969 | 77.3067 | 81.76577 | 125.5467 | 64.41009 | 12.09699 | 61.86952 | NA | 64.82649 | NA | 35.40201 | 15.33784 | 16.03098 | 18.6269 | 24.25419 | 5.842679 | 51.02123 | 10.37567 | 31.00851 | 98.47983 | NA | 59.7424 | NA |
| CUST_18922_PI429010886 | N40661 | 0.006023 | 21 | 0 | | | | | | 64.56992 | 62.99752 | 81.84143 | 44.87442 | 49.34559 | 45.89685 | 55.47209 | 37.87276 | 16.83375 | 53.22359 | 57.32094 | 56.07774 | 32.80705 | 32.80137 | 42.69298 | 58.38163 | 66.67878 | 36.97227 | 69.56167 | 40.04686 | 78.07938 | 51.73011 | 68.38203 | 59.56411 | 59.3369 | 46.11982 | 53.79628 |
| CUST_536_PI429010886 | N5882 | 0.006031 | 21 | 3 | 270013365 | hypothetical protein TcasGA2_TC011958 [Tribolium | | | 1.63E-08 | 13.09004 | 56.88203 | 31.68997 | 70.08131 | 129.4503 | 161.6167 | 63.747 | NA | 94.54715 | 47.52868 | 30.558 | 89.98836 | 47.61652 | 65.83024 | 55.28288 | 70.34764 | 9.828551 | 69.69491 | 15.4643 | 47.57087 | 28.80979 | 84.78816 | 73.46154 | 44.04837 | 77.21633 | 57.65114 | 87.29557 |
| CUST_21544_PI429010886 | N45397 | 0.006046 | 21 | 18 | | | | | | 95.28234 | 102.3344 | 116.2535 | 95.7753 | 92.96667 | 82.39034 | 76.60117 | 81.86063 | 81.69291 | 92.73234 | 83.75206 | 104.2505 | 111.7908 | 160.9334 | 92.35924 | 128.5685 | 111.4345 | 81.98334 | 139.1034 | 102.1229 | 107.638 | 93.03197 | 98.7787 | 94.3579 | 109.4535 | 81.85243 | 135.1423 |
| CUST_13597_PI429010886 | N31441 | 0.006055 | 21 | 0 | | | | | | 23.32 | 17.05374 | 22.58452 | 19.67357 | 16.3681 | 15.81556 | 11.65987 | 12.17905 | 14.00504 | 20.38809 | 10.85866 | 13.54322 | 14.25137 | 14.67638 | 17.65061 | 19.10728 | 16.54538 | 16.04761 | 16.8197 | 11.86639 | 14.76986 | 14.80809 | 19.18935 | 24.0077 | 18.26586 | 7.626632 | 17.91994 |
| CUST_38175_PI429010886 | M23892 | 0.006068 | 15 | 0 | | | | | | 162.4501 | 166.0617 | 147.7898 | 169.4295 | 125.0791 | 156.0301 | 158.317 | 144.1009 | 118.3064 | 143.8657 | 124.6867 | 131.7874 | 142.3214 | 132.6733 | 142.124 | 175.6552 | 148.7626 | 111.9784 | 170.4477 | 128.969 | 148.0647 | 137.759 | 127.5485 | 139.8628 | 99.71807 | 110.2159 | 104.6528 |
| CUST_7935_PI429010886 | N22294 | 0.006073 | 24 | 15 | | | | | | 10.89455 | 6.928497 | 7.758109 | NA | 14.32267 | 10.85125 | 9.202277 | 9.642662 | 13.295 | 10.14304 | 12.73246 | 16.22239 | 15.05158 | 8.194751 | NA | 17.07004 | 27.52267 | 10.11773 | 8.174796 | 51.0829 | 19.46833 | 10.35023 | NA | 11.41844 | 6.426783 | 3.782481 | 3.647056 |
| CUST_11466_PI429010886 | N27858 | 0.006074 | 18 | 3 | | | | | | 140.7918 | 143.2572 | 126.3583 | 121.6208 | 165.7374 | 144.1452 | 163.5913 | 151.8403 | 139.2501 | 94.88698 | 116.2211 | 126.1763 | 115.284 | 138.2244 | 130.3328 | 142.4348 | 118.6098 | 130.2863 | 144.9326 | 101.9474 | 115.3926 | 165.7374 | 190.8604 | 175.4895 | 148.0276 | 77.99438 | 111.2819 |
| CUST_15660_PI429010886 | N34935 | 0.006075 | 24 | 15 | | | | | | 22.82685 | 13.60949 | 28.99027 | 16.90667 | 19.3587 | 52.91645 | 7.375685 | 17.52814 | 7.39706 | 30.6438 | 31.23744 | 15.98734 | 15.34644 | 32.62765 | 28.57032 | 29.71386 | 41.15689 | 36.57035 | 26.01055 | 25.51724 | 34.99003 | 25.68978 | 40.94647 | 19.82681 | 21.31694 | 35.12625 | 17.00366 |
| CUST_8531_PI429010886 | N23181 | 0.00608 | 18 | 12 | | | | | | 138.133 | 153.6236 | 117.8888 | 110.0373 | 130.1545 | 124.7428 | 151.1592 | 148.2643 | 185.168 | 167.4943 | 96.06297 | 227.9427 | 180.9317 | 215.4042 | 208.2838 | 200.5697 | 125.3551 | 146.4472 | 266.0394 | 139.0161 | 207.3956 | 113.7193 | 128.8934 | 139.1997 | 171.427 | 127.8639 | 184.3634 |
| CUST_31644_PI429010886 | M2043 | 0.00608 | 15 | 0 | | | | | | 15.38835 | 30.112 | 20.66296 | 42.43668 | 9.165228 | 23.04785 | 6.725658 | 13.04417 | 8.241846 | 19.43895 | 14.73365 | 19.48105 | 7.204193 | 13.81188 | 19.22831 | 42.89937 | 15.94381 | 27.69749 | 24.8301 | 18.02463 | 12.42474 | 17.30092 | 12.30868 | 11.22442 | 13.60227 | 12.90988 | 13.00912 |
| CUST_37265_PI429010886 | M18434 | 0.006081 | 15 | 9 | 13898841 | CUB-serine protease [Panulirus argus] | Protein metabolism | Proteolysis | 8.86E-31 | 81.87168 | 142.3547 | 120.363 | 87.60236 | 48.46285 | 51.17489 | 528.8856 | 300.9186 | 364.9554 | 342.9573 | 296.5712 | 178.5939 | 75.1888 | 579.7012 | 371.054 | 413.1358 | 136.5699 | 220.1671 | 77.34848 | 281.5398 | 131.9783 | 258.7295 | 129.3708 | 260.9414 | 378.4398 | 416.1299 | 459.2637 |
| CUST_43941_PI429010886 | M64408 | 0.006082 | 24 | 15 | | | | | | 16.57544 | 23.80171 | 25.00327 | 20.18733 | 36.69997 | 31.8356 | 15.68356 | 15.557 | 14.38028 | 27.64994 | 65.55617 | 41.2187 | 15.8658 | 26.95285 | 22.1165 | 46.56491 | 33.15625 | 45.33809 | 42.89021 | 26.41986 | 32.20521 | 37.9742 | 33.86813 | 33.07217 | 42.34616 | 24.8597 | 42.00009 |
| CUST_46185_PI429010886 | M71703 | 0.006086 | 15 | 9 | | | | | | 12.09198 | 13.54122 | 16.50418 | 9.710618 | 10.96904 | 9.761566 | 10.05433 | 13.19079 | 9.965323 | 14.36708 | 17.95452 | 27.15358 | 11.0938 | 19.62917 | 14.94106 | 12.98903 | 11.71206 | 24.72926 | 10.7559 | 13.63622 | 7.940312 | 17.80913 | 8.148866 | NA | 18.16468 | 11.8786 | 20.37597 |
| CUST_2695_PI429010886 | N12921 | 0.006096 | 18 | 3 | | | | | | 171.2286 | 112.0535 | 96.07604 | 133.4505 | 134.79 | 138.094 | 122.3475 | 131.6163 | 108.7711 | 103.5071 | 113.2498 | 96.17817 | 92.48444 | 90.85633 | 92.02079 | 109.4422 | 166.8192 | 112.1381 | 143.3816 | 79.94364 | 100.8596 | 116.4584 | 122.4345 | 114.6944 | 104.7823 | 90.28628 | 107.174 |
| CUST_52000_PI429010886 | M96019 | 0.006103 | 15 | 3 | | | | | | 8.796716 | 5.852448 | NA | 7.509358 | 16.63144 | 22.22394 | 17.65061 | 8.474659 | NA | NA | 11.10882 | 8.013121 | 3.566833 | 3.738434 | 6.734253 | 6.932883 | 18.69696 | 15.32313 | 16.34764 | NA | 12.03745 | 3.585654 | 16.11615 | 11.46533 | 7.046406 | 10.81563 | 9.012961 |
| CUST_31547_PI429010886 | M1802 | 0.006104 | 15 | 9 | | | | | | 93.33448 | 1583.066 | 1809.996 | NA | 134.0744 | 43.93404 | 171.0777 | 1863.799 | 5744.456 | 4221.337 | 3893.391 | 10200.91 | 2119.433 | 11747.34 | 9207.498 | 1967.236 | 597.7736 | 1414.097 | 1458.732 | 6986.943 | 1898.136 | 14600.6 | 7395.598 | 140.4954 | 14471.07 | 7175.165 | 17268.09 |
| CUST_27839_PI429010886 | N56787 | 0.006109 | 18 | 15 | | | | | | 7.871973 | 12.79677 | 8.221669 | 9.688636 | NA | 13.31013 | NA | NA | 3.543779 | 14.76626 | 6.949308 | 11.58812 | 4.559331 | 16.69153 | 16.89352 | 13.06814 | 15.20876 | 17.72667 | 10.64168 | 9.72694 | NA | 12.60293 | 9.71829 | 9.647019 | 8.621822 | 3.467539 | 8.44541 |
| CUST_21940_PI429010886 | N46129 | 0.006109 | 21 | 15 | | | | | | 47.61652 | 58.37612 | 49.05418 | 35.50382 | 45.17236 | 34.02396 | 38.51373 | 32.86117 | 22.43497 | 41.42947 | 62.61352 | 31.59756 | 39.13386 | 43.57431 | 40.14648 | 58.8975 | 86.85226 | 60.49322 | 24.84355 | 36.04028 | 46.20496 | 58.86655 | 43.83694 | 48.52803 | 55.62148 | 24.73831 | 40.91962 |
| CUST_24875_PI429010886 | N51401 | 0.006129 | 21 | 15 | | | | | | 20.5721 | 21.98642 | 23.6887 | 9.838949 | 13.65469 | NA | 12.77138 | 15.60331 | 15.05605 | 30.99832 | 15.57695 | 24.77587 | 18.54527 | 23.531 | 19.64586 | 31.78356 | 22.23059 | 24.76116 | 23.78457 | 16.53513 | 15.8511 | 29.06247 | 10.29533 | 20.98522 | 23.01368 | 10.91255 | 13.00717 |
| CUST_11225_PI429010886 | N27474 | 0.006132 | 21 | 18 | | | | | | 39.98138 | 31.33456 | 38.18132 | 48.13345 | 37.36976 | 52.95164 | 23.67253 | 26.1596 | 30.27191 | 33.68076 | 38.13489 | 40.04686 | 45.44604 | 26.82709 | 21.24309 | 33.5333 | 109.5048 | 69.94604 | 79.47713 | 40.28049 | 55.6042 | 35.43275 | 66.67878 | 76.17525 | 35.74774 | 42.42487 | 31.01661 |

| Probe ID | ID | p-value | Period | Phase | EBI ID | Annotation | GO category | Go term | E-value | 0 | 0 | 0 | 3 | 3 | 3 | 6 | 6 | 6 | 9 | 9 | 9 | 12 | 12 | 12 | 15 | 15 | 15 | 18 | 18 | 18 | 21 | 21 | 21 | 24 | 24 | 24 |
|---|---|---|---|---|---|---|---|---|---|---|---|---|---|---|---|---|---|---|---|---|---|---|---|---|---|---|---|---|---|---|---|---|---|---|---|---|
| CUST_20866_PI429010886 | N44213 | 0.006137 | 21 | 0 | | | | | | 173.1695 | 193.6314 | 171.6689 | 140.228 | 128.8011 | 143.2947 | 123.794 | 144.7517 | 115.5578 | 166.9208 | 146.6394 | 169.0651 | 158.2442 | 162.1787 | 129.1352 | 228.9547 | 144.9545 | 150.2666 | 162.98 | 144.3246 | 150.8016 | 174.6176 | 159.5233 | 186.3412 | 169.802 | 155.5106 | 177.0624 |
| CUST_52055_PI429010886 | M96259 | 0.00614 | 12 | 3 | | | | | | 12.10565 | 8.261277 | NA | 42.60531 | 37.03706 | 53.1338 | 43.10871 | 29.28327 | 12.89985 | 20.61883 | 30.66153 | 9.891852 | 27.1196 | 8.155994 | 9.248628 | 9.093219 | 69.66945 | 38.71497 | 44.27177 | NA | 21.4795 | 8.978228 | 40.04237 | 65.56878 | 5.143659 | 10.22197 | 7.608505 |
| CUST_56221_PI429010886 | M113169 | 0.006152 | 15 | 9 | | | | | | 369.8403 | 541.0041 | 441.149 | 198.4267 | 492.8667 | 343.6779 | 370.0362 | 294.4572 | 451.3065 | 655.5465 | 611.5405 | 780.1203 | 306.6802 | 531.2482 | 482.6309 | 596.0223 | 475.9462 | 365.3412 | 399.93 | 379.882 | 385.3789 | 366.5663 | 514.3336 | 356.8337 | 623.5116 | 490.65 | 589.9381 |
| CUST_43961_PI429010886 | M64469 | 0.006152 | 15 | 9 | | | | | | 69.58233 | 83.62457 | 90.24634 | 58.28332 | 56.63828 | 82.48421 | 63.48536 | 74.08523 | 69.2413 | 91.96613 | 76.42859 | 121.1966 | 78.33196 | 84.74127 | 102.5106 | 90.73889 | 65.21686 | 88.37144 | 66.88544 | 86.54045 | 76.353 | 73.62986 | 74.29802 | 73.39754 | 91.25978 | 99.68173 | 99.70619 |
| CUST_48577_PI429010886 | M81122 | 0.006152 | 15 | 9 | | | | | | 16.94914 | 14.82674 | 27.14824 | 14.88157 | 16.46383 | 8.07676 | 18.20163 | 19.26372 | 26.15676 | 29.34549 | 23.74624 | 27.89184 | 13.56721 | 38.48153 | 18.53904 | 18.42118 | 22.54321 | 17.55275 | 18.59558 | 20.01149 | 23.85972 | 22.29224 | 19.48871 | 20.09233 | 29.11133 | 26.41719 | 29.12589 |
| CUST_52568_PI429010886 | M97992 | 0.006152 | 15 | 3 | | | | | | 823.5514 | 285.8071 | 945.2395 | 1475.875 | 1571.524 | 1698.659 | 1480.417 | 840.1524 | 391.9101 | 144.7304 | 1030.293 | 73.30133 | 253.025 | 87.17451 | 216.8379 | 146.0723 | 1038.465 | 928.4158 | 1008.511 | 60.54854 | 1492.073 | 96.71335 | 1054.702 | 1409.133 | 54.058 | 860.1543 | 66.26094 |
| CUST_8763_PI429010886 | N23553 | 0.006152 | 15 | 9 | | | | | | 212.046 | 226.3317 | 309.4606 | 236.0009 | 206.9171 | 239.521 | 215.8652 | 265.2111 | 256.5714 | 252.8872 | 275.7295 | 271.2785 | 236.8108 | 310.7615 | 317.3299 | 298.3902 | 264.1236 | 214.4171 | 237.9578 | 245.7815 | 248.2061 | 295.4403 | 264.7596 | 242.7679 | 370.4089 | 310.6611 | 339.6585 |
| CUST_17701_PI429010886 | N38428 | 0.006152 | 15 | 9 | 195162849 | GL26040 [Drosophila persimilis] >gi\|194104227\|gb | | | 2.9E-22 | 32.19898 | 33.80408 | 68.00621 | 34.29722 | 27.50907 | 29.96643 | 48.23603 | 41.68991 | 46.29572 | 55.81208 | 53.64297 | 42.6374 | 37.22562 | 51.97626 | 40.54674 | 55.76859 | 38.07238 | 28.40026 | 44.65427 | 35.82359 | 44.0999 | 49.30624 | 34.70757 | 40.7292 | 72.8742 | 47.62717 | 59.07548 |
| CUST_45488_PI429010886 | M69269 | 0.006158 | 12 | 0 | | | | | | 59.93656 | 63.06953 | 63.80077 | 40.75297 | 34.93063 | 36.42724 | 26.62834 | 52.04012 | 45.85649 | 53.33947 | 52.49852 | 79.12414 | 49.95028 | 76.44833 | 57.74713 | 61.15955 | 49.81881 | 55.53245 | 59.55765 | 58.01161 | 47.16593 | 55.25124 | 61.97713 | 45.30656 | 70.66973 | 64.45055 | 82.95644 |
| CUST_4154_PI429010886 | N16121 | 0.006158 | 21 | 18 | 321463817 | hypothetical protein DAPPUDRAFT_306990 [Daphn | Signal transduction | Other | 1.47E-33 | 16.78585 | 17.39038 | 16.72128 | NA | 10.56393 | 8.198298 | 7.198406 | 4.009454 | 6.535693 | 8.772272 | 11.58371 | NA | 12.38228 | NA | 6.098401 | 13.45887 | 15.33118 | 22.97006 | 6.27335 | 28.62243 | 18.4393 | 13.86169 | 13.51055 | 14.53096 | 23.1605 | 14.4666 | 12.73246 |
| CUST_12488_PI429010886 | N29547 | 0.006159 | 18 | 12 | 550486 | pyruvate carboxylase [Aedes aegypti] | Metabolic process | Lipid metabolic process | 1.53E-33 | 29.74822 | 57.28119 | 59.01989 | 24.50817 | 31.7359 | 15.37508 | 32.05856 | 52.95745 | 72.8194 | 81.9929 | 45.03054 | 66.40412 | 61.03938 | 102.3996 | 88.54248 | 71.11204 | 61.47627 | 30.66462 | 52.21965 | 80.91461 | 57.58322 | 49.42389 | 62.59848 | 17.86761 | 104.7208 | 34.78102 | 103.2289 |
| CUST_14944_PI429010886 | N33733 | 0.006166 | 12 | 0 | | | | | | 22.2285 | 20.29379 | 30.49715 | 21.84546 | 19.75734 | 71.99567 | 16.73391 | 19.44948 | 13.67832 | 18.49721 | 18.76152 | 27.09043 | 19.34391 | 27.97946 | 24.66568 | 23.91343 | 18.02463 | 20.60841 | 13.96399 | 16.73527 | 16.45281 | 21.99572 | 31.77138 | 15.60189 | 35.05877 | 17.39698 | 35.20228 |
| CUST_26442_PI429010886 | N54251 | 0.006166 | 12 | 0 | | | | | | 52.38415 | 100.4445 | 64.45055 | 60.77185 | 54.63526 | 88.58239 | 40.38893 | 50.32801 | 55.19772 | 62.25774 | 40.55619 | 69.16053 | 69.05841 | 57.65114 | 67.68545 | 72.06797 | 49.43857 | 59.63236 | 43.88802 | 47.51659 | 53.41262 | 59.88574 | 46.55609 | 44.79489 | 63.06213 | 83.88709 | 79.32298 |
| CUST_38739_PI429010886 | M29814 | 0.006174 | 12 | 9 | | | | | | 146.9588 | 466.1947 | 470.5429 | 39.83835 | 88.58239 | 102.9429 | 305.8476 | 566.2341 | 246.8318 | 221.5886 | 413.616 | 518.0097 | 303.6686 | 656.3922 | 381.498 | 124.0862 | 140.194 | 152.8418 | 255.5791 | 171.2801 | 164.7416 | 167.9263 | 485.2905 | 172.831 | 233.1687 | 216.6058 | 172.1829 |
| CUST_2498_PI429010886 | N12506 | 0.006178 | 18 | 12 | | | | | | 276.7604 | 255.6621 | 263.6735 | 212.7113 | 254.5885 | 200.041 | 278.8318 | 217.0982 | 225.2357 | 332.8163 | 228.1824 | 341.396 | 287.8054 | 298.6335 | 312.5863 | 408.9972 | 262.7247 | 321.4904 | 269.9093 | 300.7234 | 208.9685 | 327.471 | 215.7896 | 182.7865 | 324.4624 | 247.2726 | 283.5999 |
| CUST_42781_PI429010886 | M61244 | 0.006178 | 15 | 9 | 321465515 | hypothetical protein DAPPUDRAFT_213935 [Daphn | Metabolic process | Cellular amino acid metabolic process | 1.01E-63 | 395.4574 | 478.2055 | 478.074 | 357.2284 | 348.5888 | 308.8474 | 334.9967 | 347.9148 | 409.8763 | 533.2467 | 413.1358 | 689.8687 | 527.2284 | 505.7885 | 420.1077 | 545.6531 | 325.4382 | 369.6113 | 418.9595 | 404.5388 | 374.9864 | 579.3813 | 371.4969 | 364.7093 | 480.4111 | 447.7446 | 703.4699 |
| CUST_11943_PI429010886 | N28668 | 0.00618 | 15 | 3 | 506968565 | glucosyl/glucuronosyl transferase, partial [Coptoter | | | 2.04E-13 | 7.08513 | NA | 11.96331 | 16.53332 | 15.82118 | 44.12842 | 28.64876 | 32.02557 | 11.32653 | 7.058007 | 13.63796 | NA | 3.855888 | NA | 4.36281 | 4.985952 | 41.47082 | NA | 13.7932 | NA | 18.53311 | 5.849038 | 9.898027 | 14.74027 | 4.281876 | 14.75975 | 10.1858 |
| CUST_36255_PI429010886 | M15192 | 0.006182 | 24 | 6 | | | | | | 90.33731 | 88.51347 | 89.06701 | 77.02903 | 90.16303 | 92.71397 | 113.525 | 105.1255 | 106.0522 | 111.4235 | 83.77617 | 86.9841 | 88.47454 | 94.95262 | 81.89181 | 71.47658 | 89.24114 | 88.22213 | 129.3708 | 72.24742 | 69.20227 | 74.19557 | 70.7847 | 75.11707 | 134.2151 | 97.89619 | 98.10524 |
| CUST_55491_PI429010886 | M110344 | 0.006186 | 18 | 12 | | | | | | 103.7863 | 108.8762 | 106.872 | 72.58192 | 93.17664 | 109.4643 | 75.32357 | 124.1467 | 103.6413 | 113.1145 | 101.9474 | 186.2582 | 118.1291 | 122.1911 | 132.0478 | 111.2335 | 98.31044 | 103.2411 | 85.75328 | 100.999 | 136.4264 | 73.06271 | 96.09446 | 100.7206 | 126.2324 | 96.24145 | 125.6996 |
| CUST_43543_PI429010886 | M63316 | 0.006186 | 18 | 12 | 321479162 | hypothetical protein DAPPUDRAFT_300149 [Daphn | Metabolic process | Lipid metabolic process | 8.34E-35 | 13.95459 | 19.94541 | 10.27406 | 5.070641 | 8.39917 | 6.446625 | 12.83593 | 10.19353 | 13.69809 | 5.65908 | 17.07869 | 29.42815 | 23.2298 | 14.68459 | 15.62557 | 14.07729 | 16.7648 | 15.61612 | 9.183116 | 31.01661 | 15.82118 | 10.17778 | 14.02882 | 8.724159 | 9.03621 | 11.23137 | 22.25705 |
| CUST_12686_PI429010886 | N29871 | 0.006204 | 24 | 15 | 405953771 | Proapoptotic caspase adapter protein [Crassostrea | | | 9.35E-07 | 112.0385 | 106.3073 | 93.68609 | 87.21952 | 84.19357 | 112.9419 | 92.24128 | 89.58206 | 85.39372 | 127.001 | 110.0483 | 122.7676 | 102.0005 | 130.5118 | 148.6917 | 136.023 | 101.7859 | 142.0255 | 94.34866 | 133.6264 | 94.62564 | 141.3142 | 115.1051 | 109.0299 | 103.1223 | 91.17869 | 104.8909 |
| CUST_44960_PI429010886 | M67539 | 0.006223 | 15 | 9 | | | Protein metabolism | Proteolysis | | 68.64401 | 56.60227 | 112.9777 | 48.26954 | 43.60712 | 66.48727 | 92.95468 | 66.37927 | 77.12763 | 100.1395 | 64.3699 | 89.07632 | 65.36594 | 100.9197 | 73.21264 | 76.73952 | 82.03238 | 68.77033 | 55.0673 | 65.99023 | 63.70797 | 69.43885 | 89.60537 | 62.34386 | 67.95716 | 113.6765 | 113.4502 |
| CUST_11946_PI429010886 | N28671 | 0.006223 | 15 | 9 | | | | | | 13.0523 | 12.19257 | 22.94537 | 8.548078 | 11.44011 | 10.54982 | 10.80445 | 12.49899 | 15.88699 | 23.38375 | 23.74079 | 24.04687 | 8.343945 | 26.21095 | 6.459201 | 17.22867 | 15.73231 | 18.21981 | 15.35498 | 12.37644 | 16.07261 | 14.83326 | 14.66496 | 17.37482 | 21.92896 | 20.54425 | 20.34669 |
| CUST_36105_PI429010886 | M14643 | 0.006234 | 24 | 18 | | | Metabolic process | Other | | 11.55545 | 20.93093 | 15.82289 | 8.833142 | 10.35656 | 10.57226 | 5.560048 | 16.62548 | 9.262293 | 18.84012 | 10.05594 | 18.11507 | 13.92172 | 17.18186 | 12.1506 | 11.87443 | 12.85382 | 11.38971 | 20.27043 | 24.38309 | 23.28835 | 24.2722 | 19.19934 | 9.216332 | 12.75137 | NA | 16.20103 |
| CUST_33959_PI429010886 | M8365 | 0.006246 | 15 | 9 | | | | | | 11632.77 | 5162.076 | 20339.21 | 705.3837 | 1606.601 | 957.2333 | 16451.67 | 29696.39 | 27353.46 | 35362.62 | 13487.6 | 12465.11 | 25546.19 | 37561.18 | 19773.16 | 5811.899 | 21690.05 | 4150.569 | 10341.13 | 14744.71 | 7654.795 | 24915.2 | 25081.91 | 3546.259 | 42140.05 | 10044.17 | 38256.92 |
| CUST_42230_PI429010886 | M59742 | 0.006265 | 24 | 18 | | | | | | 70.52611 | 73.74514 | 70.96812 | 62.83219 | 62.79742 | 52.44678 | 55.16975 | 42.61497 | 41.44605 | 73.05511 | 70.80885 | 71.45222 | 56.57261 | 74.08523 | 64.49483 | 90.77809 | 48.68836 | 82.00472 | 84.36786 | 78.43466 | 70.90857 | 75.84454 | 67.59725 | 42.47769 | 84.56429 | 61.93065 | 72.37554 |
| CUST_32628_PI429010886 | M4628 | 0.006274 | 18 | 6 | | | | | | 300.8257 | 235.9095 | 265.8178 | 276.5189 | 292.258 | 205.7973 | 320.6371 | 294.596 | 329.4092 | 208.552 | 213.3434 | 264.7036 | 165.7579 | 178.2839 | 185.2639 | 381.2578 | 190.0374 | 177.7855 | 202.7615 | 186.1374 | 185.9383 | 529.0811 | 74.7945 | 320.1726 | 376.9333 | 204.0536 | 307.9072 |
| CUST_46243_PI429010886 | M71924 | 0.00628 | 15 | 12 | | | | | | 26.82962 | 31.47236 | 26.08148 | 19.63631 | 25.45721 | 20.37385 | 21.03821 | 27.90958 | 37.13409 | 34.47823 | 25.53913 | 55.48383 | 31.49741 | 45.83957 | 48.83879 | 55.2283 | 11.11646 | 28.99859 | 23.88015 | 27.99312 | 32.55113 | 33.1071 | 34.02862 | 12.03892 | 38.58794 | 37.46264 | 52.63381 |
| CUST_17420_PI429010886 | N37933 | 0.006282 | 24 | 18 | | | | | | 5.104542 | 77.85613 | 37.32162 | 25.36775 | 23.34857 | 7.424309 | 5.79109 | NA | 6.199171 | 16.55361 | 18.83592 | 32.40014 | 35.00193 | 63.62137 | 53.02384 | 42.28532 | NA | 38.93621 | 48.99962 | 40.95384 | 52.8351 | 45.45196 | 24.03945 | NA | 56.52083 | NA | 15.3003 |
| CUST_37841_PI429010886 | M21826 | 0.006295 | 15 | 9 | | | | | | 271.2223 | 972.4433 | 827.8275 | 397.2225 | 400.5132 | 8.516835 | 93.01036 | 655.2712 | 2782.19 | 2618.901 | 1660.585 | 3446.227 | 1325.963 | 5197.227 | 2659.902 | 877.9874 | 286.8668 | 598.2934 | 608.8662 | 3783.178 | 824.1385 | 2321.278 | 3876.188 | 119.6528 | 4464.413 | 3597.224 | 6779.053 |
| CUST_1841_PI429010886 | N10861 | 0.006295 | 15 | 9 | 321466121 | hypothetical protein DAPPUDRAFT_305875 [Daphn | | | 9.61E-09 | 265.8178 | 568.2036 | 685.806 | 14.49923 | 85.19825 | 27.71788 | 127.2103 | 385.3789 | 669.0767 | 1122.017 | 443.5662 | 667.377 | 857.1846 | 550.4344 | 634.6129 | 581.4141 | 281.5398 | 293.8787 | 384.8393 | 1278.963 | 372.1299 | 1025.99 | 645.7634 | 30.45027 | 1265.003 | 829.8828 | 1401.401 |
| CUST_5124_PI429010886 | N18289 | 0.006295 | 15 | 9 | | | | | | 105.3783 | 119.0396 | 121.7904 | 79.92252 | 122.0182 | 87.13975 | 97.00878 | 117.5061 | 108.4414 | 146.722 | 132.8611 | 167.982 | 97.63395 | 144.3781 | 115.5186 | 118.3356 | 97.29292 | 119.2118 | 87.2755 | 133.355 | 118.1291 | 122.1353 | 153.7764 | 118.2899 | 133.6264 | 133.9495 | 147.3934 |
| CUST_9618_PI429010886 | N24937 | 0.006295 | 15 | 9 | | | | | | 41.16894 | 47.11613 | 34.61308 | 28.41001 | 32.3288 | 29.8401 | 35.42916 | 30.3584 | 34.52588 | 41.73275 | 39.91268 | 45.488 | 33.64032 | 31.59756 | 22.3581 | 36.69017 | 31.23112 | 35.31167 | 22.29224 | 39.31465 | 30.40899 | 36.63495 | 39.8043 | 19.07608 | 51.21719 | 38.83398 | 43.33061 |
| CUST_39980_PI429010886 | M47787 | 0.006306 | 12 | 0 | | | | | | 2506.708 | 7950.432 | 3053.471 | 2423.127 | 2235.234 | 1366.468 | 1333.908 | 1665.324 | 2303.489 | 4998.753 | 3074.469 | 6709.279 | 4715.889 | 5868.528 | 6511.817 | 6388.757 | 2179.192 | 2997.206 | 2935.657 | 5830.309 | 2176.36 | 6189.048 | 3134.449 | 2430.624 | 4887.374 | 7617.588 | 6536.631 |
| CUST_21024_PI429010886 | N44494 | 0.006314 | 15 | 9 | 351699829 | Glucose-dependent insulinotropic receptor, partial | | | 2.77E-09 | 78.88657 | 154.5663 | 155.1312 | 15.24921 | 42.70658 | NA | 35.28275 | 152.8418 | 153.5797 | 309.6337 | 146.0723 | 252.7105 | 201.9396 | 196.0648 | 156.0301 | 213.366 | 56.15273 | 90.18249 | 61.53357 | 235.7566 | 62.00733 | 300.0118 | 284.3166 | 15.96673 | 297.4174 | 189.3668 | 313.7524 |
| CUST_21365_PI429010886 | N45089 | 0.006317 | 18 | 3 | 443687473 | hypothetical protein CAPTEDRAFT_197462 [Capitell | | | 1.31E-08 | 14.85935 | 22.04311 | 9.303154 | 20.08306 | 22.85484 | 16.72995 | 13.72321 | 19.56884 | 10.80561 | 18.40232 | 9.125404 | 16.1574 | 8.381146 | 6.834339 | 9.47114 | 18.90775 | 19.12864 | 15.73595 | 11.64631 | 9.508624 | 7.895988 | 20.04961 | 15.24563 | 25.60611 | 17.14965 | 11.9615 | 22.86232 |
| CUST_47847_PI429010886 | M77826 | 0.006322 | 24 | 21 | | | | | | 79.45781 | 76.15742 | 68.6762 | 88.23558 | 83.71896 | 75.09478 | 65.94453 | 62.10992 | 88.4462 | 79.66167 | 80.43582 | 102.3996 | 62.77579 | 63.97505 | 64.14066 | 119.5708 | 87.67204 | 105.779 | 89.31811 | 107.4983 | 102.17 | 116.5044 | 93.83775 | 103.2645 | 102.1823 | 90.74799 | 93.6434 |
| CUST_39822_PI429010886 | M45222 | 0.006324 | 24 | 18 | | | | | | 936.7815 | 1407.359 | 1199.593 | 1045.15 | 1073.06 | 892.3589 | 857.3719 | 837.1072 | 928.6222 | 1073.06 | 1129.308 | 2142.216 | 1135.012 | 1246.008 | 1407.359 | 2319.618 | 826.6639 | 1256.084 | 1350.099 | 1593.287 | 1417.942 | 1705.674 | 1045.788 | 1092.486 | 1541.439 | 1614.966 | 1565.628 |
| CUST_13755_PI429010886 | N31716 | 0.006333 | 24 | 21 | 291235351 | PREDICTED: zinc finger protein 347-like [Saccoglossu | | | 2.25E-22 | 46.06229 | 58.23835 | 45.215 | 36.02039 | 39.83487 | 30.8467 | 28.73729 | 23.22056 | 34.0469 | 42.85678 | 43.05742 | 73.42972 | 48.50963 | 55.26178 | 47.73713 | 58.67101 | 24.65264 | 49.34012 | 54.58654 | 46.84888 | 40.63191 | 53.13993 | 53.08483 | 53.68104 | 49.07171 | 48.80606 | 52.04647 |
| CUST_25654_PI429010886 | N52818 | 0.006333 | 24 | 21 | | | | | | 165.3955 | 113.0468 | 119.6528 | 91.94088 | 95.33691 | 124.009 | 100.566 | 101.8114 | 112.6306 | 125.6844 | 112.5793 | 84.11167 | 139.0764 | 95.32771 | 38.59967 | 139.7005 | 141.4128 | 112.4908 | 155.2361 | 114.6697 | 134.659 | 171.7375 | 150.8963 | 141.1734 | 109.2686 | 84.46837 | 135.5002 |
| CUST_5288_PI429010886 | N18496 | 0.006343 | 21 | 15 | 321473690 | hypothetical protein DAPPUDRAFT_209558 [Daphn | | | 1.33E-47 | 58.01793 | 47.25743 | 32.94683 | 31.96544 | 40.1699 | 25.13331 | 38.91166 | 43.77433 | 48.17521 | 39.71396 | 48.51868 | 49.07171 | 44.81799 | 45.78676 | 48.84923 | 58.51806 | 45.18256 | 54.93851 | 50.00256 | 65.02562 | 35.30842 | 52.47732 | 39.91731 | 41.99242 | 53.8203 | 32.10055 | 22.9227 |
| CUST_6510_PI429010886 | N20195 | 0.006344 | 21 | 0 | 225711550 | U7 snRNA-associated Sm-like protein LSm11 [Caligu | | | 2.39E-10 | 9.209552 | 26.21095 | 19.4114 | 38.57925 | 16.28372 | 11.64186 | 13.06014 | 8.650339 | 14.11041 | 21.84747 | 7.93225 | 14.41094 | 7.941557 | 7.979138 | 6.235994 | 18.91214 | 9.365159 | 10.17778 | 4.99995 | 18.97668 | 22.27554 | 21.9208 | 22.40881 | 14.53278 | 21.66199 | 21.86875 | 9.200881 |
| CUST_26635_PI429010886 | N54587 | 0.006347 | 15 | 12 | | | | | | 94.1523 | 183.8412 | 192.0215 | 92.38118 | 110.5098 | 62.68635 | 113.6358 | 161.2704 | 196.5633 | 285.3755 | 115.5578 | 281.874 | 219.9262 | 214.7331 | 189.3407 | 116.1614 | 136.6377 | 133.298 | 121.5266 | 195.8383 | 88.34755 | 168.0579 | 145.1035 | 109.0449 | 143.0236 | 103.2645 | 177.9987 |
| CUST_38346_PI429010886 | M25696 | 0.006347 | 24 | 21 | | | | | | 162.1995 | 252.2065 | 210.2883 | 207.7149 | 254.1554 | 262.9091 | 160.2794 | 138.6787 | 150.0945 | 204.7885 | 170.8572 | 188.9774 | 187.2169 | 277.0077 | 220.1671 | 243.7531 | 212.9538 | 235.3617 | 185.5006 | 285.5481 | 272.6795 | 310.0812 | 226.2023 | 233.9751 | 238.6573 | 189.7386 | 202.6087 |
| CUST_36577_PI429010886 | M16281 | 0.006355 | 18 | 15 | | | | | | 76.06574 | 137.1593 | 25.0475 | 115.6759 | 144.5809 | 48.8316 | 6.453334 | NA | 7.004476 | 37.88861 | 77.39915 | 55.07968 | 104.4054 | 349.0566 | 196.129 | 207.2391 | NA | 178.6841 | NA | 539.1803 | 135.061 | 631.4343 | 25.09429 | 114.4015 | 140.0012 | 7.648203 | 6.054897 |
| CUST_4345_PI429010886 | N16556 | 0.006355 | 15 | 12 | | | | | | 586.0335 | 307.6894 | 6490.163 | 25.73562 | 20.33108 | 7.634015 | 1522.93 | 10597.5 | 9638.897 | 9358.113 | 1890.591 | 3048.703 | 6247.817 | 16607.59 | 9433.107 | 821.0172 | 4711.518 | 291.2799 | 1119.759 | 3879.679 | 422.2989 | 12273.46 | 11089.86 | 273.733 | 14629.47 | 1709.2 | 15133.65 |
| CUST_17655_PI429010886 | N38355 | 0.006355 | 15 | 12 | | | | | | 279.0433 | 69.56934 | 3084.768 | 33.79348 | 58.51806 | 48.68836 | 867.8685 | 3022.502 | 2651.764 | 3111.72 | 360.7942 | 558.6606 | 2818.479 | 7383.427 | 2287.157 | 205.5955 | 1388.938 | 91.97543 | 445.8485 | 544.3999 | 290.2221 | 2370.125 | 4627.233 | 167.9263 | 5848.791 | 581.1144 | 5167.16 |
| CUST_18013_PI429010886 | N38997 | 0.006355 | 15 | 12 | 195572001 | GD20723 [Drosophila simulans] >gi\|194199915\|gb | Cellular Process | Cell differentiation | 1.67E-14 | 38.40419 | 30.27191 | 45.53479 | 38.58369 | 31.86956 | 25.59155 | 41.44605 | 31.24239 | 37.34183 | 42.53339 | 34.91364 | 39.98138 | 37.14124 | 54.21273 | 48.70779 | 43.72859 | 33.33635 | 34.59277 | 33.53684 | 35.11564 | 32.35284 | 41.66889 | 30.97619 | 36.63495 | 56.46018 | 41.80062 | 66.57113 |
| CUST_7271_PI429010886 | N21297 | 0.006356 | 21 | 0 | | | | | | 14.68971 | 22.01737 | 17.08729 | 14.8732 | 12.76972 | 3.782841 | 10.28851 | 7.480574 | 7.902818 | 21.18145 | 7.920199 | 8.608528 | 6.509926 | 21.32852 | 11.81798 | 9.861478 | 14.64501 | 11.30649 | 7.623642 | 12.28913 | 15.87862 | 43.44238 | 24.28972 | 13.36364 | 13.5281 | NA | 14.01751 |
| CUST_33721_PI429010886 | M7706 | 0.006364 | 18 | 3 | 342326264 | dystroglycan [Cherax quadricarinatus] | Developmental process | Nervous system development | 1.64E-35 | 74.06105 | 65.46343 | 90.97139 | 72.72106 | 82.67004 | 85.64499 | 68.88215 | 85.36118 | 116.4584 | 70.11707 | 72.52738 | 82.69106 | 61.95712 | 93.37534 | 66.7414 | 60.53815 | 70.83788 | 58.90324 | 61.19321 | 88.20739 | 81.86063 | 101.7598 | 86.15616 | 328.0964 | 75.46943 | 108.4668 | 111.6027 |
| CUST_53493_PI429010886 | M101958 | 0.006366 | 15 | 9 | 321464775 | miniature spindles-like protein [Daphnia pulex] | | | 3.97E-21 | 24.85691 | 26.48449 | 19.91752 | 16.18956 | 17.93968 | 18.64838 | 24.7458 | 20.22864 | 19.73697 | 28.53867 | 28.32194 | 20.05897 | 21.56178 | 22.93702 | 24.6038 | 24.4538 | 19.03542 | 18.69291 | 30.24809 | 18.34511 | 17.36105 | 22.11953 | 25.04386 | 20.07409 | 29.59116 | 20.16106 | 26.92163 |
| CUST_4852_PI429010886 | N17616 | 0.006366 | 15 | 9 | | | | | | 69.06525 | 60.5193 | 78.43466 | 57.51717 | 58.01161 | 52.88081 | 59.81399 | 70.02583 | 60.07014 | 66.22546 | 57.16139 | 83.48682 | 54.87756 | 76.19351 | 80.32797 | 59.50688 | 64.73185 | 76.48318 | 58.4895 | 64.88975 | 58.0658 | 63.37918 | 63.13278 | 60.12109 | 80.89652 | 70.98423 | 98.05949 |
| CUST_15736_PI429010886 | N35052 | 0.006366 | 15 | 3 | | | | | | 16.0016 | 10.24643 | 19.99534 | 19.87525 | 23.92874 | 21.71424 | 16.26691 | 25.80044 | 12.68586 | 17.07869 | 14.45421 | 7.341025 | 17.2249 | 17.54669 | 3.621244 | 17.64732 | 22.36394 | 7.753636 | 26.9442 | 23.84339 | 19.29786 | 18.64626 | 10.95668 | 9.207048 | 12.62711 | 15.14895 | 16.70546 |
| CUST_11889_PI429010886 | N28579 | 0.006375 | 18 | 15 | | | | | | 1276.669 | 1548.599 | 2323.132 | 786.706 | 953.2915 | 921.6949 | 1321.79 | 1557.483 | 1955.85 | 1888.53 | 1975.879 | 2596.508 | 2035.189 | 2462.824 | 1781.492 | 3474.89 | 1903.519 | 2155.605 | 1837.082 | 3296.897 | 1092.036 | 2115.987 | 1335.109 | 1138.397 | 2826.022 | 1542.262 | 3456.298 |
| CUST_3138_PI429010886 | N13881 | 0.006391 | 18 | 6 | | | | | | 41.15689 | 19.27537 | 350.7295 | 26.60971 | 50.57001 | 47.01326 | 104.4054 | 490.65 | 335.5871 | 360.5617 | 73.0191 | 79.82568 | 303.1495 | 695.7952 | 142.8727 | 45.18835 | 188.4563 | 64.87589 | 29.71896 | 57.76414 | 36.08462 | 174.0729 | 660.0703 | 41.20843 | 499.6354 | 76.26718 | 594.8858 |
| CUST_27738_PI429010886 | N56611 | 0.006398 | 18 | 12 | | | | | | 6.105375 | 20.79184 | 23.94925 | NA | 7.107009 | NA | 18.61312 | 16.00911 | 22.59879 | 15.75973 | 7.608505 | 21.41052 | 145.4633 | 129.4911 | 87.46258 | 109.812 | 37.81058 | 3.58299 | 16.02173 | NA | 11.74472 | 6.797558 | 10.16422 | NA | 43.1379 | 15.29079 | 85.0717 |
| CUST_28506_PI429010886 | N58079 | 0.006401 | 21 | 9 | | | | | | 10.00867 | NA | 12.17439 | 14.09753 | 9.911889 | 11.50819 | 5.921666 | 14.94106 | 16.51187 | 15.05054 | 10.37845 | 21.08802 | 8.395664 | 21.36888 | 18.47842 | 12.45001 | 8.405253 | 10.84532 | 5.960341 | 4.695828 | 7.325414 | 9.583323 | 5.075645 | 11.77781 | 15.7789 | 14.09025 | 23.05184 |
| CUST_25833_PI429010886 | N53137 | 0.006403 | 12 | 9 | | | | | | 65.13839 | 41.22377 | 65.04995 | 49.28537 | 51.5698 | 44.58572 | 51.32807 | 57.84331 | 51.14292 | 83.32632 | 52.32895 | 79.41411 | 64.64151 | 82.50766 | 58.17945 | 61.8504 | 52.35985 | 57.1457 | 67.85099 | 63.60606 | 64.09512 | 70.88996 | 68.55373 | 73.803 | 55.48383 | 59.48243 | 83.63285 |
| CUST_1315_PI429010886 | N9258 | 0.006419 | 21 | 3 | 8134678 | RecName: Full=Semaphorin-1A; AltName: Full=Fasc | Developmental process | Nervous system development | 2.26E-07 | 45.65065 | 49.69905 | 46.46451 | 59.57796 | 40.59908 | 66.32011 | 46.42845 | 57.46153 | 56.03701 | 48.41112 | 61.05172 | 44.89448 | 30.72781 | 42.26034 | 40.42785 | 46.64388 | 45.60489 | 64.40125 | 38.41931 | 42.15075 | 45.01151 | 44.36476 | 57.14083 | 26.8214 | 64.42438 | 65.53578 | 60.61979 |
| CUST_45547_PI429010886 | M69440 | 0.006421 | 24 | 6 | | | | | | 46.67338 | 33.8524 | 84.89465 | 46.05816 | 55.784 | 36.34355 | 72.1095 | 154.7269 | 90.95085 | 60.29973 | 74.57029 | 89.4807 | 84.27092 | 83.19226 | 63.43062 | 33.32019 | 61.88462 | 34.61308 | 42.65121 | 140.9164 | 49.54555 | 23.9514 | 39.36593 | 39.86189 | 44.64991 | 62.74739 | 78.54463 |
| CUST_17920_PI429010886 | N38834 | 0.006426 | 21 | 15 | | | | | | 23.34607 | 17.96838 | 19.51952 | 9.588389 | 11.66524 | 9.118641 | 18.79454 | 10.54492 | 15.23856 | 19.79173 | 13.8081 | 17.95297 | 12.10209 | 12.1856 | 23.44542 | 23.04237 | 21.55311 | 21.58663 | 9.250124 | 17.85771 | 17.07004 | 17.89993 | 16.9342 | 24.04687 | 15.21068 | 14.34109 | 8.334073 |
| CUST_52629_PI429010886 | M98230 | 0.006434 | 24 | 0 | | | | | | 14.69744 | 68.93293 | 10.39612 | 14.54491 | 7.462253 | 13.52517 | 10.98836 | 8.306883 | 11.0861 | 11.42997 | 14.73662 | 6.920375 | 6.115219 | 9.227326 | 5.526487 | 44.41825 | NA | 9.591065 | 5.032873 | 17.73942 | 7.090444 | 16.3293 | 10.83121 | 8.14537 | 22.43497 | 28.03356 | 19.92894 |
| CUST_49532_PI429010886 | M85988 | 0.006438 | 15 | 9 | | | | | | 42.34931 | 42.21564 | 78.60763 | 27.23432 | 30.4923 | 27.77246 | 38.57925 | 51.14292 | 65.11259 | 58.99785 | 43.72098 | 62.90962 | 46.06229 | 49.42389 | 52.73715 | 55.58103 | 79.84151 | 27.28673 | 41.61311 | 36.05134 | 27.34913 | 69.28978 | 72.06797 | 42.82987 | 99.79034 | 37.22874 | 81.92913 |
| CUST_6429_PI429010886 | N20090 | 0.006438 | 15 | 9 | | | | | | 11545.02 | 19438.74 | 13887.31 | 10915.56 | 10866.21 | 8327.109 | 8675.932 | 13602.1 | 14970.57 | 18150.31 | 12884.64 | 19626.64 | 13124.49 | 14950.27 | 14446.35 | 18661.44 | 9046.466 | 12762.11 | 12313.28 | 16841.23 | 11122.77 | 19067.23 | 13438.24 | 7985.61 | 20967.97 | 19491.76 | 17752.88 |
| CUST_23457_PI429010886 | N48864 | 0.006438 | 15 | 9 | | | | | | 45.25874 | 60.8738 | 62.46895 | 46.44979 | 37.15259 | 50.65259 | 50.8348 | 72.51875 | 43.73443 | 69.72006 | 41.82009 | 73.65478 | 45.80703 | 49.01735 | 53.10218 | 50.67859 | 53.26328 | 53.84859 | 32.94363 | 32.21966 | 44.0607 | 80.15845 | 47.86055 | 56.9818 | 68.25442 | 57.55495 | 85.27513 |
| CUST_29490_PI429010886 | N59966 | 0.006438 | 15 | 9 | | | | | | 67.89962 | 83.70947 | 85.62903 | 63.37317 | 60.24939 | 65.04228 | 66.35653 | 69.15357 | 90.28628 | 111.1756 | 82.55661 | 91.51834 | 76.20212 | 106.023 | 58.93488 | 76.07766 | 72.64262 | 76.69694 | 69.44924 | 78.79452 | 95.60632 | 80.91461 | 102.3423 | 84.56429 | 110.5811 | 91.11943 | 121.9844 |
| CUST_20950_PI429010886 | N44359 | 0.006443 | 18 | 3 | 306478625 | ficolin-like protein 1 [Pacifastacus leniusculus] | | | 3.66E-25 | 267.5328 | 511.7062 | 172.3811 | 328.807 | 365.9997 | 278.3016 | 360.1121 | 387.779 | 105.1778 | 198.0625 | 312.5863 | 218.636 | 262.5661 | 188.658 | 81.26685 | 255.8316 | 186.4327 | 179.5282 | 344.7288 | 105.7372 | 200.3686 | 420.4304 | 288.4469 | 427.9524 | 345.7818 | 195.0054 | 267.4897 |
| CUST_32567_PI429010886 | M4449 | 0.006456 | 18 | 9 | 2290213 | Lian-Aa1 retrotransposon protein [Aedes aegypti] | Nucleic acid metabolism | Regulation of gene expression | 2.74E-40 | 2828.097 | 4575.004 | 4064.703 | 2871.394 | 4186.218 | 3177.58 | 2504.679 | 5050.626 | 5130.907 | 4524.584 | 6136.091 | 11122.77 | 5780.138 | 4854.251 | 5086.964 | 4484.875 | 3105.643 | 3456.298 | 3118.308 | 6949.82 | 3968.239 | 4289.879 | 3013.914 | 2788.353 | 4778.44 | 3981.392 | 6587.101 |
| CUST_13877_PI429010886 | N31925 | 0.006458 | 21 | 3 | 405973388 | Transcription termination factor 2 [Crassostrea giga | | | 1.05E-12 | 1630.188 | 4135.156 | 2487.623 | 5492.691 | 2935.657 | 3051.185 | 1904.883 | 2019.046 | 1914.945 | 2331.325 | 1185.368 | 1198.309 | 1370.195 | 2441.116 | 1424.308 | 1733.695 | 917.7643 | 1917.136 | 1323.05 | 1172.595 | 3103.161 | 2296.235 | 3029.514 | 1787.33 | 3644.859 | 3318.146 | 1752.574 |
| CUST_37056_PI429010886 | M17712 | 0.006458 | 12 | 0 | | | | | | 1025.258 | 1289.943 | 1050.102 | 521.397 | 878.2313 | 836.7397 | 685.806 | 895.8021 | 872.2022 | 1133.519 | 862.6591 | 1282.355 | 922.8399 | 1133.519 | 1125.765 | 1045.788 | 818.0738 | 786.9887 | 755.8427 | 950.5113 | 954.3357 | 1183.738 | 887.8552 | 551.706 | 1207.044 | 1080.128 | 1321.214 |
| CUST_4680_PI429010886 | N17267 | 0.006458 | 12 | 3 | | | | | | 34.0788 | 44.30287 | 15.02774 | 43.06915 | 47.40881 | 41.65038 | 34.35393 | 13.85759 | 37.19186 | 21.97161 | 18.32738 | 26.59751 | 23.78457 | 24.26118 | 21.74647 | 45.10479 | 37.86895 | 39.46953 | 33.01824 | 28.19347 | 28.32194 | 30.58011 | 25.51724 | 37.60419 | 28.48104 | 24.81825 | 20.40333 |
| CUST_22608_PI429010886 | N47322 | 0.00646 | 18 | 0 | | | | | | 26.73374 | 14.46495 | 15.42925 | 12.4096 | 8.387454 | 20.67573 | 18.59558 | 15.44569 | 7.91474 | 8.974986 | 10.07591 | 7.240943 | 10.39612 | 3.624124 | 13.77941 | 17.46064 | 15.22654 | 26.02628 | 21.16443 | 11.96649 | 22.99548 | 12.30553 | 17.25337 | 27.34334 | 10.3727 | 11.95734 | 15.60693 |
| CUST_21633_PI429010886 | N45560 | 0.00646 | 21 | 0 | | | | | | 54.42343 | 42.81625 | 40.53079 | 41.7241 | 27.22484 | 20.8155 | 37.78545 | 38.23666 | 35.53394 | 24.65264 | 20.66537 | 27.24092 | 13.24259 | 37.45905 | 19.01762 | 35.9416 | 28.3608 | 41.26062 | 24.12066 | 38.42634 | 36.07548 | 43.95633 | 26.4059 | 39.11234 | 33.13396 | 45.82004 | 26.58995 |
| CUST_27721_PI429010886 | N56579 | 0.006475 | 15 | 9 | | | | | | 136.1436 | 234.7465 | 164.7626 | 88.63339 | 105.5931 | 118.233 | 411.8683 | 179.9528 | 504.2482 | 113.9999 | 98.82662 | 242.1667 | 262.4225 | 221.5477 | 207.2788 | 165.1069 | 99.53996 | 139.1812 | 98.47983 | 98.67215 | 125.9169 | 166.6809 | 199.0047 | 279.6215 | 376.5573 | 171.8712 | 613.8279 |
| CUST_33328_PI429010886 | M6612 | 0.006494 | 15 | 9 | | | | | | 49.3486 | 53.46776 | 63.79444 | 57.23868 | 58.12189 | 52.92115 | 54.6811 | 52.93772 | 53.72529 | 71.79355 | 47.55222 | 76.42859 | 69.27267 | 78.90095 | 69.59584 | 54.87756 | 57.76414 | 53.71904 | 41.46468 | 48.70779 | 55.3496 | 78.57748 | 60.41389 | 62.29412 | 65.84223 | 79.33173 | 79.50596 |
| CUST_56617_PI429010886 | M114225 | 0.006515 | 21 | 15 | 51104825 | Euphausia superba small subunit ribosomal RNA ge | | | 2.33E-88 | 944.3898 | 847.0661 | 1038.465 | 457.6266 | 1678.528 | 898.2047 | 1430.089 | 1095.89 | 1011.26 | 2103.685 | 887.8552 | 1357.763 | 1730.823 | 1425.514 | 1059.331 | 1321.79 | 1490.779 | 1631.709 | 1250.32 | 1238.144 | 1202.334 | 1385.628 | 613.4417 | 415.5762 | 494.5663 | 886.1313 | 1168.631 |
| CUST_46699_PI429010886 | M73447 | 0.006521 | 24 | 0 | | | | | | 67.12072 | 47.44967 | 63.14802 | 61.36332 | 41.89305 | 49.66578 | 41.48865 | 68.9445 | 51.01709 | 48.65694 | 39.6493 | 39.99701 | 38.35127 | 53.39323 | 40.90818 | 51.75764 | 54.46899 | 63.16045 | 58.90324 | 46.56491 | 40.32605 | 59.13459 | 51.02755 | 62.31885 | 65.61755 | 51.89227 | 66.81883 |
| CUST_6265_PI429010886 | N19870 | 0.006521 | 24 | 0 | 383865898 | PREDICTED: stress-induced-phosphoprotein 1-like [ | | | 3E-117 | 37.37861 | 44.05203 | 42.28532 | 33.82224 | 38.76201 | 22.52813 | 34.48473 | 26.89586 | 40.11037 | 34.13077 | 25.12351 | 22.51683 | 34.75551 | 23.9514 | 30.71736 | 40.81934 | 36.49052 | 34.15487 | 27.56643 | 36.27644 | 27.83166 | 39.78898 | 34.35609 | 31.01397 | 43.30223 | 29.09314 | 45.38957 |
| CUST_31887_PI429010886 | M2667 | 0.006533 | 12 | 0 | | | | | | 280.8839 | 249.1725 | 359.6577 | 209.1269 | 185.0177 | 149.9119 | 200.7304 | 275.8443 | 245.9376 | 305.6076 | 210.2883 | 255.4957 | 223.8346 | 292.7227 | 220.5819 | 214.7331 | 256.1926 | 188.3473 | 231.8976 | 238.7009 | 203.9899 | 289.5961 | 267.8414 | 236.4903 | 315.6417 | 253.1078 | 282.2712 |
| CUST_45207_PI429010886 | M68318 | 0.006551 | 21 | 3 | 399936392 | voltage-gated calcium channel beta subunit transcri | Transport | Ion transport | 1.73E-34 | 41.59399 | 68.95198 | 46.65359 | 49.81001 | 54.46306 | 60.01583 | 46.35772 | 57.07016 | 34.05805 | 34.44235 | 18.7912 | 41.41188 | 29.80111 | 40.66087 | 36.61393 | 49.28963 | 31.67438 | 41.97898 | 31.38593 | 34.71929 | 65.48233 | 42.65591 | 47.42461 | 48.5992 | 74.22931 | 73.52413 | 30.35559 |
| CUST_7518_PI429010886 | N21665 | 0.006555 | 21 | 18 | | | | | | 13.24115 | 6.428186 | 13.55928 | 10.74056 | 7.943105 | 9.3513 | 5.693385 | NA | 4.553482 | 12.24341 | 8.07058 | NA | 4.612209 | 4.035888 | 3.680032 | 11.35808 | 15.86933 | 8.069484 | 36.80914 | 17.21299 | 6.410607 | 6.078713 | 11.57286 | 14.45246 | 8.48246 | 5.110828 | 8.568969 |
| CUST_53975_PI429010886 | M103943 | 0.006555 | 12 | 3 | | | | | | 196.5989 | 159.0029 | 104.6277 | 217.0054 | 208.2116 | 295.4403 | 238.1723 | 161.5676 | 107.9913 | 130.0411 | 156.1462 | 88.95458 | 154.2676 | 127.916 | 105.6711 | 158.496 | 221.2444 | 235.1413 | 186.5931 | 183.6032 | 158.4321 | 186.3412 | 112.7849 | 126.7767 | 164.7862 | 189.7994 | 91.02902 |
| CUST_14628_PI429010886 | N33198 | 0.006576 | 21 | 18 | | | | | | 183.6281 | 234.3391 | 157.8626 | 138.4086 | 135.3099 | 135.1969 | 138.5998 | 120.3747 | 142.9378 | 217.2745 | 129.772 | 162.141 | 130.7837 | 143.517 | 145.0486 | 257.237 | 132.551 | 190.7563 | 189.2302 | 198.2178 | 196.4719 | 120.9579 | 169.3616 | 185.7909 | 117.6303 | 147.1104 | 155.5527 |
| CUST_2437_PI429010886 | N12349 | 0.006581 | 24 | 18 | | | | | | 25.62613 | 10.1832 | 15.41864 | 6.509926 | 6.075781 | NA | 11.5947 | 26.61439 | 28.36576 | 8.518007 | 35.78224 | 27.6707 | 10.7039 | 11.21201 | NA | 7.91474 | 51.39386 | 28.16499 | 62.99128 | 77.00574 | 73.73837 | 8.019973 | 16.78445 | 27.01961 | 47.55714 | 26.19664 | 22.17042 |
| CUST_44678_PI429010886 | M66656 | 0.006582 | 12 | 3 | | | | | | 1565.179 | 1178.063 | 932.1915 | 2142.216 | 1643.929 | 2206.497 | 2021.454 | 1257.974 | 1058.251 | 843.1506 | 1740.855 | 914.6851 | 1320.152 | 862.42 | 942.3167 | 1477.505 | 1865.638 | 1378.434 | 1542.262 | 1059.917 | 1363.618 | 1191.093 | 1091.604 | 1748.457 | 855.218 | 1316.22 | 807.9372 |
| CUST_3405_PI429010886 | N14443 | 0.006585 | 15 | 12 | 242013098 | protein groucho, putative [Pediculus humanus corp | Nucleic acid metabolism | Regulation of gene expression | 1.58E-74 | 22.16397 | 18.95813 | 36.05134 | 23.81072 | 18.92827 | 10.3662 | 21.01141 | 35.04798 | 28.00895 | 33.51766 | 33.04177 | 28.2496 | 35.54408 | 35.21995 | 23.34418 | 26.08713 | 29.10818 | 22.69042 | 22.51683 | 22.92529 | 18.66441 | 34.84639 | 27.77246 | 18.38425 | 31.83203 | 24.71578 | 29.8401 |
| CUST_50152_PI429010886 | M88841 | 0.006591 | 21 | 0 | 512816354 | PREDICTED: protocadherin Fat 1 isoform X5 [Xenop | Cellular Process | Cell adhesion | 6.87E-16 | 28.14284 | 18.82741 | 31.33095 | 26.08326 | 15.46051 | 21.22218 | 22.60935 | 22.74775 | 14.72582 | 11.54241 | 11.71536 | 11.29494 | 15.23856 | 30.0795 | 20.49506 | 15.95324 | 16.5954 | 15.15808 | 26.59751 | 15.48139 | 13.63311 | 38.24749 | 21.21095 | 22.32503 | 18.80919 | 20.59244 | 11.40993 |
| CUST_42683_PI429010886 | M60960 | 0.006595 | 24 | 0 | | | | | | 111.625 | 137.4615 | 124.5589 | 137.351 | 113.5137 | 103.5678 | 99.36845 | 75.13415 | 96.43909 | 105.798 | 99.10652 | 108.8877 | 104.5579 | 93.92221 | 103.0243 | 105.3648 | 97.63395 | 110.8879 | 93.45456 | 101.7238 | 121.2986 | 134.0969 | 110.2442 | 109.0963 | 131.1403 | 103.7315 | 139.3127 |
| CUST_2466_PI429010886 | N12436 | 0.006595 | 24 | 12 | 71159516 | C-type lectin [Penaeus monodon] | | | 5.02E-10 | 563.6523 | 1107.396 | 803.5259 | 716.6989 | 1181.018 | 675.7327 | 626.2648 | 933.4785 | 1272.526 | 1376.074 | 1104.361 | 1232.019 | 1478.956 | 1306.431 | 1333.908 | 1443.25 | 200.8936 | 707.905 | 1153.433 | 1031.749 | 819.1664 | 1164.641 | 843.8131 | 793.8063 | 502.6158 | 711.0836 | 716.6989 |
| CUST_33009_PI429010886 | M5713 | 0.006603 | 15 | 9 | 291220823 | PREDICTED: Saccoglossus kowalevskii hypothetical | | | | 98.59007 | 65.31258 | 70.65347 | 75.61377 | 74.36967 | 56.46834 | 82.72689 | 109.8902 | 99.79838 | 121.2504 | 108.9753 | 100.0408 | 80.29877 | 106.2285 | 84.2393 | 79.48728 | 92.52049 | 61.27293 | 96.33377 | 86.67105 | 67.36225 | 105.2954 | 97.72266 | 58.4329 | 129.772 | 92.09827 | 84.25035 |
| CUST_26368_PI429010886 | N54120 | 0.006605 | 24 | 21 | | | | | | 13.46279 | 6.906185 | 11.82579 | 15.54199 | 11.30336 | NA | 3.819537 | 7.533977 | 6.448841 | 17.70878 | 8.459432 | 7.0824 | 12.71344 | 53.98541 | 5.826164 | 11.0429 | 10.31819 | 9.148382 | 26.16897 | 13.83685 | 10.9288 | 14.67308 | 15.61433 | 20.72467 | 9.47114 | 8.821462 | 18.93228 |
| CUST_45045_PI429010886 | M67833 | 0.006608 | 12 | 0 | | | | | | 18.47667 | 84.95079 | 52.11124 | 24.04687 | 14.43921 | 9.301993 | 12.79677 | 44.29926 | 35.88091 | 61.35068 | 29.64495 | 58.05303 | 81.85243 | 93.99876 | 51.86061 | 8.573429 | 36.82067 | 19.72906 | 48.15042 | 52.66271 | 34.84639 | 20.06405 | 34.73538 | 10.95786 | 117.1089 | 41.26402 | 126.4216 |
| CUST_3919_PI429010886 | N15567 | 0.006617 | 18 | 0 | | | | | | 44.48344 | 41.17841 | 74.83385 | 40.69465 | 11.75483 | 46.66161 | 60.5193 | 58.39472 | 27.71495 | 33.16347 | 15.50711 | 12.68424 | 6.229675 | 30.73873 | 14.48944 | 42.47769 | 18.73257 | 23.89642 | 52.70775 | 58.29022 | 60.24307 | 35.38465 | 86.67105 | 21.87774 | 28.64876 | 15.44039 | 25.352 |
| CUST_33321_PI429010886 | M6590 | 0.006628 | 21 | 3 | 156550289 | PREDICTED: ileal sodium/bile acid cotransporter-lik | Transport | Ion transport | 9.59E-33 | 28.90402 | 26.52817 | 16.90254 | 55.30825 | 31.17481 | 63.67102 | 32.48585 | 40.66714 | 25.45224 | 30.75644 | 29.69161 | 22.49197 | 31.46479 | 16.27412 | 27.35753 | 25.89716 | 44.71939 | 32.78743 | 26.09805 | 22.50936 | 20.58016 | 31.00851 | 20.66537 | 36.115 | 32.55416 | 45.66434 | 38.7668 |
| CUST_17913_PI429010886 | N38822 | 0.00663 | 18 | 3 | | | | | | 113.0821 | 132.2831 | 131.513 | 199.1974 | 119.7503 | 138.9538 | 108.9753 | 105.8718 | 99.23417 | 136.3233 | 84.64574 | 146.8141 | 75.71395 | 117.0097 | 91.31323 | 105.322 | 77.02903 | 96.689 | 80.66119 | 95.66411 | 152.0093 | 136.8846 | 169.4032 | 111.6728 | 156.6286 | 119.9123 | 106.5597 |
| CUST_37090_PI429010886 | M17827 | 0.006632 | 12 | 3 | | | | | | 43.53241 | 38.24021 | 24.83878 | 33.49853 | 42.76605 | 57.36487 | 35.70676 | 24.06718 | 33.62817 | 21.53652 | 17.96838 | 29.67132 | 26.07154 | 32.6566 | 33.69868 | 35.00866 | 26.58056 | 46.19529 | 33.15625 | 19.25722 | 24.35448 | 23.25478 | 21.36429 | 34.83322 | 31.48729 | 34.8364 | 35.1893 |
| CUST_43824_PI429010886 | M64078 | 0.006632 | 12 | 3 | | | | | | 1276.133 | 3453.79 | 95.08289 | 3607.364 | 2073.005 | 2457.891 | 2431.925 | 265.7722 | 3466.9 | 78.93037 | 50.46214 | 70.70475 | 857.5648 | 255.4957 | 2455.41 | 4797.809 | 324.8235 | 4165.771 | 191.9895 | 52.42324 | 56.40861 | 1504.155 | 75.46943 | 1510.415 | 810.6402 | 269.9577 | 241.9211 |
| CUST_16611_PI429010886 | N36566 | 0.006632 | 12 | 9 | | | | | | 46.25392 | 20.56557 | 16.69997 | 20.75541 | 39.81638 | 23.32806 | 26.19664 | 22.26601 | 18.34756 | 108.8637 | 31.46479 | 35.17156 | 23.16293 | 17.43083 | 30.27449 | 14.50604 | 16.28788 | 21.7647 | 73.81016 | 20.27847 | 30.31987 | 45.36871 | 85.47277 | 46.60859 | 19.01959 | 25.8352 | 40.58787 |
| CUST_29344_PI429010886 | N59680 | 0.00664 | 18 | 9 | | | | | | 17.62699 | 18.42271 | 21.68419 | 15.84566 | 19.78599 | 14.93734 | 23.29073 | 15.90941 | 20.41093 | 24.62608 | 32.85525 | 28.05779 | 24.0728 | 27.06658 | 17.36312 | 19.90626 | 27.2627 | 24.91507 | 28.74055 | 12.6412 | 12.35487 | 14.79848 | 19.29325 | 17.64932 | 24.14877 | 16.01307 | 16.85432 |
| CUST_41966_PI429010886 | M59022 | 0.006643 | 12 | 3 | 443726219 | hypothetical protein CAPTEDRAFT_183535 [Capitell | | | 4.39E-34 | 9.45038 | 12.20709 | 10.88211 | 8.5745 | 10.92516 | 13.1339 | 13.65887 | 6.706567 | 4.472824 | NA | 4.176987 | 8.927589 | 10.61451 | 3.662216 | 6.43253 | 12.08902 | 14.90751 | 18.7049 | 7.361352 | 8.518007 | 5.812789 | 7.316619 | 3.682358 | 8.275583 | 9.388689 | 12.25276 | 11.75483 |
| CUST_50340_PI429010886 | M89506 | 0.006643 | 12 | 3 | | | | | | 12.49757 | 39.84998 | 25.99897 | 318.02 | 73.51884 | 179.8682 | 24.2001 | 96.15886 | 9.282132 | 12.8699 | NA | 16.38475 | 46.52112 | 37.34877 | 52.95745 | 155.2181 | 23.82763 | 102.3344 | 7.040982 | 212.501 | 31.43539 | 6.652276 | 36.55124 | 23.12065 | 130.8588 | 57.72307 | 13.532 |
| CUST_8446_PI429010886 | N23049 | 0.006646 | 21 | 12 | | | | | | 76.57774 | 66.99196 | 87.9523 | 46.54874 | 41.30417 | 47.95704 | 52.5224 | 74.37782 | 81.47819 | 90.03399 | 53.81515 | 74.57029 | 82.19075 | 77.55826 | 77.92082 | 97.5299 | 69.29923 | 61.60388 | 69.61944 | 72.22304 | 54.6811 | 71.96189 | 76.36771 | 56.67806 | 60.34766 | 69.1026 | 64.85632 |
| CUST_17925_PI429010886 | N38844 | 0.006654 | 24 | 15 | | | | | | 47.42461 | 58.39472 | 68.92299 | 54.08177 | 43.9859 | 48.67882 | 48.85338 | 34.50298 | 35.41295 | 70.69633 | 57.22832 | 83.97119 | 64.62304 | 70.36778 | 49.97285 | 64.52813 | 62.23219 | 77.59246 | 58.10931 | 58.78466 | 72.66838 | 63.51336 | 54.32801 | 54.36604 | 60.6915 | 53.54974 | 86.83335 |
| CUST_22148_PI429010886 | N46517 | 0.006654 | 18 | 3 | | | | | | 24.25419 | 24.26769 | 24.23621 | 32.05062 | 62.11683 | 52.48792 | 48.43988 | 15.2714 | 4.650188 | 8.284158 | 37.94571 | 8.364324 | 8.805551 | NA | 15.38978 | 7.955028 | 18.60109 | 18.70704 | 21.29581 | 8.3032 | 69.97891 | 19.86296 | 28.03992 | 52.70775 | 10.74821 | 23.5068 | 7.447234 |
| CUST_51772_PI429010886 | M95000 | 0.006658 | 12 | 9 | | | | | | 96.74817 | 224.5779 | 228.3459 | 36.16806 | 64.56992 | 10.42158 | 31.90127 | 167.776 | 291.6879 | 741.4101 | 451.6758 | 738.6637 | 275.6931 | 547.6349 | 285.9079 | 407.3532 | 119.6528 | 273.5534 | 490.2884 | 421.8908 | 413.616 | 744.0064 | 470.2955 | 65.01299 | 640.4514 | 461.6522 | 714.9465 |
| CUST_10479_PI429010886 | N26299 | 0.006658 | 12 | 0 | | | | | | 87.26148 | 73.53135 | 99.81229 | 79.35074 | 74.66117 | 69.03435 | 65.86348 | 74.94569 | 82.83897 | 104.2212 | 84.81787 | 93.25437 | 86.75819 | 109.3629 | 75.7764 | 81.10241 | 72.6524 | 80.12587 | 74.42211 | 80.86611 | 81.36657 | 106.7511 | 85.62172 | 80.26839 | 119.0071 | 82.50766 | 100.3587 |
| CUST_41565_PI429010886 | M58043 | 0.006678 | 12 | 0 | | | | | | 189.2062 | 118.9935 | 166.2981 | 153.5041 | 190.62 | 146.2811 | 138.0366 | 149.2083 | 132.0113 | 172.7143 | 129.8621 | 222.3674 | 168.032 | 223.8084 | 198.5726 | 151.2427 | 110.2887 | 128.5525 | 108.2334 | 152.263 | 128.5363 | 196.8724 | 143.1896 | 132.2443 | 237.1988 | 296.4747 | 260.2651 |
| CUST_32550_PI429010886 | M4409 | 0.006683 | 12 | 3 | 270004137 | hypothetical protein TcasGA2_TC003455 [Tribolium | Transport | Other | 1.04E-51 | 135.1811 | 131.1403 | 101.1723 | 520.9934 | 312.3161 | 945.8187 | 530.1566 | 359.1604 | 130.1396 | 35.34065 | 271.7719 | 101.9719 | 192.1486 | 120.446 | 170.5502 | 132.5919 | 744.0064 | 584.5557 | 468.822 | 111.3637 | 216.1164 | 210.8661 | 182.7865 | 214.0659 | 113.2721 | 284.9694 | 71.51375 |
| CUST_7800_PI429010886 | N22098 | 0.006683 | 12 | 0 | | | | | | 148.6082 | 198.4783 | 187.3568 | 119.1637 | 137.719 | 110.4435 | 126.8628 | 111.9496 | 155.1093 | 177.3442 | 126.2993 | 203.8966 | 162.3514 | 185.4691 | 153.8434 | 139.1034 | 113.8304 | 127.8042 | 120.579 | 152.0093 | 120.5667 | 174.356 | 175.1291 | 114.1191 | 213.5411 | 130.7243 | 161.4133 |
| CUST_53149_PI429010886 | M100409 | 0.006696 | 24 | 3 | | | | | | 45.51822 | 22.99548 | NA | 88.4462 | 50.55173 | 59.6812 | 34.48913 | 9.473492 | 35.02438 | 13.99779 | 12.99803 | 7.619757 | 12.63519 | 32.60462 | 33.68076 | 26.22091 | 13.84341 | 29.21516 | 8.435831 | 8.488945 | 8.007045 | 32.58533 | 6.869381 | 33.78634 | 10.01939 | 14.10504 | 6.410607 |
| CUST_42119_PI429010886 | M59457 | 0.006708 | 12 | 3 | 345493311 | PREDICTED: abhydrolase domain-containing protei | | | 1.35E-69 | 32.86616 | 23.94659 | 43.80188 | 28.6271 | 50.05524 | 63.68458 | 37.40087 | 24.13249 | 44.82913 | 31.33456 | 26.51493 | 26.32456 | 25.16709 | 33.82224 | 20.71979 | 40.73202 | 54.22335 | 39.49607 | 22.20953 | 23.17075 | 37.39567 | 31.79024 | 21.02215 | 22.45136 | 39.34212 | 40.3031 | 34.8131 |
| CUST_3046_PI429010886 | N13681 | 0.006708 | 12 | 3 | 405968456 | Multiple epidermal growth factor-like domains 6 [C | | | 5.86E-08 | 3464.749 | 2745.288 | 149.3268 | 7490.614 | 3339.741 | 7819.87 | 2706.735 | 3040.688 | 2352.559 | 331.4379 | 809.6878 | 373.4486 | 3269.374 | 1395.696 | 2684.729 | 4540.69 | 4584.946 | 7794.866 | 5125.683 | 967.496 | 669.0767 | 3350.556 | 197.9679 | 3576.101 | 1195.978 | 5130.907 | 30.0571 |
| CUST_37250_PI429010886 | M18365 | 0.00671 | 21 | 3 | | | | | | 9418.183 | 44080.52 | 16607.59 | 47564.51 | 27142.29 | 32962.39 | 13858.59 | 14375.97 | 14744.71 | 19401.18 | 13044.56 | 36281.72 | 19401.18 | 14160.61 | 11122.77 | 17110.44 | 7711.688 | 13177.04 | 10542.53 | 21721.24 | 21190.18 | 22231.21 | 16062.45 | 21906.22 | 22307.76 | 31314.74 | 15704.56 |
| CUST_1672_PI429010886 | N10407 | 0.00671 | 24 | 0 | | | | | | 69.58984 | 25.59155 | 47.74344 | 63.42029 | 11.24998 | 31.0625 | 9.258755 | 20.70807 | 8.386399 | 15.2714 | 20.88612 | 25.86025 | 5.411858 | 29.27339 | 10.71609 | 22.99783 | 28.60828 | 27.51169 | 21.38839 | 42.90864 | 32.63054 | 35.35004 | 34.34807 | 9.163319 | 30.48036 | 46.54874 | 35.77142 |
| CUST_20873_PI429010886 | N44223 | 0.006716 | 21 | 18 | 321468459 | hypothetical protein DAPPUDRAFT_23494 [Daphnia | Metabolic process | Cellular amino acid metabolic process | 7.16E-29 | 17.64263 | 11.86309 | 34.05805 | 12.69853 | 12.86372 | 13.2803 | 26.01055 | 13.82413 | 11.36992 | 5.755096 | 12.47348 | 10.43073 | 8.410809 | 8.053924 | 10.17638 | 4.490174 | 21.61669 | 17.06111 | 16.71152 | 19.31761 | 19.75359 | 16.76094 | 23.57465 | 17.78619 | 20.27043 | 16.70757 | 5.53642 |
| CUST_5668_PI429010886 | N19015 | 0.006744 | 24 | 15 | 405966738 | Eukaryotic translation initiation factor 4 gamma 3 [ | | | 3.87E-14 | 72.44795 | 80.88726 | 69.05841 | 78.83809 | 85.62903 | 78.1117 | 68.53497 | 57.83187 | 86.58356 | 85.90922 | 70.41805 | 75.98951 | 91.79308 | 94.30105 | 91.85305 | 99.03782 | 97.40947 | 100.336 | 89.34718 | 95.81155 | 65.40248 | 96.70093 | 74.3064 | 85.55586 | 75.58303 | 93.1664 | 95.46826 |
| CUST_39707_PI429010886 | M43222 | 0.006745 | 21 | 15 | | | | | | 74.98811 | 66.40981 | 101.6252 | 31.47618 | 45.58985 | 60.64562 | 70.69633 | 51.30486 | 68.38918 | 61.47627 | 58.85973 | 54.26471 | 96.52778 | 74.60621 | 74.20243 | 69.85356 | 82.05363 | 83.78522 | 48.74075 | 77.18047 | 70.76377 | 66.61406 | 72.45575 | 68.70977 | 50.30273 | 41.23292 | 83.2292 |
| CUST_32449_PI429010886 | M4144 | 0.006758 | 12 | 3 | 241842796 | monocarboxylate transporter, putative [Ixodes scap | Transport | Transmembrane transport | 1.37E-47 | 119.7503 | 81.22317 | 80.9845 | 143.6702 | 143.8657 | 322.2737 | 132.8034 | 148.8262 | 101.5396 | 88.10192 | 147.9921 | 106.4056 | 105.0471 | 112.7849 | 104.2639 | 109.1296 | 190.5242 | 184.8748 | 203.453 | 150.5899 | 146.0474 | 102.1229 | 114.9603 | 121.4004 | 69.57456 | 169.3778 | 97.89619 |
| CUST_18636_PI429010886 | N40146 | 0.006763 | 15 | 6 | | | | | | 22.99783 | 13.46619 | 19.45845 | 17.37911 | 14.69352 | 13.11816 | 17.61748 | 23.11042 | 17.92211 | 23.04991 | 13.64597 | 12.24341 | 13.33386 | 12.49899 | 13.03254 | 15.35034 | 15.9031 | 16.06632 | 26.72094 | 13.32189 | 20.51687 | 23.49179 | 22.11226 | 26.50702 | 14.82674 | 10.97186 | 21.07701 |
| CUST_50777_PI429010886 | M90967 | 0.006773 | 15 | 0 | | | | | | 335.9236 | 329.9922 | 357.7037 | 224.3549 | 307.8404 | 306.6802 | 223.1189 | 221.2444 | 254.4207 | 330.6772 | 295.8552 | 350.9859 | 246.0582 | 372.6528 | 236.4903 | 342.5512 | 305.3469 | 338.5409 | 282.4175 | 275.7295 | 285.4947 | 240.9894 | 261.3766 | 252.2065 | 436.6018 | 305.7949 | 351.1194 |

| Probe ID | ID | p-value | Period | Phase | EBI ID | Annotation | GO category | Go term | E-value | 0 | 0 | 0 | 3 | 3 | 3 | 6 | 6 | 6 | 9 | 9 | 9 | 12 | 12 | 12 | 15 | 15 | 15 | 18 | 18 | 18 | 21 | 21 | 21 | 24 | 24 | 24 |
|---|---|---|---|---|---|---|---|---|---|---|---|---|---|---|---|---|---|---|---|---|---|---|---|---|---|---|---|---|---|---|---|---|---|---|---|---|
| CUST_53729_PI429010886 | M102951 | 0.006778 | 21 | 0 | | | | | | 25.82466 | 24.59907 | 27.32319 | 17.53198 | 17.37759 | 20.92107 | 19.34576 | 14.07257 | 18.84012 | 30.14891 | 14.49351 | 17.73942 | 16.02003 | 20.63783 | 13.35309 | 17.35771 | 15.2558 | 16.69775 | 20.18733 | 11.12231 | 27.39567 | 27.24092 | 45.31583 | NA | 23.82763 | 16.4962 | 20.55453 |
| CUST_55517_PI429010886 | M110493 | 0.006784 | 12 | 3 | | | | | | 23.35223 | 22.69042 | 12.35967 | 54.0716 | 17.77636 | 47.8959 | 18.72259 | 17.21639 | 17.86172 | 14.27753 | 21.76863 | 16.37326 | 16.35182 | 13.90998 | 11.03355 | 56.72941 | 25.6683 | 27.71495 | 17.51958 | 20.54194 | 23.52604 | 9.748744 | 23.063 | 13.39856 | 16.58396 | 17.96537 | 17.68838 |
| CUST_3568_PI429010886 | N14776 | 0.006784 | 12 | 3 | | | | | | 60.53435 | 53.84406 | 49.06607 | 70.29196 | 80.12587 | 98.4006 | 90.38098 | 81.04828 | 60.15877 | 45.05092 | 56.84346 | 36.06457 | 55.49391 | 36.85933 | 51.18041 | 55.55874 | 111.812 | 70.13143 | 91.04016 | 37.68293 | 79.18359 | 33.20988 | 50.52879 | 60.07749 | 49.39411 | 57.06419 | 51.75281 |
| CUST_13388_PI429010886 | N31097 | 0.006784 | 12 | 3 | | | | | | 64.56424 | 36.00905 | 27.76975 | 48.59467 | 36.29122 | 47.25743 | 45.10479 | 50.41387 | 33.23976 | 22.04791 | 22.38395 | 20.08892 | 32.49365 | 28.59732 | 43.60712 | 43.15798 | 44.58945 | 45.24787 | 51.62109 | 22.93702 | 26.0929 | 40.62219 | 19.6024 | 35.34065 | 33.72145 | 22.82168 | 28.33698 |
| CUST_1239_PI429010886 | N8980 | 0.006784 | 12 | 9 | 117067248 | ribosomal protein S26 [Branchiostoma belcheri] | Protein metabolism | Ribosome biogenesis | 2.29E-33 | 23.74918 | 15.70949 | 28.6993 | 10.43918 | 16.81213 | 9.857121 | 19.75359 | 13.22099 | 26.2515 | 23.45456 | 23.09298 | 30.30557 | 19.08808 | 24.99293 | 23.94321 | 20.9065 | 15.74591 | 17.05513 | 84.2393 | 24.03945 | 27.21328 | 23.73784 | 44.03947 | 27.67304 | 22.53922 | 19.48765 | 29.01971 |
| CUST_33001_PI429010886 | M5680 | 0.006786 | 18 | 0 | | | | | | 18.62971 | 24.97756 | 22.58247 | 18.59144 | 18.94364 | 16.5596 | 7.857013 | 13.45546 | 7.329225 | 8.00182 | 6.962498 | 16.60676 | 12.63007 | 9.188895 | 15.96275 | 7.591069 | 6.263837 | 14.05746 | 23.52324 | 17.5614 | 17.63842 | 15.99247 | NA | 5.929425 | 22.47468 | 14.75649 | 10.38108 |
| CUST_37566_PI429010886 | M19521 | 0.006787 | 15 | 9 | | | | | | NA | 77.20798 | 23.20138 | 111.7362 | 116.516 | 32.69174 | 60.17363 | 296.6828 | 221.8177 | 52.801 | 512.1412 | 633.1858 | 338.7294 | 51.83879 | 243.8404 | 24.73831 | 89.57185 | 10.78772 | 42.42919 | 787.1975 | 178.7689 | NA | 123.8883 | 140.2611 | NA | 340.3246 | 162.6703 |
| CUST_27331_PI429010886 | N55858 | 0.00679 | 18 | 12 | | | | | | 618.3611 | 367.1896 | 448.4129 | 207.128 | 279.387 | 316.8471 | 320.2376 | 256.6496 | 390.7936 | 353.0021 | 327.471 | 236.4903 | 370.9949 | 480.8955 | 423.679 | 626.4662 | 204.7885 | 314.3091 | 309.8242 | 344.803 | 354.0483 | 252.4954 | 194.605 | 222.0782 | 248.0331 | 344.8512 | 400.6975 |
| CUST_50150_PI429010886 | M88826 | 0.006802 | 18 | 3 | | | | | | 67.1407 | 44.01861 | 69.25418 | 113.8871 | 29.58843 | 156.6764 | 17.75695 | 36.07274 | 13.87032 | 15.99616 | 54.35449 | 13.65696 | 17.15304 | 8.732125 | 23.38375 | 57.05842 | 114.9975 | 93.99876 | 85.0717 | 26.11231 | 92.18368 | 69.65017 | 38.67688 | 138.7005 | 45.23797 | 27.09726 | 32.08077 |
| CUST_45946_PI429010886 | M70838 | 0.006805 | 15 | 9 | | | | | | 39.49607 | 48.49058 | 50.02767 | 55.95565 | 44.64991 | 68.92299 | 43.48424 | 63.40813 | 79.21035 | 66.51353 | 60.03661 | 107.0039 | 64.70488 | 92.07382 | 44.88618 | 80.42722 | 59.49267 | 89.01214 | 36.03165 | 56.03237 | 49.96135 | 55.98779 | 70.46014 | 84.30944 | 94.91653 | 84.09478 | 89.65503 |
| CUST_36314_PI429010886 | M15551 | 0.006811 | 21 | 3 | | | | | | 529.7833 | 974.9401 | 778.2204 | 662.7679 | 914.9994 | 1076.18 | 679.1732 | 514.6723 | 600.4305 | 586.9437 | 273.965 | 716.0786 | 207.4294 | 713.4223 | 286.6682 | 448.9486 | 292.413 | 512.6993 | 113.6252 | 511.9204 | 1019.767 | 592.3414 | 1044.227 | 477.5078 | 962.2957 | 1032.594 | 564.2409 |
| CUST_18902_PI429010886 | N40617 | 0.006818 | 21 | 18 | | | | | | 32.42053 | 31.721 | 42.87529 | 38.57556 | 27.83166 | 35.82359 | 20.97008 | 19.78047 | 24.3684 | 30.96855 | 33.61051 | 27.49426 | 17.84645 | 30.29627 | 15.39724 | 32.01382 | 42.28532 | 13.52947 | 38.81815 | 47.26147 | 55.07968 | 26.30031 | 43.31707 | 24.76468 | 41.62253 | 38.78083 | 31.43244 |
| CUST_32833_PI429010886 | M5219 | 0.006825 | 18 | 9 | 512899738 | PREDICTED: uncharacterized protein LOC10174138 | | | 6.72E-20 | 426.2225 | 150.2252 | 243.5502 | 93.09068 | 120.7611 | 132.6901 | 148.3091 | 153.8239 | 186.2582 | 157.5293 | 164.4877 | 170.8572 | 137.8813 | 154.7047 | 157.7661 | 146.8304 | 102.9667 | 134.7073 | 119.4111 | 114.0301 | 133.4052 | 126.5253 | 123.6853 | 126.1505 | 140.9929 | 102.1933 | 139.9664 |
| CUST_13796_PI429010886 | N31789 | 0.006829 | 24 | 21 | | | | | | 20.39383 | 33.18183 | 27.30432 | 20.00172 | 19.46833 | 15.68356 | 12.99168 | 19.65423 | 19.03935 | 19.42074 | 15.35699 | 40.34341 | 19.17918 | 22.72662 | 19.48977 | 20.30399 | 18.54361 | 27.88257 | 20.99763 | 14.68043 | 24.56592 | 26.10123 | 28.46316 | 27.80573 | 27.25936 | 20.94928 | 26.07451 |
| CUST_29013_PI429010886 | N59027 | 0.006837 | 24 | 15 | 241569831 | conserved hypothetical protein [Ixodes scapularis] > | | | 9.51E-10 | 31.6312 | 24.52916 | 33.26161 | 21.70241 | 21.63197 | 25.1358 | 20.85646 | 26.01055 | 25.67033 | 32.10959 | 27.30762 | 33.23109 | 27.10885 | 44.36476 | 40.16678 | 37.32162 | 45.49623 | 30.47678 | 28.34801 | 30.10596 | 24.69322 | 27.28431 | 34.18144 | 35.95633 | 32.73413 | 23.84861 | 37.72041 |
| CUST_35597_PI429010886 | M12985 | 0.006852 | 15 | 12 | | | | | | 366.8876 | 303.2078 | 441.9667 | 215.0865 | 290.5429 | 375.8398 | 394.7994 | 296.8833 | 340.4766 | 376.7168 | 340.9466 | 467.665 | 406.7395 | 535.4947 | 431.462 | 432.0274 | 392.8804 | 498.1212 | 296.3167 | 276.0613 | 332.0008 | 497.8615 | 453.8761 | 403.8464 | 398.6724 | 245.2785 | 379.2136 |
| CUST_5305_PI429010886 | N18519 | 0.006854 | 21 | 15 | | | Metabolic process | Cellular amino acid metabolic process | | 1762.677 | 1726.714 | 1383.212 | 1464.638 | 1926.251 | 1433.182 | 1603.184 | 1150.815 | 1023.389 | 1569.266 | 2077.773 | 1325.963 | 1313.707 | 1349.576 | 1465.818 | 2046.644 | 2368.476 | 1593.287 | 1909.916 | 1738.403 | 1747.319 | 1870.503 | 1546.55 | 1818.602 | 1472.72 | 1081.105 | 1278.963 |
| CUST_32393_PI429010886 | M3994 | 0.00686 | 12 | 9 | 321465430 | hypothetical protein DAPPUDRAFT_55135 [Daphnia | | | 2.59E-32 | 83.34341 | 66.70229 | 136.3105 | 49.39411 | 31.82653 | 32.15824 | 93.03197 | 85.65603 | 106.7397 | 97.55406 | 85.72992 | 86.5251 | 100.2495 | 102.9429 | 73.51179 | 75.5762 | 65.08584 | 38.2775 | 47.61035 | 80.75638 | 71.49639 | 130.3093 | 138.2766 | 54.90496 | 85.59472 | 64.55134 | 133.0251 |
| CUST_33072_PI429010886 | M5899 | 0.00686 | 12 | 9 | | | | | | 446.4087 | 485.0905 | 988.4432 | 261.767 | 358.4672 | 322.9938 | 437.1883 | 375.4169 | 399.93 | 746.6817 | 591.2615 | 533.4173 | 386.3769 | 372.7154 | 595.1496 | 628.4644 | 340.1148 | 385.2863 | 279.2335 | 487.5504 | 321.803 | 477.2259 | 690.4518 | 683.9239 | 571.296 | 360.7127 | 669.6491 |
| CUST_4255_PI429010886 | N16333 | 0.006861 | 24 | 21 | 242023499 | conserved hypothetical protein [Pediculus humanus | | | 2.55E-44 | 60.28718 | 70.41805 | 72.78887 | 50.78563 | 59.04082 | 77.36396 | 54.5227 | 41.07674 | 57.10862 | 55.04733 | 56.86246 | 65.77588 | 63.25496 | 73.84429 | 59.18336 | 69.08039 | 75.43095 | 84.48505 | 67.69277 | 69.44924 | 44.22632 | 69.11088 | 71.31512 | 79.86307 | 70.35427 | 73.71983 | 65.08 |
| CUST_15118_PI429010886 | N34038 | 0.006861 | 24 | 21 | | | | | | 238.5792 | 216.1856 | 203.6685 | 165.7374 | 220.6157 | 230.7819 | 130.3093 | 140.6597 | 142.7994 | 200.6981 | 183.7339 | 233.6523 | 158.5656 | 214.2583 | 258.8229 | 279.4425 | 204.4226 | 305.0519 | 211.9851 | 247.3742 | 218.9157 | 313.7524 | 237.9979 | 204.1442 | 289.5961 | 196.8321 | 225.9932 |
| CUST_47272_PI429010886 | M75421 | 0.006871 | 24 | 9 | 321476689 | hypothetical protein DAPPUDRAFT_306461 [Daphn | | | 2.8E-11 | 46.04576 | 52.35268 | 34.97535 | 66.78152 | 102.7268 | 69.19616 | 69.72006 | 72.14089 | 60.62589 | 112.8577 | 73.89571 | 69.65899 | 106.064 | 63.00905 | 54.50124 | 44.01036 | 48.45867 | 50.17076 | 53.38747 | 59.46938 | 70.20247 | 85.84413 | 40.77403 | 65.31793 | 67.74837 | 36.08462 | 29.43141 |
| CUST_24992_PI429010886 | N51598 | 0.006894 | 21 | 18 | | | | | | 9.624456 | 12.54217 | NA | 13.91491 | 6.852264 | 19.31076 | 5.612977 | 5.927029 | 4.586506 | 6.310118 | 3.474911 | 13.29917 | 9.002381 | 7.874648 | 9.096353 | 14.35036 | 5.880062 | 16.87359 | 96.97877 | 7.15372 | NA | 10.54822 | NA | 12.58519 | 5.986624 | 6.040843 | 6.725658 |
| CUST_9895_PI429010886 | N25368 | 0.006894 | 12 | 9 | | | | | | 8.306883 | 7.090444 | 8.559956 | 5.906131 | 4.111178 | 7.497984 | 7.215021 | 5.257423 | 11.93851 | 8.956327 | 8.051681 | 11.3111 | 4.210974 | 9.376909 | 5.632763 | 7.490316 | 3.571577 | 4.140048 | 12.46576 | NA | NA | 15.56439 | 11.51059 | 9.078323 | 8.672182 | 10.15583 | 4.696595 |
| CUST_15515_PI429010886 | N34707 | 0.006899 | 21 | 3 | | | | | | 7.520119 | 6.486483 | 12.32751 | NA | 9.040661 | 7.501961 | 13.60386 | 9.889085 | 5.861533 | 5.36304 | 6.759208 | 13.97554 | 7.599564 | 8.73928 | 6.454629 | 5.765307 | 6.243139 | NA | 6.321742 | 5.051185 | NA | 6.42121 | 8.486348 | NA | 12.36405 | 14.17029 | 11.82406 |
| CUST_40873_PI429010886 | M56275 | 0.006909 | 12 | 0 | | | | | | 267.4897 | 263.1737 | 286.2536 | 210.3141 | 190.201 | 187.4537 | 221.6459 | 229.9104 | 254.7271 | 254.0567 | 227.4855 | 264.5866 | 209.0287 | 282.6926 | 276.1481 | 230.4163 | 221.1299 | 228.6997 | 232.8505 | 290.3277 | 258.8731 | 254.3377 | 262.1121 | 230.8944 | 314.7546 | 213.366 | 336.4126 |
| CUST_4787_PI429010886 | N17476 | 0.006917 | 18 | 15 | | | | | | 138.2104 | 186.8543 | 166.4042 | 124.3644 | 165.9249 | 182.9432 | 129.1185 | 119.9711 | 118.5941 | 150.2035 | 203.1221 | 247.961 | 149.7495 | 173.1369 | 164.9826 | 214.6269 | 203.4225 | 145.9962 | 187.3568 | 185.2639 | 186.6799 | 137.3696 | 178.147 | 117.5843 | 120.0958 | 166.5598 | 160.2379 |
| CUST_25340_PI429010886 | N52227 | 0.006917 | 18 | 3 | | | | | | 120.9878 | 64.38672 | 115.9016 | 102.5891 | 109.3451 | 130.357 | 120.446 | 91.80446 | 87.03787 | 97.42055 | 83.63981 | 61.5685 | 74.18015 | 91.05924 | 101.7598 | 112.1982 | 117.2885 | 100.8857 | 97.51669 | 89.87648 | 87.10078 | 102.0348 | 117.3642 | 132.9869 | 93.11072 | 87.31709 | 101.5804 |
| CUST_3270_PI429010886 | N14151 | 0.006918 | 15 | 6 | 321463133 | hypothetical protein DAPPUDRAFT_307488 [Daphn | | | 7.32E-16 | 73.53135 | 39.93503 | 59.18336 | 61.45036 | 52.92115 | 89.13187 | 136.3625 | 143.1502 | 99.15143 | 228.3136 | 109.2818 | 123.8729 | 106.6494 | 104.474 | 109.3629 | 69.18246 | 106.5468 | 89.82702 | 125.5654 | 135.3289 | 205.6958 | 182.2638 | 136.7912 | 97.71155 | 66.86178 | 95.13953 | 117.3497 |
| CUST_14551_PI429010886 | N33066 | 0.006922 | 15 | 3 | 328791459 | PREDICTED: Apis mellifera tricornered (trc), mRNA | Protein metabolism | Cellular protein modification process | 1E-72 | 67.97289 | 53.88401 | 37.72041 | 72.62704 | 63.59288 | 61.40092 | 51.70242 | 30.65659 | 45.75251 | 52.79221 | 52.7291 | 42.02786 | 43.15798 | 48.9688 | 51.24109 | 59.81399 | 43.93802 | 58.27728 | 56.14691 | 77.06228 | 60.24939 | 86.9224 | 54.77183 | 35.15108 | 53.44251 | 31.07494 | 40.46782 |
| CUST_32315_PI429010886 | M3783 | 0.006937 | 12 | 9 | 48130104 | PREDICTED: enhancer of split mgamma protein-like | Nucleic acid metabolism | Regulation of gene expression | 3.7E-48 | 208.9109 | 439.0329 | 392.9826 | 264.6314 | 172.8115 | 140.7358 | 211.2718 | 319.6424 | 369.6113 | 381.2578 | 282.3655 | 450.9914 | 249.2507 | 373.6035 | 291.5938 | 285.421 | 312.9412 | 235.4879 | 232.5216 | 356.7855 | 319.007 | 394.4542 | 464.198 | 344.5306 | 284.7376 | 347.4939 | 443.1374 |
| CUST_12501_PI429010886 | N29563 | 0.00694 | 12 | 3 | | | | | | 65.66431 | 80.57644 | 27.11255 | 195.7607 | 161.2028 | 283.6942 | 81.25969 | 79.96242 | 53.50545 | 49.60877 | 63.07552 | 44.11042 | 71.20471 | 90.64646 | 52.54081 | 132.7469 | 122.4345 | 194.9822 | 141.3582 | 98.6386 | 60.19936 | 153.0541 | 38.12809 | 147.0578 | 71.9913 | 111.4345 | 37.68617 |
| CUST_13121_PI429010886 | N30610 | 0.006949 | 24 | 21 | | | | | | 53.03364 | 105.798 | 122.3475 | 47.32005 | 61.75898 | 59.80641 | 53.02384 | 54.78436 | 47.29592 | 72.73775 | 65.9084 | 69.28978 | 66.1724 | 62.23675 | 66.77508 | 75.7868 | 64.83984 | 67.94324 | 62.85713 | 75.33028 | 72.3532 | 80.21197 | 85.77984 | 69.69491 | 101.6948 | 71.73214 | 97.95028 |
| CUST_1180_PI429010886 | N8763 | 0.006949 | 24 | 21 | | | | | | 90.3266 | 140.0797 | 131.7341 | 77.70756 | 113.0678 | 81.89953 | 77.52659 | 100.3487 | 73.57494 | 144.3781 | 76.73952 | 92.48444 | 80.61162 | 124.1278 | 106.298 | 106.6808 | 94.1523 | 112.7504 | 98.15841 | 103.9891 | 125.099 | 107.955 | 137.1423 | 130.4682 | 123.7403 | 129.772 | 127.0219 |
| CUST_43090_PI429010886 | M62047 | 0.006964 | 21 | 0 | | | | | | 66.96201 | 65.6996 | 68.41788 | 53.88876 | 63.77772 | 69.58233 | 63.39049 | 50.76541 | 57.39031 | 64.73839 | 63.77772 | 64.64151 | 40.96307 | 58.88281 | 46.17776 | 58.66026 | 53.9397 | 51.32239 | 55.87623 | 51.82417 | 55.21772 | 63.70207 | 69.45519 | 72.41676 | 69.25418 | 46.78356 | 59.9559 |
| CUST_25501_PI429010886 | N52533 | 0.006972 | 21 | 0 | | | | | | 118.7356 | 86.66049 | 80.61916 | 82.929 | 128.3615 | 91.6735 | 70.99743 | 59.86271 | 22.29454 | 96.58099 | 54.87191 | 58.49978 | 48.53823 | 44.09424 | 42.42487 | 98.87521 | 103.0243 | 77.3067 | 85.90922 | 39.20847 | 59.17148 | 110.1779 | 75.75327 | 119.6154 | 136.2681 | 67.82256 | 68.66167 |
| CUST_50998_PI429010886 | M91728 | 0.006993 | 21 | 12 | | | | | | 771.6454 | 977.0262 | 1033.757 | 983.2431 | 1409.58 | 789.4136 | 1050.96 | 3917.614 | 3511.533 | 869.992 | 3269.374 | 5609.183 | 3790.041 | 1099.991 | 2593.467 | 1206.475 | 1703.115 | 1154.447 | 853.6849 | 8515.08 | 3202.001 | 841.7695 | 938.6625 | 992.543 | 1022.691 | 2232.866 | 2500.44 |
| CUST_31456_PI429010886 | M1564 | 0.007009 | 18 | 15 | | | | | | 87.99607 | 79.41411 | 57.07573 | 50.68803 | 68.17959 | 52.17878 | 58.22303 | 67.16356 | 73.35296 | 87.6188 | 74.63865 | 73.39754 | 63.98224 | 60.66262 | 80.4086 | 101.7048 | 70.7166 | 71.07061 | 66.57113 | 100.2724 | 49.16013 | 52.99425 | 48.33175 | 43.21618 | 83.63981 | 45.3248 | 58.09597 |
| CUST_32674_PI429010886 | M4771 | 0.007009 | 18 | 3 | | | | | | 92.90826 | 39.0714 | 45.82436 | 109.5173 | 93.96395 | 54.28784 | 103.9053 | 52.18439 | 20.31875 | 27.9603 | 41.73826 | 24.25592 | 39.02375 | 36.64988 | 28.02553 | 39.73588 | 132.7602 | 54.21273 | 103.1763 | 16.24458 | 51.31053 | 53.41869 | 84.54494 | 89.81869 | 34.91043 | 43.47138 | 25.55814 |
| CUST_30583_PI429010886 | N62169 | 0.007013 | 12 | 9 | | | | | | 27.55378 | 12.87509 | 27.42267 | 14.05923 | 19.66959 | 14.24194 | 16.06834 | 10.11651 | 20.5737 | 21.70044 | 28.04755 | 31.7268 | 13.35477 | 21.41546 | 26.82962 | 16.1391 | 17.04499 | 13.10657 | 21.08444 | 26.10969 | 22.3788 | 34.30053 | 27.15358 | 16.75121 | 25.08131 | 19.56278 | 25.2475 |
| CUST_14369_PI429010886 | N32769 | 0.007014 | 24 | 15 | | | | | | 160.5552 | 155.0084 | 128.1757 | 111.8008 | 148.0995 | 151.9691 | 113.976 | 113.6504 | 96.77635 | 144.2982 | 160.6917 | 130.3093 | 132.7287 | 144.3143 | 166.7568 | 171.5147 | 168.3796 | 157.7275 | 152.2451 | 157.7092 | 134.6919 | 168.3529 | 124.3983 | 156.5024 | 231.4164 | 156.1886 | 201.6936 |
| CUST_17917_PI429010886 | N38828 | 0.007023 | 24 | 0 | 357627527 | hypothetical protein KGM_16933 [Danaus plexippu | | | 2.83E-09 | 57.33188 | 24.26118 | 33.76983 | 38.59321 | 21.18827 | 19.72298 | 52.58792 | 29.23024 | 35.15108 | 53.62183 | 20.3649 | 31.85967 | 16.84134 | 18.6269 | 5.78191 | 28.59732 | 27.64478 | 15.75401 | 25.64065 | 21.83532 | 51.11982 | 36.75997 | 49.38456 | 33.71856 | 46.55207 | 48.01961 | 52.7291 |
| CUST_44725_PI429010886 | M66795 | 0.007026 | 12 | 3 | | | | | | 30.07667 | 28.65357 | 26.16897 | 71.3655 | 37.07575 | 47.5061 | 36.00037 | 25.86296 | 96.47975 | 5.286981 | 15.10731 | NA | 28.04382 | 8.108101 | 19.56461 | 15.57864 | 36.64988 | 30.30845 | 22.80928 | 4.643844 | 13.76206 | 12.142 | NA | 20.88813 | 16.35532 | 23.4129 | 10.07219 |
| CUST_40928_PI429010886 | M56407 | 0.00703 | 15 | 12 | | | | | | 162.775 | 118.905 | 118.5121 | 78.30552 | 74.01735 | 77.12763 | 133.6865 | 212.8057 | 210.3675 | 137.9332 | 45.88252 | 101.0126 | 166.0195 | 219.0379 | 189.0327 | 97.20572 | 140.6181 | 82.27324 | 120.905 | 66.92364 | 58.00017 | 160.804 | 93.95638 | 72.88198 | 128.4938 | 97.16739 | 132.3017 |
| CUST_14076_PI429010886 | N32287 | 0.00703 | 15 | 12 | | | | | | 107.3346 | 338.2555 | 214.4445 | 35.70676 | 139.0616 | 17.56933 | 74.18777 | 609.6635 | 407.4423 | 278.8318 | 369.0374 | 644.4395 | 359.57 | 911.683 | 512.1412 | 62.74739 | 592.1986 | 69.03435 | 92.31619 | 183.4152 | 133.9495 | 168.6715 | 767.5196 | 67.65513 | 183.5489 | 306.4375 | 503.6617 |
| CUST_19218_PI429010886 | N41195 | 0.00703 | 15 | 12 | 321459389 | hypothetical protein DAPPUDRAFT_93478 [Daphnia | | | 7.15E-13 | 48.28635 | 48.99962 | 51.61085 | 45.49238 | 37.9894 | 39.6493 | 36.62028 | 40.46037 | 50.06107 | 56.92511 | 31.66159 | 47.05803 | 53.02941 | 58.53699 | 62.70075 | 61.60941 | 41.72747 | 63.39728 | 50.44452 | 42.73401 | 31.10257 | 51.89227 | 43.17145 | 30.04952 | 52.60357 | 47.02203 | 60.07014 |
| CUST_25524_PI429010886 | N52570 | 0.00703 | 15 | 12 | | | | | | 685.3204 | 654.914 | 631.8289 | 501.8237 | 603.6169 | 648.9401 | 689.0956 | 666.6962 | 663.9332 | 754.1104 | 563.0019 | 818.4343 | 859.0461 | 1051.932 | 912.7145 | 739.338 | 871.62 | 720.48 | 667.7123 | 751.2737 | 530.6869 | 847.8005 | 932.5265 | 671.5411 | 1074.496 | 633.3531 | 1422.832 |
| CUST_29742_PI429010886 | N60458 | 0.007031 | 21 | 12 | 346654464 | elongation factor 1 alpha [Barnardiorum shadini] | | | 9.72E-18 | NA | 73.85809 | 26.41485 | 248.1135 | 184.0038 | 21.6649 | 137.4373 | 1187.536 | 230.8271 | 118.6617 | 871.9238 | 1147.595 | 2615.876 | 43.75138 | 1321.214 | NA | 448.8586 | NA | 43.68024 | 20926.24 | 411.8683 | 5.701937 | 23.37682 | 11.11646 | NA | 450.7936 | 825.1657 |
| CUST_15441_PI429010886 | N34584 | 0.007037 | 24 | 3 | | | | | | 531.6819 | 707.905 | 506.5013 | 834.6224 | 578.2355 | 683.3484 | 704.1186 | 601.5918 | 672.9863 | 710.9044 | 439.2849 | 592.481 | 561.2769 | 528.618 | 533.2467 | 573.6965 | 611.8341 | 509.3966 | 539.4382 | 477.7327 | 367.8562 | 515.8865 | 419.0394 | 602.272 | 656.1679 | 639.3223 | 453.5801 |
| CUST_30168_PI429010886 | N61313 | 0.007037 | 24 | 21 | | | | | | 17.23467 | 21.13052 | 37.59059 | 23.94075 | 25.38784 | 25.2475 | 9.378139 | 17.80424 | 21.4565 | 22.48019 | 18.58833 | 23.33262 | 18.42271 | 31.77439 | 21.01141 | 31.43005 | 23.24391 | 32.80137 | 22.35984 | 43.63321 | 22.98966 | 40.46782 | 26.57271 | 28.7982 | 27.63853 | 31.40893 | 34.66525 |
| CUST_14811_PI429010886 | N33507 | 0.007059 | 21 | 0 | | | | | | 11.39726 | 16.11003 | 12.90472 | 25.78835 | 10.52136 | 5.124238 | 10.97059 | NA | NA | 4.744181 | 8.421448 | NA | 6.366804 | 12.82338 | 7.213856 | 12.31427 | 8.962125 | 11.95869 | 10.6654 | 8.363068 | 9.627312 | 16.69534 | 18.15993 | NA | 8.535901 | 5.356366 | 11.21201 |
| CUST_41927_PI429010886 | M58897 | 0.007073 | 18 | 9 | | | | | | 61.06386 | 77.3067 | 78.32339 | 85.41552 | 205.909 | 210.6402 | 62.3009 | 70.29878 | 121.3175 | 201.9745 | 190.0374 | 183.8985 | 93.40468 | 147.0019 | 135.0274 | 92.54562 | 66.07282 | 57.07016 | 64.8125 | 82.83897 | 84.07185 | 147.4281 | 110.1036 | 89.29805 | 130.8343 | 104.0454 | 91.11111 |
| CUST_12546_PI429010886 | N29633 | 0.007083 | 18 | 3 | | | | | | 16.9342 | 22.28394 | 22.04311 | 49.46731 | 25.76451 | 25.38172 | 25.37 | 20.47876 | 16.61069 | 16.14158 | 12.11329 | 15.92196 | 21.3985 | 26.68081 | 27.01961 | 14.08649 | 19.9496 | 26.22286 | 22.36394 | 29.49942 | 5.85097 | 29.19814 | 27.80368 | 29.64495 | 28.17477 | 53.53068 | 23.38375 |
| CUST_35264_PI429010886 | M12082 | 0.007088 | 21 | 18 | | | | | | 556.2338 | 709.1206 | 503.0719 | 513.2283 | 518.0939 | 539.1803 | 485.9211 | 487.6477 | 497.2573 | 573.2606 | 477.7327 | 613.8279 | 559.5018 | 572.8694 | 566.0346 | 814.5413 | 488.0718 | 537.3115 | 583.066 | 697.8167 | 594.8858 | 665.6609 | 503.8676 | 550.6217 | 553.2164 | 472.3127 | 489.9462 |
| CUST_53940_PI429010886 | M103791 | 0.007098 | 24 | 3 | | | | | | 53.15159 | 46.41206 | NA | 124.9376 | 70.58339 | 66.97079 | 97.36237 | 15.71416 | 49.89799 | NA | 17.13668 | NA | 36.44639 | 30.25566 | 30.06738 | 24.34006 | 27.91206 | 30.86285 | 8.773525 | 14.18148 | 16.53513 | 24.70162 | 15.02774 | 54.5276 | 6.373482 | 5.981371 | NA |
| CUST_34609_PI429010886 | M10141 | 0.007098 | 18 | 15 | | | | | | 19.34025 | 10.72947 | 14.68167 | 11.55147 | NA | 3.878174 | 13.4823 | 21.40624 | 21.41385 | 14.31543 | 9.165228 | 14.9294 | 21.49416 | 19.22831 | 15.82858 | 24.14372 | 10.76694 | 21.98642 | 4.647861 | 18.49721 | 12.61125 | 5.931156 | 8.743791 | 14.33423 | 6.560974 | NA | 5.740207 |
| CUST_53973_PI429010886 | M103936 | 0.007101 | 18 | 15 | | | | | | 60.41993 | 59.61145 | 70.02979 | 41.79247 | 38.35721 | 48.76564 | 53.61134 | 46.46836 | 41.50677 | 69.9145 | 48.72653 | 54.01898 | 50.39753 | 55.50473 | 60.61424 | 61.38183 | 62.76739 | 54.20454 | 74.95386 | 56.53393 | 48.8169 | 46.75809 | 52.78112 | 36.65319 | 64.78587 | 33.72715 | 57.80685 |
| CUST_27512_PI429010886 | N56189 | 0.007101 | 18 | 9 | | | | | | 262.76 | 287.1573 | 301.2285 | 215.2857 | 278.7196 | 217.8838 | 232.766 | 235.8718 | 232.766 | 395.1607 | 331.6154 | 335.677 | 271.5235 | 347.1114 | 248.2496 | 352.188 | 316.5936 | 319.2639 | 293.013 | 334.6678 | 256.9966 | 267.2122 | 226.8738 | 265.1519 | 366.8876 | 262.3746 | 384.5478 |
| CUST_19233_PI429010886 | N41223 | 0.007107 | 18 | 15 | | | | | | 117.3497 | 113.3258 | 85.25376 | 115.1051 | 107.3414 | 113.9832 | 90.83511 | 123.794 | 71.11204 | 78.44378 | 89.50659 | 76.39741 | 95.62622 | 95.09391 | 72.59952 | 126.8764 | 135.2525 | 103.3395 | 125.1948 | 97.49057 | 127.4456 | 119.7354 | 105.0857 | 102.9204 | 97.50342 | 92.50739 | 105.8252 |
| CUST_41619_PI429010886 | M58166 | 0.007115 | 18 | 6 | | | | | | 54.97901 | 48.91209 | 76.42108 | 46.33994 | 72.48039 | 51.56072 | 73.28621 | 87.67204 | 112.1069 | 52.65717 | 85.30129 | 77.41461 | 80.39867 | 82.42534 | 76.88221 | 66.50022 | 102.022 | 76.60117 | 54.25943 | 61.07112 | 20.86742 | 77.29645 | 48.67019 | 59.9058 | 112.8928 | 62.09543 | 93.05164 |
| CUST_8639_PI429010886 | N23340 | 0.007115 | 18 | 9 | 91092464 | PREDICTED: similar to CG3999 CG3999-PA [Triboliu | Metabolic process | Cellular amino acid metabolic process | 2.18E-86 | 30.08532 | 38.18132 | 62.76739 | 17.63614 | 28.75284 | 16.01505 | 43.70884 | 53.84859 | 45.18835 | 51.24109 | 42.6374 | 49.3486 | 45.70165 | 53.90994 | 41.68991 | 42.68895 | 35.38892 | 24.54416 | 30.74148 | 43.76564 | 39.84188 | 31.99053 | 45.24787 | 23.00078 | 48.43308 | 37.86509 | 68.17959 |
| CUST_48000_PI429010886 | M78466 | 0.007119 | 15 | 12 | | | | | | 108.9753 | 108.4917 | 222.3465 | 102.1576 | 110.1779 | 130.8991 | 128.7791 | 156.1572 | 142.4549 | 169.2967 | 115.9538 | 83.03102 | 170.7891 | 199.3094 | 143.069 | 114.2228 | 128.6369 | 111.6142 | 101.5112 | 117.2582 | 108.8415 | 138.7373 | 116.3712 | 118.7528 | 159.6853 | 135.2886 | 158.7326 |
| CUST_13990_PI429010886 | N32135 | 0.007125 | 24 | 18 | 321466191 | hypothetical protein DAPPUDRAFT_305911 [Daphn | Metabolic process | Nucleobase-containing compound metabolic process | 1.79E-34 | 154.0296 | 105.9691 | 149.147 | 102.3871 | 79.03234 | 63.99533 | 105.3949 | 94.97923 | 99.32217 | 112.304 | 71.52214 | 101.5527 | 126.2074 | 117.7597 | 112.0774 | 180.6894 | 66.33801 | 125.9169 | 191.7287 | 120.6382 | 198.2178 | 123.2567 | 86.27625 | 125.9453 | 128.9813 | 91.91837 | 164.7416 |
| CUST_16244_PI429010886 | N35906 | 0.007128 | 21 | 15 | | | | | | 68.56023 | 41.36789 | 35.56458 | 27.36061 | 40.17403 | 25.25393 | 27.30026 | 30.67698 | 33.12696 | 28.58747 | 27.80368 | 27.70404 | 35.37524 | 36.387 | 65.77588 | 44.99497 | 34.06071 | 40.34891 | 44.02718 | 40.18446 | 34.39152 | 42.1461 | 30.82595 | 29.30417 | 31.59119 | 34.05076 | 36.83629 |
| CUST_17983_PI429010886 | N38948 | 0.00713 | 24 | 15 | | | | | | 326.4381 | 378.7944 | 381.1947 | 225.0823 | 277.0077 | 289.2197 | 187.5065 | 337.0926 | 336.8185 | 483.8764 | 373.6828 | 547.117 | 331.3738 | 570.2153 | 409.385 | 581 | 439.2082 | 464.3001 | 342.8578 | 443.9779 | 333.1788 | 432.8549 | 522.6581 | 328.807 | 501.4801 | 354.1413 | 466.9714 |
| CUST_45979_PI429010886 | M70982 | 0.007132 | 15 | 12 | | | | | | 8.642479 | 12.46997 | 14.07874 | 6.019113 | 8.306883 | 9.678005 | 15.74798 | 24.30558 | 20.59519 | 16.24815 | 4.673008 | 14.66496 | 18.38243 | 27.39567 | 21.3372 | 9.958012 | 16.63365 | NA | 7.889839 | 9.035702 | 10.69461 | 24.24219 | 17.61531 | 8.317554 | 17.78988 | 10.78602 | 26.71388 |
| CUST_15651_PI429010886 | N34921 | 0.00714 | 21 | 9 | | | | | | 4.862972 | 585.6944 | 235.6934 | 855.8158 | 662.0948 | 366.1623 | 506.24 | 2805.418 | 2057.769 | 583.8403 | 2098.72 | 3187.857 | 2459.721 | 350.7295 | 1556.767 | NA | 821.0172 | NA | 337.2385 | 6454.955 | 1306.431 | NA | 426.6051 | 249.3051 | NA | 1880.44 | 1809.996 |
| CUST_52593_PI429010886 | M98110 | 0.007149 | 21 | 0 | | | | | | 47.79729 | 30.57426 | 46.14783 | 25.07182 | 49.59376 | 30.76441 | 68.17174 | 33.59767 | 16.88307 | 28.32194 | 39.07685 | 28.48354 | 21.89788 | 21.97852 | 17.91014 | 32.65296 | 30.06738 | 20.35771 | 55.73505 | 23.00326 | 34.33169 | 40.76795 | 77.77973 | 51.34688 | 29.53468 | 19.86296 | 22.32503 |
| CUST_9869_PI429010886 | N25327 | 0.007169 | 24 | 15 | | | | | | 38.7854 | 57.72819 | 68.15284 | 38.92288 | 60.00971 | 46.08794 | 53.71904 | 42.44777 | 30.84259 | 57.12032 | 56.03237 | 54.5227 | 42.09086 | 77.6254 | 49.20084 | 64.36368 | 66.2145 | 86.83335 | 64.56424 | 76.84997 | 62.20164 | 78.81351 | 60.17363 | 63.56474 | 73.51179 | 67.92832 | 65.26348 |
| CUST_14068_PI429010886 | N32276 | 0.007169 | 24 | 15 | 262401121 | TIA-1-like protein [Scylla paramamosain] | | | 6.5E-45 | 10.32521 | 22.78539 | 17.92595 | 16.80068 | 12.78607 | 14.51836 | 17.18186 | 8.272098 | 13.58403 | 30.57426 | 21.7647 | 19.03731 | 18.5156 | 23.6887 | 22.69042 | 24.76741 | 26.0691 | 20.6809 | 22.7759 | 42.19897 | 17.29005 | 23.79915 | 23.20138 | 21.62724 | 23.24391 | 18.46027 | 25.24216 |
| CUST_25683_PI429010886 | N52876 | 0.007175 | 21 | 3 | | | | | | 2480.699 | 5681.469 | 3503.673 | 6630.238 | 3914.042 | 4102.292 | 2533.526 | 2344.689 | 3168.052 | 3517.336 | 1637.238 | 3683.38 | 1923.113 | 3380.982 | 1441.16 | 2911.974 | 1447.664 | 2346.537 | 522.1156 | 2219.95 | 4647.339 | 3281.126 | 4748.861 | 2457.891 | 4579.426 | 4647.339 | 2741.645 |
| CUST_6982_PI429010886 | N20866 | 0.00718 | 24 | 18 | 156553318 | PREDICTED: 39S ribosomal protein L3, mitochondria | Protein metabolism | Ribosome biogenesis | 2.6E-90 | 220.8472 | 274.0681 | 228.7951 | 183.3031 | 204.5562 | 153.7099 | 153.734 | 190.201 | 216.8379 | 269.1618 | 216.65 | 285.4569 | 201.1997 | 241.7686 | 226.0846 | 273.8248 | 195.9916 | 258.1237 | 224.015 | 293.1844 | 246.1025 | 296.3167 | 216.9071 | 246.2665 | 221.4718 | 215.6089 | 217.37 |
| CUST_44879_PI429010886 | M67298 | 0.00718 | 12 | 0 | 242010869 | coiled-coil domain-containing protein, putative [Pec | | | 3.1E-10 | 48.98952 | 50.24503 | 48.65694 | 28.03123 | 35.27392 | 42.58941 | 32.4969 | 44.87166 | 49.8924 | 47.19565 | 50.90018 | 49.52577 | 37.41038 | 49.95028 | 45.83381 | 37.54361 | 29.85007 | 36.78858 | 45.49623 | 45.08487 | 42.16099 | 43.00099 | 42.56152 | 49.76383 | 58.4208 | 39.94582 | 46.95122 |
| CUST_36646_PI429010886 | M16449 | 0.007193 | 18 | 3 | | | | | | 4.565495 | 16.65372 | 14.35419 | 36.17759 | 18.47667 | 17.11553 | 10.33682 | NA | 13.83134 | 13.35829 | 8.844206 | 15.57429 | NA | 8.045043 | NA | 4.792634 | 6.235044 | NA | 13.91491 | 14.5578 | 25.57234 | 8.185415 | 21.6649 | NA | 14.08468 | 18.35945 | NA |
| CUST_43072_PI429010886 | M61997 | 0.007194 | 18 | 15 | 345486690 | PREDICTED: phosphatidylinositol 3-kinase regulator | | | 5.18E-37 | 101.637 | 97.55406 | 173.6414 | 82.00472 | 81.09417 | 90.29804 | 86.56723 | 124.3644 | 112.9777 | 120.0318 | 116.3563 | 102.6905 | 104.363 | 130.5455 | 127.5221 | 122.1524 | 138.9538 | 122.6448 | 129.9123 | 110.0194 | 91.32302 | 110.1333 | 109.3451 | 87.90722 | 126.2993 | 101.1196 | 135.2716 |
| CUST_30040_PI429010886 | N61059 | 0.007194 | 18 | 15 | | | | | | 137.351 | 116.4037 | 113.8579 | 97.50342 | 131.2743 | 121.2504 | 145.726 | 102.9589 | 138.6787 | 135.061 | 150.1517 | 139.9854 | 121.7341 | 145.8455 | 154.8485 | 160.8227 | 153.6022 | 158.1524 | 148.4609 | 130.6215 | 92.96667 | 149.9119 | 112.9528 | 93.90071 | 152.7823 | 129.6012 | 151.0826 |
| CUST_41783_PI429010886 | M58550 | 0.007199 | 15 | 3 | | | | | | 60.54854 | 59.57181 | 49.20084 | 71.55597 | 54.0232 | 52.55999 | 62.74069 | 40.36651 | 36.6668 | 40.28049 | 37.56967 | 31.23112 | 47.90583 | 28.47733 | 43.25525 | 55.01225 | 43.40065 | 40.28049 | 49.46138 | 44.81188 | 51.04482 | 42.38554 | 35.95633 | 62.66858 | 46.99705 | 32.24593 | 39.19727 |
| CUST_12075_PI429010886 | N28889 | 0.007199 | 15 | 0 | 157119253 | zinc finger protein, putative [Aedes aegypti] >gi\|10 | | | 1.38E-74 | 32.60462 | 25.34926 | 42.92316 | 22.47267 | 19.43342 | 25.89483 | 18.689 | 22.21682 | 21.86875 | 30.50065 | 25.8352 | 34.06396 | 22.65536 | 31.50476 | 21.5971 | 30.60166 | 41.99643 | 33.59767 | 28.88408 | 30.01858 | 30.95206 | 18.85554 | 30.74148 | 22.14395 | 42.77863 | 23.61236 | 22.67516 |
| CUST_18750_PI429010886 | N40349 | 0.007203 | 12 | 9 | | | Developmental process | Nervous system development | | 9.515358 | NA | 7.034635 | NA | 5.986055 | 3.776983 | 13.16084 | 12.6532 | 10.63992 | NA | 11.92505 | 12.46576 | 19.01271 | 11.66818 | 8.881606 | 10.60724 | 5.013897 | 4.247059 | 17.5491 | 20.86543 | 14.34446 | NA | 24.2514 | NA | NA | 20.48821 | 8.140928 |
| CUST_41604_PI429010886 | M58126 | 0.007208 | 15 | 12 | | | | | | 666.3149 | 2634.615 | 1682.368 | 700.4893 | 1116.604 | 904.8603 | 586.3578 | 598.8088 | 2244.992 | 1103.847 | 1226.144 | 1478.956 | 2011.77 | 2016.816 | 2249.222 | 1747.319 | 1187.991 | 1729.171 | 1199.593 | 1407.859 | 813.9324 | 1215.238 | 1166.811 | 850.2192 | 2278.385 | 1437.865 | 1490.779 |
| CUST_53653_PI429010886 | M102654 | 0.007209 | 21 | 0 | | | | | | 17.64932 | 10.31997 | NA | 13.5897 | 9.74182 | 16.11829 | 6.987277 | 8.009329 | 8.181079 | 8.434609 | 12.63783 | 8.89926 | 7.266326 | 3.946423 | 4.752288 | 14.93407 | 19.2383 | 13.52004 | 11.68424 | 8.173644 | 10.05851 | 13.56226 | NA | 16.33068 | 15.10512 | 7.426569 | 8.832139 |
| CUST_48817_PI429010886 | M82320 | 0.007209 | 21 | 0 | | | | | | 27.25419 | 25.98413 | 21.54677 | 8.053924 | 14.98214 | 10.36474 | 7.120428 | 13.19962 | 6.610857 | 16.80616 | 9.34052 | 29.9579 | 15.35382 | 19.51691 | 17.46532 | 30.76819 | 9.099665 | 14.94602 | 25.12351 | 17.32667 | 17.59447 | 41.57514 | 21.57256 | 12.90472 | 20.70392 | 12.60946 | 16.30067 |
| CUST_5900_PI429010886 | N19349 | 0.007212 | 24 | 18 | 541044141 | serum response factor-binding protein 1 [Ascaris su | | | 8.84E-15 | 44.80736 | 45.95551 | 44.4641 | 28.93334 | 28.46598 | 26.9442 | 24.49305 | 31.50476 | 44.70933 | 46.19013 | 42.81625 | 40.94065 | 37.77438 | 53.56751 | 45.34827 | 54.6573 | 36.70932 | 43.57816 | 51.85569 | 52.43379 | 42.17788 | 49.00501 | 51.92326 | 28.80614 | 48.17521 | 41.48365 | 43.10871 |
| CUST_56006_PI429010886 | M112523 | 0.007213 | 18 | 15 | | | | | | 1809.996 | 1424.855 | 1544.651 | 579.7012 | 2250.529 | 1307.203 | 2020.188 | 1519.583 | 1565.628 | 3292.379 | 1320.618 | 1800.24 | 2831.641 | 2075.258 | 2500.44 | 2553.637 | 2054.295 | 2873.023 | 1767.465 | 2237.5 | 1677.906 | 2623.017 | 967.496 | 784.6927 | 924.5282 | 1615.873 | 1827.911 |
| CUST_3580_PI429010886 | N14813 | 0.007222 | 21 | 6 | | | | | | 232.1034 | 210.1212 | 372.9677 | 55.52574 | NA | NA | 235.6566 | 603.4589 | 595.028 | 439.659 | 47.05803 | 50.18155 | 17.74325 | 42.09877 | 19.02605 | 3.75448 | 135.78 | NA | 60.36203 | 44.1892 | 25.62613 | NA | 465.9125 | 83.98715 | NA | 33.17446 | 84.99622 |
| CUST_7959_PI429010886 | N22333 | 0.007227 | 24 | 3 | | | | | | 101.2088 | 68.77778 | 148.2643 | 155.5106 | 112.9419 | 153.8888 | 129.2719 | 159.878 | 78.04542 | 52.96132 | 69.58984 | 62.25774 | 58.97452 | 57.16741 | 65.75053 | 30.06738 | 41.68991 | 24.97999 | 107.4719 | 12.93141 | 222.3148 | 18.08454 | 120.4829 | 116.3422 | 37.58201 | 78.66048 | 39.15692 |
| CUST_8969_PI429010886 | N23868 | 0.007227 | 24 | 15 | | | | | | 108.9872 | 166.2291 | 134.79 | 80.68026 | 142.3214 | 155.7638 | 159.338 | 170.2702 | 149.0336 | 239.8633 | 155.4485 | 298.0928 | 176.2225 | 177.3713 | 184.2011 | 266.6447 | 177.9272 | 204.629 | 169.2316 | 234.3704 | 164.0579 | 180.2368 | 204.4226 | 162.0946 | 189.6264 | 231.1023 | 190.5574 |
| CUST_2497_PI429010886 | N12505 | 0.007254 | 24 | 12 | | | | | | 251.0141 | 235.4879 | 295.3248 | 213.51 | 231.1023 | 198.3971 | 259.848 | 182.9909 | 262.8518 | 276.2557 | 201.9074 | 321.374 | 287.8668 | 285.421 | 320.8355 | 432.5053 | 211.0801 | 313.2163 | 283.6461 | 260.0898 | 247.0952 | 380.7018 | 258.6664 | 204.5562 | 257.237 | 221.852 | 243.8404 |
| CUST_54991_PI429010886 | M108349 | 0.007269 | 24 | 0 | | | | | | 201.9396 | 186.5931 | 222.4725 | 206.3446 | 200.5357 | 220.685 | 199.5478 | 162.473 | 169.4032 | 179.6526 | 173.1201 | 171.5942 | 129.065 | 219.3055 | 149.3697 | 109.0854 | 205.392 | 168.6715 | 143.0236 | 172.408 | 225.0823 | 193.5749 | 210.8661 | 215.7123 | 216.3391 | 224.0801 | 213.5752 |
| CUST_42149_PI429010886 | M59541 | 0.007283 | 24 | 15 | | | | | | 120.446 | 111.4345 | 111.812 | 115.6651 | 112.1381 | 156.584 | 97.40947 | 105.86 | 78.48034 | 125.719 | 107.6007 | 103.4079 | 100.9197 | 127.5485 | 110.6854 | 146.4231 | 145.308 | 151.5629 | 119.8572 | 141.5508 | 109.2515 | 133.4937 | 139.1216 | 117.4728 | 143.2327 | 120.363 | 137.2358 |
| CUST_8632_PI429010886 | N23331 | 0.007292 | 15 | 0 | 321456752 | hypothetical protein DAPPUDRAFT_229122 [Daphn | | | 5.76E-31 | 139.3321 | 155.3371 | 119.1637 | 98.2447 | 113.9999 | 122.5622 | 104.46 | 82.62874 | 110.9853 | 148.3671 | 125.0002 | 137.7352 | 135.1423 | 116.1614 | 130.7948 | 149.811 | 136.1666 | 129.2544 | 114.8344 | 166.2981 | 114.649 | 118.5941 | 114.0935 | 112.9528 | 127.2574 | 135.3575 | 150.5497 |
| CUST_31994_PI429010886 | M2941 | 0.007297 | 15 | 12 | 328784984 | PREDICTED: neutral and basic amino acid transport | | | 6.27E-17 | 2144.378 | 2176.36 | 1523.414 | 1582.373 | 2189.348 | 1331.49 | 924.2614 | 938.9786 | 1760.95 | 2267.686 | 1523.414 | 1955.85 | 2410.357 | 2411.696 | 1938.653 | 3079.902 | 1792.391 | 1779.751 | 1426.735 | 1757.983 | 694.9126 | 2225.59 | 845.6802 | 679.5311 | 1952.727 | 1295.233 | 2461.297 |
| CUST_39663_PI429010886 | M42411 | 0.007297 | 15 | 12 | 493789484 | hypothetical protein, partial [Lactobacillus iners] >g | | | 6.55E-09 | 2033.91 | 2854.292 | 3644.859 | 857.8725 | 1520.67 | 1162.112 | 6073.086 | 4594.669 | 6779.053 | 3653.581 | 2291.102 | 3120.564 | 5402.577 | 5807.129 | 3965.108 | 5239.377 | 4068.874 | 3060.282 | 3048.703 | 2235.234 | 2529.511 | 3952.869 | 5515.427 | 2877.641 | 8245.915 | 2970.275 | 11545.02 |
| CUST_41742_PI429010886 | M58437 | 0.007297 | 15 | 3 | | | | | | 33.72458 | 34.06396 | 24.52204 | 73.49284 | 57.07016 | 97.99145 | 51.53885 | 48.62912 | 38.121 | 36.13374 | 45.74288 | 48.10487 | 51.50234 | 21.08658 | 26.79789 | 40.67008 | 26.58577 | 37.83427 | 42.24809 | 57.79515 | 48.67472 | 32.41392 | 30.86679 | 82.19075 | 43.60712 | 40.2482 | 25.86598 |
| CUST_47728_PI429010886 | M77338 | 0.007297 | 15 | 12 | | | | | | 76.73952 | 98.01409 | 99.91859 | 93.92221 | 77.53281 | 110.6986 | 96.5915 | 84.14675 | 91.81369 | 108.1306 | 89.13187 | 131.0159 | 106.1197 | 130.4838 | 179.5942 | 100.9074 | 82.39034 | 82.03238 | 84.67784 | 86.55946 | 77.20798 | 107.7922 | 107.884 | 99.91859 | 105.2326 | 126.9262 | 116.0217 |
| CUST_50200_PI429010886 | M89007 | 0.007297 | 15 | 12 | | | | | | 40.55619 | 14.10863 | 117.4327 | 14.8118 | 11.10317 | 17.98995 | 46.1038 | 76.58426 | 67.92832 | 89.43652 | 17.67128 | 22.89148 | 79.22919 | 182.4687 | 33.22847 | 18.19605 | 40.84912 | 17.86958 | 20.26494 | 11.08478 | 12.45838 | 28.24069 | 93.68609 | 19.43895 | 52.74988 | 17.15702 | 78.83085 |
| CUST_1231_PI429010886 | N8939 | 0.007297 | 15 | 12 | | | | | | 107.7242 | 178.9755 | 108.064 | 123.4029 | 101.3025 | 81.7755 | 115.7617 | 111.6628 | 117.9048 | 175.8509 | 127.1757 | 169.1571 | 132.4059 | 189.2302 | 176.1772 | 85.36118 | 116.1482 | 97.15481 | 119.5328 | 94.40103 | 112.8267 | 140.2989 | 145.1631 | 88.01513 | 173.4253 | 111.8634 | 152.7363 |
| CUST_19115_PI429010886 | N41001 | 0.0073 | 24 | 18 | | | | | | 219.5427 | 187.5572 | 239.9876 | 184.3634 | 165.8727 | 108.5779 | 187.4758 | 169.3616 | 195.689 | 167.9037 | 217.7055 | 322.3624 | 160.5552 | 292.258 | 143.609 | 279.0812 | 202.0404 | 237.2944 | 292.8985 | 267.4223 | 261.7284 | 228.2235 | 232.1034 | 279.0812 | 258.6304 | 265.1519 | 240.474 |
| CUST_11083_PI429010886 | N27237 | 0.007317 | 15 | 0 | | | | | | 116.2092 | 454.2849 | 437.8204 | 61.57414 | 56.27319 | 25.90233 | 234.0614 | 144.8456 | 160.9168 | 59.25275 | 59.15695 | 34.44235 | 157.8167 | 175.6284 | 108.1985 | 299.0341 | 481.7893 | 88.46488 | 195.9916 | NA | 31.03981 | 18.47842 | 42.6726 | 54.1974 | 22.35568 | 44.68023 | 32.47256 |
| CUST_9600_PI429010886 | N24902 | 0.007325 | 15 | 6 | | | | | | 220.9369 | 120.496 | 361.9781 | 155.2361 | 227.9715 | 305.1659 | 398.0093 | 232.3859 | 157.283 | 118.275 | 255.9101 | 97.74764 | 114.0443 | 93.68609 | 101.9838 | 160.565 | 194.4273 | 138.3597 | 302.9535 | 91.37896 | 196.6343 | 141.3981 | 419.3343 | 479.0678 | 86.70732 | 123.4738 | 104.1514 |
| CUST_39357_PI429010886 | M38284 | 0.007326 | 21 | 9 | | | | | | 1940.549 | 2247.598 | 2526.384 | 2358.256 | 3190.091 | 2571.52 | 2474.132 | 4864.618 | 3802.906 | 2270.606 | 5980.233 | 7960.065 | 5265.647 | 2845.968 | 3946.907 | 2549.933 | 2828.097 | 2373.328 | 1986.907 | 6289.971 | 2289.879 | 2716.899 | 2356.592 | 2421.023 | 2352.559 | 3783.178 | 4553.274 |
| CUST_1297_PI429010886 | N9167 | 0.007337 | 24 | 3 | | | | | | 44.84606 | 24.03557 | 17.7744 | 7.863762 | 111.5879 | 72.64262 | 47.37022 | 19.59595 | 32.8503 | 10.26907 | 6.821529 | 11.73735 | 16.8101 | 6.988725 | 11.67368 | 16.0166 | NA | NA | 5.701937 | 6.604961 | 10.80877 | 25.2475 | 9.082334 | 7.388302 | 14.8651 | NA | 17.19687 |
| CUST_52575_PI429010886 | M98009 | 0.007337 | 15 | 3 | 321463341 | sodium-dependent phosphate transporter [Daphnia | | | 3.39E-31 | 11.9176 | 5.914907 | 9.896666 | 14.34646 | 107.354 | 68.21242 | 229.8288 | 12.49156 | 5.288214 | 10.65864 | 127.4456 | 6.030949 | 6.681729 | 6.606034 | 5.88154 | 7.39029 | 202.9774 | 16.94233 | 37.11592 | NA | 195.3201 | 18.50987 | 127.8146 | 152.3001 | 8.215837 | 12.30553 | 14.01249 |
| CUST_50387_PI429010886 | M89664 | 0.00734 | 24 | 21 | | | | | | 77.7529 | 83.35473 | 85.8526 | 54.5276 | 53.5429 | 88.78018 | 52.2877 | 74.7826 | 65.02562 | 45.77928 | 76.13058 | 70.11707 | 56.84346 | 81.8019 | 58.11529 | 83.29202 | 56.31693 | 67.67314 | 69.82119 | 89.97695 | 94.20676 | 110.8364 | 73.77338 | 96.44756 | 58.42676 | 76.38127 | 98.01409 |
| CUST_4768_PI429010886 | N17441 | 0.007363 | 24 | 21 | | | | | | 8.090428 | 4.811015 | NA | 12.75631 | 23.41982 | 12.80802 | 9.645798 | 5.422731 | 5.718612 | 9.274758 | 11.16458 | 6.964774 | NA | 10.05851 | 15.21532 | 13.14489 | 16.11406 | 13.46619 | 8.57568 | 8.218065 | 15.50269 | 17.91166 | 37.24643 | 16.35722 | 15.64506 | 11.26345 | 17.64932 |
| CUST_32997_PI429010886 | M5668 | 0.007377 | 15 | 0 | 321470935 | hypothetical protein DAPPUDRAFT_241712 [Daphn | | | 3.29E-97 | 34.57676 | 28.16847 | 30.03204 | 23.22371 | 25.29876 | 28.23631 | 14.67098 | 21.45407 | 19.1106 | 27.80787 | 31.6312 | 27.77642 | 30.57426 | 36.83973 | 23.89866 | 42.41265 | 28.47733 | 31.24239 | 33.00505 | 28.14284 | 25.34926 | 31.721 | 21.42649 | 26.62834 | 36.14912 | 31.81697 | 27.50644 |
| CUST_38940_PI429010886 | M32278 | 0.00738 | 15 | 6 | | | | | | 4.604286 | 32.79731 | 24.23014 | 50.20045 | 83.29202 | 38.44815 | 336.2223 | 498.7637 | 388.2685 | 92.13028 | 700.4893 | 696.5534 | 167.2734 | 25.35894 | 87.83251 | NA | 140.8068 | NA | 177.7238 | 1373.566 | 118.7227 | NA | 191.2063 | 142.0582 | 22.13925 | 278.7763 | 151.2216 |
| CUST_40047_PI429010886 | M49238 | 0.007384 | 15 | 9 | | | | | | 170.317 | 578.1088 | 547.8035 | 255.6195 | 94.69116 | 37.9853 | 268.545 | 653.1387 | 4161.995 | 1180.515 | 1954.96 | 4887.374 | 2497.013 | 2509.921 | 1746.637 | 1263.557 | 442.852 | 483.8764 | 609.4542 | 4476.427 | 774.2499 | 1529.041 | 5396.825 | 287.5478 | 1756.162 | 4803.756 | 4873.025 |
| CUST_34331_PI429010886 | M9345 | 0.007388 | 24 | 21 | 321468460 | hypothetical protein DAPPUDRAFT_52407 [Daphnia | Protein metabolism | Cellular protein modification process | 8E-83 | 85.48477 | 78.1117 | 104.1327 | 59.18762 | 71.71135 | 84.03407 | 88.49666 | 65.01299 | 86.44204 | 100.965 | 113.7893 | 108.5658 | 51.43059 | 121.8904 | 89.9999 | 102.3996 | 138.6787 | 83.06141 | 134.79 | 101.2274 | 125.408 | 136.023 | 151.2877 | 133.006 | 162.7472 | 124.6243 | 195.2252 |
| CUST_22222_PI429010886 | N46647 | 0.007406 | 24 | 3 | | | | | | 5075.28 | 4647.339 | 6511.817 | 7026.307 | 7426.728 | 5086.964 | 5528.982 | 2437.103 | 5903.55 | 5836.719 | 3184.108 | 5515.427 | 2896.285 | 4062.216 | 1322.445 | 4955.555 | 2129.816 | 1356.986 | 1569.266 | 2213.328 | 7628.757 | 4575.004 | 7373.59 | 2886.71 | 4540.69 | 3742.075 | 5009.146 |
| CUST_5622_PI429010886 | N18959 | 0.007416 | 12 | 0 | 351542215 | exonuclease 3'-5' domain containing 2 [Xenopus lae | Metabolic process | Nucleobase-containing compound metabolic process | 7.41E-77 | 21.01979 | 14.71509 | 12.69121 | NA | 7.327597 | NA | 6.243139 | 11.12353 | 3.943889 | 11.0429 | 9.344321 | 10.60542 | 11.21025 | 31.50694 | 14.72582 | 12.25586 | 5.709735 | 12.4022 | 9.743013 | 7.983639 | 14.97 | 8.858469 | 8.826373 | 14.63359 | 13.69107 | 11.3725 | 19.28994 |
| CUST_39030_PI429010886 | M33656 | 0.007422 | 24 | 3 | | | | | | 17.2249 | 15.07014 | 8.656419 | 21.16443 | 22.30073 | 18.53102 | 14.65894 | 13.44283 | 8.530807 | 6.387554 | 13.0885 | 13.27837 | 7.338708 | 10.28696 | 8.906902 | 14.87074 | 13.71331 | 8.710242 | 13.22099 | 15.51946 | 12.87174 | 13.00912 | NA | 12.92974 | 16.43668 | 7.105175 | 13.84831 |
| CUST_18683_PI429010886 | N40221 | 0.007429 | 21 | 0 | | | | | | 11.91263 | 12.79089 | 20.93093 | 4.275828 | 12.33182 | 12.20868 | 15.31496 | 10.06713 | 7.665956 | 12.53746 | 9.787782 | 10.81563 | 8.265097 | 8.925496 | 3.858845 | 15.16412 | 9.522138 | 9.610816 | 11.62614 | 9.374459 | 13.93866 | 18.19988 | 14.31222 | NA | 9.722947 | NA | 14.32775 |
| CUST_43812_PI429010886 | M64051 | 0.00744 | 24 | 0 | | | | | | 58.03638 | 72.22304 | 72.61388 | 57.00887 | 55.70806 | 60.3963 | 72.16312 | 55.15882 | 57.30764 | 49.40423 | 60.72482 | 48.22578 | 44.95443 | 32.91621 | 38.44815 | 47.5011 | 52.88081 | 40.03759 | 71.78694 | 28.06937 | 34.48125 | 38.71497 | 55.32113 | 67.87327 | 50.91495 | 57.78122 | 55.53245 |
| CUST_31594_PI429010886 | M1922 | 0.007446 | 18 | 12 | | | | | | 224.7094 | 9.603713 | 623.8166 | 38.121 | 35.94612 | 84.09478 | 133.5801 | 348.5888 | 103.654 | 517.6852 | 97.33778 | 119.228 | 651.0924 | 794.4801 | 370.5671 | NA | 313.8184 | 83.33435 | 40.48072 | 206.9171 | 123.6053 | 196.9594 | 228.0795 | 8.383502 | 84.10524 | 58.41483 | 224.5779 |
| CUST_38227_PI429010886 | M24366 | 0.007458 | 15 | 9 | | | | | | 237.4261 | 216.2628 | 357.9328 | 250.7333 | 240.0651 | 300.9186 | 223.8787 | 272.6021 | 281.9612 | 343.216 | 257.6826 | 407.6622 | 239.8387 | 408.4945 | 250.7705 | 262.2997 | 202.438 | 248.9182 | 235.5325 | 240.0259 | 330.3612 | 321.696 | 310.6611 | 286.0114 | 375.1955 | 382.5164 | 448.8586 |
| CUST_29152_PI429010886 | N59293 | 0.00746 | 24 | 15 | | | | | | 6.64085 | 5.190121 | 7.854825 | 6.589476 | 7.011247 | 11.74757 | 7.550196 | 10.12652 | 10.47683 | 21.09489 | 7.021601 | 22.55627 | 5.316896 | 10.19739 | 25.17336 | 29.01971 | 16.20777 | 17.77803 | 10.73752 | 5.623858 | 14.4005 | 9.74748 | 12.58679 | 12.12977 | 5.210194 | 5.58927 | 18.65671 |
| CUST_33148_PI429010886 | M6093 | 0.007469 | 12 | 0 | | | | | | 230.9236 | 275.6487 | 265.7722 | 232.9495 | 250.6384 | 233.3626 | 186.9145 | 192.7007 | 200.8936 | 265.2639 | 206.6019 | 252.8872 | 269.9577 | 332.964 | 273.393 | 339.8879 | 220.4105 | 228.3459 | 229.2068 | 272.7482 | 231.3049 | 312.3654 | 271.2785 | 210.5448 | 332.5951 | 254.1554 | 297.3146 |
| CUST_4560_PI429010886 | N17029 | 0.007469 | 12 | 0 | | | | | | 56.60803 | 49.41638 | 61.50815 | 55.00728 | 56.37263 | 57.84331 | 55.58103 | 38.74854 | 45.73272 | 60.64562 | 53.8998 | 76.07191 | 59.69745 | 73.30133 | 63.11741 | 51.25727 | 55.29319 | 41.96064 | 43.92643 | 61.06386 | 50.24503 | 62.56969 | 57.05842 | 47.02813 | 75.19772 | 65.42078 | 80.88726 |
| CUST_27897_PI429010886 | N56901 | 0.007469 | 12 | 0 | | | | | | 88.50576 | 97.96017 | 79.68658 | 72.23286 | 62.98504 | 79.21035 | 52.11537 | 48.85338 | 77.82271 | 66.79779 | 52.22471 | 82.02402 | 69.65899 | 66.53867 | 100.5781 | 88.98374 | 57.34357 | 95.54961 | 55.09266 | 68.89697 | 61.90324 | 94.5989 | 64.2224 | 77.77973 | 93.39666 | 91.90962 | 78.21989 |

| Probe ID | ID | p-value | Period | Phase | EBI ID | Annotation | GO category | Go term | E-value | 0 | 0 | 0 | 3 | 3 | 3 | 6 | 6 | 6 | 9 | 9 | 9 | 12 | 12 | 12 | 15 | 15 | 15 | 18 | 18 | 18 | 21 | 21 | 21 | 24 | 24 | 24 |
|---|---|---|---|---|---|---|---|---|---|---|---|---|---|---|---|---|---|---|---|---|---|---|---|---|---|---|---|---|---|---|---|---|---|---|---|---|
| CUST_32275_PI429010886 | M3684 | 0.007474 | 18 | 12 | 443695741 | hypothetical protein CAPTEDRAFT_168216 [Capitell | | | 2.8E-62 | 81.44839 | 82.32602 | 76.22636 | 67.40392 | 72.37554 | 66.64899 | 69.77447 | 54.73744 | 49.38887 | 83.34341 | 64.97549 | 73.42232 | 75.90597 | 95.74719 | 98.91679 | 95.44 | 54.12753 | 75.04971 | 71.45222 | 73.38298 | 65.34071 | 110.9726 | 69.22629 | 64.96286 | 73.13314 | 60.04192 | 65.23612 |
| CUST_40975_PI429010886 | M56521 | 0.007476 | 15 | 9 | | | | | | 191.5917 | 216.4474 | 212.501 | 178.0175 | 293.1271 | 172.2644 | 208.138 | 178.147 | 259.6731 | 284.9694 | 232.4315 | 355.9452 | 303.2078 | 324.2442 | 222.3674 | 354.2035 | 195.689 | 312.5863 | 277.7527 | 239.3938 | 159.6853 | 215.3368 | 246.9496 | 199.4834 | 540.1617 | 342.0536 | 522.2489 |
| CUST_5855_PI429010886 | N19298 | 0.007476 | 15 | 9 | | | Protein metabolism | Protein folding | | 60.69824 | 74.38643 | 52.22902 | 54.90496 | 59.052 | 39.27986 | 39.69364 | 46.59363 | 67.51475 | 85.82609 | 75.03476 | 119.885 | 61.97713 | 93.59991 | 64.74408 | 100.397 | 50.28834 | 78.94301 | 41.48365 | 68.86603 | 59.76845 | 85.10115 | 64.88975 | 46.00876 | 69.39137 | 65.62571 | 111.5216 |
| CUST_6927_PI429010886 | N20790 | 0.007477 | 18 | 12 | | | | | | 776.8406 | 1356.986 | 1081.105 | 952.6524 | 712.5461 | 764.0731 | 489.9462 | 814.5413 | 616.9292 | 1036.891 | 651.8188 | 1387.428 | 962.6096 | 984.9879 | 1246.44 | 947.592 | 800.0152 | 755.8427 | 872.8348 | 752.5982 | 832.3284 | 768.7795 | 984.2764 | 849.9143 | 686.5061 | 717.957 | 568.0071 |
| CUST_28051_PI429010886 | N57180 | 0.007478 | 15 | 0 | 156404234 | predicted protein [Nematostella vectensis] >gi\|156 | Metabolic process | Carbohydrate metabolic process | 1.35E-27 | 30.38651 | 128.8011 | 23.16574 | 4.597228 | 23.97181 | 16.3435 | 24.26769 | 20.34669 | 34.76682 | 8.654424 | 14.73565 | 11.46694 | 11.47303 | NA | 18.19204 | 29.10242 | 26.32739 | 38.20891 | 19.29325 | 26.17436 | 18.12511 | 37.37861 | NA | 9.343228 | 13.40566 | 14.33928 | 14.33423 |
| CUST_52916_PI429010886 | M99417 | 0.007485 | 24 | 18 | | | | | | 90.25676 | 91.23322 | 90.13978 | 70.47896 | 68.48249 | 96.80182 | 85.89616 | 67.08317 | 79.97877 | 87.87096 | 71.49639 | 76.55655 | 82.67802 | 178.833 | 125.6172 | 80.70446 | 80.21909 | 80.33892 | 86.7376 | 156.2777 | 173.0713 | 99.38154 | 150.0225 | 91.05924 | 88.11918 | 147.4899 | 94.90628 |
| CUST_31528_PI429010886 | M1725 | 0.007501 | 18 | 15 | | | | | | 161.4564 | 271.5235 | 204.8478 | 154.793 | 207.9553 | 126.6371 | 137.5651 | 131.2903 | 141.2301 | 207.4667 | 212.7113 | 241.638 | 186.9387 | 185.3559 | 191.7997 | 289.3248 | 195.9585 | 240.9019 | 181.7853 | 277.0077 | 187.1216 | 241.9211 | 169.802 | 135.4402 | 191.6146 | 145.308 | 184.0345 |
| CUST_10155_PI429010886 | N25783 | 0.007505 | 21 | 15 | | | | | | 819.4278 | 869.0196 | 790.1209 | 552.7878 | 779.5608 | 819.682 | 602.1279 | 576.8367 | 661.9537 | 692.0661 | 746.1542 | 1024.31 | 661.523 | 739.8087 | 767.811 | 1073.921 | 1019.446 | 815.3757 | 871.2706 | 850.2192 | 637.0757 | 849.3126 | 863.4186 | 734.9802 | 973.2028 | 611.3709 | 750.1475 |
| CUST_53020_PI429010886 | M99783 | 0.007514 | 18 | 3 | 321453851 | putative TGF-beta receptor type I baboon protein [I | Signal transduction | Other | 4.59E-14 | 72.14089 | 111.6728 | 86.4248 | 179.678 | 75.45183 | 129.1352 | 77.95844 | 71.25267 | 76.71565 | 68.81509 | 52.60928 | 97.73561 | 59.62185 | 80.37038 | 57.47681 | 84.09478 | 57.96698 | 72.3636 | 60.71887 | 71.55597 | 111.3143 | 80.59405 | 120.3098 | 78.53416 | 101.867 | 89.9999 | 72.8742 |
| CUST_20234_PI429010886 | N43069 | 0.007517 | 24 | 21 | | | | | | 9.427695 | 10.19195 | 16.67239 | 6.693409 | 5.931156 | 9.879248 | 5.711785 | 8.319795 | NA | 5.090401 | 12.55563 | 15.49433 | 6.271198 | NA | 8.496964 | 8.176012 | 12.43952 | 9.496558 | 11.01558 | 8.437018 | 10.9756 | 19.66703 | 14.03672 | 16.5713 | NA | 16.92856 | 13.64597 |
| CUST_19773_PI429010886 | N42166 | 0.007517 | 15 | 0 | | | | | | 34.18999 | 36.16119 | NA | 68.1165 | 21.07701 | 27.84642 | 59.19392 | 11.51059 | 45.65948 | 9.47114 | 6.651311 | NA | 8.917899 | 12.03892 | 26.03397 | 20.97805 | 20.38984 | 31.89471 | 29.80754 | NA | 8.502113 | 16.58396 | NA | 18.03617 | 8.402814 | 6.546282 | 9.004162 |
| CUST_53580_PI429010886 | M102352 | 0.00752 | 24 | 18 | | | | | | 38.73964 | 27.48409 | 35.18679 | 21.41937 | 29.72539 | 33.03027 | 34.14561 | 26.47171 | 19.13867 | 45.38957 | 31.6082 | 25.5511 | 30.12445 | 20.79607 | 29.57766 | 27.07492 | 39.55733 | 26.87231 | 40.15414 | 39.54839 | 40.71531 | 35.75095 | 36.12998 | 35.85975 | 40.41641 | 28.27971 | 34.28772 |
| CUST_51548_PI429010886 | M93948 | 0.007528 | 21 | 3 | 383863669 | PREDICTED: kinesin-like protein KIF21A-like isoform | | | 6.73E-11 | 5515.427 | 15271.23 | 8876.599 | 19357.18 | 11104 | 14406.9 | 5572.884 | 7404.352 | 6380.497 | 7462.719 | 4817.775 | 9739.086 | 7197.042 | 6073.086 | 5911.575 | 10401.6 | 3077.216 | 5940.317 | 2834.55 | 8721.497 | 9571.885 | 11044.29 | 10117.01 | 7470.617 | 11366.05 | 15567.33 | 6962.573 |
| CUST_7223_PI429010886 | N21214 | 0.007533 | 21 | 9 | 321463784 | hypothetical protein DAPPUDRAFT_306991 [Daphn | Metabolic process | Other | 2.5E-133 | 57.32094 | 61.34598 | 61.21559 | 68.50419 | 55.87064 | 64.85632 | 62.3113 | 68.86603 | 89.96525 | 81.18707 | 77.66626 | 77.01708 | 75.85953 | 70.02583 | 77.88301 | 58.19173 | 55.42699 | 52.84586 | 93.41435 | 67.02702 | 59.68907 | 66.32627 | 58.988 | 41.28276 | 73.47743 | 42.09528 | 66.59221 |
| CUST_43673_PI429010886 | M63643 | 0.007539 | 21 | 3 | 405957597 | Reelin [Crassostrea gigas] | | | 3.38E-47 | 89.85114 | 47.48523 | 73.51884 | 119.245 | 88.95458 | 89.37758 | 89.70412 | 62.66235 | 60.69824 | 128.0667 | 104.7208 | 104.9497 | 62.3113 | 71.20471 | 56.60227 | 42.19441 | 42.84203 | 35.30186 | 139.3127 | 60.26272 | 80.41737 | 48.29446 | 51.29496 | 61.23542 | 114.5445 | 62.45524 | 117.8738 |
| CUST_18172_PI429010886 | N39285 | 0.007545 | 21 | 3 | | | | | | 2459.721 | 6336.037 | 3459.777 | 8245.915 | 3696.299 | 4031.594 | 2475.35 | 2972.831 | 2881.575 | 2015.591 | 1950.603 | 5009.146 | 2321.278 | 3345.256 | 388.2685 | 3653.581 | 1288.361 | 1232.019 | 1508.69 | 2520.419 | 3972.597 | 1192.165 | 3419.141 | 2984.905 | 4120.1 | 4407.135 | 2764.643 |
| CUST_47388_PI429010886 | M75923 | 0.007546 | 15 | 9 | | | | | | 9.391276 | 24.74877 | 10.64286 | 14.35419 | 13.57654 | 16.99403 | 14.20484 | 61.46953 | 16.62231 | 26.05952 | 13.92315 | 29.81522 | 16.67016 | 19.02839 | 18.84701 | 16.21551 | 16.38019 | 14.70379 | 25.19237 | 13.65696 | 16.57742 | 18.4928 | 22.32739 | 25.63163 | 26.97315 | 29.87699 | 23.17075 |
| CUST_46158_PI429010886 | M71597 | 0.007548 | 21 | 18 | | | | | | 10.00344 | 26.09533 | 24.43879 | 9.927073 | 15.15241 | 12.92501 | 16.45644 | 4.86085 | 4.66886 | 13.10821 | 7.654255 | 17.09566 | 13.09531 | 11.54995 | 17.73394 | 20.66741 | 4.671082 | 14.97661 | 30.112 | 24.35448 | 39.15692 | 18.22302 | 7.886773 | 12.11676 | 21.91471 | 6.608238 | 20.38984 |
| CUST_53069_PI429010886 | M100006 | 0.00755 | 18 | 0 | 321460146 | hypothetical protein DAPPUDRAFT_93395 [Daphnia | | | 1.3E-22 | 51.49762 | 56.18196 | 53.97066 | 80.97211 | 37.52322 | 77.95844 | 47.82182 | 35.20228 | 47.52868 | 28.06124 | 28.61065 | 37.47945 | 43.37513 | 44.14193 | 50.23482 | 29.76829 | 25.36437 | 24.95013 | 101.4018 | 47.51073 | 53.52565 | 84.92511 | 55.43705 | 29.99986 | 31.08481 | 30.9626 | 44.76277 |
| CUST_56172_PI429010886 | M113022 | 0.007556 | 21 | 0 | | | | | | 15.4643 | 9.163319 | 15.17796 | 10.23633 | 4.033847 | 12.13343 | 14.07075 | 11.74252 | 12.99803 | 9.011589 | 4.323775 | 3.917902 | 4.72981 | 3.707948 | 14.62058 | 10.48808 | 4.941692 | 13.48112 | 12.06387 | 8.988276 | NA | 13.04571 | 23.85638 | 14.37169 | 14.33928 | 11.19977 | 6.782422 |
| CUST_32417_PI429010886 | M4069 | 0.007557 | 12 | 0 | | | | | | 29.18761 | 30.9722 | 26.36533 | 14.04228 | 20.19405 | 16.32704 | 11.4368 | 17.12833 | 14.67308 | 25.70084 | 18.01506 | 20.65497 | 20.37385 | 15.88543 | 19.05757 | 27.64478 | 15.7841 | 25.62877 | 12.69324 | 17.64732 | 17.40899 | 25.42895 | 37.9853 | 16.24253 | 32.67128 | 43.5699 | 21.15997 |
| CUST_45291_PI429010886 | M68616 | 0.007559 | 24 | 0 | | | | | | 59.98757 | 76.21064 | 61.03108 | 63.23408 | 63.23408 | 50.57708 | 52.40666 | 47.45553 | 55.37064 | 64.45055 | 45.30656 | 64.74408 | 46.71096 | 45.07578 | 49.83701 | 57.23868 | 26.05504 | 32.80956 | 50.17076 | 60.6558 | 46.71096 | 56.52083 | 66.71625 | 42.69742 | 74.06105 | 69.11088 | 47.16593 |
| CUST_11957_PI429010886 | N28689 | 0.007563 | 18 | 3 | | | | | | 58.94983 | 40.74811 | 75.61377 | 95.04998 | 60.97776 | 94.67269 | 104.0978 | 89.27715 | 61.77428 | 43.73443 | 37.13409 | 17.54073 | 69.85356 | 59.50688 | 63.46235 | 52.40185 | 110.7787 | 73.17053 | 80.37733 | 6.162725 | 125.2532 | 83.39175 | 138.4302 | 124.1661 | 32.09701 | 42.68003 | 48.5992 |
| CUST_37969_PI429010886 | M22508 | 0.007564 | 15 | 12 | 159078854 | serine protease [Penaeus monodon] | Protein metabolism | Proteolysis | 1.69E-42 | 1218.887 | 1334.604 | 1351.148 | 1119.369 | 858.518 | 738.4538 | 1473.383 | 1356.986 | 1890.591 | 1205.12 | 825.7659 | 1392.18 | 1682.368 | 1804.906 | 1581.923 | 1659.051 | 1198.309 | 971.1081 | 1200.709 | 950.2628 | 848.334 | 1413.053 | 1233.197 | 920.7129 | 2288.66 | 842.9384 | 2629.007 |
| CUST_39480_PI429010886 | M39996 | 0.007564 | 15 | 12 | | | | | | 1436.981 | 595.4395 | 10190.76 | 43.41776 | 50.87205 | 16.92856 | 3330.039 | 18620.02 | 18586.47 | 17362.34 | 4077.008 | 6767.816 | 15662.95 | 33895.99 | 19541.87 | 1463.822 | 8827.561 | 574.2435 | 2023.529 | 10401.6 | 747.806 | 28694.35 | 21119.13 | 491.272 | 28866.67 | 4289.879 | 29068.55 |
| CUST_49205_PI429010886 | M84665 | 0.007564 | 15 | 12 | | | | | | 33.33635 | 31.34763 | 31.09152 | 16.72128 | 18.21393 | 28.41001 | 19.80461 | 26.67812 | 32.09701 | 39.54839 | 20.00677 | 30.65915 | 37.15687 | 42.35334 | 49.46138 | 42.99343 | 18.65035 | 28.45405 | 30.3611 | 25.95233 | 24.81825 | 46.53178 | 19.44365 | 11.05143 | 34.76295 | 23.2714 | 41.37211 |
| CUST_7194_PI429010886 | N21177 | 0.007564 | 24 | 18 | 443711213 | hypothetical protein CAPTEDRAFT_147624 [Capitell | Protein metabolism | Cellular protein modification process | 2.77E-13 | 12.51404 | 17.23883 | 21.34584 | 16.2136 | 19.37598 | 17.48182 | 9.653182 | 21.86589 | 20.07638 | 21.50916 | 23.19911 | 17.73142 | 26.22826 | 30.26479 | 11.70759 | 18.54361 | 23.49179 | 27.09043 | 22.41033 | 39.74462 | 28.60108 | 20.32136 | 23.28108 | 21.76618 | 31.90512 | 19.95634 | 24.74877 |
| CUST_7524_PI429010886 | N21674 | 0.007569 | 15 | 3 | | | | | | 993.3629 | 870.3572 | 888.436 | 1137.109 | 991.907 | 1057.219 | 1204.562 | 757.6325 | 619.3802 | 595.4395 | 793.0472 | 552.0613 | 709.6744 | 656.5643 | 634.1523 | 737.0066 | 1145.481 | 794.9156 | 1082.038 | 477.4078 | 755.2577 | 694.3101 | 835.1893 | 1156.728 | 592.8448 | 586.0335 | 530.8519 |
| CUST_25909_PI429010886 | N53283 | 0.007569 | 15 | 0 | | | | | | 38.63759 | 41.96975 | 51.51243 | 34.57302 | 36.23666 | 46.4361 | 34.70757 | 29.30762 | 27.41533 | 45.89685 | 38.20891 | 36.91363 | 29.42444 | 39.25017 | 30.09768 | 46.38676 | 43.05742 | 43.07866 | 37.26633 | 41.50205 | 38.21245 | 31.6776 | 39.43891 | 30.02077 | 41.8585 | 40.66087 | 54.63011 |
| CUST_19377_PI429010886 | N41475 | 0.007574 | 15 | 9 | 443727921 | hypothetical protein CAPTEDRAFT_133965 [Capitell | Metabolic process | Cellular amino acid metabolic process | 9.8E-31 | 23.95732 | 36.64631 | 25.53642 | 22.29671 | 18.72259 | 23.32 | 24.23621 | 47.00702 | 38.79996 | 36.4309 | 48.59055 | 48.63366 | 37.44113 | 39.84998 | 34.7228 | 27.14607 | 19.20292 | 20.70543 | 26.07711 | 62.34386 | 32.90555 | 33.76323 | 30.11515 | 25.52007 | 33.09246 | 36.25859 | 51.11368 |
| CUST_34352_PI429010886 | M9399 | 0.007597 | 24 | 15 | | | | | | 493.215 | 609.869 | 437.7044 | 472.5516 | 537.701 | 486.1656 | 454.184 | 427.8624 | 455.4031 | 526.943 | 515.4981 | 624.47 | 472.997 | 533.5457 | 498.2267 | 747.636 | 592.7227 | 593.968 | 603.6169 | 635.813 | 495.3471 | 722.796 | 422.2989 | 499.2279 | 578.6895 | 456.7992 | 516.3899 |
| CUST_35063_PI429010886 | M11462 | 0.007618 | 24 | 9 | 123507472 | ankyrin repeat protein [Trichomonas vaginalis G3] > | | | 1.42E-12 | 317.6417 | 94.91653 | 384.9318 | 122.1728 | 328.5456 | 149.5977 | 384.8393 | 481.2318 | 409.277 | 848.6156 | 508.0799 | 830.6821 | 171.2286 | 78.77563 | 782.2398 | 739.0931 | 58.41483 | 231.9868 | 370.8487 | 111.5107 | 267.7928 | 87.40211 | 292.8985 | 157.231 | 127.8459 | 114.649 | 140.2989 |
| CUST_40008_PI429010886 | M48301 | 0.007625 | 21 | 3 | | | | | | 1422.337 | 4179.242 | 1876.716 | 7426.728 | 2818.479 | 3158.604 | 1596.656 | 1555.197 | 1868.48 | 2203.176 | 1059.331 | 4094.84 | 1628.664 | 1081.506 | 162.5022 | 2299.417 | 650.0167 | 1830.724 | 969.5225 | 592.3414 | 2701.759 | 3181.861 | 2456.459 | 1960.986 | 3136.621 | 3869.78 | 1522.087 |
| CUST_3685_PI429010886 | N15051 | 0.007626 | 15 | 9 | | | | | | NA | 70.96812 | 39.25313 | 102.0005 | 89.40029 | 51.51243 | 134.5271 | 313.9465 | 242.3388 | 64.56992 | 528.3775 | 694.5441 | 204.946 | 55.38718 | 116.9761 | NA | 64.125 | 7.413154 | 41.23292 | 569.1252 | 379.5119 | NA | 120.8319 | 90.68288 | NA | 341.1708 | 296.6828 |
| CUST_23832_PI429010886 | N49552 | 0.007638 | 21 | 3 | 457871791 | 40S ribosomal protein S9 [Plasmodium cynomolgi st | Protein metabolism | Ribosome biogenesis | 7.65E-27 | 2108.426 | 3330.039 | 2727.828 | 5197.227 | 3027.574 | 2945.87 | 2287.157 | 1715.766 | 2704.165 | 3147.989 | 1299.658 | 2995.003 | 1364.277 | 2939.878 | 669.2807 | 1923.113 | 1002.084 | 1608.297 | 1190.301 | 1193.941 | 3960.835 | 2073.005 | 3597.224 | 1857.991 | 3653.581 | 3126.195 | 2234.111 |
| CUST_52412_PI429010886 | M97356 | 0.007642 | 24 | 15 | | | | | | 19.67357 | 19.61572 | 23.82334 | 14.57268 | 19.50921 | 22.95471 | 32.97999 | 19.98856 | 31.24487 | 27.37719 | 33.73552 | 25.9425 | 26.98494 | 23.81911 | 19.41296 | 23.92396 | 36.54664 | 33.42828 | 47.63195 | 29.36188 | 26.23793 | 29.60276 | 36.75997 | 28.91721 | 19.31761 | 21.7344 | 25.73137 |
| CUST_43193_PI429010886 | M62369 | 0.007645 | 12 | 0 | | | | | | 371.6995 | 325.5645 | 503.8676 | 198.6688 | 227.0976 | 400.1681 | 280.3132 | 189.5057 | 260.0114 | 315.9615 | 314.9323 | 526.5682 | 342.1732 | 515.4056 | 471.3393 | 416.5528 | 303.7146 | 287.9369 | 249.0909 | 422.3809 | 312.7841 | 465.9125 | 257.9765 | 260.373 | 367.5439 | 677.3854 | 508.0799 |
| CUST_16308_PI429010886 | N36017 | 0.007652 | 15 | 12 | 459186163 | PREDICTED: P2X purinoceptor 7-like [Ciona intestina | | | 2.72E-15 | 56.7825 | 11.70937 | 32.75767 | 29.71386 | 38.70169 | 33.98233 | 18.69696 | 28.31689 | 13.08017 | 25.4983 | 47.90107 | 123.6853 | 78.23845 | 69.49923 | 99.35803 | 30.85363 | 49.16869 | 41.36789 | 28.78017 | 23.06016 | 55.56489 | 35.08626 | 62.82655 | 21.71424 | 58.00017 | 68.02073 | 62.42019 |
| CUST_13326_PI429010886 | N30994 | 0.007652 | 24 | 21 | | | | | | 126.8312 | 126.3583 | 135.9627 | 120.0167 | 109.5638 | 155.9176 | 97.32524 | 81.22317 | 89.77998 | 161.2431 | 131.0636 | 125.408 | 94.18675 | 90.29804 | 137.7989 | 147.3218 | 143.3629 | 130.2691 | 129.8789 | 106.908 | 144.6491 | 171.8979 | 139.9485 | 146.0878 | 192.898 | 104.5344 | 123.5093 |
| CUST_24596_PI429010886 | N50908 | 0.007668 | 18 | 6 | 321472175 | hypothetical protein DAPPUDRAFT_21313 [Daphnia | | | 2.35E-24 | 37.24189 | 23.14834 | 49.74933 | 27.9952 | 26.43455 | 35.52357 | 98.22103 | 49.3195 | 58.29022 | 27.61589 | 33.40373 | 20.38654 | 24.70162 | 28.01868 | 14.58166 | 27.92689 | 42.05204 | 26.90259 | 24.91507 | 28.8964 | 41.8887 | 28.36576 | 58.18474 | 35.10761 | 43.439 | 32.06599 | 46.19956 |
| CUST_17289_PI429010886 | N37716 | 0.007671 | 12 | 3 | | | | | | 11.97517 | 62.6571 | 10.45086 | 58.85113 | 158.9341 | 146.7019 | 66.20577 | 4.762239 | 50.08673 | 3.812203 | 6.462365 | 8.758494 | 5.287676 | 17.76635 | 38.1318 | 99.93216 | NA | 76.75659 | NA | 18.91365 | 8.690781 | 40.01491 | NA | 41.02914 | 12.53208 | NA | NA |
| CUST_35583_PI429010886 | M12949 | 0.007671 | 18 | 6 | | | | | | 335.0674 | 120.7928 | 225.9932 | 202.3386 | 335.333 | 312.5863 | 365.2576 | 165.2432 | 379.3496 | 260.3136 | 244.0822 | 405.9331 | 123.349 | 184.458 | 187.0803 | 173.9115 | 350.1387 | 284.2642 | 213.4377 | 129.5554 | 162.9465 | 570.3497 | 282.5938 | 324.5263 | 575.5153 | 342.3066 | 625.484 |
| CUST_12094_PI429010886 | N28913 | 0.007673 | 21 | 12 | | | | | | 86.9841 | 56.03237 | 73.38298 | 42.5506 | 58.92815 | 44.81799 | 75.98951 | 66.75063 | 41.52003 | 97.25472 | 63.54061 | 47.51073 | 78.94301 | 83.98715 | 96.29479 | 76.42108 | 91.11111 | 55.73505 | 120.8183 | 70.88996 | 81.81059 | 55.47668 | 51.93952 | 63.15525 | 47.91134 | 48.64598 | 83.43282 |
| CUST_47908_PI429010886 | M78121 | 0.007686 | 21 | 12 | 270007545 | hypothetical protein TcasGA2_TC014142 [Tribolium | Metabolic process | Nucleobase-containing compound metabolic process | 1.87E-21 | 41.94819 | NA | 126.9262 | 4.819516 | 4.90692 | 14.39787 | 19.34025 | 61.09062 | 27.53274 | 104.3119 | 20.56557 | 19.44948 | 119.0396 | 178.4723 | 53.77384 | NA | 60.57138 | 14.43213 | 15.83738 | 34.92634 | 31.13099 | 50.6298 | 40.84912 | NA | 17.69892 | NA | 25.56371 |
| CUST_14847_PI429010886 | N33568 | 0.007692 | 15 | 12 | | | | | | 15.32925 | 16.79903 | 39.81145 | NA | 4.042685 | NA | 8.416999 | 53.27432 | 25.23726 | 47.15148 | 12.09871 | 19.22009 | 27.49118 | 50.29336 | 35.4058 | 14.06114 | 12.5724 | 17.86761 | 5.907047 | 18.89448 | 11.42482 | 22.59879 | 18.99123 | NA | 35.05495 | 19.44365 | 43.44981 |
| CUST_50625_PI429010886 | M90466 | 0.007693 | 24 | 15 | | | | | | 193.8296 | 173.3481 | 138.3597 | 123.6533 | 137.687 | 150.7391 | 130.8991 | 125.4528 | 119.0242 | 177.3959 | 160.6701 | 150.8963 | 111.7143 | 165.4769 | 157.4409 | 215.5744 | 198.7167 | 196.032 | 166.4887 | 164.1481 | 136.25 | 210.4274 | 147.098 | 137.0179 | 218.1405 | 135.1495 | 166.5412 |
| CUST_9293_PI429010886 | N24387 | 0.0077 | 12 | 3 | 242011913 | Pediculus humanus corporis Actin, muscle, mRNA | Developmental process | Multicellular organismal development | 2.4E-130 | 26.05696 | 10.31326 | NA | 12.74222 | 22.46339 | 19.20292 | 8.647376 | 10.2062 | 8.723307 | 8.912458 | 8.093925 | 7.287651 | 8.072291 | NA | NA | 13.56226 | 18.47168 | 17.44361 | 9.550729 | 9.72827 | 17.17022 | 4.251901 | 7.414036 | 22.36658 | 15.69662 | 7.30902 | NA |
| CUST_33666_PI429010886 | M7550 | 0.007705 | 15 | 9 | | | | | | 76.61613 | 107.073 | 118.9329 | 59.98068 | 84.04395 | 62.506 | 102.639 | 91.25978 | 84.38957 | 134.7752 | 79.32298 | 115.202 | 76.70634 | 115.0699 | 97.43479 | 82.93626 | 67.84362 | 71.43726 | 80.842 | 84.34439 | 90.01113 | 128.4766 | 91.56459 | 81.1623 | 141.9566 | 93.35337 | 118.04 |
| CUST_14659_PI429010886 | N33247 | 0.007711 | 12 | 6 | | | | | | 60.66856 | 71.63476 | 18.56427 | 86.89271 | 105.8358 | 55.85751 | 143.7672 | 48.67019 | 112.0535 | 42.07479 | 49.40038 | 108.1674 | 31.0493 | 19.22009 | 26.10123 | 308.5953 | 27.44977 | 231.0331 | NA | 140.5313 | 60.30436 | NA | 35.22469 | 33.7142 | 9.011589 | 17.05056 | 45.93669 |
| CUST_38894_PI429010886 | M31722 | 0.007712 | 18 | 15 | | | | | | 62.21051 | 73.36115 | 109.6686 | 62.60675 | 80.5678 | 96.77635 | 78.28594 | 91.51834 | 75.69546 | 87.86031 | 92.02079 | 94.88698 | 92.68449 | 89.56339 | 98.61381 | 105.5208 | 104.302 | 84.97695 | 81.87168 | 95.67336 | 78.56402 | 73.59863 | 74.86726 | 77.31411 | 81.00454 | 78.38473 | 103.0243 |
| CUST_43454_PI429010886 | M63102 | 0.007723 | 21 | 12 | | | | | | 224.9679 | 214.2583 | 192.8746 | 145.8825 | 163.7445 | 132.9869 | 251.6265 | 184.8973 | 220.2915 | 172.213 | 173.7221 | 197.1816 | 271.6132 | 243.718 | 257.6478 | 293.4659 | 220.2915 | 180.3864 | 373.4486 | 96.26654 | 229.245 | 240.8224 | 161.0453 | 168.2847 | 195.5884 | 147.1503 | 200.8366 |
| CUST_24047_PI429010886 | N49938 | 0.007723 | 24 | 12 | | | | | | 356.7158 | 334.0181 | 262.1121 | 256.8125 | 522.2489 | 347.2399 | 476.5945 | 306.1523 | 418.1872 | 463.2589 | 386.8992 | 463.1518 | 562.4407 | 528.5021 | 458.5323 | 712.5461 | 493.9819 | 457.1179 | 454.9277 | 522.4048 | 448.0031 | 685.9612 | 455.9675 | 367.1141 | 346.6366 | 432.6248 | 483.6862 |
| CUST_56746_PI429010886 | M114568 | 0.007733 | 12 | 0 | | | | | | 307.0541 | 363.7161 | 407.6622 | 301.0265 | 315.9925 | 355.3482 | 299.2346 | 315.6417 | 334.0181 | 338.6596 | 313.3535 | 404.2573 | 334.379 | 431.3053 | 437.9165 | 381.8035 | 334.9255 | 332.1642 | 314.8276 | 355.2567 | 312.3161 | 348.6809 | 336.5091 | 321.374 | 380.7018 | 362.8276 | 421.2064 |
| CUST_31297_PI429010886 | M1163 | 0.007733 | 12 | 0 | 156543561 | PREDICTED: ubiquitin-conjugating enzyme E2 C-like | Cellular Process | Cell cycle | 1.28E-60 | 37.22147 | 46.92598 | 48.99962 | 49.38456 | 35.60511 | 47.75744 | 32.41866 | 35.1434 | 30.16704 | 39.08441 | 37.4516 | 43.12293 | 46.61273 | 42.10755 | 62.67964 | 59.0152 | 25.87167 | 29.36585 | 38.93338 | 26.7087 | 34.45395 | 54.56998 | 36.0581 | 34.26633 | 48.46881 | 42.07479 | 61.59565 |
| CUST_39537_PI429010886 | M40940 | 0.007737 | 18 | 15 | | | | | | 53.69064 | 50.11342 | 63.30844 | 40.02682 | 46.05816 | 53.16758 | 46.0364 | 33.61445 | 57.45413 | 63.68458 | 56.11071 | 63.7264 | 53.04479 | 60.8738 | 57.83783 | 62.31885 | 66.13621 | 75.92185 | 63.26276 | 56.55685 | 60.60207 | 60.22941 | 49.60877 | 49.04708 | 68.26569 | 59.3952 | 65.83727 |
| CUST_26930_PI429010886 | N55144 | 0.007742 | 15 | 12 | | | | | | 139.7302 | 128.9944 | 149.3076 | 102.0907 | 112.2413 | 130.7243 | 153.6441 | 171.5473 | 177.0904 | 183.528 | 123.2567 | 105.242 | 144.2774 | 266.1879 | 200.9243 | 176.4808 | 182.8782 | 172.1318 | 121.8599 | 165.5224 | 109.5988 | 215.0582 | 175.4136 | 128.3042 | 227.5634 | 227.0035 | 202.301 |
| CUST_30541_PI429010886 | N62088 | 0.007742 | 15 | 12 | | | | | | 765.7162 | 1093.408 | 1052.869 | 557.6662 | 734.2085 | 637.9884 | 592.1986 | 685.3204 | 838.4693 | 944.6524 | 712.1447 | 1291.558 | 800.0152 | 1279.6 | 1173.656 | 893.461 | 671.0656 | 1027.258 | 818.6448 | 792.4242 | 775.6671 | 807.9372 | 1002.452 | 702.9226 | 1068.926 | 821.7869 | 1075.686 |
| CUST_45763_PI429010886 | M70213 | 0.007744 | 24 | 18 | 345304903 | PREDICTED: neuroblastoma-amplified sequence, pa | | | 4.72E-14 | 5.719759 | 8.70488 | 10.65706 | 5.371032 | 5.895001 | 4.813354 | 5.59552 | 7.035948 | 6.260184 | 13.81863 | 7.090444 | NA | 8.060614 | 14.70379 | 7.31283 | 13.81689 | 7.305705 | 9.691153 | 9.745966 | 14.56893 | 6.113641 | 13.80474 | 13.05441 | NA | 10.4406 | NA | 11.32307 |
| CUST_9057_PI429010886 | N23998 | 0.007746 | 24 | 15 | | | | | | 43.46213 | 48.65088 | 49.51746 | 30.21036 | 35.51342 | 51.46537 | 41.70879 | 37.29054 | 34.08686 | 45.74744 | 38.34336 | 60.71325 | 39.62708 | 50.22468 | 68.33792 | 54.78436 | 65.53578 | 62.63918 | 58.29649 | 61.08538 | 52.40185 | 39.38726 | 56.68336 | 38.35721 | 76.6588 | 37.18492 | 74.7726 |
| CUST_842_PI429010886 | N7506 | 0.007747 | 18 | 15 | 442769035 | glycosyl-phosphatidylinositol-linked carbonic anhyd | | | 5.7E-62 | 3595.062 | 3872.65 | 2166.108 | 564.719 | 9020.979 | 3759.753 | 1523.414 | 2338.281 | 380.2921 | 3946.907 | 328.0964 | 3144.897 | 2050.871 | 2599.94 | 1498.845 | 93467.7 | 4340.493 | 5590.669 | 2845.968 | 451.916 | 2736.774 | 2729.519 | 2123.129 | 3564.266 | 2223.051 | 118.5776 | 1895.191 |
| CUST_38919_PI429010886 | M31957 | 0.007753 | 15 | 9 | | | | | | 1199.593 | 557.82 | 9697.645 | 34.15181 | 43.02941 | 18.81568 | 2536.388 | 16209.81 | 15743.72 | 15743.72 | 3323.085 | 5477.513 | 12021.62 | 26713.02 | 14600.6 | 1736.508 | 7026.307 | 412.5292 | 1776.211 | 8924.795 | 685.5063 | 18337.68 | 18774.91 | 544.2536 | 29281.85 | 2672.36 | 20967.97 |
| CUST_44884_PI429010886 | M67313 | 0.007753 | 15 | 9 | 309099428 | elongation factor 1-alpha [Litopenaeus vannamei] | | | 4.39E-12 | 56.66591 | 125.4528 | 85.21423 | 77.78755 | 95.65165 | 93.72794 | 100.3711 | 120.6692 | 120.8512 | 94.76564 | 103.7009 | 171.0565 | 89.91874 | 136.1436 | 100.8857 | 63.58566 | 97.97139 | 147.2361 | 75.19772 | 81.93796 | 111.7257 | 124.0734 | 132.7602 | 141.6171 | 138.0748 | 148.544 | 170.6145 |
| CUST_12362_PI429010886 | N29348 | 0.007753 | 15 | 0 | | | | | | 59.36711 | 82.70078 | 60.98516 | 56.84346 | 44.91149 | 53.68104 | 49.58479 | 39.8966 | 37.76259 | 69.79294 | 56.65296 | 69.71071 | 74.16318 | 50.11711 | 46.63569 | 67.77787 | 73.31065 | 59.96262 | 44.40996 | 65.54862 | 52.20238 | 61.11567 | 48.40695 | 43.23619 | 69.20227 | 53.25946 | 48.5802 |
| CUST_13698_PI429010886 | N31614 | 0.007753 | 15 | 0 | | | | | | 52.12912 | 34.05076 | 32.82642 | 45.43527 | 32.24298 | 59.45837 | 31.8793 | 25.06406 | 20.51967 | 37.50798 | 44.76886 | 32.42452 | 15.2714 | 43.97432 | 25.1646 | 71.56769 | 73.83558 | 57.70597 | 40.93552 | 51.53321 | 31.07173 | 41.5428 | 17.98995 | 19.2789 | 43.85634 | 31.82653 | 24.43879 |
| CUST_31623_PI429010886 | M1992 | 0.007755 | 18 | 0 | | | | | | 427.0325 | 503.7682 | 197.5737 | 160.6701 | 225.4439 | 173.0442 | 56.2163 | 50.94392 | 177.7855 | 360.9417 | 269.3899 | 378.6086 | 103.397 | 293.4659 | 185.5006 | 233.8998 | 162.6071 | 209.2891 | 328.4337 | 385.2863 | 377.56 | 285.5481 | 518.8148 | 136.023 | 209.5944 | 171.1636 | 80.65083 |
| CUST_31583_PI429010886 | M1902 | 0.007783 | 24 | 21 | 157128145 | hypothetical protein AaeL_AAEL011048 [Aedes aeg | | | 7.02E-48 | 178.2181 | 155.649 | 165.5902 | 152.5473 | 151.386 | 191.2388 | 176.6512 | 149.3496 | 140.194 | 155.9586 | 144.3246 | 147.2361 | 110.0909 | 148.8486 | 163.8946 | 167.6024 | 186.4927 | 173.7958 | 149.8948 | 164.7626 | 146.9814 | 197.0618 | 178.3785 | 169.2006 | 156.9601 | 170.0428 | 153.3961 |
| CUST_35557_PI429010886 | M12879 | 0.007789 | 21 | 12 | | | | | | 45.02093 | 79.71189 | 52.32373 | 26.01055 | 60.7605 | 49.36528 | 54.73192 | 49.37485 | 36.82067 | 70.59503 | 32.39452 | 56.03237 | 88.04251 | 55.04289 | 80.42722 | 70.80885 | 56.21187 | 70.81749 | 77.93692 | 72.50841 | 52.96132 | 70.09922 | 74.10963 | 59.20737 | 52.39551 | 44.81799 | 53.34613 |
| CUST_20147_PI429010886 | N42888 | 0.007805 | 24 | 18 | | | | | | 20.54044 | 40.34891 | 43.18546 | 15.31326 | 19.68268 | 7.831079 | 6.401811 | 3.880252 | 18.45187 | 41.94819 | 39.0489 | 45.64241 | 20.52725 | 22.05844 | 18.79454 | 21.94847 | 10.91146 | 38.05146 | 44.51031 | 23.8404 | 71.61792 | 64.28764 | 28.33698 | NA | 43.12973 | 44.73597 | 43.48424 |
| CUST_3728_PI429010886 | N15145 | 0.007805 | 15 | 3 | | | | | | 740.0272 | 746.6817 | 259.8087 | 1650.747 | 729.1026 | 2293.562 | 1122.017 | 943.3673 | 843.1506 | 832.3284 | 540.1617 | 321.4467 | 624.8531 | 722.796 | 708.1055 | 507.983 | 1279.6 | 1182.845 | 1024.31 | 870.876 | 1305.146 | 1538.611 | 321.5473 | 1026.418 | 395.1607 | 889.3445 | 207.0705 |
| CUST_3731_PI429010886 | N15149 | 0.007813 | 24 | 15 | 524878197 | PREDICTED: polypyrimidine tract-binding protein 2- | | | 2E-24 | 21.41937 | 25.63163 | 32.58962 | 17.91014 | 15.35034 | 24.91328 | 23.96487 | 18.71104 | 25.48335 | 22.25206 | 27.55645 | 29.02633 | 25.97958 | 22.46676 | 27.90958 | 36.81255 | 31.18382 | 31.36762 | 25.13071 | 33.8457 | 23.74918 | 27.38976 | 25.02094 | 29.67419 | 26.12824 | 13.6708 | 49.11669 |
| CUST_1215_PI429010886 | N8881 | 0.007813 | 21 | 3 | | | | | | 1402.469 | 456.6758 | 12.37483 | 2547.522 | 315.3064 | 2485.559 | 1163.545 | 1069.514 | 1196.575 | 13.40566 | 35.75835 | NA | 1124.753 | NA | 580.6994 | 111.7257 | 377.7333 | 526.943 | 47.92719 | NA | 30.33968 | 97.38686 | 33.96909 | 1578.633 | NA | 1997.22 | NA |
| CUST_31195_PI429010886 | M948 | 0.007814 | 21 | 3 | | | | | | 14.28677 | 22.3538 | 18.48442 | 27.85465 | 18.58498 | 29.27092 | 16.6126 | 19.23258 | 19.18487 | 16.31327 | 16.51009 | 26.14965 | 16.23717 | 15.37649 | 9.165228 | 18.58338 | 22.67743 | 23.72179 | 22.41474 | 17.4103 | 17.10365 | 25.13071 | 12.52538 | 24.0705 | 30.91745 | 21.69885 | 23.43097 |
| CUST_54213_PI429010886 | M105109 | 0.00782 | 12 | 0 | | | | | | 14.64036 | 14.20655 | 12.11329 | 7.418431 | 9.82119 | 16.29403 | 8.988276 | 10.62806 | 9.930283 | 11.88893 | 11.19024 | 8.520664 | 20.78959 | 11.78596 | 16.97579 | 12.68236 | 6.71361 | 12.81122 | 9.108247 | 10.33535 | 13.77121 | 12.59903 | 12.44359 | 15.0972 | 16.53993 | 13.83685 | 19.13431 |
| CUST_2935_PI429010886 | N13423 | 0.00782 | 12 | 0 | 260817398 | hypothetical protein BRAFLDRAFT_93219 [Branchio | | | 8.23E-19 | 29.9146 | 54.058 | 24.59907 | 20.53586 | 23.83766 | 17.29005 | 20.47876 | 18.29753 | 6.446625 | 35.05495 | 16.82346 | 28.25151 | 15.1027 | 48.41716 | 38.78884 | 28.05779 | 10.49992 | 21.18978 | 11.72075 | 28.14021 | 16.70546 | 32.33619 | 7.715488 | 19.04367 | 28.31129 | 29.42815 | 30.89913 |
| CUST_21808_PI429010886 | N45889 | 0.00782 | 12 | 0 | | | | | | 77.26285 | 78.89557 | 73.69227 | 65.85741 | 73.54244 | 61.64915 | 49.08991 | 59.43662 | 46.54874 | 80.18725 | 59.55084 | 79.26193 | 68.37525 | 84.47788 | 65.40248 | 94.53569 | 58.97452 | 65.07248 | 60.7605 | 54.07518 | 46.51613 | 78.1117 | 58.41483 | 50.38632 | 97.70002 | 52.57619 | 76.01313 |
| CUST_56644_PI429010886 | M114291 | 0.00782 | 18 | 15 | 56968003 | Anaspides tasmaniae internal transcribed spacer 2 a | | | 6.6E-100 | 196.359 | 209.3879 | 304.6076 | 89.34718 | 331.8685 | 248.7474 | 356.7158 | 245.7365 | 218.6003 | 524.2243 | 270.5265 | 186.7835 | 438.3419 | 359.3716 | 219.5144 | 339.6585 | 537.9358 | 388.8828 | 410.4134 | 313.0924 | 281.1462 | 275.7295 | 170.531 | 160.804 | 186.0312 | 269.1187 | 348.5168 |
| CUST_56182_PI429010886 | M113044 | 0.007827 | 21 | 3 | | | Protein metabolism | Proteolysis | | 155.9586 | 481.1352 | 195.1881 | 735.8213 | 264.0635 | 414.2851 | 168.1346 | 241.638 | 167.9521 | 183.1272 | 91.83515 | 284.3166 | 125.5467 | 205.4212 | 94.91653 | 128.8011 | 71.66601 | 205.7682 | 87.80183 | 112.7849 | 354.4714 | 171.6961 | 331.1092 | 177.0904 | 322.9938 | 399.475 | 160.6252 |
| CUST_41054_PI429010886 | M56772 | 0.007831 | 15 | 3 | | | | | | 24.2954 | 30.50065 | 19.37009 | 39.94582 | 42.42067 | 33.4065 | 14.9321 | 20.59519 | 25.70084 | 28.99859 | 33.06433 | 22.74008 | 6.411759 | 10.47246 | 8.572306 | 27.85193 | 26.61223 | 28.03123 | 30.53018 | 18.31723 | 36.38283 | 21.29021 | 9.145807 | 19.6198 | 29.61528 | 14.37805 | 22.29454 |
| CUST_42300_PI429010886 | M59949 | 0.007845 | 15 | 9 | 242007493 | large neutral amino acids transporter, putative [Pec | Transport | Other | 4.26E-24 | 61.63578 | 135.5426 | 109.3451 | 67.50199 | 63.80913 | 32.54901 | 26.9442 | 90.89001 | 136.4739 | 175.5219 | 111.5879 | 223.9394 | 100.4818 | 212.1657 | 136.0004 | 185.3252 | 72.11758 | 78.33196 | 58.85113 | 160.598 | 90.59403 | 128.9362 | 131.046 | 87.50342 | 152.5355 | 95.25723 | 220.6157 |
| CUST_8129_PI429010886 | N22579 | 0.007848 | 24 | 3 | 478253172 | hypothetical protein YQE_09794, partial [Dendrocto | | | 1.58E-16 | 115.3266 | 663.9332 | 130.0411 | 992.2058 | 621.7238 | 639.6792 | 465.7878 | 509.1564 | 120.0446 | 122.7974 | 505.6922 | 170.9469 | 108.8637 | 227.8356 | 342.8578 | 125.7681 | 92.02079 | 116.9387 | 117.8738 | 145.1303 | 303.854 | 146.5035 | 278.3606 | 321.3108 | 142.216 | 175.8715 | 115.8195 |
| CUST_13124_PI429010886 | N30622 | 0.007849 | 18 | 0 | | | | | | 209.0603 | 216.1505 | 169.0411 | 175.4567 | 181.8579 | 155.5309 | 116.5651 | 158.5396 | 139.7302 | 194.2624 | 174.126 | 198.4542 | 131.2193 | 182.318 | 161.7305 | 193.6603 | 146.3399 | 213.8921 | 185.4691 | 224.9966 | 207.1975 | 150.8963 | 142.216 | 160.2794 | 155.3371 | 161.5046 | 176.2005 |
| CUST_18819_PI429010886 | N40474 | 0.007853 | 24 | 9 | | | | | | 239.3938 | 292.3195 | 268.4877 | 410.7156 | 382.8597 | 178.6581 | 304.1069 | 346.1873 | 379.7153 | 668.1948 | 359.9556 | 589.6619 | 348.906 | 425.9278 | 440.0791 | 938.0188 | 98.69728 | 421.7857 | 299.9648 | 212.3072 | 309.2252 | 336.7538 | 441.0416 | 298.5904 | 307.3807 | 222.0782 | 268.82 |
| CUST_4811_PI429010886 | N17529 | 0.007862 | 12 | 0 | | | | | | 22.46932 | 12.6233 | 17.53198 | 11.55275 | 16.56157 | 11.50536 | 8.361603 | 11.13485 | 11.30106 | 7.416629 | 8.064111 | 16.90499 | 15.01118 | 15.32123 | 13.28314 | 18.14309 | 11.95734 | 12.54381 | 5.60979 | 9.202277 | 9.776853 | 16.28788 | 17.85496 | NA | 15.94563 | 16.88307 | 13.52681 |
| CUST_10477_PI429010886 | N26295 | 0.007863 | 18 | 6 | 290561783 | Gamma-crystallin A [Lepeophtheirus salmonis] | | | 3.3E-19 | NA | 123.4117 | 30.64706 | 212.8057 | 280.9299 | 161.2431 | 225.9175 | 1329.318 | 1020.69 | 146.882 | 379.882 | 647.6977 | 512.8422 | 159.3655 | 363.0024 | 6.630038 | 159.32 | NA | 74.14418 | 868.721 | 763.4512 | NA | 356.1302 | 275.2295 | NA | 854.4458 | 725.6338 |
| CUST_39500_PI429010886 | M40528 | 0.007884 | 15 | 12 | | | | | | 736.1144 | 1964.934 | 1160.295 | 669.4372 | 797.54 | 323.8163 | 1909.916 | 1099.35 | 2271.87 | 2280.777 | 1301.471 | 2384.394 | 2766.721 | 2789.972 | 2617.426 | 4243.297 | 1732.866 | 1630.188 | 1053.785 | 3288.575 | 1186.768 | 3785.297 | 3656.575 | 2154.443 | 10491.93 | 2382.34 | 10899.12 |
| CUST_19225_PI429010886 | N41209 | 0.007884 | 15 | 9 | | | | | | 20.6453 | 14.51104 | 18.6269 | NA | 5.606473 | 12.26536 | 11.16121 | 13.31508 | 12.91769 | 14.67882 | 10.19605 | 18.88563 | 10.12281 | 20.63276 | 12.01881 | 14.20169 | 10.60153 | 13.24373 | 8.187128 | 12.00862 | 10.21556 | 13.30523 | 14.08649 | 14.48944 | 20.41547 | 20.49973 | 22.92529 |
| CUST_4619_PI429010886 | N17150 | 0.007893 | 18 | 3 | | | | | | 38.61125 | 132.0478 | 68.18607 | 232.1034 | 82.06433 | 125.8598 | 53.92569 | 59.12871 | 63.04603 | 60.89252 | 34.33169 | 81.98334 | 60.58205 | 58.61502 | 43.68382 | 67.71736 | 30.59469 | 66.63641 | 49.52106 | 51.22479 | 112.9777 | 82.3472 | 97.90896 | 88.11918 | 89.01214 | 112.3305 | 42.15075 |
| CUST_56011_PI429010886 | M112531 | 0.007908 | 12 | 0 | | | | | | 2539.516 | 6516.822 | 4499.121 | 4048.081 | 4896.997 | 4128.792 | 3237.592 | 3350.556 | 3949.649 | 3722.852 | 4051.321 | 5969.266 | 4228.899 | 6240.976 | 5705.785 | 5119.45 | 2684.729 | 3938.093 | 3339.741 | 4182.62 | 3380.982 | 5004.678 | 4471.709 | 3687.08 | 6426.682 | 4174.241 | 7560.283 |
| CUST_1680_PI429010886 | N10420 | 0.007908 | 12 | 0 | 195425743 | GK10316 [Drosophila willistoni] >gi\|194157215\|gb | | | 9.74E-16 | 59.80641 | 69.64081 | 58.16774 | 44.1892 | 70.31729 | 45.28763 | 37.23147 | 38.79996 | 37.95272 | 71.14697 | 56.09338 | 69.40293 | 52.46279 | 54.50124 | 53.5429 | 109.9853 | 44.28069 | 57.42327 | 41.05859 | 41.08623 | 43.11747 | 73.49284 | 51.86061 | 44.94616 | 85.67865 | 51.38396 | 63.36658 |
| CUST_22233_PI429010886 | N46665 | 0.007908 | 12 | 0 | | | | | | 58.84482 | 121.6959 | 68.37004 | 125.2228 | 77.76127 | 58.92815 | 58.01161 | 27.76638 | 52.99425 | 128.5363 | 47.28217 | 76.82831 | 76.27537 | 84.76204 | 77.72793 | 89.43652 | 58.92238 | 73.1906 | 45.87335 | 73.04458 | 40.53465 | 86.01753 | 63.21853 | 62.06295 | 131.4711 | 86.59518 | 108.3189 |
| CUST_4138_PI429010886 | N16095 | 0.007911 | 24 | 0 | | | | | | 207.3277 | 375.4995 | 269.0643 | 180.3315 | 202.005 | 202.4642 | 170.596 | 157.0892 | 206.3446 | 180.5428 | 197.989 | 210.1562 | 157.7661 | 173.5554 | 190.62 | 246.5093 | 132.1335 | 201.294 | 173.4432 | 262.1121 | 227.0035 | 272.9711 | 214.4171 | 174.8316 | 250.4057 | 206.8694 | 220.1671 |
| CUST_18734_PI429010886 | N40321 | 0.007914 | 15 | 6 | 62126070 | C-type lectin protein [Fenneropenaeus chinensis] | | | 4.45E-07 | 16.04975 | 10.94786 | 7.94792 | NA | 25.87828 | 19.94541 | 27.62987 | 19.95383 | 19.90164 | 16.8955 | 4.270144 | 14.13977 | 6.763654 | NA | NA | 17.85496 | 6.452276 | NA | 12.36089 | 8.092867 | 23.47248 | 16.93603 | NA | NA | 7.119665 | 13.96315 | 14.82548 |
| CUST_33583_PI429010886 | M7332 | 0.007917 | 15 | 9 | | | | | | 9.611855 | 24.48759 | NA | 6.496126 | 4.331503 | NA | 36.32974 | 6.080738 | 27.88621 | 36.21632 | NA | 20.30399 | 11.08304 | 8.400369 | 13.51311 | 37.47537 | 10.28962 | 28.4998 | 5.436916 | 4.704971 | 5.026041 | 18.23052 | 25.29876 | 34.82944 | 37.28203 | 21.53219 | 24.11521 |
| CUST_38162_PI429010886 | M23774 | 0.00792 | 15 | 12 | 164632863 | masquerade-like protein, partial [Armadillidium vul | Protein metabolism | Proteolysis | 1.97E-65 | 9697.645 | 12651.8 | 8845.346 | 5819.009 | 4064.703 | 2017.499 | 11762.42 | 7107.78 | 11583.53 | 14529.33 | 4427.545 | 10282.77 | 16607.59 | 13806.99 | 17137.67 | 18556.73 | 8709.952 | 6142.292 | 6759.855 | 10610.68 | 6448.481 | 14684.57 | 12313.28 | 5330.558 | 29764.17 | 6234.905 | 30520.02 |
| CUST_38721_PI429010886 | M29587 | 0.00792 | 15 | 12 | 7320913 | masquerade-like protein [Pacifastacus leniusculus] | Protein metabolism | Proteolysis | 5.06E-19 | 7347.807 | 9638.897 | 7197.042 | 4232.305 | 2805.418 | 1318.564 | 8802.545 | 5162.076 | 8854.389 | 9686 | 3259.251 | 7415.798 | 14274.58 | 12021.62 | 11161.59 | 14182.86 | 7083.117 | 4759.605 | 5888.291 | 8024.987 | 4817.775 | 11327.23 | 10282.77 | 3872.65 | 22054.78 | 4239.143 | 21552.65 |
| CUST_51950_PI429010886 | M95795 | 0.00792 | 15 | 12 | | | | | | 23.81358 | 32.18854 | 25.55311 | 13.74381 | 23.11258 | 7.90501 | 24.42522 | 24.69848 | 16.23539 | 32.99565 | 19.39216 | 17.65649 | 24.42322 | 27.97387 | 25.7937 | 29.18503 | 23.14834 | 26.14965 | 13.31154 | 17.13916 | 11.4538 | 29.8311 | 17.61145 | 23.15281 | 30.16704 | 15.91075 | 21.94847 |
| CUST_41461_PI429010886 | M57788 | 0.007934 | 12 | 9 | | | | | | 458.9977 | 1369.558 | 1764.536 | 109.4422 | 73.71157 | 47.06225 | 626.8612 | 2124.898 | 2779.017 | 2474.132 | 1323.469 | 2896.285 | 1154.447 | 1921.833 | 1380.113 | 488.7111 | 867.1839 | 155.0724 | 426.1051 | 2459.721 | 468.3619 | 1805.829 | 1684.561 | 78.21131 | 1247.588 | 946.4318 | 1371.865 |
| CUST_38383_PI429010886 | M26081 | 0.007938 | 15 | 9 | | | | | | 11690.52 | 23934.78 | 24777.72 | 6005.835 | 14928.07 | 4988.507 | 7490.614 | 9882.018 | 33611.5 | 41702.57 | 33148.92 | 46673.5 | 27142.29 | 40354.05 | 32430.96 | 36281.72 | 7470.617 | 27018.31 | 26153.6 | 46346.16 | 33529.19 | 45272.91 | 31314.74 | 13579.45 | 38394.55 | 47369.29 | 58446.35 |
| CUST_31966_PI429010886 | M2853 | 0.007938 | 15 | 9 | 321479500 | hypothetical protein DAPPUDRAFT_39582 [Daphnia | | | 6.6E-28 | 381.2578 | 356.7158 | 473.5692 | 265.4892 | 419.8201 | 365.0271 | 300.7234 | 574.4912 | 553.514 | 874.1627 | 324.7416 | 647.5069 | 436.1525 | 990.9102 | 536.0428 | 766.9049 | 432.1375 | 425.3087 | 326.4381 | 468.4803 | 285.5481 | 1371.349 | 707.3962 | 266.7997 | 1286.564 | 473.6842 | 1498.845 |
| CUST_50260_PI429010886 | M89214 | 0.007938 | 15 | 3 | | | | | | 131.9942 | 58.52324 | 93.51914 | 139.7005 | 242.7679 | 206.8694 | 287.1573 | 62.66858 | 59.234 | 26.61223 | 194.3859 | 21.84275 | 57.20099 | 47.62717 | 55.76383 | 57.78122 | 317.5778 | 156.3242 | 256.281 | 21.59129 | 206.5059 | 58.02431 | 201.7937 | 266.9855 | 46.83015 | 55.16975 | 27.2017 |
| CUST_10029_PI429010886 | N25589 | 0.007938 | 15 | 9 | 157118755 | hypothetical protein AaeL_AAEL008394 [Aedes aeg | | | 2.68E-30 | 548.2244 | 698.6187 | 658.8544 | 555.8637 | 529.2101 | 520.2836 | 501.702 | 606.9446 | 598.2934 | 847.0661 | 633.0392 | 842.6525 | 682.7483 | 767.2232 | 627.8304 | 835.4969 | 648.6302 | 580.161 | 504.5252 | 754.3579 | 544.5308 | 810.6402 | 628.4644 | 467.9877 | 893.1457 | 581.2638 | 815.0535 |
| CUST_13052_PI429010886 | N30501 | 0.007938 | 15 | 0 | | | | | | 256.4303 | 207.4294 | 197.8235 | 167.8299 | 195.1137 | 209.9951 | 133.9495 | 169.7046 | 138.133 | 215.5744 | 186.1103 | 180.6083 | 158.0561 | 171.1636 | 202.7615 | 217.8838 | 181.649 | 207.9222 | 196.5989 | 153.8044 | 203.006 | 215.7123 | 141.3981 | 137.2358 | 203.0375 | 148.9832 | 157.2555 |
| CUST_31627_PI429010886 | M2007 | 0.007941 | 21 | 15 | | | | | | 610.8058 | 497.8615 | 526.019 | 506.7573 | 513.9071 | 659.4054 | 406.1396 | 416.5528 | 407.2671 | 425.8413 | 427.0325 | 444.7159 | 420.5887 | 515.7103 | 528.026 | 558.0588 | 561.2769 | 622.1973 | 549.5559 | 553.3502 | 490.7702 | 553.3502 | 442.5257 | 572.8694 | 478.8398 | 599.1678 | 505.7885 |
| CUST_15548_PI429010886 | N34767 | 0.007951 | 24 | 15 | | | | | | 13.92993 | 12.46111 | 20.69967 | 42.03557 | 30.78794 | 26.01832 | 35.95633 | 29.50356 | 16.39504 | 24.22757 | 19.59595 | 17.78423 | 35.91157 | 72.9595 | 51.12395 | 43.62743 | 210.1212 | 58.10187 | 24.0705 | 10.23206 | 24.9077 | 79.44802 | 18.34756 | 32.38846 | 14.56514 | 13.60386 | 42.00978 |
| CUST_15600_PI429010886 | N34849 | 0.007951 | 21 | 0 | | | | | | 347.1114 | 355.2567 | 333.0328 | 198.9664 | 309.7678 | 220.1671 | 252.2638 | 200.3686 | 198.3401 | 326.7477 | 316.5285 | 268.9771 | 215.4716 | 305.4951 | 187.387 | 278.0994 | 261.2237 | 245.8985 | 326.6831 | 403.3076 | 332.1138 | 405.7694 | 313.2163 | 254.4554 | 382.2635 | 217.1338 | 312.9412 |
| CUST_46311_PI429010886 | M72165 | 0.007959 | 15 | 9 | | | | | | 240.474 | 201.7937 | 267.5678 | 222.2769 | 219.1881 | 190.9127 | 207.5948 | 209.1625 | 257.5943 | 281.1462 | 236.7279 | 282.6926 | 228.3928 | 301.7927 | 300.1314 | 272.7482 | 269.3458 | 251.2251 | 239.1179 | 233.1687 | 263.9219 | 254.8434 | 285.8071 | 244.3099 | 314.3091 | 278.2072 | 295.3859 |
| CUST_42385_PI429010886 | M60156 | 0.007979 | 21 | 15 | | | | | | 216.807 | 163.1486 | 192.4758 | 67.39787 | 141.3308 | 169.3778 | 213.0864 | 147.7228 | 144.7304 | 256.0552 | 74.68625 | 205.5305 | 232.1034 | 191.886 | 150.2252 | 193.8618 | 287.2755 | 187.4537 | 91.11943 | 253.9783 | 195.4506 | 146.0101 | 146.0295 | 133.0951 | 122.3764 | 120.9878 | 110.9478 |
| CUST_31079_PI429010886 | M651 | 0.007979 | 21 | 9 | 528767107 | hypothetical protein CB1_000300004 [Camelus feru | Transport | Vesicle-mediated transport | 5.57E-21 | 19.74682 | 32.43294 | 17.68083 | 54.1974 | 116.4359 | 33.68931 | 64.8815 | 180.1028 | 118.7227 | 36.32136 | 257.3603 | 392.5464 | 147.4496 | 44.12317 | 90.53385 | 17.51738 | 61.6776 | 21.36688 | 57.83783 | 492.5912 | 176.0261 | 7.222005 | 34.86394 | 55.33618 | 27.40178 | 173.6414 | 112.4734 |
| CUST_13236_PI429010886 | N30828 | 0.007992 | 18 | 6 | | | | | | 39.20036 | 33.14786 | 42.10422 | 59.6812 | 51.65313 | 39.18988 | 88.17592 | 39.68929 | 61.94492 | 70.36778 | 40.59153 | 33.40072 | 38.91585 | 25.32849 | 21.73089 | 34.89593 | 20.13696 | 45.02093 | 31.90512 | 45.31985 | 36.53489 | 61.25018 | 24.78008 | 24.09426 | 52.15419 | 40.98703 | 60.01583 |
| CUST_30898_PI429010886 | M152 | 0.008003 | 15 | 9 | 195969368 | chitinase 1 precursor [Litopenaeus vannamei] | Metabolic process | Carbohydrate metabolic process | 1.4E-154 | 24.01545 | 37.45529 | 19.23364 | 58.54952 | 153.376 | 16.75298 | 118.3504 | 307.9072 | 219.2633 | 94.37733 | 307.6262 | 416.7544 | 188.3767 | 27.28431 | 115.3125 | 16.31556 | 89.85114 | 9.700584 | 29.49276 | 504.1608 | 196.5334 | 41.56705 | 100.3848 | 104.9922 | 56.54516 | 214.361 | 166.0617 |
| CUST_49986_PI429010886 | M88105 | 0.008008 | 24 | 0 | 291236404 | PREDICTED: hypothetical protein [Saccoglossus kow | Nucleic acid metabolism | DNA metabolic process | 2.49E-10 | 13.19581 | 14.09213 | 21.43684 | 27.87027 | 11.83887 | 13.62607 | 8.297472 | 5.5047 | 11.90796 | 13.00185 | 7.541361 | 18.57889 | 4.550924 | NA | 13.39537 | 12.3071 | 12.16799 | 13.02465 | 15.00483 | 5.224773 | 10.97339 | 13.60023 | 13.53636 | 15.42553 | 19.98576 | 14.67098 | 29.3391 |
| CUST_14112_PI429010886 | N32345 | 0.008009 | 15 | 12 | | | | | | 23.56736 | 16.42915 | 26.43455 | 13.24115 | 24.95301 | 13.58403 | 7.43529 | 19.05569 | 19.07106 | 26.84667 | 17.46532 | 22.9868 | 19.676 | 30.18791 | 30.58787 | 23.52815 | 15.7975 | 16.31744 | 17.76635 | 13.91491 | 14.51263 | 19.98178 | 23.83451 | 20.28061 | 29.8401 | 14.80182 | 29.32288 |
| CUST_25411_PI429010886 | N52364 | 0.008009 | 15 | 3 | | | | | | 7.792392 | 5.000861 | 22.93439 | 26.10123 | 13.96931 | 40.40197 | 19.36095 | 11.06303 | 9.837542 | 12.98302 | 23.33967 | 7.426569 | 16.31198 | 8.330776 | 6.982708 | 11.41469 | 29.13814 | 12.63519 | 29.99986 | 13.39116 | 23.39301 | 9.884881 | 13.16888 | 11.19696 | 12.43952 | 9.106984 | 10.25766 |
| CUST_6833_PI429010886 | N20657 | 0.008015 | 18 | 3 | 321461683 | hypothetical protein DAPPUDRAFT_325988 [Daphn | Nucleic acid metabolism | RNA metabolic process | 1.32E-62 | 10.78913 | 13.48403 | 8.401293 | 12.49899 | 10.35023 | 21.40056 | 15.41864 | 13.48403 | 5.439411 | 5.51343 | 5.730029 | 5.165507 | 4.312161 | 7.731153 | 6.849092 | 15.53607 | 16.87203 | NA | 7.892117 | 11.18584 | 8.994284 | 19.47107 | 7.880481 | 16.37858 | 10.7968 | 11.50819 | 7.472276 |
| CUST_8915_PI429010886 | N23789 | 0.008024 | 18 | 9 | 270003026 | hypothetical protein TcasGA2_TC000046 [Tribolium | | | 3.54E-30 | 236.0508 | 262.4649 | 253.5447 | 237.8356 | 257.772 | 248.9182 | 232.8026 | 246.3941 | 292.7227 | 344.4708 | 252.7105 | 467.9877 | 271.1281 | 325.3686 | 280.8226 | 366.3446 | 241.0846 | 311.394 | 297.8606 | 296.4182 | 280.9299 | 234.2305 | 224.6396 | 259.0023 | 301.5634 | 264.0635 | 336.4536 |
| CUST_54300_PI429010886 | M105481 | 0.008029 | 18 | 15 | | | | | | 38.58794 | 35.13913 | 41.26402 | 36.49315 | 36.96758 | 38.96658 | 40.10273 | 36.51599 | 41.60369 | 40.26663 | 42.46399 | 44.55872 | 44.87166 | 40.53079 | 46.75809 | 43.12662 | 47.3594 | 47.40164 | 42.06392 | 46.58164 | 39.72298 | 36.40505 | 46.33994 | 30.68445 | 58.66467 | 43.24561 | 49.41092 |
| CUST_50250_PI429010886 | M89164 | 0.00803 | 15 | 3 | | | | | | 52.75651 | 42.79014 | 22.55376 | 69.35049 | 43.20136 | 62.84333 | 34.95753 | 42.92316 | 30.02913 | 24.69322 | 24.76116 | 30.13158 | 28.87402 | 41.01917 | 42.84657 | 70.18575 | 28.53623 | 50.56281 | 38.09997 | 33.95306 | 40.06113 | 55.65323 | 32.73752 | 35.98369 | 33.75052 | 27.25419 | 16.46586 |
| CUST_52067_PI429010886 | M96303 | 0.00803 | 15 | 3 | | | | | | 259.2819 | 280.4106 | 169.9489 | 472.4043 | 261.3191 | 432.5053 | 324.3223 | 287.4102 | 249.8541 | 275.3091 | 191.2652 | 155.649 | 249.8117 | 278.3606 | 239.3501 | 256.1116 | 378.7944 | 277.9987 | 293.1271 | 229.3656 | 337.6916 | 410.508 | 230.1517 | 289.8469 | 252.4045 | 214.0659 | 208.027 |
| CUST_11207_PI429010886 | N27444 | 0.00803 | 15 | 9 | | | | | | 461.7538 | 547.465 | 1284.605 | 33.18183 | 207.6194 | 127.6161 | 361.2261 | 1769.215 | 1931.541 | 1928.541 | 1072.188 | 1497.212 | 996.654 | 3219.948 | 1321.79 | 699.6538 | 1088.645 | 562.4407 | 1030.293 | 1606.044 | 781.185 | 3584.321 | 1706.496 | 125.1102 | 4407.135 | 1393.263 | 4506.751 |
| CUST_13860_PI429010886 | N31902 | 0.00803 | 15 | 9 | | | | | | 211.5909 | 304.6076 | 357.6255 | 205.5955 | 218.1405 | 141.9566 | 150.0374 | 222.7049 | 271.9546 | 264.5273 | 285.6058 | 292.6086 | 197.384 | 367.4512 | 248.5974 | 366.2536 | 255.8665 | 253.2546 | 107.5704 | 263.4861 | 143.3629 | 242.5125 | 186.3412 | 141.6567 | 489.8149 | 256.1926 | 409.1687 |

| Probe ID | ID | p-value | Period | Phase | EBI ID | Annotation | GO category | Go term | E-value | 0 | 0 | 0 | 3 | 3 | 3 | 6 | 6 | 6 | 9 | 9 | 9 | 12 | 12 | 12 | 15 | 15 | 15 | 18 | 18 | 18 | 21 | 21 | 21 | 24 | 24 | 24 |
|---|---|---|---|---|---|---|---|---|---|---|---|---|---|---|---|---|---|---|---|---|---|---|---|---|---|---|---|---|---|---|---|---|---|---|---|---|
| CUST_14237_PI429010886 | N32548 | 0.008033 | 24 | 6 | | | | | | 36.08065 | 24.07736 | 21.05341 | 26.19371 | 23.47248 | 23.94075 | 50.403 | 36.08462 | 57.7399 | 31.86259 | 27.14824 | 15.4125 | 24.34759 | 21.80739 | 12.81767 | 21.0027 | 25.84268 | 16.62548 | 23.79492 | 20.35525 | 18.21981 | 35.85651 | 10.35144 | 13.24115 | 31.02055 | 20.11049 | 25.80821 |
| CUST_11670_PI429010886 | N28211 | 0.008033 | 12 | 9 | | | | | | 86.94867 | 91.00006 | 100.4962 | 76.53451 | 78.59764 | 81.82047 | 69.04997 | 67.89962 | 84.06167 | 106.3603 | 101.7238 | 105.5539 | 87.46258 | 110.2442 | 80.9845 | 79.44029 | 94.42153 | 91.60957 | 82.46901 | 74.74125 | 93.95638 | 101.2088 | 104.4333 | 95.12719 | 91.6735 | 81.87922 | 86.23591 |
| CUST_52180_PI429010886 | M96666 | 0.008034 | 15 | 3 | | | | | | 30.92489 | 25.87828 | NA | 70.85223 | 40.69465 | 73.51179 | 33.80016 | 40.94647 | 13.53805 | 6.698064 | 25.15661 | NA | 20.97996 | 3.792996 | 34.65808 | 5.85187 | 115.5 | 47.98542 | 48.17972 | NA | NA | 3.498537 | 8.026683 | 29.18761 | 5.11464 | 14.35201 | NA |
| CUST_25186_PI429010886 | N51935 | 0.008035 | 18 | 6 | | | | | | NA | 6.810134 | NA | 56.49623 | 77.15963 | 15.78638 | 14.20045 | 210.2883 | 177.5678 | 17.64058 | 26.54031 | 24.87802 | 15.86385 | 16.57742 | 15.12292 | NA | 4.969785 | 5.207994 | 29.28327 | 118.905 | 172.7406 | NA | 54.50124 | 44.16686 | NA | 61.84039 | 234.017 |
| CUST_43096_PI429010886 | M62058 | 0.008045 | 21 | 18 | | | | | | 219.0917 | 121.6835 | 164.8518 | 112.0535 | 96.32091 | 275.8443 | 301.7456 | 241.8731 | 185.1373 | 624.6679 | 207.4294 | 403.6614 | 269.2314 | 213.7556 | 304.8602 | 275.6931 | 218.2859 | 368.3133 | 420.025 | 280.5674 | 380.124 | 686.1452 | 316.5936 | 213.0233 | 176.1434 | 207.7747 | 166.6117 |
| CUST_54556_PI429010886 | M106513 | 0.008047 | 12 | 9 | | | | | | 35.5818 | 27.4656 | 47.59957 | 19.92242 | 58.00667 | 60.06147 | 70.98423 | 22.98483 | 81.60393 | 84.60083 | 87.84073 | 100.1395 | 26.63149 | 77.06228 | 58.86655 | 61.28936 | 33.33921 | 59.7424 | 64.96286 | 54.91034 | 60.77736 | 63.86613 | 87.30486 | 72.69379 | 15.79313 | 79.47713 | 59.52835 |
| CUST_50779_PI429010886 | M90977 | 0.008048 | 18 | 12 | 156403087 | predicted protein [Nematostella vectensis] >gi\|156 | | | 8.85E-32 | 159.338 | 99.7668 | 160.9334 | 81.74728 | 94.07093 | 73.77338 | 166.3805 | 131.2414 | 171.427 | 209.4461 | 101.6252 | 112.9234 | 202.4041 | 208.936 | 168.9486 | 271.0212 | 108.3614 | 149.2467 | 170.596 | 92.66541 | 143.8077 | 175.1526 | 102.4176 | 96.24145 | 109.8428 | 100.64 | 172.5751 |
| CUST_38142_PI429010886 | M23621 | 0.008049 | 24 | 21 | | | | | | 4854.251 | 3496.725 | 3604.11 | 1789.616 | 2170.679 | 2706.735 | 2214.33 | 1937.537 | 1340.303 | 4579.426 | 2565.951 | 4008.447 | 4915.943 | 5515.427 | 4817.775 | 4944.792 | 2271.87 | 2638.062 | 1485.965 | 3704.973 | 2823.396 | 5585.575 | 4589.406 | 3983.683 | 3116.138 | 2417.914 | 3040.688 |
| CUST_468_PI429010886 | N5482 | 0.008056 | 15 | 3 | 460838092 | strongly chitin-binding protein-1 [Procambarus clarl | | | 1.13E-16 | 4318.11 | 1338.953 | 2417.914 | 8686.323 | 9919.546 | 9341.534 | 8760.24 | 3013.914 | 2363.842 | 287.0591 | 7145.704 | 103.7315 | 2164.947 | 235.8718 | 1081.506 | 448.199 | 11510.47 | 4887.374 | 8974.042 | 140.8238 | 12673.02 | 534.4051 | 6894.743 | 9371.785 | 273.3216 | 3861.408 | 139.9278 |
| CUST_36731_PI429010886 | M16687 | 0.008062 | 18 | 6 | | | | | | 20.22094 | 12.85718 | 18.49894 | 34.06396 | 16.70546 | 12.97507 | 24.50817 | 15.50269 | 27.41751 | 14.78546 | 21.53411 | 10.59051 | 5.485812 | 15.44569 | 15.26938 | 14.96828 | 17.14756 | 7.731153 | 24.66785 | 9.5748 | 15.21234 | 23.59336 | 17.29417 | 16.51743 | 21.87538 | 35.86991 | 24.42075 |
| CUST_19156_PI429010886 | N41069 | 0.008062 | 18 | 12 | | | | | | 47.21974 | 57.37859 | 69.41655 | 73.9797 | 60.15877 | 81.29483 | 47.30806 | 60.87936 | 57.69532 | 62.406 | 68.2359 | 100.1493 | 76.50308 | 87.92471 | 102.1126 | 84.1757 | 70.15478 | 74.17182 | 52.15768 | 64.95763 | 55.87623 | 70.99058 | 63.72139 | 58.45028 | 72.09388 | 84.43869 | 70.74029 |
| CUST_49525_PI429010886 | M85975 | 0.008065 | 15 | 6 | 195999790 | hypothetical protein TRIADDRAFT_21514 [Trichopla | Transport | Ion transport | 1.06E-08 | 11.12035 | 4.5493 | 7.31283 | 7.873199 | 10.01285 | 29.50649 | 11.27249 | 13.67972 | 4.640181 | 9.183116 | 21.09489 | 8.847678 | 5.835764 | 3.607383 | 8.475934 | 7.097098 | 8.724159 | 3.934182 | 12.5052 | 12.24457 | 13.17056 | 12.36089 | 12.89788 | 13.74195 | 8.389601 | 11.30463 | 11.9678 |
| CUST_21270_PI429010886 | N44922 | 0.008068 | 21 | 6 | | | | | | 16.26691 | NA | 28.67208 | NA | NA | NA | 30.8467 | 57.16139 | 21.82183 | 24.03089 | 10.56773 | 13.16396 | 9.258755 | 6.680971 | 3.761954 | NA | 10.03463 | 8.338462 | 23.4129 | 10.64535 | 21.44879 | 7.868714 | 32.05527 | 13.19382 | 22.12582 | 9.341797 | 20.36028 |
| CUST_38634_PI429010886 | M28706 | 0.008084 | 12 | 0 | | | | | | 19.54896 | 21.89378 | 10.9225 | 18.92827 | 17.98785 | 22.89389 | 17.04303 | 16.71152 | 16.66622 | 20.80307 | 19.01018 | 18.59683 | 20.9473 | 19.54896 | 28.34529 | 24.32155 | 18.50047 | 17.0998 | 18.70952 | 17.01511 | 17.76311 | 17.2758 | 20.10727 | 33.40373 | 24.80851 | 19.41782 | 29.58843 |
| CUST_46067_PI429010886 | M71245 | 0.008089 | 18 | 0 | | | | | | 6.490205 | 19.82066 | 7.648203 | 13.36717 | 10.89455 | 10.40028 | 4.773638 | 5.603976 | 5.88771 | 6.170344 | 4.710505 | 7.393906 | 7.399323 | 5.896612 | NA | 17.59108 | 11.08171 | 7.418431 | NA | 8.010366 | NA | 7.284707 | 18.21981 | 5.743146 | 6.995543 | NA | 5.719759 |
| CUST_39562_PI429010886 | M41231 | 0.008102 | 15 | 12 | 51104825 | Euphausia superba small subunit ribosomal RNA ge | | | | 46.89105 | 26.73668 | 40.20059 | 29.04226 | 25.58065 | 39.76167 | 23.04785 | 17.74945 | 30.23517 | 55.21772 | 19.14778 | 26.70117 | 33.01533 | 38.73055 | 41.30947 | 39.37435 | 40.1229 | 30.45321 | 31.8918 | 26.58056 | 22.94743 | 20.82207 | 33.48474 | 25.38172 | 46.28376 | 32.2878 | 38.31256 |
| CUST_29232_PI429010886 | N59440 | 0.008106 | 24 | 18 | | | | | | 19.95854 | 39.62708 | 39.83835 | 46.00478 | 30.12077 | 64.66893 | 18.24285 | 53.68104 | 16.11615 | 20.53839 | 61.89778 | 57.2685 | 20.73391 | 161.2028 | 129.1185 | 41.55626 | 164.5463 | 63.57842 | 108.6016 | 148.1764 | 107.0416 | 159.8607 | 140.0198 | 65.19644 | 189.4794 | 21.98819 | 95.91781 |
| CUST_35065_PI429010886 | M11466 | 0.008116 | 18 | 15 | | | | | | 153.5041 | 168.0579 | 135.1252 | 136.8075 | 134.4388 | 174.2722 | 83.76494 | 91.98586 | 105.2326 | 179.5942 | 144.2098 | 180.4643 | 102.8545 | 127.7418 | 121.1638 | 177.1551 | 236.1903 | 143.9489 | 119.0071 | 196.4409 | 121.2116 | 135.5951 | 131.2743 | 120.9219 | 149.2835 | 116.9236 | 100.3848 |
| CUST_21002_PI429010886 | N44456 | 0.008122 | 24 | 0 | 321477956 | hypothetical protein DAPPUDRAFT_304762 [Daphn | Protein metabolism | Cellular protein modification process | 1.48E-16 | 14.93734 | 15.04347 | 10.21387 | 8.796716 | 11.3957 | 18.19605 | 12.20426 | 9.576049 | 7.899647 | 11.03187 | 9.129182 | 12.03892 | 7.732289 | 9.605462 | 4.877934 | 27.4871 | 4.799209 | 10.69036 | 13.0306 | 17.04861 | 10.88684 | 24.45097 | NA | 8.092867 | 26.99397 | 28.40282 | 20.44752 |
| CUST_31975_PI429010886 | M2883 | 0.008122 | 15 | 9 | 328702773 | PREDICTED: hypothetical protein LOC100575450 [A | | | 4.36E-20 | 80.72119 | 107.0039 | 107.3414 | 76.88221 | 100.9197 | 72.32108 | 72.32108 | 97.10121 | 108.3037 | 153.6236 | 99.19224 | 118.1469 | 78.97339 | 108.064 | 110.0483 | 122.6448 | 118.5121 | 109.2372 | 92.5954 | 98.19642 | 94.69116 | 139.8337 | 106.8412 | 60.3241 | 149.1909 | 113.8702 | 131.1403 |
| CUST_32007_PI429010886 | M2980 | 0.008122 | 15 | 9 | 321475050 | hypothetical protein DAPPUDRAFT_127519 [Daphn | Metabolic process | Cellular amino acid metabolic process | 6.02E-51 | 300.0118 | 589.4448 | 428.865 | 197.0135 | 282.3655 | 118.3504 | 207.1617 | 472.3127 | 325.5645 | 732.077 | 528.618 | 509.4935 | 486.0261 | 428.1275 | 369.8842 | 464.5441 | 290.5996 | 319.5171 | 342.6195 | 613.8279 | 322.9938 | 554.4782 | 412.6269 | 133.6452 | 756.9801 | 377.8878 | 643.3515 |
| CUST_44659_PI429010886 | M66592 | 0.008122 | 15 | 9 | | | | | | 22.86023 | 42.73857 | 84.55489 | 10.6773 | 25.33989 | 14.17331 | 10.63784 | 60.82114 | 92.92758 | 108.7588 | 42.26034 | 144.5244 | 42.05465 | 95.11624 | 46.30373 | 87.41325 | 30.75954 | 17.95097 | 39.07685 | 52.85191 | 33.15216 | 70.15478 | 99.2894 | 14.03086 | 182.9432 | 40.56586 | 108.6974 |
| CUST_9613_PI429010886 | N24924 | 0.008122 | 15 | 9 | | | | | | 2450.654 | 3372.782 | 2874.609 | 1542.262 | 2031.538 | 1137.917 | 1315.839 | 2542.502 | 2961.93 | 4381.739 | 2459.721 | 3905.483 | 3018.553 | 3905.483 | 3429.061 | 4901.718 | 1973.742 | 2563.253 | 2680.458 | 4377.65 | 2509.921 | 4561.712 | 3124.162 | 1565.628 | 6122.745 | 5023.801 | 5848.791 |
| CUST_10721_PI429010886 | N26684 | 0.008122 | 15 | 0 | | | | | | 1971.63 | 2306.592 | 1883.709 | 1795.627 | 1645.771 | 1914.945 | 1195.978 | 1277.616 | 1338.117 | 2065.573 | 1663.806 | 2253.027 | 1276.133 | 1859.87 | 1924.978 | 2665.55 | 2016.816 | 1951.832 | 1770.146 | 2340.111 | 1619.494 | 1911.091 | 1481.201 | 1013.924 | 2108.426 | 1924.978 | 2098.72 |
| CUST_12556_PI429010886 | N29646 | 0.008122 | 15 | 9 | 220172371 | putative elastin a [Rimicaris exoculata] | | | 5.1E-16 | 408.0489 | 3013.914 | 2316.991 | 490.7702 | 416.1299 | 82.19075 | 249.7604 | 1154.447 | 4073.081 | 4778.44 | 2966.269 | 6885.41 | 1590.414 | 5917.438 | 3673.827 | 4519.983 | 504.8164 | 1818.602 | 2022.724 | 3994.868 | 2472.114 | 7221.522 | 3616.168 | 248.7014 | 7669.986 | 4098.525 | 7457.426 |
| CUST_16771_PI429010886 | N36844 | 0.008122 | 15 | 9 | | | | | | 17.21816 | 31.40553 | 22.92529 | 10.05721 | 16.36491 | 14.59087 | 14.12299 | 40.11789 | 48.9358 | 27.0727 | 20.11768 | 25.16709 | 38.09087 | 48.3123 | 22.092 | 39.92228 | 17.52398 | 20.83504 | 21.0901 | 17.83033 | 20.76905 | 38.08235 | 22.70929 | 14.8732 | 95.50344 | 38.78884 | 164.4877 |
| CUST_14486_PI429010886 | N32966 | 0.008136 | 12 | 6 | | | | | | 4.940431 | 8.075584 | NA | NA | 4.223313 | 8.300728 | 8.982813 | 10.97825 | 16.74589 | NA | 7.415398 | 8.448821 | 6.349977 | 8.914093 | 8.752945 | 14.83888 | 8.970081 | 16.12591 | 9.41784 | 17.06111 | NA | 6.860297 | 12.98669 | NA | 4.052238 | NA | 5.320278 |
| CUST_50938_PI429010886 | M91511 | 0.008137 | 24 | 18 | | | | | | 152.9711 | 211.5605 | 241.0332 | 195.5884 | 203.9899 | 207.9906 | 183.2048 | 176.1434 | 204.0536 | 180.3864 | 188.2417 | 193.8618 | 197.4111 | 220.7455 | 212.4411 | 254.7626 | 212.9083 | 198.7745 | 194.0352 | 256.8944 | 234.1059 | 205.8446 | 223.9394 | 221.7682 | 238.5428 | 194.1209 | 229.9564 |
| CUST_40529_PI429010886 | M55381 | 0.008141 | 12 | 0 | | | | | | 120.1371 | 121.6348 | 132.7287 | 84.53623 | 92.67278 | 74.51983 | 81.7755 | 95.63731 | 103.0243 | 148.2477 | 114.5736 | 119.5504 | 114.1048 | 136.8231 | 124.6057 | 136.9776 | 104.2505 | 96.24145 | 111.7487 | 137.1593 | 107.4857 | 114.5611 | 151.6013 | 74.25035 | 149.7134 | 148.0995 | 155.5733 |
| CUST_4241_PI429010886 | N16311 | 0.008141 | 12 | 0 | 321468390 | hypothetical protein DAPPUDRAFT_304841 [Daphn | | | 1.51E-23 | 45.84834 | 57.91335 | 66.70898 | 31.78356 | 36.78858 | 47.32005 | 48.96007 | 56.94451 | 46.96643 | 56.47886 | 38.82583 | 53.36598 | 53.12043 | 61.04516 | 39.63103 | 43.70884 | 32.7674 | 42.50508 | 42.61032 | 52.06984 | 53.57981 | 43.38005 | 62.5758 | 52.86776 | 56.72265 | 60.61979 | 56.50237 |
| CUST_5866_PI429010886 | N19311 | 0.008141 | 12 | 0 | | | | | | 274.5343 | 282.9535 | 289.8469 | 232.8505 | 251.2548 | 237.2477 | 269.5259 | 247.0088 | 275.2679 | 283.788 | 270.2125 | 280.5129 | 233.4512 | 272.4885 | 252.4045 | 258.545 | 239.1179 | 229.245 | 242.9037 | 267.9382 | 271.0795 | 247.8273 | 350.314 | 232.8922 | 308.2184 | 324.8963 | 310.2726 |
| CUST_29920_PI429010886 | N60819 | 0.008141 | 12 | 0 | | | | | | 63.12362 | 99.89328 | 104.8392 | 48.2481 | 51.59735 | 53.50545 | 60.85469 | 64.55964 | 63.96144 | 85.19825 | 64.48135 | 90.29804 | 55.5381 | 117.1213 | 102.7268 | 111.4592 | 59.32225 | 63.16675 | 70.31729 | 83.74259 | 68.68381 | 72.72106 | 90.83511 | 94.44207 | 91.84848 | 72.41676 | 91.66231 |
| CUST_54057_PI429010886 | M104306 | 0.008153 | 18 | 12 | | | | | | 12.51969 | 16.87735 | 21.28123 | 8.376213 | 7.929534 | 4.290274 | 10.4406 | 8.730965 | 15.27765 | 19.14544 | 15.63271 | 13.17199 | 15.30623 | 19.44722 | 15.37156 | 14.17166 | 18.47842 | 7.911129 | 9.284755 | 17.78619 | 9.325475 | 15.44955 | 5.964046 | 12.17281 | 16.0983 | 14.28453 | 11.78768 |
| CUST_38616_PI429010886 | M28570 | 0.008173 | 15 | 12 | | | | | | 7853.842 | 4094.84 | 16184.83 | 366.4854 | 993.3629 | 542.1254 | 12089.86 | 23580.44 | 16394.35 | 24081.12 | 7498.771 | 7938.371 | 17482.08 | 31581.61 | 16238.88 | 2906.073 | 17506.5 | 2462.824 | 7273.892 | 8814.529 | 4411.84 | 19037.21 | 19666.57 | 1909.916 | 28919.9 | 5080.996 | 27577.88 |
| CUST_46024_PI429010886 | M71100 | 0.008215 | 15 | 9 | | | | | | 511.7062 | 347.4375 | 542.7169 | 334.9967 | 326.9766 | 239.3938 | 299.9648 | 291.0552 | 361.9781 | 748.2524 | 350.314 | 661.523 | 368.0062 | 395.3575 | 452.9912 | 395.8483 | 381.6649 | 396.481 | 260.9414 | 419.7481 | 437.9769 | 472.773 | 559.2711 | 292.413 | 552.1675 | 652.581 | 619.0377 |
| CUST_49824_PI429010886 | M87332 | 0.008215 | 15 | 3 | 391329623 | PREDICTED: uncharacterized protein LOC10090138 | | | 2.24E-07 | 506.1361 | 642.6469 | 344.4277 | 1734.303 | 2040.559 | 1306.431 | 981.4865 | 1380.113 | 114.529 | 17.85258 | 960.7453 | 14.05335 | 886.3986 | 34.6687 | 499.1257 | 75.67767 | 1209.365 | 369.2549 | 1109.205 | 18.18793 | 753.2014 | 42.29494 | 376.5573 | 1426.735 | 7.409357 | 197.989 | 10.2728 |
| CUST_4151_PI429010886 | N16116 | 0.008215 | 15 | 9 | 255653874 | crustin 4 [Panulirus japonicus] | | | 2.37E-16 | 489.9462 | 1607.248 | 1483.13 | 421.3888 | 768.0399 | 409.9605 | 452.1056 | 690.2519 | 1530.336 | 958.279 | 1287.748 | 3272.814 | 1062.278 | 2203.176 | 1709.2 | 3013.914 | 532.9396 | 1246.008 | 554.3622 | 1314.491 | 717.1047 | 3071.798 | 999.7477 | 561.1634 | 2701.759 | 1719.247 | 3380.982 |
| CUST_42719_PI429010886 | M61053 | 0.008215 | 24 | 0 | | | | | | 10.70732 | 15.57864 | 17.23883 | 22.63948 | 12.71524 | 16.44013 | 12.25276 | 8.833142 | 5.365255 | 13.73363 | 9.3513 | 9.87605 | 4.267713 | 7.597404 | 9.559604 | 16.23932 | 13.47393 | 21.04144 | 22.30272 | 5.514158 | 11.97367 | 11.11786 | 40.61218 | 9.495063 | 26.17436 | 21.15997 | 25.4312 |
| CUST_7466_PI429010886 | N21587 | 0.008233 | 12 | 9 | 156540796 | PREDICTED: transcription initiation factor IIE subuni | | | 3.89E-41 | 59.33007 | 57.71214 | 65.8929 | 43.20136 | 49.14494 | 58.86655 | 52.5097 | 45.76846 | 52.92115 | 90.43345 | 65.38012 | 86.32314 | 72.05058 | 59.40092 | 71.94024 | 68.44169 | 50.49286 | 64.94243 | 66.36423 | 84.25035 | 94.37733 | 77.36396 | 73.01018 | 79.46381 | 126.3583 | 56.19981 | 80.24894 |
| CUST_4563_PI429010886 | N17035 | 0.008234 | 24 | 9 | | | | | | 45.30213 | 205.078 | 109.0299 | 67.55015 | NA | 4.518931 | 250.8965 | 149.8536 | 181.4625 | 387.589 | 349.4134 | 429.6585 | 35.07632 | 100.0986 | 79.19112 | 67.04072 | NA | 58.56755 | 26.36533 | 23.73784 | 20.42712 | 40.24278 | 237.1988 | 169.8718 | 7.88886 | NA | 15.73231 |
| CUST_33927_PI429010886 | M8267 | 0.008235 | 12 | 0 | | | | | | 3090.22 | 2292.412 | 3060.282 | 993.3629 | 1600.359 | 2065.573 | 2288.66 | 1947.429 | 2163.813 | 2873.023 | 1990.565 | 2921.018 | 2770.165 | 2612.527 | 2402.318 | 1612.797 | 1815.297 | 2325.953 | 893.1457 | 2448.925 | 1920.006 | 3852.498 | 1865.638 | 1860.994 | 2863.818 | 2258.839 | 2789.972 |
| CUST_3088_PI429010886 | N13771 | 0.008235 | 12 | 3 | 156552406 | PREDICTED: transaldolase-like [Nasonia vitripennis] | Metabolic process | Carbohydrate metabolic process | 3.3E-127 | 3558.109 | 2869.202 | 2238.739 | 4868.886 | 3783.178 | 4515.16 | 4303.084 | 2774.636 | 2336.801 | 1850.903 | 3480.516 | 2033.91 | 2729.519 | 1741.833 | 2299.417 | 3371.152 | 3762.541 | 3590.082 | 3266.951 | 2395.771 | 2595.092 | 2692.301 | 2219.95 | 3469.507 | 2049.694 | 2661.313 | 1818.602 |
| CUST_7064_PI429010886 | N20989 | 0.00825 | 18 | 12 | 374079276 | GalNAc/Gal-specific lectin [Crenomytilus grayanus] | Cellular Process | Cell death | 1.76E-21 | 566.6261 | 607.4385 | 932.5265 | 358.225 | 482.8729 | 368.0062 | 673.5866 | 738.859 | 600.7483 | 980.1159 | 614.705 | 659.4054 | 748.4841 | 930.2399 | 663.9332 | 735.8213 | 704.3489 | 524.852 | 596.0223 | 714.0634 | 588.4339 | 834.0915 | 507.1661 | 527.6079 | 894.2724 | 538.762 | 732.077 |
| CUST_13649_PI429010886 | N31528 | 0.008258 | 24 | 0 | | | | | | 301.5634 | 284.9694 | 312.4226 | 173.0713 | 243.3595 | 316.8071 | 209.9677 | 178.5939 | 223.7779 | 270.8602 | 225.8926 | 207.3956 | 127.0345 | 209.3167 | 190.1335 | 325.3231 | 295.5774 | 269.9974 | 293.7629 | 237.0939 | 301.9881 | 244.4939 | 252.4045 | 231.931 | 391.4581 | 286.4677 | 337.0265 |
| CUST_18671_PI429010886 | N40199 | 0.008266 | 24 | 0 | | | | | | 198.2503 | 184.1659 | 189.8316 | 195.2959 | 155.7638 | 170.2922 | 176.2631 | 148.9832 | 136.1246 | 176.5188 | 161.54 | 184.5131 | 123.5719 | 172.7406 | 142.069 | 163.6107 | 150.286 | 156.8624 | 170.9831 | 126.7579 | 125.2116 | 186.8543 | 195.0326 | 135.061 | 206.9171 | 167.6742 | 204.0536 |
| CUST_43910_PI429010886 | M64308 | 0.00828 | 18 | 0 | | | | | | 17.74325 | 22.88357 | 25.40325 | 16.79088 | 13.14638 | 22.71614 | 11.30336 | 10.65167 | 20.74695 | 17.36679 | 19.03935 | 11.94281 | 8.637107 | 9.656691 | 13.89481 | 12.10865 | 25.05489 | 20.40111 | 20.89073 | 19.37939 | 29.93503 | 12.46997 | 19.13027 | 12.27925 | 14.38683 | 17.93968 | 20.17771 |
| CUST_782_PI429010886 | N7294 | 0.008287 | 21 | 3 | 67625710 | TPA: endonuclease-reverse transcriptase [Schistoso | | | 6.53E-23 | NA | 16.5713 | 7.454608 | 22.76882 | NA | 14.9512 | NA | 5.339308 | 4.487534 | 16.14788 | 4.300882 | 20.09393 | 4.634681 | NA | NA | 9.180913 | 4.79181 | 14.77827 | 7.535575 | 6.774547 | 11.62495 | 8.393397 | NA | 12.29952 | 33.86312 | 16.57372 | 16.77475 |
| CUST_22738_PI429010886 | N47579 | 0.008303 | 21 | 9 | | | | | | 23.86861 | 21.93061 | 21.60172 | 25.62613 | 25.01541 | 28.96949 | 22.82362 | 31.19161 | 29.68097 | 41.16894 | 34.43474 | 31.94051 | 29.29243 | 30.32196 | 33.68931 | 49.73773 | 22.63247 | 32.83309 | 24.316 | 32.54901 | 32.64929 | 24.46354 | 32.95758 | 18.74405 | 31.89828 | 25.5267 | 39.50189 |
| CUST_53956_PI429010886 | M103868 | 0.008307 | 15 | 9 | 321462209 | hypothetical protein DAPPUDRAFT_325521 [Daphn | | | 1.93E-11 | 175.5504 | 214.6655 | 221.873 | 184.0938 | 189.5352 | 164.0384 | 183.4152 | 155.4149 | 184.1431 | 270.3602 | 184.0345 | 227.5328 | 139.5098 | 214.1014 | 216.6058 | 210.1212 | 197.0618 | 172.5221 | 156.3501 | 176.0261 | 172.8801 | 235.8718 | 204.4512 | 176.2423 | 281.599 | 206.2668 | 277.9987 |
| CUST_7781_PI429010886 | N22067 | 0.008307 | 15 | 0 | 541984961 | PREDICTED: FAD-linked sulfhydryl oxidase ALR [Falc | Metabolic process | Oxidation-reduction process | 6.14E-38 | 25.34926 | 28.10273 | 22.90764 | 26.12486 | 20.2329 | 15.42005 | 14.46778 | 14.05335 | 9.432782 | 22.76479 | 12.23461 | 22.41033 | 19.11878 | 19.69401 | 23.84605 | 34.87758 | 11.26345 | 14.46778 | 15.01453 | 18.21241 | 19.32542 | 14.71926 | 8.293632 | 11.70259 | 21.3985 | 22.5314 | 13.54122 |
| CUST_15818_PI429010886 | N35198 | 0.008307 | 15 | 0 | | | | | | 55.30825 | 81.44839 | 91.30112 | 90.31563 | 77.02903 | 110.3616 | 38.45723 | 30.69467 | 33.05172 | 50.3553 | 75.11707 | 94.07093 | 41.68251 | 62.12557 | 63.39049 | 94.127 | 74.6525 | 104.6964 | 67.32283 | 52.57619 | 61.15955 | 61.911 | 45.6007 | 49.64767 | 97.73561 | 68.1364 | 70.4935 |
| CUST_45470_PI429010886 | M69207 | 0.008328 | 12 | 0 | | | | | | 155.7012 | 193.9691 | 254.3377 | 156.2332 | 159.4512 | 131.879 | 152.6964 | 155.9427 | 201.9074 | 213.8072 | 177.1086 | 210.9393 | 178.4456 | 271.1821 | 220.7134 | 202.9774 | 127.3036 | 159.338 | 198.6919 | 180.8997 | 174.1781 | 181.9242 | 232.6257 | 173.8312 | 229.9104 | 201.5551 | 252.6775 |
| CUST_29457_PI429010886 | N59901 | 0.008328 | 12 | 0 | | | | | | 129.0466 | 124.6537 | 160.2021 | 73.48596 | 99.31284 | 102.022 | 72.73041 | 107.8324 | 105.1478 | 139.4961 | 107.7787 | 147.2543 | 110.6854 | 162.1184 | 143.0445 | 161.6619 | 95.66411 | 112.1834 | 120.0318 | 103.9053 | 123.0412 | 156.1886 | 141.5508 | 82.01377 | 147.2771 | 143.002 | 164.2928 |
| CUST_15392_PI429010886 | N34507 | 0.008344 | 24 | 0 | | | | | | 188.1161 | 176.5718 | 241.4042 | 165.2432 | 185.3252 | 120.7928 | 163.2888 | 150.4655 | 180.9857 | 235.1413 | 172.3811 | 110.0483 | 178.4123 | 138.7197 | 97.93806 | 96.64522 | 164.4613 | 93.9354 | 124.8573 | 141.3308 | 232.1818 | 89.6226 | 168.2221 | 172.408 | 207.2788 | 160.1816 | 267.177 |
| CUST_35364_PI429010886 | M12382 | 0.008344 | 15 | 3 | 321459829 | hypothetical protein DAPPUDRAFT_217067 [Daphn | Metabolic process | Cellular amino acid metabolic process | 1.8E-125 | 215.4042 | 207.3277 | 13.76031 | 339.6585 | 385.0916 | 285.4947 | 258.6664 | 85.10975 | 123.7403 | 24.929 | 30.77501 | 9.055842 | 130.0689 | 60.49322 | 121.9844 | 176.1772 | 74.27647 | 119.4425 | 145.2206 | 85.51349 | 26.81787 | 236.856 | 7.482892 | 136.597 | 112.6604 | 47.72724 | 11.91408 |
| CUST_50299_PI429010886 | M89359 | 0.008347 | 12 | 0 | | | | | | 92.04256 | 67.59725 | 105.0612 | 99.52485 | 90.06088 | 137.6307 | 72.01712 | 78.40746 | 83.44238 | 101.6676 | 87.35248 | 135.78 | 107.4484 | 145.2206 | 154.0002 | 117.9689 | 86.4151 | 103.802 | 91.85305 | 86.13317 | 101.2088 | 109.8741 | 96.66502 | 75.93064 | 119.4271 | 158.9341 | 136.9283 |
| CUST_6622_PI429010886 | N20362 | 0.008365 | 18 | 6 | | | | | | 25.78835 | 63.86613 | 29.94885 | 57.74713 | 38.40419 | 32.8295 | 86.27625 | 60.23492 | 97.84604 | 60.68591 | 36.69324 | 31.32056 | 42.44469 | 65.23612 | 38.63759 | 59.52293 | 26.28688 | 28.39147 | 61.27293 | 41.62677 | 49.41092 | 62.1467 | 39.0152 | 66.19578 | 59.80641 | 76.77824 | 62.38529 |
| CUST_27230_PI429010886 | N55676 | 0.00837 | 21 | 0 | | | | | | 44.58058 | 32.10959 | 40.18883 | 13.68648 | 18.48442 | 26.54903 | 14.68799 | 21.9058 | 24.93189 | 11.0059 | 14.11808 | 17.67291 | 20.94544 | 26.66659 | 22.03473 | 33.32679 | 39.32188 | 25.86598 | 18.90775 | 18.74405 | 8.383502 | 31.82074 | 20.36313 | 40.13494 | 42.47769 | 17.13473 | 30.18478 |
| CUST_19976_PI429010886 | N42546 | 0.008373 | 18 | 0 | | | | | | 14.58939 | 22.76207 | 20.89073 | 38.88089 | 10.44304 | 20.4401 | 20.53338 | 4.816403 | 10.6773 | 11.92505 | 13.94276 | 10.17939 | 14.48423 | 8.267442 | 12.96066 | 18.69088 | 12.34868 | 14.33423 | 20.81747 | 14.8801 | 14.80005 | 17.132 | 10.10703 | 14.11041 | 6.62121 | 12.39561 | NA |
| CUST_8558_PI429010886 | N23221 | 0.008376 | 15 | 12 | | | | | | 13.9828 | 26.62182 | NA | 9.015517 | 7.82506 | 10.13526 | 6.613886 | 11.40101 | 7.991765 | 13.3566 | 13.40566 | 7.101515 | 12.85526 | 13.36577 | 18.6803 | 12.01254 | 9.569411 | 14.00188 | 18.56579 | 14.9321 | 16.60305 | 6.538957 | 6.987277 | 7.426569 | 16.49199 | 12.75631 | 16.07261 |
| CUST_55909_PI429010886 | M112083 | 0.008383 | 18 | 15 | | | | | | 26.80742 | 34.00967 | 25.4154 | 21.97852 | 26.4818 | 21.15749 | 12.75297 | 29.07242 | 17.95097 | 23.17379 | 23.29073 | 34.88888 | 29.82235 | 31.66434 | 26.03903 | 40.10273 | 22.69285 | 64.95763 | 37.40747 | 38.29927 | 29.76585 | 23.28379 | 23.45456 | 20.05201 | 44.70933 | 19.88004 | 15.50857 |
| CUST_17810_PI429010886 | N38634 | 0.008383 | 18 | 9 | 383860456 | PREDICTED: probable deoxyhypusine synthase-like | Protein metabolism | Cellular protein modification process | 9.37E-35 | 22.10198 | 34.6933 | 23.68051 | 18.66111 | 23.32806 | 17.7744 | 22.42169 | 22.04311 | 16.45817 | 42.21564 | 30.41187 | 34.15487 | 28.25834 | 28.25834 | 28.49553 | 37.81392 | 22.62086 | 25.42315 | 23.4104 | 36.99652 | 29.72938 | 27.84094 | 15.59123 | 20.1154 | 40.2482 | 15.41864 | 25.59863 |
| CUST_11578_PI429010886 | N28063 | 0.008399 | 15 | 9 | | | | | | 77.01708 | 39.22918 | 62.48649 | 35.00866 | 36.73877 | 27.51707 | 55.72398 | 55.05996 | 45.24266 | 57.1457 | 66.99196 | 81.01356 | 44.14799 | 84.48505 | 30.59095 | 65.29553 | 59.82242 | 55.45871 | 54.08177 | 52.05749 | 47.39274 | 47.16106 | 82.46901 | 41.8722 | 74.3064 | 59.58353 | 85.22504 |
| CUST_1085_PI429010886 | N8443 | 0.008399 | 24 | 18 | | | | | | 566.7662 | 1021.127 | 795.1421 | 701.8561 | 777.7644 | 775.4257 | 730.0267 | 758.445 | 698.2239 | 858.518 | 810.9217 | 1206.475 | 889.7966 | 628.1675 | 765.2983 | 854.951 | 855.8158 | 946.2082 | 996.2576 | 1147.081 | 917.7643 | 861.813 | 908.1498 | 717.1047 | 966.7908 | 854.7156 | 909.757 |
| CUST_4530_PI429010886 | N16976 | 0.008412 | 18 | 3 | | | | | | 269.6735 | 82.48421 | 198.1505 | 279.5586 | 377.4127 | 169.2967 | 355.6114 | 41.72747 | 23.46287 | 57.10423 | 216.2249 | 64.47443 | 25.01145 | 51.51243 | 20.3487 | 83.07834 | 74.63865 | 43.46708 | 196.1855 | 39.46416 | 326.7925 | 44.29926 | 474.8115 | 430.573 | 180.2368 | 25.46248 | 19.18718 |
| CUST_7551_PI429010886 | N21715 | 0.00843 | 18 | 12 | | | | | | 15.56605 | 16.83941 | 5.028291 | 19.06154 | 15.51227 | 11.50042 | 17.45107 | 15.45106 | 17.11553 | 19.676 | 12.8607 | 30.0232 | 23.02438 | 22.39033 | 26.89938 | 28.98446 | 19.91373 | 21.96744 | 21.83723 | 23.1075 | 24.0077 | 14.37805 | 11.10317 | 6.424398 | 26.42807 | 36.30688 | 28.41001 |
| CUST_1026_PI429010886 | N8260 | 0.008444 | 15 | 3 | | | | | | 184.9825 | 227.0035 | 60.3963 | 583.9827 | 219.5144 | 468.4803 | 279.8319 | 325.6779 | 197.86 | 176.3863 | 85.86351 | 9.197112 | 181.4398 | 224.5446 | 158.635 | 178.0431 | 259.2453 | 211.9851 | 366.9733 | 43.60346 | 319.0748 | 569.6586 | 109.4535 | 283.4361 | 105.4471 | 256.6496 | 45.11398 |
| CUST_54269_PI429010886 | M105340 | 0.008444 | 15 | 9 | | | | | | 136.8476 | 162.719 | 186.5931 | 134.7571 | 120.5234 | 125.6172 | 123.2882 | 132.4059 | 154.2676 | 170.0196 | 136.0526 | 210.7453 | 155.979 | 201.9745 | 170.8572 | 171.0319 | 140.0012 | 162.98 | 152.6523 | 158.317 | 144.8798 | 180.661 | 150.1848 | 143.4489 | 182.6771 | 199.0699 | 222.9397 |
| CUST_36290_PI429010886 | M15451 | 0.008444 | 12 | 0 | | | | | | 26.46581 | 31.86571 | 24.79214 | 20.58435 | 20.68548 | 35.86299 | 25.2475 | 23.02707 | 20.03687 | 21.01141 | 31.80323 | 33.20988 | 26.84327 | 35.49911 | 33.80907 | 22.9575 | 33.97352 | 25.73562 | 19.64187 | 25.67466 | 18.58833 | 33.27151 | 23.29812 | 21.9039 | 26.58577 | 37.10254 | 28.68856 |
| CUST_33993_PI429010886 | M8449 | 0.008444 | 15 | 9 | | | | | | 793.5884 | 2109.682 | 1318.096 | 490.65 | 699.6538 | 327.7802 | 2002.228 | 1667.846 | 2526.384 | 2375.935 | 1028.799 | 1905.993 | 2437.103 | 2454.105 | 1814.412 | 3464.749 | 1569.266 | 1065.852 | 1154.447 | 1664.492 | 1031.749 | 3380.982 | 1644.909 | 735.196 | 4381.739 | 1581.192 | 5843.06 |
| CUST_2449_PI429010886 | N12371 | 0.008444 | 15 | 9 | | | | | | 328.6838 | 379.1503 | 444.0863 | 309.1695 | 301.7456 | 366.7345 | 280.2255 | 419.4237 | 413.0515 | 412.7593 | 316.2028 | 499.9431 | 362.4143 | 499.1257 | 470.9976 | 392.0959 | 337.3994 | 422.1729 | 344.8512 | 398.7713 | 354.2504 | 426.6051 | 391.819 | 368.0062 | 457.7503 | 461.2479 | 475.1468 |
| CUST_8715_PI429010886 | N23469 | 0.008444 | 12 | 0 | | | | | | 148.3869 | 134.8756 | 157.8347 | 109.6802 | 120.2652 | 117.4327 | 92.38118 | 118.4632 | 142.5643 | 166.3805 | 138.7005 | 159.5837 | 123.056 | 174.5081 | 178.2839 | 155.4298 | 114.3333 | 133.7417 | 117.7161 | 149.4856 | 129.3708 | 166.9208 | 145.9801 | 127.3248 | 171.5722 | 139.6592 | 160.9512 |
| CUST_31620_PI429010886 | M1983 | 0.008445 | 15 | 9 | | | | | | 17.86172 | 17.72933 | 40.25214 | NA | NA | NA | NA | 8.52188 | 111.027 | 52.88081 | 110.4846 | 124.0489 | 22.52813 | 97.90896 | 78.78492 | 36.08462 | NA | 96.07604 | 17.80246 | 83.87586 | 14.98994 | 52.91645 | 35.81535 | NA | 102.8187 | 106.298 | 100.2579 |
| CUST_42494_PI429010886 | M60414 | 0.008448 | 15 | 0 | | | | | | 137.2919 | 106.3929 | 106.8104 | 118.6098 | 71.02655 | 107.638 | 109.4779 | 86.7376 | 84.59116 | 82.94784 | 52.21271 | 32.58197 | 73.93957 | 61.88462 | 51.20753 | 92.57501 | 111.485 | 89.18534 | 86.66049 | 65.76942 | 67.14861 | 56.53962 | 55.5206 | 99.4135 | 85.8526 | 71.63476 | 63.15525 |
| CUST_16911_PI429010886 | N37065 | 0.008451 | 21 | 12 | | | | | | 7.117252 | 35.48686 | NA | 3.766314 | 14.91728 | 11.56849 | 19.73697 | 22.16629 | 6.574274 | 27.37091 | 20.33108 | 18.28609 | 61.71609 | 44.11518 | 24.34006 | 22.7667 | 5.442479 | 13.28864 | 17.06111 | 52.16399 | 5.070144 | 15.76954 | 19.32542 | NA | 6.007021 | 10.13043 | 9.163319 |
| CUST_50468_PI429010886 | M89932 | 0.008469 | 15 | 12 | | | | | | 47.20161 | 57.54706 | 54.95061 | 27.43923 | 34.51534 | 50.61812 | 48.61196 | 36.8491 | 32.21416 | 37.70021 | 39.414 | 53.83853 | 48.3123 | 50.21157 | 55.96041 | 65.57569 | 50.25117 | 52.06984 | 46.45397 | 47.96198 | 43.88387 | 38.87395 | 31.31664 | 45.50116 | 53.17877 | 56.14691 | 70.02979 |
| CUST_41401_PI429010886 | M57652 | 0.008472 | 15 | 12 | | | | | | 154.5009 | 139.1588 | 204.629 | 139.1216 | 152.147 | 150.4117 | 142.9378 | 141.3792 | 152.3559 | 177.0904 | 168.9486 | 190.588 | 154.7492 | 237.9203 | 204.2578 | 177.3713 | 202.3386 | 195.1331 | 152.0579 | 173.8566 | 166.4887 | 169.3227 | 178.1191 | 162.0241 | 165.2714 | 192.1754 | 211.6792 |
| CUST_25678_PI429010886 | N52866 | 0.008472 | 15 | 12 | | | | | | 47.91134 | 32.77524 | 72.59038 | 35.46793 | 24.6038 | 35.33784 | 21.10951 | 33.67217 | 34.46151 | 35.35407 | 37.6475 | 49.64123 | 47.23018 | 108.4775 | 48.9358 | 54.86156 | 45.46745 | 47.45553 | 45.31985 | 44.63407 | 46.55609 | 46.32451 | 46.22951 | 37.10254 | 61.11567 | 41.44167 | 62.44513 |
| CUST_36761_PI429010886 | M16773 | 0.008484 | 21 | 9 | 349502991 | O-methyltransferase [Penaeus monodon] | | | 3.15E-85 | 52.95745 | 67.11047 | 46.35165 | 72.92837 | 100.7905 | 53.63782 | 76.06574 | 284.3166 | 223.0503 | 84.722 | 198.7421 | 171.8979 | 148.6082 | 69.0257 | 89.89197 | 31.62413 | 111.1116 | 33.94434 | 80.90583 | 322.9938 | 104.9922 | 44.15772 | 73.69227 | 52.89103 | 51.69046 | 120.496 | 70.00892 |
| CUST_52738_PI429010886 | M98728 | 0.008484 | 18 | 0 | | | | | | 69.14635 | 81.39522 | 84.98778 | 63.89787 | 88.37144 | 21.89117 | 36.01731 | 12.51235 | 16.38019 | 30.2284 | 41.84077 | 51.75281 | 50.28834 | 112.457 | 65.39564 | 86.60546 | 22.16629 | 60.6077 | 26.66065 | 138.8758 | 74.97194 | 197.2228 | 45.35228 | 75.64933 | 68.14294 | 8.176012 | 17.59799 |
| CUST_9477_PI429010886 | N24697 | 0.008484 | 24 | 15 | 328899290 | receptor accessory protein 5 [Procambarus clarkii] | | | 1.24E-39 | 398.3325 | 570.6164 | 244.4939 | 94.61126 | 762.7979 | 546.8107 | 403.4054 | 307.6894 | 506.7573 | 685.3204 | 563.3684 | 1303.313 | 877.4226 | 641.17 | 717.1047 | 566.4251 | 994.0668 | 844.933 | 468.3619 | 546.8107 | 412.7593 | 592.9907 | 381.3389 | 855.518 | 519.6135 | 318.8664 | 450.9914 |
| CUST_4301_PI429010886 | N16451 | 0.008492 | 15 | 9 | | | | | | 12.81928 | 15.25972 | 14.71698 | 8.928641 | 9.947764 | 11.8786 | 15.04503 | 8.727631 | 9.682017 | 17.26689 | 14.91728 | 49.51255 | 15.15061 | 12.7372 | 9.564156 | 18.08937 | 12.09063 | 19.72298 | 9.402874 | 11.74252 | 9.25417 | 19.99756 | 10.81563 | 18.81916 | 21.09286 | 13.22835 | 15.66989 |
| CUST_34294_PI429010886 | M9259 | 0.008498 | 18 | 12 | | | | | | 987.1593 | 1249.348 | 803.7954 | 1992.587 | 982.4374 | 1339.496 | 1486.604 | 1179.987 | 1673.62 | 1601.013 | 1053.785 | 2224.226 | 2286.095 | 1962.83 | 2626.728 | 2963.617 | 898.6441 | 1618.557 | 1581.923 | 1027.997 | 1081.506 | 1629.467 | 731.2482 | 1152.01 | 1731.857 | 1609.005 | 1982.266 |
| CUST_43838_PI429010886 | M64128 | 0.008508 | 24 | 15 | 521034512 | Zinc finger protein 212 [Myotis brandtii] | | | 1.22E-07 | NA | 13.39381 | 7.97358 | 12.96601 | 7.761493 | 6.559123 | 7.162712 | 4.987261 | 5.180096 | 6.618021 | 7.092466 | 10.05721 | 10.8676 | 3.281911 | 11.36478 | 13.74963 | 12.36224 | 13.66405 | 11.22575 | 5.642111 | NA | 17.51738 | 8.597478 | NA | 13.34056 | NA | 8.898126 |
| CUST_1550_PI429010886 | N10034 | 0.008515 | 12 | 0 | | | | | | 33.67217 | 35.34065 | 32.29627 | 22.14979 | 26.45134 | 21.99795 | 25.88089 | 24.89895 | 29.41419 | 31.41586 | 26.44892 | 28.39426 | 28.57291 | 26.85111 | 30.8467 | 32.48228 | 15.96955 | 22.51683 | 26.42807 | 23.01614 | 30.00897 | 26.4245 | 33.29815 | 23.61236 | 32.88145 | 23.23227 | 35.6284 |
| CUST_25940_PI429010886 | N53341 | 0.008529 | 12 | 9 | | | | | | 52.25211 | 79.0521 | 71.6044 | 39.00007 | 43.06591 | 60.53815 | 47.72724 | 39.19383 | 61.90324 | 95.30462 | 57.62155 | 88.10192 | 64.2224 | 87.54624 | 75.86883 | 52.80847 | 58.94211 | 54.41242 | 80.42722 | 72.3461 | 75.44459 | 112.9419 | 83.45905 | 52.95745 | 72.69379 | 56.97163 | 72.00003 |
| CUST_31403_PI429010886 | M1446 | 0.00853 | 21 | 15 | | | | | | 232.0593 | 156.3242 | 153.5797 | 66.56405 | 121.8439 | 83.99654 | 112.3653 | 89.49881 | 117.0097 | 196.359 | 144.3428 | 130.2384 | 142.8727 | 146.3399 | 75.19772 | 153.7764 | 150.3479 | 159.2768 | 166.7068 | 146.1758 | 146.2229 | 106.4846 | 147.8312 | 164.4613 | 148.2854 | 127.0219 | 108.9599 |
| CUST_22625_PI429010886 | N47352 | 0.008531 | 15 | 0 | | | | | | 361.7651 | 374.9864 | 316.4275 | 270.3119 | 301.0265 | 332.8163 | 258.4381 | 262.0228 | 230.1517 | 295.3859 | 276.5189 | 269.2795 | 201.7309 | 262.9561 | 278.6013 | 459.7686 | 347.794 | 322.8571 | 299.285 | 319.9685 | 293.3716 | 396.7445 | 256.8944 | 274.296 | 423.8598 | 297.5373 | 318.1453 |
| CUST_6636_PI429010886 | N20381 | 0.008534 | 21 | 18 | | | | | | 150.5251 | 206.5428 | 86.06426 | 161.4133 | 77.72793 | 77.90078 | 147.687 | 161.9593 | 123.3865 | 143.8077 | 107.638 | 152.3775 | 171.5473 | 111.9364 | 179.0014 | 331.5388 | 133.5378 | 170.3686 | 181.4398 | 227.9715 | 148.5892 | 141.5508 | 68.90417 | 122.7332 | 74.7266 | 124.6404 | 95.82559 |
| CUST_15353_PI429010886 | N34445 | 0.008534 | 24 | 0 | 383864629 | PREDICTED: uncharacterized protein LOC10088293 | | | 4.44E-07 | 13.7877 | 9.606473 | 21.82677 | 12.99442 | 12.08102 | 17.3497 | 6.21115 | 8.027473 | 16.42197 | 11.58688 | 6.228306 | 9.338175 | 3.208599 | 14.07538 | 10.70604 | 7.110255 | 10.14463 | 8.48246 | 8.2512 | 9.200881 | 7.50844 | 11.44906 | 15.64945 | 8.947846 | 16.31198 | 12.89288 | 15.44569 |
| CUST_32140_PI429010886 | M3339 | 0.008537 | 21 | 15 | | | | | | 28.13335 | 33.67567 | 31.47618 | 30.91161 | 26.37038 | 30.98507 | 25.47479 | 31.44965 | 19.2347 | 42.11302 | 23.73387 | 35.30186 | 27.40752 | 25.76451 | 33.3136 | 56.40373 | 32.29627 | 37.06438 | 34.04318 | 35.95633 | 37.60789 | 28.78903 | 19.73888 | 35.50744 | 34.75551 | 18.0096 | 29.99408 |
| CUST_51916_PI429010886 | M95682 | 0.008541 | 12 | 0 | 321470737 | putative histone deacetylase silent information regu | | | 2.88E-34 | 47.15656 | 44.42212 | 46.01155 | 38.05146 | 23.63855 | 21.55161 | 29.55843 | 34.01746 | 34.59277 | 38.79122 | 32.29997 | 35.50744 | 37.06061 | 61.32673 | 49.26562 | 45.40667 | 31.73369 | 29.6364 | 44.20422 | 35.30186 | 33.60838 | 32.83834 | 33.35513 | 31.19434 | 50.67859 | 33.17839 | 54.96227 |
| CUST_44740_PI429010886 | M66845 | 0.008556 | 18 | 3 | | | | | | 19.3883 | 37.74031 | 23.38605 | 53.66581 | 22.36658 | 39.90155 | 24.52916 | 19.11453 | 29.91175 | 20.19166 | 7.26746 | 16.56157 | 15.85862 | 28.99027 | 16.25775 | 22.34365 | 5.698946 | 29.89732 | 18.12837 | 14.63523 | 23.04991 | 41.20095 | 22.64343 | 34.32332 | 19.52364 | 45.49238 | 16.60676 |
| CUST_51844_PI429010886 | M95369 | 0.008562 | 18 | 6 | | | | | | 5.792463 | 7.641633 | 8.337419 | 6.101292 | 6.201123 | 18.29602 | 8.242842 | 8.224052 | NA | 8.135439 | 5.820473 | 6.678981 | 6.11207 | 10.19605 | 8.213559 | 7.151549 | 4.64454 | 7.90068 | 4.272415 | NA | 5.049855 | 9.606473 | 18.18164 | 11.25926 | 11.60477 | 20.79184 | 12.05344 |
| CUST_49240_PI429010886 | M84868 | 0.008588 | 24 | 21 | | | | | | 44.4501 | 36.8676 | 40.85343 | 40.65477 | 33.81235 | 45.49238 | 29.52396 | 21.26565 | 31.15277 | 35.96223 | 33.50292 | 26.10687 | 28.89154 | 29.64988 | 43.00468 | 55.22248 | 30.66153 | 50.0808 | 77.06228 | 43.7836 | 33.7538 | 61.02204 | 34.21609 | 44.55249 | 54.34709 | 23.59085 | 40.74434 |
| CUST_2880_PI429010886 | N13303 | 0.008588 | 15 | 12 | 260803744 | hypothetical protein BRAFLDRAFT_211873 [Branchi | | | 8.49E-42 | 126.6371 | 176.3364 | 119.3486 | 129.6562 | 95.84863 | 134.1962 | 156.539 | 126.4569 | 153.6882 | 162.4501 | 139.545 | 193.6848 | 213.5752 | 216.1505 | 215.9452 | 239.571 | 125.2377 | 167.5706 | 119.585 | 194.9477 | 91.24943 | 209.3879 | 126.7035 | 96.42858 | 162.9465 | 123.5279 | 159.6044 |
| CUST_24707_PI429010886 | N51124 | 0.008589 | 18 | 0 | | | | | | 84.22907 | 59.65312 | 52.58792 | 37.10843 | 38.78083 | 53.69723 | 65.01299 | 48.9688 | 43.87581 | 35.72924 | 32.09701 | 23.49179 | 34.9676 | 38.68605 | 32.81302 | 42.56152 | 57.54706 | 55.38718 | 50.01173 | 40.50555 | 52.69412 | 45.26792 | 29.76053 | 61.84039 | 42.52046 | 42.18909 | 37.49092 |
| CUST_32126_PI429010886 | M3294 | 0.008609 | 12 | 3 | 321471368 | hypothetical protein DAPPUDRAFT_316896 [Daphn | Protein metabolism | Proteolysis | 1.08E-28 | 197.0951 | 204.7416 | 257.5442 | 262.997 | 372.1299 | 391.346 | 387.0723 | 224.1509 | 299.389 | 347.7401 | 217.8838 | 215.7523 | 200.9848 | 262.2997 | 197.9029 | 455.8444 | 627.6184 | 414.9669 | 255.5333 | 142.5067 | 228.4677 | 239.9392 | 304.2796 | 233.2471 | 375.7452 | 212.8309 | 220.8169 |
| CUST_32172_PI429010886 | M3428 | 0.00862 | 18 | 3 | | | | | | 946.2082 | 830.6821 | 964.2217 | 939.7655 | 999.4148 | 926.1166 | 611.9605 | 737.0066 | 877.6784 | 1383.212 | 709.6744 | 936.7815 | 476.9743 | 793.8063 | 771.3694 | 566.9632 | 796.7421 | 828.1362 | 872.488 | 624.2921 | 492.0126 | 1589.601 | 1063.638 | 857.8725 | 784.2356 | 812.8751 | 711.9048 |
| CUST_34659_PI429010886 | M10269 | 0.008628 | 12 | 3 | | | | | | 622.7518 | 436.3193 | 340.867 | 358.4672 | 876.1774 | 890.3835 | 426.7007 | 398.993 | 392.8804 | 351.8276 | 277.9328 | 326.9265 | 369.8403 | 365.9198 | 175.7782 | 549.6818 | 523.4011 | 521.7265 | 336.4536 | 327.9176 | 387.0723 | 582.8028 | 298.3253 | 311.9883 | 520.1128 | 250.7705 | 403.0406 |
| CUST_40370_PI429010886 | M54605 | 0.008638 | 12 | 0 | | | | | | 211.9851 | 258.2663 | 271.4065 | 242.9704 | 202.4041 | 252.4954 | 169.9162 | 218.9512 | 199.1321 | 225.5499 | 172.436 | 336.4536 | 239.6393 | 288.1636 | 267.8414 | 285.1566 | 219.0917 | 231.8541 | 208.8792 | 259.2819 | 198.1233 | 242.3388 | 209.1269 | 206.3065 | 248.7474 | 289.8985 | 259.3271 |
| CUST_41768_PI429010886 | M58509 | 0.008643 | 18 | 0 | 255653874 | crustin 4 [Panulirus japonicus] | | | 9.33E-27 | 3534.122 | NA | 3225.426 | 546.5203 | 2608.992 | 1672.661 | 5162.076 | 201.008 | 40.48521 | 23.29568 | 2951.483 | 7.943105 | 104.7962 | 41.80062 | 38.04684 | NA | 6727.843 | 1338.117 | 5445.033 | NA | 3067.321 | NA | 4144.215 | 4110.832 | NA | 164.4613 | 5.263094 |
| CUST_41699_PI429010886 | M58342 | 0.008664 | 21 | 0 | | | | | | 113.9999 | 87.30486 | 97.70002 | 70.80146 | 84.08227 | 76.17525 | 77.39037 | 85.93471 | 67.5589 | 112.5615 | 75.31286 | 84.1757 | 85.30129 | 114.1927 | 87.68051 | 84.31618 | 91.21234 | 72.73775 | 93.73888 | 97.00878 | 94.63457 | 96.9916 | 143.2327 | 118.2591 | 120.0792 | 46.81599 | 69.55824 |
| CUST_53790_PI429010886 | M103204 | 0.008668 | 21 | 9 | | | | | | 1097.209 | 1296.822 | 1705.674 | 1350.099 | 1456.136 | 1461.877 | 1045.476 | 1943.851 | 1923.113 | 1736.508 | 2246.519 | 3332.072 | 1872.445 | 1787.33 | 1503.323 | 1506.006 | 1421.097 | 1407.359 | 1109.944 | 2199.321 | 1479.619 | 1564.24 | 1416.05 | 1287.748 | 1850.903 | 1628.664 | 2340.111 |
| CUST_3642_PI429010886 | N14949 | 0.00867 | 18 | 3 | | | | | | 491.1625 | 449.6922 | 490.2884 | 466.0826 | 677.5621 | 486.0261 | 535.1571 | 360.4157 | 360.5617 | 366.5663 | 507.4019 | 383.5597 | 322.4251 | 379.7153 | 405.3248 | 583.7272 | 353.689 | 474.912 | 504.6329 | 521.963 | 423.3887 | 668.1948 | 659.4054 | 738.6637 | 446.4087 | 287.6352 | 318.2412 |
| CUST_37135_PI429010886 | M17980 | 0.00867 | 15 | 0 | | | | | | 38.33099 | 56.8218 | 49.13878 | 56.34108 | 42.49492 | 76.353 | 46.85243 | 28.14633 | 44.07915 | 30.30557 | 41.71517 | 23.94321 | 32.01382 | 47.90107 | 17.04861 | 48.25771 | 56.76781 | 50.53472 | 40.44714 | 32.15263 | 50.34321 | 53.48774 | 35.07318 | 46.25026 | 35.01718 | 36.63495 | 28.37529 |
| CUST_43401_PI429010886 | M62945 | 0.008677 | 18 | 12 | | | | | | 107.319 | 102.2185 | 122.7034 | 93.60773 | 74.6525 | 50.98064 | 73.59863 | 59.25893 | 85.00815 | 81.87168 | 78.07189 | 114.3485 | 117.746 | 133.1932 | 126.3583 | 107.884 | 64.56424 | 128.623 | 130.5635 | 84.73278 | 104.5131 | 75.49081 | 147.0211 | 65.89913 | 100.64 | 79.043 | 61.40781 |
| CUST_13260_PI429010886 | N30867 | 0.008685 | 18 | 3 | 242016769 | pre-mRNA-splicing factor cwc25, putative [Pediculu | | | 4.94E-52 | 246.7846 | 347.9148 | 311.6128 | 498.8709 | 282.2712 | 349.8347 | 218.4011 | 174.3314 | 202.1175 | 271.0795 | 156.8398 | 308.3369 | 197.0135 | 258.6664 | 171.4799 | 153.6022 | 139.2667 | 256.333 | 161.6447 | 177.5136 | 347.3822 | 291.6879 | 406.5948 | 244.6702 | 322.3624 | 184.9825 | 194.5472 |
| CUST_14075_PI429010886 | N32286 | 0.00869 | 21 | 18 | | | | | | 16.7648 | 10.81881 | 15.66178 | 11.17512 | 14.94106 | 12.51404 | NA | 11.78251 | 9.25417 | 15.95324 | 6.88044 | 11.04906 | 11.4476 | 8.613049 | 12.86683 | 9.60066 | 10.644 | 13.36577 | 16.23152 | 32.363 | 14.43375 | 12.44135 | 16.64794 | 17.81187 | 10.49518 | 13.93866 | 15.42195 |
| CUST_49817_PI429010886 | M87287 | 0.008691 | 24 | 15 | | | | | | 163.162 | 394.958 | 136.8846 | 108.6787 | 189.0589 | 77.29645 | 16.31744 | 25.39871 | 67.44354 | 248.0682 | 173.3481 | 279.6215 | 152.0345 | 184.8121 | 185.7109 | 329.7729 | NA | 223.676 | 99.77775 | 338.1543 | 204.3871 | 314.3091 | 164.0893 | 112.8928 | 241.0332 | 141.4293 | 160.1541 |
| CUST_44267_PI429010886 | M65450 | 0.008701 | 24 | 15 | | | | | | 19.3376 | 19.68474 | 32.44398 | 12.77633 | 20.04541 | 23.30987 | 16.87203 | 14.82853 | 10.53483 | 20.84115 | 14.80809 | 25.97092 | 25.68589 | 30.91453 | 19.4114 | 34.98674 | 29.21516 | 25.91941 | 30.3942 | 17.9732 | 28.41298 | 17.74547 | 22.16055 | 36.91363 | 26.37834 | 24.00983 | 22.26808 |
| CUST_40813_PI429010886 | M56146 | 0.008707 | 21 | 18 | | | Nucleic acid metabolism | DNA metabolic process | | 11.69586 | 7.889839 | NA | 8.167815 | 8.435831 | 7.859342 | NA | 3.876515 | 11.54084 | 7.707868 | 4.128195 | 4.442782 | 5.4403 | 11.16751 | NA | 9.569411 | 5.38289 | 14.926 | NA | 11.60948 | 12.62711 | 13.79851 | 10.34377 | NA | NA | 11.14537 | 4.931371 |
| CUST_8878_PI429010886 | N23738 | 0.008709 | 24 | 3 | | | | | | 275.6931 | 10310.79 | 533.6685 | 18410.6 | 7950.432 | 8675.932 | 5477.513 | 6587.101 | 28.59435 | 308.7172 | 6511.817 | 414.1007 | 437.5902 | 3168.052 | 6085.653 | 126.1032 | 41.18311 | 124.5707 | 312.7841 | 981.8641 | 3132.563 | NA | 3384.084 | 3108.301 | NA | NA | 10.99269 |
| CUST_9746_PI429010886 | N25132 | 0.008715 | 15 | 9 | 546682401 | hypothetical protein D910_09660 [Dendroctonus p | | | 1.33E-43 | 43.27178 | 64.76438 | 64.95763 | 49.08084 | 69.79294 | 63.66464 | 57.81384 | 99.23417 | 110.5098 | 82.10096 | 98.08436 | 108.8762 | 83.92582 | 91.25978 | 52.93772 | 47.69448 | 62.53849 | 91.21234 | 45.53002 | 75.3452 | 72.7025 | 43.97029 | 69.63319 | 95.56359 | 79.20223 | 100.6058 | 113.9294 |
| CUST_36824_PI429010886 | M16956 | 0.008728 | 12 | 9 | | | | | | 46.19529 | 33.03817 | 44.16197 | 38.66452 | 32.42452 | 31.30264 | 41.06137 | 33.77253 | 41.48865 | 30.90472 | 47.04186 | 68.61072 | 62.54934 | 66.23254 | 35.77567 | 44.29399 | 36.02039 | 27.4118 | 20.42712 | 57.19607 | 61.08538 | 141.2899 | 109.2372 | 125.4528 | 51.24529 | 51.67197 | 53.02384 |
| CUST_15559_PI429010886 | N34783 | 0.008733 | 24 | 6 | | | | | | 54.35449 | 13.5398 | 98.41575 | 64.90973 | 22.51238 | 59.06999 | 93.35337 | 154.0476 | 126.3646 | 112.0774 | 12.74947 | 9.71557 | 58.60996 | 50.11342 | 38.91955 | 7.264223 | 45.15533 | 47.07665 | 33.11438 | 8.186201 | 8.194751 | 24.5772 | 71.16635 | 64.18045 | 53.86098 | 24.04426 | 35.43275 |
| CUST_18229_PI429010886 | N39391 | 0.008733 | 24 | 18 | | | | | | 51.2626 | 49.52106 | 26.50986 | 55.9772 | 44.58058 | 43.77809 | 42.37752 | 37.42936 | 30.85032 | 56.41974 | 40.29977 | 40.53866 | 43.34938 | 51.36601 | 38.91585 | 60.66856 | 48.09045 | 47.12699 | 50.32801 | 73.25256 | 77.7529 | 40.78513 | 50.77029 | 50.11342 | 51.47755 | 39.38726 | 50.65259 |
| CUST_25386_PI429010886 | N52326 | 0.008736 | 12 | 0 | 170819724 | reverse transcriptase [Daphnia pulex] | | | 1.69E-08 | 73.67107 | 64.10151 | 93.94852 | 67.47589 | 57.70169 | 67.73286 | 71.67319 | 76.11306 | 69.82734 | 86.61515 | 67.21241 | 66.99196 | 71.26057 | 99.57087 | 110.6986 | 56.13335 | 65.40248 | 93.43338 | 55.80731 | 76.43974 | 59.98068 | 82.9954 | 87.02844 | 84.87934 | 89.36758 | 103.4079 | 102.1823 |
| CUST_10784_PI429010886 | N26786 | 0.008757 | 21 | 18 | | | | | | 162.7985 | 197.2228 | 221.3126 | 221.06 | 178.147 | 254.5885 | 121.9844 | 197.8235 | 164.8298 | 183.6909 | 154.2025 | 298.7909 | 201.6936 | 248.2927 | 228.1106 | 331.4917 | 180.1444 | 256.5714 | 253.3366 | 257.7314 | 382.5164 | 295.5774 | 190.6724 | 210.9393 | 201.8776 | 158.6824 | 250.3612 |
| CUST_17114_PI429010886 | N37384 | 0.008757 | 24 | 15 | | | | | | 318.2985 | 290.8448 | 450.4975 | 381.4199 | 341.2566 | 369.8842 | 296.6312 | 405.6393 | 353.1722 | 484.5005 | 377.2506 | 472.4043 | 423.5928 | 489.338 | 356.7506 | 493.215 | 465.4504 | 559.6795 | 459.5005 | 430.2513 | 362.6784 | 444.0863 | 465.7878 | 479.5081 | 351.983 | 321.6396 | 478.2055 |

| Probe ID | ID | p-value | Period | Phase | EBI ID | Annotation | GO category | Go term | E-value | 0 | 0 | 0 | 3 | 3 | 3 | 6 | 6 | 6 | 9 | 9 | 9 | 12 | 12 | 12 | 15 | 15 | 15 | 18 | 18 | 18 | 21 | 21 | 21 | 24 | 24 | 24 |
|---|---|---|---|---|---|---|---|---|---|---|---|---|---|---|---|---|---|---|---|---|---|---|---|---|---|---|---|---|---|---|---|---|---|---|---|---|
| CUST_8141_PI429010886 | N22594 | 0.008759 | 18 | 9 | 328506975 | RuvB-like protein 2 [Penaeus monodon] | Nucleic acid metabolism | DNA metabolic process | 1.8E-93 | 39.58144 | 39.18988 | 37.0569 | 25.50065 | 54.24368 | 23.13968 | 38.09087 | 86.55946 | 110.3513 | 58.597 | 97.97139 | 139.7174 | 81.73063 | 40.59908 | 76.95909 | 56.39304 | 35.38465 | 43.81829 | 36.85933 | 50.42986 | 40.37514 | 70.36056 | 50.4975 | 28.64267 | 59.2457 | 36.42394 | 52.17339 |
| CUST_15797_PI429010886 | N35166 | 0.008767 | 15 | 0 | | | | | | 144.1866 | 163.1012 | 161.355 | 118.1115 | 122.3609 | 97.42055 | 119.1955 | 119.0242 | 108.2745 | 148.5892 | 106.1754 | 118.378 | 137.8924 | 121.0016 | 121.0512 | 160.9334 | 157.0634 | 125.0127 | 152.4677 | 157.1911 | 102.5545 | 134.8244 | 104.4835 | 113.475 | 139.7174 | 98.75692 | 128.7791 |
| CUST_48348_PI429010886 | M79948 | 0.008768 | 21 | 6 | 378556263 | O-methyltransferase [Litopenaeus vannamei] | Metabolic process | Other | 9.93E-27 | 116.3712 | 159.6044 | 127.0638 | 169.0201 | 250.0775 | 132.2265 | 184.6775 | 417.2965 | 359.1604 | 152.9711 | 301.7456 | 365.9198 | 244.2054 | 190.4186 | 179.2612 | 126.6828 | 167.2066 | 110.6336 | 184.4143 | 479.3073 | 230.2846 | 136.7329 | 140.3314 | 159.6292 | 143.1502 | 252.1291 | 170.1249 |
| CUST_21659_PI429010886 | N45609 | 0.008769 | 21 | 18 | | | | | | 12.67545 | 12.18439 | 20.35525 | NA | 10.82328 | 26.34227 | 5.889332 | 5.779406 | NA | 10.39055 | 3.439819 | 10.21711 | 9.731006 | 6.355532 | 20.55297 | 16.11943 | 8.052649 | 13.23323 | 24.41741 | 16.37858 | 20.02647 | 20.72239 | 7.034635 | 17.16641 | 7.807289 | 10.21823 | 13.04417 |
| CUST_50496_PI429010886 | M90011 | 0.008818 | 21 | 3 | | | | | | 147.2113 | 94.1523 | 143.9014 | 137.7352 | 110.9478 | 156.6286 | 135.061 | 132.4891 | 113.0059 | 139.47 | 92.74338 | 110.6199 | 93.81682 | 117.9048 | 123.2423 | 104.3886 | 90.22371 | 116.2211 | 131.9637 | 89.49881 | 100.1395 | 105.7372 | 182.4397 | 130.1396 | 136.7329 | 164.3174 | 135.5426 |
| CUST_11000_PI429010886 | N27109 | 0.008823 | 24 | 18 | | | | | | 154.6585 | 157.7092 | 206.9547 | 99.89328 | 104.6405 | 142.0394 | 161.1033 | 150.7034 | 159.5233 | 91.36505 | 189.3158 | 244.7282 | 134.1962 | 233.3995 | 179.7697 | 249.1317 | 170.6697 | 207.0705 | 254.095 | 190.6989 | 217.0982 | 200.5357 | 154.2903 | 123.8281 | 230.8271 | 132.1519 | 194.8405 |
| CUST_30952_PI429010886 | M294 | 0.008827 | 12 | 3 | | | | | | 843.1506 | 843.5117 | 463.3861 | 880.6305 | 715.6525 | 987.5052 | 748.0078 | 567.2204 | 621.5625 | 530.8519 | 665.511 | 542.7169 | 715.8969 | 522.9557 | 563.9632 | 844.642 | 1005.713 | 776.1766 | 879.3705 | 607.9318 | 544.6485 | 653.8395 | 503.4817 | 689.3131 | 695.7952 | 598.9907 | 412.5292 |
| CUST_53445_PI429010886 | M101732 | 0.008859 | 12 | 0 | | | | | | 75.70522 | 73.64901 | 53.63782 | 83.383 | 77.21633 | 71.81025 | 41.47082 | 63.66464 | 58.36968 | 83.39175 | 61.32192 | 60.97776 | 96.05386 | 73.15736 | 62.5561 | 68.95198 | 64.20475 | 46.11558 | 46.58823 | 43.62391 | 61.41511 | 103.6651 | 66.97079 | 74.15113 | 113.2976 | 78.55433 | 111.0395 |
| CUST_46593_PI429010886 | M73096 | 0.008862 | 21 | 15 | | | | | | 36.6789 | 46.37163 | 35.36774 | 31.60035 | 46.66745 | 34.82515 | 21.55161 | 23.16293 | 26.15331 | 56.71651 | 41.30947 | 34.62398 | 29.88134 | 38.02052 | 32.45773 | 70.3766 | 43.70451 | 48.08498 | 35.53033 | 61.46235 | 40.57649 | 31.277 | 41.87571 | 26.27126 | 55.99321 | 22.64182 | 41.41686 |
| CUST_18704_PI429010886 | N40256 | 0.008862 | 18 | 3 | | | | | | 3711.262 | 5769.917 | 5125.683 | 9864.87 | 5717.187 | 6026.937 | 4036.57 | 3218.113 | 4116.427 | 3092.843 | 2304.824 | 5133.687 | 2506.708 | 5853.91 | 2743.533 | 3876.188 | 1846.987 | 2604.753 | 2354.203 | 2975.526 | 6468.264 | 5819.009 | 6314.548 | 2890.576 | 5888.291 | 4031.594 | 3713.677 |
| CUST_30586_PI429010886 | N62174 | 0.00887 | 24 | 18 | | | | | | 17.83632 | 4.590232 | 38.33099 | 4.39117 | 5.853359 | 31.90127 | 33.84879 | 9.960996 | 8.00182 | 48.31638 | 49.74933 | 46.36775 | 19.07608 | 20.93093 | 23.96204 | 20.14785 | 24.83878 | 32.45453 | 42.96811 | 75.96227 | 76.59109 | 77.91084 | 53.21879 | 46.69563 | 30.40511 | 56.50237 | 22.14748 |
| CUST_40276_PI429010886 | M52376 | 0.008872 | 21 | 3 | | | | | | 17.69032 | 30.37957 | 20.1154 | 22.6112 | 35.55848 | 30.50322 | 40.50125 | 7.139757 | 25.03495 | 10.15754 | 18.05344 | 36.387 | 10.9288 | 16.50096 | 6.593457 | 9.02447 | 6.531816 | 44.95443 | 10.89307 | 14.40255 | 29.88417 | 20.35525 | 40.71531 | 48.53823 | 31.93333 | 35.48686 | 54.24943 |
| CUST_22030_PI429010886 | N46299 | 0.008885 | 21 | 0 | | | | | | 47.06225 | 52.71843 | 62.12557 | 46.64901 | 40.13905 | 34.95466 | 29.24887 | 14.91902 | 27.49986 | 24.30558 | 35.88091 | 23.05482 | 35.02438 | 27.97666 | 28.743 | 41.34285 | 13.79507 | 26.01832 | 22.18079 | 26.55476 | 27.42777 | 44.91724 | 30.112 | 38.32192 | 36.07548 | 16.19477 | 35.56458 |
| CUST_17444_PI429010886 | N37974 | 0.008886 | 15 | 0 | | | | | | 72.79951 | 88.3275 | 57.48318 | 78.93037 | 43.43161 | 37.02998 | 39.00627 | 64.29604 | 36.81664 | 61.5685 | 57.18723 | 68.25944 | 70.54693 | 70.42582 | 67.71039 | 74.08523 | 58.57388 | 81.2413 | 48.74628 | 86.05551 | 68.29796 | 61.45036 | 51.04982 | 54.17626 | 49.72048 | 52.26577 | 69.36484 |
| CUST_57242_PI429010886 | E2832 | 0.00891 | 18 | 9 | 56118690 | ribonucleotide reductase M2, gene 2 [Xenopus (Silu | Metabolic process | Nucleobase-containing compound metabolic process | 8.78E-19 | 34.93327 | 57.12722 | 21.96294 | 41.57514 | 34.25171 | NA | 46.98145 | 67.68545 | 63.67902 | 97.30444 | 45.7637 | 64.15379 | 90.23885 | 52.95164 | 59.21305 | 82.53975 | 48.42693 | 25.72198 | 42.09877 | 47.95255 | 44.62034 | 43.363 | 39.40978 | 28.87669 | 96.36676 | 14.66602 | 71.45222 |
| CUST_24802_PI429010886 | N51272 | 0.008917 | 15 | 0 | | | | | | 24.51944 | NA | 20.45683 | 7.61532 | 9.59598 | 20.06979 | 11.12035 | 9.558027 | 7.42944 | 9.678005 | 7.393056 | 7.109363 | 12.43401 | 8.788925 | 13.45887 | 17.99612 | 21.17293 | 23.15281 | 10.49848 | 11.10065 | 8.865999 | 6.479275 | 20.22452 | 16.86851 | 9.142203 | 9.853418 | 13.48586 |
| CUST_15885_PI429010886 | N35297 | 0.008931 | 21 | 12 | 444707405 | Fez family zinc finger protein 2 [Tupaia chinensis] | | | 4.69E-09 | 46.71096 | 23.74918 | 26.34508 | 10.62697 | 11.51911 | 20.35525 | 19.78961 | 29.02946 | 14.22674 | 36.49611 | 27.26909 | 24.79862 | 33.5333 | 20.84115 | 29.0899 | 27.50644 | 23.82334 | 17.21639 | 30.21631 | 44.52812 | 18.09635 | 17.07004 | 37.56088 | 13.30229 | 15.71416 | 13.49109 | 15.15061 |
| CUST_30350_PI429010886 | N61689 | 0.008931 | 18 | 12 | | | | | | 95.63731 | 113.8196 | 101.3667 | 61.50815 | 73.3681 | 68.95801 | 80.38356 | 86.07302 | 88.66033 | 106.3744 | 62.63151 | 93.71752 | 114.787 | 104.0321 | 116.332 | 123.0707 | 79.66167 | 64.6187 | 96.74817 | 85.41552 | 63.99533 | 104.8657 | 44.14799 | 72.22304 | 78.59764 | 106.7749 | 77.37108 |
| CUST_14726_PI429010886 | N33358 | 0.008934 | 21 | 3 | 405976720 | Terminal uridylyltransferase 4 [Crassostrea gigas] | | | 1.7E-19 | 20.53115 | 11.62339 | NA | 30.43159 | 19.91373 | 10.16697 | 24.35938 | 17.71272 | 11.97234 | 20.72239 | 10.41586 | 9.027092 | 17.25976 | 23.46752 | 10.72726 | 10.35813 | 8.198298 | 10.65599 | 16.27412 | 15.8263 | 17.18028 | 19.76503 | 9.672443 | 17.24927 | 23.16735 | 24.17916 | 21.25627 |
| CUST_31253_PI429010886 | M1084 | 0.008944 | 21 | 3 | 321478941 | hypothetical protein DAPPUDRAFT_219955 [Daphni | | | 6.14E-28 | 212.8057 | 350.6566 | 249.3844 | 372.2873 | 225.7224 | 263.4861 | 221.7682 | 207.1975 | 195.9288 | 260.9034 | 183.2331 | 328.4958 | 160.6252 | 249.4607 | 161.4564 | 244.4592 | 137.4175 | 177.2148 | 177.7855 | 250.7705 | 329.0813 | 249.3844 | 298.8597 | 202.438 | 288.1636 | 322.2737 | 272.3366 |
| CUST_133_PI429010886 | N1323 | 0.00895 | 21 | 0 | 321464825 | hypothetical protein DAPPUDRAFT_306600 [Daphn | Protein metabolism | Cellular protein modification process | 1.8E-97 | 519.4558 | 514.8406 | 499.7486 | 421.6972 | 425.4184 | 496.7325 | 475.032 | 414.8654 | 391.346 | 421.3888 | 412.0745 | 391.4581 | 415.3866 | 449.5149 | 509.7336 | 411.1038 | 502.3399 | 445.7286 | 464.6636 | 332.753 | 361.4417 | 495.7993 | 490.9356 | 504.949 | 452.6313 | 436.8338 | 363.0918 |
| CUST_42132_PI429010886 | M59493 | 0.008971 | 12 | 0 | | | | | | 71.80124 | 108.8513 | 131.6163 | 116.1332 | 103.0771 | 95.04998 | 68.82889 | 89.90347 | 84.51934 | 77.57474 | 89.59424 | 83.32632 | 93.62063 | 120.6514 | 101.6576 | 97.17307 | 86.57696 | 90.08902 | 94.69116 | 80.49518 | 76.83746 | 98.41575 | 104.4202 | 102.6772 | 126.7035 | 102.2659 | 102.4647 |
| CUST_14539_PI429010886 | N33048 | 0.008974 | 21 | 18 | | | | | | 33.19476 | 42.29841 | 33.00184 | 19.3883 | 24.88909 | 35.63872 | 20.73682 | 20.62099 | 18.41342 | 29.44853 | 14.79157 | 112.064 | 20.49973 | 26.19976 | 38.24021 | 30.53018 | 30.17815 | 27.33369 | 40.66714 | 34.94529 | 29.41742 | 24.84953 | 30.06426 | 33.1299 | 26.8901 | 29.04567 | 29.32849 |
| CUST_46524_PI429010886 | M72897 | 0.008979 | 12 | 3 | 131573117 | Mesenchytraeus solifugus alpha tubulin a2 mRNA, | Cellular component organization | Cytoskeleton organization | 3.16E-67 | 661.6789 | 611.3709 | 331.8125 | 906.5131 | 728.4044 | 789.7368 | 648.9401 | 562.1725 | 528.618 | 484.2538 | 630.1031 | 332.1642 | 581.1144 | 449.1802 | 318.6212 | 602.4628 | 733.9745 | 778.2204 | 744.5402 | 383.1748 | 622.5542 | 725.0021 | 377.6304 | 766.1345 | 472.4043 | 435.3919 | 390.5304 |
| CUST_9243_PI429010886 | N24289 | 0.008982 | 18 | 3 | 157169410 | dopamine beta hydroxylase [Aedes aegypti] >gi\|10 | Metabolic process | Cellular amino acid metabolic process | 7.77E-24 | 386.5159 | 320.3749 | 340.1906 | 483.4448 | 402.4861 | 327.9176 | 401.3173 | 324.0592 | 281.735 | 312.6532 | 233.6069 | 207.0425 | 241.5575 | 335.74 | 270.0349 | 417.2023 | 420.9194 | 311.4551 | 443.7417 | 233.3267 | 319.1396 | 600.9036 | 324.4624 | 345.8615 | 356.6027 | 260.9414 | 210.3675 |
| CUST_44746_PI429010886 | M66862 | 0.008985 | 21 | 18 | | | | | | 112.5793 | 95.28234 | 105.7372 | 98.39109 | 109.5048 | 75.3452 | 83.27348 | 94.69116 | 76.22636 | 106.097 | 90.54311 | 90.62378 | 84.63515 | 124.9376 | 99.17539 | 125.9169 | 101.7152 | 91.05924 | 103.5565 | 137.4175 | 129.1185 | 104.7461 | 109.0449 | 85.05317 | 128.6038 | 76.61613 | 108.6469 |
| CUST_33302_PI429010886 | M6543 | 0.009007 | 24 | 18 | | | | | | 337.1609 | 362.6784 | 336.2223 | 270.8602 | 314.0114 | 291.3327 | 246.1025 | 256.1116 | 273.2194 | 345.2409 | 293.0543 | 360.7942 | 321.1502 | 426.1051 | 404.2573 | 439.659 | 298.2497 | 313.8962 | 319.448 | 482.8729 | 312.9412 | 456.0552 | 358.55 | 310.2077 | 390.1389 | 295.5186 | 326.8661 |
| CUST_30721_PI429010886 | N62475 | 0.009012 | 18 | 9 | | | | | | 95.9511 | 117.9497 | 108.5779 | 79.62582 | 93.94852 | 78.90095 | 123.0412 | 107.955 | 136.3105 | 55.14182 | 126.6371 | 129.9538 | 109.6686 | 128.1445 | 123.1377 | 118.3064 | 104.7362 | 120.4315 | 93.81682 | 122.949 | 81.03057 | 97.24592 | 108.9872 | 64.6187 | 116.3712 | 79.15691 | 127.3036 |
| CUST_5294_PI429010886 | N18504 | 0.009025 | 12 | 3 | | | | | | 755.8427 | 2541.147 | 101.9359 | 4131.811 | 2229.117 | 1856.885 | 1847.968 | 123.2423 | 1715.043 | 116.9944 | 71.09841 | 81.42898 | 396.0145 | 752.1706 | 1853.071 | 3883.492 | 163.051 | 2615.876 | 161.2028 | 299.389 | 109.2818 | 2306.592 | 66.022 | 1228.27 | 1040.536 | 183.6281 | 291.7598 |
| CUST_56701_PI429010886 | M114471 | 0.009039 | 24 | 0 | | | | | | 476.7276 | 407.3532 | 601.0454 | 364.6302 | 483.2456 | 318.7622 | 424.5435 | 373.896 | 448.0031 | 498.6758 | 428.6284 | 422.3809 | 374.5146 | 359.8853 | 380.0505 | 476.8506 | 393.8505 | 371.9875 | 406.6708 | 350.2055 | 505.2828 | 446.8256 | 576.6597 | 413.3489 | 635.6512 | 512.9488 | 648.287 |
| CUST_51499_PI429010886 | M93736 | 0.009039 | 24 | 0 | | | | | | 102.1933 | 124.4949 | 121.6835 | 94.25565 | 111.4345 | 101.2406 | 79.61927 | 59.55084 | 100.6811 | 80.61916 | 74.97194 | 97.40947 | 39.80101 | 64.47443 | 46.19956 | 108.3037 | 58.81306 | 50.21157 | 98.62916 | 70.00254 | 135.8973 | 47.41387 | 75.85953 | 65.84223 | 147.4736 | 61.96906 | 79.36014 |
| CUST_1713_PI429010886 | N10509 | 0.009053 | 15 | 12 | | | | | | 914.6851 | 1761.883 | 1325.228 | 690.6945 | 790.3406 | 860.1543 | 610.2139 | 637.6126 | 635.6512 | 1269.191 | 920.0256 | 1165.381 | 1133.023 | 1459.126 | 1599.783 | 1261.977 | 867.8685 | 877.6784 | 1462.44 | 1062.278 | 1220.236 | 1012.95 | 1155.687 | 1015.362 | 1373.036 | 1076.944 | 1428.874 |
| CUST_24989_PI429010886 | N51593 | 0.009053 | 15 | 12 | | | | | | 16.22239 | 26.98251 | 25.06956 | 16.39729 | 21.62429 | 17.97978 | 18.24494 | 15.39298 | 19.33417 | 28.49553 | 25.16709 | 36.04028 | 27.92219 | 35.82359 | 36.04028 | 29.32033 | 19.80461 | 30.25328 | 22.8759 | 17.03078 | 31.02055 | 32.00727 | 19.37234 | 22.06655 | 23.30384 | 25.41357 | 32.75472 |
| CUST_33576_PI429010886 | M7318 | 0.009057 | 21 | 0 | | | | | | 56.30906 | 46.71096 | 33.3468 | 43.56595 | 24.8201 | 30.9626 | 30.72063 | 36.53812 | 26.97875 | 45.22744 | 28.01868 | 27.384 | 40.42785 | 21.61291 | 24.72623 | 48.72124 | 37.52322 | 27.07492 | 40.76277 | 47.32005 | 30.88328 | 56.91814 | 39.08829 | 49.67248 | 36.90619 | 39.71872 | 30.26846 |
| CUST_55221_PI429010886 | M109172 | 0.009058 | 24 | 21 | 431895738 | 5'-AMP-activated protein kinase subunit gamma-2 [ | | | 5.25E-08 | 49.81001 | 30.96855 | 39.75659 | 39.61454 | 35.1685 | 37.48336 | 34.4458 | 29.54213 | 27.45261 | 103.7009 | 32.70652 | 33.72458 | 32.9302 | 48.99962 | 48.80089 | 39.45879 | 26.48449 | 32.3787 | 35.11564 | 66.13621 | 37.40747 | 73.35296 | 54.94382 | 70.02979 | 72.44795 | 93.56818 | 44.02245 |
| CUST_11253_PI429010886 | N27517 | 0.009058 | 24 | 21 | | | | | | 28.58408 | 17.74945 | 19.3208 | 13.85139 | 23.13337 | 8.73506 | 14.70218 | 13.94862 | 14.47943 | 13.72321 | 19.07608 | 26.33364 | 20.69763 | 16.08792 | 18.20618 | 19.26372 | 14.89947 | 24.78255 | 24.17334 | 24.69594 | 18.89688 | 29.9042 | 20.80589 | 24.16612 | 23.02707 | 20.05683 | 16.95326 |
| CUST_55735_PI429010886 | M111521 | 0.009075 | 18 | 9 | | | | | | 22.61839 | 37.78218 | 18.83139 | 66.46046 | 83.87586 | 37.0175 | 91.66231 | 214.7684 | 137.2358 | 62.75575 | 157.4775 | 140.8976 | 116.1033 | 40.53866 | 50.12589 | 8.786108 | 68.93293 | 3.958611 | 12.41359 | 259.2052 | 82.29118 | 25.30072 | 87.53485 | 85.24568 | 9.593653 | 99.22324 | 110.2887 |
| CUST_19694_PI429010886 | N42023 | 0.009098 | 21 | 15 | | | | | | 59.48243 | 67.69277 | 65.88576 | 43.81481 | 46.65817 | 43.67532 | 49.21663 | 57.60743 | 56.94451 | 76.03079 | 62.02406 | 87.88231 | 50.5231 | 43.82673 | 59.65312 | 67.45213 | 77.98525 | 69.04216 | 73.12656 | 44.24444 | 60.38361 | 69.93057 | 87.6112 | 80.38356 | 60.13681 | 44.18564 | 36.61393 |
| CUST_34689_PI429010886 | M10357 | 0.009101 | 18 | 15 | | | | | | 192.8746 | 197.1816 | 196.9798 | 156.4282 | 167.2309 | 179.5282 | 182.965 | 177.4241 | 141.5508 | 186.3153 | 175.1085 | 234.8913 | 229.245 | 213.6068 | 195.4236 | 261.1858 | 217.7746 | 220.7455 | 218.804 | 257.3277 | 116.6217 | 228.5019 | 171.9322 | 139.6239 | 235.7566 | 172.9784 | 211.0494 |
| CUST_4724_PI429010886 | N17348 | 0.009101 | 18 | 15 | | | | | | 167.8053 | 120.3098 | 113.7768 | 107.8082 | 74.40209 | 77.33177 | 104.3742 | 81.2413 | 121.9963 | 117.4327 | 90.02313 | 88.05483 | 125.8218 | 98.98439 | 112.1069 | 157.6076 | 117.4728 | 114.2883 | 102.639 | 99.31284 | 105.6351 | 80.75638 | 77.3067 | 101.3443 | 100.2191 | 119.2791 | 69.09575 |
| CUST_18440_PI429010886 | N39804 | 0.009118 | 21 | 0 | | | | | | 37.84521 | 22.16225 | 29.63396 | 21.29581 | 40.67008 | 21.79421 | 45.7065 | 5.269474 | 15.42195 | 14.4735 | 11.82579 | 20.86339 | 11.53244 | 13.95459 | 23.21071 | 36.69997 | 24.07489 | 19.90878 | 28.48649 | 13.07517 | 20.14149 | 31.25517 | 45.3644 | 27.28673 | 20.66168 | 34.71929 | 28.66257 |
| CUST_10747_PI429010886 | N26721 | 0.00912 | 15 | 12 | | | | | | 252.0389 | 233.0458 | 358.4672 | 180.8117 | 213.4663 | 173.3934 | 250.587 | 369.1054 | 407.6622 | 399.8374 | 244.9337 | 308.0149 | 331.1092 | 473.1073 | 345.639 | 285.2001 | 327.6399 | 225.5895 | 262.4225 | 277.9328 | 269.7651 | 299.389 | 317.9758 | 178.1983 | 593.1266 | 271.4416 | 479.6761 |
| CUST_23520_PI429010886 | N48973 | 0.009123 | 15 | 0 | | | | | | 114.7256 | 141.2899 | 124.2426 | 123.7165 | 127.5671 | 110.5384 | 68.69041 | 82.47663 | 68.25442 | 77.84895 | 127.1579 | 106.872 | 85.50355 | 78.00387 | 64.20475 | 212.0939 | 146.0474 | 97.25472 | 131.1671 | 91.58478 | 84.1835 | 58.81306 | 98.95253 | 96.37601 | 102.8709 | 86.3602 | 81.72278 |
| CUST_15010_PI429010886 | N33856 | 0.009124 | 21 | 3 | | | | | | 13.30659 | 14.15692 | 36.85229 | 26.14767 | 31.28349 | 39.06616 | 19.42689 | 20.56778 | 28.92063 | 25.97958 | 26.99024 | 43.16246 | 19.84447 | 25.26607 | 24.61812 | 16.81795 | 19.73697 | 18.30186 | 20.21114 | 24.97999 | 14.38818 | 26.78191 | 24.80361 | 13.36864 | 25.37889 | 25.62143 | 42.09877 |
| CUST_47871_PI429010886 | M77961 | 0.009124 | 12 | 9 | 213950420 | nitric oxide synthase [Penaeus monodon] | Metabolic process | Oxidation-reduction process | 2.87E-43 | 69.64081 | 50.97047 | 79.26902 | 44.47869 | 43.44981 | 10.27942 | 28.80255 | 53.02384 | 61.82662 | 73.803 | 48.02359 | 94.63457 | 54.28784 | 65.95253 | 64.04953 | 46.72383 | 39.14518 | 45.00065 | 64.40125 | 60.0207 | 69.19616 | 71.32452 | 87.94324 | 31.52633 | 96.55629 | 45.45728 | 65.39564 |
| CUST_19307_PI429010886 | N41357 | 0.00915 | 24 | 0 | 322779472 | hypothetical protein SINV_07997 [Solenopsis invict | | | 1.04E-11 | 46.27883 | 35.98369 | 49.81358 | 32.68104 | 34.46849 | 44.61606 | 38.35721 | 29.63867 | 22.49197 | 51.28378 | 27.9866 | 21.7968 | 31.19434 | 20.55898 | 28.64539 | 35.98798 | 44.05675 | 35.11191 | 21.44644 | 37.35904 | 44.331 | 52.034 | 33.49853 | 28.93067 | 55.90718 | 46.20996 | 34.86394 |
| CUST_43874_PI429010886 | M64208 | 0.009157 | 15 | 12 | 321463591 | hypothetical protein DAPPUDRAFT_307190 [Daphn | | | 1.84E-29 | 10.96072 | 14.95429 | 11.36478 | 12.77316 | 9.428784 | NA | 5.732347 | 6.779829 | 4.591596 | 10.38108 | 11.21679 | 10.84227 | 24.97999 | NA | 10.42904 | 16.07261 | 8.771173 | 9.928945 | 5.920001 | 13.83521 | NA | 10.41462 | NA | 5.069505 | 8.687252 | 11.22853 | 15.27919 |
| CUST_29077_PI429010886 | N59152 | 0.009165 | 24 | 0 | | | | | | 38.23666 | 42.50508 | 39.18988 | 51.50234 | 30.72417 | 32.68104 | 27.61589 | 27.71021 | 25.92266 | 47.27187 | 42.41594 | 36.7523 | 25.63163 | 21.61979 | 18.12837 | 36.50859 | 22.89148 | 29.9971 | 14.20045 | 27.56643 | 29.61255 | 35.04798 | 34.51949 | 29.00151 | 47.57729 | 26.38969 | 45.77355 |
| CUST_41687_PI429010886 | M58310 | 0.009172 | 24 | 15 | | | | | | 169.1571 | 183.6032 | 146.902 | 135.6966 | 155.5309 | 139.3127 | 100.5206 | 125.4314 | 129.3867 | 196.9122 | 146.585 | 135.0274 | 121.1828 | 156.4704 | 163.7139 | 238.5428 | 169.275 | 192.7273 | 157.8626 | 179.5033 | 146.0295 | 188.1392 | 154.0476 | 141.0077 | 232.2704 | 162.3514 | 214.1014 |
| CUST_25966_PI429010886 | N53401 | 0.009172 | 24 | 12 | | | | | | 209.1953 | 251.404 | 333.9444 | 187.276 | 260.1739 | 318.4396 | 219.0379 | 328.0426 | 396.0145 | 349.8347 | 261.922 | 323.8952 | 450.7936 | 496.875 | 421.125 | 276.7604 | 509.3966 | 431.3053 | 376.1162 | 279.6215 | 462.321 | 448.7865 | 448.199 | 233.2127 | 392.2104 | 308.2184 | 352.7559 |
| CUST_37968_PI429010886 | M22502 | 0.009175 | 18 | 0 | | | | | | 89.56339 | NA | 117.9497 | 41.78277 | 56.19324 | 82.50766 | 65.3527 | 31.84731 | 12.6619 | 15.09234 | 59.94958 | NA | 12.14661 | 8.624589 | 6.20008 | 5.453763 | 102.7028 | 35.23447 | 94.50596 | NA | 36.05134 | NA | 43.46213 | 50.32404 | NA | 11.55275 | 4.262104 |
| CUST_39399_PI429010886 | M38859 | 0.009182 | 21 | 18 | | | | | | 210.676 | 339.8212 | 206.7246 | 164.1481 | 243.718 | 188.3767 | 144.9176 | 101.2406 | 175.8238 | 206.1326 | 186.6799 | 365.1838 | 206.6019 | 232.3859 | 265.0674 | 391.0528 | 115.2692 | 297.7533 | 235.4286 | 244.1166 | 241.0846 | 302.4151 | 203.453 | 199.2344 | 240.2928 | 186.0312 | 209.7275 |
| CUST_5758_PI429010886 | N19159 | 0.009213 | 18 | 12 | | | | | | 56.99104 | 51.74182 | 55.16975 | 45.63167 | 55.33057 | 46.41914 | 54.04881 | 38.54256 | 28.99859 | 76.04812 | 52.70775 | 55.16975 | 89.20945 | 58.83776 | 71.77967 | 81.89181 | 53.89507 | 56.19324 | 51.04482 | 74.34861 | 41.02914 | 83.20205 | 49.09733 | 39.21751 | 67.1799 | 38.34336 | 49.21104 |
| CUST_28096_PI429010886 | N57291 | 0.009214 | 18 | 15 | | | | | | 10.90286 | 14.29674 | 9.035702 | 8.772272 | NA | 9.593653 | 3.936096 | 6.95796 | 6.374853 | 10.6198 | 12.3974 | NA | 6.022117 | 18.94687 | NA | 7.585958 | 20.20612 | 11.05595 | 8.883037 | 10.44304 | 6.115219 | 6.530509 | 8.045043 | NA | 4.53374 | NA | 6.21618 |
| CUST_5196_PI429010886 | N18387 | 0.009214 | 15 | 9 | 383860504 | PREDICTED: 7SK snRNA methylphosphate capping e | | | 3.18E-82 | 155.979 | 162.2752 | 160.3693 | 111.4345 | 115.4469 | 134.5558 | 115.8704 | 159.2049 | 164.3967 | 168.9219 | 150.5005 | 227.4117 | 149.8536 | 229.0179 | 181.7953 | 163.7445 | 124.9376 | 144.6167 | 133.4505 | 167.3467 | 144.77 | 181.5403 | 208.3478 | 118.3504 | 221.5021 | 160.5552 | 204.629 |
| CUST_34443_PI429010886 | M9692 | 0.009221 | 18 | 9 | | | | | | 86.67105 | 100.2834 | 97.63395 | 78.78492 | 89.70412 | 80.62509 | 90.45788 | 244.8254 | 157.7938 | 95.13953 | 511.4224 | 563.7899 | 187.0999 | 84.85912 | 170.7553 | 100.6058 | 123.2747 | 73.15736 | 81.12186 | 613.2651 | 134.3489 | 87.19276 | 95.81155 | 74.69976 | 114.1483 | 108.2888 | 180.9607 |
| CUST_16086_PI429010886 | N35631 | 0.009221 | 18 | 15 | | | | | | 734.7434 | 1051.932 | 625.8341 | 911.24 | 678.7202 | 716.5268 | 643.9207 | 640.6647 | 553.6437 | 656.7764 | 665.3114 | 799.8512 | 709.3475 | 554.3622 | 715.6525 | 1227.773 | 774.0266 | 850.7537 | 789.4136 | 913.4609 | 604.5341 | 1111.525 | 581.9863 | 746.1542 | 690.4518 | 635.2846 | 538.4051 |
| CUST_27476_PI429010886 | N56125 | 0.009221 | 18 | 15 | | | | | | 26.51868 | 31.70016 | 31.78686 | 22.43908 | 22.59328 | 22.19563 | 23.59085 | 18.02463 | 23.91945 | 24.24502 | 32.62278 | 27.96355 | 21.2878 | 27.0727 | 24.983 | 34.40003 | 68.05219 | 28.19347 | 32.87841 | 25.80821 | 13.31013 | 24.35681 | 17.64462 | 22.42981 | 38.28633 | 20.86742 | 21.33598 |
| CUST_28642_PI429010886 | N58342 | 0.009221 | 18 | 9 | 260833120 | hypothetical protein BRAFLDRAFT_63860 [Branchio | | | 2.51E-10 | 82.65719 | 25.41871 | 79.55126 | 34.04318 | 49.5373 | 40.13905 | 36.00421 | 78.101 | 85.17215 | 152.8609 | 31.99517 | 90.08902 | 69.08039 | 56.38454 | 77.5823 | 51.67859 | 53.46776 | 71.91814 | 31.65825 | 58.40782 | 62.3113 | 40.43694 | 23.1013 | 23.94659 | 69.82119 | 57.59047 | 75.56946 |
| CUST_30297_PI429010886 | N61574 | 0.009221 | 18 | 15 | | | | | | 18.52264 | 31.95868 | 52.15768 | 16.35532 | 13.96684 | 14.06235 | 13.68648 | 32.42053 | 14.71926 | 32.44657 | 20.07209 | 27.22484 | 30.18478 | 41.22879 | 30.57659 | 31.30771 | 33.97728 | 27.9603 | 32.79355 | 18.37554 | 28.17154 | 22.96293 | 28.43068 | 14.93734 | 29.81183 | 22.55992 | 27.8201 |
| CUST_3308_PI429010886 | N14229 | 0.009241 | 15 | 12 | | | | | | 101.4752 | 108.048 | 134.4594 | 87.97637 | 107.7388 | 87.10078 | 90.31563 | 105.0471 | 114.4714 | 117.847 | 113.9832 | 185.0712 | 106.8104 | 136.4498 | 137.8443 | 132.88 | 119.1161 | 145.4832 | 96.92806 | 91.90153 | 118.3064 | 132.9281 | 108.4314 | 94.85349 | 160.0108 | 102.7524 | 143.6702 |
| CUST_20422_PI429010886 | N43410 | 0.009241 | 15 | 9 | | | | | | 75.62563 | 88.40608 | 70.43457 | 34.29142 | 69.72451 | 67.79099 | 85.60322 | 71.32452 | 77.49116 | 86.46205 | 70.52611 | 77.3067 | 66.33339 | 73.54244 | 74.3404 | 83.66854 | 59.90035 | 63.04603 | 61.69829 | 59.84297 | 67.02702 | 78.73486 | 74.99621 | 71.38963 | 84.46837 | 68.34468 | 93.38696 |
| CUST_10539_PI429010886 | N26390 | 0.00927 | 21 | 15 | | | | | | 24.04426 | 18.69874 | 23.01108 | 14.1847 | 22.22589 | 23.26561 | 16.50225 | 9.606473 | 15.78638 | 16.01307 | 13.43446 | 6.598712 | 20.71812 | 14.19691 | 15.79004 | 21.25027 | 28.09437 | 29.82479 | 25.71973 | 19.96799 | 23.62598 | 18.88759 | 19.15208 | 13.532 | 39.0395 | 18.53665 | 33.39183 |
| CUST_27164_PI429010886 | N55561 | 0.009273 | 24 | 0 | | | | | | 27.60563 | 24.58762 | 12.73533 | 21.14763 | 24.09666 | 21.18978 | 15.31157 | 18.65486 | 14.18148 | 9.179429 | 13.04197 | 6.599438 | 13.74195 | 11.54499 | 21.70738 | 23.20138 | 28.28914 | 14.72118 | 20.69091 | 24.135 | 14.90361 | 34.15487 | 14.90751 | 16.59282 | 31.98439 | 16.90499 | 15.56439 |
| CUST_617_PI429010886 | N6342 | 0.009288 | 21 | 6 | 528746117 | C-type lectin 3 [Fenneropenaeus merguiensis] | | | 1.72E-21 | 4.463887 | 170.2053 | 76.00655 | 297.3146 | 294.596 | 373.1186 | 439.0329 | 2254.33 | 1650.747 | 113.525 | 2030.719 | 2428.103 | 346.6366 | 136.2681 | 256.6122 | NA | 332.0505 | NA | 49.69905 | 4001.148 | 1486.604 | NA | 206.7552 | 108.2468 | NA | 1308.744 | 834.3363 |
| CUST_55320_PI429010886 | M109553 | 0.009294 | 24 | 21 | | | | | | 233.7917 | 252.0883 | 230.4393 | 221.852 | 265.3779 | 254.189 | 215.9006 | 207.9906 | 205.2221 | 258.4896 | 222.3465 | 231.931 | 202.005 | 242.5125 | 145.8314 | 312.052 | 239.4694 | 222.9689 | 220.0411 | 268.7796 | 237.1988 | 292.258 | 263.6263 | 271.0212 | 292.6509 | 217.6655 | 266.0394 |
| CUST_29188_PI429010886 | N59357 | 0.009294 | 24 | 21 | | | | | | 78.29209 | 92.35924 | 108.5223 | 55.76383 | 78.01256 | 83.83314 | 52.16399 | 73.43455 | 76.69694 | 69.93057 | 62.83219 | 132.8222 | 89.26714 | 92.38118 | 70.91513 | 101.9359 | 81.08583 | 93.55006 | 93.56818 | 77.82271 | 94.70207 | 112.0774 | 101.7455 | 79.50596 | 89.63044 | 106.8281 | 118.4789 |
| CUST_51943_PI429010886 | M95758 | 0.009325 | 15 | 3 | 242016983 | hypothetical protein Phum_PHUM412790 [Pediculu | | | 3.43E-07 | 75.65779 | 27.90015 | NA | 72.96725 | 33.54956 | 66.75988 | 58.01793 | 52.93292 | 22.73586 | 4.853252 | 9.823191 | NA | 63.105 | 9.87605 | 28.99306 | 194488.2 | 37.37431 | 23.37181 | 31.29367 | NA | NA | 13.29348 | 7.792392 | 41.76704 | 9.814804 | 30.94893 | 6.969672 |
| CUST_30467_PI429010886 | N61954 | 0.009341 | 18 | 9 | | | | | | 42.71853 | 41.53959 | 33.47045 | 26.78662 | 30.20292 | 36.10574 | 33.35026 | 40.66087 | 55.00728 | 64.80696 | 44.27177 | 63.80077 | 56.22954 | 59.57796 | 41.26062 | 50.36639 | 39.82861 | 31.70959 | 41.53622 | 75.37057 | 39.18988 | 34.28424 | 56.88497 | 32.2878 | 47.29164 | 40.05116 | 55.36449 |
| CUST_14756_PI429010886 | N33411 | 0.009343 | 21 | 18 | | | | | | 183.4397 | 261.767 | 49.63764 | 113.475 | 245.4514 | 81.02279 | 15.04347 | 4.199088 | 6.219397 | 111.4345 | 78.30025 | 138.3904 | 119.2945 | 309.3892 | 168.8972 | 226.2023 | 17.35915 | 209.6338 | 27.25206 | 454.8231 | 169.1739 | 376.1162 | 38.29258 | 154.3172 | 115.3546 | 29.1329 | 25.00635 |
| CUST_35020_PI429010886 | M11354 | 0.00936 | 15 | 0 | | | | | | 1230.999 | 1336.854 | 1084.753 | 986.0892 | 989.5639 | 1085.118 | 915.8521 | 796.983 | 806.0846 | 1220.626 | 939.7655 | 1042.897 | 715.3982 | 1135.995 | 1453.962 | 1748.457 | 1136.737 | 1106.085 | 986.4774 | 1272.526 | 957.5442 | 1280.61 | 797.3133 | 867.1839 | 1267.018 | 977.4233 | 1124.348 |
| CUST_597_PI429010886 | N6283 | 0.009364 | 21 | 3 | | | | | | 37.76259 | 36.43958 | 41.05122 | 55.14628 | 37.66305 | 41.27857 | 38.11258 | 38.61125 | 26.66065 | 38.23666 | 35.56782 | 41.14845 | 27.17268 | 38.24021 | 38.14533 | 33.59506 | 31.08214 | 41.57987 | 36.07274 | 45.24266 | 39.667 | 44.48912 | 46.68828 | 44.66782 | 55.78991 | 56.40373 | 49.64123 |
| CUST_31860_PI429010886 | M2592 | 0.009365 | 15 | 12 | 156547271 | PREDICTED: guanine nucleotide exchange factor MS | Signal transduction | Other | 2.48E-23 | 20.24846 | 6.566142 | 11.40707 | NA | 9.819588 | 13.81321 | 14.21847 | 4.913255 | 8.379946 | 18.35021 | 5.249409 | 15.24741 | 28.40026 | 27.3183 | 16.8197 | 13.52681 | 17.69248 | 9.72694 | 15.8263 | 4.970591 | 8.613965 | 8.152575 | 8.479761 | NA | 28.53359 | 8.699683 | 25.70952 |
| CUST_18344_PI429010886 | N39620 | 0.00937 | 18 | 12 | | | | | | 31.8793 | 35.10761 | 63.17657 | 82.73488 | 71.89357 | 68.19793 | 70.3852 | 94.03801 | 100.0618 | 83.19226 | 49.63154 | 41.34656 | 91.13848 | 135.1423 | 132.6901 | 91.84392 | 51.79974 | 75.62563 | 38.74344 | 55.34146 | 47.90583 | 123.9078 | 49.36928 | 58.63339 | 132.5094 | 54.8287 | 116.2535 |
| CUST_8667_PI429010886 | N23386 | 0.009373 | 24 | 0 | | | | | | 145.6462 | 109.0963 | 152.8002 | 107.2744 | 112.7849 | 127.2103 | 118.8413 | 98.96023 | 100.7693 | 124.3983 | 125.9794 | 125.5232 | 95.84863 | 118.1469 | 86.88496 | 109.2372 | 134.1962 | 104.9053 | 94.127 | 100.0068 | 139.6592 | 106.1954 | 142.5512 | 118.3239 | 131.3416 | 131.327 | 158.7737 |
| CUST_24123_PI429010886 | N50072 | 0.009373 | 24 | 0 | | | | | | 29.06514 | 17.82405 | 26.8214 | 29.9971 | 21.45934 | 16.14788 | 23.30079 | 16.00542 | 23.77247 | 16.59828 | 26.88741 | 13.59282 | 10.71453 | 10.26589 | 6.524534 | 19.14299 | 27.18447 | 38.18132 | 21.5895 | 27.14607 | 30.07667 | 25.51149 | 23.49179 | 14.72238 | 34.0757 | 28.24659 | 38.93338 |
| CUST_12761_PI429010886 | N29995 | 0.009389 | 18 | 3 | | | | | | 117.1784 | 122.404 | 113.1265 | 143.2947 | 114.0301 | 135.1036 | 129.4353 | 96.63027 | 108.6469 | 142.418 | 116.2372 | 86.4151 | 81.55572 | 86.62519 | 46.65359 | 117.6032 | 213.636 | 103.1588 | 148.3869 | 99.35803 | 143.7439 | 164.9397 | 168.9219 | 123.7589 | 169.8718 | 126.2074 | 94.50596 |
| CUST_3073_PI429010886 | N13735 | 0.009389 | 21 | 0 | 260813707 | hypothetical protein BRAFLDRAFT_127740 [Branchi | Metabolic process | Oxidation-reduction process | 4.67E-38 | 8.081076 | 11.84044 | 14.57386 | 12.64389 | 13.43914 | 12.17117 | 4.940431 | 6.756825 | 4.453369 | 6.037096 | 5.687945 | 8.385021 | 10.03833 | 9.170391 | 15.21068 | 7.436413 | 5.361029 | 15.64186 | 7.743875 | 10.87917 | 16.14981 | 23.59869 | 11.98265 | NA | 9.064427 | 9.366783 | 12.12977 |
| CUST_4330_PI429010886 | N16525 | 0.009394 | 18 | 12 | | | | | | 170.6936 | 165.7579 | 192.1486 | 258.4027 | 151.4764 | 186.7835 | 217.1338 | 179.2308 | 320.3175 | 270.8273 | 236.2204 | 317.3817 | 272.2987 | 401.9872 | 207.1975 | 237.0939 | 253.3806 | 152.5473 | 138.8211 | 140.8807 | 268.1153 | 156.2584 | 202.5771 | 174.126 | 308.7172 | 167.6463 | 333.0328 |
| CUST_23243_PI429010886 | N48500 | 0.009394 | 18 | 12 | | | | | | 31.18382 | 33.83173 | 24.78858 | 28.85616 | 18.58833 | 28.40026 | 27.17008 | 35.40201 | 40.21556 | 34.8364 | 36.31618 | 47.37022 | 36.92762 | 39.83835 | 52.74471 | 37.72421 | 34.26964 | 19.66187 | 26.23528 | 38.81418 | 31.71188 | 31.64126 | 33.21431 | 25.33083 | 49.69905 | 28.27144 | 37.71676 |
| CUST_37794_PI429010886 | M21622 | 0.009401 | 15 | 12 | 242007350 | 15 kDa selenoprotein precursor, putative [Pediculus | | | 4.84E-43 | 3296.897 | 5848.791 | 3172.097 | 3103.161 | 3530.217 | 2823.396 | 2416.782 | 2331.325 | 2474.132 | 3847.06 | 2961.93 | 4549.28 | 3676.785 | 3869.78 | 4965.834 | 5278.442 | 2189.348 | 3638.002 | 2970.275 | 5119.45 | 3206.496 | 4297.995 | 2966.269 | 2747.282 | 3077.216 | 3197.843 | 3459.777 |
| CUST_48521_PI429010886 | M80777 | 0.009401 | 15 | 12 | | | | | | 478.8398 | 618.3611 | 700.0017 | 512.0216 | 531.8244 | 673.8089 | 510.1908 | 595.028 | 593.331 | 597.7736 | 541.0041 | 700.9759 | 601.0454 | 831.7121 | 773.6682 | 643.3515 | 680.1392 | 620.4611 | 551.706 | 552.8917 | 544.0717 | 569.7753 | 626.2648 | 541.4466 | 613.6489 | 705.3837 | 717.4734 |
| CUST_462_PI429010886 | N5434 | 0.009401 | 15 | 12 | | | | | | 227.5634 | 86.28978 | 3569.653 | 30.78076 | 29.93929 | 54.8287 | 598.9907 | 5391.461 | 3246.287 | 4420.361 | 115.5444 | 215.8652 | 3150.433 | 7909.769 | 3315.042 | 53.45236 | 2070.325 | 47.2395 | 147.8312 | 191.9229 | 97.77159 | 1623.972 | 4365.095 | 122.0703 | 5349.606 | 200.9848 | 4743.467 |
| CUST_57_PI429010886 | N262 | 0.009411 | 24 | 21 | | | | | | 143.5804 | 118.246 | 195.7902 | 149.0167 | 138.8541 | 135.78 | 123.6359 | 140.5991 | 129.4353 | 182.5193 | 138.6617 | 132.3017 | 151.2216 | 192.0915 | 218.1405 | 183.3645 | 157.1144 | 156.4118 | 162.3689 | 173.6164 | 147.3218 | 186.6438 | 192.6124 | 170.6697 | 184.8748 | 167.0167 | 178.3785 |
| CUST_33223_PI429010886 | M6326 | 0.00942 | 12 | 0 | | | | | | 629.2275 | 622.5542 | 452.4018 | 348.5168 | 468.7286 | 317.9044 | 438.1052 | 299.502 | 414.3494 | 457.7503 | 311.2463 | 415.8541 | 661.0472 | 694.9126 | 487.8771 | 522.9557 | 362.5859 | 450.649 | 505.0665 | 433.0579 | 453.8761 | 771.6454 | 436.0745 | 456.6758 | 594.2743 | 490.5451 | 919.3319 |
| CUST_31099_PI429010886 | M695 | 0.009422 | 18 | 15 | 260831390 | hypothetical protein BRAFLDRAFT_117885 [Branchi | Metabolic process | Cellular amino acid metabolic process | | 46.64388 | 70.02583 | 40.54346 | 56.77783 | 44.08227 | 38.33878 | 36.77308 | 35.1468 | 44.90621 | 46.90544 | 46.30865 | 80.74596 | 51.63758 | 48.53823 | 61.67082 | 60.49322 | 55.50038 | 63.59288 | 43.62391 | 74.07672 | 44.00078 | 57.12722 | 32.44398 | 33.05172 | 49.02476 | 39.39544 | 41.89757 |
| CUST_10305_PI429010886 | N26032 | 0.009424 | 21 | 9 | | | | | | NA | 5.626214 | NA | 7.375685 | 18.65246 | 7.579956 | 6.131047 | 37.88493 | 34.25879 | NA | 36.01731 | 55.42181 | 32.03971 | NA | 17.62699 | 12.6532 | 13.09004 | NA | 10.33682 | 62.92532 | 35.29256 | NA | 5.993779 | 10.16864 | 4.289055 | 25.21391 | 25.68978 |
| CUST_52355_PI429010886 | M97174 | 0.009434 | 21 | 3 | 397881230 | crustin [Macrobrachium rosenbergii] | | | 1.07E-09 | 8.322322 | 130.0555 | 82.62874 | 89.03619 | 95.96344 | NA | 15.20148 | 117.8888 | 127.8042 | 93.02061 | 28.93983 | 58.08697 | 123.4881 | 161.6849 | 76.0951 | 64.17425 | 12.08902 | 46.7059 | 5.007946 | 17.9021 | 13.32018 | 32.61136 | 75.89665 | 31.92079 | 47.30806 | 209.8989 | 213.8072 |
| CUST_35156_PI429010886 | M11751 | 0.009435 | 18 | 0 | | | | | | 40.93552 | 67.52161 | 49.31188 | 45.58039 | 51.78377 | 41.75433 | 36.50859 | 31.79359 | 33.17041 | 51.01709 | 30.83099 | 44.4293 | 51.80467 | 38.7668 | 49.59376 | 57.10862 | 36.18187 | 50.22122 | 57.87372 | 45.43527 | 55.28753 | 43.17605 | 33.42509 | 41.46468 | 43.02941 | 26.64032 | 45.40667 |
| CUST_40603_PI429010886 | M55599 | 0.009448 | 15 | 9 | | | | | | 77.97597 | 70.05382 | 112.2549 | 74.54008 | 148.7285 | 121.1456 | 66.50022 | 181.2996 | 145.5578 | 210.7973 | 209.83 | 140.9929 | 43.06915 | 121.6208 | 80.72822 | 71.97535 | 90.79757 | 100.4962 | 140.4504 | 138.7005 | 132.5328 | 170.8864 | 196.4155 | 42.242 | 152.988 | 93.82886 | 201.6411 |
| CUST_22362_PI429010886 | N46893 | 0.00946 | 12 | 3 | 121484216 | cuticle protein CB6 [Portunus pelagicus] | | | 3.08E-16 | 322.0841 | 65.36594 | 593.6703 | 685.806 | 347.9148 | 1047.894 | 634.7435 | 340.1148 | 166.7787 | 58.85973 | 339.4134 | NA | 257.5143 | 106.5084 | 153.0541 | NA | 876.7586 | 509.4935 | 737.2359 | NA | 302.9535 | 4.581089 | 326.7925 | 634.4094 | 39.78059 | 329.7729 | 32.21416 |
| CUST_37960_PI429010886 | M22470 | 0.009475 | 21 | 12 | 321478058 | hypothetical protein DAPPUDRAFT_230231 [Daphni | | | 1.31E-29 | 142.0799 | 182.2299 | 153.7764 | 138.2562 | 160.6473 | 125.4876 | 98.07031 | 125.719 | 158.2183 | 169.3396 | 137.1811 | 214.9497 | 219.5762 | 190.3637 | 240.0651 | 220.511 | 141.6752 | 188.1904 | 161.0453 | 200.7582 | 160.5748 | 206.0656 | 159.0362 | 140.8976 | 176.7077 | 179.6526 | 198.2503 |
| CUST_55000_PI429010886 | M108367 | 0.00951 | 18 | 12 | | | | | | 23.91945 | 24.38309 | 11.18296 | 18.88046 | 18.48864 | 13.89615 | 28.43336 | 28.48875 | 30.26846 | 32.82642 | 24.56849 | 24.31347 | 29.06514 | 70.31729 | 26.57271 | 34.38336 | 21.06828 | 24.77058 | 30.91453 | 21.86356 | 21.02813 | 22.20953 | 24.89184 | 20.06061 | 39.20447 | 17.04861 | 21.72694 |
| CUST_7144_PI429010886 | N21103 | 0.00951 | 21 | 15 | | | | | | 33.1299 | 37.87644 | 40.63456 | 30.12077 | 38.32192 | 26.14633 | 33.81876 | 27.95174 | 36.39578 | 48.46881 | 28.1812 | 35.5525 | 42.58462 | 33.08345 | 38.37992 | 50.403 | 42.78573 | 40.42007 | 41.5428 | 35.76744 | 36.41327 | 39.36593 | 41.84077 | 26.28334 | 38.31256 | 28.82637 | 32.44055 |
| CUST_12325_PI429010886 | N29289 | 0.009516 | 21 | 15 | | | | | | 26.59511 | 30.27449 | 13.70338 | 17.64932 | 23.47003 | 18.36555 | 13.70505 | 10.85446 | 16.2136 | 26.03036 | 22.65189 | 32.13434 | 16.65641 | 30.73213 | 32.70344 | 31.73369 | 21.97651 | 35.83044 | 13.59444 | 30.59854 | 31.40253 | 36.49611 | 19.24305 | 20.52907 | 23.02215 | 18.37212 | 20.99955 |
| CUST_52801_PI429010886 | M98971 | 0.009517 | 15 | 12 | | | | | | 66.97079 | 81.97482 | 73.57494 | 62.06295 | 60.17363 | 51.90738 | 53.57478 | 55.53245 | 69.80469 | 87.05061 | 76.924 | 84.08227 | 67.80419 | 90.67241 | 92.92758 | 76.42859 | 67.83607 | 61.3315 | 57.664 | 73.73041 | 60.53815 | 93.87602 | 68.07541 | 58.35369 | 64.80137 | 66.06376 | 85.79457 |
| CUST_11311_PI429010886 | N27605 | 0.009517 | 15 | 3 | | | | | | 232.6627 | 65.61755 | 171.5942 | 249.422 | 296.4182 | 481.413 | 243.7531 | 159.9839 | 77.57474 | 71.43726 | 167.982 | 66.83042 | 95.31428 | 57.60056 | 83.91514 | 66.62755 | 298.9754 | 237.0425 | 490.4258 | 81.60393 | 233.7145 | 65.48233 | 235.2181 | 368.8573 | 97.38686 | 210.2559 | 74.86136 |
| CUST_27237_PI429010886 | N55692 | 0.009517 | 15 | 12 | | | | | | 580.3063 | 736.8107 | 770.1948 | 563.3684 | 696.9471 | 687.0726 | 537.701 | 605.0923 | 575.3599 | 730.4799 | 594.1197 | 642.8014 | 635.6512 | 815.3757 | 838.4693 | 789.7368 | 748.4841 | 684.3658 | 733.5595 | 720.7013 | 599.7852 | 730.0267 | 610.3506 | 616.0789 | 781.185 | 662.7679 | 794.1984 |
| CUST_12547_PI429010886 | N29635 | 0.009524 | 24 | 15 | | | | | | 214.2583 | 238.7409 | 246.2258 | 200.9848 | 188.3767 | 205.7682 | 195.392 | 182.2638 | 229.9564 | 267.0284 | 179.5282 | 253.4728 | 204.0536 | 254.4916 | 223.6052 | 290.3277 | 244.8254 | 248.7474 | 208.7066 | 251.4731 | 242.6822 | 286.423 | 226.6106 | 230.7517 | 233.9335 | 246.6846 | 220.511 |
| CUST_25373_PI429010886 | N52296 | 0.009524 | 24 | 15 | | | | | | 10.40388 | 22.8759 | 16.85815 | 11.29639 | 14.85731 | 13.27837 | 4.966684 | 6.763654 | 7.002324 | 15.35382 | 10.44754 | 21.77287 | 12.21701 | 18.89688 | 32.02169 | 22.40379 | 37.77438 | 15.7975 | 9.762969 | 22.97432 | 17.6292 | 23.40827 | 21.09759 | 12.91769 | 14.71224 | 13.94454 | 26.51868 |
| CUST_29125_PI429010886 | N59247 | 0.009525 | 21 | 3 | | | | | | 11.83706 | 16.7648 | 7.125214 | NA | 13.87032 | 14.05746 | 9.454588 | 11.23684 | 15.00845 | 7.41743 | 6.503335 | 11.55147 | 7.624707 | 7.163947 | 8.305626 | 10.0073 | 6.725658 | 11.37935 | 8.128829 | 18.57528 | 8.419112 | 16.50813 | 14.89292 | 9.784863 | 12.22313 | 15.88543 | 18.18378 |
| CUST_21428_PI429010886 | N45202 | 0.009529 | 24 | 21 | | | | | | 41.65038 | 38.76201 | 70.33128 | 36.77308 | 47.90583 | 57.41889 | 46.93642 | 33.79631 | 39.96875 | 40.53465 | 46.91616 | 59.776 | 44.37026 | 48.30896 | 49.55874 | 56.43628 | 48.34221 | 52.02242 | 47.41918 | 55.43705 | 59.3369 | 65.0047 | 51.24529 | 58.58633 | 68.53497 | 45.37412 | 47.25743 |
| CUST_19925_PI429010886 | N42461 | 0.009559 | 15 | 0 | | | | | | 323.754 | 230.5922 | 263.3865 | 245.1598 | 209.6694 | 273.6024 | 338.6596 | 236.9243 | 223.377 | 186.6438 | 196.6789 | 125.0127 | 189.6264 | 205.2221 | 234.141 | 234.4818 | 286.0749 | 210.2559 | 287.7468 | 167.8576 | 204.7885 | 232.6627 | 209.5249 | 254.2781 | 208.4275 | 157.7661 | 171.5473 |
| CUST_31015_PI429010886 | M464 | 0.009563 | 21 | 0 | | | | | | 4051.321 | 4451.275 | 4303.084 | 3204.304 | 2833.332 | 2766.721 | 3074.469 | 2908.57 | 3071.798 | 3759.753 | 3116.138 | 3053.471 | 3027.574 | 3421.62 | 3296.897 | 4833.67 | 3670.658 | 3208.657 | 4208.899 | 2980.843 | 3283.608 | 5125.683 | 3339.741 | 3384.084 | 4330.695 | 3225.426 | 3202.001 |
| CUST_30932_PI429010886 | M252 | 0.00958 | 18 | 9 | 548352422 | PREDICTED: nuclear pore complex protein Nup153- | | | 5.09E-47 | 540.8491 | 562.4407 | 574.2435 | 417.4014 | 432.8549 | 406.7395 | 422.1729 | 552.4266 | 473.9584 | 682.478 | 523.2717 | 627.6184 | 515.8865 | 629.802 | 482.5207 | 580.8644 | 577.0235 | 564.3927 | 535.6554 | 594.1197 | 499.1257 | 493.9819 | 541.117 | 441.6462 | 685.9612 | 483.4448 | 603.6169 |
| CUST_40793_PI429010886 | M56092 | 0.009597 | 15 | 9 | | | | | | 212.2526 | 284.093 | 314.1625 | 258.602 | 191.9558 | 225.6591 | 209.2252 | 211.6272 | 242.8033 | 312.3654 | 287.6352 | 357.0544 | 281.652 | 332.3269 | 440.5078 | 316.971 | 229.245 | 295.1449 | 259.439 | 285.4569 | 311.1732 | 335.1161 | 275.4021 | 315.7757 | 336.8758 | 398.2452 | 346.3085 |
| CUST_32981_PI429010886 | M5626 | 0.009597 | 15 | 0 | | | | | | 222.6599 | 212.7113 | 188.8906 | 160.2216 | 187.6525 | 183.1272 | 98.84179 | 84.36786 | 73.38298 | 177.9616 | 286.423 | 180.7887 | 76.43974 | 140.9566 | 85.4443 | 254.7271 | 200.2396 | 208.39 | 156.0552 | 240.6035 | 151.9294 | 200.3982 | 71.17303 | 93.27103 | 200.3982 | 88.69099 | 146.5035 |
| CUST_11777_PI429010886 | N28386 | 0.009597 | 15 | 0 | | | | | | 508.1895 | 503.6617 | 496.2657 | 467.7939 | 433.3733 | 524.852 | 348.3321 | 370.9223 | 345.9993 | 459.6219 | 399.3695 | 501.4801 | 355.3996 | 474.4772 | 465.1151 | 745.3969 | 498.525 | 565.5557 | 474.1045 | 535.1571 | 467.7939 | 519.9761 | 336.4536 | 465.1151 | 545.6531 | 404.0517 | 544.0717 |
| CUST_14765_PI429010886 | N33434 | 0.009626 | 24 | 12 | 270012890 | hypothetical protein TcasGA2_TC001664 [Tribolium | | | 2.07E-15 | 241.8731 | 219.3834 | 283.5999 | 220.2915 | 199.8756 | 200.3686 | 198.9664 | 208.8382 | 235.0953 | 280.2656 | 235.8014 | 278.5157 | 231.6859 | 366.8204 | 344.24 | 315.7204 | 245.3745 | 288.5922 | 256.9474 | 343.5515 | 202.7268 | 290.995 | 329.2355 | 229.2068 | 307.5607 | 217.9412 | 281.1462 |
| CUST_12935_PI429010886 | N30294 | 0.009632 | 18 | 6 | | | | | | 189.5057 | 207.6805 | 155.8094 | 118.6343 | 344.24 | 379.6269 | 279.2899 | 244.5842 | 303.7146 | 214.5095 | 106.6808 | 172.4652 | 136.8618 | 41.70336 | 132.109 | 218.4835 | 126.9262 | 36.49052 | 191.4647 | 228.8706 | 175.4895 | 270.6659 | 145.0486 | 62.4936 | 149.0336 | 274.3455 | 330.8112 |
| CUST_36135_PI429010886 | M14756 | 0.00964 | 21 | 3 | | | | | | 25.83289 | 12.84641 | 16.18083 | 24.46901 | 14.22183 | 27.35532 | 33.38032 | 16.83181 | 9.082334 | 26.84327 | 11.52569 | 12.70718 | 4.693023 | 24.33679 | NA | 10.25912 | 9.516775 | 13.82753 | 13.18436 | 9.220146 | 24.80851 | 25.85777 | 21.98642 | 16.89803 | 25.71165 | 26.24578 | 31.11066 |
| CUST_46101_PI429010886 | M71369 | 0.009642 | 12 | 0 | 332026908 | hypothetical protein G5I_04441 [Acromyrmex echir | | | 5.79E-07 | 34.7228 | 39.61101 | 55.75375 | 21.87979 | 15.92987 | 7.704662 | 19.3208 | 22.85484 | 23.39071 | 34.77329 | 15.88308 | 13.19006 | 24.94331 | 23.44259 | 27.31066 | 25.56096 | 12.30417 | 14.79665 | 19.33574 | 21.24309 | 29.24532 | 28.10538 | 39.86606 | 27.19088 | 32.98635 | 20.98522 | 37.64513 |
| CUST_44780_PI429010886 | M66967 | 0.009644 | 12 | 0 | 2576297 | ZNF6 [Eublepharis macularius] | | | 2.29E-11 | 30.558 | 28.76281 | 35.44546 | 26.39223 | 31.37784 | 25.01541 | 14.61093 | 23.62815 | 25.65833 | 35.52357 | 25.05489 | 32.64929 | 27.04395 | 41.19711 | 38.89725 | 37.2973 | 24.47717 | 39.27986 | 26.44212 | 35.24411 | 22.12374 | 32.38272 | 34.68251 | 19.21762 | 38.0304 | 338.2007 | 38.76201 |
| CUST_44442_PI429010886 | M65999 | 0.009644 | 24 | 15 | | | | | | 106.2054 | 147.1503 | 170.3963 | 120.3899 | 104.9253 | 122.9126 | 119.6777 | 130.0042 | 115.1745 | 139.8135 | 117.5697 | 159.8336 | 120.8512 | 147.7682 | 140.1347 | 152.7576 | 168.5577 | 172.7406 | 150.242 | 132.4059 | 167.5217 | 141.9384 | 158.8514 | 102.7727 | 147.6124 | 108.3037 | 157.6619 |
| CUST_49201_PI429010886 | M84656 | 0.009644 | 24 | 18 | | | | | | 31.57401 | 29.12589 | 42.54336 | 25.88998 | 20.24475 | 25.90815 | 22.56226 | 19.78961 | 15.40573 | 34.03541 | 20.53839 | 23.96699 | 18.76711 | 32.43294 | 29.34549 | 32.47256 | 27.04148 | 40.86821 | 37.03334 | 35.02024 | 32.07184 | 29.5983 | 37.49843 | 19.84686 | 34.07208 | 36.74882 | 44.04347 |
| CUST_2063_PI429010886 | N11469 | 0.009644 | 24 | 9 | | | | | | 19.93306 | 30.59854 | 61.23542 | 58.03044 | 34.80311 | 25.50065 | 59.1427 | 40.09922 | 26.33847 | 42.92316 | 70.74029 | 46.52715 | 36.68252 | 42.14262 | 35.02024 | 22.90764 | 30.18478 | 16.0166 | 24.9855 | 48.71731 | 24.4716 | 18.83394 | 27.95174 | 9.761566 | 21.74369 | 54.80919 | 40.66388 |
| CUST_642_PI429010886 | N6507 | 0.009644 | 24 | 15 | | | | | | 191.886 | 230.2231 | 140.5657 | 151.707 | 129.6714 | 179.0372 | 235.2181 | 135.7103 | 215.8652 | 143.1678 | 139.7302 | 175.1085 | 186.7272 | 234.3704 | 210.4017 | 242.6123 | 278.8959 | 206.8026 | 227.4444 | 242.8547 | 200.8116 | 156.9336 | 179.7058 | 198.008 | 246.6846 | 138.059 | 163.2051 |
| CUST_36501_PI429010886 | M16078 | 0.009645 | 18 | 0 | | | | | | 20.74508 | 25.03495 | 12.24661 | 11.0861 | 24.08352 | 8.185415 | 12.24661 | 15.23856 | 8.541205 | 22.31012 | 15.65986 | NA | 17.88508 | 11.60175 | NA | 17.78225 | 18.17647 | 13.86795 | 17.84197 | 31.97403 | 21.96101 | 17.21639 | 12.55922 | 6.529408 | 13.11125 | 4.39723 | 7.050922 |
| CUST_18453_PI429010886 | N39824 | 0.009646 | 24 | 18 | | | | | | 213.8921 | 251.6265 | 189.4251 | 143.2327 | 150.5497 | 132.033 | 177.0286 | 166.8022 | 195.0743 | 219.4603 | 171.8979 | 207.8011 | 217.1953 | 229.0599 | 180.2584 | 275.0769 | 162.0477 | 216.5751 | 234.7465 | 246.1869 | 188.7518 | 276.4683 | 172.5998 | 160.0314 | 264.6314 | 158.2183 | 179.7342 |
| CUST_49350_PI429010886 | M85316 | 0.009648 | 21 | 15 | 156541932 | PREDICTED: probable GDP-L-fucose synthase-like [N | Metabolic process | Other | 1.17E-31 | 35.71639 | 18.39397 | 50.54005 | 30.02913 | 7.450142 | 19.46833 | 10.70821 | 32.38272 | 7.198406 | 38.39957 | 41.09065 | 35.41597 | 39.61454 | 19.62649 | 38.12809 | 60.88717 | 22.70256 | 40.94065 | 27.61834 | 17.20661 | 20.03192 | 48.12746 | 25.75169 | 40.07867 | 7.481739 | 7.936545 | 13.41841 |

| Probe ID | ID | p-value | Period | Phase | EBI ID | Annotation | GO category | Go term | E-value | 0 | 0 | 0 | 3 | 3 | 3 | 6 | 6 | 6 | 9 | 9 | 9 | 12 | 12 | 12 | 15 | 15 | 15 | 18 | 18 | 18 | 21 | 21 | 21 | 24 | 24 | 24 |
|---|---|---|---|---|---|---|---|---|---|---|---|---|---|---|---|---|---|---|---|---|---|---|---|---|---|---|---|---|---|---|---|---|---|---|---|---|
| CUST_14047_PI429010886 | N32245 | 0.009648 | 18 | 12 | | | | | | 97.84604 | 135.061 | 108.7487 | 111.0532 | 97.6483 | 100.8047 | 97.70002 | 121.3175 | 108.657 | 170.596 | 88.19572 | 138.6217 | 108.5223 | 149.2244 | 143.2735 | 140.7156 | 85.62172 | 105.0723 | 98.97177 | 102.4425 | 102.8301 | 90.27604 | 96.38791 | 86.47473 | 135.1811 | 137.2358 | 116.6682 |
| CUST_33926_PI429010886 | M8257 | 0.009656 | 24 | 21 | 291225731 | PREDICTED: kinesin family member 21A-like [Saccog | Cellular component organization | Cytoskeleton organization | 2.26E-94 | 85.40569 | 89.02227 | 88.62563 | 83.66854 | 101.6806 | 100.651 | 74.66823 | 57.51717 | 57.3007 | 76.74889 | 87.2868 | 86.63308 | 47.3028 | 83.78522 | 36.45907 | 108.6016 | 101.3025 | 111.5216 | 99.94463 | 74.04651 | 93.56818 | 89.70412 | 106.9212 | 121.8904 | 97.40947 | 83.67885 | 84.1757 |
| CUST_26931_PI429010886 | N55145 | 0.009677 | 15 | 0 | | | | | | 32.22977 | 8.812213 | 26.47532 | 20.62307 | 18.15465 | 12.95945 | 13.9022 | 11.96331 | 13.33386 | 8.195858 | 13.15317 | 19.89384 | 10.7921 | 7.915611 | 23.43097 | 25.56371 | 17.78988 | 27.66624 | 28.29331 | 21.72163 | 20.11768 | 16.79517 | 19.85432 | 10.63417 | 11.58688 | 8.372351 | 16.36491 |
| CUST_20424_PI429010886 | N43412 | 0.009687 | 21 | 0 | | | | | | 37.81392 | 32.21099 | 36.89165 | 40.1303 | 26.0691 | 31.74172 | 27.33369 | 21.79918 | 48.26954 | 30.68719 | 22.74317 | 19.95634 | 25.35443 | 30.16973 | 18.55113 | 40.59586 | 27.38684 | 31.00851 | 31.16892 | 23.94321 | 27.75759 | 31.70409 | 36.57353 | 31.11066 | 27.75759 | 27.4692 | 24.5556 |
| CUST_22316_PI429010886 | N46801 | 0.009688 | 24 | 21 | 395751682 | PREDICTED: zinc finger protein 528-like [Pongo abel | | | 8.2E-09 | 20.50772 | 16.82979 | 18.14494 | 11.96496 | 32.55113 | 33.13396 | 19.83923 | 20.10327 | 11.89545 | 14.47801 | 22.56723 | 14.21003 | 15.45319 | 19.56129 | 9.641424 | 33.30259 | 29.79322 | 16.12393 | 23.05482 | 37.86895 | 20.15023 | 35.32273 | 25.10042 | 40.99957 | 23.86522 | 16.10523 | 15.51619 |
| CUST_7057_PI429010886 | N20977 | 0.009697 | 15 | 3 | | | | | | 120.3504 | 129.1009 | 109.1565 | 142.194 | 137.0338 | 151.1369 | 105.7587 | 112.2867 | 110.0909 | 115.1182 | 116.4584 | 119.4919 | 88.01513 | 90.13978 | 108.3885 | 103.3395 | 140.036 | 118.04 | 112.7046 | 129.6012 | 112.304 | 154.0296 | 100.965 | 119.5708 | 130.8588 | 96.25237 | 99.18362 |
| CUST_19135_PI429010886 | N41028 | 0.009701 | 21 | 15 | | | | | | 12.87351 | 20.9065 | 18.4372 | 15.10512 | 16.00911 | 3.813663 | 23.75572 | 12.40589 | 17.63322 | 22.50429 | 52.645 | 16.03532 | 19.12346 | 12.19407 | NA | 17.33696 | 36.80555 | 23.30987 | 17.12595 | NA | 20.72678 | 17.68291 | 17.08381 | 7.26746 | 16.59282 | 12.01881 | 9.032579 |
| CUST_37885_PI429010886 | M22046 | 0.009715 | 15 | 9 | 149675211 | cuticle proprotein proCP5.2 [Callinectes sapidus] | | | 8.11E-41 | 3190.091 | 1237.49 | 4803.756 | 1145.481 | 793.8063 | 1035.008 | 2949.668 | 13070.61 | 11454.19 | 12916.14 | 5911.575 | 7536.588 | 10130.67 | 20208.21 | 11710.39 | 2130.831 | 8165.772 | 840.3682 | 3407.322 | 10760.71 | 2934.424 | 23420.74 | 13275.24 | 1200.709 | 25677.47 | 5572.884 | 26218.61 |
| CUST_36528_PI429010886 | M16147 | 0.009734 | 21 | 0 | | | | | | 289.7899 | 281.1462 | 247.4599 | 137.0338 | 229.6175 | 228.9161 | 188.2724 | 184.5783 | 126.8447 | 196.9798 | 169.7046 | 139.6056 | 123.5431 | 181.2412 | 145.6295 | 290.0281 | 305.5566 | 269.8655 | 62.31885 | 171.7375 | 148.9405 | 273.3216 | 149.4495 | 299.285 | 235.3127 | 210.8661 | 231.2637 |
| CUST_39355_PI429010886 | M38223 | 0.009749 | 15 | 12 | | | | | | 5471.152 | 442.9673 | 15922.04 | 245.3246 | 525.2083 | 394.8342 | 8094.902 | 21119.13 | 17110.44 | 14229.78 | 2667.238 | 3849.875 | 17837.74 | 25136.37 | 12630.84 | 1066.114 | 9670.846 | 530.3833 | 4676.187 | 4415.4 | 2065.573 | 11983.26 | 15813 | 2607.508 | 24777.72 | 2370.125 | 23818.16 |
| CUST_1663_PI429010886 | N10381 | 0.009749 | 15 | 12 | | | | | | 211.7401 | 226.8346 | 367.6066 | 210.6087 | 222.9038 | 241.638 | 227.6752 | 239.521 | 278.3606 | 278.397 | 249.422 | 330.3127 | 276.9079 | 386.6669 | 289.4881 | 288.0337 | 247.2726 | 268.9771 | 244.3511 | 252.5323 | 267.1348 | 296.266 | 314.9323 | 270.2125 | 339.0413 | 265.1181 | 367.27 |
| CUST_7354_PI429010886 | N21421 | 0.009749 | 15 | 12 | 328782794 | PREDICTED: HEAT repeat-containing protein 3-like [ | | | 2.16E-24 | 33.32679 | 28.49131 | 28.42343 | 23.43812 | 29.97696 | 34.15487 | 24.02357 | 17.67943 | 17.08729 | 58.4208 | 37.21299 | 33.45717 | 27.13002 | 48.80089 | 46.991 | 37.74785 | 20.09393 | 21.10951 | 26.40308 | 34.93327 | 27.71267 | 28.07949 | 17.25469 | 18.42696 | 19.8522 | 20.64798 | 44.92544 |
| CUST_18549_PI429010886 | N39994 | 0.009749 | 15 | 12 | | | | | | 58.50555 | 53.14405 | 64.60382 | 52.74988 | 43.5699 | 63.67902 | 51.0829 | 67.53545 | 69.87848 | 68.1364 | 64.50807 | 87.99607 | 44.84989 | 90.18249 | 91.79308 | 74.14418 | 52.96132 | 69.76028 | 52.2877 | 47.14504 | 62.5758 | 86.46205 | 61.59565 | 55.11221 | 64.19796 | 80.71386 | 77.33177 |
| CUST_2334_PI429010886 | N12126 | 0.009755 | 12 | 3 | 383862421 | PREDICTED: uncharacterized protein LOC10087722( | | | 4.07E-28 | 145.794 | 150.0225 | 147.3218 | 182.4397 | 183.4152 | 220.485 | 167.0802 | 135.5237 | 97.58263 | 153.0166 | 158.5157 | 109.9447 | 129.4503 | 131.5799 | 166.1459 | 176.2631 | 174.3026 | 186.0881 | 184.458 | 153.2544 | 160.5038 | 177.3713 | 141.0258 | 131.39 | 119.8572 | 157.1144 | 142.124 |
| CUST_36342_PI429010886 | M15663 | 0.009756 | 12 | 0 | | | | | | 26.07711 | 43.48893 | 23.83766 | 17.76083 | 17.29005 | 19.47376 | 13.85598 | 9.855895 | 14.41572 | 24.43879 | 19.0844 | 34.61592 | 15.17932 | 21.33841 | 30.91745 | 32.63646 | 17.81636 | 21.11131 | 15.71416 | 23.65146 | 19.50301 | 23.6747 | 20.93304 | 16.49199 | 21.35072 | 32.08763 | 28.63701 |
| CUST_21448_PI429010886 | N45241 | 0.009756 | 12 | 0 | | | | | | 106.298 | 116.516 | 105.1478 | 102.8709 | 71.14036 | 101.5014 | 83.57768 | 85.67865 | 78.25555 | 102.3532 | 79.48728 | 102.0104 | 80.71386 | 81.60393 | 110.9726 | 131.4266 | 77.43118 | 94.02768 | 79.91242 | 76.674 | 97.05352 | 104.1211 | 95.84863 | 76.95909 | 123.5563 | 145.5389 | 123.7589 |
| CUST_7688_PI429010886 | N21924 | 0.009764 | 24 | 21 | | | | | | 1125.765 | 929.9665 | 1225.452 | 689.6848 | 970.6979 | 898.9921 | 1061.463 | 771.0971 | 983.8663 | 1054.702 | 907.1459 | 1178.991 | 659.1977 | 1245.02 | 1065.852 | 1144.662 | 1233.738 | 962.8744 | 1085.565 | 1052.869 | 1267.999 | 1310.544 | 1422.337 | 1324.694 | 1616.675 | 1407.859 | 1932.967 |
| CUST_17553_PI429010886 | N38167 | 0.009764 | 24 | 18 | | | | | | 49.57451 | 52.89696 | 52.45794 | 47.02813 | 40.40197 | 39.78059 | 49.67248 | 39.97268 | 55.74249 | 52.2877 | 52.53463 | 47.24387 | 49.2211 | 47.21974 | 46.56491 | 63.105 | 62.66235 | 50.70349 | 67.45213 | 58.84482 | 54.37737 | 62.07028 | 49.54106 | 44.59831 | 73.91159 | 41.99242 | 56.53962 |
| CUST_22419_PI429010886 | N46994 | 0.009764 | 24 | 18 | | | | | | 23.12065 | 15.73405 | 28.79502 | 13.21125 | 15.60693 | 22.9227 | 20.96144 | 25.31087 | 18.03368 | 27.99024 | 18.74035 | 18.19015 | 19.16281 | 22.33151 | 21.92688 | 27.51937 | 27.76033 | 26.27382 | 35.39506 | 24.87526 | 30.6513 | 20.41547 | 22.62281 | 24.88119 | 22.66333 | 32.44055 | 28.05565 |
| CUST_8783_PI429010886 | N23581 | 0.009781 | 24 | 3 | 321459261 | hypothetical protein DAPPUDRAFT_300520 [Daphn | | | 5.62E-07 | 247.4184 | 198.7421 | 183.528 | 311.8796 | 169.3396 | 289.2842 | 261.4208 | 203.7918 | 270.1288 | 192.1174 | 135.5426 | 124.7428 | 226.5039 | 200.85 | 223.7478 | 188.591 | 157.9028 | 176.1772 | 167.2734 | 138.2244 | 148.65 | 174.4245 | 162.3689 | 218.5606 | 184.3061 | 220.8169 | 137.6717 |
| CUST_17426_PI429010886 | N37941 | 0.009782 | 15 | 9 | | | | | | 26.19664 | 34.02396 | 36.22069 | 28.79209 | 38.80417 | 42.1461 | 28.00512 | 37.48336 | 41.18311 | 47.7103 | 36.41327 | 62.86517 | 35.9369 | 37.69724 | 39.56391 | 36.07548 | 34.78102 | 55.41559 | 38.63168 | 39.27214 | 36.29774 | 54.89949 | 39.49607 | 70.15478 | 60.79572 | 41.78704 | 58.27728 |
| CUST_55269_PI429010886 | M109401 | 0.009786 | 24 | 21 | | | | | | 98.92872 | 94.24344 | 98.52867 | 87.32671 | 96.75892 | 83.85047 | 86.30175 | 92.96667 | 77.48229 | 94.30105 | 79.36987 | 71.37464 | 106.3173 | 90.09804 | 68.70977 | 81.44042 | 98.45237 | 89.33478 | 79.59942 | 91.49463 | 97.37669 | 108.6304 | 90.97139 | 106.4305 | 122.2713 | 90.75806 | 103.3291 |
| CUST_32762_PI429010886 | M5057 | 0.009813 | 12 | 0 | 332376103 | unknown [Dendroctonus ponderosae] >gi\|4782596 | Nucleic acid metabolism | Regulation of gene expression | 2.12E-76 | NA | 11.73913 | 7.895988 | 8.205981 | 5.032873 | 7.352114 | 4.285592 | 6.132189 | 6.212154 | 9.145807 | 8.892843 | 8.286303 | 7.553772 | 15.52381 | 4.630769 | NA | 6.982708 | 11.01284 | NA | 4.973007 | 5.195945 | NA | 8.997185 | 9.188895 | 18.66273 | 11.01084 | 19.11453 |
| CUST_34791_PI429010886 | M10681 | 0.00982 | 24 | 0 | | | | | | 257.4087 | 237.506 | 242.9704 | 200.041 | 227.0266 | 224.9139 | 241.3477 | 190.0092 | 206.5059 | 325.5645 | 220.7455 | 232.8026 | 191.0327 | 219.2633 | 176.7077 | 222.3465 | 231.2637 | 215.0582 | 212.606 | 247.0088 | 210.0239 | 287.3657 | 217.0351 | 202.262 | 321.5473 | 251.2548 | 260.9776 |
| CUST_57181_PI429010886 | E2164 | 0.009834 | 15 | 9 | 260834581 | hypothetical protein BRAFLDRAFT_221908 [Branchi | | | 6.18E-25 | 1483.935 | 2218.572 | 1347.753 | 947.8741 | 756.4835 | 163.5179 | 584.5557 | 966.1688 | 1415.482 | 3477.696 | 2042.507 | 3197.843 | 1742.655 | 1601.861 | 2306.592 | 4190.35 | 669.0767 | 2047.628 | 1834.08 | 1497.781 | 2020.188 | 2770.165 | 1464.638 | 392.0959 | 3641.417 | 2373.328 | 4144.215 |
| CUST_43004_PI429010886 | M61827 | 0.009834 | 15 | 0 | | | | | | 143.3816 | 120.4034 | 120.6113 | 116.7957 | 109.2686 | 91.81369 | 124.7213 | 55.63288 | 80.31713 | 95.61724 | 49.96135 | 94.09795 | 103.2145 | 108.3441 | 95.9293 | 186.1374 | 152.8609 | 138.1473 | 82.39034 | 121.1638 | 66.59221 | 129.7547 | 32.98635 | 64.99075 | 185.7639 | 86.2674 | 154.6585 |
| CUST_14835_PI429010886 | N33548 | 0.009834 | 15 | 9 | | | | | | 295.3859 | 785.2299 | 974.3808 | 622.9374 | 848.334 | 395.2528 | 135.074 | 1639.617 | 2588.897 | 3074.469 | 1729.171 | 3597.224 | 1989.387 | 4955.555 | 3713.677 | 2821.243 | 617.9947 | 1588.254 | 1230.543 | 2435.819 | 1103.847 | 7347.807 | 2464.001 | 395.6626 | 6802.929 | 2648.384 | 8046.364 |
| CUST_30311_PI429010886 | N61603 | 0.009838 | 15 | 12 | 405951572 | 1-phosphatidylinositol-4,5-bisphosphate phosphodi | Signal transduction | Intracellular signal transduction | 1.57E-12 | 39.90155 | 40.68218 | 33.4065 | 33.38385 | 23.43377 | 28.53623 | 26.04444 | 33.57067 | 27.64478 | 30.56193 | 35.76744 | 37.69724 | 41.56705 | 48.01961 | 42.29018 | 59.25275 | 37.81392 | 40.24278 | 45.03054 | 38.37256 | 25.48335 | 36.14912 | 36.82802 | 27.95506 | 46.66745 | 23.95732 | 49.25815 |
| CUST_24230_PI429010886 | N50268 | 0.009861 | 24 | 0 | | | | | | 7.467895 | 11.412 | NA | 11.84887 | 4.678772 | 4.439445 | 3.683135 | 7.284707 | 5.958556 | 4.091511 | 4.238098 | 4.920313 | 5.965808 | 4.482425 | 4.637609 | 10.49992 | 5.622844 | NA | 8.964575 | NA | NA | 37.81392 | NA | 5.327 | 5.97707 | NA | 12.75631 |
| CUST_56917_PI429010886 | M114898 | 0.009865 | 15 | 12 | | | | | | 647.5069 | 874.5756 | 980.1159 | 572.0401 | 663.9332 | 947.2744 | 609.6635 | 647.6977 | 747.2269 | 794.1984 | 778.415 | 1328.909 | 756.1137 | 1236.95 | 972.1119 | 979.2899 | 753.9359 | 860.1543 | 745.5335 | 762.7979 | 778.2204 | 702.7008 | 756.7863 | 678.7202 | 895.8021 | 924.0246 | 996.2576 |
| CUST_32908_PI429010886 | M5443 | 0.009865 | 15 | 12 | 242007544 | Pediculus humanus corporis protein Mo25, putative | | | 2.03E-63 | 149.811 | 155.8611 | 167.6024 | 110.1779 | 117.9302 | 123.2882 | 141.7485 | 110.1036 | 139.419 | 179.0372 | 138.6419 | 192.922 | 155.5309 | 186.4927 | 230.4843 | 193.108 | 117.5843 | 144.4224 | 149.5538 | 170.4197 | 112.4193 | 166.0195 | 126.3312 | 130.5635 | 192.4758 | 138.2381 | 222.2769 |
| CUST_52611_PI429010886 | M98162 | 0.009865 | 15 | 12 | | | | | | 13.93866 | 10.86536 | 16.77789 | 7.637236 | 11.11474 | 12.05879 | 10.04473 | 12.52875 | 17.99612 | 17.4849 | 16.59129 | 11.99091 | 9.381836 | 23.2298 | 19.49659 | 19.47107 | 5.148826 | 21.23667 | 7.30278 | 8.919059 | 13.80323 | 12.55445 | 11.56849 | 14.36186 | 19.28281 | 20.15665 | 16.26559 |
| CUST_49303_PI429010886 | M85100 | 0.009867 | 21 | 12 | 307212786 | Myosin heavy chain, muscle [Harpegnathos saltator | | | 4.46E-47 | 9.956467 | 7.980175 | 3.25959 | 7.234602 | 8.128829 | 9.86011 | 19.20795 | NA | 9.033618 | 12.66733 | 13.15835 | 13.19962 | 12.07145 | 16.19744 | 8.490289 | 8.339436 | 10.65864 | 17.85771 | 13.44053 | 7.010504 | 16.16705 | NA | 8.236094 | 5.542516 | 10.2728 | 7.208745 | 6.724594 |
| CUST_29907_PI429010886 | N60797 | 0.009868 | 12 | 0 | | | | | | 11.86764 | 28.53867 | 37.47945 | 9.071169 | 18.46255 | 10.9756 | 14.29674 | 11.4448 | 9.375364 | 21.03267 | 15.00483 | 15.51227 | 19.42289 | 20.77162 | 21.64331 | 18.12837 | 15.9198 | 19.28584 | 8.800802 | 21.7761 | 20.5469 | 12.04026 | 19.93091 | 12.68973 | 25.20056 | 28.72783 | 30.81978 |
| CUST_44847_PI429010886 | M67189 | 0.009873 | 15 | 9 | 260829971 | hypothetical protein BRAFLDRAFT_114926 [Branchi | Nucleic acid metabolism | Transcription, DNA-dependent | 2.94E-19 | 5.885176 | 14.92766 | 9.636382 | NA | 9.945177 | 7.034635 | 5.9437 | 10.50262 | 8.707775 | 19.14778 | 9.573493 | 38.02365 | 4.996795 | 13.05889 | 15.74407 | 10.5119 | 6.105375 | NA | 7.127057 | 8.207161 | 6.507788 | 15.38273 | 10.22803 | 12.73533 | 14.2983 | 17.34168 | 15.55875 |
| CUST_25801_PI429010886 | N53086 | 0.009874 | 12 | 3 | 157125446 | four and a half lim domains [Aedes aegypti] >gi\|108 | | | 6.67E-64 | 43.9859 | 46.56491 | 44.4293 | 60.84782 | 50.69191 | 59.18336 | 54.59618 | 61.70387 | 47.27187 | 45.06422 | 40.33441 | 36.94944 | 37.35358 | 31.76715 | 38.0368 | 50.56281 | 84.70201 | 64.85632 | 140.7918 | 41.28276 | 43.22574 | 52.46763 | 33.60101 | 49.18419 | 40.74434 | 63.48536 | 36.59066 |
| CUST_12944_PI429010886 | N30321 | 0.009882 | 24 | 3 | 488511459 | PREDICTED: rho GTPase-activating protein 18 [Dasy | | | 3.53E-08 | 24.99534 | 20.04708 | 22.3415 | 25.25647 | 19.19334 | 32.97999 | 19.13174 | 18.15141 | 13.58003 | 13.00717 | 16.99042 | 14.1264 | 11.21679 | 14.09213 | 22.68707 | 12.54217 | 18.41753 | 17.23883 | 14.06114 | 6.220117 | 11.11331 | 14.98523 | 21.13052 | 21.21967 | 9.739447 | 18.74405 | 11.04457 |
| CUST_9516_PI429010886 | N24768 | 0.00989 | 18 | 12 | | | | | | 52.40666 | 59.52835 | 49.03345 | 38.46426 | 44.59831 | 44.56286 | 44.06771 | 55.82952 | 38.30957 | 66.66314 | 53.00119 | 61.2231 | 66.55641 | 62.45053 | 64.62304 | 58.20859 | 65.74131 | 46.35994 | 70.59503 | 53.38382 | 52.82166 | 59.18762 | 51.53321 | 44.85517 | 55.55289 | 46.62146 | 72.98413 |
| CUST_48967_PI429010886 | M83231 | 0.009893 | 24 | 21 | 307715368 | arginine kinase 1 [Macrobrachium rosenbergii] | | | 9.16E-29 | 29.44199 | 24.135 | 18.94519 | 28.08525 | 18.25337 | 24.84355 | 12.82706 | 17.26917 | 13.99028 | 15.4643 | 28.70795 | 20.79184 | 12.05188 | 23.67253 | 16.13004 | 13.85598 | 25.50611 | 16.26898 | 23.63048 | 19.7129 | 23.78208 | 25.92862 | 27.84371 | 28.11334 | 36.75622 | 23.24144 | 19.4153 |
| CUST_3623_PI429010886 | N14909 | 0.009905 | 24 | 15 | | | | | | 37.58201 | 64.68215 | 46.20996 | 29.56124 | 38.26462 | 23.13699 | 36.93108 | 42.15632 | 44.42567 | 64.31019 | 51.17489 | 46.81158 | 45.03911 | 48.84923 | 44.24444 | 52.97067 | 59.6812 | 53.31549 | 36.606 | 66.20577 | 46.06229 | 55.71823 | 42.15632 | 42.49968 | 54.10775 | 35.9589 | 48.6393 |
| CUST_19269_PI429010886 | N41278 | 0.009905 | 24 | 18 | 443735033 | hypothetical protein CAPTEDRAFT_226782 [Capitell | | | 1.76E-11 | 29.09612 | 22.37068 | 27.00978 | 35.54827 | 25.94889 | 28.743 | 15.82481 | 36.52268 | 25.89716 | 27.27234 | 30.37057 | 28.39724 | 29.47231 | 39.67041 | 37.57841 | 44.4641 | 32.16133 | 35.06879 | 40.43694 | 53.58478 | 33.70252 | 33.95872 | 37.28683 | 28.99306 | 38.92288 | 27.27234 | 33.63068 |
| CUST_30266_PI429010886 | N61518 | 0.00991 | 12 | 6 | | | | | | 8.692145 | 6.239269 | 11.44011 | 27.02172 | 9.004162 | 13.24259 | 15.07523 | 35.21227 | 22.40379 | 5.744855 | 13.23183 | 11.97517 | 12.60293 | 7.893242 | 7.492805 | NA | 25.85184 | 16.03903 | 27.36061 | 12.21352 | 43.95205 | 9.460042 | 19.07608 | 14.66408 | 10.32671 | 13.97235 | 17.37315 |
| CUST_47653_PI429010886 | M77058 | 0.009918 | 21 | 3 | | | | | | 47.11037 | 49.40038 | 62.71939 | 46.11982 | 57.7399 | 67.7616 | 59.12378 | 45.4417 | 54.7161 | 54.99653 | 45.6007 | 53.57981 | 46.30865 | 45.77928 | 42.99716 | 57.96208 | 50.10697 | 51.49762 | 48.68295 | 51.95916 | 57.85675 | 48.95474 | 63.29578 | 48.75681 | 90.44569 | 66.26094 | 83.66854 |
| CUST_13911_PI429010886 | N31991 | 0.009919 | 18 | 0 | | | | | | 238.5008 | 209.1269 | 264.3268 | 165.2714 | 202.7615 | 204.6898 | 246.5795 | 183.091 | 162.8265 | 249.8541 | 155.3897 | 154.1139 | 205.8446 | 231.0331 | 194.1492 | 198.8064 | 191.886 | 205.3314 | 274.7936 | 192.363 | 241.9617 | 206.0055 | 218.7669 | 196.7037 | 159.9648 | 140.9396 | 159.1708 |
| CUST_20955_PI429010886 | N44368 | 0.009936 | 21 | 9 | | | | | | 109.3295 | 89.94573 | 113.8871 | 110.9326 | 91.12979 | 113.8702 | 96.84604 | 119.0883 | 120.1692 | 121.5472 | 114.0787 | 148.0276 | 98.92872 | 142.0394 | 129.6714 | 110.8741 | 113.1942 | 128.5685 | 111.1891 | 142.4549 | 97.65941 | 109.7631 | 105.8984 | 76.61613 | 129.9538 | 95.5926 | 118.275 |
| CUST_6808_PI429010886 | N20627 | 0.00994 | 18 | 15 | 546683481 | hypothetical protein D910_10583 [Dendroctonus pe | | | 7.31E-48 | 37.2354 | 50.80674 | 71.01876 | 31.20492 | 25.79616 | 36.08065 | 43.86454 | 45.03054 | 53.25946 | 35.39506 | 57.71752 | 71.18045 | 40.1276 | 48.6393 | 59.94215 | 69.74095 | 56.74919 | 61.69829 | 63.54619 | 43.56037 | 51.42128 | 55.62798 | 36.45455 | 27.18226 | 63.11741 | 60.73689 | 55.24489 |
| CUST_51759_PI429010886 | M94916 | 0.009952 | 15 | 9 | | | | | | 69.15357 | 87.08384 | 117.0501 | 47.05803 | 76.04812 | 64.4873 | 38.85712 | 117.1353 | 189.9402 | 151.0368 | 148.4035 | 184.9825 | 96.44756 | 161.5046 | 134.8627 | 188.8906 | 113.6118 | 90.43345 | 88.10192 | 131.2193 | 75.99857 | 203.5264 | 128.6574 | 48.64598 | 204.2904 | 97.10121 | 188.1392 |
| CUST_3695_PI429010886 | N15069 | 0.009952 | 15 | 0 | | | | | | 294.7498 | 413.9155 | 355.9452 | 357.2936 | 338.5409 | 322.0219 | 325.8124 | 290.4297 | 279.0433 | 335.1917 | 283.9501 | 341.5068 | 327.8511 | 347.5866 | 339.7564 | 447.7446 | 352.1132 | 369.3306 | 308.3972 | 304.1627 | 317.3817 | 387.1393 | 272.8585 | 283.0254 | 363.2398 | 289.7899 | 324.3223 |
| CUST_3754_PI429010886 | N15196 | 0.009952 | 15 | 9 | 321463404 | hypothetical protein DAPPUDRAFT_324399 [Daphn | Transport | Ion transport | 7.88E-09 | 87.15435 | 83.26471 | 142.5512 | 72.54647 | 76.07766 | 72.85867 | 89.26714 | 81.8019 | 115.9016 | 141.9566 | 106.5084 | 97.3486 | 86.18635 | 136.2095 | 104.1068 | 113.8579 | 91.56459 | 94.5785 | 85.53529 | 92.37125 | 68.2359 | 110.3616 | 122.8849 | 68.85144 | 112.9069 | 87.26858 | 139.1034 |
| CUST_4940_PI429010886 | N17851 | 0.009952 | 15 | 0 | | | | | | 36.80914 | 40.29977 | 57.84331 | 28.83874 | 31.18382 | 28.44656 | 24.8962 | 25.11484 | 32.11685 | 41.68991 | 31.28664 | 47.17274 | 34.21062 | 33.96909 | 30.86904 | 50.7913 | 45.48365 | 48.65088 | 29.97696 | 38.14533 | 39.44223 | 23.74339 | 34.12708 | 29.30762 | 49.24687 | 56.09789 | 51.13662 |
| CUST_4950_PI429010886 | N17877 | 0.009965 | 21 | 0 | | | | | | 22.32503 | 47.35331 | 40.44128 | NA | 21.3985 | 18.69696 | 8.804214 | 41.97456 | 11.25441 | 14.66408 | 17.91608 | NA | 15.01303 | 17.96131 | 6.444219 | 62.64364 | 33.69201 | 28.10839 | 45.3426 | 13.37036 | 22.39942 | 31.84942 | 50.26445 | 44.92544 | 33.83579 | 12.44135 | 15.24223 |
| CUST_27471_PI429010886 | N56116 | 0.009971 | 15 | 12 | | | | | | 75.09478 | 167.9521 | 109.5638 | 104.8657 | 85.72262 | 146.939 | 132.4515 | 109.4106 | 138.2766 | 137.3935 | 145.4402 | 254.6874 | 126.6371 | 193.3576 | 222.5078 | 121.9257 | 90.51231 | 238.8964 | 81.07113 | 87.88231 | 141.5325 | 119.1637 | 183.2331 | 108.1306 | 159.8607 | 161.355 | 205.9317 |
| CUST_46181_PI429010886 | M71693 | 0.00998 | 21 | 3 | | | | | | 11.11919 | 95.74719 | 12.39561 | 15.92196 | 14.99955 | 19.84686 | 13.39711 | 18.16906 | 12.7372 | 14.37805 | 12.31007 | 9.650569 | 6.829705 | 13.33577 | 8.446645 | 14.94965 | 12.05879 | 15.06711 | 15.04503 | 14.72748 | 11.33051 | 8.910931 | 7.210827 | 16.09243 | 16.8622 | 19.05569 | 15.15645 |
| CUST_33577_PI429010886 | M7319 | 0.00998 | 21 | 12 | | | | | | 201.7937 | 150.5251 | 167.321 | 182.5915 | 171.8712 | 176.3364 | 275.0769 | 166.519 | 226.3317 | 208.515 | 159.1135 | 191.0009 | 345.9305 | 390.8735 | 289.9742 | 221.0933 | 121.2272 | 174.4245 | 289.9742 | 176.1138 | 207.7427 | 149.6919 | 169.4541 | 132.7894 | 358.3575 | 168.7266 | 201.3634 |
| CUST_24932_PI429010886 | N51503 | 0.009981 | 12 | 0 | | | | | | 37.64162 | 30.17815 | 28.75932 | 35.67442 | 17.03915 | 40.34574 | 7.3856 | 19.20795 | 17.31897 | 23.4957 | 17.59447 | 55.38216 | 37.99673 | 40.23032 | 30.29871 | 40.36236 | 14.30234 | 30.23833 | 19.42074 | 28.69415 | 21.59129 | 27.49426 | 22.66333 | 21.70445 | 30.37957 | 51.82417 | 33.4065 |
| CUST_11258_PI429010886 | N27522 | 0.009981 | 15 | 12 | | | | | | 18.31307 | 16.43668 | 23.0986 | 12.60126 | 14.52959 | 17.6966 | 14.52048 | 14.25604 | 23.2348 | 18.98962 | 15.71246 | 45.01588 | 22.61839 | 25.22969 | 35.34739 | 20.1154 | 18.74405 | 30.67698 | 13.09004 | 17.17022 | 18.95062 | 13.52947 | 31.30771 | 19.76039 | 15.72851 | 37.71676 | 111.7758 |
| CUST_12257_PI429010886 | N29175 | 0.009981 | 15 | 12 | | | | | | 497.8615 | 554.0852 | 542.3878 | 366.8204 | 401.2357 | 338.0013 | 460.7596 | 493.215 | 442.7732 | 611.5405 | 421.6972 | 443.5662 | 531.5557 | 648.0827 | 607.4385 | 589.4448 | 451.5463 | 404.1368 | 367.5439 | 552.7878 | 328.8667 | 625.1793 | 480.999 | 310.9824 | 591.0201 | 341.7763 | 513.2283 |
| CUST_19073_PI429010886 | N40935 | 0.009981 | 15 | 12 | 295919777 | G protein-coupled receptor 89 [Scylla paramamosai | | | 6.21E-57 | 66.54333 | 51.58632 | 79.83563 | 49.355 | 54.84373 | 57.028 | 65.34071 | 47.13629 | 60.78952 | 55.50038 | 63.5725 | 74.94569 | 66.10733 | 63.43062 | 76.674 | 72.07584 | 43.73764 | 62.81643 | 44.74623 | 64.07157 | 42.16986 | 62.97162 | 55.20237 | 52.35985 | 68.79393 | 57.77025 | 67.64754 |
| CUST_55132_PI429010886 | M108827 | 0.00999 | 12 | 9 | | | | | | 51.37093 | 77.98525 | 45.79191 | 73.55011 | 61.70387 | 78.81351 | 57.75281 | 77.67565 | 43.66792 | 66.61406 | 94.75367 | 96.05386 | 50.34321 | 39.55173 | 25.08131 | 80.92356 | 93.43338 | 39.6493 | 70.3928 | 143.3153 | 69.49923 | 98.04327 | 82.91863 | 94.41213 | 64.67521 | 67.47589 | 56.11599 |
| CUST_17295_PI429010886 | N37729 | 0.00999 | 12 | 0 | | | | | | 53.48278 | 76.90742 | 48.42693 | 38.73495 | 33.87601 | 28.27702 | 44.28069 | 37.37861 | 69.216 | 153.7764 | 49.01378 | 46.07745 | 40.55619 | 96.37601 | 63.66464 | 50.78152 | 21.87538 | 31.71523 | 55.58103 | 14.19367 | 59.07909 | 99.50846 | 39.87225 | 31.95868 | 199.281 | 61.57414 | 156.539 |
| CUST_52001_PI429010886 | M96020 | 0.009999 | 24 | 21 | | | | | | 30.40899 | 25.33083 | 54.91632 | 27.0513 | 27.92219 | 23.85453 | 21.19536 | 23.25228 | 34.73965 | 44.45813 | 31.39871 | 41.33159 | 29.93088 | 43.43161 | 32.51998 | 44.42212 | 26.97522 | 30.26479 | 52.33357 | 41.88371 | 45.09974 | 45.14299 | 45.1097 | 43.79745 | 53.35103 | 36.64169 | 62.07028 |
| CUST_41584_PI429010886 | M58090 | 0.010023 | 18 | 15 | | | | | | 66.08238 | 65.87593 | 79.52449 | 49.69905 | 55.79937 | 56.49623 | 52.38937 | 49.49037 | 77.83266 | 68.89697 | 58.40039 | 81.22317 | 82.31536 | 75.03476 | 73.83558 | 106.8828 | 72.04379 | 76.18529 | 75.29438 | 63.83994 | 62.54347 | 60.15877 | 81.92913 | 61.95712 | 74.93674 | 65.26348 | 71.07701 |
| CUST_56031_PI429010886 | M112588 | 0.010023 | 24 | 15 | | | | | | 64.41009 | 72.78118 | 78.75847 | 68.08199 | 78.52355 | 69.14635 | 118.246 | 111.1756 | 108.9599 | 62.62111 | 130.7638 | 190.3369 | 121.2986 | 83.49596 | 120.4315 | 129.1685 | 88.47454 | 119.8693 | 96.60043 | 180.3556 | 91.96613 | 120.8512 | 84.60749 | 84.95079 | 79.48728 | 77.74288 | 153.5797 |
| CUST_46076_PI429010886 | M71293 | 0.010039 | 18 | 0 | | | | | | 38.56543 | 37.55188 | 22.94293 | 31.99941 | 25.21391 | 32.14209 | 20.49313 | 21.71424 | 19.36786 | 26.54031 | 27.56643 | 27.66265 | 24.27705 | 17.96838 | 28.88408 | 43.07866 | 25.03208 | 22.51476 | 25.13331 | 35.65407 | 34.45011 | 32.10959 | 18.11507 | 18.82741 | 19.64586 | 11.06303 | 23.4177 |
| CUST_39545_PI429010886 | M41087 | 0.010046 | 21 | 12 | 292384631 | Meganyctiphanes norvegica isolate K13e 18S ribosc | | | 9.71E-77 | 921.275 | 618.6744 | 767.5196 | 355.116 | 1280.61 | 797.3133 | 1016.851 | 692.0661 | 837.3907 | 1746.637 | 607.116 | 987.1593 | 1507.459 | 1029.814 | 991.6341 | 864.2169 | 993.3629 | 1626.337 | 1043.787 | 965.4945 | 749.487 | 880.2112 | 452.1056 | 379.8142 | 328.807 | 484.8203 | 867.8685 |
| CUST_7473_PI429010886 | N21597 | 0.010047 | 21 | 0 | 344307582 | PREDICTED: zinc finger protein 623 [Loxodonta afric | | | 1.38E-08 | 32.40747 | 8.781809 | 10.70092 | NA | 7.561772 | NA | NA | 6.801891 | 3.48209 | 5.466194 | 5.091571 | 5.662031 | 5.322871 | 6.599438 | 5.813584 | 12.58824 | 6.006114 | 14.43577 | 3.957054 | 4.951002 | 7.594313 | 11.74368 | 14.70578 | 8.208279 | 6.429353 | 11.13787 | 5.253549 |
| CUST_38033_PI429010886 | M22931 | 0.010071 | 15 | 9 | 46240810 | crustin-like peptide [Marsupenaeus japonicus] | | | 2.91E-08 | 137.7352 | 390.4272 | 328.4958 | 101.1468 | 121.5738 | 50.95506 | 326.7477 | 136.5503 | 427.6746 | 442.9673 | 170.0985 | 455.8444 | 467.4407 | 773.6682 | 322.9274 | 940.8909 | 663.1345 | 310.4773 | 69.35049 | 568.2036 | 143.517 | 396.0145 | 1512.011 | 449.1802 | 3126.195 | 527.7394 | 3405.484 |
| CUST_40237_PI429010886 | M51959 | 0.010071 | 15 | 0 | | | | | | 170.2922 | 188.5235 | 167.8299 | 121.3307 | 134.2279 | 143.7864 | 113.6765 | 97.05352 | 101.6676 | 156.7058 | 153.5041 | 149.7495 | 108.2155 | 94.75367 | 134.1962 | 166.4251 | 136.7126 | 155.3616 | 135.6641 | 149.3268 | 124.1278 | 148.5046 | 122.5004 | 126.1983 | 172.831 | 129.9323 | 109.2144 |
| CUST_13367_PI429010886 | N31065 | 0.010071 | 15 | 3 | 321478280 | hypothetical protein DAPPUDRAFT_40908 [Daphnia | Transport | Ion transport | 1.5E-24 | 96.37601 | 63.12362 | 102.639 | 91.42252 | 72.10026 | 103.3151 | 98.36103 | 86.67899 | 69.93057 | 57.76074 | 51.54875 | 57.09554 | 52.07596 | 75.45906 | 90.85633 | 55.55289 | 102.3996 | 66.64273 | 100.0618 | 59.18336 | 98.86383 | 86.38663 | 84.22055 | 74.88 | 53.37885 | 65.59604 | 70.6776 |
| CUST_14344_PI429010886 | N32731 | 0.010071 | 15 | 3 | | | | | | 328.9809 | 421.6972 | 170.8077 | 544.7766 | 454.8231 | 653.6532 | 274.404 | 248.0682 | 178.3288 | 222.7411 | 214.8393 | 179.4478 | 213.1195 | 310.3428 | 397.7001 | 604.9689 | 346.5745 | 503.7682 | 442.1751 | 403.1472 | 224.015 | 471.0971 | 177.0624 | 372.3592 | 219.5762 | 351.2576 | 137.759 |
| CUST_44130_PI429010886 | M65029 | 0.010073 | 18 | 12 | 307202295 | Segmentation protein cap'n'collar [Harpegnathos sa | | | 3.43E-07 | 144.6491 | 223.9141 | 231.5694 | 177.9272 | 179.1322 | 180.7887 | 171.451 | 214.921 | 201.6936 | 254.095 | 214.8824 | 267.1348 | 239.8633 | 246.8808 | 262.5661 | 271.7719 | 209.1269 | 174.3884 | 193.2088 | 264.6685 | 192.2319 | 199.8275 | 201.2454 | 207.5948 | 236.4903 | 192.7647 | 250.2782 |
| CUST_48396_PI429010886 | M80129 | 0.010073 | 15 | 0 | | | | | | 265.9049 | 277.7994 | 245.7365 | 256.6496 | 257.6826 | 282.3655 | 254.0209 | 245.5292 | 217.8082 | 236.7681 | 232.7081 | 233.9335 | 230.6606 | 294.1484 | 297.5859 | 331.2853 | 307.6262 | 248.959 | 264.269 | 299.4458 | 214.5304 | 276.2073 | 281.874 | 250.3612 | 247.1436 | 225.7607 | 197.122 |
| CUST_37467_PI429010886 | M19131 | 0.010081 | 15 | 0 | 321455420 | hypothetical protein DAPPUDRAFT_218978 [Daphn | Metabolic process | Carbohydrate metabolic process | 9.82E-92 | 2582.117 | 2368.476 | 2125.97 | 2329.908 | 1253.921 | 2269.213 | 615.066 | 1170.459 | 519.0655 | 1474.825 | 1494.238 | 1571.524 | 405.4301 | 1522.087 | 989.1564 | 2125.97 | 1390.385 | 1815.297 | 1197.011 | 3727.046 | 1745.549 | 2009.101 | 805.5548 | 938.9786 | 1555.864 | 1445.794 | 1369.558 |
| CUST_14246_PI429010886 | N32562 | 0.010093 | 12 | 0 | | | | | | 50.74126 | 53.11468 | 72.2954 | 40.35674 | 39.93503 | 29.62583 | 33.82224 | 39.8043 | 38.68077 | 64.19796 | 49.14895 | 52.85191 | 37.26268 | 71.35987 | 47.2535 | 54.22335 | 48.26458 | 44.83858 | 44.31012 | 37.08279 | 51.37508 | 63.30844 | 52.22902 | 40.73202 | 71.23473 | 63.70797 | 55.20849 |
| CUST_12531_PI429010886 | N29609 | 0.010094 | 21 | 0 | | | | | | 306.5332 | 138.7812 | 329.0813 | 123.7772 | 201.1709 | 132.109 | 358.4672 | 73.26024 | 97.75958 | 112.5022 | 210.5151 | 138.3764 | 90.31563 | 85.03701 | 86.15616 | 118.5367 | 300.8257 | 126.1763 | 296.6828 | 112.0953 | 237.2477 | 153.9132 | 289.5463 | 271.6624 | 121.8904 | 77.44837 | 98.69728 |
| CUST_19142_PI429010886 | N41040 | 0.010096 | 21 | 0 | | | | | | 12.12459 | 10.93941 | 14.04977 | NA | 6.074124 | NA | 6.939821 | 4.995434 | 6.856722 | 10.49349 | 4.092773 | 8.123884 | 4.237809 | NA | 6.630788 | 4.839971 | 8.492959 | 7.511797 | NA | 8.902179 | 5.38382 | 6.818591 | 15.88827 | 9.165228 | 7.018038 | 7.523965 | 12.35815 |
| CUST_38132_PI429010886 | M23524 | 0.010106 | 12 | 0 | | | | | | 754.5887 | 502.7361 | 1474.289 | 69.82119 | 168.6416 | 118.5367 | 741.7837 | 1661.324 | 1009.233 | 2070.325 | 480.999 | 602.4628 | 1292.191 | 1846.031 | 857.5648 | 456.4562 | 705.2005 | 388.4161 | 834.3363 | 711.0836 | 533.4173 | 498.1212 | 1094.409 | 408.8989 | 2227.948 | 712.3784 | 2003.234 |
| CUST_13094_PI429010886 | N30563 | 0.010106 | 12 | 3 | | | | | | 145.0813 | 139.2323 | 103.397 | 217.4459 | 316.5936 | 302.8941 | 308.5953 | 128.3894 | 120.8183 | 97.29292 | 181.1767 | 108.3441 | 132.1901 | 122.3475 | 153.1511 | 200.6114 | 194.777 | 227.5328 | 167.0167 | 108.657 | 156.7999 | 153.4856 | 149.731 | 206.1326 | 112.9234 | 152.7823 | 119.9288 |
| CUST_16109_PI429010886 | N35671 | 0.010106 | 12 | 0 | 307204462 | ATP-dependent RNA helicase DDX42 [Harpegnathos | | | 1.68E-22 | 683.3484 | 791.0227 | 789.0995 | 665.3114 | 604.2963 | 574.2435 | 599.7852 | 644.3019 | 613.4417 | 772.4833 | 661.6789 | 886.3986 | 696.7816 | 824.9455 | 875.926 | 921.6949 | 566.6261 | 611.8341 | 631.2945 | 779.1452 | 650.8924 | 732.077 | 682.478 | 501.9627 | 817.1902 | 749.7018 | 809.6878 |
| CUST_18463_PI429010886 | N39837 | 0.010116 | 18 | 12 | 391348270 | PREDICTED: Metaseiulus occidentalis histone H3.3-l | Cellular component organization | Chromosome organization | 6.67E-54 | 6.491213 | 45.79191 | 7.348282 | 9.887988 | 7.41743 | 3.921463 | 11.98424 | 12.44359 | 13.93254 | 17.67943 | 6.561972 | 17.18917 | 14.21644 | 13.51511 | 18.66627 | 13.10341 | 9.080702 | 10.63992 | 13.32448 | 9.854344 | 12.45527 | 9.373204 | 6.357899 | NA | 16.69775 | 7.332766 | 4.514521 |
| CUST_31668_PI429010886 | M2128 | 0.010117 | 24 | 21 | 519767752 | cyclin H [Penaeus monodon] | Cellular Process | Cell cycle | 2.52E-91 | 35.00193 | 37.08698 | 25.04089 | 28.06711 | 43.93802 | 31.77138 | 33.1987 | 26.25499 | 34.64486 | 40.72449 | 37.62291 | 54.45016 | 28.83874 | 24.21006 | 37.09113 | 62.83869 | 35.05162 | 67.27473 | 33.81529 | 41.78277 | 43.37513 | 64.18609 | 62.18234 | 68.0898 | 36.31145 | 54.86684 | 58.78466 |
| CUST_24213_PI429010886 | N50237 | 0.010122 | 24 | 0 | | | | | | 81.00454 | 78.39028 | 58.62419 | 47.93244 | 55.39951 | 41.97456 | 38.02365 | 42.29018 | 37.62291 | 49.66096 | 73.84429 | 38.83801 | 42.81625 | 39.82447 | 14.59227 | 74.51983 | 59.34665 | 40.35311 | 55.16431 | 51.62109 | 59.76158 | 48.41112 | 56.30291 | 38.26903 | 85.19825 | 52.20745 | 69.56167 |
| CUST_49865_PI429010886 | M87501 | 0.010143 | 21 | 3 | | | | | | 115.9278 | 137.8443 | 105.9793 | 125.1284 | 118.802 | 139.1997 | 110.9624 | 88.13649 | 80.94247 | 117.9689 | 104.0648 | 100.2834 | 101.565 | 112.1218 | 109.6802 | 109.4643 | 132.6901 | 118.2899 | 112.7504 | 120.4829 | 119.4111 | 115.7889 | 118.5941 | 99.31284 | 141.3142 | 122.949 | 134.0969 |
| CUST_33790_PI429010886 | M7891 | 0.010145 | 21 | 0 | | | | | | 58.04233 | 83.4507 | 61.64915 | 57.97853 | 44.11042 | 52.9063 | 37.88493 | 27.90015 | 34.00581 | 52.04647 | 50.21157 | 76.62236 | 51.35337 | 54.22335 | 36.17759 | 66.25397 | 41.67274 | 40.99957 | 63.76139 | 75.7764 | 46.11558 | 82.55661 | 69.47796 | 45.72547 | 69.63319 | 47.68335 | 58.47643 |
| CUST_6065_PI429010886 | N19580 | 0.010156 | 15 | 9 | 405969462 | hypothetical protein CGI_10025795 [Crassostrea gig | | | 4.46E-29 | 125.5467 | 140.8613 | 109.5988 | 96.8323 | 120.8512 | 94.01656 | 76.01313 | 108.3614 | 99.7668 | 194.4818 | 150.4117 | 179.3756 | 135.5775 | 157.7938 | 127.0971 | 177.8158 | 129.4038 | 115.2365 | 133.2442 | 166.0617 | 117.9048 | 127.2574 | 137.1215 | 80.51303 | 213.0626 | 156.4118 | 166.8689 |
| CUST_43942_PI429010886 | M64411 | 0.010164 | 24 | 21 | 308220078 | LIM class homeobox transcription factor Lmx [Mner | | | 1.63E-19 | 6.385249 | 7.352114 | 17.87837 | 14.05599 | 19.00199 | 7.518909 | 18.76152 | 12.08776 | 9.300708 | 19.34576 | 18.12711 | 9.895282 | 18.09887 | 26.16386 | 26.38499 | 21.37396 | 35.50382 | 20.83234 | 20.05897 | 14.78359 | 33.05506 | 28.16035 | 42.72999 | 27.89681 | 25.1646 | 12.20042 | 15.33595 |
| CUST_56087_PI429010886 | M112734 | 0.010166 | 24 | 15 | | | | | | 157589.4 | 177655.8 | 168734.7 | 165207 | 161558.4 | 161030.7 | 153078.2 | 193371.5 | 170126.9 | 158283.5 | 168734.7 | 167209.6 | 185961.4 | 174460.2 | 201745.5 | 180118 | 184810.5 | 226545.4 | 180118 | 171853.4 | 163136 | 200261.3 | 177655.8 | 204770.3 | 144434.8 | 175370.5 | 171853.4 |
| CUST_2756_PI429010886 | N13042 | 0.010166 | 24 | 15 | | | | | | 98.52867 | 89.25924 | 119.4557 | 62.64364 | 83.63981 | 89.5312 | 109.2258 | 98.80707 | 92.98929 | 78.05131 | 89.35703 | 76.98892 | 104.1211 | 119.3486 | 117.3642 | 109.3742 | 196.7621 | 129.3867 | 134.02 | 103.0385 | 74.79904 | 133.3139 | 110.4991 | 110.0909 | 120.7762 | 62.33105 | 99.4422 |
| CUST_30937_PI429010886 | M264 | 0.010175 | 24 | 15 | | | | | | 142.1431 | 176.2005 | 152.9711 | 133.0821 | 143.5925 | 118.802 | 94.44207 | 108.6016 | 120.9334 | 172.2354 | 147.5068 | 170.4197 | 121.8132 | 137.9192 | 140.8613 | 232.9884 | 160.4822 | 163.8724 | 158.91 | 150.4655 | 132.7287 | 201.1709 | 180.4354 | 124.8931 | 172.7406 | 130.8991 | 151.1026 |
| CUST_8475_PI429010886 | N23096 | 0.010175 | 24 | 15 | | | | | | 517.4057 | 675.2822 | 508.1895 | 425.0392 | 461.7538 | 496.1582 | 361.8439 | 356.9822 | 401.9872 | 540.3051 | 446.6411 | 595.7445 | 390.4272 | 542.5507 | 483.3249 | 703.6838 | 454.7048 | 638.2757 | 519.4558 | 684.5685 | 443.7417 | 638.1084 | 509.2578 | 431.3053 | 560.3037 | 492.7259 | 554.3622 |
| CUST_34777_PI429010886 | M10622 | 0.010179 | 18 | 9 | | | | | | 166.6117 | 197.4256 | 206.1701 | 178.862 | 188.1161 | 160.8227 | 141.9384 | 245.5656 | 218.7669 | 238.455 | 291.3327 | 335.8595 | 246.2665 | 209.8084 | 218.7209 | 200.4624 | 173.7422 | 196.8321 | 168.3078 | 362.4934 | 260.0898 | 175.3395 | 222.6053 | 163.4482 | 217.0054 | 253.9339 | 273.6903 |
| CUST_20377_PI429010886 | N43336 | 0.010179 | 18 | 9 | | | | | | 19.78441 | 35.93002 | 48.69326 | 11.66976 | 18.04709 | 7.822452 | 27.35532 | 18.23695 | 33.47616 | 28.7462 | 29.5273 | 31.94735 | 19.88004 | 20.3394 | 31.30264 | 17.75139 | 23.74624 | 16.73905 | 16.45817 | 19.83698 | 28.65128 | 8.253508 | 27.95506 | 8.213559 | 22.39477 | 16.42533 | 19.57848 |
| CUST_42345_PI429010886 | M60055 | 0.010189 | 15 | 9 | | | | | | 115.9863 | 112.304 | 146.4805 | 84.03407 | 83.49139 | 63.77772 | 111.7362 | 153.5334 | 167.5217 | 119.1803 | 149.1265 | 143.4699 | 113.976 | 183.574 | 119.4111 | 129.357 | 56.22273 | 96.75892 | 107.1532 | 113.0933 | 127.2103 | 138.9538 | 174.4543 | 95.52923 | 179.616 | 125.5467 | 175.2355 |
| CUST_423_PI429010886 | N5141 | 0.010189 | 15 | 9 | 220172371 | putative elastin a [Rimicaris exoculata] | | | 6.1E-13 | 5435.626 | 19269.36 | 12296.87 | 1092.486 | 3480.516 | 274.6972 | 650.7381 | 8196.001 | 19571.84 | 33059.64 | 26037.41 | 44080.52 | 19120.04 | 29210.68 | 30145.84 | 34979.34 | 4696.717 | 18524.86 | 23632.71 | 30360.4 | 24543.78 | 38834.33 | 20510.07 | 1192.165 | 43935.4 | 30232.55 | 49654.41 |
| CUST_23843_PI429010886 | N49571 | 0.010195 | 21 | 0 | | | | | | 103.4856 | 103.0871 | 93.9354 | 92.63222 | 73.21264 | 93.76216 | 69.80023 | 72.28479 | 78.61543 | 92.90826 | 69.93933 | 93.76216 | 87.93229 | 100.2191 | 91.98586 | 101.2088 | 71.18045 | 114.4136 | 80.94247 | 105.9129 | 98.21029 | 103.109 | 94.19343 | 91.01032 | 96.60043 | 79.26902 | 102.6264 |
| CUST_2177_PI429010886 | N11800 | 0.010213 | 15 | 12 | | | | | | 254.9978 | 296.0085 | 303.3224 | 239.9082 | 163.791 | 192.39 | 177.9272 | 299.4458 | 278.397 | 368.614 | 192.1973 | 392.8804 | 283.5419 | 522.4048 | 416.9251 | 403.7411 | 185.0177 | 278.7196 | 224.3925 | 361.2861 | 241.5998 | 377.0163 | 372.9677 | 236.394 | 412.2515 | 271.7719 | 403.4054 |
| CUST_6229_PI429010886 | N19809 | 0.010213 | 15 | 12 | 91089189 | PREDICTED: similar to gamma glutamyl transpeptid | Metabolic process | Lipid metabolic process | 6.41E-75 | 14.47659 | 9.461053 | 19.08628 | 7.484947 | 7.423086 | 11.2694 | 14.84745 | 15.50711 | 10.9664 | 21.27079 | 6.705496 | 10.99994 | 15.37785 | 25.99654 | 24.77587 | 13.89481 | 18.94687 | 10.37139 | 6.170344 | 5.844471 | 16.98682 | 14.35579 | 16.69534 | 8.634653 | 13.8498 | 13.94032 | 22.78539 |
| CUST_42898_PI429010886 | M61545 | 0.010223 | 12 | 0 | | | | | | 225.1942 | 279.6762 | 287.8668 | 210.9393 | 195.0743 | 243.4729 | 225.2988 | 214.361 | 227.9427 | 237.9979 | 246.2258 | 321.0207 | 241.638 | 326.8661 | 315.5852 | 239.6937 | 229.2068 | 223.1512 | 229.0179 | 261.4208 | 241.638 | 289.8469 | 256.9474 | 213.6947 | 274.0681 | 257.455 | 293.6394 |
| CUST_12212_PI429010886 | N29111 | 0.010227 | 21 | 0 | 260806468 | hypothetical protein BRAFLDRAFT_59551 [Branchio | | | 1.31E-09 | 157.231 | 162.5301 | 97.67416 | 63.77021 | 73.48596 | 71.13139 | 88.07214 | 126.1892 | 70.66226 | 80.4086 | 62.82655 | 90.85633 | 55.22248 | 73.57494 | 70.58805 | 73.44541 | 110.4208 | 148.5892 | 111.9132 | 56.02183 | 135.5002 | 89.88488 | 260.6408 | 189.3905 | 145.0185 | 51.76265 | 149.3076 |
| CUST_4649_PI429010886 | N17209 | 0.010246 | 21 | 3 | | | | | | 117.0501 | 106.002 | 122.4345 | 141.1131 | 130.357 | 137.5108 | 113.9294 | 95.73603 | 121.0374 | 166.6587 | 87.91683 | 146.8587 | 90.06996 | 113.976 | 120.2505 | 106.4489 | 102.2907 | 130.4407 | 101.8776 | 83.03102 | 103.3291 | 172.1642 | 98.73097 | 122.949 | 146.3194 | 123.2567 | 153.961 |
| CUST_12440_PI429010886 | N29466 | 0.010246 | 21 | 0 | | | | | | 237.6429 | 239.3501 | 209.4212 | 174.7413 | 193.6848 | 150.5251 | 158.7737 | 145.0637 | 175.6552 | 187.1216 | 189.4794 | 224.6063 | 189.3407 | 220.6157 | 179.8948 | 284.3688 | 167.9037 | 219.4848 | 175.5769 | 193.4196 | 206.2372 | 216.1505 | 236.1561 | 205.2623 | 174.5672 | 150.2666 | 199.6316 |
| CUST_47755_PI429010886 | M77481 | 0.010264 | 24 | 18 | | | | | | 45.85232 | 47.5835 | 55.34146 | 51.25727 | 42.83757 | 48.01455 | 38.6903 | 53.43689 | 41.35575 | 57.71752 | 42.23748 | 69.49923 | 48.01961 | 59.81399 | 60.94428 | 62.88366 | 47.17722 | 72.2558 | 56.58363 | 69.81278 | 65.49092 | 54.27461 | 56.69148 | 45.43013 | 62.70075 | 36.62775 | 55.74802 |
| CUST_55748_PI429010886 | M111584 | 0.010265 | 18 | 3 | | | | | | 47.55222 | 45.54446 | 39.95554 | 60.47407 | 45.63167 | 61.07679 | 37.93755 | 40.71208 | 37.77068 | 33.63387 | 33.03817 | 56.02183 | 40.77106 | 42.23748 | 31.61657 | 45.21982 | 42.42919 | 60.07014 | 63.62137 | 41.0817 | 43.40546 | 63.58566 | 42.81164 | 52.20238 | 38.18132 | 46.25026 | 47.93942 |
| CUST_11885_PI429010886 | N28572 | 0.010265 | 15 | 12 | | | | | | 29.07242 | 26.6839 | 32.73752 | 17.5491 | 19.61118 | 40.84069 | 22.38395 | 38.6196 | 33.26796 | 43.01496 | 33.46044 | 42.33801 | 43.38432 | 54.6863 | 53.88401 | 34.76984 | 15.60331 | 30.77501 | 28.68237 | 25.24001 | 33.69477 | 43.48893 | 25.25069 | 20.22452 | 38.45723 | 25.79784 | 40.23032 |
| CUST_8662_PI429010886 | N23376 | 0.01028 | 24 | 15 | 242019922 | Neuroglian precursor, putative [Pediculus humanus | | | 4.98E-89 | 29.02946 | 25.37501 | 38.40419 | 28.61065 | 24.77587 | 27.38976 | 32.86927 | 23.81911 | 26.72094 | 32.46954 | 35.06879 | 37.03706 | 33.08345 | 38.85712 | 41.47521 | 43.67532 | 35.0422 | 39.54474 | 43.45754 | 47.2535 | 22.92066 | 48.76564 | 30.86904 | 26.93871 | 41.75113 | 29.08151 | 30.62163 |
| CUST_5948_PI429010886 | N19415 | 0.010281 | 18 | 0 | | | | | | 805.8232 | 1035.582 | 696.3149 | 686.5061 | 764.5382 | 684.1373 | 518.0097 | 544.9594 | 564.2409 | 754.5887 | 645.5341 | 972.7549 | 617.6618 | 592.3414 | 751.2737 | 931.1392 | 572.181 | 788.8563 | 659.8695 | 876.4661 | 623.8166 | 959.6386 | 582.1613 | 696.9471 | 566.9632 | 506.5013 | 637.7954 |
| CUST_30682_PI429010886 | N62385 | 0.010296 | 24 | 15 | | | | | | 46.18431 | 44.15212 | 57.99026 | 34.39507 | 40.91581 | 40.50555 | 46.29899 | 44.30287 | 42.71853 | 27.45965 | 41.80062 | 56.24014 | 45.85232 | 42.78187 | 47.44008 | 62.16793 | 58.15467 | 61.75222 | 56.26704 | 51.40578 | 30.77807 | 64.34064 | 49.52106 | 48.34221 | 62.17487 | 38.16824 | 52.83011 |
| CUST_38632_PI429010886 | M28703 | 0.010308 | 15 | 9 | | | | | | 386.5802 | 526.8072 | 548.5092 | 378.265 | 447.7446 | 461.882 | 399.7554 | 441.0416 | 497.9965 | 581.1144 | 486.3734 | 607.4385 | 438.9413 | 503.0719 | 414.3494 | 494.5663 | 405.9943 | 460.3656 | 412.4272 | 479.4117 | 483.7826 | 511.5434 | 506.9726 | 432.5053 | 656.946 | 540.1617 | 615.7661 |
| CUST_40695_PI429010886 | M55882 | 0.010308 | 15 | 3 | | | | | | 5.946298 | 24.33153 | 8.652112 | 602.798 | 1257.529 | 832.3284 | 38.121 | 7.801914 | 22.66333 | 8.767176 | 5.546661 | 16.43063 | 62.3009 | 8.373525 | 27.17605 | 12.51827 | 808.9549 | 658.8544 | 14.23639 | 13.32908 | 16.4683 | 12.84456 | 14.81603 | 17.06824 | 7.118562 | 4.209782 | 6.835739 |
| CUST_52937_PI429010886 | M99525 | 0.010308 | 15 | 9 | | | | | | 161.4133 | 156.4704 | 215.4042 | 126.4216 | 142.821 | 175.9214 | 151.9486 | 149.5758 | 180.661 | 189.0068 | 180.8997 | 263.345 | 149.6388 | 211.1514 | 206.3446 | 192.9815 | 145.4206 | 160.5038 | 138.0091 | 164.2007 | 191.886 | 162.141 | 176.4634 | 151.5201 | 211.0494 | 202.005 | 215.3368 |
| CUST_3235_PI429010886 | N14075 | 0.010308 | 15 | 3 | | | | | | 460.8905 | 8845.346 | 675.0461 | 18051.51 | 12999.66 | 12762.11 | 7876.866 | 13400.98 | 65.37264 | 10.6464 | 11690.52 | 34.58379 | 41.9571 | 1451.905 | 4724.809 | 203.2288 | 28.99027 | 256.8944 | 1608.297 | 163.162 | 3835.814 | 41.53296 | 3569.653 | 2682.549 | 10.12411 | 19.95383 | 18.11698 |
| CUST_56069_PI429010886 | M112672 | 0.010311 | 24 | 21 | | | | | | 19.55556 | 10.05851 | 12.1001 | 8.475297 | 6.148262 | 10.41753 | 9.04725 | 9.581554 | 7.404114 | 7.546453 | 7.44504 | 6.628016 | 15.73595 | 7.090444 | 7.358125 | 12.61125 | 14.37562 | 17.93802 | NA | 7.137459 | 9.959454 | 14.84035 | 16.5818 | NA | 17.50524 | 10.4869 | 9.148382 |
| CUST_686_PI429010886 | N6751 | 0.010317 | 12 | 0 | | | | | | 137.6307 | 149.9119 | 141.5325 | 142.5887 | 141.1734 | 161.4133 | 121.5738 | 128.3042 | 127.5221 | 160.8449 | 123.9463 | 191.1131 | 136.3426 | 172.6317 | 198.008 | 154.8716 | 135.6641 | 135.6803 | 134.9154 | 140.7358 | 134.3159 | 174.8316 | 153.6236 | 131.4266 | 168.6291 | 175.9719 | 156.2777 |
| CUST_2164_PI429010886 | N11743 | 0.01032 | 18 | 12 | 195618180 | hypothetical protein [Zea mays] | Cellular component organization | Chromosome organization | 1.07E-08 | 200.0082 | 248.428 | 293.7629 | 186.6799 | 228.1106 | 156.4704 | 192.6124 | 188.8906 | 387.9318 | 240.734 | 284.7868 | 527.8694 | 375.9369 | 416.9251 | 418.2809 | 594.7631 | 213.366 | 304.1069 | 340.867 | 395.2528 | 336.4126 | 313.0231 | 226.1214 | 185.8424 | 625.8341 | 348.906 | 541.7506 |
| CUST_17595_PI429010886 | N38245 | 0.010329 | 15 | 3 | | | | | | 107.7492 | 125.5811 | 98.03383 | 132.2661 | 171.7093 | 115.9863 | 146.2634 | 105.711 | 98.02439 | 111.8993 | 156.584 | 78.68384 | 86.5251 | 115.2365 | 76.93173 | 87.90722 | 138.3764 | 110.8479 | 156.2584 | 114.5611 | 153.4856 | 103.9891 | 148.544 | 92.79213 | 122.6882 | 91.60957 | 81.97482 |
| CUST_41122_PI429010886 | M56918 | 0.010332 | 24 | 0 | | | | | | 38.66828 | 41.76704 | 18.42911 | 33.68076 | 34.63137 | 30.27835 | 32.67128 | 30.81346 | 14.14626 | 18.5156 | 19.21762 | 31.1045 | 15.28811 | 15.70628 | 19.29325 | 28.99859 | 34.39507 | 14.41764 | 29.81183 | 24.08352 | 18.27067 | 36.10883 | 30.01154 | 9.370503 | 39.82447 | 116.73 | 27.9866 |
| CUST_15299_PI429010886 | N34355 | 0.010333 | 24 | 15 | 328709829 | PREDICTED: heterogeneous nuclear ribonucleoprot | | | 5.05E-27 | 12.34516 | 24.28 | 26.93068 | 18.27526 | 20.16852 | 10.86638 | 9.928249 | 18.29426 | 15.58239 | 11.75973 | 23.45219 | 35.01361 | 20.05897 | 34.00967 | 30.00667 | 34.70757 | 20.29845 | 26.4818 | 29.56124 | 34.1704 | 21.14959 | 24.80133 | 18.31723 | 18.43075 | 21.71424 | 16.94406 | 31.21185 |
| CUST_37549_PI429010886 | M19446 | 0.010339 | 12 | 0 | | | | | | 1717.641 | 1785.596 | 1554.539 | 841.0533 | 992.543 | 1060.588 | 1339.496 | 999.0406 | 1634.318 | 1323.469 | 722.796 | 1517.009 | 1220.626 | 1884.761 | 1780.629 | 2124.898 | 644.1161 | 1233.197 | 1077.802 | 982.8541 | 1135.995 | 2302.386 | 1762.677 | 1320.618 | 1376.074 | 2362.762 | 2129.816 |
| CUST_15329_PI429010886 | N34414 | 0.010339 | 12 | 3 | 332021656 | DOMON domain-containing protein [Acromyrmex e | | | 2.76E-41 | 50.36639 | 24.96359 | 20.28841 | 105.3949 | 78.44378 | 191.0009 | 91.14746 | 53.41262 | 45.58985 | 15.4125 | 72.28479 | 5.990926 | 51.24109 | 17.77043 | 39.78059 | 27.40178 | 137.9192 | 81.79478 | 89.66356 | 26.62182 | 52.89103 | 22.67743 | 34.80955 | 82.74334 | 14.48423 | 63.08199 | 8.199563 |
| CUST_45680_PI429010886 | M69891 | 0.010345 | 15 | 0 | 321474894 | hypothetical protein DAPPUDRAFT_309115 [Daphn | | | 1.24E-17 | 672.763 | 884.6458 | 613.0465 | 605.6379 | 635.0709 | 652.3862 | 497.2573 | 282.4175 | 368.6847 | 341.0355 | 488.8934 | 576.6597 | 531.2482 | 690.6945 | 628.3128 | 1389.409 | 503.8676 | 877.4226 | 620.7934 | 568.0071 | 365.6206 | 1126.187 | 528.618 | 414.6925 | 620.1455 | 351.7659 | 482.6309 |

| Probe ID | ID | p-value | Period | Phase | EBI ID | Annotation | GO category | Go term | E-value | 0 | 0 | 0 | 3 | 3 | 3 | 6 | 6 | 6 | 9 | 9 | 9 | 12 | 12 | 12 | 15 | 15 | 15 | 18 | 18 | 18 | 21 | 21 | 21 | 24 | 24 | 24 |
|---|---|---|---|---|---|---|---|---|---|---|---|---|---|---|---|---|---|---|---|---|---|---|---|---|---|---|---|---|---|---|---|---|---|---|---|---|
| CUST_35608_PI429010886 | M13011 | 0.010352 | 24 | 18 | 291232275 | PREDICTED: eukaryotic translation initiation factor | | | 1.4E-16 | 71.46149 | 62.12557 | 49.355 | 46.09323 | 46.991 | 60.35512 | 36.20273 | 55.07968 | 59.33007 | 78.05922 | 55.77862 | 72.60868 | 68.1364 | 73.21949 | 63.89005 | 81.51675 | 60.66856 | 64.77801 | 75.93804 | 96.85587 | 76.05543 | 83.23552 | 62.46096 | 30.37057 | 82.46049 | 82.44448 | 92.97872 |
| CUST_45682_PI429010886 | M69895 | 0.010354 | 21 | 15 | | | | | | 49.62512 | 38.38764 | 48.55278 | 19.91592 | 30.94893 | 28.84183 | 28.27144 | 28.02151 | 21.93061 | 36.64631 | 55.10664 | 31.267 | 25.05744 | 28.50866 | 32.3494 | 62.75575 | 48.76564 | 44.70035 | 44.94077 | 29.30417 | 38.36098 | 33.00843 | 22.40634 | 42.83757 | 51.30066 | 32.50043 | 31.54083 |
| CUST_34841_PI429010886 | M10861 | 0.010374 | 21 | 3 | | | Signal transduction | Other | | 26902.5 | 36398.63 | 28551.51 | 48296.18 | 40755.59 | 37322.31 | 26343.04 | 21478.53 | 27142.29 | 32869.85 | 20208.21 | 28306.41 | 21950.73 | 28551.51 | 26343.04 | 26474.55 | 14471.07 | 30145.84 | 20443.66 | 21597.2 | 39918.27 | 26858.78 | 33813.8 | 22140.21 | 37322.31 | 36281.72 | 27018.31 |
| CUST_49051_PI429010886 | M83811 | 0.010383 | 21 | 0 | | | | | | 26.13969 | 27.64723 | 30.58011 | 19.98576 | 16.42915 | 21.26565 | 22.29887 | 13.67691 | 16.12222 | 25.27723 | 23.3505 | 15.36478 | 21.41937 | 25.6683 | 18.31091 | 18.77363 | 18.73634 | 16.55361 | 26.14965 | 23.81072 | 26.12486 | 34.93733 | 24.20353 | 17.9819 | 22.26808 | 16.91321 | 30.01858 |
| CUST_42030_PI429010886 | M59200 | 0.010392 | 24 | 0 | | | | | | 13.37205 | 14.18148 | 3.608723 | 10.14901 | 12.02328 | 16.12591 | 8.440373 | 10.17045 | 7.874648 | 7.555334 | 6.86152 | 10.13526 | 10.23084 | 10.87507 | 7.096174 | 13.31814 | 10.90286 | 12.66916 | 11.3438 | 9.77251 | 13.02465 | 11.38971 | 15.06859 | 7.624707 | 24.54416 | 10.47683 | 16.0073 |
| CUST_2903_PI429010886 | N13357 | 0.010402 | 18 | 0 | 321455420 | hypothetical protein DAPPUDRAFT_218978 [Daphn | Metabolic process | Carbohydrate metabolic process | 4.73E-96 | 2170.679 | 1901.328 | 2127.118 | 2299.417 | 1053.785 | 2538.071 | 480.5601 | 713.1739 | 622.024 | 1257.529 | 1518.655 | 1669.785 | 173.7422 | 1711.88 | 805.0049 | 1344.053 | 1269.191 | 1691.46 | 1249.348 | 3292.379 | 1529.041 | 1818.602 | 699.845 | 785.2299 | 1138.766 | 1392.18 | 804.2481 |
| CUST_25121_PI429010886 | N51816 | 0.010404 | 21 | 15 | | | | | | 113.6118 | 99.75328 | 152.8418 | 144.2774 | 135.1969 | 178.8016 | 95.39206 | 125.0614 | 96.33377 | 122.6882 | 151.9691 | 142.124 | 117.1499 | 128.5885 | 131.1802 | 191.7997 | 173.9876 | 170.8572 | 143.2115 | 163.6107 | 145.726 | 172.3518 | 107.1844 | 141.0429 | 144.1866 | 120.1854 | 133.8101 |
| CUST_38514_PI429010886 | M27586 | 0.010419 | 18 | 3 | | | | | | 17.99612 | 19.05357 | 20.4413 | 29.69991 | 28.37529 | 40.37514 | 30.00897 | 14.83018 | 22.12582 | 23.00078 | 17.75139 | 17.71272 | 17.10987 | 27.4656 | 19.03542 | 26.53218 | 16.08632 | 25.20056 | 26.51868 | 18.97898 | 25.03856 | 27.12475 | 318.02 | 29.48584 | 15.99431 | 21.17737 | 14.4635 |
| CUST_49705_PI429010886 | M86793 | 0.010419 | 18 | 3 | 343131445 | Ichthyophthirius multifiliis strain G5 mitochondrion, | | | 5.89E-65 | 781.185 | 573.2606 | 227.0035 | 5152.319 | 2457.891 | 5065.56 | 1114.646 | 852.3106 | 532.8085 | 326.7925 | 1926.251 | 557.0435 | 727.4325 | 1523.414 | 1653.841 | 1394.051 | 2024.906 | 1169.079 | 2325.953 | 1492.759 | 834.0915 | 2906.073 | 2763.052 | 3861.408 | 245.1154 | 2204.613 | 193.267 |
| CUST_14585_PI429010886 | N33118 | 0.010419 | 18 | 9 | | | | | | 221.3847 | 330.4301 | 260.4155 | 305.1009 | 242.0784 | 86.03759 | 125.3551 | 333.6383 | 247.1436 | 390.2131 | 412.703 | 511.8133 | 295.2655 | 94.53569 | 240.5734 | 258.7017 | 221.165 | 347.8665 | 318.7622 | 185.6237 | 263.6263 | 175.0831 | 92.05581 | 220.2915 | 311.9883 | 292.0563 | 391.2629 |
| CUST_45443_PI429010886 | M69127 | 0.010421 | 24 | 18 | | | | | | 21.92246 | 20.95946 | 20.59244 | 18.81343 | 20.83003 | 31.31098 | 12.46997 | 19.78239 | 16.68781 | 13.36577 | 18.45401 | 8.459432 | 22.83945 | 18.71104 | 25.6517 | 19.01762 | 27.5887 | 15.05605 | 23.44259 | 21.64655 | 27.38976 | 33.90948 | 17.13916 | 25.47479 | 23.5585 | 17.99221 | 30.04719 |
| CUST_53100_PI429010886 | M100139 | 0.010426 | 15 | 3 | | | | | | 81.60393 | 53.21879 | 56.91101 | 72.11758 | 79.06168 | 88.08405 | 75.14455 | 43.81006 | 39.91731 | 62.36898 | 101.1196 | 31.48252 | 64.11406 | 40.2115 | 47.01326 | 57.56812 | 105.8112 | 64.45839 | 90.02313 | 62.27229 | 78.95924 | 65.71273 | 64.11406 | 61.41511 | 47.79729 | 37.49843 | 32.98635 |
| CUST_38027_PI429010886 | M22886 | 0.010426 | 15 | 9 | | | | | | 6026.937 | 18150.31 | 12840.33 | 3572.519 | 5342.716 | 2346.537 | 5620.472 | 6217.899 | 18620.02 | 17900.98 | 11545.02 | 22926.29 | 11673.13 | 20967.97 | 14928.07 | 15327.79 | 5471.152 | 10866.21 | 10102.29 | 18896.93 | 15604.09 | 20010.45 | 15971.11 | 4635.589 | 19822.05 | 22662.24 | 30443.79 |
| CUST_39583_PI429010886 | M41403 | 0.010426 | 15 | 9 | | | | | | 1660.585 | 1155.687 | 2688.606 | 430.8818 | 802.2989 | 326.9766 | 1633.35 | 4161.995 | 2949.668 | 4480.043 | 1089.128 | 1478.287 | 2863.818 | 2838.594 | 1014.347 | 1406.196 | 2214.33 | 980.4603 | 1459.126 | 1021.127 | 909.4624 | 2138.481 | 1756.971 | 338.6596 | 4769.116 | 1110.444 | 4965.834 |
| CUST_43912_PI429010886 | M64311 | 0.010426 | 15 | 6 | | | | | | 45.02788 | 51.90291 | 46.86207 | 194.1492 | 69.07358 | 64.9677 | 69.51764 | 60.54253 | 85.65603 | 87.15435 | 65.89913 | 91.95171 | 69.31912 | 74.0547 | 80.95222 | 67.79763 | 54.37737 | 67.89063 | 82.67802 | 126.9262 | 71.91218 | 88.4462 | 112.5941 | 120.7487 | 48.98001 | 82.21549 | 74.66823 |
| CUST_26306_PI429010886 | N54029 | 0.010426 | 24 | 21 | | | | | | 38.63168 | 44.16686 | 66.09037 | 45.18256 | 34.36888 | 40.883 | 29.29583 | 37.42124 | 48.21189 | 22.80672 | 36.8491 | 39.45879 | 41.41686 | 54.5454 | 46.27475 | 46.46836 | 36.87107 | 68.02926 | 60.28718 | 37.93423 | 58.72685 | 59.72331 | 49.37977 | 51.81515 | 52.93772 | 43.6379 | 66.70898 |
| CUST_50544_PI429010886 | M90210 | 0.010429 | 21 | 18 | | | | | | 9.267153 | 7.272559 | 16.07659 | 3.803141 | 5.995731 | NA | 6.072871 | 5.441883 | NA | 7.621463 | 9.576049 | 3.798968 | 7.22526 | 8.451827 | 3.897995 | 9.74748 | 5.448479 | 7.816139 | 17.20528 | 10.62697 | 9.922626 | 6.404533 | NA | NA | 9.171762 | 3.374359 | 10.78772 |
| CUST_812_PI429010886 | N7388 | 0.010446 | 24 | 18 | | | | | | 76.18529 | 61.41511 | 68.1364 | 65.68089 | 60.3963 | 60.99125 | 43.0609 | 58.54952 | 67.82991 | 71.62646 | 71.33278 | 69.751 | 64.48135 | 87.63509 | 66.52424 | 80.21909 | 82.26332 | 82.04247 | 79.24559 | 86.80804 | 72.1095 | 76.72395 | 91.12979 | 57.17501 | 85.87525 | 57.79515 | 86.7788 |
| CUST_35309_PI429010886 | M12199 | 0.010455 | 12 | 3 | | | | | | 49.64123 | 41.30417 | 38.86122 | 52.60928 | 57.13414 | 69.29923 | 79.39521 | 45.54446 | 31.0493 | 62.79742 | 47.84187 | 41.41686 | 31.64376 | 38.87395 | 37.7275 | 47.93244 | 73.1906 | 55.55874 | 49.53059 | 32.98295 | 67.30267 | 38.79996 | 47.9222 | 54.25425 | 44.07915 | 29.59116 | 31.56711 |
| CUST_31633_PI429010886 | M2024 | 0.010455 | 12 | 0 | | | | | | 234.8913 | 363.6175 | 260.5083 | 228.3459 | 215.3723 | 174.7413 | 200.6114 | 216.4729 | 296.3588 | 317.1399 | 252.4045 | 233.124 | 255.1161 | 256.281 | 309.8242 | 292.0563 | 139.9854 | 228.0795 | 232.5572 | 231.3759 | 186.167 | 351.5336 | 208.515 | 200.184 | 404.4491 | 287.1573 | 282.1142 |
| CUST_4264_PI429010886 | N16359 | 0.010455 | 12 | 3 | | | | | | 62.70075 | 25.8104 | 24.95301 | 153.237 | 105.3648 | 316.4852 | 95.07199 | 96.55629 | 28.71325 | 23.68272 | 89.54002 | 26.00775 | 39.61818 | 21.93695 | 42.6374 | 31.32413 | 162.7985 | 162.0477 | 191.6398 | 29.11372 | 51.82417 | 26.37273 | 63.04603 | 86.58356 | 23.56736 | 157.1328 | 37.51655 |
| CUST_50839_PI429010886 | M91195 | 0.010457 | 12 | 0 | | | | | | 88.3275 | 88.99049 | 105.7694 | 56.24668 | 66.10733 | 79.41411 | 67.64754 | 67.45213 | 81.59575 | 145.5994 | 89.87648 | 104.5579 | 80.9345 | 117.3497 | 118.4488 | 96.34482 | 63.73904 | 90.64646 | 80.61162 | 97.5983 | 101.3168 | 101.3567 | 90.54311 | 72.37554 | 92.70294 | 133.2264 | 122.5481 |
| CUST_15865_PI429010886 | N35267 | 0.010457 | 15 | 12 | | | | | | 65.86348 | 178.6287 | 162.5891 | 150.3004 | 113.8053 | 131.259 | 145.0637 | 155.1509 | 172.0275 | 153.734 | 142.1826 | 312.052 | 119.7932 | 258.1237 | 220.685 | 160.804 | 124.3983 | 249.7124 | 123.1229 | 98.129 | 124.5927 | 136.0526 | 227.8652 | 168.0579 | 216.2628 | 198.5364 | 193.714 |
| CUST_16209_PI429010886 | N35846 | 0.010471 | 24 | 21 | | | | | | 77.55036 | 91.90153 | 118.3064 | 106.7278 | 86.28978 | 144.8962 | 101.8407 | 70.46014 | 65.52616 | 80.38938 | 86.43501 | 78.36561 | 85.60322 | 91.11111 | 94.55772 | 89.22919 | 131.1249 | 117.1631 | 107.2744 | 100.566 | 106.8828 | 110.8992 | 128.6369 | 110.3421 | 77.99438 | 88.14739 | 63.94718 |
| CUST_55419_PI429010886 | M110013 | 0.010481 | 18 | 6 | 270009780 | hypothetical protein TcasGA2_TC009077 [Tribolium | | | 5.59E-10 | 949.8478 | 1428.874 | 1637.238 | 736.1144 | 961.5038 | 925.4363 | 2582.117 | 2286.095 | 3899.401 | 1762.677 | 1251.807 | 1520.198 | 1792.391 | 1755.419 | 1826.751 | 2243.877 | 1636.442 | 1502.524 | 1464.638 | 811.2478 | 1212.885 | 977.4233 | 1846.031 | 1444.613 | 2877.641 | 1070.988 | 4643.325 |
| CUST_2394_PI429010886 | N12258 | 0.010482 | 24 | 18 | | | | | | 24.67398 | 25.59587 | 14.12299 | 8.876178 | 8.793342 | 10.32292 | 6.746227 | 5.696732 | 10.32292 | 22.42169 | 13.98352 | 23.063 | 14.01595 | 21.036 | 20.25261 | 24.52204 | 12.0248 | 18.5867 | 21.49202 | 35.52357 | 17.56531 | 25.4983 | 17.33078 | 13.53466 | 23.63331 | 12.49757 | 32.18334 |
| CUST_38950_PI429010886 | M32421 | 0.010494 | 24 | 15 | 321449602 | hypothetical protein DAPPUDRAFT_120690 [Daphn | | | 4.09E-21 | 197.7545 | 160.0546 | 180.661 | 298.1939 | 163.7139 | 182.8109 | 284.9694 | 281.652 | 253.7351 | 250.8554 | 154.1562 | 281.447 | 259.6247 | 333.6383 | 254.8434 | 319.8933 | 270.3602 | 304.9967 | 239.0491 | 251.8524 | 220.2915 | 216.5095 | 293.8285 | 231.3422 | 221.4446 | 129.5087 | 264.269 |
| CUST_44747_PI429010886 | M66866 | 0.010494 | 24 | 0 | | | | | | 32.95758 | 27.11255 | 42.38554 | 30.95577 | 26.2515 | 32.7846 | 16.15147 | 26.24834 | 30.24809 | 42.03093 | 21.72395 | 19.63939 | 22.16225 | 29.75464 | 27.99024 | 16.7895 | 23.26561 | 30.13575 | 26.78662 | 23.53859 | 24.27461 | 24.8275 | 23.20447 | 25.20545 | 33.75702 | 34.33559 | 31.29954 |
| CUST_7119_PI429010886 | N21069 | 0.010494 | 24 | 0 | | | | | | 40.27161 | 55.5381 | 40.95911 | 58.01793 | 24.14057 | 36.24706 | 34.05076 | 42.36335 | 27.78206 | 38.1599 | 31.43539 | 44.95044 | 40.58787 | 36.76731 | 29.94487 | 23.85972 | 25.91041 | 31.10751 | 36.04028 | 34.27356 | 37.69054 | 35.78629 | 36.86394 | 29.91175 | 36.71638 | 47.44495 | 41.28609 |
| CUST_26243_PI429010886 | N53904 | 0.010494 | 24 | 0 | | | | | | 16.75931 | 19.26784 | 23.75128 | 15.63968 | 10.79562 | 29.94885 | 18.98962 | 15.91395 | 14.08785 | 15.74217 | 14.46162 | 13.95627 | 14.28302 | 18.46537 | 11.33971 | 14.46495 | 22.44818 | 25.02916 | 18.24055 | 15.9498 | 19.97907 | 27.68313 | 19.90278 | 16.35182 | 20.95213 | 23.1605 | 35.57263 |
| CUST_24337_PI429010886 | N50443 | 0.010521 | 12 | 9 | | | | | | 124.5429 | 179.5033 | 102.5637 | 101.218 | 73.06271 | 72.86527 | 73.81964 | 117.0236 | 117.621 | 195.1881 | 131.3416 | 219.061 | 178.4723 | 125.3551 | 59.61145 | 179.7938 | 81.26685 | 131.046 | 144.1009 | 125.9625 | 124.7893 | 166.8022 | 141.6567 | 258.8229 | 119.5708 | 117.2337 | 143.9244 |
| CUST_39649_PI429010886 | M42181 | 0.01053 | 15 | 3 | | | | | | 68.5998 | 5.711785 | 93.5311 | 14.67638 | 73.14833 | 73.16394 | 39.31816 | 64.59746 | 37.84521 | 11.36992 | 15.13462 | NA | 16.69384 | 7.149572 | 4.187076 | NA | 94.64608 | 45.70165 | 81.67574 | NA | 43.18546 | NA | 17.92211 | 15.02569 | 7.702551 | 13.12172 | 4.39723 |
| CUST_40204_PI429010886 | M51719 | 0.010532 | 15 | 3 | | | | | | NA | 3.972829 | 11.40246 | 43.51593 | 44.5467 | 56.18729 | 56.80303 | 19.26784 | NA | 10.18097 | 27.88984 | NA | 4.680323 | NA | 3.514447 | 7.972595 | 32.28425 | 43.09974 | 56.47376 | 7.017135 | 72.89846 | 5.704319 | 84.48505 | 55.54166 | 10.19943 | 35.70676 | 4.866661 |
| CUST_57276_PI429010886 | E3651 | 0.010545 | 15 | 9 | 4585366 | extracellular superoxide dismutase precursor [Pacif | Metabolic process | Oxidation-reduction process | 2.4E-21 | 86.35271 | 69.38283 | 253.8833 | 35.02438 | 33.92452 | 47.26675 | 112.0225 | 328.6116 | 316.0527 | 448.0873 | 75.94687 | 112.8928 | 180.8117 | 512.8422 | 236.9243 | 62.5758 | 272.4885 | 60.40681 | 81.74728 | 235.2753 | 69.13041 | 358.4672 | 372.1299 | 61.72335 | 615.211 | 112.9419 | 557.6662 |
| CUST_39875_PI429010886 | M46060 | 0.010545 | 15 | 9 | | | | | | 889.0555 | 637.2498 | 667.1794 | 429.0337 | 539.016 | 560.3037 | 684.8725 | 519.9761 | 707.905 | 1592.642 | 776.1766 | 984.9879 | 803.5259 | 858.7607 | 578.8676 | 1163.104 | 629.6206 | 777.0504 | 674.2322 | 638.9314 | 752.9771 | 834.6224 | 692.7803 | 877.6784 | 1067.136 | 658.0264 | 1148.695 |
| CUST_18366_PI429010886 | N39657 | 0.010545 | 15 | 9 | | | | | | 55.79477 | 61.79791 | 67.83607 | 49.07171 | 52.53463 | 22.63247 | 51.33619 | 60.69824 | 69.32875 | 95.17141 | 62.05533 | 55.99321 | 53.0654 | 68.72963 | 57.82155 | 60.69824 | 62.74739 | 60.9102 | 59.96262 | 60.72981 | 58.38163 | 53.63782 | 63.1995 | 38.73495 | 78.30552 | 76.80142 | 90.04123 |
| CUST_1921_PI429010886 | N11080 | 0.010549 | 15 | 3 | | | | | | 21.97651 | 10.38437 | 23.52815 | 10729.62 | 11747.34 | 20248.42 | 5197.227 | 1794.046 | 24.08567 | 12.6412 | 5953.512 | 18.44505 | 22.20218 | NA | 8.626596 | 8.215837 | 10310.79 | 9341.534 | 15029.96 | 10.38108 | 2667.238 | 9.290785 | 2029.752 | 2365.487 | 10.10287 | 1853.071 | 10.22324 |
| CUST_50566_PI429010886 | M90295 | 0.010549 | 24 | 15 | | | | | | 221.4718 | 222.7411 | 233.0458 | 149.4288 | 220.9933 | 286.2536 | 187.6525 | 183.9501 | 162.6071 | 227.9427 | 221.3847 | 168.99 | 104.3393 | 168.9219 | 168.5577 | 306.479 | 275.547 | 337.2385 | 275.4021 | 266.9855 | 272.8585 | 235.7212 | 201.7309 | 244.1166 | 298.9214 | 189.4251 | 324.5263 |
| CUST_17671_PI429010886 | N38386 | 0.010555 | 21 | 3 | | | | | | 12.17117 | 13.69236 | 13.95744 | 14.86246 | 16.74929 | 14.80005 | 13.69107 | 6.492705 | 6.928497 | 18.80396 | 8.994284 | 30.04952 | 11.87443 | 9.568409 | 9.745966 | 10.29246 | 10.25912 | 9.77251 | 13.28601 | 17.15538 | 10.25912 | 10.72561 | 24.03363 | NA | 16.19477 | 20.00677 | 22.37611 |
| CUST_32122_PI429010886 | M3286 | 0.010557 | 24 | 15 | 260824760 | hypothetical protein BRAFLDRAFT_113907 [Branchi | | | 1E-48 | 49.11313 | 61.0979 | 55.42699 | 46.53779 | 40.15414 | 42.19897 | 62.54347 | 40.4721 | 54.25425 | 84.98778 | 46.1565 | 68.8749 | 72.66838 | 53.1957 | 57.32582 | 89.67555 | 35.1685 | 88.01513 | 51.79552 | 74.96433 | 67.17456 | 71.18669 | 72.14089 | 62.63151 | 60.69824 | 48.84923 | 68.56896 |
| CUST_11740_PI429010886 | N28331 | 0.010557 | 24 | 18 | 242013211 | conserved hypothetical protein [Pediculus humanus | | | 9.77E-43 | 194.4538 | 311.0329 | 237.1286 | 264.5866 | 228.586 | 266.6935 | 138.7624 | 217.5495 | 146.1977 | 183.6032 | 189.5352 | 277.2458 | 273.733 | 263.7458 | 285.1566 | 303.9813 | 186.9949 | 195.3573 | 303.4405 | 297.6952 | 280.8839 | 294.5278 | 206.0656 | 231.6859 | 271.5735 | 269.7173 | 241.9617 |
| CUST_34731_PI429010886 | M10488 | 0.010572 | 21 | 3 | | | | | | 538.0354 | 1046.691 | 785.2299 | 1746.637 | 723.6629 | 924.8591 | 497.3582 | 532.9396 | 632.2521 | 669.8776 | 367.7937 | 959.9717 | 466.8917 | 820.2371 | 359.4804 | 500.6766 | 251.4349 | 574.8968 | 275.1358 | 352.0546 | 907.7978 | 756.3176 | 1030.293 | 595.5631 | 992.543 | 1055.576 | 559.2711 |
| CUST_47888_PI429010886 | M78029 | 0.010573 | 18 | 3 | | | | | | 1013.924 | 2764.643 | 1394.642 | 4365.095 | 1846.031 | 1941.576 | 1202.334 | 965.4945 | 1358.264 | 1380.785 | 673.3658 | 2144.378 | 783.5804 | 1050.102 | 360.037 | 1324.694 | 427.7928 | 417.4973 | 267.4223 | 558.2631 | 1942.982 | 1562.541 | 1975.879 | 1067.598 | 2073.005 | 972.4433 | 1031.749 |
| CUST_47103_PI429010886 | M74806 | 0.010583 | 12 | 0 | 339232928 | putative zinc finger protein [Trichinella spiralis] >gi\| | | | 7.06E-21 | 957.5442 | 999.7477 | 1122.467 | 797.3133 | 581 | 735.8213 | 681.1329 | 630.6794 | 730.0267 | 986.7668 | 847.5292 | 1217.47 | 764.3328 | 1012.516 | 996.2576 | 1119.759 | 839.2544 | 771.9192 | 706.0046 | 999.7477 | 825.4395 | 932.5265 | 732.314 | 704.3489 | 917.7643 | 1710.023 | 1109.205 |
| CUST_18482_PI429010886 | N39879 | 0.010588 | 15 | 9 | 260822597 | hypothetical protein BRAFLDRAFT_209586 [Branchi | Nucleic acid metabolism | Regulation of gene expression | 1.57E-23 | 10.39491 | 9.264427 | 10.88348 | 13.32189 | 17.08729 | 12.1537 | 6.194786 | 16.74068 | 22.32739 | 15.30361 | 24.95635 | 30.76441 | 20.85366 | 19.90878 | 20.06593 | 24.10675 | 12.12816 | 13.93595 | 25.53642 | 24.12979 | 14.75825 | 26.86268 | 19.70553 | NA | 19.68474 | 37.45529 | 32.64658 |
| CUST_50699_PI429010886 | M90692 | 0.010594 | 21 | 9 | | | | | | 48.67472 | 53.16284 | 73.5024 | 52.01848 | 66.75063 | 59.85492 | 71.75603 | 67.1407 | 60.26272 | 73.77697 | 85.59472 | 66.39266 | 52.55999 | 85.62903 | 78.62359 | 55.21295 | 62.23675 | 64.62304 | 65.75053 | 68.21242 | 73.62986 | 58.69441 | 51.4111 | 57.05184 | 65.27575 | 62.67964 | 68.08199 |
| CUST_45539_PI429010886 | M69422 | 0.010605 | 15 | 0 | | | Metabolic process | Other | | 15.61612 | 16.27057 | 14.51392 | 10.94248 | 10.21823 | 13.39381 | 14.92301 | 4.510564 | 5.846111 | 11.12035 | 7.183846 | 10.14992 | 9.140795 | 4.692488 | 6.115825 | 28.48875 | 15.66371 | 16.36632 | 17.7958 | 8.970081 | 10.76236 | 12.11037 | 8.477051 | 6.342428 | 10.95578 | 6.314907 | 10.73339 |
| CUST_12580_PI429010886 | N29690 | 0.010609 | 24 | 21 | 156539569 | PREDICTED: SET and MYND domain-containing prot | | | 4.47E-07 | 13.55928 | 12.90472 | 10.12411 | 5.507769 | 15.96275 | 21.26038 | 9.268485 | 10.53799 | 6.339538 | 8.827909 | 5.57472 | NA | 5.993779 | 8.339436 | 4.93837 | 14.41386 | 17.57242 | 13.66257 | 13.95459 | 9.207048 | 11.65987 | 15.23556 | 11.74047 | 18.50987 | 10.28851 | NA | 11.14537 |
| CUST_40419_PI429010886 | M55031 | 0.010614 | 15 | 9 | | | | | | 1223.156 | 1770.146 | 1390.385 | 1230.543 | 860.1543 | 1216.535 | 916.8157 | 1171.568 | 1101.197 | 1654.803 | 1164.105 | 1779.751 | 1244.647 | 1686.366 | 1617.488 | 1670.981 | 985.7913 | 1417.942 | 1309.194 | 1392.643 | 1302.095 | 1444.045 | 1426.122 | 1562.541 | 1888.53 | 2343.419 | 1873.43 |
| CUST_27904_PI429010886 | N56913 | 0.010617 | 15 | 0 | 321458259 | hypothetical protein DAPPUDRAFT_202914 [Daphn | | | 9.67E-26 | 59.76845 | 57.96698 | 41.73275 | 25.20545 | 56.61232 | 44.63016 | 52.89103 | 35.49419 | 40.96307 | 41.86651 | 38.64078 | 34.90297 | 51.04982 | 40.37133 | 34.65468 | 54.5227 | 50.36639 | 38.67688 | 56.76191 | 52.47261 | 43.48893 | 47.45909 | 38.57925 | 36.4309 | 38.91166 | 35.74458 | 28.5643 |
| CUST_18491_PI429010886 | N39891 | 0.010627 | 21 | 3 | | | | | | 24.43879 | 22.49197 | 18.67439 | 29.65821 | 17.46899 | 26.23528 | 26.50391 | 15.07352 | 21.90983 | 28.35096 | 9.813406 | 23.30079 | 16.77252 | 16.94555 | 10.7559 | 14.08315 | 14.45246 | 14.55066 | 20.02885 | 49.65196 | 33.72145 | 25.03495 | 28.08839 | 22.61376 | 27.74158 | 36.76396 | 32.46401 |
| CUST_1501_PI429010886 | N9836 | 0.010633 | 12 | 0 | | | | | | 2786.84 | 4239.143 | 3815.349 | 2027.433 | 2380.879 | 2064.29 | 2312.077 | 2076.382 | 2092.578 | 3667.985 | 2978.783 | 3858.586 | 2888.695 | 4330.695 | 4169.34 | 5256.29 | 2321.278 | 2408.505 | 2475.35 | 4263.359 | 2485.559 | 3661.505 | 3057.78 | 2873.023 | 3972.597 | 2740.182 | 4008.447 |
| CUST_13192_PI429010886 | N30754 | 0.010649 | 12 | 3 | | | | | | 61.04516 | 45.45196 | 20.06405 | 88.60515 | 38.25818 | 42.43356 | 37.24643 | 35.37524 | 28.56717 | 29.27728 | 21.04609 | 16.70318 | 27.32872 | 37.9853 | 20.54194 | 43.0609 | 43.22077 | 34.96061 | 26.37273 | 49.72682 | 23.04237 | 31.00211 | 22.21962 | 23.25478 | 48.52803 | 36.42724 | 25.79784 |
| CUST_47201_PI429010886 | M75178 | 0.010656 | 21 | 3 | | | | | | 7.548864 | 14.89135 | 35.37524 | 20.37797 | 76.99966 | 82.6196 | 51.37925 | 4.189741 | 55.84117 | 26.9152 | 21.51397 | 11.89991 | 17.60822 | 172.3518 | 32.77063 | 8.698364 | 12.68111 | 46.59363 | 5.908014 | 7.129573 | 13.37557 | 26.50391 | 16.88307 | 21.85231 | 126.9841 | 28.4998 | 39.78898 |
| CUST_9506_PI429010886 | N24752 | 0.010658 | 21 | 15 | 195490130 | Drosophila yakuba GE21090 (Dyak\GE21090), mRN | Protein metabolism | Cellular protein modification process | 1.9E-106 | 62.38529 | 56.58363 | 68.2813 | 79.47713 | 57.03895 | 49.8819 | 49.47471 | 52.801 | 45.52189 | 75.4087 | 63.64323 | 71.57902 | 49.38217 | 82.04247 | 62.5758 | 84.07185 | 74.04651 | 69.31235 | 70.68641 | 54.05301 | 69.82734 | 75.07319 | 64.23886 | 56.23475 | 66.04832 | 57.30764 | 51.1689 |
| CUST_53910_PI429010886 | M103670 | 0.010663 | 24 | 15 | 260830643 | hypothetical protein BRAFLDRAFT_126836 [Branchi | Metabolic process | Other | 1.68E-21 | 13.35156 | 16.37493 | 12.31007 | 5.859043 | 12.83173 | 7.925956 | 12.68722 | 21.70738 | 10.14992 | 18.36323 | 38.28633 | 13.16888 | 12.75631 | 18.75687 | 35.30492 | 24.26118 | 18.03368 | 19.18935 | 25.44661 | 10.62249 | NA | 18.50047 | 17.80566 | 18.60109 | 19.2383 | 11.81616 | 11.03039 |
| CUST_47089_PI429010886 | M74771 | 0.010671 | 24 | 15 | 321467470 | hypothetical protein DAPPUDRAFT_320464 [Daphn | Protein metabolism | Cellular protein modification process | 2.77E-08 | 63.40267 | 51.80926 | 79.20223 | 61.63016 | 67.59014 | 78.12041 | 50.26445 | 50.13498 | 45.84492 | 73.64901 | 51.77357 | 70.80885 | 43.71768 | 51.30066 | 74.7726 | 79.26902 | 84.92511 | 94.09795 | 75.7868 | 66.2145 | 59.96834 | 68.2359 | 59.22654 | 74.63865 | 68.36198 | 67.10317 | 71.94024 |
| CUST_46632_PI429010886 | M73232 | 0.010698 | 15 | 9 | | | | | | 93.21704 | 98.28699 | 119.1476 | 76.08615 | 91.42252 | 98.71598 | 100.7693 | 88.87538 | 104.9421 | 127.601 | 94.27998 | 122.4667 | 103.1763 | 105.6225 | 57.48318 | 104.164 | 77.68249 | 55.14182 | 79.35074 | 106.7278 | 79.50596 | 125.9453 | 119.9009 | 87.80183 | 127.9952 | 116.3867 | 147.2361 |
| CUST_55665_PI429010886 | M111192 | 0.0107 | 12 | 0 | | | | | | 19.33574 | 23.81072 | 31.34117 | 16.12222 | 18.61046 | 25.37 | 15.71416 | 19.77641 | 27.87925 | 21.27912 | 26.35211 | 25.16199 | 24.47717 | 25.0475 | 26.4083 | 18.86017 | 21.12887 | 19.39031 | 16.91691 | 23.64437 | 21.91188 | 23.63583 | 25.67033 | 16.85432 | 30.68719 | 30.59854 | 36.35659 |
| CUST_24620_PI429010886 | N50958 | 0.010707 | 21 | 15 | 338809146 | hemocyanin [Exopalaemon carinicauda] | | | 4.6E-12 | NA | 11.73735 | 9.84998 | 16.4346 | 96.8234 | NA | 61.11567 | 199.9828 | 36.99055 | 20.58435 | 147.3674 | 184.1659 | 1173.091 | 24.63851 | 567.7179 | NA | 236.4903 | NA | 13.04571 | 10597.5 | 273.733 | NA | 11.74757 | 8.754916 | 4.034985 | 57.42327 | 239.0792 |
| CUST_40800_PI429010886 | M56112 | 0.010707 | 12 | 6 | | | | | | 198.4267 | 170.4477 | 209.5249 | 134.7073 | 170.596 | 207.6455 | 224.3204 | 229.8288 | 183.786 | 189.9402 | 161.711 | 187.6525 | 155.2181 | 149.731 | 135.6966 | 179.1099 | 186.8185 | 154.3982 | 213.6068 | 173.4981 | 192.2865 | 171.9761 | 275.7928 | 201.1422 | 95.91781 | 139.5229 | 103.0015 |
| CUST_31901_PI429010886 | M2706 | 0.010713 | 24 | 9 | | | | | | 24.17061 | 31.72455 | 35.26822 | 39.76167 | 43.01496 | 20.61726 | 37.22147 | 85.01801 | 44.63765 | 41.74772 | 58.17945 | 58.44181 | 53.20866 | 31.52633 | 47.75268 | 32.80956 | 29.64988 | 22.88862 | 22.24289 | 95.84863 | 42.38554 | 27.22484 | 36.47407 | 23.42495 | 20.53839 | 28.27702 | 24.62928 |
| CUST_43336_PI429010886 | M62762 | 0.010727 | 18 | 12 | | | | | | 36.98363 | 42.09877 | 57.78767 | 37.52021 | 24.47403 | 41.66889 | 40.95098 | 44.83381 | 67.01506 | 38.93338 | 34.10794 | 59.57796 | 54.38291 | 64.83329 | 56.11071 | 50.92195 | 50.64584 | 55.6145 | 37.96902 | 52.79712 | 38.22525 | 41.96975 | 33.87601 | 37.35904 | 63.13278 | 42.19441 | 69.18954 |
| CUST_43738_PI429010886 | M63829 | 0.010755 | 21 | 9 | | | | | | 10.04605 | 50.3484 | 10.85593 | 66.2145 | 98.11651 | 29.38309 | 42.0037 | 89.96525 | 74.74764 | 42.38554 | 173.3024 | 191.3655 | 65.33287 | 18.86217 | 23.67732 | 4.934877 | 72.03066 | NA | 14.39924 | 362.9082 | 40.89152 | 9.576049 | 22.97006 | 8.557801 | NA | 73.55823 | 83.89746 |
| CUST_25608_PI429010886 | N52736 | 0.010755 | 15 | 0 | | | | | | 13.53805 | 12.20868 | NA | 14.38182 | 4.988225 | 8.541205 | 4.545884 | 4.976399 | 5.590644 | NA | 4.098845 | 6.927136 | 8.263899 | 6.105375 | 8.178429 | 15.75243 | 11.83012 | 6.477097 | 9.326745 | NA | 5.310263 | 5.116273 | NA | 6.104204 | 12.84324 | NA | 11.83225 |
| CUST_55092_PI429010886 | M108692 | 0.010757 | 18 | 9 | | | | | | 21.58663 | 19.4542 | 22.64587 | 29.24265 | 20.63988 | 32.47618 | 16.03098 | 21.12385 | 25.42066 | 28.02553 | 21.74647 | 41.5428 | 27.88257 | 26.82962 | 36.8021 | 26.22564 | 27.95506 | 24.08567 | 18.0662 | 15.18848 | 18.88381 | 24.18144 | 11.51207 | 14.33928 | 39.61101 | 46.81158 | 27.75482 |
| CUST_38598_PI429010886 | M28437 | 0.010777 | 18 | 0 | | | | | | 82.18107 | 28.08839 | 74.97848 | 35.78224 | 46.47908 | 44.23444 | 56.96512 | 26.4818 | 22.35984 | 23.02215 | 47.79169 | 14.46019 | 29.21516 | 26.57549 | 40.01491 | 35.45648 | 88.40608 | 37.03334 | 61.75222 | 42.33801 | 47.36255 | 39.78059 | 71.38156 | 63.39728 | 35.22807 | 29.16954 | 33.14406 |
| CUST_22034_PI429010886 | N46303 | 0.010782 | 15 | 0 | | | | | | 112.3653 | 95.19444 | 119.3258 | 49.44344 | 110.8879 | 113.4862 | 103.8326 | 64.58903 | 67.02702 | 112.0535 | 91.62991 | 76.03079 | 87.55497 | 63.04603 | 89.97695 | 118.3919 | 112.0953 | 115.5867 | 81.79478 | 97.50342 | 80.0595 | 100.3848 | 41.69983 | 54.37737 | 112.4734 | 95.18251 | 79.84889 |
| CUST_10094_PI429010886 | N25683 | 0.010794 | 15 | 9 | | | | | | 52.35985 | 92.10717 | 100.1395 | 73.91931 | 98.23281 | 91.51834 | 137.351 | 80.04179 | 110.7887 | 101.6485 | 102.0104 | 153.4856 | 74.42211 | 133.8452 | 101.3304 | 65.13281 | 74.97194 | 153.0835 | 55.21772 | 60.71887 | 125.6428 | 99.77775 | 139.3127 | 126.539 | 109.0596 | 145.2206 | 183.5489 |
| CUST_8040_PI429010886 | N22456 | 0.010811 | 21 | 18 | 332030550 | DNA-directed RNA polymerases I and III subunit RP/ | | | 8.2E-21 | 90.45788 | 94.54715 | 64.76438 | 67.78689 | 67.29642 | 58.13115 | 25.32554 | 17.98635 | 26.37273 | 52.4826 | 48.39289 | 75.13415 | 39.288 | 92.00286 | 92.39293 | 123.321 | 22.66107 | 89.17313 | 50.49286 | 139.0376 | 76.90742 | 114.2341 | 38.01489 | 85.09002 | 80.85722 | 44.64991 | 31.73369 |
| CUST_22155_PI429010886 | N46532 | 0.010824 | 24 | 0 | 241735164 | phosphoglucomutase/phosphomannomutase, puta | Metabolic process | Carbohydrate metabolic process | 2.2E-22 | 14.73565 | 20.09671 | 8.149809 | 15.1999 | 10.52648 | 5.921666 | 11.54084 | 15.31913 | 8.999498 | 12.49275 | 5.328263 | 16.26559 | 8.802891 | 5.880062 | 7.14839 | 14.39574 | 4.438084 | 7.801914 | 12.79498 | 16.61727 | 10.93346 | 13.73929 | 10.99803 | NA | 18.69696 | 23.14834 | 15.89427 |
| CUST_31734_PI429010886 | M2270 | 0.010826 | 18 | 15 | 543735440 | PREDICTED: conserved oligomeric Golgi complex su | Transport | Protein transport | 5.07E-28 | 67.47589 | 81.17779 | 65.11259 | 62.62665 | 61.42421 | 68.69041 | 75.91487 | 64.42438 | 72.24742 | 58.75213 | 67.59014 | 76.84434 | 74.50049 | 92.25373 | 81.08583 | 116.4037 | 84.80692 | 98.67215 | 65.59604 | 90.56267 | 45.92841 | 91.56459 | 51.71264 | 61.5801 | 80.79066 | 56.08637 | 68.06658 |
| CUST_55480_PI429010886 | M110307 | 0.010847 | 18 | 3 | 155966288 | putative cuticle protein [Lepeophtheirus salmonis] | | | 1.73E-22 | 10.3075 | 6.730364 | 8.15397 | 16.26432 | 8.783511 | 18.65246 | 17.12411 | 8.382851 | 4.358673 | 5.298018 | 5.023732 | 3.806553 | 5.818644 | 4.619267 | NA | 6.271198 | 5.22802 | NA | 10.46161 | 6.060974 | 15.81194 | NA | 3.692253 | 23.23746 | NA | NA | 9.067263 |
| CUST_29438_PI429010886 | M59863 | 0.010863 | 21 | 18 | | | | | | 12.71524 | 13.18143 | 10.46975 | NA | 14.25137 | 10.19353 | 10.71453 | 4.222969 | 8.617079 | 9.787782 | 6.307552 | 13.94176 | 9.156873 | 6.626935 | 7.96115 | 20.67573 | 18.74861 | 11.26503 | 13.96065 | 12.45151 | 24.54416 | 22.05551 | NA | 9.963882 | 9.941638 | NA | NA |
| CUST_22270_PI429010886 | N46722 | 0.010865 | 21 | 15 | | | | | | 43.47138 | 56.77783 | 72.39117 | 43.45323 | 45.57502 | 83.31796 | 18.60109 | 12.42375 | 35.17156 | 44.8896 | 63.42029 | 49.41092 | 10.56079 | 55.784 | 42.28532 | 100.8175 | 56.78823 | 68.85742 | 47.19565 | 63.89005 | 75.50768 | 57.41489 | 35.70676 | 24.70791 | 57.07573 | 41.52389 | 49.07171 |
| CUST_18930_PI429010886 | N40673 | 0.010886 | 24 | 0 | | | | | | 5.163161 | 17.82561 | NA | NA | 10.66414 | 8.017906 | 12.16314 | 9.126873 | 11.39273 | 8.450172 | 10.78464 | NA | 6.791617 | 7.474432 | 6.836925 | 11.00785 | 5.088742 | 8.709062 | 4.45976 | 8.650339 | 10.24027 | 14.79665 | 11.22442 | 17.07869 | 15.28811 | 11.50536 | 14.84035 |
| CUST_3012_PI429010886 | N13602 | 0.010887 | 15 | 9 | | | | | | 37.70458 | 184.7859 | 460.255 | 9.165228 | 6.64085 | NA | 69.78353 | 85.41552 | 214.1014 | 83.79573 | 198.0363 | 244.0822 | 70.74029 | 260.1739 | 177.6974 | 153.1912 | 17.67558 | 159.4972 | 94.40103 | 113.0678 | 22.2368 | 498.8709 | 309.8242 | 36.50859 | 504.1608 | 187.251 | 920.0256 |
| CUST_23588_PI429010886 | N49078 | 0.010923 | 15 | 12 | | | | | | 53.5429 | 63.43828 | 51.26857 | 31.82901 | 43.57816 | 40.5109 | 31.87606 | 36.37581 | 56.07148 | 54.63875 | 51.02755 | 69.63319 | 40.48072 | 69.86983 | 55.37764 | 63.32035 | 40.74434 | 63.21853 | 54.51541 | 54.77637 | 45.75251 | 35.18679 | 38.61125 | 39.54096 | 62.5758 | 61.64233 | 78.65203 |
| CUST_43960_PI429010886 | M64466 | 0.010924 | 15 | 9 | | | | | | 48.98001 | 20.64798 | 27.08074 | NA | 4.545299 | 4.350583 | 6.66543 | 4.474106 | 4.89178 | 712.9294 | 140.8407 | 45.29693 | 225.8926 | 135.7577 | 143.7439 | 30.82801 | 10.12007 | NA | 227.0558 | NA | NA | 13.20951 | 37.4709 | 472.0832 | 52.76183 | 249.6087 | 344.0991 |
| CUST_47488_PI429010886 | M76319 | 0.010927 | 18 | 3 | 242022977 | RING finger-containing protein, putative [Pediculus | | | 9.83E-07 | 14.00188 | 8.683756 | NA | 42.88574 | 6.239269 | 55.01668 | 26.11497 | 5.097353 | 16.78757 | 6.616037 | 5.346894 | 4.122127 | 8.00182 | 7.678477 | 6.515981 | 12.05879 | 11.8448 | 34.20386 | 5.674565 | 8.768772 | 12.07461 | 15.29079 | NA | 23.89642 | 9.633666 | 37.29408 | 12.23618 |
| CUST_52872_PI429010886 | M99276 | 0.010934 | 18 | 12 | | | | | | 90.70315 | 119.7503 | 102.6011 | 64.93326 | 60.06147 | 89.66356 | 140.9396 | 89.9999 | 112.609 | 107.1133 | 64.63524 | 108.4414 | 91.16786 | 121.8599 | 123.444 | 117.0962 | 111.4235 | 110.7482 | 95.03994 | 118.3356 | 116.2781 | 95.82559 | 80.9345 | 103.9053 | 96.67753 | 100.9074 | 95.34993 |
| CUST_24233_PI429010886 | N50274 | 0.010941 | 15 | 0 | | | | | | 23.78457 | 27.52267 | 20.62307 | 18.98793 | 25.33406 | 3.862861 | 4.828419 | 9.080702 | 11.68238 | 17.14547 | 17.39038 | 16.44239 | 27.35173 | 13.33763 | NA | 19.38567 | 21.67714 | 23.93544 | 7.714116 | 14.52048 | NA | 15.61433 | 16.39504 | 6.02125 | 33.12364 | 4.714099 | 16.35941 |
| CUST_16324_PI429010886 | N36046 | 0.010946 | 21 | 0 | | | | | | 24.69848 | 20.05201 | 28.56717 | 18.90775 | 6.312211 | 20.47372 | 28.28611 | 16.52278 | 14.17029 | 15.58738 | 11.29639 | 20.00677 | 17.15702 | 17.57797 | 20.09671 | 12.56927 | 12.46576 | 16.75738 | 25.76975 | 19.11878 | 19.22831 | 25.27217 | 23.02707 | 26.16563 | 10.70265 | 17.86315 | 20.31618 |
| CUST_56540_PI429010886 | M114046 | 0.010956 | 15 | 3 | | | | | | 505.2828 | 367.27 | 470.2 | 5113.292 | 6833.331 | 5596.154 | 2995.003 | 3752.095 | 231.6859 | 27.77944 | 3048.703 | 23.01368 | 541.7506 | 26.89381 | 478.9465 | 44.27177 | 2452.337 | 506.3576 | 2015.591 | 39.87225 | 4660.817 | 66.7414 | 2886.71 | 3921.02 | 38.4769 | 1103.397 | 17.98635 |
| CUST_36091_PI429010886 | M14594 | 0.010982 | 21 | 9 | 330434836 | hypothetical protein [Macrobrachium nipponense] | | | 1.44E-12 | 6.742836 | 291.7598 | 15.09563 | 64.56992 | 332.0505 | 339.7564 | 13.18143 | 601.3413 | 346.5745 | 313.1633 | 683.5585 | 630.6794 | 220.2522 | 290.9496 | 162.251 | 8.167031 | 54.79286 | 32.99565 | 32.56422 | 173.3481 | 118.1469 | 202.0834 | 61.67082 | 261.3766 | 72.13281 | NA | 20.09671 |
| CUST_21816_PI429010886 | N45906 | 0.010984 | 15 | 12 | | | | | | 12.89444 | 14.09963 | 21.9039 | 17.39038 | 8.838077 | 3.536544 | 8.00826 | 15.05158 | 13.70505 | 9.939882 | 11.74368 | 21.6649 | 13.0306 | 18.72449 | 21.26565 | 11.8227 | 14.83746 | 18.10067 | 9.786144 | 9.837542 | 9.962271 | 17.66287 | 12.48715 | NA | 23.51373 | 15.07677 | 17.96838 |
| CUST_23899_PI429010886 | N49673 | 0.010999 | 18 | 3 | 321466046 | hypothetical protein DAPPUDRAFT_321848 [Daphn | | | 8.88E-08 | 309.8242 | 1753.366 | 333.6383 | 2390.205 | 796.5277 | 930.2399 | 476.8506 | 588.4339 | 369.6879 | 403.0406 | 282.9535 | 1012.95 | 498.525 | 273.5534 | 202.5771 | 636.1084 | 129.6012 | 440.428 | 162.3872 | 496.6049 | 579.3813 | 823.8136 | 566.9632 | 606.6133 | 709.528 | 356.4277 | 286.0114 |
| CUST_47512_PI429010886 | M76418 | 0.011 | 18 | 0 | | | | | | 789.9286 | 1027.698 | 435.6005 | 733.3828 | 723.4421 | 498.3855 | 532.8085 | 303.0793 | 566.4251 | 406.5096 | 419.7481 | 429.4606 | 375.2702 | 294.2777 | 423.4903 | 857.5648 | 260.0898 | 631.2945 | 551.5652 | 600.1331 | 509.8767 | 627.6184 | 400.5981 | 467.5419 | 448.0873 | 424.9332 | 230.1158 |
| CUST_13343_PI429010886 | N31026 | 0.011004 | 24 | 15 | | | | | | 11.43429 | 13.33083 | 15.19477 | 14.18775 | 15.18687 | 6.980991 | 6.247898 | 12.7217 | 11.54241 | 26.05696 | 17.98401 | 14.22674 | 17.33078 | 14.54491 | 18.09247 | 18.554 | 25.28193 | 20.13319 | 13.35829 | 16.26559 | 11.11044 | 15.83559 | 14.45543 | 18.67657 | 22.28394 | 7.840127 | 14.57268 |
| CUST_24133_PI429010886 | N50092 | 0.011014 | 24 | 21 | | | | | | 17.04092 | 25.88998 | NA | 9.731006 | 7.497984 | NA | 6.911676 | 5.903765 | 11.74904 | 15.49998 | 5.426738 | 11.36205 | 23.12065 | 8.998452 | 15.45319 | 12.94169 | NA | 9.960996 | 15.64186 | NA | 6.630788 | 26.35743 | NA | 52.53115 | 11.79036 | 38.9709 | 21.24533 |
| CUST_23992_PI429010886 | N49832 | 0.011017 | 18 | 3 | 383862055 | PREDICTED: aminopeptidase N-like [Megachile rotu | | | 2.36E-08 | 41.13272 | 40.19775 | 41.09417 | 95.67336 | 76.76597 | 57.47681 | 54.6573 | 23.49374 | 34.82944 | 31.1045 | 32.86388 | 9.573493 | 35.76089 | 59.776 | 57.5238 | 58.17945 | 22.06407 | 66.25397 | 34.98328 | 42.15632 | 41.19488 | 66.78152 | 40.6037 | 76.96996 | 56.53962 | 12.08465 | 19.78047 |
| CUST_30174_PI429010886 | N61327 | 0.011018 | 18 | 15 | 242013531 | conserved hypothetical protein [Pediculus humanus | | | 7.13E-16 | 18.53311 | 12.85919 | 20.75935 | 10.33003 | 19.12346 | NA | 13.31013 | 4.896171 | 20.13934 | 30.62163 | 19.91373 | 27.85465 | 16.94046 | 23.02215 | 23.28835 | 26.77489 | NA | 27.34621 | 13.01679 | 27.86411 | 17.40133 | 15.92987 | 9.425811 | 13.87948 | 24.19734 | 20.37797 | 17.94343 |
| CUST_16075_PI429010886 | N35612 | 0.01102 | 21 | 0 | | | | | | 5.055818 | 225.6591 | 94.26766 | NA | 32.89038 | 45.3248 | 16.54329 | 33.58859 | 24.03089 | 28.77687 | 23.79718 | 62.16132 | 15.59665 | 16.35354 | 8.773525 | 5.932286 | NA | 6.066547 | NA | 71.15715 | NA | 133.4505 | 63.58566 | 35.00866 | 7.399323 | 57.84331 | 67.08962 |
| CUST_23253_PI429010886 | N48514 | 0.011034 | 12 | 3 | | | | | | 1972.844 | 1299.165 | 425.9278 | 4579.426 | 2545.683 | 6297.225 | 2340.111 | 1324.694 | 1595.711 | 109.6246 | 1051.549 | 88.63339 | 1465.294 | 840.6106 | 1532.545 | 2137.444 | 2771.702 | 3651.445 | 959.2541 | 394.3816 | 1534.856 | 1947.429 | 922.2736 | 3914.042 | 448.7865 | 2044.563 | 35.38465 |
| CUST_16670_PI429010886 | N36660 | 0.011046 | 24 | 3 | 321479334 | hypothetical protein DAPPUDRAFT_39648 [Daphnia | Protein metabolism | Proteolysis | 1.82E-61 | 38.41931 | 37.83427 | 26.78992 | 97.44558 | 39.06616 | 93.1869 | 44.0607 | 27.81087 | 38.49435 | 35.93356 | 11.7971 | 45.7289 | 27.16436 | 45.73763 | 28.87402 | 35.16453 | 12.63126 | 35.70225 | 19.35261 | 27.15737 | 26.56221 | 35.11845 | 56.72265 | 42.80091 | 42.77108 | 82.8076 | 23.45771 |
| CUST_56882_PI429010886 | M114825 | 0.011048 | 18 | 12 | | | | | | 54.61808 | 48.45297 | 53.38747 | 54.21774 | 46.34675 | 54.60219 | 47.20161 | 54.56998 | 51.63118 | 64.42438 | 61.30991 | 57.10423 | 68.25944 | 62.45524 | 63.00905 | 60.12977 | 57.82155 | 57.07573 | 52.54728 | 62.76739 | 51.80467 | 61.81309 | 39.0395 | 55.27747 | 55.85751 | 41.15368 | 75.93804 |
| CUST_56152_PI429010886 | M112977 | 0.011066 | 21 | 3 | | | | | | 103.0505 | 134.3489 | 124.4616 | 131.2414 | 118.4488 | 277.2896 | 99.63032 | 123.444 | 123.0174 | 125.4876 | 121.5886 | 138.4086 | 137.0908 | 129.1875 | 121.7904 | 147.4736 | 114.4264 | 123.7772 | 90.43345 | 97.33778 | 88.69099 | 109.7061 | 114.2465 | 132.7049 | 137.865 | 158.6525 | 153.8044 |
| CUST_39524_PI429010886 | M40813 | 0.011076 | 15 | 9 | | | | | | 774.0266 | 757.6325 | 766.6399 | 811.2478 | 629.802 | 773.1832 | 801.5312 | 715.6525 | 1055.909 | 830.4425 | 824.9455 | 1226.144 | 917.4556 | 1388.367 | 819.682 | 665.6609 | 768.7795 | 699.0502 | 847.9888 | 654.1515 | 946.7835 | 1090.829 | 713.1739 | 670.3311 | 1118.972 | 981.4865 | 1263.557 |
| CUST_35751_PI429010886 | M13413 | 0.011078 | 24 | 18 | | | | | | 760.5296 | 926.1166 | 680.3748 | 625.8341 | 703.4699 | 661.3119 | 512.1412 | 568.6876 | 590.4824 | 812.5634 | 718.917 | 1042.025 | 614.2182 | 817.1902 | 847.8005 | 1194.964 | 647.3023 | 834.6224 | 836.7397 | 977.4233 | 737.7582 | 1147.081 | 821.28 | 623.6818 | 897.1177 | 774.2499 | 951.9745 |
| CUST_48349_PI429010886 | M79954 | 0.011081 | 12 | 0 | | | | | | 113.6011 | 129.357 | 86.27625 | 95.24605 | 101.8114 | 87.24093 | 85.79457 | 90.34874 | 95.65165 | 106.9318 | 99.36845 | 109.4535 | 99.06334 | 115.9538 | 137.5442 | 130.2863 | 84.1647 | 128.8934 | 77.5823 | 103.457 | 70.77093 | 99.99373 | 119.0689 | 77.79872 | 125.9169 | 107.073 | 125.0791 |
| CUST_5185_PI429010886 | N18371 | 0.011081 | 15 | 0 | 345494023 | PREDICTED: Nasonia vitripennis hypothetical protei | Transport | Protein transport | 3.3E-104 | 163.5179 | 204.7693 | 142.8394 | 140.7156 | 144.9989 | 175.6284 | 105.1778 | 99.90716 | 141.8965 | 163.1486 | 125.8769 | 172.1829 | 146.4472 | 157.1144 | 172.7406 | 198.3401 | 103.0771 | 151.0826 | 131.513 | 185.414 | 158.7326 | 105.4208 | 134.6396 | 93.25437 | 136.8618 | 127.2423 | 133.7064 |
| CUST_8368_PI429010886 | N22945 | 0.011091 | 21 | 18 | 242023867 | tyrosine-protein phosphatase non-receptor type, pu | Metabolic process | Cellular amino acid metabolic process | 3.63E-47 | 18.70704 | 26.66065 | 28.93067 | 19.50301 | 27.68808 | 25.46747 | 17.04092 | 16.95796 | 15.02569 | 30.65659 | 26.55476 | 20.32136 | 19.6064 | 21.01141 | 22.01419 | 25.48959 | 20.53338 | 32.71054 | 38.1599 | 25.53642 | 24.95635 | 37.22354 | 26.09533 | 22.39033 | 29.58843 | 17.31897 | 17.24279 |
| CUST_40572_PI429010886 | M55503 | 0.011107 | 21 | 0 | | | | | | 139.8961 | 148.7085 | 159.7611 | 112.1834 | 150.5702 | 122.1188 | 100.64 | 87.29557 | 115.2179 | 151.4764 | 119.8071 | 83.30915 | 117.7023 | 98.05949 | 103.1588 | 121.3613 | 160.9334 | 127.6888 | 117.9048 | 134.3863 | 90.71122 | 134.5271 | 152.988 | 144.5986 | 214.4445 | 108.9872 | 142.7716 |
| CUST_30301_PI429010886 | N61582 | 0.011113 | 21 | 15 | 338809146 | hemocyanin [Exopalaemon carinicauda] | | | 3.17E-13 | 5.951528 | 21.39067 | NA | 38.42267 | 125.3376 | 6.049495 | 108.1674 | 542.1254 | 185.0712 | 61.30991 | 381.1947 | 385.9721 | 1669.785 | 25.01145 | 638.6578 | NA | 483.8764 | NA | 70.24827 | 14684.57 | 525.2083 | NA | 24.38309 | 19.64813 | NA | 110.2326 | 500.6766 |
| CUST_28134_PI429010886 | N57357 | 0.011117 | 18 | 3 | | | | | | 172.3293 | 290.7302 | 238.5792 | 427.3564 | 203.2739 | 217.2477 | 164.2501 | 89.21981 | 250.7333 | 209.6694 | 120.9878 | 283.5999 | 90.89001 | 224.668 | 109.6246 | 50.546 | 83.91514 | 106.3073 | 36.85539 | 110.3513 | 310.7127 | 220.3651 | 377.4878 | 188.2417 | 222.2031 | 293.7629 | 172.054 |
| CUST_33932_PI429010886 | M8281 | 0.011135 | 18 | 12 | | | | | | 105.005 | 119.2623 | 99.82512 | 96.29479 | 84.86918 | 69.84364 | 100.1493 | 112.1834 | 112.7651 | 126.7579 | 85.38814 | 123.9921 | 130.8512 | 122.4782 | 142.069 | 145.1303 | 113.7623 | 98.82662 | 109.0854 | 163.329 | 104.6277 | 109.5287 | 110.5384 | 98.7787 | 149.0167 | 110.5384 | 141.6025 |
| CUST_46269_PI429010886 | M72019 | 0.01114 | 24 | 9 | 292807621 | ADP-ribosylation factor [Marsupenaeus japonicus] | Signal transduction | Other | 1.64E-09 | 8.201884 | 12.91624 | 12.9342 | 14.35201 | 12.15636 | 18.40021 | 18.58498 | 4.419338 | 21.38369 | 32.49365 | 59.57181 | 72.40904 | 13.94862 | 33.45717 | 23.95732 | 12.20868 | 17.73942 | 30.52136 | 22.29671 | 83.2292 | 27.93228 | 24.97464 | NA | 20.28484 | 20.85111 | 16.55113 | 11.88135 |
| CUST_47606_PI429010886 | M76832 | 0.01114 | 24 | 9 | | | | | | 5.227424 | 9.457848 | 4.355206 | 25.98413 | 12.07461 | 17.39698 | 10.02441 | 58.13824 | 20.18328 | 19.97907 | 27.0727 | 23.02707 | 12.72475 | 12.43952 | 8.286303 | 4.583391 | 8.839067 | 12.54687 | 12.45001 | 15.2618 | 9.488473 | NA | 12.45151 | 10.61167 | 11.19559 | 12.04026 | 8.236094 |
| CUST_4752_PI429010886 | N17406 | 0.01116 | 21 | 18 | | | | | | 494.2193 | 421.8908 | 375.7452 | 290.5429 | 233.6523 | 338.2555 | 302.2127 | 367.6066 | 196.2921 | 299.7277 | 392.7909 | 390.4272 | 166.4452 | 393.1602 | 309.3892 | 531.5557 | 315.2378 | 544.2536 | 465.4504 | 411.4634 | 536.0428 | 383.7251 | 338.3238 | 336.6312 | 481.413 | 317.5249 | 506.1361 |
| CUST_52388_PI429010886 | M97262 | 0.011171 | 18 | 3 | | | | | | 8.342648 | 27.79102 | 12.90145 | 46.44351 | 54.32031 | 78.39762 | 12.8903 | NA | 10.16864 | 22.16055 | 10.84809 | 11.33312 | 7.279581 | 29.2332 | 13.89928 | 10.54091 | 15.05287 | 22.96785 | 22.82362 | 26.93068 | 16.96167 | 60.63941 | NA | 45.18835 | 21.53652 | 54.14583 | 6.391082 |
| CUST_51996_PI429010886 | M95996 | 0.011187 | 21 | 18 | | | | | | 32.82642 | 32.54901 | 27.34913 | 27.97056 | 18.63533 | 10.58033 | 11.37935 | 10.96904 | 13.81188 | 28.10538 | 25.21391 | 16.17384 | 11.79386 | 24.04991 | 16.52754 | 18.3198 | 12.20158 | 46.00478 | 17.70313 | 50.58779 | 28.40282 | 27.59392 | 37.55636 | 15.4125 | 25.47915 | 19.03069 | 24.47717 |
| CUST_38398_PI429010886 | M26203 | 0.011194 | 12 | 0 | | | | | | 5.614366 | 9.864571 | 12.34516 | 8.614965 | 12.76656 | 10.67318 | NA | 9.515358 | NA | 7.496731 | 5.21274 | 15.06711 | 13.68485 | 13.70338 | 24.09666 | 11.76313 | 9.250124 | 12.59071 | 6.540321 | 6.875138 | NA | 5.485036 | NA | 8.474659 | 22.81381 | 14.39787 | 25.93979 |
| CUST_37355_PI429010886 | M18773 | 0.011205 | 12 | 0 | 56121719 | ribosomal protein L26 [Penaeus monodon] >gi\|124 | Protein metabolism | Ribosome biogenesis | 2.31E-60 | 613.6489 | 849.9143 | 913.4609 | 403.5814 | 439.1396 | 500.9078 | 666.6962 | 705.2005 | 792.7128 | 731.0066 | 616.4707 | 1298.64 | 687.5678 | 962.6096 | 883.6545 | 867.1839 | 453.2475 | 457.5237 | 667.5342 | 695.5556 | 597.1763 | 853.9995 | 728.235 | 740.4281 | 716.9224 | 714.0634 | 759.8078 |
| CUST_25150_PI429010886 | N51875 | 0.011205 | 12 | 0 | 548350131 | PREDICTED: zinc finger and BTB domain-containing | | | 1.24E-09 | 31.88231 | 26.77181 | 49.3195 | 16.64136 | 28.39426 | 19.14299 | 55.52574 | 24.21798 | 31.82074 | 30.75954 | 24.71578 | 35.20829 | 32.19497 | 54.60894 | 40.83694 | 43.33061 | 24.70791 | 21.80739 | 25.50611 | 26.64032 | 26.48449 | 42.58941 | 31.36122 | 22.1415 | 46.78356 | 35.66075 | 52.37245 |
| CUST_57300_PI429010886 | E4642 | 0.011215 | 21 | 15 | 548856974 | hypothetical protein AMTR_s00032p00056500 [Am | Cellular component organization | Cytoskeleton organization | 9.02E-08 | 342.2472 | 616.0789 | 497.0198 | 445.0547 | 423.767 | 579.8544 | 383.093 | 284.0133 | 397.1398 | 307.8404 | 337.0926 | 376.1818 | 405.8466 | 410.9035 | 446.9611 | 559.8475 | 520.9934 | 656.3922 | 386.1193 | 649.5445 | 442.5257 | 824.3794 | 247.6419 | 595.4395 | 460.1631 | 504.2482 | 427.9524 |
| CUST_1866_PI429010886 | N10910 | 0.011225 | 12 | 3 | | | | | | 93.65476 | 581.2638 | 20.92287 | 1247.588 | 861.1597 | 884.6458 | 1119.759 | NA | 1244.117 | 19.98576 | 5.094187 | 14.30234 | 76.96996 | 129.772 | 1479.619 | 3872.65 | 33.46305 | 2961.93 | 20.09831 | 25.61779 | 10.52275 | 632.2521 | 6.411759 | 473.1073 | 57.22072 | NA | 20.49729 |
| CUST_41884_PI429010886 | M58788 | 0.011236 | 24 | 0 | 449471748 | PREDICTED: cartilage intermediate layer protein 1 [ | | | 3.8E-12 | 1694.466 | 1543.721 | 1340.303 | 1455.397 | 1541.439 | 1720.979 | 1355.659 | 1240.437 | 1308.281 | 1491.426 | 1415.482 | 1697.824 | 1118.208 | 1257.974 | 1042.897 | 2217.377 | 1395.696 | 1463.822 | 1310.544 | 1578.633 | 1246.008 | 1672.661 | 991.3315 | 1294.709 | 2067.695 | 1905.993 | 1731.857 |
| CUST_38650_PI429010886 | M28840 | 0.011248 | 15 | 3 | | | | | | 242.302 | 212.3302 | 167.5472 | 305.3469 | 456.6758 | 368.397 | 476.472 | 204.658 | 160.8644 | 162.0779 | 387.5117 | 222.3465 | 187.9283 | 155.8377 | 169.6125 | 184.9236 | 661.8313 | 254.8048 | 610.9693 | 192.8311 | 295.2655 | 191.3362 | 355.3996 | 363.6175 | 146.4805 | 182.9296 | 111.2168 |
| CUST_47435_PI429010886 | M76103 | 0.01125 | 12 | 0 | | | | | | 21.75394 | 14.82853 | 17.85496 | 11.73206 | 10.88348 | 9.154209 | 13.73069 | 10.38786 | NA | 10.25442 | 11.15425 | 17.14547 | 14.53785 | 11.82579 | 25.0475 | 8.800802 | 20.74508 | 13.58792 | 12.27649 | 12.90802 | 6.821529 | 9.534612 | 10.59173 | NA | 23.09298 | 22.67516 | 19.58405 |
| CUST_24669_PI429010886 | N51049 | 0.011256 | 15 | 3 | | | | | | 15.97964 | 6.401811 | 11.61773 | 25.57942 | 16.61727 | 27.55992 | 23.53335 | 16.74589 | 12.96066 | NA | 8.914093 | NA | 8.073322 | NA | 7.345898 | NA | 35.83044 | 19.28994 | 27.48907 | NA | 12.32189 | 25.56371 | 9.873369 | 17.42826 | 6.351061 | 16.0166 | 5.870883 |

| Probe ID | ID | p-value | Period | Phase | EBI ID | Annotation | GO category | Go term | E-value | 0 | 0 | 0 | 3 | 3 | 3 | 6 | 6 | 6 | 9 | 9 | 9 | 12 | 12 | 12 | 15 | 15 | 15 | 18 | 18 | 18 | 21 | 21 | 21 | 24 | 24 | 24 |
|---|---|---|---|---|---|---|---|---|---|---|---|---|---|---|---|---|---|---|---|---|---|---|---|---|---|---|---|---|---|---|---|---|---|---|---|---|
| CUST_38426_PI429010886 | M26505 | 0.011263 | 18 | 6 | | | | | | 139.2832 | 234.3704 | 255.3857 | 167.8053 | 196.359 | 215.6344 | 204.0863 | 201.9396 | 217.7055 | 201.6148 | 200.1336 | 224.5142 | 204.629 | 162.473 | 189.684 | 177.896 | 186.1103 | 154.8105 | 195.4506 | 189.144 | 179.8447 | 197.7545 | 300.0118 | 214.7331 | 210.0511 | 206.0656 | 257.0697 |
| CUST_47229_PI429010886 | M75292 | 0.011278 | 24 | 3 | 390346858 | PREDICTED: N-acetylated-alpha-linked acidic dipept | Protein metabolism | Proteolysis | 7.17E-11 | 44.12842 | 33.23109 | 37.89385 | 29.71169 | 29.22246 | 32.99848 | 20.87902 | 22.31012 | 13.15128 | 28.33698 | 22.67516 | 5.193441 | 13.89131 | 17.99464 | 15.25416 | 14.84618 | 12.15878 | 7.624707 | 33.54623 | 11.90477 | 20.74695 | 17.06824 | 13.82074 | 10.83296 | 19.07608 | 14.72394 | 17.55722 |
| CUST_9668_PI429010886 | N25020 | 0.011279 | 15 | 0 | | | | | | 55.15261 | 164.9397 | 152.7576 | 23.12297 | 129.9538 | 134.5271 | 94.49669 | 87.16389 | 80.13478 | 78.93556 | 103.4079 | 127.3882 | 138.3764 | 213.1567 | 103.8441 | 87.03787 | 253.9783 | 155.8809 | 98.70636 | 208.1099 | 163.6601 | 85.03701 | 44.19495 | 72.4658 | 127.601 | 88.78823 | 110.7189 |
| CUST_24289_PI429010886 | N50368 | 0.01129 | 18 | 15 | | | | | | 36.98717 | 35.97953 | 20.23937 | 26.49443 | 28.888 | 25.03495 | 22.52377 | 30.19656 | 19.17918 | 43.33061 | 21.98819 | 39.0152 | 22.21682 | 43.15798 | 34.53803 | 36.11849 | 38.04684 | 35.1434 | 30.78794 | 31.95131 | 34.56933 | 40.54346 | 27.23134 | 33.23976 | 27.59392 | 19.9496 | 24.72309 |
| CUST_4322_PI429010886 | N16509 | 0.011291 | 12 | 3 | 242019973 | conserved hypothetical protein [Pediculus humanus | | | 2.47E-21 | 20.44923 | 12.89985 | 22.74317 | 29.29243 | 27.36205 | 30.31727 | 17.24279 | 27.59937 | 25.60312 | 18.29426 | 9.02447 | 23.95916 | 33.41929 | 29.50356 | 28.82923 | 43.47746 | 14.15156 | 73.76113 | 22.66789 | 25.95987 | 20.28929 | 19.81449 | 12.16799 | 25.22098 | 25.06723 | 22.57964 | 24.12979 |
| CUST_1165_PI429010886 | N8715 | 0.011291 | 12 | 9 | 54042606 | arthrodial cuticle protein AMP8.1 [Callinectes sapid | | | 1.23E-13 | 51.33619 | 43.77809 | 112.5446 | 54.71071 | 91.91837 | 51.92939 | 219.6868 | 91.16786 | 277.9987 | 306.736 | 241.1275 | 374.9159 | 92.96667 | 528.3775 | 14.23976 | 113.4862 | 125.3173 | 29.48584 | 36.28805 | 267.4607 | 148.196 | 125.9625 | 486.0261 | 312.8606 | 150.0759 | 66.47359 | 240.9019 |
| CUST_44064_PI429010886 | M64830 | 0.011292 | 18 | 0 | 91076314 | PREDICTED: similar to spliceosome associated prote | Nucleic acid metabolism | RNA metabolic process | 3.6E-60 | 19.81178 | 29.62034 | 28.85616 | 28.15466 | 24.59542 | 30.41187 | 27.79102 | 10.09605 | 16.5954 | 13.25777 | 18.56427 | 18.01314 | 13.8859 | 16.75931 | 11.69128 | 23.66453 | 31.20174 | 25.77541 | 35.74458 | 14.81994 | 21.03821 | 22.24561 | 24.90221 | 25.97297 | 25.30072 | 18.89922 | 19.676 |
| CUST_11019_PI429010886 | N27135 | 0.011296 | 18 | 0 | 299782457 | kinesin 2B [Nasonia vitripennis] >gi\|299782459\|ref | Cellular component organization | Cytoskeleton organization | 4.87E-30 | 29.1329 | 24.20353 | 24.24945 | 12.47875 | 22.93439 | 14.00339 | NA | 15.78805 | NA | 10.05851 | 7.437557 | NA | 3.566988 | 17.79901 | 3.901825 | 34.94119 | 12.33047 | 12.39561 | 35.41597 | 21.71687 | 11.04742 | 14.77486 | 12.98781 | 6.699155 | 4.439948 | NA | NA |
| CUST_23621_PI429010886 | N49152 | 0.011299 | 18 | 15 | | | | | | 12.1506 | 16.51414 | 23.31493 | 10.87645 | 25.81311 | NA | 16.80437 | 14.04854 | 5.23284 | 29.46873 | 12.81434 | 29.95513 | 16.29078 | 18.67657 | 28.84183 | 36.41652 | 19.65423 | 18.13841 | 17.09174 | 17.61145 | 18.11108 | 8.316685 | 15.0972 | 4.010865 | 14.64693 | 14.73365 | 15.55244 |
| CUST_4769_PI429010886 | N17443 | 0.0113 | 24 | 21 | | | | | | 8.148866 | 6.169502 | 8.472888 | 13.7972 | 5.358734 | NA | 5.49933 | 8.136532 | 6.120324 | 5.747789 | 3.86495 | 10.27406 | 15.31496 | 11.94615 | 11.85797 | NA | NA | 11.90309 | 5.552969 | 13.1339 | NA | 14.02745 | 11.92305 | 18.88215 | 9.049354 | 7.02021 | 13.94176 |
| CUST_54434_PI429010886 | M106010 | 0.01131 | 24 | 9 | | | | | | 25.58242 | 26.61439 | 18.42271 | 18.42911 | 38.70657 | 12.79225 | 22.85699 | 74.84295 | 35.72108 | 26.07451 | 65.36011 | 46.81599 | 52.68897 | 17.84395 | 19.63631 | 12.7372 | 25.67234 | NA | 25.68021 | 92.52049 | 22.04537 | 15.15984 | 11.27249 | 11.89991 | 19.92894 | 12.55922 | 38.04057 |
| CUST_6935_PI429010886 | N20802 | 0.011315 | 12 | 3 | | | | | | 18.08051 | 31.44965 | 15.09377 | 71.59535 | 21.7873 | 98.94163 | 34.15487 | 17.02832 | 20.859 | 4.888509 | 14.47659 | 6.645675 | 22.73586 | 13.45283 | 17.74547 | 21.71687 | 32.89038 | 47.53841 | 5.447078 | 18.63301 | 19.57585 | 18.24494 | 5.957887 | 64.01814 | 5.677532 | 36.53021 | NA |
| CUST_39624_PI429010886 | M41918 | 0.011324 | 21 | 15 | | | | | | 866.5099 | 595.7445 | 787.1975 | 251.1828 | 1025.258 | 710.9044 | 1051.932 | 733.1697 | 710.6638 | 1358.952 | 539.2831 | 703.8812 | 1525.167 | 1048.248 | 827.5431 | 1104.361 | 1267.999 | 1404.631 | 909.757 | 929.5412 | 834.3363 | 1262.411 | 503.9702 | 477.3001 | 374.5146 | 684.7245 | 914.1643 |
| CUST_30170_PI429010886 | N61316 | 0.011339 | 24 | 15 | | | | | | 26.89381 | 16.0587 | 22.03665 | 9.694084 | 13.97554 | 14.36708 | 27.24651 | 18.55819 | 20.80086 | 15.79548 | 24.20353 | 25.21717 | 18.50047 | 30.55085 | 28.84855 | 35.51693 | 22.10673 | 40.29023 | 18.93518 | 25.49499 | 18.59923 | 29.36188 | 34.35136 | 15.97633 | 26.61439 | 24.62608 | 32.53107 |
| CUST_53699_PI429010886 | M102832 | 0.011349 | 15 | 0 | | | | | | 196.9351 | 197.9029 | 213.5752 | 150.2252 | 145.8825 | 180.4095 | 130.3469 | 156.4871 | 135.6641 | 181.2996 | 176.2423 | 232.1818 | 150.9664 | 193.1525 | 225.1125 | 246.1869 | 156.4871 | 193.3866 | 166.8689 | 171.1636 | 216.1505 | 191.886 | 163.6601 | 148.9832 | 172.436 | 177.8716 | 173.6967 |
| CUST_672_PI429010886 | N6680 | 0.011361 | 15 | 0 | 290462391 | Cuticle protein 6 [Lepeophtheirus salmonis] | | | 4.4E-19 | 9950.402 | 428.7157 | 8165.772 | 4467.857 | 2090.413 | 7154.842 | 7017.655 | 4476.427 | 2110.744 | 1238.697 | 3772.58 | 3.849046 | 2454.105 | 1155.687 | 1260.157 | NA | 10341.13 | 7355.509 | 6005.835 | 18.1208 | 5181.094 | 59.92145 | 3514.364 | 5681.469 | 210.4274 | 2089.376 | 148.1409 |
| CUST_8328_PI429010886 | N22878 | 0.011362 | 24 | 15 | 66499523 | PREDICTED: peroxisomal membrane protein PMP34 | Transport | Mitochondrial transport | 5.73E-85 | 26.97315 | 32.23977 | 30.76441 | 15.7022 | 23.58007 | 23.60076 | 28.36368 | 33.08815 | 22.21962 | 34.96061 | 32.9302 | 32.19898 | 27.025 | 44.8896 | 32.80956 | 44.03536 | 36.09146 | 41.72747 | 27.01961 | 65.56878 | 35.36241 | 45.40667 | 45.17801 | 20.68784 | 38.56175 | 33.03027 | 54.09438 |
| CUST_46296_PI429010886 | M72110 | 0.011367 | 15 | 0 | | | | | | 156.7272 | 108.0186 | 181.5662 | 143.5357 | 92.35924 | 150.6325 | 140.194 | 94.02768 | 94.01656 | 120.8512 | 100.9304 | 73.37448 | 121.3759 | 140.1347 | 121.7341 | 165.5224 | 147.2543 | 155.9427 | 97.05352 | 145.5994 | 129.1685 | 206.3065 | 158.7737 | 106.5216 | 118.1115 | 104.2347 | 110.7787 |
| CUST_93_PI429010886 | N737 | 0.011376 | 24 | 21 | 532759730 | antimicrobial peptide type 2 precursor IIc [Pandalop | | | 1.31E-26 | 9096.106 | 6879.978 | 8955.736 | 4676.187 | 9358.113 | 6461.792 | 11276.69 | 4584.946 | 6863.631 | 5674.79 | 6314.548 | 5251.069 | 5498.252 | 8667.659 | 6434.019 | 8550.193 | 6247.817 | 5800.891 | 9992.798 | 4787.683 | 11259.43 | 10760.71 | 10831.29 | 16271.02 | 7107.78 | 4748.861 | 7773.881 |
| CUST_32690_PI429010886 | M4820 | 0.011388 | 24 | 9 | 321472416 | hypothetical protein DAPPUDRAFT_240263 [Daphni | | | 9.99E-25 | 93.04202 | 110.3421 | 84.78816 | 176.4634 | 253.5447 | 93.71752 | 134.0378 | 688.7233 | 428.4141 | 99.19224 | 655.0692 | 675.2822 | 745.8794 | 89.22919 | 368.0783 | 47.55222 | 302.3102 | 21.86356 | 108.7243 | 1063.907 | 369.7785 | 39.44223 | 75.60669 | 52.29382 | 53.94407 | 306.0346 | 329.5353 |
| CUST_28830_PI429010886 | N58695 | 0.011394 | 12 | 0 | 395508757 | PREDICTED: zinc finger protein 569-like [Sarcophilus | | | 7.35E-10 | 29.47649 | 34.51949 | 28.66914 | 21.04144 | 29.63396 | 20.58016 | 17.28605 | 16.36148 | 20.16852 | 15.57864 | 22.5725 | 23.01614 | 29.46496 | 24.17334 | 20.97223 | 25.41068 | 18.00505 | 22.23809 | 20.72239 | 29.61833 | 18.81158 | 40.4329 | 17.87105 | 21.5971 | 33.57067 | 30.3023 | 24.0044 |
| CUST_13954_PI429010886 | N32079 | 0.011419 | 12 | 3 | | | | | | 61.8504 | 56.81475 | 33.52618 | 79.19112 | 48.29446 | 88.16694 | 40.36236 | 57.52971 | 45.3118 | 43.83694 | 47.5011 | 29.17612 | 48.67472 | 28.3805 | 35.9589 | 59.96262 | 80.30929 | 61.35068 | 72.07584 | 46.47908 | 47.77962 | 69.80023 | 36.72024 | 29.04567 | 77.50869 | 52.60357 | 53.0654 |
| CUST_19056_PI429010886 | N40910 | 0.011428 | 21 | 6 | | | | | | 36.8021 | 46.69134 | 47.3594 | 41.96975 | 63.67902 | 50.06979 | 76.25978 | 71.47658 | 75.87721 | 68.25944 | 47.49605 | 63.8461 | 46.94189 | 47.97063 | 55.07381 | 64.04953 | 43.19614 | 44.21249 | 107.0314 | 46.48282 | 60.89775 | 48.42693 | 47.88363 | 47.9222 | 48.19648 | 60.89775 | 70.74696 |
| CUST_12882_PI429010886 | N30202 | 0.011438 | 18 | 9 | | | | | | 19.47107 | 31.2144 | 22.27756 | 22.64961 | 24.08567 | 18.39397 | 27.47445 | 30.41942 | 22.13675 | 26.96538 | 29.78998 | 29.1329 | 31.88231 | 28.81252 | 25.09429 | 28.89154 | 25.16709 | 26.64032 | 23.77465 | 27.84371 | 30.37957 | 19.82309 | 20.6453 | 19.17708 | 37.52679 | 8.212232 | 22.07569 |
| CUST_34262_PI429010886 | M9188 | 0.011469 | 24 | 18 | | | | | | 11.67245 | 18.47168 | 8.48246 | 14.49726 | 12.40735 | 11.88135 | 14.20169 | 9.684471 | 9.105658 | 35.8336 | 11.92046 | 23.15048 | 15.27765 | 27.92065 | 16.91106 | 16.97579 | 7.062308 | 21.96895 | 43.37513 | 26.43248 | 32.44055 | 20.09671 | 20.21114 | 19.68474 | 104.2917 | 8.317554 | 14.75394 |
| CUST_2893_PI429010886 | N13335 | 0.011469 | 24 | 18 | 186969143 | gastrolith protein [Cherax quadricarinatus] | Metabolic process | Carbohydrate metabolic process | | 36.85229 | 35.96518 | 22.31957 | 30.33968 | 36.35659 | 38.97838 | 27.99312 | 28.04755 | 35.08626 | 53.71399 | 23.72867 | 42.68463 | 35.33485 | 48.2481 | 55.21772 | 63.73904 | 28.42343 | 33.16347 | 64.4873 | 79.36987 | 44.78041 | 52.52687 | 35.40982 | 57.53634 | 44.05203 | 68.95198 | 57.58322 |
| CUST_14926_PI429010886 | N33701 | 0.011469 | 24 | 18 | | | | | | 15.54715 | 17.16843 | 12.10746 | 11.67672 | 15.37925 | 15.13091 | 13.55467 | 4.209318 | 11.64935 | 17.33333 | 9.877583 | 23.50136 | 12.51969 | 14.97314 | 21.5895 | 17.96665 | 10.84947 | 25.9425 | 15.73405 | 29.0109 | 27.10054 | 22.21172 | 11.87577 | 20.11768 | 34.01329 | 21.58196 | 24.96359 |
| CUST_20204_PI429010886 | N43016 | 0.011469 | 24 | 18 | | | | | | 62.76241 | 69.82119 | 74.4613 | 44.57334 | 48.069 | 52.40666 | 33.37654 | 40.48521 | 42.81164 | 92.09827 | 50.77029 | 122.7515 | 40.88839 | 57.80004 | 71.71135 | 147.5581 | 39.64114 | 63.42029 | 71.18669 | 78.59764 | 85.93471 | 66.37927 | 78.30025 | 59.82242 | 68.89154 | 78.33945 | 101.5527 |
| CUST_47056_PI429010886 | M74671 | 0.011471 | 24 | 3 | | | | | | 93.83775 | 69.24723 | 110.9478 | 121.0374 | 110.7887 | 125.9625 | 87.14788 | 98.36103 | 112.9069 | 133.8722 | 92.08467 | 87.71808 | 85.59472 | 126.6662 | 95.28234 | 87.55497 | 88.4462 | 105.1255 | 86.5251 | 59.36711 | 77.10171 | 85.01801 | 79.86307 | 114.7111 | 115.478 | 82.19809 | 143.8832 |
| CUST_11493_PI429010886 | N27897 | 0.011482 | 18 | 3 | 321477834 | hypothetical protein DAPPUDRAFT_311166 [Daphn | Protein metabolism | Proteolysis | 1.05E-11 | 22.9868 | 31.08214 | NA | 33.0225 | 39.44972 | 25.45721 | 25.5511 | 19.37939 | 16.65641 | 13.732 | 9.719917 | 6.395897 | 23.59336 | NA | 22.69512 | 22.90466 | 20.18978 | 19.90626 | 13.8536 | 13.83327 | 9.665421 | 32.01033 | NA | 19.77641 | 25.07963 | 13.96779 | 7.664993 |
| CUST_6087_PI429010886 | N19613 | 0.011508 | 21 | 9 | 321471995 | hypothetical protein DAPPUDRAFT_230717 [Daphn | Metabolic process | Other | 1.2E-156 | 35.88462 | 126.3916 | 39.25762 | 128.037 | 131.2193 | 29.43648 | 71.71135 | 237.8874 | 143.609 | 102.3183 | 224.8249 | 335.74 | 183.3903 | 78.15144 | 93.90071 | 60.12109 | 89.5312 | 56.70249 | 51.78005 | 485.2905 | 171.1195 | 58.96932 | 80.55811 | 56.53962 | 59.67663 | 160.2021 | 129.3018 |
| CUST_19229_PI429010886 | N41219 | 0.011508 | 18 | 12 | | | | | | 11.71083 | 14.5219 | NA | 11.88603 | 16.04761 | 5.867579 | 15.19655 | 14.51697 | 20.87033 | 15.17932 | 16.11406 | 20.02885 | 22.92991 | 43.39175 | 23.26041 | 20.6694 | 14.40694 | 9.365159 | 19.3587 | 22.47719 | 19.64813 | 6.911676 | NA | 13.35309 | 23.72626 | 30.52691 | 29.5483 |
| CUST_26697_PI429010886 | N54721 | 0.011516 | 18 | 3 | | | | | | 25.62877 | 5.880926 | 4.491993 | NA | 28.7462 | 30.5069 | NA | 12.96601 | 24.84953 | NA | 13.26743 | 8.163573 | 5.503527 | 11.45611 | 3.874194 | 19.17708 | NA | 9.124 | 13.95744 | 12.94469 | NA | 14.46495 | 17.73142 | NA | 9.093219 | 7.223501 | 25.02916 |
| CUST_33345_PI429010886 | M6658 | 0.011525 | 21 | 12 | | | | | | 523.093 | 594.1197 | 723.4421 | 637.2498 | 991.3315 | 479.3073 | 745.3969 | 2777.472 | 2279.672 | 617.029 | 2348.331 | 3390.638 | 2722.22 | 746.9886 | 2076.382 | 819.4278 | 1302.095 | 737.0066 | 567.9112 | 6359.374 | 2365.487 | 475.6507 | 764.0731 | 653.1387 | 740.4281 | 1531.812 | 1883.709 |
| CUST_53451_PI429010886 | M101760 | 0.011547 | 12 | 9 | 328899820 | ribosomal protein S7 [Procambarus clarkii] | Protein metabolism | Ribosome biogenesis | 3.27E-35 | 527.7394 | 674.2322 | 552.5713 | 253.7351 | 464.6636 | 458.8168 | 438.6413 | 504.949 | 496.7325 | 521.1091 | 545.2224 | 511.0593 | 490.0839 | 536.7924 | 443.1374 | 257.1155 | 477.9922 | 470.4395 | 450.2218 | 602.4628 | 419.0394 | 588.9896 | 528.7455 | 569.4459 | 479.189 | 562.0583 | 454.0927 |
| CUST_4354_PI429010886 | N16579 | 0.011547 | 12 | 3 | | | | | | 236.1246 | 489.583 | 34.97152 | 779.5608 | 448.7865 | 429.4606 | 290.3758 | 51.10924 | 234.3391 | 38.74854 | 30.59854 | 23.46033 | 163.3967 | 157.7092 | 379.2136 | 690.0315 | 56.67223 | 400.3298 | 69.4087 | 135.6803 | 55.04733 | 429.4606 | 26.58056 | 267.619 | 152.7158 | 44.29926 | 72.49991 |
| CUST_37852_PI429010886 | M21875 | 0.011561 | 12 | 0 | | | | | | 374.682 | 1621.924 | 1007.936 | 222.6053 | 505.8775 | 77.38462 | 172.3518 | 679.7235 | 1273.05 | 2075.258 | 810.2943 | 1535.786 | 1689.395 | 2133.627 | 1762.677 | 726.7504 | 381.9407 | 1647.631 | 771.0971 | 1793.14 | 828.5874 | 1968.333 | 2130.831 | 1072.593 | 3124.162 | 1227.15 | 3453.79 |
| CUST_15496_PI429010886 | N34676 | 0.011565 | 24 | 18 | | | | | | 27.085 | 54.60894 | 23.74918 | 14.90976 | 24.28604 | 10.43309 | 25.38784 | 20.41093 | 14.571 | 16.31744 | 21.8498 | 36.22709 | 26.30532 | 26.12256 | 26.05952 | 34.90297 | 23.21566 | 39.98555 | 26.8539 | 43.43161 | 33.75702 | 27.17605 | 24.52447 | 12.91624 | 33.37654 | 18.59683 | 37.00901 |
| CUST_35461_PI429010886 | M12627 | 0.011575 | 15 | 3 | | | Protein metabolism | Regulation of translational elongation | | 28.77687 | 27.57296 | 39.08441 | 37.32162 | 187.4758 | 130.0411 | 121.5886 | 30.02625 | 34.35136 | 24.80361 | 109.7974 | 27.47445 | 19.19575 | 29.5457 | 22.33694 | 26.44212 | 273.4298 | 88.40608 | 92.37125 | 19.96165 | 124.6057 | 29.02633 | 109.902 | 83.6912 | 26.27126 | 29.11768 | 20.4401 |
| CUST_25757_PI429010886 | N53007 | 0.011584 | 21 | 0 | | | | | | 34.97535 | 24.4797 | 18.48046 | 26.23964 | 27.08074 | 17.34784 | 27.35753 | 20.10077 | 11.06303 | 19.15374 | 20.00172 | 23.27942 | 18.56579 | 15.1109 | 15.44039 | 22.49197 | 16.81454 | 22.36658 | 11.92991 | 33.87876 | 24.32948 | 22.97663 | 35.62064 | 32.04673 | 21.38369 | 18.94172 | 24.70329 |
| CUST_1543_PI429010886 | N10001 | 0.011587 | 12 | 9 | | | | | | 138.1676 | 158.8004 | 59.05765 | 7.164995 | 18.66441 | 4.129887 | 27.5887 | 7.530653 | 5.392196 | 121.649 | 78.32339 | 532.9396 | 23.69143 | 37.76259 | 21.55311 | 50.76541 | NA | 22.55992 | 29.54213 | 20.43255 | 39.57761 | 16.91321 | 249.0021 | 278.469 | 52.31892 | 11.58977 | 45.68644 |
| CUST_49934_PI429010886 | M87791 | 0.01159 | 15 | 12 | | | | | | 19.32322 | 11.52212 | 21.33598 | 9.220146 | 13.71042 | 13.36717 | 19.01531 | 7.590552 | 14.66496 | 10.6028 | 17.16312 | 23.06972 | 26.80106 | 30.80046 | 19.38333 | 9.347162 | 18.71899 | 20.09393 | 16.57544 | 7.959892 | 14.2983 | 6.606034 | 17.21299 | 17.69892 | 13.72475 | 16.72128 | 14.86659 |
| CUST_41031_PI429010886 | M56686 | 0.011612 | 21 | 6 | | | | | | 14.8651 | 11.45904 | 8.931253 | 23.90824 | 23.72626 | 14.28453 | 16.79517 | 39.79718 | 40.63765 | 35.27392 | 15.75594 | 31.18147 | 21.12887 | 12.32751 | 16.36343 | 28.68501 | 21.62904 | 20.71979 | 17.51574 | 45.70165 | 36.27265 | 15.48139 | 12.24341 | 12.05879 | 32.19156 | 18.46255 | 25.64589 |
| CUST_54900_PI429010886 | M108126 | 0.011615 | 21 | 3 | | | | | | 16.37858 | 16.74929 | 16.60305 | 16.96368 | 27.15098 | 14.48619 | 5.260014 | 16.25197 | 12.8607 | 15.11963 | 12.59239 | 22.03875 | NA | 9.710618 | 6.967135 | 16.94046 | NA | 6.794828 | 5.449338 | 13.63953 | 11.57662 | 13.54322 | 24.3684 | 10.3075 | 16.27412 | 16.98325 | 27.71267 |
| CUST_4633_PI429010886 | N17176 | 0.011644 | 15 | 12 | | | | | | 300.6766 | 11.71707 | 1720.979 | 13.37205 | 3.977016 | NA | 750.3627 | 3079.902 | 1486.604 | 1834.08 | 21.18631 | 41.11909 | 3008.795 | 5590.669 | 2044.563 | 15.58239 | 1414.763 | NA | 89.35703 | 31.58406 | 9.829719 | 329.7729 | 2065.573 | 44.85914 | 1243.416 | 21.80535 | 1986.907 |
| CUST_42507_PI429010886 | M60457 | 0.011651 | 15 | 3 | | | | | | 332.753 | 249.3844 | 207.9906 | 1174.011 | 199.9465 | 845.457 | 502.3399 | 1043.343 | 157.1911 | 77.35795 | 137.759 | 11.03039 | 118.4789 | 61.40092 | 140.974 | 42.96811 | 134.1419 | 60.10166 | 166.0617 | 63.40267 | 437.9165 | 32.81302 | 334.1776 | 483.7826 | 8.810922 | 69.65017 | 6.115825 |
| CUST_53990_PI429010886 | M103998 | 0.011667 | 24 | 15 | 189234352 | PREDICTED: similar to spectrin [Tribolium castaneur | | | 1.04E-83 | 98.55351 | 93.67647 | 83.19226 | 111.5879 | 118.4632 | 108.0186 | 99.22324 | 78.86687 | 109.2686 | 97.78298 | 110.9478 | 99.84559 | 91.49463 | 116.8917 | 146.2438 | 132.9489 | 127.4645 | 104.4202 | 148.7446 | 127.9641 | 99.65085 | 123.0174 | 101.487 | 101.281 | 99.67314 | 93.3114 | 88.22213 |
| CUST_51666_PI429010886 | M94497 | 0.011673 | 24 | 0 | | | | | | 39.24656 | 55.18575 | 67.04779 | NA | 32.53672 | 19.28281 | 24.28 | 6.469129 | 37.58201 | 47.76946 | 38.85028 | 59.55765 | 20.23937 | 32.91264 | 26.37038 | 5.05047 | 29.9042 | 32.52687 | 15.25038 | 40.79687 | 23.01614 | 29.26565 | 32.31627 | 41.55928 | 48.44993 | 71.54826 | 42.50976 |
| CUST_10969_PI429010886 | N27064 | 0.011676 | 12 | 3 | 242007122 | Aminopeptidase N precursor, putative [Pediculus hu | Protein metabolism | Proteolysis | 5.98E-12 | 31.11356 | 20.86543 | 15.08742 | 68.45737 | 36.21632 | 83.66854 | 47.91527 | 34.88082 | 14.13262 | 13.31013 | 23.22371 | 18.7912 | 35.35004 | 21.68681 | 29.2332 | 29.9146 | 51.56072 | 45.89316 | 46.99705 | 13.56063 | 33.38385 | 14.32141 | 10.5422 | 58.82472 | 16.48468 | 41.16094 | 16.38885 |
| CUST_7913_PI429010886 | N22265 | 0.011679 | 12 | 0 | 241999648 | src tyrosine kinase, putative [Ixodes scapularis] >gi\| | Developmental process | Other | 4.6E-104 | 38.2551 | 40.45279 | 30.39192 | 35.27392 | 34.06071 | 35.19263 | 27.57506 | 28.60108 | 29.21913 | 36.02623 | 29.71169 | 23.99191 | 36.96378 | 30.42832 | 47.00108 | 31.82901 | 34.35136 | 37.52021 | 37.63465 | 26.0929 | 27.39302 | 37.04031 | 39.83835 | 37.6674 | 42.19441 | 35.05495 | 40.34574 |
| CUST_19557_PI429010886 | N41776 | 0.011679 | 12 | 0 | | | | | | 104.0321 | 79.45781 | 108.8637 | 76.46353 | 81.7755 | 69.26327 | 86.67899 | 70.24276 | 68.24289 | 82.62874 | 89.24944 | 85.83424 | 99.19224 | 96.41487 | 100.999 | 99.45401 | 93.03197 | 78.41704 | 77.0714 | 108.5223 | 65.84868 | 98.56528 | 83.383 | 87.98669 | 110.9171 | 79.44802 | 120.905 |
| CUST_8571_PI429010886 | N23236 | 0.011689 | 18 | 3 | | | | | | 8.011679 | 3.778033 | 8.904805 | 14.42559 | 12.62191 | 14.13431 | 5.226041 | 5.914907 | NA | 10.76376 | 6.564241 | 6.701803 | 8.225765 | 3.898969 | 4.030756 | 7.202889 | 11.90309 | NA | 12.10209 | 5.183066 | NA | 10.66649 | 14.68311 | NA | 30.92489 | NA | 11.11786 |
| CUST_569_PI429010886 | N6098 | 0.011695 | 12 | 0 | 358442122 | Broad-complex protein isoform 4 [Penaeus monodc | | | 3.5E-45 | 85.17905 | 79.32298 | 113.1518 | 73.23407 | 76.45771 | 72.92837 | 75.7673 | 77.48229 | 75.68737 | 113.1942 | 60.46074 | 88.24844 | 91.066 | 100.8596 | 104.6153 | 77.5823 | 77.3067 | 73.01018 | 63.90408 | 102.2773 | 74.19557 | 91.29321 | 76.24971 | 64.27512 | 106.5981 | 87.16389 | 118.55 |
| CUST_42155_PI429010886 | M59566 | 0.011705 | 21 | 18 | | | | | | 155.4298 | 143.4699 | 190.3637 | 115.4049 | 137.6307 | 123.7264 | 117.2428 | 103.4451 | 126.8079 | 152.7823 | 133.5211 | 194.8606 | 120.2323 | 141.4486 | 170.0663 | 146.0101 | 138.4649 | 169.7841 | 159.2768 | 152.988 | 155.9986 | 146.0723 | 125.3899 | 132.2049 | 146.1343 | 130.9454 | 154.5663 |
| CUST_20146_PI429010886 | N42887 | 0.011728 | 18 | 0 | | | | | | 24.41426 | 13.532 | 17.50149 | 8.099309 | 4.346382 | NA | NA | 6.095505 | 11.33051 | 8.29257 | 4.693553 | NA | 5.034956 | 9.434112 | NA | 11.87577 | NA | NA | 13.69985 | 10.36906 | 9.684471 | 15.21234 | 9.416182 | 8.205981 | 6.326533 | 9.589321 | 3.522616 |
| CUST_27260_PI429010886 | N55722 | 0.011734 | 18 | 15 | 301791387 | PREDICTED: BET1-like protein-like [Ailuropoda mela | | | 7.14E-13 | 8.069484 | 10.61167 | NA | 20.41758 | 6.394773 | 10.46694 | 7.274775 | 9.407802 | 4.838598 | 10.78602 | 19.88479 | 7.966321 | 6.431841 | 14.99598 | 16.51607 | 10.49349 | NA | NA | 9.84998 | 16.21863 | 10.75755 | 7.702551 | 8.571028 | NA | 4.832909 | 3.899641 | 6.272559 |
| CUST_46035_PI429010886 | M71142 | 0.01174 | 15 | 12 | | | | | | 28.83874 | 20.6453 | 29.28006 | 18.02234 | 17.01911 | 12.88285 | 17.3028 | 11.07279 | 22.21682 | 38.94819 | 18.71899 | 29.23024 | 39.09942 | 17.67558 | 34.50768 | 25.48335 | 23.08947 | 35.69567 | 18.53665 | 15.21915 | 17.77636 | 19.07331 | 17.46064 | 17.91014 | 37.41548 | 8.754916 | 27.86411 |
| CUST_22060_PI429010886 | N46350 | 0.01174 | 15 | 12 | | | | | | 39.56122 | 36.21024 | 34.0026 | 23.6747 | 39.55733 | 33.65406 | 27.91811 | 28.84855 | 17.74945 | 44.75473 | 30.87561 | 45.87664 | 38.70169 | 44.75093 | 53.6056 | 46.02417 | 14.05746 | 52.21271 | 28.51882 | 39.78898 | 43.8795 | 36.61393 | 19.95383 | 30.87202 | 34.42302 | 45.33809 | 47.13105 |
| CUST_56892_PI429010886 | M114849 | 0.011755 | 24 | 18 | | | | | | 719.8537 | 1148.187 | 1004.727 | 561.5311 | 678.2021 | 875.926 | 602.4628 | 791.0227 | 861.1597 | 948.7418 | 736.1144 | 1287.066 | 816.3533 | 1140.952 | 956.1852 | 1126.811 | 892.629 | 1090.829 | 952.9126 | 1139.583 | 1153.924 | 1094.71 | 967.9514 | 1022.691 | 1099.991 | 1082.038 | 1077.802 |
| CUST_52807_PI429010886 | M98997 | 0.011755 | 21 | 0 | | | | | | 24.1047 | 43.09974 | 36.97731 | 28.66257 | 22.03473 | 19.91373 | 18.98793 | 23.18582 | 25.97092 | 26.11989 | 16.53332 | 17.03233 | 25.75936 | 32.63377 | 33.91698 | 39.46416 | 16.41397 | 26.1771 | 19.81911 | 26.56771 | 28.11091 | 38.03336 | 27.91811 | 35.66685 | 32.98295 | 28.20989 | 25.06723 |
| CUST_23793_PI429010886 | N49480 | 0.011765 | 12 | 0 | | | | | | 44.87442 | 179.7697 | 109.5173 | 3.436392 | 11.65798 | NA | 9.949258 | 62.27945 | 62.67964 | 80.39867 | 53.20016 | 100.9197 | 111.3283 | 115.9278 | 52.31892 | 29.75085 | 18.43075 | 31.32056 | 71.83386 | 107.2518 | 79.84889 | 31.29617 | 178.5448 | NA | 115.9863 | 47.23018 | 140.2437 |
| CUST_28910_PI429010886 | N58827 | 0.011775 | 15 | 9 | 242001898 | ankyrin repeat containing protein [Ixodes scapularis | Signal transduction | Other | 3.22E-11 | 15.68573 | 64.85632 | 18.36323 | 13.71331 | 9.72827 | 11.52078 | 5.311014 | 23.46287 | 8.982813 | 23.74624 | 16.69384 | 20.88612 | 14.63709 | 13.69107 | 18.60572 | 18.09635 | 11.20426 | 9.059534 | 10.43208 | 15.17161 | 8.191948 | 11.20426 | 18.56427 | 7.111485 | 19.56685 | 37.7275 | 27.42267 |
| CUST_31070_PI429010886 | M607 | 0.011777 | 15 | 9 | 405960855 | Charged multivesicular body protein 2b [Crassostre | Transport | Protein transport | 1.98E-50 | 58.30365 | 65.6728 | 42.242 | 64.26189 | 52.67412 | 66.37222 | 58.99785 | 66.15125 | 66.70898 | 118.3632 | 99.83489 | 150.3647 | 61.5685 | 68.19793 | 64.26189 | 105.3388 | 57.20678 | 62.42019 | 67.87327 | 74.79904 | 46.39793 | 72.96725 | 45.65065 | 68.6262 | 88.99049 | 81.34562 | 67.26789 |
| CUST_32666_PI429010886 | M4754 | 0.011788 | 21 | 9 | 260791579 | hypothetical protein BRAFLDRAFT_125738 [Branchi | Metabolic process | Oxidation-reduction process | 6.3E-115 | 28.80979 | 50.403 | 35.24786 | 35.43275 | 76.53451 | 38.65189 | 38.38764 | 127.9952 | 85.90922 | 60.56579 | 99.52485 | 79.97123 | 85.75328 | 29.57087 | 47.97063 | 53.32755 | 56.30291 | 38.34336 | 43.28157 | 200.2396 | 61.80458 | 33.54623 | 44.39447 | 29.83725 | 36.40199 | 66.10733 | 53.12043 |
| CUST_46552_PI429010886 | M72964 | 0.011791 | 24 | 21 | | | | | | 333.6383 | 289.5961 | 300.1314 | 283.5999 | 263.2877 | 283.4361 | 214.4445 | 221.3497 | 204.7693 | 319.448 | 260.2651 | 247.4184 | 235.6197 | 267.7292 | 252.6775 | 415.5762 | 302.8326 | 303.0793 | 258.0303 | 331.8125 | 269.8655 | 346.965 | 266.851 | 258.2202 | 346.1184 | 273.0127 | 290.7302 |
| CUST_9215_PI429010886 | N24247 | 0.0118 | 21 | 0 | | | | | | 81.85243 | 93.84895 | 89.74363 | 70.30533 | 79.65072 | 59.99556 | 60.9102 | 60.72482 | 47.90107 | 88.86362 | 58.15995 | 61.06386 | 66.45252 | 67.15618 | 66.13621 | 70.47227 | 69.54952 | 76.16727 | 60.44797 | 64.62929 | 67.16912 | 72.23286 | 72.8924 | 67.08317 | 101.1196 | 62.0828 | 88.11918 |
| CUST_54693_PI429010886 | M107184 | 0.011821 | 12 | 0 | | | | | | 73.44541 | 57.58322 | 72.55294 | 54.7161 | 38.39579 | 70.85223 | 39.33849 | 41.18849 | 47.90583 | 54.83755 | 47.00108 | 56.74499 | 69.56934 | 72.44248 | 59.56411 | 71.6044 | 51.05906 | 70.77093 | 53.41869 | 63.42473 | 60.12977 | 64.22812 | 79.80797 | 47.40597 | 87.42512 | 62.29412 | 75.64217 |
| CUST_50193_PI429010886 | M88985 | 0.011821 | 12 | 6 | 146217394 | cathepsin L [Penaeus monodon] | | | 1.5E-21 | 90.73889 | 22.0053 | 43.50773 | 26.87814 | 55.09927 | 24.6471 | 199.3094 | 85.54854 | 175.1768 | 109.19 | 81.00454 | 133.2798 | 96.72232 | 47.76946 | 38.12468 | 172.9784 | 91.55434 | 113.3117 | 135.5951 | 106.2858 | 115.4049 | 95.66411 | 89.86196 | 150.9399 | 102.7422 | 64.46821 | 49.54106 |
| CUST_37753_PI429010886 | M21399 | 0.011833 | 15 | 12 | 506968295 | 60S ribosomal protein L6 [Coptotermes formosanus | Protein metabolism | Ribosome biogenesis | 4.65E-61 | 48.63366 | 99.59438 | 106.908 | 69.15357 | 54.96227 | 24.45653 | 44.03128 | 89.5312 | 150.5005 | 193.1525 | 133.6116 | 257.1613 | 194.3207 | 320.9097 | 264.269 | 326.0308 | 44.014 | 183.3903 | 104.8753 | 155.1509 | 106.9501 | 308.3369 | 183.1272 | 30.41942 | 264.8381 | 141.0077 | 261.7284 |
| CUST_52194_PI429010886 | M96702 | 0.011833 | 21 | 15 | | | | | | 106.1639 | 68.30614 | 67.74201 | 39.94945 | 68.27282 | 64.56992 | 51.25067 | 55.6932 | 44.0607 | 80.73902 | 59.40092 | 59.34275 | 31.68444 | 63.3148 | 39.04491 | 89.44557 | 84.52872 | 89.31811 | 88.23558 | 80.30929 | 81.09417 | 87.6112 | 58.80718 | 54.78436 | 109.5894 | 47.98542 | 80.87419 |
| CUST_37922_PI429010886 | M22256 | 0.011858 | 24 | 0 | 152013691 | selenoprotein W2 [Artemia franciscana] | Metabolic process | Oxidation-reduction process | 7.82E-20 | 37.38785 | 39.54839 | 25.70664 | 29.35151 | 28.81252 | 28.48649 | 20.2137 | 28.03356 | 27.85864 | 34.55538 | 25.47915 | 56.78823 | 19.40477 | 19.83251 | 22.86827 | 25.34926 | 27.36205 | 22.45787 | 26.74986 | 35.6576 | 30.69887 | 26.63383 | 22.45136 | 24.83589 | 31.82653 | 29.0109 | 33.04443 |
| CUST_9806_PI429010886 | N25234 | 0.011878 | 24 | 21 | 157125424 | mitochondrial ribosomal protein, S34, putative [Aec | | | 3.91E-32 | 72.84145 | 101.7734 | 71.86244 | 56.61232 | 61.01573 | 56.46018 | 45.98092 | 44.42212 | 55.62148 | 83.56858 | 58.93488 | 77.65762 | 55.37064 | 75.12567 | 57.95385 | 109.9562 | 57.256 | 58.22987 | 61.58977 | 82.16288 | 72.53579 | 95.65165 | 76.81043 | 66.23901 | 100.1395 | 71.07061 | 72.50841 |
| CUST_33590_PI429010886 | M7361 | 0.01188 | 21 | 15 | | | | | | 13.54494 | 14.12148 | 11.55147 | 4.471973 | 18.84012 | NA | 12.67037 | NA | 9.040661 | 14.79509 | 13.62943 | 34.51534 | 15.90074 | 15.5539 | 16.91321 | 18.5156 | 13.06814 | 20.61073 | 15.06711 | 11.83706 | 21.17737 | 19.26978 | 12.73861 | 9.477835 | 9.81794 | 7.496731 | 12.53922 |
| CUST_2218_PI429010886 | N11894 | 0.011909 | 18 | 15 | | | | | | 2800.799 | 3590.082 | 2720.754 | 2838.594 | 3036.431 | 3659.313 | 2667.238 | 2457.891 | 2720.754 | 2764.643 | 2638.062 | 3756.632 | 2338.281 | 2506.708 | 2978.783 | 3582.089 | 3456.298 | 3514.364 | 3355.686 | 3219.948 | 2898.401 | 2831.641 | 2669.53 | 3040.688 | 2370.125 | 2495.356 | 2892.813 |
| CUST_51754_PI429010886 | M94889 | 0.011917 | 15 | 0 | | | | | | 94.47657 | 69.05841 | 61.02204 | 80.37038 | 53.25416 | 55.79477 | 84.34439 | 29.73496 | 50.1495 | 59.73525 | 39.39544 | 41.68626 | 63.03943 | 47.29592 | 63.81737 | 70.28439 | 65.72077 | 64.04369 | 58.50555 | 41.82918 | 59.71534 | 45.55401 | 71.35403 | 77.1122 | 48.51868 | 45.80703 | 41.11502 |
| CUST_36326_PI429010886 | M15606 | 0.011925 | 18 | 9 | | | | | | 4.842579 | 3.645673 | 3.522772 | 15.0972 | 4.006373 | NA | 8.932629 | 6.186749 | 4.922559 | 18.62971 | 15.90941 | 9.717046 | 8.055935 | 12.28249 | 17.27141 | 19.20545 | 9.068712 | 14.33928 | 10.34377 | 5.548243 | NA | NA | NA | NA | 11.55822 | 6.304011 | 16.27057 |
| CUST_51124_PI429010886 | M92286 | 0.011925 | 15 | 0 | | | | | | 8.807989 | 20.40692 | NA | 16.75931 | 10.53088 | 6.886089 | 6.775327 | NA | NA | 9.448882 | 4.62701 | NA | 5.363869 | 6.277631 | 5.283347 | 18.34511 | 21.71104 | 16.20611 | 9.645798 | 10.78464 | 12.47112 | 13.06649 | 4.66052 | NA | 10.44576 | NA | 6.092564 |
| CUST_1872_PI429010886 | N10927 | 0.011938 | 18 | 3 | | | | | | 4873.025 | 1061.463 | 6247.817 | 10559.01 | 4876.999 | 11013.97 | 8888.62 | 4284.782 | 970.0099 | 257.455 | 3477.696 | 16.77607 | 645.7634 | 277.7171 | 740.0272 | 77.52659 | 4440.858 | 1512.912 | 3997.898 | 31.35346 | 6630.238 | 21.87774 | 6654.81 | 7479.639 | 37.50798 | 2067.695 | 63.23998 |
| CUST_6492_PI429010886 | N20173 | 0.011944 | 21 | 18 | 328780007 | PREDICTED: major antigen [Apis mellifera] | | | 1.3E-10 | 168.58 | 219.3496 | 186.2287 | 126.5253 | 89.95558 | 103.1666 | 166.1287 | 95.54961 | 107.4484 | 191.145 | 152.8418 | 131.4056 | 112.457 | 166.186 | 173.6625 | 154.3834 | 71.23473 | 129.9123 | 175.1526 | 199.6816 | 187.6525 | 133.5047 | 160.804 | 152.7823 | 95.39909 | 149.6388 | 160.598 |
| CUST_42292_PI429010886 | M59923 | 0.011954 | 24 | 21 | 260833078 | hypothetical protein BRAFLDRAFT_63881 [Branchio | Protein metabolism | Cellular protein modification process | 5.8E-07 | 15.2636 | 15.71246 | NA | 37.03706 | 53.11468 | 55.45871 | 6.1333 | NA | 5.824752 | 4.198646 | 5.262289 | 13.14971 | 6.303067 | 32.15824 | 47.70026 | 66.69348 | NA | 64.5831 | NA | 15.00987 | 8.932629 | 74.6925 | NA | 55.90028 | 36.90877 | NA | 12.80617 |
| CUST_35149_PI429010886 | M11726 | 0.011954 | 15 | 9 | 334310856 | PREDICTED: FAS-associated factor 2 [Monodelphis c | | | 3.93E-62 | 26.09533 | 25.12574 | 11.29494 | 23.23883 | 20.92107 | 19.03542 | 27.8344 | 21.29353 | 22.4878 | 34.54086 | 25.82998 | 30.57659 | 25.50611 | 29.10242 | 24.63851 | 21.62429 | 18.81343 | 27.15737 | 21.05599 | 39.47942 | 7.682987 | 14.22354 | 27.52478 | 24.45097 | 28.50866 | 25.40325 | 29.23595 |
| CUST_51245_PI429010886 | M92787 | 0.011954 | 15 | 0 | | | | | | 201.5302 | 238.3252 | 172.3518 | 162.3689 | 167.732 | 152.1844 | 140.0584 | 121.6835 | 134.5558 | 221.1299 | 135.5002 | 164.4185 | 164.2756 | 201.5302 | 176.4634 | 274.6475 | 162.719 | 222.0782 | 164.6258 | 164.4185 | 144.4421 | 250.5389 | 144.5986 | 146.0474 | 202.438 | 143.5589 | 175.4895 |
| CUST_34335_PI429010886 | M9353 | 0.011969 | 21 | 12 | 321478997 | hypothetical protein DAPPUDRAFT_309622 [Daphn | | | 5.4E-40 | 289.5463 | 359.57 | 349.7536 | 342.0536 | 295.8079 | 325.619 | 367.921 | 267.0878 | 303.6176 | 377.56 | 326.5696 | 408.5798 | 350.793 | 370.0362 | 431.7288 | 491.0819 | 304.8602 | 358.225 | 410.7156 | 406.415 | 325.6779 | 450.4975 | 351.4461 | 317.3817 | 307.6894 | 293.5772 | 292.7654 |
| CUST_934_PI429010886 | N7867 | 0.011994 | 18 | 12 | | | | | | 48.68836 | 63.40267 | 66.29488 | 69.12024 | 47.23493 | 53.57478 | 48.41112 | 72.96725 | 56.61232 | 89.34718 | 45.40667 | 90.41498 | 56.16292 | 126.8944 | 78.68384 | 121.2986 | 72.23286 | 65.11921 | 61.85594 | 36.387 | 45.61861 | 39.58944 | 55.99321 | 33.04717 | 54.95061 | 41.42564 | 65.75053 |
| CUST_20504_PI429010886 | N43573 | 0.011997 | 18 | 15 | | | | | | 8.897078 | 20.33623 | 13.43047 | NA | 18.28186 | 9.47114 | 6.244116 | 6.367638 | 6.093268 | 5.371032 | 5.479138 | 14.94458 | 11.99091 | 7.495131 | 13.92315 | 15.3428 | 24.11521 | 17.30489 | 11.0392 | 8.013121 | 19.25031 | 16.26104 | 5.920001 | 6.510984 | 8.812213 | NA | 16.44885 |
| CUST_54822_PI429010886 | M107833 | 0.012008 | 18 | 0 | | | | | | 194.2907 | 198.6505 | 90.91352 | 99.18362 | 148.5046 | 99.05161 | 62.34386 | 60.8738 | 93.6434 | 88.98374 | 92.90826 | 129.0306 | 125.9794 | 195.555 | 161.1809 | 144.0267 | 40.16678 | 100.9197 | 196.3252 | 187.3568 | 147.5412 | 149.4128 | 50.9755 | 89.09879 | 83.0149 | 115.6902 | 98.56528 |
| CUST_52313_PI429010886 | M97028 | 0.012041 | 15 | 12 | 309356629 | Protein CBR-NAS-4 [Caenorhabditis briggsae] | Protein metabolism | Proteolysis | 2.29E-19 | 147.1104 | 83.44238 | 43.92643 | 19.32948 | 36.62028 | 44.66782 | 44.97088 | 46.00876 | 30.76441 | 86.91384 | 66.5854 | 74.89819 | 82.09306 | 82.9954 | 62.87324 | 68.88215 | 42.82987 | 26.36033 | 65.25047 | 20.58435 | 37.82192 | 39.64527 | 18.51403 | 64.56992 | 78.86687 | 24.42522 | 76.38976 |
| CUST_29553_PI429010886 | N60094 | 0.012041 | 15 | 12 | 549834 | RecName: Full=Zinc finger Y-chromosomal protein 1 | Nucleic acid metabolism | Regulation of gene expression | 2.16E-09 | 11.07586 | 12.75753 | 16.96368 | 9.057109 | 8.829376 | 6.791617 | 5.296822 | 8.173644 | 13.95091 | 15.32584 | 14.91902 | 18.4246 | 11.31528 | 19.82066 | 15.36262 | 23.07266 | 6.675568 | 10.85866 | 8.100321 | 9.252049 | 5.656475 | 10.73339 | 12.63126 | 4.553482 | 15.23056 | 4.075336 | 22.75029 |
| CUST_1099_PI429010886 | N8496 | 0.01205 | 15 | 9 | | | | | | 9.269541 | 10.49518 | 10.03543 | 7.92158 | 5.532517 | NA | 16.67558 | 7.683919 | 7.076703 | 9.538641 | 11.91129 | NA | 10.72561 | 10.41753 | 9.598505 | 10.99994 | 7.526258 | 8.93642 | 9.213601 | 5.807016 | 6.739431 | 11.41581 | 12.04453 | 12.53403 | 10.11317 | NA | 12.59239 |
| CUST_42296_PI429010886 | M59941 | 0.012052 | 21 | 0 | | | | | | 98.49703 | 94.46416 | 100.3232 | 81.75759 | 60.75286 | 118.4488 | 84.69362 | 62.61352 | 90.23885 | 57.62155 | 52.56971 | 95.17141 | 60.68591 | 148.4035 | 69.38283 | 43.72098 | 86.09048 | 102.7422 | 79.36987 | 95.29429 | 89.49881 | 109.3451 | 114.8221 | 126.2208 | 75.7764 | 97.80592 | 61.25018 |
| CUST_3915_PI429010886 | N15556 | 0.012052 | 21 | 18 | | | | | | 7.487023 | 17.58162 | 12.91494 | 6.125711 | 11.88011 | 13.38206 | 7.368786 | 6.050323 | 6.877297 | 16.06834 | 53.72529 | 8.314282 | 8.213559 | 17.86172 | 10.02996 | 13.40222 | 12.97654 | 13.66601 | 15.93948 | 15.37345 | 20.52907 | 12.394 | 6.346476 | 13.57505 | 16.54136 | 10.11651 | 12.41359 |
| CUST_9151_PI429010886 | N24141 | 0.012054 | 21 | 0 | | | | | | 162.6071 | 180.4643 | 126.9474 | 144.4224 | 131.7576 | 117.3985 | 129.6562 | 111.812 | 121.7904 | 165.2222 | 142.8727 | 134.79 | 120.2505 | 109.7501 | 121.2986 | 169.4541 | 146.8304 | 137.5853 | 134.6919 | 139.8135 | 130.2691 | 173.3481 | 158.2669 | 160.259 | 161.5934 | 130.0885 | 142.2748 |
| CUST_33986_PI429010886 | M8431 | 0.012058 | 15 | 9 | 124245110 | tetraspanins-like protein-8 [Fenneropenaeus chiner | | | 5.03E-08 | 148.8262 | 100.999 | 72.96725 | 143.517 | 112.8702 | 53.04998 | 126.4765 | 319.0748 | 217.4803 | 208.3478 | 217.4459 | 282.166 | 194.4818 | 79.71189 | 118.378 | 106.2285 | 205.8829 | 92.12003 | 148.5892 | 522.8025 | 175.3395 | 262.76 | 106.002 | 206.8694 | 272.8585 | 194.0645 | 343.0578 |
| CUST_5072_PI429010886 | N18224 | 0.012095 | 12 | 3 | 321468990 | hypothetical protein DAPPUDRAFT_318992 [Daphn | Signal transduction | Other | | 246.6234 | 204.5915 | 120.7928 | 412.0745 | 303.9813 | 540.3776 | 325.619 | 275.6931 | 187.8401 | 156.2928 | 215.9695 | 114.6697 | 226.151 | 121.4004 | 162.3309 | 210.5448 | 373.3663 | 368.2489 | 398.132 | 173.2007 | 156.0301 | 208.3184 | 122.2713 | 231.5437 | 164.4877 | 194.6574 | 128.8389 |
| CUST_32560_PI429010886 | M4435 | 0.012097 | 21 | 18 | 443717635 | hypothetical protein CAPTEDRAFT_220697 [Capitell | Nucleic acid metabolism | RNA metabolic process | 2.15E-50 | 21.4174 | 25.26334 | 32.24939 | 19.5508 | 13.68318 | 19.00616 | 11.23684 | 12.87701 | 7.605624 | 18.15598 | 17.94343 | 17.15538 | 28.02833 | 32.04305 | 17.55503 | 28.31689 | 9.082334 | 38.69682 | 9.427695 | 25.33083 | 35.00516 | 20.62529 | 24.81825 | 13.32601 | 23.64095 | 18.67218 | 17.83843 |
| CUST_15359_PI429010886 | N34457 | 0.012107 | 15 | 3 | | | | | | 203.8966 | 189.5687 | 180.3556 | 248.3781 | 210.9393 | 286.1419 | 189.4567 | 168.58 | 189.0589 | 207.6805 | 200.8635 | 203.8319 | 165.9754 | 168.5282 | 123.1997 | 201.1997 | 299.1597 | 215.3103 | 170.5765 | 210.7637 | 253.1914 | 230.9236 | 184.6775 | 226.4213 | 203.8319 | 161.7779 | 189.8916 |
| CUST_45274_PI429010886 | M68551 | 0.01211 | 15 | 9 | | | | | | 22.19273 | 21.94249 | 21.67212 | 17.92595 | 19.40757 | 21.87538 | 16.22034 | 30.90472 | 27.70404 | 43.71768 | 26.22826 | 40.39881 | 41.59399 | 35.01718 | 27.4871 | 33.53978 | 14.73015 | 29.43141 | 24.80851 | 30.43439 | 25.51724 | 32.9302 | 25.38492 | 25.82739 | 29.93929 | 28.88408 | 32.13794 |
| CUST_25010_PI429010886 | N51627 | 0.01212 | 15 | 0 | | | | | | 15.07677 | 16.3293 | 15.83738 | 10.98618 | 9.171762 | 16.94914 | 8.073322 | 9.451754 | 9.218873 | 15.51227 | 10.35316 | 11.25441 | 10.77921 | 14.75103 | 13.71331 | 19.56129 | 9.736405 | 18.86766 | 12.20709 | 14.00731 | 15.57429 | 13.96684 | 13.09162 | NA | 14.4465 | 13.49282 | 11.65277 |
| CUST_25406_PI429010886 | N52357 | 0.012144 | 15 | 0 | | | | | | 87.00147 | 83.46951 | 102.9667 | 56.7825 | 64.44572 | 75.38236 | 68.96573 | 52.76183 | 57.09018 | 59.052 | 61.10331 | 54.80426 | 64.35394 | 125.7681 | 66.75988 | 83.21112 | 75.68737 | 84.42877 | 73.07849 | 53.28087 | 66.56405 | 82.71117 | 69.59584 | 61.45036 | 81.85243 | 64.98263 | 81.25969 |
| CUST_51001_PI429010886 | M91756 | 0.012153 | 15 | 9 | | | | | | 19.74119 | 30.00329 | 35.29716 | 15.59485 | 21.27912 | 26.58056 | 19.10453 | 21.5046 | 25.07963 | 28.92395 | 26.39223 | 30.68045 | 22.61376 | 30.63064 | 26.72669 | 27.33674 | 23.47125 | 29.33547 | 14.83746 | 26.21095 | 18.45614 | 22.5314 | 18.07887 | 25.37889 | 24.12427 | 168.0847 | 34.91043 |
| CUST_11363_PI429010886 | N27690 | 0.012155 | 24 | 0 | | | | | | 113.8702 | 118.2155 | 148.9832 | 95.32771 | 89.92723 | 95.30462 | 87.16389 | 77.39915 | 91.85305 | 114.4563 | 94.18675 | 97.92701 | 87.62612 | 108.7243 | 97.282 | 138.133 | 105.242 | 110.7189 | 95.60632 | 116.1174 | 102.6532 | 107.3035 | 106.7139 | 105.0612 | 134.8756 | 116.7797 | 122.7034 |
| CUST_4535_PI429010886 | N16981 | 0.012158 | 12 | 3 | | | | | | 128.6574 | 58.81306 | 21.93409 | 100.5915 | 166.0195 | 179.3194 | 41.43739 | 44.90621 | 26.24834 | 31.51891 | 20.96144 | 11.69456 | 4.882948 | 28.75932 | 18.59345 | 46.55207 | 94.11857 | 74.87405 | 51.21719 | 4.845875 | 22.25913 | 34.59885 | 12.01567 | 83.90478 | 55.23983 | 47.3594 | 23.79718 |
| CUST_49033_PI429010886 | M83708 | 0.012162 | 18 | 6 | 321471093 | hypothetical protein DAPPUDRAFT_241093 [Daphn | Signal transduction | Other | 1.98E-15 | 54.89215 | 38.95233 | 54.27461 | 64.65683 | 68.47784 | 76.24971 | 87.6112 | 87.69732 | 79.26902 | 69.68123 | 47.75744 | 68.55373 | 90.62378 | 71.91814 | 67.64754 | 66.44095 | 89.13187 | 47.13105 | 75.27932 | 45.62788 | 46.7059 | 70.50114 | 61.22888 | 69.00002 | 96.81237 | 40.01136 | 86.3406 |
| CUST_13339_PI429010886 | N31020 | 0.01217 | 15 | 3 | | | | | | 225.8046 | 202.7794 | 151.8121 | 692.7803 | 1413.603 | 828.8539 | 947.2744 | 679.5311 | 24.00128 | 4.822002 | 1103.397 | NA | 295.8552 | 7.556212 | 119.4919 | NA | 1242.81 | NA | 950.5113 | NA | 951.2956 | NA | 713.8389 | 1017.693 | 4.490174 | 91.35555 | 4.898275 |
| CUST_31367_PI429010886 | M1361 | 0.012191 | 24 | 21 | 443689293 | hypothetical protein CAPTEDRAFT_202452 [Capitell | Protein metabolism | Regulation of translational initiation | 2.47E-36 | 119.885 | 140.0198 | 99.42759 | 102.9064 | 102.0907 | 105.9691 | 78.75847 | 80.42722 | 89.43652 | 99.4422 | 90.57431 | 120.7611 | 106.2054 | 123.056 | 118.5239 | 108.1857 | 87.65825 | 106.4577 | 88.02287 | 116.4869 | 114.2745 | 152.0834 | 111.9784 | 110.9478 | 112.0385 | 106.8104 | 106.6656 |
| CUST_1194_PI429010886 | N8797 | 0.012192 | 15 | 12 | | | | | | 2204.613 | 5897.382 | 8255.78 | 2951.483 | 4077.008 | 1935.871 | 2731.57 | 9235.263 | 9670.846 | 13400.98 | 2306.592 | 7692.588 | 7662.198 | 15845.68 | 11289.07 | 6329.544 | 1705.674 | 4396.276 | 1621.924 | 5492.691 | 2848.734 | 13355.9 | 6416.964 | 2944.104 | 11942.94 | 4165.771 | 10559.01 |
| CUST_19663_PI429010886 | N41970 | 0.012201 | 24 | 15 | | | | | | 30.73521 | 40.42007 | 32.09701 | 22.28112 | 32.83309 | 21.19908 | 22.35102 | 22.92529 | 30.21934 | 38.4072 | 28.27144 | 39.96875 | 36.72024 | 60.25585 | 28.59732 | 43.46213 | 44.25982 | 38.80762 | 45.47762 | 36.66046 | 30.95577 | 40.63191 | 39.45879 | 30.54779 | 42.93476 | 31.51141 | 41.78277 |
| CUST_23597_PI429010886 | N49090 | 0.012201 | 24 | 6 | | | | | | 77.66626 | 149.8301 | 107.7642 | 59.44434 | 20.66168 | 15.76954 | 124.8931 | 116.8209 | 76.23608 | 138.8541 | 116.4869 | 89.419 | 77.4993 | 25.09429 | 46.65817 | 62.83219 | 27.87322 | 26.63815 | 25.39336 | 61.12874 | 17.04092 | 7.070433 | 56.61759 | 25.72813 | 14.76794 | 9.246707 | 46.11558 |
| CUST_10103_PI429010886 | N25699 | 0.012208 | 21 | 3 | 524889431 | PREDICTED: uncharacterized protein LOC10185317 | Nucleic acid metabolism | DNA replication | 1.69E-45 | 13.00185 | 7.873199 | 12.6884 | 13.31647 | 12.18023 | 8.805551 | 20.02885 | 7.950059 | 8.510732 | 8.330776 | 6.786633 | 11.44313 | 4.165633 | 10.76694 | 4.876287 | 11.85674 | 12.88124 | NA | 12.07646 | 8.506994 | 7.353206 | 3.89041 | 15.61967 | 9.41784 | 22.00022 | 15.58392 | 22.5725 |
| CUST_31600_PI429010886 | M1940 | 0.012213 | 12 | 0 | | | | | | 1382.607 | 1556.767 | 1464.638 | 1172.181 | 948.7418 | 1010.24 | 1075.686 | 1179.34 | 1279.6 | 1509.413 | 1042.392 | 1467.586 | 1355.659 | 1573.105 | 1598.927 | 1682.988 | 1090.829 | 1128.807 | 1219.823 | 1236.256 | 1248.908 | 1573.105 | 1322.445 | 1081.506 | 1784.695 | 1190.624 | 1452.711 |
| CUST_52844_PI429010886 | M99182 | 0.012214 | 18 | 12 | | | | | | 14.13977 | 36.06457 | 10.74056 | 19.66479 | 18.67218 | NA | 76.08615 | 5.473065 | 63.00378 | 28.73136 | 14.99598 | 35.51007 | 74.04651 | 69.98559 | 45.70165 | 19.11878 | 42.71137 | 18.06186 | 14.80507 | 12.43272 | 10.99038 | 25.61779 | 53.99759 | 42.53014 | 33.24397 | 28.69117 | 31.09756 |
| CUST_28288_PI429010886 | N57673 | 0.012244 | 18 | 15 | | | | | | 52.89103 | 75.85108 | 52.88081 | 62.25774 | 55.61005 | 50.38632 | 47.18622 | 55.50038 | 39.67691 | 47.88363 | 58.58074 | 56.65877 | 60.53435 | 49.81001 | 56.80303 | 66.80323 | 59.01989 | 57.19607 | 56.74499 | 66.26607 | 43.75577 | 57.15725 | 42.21202 | 48.61666 | 55.26178 | 51.63758 | 48.53823 |
| CUST_23966_PI429010886 | N49786 | 0.012249 | 12 | 0 | | | | | | 207.128 | 308.3972 | 220.8169 | 184.6172 | 170.2454 | 199.3801 | 149.4828 | 167.1839 | 175.8023 | 185.5006 | 117.9302 | 275.8443 | 156.6286 | 230.5922 | 235.838 | 230.4163 | 130.7243 | 232.1489 | 151.707 | 173.8312 | 200.0776 | 192.2319 | 157.0892 | 202.7268 | 237.6429 | 218.6003 | 245.9792 |
| CUST_35086_PI429010886 | M11534 | 0.012257 | 15 | 3 | | | | | | 200.9243 | 77.92082 | 42.82987 | 959.9717 | 697.8167 | 2073.005 | 1640.228 | 194.1209 | 65.9084 | 9.692914 | 1278.963 | 13.5897 | 191.3655 | 34.70757 | 110.765 | 57.6895 | 2733.383 | 1210.833 | 2624.92 | 72.21756 | 3983.683 | 57.41889 | 1423.398 | 1561.567 | 22.47719 | 462.7955 | 16.09243 |
| CUST_6973_PI429010886 | N20854 | 0.012257 | 15 | 9 | 291236570 | PREDICTED: coagulation factor VIII-associated prote | | | 4.11E-58 | 182.2902 | 175.1291 | 197.6076 | 181.1348 | 170.2284 | 154.6801 | 146.1758 | 202.3386 | 171.4068 | 244.4297 | 193.8995 | 273.1684 | 166.7068 | 161.7557 | 160.804 | 184.3061 | 161.4133 | 167.9521 | 129.7027 | 105.8358 | 134.8934 | 188.0879 | 163.4482 | 118.675 | 255.8316 | 161.7557 | 208.6342 |
| CUST_34018_PI429010886 | M8518 | 0.012266 | 18 | 3 | 242003551 | Tob1 protein, putative [Pediculus humanus corporis | | | 9.83E-43 | 15.56261 | 42.35334 | 24.8275 | 25.71973 | 33.99576 | 36.79834 | 19.86296 | 19.24807 | 10.96329 | 12.16991 | 16.04256 | 22.07207 | 12.01444 | 22.20953 | 11.38323 | 13.35477 | 16.05605 | 22.85266 | 13.23813 | 20.49729 | 35.39506 | 15.81556 | 39.87971 | 34.09884 | 33.9856 | 12.92974 | 16.56922 |
| CUST_38214_PI429010886 | M24181 | 0.012278 | 24 | 6 | | | | | | 45.16608 | 14.84953 | 113.6011 | 17.31232 | NA | NA | 141.5508 | 239.0491 | 175.8509 | 238.3762 | 78.9119 | 125.099 | 55.44212 | 58.22303 | 25.7467 | 5.312395 | 102.1126 | NA | 6.995543 | 57.56812 | 5.603976 | 13.14971 | 293.8787 | 18.44505 | 6.588073 | 15.65986 | 114.3743 |
| CUST_11412_PI429010886 | N27764 | 0.012289 | 24 | 6 | | | | | | 11.50819 | 24.39326 | NA | 785.2299 | 34.22484 | 280.8839 | NA | 505.0665 | 445.2354 | 17.91166 | 363.2398 | 14.38818 | 4.1157 | 15.9031 | 26.75848 | 4.527192 | NA | NA | 6.714373 | 28.90402 | 68.89154 | NA | 595.028 | 10.82172 | 17.78619 | NA | 131.1004 |
| CUST_36556_PI429010886 | M16224 | 0.012291 | 24 | 0 | | | | | | 93.50952 | 99.17539 | 142.6158 | 73.42232 | 90.90134 | 112.7145 | 104.1068 | 80.61916 | 82.86721 | 83.12577 | 85.15981 | 101.9095 | 81.84143 | 93.42325 | 70.3928 | 88.04251 | 93.20644 | 111.5705 | 96.94706 | 85.81376 | 91.62991 | 90.92371 | 113.3258 | 95.07199 | 132.5328 | 117.1499 | 96.8234 |
| CUST_42545_PI429010886 | M60556 | 0.012294 | 12 | 9 | 321463530 | hypothetical protein DAPPUDRAFT_307215 [Daphn | Metabolic process | Carbohydrate metabolic process | 1.35E-16 | 47.32005 | 14.31728 | 16.09243 | NA | 23.30987 | 29.90756 | 63.70797 | 40.32605 | 36.77765 | 105.6617 | 45.3426 | 36.59373 | 14.90361 | 7.765456 | 7.544684 | 13.61134 | 35.23219 | 50.01795 | 73.45438 | 38.18452 | 47.61035 | 39.31465 | 41.68626 | 63.94718 | 44.63407 | 26.9152 | 37.06438 |

| Probe ID | ID | p-value | Period | Phase | EBI ID | Annotation | GO category | Go term | E-value | 0 | 0 | 0 | 3 | 3 | 3 | 6 | 6 | 6 | 9 | 9 | 9 | 12 | 12 | 12 | 15 | 15 | 15 | 18 | 18 | 18 | 21 | 21 | 21 | 24 | 24 | 24 |
|---|---|---|---|---|---|---|---|---|---|---|---|---|---|---|---|---|---|---|---|---|---|---|---|---|---|---|---|---|---|---|---|---|---|---|---|---|
| CUST_14359_PI429010886 | N32756 | 0.01232 | 24 | 18 | | | | | | 46.40223 | 33.47045 | 36.92465 | 22.03473 | 39.00381 | 32.52687 | 24.62111 | 34.00581 | 31.20174 | 43.87581 | 37.63897 | 45.3426 | 28.34801 | 48.05138 | 38.3841 | 49.66096 | 27.85193 | 64.18045 | 48.25178 | 42.16986 | 45.35819 | 41.28967 | 35.44133 | 34.95753 | 45.82004 | 35.53033 | 39.8936 |
| CUST_29336_PI429010886 | N59668 | 0.01232 | 24 | 0 | | | | | | 286.997 | 419.1452 | 513.797 | 362.4934 | 402.9446 | 390.7246 | 374.1535 | 403.2396 | 325.0806 | 492.5912 | 383.7251 | 390.6066 | 380.9707 | 307.1797 | 191.528 | 445.5129 | 371.2619 | 345.9305 | 383.351 | 368.7924 | 438.2102 | 461.1208 | 478.074 | 413.4726 | 571.7132 | 419.8201 | 464.4064 |
| CUST_22786_PI429010886 | N47668 | 0.012322 | 21 | 15 | | | | | | 11.46694 | 16.85623 | 16.39936 | 15.38835 | 16.45281 | 18.76711 | 8.437018 | 15.96811 | 11.69456 | 18.31489 | 11.82115 | 10.93941 | 19.30028 | 12.04133 | 11.43831 | 30.29871 | 20.13934 | 19.88004 | 14.47943 | 41.65923 | 17.5674 | 13.23323 | 19.30381 | 18.48046 | 23.95482 | 3.797811 | 10.27148 |
| CUST_53293_PI429010886 | M101151 | 0.012347 | 24 | 18 | 405965492 | Putative aquaporin-10 [Crassostrea gigas] | | | 1.11E-13 | 13.77798 | 19.95159 | 15.5539 | 10.03222 | 12.46576 | 8.011679 | 14.06235 | 14.45421 | 21.248 | 40.97837 | 23.13514 | 30.56491 | 20.84115 | 28.39575 | 23.17992 | 23.97181 | 14.56893 | 21.14025 | 33.76678 | 27.57506 | 26.67547 | 27.09726 | 15.23223 | 17.26487 | 42.38165 | 22.89976 | 41.94819 |
| CUST_51443_PI429010886 | M93553 | 0.012347 | 24 | 21 | | | | | | 53.2143 | 59.16475 | 79.09571 | 39.64527 | 59.91437 | 51.11982 | 49.03345 | 42.80624 | 48.75681 | 73.49284 | 59.11379 | 46.89592 | 47.58792 | 63.70207 | 42.58462 | 46.93128 | 59.37489 | 65.56878 | 55.6042 | 65.69324 | 68.83612 | 58.544 | 86.43501 | 70.65347 | 58.32457 | 69.08039 | 67.19434 |
| CUST_7398_PI429010886 | N21482 | 0.012347 | 24 | 18 | 530636057 | PREDICTED: cyclin-D1-binding protein 1 homolog [C | | | 4.23E-35 | 95.18251 | 104.2917 | 112.8267 | 66.78979 | 63.93448 | 56.07148 | 64.648 | 78.15144 | 83.383 | 120.7015 | 88.96346 | 130.4682 | 100.7779 | 104.6277 | 91.90962 | 110.737 | 55.53245 | 78.95924 | 105.8112 | 122.0182 | 126.727 | 103.457 | 101.4116 | 70.68641 | 105.1778 | 80.12587 | 110.4732 |
| CUST_47408_PI429010886 | M75989 | 0.012363 | 21 | 3 | | | | | | 99.06334 | 236.2907 | 92.47211 | 429.8795 | 155.8809 | 181.1767 | 116.4869 | 109.3629 | 101.2088 | 117.2732 | 63.81737 | 125.8769 | 77.06228 | 110.6199 | 59.68907 | 119.5328 | 58.44181 | 134.1419 | 69.08941 | 62.94659 | 188.5235 | 152.3559 | 194.1209 | 117.7597 | 200.184 | 262.8518 | 71.32452 |
| CUST_1264_PI429010886 | N9070 | 0.01237 | 12 | 3 | 156349174 | hypothetical protein NEMVEDRAFT_v1g143109 [Ne | | | 8.25E-11 | 3055.899 | 751.4303 | 970.0099 | 14629.47 | 6065.211 | 9501.999 | 7479.639 | 9371.785 | 1901.328 | 19.99991 | 4.700761 | 3.832017 | 4171.713 | NA | 1520.67 | NA | 17966.78 | 19120.04 | 8582.669 | NA | 6787.522 | NA | 4157.716 | 12983.85 | NA | 11289.07 | NA |
| CUST_9066_PI429010886 | N24011 | 0.012373 | 18 | 15 | | | | | | 16000.49 | 12445.58 | 4440.858 | 11510.47 | 19571.84 | 6247.817 | 12.5052 | 28.29331 | 771.6454 | 3651.445 | 7909.769 | 9919.546 | 6033.954 | 14406.9 | 19357.18 | 29142.09 | 5.14605 | 18217.03 | 2740.182 | 27412.06 | 7853.842 | 22767.31 | 4524.584 | 7890.624 | 4028.762 | 1943.851 | 2212.175 |
| CUST_40594_PI429010886 | M55562 | 0.01238 | 24 | 21 | 321477285 | hypothetical protein DAPPUDRAFT_305537 [Daphn | | | 5.39E-38 | 140.6991 | 123.2134 | 170.6324 | 103.4225 | 152.565 | 128.7368 | 103.4451 | 114.2883 | 143.9244 | 109.5408 | 198.008 | 114.6015 | 114.1326 | 96.66502 | 66.73255 | 159.338 | 118.246 | 89.45826 | 140.0797 | 113.3559 | 136.6977 | 155.9986 | 142.9167 | 144.1009 | 216.2249 | 142.4863 | 134.3863 |
| CUST_37718_PI429010886 | M21212 | 0.012391 | 24 | 18 | 77743875 | Euphausia superba isolate DGGE band AntEuk13-5 | | | 4.5E-156 | 1035.582 | 557.9517 | 1000.153 | 629.802 | 760.0455 | 1175.839 | 440.6954 | 532.8085 | 696.1501 | 1059.917 | 1186.768 | 923.7156 | 954.6687 | 1706.496 | 1434.403 | 1192.582 | 1328.52 | 1309.784 | 1317.338 | 1949.421 | 1581.923 | 879.939 | 713.4223 | 523.4011 | 2132.178 | 1471.445 | 2452.337 |
| CUST_37124_PI429010886 | M17927 | 0.012391 | 12 | 0 | 340722096 | PREDICTED: Bombus terrestris dual specificity mitog | Protein metabolism | Cellular protein modification process | 2E-84 | 85.32395 | 89.24114 | 94.68098 | 80.22855 | 78.87647 | 95.83965 | 66.22546 | 52.05749 | 69.56167 | 93.94852 | 79.49534 | 88.37144 | 89.49881 | 107.823 | 102.5272 | 118.7528 | 66.11527 | 81.09417 | 68.41788 | 68.93293 | 80.73902 | 83.61604 | 84.27092 | 93.62063 | 90.21463 | 74.17182 | 79.5827 |
| CUST_49189_PI429010886 | M84589 | 0.012391 | 12 | 0 | | | | | | 167.0511 | 138.8758 | 121.5738 | 290.0281 | 120.1371 | 120.5518 | 91.77189 | 118.9193 | 96.74817 | 130.3743 | 105.0723 | 137.5108 | 125.1637 | 146.9588 | 154.2903 | 147.7898 | 89.48953 | 116.8603 | 103.1477 | 91.05924 | 101.5014 | 115.4469 | 108.4414 | 118.8737 | 127.0833 | 103.2145 | 110.3616 |
| CUST_32655_PI429010886 | M4707 | 0.012408 | 15 | 0 | 242025576 | alpha/beta hydrolase domain containing protein 1, | | | 1.37E-59 | 557.6662 | 700.4893 | 550.4344 | 338.0611 | 553.514 | 525.5752 | 443.4781 | 478.5158 | 350.3639 | 528.618 | 566.6261 | 489.338 | 293.6394 | 371.9875 | 493.8599 | 914.1643 | 499.1257 | 615.5507 | 547.2331 | 570.2153 | 493.7491 | 455.2984 | 402.2817 | 496.875 | 515.1721 | 513.0951 | 573.9804 |
| CUST_32685_PI429010886 | M4810 | 0.012408 | 15 | 9 | 149675211 | cuticle proprotein proCP5.2 [Callinectes sapidus] | | | 5.32E-71 | 4436.798 | 2077.773 | 7930.333 | 1764.536 | 1014.347 | 1649.441 | 4882.08 | 15892.67 | 18496.67 | 15604.09 | 12053.21 | 14493.88 | 15845.68 | 32528.46 | 18257.85 | 4263.359 | 11143.54 | 1531.812 | 5959.328 | 15060.57 | 3934.354 | 31396.1 | 18524.86 | 1581.192 | 36785.94 | 9660.218 | 33985.8 |
| CUST_46265_PI429010886 | M71998 | 0.012408 | 15 | 9 | | | | | | 187.181 | 229.6885 | 234.6243 | 153.7558 | 178.833 | 141.6349 | 136.1246 | 158.7119 | 208.2116 | 345.4444 | 235.6566 | 284.6976 | 185.4432 | 256.7514 | 243.4729 | 288.1636 | 200.9243 | 215.1259 | 208.138 | 274.0115 | 203.5264 | 283.9501 | 256.6122 | 160.5552 | 339.3078 | 217.7746 | 306.1523 |
| CUST_48606_PI429010886 | M81242 | 0.012408 | 15 | 0 | | | | | | 33.68381 | 48.90708 | 37.40087 | 39.28519 | 29.61255 | 49.42389 | 22.13275 | 27.58029 | 25.95987 | 32.55113 | 26.21698 | 39.02972 | 33.54956 | 38.22991 | 41.03316 | 48.40695 | 31.82901 | 49.54555 | 31.09423 | 43.40065 | 23.84339 | 29.94885 | 31.63804 | 35.82003 | 30.41187 | 36.14137 | 42.06392 |
| CUST_31971_PI429010886 | M2871 | 0.012432 | 21 | 0 | | | | | | 891.7466 | 943.3673 | 808.4761 | 827.8275 | 710.9044 | 785.0368 | 635.813 | 598.6499 | 669.4372 | 761.7734 | 681.7268 | 808.4761 | 683.9239 | 761.273 | 800.0152 | 1167.349 | 632.4528 | 832.0351 | 818.0738 | 741.2403 | 733.3828 | 930.2399 | 671.5411 | 818.4343 | 792.4242 | 804.8286 | 694.7611 |
| CUST_24723_PI429010886 | N51149 | 0.012436 | 24 | 0 | 226693231 | arginine kinase 1 [Neocaridina denticulata] | | | 3.5E-52 | 130.2384 | 129.2544 | 152.5473 | 81.42898 | 136.6377 | 124.6057 | 114.7609 | 79.45781 | 97.88296 | 125.1102 | 114.3617 | 107.638 | 63.53447 | 101.5112 | 87.31709 | 125.3551 | 128.1445 | 150.4806 | 122.2551 | 97.5299 | 102.7028 | 116.7168 | 94.01656 | 100.542 | 188.5235 | 116.0676 | 177.6428 |
| CUST_39249_PI429010886 | M36704 | 0.012436 | 15 | 9 | 328899610 | ribosomal protein L35 [Procambarus clarkii] | Protein metabolism | Ribosome biogenesis | 2.39E-20 | 3227.523 | 7637.386 | 4561.712 | 5367.717 | 6474.137 | 7355.509 | 5654.855 | 4399.213 | 7605.917 | 7207.278 | 5693.449 | 10790.28 | 6273.384 | 5289.144 | 5787.347 | 5181.094 | 3572.519 | 3960.835 | 4083.732 | 4532.248 | 5663.977 | 8627.68 | 5125.683 | 5283.671 | 6253.288 | 5794.422 | 9106.522 |
| CUST_9689_PI429010886 | N25054 | 0.012457 | 21 | 3 | | | | | | 66663.04 | 54992.02 | 72295.32 | 78081.9 | 75608.53 | 76476.71 | 68283.18 | 62198.15 | 74829.64 | 76141.25 | 54279.51 | 53582.06 | 60338.91 | 69607.7 | 52133.5 | 54708.78 | 52551.15 | 65872.24 | 57482.98 | 57772.94 | 83813.84 | 71255.08 | 79681.93 | 45401.69 | 74289.93 | 71527.46 | 70048.09 |
| CUST_49972_PI429010886 | M88018 | 0.012459 | 15 | 0 | | | | | | 86.95691 | NA | 151.3078 | 42.2764 | 58.33665 | 109.6112 | 94.18675 | 83.92582 | 42.54782 | 55.15261 | 39.59771 | NA | 52.59262 | 54.5396 | 31.8423 | NA | 109.9447 | 87.26148 | 166.7568 | NA | 85.52397 | NA | 98.59007 | 93.81682 | 5.119427 | 81.8019 | 6.760169 |
| CUST_32147_PI429010886 | M3364 | 0.012462 | 24 | 21 | | | | | | 84.20531 | 90.33731 | 63.82351 | 70.52611 | 68.2813 | 46.38212 | 56.31693 | 40.25661 | 59.29234 | 67.21241 | 65.38596 | 85.60322 | 59.09902 | 83.87586 | 89.35703 | 110.2048 | 52.21965 | 102.8432 | 61.25792 | 109.0449 | 74.48926 | 99.00087 | 82.84904 | 79.043 | 79.33173 | 62.81643 | 78.82383 |
| CUST_46174_PI429010886 | M71678 | 0.012477 | 24 | 0 | | | | | | 40.33076 | 28.49366 | 21.51643 | 16.30792 | 16.50225 | 21.49674 | 34.08252 | 15.98525 | 17.3861 | 19.61727 | 17.40574 | 13.78438 | 18.46967 | 11.74368 | 21.91188 | 33.27763 | 20.45436 | 27.95506 | 27.4201 | 22.77856 | 24.19734 | 40.05517 | 19.83086 | 26.13969 | 43.14351 | 18.50772 | 37.31704 |
| CUST_31120_PI429010886 | M739 | 0.012483 | 24 | 15 | 291231880 | PREDICTED: predicted protein-like [Saccoglossus ko | | | 1.48E-41 | 144.1199 | 169.9162 | 243.3595 | 135.5775 | 156.5024 | 160.4822 | 133.8452 | 157.4172 | 149.2835 | 239.2718 | 225.6208 | 329.5922 | 280.6238 | 233.9335 | 266.9045 | 267.2735 | 267.9382 | 166.2981 | 251.1828 | 161.7305 | 169.9162 | 234.443 | 327.9176 | 265.6599 | 211.6792 | 198.7745 | 260.5083 |
| CUST_36014_PI429010886 | M14292 | 0.012493 | 15 | 3 | | | | | | 30.43732 | 52.53115 | 49.69905 | 48.2905 | 54.15061 | 44.90621 | 46.19529 | 33.64659 | 36.2246 | 49.95028 | 43.82673 | 31.35806 | 27.81399 | 20.49313 | 22.59328 | 44.7891 | 50.8348 | 37.53618 | 47.47988 | 45.07578 | 39.0395 | 46.07745 | 39.7525 | 30.19656 | 44.01036 | 22.19563 | 32.60782 |
| CUST_30924_PI429010886 | M231 | 0.012493 | 15 | 12 | | | | | | 771.9192 | 978.9007 | 885.7556 | 847.0661 | 654.7049 | 793.9994 | 539.4382 | 638.47 | 758.8197 | 943.689 | 688.7233 | 1070.582 | 765.2983 | 1083.296 | 1192.582 | 1114.646 | 759.2915 | 974.6113 | 727.1859 | 1160.888 | 821.4933 | 915.8521 | 767.2232 | 688.5897 | 1064.349 | 1013.272 | 1095.532 |
| CUST_11821_PI429010886 | N28458 | 0.012493 | 15 | 12 | 198435252 | PREDICTED: costars family protein ABRACL-like [Cio | | | 8.84E-25 | 81.51675 | 60.45419 | 97.92701 | 80.72822 | 66.4925 | 56.57948 | 48.65694 | 70.33128 | 61.45036 | 135.9425 | 71.91218 | 92.9186 | 110.5384 | 107.8324 | 110.7482 | 124.732 | 55.2283 | 80.9845 | 76.57062 | 91.60957 | 104.4333 | 91.28115 | 78.01974 | 52.79712 | 107.7388 | 83.79573 | 130.7098 |
| CUST_27777_PI429010886 | N56676 | 0.012499 | 24 | 6 | | | | | | 19.19934 | 12.502 | 21.74369 | 11.54685 | 20.81295 | 31.08481 | 32.68104 | 27.71267 | 30.34438 | 19.04736 | 10.8172 | 6.702805 | 14.54325 | 17.8888 | 16.8263 | 7.47347 | 15.81556 | 9.057109 | 27.45965 | NA | 8.648968 | 10.71609 | 9.055842 | 19.28994 | 12.04453 | 9.69944 | NA |
| CUST_35451_PI429010886 | M12607 | 0.012499 | 21 | 18 | 295639972 | chitinase 2 [Penaeus monodon] | Metabolic process | Carbohydrate metabolic process | 5.4E-143 | 69.19616 | 44.40267 | 18.20618 | 77.84895 | 87.08384 | 47.42461 | 22.70524 | 5.113279 | 20.08095 | 42.17788 | 51.57463 | 46.3344 | 77.95158 | 61.08538 | 54.13488 | 106.7397 | 8.545589 | 52.4135 | 58.32968 | 87.7947 | 101.2912 | 148.4834 | 33.8524 | 56.43628 | 120.9579 | 31.15965 | 23.2088 |
| CUST_38595_PI429010886 | M28404 | 0.012499 | 21 | 9 | | | | | | 63.51336 | 140.2062 | 91.59828 | 97.87109 | 438.2102 | 506.24 | 141.3582 | 222.9689 | 71.65614 | 248.7014 | 338.7848 | 451.3065 | 86.3406 | 135.5426 | 178.5448 | 122.7332 | 540.3776 | 227.4117 | 150.5005 | 214.6655 | 62.11683 | 58.597 | 94.55772 | 56.69692 | 87.10078 | 574.2435 | 173.5554 |
| CUST_4381_PI429010886 | N16634 | 0.0125 | 12 | 0 | | | | | | 34.7029 | 32.80137 | 54.46306 | 24.07489 | 24.58762 | 25.05489 | 32.30965 | 28.93983 | 30.53018 | 18.23858 | 26.83276 | 33.12364 | 42.6663 | 51.20005 | 31.43005 | 29.06792 | 26.11989 | 41.29432 | 36.12242 | 32.50599 | 28.04755 | 34.58756 | 31.2636 | 21.26038 | 52.32895 | 37.84181 | 36.21024 |
| CUST_16595_PI429010886 | N36525 | 0.0125 | 12 | 3 | | | | | | 59.25275 | 36.53812 | 40.36236 | 63.3148 | 86.08273 | 101.0393 | 100.9074 | 59.234 | 33.25728 | 24.18144 | 75.30403 | 27.59392 | 42.18155 | 42.95918 | 37.60419 | 121.1638 | 109.0854 | 62.82655 | 56.22273 | 31.89828 | 66.23254 | 50.72999 | 57.94787 | 81.92166 | 40.17935 | 37.50798 | 30.40149 |
| CUST_25872_PI429010886 | N53213 | 0.0125 | 12 | 0 | | | | | | 31.68203 | 73.1906 | 60.69824 | 28.83204 | 59.57796 | 50.37311 | 29.64209 | 45.27903 | 58.28332 | 26.62182 | 27.29125 | 57.49062 | 69.22629 | 37.67574 | 48.62912 | 65.11921 | 91.22284 | 57.56812 | 34.97152 | 34.45011 | 47.59295 | 32.45773 | 27.47176 | 23.29812 | 33.19093 | 60.79572 | 166.0195 |
| CUST_27884_PI429010886 | N56874 | 0.012503 | 24 | 18 | | | | | | 40.39881 | 32.15505 | 67.59725 | 16.25929 | 31.03692 | 28.30863 | 40.77403 | 42.94589 | 35.4058 | 34.87551 | 44.94077 | 49.86266 | 43.95633 | 55.96041 | 57.22832 | 60.09613 | 51.71837 | 36.70412 | 65.83024 | 51.4111 | 38.24304 | 49.38456 | 41.85446 | 44.55872 | 53.20866 | 43.09035 | 58.29649 |
| CUST_12073_PI429010886 | N28887 | 0.012508 | 18 | 0 | | | | | | 635.6512 | 381.8763 | 636.1084 | 446.8256 | 296.1446 | 377.8878 | 326.7925 | 334.1058 | 342.5053 | 446.2007 | 363.0918 | 428.0243 | 332.3269 | 396.2899 | 393.8505 | 583.4081 | 621.7238 | 422.6396 | 470.0153 | 554.9764 | 438.1052 | 377.4127 | 578.1088 | 431.842 | 789.0995 | 302.3102 | 340.1906 |
| CUST_28206_PI429010886 | N57503 | 0.012508 | 21 | 6 | | | | | | 24.54709 | 34.52311 | 47.3028 | 30.94543 | 41.44167 | 47.49605 | 40.28418 | 42.05465 | 47.48523 | 38.26462 | 47.07001 | 59.2457 | 41.61311 | 38.18132 | 39.50511 | 49.85667 | 16.8666 | 37.52322 | 35.678 | 34.58379 | 35.41597 | 33.62524 | 38.0269 | 34.29142 | 43.33449 | 43.52847 | 55.01225 |
| CUST_14429_PI429010886 | N32869 | 0.012527 | 18 | 15 | | | | | | 36.94632 | 37.6014 | 28.68501 | 30.73521 | 31.30264 | 27.59147 | 17.94517 | 18.57528 | 27.91206 | 29.43393 | 22.72881 | 33.04717 | 22.60454 | 15.2714 | 30.56193 | 38.45723 | 26.37038 | 41.38719 | 21.55161 | 31.32056 | 23.30987 | 41.36078 | 20.63988 | 36.43958 | 19.62649 | 14.01751 | 22.13925 |
| CUST_53148_PI429010886 | M100408 | 0.012534 | 12 | 0 | | | | | | 17.63183 | 23.13968 | 21.35972 | 8.994284 | 19.98576 | 20.16285 | 12.27925 | 17.15538 | 17.71709 | 22.9275 | 15.74407 | 20.02426 | 20.45832 | 19.27695 | 26.97522 | 19.35495 | 14.34109 | 17.37163 | 15.32781 | 13.01381 | 22.89976 | 22.01419 | 14.68592 | 11.30106 | 27.34621 | 29.9146 | 37.03334 |
| CUST_10596_PI429010886 | N26486 | 0.012534 | 12 | 0 | 260796835 | hypothetical protein BRAFLDRAFT_119547 [Branchi | | | 5.59E-56 | 62.99752 | 76.18529 | 69.30744 | 41.37211 | 58.2166 | 59.72927 | 41.66498 | 43.73443 | 51.04982 | 71.71135 | 44.70035 | 75.4087 | 45.33809 | 69.46349 | 69.68816 | 68.95801 | 49.44854 | 68.73669 | 50.65259 | 53.88876 | 64.80137 | 65.26948 | 48.76067 | 54.69881 | 101.218 | 65.88576 | 80.0595 |
| CUST_35700_PI429010886 | M13254 | 0.012562 | 18 | 3 | | | | | | 916.49 | 222.0782 | 663.9332 | 527.7394 | 398.132 | 721.1961 | 633.3531 | 364.2987 | 172.1642 | 98.38324 | 313.0924 | 55.03461 | 250.587 | 156.2332 | 178.3086 | 150.8963 | 649.3217 | 419.9089 | 837.1072 | 59.70983 | 443.8496 | 200.2396 | 648.4582 | 813.4135 | 108.3885 | 397.7744 | 99.01075 |
| CUST_43043_PI429010886 | M61933 | 0.012563 | 18 | 15 | 117414788 | Euphausia mutica 18S small subunit ribosomal RNA | | | 5.73E-79 | 101.2274 | 62.94659 | 96.25237 | 21.32603 | 116.2211 | 58.4208 | 78.32339 | 76.57774 | 48.02842 | 145.899 | 79.72112 | 72.8742 | 143.2735 | 128.8011 | 61.25018 | 134.162 | 99.40267 | 94.00857 | 98.86383 | 90.8645 | 91.04966 | 48.03245 | 68.73669 | 44.22142 | 38.91955 | 50.04478 | 103.2145 |
| CUST_47806_PI429010886 | M77682 | 0.012581 | 24 | 0 | | | | | | 658.6556 | 892.8593 | 796.2928 | 678.2021 | 676.1626 | 708.7623 | 533.8243 | 639.8175 | 634.9072 | 791.8244 | 648.4582 | 930.8848 | 668.1948 | 492.4839 | 561.2769 | 862.1393 | 586.3578 | 789.7368 | 643.2335 | 786.1873 | 814.8201 | 888.436 | 700.9759 | 662.2599 | 1061.463 | 831.0172 | 1112.909 |
| CUST_6451_PI429010886 | N20119 | 0.012581 | 24 | 0 | 410902708 | PREDICTED: Takifugu rubripes tubulin gamma-1 cha | Cellular component organization | Cytoskeleton organization | 2.65E-97 | 43.83106 | 49.13393 | 43.21618 | 23.48661 | 21.17943 | 30.61198 | 11.56285 | 32.06599 | 26.16206 | 44.1334 | 30.79365 | 42.84203 | 18.44318 | 21.19291 | 36.94269 | 44.48344 | 26.30221 | 37.86103 | 40.36651 | 21.48446 | 30.94122 | 28.53253 | 37.27042 | 33.57067 | 46.87582 | 45.87664 | 44.7891 |
| CUST_47455_PI429010886 | M76202 | 0.012589 | 15 | 12 | | | | | | 61.80458 | 71.33278 | 108.8415 | NA | 20.22682 | NA | 24.55066 | 90.41498 | 170.2284 | 138.7812 | 96.77635 | 183.266 | 170.0985 | 409.7595 | 225.4439 | 50.77711 | 189.9152 | 48.12117 | 6.51898 | 180.9857 | 14.69551 | 93.04202 | 177.3959 | 3.414082 | 159.3888 | 80.32797 | 123.9463 |
| CUST_42578_PI429010886 | M60633 | 0.012592 | 18 | 0 | | | | | | 26.22564 | 12.93967 | 14.59396 | NA | 7.090444 | NA | 9.194369 | 11.92505 | 13.5817 | 7.766831 | 5.394188 | 6.396975 | 7.393906 | 7.521524 | 8.21709 | 8.462821 | 11.38971 | 13.33083 | 6.301983 | 18.08454 | 23.40583 | 6.948268 | 9.666802 | 19.37939 | 7.681885 | NA | 10.49518 |
| CUST_1333_PI429010886 | N9318 | 0.012602 | 24 | 21 | | | | | | 9.407802 | 10.25912 | 13.0885 | 6.313975 | 12.24661 | 11.54372 | 7.884889 | 7.670181 | 10.97938 | 6.362315 | 11.98592 | 10.4406 | 6.147413 | 10.31476 | 5.05047 | 5.308585 | 5.858189 | 13.89615 | 6.938783 | 6.727538 | NA | 13.47559 | 12.08102 | 17.61017 | 10.31018 | 13.3326 | 11.09833 |
| CUST_34953_PI429010886 | M11153 | 0.012607 | 21 | 0 | | | | | | 60.08957 | 67.79763 | 84.28941 | 78.60763 | 45.21982 | 49.96135 | 48.75136 | 45.50557 | 42.79014 | 54.10071 | 50.88842 | 43.8479 | 47.02813 | 62.4936 | 40.66087 | 56.50947 | 64.40125 | 55.28753 | 62.38529 | 41.19711 | 64.73185 | 54.5089 | 75.4087 | 53.76018 | 64.07157 | 47.21351 | 48.75136 |
| CUST_36513_PI429010886 | M16102 | 0.012632 | 12 | 9 | | | | | | 9.827509 | 4.451004 | 17.18399 | 9.447649 | 9.224472 | 7.07379 | 10.39792 | 26.92163 | 22.71838 | 24.09176 | 23.47811 | 22.16225 | 10.35487 | 31.6343 | 20.6453 | 20.08697 | 16.27803 | 13.79131 | 10.43455 | 22.40634 | 16.83531 | 29.02946 | 25.08853 | NA | 37.5742 | NA | 18.76417 |
| CUST_22399_PI429010886 | N46965 | 0.012634 | 21 | 15 | | | | | | 170.3963 | 181.2698 | 207.5722 | 121.6348 | 199.4211 | 189.144 | 186.9949 | 190.8604 | 178.4723 | 196.8321 | 186.4327 | 216.4474 | 195.7902 | 228.5019 | 202.8541 | 180.7887 | 260.7973 | 220.5435 | 225.8046 | 209.7275 | 179.2012 | 198.7745 | 200.3982 | 216.5751 | 156.2928 | 143.3418 | 196.4155 |
| CUST_23075_PI429010886 | N48215 | 0.012643 | 15 | 12 | | | | | | 38.20891 | 37.63465 | 73.70231 | 25.75936 | 27.00493 | 41.71124 | 41.52003 | 52.27598 | 45.26792 | 43.34938 | 41.6172 | 52.59262 | 51.2779 | 63.82351 | 56.57261 | 46.02927 | 46.52715 | 38.94819 | 57.08439 | 44.02718 | 27.73533 | 45.23797 | 45.73272 | 30.48948 | 59.76158 | 54.6573 | 62.01223 |
| CUST_56030_PI429010886 | M112586 | 0.012644 | 12 | 3 | 321478880 | hypothetical protein DAPPUDRAFT_310106 [Daphn | Metabolic process | Carbohydrate metabolic process | 2.01E-20 | 524.4761 | 326.1708 | 97.31477 | 1616.675 | 1316.22 | 1895.191 | 1137.521 | 636.1084 | 342.0536 | 54.97901 | 1041.548 | 115.8594 | 525.7241 | 102.4425 | 366.4064 | 153.9392 | 1978.873 | 1275.086 | 551.3995 | 120.5667 | 625.332 | 290.2221 | 572.7376 | 1492.759 | 72.60868 | 864.2169 | 34.31858 |
| CUST_11818_PI429010886 | N28453 | 0.012644 | 12 | 3 | | | | | | 156.4871 | 32.16133 | 68.25442 | 426.3841 | 386.6669 | 857.8725 | 408.1397 | 212.1657 | 52.01256 | 9.750393 | 303.854 | 19.56461 | 128.8011 | 10.70994 | 51.50855 | 14.69352 | 913.4609 | 700.4893 | 524.9601 | 14.6546 | 189.5687 | 13.61539 | 179.5282 | 351.1934 | 12.61292 | 303.2078 | 7.885901 |
| CUST_21397_PI429010886 | N45146 | 0.012644 | 12 | 3 | 260813040 | hypothetical protein BRAFLDRAFT_95006 [Branchio | Metabolic process | Nitrogen compound metabolic process | 2.35E-07 | 26.14633 | 9.02447 | 7.987477 | 53.18376 | 40.85343 | 159.4135 | 16.32267 | 50.42448 | 5.749389 | 15.74591 | 57.18099 | 5.175611 | 22.39942 | 11.09075 | 13.86905 | 19.31761 | 65.96571 | 80.53946 | 119.9288 | 18.42271 | 19.2347 | 11.26061 | 26.06265 | 23.26285 | 7.441066 | 15.67417 | 13.40721 |
| CUST_2442_PI429010886 | N12359 | 0.012659 | 24 | 21 | | | | | | 32.90178 | 34.12708 | 34.1704 | 26.88229 | 21.16222 | 33.44293 | 21.93695 | 16.95326 | 27.25936 | 26.2903 | 31.12088 | 30.10233 | 40.3065 | 23.68579 | 29.25886 | 35.09563 | 30.20292 | 35.66075 | 26.36365 | 21.78224 | 35.55553 | 35.07318 | 41.36078 | 41.36789 | 41.62677 | 42.41594 | 58.13824 |
| CUST_36877_PI429010886 | M17119 | 0.012676 | 12 | 0 | | | | | | 15.16642 | 43.58778 | 31.33456 | 26.32739 | 22.79581 | 24.25419 | 9.257427 | 21.32603 | 26.63815 | 30.71736 | 24.69322 | 39.11677 | 27.21982 | 61.91569 | 43.83694 | 35.22807 | 14.21847 | 29.43926 | 14.52793 | 33.03817 | 18.7709 | 43.79745 | 27.14155 | 15.57695 | 37.63897 | 40.77106 | 36.34355 |
| CUST_41821_PI429010886 | M58632 | 0.012676 | 12 | 0 | 383850170 | PREDICTED: kaptin-like [Megachile rotundata] | | | 1.72E-21 | 29.49942 | 56.70973 | 23.30384 | 23.38781 | 18.11915 | 17.92979 | 16.07261 | 15.83863 | 25.15091 | 28.70795 | 19.95383 | 19.64381 | 24.23621 | 25.888 | 49.02131 | 35.27881 | 20.12612 | 17.47239 | 11.85953 | 23.94925 | 15.24921 | 42.12286 | 15.05158 | 22.90764 | 22.74317 | 23.07793 | 33.03817 |
| CUST_52929_PI429010886 | M99498 | 0.012676 | 12 | 0 | | | | | | 1086.536 | 856.1081 | 768.2695 | 1310.544 | 689.6848 | 660.0703 | 556.3775 | 585.2479 | 666.1474 | 992.2058 | 957.8471 | 1124.348 | 719.8537 | 2060.137 | 1527.118 | 1629.467 | 680.741 | 684.7245 | 674.6226 | 1165.381 | 734.477 | 1210.418 | 939.7655 | 722.0011 | 1649.441 | 1225.452 | 1392.18 |
| CUST_3144_PI429010886 | N13892 | 0.012676 | 12 | 0 | | | | | | 29.68336 | 57.33863 | 60.6077 | 41.2427 | 18.1616 | 19.0657 | 18.86766 | 23.85638 | 26.8539 | 35.74774 | 33.09643 | 28.82923 | 37.62657 | 55.29319 | 54.83755 | 39.65895 | 22.59328 | 44.03947 | 21.51773 | 32.86927 | 21.49928 | 65.49092 | 47.08024 | 38.74854 | 45.04433 | 32.51998 | 36.2246 |
| CUST_11341_PI429010886 | N27651 | 0.01268 | 18 | 12 | | | | | | 38.11258 | 28.42752 | 25.77821 | 19.44155 | 24.24502 | 20.34203 | 18.06186 | 16.14981 | 22.62281 | 31.91824 | 22.74775 | 28.229 | 26.69053 | 33.54956 | 32.56046 | 30.31727 | 34.56663 | 23.21071 | 23.81072 | 17.33333 | 25.59587 | 23.72179 | 36.19875 | 25.14633 | 27.7492 | 22.70524 | 22.18079 |
| CUST_39874_PI429010886 | M46058 | 0.01268 | 21 | 12 | 148841019 | hemocyanin subunit L [Marsupenaeus japonicus] | Transport | Other | 4.27E-24 | 56.78823 | 80.51303 | 70.58339 | 129.4353 | 260.5083 | 40.58399 | 224.4715 | 765.7162 | 281.4899 | 113.0468 | 577.5152 | 699.4291 | 2850.368 | 133.2626 | 1320.618 | 67.46884 | 873.1071 | 76.24385 | 148.2477 | 25496.93 | 916.49 | 74.40209 | 124.6404 | 65.87006 | 60.88717 | 237.506 | 859.4575 |
| CUST_14371_PI429010886 | N32771 | 0.012683 | 21 | 0 | | | | | | 72.47293 | 78.66048 | 65.77588 | 50.30627 | 30.21036 | 31.82901 | 22.66333 | 46.8066 | 30.62818 | 40.03126 | 25.25069 | 33.1071 | 43.33449 | 59.78571 | 41.06999 | 59.00313 | 53.68104 | 38.54575 | 29.88134 | 51.192 | 59.06419 | 49.99255 | 37.93755 | 59.46938 | 59.61145 | 27.78206 | 63.08907 |
| CUST_5046_PI429010886 | N18191 | 0.012686 | 24 | 15 | 380028927 | PREDICTED: ubiquitin carboxyl-terminal hydrolase 7 | | | | 118.8596 | 117.2885 | 95.21856 | 84.47788 | 83.34341 | 107.9692 | 85.75328 | 86.93205 | 83.32632 | 141.72 | 104.4333 | 150.4806 | 96.029 | 114.2091 | 104.8753 | 152.8609 | 106.002 | 149.3697 | 117.0962 | 116.6405 | 123.0016 | 113.6358 | 122.2551 | 100.1974 | 149.5758 | 122.6587 | 107.6119 |
| CUST_20508_PI429010886 | N43577 | 0.012687 | 21 | 3 | | | | | | 21.89117 | 35.37524 | 27.0028 | 59.6055 | 26.98754 | 43.78734 | 15.38835 | 21.35072 | 21.7647 | 20.83704 | 11.18163 | 36.23362 | 14.44097 | 24.63224 | 11.17512 | 19.73096 | 5.401786 | 29.34549 | 12.98486 | 5.555758 | 27.73257 | 22.27045 | 31.71799 | 28.57032 | 28.46316 | 49.04149 | 15.444 |
| CUST_49282_PI429010886 | M85009 | 0.012691 | 18 | 0 | | | | | | 37.97967 | 28.88138 | 34.9503 | 11.12035 | 20.30807 | 12.65789 | 27.41533 | 15.19655 | 13.27381 | 17.34013 | 14.84745 | 24.77801 | 21.13287 | 19.84447 | 28.96358 | 51.4 | 27.2271 | 16.63564 | 29.09612 | 28.49131 | 26.70421 | 21.23199 | 20.9473 | 10.34377 | 23.46033 | 17.4527 | 13.5521 |
| CUST_20653_PI429010886 | N43824 | 0.012701 | 18 | 12 | 321458840 | hypothetical protein DAPPUDRAFT_228516 [Daphn | | | 1.2E-15 | 11.37412 | 16.41995 | 9.824879 | 4.418861 | 9.146938 | NA | 3.952449 | 10.20734 | 11.73913 | 19.57173 | 11.78894 | 8.174796 | 14.1603 | 12.5052 | 17.02082 | 12.43401 | 12.34338 | 14.28302 | 10.69655 | 6.261173 | 10.94929 | 9.525782 | 14.8977 | 5.933128 | 6.541514 | NA | 12.79367 |
| CUST_38646_PI429010886 | M28818 | 0.012711 | 15 | 9 | 395537647 | PREDICTED: zinc finger protein 160-like, partial [Sar | Nucleic acid metabolism | Regulation of gene expression | 3.21E-29 | 13.47949 | 21.03267 | 21.94699 | 16.87735 | 14.54005 | 7.591069 | 8.680488 | 14.96828 | 22.17314 | 38.55767 | 21.29353 | 26.43937 | 17.52398 | 23.04991 | 18.56257 | 17.95875 | 15.5304 | 21.3795 | 14.80676 | 13.60767 | 15.03992 | 25.28773 | 24.04426 | 8.283166 | 16.24815 | 15.96492 | 27.70733 |
| CUST_3990_PI429010886 | N15737 | 0.012711 | 15 | 9 | | | | | | 1357.763 | 1854.457 | 1599.783 | 1267.999 | 1336.854 | 1253.307 | 1177.081 | 1264.096 | 1620.39 | 2069.143 | 1451.1 | 2526.384 | 1447.664 | 1868.48 | 1964.934 | 2543.989 | 1400.033 | 1680.099 | 1356.986 | 1747.319 | 1597.443 | 1869.455 | 1545.528 | 1326.638 | 2063.321 | 1727.428 | 2300.758 |
| CUST_13730_PI429010886 | N31675 | 0.012711 | 15 | 9 | 532759728 | antimicrobial peptide type 2 precursor IIb [Pandalo | | | 3.06E-32 | 305.4951 | 822.0802 | 637.0757 | 569.6586 | 363.8038 | 151.9486 | 468.2572 | 491.9013 | 854.2242 | 1400.624 | 948.388 | 1333.433 | 1118.972 | 1504.155 | 950.5113 | 1184.785 | 625.332 | 1083.765 | 427.8624 | 2168.863 | 777.0504 | 2128.599 | 2300.758 | 1099.35 | 2900.662 | 879.3705 | 3256.546 |
| CUST_28848_PI429010886 | N58721 | 0.012711 | 15 | 9 | | | | | | 772.946 | 2092.578 | 1312.47 | 1433.821 | 941.2341 | 326.7925 | 1535.786 | 971.1081 | 2101.303 | 3464.749 | 2321.278 | 4110.832 | 2937.772 | 3517.336 | 3029.514 | 2266.195 | 1370.195 | 2368.476 | 1049.625 | 4896.997 | 1717.641 | 4480.043 | 4617.753 | 2379.147 | 6189.048 | 2027.433 | 6247.817 |
| CUST_14163_PI429010886 | N32415 | 0.012711 | 18 | 15 | | | | | | 103.5071 | 116.178 | 120.6877 | 64.9512 | 75.98951 | 105.0857 | 72.05058 | 81.30299 | 104.5244 | 120.3338 | 102.9808 | 98.04327 | 110.8364 | 141.4697 | 115.8195 | 136.3926 | 113.0821 | 117.6303 | 91.24255 | 129.9538 | 96.20672 | 104.6528 | 92.61954 | 112.0535 | 113.2396 | 104.976 | 102.9667 |
| CUST_18903_PI429010886 | N40618 | 0.012723 | 18 | 3 | | | | | | 5980.233 | 13245.53 | 8336.973 | 19541.87 | 9341.534 | 11400.82 | 6108.092 | 4743.467 | 7930.333 | 8783.054 | 4399.213 | 7017.655 | 6148.012 | 6307.408 | 6621.858 | 9855.517 | 3701.465 | 5471.152 | 3120.564 | 6511.817 | 11202.95 | 10884.33 | 10542.53 | 7228.251 | 10831.29 | 12884.64 | 6969.97 |
| CUST_30549_PI429010886 | N62105 | 0.012723 | 24 | 3 | | | | | | 24.6471 | 41.38719 | 31.2636 | 71.03479 | 27.58334 | 41.01917 | 27.79833 | 21.24048 | 15.55047 | 18.13226 | 8.110471 | 24.48268 | 15.70408 | 29.31162 | 16.87735 | 14.65658 | 13.00717 | 22.43724 | 9.627312 | 15.54199 | 27.63304 | 28.98096 | 52.34171 | 12.07461 | 29.09978 | 32.60462 | 18.04283 |
| CUST_38309_PI429010886 | M25217 | 0.012736 | 15 | 6 | | | | | | 42.68463 | 33.79348 | 36.54259 | 50.06533 | 47.91134 | 25.77541 | 62.85005 | 92.5954 | 79.6699 | 51.14292 | 47.67267 | 31.33759 | 37.52679 | 60.29484 | 43.439 | 30.76441 | 45.61505 | 35.31845 | 24.94778 | 43.03291 | 58.16774 | 91.1558 | 41.92389 | 34.1956 | 46.76971 | 46.74367 | 50.51081 |
| CUST_12192_PI429010886 | N29077 | 0.01275 | 15 | 9 | | | | | | 40.18883 | 84.92511 | 42.86965 | 47.75744 | 42.57633 | 54.14016 | 42.41265 | 44.59476 | 56.16292 | 63.82351 | 64.07997 | 97.36237 | 50.17076 | 45.98092 | 52.55217 | 64.50807 | 39.52535 | 64.03011 | 30.12445 | 30.69887 | 41.78277 | 112.3515 | 43.8795 | 31.96544 | 59.51435 | 47.11037 | 104.9497 |
| CUST_3009_PI429010886 | N13595 | 0.012754 | 21 | 9 | 189240203 | PREDICTED: similar to cytochrome P450 [Tribolium | Metabolic process | Oxidation-reduction process | 4.45E-70 | 385.5507 | 1181.018 | 1001.156 | 1640.228 | 1124.348 | 634.6129 | 627.4273 | 1407.359 | 717.7368 | 1710.023 | 2361.621 | 2123.129 | 1738.403 | 1332.224 | 1262.411 | 1454.733 | 1150.815 | 558.6606 | 1179.987 | 2016.816 | 1335.109 | 1106.54 | 748.0078 | 727.7317 | 1066.79 | 1001.62 | 1206.475 |
| CUST_11382_PI429010886 | N27723 | 0.012796 | 21 | 18 | | | | | | 321.0207 | 690.6945 | 343.9276 | 310.1413 | 367.7937 | 306.9203 | 243.8404 | 191.6398 | 173.5211 | 491.4278 | 381.8763 | 533.6685 | 351.9054 | 284.2642 | 391.5964 | 446.0696 | 338.7294 | 408.1397 | 384.9318 | 504.3528 | 493.5731 | 361.4417 | 519.0655 | 285.3112 | 299.0341 | 415.6575 | 450.2218 |
| CUST_8364_PI429010886 | N22940 | 0.012796 | 24 | 12 | | | | | | 23.04991 | 18.87315 | 19.36347 | 29.19814 | 72.20186 | 14.98523 | 33.02612 | 99.24333 | 50.80203 | 9.154209 | 227.8356 | 221.165 | 82.94784 | NA | 46.49731 | 8.173644 | 37.04826 | 15.27765 | 20.80589 | 333.2881 | 73.16394 | 8.203406 | 12.32047 | 10.21823 | 12.18711 | 47.7103 | 22.97943 |
| CUST_45631_PI429010886 | M69725 | 0.012807 | 21 | 12 | 209418466 | serpin 8 [Branchiostoma lanceolatum] | | | 5.77E-31 | 2426.131 | 3456.298 | 2362.762 | 1609.757 | 2201.928 | 1646.767 | 1832.42 | 2282.54 | 2256.115 | 2818.479 | 2237.5 | 3659.313 | 2478.692 | 2310.525 | 2489.582 | 3421.62 | 1981.218 | 2441.116 | 2123.129 | 3240.576 | 2014.561 | 2845.968 | 2015.591 | 1786.373 | 2232.866 | 1551.584 | 2005.797 |
| CUST_48414_PI429010886 | M80205 | 0.012816 | 24 | 21 | | | | | | 40.87212 | 68.50419 | 75.7673 | 70.93175 | 76.62962 | 28.27971 | 49.10355 | 32.05527 | 41.28276 | 46.00478 | 61.47627 | 82.77162 | 52.91645 | 72.7025 | 13.52366 | 61.46953 | 40.50555 | 48.03245 | 63.40813 | 59.17148 | 89.4807 | 101.2406 | 80.96373 | 77.76831 | 75.12567 | 73.77697 | 70.13143 |
| CUST_40907_PI429010886 | M56361 | 0.012818 | 12 | 0 | 443696893 | hypothetical protein CAPTEDRAFT_171008 [Capitell | | | 1.1E-09 | 53.73593 | 220.9066 | 204.885 | 87.44346 | 57.20099 | 17.53908 | 14.41572 | 34.35393 | 50.46214 | 39.06311 | 84.15456 | 91.0847 | 65.07248 | 99.54848 | 84.89465 | 51.273 | 59.22278 | 53.37349 | 75.24399 | 79.13143 | 38.14903 | 111.4345 | 146.3509 | 46.67338 | 66.32627 | 107.512 | 144.3143 |
| CUST_45635_PI429010886 | M69746 | 0.012818 | 12 | 0 | | | | | | 195.5884 | 162.4501 | 165.8485 | 145.6462 | 163.3744 | 169.4763 | 144.6317 | 140.5313 | 157.1144 | 216.5751 | 126.7579 | 188.2189 | 186.1103 | 187.3568 | 193.6016 | 281.599 | 135.5775 | 178.0976 | 133.8101 | 152.3559 | 135.5775 | 201.1076 | 147.1104 | 181.2186 | 194.3549 | 211.3587 | 211.8227 |
| CUST_4978_PI429010886 | N18011 | 0.012818 | 12 | 0 | | | | | | 26.93627 | 18.97492 | 22.22394 | 18.27296 | 10.92086 | 15.05605 | 14.41094 | 12.36405 | 18.78752 | 18.50987 | 14.5269 | 14.32411 | 9.310436 | 34.61308 | 44.55872 | 29.34549 | 18.97668 | 16.29403 | 12.87351 | 14.84209 | 13.64597 | 29.15809 | 15.29798 | 11.93548 | 22.97663 | 21.20384 | 32.23691 |
| CUST_27917_PI429010886 | N56936 | 0.012827 | 18 | 3 | | | | | | 12.96775 | 10.00209 | 9.020299 | 15.55875 | 8.117917 | 15.2558 | 9.786144 | 51.20393 | 7.842686 | 11.89031 | 6.91834 | 8.546868 | 7.442152 | 3.66566 | 6.342428 | 4.497578 | 10.67318 | 7.44504 | 9.570885 | 10.87784 | 8.687252 | 8.533663 | 16.64328 | 9.680237 | 10.77657 | NA | 7.822452 |
| CUST_27100_PI429010886 | N55448 | 0.012836 | 21 | 3 | | | | | | 88.13649 | 174.9558 | 74.76616 | 367.5439 | 107.0816 | 179.5942 | 102.1576 | 76.88757 | 92.97872 | 76.8667 | 44.91724 | 115.1745 | 55.45229 | 86.09048 | 69.68123 | 77.79872 | 35.78945 | 92.69475 | 30.18478 | 76.59109 | 178.7072 | 92.70294 | 158.0759 | 80.4611 | 143.2572 | 212.1657 | 52.30377 |
| CUST_5202_PI429010886 | N18393 | 0.012837 | 18 | 9 | | | Transport | Other | | 53.17877 | 41.59399 | 45.87335 | 26.85728 | 33.23404 | 35.32948 | 47.71977 | 77.78755 | 66.23254 | 40.86391 | 74.80865 | 67.79763 | 77.16796 | 42.49492 | 43.92643 | 37.30131 | 55.23417 | 24.83327 | 43.97896 | 110.5233 | 39.00381 | 39.33444 | 33.40072 | 27.49118 | 44.90024 | 40.66714 | 51.07827 |
| CUST_31378_PI429010886 | M1395 | 0.012843 | 24 | 15 | 321461113 | hypothetical protein DAPPUDRAFT_216285 [Daphn | | | 5.5E-65 | 255.4957 | 227.4117 | 168.4127 | 127.3036 | 191.3362 | 171.3142 | 164.2252 | 180.7626 | 185.5201 | 297.4174 | 213.51 | 318.6961 | 168.0847 | 256.8125 | 252.3062 | 328.807 | 243.6339 | 264.7596 | 192.4758 | 268.4877 | 174.9558 | 243.4369 | 132.4401 | 243.6699 | 214.9913 | 211.9851 | 191.7287 |
| CUST_37910_PI429010886 | M22209 | 0.012856 | 15 | 12 | | | | | | 6759.855 | 13937.5 | 10915.56 | 2770.165 | 5699.333 | 2999.441 | 5693.449 | 6885.41 | 13937.5 | 12884.64 | 8550.193 | 19004.47 | 9825.362 | 11472.39 | 13959.79 | 16762.52 | 5013.208 | 11313.76 | 7470.617 | 8483.725 | 7950.432 | 16638.44 | 7938.371 | 6005.835 | 13245.53 | 9261.186 | 15892.67 |
| CUST_24655_PI429010886 | N51025 | 0.012861 | 24 | 21 | | | | | | 21.34584 | 19.84686 | 32.63054 | 31.71799 | 21.55311 | 32.43067 | 30.5069 | 24.98792 | 29.27339 | 19.42496 | 17.67558 | 19.6024 | 17.62325 | 34.50298 | 23.15839 | 27.14824 | 27.16436 | 27.04395 | 31.54453 | 18.3679 | 35.89648 | 26.9152 | 35.23447 | 34.6933 | 45.27903 | 39.91268 | 33.51766 |
| CUST_9218_PI429010886 | N24253 | 0.012862 | 15 | 9 | | | | | | 107.6537 | 109.1135 | 95.91036 | 64.09512 | 80.82297 | 75.10926 | 92.09827 | 90.82199 | 84.49247 | 121.2986 | 88.53305 | 110.7887 | 103.8326 | 98.61381 | 108.4222 | 122.9126 | 95.01446 | 117.2337 | 106.2285 | 103.0974 | 83.41229 | 109.3629 | 90.02313 | 107.4618 | 119.7354 | 152.5473 | 106.872 |
| CUST_13405_PI429010886 | N31122 | 0.01287 | 24 | 0 | | | | | | 30.40511 | 33.17446 | 26.23964 | 15.53405 | 17.96396 | 34.31858 | 30.22164 | 20.94079 | 24.78858 | 22.56226 | 23.76609 | 25.64065 | 12.08465 | 22.17526 | 15.5384 | 46.54874 | 21.12654 | 49.02131 | 24.55823 | 20.81747 | 29.01971 | 25.90233 | 25.78835 | 24.12233 | 34.36064 | 31.37421 | 36.78203 |
| CUST_17637_PI429010886 | N38320 | 0.012888 | 24 | 3 | | | | | | 37.9742 | 5.051185 | 18.7688 | 76.01313 | 51.75764 | 22.92529 | 35.54095 | 30.40899 | 73.47743 | 15.2034 | 23.52815 | NA | 15.01814 | 20.84589 | 16.18956 | 10.96329 | 17.64462 | 29.37298 | 5.416008 | 23.30079 | 11.16751 | 12.03745 | 18.08051 | 8.415892 | 5.271736 | 78.86687 | 28.87402 |
| CUST_52562_PI429010886 | M97957 | 0.012888 | 12 | 9 | | | | | | 59.46938 | 65.36011 | 76.81043 | 8.774888 | 14.83746 | 11.63473 | 23.75855 | 10.88211 | 13.13981 | 85.17905 | 53.04998 | 136.2319 | 6.463315 | 152.4677 | 52.45794 | 32.88145 | NA | 24.65264 | 17.96396 | 24.99108 | 47.58792 | 20.86179 | 79.07776 | 102.1229 | 35.78224 | 21.47442 | 37.08279 |
| CUST_5724_PI429010886 | N19102 | 0.012893 | 18 | 15 | 242023540 | ATP-dependent RNA helicase Ddx1, putative [Pedicu | | | 3.1E-154 | 173.5096 | 205.1968 | 173.5211 | 164.2007 | 184.8748 | 137.687 | 98.59007 | 149.2677 | 130.8991 | 227.4117 | 194.1209 | 215.9695 | 208.7767 | 168.8733 | 136.2923 | 308.0795 | 171.4068 | 198.008 | 198.3716 | 203.5636 | 145.1427 | 210.7138 | 159.6853 | 172.054 | 169.847 | 140.436 | 159.0925 |
| CUST_25448_PI429010886 | N52441 | 0.012902 | 18 | 15 | | | | | | 23.68272 | 24.62608 | 29.66792 | 15.46051 | 22.78329 | 16.25426 | 8.945536 | 18.10904 | 23.59869 | 63.93448 | 28.44308 | 23.49374 | 25.59587 | 38.33878 | 31.56943 | 37.56967 | 29.30762 | 43.5699 | 23.1605 | 34.01746 | 25.40127 | 38.3841 | 21.73902 | 15.07014 | 43.24561 | 36.07274 | 40.94065 |
| CUST_50117_PI429010886 | M88687 | 0.012929 | 24 | 3 | | | | | | 22.69285 | 21.80116 | 18.51403 | 20.09671 | 25.1894 | 31.29367 | 22.63022 | 14.23448 | 13.19382 | 15.50711 | 20.77685 | 24.52679 | 7.463527 | NA | 18.37554 | 13.295 | 15.83738 | 13.63096 | 9.703156 | 16.72995 | 14.73662 | 15.54567 | 10.75121 | 13.44588 | 22.6017 | 25.02916 | 12.98486 |
| CUST_50421_PI429010886 | M89755 | 0.012931 | 12 | 3 | | | | | | 67.33602 | 16.94759 | 36.08462 | 166.3529 | 167.0167 | 330.6772 | 113.6906 | 132.8222 | 49.50652 | 17.11711 | 88.50576 | 44.76277 | 46.49731 | 15.83863 | 66.92953 | 49.19582 | 168.4961 | 158.0561 | 193.556 | 10.71996 | 75.83538 | 29.13595 | 22.33694 | 112.0774 | 20.14149 | 159.7175 | 12.81122 |
| CUST_32731_PI429010886 | M4950 | 0.012938 | 15 | 9 | | | | | | 46.80174 | 207.5012 | 120.2323 | 404.2573 | 325.9737 | 408.2171 | 427.0325 | 892.8593 | 712.9294 | 305.0519 | 1630.188 | 2264.822 | 429.2473 | 241.4042 | 256.8944 | NA | 175.9447 | 43.34938 | 102.0461 | 1085.118 | 1494.238 | NA | 403.8464 | 122.1353 | 114.8344 | 791.2722 | 1008.511 |
| CUST_31624_PI429010886 | M1994 | 0.012944 | 15 | 12 | 380026333 | PREDICTED: uncharacterized protein LOC10086635( | | | 1.75E-35 | 50.01795 | 51.36009 | 61.25018 | 36.85539 | 45.67726 | 22.85266 | 26.07711 | 57.80685 | 47.64282 | 56.50237 | 34.12241 | 68.14294 | 63.34141 | 67.74837 | 50.74126 | 52.16399 | 49.97285 | 48.07404 | 35.72108 | 51.51637 | 44.3588 | 58.5117 | 46.89105 | 50.75355 | 51.14708 | 44.11518 | 59.23972 |
| CUST_24982_PI429010886 | N51582 | 0.012944 | 15 | 12 | | | | | | 61.16623 | 75.19772 | 68.66167 | 61.11029 | 38.08235 | 57.5238 | 49.33073 | 47.82182 | 60.68127 | 71.02655 | 55.76383 | 83.2292 | 53.23845 | 79.55126 | 81.96733 | 84.57304 | 59.52835 | 74.55697 | 52.77501 | 60.62589 | 58.67101 | 67.45213 | 61.57414 | 57.5416 | 78.35123 | 71.49639 | 69.76759 |
| CUST_23769_PI429010886 | N49434 | 0.012945 | 24 | 15 | 332024498 | Uncharacterized protein [Acromyrmex echinatior] | | | 2.05E-14 | 8.66034 | 10.76087 | 7.042021 | 8.378893 | 8.357942 | 13.39222 | 8.504674 | 5.439411 | 7.746908 | 16.97579 | 10.19501 | 9.407802 | 4.793918 | 10.22437 | 10.34912 | 17.77235 | 10.45833 | 13.06343 | 14.97861 | 12.30261 | 11.30463 | 16.04256 | 8.018938 | 10.14604 | 17.16843 | 10.2343 | 8.775837 |
| CUST_32182_PI429010886 | M3459 | 0.012958 | 21 | 0 | | | | | | 226.768 | 223.3365 | 210.7453 | 188.8906 | 200.7871 | 236.4243 | 177.5136 | 163.5913 | 155.8927 | 206.9847 | 169.9489 | 208.515 | 182.8475 | 186.7835 | 166.5412 | 229.6175 | 204.4512 | 226.5039 | 218.0605 | 194.777 | 190.7563 | 239.3938 | 194.4818 | 193.6314 | 208.5959 | 202.7794 | 193.6603 |
| CUST_38557_PI429010886 | M27976 | 0.012965 | 15 | 3 | | | | | | 95.70219 | 35.22807 | 98.39109 | 398.0093 | 325.2563 | 529.4905 | 290.7302 | 143.5804 | 125.5467 | 6.075781 | 185.6237 | NA | 170.2284 | 9.363982 | 67.59725 | 6.616835 | 745.5335 | 512.8422 | 736.5339 | NA | 461.9687 | NA | 237.9578 | 348.5888 | 4.708846 | 345.2409 | NA |
| CUST_44706_PI429010886 | M66737 | 0.012972 | 24 | 21 | | | | | | 27.95797 | 22.99783 | 33.30987 | 26.26013 | 24.53193 | 24.93429 | 16.75738 | 17.15304 | 29.65277 | 30.27835 | 38.63759 | 33.03817 | 28.25151 | 27.05448 | 26.98034 | 32.06861 | 28.14021 | 31.0625 | 35.99615 | 27.95506 | 31.43817 | 38.53966 | 35.18059 | 32.55631 | 29.70687 | 27.74359 | 30.81346 |
| CUST_50956_PI429010886 | M91568 | 0.012972 | 24 | 21 | | | | | | 29.63396 | 50.67859 | 53.48774 | 29.16623 | 21.41052 | 38.79122 | 35.53394 | 6.515981 | 19.40005 | 35.9416 | 25.52007 | 48.32025 | 26.25702 | 34.09884 | 26.46898 | 77.44015 | 37.22874 | 54.73192 | 34.0026 | 41.29944 | 32.72316 | 46.35165 | 43.74674 | 55.43705 | 68.1923 | 37.6475 | 55.37764 |
| CUST_30542_PI429010886 | N62090 | 0.012972 | 24 | 3 | | | | | | 86.18635 | 71.6044 | 70.34764 | 72.32108 | 79.36014 | 104.9178 | 70.58805 | 65.16761 | 61.52513 | 66.47359 | 65.92191 | 77.40739 | 62.5636 | 60.78457 | 82.80093 | 62.89021 | 100.2537 | 55.1911 | 61.61655 | 42.82196 | 42.57633 | 65.40248 | 69.76759 | 88.08405 | 118.7528 | 70.97584 | 52.2983 |
| CUST_38071_PI429010886 | M23193 | 0.012991 | 18 | 15 | | | | | | 2054.295 | 1807.03 | 1294.709 | 1007.032 | 2567.686 | 1954.96 | 1253.307 | 1239.225 | 1049.625 | 2487.623 | 1544.651 | 2218.572 | 1504.846 | 1808.217 | 1670.5 | 2771.702 | 2834.55 | 2777.472 | 1533.433 | 1361.981 | 1447.664 | 2587.199 | 1149.353 | 1710.023 | 1678.528 | 1458.041 | 1492.759 |
| CUST_36480_PI429010886 | M16026 | 0.012991 | 24 | 15 | | | | | | 58.78466 | 46.82282 | 47.01326 | 51.04482 | 40.19376 | 45.39895 | 46.58823 | 41.85446 | 39.87566 | 50.20045 | 60.26917 | 61.30991 | 36.23123 | 41.66889 | 46.08198 | 66.99867 | 60.94428 | 64.42438 | 42.64756 | 59.61145 | 51.16457 | 58.14321 | 67.60441 | 46.41206 | 56.58363 | 48.90708 | 49.67787 |
| CUST_13966_PI429010886 | N32097 | 0.012997 | 21 | 3 | | | | | | 930.2399 | 3790.041 | 1890.591 | 5738.426 | 2325.953 | 2747.282 | 1652.112 | 1577.693 | 1531.812 | 2002.228 | 795.9721 | 2472.114 | 1107.396 | 1071.523 | 1224.871 | 2088.06 | 620.6433 | 1665.986 | 732.8997 | 1150.482 | 2558.435 | 1311.23 | 2511.522 | 1527.118 | 3022.502 | 3134.449 | 1305.705 |
| CUST_11886_PI429010886 | N28574 | 0.013009 | 15 | 12 | 46240812 | crustin-like peptide type 2 [Marsupenaeus japonicu | | | 1.52E-08 | 341.396 | 861.813 | 917.1171 | 290.4858 | 379.882 | 163.0075 | 1152.374 | 761.0622 | 1483.935 | 1505.495 | 801.0442 | 1777.79 | 1813.091 | 2011.77 | 1647.631 | 2495.356 | 1086.536 | 929.0194 | 639.5245 | 2524.383 | 794.7159 | 1920.945 | 3218.113 | 1352.582 | 6621.858 | 1478.956 | 7426.728 |
| CUST_2148_PI429010886 | N11685 | 0.013013 | 15 | 9 | | | | | | 218.1069 | 197.384 | 304.5092 | 223.9141 | 211.1942 | 182.5451 | 237.166 | 174.2228 | 229.6885 | 258.4381 | 253.025 | 243.4729 | 197.1816 | 251.9218 | 191.886 | 238.7009 | 259.2052 | 204.1086 | 179.3955 | 180.0766 | 221.6212 | 187.4537 | 251.0607 | 137.0908 | 260.7973 | 269.2314 | 326.7925 |
| CUST_12632_PI429010886 | N29778 | 0.013013 | 15 | 0 | | | | | | 212.4692 | 340.057 | 178.1697 | 236.0508 | 224.3925 | 162.6994 | 130.6431 | 199.5921 | 133.9495 | 332.54 | 189.0327 | 209.9313 | 210.7138 | 176.6512 | 185.5201 | 374.2323 | 256.2441 | 192.2319 | 267.8414 | 241.1275 | 165.4234 | 182.6771 | 147.073 | 124.1278 | 274.3455 | 134.6919 | 161.8434 |
| CUST_391_PI429010886 | N4800 | 0.013013 | 15 | 9 | 225712386 | Cuticle protein 6 [Lepeophtheirus salmonis] | | | 4.51E-21 | 6448.481 | 15353.74 | 23420.74 | 1210.833 | 1882.712 | 211.5909 | 7099.277 | 24647.46 | 29816.7 | 38496.63 | 15060.57 | 26153.6 | 31231.03 | 42714.39 | 29468.93 | 27703.57 | 10178.43 | 9061.383 | 19960.08 | 28043.16 | 21773.32 | 45084.56 | 33351.13 | 1410.909 | 61640.76 | 25546.19 | 62522.62 |
| CUST_36813_PI429010886 | M16931 | 0.013016 | 21 | 0 | | | | | | 79.96242 | 140.4751 | 87.17451 | 65.08 | 80.74596 | 105.0612 | 88.66033 | 73.61433 | 68.1054 | 86.70732 | 81.04828 | 89.93577 | 47.51659 | 82.36794 | 53.50062 | 85.76295 | 40.58787 | 67.85099 | 71.70446 | 96.72232 | 94.49669 | 89.06701 | 97.84604 | 113.0468 | 107.4719 | 76.24971 | 107.9121 |
| CUST_23726_PI429010886 | N49357 | 0.013054 | 12 | 3 | 321461663 | hypothetical protein DAPPUDRAFT_605 [Daphnia p | | | 1.86E-28 | 14.35036 | 6.874216 | 29.86288 | 37.06882 | 12.8856 | 56.47376 | 32.50331 | 13.21272 | 21.44476 | NA | 5.954939 | 3.828908 | 6.115825 | 11.70041 | 10.09463 | NA | 67.30267 | 42.99343 | 23.06016 | 4.83618 | 16.16098 | 5.24251 | 12.04261 | 18.45401 | 4.922559 | 16.02669 | 3.577113 |
| CUST_10247_PI429010886 | N25942 | 0.013075 | 12 | 9 | | | | | | 53.54974 | 48.00964 | 60.78952 | 57.72307 | 42.9046 | 56.02183 | 50.69191 | 58.67766 | 58.38163 | 74.19557 | 65.61007 | 87.06371 | 52.39551 | 41.39181 | 43.91811 | 57.62155 | 36.48665 | 57.96208 | 44.19983 | 54.6863 | 60.61424 | 166.3529 | 64.8125 | 55.84573 | 45.88881 | 59.51435 | 40.55619 |
| CUST_13735_PI429010886 | N31685 | 0.013075 | 12 | 3 | | | | | | 461.882 | 320.1082 | 327.144 | 545.3681 | 446.4087 | 659.0457 | 395.7641 | 462.6916 | 346.7104 | 349.6083 | 382.663 | 383.8497 | 352.2533 | 337.2385 | 374.4434 | 444.8946 | 466.0044 | 550.6217 | 516.3899 | 429.3715 | 345.9305 | 430.573 | 301.7927 | 392.2104 | 377.8098 | 482.8729 | 350.0802 |
| CUST_54045_PI429010886 | M104222 | 0.013085 | 21 | 3 | 241849002 | alternative splicing factor ASF/SF2, putative [Ixodes | | | 5.13E-38 | 33.14406 | 34.00581 | 37.42448 | 36.59373 | 41.28276 | 30.5127 | 30.04836 | 32.94683 | 36.03688 | 17.59799 | 27.16077 | 31.52203 | 31.35806 | 23.19661 | 31.90512 | 39.82861 | 34.60204 | 24.40411 | 36.63133 | 28.23857 | 24.15872 | 47.62012 | 28.65357 | 27.79511 | 36.87579 | 38.29585 | 38.34742 |
| CUST_41419_PI429010886 | M57685 | 0.013095 | 15 | 12 | | | | | | 33.62105 | 25.55609 | 33.47616 | 24.11521 | 13.48403 | 18.25166 | 31.72455 | 18.66273 | 23.22371 | 27.14824 | 18.92468 | 18.31091 | 30.48251 | 32.89329 | 36.67205 | 40.27597 | 24.93429 | 22.17526 | 17.98401 | 21.63352 | 19.18718 | 40.71208 | 21.59499 | 15.87452 | 25.69829 | 22.8759 | 27.58334 |
| CUST_5005_PI429010886 | N18118 | 0.013095 | 15 | 12 | | | | | | 118.0563 | 35.42374 | 53.87443 | 38.90216 | 30.24099 | 54.53458 | 74.71666 | 60.39123 | 63.50632 | 39.49159 | 72.03576 | 35.54408 | 70.3766 | 432.1375 | 194.605 | 74.47085 | 45.52612 | 100.3487 | 47.57729 | 46.50141 | 49.83701 | 87.06371 | 62.96663 | 36.25025 | 78.17016 | 96.40299 | 80.68026 |

| Probe ID | ID | p-value | Period | Phase | EBI ID | Annotation | GO category | Go term | E-value | 0 | 0 | 0 | 3 | 3 | 3 | 6 | 6 | 6 | 9 | 9 | 9 | 12 | 12 | 12 | 15 | 15 | 15 | 18 | 18 | 18 | 21 | 21 | 21 | 24 | 24 | 24 |
|---|---|---|---|---|---|---|---|---|---|---|---|---|---|---|---|---|---|---|---|---|---|---|---|---|---|---|---|---|---|---|---|---|---|---|---|---|
| CUST_72_PI429010886 | N395 | 0.013103 | 12 | 0 | | | | | | 61.38847 | 66.81883 | 82.59837 | 74.69976 | 79.80797 | 79.97877 | 69.68123 | 58.99785 | 59.27376 | 74.57029 | 60.44797 | 69.81278 | 78.19261 | 101.2666 | 86.7497 | 73.90307 | 71.6044 | 62.68635 | 72.11758 | 66.34998 | 62.44513 | 87.30486 | 81.03057 | 60.14737 | 94.47657 | 83.35473 | 74.55697 |
| CUST_43651_PI429010886 | M63583 | 0.013128 | 24 | 18 | | | | | | 20.89343 | 51.34688 | 60.30436 | 36.12998 | 27.08746 | 19.21762 | 45.05595 | 41.42564 | 25.05744 | 27.72962 | 20.15879 | 42.91838 | 36.07274 | 54.8287 | 37.91955 | 74.43452 | 26.22564 | 33.65009 | 80.50367 | 64.61143 | 62.48029 | 62.04074 | 33.28088 | 32.45148 | 39.83835 | 28.7982 | 60.82746 |
| CUST_12627_PI429010886 | N29768 | 0.013146 | 18 | 9 | | | | | | 176.2795 | 207.2391 | 187.9002 | 144.3246 | 138.4789 | 117.0641 | 111.3943 | 155.8611 | 157.8167 | 255.1996 | 165.3446 | 208.6342 | 144.9176 | 186.2287 | 187.9283 | 249.8117 | 161.711 | 178.4456 | 153.5797 | 195.2252 | 144.0774 | 151.0154 | 175.1768 | 109.1768 | 226.6353 | 146.9814 | 211.3962 |
| CUST_52858_PI429010886 | M99237 | 0.013159 | 24 | 0 | 533148364 | PREDICTED: mitotic spindle-associated MMXD comp | | | 1.81E-25 | 48.41112 | 51.02123 | 43.81006 | 19.68628 | 38.39166 | 25.73774 | 39.40002 | 26.46898 | 47.32005 | 39.99252 | 23.96204 | 29.93088 | 21.16716 | 28.65923 | 23.66736 | 32.7674 | 13.56063 | 34.91043 | 42.8661 | 24.23967 | 37.80681 | 40.87944 | 16.88136 | 23.76056 | 50.04478 | 27.0144 | 45.34827 |
| CUST_56111_PI429010886 | M112803 | 0.013162 | 15 | 3 | | | | | | 289.2842 | 319.007 | 149.8948 | 467.9877 | 340.5982 | 349.5009 | 333.5908 | 168.4961 | 112.8577 | 179.7938 | 269.2314 | 155.2361 | 204.3252 | 140.1704 | 135.2179 | 193.2088 | 225.7224 | 204.4866 | 190.6724 | 262.1655 | 208.39 | 333.708 | 142.5067 | 194.777 | 172.9267 | 165.9977 | 65.65578 |
| CUST_14420_PI429010886 | N32858 | 0.013162 | 18 | 9 | | | | | | 171.5473 | 99.62003 | 123.4117 | 106.7397 | 211.0494 | 137.2513 | 256.5714 | 158.8514 | 167.1292 | 233.2471 | 227.8652 | 200.7582 | 99.53996 | 165.7972 | 186.7272 | 232.7081 | 183.9787 | 252.7637 | 146.765 | 141.3142 | 136.4108 | 189.5352 | 122.5181 | 198.2178 | 192.9519 | 294.4572 | 316.122 |
| CUST_33999_PI429010886 | M8467 | 0.013165 | 15 | 9 | 270004843 | cubitus interruptus [Tribolium castaneum] | | | 6.33E-11 | 41.59799 | 59.76845 | 55.00033 | 42.64756 | 66.00564 | 50.9755 | 44.79489 | 38.45723 | 60.68591 | 61.5 | 50.23482 | 75.17352 | 73.16394 | 70.108 | 49.02873 | 68.44956 | 34.40352 | 69.74095 | 32.00727 | 54.1859 | 52.18439 | 70.02583 | 54.78436 | 80.19532 | 80.6942 | 81.07113 | 103.6651 |
| CUST_1578_PI429010886 | N10134 | 0.013165 | 15 | 9 | | | | | | 2288.66 | 2939.878 | 2773.22 | 2747.282 | 2593.467 | 2811.816 | 2629.007 | 2092.578 | 2826.022 | 3303.552 | 2491.849 | 3469.507 | 2524.383 | 2945.87 | 3022.502 | 2863.818 | 2363.842 | 2632.403 | 2169.749 | 2736.774 | 2834.55 | 2949.668 | 2838.594 | 2925.159 | 3358.264 | 3368.325 | 3222.703 |
| CUST_6952_PI429010886 | N20825 | 0.013165 | 15 | 0 | | | | | | 663.1345 | 739.338 | 783.8018 | 659.8695 | 688.7233 | 878.7545 | 279.0433 | 238.8964 | 261.0789 | 634.1523 | 882.153 | 698.8251 | 190.8326 | 561.1634 | 355.5348 | 745.8794 | 735.5143 | 704.1186 | 527.0813 | 959.6386 | 644.1161 | 525.5752 | 453.79 | 375.4169 | 820.0337 | 505.2828 | 519.1962 |
| CUST_8012_PI429010886 | N22415 | 0.013165 | 15 | 9 | 383859710 | PREDICTED: transcription elongation factor SPT5-lik | Nucleic acid metabolism | Regulation of gene expression | 5.52E-40 | 22.44818 | 41.40356 | 23.8296 | 26.44421 | 24.78255 | 19.08808 | 33.91251 | 26.36808 | 30.6709 | 36.48332 | 37.80681 | 35.29716 | 22.59655 | 42.68463 | 32.52687 | 41.09904 | 21.70044 | 29.69991 | 25.11853 | 23.13968 | 27.21982 | 37.62657 | 32.80956 | 36.08743 | 43.13359 | 26.98251 | 38.51022 |
| CUST_17137_PI429010886 | N37415 | 0.013165 | 15 | 6 | | | | | | 25.15661 | 19.40925 | 41.62253 | 27.95506 | 28.45961 | 26.26779 | 30.5332 | 34.99003 | 45.05092 | 25.24001 | 29.11372 | 30.41595 | 31.44965 | 40.28418 | 26.75604 | 25.22098 | 24.14372 | 21.40881 | 20.6935 | 32.17296 | 31.66159 | 41.07674 | 28.87402 | 32.53107 | 32.90178 | 30.06877 | 25.73345 |
| CUST_48601_PI429010886 | M81218 | 0.01317 | 24 | 15 | | | | | | 9.778317 | 4.364625 | 8.143445 | 3.878858 | 11.08304 | NA | NA | 5.135124 | 4.799639 | 5.009581 | 10.50787 | NA | 9.388689 | 7.656642 | 21.34584 | 44.33871 | 20.6434 | 17.00562 | 5.490342 | 4.508565 | 19.61118 | 11.06582 | NA | 10.52648 | 9.343228 | 3.961673 | NA |
| CUST_4726_PI429010886 | N17351 | 0.013188 | 18 | 3 | | | | | | 5.191851 | 18.85135 | NA | 31.43244 | 10.29246 | 26.60787 | 16.95796 | 4.413972 | 9.23673 | 5.973441 | 10.32671 | 11.89545 | 4.415485 | 3.288492 | 9.323293 | 11.71408 | NA | 12.6884 | NA | 8.201884 | 16.13535 | 16.96853 | 28.76281 | 10.79019 | 19.5882 | 18.17081 | 5.675402 |
| CUST_43567_PI429010886 | M63362 | 0.013188 | 15 | 12 | | | | | | 10.82657 | 6.261173 | 20.47876 | 7.20793 | 6.474157 | 7.342643 | 4.996297 | 8.499443 | 12.74222 | 16.46215 | 4.182504 | 14.29027 | 59.70379 | 29.55524 | 15.15645 | 9.750393 | 10.25079 | 9.834889 | 8.07676 | 11.51778 | 9.384386 | 9.192825 | 13.52202 | NA | 12.27296 | 6.740179 | 13.94276 |
| CUST_14459_PI429010886 | N32924 | 0.013189 | 21 | 18 | 321458586 | hypothetical protein DAPPUDRAFT_300887 [Daphn | Transport | Ion transport | 4.96E-34 | 67.43306 | 75.99857 | 51.87045 | 61.60388 | 51.56072 | 33.69477 | 38.33525 | 24.16825 | 28.09147 | 70.28439 | 55.29319 | 44.5467 | 58.84482 | 61.12299 | 50.17076 | 83.76494 | 32.66548 | 39.93503 | 47.18136 | 86.08273 | 69.29923 | 102.7622 | 46.88603 | 42.22198 | 55.08741 | 46.58823 | 41.55626 |
| CUST_24396_PI429010886 | N50554 | 0.013192 | 24 | 0 | | | | | | 40.03501 | 26.19976 | 65.53578 | 26.51493 | 17.61362 | 8.418027 | 22.0796 | 43.22574 | 45.50557 | 22.71614 | 19.62227 | 14.53418 | 16.80616 | 3.85977 | 13.4766 | 19.92471 | 23.89866 | 15.42734 | 25.57573 | 27.43563 | 23.35433 | 24.29286 | 31.43817 | 16.79903 | 27.18775 | 16.65372 | 24.84105 |
| CUST_33767_PI429010886 | M7837 | 0.013211 | 24 | 15 | | | | | | 521.3278 | 626.4662 | 504.6329 | 474.2296 | 611.9605 | 595.7445 | 389.7993 | 495.3471 | 338.892 | 649.8854 | 576.6597 | 774.5083 | 473.2302 | 488.0718 | 486.7621 | 652.2033 | 781.185 | 585.0822 | 672.3398 | 525.5752 | 504.7239 | 638.7779 | 635.9764 | 615.211 | 627.2172 | 445.7286 | 509.7336 |
| CUST_28241_PI429010886 | N57575 | 0.013227 | 24 | 18 | | | | | | 242.4714 | 209.7275 | 255.4386 | 189.6625 | 158.8514 | 163.8459 | 185.8972 | 220.485 | 172.9097 | 245.4886 | 205.2221 | 207.3573 | 205.909 | 217.7746 | 164.1721 | 207.0425 | 249.8933 | 192.586 | 208.9955 | 267.5678 | 225.5895 | 224.4715 | 217.7483 | 179.678 | 211.4366 | 182.2638 | 192.442 |
| CUST_10495_PI429010886 | N26324 | 0.013235 | 18 | 3 | | | | | | 73.34493 | 114.0443 | 81.65401 | 171.5147 | 142.8394 | 90.11072 | 104.8909 | 88.92819 | 38.4072 | 47.31501 | 120.9718 | 109.4106 | 45.35819 | 53.50062 | 83.0149 | 57.45413 | 65.23612 | 66.59221 | 40.23874 | 60.74611 | 164.7862 | 105.798 | 114.6834 | 117.621 | 106.3333 | 81.54717 | 62.04982 |
| CUST_50020_PI429010886 | M88284 | 0.013239 | 18 | 12 | | | | | | 43.65248 | 27.61208 | 50.131 | 21.05599 | 33.56101 | 38.4072 | 43.44238 | 37.47945 | 35.30186 | 36.387 | 26.93068 | 70.41805 | 43.58328 | 63.78913 | 89.02227 | 81.76577 | 41.05122 | 50.33169 | 50.29336 | 48.44993 | 47.37022 | 40.17403 | 39.08441 | 30.71736 | 59.22654 | 79.17523 | 63.1929 |
| CUST_25322_PI429010886 | N52197 | 0.01324 | 18 | 0 | 409892959 | glycosyl-phosphatidylinositol-linked carbonic anhyd | | | 4.35E-18 | 100.0408 | 64.87589 | 90.01113 | 114.5149 | 80.63947 | 116.0217 | 30.16372 | 38.06764 | 22.74008 | 79.37795 | 39.14912 | 79.5827 | 53.56751 | 49.46731 | 50.546 | 79.79104 | 42.59502 | 65.09219 | 106.064 | 43.27701 | 72.58192 | 38.11743 | 17.08222 | 71.52967 | 58.11529 | 29.30133 | 28.1226 |
| CUST_35755_PI429010886 | M13427 | 0.013245 | 15 | 12 | 260786739 | hypothetical protein BRAFLDRAFT_63363 [Branchio | | | 1.39E-10 | 40.14284 | 38.58369 | 59.15067 | 38.2326 | 38.60384 | 36.60129 | 46.17776 | 50.26232 | 49.36528 | 65.24322 | 31.66434 | 49.77895 | 41.47521 | 61.15955 | 54.01314 | 57.94186 | 43.23619 | 50.94926 | 31.85271 | 44.10538 | 46.71536 | 46.83015 | 46.54874 | 33.37654 | 49.84204 | 52.2983 | 50.93524 |
| CUST_38378_PI429010886 | M26020 | 0.013245 | 15 | 12 | | | | | | 42441.75 | 4205.626 | 81077.7 | 1492.073 | 3390.638 | 2379.147 | 48465.49 | 103589.1 | 78081.9 | 78574.02 | 21220.67 | 21906.22 | 82964.44 | 107232.5 | 78574.02 | 10524.41 | 58209.54 | 3787.685 | 37739.19 | 33611.5 | 15246.84 | 70816.79 | 93952.87 | 16000.49 | 101136 | 15778.6 | 86620.4 |
| CUST_34287_PI429010886 | M9245 | 0.013245 | 15 | 12 | | | | | | 16.93833 | 22.89389 | 32.17021 | 18.69493 | 9.36909 | 13.99779 | 12.17715 | 15.26731 | 22.19273 | 19.99051 | 22.46339 | 21.45407 | 25.85777 | 22.7667 | 14.70379 | 19.83698 | 22.86472 | 14.51836 | 11.7971 | 22.1165 | 10.82851 | 14.06114 | 18.91018 | 18.02985 | 32.76173 | 14.33789 | 23.43812 |
| CUST_9733_PI429010886 | N25116 | 0.013245 | 15 | 12 | | | | | | 63.00378 | 47.82652 | 78.33945 | 52.23527 | 41.45045 | 39.62195 | 38.66828 | 50.15777 | 56.60803 | 55.92013 | 52.93292 | 67.12853 | 49.27153 | 55.80731 | 88.07214 | 54.45016 | 63.80913 | 49.3195 | 44.36476 | 48.98001 | 35.4058 | 65.93703 | 51.15902 | 40.62506 | 62.89604 | 51.50234 | 61.03938 |
| CUST_11789_PI429010886 | N28406 | 0.013245 | 15 | 12 | | | | | | 67.98118 | 41.65038 | 57.16139 | 34.74405 | 33.21734 | 33.19093 | 53.01337 | 68.30614 | 34.20386 | 79.36014 | 38.19149 | 42.6663 | 48.90211 | 70.61376 | 49.39411 | 37.07935 | 67.7616 | 52.5097 | 52.55999 | 31.97801 | 38.25121 | 37.20514 | 42.65591 | 37.03334 | 83.23552 | 37.9742 | 57.65633 |
| CUST_51744_PI429010886 | M94823 | 0.013265 | 21 | 3 | 465952717 | hypothetical protein UY3_17887 [Chelonia mydas] | | | 3.54E-23 | 1218.367 | 3730.34 | 1787.33 | 4755.263 | 2236.34 | 2404.795 | 1453.376 | 1693.628 | 1327.226 | 1929.904 | 795.7542 | 2108.426 | 1159.33 | 1736.508 | 1251.807 | 1634.318 | 679.5311 | 1412.634 | 858.518 | 1382.607 | 2298.025 | 2511.522 | 1895.191 | 1606.044 | 2698.741 | 2877.641 | 1178.063 |
| CUST_8563_PI429010886 | N23226 | 0.013268 | 15 | 0 | 307184380 | Carbohydrate sulfotransferase 14 [Camponotus flor | Metabolic process | Carbohydrate metabolic process | 1.37E-19 | 296.8223 | 335.74 | 426.0225 | 185.8187 | 169.5826 | 281.9234 | 222.2769 | 252.4492 | 244.9911 | 285.1566 | 221.425 | 295.0205 | 280.7168 | 407.6622 | 285.6058 | 379.2779 | 310.0812 | 332.8858 | 199.1036 | 351.2576 | 283.9501 | 343.4753 | 250.8185 | 262.2092 | 285.2578 | 274.9043 | 321.803 |
| CUST_55589_PI429010886 | M110871 | 0.013285 | 24 | 21 | | | | | | 68.78674 | 76.28297 | 89.72544 | 59.91437 | 63.1995 | 73.47109 | 63.17657 | 66.50645 | 65.71273 | 93.62063 | 63.1929 | 79.07032 | 54.40561 | 79.01755 | 71.30124 | 65.36594 | 74.51037 | 78.16064 | 81.63335 | 58.7202 | 81.03965 | 81.30299 | 108.6304 | 90.3035 | 69.80023 | 71.93323 | 79.4217 |
| CUST_36242_PI429010886 | M15142 | 0.013285 | 24 | 21 | | | | | | 569.2573 | 563.6523 | 516.5602 | 486.3734 | 359.3716 | 459.6219 | 380.8862 | 529.7833 | 394.5293 | 605.0923 | 490.2884 | 616.2727 | 526.8072 | 667.7123 | 441.0416 | 617.029 | 571.296 | 451.4433 | 574.3589 | 535.6554 | 537.4529 | 698.4337 | 582.9606 | 628.3128 | 780.9267 | 604.4329 | 696.015 |
| CUST_44597_PI429010886 | M66428 | 0.013285 | 24 | 3 | | | | | | 1407.859 | 964.2217 | 663.1345 | 1884.761 | 1444.045 | 2208.121 | 1364.277 | 1118.972 | 1165.934 | 908.1498 | 813.1389 | 1072.593 | 944.6524 | 829.8828 | 711.9048 | 1037.639 | 1953.939 | 1696.249 | 669.6491 | 776.6013 | 800.3484 | 847.0661 | 930.2399 | 1914.117 | 910.1372 | 1912.382 | 838.4693 |
| CUST_19887_PI429010886 | N42390 | 0.013285 | 24 | 21 | 345498241 | PREDICTED: transportin-1 [Nasonia vitripennis] | Transport | Protein transport | 1.51E-54 | 24.53942 | 21.48663 | 24.4716 | 23.99191 | 18.57528 | 16.70757 | 9.086428 | 20.07638 | 24.0618 | 28.1226 | 17.33858 | 25.80044 | 17.92364 | 33.72458 | 22.81875 | 33.56428 | 9.23209 | 28.94202 | 23.06972 | 30.52691 | 26.93627 | 43.5925 | 31.39582 | 25.88533 | 28.31689 | 28.57291 | 29.80377 |
| CUST_10600_PI429010886 | N26492 | 0.013297 | 24 | 15 | | | | | | 31.97801 | 20.87902 | 19.70849 | 23.25478 | 34.0946 | 22.54321 | 15.77707 | 25.352 | 20.91887 | 32.34622 | 29.54213 | 33.96909 | 28.13108 | 33.69477 | 32.29627 | 34.68835 | 55.15261 | 38.121 | 35.09563 | 88.18553 | 18.554 | 38.0368 | 32.24939 | 33.69477 | 45.98571 | 32.03583 | 35.28945 |
| CUST_39003_PI429010886 | M33285 | 0.013301 | 18 | 3 | | | | | | 30.52136 | 38.74344 | 37.26633 | 42.01729 | 59.98757 | 49.45564 | 27.28431 | 28.20146 | 28.78291 | 21.8123 | 28.62456 | 25.31087 | 27.54647 | 41.17841 | 33.42828 | 47.41387 | 26.22091 | 38.0116 | 16.11943 | 38.99636 | 30.46421 | 55.9772 | 27.08746 | 51.46072 | 38.19149 | 23.74918 | 27.4656 |
| CUST_3734_PI429010886 | N15157 | 0.013301 | 18 | 15 | | | | | | 756.9801 | 1092.918 | 1665.986 | 331.4917 | 114.7256 | 356.7855 | 825.4395 | 1298.051 | 1608.297 | 206.0656 | 1210.418 | 846.4177 | 784.2356 | 1094.71 | 660.3398 | 1238.144 | 945.5514 | 1040.536 | 767.5196 | 473.4648 | 587.9777 | 658.8544 | 483.4448 | 258.1237 | 329.473 | 703.6838 | 698.4337 |
| CUST_278_PI429010886 | N3272 | 0.013301 | 18 | 3 | 307194443 | hypothetical protein EAI_04489 [Harpegnathos salt | | | 6.38E-74 | 5167.16 | 7347.807 | 522.8025 | 9501.999 | 6321.861 | 7443.214 | 9541.284 | 2158.318 | 5590.669 | 293.013 | 2309.39 | 235.2484 | 3134.449 | 634.2891 | 3259.251 | 3309.537 | 4639.445 | 6794.71 | 3722.852 | 253.3806 | 2318.551 | 1798.294 | 4854.251 | 8888.62 | 563.0019 | 3419.141 | 507.4019 |
| CUST_7215_PI429010886 | N21203 | 0.013301 | 24 | 15 | | | | | | 84.62693 | 100.7517 | 93.06352 | 69.4087 | 86.13317 | 85.10115 | 96.64522 | 119.9827 | 98.6608 | 120.7611 | 95.39909 | 113.6641 | 83.04083 | 118.3632 | 134.2279 | 130.2006 | 123.321 | 100.999 | 98.59007 | 127.6673 | 94.11857 | 105.0857 | 106.5352 | 87.65825 | 111.2059 | 80.23994 | 109.812 |
| CUST_1101_PI429010886 | N8499 | 0.013301 | 24 | 15 | | | | | | 106.8563 | 81.41269 | 93.44686 | 73.60851 | 66.86178 | 93.84895 | 79.44802 | 81.31167 | 104.5986 | 67.48171 | 83.53882 | 98.11651 | 94.01656 | 114.0787 | 119.9827 | 124.672 | 119.7198 | 115.3001 | 94.01656 | 111.8993 | 65.13281 | 122.3268 | 76.13932 | 86.62519 | 110.4991 | 89.34718 | 108.9026 |
| CUST_38561_PI429010886 | M28009 | 0.013316 | 15 | 3 | | | | | | 2536.388 | 983.2431 | 1534.021 | 6297.225 | 7364.493 | 6925.74 | 5830.309 | 2086.783 | 1423.398 | 166.9427 | 5602.191 | 60.84139 | 1594.841 | 274.404 | 698.0293 | 285.6058 | 7853.842 | 3172.097 | 6495.601 | 85.22504 | 6528.23 | 390.7246 | 4594.669 | 5271.276 | 320.9666 | 2366.854 | 111.1643 |
| CUST_44483_PI429010886 | M66113 | 0.013316 | 15 | 9 | | | | | | 29.76053 | 19.88671 | 32.18854 | 21.55311 | 17.7534 | 20.28484 | 26.41485 | 18.57367 | 25.56096 | 29.14509 | 20.53338 | 32.76173 | 23.88015 | 25.45721 | 25.33771 | 18.7895 | 22.18079 | 27.43011 | 24.72926 | 16.13535 | 28.43336 | 28.1812 | 22.39942 | 18.98392 | 33.8457 | 37.05276 | 35.38892 |
| CUST_46230_PI429010886 | M71871 | 0.013316 | 15 | 9 | | | | | | 772.1398 | 951.9745 | 1008.511 | 548.5092 | 803.077 | 834.0915 | 710.4011 | 779.1452 | 795.1421 | 1337.58 | 781.4539 | 1404.631 | 806.3497 | 1148.187 | 1181.87 | 1133.023 | 742.4096 | 762.7979 | 907.7978 | 757.419 | 817.7915 | 957.8471 | 967.1284 | 846.1561 | 1041.548 | 1091.244 | 1271.51 |
| CUST_16033_PI429010886 | N35540 | 0.013316 | 15 | 3 | | | | | | 157.17 | 41.17264 | 111.3943 | 184.7518 | 158.5396 | 197.989 | 241.0846 | 116.4359 | 65.23612 | 53.70396 | 79.93349 | 48.45867 | 98.90127 | 97.00878 | 92.29442 | 65.26948 | 244.8705 | 141.8325 | 268.2743 | 60.63321 | 95.56359 | 90.74799 | 77.89081 | 155.4485 | 54.75352 | 105.779 | 33.69201 |
| CUST_21927_PI429010886 | N46107 | 0.013316 | 15 | 3 | | | | | | 847.9888 | 331.5388 | 527.3498 | 895.455 | 954.6687 | 1486.604 | 1084.753 | 733.9745 | 433.8707 | 172.2644 | 493.7491 | 81.87168 | 433.59 | 136.3426 | 296.3588 | 218.2859 | 1492.759 | 885.3464 | 1138.766 | 119.3119 | 519.4558 | 191.4981 | 393.8505 | 690.0315 | 178.3785 | 492.9704 | 112.0774 |
| CUST_22007_PI429010886 | N46252 | 0.013316 | 15 | 3 | | | | | | 113.0678 | 79.35074 | 71.52214 | 174.126 | 110.9974 | 166.2545 | 177.2148 | 101.0493 | 105.779 | 37.51655 | 64.44572 | 34.53064 | 87.77632 | 58.40782 | 100.2191 | 73.59863 | 115.0699 | 68.35087 | 101.6252 | 31.95513 | 158.8514 | 38.68077 | 84.51934 | 143.9903 | 39.00627 | 88.01513 | 48.12746 |
| CUST_20664_PI429010886 | N43840 | 0.013344 | 15 | 9 | | | | | | 258.4896 | 268.1939 | 323.1264 | 224.858 | 237.4703 | 212.1657 | 179.6526 | 239.8633 | 286.997 | 368.494 | 250.4969 | 403.2396 | 276.8504 | 355.6114 | 301.7927 | 393.5988 | 189.6625 | 260.5083 | 233.8649 | 311.9883 | 234.0614 | 271.8242 | 350.793 | 231.648 | 349.3181 | 275.9866 | 350.1387 |
| CUST_15535_PI429010886 | N34748 | 0.01337 | 12 | 3 | | | | | | 115.0806 | 76.68397 | 91.33174 | 85.61256 | 91.22284 | 126.4765 | 88.43444 | 80.26839 | 77.89081 | 74.7945 | 76.96996 | 82.49999 | 83.45905 | 92.87668 | 87.53485 | 122.2019 | 99.45401 | 138.5572 | 91.7472 | 87.99607 | 86.7497 | 87.82151 | 74.54819 | 97.46537 | 95.80114 | 78.62359 | 105.6827 |
| CUST_31762_PI429010886 | M2343 | 0.013396 | 15 | 12 | 288188858 | serine proteinase inhibitor 8 [Penaeus monodon] | | | 7.59E-44 | 3262.349 | 5055.748 | 4427.545 | 2038.918 | 2596.508 | 1786.373 | 2747.282 | 4120.1 | 4672.866 | 4769.116 | 2547.522 | 3549.404 | 4803.756 | 5539.858 | 5299.221 | 5613.6 | 2748.983 | 2921.018 | 2948.044 | 4635.589 | 2303.489 | 6812.048 | 3332.072 | 2023.529 | 6448.481 | 2995.003 | 5699.333 |
| CUST_55560_PI429010886 | M110721 | 0.013418 | 21 | 0 | | | | | | 88.83369 | 104.0193 | 103.8532 | 67.97289 | 55.38216 | 56.76191 | 64.10673 | 54.5089 | 46.42413 | 79.62582 | 91.98586 | 80.06962 | 68.07541 | 69.59584 | 82.43374 | 95.60632 | 75.39347 | 102.2534 | 91.71625 | 64.41009 | 79.78328 | 53.72529 | 96.0412 | 112.7504 | 70.5402 | 79.1048 | 65.68694 |
| CUST_40548_PI429010886 | M55428 | 0.013441 | 24 | 3 | | | | | | 13.2768 | 18.2155 | 12.86372 | 18.41342 | 22.69834 | 23.08011 | 10.41586 | 8.247776 | 11.86764 | 22.4606 | 16.56648 | 22.29454 | 7.480574 | 14.92301 | 10.26272 | 11.46533 | 16.40268 | 15.90074 | 12.57863 | 8.44541 | 10.96904 | 11.02819 | 5.289158 | 13.30659 | 15.18848 | 14.59396 | 14.09025 |
| CUST_48018_PI429010886 | M78558 | 0.013447 | 15 | 12 | | | | | | 15.00987 | 11.22853 | 18.79959 | NA | 6.772515 | 7.758109 | 8.886857 | 10.12787 | 10.32153 | 9.432782 | 4.693023 | 14.07399 | 21.53411 | 13.54322 | 16.42533 | 11.85349 | 7.58227 | 15.50146 | 11.49926 | 4.86774 | 6.327324 | 9.15176 | 10.56222 | 12.30261 | 13.33386 | 7.818797 | 9.108247 |
| CUST_54824_PI429010886 | M107846 | 0.013466 | 18 | 12 | | | | | | 181.5941 | 261.6019 | 198.0625 | 147.5068 | 137.2699 | 152.3559 | 280.8226 | 172.408 | 220.0942 | 192.324 | 161.6167 | 281.0282 | 265.0674 | 294.0478 | 307.7422 | 207.1022 | 147.8755 | 144.8631 | 492.0126 | 155.5527 | 209.9677 | 225.2614 | 155.7397 | 160.6701 | 271.918 | 166.8948 | 254.5885 |
| CUST_55036_PI429010886 | M108454 | 0.013467 | 15 | 9 | | | | | | 238.2441 | 252.9368 | 306.8772 | 208.027 | 224.8249 | 196.5038 | 207.9553 | 248.2061 | 268.1467 | 348.5888 | 208.9685 | 330.1788 | 233.5227 | 272.25 | 268.9268 | 276.8504 | 312.4689 | 267.5328 | 248.428 | 263.5218 | 205.6287 | 280.0685 | 265.8178 | 208.2464 | 322.1425 | 251.8789 | 336.9449 |
| CUST_56823_PI429010886 | M114716 | 0.013467 | 15 | 9 | | | | | | 221.6459 | 365.0271 | 328.5456 | 302.7759 | 255.8665 | 333.5081 | 267.1348 | 252.8872 | 330.6772 | 374.7559 | 334.6678 | 515.5917 | 273.1122 | 340.7478 | 386.742 | 342.5053 | 256.3774 | 404.9556 | 245.3745 | 318.8167 | 316.0527 | 348.906 | 421.6972 | 326.9265 | 388.7082 | 338.6596 | 456.4562 |
| CUST_9861_PI429010886 | N25315 | 0.013467 | 15 | 0 | | | | | | 43.5699 | 39.77048 | 38.11743 | 39.31816 | 33.11064 | 45.58535 | 25.37501 | 23.41982 | 34.95753 | 42.82987 | 28.93678 | 37.20061 | 24.43342 | 36.83629 | 37.69054 | 36.04391 | 40.65477 | 40.50555 | 22.33422 | 25.79616 | 36.74157 | 35.30186 | 19.84686 | 20.53338 | 49.36928 | 28.99027 | 38.82583 |
| CUST_26385_PI429010886 | N54152 | 0.013467 | 15 | 3 | 194756716 | GF11408 [Drosophila ananassae] >gi\|190621919\|gl | | | 1.36E-07 | 215.3103 | 228.2456 | 54.66707 | 470.7834 | 380.4688 | 439.2082 | 425.1252 | 82.95644 | 473.5692 | 12.49592 | 66.18886 | 35.89324 | 195.555 | 106.4697 | 233.8998 | 170.1249 | 116.3059 | 235.0591 | 72.41676 | 96.29479 | 103.7863 | 229.9104 | 71.18045 | 303.1495 | 65.18101 | 71.10646 | 20.27847 |
| CUST_1340_PI429010886 | N9338 | 0.013468 | 15 | 12 | | | | | | 5197.227 | 11074.44 | 8504.473 | 2026.043 | 4263.359 | 2750.47 | 4557.188 | 5744.456 | 10575.43 | 10296.63 | 6091.908 | 12273.46 | 7995.416 | 9061.383 | 12422.81 | 13649.38 | 3981.392 | 8176.608 | 5265.647 | 7145.704 | 6091.908 | 12916.14 | 5998.97 | 3696.299 | 11202.95 | 7628.757 | 14355.49 |
| CUST_22878_PI429010886 | N47840 | 0.013475 | 15 | 9 | | | | | | 9.020299 | 17.23326 | 20.88392 | NA | 8.454339 | 7.411834 | 13.11125 | 6.488863 | 13.94176 | 17.43225 | 8.746322 | 13.86795 | 11.28527 | 36.70412 | 13.05441 | 11.69586 | 11.01907 | NA | 8.774888 | 9.152945 | 6.646603 | 22.82685 | NA | 9.220146 | 25.03856 | 14.45421 | 22.28112 |
| CUST_6878_PI429010886 | N20723 | 0.013494 | 24 | 21 | | | | | | 3551.684 | 3408.807 | 3169.636 | 3208.657 | 3627.549 | 3699.019 | 3838.985 | 2918.106 | 2903.61 | 2869.202 | 3306.501 | 1790.532 | 2756.174 | 2424.452 | 2800.799 | 3893.391 | 4424.066 | 3696.299 | 5572.884 | 2472.114 | 3446.227 | 3342.501 | 4114.35 | 5293.128 | 3222.703 | 3150.433 | 3472.182 |
| CUST_35208_PI429010886 | M11904 | 0.013503 | 12 | 3 | 328784017 | PREDICTED: LOW QUALITY PROTEIN: TBC1 domain f | Signal transduction | Other | 9.07E-66 | 41.45965 | 18.11915 | 28.24369 | 41.50969 | 37.95272 | 50.39093 | 35.5525 | 24.21503 | 21.84546 | 28.21268 | 30.35236 | 23.06016 | 29.92689 | 20.95699 | 17.33696 | 26.01379 | 53.07973 | 44.80736 | 45.57 | 30.07017 | 31.46943 | 20.25895 | 26.97522 | 33.13714 | 26.97522 | 24.316 | 21.98642 |
| CUST_50946_PI429010886 | M91525 | 0.013503 | 15 | 3 | | | | | | 214.6269 | 132.1519 | 118.378 | 253.6933 | 245.8233 | 409.385 | 287.5857 | 188.9473 | 113.3366 | 74.94569 | 193.4435 | 68.63241 | 134.0378 | 54.63526 | 111.9958 | 112.1531 | 284.0133 | 202.6446 | 312.9412 | 76.82831 | 147.2543 | 92.03214 | 175.8238 | 242.3824 | 77.59246 | 147.4736 | 40.54674 |
| CUST_48365_PI429010886 | M80007 | 0.013528 | 21 | 0 | 543719922 | PREDICTED: 40S ribosomal protein S27-like, partial \| | Protein metabolism | Ribosome biogenesis | 6.15E-09 | 24.61539 | 27.9603 | 15.7841 | 13.34705 | 29.64209 | 22.73586 | 16.79729 | 12.02328 | 13.23813 | 17.53908 | 13.35309 | 13.40926 | 19.28584 | 13.06343 | 14.36708 | 18.80396 | 20.98522 | 22.38584 | 15.62557 | 18.98392 | 6.994251 | 34.63494 | 25.05489 | 22.15622 | 16.42915 | 14.24812 | 30.46421 |
| CUST_24508_PI429010886 | N50741 | 0.013537 | 15 | 9 | | | | | | 77.2785 | 74.25631 | 106.4577 | 62.15321 | 72.72106 | 53.09676 | 63.09759 | 91.21234 | 78.59764 | 135.6184 | 91.55434 | 95.96344 | 81.38307 | 102.8301 | 116.3712 | 106.5981 | 90.60771 | 82.47663 | 73.76113 | 105.6941 | 84.87934 | 87.22914 | 113.6358 | 67.95716 | 103.6762 | 81.2413 | 126.0286 |
| CUST_8664_PI429010886 | N23381 | 0.013546 | 15 | 12 | | | | | | 174.6884 | 201.9074 | 202.9438 | 177.2677 | 115.9694 | 129.2905 | 116.4584 | 185.0427 | 183.8217 | 181.1566 | 143.8832 | 193.3073 | 170.8315 | 266.5285 | 220.3236 | 241.4042 | 156.4459 | 144.7064 | 151.7333 | 178.2181 | 151.8603 | 211.4366 | 192.8746 | 135.1811 | 200.8936 | 208.8792 | 216.807 |
| CUST_21459_PI429010886 | N45261 | 0.013546 | 15 | 12 | | | | | | 9686 | 8854.389 | 12916.14 | 10165.13 | 8165.772 | 7590.588 | 6976.379 | 6767.816 | 12374.53 | 11619.8 | 9418.183 | 19711.48 | 13988.83 | 13177.04 | 23934.78 | 8436.312 | 6528.23 | 17078.69 | 11366.05 | 7853.842 | 10415.22 | 18116.83 | 11583.53 | 10915.56 | 16762.52 | 12840.33 | 12523.21 |
| CUST_41179_PI429010886 | M57074 | 0.013559 | 24 | 0 | | | | | | 59.52835 | 57.028 | 53.75298 | 47.88942 | 42.06392 | 63.03943 | 66.54333 | 55.59388 | 50.87205 | 38.37256 | 36.83629 | 36.8676 | 52.5224 | 36.42724 | 39.52923 | 54.90496 | 36.83973 | 36.78203 | 41.909 | 45.33809 | 49.81001 | 41.26062 | 48.51868 | 62.406 | 63.00905 | 54.6863 | 42.56152 |
| CUST_17967_PI429010886 | N38918 | 0.013559 | 15 | 12 | | | | | | 81.86063 | 54.93259 | 75.70522 | 52.8747 | 65.14347 | 54.30012 | 71.09226 | 60.26272 | 58.98538 | 69.56167 | 71.18669 | 66.5854 | 68.06658 | 67.32952 | 86.22581 | 81.12186 | 67.18762 | 67.63514 | 62.10992 | 52.58792 | 63.64812 | 61.31684 | 63.11741 | 66.07282 | 68.4981 | 53.09159 | 57.29447 |
| CUST_55563_PI429010886 | M110752 | 0.013592 | 18 | 12 | | | | | | 1027.997 | 705.3837 | 844.642 | 317.4402 | 1295.233 | 725.8016 | 1028.397 | 876.7586 | 660.5155 | 1892.721 | 734.2085 | 937.1457 | 1361.387 | 1155.257 | 978.4784 | 831.7121 | 935.3105 | 1557.483 | 992.543 | 805.2979 | 944.0597 | 785.0368 | 481.6846 | 399.1635 | 450.1209 | 675.7327 | 1080.128 |
| CUST_24911_PI429010886 | N51468 | 0.013592 | 18 | 3 | | | | | | 83.09489 | 76.88757 | 44.80164 | 119.4425 | 73.86807 | 99.22324 | 50.01173 | 63.72139 | 65.96571 | 72.49991 | 35.6143 | 79.32298 | 60.42655 | 54.99653 | 60.42655 | 88.85357 | 58.38163 | 73.39754 | 49.2211 | 71.46757 | 130.0555 | 89.75292 | 87.00147 | 105.5089 | 68.43089 | 88.11918 | 106.5216 |
| CUST_30772_PI429010886 | N62630 | 0.013593 | 18 | 3 | | | | | | 48.069 | 15.02774 | 3.459417 | 61.96298 | 29.69161 | 51.06646 | 30.46421 | 24.75921 | 24.46151 | 9.067263 | NA | 7.627544 | 23.82334 | 10.17939 | 27.01753 | 20.34669 | NA | 30.55085 | NA | 91.87634 | 9.418869 | 31.68743 | NA | 62.23675 | 19.09218 | 30.94122 | 5.744855 |
| CUST_25835_PI429010886 | N53140 | 0.013597 | 24 | 21 | | | | | | 215.5744 | 281.3948 | 219.7749 | 186.1374 | 226.6782 | 133.5047 | 181.5127 | 193.0602 | 191.0009 | 324.1857 | 232.2704 | 214.8824 | 229.4944 | 239.1179 | 226.6782 | 241.3477 | 188.658 | 215.9452 | 242.8033 | 233.9335 | 269.6735 | 273.8248 | 249.7124 | 226.768 | 273.6399 | 246.7846 | 244.1579 |
| CUST_34346_PI429010886 | M9390 | 0.013603 | 24 | 15 | | | | | | 228.0216 | 226.1214 | 202.5331 | 211.4366 | 212.4051 | 261.6861 | 189.7693 | 189.7138 | 171.1038 | 230.2231 | 241.3085 | 256.7514 | 138.7005 | 210.5448 | 174.7134 | 305.3469 | 262.8116 | 282.3228 | 245.9792 | 235.3617 | 224.5779 | 258.545 | 215.2013 | 296.8223 | 347.4939 | 245.6075 | 228.8706 |
| CUST_8461_PI429010886 | N23075 | 0.013605 | 12 | 9 | | | | | | 26.32207 | 27.08746 | 27.58634 | 14.38028 | 23.78457 | 8.833142 | NA | 20.25021 | 38.98332 | 58.4895 | 29.03344 | 35.50382 | 37.03334 | 45.46745 | 26.22826 | 37.45529 | 21.76203 | 17.43627 | 40.06518 | 48.8316 | 25.13071 | 38.75385 | 49.35995 | NA | 98.03383 | 25.75422 | 80.26839 |
| CUST_20483_PI429010886 | N43539 | 0.01361 | 18 | 3 | | | | | | 245.2785 | 198.2869 | 206.7552 | 371.6228 | 214.7684 | 458.0989 | 278.9452 | 255.7416 | 258.0874 | 199.7913 | 150.434 | 116.6557 | 300.8257 | 216.807 | 218.4381 | 206.3446 | 219.7749 | 235.1729 | 217.3065 | 132.2265 | 128.4226 | 624.6679 | 284.8452 | 311.8796 | 175.2126 | 250.3151 | 168.6965 |
| CUST_31851_PI429010886 | M2575 | 0.013615 | 24 | 21 | | | | | | 110.8879 | 96.05386 | 140.5141 | 113.8196 | 115.7238 | 130.2384 | 87.75721 | 119.4111 | 96.56866 | 133.1932 | 98.02439 | 113.2085 | 88.40608 | 121.5472 | 103.1898 | 134.4739 | 134.9686 | 132.4401 | 111.7143 | 146.5131 | 126.3708 | 139.9854 | 152.3001 | 171.1636 | 106.2285 | 112.7849 | 86.82244 |
| CUST_37383_PI429010886 | M18847 | 0.013618 | 15 | 0 | 321455420 | hypothetical protein DAPPUDRAFT_218978 [Daphn | Metabolic process | Carbohydrate metabolic process | 5.49E-92 | 10491.93 | 12584.81 | 8611.654 | 8504.473 | 11729.06 | 13764.3 | 3576.101 | 2507.968 | 4706.053 | 8006.158 | 11495.14 | 12214.41 | 3656.575 | 8228.765 | 7794.866 | 16062.45 | 7864.497 | 13400.98 | 7154.842 | 12422.81 | 9169.5 | 9096.106 | 5188.094 | 5487.234 | 10259.45 | 7306.322 | 9382.703 |
| CUST_33033_PI429010886 | M5780 | 0.013618 | 15 | 9 | | | | | | 307.2905 | 1274.119 | 972.1119 | 263.6263 | 568.8782 | 131.9783 | 304.0369 | 604.7306 | 1214.782 | 1528.209 | 845.9343 | 1452.711 | 1120.205 | 1728.225 | 816.9007 | 1317.659 | 1061.893 | 705.3837 | 859.9982 | 950.8038 | 977.0262 | 2485.559 | 1661.324 | 224.6396 | 3197.843 | 1137.917 | 3276.015 |
| CUST_45774_PI429010886 | M70244 | 0.013618 | 15 | 9 | | | | | | 667.8908 | 477.6391 | 685.3204 | 484.8203 | 582.1613 | 579.5177 | 537.4529 | 562.0583 | 577.6505 | 705.2005 | 574.4912 | 700.208 | 586.8079 | 724.6164 | 670.3311 | 774.7379 | 515.8865 | 641.3583 | 561.1634 | 537.9358 | 604.7306 | 620.3013 | 772.4833 | 543.6335 | 783.8018 | 828.3608 | 821.7869 |
| CUST_48548_PI429010886 | M80944 | 0.013618 | 15 | 9 | | | | | | 145.4206 | 106.1066 | 145.9801 | 113.5868 | 121.5472 | 87.70762 | 127.8373 | 127.8287 | 117.5843 | 189.267 | 135.6803 | 189.5057 | 101.5278 | 212.2276 | 113.2498 | 140.1704 | 106.4186 | 163.791 | 113.7893 | 142.7994 | 140.1704 | 178.0648 | 156.2928 | 150.2035 | 227.4444 | 164.2756 | 218.8861 |
| CUST_20017_PI429010886 | N42655 | 0.013618 | 15 | 0 | | | | | | 7509.168 | 12114.66 | 7828.426 | 6240.976 | 9770.164 | 9046.466 | 3003.506 | 1715.043 | 3124.162 | 8363.4 | 9140.621 | 10649.47 | 2189.348 | 6516.822 | 5693.449 | 12744.97 | 7355.509 | 8627.68 | 5596.154 | 10217.96 | 6794.71 | 7083.117 | 5217.309 | 4094.84 | 6969.97 | 4396.276 | 6812.048 |
| CUST_28598_PI429010886 | N58265 | 0.013618 | 15 | 9 | | | | | | 160.2794 | 147.1104 | 163.2051 | 117.2072 | 110.0483 | 91.6409 | 148.5892 | 207.4667 | 144.5809 | 161.6447 | 159.338 | 202.005 | 200.0776 | 134.5271 | 128.1445 | 160.2968 | 163.0297 | 121.2272 | 154.595 | 134.9314 | 108.077 | 168.032 | 176.68 | 121.2504 | 227.0976 | 122.3027 | 186.883 |
| CUST_30137_PI429010886 | N61240 | 0.013618 | 15 | 0 | | | | | | 31.87606 | 38.45312 | 60.13681 | 42.70151 | 44.00542 | 54.02913 | 31.25971 | 21.15997 | 31.58406 | 30.01154 | 43.59959 | 37.65478 | 34.41491 | 46.29899 | 26.02914 | 53.11468 | 45.73763 | 44.54185 | 39.62195 | 28.888 | 45.03591 | 26.78662 | 21.90202 | 29.09314 | 44.1892 | 49.71501 | 46.24589 |
| CUST_45580_PI429010886 | M69570 | 0.013631 | 21 | 3 | | | | | | 89.07632 | 57.14083 | 69.47796 | 82.89486 | 84.53623 | 77.1366 | 77.48229 | 51.13109 | 55.27747 | 86.64513 | 55.37764 | 79.30391 | 56.24668 | 53.15786 | 57.84331 | 79.71189 | 73.08468 | 76.31563 | 59.49267 | 67.15618 | 70.01711 | 73.28621 | 40.70657 | 79.99032 | 100.397 | 119.2945 | 115.7331 |
| CUST_8149_PI429010886 | N22604 | 0.013633 | 18 | 12 | | | | | | 16.68621 | 23.47003 | 13.07347 | 14.56514 | 15.41112 | NA | 14.75975 | 16.66286 | 20.27847 | 13.52202 | 23.97713 | 24.8221 | 25.62143 | 29.80754 | 29.41046 | 24.62356 | 13.54942 | 25.69538 | 22.74902 | 29.6237 | 20.89073 | 19.3955 | 10.47683 | 16.56559 | 31.16623 | 9.11334 | 25.33989 |
| CUST_35350_PI429010886 | M12339 | 0.013637 | 15 | 0 | | | | | | 23.96204 | 30.34164 | 17.06586 | 17.79403 | 4.524037 | 5.185762 | 9.275747 | 4.522263 | 6.153881 | 12.0298 | 17.92211 | 18.06411 | 22.42981 | 14.52373 | 12.13896 | 26.10969 | 22.10198 | 8.616285 | 25.00056 | 10.90993 | NA | 20.89343 | 7.631181 | 10.8963 | 11.71536 | 19.47107 | 5.153026 |
| CUST_43129_PI429010886 | M62139 | 0.013642 | 21 | 15 | 338809146 | hemocyanin [Exopalaemon carinicauda] | | | 2.6E-15 | NA | 14.25924 | NA | 52.26577 | 116.2937 | 6.956638 | 111.625 | 547.117 | 182.1159 | 59.46444 | 345.4444 | 434.5491 | 1606.601 | 20.83234 | 637.0757 | NA | 534.8364 | NA | 86.72657 | 16480.39 | 540.0536 | 10.68951 | 29.20384 | 19.29786 | NA | 99.2894 | 515.307 |
| CUST_42744_PI429010886 | M61140 | 0.013647 | 12 | 9 | 345481856 | PREDICTED: ras-like GTP-binding protein Rho1 [Nas | Transport | Protein transport | 5.37E-55 | 11.73735 | 30.91453 | 18.56806 | 6.959325 | 4.142037 | 5.850077 | 10.86868 | 11.19024 | 18.89688 | 15.1109 | 10.89455 | 26.0691 | 16.14566 | 9.278391 | 10.43309 | 12.74804 | 5.82395 | NA | 15.4798 | 25.5315 | 10.28467 | NA | 34.06071 | 18.82539 | 29.83449 | 9.194369 | 27.92065 |
| CUST_56761_PI429010886 | M114598 | 0.013647 | 15 | 12 | | | | | | 1721.92 | 1802.411 | 1780.629 | 1302.682 | 1231.516 | 1039.457 | 1409.58 | 1421.097 | 1262.411 | 1680.099 | 1493.465 | 1811.851 | 1778.896 | 1914.945 | 2217.377 | 2119.433 | 1796.352 | 1307.203 | 1664.492 | 1857.991 | 1555.864 | 2595.092 | 1627.141 | 1582.373 | 2195.833 | 1498.316 | 1751.759 |
| CUST_31663_PI429010886 | M2104 | 0.013653 | 15 | 0 | | | | | | 1666.923 | 1688.453 | 1366.468 | 898.9921 | 1265.474 | 1299.165 | 855.218 | 987.5052 | 765.0242 | 1382.607 | 1277.111 | 1132.649 | 628.4644 | 1181.018 | 1024.808 | 2042.507 | 1392.643 | 1485.965 | 1381.449 | 1555.197 | 1124.753 | 1675.386 | 894.2724 | 1020.365 | 1885.749 | 1249.958 | 1436.981 |
| CUST_46173_PI429010886 | M71666 | 0.013658 | 21 | 0 | | | | | | 31.75251 | 40.46037 | 34.94529 | 37.02581 | 32.03971 | 29.66445 | 16.56322 | 28.65357 | 22.85484 | 29.75085 | 28.07949 | 48.80089 | 28.48354 | 28.03701 | 33.83579 | 32.17021 | 23.09604 | 31.7268 | 30.70184 | 58.98538 | 30.85032 | 33.69477 | 44.29926 | 31.96946 | 29.49942 | 32.58962 | 32.26619 |
| CUST_3513_PI429010886 | N14661 | 0.013659 | 18 | 6 | | | | | | 222.4378 | 195.1881 | 79.91242 | 269.6262 | 245.1154 | 33.33046 | 257.5143 | 441.3844 | 589.6619 | 400.3298 | 298.7909 | 350.0282 | 259.5337 | 422.0878 | 216.3677 | 334.5946 | 611.3709 | 108.4414 | 116.8442 | 253.3366 | 131.5965 | 395.8483 | 99.95963 | 117.5373 | 554.3622 | 172.4652 | 454.6218 |
| CUST_33066_PI429010886 | M5880 | 0.013661 | 24 | 3 | 358442122 | Broad-complex protein isoform 4 [Penaeus monodo | | | 1.63E-24 | 94.51496 | 213.1848 | 143.6492 | 215.9006 | 169.8718 | 265.7722 | 111.9784 | 125.0002 | 121.0512 | 87.68861 | 40.27161 | 173.8566 | 88.65038 | 80.70446 | 35.51693 | 88.49089 | 29.53078 | 110.0025 | 45.73272 | 91.02196 | 205.8829 | 108.6889 | 275.7295 | 14.74948 | 106.5468 | 239.9392 | 79.08585 |
| CUST_15564_PI429010886 | N34794 | 0.013662 | 15 | 9 | | | | | | 138.2381 | 181.7533 | 167.0511 | 157.0763 | 159.948 | 162.7472 | 152.0579 | 175.7472 | 147.8312 | 177.2148 | 171.451 | 200.337 | 185.1373 | 202.4996 | 169.7593 | 181.075 | 145.5994 | 165.6398 | 174.1985 | 162.473 | 153.0166 | 167.6742 | 183.266 | 176.8527 | 188.0879 | 170.6324 | 198.1863 |
| CUST_49685_PI429010886 | M86700 | 0.013679 | 18 | 15 | | | | | | 28.65923 | 39.63103 | 36.83973 | 23.37424 | 20.67573 | 15.40711 | 24.03089 | 9.895282 | 18.76417 | 50.79816 | 33.08815 | 57.1457 | 31.94735 | 35.24786 | 34.80955 | 46.682 | 48.17007 | 34.13077 | 48.26954 | 6.343801 | 28.46803 | 19.40925 | 45.25329 | 22.21172 | 26.51217 | 22.18561 | 32.16133 |
| CUST_24177_PI429010886 | N50173 | 0.013698 | 18 | 3 | 219809644 | Kazal-type serine proteinase inhibitor 1 [Fennerope | Protein metabolism | Proteolysis | 1.33E-11 | 16.33892 | 28.75284 | NA | 114.845 | 183.1759 | 199.9107 | 60.95084 | 12.43137 | 50.26445 | 7.103537 | 5.697389 | 8.256397 | 13.43228 | 54.03548 | 88.08405 | 194.6296 | 12.09352 | 171.6462 | 21.32603 | 19.80974 | 14.31222 | 174.8583 | 11.22009 | 128.8389 | 74.66823 | 6.506768 | 6.606034 |
| CUST_44051_PI429010886 | M64767 | 0.01375 | 24 | 21 | | | | | | 77.38027 | 104.7731 | 110.1494 | 85.06299 | 89.22919 | 106.9663 | 85.03701 | 101.1328 | 84.97695 | 86.20359 | 97.82533 | 103.1588 | 79.18359 | 81.69291 | 92.77688 | 105.0857 | 106.9663 | 97.13391 | 88.52351 | 109.9302 | 95.91781 | 121.2504 | 105.2542 | 111.4706 | 96.21869 | 89.30785 | 99.06334 |
| CUST_43021_PI429010886 | M61878 | 0.013753 | 24 | 21 | 242007661 | conserved hypothetical protein [Pediculus humanus | | | 1.2E-29 | 71.31512 | 90.53385 | 69.4087 | 78.48034 | 63.15525 | 73.84429 | 65.56207 | 69.16983 | 61.34598 | 88.00476 | 67.80419 | 91.14746 | 78.12789 | 79.875 | 66.2145 | 92.98929 | 59.59032 | 69.17707 | 86.27625 | 77.2785 | 74.92755 | 97.30444 | 81.61454 | 108.4314 | 93.58403 | 72.27253 | 80.29008 |
| CUST_43097_PI429010886 | M62059 | 0.013753 | 24 | 21 | | | | | | 208.3184 | 252.8575 | 245.2402 | 213.7802 | 192.898 | 225.9175 | 170.3686 | 196.157 | 213.1567 | 207.0153 | 211.1942 | 263.2877 | 216.6058 | 258.3105 | 247.9064 | 252.3543 | 185.7639 | 233.5692 | 196.6789 | 215.6344 | 265.532 | 230.2231 | 275.9326 | 291.8868 | 269.0306 | 244.4297 | 271.6624 |
| CUST_660_PI429010886 | N6611 | 0.013753 | 24 | 3 | | | | | | 190.62 | 150.434 | 117.6303 | 353.1722 | 154.1139 | 137.7352 | 279.8719 | 102.0005 | 182.0202 | 116.2092 | 109.002 | 109.7061 | 134.6919 | 115.5 | 125.5232 | 123.8447 | 96.5167 | 115.1567 | 98.33611 | 95.41159 | 88.52351 | 140.147 | 129.5737 | 102.2534 | 75.32357 | 95.70219 | 86.02859 |
| CUST_37523_PI429010886 | M19361 | 0.013756 | 21 | 9 | 333470610 | Cherax quadricarinatus alpha-I tubulin mRNA, comp | Cellular component organization | Cytoskeleton organization | | 11.91408 | 46.32451 | 9.467102 | 66.73255 | 166.4042 | 51.44726 | 52.99425 | 362.4143 | 268.079 | 37.71676 | 225.4823 | 416.1299 | 295.1449 | 21.81525 | 152.0345 | 9.585988 | 96.26654 | NA | 47.04186 | 269.9093 | NA | NA | 41.23292 | 29.53078 | 6.19085 | 172.1642 | 183.574 |
| CUST_30552_PI429010886 | N62113 | 0.01376 | 15 | 3 | | | | | | 9.175781 | 5.994911 | 10.73906 | 16.78198 | 58.67766 | 31.73942 | 62.77579 | 10.45833 | 8.060022 | 5.389345 | 27.01225 | 17.26917 | 11.9678 | 3.954561 | 4.207245 | 5.140205 | 40.79309 | 12.56396 | 49.51255 | NA | 16.15388 | 4.182504 | 26.64032 | 20.81168 | 10.04605 | 9.291998 | 4.688175 |
| CUST_13613_PI429010886 | N31470 | 0.013761 | 24 | 0 | | | | | | 23.53335 | 21.96744 | 27.47176 | 10.05995 | 17.58162 | 7.182647 | 26.8539 | 12.03065 | 12.4643 | 20.52907 | 14.64693 | 18.65486 | 9.889085 | 14.1623 | 13.64415 | 17.76828 | 13.44859 | 15.56439 | 8.950802 | 13.34056 | 10.98836 | 19.44365 | 19.02605 | NA | 19.07791 | 29.44539 | 31.25517 |
| CUST_14937_PI429010886 | N33725 | 0.013768 | 18 | 12 | | | | | | 187.8401 | 224.9395 | 210.3675 | 159.0925 | 172.7709 | 182.6397 | 199.1321 | 244.3099 | 174.8815 | 220.9066 | 196.1002 | 249.7124 | 242.041 | 231.1023 | 272.4885 | 282.5938 | 236.9573 | 193.8995 | 218.636 | 277.3932 | 182.8782 | 270.6283 | 197.4721 | 162.6286 | 214.3921 | 192.0579 | 285.0949 |
| CUST_28668_PI429010886 | N58395 | 0.013774 | 18 | 9 | | | | | | 53.57981 | 68.17959 | 81.30299 | 51.31578 | 52.27055 | 31.02908 | 26.39223 | 72.6348 | 89.07632 | 103.6887 | 96.9916 | 133.2442 | 64.42438 | 70.53195 | 81.93796 | 78.00387 | 71.43726 | 63.08907 | 70.57795 | 123.9078 | 89.21981 | 76.29064 | 76.95909 | 39.92653 | 116.4869 | 87.39325 | 113.5017 |
| CUST_44503_PI429010886 | M66158 | 0.01379 | 15 | 3 | | | | | | 69.27267 | 70.09922 | 63.96144 | 78.38473 | 78.73486 | 73.62986 | 66.70229 | 64.25402 | 55.72785 | 73.0191 | 68.85742 | 81.20466 | 53.46055 | 54.076 | 49.21663 | 180.8735 | 82.55028 | 67.81299 | 77.45737 | 85.62903 | 61.27293 | 75.37057 | 64.27512 | 69.00612 | 68.56896 | 57.24358 | 59.86271 |
| CUST_2037_PI429010886 | N11382 | 0.013812 | 12 | 0 | | | | | | 348.906 | 456.3557 | 435.9034 | 249.8933 | 331.3738 | 323.4981 | 300.6766 | 320.1726 | 320.7508 | 423.1161 | 335.1917 | 339.2296 | 355.193 | 422.3809 | 405.9943 | 432.25 | 317.3817 | 233.7572 | 356.0402 | 435.6005 | 381.737 | 426.3841 | 367.7937 | 346.5745 | 396.3895 | 362.1966 | 511.9204 |
| CUST_34874_PI429010886 | M10960 | 0.013816 | 21 | 9 | 543742248 | PREDICTED: UDP-glucuronosyltransferase 2A2-like i | Metabolic process | Other | 1.19E-12 | 4.783584 | 26.84667 | 7.710119 | 30.70184 | 65.95253 | 14.87518 | 41.36078 | 233.0458 | 124.5589 | 26.08978 | 269.9974 | 235.2753 | 171.9322 | 13.21509 | 94.127 | NA | 43.85239 | NA | 8.423847 | 507.983 | 198.3401 | NA | 17.68374 | 16.68994 | NA | 112.9528 | 106.8828 |
| CUST_23979_PI429010886 | N49811 | 0.013816 | 21 | 12 | | | | | | 100.7779 | 122.84 | 115.8883 | 94.69116 | 91.30112 | 101.7859 | 72.09388 | 95.20545 | 70.50114 | 87.75721 | 75.66735 | 106.2054 | 127.112 | 119.2623 | 119.7198 | 119.3486 | 97.79572 | 93.51914 | 107.6458 | 116.1482 | 103.1588 | 124.4949 | 68.5444 | 119.1637 | 105.3648 | 75.51588 | 95.47811 |
| CUST_46450_PI429010886 | M72682 | 0.01382 | 18 | 12 | | | | | | 15.99431 | 6.107648 | 9.748744 | 12.35487 | 15.22485 | 12.87351 | 11.57436 | 18.52719 | 15.81392 | 22.57964 | 14.1684 | 11.14181 | 15.55875 | 14.80364 | 21.10951 | 18.54155 | 16.36491 | 15.07014 | 11.09239 | 9.775801 | 6.883094 | 12.41217 | 12.52875 | 11.21554 | 16.75121 | 16.51414 | 10.52136 |
| CUST_49531_PI429010886 | M85986 | 0.013821 | 18 | 12 | | | | | | 2811.816 | 2926.98 | 4225.122 | 1230.999 | 1613.882 | 867.8685 | 3126.195 | 4499.121 | 3503.673 | 4293.225 | 2166.108 | 2343.419 | 4038.67 | 4839.291 | 2740.182 | 3483.315 | 3713.677 | 1783.909 | 2908.57 | 2543.989 | 2013.592 | 2491.849 | 3477.696 | 1750.319 | 4123.826 | 1935.871 | 3977.496 |
| CUST_6680_PI429010886 | N20453 | 0.013838 | 15 | 9 | 405966469 | Regucalcin [Crassostrea gigas] | Metabolic process | Other | 3.88E-62 | 26.45722 | 66.06376 | 57.94186 | 53.35562 | 91.72554 | 31.36122 | 60.87936 | 210.7138 | 181.3801 | 64.85632 | 142.4549 | 129.3352 | 176.94 | 69.15357 | 88.11918 | 23.69143 | 45.64241 | 31.68203 | 54.01314 | 224.1286 | 45.81043 | 56.38454 | 79.60953 | 66.50645 | 58.26543 | 79.60953 | 131.7874 |
| CUST_2954_PI429010886 | N13465 | 0.013851 | 18 | 3 | 270046092 | serine proteinase inhibitor [Marsupenaeus japonicu | | | 1.5E-90 | 1201.959 | 348.178 | 711.6467 | 1265.003 | 610.0761 | 1007.936 | 1542.262 | 841.0533 | 1002.084 | 436.8338 | 506.9726 | 524.9601 | 934.2348 | 416.025 | 430.6917 | 659.6239 | 643.3515 | 540.5402 | 956.7714 | 232.1489 | 659.4054 | 706.7803 | 990.5143 | 1259.079 | 679.5311 | 841.7695 | 720.0434 |
| CUST_45500_PI429010886 | M69310 | 0.013853 | 24 | 3 | | | | | | 39.36593 | 53.13993 | 79.45781 | 79.73386 | 103.1763 | 52.38937 | 94.89615 | 37.18815 | 51.91715 | 90.33731 | 27.18775 | 48.40257 | 93.55728 | 51.53321 | 76.2979 | 30.17815 | 17.61748 | 17.01718 | 18.88981 | 34.67355 | 26.10687 | 5.500018 | 132.6901 | 27.2627 | NA | 38.16452 | 18.32738 |
| CUST_7127_PI429010886 | N21078 | 0.013877 | 18 | 3 | 307213045 | HemK methyltransferase family member 1 [Harpegn | Protein metabolism | Cellular protein modification process | 2.52E-57 | 30.79365 | 26.16563 | 22.59879 | 38.05463 | 29.55843 | 22.82168 | 23.4832 | 23.78915 | 21.96101 | 24.01772 | 17.47076 | 19.24611 | 19.78047 | 24.62111 | 22.1046 | 29.74413 | 18.14179 | 33.76983 | 17.22225 | 15.79004 | 21.7968 | 30.95933 | 31.17772 | 33.91698 | 27.69389 | 29.18761 | 24.34275 |
| CUST_19896_PI429010886 | N42409 | 0.013878 | 12 | 9 | | | | | | 232.1818 | 224.6063 | 218.804 | 201.5302 | 194.4818 | 207.4667 | 153.8729 | 183.6909 | 199.1974 | 254.0209 | 236.9951 | 238.5008 | 182.9016 | 245.3246 | 170.3686 | 214.9913 | 148.5892 | 183.4152 | 204.4866 | 216.4729 | 235.6934 | 226.6106 | 260.3136 | 263.1737 | 274.9598 | 270.4039 | 278.7763 |
| CUST_21306_PI429010886 | N44988 | 0.013894 | 24 | 0 | 312371207 | hypothetical protein AND_22408 [Anopheles darlin | | | 1.89E-21 | 13.4766 | 15.70408 | 7.093765 | 4.956134 | 7.810859 | NA | 9.838949 | 4.210974 | 6.843848 | 10.11651 | 9.613143 | NA | 4.734116 | 6.975434 | 6.256959 | 7.715488 | 12.32915 | 10.38108 | 8.724159 | 8.443998 | 12.57101 | 12.08102 | 8.260264 | 10.12151 | 16.29644 | 10.90827 | 15.51399 |
| CUST_39112_PI429010886 | M34694 | 0.013905 | 18 | 15 | | | | | | 818.4343 | 457.1179 | 571.4497 | 192.1973 | 953.9498 | 558.7985 | 643.7576 | 511.8133 | 463.7968 | 1214.782 | 525.1056 | 625.484 | 1016.074 | 944.6524 | 667.8908 | 664.1086 | 1146.628 | 1112.909 | 780.6851 | 979.7027 | 665.3114 | 854.2242 | 416.2434 | 319.007 | 255.0348 | 611.2062 | 677.5621 |
| CUST_8168_PI429010886 | N22637 | 0.01391 | 24 | 21 | | | | | | 32.35941 | 39.43541 | 51.192 | 39.79339 | 36.65319 | 41.92727 | 29.92689 | 25.71698 | 29.68582 | 30.44094 | 51.80467 | 41.45965 | 27.52478 | 49.02873 | 33.31614 | 39.93142 | 43.44238 | 46.46836 | 36.09146 | 33.17446 | 36.18556 | 46.58823 | 45.4027 | 63.52108 | 42.83757 | 40.79309 | 54.01314 |
| CUST_10772_PI429010886 | N26765 | 0.013921 | 15 | 9 | | | | | | 26.22826 | 22.53631 | 27.80787 | 22.3788 | 22.12374 | 18.41342 | 16.16705 | 28.23315 | 13.92315 | 32.66922 | 30.00897 | 31.277 | 23.07266 | 16.79285 | 23.49374 | 35.67075 | 23.99863 | 26.13969 | 19.82681 | 28.45711 | 19.22658 | 30.79653 | 20.00677 | 28.46316 | 28.59435 | 40.87555 | 30.17169 |
| CUST_27024_PI429010886 | N55314 | 0.013921 | 15 | 9 | | | | | | 50.59835 | 61.8328 | 53.99759 | 35.73252 | 43.20694 | 38.30957 | 54.35965 | 48.8316 | 42.68463 | 68.78674 | 48.84923 | 85.46155 | 47.30806 | 62.63151 | 51.7068 | 68.65348 | 48.38349 | 68.82274 | 65.09219 | 56.4484 | 43.19279 | 81.19526 | 65.62571 | 31.70409 | 84.89465 | 80.75638 | 83.78522 |
| CUST_25720_PI429010886 | N52938 | 0.013925 | 21 | 15 | | | | | | 57.75281 | 69.72451 | 86.48234 | 60.79572 | 78.13459 | 93.34264 | 67.95716 | 64.28764 | 78.07938 | 61.44485 | 51.9493 | 67.19434 | 68.99159 | 96.73765 | 73.48596 | 109.6567 | 104.6528 | 83.89746 | 72.76448 | 86.66049 | 108.6469 | 87.20925 | 80.33892 | 81.10241 | 109.6802 | 77.98525 | 74.29802 |

| Probe ID | ID | p-value | Period | Phase | EBI ID | Annotation | GO category | Go term | E-value | 0 | 0 | 0 | 3 | 3 | 3 | 6 | 6 | 6 | 9 | 9 | 9 | 12 | 12 | 12 | 15 | 15 | 15 | 18 | 18 | 18 | 21 | 21 | 21 | 24 | 24 | 24 |
|---|---|---|---|---|---|---|---|---|---|---|---|---|---|---|---|---|---|---|---|---|---|---|---|---|---|---|---|---|---|---|---|---|---|---|---|---|
| CUST_33324_PI429010886 | M6599 | 0.013926 | 24 | 15 | | | | | | 326.9766 | 545.1015 | 508.6284 | 195.9288 | 362.9082 | 285.5481 | 482.976 | 324.1857 | 409.7595 | 410.9902 | 396.9018 | 266.4363 | 418.9595 | 530.2702 | 397.5988 | 780.9267 | 496.5233 | 567.3609 | 694.7611 | 361.0653 | 496.5233 | 629.9728 | 639.1213 | 447.5499 | 879.6665 | 440.6954 | 581.2638 |
| CUST_29930_PI429010886 | N60836 | 0.013928 | 21 | 9 | | | | | | 35.98798 | 34.8364 | 46.69134 | 13.85986 | 36.12687 | 29.65821 | 35.47721 | 44.94616 | 23.46033 | 53.00119 | 50.02767 | 59.42335 | 29.02633 | 52.26577 | 34.90742 | 46.0983 | 41.65544 | 29.53078 | 38.22525 | 45.66434 | 18.47407 | 34.57676 | 36.40505 | 28.22043 | 31.13099 | 26.60013 | 31.03692 |
| CUST_46427_PI429010886 | M72634 | 0.013976 | 24 | 0 | | | | | | 94.127 | 81.87168 | 105.0857 | 93.96395 | 77.10171 | 84.96672 | 78.53416 | 78.57092 | 70.63158 | 86.65167 | 79.74735 | 52.77021 | 90.77809 | 65.54211 | 79.89836 | 109.5173 | 88.05483 | 65.17572 | 73.79476 | 52.56971 | 62.67301 | 177.6145 | 92.31619 | 110.5384 | 154.6176 | 143.4291 | 156.8624 |
| CUST_15321_PI429010886 | N34396 | 0.01398 | 15 | 9 | | | | | | 14.10671 | 28.38786 | 25.4312 | 5.920956 | 12.00862 | 13.3861 | 14.32917 | 18.56579 | 19.50301 | 27.60111 | 14.17989 | 31.53429 | 18.32343 | 26.43248 | 26.06265 | 23.29568 | 16.77475 | 20.74695 | 18.91018 | 15.91395 | 18.01173 | 30.27835 | 14.58718 | 25.98413 | 24.37698 | 19.74282 | 27.23134 |
| CUST_17764_PI429010886 | N38551 | 0.013986 | 24 | 12 | 325303532 | TPA_inf: hypothetical secreted protein 1935 [Ambly | | | 1.22E-12 | 22.94293 | 20.35771 | 18.44972 | 17.63322 | 20.08306 | 6.080738 | 18.08051 | 12.29647 | 20.11281 | 22.72881 | 15.14331 | 19.3376 | 27.43309 | 25.69293 | 29.48277 | 22.95471 | 24.8962 | 19.58231 | 16.00542 | 13.25777 | 21.07965 | 23.94925 | 18.98392 | 26.62182 | 26.39223 | 12.75753 | 14.62779 |
| CUST_36828_PI429010886 | M16972 | 0.013992 | 24 | 18 | | | | | | 22.97432 | 26.95285 | 19.57848 | 33.07929 | 20.19663 | 36.46662 | 19.67133 | 12.22639 | 27.70733 | 24.42522 | 18.07527 | 23.5585 | 31.2636 | 14.97 | 46.43129 | 35.15108 | 18.53102 | 41.96451 | 50.78563 | 40.13905 | 54.74747 | 25.55311 | 30.9788 | 39.87971 | 23.5068 | 72.44795 | 22.4606 |
| CUST_35668_PI429010886 | M13197 | 0.013998 | 18 | 15 | 291242803 | PREDICTED: dehydrogenase/reductase (SDR family) | Metabolic process | Other | 3.5E-45 | 82.76433 | 84.722 | 55.9701 | 47.63195 | 56.69148 | 55.07968 | 56.31266 | 85.04454 | 70.42582 | 95.42654 | 73.35296 | 81.51675 | 79.30391 | 77.2245 | 78.92088 | 97.73561 | 65.03693 | 95.96344 | 74.27647 | 103.3023 | 73.09177 | 95.98918 | 53.56751 | 59.75541 | 61.32192 | 79.27531 | 69.00002 |
| CUST_7766_PI429010886 | N22043 | 0.014002 | 21 | 12 | | | | | | 13.4001 | 6.954585 | NA | 7.190888 | 4.542348 | NA | 5.099536 | 4.263728 | 7.11441 | 13.5705 | 9.679037 | 9.77251 | 13.86613 | NA | NA | 11.31226 | 7.895988 | 12.69552 | 19.3658 | 8.323544 | 9.180913 | 13.72174 | 14.79848 | NA | 8.982813 | 3.701795 | 6.836925 |
| CUST_3799_PI429010886 | N15294 | 0.014009 | 15 | 9 | | | | | | 46.25026 | 134.282 | 155.6229 | 6.9339 | 13.81863 | NA | 33.04717 | 139.545 | 287.3115 | 194.3859 | 229.245 | 333.5908 | 107.9692 | 432.5053 | 214.3213 | 211.5909 | 30.98764 | 63.91986 | 210.9062 | 210.3141 | 186.4588 | 254.2335 | 144.6868 | 43.5437 | 659.1977 | 235.4879 | 534.5603 |
| CUST_9980_PI429010886 | N25505 | 0.014022 | 24 | 0 | 395856453 | PREDICTED: E3 ubiquitin-protein ligase RNF123 [Otc | | | 5.12E-21 | 127.9641 | 144.5473 | 162.9465 | 144.9176 | 122.7034 | 95.61724 | 106.1639 | 108.5374 | 101.3914 | 167.9263 | 126.7933 | 116.1174 | 76.01313 | 93.19487 | 75.65779 | 120.0446 | 104.7461 | 87.45287 | 131.4476 | 167.3634 | 133.5378 | 139.359 | 104.6528 | 120.9334 | 185.2303 | 101.9095 | 130.5635 |
| CUST_52043_PI429010886 | M96183 | 0.014025 | 21 | 0 | | | | | | 18.09414 | 7.147189 | 11.60175 | 9.854344 | 6.397942 | NA | 8.279758 | 5.744005 | 9.692914 | 14.07538 | 4.282546 | 4.453369 | NA | 5.089752 | 3.911057 | 9.833527 | 3.536967 | 10.27034 | 5.950777 | 3.672976 | 14.39574 | NA | 22.64182 | 8.949545 | 10.44304 | 11.18429 | 9.771392 |
| CUST_19282_PI429010886 | N41306 | 0.014038 | 15 | 0 | | | | | | 83.26471 | 89.96525 | 79.27531 | 72.81129 | 32.26938 | 50.34321 | 46.73511 | 67.46884 | 30.73873 | 67.42469 | 59.0152 | 46.1807 | 69.56167 | 66.32627 | 63.6369 | 76.55655 | 51.4 | 73.82839 | 65.93142 | 67.60441 | 59.2457 | 67.98118 | 52.63886 | 48.77592 | 73.803 | 49.9806 | 81.30299 |
| CUST_14511_PI429010886 | N33000 | 0.014066 | 24 | 21 | | | | | | 107.6007 | 75.58303 | 103.1666 | 116.3563 | 75.44459 | 137.0338 | 73.67904 | 91.59828 | 70.3766 | 98.21029 | 103.202 | 129.896 | 87.42512 | 120.1692 | 111.637 | 105.4997 | 115.4344 | 112.8118 | 104.1782 | 100.5206 | 120.5952 | 119.3927 | 125.4203 | 130.0689 | 122.5827 | 81.51675 | 107.8556 |
| CUST_10470_PI429010886 | N26286 | 0.014073 | 15 | 12 | | | | | | 8.377586 | 6.474974 | 10.037 | 3.878647 | 4.476331 | NA | 9.754482 | 4.621301 | 4.72981 | 8.345317 | 6.075781 | 14.73565 | 6.770324 | 6.763654 | 15.93149 | 10.17638 | 4.756376 | 8.876178 | 5.505595 | 4.969785 | 5.824752 | 6.806239 | 6.264624 | 8.381146 | 11.8751 | 8.617079 | 5.922622 |
| CUST_10586_PI429010886 | N26471 | 0.014087 | 15 | 9 | | | | | | 11.36205 | 13.22835 | 3.591603 | 11.19977 | 21.18978 | 9.664283 | 13.76394 | 36.387 | 24.74137 | 8.931253 | 26.8214 | 36.58254 | 16.50225 | 17.31011 | 4.51755 | 4.179974 | 14.07874 | 13.06014 | 3.700971 | 17.00755 | 26.70117 | 4.280152 | 23.74339 | 44.67201 | 17.19336 | 30.59095 | 45.62788 |
| CUST_55247_PI429010886 | M109282 | 0.014098 | 24 | 0 | | | | | | 1395.016 | 1769.215 | 1870.503 | 1606.044 | 1419.795 | 1410.296 | 1144.142 | 1227.15 | 1408.714 | 1531.812 | 1315.839 | 2466.842 | 1245.02 | 1914.117 | 1468.172 | 1215.238 | 1152.374 | 1201.959 | 1248.908 | 1417.942 | 1585.767 | 1764.536 | 1371.349 | 1248.908 | 1622.403 | 1604.147 | 2075.258 |
| CUST_42128_PI429010886 | M59486 | 0.014103 | 24 | 18 | 242003110 | zinc finger protein DHHC domain containing protein | Metabolic process | Oxidation-reduction process | 2.44E-26 | 61.2655 | 50.98504 | 40.88839 | 38.36859 | 47.64282 | 36.8676 | 40.90354 | 30.3942 | 48.03245 | 50.89404 | 34.58379 | 48.84271 | 44.91724 | 59.06419 | 52.88574 | 70.90857 | 36.40199 | 49.12303 | 54.91034 | 71.33949 | 57.96698 | 58.22303 | 54.95061 | 34.81682 | 69.94604 | 45.12848 | 51.13109 |
| CUST_41586_PI429010886 | M58092 | 0.014159 | 24 | 3 | | | | | | 28.94785 | 48.50046 | 7.581211 | 34.36064 | 51.01709 | 61.50815 | 31.46479 | 12.51969 | 16.3681 | 10.29246 | 38.45312 | 8.360428 | 8.110471 | 3.68409 | 13.49626 | 12.79367 | 25.62877 | 11.64631 | 37.07575 | 12.70194 | 27.61589 | 6.131047 | 29.33547 | 23.13968 | 38.06259 | 19.3376 | 11.62851 |
| CUST_46682_PI429010886 | M73403 | 0.014165 | 24 | 21 | | | | | | 94.00857 | 67.32952 | 80.08977 | 64.2224 | 66.6859 | 77.94557 | 71.74926 | 62.94032 | 78.16064 | 85.32395 | 59.60108 | 61.2655 | 61.86332 | 100.4818 | 85.80553 | 99.94463 | 80.5678 | 86.27625 | 64.19796 | 85.96929 | 67.26789 | 95.25723 | 101.1468 | 95.08289 | 97.98092 | 66.15125 | 81.5369 |
| CUST_7484_PI429010886 | N21613 | 0.014165 | 24 | 15 | 157106769 | ankyrin 2,3/unc44 [Aedes aegypti] >gi\|108868779\| | | | 1.98E-29 | 16.88691 | 16.37668 | 7.349454 | 11.81981 | 14.10863 | 8.08569 | 35.51342 | 21.26849 | 21.46189 | 21.94448 | 26.8901 | 8.225765 | 22.09437 | 24.71578 | 23.31281 | 27.52267 | 31.35806 | 28.40282 | 28.9598 | 14.25774 | 16.64794 | 22.37068 | 17.70313 | 12.99442 | 29.94174 | 12.98486 | 40.30994 |
| CUST_43421_PI429010886 | M63002 | 0.014171 | 15 | 12 | | | | | | 31.36464 | 79.21035 | 63.51336 | 69.61278 | 41.73826 | 89.84183 | 53.51858 | 54.98545 | 61.40781 | 76.18529 | 75.10177 | 126.2993 | 89.6226 | 85.95 | 130.1677 | 75.05721 | 56.65296 | 69.09575 | 41.91362 | 66.73255 | 58.96311 | 61.74634 | 52.8747 | 65.87593 | 84.87934 | 57.49062 | 67.98998 |
| CUST_11519_PI429010886 | N27955 | 0.014171 | 15 | 12 | | | | | | 143.9489 | 132.7287 | 175.2864 | 123.349 | 129.7547 | 128.0141 | 124.0296 | 133.3318 | 121.7165 | 171.1195 | 127.0219 | 138.2766 | 157.9764 | 156.7058 | 171.0777 | 197.9029 | 137.9867 | 142.5067 | 140.8807 | 148.886 | 144.8962 | 154.8485 | 132.033 | 136.6618 | 156.0758 | 148.5046 | 163.3073 |
| CUST_11634_PI429010886 | N28156 | 0.014178 | 21 | 18 | 241726004 | mrg-binding protein, putative [Ixodes scapularis] >g | Nucleic acid metabolism | Regulation of gene expression | 1.92E-22 | 68.95801 | 72.32108 | 53.53068 | 56.95101 | 77.56897 | 52.66699 | 34.87758 | 39.41947 | 46.61273 | 81.13979 | 50.39093 | 75.43644 | 52.78112 | 70.80885 | 62.85713 | 72.9942 | 40.43694 | 71.52967 | 58.15467 | 83.27348 | 75.52934 | 57.9197 | 55.63288 | 53.83853 | 56.51605 | 52.46763 | 64.96286 |
| CUST_1140_PI429010886 | N8622 | 0.014187 | 15 | 12 | | | | | | 94.38745 | 58.73387 | 62.16793 | 14.23571 | 32.2878 | 20.72901 | 99.01075 | 44.33537 | 103.1763 | 93.28305 | 47.72724 | 92.10717 | 69.46349 | 129.8789 | 87.36382 | 40.74077 | 65.81652 | 33.08345 | 55.49391 | 52.801 | 50.65756 | 54.32801 | 104.964 | 10.25079 | 127.6888 | 61.16623 | 79.21035 |
| CUST_36059_PI429010886 | M14489 | 0.014187 | 24 | 21 | 504155460 | PREDICTED: EP300-interacting inhibitor of different | Response to stress | DNA repair | 1.01E-14 | 3.685407 | 6.964774 | 6.849092 | NA | 4.463887 | 6.668324 | 4.75422 | 3.410585 | 5.79109 | NA | 7.281858 | 8.189309 | NA | | 3.437117 | 14.80809 | 6.674655 | NA | 8.681407 | 11.13787 | 6.178456 | 37.0569 | 6.999938 | 24.05186 | 14.91902 | 9.286054 | 3.444707 |
| CUST_54026_PI429010886 | M104151 | 0.014189 | 15 | 3 | 321458008 | hypothetical protein DAPPUDRAFT_301164 [Daphn | Transport | Transmembrane transport | 1.34E-46 | 48.16109 | 42.59897 | 57.38433 | 40.99424 | 30.36684 | 81.00454 | 56.10477 | 31.32056 | 45.3248 | 36.46662 | 50.08673 | 36.85229 | 21.64916 | 22.94537 | 21.71424 | 24.07489 | 50.20569 | 86.97645 | 46.45766 | 72.32108 | 61.70387 | 31.09152 | 44.62585 | 37.5119 | 42.05465 | 19.01531 | 28.3053 |
| CUST_1025_PI429010886 | N8259 | 0.014213 | 24 | 3 | | | | | | 657.4366 | 19.82899 | 73.73041 | 1446.944 | 1094.71 | 2665.55 | 2203.176 | 922.8399 | 2162.684 | 10.72726 | 977.7803 | 119.7645 | 12.84456 | 27.00493 | 10.01115 | 37.2354 | 424.7309 | 499.859 | 874.1627 | 5.194327 | 7.867078 | NA | 194.9182 | 630.1031 | 6.31804 | 3118.308 | 31.69618 |
| CUST_16733_PI429010886 | N36772 | 0.014231 | 24 | 18 | | | | | | 108.2633 | 274.2312 | 187.0803 | 83.48026 | 99.93216 | 45.87335 | 4.8159 | 13.92172 | 60.25585 | 183.021 | 190.3637 | 186.3956 | 74.84295 | 120.4628 | 76.90742 | 347.1114 | 5.085315 | 246.9496 | 146.3399 | 315.3784 | 158.1305 | 256.4303 | 141.6025 | 54.27461 | 214.7985 | 115.202 | 122.4191 |
| CUST_37118_PI429010886 | M17907 | 0.014245 | 24 | 0 | | | | | | 16.19147 | 24.2001 | 15.84192 | 8.219245 | 16.35182 | 15.80056 | 16.07034 | 17.46532 | 25.78835 | 12.22036 | 13.4823 | 7.37785 | 6.832319 | 3.724963 | 14.60042 | 14.66496 | 14.24976 | 23.02215 | 17.29657 | 18.07887 | 16.69384 | 14.74259 | 12.56396 | 11.96331 | 25.10875 | 17.9732 | 18.31723 |
| CUST_42258_PI429010886 | M59819 | 0.014251 | 21 | 6 | | | | | | 27.53501 | 22.32739 | 15.98734 | 26.38969 | 48.40257 | 17.21816 | 40.53866 | 27.89422 | 38.02052 | 31.01397 | 30.13914 | 27.52766 | 24.41741 | 16.38475 | 27.17268 | 32.34301 | 20.33623 | 19.40925 | 15.1027 | 25.97092 | 29.14178 | 25.6517 | 23.4957 | 22.11953 | 30.3731 | 26.23528 | 33.24682 |
| CUST_1512_PI429010886 | N9885 | 0.014256 | 21 | 9 | | | | | | 5.717554 | 4.994508 | NA | NA | 15.83087 | 11.76447 | 4.11313 | 30.68719 | 16.06241 | NA | 32.23691 | 33.40939 | 20.28484 | 6.374853 | 17.76635 | 9.477835 | 12.50951 | 6.215347 | 4.126369 | 37.81058 | 10.15583 | NA | NA | 6.347106 | 5.130814 | 10.79019 | 8.622966 |
| CUST_56246_PI429010886 | M113258 | 0.014264 | 21 | 12 | | | | | | 764.0731 | 428.865 | 584.1415 | 235.6934 | 899.2493 | 584.5557 | 788.0347 | 559.1604 | 632.0615 | 1140.062 | 504.2482 | 628.8832 | 980.1159 | 747.2269 | 797.7975 | 633.9622 | 732.077 | 1057.689 | 698.0293 | 718.6829 | 590.4824 | 666.1474 | 346.6366 | 260.0898 | 244.904 | 560.5893 | 619.5429 |
| CUST_49538_PI429010886 | M86024 | 0.014279 | 24 | 18 | | | | | | 89.24114 | 114.2341 | 101.6113 | 80.63305 | 87.60236 | 76.924 | 81.42898 | 79.07032 | 69.9232 | 95.76592 | 94.02768 | 106.6154 | 80.49518 | 100.4233 | 125.0127 | 105.2191 | 99.74324 | 83.51601 | 94.36809 | 122.7816 | 104.8657 | 114.6944 | 103.6146 | 97.40947 | 117.847 | 102.2413 | 161.6447 |
| CUST_6126_PI429010886 | N19661 | 0.014298 | 18 | 12 | 118405102 | notchless homolog 1 [Xenopus (Silurana) tropicalis] | Developmental process | Embryo development | 1.1E-178 | 149.5758 | 197.2996 | 190.0964 | 115.2365 | 133.0452 | 93.46673 | 116.9387 | 137.2699 | 183.3264 | 187.5869 | 140.8068 | 206.8026 | 177.9616 | 204.3252 | 172.831 | 210.4575 | 116.9761 | 170.7891 | 143.2327 | 214.7684 | 137.9867 | 179.678 | 130.0885 | 107.1033 | 181.2186 | 154.9129 | 182.8109 |
| CUST_31989_PI429010886 | M2931 | 0.014305 | 18 | 15 | 117959988 | clottable protein [Marsupenaeus japonicus] | Transport | Lipid transport | 7.44E-30 | 33.24682 | 29.61255 | 28.11091 | 29.10818 | 38.04057 | 39.41947 | 29.95513 | 25.33083 | 22.19942 | 29.29818 | 46.00478 | 47.16106 | 32.82642 | 35.53394 | 28.35096 | 38.42916 | 68.56896 | 56.80732 | 30.68719 | 171.5147 | 28.71325 | 19.16725 | 34.06729 | 15.32313 | 30.89255 | 25.58851 | 13.90384 |
| CUST_44887_PI429010886 | M67322 | 0.014328 | 24 | 0 | | | | | | 58.51806 | 61.65538 | 60.93069 | 40.1071 | 30.77807 | 43.7253 | 37.33468 | 30.29627 | 42.13297 | 25.31882 | 31.8356 | 36.87579 | 28.83204 | 34.34807 | 44.41825 | 42.29841 | 26.38163 | 26.57549 | 43.90259 | 20.53839 | 60.66262 | 29.57425 | 48.23121 | 38.34742 | 41.83506 | 38.18132 | 42.65591 |
| CUST_19210_PI429010886 | N41175 | 0.014343 | 21 | 0 | | | | | | 71.38963 | 54.73744 | 72.73041 | 37.77438 | 58.15467 | 48.00964 | 55.10664 | 42.04002 | 30.60166 | 56.17296 | 56.40861 | 47.27714 | 30.06738 | 50.84587 | 39.1027 | 62.0828 | 64.88975 | 71.44429 | 56.56684 | 55.28288 | 67.13373 | 78.18464 | 51.45306 | 65.6728 | 64.05654 | 34.96354 | 32.26938 |
| CUST_36339_PI429010886 | M15646 | 0.01436 | 12 | 3 | | | | | | 1487.266 | 895.8021 | 1363.213 | 1369.558 | 1874.82 | 2900.662 | 1238.697 | 706.4958 | 132.8222 | 488.7111 | 485.4409 | 144.3143 | 1829.647 | 692.7803 | 1336.322 | 1098.749 | 1486.604 | 1786.373 | 810.6402 | 512.0216 | 743.1041 | 308.528 | 930.8848 | 1558.572 | 622.9374 | 2203.176 | 414.4964 |
| CUST_25558_PI429010886 | N52643 | 0.014408 | 21 | 12 | | | | | | 16.66456 | 33.99233 | 17.89329 | 20.63001 | 6.099554 | 9.135099 | 23.51171 | 33.5432 | 11.07141 | 8.613049 | 24.12233 | 22.72441 | 38.20449 | 28.97523 | 33.35026 | 43.60346 | NA | 20.97996 | 8.182672 | 17.93568 | 12.35967 | 20.98763 | 18.02234 | 15.39724 | 5.003893 | NA | 11.22153 |
| CUST_26448_PI429010886 | N54261 | 0.014412 | 21 | 0 | | | | | | 15.58918 | 13.42229 | 13.50907 | 6.974362 | 11.12902 | NA | 11.66655 | 4.717037 | 10.27148 | 3.599801 | 4.955498 | 8.974986 | NA | 21.69169 | 9.375364 | 10.91255 | 9.504182 | 14.28145 | 9.376909 | 7.742712 | 14.20342 | 9.745966 | 19.75359 | 20.82009 | 19.38567 | 8.229169 | 6.574274 |
| CUST_43719_PI429010886 | M63788 | 0.014418 | 24 | 15 | | | | | | 11.9801 | 15.48667 | 17.38288 | 7.578825 | 12.49757 | 12.41645 | 4.739251 | 15.13956 | 9.312357 | 13.64945 | 7.521524 | 15.60949 | 13.61539 | 20.6453 | 23.1605 | 22.67085 | 17.7649 | 20.11768 | 48.89187 | 14.01909 | 13.80666 | 19.44494 | 18.18793 | 12.27788 | 19.54896 | 19.81675 | 16.99042 |
| CUST_33859_PI429010886 | M8063 | 0.014418 | 21 | 12 | 293629632 | hemocyanin HC2 [Limnoria quadripunctata] | | | | NA | 112.5301 | 42.41265 | 373.6035 | 219.0917 | 38.79996 | 136.9448 | 1320.152 | 275.7295 | 160.8644 | 1174.994 | 1426.735 | 3046.724 | 60.68591 | 2012.591 | NA | 544.3999 | NA | 47.56097 | 22140.21 | 373.2026 | NA | 27.08074 | 30.48036 | NA | 678.2021 | 883.6545 |
| CUST_32019_PI429010886 | M3013 | 0.01443 | 15 | 9 | | | | | | 1574.342 | 1872.445 | 1674.38 | 1120.861 | 1296.822 | 1050.102 | 1302.095 | 1300.946 | 1354.066 | 1817.335 | 1355.174 | 1821.885 | 1382.151 | 1752.574 | 1770.146 | 2095.03 | 1419.195 | 1314.491 | 1434.403 | 1582.373 | 1268.692 | 1892.721 | 1645.771 | 1243.416 | 2096.527 | 1520.67 | 1878.86 |
| CUST_6843_PI429010886 | N20671 | 0.01444 | 15 | 9 | | | | | | 206.4448 | 288.2204 | 303.854 | 227.7485 | 267.7292 | 345.4444 | 298.8597 | 247.2314 | 347.9148 | 379.8142 | 321.753 | 349.6083 | 275.3091 | 379.3496 | 268.3811 | 248.1506 | 284.093 | 300.9186 | 294.1484 | 265.6599 | 345.1018 | 99.74324 | 330.2472 | 309.3892 | 417.9411 | 401.4797 | 375.3397 |
| CUST_7274_PI429010886 | N21303 | 0.01444 | 24 | 21 | | | | | | 29.49638 | 37.82706 | 24.71814 | 21.14577 | 28.06937 | 38.121 | 35.24075 | 23.37682 | 22.44547 | 23.63583 | 28.26309 | 26.35743 | 30.43732 | 21.40881 | 28.44656 | 28.24369 | 37.27794 | 31.04579 | 28.45711 | 38.77656 | 32.80137 | 37.25844 | 43.0609 | 40.23396 | 17.95297 | 30.08229 | 19.31282 |
| CUST_50759_PI429010886 | M90912 | 0.014443 | 15 | 9 | | | | | | 5.367511 | 16.8666 | 35.78629 | NA | NA | NA | NA | 19.56129 | 44.22939 | 18.07527 | 49.49452 | 65.36011 | 24.32573 | 65.86348 | 44.42567 | 20.39383 | NA | 11.44313 | 13.34528 | 46.44351 | 14.46019 | 69.90395 | 37.88493 | NA | 94.71285 | 43.50773 | 79.72112 |
| CUST_23513_PI429010886 | N48957 | 0.014452 | 21 | 3 | 356541209 | PREDICTED: uncharacterized protein LOC10079005: | | | 4.44E-07 | 691.1905 | 1376.551 | 854.951 | 2042.507 | 997.0196 | 1151.226 | 717.1047 | 672.5182 | 756.3176 | 852.3106 | 482.287 | 795.7542 | 596.3171 | 836.1144 | 573.39 | 799.5941 | 430.7898 | 711.6467 | 500.086 | 683.5585 | 1085.118 | 890.6855 | 1059.331 | 694.9126 | 1079.829 | 1268.692 | 618.8739 |
| CUST_6443_PI429010886 | N20106 | 0.014453 | 21 | 15 | 260803443 | hypothetical protein BRAFLDRAFT_219212 [Branchi | | | 9.28E-30 | 67.17456 | 115.9278 | 66.64273 | 58.56187 | 64.09512 | 40.75734 | 29.87357 | 48.52803 | 50.59331 | 69.53162 | 52.24698 | 75.80349 | 65.86348 | 76.70634 | 64.21464 | 104.2212 | 65.64906 | 78.89557 | 60.65096 | 60.80176 | 69.45519 | 107.9121 | 64.07157 | 54.75352 | 78.84971 | 48.66621 | 68.90417 |
| CUST_32074_PI429010886 | M3166 | 0.014455 | 24 | 15 | 332376270 | unknown [Dendroctonus ponderosae] | | | 9.19E-45 | 29.2558 | 22.42598 | 25.39002 | 15.32584 | 17.58482 | 27.60309 | 23.57785 | 25.17616 | 23.70291 | 41.10188 | 31.43005 | 30.95577 | 27.70733 | 28.42343 | 28.40282 | 35.49419 | 38.85712 | 29.40744 | 34.91643 | 36.89793 | 26.60537 | 32.18039 | 39.32573 | 30.16372 | 37.86895 | 23.70771 | 44.48344 |
| CUST_54362_PI429010886 | M105728 | 0.014469 | 15 | 3 | | | | | | 88.57155 | 49.05418 | 76.28297 | 71.38156 | 74.86726 | 121.5266 | 62.64364 | 100.7779 | 46.05816 | 33.93351 | 68.98284 | 34.2288 | 93.49845 | 47.40164 | 45.2026 | 47.67267 | 219.5427 | 125.3551 | 207.7427 | 59.65312 | 108.4668 | 64.15379 | 208.5959 | 164.9098 | 49.8518 | 46.86688 | 43.47746 |
| CUST_40523_PI429010886 | M55361 | 0.014474 | 18 | 12 | 270013391 | hypothetical protein TcasGA2_TC011986 [Tribolium | Cellular Process | Cell proliferation | 1.81E-93 | 19.05109 | 15.67634 | 26.56221 | 16.5578 | 14.2983 | 19.59849 | 29.11372 | 29.21058 | 21.01736 | 21.25435 | 24.32948 | 9.873369 | 25.32554 | 35.09563 | 26.21415 | 17.39698 | 30.27835 | 15.86747 | 37.83427 | 15.79894 | 18.01669 | 8.796716 | 18.59345 | 12.83026 | 20.52725 | 19.25276 | 20.53338 |
| CUST_36990_PI429010886 | M17473 | 0.014479 | 15 | 0 | | | | | | 20.4859 | 26.13131 | 20.23721 | 22.82685 | 10.8172 | 31.15277 | 13.29711 | 11.79794 | 7.138401 | NA | 11.24847 | 25.68743 | 16.82979 | 19.29593 | 11.55948 | 24.23967 | 7.663615 | 16.20429 | 11.95482 | 16.8955 | 19.6916 | 11.13485 | 9.999252 | 9.712939 | 14.35419 | 14.1198 | 15.47412 |
| CUST_4479_PI429010886 | N16859 | 0.014479 | 15 | 9 | 332372624 | unknown [Dendroctonus ponderosae] | Cellular component organization | Chromosome organization | 1.17E-39 | 22.91473 | 16.57948 | 12.11676 | 10.17638 | 11.26822 | 13.072 | 6.398941 | 13.58403 | 18.3383 | 22.88601 | 18.28609 | 21.9738 | 8.789967 | 12.11941 | 13.7932 | 21.09759 | NA | 15.61967 | 10.08948 | 12.24024 | 15.7734 | 15.13318 | 18.16713 | 14.95825 | 22.42169 | 18.24734 | 16.63365 |
| CUST_19856_PI429010886 | N42326 | 0.014504 | 24 | 9 | 149409130 | PREDICTED: COP9 signalosome complex subunit 3 [( | Developmental process | Embryo development | 5.39E-45 | 11.22575 | 6.522752 | NA | 9.748744 | 7.488246 | NA | 9.413901 | NA | 10.87507 | 21.45934 | 9.858236 | 12.64557 | 9.441058 | 10.86536 | 9.049354 | 12.04909 | 3.961673 | 7.490316 | 10.23894 | 4.495511 | 7.753636 | 6.143836 | NA | NA | 5.049338 | 5.888357 | 5.710989 |
| CUST_36812_PI429010886 | M16930 | 0.014506 | 15 | 9 | | | | | | 154.2483 | 112.3805 | 140.6429 | 101.8776 | 50.47205 | 72.53579 | 63.83994 | 86.2674 | 124.3005 | 142.6158 | 84.06167 | 144.77 | 104.6828 | 76.2979 | 103.1477 | 88.22213 | 93.62063 | 101.3168 | 58.36485 | 62.54347 | 83.49596 | 66.88544 | 93.19487 | 59.22654 | 105.5208 | 95.94174 | 144.2346 |
| CUST_34135_PI429010886 | M8857 | 0.014511 | 24 | 21 | | | | | | 12.76373 | 25.00056 | 9.995172 | 38.00715 | 14.51836 | 9.809268 | 14.85609 | 21.33093 | 30.3942 | 14.53785 | 9.717046 | 14.48109 | 19.24305 | 9.99769 | 16.53332 | 29.50933 | 15.06396 | 20.66296 | 33.56428 | 20.68548 | 18.93949 | 25.04089 | 36.72937 | 31.12392 | 18.48046 | 33.84232 | 41.16431 |
| CUST_25675_PI429010886 | N52859 | 0.014511 | 24 | 18 | | | | | | 34.42302 | 28.77383 | 48.97519 | 38.46752 | 44.83381 | 42.20313 | 44.15212 | 35.81064 | 30.15647 | 42.59502 | 43.97029 | 54.86684 | 38.35721 | 68.80103 | 64.64151 | 42.11724 | 30.44767 | 56.7825 | 65.41017 | 50.39093 | 71.30124 | 82.23746 | 59.06419 | 56.74499 | 40.11789 | 45.00065 | 50.89404 |
| CUST_52255_PI429010886 | M96865 | 0.014525 | 12 | 3 | | | | | | 38.04406 | 40.84477 | 44.08227 | 106.7511 | 82.73488 | 113.3684 | 89.37758 | 62.53094 | 128.037 | 52.51409 | 13.88785 | 27.0144 | 28.53623 | 55.42181 | 62.71467 | 114.6191 | 40.33441 | 43.35936 | 87.68861 | 54.73744 | 90.06088 | 32.40014 | 39.50511 | 41.20481 | 60.66262 | 71.31512 | 103.654 |
| CUST_5861_PI429010886 | N19306 | 0.014525 | 12 | 3 | 405976099 | Annexin A7 [Crassostrea gigas] | | | 5.03E-73 | 589.9381 | 577.0235 | 353.962 | 545.9123 | 608.8662 | 692.5393 | 498.7637 | 424.2638 | 365.7398 | 358.7125 | 285.5481 | 338.5409 | 463.9991 | 525.2083 | 504.3528 | 615.7661 | 328.5456 | 544.5308 | 477.6391 | 392.3971 | 400.7798 | 438.5463 | 264.5866 | 569.7753 | 432.8549 | 407.1309 | 459.0733 |
| CUST_3578_PI429010886 | N14809 | 0.014531 | 21 | 9 | 241999376 | ubiquitin protein ligase [Ixodes scapularis] >gi\|2154 | Cellular Process | Cell cycle | 6.57E-33 | 82.74334 | 138.4086 | 101.6485 | 141.3981 | 144.7965 | 111.7362 | 97.58263 | 257.237 | 218.9512 | 145.3839 | 272.638 | 335.1161 | 170.3963 | 153.5334 | 134.0744 | 91.21234 | 145.3456 | 116.2781 | 149.0167 | 455.8444 | 157.8167 | 123.5719 | 114.1483 | 113.4502 | 83.54736 | 205.9317 | 187.9283 |
| CUST_36042_PI429010886 | M14411 | 0.014546 | 18 | 3 | | | | | | 21.97852 | 18.78752 | NA | 22.58566 | 37.41548 | 39.78497 | 25.78835 | 12.14661 | 20.9065 | 7.31283 | 11.49926 | NA | 6.292935 | 15.228 | 17.4145 | 25.9871 | 34.6609 | 33.9856 | 40.42785 | 6.398941 | 20.45436 | 46.9109 | 12.028 | 41.97456 | 11.62339 | 21.55311 | 6.695841 |
| CUST_43311_PI429010886 | M62710 | 0.014577 | 24 | 3 | | | | | | 27.86411 | 18.27526 | 11.55545 | 43.55575 | 28.51242 | 26.77939 | 43.49365 | 22.7209 | 37.06882 | 21.40881 | 26.24303 | NA | 19.1562 | 6.042681 | 16.63804 | 30.48948 | 11.48646 | 25.68978 | 10.90408 | 5.795968 | 13.75707 | 41.16894 | 19.55335 | 19.38567 | 27.66265 | 6.639862 | 6.530509 |
| CUST_18509_PI429010886 | N39923 | 0.014577 | 24 | 0 | | | | | | 51.40578 | 45.61505 | 60.36203 | 57.82155 | 39.90543 | 48.65088 | 39.49607 | 55.85751 | 51.84431 | 40.85343 | 32.08763 | 29.40344 | 28.51585 | 51.04482 | 49.29932 | 40.25661 | 36.22069 | 48.66243 | 47.10091 | 26.63815 | 38.64078 | 48.17521 | 54.89215 | 41.49369 | 54.19041 | 60.71325 | 50.20045 |
| CUST_31474_PI429010886 | M1616 | 0.014578 | 12 | 3 | | | | | | 156.0552 | 33.01533 | 10.59802 | 197.2642 | 151.6234 | 332.964 | 136.6377 | 193.0385 | 64.56992 | 4.821627 | 54.41833 | 6.337987 | 114.3208 | 12.47694 | 54.93259 | 20.70543 | 218.6003 | 163.6892 | 129.1538 | 3.58777 | 82.82132 | 31.82653 | 18.39223 | 149.2083 | 11.61773 | 143.3153 | NA |
| CUST_1485_PI429010886 | N9777 | 0.014598 | 24 | 6 | 501293550 | non-specific lipid-transfer protein-like [Riptortus pe | Metabolic process | Other | | 11.89545 | 12.17117 | 14.21644 | 25.03208 | 25.58535 | 10.67575 | 13.38957 | 66.65717 | 48.40257 | 11.60175 | 39.21347 | 32.8525 | 34.63137 | NA | 22.87112 | 15.48139 | 32.54901 | 9.3513 | 12.33182 | 74.4613 | 25.71503 | 9.04617 | 8.695689 | NA | 14.06235 | 10.52648 | 15.44211 |
| CUST_53575_PI429010886 | M102320 | 0.014602 | 12 | 6 | | | | | | 25.70952 | 16.56922 | 8.272098 | 24.95635 | 15.97964 | 18.11108 | 24.53942 | 21.72859 | 19.50736 | 27.9397 | 14.18297 | 25.32554 | 15.56031 | 15.46627 | 7.786537 | 24.28324 | 24.0648 | 14.62285 | 31.61657 | 19.76503 | 27.58334 | 27.07724 | 21.26565 | 21.20384 | 18.40021 | 10.6044 | 10.95916 |
| CUST_57149_PI429010886 | E1610 | 0.014616 | 15 | 0 | 2098753 | alpha-2-tubulin [Gecarcinus lateralis] | Cellular component organization | Cytoskeleton organization | 1.78E-56 | 7784.736 | 10341.13 | 4340.493 | 6607.963 | 7071.027 | 10015.79 | 5800.891 | 3485.611 | 3883.492 | 3701.465 | 5819.009 | 3411.47 | 3965.108 | 3051.185 | 3469.507 | 7637.386 | 5009.146 | 5762.468 | 6571.189 | 3734.025 | 5176.163 | 3443.242 | 3077.216 | 6197.441 | 3278.647 | 5705.785 | 2423.127 |
| CUST_54330_PI429010886 | M105618 | 0.014617 | 18 | 0 | | | | | | 42.50508 | 42.29018 | 29.52396 | 39.30201 | 22.94962 | 27.36801 | 21.2878 | 31.27363 | 33.15216 | 10.09227 | 19.12346 | 17.05056 | 22.15217 | 24.5797 | 33.53978 | 35.85651 | 22.41939 | 43.65248 | 48.79699 | 44.72593 | 20.89839 | 34.50513 | 16.19477 | 35.96056 | 40.13905 | 40.69827 | 16.64978 |
| CUST_51261_PI429010886 | M92858 | 0.014651 | 18 | 3 | 260828125 | beta-transducin repeat containing protein, isoform | | | 1.23E-33 | 22.43497 | 15.05605 | 14.7136 | 23.14492 | 23.24949 | 32.79034 | 26.58995 | 12.12977 | 19.86296 | 21.05993 | 23.1013 | 17.13473 | 14.16669 | 17.52653 | 12.54217 | 22.9097 | 28.06937 | 17.5614 | 21.19536 | 18.36555 | 32.45148 | 25.06105 | 20.859 | 42.68463 | 20.23721 | 21.80317 | 12.18439 |
| CUST_25267_PI429010886 | N52092 | 0.014653 | 24 | 18 | | | | | | 21.2243 | 16.10718 | 23.51614 | 7.819931 | 6.811352 | 16.21551 | 12.55145 | 14.02882 | 4.548326 | 23.4129 | 16.68781 | 24.56849 | 24.53942 | 35.80705 | 20.9954 | 13.91147 | 19.9741 | 21.45154 | 20.88122 | NA | 37.04426 | 19.90878 | 31.97403 | 31.09756 | 23.30725 | NA | 25.78586 |
| CUST_35336_PI429010886 | M12304 | 0.014678 | 15 | 0 | 543727442 | PREDICTED: hydroxysteroid dehydrogenase-like pro | Metabolic process | Other | 7.45E-18 | 16.38676 | 14.71224 | NA | 19.72601 | 14.70218 | 9.0794 | 12.7161 | 4.525481 | 10.88348 | 12.07272 | 4.858012 | 14.50091 | 13.75707 | 15.40925 | 11.12902 | 16.04554 | NA | NA | NA | 15.70949 | 8.568969 | 8.00826 | 6.698064 | 14.46162 | 11.98265 | 8.033791 | 13.45546 |
| CUST_40754_PI429010886 | M56007 | 0.014685 | 24 | 12 | | | | | | 323.8163 | 495.4416 | 508.4411 | 270.6283 | 368.1562 | 364.1216 | 354.9858 | 408.7569 | 401.7586 | 630.4701 | 405.3248 | 625.7026 | 480.4111 | 910.5801 | 594.4635 | 487.3364 | 440.428 | 414.1007 | 619.8196 | 466.4129 | 481.9422 | 411.1038 | 656.3922 | 473.2302 | 385.8989 | 474.912 | 445.0547 |
| CUST_47309_PI429010886 | M75561 | 0.014686 | 21 | 0 | | | | | | 60.36203 | 44.29926 | 53.18376 | 43.43161 | 28.78291 | 18.97043 | 33.70252 | 28.84595 | 30.85708 | 86.57696 | 37.30131 | 41.25188 | 29.92416 | 54.81682 | 50.03352 | 43.99167 | 32.36622 | 35.81064 | 36.47986 | 57.15725 | 36.69997 | 48.48483 | 55.67871 | 73.25256 | 37.74031 | 34.29142 | 52.06984 |
| CUST_33783_PI429010886 | M7878 | 0.01469 | 12 | 9 | 524881835 | PREDICTED: MIT domain-containing protein 1-like is | | | 1.7E-55 | 66.46786 | 68.25944 | 78.17526 | 47.55714 | 52.48792 | 45.57502 | 47.63767 | 54.96227 | 68.47784 | 60.43972 | 56.13335 | 61.93566 | 69.216 | 55.11221 | 53.89507 | 83.56858 | 46.41914 | 49.68201 | 54.94382 | 53.32175 | 56.12021 | 80.38938 | 72.85867 | 64.3589 | 57.53634 | 53.6056 | 58.19173 |
| CUST_962_PI429010886 | N8002 | 0.014699 | 24 | 3 | | | | | | 15.46627 | 11.0059 | 22.81381 | 30.49715 | 12.4451 | 13.66038 | 15.444 | 7.133972 | 13.43047 | NA | 11.39726 | 11.69586 | 4.764736 | 8.655368 | NA | NA | 6.192383 | 8.386399 | NA | 6.748738 | 19.02231 | 8.207161 | 11.83103 | 20.71004 | 11.9615 | 11.93962 | 10.39491 |
| CUST_5436_PI429010886 | N18698 | 0.014702 | 24 | 18 | | | | | | 71.93323 | 58.49978 | 76.01313 | 63.46985 | 67.16356 | 66.31361 | 77.01708 | 62.26361 | 72.75459 | 106.872 | 83.85047 | 69.65017 | 85.02582 | 77.98525 | 121.3307 | 94.99099 | 80.65083 | 84.15456 | 116.2372 | 91.77189 | 70.87083 | 94.30937 | 70.06431 | 79.23712 | 107.533 | 91.22284 | 84.52872 |
| CUST_11287_PI429010886 | N27562 | 0.014702 | 24 | 0 | 321476705 | hypothetical protein DAPPUDRAFT_43248 [Daphnia | Developmental process | Multicellular organismal development | 1.21E-30 | 106.0819 | 97.01852 | 85.30129 | 87.11006 | 101.281 | 78.55433 | 92.06564 | 68.24289 | 79.5425 | 87.12746 | 69.00612 | 70.77093 | 78.62359 | 91.34253 | 78.38473 | 98.7503 | 82.71117 | 101.637 | 92.03214 | 86.70732 | 90.73889 | 87.35248 | 106.4846 | 93.92221 | 114.0301 | 79.5425 | 119.0883 |
| CUST_22140_PI429010886 | N46504 | 0.014742 | 21 | 3 | 321479256 | adenylyl cyclase [Daphnia pulex] | Signal transduction | Intracellular signal transduction | 6.12E-57 | 67.82256 | 114.4015 | 73.91931 | 162.1587 | 84.25035 | 112.304 | 71.65614 | 61.25018 | 63.58566 | 59.20737 | 55.07381 | 71.51375 | 40.59586 | 65.08584 | 81.39522 | 82.10096 | 41.98854 | 72.05819 | 53.10839 | 63.4424 | 108.1857 | 85.22504 | 96.8323 | 65.81652 | 108.0186 | 100.7905 | 52.73715 |
| CUST_10604_PI429010886 | N26497 | 0.014742 | 24 | 0 | | | | | | 98.73097 | 62.17487 | 131.1671 | 46.02417 | 66.34998 | 47.97564 | 117.6032 | 30.36684 | 59.25275 | 73.1075 | 54.95718 | 51.04982 | 65.66431 | 39.62195 | 58.05303 | 73.63983 | 49.5373 | 59.50102 | 20.98822 | 91.55434 | 56.71651 | 70.7113 | 93.41435 | 54.14016 | 126.4216 | 123.5431 | 95.689 |
| CUST_11154_PI429010886 | N27357 | 0.014744 | 24 | 0 | | | | | | 19.56884 | 13.37697 | 22.61839 | 16.5818 | 20.60646 | 11.7971 | 16.18253 | 12.34688 | NA | 19.49405 | 14.29372 | 11.35064 | 6.554733 | 11.17818 | 7.327597 | 16.62775 | 17.83033 | 10.04473 | 11.35281 | 11.43263 | 14.32775 | 18.57528 | 12.41359 | 10.71844 | 21.33841 | 15.0068 | 11.74612 |
| CUST_38085_PI429010886 | M23271 | 0.014752 | 21 | 3 | | | | | | 470.9015 | 580.8644 | 466.0826 | 434.9301 | 569.7753 | 560.1437 | 331.2853 | 351.983 | 433.7721 | 506.5013 | 457.5237 | 600.5562 | 286.8668 | 370.9949 | 410.9902 | 435.6005 | 419.5293 | 432.25 | 351.8276 | 473.9584 | 394.8691 | 482.6309 | 389.4334 | 397.5988 | 645.5341 | 498.1212 | 465.6558 |
| CUST_6486_PI429010886 | N20163 | 0.014753 | 15 | 12 | 123400230 | ankyrin repeat protein [Trichomonas vaginalis G3] > | | | 1.4E-23 | 509.2578 | 711.0836 | 489.9462 | 755.5617 | 503.7682 | 419.0394 | 569.1252 | 597.3714 | 556.2338 | 617.8466 | 745.5335 | 616.6013 | 902.7962 | 789.7368 | 802.0583 | 1120.861 | 504.7239 | 639.3223 | 572.5618 | 484.2538 | 552.3175 | 790.1209 | 552.7878 | 385.0916 | 774.9868 | 461.9687 | 981.8641 |
| CUST_43859_PI429010886 | M64171 | 0.014763 | 12 | 0 | | | | | | 83.2292 | 96.54198 | 96.20672 | 55.19772 | 65.8929 | 86.30175 | 64.3345 | 44.68023 | 65.81652 | 92.46177 | 55.44777 | 76.50308 | 54.08177 | 74.86726 | 67.19434 | 73.85269 | 68.96573 | 76.84434 | 67.37108 | 60.07014 | 63.79444 | 90.13978 | 67.50801 | 69.72006 | 93.17664 | 72.91578 | 105.2954 |
| CUST_7812_PI429010886 | N22119 | 0.014764 | 18 | 3 | 321474268 | hypothetical protein DAPPUDRAFT_45850 [Daphnia | | | 1.17E-81 | 190.0374 | 102.7139 | 158.1305 | 159.878 | 217.2477 | 136.4264 | 245.7815 | 113.3684 | 81.64235 | 80.99667 | 197.4721 | 68.64401 | 86.94051 | 88.39516 | 96.63027 | 129.2382 | 224.9395 | 120.2652 | 183.6687 | 97.96017 | 173.3233 | 133.0251 | 187.2169 | 207.7149 | 101.3168 | 73.14833 | 81.78469 |
| CUST_670_PI429010886 | N6666 | 0.014776 | 21 | 6 | 139000435 | tick legumain [Haemaphysalis longicornis] | Protein metabolism | Proteolysis | 4.44E-73 | NA | 166.0384 | 77.78755 | 708.7623 | 445.6175 | 270.9645 | 364.9554 | 2731.57 | 1434.403 | 287.6352 | 1935.871 | 2053.321 | 866.2027 | 216.1164 | 420.7782 | 8.136532 | 533.2467 | NA | 166.585 | 3371.152 | 221.5886 | NA | 277.0077 | 205.078 | NA | 988.0968 | 656.5643 |
| CUST_9470_PI429010886 | N24684 | 0.014779 | 24 | 15 | | | | | | 36.69997 | 43.9859 | 36.24706 | 30.90791 | 23.74624 | 20.89839 | 20.80589 | 32.55113 | 28.73136 | 43.61983 | 23.77247 | 37.42124 | 34.45811 | 46.4361 | 35.70225 | 38.723 | 45.3118 | 35.25625 | 37.17994 | 46.34675 | 25.49499 | 45.93985 | 34.23531 | 36.57353 | 35.29716 | 35.11845 | 40.2482 |
| CUST_2134_PI429010886 | N11654 | 0.01478 | 24 | 3 | | | | | | 6.063329 | 21.8498 | 20.22452 | NA | 12.45001 | NA | NA | 10.03833 | 12.42241 | 7.862605 | 11.6401 | 10.17638 | 5.807016 | 8.958591 | NA | 5.929425 | 9.407802 | 10.34266 | 6.446625 | 4.82051 | 3.417106 | 8.310857 | 8.143445 | NA | 13.49109 | 13.57654 | 5.678179 |
| CUST_9601_PI429010886 | N24903 | 0.014792 | 15 | 9 | 24666141 | CG5567 [Drosophila melanogaster] >gi\|7293934\|gb | Metabolic process | Other | 4.76E-36 | 7.886773 | 14.91553 | 7.408115 | 11.67245 | 17.4398 | 13.04197 | 5.965808 | 14.05746 | 14.9512 | 12.83923 | 38.19855 | 18.69696 | 12.52538 | 14.36708 | 15.66502 | 14.29202 | 12.63519 | 11.49258 | 11.56124 | 11.8101 | 10.41346 | 39.30201 | 13.49842 | 18.75909 | 19.48299 | 14.4879 | 23.33967 |
| CUST_36562_PI429010886 | M16240 | 0.014802 | 18 | 3 | | | | | | 19.34811 | 17.50149 | 8.304372 | 141.6867 | 93.67647 | 99.2772 | 44.61206 | 16.19744 | 29.48584 | 3.965832 | 4.33335 | NA | 8.699683 | 81.9093 | 67.66412 | 71.46757 | NA | 83.63981 | NA | 26.53007 | 25.11484 | 88.82378 | NA | 40.88839 | 28.42343 | NA | NA |
| CUST_36473_PI429010886 | M16004 | 0.014855 | 12 | 9 | | | | | | 40.69827 | 50.73538 | 71.70446 | 39.17072 | 34.07208 | 40.83264 | 32.13434 | 63.70207 | 51.95466 | 66.3431 | 62.90335 | 58.94211 | 65.56878 | 67.54243 | 51.60744 | 50.2407 | 35.52357 | 38.26462 | 49.38887 | 88.40608 | 44.33537 | 82.82848 | 73.37448 | 49.29515 | 45.30656 | 82.51706 | 76.21767 |
| CUST_34464_PI429010886 | M9749 | 0.014855 | 12 | 3 | | | | | | 1092.918 | 651.2944 | 271.4917 | 1337.58 | 663.5689 | 2108.426 | 847.8005 | 1442.354 | 875.643 | 262.76 | 338.4841 | 163.8253 | 557.9517 | 369.3306 | 541.6023 | 567.0781 | 1134.542 | 1189.668 | 1317.659 | 288.3422 | 255.8665 | 457.1179 | 164.8084 | 1181.018 | 439.2082 | 1210.418 | 320.0372 |
| CUST_2482_PI429010886 | N12477 | 0.014855 | 12 | 9 | | | | | | 95.41159 | 200.0776 | 126.6371 | 68.56896 | 98.05949 | 64.26853 | 80.01037 | 118.8413 | 107.4857 | 141.8494 | 124.3983 | 148.3475 | 94.79905 | 173.5738 | 123.1846 | 79.0243 | 163.569 | 63.86613 | 120.8888 | 67.97289 | 68.05219 | 109.9562 | 171.0565 | 140.7563 | 115.8594 | 85.94103 | 98.43882 |
| CUST_1201_PI429010886 | N8817 | 0.014861 | 24 | 12 | | | | | | 12.53064 | 11.87443 | 8.893352 | NA | 7.65571 | 13.63311 | 7.563974 | 12.6233 | 10.01285 | 10.86011 | 6.44346 | 20.34336 | 25.31087 | 18.07682 | 23.4129 | 20.19663 | 9.370503 | 24.91723 | 21.10225 | 15.29468 | 23.65146 | 16.74792 | 20.45683 | 11.79192 | 8.89926 | 20.07855 | 16.05605 |
| CUST_20106_PI429010886 | N42821 | 0.014863 | 24 | 15 | 242023524 | Heterogeneous nuclear ribonucleoprotein A1, putat | | | 6.17E-25 | 75.2095 | 111.637 | 93.92221 | 63.52108 | 94.18675 | 69.82734 | 69.2413 | 77.65762 | 79.53245 | 95.33691 | 93.66147 | 118.4142 | 83.28321 | 104.4971 | 119.0883 | 114.5044 | 86.46205 | 113.3684 | 109.6802 | 115.0699 | 83.97119 | 105.5375 | 78.76718 | 85.18737 | 98.43882 | 84.44547 | 91.46281 |
| CUST_47803_PI429010886 | M77671 | 0.01487 | 24 | 3 | | | | | | 199.9107 | 392.4795 | 220.5435 | 620.3013 | 250.3151 | 344.4277 | 244.0822 | 197.2996 | 240.1049 | 221.425 | 120.114 | 270.2125 | 148.2477 | 263.1362 | 117.1213 | 215.2013 | 93.14117 | 135.8061 | 67.48761 | 185.5006 | 357.7037 | 172.1829 | 414.9669 | 211.2278 | 262.2522 | 365.0271 | 180.8735 |
| CUST_52151_PI429010886 | M96572 | 0.014874 | 12 | 0 | | | | | | 58.5117 | 36.11849 | 50.00676 | 29.6775 | 35.71333 | 30.3023 | 20.89073 | 46.42845 | 25.68743 | 33.00184 | 42.62361 | 46.18431 | 41.01337 | 60.85469 | 55.71375 | 43.82673 | 34.80653 | 39.55173 | 38.05146 | 48.70302 | 37.27042 | 46.78356 | 40.54346 | 41.37701 | 46.8066 | 50.31204 | 39.80101 |
| CUST_10794_PI429010886 | N26801 | 0.014874 | 12 | 0 | | | | | | 512.1412 | 523.4011 | 517.2906 | 451.916 | 302.092 | 256.6122 | 310.1413 | 406.3292 | 417.2965 | 629.4397 | 450.9914 | 465.6558 | 571.5932 | 734.477 | 527.0813 | 860.82 | 333.708 | 319.8933 | 504.7239 | 572.4248 | 455.2984 | 637.6126 | 473.9584 | 304.8063 | 526.6917 | 625.9634 | 659.4054 |
| CUST_10546_PI429010886 | N26404 | 0.014877 | 18 | 15 | | | | | | 52.77021 | 48.03245 | 51.33151 | 43.60346 | 49.29515 | 65.69324 | 45.3118 | 27.34334 | 35.07632 | 37.64162 | 70.30533 | 39.28519 | 50.546 | 61.79791 | 68.16131 | 54.2941 | 66.87005 | 54.45697 | 62.37619 | 73.31065 | 41.20843 | 45.53479 | 64.45055 | 52.26577 | 58.64138 | 44.16686 | 33.51061 |
| CUST_33556_PI429010886 | M7268 | 0.014886 | 15 | 12 | | | | | | 13.64597 | 8.08569 | NA | NA | 15.32925 | 21.14025 | 5.555758 | 7.349454 | 7.54037 | 22.39033 | 8.572306 | 13.29029 | 13.91309 | 20.68333 | 13.7828 | 13.48403 | 12.36089 | 48.63366 | 11.55416 | 3.724456 | 6.397942 | 11.60948 | 5.255245 | 10.21387 | 11.22442 | 7.674635 | 9.758601 |
| CUST_1258_PI429010886 | N9051 | 0.014894 | 21 | 9 | 321453628 | hypothetical protein DAPPUDRAFT_333779 [Daphn | | | 3.7E-102 | 28.1812 | 71.92462 | 39.54096 | 107.2622 | 186.2582 | 64.24619 | 102.7727 | 451.3065 | 275.6931 | 88.75833 | 490.166 | 498.8709 | 288.9328 | 49.1739 | 142.7507 | 69.42462 | 142.216 | 29.78998 | 33.49626 | 650.3692 | 391.4581 | 30.83339 | 78.25555 | 52.93292 | 33.22847 | 140.5991 | 426.9229 |
| CUST_56290_PI429010886 | M113390 | 0.014896 | 18 | 3 | 77743875 | Euphausia superba isolate DGGE band AntEuk13-5 : | | | | 226545.4 | 234264.9 | 223244.4 | 260608.7 | 234264.9 | 223244.4 | 226545.4 | 260608.7 | 217233 | 260608.7 | 214624.2 | 204770.3 | 217233 | 212288.9 | 212288.9 | 207938.5 | 200261.3 | 204770.3 | 220173.8 | 203520.1 | 234264.9 | 220173.8 | 260608.7 | 220173.8 | 260608.7 | 220173.8 | 217233 |
| CUST_38626_PI429010886 | M28655 | 0.014896 | 12 | 3 | | | | | | 165.1069 | 1164.641 | 35.73798 | 1276.669 | 777.3766 | 948.7418 | 564.3927 | 38.35127 | 587.6341 | 9.273731 | 13.70505 | NA | 74.47085 | 73.87582 | 422.8357 | 943.689 | 65.04995 | 679.9331 | 49.57451 | 14.6922 | 20.50947 | 217.7746 | 28.93067 | 219.8889 | 109.5748 | 76.36771 | 89.02227 |
| CUST_39364_PI429010886 | M38412 | 0.014896 | 12 | 9 | | | | | | 22.48304 | 26.30031 | 31.23112 | 23.17379 | 31.56432 | NA | 23.27942 | 31.40893 | 19.76503 | 56.74919 | 31.59119 | 53.07285 | 28.29864 | 28.82637 | 12.7744 | 30.92026 | 8.326131 | 16.75931 | 20.56111 | 34.26311 | 26.08326 | 38.29585 | 29.24887 | 33.86006 | 18.54724 | 33.03437 | 35.03861 |
| CUST_53484_PI429010886 | M101902 | 0.014901 | 18 | 9 | | | | | | 17.12595 | 19.26616 | 23.22371 | 13.29711 | 14.71509 | 10.44304 | 13.83134 | 18.77363 | 14.60951 | 35.85651 | 23.53859 | 21.80739 | 22.71614 | 23.5415 | 19.53485 | 21.38369 | 16.72128 | 19.35656 | 22.55376 | 25.21391 | 18.75909 | 26.9442 | 14.25924 | 14.83746 | 29.64495 | 17.67558 | 22.15217 |
| CUST_36519_PI429010886 | M16116 | 0.014901 | 18 | 9 | 443707979 | hypothetical protein CAPTEDRAFT_228167 [Capitell | | | 3.8E-36 | 28.44656 | 24.21253 | 33.18183 | 67.12853 | 28.66914 | 36.91363 | 34.06396 | 55.29319 | 32.46691 | 40.1276 | 40.63765 | 41.78277 | 29.89152 | 34.50768 | 31.55142 | 30.43159 | 37.16922 | 29.06247 | 28.31689 | 36.79834 | 27.3183 | 41.45045 | 27.07911 | 35.6143 | 38.6196 | 30.63369 | 36.17759 |
| CUST_43444_PI429010886 | M63073 | 0.014933 | 18 | 12 | 242013405 | class B secretin-like G-protein coupled receptor GPI | Signal transduction | Other | 8.53E-09 | 45.90609 | 83.31796 | 42.11302 | 46.83381 | 31.23112 | 51.71264 | 62.07681 | 89.63044 | 92.04256 | 108.9872 | 55.84117 | 64.32323 | 86.17533 | 90.31563 | 66.48237 | 77.0714 | 34.82147 | 42.27106 | 31.63804 | 77.45737 | 28.02553 | 38.92288 | 81.27628 | 46.13321 | 70.80885 | 55.784 | 83.48026 |
| CUST_3392_PI429010886 | N14417 | 0.014939 | 15 | 3 | | | | | | 2365.487 | 2148.593 | 1834.08 | 20010.45 | 24081.12 | 22767.31 | 11182.42 | 12354.92 | 684.5685 | 48.85705 | 11632.77 | 66.32011 | 2349.941 | 175.1085 | 2447.295 | 120.8888 | 8470.008 | 2366.854 | 7415.798 | 128.2179 | 16925.4 | 424.8243 | 10491.93 | 16762.52 | 126.2631 | 4733.383 | 72.10026 |
| CUST_34055_PI429010886 | M8613 | 0.014943 | 12 | 6 | 241566044 | peroxisomal membrane protein, putative [Ixodes sc | Transport | Transmembrane transport | 1.13E-82 | 27.54447 | 21.31486 | 21.17074 | 28.26641 | 24.14877 | 36.99652 | 42.63354 | 48.35952 | 39.57761 | 26.25499 | 33.3468 | 33.14406 | 27.31066 | 23.46033 | 35.33784 | 31.93333 | 26.56221 | 24.86718 | 28.17746 | 44.05675 | 20.27847 | 26.92163 | 27.43563 | 30.705 | 21.92246 | 23.84339 | 25.07963 |
| CUST_18286_PI429010886 | N39502 | 0.014943 | 15 | 9 | | | | | | 59.75541 | 51.09668 | 52.09002 | 32.61875 | 49.3195 | 43.55173 | 43.59959 | 39.00381 | 41.81244 | 110.4435 | 56.69692 | 97.78298 | 81.56419 | 70.77767 | 68.5761 | 98.6608 | 63.70797 | 78.66048 | 42.11302 | 79.29506 | 52.43827 | 97.93806 | 47.38756 | 55.36009 | 80.4611 | 51.09155 | 115.1182 |
| CUST_36520_PI429010886 | M16117 | 0.014954 | 15 | 12 | | | | | | NA | 17.90806 | 18.66441 | NA | 8.270829 | NA | 18.01905 | 33.84232 | 26.44212 | 49.80346 | 10.13438 | 21.91188 | 113.7364 | 106.3603 | 61.89112 | 44.02718 | 86.4248 | 19.37939 | 5.017204 | 29.6775 | 5.535611 | 89.63044 | 73.18236 | 10.15439 | 157.4606 | 19.7129 | 159.0029 |
| CUST_27849_PI429010886 | N56808 | 0.014968 | 15 | 0 | | | | | | 18.74861 | 14.31095 | 19.34225 | 10.59802 | 20.77685 | 17.27372 | 53.51251 | 10.10703 | 26.03629 | 13.65887 | 13.87826 | 11.20539 | 12.22747 | 13.2165 | 16.31911 | 28.60372 | 34.51101 | 84.40117 | 16.02003 | 8.083245 | 16.7895 | 14.95656 | 12.23461 | 11.81299 | 9.278391 | 6.277631 | 14.2983 |
| CUST_10147_PI429010886 | N25771 | 0.014973 | 15 | 3 | 313881430 | ankyrin repeat protein [synthetic construct] | | | 1.48E-09 | 48.76564 | 66.89269 | 8.620684 | 118.6904 | 136.597 | 143.1896 | 70.05382 | 11.64471 | 46.18431 | 8.095294 | 13.13981 | 7.222005 | 66.19578 | 140.6816 | 76.02137 | 124.3489 | 27.63304 | 150.5899 | 15.09377 | 98.70636 | 37.0569 | 88.58239 | 8.492959 | 96.81237 | 64.99075 | 6.652893 | 8.128829 |
| CUST_23224_PI429010886 | N48472 | 0.014973 | 12 | 0 | | | | | | 33.12696 | 26.71164 | 29.12589 | 6.999938 | 7.704662 | 11.99536 | 24.72926 | 11.9716 | 18.28397 | 38.49903 | 17.53384 | 28.03123 | 20.54425 | 17.07869 | 14.1847 | 5.123692 | 17.20395 | 14.20655 | 29.75085 | 17.80745 | 22.50429 | 24.99293 | 14.64693 | 25.25069 | 28.16847 | 17.96665 | 35.64771 |
| CUST_53477_PI429010886 | M101879 | 0.014992 | 21 | 9 | 225713522 | 2-acylglycerol O-acyltransferase 1 [Lepeophtheirus : | | | 1.28E-09 | 4.1182 | 5.285615 | NA | 19.83493 | 24.61539 | 13.36864 | 11.49378 | 94.94128 | 46.82282 | NA | 54.05301 | 59.6278 | 25.53352 | NA | 11.94414 | 7.463527 | 19.39824 | 8.546868 | 7.72615 | 104.6652 | 34.43117 | 9.520915 | 6.324533 | 24.40654 | NA | 18.12277 | 13.25918 |
| CUST_45956_PI429010886 | M70869 | 0.015004 | 18 | 3 | 77743875 | Euphausia superba isolate DGGE band AntEuk13-5 : | | | 4.5E-176 | 65.52616 | 63.25496 | 86.57696 | 97.09132 | 102.5272 | 100.999 | 61.01573 | 77.61759 | 71.18669 | 78.46976 | 72.11758 | 67.31728 | 61.36332 | 96.94706 | 39.93142 | 69.31235 | 74.7826 | 92.03214 | 84.7131 | 57.85675 | 67.16356 | 95.07199 | 96.08349 | 87.75721 | 84.1647 | 72.14089 | 136.7775 |
| CUST_31035_PI429010886 | M525 | 0.01501 | 24 | 15 | | | | | | 231.7343 | 283.4361 | 244.0822 | 178.2181 | 177.6145 | 237.8874 | 169.6552 | 222.818 | 205.7973 | 219.6484 | 269.3458 | 260.0114 | 206.9171 | 263.8365 | 256.9966 | 305.8476 | 265.6039 | 277.6024 | 223.4595 | 255.3857 | 251.3539 | 225.7607 | 238.0396 | 275.3515 | 311.0329 | 259.2052 | 251.6265 |
| CUST_36506_PI429010886 | M16090 | 0.015015 | 24 | 21 | | | | | | 17.3946 | 18.61514 | 22.50936 | 13.027 | 12.66387 | 29.69737 | 13.46995 | 14.29674 | 9.529604 | 22.12824 | 14.41938 | 14.88157 | 15.21358 | 14.73015 | 15.84192 | 14.70096 | 28.5055 | 17.91813 | 17.96537 | 15.08066 | 15.84016 | 16.59828 | 36.7953 | 16.59828 | 32.75767 | 18.65035 | 20.04296 |
| CUST_29279_PI429010886 | N59536 | 0.015018 | 18 | 12 | | | | | | 66.85557 | 46.4361 | 43.02039 | 89.63044 | 19.97113 | 38.18832 | 44.19495 | 15.60562 | 50.68297 | 32.98902 | 18.02463 | 193.4709 | 267.983 | 348.0084 | 268.6688 | 86.55946 | 71.26057 | 71.3655 | 671.3234 | 25.87358 | 621.8584 | 41.99242 | 43.23619 | 24.36596 | 37.21745 | 40.78513 | 44.10538 |
| CUST_32582_PI429010886 | M4478 | 0.015024 | 18 | 3 | | | | | | 10.63992 | 18.90775 | 8.834126 | 19.52364 | 11.26684 | 14.14994 | 17.36105 | 14.49351 | 5.322036 | 15.97793 | 6.675568 | 9.877583 | 4.26798 | NA | NA | 9.301993 | 10.18958 | 9.108247 | 16.08632 | 12.08465 | 18.09247 | 20.0846 | NA | NA | 11.08939 | 5.842679 | 6.768573 |
| CUST_19167_PI429010886 | N41095 | 0.015025 | 21 | 15 | | | | | | 15.30179 | 16.59282 | 13.70765 | 18.08937 | 14.82167 | 13.20796 | 9.750393 | 11.34865 | 18.40232 | 11.98753 | 6.304011 | 12.47694 | 15.49998 | 8.617079 | 17.72667 | 21.33598 | 115.0806 | 25.74156 | 16.21863 | 6.43253 | 16.92637 | 22.35568 | 23.37424 | 14.86823 | 11.66655 | 20.13696 | 13.76981 |

| Probe ID | ID | p-value | Period | Phase | EBI ID | Annotation | GO category | Go term | E-value | 0 | 0 | 0 | 3 | 3 | 3 | 6 | 6 | 6 | 9 | 9 | 9 | 12 | 12 | 12 | 15 | 15 | 15 | 18 | 18 | 18 | 21 | 21 | 21 | 24 | 24 | 24 |
|---|---|---|---|---|---|---|---|---|---|---|---|---|---|---|---|---|---|---|---|---|---|---|---|---|---|---|---|---|---|---|---|---|---|---|---|---|
| CUST_1064_PI429010886 | N8384 | 0.015035 | 15 | 9 | | | | | | 33.82224 | 77.82271 | 64.8815 | 55.93124 | 68.01317 | 83.7299 | 83.99654 | 61.5685 | 114.2228 | 80.01037 | 83.83973 | 149.3268 | 48.10487 | 95.50344 | 57.72307 | 35.41295 | 56.0662 | 104.3119 | 40.98319 | 55.03461 | 70.72361 | 62.62665 | 92.18368 | 103.6255 | 102.5545 | 83.02241 | 139.9854 |
| CUST_38084_PI429010886 | M23266 | 0.015036 | 21 | 6 | | | | | | 456.9093 | 470.7834 | 242.302 | 500.3626 | 338.5891 | 509.7336 | 587.2428 | 468.7286 | 508.5068 | 256.1926 | 149.974 | 267.5678 | 102.7622 | 142.3547 | 167.4717 | 183.1759 | 398.5298 | 402.6962 | 425.0392 | 69.72451 | 260.7005 | 122.4782 | 184.0938 | 434.7605 | 87.01627 | 728.0084 | 228.274 |
| CUST_34606_PI429010886 | M10133 | 0.015051 | 12 | 0 | | | | | | 332.4166 | 457.234 | 353.8817 | 247.8273 | 346.5286 | 246.2258 | 298.7079 | 281.5398 | 303.2078 | 363.7161 | 333.6383 | 392.3971 | 359.0068 | 304.437 | 275.0389 | 280.3633 | 260.4155 | 233.5227 | 302.4916 | 415.3047 | 311.8189 | 450.2218 | 354.3972 | 246.1531 | 465.4504 | 365.9997 | 403.1472 |
| CUST_15455_PI429010886 | N34608 | 0.015051 | 12 | 0 | | | | | | 72.52738 | 60.3963 | 70.14592 | 47.91134 | 54.73192 | 53.78964 | 45.71121 | 42.47169 | 60.5059 | 57.80685 | 52.74988 | 59.06419 | 55.29787 | 104.2347 | 62.29412 | 51.61587 | 62.48649 | 41.59029 | 43.66792 | 64.04369 | 59.45067 | 62.11683 | 61.91569 | 61.14077 | 57.37114 | 73.94862 | 67.52947 |
| CUST_22418_PI429010886 | N46992 | 0.015058 | 24 | 3 | 405960827 | Anoctamin-8 [Crassostrea gigas] | | | 9.33E-24 | 56.73383 | 39.71396 | 35.02024 | 61.12874 | 35.57579 | 52.97579 | 48.77148 | 40.69465 | 38.59967 | 44.82294 | 33.36548 | 36.06105 | 33.46666 | 28.34801 | 30.0571 | 38.41931 | 35.79389 | 37.08279 | 30.02625 | 24.29748 | 31.6312 | 32.09117 | 61.72981 | 50.9653 | 36.7523 | 80.51303 | 30.60166 |
| CUST_57012_PI429010886 | M115117 | 0.015059 | 24 | 18 | | | | | | 260.0114 | 343.7569 | 321.9594 | 247.587 | 232.0593 | 230.9236 | 205.5955 | 234.6626 | 230.0814 | 352.4518 | 228.9161 | 417.9411 | 306.3807 | 311.8189 | 398.6724 | 551.8489 | 202.4996 | 352.4518 | 278.1576 | 446.4087 | 379.0729 | 434.4861 | 306.6185 | 221.0323 | 268.82 | 270.1737 | 299.502 |
| CUST_4173_PI429010886 | N16163 | 0.015099 | 18 | 9 | 354990989 | anti-lipopolysaccharide factor isoform 6 [Penaeus m | | | 5.61E-26 | 1560.88 | 1614.376 | 559.0577 | 593.1266 | 702.0078 | 607.7896 | 1960.986 | 585.6944 | 2580.486 | 2908.57 | 1004.727 | 4165.771 | 2945.87 | 2054.295 | 1692.652 | 2012.591 | 943.689 | 991.3315 | 3345.256 | 346.9119 | 1650.747 | 697.8167 | 1178.063 | 831.0172 | 1787.33 | 660.0703 | 3269.374 |
| CUST_4437_PI429010886 | N16755 | 0.015099 | 18 | 3 | | | | | | 496.6049 | 113.475 | 335.1161 | 440.428 | 299.1597 | 674.0173 | 457.6266 | 229.2068 | 139.8337 | 78.77563 | 223.7779 | 92.07382 | 128.7232 | 104.0454 | 157.7275 | 97.01852 | 330.8112 | 245.4514 | 381.1947 | 72.08475 | 442.6624 | 172.5751 | 423.767 | 450.4975 | 72.21046 | 309.7678 | 64.50807 |
| CUST_19719_PI429010886 | N42065 | 0.015099 | 18 | 15 | 321458591 | hypothetical protein DAPPUDRAFT_61988 [Daphnia | Metabolic process | Nucleobase-containing compound metabolic process | 3.36E-07 | 187.1216 | 208.7066 | 140.0797 | 96.40299 | 197.0393 | 179.2012 | 169.1571 | 136.8231 | 148.0082 | 210.0878 | 156.7272 | 130.6959 | 187.1216 | 139.8628 | 147.6124 | 256.1926 | 189.6264 | 190.0374 | 201.7725 | 206.469 | 134.162 | 166.2545 | 150.4806 | 166.1459 | 144.2346 | 137.33 | 89.30785 |
| CUST_21247_PI429010886 | N44885 | 0.015099 | 18 | 9 | 170285555 | putative tetratricopeptide repeat domain 11 protei | | | 1.31E-30 | 119.0689 | 172.8115 | 233.6523 | 156.5024 | 148.3869 | 150.914 | 166.1459 | 176.5188 | 195.5222 | 216.65 | 193.4709 | 236.856 | 157.9551 | 240.8224 | 200.041 | 205.6287 | 129.9123 | 175.1291 | 186.3956 | 161.4564 | 169.3616 | 193.0913 | 164.7626 | 151.1785 | 230.2846 | 151.4536 | 191.6952 |
| CUST_7922_PI429010886 | N22278 | 0.015109 | 21 | 15 | | | | | | 116.8442 | 120.4829 | 118.4299 | 142.124 | 145.2206 | 125.3551 | 74.29802 | 99.8601 | 98.6386 | 112.6431 | 101.7048 | 117.6882 | 100.1858 | 157.4606 | 156.5024 | 135.6184 | 132.8611 | 166.8192 | 90.22371 | 147.9354 | 125.8034 | 189.1715 | 127.112 | 124.447 | 117.2337 | 105.3086 | 99.79034 |
| CUST_8684_PI429010886 | N23413 | 0.015123 | 12 | 0 | 347968037 | AGAP002539-PB [Anopheles gambiae str. PEST] >gi | | | 4.07E-35 | 137.8443 | 190.6989 | 167.3841 | 151.9866 | 151.6013 | 165.9977 | 105.798 | 142.2748 | 171.5473 | 200.8936 | 148.1205 | 171.1195 | 148.886 | 193.4196 | 192.1973 | 182.8109 | 158.9341 | 162.5891 | 138.2766 | 147.2999 | 133.5211 | 157.7661 | 151.1369 | 134.8627 | 200.6692 | 168.7503 | 187.6525 |
| CUST_19155_PI429010886 | N41068 | 0.015125 | 12 | 0 | | | | | | 13.57199 | 13.42609 | 12.46111 | 8.887892 | 7.841585 | NA | 4.093048 | 5.51835 | NA | 6.715766 | 8.509556 | 8.442875 | 7.39029 | 14.16669 | 11.50377 | 18.33589 | 4.73084 | 9.273731 | 8.304372 | 8.466065 | NA | 8.943967 | NA | 7.52281 | 21.79918 | NA | NA |
| CUST_32383_PI429010886 | M3980 | 0.015142 | 15 | 12 | | | | | | 414.3494 | 662.2599 | 571.9057 | 340.1906 | 505.2828 | 469.6397 | 358.4083 | 395.2528 | 430.3425 | 528.1283 | 462.4656 | 661.0472 | 503.8676 | 586.8079 | 526.5682 | 644.1161 | 554.2309 | 486.8632 | 446.0696 | 340.867 | 384.3454 | 479.3073 | 492.3863 | 505.6922 | 507.4019 | 359.7386 | 526.3482 |
| CUST_47242_PI429010886 | M75323 | 0.015142 | 15 | 12 | 321469983 | hypothetical protein DAPPUDRAFT_303785 [Daphn | Protein metabolism | Proteolysis | 5.75E-61 | 301.9881 | 253.0671 | 193.0075 | 172.213 | 209.4212 | 168.2678 | 323.3503 | 254.8048 | 378.1407 | 444.5828 | 146.939 | 314.3091 | 392.0026 | 323.4046 | 256.1116 | 269.8655 | 171.0028 | 167.0167 | 138.9365 | 189.1194 | 164.9098 | 272.1441 | 252.3543 | 274.404 | 220.9066 | 205.2221 | 272.6795 |
| CUST_49687_PI429010886 | M86705 | 0.015147 | 15 | 12 | | | | | | 214.7684 | 279.1889 | 233.1687 | 224.0471 | 199.344 | 262.6197 | 223.5686 | 234.823 | 200.4301 | 293.1271 | 251.745 | 387.5117 | 262.3746 | 265.9437 | 328.3818 | 294.8062 | 226.6353 | 276.0613 | 233.2127 | 186.1103 | 229.7558 | 243.0281 | 290.5429 | 182.494 | 281.2019 | 245.8985 | 277.8925 |
| CUST_34247_PI429010886 | M9159 | 0.015147 | 21 | 15 | | | | | | 82.90947 | 60.3241 | 99.87407 | 38.87013 | 46.62471 | 39.74876 | 36.79834 | 71.00916 | 44.49519 | 49.54106 | 44.73069 | 58.77193 | 48.28635 | 81.96733 | 57.12722 | 82.06433 | 53.3996 | 64.56424 | 43.45323 | 83.08523 | 44.87166 | 65.64906 | 73.20424 | 80.57644 | 50.37741 | 31.07874 | 39.55173 |
| CUST_25172_PI429010886 | N51910 | 0.015148 | 21 | 12 | 156552305 | PREDICTED: condensin complex subunit 3-like [Nas | | | 1.64E-09 | 110.7482 | 131.3569 | 102.0005 | 97.31477 | 87.99607 | 114.6578 | 63.46235 | 83.12577 | 83.21112 | 102.9324 | 90.97139 | 139.8446 | 111.6628 | 122.7974 | 117.7722 | 139.8337 | 88.66033 | 135.5951 | 120.7015 | 109.0596 | 103.5678 | 112.4033 | 115.2692 | 88.38108 | 106.6808 | 91.01032 | 164.8298 |
| CUST_44834_PI429010886 | M67144 | 0.015159 | 21 | 0 | | | | | | 20.33623 | 6.092564 | 18.11108 | 9.048255 | 8.069484 | 14.79157 | 13.3566 | 9.44636 | 8.588693 | 5.268714 | 5.29544 | NA | 8.778682 | NA | 7.378942 | 8.540006 | 16.96557 | 10.36906 | 10.9756 | 10.4013 | 4.844968 | 15.64945 | 14.10338 | 13.25918 | 11.94414 | 8.967045 | 9.257427 |
| CUST_56527_PI429010886 | M114015 | 0.01516 | 24 | 18 | | | | | | 79.38753 | 99.77775 | 120.6382 | 88.41726 | 86.00719 | 78.05922 | 67.80419 | 82.15481 | 79.61927 | 114.5445 | 94.17604 | 164.2756 | 90.60771 | 113.3918 | 74.67944 | 107.4857 | 80.92356 | 89.25924 | 125.6844 | 119.1476 | 100.3042 | 116.531 | 122.8182 | 102.205 | 99.93216 | 96.77635 | 126.5253 |
| CUST_46948_PI429010886 | M74272 | 0.015167 | 21 | 15 | 380743551 | plasma membrane calcium ATPase [Callinectes sapi | Transport | Ion transport | 4.8E-25 | 13.87348 | 9.510155 | 12.4672 | 5.95738 | 9.148382 | 10.53669 | 5.285615 | 6.611992 | 9.132253 | 7.005798 | 7.342643 | 16.23152 | 9.768636 | 21.00749 | 7.388302 | 15.01997 | 11.80273 | 10.83121 | 14.17556 | 14.03086 | 8.801809 | 9.117443 | 12.07791 | 16.06458 | 14.01595 | 8.658987 | 9.002381 |
| CUST_33145_PI429010886 | M6080 | 0.015185 | 12 | 9 | | | | | | 132.0609 | 99.60857 | 168.1071 | 82.97709 | 89.13187 | 82.1182 | 121.3307 | 101.281 | 91.6409 | 136.6618 | 131.6883 | 104.8753 | 85.13186 | 119.2945 | 90.90134 | 78.01256 | 115.8883 | 86.9224 | 113.1045 | 92.1489 | 110.765 | 119.5328 | 107.1382 | 93.05164 | 88.6126 | 86.54824 | 102.5774 |
| CUST_48237_PI429010886 | M79471 | 0.015185 | 12 | 3 | 325974185 | Botryotinia fuckeliana isolate T4 SuperContig_34_1 | Cellular component organization | Cytoskeleton organization | 4.73E-51 | 9287.843 | 9647.722 | 5229.119 | 12485.87 | 9096.106 | 10899.12 | 7737.591 | 4920.271 | 7974.043 | 5326.176 | 5868.528 | 7373.59 | 7678.532 | 7282.171 | 8904.357 | 12422.81 | 6536.631 | 10899.12 | 7450.764 | 6894.743 | 6879.978 | 6490.163 | 5319.238 | 8421.341 | 6217.899 | 10964.51 | 6932.514 |
| CUST_42763_PI429010886 | M61184 | 0.015191 | 15 | 0 | | | | | | 55.14628 | 42.22821 | 56.0662 | 32.25357 | 35.9416 | 54.73192 | 28.27971 | 28.8964 | 33.08345 | 45.58985 | 42.53014 | 48.53823 | 22.97663 | 31.99053 | 39.68514 | 52.44678 | 52.83011 | 54.00554 | 40.40681 | 39.68514 | 49.89799 | 39.7396 | 42.93894 | 29.46206 | 50.69191 | 55.37764 | 48.00964 |
| CUST_42908_PI429010886 | M61569 | 0.015191 | 15 | 0 | | | | | | 786.706 | 236.7681 | 302.2127 | 98.21029 | 651.9918 | 214.4744 | 110.7189 | 142.8582 | 129.9706 | 452.9164 | 117.3642 | 86.67899 | 152.8609 | 201.3287 | 186.6438 | 344.6094 | 345.4444 | 446.8256 | 150.4655 | 229.4529 | 187.746 | 159.1135 | 111.7039 | 531.1131 | 181.0004 | 129.7943 | 204.2199 |
| CUST_27140_PI429010886 | N55515 | 0.015191 | 15 | 9 | | | | | | 49.75939 | 76.44833 | 38.86489 | 51.24109 | 27.29125 | 45.05092 | 41.95242 | 39.18602 | 52.43379 | 76.07766 | 42.06993 | 49.08702 | 38.36098 | 34.90893 | 40.68573 | 39.64114 | 51.89227 | 54.41833 | 37.09799 | 35.34739 | 31.70016 | 51.7068 | 48.3123 | 53.86098 | 83.98715 | 62.43324 | 95.84863 |
| CUST_52039_PI429010886 | M96174 | 0.015194 | 21 | 3 | 345486208 | PREDICTED: neither inactivation nor afterpotential | | | 6.94E-08 | 24.83878 | 12.2288 | 32.6566 | 36.41327 | 27.24397 | 33.30691 | 17.3946 | 19.35495 | 15.23056 | 15.79172 | 23.46287 | 21.17074 | 14.09213 | 23.24949 | 11.97517 | 13.94276 | 20.71004 | 32.65993 | 13.42609 | 14.1684 | 24.84953 | 16.48057 | 9.86011 | 18.8381 | 27.68313 | 43.44981 | 32.26103 |
| CUST_3390_PI429010886 | N14413 | 0.015201 | 24 | 18 | | | | | | 167.1839 | 173.4981 | 148.3475 | 203.1953 | 169.8899 | 155.2537 | 110.0483 | 135.8973 | 148.6917 | 226.3675 | 130.3328 | 190.3637 | 161.3901 | 220.3651 | 195.0743 | 206.4448 | 157.7092 | 196.359 | 281.652 | 212.3302 | 187.5374 | 219.9571 | 160.3693 | 176.0053 | 270.3602 | 178.6287 | 211.1137 |
| CUST_22673_PI429010886 | N47435 | 0.015201 | 24 | 18 | | | | | | 36.25859 | 44.68637 | 48.44993 | 46.26909 | 52.31329 | 49.05418 | 33.76678 | 40.06518 | 45.77928 | 58.73387 | 53.6056 | 60.21705 | 53.10839 | 42.91318 | 49.98628 | 67.33602 | 48.93138 | 63.3033 | 52.95745 | 75.32357 | 91.29321 | 63.88437 | 55.25124 | 41.41188 | 71.43104 | 59.93656 | 54.26471 |
| CUST_27492_PI429010886 | N56161 | 0.015201 | 24 | 18 | | | | | | 26.60013 | 36.37866 | 46.52715 | 40.61218 | 35.30842 | 36.53812 | 18.52484 | 24.17916 | 25.44437 | 44.61206 | 23.20138 | 39.00381 | 35.82698 | 59.21866 | 36.05134 | 43.2321 | 31.40893 | 39.69364 | 40.35674 | 56.52669 | 45.53002 | 41.53959 | 44.22939 | 27.27496 | 45.3248 | 40.35311 | 62.96106 |
| CUST_53124_PI429010886 | M100305 | 0.015208 | 24 | 21 | | | | | | 18.04283 | 24.23014 | 29.5983 | 43.27178 | 24.01772 | 20.61073 | 18.98105 | 11.51639 | 22.40881 | 28.62456 | 21.3486 | 21.66748 | 17.82405 | 21.27912 | 21.01736 | 34.14111 | 13.26048 | 18.45819 | 32.45148 | 21.36429 | 21.36688 | 29.74413 | 33.10045 | 32.47256 | 23.77729 | 29.0776 | 28.54216 |
| CUST_34403_PI429010886 | M9570 | 0.015208 | 24 | 12 | 465958871 | Gamma-butyrobetaine dioxygenase [Chelonia myda | Metabolic process | Oxidation-reduction process | 1.06E-30 | 75.39347 | 48.3123 | 79.60953 | 56.64713 | 50.00256 | 66.33339 | 66.46046 | 67.80419 | 111.485 | 48.22079 | 84.25035 | 128.7232 | 84.1757 | 94.45361 | 86.61515 | 112.7975 | 43.27178 | 60.77736 | 85.61256 | 75.67767 | 50.46214 | 62.87881 | 39.0489 | 47.26675 | 62.45524 | 44.56843 | 55.33057 |
| CUST_24968_PI429010886 | N51555 | 0.015208 | 24 | 15 | | | | | | 84.42877 | 86.25852 | 74.0547 | 66.53186 | 53.00673 | 82.33655 | 63.99533 | 79.07776 | 68.4981 | 110.6336 | 76.08615 | 73.34493 | 38.2551 | 49.49896 | 64.34064 | 146.4472 | 94.30937 | 128.1087 | 91.34253 | 81.98334 | 85.00815 | 84.85912 | 113.7768 | 124.0296 | 130.3093 | 68.91301 | 75.82559 |
| CUST_16503_PI429010886 | N36346 | 0.015209 | 24 | 15 | 321473698 | hypothetical protein DAPPUDRAFT_194445 [Daphn | | | 2.89E-22 | 87.2755 | 109.1768 | 86.49079 | 79.88802 | 85.18737 | 73.1185 | 69.86983 | 86.08273 | 74.3064 | 94.26766 | 93.59991 | 105.3949 | 97.47845 | 99.83489 | 99.15143 | 102.3532 | 100.7517 | 93.72794 | 111.2168 | 92.43125 | 84.722 | 104.8392 | 91.09799 | 73.93015 | 117.2072 | 60.05679 | 90.49352 |
| CUST_39442_PI429010886 | M39542 | 0.015228 | 12 | 0 | | | | | | 292.8985 | 355.6114 | 357.2284 | 270.2714 | 267.619 | 299.502 | 263.8365 | 264.1731 | 276.6094 | 403.3076 | 274.404 | 389.3563 | 286.3672 | 358.225 | 326.2404 | 287.1573 | 220.511 | 294.2777 | 281.599 | 294.3403 | 318.4396 | 369.2549 | 350.4435 | 285.0395 | 453.3672 | 340.6846 | 436.9493 |
| CUST_12272_PI429010886 | N29205 | 0.015228 | 12 | 3 | | | | | | 197.3524 | 170.8315 | 182.2638 | 327.2675 | 188.9774 | 223.0044 | 249.0021 | 289.0715 | 219.6484 | 239.8387 | 201.9396 | 163.9899 | 234.521 | 201.1076 | 169.2006 | 289.7475 | 262.9091 | 222.9038 | 188.8504 | 202.262 | 186.3956 | 277.8488 | 140.8613 | 148.6709 | 182.2299 | 112.4311 | 138.1939 |
| CUST_28400_PI429010886 | N57874 | 0.01523 | 21 | 3 | | | | | | 6.393088 | 18.02828 | 13.57199 | 20.4413 | 9.08762 | 11.51639 | 9.757187 | 6.994251 | 7.012133 | 10.73106 | 10.0073 | 16.73161 | 5.282757 | 4.4993 | 6.854646 | 7.190888 | 4.8159 | NA | 11.05702 | 14.66789 | NA | 9.639955 | NA | NA | 21.60669 | 19.67774 | 9.947764 |
| CUST_16746_PI429010886 | N36795 | 0.015237 | 21 | 3 | | | | | | 6.186749 | 25.86961 | 10.53088 | 16.34119 | 8.70488 | 11.81299 | 9.316937 | 4.790284 | NA | 10.99269 | NA | 6.597762 | 3.907357 | 10.06452 | NA | 6.62024 | NA | NA | 7.509358 | 12.89288 | 6.991817 | NA | 7.865975 | 10.80877 | 28.27339 | 16.76094 | 18.50772 |
| CUST_6092_PI429010886 | N19620 | 0.015241 | 18 | 0 | 386376745 | prostaglandin E synthase 2 [Penaeus monodon] | Metabolic process | Oxidation-reduction process | 6.9E-99 | 219.3055 | 319.0748 | 423.3887 | 157.8167 | 208.9685 | 229.2068 | 169.5547 | 210.6402 | 263.5785 | 121.2697 | 185.2303 | 316.2028 | 203.7918 | 256.4811 | 276.7604 | 300.0118 | 237.7359 | 238.5428 | 308.528 | 303.6686 | 278.9995 | 321.6396 | 233.3267 | 174.5449 | 208.8382 | 201.1422 | 210.4862 |
| CUST_39936_PI429010886 | M47037 | 0.015251 | 21 | 9 | 321455300 | hypothetical protein DAPPUDRAFT_64687 [Daphnia | | | 2.52E-55 | NA | 379.2136 | 112.1982 | 729.5711 | 787.1975 | 310.1413 | 587.6341 | 2189.348 | 1209.365 | 310.9824 | 1963.68 | 2491.849 | 1867.343 | 371.6995 | 1139.145 | NA | 928.6222 | NA | 266.5285 | 5528.982 | 1325.963 | 6.925886 | 347.6562 | 210.0239 | NA | 1165.934 | 1261.41 |
| CUST_10362_PI429010886 | N26120 | 0.015251 | 21 | 18 | | | | | | 293.5299 | 156.4282 | 378.4398 | 140.8976 | 158.1732 | 114.5044 | 322.1425 | 162.0779 | 164.6012 | 243.2097 | 238.1723 | 176.3364 | 215.0865 | 310.7127 | 158.0759 | 253.7351 | 263.9219 | 179.8447 | 352.5288 | 170.5103 | 212.1657 | 237.506 | 297.9644 | 269.9577 | 172.5457 | 100.1974 | 148.0082 |
| CUST_10673_PI429010886 | N26604 | 0.015257 | 21 | 3 | 488516396 | PREDICTED: ectonucleotide pyrophosphatase/phos | | | 1.17E-21 | 290.175 | 777.3766 | 510.76 | 973.4615 | 774.7379 | 749.7018 | 555.8637 | 674.2322 | 688.7233 | 561.5311 | 516.0613 | 887.8552 | 538.5366 | 607.5979 | 474.2296 | 423.8598 | 308.7717 | 381.8035 | 280.6713 | 973.2028 | 1089.128 | 408.8064 | 886.7923 | 472.651 | 750.1475 | 1011.687 | 554.4782 |
| CUST_47462_PI429010886 | M76220 | 0.015263 | 24 | 18 | | | | | | 42.13297 | 21.98819 | 29.05988 | 17.70878 | 3.422033 | 11.39415 | 28.23315 | 9.323293 | 5.902502 | 21.78224 | 9.498899 | 8.720501 | 16.51607 | 38.94819 | 23.97462 | 78.70239 | 14.97861 | 11.59993 | 19.5882 | 126.1032 | 105.322 | 49.07171 | 23.45219 | 18.63533 | 25.68589 | 62.07681 | 5.302715 |
| CUST_29155_PI429010886 | N59303 | 0.015263 | 24 | 15 | | | | | | 61.05832 | 72.21756 | 81.29483 | 53.15786 | 54.7161 | 72.22304 | 52.31892 | 67.42469 | 55.47209 | 66.92364 | 56.15273 | 77.63254 | 61.60941 | 110.8741 | 84.015 | 88.53305 | 75.11707 | 91.02196 | 66.03995 | 96.4587 | 80.47827 | 93.51914 | 72.1095 | 69.46349 | 87.97637 | 91.84392 | 63.06953 |
| CUST_46323_PI429010886 | M72220 | 0.015265 | 24 | 18 | | | | | | 186.5931 | 168.2432 | 212.8755 | 197.4721 | 176.601 | 179.7342 | 207.6194 | 196.8724 | 223.8084 | 204.946 | 223.1811 | 261.4208 | 281.0282 | 255.4386 | 333.708 | 367.1896 | 199.1618 | 235.838 | 262.8116 | 288.8593 | 231.9868 | 360.2672 | 226.6782 | 215.028 | 316.2028 | 306.093 | 322.9938 |
| CUST_30749_PI429010886 | N62553 | 0.015266 | 21 | 6 | | | | | | 5.341143 | 4.358079 | 9.840112 | 6.209359 | 7.15372 | 9.712939 | 8.8792 | 17.85771 | 12.4831 | 5.60979 | 6.404533 | NA | 18.99317 | 8.556731 | 5.328263 | 8.656419 | 6.439909 | 12.15636 | 5.153026 | 6.023483 | 4.559704 | NA | 5.558018 | 8.549314 | 6.230319 | 8.94682 | 14.26692 |
| CUST_43219_PI429010886 | M62437 | 0.015268 | 18 | 3 | | | | | | 20.09233 | 8.326131 | 20.36949 | 23.0986 | 25.43424 | 15.74407 | 15.67417 | 5.946298 | 12.75871 | 17.91398 | 10.25912 | 8.597478 | 4.913939 | 10.05217 | 6.276753 | 12.6487 | 21.03086 | 18.44972 | 12.31182 | 8.0157 | 5.538092 | 16.27976 | 20.31618 | NA | 15.60693 | 9.669901 | 19.35261 |
| CUST_2257_PI429010886 | N11972 | 0.015283 | 21 | 18 | 321460374 | hypothetical protein DAPPUDRAFT_327214 [Daphn | | | 9.6E-68 | 34.96354 | 39.83835 | 12.34182 | 33.01349 | 24.41741 | 27.46314 | 19.66187 | 23.74079 | 17.72933 | 30.38651 | 37.16398 | 29.32849 | 24.28972 | 28.77383 | 42.51309 | 55.13629 | 26.84327 | 35.68582 | 46.39268 | 49.55412 | 30.3471 | 34.83322 | 27.72017 | 18.57026 | 32.66922 | 36.10574 | 27.68808 |
| CUST_39423_PI429010886 | M39190 | 0.015288 | 24 | 15 | 255653868 | crustin 1 [Panulirus japonicus] | | | 1.4E-08 | 64.3345 | 123.5431 | 137.649 | 48.66621 | 38.23666 | 6.97251 | 68.33006 | 84.79763 | 113.8579 | 131.6679 | 126.4569 | 98.07031 | 210.2559 | 178.7372 | 131.39 | 173.4981 | 216.9753 | 122.0703 | 91.94088 | 203.1495 | 126.1505 | 158.7737 | 499.1257 | 94.02768 | 1316.22 | 130.1208 | 947.8741 |
| CUST_15846_PI429010886 | N35237 | 0.015288 | 24 | 15 | | | | | | 64.43856 | 68.64401 | 78.73486 | 53.80266 | 96.36676 | 43.83106 | 67.47589 | 87.80183 | 55.9701 | 105.4884 | 46.11029 | 64.95763 | 51.273 | 53.8203 | 70.43457 | 125.8598 | 102.2773 | 149.0785 | 163.4482 | 69.11088 | 120.7015 | 69.9232 | 89.28732 | 119.1299 | 71.87199 | 60.12977 | 107.4359 |
| CUST_42883_PI429010886 | M61508 | 0.015289 | 18 | 3 | 28573624 | retained, isoform A [Drosophila melanogaster] >gi\| | Reproduction | Reproduction | 6.28E-53 | 10.77657 | 11.0938 | 7.423086 | 22.8004 | 13.44859 | 41.67274 | 13.10341 | 4.72626 | 19.9835 | 4.810355 | 11.64471 | 6.042681 | 10.9956 | 11.27249 | 4.229484 | 4.560304 | 6.600465 | 16.00542 | 8.017906 | 13.22714 | 13.63459 | 15.18532 | 11.49641 | NA | 9.28711 | 13.47203 | 4.959572 |
| CUST_361_PI429010886 | N4431 | 0.015295 | 18 | 3 | | | | | | 1725.627 | 510.76 | 3607.364 | 2809.847 | 2287.157 | 2890.576 | 3929.651 | 3534.122 | 929.2639 | 538.762 | 2010.691 | 49.48526 | 441.5814 | 385.0916 | 384.9318 | 31.71523 | 1373.566 | 52.98961 | 2102.543 | 35.63872 | 1875.725 | NA | 1988.345 | 2518.59 | 39.52248 | 65.12567 | 54.25943 |
| CUST_1773_PI429010886 | N10666 | 0.015298 | 18 | 15 | | | | | | 20.06061 | 14.49351 | 20.15261 | 13.63953 | 6.130259 | 15.88403 | 19.50921 | 9.439346 | 8.373525 | 17.02082 | 18.98962 | 20.41758 | 15.03377 | 15.99247 | 20.03192 | 20.36949 | 19.51691 | 16.53843 | 12.90472 | 16.94406 | 19.52762 | 11.23514 | 16.31327 | 8.371508 | 13.73772 | 19.26372 | 10.22197 |
| CUST_55923_PI429010886 | M112145 | 0.015303 | 12 | 0 | | | | | | 155.4719 | 129.896 | 174.3314 | 108.0008 | 126.1032 | 145.6295 | 78.95924 | 92.09827 | 123.1656 | 115.5186 | 115.5867 | 98.45237 | 108.6177 | 132.7049 | 128.6369 | 193.9691 | 113.9832 | 162.2752 | 97.29292 | 94.88698 | 130.6959 | 162.2159 | 93.39666 | 132.5094 | 239.8387 | 127.0483 | 203.6001 |
| CUST_6223_PI429010886 | N19798 | 0.01532 | 15 | 0 | 321477834 | hypothetical protein DAPPUDRAFT_311166 [Daphn | Protein metabolism | Proteolysis | 6.48E-35 | 214.2348 | 147.6693 | 121.4237 | 66.89269 | 146.8141 | 143.4489 | 106.9212 | 161.9364 | 124.4126 | 104.7362 | 135.9242 | 101.2406 | 128.1757 | 146.2229 | 120.3747 | 164.572 | 150.8016 | 113.1427 | 132.7741 | 85.19825 | 120.2652 | 99.20203 | 127.8042 | 106.5844 | 82.71912 | 55.9772 | 86.04961 |
| CUST_9051_PI429010886 | N23990 | 0.01532 | 15 | 9 | | | | | | 30.85032 | 62.69277 | 57.23868 | 60.3241 | 70.56572 | 73.46154 | 69.25418 | 77.1205 | 113.7768 | 56.56684 | 96.28021 | 155.9986 | 73.61433 | 99.90716 | 72.13281 | 32.08763 | 54.5089 | 85.3343 | 36.115 | 114.0935 | 83.13765 | 72.3636 | 117.746 | 98.32258 | 76.16727 | 95.80114 | 146.8499 |
| CUST_23261_PI429010886 | N48529 | 0.01532 | 15 | 9 | | | | | | 77.10171 | 68.44169 | 124.009 | 128.6574 | 128.0141 | 79.45781 | 82.69106 | 105.1367 | 84.41305 | 163.7665 | 142.34 | 130.9859 | 115.7071 | 114.443 | 125.4203 | 102.9808 | 96.75892 | 89.75292 | 111.6499 | 90.92371 | 117.2885 | 154.4215 | 130.2006 | 120.2042 | 146.8499 | 86.7972 | 126.5542 |
| CUST_52095_PI429010886 | M96397 | 0.015325 | 15 | 9 | 189233835 | PREDICTED: similar to trypsin-like serine protease [T | Protein metabolism | Proteolysis | 1.15E-18 | 19.88004 | 17.71272 | 51.84431 | 25.17336 | 27.3183 | 16.96708 | 20.86742 | 41.28967 | 52.57619 | 62.89604 | 32.01858 | 30.6513 | 36.88342 | 64.75716 | 40.82788 | 47.44495 | 24.23967 | 20.71812 | 16.48468 | 31.33095 | 18.75687 | 62.82655 | 26.01607 | 13.35477 | 66.578 | 32.94683 | 62.28748 |
| CUST_22451_PI429010886 | N47054 | 0.015336 | 15 | 12 | | | | | | 21.16443 | 7.28896 | 9.488473 | 6.089115 | 5.823418 | 7.675832 | 22.26601 | 12.52538 | 8.12515 | 6.205172 | 9.362608 | 7.555334 | 26.06598 | 15.78045 | NA | 12.09352 | 6.99794 | NA | 11.14537 | 5.977954 | 6.778155 | 13.68809 | 7.183846 | 9.156873 | 15.02774 | 10.51343 | 13.65696 |
| CUST_15424_PI429010886 | N34558 | 0.015336 | 15 | 6 | | | | | | 656.7764 | 96.15886 | 863.9077 | 273.4925 | 192.4131 | 285.7468 | 728.0084 | 1664.492 | 1758.855 | 756.9801 | 242.7679 | 103.0015 | 85.51349 | 85.89616 | 52.08136 | 16.08632 | 284.6976 | 44.08735 | 227.8356 | 703.8812 | 548.3934 | 22.05258 | 1336.322 | 384.3454 | 104.164 | 165.7972 | 62.73269 |
| CUST_11921_PI429010886 | N28631 | 0.015339 | 12 | 3 | | | | | | 435.8026 | 606.1944 | 328.6838 | 670.112 | 373.2026 | 441.5814 | 308.9771 | 341.3294 | 313.8962 | 376.8596 | 313.2862 | 257.9077 | 337.3994 | 382.1801 | 428.1275 | 496.875 | 408.8064 | 453.7002 | 358.2812 | 545.8082 | 353.5438 | 331.2853 | 319.5751 | 375.3397 | 359.6577 | 460.7596 | 310.9824 |
| CUST_53433_PI429010886 | M101684 | 0.015344 | 21 | 9 | | | | | | 10.35656 | 15.28107 | NA | 12.66068 | 67.53545 | 11.7224 | 22.9227 | 144.2982 | 96.75892 | 13.8928 | 158.4583 | 197.2642 | 80.63305 | 13.10516 | 21.03086 | NA | 68.30614 | 15.7177 | 14.30732 | 254.095 | 14.71509 | 6.951132 | 20.53115 | 12.35337 | 18.57528 | 41.28967 | 63.50632 |
| CUST_42756_PI429010886 | M61169 | 0.015347 | 18 | 6 | | | | | | 4.370166 | 73.93957 | 9.339132 | 23.37953 | 18.40839 | 17.55722 | 6.869381 | 65.17572 | 93.97578 | 24.44489 | NA | 53.1338 | 13.2544 | 28.51585 | 25.10548 | 33.22847 | NA | 8.919059 | 10.92713 | NA | 4.312161 | 18.02091 | 15.13091 | 10.46038 | 104.5737 | 120.1371 | 30.88587 |
| CUST_35658_PI429010886 | M13163 | 0.015357 | 24 | 12 | 530588606 | PREDICTED: vesicle transport protein SFT2A [Chryse | Transport | Vesicle-mediated transport | 4.48E-31 | 107.638 | 87.68861 | 122.2181 | 88.13649 | 96.90956 | 82.85967 | 92.48444 | 101.6113 | 103.8804 | 111.0963 | 124.3147 | 120.2652 | 91.48208 | 124.9049 | 124.5429 | 115.7777 | 116.2656 | 106.023 | 106.2399 | 124.6404 | 114.5149 | 110.2326 | 109.7731 | 97.13391 | 95.72441 | 108.6304 | 117.847 |
| CUST_27093_PI429010886 | N55435 | 0.015367 | 21 | 3 | | | | | | 6.939821 | 7.499239 | 10.67013 | 11.01084 | 20.31071 | NA | 12.00666 | 6.099554 | 34.7772 | 7.563005 | 6.779829 | 21.75394 | 10.09707 | 6.578887 | 4.884781 | 3.6657 | 10.07591 | 9.255767 | NA | 10.50509 | 6.794828 | 14.10338 | NA | 10.07029 | 17.72667 | 13.04417 | NA |
| CUST_56511_PI429010886 | M113967 | 0.015374 | 24 | 18 | | | | | | 1404.631 | 1293.664 | 1541.439 | 1581.923 | 1076.944 | 1097.654 | 945.8187 | 909.1015 | 1050.102 | 1410.296 | 1182.29 | 2114.712 | 1136.737 | 1480.417 | 2081.941 | 2873.023 | 851.4085 | 1175.49 | 1560.159 | 2062.191 | 1848.861 | 2127.118 | 1343.537 | 1424.308 | 1609.005 | 1595.711 | 1749.353 |
| CUST_21350_PI429010886 | N45059 | 0.01538 | 12 | 0 | | | | | | 196.9798 | 161.0222 | 156.3242 | 140.3314 | 111.027 | 125.3173 | 125.0127 | 118.4299 | 139.7174 | 160.0546 | 135.3099 | 152.5355 | 152.7576 | 160.531 | 193.6848 | 196.2537 | 142.2988 | 140.6181 | 180.3864 | 175.1526 | 135.1566 | 160.6252 | 150.6325 | 148.5046 | 192.5048 | 156.4704 | 195.0326 |
| CUST_19283_PI429010886 | N41308 | 0.015384 | 15 | 0 | | | | | | 114.6191 | 101.6576 | 134.3348 | 127.4128 | 118.378 | 174.1985 | 44.42212 | 43.9859 | 49.97285 | 85.35234 | 140.5313 | 112.3653 | 29.56761 | 98.68398 | 58.23374 | 123.5719 | 119.2945 | 143.1678 | 73.62026 | 189.2921 | 88.50576 | 94.48599 | 63.73904 | 76.57062 | 92.90826 | 92.00286 | 78.17016 |
| CUST_40616_PI429010886 | M55648 | 0.015391 | 15 | 12 | | | | | | 154.8889 | 189.8565 | 182.709 | 141.7869 | 163.7665 | 210.0511 | 112.1531 | 155.5733 | 147.8481 | 183.7339 | 167.6742 | 210.3675 | 187.0803 | 186.0312 | 202.5331 | 189.2302 | 172.9784 | 184.3634 | 134.4739 | 189.4251 | 167.4539 | 158.6824 | 182.318 | 147.073 | 207.853 | 208.2116 | 208.515 |
| CUST_35000_PI429010886 | M11295 | 0.015405 | 12 | 9 | | | | | | 63.46985 | 49.13878 | 50.277 | 56.52669 | 62.61352 | 62.89021 | 62.27945 | 68.84477 | 56.56684 | 72.59038 | 65.56207 | 63.13991 | 64.80137 | 52.44678 | 42.71137 | 61.33812 | 62.41453 | 64.83329 | 88.7408 | 64.55964 | 60.72981 | 55.4319 | 76.77824 | 62.68635 | 47.45909 | 50.79816 | 49.43857 |
| CUST_6124_PI429010886 | N19659 | 0.015405 | 12 | 0 | | | | | | 2098.72 | 3154.98 | 2421.023 | 1424.308 | 1540.227 | 1300.415 | 1415.482 | 1784.695 | 1915.9 | 2465.06 | 1564.24 | 2595.092 | 2480.699 | 1871.332 | 2474.132 | 2607.508 | 1407.859 | 1869.455 | 1554.539 | 2041.736 | 1837.915 | 2569.563 | 1884.761 | 1409.58 | 2236.34 | 2175.001 | 3156.693 |
| CUST_7659_PI429010886 | N21882 | 0.015406 | 18 | 6 | | | | | | 116.1482 | 287.8668 | 319.6424 | 373.896 | 161.1033 | 156.3501 | 401.6619 | 424.4597 | 572.181 | 537.3115 | 328.8667 | 419.0394 | 228.547 | 423.2024 | 323.9834 | 502.3399 | 186.0881 | 360.9417 | 91.04966 | 258.2202 | 68.97204 | 738.2515 | 430.0642 | 108.3189 | 723.9047 | 155.5309 | 462.1839 |
| CUST_47367_PI429010886 | M75816 | 0.01541 | 24 | 21 | 345488503 | PREDICTED: DENN domain-containing protein 5A-lik | | | 1.22E-59 | 17.67128 | 12.54832 | 16.07923 | NA | 7.485838 | 5.862531 | 11.85797 | 4.571104 | 5.707653 | 12.62032 | 12.92194 | 8.238355 | 7.565304 | 6.586963 | 7.894781 | 10.47118 | 22.37611 | 10.76549 | 21.03821 | 6.251086 | 18.57232 | 16.57643 | 17.59799 | 13.37697 | 12.36577 | NA | 9.907267 |
| CUST_13900_PI429010886 | N31969 | 0.01541 | 24 | 0 | | | | | | 20.78572 | 28.48649 | 21.04609 | 20.69763 | 17.72933 | 23.8296 | 12.24457 | 7.505181 | 20.06979 | 15.45471 | 22.3415 | 6.578227 | 11.31368 | 12.60946 | 16.65148 | 23.47248 | 18.38957 | 17.40769 | 19.46629 | 24.37441 | 18.7049 | 18.98793 | 15.557 | 13.03592 | 20.80307 | 32.33619 | 16.66286 |
| CUST_52129_PI429010886 | M96490 | 0.015436 | 15 | 0 | | | | | | 110.8479 | 79.44029 | 71.64903 | 58.11529 | 81.25969 | 109.7631 | 49.65587 | 54.48925 | 62.16793 | 47.75268 | 64.87589 | 65.9084 | 100.542 | 66.40412 | 75.84454 | 109.9302 | 72.52738 | 90.55235 | 78.18464 | 70.65347 | 50.98504 | 97.01852 | 34.00967 | 106.0819 | 63.76139 | 65.63314 | 47.77962 |
| CUST_46709_PI429010886 | M73478 | 0.015448 | 21 | 12 | | | | | | 54.89562 | 44.61606 | 56.76191 | 31.35806 | 37.27794 | 30.40149 | 58.60431 | 47.45909 | 59.02601 | 53.03364 | 58.36485 | 66.66314 | 63.21853 | 71.5379 | 54.78436 | 66.43118 | 43.15798 | 51.04982 | 48.48483 | 50.22974 | 59.44434 | 53.20866 | 61.55497 | 52.8414 | 52.53115 | 40.73202 | 58.22987 |
| CUST_32004_PI429010886 | M2975 | 0.015468 | 21 | 0 | | | | | | 12.07461 | 36.50859 | NA | 14.8771 | 9.55327 | 6.723673 | 6.130259 | 4.740581 | 6.81575 | 13.46088 | 9.320692 | NA | 6.561972 | NA | 3.689496 | 15.8658 | 4.506846 | 11.13787 | 14.97661 | 5.907047 | 6.176204 | 11.56285 | 27.4471 | 14.46988 | 16.06241 | 10.39612 | NA |
| CUST_41303_PI429010886 | M57406 | 0.01547 | 24 | 12 | | | | | | 81.56419 | 296.5251 | 155.8809 | 556.2338 | 352.4518 | 494.0702 | 425.4184 | 187.9283 | 215.167 | 709.3475 | 162.3689 | 676.1626 | 1841.426 | 2121.44 | 1966.099 | 369.8842 | 1970.653 | 1142.155 | 611.3709 | 43.10477 | 161.3901 | 393.1602 | 706.4958 | 1614.376 | 636.5467 | 216.9753 | 245.4886 |
| CUST_16832_PI429010886 | N36935 | 0.01547 | 24 | 0 | | | | | | 489.1062 | 537.0505 | 431.3819 | 394.3244 | 415.4709 | 404.7043 | 366.7345 | 338.7294 | 296.8223 | 426.3129 | 341.0355 | 431.1968 | 269.4403 | 403.3076 | 369.3306 | 551.2455 | 327.471 | 379.8142 | 327.535 | 517.2906 | 418.4668 | 482.1854 | 389.0546 | 369.9548 | 442.7732 | 444.0863 | 461.5523 |
| CUST_36871_PI429010886 | M17102 | 0.015471 | 24 | 21 | | | | | | 40.90354 | 46.71536 | 44.97678 | 32.8525 | 35.07632 | 25.50611 | 31.01397 | 26.20316 | 21.63352 | 51.36009 | 19.25276 | 36.65319 | 44.6585 | 36.03165 | 37.48716 | 44.19983 | 25.26607 | 36.8879 | 36.92762 | 51.14708 | 48.75681 | 52.8351 | 46.44979 | 41.29944 | 57.89544 | 20.31401 | 53.5429 |
| CUST_25779_PI429010886 | N53049 | 0.015476 | 24 | 6 | | | | | | 18.77363 | 27.24092 | 27.87322 | 31.0493 | 21.64331 | 42.40387 | 43.1527 | 44.52812 | 32.32519 | 40.31382 | 31.52203 | 21.62181 | 28.17477 | 30.48607 | 25.99654 | 21.90983 | 21.9058 | 16.58905 | 21.99145 | 21.4795 | 27.00762 | 31.8423 | 26.00775 | 14.27406 | 18.86439 | 10.39893 | 10.5119 |
| CUST_44300_PI429010886 | M65548 | 0.015483 | 12 | 0 | | | | | | 239.4694 | 341.5068 | 289.679 | 274.5343 | 281.735 | 340.2645 | 213.366 | 261.9745 | 242.041 | 381.9407 | 248.1135 | 451.1707 | 293.5772 | 392.9826 | 492.7259 | 288.8593 | 191.9558 | 249.5102 | 225.3596 | 249.2507 | 290.3758 | 309.2864 | 296.0927 | 286.2536 | 297.8198 | 359.2285 | 347.3329 |
| CUST_12957_PI429010886 | N30348 | 0.015485 | 21 | 9 | | | | | | NA | 5.390025 | 8.429067 | 9.988677 | 11.81158 | 16.52278 | 4.354924 | 35.29256 | 29.65544 | NA | 42.82987 | 43.65248 | 33.49263 | 7.50844 | 24.21798 | 7.380862 | 12.31182 | NA | 4.800213 | 93.6434 | 14.89292 | NA | 6.903956 | NA | NA | 15.67918 | 22.82948 |
| CUST_42890_PI429010886 | M61527 | 0.015492 | 15 | 12 | 291234661 | PREDICTED: H2A histone family, member Y-like [Sac | | | 1.19E-51 | 26.46898 | 32.22274 | 54.06396 | 25.38492 | 25.71503 | 28.7982 | 14.61919 | 23.33499 | 32.74461 | 28.3805 | 31.52633 | 32.81991 | 32.80342 | 47.23493 | 50.36094 | 39.15692 | 69.53162 | 47.33315 | 34.1956 | 30.45779 | 25.55609 | 38.59967 | 39.1747 | 20.52466 | 41.59799 | 40.42484 | 23.91073 |
| CUST_21956_PI429010886 | N46155 | 0.015492 | 15 | 0 | | | | | | 57.64388 | 60.23492 | 53.37349 | 38.58794 | 44.31783 | 48.5992 | 39.1747 | 41.6388 | 39.28519 | 43.60346 | 48.78617 | 49.23486 | 38.95593 | 55.54166 | 58.71363 | 50.39753 | 59.9559 | 76.83746 | 61.33812 | 37.62657 | 66.98492 | 50.36639 | 42.88135 | 47.30806 | 53.41869 | 55.4319 | 58.92815 |
| CUST_45961_PI429010886 | M70909 | 0.015497 | 18 | 15 | 170057904 | phd finger protein [Culex quinquefasciatus] >gi\|167 | | | 6.48E-36 | 9.615739 | 12.78901 | 17.64932 | 10.54492 | 8.8792 | 9.638679 | 11.0429 | 7.957708 | 5.490342 | 15.20148 | 5.97138 | 15.7161 | 10.85866 | 11.73206 | 8.118926 | 15.29079 | 14.00927 | 16.46383 | 20.5469 | 10.35656 | 11.06786 | 8.205981 | 16.84886 | 8.199563 | 9.648135 | 10.77469 | 6.478519 |
| CUST_15729_PI429010886 | N35041 | 0.015497 | 18 | 3 | | | | | | 143.7068 | 126.0646 | 135.6803 | 158.5656 | 124.8492 | 224.858 | 206.5428 | 115.4907 | 96.58099 | 83.4507 | 198.4267 | 92.92758 | 83.8218 | 111.5107 | 99.65085 | 172.1318 | 134.3489 | 213.4377 | 216.0426 | 93.84895 | 108.7243 | 162.8992 | 231.0649 | 221.5886 | 105.1 | 108.4222 | 68.65348 |
| CUST_15779_PI429010886 | N35137 | 0.015497 | 18 | 9 | | | | | | 224.2596 | 217.3065 | 261.9745 | 213.9895 | 253.8833 | 256.2441 | 198.7421 | 271.6132 | 253.2546 | 296.7454 | 286.3036 | 325.1931 | 256.4303 | 300.4403 | 286.0749 | 269.9093 | 246.7296 | 269.1187 | 240.8565 | 294.8062 | 256.8944 | 252.9368 | 270.2125 | 212.501 | 303.7719 | 225.1487 | 333.5081 |
| CUST_19266_PI429010886 | N41274 | 0.015497 | 18 | 15 | | | | | | 84.22055 | 93.69637 | 45.04433 | 121.1456 | 88.01513 | 105.57 | 83.46951 | 54.50124 | 57.96698 | 63.32035 | 90.22371 | 60.62589 | 68.17959 | 103.109 | 90.67241 | 91.24599 | 113.9625 | 110.8992 | 98.21029 | 58.77783 | 69.89586 | 85.50355 | 57.49062 | 85.04454 | 65.4438 | 60.80176 | 53.35103 |
| CUST_20862_PI429010886 | N44205 | 0.015497 | 18 | 15 | 507973683 | PREDICTED: putative tRNA pseudouridine synthase | Metabolic process | Nucleobase-containing compound metabolic process | 6.29E-17 | 39.26587 | 46.95662 | 44.61606 | 34.27813 | 41.68626 | 29.74088 | 39.63103 | 35.7998 | 33.14087 | 60.4668 | 40.94065 | 41.56705 | 36.27978 | 29.09978 | 38.28975 | 53.03364 | 52.44678 | 43.59595 | 37.06882 | 44.74185 | 36.60129 | 43.81006 | 36.91363 | 40.78513 | 40.59153 | 31.05167 | 31.54453 |
| CUST_23227_PI429010886 | N48477 | 0.01553 | 15 | 12 | | | | | | 32.59218 | 50.01795 | 44.28888 | 26.5649 | 26.70117 | 39.59771 | 23.11258 | 34.119 | 32.73103 | 47.94376 | 38.05146 | 61.42421 | 34.5638 | 59.9559 | 56.58921 | 86.80804 | 49.73229 | 42.99716 | 41.19711 | 40.53465 | 37.49843 | 46.37649 | 39.57456 | 24.87802 | 43.07866 | 55.23417 | 62.46895 |
| CUST_46622_PI429010886 | M73197 | 0.015547 | 24 | 6 | | | | | | 271.6624 | 169.2493 | 23.08457 | 308.6668 | 48.94831 | 69.89586 | 296.0384 | 40.40681 | 315.7757 | 23.93394 | 26.53793 | 32.51731 | 211.6272 | 22.82362 | 163.6107 | 68.17959 | 10.55308 | 74.45078 | 9.712939 | 14.97861 | 22.46676 | 60.76524 | 33.44293 | 154.3982 | 28.78903 | 37.36976 | 6.018145 |
| CUST_38388_PI429010886 | M26165 | 0.015552 | 21 | 9 | | | | | | 5.131288 | 13.70846 | 13.68648 | 47.99403 | 75.66735 | 26.82709 | 40.97284 | 186.883 | 93.11072 | 16.92026 | 224.9139 | 316.5936 | 119.2791 | 36.15703 | 71.86244 | 5.285615 | 60.36203 | NA | 6.572882 | 794.1984 | 114.5611 | NA | 21.21967 | 6.534722 | 6.13531 | 98.67215 | 59.15067 |
| CUST_3942_PI429010886 | N15624 | 0.015553 | 15 | 3 | | | | | | 13.47795 | 19.13027 | 15.71416 | 12.97798 | 17.9732 | 22.83161 | 18.7509 | 11.99825 | 13.86905 | 10.03383 | 9.884881 | 10.34781 | 11.72403 | 13.3483 | 12.65789 | 173.2007 | 14.04228 | 13.52517 | 10.35656 | 18.28861 | 23.30725 | 15.81822 | NA | 7.871072 | 13.63096 | 14.44266 | 6.947339 |
| CUST_28490_PI429010886 | N58044 | 0.015564 | 15 | 0 | | | | | | 24.35068 | 24.69594 | 26.40308 | 24.69054 | 20.59775 | 26.43455 | 12.69324 | 10.23296 | 11.29332 | 25.13071 | 15.35258 | 18.51256 | 10.41181 | 140.0012 | 30.07667 | 25.06723 | 23.25736 | 26.11497 | 13.45887 | 18.00505 | 21.04144 | 21.97161 | 20.07638 | NA | 21.7647 | 21.5971 | 18.46372 |
| CUST_44438_PI429010886 | M65988 | 0.015568 | 24 | 0 | 156539569 | PREDICTED: SET and MYND domain-containing prot | | | 5.08E-09 | 145.8455 | 128.7498 | 110.0909 | 53.02941 | 107.8556 | 60.90428 | 104.9421 | 86.75819 | 49.02131 | 84.52872 | 76.74889 | 86.65167 | 85.73944 | 90.02313 | 77.53281 | 128.1757 | 80.63305 | 88.3275 | 82.14765 | 70.36778 | 55.28288 | 133.9495 | 51.11368 | 100.6811 | 158.1524 | 114.9603 | 136.0755 |
| CUST_52249_PI429010886 | M96844 | 0.015568 | 15 | 12 | | | | | | 590.149 | 711.9048 | 792.4242 | 538.667 | 602.798 | 760.7539 | 579.3813 | 534.2439 | 583.066 | 697.3779 | 644.7638 | 934.9001 | 648.0827 | 922.48 | 1066.114 | 870.876 | 618.8739 | 653.8395 | 604.7306 | 713.4223 | 649.5445 | 761.273 | 669.2807 | 585.5822 | 731.0066 | 845.457 | 875.926 |
| CUST_7341_PI429010886 | N21404 | 0.015576 | 15 | 9 | 321460896 | hypothetical protein DAPPUDRAFT_216354 [Daphn | Protein metabolism | Proteolysis | 1.26E-24 | 179.1322 | 263.2372 | 195.3201 | 179.7938 | 165.4234 | 177.3713 | 280.165 | 210.3335 | 280.5129 | 528.8856 | 305.2258 | 242.302 | 178.9487 | 175.9447 | 231.4164 | 313.5825 | 212.8057 | 213.9895 | 227.642 | 160.259 | 150.0759 | 206.6326 | 159.948 | 291.2799 | 194.777 | 262.6197 | 398.2452 |
| CUST_15186_PI429010886 | N34153 | 0.015582 | 12 | 0 | | | | | | 58.40782 | 53.6538 | 58.27233 | 41.93775 | 33.64032 | 40.4329 | 38.71497 | 52.75651 | 46.40711 | 67.39787 | 45.1918 | 55.84117 | 54.11265 | 72.14746 | 47.40881 | 54.66707 | 48.13849 | 46.36775 | 41.56303 | 48.24061 | 58.12612 | 54.01314 | 61.04516 | 43.0244 | 61.16623 | 49.16869 | 63.27865 |
| CUST_22269_PI429010886 | N46721 | 0.015582 | 12 | 3 | | | | | | 1354.655 | 730.7862 | 425.7405 | 2595.092 | 2624.92 | 5991.557 | 3186.032 | 1143.075 | 1986.907 | 57.59047 | 1966.099 | 15.7789 | 1475.875 | 520.2836 | 765.4435 | 992.543 | 5430.703 | 6281.928 | 1735.328 | 282.0094 | 2076.382 | 1254.757 | 1516.22 | 4001.148 | 195.7902 | 2215.807 | 14.32917 |
| CUST_51864_PI429010886 | M95451 | 0.015653 | 24 | 3 | | | | | | 11.21679 | 5.193441 | NA | 22.71838 | 13.58003 | 24.91723 | 10.75291 | 8.5745 | 20.30399 | 12.28582 | NA | 3.863772 | 4.315859 | 6.474157 | 5.394984 | 41.38053 | 7.156953 | NA | NA | NA | 9.118641 | 8.416999 | 9.654274 | 16.52278 | 10.29115 | 11.04144 | 15.75594 |
| CUST_25355_PI429010886 | N52253 | 0.015658 | 18 | 3 | | | | | | 29.79322 | 18.2618 | 19.80461 | 31.68203 | 30.28193 | 22.98207 | 18.9048 | 13.92624 | 15.6362 | 19.9426 | 20.13319 | 16.47383 | 16.98819 | 17.48685 | 17.77803 | 29.62583 | 15.24047 | 24.0705 | 19.22009 | 18.00284 | 20.98763 | 25.97297 | 20.37942 | 22.26808 | 29.51436 | 17.91813 | 24.4797 |
| CUST_23710_PI429010886 | N49332 | 0.015661 | 18 | 15 | | | | | | 35.03294 | 19.51092 | 20.52907 | 30.19994 | 12.84177 | 32.82642 | 16.1321 | 12.80086 | 10.11533 | 11.06303 | 9.452942 | 11.95482 | 8.670788 | 23.89355 | 20.07855 | 24.28972 | 21.38676 | 24.65512 | 19.51465 | 8.403892 | 35.27881 | 22.58566 | 12.5608 | 19.04191 | 20.45683 | 17.02546 | 14.52793 |
| CUST_53347_PI429010886 | M101351 | 0.015665 | 24 | 0 | | | | | | 38.87013 | 31.51891 | 32.90178 | 27.09726 | 27.35532 | 19.18718 | 13.295 | 14.69551 | NA | 32.816 | 20.73834 | 19.9356 | 12.0793 | 13.87826 | 7.3856 | 28.72316 | 21.26565 | 16.62053 | 20.82009 | 18.16906 | 14.02096 | 17.91608 | 6.261173 | 34.0757 | 39.32188 | 14.80982 | 21.49202 |
| CUST_41331_PI429010886 | M57465 | 0.01567 | 12 | 0 | | | | | | 22.93439 | 20.61726 | 23.2348 | 12.75753 | 9.74748 | 20.07209 | 18.43075 | 8.853279 | 18.98793 | 13.01679 | 10.93186 | 16.94555 | 14.91553 | 29.58077 | 22.88862 | 24.46354 | 3.836556 | 19.22009 | 18.34116 | 6.141389 | 14.10863 | 24.1098 | 32.15824 | 21.07244 | 26.19664 | 21.80952 | 31.32413 |
| CUST_19971_PI429010886 | N42537 | 0.01567 | 24 | 15 | 307211213 | Uncharacterized protein KIAA0241 [Harpegnathos s | | | 1.28E-22 | 18.92641 | 14.85125 | 16.05605 | 13.33577 | 14.571 | 10.30492 | 17.4527 | 13.8081 | 12.97507 | 21.14577 | 13.17601 | 14.77827 | 9.599473 | 29.32033 | 19.62649 | 19.00448 | 22.4606 | 26.43024 | 17.59108 | 23.9514 | 9.473492 | 20.74295 | 18.52484 | 20.27214 | 18.81492 | 21.30938 | 17.6024 |
| CUST_22926_PI429010886 | N47931 | 0.015671 | 24 | 21 | 332857184 | PREDICTED: zinc finger protein 845 [Pan troglodytes | | | 3.37E-07 | 5.026041 | 10.17638 | NA | 15.13091 | 4.001936 | 7.350712 | NA | 8.587225 | NA | 6.763654 | NA | 7.098337 | 5.645769 | 3.873898 | 5.24251 | 6.370435 | 3.570227 | NA | 7.833526 | NA | 14.23269 | 12.0315 | 10.04937 | 13.69236 | 9.890597 | 14.046 | 10.24258 |
| CUST_5533_PI429010886 | N18827 | 0.01568 | 24 | 15 | | | | | | 729.5711 | 740.2282 | 732.8997 | 541.31 | 560.7028 | 689.4854 | 547.117 | 668.4719 | 724.6164 | 795.552 | 719.1308 | 992.543 | 628.6974 | 831.0172 | 737.499 | 830.2031 | 751.4303 | 789.0995 | 927.1088 | 760.5296 | 717.4734 | 810.0074 | 792.1417 | 771.0971 | 778.415 | 626.1001 | 822.9643 |
| CUST_52323_PI429010886 | M97056 | 0.015695 | 18 | 15 | | | | | | 32.02812 | 27.25936 | 45.80703 | 23.38605 | 35.96223 | 36.98717 | 19.32322 | 20.06061 | 24.37698 | 33.08815 | 27.00762 | 34.85719 | 27.76975 | 35.69567 | 37.20514 | 30.48251 | 43.5699 | 40.03126 | 31.78686 | 36.52603 | 30.99487 | 42.33366 | 31.27363 | 22.25206 | 28.39147 | 24.24945 | 32.45773 |
| CUST_26774_PI429010886 | N54855 | 0.015695 | 18 | 15 | | | | | | 15.5215 | 17.62531 | 28.64876 | 15.92833 | 14.84209 | 6.406079 | 15.2558 | 23.56991 | 19.64381 | 23.39301 | 14.49923 | 19.79173 | 19.97113 | 29.98761 | 17.97064 | 35.69903 | 26.76358 | 32.21966 | 12.19897 | 22.71838 | 14.98356 | 16.48255 | 22.38584 | 13.12172 | 33.45717 | 11.70432 | 15.57429 |
| CUST_29393_PI429010886 | N59775 | 0.015695 | 18 | 15 | | | | | | 29.62034 | 15.74407 | 33.42509 | 26.77181 | 17.02313 | 25.82739 | 19.87086 | 29.05431 | 25.62143 | 24.28604 | 30.92757 | 28.07255 | 28.37529 | 45.57 | 26.1771 | 39.29828 | 28.78598 | 32.93338 | 20.28225 | 27.77642 | 26.72384 | 22.55627 | 27.63304 | 19.3508 | 31.90818 | 27.5887 | 21.56963 |
| CUST_41756_PI429010886 | M58482 | 0.015716 | 24 | 18 | | | | | | 109.4643 | 189.3158 | 113.5525 | 141.4293 | 157.4172 | 131.4929 | 150.5497 | 98.59007 | 92.8298 | 149.8948 | 184.8748 | 96.76761 | 110.6582 | 110.5931 | 28.78291 | 204.7693 | 182.9909 | 101.7342 | 221.852 | 204.946 | 167.6977 | 198.7167 | 136.9448 | 162.3309 | 189.1715 | 119.7777 | 120.1521 |
| CUST_6059_PI429010886 | N19573 | 0.015717 | 21 | 9 | 321477830 | hypothetical protein DAPPUDRAFT_230283 [Daphn | | | 6.5E-69 | 19.54386 | 17.83843 | 46.27883 | 32.55113 | 38.2551 | 27.13299 | 48.63366 | 90.04123 | 77.38462 | 62.39032 | 69.16053 | 57.05842 | 92.79213 | 32.50599 | 54.91632 | 24.27705 | 36.66046 | 34.7228 | 81.44839 | 164.4613 | 92.93954 | 17.49601 | 27.44183 | 30.57426 | 43.76947 | 62.10251 | 72.4241 |
| CUST_20087_PI429010886 | N42786 | 0.015717 | 18 | 3 | 242009353 | conserved hypothetical protein [Pediculus humanus | | | 9.83E-15 | 124.7587 | 67.09564 | 140.6181 | 438.2102 | 172.831 | 283.6461 | 140.7563 | 48.04331 | 120.9219 | 55.25124 | 71.01876 | 195.4506 | 89.82702 | 155.3616 | 76.16727 | 127.4456 | 45.89316 | 140.9396 | 67.82256 | 71.49639 | 97.79572 | 144.3614 | 212.9083 | 147.687 | 158.101 | 227.6752 | 106.6154 |
| CUST_37961_PI429010886 | M22481 | 0.015718 | 21 | 12 | | | | | | 34.75963 | 2851.724 | 2024.906 | 27.21982 | 26.41986 | 31.85967 | 18.96468 | 17.11368 | 26.87538 | 57.53634 | 2886.71 | 4965.834 | 208.3478 | 4791.679 | 5528.982 | 13959.79 | 170.2454 | 9.083511 | 13.78635 | 5009.146 | 4345.199 | 2663.095 | 3230.715 | 300.2764 | 29.20384 | 15.10731 | 45.30213 |
| CUST_2836_PI429010886 | N13211 | 0.015721 | 15 | 9 | 380023990 | PREDICTED: major facilitator superfamily domain-cc | | | 8.84E-30 | 29.38309 | 23.12912 | 17.31674 | 10.66012 | 27.61589 | 21.24533 | 49.97285 | 43.41776 | 33.03027 | 152.0579 | 27.89681 | 95.65165 | 43.12293 | 34.21339 | 32.47618 | 109.4535 | 52.04012 | 38.92892 | 26.9466 | 21.32603 | 39.45879 | 34.71503 | 55.59887 | 19.33417 | 97.30444 | 30.71736 | 56.27319 |
| CUST_38819_PI429010886 | M30933 | 0.015724 | 15 | 12 | | | | | | 4579.426 | 4651.575 | 5029.59 | 1675.386 | 3630.71 | 3105.643 | 3582.089 | 2727.828 | 3858.586 | 4110.832 | 3656.575 | 4988.507 | 4685.303 | 6174.548 | 4044.665 | 5510.561 | 3192.934 | 4519.983 | 3717.434 | 4404.238 | 3259.251 | 5769.917 | 3158.604 | 3251.962 | 4257.291 | 3849.875 | 5780.138 |
| CUST_33109_PI429010886 | M5992 | 0.015724 | 15 | 6 | | | | | | 18.75687 | 24.17916 | 23.35223 | 22.33694 | 24.03089 | 25.5315 | 27.38684 | 18.05975 | 34.54086 | 25.32554 | 11.52212 | 18.01506 | 17.80246 | 20.58016 | 18.65246 | 2328.414 | 21.82911 | 21.12887 | 14.43577 | 26.69852 | 24.929 | 35.07942 | 28.3838 | 33.33046 | 32.70344 | 24.66303 | 18.65246 |
| CUST_22067_PI429010886 | N46360 | 0.015724 | 15 | 12 | | | | | | 1394.051 | 2139.684 | 1914.945 | 1339.496 | 1465.294 | 1601.861 | 1324.078 | 1395.016 | 1573.105 | 1842.625 | 1594.841 | 2478.692 | 1648.471 | 2205.198 | 2482.039 | 2184.967 | 1576.839 | 1864.705 | 1441.16 | 1771.34 | 1539.294 | 2168.863 | 1978.873 | 1696.945 | 1951.832 | 1588.254 | 2101.303 |
| CUST_47635_PI429010886 | M76979 | 0.015748 | 15 | 12 | | | | | | 186.1103 | 199.1321 | 209.1269 | 207.0705 | 158.7119 | 166.0617 | 129.8119 | 159.5233 | 180.634 | 229.178 | 190.8326 | 303.4405 | 213.1848 | 203.8966 | 258.3105 | 201.8347 | 172.3293 | 201.5302 | 177.5459 | 196.4155 | 184.3061 | 205.1195 | 200.2689 | 158.3361 | 238.3762 | 179.9528 | 230.0814 |

| Probe ID | ID | p-value | Period | Phase | EBI ID | Annotation | GO category | Go term | E-value | 0 | 0 | 0 | 3 | 3 | 3 | 6 | 6 | 6 | 9 | 9 | 9 | 12 | 12 | 12 | 15 | 15 | 15 | 18 | 18 | 18 | 21 | 21 | 21 | 24 | 24 | 24 |
|---|---|---|---|---|---|---|---|---|---|---|---|---|---|---|---|---|---|---|---|---|---|---|---|---|---|---|---|---|---|---|---|---|---|---|---|---|
| CUST_41462_PI429010886 | M57790 | 0.015759 | 12 | 3 | | | | | | 14.58359 | 14.8771 | 11.52212 | 26.75848 | 27.77944 | 20.59992 | 36.47986 | 16.22526 | 14.93582 | 16.49199 | 19.56278 | 24.09915 | 17.08079 | 13.23043 | 14.12813 | 21.35291 | 31.34117 | 15.45873 | 17.34415 | 17.70164 | 25.16709 | 13.01381 | 36.66046 | 22.66107 | 14.39261 | 17.43627 | 14.75025 |
| CUST_34187_PI429010886 | M8997 | 0.015759 | 12 | 0 | | | | | | 202.9438 | 358.4672 | 297.4174 | 78.28594 | 97.85969 | 110.8588 | 90.16303 | 186.6799 | 133.4052 | 105.1478 | 183.0597 | 172.957 | 173.2522 | 259.1091 | 127.3248 | 224.8249 | 111.637 | 76.78516 | 118.4632 | 324.5803 | 151.2035 | 201.085 | 224.0801 | 128.1445 | 215.028 | 282.3228 | 169.9489 |
| CUST_30432_PI429010886 | N61884 | 0.015785 | 15 | 6 | | | | | | 64.71156 | 70.51648 | 90.60771 | 243.4729 | 329.5353 | 597.1763 | 306.2716 | 848.6156 | 517.1879 | 538.1956 | 174.2722 | 126.2324 | NA | 130.8168 | 58.13115 | 651.8188 | 332.54 | 190.62 | 11.14854 | 218.3592 | 303.9241 | 553.514 | 313.4993 | 612.5844 | 515.4056 | 410.594 | 236.8108 |
| CUST_7181_PI429010886 | N21156 | 0.015796 | 18 | 0 | 321456676 | hypothetical protein DAPPUDRAFT_330720 [Daphn | Metabolic process | Oxidation-reduction process | 6.87E-63 | 275.8443 | 309.4606 | 338.7848 | 200.0082 | 247.1436 | 185.3847 | 223.4939 | 144.1866 | 220.2228 | 266.7997 | 202.7268 | 247.8745 | 190.4186 | 260.0114 | 258.545 | 255.6195 | 177.0624 | 282.9535 | 224.6063 | 235.7212 | 315.0245 | 312.2468 | 214.6655 | 280.2656 | 221.5477 | 190.0092 | 187.4758 |
| CUST_55975_PI429010886 | M112389 | 0.0158 | 24 | 15 | | | | | | 983.5467 | 1211.313 | 1153.924 | 1095.532 | 1085.565 | 1186.311 | 803.7954 | 944.3898 | 1003.294 | 1413.053 | 1065.113 | 1447.664 | 1225.452 | 1394.642 | 1149.353 | 1620.39 | 1162.648 | 1366.468 | 1275.086 | 1257.529 | 1100.556 | 1451.1 | 1286.564 | 1357.763 | 1172.181 | 916.176 | 1385.628 |
| CUST_11501_PI429010886 | N27917 | 0.0158 | 24 | 6 | | | | | | 613.0465 | 150.5702 | 59.86271 | 218.8408 | 60.08957 | 232.1034 | 403.4755 | 541.9679 | 432.0274 | 16.42728 | 38.04057 | 37.77068 | 440.0791 | 25.24001 | 317.6417 | 41.17264 | 44.331 | 49.9806 | 44.64586 | 54.11265 | 54.70385 | 64.37974 | 17.65649 | 358.6229 | 48.93138 | 415.3047 | 66.98492 |
| CUST_17297_PI429010886 | N37731 | 0.0158 | 24 | 15 | 345482168 | PREDICTED: kinesin-like protein unc-104 [Nasonia v | | | 1.95E-38 | 39.56391 | 39.31065 | 42.08627 | 39.08441 | 41.43272 | 23.45771 | 30.66462 | 28.08525 | 30.81693 | 38.73055 | 43.0244 | 51.58632 | 26.59751 | 57.40224 | 44.30287 | 48.80606 | 53.35562 | 45.88881 | 46.64002 | 65.42078 | 30.9788 | 44.7891 | 42.79462 | 38.69682 | 50.20045 | 38.70657 | 58.88281 |
| CUST_32141_PI429010886 | M3340 | 0.015805 | 21 | 9 | 383852569 | PREDICTED: uncharacterized protein LOC10088243 | | | 2.46E-37 | 57.55495 | 67.9638 | 25.22384 | 16.0983 | 23.99602 | 20.8433 | 73.39754 | 279.5586 | 31.9232 | 42.53014 | 130.1208 | 88.74926 | 80.37733 | 108.3885 | 33.80408 | 115.0086 | 76.62599 | 45.52189 | 126.5833 | 24.57116 | 58.17407 | 40.98319 | 51.25727 | 72.30449 | 25.33083 | 48.41112 | 62.89604 |
| CUST_8224_PI429010886 | N22737 | 0.01582 | 21 | 15 | | | | | | 19.09941 | 16.22392 | 20.95699 | 14.19203 | 13.62607 | 12.14326 | 9.529604 | 15.35498 | 13.89365 | 24.02102 | 20.65497 | 18.55589 | 27.50275 | 12.91176 | 16.33291 | 21.96101 | 14.14626 | 32.69174 | 21.87774 | 22.06407 | 17.53688 | 19.01531 | 17.26803 | 15.17796 | 26.88229 | 9.002381 | 16.67423 |
| CUST_42256_PI429010886 | M59815 | 0.015828 | 21 | 0 | | | | | | 31.43539 | 8.174796 | 32.61136 | NA | 4.625348 | NA | 4.404187 | 5.807016 | NA | 7.819931 | 6.146383 | 6.089115 | 4.69711 | 7.953838 | 3.705602 | 5.906131 | 46.31902 | 6.938783 | 5.72427 | 6.415718 | NA | 19.1106 | 30.33968 | NA | 11.85797 | 7.144042 | 15.5488 |
| CUST_8724_PI429010886 | N23481 | 0.015843 | 24 | 0 | 148706233 | protease, serine, 15, isoform CRA_b [Mus musculus | Response to stress | Other | 1.2E-113 | 23.02707 | 18.04537 | 12.20709 | 16.11142 | 12.09352 | 16.1997 | 10.56079 | 9.279943 | 11.52078 | 6.952548 | 17.5216 | 15.91213 | 9.462739 | 8.207161 | 12.51827 | 15.38675 | 13.42458 | 22.498 | 13.42877 | 14.96682 | 9.900278 | 14.69744 | 13.41264 | 8.824107 | 17.61362 | 17.77636 | 18.19818 |
| CUST_17255_PI429010886 | N37659 | 0.015845 | 24 | 21 | | | | | | 17.98995 | 9.447649 | 14.08468 | NA | 7.042021 | 11.32516 | 9.444187 | 5.369319 | 6.006114 | 3.886115 | 14.57386 | 11.06447 | NA | 5.251174 | 9.265801 | 10.95786 | 25.63785 | 14.02745 | 10.8963 | 6.808243 | 14.40546 | 15.72851 | 13.73929 | 14.77643 | 13.26743 | 6.158206 | 11.05595 |
| CUST_37636_PI429010886 | M20050 | 0.015857 | 24 | 15 | 77743875 | Euphausia superba isolate DGGE band AntEuk13-5 | | | | 131916.1 | 131916.1 | 119417.1 | 139148.1 | 118088.6 | 130067.6 | 135996.9 | 63412.19 | 130067.6 | 139845.6 | 148780.5 | 143243.9 | 117180.4 | 127883.9 | 145034.3 | 154243.9 | 148206.7 | 159657.9 | 140655.5 | 160411.4 | 127883.9 | 141355.5 | 137174.3 | 169441.2 | 107761.9 | 81423.55 | 143243.9 |
| CUST_18763_PI429010886 | N40367 | 0.015863 | 24 | 21 | | | | | | 17.18399 | 21.62724 | 17.00952 | 19.08147 | 20.92287 | 17.58162 | 16.24039 | 21.50201 | 13.14309 | 20.27214 | 13.43047 | 21.8729 | 16.30995 | 24.48268 | 13.62433 | 19.43524 | 39.36593 | 17.96131 | 24.20666 | 28.72316 | 23.38605 | 20.89839 | 33.14087 | 28.888 | 20.26748 | 25.33083 | 17.19879 |
| CUST_45171_PI429010886 | M68219 | 0.015877 | 24 | 0 | | | | | | 13.89481 | 11.40101 | 14.58718 | 14.08468 | 10.59651 | 16.53993 | 9.477835 | 6.586963 | 9.939882 | 6.336048 | 9.796108 | NA | 10.78913 | 6.968129 | 11.26503 | 9.631079 | 6.244116 | 12.02167 | 11.00948 | 9.384386 | 6.936041 | 7.08513 | 24.84656 | 10.74554 | 14.98047 | 10.10842 | 28.91721 |
| CUST_48247_PI429010886 | M79511 | 0.015883 | 21 | 0 | | | | | | 9.661698 | 17.86001 | 13.24553 | NA | 8.045043 | 9.304008 | 6.62304 | 8.289001 | 4.502545 | 11.7971 | 10.18958 | 11.15122 | 8.267442 | 29.44853 | 5.119427 | 9.148382 | 8.910931 | NA | NA | 7.453496 | 12.01254 | 12.35487 | 18.12511 | 16.7373 | 15.3592 | 6.738368 | NA |
| CUST_4867_PI429010886 | N17651 | 0.015884 | 24 | 18 | | | | | | 188.0537 | 196.5038 | 259.4845 | 196.129 | 198.308 | 209.4212 | 227.3812 | 240.2928 | 223.2546 | 219.0917 | 260.4633 | 275.5962 | 315.6417 | 314.7546 | 378.7197 | 423.4903 | 205.909 | 275.6931 | 289.2842 | 344.5306 | 286.7556 | 428.9575 | 303.6176 | 284.7868 | 341.3294 | 367.4512 | 354.7389 |
| CUST_26963_PI429010886 | N55193 | 0.015884 | 24 | 21 | 321471668 | hypothetical protein DAPPUDRAFT_316454 [Daphn | Metabolic process | Other | 6.89E-32 | 60.66262 | 95.21856 | 94.43038 | 60.08391 | 117.3985 | 106.6154 | 64.80696 | 47.73188 | 73.48596 | 77.42171 | 49.21104 | 73.15736 | 64.85632 | 126.0286 | 86.65167 | 96.689 | 103.6058 | 82.26332 | 82.19809 | 104.964 | 74.43452 | 199.9828 | 87.70762 | 125.9958 | 81.91685 | 50.50708 | 78.20249 |
| CUST_28852_PI429010886 | N58727 | 0.015884 | 24 | 21 | | | | | | 56.65296 | 74.0547 | 58.34728 | 54.40089 | 51.49206 | 43.80188 | 32.51731 | 34.05805 | 43.09035 | 64.57467 | 40.44483 | 69.68816 | 52.52687 | 68.82274 | 83.88709 | 97.25472 | 42.21564 | 60.79572 | 51.30486 | 114.5891 | 54.25425 | 82.28242 | 63.73239 | 57.49879 | 82.17099 | 47.82182 | 55.92013 |
| CUST_52265_PI429010886 | M96905 | 0.015885 | 18 | 3 | 124487752 | hypothetical protein [Maconellicoccus hirsutus] | | | 1.65E-11 | 129.6012 | 161.2431 | 101.8776 | 229.0179 | 149.9119 | 219.8055 | 149.3496 | 143.2327 | 107.9821 | 114.6697 | 121.0016 | 107.2229 | 112.4452 | 100.5206 | 115.7617 | 104.351 | 158.2669 | 152.424 | 152.1158 | 114.0624 | 140.4751 | 150.5899 | 150.449 | 140.3134 | 130.9642 | 164.4877 | 91.85305 |
| CUST_20878_PI429010886 | N44234 | 0.015891 | 24 | 0 | | | | | | 70.88181 | 69.42462 | 83.83314 | 50.7913 | 86.48234 | 93.59991 | 65.76284 | 40.16678 | 69.27267 | 54.8287 | 41.52003 | 78.56402 | 36.98363 | 61.02614 | 49.25162 | 62.69277 | 32.69174 | 65.04995 | 42.70151 | 32.11242 | 75.43644 | 52.19516 | 119.228 | 69.13041 | 92.31619 | 88.62563 | 59.80108 |
| CUST_49448_PI429010886 | M85664 | 0.015905 | 15 | 3 | | | | | | 35.11564 | 15.1027 | 7.528845 | 46.54214 | 18.91018 | 57.28882 | 36.92762 | 29.31792 | 20.74032 | 4.813825 | 9.833527 | 10.9768 | 20.70807 | 4.019481 | 8.995799 | 11.93851 | 35.31634 | 18.10692 | 18.69696 | NA | NA | 13.40926 | NA | 25.03856 | 4.625815 | 24.18723 | NA |
| CUST_50517_PI429010886 | M90116 | 0.015918 | 15 | 12 | | | | | | 22.42354 | 17.47664 | 14.77179 | 11.48488 | 11.52352 | 13.14638 | 14.75394 | 63.98783 | 10.76813 | 15.48667 | 17.1412 | 18.04108 | 30.39192 | 18.59451 | 29.21516 | 22.73586 | 20.00172 | 9.672443 | 8.215837 | 19.19575 | 11.66524 | 15.64186 | 15.62557 | 24.75161 | 15.31913 | 12.59239 | 24.67666 |
| CUST_13750_PI429010886 | N31705 | 0.015918 | 15 | 12 | | | | | | 98.50813 | 175.6726 | 225.693 | 140.7358 | 140.2611 | 185.3559 | 195.555 | 203.8732 | 219.1432 | 172.436 | 208.7767 | 292.1904 | 201.3634 | 396.6524 | 249.3051 | 149.3929 | 187.276 | 291.1029 | 130.4101 | 153.2997 | 190.201 | 175.4136 | 286.8668 | 212.0119 | 256.281 | 177.758 | 258.8731 |
| CUST_15429_PI429010886 | N34568 | 0.015918 | 15 | 12 | | | | | | 1093.92 | 1418.381 | 1373.566 | 1096.334 | 1221.884 | 1213.834 | 1081.105 | 1061.463 | 1304.617 | 1405.495 | 1168.631 | 1607.248 | 1307.703 | 1680.099 | 1507.459 | 1727.428 | 1428.874 | 1410.296 | 1230.999 | 1310.544 | 1042.392 | 1488.789 | 1249.958 | 1393.263 | 1845.048 | 1268.323 | 1884.761 |
| CUST_30562_PI429010886 | N62125 | 0.01592 | 15 | 0 | | | | | | 18.51801 | 22.1833 | 19.51092 | 14.34446 | 16.32095 | 13.63622 | 18.37346 | 19.00616 | 12.81312 | 15.98933 | 11.55545 | 18.94868 | 18.09247 | 16.29644 | 6.319994 | 20.06405 | 39.20036 | 28.62456 | 19.61118 | 13.5398 | 15.67763 | 18.02828 | NA | 15.69237 | 16.42376 | 12.87509 | 11.33051 |
| CUST_8128_PI429010886 | N22576 | 0.015921 | 15 | 9 | 391333152 | PREDICTED: uncharacterized protein LOC10090325 | | | 2.82E-12 | 339.4134 | 562.8166 | 515.0601 | 424.6374 | 452.4978 | 434.8523 | 367.5439 | 425.4184 | 405.9943 | 607.7896 | 453.5801 | 583.4081 | 433.0579 | 594.2743 | 503.9702 | 646.2272 | 428.7852 | 497.7449 | 392.0959 | 453.79 | 300.5825 | 676.7937 | 406.9652 | 391.346 | 631.2945 | 723.9047 | 784.6927 |
| CUST_33267_PI429010886 | M6444 | 0.015925 | 24 | 18 | | | | | | 27.2017 | 40.87944 | 20.13119 | 20.25455 | 16.1574 | 12.78901 | 16.89803 | 9.54713 | 23.28835 | 15.04869 | 18.96046 | 44.49519 | 11.48488 | 26.71614 | 26.61223 | 27.44439 | 11.30247 | 26.86268 | 24.62356 | 35.13017 | 33.07929 | 33.88263 | 24.45653 | 31.64126 | 29.47231 | 23.06016 | 26.09533 |
| CUST_1727_PI429010886 | N10549 | 0.015925 | 24 | 18 | | | | | | 3206.496 | 3792.107 | 3347.664 | 4680.081 | 4357.297 | 4992.636 | 3161.551 | 3172.097 | 3100.565 | 2929.717 | 4263.359 | 3616.168 | 3266.951 | 3624.616 | 3821.032 | 4058.299 | 5439.997 | 4297.995 | 5602.191 | 3451.374 | 4424.066 | 3554.679 | 4808.982 | 4711.518 | 3355.686 | 3991.112 | 3347.664 |
| CUST_19202_PI429010886 | N41159 | 0.01593 | 15 | 0 | 307189811 | Protein piccolo [Camponotus floridanus] | | | 1.16E-07 | 29.90107 | 34.70757 | 36.69324 | 25.79616 | 27.89681 | 19.82899 | 17.19461 | 20.25895 | 15.4798 | 38.78884 | 35.23219 | 26.11231 | 12.06262 | 49.10355 | 28.43336 | 35.60511 | 30.23833 | 47.3396 | 27.22266 | 25.92533 | 30.34438 | 29.48584 | 23.93394 | 20.70807 | 49.33073 | 19.56129 | 35.1468 |
| CUST_39600_PI429010886 | M41641 | 0.015937 | 12 | 0 | 77743875 | Euphausia superba isolate DGGE band AntEuk13-5 | | | 7.6E-174 | 74.34861 | 87.6112 | 72.40334 | 43.87581 | 51.82417 | 62.44513 | 55.55874 | 58.70799 | 83.13765 | 68.14294 | 55.95565 | 85.65603 | 65.9853 | 80.1769 | 62.53849 | 59.49267 | 58.25978 | 74.89819 | 53.24987 | 69.20227 | 60.81378 | 62.46096 | 89.75292 | 71.80124 | 83.83973 | 70.7847 | 93.17664 |
| CUST_19294_PI429010886 | N41329 | 0.015937 | 12 | 3 | | | | | | 25.45721 | 24.316 | 23.00815 | 33.77253 | 70.27178 | 45.05944 | 56.57261 | 43.64196 | 29.78164 | 27.0144 | 37.92604 | 18.50615 | 29.38309 | 18.08051 | 38.71497 | 32.42452 | 79.85552 | 54.79813 | 84.32383 | 9.751886 | 40.8947 | 33.3136 | 28.68501 | 47.1063 | 30.0232 | 23.85453 | 31.77739 |
| CUST_31670_PI429010886 | M2133 | 0.015961 | 15 | 9 | | | | | | 376.8596 | 388.7869 | 283.2818 | 380.8862 | 336.3354 | 266.1283 | 367.0612 | 344.6094 | 340.867 | 557.9517 | 383.4448 | 428.9575 | 405.9331 | 385.3789 | 327.9867 | 494.3438 | 370.4089 | 309.1695 | 316.0527 | 367.921 | 315.3784 | 592.5758 | 349.6083 | 282.2114 | 629.4397 | 368.7924 | 391.5964 |
| CUST_39348_PI429010886 | M38124 | 0.015961 | 15 | 9 | 390362924 | PREDICTED: zinc finger protein 84-like [Strongylocen | | | 6.86E-17 | 29.81837 | 38.91166 | 39.70082 | 30.74351 | 38.91166 | 27.91558 | 27.71021 | 34.55995 | 36.04752 | 41.52003 | 52.33717 | 49.49037 | 39.8885 | 48.36598 | 40.27161 | 48.1847 | 21.7647 | 38.09477 | 28.10839 | 37.54075 | 48.94359 | 47.13105 | 39.55733 | 37.25447 | 48.17972 | 40.20429 | 45.69115 |
| CUST_3763_PI429010886 | N15223 | 0.015961 | 15 | 0 | | | | | | 2929.717 | 3493.781 | 3213.851 | 2137.444 | 2435.819 | 3029.514 | 2114.712 | 1656.441 | 2416.782 | 2988.348 | 2472.114 | 3722.852 | 2444.229 | 2731.57 | 3443.242 | 3398.882 | 2712.01 | 3187.857 | 2459.721 | 3429.061 | 2698.741 | 3332.072 | 2448.925 | 1555.864 | 2718.679 | 2656.499 | 3100.565 |
| CUST_21460_PI429010886 | N45262 | 0.015967 | 21 | 18 | | | | | | 14.66789 | 16.55361 | 15.10927 | 7.049754 | 8.446645 | 10.20122 | 4.101557 | 9.403927 | 9.602601 | 17.10987 | 9.851879 | 16.8197 | 7.425323 | 8.949545 | 9.202277 | 11.34865 | 19.88848 | 24.93734 | 17.68083 | 17.64932 | 13.03254 | 14.76465 | 14.84035 | 6.474974 | 32.72017 | 11.8505 | 12.94753 |
| CUST_20461_PI429010886 | N43485 | 0.015984 | 24 | 21 | | | | | | 11.10641 | 11.87142 | 16.18956 | 7.788223 | 8.733788 | 11.19977 | 12.07646 | 5.707653 | 8.905799 | 4.241882 | 8.656419 | 10.70604 | 16.86851 | 14.07257 | 21.83957 | 11.27249 | 14.28677 | 18.73831 | 11.6136 | 17.17252 | 9.136468 | 14.58939 | 14.63109 | 19.34025 | 15.78805 | 8.800802 | 12.68722 |
| CUST_5657_PI429010886 | N19000 | 0.015991 | 18 | 0 | 195484963 | GE12547 [Drosophila yakuba] >gi\|194176995\|gb\|E | Cellular Process | Cell proliferation | | 68.34468 | 90.78956 | 43.9135 | 28.53148 | 64.11406 | 38.35127 | 44.03947 | 41.92389 | 44.40636 | 40.36236 | 44.0607 | 48.82597 | 38.2326 | 28.15071 | 40.74077 | 47.62012 | 55.70806 | 52.99425 | 63.24801 | 79.23712 | 46.93128 | 49.55412 | 44.61606 | 50.30627 | 39.55173 | 36.4309 | 59.22278 |
| CUST_51508_PI429010886 | M93784 | 0.015998 | 24 | 15 | | | | | | 29.67419 | 31.30264 | 26.01379 | 33.48853 | 18.60353 | 24.26769 | 22.20218 | 22.24289 | 26.01832 | 23.52324 | 39.52248 | 24.16344 | 18.73049 | 38.1599 | 26.11989 | 44.80736 | 31.56077 | 43.50773 | 28.85946 | 37.02581 | 29.9042 | 44.45342 | 39.84998 | 24.5556 | 35.51693 | 33.94751 | 34.57302 |
| CUST_6775_PI429010886 | N20585 | 0.016 | 15 | 0 | | | | | | 16.51743 | 21.65726 | NA | 17.2758 | 19.54896 | 8.883037 | 11.56409 | 7.088352 | 11.55545 | 14.39787 | 13.95627 | 7.931172 | 15.39724 | 8.447696 | 14.65658 | 32.8723 | 21.3985 | 22.78329 | 14.49586 | 9.495063 | 12.35337 | 10.20974 | 28.90009 | NA | 18.42696 | 7.666723 | 12.79677 |
| CUST_19445_PI429010886 | N41594 | 0.016004 | 18 | 12 | 196002303 | hypothetical protein TRIADDRAFT_14880 [Trichopla | Metabolic process | Other | 6.71E-19 | 36.33605 | 28.20406 | 58.90324 | 37.20061 | 11.82852 | 32.84559 | 14.69551 | 25.96531 | 16.30613 | 53.02384 | 68.41788 | 43.18546 | 97.56553 | 50.33907 | 40.9233 | 115.837 | 25.19237 | 45.76846 | 44.37542 | 13.90672 | 34.37569 | 70.16242 | 45.52612 | 48.33618 | 18.32163 | 15.08227 | 9.865884 |
| CUST_36601_PI429010886 | M16331 | 0.016025 | 21 | 0 | | | | | | 145.8124 | 192.1754 | 133.2798 | 117.746 | 136.1666 | 150.5702 | 111.1891 | 110.4435 | 122.2713 | 131.4476 | 141.4293 | 169.9686 | 99.69352 | 114.5611 | 114.0624 | 121.8291 | 138.2104 | 110.6759 | 137.4942 | 146.4651 | 123.8281 | 172.7927 | 138.2955 | 138.5342 | 156.2777 | 130.2006 | 111.151 |
| CUST_51231_PI429010886 | M92710 | 0.01603 | 24 | 0 | | | | | | 6949.82 | 9670.846 | 7470.617 | 5787.347 | 6941.938 | 5539.858 | 4909.967 | 5256.29 | 7188.247 | 6536.631 | 6388.757 | 9261.186 | 5744.456 | 4208.899 | 4778.44 | 9219.301 | 5119.45 | 6366.193 | 5336.786 | 8876.599 | 7175.165 | 7737.591 | 6779.053 | 5738.426 | 7306.322 | 7318.038 | 7516.748 |
| CUST_53987_PI429010886 | M103989 | 0.016033 | 21 | 18 | | | | | | 78.93037 | 62.46895 | 64.65683 | 52.70775 | 55.34146 | 56.39304 | 53.95069 | 43.37513 | 46.41206 | 56.76191 | 65.88576 | 63.68912 | 50.04478 | 61.58977 | 66.22546 | 90.69277 | 53.2143 | 60.11602 | 70.56572 | 62.6571 | 55.37764 | 72.74687 | 62.71467 | 55.57729 | 60.07749 | 43.59959 | 56.88203 |
| CUST_25454_PI429010886 | N52447 | 0.016038 | 24 | 15 | | | | | | 119.1803 | 114.2465 | 143.2572 | 93.44686 | 87.14788 | 130.7098 | 75.92185 | 80.86611 | 94.99099 | 113.2085 | 88.7408 | 157.7275 | 99.49562 | 139.8015 | 98.86383 | 140.9566 | 144.7064 | 132.2265 | 102.9429 | 116.5927 | 118.0563 | 120.8052 | 111.8739 | 129.2719 | 121.2272 | 101.4644 | 133.9011 |
| CUST_33645_PI429010886 | M7500 | 0.016053 | 21 | 18 | | | | | | 237.3703 | 243.2786 | 261.1858 | 228.6997 | 211.4751 | 257.4087 | 212.8309 | 202.3705 | 180.1966 | 199.1036 | 242.5645 | 258.4381 | 190.62 | 249.3051 | 211.8227 | 265.6039 | 238.1723 | 224.884 | 264.7036 | 272.7999 | 238.1723 | 282.4175 | 293.704 | 200.5357 | 252.5323 | 223.377 | 198.1505 |
| CUST_6693_PI429010886 | N20470 | 0.016053 | 21 | 9 | 156546442 | PREDICTED: 1-acyl-sn-glycerol-3-phosphate acyltrar | Metabolic process | Other | 8.34E-75 | 54.77637 | 76.30835 | 78.76718 | 82.56662 | 83.89746 | 91.69639 | 57.51717 | 100.6811 | 123.4738 | 81.51675 | 97.5299 | 141.7648 | 93.13261 | 83.25716 | 75.58303 | 77.1366 | 46.12593 | 62.29412 | 72.5683 | 88.77007 | 107.9228 | 63.65227 | 75.40046 | 29.21516 | 101.6806 | 97.81711 | 79.81539 |
| CUST_40411_PI429010886 | M55005 | 0.016055 | 21 | 12 | | | | | | 195.7902 | 202.1751 | 286.9542 | 197.1531 | 231.3049 | 242.1214 | 211.2278 | 147.3218 | 220.0411 | 269.5045 | 209.9313 | 412.7593 | 270.6659 | 284.536 | 253.1914 | 252.6775 | 246.2258 | 299.7832 | 293.5299 | 272.6795 | 275.0029 | 243.5502 | 269.4403 | 220.6157 | 219.0917 | 226.151 | 283.3904 |
| CUST_48178_PI429010886 | M79245 | 0.016059 | 15 | 3 | | | | | | 42.51309 | 3.940183 | 53.99181 | 30.77501 | 84.41305 | 106.2586 | 116.0813 | 35.30492 | 17.52398 | 12.56927 | 56.55039 | NA | 24.94778 | 8.854627 | 11.36366 | NA | 168.6965 | 49.52106 | 170.0663 | NA | 46.23898 | NA | 84.90321 | 90.92371 | 10.65599 | 25.51724 | 12.95606 |
| CUST_18243_PI429010886 | N39421 | 0.016084 | 15 | 12 | | | | | | 67.21241 | 204.8186 | 169.3227 | 40.39432 | 69.4087 | 41.50205 | 7.279581 | 22.63755 | 30.96855 | 77.49116 | 80.75638 | 211.0236 | 110.1779 | 236.2907 | 209.2252 | 110.4846 | 8.799195 | 77.15963 | 40.64728 | 96.81237 | 56.59481 | 304.2796 | 97.6483 | 27.65747 | 79.07032 | 35.64343 | 66.67032 |
| CUST_7899_PI429010886 | N22242 | 0.01609 | 24 | 21 | 71159516 | C-type lectin [Penaeus monodon] | | | 9.67E-12 | 1318.564 | 1652.112 | 1436.981 | 917.1171 | 1376.074 | 1755.419 | 1038.033 | 1173.091 | 935.3105 | 1210.626 | 1173.656 | 1474.825 | 845.2412 | 1249.958 | 1244.117 | 2474.132 | 1312.47 | 1825.909 | 1324.694 | 1618.557 | 1477.505 | 1542.262 | 1299.658 | 2035.189 | 2101.303 | 2155.605 | 2169.749 |
| CUST_13940_PI429010886 | N32052 | 0.016093 | 18 | 15 | | | | | | 66.53867 | 69.18246 | 57.83783 | 67.1407 | 52.63381 | 42.30947 | 45.97157 | 46.37649 | 38.93621 | 52.68897 | 92.88396 | 60.02795 | 62.89604 | 59.37489 | 49.21104 | 85.39372 | 51.04982 | 76.61613 | 42.41594 | 66.7414 | 56.31693 | 66.28856 | 50.98064 | 55.40965 | 65.61007 | 31.29367 | 50.10243 |
| CUST_32200_PI429010886 | M3505 | 0.016098 | 15 | 12 | | | | | | 84.03407 | 118.6098 | 99.98303 | 63.83268 | 80.842 | 80.12587 | 81.9093 | 57.56078 | 92.65529 | 110.5556 | 71.89357 | 94.71285 | 69.94604 | 159.2768 | 97.15481 | 107.3764 | 134.8756 | 114.5736 | 71.44429 | 59.1427 | 87.12746 | 96.19258 | 100.4233 | 97.49057 | 146.722 | 103.0871 | 130.4101 |
| CUST_84_PI429010886 | N558 | 0.016106 | 15 | 12 | | | | | | 42284.08 | 4169.34 | 56447.03 | 1403.267 | 3011.822 | 2423.127 | 51007.02 | 104856.2 | 76476.71 | 79324.15 | 18586.47 | 21067.41 | 60028.43 | 81892.6 | 79681.93 | 9341.534 | 54708.78 | 4041.003 | 37163.2 | 32962.39 | 16762.52 | 56447.03 | 74289.93 | 19923.01 | 82404.75 | 17137.67 | 87164.76 |
| CUST_8167_PI429010886 | N22636 | 0.016114 | 12 | 0 | | | | | | 3079.902 | 3332.072 | 4038.67 | 1431.436 | 1920.006 | 2214.33 | 2278.385 | 1760.95 | 2794.005 | 2229.117 | 1942.982 | 3651.445 | 2786.84 | 4471.709 | 2731.57 | 2514.893 | 1938.653 | 2720.754 | 1629.467 | 3195.434 | 2579.022 | 3323.085 | 2873.023 | 2506.708 | 2434.013 | 2729.519 | 2953.556 |
| CUST_45403_PI429010886 | M69002 | 0.016122 | 18 | 9 | | | | | | 71.95612 | 78.79452 | 90.65923 | 34.9939 | 35.89324 | 23.29568 | 72.92837 | 82.27324 | 85.38257 | 195.1137 | 71.68684 | 80.60147 | 89.76549 | 57.664 | 106.9908 | 86.67105 | 45.26245 | 56.9818 | 135.1969 | 49.20588 | 76.51274 | 70.7847 | 50.91495 | 54.72113 | 44.58058 | 75.82559 | 135.3575 |
| CUST_1689_PI429010886 | N10447 | 0.01613 | 18 | 3 | 123464707 | ankyrin repeat protein [Trichomonas vaginalis G3] > | | | 4.62E-42 | 91.95171 | 151.0563 | 76.41163 | 232.8026 | 117.9497 | 125.751 | 73.83558 | 40.50555 | 85.51349 | 82.19809 | 60.13681 | 56.61232 | 51.40578 | 98.41575 | 46.49334 | 211.5605 | 29.9971 | 162.1587 | 53.07285 | 76.40353 | 144.1199 | 244.3099 | 172.9946 | 115.0806 | 124.8716 | 54.69881 | 61.95712 |
| CUST_9074_PI429010886 | N24021 | 0.016133 | 18 | 3 | | | | | | 7.127057 | 11.29768 | 8.783511 | NA | 10.88211 | NA | NA | 8.415892 | 16.53843 | 7.330555 | 6.805373 | 13.23962 | 3.762808 | 3.60732 | 10.86396 | 4.599604 | 5.625419 | NA | 10.73106 | 10.98742 | 5.414583 | NA | 14.74805 | NA | 13.20437 | 5.698164 | 16.29756 |
| CUST_35746_PI429010886 | M13385 | 0.016135 | 21 | 15 | | | | | | 292.7654 | 276.6094 | 288.5334 | 315.6417 | 275.8944 | 371.7567 | 247.4599 | 243.3133 | 211.7142 | 345.3911 | 245.0284 | 250.8185 | 251.0141 | 204.0225 | 307.9665 | 389.3563 | 317.9758 | 316.3545 | 377.4878 | 321.374 | 310.8131 | 309.1008 | 263.4861 | 254.6455 | 294.1484 | 352.5288 | 270.4039 |
| CUST_53088_PI429010886 | M100103 | 0.016137 | 15 | 3 | | | | | | 20.88122 | 7.213856 | 14.86396 | 18.2356 | 25.60312 | 47.17274 | 25.39336 | 7.789768 | 4.72626 | 5.038317 | 26.77489 | NA | 3.996664 | NA | 6.770324 | NA | 74.0547 | 22.04079 | 55.74802 | NA | 35.98798 | NA | 30.48251 | 36.606 | 4.438412 | 17.02696 | NA |
| CUST_46957_PI429010886 | M74308 | 0.016153 | 15 | 9 | | | | | | 37.79822 | 38.91585 | 34.85305 | 28.23631 | 36.55801 | 36.64169 | 26.64257 | 33.75702 | 41.14845 | 42.69298 | 39.05183 | 43.11192 | 31.51484 | 40.88839 | 37.77438 | 32.3494 | 28.93678 | 37.38785 | 32.93575 | 31.40893 | 26.7087 | 36.9176 | 32.37332 | 48.69863 | 41.87987 | 30.98188 | 45.43013 |
| CUST_5730_PI429010886 | N19114 | 0.016153 | 15 | 3 | | | | | | 769.8974 | 135.403 | 565.0414 | 4859.11 | 10233.27 | 8378.604 | 9407.387 | 681.3848 | 104.0821 | 50.69847 | 8285.528 | 9.910844 | 186.0881 | 105.57 | 153.2544 | 111.6027 | 11566.7 | 3095.586 | 15221.8 | 52.70775 | 11908.36 | 37.59821 | 8086.505 | 9950.402 | 70.91513 | 1333.908 | 20.37181 |
| CUST_9135_PI429010886 | N24119 | 0.016153 | 15 | 9 | | | | | | 25.95233 | 26.30532 | 42.46136 | 11.28708 | 25.04907 | 17.07869 | 26.09533 | 19.09504 | 28.51242 | 27.32084 | 30.29325 | 47.71977 | 22.34365 | 41.09904 | 39.68929 | 37.54698 | 29.45836 | 27.50907 | 28.35096 | 27.78206 | 23.83451 | 30.3731 | 54.13488 | 28.37201 | 35.06362 | 32.67128 | 43.898 |
| CUST_15733_PI429010886 | N35047 | 0.016153 | 15 | 0 | | | | | | 187.6138 | 173.4432 | 216.0765 | 175.5504 | 172.5457 | 174.5081 | 142.5887 | 120.5115 | 152.9004 | 157.0892 | 157.0455 | 178.3785 | 143.1502 | 222.578 | 100.64 | 217.4042 | 202.9175 | 232.2704 | 147.2361 | 167.4108 | 168.6014 | 174.0405 | 170.0196 | 158.496 | 212.9538 | 152.9208 | 193.9691 |
| CUST_22438_PI429010886 | N47031 | 0.016153 | 15 | 3 | | | | | | 1621.924 | 279.1338 | 1614.376 | 2415.728 | 1634.318 | 4787.683 | 3206.496 | 660.3398 | 665.511 | 164.1721 | 2162.684 | 8.438285 | 632.8192 | 163.2245 | 434.8523 | 165.5902 | 3667.985 | 2208.121 | 2856.456 | 133.561 | 3604.11 | 334.7375 | 2348.331 | 3365.303 | 77.84223 | 1143.075 | 37.24189 |
| CUST_26490_PI429010886 | N54335 | 0.016153 | 15 | 9 | | | | | | 20.5469 | 12.71046 | 17.25337 | 15.45106 | 15.08066 | 13.27508 | 12.33047 | 21.6649 | 19.99051 | 22.83161 | 17.90404 | 26.12256 | 24.71374 | 14.17659 | 15.22654 | 22.20487 | 10.86137 | 13.94862 | 10.53483 | 15.49817 | 21.90983 | 18.83592 | 18.32738 | 11.76447 | 30.21631 | 22.89148 | 23.97713 |
| CUST_6606_PI429010886 | N20339 | 0.016186 | 18 | 15 | | | | | | 941.2341 | 955.4895 | 888.7344 | 886.3986 | 855.8158 | 857.3719 | 649.7314 | 595.7445 | 778.5698 | 762.7979 | 719.37 | 845.9343 | 649.7314 | 957.8471 | 841.7695 | 1183.341 | 782.2398 | 910.5801 | 906.8473 | 825.7659 | 708.1055 | 1089.128 | 806.3497 | 723.1895 | 851.4085 | 650.5246 | 745.5335 |
| CUST_45528_PI429010886 | M69390 | 0.016186 | 18 | 0 | | | | | | 106.5216 | 92.73234 | 70.64745 | 78.40746 | 98.90127 | 82.57755 | 52.19516 | 54.42343 | 63.21301 | 98.94163 | 71.43726 | 58.99061 | 72.24742 | 89.80969 | 78.83809 | 132.7894 | 57.13414 | 72.43161 | 86.93205 | 152.7363 | 70.44162 | 106.6808 | 71.56769 | 53.6538 | 68.80724 | 64.89602 | 54.27879 |
| CUST_17743_PI429010886 | N38517 | 0.016204 | 12 | 0 | 321468531 | hypothetical protein DAPPUDRAFT_304474 [Daphn | Nucleic acid metabolism | Regulation of gene expression | 2.41E-21 | 31.48445 | 41.22879 | 37.54075 | 31.50476 | 35.00193 | 35.78945 | 29.11133 | 29.48584 | 23.99863 | 38.55322 | 27.37419 | 41.05859 | 26.30221 | 42.02786 | 39.46953 | 33.01166 | 34.60947 | 39.88324 | 23.23883 | 28.60372 | 40.17935 | 37.70458 | 27.01753 | 32.01033 | 47.76338 | 52.09002 | 46.13321 |
| CUST_29720_PI429010886 | N60410 | 0.016221 | 21 | 18 | | | | | | 39.46953 | 29.46496 | 24.42797 | 26.94977 | 29.79862 | 40.87555 | 20.08892 | 26.54351 | 23.87245 | 24.80133 | 27.08746 | 16.64978 | 18.12511 | 32.68431 | 38.13489 | 43.30808 | 32.91963 | 32.4042 | 38.92558 | 34.22484 | 36.04752 | 34.50768 | 23.90354 | 36.72024 | 41.99242 | 27.70404 | 27.7492 |
| CUST_52974_PI429010886 | M99643 | 0.016228 | 21 | 3 | | | | | | 19.30028 | 33.11951 | 22.86023 | 40.34341 | 30.56193 | 45.4417 | 29.09612 | 25.10042 | 32.30965 | 39.98555 | 41.7241 | 16.87967 | 22.23551 | 18.4928 | 26.46256 | 29.32288 | 27.86689 | 22.84941 | 35.73798 | 23.30384 | 18.97898 | 21.65726 | 29.40344 | 33.44743 | 55.85206 | 30.92489 | 34.56663 |
| CUST_18543_PI429010886 | N39985 | 0.016228 | 21 | 0 | | | | | | 24.61213 | 25.95233 | 36.3679 | 18.8381 | 23.77247 | 19.45678 | 25.05744 | 29.71613 | 13.31814 | 27.18775 | 16.50418 | 22.88115 | 12.27121 | 15.96673 | 16.17624 | 17.39272 | 26.4818 | 17.30092 | 26.97522 | 19.38108 | 18.31723 | 21.36688 | 28.42135 | 21.81525 | 19.3376 | 17.91014 | 27.06658 |
| CUST_27474_PI429010886 | N56122 | 0.016233 | 24 | 18 | | | | | | 64.55134 | 44.0607 | 57.52971 | 62.56969 | 48.66621 | 53.32755 | 60.72482 | 29.72539 | 52.31892 | 50.73538 | 85.61256 | 51.48154 | 63.39728 | 56.39846 | 52.801 | 87.53485 | 150.2035 | 56.74499 | 79.70393 | 132.2443 | 64.02386 | 71.57464 | 66.82399 | 45.00666 | 76.84997 | 45.11939 | 57.7399 |
| CUST_21155_PI429010886 | N44728 | 0.016241 | 21 | 18 | | | | | | 152.4486 | 150.7201 | 156.5024 | 145.794 | 157.0455 | 114.9742 | 96.86826 | 114.1927 | 138.4649 | 149.5758 | 139.0161 | 150.78 | 120.4315 | 169.7046 | 138.059 | 175.4567 | 101.92 | 162.5528 | 155.2787 | 159.3655 | 155.7397 | 204.4866 | 159.0669 | 136.9094 | 152.2451 | 138.5998 | 143.1502 |
| CUST_14495_PI429010886 | N32980 | 0.016252 | 21 | 3 | | | | | | 33.70683 | 29.87357 | 24.97464 | 33.1299 | 28.48104 | 29.98761 | 23.04991 | 17.61531 | 22.96015 | 30.39055 | 26.38163 | 24.59251 | 18.41069 | 17.98401 | 24.18144 | 29.96361 | 22.3581 | 30.20292 | 23.59085 | 19.03542 | 28.37529 | 18.50615 | 25.03495 | 23.65678 | 31.94051 | 28.45405 | 38.83398 |
| CUST_33085_PI429010886 | M5928 | 0.016253 | 12 | 3 | | | | | | 250.3151 | 20.67779 | 42.42067 | 917.9837 | 524.2988 | 2267.686 | 518.627 | 714.2963 | 100.0857 | 5.828885 | 518.5036 | 8.8792 | 184.2793 | NA | 71.35403 | NA | 1091.244 | 1432.157 | 1016.074 | 7.395008 | 305.6076 | NA | 230.5922 | 565.2058 | 5.789425 | 1248.552 | 4.075336 |
| CUST_36136_PI429010886 | M14761 | 0.016264 | 18 | 9 | | | | | | 34.67355 | 23.26561 | 53.80851 | 12.93967 | 20.37797 | 11.05143 | 21.74146 | 37.0175 | 16.72767 | 29.86056 | 73.81964 | 32.83834 | 28.52476 | 34.13801 | 23.21318 | 33.82224 | 28.12721 | 23.36677 | 21.71104 | 20.39186 | 33.64659 | 22.28917 | 30.88587 | 20.50362 | 25.43742 | 14.32267 | 22.94293 |
| CUST_12896_PI429010886 | N30226 | 0.016268 | 21 | 3 | | | | | | 17.44803 | 29.82479 | 37.50798 | 67.74201 | 23.53859 | 42.39566 | 19.1106 | 10.57913 | 21.47046 | 12.7744 | 12.18324 | 24.63851 | 8.984223 | 21.10435 | 37.21745 | 14.29027 | NA | 19.22658 | 7.818797 | 11.18163 | 26.85728 | 21.82435 | 32.83309 | 19.73697 | 24.93429 | 38.29927 | 24.79214 |
| CUST_36495_PI429010886 | M16064 | 0.016272 | 24 | 21 | | | | | | 120.3098 | 128.1244 | 87.43516 | 119.0689 | 117.3341 | 106.1197 | 78.93556 | 122.452 | 78.67424 | 99.69352 | 70.58339 | 130.8168 | 96.8323 | 100.734 | 97.75958 | 126.9262 | 80.29877 | 118.6493 | 103.9053 | 101.6676 | 138.4518 | 107.4359 | 171.2587 | 180.8415 | 168.154 | 150.4117 | 103.1666 |
| CUST_3914_PI429010886 | N15555 | 0.016275 | 15 | 12 | | | | | | 44.98472 | 56.97163 | 55.37764 | 49.63764 | 42.41594 | 63.33459 | 37.45905 | 40.03126 | 42.35962 | 54.94382 | 40.08825 | 63.72139 | 154.4215 | 62.90962 | 63.1929 | 55.90028 | 39.62195 | 50.29336 | 40.33441 | 48.95474 | 53.78964 | 69.78353 | 37.65902 | 43.90905 | 57.37859 | 65.11259 | 45.35819 |
| CUST_38878_PI429010886 | M31485 | 0.016291 | 12 | 0 | | | | | | 2232.073 | 2892.813 | 1373.036 | 330.4301 | 463.3861 | 153.2544 | 4054.765 | 582.9606 | 616.6013 | 857.3719 | 886.3986 | 946.2082 | 915.3775 | 639.8175 | 2435.819 | 477.6391 | 434.0155 | 510.5227 | 2200.508 | 907.6325 | 1344.532 | 283.5419 | 458.217 | 1569.952 | 839.8931 | 1581.923 | 2351.436 |
| CUST_46475_PI429010886 | M72753 | 0.016291 | 18 | 15 | | | | | | 96.33377 | 90.03399 | 94.81013 | 60.7605 | 75.25456 | 78.02809 | 77.2245 | 99.2894 | 67.52947 | 93.09068 | 93.50952 | 87.96508 | 85.36118 | 92.69475 | 99.98303 | 101.4116 | 103.6887 | 98.08436 | 89.09879 | 117.6603 | 59.43076 | 78.07938 | 80.21909 | 80.9345 | 118.04 | 93.65476 | 128.6916 |
| CUST_18705_PI429010886 | N40257 | 0.016294 | 21 | 12 | | | | | | 74.48084 | 42.30947 | 70.60594 | 24.82512 | 45.83381 | 97.54257 | 48.2905 | 43.8795 | 48.91662 | 44.31012 | 43.29738 | 122.3475 | 111.2059 | 117.0813 | 116.6057 | 95.7753 | 82.44815 | 122.7816 | 15.75401 | 81.03057 | 60.89252 | 102.9204 | 99.03782 | 70.28439 | 31.5798 | 57.74713 | 50.11711 |
| CUST_13691_PI429010886 | N31601 | 0.016296 | 24 | 21 | | | | | | 38.53689 | 53.56751 | 48.51868 | 35.75095 | 31.7268 | 36.93404 | 25.41068 | 43.24186 | 31.67165 | 35.56458 | 30.66462 | 46.01632 | 37.90169 | 47.72724 | 39.61101 | 45.08487 | 52.801 | 29.99986 | 50.74126 | 45.87664 | 40.75297 | 33.76025 | 58.7202 | 57.2685 | 51.93952 | 39.06616 | 49.3358 |
| CUST_5478_PI429010886 | N18757 | 0.016297 | 18 | 9 | 321476119 | hypothetical protein DAPPUDRAFT_312603 [Daphn | | | 1.02E-79 | 128.7498 | 162.9606 | 141.4904 | 122.1524 | 126.9103 | 100.9074 | 130.4101 | 127.4851 | 145.726 | 213.51 | 143.2115 | 173.6164 | 143.5589 | 182.204 | 145.9616 | 190.2377 | 102.9204 | 146.8007 | 142.3777 | 220.4105 | 136.1838 | 172.5221 | 127.3882 | 114.6323 | 175.4362 | 205.7316 | 190.9462 |
| CUST_1195_PI429010886 | N8800 | 0.016297 | 15 | 12 | | | | | | 1150.815 | 4499.121 | 4693.3 | 2217.377 | 2406.498 | 1197.011 | 1600.359 | 5278.442 | 5113.292 | 10575.43 | 1482.523 | 6461.792 | 5824.829 | 10401.6 | 5819.009 | 3126.195 | 837.3907 | 2328.414 | 1055.909 | 3130.59 | 1670.5 | 7568.915 | 3823.851 | 1869.455 | 6986.943 | 2266.195 | 6941.938 |
| CUST_46871_PI429010886 | M74019 | 0.016306 | 15 | 12 | | | | | | 132.9489 | 262.8518 | 173.4981 | 163.9694 | 164.3967 | 165.0319 | 131.7044 | 118.3504 | 124.6243 | 252.1679 | 95.16133 | 212.0119 | 167.982 | 194.2624 | 184.1659 | 184.4849 | 151.752 | 151.8121 | 145.7727 | 97.81711 | 154.7492 | 126.6828 | 120.3338 | 86.54045 | 189.2062 | 85.93471 | 79.29506 |
| CUST_787_PI429010886 | N7317 | 0.016314 | 18 | 12 | | | | | | 124.5285 | 144.2098 | 170.5502 | 107.1617 | 103.3649 | 121.6679 | 105.1 | 99.66003 | 134.0378 | 185.1977 | 116.3712 | 174.5672 | 127.9775 | 196.9122 | 178.7689 | 187.181 | 96.10628 | 173.0442 | 199.0047 | 160.3693 | 165.2432 | 117.3776 | 128.4766 | 116.1911 | 154.5663 | 122.5319 | 157.3097 |
| CUST_43353_PI429010886 | M62817 | 0.016343 | 15 | 3 | 242012953 | conserved hypothetical protein [Pediculus humanus | | | 2.05E-31 | 32.23977 | 34.96061 | NA | 67.21241 | 13.83803 | 41.09904 | 24.19734 | 25.07677 | 10.97389 | NA | 9.72432 | NA | 29.60942 | 3.652572 | 17.74139 | 10.42063 | 32.82642 | 27.52267 | 19.50089 | NA | NA | 12.99803 | NA | 22.41298 | 11.68424 | 9.782316 | 4.62816 |
| CUST_1658_PI429010886 | N10362 | 0.016345 | 15 | 3 | | | | | | 175.2604 | 84.60749 | 102.9324 | 125.4314 | 103.2145 | 126.5686 | 175.6726 | 89.29805 | 67.53545 | 71.81025 | 87.09496 | 56.41396 | 92.61954 | 82.24759 | 76.7303 | 85.17905 | 144.8456 | 101.9359 | 144.0267 | 71.66601 | 90.34874 | 88.83369 | 86.13317 | 112.1069 | 89.43652 | 61.56139 | 49.70586 |
| CUST_2326_PI429010886 | N12109 | 0.016345 | 15 | 9 | 332183191 | sodium potassium-transporting ATPase subunit bet | Transport | Ion transport | 1.61E-65 | 536.4898 | 591.3951 | 371.903 | 267.5678 | 420.504 | 226.6106 | 486.7621 | 333.1118 | 483.4448 | 600.2864 | 510.3855 | 522.1156 | 422.4541 | 482.6309 | 376.8596 | 466.0044 | 337.6237 | 422.0878 | 358.7125 | 282.9535 | 338.4841 | 597.7736 | 350.9859 | 322.2147 | 859.4575 | 256.52 | 466.0826 |
| CUST_4210_PI429010886 | N16244 | 0.016365 | 24 | 21 | 242023907 | C-jun-amino-terminal kinase-interacting protein, pu | Metabolic process | Other | 1.54E-07 | 61.03108 | 59.052 | 60.99798 | 51.72418 | 53.73015 | 74.92755 | 47.7103 | 48.49494 | 54.5396 | 60.53815 | 55.45229 | 56.39846 | 45.215 | 54.63875 | 50.77711 | 68.22752 | 61.40781 | 48.22578 | 60.22346 | 69.42462 | 59.64714 | 60.86143 | 65.02562 | 62.42019 | 60.58205 | 51.35337 | 63.23408 |
| CUST_1097_PI429010886 | N8487 | 0.016381 | 18 | 0 | 321470919 | hypothetical protein DAPPUDRAFT_302851 [Daphn | Metabolic process | Oxidation-reduction process | | 212.1657 | 230.9612 | 162.3309 | 189.6264 | 190.4443 | 185.8972 | 189.144 | 141.5789 | 124.447 | 171.8712 | 158.3894 | 168.2432 | 154.8889 | 145.5578 | 185.414 | 211.8754 | 168.7959 | 187.3568 | 181.1566 | 217.6655 | 148.1205 | 207.0425 | 123.5093 | 202.0404 | 136.491 | 134.02 | 128.4226 |
| CUST_11871_PI429010886 | N28544 | 0.016384 | 12 | 0 | | | | | | 48.72124 | 52.08136 | 25.48601 | 27.18447 | 49.81001 | 30.29627 | 12.60802 | 23.70044 | 23.57221 | 29.56761 | 23.58557 | 42.81625 | 36.68661 | 34.15487 | 36.52268 | 26.74243 | 17.43083 | 37.74522 | 22.46339 | 25.75169 | 23.64962 | 55.37764 | 25.50881 | 21.97852 | 52.84586 | 26.24834 | 35.89984 |
| CUST_8492_PI429010886 | N23121 | 0.016402 | 24 | 0 | 154416763 | viral A-type inclusion protein [Trichomonas vaginali | | | 5.32E-11 | 69.87848 | 95.7582 | 82.60943 | 78.21131 | 63.30844 | 79.5425 | 54.45697 | 60.95084 | 63.56474 | 84.38957 | 70.40792 | 91.87634 | 52.66271 | 65.71273 | 48.03764 | 72.28479 | 49.47977 | 80.74596 | 52.71293 | 65.41017 | 74.51037 | 55.00728 | 72.96725 | 51.64328 | 82.23746 | 89.51747 | 92.10717 |
| CUST_56589_PI429010886 | M114176 | 0.016427 | 18 | 15 | | | | | | 256.2441 | 170.8572 | 233.3995 | 48.00546 | 296.5712 | 167.4539 | 220.1939 | 228.6362 | 131.6679 | 434.2638 | 153.7099 | 152.988 | 282.5938 | 270.6283 | 191.7287 | 251.0973 | 296.6312 | 293.5299 | 292.1061 | 263.2372 | 195.9585 | 179.4229 | 130.8168 | 80.21197 | 77.3067 | 158.5869 | 228.1824 |
| CUST_43461_PI429010886 | M63123 | 0.016451 | 21 | 0 | | | | | | 22.41474 | 39.126 | 32.56046 | 15.45873 | 28.69117 | 22.64182 | 18.21746 | 14.18148 | 22.23551 | 25.87358 | 18.84163 | 33.40373 | 15.99247 | 8.79557 | 17.37482 | 13.13514 | 15.18532 | 14.27944 | 14.35905 | 21.54181 | 20.37942 | 17.12595 | 28.05779 | 21.80739 | 26.84667 | 14.54705 | 17.52979 |
| CUST_25026_PI429010886 | N51656 | 0.016463 | 12 | 3 | | | | | | 11.81798 | 9.132253 | 12.37319 | 15.82289 | 22.19942 | 33.7538 | 18.37743 | NA | 12.66387 | 11.5308 | 8.205981 | 5.426738 | 9.252049 | 18.17736 | 6.570072 | 20.47372 | 21.63352 | 39.59771 | 13.67691 | 9.570885 | 9.427695 | 26.69563 | 13.0885 | 28.51882 | 15.57695 | 10.5296 | 4.48919 |
| CUST_30543_PI429010886 | N62093 | 0.016483 | 18 | 3 | 442630141 | masquerade, isoform C [Drosophila melanogaster] : | Protein metabolism | Proteolysis | 6.41E-14 | 11.02964 | 18.32738 | NA | 51.74182 | 99.89328 | 127.3371 | 10.10419 | NA | 10.91384 | 3.306337 | 4.266004 | NA | 8.707775 | 42.89937 | 23.08947 | 35.54095 | 5.006703 | 61.45704 | 4.178879 | 40.87944 | 5.029969 | 100.2406 | NA | 49.77447 | 29.56366 | NA | NA |
| CUST_911_PI429010886 | N7801 | 0.01649 | 18 | 9 | | | | | | 35.44546 | 43.17145 | 50.58779 | 51.67197 | 42.19897 | 59.76158 | 46.02417 | 44.98472 | 62.80799 | 50.6298 | 53.3996 | 70.12587 | 57.8814 | 52.1905 | 60.61979 | 49.60341 | 45.01151 | 58.597 | 49.11313 | 36.4309 | 33.78634 | 56.77783 | 67.53545 | 40.06816 | 56.27804 | 56.21187 | 63.73904 |
| CUST_6816_PI429010886 | N20638 | 0.0165 | 15 | 12 | | | | | | 472.5516 | 567.2204 | 559.1604 | 420.025 | 375.9369 | 363.8038 | 344.3603 | 382.1801 | 450.5804 | 531.5557 | 373.4486 | 603.2889 | 463.3861 | 659.6239 | 580.3063 | 593.1266 | 342.0536 | 519.9761 | 424.2001 | 486.0261 | 420.025 | 504.2482 | 401.7586 | 358.2812 | 598.9907 | 433.7721 | 578.8676 |
| CUST_16521_PI429010886 | N36383 | 0.0165 | 15 | 12 | | | | | | 448.0031 | 610.3506 | 601.962 | 469.4022 | 437.8204 | 566.9632 | 377.1617 | 386.1193 | 453.7002 | 586.8079 | 430.7898 | 835.1893 | 494.4534 | 682.0753 | 703.0939 | 625.7026 | 411.751 | 568.9937 | 528.3775 | 519.3194 | 486.2625 | 596.8255 | 508.3277 | 390.7246 | 555.136 | 599.656 | 638.6578 |
| CUST_25988_PI429010886 | N53444 | 0.0165 | 15 | 12 | | | | | | 1140.952 | 1500.831 | 1460.033 | 1136.737 | 1058.251 | 1049.625 | 1059.917 | 1166.392 | 1239.225 | 1378.873 | 1156.123 | 1751.202 | 1183.738 | 1558.572 | 1594.841 | 1623.972 | 1234.796 | 1326.638 | 1136.737 | 1570.987 | 1144.662 | 1556.767 | 1161.62 | 1046.312 | 1581.923 | 1453.376 | 1575.017 |
| CUST_26766_PI429010886 | N54837 | 0.0165 | 15 | 12 | | | | | | 1032.186 | 1489.438 | 1386.924 | 1106.54 | 1222.467 | 1241.637 | 960.3576 | 1280.61 | 1390.385 | 1464.638 | 1156.728 | 1838.739 | 1265.474 | 1673.62 | 1644.909 | 1553.02 | 1211.313 | 1539.294 | 1183.738 | 1363.618 | 1209.365 | 1566.438 | 1146.628 | 1251.807 | 1629.467 | 1424.855 | 1491.426 |
| CUST_10751_PI429010886 | N26730 | 0.016522 | 21 | 18 | 498984877 | PREDICTED: protein FAM32A-like [Ceratitis capitata | Cellular Process | Cell death | 1.77E-14 | 366.3446 | 319.2639 | 438.2102 | 246.7296 | 249.6087 | 287.8054 | 281.447 | 324.4624 | 322.7291 | 277.3264 | 258.545 | 331.5388 | 411.1038 | 306.5332 | 312.9412 | 313.2163 | 349.4134 | 270.8273 | 413.0515 | 276.4162 | 346.2493 | 412.6269 | 335.9972 | 329.1684 | 280.12 | 256.8548 | 248.0682 |
| CUST_6455_PI429010886 | N20123 | 0.016532 | 24 | 15 | | | | | | 66.88544 | 71.11942 | 75.16329 | 85.926 | 88.40608 | 83.89746 | 69.83541 | 80.59405 | 59.40092 | 85.69951 | 65.36594 | 109.0854 | 90.84494 | 80.02167 | 84.57831 | 111.7621 | 102.3532 | 115.5578 | 131.7576 | 75.07319 | 61.95208 | 104.5737 | 84.47788 | 92.13928 | 80.24894 | 65.6728 | 64.48135 |
| CUST_36214_PI429010886 | M15031 | 0.016538 | 15 | 0 | | | | | | 13.91626 | 32.44657 | 15.33921 | 5.942845 | 11.73596 | 12.16991 | 6.454629 | 7.094999 | 9.428784 | 18.00284 | 24.00983 | 11.65145 | 11.7224 | 10.73592 | 8.656419 | 16.27803 | 21.93936 | 16.02537 | 12.78901 | 10.22197 | 22.57524 | 5.780206 | 10.50262 | 14.70379 | 17.40574 | 10.28563 | 15.90941 |
| CUST_4271_PI429010886 | N16373 | 0.016538 | 15 | 0 | | | | | | 3325.521 | 4613.187 | 3360.345 | 1995.819 | 2884.034 | 2929.717 | 1637.238 | 1606.601 | 2012.591 | 3283.608 | 2836.89 | 3572.519 | 1875.725 | 2759.437 | 3249.343 | 5985.533 | 2747.282 | 3899.401 | 2763.052 | 3899.401 | 2583.846 | 3402.667 | 2377.614 | 1863.799 | 4399.213 | 2385.821 | 3722.852 |
| CUST_8137_PI429010886 | N22589 | 0.016538 | 15 | 9 | 260823354 | hypothetical protein BRAFLDRAFT_208157 [Branchi | Metabolic process | Other | 9.13E-57 | 359.4804 | 417.6101 | 465.6558 | 299.502 | 315.9615 | 197.3275 | 196.129 | 294.1484 | 604.7306 | 745.0756 | 410.9902 | 745.3969 | 544.6485 | 431.3819 | 537.3115 | 421.0101 | 223.8084 | 158.2045 | 148.1205 | 582.2842 | 138.4956 | 572.8694 | 481.5308 | 137.719 | 795.1421 | 252.5323 | 891.7466 |
| CUST_12016_PI429010886 | N28781 | 0.016538 | 15 | 0 | | | | | | 66.39938 | 65.26348 | 60.26272 | 46.61273 | 59.776 | 57.71752 | 37.44766 | 32.61875 | 43.77433 | 53.88876 | 49.46731 | 76.76597 | 56.99104 | 45.48365 | 70.7113 | 77.55036 | 53.44251 | 62.33854 | 54.86684 | 69.85985 | 52.27598 | 58.29022 | 46.55991 | 50.4763 | 63.89787 | 72.23286 | 60.05019 |
| CUST_14228_PI429010886 | N32524 | 0.016564 | 12 | 0 | | | | | | 34.70065 | 32.3288 | 38.44815 | 28.48104 | 26.15676 | 46.01155 | 25.86296 | 26.47532 | 18.56427 | 30.3611 | 28.41298 | 55.77394 | 29.3391 | 32.23691 | 47.31501 | 34.0026 | 25.09429 | 28.81481 | 29.14874 | 23.96961 | 45.80326 | 36.85933 | 35.76089 | 28.85946 | 46.11029 | 49.56436 | 39.45385 |
| CUST_10047_PI429010886 | N25613 | 0.016589 | 24 | 0 | | | | | | 54.24943 | 48.25771 | 50.49286 | 37.95647 | 44.4501 | 33.66515 | 30.93394 | 27.49986 | 48.61666 | 65.0047 | 42.92761 | 51.0383 | 30.70822 | 40.3031 | 41.41686 | 48.04331 | 24.31121 | 43.32293 | 43.35404 | 39.49607 | 48.38349 | 32.60782 | 45.0701 | 42.52496 | 61.21559 | 51.79974 | 72.97459 |
| CUST_15126_PI429010886 | N34049 | 0.016589 | 24 | 0 | | | | | | 116.5533 | 104.3886 | 119.5708 | 67.03346 | 76.54184 | 107.0545 | 91.41127 | 97.06276 | 70.13143 | 133.2074 | 99.88549 | 80.21909 | 72.3532 | 82.56662 | 58.55654 | 87.68051 | 80.07884 | 105.2081 | 102.7727 | 84.2989 | 96.44756 | 51.01709 | 93.99876 | 132.2265 | 135.061 | 110.2887 | 99.66003 |
| CUST_21729_PI429010886 | N45746 | 0.016595 | 15 | 12 | | | | | | 9.24163 | 8.533663 | 11.38323 | 11.71707 | 11.17076 | NA | 6.964774 | NA | 6.160853 | 7.889839 | 18.40425 | 15.47775 | 8.906902 | 20.23079 | 15.2073 | 13.48112 | 11.86129 | 9.432782 | 11.01728 | 13.07347 | 10.46578 | 7.861738 | 7.193295 | 8.607023 | 17.53198 | 14.47801 | 15.98363 |
| CUST_18913_PI429010886 | N40641 | 0.016609 | 18 | 3 | | | | | | 10.42063 | 20.7312 | 14.69352 | 23.20138 | 26.54593 | 18.98615 | 17.25469 | 13.84341 | 24.23621 | 17.98635 | 5.906131 | 14.21999 | 11.09945 | 8.470751 | 17.84395 | 23.55239 | 12.33539 | 14.54325 | 14.75649 | 12.13161 | 18.37554 | 14.40694 | 28.21862 | 18.51985 | 18.48442 | 20.44316 | 17.66287 |
| CUST_28259_PI429010886 | N57617 | 0.016609 | 21 | 18 | | | | | | 235.5325 | 902.1996 | 814.2205 | 427.7928 | 97.44558 | 33.5333 | 138.6419 | 640.8065 | 556.906 | 1248.106 | 228.2235 | 797.54 | 781.4539 | 1158.222 | 940.8909 | 430.6917 | 923.2313 | 96.61745 | 892.3589 | 1839.737 | 1153.433 | 329.9922 | 1156.123 | 94.87745 | 84.90321 | 574.6243 | 771.6454 |
| CUST_25431_PI429010886 | N52406 | 0.016636 | 24 | 0 | | | | | | 13.90091 | 11.24522 | 17.67128 | NA | 5.791904 | 11.19204 | 5.374782 | 10.27942 | 9.44636 | 4.243821 | 4.683701 | NA | 8.878575 | 8.348712 | 4.454348 | 13.45283 | 4.950386 | NA | 7.400394 | 9.357193 | NA | 5.685055 | 6.503335 | NA | 7.272559 | 21.77078 | 8.578143 |
| CUST_42261_PI429010886 | M59827 | 0.016637 | 21 | 18 | | | | | | 116.8757 | 163.9899 | 145.7727 | 124.9049 | 123.5431 | 168.6416 | 113.1045 | 97.49057 | 89.86196 | 138.2955 | 160.7094 | 164.8776 | 111.4954 | 123.2423 | 128.7498 | 216.7674 | 142.727 | 135.6517 | 139.8446 | 155.5733 | 173.2007 | 155.2537 | 144.8798 | 134.9314 | 122.1524 | 146.5479 | 137.5279 |
| CUST_57053_PI429010886 | E254 | 0.01664 | 15 | 0 | | | | | | 9072.043 | 10575.43 | 5139.37 | 13339.53 | 8550.193 | 11243.07 | 6767.816 | 5712.144 | 4515.16 | 4250.591 | 6833.331 | 3942.272 | 4937.213 | 3586.445 | 4174.241 | 8597.6 | 5917.438 | 7490.614 | 7338.425 | 4528.171 | 5811.899 | 4944.792 | 3785.297 | 7637.386 | 4537.099 | 6544.343 | 2667.238 |
| CUST_10195_PI429010886 | N25853 | 0.016647 | 24 | 15 | 260784622 | hypothetical protein BRAFLDRAFT_231367 [Branchi | | | 3.18E-28 | 163.7139 | 296.8223 | 190.9127 | 187.0261 | 227.642 | 134.0744 | 145.3456 | 145.794 | 166.9686 | 351.4461 | 178.0976 | 318.3844 | 155.4878 | 185.7909 | 206.1701 | 378.5438 | 204.1851 | 259.4845 | 222.9397 | 237.9578 | 206.5724 | 293.1844 | 196.9351 | 182.5193 | 201.6411 | 160.0314 | 250.4345 |

| Probe ID | ID | p-value | Period | Phase | EBI ID | Annotation | GO category | Go term | E-value | 0 | 0 | 0 | 3 | 3 | 3 | 6 | 6 | 6 | 9 | 9 | 9 | 12 | 12 | 12 | 15 | 15 | 15 | 18 | 18 | 18 | 21 | 21 | 21 | 24 | 24 | 24 |
|---|---|---|---|---|---|---|---|---|---|---|---|---|---|---|---|---|---|---|---|---|---|---|---|---|---|---|---|---|---|---|---|---|---|---|---|---|
| CUST_26311_PI429010886 | N54035 | 0.016665 | 24 | 21 | | | | | | 4.886513 | 5.107128 | NA | 6.422087 | 4.025282 | NA | NA | NA | 4.478759 | 8.047455 | 4.203397 | 6.330913 | 6.576173 | 7.855725 | 5.774825 | 14.19367 | 5.913315 | NA | 8.373525 | 9.462739 | 12.90641 | 9.532078 | 12.72931 | NA | 13.85759 | 6.724594 | 6.597762 |
| CUST_23639_PI429010886 | N49188 | 0.016684 | 21 | 15 | | | | | | 68.91301 | 45.39483 | 42.09877 | 37.70765 | 54.20454 | 36.94944 | 37.00144 | 34.41491 | 48.80606 | 48.35952 | 54.3134 | 52.23527 | 34.32332 | 38.0116 | 57.6895 | 99.40267 | 61.38183 | 69.07358 | 55.23983 | 51.37508 | 49.09733 | 56.16844 | 35.11191 | 40.2964 | 68.21242 | 42.6726 | 48.46285 |
| CUST_56331_PI429010886 | M113518 | 0.016685 | 18 | 3 | 546682942 | hypothetical protein D910_10129 [Dendroctonus p | | | 6.83E-08 | 405.3248 | 491.0819 | 297.209 | 3536.586 | 3599.759 | 4627.233 | 2118.286 | 3292.379 | 300.6388 | 65.63314 | 1500.831 | 87.40211 | 513.0951 | 179.2925 | 288.5922 | 107.9175 | 1317.338 | 586.9437 | 847.5292 | 45.35228 | 1720.101 | 157.5747 | 1628.664 | 2992.796 | 108.6787 | 529.4905 | 117.0236 |
| CUST_51572_PI429010886 | M94038 | 0.016686 | 18 | 9 | 260813697 | hypothetical protein BRAFLDRAFT_60838 [Branchio | | | 1.66E-09 | 51.17489 | 81.34562 | 53.9331 | 50.24503 | 62.16793 | 49.9806 | 40.65724 | 60.54854 | 51.07827 | 75.11707 | 65.51399 | 84.83474 | 60.86143 | 67.09564 | 82.62874 | 83.85047 | 54.43209 | 63.89005 | 42.27106 | 83.11485 | 54.10775 | 61.5685 | 53.04998 | 39.414 | 82.25514 | 50.39753 | 55.14628 |
| CUST_38411_PI429010886 | M26337 | 0.016689 | 18 | 9 | | | | | | 2001.122 | 792.1417 | 690.9208 | 493.8599 | 375.5434 | 161.1809 | 718.2136 | 1687.609 | 1321.214 | 3835.814 | 698.4337 | 2913.591 | 1557.99 | 1490.779 | 1794.046 | 1851.755 | 472.773 | 562.8166 | 1924.978 | 484.9716 | 761.7734 | 1187.536 | 495.8952 | 376.0453 | 174.8583 | 807.4639 | 1692.067 |
| CUST_54786_PI429010886 | M107677 | 0.016695 | 15 | 12 | | | | | | 1187.094 | 1137.521 | 993.7035 | 730.6182 | 993.7035 | 1075.314 | 2361.621 | 1308.281 | 2067.695 | 1309.194 | 862.8974 | 1112.549 | 2370.125 | 2469.728 | 1984.848 | 1479.619 | 1173.656 | 1054.702 | 1768.416 | 734.9802 | 945.2395 | 1317.659 | 759.8078 | 1268.323 | 1156.728 | 1640.228 | 3144.897 |
| CUST_36604_PI429010886 | M16336 | 0.016695 | 15 | 12 | | | | | | 133.1553 | 148.5892 | 142.963 | 113.9625 | 100.8435 | 134.3159 | 148.544 | 119.228 | 204.1442 | 132.7741 | 139.1216 | 195.0054 | 157.7478 | 180.2796 | 184.5131 | 175.4895 | 132.8952 | 148.1409 | 148.0447 | 128.4554 | 113.0933 | 177.9616 | 170.1693 | 124.2716 | 151.6596 | 102.7268 | 184.458 |
| CUST_4647_PI429010886 | N17205 | 0.016695 | 15 | 12 | | | | | | 275.5962 | 210.5891 | 393.9841 | 285.2001 | 177.8716 | 292.1904 | 221.873 | 263.6735 | 256.8125 | 350.9859 | 288.2204 | 456.2722 | 426.0225 | 475.7608 | 542.3878 | 396.9018 | 256.0552 | 347.1114 | 248.1135 | 322.2147 | 354.7905 | 571.296 | 376.4458 | 286.19 | 420.2091 | 362.4143 | 507.8519 |
| CUST_53098_PI429010886 | M100133 | 0.016706 | 24 | 18 | | | | | | 43.38005 | 46.07745 | 39.90155 | 36.74157 | 43.74674 | 27.93572 | 36.7129 | 56.09338 | 31.21873 | 77.38027 | 40.40681 | 40.96307 | 28.48875 | 56.47886 | 34.77329 | 82.25514 | 54.11265 | 128.2056 | 131.1966 | 40.28418 | 159.2366 | 45.16011 | 87.42512 | 91.49463 | 36.29351 | 25.39589 | 89.5493 |
| CUST_30944_PI429010886 | M283 | 0.016709 | 24 | 21 | 172051148 | putative mitochondrial ribosomal protein S24 [Orni | | | 2.04E-51 | 185.414 | 208.2464 | 203.2288 | 151.2427 | 188.1392 | 149.7134 | 146.765 | 156.3748 | 150.6133 | 220.8169 | 163.9694 | 234.141 | 235.8718 | 177.5678 | 218.8408 | 265.7201 | 145.9616 | 232.9495 | 180.731 | 188.2724 | 198.0625 | 253.8408 | 223.7779 | 203.6001 | 187.4537 | 201.1997 | 192.1486 |
| CUST_2668_PI429010886 | N12871 | 0.016709 | 24 | 21 | 242024523 | pdz domain containing guanine nucleotide exchang | Signal transduction | Other | 1.5E-175 | 31.6776 | 36.41327 | 47.01793 | 26.53469 | 25.47702 | 31.58406 | 30.11859 | 23.75346 | 19.91992 | 39.27214 | 32.03583 | 26.23528 | 22.17757 | 32.98902 | 19.11878 | 27.13842 | 43.55575 | 45.24266 | 34.32332 | 32.41678 | 47.49104 | 28.46598 | 64.99854 | 39.0714 | 56.93106 | 55.38216 | 49.25162 |
| CUST_7933_PI429010886 | N22292 | 0.016709 | 24 | 21 | 158292095 | AGAP004370-PA [Anopheles gambiae str. PEST] >gi | Nucleic acid metabolism | Regulation of gene expression | 1.08E-39 | 316.4275 | 302.7759 | 286.3036 | 246.1531 | 203.6001 | 284.0133 | 190.2377 | 265.532 | 273.6399 | 364.2104 | 222.8598 | 405.2638 | 241.7291 | 277.2458 | 440.6954 | 466.6293 | 278.0457 | 348.7613 | 262.0228 | 289.4881 | 283.3904 | 315.7757 | 322.9938 | 416.4515 | 303.2741 | 307.2464 | 284.9694 |
| CUST_14889_PI429010886 | N33640 | 0.016716 | 24 | 3 | | | | | | 1065.529 | 3312.37 | 1304.617 | 4876.999 | 1686.366 | 2133.627 | 1095.161 | 1317.659 | 994.4459 | 1297.484 | 595.1496 | 2189.348 | 1036.891 | 1131.289 | 948.7418 | 1182.29 | 394.7279 | 1167.349 | 699.845 | 1139.145 | 1797.174 | 1938.653 | 1781.492 | 1167.349 | 2115.987 | 1948.38 | 811.2478 |
| CUST_11784_PI429010886 | N28398 | 0.016723 | 15 | 9 | | | | | | 759.5836 | 887.8552 | 968.7311 | 685.3204 | 722.796 | 718.917 | 707.905 | 791.8244 | 882.5331 | 878.6172 | 749.487 | 1205.12 | 828.5874 | 978.4784 | 988.77 | 1099.35 | 691.1905 | 828.3608 | 810.9217 | 936.0363 | 768.5376 | 953.9498 | 743.4007 | 720.2695 | 1126.811 | 967.9514 | 1055.576 |
| CUST_50107_PI429010886 | M88649 | 0.016727 | 24 | 21 | | | | | | 5.032346 | 12.37644 | NA | 5.895001 | 10.36474 | NA | 5.211167 | 3.355064 | 5.088083 | 8.688469 | 6.55575 | NA | 4.065161 | 9.058184 | 12.31427 | 17.46899 | 6.652276 | 23.90131 | 5.302715 | 10.08636 | NA | 21.02638 | 8.046291 | 21.93409 | 16.15147 | 6.760169 | 9.373204 |
| CUST_42449_PI429010886 | M60312 | 0.01673 | 15 | 9 | | | | | | 62.91706 | 60.47407 | 61.28097 | 34.99807 | 63.26276 | 38.96253 | 45.41664 | 48.13345 | 54.56998 | 77.31411 | 86.03759 | 55.42699 | 49.11313 | 64.125 | 63.48998 | 78.97339 | 52.77021 | 33.93687 | 56.69692 | 58.37612 | 41.32421 | 56.57948 | 44.9603 | 47.59957 | 67.08317 | 69.90395 | 67.03346 |
| CUST_9915_PI429010886 | N25398 | 0.01673 | 15 | 9 | | | | | | 60.80817 | 85.3343 | 138.7197 | 67.77787 | 92.81476 | 66.41721 | 82.49999 | 204.3871 | 190.0374 | 222.2406 | 138.2766 | 187.181 | 104.9178 | 314.9825 | 141.6349 | 181.4858 | 131.5799 | 128.3451 | 153.1027 | 105.0723 | 126.6474 | 213.366 | 252.4492 | 57.04658 | 266.4363 | 188.2189 | 366.4064 |
| CUST_30462_PI429010886 | N61947 | 0.01673 | 15 | 0 | | | | | | 24.23621 | 11.74252 | 44.1761 | 16.24458 | 29.06247 | 24.34006 | 12.10865 | 10.40388 | 13.65469 | 19.27159 | 38.90805 | 14.00339 | 11.30336 | 19.68268 | 13.4823 | 30.8467 | 33.80907 | 36.4354 | 25.33406 | 27.37091 | 25.44661 | 13.68318 | 12.01073 | 12.83461 | 24.61213 | 29.53988 | 26.45407 |
| CUST_45022_PI429010886 | M67737 | 0.016739 | 24 | 15 | | | | | | 58.27233 | 42.22198 | 59.052 | 59.00876 | 52.063 | 81.46299 | 71.35987 | 54.1974 | 59.61145 | 65.83727 | 71.19859 | 67.22077 | 60.82114 | 69.07358 | 89.86196 | 196.6343 | 77.53281 | 65.6996 | 65.50557 | 62.70075 | 86.35271 | 79.72112 | 67.32283 | 86.4072 | 64.31677 | 66.53867 | 51.37093 |
| CUST_16657_PI429010886 | N36644 | 0.016742 | 15 | 0 | | | | | | 259.6731 | 298.1495 | 209.2891 | 201.008 | 263.7999 | 257.8674 | 161.0687 | 135.4402 | 181.4398 | 224.3549 | 156.6593 | 286.6682 | 199.6816 | 207.128 | 291.5581 | 272.7999 | 222.8598 | 292.1061 | 209.0873 | 236.5825 | 187.9002 | 223.7779 | 184.0038 | 242.9704 | 275.1358 | 177.0904 | 181.7953 |
| CUST_48947_PI429010886 | M83096 | 0.016755 | 15 | 6 | | | | | | 5.089219 | 6.829705 | NA | 23.57465 | 9.252049 | 7.188579 | NA | NA | 10.34644 | 8.832139 | 7.348282 | 4.461614 | 4.89178 | 7.297269 | 7.034635 | 6.669172 | 8.97147 | NA | 10.66241 | 9.609115 | 9.416182 | 13.69107 | NA | 7.988553 | 8.629296 | 10.12652 | 6.533554 |
| CUST_48981_PI429010886 | M83349 | 0.016764 | 24 | 18 | 546673612 | hypothetical protein D910_02598 [Dendroctonus p | Protein metabolism | Cellular protein modification process | 1.61E-18 | 38.69682 | 24.31817 | 32.06861 | 25.44437 | 28.14284 | 19.87757 | 20.91887 | 27.42267 | 29.3391 | 27.84371 | 48.35952 | 17.85496 | 24.15872 | 38.66452 | 25.28193 | 29.73496 | 58.40039 | 26.10687 | 42.40387 | 48.35331 | 33.21734 | 34.88582 | 31.36464 | 28.15071 | 37.60789 | 29.69394 | 30.13914 |
| CUST_51337_PI429010886 | M93143 | 0.016771 | 18 | 6 | 260800466 | hypothetical protein BRAFLDRAFT_118609 [Branchi | | | 6.66E-28 | 79.81539 | 90.48379 | 112.3515 | 117.1089 | 113.7469 | 104.6828 | 129.9842 | 113.1045 | 138.0091 | 101.0858 | 105.3783 | 97.31477 | 113.1785 | 109.8598 | 118.6904 | 125.751 | 107.4719 | 74.88917 | 112.9528 | 90.37187 | 107.543 | 126.8192 | 73.31065 | 87.30486 | 139.8337 | 130.3926 | 159.5684 |
| CUST_32414_PI429010886 | M4058 | 0.016785 | 18 | 0 | 321478511 | hypothetical protein DAPPUDRAFT_40294 [Daphnia | | | 2.27E-18 | 35.1468 | 36.21632 | 30.62163 | 14.46778 | 25.59587 | 16.90499 | 11.41324 | 17.27372 | 22.41939 | 28.14633 | 21.4691 | 34.91643 | 32.66922 | 27.77944 | 31.95513 | 41.19265 | 15.54357 | 29.57425 | 35.0422 | 33.78308 | 19.05109 | 28.92395 | 27.6707 | 21.32603 | 26.83617 | 21.12654 | 27.03844 |
| CUST_9378_PI429010886 | N24517 | 0.016788 | 24 | 15 | | | | | | 186.2827 | 190.4186 | 164.1178 | 137.2919 | 146.2634 | 135.3397 | 130.6585 | 149.2835 | 106.5708 | 224.7802 | 174.5672 | 130.1396 | 125.3376 | 169.9973 | 150.9847 | 240.7867 | 227.1414 | 212.1657 | 142.7716 | 162.2752 | 150.1517 | 222.5078 | 191.5917 | 174.5672 | 281.1462 | 138.9365 | 189.6264 |
| CUST_6892_PI429010886 | N20741 | 0.016793 | 15 | 3 | 321476027 | hypothetical protein DAPPUDRAFT_312473 [Daphn | | | 1.19E-34 | 13.1549 | 8.681407 | 10.66012 | 14.36868 | 24.56849 | 13.66784 | 17.16495 | 9.512792 | NA | 12.69324 | 14.36186 | 8.712785 | 5.038317 | 6.794828 | 6.975434 | 6.012808 | 19.35656 | 12.31007 | 6.039035 | NA | 41.55928 | 6.140199 | 17.92595 | 20.57577 | 8.587225 | 8.634653 | 5.840732 |
| CUST_31969_PI429010886 | M2866 | 0.016802 | 24 | 18 | | | | | | 116.0529 | 174.7413 | 137.4942 | 84.93221 | 120.4034 | 97.20572 | 81.52938 | 92.90826 | 103.0871 | 137.0908 | 97.98092 | 163.162 | 107.2229 | 141.0258 | 113.6252 | 147.9625 | 119.0396 | 159.3655 | 132.9657 | 163.2245 | 134.7571 | 160.8644 | 117.5244 | 84.97695 | 127.9775 | 139.8015 | 132.9869 |
| CUST_12382_PI429010886 | N29379 | 0.016802 | 21 | 15 | | | | | | 275.6487 | 297.14 | 258.4381 | 239.2718 | 282.651 | 246.0582 | 210.7138 | 249.0021 | 214.4445 | 301.0804 | 249.3844 | 245.1598 | 224.668 | 286.4677 | 257.455 | 452.0017 | 329.1684 | 293.1271 | 307.2905 | 318.9508 | 255.1161 | 411.2669 | 212.8309 | 199.4211 | 398.0093 | 196.4409 | 273.2835 |
| CUST_49318_PI429010886 | M85180 | 0.016803 | 24 | 0 | | | | | | 8.195858 | 8.893352 | NA | NA | 7.419678 | 10.00867 | 5.475709 | 8.498279 | NA | 6.31321 | 7.355758 | 6.319033 | 5.961141 | NA | 3.669957 | 4.478759 | 9.279943 | 12.73078 | 8.512073 | NA | 6.287022 | 8.315583 | 7.466636 | 9.175781 | 13.41579 | 9.017882 | 20.72678 |
| CUST_1503_PI429010886 | N9845 | 0.016856 | 24 | 6 | | | Metabolic process | Carbohydrate metabolic process | | 1224.331 | 5144.095 | 2169.749 | 2493.282 | 1076.18 | 1014.347 | 1580.29 | 2491.849 | 1292.668 | 1091.244 | 844.642 | 981.4865 | 756.4835 | 1069.514 | 975.9966 | 752.3295 | 219.9262 | 348.0871 | 113.3559 | 1319.125 | 645.5341 | 160.778 | 2215.807 | 900.4832 | 42.46399 | 777.3766 | 581.8912 |
| CUST_35109_PI429010886 | M11610 | 0.016873 | 12 | 0 | 443722965 | hypothetical protein CAPTEDRAFT_136995, partial [ | | | 1.25E-09 | 504.2482 | 559.8475 | 665.3114 | 381.498 | 339.8212 | 306.2123 | 307.7422 | 418.2809 | 568.2975 | 757.2157 | 397.4231 | 642.6469 | 509.4935 | 711.6467 | 546.956 | 528.618 | 412.1707 | 390.3686 | 403.8464 | 562.4407 | 515.1721 | 566.6261 | 576.8367 | 350.793 | 609.869 | 519.6135 | 683.9239 |
| CUST_9148_PI429010886 | N24136 | 0.016873 | 12 | 3 | | | | | | 10866.21 | 4589.406 | 3764.548 | 19773.16 | 14600.6 | 28306.41 | 17436.39 | 5233.779 | 7617.588 | 213.4066 | 9839.748 | 130.1545 | 7457.426 | 2697.082 | 6630.238 | 7239.058 | 17943.08 | 21597.2 | 11882.91 | 986.7668 | 11161.59 | 6863.631 | 10348.94 | 20686.07 | 1295.841 | 10884.33 | 77.03965 |
| CUST_48340_PI429010886 | M79906 | 0.016884 | 18 | 12 | | | | | | 182.8475 | 176.7754 | 200.7582 | 154.222 | 153.1912 | 149.0167 | 165.9977 | 145.4206 | 156.3918 | 215.167 | 154.3563 | 211.5135 | 161.6447 | 241.2571 | 277.2458 | 238.9663 | 150.3171 | 210.7973 | 165.9503 | 238.1333 | 206.0055 | 176.94 | 168.4961 | 155.9586 | 197.3275 | 196.5989 | 228.3928 |
| CUST_18504_PI429010886 | N39916 | 0.016885 | 24 | 0 | | | | | | 10.50912 | 13.58003 | 10.48357 | NA | 8.670788 | 6.041641 | 4.412198 | 7.538194 | 8.073322 | NA | 7.061176 | 7.329225 | 7.818797 | 8.886857 | 7.862605 | 11.12231 | NA | 3.963741 | 8.329617 | 11.89878 | 6.11892 | 14.46019 | 7.112561 | 9.504182 | 11.81299 | 11.62614 | 9.399234 |
| CUST_45127_PI429010886 | M68077 | 0.016889 | 15 | 12 | 260791400 | hypothetical protein BRAFLDRAFT_89523 [Branchio | | | 3.93E-27 | 54.28784 | 45.71627 | 77.39037 | 38.56543 | 38.24021 | 45.07578 | 46.02927 | 73.65478 | 64.86794 | 63.65227 | 57.49062 | 57.34886 | 85.01801 | 113.525 | 58.0658 | 42.85678 | 72.78887 | 33.84232 | 40.55137 | 48.35952 | 58.36102 | 54.04881 | 89.10973 | 55.32113 | 70.02583 | 42.12575 | 75.27932 |
| CUST_13723_PI429010886 | N31661 | 0.016889 | 15 | 12 | | | | | | 75.49979 | 56.48952 | 97.21485 | 62.04982 | 51.41614 | 57.28119 | 111.5352 | 272.1063 | 100.5781 | 331.1092 | 57.00393 | 23.28835 | 379.8142 | 345.1018 | 164.0153 | 88.8824 | 287.2312 | 63.98783 | 69.24723 | 94.76564 | 40.66388 | 114.5891 | 148.6082 | 43.48424 | 245.8985 | 59.12378 | 115.8059 |
| CUST_10267_PI429010886 | N25975 | 0.016892 | 12 | 3 | | | | | | 34.60947 | 10.037 | 9.325475 | 55.72398 | 48.3768 | 56.93106 | 35.05495 | 14.21847 | 26.59511 | NA | 5.210194 | NA | 49.55005 | 22.89389 | 34.78474 | 69.51764 | 28.85946 | 91.6409 | 41.10188 | 8.661553 | 20.56296 | 16.87203 | 10.80877 | 42.85678 | 5.972532 | 3.635461 | NA |
| CUST_2920_PI429010886 | N13391 | 0.016915 | 24 | 21 | | | | | | 18.81916 | 27.1975 | 36.27265 | 38.16452 | 23.16735 | 53.84859 | 24.84105 | 25.18558 | 17.37911 | 41.9189 | 25.77821 | 15.40382 | 59.6055 | 50.55173 | 54.86156 | 34.36888 | 53.47338 | 28.3053 | 91.72554 | 24.9855 | 51.89227 | 57.35641 | 53.64846 | 57.77025 | 29.63396 | 34.23531 | 33.37654 |
| CUST_31650_PI429010886 | M2054 | 0.016922 | 15 | 3 | 123474370 | ankyrin repeat protein [Trichomonas vaginalis G3] > | | | 3.79E-27 | 1156.728 | 997.0196 | 634.4094 | 1724.959 | 1079.829 | 1631.11 | 1928.541 | 989.9215 | 1047.894 | 274.2312 | 807.9372 | 291.9341 | 964.5529 | 517.2906 | 671.0656 | 882.5331 | 1550.902 | 1051.549 | 1377.193 | 400.8956 | 675.5498 | 646.4459 | 614.705 | 1380.785 | 561.029 | 712.7429 | 460.1631 |
| CUST_51362_PI429010886 | M93264 | 0.016922 | 15 | 9 | | | | | | 219.7749 | 135.0089 | 164.4877 | 151.2877 | 114.4136 | 139.47 | 111.1235 | 265.0674 | 181.9242 | 235.9095 | 113.8053 | 247.9064 | 133.1101 | 270.2125 | 279.8319 | 192.1174 | 144.4836 | 166.6587 | 164.4613 | 158.5656 | 137.5853 | 192.0215 | 175.7472 | 212.0939 | 419.6576 | 233.484 | 390.2131 |
| CUST_24758_PI429010886 | N51205 | 0.016938 | 15 | 9 | | | | | | 109.4262 | 99.31284 | 130.8664 | 116.2092 | 104.8539 | 83.33435 | 120.0318 | 94.77604 | 118.6098 | 139.1382 | 117.621 | 149.6388 | 107.0174 | 147.1104 | 166.2726 | 138.4302 | 105.1255 | 127.7612 | 89.5312 | 114.7609 | 113.2243 | 134.8111 | 159.3655 | 94.24344 | 139.0161 | 107.9228 | 163.8253 |
| CUST_42179_PI429010886 | M59617 | 0.016939 | 15 | 9 | | | | | | 8.228156 | 10.82172 | 13.26351 | 12.46576 | 4.669465 | NA | 5.668195 | 6.15687 | 11.68424 | NA | 11.24362 | 22.94051 | 10.78464 | 17.64932 | 14.65028 | 18.53665 | 10.46038 | 11.89374 | 5.389345 | 11.65447 | 6.100648 | 16.68621 | 9.943996 | 10.22803 | 15.557 | 18.7049 | 13.07663 |
| CUST_27658_PI429010886 | N56460 | 0.016946 | 24 | 0 | | | | | | 26.66659 | 34.0788 | 30.558 | 26.6361 | 23.82334 | 19.22658 | 14.32775 | 18.59923 | 25.11237 | 25.43742 | 17.94904 | 22.72662 | 11.9801 | 27.16436 | 27.0028 | 39.14518 | 23.91586 | 27.35753 | 23.40051 | 13.76394 | 27.18775 | 31.43539 | 27.04728 | 18.47667 | 31.50694 | 36.14573 | 31.41951 |
| CUST_12905_PI429010886 | N30244 | 0.016946 | 18 | 12 | 391325031 | PREDICTED: zinc finger protein 484-like [Metaseiulu | | | 2.3E-11 | 21.42649 | 24.97464 | 27.54647 | 15.37508 | 14.97314 | 17.7534 | 15.16804 | 22.54108 | 18.78315 | 44.51031 | 17.25337 | 31.06856 | 24.79214 | 31.04302 | 29.9146 | 41.65544 | 15.39298 | 22.31957 | 21.61386 | 20.71979 | 46.0983 | 21.78224 | 21.3985 | 10.14024 | 34.06729 | 17.18634 | 29.50356 |
| CUST_46424_PI429010886 | M72631 | 0.016957 | 12 | 0 | 77743875 | Euphausia superba isolate DGGE band AntEuk13-5 | | | 5.7E-125 | 119.6384 | 193.5271 | 130.2535 | 120.4315 | 92.88396 | 68.66167 | 52.90229 | 84.722 | 104.8392 | 173.2522 | 111.5107 | 154.0296 | 245.4514 | 146.0723 | 129.6855 | 101.1328 | 106.941 | 88.25864 | 80.77462 | 117.2072 | 101.487 | 129.735 | 113.7893 | 87.9523 | 115.5578 | 69.49122 | 146.1977 |
| CUST_47896_PI429010886 | M78080 | 0.016957 | 12 | 0 | | | | | | 23.61845 | 25.08131 | 19.05569 | 23.4104 | 16.52475 | 12.68586 | 15.2174 | 9.87019 | 13.55355 | 14.03531 | 18.9048 | 29.70337 | 17.38464 | 25.92266 | 18.97043 | 23.46287 | 10.8676 | 12.80802 | 18.73459 | 17.84645 | 23.08011 | 23.28108 | 21.11131 | 14.48423 | 25.32849 | 25.47479 | 24.17916 |
| CUST_22958_PI429010886 | N48006 | 0.016961 | 24 | 0 | | | | | | 55.64622 | 82.67802 | 93.78702 | 48.36598 | 52.45794 | 53.62643 | 52.81394 | 65.02052 | 54.5089 | 86.72657 | 51.5698 | 73.35296 | 40.74434 | 49.40423 | 41.87571 | 70.74029 | 74.90972 | 54.92234 | 42.02191 | 63.13278 | 54.63526 | 56.99751 | 93.96395 | 70.24827 | 80.23994 | 66.18886 | 77.82271 |
| CUST_7911_PI429010886 | N22261 | 0.016968 | 21 | 6 | 321476498 | hypothetical protein DAPPUDRAFT_96952 [Daphnia | | | 3.81E-29 | 292.1061 | 385.7092 | 385.3789 | 315.1705 | 513.5475 | 429.1228 | 489.1062 | 440.3254 | 605.3029 | 474.8115 | 416.1299 | 475.2865 | 375.6267 | 547.2331 | 449.0628 | 443.8496 | 297.9644 | 375.2702 | 469.6397 | 346.8405 | 469.28 | 415.0913 | 411.5645 | 408.3975 | 427.0325 | 431.842 | 588.751 |
| CUST_16582_PI429010886 | N36503 | 0.016968 | 21 | 0 | 47219760 | unnamed protein product [Tetraodon nigroviridis] | | | 6.93E-08 | 15.50857 | 15.8263 | 13.44859 | 8.342648 | 8.92426 | 7.374786 | 6.633519 | 8.905799 | 7.944424 | 15.5215 | 10.57449 | 13.71506 | 8.5745 | 10.46578 | 15.12683 | 15.14331 | 9.363982 | 19.91752 | 8.712785 | 10.58195 | 9.144683 | 11.80446 | 15.29306 | 20.71812 | 8.378893 | 9.476285 | 12.18324 |
| CUST_11743_PI429010886 | N28334 | 0.016985 | 15 | 3 | | | | | | 8.771173 | 4.733482 | 22.8042 | 29.67132 | 28.66586 | 25.00056 | 40.07222 | 4.398494 | 15.12127 | 4.125002 | 34.05076 | 6.343801 | 8.851169 | 8.095294 | 6.391082 | 6.425284 | 94.89615 | 86.88496 | 42.61497 | 29.99986 | 46.11029 | 18.35945 | 20.90912 | 23.58557 | 5.007287 | NA | NA |
| CUST_47983_PI429010886 | M78387 | 0.016991 | 15 | 12 | 148226561 | coiled-coil domain-containing protein 93 [Xenopus l | | | 6.33E-30 | 9.992565 | 9.611855 | 9.694084 | 7.293335 | 7.96115 | NA | 12.11941 | 9.228812 | 7.36754 | NA | 8.781809 | 9.979521 | 11.28708 | 11.65277 | 14.05481 | 28.9272 | 8.629296 | 14.86823 | 5.231414 | 7.871072 | 10.48357 | 11.89031 | 8.378893 | 6.548124 | 15.37156 | 8.973721 | 13.46425 |
| CUST_34037_PI429010886 | M8566 | 0.017064 | 12 | 0 | | | | | | 102.1576 | 95.98918 | 109.7501 | 78.93556 | 60.41389 | 97.282 | 66.27543 | 77.1366 | 84.1757 | 112.4033 | 78.26326 | 115.3428 | 86.54045 | 112.3653 | 116.6682 | 128.4226 | 75.3452 | 101.5278 | 91.54224 | 90.27604 | 94.47657 | 101.9359 | 102.8432 | 76.73952 | 124.2716 | 129.8621 | 128.5885 |
| CUST_3947_PI429010886 | N15639 | 0.017064 | 18 | 9 | 402768955 | pacifastin heavy chain [Macrobrachium rosenbergii | Transport | Ion transport | 4.65E-21 | 177.9616 | 593.968 | 535.1571 | 185.6237 | 118.0091 | 160.7305 | 350.9859 | 281.8261 | 143.8832 | 448.8586 | 382.7566 | 382.8597 | 356.9822 | 345.5828 | 315.8463 | 137.3083 | 358.1231 | 301.0265 | 453.79 | 136.4498 | 244.4592 | 273.4298 | 220.511 | 128.3451 | 613.2651 | 252.6253 | 503.4817 |
| CUST_29854_PI429010886 | N60694 | 0.017064 | 12 | 0 | | | | | | 39.19727 | 41.9571 | 46.35772 | 37.27042 | 39.81145 | 47.94823 | 33.51452 | 30.35236 | 35.66432 | 34.71503 | 31.33759 | 37.30131 | 34.2205 | 40.83264 | 35.53745 | 35.39935 | 73.71157 | 36.32974 | 28.7018 | 28.08839 | 38.05463 | 32.78743 | 23.34144 | 32.18557 | 46.82282 | 35.27881 | 56.65296 |
| CUST_659_PI429010886 | N6603 | 0.017066 | 15 | 12 | | | | | | 784.4175 | 918.9933 | 845.2412 | 714.2963 | 684.7245 | 654.4842 | 669.2807 | 715.1399 | 738.859 | 919.3319 | 743.1041 | 834.6224 | 882.153 | 936.398 | 1071.523 | 1064.349 | 745.0756 | 793.0472 | 718.4589 | 1156.123 | 793.3041 | 1048.763 | 752.1706 | 628.1675 | 927.8138 | 864.8993 | 1087.079 |
| CUST_33895_PI429010886 | M8172 | 0.017068 | 21 | 9 | | | | | | 10.08763 | 62.3009 | 43.28801 | 91.78258 | 90.26625 | 74.38643 | 101.6806 | 280.165 | 254.8048 | 56.61759 | 372.7154 | 529.6585 | 190.3851 | 79.74735 | 131.0034 | 50.22122 | 135.6374 | 44.04837 | 42.28532 | 632.4528 | 305.2258 | 3.962377 | 124.6867 | 68.51154 | 5.123205 | 225.1125 | 162.719 |
| CUST_11131_PI429010886 | N27313 | 0.017079 | 21 | 3 | | | | | | 617.9947 | 1550.226 | 890.3835 | 1011.687 | 974.3808 | 1156.123 | 694.3101 | 794.1984 | 741.2403 | 814.5413 | 410.3497 | 1338.953 | 667.5342 | 684.8725 | 486.9644 | 850.2192 | 299.4458 | 431.6488 | 114.1927 | 394.5293 | 1039.842 | 962.8744 | 1251.3 | 760.0455 | 1165.064 | 1436.981 | 616.2727 |
| CUST_17529_PI429010886 | N38125 | 0.017082 | 21 | 12 | 321451689 | hypothetical protein DAPPUDRAFT_335707 [Daphn | Metabolic process | Other | 2.07E-15 | 59.30066 | 64.3345 | 52.32895 | 59.76845 | 44.99497 | 53.74643 | 63.6301 | 53.55422 | 61.49124 | 75.10926 | 57.23179 | 71.07061 | 69.76759 | 72.13281 | 68.6262 | 53.42416 | 59.04082 | 65.59604 | 69.22629 | 74.52928 | 69.22629 | 81.85243 | 67.32952 | 52.0001 | 45.68644 | 51.22479 | 65.95253 |
| CUST_49967_PI429010886 | M87978 | 0.017083 | 15 | 12 | | | | | | 29.05988 | 20.23489 | 86.31209 | 13.57654 | 12.93585 | 20.08697 | 38.81815 | 160.2968 | 81.42045 | 63.02348 | 22.6112 | 28.32496 | 120.2195 | 153.0835 | 47.9222 | 20.68548 | 88.85357 | 19.79375 | 22.41298 | 21.07965 | 23.1605 | 53.9331 | 82.72689 | 28.77061 | 106.6494 | 13.95252 | 106.8281 |
| CUST_50345_PI429010886 | M89523 | 0.017083 | 15 | 12 | | | | | | 39.36991 | 24.21798 | 32.84559 | 14.05335 | 16.61483 | 33.99233 | 11.3912 | 25.64589 | 21.33294 | 25.09429 | 34.95466 | 43.1379 | 30.31136 | 29.77206 | 35.71039 | 35.05162 | 22.84941 | 18.4928 | 16.10051 | 23.21852 | 21.04609 | 37.36325 | 19.5463 | 21.036 | 28.97255 | 29.01971 | 26.96182 |
| CUST_8432_PI429010886 | N23030 | 0.017083 | 15 | 12 | 405960555 | Fanconi anemia group J protein [Crassostrea gigas] | Response to stress | DNA repair | 1.35E-21 | 20.51967 | 20.8959 | 7.149572 | 12.05708 | 4.287357 | 14.02288 | 17.61934 | 15.79894 | 22.01914 | 19.11687 | 5.695981 | 22.25206 | 25.64065 | 23.22056 | 24.71374 | 12.70027 | 10.95786 | 19.66703 | 13.27381 | 16.33291 | 9.105658 | 12.89788 | 17.33333 | 25.80044 | 17.34629 | 8.956327 | 20.77162 |
| CUST_15139_PI429010886 | N34068 | 0.017083 | 15 | 12 | | | | | | 39.45879 | 44.84166 | 57.10423 | 31.01112 | 48.39289 | 46.24589 | 36.7953 | 27.58334 | 36.40199 | 44.45342 | 46.9717 | 47.78664 | 41.09904 | 62.96106 | 45.18256 | 36.85229 | 44.93417 | 49.27786 | 38.84241 | 49.39411 | 44.20422 | 35.29256 | 35.59734 | 44.15772 | 47.2535 | 41.75113 | 51.6678 |
| CUST_53537_PI429010886 | M102158 | 0.017086 | 18 | 9 | | | | | | 75.46943 | 45.37412 | 68.03939 | 60.99798 | 49.07593 | 34.27356 | 49.89799 | 51.93461 | 72.38234 | 81.38307 | 82.67802 | 60.96666 | 72.90791 | 76.60738 | 50.277 | 69.9232 | 55.66641 | 46.64901 | 75.30403 | 71.3655 | 52.65717 | 43.45754 | 63.98224 | 30.558 | 67.51475 | 62.15321 | 87.59441 |
| CUST_11719_PI429010886 | N28294 | 0.017096 | 21 | 0 | 545182363 | PREDICTED: LOW QUALITY PROTEIN: integrator con | | | 1.65E-13 | 41.42947 | 14.39019 | 39.60553 | 18.40232 | 16.68781 | 19.59067 | 29.42101 | 14.5414 | 25.00327 | 26.76517 | 15.8658 | 11.09741 | 11.94752 | 19.34811 | 10.38437 | 16.24815 | 69.28034 | 25.09128 | 20.49048 | 8.140015 | 19.95159 | 31.69618 | 47.95255 | 40.53465 | 29.63867 | 20.52466 | 12.19407 |
| CUST_35096_PI429010886 | M11564 | 0.017103 | 21 | 0 | 91079022 | PREDICTED: similar to DnaJ (Hsp40) homolog, subfa | Protein metabolism | Protein folding | 6.44E-27 | 14.52793 | 17.15146 | 21.13052 | 13.82239 | 15.70949 | NA | 5.75743 | NA | 14.74649 | 10.72561 | 13.54122 | 10.23633 | 9.322057 | 12.73533 | 8.954946 | 14.42999 | 16.58396 | 15.44211 | 11.30336 | 19.36786 | 12.43137 | 12.90472 | NA | 15.58918 | 11.92046 | 6.370435 | 15.43128 |
| CUST_9588_PI429010886 | N24880 | 0.017112 | 18 | 12 | 321456662 | hypothetical protein DAPPUDRAFT_63727 [Daphnia | Protein metabolism | Proteolysis | 3.36E-73 | 344.3603 | 455.6129 | 310.518 | 253.3046 | 435.7052 | 286.8104 | 493.215 | 457.234 | 581.5974 | 476.4015 | 427.7928 | 505.3693 | 641.3583 | 508.1895 | 506.1361 | 567.608 | 396.1913 | 240.7867 | 503.6617 | 441.5814 | 378.3563 | 414.6092 | 343.216 | 336.4126 | 545.9123 | 330.1179 | 742.2219 |
| CUST_50710_PI429010886 | M90725 | 0.017114 | 15 | 3 | 152949487 | calcification associated soluble matrix protein 2 [Pr | | | 6.88E-23 | 1233.738 | 661.523 | 797.54 | 1517.009 | 3126.195 | 3008.795 | 2653.866 | 988.0968 | 694.3101 | 60.49322 | 2516.877 | 47.85374 | 743.1041 | 239.2718 | 416.8412 | 393.0672 | 2223.051 | 1837.915 | 2818.479 | 84.27965 | 2164.947 | 335.8595 | 1770.146 | 2710.041 | 301.849 | 1212.358 | 49.36528 |
| CUST_3394_PI429010886 | N14421 | 0.017114 | 15 | 9 | | | | | | 483.597 | 961.1254 | 754.7954 | 403.9415 | 572.8694 | 457.7503 | 894.9707 | 887.5191 | 1029.372 | 1012.95 | 1056.762 | 768.2695 | 465.6558 | 765.9632 | 1011.26 | 1458.732 | 830.2031 | 901.5294 | 1065.529 | 563.9632 | 577.5152 | 1321.214 | 799.1588 | 582.8028 | 1880.44 | 920.7129 | 1926.251 |
| CUST_24202_PI429010886 | N50220 | 0.017114 | 15 | 9 | | | | | | 20.70543 | 25.99299 | 20.03687 | 20.26285 | 15.55244 | 21.8123 | 15.11766 | 23.43812 | 32.26335 | 20.4413 | 24.97756 | 33.49626 | 22.12202 | 30.98507 | 19.78047 | 24.15101 | 26.46581 | 19.03069 | 21.27079 | 22.42981 | 18.87721 | 22.31012 | 26.87231 | 24.69322 | 30.12077 | 33.7899 | 28.72783 |
| CUST_27147_PI429010886 | N55524 | 0.017114 | 15 | 0 | | | | | | 1148.695 | 1720.101 | 1630.188 | 977.7803 | 1504.846 | 1015.362 | 810.9217 | 658.4007 | 1239.798 | 1458.732 | 1412.634 | 1282.889 | 841.4264 | 798.8942 | 1142.155 | 2671.216 | 1854.457 | 1901.328 | 1050.96 | 1338.953 | 1696.945 | 1284.158 | 836.4238 | 1058.786 | 2076.382 | 1468.172 | 1438.603 |
| CUST_624_PI429010886 | N6383 | 0.017114 | 15 | 3 | | | Developmental process | Muscle structure development | | 9459.703 | 9588.886 | 6160.933 | 13887.31 | 11864.38 | 8974.042 | 11143.54 | 7383.427 | 6879.978 | 4755.263 | 9020.979 | 4297.995 | 7266.955 | 4186.218 | 5811.899 | 7188.247 | 10559.01 | 9726.619 | 13085.78 | 4289.879 | 6321.861 | 7175.165 | 5561.701 | 9131.511 | 4174.241 | 6203.668 | 2723.994 |
| CUST_1143_PI429010886 | N8626 | 0.017114 | 15 | 9 | 17066696 | reverse transcriptase/ribonuclease H/putative meth | | | 8.82E-08 | 36.87107 | 31.19824 | 37.34183 | 32.76173 | 30.43159 | 36.77308 | 29.28593 | 33.98937 | 40.61781 | 48.40257 | 33.98233 | 42.35648 | 35.33784 | 51.13662 | 40.28418 | 41.73826 | 36.75622 | 37.16398 | 28.33698 | 38.58369 | 22.91619 | 52.33357 | 29.9579 | 33.93687 | 51.65313 | 38.55767 | 43.94789 |
| CUST_42329_PI429010886 | M60020 | 0.017121 | 24 | 21 | | | | | | 577.0235 | 715.1399 | 661.6789 | 513.344 | 485.2905 | 661.8313 | 473.4648 | 536.4898 | 498.3855 | 520.2836 | 528.8856 | 744.8667 | 582.8028 | 531.9567 | 559.0577 | 756.7863 | 523.6559 | 508.5068 | 574.2435 | 637.6126 | 652.2033 | 641.17 | 670.6469 | 679.7235 | 775.9372 | 666.3149 | 783.3097 |
| CUST_10067_PI429010886 | N25641 | 0.017121 | 24 | 6 | | | | | | 12.19257 | 20.93627 | 21.61291 | 8.848997 | 22.26173 | 36.81255 | 25.00056 | 37.40452 | 26.96182 | 22.37611 | 13.47949 | 19.6594 | 18.50256 | 21.79918 | 10.62806 | 15.74407 | 17.06111 | 20.88612 | 9.828551 | 28.06449 | 17.05513 | 10.28142 | 17.85063 | 8.321397 | 12.09699 | 43.12662 | 23.14834 |
| CUST_4465_PI429010886 | N16814 | 0.017129 | 24 | 21 | | | | | | 42.96262 | 35.65161 | 52.10631 | 42.59502 | 24.11276 | 36.83973 | 53.20866 | 47.76946 | 36.61393 | 41.26062 | 27.36512 | 38.2326 | 28.90976 | 32.70344 | 32.8295 | 42.65121 | 46.77465 | 31.34507 | 34.9503 | 38.71949 | 35.29716 | 42.70151 | 55.42181 | 48.22079 | 40.09578 | 43.50197 | 45.51822 |
| CUST_28790_PI429010886 | N58624 | 0.017154 | 15 | 9 | | | | | | 163.5397 | 161.4564 | 177.9987 | 132.5328 | 114.2745 | 110.5811 | 125.8442 | 131.1802 | 127.4456 | 196.6789 | 123.9921 | 185.6524 | 128.4554 | 163.3744 | 160.4594 | 150.6728 | 107.4484 | 147.3674 | 134.6396 | 154.0476 | 129.6855 | 170.1249 | 169.8899 | 105.3783 | 205.9692 | 170.8864 | 186.4588 |
| CUST_29690_PI429010886 | N60353 | 0.017154 | 15 | 3 | | | | | | 17197.8 | 6925.74 | 5627.009 | 31923.04 | 21721.24 | 48107.91 | 27412.06 | 7794.866 | 10469.53 | 373.826 | 15743.72 | 126.9474 | 12175.63 | 4864.618 | 10760.71 | 11259.43 | 30082.79 | 37972.43 | 17786.81 | 1837.915 | 17299.35 | 10777.69 | 14970.57 | 31492.49 | 1818.602 | 17674.75 | 151.6449 |
| CUST_644_PI429010886 | N6518 | 0.017154 | 15 | 3 | | | | | | 984.9879 | 352.327 | 549.794 | 1846.987 | 1396.326 | 2740.182 | 1665.986 | 695.1115 | 469.825 | 61.30366 | 1138.397 | 37.17503 | 490.5451 | 164.9826 | 366.089 | 283.7427 | 2563.253 | 1724.959 | 1378.434 | 90.47199 | 1148.187 | 332.8858 | 975.7147 | 1672.079 | 84.015 | 1059.331 | 6.589476 |
| CUST_14791_PI429010886 | N33473 | 0.017162 | 21 | 0 | | | | | | 21.82435 | 45.30213 | 29.60636 | 17.81636 | 27.62424 | 37.81058 | 16.5818 | 23.00815 | 22.13024 | 14.42793 | 13.26174 | 28.30863 | 12.57535 | 29.45562 | 8.296408 | 15.65642 | 10.33897 | 10.79562 | 10.24643 | 5.670388 | 43.68024 | 21.17293 | 37.13409 | 30.66153 | 26.44421 | 38.54962 | 12.93269 |
| CUST_22003_PI429010886 | N46242 | 0.017163 | 18 | 3 | 241598583 | rassf1, putative [Ixodes scapularis] >gi\|215500481\| | Signal transduction | Intracellular signal transduction | 1.23E-33 | 76.34394 | 54.58347 | 55.05355 | 72.43161 | 60.58205 | 92.04256 | 68.33792 | 61.52513 | 58.4576 | 44.70035 | 60.12109 | 47.08024 | 54.67468 | 55.78991 | 50.07576 | 69.45519 | 54.75352 | 63.43828 | 98.19642 | 63.62137 | 46.09323 | 68.27282 | 55.59388 | 87.73688 | 62.22607 | 65.28922 | 68.16131 |
| CUST_33578_PI429010886 | M7321 | 0.017183 | 24 | 9 | | | | | | 53.10218 | 70.89744 | 44.02718 | 76.33468 | 44.63016 | 27.53905 | 74.88 | 37.20061 | 50.11711 | 50.9653 | 60.80176 | 51.64328 | 34.26311 | 42.29018 | 37.13071 | 55.85751 | 42.37343 | 57.02142 | 31.9232 | 25.0272 | 20.79184 | 34.21339 | 35.50744 | 38.09997 | 50.26445 | 49.44854 | 38.07688 |
| CUST_47188_PI429010886 | M75111 | 0.017191 | 21 | 0 | | | | | | 69.79294 | 97.63395 | 81.25969 | 64.44572 | 67.21241 | 58.29022 | 49.96135 | 43.00099 | 53.32175 | 83.96253 | 47.5835 | 57.28119 | 56.02183 | 81.42045 | 26.4818 | 73.31065 | 60.08957 | 59.79177 | 60.93646 | 68.17174 | 68.19793 | 96.6566 | 79.14043 | 55.33057 | 88.59096 | 46.82282 | 87.12746 |
| CUST_27047_PI429010886 | N55353 | 0.017243 | 21 | 3 | 91078062 | PREDICTED: similar to conserved hypothetical prote | Cellular Process | Cell adhesion | 8.92E-24 | 15.1699 | 16.26104 | 8.779513 | 27.77246 | 20.46944 | 21.69445 | 13.97235 | 4.387938 | 9.205662 | 11.38156 | 7.841585 | 5.669781 | 7.255725 | 10.25912 | 11.46217 | 17.27372 | 13.20597 | 22.44161 | 22.52113 | 9.933074 | 14.77179 | 18.86937 | 13.38764 | 10.05851 | 15.66371 | 18.77363 | NA |
| CUST_43624_PI429010886 | M63528 | 0.017256 | 12 | 3 | | | | | | 93.96395 | 100.566 | 136.3426 | 109.8598 | 97.21485 | 146.4805 | 100.2495 | 91.76014 | 78.31562 | 105.57 | 83.4507 | 93.60773 | 86.15616 | 105.7372 | 107.7492 | 134.9154 | 106.8828 | 103.1223 | 101.5527 | 91.95171 | 89.24114 | 84.03407 | 88.60515 | 98.62916 | 106.3446 | 104.322 | 98.7787 |
| CUST_45933_PI429010886 | M70788 | 0.017256 | 18 | 15 | | | | | | 786.4352 | 1118.208 | 648.6302 | 643.3515 | 778.015 | 753.467 | 448.6531 | 520.1128 | 555.3104 | 870.5556 | 710.22 | 913.0418 | 654.3393 | 577.8225 | 631.8289 | 766.1345 | 893.1457 | 689.6848 | 737.0066 | 667.7123 | 610.0761 | 695.332 | 611.3709 | 590.3441 | 844.933 | 519.3194 | 513.6722 |
| CUST_32460_PI429010886 | M4180 | 0.017262 | 15 | 0 | | | | | | 86.32314 | 79.13143 | 73.73837 | 61.89112 | 59.78571 | 26.41485 | 64.91729 | 32.39121 | 31.99941 | 58.544 | 47.80199 | 61.08538 | 67.42469 | 72.92262 | 65.46343 | 94.76564 | 56.09338 | 55.31379 | 63.98783 | 70.34764 | 84.75109 | 41.05859 | 65.99023 | 37.74031 | 51.7068 | 48.40695 | 86.76715 |
| CUST_57251_PI429010886 | E2956 | 0.017277 | 15 | 12 | | | Protein metabolism | Ribosome biogenesis | | 29537.19 | 30891.2 | 26101.79 | 30706.69 | 30706.69 | 28752.77 | 19870.47 | 24244.07 | 28378.02 | 25546.19 | 29610.14 | 33418.81 | 29610.14 | 31396.1 | 44794.66 | 30706.69 | 19626.64 | 31148.55 | 24915.2 | 38256.92 | 26594.86 | 29468.93 | 23368.13 | 21011.69 | 24425.67 | 27353.46 | 29281.85 |
| CUST_45091_PI429010886 | M67978 | 0.017277 | 15 | 12 | | | | | | 45.43013 | 77.34 | 85.79457 | 56.10477 | 43.27178 | 41.57079 | 52.49227 | 63.747 | 66.30772 | 49.14895 | 51.83879 | 60.6558 | 61.53942 | 145.4981 | 98.19642 | 69.94604 | 54.33444 | 59.03159 | 63.42473 | 46.45766 | 44.85517 | 45.95551 | 65.52115 | 66.04832 | 94.23139 | 47.65996 | 156.8837 |
| CUST_49599_PI429010886 | M86355 | 0.017277 | 15 | 12 | | | | | | 301.0804 | 267.7292 | 424.5435 | 166.1651 | 154.9491 | 128.8011 | 260.373 | 359.8853 | 335.8009 | 431.0477 | 240.474 | 330.9233 | 308.0149 | 452.716 | 296.8833 | 271.5735 | 285.6058 | 208.515 | 286.6682 | 242.3824 | 254.2335 | 243.2786 | 384.1514 | 192.2628 | 394.2069 | 242.9704 | 344.8512 |
| CUST_26454_PI429010886 | N54271 | 0.017277 | 15 | 12 | 321470711 | hypothetical protein DAPPUDRAFT_317365 [Daphn | | | 1.13E-10 | 27.75224 | 24.22062 | 57.79515 | 23.10525 | 9.706284 | 17.69248 | 28.50866 | 11.31889 | 41.10579 | 23.05184 | 19.12737 | 41.15103 | 41.01917 | 38.80417 | 28.03701 | 29.24887 | 11.54241 | 43.60712 | 12.96601 | 14.31222 | 16.86404 | 26.98034 | 30.37957 | 16.97048 | 18.93773 | 35.18679 | 21.49416 |
| CUST_13603_PI429010886 | N31448 | 0.017285 | 18 | 15 | | | | | | 191.6952 | 245.6875 | 136.4108 | 183.6281 | 190.2377 | 125.8218 | 148.6917 | 130.8512 | 87.55497 | 202.7794 | 226.8346 | 224.8249 | 167.6463 | 123.6359 | 209.5249 | 207.4294 | 208.0738 | 206.469 | 207.9553 | 148.7285 | 155.7638 | 238.8193 | 182.5658 | 149.4128 | 155.7184 | 126.0646 | 97.32524 |
| CUST_38572_PI429010886 | M28140 | 0.017297 | 21 | 0 | | | | | | 436.8338 | 434.7605 | 389.871 | 353.25 | 386.3769 | 373.896 | 371.4417 | 333.708 | 353.3873 | 342.6916 | 423.5928 | 403.8464 | 349.6083 | 408.8989 | 346.965 | 378.3563 | 411.3644 | 390.2834 | 374.8258 | 403.7411 | 440.9199 | 432.1375 | 435.8026 | 361.9091 | 372.8945 | 353.4537 | 417.1018 |
| CUST_35224_PI429010886 | M11957 | 0.017298 | 24 | 18 | | | | | | 22.33422 | 22.10673 | 23.52604 | 22.20218 | 8.935164 | 6.528004 | 23.09604 | 16.8622 | 23.11042 | 27.57791 | 17.81187 | 18.11108 | 20.40333 | 21.60382 | 15.90941 | 34.23165 | 20.49506 | 27.21982 | 31.5798 | 26.80742 | 26.71847 | 39.61454 | 23.51171 | 32.80137 | 23.56991 | 28.75932 | 38.93982 |
| CUST_44734_PI429010886 | M66830 | 0.017307 | 15 | 9 | 478250366 | hypothetical protein YQE_12440, partial [Dendroct | Nucleic acid metabolism | Regulation of gene expression | 2.21E-14 | 55.51379 | 44.58572 | 87.13975 | 44.14799 | 57.15078 | 49.53059 | 50.45103 | 58.57388 | 66.07282 | 65.9853 | 59.1427 | 66.63641 | 47.15148 | 66.99196 | 55.01668 | 80.08977 | 64.48135 | 54.86684 | 65.26348 | 52.32895 | 51.53885 | 67.55015 | 75.4776 | 59.87756 | 109.5287 | 102.0816 | 87.73688 |
| CUST_47506_PI429010886 | M76394 | 0.017307 | 15 | 0 | | | | | | 491.5609 | 460.5023 | 451.1707 | 450.9914 | 461.9687 | 595.1496 | 447.5499 | 426.5023 | 388.8828 | 499.859 | 413.0515 | 455.745 | 389.0546 | 423.8598 | 414.9669 | 567.9112 | 602.798 | 534.723 | 478.7395 | 439.9985 | 331.3738 | 414.2198 | 428.9575 | 317.4719 | 433.4824 | 358.6229 | 476.4015 |
| CUST_51545_PI429010886 | M93940 | 0.017307 | 15 | 9 | 91089021 | PREDICTED: similar to CG5808 CG5808-PA [Triboliu | | | 4.78E-08 | 412.5292 | 915.6162 | 472.5516 | 625.1793 | 337.7668 | 225.9576 | 150.8016 | 282.6926 | 460.7596 | 637.9884 | 961.1254 | 534.723 | 610.6415 | 807.9372 | 613.1363 | 801.5312 | 473.1073 | 381.498 | 353.3873 | 565.8837 | 435.2247 | 869.29 | 689.8687 | 308.2184 | 1803.696 | 634.4094 | 1311.842 |
| CUST_11500_PI429010886 | N27915 | 0.017307 | 15 | 0 | | | | | | 379.2136 | 698.8251 | 568.0071 | 526.3482 | 560.3037 | 802.2989 | 269.6735 | 174.0729 | 376.4458 | 556.2338 | 731.9128 | 731.4443 | 229.5365 | 549.9307 | 329.3607 | 747.4574 | 581.8912 | 785.705 | 460.5023 | 481.5308 | 528.618 | 468.3619 | 341.9848 | 376.8596 | 684.3658 | 446.3108 | 528.618 |
| CUST_17532_PI429010886 | N38132 | 0.017307 | 15 | 9 | 390352602 | PREDICTED: zinc finger protein 84-like [Strongylocei | | | 2.25E-13 | 142.194 | 140.228 | 147.1104 | 100.4336 | 112.3183 | 115.0228 | 135.4807 | 103.8673 | 129.2002 | 186.9949 | 128.4766 | 159.2127 | 110.192 | 158.5869 | 133.6452 | 151.2216 | 129.0306 | 150.5702 | 135.1252 | 129.7547 | 122.5181 | 206.5428 | 308.5953 | 137.1215 | 159.4972 | 150.2252 | 159.5233 |
| CUST_27123_PI429010886 | N55487 | 0.017307 | 15 | 9 | | | | | | 84.99622 | 77.24703 | 74.7266 | 54.74747 | 60.41993 | 60.4668 | 42.93137 | 67.70114 | 74.44282 | 81.61454 | 75.15426 | 112.4193 | 61.96298 | 71.67319 | 84.70201 | 98.28699 | 57.92486 | 78.07189 | 67.34293 | 64.6187 | 78.18464 | 70.02979 | 89.95558 | 85.79457 | 112.6894 | 75.06598 | 116.3712 |
| CUST_52366_PI429010886 | M97214 | 0.017317 | 15 | 12 | | | | | | 34.27356 | 27.94276 | 36.93108 | 14.22183 | 21.38108 | 30.65462 | 26.38687 | 20.16285 | 35.8336 | 14.9864 | 26.16897 | 32.74781 | 29.02946 | 41.78704 | 37.13409 | 34.64486 | 30.88907 | 39.33067 | 27.26909 | 22.3138 | 24.58529 | 34.09109 | 6.664096 | 16.03098 | 43.0609 | 27.97666 | 27.97387 |
| CUST_42577_PI429010886 | M60630 | 0.017329 | 15 | 0 | | | | | | 107.9228 | 118.8596 | 89.45826 | 94.50596 | 130.8168 | 133.1932 | 67.51475 | 82.71912 | 70.36056 | 82.46049 | 97.71155 | 85.04454 | 97.72266 | 80.54851 | 74.89819 | 149.1909 | 107.0931 | 116.9761 | 113.2721 | 116.1614 | 87.53485 | 119.245 | 85.87525 | 91.20024 | 100.7206 | 86.51241 | 64.25402 |
| CUST_53979_PI429010886 | M103956 | 0.017337 | 24 | 9 | | | | | | 46.11982 | 30.10233 | 15.41663 | 31.1168 | 48.98425 | 41.70336 | 31.16623 | 40.80459 | 40.23032 | 49.75477 | 45.36871 | 51.79974 | 38.86489 | 47.57729 | 43.76564 | 40.82323 | 43.35404 | 39.53295 | 68.06012 | 36.85229 | 35.11191 | 29.88865 | 43.24561 | 25.72523 | 63.37317 | 17.69032 | 29.12321 |
| CUST_36370_PI429010886 | M15731 | 0.017341 | 15 | 9 | | | | | | 33.14087 | 46.64388 | 45.08487 | 20.75304 | 38.16824 | 29.62034 | 27.13002 | 31.53429 | 38.12468 | 46.75333 | 45.3644 | 55.3496 | 48.2481 | 52.96595 | 47.44967 | 54.6863 | 34.73965 | 33.31614 | 42.68463 | 40.4721 | 34.9676 | 42.01729 | 49.02131 | 30.86679 | 65.15734 | 34.08252 | 67.73286 |
| CUST_4027_PI429010886 | N15829 | 0.017341 | 15 | 12 | | | | | | 136.0526 | 96.74817 | 111.7143 | 80.33892 | 82.76433 | 115.8195 | 82.67004 | 97.75958 | 84.05405 | 103.2411 | 90.74799 | 161.1033 | 119.9123 | 159.9242 | 185.3559 | 105.9305 | 95.16133 | 130.6585 | 94.54715 | 101.92 | 101.0721 | 126.1763 | 115.1182 | 68.73669 | 99.06334 | 109.7874 | 122.2373 |
| CUST_16997_PI429010886 | N37207 | 0.017351 | 15 | 12 | | | | | | 379.4393 | 449.2825 | 400.0206 | 323.4046 | 314.8276 | 330.527 | 226.3317 | 249.422 | 277.9328 | 387.779 | 309.2864 | 352.6729 | 410.239 | 402.4861 | 334.6678 | 398.4336 | 291.9341 | 309.7028 | 328.7379 | 416.5528 | 331.8685 | 343.5515 | 356.4277 | 301.7456 | 382.9442 | 307.3807 | 356.9822 |
| CUST_53327_PI429010886 | M101290 | 0.017351 | 12 | 0 | | | | | | 288.1296 | 332.54 | 336.5091 | 254.0567 | 224.9395 | 251.662 | 222.3674 | 238.5792 | 268.4877 | 313.2163 | 250.2477 | 379.882 | 270.3119 | 371.2619 | 704.5835 | 315.7757 | 286.997 | 299.9648 | 280.2656 | 299.7277 | 325.5645 | 389.0546 | 332.5951 | 254.0209 | 336.9449 | 367.921 | 449.8886 |
| CUST_17332_PI429010886 | N37789 | 0.017351 | 15 | 12 | 537266861 | RE1-silencing transcription factor-like protein [Crice | Response to stress | Other | 5.09E-11 | 45.11398 | 24.70497 | 25.8162 | 9.413901 | 12.68236 | 17.0998 | 18.89688 | 26.23793 | 18.46027 | 27.05448 | 18.50772 | 32.53672 | 18.65486 | 25.44437 | 88.66033 | 103.6887 | 13.45546 | 16.81632 | 24.57313 | 13.28477 | 20.6694 | 22.86827 | 14.7136 | 20.51967 | 27.85864 | 24.09666 | 20.93627 |
| CUST_34329_PI429010886 | M9342 | 0.017359 | 24 | 9 | | | | | | 88.4815 | 122.8574 | 115.9131 | 111.8739 | 127.2423 | 115.3001 | 97.96017 | 146.622 | 140.5991 | 162.6703 | 146.1758 | 166.6117 | 102.2659 | 69.08039 | 144.7965 | 109.2515 | 137.687 | 155.2181 | 116.4744 | 127.8146 | 70.58339 | 131.7576 | 137.9332 | 87.60236 | 93.20644 | 100.9304 | 67.54243 |
| CUST_2252_PI429010886 | N11966 | 0.017359 | 24 | 9 | 225714106 | Cytosolic non-specific dipeptidase [Lepeophtheirus | Protein metabolism | Proteolysis | 5.3E-177 | 95.56359 | 168.6167 | 97.71155 | 120.9878 | 149.7495 | 96.07604 | 191.4981 | 200.108 | 151.8121 | 117.6403 | 243.6339 | 216.304 | 182.4397 | 87.21952 | 130.0885 | 120.4315 | 99.17539 | 106.0819 | 72.13281 | 321.3108 | 157.5293 | 101.3667 | 115.8704 | 96.36676 | 75.66735 | 88.20739 | 88.49089 |
| CUST_7984_PI429010886 | N22370 | 0.01738 | 24 | 15 | | | | | | 223.1189 | 250.4345 | 208.515 | 192.6463 | 204.2578 | 262.2522 | 203.324 | 214.4744 | 123.9578 | 311.9883 | 221.4446 | 311.9883 | 168.58 | 183.9787 | 224.1286 | 370.0362 | 267.8414 | 322.2737 | 286.3036 | 287.4768 | 294.4572 | 259.0023 | 209.6694 | 289.3248 | 372.5738 | 211.6792 | 407.0568 |
| CUST_17708_PI429010886 | N38447 | 0.017396 | 12 | 3 | 260813816 | hypothetical protein BRAFLDRAFT_114918 [Branchi | Protein metabolism | Protein folding | 1.76E-27 | 51.42128 | 52.77021 | 43.72098 | 53.75298 | 70.08934 | 60.38361 | 84.2614 | 49.39411 | 25.07401 | 50.14442 | 60.85469 | 40.28049 | 41.53622 | 31.46674 | 40.2482 | 72.84986 | 96.19258 | 65.81652 | 85.88841 | 33.59767 | 57.40224 | 62.27945 | 61.94492 | 64.77051 | 45.07578 | 39.16114 | 17.97736 |
| CUST_33842_PI429010886 | M8004 | 0.017401 | 18 | 15 | 189237826 | PREDICTED: similar to laminin subunit gamma-3 [Tri | | | 3.52E-30 | 8.685936 | 16.49404 | 14.15536 | 7.410795 | 11.54827 | 16.35722 | 4.443277 | 5.211167 | 12.27649 | 7.770497 | 10.81157 | NA | 11.09741 | 8.727631 | 5.588347 | 33.03027 | NA | 16.75121 | 12.76773 | 10.14604 | 14.98047 | 16.11142 | 11.36093 | 13.30863 | 11.40559 | 8.036889 | 10.80561 |
| CUST_8634_PI429010886 | N23333 | 0.017405 | 15 | 0 | | | | | | 72.03576 | 64.26189 | 50.06533 | 32.99167 | 29.47231 | 10.06856 | 31.05167 | 29.97999 | 30.60425 | 35.72108 | 38.89725 | 39.05552 | 52.93292 | 42.12286 | 32.06861 | 40.78513 | 42.51309 | 25.03495 | 36.12998 | 35.21227 | 26.63815 | 35.1468 | 26.61723 | 38.61391 | 41.03316 | 23.36932 | 41.6388 |
| CUST_8519_PI429010886 | N23161 | 0.017407 | 15 | 0 | | | | | | 10.84659 | 12.5052 | NA | 11.40246 | 8.639749 | 9.477835 | 6.981775 | 4.372774 | 6.271198 | 10.27034 | 9.861478 | 8.492959 | 12.77633 | 11.7952 | 6.43023 | 6.191768 | 15.28613 | 16.60048 | 12.61125 | 7.60417 | 11.2994 | 9.864571 | NA | 7.009314 | 9.082334 | 10.48257 | NA |
| CUST_25704_PI429010886 | N52914 | 0.017422 | 21 | 18 | | | | | | 179.2612 | 162.473 | 128.0902 | 166.0384 | 177.1792 | 191.6398 | 112.2413 | 127.0219 | 123.9272 | 157.0244 | 129.4038 | 220.5819 | 123.5279 | 163.0297 | 189.9733 | 201.085 | 188.0209 | 234.141 | 224.0471 | 229.6885 | 172.3518 | 266.2431 | 161.084 | 152.565 | 164.1481 | 247.9064 | 162.1787 |
| CUST_23560_PI429010886 | N49032 | 0.017433 | 18 | 3 | | | | | | 21.07965 | 22.57752 | 9.44636 | 23.41982 | 47.42461 | 32.51998 | 8.113982 | NA | 8.526397 | 17.51307 | 8.962125 | 5.738619 | 13.46425 | 6.43429 | 13.99986 | 41.52389 | 13.07843 | 22.95261 | NA | 38.28975 | 12.82338 | 46.70127 | NA | 34.8398 | 14.57386 | 6.461537 | NA |
| CUST_45248_PI429010886 | M68481 | 0.017438 | 24 | 21 | 215422331 | adenosine deaminase, tRNA-specific 2, TAD2 homol | | | 1.97E-15 | 8.582555 | 16.94914 | 9.653182 | 6.560974 | 11.50698 | 6.402614 | 3.574869 | NA | 4.581089 | 14.22529 | 4.416972 | 15.80722 | 4.053556 | 9.173022 | 26.87538 | 17.89801 | 4.86484 | 10.77469 | NA | 9.22307 | 9.188895 | 34.69687 | 12.41217 | 22.80223 | 13.23183 | 14.18973 | 5.789425 |
| CUST_31580_PI429010886 | M1890 | 0.017459 | 24 | 15 | 374842188 | opsin protein [Charybdis japonica] | Signal transduction | Other | 1.91E-43 | 263.8863 | 414.2198 | 355.193 | 187.8401 | 372.3592 | 343.0578 | 228.1824 | 196.9594 | 223.7478 | 342.1102 | 417.6101 | 278.3016 | 184.8973 | 339.6011 | 265.6039 | 546.6515 | 428.6284 | 450.7936 | 385.5507 | 450.2218 | 354.3972 | 385.1644 | 179.8168 | 259.5337 | 454.6218 | 277.9328 | 394.3244 |
| CUST_37087_PI429010886 | M17810 | 0.017471 | 15 | 12 | | | | | | 172.5751 | 160.8644 | 533.8243 | 160.2021 | 152.0093 | 185.7359 | 163.4951 | 149.5353 | 485.9211 | 194.747 | 263.7999 | 342.1732 | 350.0802 | 581.2638 | 338.0611 | 353.6127 | 297.2502 | 235.7212 | 153.1912 | 275.3515 | 140.3134 | 732.5018 | 464.8665 | 213.0864 | 866.8694 | 183.4397 | 542.1254 |
| CUST_1865_PI429010886 | N10909 | 0.017474 | 15 | 0 | | | | | | 163.3073 | 2181.474 | NA | 4309.606 | 1412.004 | 1426.735 | 831.7121 | 8.804214 | 847.5292 | 14.39019 | 5.102784 | 6.706567 | 76.15742 | 537.9358 | 689.8687 | 1596.656 | 17.10625 | 1167.756 | 15.7022 | 31.33456 | 9.748744 | 1274.119 | 11.42142 | 789.7368 | 85.02582 | 3.770111 | 23.78915 |

| Probe ID | ID | p-value | Period | Phase | EBI ID | Annotation | GO category | Go term | E-value | 0 | 0 | 0 | 3 | 3 | 3 | 6 | 6 | 6 | 9 | 9 | 9 | 12 | 12 | 12 | 15 | 15 | 15 | 18 | 18 | 18 | 21 | 21 | 21 | 24 | 24 | 24 |
|---|---|---|---|---|---|---|---|---|---|---|---|---|---|---|---|---|---|---|---|---|---|---|---|---|---|---|---|---|---|---|---|---|---|---|---|---|
| CUST_52280_PI429010886 | M96945 | 0.017481 | 15 | 12 | | | | | | 19.36347 | 13.86795 | 15.42005 | 12.06387 | 11.30106 | 5.870883 | 11.57836 | 7.833526 | 10.39312 | 12.93269 | 17.3225 | 8.567471 | 18.16906 | 18.7709 | 17.31011 | 13.21509 | 7.894781 | 21.05098 | 17.05678 | 19.67357 | 20.23489 | 12.6412 | 12.17905 | 6.616835 | 12.8856 | 30.04719 | 19.45678 |
| CUST_6716_PI429010886 | N20502 | 0.017483 | 18 | 15 | 117959988 | clottable protein [Marsupenaeus japonicus] | | | 1.88E-32 | 209.2891 | 246.3068 | 154.7492 | 223.5364 | 159.878 | 155.4878 | 109.0299 | 102.6264 | 110.9127 | 268.6359 | 185.104 | 208.674 | 144.6688 | 159.4972 | 110.3513 | 175.39 | 168.7503 | 268.588 | 152.9208 | 612.4623 | 141.4128 | 144.2537 | 198.6919 | 127.5355 | 147.911 | 145.0637 | 116.9944 |
| CUST_5040_PI429010886 | N18185 | 0.017499 | 15 | 9 | | | | | | 3402.667 | 3222.703 | 3421.62 | 2371.653 | 3315.042 | 2209.536 | 3053.471 | 2509.921 | 3670.658 | 4197.681 | 3514.364 | 3546.259 | 3152.492 | 4309.606 | 2431.925 | 4733.383 | 3350.556 | 2218.572 | 3246.287 | 3103.161 | 2900.662 | 4094.84 | 3098.049 | 1880.44 | 3921.02 | 4322.611 | 5060.995 |
| CUST_7809_PI429010886 | N22116 | 0.017499 | 15 | 9 | | | | | | 25.71698 | 39.09341 | 29.61255 | 20.72239 | 31.67165 | 15.65479 | 25.80821 | 14.60539 | 32.14209 | 34.14111 | 34.46491 | 37.6014 | 31.87279 | 36.56569 | 29.49638 | 42.77863 | 19.52138 | 53.1957 | 21.00534 | 27.51414 | 31.13983 | 27.53501 | 37.16398 | 32.13434 | 40.98995 | 47.77531 | 36.25859 |
| CUST_7250_PI429010886 | N21262 | 0.017513 | 21 | 9 | 291229644 | PREDICTED: SOrbitol DeHydrogenase family membe | Metabolic process | Oxidation-reduction process | 2.01E-71 | 50.19147 | 40.17403 | 58.28332 | 70.94036 | 100.8686 | 44.29926 | 57.39701 | 218.5606 | 155.3131 | 70.5402 | 154.5663 | 205.392 | 140.8976 | 68.52038 | 94.68098 | 46.89105 | 63.52784 | 47.02813 | 113.0567 | 393.4936 | 113.0325 | 66.3431 | 45.72037 | 55.55874 | 52.5224 | 105.7587 | 107.409 |
| CUST_13357_PI429010886 | N31050 | 0.017515 | 24 | 9 | 282403505 | fumble isoform 2 [Tribolium castaneum] | Metabolic process | Cellular amino acid metabolic process | 3.85E-49 | 9.288423 | 15.036 | 13.0288 | 14.10202 | 13.03592 | 5.409501 | 18.41753 | 16.97468 | 9.318343 | 18.76711 | 20.54194 | 18.56257 | 11.85185 | 16.13721 | 10.22803 | 15.25738 | 9.577239 | 12.01567 | 15.78237 | 22.39477 | 11.27882 | 7.675832 | 7.989468 | 14.61408 | 11.29639 | 9.273731 | 12.73078 |
| CUST_25106_PI429010886 | N51792 | 0.017525 | 18 | 3 | | | | | | 7.58227 | 4.993679 | 9.528362 | 18.0732 | 16.11003 | 16.23539 | 12.50339 | 16.37668 | 15.62192 | 6.327324 | 13.64729 | 14.79313 | 7.974486 | 13.56063 | 8.120251 | 6.723673 | 12.06387 | 14.47659 | 15.26731 | 7.387333 | 10.60724 | 9.055842 | 17.34415 | 14.68971 | 14.85935 | 11.88743 | 16.23324 |
| CUST_22146_PI429010886 | N46514 | 0.017529 | 12 | 3 | | | | | | 201.085 | 189.1715 | 112.8118 | 176.3863 | 208.39 | 207.1617 | 202.7794 | 192.586 | 216.3391 | 113.8702 | 172.0048 | 141.8325 | 200.5697 | 261.2742 | 247.1436 | 317.6987 | 190.9462 | 233.6523 | 236.2204 | 275.547 | 170.6697 | 163.5913 | 170.0985 | 104.0004 | 219.9262 | 162.6994 | 183.4917 |
| CUST_1199_PI429010886 | N8812 | 0.017529 | 12 | 9 | | | | | | 11.85185 | 16.0073 | 13.5897 | 5.112535 | 10.50787 | 9.260769 | 13.13632 | 7.063727 | 9.665421 | 21.64092 | 11.63473 | 25.50611 | 11.62851 | 11.46096 | 22.71614 | 18.21746 | 7.266326 | 11.12353 | 16.0983 | 13.19962 | 13.58003 | 21.19908 | 13.11618 | 16.7444 | 15.65642 | 10.41346 | 13.50761 |
| CUST_36534_PI429010886 | M16163 | 0.017534 | 24 | 3 | 270004403 | hypothetical protein TcasGA2_TC003754 [Tribolium | Signal transduction | Other | 2.15E-76 | 20.83003 | 15.42195 | 17.27141 | 12.54513 | 25.95987 | 15.7577 | 17.69032 | 22.70929 | 6.230319 | 18.78315 | 9.490926 | 8.812213 | 17.73142 | 15.48139 | 8.2512 | 12.59383 | 4.365387 | 15.55534 | 7.17066 | 8.496964 | 5.426116 | 9.017882 | 13.0523 | 13.63622 | 15.58555 | 8.796716 | 15.37508 |
| CUST_20436_PI429010886 | N43432 | 0.017534 | 24 | 21 | | | | | | 35.19889 | 126.7437 | 43.33061 | 54.95061 | 21.44277 | 24.13249 | 15.21234 | 13.18436 | 28.37529 | 48.69863 | 61.2083 | 16.25623 | 30.59469 | 92.04256 | 62.62111 | 102.6011 | 4.743067 | 21.7873 | 34.80653 | 108.0345 | 67.27473 | 117.4327 | 80.07884 | 32.79034 | 72.16939 | 64.23886 | 46.6 |
| CUST_30391_PI429010886 | N61781 | 0.017534 | 24 | 21 | 157129622 | hypothetical protein AaeL_AAEL011552 [Aedes aeg | | | 1.12E-18 | 12.71344 | 9.031084 | 16.01839 | 10.79319 | 11.80809 | 4.752288 | 6.632723 | 7.689251 | 11.22853 | 9.492295 | 13.30659 | 14.67308 | 9.389836 | 9.775801 | 16.72995 | 10.67448 | 9.915982 | 16.63144 | 15.02774 | 6.201123 | 19.83923 | 13.42609 | 27.92219 | 33.99233 | 16.25623 | 15.85862 | 7.189683 |
| CUST_14183_PI429010886 | N32448 | 0.017534 | 24 | 0 | | | | | | 20.10327 | 52.58792 | 28.7982 | 23.89642 | 28.58102 | 19.99051 | 22.26367 | 27.43563 | NA | 18.22532 | 11.4538 | 7.694991 | 7.43529 | 11.34865 | 6.953549 | 14.39423 | 22.19008 | 9.915982 | 25.8104 | 5.130091 | NA | 19.12864 | 12.30553 | 9.017882 | 25.21131 | NA | 8.894586 |
| CUST_55243_PI429010886 | M109269 | 0.017543 | 12 | 0 | | | | | | 1368.903 | 1969.415 | 1590.414 | 1444.613 | 1287.066 | 1382.607 | 1281.734 | 1319.603 | 1382.607 | 1670.981 | 1434.403 | 1913.282 | 1481.201 | 1968.333 | 2075.258 | 1856.885 | 1393.263 | 1544.651 | 1489.438 | 1601.013 | 1310.544 | 1781.492 | 1563.475 | 1534.856 | 1975.879 | 1524.206 | 1917.136 |
| CUST_3853_PI429010886 | N15407 | 0.017543 | 15 | 9 | | | | | | 225.5174 | 189.144 | 267.4607 | 273.965 | 178.3086 | 222.7049 | 183.4397 | 192.7273 | 232.9495 | 274.1119 | 267.4223 | 385.2863 | 190.5574 | 393.4936 | 421.7857 | 274.0115 | 258.3105 | 298.1939 | 194.9822 | 227.3812 | 204.9855 | 328.4337 | 244.3511 | 222.7049 | 468.2572 | 257.3603 | 342.6916 |
| CUST_7158_PI429010886 | N21123 | 0.017543 | 15 | 9 | 345484364 | PREDICTED: DNA-directed RNA polymerase I subuni | Nucleic acid metabolism | Transcription, DNA-dependent | 5.43E-20 | 81.42045 | 81.04828 | 86.67105 | 52.63886 | 67.71039 | 56.40373 | 68.48249 | 82.73488 | 80.55811 | 86.67105 | 82.02402 | 81.67574 | 74.22065 | 100.6811 | 66.37927 | 88.51347 | 86.28429 | 69.20227 | 60.98516 | 78.89557 | 61.76631 | 80.66968 | 71.48709 | 81.72278 | 69.08039 | 71.83981 | 118.5634 |
| CUST_14711_PI429010886 | N33335 | 0.017551 | 18 | 6 | | | | | | 106.4989 | 72.10026 | 76.06574 | 88.3593 | 110.2618 | 123.9272 | 229.4529 | 130.6585 | 179.8682 | 151.0563 | 78.37536 | 93.97578 | 125.8598 | 184.6172 | 114.2091 | 150.3004 | 92.03214 | 141.1131 | 162.3309 | 87.5248 | 169.9973 | 107.9401 | 127.001 | 80.83362 | 236.9573 | 238.9663 | 198.8064 |
| CUST_3630_PI429010886 | N14921 | 0.017559 | 15 | 12 | 67083873 | pre-mRNA splicing factor SF3b 10 kDa subunit [Ixod | | | 5.55E-35 | 302.0506 | 311.8189 | 299.837 | 235.5325 | 307.1296 | 239.2906 | 265.6599 | 183.4397 | 291.8279 | 337.6237 | 280.5129 | 367.3426 | 309.9578 | 328.9809 | 201.1997 | 316.122 | 303.6686 | 306.6802 | 301.291 | 232.3526 | 261.0269 | 280.8226 | 229.4529 | 218.1069 | 317.3817 | 292.413 | 289.7899 |
| CUST_38073_PI429010886 | M23205 | 0.017568 | 18 | 12 | 223588323 | anti-lipopolysaccharide factor 2 [Marsupenaeus jap | | | 3.2E-25 | 142.8955 | 129.8621 | 34.16228 | 25.86025 | 68.09627 | 50.59331 | 122.7676 | 63.3033 | 184.0938 | 191.3918 | 59.90035 | 320.5083 | 238.7409 | 195.4236 | 119.7645 | 95.18251 | 84.32383 | 93.47603 | 286.6201 | 20.97422 | 156.8398 | 59.34665 | 62.95412 | 72.11758 | 76.49245 | 49.89799 | 168.1346 |
| CUST_11420_PI429010886 | N27776 | 0.017609 | 24 | 15 | | | | | | 385.9721 | 397.525 | 307.9665 | 331.3738 | 291.5938 | 317.6987 | 272.8585 | 276.1481 | 288.1636 | 394.6245 | 340.3246 | 349.4134 | 344.5306 | 376.7168 | 381.6649 | 501.3373 | 342.4231 | 385.8062 | 322.5056 | 399.0919 | 331.1092 | 459.7686 | 312.7398 | 299.5774 | 427.4471 | 361.4417 | 332.964 |
| CUST_299_PI429010886 | N3597 | 0.017612 | 12 | 0 | 321465515 | hypothetical protein DAPPUDRAFT_213935 [Daphn | Metabolic process | Cellular amino acid metabolic process | 5.57E-32 | 356.3531 | 322.0841 | 408.5798 | 246.5795 | 216.6058 | 200.3064 | 233.484 | 310.0283 | 284.7868 | 318.7622 | 331.4917 | 404.8823 | 319.2639 | 236.2204 | 332.964 | 348.7613 | 245.0768 | 248.1135 | 230.36 | 286.0749 | 236.613 | 300.5238 | 331.7667 | 300.0603 | 286.6682 | 248.3342 | 362.5859 |
| CUST_6937_PI429010886 | N20804 | 0.017627 | 21 | 9 | | | | | | 8.405253 | 35.51693 | 33.77621 | 68.68381 | 129.3158 | 39.08137 | 44.83381 | 326.0308 | 191.886 | 43.97029 | 396.2899 | 430.7898 | 207.2391 | 32.21966 | 87.49111 | 9.748744 | 91.27166 | NA | 53.57478 | 679.9331 | 267.2735 | 9.845679 | 61.65538 | 32.91621 | 11.86639 | 141.1498 | 191.0009 |
| CUST_45949_PI429010886 | M70845 | 0.017635 | 18 | 15 | | | | | | 19.46629 | 30.54779 | 15.38835 | 14.04854 | 15.30965 | 22.13024 | 18.58096 | 15.66989 | 13.92624 | 33.96314 | 22.01737 | 25.17015 | 15.5721 | 17.11186 | 13.34357 | 40.4721 | 27.02172 | 30.54342 | 15.79894 | 48.71321 | 29.93088 | 26.49815 | 9.524556 | 23.86269 | 22.0504 | 9.863022 | 10.29115 |
| CUST_52444_PI429010886 | M97467 | 0.017646 | 15 | 9 | | | | | | 16.27976 | 15.40382 | 8.820463 | 8.543285 | 11.33312 | 14.67473 | 9.73488 | 20.98763 | 18.24285 | NA | 21.3985 | 27.67518 | 19.49405 | 14.89292 | 17.79403 | 22.94962 | 15.88699 | 17.62699 | 20.859 | 16.61483 | 9.972812 | 23.57785 | 10.68037 | 14.65365 | 19.74451 | NA | 23.95732 |
| CUST_918_PI429010886 | N7824 | 0.017674 | 21 | 9 | 390364315 | PREDICTED: poly(U)-specific endoribonuclease-like i | Signal transduction | Other | 6.29E-50 | 8.56209 | 1205.667 | 492.7259 | 1272.526 | 1271.51 | 464.7526 | 788.8563 | 3493.781 | 2558.435 | 1055.576 | 4368.248 | 6289.971 | 2244.992 | 598.8088 | 1652.112 | NA | 1298.051 | NA | 450.2983 | 11816.95 | 3172.097 | 9.949298 | 902.5128 | 681.7268 | 10.55129 | 2324.619 | 2939.878 |
| CUST_34320_PI429010886 | M9318 | 0.017682 | 18 | 15 | 242017663 | anaphase-promoting complex subunit, putative [Pe | Protein metabolism | Proteolysis | 3.5E-41 | 53.80851 | 57.3007 | 45.58039 | 43.90905 | 33.65009 | 29.65544 | 41.78277 | 47.29592 | 42.48584 | 64.77051 | 41.18311 | 55.05355 | 46.7059 | 42.11724 | 46.16699 | 59.04714 | 56.72941 | 48.5992 | 62.39032 | 36.46662 | 46.8066 | 43.57431 | 37.43361 | 38.51713 | 70.04597 | 56.41396 | 41.97898 |
| CUST_54935_PI429010886 | M108205 | 0.017691 | 15 | 0 | | | Metabolic process | Carbohydrate metabolic process | | 452.716 | 510.9063 | 696.1501 | 480.2626 | 624.13 | 882.8865 | 238.6573 | 161.827 | 310.5967 | 509.0016 | 628.8832 | 659.0457 | 164.5164 | 424.6374 | 380.8862 | 512.2788 | 574.2435 | 748.4841 | 364.2104 | 424.6374 | 478.7395 | 357.7936 | 356.6027 | 413.2538 | 606.4913 | 452.716 | 562.6249 |
| CUST_35093_PI429010886 | M11556 | 0.017691 | 15 | 9 | | | | | | 24.38309 | 19.51691 | 22.46339 | 14.18148 | 18.50256 | 13.86795 | 19.10864 | 24.6789 | 21.98819 | 20.60841 | 17.69032 | 30.11515 | 20.42397 | 20.74508 | 20.34669 | 33.00184 | 26.62834 | 19.51691 | 16.4962 | 21.7799 | 17.89329 | 23.99602 | 23.44768 | 15.57429 | 32.61136 | 25.55311 | 28.10273 |
| CUST_14233_PI429010886 | N32541 | 0.017691 | 15 | 9 | 392340849 | PREDICTED: zinc finger protein 888-like [Rattus norv | | | 3.8E-31 | 31.17481 | 32.05062 | 30.45027 | 8.500792 | 27.54224 | 24.02788 | 19.676 | 25.95233 | 29.8401 | 36.50859 | 38.76201 | 36.23123 | 27.13598 | 25.63451 | 30.30845 | 39.3477 | 14.17556 | 37.58201 | 19.97907 | 26.45134 | 26.2903 | 44.55872 | 27.43923 | 21.98126 | 39.44223 | 30.5127 | 37.12271 |
| CUST_44477_PI429010886 | M66099 | 0.01774 | 24 | 18 | | | | | | 39.55173 | 18.84701 | 22.48589 | 7.787579 | 22.33422 | 15.67763 | 37.29408 | 11.93292 | 21.4174 | 40.39881 | 37.21745 | 17.91994 | 35.39935 | 24.62608 | 12.18164 | 43.95633 | 54.91034 | 15.01118 | 49.08702 | 51.24109 | 46.68828 | 44.64106 | 8.545589 | 12.04026 | 34.71503 | 19.15374 | 26.9152 |
| CUST_48524_PI429010886 | M80801 | 0.01774 | 24 | 21 | | | | | | 144.3428 | 151.4536 | 186.0312 | 161.4808 | 149.9961 | 148.0647 | 140.9164 | 147.2113 | 147.6505 | 183.8217 | 125.0614 | 220.6157 | 127.7879 | 201.6148 | 177.2677 | 187.9811 | 161.6167 | 238.0396 | 157.4172 | 172.1642 | 150.3647 | 220.7134 | 205.6287 | 200.108 | 192.7273 | 156.2584 | 182.3659 |
| CUST_34491_PI429010886 | M9826 | 0.01774 | 24 | 18 | 537079664 | zinc finger protein, partial [Cricetulus griseus] | | | 4.33E-45 | 58.8975 | 85.09002 | 84.08227 | 57.54706 | 67.01506 | 54.5396 | 67.11047 | 62.406 | 79.93349 | 50.70831 | 78.02809 | 114.3333 | 68.09627 | 92.77688 | 72.84986 | 90.21463 | 77.6254 | 88.07214 | 94.61126 | 129.1185 | 81.5806 | 111.812 | 80.91461 | 52.645 | 88.49089 | 87.83251 | 83.30915 |
| CUST_18595_PI429010886 | N40075 | 0.017753 | 15 | 0 | | | | | | 49.49452 | 43.59959 | 24.50538 | 23.51614 | 23.08011 | 42.58462 | 23.99379 | 15.14895 | 20.59992 | 31.43005 | 30.05351 | 22.17314 | 19.04367 | 18.30186 | 32.30335 | 39.05183 | 36.387 | 31.67438 | 29.77528 | 41.02447 | 38.92288 | 28.22595 | 29.23595 | 15.69662 | 34.46151 | 32.75095 | 32.2878 |
| CUST_6599_PI429010886 | N20330 | 0.017763 | 18 | 15 | 242011385 | conserved hypothetical protein [Pediculus humanus | | | 5.14E-37 | 27.99024 | 33.3136 | 32.59218 | 19.41296 | 20.28061 | 14.61408 | 26.43705 | 23.86269 | 27.37091 | 31.95131 | 29.23595 | 31.29367 | 27.15098 | 16.81632 | 25.36437 | 25.01145 | 27.56341 | 39.36991 | 28.60108 | 40.38558 | 19.16559 | 22.76479 | 23.27942 | 16.51607 | 26.77723 | 29.14509 | 20.29574 |
| CUST_42938_PI429010886 | M61668 | 0.017764 | 24 | 18 | | | | | | 9.102009 | 7.799519 | 10.39491 | 4.552819 | 4.839971 | NA | 5.573433 | 7.869843 | 5.86895 | NA | 7.467895 | 12.84324 | 8.163573 | 7.781741 | 16.63365 | 9.717046 | 8.542736 | 8.29257 | 10.30907 | 8.974986 | NA | 6.577338 | 11.4768 | NA | 15.08925 | NA | 5.538908 |
| CUST_6679_PI429010886 | N20451 | 0.01777 | 15 | 0 | 405950372 | Histone-lysine N-methyltransferase PRDM9 [Crasso | | | 5.65E-58 | 23.32806 | 16.73527 | NA | 17.13916 | 16.72995 | 14.39261 | 7.990456 | 7.719905 | 9.851879 | 15.71246 | 14.31543 | 13.98209 | 14.17556 | 9.243743 | 10.05309 | 18.29753 | 14.56514 | 10.49992 | 9.661698 | 11.96331 | 17.32049 | 14.84745 | 7.82601 | 8.70488 | 15.036 | NA | 12.57101 |
| CUST_7190_PI429010886 | N21173 | 0.017797 | 21 | 12 | 403331926 | hypothetical protein OXYTRI_14913 [Oxytricha trifa | | | 2.22E-10 | 9.445353 | 14.48109 | 9.061963 | 7.63023 | 14.27247 | NA | 11.73913 | 29.42815 | 37.15259 | 18.76152 | 26.95518 | 32.94969 | 37.75935 | 19.34225 | 30.75644 | 78891.09 | 17.35176 | 28.20989 | 11.14656 | 82.74334 | 46.00056 | 3.378954 | 19.64187 | 7.665956 | 4.936158 | 35.73798 | 30.73521 |
| CUST_41606_PI429010886 | M58128 | 0.017811 | 21 | 3 | | | | | | 165.5416 | 65.83727 | 156.3748 | 198.6919 | 159.1135 | 353.1722 | 224.1873 | 189.0068 | 106.3333 | 139.9664 | 134.3863 | 85.89616 | 105.322 | 130.9454 | 54.2941 | 50.46685 | 390.5304 | 171.6223 | 87.25084 | 51.4872 | 108.9165 | 99.69352 | 132.7049 | 227.3449 | 196.032 | 191.5917 | 133.3318 |
| CUST_48102_PI429010886 | M78890 | 0.017811 | 24 | 18 | | | | | | 70.64745 | 74.32582 | 72.18474 | 58.76571 | 67.41072 | 64.25402 | 52.04647 | 57.48318 | 73.78486 | 72.98413 | 71.95612 | 95.23078 | 67.28983 | 66.32011 | 73.35296 | 82.26332 | 60.89775 | 77.80891 | 84.76204 | 85.29111 | 76.36771 | 64.50807 | 80.37038 | 71.96791 | 83.39175 | 61.13394 | 82.93626 |
| CUST_34982_PI429010886 | M11238 | 0.01787 | 21 | 12 | 328717649 | PREDICTED: Y+L amino acid transporter 2-like isofor | Transport | Ion transport | 8.25E-27 | 5.222221 | 13.28694 | 9.559604 | 14.31222 | 9.510155 | 16.0514 | NA | 15.25233 | 7.324098 | 17.4103 | 10.97186 | 16.28372 | 19.78599 | 23.4129 | 22.27295 | 9.754482 | 12.70528 | 11.7224 | 16.07923 | 18.03173 | 6.924715 | 12.85526 | 11.32033 | 5.636736 | 17.97978 | 16.59828 | 24.46354 |
| CUST_36548_PI429010886 | M16206 | 0.017876 | 15 | 9 | 223673192 | Salmo salar clone ssal-rgh-519-317 Ubiquitin-conjug | Cellular Process | Cell cycle | 2.53E-71 | 289.9742 | 345.9305 | 310.7127 | 360.1952 | 358.4083 | 320.0372 | 302.7759 | 400.5132 | 386.742 | 384.4603 | 453.8761 | 510.3855 | 390.2131 | 419.7481 | 363.8764 | 373.6035 | 270.5265 | 277.7527 | 359.57 | 541.4466 | 326.5696 | 432.3349 | 261.5579 | 281.9234 | 395.2528 | 378.5438 | 393.405 |
| CUST_34947_PI429010886 | M11140 | 0.017881 | 18 | 9 | | | | | | 57.99026 | 48.32558 | 92.96667 | 42.77108 | 50.26445 | 45.83381 | 31.82901 | 87.46258 | 98.82662 | 79.03234 | 96.4587 | 106.1639 | 58.22303 | 69.58233 | 87.68861 | 94.33627 | 45.01588 | 54.24943 | 53.36598 | 112.6761 | 65.13839 | 57.62563 | 60.37722 | 40.53866 | 51.5653 | 97.87109 | 77.66626 |
| CUST_34167_PI429010886 | M8933 | 0.017893 | 21 | 15 | | | | | | 616.9292 | 752.803 | 568.2036 | 614.3849 | 566.7662 | 541.8489 | 495.2283 | 497.6528 | 444.4951 | 777.7644 | 598.9907 | 625.9634 | 514.8406 | 665.6609 | 529.2101 | 785.2299 | 702.4802 | 636.6917 | 682.2742 | 665.035 | 558.7985 | 841.4264 | 489.338 | 553.7439 | 1004.987 | 518.2235 | 567.9112 |
| CUST_21856_PI429010886 | N45981 | 0.017895 | 15 | 3 | | | | | | 135.8973 | 119.508 | 120.8052 | 172.7143 | 190.9766 | 312.4226 | 228.9161 | 201.8347 | 153.3961 | 162.6527 | 189.2921 | 120.8052 | 147.0019 | 113.475 | 119.8572 | 104.6528 | 320.5083 | 182.6771 | 226.5808 | 137.2699 | 247.2726 | 140.3976 | 202.4041 | 247.7794 | 145.899 | 183.266 | 127.3371 |
| CUST_22623_PI429010886 | N47347 | 0.017896 | 12 | 0 | | | | | | 173.0247 | 204.7885 | 205.909 | 188.2993 | 166.3529 | 136.3926 | 128.4226 | 149.5538 | 165.1224 | 192.39 | 161.7557 | 185.414 | 190.8837 | 249.1725 | 218.2859 | 197.44 | 180.3315 | 219.3834 | 148.8666 | 247.8745 | 181.0372 | 209.9951 | 206.9171 | 171.4068 | 278.397 | 206.9171 | 219.3834 |
| CUST_30022_PI429010886 | N61021 | 0.017903 | 21 | 18 | | | | | | 73.16394 | 106.8281 | 76.17525 | 59.59632 | 67.7681 | 49.69905 | 50.84005 | 47.01793 | 55.48994 | 93.2268 | 68.82274 | 71.87825 | 64.6187 | 65.03148 | 48.89187 | 111.1756 | 60.10166 | 71.6044 | 81.78469 | 91.07591 | 70.21778 | 75.12567 | 66.39938 | 63.08199 | 108.8762 | 62.11683 | 65.11259 |
| CUST_34688_PI429010886 | M10348 | 0.017925 | 15 | 9 | 75022291 | RecName: Full=Oplophorus-luciferin 2-monooxyger | | | 7.95E-36 | 183.7194 | 252.4045 | 339.8879 | 293.3716 | 392.2104 | 196.7297 | 406.3292 | 863.4186 | 828.5874 | 319.2197 | 615.3731 | 790.7325 | 691.1905 | 388.5645 | 461.5523 | 190.6724 | 254.7626 | 219.1432 | 312.4689 | 1271.998 | 454.0927 | 342.6916 | 370.6555 | 235.5779 | 289.131 | 672.763 | 737.7582 |
| CUST_56469_PI429010886 | M113878 | 0.017946 | 24 | 21 | | | | | | 82.26332 | 94.27998 | 89.50659 | 103.9666 | 80.37733 | 82.23746 | 84.38957 | 75.45183 | 63.13278 | 96.32091 | 69.05841 | 108.0902 | 95.09391 | 101.0858 | 63.76139 | 85.00815 | 79.09571 | 88.04251 | 114.165 | 71.62646 | 86.32314 | 117.1631 | 108.7243 | 107.1382 | 91.23322 | 116.5651 | 98.59007 |
| CUST_12233_PI429010886 | N29144 | 0.017946 | 24 | 18 | | | | | | 158.0759 | 159.5474 | 230.1158 | 91.50805 | 101.0126 | 95.47811 | 112.4908 | 109.5173 | 116.2211 | 189.6625 | 141.0429 | 124.3334 | 123.0707 | 161.0687 | 125.6016 | 139.47 | 210.6087 | 116.6405 | 181.2698 | 164.6012 | 147.0019 | 156.6593 | 160.4822 | 95.7582 | 264.269 | 116.0217 | 239.9082 |
| CUST_49898_PI429010886 | M87657 | 0.017954 | 24 | 0 | | | | | | 93.75113 | 108.7588 | 107.6007 | 99.97208 | 72.71237 | 70.35427 | 92.19432 | 76.40353 | 76.00655 | 76.23608 | 63.95336 | 82.11078 | 57.96698 | 75.7764 | 81.0784 | 95.45887 | 68.92299 | 89.49881 | 70.87083 | 56.86246 | 98.50813 | 102.6905 | 78.07938 | 79.47713 | 89.47031 | 98.68398 | 84.20531 |
| CUST_28714_PI429010886 | N58477 | 0.017954 | 24 | 0 | | | | | | 52.22471 | 44.9603 | 57.03251 | 23.83176 | 26.53218 | 27.16436 | 30.21934 | 48.22578 | 40.59153 | 32.7846 | 44.73597 | 27.72962 | 27.30026 | 29.02946 | 16.46028 | 35.20829 | 47.95255 | 29.62583 | 42.96262 | 52.31892 | 29.68097 | 46.20496 | 45.3118 | 29.9971 | 37.13804 | 44.90024 | 49.49037 |
| CUST_44831_PI429010886 | M67137 | 0.017961 | 21 | 3 | 443690866 | hypothetical protein CAPTEDRAFT_204931 [Capitell | | | 7.37E-26 | 15.66178 | 30.71736 | 22.15408 | 44.1334 | 30.39192 | 31.86571 | 26.68081 | 12.20426 | 19.72601 | 16.37493 | 7.923943 | 14.89135 | 9.060793 | 20.62099 | 19.00616 | 19.17918 | NA | 25.35894 | 11.73206 | 8.238355 | 22.63247 | 27.19369 | 18.5156 | 13.52004 | 36.87107 | 30.80438 | 12.46111 |
| CUST_49633_PI429010886 | M86465 | 0.017966 | 15 | 3 | 291235783 | PREDICTED: galactose-3-O-sulfotransferase 1-like [S | Metabolic process | Lipid metabolic process | 8.07E-13 | 34.54086 | 33.59767 | 7.23801 | 81.57207 | 50.70831 | 35.63135 | 37.92604 | 3.791006 | 32.42452 | 21.06491 | 5.956873 | 19.91373 | 25.57234 | 25.82998 | 55.08741 | 34.84989 | NA | 40.16678 | NA | 61.87735 | 13.49109 | 92.55609 | 12.35021 | 62.43324 | 13.14806 | 5.274791 | 9.485366 |
| CUST_10217_PI429010886 | N25891 | 0.017969 | 24 | 15 | | | | | | 128.1244 | 167.3634 | 138.6217 | 86.24828 | 129.2544 | 138.2244 | 112.4193 | 140.3976 | 159.3003 | 176.94 | 147.9354 | 202.6087 | 137.2919 | 183.528 | 172.6317 | 204.0225 | 151.5005 | 191.7619 | 162.2752 | 182.2299 | 171.784 | 184.9236 | 136.2923 | 169.814 | 176.1138 | 128.1595 | 210.2883 |
| CUST_8073_PI429010886 | N22500 | 0.017981 | 15 | 6 | 478258829 | hypothetical protein YQE_04700, partial [Dendroct | Cellular Process | Cell proliferation | 8.39E-24 | 51.03287 | 61.12299 | 57.16741 | 89.43652 | 61.48326 | 41.10188 | 88.11918 | 103.2771 | 83.03102 | 75.13415 | 62.04074 | 99.2772 | 62.48029 | 62.36898 | 66.96201 | 92.28356 | 44.05203 | 62.06295 | 64.77801 | 34.73053 | 68.56896 | 87.02844 | 78.65203 | 69.17707 | 72.96725 | 57.45413 | 88.95458 |
| CUST_35770_PI429010886 | M13463 | 0.018004 | 15 | 3 | | | | | | 905.952 | 652.2033 | 721.3883 | 795.1421 | 779.7877 | 1338.117 | 972.4433 | 736.3462 | 464.4064 | 431.462 | 661.3119 | 453.0933 | 640.4514 | 457.3191 | 617.1846 | 727.1859 | 1183.341 | 990.9102 | 1265.003 | 499.4614 | 700.0017 | 535.9164 | 663.5689 | 894.2724 | 463.4816 | 958.279 | 460.7596 |
| CUST_1535_PI429010886 | N9969 | 0.018017 | 21 | 0 | | | | | | 97.30444 | 81.98334 | 94.70207 | 32.46691 | 71.91814 | 130.1545 | 63.03943 | 75.17352 | 38.16824 | 58.02431 | 76.24971 | 42.47769 | 61.99224 | 91.17869 | 62.5636 | 73.03522 | 104.8909 | 113.2085 | 97.6483 | 77.89081 | 76.45771 | 71.74241 | 98.94163 | 170.317 | 51.83037 | 96.08349 | 56.69148 |
| CUST_22162_PI429010886 | N46541 | 0.018025 | 21 | 0 | 85677399 | pigment-dispersing hormone 3 [Marsupenaeus japc | Signal transduction | Other | 3.42E-07 | 51.63758 | 14.60389 | 105.3783 | 42.53014 | 65.75053 | 71.45222 | 32.02169 | 28.86885 | 68.91937 | 19.689 | 39.44972 | 22.25913 | 12.76972 | 50.24503 | 27.99312 | 81.68427 | 32.58962 | 19.92242 | 47.93942 | 9.74748 | 28.9143 | 105.0313 | NA | 85.46155 | 54.67468 | 46.00056 | 100.4062 |
| CUST_46516_PI429010886 | M72875 | 0.018027 | 24 | 15 | | | | | | 7.592295 | 8.997185 | 4.250272 | 8.830666 | 8.59214 | 7.173073 | 13.43047 | 8.855559 | 8.625409 | 9.714422 | NA | 6.387554 | 9.720983 | NA | 5.267619 | 20.88122 | 13.40406 | 13.75707 | 6.914807 | 4.698051 | 10.66649 | 10.38575 | 6.362315 | 15.95746 | 5.710989 | 3.77223 | 8.051681 |
| CUST_37339_PI429010886 | M18693 | 0.018081 | 24 | 0 | 9367086 | Euphausia superba large subunit ribosomal RNA gei | Metabolic process | Generation of precursor metabolites and energy | | 88771.33 | 99042.74 | 90253.54 | 99042.74 | 91360.38 | 72295.32 | 95014.94 | 89222.73 | 96143.7 | 90253.54 | 90794.74 | 55642.21 | 85436.14 | 82404.75 | 40986.13 | 56627.52 | 80636.04 | 71869.37 | 88771.33 | 95621.16 | 88771.33 | 47937.32 | 81077.7 | 89222.73 | 84866.33 | 96143.7 | 96143.7 |
| CUST_16460_PI429010886 | N36270 | 0.018132 | 12 | 0 | | | | | | 24.16344 | 20.41093 | 17.88028 | 14.76986 | 12.29489 | 18.38014 | 10.77657 | 11.12791 | 15.28371 | 19.69856 | 9.253041 | 17.73598 | 21.08802 | 19.72906 | 20.96593 | 25.56608 | 17.87105 | 16.8666 | 9.522138 | 12.96601 | 10.27804 | 13.15835 | 19.51691 | 14.10082 | 12.65013 | 9.915982 | 17.06111 |
| CUST_38459_PI429010886 | M26950 | 0.018146 | 18 | 6 | | | | | | 37.09799 | 37.72421 | 41.8496 | 22.98966 | 28.22595 | 47.39274 | 66.52424 | 282.8614 | 52.85888 | 21.32406 | 47.90583 | 45.67726 | 25.09128 | 57.09554 | 12.64389 | 23.565 | 27.60836 | 35.24075 | 53.64846 | 25.15936 | 68.47784 | 28.79502 | 56.87008 | 98.43882 | 48.98425 | 51.73011 | 69.13832 |
| CUST_56458_PI429010886 | M113843 | 0.018148 | 12 | 3 | | | | | | 73.86349 | 77.07852 | 36.57353 | 93.77397 | 81.29483 | 76.64595 | 79.67757 | 56.09789 | 49.17946 | 55.96041 | 80.62509 | 65.28922 | 59.6416 | 47.37598 | 43.71768 | 70.7847 | 69.43885 | 77.47192 | 72.98413 | 63.07552 | 71.72159 | 81.48802 | 56.70973 | 56.55039 | 52.85191 | 53.99181 | 35.53033 |
| CUST_48725_PI429010886 | M81866 | 0.018148 | 15 | 3 | | | | | | 24.86718 | 21.10225 | 19.05954 | 36.02623 | 31.43244 | 43.05396 | 26.70117 | 24.26118 | 15.65092 | 23.04508 | 28.50232 | 15.1736 | 21.94699 | 19.7129 | 20.26494 | 18.59923 | 32.86117 | 24.72089 | 46.42845 | 23.40412 | 38.86122 | 21.30062 | 13.89928 | 16.02537 | 14.49586 | 29.66445 | 13.77121 |
| CUST_20754_PI429010886 | N44001 | 0.018153 | 24 | 21 | | | | | | 26.24303 | 23.59085 | 18.04108 | 40.73672 | 21.61111 | 22.92066 | 8.167815 | 8.488945 | 15.32478 | 24.1375 | 18.19204 | 23.37181 | 18.04709 | 36.74157 | 23.23883 | 33.61445 | 15.04717 | 32.99848 | 7.198406 | 30.75954 | 34.67799 | 56.35545 | 28.61965 | 40.91962 | 43.57431 | 13.34056 | 16.19313 |
| CUST_54803_PI429010886 | M107750 | 0.018156 | 18 | 9 | | | | | | 16.97048 | 15.22088 | 21.45154 | NA | 15.59286 | 10.02222 | 17.76828 | 37.21299 | 33.00505 | 24.28324 | 26.7682 | 25.88089 | 25.53642 | 20.08306 | 14.58718 | 33.11438 | 20.49729 | 19.13027 | 25.01541 | 34.02396 | 11.34226 | 17.02313 | 9.528362 | 9.357193 | 20.22864 | 16.61891 | 28.23631 |
| CUST_14023_PI429010886 | N32193 | 0.018167 | 18 | 9 | 242008519 | UDP-GalNAc:polypeptide N-acetylgalactosaminyltra | Metabolic process | Carbohydrate metabolic process | 1.39E-24 | 211.2278 | 129.2905 | 240.0651 | 88.57155 | 138.7624 | 164.2501 | 221.0933 | 252.5323 | 204.8186 | 336.2744 | 279.5177 | 337.3994 | 191.6146 | 178.1191 | 151.3368 | 127.723 | 145.2433 | 96.1439 | 219.2633 | 123.5719 | 245.5656 | 173.7221 | 176.1772 | 105.7694 | 298.6335 | 111.9784 | 212.4051 |
| CUST_35902_PI429010886 | M13860 | 0.018175 | 18 | 12 | | | | | | 171.8979 | 143.6492 | 127.601 | 153.6441 | 110.9326 | 87.15435 | 90.6343 | 120.2323 | 98.47983 | 164.2252 | 159.948 | 154.0684 | 154.6378 | 168.0847 | 160.5748 | 245.2402 | 113.3918 | 178.5686 | 131.39 | 132.8034 | 122.7194 | 185.2639 | 131.3416 | 116.7957 | 163.5179 | 112.5941 | 119.6887 |
| CUST_37584_PI429010886 | M19621 | 0.018184 | 18 | 0 | | | | | | 3893.391 | 18.35945 | 4169.34 | 944.3898 | 1631.11 | 2462.824 | 4579.426 | 1684.561 | 702.2549 | 505.5887 | 1269.191 | 5.344486 | 808.9549 | 543.3625 | 209.5686 | NA | 3116.138 | 1149.353 | 3720.06 | NA | 2745.288 | NA | 1701.136 | 2086.783 | 38.46426 | 1479.619 | 73.67904 |
| CUST_36075_PI429010886 | M14547 | 0.018186 | 24 | 0 | | | | | | 310.3428 | 339.9729 | 198.8522 | 278.5705 | 386.9736 | 160.5748 | 146.585 | 210.2559 | 122.6587 | 181.9024 | 312.9412 | 176.7077 | 176.8268 | 136.0755 | 121.3454 | 129.0136 | 170.6936 | 63.52108 | 189.9733 | 303.55 | 147.9625 | 142.34 | 70.41805 | 39.93503 | 193.2088 | 224.4715 | 218.2149 |
| CUST_50785_PI429010886 | M91005 | 0.018196 | 15 | 0 | | | | | | 18.0708 | 7.745791 | 8.821462 | 6.503335 | 7.756103 | 9.598505 | 4.159629 | 3.633312 | NA | 4.634681 | 7.408115 | NA | 7.175424 | 9.722181 | 6.833163 | 7.875532 | 3.534566 | 11.23684 | 9.344321 | 6.927136 | NA | 3.869383 | 3.999953 | NA | 10.89944 | 4.643844 | 5.313502 |
| CUST_13301_PI429010886 | N30943 | 0.018253 | 18 | 3 | | | | | | 28.16847 | 47.14099 | 31.22768 | 46.45766 | 46.84888 | 46.09323 | 29.41742 | 22.11226 | 25.75668 | 38.56175 | 15.08066 | 43.19614 | 29.69737 | 26.78992 | 28.12721 | 43.57431 | 27.25419 | 41.26402 | 33.23404 | 25.7937 | 39.59389 | 42.16986 | 46.35772 | 53.34613 | 31.87606 | 60.62589 | 29.5952 |
| CUST_41982_PI429010886 | M59063 | 0.01828 | 15 | 9 | | | | | | 46.06884 | 38.20449 | 25.78047 | 41.81668 | 49.10837 | 22.28917 | 38.0368 | 58.32457 | 47.9222 | 65.08584 | 61.21559 | 78.85902 | 53.57981 | 38.67242 | 44.02718 | 34.27813 | 31.15965 | 34.71088 | 45.99667 | 72.66838 | 49.28963 | 58.92238 | 29.62034 | 34.03924 | 31.70959 | 76.11306 | 72.03576 |
| CUST_15368_PI429010886 | N34466 | 0.01828 | 15 | 0 | | | | | | 164.9397 | 164.7416 | 167.4943 | 151.0826 | 180.4643 | 176.6512 | 181.4093 | 138.4649 | 95.86282 | 120.5115 | 177.5678 | 104.4333 | 132.8952 | 119.841 | 158.101 | 182.0202 | 206.0656 | 141.2899 | 180.4919 | 148.6276 | 151.4764 | 167.776 | 160.2794 | 127.8287 | 127.2704 | 119.8693 | 116.2372 |
| CUST_20825_PI429010886 | N44133 | 0.01832 | 12 | 9 | 321476816 | hypothetical protein DAPPUDRAFT_42950 [Daphnia | Metabolic process | Oxidation-reduction process | 3.02E-36 | 11.40101 | 30.85363 | 71.72709 | 10.89022 | 13.86613 | NA | 29.22688 | 14.51263 | 15.3003 | 19.19575 | 25.51449 | 31.50111 | 19.86296 | 33.45456 | 19.79375 | 12.06652 | 14.70578 | 8.298481 | 14.08468 | 39.07685 | 10.06452 | 29.27339 | 68.01317 | NA | 25.7937 | 18.14003 | 35.69319 |
| CUST_14740_PI429010886 | N33385 | 0.018327 | 15 | 9 | | | | | | 68.75407 | 194.8606 | 1879.633 | 20.16106 | 29.30762 | NA | 56.87504 | 2247.598 | 3876.188 | 5133.687 | 1318.096 | 2300.758 | 2493.282 | 9305.385 | 4557.188 | 741.589 | 863.9077 | 348.906 | 704.3489 | 3690.445 | 891.0101 | 10524.41 | 4537.099 | 108.3885 | 9106.522 | 3360.345 | 11385.83 |
| CUST_34124_PI429010886 | M8827 | 0.01834 | 21 | 18 | | | | | | 318.6961 | 458.895 | 286.7556 | 296.208 | 438.7455 | 238.941 | 230.36 | 229.3254 | 244.2054 | 467.7939 | 294.6436 | 424.5435 | 342.5512 | 291.3805 | 329.7729 | 568.2036 | 237.5773 | 341.713 | 402.9446 | 500.7784 | 356.9822 | 458.8168 | 281.1462 | 254.2335 | 354.3068 | 257.0697 | 307.2905 |
| CUST_53476_PI429010886 | M101877 | 0.018355 | 18 | 15 | | | | | | 681.1329 | 365.8372 | 581.5974 | 135.5426 | 593.331 | 388.0401 | 723.6629 | 485.7919 | 434.7605 | 873.8827 | 348.4045 | 391.819 | 870.876 | 637.7954 | 541.8489 | 610.5161 | 702.2549 | 868.721 | 595.8659 | 548.3934 | 468.2572 | 625.8341 | 236.0009 | 243.3133 | 245.1937 | 410.8257 | 627.8304 |
| CUST_23593_PI429010886 | N49084 | 0.018355 | 21 | 3 | | | | | | 83.86935 | 86.70732 | 119.585 | 108.1674 | 103.7863 | 110.2159 | 122.1037 | 86.47473 | 78.69319 | 129.3018 | 73.51179 | 87.59441 | 81.58984 | 98.4006 | 57.23868 | 82.24759 | 87.26148 | 89.09879 | 96.73765 | 74.94569 | 99.91859 | 163.9462 | 102.6772 | 91.24943 | 117.746 | 102.5545 | 99.45401 |
| CUST_27060_PI429010886 | N55379 | 0.018361 | 15 | 3 | | | | | | 3.575435 | 9.126873 | 13.71331 | 6.446625 | 10.73459 | 14.80676 | 4.085521 | 8.954946 | 9.020299 | 10.09872 | 3.996805 | 7.679502 | 4.977762 | 3.666764 | 3.557249 | 5.959516 | 9.055842 | 8.514476 | 4.304478 | 20.86742 | 10.20734 | 10.33417 | 8.103168 | NA | 5.93716 | NA | 9.50058 |
| CUST_42364_PI429010886 | M60103 | 0.018374 | 12 | 3 | | | | | | 182.0483 | 218.3592 | 172.9784 | 278.7763 | 286.5169 | 219.2135 | 193.5749 | 159.4512 | 185.6237 | 177.1267 | 183.6687 | 165.7374 | 152.2767 | 177.8158 | 165.2714 | 229.8746 | 178.7689 | 240.9417 | 190.2958 | 175.9719 | 190.0964 | 191.3655 | 164.6734 | 164.6012 | 274.296 | 179.678 | 166.519 |
| CUST_23664_PI429010886 | N49231 | 0.018391 | 12 | 6 | | | | | | 29.5952 | 11.70566 | 13.92315 | 113.2835 | 40.32087 | 433.8707 | 154.3359 | 260.0898 | 97.19605 | NA | 105.8252 | NA | 81.87168 | NA | 41.7957 | NA | 171.2286 | 129.9706 | 948.388 | NA | 32.68774 | NA | 27.12475 | 179.1629 | 6.833163 | 62.61352 | 4.580393 |
| CUST_14391_PI429010886 | N32805 | 0.018403 | 24 | 0 | | | | | | 61.72981 | 64.48135 | 55.36009 | 53.30382 | 51.0829 | 58.04233 | 52.71293 | 53.01853 | 42.89451 | 47.83781 | 40.99957 | 46.76098 | 43.53241 | 47.43502 | 29.43926 | 42.35962 | 56.79499 | 51.9493 | 61.44485 | 50.88285 | 30.89566 | 59.07909 | 33.26161 | 37.93755 | 63.37918 | 47.39715 | 49.2211 |
| CUST_13186_PI429010886 | N30745 | 0.018424 | 24 | 3 | | | | | | 103.457 | 165.5224 | 123.3713 | 235.9573 | 126.9474 | 144.4632 | 102.6532 | 98.11651 | 118.4488 | 107.898 | 83.93301 | 121.9963 | 92.08467 | 111.7257 | 80.03068 | 114.3617 | 58.46928 | 58.544 | 66.12938 | 65.25641 | 163.9462 | 94.91653 | 169.0881 | 111.6027 | 160.7305 | 66.09037 | 99.75328 |
| CUST_28860_PI429010886 | N58742 | 0.018426 | 24 | 15 | | | | | | 10.12547 | 8.52275 | 7.785373 | 10.77337 | 11.99992 | 10.25265 | 4.207388 | 6.703633 | 6.563098 | 17.64462 | 4.858604 | 23.83608 | 6.126585 | 8.618402 | 8.94682 | 18.75909 | 10.6044 | 17.68458 | 14.79313 | 10.32397 | NA | 16.45078 | NA | 13.29711 | 15.24741 | 9.457848 | 10.90286 |
| CUST_28174_PI429010886 | N57420 | 0.018428 | 18 | 12 | | | | | | 7.876336 | 6.881575 | 8.051681 | NA | 12.20158 | 15.23556 | 14.49656 | 9.328195 | 12.88124 | 11.17076 | 13.68935 | 15.08925 | 11.27574 | 22.14979 | 27.80368 | 12.92383 | 13.37838 | 15.2034 | 9.274758 | 12.14502 | 10.26743 | 16.68253 | 8.116528 | 7.311535 | 18.62473 | 62.46895 | 15.14895 |
| CUST_43490_PI429010886 | M63184 | 0.018436 | 15 | 12 | | | | | | 234.9764 | 239.3938 | 275.7928 | 217.7055 | 250.4345 | 231.6859 | 174.6614 | 230.7819 | 237.9203 | 332.964 | 247.0088 | 300.2063 | 258.0874 | 365.3412 | 293.414 | 289.5961 | 259.5337 | 282.651 | 245.5656 | 296.5712 | 276.5189 | 267.9382 | 258.7295 | 188.9157 | 326.6831 | 210.5891 | 321.6396 |
| CUST_10213_PI429010886 | N25884 | 0.01846 | 18 | 15 | | | | | | 3869.78 | 4627.233 | 2997.206 | 3595.062 | 3443.242 | 3509.529 | 3179.631 | 2431.925 | 2452.337 | 3466.9 | 2828.097 | 3386.414 | 2456.459 | 3100.565 | 3503.673 | 4693.3 | 3292.379 | 3701.465 | 3739.652 | 3602.263 | 2992.796 | 4205.626 | 2569.563 | 2968.006 | 3586.445 | 2551.399 | 2879.829 |
| CUST_40471_PI429010886 | M55224 | 0.018461 | 18 | 0 | | | | | | 37.31704 | 35.83044 | 25.20545 | 36.3262 | 28.8539 | 36.82802 | 34.98328 | 27.09386 | 23.02215 | 24.19734 | 38.04406 | 20.15879 | 21.35072 | 27.27727 | 17.3497 | 35.07632 | 45.26792 | 24.1098 | 39.18602 | 34.31858 | 37.99335 | 27.33674 | 29.04567 | 38.87395 | 31.2907 | 32.816 | 31.30264 |
| CUST_3814_PI429010886 | N15333 | 0.018461 | 18 | 0 | | | | | | 22.82948 | 18.19015 | 19.6064 | 15.2558 | 18.55589 | 28.80255 | 11.73596 | 17.50689 | 18.87315 | 16.40616 | 13.77293 | 18.16468 | 14.8651 | 14.54705 | 17.17022 | 17.73142 | 19.88268 | 21.8588 | 27.05448 | 19.27537 | 21.70241 | 15.77161 | 15.33595 | 20.63988 | 20.13562 | 18.04283 | 21.02813 |
| CUST_11495_PI429010886 | N27899 | 0.018477 | 12 | 3 | | | | | | 30.95933 | 18.92641 | 22.62792 | 33.8457 | 46.63569 | 49.07593 | 28.25834 | 28.36898 | 15.09075 | 22.38827 | 23.75128 | 15.95746 | 18.25337 | 15.0164 | NA | 33.65406 | 33.52618 | 34.3148 | 29.17357 | 27.98319 | 30.03474 | 29.68097 | 13.57199 | 37.8309 | 35.57579 | 23.75346 | 22.73782 |
| CUST_18028_PI429010886 | N39022 | 0.0185 | 15 | 0 | | | | | | 538.762 | 330.3612 | 201.6148 | 123.2567 | 478.5158 | 236.4903 | 356.0402 | 224.4715 | 161.4133 | 350.5238 | 212.0119 | 157.3307 | 284.536 | 192.0215 | 162.9465 | 474.5547 | 535.1571 | 290.0281 | 304.7525 | 214.2583 | 249.1725 | 440.9199 | 110.2887 | 96.85587 | 160.4181 | 112.8438 | 106.3446 |
| CUST_37582_PI429010886 | M19596 | 0.0185 | 15 | 3 | | | | | | 90.61609 | 34.99807 | 21.35775 | 1251.807 | 4068.874 | 3411.47 | 2850.368 | 575.8176 | 19.26372 | 4.911986 | 2786.84 | 18.01173 | 49.61919 | 9.001207 | 35.43275 | 9.352687 | 2932.434 | 1648.471 | 1266.654 | NA | 2244.992 | NA | 2469.728 | 2650.517 | 6.733142 | 416.025 | NA |
| CUST_215_PI429010886 | N2681 | 0.018515 | 24 | 18 | 459217370 | cyclin A [Litopenaeus vannamei] >gi\|459217372\|gb | Cellular Process | Cell cycle | 1.1E-166 | 191.528 | 233.6842 | 197.4721 | 153.961 | 156.7058 | 160.1083 | 152.0093 | 153.2997 | 149.8301 | 224.9679 | 198.2869 | 211.85 | 178.5202 | 224.6396 | 184.3061 | 287.4768 | 166.2545 | 201.085 | 197.5074 | 276.6094 | 232.5962 | 232.766 | 171.0777 | 143.609 | 211.6272 | 172.957 | 247.0952 |
| CUST_16345_PI429010886 | N36080 | 0.018524 | 24 | 15 | | | | | | 24.92176 | 24.35681 | 19.42496 | 22.69042 | 15.99247 | 30.69155 | 18.71576 | 10.30492 | 14.49726 | 20.11281 | 16.00911 | 21.60941 | 19.6024 | 16.66793 | 29.39265 | 42.9427 | 28.9598 | 31.24843 | 27.01225 | 85.8526 | 20.56557 | 23.51171 | 20.91226 | 16.67423 | 27.34334 | 28.82923 | 27.45965 |
| CUST_42960_PI429010886 | M61730 | 0.018531 | 21 | 0 | 133916460 | hypothetical protein [Thermobia domestica] | | | 9.35E-38 | 26.47171 | 57.22832 | 29.34549 | 20.61883 | 30.41595 | 35.69319 | 21.45407 | 19.88268 | 26.6839 | 25.33083 | 15.61967 | 33.94054 | 26.04992 | 22.12374 | 25.80322 | 25.88089 | 22.94962 | 29.3424 | 33.87211 | 27.9952 | 20.77685 | 29.44539 | 35.63872 | 39.40978 | 27.54224 | 23.89866 | 7.908427 |
| CUST_51081_PI429010886 | M92086 | 0.018565 | 24 | 21 | | | | | | 107.4719 | 115.5186 | 126.0646 | 111.9784 | 100.397 | 108.7588 | 90.25676 | 94.23139 | 80.57644 | 120.7928 | 113.525 | 110.2048 | 89.56339 | 114.6578 | 122.9865 | 116.6057 | 95.42654 | 91.23322 | 116.3563 | 111.4592 | 116.7026 | 126.9651 | 108.9403 | 123.8085 | 133.1717 | 103.8441 | 112.4033 |
| CUST_52084_PI429010886 | M96360 | 0.018565 | 24 | 21 | 321466007 | hypothetical protein DAPPUDRAFT_305992 [Daphn | Nucleic acid metabolism | RNA metabolic process | 4.76E-39 | 55.95035 | 82.3472 | 60.47407 | 57.92964 | 72.84145 | 45.20819 | 34.26633 | 36.41327 | 54.28784 | 70.6776 | 59.41199 | 67.33602 | 47.01793 | 76.62236 | 62.64364 | 60.64562 | 44.67525 | 70.03699 | 66.20577 | 70.54693 | 59.58353 | 96.58099 | 61.36946 | 86.07302 | 66.03172 | 51.23152 | 81.04828 |
| CUST_2404_PI429010886 | N12279 | 0.018565 | 24 | 21 | | | | | | 116.962 | 96.06297 | 77.2245 | 130.6003 | 130.5118 | 67.34873 | 68.91301 | 23.0986 | 87.78461 | 175.2126 | 114.1048 | 79.07776 | 142.9167 | 80.72119 | 107.955 | 83.7299 | 26.72979 | 179.1099 | 112.5793 | 228.9161 | 119.2945 | 231.235 | 149.0785 | 136.4739 | 24.88398 | 199.6316 | 131.513 |
| CUST_3331_PI429010886 | N14283 | 0.018585 | 12 | 3 | 242012953 | conserved hypothetical protein [Pediculus humanus | | | 6.37E-10 | 1322.445 | 4171.713 | 148.8666 | 3799.249 | 2569.563 | 1915.9 | 2556.905 | 138.2955 | 2435.819 | 91.42252 | 52.21965 | 60.37722 | 826.9906 | 409.0918 | 1909.003 | 4107.091 | 151.12 | 2413.596 | 161.3901 | 203.006 | 65.57569 | 2148.593 | 70.169 | 1120.205 | 929.0194 | 139.2323 | 313.2862 |
| CUST_3661_PI429010886 | N14985 | 0.018585 | 12 | 3 | | | | | | 1378.873 | 3036.431 | 171.4799 | 2851.724 | 1515.268 | 1280.61 | 2092.578 | 168.4127 | 2004.816 | 79.5827 | 81.89953 | 51.32239 | 942.6383 | 229.7242 | 1188.466 | 2587.199 | 234.4127 | 1634.318 | 199.9828 | 83.40091 | 59.40544 | 1251.3 | 90.99167 | 822.5821 | 624.6679 | 236.9951 | 237.1286 |
| CUST_956_PI429010886 | N7972 | 0.018585 | 12 | 9 | | | | | | 28.05565 | 23.43377 | 27.40178 | 16.98462 | 23.44093 | 13.35156 | 41.60845 | 24.05844 | 17.57463 | 37.03334 | 31.41586 | 32.38846 | 31.23112 | 26.38499 | 33.41208 | 30.30845 | 26.69312 | 35.53394 | 33.40939 | 26.22564 | 30.90181 | 39.03429 | 38.32695 | 36.48665 | 34.26964 | 34.28772 | 37.70021 |
| CUST_50166_PI429010886 | M88898 | 0.018624 | 21 | 0 | | | | | | 16.51187 | 22.87112 | 21.65521 | 26.71164 | 22.50662 | 13.75107 | 7.458433 | 9.138723 | 15.30179 | 18.90668 | 16.5492 | 21.37396 | 15.74925 | 7.274775 | 13.76394 | 21.25854 | 18.03887 | 18.20618 | 21.35291 | 17.25976 | 15.30623 | 21.5495 | 24.55823 | 19.78441 | 28.73729 | 17.72211 | 18.38425 |
| CUST_38619_PI429010886 | M28599 | 0.018642 | 24 | 0 | | | | | | 508.0314 | 378.3563 | 448.0031 | 411.957 | 426.8105 | 504.8164 | 587.1049 | 351.0419 | 290.2748 | 371.903 | 615.7661 | 236.2907 | 284.2642 | 290.0281 | 161.9364 | 336.4536 | 449.8886 | 247.5005 | 482.4063 | 351.5336 | 515.8865 | 375.0345 | 433.1734 | 425.7405 | 512.2788 | 545.3681 | 402.7742 |
| CUST_11270_PI429010886 | N27541 | 0.018643 | 18 | 3 | 225714206 | Cuticle protein 7 [Lepeophtheirus salmonis] | | | 1.88E-23 | 1290.993 | 493.215 | 7.338708 | 1280.61 | 349.1356 | 1077.327 | 690.6945 | 696.7816 | 768.7795 | 22.64343 | 20.55453 | 29.46496 | 909.1015 | 70.03699 | 268.1467 | 307.6894 | 196.9798 | 546.5203 | NA | 126.9841 | 80.82297 | 965.2192 | NA | 3790.041 | 71.61132 | 1416.05 | 8.56112 |
| CUST_13111_PI429010886 | N30594 | 0.018652 | 24 | 18 | | | | | | 33.37016 | 41.26062 | 33.95071 | 40.59908 | 26.62834 | 32.05527 | 16.83941 | 28.33698 | 28.88408 | 33.97352 | 30.85032 | 41.29432 | 32.46401 | 42.38554 | 31.6776 | 57.00393 | 27.15358 | 39.66277 | 43.1379 | 54.74747 | 33.53684 | 53.33947 | 33.01824 | 33.05858 | 45.61505 | 36.3679 | 39.7396 |
| CUST_7047_PI429010886 | N20959 | 0.01866 | 15 | 12 | 321467326 | GST-N-Metaxin-like protein [Daphnia pulex] | | | 1.09E-63 | 350.1387 | 604.2963 | 382.2635 | 344.4277 | 217.37 | 202.262 | 393.9841 | 551.3995 | 449.7775 | 593.6703 | 454.0927 | 667.8908 | 489.1062 | 1046.057 | 588.8612 | 379.882 | 90.31563 | 296.7454 | 366.7345 | 472.5516 | 316.3545 | 393.782 | 494.8206 | 174.8815 | 188.6896 | 458.5323 | 550.9319 |
| CUST_54253_PI429010886 | M105300 | 0.018667 | 21 | 3 | | | | | | 78.80354 | 104.6405 | 85.37214 | 93.35337 | 99.13646 | 105.6941 | 77.61759 | 38.0269 | 64.28336 | 76.54184 | 77.51881 | 98.55351 | 100.3487 | 54.59618 | 63.54619 | 101.7859 | 86.83335 | 82.76433 | 65.82296 | 100.468 | 66.938 | 88.46488 | 103.2289 | 124.7587 | 99.17539 | 104.1211 | 121.8737 |
| CUST_42895_PI429010886 | M61540 | 0.018676 | 15 | 12 | | | | | | 83.62457 | 114.7999 | 98.62916 | 71.02655 | 76.98006 | 87.58299 | 71.05715 | 72.9477 | 61.25018 | 96.35645 | 85.13186 | 85.63673 | 81.5369 | 120.7762 | 111.5529 | 97.61331 | 92.47211 | 95.97714 | 86.96346 | 109.3042 | 80.55811 | 115.202 | 81.56419 | 73.37448 | 98.31044 | 97.07573 | 86.67899 |
| CUST_19552_PI429010886 | N41765 | 0.018676 | 15 | 12 | | | | | | 19.3658 | 7.297269 | 23.38375 | 16.28372 | 22.57964 | 46.75333 | 14.58359 | 18.88215 | 18.21042 | 25.27977 | 20.08306 | 23.1605 | 22.7759 | 41.11502 | 31.4416 | 27.8344 | 33.6052 | 27.19088 | 26.2515 | 36.8021 | 29.1329 | 18.0732 | 9.316937 | 10.75121 | 29.0109 | 24.86526 | 33.20597 |
| CUST_10006_PI429010886 | N25554 | 0.01868 | 18 | 12 | | | | | | 558.4057 | 924.8591 | 578.3985 | 552.5713 | 588.9896 | 506.9726 | 441.6462 | 502.0515 | 490.5451 | 689.4854 | 497.5175 | 748.9581 | 618.8739 | 777.7644 | 658.8544 | 991.6341 | 630.6794 | 602.9273 | 665.8633 | 772.4833 | 514.6723 | 914.3958 | 571.4497 | 530.5435 | 647.6977 | 452.8393 | 688.1653 |
| CUST_56580_PI429010886 | M114154 | 0.01868 | 21 | 3 | | | | | | 461.0025 | 61.93566 | 10.72419 | 375.7452 | 114.649 | 381.737 | 217.3402 | 275.3515 | 130.4232 | NA | 6.98493 | NA | 149.7694 | 6.65508 | 58.87201 | 58.34154 | 167.1292 | 183.9247 | 3.699533 | 9.435368 | 13.99028 | 29.69991 | 10.32811 | 557.0435 | NA | 325.0806 | NA |
| CUST_23345_PI429010886 | N48672 | 0.0187 | 12 | 9 | | | | | | 39.90543 | 43.56037 | 41.35575 | 35.4902 | 41.02447 | 54.10071 | 47.10091 | 37.65478 | 29.64988 | 44.46924 | 50.14442 | 60.48684 | 36.8676 | 33.70683 | 37.17503 | 38.33878 | 47.01326 | 46.12938 | 45.76846 | 43.98243 | 45.97157 | 75.61377 | 57.84964 | 55.52574 | 43.51237 | 44.80736 | 42.73401 |
| CUST_7237_PI429010886 | N21237 | 0.018704 | 21 | 9 | 443717291 | hypothetical protein CAPTEDRAFT_20779 [Capitella | | | 1.59E-40 | 117.3039 | 135.3886 | 112.1381 | 184.3384 | 208.4785 | 84.51934 | 155.2361 | 346.5286 | 281.2623 | 200.184 | 464.5441 | 507.2776 | 234.141 | 92.39293 | 175.9719 | 178.7072 | 165.5714 | 76.32596 | 123.4578 | 411.1038 | 257.3277 | 103.397 | 140.7358 | 108.4222 | 103.0135 | 157.4172 | 331.029 |
| CUST_13471_PI429010886 | N31246 | 0.018704 | 21 | 9 | 241695265 | integral membrane protein GPR177-B, putative [Ixo | Signal transduction | Other | 1.97E-42 | 18.4821 | 21.96556 | 20.87033 | 30.0232 | 28.5608 | 24.68523 | 21.65997 | 27.15358 | 36.19875 | 40.18883 | 22.98207 | 37.86895 | 20.06593 | 44.55872 | 28.37529 | 33.83173 | 22.28718 | 32.34301 | 22.15622 | 28.14805 | 24.12233 | 29.72539 | 21.1696 | 15.94162 | 34.30707 | 19.46131 | 38.31739 |
| CUST_15531_PI429010886 | N34741 | 0.018709 | 18 | 12 | | | | | | 71.73214 | 70.20962 | 71.62646 | 61.79791 | 53.27432 | 42.95145 | 51.02123 | 66.70229 | 70.88181 | 92.40099 | 58.88281 | 80.63947 | 71.10646 | 86.3602 | 73.45438 | 99.67314 | 43.14736 | 74.316 | 72.92837 | 57.66915 | 64.28336 | 76.13058 | 56.19324 | 56.11599 | 65.30643 | 58.30365 | 70.73325 |
| CUST_16379_PI429010886 | N36134 | 0.018716 | 18 | 6 | 75022291 | RecName: Full=Oplophorus-luciferin 2-monooxyger | | | 6.9E-08 | NA | 5.228734 | 10.80175 | 22.77196 | 25.10875 | 12.05344 | 27.77642 | 75.88452 | 70.28439 | 6.594234 | 69.56167 | 74.74125 | 13.24115 | 13.70505 | 12.28913 | NA | 28.39724 | NA | 4.912682 | 67.34873 | 18.29602 | NA | 21.70738 | 18.69291 | NA | 57.19607 | 38.83801 |
| CUST_22482_PI429010886 | N47112 | 0.01872 | 15 | 0 | | | | | | 42.90864 | 42.40805 | 46.83381 | 21.01435 | 18.01905 | 24.31347 | 30.48948 | 27.40469 | 22.67277 | 22.0504 | 16.22526 | 20.95946 | 18.9175 | 27.7492 | 107.0545 | 27.36061 | 43.58328 | 22.10673 | 28.57866 | 20.55089 | 24.18723 | 23.35652 | 14.1603 | 22.46932 | 23.62378 | 12.54065 | 20.08892 |
| CUST_22264_PI429010886 | N46713 | 0.018734 | 24 | 21 | | | | | | 156.8624 | 83.63981 | 193.6314 | 194.9477 | 105.242 | 184.2011 | 176.9135 | 111.485 | 171.8979 | 162.6071 | 154.6176 | 141.5625 | 94.90628 | 170.9831 | 149.0992 | 131.1671 | 160.3693 | 163.0075 | 158.6525 | 167.8053 | 201.9396 | 184.3634 | 202.8821 | 184.1431 | 206.6657 | 177.0286 | 180.8997 |
| CUST_35390_PI429010886 | M12455 | 0.018747 | 24 | 21 | | | | | | 547.6349 | 676.5905 | 580.161 | 500.2189 | 540.6649 | 530.042 | 470.7834 | 493.7491 | 466.9714 | 622.5542 | 454.184 | 638.7779 | 499.1257 | 640.4514 | 522.4048 | 652.581 | 519.9761 | 544.6485 | 547.8035 | 704.9925 | 534.723 | 688.5897 | 577.0235 | 515.5917 | 671.8789 | 552.3175 | 745.0756 |

| Probe ID | ID | p-value | Period | Phase | EBI ID | Annotation | GO category | Go term | E-value | 0 | 0 | 0 | 3 | 3 | 3 | 6 | 6 | 6 | 9 | 9 | 9 | 12 | 12 | 12 | 15 | 15 | 15 | 18 | 18 | 18 | 21 | 21 | 21 | 24 | 24 | 24 |
|---|---|---|---|---|---|---|---|---|---|---|---|---|---|---|---|---|---|---|---|---|---|---|---|---|---|---|---|---|---|---|---|---|---|---|---|---|
| CUST_24170_PI429010886 | N50160 | 0.018747 | 24 | 18 | | | | | | 8.29527 | 35.03522 | 50.76541 | 16.21863 | 29.83725 | 17.85771 | 31.52203 | 30.43159 | 20.51687 | 45.90172 | 33.29815 | 51.05482 | 36.387 | 67.89063 | 41.70336 | 60.57719 | 54.80426 | 32.45773 | 31.00505 | 90.28628 | 46.66745 | 31.36927 | 64.77051 | 19.28099 | 65.9729 | 25.04386 | 60.5947 |
| CUST_43873_PI429010886 | M64207 | 0.018749 | 15 | 6 | | | Metabolic process | Carbohydrate metabolic process | | NA | 20.68333 | 14.96269 | 108.8513 | 69.24723 | 59.98068 | 42.39985 | 394.3244 | 310.2726 | 29.24532 | 207.853 | 264.7596 | 35.21995 | 42.44469 | 29.5179 | NA | 14.72582 | NA | 14.84838 | 203.2991 | 291.8868 | NA | 101.7859 | 80.75638 | NA | 274.7936 | 51.65313 |
| CUST_39611_PI429010886 | M41753 | 0.018772 | 12 | 0 | | | | | | 17.42213 | 23.97713 | 16.29894 | 15.76192 | 17.90404 | 10.89022 | 11.77319 | 12.28582 | 9.981072 | 28.85946 | 17.49801 | 26.04672 | 19.29593 | 19.45678 | 14.69352 | 22.81381 | 13.57199 | 14.04977 | 15.7577 | 12.74947 | 16.77093 | 17.38464 | 14.13431 | 10.17778 | 23.90131 | 23.80754 | 21.89788 |
| CUST_24414_PI429010886 | N50582 | 0.018778 | 24 | 9 | | | | | | 710.22 | 1197.011 | 1181.018 | 752.3295 | 666.8781 | 852.6077 | 1020.69 | 918.9933 | 1073.527 | 1270.943 | 897.1177 | 1412.004 | 860.82 | 1186.768 | 913.0418 | 1216.97 | 447.8279 | 751.781 | 558.0588 | 494.6999 | 747.636 | 1219.823 | 701.1457 | 382.663 | 793.0472 | 588.8612 | 883.3994 |
| CUST_48122_PI429010886 | M78981 | 0.01879 | 24 | 21 | | | | | | 8.282005 | 7.565304 | 17.46064 | 4.192346 | 3.951927 | 13.12407 | 3.580954 | NA | 6.068824 | NA | 6.207162 | 4.448023 | 4.354588 | 3.459769 | 4.942301 | 7.598655 | 5.97707 | 22.53631 | NA | NA | 10.12787 | 8.680488 | 10.32671 | 9.650569 | 8.10577 | NA | 10.87355 |
| CUST_34898_PI429010886 | M11020 | 0.018794 | 21 | 0 | 332022382 | Anaphase-promoting complex subunit 5 [Acromyrm | | | 3.58E-17 | 83.74259 | 69.26327 | 98.23281 | 55.26178 | 59.33007 | 54.96916 | 49.688 | 62.65132 | 55.30305 | 81.35601 | 57.256 | 80.63305 | 64.83984 | 56.19324 | 50.42448 | 69.40293 | 62.66858 | 63.61419 | 69.28978 | 70.77093 | 71.34779 | 78.93556 | 66.33339 | 64.31019 | 70.88181 | 45.46745 | 65.40248 |
| CUST_35363_PI429010886 | M12380 | 0.018796 | 12 | 3 | | | | | | 30.45779 | 47.67821 | 31.15588 | 66.09773 | 50.19147 | 71.65614 | 35.96518 | 54.69269 | 32.90861 | 34.22484 | 30.9722 | 41.26402 | 47.2535 | 35.18059 | 33.49626 | 44.84989 | 52.55217 | 54.69881 | 46.41914 | 49.01378 | 48.50046 | 49.9806 | 28.94785 | 44.64991 | 38.34742 | 65.11921 | 40.36236 |
| CUST_30894_PI429010886 | M135 | 0.018796 | 12 | 3 | 307204444 | Limkain-b1 [Harpegnathos saltator] | | | | 51.0383 | 35.73798 | 52.70775 | 52.76535 | 42.77108 | 72.28479 | 58.09597 | 36.80914 | 51.81515 | 36.5984 | 45.46228 | 23.64962 | 30.43159 | 41.81244 | 41.8585 | 50.48246 | 61.51764 | 58.86655 | 56.55039 | 33.80016 | 50.48761 | 38.50305 | 43.11747 | 39.78059 | 41.79247 | 37.04426 | 40.84912 |
| CUST_2336_PI429010886 | N12130 | 0.018796 | 12 | 3 | | | | | | 249.9317 | 61.45704 | 181.8579 | 382.7566 | 158.91 | 786.1873 | 219.715 | 364.2104 | 119.1476 | 50.19147 | 172.4652 | 44.51031 | 173.6967 | 77.51881 | 115.8883 | 200261.3 | 521.963 | 402.5662 | 569.1252 | 47.11037 | 127.8639 | 65.38596 | 162.8992 | 323.2711 | 58.76571 | 330.4301 | 48.67019 |
| CUST_909_PI429010886 | N7786 | 0.018796 | 12 | 3 | 321460066 | hypothetical protein DAPPUDRAFT_112094 [Daphni | | | 8.92E-16 | 1950.603 | 1478.287 | 467.665 | 5539.858 | 2543.989 | 7794.866 | 2520.419 | 1453.962 | 1550.226 | 125.7351 | 975.9966 | 103.2892 | 1828.604 | 1197.011 | 2182.975 | 2211.182 | 3103.161 | 4415.4 | 994.0668 | 477.2259 | 1544.651 | 2214.33 | 1128.352 | 4847.521 | 495.8952 | 2638.062 | 41.15368 |
| CUST_11736_PI429010886 | N28326 | 0.018843 | 21 | 12 | 157124198 | papilin [Aedes aegypti] >gi\|108874079\|gb\|EAT383( | | | 1.18E-55 | 57.24358 | 77.15161 | 79.11601 | 68.71451 | 89.65503 | 61.74634 | 100.5554 | 85.21423 | 92.45305 | 105.8252 | 97.79572 | 90.93451 | 92.25373 | 110.2618 | 86.6967 | 80.77462 | 97.68675 | 49.688 | 129.357 | 116.6057 | 77.23965 | 47.67821 | 90.15151 | 78.37536 | 86.08273 | 78.75847 | 99.09557 |
| CUST_17986_PI429010886 | N38955 | 0.01886 | 21 | 15 | | | | | | 481.413 | 465.1151 | 386.3769 | 290.4297 | 384.5478 | 326.1708 | 280.5674 | 277.8925 | 239.1179 | 434.0155 | 379.6269 | 432.1375 | 266.851 | 326.0308 | 355.6114 | 544.2536 | 506.3576 | 420.8347 | 422.5155 | 421.3888 | 320.9666 | 457.4348 | 337.2385 | 358.4083 | 555.454 | 330.6772 | 368.397 |
| CUST_56241_PI429010886 | M113241 | 0.018862 | 12 | 0 | | | | | | 1112.064 | 811.9464 | 527.7394 | 438.9413 | 263.3865 | 140.1704 | 532.3882 | 523.4011 | 331.8685 | 2555.115 | 690.4518 | 1580.29 | 1081.105 | 750.8373 | 1090.456 | 471.8606 | 246.9496 | 355.5348 | 890.6855 | 587.2428 | 681.5488 | 1361.981 | 351.8276 | 424.3711 | 137.9332 | 845.2412 | 2282.54 |
| CUST_40424_PI429010886 | M55047 | 0.018879 | 24 | 18 | | | | | | 891.3926 | 1364.868 | 1134.542 | 1128.807 | 1023.799 | 941.2341 | 842.6525 | 721.8143 | 791.8244 | 897.9401 | 1057.689 | 1999.641 | 1277.111 | 1248.908 | 1749.353 | 2159.598 | 757.8647 | 1223.726 | 1251.807 | 1758.855 | 1255.497 | 1422.832 | 1065.529 | 1210.833 | 1308.744 | 1546.55 | 1518.655 |
| CUST_47098_PI429010886 | M74785 | 0.018879 | 24 | 18 | 270010451 | hypothetical protein TcasGA2_TC009846 [Tribolium | | | 1.76E-25 | 17.61362 | 29.76053 | 21.72163 | 17.83843 | 26.81319 | 19.28099 | 20.22094 | 8.230173 | 18.69493 | 21.32116 | 22.75029 | 29.57766 | 17.39698 | 21.27079 | 25.01801 | 24.55823 | 26.79307 | 22.0053 | 23.04785 | 32.29627 | 27.62424 | 31.40893 | 24.99108 | 19.81178 | 23.19661 | 19.81178 | 23.531 |
| CUST_34007_PI429010886 | M8491 | 0.018907 | 21 | 9 | | | | | | 438.2102 | 550.6217 | 515.4056 | 346.3085 | 725.0021 | 690.6945 | 513.4234 | 464.3001 | 701.8561 | 2050.871 | 588.4339 | 1202.334 | 793.3041 | 1096.765 | 647.3023 | 1129.308 | 504.6329 | 756.9801 | 695.332 | 205.5305 | 580.436 | 489.1062 | 523.5388 | 445.6175 | 383.4448 | 631.2945 | 1037.274 |
| CUST_23919_PI429010886 | N49700 | 0.018914 | 15 | 12 | | | | | | 20.83504 | 22.89626 | 34.37569 | 15.28107 | 12.71188 | 6.905105 | 19.66703 | 35.61182 | 23.83766 | 31.28349 | 20.26494 | 21.16716 | 21.52428 | 33.57754 | 22.19942 | 25.28193 | 11.89374 | 9.277187 | 20.25895 | 12.91494 | 19.24611 | 20.72678 | 25.57573 | NA | 30.91161 | 20.72678 | 28.45405 |
| CUST_36036_PI429010886 | M14380 | 0.018919 | 15 | 0 | | | | | | 131.9637 | 138.8541 | 117.487 | 115.0894 | 109.7371 | 94.18675 | 107.5704 | 97.63395 | 84.04395 | 126.9651 | 85.67865 | 86.49079 | 107.9401 | 103.397 | 94.18675 | 147.341 | 117.5373 | 112.609 | 114.4836 | 94.23139 | 83.0149 | 119.4271 | 77.03965 | 92.46177 | 127.7879 | 87.91683 | 75.17352 |
| CUST_43464_PI429010886 | M63127 | 0.018927 | 15 | 12 | | | | | | 134.5033 | 145.1631 | 154.4419 | 117.3985 | 127.7612 | 94.76564 | 105.6827 | 131.4266 | 124.1861 | 168.0847 | 123.349 | 136.9776 | 159.2768 | 179.5033 | 141.1131 | 155.9986 | 146.5035 | 145.9616 | 134.4036 | 142.6543 | 172.1063 | 189.144 | 135.3397 | 113.0325 | 191.9895 | 141.6867 | 140.1157 |
| CUST_35500_PI429010886 | M12728 | 0.018932 | 15 | 12 | 260813390 | hypothetical protein BRAFLDRAFT_103418 [Branchio | | | 1.78E-38 | 172.0275 | 193.8995 | 196.359 | 149.7495 | 140.8068 | 134.6396 | 142.6543 | 154.8105 | 157.3603 | 205.909 | 151.5629 | 172.1063 | 158.6525 | 201.008 | 206.3065 | 223.0503 | 148.2477 | 204.8186 | 198.7421 | 168.5577 | 138.8211 | 203.7035 | 148.196 | 162.473 | 156.6593 | 165.2432 | 176.2225 |
| CUST_43137_PI429010886 | M62169 | 0.018937 | 18 | 12 | 66524904 | PREDICTED: membrane-associated protein Hem [Ap | | | 4.55E-57 | 102.205 | 77.39915 | 87.67204 | 75.87721 | 70.23023 | 84.57831 | 89.49881 | 97.47845 | 85.72992 | 87.84073 | 78.58831 | 86.59518 | 90.09804 | 106.872 | 94.85349 | 100.0727 | 73.59863 | 88.94052 | 88.4462 | 85.75328 | 87.20925 | 92.50739 | 80.46995 | 66.78152 | 94.20676 | 72.96725 | 84.70201 |
| CUST_49928_PI429010886 | M87776 | 0.018976 | 21 | 0 | | | | | | 347.4375 | 387.589 | 428.9575 | 260.9776 | 239.9876 | 272.5517 | 240.8565 | 306.3807 | 261.922 | 329.4092 | 238.455 | 253.0671 | 312.5243 | 379.0179 | 377.3372 | 393.4936 | 302.7176 | 329.9108 | 336.9449 | 321.753 | 281.735 | 408.1397 | 333.8649 | 316.8071 | 362.1966 | 258.0303 | 347.1114 |
| CUST_799_PI429010886 | N7359 | 0.018986 | 12 | 0 | | | | | | 212.7113 | 230.36 | 273.4925 | 183.6032 | 205.2623 | 262.8518 | 185.9766 | 223.377 | 186.2287 | 261.922 | 216.6058 | 299.285 | 250.036 | 301.4214 | 226.4554 | 269.5764 | 212.1377 | 234.7048 | 184.5783 | 209.8989 | 254.4916 | 235.0953 | 230.4843 | 226.5808 | 264.9246 | 270.3119 | 285.8071 |
| CUST_32292_PI429010886 | M3709 | 0.01899 | 15 | 0 | | | | | | 658.8544 | 733.1697 | NA | 1485.346 | 353.4537 | 431.93 | 746.1542 | NA | 839.8931 | 5.327653 | 4.143787 | 3.754246 | 308.2627 | 79.14043 | 548.077 | 505.7885 | NA | 454.2849 | NA | 28.60828 | 5.950176 | 236.8954 | 5.777163 | 247.4184 | 104.1985 | NA | NA |
| CUST_45454_PI429010886 | M69161 | 0.018997 | 15 | 3 | | | | | | 729.346 | 127.8287 | 831.7121 | 3425.229 | 19773.16 | 11001.7 | 17482.08 | 1759.978 | 245.2402 | 44.42567 | 24707.51 | 8.657735 | 204.6898 | 123.6853 | 147.523 | 9.796108 | 24175.16 | 6042.693 | 31801.7 | NA | 26343.04 | 22.46932 | 20926.24 | 28378.02 | 80.70446 | 1771.34 | 13.78911 |
| CUST_52730_PI429010886 | M98691 | 0.018997 | 15 | 9 | | | | | | 175.39 | 148.9131 | 1837.915 | 10.05433 | 11.31627 | NA | 532.9396 | 2951.483 | 2988.348 | 3222.703 | 717.957 | 1135.995 | 2284.352 | 6863.631 | 2805.418 | 365.0271 | 1507.459 | 144.5986 | 361.2861 | 1721.92 | 172.2354 | 5113.292 | 4116.427 | 80.68886 | 5980.233 | 731.4443 | 5105.143 |
| CUST_8708_PI429010886 | N22547 | 0.018997 | 15 | 15 | 241998974 | conserved hypothetical protein [Ixodes scapularis] : | | | 3.86E-31 | 16.70546 | 25.51149 | 26.83276 | 15.08742 | 17.04499 | 26.80106 | 17.95097 | 18.40232 | 26.49815 | 22.10673 | 27.78747 | 28.55766 | 21.94847 | 34.00581 | 30.02625 | 30.37645 | 21.28517 | 61.45036 | 22.63948 | 48.87456 | 20.50176 | 29.62583 | 22.30272 | 28.84855 | 26.05696 | 17.51574 | 21.71424 |
| CUST_2599_PI429010886 | N12731 | 0.019003 | 24 | 18 | 345483293 | PREDICTED: LOW QUALITY PROTEIN: myosin-IIIa [Na | | | | 1912.382 | 1921.833 | 1392.18 | 1168.631 | 1474.825 | 1669.785 | 1063.638 | 954.6687 | 985.3973 | 2024.906 | 1516.22 | 1685.531 | 803.077 | 1550.226 | 1485.346 | 2112.767 | 1682.988 | 1932.967 | 1689.395 | 2331.325 | 1724.056 | 1809.996 | 1323.469 | 1598.927 | 2351.436 | 1338.953 | 1990.565 |
| CUST_49492_PI429010886 | M85858 | 0.019007 | 12 | 0 | | | | | | 264.8881 | 768.2695 | 17.2249 | 1412.634 | 702.7008 | 391.7449 | 574.8968 | 38.98332 | 349.1356 | 26.42807 | 31.59756 | 7.266326 | 127.5221 | 96.4587 | 275.6931 | 1076.944 | 67.12072 | 561.9199 | 48.05852 | 77.47192 | 24.24502 | 815.8741 | 5.103744 | 257.9765 | 323.0511 | 52.28302 | 32.42452 |
| CUST_4180_PI429010886 | N16179 | 0.019007 | 12 | 3 | | | | | | 145.1427 | 59.86271 | 27.05759 | 155.8809 | 105.4208 | 249.422 | 147.895 | 144.3925 | 99.94463 | 24.0422 | 33.38385 | 6.924715 | 90.17193 | 57.21451 | 102.9204 | 100.3128 | 168.0847 | 146.3045 | 126.3916 | 31.01397 | 28.25151 | 76.83746 | 56.88203 | 207.3956 | 51.37925 | 108.2468 | 38.19514 |
| CUST_10323_PI429010886 | N26059 | 0.019007 | 12 | 3 | | | | | | 680.9808 | 778.015 | 661.3119 | 897.9401 | 622.9374 | 943.689 | 861.1597 | 600.7483 | 844.642 | 296.3167 | 530.8519 | 632.6214 | 636.3168 | 700.208 | 822.3869 | 849.5817 | 901.9536 | 810.6402 | 830.2031 | 533.4173 | 572.181 | 633.9622 | 694.099 | 801.7718 | 731.2482 | 614.2182 | 646.5567 |
| CUST_19849_PI429010886 | N42316 | 0.019007 | 12 | 9 | | | | | | 33.05858 | 36.06929 | 36.52603 | 24.63631 | 26.37273 | 31.66434 | 33.77621 | 35.65161 | 30.0571 | 48.23603 | 32.80705 | 39.21347 | 36.52603 | 33.81876 | 32.72316 | 39.07685 | 18.82343 | 26.94089 | 24.9077 | 31.20492 | 34.21062 | 44.22939 | 45.33809 | 31.2089 | 41.56705 | 26.43705 | 44.81188 |
| CUST_24374_PI429010886 | N50505 | 0.019007 | 12 | 9 | | | | | | 56.28989 | 90.37187 | 52.96132 | 38.723 | 43.56595 | 34.10794 | 52.88574 | 65.81652 | 38.39166 | 88.4462 | 56.28307 | 85.141 | 51.48154 | 69.17707 | 72.2558 | 115.7331 | 34.62398 | 37.73674 | 43.02941 | 38.93338 | 54.89949 | 59.04082 | 65.16761 | 80.08977 | 72.00003 | 69.751 | 48.79079 |
| CUST_30750_PI429010886 | N62557 | 0.019007 | 12 | 9 | | | | | | 125.2228 | 129.4038 | 165.324 | 104.1782 | 82.25514 | 122.3609 | 122.6119 | 120.4034 | 212.8309 | 186.5931 | 109.6686 | 158.1524 | 138.8541 | 135.3099 | 161.1033 | 100.965 | 112.4452 | 140.6816 | 92.05581 | 142.0799 | 155.2537 | 168.2221 | 169.4295 | 143.124 | 157.3996 | 164.7204 | 193.2088 |
| CUST_711_PI429010886 | N6883 | 0.019007 | 12 | 3 | 321472705 | hypothetical protein DAPPUDRAFT_239811 [Daphn | | | 1.27E-26 | 6434.019 | 8015.915 | 989.1564 | 19163.99 | 12445.58 | 24915.2 | 9448.983 | 3886.072 | 7890.624 | 1949.421 | 4365.095 | 3218.113 | 8237.502 | 4415.4 | 5757.178 | 8696.127 | 12804.38 | 19067.23 | 3667.985 | 3572.519 | 3200.017 | 8165.772 | 2302.386 | 13202.87 | 3095.586 | 12021.62 | 1486.604 |
| CUST_38588_PI429010886 | M28347 | 0.019031 | 24 | 15 | | | | | | 512.6993 | 655.9864 | 693.5345 | 576.3102 | 655.5465 | 591.5595 | 541.31 | 543.8134 | 646.7171 | 671.5411 | 586.9437 | 705.5663 | 658.4007 | 748.9581 | 676.3702 | 708.9187 | 722.474 | 675.9235 | 671.7237 | 660.7381 | 671.3234 | 656.5643 | 710.4011 | 582.1613 | 844.11 | 589.4448 | 731.4443 |
| CUST_41034_PI429010886 | M56694 | 0.019031 | 24 | 15 | | | | | | 638.6578 | 614.5524 | 715.8969 | 599.7852 | 524.4761 | 566.2341 | 487.8771 | 507.983 | 568.3832 | 657.2757 | 624.8531 | 847.8005 | 630.9156 | 848.334 | 844.3643 | 865.4579 | 657.8288 | 664.1086 | 631.8289 | 722.2644 | 655.2712 | 902.1996 | 609.1465 | 540.3051 | 801.3257 | 719.37 | 874.1627 |
| CUST_7485_PI429010886 | N21614 | 0.019031 | 12 | 15 | 321463650 | hypothetical protein DAPPUDRAFT_324056 [Daphn | | | 5.37E-59 | 62.10992 | 53.57478 | 41.59399 | 57.19607 | 41.99643 | 25.53913 | 30.70822 | 37.62657 | 62.45524 | 75.51588 | 48.36598 | 38.53689 | 66.08238 | 79.27531 | 70.46014 | 122.4345 | 23.4177 | 67.60441 | 58.81306 | 62.21051 | 57.5238 | 68.44956 | 55.93124 | 28.11091 | 53.41262 | 58.4576 | 48.93138 |
| CUST_33292_PI429010886 | M6518 | 0.019042 | 18 | 9 | 223588323 | anti-lipopolysaccharide factor 2 [Marsupenaeus jap | | | 2.12E-27 | 66.75988 | 72.81129 | 15.67417 | 17.43225 | 28.97523 | 28.75284 | 77.93692 | 18.4372 | 88.04251 | 123.5891 | 50.56281 | 144.3781 | 129.6012 | 74.7726 | 51.96426 | 70.24827 | 50.11711 | 37.86509 | 169.9489 | 3.992492 | 62.53849 | 22.63755 | 32.99167 | 33.22847 | 75.33028 | 20.75737 | 71.65614 |
| CUST_51185_PI429010886 | M92511 | 0.019055 | 24 | 15 | | | | | | 108.8172 | 144.5655 | 174.6884 | 76.11306 | 113.5681 | 126.0646 | 89.85114 | 87.82151 | 101.0721 | 144.8798 | 96.70093 | 142.8955 | 98.33611 | 158.5157 | 163.7445 | 194.3549 | 120.5952 | 161.0763 | 120.496 | 161.8906 | 125.6844 | 142.0255 | 157.283 | 109.19 | 144.6491 | 172.5221 | 105.3648 |
| CUST_19815_PI429010886 | N42244 | 0.019076 | 12 | 9 | 332018661 | Stathmin-4 [Acromyrmex echinatior] | Response to stress | Defence responce | 1.16E-07 | 34.02396 | 25.94889 | 38.02052 | 24.5371 | 24.11784 | 35.992 | 35.86991 | 29.45107 | 36.47986 | 44.87442 | 37.19463 | 51.89822 | 35.54827 | 32.03181 | 31.28349 | 36.6789 | 50.52879 | 34.872 | 39.24212 | 36.70932 | 35.7959 | 38.44451 | 43.27178 | 46.41914 | 38.08235 | 25.68978 | 36.32974 |
| CUST_28943_PI429010886 | N58890 | 0.019079 | 24 | 18 | | | | | | 20.74944 | 18.49894 | 3.554665 | 11.07279 | 16.53144 | 13.37557 | 13.08205 | 10.95786 | 6.947339 | 17.05056 | 14.10504 | 15.75112 | 11.89729 | 19.19934 | 19.44365 | 26.88416 | 15.06859 | 15.94162 | 18.8295 | 15.65479 | 25.1646 | 28.49553 | 13.44283 | 13.40566 | 20.78572 | 10.04473 | 13.90672 |
| CUST_41845_PI429010886 | M58684 | 0.019084 | 18 | 0 | 321468138 | hypothetical protein DAPPUDRAFT_319730 [Daphn | | | 5.87E-15 | 45.3248 | 36.08065 | 31.68203 | 15.42005 | 26.02411 | 14.54005 | 24.15101 | 18.78315 | 20.60841 | 37.37431 | 23.92396 | 39.99252 | 33.52987 | 24.56345 | 26.45984 | 27.80573 | 15.59286 | 21.15229 | 27.26616 | 32.60067 | 36.31145 | 30.42832 | 28.42343 | 14.25774 | 23.89642 | 8.724159 | 15.57695 |
| CUST_38905_PI429010886 | M31812 | 0.01909 | 24 | 3 | | | | | | 5917.438 | 227.604 | 21.248 | 3621.392 | 207.128 | 1129.849 | 756.9801 | 200.041 | 696.3149 | NA | 59.22654 | 157.4606 | 360.3606 | 119.5708 | 498.1212 | 86.02859 | 50.67294 | 36.34355 | 108.8006 | 22.02831 | 43.86105 | 465.6558 | 27.25936 | 6818.919 | NA | 68.29075 | 30.86679 |
| CUST_10504_PI429010886 | N26336 | 0.019107 | 24 | 12 | 156541012 | PREDICTED: DOMON domain-containing protein CG | | | 6.2E-47 | 150.5702 | 270.4039 | 199.6816 | 465.9125 | 285.6058 | 236.8108 | 294.9448 | 248.9182 | 121.4004 | 204.4226 | 304.437 | 325.9737 | 206.8026 | 193.6314 | 254.8048 | 144.9989 | 144.0441 | 139.47 | 168.154 | 153.3324 | 163.5397 | 181.1004 | 294.596 | 170.3395 | 139.0376 | 165.1872 | 171.1406 |
| CUST_36027_PI429010886 | M14326 | 0.019135 | 12 | 3 | | | | | | 81.29483 | 125.099 | 58.76571 | 226.8032 | 165.5416 | 619.9697 | 154.6378 | 97.73561 | 122.0703 | 44.59476 | 150.8706 | 50.08673 | 86.68692 | 63.65227 | 78.97339 | 92.29442 | 208.2464 | 583.8403 | 120.7327 | 54.08177 | 135.1252 | 86.88496 | 98.38324 | 305.4399 | 50.5231 | 175.2355 | 39.49607 |
| CUST_24556_PI429010886 | N50833 | 0.019135 | 12 | 3 | | | | | | 25.2086 | 24.16612 | 27.2017 | 42.93894 | 36.67483 | 89.30785 | 66.89269 | 19.16559 | 32.78743 | 28.24069 | 39.97721 | 30.72781 | 20.65497 | 23.37181 | 17.21077 | 29.27728 | 68.36198 | 62.01223 | 27.49118 | 24.95301 | 56.16292 | 25.26607 | 69.16983 | 64.33014 | 23.08011 | 36.66046 | 27.61589 |
| CUST_3019_PI429010886 | N22427 | 0.019165 | 21 | 9 | 260833760 | hypothetical protein BRAFLDRAFT_83094 [Branchio | | | 6.82E-23 | 233.6069 | 353.4537 | 337.7668 | 100.4233 | 241.4042 | 200.9492 | 477.3001 | 356.2218 | 328.4337 | 989.9215 | 379.4393 | 450.649 | 366.8876 | 350.5238 | 304.2796 | 277.4972 | 201.6148 | 186.883 | 626.1001 | 88.66033 | 174.2465 | 181.8416 | 259.5794 | 389.9711 | 178.147 | 222.7411 | 207.853 |
| CUST_1040_PI429010886 | N26192 | 0.019172 | 21 | 3 | | | | | | 38.85028 | 21.43684 | 28.73411 | 40.23874 | 33.77253 | 35.20228 | 26.86002 | 29.49942 | 24.80133 | 33.85642 | 27.71267 | 24.44247 | 35.84962 | 21.71104 | 29.30417 | 25.50881 | 27.83815 | 24.02357 | 28.92063 | 31.90512 | 27.03844 | 38.00094 | 33.84879 | 21.4174 | 41.63072 | 35.36774 | 33.46666 |
| CUST_43286_PI429010886 | M62631 | 0.019174 | 15 | 12 | 350426664 | PREDICTED: putative polypeptide N-acetylgalactosa | | | 9.11E-41 | 27.34621 | 26.64821 | 49.52106 | 29.77814 | 14.36186 | 21.48446 | 22.20487 | 30.43732 | 33.32019 | 44.14193 | 44.29399 | 36.73446 | 31.85611 | 46.32451 | 41.21277 | 22.01737 | 20.90506 | 27.91811 | 20.58435 | 23.44542 | 30.558 | 32.07598 | 32.06361 | 17.98635 | 41.71124 | 21.94847 | 22.35984 |
| CUST_7293_PI429010886 | N21328 | 0.019174 | 15 | 12 | 321476561 | hypothetical protein DAPPUDRAFT_43139 [Daphnia | | | 1.4E-118 | 110.7887 | 116.7168 | 121.4973 | 91.20024 | 79.32298 | 80.94247 | 72.3404 | 83.20205 | 111.7143 | 154.6176 | 112.6604 | 130.7466 | 124.4126 | 147.0211 | 132.7602 | 159.1972 | 85.25376 | 113.2396 | 109.8902 | 125.9794 | 98.65062 | 153.8239 | 117.5373 | 96.029 | 152.675 | 100.2916 | 125.6172 |
| CUST_30345_PI429010886 | N61677 | 0.019174 | 15 | 12 | | | | | | 66.14351 | 11.95734 | 98.56528 | 41.75113 | 4.405905 | 32.32032 | 134.0969 | 94.27998 | 95.14966 | 96.63027 | 21.21967 | 25.93686 | 126.2448 | 220.8734 | 88.7086 | 31.45667 | 119.0689 | 65.31793 | 46.0364 | 22.87323 | 24.97999 | 29.91175 | 46.47908 | 36.01332 | 82.74334 | 67.88326 | 69.34114 |
| CUST_29161_PI429010886 | N59310 | 0.019197 | 15 | 3 | | | | | | 4.451004 | 4.004614 | 7.032309 | 8.850436 | 12.78761 | 14.61919 | 4.869685 | 7.795868 | 11.28834 | 7.366514 | 6.212927 | 7.712934 | 6.689971 | 4.521715 | 6.25555 | 6.827991 | 4.691227 | NA | 6.470119 | 16.80832 | NA | 6.351061 | 10.76236 | 6.799702 | 4.45976 | NA | 5.588347 |
| CUST_19182_PI429010886 | N41125 | 0.019199 | 12 | 0 | | | | | | 106.9908 | 105.8718 | 105.4471 | 87.8914 | 86.4248 | 72.78118 | 62.25774 | 101.5961 | 85.86351 | 110.8154 | 90.71122 | 100.5206 | 108.6787 | 100.0408 | 99.60857 | 139.562 | 90.11953 | 84.50068 | 93.72794 | 85.86351 | 74.70996 | 89.16253 | 131.4266 | 96.46972 | 116.6557 | 92.19432 | 101.1723 |
| CUST_15451_PI429010886 | N34601 | 0.019208 | 18 | 0 | | | | | | 58.36102 | 71.5379 | 52.52687 | 56.87008 | 52.74471 | 52.24698 | 47.17274 | 45.35228 | 44.18048 | 41.38053 | 50.43461 | 35.37802 | 54.27879 | 60.19936 | 62.74069 | 80.19532 | 40.08825 | 57.14083 | 54.6573 | 53.78486 | 64.04953 | 75.69546 | 42.06392 | 51.09668 | 60.89252 | 42.92761 | 44.31783 |
| CUST_41988_PI429010886 | M59084 | 0.019212 | 15 | 9 | 67625699 | TPA: endonuclease-reverse transcriptase [Schistoso | Nucleic acid metabolism | DNA replication | 4.81E-56 | 136.9633 | 191.3362 | 152.9004 | 115.8195 | 113.2498 | 107.1617 | 124.5707 | 107.6827 | 142.2559 | 291.8279 | 153.3961 | 219.7749 | 134.577 | 232.9884 | 203.9202 | 227.5634 | 125.6673 | 150.6133 | 141.9837 | 172.2644 | 176.7754 | 194.8105 | 163.0075 | 150.3479 | 199.4521 | 153.0166 | 231.3759 |
| CUST_18988_PI429010886 | N40777 | 0.019212 | 15 | 9 | | | | | | 15.11766 | 14.23976 | 19.81178 | 15.28613 | 13.14806 | 13.74525 | 16.16294 | 18.94519 | 27.58334 | 31.02908 | 24.08352 | 32.89038 | 25.50317 | 21.10951 | 17.85771 | 28.20406 | 21.48446 | 36.4309 | 15.92833 | 29.09978 | 13.65114 | 37.07575 | 21.91471 | 19.27396 | 52.34171 | 16.06241 | 27.16781 |
| CUST_39184_PI429010886 | M35789 | 0.019218 | 12 | 9 | 115291340 | oncoprotein nm23 [Litopenaeus vannamei] | Metabolic process | Nucleobase-containing compound metabolic process | 1.04E-34 | 72.38234 | 79.56143 | 78.42487 | 45.31583 | 51.37093 | 39.82861 | 62.42674 | 66.4925 | 72.45575 | 100.3232 | 73.87582 | 73.28621 | 75.64933 | 69.00002 | 46.42845 | 75.2095 | 46.52112 | 50.67859 | 52.28302 | 77.40739 | 64.80696 | 87.76889 | 59.12871 | 69.54952 | 76.32596 | 59.74927 | 66.29488 |
| CUST_31194_PI429010886 | M947 | 0.019218 | 21 | 9 | | | | | | NA | 20.35072 | 6.85925 | 40.27597 | 35.08291 | 20.91693 | 35.19595 | 273.2835 | 236.9951 | 25.47915 | 312.052 | 309.0322 | 49.66096 | 52.90229 | 44.59476 | NA | 28.49366 | NA | 3.358666 | 465.4504 | 214.9497 | NA | 25.56884 | 6.521689 | NA | 179.3955 | 78.81351 |
| CUST_43920_PI429010886 | M66331 | 0.019249 | 21 | 3 | 321458955 | hypothetical protein DAPPUDRAFT_328543 [Daphn | | | 6.26E-64 | 57.8814 | 48.12117 | 43.05742 | 71.73214 | 49.02131 | 71.43726 | 50.9755 | 46.7059 | 43.00832 | 35.04514 | 50.74126 | 36.16806 | 40.1147 | 52.20238 | 34.86394 | 51.09155 | 37.59059 | 59.07909 | 58.14864 | 43.93802 | 45.46745 | 58.78466 | 61.46235 | 41.68991 | 54.02913 | 49.24106 | 61.42421 |
| CUST_43887_PI429010886 | M64246 | 0.019249 | 21 | 18 | | | | | | 90.58155 | 79.15691 | 55.57175 | 78.32339 | 70.84411 | 55.49391 | 53.9397 | 44.13738 | 66.34998 | 68.77778 | 65.58458 | 51.72418 | 61.45704 | 60.69824 | 85.43667 | 101.8114 | 58.53699 | 75.3452 | 74.93674 | 76.19351 | 91.14746 | 88.78823 | 67.45213 | 74.38643 | 91.11111 | 59.26817 | 68.93293 |
| CUST_8796_PI429010886 | N23604 | 0.01925 | 21 | 3 | 471414688 | PREDICTED: zinc finger protein 235 [Trichechus mar | | | 3.13E-38 | 7.472276 | 7.525118 | NA | 6.812888 | 5.183066 | 28.18417 | 5.803366 | 7.213856 | 12.87701 | 7.626632 | 4.162252 | 5.793257 | 4.170866 | 6.575352 | 6.540321 | 6.563098 | 4.093706 | NA | 6.486483 | 3.916855 | NA | NA | 8.669897 | 5.98749 | 10.33417 | 9.133825 | 10.94103 |
| CUST_31432_PI429010886 | M1512 | 0.019255 | 12 | 0 | 241696779 | conserved hypothetical protein [Ixodes scapularis] : | | | 1.5E-08 | 2232.866 | 2158.318 | 1901.328 | 2095.03 | 1691.46 | 1853.071 | 1658.285 | 1589.601 | 1576.033 | 1818.602 | 1488.789 | 1966.099 | 1949.421 | 2384.394 | 1854.457 | 2576.961 | 1708.455 | 1744.83 | 1642.187 | 2206.497 | 1580.775 | 2430.624 | 1874.82 | 1777.044 | 2167.205 | 1840.589 | 1904.883 |
| CUST_8674_PI429010886 | N23396 | 0.019264 | 21 | 15 | | | | | | 20.78351 | 11.95622 | 18.88759 | 10.26272 | 16.08188 | 13.32601 | 12.63007 | 6.112624 | 6.517889 | 6.537253 | 15.7734 | 17.61145 | 10.13183 | 7.075697 | 13.3483 | 15.77573 | 21.35072 | 25.71503 | 18.73634 | 16.22034 | 4.672014 | 13.85986 | 15.82481 | 14.44266 | 17.49952 | 13.3739 | 12.01881 |
| CUST_54423_PI429010886 | M105937 | 0.01929 | 12 | 0 | | | | | | 213.5411 | 149.4702 | 279.8719 | 120.5667 | 123.1656 | 138.1473 | 184.8426 | 196.1855 | 195.3573 | 191.5917 | 118.0864 | 221.1299 | 179.3477 | 238.7756 | 184.1431 | 128.3731 | 157.7661 | 153.8729 | 155.8094 | 141.8752 | 147.4736 | 207.1022 | 269.2795 | 181.0152 | 205.6958 | 191.2063 | 189.3905 |
| CUST_27420_PI429010886 | N56030 | 0.019293 | 21 | 15 | 242002324 | sugar transporter, putative [Ixodes scapularis] >gi\|2 | Transport | Carbohydrate transport | 2.1E-20 | 17.02696 | 12.16044 | NA | 10.86137 | 6.729385 | NA | NA | NA | 9.297397 | 6.98368 | 4.562691 | 10.81295 | 4.258091 | 8.474659 | 16.4962 | 21.70445 | NA | 20.60423 | 5.191851 | 21.19713 | 15.35699 | 18.14702 | NA | 12.04909 | 15.58239 | 5.098041 | 5.014559 |
| CUST_40565_PI429010886 | M55473 | 0.019296 | 18 | 15 | | | | | | 109.812 | 143.6702 | 95.9293 | 149.2467 | 133.135 | 136.1838 | 96.41487 | 101.0721 | 88.66977 | 115.3926 | 107.2382 | 122.6587 | 100.1156 | 124.513 | 117.9497 | 203.1014 | 133.3923 | 147.9921 | 133.0821 | 138.3597 | 119.9589 | 148.0995 | 111.485 | 126.8764 | 134.02 | 85.12067 | 97.282 |
| CUST_20170_PI429010886 | N42941 | 0.019296 | 18 | 12 | | | | | | 7.635215 | 14.33789 | 16.02003 | 13.61539 | 9.288423 | 9.203374 | 6.951132 | 12.4672 | 10.51063 | 20.45832 | 10.50509 | 14.91728 | 14.94458 | 30.58787 | 25.38784 | 23.43573 | 10.70994 | 18.41342 | 11.34865 | 56.91814 | 17.97978 | 12.66733 | 15.73595 | 8.813762 | 14.21847 | 19.63157 | 12.73395 |
| CUST_5829_PI429010886 | N19257 | 0.019309 | 15 | 12 | 380016801 | PREDICTED: uncharacterized protein LOC10086977 | | | 2.08E-53 | 219.6868 | 267.4607 | 285.1566 | 253.025 | 224.4296 | 205.6287 | 230.2231 | 243.166 | 323.9834 | 247.961 | 234.5938 | 372.3592 | 310.1413 | 328.6838 | 309.5934 | 386.0388 | 186.3153 | 309.8242 | 237.1988 | 229.2792 | 245.8548 | 295.1449 | 218.173 | 219.2135 | 335.5871 | 258.9285 | 292.6086 |
| CUST_25270_PI429010886 | N52095 | 0.019316 | 21 | 3 | | | | | | 22.91836 | 22.9575 | 9.181579 | 58.22303 | 12.01996 | 83.80399 | 56.15718 | 17.50878 | 28.18833 | NA | 7.02756 | NA | 42.11302 | NA | 32.37332 | 16.10308 | 18.44318 | 27.28895 | 11.99825 | 4.943292 | 7.638915 | 13.29711 | NA | 61.56139 | NA | 47.19112 | NA |
| CUST_40682_PI429010886 | M55853 | 0.019325 | 24 | 0 | | | | | | 8.055145 | 20.92823 | 18.13226 | 4.634681 | 11.37412 | 10.43073 | 5.168709 | 10.12281 | 7.219732 | 9.761566 | 9.275747 | 11.78894 | 6.56829 | 10.54822 | 7.311535 | 11.29494 | 10.34523 | 7.395008 | 9.104602 | 10.78228 | 8.361603 | 9.277187 | NA | 11.37788 | 13.38764 | 11.82406 | 12.7161 |
| CUST_33634_PI429010886 | M7476 | 0.019326 | 24 | 21 | | | | | | 33.41929 | 43.65248 | 41.15368 | 30.54342 | 27.13002 | 31.51891 | 26.35513 | 42.69742 | 31.19434 | 42.70151 | 23.51614 | 27.59937 | 28.20406 | 37.63001 | 34.119 | 40.82788 | 35.58457 | 32.68774 | 34.84252 | 39.91268 | 39.96079 | 43.10477 | 45.77928 | 37.80277 | 40.34574 | 30.50065 | 44.78041 |
| CUST_4012_PI429010886 | N15787 | 0.019334 | 24 | 18 | | | | | | 138.6419 | 142.6543 | 141.5103 | 131.6163 | 120.3747 | 120.8888 | 111.8858 | 105.3783 | 139.9078 | 183.1759 | 126.0646 | 144.5043 | 133.7926 | 168.4127 | 183.9787 | 191.7619 | 101.4215 | 148.7855 | 168.2847 | 197.7844 | 150.2252 | 209.2891 | 131.9942 | 139.419 | 175.1768 | 153.2103 | 174.4543 |
| CUST_55888_PI429010886 | M112016 | 0.019353 | 12 | 0 | | | | | | 875.295 | 1073.06 | 946.4318 | 827.5431 | 679.9331 | 785.9549 | 671.7237 | 753.9359 | 867.8685 | 1037.639 | 752.803 | 1123.855 | 816.6897 | 1177.547 | 1244.647 | 984.6642 | 711.9048 | 891.0101 | 906.266 | 994.7785 | 906.5131 | 992.2058 | 903.546 | 716.2921 | 1043.343 | 1216.535 | 1289.132 |
| CUST_44701_PI429010886 | M66722 | 0.019353 | 12 | 0 | | | | | | 113.8579 | 11.49086 | 157.0455 | 361.7651 | 292.0038 | 676.7937 | 615.9204 | 108.0345 | 70.19365 | 22.03875 | 727.1859 | 7.277507 | 11.55545 | 17.3225 | 12.50339 | 22.3538 | 863.4186 | 453.5801 | 125.8218 | 20.08892 | 473.5692 | 14.95825 | 285.0949 | 468.7286 | 7.993052 | 71.98272 | 9.79486 |
| CUST_4326_PI429010886 | N16518 | 0.019353 | 12 | 0 | | | | | | 51.32807 | 51.58072 | 70.2629 | 39.40451 | 29.58412 | 28.85946 | 29.62034 | 22.70056 | 41.44167 | 65.4438 | 46.55609 | 59.9743 | 34.85305 | 56.05475 | 42.9427 | 49.52106 | 36.64169 | 51.23628 | 42.72555 | 53.84406 | 44.94616 | 54.45697 | 48.78129 | 37.08279 | 60.15341 | 65.30029 | 66.11527 |
| CUST_17351_PI429010886 | N37819 | 0.019353 | 12 | 0 | | | | | | 239.571 | 577.9827 | 578.8676 | 199.0047 | 253.2546 | 327.0942 | 240.3331 | 404.7043 | 473.6842 | 470.2955 | 317.4719 | 597.3714 | 530.042 | 852.9478 | 565.2058 | 279.0433 | 318.0852 | 320.5709 | 520.6248 | 488.4404 | 478.8398 | 446.7333 | 512.0216 | 200.1336 | 466.9714 | 486.7621 | 778.015 |
| CUST_23679_PI429010886 | N49268 | 0.019353 | 12 | 0 | | | | | | 31.81303 | 39.29828 | 46.75809 | 39.92653 | 25.46248 | 23.77956 | 20.97996 | 28.26309 | 30.40899 | 40.68988 | 29.5983 | 40.14648 | 29.8401 | 43.36723 | 41.4213 | 24.26769 | 20.5469 | 31.83203 | 29.05988 | 19.2347 | 39.12985 | 52.32895 | 51.2626 | 22.74775 | 64.56992 | 39.82447 | 45.45196 |
| CUST_26838_PI429010886 | N54966 | 0.019353 | 12 | 3 | | | | | | 4680.081 | 2558.435 | 1696.945 | 10899.12 | 8391.959 | 18496.67 | 10282.77 | 4102.292 | 6091.908 | 136.9776 | 5435.626 | 139.8337 | 4808.982 | 1382.151 | 3785.297 | 3762.541 | 14274.58 | 18377.26 | 7764.128 | 929.9665 | 4627.233 | 3680.553 | 3921.02 | 11400.82 | 681.5488 | 7692.588 | 103.7577 |
| CUST_25096_PI429010886 | N51777 | 0.019356 | 15 | 0 | | | | | | 23.50136 | 7.621463 | 29.70337 | 20.54194 | 17.71272 | 19.95634 | 15.04174 | 13.8498 | 14.97861 | 12.3155 | 19.61118 | 10.91844 | 7.906354 | 13.8828 | NA | 29.82479 | 20.52725 | 17.81453 | 18.66627 | 17.08982 | 32.92673 | 17.34629 | 9.033618 | 10.62806 | 13.80474 | 10.94103 | 12.33047 |
| CUST_23745_PI429010886 | N49388 | 0.01939 | 15 | 0 | | | | | | 27.85465 | 21.44277 | 19.47912 | 17.25469 | 17.17494 | 29.01971 | 6.253831 | 11.51496 | 7.353206 | 28.87134 | 11.77319 | 10.8676 | NA | 24.50817 | 10.58195 | 20.15665 | 15.55047 | 29.47231 | 15.50146 | 10.88211 | 14.92766 | 15.92833 | 11.24259 | 17.76311 | 18.82539 | 15.50711 | 18.77555 |
| CUST_26418_PI429010886 | N54212 | 0.019393 | 18 | 12 | 307207938 | Protein polybromo-1 [Harpegnathos saltator] | | | 2.32E-12 | 12.60615 | 10.28317 | 21.54181 | 8.235102 | 5.697389 | 6.699155 | 15.16412 | 16.40616 | 13.23183 | 15.80258 | 13.92025 | 9.441058 | 34.12708 | 17.41182 | 11.90796 | 6.007021 | 23.83451 | 8.178429 | 13.79131 | 12.0863 | 9.559604 | 13.1549 | 5.685055 | 3.923061 | 17.4145 | 5.852448 | 9.451754 |
| CUST_33364_PI429010886 | N31059 | 0.019403 | 18 | 15 | | | | | | 147.523 | 119.7777 | 151.582 | 77.69279 | 87.75721 | 111.4592 | 123.5719 | 99.1645 | 107.3346 | 51.05906 | 131.9084 | 115.7448 | 118.0238 | 124.8107 | 134.0609 | 189.7994 | 128.1087 | 138.5194 | 142.821 | 165.8485 | 100.3711 | 152.4677 | 96.32091 | 78.52355 | 186.0597 | 84.90321 | 124.5927 |
| CUST_23071_PI429010886 | N48207 | 0.019405 | 18 | 3 | | | | | | 5.97138 | 9.106984 | 3.948807 | NA | 12.09198 | 13.49282 | 9.23673 | 8.066277 | NA | 6.460606 | 4.764203 | 8.871706 | 5.802569 | 8.149809 | 3.739398 | 4.93415 | 5.710989 | 8.935164 | 6.040843 | 9.319645 | 5.934873 | NA | 9.010507 | NA | 7.837273 | 10.04605 | 5.161394 |
| CUST_41809_PI429010886 | M58603 | 0.019423 | 15 | 12 | 307183303 | AP-2 complex subunit alpha [Camponotus floridanu | Transport | Protein transport | 3.75E-74 | 34.71503 | 47.88942 | 44.63016 | 30.38917 | 38.49903 | 35.1893 | 33.99968 | 37.85261 | 33.1299 | 62.66858 | 37.48716 | 56.11599 | 55.90718 | 84.62693 | 68.41131 | 59.28015 | 29.44853 | 37.30837 | 45.488 | 45.74744 | 42.24809 | 48.39289 | 44.07091 | 48.50963 | 50.43862 | 38.6481 | 48.97519 |
| CUST_2594_PI429010886 | N12719 | 0.019423 | 15 | 0 | 91082911 | PREDICTED: similar to dre4 CG1828-PA [Tribolium c | Cellular Process | Other | | 306.6185 | 404.8823 | 301.1198 | 275.0029 | 287.7468 | 235.6934 | 219.3055 | 263.0553 | 291.7598 | 418.4668 | 312.3654 | 443.0418 | 319.8933 | 444.8018 | 460.0363 | 459.5005 | 238.8599 | 362.6784 | 275.0389 | 409.1687 | 303.6176 | 432.6248 | 349.4134 | 258.6664 | 452.9912 | 326.5696 | 430.2513 |
| CUST_52203_PI429010886 | M96718 | 0.019424 | 18 | 0 | | | | | | 40.11037 | 23.02707 | 28.78598 | 17.89329 | 25.37501 | 18.66273 | 22.98207 | 15.47604 | 11.86309 | 26.34227 | 16.5818 | 22.09691 | 18.59683 | 19.59409 | 11.98424 | 20.76696 | 22.75029 | 21.39067 | 29.20057 | 20.13319 | 27.34621 | 36.30688 | 20.34203 | 15.29619 | 24.40888 | 15.29619 | 15.91075 |
| CUST_924_PI429010886 | N7840 | 0.019425 | 18 | 0 | | | | | | 5953.512 | 2929.717 | 5774.702 | 4208.899 | 3235.532 | 3569.653 | 4440.858 | 2125.97 | 1073.921 | 1538.611 | 2888.695 | 1892.721 | 1373.566 | 1386.924 | 1183.341 | 1593.287 | 3469.507 | 2137.444 | 5969.266 | 1054.702 | 2809.847 | 1672.079 | 3917.614 | 2865.864 | 2315.282 | 2890.576 | 1038.971 |
| CUST_32325_PI429010886 | M3807 | 0.01943 | 12 | 9 | 383861551 | PREDICTED: cytochrome c oxidase assembly proteir | | | 4.18E-24 | 11.00785 | 15.29798 | 17.2758 | 13.71506 | 12.82499 | 6.234005 | 8.552148 | 16.21736 | 9.757187 | 20.28753 | 20.52466 | 19.43095 | 13.072 | 16.15878 | 7.052829 | 15.4643 | 13.63622 | 14.88541 | 16.33068 | 17.72667 | 13.85598 | 21.33093 | 19.51255 | 12.25586 | 14.30234 | 14.50604 | 17.15146 |
| CUST_45458_PI429010886 | M69171 | 0.01943 | 12 | 3 | 443698035 | hypothetical protein CAPTEDRAFT_185094 [Capitell | | | 2.09E-27 | 20.36028 | 22.27045 | 26.88229 | 26.0691 | 24.05603 | 56.03237 | 27.52478 | 31.04302 | 17.20661 | 24.27705 | 18.40232 | 23.61475 | 20.27642 | 27.41751 | 28.11334 | 27.14357 | 43.02941 | 40.21556 | 30.32872 | 19.87525 | 26.46256 | 25.93469 | 18.32541 | 15.97144 | 32.80956 | 30.47678 | 28.11972 |
| CUST_12849_PI429010886 | N30152 | 0.01943 | 15 | 3 | 497853828 | transcriptional regulator [Pseudomonas sp. PAMC 2 | Metabolic process | Other | 7.11E-09 | 321.9594 | 242.1982 | 257.3277 | 433.1734 | 361.5394 | 446.5232 | 317.2087 | 239.146 | 251.404 | 259.0619 | 343.1257 | 225.4439 | 257.3277 | 282.0094 | 306.5697 | 263.2877 | 362.9082 | 460.1631 | 440.9199 | 245.8985 | 260.9414 | 294.7498 | 203.1221 | 257.1155 | 295.2655 | 385.7092 | 279.0433 |
| CUST_52047_PI429010886 | M96219 | 0.019435 | 21 | 3 | | | | | | 36.04391 | 26.72094 | 34.89593 | 42.17788 | 26.26013 | 43.12293 | 29.34792 | 22.65189 | 29.53078 | 22.45136 | 24.01772 | 26.38163 | 27.86411 | 23.40051 | 18.59144 | 27.43923 | 33.90631 | 39.67041 | 30.41187 | 23.64095 | 25.75936 | 31.09423 | 29.19814 | 25.89716 | 41.49369 | 27.2017 | 35.73798 |
| CUST_49117_PI429010886 | M84180 | 0.019455 | 15 | 0 | | | | | | 27.37091 | 19.55335 | 33.88263 | 20.35525 | 17.96131 | 17.88028 | 18.94868 | 13.295 | 10.63659 | 24.73831 | 23.14834 | 18.81645 | 19.45997 | 18.2155 | 24.01772 | 22.54108 | 29.55843 | 27.63541 | 16.37181 | 29.31792 | 25.40325 | 20.84115 | 19.63631 | 14.42559 | 27.89184 | 19.90393 | 24.03945 |
| CUST_53316_PI429010886 | M101239 | 0.01948 | 15 | 3 | | | | | | 218.7669 | 157.4172 | 229.7558 | 355.0658 | 357.4809 | 498.7637 | 311.1732 | 189.4251 | 152.8184 | 133.4278 | 354.0483 | 151.8121 | 161.1553 | 167.982 | 125.1102 | 154.2903 | 505.7885 | 276.5189 | 405.6393 | 152.5966 | 358.7125 | 162.0241 | 381.1947 | 474.5547 | 155.4298 | 279.5586 | 128.195 |
| CUST_32626_PI429010886 | M4626 | 0.019482 | 12 | 0 | | | | | | 140.6181 | 167.3841 | 131.2903 | 140.4751 | 124.1092 | 122.7034 | 103.8441 | 113.2721 | 122.2181 | 130.9642 | 128.3244 | 151.7333 | 140.3314 | 154.1768 | 179.0604 | 131.3569 | 118.905 | 140.974 | 135.5002 | 162.141 | 130.0411 | 177.2677 | 137.0769 | 130.9315 | 145.8124 | 136.3782 | 167.1087 |
| CUST_45452_PI429010886 | M69155 | 0.019482 | 12 | 0 | | | | | | 127.5818 | 138.5786 | 180.8997 | 127.5485 | 125.6172 | 117.2428 | 112.457 | 122.6882 | 106.4489 | 167.6463 | 112.8702 | 135.1811 | 135.6517 | 157.7661 | 78.72104 | 145.0185 | 121.3759 | 118.1621 | 117.7023 | 128.2673 | 109.2258 | 142.4549 | 133.8452 | 141.8752 | 151.7723 | 134.9903 | 145.7071 |
| CUST_31283_PI429010886 | M1136 | 0.019515 | 24 | 9 | 512875275 | PREDICTED: hepatic lectin-like [Xenopus (Silurana) t | | | 2.02E-07 | NA | 54.8287 | NA | 107.7388 | 22.88601 | 24.15101 | 27.80368 | 335.1161 | 230.1158 | NA | 782.2398 | 953.9498 | 195.3573 | 114.7256 | 146.3743 | NA | 70.14592 | NA | 8.425113 | 636.3168 | 89.60537 | NA | 103.2645 | 102.1576 | 4.132397 | 256.1518 | 12.10746 |
| CUST_42022_PI429010886 | M59181 | 0.019539 | 21 | 15 | 546685505 | hypothetical protein D910_12274 [Dendroctonus p | | | 5.12E-14 | 11.24123 | 12.03392 | 13.98352 | 7.052829 | 7.117252 | 10.37992 | 10.69036 | 5.753092 | 7.287651 | 9.035702 | 11.03187 | 8.968773 | 5.109231 | 11.78251 | 3.709951 | 24.48268 | 17.98995 | 12.51235 | 10.28696 | 8.75735 | 15.60693 | 12.06387 | 11.81158 | 8.241846 | 18.31489 | 5.940285 | 7.671645 |
| CUST_54232_PI429010886 | M105201 | 0.019547 | 15 | 9 | | | | | | 676.9355 | 832.3284 | 878.7545 | 621.5625 | 661.3119 | 664.1086 | 584.1415 | 579.5177 | 733.9745 | 865.6927 | 635.2846 | 971.1081 | 670.6469 | 761.5045 | 950.2628 | 884.6458 | 682.2742 | 678.5082 | 602.798 | 811.5226 | 680.741 | 824.5491 | 829.4016 | 631.8289 | 962.8744 | 831.7121 | 1010.922 |
| CUST_56636_PI429010886 | M114267 | 0.019549 | 18 | 3 | | | | | | 361.1547 | 174.356 | 330.882 | 80.07884 | 414.1007 | 286.9075 | 406.7395 | 288.0337 | 245.7365 | 940.5709 | 237.9578 | 296.4747 | 440.3254 | 382.0118 | 344.8512 | 359.6577 | 523.093 | 660.7381 | 277.7994 | 400.1052 | 304.5545 | 328.3197 | 190.0374 | 141.5103 | 200.337 | 291.1632 | 364.1216 |
| CUST_21119_PI429010886 | N44668 | 0.019549 | 18 | 3 | | | | | | 38.28633 | 14.94965 | 26.12486 | 29.22688 | 30.61606 | 37.36668 | 34.60204 | 22.10198 | 20.50772 | 8.10145 | 18.2356 | 13.62763 | 8.21709 | 38.05769 | 20.12612 | 87.29557 | 36.39221 | 26.95518 | 39.82861 | 13.15672 | 37.77885 | 53.44719 | 41.69502 | 27.35532 | 24.929 | 24.28972 | 16.12785 |
| CUST_8538_PI429010886 | N23189 | 0.019562 | 24 | 18 | 443688395 | hypothetical protein CAPTEDRAFT_162594 [Capitell | | | 3.59E-44 | 22.9227 | 17.37911 | 36.09309 | 16.25197 | 21.06491 | 20.68333 | 20.93443 | 19.32322 | 16.20611 | 25.89716 | 16.87055 | 33.70683 | 20.69556 | 22.30272 | 27.36801 | 27.16781 | 17.9819 | 36.55801 | 23.97181 | 43.94258 | 30.21631 | 34.36888 | 23.09604 | 21.25854 | 25.48959 | 36.93404 | 25.35696 |
| CUST_20645_PI429010886 | N43808 | 0.019568 | 15 | 12 | | | | | | 38.20162 | 35.36774 | 30.14577 | 24.12658 | 26.84327 | 16.39504 | 18.39819 | 25.80322 | 20.6935 | 36.18556 | 22.27045 | 36.28805 | 30.66153 | 33.68931 | 33.0759 | 39.81145 | 44.87442 | 31.60035 | 18.05089 | 28.00512 | 31.1168 | 35.97533 | 29.0776 | 22.13675 | 31.30264 | 36.99652 | 32.55416 |
| CUST_57114_PI429010886 | E1122 | 0.01957 | 12 | 0 | 383857078 | PREDICTED: LOW QUALITY PROTEIN: nucleosome-re | | | 9.14E-51 | 285.7004 | 287.0591 | 411.751 | 251.9218 | 261.922 | 250.0775 | 193.714 | 268.4354 | 322.0841 | 356.3531 | 258.0034 | 372.5738 | 236.9951 | 390.973 | 391.346 | 330.1179 | 206.469 | 311.5276 | 263.7458 | 285.0949 | 339.1595 | 386.5802 | 345.639 | 238.2716 | 374.9864 | 388.2685 | 411.8683 |
| CUST_34585_PI429010886 | M10080 | 0.01957 | 12 | 3 | 4433540 | 5-aminolevulinate synthase [Limulus polyphemus] | Metabolic process | Other | 1.3E-129 | 93.9354 | 84.40117 | 120.5371 | 130.9315 | 150.7034 | 378.265 | 230.5922 | 136.0755 | 128.8389 | 44.75892 | 202.005 | 119.6528 | 118.6098 | 83.33435 | 114.4136 | 107.6537 | 329.9108 | 230.8271 | 270.4883 | 107.884 | 108.6889 | 88.05483 | 104.8909 | 140.9929 | 61.00433 | 140.0584 | 95.13953 |
| CUST_35620_PI429010886 | M102512 | 0.019572 | 15 | 3 | 443716906 | hypothetical protein CAPTEDRAFT_3285 [Capitella t | Transport | Protein transport | 3.34E-35 | 5308.348 | 8006.158 | 3790.041 | 1181.018 | 579.5177 | 420.2091 | 5627.009 | 1975.879 | 1757.983 | 3144.897 | 2710.041 | 4467.857 | 1979.892 | 1789.616 | 8165.772 | 1532.545 | 857.8725 | 1351.148 | 5152.319 | 2854.292 | 2306.592 | 594.2743 | 1915.9 | 3092.843 | 643.5529 | 3785.297 | 6802.929 |
| CUST_36340_PI429010886 | M15649 | 0.01957 | 12 | 0 | | | | | | 17.78423 | 19.42864 | 23.4104 | 16.21134 | 19.34576 | 12.84883 | 14.77313 | 15.12966 | 15.18342 | 18.65856 | 17.04303 | 24.0618 | 17.65649 | 17.38288 | 22.29887 | 14.35732 | 12.40735 | 12.0863 | 20.16106 | 13.34528 | 18.36555 | 21.10951 | 17.88028 | 12.96349 | 17.08982 | 18.73831 | 34.91364 |
| CUST_42360_PI429010886 | M60095 | 0.01957 | 12 | 3 | 260811974 | hypothetical protein BRAFLDRAFT_67761 [Branchio | | | 3.95E-17 | 135.0089 | 151.2216 | 116.3867 | 158.8004 | 204.8478 | 197.4721 | 145.8455 | 133.0951 | 133.1553 | 143.9014 | 143.609 | 95.02448 | 118.0091 | 124.672 | 115.4652 | 162.719 | 233.0195 | 179.1322 | 188.3253 | 68.40354 | 115.3125 | 122.5827 | 99.57087 | 161.9804 | 74.3942 | 141.7485 | 82.84904 |
| CUST_43462_PI429010886 | M63124 | 0.01957 | 12 | 3 | 157130852 | myosin i [Aedes aegypti] >gi\|108871741\|gb\|EAT35 | | | 2.58E-58 | 30.3942 | 29.63867 | 23.03149 | 41.49369 | 33.95872 | 51.44191 | 54.63011 | 36.49183 | 29.82743 | 25.28525 | 49.79836 | 23.76325 | 29.42101 | 28.17911 | 29.6316 | 32.29627 | 55.66127 | 48.069 | 86.75819 | 36.17443 | 25.87167 | 21.88587 | 32.97611 | 43.00099 | 9.949298 | 35.88829 | 22.00276 |
| CUST_2290_PI429010886 | N12033 | 0.01957 | 12 | 0 | | | | | | 66.52424 | 73.34493 | 68.53497 | 44.3588 | 56.8218 | 35.60906 | 54.01314 | 39.76167 | 57.34886 | 68.22048 | 51.2779 | 79.64311 | 60.09613 | 79.5827 | 74.04651 | 62.67301 | 49.92232 | 53.27432 | 59.12378 | 74.40209 | 64.38672 | 82.77162 | 67.74201 | 45.17236 | 83.42195 | 49.96135 | 83.67885 |
| CUST_1741_PI429010886 | N10580 | 0.019582 | 24 | 3 | 225714082 | Troponin T [Lepeophtheirus salmonis] | | | 3.7E-39 | 267.1348 | 365.9198 | 258.545 | 356.2218 | 336.5091 | 217.7483 | 246.4698 | 285.421 | 266.9446 | 281.4899 | 322.7291 | 254.0209 | 182.318 | 221.165 | 187.954 | 198.0625 | 228.0795 | 218.9512 | 245.6501 | 260.4155 | 317.2703 | 308.5953 | 289.423 | 245.408 | 279.4425 | 272.2987 | 243.1192 |
| CUST_42181_PI429010886 | M59619 | 0.019598 | 24 | 0 | 156381428 | predicted protein [Nematostella vectensis] >gi\|156 | | | 1.41E-08 | 31.9127 | 37.66305 | 40.47688 | 28.42343 | 23.15281 | 27.03336 | 23.6747 | 20.55898 | 20.02214 | 41.02447 | 27.75759 | 24.35938 | 15.96673 | 26.54031 | 21.42649 | 31.22768 | 34.83322 | 46.48282 | 29.99067 | 28.59095 | 31.24843 | 35.0422 | 28.00132 | 23.47569 | 38.60692 | 33.96314 | 47.26147 |
| CUST_43453_PI429010886 | M63101 | 0.019598 | 24 | 0 | | | | | | 87.42512 | 64.96528 | 42.85091 | 41.63072 | 37.68617 | 52.43827 | 44.08227 | 49.59839 | 32.78199 | 30.74148 | 30.84259 | 40.92745 | 37.0175 | 31.73044 | 35.42916 | 41.94819 | 36.76396 | 52.2983 | 21.7968 | 23.19911 | 28.64539 | 43.88802 | 29.95513 | 41.69502 | 54.72113 | 47.43001 | 39.61818 |
| CUST_18316_PI429010886 | N39565 | 0.019598 | 24 | 0 | | | | | | 107.7787 | 94.81013 | 103.5071 | 108.3734 | 89.36758 | 84.92511 | 70.88181 | 69.751 | 92.90826 | 94.58929 | 101.6485 | 78.14128 | 68.78674 | 68.74728 | 83.16839 | 123.2423 | 85.46155 | 98.45237 | 87.5621 | 102.5106 | 85.79457 | 131.9942 | 91.36505 | 76.82089 | 113.8196 | 86.6967 | 118.6098 |
| CUST_1541_PI429010886 | N9996 | 0.019618 | 24 | 0 | | | | | | 76.84434 | 92.34903 | 97.29292 | 71.46757 | 76.47488 | 69.94604 | 72.76448 | 70.34764 | 67.75526 | 74.90972 | 66.81883 | 88.65038 | 61.90324 | 89.57185 | 62.48649 | 82.51706 | 67.59725 | 70.73325 | 87.93229 | 85.78724 | 105.1159 | 85.04454 | 78.21989 | 78.84468 | 101.7859 | 83.99654 | 110.0751 |
| CUST_28236_PI429010886 | N57566 | 0.019636 | 21 | 0 | | | | | | 57.46153 | 32.17021 | 43.08722 | 46.43129 | 33.24397 | 58.09597 | 25.14408 | 31.59448 | 28.07255 | 46.81599 | 21.5046 | 12.53584 | 28.71325 | 41.68626 | 31.17167 | 40.67441 | 45.3248 | 41.06999 | 35.60511 | 33.97728 | 31.77739 | 45.4417 | 43.66151 | 51.21719 | 53.61735 | 35.32948 | 40.50125 |
| CUST_22420_PI429010886 | N46995 | 0.019654 | 21 | 15 | | | | | | 58.16774 | 41.81668 | 45.86806 | 16.58905 | 38.62767 | 31.14323 | 60.21415 | 16.5596 | 31.23112 | 55.29319 | 42.38554 | 46.31285 | 28.32194 | 48.71731 | 27.39792 | 67.64754 | 47.56097 | 49.57895 | 15.91213 | 43.41027 | 30.47678 | 41.71124 | 40.08304 | 42.58108 | 20.81295 | 21.67714 | 27.0727 |
| CUST_5492_PI429010886 | N18773 | 0.019659 | 18 | 3 | | | Metabolic process | Nucleobase-containing compound metabolic process | | 340.8239 | 396.481 | 316.971 | 229.245 | 350.314 | 369.4961 | 248.5464 | 236.449 | 198.5726 | 346.4727 | 256.9966 | 343.6779 | 249.5597 | 275.7295 | 350.8381 | 356.9822 | 233.9751 | 379.1503 | 254.9115 | 368.0062 | 253.7351 | 399.641 | 270.3119 | 281.8261 | 246.6234 | 233.5227 | 197.1531 |
| CUST_13825_PI429010886 | N31840 | 0.019698 | 15 | 9 | | | | | | 44.05675 | 41.8722 | 46.47908 | 48.10487 | 39.88324 | 37.44766 | 32.01382 | 42.84657 | 61.99224 | 83.48682 | 45.50116 | 79.81539 | 46.58823 | 82.75397 | 68.31995 | 61.30366 | 48.85338 | 64.01028 | 51.78377 | 50.24503 | 62.97836 | 70.46616 | 68.18607 | 51.14292 | 72.1095 | 70.56572 | 72.92262 |
| CUST_5642_PI429010886 | N18982 | 0.019708 | 12 | 0 | | | | | | 446.0696 | 490.7702 | 502.0515 | 526.5682 | 470.2955 | 387.9318 | 366.089 | 373.4486 | 389.871 | 467.8704 | 376.3756 | 449.6075 | 396.481 | 529.2101 | 430.7898 | 417.6101 | 344.803 | 438.9413 | 432.3349 | 346.5286 | 414.3494 | 492.7259 | 384.9318 | 363.9558 | 681.8773 | 420.4304 | 494.5663 |

| Probe ID | ID | p-value | Period | Phase | EBI ID | Annotation | GO category | Go term | E-value | 0 | 0 | 0 | 3 | 3 | 3 | 6 | 6 | 6 | 9 | 9 | 9 | 12 | 12 | 12 | 15 | 15 | 15 | 18 | 18 | 18 | 21 | 21 | 21 | 24 | 24 | 24 |
|---|---|---|---|---|---|---|---|---|---|---|---|---|---|---|---|---|---|---|---|---|---|---|---|---|---|---|---|---|---|---|---|---|---|---|---|---|
| CUST_9235_PI429010886 | N24278 | 0.019708 | 12 | 0 | | | | | | 27.84094 | 92.07382 | 58.4329 | 31.8356 | 17.49601 | 14.11461 | 16.20777 | 12.82499 | 5.767846 | 48.29446 | 6.541514 | 14.72118 | 26.3141 | 32.52687 | 21.57953 | 7.986139 | 31.57619 | 18.46372 | 17.21816 | 31.8423 | 11.94615 | 13.44962 | 27.39792 | 16.29756 | 41.29432 | 15.4643 | 33.45717 |
| CUST_34249_PI429010886 | M9164 | 0.019727 | 21 | 9 | | | | | | 15.32584 | 17.42392 | 11.88414 | 22.31012 | 26.04672 | 14.50945 | 19.95854 | 66.18886 | 42.78573 | 24.17061 | 48.3882 | 61.35668 | 54.34709 | 32.96039 | 30.02913 | 13.86466 | 13.46781 | 8.668476 | 17.41182 | 196.359 | 60.95703 | 14.15692 | 10.48086 | NA | 5.924102 | 23.69143 | 36.26174 |
| CUST_56800_PI429010886 | M114674 | 0.019747 | 24 | 18 | | | | | | 250.1572 | 211.1514 | 264.7596 | 206.469 | 227.4855 | 183.021 | 203.1014 | 204.946 | 228.7951 | 289.4881 | 230.9236 | 231.648 | 221.165 | 300.0118 | 289.131 | 298.2497 | 242.3388 | 212.5688 | 262.9561 | 341.3294 | 271.6624 | 268.9268 | 260.5389 | 207.2788 | 345.3079 | 206.0055 | 293.7629 |
| CUST_33268_PI429010886 | M6449 | 0.019787 | 12 | 3 | | | | | | 254.2335 | 195.5884 | 290.9496 | 934.5759 | 1048.248 | 1881.402 | 916.176 | 730.7862 | 336.682 | 345.1018 | 926.1166 | 127.4128 | 349.1356 | 331.9382 | 299.389 | 162.9465 | 1879.633 | 1027.997 | 977.0262 | 149.7905 | 872.488 | 98.00231 | 808.9549 | 913.4609 | 174.2722 | 583.7272 | 188.3123 |
| CUST_19641_PI429010886 | N41926 | 0.019791 | 18 | 12 | | | | | | 18.70952 | 19.58019 | 22.41298 | 17.43453 | 20.70807 | 17.34415 | 19.84931 | 29.10818 | 26.26013 | 30.15647 | 20.90912 | 34.96061 | 29.27728 | 27.88984 | 31.78356 | 30.17589 | 21.02638 | 23.45219 | 23.4501 | 52.83011 | 26.19371 | 37.56417 | 8.709062 | 15.85862 | 55.11957 | 16.74268 | 26.79307 |
| CUST_24468_PI429010886 | N50678 | 0.019806 | 21 | 3 | 390347449 | PREDICTED: zinc finger protein 84-like [Strongylocer | | | 7.68E-15 | 547.2331 | 770.419 | 568.5491 | 757.6325 | 778.2204 | 490.7702 | 579.5177 | 469.9116 | 622.7518 | 568.6876 | 330.2472 | 832.9715 | 333.2881 | 645.7634 | 226.242 | 483.2456 | 179.9161 | 282.8059 | 287.3115 | 441.2532 | 1091.604 | 339.9729 | 1216.535 | 333.2348 | 952.9126 | 976.3736 | 549.794 |
| CUST_39982_PI429010886 | M47801 | 0.01983 | 21 | 15 | | | | | | 16.16875 | 32.01858 | 31.83796 | 34.84252 | 26.27126 | 37.34183 | 64.38672 | 33.6052 | 30.66462 | 22.72441 | 20.90506 | 11.78106 | 41.78277 | 166.9686 | 103.2289 | 104.4433 | 95.44953 | 95.51753 | 64.15379 | 378.9452 | 71.09841 | 30.16973 | 28.31129 | 54.50124 | 60.99798 | 12.03276 | 53.53068 |
| CUST_17878_PI429010886 | N38756 | 0.019837 | 24 | 0 | 317165637 | Mesorhizobium ciceri biovar biserrulae WSM1271, ( | Metabolic process | Cellular amino acid metabolic process | 3E-92 | 30.32568 | 21.93409 | 29.19814 | 10.25265 | 19.92665 | 18.98793 | 9.253041 | 8.931253 | 16.01505 | 18.7912 | 14.12148 | 27.80573 | 6.891974 | 11.63473 | 6.503335 | 11.92991 | 30.52136 | 12.16205 | 17.94343 | 20.58016 | 17.8888 | 16.82346 | 7.575226 | 16.06632 | 26.57271 | 13.09346 | 18.71576 |
| CUST_47429_PI429010886 | M76077 | 0.019846 | 15 | 12 | 241751603 | G-protein coupled receptor, putative [Ixodes scapul | Signal transduction | Other | 9.67E-10 | 221.9188 | 216.9753 | 284.8452 | 174.8053 | 175.1291 | 198.308 | 199.0699 | 183.786 | 200.5357 | 280.5129 | 211.9082 | 251.1828 | 214.6269 | 287.6725 | 272.1926 | 291.0552 | 222.2031 | 215.2013 | 220.3236 | 231.4586 | 186.3412 | 280.2656 | 225.8046 | 180.7626 | 334.6678 | 227.2732 | 289.2197 |
| CUST_4438_PI429010886 | N16756 | 0.019846 | 18 | 3 | | | | | | 459.6219 | 103.1342 | 347.1114 | 473.6842 | 292.5508 | 690.2519 | 451.7787 | 219.8489 | 141.5625 | 111.2973 | 246.5478 | 86.76715 | 117.7722 | 109.2818 | 156.3082 | 98.57526 | 317.5249 | 255.4957 | 346.2493 | 67.89063 | 462.5707 | 137.4942 | 356.5042 | 544.6485 | 81.49546 | 220.0411 | 59.63236 |
| CUST_11290_PI429010886 | N27570 | 0.019853 | 21 | 15 | | | | | | 79.08585 | 72.21046 | 74.01735 | 55.57175 | 68.40354 | 50.8348 | 49.11313 | 58.59263 | 35.34739 | 71.09226 | 136.3625 | 70.89744 | 44.40267 | 51.94426 | 35.73798 | 95.81155 | 100.2082 | 69.37437 | 87.65825 | 68.92299 | 76.49245 | 66.89269 | 58.15995 | 64.08758 | 142.2748 | 64.78587 | 58.28332 |
| CUST_48067_PI429010886 | M78753 | 0.019872 | 18 | 3 | | | | | | 46.85704 | 59.34275 | 38.85028 | 74.51983 | 47.57087 | 73.53135 | 53.65679 | 48.06558 | 45.85232 | 45.60489 | 40.10273 | 50.01795 | 39.47942 | 43.28157 | 40.33441 | 53.36025 | 48.08 | 36.47059 | 40.85343 | 31.19824 | 48.33175 | 44.2637 | 62.33105 | 62.45053 | 56.17296 | 70.53195 | 38.153 |
| CUST_49016_PI429010886 | M83639 | 0.01991 | 18 | 0 | | | | | | 13.23625 | NA | 20.55089 | 9.121241 | 11.92046 | 23.17075 | 11.21303 | 11.9615 | 5.849038 | 5.839754 | 17.21299 | NA | 7.594313 | 8.156831 | 3.964554 | 4.693553 | 20.61255 | 14.78172 | 18.48442 | NA | 17.48685 | 9.34052 | 13.11271 | 19.13431 | 5.530952 | 9.434112 | NA |
| CUST_53703_PI429010886 | M102845 | 0.019921 | 15 | 3 | | | | | | 86.66049 | 70.92151 | 70.31729 | 82.91863 | 97.23707 | 128.5685 | 84.96672 | 78.70239 | 68.89697 | 72.41676 | 93.77397 | 60.99125 | 80.68886 | 65.17572 | 62.83869 | 77.53281 | 135.4807 | 74.3404 | 95.19444 | 106.4489 | 85.47277 | 88.14739 | 75.3452 | 89.34718 | 88.71715 | 88.96346 | 78.0365 |
| CUST_40589_PI429010886 | M55550 | 0.019921 | 15 | 12 | | | | | | 126.8944 | 100.0527 | 117.3985 | 89.15191 | 80.33892 | 66.63641 | 208.8093 | 198.0625 | 203.7326 | 142.2559 | 74.01735 | 94.30105 | 162.6527 | 256.6122 | 147.1104 | 86.15616 | 120.4142 | 83.65751 | 116.4203 | 55.58103 | 88.42631 | 155.4485 | 145.578 | 102.7524 | 257.0697 | 141.4486 | 131.1671 |
| CUST_52828_PI429010886 | M99148 | 0.019935 | 12 | 0 | 336944 | Euphausia superba 16S ribosomal RNA gene, partial | | | 2.5E-71 | 439.1396 | 494.9533 | 520.8337 | 425.9278 | 423.8598 | 472.997 | 384.7174 | 363.8764 | 386.5802 | 502.7361 | 419.1452 | 524.0613 | 409.9605 | 555.136 | 523.7505 | 609.0182 | 405.0461 | 504.3528 | 383.7894 | 477.1155 | 421.6001 | 577.5152 | 501.702 | 436.4909 | 548.6331 | 481.413 | 574.1082 |
| CUST_54288_PI429010886 | M105455 | 0.019942 | 15 | 0 | | | | | | 139.9078 | 137.9192 | 132.2443 | 104.4333 | 127.3882 | 116.7168 | 99.47188 | 89.64325 | 119.228 | 131.771 | 114.0301 | 120.3899 | 108.3285 | 118.275 | 134.162 | 138.9538 | 120.2195 | 117.9302 | 130.6959 | 133.6452 | 116.3059 | 110.652 | 128.8206 | 102.5545 | 140.8613 | 109.3295 | 102.8545 |
| CUST_36084_PI429010886 | M14583 | 0.019957 | 18 | 3 | | | | | | 21.14283 | 12.19407 | NA | 18.07527 | 15.20148 | 28.23857 | 11.76966 | 8.683756 | 7.382145 | 6.136953 | 13.10821 | 5.548767 | 14.90147 | NA | 6.117837 | 10.44442 | 20.31071 | 20.79184 | 17.91994 | 9.243743 | 21.37183 | 9.651747 | 25.8352 | 15.88012 | 12.74222 | 8.199563 | 13.97554 |
| CUST_14508_PI429010886 | N32997 | 0.019972 | 15 | 9 | 194246065 | putative legumain-like protease precursor [Dermac | | | 6.91E-09 | NA | 8.442875 | 7.885901 | 12.9382 | 36.36062 | 9.462739 | 20.144 | 55.49391 | 7.654255 | 13.16535 | 63.80913 | 48.62311 | 72.17688 | 10.09344 | 32.68104 | NA | 11.88893 | 4.75175 | 8.366386 | 106.9212 | 17.36312 | NA | 22.62556 | 18.40425 | NA | 72.65998 | 41.70879 |
| CUST_13582_PI429010886 | N31417 | 0.019997 | 24 | 21 | | | | | | 150.286 | 196.8017 | 197.44 | 125.9453 | 125.2805 | 90.93451 | 85.926 | 107.533 | 139.2667 | 237.4703 | 122.1353 | 204.7153 | 203.006 | 220.2228 | 195.3201 | 171.8979 | 145.0486 | 197.6731 | 180.1444 | 201.008 | 247.5396 | 215.9006 | 222.0782 | 200.0082 | 224.5779 | 134.9479 | 253.4186 |
| CUST_47840_PI429010886 | M77802 | 0.020005 | 12 | 0 | | | | | | 426.5023 | 418.8645 | 512.2788 | 215.5318 | 231.0331 | 308.4843 | 418.7921 | 305.9676 | 332.4166 | 326.6284 | 380.124 | 397.9322 | 381.8763 | 551.1 | 304.942 | 272.9711 | 320.3749 | 376.7168 | 240.8224 | 416.2434 | 376.9333 | 453.5801 | 446.8256 | 303.6176 | 387.589 | 378.8717 | 379.6269 |
| CUST_52970_PI429010886 | M99628 | 0.020005 | 12 | 0 | | | | | | 288.0844 | 341.5855 | 342.4231 | 130.1875 | 251.9218 | 221.852 | 220.485 | 258.1237 | 296.6828 | 291.1029 | 269.7173 | 359.6577 | 188.56 | 378.265 | 315.9066 | 189.684 | 251.745 | 268.0379 | 253.1078 | 336.7538 | 301.1632 | 330.6772 | 292.6509 | 295.8552 | 371.3509 | 220.9066 | 399.475 |
| CUST_13466_PI429010886 | N31238 | 0.020005 | 12 | 9 | | | | | | 128.2056 | 200.6114 | 141.7029 | 151.7898 | 129.0306 | 114.9376 | 156.0949 | 150.7548 | 132.0609 | 214.7331 | 138.6787 | 168.58 | 125.4876 | 134.02 | 114.165 | 250.6384 | 129.0466 | 153.0541 | 163.791 | 126.2993 | 142.8727 | 176.4102 | 190.4443 | 168.154 | 141.2301 | 89.73481 | 87.36382 |
| CUST_31125_PI429010886 | M756 | 0.020011 | 21 | 15 | 291244918 | PREDICTED: hypothetical protein [Saccoglossus kow | | | 1.41E-26 | 6.883094 | 5.912581 | 10.13796 | 9.308024 | 7.50844 | 5.617002 | 6.739431 | 4.398975 | 17.15304 | 8.585014 | 6.257639 | 8.167031 | 7.183846 | NA | 8.355196 | 11.58371 | 10.43208 | 13.62763 | 10.06452 | 8.695689 | 7.591069 | 15.55047 | 8.75735 | 3.349167 | 5.030883 | 6.646603 | 5.185088 |
| CUST_9226_PI429010886 | N24267 | 0.020017 | 24 | 18 | | | | | | 331.5388 | 592.9907 | 346.6366 | 350.9859 | 353.25 | 220.6157 | 203.3538 | 187.5869 | 285.7004 | 488.5796 | 364.2987 | 627.0644 | 356.2894 | 318.5558 | 359.6577 | 667.377 | 222.1142 | 372.1299 | 383.9376 | 510.1908 | 427.9524 | 478.2055 | 378.6086 | 233.5227 | 375.2702 | 383.017 | 426.8105 |
| CUST_48653_PI429010886 | M81489 | 0.020034 | 21 | 18 | | | | | | 269.2795 | 321.753 | 178.6841 | 256.4811 | 193.6314 | 207.885 | 168.6416 | 190.7772 | 177.8416 | 213.1848 | 185.1977 | 198.6919 | 206.9547 | 227.312 | 236.856 | 338.4186 | 199.0699 | 257.0697 | 225.5499 | 264.5866 | 208.4785 | 338.9665 | 174.1985 | 223.419 | 301.4214 | 197.3524 | 186.0881 |
| CUST_42776_PI429010886 | M61225 | 0.020035 | 21 | 15 | | | | | | 37.65902 | 26.44706 | 31.68444 | 40.72449 | 21.17508 | 36.21438 | 19.43095 | 24.24945 | 27.69749 | 33.42509 | 30.3611 | 18.10904 | 28.33335 | 28.82389 | 33.85642 | 47.20692 | 34.48125 | 46.95662 | 50.16596 | 30.12812 | 37.93755 | 32.15505 | 31.98439 | 30.62818 | 30.74351 | 48.06558 | 18.30638 |
| CUST_3714_PI429010886 | N15115 | 0.020037 | 24 | 0 | | | | | | 339.6585 | 301.3697 | 9.246707 | 160.2968 | 91.28115 | 191.5917 | 86.80804 | 338.3238 | 73.15736 | 136.9448 | 72.17688 | 45.22377 | 8.695689 | 32.46401 | 82.33655 | 354.0483 | 7.360126 | 3.431994 | 228.3459 | 76.21064 | 203.1221 | 5.39571 | 26.05696 | 415.7405 | 214.0659 | 462.4656 | 275.8944 |
| CUST_14901_PI429010886 | N33663 | 0.020043 | 24 | 15 | | | | | | 175.8023 | 183.4917 | 140.4751 | 160.531 | 133.0951 | 148.65 | 105.4619 | 109.5287 | 131.5284 | 184.2307 | 150.2666 | 213.4377 | 208.8792 | 181.6229 | 169.275 | 293.8285 | 128.8206 | 206.1701 | 170.8864 | 167.5472 | 163.1486 | 223.419 | 152.9711 | 178.3086 | 169.6296 | 165.716 | 167.6024 |
| CUST_42990_PI429010886 | M61800 | 0.020045 | 21 | 6 | 72094941 | PREDICTED: protein SpAN-like [Strongylocentrotus | Protein metabolism | Proteolysis | 8.66E-34 | 35.36241 | 29.49276 | 28.51882 | 65.34609 | 13.98077 | 51.63118 | 77.0714 | 96.76761 | 54.0716 | 20.70543 | 56.37263 | 47.84739 | 68.97204 | 25.7467 | 48.09045 | 29.31162 | 25.38172 | 13.2768 | 15.64945 | 91.16786 | 26.44212 | 29.43648 | 38.24021 | 9.845679 | 38.67688 | 40.52511 | 14.79848 |
| CUST_19381_PI429010886 | N41482 | 0.020055 | 15 | 9 | | | | | | 86.99215 | 64.80696 | 87.09496 | 58.15995 | 73.87582 | 61.86952 | 65.81652 | 69.18246 | 90.24634 | 77.34848 | 60.19028 | 75.02406 | 71.2117 | 79.9054 | 76.13932 | 75.37057 | 74.52928 | 54.95061 | 66.06376 | 53.8203 | 64.31677 | 84.49247 | 72.16939 | 70.02583 | 88.91586 | 82.06433 | 95.25723 |
| CUST_1168_PI429010886 | N8722 | 0.020057 | 12 | 0 | | | | | | 255.0746 | 296.266 | 311.8796 | 241.2255 | 229.7558 | 226.0846 | 254.6455 | 276.3669 | 283.3333 | 375.5434 | 269.2795 | 342.6916 | 301.5634 | 443.1374 | 370.2985 | 359.4272 | 232.3526 | 284.8452 | 285.2001 | 331.7667 | 340.4766 | 319.007 | 343.3378 | 271.8242 | 389.4334 | 330.0503 | 377.8098 |
| CUST_6813_PI429010886 | N20634 | 0.020078 | 18 | 12 | 380026333 | PREDICTED: uncharacterized protein LOC10086635( | | | 5.44E-35 | 105.0313 | 135.2886 | 167.3467 | 71.70446 | 97.11051 | 52.07596 | 113.5868 | 155.0502 | 132.7287 | 253.025 | 121.6208 | 111.1643 | 168.0101 | 214.3921 | 125.6844 | 147.911 | 109.2818 | 89.60537 | 123.056 | 172.8115 | 122.8849 | 138.6617 | 109.2686 | 88.22213 | 157.4172 | 113.9832 | 194.9182 |
| CUST_48970_PI429010886 | M83247 | 0.020085 | 18 | 9 | 340384903 | PREDICTED: hypothetical protein LOC100634292 [A | | | 8.51E-22 | 641.3583 | 644.4395 | 790.5889 | 573.0502 | 596.4585 | 575.6787 | 530.2702 | 698.0293 | 634.2891 | 885.0858 | 704.5835 | 879.0511 | 650.7381 | 827.5431 | 803.3312 | 920.0256 | 650.2354 | 660.3398 | 665.6609 | 858.518 | 628.3128 | 801.5312 | 676.3702 | 494.8206 | 980.4603 | 635.437 | 897.6332 |
| CUST_13278_PI429010886 | N30901 | 0.020094 | 24 | 18 | | | | | | 122.4191 | 163.162 | 113.475 | 115.4907 | 102.8432 | 108.7377 | 89.72544 | 90.37187 | 85.40569 | 165.7579 | 118.905 | 147.7053 | 109.6904 | 130.1208 | 174.4075 | 188.0537 | 109.7061 | 131.7223 | 130.7638 | 172.9097 | 140.6597 | 144.7304 | 132.8409 | 146.8304 | 180.1444 | 149.2083 | 164.8518 |
| CUST_32363_PI429010886 | M3936 | 0.020096 | 21 | 18 | 321479207 | hypothetical protein DAPPUDRAFT_299967 [Daphn | Nucleic acid metabolism | RNA metabolic process | 1.3E-133 | 69.29923 | 89.41173 | 71.37464 | 41.909 | 66.4223 | 50.546 | 43.88387 | 50.91495 | 48.1847 | 89.80969 | 67.9344 | 79.39521 | 66.50022 | 61.69829 | 69.43885 | 103.5194 | 48.51868 | 98.68398 | 68.15284 | 106.1871 | 72.31183 | 103.8205 | 63.32755 | 43.25052 | 66.24577 | 71.20471 | 64.56424 |
| CUST_10943_PI429010886 | N27029 | 0.020107 | 24 | 12 | | | | | | 68.56896 | 70.59503 | 63.88437 | 56.40861 | 51.81515 | 39.67041 | 55.45229 | 43.7836 | 67.56871 | 82.41691 | 61.34598 | 79.85552 | 71.83981 | 80.25744 | 69.17707 | 84.51009 | 42.00009 | 74.14418 | 75.2717 | 72.89846 | 49.25162 | 72.10026 | 67.66412 | 29.06247 | 70.61832 | 54.67468 | 63.65837 |
| CUST_2275_PI429010886 | N12009 | 0.020111 | 24 | 6 | | | | | | NA | 218.3262 | 22.78102 | 381.8035 | 77.94557 | 353.5438 | 683.7716 | 31.46178 | 592.8448 | 6.326533 | 60.75286 | NA | NA | NA | 19.50301 | 8.00826 | 18.28186 | 29.53645 | 172.054 | 3.667035 | 13.36104 | 11.50923 | NA | 60.57138 | 40.84069 | NA | 17.67943 |
| CUST_34194_PI429010886 | M9018 | 0.020124 | 24 | 0 | 225714082 | Troponin T [Lepeophtheirus salmonis] | | | 2.9E-09 | 17.24279 | 17.69248 | 17.09735 | 14.98356 | NA | 8.383502 | NA | 23.31281 | 12.42474 | 10.55729 | 11.02819 | 12.08102 | 11.70566 | 14.47943 | 9.192825 | 6.791617 | 5.330779 | 14.30893 | 7.761493 | 40.00557 | 17.29417 | 12.53746 | 9.341797 | NA | 14.70984 | 14.70578 | 17.70509 |
| CUST_35153_PI429010886 | M11735 | 0.020127 | 18 | 3 | | | Nucleic acid metabolism | RNA metabolic process | | 41.44605 | 40.67771 | 32.74461 | 88.58239 | 42.82196 | 71.03904 | 32.69544 | 37.72421 | 22.64182 | 32.8525 | 31.08775 | 37.11196 | 26.42807 | 23.86522 | 34.53375 | 20.80086 | 26.81554 | 51.42712 | 23.47569 | 65.44799 | 29.86288 | 63.40813 | 28.54216 | 49.11313 | 38.87395 | 65.32582 | 26.61223 |
| CUST_56100_PI429010886 | M112765 | 0.020148 | 21 | 3 | | | | | | 115.0699 | 177.758 | 130.2006 | 147.3848 | 146.3399 | 156.9601 | 112.2867 | 116.8917 | 111.685 | 124.8269 | 130.8991 | 146.5292 | 130.4838 | 138.1676 | 114.6944 | 144.3428 | 110.605 | 131.863 | 117.1213 | 147.1881 | 144.1656 | 139.1034 | 144.3428 | 151.9486 | 172.054 | 156.8398 | 150.2035 |
| CUST_43214_PI429010886 | M62421 | 0.020165 | 18 | 12 | | | | | | 49.00501 | 44.93417 | 49.68201 | 29.87357 | 47.3396 | 25.14839 | 98.81579 | 48.03764 | 42.83336 | 60.89775 | 34.89251 | 43.52847 | 65.71273 | 61.57414 | 61.03938 | 102.5774 | 11.85349 | 75.89665 | 52.06984 | 41.14845 | 41.80456 | 40.3065 | 40.05517 | NA | 27.97387 | 43.11192 | 62.33105 |
| CUST_35521_PI429010886 | M12775 | 0.02018 | 21 | 3 | 478256032 | hypothetical protein YQE_07197, partial [Dendroctc | | | 1.47E-45 | 75.58303 | 109.399 | 78.95217 | 119.7645 | 88.06388 | 125.9322 | 72.66838 | 85.2824 | 79.62582 | 100.5554 | 54.8287 | 55.26178 | 70.02583 | 93.73888 | 52.04647 | 94.88698 | 71.5379 | 105.6711 | 78.90095 | 62.61352 | 85.2824 | 83.98715 | 185.1977 | 88.96346 | 83.40091 | 137.9192 | 89.22919 |
| CUST_57285_PI429010886 | E4142 | 0.020185 | 15 | 9 | | | | | | 7985.61 | 1777.044 | 15133.65 | 1303.313 | 1732.866 | 2096.527 | 12273.46 | 20443.66 | 18337.68 | 28226.57 | 8015.915 | 8574.409 | 13806.99 | 22574.76 | 13400.98 | 4128.792 | 14054.76 | 3152.492 | 10348.94 | 10435.28 | 5911.575 | 18524.86 | 17966.78 | 4992.636 | 29896.62 | 8391.959 | 24341.67 |
| CUST_24552_PI429010886 | N50828 | 0.020185 | 15 | 0 | | | | | | 81.25969 | 79.74735 | 56.36185 | 73.57494 | 55.42181 | 64.83329 | 58.00667 | 35.51007 | 46.39268 | 63.39049 | 71.56769 | 36.00037 | 53.34613 | 51.00626 | 66.36423 | 99.20203 | 57.15078 | 80.61162 | 55.38718 | 57.24358 | 68.50419 | 64.90973 | 49.688 | 54.60219 | 65.58458 | 61.58977 | 48.81237 |
| CUST_47865_PI429010886 | M77925 | 0.020191 | 15 | 3 | | | | | | 11.38637 | 16.43851 | NA | 22.40634 | 44.73069 | 83.45905 | 7.182647 | 10.49518 | 12.27788 | 12.1001 | 7.463527 | NA | 11.11786 | 13.82413 | 29.73209 | 40.87212 | 6.15687 | 43.5437 | NA | 27.72962 | 12.96451 | 12.51235 | NA | 12.62537 | 14.85609 | 5.998353 | 7.54037 |
| CUST_10536_PI429010886 | N26386 | 0.020207 | 24 | 21 | | | | | | 30.59469 | 42.69298 | 21.18391 | 13.31366 | 22.74587 | 14.90147 | 19.34225 | 13.45079 | 13.26879 | 28.14284 | 29.04798 | 21.51773 | 20.79384 | 12.53746 | 20.59244 | 31.73942 | 16.90254 | 30.95577 | 21.35072 | 18.68272 | 14.48109 | 51.62562 | 43.9229 | 32.858 | 33.4065 | 8.710242 | 22.82685 |
| CUST_13347_PI429010886 | N31030 | 0.020221 | 24 | 18 | | | | | | 340.867 | 420.025 | 400.6975 | 265.8546 | 411.751 | 270.8273 | 200.9243 | 251.7868 | 260.373 | 524.852 | 305.6076 | 488.97 | 361.8439 | 464.3001 | 399.5497 | 497.7449 | 242.6822 | 330.527 | 362.3529 | 562.6249 | 448.8586 | 496.6049 | 367.1896 | 282.7521 | 366.4854 | 358.8712 | 412.703 |
| CUST_2653_PI429010886 | N12841 | 0.020221 | 24 | 0 | 307204018 | Protein tumorous imaginal discs, mitochondrial [Ha | Response to stress | Other | 9.6E-144 | 83.30915 | 90.11953 | 81.36657 | 81.87168 | 86.38663 | 66.54991 | 78.44959 | 82.71117 | 61.71189 | 73.99757 | 67.39787 | 84.67784 | 74.21285 | 72.1095 | 61.12299 | 71.94962 | 56.0501 | 69.81278 | 86.4151 | 69.49923 | 60.20751 | 97.05352 | 104.302 | 58.20859 | 94.11857 | 75.11707 | 90.72052 |
| CUST_893_PI429010886 | N7724 | 0.020223 | 24 | 12 | | | | | | 74.64567 | 93.09068 | 77.29645 | 63.1929 | 61.93065 | 66.73255 | 49.84719 | 72.84986 | 83.93301 | 101.5527 | 81.64235 | 94.65769 | 71.9016 | 125.8598 | 127.5671 | 107.955 | 82.42534 | 96.76761 | 65.66431 | 104.4333 | 84.55489 | 97.62464 | 87.58299 | 70.54693 | 97.18779 | 86.4072 | 96.36676 |
| CUST_56615_PI429010886 | M114222 | 0.02023 | 18 | 15 | | | | | | 418.2809 | 296.6828 | 360.2672 | 105.6617 | 502.4781 | 275.2679 | 326.5696 | 394.3816 | 219.2135 | 707.6225 | 251.4349 | 285.1566 | 614.5524 | 527.6079 | 250.4057 | 513.4234 | 458.9977 | 463.5935 | 450.5804 | 476.1852 | 373.4486 | 298.5904 | 202.262 | 184.0038 | 170.0663 | 263.8365 | 446.7333 |
| CUST_5505_PI429010886 | N18789 | 0.020239 | 15 | 12 | | | | | | 71.22702 | 78.68384 | 88.04251 | 36.87579 | 62.4936 | 50.48761 | 68.26569 | 64.8492 | 89.33478 | 102.9204 | 95.36046 | 89.67555 | 102.9919 | 130.6959 | 93.45456 | 104.2766 | 54.71071 | 56.47886 | 90.90134 | 100.1729 | 69.94604 | 108.2254 | 72.16312 | 53.03978 | 104.0004 | 70.66973 | 104.5873 |
| CUST_11689_PI429010886 | N28242 | 0.020244 | 24 | 18 | | | | | | 36.23666 | 36.64169 | 31.31664 | 36.24706 | 37.99335 | 25.99299 | 27.28016 | 26.91205 | 27.9603 | 32.26335 | 35.50744 | 43.58328 | 28.58747 | 43.11192 | 52.42987 | 39.03429 | 26.02914 | 40.01136 | 56.30906 | 42.40805 | 46.42413 | 46.74774 | 31.76715 | 29.58412 | 46.25392 | 38.87395 | 44.41825 |
| CUST_28125_PI429010886 | N57342 | 0.020247 | 24 | 12 | | | | | | 26.4818 | 48.97519 | 48.78617 | 72.41676 | 33.42247 | 28.66586 | 40.04237 | 39.46416 | 36.85539 | 50.37741 | 64.09512 | 59.0899 | 54.12178 | 65.79499 | 56.24668 | 27.63304 | 33.09246 | 11.56409 | 39.71872 | 58.61502 | 39.24212 | 35.71039 | 21.79918 | 28.65923 | 20.08306 | 45.41077 | 28.11334 |
| CUST_7019_PI429010886 | N20924 | 0.020299 | 15 | 9 | 433335621 | ecdysone receptor isoform 2a [Callinectes sapidus] | | | 5.24E-35 | 19.93091 | 23.31758 | 51.59241 | 38.67242 | 31.2089 | 57.01626 | 29.81837 | 41.03316 | 42.39566 | 40.25214 | 49.15395 | 43.1527 | 17.47454 | 25.22662 | 41.31796 | 30.22418 | 29.49276 | 36.20575 | 32.89329 | 31.51141 | 34.80311 | 35.57881 | 36.83973 | 27.38684 | 41.35575 | 57.5238 | 64.71156 |
| CUST_476_PI429010886 | N5552 | 0.020306 | 18 | 3 | | | | | | 50.54005 | 36.20273 | 47.83781 | 39.68132 | 70.09922 | 60.6558 | 56.74919 | 45.53002 | 46.25868 | 45.43527 | 57.10423 | 29.03344 | 34.48473 | 42.99343 | 28.7253 | 41.4548 | 55.59388 | 52.91094 | 29.11768 | 41.00395 | 75.66735 | 56.95101 | 83.27348 | 62.07028 | 63.36658 | 40.3065 | 61.3315 |
| CUST_53557_PI429010886 | M102241 | 0.020315 | 24 | 12 | | | | | | 29.56124 | 29.80111 | 17.83843 | 34.96354 | 18.86766 | 46.51613 | 36.68661 | 33.56428 | 22.84721 | 54.24368 | 29.19475 | 36.18187 | 46.38212 | 35.08626 | 43.50773 | 45.64241 | 23.75346 | 36.07274 | 39.53783 | 33.70683 | 38.78884 | 29.0899 | 31.16623 | 36.09309 | 30.72417 | 27.05448 | 32.86388 |
| CUST_13411_PI429010886 | N31130 | 0.020335 | 18 | 12 | | | | | | 296.6828 | 277.3932 | 223.2546 | 290.2748 | 295.2206 | 257.1155 | 298.1939 | 239.2718 | 214.4171 | 385.8989 | 281.692 | 368.0783 | 332.964 | 318.3844 | 344.0193 | 386.8992 | 269.3899 | 317.2087 | 362.6784 | 288.0844 | 310.2077 | 356.7158 | 215.1465 | 341.0906 | 272.019 | 248.5464 | 246.0582 |
| CUST_9722_PI429010886 | N25096 | 0.02034 | 18 | 3 | 260820904 | hypothetical protein BRAFLDRAFT_218285 [Branchi | | | 1.57E-07 | 297.2502 | 956.4623 | 476.7276 | 927.1088 | 489.1062 | 495.0933 | 368.9642 | 501.3373 | 535.2858 | 421.6972 | 277.7994 | 1055.17 | 447.7446 | 206.6019 | 215.167 | 323.6761 | 160.7094 | 374.607 | 84.95079 | 463.0515 | 772.946 | 473.4648 | 623.6818 | 519.0655 | 513.0951 | 843.8131 | 377.0163 |
| CUST_23651_PI429010886 | N49204 | 0.020388 | 12 | 0 | | | | | | 63.01798 | 57.62155 | 67.49489 | 34.86394 | 64.22812 | 65.51399 | 46.30865 | 46.13849 | 58.81878 | 65.70437 | 55.21295 | 60.9102 | 59.81399 | 59.11379 | 82.75397 | 78.08745 | 41.47981 | 61.86952 | 55.07381 | 43.04692 | 49.998 | 76.12074 | 62.11683 | 58.38163 | 72.32108 | 65.49916 | 81.67574 |
| CUST_14833_PI429010886 | N33546 | 0.020391 | 21 | 18 | | | | | | 22.02142 | 43.68382 | 13.99028 | 15.5705 | 25.12826 | 17.01358 | 7.632732 | 10.2112 | 12.23011 | 16.75298 | 16.33477 | 7.52281 | 16.26432 | 23.41982 | 17.70164 | 25.69829 | 15.444 | 18.54906 | 22.19942 | 24.18144 | 17.9021 | 19.50921 | 14.63359 | 16.58665 | 29.14178 | 16.93603 | 14.39019 |
| CUST_10175_PI429010886 | N25815 | 0.020392 | 21 | 0 | | | | | | 49.11669 | 56.04356 | 47.69004 | 34.47823 | 33.06846 | 42.53014 | 30.86904 | 33.41208 | 29.83725 | 43.21123 | 32.79355 | 35.05495 | 30.46627 | 43.87014 | 41.06525 | 51.21188 | 39.02375 | 31.30264 | 58.93488 | 32.3787 | 46.63569 | 51.67197 | 33.22111 | 35.12261 | 47.82182 | 32.03971 | 43.84173 |
| CUST_7343_PI429010886 | N21406 | 0.020401 | 15 | 12 | 339896293 | cytochrome P450 [Bemisia tabaci] | Metabolic process | Oxidation-reduction process | 2.79E-48 | 377.7333 | 1045.788 | 644.1161 | 812.5634 | 280.5674 | 330.3127 | 496.1582 | 316.7444 | 440.0791 | 805.5548 | 603.9714 | 637.7954 | 809.3886 | 1001.156 | 856.8878 | 626.2648 | 426.3129 | 511.4224 | 791.2722 | 1010.552 | 765.2983 | 563.0019 | 505.4528 | 387.2348 | 589.6619 | 338.3238 | 650.2354 |
| CUST_12443_PI429010886 | N29469 | 0.020419 | 15 | 12 | | | | | | 45.98092 | 32.63905 | 42.5721 | 45.02093 | 25.26092 | 29.03947 | 41.96451 | 60.92331 | 44.67201 | 42.15075 | 39.67041 | 39.667 | 59.04714 | 37.47945 | 54.99254 | 34.91043 | 47.12699 | 52.24698 | 38.83398 | 32.30669 | 29.85387 | 36.47407 | 32.87841 | 40.27597 | 54.04063 | 50.82151 | 37.32614 |
| CUST_21263_PI429010886 | N44909 | 0.020419 | 15 | 12 | | | | | | 64.8125 | 67.44354 | 66.2145 | 46.67338 | 59.3369 | 59.36711 | 53.80266 | 40.81576 | 30.8467 | 73.46154 | 69.77447 | 66.48237 | 66.35653 | 72.03576 | 48.05138 | 64.04369 | 69.58984 | 96.86826 | 66.83042 | 47.56602 | 56.96512 | 67.04072 | 50.6408 | 43.6379 | 97.90896 | 54.61808 | 62.97836 |
| CUST_56825_PI429010886 | M114721 | 0.020428 | 15 | 9 | | | | | | 720.48 | 691.5947 | 884.6458 | 675.2822 | 683.5585 | 619.5429 | 729.1026 | 700.4893 | 776.3963 | 1072.593 | 802.0583 | 884.4098 | 729.8175 | 983.2431 | 1051.932 | 1049.194 | 728.4044 | 776.6013 | 784.6927 | 970.6979 | 785.9549 | 979.2899 | 898.2047 | 682.478 | 1121.554 | 846.1561 | 1056.762 |
| CUST_31595_PI429010886 | M1924 | 0.020428 | 15 | 9 | 270007800 | hypothetical protein TcasGA2_TC014502 [Tribolium | | | 5.11E-07 | 595.028 | 783.9719 | 1811.851 | 297.7533 | 716.0786 | 620.4611 | 803.3312 | 1603.184 | 1372.611 | 2406.498 | 829.1909 | 1187.991 | 1320.618 | 2287.157 | 1228.27 | 920.7129 | 902.7962 | 554.9764 | 782.2398 | 1055.576 | 818.0738 | 2343.419 | 1808.217 | 604.2963 | 2146.135 | 885.3464 | 2706.735 |
| CUST_11995_PI429010886 | N28748 | 0.020428 | 15 | 9 | | | | | | 1106.085 | 1739.21 | 1756.162 | 990.9102 | 925.8412 | 891.0101 | 1053.785 | 1272.526 | 1522.93 | 2299.417 | 1589.601 | 1869.455 | 1289.132 | 1818.602 | 1504.155 | 2858.942 | 1316.85 | 1581.192 | 1186.768 | 2058.855 | 1499.492 | 1995.819 | 1278.309 | 1192.582 | 2090.413 | 1493.465 | 2318.551 |
| CUST_50918_PI429010886 | M91455 | 0.020428 | 24 | 6 | | | | | | 40.61781 | 20.63783 | NA | 41.36789 | 7.500434 | 113.976 | 55.3496 | 22.3415 | 52.31329 | 46.25026 | 14.03531 | 56.02742 | 49.23486 | 11.94414 | 23.11042 | 8.110471 | 25.07182 | 9.768636 | 14.46019 | 4.640847 | 7.743875 | 20.03687 | 4.763542 | 78.15144 | 11.27882 | 17.51307 | 10.5296 |
| CUST_331_PI429010886 | N3958 | 0.020485 | 24 | 21 | 117067248 | ribosomal protein S26 [Branchiostoma belcheri] | Protein metabolism | Ribosome biogenesis | 2.62E-30 | 79.14043 | 94.20676 | 43.12293 | 69.96115 | 122.7194 | 84.96006 | 49.47977 | 84.34439 | 40.02682 | 70.51648 | 135.1036 | 63.52108 | 77.90078 | 63.46985 | 17.01911 | 54.85716 | 74.97848 | 38.19514 | 94.27998 | 87.08384 | 209.3167 | 98.53734 | 155.8611 | 121.9844 | 176.2423 | 116.1911 | 104.7962 |
| CUST_29053_PI429010886 | N59114 | 0.02049 | 24 | 3 | | | | | | 8.546868 | 10.90827 | 18.1574 | 17.35176 | 11.87142 | 54.92234 | 25.4312 | 22.28718 | 5.510093 | 11.7505 | NA | 16.29403 | 14.78673 | 11.8786 | 15.4643 | 20.16285 | 9.227326 | 10.19086 | 8.250428 | 4.602645 | 7.660234 | 5.924868 | 22.51476 | NA | 10.70821 | 19.02839 | 11.60633 |
| CUST_45076_PI429010886 | M67920 | 0.020492 | 18 | 15 | 506965395 | troponin T [Coptotermes formosanus] | | | 6.35E-28 | 441.5814 | 569.4459 | 717.2947 | 436.1525 | 515.4056 | 557.1607 | 559.6795 | 423.3111 | 515.7103 | 502.0515 | 629.4397 | 665.3114 | 557.1607 | 760.2724 | 604.4329 | 679.1732 | 744.5402 | 574.3589 | 635.437 | 567.7179 | 459.5005 | 538.762 | 559.0577 | 400.5981 | 708.1055 | 609.869 | 725.8016 |
| CUST_36249_PI429010886 | M15170 | 0.020496 | 21 | 6 | 345319844 | PREDICTED: solute carrier family 22 member 8-like | Transport | Ion transport | 3.21E-17 | 112.6894 | 79.31367 | 63.05647 | 92.12003 | 53.86098 | 134.0969 | 125.751 | 104.4835 | 128.6916 | 87.19276 | 91.37896 | 93.04202 | 85.06299 | 129.065 | 98.14502 | 77.6254 | 69.9701 | 75.12567 | 76.89998 | 84.57304 | 48.13345 | 86.75819 | 103.6413 | 101.1328 | 63.46985 | 139.8135 | 80.27864 |
| CUST_46757_PI429010886 | M73623 | 0.020539 | 15 | 12 | | | | | | 72.23286 | 45.80703 | 170.8572 | 34.78955 | 24.21006 | 38.22142 | 106.1322 | 178.7372 | 71.63476 | 145.3261 | 46.22951 | 70.74696 | 106.8828 | 171.7093 | 117.3157 | 50.58779 | 79.37795 | 44.21756 | 66.87005 | 77.76831 | 64.48135 | 75.43644 | 154.0002 | 48.28635 | 177.2677 | 86.02859 | 129.4911 |
| CUST_2891_PI429010886 | N13328 | 0.020544 | 21 | 9 | 170066054 | creatine amidinohydrolase [Bacillus sp. BSD-8] | Cellular Process | Other | 2.9E-129 | NA | 145.6295 | 56.00982 | 92.50008 | 177.4848 | 48.5148 | 97.68675 | 467.665 | 422.9234 | 146.6794 | 403.7411 | 407.0568 | 403.3076 | 39.92228 | 163.0297 | NA | 123.5431 | NA | 77.92872 | 1580.775 | 197.9679 | 3.812042 | 77.15161 | 54.98545 | NA | 356.2894 | 212.1159 |
| CUST_15395_PI429010886 | N34511 | 0.020549 | 18 | 15 | | | | | | 1347.753 | 1187.991 | 1315.293 | 1152.92 | 712.9294 | 739.8087 | 697.0634 | 883.1215 | 556.543 | 810.0074 | 1145.003 | 2012.591 | 537.5555 | 1492.759 | 3130.59 | 1332.781 | 2004.816 | 2398.993 | 1388.367 | 827.2898 | 804.0124 | 1652.941 | 801.3257 | 693.7505 | 1710.741 | 1076.531 | 609.1465 |
| CUST_37059_PI429010886 | M17722 | 0.020554 | 24 | 15 | | | | | | 901.5294 | 967.496 | 1106.54 | 921.6949 | 916.8157 | 1001.156 | 764.3328 | 859.7051 | 900.857 | 1132.32 | 874.1627 | 1223.156 | 899.5738 | 1240.783 | 1432.157 | 1564.24 | 1009.233 | 1106.54 | 993.7035 | 1112.909 | 1064.697 | 1311.842 | 1003.294 | 893.461 | 1289.943 | 945.2395 | 1286.564 |
| CUST_50374_PI429010886 | M89616 | 0.020554 | 24 | 15 | | | | | | 681.3848 | 785.9549 | 778.8569 | 666.6962 | 679.1732 | 785.705 | 623.9709 | 588.5777 | 665.6609 | 927.8138 | 632.0615 | 832.0351 | 652.7665 | 929.5412 | 980.1159 | 937.6859 | 745.8794 | 892.0751 | 824.1385 | 815.3757 | 742.4096 | 883.3994 | 722.796 | 663.9332 | 956.4623 | 715.8969 | 888.436 |
| CUST_3103_PI429010886 | N13797 | 0.02056 | 24 | 18 | | | | | | 50.22468 | 62.1958 | 63.4424 | 25.31882 | 36.76731 | 38.78083 | 39.58944 | 38.95593 | 39.39152 | 62.4936 | 41.15368 | 45.97615 | 40.20059 | 54.4284 | 43.20136 | 93.12227 | 34.50768 | 69.87848 | 67.70114 | 53.48774 | 47.86881 | 53.71399 | 52.22471 | 42.84657 | 78.29209 | 47.35331 | 50.14002 |
| CUST_24322_PI429010886 | N50415 | 0.020562 | 15 | 0 | 283854609 | DEAD(Asp-Glu-Ala-Asp) box polypeptide 5 [Macrobı | | | 1.8E-08 | 69.85985 | 67.20205 | 87.44346 | 51.50855 | 70.36778 | 59.27376 | 47.64968 | 44.50009 | 56.64379 | 70.77767 | 69.89586 | 73.73837 | 65.64051 | 59.28015 | 74.96433 | 92.89135 | 59.79177 | 81.01356 | 65.55617 | 81.11384 | 60.78952 | 89.02227 | 53.48278 | 51.07827 | 65.54211 | 58.597 | 70.33128 |
| CUST_586_PI429010886 | N6225 | 0.020563 | 12 | 6 | | | | | | 138.323 | 11.63473 | 171.7375 | 184.9236 | 152.3983 | 284.1504 | 581.9863 | 345.7017 | 130.6431 | 65.30643 | 72.58192 | NA | 57.256 | 40.19775 | 23.87529 | 20.97008 | 503.9702 | 115.4184 | 354.7905 | NA | 151.707 | 5.519534 | 170.5765 | 258.0303 | 15.99774 | 143.1896 | 5.91164 |
| CUST_913_PI429010886 | N7810 | 0.020563 | 15 | 12 | 255653872 | crustin 3 [Panulirus japonicus] | | | 4.79E-08 | 63.14802 | 178.0976 | 151.0368 | 28.888 | 47.54347 | 13.56721 | 34.88582 | 102.0461 | 118.675 | 208.8382 | 101.5396 | 143.4489 | 157.7092 | 190.1335 | 180.016 | 152.6066 | 426.0225 | 123.3713 | 60.18184 | 329.9108 | 72.41676 | 230.6606 | 508.7091 | 137.3935 | 771.0971 | 151.707 | 838.9983 |
| CUST_34259_PI429010886 | M9184 | 0.020567 | 24 | 15 | 242016957 | tubulin-specific chaperone D, putative [Pediculus hu | Metabolic process | Other | 3.33E-36 | 12.67185 | 13.99986 | 13.78635 | 4.915853 | 7.173073 | 8.667042 | 6.937577 | 5.884318 | 12.63519 | 11.55416 | 9.554371 | 12.21352 | 8.154793 | 20.99337 | 4.45976 | 20.93093 | 21.49416 | NA | 13.92025 | 31.74877 | 14.90751 | 6.559984 | 14.59759 | 9.44636 | 18.59558 | NA | 21.32852 |
| CUST_24612_PI429010886 | N50942 | 0.020572 | 24 | 6 | | | | | | 540.3051 | 659.1977 | 574.1082 | 450.3936 | 480.2626 | 1364.868 | 627.2172 | 525.3464 | 719.1308 | 241.8731 | 469.0438 | 485.5971 | 546.0726 | 199.3801 | 435.3919 | 310.0812 | 169.7046 | 306.736 | 428.6284 | 272.9711 | 283.6461 | 118.55 | 378.9452 | 450.7936 | 138.2955 | 1416.429 | 166.2545 |
| CUST_32333_PI429010886 | M3847 | 0.020588 | 24 | 15 | 321462981 | hypothetical protein DAPPUDRAFT_307524 [Daphn | | | 8.88E-34 | 95.57811 | 114.4563 | 111.1891 | 112.1381 | 119.7777 | 119.228 | 115.4469 | 144.5986 | 94.84242 | 106.2858 | 128.4554 | 112.3515 | 151.1785 | 125.4203 | 173.2308 | 215.8652 | 131.7576 | 235.2484 | 141.5325 | 152.3001 | 105.3086 | 149.4495 | 133.7417 | 100.566 | 116.0087 | 129.8259 | 124.3644 |
| CUST_33520_PI429010886 | M7143 | 0.020614 | 12 | 0 | 528920082 | PREDICTED: LOW QUALITY PROTEIN: zinc finger pro | | | 3.41E-51 | 16.53993 | 27.61834 | 20.92648 | 14.12813 | 20.69556 | 21.53411 | 9.461053 | 18.36165 | 18.94172 | 16.34119 | 18.49721 | 27.72017 | 21.25435 | 26.73153 | 27.86117 | 58.54952 | 14.68592 | 23.54424 | 13.60583 | 18.53311 | 17.88028 | 24.26769 | 21.32852 | 21.60669 | 27.10385 | 30.79135 | 22.81381 |
| CUST_13450_PI429010886 | N31211 | 0.020614 | 12 | 6 | | | | | | 28.23857 | 23.5201 | 38.91166 | 24.48759 | 28.59435 | 30.25328 | 44.64991 | 35.01361 | 29.17928 | 31.51141 | 34.52588 | 20.39186 | 21.15749 | 19.42496 | 15.02374 | 28.31129 | 33.82224 | 21.04421 | 35.71639 | 13.8397 | 36.65319 | 27.56341 | 33.73933 | 136.3926 | 14.69352 | 14.00339 | 11.70759 |
| CUST_39211_PI429010886 | M36163 | 0.020627 | 15 | 9 | | | | | | 4540.69 | 730.2123 | 6621.858 | 597.939 | 778.5698 | 1225.452 | 5092.903 | 10247.75 | 10746.13 | 11673.13 | 4154.657 | 4561.712 | 7415.798 | 12334.51 | 6307.408 | 1322.445 | 5769.917 | 1441.828 | 4080.667 | 4868.886 | 2913.591 | 10935.67 | 9611.158 | 2513.183 | 17966.78 | 3411.47 | 10102.29 |
| CUST_9397_PI429010886 | N24549 | 0.020627 | 18 | 12 | | | | | | 146.3194 | 75.49081 | 92.05581 | 82.72689 | 166.8192 | 112.6604 | 165.7374 | 177.2148 | 192.6463 | 191.1723 | 119.1803 | 190.62 | 531.8244 | 447.2563 | 308.528 | 206.9171 | 173.8566 | 159.32 | 298.4347 | 139.8446 | 103.0771 | 207.9553 | 183.091 | 220.685 | 310.3428 | 109.4262 | 398.993 |
| CUST_1515_PI429010886 | N9895 | 0.020653 | 21 | 0 | | | | | | 127.7418 | 201.2707 | 225.3596 | 143.2115 | 151.2427 | 173.4253 | 123.4235 | 132.0735 | 113.6641 | 163.329 | 125.2677 | 143.2735 | 117.0097 | 138.8365 | 151.1785 | 165.2222 | 125.408 | 129.9538 | 121.4973 | 173.1695 | 144.9791 | 159.0029 | 168.2432 | 201.7309 | 161.9153 | 129.5373 | 192.0915 |
| CUST_31232_PI429010886 | M1034 | 0.020671 | 15 | 0 | 157134870 | low molecular weight protein-tyrosine-phosphatase | Metabolic process | Cellular amino acid metabolic process | 4.56E-48 | 261.9745 | 268.9771 | 234.443 | 248.959 | 206.8341 | 192.442 | 104.4433 | 153.7558 | 146.3889 | 217.7746 | 171.9647 | 371.6995 | 211.0236 | 200.1594 | 213.4377 | 275.1873 | 143.2947 | 264.3268 | 219.8055 | 266.2431 | 192.6772 | 253.1078 | 150.9847 | 190.8326 | 255.6195 | 181.075 | 210.7453 |
| CUST_54647_PI429010886 | M107019 | 0.020671 | 15 | 9 | | | | | | 10.05721 | 13.64945 | 16.5596 | 7.902818 | 11.34865 | 9.965323 | 11.01416 | 13.81689 | 12.31693 | 27.47176 | 16.02669 | 29.8705 | 13.79507 | 25.68589 | 15.32925 | 30.40511 | 12.11176 | 16.89118 | 16.38286 | 24.02102 | 9.486543 | 26.90259 | 31.40253 | 13.50586 | 20.07409 | 20.74695 | 25.53913 |
| CUST_35259_PI429010886 | M12063 | 0.020671 | 15 | 9 | 291237055 | PREDICTED: predicted protein-like [Saccoglossus ko | | | 8.14E-28 | 46.61752 | 47.3028 | 59.27376 | 40.09578 | 44.70504 | 54.03548 | 48.30896 | 51.96426 | 62.37619 | 62.69277 | 54.0232 | 83.87586 | 47.43001 | 55.37764 | 86.67105 | 49.86266 | 54.59618 | 59.38984 | 44.78041 | 54.46306 | 65.68089 | 51.1689 | 47.47594 | 52.1682 | 63.30844 | 64.56424 | 77.69279 |
| CUST_37428_PI429010886 | M18986 | 0.020671 | 15 | 9 | | | | | | 1056.762 | 1068.447 | 908.5337 | 642.6469 | 748.7335 | 700.0017 | 1024.808 | 702.4802 | 824.5491 | 2106.309 | 947.2744 | 1411.354 | 1106.942 | 1157.228 | 846.752 | 1843.461 | 913.8189 | 902.1996 | 921.954 | 879.6665 | 945.5514 | 1245.02 | 821.0172 | 1117.033 | 1293.201 | 813.4135 | 1490.148 |
| CUST_567_PI429010886 | N6090 | 0.020671 | 15 | 3 | 1272239 | Homarus americanus alpha-I tubulin mRNA, comple | Cellular component organization | Cytoskeleton organization | | 11030.7 | 13887.31 | 4873.025 | 18830.56 | 11327.23 | 18410.6 | 9950.402 | 7373.59 | 7197.042 | 3453.79 | 7347.807 | 4553.274 | 7404.352 | 4404.238 | 7737.591 | 7669.986 | 9106.522 | 13245.53 | 10721.33 | 4123.826 | 7415.798 | 5206.134 | 5407.054 | 13555.43 | 3175.174 | 10760.71 | 2612.527 |
| CUST_1161_PI429010886 | N8703 | 0.020671 | 15 | 9 | | | | | | 23.42495 | 7.181648 | 13.29917 | 15.98734 | 19.01271 | 11.90992 | 17.21639 | 13.35156 | 10.94929 | 17.44803 | 17.96537 | 22.53631 | 13.9022 | 33.26161 | 15.13796 | 14.68592 | 15.3428 | 14.71926 | 7.292253 | 14.23976 | 11.74047 | 20.58696 | 12.49592 | 13.21926 | 26.91798 | 25.4055 | 21.05098 |
| CUST_22143_PI429010886 | N46511 | 0.020676 | 21 | 6 | 242019815 | hemolectin, putative [Pediculus humanus corporis] | Cellular Process | Cell adhesion | 4.73E-22 | 119.7777 | 131.0159 | 140.7156 | 154.1768 | 195.6184 | 145.726 | 193.0913 | 182.0202 | 163.5179 | 160.531 | 183.8709 | 132.0609 | 164.9397 | 168.032 | 201.1076 | 181.8837 | 156.8398 | 98.04327 | 169.4763 | 149.3076 | 117.7859 | 172.854 | 107.0314 | 93.29834 | 158.91 | 157.4409 | 185.2639 |
| CUST_36254_PI429010886 | M15189 | 0.020711 | 15 | 3 | | | | | | 28.11334 | NA | 28.24069 | 19.16079 | 46.69563 | 35.01718 | 41.47521 | 19.48537 | 8.495784 | 8.657735 | 26.28334 | NA | NA | 4.595081 | 9.524556 | 8.366386 | 46.49731 | 23.60284 | 61.89112 | NA | 42.28532 | 3.557533 | 41.75113 | 30.79365 | 12.53584 | 11.54995 | NA |
| CUST_47709_PI429010886 | M77278 | 0.020717 | 21 | 15 | | | | | | 17.81636 | 27.43011 | 32.91963 | NA | 15.36478 | 16.66286 | 28.55509 | 31.46674 | 20.14785 | 24.91723 | 19.28584 | 22.79797 | 29.09978 | 42.33801 | 20.18328 | 35.94612 | 34.14862 | 32.64658 | 14.76122 | 11.98928 | 5.160214 | 45.51332 | 20.04708 | 24.22757 | 9.267153 | 17.64462 | 12.95283 |
| CUST_10447_PI429010886 | N26250 | 0.020717 | 21 | 0 | 495404433 | transcriptional regulator [Phyllobacterium sp. YR53 | Nucleic acid metabolism | Regulation of gene expression | 1.5E-99 | 8.034981 | 13.55063 | 17.86001 | 7.659037 | 15.13091 | 13.73929 | 8.746322 | 4.831608 | 6.513918 | 8.914093 | 10.7807 | NA | 7.727602 | NA | 8.245374 | 13.42033 | 5.980599 | 13.38764 | 17.55108 | 11.33171 | 13.07005 | 23.2088 | NA | NA | 7.456344 | NA | 11.30463 |
| CUST_15699_PI429010886 | N34994 | 0.020739 | 18 | 9 | 321454608 | hypothetical protein DAPPUDRAFT_303518 [Daphn | Metabolic process | Carbohydrate metabolic process | 3.8E-14 | 23.58007 | 24.67089 | 35.8731 | 15.11963 | 15.18532 | 17.03814 | 23.46033 | 19.47912 | 24.06718 | 29.70907 | 23.59869 | 22.32946 | 17.18634 | 25.4154 | 27.80089 | 19.28994 | 19.81178 | 21.19536 | 32.10376 | 21.7344 | 10.03383 | 18.48442 | 11.53244 | 11.05993 | 18.21042 | 23.58007 | 28.30863 |
| CUST_32134_PI429010886 | M3324 | 0.020745 | 18 | 9 | 321453647 | hypothetical protein DAPPUDRAFT_65881 [Daphnia | Transport | Protein transport | 3.16E-42 | 91.66231 | 111.2474 | 165.6901 | 83.79573 | 74.08523 | 96.13039 | 129.8621 | 87.03787 | 150.6483 | 188.7198 | 106.3847 | 154.2025 | 115.1182 | 170.0428 | 134.4036 | 131.513 | 91.23322 | 119.5504 | 112.3515 | 103.7577 | 120.2505 | 101.0721 | 117.2199 | 112.5022 | 156.4871 | 88.15874 | 138.6617 |
| CUST_39057_PI429010886 | M34033 | 0.020747 | 21 | 18 | | | | | | 50.25117 | 77.93692 | 62.98504 | 57.5416 | 66.62115 | 55.62798 | 38.22142 | 40.88839 | 45.04433 | 65.69324 | 55.62148 | 64.99854 | 57.45413 | 63.39049 | 67.87327 | 80.29877 | 49.2211 | 54.84373 | 65.19644 | 72.57482 | 68.1364 | 72.05819 | 58.86655 | 53.77942 | 77.15963 | 52.43827 | 65.54862 |
| CUST_29789_PI429010886 | N60551 | 0.020749 | 21 | 0 | | | | | | 17.42054 | 14.70379 | 22.57524 | NA | 6.903956 | 6.950466 | 5.314464 | NA | 9.966503 | 14.23976 | 10.63106 | 10.8979 | 12.3071 | 6.400904 | 9.72694 | 6.823408 | 5.577844 | NA | 8.810922 | 11.45611 | NA | 15.75401 | 14.91181 | NA | 11.42857 | 6.995543 | 16.39936 |
| CUST_16954_PI429010886 | N37136 | 0.020753 | 12 | 0 | | | | | | 19.4542 | 21.14283 | 17.36679 | 16.07442 | 5.81075 | NA | 11.31757 | 13.32189 | 14.68311 | 16.80437 | 16.72767 | 15.5488 | 15.40573 | 18.62216 | 16.49958 | 19.16951 | 12.28413 | 13.32307 | 12.00128 | 18.99745 | 9.753321 | 26.64257 | 14.30732 | 15.9498 | 25.05018 | 15.61118 | 23.93813 |
| CUST_38837_PI429010886 | M31074 | 0.020769 | 21 | 3 | 156938905 | profilin [Penaeus monodon] | Cellular component organization | Cytoskeleton organization | 2.42E-30 | 100.1493 | 106.3744 | 138.3413 | 128.7368 | 122.5481 | 194.9477 | 127.2574 | 117.9167 | 138.323 | 96.4587 | 108.2468 | 147.7898 | 121.3175 | 103.2289 | 116.7605 | 97.32524 | 106.1954 | 113.2835 | 105.6505 | 101.8407 | 124.4818 | 130.0555 | 93.43338 | 134.1792 | 155.1509 | 141.5508 | 112.7849 |
| CUST_2827_PI429010886 | N13191 | 0.020803 | 18 | 15 | | | | | | 175.6005 | 228.4344 | 162.6994 | 155.8927 | 172.2354 | 141.6867 | 130.9315 | 120.7407 | 120.7487 | 304.8602 | 142.3777 | 348.4045 | 202.301 | 168.329 | 211.6792 | 259.4845 | 84.97695 | 254.7271 | 153.9132 | 228.8282 | 175.8911 | 263.0952 | 150.8429 | 146.2438 | 133.9999 | 200.6981 | 123.2423 |
| CUST_31933_PI429010886 | M2770 | 0.020808 | 24 | 15 | | | | | | 636.6917 | 699.0502 | 643.7576 | 418.6867 | 561.5311 | 593.8074 | 424.4597 | 491.0083 | 463.5935 | 653.1387 | 703.3407 | 602.9273 | 339.9729 | 570.6164 | 492.8667 | 787.4832 | 699.2311 | 821.0172 | 682.9708 | 664.8684 | 583.2176 | 775.4257 | 488.7111 | 605.0923 | 1025.618 | 621.0146 | 744.2633 |
| CUST_43000_PI429010886 | M61816 | 0.020817 | 12 | 0 | | | | | | 42.76605 | 321.0207 | 66.31361 | 54.61808 | 81.9929 | NA | NA | 36.10137 | 12.83461 | 38.59666 | 270.8273 | 30.35559 | 239.571 | 198.3401 | 137.0908 | 38.19149 | 8.323544 | 35.22469 | 11.85674 | NA | NA | 191.7619 | 125.6499 | NA | 222.1445 | 50.23482 | 101.637 |
| CUST_31445_PI429010886 | M1544 | 0.020819 | 18 | 12 | 340720305 | PREDICTED: uridine-cytidine kinase-like 1-like isofor | Metabolic process | Nucleobase-containing compound metabolic process | 5.32E-78 | 39.98991 | 79.08585 | 42.23748 | 18.16809 | 22.66107 | 15.73231 | 25.97092 | 33.2742 | 28.26641 | 33.86006 | 25.2086 | 21.71424 | 46.40711 | 19.20795 | 163.6362 | 28.72783 | 30.99487 | 32.54445 | 29.69737 | 18.2155 | 36.20575 | 25.22384 | 17.92979 | 23.05482 | 27.24651 | 21.43485 | 28.64039 |
| CUST_7419_PI429010886 | N21516 | 0.020819 | 18 | 6 | | | | | | 20.0133 | 17.16312 | 15.97633 | 15.6436 | 24.983 | 10.94103 | 18.23052 | 37.53024 | 18.98105 | 19.37414 | 8.190659 | 10.33417 | 21.02638 | 11.08749 | 17.19687 | 16.0983 | 4.620483 | 10.59996 | 12.67185 | 11.62495 | 23.4177 | 15.6362 | 20.34669 | 13.87348 | 39.48748 | 22.00276 | 22.31957 |
| CUST_14426_PI429010886 | N32865 | 0.020819 | 18 | 15 | | | | | | 423.5928 | 370.7278 | 331.1696 | 320.693 | 372.2873 | 377.0882 | 366.4854 | 299.285 | 267.7292 | 303.3749 | 378.5438 | 293.3227 | 327.6399 | 334.0181 | 306.9203 | 431.1968 | 417.0088 | 429.9553 | 438.3419 | 350.3639 | 286.6201 | 382.3424 | 283.5032 | 313.3535 | 351.1934 | 329.4092 | 266.851 |
| CUST_8313_PI429010886 | N22858 | 0.020831 | 21 | 0 | | | | | | 49.81881 | 56.52669 | 55.35407 | 77.87542 | 33.03027 | 42.61497 | 11.54307 | 4.031757 | NA | 40.57245 | 5.730898 | 62.03099 | 44.63016 | NA | 22.11953 | 45.09026 | 25.29581 | 39.09942 | 19.46511 | 63.03943 | 77.69279 | 153.1912 | 21.49011 | 93.73888 | 21.20384 | 23.565 | 24.5772 |
| CUST_30983_PI429010886 | M378 | 0.020841 | 12 | 0 | 242021897 | conserved hypothetical protein [Pediculus humanus | Metabolic process | Cellular amino acid metabolic process | 4.1E-150 | 752.3295 | 731.6805 | 907.4672 | 772.6877 | 734.7434 | 825.1657 | 707.6225 | 660.0703 | 695.332 | 829.1909 | 655.3909 | 793.3041 | 583.4081 | 1002.084 | 775.9372 | 722.2644 | 786.4352 | 741.4101 | 709.6744 | 765.7162 | 720.7013 | 813.6581 | 763.2137 | 770.9286 | 854.2242 | 885.7556 | 941.6865 |
| CUST_11981_PI429010886 | N28724 | 0.020841 | 12 | 0 | | | | | | 26.92163 | 44.47869 | 33.72715 | 41.24587 | 40.17935 | 39.43891 | 20.03687 | 22.56723 | 34.0946 | 28.99536 | 25.93179 | 41.4213 | 44.53615 | 50.1495 | 57.43585 | 34.2205 | 19.88671 | 38.91166 | 27.65371 | 34.34564 | 28.67208 | 38.91585 | 34.65808 | 36.49611 | 47.02813 | 34.44235 | 36.27265 |
| CUST_41089_PI429010886 | M56850 | 0.020858 | 18 | 15 | | | | | | 342.3066 | 330.4772 | 258.7295 | 228.4677 | 316.0527 | 299.837 | 204.5255 | 259.848 | 191.5917 | 271.5735 | 301.9881 | 296.4182 | 241.8211 | 258.7295 | 274.4437 | 400.1052 | 280.4106 | 286.19 | 342.3707 | 384.1514 | 258.4381 | 387.3252 | 209.5944 | 253.5447 | 251.404 | 201.085 | 282.8059 |
| CUST_37413_PI429010886 | M18944 | 0.020881 | 15 | 9 | | | | | | 10524.41 | 2065.573 | 15631.77 | 1648.471 | 1863.799 | 3466.9 | 13085.78 | 25546.19 | 24341.67 | 25830.1 | 10949.63 | 12898.01 | 22818.38 | 34379.32 | 19491.76 | 4864.618 | 14529.33 | 5167.16 | 13375.82 | 13245.53 | 8006.158 | 23514.49 | 24425.67 | 7107.78 | 43024.52 | 9625.043 | 26858.78 |
| CUST_39901_PI429010886 | M46554 | 0.020899 | 18 | 6 | 532759728 | antimicrobial peptide type 2 precursor IIb [Pandalo | | | 4.6E-20 | 24.03945 | 42.04788 | 13.58569 | 22.27295 | 21.80739 | 8.363068 | 142.1431 | 65.43441 | 204.1086 | 51.30486 | 14.265 | 163.5913 | 63.87879 | 34.58379 | 54.22335 | 97.13391 | 86.94867 | 114.813 | 24.70162 | 70.33128 | 36.64169 | 30.43159 | 59.37489 | 45.36871 | 171.6223 | 118.1855 | 257.8674 |
| CUST_15997_PI429010886 | N35480 | 0.020907 | 12 | 3 | | | | | | 89.34718 | 173.7422 | 13.10657 | 971.7833 | 391.9101 | 475.9462 | 652.581 | NA | 602.9273 | 12.00862 | 5.993779 | 8.030742 | 1528.209 | 156.3082 | 1237.49 | 365.3992 | NA | 356.8925 | 23.64962 | 6.180449 | 12.73395 | 18.09247 | NA | 126.3916 | 60.4668 | 64.20475 | NA |
| CUST_35360_PI429010886 | M12376 | 0.020914 | 15 | 9 | | | | | | 112.5022 | 237.5448 | 187.746 | 111.9958 | 149.2677 | 75.72117 | 82.18107 | 189.0589 | 261.3766 | 377.4127 | 210.676 | 398.8868 | 230.2231 | 336.1486 | 268.8761 | 353.4537 | 85.77984 | 200.6981 | 197.2996 | 281.3522 | 219.3496 | 333.5908 | 290.4858 | 89.419 | 293.7629 | 245.1598 | 379.882 |

| Probe ID | ID | p-value | Period | Phase | EBI ID | Annotation | GO category | Go term | E-value | 0 | 0 | 0 | 3 | 3 | 3 | 6 | 6 | 6 | 9 | 9 | 9 | 12 | 12 | 12 | 15 | 15 | 15 | 18 | 18 | 18 | 21 | 21 | 21 | 24 | 24 | 24 |
|---|---|---|---|---|---|---|---|---|---|---|---|---|---|---|---|---|---|---|---|---|---|---|---|---|---|---|---|---|---|---|---|---|---|---|---|---|
| CUST_38149_PI429010886 | M23683 | 0.020914 | 15 | 0 | 321475489 | hypothetical protein DAPPUDRAFT_208220 [Daphni | | | 5.02E-56 | 98.51875 | 112.1381 | 84.722 | 98.68398 | 90.41498 | 83.44238 | 79.74735 | 79.1048 | 68.44956 | 100.3487 | 91.40169 | 103.654 | 104.2917 | 79.043 | 90.04123 | 94.95262 | 91.58478 | 82.21549 | 113.1145 | 65.94453 | 87.59441 | 69.35049 | 73.47743 | 63.07552 | 77.93692 | 83.64909 | 84.93221 |
| CUST_39504_PI429010886 | M40586 | 0.020917 | 15 | 12 | | | | | | 1424.308 | 204.5562 | 12563.99 | 14.85468 | 43.21123 | 32.45773 | 2601.307 | 27219.28 | 13988.83 | 17000.76 | 402.4861 | 867.4252 | 15389.68 | 27703.57 | 10296.63 | 168.2221 | 8536.698 | 120.0318 | 953.5865 | 860.5065 | 309.6337 | 5477.513 | 18661.44 | 410.594 | 20208.21 | 304.0369 | 19739.66 |
| CUST_15556_PI429010886 | N34778 | 0.020917 | 15 | 6 | | | | | | 297.6952 | 317.5778 | 231.8131 | 327.8511 | 427.3564 | 384.9318 | 367.4512 | 430.0642 | 318.0852 | 332.753 | 340.6846 | 487.1736 | 278.8318 | 289.5961 | 303.3224 | 377.0882 | 280.8226 | 324.0592 | 350.2055 | 501.5691 | 309.7678 | 447.9038 | 304.5545 | 427.7928 | 402.7742 | 348.0084 | 299.3397 |
| CUST_16237_PI429010886 | N35893 | 0.020917 | 15 | 12 | | | | | | 28306.41 | 3448.497 | 63067.91 | 2854.292 | 2894.613 | 5162.076 | 29610.14 | 78574.02 | 64277.46 | 59501.35 | 18150.31 | 20168.54 | 69607.7 | 88276.1 | 53278.3 | 10575.43 | 43151.77 | 5717.187 | 29468.93 | 22818.38 | 16297.18 | 45401.69 | 65559.81 | 11650.8 | 68283.18 | 15872.37 | 64571.61 |
| CUST_20128_PI429010886 | N42856 | 0.020917 | 15 | 12 | | | | | | 126.5542 | 123.3713 | 147.5581 | 190.0092 | 184.3384 | 153.3961 | 87.96508 | 106.1197 | 118.0864 | 145.5389 | 192.363 | 255.4386 | 198.4267 | 228.1106 | 280.0685 | 253.2546 | 130.6774 | 150.8706 | 106.5468 | 213.0233 | 132.6337 | 143.4947 | 123.9921 | 174.4245 | 163.9462 | 120.6514 | 144.9545 |
| CUST_3362_PI429010886 | N14361 | 0.020939 | 24 | 21 | | | | | | 42.43668 | 58.12189 | 74.66823 | 46.71536 | 33.83579 | 39.02375 | 35.50744 | 40.91581 | 49.99255 | 32.41678 | 32.78199 | 49.75477 | 50.88285 | 71.70446 | 41.80808 | 53.43124 | 49.51746 | 52.59262 | 38.87769 | 42.44034 | 58.60431 | 52.09599 | 73.99757 | 53.57478 | 43.97896 | 72.13281 | 72.72106 |
| CUST_10097_PI429010886 | N25686 | 0.020952 | 24 | 18 | | | | | | 38.09997 | 50.98064 | 37.9742 | 26.93627 | 46.61752 | 30.9788 | 25.08483 | 26.56771 | 28.94785 | 75.67767 | 38.1318 | 36.19875 | 37.09496 | 40.29023 | 39.0152 | 51.92326 | 30.4923 | 39.414 | 41.69983 | 64.38672 | 42.18503 | 51.0383 | 25.0475 | 38.53317 | 55.784 | 16.9221 | 39.92653 |
| CUST_3432_PI429010886 | N14487 | 0.020964 | 24 | 21 | | | | | | 6.734253 | 11.91129 | 18.28004 | 7.40613 | 12.028 | 18.09635 | 11.7224 | 5.865878 | 7.762745 | 9.007525 | 9.949298 | 13.36364 | 7.319807 | NA | 12.33822 | 11.38637 | 11.8016 | 11.05033 | 11.97713 | 7.092466 | 12.39128 | 22.88357 | 13.63096 | 26.45407 | 15.25038 | 12.67376 | 9.166269 |
| CUST_46465_PI429010886 | M72731 | 0.020977 | 18 | 9 | | | | | | 20.71154 | 28.71654 | 33.71073 | 24.11276 | 17.32049 | 26.45722 | 24.40888 | 27.80573 | 25.21717 | 34.01329 | 25.02916 | 41.62677 | 27.42544 | 35.03294 | 35.59331 | 33.1071 | 22.00022 | 24.29748 | 26.87231 | 32.39121 | 26.78191 | 26.8663 | 24.76741 | 21.21393 | 41.28967 | 26.85728 | 40.58399 |
| CUST_38352_PI429010886 | M25770 | 0.020982 | 24 | 9 | | | | | | 312.6532 | 445.6175 | 199.5921 | 699.4291 | 343.1257 | 415.3866 | 355.7822 | 488.0718 | 403.4054 | 892.0751 | 815.5774 | 539.016 | 429.3715 | 966.7908 | 586.2213 | 467.3451 | 130.1875 | 218.4381 | 338.2007 | 583.4081 | 326.4884 | 441.5046 | 536.4898 | 206.5428 | 231.3049 | 451.916 | 173.5738 |
| CUST_20611_PI429010886 | N43747 | 0.021016 | 21 | 12 | | | | | | 190.588 | 189.7386 | 192.557 | 188.9774 | 136.8476 | 195.2959 | 171.1038 | 176.4808 | 142.0394 | 288.5334 | 143.7253 | 233.6842 | 221.7247 | 368.0062 | 327.7237 | 283.3333 | 214.1332 | 253.0671 | 137.8443 | 266.2859 | 246.9496 | 237.5773 | 201.6936 | 236.2907 | 332.753 | 176.1772 | 356.1302 |
| CUST_35415_PI429010886 | M12519 | 0.021017 | 15 | 3 | | | | | | 506.7573 | 263.345 | 90.84494 | 1012.95 | 662.2599 | 1226.638 | 826.9906 | 407.2671 | 256.4303 | 66.46786 | 457.038 | 37.45529 | 312.8606 | 150.1517 | 264.1731 | 262.3746 | 991.907 | 740.0272 | 686.6887 | 124.4616 | 593.6703 | 253.5058 | 679.5311 | 998.694 | 93.96395 | 451.3065 | 27.71788 |
| CUST_11851_PI429010886 | N28513 | 0.021031 | 24 | 0 | | | | | | 81.22317 | 82.60943 | 113.3918 | 83.53882 | 57.09554 | 66.1724 | 83.29887 | 50.28834 | 65.40248 | 87.09496 | 62.51292 | 77.64933 | 53.75795 | 72.16312 | 55.11957 | 83.28321 | 59.93656 | 73.14043 | 56.02183 | 59.82999 | 87.16389 | 90.3035 | 92.93954 | 54.26471 | 109.3881 | 87.34049 | 84.03407 |
| CUST_50102_PI429010886 | M88632 | 0.021035 | 15 | 0 | | | | | | 1269.191 | 1164.105 | 1109.205 | 1211.313 | 1187.536 | 1325.228 | 1047.894 | 1012.109 | 857.5648 | 1109.612 | 1066.79 | 926.7413 | 947.592 | 1008.511 | 1069.514 | 1594.166 | 1256.084 | 1218.887 | 1256.084 | 1080.655 | 921.954 | 1377.193 | 856.1081 | 1027.997 | 1103.847 | 862.1393 | 926.1166 |
| CUST_17303_PI429010886 | N37744 | 0.021039 | 18 | 12 | | | | | | 8.185415 | 8.419112 | 12.37856 | 7.795868 | 10.15583 | 10.88915 | 14.60539 | 7.917706 | NA | 6.406079 | 11.12231 | 21.35972 | 11.19024 | 18.13226 | 17.34629 | 11.30787 | 15.80258 | NA | 11.58688 | 12.54381 | 16.00911 | 6.31321 | 7.049754 | 10.02549 | 18.17081 | 11.87728 | 15.95324 |
| CUST_2307_PI429010886 | N12070 | 0.021046 | 21 | 0 | | | | | | 42.26034 | 53.78964 | 61.06386 | 27.17268 | 22.41939 | 22.83694 | 25.17616 | 23.00521 | 39.16114 | 31.41271 | 35.51342 | 27.10054 | 33.49853 | 27.57791 | 36.19062 | 43.76947 | 28.50232 | 39.06616 | 39.6493 | 37.9894 | 23.72626 | 38.51373 | 43.35404 | 29.42101 | 28.89154 | 21.41937 | 30.94893 |
| CUST_565_PI429010886 | N6077 | 0.021047 | 24 | 9 | 380853796 | protaglandin reductase 1 [Penaeus monodon] | Metabolic process | Oxidation-reduction process | 5.89E-89 | 26.78447 | 36.8462 | 36.96378 | 29.50933 | 39.0395 | 8.60504 | 32.50993 | 133.5378 | 65.22887 | 40.57649 | 157.7478 | 130.1208 | 101.8776 | 7.359153 | 48.76564 | 12.85234 | 47.39715 | NA | 25.27977 | 151.6596 | 77.2785 | 21.71424 | 17.12281 | 3.439984 | 13.2803 | 36.12998 | 62.24459 |
| CUST_13415_PI429010886 | N31139 | 0.02106 | 21 | 12 | | | | | | 523.5388 | 648.6302 | 707.905 | 611.2062 | 974.6113 | 538.1956 | 811.9464 | 2684.729 | 2287.157 | 630.6794 | 2260.474 | 3818.838 | 2771.702 | 665.3114 | 2115.987 | 793.8063 | 1135.995 | 667.8908 | 645.5341 | 5875.497 | 2032.79 | 610.3506 | 772.6877 | 637.2498 | 692.5393 | 1465.294 | 1627.802 |
| CUST_886_PI429010886 | N7700 | 0.021069 | 18 | 15 | | | | | | 17.39906 | 12.10209 | NA | 15.85664 | NA | 5.671066 | 10.69771 | 10.49349 | NA | 4.99831 | NA | 3.538902 | 18.90668 | 21.47442 | 13.78438 | 17.93968 | NA | 16.79729 | 17.04658 | NA | 15.38978 | 11.2694 | 14.80182 | 15.80258 | 8.674882 | 7.457269 | 22.18561 |
| CUST_55284_PI429010886 | M109440 | 0.021073 | 18 | 9 | 19921528 | Asparaginyl-tRNA synthetase, isoform A [Drosophila | Metabolic process | Cellular amino acid metabolic process | 7.71E-13 | 13.04874 | 29.05988 | 26.18577 | 19.0691 | 17.06111 | 8.685006 | 9.621618 | 20.82009 | 21.89378 | 29.0776 | 22.53922 | 23.4104 | 17.02082 | 16.43274 | 40.43694 | 21.9039 | 16.84517 | 22.32278 | 7.13523 | 17.26212 | 19.99991 | 19.01959 | 6.927136 | 12.10746 | 23.2088 | 20.65041 | 26.1771 |
| CUST_52479_PI429010886 | M97580 | 0.021073 | 18 | 6 | | | | | | 101.3304 | 69.44924 | 123.4881 | 120.9454 | 82.03238 | 94.45361 | 119.5229 | 148.0276 | 137.9332 | 89.5312 | 89.02227 | 65.41017 | 99.7668 | 119.3795 | 98.05949 | 77.23965 | 50.16596 | 45.7065 | 96.09446 | 271.2785 | 50.9653 | 134.2991 | 102.4301 | 117.9048 | 122.2898 | 90.81223 | 111.7487 |
| CUST_5840_PI429010886 | N19275 | 0.021073 | 18 | 9 | 321468789 | hypothetical protein DAPPUDRAFT_188248 [Daphn | Metabolic process | Oxidation-reduction process | 1.37E-87 | 314.7546 | 592.1986 | 396.0145 | 341.2566 | 325.8124 | 176.5718 | 209.7628 | 606.1944 | 657.4366 | 680.1392 | 600.1331 | 806.3497 | 545.2224 | 667.377 | 502.7361 | 588.4339 | 320.9666 | 296.266 | 413.5257 | 844.933 | 581 | 447.8279 | 419.1452 | 190.4443 | 750.8373 | 385.3789 | 793.3041 |
| CUST_10676_PI429010886 | N26608 | 0.021073 | 18 | 3 | 169931080 | cuticular protein [Artemia franciscana] | Metabolic process | Carbohydrate metabolic process | 2.48E-16 | 1259.651 | 316.2028 | 891.0101 | 1897.37 | 1760.95 | 4289.879 | 1737.368 | 1216.535 | 581 | 212.7113 | 788.6009 | 146.1555 | 1011.26 | 453.8761 | 678.7202 | 283.5999 | 1591.999 | 874.9124 | 1290.993 | 117.0097 | 913.0418 | 452.8393 | 995.7921 | 2701.759 | 220.3236 | 2263.289 | 234.2305 |
| CUST_5825_PI429010886 | N19252 | 0.021076 | 21 | 6 | 189235312 | PREDICTED: similar to AGAP007340-PB [Tribolium c | Transport | Transmembrane transport | 1.42E-55 | 104.351 | 80.63305 | 156.0949 | 157.6456 | 85.82609 | 238.8964 | 167.2066 | 227.604 | 161.8008 | 129.2244 | 90.68288 | 111.8008 | 115.4907 | 107.3764 | 131.4266 | 83.16839 | 98.90127 | 92.9186 | 158.7513 | 74.22931 | 95.51753 | 74.70996 | 121.4004 | 128.1757 | 93.76216 | 183.8217 | 93.60773 |
| CUST_26146_PI429010886 | N53749 | 0.021076 | 18 | 3 | | | | | | 15.03992 | 21.68921 | 14.97143 | 56.48952 | 21.58422 | 43.69907 | 12.60453 | 10.16422 | 10.47564 | 11.23684 | 14.4879 | 18.12837 | 7.729578 | 12.03567 | 19.36347 | 20.93304 | 10.2343 | 11.4368 | 18.18555 | 11.10462 | 9.278391 | 28.26309 | 18.63533 | 31.56077 | 27.08074 | 10.338 | 8.423847 |
| CUST_50145_PI429010886 | M88809 | 0.021087 | 21 | 15 | | | | | | 264.2251 | 292.6509 | 288.0337 | 239.6059 | 292.4718 | 261.6019 | 263.2372 | 222.3674 | 212.2832 | 330.3127 | 397.7744 | 318.5103 | 263.5218 | 250.2477 | 265.1519 | 342.2472 | 403.7411 | 304.7525 | 356.8925 | 279.5177 | 245.1598 | 231.0331 | 294.9448 | 332.2552 | 347.5866 | 242.6822 | 252.1291 |
| CUST_26132_PI429010886 | N53725 | 0.021087 | 21 | 15 | | | | | | 8.084402 | 5.96252 | 12.0262 | 8.670788 | 7.316619 | 13.6708 | 15.4721 | 13.93254 | 9.774844 | NA | 8.027473 | 9.988677 | 9.465987 | 16.73527 | 17.63183 | 11.95482 | NA | 20.14149 | 8.221669 | 15.25416 | 11.78768 | 6.076503 | 5.817842 | NA | 7.23801 | 16.44746 | 15.12292 |
| CUST_1609_PI429010886 | N10225 | 0.021088 | 21 | 12 | | | Metabolic process | Oxidation-reduction process | | 1918.897 | 2663.095 | 2014.561 | 1569.952 | 1818.602 | 1835.374 | 1561.567 | 1631.709 | 1863.799 | 2514.893 | 1886.936 | 2675.265 | 2042.507 | 2511.522 | 2701.759 | 3219.948 | 1869.455 | 1972.844 | 2091.598 | 2757.444 | 1964.934 | 2858.942 | 1926.251 | 1702.054 | 2456.459 | 1751.202 | 2513.183 |
| CUST_38636_PI429010886 | M28713 | 0.021091 | 12 | 3 | | | | | | 89.32488 | 108.2254 | 102.5545 | 131.2743 | 100.8596 | 99.15143 | 85.30129 | 84.47788 | 90.15151 | 98.87521 | 74.3942 | 85.37214 | 99.17539 | 107.9821 | 107.1241 | 177.1792 | 110.9478 | 128.0504 | 122.4191 | 93.69637 | 91.95171 | 110.3138 | 98.6608 | 72.4241 | 120.7611 | 75.2875 | 108.7377 |
| CUST_35246_PI429010886 | M12038 | 0.021101 | 24 | 21 | | | | | | 26.41115 | 28.5643 | 32.45773 | 17.5796 | 19.689 | 30.51803 | 12.42375 | 14.23269 | 25.5267 | 18.75909 | 19.20292 | 29.64209 | 16.64328 | 20.144 | 31.04302 | 32.26335 | 28.13722 | 40.69827 | 18.59144 | 27.18447 | 34.06071 | 41.52389 | 33.26796 | 21.07029 | 36.35659 | 14.44097 | 37.37861 |
| CUST_41523_PI429010886 | M57957 | 0.021101 | 24 | 21 | | | | | | 27.22941 | 28.55509 | 27.10885 | 25.10875 | 27.31066 | 26.11497 | 18.74202 | 26.00383 | 26.64257 | 28.23315 | 23.60499 | 13.19581 | 29.89381 | 27.37419 | 28.89154 | 33.45456 | 26.67812 | 32.86388 | 27.22484 | 36.8462 | 12.58067 | 36.64631 | 29.87357 | 28.27339 | 27.5502 | 21.60382 | 31.48252 |
| CUST_54301_PI429010886 | M105482 | 0.021111 | 21 | 6 | | | | | | 18.55589 | 6.364227 | 15.01303 | 22.13675 | 5.423434 | 5.338547 | 69.46903 | 44.51597 | 69.90395 | 6.720477 | NA | NA | 27.28895 | 30.28884 | 20.74295 | 32.56422 | 14.85609 | 42.05465 | 19.82066 | 10.63784 | NA | 3.958033 | 14.73176 | 19.00858 | 52.25211 | 12.30062 | 26.25499 |
| CUST_10929_PI429010886 | N27010 | 0.021122 | 15 | 0 | | | | | | 1267.407 | 1650.747 | 1205.667 | 1006.198 | 1168.111 | 1209.365 | 1150.482 | 974.9401 | 956.7714 | 713.4223 | 998.694 | 1388.367 | 1155.687 | 1123.432 | 1241.135 | 1857.991 | 1081.506 | 1556.767 | 1435.814 | 1205.12 | 999.0406 | 2056.54 | 795.9721 | 1075.314 | 1217.47 | 750.3627 | 831.7121 |
| CUST_8280_PI429010886 | N22811 | 0.021144 | 15 | 9 | 260813792 | hypothetical protein BRAFLDRAFT_124342 [Branchi | Metabolic process | Other | 7.03E-38 | 15.3592 | 16.58396 | 17.53688 | 13.30523 | 20.88612 | 16.24039 | 25.09705 | 50.85184 | 27.84371 | 33.05858 | 39.7396 | 36.63133 | 24.72309 | 21.05993 | 20.28484 | 28.40026 | 24.39326 | 28.40654 | 18.19605 | 60.45419 | 25.46966 | 19.98856 | 28.13722 | 29.82479 | 25.3441 | 33.88658 | 41.94521 |
| CUST_17778_PI429010886 | N38572 | 0.021145 | 18 | 6 | | | | | | 36.39221 | 50.92195 | 36.54664 | 50.67294 | 38.87395 | 28.98746 | 61.03938 | 94.34866 | 65.43441 | 51.73816 | 47.66523 | 61.34598 | 55.86399 | 87.71808 | 37.04826 | 43.61355 | 63.03207 | 59.91437 | 52.79221 | 36.48332 | 53.47338 | 43.00832 | 57.71752 | 60.43972 | 49.59376 | 77.1205 | 64.29604 |
| CUST_56498_PI429010886 | M113940 | 0.021158 | 15 | 3 | | | | | | 223.6052 | 260.7973 | 165.7579 | 250.2094 | 259.7703 | 305.3843 | 191.6952 | 189.8916 | 169.0201 | 192.7945 | 209.6338 | 198.6003 | 181.649 | 206.9171 | 247.1825 | 239.7427 | 228.547 | 228.4344 | 245.7365 | 263.8365 | 182.1441 | 253.8833 | 163.4134 | 208.0738 | 166.1109 | 169.9973 | 169.9973 |
| CUST_33469_PI429010886 | M6969 | 0.021158 | 15 | 0 | | | | | | 275.9326 | 322.9274 | 339.6585 | 215.7523 | 268.545 | 286.19 | 203.3901 | 246.5478 | 213.0233 | 251.2251 | 225.0823 | 358.55 | 221.0933 | 268.545 | 236.1561 | 305.4951 | 293.9351 | 299.6232 | 275.0029 | 205.9317 | 233.2471 | 240.682 | 273.1684 | 262.76 | 303.7146 | 263.345 | 290.0281 |
| CUST_9078_PI429010886 | N24028 | 0.021158 | 15 | 3 | | | | | | 313.5825 | 207.0425 | 184.7518 | 277.7171 | 299.6797 | 328.2844 | 333.0328 | 295.5774 | 206.8026 | 176.1772 | 254.4916 | 158.2442 | 245.9376 | 165.4521 | 198.6919 | 225.2614 | 359.297 | 276.1481 | 472.3127 | 283.7427 | 228.8706 | 157.0047 | 247.0492 | 304.2796 | 132.2661 | 297.361 | 143.7672 |
| CUST_29476_PI429010886 | N59943 | 0.021158 | 15 | 9 | | | | | | 209.1269 | 402.5662 | 281.874 | 216.6058 | 240.734 | 138.9124 | 179.8682 | 281.599 | 269.0643 | 493.7491 | 325.0318 | 366.4854 | 359.3716 | 330.6123 | 337.3367 | 411.8683 | 283.8866 | 244.7282 | 315.0245 | 445.8485 | 248.8723 | 537.3115 | 330.1179 | 180.9317 | 664.4926 | 409.1687 | 485.2041 |
| CUST_29511_PI429010886 | N60010 | 0.021158 | 15 | 9 | | | | | | 386.8992 | 467.8704 | 514.1495 | 419.4237 | 391.4581 | 406.809 | 391.7449 | 435.3919 | 465.4504 | 649.3217 | 414.9669 | 580.3063 | 467.665 | 607.2425 | 512.8422 | 560.3037 | 442.2873 | 454.6218 | 460.255 | 585.5822 | 388.3479 | 581.2638 | 487.0643 | 441.3844 | 601.0454 | 528.3775 | 655.0692 |
| CUST_30076_PI429010886 | N61136 | 0.021158 | 15 | 3 | 343481903 | unknown [Pectinophora gossypiella] | | | 1.28E-14 | 79.79104 | 50.64584 | 62.583 | 110.1036 | 129.7943 | 251.5259 | 119.3486 | 72.75459 | 66.60001 | 45.55401 | 66.82399 | 35.01718 | 70.48615 | 53.9049 | 50.93999 | 52.54728 | 189.7994 | 164.7204 | 160.2968 | 44.91724 | 78.08745 | 53.80851 | 71.57902 | 137.0769 | 43.94789 | 71.75603 | 47.01793 |
| CUST_9929_PI429010886 | N25427 | 0.021166 | 15 | 0 | | | | | | 97.1202 | 126.3708 | 86.25852 | 76.24385 | 63.16675 | 89.60537 | 57.38433 | 78.61543 | 55.32113 | 73.54244 | 68.05219 | 67.10317 | 74.82753 | 84.90321 | 114.4015 | 103.1223 | 88.16694 | 72.58192 | 89.68808 | 62.24459 | 78.28594 | 85.67865 | 70.80146 | 80.42722 | 87.49111 | 73.48596 | 62.16132 |
| CUST_42211_PI429010886 | M59699 | 0.021176 | 21 | 15 | | | | | | 41.80062 | 43.00468 | 31.87606 | 41.40709 | 38.31256 | 33.31614 | 30.36684 | 23.71505 | 26.11716 | 42.76199 | 38.10393 | 37.50197 | 33.95306 | 35.38465 | 30.74654 | 44.71419 | 44.8896 | 38.15596 | 37.42936 | 46.1565 | 38.09087 | 54.09438 | 22.93214 | 42.33366 | 38.68605 | 32.18186 | 33.86813 |
| CUST_41119_PI429010886 | M56912 | 0.021181 | 21 | 3 | | | | | | 165.5714 | 170.3963 | 153.3563 | 144.1452 | 727.7317 | 441.9667 | 146.4472 | 223.2901 | 179.0874 | 164.5463 | 480.2626 | 297.0259 | 134.9903 | 142.0014 | 140.7918 | 470.4395 | 206.5428 | 95.63731 | 128.673 | 292.4718 | 164.4185 | 164.3967 | 402.2126 | 121.1828 | 511.0593 | 868.0572 | 1033.757 |
| CUST_2228_PI429010886 | N11917 | 0.021195 | 24 | 18 | | | | | | 190.9127 | 217.37 | 228.2456 | 164.2501 | 183.786 | 150.2666 | 148.4479 | 168.6416 | 149.7495 | 233.2127 | 166.6117 | 240.9894 | 192.898 | 199.1618 | 194.0645 | 180.7626 | 136.6153 | 170.9183 | 199.7432 | 246.5478 | 224.9395 | 237.5448 | 201.6148 | 173.7706 | 225.6591 | 171.6462 | 205.078 |
| CUST_50435_PI429010886 | M89803 | 0.021222 | 18 | 3 | | | | | | 77.00574 | 50.60456 | 14.53622 | 98.56528 | 29.28327 | 59.73525 | NA | NA | 4.881906 | 46.83826 | 39.68514 | 31.36122 | 23.69356 | 11.10641 | 23.38175 | 22.70929 | 24.69848 | 30.44094 | 15.66738 | 24.0618 | 25.61415 | 97.2708 | 33.66515 | 77.7747 | 36.48665 | NA | NA |
| CUST_31189_PI429010886 | M931 | 0.021232 | 21 | 15 | 60652230 | protease [Homarus americanus] | Protein metabolism | Proteolysis | 1.11E-24 | 1193.941 | 1420.397 | 1089.128 | 950.2628 | 1164.641 | 941.6865 | 1816.167 | 1258.369 | 1065.529 | 2348.331 | 1341.448 | 1296.243 | 1300.415 | 1447.664 | 1063.11 | 2098.72 | 1601.013 | 1114.646 | 1256.525 | 1287.066 | 876.1774 | 1522.087 | 1255.497 | 1570.987 | 1057.219 | 810.6402 | 1198.309 |
| CUST_36207_PI429010886 | M15017 | 0.021247 | 24 | 21 | | | | | | 26.56771 | 31.20174 | 20.66168 | 17.61531 | 20.25261 | 19.37598 | 17.10186 | 12.08465 | 18.32343 | 33.79631 | 16.73391 | 25.76975 | 17.27797 | 27.16781 | 29.32849 | 31.01397 | 24.85164 | 20.43418 | 28.1987 | 27.69389 | 23.30079 | 41.55928 | 31.48445 | 15.83311 | 27.45965 | 25.888 | 23.59336 |
| CUST_16717_PI429010886 | N36746 | 0.021247 | 24 | 15 | | | | | | 54.12753 | 55.17531 | 36.60129 | 50.42986 | 40.62219 | 31.64722 | 32.6735 | 28.24069 | 36.35659 | 46.48282 | 40.62219 | 66.13621 | 56.44254 | 47.77962 | 43.94258 | 65.97871 | 46.64002 | 68.78674 | 51.1868 | 58.35369 | 56.92511 | 62.44095 | 45.84834 | 36.3262 | 70.02979 | 63.36658 | 61.44485 |
| CUST_19647_PI429010886 | N41937 | 0.021248 | 21 | 9 | | | | | | 15.30761 | 12.1537 | 13.25045 | 22.1046 | 18.79291 | 12.6884 | 21.01736 | 36.03688 | 23.64095 | 20.91496 | 25.94523 | 19.11687 | 38.95233 | 15.36478 | 20.02885 | 11.24362 | 17.5491 | NA | 26.96182 | 71.31512 | 13.82074 | 17.11553 | 19.71085 | 10.48943 | 17.81636 | 13.60767 | 17.80075 |
| CUST_56242_PI429010886 | M113245 | 0.021256 | 15 | 0 | | | | | | 25.26918 | 18.70053 | 36.8491 | 13.75242 | 20.63001 | 14.0442 | 14.94106 | 14.55595 | 18.42271 | 19.84686 | 17.29005 | 11.05263 | 19.32763 | 25.2475 | 11.08171 | 13.39711 | 20.77886 | 23.05797 | 17.86554 | 14.64036 | 15.20876 | 14.56343 | 9.991254 | 10.37139 | 20.06405 | 18.91214 | 23.58557 |
| CUST_8059_PI429010886 | N22479 | 0.021264 | 15 | 12 | | | | | | 83.383 | 79.01755 | 84.66815 | 63.77772 | 66.33801 | 64.28764 | 47.79169 | 60.67368 | 76.91503 | 79.97877 | 75.24399 | 116.2372 | 89.29805 | 97.05352 | 94.24344 | 67.59014 | 77.01708 | 68.22752 | 73.0191 | 93.91055 | 45.69115 | 77.12763 | 99.79838 | 58.79065 | 95.86282 | 61.48326 | 87.90722 |
| CUST_50888_PI429010886 | M91350 | 0.021276 | 15 | 12 | | | | | | 6.288505 | 24.5556 | 33.90175 | 15.15061 | 7.475526 | NA | 48.91209 | 27.21684 | 129.5236 | 90.01113 | 3.782381 | 29.87699 | 355.9452 | 346.6366 | 146.902 | 124.3147 | 64.82649 | 29.97696 | 15.97964 | 56.99751 | 26.75848 | 76.36771 | 93.82886 | 8.6774 | 704.9925 | 37.38397 | 789.9286 |
| CUST_216_PI429010886 | N2716 | 0.02128 | 12 | 3 | | | | | | 9756.741 | 6949.82 | 396.1913 | 27088.59 | 21431.7 | 27142.29 | 11926.23 | 15000.31 | 6049.937 | 348.6809 | 5596.154 | 214.4171 | 8986.011 | 431.0477 | 6329.544 | 3138.529 | 11413.33 | 12089.86 | 15922.04 | 336.2223 | 3296.897 | 653.9628 | 2179.192 | 10866.21 | 422.6396 | 15133.65 | 99.03782 |
| CUST_25440_PI429010886 | N52426 | 0.02128 | 12 | 3 | 493636260 | hypothetical protein, partial [Lactobacillus jensenii] | | | 1.11E-10 | 32.06361 | 28.79209 | 12.22639 | 121.2504 | 108.3441 | 185.0427 | 82.02402 | 42.51692 | 11.12035 | 7.57659 | 106.5352 | 7.593357 | 32.01033 | 29.61255 | 40.62219 | 22.37306 | 212.8309 | 173.4253 | 146.2229 | 55.91435 | 70.30533 | 53.07973 | 93.36286 | 135.8061 | 6.278329 | 109.399 | 10.13043 |
| CUST_52538_PI429010886 | M97857 | 0.021289 | 21 | 3 | | | | | | 29.39991 | 55.23983 | 35.20228 | 84.11167 | 35.24411 | 45.52612 | 32.63377 | 27.47176 | 29.77814 | 28.92063 | 23.81358 | 40.70276 | 24.28 | 26.73933 | 29.12856 | 29.29818 | 12.83327 | 66.23901 | 14.60389 | 27.0144 | 36.80555 | 29.53078 | 42.53014 | 39.56122 | 41.86651 | 53.40548 | 22.35568 |
| CUST_931_PI429010886 | N7861 | 0.021315 | 24 | 21 | | | | | | 58.46315 | 52.01256 | 45.81043 | 39.21751 | 31.04579 | 64.41009 | 50.52879 | 30.558 | 18.94868 | 71.3655 | 70.88996 | 54.08762 | 17.32049 | 29.96972 | 17.45713 | 93.3114 | 43.09974 | 62.54934 | 84.12226 | 44.93417 | 50.74837 | 62.406 | 74.93674 | 74.12883 | 130.1208 | 73.29485 | 100.7779 |
| CUST_37616_PI429010886 | M19862 | 0.021325 | 21 | 9 | | | | | | 1524.206 | 482.0705 | 406.5096 | 468.6028 | 167.1292 | 81.62354 | 457.1179 | 1070.988 | 786.1873 | 3680.553 | 423.679 | 1872.445 | 880.2112 | 951.6021 | 1467.586 | 982.8541 | 389.9711 | 345.2409 | 900.1864 | 270.8273 | 541.4466 | 726.9909 | 311.1732 | 554.3622 | 140.3134 | 386.742 | 766.9049 |
| CUST_13179_PI429010886 | N30726 | 0.021327 | 18 | 3 | | | | | | 113.475 | 89.14278 | 54.73744 | 148.3243 | 97.4551 | 102.1126 | 108.064 | 57.40815 | 89.88488 | 60.5193 | 56.32248 | 77.50869 | 93.01036 | 52.37245 | 77.83266 | 70.04597 | 41.69983 | 66.50645 | 63.3033 | 70.02583 | 71.07061 | 105.9444 | 61.08538 | 164.5164 | 73.23407 | 83.56858 | 65.9729 |
| CUST_33207_PI429010886 | M6272 | 0.021333 | 15 | 3 | | | | | | 726.086 | 1756.971 | 301.467 | 5969.266 | 4729.674 | 4882.08 | 2551.399 | 3456.298 | 414.4964 | 19.43095 | 2560.213 | 16.43851 | 334.1058 | 70.47896 | 658.1422 | 139.545 | 1886.936 | 563.9632 | 792.4242 | 23.97968 | 1998.595 | 67.15618 | 1819.925 | 3105.643 | 136.9989 | 334.2433 | 111.4345 |
| CUST_21239_PI429010886 | N44873 | 0.021333 | 18 | 3 | | | | | | 16.4962 | 15.04174 | 10.67448 | 27.60111 | 19.28099 | 27.2627 | 14.60716 | 14.73365 | 6.706567 | 10.15159 | 14.76794 | 14.55066 | 10.8963 | 9.322057 | 17.67943 | 15.40175 | 17.41767 | 13.47393 | 21.88907 | 12.47348 | 8.947846 | 21.84546 | 17.4145 | 20.81747 | 11.26503 | 59.78571 | 17.89088 |
| CUST_32231_PI429010886 | M3589 | 0.021336 | 18 | 15 | | | | | | 67.39787 | 70.74029 | 59.38223 | 51.25067 | 62.00733 | 64.32323 | 50.67859 | 45.6737 | 35.30842 | 59.59032 | 88.78018 | 55.98343 | 48.84923 | 57.89544 | 52.46763 | 89.50659 | 65.8929 | 80.72822 | 89.30785 | 89.27715 | 56.07148 | 106.4397 | 25.62386 | 46.59363 | 93.59991 | 38.49903 | 37.95647 |
| CUST_38779_PI429010886 | M30303 | 0.021347 | 21 | 15 | | | | | | 728.8915 | 1016.851 | 675.9235 | 715.6525 | 676.9355 | 649.1033 | 701.3851 | 603.6169 | 587.4356 | 903.546 | 975.2952 | 911.683 | 851.0651 | 914.6851 | 398.8868 | 1044.227 | 1557.99 | 744.5402 | 874.9124 | 1531.156 | 637.6126 | 889.3445 | 706.7803 | 731.2482 | 705.5663 | 490.9356 | 1183.738 |
| CUST_3598_PI429010886 | N14851 | 0.021372 | 24 | 21 | | | | | | 74.58008 | 110.1036 | 124.3147 | 76.60117 | 111.1756 | 88.27274 | 62.16793 | 85.34367 | 82.24759 | 114.9498 | 127.5355 | 82.929 | 93.91055 | 107.2112 | 118.3919 | 102.4301 | 165.7787 | 75.33028 | 126.6031 | 131.6883 | 105.57 | 157.9764 | 145.3984 | 116.5927 | 110.8044 | 83.29202 | 63.83994 |
| CUST_31783_PI429010886 | M2403 | 0.021401 | 15 | 9 | 321458775 | hypothetical protein DAPPUDRAFT_300656 [Daphn | Nucleic acid metabolism | Regulation of gene expression | 6.3E-29 | 366.5663 | 600.5562 | 505.0665 | 348.0084 | 344.9163 | 304.1627 | 302.8941 | 493.9819 | 568.2036 | 609.0182 | 388.5645 | 702.7008 | 528.1283 | 634.9072 | 522.8025 | 586.9437 | 380.124 | 418.7921 | 388.1952 | 526.3482 | 443.4781 | 686.5061 | 561.9199 | 359.0948 | 632.4528 | 417.4973 | 685.5063 |
| CUST_44807_PI429010886 | M67058 | 0.021401 | 15 | 9 | 319738740 | Paracyclopina nana heat shock protein 90 mRNA, cc | Protein metabolism | Protein folding | 1.64E-66 | 775.6671 | 1132.649 | 997.0196 | 623.9709 | 863.1365 | 562.3412 | 712.7429 | 863.6121 | 996.654 | 1569.952 | 929.5412 | 1895.191 | 1175.839 | 1431.436 | 919.3319 | 1594.841 | 616.2727 | 974.3808 | 773.4201 | 1284.605 | 953.9498 | 1401.401 | 1115.083 | 695.5556 | 1626.337 | 942.3167 | 2118.286 |
| CUST_47297_PI429010886 | M75510 | 0.021401 | 15 | 9 | | | | | | 157.7661 | 169.1739 | 175.3395 | 129.9842 | 131.6679 | 170.5765 | 127.112 | 153.376 | 155.0808 | 205.1599 | 113.9031 | 198.5143 | 151.12 | 183.021 | 186.167 | 170.0985 | 154.0879 | 178.9173 | 106.6154 | 177.0286 | 141.5625 | 125.5467 | 162.3514 | 166.9427 | 283.8348 | 187.4537 | 218.8408 |
| CUST_48783_PI429010886 | M82183 | 0.021401 | 15 | 9 | | | | | | 52.1682 | 48.26458 | 57.37114 | 48.96007 | 32.15505 | 40.02231 | 65.20347 | 55.37764 | 55.04289 | 24.32948 | 61.7839 | 78.84971 | 48.63366 | 65.65578 | 73.37448 | 55.52574 | 47.13629 | 59.15067 | 58.38163 | 43.58778 | 41.59399 | 64.32323 | 64.17425 | 48.75136 | 81.33169 | 52.51409 | 73.02744 |
| CUST_11372_PI429010886 | N27709 | 0.021401 | 15 | 0 | | | | | | 123.2134 | 192.557 | 155.9427 | 120.0446 | 167.6024 | 218.636 | 81.08583 | 131.9637 | 108.8513 | 165.716 | 242.0042 | 137.616 | 73.54244 | 108.7837 | 139.0764 | 222.0782 | 186.7032 | 192.557 | 155.1093 | 140.5892 | 144.3143 | 130.8664 | 96.28021 | 95.03994 | 173.3934 | 96.21869 | 150.1517 |
| CUST_15229_PI429010886 | N34231 | 0.021401 | 15 | 3 | 321458553 | hypothetical protein DAPPUDRAFT_62084 [Daphnia | Metabolic process | Carbohydrate metabolic process | 1.37E-24 | 1355.174 | 358.1231 | 716.2921 | 1947.429 | 2975.526 | 2500.44 | 2274.622 | 973.9722 | 304.1627 | 33.54623 | 1929.904 | 12.11785 | 680.741 | 56.60803 | 343.3863 | 130.8825 | 3074.469 | 1159.33 | 1845.048 | 35.992 | 1405.495 | 163.9694 | 1091.244 | 1934.877 | 22.55101 | 1259.079 | 9.548233 |
| CUST_543_PI429010886 | N5909 | 0.021401 | 15 | 9 | | | | | | 354.2504 | 118.0238 | 4506.751 | 15.58064 | 23.84605 | 10.72726 | 1285.231 | 6879.978 | 8036.745 | 6407.392 | 1015.729 | 1367.805 | 3630.71 | 12916.14 | 5545.717 | 231.1968 | 4077.008 | 128.4226 | 744.0064 | 1794.046 | 275.6931 | 9131.511 | 10247.75 | 238.1333 | 13214.51 | 895.8021 | 10729.62 |
| CUST_24413_PI429010886 | N50580 | 0.021409 | 18 | 12 | | | | | | 249.3844 | 174.5672 | 219.0379 | 186.3956 | 209.4461 | 205.9317 | 205.4682 | 208.6342 | 266.5285 | 295.8552 | 288.6485 | 261.3191 | 337.0926 | 267.619 | 308.7717 | 448.4129 | 201.7309 | 205.0143 | 499.4614 | 202.6446 | 287.3115 | 213.0864 | 270.3602 | 162.7472 | 249.3051 | 228.1824 | 245.1937 |
| CUST_41779_PI429010886 | M58539 | 0.021415 | 15 | 12 | 225710954 | UPF0351 protein C9orf32 homolog [Caligus rogercr | | | 2.26E-38 | 116.7026 | 136.3233 | 122.7194 | 95.42654 | 107.4216 | 92.19432 | 86.7376 | 93.82886 | 88.57155 | 138.3597 | 108.6304 | 157.1144 | 119.9288 | 113.9999 | 122.7816 | 150.8429 | 92.26296 | 107.354 | 90.23885 | 126.1331 | 109.8598 | 119.3258 | 94.62564 | 59.26817 | 125.8931 | 110.3957 | 132.3624 |
| CUST_6751_PI429010886 | N20555 | 0.021415 | 15 | 12 | | | | | | 520.4614 | 575.6787 | 548.077 | 498.3855 | 516.9528 | 654.3393 | 433.1734 | 565.6978 | 444.5828 | 610.3506 | 593.968 | 606.3721 | 521.8521 | 634.7435 | 614.5524 | 709.528 | 556.3775 | 483.6862 | 536.6546 | 653.4911 | 551.706 | 423.767 | 514.8406 | 458.4442 | 766.9049 | 574.8968 | 566.7662 |
| CUST_8614_PI429010886 | N23304 | 0.021415 | 15 | 12 | 524999176 | PREDICTED: AP-4 complex subunit sigma-1 isoform | Transport | Protein transport | 3.4E-23 | 42.4817 | 43.41027 | 47.3396 | 32.9302 | 31.40893 | 40.27597 | 40.91962 | 27.72017 | 42.29018 | 41.20843 | 35.06362 | 46.682 | 34.93327 | 46.02004 | 46.12593 | 44.87845 | 34.0788 | 59.35924 | 26.53007 | 34.16739 | 30.75954 | 61.20199 | 28.73136 | 32.11685 | 45.15073 | 40.76277 | 45.66434 |
| CUST_26441_PI429010886 | N54249 | 0.021415 | 15 | 12 | | | | | | 105.3539 | 119.245 | 65.23612 | 136.7912 | 104.7461 | 72.10026 | 67.77787 | 41.25578 | 48.46285 | 83.88709 | 144.9989 | 131.7223 | 103.1588 | 124.7893 | 88.17592 | 101.7238 | 113.2835 | 65.72077 | 71.09841 | 176.0509 | 73.91159 | 68.89154 | 48.98425 | 49.20588 | 66.70898 | 111.4084 | 72.79951 |
| CUST_1043_PI429010886 | N8304 | 0.021415 | 15 | 12 | | | | | | 699.2311 | 872.488 | 959.6386 | 666.5369 | 710.22 | 880.2112 | 705.2005 | 793.8063 | 770.1948 | 906.266 | 760.5296 | 1183.738 | 846.752 | 1094.409 | 1233.197 | 1033.403 | 760.0455 | 768.2695 | 740.6945 | 928.4158 | 766.9049 | 1013.272 | 905.268 | 675.9235 | 1196.575 | 1018.693 | 1021.127 |
| CUST_52167_PI429010886 | M96623 | 0.02144 | 21 | 0 | 196006295 | hypothetical protein TRIADDRAFT_26025 [Trichopla | | | 8.9E-16 | 66.50645 | 55.40579 | 66.78152 | 51.28378 | 50.17076 | 59.58353 | 55.82952 | 57.82155 | 38.7854 | 50.43862 | 50.25117 | 52.58792 | 45.66434 | 51.21188 | 38.33878 | 61.67082 | 53.00119 | 43.09035 | 42.89937 | 62.10992 | 46.43129 | 56.57261 | 64.8815 | 60.15341 | 50.32404 | 34.85305 | 39.9644 |
| CUST_14611_PI429010886 | N33171 | 0.02144 | 21 | 0 | | | | | | 44.8824 | 54.04063 | 53.91951 | 42.41265 | 38.18452 | 43.89224 | 29.08411 | 40.80851 | 36.38283 | 58.13824 | 33.90175 | 35.55553 | 27.32583 | 30.58363 | 44.92544 | 45.3118 | 29.36585 | 45.80703 | 41.96451 | 35.0422 | 46.89105 | 55.51379 | 46.682 | 39.83835 | 54.40561 | 40.17935 | 43.67133 |
| CUST_25550_PI429010886 | N52624 | 0.02144 | 21 | 0 | | | | | | 20.13562 | 20.65721 | 15.83311 | 7.298488 | 13.46995 | 23.17992 | 13.44726 | 17.15146 | 12.71188 | 6.48461 | 13.23813 | 22.84721 | 7.898568 | 7.204193 | 7.991765 | 11.95869 | 15.78805 | 21.96294 | 13.47795 | 8.093925 | 14.46162 | 25.352 | 13.58569 | 24.03363 | 13.92846 | 7.204193 | 7.080158 |
| CUST_49791_PI429010886 | M87121 | 0.021461 | 15 | 9 | | | | | | 129.5889 | 135.3575 | 168.154 | 124.0489 | 111.6628 | 131.7044 | 114.6015 | 133.7242 | 117.2428 | 152.3001 | 118.0091 | 178.833 | 149.3268 | 158.0759 | 171.1862 | 155.8611 | 114.4714 | 157.7661 | 127.7879 | 147.0578 | 154.2676 | 149.0785 | 139.47 | 119.8693 | 170.3686 | 161.2704 | 174.8053 |
| CUST_8427_PI429010886 | N23020 | 0.021462 | 24 | 15 | | | | | | 256.8125 | 403.5814 | 335.2532 | 237.4703 | 244.4939 | 197.44 | 204.1442 | 204.4866 | 234.7465 | 373.4486 | 275.3515 | 334.7375 | 280.6713 | 309.8242 | 328.0426 | 441.149 | 223.8346 | 379.6269 | 306.2716 | 280.7168 | 335.333 | 424.5435 | 293.9979 | 309.8951 | 346.5745 | 268.0379 | 356.7158 |
| CUST_31779_PI429010886 | M2387 | 0.021497 | 21 | 18 | | | | | | 262.8116 | 355.7822 | 220.7455 | 229.5365 | 229.2068 | 194.3859 | 185.0712 | 177.8416 | 193.0075 | 305.4399 | 230.1871 | 289.0715 | 220.2915 | 225.2357 | 251.3539 | 396.3895 | 194.6975 | 228.5019 | 250.1141 | 342.3707 | 282.6926 | 301.9881 | 238.7009 | 188.7198 | 266.3801 | 212.606 | 266.6935 |
| CUST_30219_PI429010886 | N61421 | 0.021497 | 24 | 21 | | | | | | 94.40103 | 95.14966 | 121.7341 | 80.03068 | 85.1516 | 128.7791 | 77.46325 | 95.33691 | 75.66735 | 117.2199 | 98.53734 | 108.5045 | 85.46155 | 107.9692 | 116.3563 | 126.1505 | 109.5408 | 119.5972 | 94.1523 | 126.9651 | 134.486 | 123.6053 | 118.1115 | 108.4515 | 180.4095 | 114.4563 | 149.6184 |
| CUST_9525_PI429010886 | N24780 | 0.021498 | 24 | 6 | | | | | | 12.31693 | 13.16261 | 7.160939 | 11.12353 | 7.499239 | 10.40254 | 18.83394 | NA | 12.75753 | NA | 7.675832 | 11.81981 | 6.551813 | 9.854344 | 12.04133 | 9.544892 | 4.812672 | 7.819931 | 5.215206 | 4.540022 | 7.097098 | 10.01379 | 15.036 | NA | 7.345898 | 10.98742 | 4.93182 |
| CUST_7137_PI429010886 | N21092 | 0.021519 | 12 | 3 | 290562605 | Haloalkane dehalogenase [Lepeophtheirus salmonis | | | 3.07E-55 | 30.73873 | 19.04932 | 9.935826 | 76.26718 | 62.04982 | 117.5697 | 64.10151 | 15.3003 | 28.29582 | 7.100449 | 32.57877 | 10.17045 | 33.07929 | 12.48715 | 23.59085 | 17.60614 | 60.68591 | 68.1054 | 60.07749 | 5.655799 | 24.68775 | 13.04046 | 14.46162 | 38.01489 | 7.865975 | 16.34592 | 5.921666 |
| CUST_47683_PI429010886 | M77166 | 0.021532 | 21 | 15 | 242017555 | presqualene diphosphate phosphatase, putative [Pe | Metabolic process | Other | 7.94E-12 | 8.729008 | 15.29306 | NA | 9.086428 | 10.99151 | 8.049312 | 15.63271 | 9.270637 | 14.27562 | 20.16612 | 13.78092 | 14.67638 | 8.732125 | 9.753321 | 16.98325 | 18.56806 | 14.70379 | 16.53144 | 17.99854 | 13.31154 | 13.32601 | 12.34688 | 12.79089 | 20.9954 | 15.7975 | 4.911986 | 6.709669 |
| CUST_6297_PI429010886 | N19916 | 0.021562 | 15 | 12 | | | | | | 354.6545 | 306.0346 | 875.926 | 272.1926 | 276.6492 | 336.8758 | 499.3294 | 634.2891 | 658.8544 | 604.7306 | 322.4251 | 418.9595 | 578.5402 | 1125.765 | 450.5804 | 381.498 | 558.5528 | 424.2638 | 348.7613 | 362.7402 | 380.3886 | 421.6001 | 602.4628 | 338.4841 | 447.7446 | 340.4766 | 515.5917 |
| CUST_46198_PI429010886 | M71738 | 0.021566 | 24 | 3 | | | | | | 11.62742 | 10.03543 | NA | 19.58619 | 13.63953 | 18.17304 | 14.28863 | 11.37559 | 15.1999 | 4.709883 | 5.369808 | NA | 6.39392 | 11.68718 | 7.965022 | 7.516724 | 10.84659 | 17.51086 | 5.72681 | 6.315643 | 5.633982 | 7.853568 | NA | 23.04508 | 6.363361 | 12.55445 | 11.17643 |
| CUST_34427_PI429010886 | M9638 | 0.021569 | 24 | 15 | 321469052 | hypothetical protein DAPPUDRAFT_212220 [Daphn | Transport | Protein transport | 8E-160 | 45.93195 | 37.13071 | 54.22335 | 38.06259 | 61.53357 | 35.23712 | 37.44113 | 26.51217 | 35.1893 | 65.6728 | 52.82166 | 46.64388 | 48.08498 | 45.39483 | 65.11921 | 69.50944 | 48.82147 | 304.437 | 41.64394 | 47.29164 | 44.09424 | 62.54347 | 39.20036 | 39.52535 | 50.78152 | 46.90121 | 49.87667 |
| CUST_28247_PI429010886 | N57590 | 0.02159 | 15 | 9 | | | | | | 140.9929 | 127.3036 | 140.3818 | 161.4564 | 130.3926 | 149.5758 | 153.8888 | 138.7197 | 149.1909 | 189.8316 | 144.5244 | 162.4256 | 133.8101 | 178.3541 | 153.5334 | 159.4972 | 144.5473 | 142.7507 | 135.6374 | 147.1104 | 146.3194 | 150.434 | 129.5373 | 151.5411 | 220.4437 | 154.6176 | 241.5289 |
| CUST_13474_PI429010886 | N31252 | 0.02159 | 15 | 0 | | | | | | 284.3688 | 245.2785 | 260.3136 | 194.9822 | 193.8296 | 178.2181 | 184.3061 | 226.9563 | 188.8906 | 232.1818 | 193.0602 | 206.2668 | 251.0607 | 283.1467 | 237.3703 | 279.6762 | 215.028 | 238.8193 | 234.6626 | 284.6531 | 226.3675 | 282.8614 | 197.5074 | 165.1872 | 269.6735 | 192.8311 | 236.613 |
| CUST_49320_PI429010886 | M85182 | 0.021592 | 24 | 18 | | | | | | 26.93342 | 9.133825 | 7.432825 | 24.14877 | 20.59244 | 15.35382 | 15.88012 | 5.85187 | 12.46283 | 18.3198 | 9.918797 | 11.16121 | 13.75845 | 6.9339 | 11.5275 | 20.85111 | 15.59665 | 16.4962 | 17.2249 | 27.81087 | 22.2368 | 17.20661 | 20.62099 | 18.06516 | 16.59828 | 186.9387 | 16.73391 |
| CUST_137_PI429010886 | N1431 | 0.021641 | 15 | 12 | 390331900 | PREDICTED: protein SpAN-like [Strongylocentrotus p | Protein metabolism | Proteolysis | 2.11E-55 | 9382.703 | 13400.98 | 8237.502 | 4944.792 | 9140.621 | 7318.038 | 12898.01 | 5585.575 | 8745.677 | 10685.37 | 6495.601 | 6059.267 | 12089.86 | 10510.22 | 8352.868 | 6932.514 | 6211.63 | 7083.117 | 5162.076 | 5367.717 | 5171.854 | 7920.23 | 2278.385 | 3815.349 | 9965.559 | 9182.945 | 6630.238 |
| CUST_56950_PI429010886 | M114985 | 0.021644 | 15 | 9 | | | | | | 197.3275 | 211.3121 | 251.204 | 156.7058 | 167.2554 | 173.6967 | 172.2644 | 178.3785 | 198.8955 | 240.646 | 164.3174 | 217.7746 | 177.1792 | 227.4444 | 189.2302 | 207.6194 | 175.2355 | 206.5724 | 161.9364 | 201.1997 | 163.0075 | 220.1334 | 187.1216 | 156.2332 | 242.1982 | 213.9187 | 210.9062 |
| CUST_31718_PI429010886 | M2239 | 0.021644 | 15 | 9 | 291239931 | PREDICTED: GK11735-like [Saccoglossus kowalevski | | | 1.65E-13 | 302.3102 | 409.9605 | 407.4423 | 324.1237 | 273.4925 | 232.1818 | 216 | 394.2069 | 560.1437 | 456.6758 | 499.1257 | 619.6845 | 351.6009 | 408.6642 | 440.22 | 612.9216 | 251.404 | 383.9376 | 428.0243 | 321.2502 | 379.0179 | 470.9015 | 391.4581 | 253.8833 | 355.8536 | 466.3395 | 674.891 |
| CUST_38584_PI429010886 | M28285 | 0.021644 | 15 | 9 | 220172371 | putative elastin a [Rimicaris exoculata] | | | 1.21E-12 | 281.447 | 1519.583 | 1329.963 | 263.5785 | 299.0341 | 39.46416 | 136.597 | 618.6744 | 2118.286 | 3034.646 | 1553.73 | 3489.908 | 820.5141 | 3432.751 | 1805.829 | 2434.013 | 274.6475 | 951.2956 | 1420.397 | 2144.378 | 1537.974 | 4080.667 | 2081.941 | 132.4207 | 4025.068 | 2119.433 | 4107.091 |
| CUST_42993_PI429010886 | M61807 | 0.021644 | 15 | 0 | | | | | | 297.14 | 297.5859 | 344.3603 | 207.0425 | 314.3652 | 336.682 | 203.5636 | 241.6881 | 214.3921 | 334.8266 | 297.361 | 326.2957 | 149.5353 | 285.7004 | 281.9612 | 363.9558 | 381.8763 | 388.2685 | 335.4811 | 339.3078 | 263.2877 | 286.997 | 233.828 | 223.0851 | 468.6028 | 276.3669 | 386.8992 |
| CUST_9824_PI429010886 | N25257 | 0.021644 | 15 | 9 | 321463706 | hypothetical protein DAPPUDRAFT_56817 [Daphnia | Metabolic process | Cellular amino acid metabolic process | 1.65E-56 | 108.4668 | 118.4299 | 102.9808 | 92.97872 | 84.88556 | 46.69134 | 81.45472 | 141.4128 | 114.6834 | 191.9895 | 148.2477 | 125.2116 | 162.3056 | 157.17 | 102.6011 | 118.2591 | 98.42661 | 111.812 | 83.16029 | 177.8158 | 93.80027 | 233.088 | 116.2535 | 70.3766 | 268.3811 | 83.40091 | 216.1505 |
| CUST_10146_PI429010886 | N25769 | 0.021644 | 15 | 9 | | | | | | 36.60913 | 41.97456 | 35.83044 | 29.97696 | 34.20386 | 21.29353 | 43.48893 | 34.43474 | 30.85363 | 48.12117 | 29.60942 | 48.73236 | 31.36464 | 46.95122 | 39.23286 | 36.7953 | 23.93544 | 32.28075 | 30.01154 | 35.25625 | 42.30429 | 42.67635 | 25.96869 | 33.24397 | 45.25329 | 44.55249 | 58.14321 |
| CUST_1488_PI429010886 | N9785 | 0.021644 | 15 | 9 | | | | | | 44.50661 | 57.6895 | 58.74569 | 48.87456 | 49.04708 | 56.95101 | 37.19805 | 43.16663 | 57.40815 | 63.28709 | 51.68456 | 76.8667 | 42.21202 | 52.55999 | 48.32025 | 64.8492 | 39.05183 | 50.32404 | 42.20313 | 45.64715 | 32.54901 | 58.27728 | 50.69847 | 41.07674 | 62.44095 | 59.57796 | 74.25631 |
| CUST_10277_PI429010886 | N25990 | 0.021664 | 15 | 12 | | | | | | 101.7048 | 93.68609 | 108.6889 | 84.57304 | 96.09446 | 92.21711 | 81.18707 | 79.44029 | 104.205 | 102.1576 | 96.08349 | 110.9726 | 110.2159 | 125.1102 | 122.3193 | 113.6765 | 94.00857 | 96.58099 | 90.81223 | 106.8281 | 101.4752 | 113.0468 | 108.3734 | 91.04016 | 125.0127 | 98.11651 | 156.8208 |
| CUST_16663_PI429010886 | N36651 | 0.021664 | 15 | 3 | | | | | | 23.26869 | 16.3703 | 33.7306 | 30.30557 | 38.88089 | 64.08758 | 54.14016 | 22.54321 | 18.85554 | 16.8197 | 42.77863 | 11.49926 | 15.29306 | 15.8511 | 12.60946 | 15.24921 | 75.44459 | 40.07867 | 52.30377 | 7.411834 | 41.36078 | 27.62695 | 35.75835 | 29.70907 | 16.97579 | 21.87774 | 15.11636 |
| CUST_18472_PI429010886 | N39862 | 0.021664 | 15 | 3 | | | | | | 193.4964 | 88.74926 | 223.7478 | 403.2396 | 356.5042 | 367.4512 | 299.6797 | 341.1708 | 98.10524 | 139.545 | 331.8125 | 138.4956 | 138.9958 | 75.1394 | 118.2001 | 126.3438 | 278.2553 | 339.2296 | 489.6976 | 92.9186 | 287.8668 | 191.2063 | 255.8316 | 221.4446 | 89.64325 | 495.8952 | 129.5373 |
| CUST_28947_PI429010886 | N58898 | 0.021664 | 15 | 12 | | | | | | 55.58103 | 55.66641 | 48.73587 | 45.29251 | 43.961 | 40.48072 | 49.7894 | 40.92745 | 44.19983 | 68.22752 | 40.50125 | 46.23463 | 53.95069 | 72.86527 | 68.29796 | 57.664 | 48.3123 | 49.37485 | 57.80004 | 37.50798 | 44.40636 | 59.44434 | 48.00964 | 47.18136 | 79.96242 | 41.72747 | 64.31677 |
| CUST_41519_PI429010886 | M57936 | 0.021671 | 15 | 0 | | | | | | 273.8248 | 295.7576 | 186.1103 | 360.037 | 225.9175 | 221.5021 | 239.8387 | 164.4877 | 179.2612 | 161.827 | 180.3315 | 164.0153 | 214.7331 | 170.5765 | 230.5203 | 329.9922 | 244.2379 | 232.5962 | 184.654 | 219.8055 | 176.8268 | 243.5153 | 195.0743 | 193.5749 | 192.442 | 274.1119 | 175.9719 |
| CUST_50835_PI429010886 | M91184 | 0.021671 | 15 | 0 | | | | | | 16.03903 | 26.44892 | 17.18028 | 21.4795 | 23.23746 | 21.25027 | 30.87202 | 18.0662 | 7.25708 | 15.1736 | 6.364227 | 9.381836 | 12.89633 | 18.62473 | 14.61619 | 25.57573 | 23.59085 | 20.58016 | 24.97756 | 13.85312 | 10.90545 | 16.24458 | 18.29194 | 27.51169 | 14.53096 | 10.41028 | 10.76376 |
| CUST_1570_PI429010886 | N10105 | 0.021672 | 21 | 9 | | | | | | 3.935641 | 28.97255 | 12.87174 | 82.14765 | 193.9282 | 27.31066 | 77.86556 | 469.1551 | 308.528 | 25.71973 | 413.7293 | 644.7638 | 270.5782 | 32.63646 | 135.3886 | NA | 121.3613 | NA | 6.616037 | 1198.73 | 328.1634 | 5.072381 | 44.15772 | 49.50242 | 12.05044 | 278.4332 | 272.2987 |
| CUST_26634_PI429010886 | N54586 | 0.021698 | 21 | 3 | | | | | | 56.19324 | 37.57841 | 36.90877 | 47.41387 | 58.59263 | 45.91808 | 29.07242 | 34.60947 | 35.44816 | 57.08439 | 46.06884 | 43.28403 | 26.80447 | 31.85967 | 32.26619 | 48.84271 | 44.66782 | 50.23482 | 40.97284 | 39.65895 | 50.18155 | 68.49231 | 49.43857 | 35.39506 | 70.21778 | 52.645 | 57.56078 |
| CUST_38820_PI429010886 | M30943 | 0.021702 | 18 | 12 | | | | | | 6350.776 | 10178.43 | 8176.608 | 4782.509 | 6600.16 | 6910.632 | 6654.81 | 5717.187 | 8597.6 | 12610.19 | 6461.792 | 10296.63 | 10247.75 | 11122.77 | 11864.38 | 12053.21 | 6234.905 | 10469.53 | 7930.333 | 10247.75 | 7794.866 | 11545.02 | 5447.86 | 5819.009 | 10685.37 | 7071.027 | 10366.97 |
| CUST_2375_PI429010886 | N12213 | 0.021702 | 18 | 3 | 156550883 | PREDICTED: hypothetical protein LOC100118283 [N | Protein metabolism | Cellular protein modification process | 4.05E-38 | 42.69298 | 47.39715 | 58.10187 | 53.67582 | 50.10697 | 49.09733 | 74.14418 | 44.53184 | 44.87845 | 35.03522 | 32.60067 | 44.39886 | 39.6493 | 32.56046 | 41.48365 | 41.76249 | 50.4763 | 36.71638 | 54.43748 | 30.69155 | 38.5218 | 46.11558 | 42.95918 | 67.55015 | 41.85446 | 51.7675 | 34.59885 |
| CUST_52727_PI429010886 | M98669 | 0.021711 | 15 | 3 | | | | | | 21.93409 | 21.37183 | 30.07342 | 27.58029 | 35.94919 | 40.45663 | 39.68929 | 32.51731 | 23.65146 | 17.80913 | 31.13099 | 15.1999 | 15.89244 | 14.52536 | 13.95627 | 11.75847 | 30.80438 | 18.9175 | 40.18883 | 13.57843 | 20.05201 | 15.06062 | 31.78686 | 25.39589 | 8.274264 | 24.88909 | 19.91992 |
| CUST_20221_PI429010886 | N43043 | 0.021724 | 24 | 18 | | | | | | 74.89819 | 142.1088 | 65.02562 | 57.56078 | 66.90864 | 36.98363 | 23.3977 | 29.41419 | 38.04057 | 102.8937 | 84.28941 | 113.0821 | 86.93205 | 84.99622 | 98.85463 | 119.3795 | 44.00542 | 71.41994 | 73.86807 | 122.8973 | 110.1494 | 99.53996 | 82.10096 | 50.93524 | 66.48727 | 100.397 | 86.60546 |
| CUST_7081_PI429010886 | N21014 | 0.021756 | 21 | 6 | 321478626 | hypothetical protein DAPPUDRAFT_303213 [Daphni | | | 1.24E-17 | 73.31065 | 78.73486 | 80.53946 | 138.9365 | 131.6288 | 92.53187 | 139.5229 | 116.6557 | 101.7598 | 76.98892 | 115.7777 | 75.12567 | 119.9827 | 81.12186 | 102.3871 | 100.3848 | 120.363 | 76.89998 | 113.525 | 79.17523 | 63.06213 | 95.47811 | 53.74643 | 57.22832 | 115.4049 | 110.1333 | 124.3822 |
| CUST_33756_PI429010886 | M7807 | 0.021756 | 21 | 9 | | | | | | 26.82709 | 19.70849 | 23.96204 | 35.96862 | 57.01626 | 81.58984 | 43.70451 | 104.0004 | 61.56139 | 50.039 | 96.47975 | 69.04216 | 105.2803 | 30.89913 | 51.00123 | 19.73697 | 90.0502 | 40.62219 | 37.64162 | 125.2677 | 143.5589 | 15.13796 | 24.58201 | 42.72555 | 23.00326 | 27.87681 | 70.3928 |
| CUST_6535_PI429010886 | N20228 | 0.021766 | 12 | 0 | | | | | | 286.3036 | 350.2055 | 315.9066 | 235.3127 | 105.7694 | 157.283 | 221.9188 | 192.5048 | 227.604 | 258.2663 | 182.6137 | 207.1975 | 233.6069 | 257.9077 | 146.1758 | 233.9335 | 135.2315 | 209.83 | 149.0574 | 220.4437 | 182.318 | 258.8229 | 218.5606 | 208.936 | 351.9054 | 226.7097 | 302.2555 |
| CUST_5487_PI429010886 | N18768 | 0.021769 | 24 | 0 | | | | | | 527.2284 | 537.1757 | 587.4356 | 479.0678 | 452.1056 | 596.4585 | 465.9125 | 373.826 | 503.9702 | 566.0346 | 297.7533 | 393.2523 | 375.1955 | 433.2734 | 483.8764 | 934.2348 | 440.6015 | 738.0296 | 476.9743 | 405.8466 | 399.2623 | 1154.959 | 431.7288 | 581 | 990.9102 | 626.6888 | 872.488 |
| CUST_57017_PI429010886 | M115131 | 0.021823 | 24 | 21 | | | | | | 21.53411 | 21.39591 | 25.23241 | 21.51171 | 24.31121 | 21.21095 | 21.54181 | 22.89389 | 17.14547 | 13.72475 | 21.0027 | 36.36062 | 10.6182 | 17.23883 | 17.01718 | 28.19099 | 21.8729 | 35.71639 | 19.25911 | 17.80246 | 12.28413 | 26.38687 | 32.86616 | 27.89184 | 20.13934 | 26.39674 | 26.64522 |
| CUST_46594_PI429010886 | M73098 | 0.021866 | 15 | 9 | | | Protein metabolism | Cellular protein modification process | | 6.626935 | 13.20272 | NA | 6.256959 | 12.13161 | 14.48944 | 7.093765 | 6.439909 | 10.24518 | 17.70509 | 11.51059 | 23.22371 | 10.05851 | 9.096353 | 7.179499 | 6.358915 | NA | 8.815034 | 11.28356 | 8.245374 | 13.62289 | 16.29894 | NA | NA | 20.29145 | 13.86169 | 22.33967 |
| CUST_10688_PI429010886 | N26631 | 0.021868 | 24 | 18 | | | | | | 106.7999 | 115.3125 | 119.5972 | 92.87668 | 108.8877 | 108.048 | 82.65719 | 90.47199 | 103.0771 | 119.9827 | 110.9624 | 129.5889 | 94.67269 | 119.6887 | 159.8607 | 149.2835 | 102.8937 | 117.7023 | 122.2713 | 116.1033 | 116.0297 | 113.9467 | 116.6845 | 133.0452 | 119.0526 | 109.2144 | 120.363 |

| Probe ID | ID | p-value | Period | Phase | EBI ID | Annotation | GO category | Go term | E-value | 0 | 0 | 0 | 3 | 3 | 3 | 6 | 6 | 6 | 9 | 9 | 9 | 12 | 12 | 12 | 15 | 15 | 15 | 18 | 18 | 18 | 21 | 21 | 21 | 24 | 24 | 24 |
|---|---|---|---|---|---|---|---|---|---|---|---|---|---|---|---|---|---|---|---|---|---|---|---|---|---|---|---|---|---|---|---|---|---|---|---|---|
| CUST_20111_PI429010886 | N42830 | 0.021868 | 21 | 0 | | | | | | 24.54149 | 27.97387 | 34.28424 | 23.70484 | 18.46027 | 25.68021 | 32.35941 | 18.59558 | 18.05541 | 34.48473 | 23.94321 | 21.45934 | 16.62399 | 21.83723 | 23.52604 | 18.62971 | 20.06061 | 31.49395 | 20.68784 | 19.07106 | 30.36684 | 28.78598 | 32.84186 | 39.13986 | 27.49118 | 24.99108 | 27.9072 |
| CUST_11438_PI429010886 | N27805 | 0.021873 | 18 | 3 | | | | | | 15.12815 | 17.02696 | 9.156873 | 17.57242 | 26.82709 | 21.51643 | 16.29159 | 9.993532 | 31.13379 | 13.35309 | 14.12974 | 10.09227 | 10.98742 | 11.88288 | 9.456396 | 28.9598 | 14.09753 | 20.54425 | 19.21762 | 18.30638 | 16.96853 | 19.9741 | 23.74624 | 17.0738 | 18.618 | 22.29224 | 5.761486 |
| CUST_34598_PI429010886 | M10120 | 0.021887 | 15 | 0 | | | | | | 144.2346 | 126.5686 | 84.04395 | 112.9639 | 101.891 | 113.8196 | 66.55641 | 69.58233 | 90.40629 | 79.85552 | 104.3886 | 82.64391 | 84.49247 | 84.13816 | 95.73603 | 125.7869 | 87.01627 | 119.1299 | 73.16394 | 100.2916 | 80.72119 | 94.69116 | 60.92331 | 88.86362 | 80.88726 | 93.59991 | 84.98778 |
| CUST_34718_PI429010886 | M10449 | 0.021887 | 15 | 0 | | | | | | 622.3874 | 463.5935 | 431.7288 | 392.0026 | 450.9914 | 437.8204 | 352.6729 | 376.0037 | 309.8242 | 402.7742 | 442.7732 | 473.5692 | 302.566 | 400.3298 | 401.3173 | 678.3407 | 531.9567 | 552.4266 | 514.6723 | 479.5081 | 359.3716 | 538.667 | 309.9578 | 338.0611 | 647.3023 | 390.6066 | 482.287 |
| CUST_35610_PI429010886 | M13016 | 0.021887 | 15 | 9 | | | | | | 228.0795 | 285.3755 | 367.1896 | 190.8604 | 140.194 | 98.75692 | 170.7553 | 296.4747 | 265.1181 | 364.0324 | 292.1904 | 340.5239 | 234.5938 | 331.6846 | 270.0917 | 294.4572 | 277.0736 | 180.7626 | 275.8944 | 363.0024 | 292.5508 | 323.8163 | 422.0003 | 247.3239 | 409.1687 | 370.3508 | 429.4606 |
| CUST_39237_PI429010886 | M36559 | 0.021887 | 15 | 9 | | | | | | 1565.628 | 4374.715 | 1013.924 | 1511.146 | 2140.816 | 1117.627 | 2346.537 | 1217.47 | 3799.249 | 4635.589 | 2368.476 | 7128.301 | 2107.396 | 2175.001 | 3222.703 | 4571.032 | 614.8997 | 3411.47 | 1617.488 | 3767.24 | 1856.885 | 5974.692 | 2189.348 | 4021.809 | 3883.492 | 4150.569 | 4411.84 |
| CUST_42778_PI429010886 | M61230 | 0.021887 | 15 | 9 | | | | | | 21.66199 | 20.71812 | 13.76981 | 20.52725 | 7.472276 | 20.55089 | 13.64945 | 25.66555 | 16.15878 | 23.50136 | 22.24949 | 26.76517 | 21.83532 | 51.93461 | 20.07638 | 28.14021 | 17.70313 | 20.60646 | 12.50339 | 28.74973 | 17.42392 | 23.063 | 33.10392 | 21.8588 | 31.28165 | 24.85393 | 31.33759 |
| CUST_33221_PI429010886 | M6323 | 0.021887 | 15 | 9 | 260794242 | hypothetical protein BRAFLDRAFT_114861 [Branchi | Nucleic acid metabolism | Regulation of gene expression | 8.67E-09 | 187.5869 | 154.2676 | 237.166 | 143.5804 | 139.562 | 114.3896 | 144.3781 | 158.6525 | 145.201 | 188.2417 | 145.4832 | 159.0925 | 134.02 | 165.0799 | 157.9764 | 181.9887 | 155.2787 | 118.7113 | 129.0466 | 152.9208 | 126.9103 | 198.6003 | 154.9827 | 78.59764 | 226.7097 | 163.2051 | 179.2012 |
| CUST_43732_PI429010886 | M63816 | 0.021887 | 15 | 9 | | | | | | 11.64471 | 20.78572 | 22.59655 | 9.203374 | 9.844287 | 11.42142 | 10.5667 | 15.94381 | 19.43342 | 28.77061 | 5.358734 | 24.92176 | 11.75847 | 24.72926 | 17.46899 | 30.93077 | 8.454339 | 17.20661 | 7.541361 | 11.64355 | 20.98935 | 27.9072 | 19.25817 | 28.20989 | 24.77058 | 34.09884 | 25.87632 |
| CUST_44095_PI429010886 | M64933 | 0.021887 | 15 | 9 | | | | | | 464.9946 | 521.7265 | 643.9207 | 308.2184 | 464.9946 | 416.7544 | 400.0206 | 364.8104 | 481.1352 | 606.3721 | 490.7702 | 542.8448 | 447.9038 | 588.4339 | 506.9726 | 570.6164 | 439.7553 | 496.5233 | 459.2637 | 478.2055 | 485.7919 | 591.8445 | 492.0126 | 451.1707 | 648.7533 | 535.2858 | 582.4599 |
| CUST_44301_PI429010886 | M65549 | 0.021887 | 15 | 9 | | | | | | 37.16398 | 34.01329 | 34.84989 | 32.71054 | 32.06861 | 25.98413 | 36.93404 | 46.52112 | 28.78598 | 69.46349 | 51.22479 | 40.22053 | 19.86843 | 22.13925 | 55.70806 | 56.85652 | 44.1892 | 31.72455 | 29.53078 | 36.65319 | 38.11743 | 45.28763 | 43.38758 | 29.31792 | 58.14864 | 139.419 | 51.30066 |
| CUST_51150_PI429010886 | M92373 | 0.021887 | 15 | 9 | | | | | | 57.22832 | 51.37093 | 65.84868 | 45.93669 | 28.71654 | 20.10077 | 29.2624 | 90.62378 | 72.31183 | 104.5737 | 63.68458 | 74.98811 | 71.48709 | 101.6576 | 58.7202 | 80.44402 | 38.31739 | 44.23444 | 42.2531 | 70.83788 | 36.19875 | 107.1532 | 73.39754 | 31.7359 | 119.5504 | 32.92673 | 78.01256 |
| CUST_51333_PI429010886 | M93109 | 0.021887 | 15 | 9 | | | | | | 337.9324 | 305.6076 | 408.8064 | 268.6688 | 345.9993 | 297.3146 | 248.5974 | 313.4226 | 363.2975 | 411.3644 | 313.8962 | 422.6396 | 330.882 | 447.4667 | 370.6555 | 442.852 | 308.8474 | 368.8573 | 280.0237 | 383.7251 | 304.8063 | 364.2104 | 413.9155 | 339.6585 | 456.0552 | 361.7087 | 474.3801 |
| CUST_4519_PI429010886 | N16955 | 0.021887 | 15 | 9 | | | | | | 152.8184 | 762.5883 | 386.0388 | 113.4862 | 197.9029 | 177.6974 | 108.3989 | 154.2483 | 416.025 | 1553.73 | 533.5457 | 2004.816 | 366.6536 | 603.7585 | 39.21751 | 783.8018 | 153.8239 | 381.2578 | 260.0114 | 286.3036 | 373.3663 | 472.1948 | 541.31 | 111.9958 | 1383.212 | 458.6579 | 2270.606 |
| CUST_21531_PI429010886 | N45380 | 0.021887 | 15 | 9 | | | | | | 84.93968 | 33.51061 | 67.02702 | 37.75183 | 27.93228 | 49.32644 | 31.84731 | 47.68335 | 57.89544 | 74.54008 | 37.83427 | 70.44162 | 40.34891 | 63.4424 | 101.891 | 65.12567 | 33.96909 | 57.49062 | 37.19463 | 50.59835 | 42.00978 | 73.40872 | 78.46976 | 41.71124 | 76.89998 | 118.9329 | 101.9838 |
| CUST_22119_PI429010886 | N46467 | 0.021887 | 15 | 0 | | | | | | 21.44277 | 19.11241 | 27.65371 | 12.93585 | 23.32 | 30.64068 | 19.92471 | 17.23883 | 13.3617 | 19.88671 | 23.82763 | 18.23052 | 19.16725 | 12.78761 | 20.57577 | 23.91586 | 19.35261 | 23.51614 | 15.24223 | 20.37942 | 17.77235 | 13.87948 | 7.670181 | 17.04861 | 14.18973 | 18.72449 | 20.48326 |
| CUST_28747_PI429010886 | N58543 | 0.021887 | 15 | 9 | | | | | | 19.75556 | 38.54575 | 39.58144 | 26.61439 | 21.42649 | 20.70807 | 18.19413 | 44.82913 | 31.33456 | 41.48365 | 28.99859 | 33.12696 | 35.40982 | 51.85122 | 28.81481 | 32.39452 | 21.65997 | 25.35696 | 19.12543 | 30.36684 | 12.47217 | 35.08291 | 61.64915 | 14.50403 | 48.06558 | 33.66812 | 47.39274 |
| CUST_30814_PI429010886 | N62791 | 0.021887 | 15 | 9 | | | | | | 181.1004 | 242.0042 | 247.8745 | 180.162 | 176.0053 | 207.3277 | 167.5706 | 200.5697 | 195.4236 | 230.9236 | 203.3538 | 276.1029 | 191.528 | 251.8524 | 193.5749 | 238.2063 | 197.384 | 230.3934 | 172.1318 | 210.676 | 215.3368 | 215.6344 | 230.2231 | 219.4136 | 287.1125 | 219.8489 | 290.175 |
| CUST_9031_PI429010886 | N23963 | 0.021892 | 21 | 9 | 115345804 | hemolymph clottable protein [Litopenaeus vannam | | | 3.11E-15 | 66.66314 | 128.2524 | 17.20887 | 196.3862 | 132.652 | 20.01489 | 159.0669 | 415.7405 | 186.6438 | 123.3055 | 402.6363 | 218.8408 | 405.2638 | 82.79408 | 104.0557 | 88.41726 | 148.3243 | 33.46305 | 94.62564 | 478.5158 | 144.0591 | 123.1229 | 53.46055 | 54.40561 | 90.38978 | 102.639 | 100.5297 |
| CUST_7802_PI429010886 | N22106 | 0.021906 | 21 | 12 | | | | | | 231.8976 | 264.2251 | 295.2655 | 300.3777 | 247.2726 | 340.5239 | 278.0994 | 256.8944 | 318.6212 | 426.0225 | 239.571 | 284.4041 | 391.346 | 430.2513 | 415.7405 | 405.5199 | 346.6366 | 402.3792 | 273.2194 | 313.7524 | 270.6283 | 306.479 | 292.0563 | 255.3857 | 402.2817 | 304.5092 | 387.4359 |
| CUST_38040_PI429010886 | M22993 | 0.021913 | 15 | 12 | | | | | | 346.5745 | 269.2314 | 351.5336 | 216.304 | 221.7247 | 266.5934 | 324.7416 | 297.6952 | 338.3238 | 260.5083 | 241.4805 | 328.1634 | 316.4852 | 315.5114 | 348.5168 | 394.7994 | 261.123 | 301.5634 | 284.3688 | 255.7416 | 189.0327 | 320.5709 | 275.0769 | 242.4336 | 341.9848 | 277.7171 | 327.2675 |
| CUST_48878_PI429010886 | M82667 | 0.021913 | 15 | 12 | 157133857 | hypothetical protein AaeL_AAEL012905 [Aedes aeg | | | 2.61E-07 | 261.6861 | 146.8499 | 311.8189 | 215.028 | 166.9686 | 260.2212 | 284.8452 | 299.6232 | 265.2639 | 295.7576 | 208.9955 | 236.7681 | 314.1625 | 375.6267 | 340.1148 | 221.9563 | 332.3269 | 252.5773 | 316.2685 | 200.21 | 178.4972 | 254.0209 | 299.5774 | 265.9437 | 270.3119 | 268.4877 | 308.6668 |
| CUST_49105_PI429010886 | M84111 | 0.021913 | 15 | 12 | | | | | | 463.3861 | 401.6619 | 542.1254 | 322.2737 | 386.4471 | 424.0774 | 382.663 | 393.782 | 475.2865 | 514.8406 | 377.4878 | 666.8781 | 506.9726 | 718.6829 | 794.1984 | 629.802 | 341.0906 | 477.2259 | 391.5301 | 541.31 | 484.2538 | 607.2425 | 574.4912 | 362.9082 | 650.7381 | 689.3131 | 635.2846 |
| CUST_33992_PI429010886 | M8448 | 0.021913 | 15 | 6 | | | | | | 47.24387 | 84.98778 | 40.18446 | 104.9421 | 74.61343 | 86.07302 | 134.6396 | 22.58247 | 133.7064 | 90.84494 | 56.96512 | 131.7341 | 50.06533 | 26.01607 | 37.96902 | 70.169 | 73.61433 | 50.54005 | 67.7681 | 68.82274 | 100.5098 | 148.0447 | 42.29841 | 107.6119 | 59.49267 | 57.34886 | 31.46479 |
| CUST_16955_PI429010886 | N37137 | 0.021946 | 21 | 9 | | | | | | 5.923247 | 30.09221 | 12.99648 | 60.94428 | 90.18249 | 26.35743 | 60.12977 | 184.3634 | 147.0019 | 29.77528 | 202.9774 | 306.5697 | 119.0071 | 61.50815 | 91.82524 | NA | 46.72053 | NA | 33.03027 | 254.7271 | 211.9851 | 3.837296 | 78.39762 | 48.45297 | 7.841585 | 142.8727 | 177.1792 |
| CUST_11137_PI429010886 | N27327 | 0.021947 | 21 | 15 | | | | | | 58.65437 | 34.36554 | 49.43857 | 20.85366 | 38.45723 | 51.79974 | 20.37942 | 14.59908 | 16.91321 | 38.77656 | 50.82151 | 24.85691 | 16.8955 | 34.56933 | 21.7761 | 43.02941 | 70.81749 | 49.81001 | 45.12448 | 44.41428 | 34.98674 | 38.04684 | 41.8249 | 24.41159 | 47.23018 | 26.95518 | 38.07238 |
| CUST_54866_PI429010886 | M108026 | 0.021956 | 21 | 12 | 390517064 | hemocyanin alpha subunit 2 [Caridina multidentata | | | 2.29E-11 | NA | 91.12979 | 25.23726 | 282.7521 | 163.569 | 28.02833 | 119.9123 | 1083.296 | 198.4267 | 126.4216 | 955.0872 | 1059.331 | 2457.891 | 77.32264 | 1138.766 | NA | 435.9034 | NA | 52.17339 | 17535.03 | 372.3592 | NA | 30.74654 | 20.97422 | NA | 414.3494 | 676.9355 |
| CUST_13056_PI429010886 | N30505 | 0.021965 | 18 | 9 | | | | | | 11.29332 | 17.74325 | 11.65987 | 7.957708 | 10.13327 | NA | 12.47348 | 13.70505 | 27.02172 | 24.89184 | 14.74027 | 21.26038 | 19.74282 | 18.53515 | 30.66462 | 14.23097 | 5.528263 | 12.54217 | 15.74591 | 24.36264 | 14.42999 | 18.39397 | 8.871706 | 6.245942 | 22.13275 | 12.77756 | 24.08827 |
| CUST_54700_PI429010886 | M107214 | 0.021987 | 24 | 0 | | | | | | 41.41686 | 40.20735 | 44.4501 | 43.29309 | 36.9383 | 16.37493 | 26.60787 | 37.77885 | 35.74458 | 40.28418 | 36.07274 | 31.68203 | 18.98615 | 25.75936 | 38.16452 | 47.75744 | 31.40553 | 45.78676 | 38.36474 | 40.84477 | 28.57593 | 40.9306 | 32.36995 | 30.93804 | 60.77185 | 42.73401 | 53.99759 |
| CUST_54725_PI429010886 | M107331 | 0.021987 | 24 | 0 | | | | | | 280.7168 | 182.3416 | 210.5448 | 133.2074 | 170.0985 | 175.4362 | 276.7086 | 201.1997 | 138.969 | 231.6859 | 173.6625 | 184.7518 | 160.4822 | 124.9715 | 79.25286 | 170.2053 | 208.5959 | 143.9734 | 146.7445 | 186.0597 | 186.2287 | 150.5497 | 193.0385 | 156.8624 | 176.0053 | 235.838 | 223.7055 |
| CUST_40189_PI429010886 | M51523 | 0.021996 | 18 | 12 | | | | | | 6240.976 | 10884.33 | 8147.956 | 4388.254 | 7099.277 | 6174.548 | 6925.74 | 5349.606 | 7516.748 | 14375.97 | 5800.891 | 8904.357 | 10200.91 | 11182.42 | 11222.08 | 12502.58 | 6687.729 | 10790.28 | 7516.748 | 10915.56 | 7701.667 | 11289.07 | 5119.45 | 6065.211 | 9382.703 | 7669.986 | 10233.27 |
| CUST_37517_PI429010886 | M19337 | 0.022011 | 18 | 0 | | | | | | 5888.291 | NA | 5050.626 | 901.9536 | 4915.943 | 4313.91 | 8313.066 | 372.6528 | 95.98918 | 31.32413 | 5565.727 | 6.097609 | 166.3173 | 104.8909 | 88.78823 | NA | 9219.301 | 2874.609 | 6587.101 | NA | 5130.907 | NA | 7568.915 | 7266.955 | 6.041641 | 348.4045 | 11.56735 |
| CUST_46680_PI429010886 | M73395 | 0.022018 | 15 | 0 | | | | | | 19.11687 | 16.57544 | 7.86472 | NA | 15.86933 | 12.00862 | 8.706462 | 15.10927 | 5.880062 | 10.44754 | 5.701937 | 9.737989 | 16.30067 | 8.648968 | NA | 18.34346 | 19.75556 | 20.97223 | 12.49275 | 6.975434 | 10.80175 | 15.80873 | 14.62912 | 10.4869 | 9.269541 | 10.5066 | 13.4573 |
| CUST_44573_PI429010886 | M66357 | 0.022022 | 24 | 15 | | | | | | 111.4235 | 111.9633 | 162.4256 | 65.93142 | 86.78972 | 84.91262 | 98.98439 | 91.14746 | 102.3423 | 126.3312 | 85.36118 | 134.7073 | 102.8432 | 147.911 | 122.452 | 135.0089 | 128.5095 | 150.5251 | 126.0646 | 106.4846 | 101.5527 | 121.6679 | 124.2426 | 102.0005 | 178.5939 | 105.1478 | 157.0455 |
| CUST_2415_PI429010886 | N12299 | 0.02203 | 15 | 9 | 303277513 | predicted protein [Micromonas pusilla CCMP1545] | | | 9.84E-13 | 80.29008 | 434.2638 | 473.6842 | 36.16119 | 69.08039 | NA | 42.72999 | 313.7524 | 594.5741 | 628.3128 | 398.0093 | 641.17 | 260.5083 | 952.6524 | 419.4237 | 451.6758 | 120.8319 | 198.5143 | 298.3902 | 487.6477 | 333.3682 | 743.802 | 467.3451 | NA | 1494.92 | 530.5435 | 1251.3 |
| CUST_9570_PI429010886 | N24855 | 0.022033 | 24 | 18 | | | | | | 30.34969 | 34.44235 | 28.92395 | 23.68579 | 30.90181 | 26.93871 | 28.11334 | 15.65642 | 31.21185 | 29.99067 | 34.46151 | 27.49986 | 25.09429 | 34.33884 | 22.81578 | 37.10843 | 27.57791 | 33.05506 | 53.22359 | 35.69567 | 35.85651 | 33.76983 | 23.44093 | 31.99517 | 35.678 | 20.54194 | 35.33485 |
| CUST_51335_PI429010886 | M93133 | 0.022039 | 15 | 3 | 443695706 | hypothetical protein CAPTEDRAFT_227200, partial [ | | | 2.48E-18 | 127.6161 | 77.31411 | 128.5363 | 91.33174 | 146.902 | 125.6996 | 197.122 | 55.30825 | 67.62103 | 42.42919 | 159.9648 | 47.80852 | 71.35403 | 83.59685 | 91.50805 | 104.0821 | 219.1432 | 90.59403 | 161.1033 | 91.36505 | 113.4075 | 78.07189 | 138.0366 | 151.8851 | 63.22526 | 50.03352 | 66.53867 |
| CUST_30626_PI429010886 | N62264 | 0.02205 | 24 | 15 | | | | | | 28.64876 | 59.84297 | 56.66591 | 23.79718 | 52.68897 | 29.14874 | 57.49062 | 38.19855 | 25.80604 | 74.48926 | 55.4319 | 83.4507 | 49.64767 | 61.5685 | 81.20466 | 89.13187 | 80.15845 | 57.65114 | 56.9818 | 101.9601 | 77.03965 | 60.57719 | 59.28015 | 20.36313 | 96.11914 | 40.1147 | 80.02167 |
| CUST_27215_PI429010886 | N55646 | 0.022076 | 24 | 15 | | | | | | 53.03978 | 45.49238 | 49.12739 | 63.50632 | 42.67635 | 57.81384 | 47.87924 | 45.72037 | 40.28418 | 62.2514 | 49.72048 | 53.78964 | 38.35427 | 35.992 | 62.32467 | 64.99075 | 77.94557 | 57.86313 | 69.00612 | 51.34112 | 61.31684 | 59.10759 | 54.58347 | 54.87191 | 66.29488 | 52.04012 | 47.7103 |
| CUST_6812_PI429010886 | N20632 | 0.022077 | 24 | 18 | 291243678 | PREDICTED: La autoantigen-like [Saccoglossus kowa | | | 1.2E-12 | 132.7894 | 120.4142 | 79.89836 | 72.3636 | 87.81308 | 64.15379 | 75.26431 | 70.108 | 76.55131 | 169.4652 | 139.9854 | 152.1158 | 61.80458 | 74.97848 | 81.81059 | 176.7991 | 113.7893 | 197.5074 | 144.3143 | 196.9594 | 172.082 | 132.7287 | 95.04998 | 92.75345 | 214.8393 | 173.1695 | 180.0766 |
| CUST_52958_PI429010886 | M99599 | 0.022085 | 12 | 3 | | | | | | 46.12593 | 53.48774 | 38.58794 | 64.31019 | 44.17135 | 90.08157 | 40.34341 | 42.83336 | 23.5201 | 50.66735 | 40.05116 | 47.61652 | 36.85229 | 37.02134 | 36.17443 | 45.87335 | 67.16912 | 54.15061 | 43.1527 | 48.76564 | 40.89955 | 54.60894 | 41.50677 | 40.00171 | 40.98703 | 25.86598 | 31.81303 |
| CUST_19688_PI429010886 | N42013 | 0.022088 | 18 | 15 | | | | | | 394.2069 | 366.7345 | 590.149 | 183.8709 | 408.6642 | 494.9533 | 356.4277 | 224.5779 | 261.8822 | 327.2675 | 318.8167 | 462.1839 | 257.5442 | 575.0804 | 307.5089 | 399.5497 | 421.0101 | 622.5542 | 214.7012 | 377.6304 | 376.0037 | 507.709 | 351.2576 | 194.8105 | 167.4539 | 238.8964 | 250.7705 |
| CUST_13184_PI429010886 | N30737 | 0.022121 | 24 | 0 | | | Signal transduction | Intracellular signal transduction | | 13.68648 | 17.28035 | 20.97008 | 13.07843 | 9.934601 | 9.855895 | NA | 9.800176 | 11.0392 | 14.63109 | 20.25455 | NA | 9.304008 | 14.73176 | 10.10287 | 11.85473 | 13.30523 | 10.6044 | 8.046291 | 7.795868 | 13.8828 | 15.57864 | 16.61069 | 16.14788 | 20.5737 | 14.15692 | 16.90254 |
| CUST_49463_PI429010886 | M85738 | 0.022128 | 24 | 6 | 242019963 | conserved hypothetical protein [Pediculus humanus | | | 1.16E-29 | 49.95685 | 43.39175 | 36.99297 | 25.45947 | 39.79718 | 43.35936 | 45.27903 | 119.7932 | 39.43541 | 56.53962 | 39.32573 | 49.73229 | 36.99297 | 42.89451 | 28.15466 | 37.65478 | 22.07569 | 38.42267 | 29.58412 | 27.12475 | 32.74781 | 35.9416 | 40.34574 | 31.10751 | 37.60419 | 24.55823 | 24.76468 |
| CUST_56229_PI429010886 | M113201 | 0.02213 | 15 | 9 | | | | | | 35.31167 | 29.98387 | 42.05465 | 22.56982 | 20.82207 | 28.6271 | 38.90805 | 27.4201 | 33.87601 | 38.2326 | 21.33841 | 37.06438 | 35.86991 | 16.7444 | 53.16758 | 30.41187 | 25.9425 | 31.00505 | 24.49566 | 26.14633 | 29.3391 | 41.17264 | 31.64722 | 33.61445 | 39.36079 | 37.41548 | 39.98138 |
| CUST_33740_PI429010886 | M7756 | 0.02213 | 15 | 9 | | | | | | 124.7428 | 89.72544 | 130.9642 | 43.88802 | 100.4062 | 95.96344 | 99.62003 | 124.3005 | 98.03383 | 126.2074 | 107.9913 | 125.9958 | 98.88783 | 140.8407 | 87.11963 | 74.29008 | 157.3097 | 110.3513 | 76.52417 | 87.2868 | 116.2656 | 119.9288 | 124.9549 | 113.9031 | 151.9294 | 116.9761 | 153.4178 |
| CUST_2852_PI429010886 | N13248 | 0.02213 | 15 | 0 | 85057633 | hypothetical protein AYWB_353 [Aster yellows witc | | | 6.11E-08 | 135.3099 | 213.6671 | 144.9791 | 140.6816 | 194.6782 | 129.8119 | 73.95574 | 62.1958 | 94.79006 | 140.8407 | 120.4829 | 159.4972 | 115.9016 | 136.25 | 107.3899 | 161.6167 | 57.18099 | 180.5686 | 93.14117 | 85.45263 | 116.531 | 109.7501 | 105.9444 | 91.70502 | 136.2095 | 108.4109 | 141.2095 |
| CUST_7346_PI429010886 | N21410 | 0.02213 | 15 | 9 | 291235045 | PREDICTED: glycine receptor, alpha 2-like [Saccoglo | Transport | Ion transport | 1.38E-25 | 68.5761 | 64.40125 | 113.5137 | 74.88 | 63.05147 | 77.74288 | 49.8518 | 45.39483 | 77.2245 | 140.0584 | 84.20531 | 142.4863 | 59.6278 | 74.44282 | 85.06299 | 150.0374 | 56.89076 | 112.1218 | 67.41072 | 43.30223 | 58.93488 | 86.93205 | 78.88657 | 101.1723 | 122.9325 | 100.1395 | 92.48444 |
| CUST_30687_PI429010886 | N62401 | 0.022147 | 21 | 12 | | | | | | 46.44351 | 40.25214 | 48.49494 | 52.44678 | 47.23018 | 51.1868 | 57.9941 | 46.16699 | 54.45697 | 48.78617 | 61.94492 | 72.9942 | 58.62419 | 71.76497 | 73.15736 | 70.86123 | 54.48925 | 55.25615 | 54.60219 | 53.00119 | 62.36156 | 57.56078 | 57.67685 | 48.23121 | 73.71983 | 48.83879 | 68.5444 |
| CUST_33902_PI429010886 | M8194 | 0.022151 | 24 | 0 | | | | | | 32.85525 | 29.32561 | 35.24411 | 32.21966 | 17.17879 | 25.39002 | 21.79167 | 27.97056 | 20.37181 | 30.69887 | 27.48409 | 33.72145 | 20.96791 | 31.08481 | 20.71589 | 24.24945 | 22.29224 | 25.53642 | 26.13341 | 20.17771 | 23.80497 | 18.40232 | 32.30669 | 31.32413 | 32.57877 | 35.24411 | 42.51692 |
| CUST_9628_PI429010886 | N24953 | 0.022157 | 15 | 12 | 478859730 | glutathione S-transferases [Macrobrachium nippon | Metabolic process | Cellular amino acid metabolic process | 2.24E-58 | 448.8586 | 1455.397 | 1029.372 | 817.4781 | 276.4162 | 362.1966 | 323.4981 | 417.0088 | 397.5988 | 1032.186 | 355.116 | 1005.284 | 1080.128 | 1070.179 | 1154.959 | 760.0455 | 363.4546 | 311.0329 | 309.3892 | 947.2744 | 324.8235 | 527.2284 | 626.4662 | 305.8476 | 182.709 | 925.4363 | 1209.913 |
| CUST_69_PI429010886 | N298 | 0.022162 | 15 | 12 | | | | | | 24.35681 | 31.8356 | 38.63168 | 40.56586 | 25.32554 | 55.29787 | 21.63352 | 25.62143 | 15.99082 | 25.37501 | 22.48019 | 44.57334 | 36.57035 | 39.50189 | 41.96451 | 28.65673 | 28.08233 | 30.02913 | 26.05504 | 31.54083 | 35.38892 | 23.531 | 29.8401 | 25.56096 | 29.72221 | 34.30407 | 29.17357 |
| CUST_15691_PI429010886 | N34983 | 0.022162 | 15 | 12 | | | | | | 65.72077 | 138.7005 | 149.974 | 72.44248 | 90.52141 | 123.0412 | 123.1997 | 90.79757 | 138.4789 | 98.34685 | 126.1505 | 192.557 | 120.6234 | 171.3867 | 141.6752 | 104.7962 | 124.2579 | 177.0624 | 88.19572 | 115.4907 | 94.67269 | 131.4929 | 163.6107 | 115.6165 | 158.2913 | 120.4315 | 172.6317 |
| CUST_27327_PI429010886 | N55852 | 0.022165 | 24 | 15 | | | | | | 33.10045 | 27.08074 | 38.92892 | 24.6789 | 25.50611 | 18.01506 | 28.09932 | 21.14959 | 23.8296 | 32.17296 | 30.18791 | 30.63064 | 20.65238 | 33.92714 | 32.28425 | 55.86399 | 38.99142 | 37.72041 | 35.03522 | 27.80787 | 13.87826 | 39.58144 | 28.20666 | 15.25972 | 35.66685 | 23.43812 | 31.27363 |
| CUST_10300_PI429010886 | N26026 | 0.022183 | 24 | 6 | 322791130 | hypothetical protein SINV_06374 [Solenopsis invict | Metabolic process | Oxidation-reduction process | 2.33E-28 | 40.92745 | 84.57831 | 71.94024 | 41.60369 | 22.41298 | NA | 60.93069 | 111.2059 | 42.32326 | 55.95035 | 41.53959 | 30.77101 | 23.05184 | 16.79088 | 23.6887 | 22.43908 | NA | 8.781809 | 8.664964 | 39.79718 | 19.42496 | NA | 104.3119 | 42.84203 | NA | 27.81087 | 16.3703 |
| CUST_45810_PI429010886 | M70366 | 0.022185 | 24 | 6 | | | | | | NA | 158.5157 | 139.1997 | 181.8416 | 342.2472 | 200.1336 | NA | 1172.181 | 1568.166 | 11.48964 | 261.1858 | 133.9495 | 30.56491 | 194.0962 | 33.99968 | NA | 163.8253 | NA | 236.7279 | 1110.444 | 161.4564 | 3.318802 | 24.87526 | 7.922723 | 3.989484 | 4.139836 | NA |
| CUST_33447_PI429010886 | M6907 | 0.022214 | 21 | 12 | | | | | | 22.89148 | 23.7918 | 20.23079 | 16.70757 | 17.69032 | 10.9225 | 24.26504 | 20.58918 | 27.384 | 37.47537 | 17.22674 | 16.16875 | 24.0077 | 82.00472 | 23.88653 | 24.44489 | 23.9514 | 19.75556 | 30.73213 | 16.26432 | 31.05637 | 28.73136 | 26.03903 | 21.2067 | 23.26041 | 14.96828 | 15.036 |
| CUST_16480_PI429010886 | N36306 | 0.022214 | 21 | 0 | | | | | | 353.689 | 363.8038 | 238.5428 | 197.4111 | 318.1453 | 207.1022 | 208.8587 | 144.3246 | 208.4785 | 299.7832 | 240.3331 | 257.2859 | 190.6989 | 157.9551 | 166.3805 | 222.7049 | 273.1122 | 156.0758 | 211.6792 | 233.7145 | 183.574 | 341.396 | 266.7622 | 339.8879 | 403.1472 | 154.6801 | 192.6463 |
| CUST_24427_PI429010886 | N50608 | 0.022214 | 21 | 3 | | | | | | 242.9704 | 223.7478 | 257.2859 | 292.3676 | 236.1246 | 423.767 | 205.0143 | 203.8319 | 244.3099 | 248.4663 | 213.1848 | 318.8664 | 157.6874 | 231.2637 | 235.6934 | 225.693 | 243.0281 | 356.7855 | 265.6599 | 207.6194 | 221.6861 | 273.965 | 289.4881 | 272.1063 | 315.0245 | 453.5801 | 296.9872 |
| CUST_37376_PI429010886 | M18825 | 0.022215 | 21 | 9 | 256539086 | fatty acids binding protein [Fenneropenaeus chinen | Symbiosis, encompassing mutualism through parasitism | Viral process | 1.79E-31 | 3610.458 | 4080.667 | 2288.66 | 2569.563 | 4842.442 | 3610.458 | 2377.614 | 6260.184 | 4882.08 | 4733.383 | 6116.034 | 10401.6 | 3908.116 | 3759.753 | 4959.867 | 3031.864 | 2915.652 | 3604.11 | 2538.071 | 11222.08 | 6544.343 | 2108.426 | 3186.032 | 1716.583 | 3977.496 | 5492.691 | 6516.822 |
| CUST_13975_PI429010886 | N32113 | 0.022215 | 24 | 18 | | | | | | 484.1582 | 533.5457 | 530.9675 | 409.8763 | 464.4064 | 410.239 | 442.7732 | 427.1137 | 464.5441 | 478.5158 | 483.2456 | 545.9123 | 459.7686 | 510.5227 | 504.1608 | 694.5441 | 510.5227 | 532.0502 | 576.5142 | 595.7445 | 478.4338 | 574.1082 | 472.8988 | 365.9997 | 678.2021 | 404.4491 | 607.4385 |
| CUST_23940_PI429010886 | N49735 | 0.022215 | 15 | 6 | | | | | | 32.34622 | 19.56461 | 50.91495 | 32.20795 | 35.23447 | 83.06141 | 73.88403 | 38.85712 | 34.37569 | 36.87579 | 21.14283 | 6.082494 | 29.00481 | 30.87561 | 15.4721 | 21.55311 | 70.35427 | 24.6038 | 49.93144 | 8.037888 | 32.33619 | 108.3037 | 52.76183 | 48.48054 | 26.65768 | 49.58479 | 20.59992 |
| CUST_44881_PI429010886 | M67306 | 0.022217 | 18 | 12 | | | | | | 16.04554 | 12.97975 | 17.96838 | 14.85935 | 20.87033 | 14.72118 | 20.06405 | 49.9806 | 42.1391 | 51.67859 | 42.09086 | 20.20177 | 39.22239 | 69.18246 | 29.44199 | 19.74282 | 22.30489 | 15.88012 | 35.62474 | 12.23764 | 21.5971 | 34.21062 | 16.8666 | 23.67732 | 62.06295 | 12.81312 | 38.32695 |
| CUST_44434_PI429010886 | M65978 | 0.022219 | 15 | 0 | | | | | | 452.0017 | 309.8951 | 364.8104 | 428.4141 | 312.5243 | 509.603 | 432.0274 | 375.0345 | 262.9091 | 292.4718 | 294.3403 | 198.0363 | 285.2001 | 255.0348 | 266.851 | 395.6626 | 560.1437 | 382.5164 | 452.0017 | 230.254 | 303.7146 | 285.4947 | 378.265 | 463.6969 | 231.4586 | 273.6399 | 220.8734 |
| CUST_7791_PI429010886 | N22079 | 0.022233 | 21 | 6 | 390355103 | PREDICTED: 2-acylglycerol O-acyltransferase 2-A-lik | | | 5.74E-20 | 9.092072 | 9.258755 | 6.546282 | 18.1208 | 16.12591 | 11.58371 | 25.03495 | 41.60369 | 28.87134 | 8.316685 | 44.45342 | 41.12787 | 27.02172 | 9.800176 | 22.72441 | 18.12996 | 9.993532 | 13.6193 | 12.32915 | 70.40792 | 20.55089 | 30.12445 | 5.69423 | 12.2919 | 13.87032 | 17.54318 | 16.85059 |
| CUST_31459_PI429010886 | M1573 | 0.022247 | 21 | 18 | | | | | | 214.1332 | 213.7556 | 150.5251 | 176.885 | 158.635 | 138.3597 | 130.4407 | 115.6651 | 115.0894 | 192.9519 | 156.7999 | 212.9853 | 132.6083 | 178.0976 | 175.8238 | 202.1751 | 139.9664 | 140.228 | 176.5718 | 232.9884 | 208.3478 | 222.2769 | 143.6248 | 126.045 | 205.9317 | 175.5504 | 184.9236 |
| CUST_33331_PI429010886 | M6616 | 0.022262 | 21 | 6 | | | | | | 357.5465 | 931.4066 | 414.3494 | 1334.604 | 841.0533 | 596.8255 | 1121.554 | 1136.737 | 1371.349 | 968.3869 | 892.629 | 1871.332 | 1114.351 | 1025.99 | 1073.527 | 1865.638 | 688.7233 | 972.4433 | 2585.542 | 886.7923 | 1693.628 | 762.3115 | 953.5865 | 467.4407 | 961.5038 | 714.7333 | 1295.233 |
| CUST_41158_PI429010886 | M57018 | 0.022275 | 21 | 0 | | | | | | 55.84117 | 46.84472 | 39.22918 | 32.51731 | 53.37349 | 27.38056 | 29.56761 | 24.23621 | 33.22506 | 51.35337 | 16.0606 | 48.3768 | 27.25419 | 30.07667 | 22.97432 | 42.60531 | 40.90818 | 25.84583 | 52.75917 | 28.22043 | 52.91645 | 44.28544 | 48.90708 | 43.62743 | 34.83322 | 32.38525 | 31.10751 |
| CUST_20012_PI429010886 | N42645 | 0.022289 | 18 | 12 | 242023449 | conserved hypothetical protein [Pediculus humanus | | | 1.99E-07 | 66.33801 | 262.5059 | 140.9929 | 66.24577 | 116.73 | 84.40117 | 98.05949 | 120.363 | 79.1048 | 173.7958 | 177.9987 | 103.3151 | 204.7153 | 301.9881 | 162.0946 | 367.7937 | 30.02077 | 114.4714 | 112.5446 | 147.0019 | 82.42534 | 108.7711 | 92.95468 | 60.98516 | 70.77093 | 226.2023 | 110.6582 |
| CUST_50793_PI429010886 | M91027 | 0.022299 | 24 | 18 | | | | | | 10.27034 | 11.09239 | 55.98343 | 9.791272 | 7.276095 | NA | 5.608378 | 6.960178 | 10.33535 | 10.90106 | 5.556495 | NA | 5.339308 | 20.7312 | 16.82979 | 12.46576 | 14.46778 | 6.962498 | 26.67261 | 13.05889 | 13.84341 | 16.97291 | 10.74664 | NA | 8.626596 | 20.55453 | 11.85473 |
| CUST_12303_PI429010886 | N29258 | 0.022301 | 24 | 6 | | | | | | 23.38375 | 16.89352 | 27.25419 | 22.57752 | 23.34144 | 32.67642 | 28.84595 | 25.33771 | 27.66624 | 9.737989 | 26.10123 | 22.88357 | 12.05492 | 25.70952 | 19.27537 | 23.74624 | 22.49442 | 38.24749 | 20.61726 | 17.69032 | 15.39724 | 22.42169 | 15.56753 | 14.20655 | 26.33364 | 24.01545 | 18.46027 |
| CUST_45648_PI429010886 | M69789 | 0.02233 | 24 | 12 | | | | | | 13.10516 | 11.13632 | 9.685625 | 3.359896 | 4.559704 | 4.029079 | 10.91573 | 15.07677 | 12.64236 | 8.189309 | 6.02125 | 7.066118 | 11.49258 | 20.40507 | 15.43128 | 17.58162 | 12.17715 | 10.56222 | 16.4346 | 4.729352 | 8.415892 | 15.12127 | 14.54874 | 14.01595 | 8.155994 | 11.40403 | 14.58718 |
| CUST_34111_PI429010886 | M8803 | 0.022334 | 18 | 0 | | | | | | 15.05605 | 16.50813 | 14.62779 | 26.9466 | 4.576266 | 5.488289 | NA | NA | 3.679299 | 9.430417 | 5.15456 | 8.538401 | 6.459201 | NA | 12.46997 | 7.668108 | 14.88541 | 6.393088 | 7.030976 | 15.49062 | 10.4869 | 9.79911 | NA | NA | 7.668108 | NA | 5.051185 |
| CUST_19724_PI429010886 | N42071 | 0.022334 | 24 | 21 | | | | | | 47.68335 | 66.92364 | 92.35924 | 34.63137 | 44.31012 | 40.06113 | 44.20781 | 31.92933 | 41.57079 | 34.29142 | 58.64138 | 36.8879 | 34.82944 | 65.50557 | 58.23835 | 60.74611 | 52.034 | 50.74837 | 61.03108 | 34.119 | 47.74853 | 73.89571 | 57.07016 | 63.41464 | 105.3783 | 55.08741 | 73.47743 |
| CUST_1784_PI429010886 | N10697 | 0.022342 | 18 | 9 | 291230131 | PREDICTED: hypothetical protein [Saccoglossus kow | Metabolic process | Other | 8.31E-13 | 52.06984 | 47.17722 | 82.01377 | 41.8722 | 28.94202 | 39.26949 | 47.46581 | 49.21104 | 74.07672 | 73.91159 | 50.37311 | 65.64051 | 50.71879 | 73.26779 | 51.75764 | 63.1832 | 35.8731 | 57.76074 | 46.00056 | 69.49122 | 46.66161 | 25.92266 | 54.80426 | 34.50768 | 57.72819 | 82.13647 | 71.06446 |
| CUST_18899_PI429010886 | N40614 | 0.022342 | 18 | 9 | | | | | | 43.80188 | 57.65114 | 36.96758 | 41.16094 | 31.13642 | 19.48537 | 29.53988 | 55.27747 | 42.51692 | 64.79479 | 48.16109 | 56.48952 | 44.68023 | 58.02431 | 43.5437 | 56.83575 | 40.54346 | 41.15103 | 42.62004 | 38.7854 | 43.07866 | 56.80732 | 41.4213 | 27.68313 | 67.33602 | 32.10959 | 50.96015 |
| CUST_41775_PI429010886 | M58532 | 0.022345 | 15 | 0 | | | | | | 348.9836 | 348.5168 | 363.2975 | 216.5438 | 266.4816 | 219.4136 | 217.9121 | 201.3634 | 210.2883 | 275.9866 | 320.5083 | 318.02 | 210.676 | 274.1119 | 265.5106 | 361.3416 | 329.8367 | 280.6713 | 244.0822 | 377.8098 | 295.9544 | 292.3676 | 255.7416 | 245.7815 | 384.9318 | 159.9242 | 314.7546 |
| CUST_48263_PI429010886 | M79590 | 0.022345 | 15 | 0 | | | | | | 202.0834 | 213.51 | 207.5948 | 146.5035 | 178.3288 | 189.5352 | 132.0478 | 137.8094 | 118.2155 | 188.7518 | 157.17 | 190.0645 | 124.009 | 191.6952 | 176.7462 | 242.6822 | 202.005 | 180.4095 | 187.5065 | 184.8973 | 159.0029 | 218.0605 | 173.944 | 159.3655 | 202.4642 | 159.9839 | 210.9764 |
| CUST_47013_PI429010886 | M74498 | 0.022367 | 15 | 3 | | | | | | 38.79646 | 28.43336 | 22.77196 | 69.46349 | 47.15656 | 113.1645 | 74.3064 | 55.42699 | 12.13896 | 14.74649 | 61.1536 | 5.825471 | 25.50065 | 3.514019 | 3.655024 | 9.297397 | 98.70636 | 54.34709 | 40.38893 | NA | 26.14633 | 24.44489 | 17.22069 | 55.1911 | 4.740148 | 26.59238 | NA |
| CUST_31264_PI429010886 | M1104 | 0.022374 | 15 | 0 | | | | | | 334.8266 | 314.5564 | 340.3246 | 204.1851 | 341.5068 | 341.9433 | 154.5247 | 78.33945 | 158.9804 | 257.237 | 354.5586 | 231.5437 | 118.6493 | 222.9689 | 227.4117 | 345.639 | 327.8511 | 322.1425 | 283.6942 | 340.7478 | 283.6942 | 255.6621 | 139.8337 | 153.6441 | 350.1387 | 252.4954 | 288.4954 |
| CUST_51711_PI429010886 | M94674 | 0.022374 | 15 | 6 | | | | | | 64.69663 | 26.00383 | 59.64714 | 76.12074 | 62.23219 | 77.4993 | 161.2704 | 91.46281 | 57.66915 | 68.27282 | 93.58403 | 17.82561 | 83.71896 | 104.8092 | 78.65203 | 65.21686 | 74.15113 | 46.22541 | 88.60515 | 77.44015 | 73.5024 | 168.99 | 93.59388 | 122.404 | 135.4807 | 37.35904 | 56.65877 |
| CUST_6788_PI429010886 | N20603 | 0.022374 | 15 | 0 | | | | | | 877.6784 | 1043.343 | 1146.628 | 621.7238 | 765.2983 | 934.2348 | 716.9224 | 492.7259 | 553.3502 | 920.7129 | 803.7954 | 967.9514 | 357.9328 | 669.0767 | 849.3126 | 1067.136 | 733.7989 | 996.2576 | 995.7921 | 822.9643 | 829.4016 | 801.7718 | 575.3599 | 688.3695 | 1048.763 | 657.1327 | 1149.353 |
| CUST_9786_PI429010886 | N25204 | 0.022374 | 15 | 0 | | | | | | 65.08 | 110.765 | 84.49247 | 78.35123 | 64.18609 | 49.88882 | 45.55401 | 45.48365 | 49.59376 | 104.0321 | 73.08468 | 103.0135 | 53.95436 | 50.71291 | 53.96058 | 80.43582 | 64.50028 | 71.25267 | 49.00501 | 76.60738 | 59.25893 | 43.39175 | 64.54153 | 32.80956 | 97.03372 | 54.07518 | 78.63344 |
| CUST_12338_PI429010886 | N29307 | 0.022375 | 21 | 0 | | | | | | 12.33182 | 22.29887 | 18.39223 | 8.90957 | 16.90108 | 20.5469 | 8.370071 | 7.081402 | 6.470119 | 11.47303 | 9.60066 | 20.208 | 10.43455 | 11.42857 | 15.60331 | 12.29489 | 11.47168 | 18.78533 | 9.737989 | 27.43923 | 10.25442 | 14.14808 | 12.72475 | 23.28835 | 12.14051 | 17.93568 | 15.78638 |
| CUST_24915_PI429010886 | N51474 | 0.022375 | 21 | 3 | | | | | | 49.14494 | 101.3443 | 89.03619 | 105.779 | 63.96858 | 91.1558 | 87.37332 | 39.12009 | 86.70732 | 63.73239 | 74.10143 | 159.7825 | 50.57194 | 103.9666 | 61.71609 | 55.56489 | 59.54485 | 145.8455 | 42.242 | 26.05504 | 69.04997 | 77.74288 | 101.7152 | 87.35248 | 107.2229 | 109.9562 | 127.7418 |
| CUST_17203_PI429010886 | N37566 | 0.022377 | 24 | 6 | | | | | | 129.357 | 357.4809 | 321.2502 | 388.966 | 181.0372 | 130.6003 | 188.0879 | 432.8549 | 130.6959 | 178.9173 | 143.6702 | 129.065 | 170.0196 | 147.1696 | 96.54198 | 46.79864 | 53.13993 | 35.72924 | 34.47396 | 144.1656 | 66.46786 | 14.21316 | 322.593 | 169.275 | 16.35354 | 130.4232 | 39.1747 |
| CUST_21795_PI429010886 | N45868 | 0.022381 | 24 | 0 | | | | | | 13.63096 | 11.81798 | 28.5608 | 6.332988 | 12.6398 | 23.51373 | 5.236771 | 15.91213 | 11.08304 | 15.51771 | 8.794657 | 12.43735 | 9.021728 | 10.5066 | 5.820473 | 15.1109 | 12.23171 | 9.485366 | 12.11941 | 17.19113 | 12.27121 | 15.24741 | 15.444 | NA | 17.55969 | 14.03086 | 15.69846 |
| CUST_50778_PI429010886 | M90968 | 0.022386 | 21 | 15 | | | | | | 19.84167 | 17.48685 | 32.17296 | 13.03592 | 46.15285 | 61.64233 | 31.98439 | 9.890597 | 14.20484 | 48.20166 | 52.11124 | 38.08235 | 65.04995 | 23.17643 | 54.42343 | 43.7836 | 78.07938 | 78.83085 | 39.90543 | 5.467236 | 6.323484 | 17.69459 | NA | 11.15682 | 31.27363 | 12.63007 | 8.73928 |
| CUST_53172_PI429010886 | M100539 | 0.022388 | 24 | 21 | | | | | | 96.30815 | 87.55497 | 121.649 | 89.60537 | 78.99159 | 86.63308 | 58.83776 | 74.61978 | 90.52141 | 92.5954 | 103.0626 | 107.1532 | 109.6246 | 98.6386 | 75.85108 | 110.6582 | 100.734 | 105.86 | 82.82848 | 119.7354 | 100.1156 | 100.7779 | 154.3834 | 97.25472 | 107.3346 | 92.13928 | 126.9474 |
| CUST_37133_PI429010886 | M17951 | 0.022402 | 18 | 15 | 345484846 | PREDICTED: glycogen debranching enzyme-like [Na | Metabolic process | Generation of precursor metabolites and energy | 2.19E-65 | 11.59565 | 9.244945 | 14.36354 | 18.49894 | 10.67877 | 20.91226 | 8.796716 | 8.357942 | 6.898311 | 15.5721 | 17.05678 | 13.15672 | 10.80725 | 6.66209 | 12.85718 | 16.24039 | 18.78041 | 19.57173 | 16.04554 | 37.52322 | 12.60946 | 13.52202 | 6.02676 | 11.68857 | 10.07219 | 12.03745 | 12.05594 |
| CUST_42534_PI429010886 | M60524 | 0.022404 | 21 | 9 | 321457450 | hypothetical protein DAPPUDRAFT_189444 [Daphn | | | 1.45E-29 | 559.3835 | 649.8854 | 664.4926 | 453.7002 | 602.4628 | 493.4379 | 559.0577 | 663.4104 | 661.8313 | 714.5213 | 691.8386 | 982.4374 | 633.8371 | 596.4585 | 474.5547 | 710.9044 | 624.47 | 691.1905 | 569.2573 | 961.8209 | 632.0615 | 728.8915 | 590.3441 | 522.5582 | 604.5341 | 501.5691 | 551.2455 |
| CUST_43060_PI429010886 | M61963 | 0.022409 | 15 | 9 | | | | | | 353.0021 | 338.5409 | 409.7595 | 330.0503 | 271.3533 | 343.3863 | 274.3455 | 344.4708 | 333.1118 | 423.3111 | 335.4811 | 503.7682 | 323.4981 | 497.1091 | 426.1051 | 368.3133 | 337.8584 | 335.5292 | 283.3904 | 335.0674 | 389.52 | 375.6267 | 310.5967 | 371.903 | 390.7246 | 462.5707 | 372.6528 |
| CUST_17399_PI429010886 | N37900 | 0.022409 | 15 | 9 | 411100745 | cation-dependent mannose-6-phosphate receptor, | Transport | Protein transport | 7.88E-23 | 26.77489 | 22.21962 | 28.8656 | 25.19447 | 16.14158 | 18.35581 | 25.26607 | 11.17643 | 23.34144 | 38.85028 | 22.51476 | 81.11384 | 29.24887 | 37.55636 | 31.51141 | 32.68431 | 16.16487 | 26.76116 | 24.10675 | 27.5502 | 16.84134 | 29.35866 | 27.71495 | 21.05341 | 31.85611 | 18.07887 | 40.68218 |
| CUST_3601_PI429010886 | N14856 | 0.022409 | 24 | 12 | | | | | | 490.4258 | 1396.326 | 625.332 | 419.5293 | 783.8018 | 208.7767 | 1361.981 | 866.2027 | 1526.305 | 1131.729 | 659.1977 | 1560.159 | 2395.771 | 2676.76 | 1951.832 | 2805.418 | 1615.873 | 1179.34 | 957.5442 | 2106.309 | 821.28 | 2273.444 | 1636.442 | 1622.403 | 5596.154 | 1072.188 | 6366.193 |
| CUST_6183_PI429010886 | N19739 | 0.022411 | 15 | 12 | | | Nucleic acid metabolism | Regulation of gene expression | | 403.4054 | 400.6975 | 497.9965 | 273.1684 | 294.8062 | 317.5249 | 277.7994 | 316.8471 | 388.4984 | 453.9938 | 326.3773 | 411.1038 | 319.1396 | 428.7157 | 411.957 | 473.1073 | 353.0802 | 370.4089 | 328.0426 | 308.1438 | 331.2319 | 376.4458 | 491.0819 | 313.1633 | 411.8683 | 373.6828 | 461.2479 |
| CUST_8341_PI429010886 | N22905 | 0.022411 | 15 | 12 | | | | | | 294.9448 | 340.5982 | 387.5117 | 292.1904 | 268.6688 | 333.1788 | 260.4633 | 282.0775 | 344.0991 | 338.0611 | 266.6447 | 541.4466 | 305.7396 | 565.0414 | 587.6341 | 394.958 | 273.0635 | 397.8439 | 297.6952 | 304.1069 | 317.5778 | 381.6649 | 397.525 | 349.9147 | 406.809 | 441.9667 | 533.0782 |
| CUST_12500_PI429010886 | N29562 | 0.022411 | 15 | 12 | | | | | | 313.8962 | 261.922 | 169.847 | 191.3362 | 231.235 | 169.8899 | 249.1317 | 251.0973 | 208.9685 | 225.693 | 173.1695 | 251.0973 | 364.1216 | 276.6492 | 288.1296 | 313.8962 | 274.9043 | 173.9115 | 204.1086 | 179.5942 | 145.749 | 286.8104 | 172.2644 | 221.7247 | 300.0603 | 218.9512 | 189.0327 |
| CUST_7811_PI429010886 | N22118 | 0.022454 | 24 | 18 | | | | | | 1050.96 | 973.4615 | 1461.877 | 1002.084 | 875.295 | 939.7655 | 822.0802 | 1072.593 | 1022.691 | 1159.875 | 1013.618 | 1276.133 | 1047.894 | 1638.031 | 1191.699 | 1396.98 | 1197.469 | 1221.884 | 1192.582 | 1465.294 | 1099.653 | 1407.859 | 1319.125 | 1027.698 | 1493.465 | 984.6642 | 1538.611 |
| CUST_17519_PI429010886 | N38106 | 0.022454 | 24 | 21 | | | | | | 31.94735 | 34.13077 | 48.16109 | 32.60462 | 23.19661 | 40.66714 | 33.10045 | 40.59586 | 23.29568 | 50.10243 | 28.00512 | 36.16119 | 16.5713 | 37.53024 | 32.79034 | 41.7785 | 39.25762 | 43.71284 | 30.76819 | 35.22807 | 47.18136 | 53.32755 | 65.41017 | 51.66322 | 36.52268 | 35.26822 | 41.14494 |
| CUST_36309_PI429010886 | M15533 | 0.022454 | 21 | 0 | 524942852 | PREDICTED: ribonucleases P/MRP protein subunit P | Nucleic acid metabolism | RNA metabolic process | 2.77E-20 | 9.105658 | 10.6154 | 8.263899 | 5.924102 | 4.969785 | NA | 4.095821 | 4.537923 | 5.00304 | 5.308585 | 6.063329 | 9.344321 | 4.74626 | NA | 6.047731 | 9.483801 | 11.98126 | 3.407621 | 9.379442 | 8.300728 | 6.033088 | 13.18006 | 6.575352 | NA | 7.14839 | 8.681407 | 4.650724 |
| CUST_49842_PI429010886 | M87391 | 0.022475 | 21 | 18 | 528746119 | C-type lectin 4 [Fenneropenaeus merguiensis] | | | 3.99E-08 | 87.11963 | 100.7109 | 77.32264 | 35.27881 | 75.70522 | 24.19346 | NA | NA | 21.23667 | 102.8784 | 49.97285 | 116.73 | 61.48326 | 49.56436 | 72.2954 | 124.5707 | NA | 71.56769 | 82.97709 | 146.622 | 87.7947 | 122.7194 | 92.767 | 23.72626 | 57.17501 | 44.21249 | 69.9232 |
| CUST_28912_PI429010886 | N58836 | 0.022482 | 24 | 21 | | | | | | 12.85919 | 8.723307 | NA | 6.971491 | 9.36819 | 10.86868 | 19.40236 | 10.97938 | 8.117917 | 6.31804 | 10.11317 | 6.483563 | 12.70194 | 9.330885 | 11.03039 | 11.48646 | 15.87262 | 8.131006 | 16.75535 | 8.07058 | 17.14965 | 15.05446 | 14.80809 | NA | 12.34338 | 12.74006 | 11.41844 |
| CUST_9923_PI429010886 | N25416 | 0.022488 | 24 | 0 | 533196316 | PREDICTED: apoptogenic protein 1, mitochondrial [ | | | 1.73E-24 | 61.54664 | 62.97836 | 67.95716 | 56.59481 | 55.79477 | 45.09026 | 35.73798 | 40.1699 | 50.72999 | 43.42703 | 45.63167 | 48.72653 | 46.17776 | 52.50431 | 58.97452 | 63.27865 | 34.66525 | 33.48853 | 47.87924 | 52.68156 | 50.82151 | 57.20678 | 52.063 | 45.84492 | 55.38216 | 46.1807 | 60.56579 |
| CUST_52930_PI429010886 | M99499 | 0.022494 | 15 | 9 | | | | | | 182.8109 | 151.582 | 199.2344 | 185.168 | 184.9236 | 154.6378 | 161.6619 | 188.4915 | 172.408 | 228.0483 | 203.7035 | 210.2559 | 157.2555 | 220.485 | 143.3153 | 154.9299 | 188.2417 | 144.1656 | 159.2127 | 193.3347 | 185.6524 | 202.6837 | 180.3864 | 120.5518 | 214.6655 | 212.9853 | 291.2128 |
| CUST_53547_PI429010886 | M102205 | 0.022504 | 21 | 9 | 260808985 | hypothetical protein BRAFLDRAFT_64355 [Branchio | | | 2.04E-12 | 7.738172 | 8.132198 | 9.010507 | NA | 8.455318 | NA | 12.55922 | 12.95753 | 11.51323 | 19.28994 | 15.63054 | 11.59565 | 12.14778 | 14.03531 | 12.40735 | 13.60767 | 7.747649 | 11.52917 | 11.15682 | 19.2011 | 6.541514 | 12.28913 | 9.593653 | 7.614141 | 15.78237 | 13.45433 | 4.927102 |
| CUST_52764_PI429010886 | M98826 | 0.02253 | 15 | 3 | | | | | | 7.681885 | 7.005798 | 13.89803 | 14.70767 | 24.68775 | 22.1684 | 17.69459 | 8.519462 | NA | 17.16843 | 4.817559 | NA | 9.53977 | 14.39924 | 5.554616 | 6.895161 | 12.21534 | 11.25926 | 18.86217 | 14.06382 | 31.74511 | 11.48811 | 14.44097 | 13.25777 | 9.599473 | 17.61531 | 20.69556 |
| CUST_55473_PI429010886 | M110281 | 0.022578 | 12 | 3 | | | | | | 35.63135 | 26.64522 | 23.69143 | 52.45794 | 50.42448 | 51.37093 | 41.12468 | 25.79784 | 32.49365 | 20.22452 | 23.59336 | 11.48646 | 20.39383 | 26.6839 | 27.72017 | 42.71853 | 25.40127 | 39.52535 | 34.0788 | 10.2558 | 19.03069 | 42.17788 | 17.96838 | 32.21099 | 29.24532 | 23.11258 | 26.84667 |
| CUST_23367_PI429010886 | N48716 | 0.022584 | 24 | 15 | | | | | | 163.6892 | 177.6724 | 182.3936 | 140.8613 | 160.4594 | 184.7859 | 130.3926 | 139.0616 | 134.8244 | 158.5396 | 167.6463 | 207.5722 | 157.0244 | 163.6601 | 200.4624 | 206.3065 | 169.2967 | 206.2372 | 171.9761 | 181.1767 | 192.1486 | 192.2628 | 189.2921 | 153.5041 | 203.6685 | 163.6107 | 269.2314 |
| CUST_48146_PI429010886 | M79093 | 0.022593 | 24 | 9 | 339239683 | putative integrase core domain protein [Trichinella | | | 1.57E-12 | 22.72662 | 35.70225 | 33.57067 | 17.7649 | 32.39121 | 32.6735 | 26.12486 | 33.38385 | 38.11258 | 28.66914 | 44.79489 | 37.2973 | 32.82252 | 32.90555 | 25.64332 | 28.30216 | 18.81916 | 20.19663 | 28.10538 | 23.04508 | 10.94377 | 28.22043 | 22.13675 | 21.49674 | 35.81064 | 30.69887 | 20.48821 |
| CUST_24511_PI429010886 | N50749 | 0.022615 | 24 | 3 | | | | | | 87.34049 | 73.87582 | 61.63578 | 71.80124 | 95.4903 | 146.1555 | 91.90962 | 74.90972 | 64.48135 | 89.86196 | 71.87199 | 68.2813 | 57.56812 | 59.69745 | 50.84587 | 75.60669 | 70.57215 | 65.20347 | 66.67032 | 60.96666 | 59.28688 | 93.48735 | 60.28242 | 77.20798 | 93.72794 | 66.63641 | 44.70933 |
| CUST_56089_PI429010886 | M112737 | 0.022617 | 24 | 18 | | | | | | 301.6859 | 257.5143 | 308.3369 | 180.5686 | 284.0133 | 269.8218 | 235.6197 | 240.9019 | 247.4599 | 297.8198 | 251.0973 | 227.0266 | 264.3268 | 266.0871 | 252.8872 | 343.1257 | 252.9891 | 275.8443 | 252.2638 | 369.1665 | 311.3229 | 335.5871 | 286.0749 | 233.0195 | 297.2502 | 287.6725 | 315.7757 |
| CUST_34481_PI429010886 | M9798 | 0.022617 | 24 | 18 | | | | | | 342.1732 | 415.6575 | 296.5251 | 281.2963 | 252.0389 | 262.2997 | 231.3759 | 232.0593 | 242.4714 | 331.2853 | 264.6685 | 332.6812 | 280.5674 | 342.2472 | 300.0118 | 417.7156 | 285.421 | 317.032 | 322.7291 | 360.4943 | 308.2184 | 367.4512 | 301.5634 | 239.6059 | 433.7019 | 294.2267 | 365.1838 |
| CUST_32992_PI429010886 | M5656 | 0.022651 | 15 | 0 | | | | | | 53.05965 | 56.30906 | 65.4438 | 32.03181 | 47.92719 | 39.68514 | 41.20481 | 46.13321 | 41.4213 | 41.37701 | 46.74367 | 52.55999 | 47.33315 | 45.34827 | 58.35369 | 52.85888 | 52.86776 | 66.34998 | 40.20429 | 48.83879 | 39.90155 | 47.07001 | 52.79712 | 45.88252 | 51.15134 | 43.46708 | 48.55278 |
| CUST_43585_PI429010886 | M63415 | 0.022658 | 21 | 6 | | | | | | 43.19279 | 65.12567 | 60.09613 | 64.48135 | 70.73325 | 58.67101 | 73.93015 | 107.3346 | 107.1945 | 66.00564 | 110.3513 | 84.66815 | 68.02926 | 28.5055 | 58.30365 | 68.68381 | 52.0001 | 48.50963 | 87.36382 | 107.3035 | 86.12374 | 43.33729 | 45.67726 | 40.26663 | 97.55406 | 76.71565 | 104.192 |
| CUST_34892_PI429010886 | M11005 | 0.02266 | 18 | 0 | 225719090 | Agglutinin isolectin 1 precursor [Caligus clemensi] | | | 6.49E-23 | 837.9656 | 1390.385 | 1256.992 | 362.1966 | 169.5334 | 305.2258 | 169.2006 | 177.5884 | NA | 524.5768 | 1293.664 | 788.0347 | NA | 109.6686 | 66.05732 | 1487.266 | 378.265 | 876.7586 | 881.8414 | 917.4556 | 1169.504 | 46.40711 | 605.3029 | 148.4195 | 539.2831 | NA | 68.64401 |
| CUST_32756_PI429010886 | M5041 | 0.02266 | 15 | 3 | | | | | | 8.545589 | 6.782422 | 13.02142 | 23.1605 | 11.15573 | 27.59392 | 18.69874 | 14.58939 | 13.31366 | 15.71949 | 16.86404 | 9.881836 | 9.04725 | 7.97358 | 8.107003 | 10.55129 | 33.01824 | 21.13597 | 12.74876 | 23.90131 | 35.54095 | 5.22546 | 20.20177 | 24.80851 | 11.30957 | 21.63606 | 8.134417 |
| CUST_2829_PI429010886 | N13195 | 0.02266 | 15 | 12 | | | | | | 114.787 | 92.06564 | 92.56423 | 107.174 | 107.1241 | 87.68861 | 81.21391 | 75.29438 | 83.95292 | 118.0698 | 88.10192 | 112.5446 | 98.91679 | 121.284 | 102.639 | 121.7504 | 81.20466 | 94.81726 | 90.73889 | 82.85967 | 89.09879 | 108.2888 | 68.6762 | 88.02287 | 107.1133 | 109.8291 | 94.44207 |
| CUST_26989_PI429010886 | N55236 | 0.02266 | 15 | 12 | | | | | | 32.92321 | 28.77687 | 30.11859 | 25.15936 | 24.91328 | 36.62478 | 10.01001 | 13.13514 | 18.3198 | 54.23167 | 26.81787 | 33.01166 | 21.70241 | 32.59686 | 32.12082 | 24.05186 | 40.1147 | 38.71949 | 28.6142 | 28.5643 | 24.17334 | 19.66959 | 25.73137 | 18.43402 | 32.79355 | 42.75192 | 33.13714 |
| CUST_53722_PI429010886 | M102926 | 0.022664 | 12 | 3 | | | | | | 412.1707 | 370.5671 | 351.1934 | 655.0692 | 473.5692 | 437.4103 | 418.8645 | 414.9669 | 304.8602 | 347.4375 | 369.1665 | 385.3789 | 338.0013 | 346.1873 | 103.9537 | 393.3268 | 437.7044 | 362.0452 | 454.2849 | 426.2225 | 336.5695 | 279.924 | 276.6094 | 344.0193 | 321.0768 | 435.9034 | 321.1502 |
| CUST_38337_PI429010886 | M25589 | 0.022673 | 12 | 0 | | | | | | 1248.908 | 1824.738 | 1614.966 | 1177.547 | 1009.6 | 670.112 | 787.1975 | 1266.139 | 1130.431 | 1188.466 | 1378.434 | 1807.03 | 1305.146 | 1054.214 | 1031.135 | 944.9878 | 991.3315 | 954.6687 | 825.7659 | 1798.294 | 1168.111 | 1115.976 | 1430.775 | 1150.482 | 1781.492 | 1368.903 | 1800.24 |

| Probe ID | ID | p-value | Period | Phase | EBI ID | Annotation | GO category | Go term | E-value | 0 | 0 | 0 | 3 | 3 | 3 | 6 | 6 | 6 | 9 | 9 | 9 | 12 | 12 | 12 | 15 | 15 | 15 | 18 | 18 | 18 | 21 | 21 | 21 | 24 | 24 | 24 |
|---|---|---|---|---|---|---|---|---|---|---|---|---|---|---|---|---|---|---|---|---|---|---|---|---|---|---|---|---|---|---|---|---|---|---|---|---|
| CUST_35731_PI429010886 | M13336 | 0.022673 | 18 | 9 | 326579693 | JHE-like carboxylesterase 2 [Pandalopsis japonica] | | | 3.44E-60 | 32.21099 | 34.14862 | 51.62109 | 25.5649 | 10.26743 | 14.72238 | 27.64994 | 22.42169 | 42.74237 | 42.83757 | 33.35513 | 32.79355 | 27.66624 | 27.80089 | 33.45023 | 33.29815 | 32.70344 | 15.10512 | 13.90831 | 34.21062 | 15.59997 | 25.68325 | 18.65856 | 20.69967 | 26.45134 | 26.19976 | 22.47267 |
| CUST_7225_PI429010886 | N21218 | 0.022691 | 15 | 9 | 283138829 | endo-beta-1,4-glucanase [Coptotermes formosanus | Metabolic process | Carbohydrate metabolic process | 3.07E-51 | 5.336261 | 6.298477 | NA | 10.44576 | 8.396527 | 26.57271 | 8.475934 | 62.60675 | 32.07598 | 11.26061 | 48.69863 | 37.83427 | 15.06396 | 7.563005 | 11.16007 | 7.593357 | 9.187257 | NA | 8.163573 | 37.19186 | 14.6546 | 9.253041 | 14.41094 | NA | NA | 9.757187 | 19.63409 |
| CUST_53716_PI429010886 | M102893 | 0.022711 | 18 | 15 | 307212792 | Sterile alpha and TIR motif-containing protein 1 [Ha | Signal transduction | Other | 2.82E-33 | 31.30771 | 22.56982 | 35.47721 | 27.22941 | 32.63905 | 26.50017 | 26.88741 | 21.4644 | 26.93627 | 28.19636 | 34.2205 | 24.79214 | 19.14111 | 42.2806 | 36.115 | 25.77541 | 31.44457 | 38.28234 | 56.2163 | 34.24386 | 28.06124 | 33.17041 | 33.11438 | 29.76829 | 20.28929 | 22.84941 | 25.53352 |
| CUST_14583_PI429010886 | N33116 | 0.022722 | 12 | 3 | | | | | | 106.5468 | 67.92832 | 23.61053 | 189.267 | 95.37902 | 298.4347 | 106.1516 | 65.79499 | 43.27701 | 4.931371 | 78.84468 | 3.685126 | 66.97799 | 13.24115 | 37.38785 | 44.48912 | 190.9766 | 166.1109 | 102.2299 | 16.27641 | 42.71853 | 38.30957 | 21.02813 | 112.8438 | 8.822611 | 62.53094 | 7.02868 |
| CUST_40817_PI429010886 | N56157 | 0.022724 | 24 | 3 | | | | | | 43.48001 | 32.61136 | 13.7828 | 59.80108 | 34.48913 | 44.38974 | 32.75767 | 34.14862 | 28.55766 | 29.20057 | 18.05975 | 29.13011 | 41.12787 | 27.18775 | 24.20666 | 31.53803 | 20.21835 | 28.39426 | 25.56884 | 20.99955 | 25.55814 | 37.26633 | 19.64586 | 33.21734 | 32.46954 | 30.86904 | 19.80974 |
| CUST_48543_PI429010886 | M80903 | 0.022724 | 24 | 21 | | | | | | 86.36905 | 84.08227 | 86.45155 | 86.58356 | 74.15763 | 67.91738 | 71.57902 | 71.08447 | 74.75705 | 98.57526 | 92.96667 | 85.75328 | 75.69546 | 122.6286 | 85.29111 | 79.82568 | 95.98918 | 81.37626 | 68.75407 | 87.86031 | 90.69277 | 109.0963 | 113.4637 | 103.6058 | 122.5319 | 102.6011 | 89.69655 |
| CUST_34069_PI429010886 | M8668 | 0.022724 | 24 | 21 | | | | | | 50.7913 | 102.9667 | 79.56143 | 66.28856 | 50.21619 | 41.06999 | 32.98295 | 61.88462 | 66.4925 | 109.6112 | 56.28989 | 81.31167 | 75.25456 | 69.31912 | 58.04233 | 97.09132 | 65.09833 | 95.10396 | 65.23612 | 73.49284 | 75.91487 | 85.74608 | 124.4949 | 88.28345 | 83.61604 | 94.32297 | 74.26685 |
| CUST_32062_PI429010886 | M3141 | 0.022751 | 21 | 9 | | | | | | 12.14326 | 19.14544 | 17.11711 | 35.33784 | 35.27881 | 24.15872 | 26.18577 | 182.2902 | 79.67757 | 16.45485 | 630.9156 | 839.8931 | 140.1157 | 23.11258 | 71.24301 | 23.96699 | 56.96512 | 9.676508 | 23.50904 | 579.2037 | 65.93703 | 21.35972 | 15.58239 | 31.16623 | 11.63852 | 66.03172 | 83.62457 |
| CUST_122_PI429010886 | N1176 | 0.022751 | 21 | 3 | 371591210 | Euphausia superba voucher 980349 mitochondrion, | | | | 84866.33 | 50702.21 | 78574.02 | 89222.73 | 98448.52 | 88276.1 | 88771.33 | 86045.29 | 78891.09 | 104141 | 88276.1 | 80636.04 | 92843.62 | 56627.52 | 53582.06 | 64571.61 | 81077.7 | 52133.5 | 47060.78 | 70048.09 | 90253.54 | 89699.25 | 87164.76 | 88276.1 | 91927.09 | 94450 | 85436.14 |
| CUST_2373_PI429010886 | N12208 | 0.022768 | 15 | 9 | | | | | | 101.6252 | 159.2127 | 124.3983 | 125.1451 | 133.1932 | 142.5887 | 145.9962 | 104.4433 | 159.5684 | 153.8044 | 152.6328 | 163.7665 | 123.3316 | 187.4537 | 129.9706 | 125.0614 | 121.9963 | 139.2323 | 104.4054 | 120.6877 | 160.3449 | 130.2006 | 169.9686 | 180.4095 | 176.3364 | 155.4298 | 200.6444 |
| CUST_326_PI429010886 | N3906 | 0.02281 | 15 | 0 | | | | | | 199.5478 | NA | 366.5663 | 95.97714 | 97.2708 | 188.9473 | 420.8347 | 162.5891 | 85.17905 | 61.68471 | 203.0375 | 8.518007 | 84.89465 | 93.72794 | 23.75346 | NA | 410.1622 | 189.8316 | 271.0212 | NA | 281.9234 | 21.25262 | 213.1195 | 239.0183 | 25.71973 | 54.15621 | 25.42895 |
| CUST_11129_PI429010886 | N27310 | 0.02281 | 21 | 15 | 242007148 | CRSP complex subunit, putative [Pediculus humanu | | | 1.36E-32 | 4.582799 | 7.708974 | NA | 7.840127 | 5.163161 | NA | NA | 3.228837 | 6.413493 | 8.673687 | 4.028405 | NA | 5.949232 | 10.02652 | 8.486348 | 8.960873 | 9.460042 | 12.32047 | NA | 15.37012 | 8.711267 | 6.884007 | NA | 6.18554 | 12.94753 | 3.160393 | 4.656197 |
| CUST_47881_PI429010886 | M77998 | 0.022816 | 24 | 0 | 170819724 | reverse transcriptase [Daphnia pulex] | | | 3.93E-11 | 32.88602 | 32.07184 | 42.61497 | 17.03915 | 24.74877 | 18.79454 | 30.50923 | 29.80377 | 31.47236 | 22.61587 | 20.96593 | 21.51643 | 18.48864 | 38.9709 | 17.87442 | 31.13099 | 23.46752 | 32.69544 | 32.363 | 26.52453 | 19.42689 | 57.84964 | 40.00557 | 24.67666 | 43.42171 | 28.71654 | 31.8 |
| CUST_3492_PI429010886 | N14613 | 0.022826 | 24 | 15 | | | | | | 47.1063 | 29.00481 | 28.09932 | 27.79511 | 32.48228 | 58.64138 | 42.38554 | 33.25306 | 34.63137 | 61.05832 | 48.65694 | 62.82655 | 52.12912 | 47.0845 | 50.76541 | 66.59221 | 64.9677 | 42.26034 | 59.68907 | 58.80718 | 59.26817 | 59.64714 | 20.10533 | NA | 51.14292 | 42.68895 | 42.47769 |
| CUST_50794_PI429010886 | M91031 | 0.022834 | 18 | 3 | | | | | | 14.86246 | 9.020299 | 16.11615 | 12.84883 | 22.39477 | 19.3658 | 20.12835 | 7.693635 | 6.051294 | 9.384386 | 8.653249 | 7.454608 | 4.944022 | NA | 10.73106 | 12.60453 | 9.464207 | NA | 16.79285 | 9.506797 | 25.78299 | 10.76813 | 13.65696 | 29.02323 | 22.4878 | 11.83225 | 15.59286 |
| CUST_38924_PI429010886 | M32005 | 0.022846 | 12 | 9 | | | | | | 101.0263 | 67.08962 | 90.16303 | 51.37508 | 53.90994 | 26.87538 | 88.90431 | 87.36382 | 92.43125 | 288.9883 | 62.42674 | 108.4775 | 146.0101 | 147.7053 | 104.976 | 86.21546 | 56.57261 | 64.37974 | 190.3154 | 83.00511 | 82.05363 | 122.0703 | 124.1661 | 77.33177 | 63.43062 | 107.7922 | 260.055 |
| CUST_1431_PI429010886 | N9606 | 0.022875 | 18 | 12 | | | | | | 279.8719 | 417.4014 | 456.6758 | 307.3807 | 273.0127 | 348.8261 | 192.7945 | 348.2614 | 419.3343 | 394.3816 | 385.0916 | 805.0049 | 512.4566 | 747.4574 | 689.0956 | 591.2615 | 352.188 | 439.5513 | 434.5491 | 552.3175 | 441.0416 | 438.7455 | 508.1895 | 264.7951 | 666.1474 | 554.0852 | 538.0354 |
| CUST_36878_PI429010886 | M17120 | 0.022883 | 12 | 3 | | | | | | 83.37454 | 64.72483 | 50.17569 | 97.73561 | 101.3914 | 148.9405 | 86.84519 | 60.26917 | 45.57502 | 58.54952 | 84.45921 | 60.04192 | 64.26189 | 68.1364 | 52.70775 | 82.71912 | 101.6113 | 106.5981 | 115.7238 | 51.30486 | 76.23608 | 67.41072 | 72.90791 | 93.42325 | 61.52513 | 54.76027 | 59.51435 |
| CUST_5452_PI429010886 | N18724 | 0.022883 | 12 | 0 | | | | | | 2213.328 | 3126.195 | 2388.967 | 1312.967 | 1378.873 | 1193.941 | 1428.874 | 1773.273 | 1834.08 | 3024.762 | 1778.896 | 2416.782 | 2012.591 | 2056.54 | 2181.474 | 3143.095 | 1622.403 | 1657.221 | 1863.078 | 2315.282 | 1948.38 | 2156.875 | 1936.604 | 1295.841 | 2368.476 | 1883.709 | 2800.799 |
| CUST_7015_PI429010886 | N20917 | 0.022886 | 21 | 6 | | | | | | 263.1737 | 117.1499 | 484.8203 | 37.55188 | 92.47211 | 12.28125 | 208.9955 | 324.7416 | 297.3146 | 217.0599 | 124.4616 | 152.8184 | 45.53002 | 7.057042 | 32.10959 | NA | 83.34341 | 8.578143 | 66.47359 | 104.46 | 58.14864 | NA | 322.4251 | 102.1471 | NA | 151.8403 | 150.5702 |
| CUST_56313_PI429010886 | M113476 | 0.022909 | 24 | 21 | | | | | | 20.01149 | 22.55992 | 35.40201 | 32.16411 | 13.04571 | 37.12735 | 9.703156 | 16.27976 | 21.86356 | 22.6017 | 23.99191 | 21.94448 | 16.15585 | 26.08713 | 34.30407 | 47.90583 | 47.96622 | 36.3007 | 23.66736 | 28.76281 | 17.23726 | 38.10884 | 35.46793 | 30.34438 | 33.7306 | 32.36622 | 41.65038 |
| CUST_35638_PI429010886 | M13080 | 0.022909 | 21 | 15 | | | | | | 265.4463 | 383.1748 | 222.7897 | 259.2052 | 301.6337 | 264.7596 | 191.7287 | 230.5542 | 187.0261 | 266.5934 | 289.7899 | 346.4727 | 283.2143 | 271.5235 | 239.1825 | 319.2639 | 386.2115 | 340.057 | 375.0345 | 294.9448 | 231.6114 | 344.6094 | 282.2712 | 200.6444 | 379.0179 | 218.242 | 277.3264 |
| CUST_19125_PI429010886 | N41015 | 0.02291 | 21 | 0 | | | | | | 95.689 | 85.95 | 72.07584 | 82.85967 | 73.66521 | 72.69379 | 51.6678 | 55.22248 | 43.21618 | 78.80354 | 58.64723 | 69.38283 | 54.15621 | 69.73102 | 48.59055 | 87.97637 | 76.15742 | 74.75705 | 72.8924 | 65.93142 | 70.64122 | 92.56423 | 63.30844 | 78.80354 | 105.3539 | 71.29059 | 75.33028 |
| CUST_37169_PI429010886 | M18077 | 0.022916 | 12 | 9 | 328700186 | PREDICTED: lactosylceramide 4-alpha-galactosyltrar | | | 6.61E-16 | 12.40087 | 6.908042 | 16.32095 | 4.675508 | 3.909968 | 6.786633 | 13.90091 | 12.29489 | 9.632488 | 11.41745 | NA | 12.28249 | 14.98837 | 18.66977 | 6.625606 | 6.413493 | 6.811352 | NA | 6.608238 | NA | NA | 8.172783 | 22.04311 | 20.00677 | 12.62191 | NA | 9.04725 |
| CUST_45706_PI429010886 | M69998 | 0.022917 | 18 | 3 | | | | | | 42.85091 | 62.79742 | 77.59246 | 56.55039 | 68.63241 | 67.19434 | 60.91532 | 44.34868 | 67.12853 | 49.10837 | 56.00276 | 37.12735 | 27.68808 | 55.20237 | 54.75352 | 33.13396 | 44.64106 | 55.80731 | 67.22077 | 14.67638 | 43.44238 | 46.55207 | 67.52161 | 68.6762 | 47.07665 | 58.94983 | 64.88975 |
| CUST_45215_PI429010886 | M68354 | 0.022917 | 24 | 15 | | | | | | 216.8689 | 224.3925 | 242.0042 | 158.8514 | 225.4823 | 281.1462 | 175.4895 | 143.4291 | 146.3399 | 272.1926 | 258.7017 | 245.0284 | 112.0385 | 171.5147 | 154.1768 | 300.2764 | 323.2711 | 313.9465 | 255.0746 | 241.4805 | 279.6215 | 269.8218 | 175.2864 | 207.6455 | 339.8879 | 206.4448 | 274.4437 |
| CUST_11829_PI429010886 | N28477 | 0.022918 | 18 | 15 | | | | | | 560.3037 | 667.7123 | 636.6917 | 463.903 | 512.9488 | 599.2901 | 553.6437 | 521.5716 | 610.8058 | 370.5671 | 543.5013 | 681.8773 | 604.5341 | 699.4291 | 717.4734 | 914.9994 | 635.9764 | 649.5445 | 677.5621 | 699.0502 | 491.0083 | 651.6164 | 465.3498 | 459.5005 | 685.1065 | 499.9413 | 543.1668 |
| CUST_31763_PI429010886 | M2345 | 0.022927 | 21 | 3 | 383856339 | PREDICTED: Megachile rotundata cyclin-dependent | Protein metabolism | Cellular protein modification process | 1.46E-51 | 2124.898 | 3929.651 | 2814.544 | 9795.872 | 4094.84 | 3968.239 | 2189.348 | 2480.699 | 2476.731 | 3156.693 | 1335.109 | 4455.764 | 1324.694 | 2306.592 | 2483.895 | 3638.002 | 1060.588 | 2630.661 | 1377.953 | 2466.842 | 3333.763 | 3968.239 | 3815.349 | 2410.357 | 4876.999 | 3459.777 | 2089.376 |
| CUST_56_PI429010886 | N259 | 0.022983 | 12 | 0 | | | | | | 1930.669 | 2955.19 | 2004.816 | 1841.426 | 1505.495 | 1543.721 | 1426.122 | 1518.655 | 1720.979 | 2020.188 | 1495.765 | 2076.382 | 1726.714 | 2211.182 | 2856.456 | 2935.657 | 1461.877 | 2114.712 | 1571.524 | 2258.839 | 1732.866 | 1883.709 | 1930.669 | 1707.181 | 2348.331 | 2428.103 | 2352.559 |
| CUST_51941_PI429010886 | M95754 | 0.02299 | 12 | 9 | | | | | | 55.13629 | 61.5685 | 60.21705 | 4.144645 | 35.03861 | NA | 18.91531 | 35.4728 | 45.28288 | 144.8143 | 57.04658 | 251.4349 | NA | 17.27797 | 30.63709 | 108.2888 | 11.22509 | 26.56221 | 41.09904 | 17.54073 | 56.8218 | NA | 61.96906 | NA | 46.30865 | 25.61197 | 34.02862 |
| CUST_36740_PI429010886 | M16714 | 0.022995 | 24 | 21 | | | Protein metabolism | Cellular protein modification process | | 230.3269 | 220.8169 | 160.4181 | 165.5224 | 198.5143 | 184.1659 | 213.4663 | 194.777 | 177.1551 | 255.6621 | 150.1195 | 198.1863 | 208.2838 | 208.3184 | 229.2792 | 270.0349 | 191.9895 | 204.5255 | 209.3879 | 249.7604 | 224.0471 | 265.7722 | 274.5343 | 250.2477 | 314.8276 | 249.7124 | 281.735 |
| CUST_32563_PI429010886 | M4439 | 0.023007 | 15 | 9 | | | | | | 21.11687 | 71.17303 | 54.56432 | 10.2415 | 4.778031 | NA | 4.371588 | 60.24939 | 91.54224 | 105.7694 | 55.74802 | 83.63285 | 37.74785 | 123.2283 | 58.5117 | 50.131 | 18.25747 | 30.3145 | 56.94451 | 77.66626 | 52.39551 | 141.0258 | 44.15212 | NA | 247.7341 | 96.09446 | 134.3159 |
| CUST_44490_PI429010886 | M66128 | 0.023024 | 15 | 9 | | | | | | 16.13721 | 16.64512 | 15.56753 | 16.7648 | 12.9711 | 12.81767 | 16.83531 | 14.96077 | 16.03304 | 18.99745 | 22.3788 | 17.02546 | 17.38288 | 23.21852 | 20.95213 | 20.84836 | 10.73906 | 15.81556 | 20.97422 | 13.47795 | 18.65486 | 19.2789 | 19.50301 | 19.22446 | 19.18487 | 25.73562 | 27.62424 |
| CUST_15577_PI429010886 | N34817 | 0.023033 | 24 | 3 | | | | | | 20.98763 | 18.85808 | 14.24976 | 20.80857 | 17.78619 | 17.64058 | 14.37012 | 13.43914 | 12.53584 | 21.13597 | 9.501422 | 15.05158 | 15.35258 | 10.90408 | 12.43735 | 12.01996 | 10.02996 | 8.663836 | 15.87862 | NA | 17.12071 | 10.68037 | 4.74894 | 5.282757 | 32.44398 | 12.56262 | 13.07843 |
| CUST_39234_PI429010886 | M36530 | 0.023039 | 18 | 12 | 77743875 | Euphausia superba isolate DGGE band AntEuk13-5 : | | | | 54.43748 | 62.71939 | 47.28217 | 52.38937 | 66.5854 | 56.31693 | 48.05138 | 61.5801 | 54.88431 | 80.27864 | 66.37927 | 85.30129 | 71.64903 | 90.25676 | 72.19392 | 71.34779 | 46.67338 | 67.44354 | 56.57261 | 57.75281 | 60.99125 | 69.85985 | 72.43161 | 48.49494 | 58.10931 | 63.87286 | 70.04597 |
| CUST_2276_PI429010886 | N12010 | 0.023039 | 24 | 3 | | | | | | NA | 252.3543 | 10.95075 | 480.8955 | 125.719 | 440.428 | 768.0399 | 35.29716 | 645.5341 | 7.489011 | 58.33665 | NA | NA | NA | 14.28453 | 9.505528 | 29.06247 | 37.68617 | 234.0614 | 5.97707 | 5.773804 | 8.925496 | NA | 65.64051 | 39.00007 | NA | 23.31758 |
| CUST_50203_PI429010886 | M89017 | 0.023079 | 15 | 12 | | | | | | 552.1675 | 675.5498 | 622.1973 | 522.4048 | 467.8704 | 529.9042 | 446.9611 | 454.4901 | 543.3625 | 698.8251 | 475.6507 | 1166.811 | 480.5601 | 573.39 | 666.3149 | 671.5411 | 533.4173 | 575.5153 | 423.8598 | 586.0335 | 495.5775 | 549.6818 | 448.4129 | 464.5441 | 608.5272 | 634.2891 | 741.7837 |
| CUST_2395_PI429010886 | N12259 | 0.023079 | 21 | 0 | | | | | | 17.77636 | 26.10463 | 26.92502 | 19.6594 | 10.65431 | 17.11883 | 11.25121 | 8.535901 | 4.636066 | 20.76453 | 19.96799 | 17.51738 | 14.78884 | 21.4565 | 14.07399 | 23.79718 | 5.161394 | 18.36165 | 15.82481 | 15.83863 | 6.727538 | 27.21328 | 20.83932 | 19.78047 | 17.03463 | 21.25435 | 25.12826 |
| CUST_7973_PI429010886 | N22355 | 0.02308 | 24 | 0 | 528494560 | PREDICTED: uncharacterized protein LOC571260 isc | | | 2.35E-63 | 55.04733 | 69.53748 | 58.544 | 52.92115 | 60.49322 | 46.26383 | 51.02755 | 44.6026 | 55.54708 | 50.87205 | 48.95474 | 90.38098 | 39.06616 | 45.39895 | 56.56158 | 43.51237 | 33.36548 | 37.30507 | 56.86246 | 106.8412 | 76.13058 | 48.06558 | 51.80926 | 48.93138 | 55.15882 | 54.99254 | 72.62704 |
| CUST_56189_PI429010886 | M113059 | 0.023084 | 24 | 12 | 308512488 | Caenorhabditis remanei hypothetical protein (CRE_ | Metabolic process | Carbohydrate metabolic process | 1.99E-53 | 7.326709 | 16.76094 | 12.55296 | 11.22853 | 12.09699 | 8.289001 | 7.335496 | 5.025247 | 14.13095 | 22.8445 | 11.59565 | 19.91122 | 17.56349 | 24.52916 | 18.6269 | 16.97468 | NA | 19.09764 | 13.07005 | 17.74945 | 10.87355 | 30.27449 | 10.73459 | 15.73055 | 13.56721 | 10.25766 | 17.99612 |
| CUST_37987_PI429010886 | M22615 | 0.023101 | 15 | 12 | 225680773 | predicted protein [Paracoccidioides brasiliensis Pb0 | | | 8.48E-07 | NA | 17.8956 | NA | 5.398604 | 5.799915 | NA | 74.43452 | 20.4413 | 84.53623 | 16.22239 | 9.460042 | 38.02052 | 145.9425 | 142.1431 | 60.07749 | 81.46986 | 131.7044 | 11.21303 | 5.795169 | 24.44087 | 10.38684 | 53.46776 | 49.18958 | 4.107908 | 134.3489 | 15.27365 | 269.1187 |
| CUST_33612_PI429010886 | M7425 | 0.023108 | 24 | 18 | | | | | | 132.3396 | 135.3397 | 119.1024 | 98.70636 | 134.9903 | 119.2791 | 126.8944 | 91.31323 | 108.8006 | 144.0267 | 119.8572 | 142.632 | 132.2443 | 129.1538 | 145.8455 | 175.3631 | 99.87407 | 129.2905 | 149.9331 | 169.8261 | 143.0236 | 158.496 | 122.2373 | 119.508 | 152.6328 | 102.022 | 150.9847 |
| CUST_7145_PI429010886 | N21105 | 0.023108 | 18 | 0 | | | | | | 1624.747 | 1990.565 | 1740.855 | 1905.993 | 1966.099 | 1627.141 | 1294.143 | 1156.123 | 1193.094 | 1866.472 | 1656.441 | 1762.677 | 1305.705 | 1743.746 | 1679.237 | 2939.878 | 1598.927 | 1736.508 | 1769.215 | 2088.06 | 1638.912 | 2408.505 | 1467.586 | 1363.213 | 1709.2 | 1328.52 | 1460.496 |
| CUST_7444_PI429010886 | N21561 | 0.023115 | 12 | 9 | 321469997 | hypothetical protein DAPPUDRAFT_303691 [Daphn | Developmental process | Nervous system development | 1.81E-54 | 131.1671 | 177.2148 | 115.3764 | 103.4856 | 96.10628 | 73.81964 | 87.90722 | 115.6759 | 121.6056 | 158.496 | 138.9958 | 144.1199 | 131.4929 | 134.595 | 125.3729 | 175.2604 | 92.32556 | 111.9496 | 128.1244 | 177.5678 | 148.0995 | 151.9691 | 148.2643 | 128.8549 | 120.8512 | 139.359 | 152.5175 |
| CUST_18717_PI429010886 | N40283 | 0.023132 | 15 | 3 | 307200827 | Uncharacterized protein C9E9.15 [Harpegnathos sal | | | 2.36E-24 | 15.33408 | 15.66502 | 25.14408 | 23.37181 | 23.194 | 23.58263 | 13.76206 | 15.83087 | 22.78329 | 11.53559 | 10.93941 | 20.01489 | 10.17467 | 13.67523 | 20.55898 | 17.43627 | 18.50256 | 20.11049 | 19.8522 | 10.37845 | 24.55823 | 20.11049 | 24.43879 | 19.3376 | 9.089436 | 17.18186 | 12.85382 |
| CUST_43699_PI429010886 | M63723 | 0.023149 | 12 | 0 | | | | | | 173.1369 | 236.856 | 234.555 | 192.6124 | 156.9336 | 187.5065 | 163.0075 | 173.9653 | 221.3497 | 226.242 | 192.1754 | 254.8048 | 193.9282 | 341.2566 | 271.6624 | 231.8131 | 201.085 | 204.7885 | 211.2278 | 239.521 | 224.6063 | 221.8177 | 235.8718 | 212.8057 | 250.2477 | 272.1441 | 265.1519 |
| CUST_2771_PI429010886 | N13073 | 0.023149 | 12 | 0 | | | | | | 289.0715 | 268.8761 | 239.6937 | 179.2925 | 210.4017 | 181.9887 | 206.2372 | 237.5448 | 230.8646 | 260.1385 | 217.2745 | 254.5885 | 235.6934 | 249.8933 | 272.7999 | 229.9104 | 243.3133 | 251.8524 | 244.8254 | 244.1579 | 249.3051 | 293.013 | 253.2546 | 259.5794 | 300.9857 | 205.4212 | 305.9676 |
| CUST_17453_PI429010886 | N37987 | 0.023157 | 24 | 6 | | | | | | 6.411759 | 5.94198 | 7.834552 | NA | 4.470524 | 22.64343 | 11.08749 | 26.11497 | 13.73574 | 7.478506 | 8.960873 | 31.93333 | 4.046793 | NA | NA | 8.155994 | 7.113361 | NA | 5.224196 | 5.890122 | 11.16121 | 8.178429 | 4.235116 | 3.687201 | 12.72278 | 6.606034 | 9.435368 |
| CUST_19291_PI429010886 | N41325 | 0.023173 | 12 | 3 | | | | | | 267.0284 | 230.7517 | 279.9837 | 306.0346 | 332.1642 | 483.4448 | 338.0013 | 299.837 | 240.2928 | 251.0973 | 282.1142 | 265.0157 | 276.2901 | 212.8057 | 232.1034 | 254.095 | 418.03 | 349.4134 | 344.1554 | 219.2633 | 309.9578 | 270.5265 | 278.2072 | 307.2464 | 305.1659 | 266.3801 | 268.545 |
| CUST_31696_PI429010886 | M2190 | 0.023186 | 21 | 9 | 321469587 | hypothetical protein DAPPUDRAFT_304152 [Daphn | Metabolic process | Generation of precursor metabolites and energy | 3.15E-21 | 12.64389 | 95.65165 | 37.65902 | 150.5497 | 212.1945 | 18.74035 | 115.3428 | 587.6341 | 517.4057 | 154.5247 | 733.1697 | 821.0172 | 353.4537 | 60.24307 | 176.0509 | 5.967104 | 195.3573 | NA | 45.61861 | 1463.822 | 526.5682 | 18.6314 | 82.85967 | 68.79393 | 4.129176 | 463.9991 | 277.2458 |
| CUST_49083_PI429010886 | M84000 | 0.023186 | 21 | 3 | | | | | | 74.73352 | 104.9497 | 66.13621 | 86.16637 | 74.92071 | 98.70636 | 46.31902 | 62.23219 | 68.1923 | 88.46488 | 49.75477 | 79.28399 | 71.82501 | 60.6558 | 50.70831 | 88.71715 | 60.15877 | 65.08584 | 76.29064 | 71.52214 | 104.3393 | 81.58984 | 76.31563 | 104.9253 | 116.1332 | 72.81129 | 103.2411 |
| CUST_34332_PI429010886 | M9346 | 0.023194 | 15 | 12 | 67625699 | TPA: endonuclease-reverse transcriptase [Schistoso | | | 2.97E-33 | 142.8582 | 152.2451 | 184.9825 | 146.585 | 126.6828 | 128.3244 | 109.812 | 147.0211 | 157.8626 | 205.5044 | 164.1721 | 185.5006 | 167.1087 | 203.3901 | 217.8838 | 208.936 | 150.434 | 162.4256 | 143.8832 | 208.3478 | 124.7213 | 191.6952 | 209.0603 | 157.0455 | 179.9875 | 142.7716 | 194.6574 |
| CUST_29116_PI429010886 | N59229 | 0.023217 | 15 | 9 | | | | | | 13.88785 | 13.15317 | NA | 9.549172 | 6.836925 | NA | 10.45244 | 12.70382 | 6.289108 | 13.58569 | 19.66703 | 18.60572 | 15.15241 | 20.75304 | 8.639749 | 24.93734 | 8.356596 | 20.31071 | 10.24027 | 13.5817 | 11.38442 | 15.5488 | 16.77607 | 13.91491 | 19.58231 | 21.62181 | 19.7129 |
| CUST_24016_PI429010886 | N49866 | 0.023225 | 21 | 0 | 241255103 | FERM domain-containing protein, putative [Ixodes s | | | 2.04E-28 | 16.79903 | 22.6112 | 29.11901 | 18.8295 | 14.68592 | 18.53311 | 10.82657 | 8.73506 | 9.655515 | 21.14025 | 9.583323 | 25.54204 | 20.29574 | 14.05923 | 23.64437 | 20.75152 | 10.46161 | 25.73774 | 19.20978 | 15.33118 | 13.80995 | 28.16499 | 20.16285 | 24.76116 | 15.54715 | 11.24691 | 20.26494 |
| CUST_42064_PI429010886 | M59300 | 0.023241 | 18 | 15 | | | | | | 224.015 | 264.9246 | 195.2959 | 245.6501 | 245.0768 | 233.7917 | 176.2005 | 200.6692 | 201.5551 | 216.8379 | 206.8341 | 238.5792 | 201.3287 | 204.8478 | 239.7427 | 273.8764 | 209.7275 | 261.511 | 254.5885 | 244.2771 | 214.1014 | 262.4649 | 217.4042 | 233.9335 | 229.6885 | 174.126 | 192.6124 |
| CUST_12001_PI429010886 | N28759 | 0.02326 | 24 | 18 | | | | | | 114.4015 | 92.95468 | 99.88549 | 73.02744 | 76.14971 | 76.94138 | 71.34779 | 60.79572 | 68.00621 | 59.22278 | 86.99215 | 120.7327 | 64.03635 | 91.04966 | 83.63981 | 98.01409 | 91.48208 | 105.0612 | 115.5186 | 110.273 | 81.08583 | 94.64608 | 81.92166 | 89.84183 | 113.6504 | 111.5705 | 112.8118 |
| CUST_37282_PI429010886 | M18493 | 0.023263 | 15 | 9 | | | | | | 435.2247 | 426.2225 | 584.5557 | 351.983 | 412.3362 | 416.2434 | 451.6758 | 503.3426 | 459.5005 | 611.8341 | 418.7921 | 506.24 | 430.3425 | 439.0329 | 447.6451 | 463.3861 | 428.865 | 375.7452 | 379.3496 | 385.8062 | 391.819 | 334.9967 | 522.8025 | 346.9119 | 463.7968 | 394.4542 | 549.5559 |
| CUST_44020_PI429010886 | M64669 | 0.023265 | 24 | 21 | | | | | | 162.0477 | 182.3659 | 232.4315 | 185.8695 | 234.2305 | 167.5706 | 165.1538 | 146.4805 | 159.32 | 207.853 | 168.329 | 224.1509 | 171.3364 | 208.515 | 190.8326 | 201.3867 | 173.1695 | 184.8121 | 202.6446 | 236.2907 | 211.3962 | 231.3759 | 244.4939 | 174.1468 | 247.8273 | 230.7819 | 258.0874 |
| CUST_38153_PI429010886 | M23700 | 0.023268 | 12 | 3 | 443701822 | hypothetical protein CAPTEDRAFT_206264 [Capitell | Protein metabolism | Proteolysis | 2.93E-08 | 144.0267 | 10.85125 | NA | 527.0813 | 108.5223 | 885.0858 | 125.6016 | 151.752 | 44.38038 | 5.701277 | 16.88136 | NA | 88.18553 | NA | 46.67853 | 15.15241 | 446.4087 | 449.1802 | 39.53295 | NA | 18.13226 | 7.065415 | 10.55447 | 363.0918 | NA | 294.5278 | NA |
| CUST_2991_PI429010886 | N13558 | 0.023272 | 24 | 21 | | | | | | 11.23989 | 7.862605 | 9.933074 | 6.834339 | 8.421448 | 6.939821 | 6.385249 | 12.91494 | 9.397921 | 6.736876 | 4.504502 | 16.1429 | NA | 3.916306 | 10.35656 | 8.801809 | 7.54037 | 13.20951 | 10.55129 | 5.975832 | 11.24522 | 14.1684 | 19.67133 | 14.62416 | 8.457009 | 12.07791 | 16.68621 |
| CUST_9662_PI429010886 | N25011 | 0.023305 | 18 | 3 | | | | | | 39.65895 | 25.60131 | 47.21351 | 91.1558 | 79.09571 | 256.0552 | 62.85713 | 47.9222 | 41.9046 | 28.6993 | 53.14405 | 14.19523 | 36.51599 | 35.02024 | 16.17931 | 33.89069 | 68.71451 | 123.9463 | 44.53615 | 20.55297 | 72.96725 | 51.49206 | 41.42947 | 99.03782 | 24.0705 | 115.9694 | 27.91558 |
| CUST_48960_PI429010886 | M83211 | 0.023316 | 15 | 9 | | | | | | 15.036 | 17.73142 | 30.78534 | 24.28604 | 21.89788 | 27.15098 | 24.66568 | 21.34327 | 21.50201 | 29.58077 | 32.5763 | 26.05952 | 15.32478 | 15.7177 | 28.6303 | 21.1696 | 28.9143 | 16.40453 | 21.86589 | 19.18101 | 14.80982 | 29.29243 | 24.56592 | 28.50866 | 39.53295 | 27.34334 | 33.48474 |
| CUST_19193_PI429010886 | N41141 | 0.023333 | 24 | 18 | | | | | | 11.20426 | 14.10504 | 16.5954 | 11.09945 | 9.779594 | 13.13833 | 3.414573 | 8.115242 | 15.228 | 11.56124 | 10.9756 | 23.32 | 12.65013 | 26.38163 | 9.121241 | 15.78045 | 13.93595 | 16.97989 | 13.26879 | 20.46944 | 17.55722 | 26.79789 | 12.91624 | 12.95283 | 12.33047 | 12.81122 | 25.68589 |
| CUST_9808_PI429010886 | N25236 | 0.023353 | 21 | 0 | | | | | | 347.9148 | 540.0536 | 397.5988 | 301.3697 | 268.3811 | 197.2996 | 292.5508 | 279.8319 | 238.4211 | 379.882 | 363.7161 | 274.3455 | 227.9427 | 262.3746 | 234.1059 | 440.3254 | 305.4951 | 308.6668 | 419.0394 | 273.0635 | 310.0283 | 590.149 | 370.7278 | 321.8946 | 510.1908 | 216.65 | 361.0091 |
| CUST_13270_PI429010886 | N30888 | 0.023373 | 12 | 3 | | | | | | 65.76284 | 74.92071 | 59.59632 | 108.6304 | 73.97012 | 145.861 | 76.69076 | 84.07185 | 51.5653 | 76.58426 | 56.67223 | 48.99561 | 62.1958 | 46.26383 | 59.04714 | 81.17779 | 95.87365 | 93.96395 | 62.65132 | 52.63381 | 62.59848 | 67.40392 | 69.25418 | 59.49267 | 61.61655 | 74.79904 | 50.33907 |
| CUST_22537_PI429010886 | N47203 | 0.023375 | 18 | 3 | | | | | | 39.7525 | 37.60789 | 49.24106 | 63.29578 | 40.70657 | 70.52611 | 70.73325 | 49.75939 | 42.0436 | 53.96058 | 35.53394 | 58.82472 | 41.80456 | 52.55217 | 49.63764 | 37.50197 | 28.1812 | 40.5937 | 27.93572 | 32.42452 | 55.18575 | 57.04658 | 65.49092 | 43.0609 | 47.22465 | 70.32547 | 39.52535 |
| CUST_12170_PI429010886 | N29040 | 0.023378 | 18 | 15 | | | | | | 43.40065 | 51.01157 | 57.60056 | 50.09589 | 43.86454 | 46.20496 | 19.77865 | 37.80277 | 47.88363 | 56.04356 | 44.62585 | 77.27026 | 47.19565 | 70.06431 | 55.50473 | 80.82297 | 46.75333 | 61.07679 | 63.22526 | 57.65114 | 62.71939 | 51.43059 | 43.05056 | 28.13108 | 45.22744 | 60.99125 | 52.79712 |
| CUST_32066_PI429010886 | M3147 | 0.023384 | 12 | 3 | 291243110 | PREDICTED: N-acetylated alpha-linked acidic dipept | Protein metabolism | Proteolysis | 4.1E-127 | 1334.604 | 1735.328 | 635.437 | 2076.382 | 1192.582 | 2024.906 | 1425.514 | 952.3475 | 916.176 | 529.2101 | 917.4556 | 598.2934 | 856.8878 | 435.3919 | 1055.17 | 1192.165 | 1756.971 | 1792.391 | 1701.136 | 662.2599 | 609.308 | 1186.311 | 546.0726 | 1537.974 | 532.0502 | 1664.492 | 559.1604 |
| CUST_19494_PI429010886 | N41669 | 0.023384 | 12 | 3 | | | | | | 86.57696 | 37.16398 | 86.82244 | 25.749 | 87.69732 | 95.62622 | 63.11008 | 25.55311 | 57.88958 | 51.49206 | 33.89069 | 28.83526 | 46.11982 | 89.09879 | 59.234 | 78.42487 | 89.38803 | 64.80696 | 55.71823 | 29.10476 | 51.83879 | 53.58897 | 26.11716 | 28.90658 | 32.77063 | 43.76564 | 41.24587 |
| CUST_13736_PI429010886 | N31686 | 0.023391 | 18 | 0 | | | | | | 612.5844 | 519.7383 | 814.5413 | 510.3855 | 769.2478 | 681.8773 | 644.6096 | 289.2197 | 375.4995 | 363.2975 | 874.5756 | 443.6521 | 377.56 | 398.3325 | 461.3593 | 536.6546 | 904.3304 | 368.6847 | 870.876 | 452.9164 | 941.2341 | 532.9396 | 751.1067 | 754.7954 | 497.8615 | 403.6614 | 433.4824 |
| CUST_56278_PI429010886 | M113357 | 0.023415 | 12 | 0 | | | | | | 126.9262 | 84.34439 | 156.8624 | 93.78702 | 91.71625 | 86.02859 | 109.1768 | 106.2054 | 117.0962 | 129.9842 | 118.6904 | 135.9425 | 122.3475 | 125.5467 | 123.8883 | 114.529 | 107.7922 | 96.41487 | 115.4184 | 151.1785 | 109.7874 | 123.0876 | 135.4402 | 100.2082 | 171.0028 | 109.5748 | 135.2525 |
| CUST_12427_PI429010886 | N29444 | 0.023415 | 12 | 3 | 478256790 | hypothetical protein YQE_06536, partial [Dendroctc | | | 1.6E-59 | 54.67468 | 36.606 | 36.34774 | 67.08962 | 106.5981 | 86.4248 | 111.0395 | 41.06137 | 40.7292 | 17.27372 | 71.18045 | 18.46967 | 36.92465 | 28.19347 | 38.59666 | 49.2211 | 136.3625 | 64.32323 | 64.65683 | 23.78727 | 46.1807 | 37.43361 | 42.46268 | 80.37733 | 43.55575 | 37.76259 | 21.12887 |
| CUST_791_PI429010886 | N7328 | 0.02342 | 15 | 3 | | | Metabolic process | Carbohydrate metabolic process | | 653.3522 | 116.6682 | 307.1797 | 705.2005 | 696.3149 | 1186.768 | 971.3866 | 305.3469 | 178.4456 | 5.705112 | 400.5981 | 121.2116 | 225.5895 | 4.946129 | 110.5556 | 71.69416 | 1700.08 | 1061.463 | 699.0502 | NA | 374.0543 | 9.71557 | 666.6962 | 1261.41 | 5.486804 | 590.6242 | NA |
| CUST_35330_PI429010886 | M12287 | 0.023437 | 24 | 15 | | | | | | 86.06426 | 111.5529 | 98.19642 | 73.70231 | 84.77843 | 81.16843 | 75.0125 | 71.87825 | 86.87439 | 94.81726 | 68.77778 | 168.329 | 91.1558 | 98.33611 | 129.2905 | 119.0526 | 96.64522 | 110.0647 | 91.51834 | 110.9171 | 111.4954 | 118.3239 | 103.8326 | 88.16694 | 110.7081 | 96.35645 | 124.1861 |
| CUST_22012_PI429010886 | N46262 | 0.023439 | 21 | 18 | | | | | | 12.06108 | 17.00167 | 13.84136 | 7.204193 | 5.347712 | 9.496558 | 9.801459 | 8.790994 | 5.092364 | 9.96806 | 10.18199 | 13.62943 | 15.83311 | 5.015833 | 16.77093 | 18.99317 | 3.692368 | 22.02831 | 28.91721 | 12.29346 | NA | 12.24661 | 7.180488 | NA | 11.71083 | 9.53977 | 15.99431 |
| CUST_37222_PI429010886 | M18256 | 0.023473 | 12 | 0 | | | | | | 178.0431 | 235.0591 | 233.3626 | 186.006 | 153.5542 | 191.5641 | 138.6217 | 156.4282 | 190.4729 | 186.8185 | 161.3901 | 260.5083 | 204.2578 | 293.6394 | 352.8273 | 253.4186 | 132.473 | 197.0618 | 138.9958 | 223.8084 | 206.6326 | 211.9082 | 226.768 | 179.1099 | 250.5389 | 233.088 | 254.095 |
| CUST_51073_PI429010886 | M92051 | 0.023475 | 24 | 0 | | | | | | 26.50017 | 31.2907 | 33.39183 | 24.21798 | 30.77101 | 33.75052 | 34.13801 | 31.16262 | 21.27347 | 27.09386 | 32.75767 | 22.30073 | 20.43255 | 27.98319 | 16.78757 | 29.13814 | 29.57425 | 29.55524 | 30.74938 | 28.99306 | 31.99517 | 34.85719 | 31.79024 | 25.93469 | 36.94944 | 29.93503 | 39.71396 |
| CUST_22814_PI429010886 | N47725 | 0.023494 | 24 | 9 | 321477586 | carbonyl reductase [Daphnia pulex] | Metabolic process | Other | 4.1E-21 | 85.20601 | 131.2743 | 87.74689 | 103.9452 | 123.6231 | 101.7048 | 112.5446 | 176.4383 | 130.3093 | 124.7036 | 133.0821 | 187.0582 | 129.5737 | 97.72266 | 133.4052 | 181.1566 | 115.5186 | 112.8752 | 132.3201 | 314.4942 | 104.192 | 131.2193 | 84.76204 | 111.0395 | 108.6974 | 73.97012 | 109.6246 |
| CUST_49050_PI429010886 | M83796 | 0.023513 | 21 | 15 | | | | | | 11.00169 | 17.96131 | 16.5818 | 14.29954 | 11.23137 | 28.62243 | 15.91598 | 10.85593 | 13.0053 | 7.627544 | 11.56124 | 13.74525 | 6.276753 | 24.40654 | 12.02328 | 30.62521 | 30.40899 | 31.15965 | 25.08483 | 22.85266 | 11.70432 | 11.6136 | 16.29403 | 19.50089 | 25.68978 | 14.1448 | 20.55297 |
| CUST_46724_PI429010886 | M73526 | 0.02352 | 24 | 6 | | | | | | 44.2637 | 40.02231 | 28.36576 | 41.23292 | 28.29331 | 64.48135 | 44.88618 | 36.97731 | 55.04733 | 19.61371 | 12.90641 | 17.67558 | 43.59959 | 19.76039 | 27.48106 | 31.43817 | 25.81311 | 29.98761 | 17.5674 | 16.02367 | 18.13609 | 30.55547 | 28.99027 | 32.89329 | 23.73591 | 32.94969 | 24.28 |
| CUST_33807_PI429010886 | M7923 | 0.023536 | 24 | 18 | 91087253 | PREDICTED: similar to GA15510-PA [Tribolium casta | | | 3.42E-25 | 20.57784 | 21.32852 | 14.07257 | 13.23043 | 14.33237 | 13.18143 | 10.86011 | 14.5269 | 18.78533 | 17.51738 | 19.67133 | 22.65868 | 15.51227 | 15.3428 | 17.95452 | 16.5596 | 18.89922 | 23.79492 | 19.44494 | 38.51022 | 25.64332 | 17.80424 | 11.93724 | 12.15636 | 25.11484 | 18.20852 | 15.76779 |
| CUST_7550_PI429010886 | N21714 | 0.023536 | 24 | 18 | | | | | | 418.3911 | 1022.013 | 552.3175 | 278.9452 | 587.9777 | 306.8772 | 448.5246 | 421.7857 | 616.4707 | 1095.161 | 680.1392 | 1040.536 | 700.6808 | 1047.894 | 803.077 | 1088.106 | 330.882 | 757.8647 | 726.6345 | 1540.227 | 856.1081 | 2354.203 | 1276.669 | 425.5183 | 1268.323 | 397.1398 | 1227.773 |
| CUST_3552_PI429010886 | N14733 | 0.023539 | 24 | 21 | | | | | | 875.926 | 897.1177 | 564.719 | 548.6331 | 586.3578 | 506.5013 | 460.6363 | 290.6552 | 470.2955 | 601.7703 | 366.5663 | 561.7404 | 955.0872 | 896.5197 | 353.6127 | 680.741 | 457.6266 | 500.086 | 785.705 | 643.9207 | 634.6129 | 969.5225 | 604.5341 | 595.1496 | 743.4007 | 816.3533 | 1158.222 |
| CUST_7132_PI429010886 | N21085 | 0.02355 | 12 | 0 | | | | | | 282.3228 | 298.4347 | 285.7468 | 221.3847 | 273.7876 | 260.5083 | 199.4834 | 213.4377 | 239.0183 | 278.7196 | 239.9082 | 318.2985 | 216.9356 | 337.4667 | 308.0795 | 345.2409 | 288.1296 | 283.7427 | 265.3779 | 248.7921 | 255.4386 | 329.1684 | 267.5328 | 274.6972 | 330.1788 | 249.6664 | 333.3682 |
| CUST_10786_PI429010886 | N26789 | 0.02355 | 12 | 0 | 224028155 | angiotensin converting enzyme [Pontastacus leptod | Protein metabolism | Proteolysis | 3.6E-23 | 783.9719 | 1724.959 | 800.6759 | 774.2499 | 870.876 | 454.3834 | 385.1644 | 448.4129 | 477.3001 | 1401.401 | 908.5337 | 1537.216 | 1278.963 | 1596.656 | 1020.365 | 1580.775 | 583.066 | 918.9933 | 894.0864 | 1383.212 | 672.9863 | 1831.59 | 765.0242 | 603.7585 | 1676.557 | 1423.398 | 1577.693 |
| CUST_7732_PI429010886 | N21989 | 0.023564 | 18 | 9 | | | | | | 80.6942 | 133.5378 | 116.1174 | 59.35346 | 106.023 | 93.62063 | 54.46899 | 129.0306 | 109.9302 | 183.7194 | 172.2644 | 287.8668 | 124.7428 | 144.7517 | 126.0286 | 176.1138 | 111.6728 | 147.4736 | 110.6458 | 224.4296 | 141.3981 | 139.2832 | 82.25514 | 117.4463 | 185.7109 | 51.78377 | 203.1014 |
| CUST_17828_PI429010886 | N38656 | 0.023591 | 15 | 9 | 291236607 | PREDICTED: zinc finger protein 197-like, partial [Sac | | | 2.45E-37 | 23.76609 | 60.86834 | 45.47327 | 52.71293 | 53.6056 | 61.99224 | 68.29075 | 51.66322 | 86.04961 | 64.09512 | 53.84406 | 105.0471 | 44.74623 | 93.35337 | 43.69907 | 25.45947 | 41.96451 | 102.1011 | 49.11669 | 38.95593 | 78.35921 | 41.16094 | 104.322 | 90.89001 | 102.5401 | 62.69277 | 135.6803 |
| CUST_11377_PI429010886 | N27715 | 0.023597 | 21 | 0 | | | | | | 17.21077 | 19.78961 | NA | 18.7464 | 21.34327 | 16.7648 | 12.51235 | 11.48488 | 6.613886 | 7.438777 | 15.31913 | 11.10317 | 17.54669 | 3.982345 | 7.516724 | 119417.1 | 7.953838 | 22.14395 | 19.72601 | 12.32751 | 23.8404 | 27.60563 | 15.73939 | 21.74923 | 31.66766 | 11.45611 | 6.176204 |
| CUST_35671_PI429010886 | M13200 | 0.02362 | 21 | 9 | 321478292 | hypothetical protein DAPPUDRAFT_303125 [Daphn | | | 3.24E-49 | 16.77093 | 18.2356 | 11.58812 | 16.56922 | 21.40624 | 9.03782 | 17.22069 | 95.0012 | 43.38005 | 24.70162 | 57.42962 | 48.59467 | 69.32875 | 17.08982 | 28.17911 | 28.80979 | 42.42487 | 4.629241 | 24.66033 | 97.95028 | 27.71495 | 6.481784 | 39.39544 | 6.328546 | 17.16641 | 20.81295 | 8.927589 |
| CUST_9703_PI429010886 | N25070 | 0.02362 | 21 | 0 | | | | | | 31.36762 | 13.56063 | 35.10761 | 16.07659 | 10.48257 | 14.65894 | 7.941557 | 17.03463 | 21.68228 | 9.837542 | 12.04453 | 23.11042 | 3.902569 | 16.2854 | 15.09075 | 16.5492 | 13.41841 | 17.98635 | 22.16629 | 16.5713 | 28.42752 | 23.68051 | 20.90297 | 24.54149 | 24.23014 | 10.34781 | 5.738619 |
| CUST_41055_PI429010886 | M56773 | 0.023621 | 24 | 6 | | | | | | 322.9938 | 278.5157 | NA | 291.9341 | 31.14323 | 32.19898 | 385.8989 | 31.23744 | 313.2163 | NA | 9.956467 | NA | 210.4862 | NA | 95.33691 | 18.10904 | 8.84632 | 32.22652 | 7.515463 | 5.956873 | NA | 11.93962 | 10.82989 | 88.57155 | 10.41028 | 7.158847 | NA |
| CUST_12350_PI429010886 | N29331 | 0.023628 | 18 | 6 | | | Metabolic process | Carbohydrate metabolic process | | 4.458762 | 28.8656 | NA | 76.77824 | 32.59218 | 55.45229 | 59.60108 | 222.0327 | 185.8187 | 35.06362 | 75.88452 | 75.39347 | 83.76494 | 22.20487 | 40.96307 | NA | 25.04089 | NA | 33.74355 | 223.377 | 35.86628 | 7.835676 | 100.2315 | 70.63158 | NA | 134.4195 | 134.8627 |
| CUST_33100_PI429010886 | M5970 | 0.023636 | 21 | 18 | | | | | | 155.0724 | 37.73674 | 150.0759 | 44.68637 | 151.7723 | 149.811 | 261.8083 | 46.84888 | 29.53988 | 36.69324 | 265.9908 | 30.38324 | 26.07711 | 30.63709 | 33.15625 | 35.6894 | 378.9452 | 74.83385 | 332.753 | 35.83833 | 278.397 | 51.16457 | 308.3972 | 251.4349 | 48.6393 | 49.57895 | 39.62195 |
| CUST_38986_PI429010886 | M33013 | 0.023646 | 21 | 3 | | | | | | 61.12299 | 16.79729 | 31.98439 | 35.75443 | 38.48636 | 52.06984 | 56.0501 | 36.08065 | 34.83322 | 30.44767 | 34.65468 | 36.63495 | 24.05844 | 33.03817 | 31.16262 | 26.51493 | 47.15656 | 42.71137 | 38.90805 | 17.61531 | 27.9072 | 72.3461 | 36.23985 | 48.2905 | 59.41199 | 64.59746 | 53.29849 |
| CUST_50165_PI429010886 | M88895 | 0.023653 | 12 | 3 | | | | | | 94.37733 | 121.0793 | 17.32475 | 210.676 | 133.2264 | 248.959 | 81.64235 | 52.24124 | 21.36429 | 10.28696 | 109.399 | 9.424573 | 68.85144 | 35.8336 | 120.6382 | 89.40029 | 238.6573 | 199.7685 | 92.52049 | 21.08658 | 48.09585 | 43.14736 | 52.25211 | 101.8407 | 19.18101 | 66.44604 | 7.03993 |
| CUST_54081_PI429010886 | M104450 | 0.023667 | 24 | 12 | 68139273 | reverse transcriptase [Nullibrotheas allenii] | Nucleic acid metabolism | DNA replication | 2.31E-08 | 233.088 | 304.8063 | 322.0219 | 298.8597 | 245.7365 | 260.1739 | 235.838 | 297.6429 | 303.2741 | 330.7539 | 331.2853 | 332.2188 | 316.8471 | 443.9779 | 458.217 | 428.5138 | 289.4881 | 305.7949 | 336.2223 | 387.0723 | 308.7717 | 363.2398 | 352.8752 | 284.4041 | 395.8483 | 286.9542 | 345.2409 |
| CUST_19417_PI429010886 | N41544 | 0.023677 | 18 | 12 | | | | | | 17.41182 | 18.94868 | 23.1605 | 18.7509 | 21.54483 | 19.39031 | 16.87055 | 22.03473 | 24.63631 | 21.86875 | 30.63369 | 28.98746 | 22.62556 | 39.67377 | 24.83878 | 21.22623 | 42.13297 | 23.37682 | 24.37698 | 16.4346 | 22.33151 | 15.79004 | 11.51323 | 26.22826 | 38.35427 | 19.4153 | 15.62361 |
| CUST_34880_PI429010886 | M10971 | 0.023681 | 12 | 0 | | | | | | 196.2247 | 224.7094 | 275.6487 | 150.7034 | 150.2666 | 148.7626 | 148.0276 | 222.5361 | 176.0053 | 202.301 | 157.1144 | 280.3633 | 190.2685 | 220.9933 | 163.8459 | 196.9594 | 180.1966 | 156.6286 | 182.6137 | 185.3559 | 155.6736 | 211.7401 | 238.3762 | 158.9341 | 221.0323 | 194.8105 | 239.0491 |
| CUST_35332_PI429010886 | M12290 | 0.023681 | 12 | 3 | | | | | | 305.3843 | 261.6861 | 216.1505 | 320.9097 | 310.7127 | 452.8393 | 271.2785 | 277.0736 | 318.5103 | 263.9219 | 246.8318 | 356.6641 | 275.5051 | 230.8271 | 282.0094 | 371.6995 | 346.5286 | 396.5647 | 457.7503 | 290.2748 | 234.9258 | 351.3648 | 216.65 | 338.0013 | 272.9711 | 255.3452 | 258.4381 |
| CUST_42223_PI429010886 | M59720 | 0.023681 | 12 | 0 | | | | | | 225.4823 | 289.679 | 311.4551 | 129.1875 | 173.0713 | 166.4251 | 140.8807 | 313.5825 | 250.9845 | 333.3682 | 180.162 | 368.0062 | 292.258 | 401.0839 | 287.2312 | 228.8706 | 161.7305 | 165.8216 | 186.6438 | 335.1917 | 167.5706 | 313.5825 | 303.7146 | 185.8695 | 205.7973 | 361.9091 | 335.0674 |
| CUST_43270_PI429010886 | M62583 | 0.023681 | 12 | 0 | | | | | | 28.32194 | 29.64988 | 38.48636 | 14.1362 | 20.26494 | 23.60499 | 22.13925 | 23.00078 | 26.0691 | 27.00762 | 21.51643 | 42.83757 | 24.77058 | 28.98446 | 33.44293 | 29.12321 | 21.58663 | 24.75161 | 25.75422 | 25.79784 | 22.34831 | 28.3838 | 26.0691 | 31.38208 | 25.35894 | 32.15263 | 25.05744 |
| CUST_45141_PI429010886 | M68114 | 0.023681 | 12 | 9 | 91090522 | PREDICTED: similar to mitochondrial NADH:ubiquin | | | 6.79E-10 | 246.2665 | 374.607 | 223.6419 | 437.9165 | 403.9415 | 446.3108 | 209.0287 | 303.7146 | 447.6451 | 545.1015 | 438.2102 | 565.8837 | 473.8216 | 422.4541 | 336.682 | 326.6284 | 373.9776 | 455.5337 | 545.8082 | 554.2309 | 426.3129 | 706.4958 | 381.1294 | 512.8422 | 342.3707 | 222.2769 | 302.092 |
| CUST_3256_PI429010886 | N14119 | 0.023681 | 12 | 0 | 9186886 | BCS-2 [Amphibalanus amphitrite] | | | 1.79E-28 | 1917.136 | 3500.164 | 4361.48 | 1257.974 | 1718.489 | 1174.011 | 983.2431 | 1795.627 | 1736.508 | 1839.737 | 1923.113 | 2497.013 | 2363.842 | 2377.614 | 1809.996 | 1991.454 | 964.5529 | 1257.974 | 897.6332 | 2761.396 | 1187.536 | 1929.904 | 1756.162 | 1062.278 | 1395.696 | 2104.851 | 2426.131 |
| CUST_3565_PI429010886 | N14765 | 0.023681 | 12 | 0 | | | | | | 53.73015 | 47.56097 | 78.63344 | 17.76311 | 18.77555 | 22.73323 | 31.41271 | 70.7166 | 64.38672 | 61.69829 | 41.37701 | 41.04712 | 59.19392 | 48.28635 | 60.84782 | 45.96465 | 22.79184 | 28.80614 | 36.13819 | 54.1974 | 38.59967 | 154.463 | 35.30492 | 20.34468 | 212.1159 | 31.14945 | 326.7477 |
| CUST_14254_PI429010886 | N32578 | 0.023681 | 12 | 0 | | | | | | 292.413 | 257.9765 | 264.1731 | 253.1914 | 205.1599 | 196.032 | 260.8562 | 190.5242 | 224.3549 | 265.9437 | 230.4393 | 288.0337 | 209.3879 | 340.3246 | 265.9437 | 260.7556 | 141.6349 | 241.5575 | 231.8541 | 223.0503 | 250.2094 | 284.3688 | 281.9612 | 263.3865 | 304.437 | 208.027 | 302.566 |
| CUST_54817_PI429010886 | M107797 | 0.023682 | 18 | 9 | 307184242 | Stromal interaction molecule-like protein [Camponc | | | 1.81E-47 | 27.59147 | 26.4784 | 21.53652 | 27.16781 | 22.80928 | 24.69594 | 29.76829 | 24.62928 | 29.08151 | 27.50275 | 28.83204 | 45.69586 | 22.35568 | 32.18334 | 31.23466 | 31.11066 | 28.97849 | 36.23362 | 35.48044 | 24.89381 | 20.43418 | 27.09386 | 24.42797 | 14.60716 | 35.84618 | 26.80742 | 28.98446 |
| CUST_34948_PI429010886 | M11141 | 0.023755 | 18 | 12 | | | | | | 94.96437 | 315.2378 | 227.4444 | 103.0135 | 120.446 | 131.7874 | 139.6056 | 173.7958 | 128.7368 | 204.5915 | 226.4554 | 167.5706 | 245.8985 | 433.1734 | 202.6446 | 270.4039 | 51.00123 | 191.6952 | 137.6307 | 233.8998 | 150.914 | 100.7517 | 168.6715 | 149.1678 | 71.06446 | 424.7309 | 180.9317 |
| CUST_13421_PI429010886 | N31151 | 0.023755 | 18 | 12 | 345489606 | PREDICTED: histone deacetylase 6-like [Nasonia vitr | | | 1.27E-20 | 94.95262 | 61.09062 | 135.2525 | 67.62921 | 77.60864 | 99.35803 | 109.19 | 79.14778 | 116.8091 | 49.58882 | 131.0159 | 108.8637 | 153.8729 | 151.9486 | 168.4127 | 208.1099 | 100.2537 | 99.05161 | 208.0738 | 96.88082 | 74.0547 | 105.5089 | 100.1858 | 50.19562 | 115.7448 | 90.02313 | 146.622 |
| CUST_27088_PI429010886 | N55427 | 0.023756 | 24 | 9 | | | | | | 157.0455 | 136.8476 | 216.8379 | 212.2276 | 301.5058 | 273.7876 | 207.1975 | 188.2189 | 269.9974 | 237.9203 | 627.2172 | 557.1607 | 175.9719 | 229.4944 | 254.955 | 557.9517 | 252.7637 | 293.8285 | 393.6847 | 136.5329 | 95.03994 | 151.7333 | 73.64901 | 178.5686 | 106.2586 | 429.3715 | 87.13975 |
| CUST_41602_PI429010886 | M58119 | 0.023758 | 21 | 6 | | | | | | 27.12757 | 21.3795 | 43.52397 | 25.58535 | 22.20218 | 49.69406 | 31.38813 | 59.54039 | 51.30066 | 53.51858 | 38.86122 | 27.9866 | 32.45453 | 40.01136 | 27.30432 | 22.85123 | 36.07274 | 14.73015 | 14.56514 | 30.3611 | 10.73459 | 21.4795 | 52.56971 | 34.17378 | 31.11066 | 26.56221 | 23.19098 |
| CUST_18694_PI429010886 | N40242 | 0.023792 | 21 | 3 | | | | | | 11.59303 | 26.97875 | 21.53411 | 21.38676 | 20.40333 | 21.78927 | 19.6916 | 24.8301 | 29.46873 | 20.07855 | 19.02231 | 21.30938 | 15.5304 | 16.16098 | 14.98837 | 27.32319 | 10.52648 | 20.53839 | 14.91349 | 22.99783 | 15.5215 | 18.81916 | 13.34989 | 15.5539 | 28.41001 | 23.5177 | 30.55085 |
| CUST_4565_PI429010886 | N17040 | 0.023806 | 24 | 21 | | | | | | 22.55992 | 21.4035 | 30.93394 | 21.9039 | 24.35681 | 28.61737 | 19.34811 | 17.40347 | 27.39302 | 24.51394 | 30.85961 | 24.87256 | 24.8201 | 22.51238 | 22.56226 | 24.61539 | 26.73933 | 32.33975 | 26.60971 | 26.64821 | 30.30845 | 27.54224 | 32.44055 | 31.0493 | 23.58007 | 27.23432 | 28.40026 |
| CUST_17665_PI429010886 | N38380 | 0.023806 | 24 | 21 | 242020404 | conserved hypothetical protein [Pediculus humanus | Signal transduction | Other | 3.81E-13 | 22.0997 | 18.71576 | 25.54204 | 15.31496 | 16.49783 | 13.28156 | 19.20545 | 18.56427 | 18.94364 | 52.21965 | 22.47719 | 22.67277 | 9.39688 | 22.92529 | 31.95131 | 24.983 | 44.70035 | 27.72317 | 25.22098 | 25.45224 | 26.45134 | 28.05779 | 33.73552 | 30.34969 | 34.62871 | 28.5055 | 35.84962 |
| CUST_54889_PI429010886 | M108095 | 0.023812 | 21 | 3 | 291404023 | PREDICTED: zinc finger protein 11B-like [Oryctolagu | Nucleic acid metabolism | Regulation of gene expression | 2.72E-37 | 48.9688 | 77.84895 | 43.49759 | 131.0289 | 42.72555 | 69.08039 | 56.13335 | 35.63559 | 42.04788 | 54.60894 | 11.71408 | 49.89799 | 27.68104 | 38.92288 | 43.25052 | 39.09942 | 18.13841 | 55.63288 | 23.84605 | 30.50923 | 76.55131 | 57.71214 | 61.95712 | 51.83879 | 58.57388 | 89.90347 | 39.16114 |
| CUST_40859_PI429010886 | M56242 | 0.023821 | 12 | 3 | 298162780 | early cuticle protein 6 [Callinectes sapidus] | | | 1.62E-14 | 3440.367 | 1184.216 | 1500.167 | 17165.71 | 11089.86 | 23241.72 | 12374.53 | 2879.829 | 3509.529 | 95.91781 | 10310.79 | 90.38978 | 2686.919 | 1010.552 | 1866.472 | 1311.842 | 16480.39 | 16969.9 | 5911.575 | 284.5979 | 14928.07 | 2417.914 | 9937.704 | 17457.06 | 355.3996 | 8436.312 | 78.07938 |
| CUST_4868_PI429010886 | N17653 | 0.023827 | 15 | 12 | | | | | | 40.10273 | 36.25388 | 60.10166 | 40.39881 | 45.28288 | 46.28376 | 24.78255 | 38.68077 | 53.56751 | 49.21663 | 37.66305 | 54.76027 | 52.19516 | 61.38847 | 63.23408 | 70.3852 | 109.2372 | 51.25727 | 48.57594 | 60.49322 | 36.14912 | 47.52868 | 36.83629 | 33.19476 | 68.05219 | 44.25018 | 66.44095 |
| CUST_33493_PI429010886 | M7047 | 0.023828 | 24 | 12 | 321463241 | hypothetical protein DAPPUDRAFT_22132 [Daphnia | Metabolic process | Cellular amino acid metabolic process | 4.97E-33 | 29.5273 | 42.9046 | 59.0851 | 20.31875 | 26.63149 | 35.53033 | 60.77185 | 53.28731 | 53.07973 | 63.3033 | 21.32603 | 23.05184 | 51.13662 | 76.72395 | 68.58333 | 35.7998 | 49.02476 | 42.89937 | 49.66578 | 46.66161 | 55.15882 | 67.47589 | 40.49183 | 49.74292 | 38.1318 | 30.11515 | 46.71096 |
| CUST_1945_PI429010886 | N11138 | 0.023843 | 15 | 9 | | | | | | 9.59598 | 11.89991 | 25.10875 | 9.28355 | 9.691153 | NA | 17.58365 | 60.34181 | 23.71308 | 25.7467 | 41.12787 | 40.66087 | 15.63271 | 29.6615 | 12.28413 | 8.342648 | 28.43336 | 4.62816 | 6.729385 | 11.18034 | 38.57925 | 8.848997 | 33.92046 | 9.044875 | 42.35334 | 17.15538 | 32.22652 |
| CUST_46264_PI429010886 | M71990 | 0.02386 | 24 | 0 | 298204333 | endonuclease-reverse transcriptase [Bombyx mori] | Nucleic acid metabolism | DNA replication | 8.43E-12 | 18.02985 | 18.81343 | 24.4665 | 21.49202 | 14.39019 | 11.6794 | 7.90501 | 14.01418 | 7.141791 | 15.81392 | 18.0662 | 20.1154 | 12.25586 | 24.03557 | 14.63709 | 14.50403 | 12.17805 | 7.306974 | 12.90269 | 22.37068 | 15.98734 | 12.02328 | 13.56887 | 12.60293 | 27.28673 | 26.52223 | 27.47752 |
| CUST_11741_PI429010886 | N28332 | 0.023865 | 15 | 9 | | | | | | 13.09531 | 14.78546 | 10.2415 | 15.07523 | 16.48057 | 16.45281 | 15.33118 | 27.34621 | 14.69551 | 18.12996 | 19.49159 | 28.16035 | 15.93948 | 22.0997 | 22.48589 | 19.9496 | 18.80396 | 9.632488 | 17.61145 | 23.75128 | 12.77756 | 13.35309 | 24.43342 | 23.81911 | 21.40624 | 31.44457 | 22.41474 |

| Probe ID | ID | p-value | Period | Phase | EBI ID | Annotation | GO category | Go term | E-value | 0 | 0 | 0 | 3 | 3 | 3 | 6 | 6 | 6 | 9 | 9 | 9 | 12 | 12 | 12 | 15 | 15 | 15 | 18 | 18 | 18 | 21 | 21 | 21 | 24 | 24 | 24 |
|---|---|---|---|---|---|---|---|---|---|---|---|---|---|---|---|---|---|---|---|---|---|---|---|---|---|---|---|---|---|---|---|---|---|---|---|---|
| CUST_21782_PI429010886 | N45845 | 0.023865 | 15 | 9 | | | | | | 22.97663 | 12.02328 | 18.24285 | 27.36512 | 18.69493 | 56.11071 | 19.97654 | 16.84886 | 12.94311 | 50.08673 | 36.06457 | 78.78492 | 15.5279 | 33.30987 | 34.74405 | 21.52428 | 9.439346 | 13.45887 | 44.9603 | 28.7018 | 15.84737 | 14.53418 | 20.75935 | 42.43668 | 28.87402 | 52.5224 | 37.16398 |
| CUST_12120_PI429010886 | N28960 | 0.023875 | 15 | 0 | | | | | | 176.2631 | 180.6083 | 138.6617 | 109.5894 | 130.8991 | 108.2633 | 101.92 | 114.8783 | 108.3989 | 140.2989 | 128.0141 | 119.9711 | 122.3027 | 119.6154 | 139.4507 | 166.4042 | 133.1717 | 133.5939 | 144.6167 | 127.0971 | 124.2426 | 133.2442 | 127.0219 | 110.2048 | 184.8973 | 138.9124 | 144.1009 |
| CUST_245_PI429010886 | N2986 | 0.023887 | 18 | 9 | | | | | | 10.22956 | 5.654326 | 22.91473 | 7.828316 | 10.00209 | 19.26372 | 13.26879 | 21.35581 | 20.6434 | 29.30417 | 29.05431 | 26.84868 | 12.96349 | 24.96534 | 28.05565 | 30.47101 | 8.922925 | 25.68021 | 17.52814 | 27.41751 | 26.72979 | 24.43342 | 19.04932 | 13.38378 | 24.19014 | 21.00917 | 28.35783 |
| CUST_34726_PI429010886 | M10472 | 0.023893 | 15 | 12 | | | | | | 20.62099 | 18.54527 | 21.55908 | 8.573429 | 10.36072 | 19.72128 | 12.17805 | 16.96167 | 20.93844 | 13.74963 | 7.948839 | 19.21466 | 17.34168 | 24.2228 | 28.21268 | 14.43734 | 19.676 | 12.71803 | 13.05013 | 13.42609 | 9.556923 | 15.90721 | 17.28035 | 8.073322 | 17.63322 | 14.78546 | 22.0325 |
| CUST_1415_PI429010886 | N9562 | 0.023909 | 12 | 3 | | | | | | 965.8744 | 265.6599 | 153.1027 | 3276.015 | 412.4272 | 4613.187 | 1017.693 | 1260.686 | 794.7159 | 6.671239 | 343.7569 | NA | 1275.086 | 20.76453 | 535.6554 | NA | 2768.781 | 2898.401 | 588.1169 | NA | 293.1844 | 5.106462 | 181.9242 | 2692.301 | NA | 2945.87 | NA |
| CUST_35270_PI429010886 | M12101 | 0.023922 | 12 | 3 | | | | | | 844.642 | 248.4954 | 9.258755 | 987.5052 | 864.2169 | 1520.67 | 551.3995 | 866.8694 | 356.8925 | 10.18722 | 287.8054 | 10.88684 | 660.5155 | 26.8663 | 306.479 | 100.3232 | 1066.114 | 896.1269 | 622.1973 | 17.18634 | 453.9938 | 97.5983 | 108.5374 | 858.7607 | 32.8723 | 813.6581 | 6.980991 |
| CUST_3134_PI429010886 | N13871 | 0.023922 | 12 | 3 | | | | | | 1581.923 | 5125.683 | 181.6996 | 5794.422 | 2573.258 | 2119.433 | 3060.282 | 171.0319 | 3613.349 | 92.30386 | 98.39109 | 95.9293 | 1046.691 | 337.0265 | 2986.925 | 4715.889 | 312.1828 | 3489.908 | 298.1939 | 69.93933 | 109.1135 | 1601.013 | 143.5589 | 1089.512 | 739.6144 | 251.8524 | 451.7787 |
| CUST_11408_PI429010886 | N27759 | 0.023922 | 12 | 9 | | | | | | 1437.865 | 2304.824 | 2260.474 | 1659.929 | 1754.186 | 1295.233 | 1345.526 | 2312.077 | 3443.242 | 3020.819 | 2147.931 | 4182.62 | 2226.703 | 1328.909 | 1920.006 | 2140.816 | 1070.988 | 1343.537 | 867.8685 | 7338.425 | 1773.273 | 4873.025 | 2858.942 | 1734.303 | 2485.559 | 2454.105 | 3578.372 |
| CUST_22986_PI429010886 | N48057 | 0.023922 | 12 | 3 | | | | | | 122.6587 | 153.4178 | 83.78522 | 210.2883 | 167.4539 | 230.9992 | 135.6966 | 93.5311 | 99.00087 | 86.71532 | 110.6199 | 81.73063 | 131.0636 | 128.8206 | 138.0216 | 198.6688 | 123.6533 | 211.6967 | 144.7517 | 133.1717 | 87.42512 | 133.9999 | 77.72793 | 227.8356 | 115.2179 | 137.0769 | 77.34 |
| CUST_27273_PI429010886 | N55750 | 0.023923 | 21 | 3 | | | | | | 50.57194 | 75.30403 | 63.52108 | 119.9123 | 66.81883 | 86.06426 | 56.28307 | 39.8936 | 48.62311 | 48.98001 | 43.26504 | 52.56971 | 37.21299 | 84.4182 | 44.03536 | 47.01793 | 61.03938 | 48.81237 | 38.87395 | 42.52496 | 76.57062 | 64.30394 | 85.80553 | 47.06657 | 83.87586 | 63.29578 | 51.192 |
| CUST_15351_PI429010886 | N34443 | 0.02393 | 21 | 3 | | | | | | 16.71152 | 24.70162 | 26.05504 | 19.39031 | 46.97546 | 44.65427 | 10.59051 | 26.61953 | 33.24682 | 27.56341 | 23.37181 | 36.02039 | NA | 39.99701 | 12.028 | 27.91811 | 13.89481 | 24.25592 | 26.11497 | 7.045477 | 9.065589 | 27.49118 | 11.42997 | 39.08441 | 42.67635 | 32.74461 | 35.98798 |
| CUST_25588_PI429010886 | N52695 | 0.023943 | 15 | 12 | | | | | | 67.37749 | 83.09489 | 88.29574 | 47.45553 | 68.24289 | 62.06295 | 42.82196 | 58.14864 | 64.70488 | 68.41788 | 59.46444 | 61.85594 | 59.59632 | 88.89139 | 80.29008 | 83.19226 | 70.3928 | 72.86527 | 69.30744 | 83.1842 | 89.87648 | 71.33949 | 57.03251 | 51.61587 | 80.19532 | 104.7731 | 86.03759 |
| CUST_55974_PI429010886 | M112387 | 0.023948 | 12 | 0 | | | | | | 176.885 | 186.9659 | 216.6952 | 147.1881 | 154.3982 | 165.5902 | 151.3368 | 173.2007 | 151.5005 | 199.5921 | 140.147 | 213.51 | 167.2309 | 189.6625 | 181.957 | 180.2368 | 138.2381 | 171.4799 | 157.6874 | 182.709 | 171.7601 | 188.427 | 182.2638 | 173.2729 | 228.1824 | 156.5208 | 219.8489 |
| CUST_1710_PI429010886 | N10504 | 0.023948 | 12 | 0 | 1764109 | pacifastin light chain precursor [Pacifastacus leniusc | | | 2.38E-14 | 901.9536 | 893.6919 | 1550.902 | 227.8652 | 655.3909 | 352.327 | 665.3114 | 1053.785 | 901.5294 | 1555.864 | 773.8104 | 1090.456 | 1143.075 | 1598.927 | 940.108 | 812.5634 | 838.4693 | 590.149 | 607.9318 | 1327.874 | 868.3571 | 1966.099 | 1228.741 | 620.3013 | 1385.628 | 600.9036 | 1594.841 |
| CUST_14480_PI429010886 | N32957 | 0.023948 | 12 | 0 | | | | | | 54.10775 | 128.0667 | 159.2768 | 49.05985 | 49.75939 | 118.5239 | 68.83612 | 78.56402 | 107.6969 | 109.1135 | 69.79294 | 126.7579 | 90.0502 | 172.7709 | 122.6587 | 53.8551 | 81.9929 | 79.77146 | 59.76845 | 99.06334 | 91.45169 | 75.93804 | 134.1249 | 49.36528 | 127.7418 | 143.4489 | 121.8132 |
| CUST_25489_PI429010886 | N52510 | 0.023948 | 12 | 0 | 383856052 | PREDICTED: ubiquitin thioesterase OTU1-like [Mega | | | 2.23E-14 | 48.69326 | 30.21389 | 52.20745 | 21.82183 | 20.19927 | 29.73851 | 22.51238 | 31.57401 | 39.74462 | 41.57514 | 25.17921 | 40.76795 | 27.41751 | 42.40805 | 41.72747 | 35.80323 | 36.24706 | 28.83526 | 28.62456 | 47.37022 | 22.58247 | 39.31816 | 36.02623 | 31.82347 | 41.74772 | 43.28801 | 36.9176 |
| CUST_30283_PI429010886 | N61549 | 0.023948 | 12 | 0 | | | | | | 263.0553 | 200.8635 | 242.1982 | 198.7167 | 167.8836 | 148.08 | 202.0404 | 211.3962 | 191.2388 | 188.9473 | 245.5292 | 230.7517 | 273.965 | 286.8668 | 155.7397 | 261.7284 | 172.0048 | 171.3867 | 127.723 | 257.5442 | 230.7819 | 228.0795 | 306.8772 | 204.946 | 352.7559 | 281.2623 | 361.3416 |
| CUST_1125_PI429010886 | N8575 | 0.023948 | 12 | 9 | 389609429 | mitochondrial NADH:ubiquinone oxidoreductase ES | | | 2E-10 | 305.7949 | 349.238 | 246.0125 | 462.1839 | 519.1962 | 417.1018 | 240.1418 | 370.9949 | 457.5237 | 569.2573 | 530.042 | 499.1257 | 468.6028 | 448.4129 | 379.0729 | 370.4877 | 376.4458 | 484.9716 | 500.9078 | 608.8662 | 512.5692 | 744.2633 | 348.7613 | 547.465 | 433.8707 | 244.3099 | 338.0611 |
| CUST_2976_PI429010886 | N13523 | 0.023972 | 18 | 12 | | | | | | 78.92088 | 90.73889 | 79.07776 | 68.02926 | 56.70249 | 53.76242 | 65.73281 | 59.07909 | 70.51648 | 89.10973 | 53.10839 | 71.72159 | 88.39516 | 126.8312 | 118.9329 | 79.62582 | 57.664 | 79.51299 | 73.54244 | 91.81369 | 65.81652 | 89.43652 | 64.56424 | 67.43306 | 70.34764 | 66.46046 | 82.44448 |
| CUST_5587_PI429010886 | N18909 | 0.023992 | 21 | 12 | 260794975 | hypothetical protein BRAFLDRAFT_68970 [Branchio | | | 1.74E-81 | 15.47031 | 10.29908 | NA | 23.87788 | 8.0157 | 3.550382 | 12.92801 | 8.281111 | 11.55275 | 24.05186 | 20.25895 | 14.03191 | 31.02643 | 13.5398 | 18.4677 | 14.74027 | 14.67098 | NA | 14.38028 | 35.45118 | 6.093268 | 13.94276 | 14.53096 | 8.613965 | 9.048255 | 10.11773 | 8.459432 |
| CUST_25509_PI429010886 | N52546 | 0.024019 | 24 | 18 | | | | | | 131.7576 | 132.2049 | 116.3059 | 100.651 | 145.4832 | 106.9212 | 136.9633 | 111.9784 | 112.8928 | 135.5951 | 149.2835 | 118.6098 | 132.8034 | 144.6491 | 100.4962 | 171.8172 | 160.9995 | 132.2443 | 155.7397 | 167.321 | 159.4972 | 157.2555 | 129.2244 | 128.8206 | 187.3307 | 121.9392 | 138.7812 |
| CUST_26430_PI429010886 | N54233 | 0.024022 | 15 | 0 | | | | | | 13.16535 | NA | 14.28677 | 11.85797 | 9.354027 | NA | 5.031644 | 9.585988 | 10.65599 | 10.56079 | 8.910931 | NA | 41.30947 | 9.315276 | 18.28861 | 18.56257 | 13.2528 | 13.86613 | 15.32313 | 17.35586 | 14.51836 | 4.965994 | 13.59282 | 8.442875 | 8.717232 | 8.692145 | 15.7577 |
| CUST_36591_PI429010886 | M16307 | 0.024022 | 15 | 3 | | | | | | 29.2624 | 21.18145 | 18.29194 | 31.98439 | 23.4129 | 32.15263 | 22.46676 | 14.99955 | 16.49404 | 19.83086 | 30.04146 | 13.7932 | 19.37939 | 15.51056 | 20.75737 | 25.57234 | 40.61781 | 29.21058 | 32.11685 | 18.60109 | 23.2859 | 17.88508 | 37.19805 | 21.37656 | 15.87262 | 14.89135 | 18.26586 |
| CUST_7582_PI429010886 | N21759 | 0.024025 | 24 | 0 | | | | | | 344.7288 | 291.2799 | 380.7812 | 149.8301 | 262.6197 | 323.0511 | 167.1583 | 206.1701 | 208.0738 | 274.5343 | 214.1332 | 303.0187 | 158.0318 | 218.7669 | 238.455 | 324.6687 | 348.4045 | 445.1412 | 317.0842 | 282.8614 | 260.0114 | 285.4569 | 249.2507 | 275.9866 | 489.9462 | 365.9198 | 442.0725 |
| CUST_53194_PI429010886 | M100662 | 0.024034 | 21 | 9 | | | | | | 34.41169 | 259.1574 | 158.1524 | 820.5141 | 1588.949 | 371.4969 | 531.1131 | 4001.148 | 2549.933 | 141.0607 | 4228.899 | 4782.509 | 3489.908 | 289.8469 | 906.5131 | NA | 1157.228 | 42.1391 | 230.7819 | 4988.507 | 2865.864 | 7.742712 | 387.6806 | 267.1348 | 19.03935 | 626.8612 | 2292.412 |
| CUST_20775_PI429010886 | N44037 | 0.024044 | 15 | 12 | | | | | | 130.3093 | 143.6875 | 129.0136 | 108.9165 | 120.8888 | 98.60004 | 103.6651 | 156.1886 | 122.7974 | 146.3743 | 142.8955 | 219.9571 | 134.4594 | 148.4195 | 193.0913 | 173.0953 | 122.5181 | 124.3822 | 158.0023 | 111.7908 | 112.2705 | 168.2432 | 162.6994 | 108.2155 | 171.0319 | 123.056 | 140.7156 |
| CUST_52090_PI429010886 | M96383 | 0.024048 | 18 | 12 | 354990989 | anti-lipopolysaccharide factor isoform 6 [Penaeus m | | | 6.06E-28 | 2147.931 | 2645.933 | 725.8016 | 674.6226 | 1335.768 | 996.2576 | 3899.401 | 927.5266 | 4655.618 | 3216.436 | 1290.993 | 5435.626 | 5361.99 | 4279.028 | 3809.024 | 2697.082 | 1748.457 | 1756.971 | 5023.801 | 538.4051 | 2706.735 | 1500.167 | 1795.627 | 1271.51 | 2178.033 | 1004.987 | 4613.187 |
| CUST_10901_PI429010886 | N26965 | 0.024048 | 18 | 3 | | | | | | 49.02476 | 50.95506 | 23.16293 | 35.22469 | 38.79122 | 104.2212 | 40.48796 | 47.70026 | 27.65747 | 31.17481 | 32.93932 | 23.16735 | 15.57864 | 20.4413 | 13.10037 | 31.78686 | 41.69502 | 46.86688 | 14.25774 | 31.83203 | 24.38566 | 58.11529 | 33.54956 | 29.29583 | 18.8381 | 37.71174 | 26.05504 |
| CUST_50265_PI429010886 | M89232 | 0.024049 | 24 | 15 | | | | | | 80.90583 | 101.7455 | 86.2674 | 86.32314 | 101.7859 | 91.59828 | 72.44248 | 74.316 | 75.82559 | 116.5927 | 93.19487 | 104.2639 | 77.80891 | 88.33792 | 99.90716 | 216.9753 | 84.83474 | 195.8687 | 92.2729 | 106.9663 | 108.5045 | 155.8809 | 86.60546 | 90.8776 | 109.902 | 79.96242 | 100.734 |
| CUST_43840_PI429010886 | M64131 | 0.024068 | 18 | 6 | 242006829 | conserved hypothetical protein [Pediculus humanus | Transport | Transmembrane transport | 9.9E-07 | 236.0009 | 214.7985 | 77.76127 | 214.921 | 284.6976 | 60.22346 | 348.0871 | 346.0687 | 233.5227 | 187.746 | 93.55006 | 241.5289 | 256.2441 | 235.2484 | 226.768 | 113.4502 | 323.4046 | 159.0925 | 141.2095 | 184.2548 | 68.80724 | 303.3749 | 110.605 | 237.4703 | 621.0146 | 204.9184 | 346.5286 |
| CUST_17539_PI429010886 | N38145 | 0.024069 | 15 | 9 | | | | | | NA | 23.30079 | NA | 42.56702 | 47.60452 | 38.54575 | 62.68635 | 162.6994 | 126.9841 | 28.29864 | 309.0322 | 359.8853 | 81.40583 | 21.70901 | 52.12333 | 4.702194 | 33.30259 | NA | 11.02488 | 227.604 | 132.551 | NA | 58.83154 | 41.2187 | NA | 163.3967 | 105.86 |
| CUST_47534_PI429010886 | M76507 | 0.024077 | 24 | 21 | | | | | | 12.73395 | 20.06593 | 19.84167 | 14.25924 | 15.1141 | 18.50256 | 7.259344 | 13.25777 | 8.752945 | 16.06241 | 9.319645 | 20.43255 | 12.23764 | 15.07352 | 15.1414 | 22.24949 | 13.11969 | 17.10987 | 12.35337 | 18.19204 | 14.1198 | 20.16852 | 17.03814 | 82.90011 | 11.56735 | 71.77101 | 26.42193 |
| CUST_43955_PI429010886 | M64456 | 0.024079 | 24 | 18 | | | | | | 14.49175 | 25.49244 | 23.40412 | 16.23047 | 13.63311 | 17.45526 | 14.51697 | 18.31489 | 12.18324 | 19.82309 | 19.0691 | 17.91608 | 23.00078 | 18.86593 | 27.71495 | 31.67438 | 13.41264 | 24.5772 | 28.03356 | 36.6789 | 15.8263 | 23.76056 | 15.24413 | 19.40005 | 25.89483 | 18.45819 | 16.88136 |
| CUST_2676_PI429010886 | N12887 | 0.024081 | 24 | 15 | | | | | | 897.1177 | 941.2341 | 897.6332 | 726.086 | 865.9484 | 854.2242 | 689.4854 | 707.1584 | 609.308 | 953.9498 | 870.876 | 869.5437 | 663.7859 | 865.4579 | 842.0657 | 1239.798 | 918.3868 | 1025.99 | 878.6172 | 1045.476 | 715.3982 | 1127.801 | 817.1902 | 708.4547 | 1071.809 | 727.4325 | 961.8209 |
| CUST_10062_PI429010886 | N25633 | 0.024118 | 12 | 0 | | | | | | 34.60204 | 32.36622 | 39.87971 | 26.56221 | 27.10661 | 28.17154 | 18.57528 | 30.19994 | 25.80821 | 32.94363 | 23.17075 | 35.78629 | 30.62818 | 46.12938 | 37.70021 | 43.10871 | 24.40888 | 41.67274 | 29.13011 | 28.44009 | 31.0325 | 35.13017 | 19.65423 | 33.33635 | 33.68076 | 52.24124 | 36.7129 |
| CUST_37058_PI429010886 | M17715 | 0.024123 | 18 | 15 | 405959130 | Elongator complex protein 1 [Crassostrea gigas] | | | 7.44E-29 | 24.29748 | 18.96468 | 25.95428 | 23.04508 | 14.62779 | 15.08419 | 20.55693 | 18.21981 | 15.83738 | 22.35568 | 16.27412 | 26.78992 | 26.51493 | 21.69885 | 68.02073 | 36.27978 | 25.32554 | 22.13275 | 27.68618 | 25.2475 | 14.9294 | 23.54965 | 18.56806 | 11.60477 | 21.92688 | 16.2136 | 33.23641 |
| CUST_17052_PI429010886 | N37286 | 0.024155 | 15 | 0 | | | | | | 250.8185 | 615.5507 | 284.9694 | 222.4725 | NA | NA | 10.03121 | 15.5721 | 54.70385 | 258.0303 | 40.1147 | 222.6599 | NA | 140.4504 | 205.2623 | 114.0935 | NA | 178.4723 | 221.873 | 216.3391 | 295.3248 | 6.734253 | 62.39032 | NA | 150.2035 | 276.2901 | 121.6208 |
| CUST_37246_PI429010886 | M18356 | 0.024172 | 24 | 21 | | | | | | 113.6504 | 92.45305 | 91.01032 | 86.25852 | 91.68314 | 94.38745 | 77.7529 | 83.4507 | 84.1835 | 88.37144 | 84.96672 | 88.50576 | 67.9638 | 76.57062 | 95.9511 | 138.7005 | 101.0858 | 102.782 | 89.0565 | 102.0348 | 95.34993 | 105.1255 | 124.1861 | 80.10966 | 135.8061 | 101.6485 | 109.3295 |
| CUST_32820_PI429010886 | M5189 | 0.02419 | 18 | 9 | | | | | | 10.23296 | 17.95097 | 10.1626 | 12.57863 | 16.31744 | 10.34912 | 21.89979 | 17.7534 | 16.14566 | 22.70735 | 22.54321 | 26.35513 | 13.13227 | 19.63409 | 19.58405 | 21.51171 | 15.72409 | 14.84209 | 7.654255 | 16.98127 | 19.9496 | 25.34638 | 18.78041 | 8.905799 | 19.91992 | 11.9014 | 29.94885 |
| CUST_54447_PI429010886 | M106057 | 0.02419 | 12 | 3 | | | | | | 17.28228 | 10.79019 | 19.05357 | 13.20123 | 19.05757 | 25.76451 | 14.65365 | 18.87721 | 11.49378 | 8.163573 | 8.525365 | 14.51632 | 12.61842 | 14.046 | 16.96368 | 19.26616 | 21.48139 | 33.00184 | 17.47867 | 16.47211 | 6.578227 | 15.10512 | 11.78768 | 17.89801 | 28.97523 | 16.20777 | 24.30007 |
| CUST_56144_PI429010886 | M112941 | 0.02419 | 12 | 9 | | | | | | 169.0881 | 202.4642 | 207.3956 | 170.8315 | 190.7563 | 174.8815 | 175.8509 | 138.094 | 211.951 | 306.8772 | 194.5783 | 237.5773 | 178.3288 | 269.8218 | 200.7582 | 189.3905 | 126.6371 | 174.7759 | 169.7593 | 195.4506 | 223.9704 | 211.9851 | 239.7925 | 251.5259 | 382.8597 | 140.8238 | 355.8536 |
| CUST_31915_PI429010886 | M2726 | 0.02419 | 12 | 3 | | | | | | 634.7435 | 580.161 | 462.1839 | 679.9331 | 714.0634 | 754.3579 | 656.3922 | 547.6349 | 376.7168 | 501.702 | 581.8912 | 455.4031 | 460.5023 | 544.9594 | 484.1582 | 688.1653 | 684.3658 | 527.3498 | 729.5711 | 565.6978 | 477.2259 | 522.6581 | 472.997 | 480.2626 | 404.0517 | 628.1675 | 545.6531 |
| CUST_1247_PI429010886 | N9014 | 0.02419 | 12 | 3 | | | | | | 6049.937 | 4190.35 | 474.8115 | 12538.73 | 4451.275 | 12175.63 | 5705.785 | 5911.575 | 4208.899 | 252.6253 | 1917.136 | 327.7237 | 5293.128 | 331.4917 | 2975.526 | 1756.162 | 6253.288 | 7306.322 | 6049.937 | 197.0393 | 1157.816 | 1157.228 | 846.752 | 5620.472 | 460.255 | 7647.424 | 533.5457 |
| CUST_3660_PI429010886 | N14982 | 0.02421 | 18 | 9 | | | | | | 269.1187 | 312.052 | 340.9466 | 262.2997 | 263.2372 | 288.2901 | 264.7951 | 256.9966 | 278.3016 | 407.1309 | 325.6779 | 384.6311 | 298.9214 | 373.0312 | 360.2672 | 377.6304 | 326.6284 | 288.4954 | 328.9809 | 354.3068 | 315.7204 | 344.0991 | 304.3497 | 235.5325 | 371.4417 | 246.1869 | 375.7452 |
| CUST_15904_PI429010886 | N35322 | 0.02421 | 18 | 3 | | | | | | 46.76098 | 61.71189 | 52.51409 | 60.40681 | 70.72361 | 50.77711 | 64.3699 | 42.35334 | 39.6493 | 36.52603 | 40.81311 | 43.56037 | 56.57948 | 27.33167 | 67.10317 | 56.64713 | 60.00077 | 58.75806 | 59.67663 | 50.28834 | 54.85716 | 66.938 | 57.97363 | 61.00433 | 44.55872 | 44.05675 | 44.44423 |
| CUST_30900_PI429010886 | M159 | 0.024214 | 12 | 0 | | | Transport | Protein transport | | 982.4374 | 1342.338 | 1097.209 | 769.0647 | 821.0172 | 838.4693 | 773.4201 | 838.4693 | 897.6332 | 1033.403 | 798.3051 | 1137.521 | 802.795 | 1163.545 | 1332.781 | 1157.816 | 782.7144 | 836.4238 | 800.6759 | 910.1372 | 845.9343 | 1042.025 | 1056.762 | 968.3869 | 1168.631 | 832.3284 | 1023.389 |
| CUST_5987_PI429010886 | N19471 | 0.024214 | 12 | 3 | 321479371 | hypothetical protein DAPPUDRAFT_300085 [Daphn | Cellular Process | Cell cycle | 1.87E-62 | 8.502113 | 10.18097 | 4.765373 | 17.67291 | 13.02465 | 12.41217 | 20.4513 | 11.17076 | 11.65277 | 27.10661 | 8.348712 | 8.180112 | 7.453496 | 10.3727 | 12.24341 | 17.14295 | 18.41069 | 25.1646 | 14.84209 | 14.20342 | 9.198308 | 13.17199 | 14.42793 | 17.86315 | 16.7648 | 4.625348 | 5.820473 |
| CUST_25577_PI429010886 | N52677 | 0.024214 | 12 | 3 | | | | | | 84.51934 | 54.13488 | 52.96595 | 119.1299 | 69.05841 | 147.6505 | 66.43118 | 93.75113 | 70.21778 | 71.18669 | 70.3928 | 65.42947 | 63.28709 | 35.37802 | 69.49923 | 89.86196 | 86.15616 | 81.82047 | 106.9501 | 68.18607 | 64.16931 | 82.17099 | 30.88907 | 52.55999 | 71.28268 | 60.85469 | 47.65494 |
| CUST_35632_PI429010886 | M13069 | 0.024224 | 21 | 6 | | | | | | 475.032 | 26.34227 | 66.4925 | 822.5821 | 1453.376 | 2134.91 | 1250.839 | 900.1864 | 1353.303 | NA | 1859.87 | 238.6573 | NA | 19.27396 | 9.588389 | 50.93524 | 821.28 | 689.3131 | 1165.064 | 24.28972 | NA | NA | 232.9174 | 1350.609 | NA | 1438.603 | 72.4658 |
| CUST_47441_PI429010886 | M76132 | 0.024231 | 12 | 0 | | | | | | 13.41075 | 10.90545 | 14.51836 | 8.793342 | 8.203406 | NA | 15.4798 | 6.303067 | 4.64454 | 14.50945 | 7.686663 | 9.482226 | 10.00867 | NA | 18.24055 | 14.34788 | 13.06343 | 15.34115 | 9.87605 | 6.221936 | 5.93818 | 13.93866 | 7.188579 | 8.368824 | 14.3364 | 11.74612 | 26.02914 |
| CUST_48011_PI429010886 | M78526 | 0.024232 | 15 | 0 | | | | | | 412.0745 | NA | 532.1598 | 178.3541 | 564.3927 | 371.4417 | 331.2319 | 433.8707 | 166.186 | 122.9711 | 207.6805 | NA | 66.75063 | 37.61954 | 20.41951 | NA | 1106.085 | 250.7333 | 214.2583 | NA | 156.6119 | NA | 140.1157 | 251.662 | 43.26125 | 154.8889 | 7.313769 |
| CUST_20848_PI429010886 | N44183 | 0.024248 | 21 | 0 | | | | | | 72.13281 | 62.29412 | 45.46745 | 44.15772 | 28.66257 | 40.71531 | 41.83506 | 56.60803 | 40.70276 | 32.97611 | 36.52268 | 40.18446 | 56.9818 | 39.288 | 48.5148 | 69.64081 | 22.71378 | 56.99104 | 53.51251 | 49.14895 | 30.61606 | 59.3952 | 49.77447 | 54.63526 | 52.77501 | 43.12293 | 40.04237 |
| CUST_16529_PI429010886 | N36400 | 0.024261 | 21 | 0 | 195162205 | GL14252 [Drosophila persimilis] >gi\|194103844\|gb | Cellular Process | Cell death | 5.61E-10 | 71.04758 | 171.1195 | 76.21767 | 60.09613 | 70.5402 | 89.88488 | 76.13932 | 58.61502 | 65.89913 | 80.65083 | 53.61735 | 64.125 | 52.25652 | 88.40608 | 52.18439 | 57.60056 | 62.96106 | 77.29645 | 75.98025 | 61.62124 | 64.14066 | 80.48768 | 70.85223 | 91.43481 | 69.41655 | 80.54851 | 87.01627 |
| CUST_23156_PI429010886 | N48350 | 0.024274 | 15 | 9 | | | | | | 1120.205 | 1485.346 | 1338.117 | 1197.916 | 1259.079 | 1278.963 | 1149.353 | 1307.203 | 1164.641 | 1425.514 | 1187.094 | 1462.44 | 1302.095 | 1347.073 | 1392.18 | 1576.839 | 1368.295 | 1145.003 | 1249.958 | 1073.527 | 1133.519 | 1419.795 | 1282.889 | 1230.543 | 1587.608 | 1289.943 | 1640.228 |
| CUST_5242_PI429010886 | N18441 | 0.024285 | 24 | 21 | 345490321 | PREDICTED: LOW QUALITY PROTEIN: ets DNA-bindi | Nucleic acid metabolism | Regulation of gene expression | 3.02E-50 | 391.2629 | 294.3403 | 427.2547 | 300.5238 | 966.4783 | 249.9317 | 1087.534 | 184.6172 | 222.3148 | 228.8282 | 1522.087 | 303.3749 | 257.8206 | 345.9305 | 252.5773 | 417.4973 | 2007.972 | 349.1356 | 690.0315 | 377.4127 | 1607.248 | 410.1622 | 1752.574 | 1587.608 | 425.8413 | 234.4818 | 288.7076 |
| CUST_35611_PI429010886 | M13019 | 0.024307 | 18 | 12 | 91079592 | PREDICTED: similar to CG12935 CG12935-PA [Tribol | | | 9.43E-48 | 112.8438 | 141.0258 | 113.9999 | 100.4445 | 104.322 | 154.4419 | 133.4052 | 123.1539 | 127.4128 | 130.4838 | 115.478 | 148.4479 | 145.4981 | 147.6293 | 178.2181 | 160.1366 | 127.5485 | 138.9958 | 104.6652 | 137.0338 | 87.75721 | 131.838 | 93.33448 | 111.2474 | 123.2567 | 86.61515 | 144.1866 |
| CUST_31799_PI429010886 | M2442 | 0.02431 | 21 | 15 | 156405142 | predicted protein [Nematostella vectensis] >gi\|156 | | | 1.05E-90 | 97.05352 | 114.1927 | 97.282 | 83.34341 | 69.9232 | 91.32302 | 83.81066 | 76.06574 | 117.1353 | 94.68098 | 91.34253 | 180.2584 | 138.094 | 118.495 | 120.446 | 178.2839 | 82.48421 | 145.1303 | 142.216 | 145.726 | 86.66049 | 138.4302 | 85.493 | 85.67865 | 201.4604 | 82.55661 | 79.37795 |
| CUST_5953_PI429010886 | N19420 | 0.024315 | 24 | 15 | 241063674 | ribosomal processing protein, putative [Ixodes scap | Metabolic process | Other | 7.2E-130 | 47.47127 | 50.53472 | 45.0701 | 47.19112 | 57.94787 | 47.59957 | 38.63759 | 47.03677 | 44.00542 | 75.54672 | 74.316 | 88.8824 | 58.45028 | 87.65825 | 68.36198 | 92.02079 | 75.49081 | 78.44959 | 55.3496 | 58.30365 | 49.82473 | 64.31677 | 63.96858 | 46.83826 | 86.13317 | 60.47979 | 98.95253 |
| CUST_13181_PI429010886 | N30729 | 0.024327 | 15 | 9 | | | Metabolic process | Carbohydrate metabolic process | | 4.580393 | 363.1719 | 144.6688 | 447.8279 | 639.5245 | 192.363 | 344.4277 | 1429.583 | 951.2956 | 291.5581 | 1494.92 | 2078.653 | 731.6805 | 215.5318 | 475.9462 | 6.486483 | 486.7621 | 9.583323 | 148.3091 | 3390.638 | 725.1936 | 11.13027 | 407.8386 | 303.854 | NA | 1040.9 | 799.8512 |
| CUST_7529_PI429010886 | N21679 | 0.024334 | 21 | 12 | | | | | | 47.29592 | 56.23475 | 39.12009 | 39.31465 | 35.8336 | 36.32136 | 24.66303 | 36.14137 | 35.12261 | 72.04379 | 47.88942 | 49.83701 | 44.94616 | 50.4975 | 79.30391 | 63.17657 | 34.34243 | 58.56187 | 45.09026 | 48.43649 | 46.54874 | 59.76845 | 45.77355 | 35.75835 | 53.14405 | 43.41027 | 42.53014 |
| CUST_41376_PI429010886 | M57585 | 0.024342 | 18 | 12 | | | | | | 77.7747 | 100.0857 | 108.8006 | 93.07732 | 65.11921 | 73.76805 | 238.455 | 111.9132 | 353.25 | 108.8877 | 82.24759 | 134.3159 | 229.7558 | 258.1697 | 146.5292 | 223.4939 | 247.8745 | 94.94128 | 125.2116 | 108.2633 | 125.9794 | 92.26296 | 132.0609 | 80.18725 | 379.0729 | 151.9116 | 448.5246 |
| CUST_18019_PI429010886 | N39007 | 0.024349 | 15 | 3 | | | | | | 13.97235 | NA | 25.86025 | 21.46638 | 23.08733 | 102.2907 | 42.00978 | 16.04554 | 16.03098 | 8.361603 | 23.46033 | NA | 7.181648 | 11.24847 | 5.557245 | NA | 79.84889 | 25.7467 | 59.83655 | NA | 18.38425 | NA | 19.43895 | 28.62243 | 4.036076 | 19.60466 | 7.64912 |
| CUST_43144_PI429010886 | M62189 | 0.024357 | 21 | 3 | | | | | | 28.10839 | 36.03688 | 17.20661 | 31.53803 | 28.97255 | 29.87357 | 28.08525 | 27.84371 | 18.54155 | 21.18978 | 25.13924 | 33.54623 | 23.13968 | 26.36533 | 19.23624 | 25.38492 | 19.74119 | 32.53107 | 19.07331 | 25.31882 | 23.42234 | 28.65923 | 23.33499 | 18.64355 | 40.83694 | 34.37569 | 34.23978 |
| CUST_33753_PI429010886 | M7799 | 0.024357 | 21 | 0 | | | | | | 197.989 | 121.7165 | 242.5125 | 133.4752 | 96.91901 | 82.72689 | 103.2771 | 80.18725 | 74.64567 | 159.4512 | 95.03994 | 76.38976 | 110.9478 | 164.7416 | 103.9766 | 102.205 | 74.02683 | 86.76715 | 128.0504 | 98.84179 | 106.3603 | 164.0579 | 128.2848 | 109.5748 | 117.5373 | 73.0191 | 76.30835 |
| CUST_35572_PI429010886 | M12912 | 0.024372 | 18 | 12 | | | | | | 63.6369 | 84.80692 | 72.27253 | 54.1859 | 66.80323 | 86.01753 | 50.38211 | 60.40681 | 47.83293 | 105.7491 | 68.69981 | 114.649 | 67.83607 | 133.4752 | 94.25565 | 109.5048 | 59.84805 | 85.9812 | 76.45771 | 69.58984 | 77.66626 | 90.24634 | 62.00083 | 39.94582 | 89.91874 | 48.29446 | 43.14351 |
| CUST_24678_PI429010886 | N51068 | 0.024373 | 21 | 3 | | | | | | 22.0851 | 13.75845 | 17.95097 | 26.4818 | 36.51599 | 12.46576 | 7.50435 | NA | 6.723673 | 5.079117 | 16.90254 | 7.527497 | 17.02313 | 12.75137 | 13.32189 | 20.28061 | 17.89993 | 14.83888 | 11.68092 | 18.42911 | 13.2803 | 19.27159 | 16.31556 | 28.02833 | 19.55757 | 17.78225 | 27.30762 |
| CUST_11117_PI429010886 | N27293 | 0.024394 | 24 | 15 | | | | | | NA | 8.201884 | 7.679502 | 7.958671 | 6.380402 | NA | 9.299202 | 11.75341 | 8.8792 | 18.56579 | 18.1208 | 13.16084 | 9.072115 | 24.26504 | 24.03557 | 22.13675 | 14.40423 | 31.40553 | 7.150597 | 10.10137 | NA | 51.28805 | 15.35382 | 21.09286 | 28.01175 | NA | 6.023483 |
| CUST_11432_PI429010886 | N27794 | 0.024402 | 24 | 21 | | | | | | 538.5366 | 525.1056 | 595.7445 | 516.9528 | 565.434 | 619.6845 | 419.1452 | 411.2669 | 318.6961 | 582.6377 | 414.6925 | 538.0354 | 450.5804 | 428.9575 | 444.0863 | 659.4054 | 548.3934 | 459.1503 | 470.6652 | 542.5507 | 558.5528 | 609.1465 | 457.4348 | 554.0852 | 735.196 | 588.4339 | 580.161 |
| CUST_44952_PI429010886 | M67514 | 0.024414 | 15 | 9 | | | | | | 103.3491 | 80.47827 | 87.57209 | 88.77007 | 97.96017 | 118.7227 | 84.7131 | 96.56866 | 91.51834 | 113.7893 | 109.4106 | 115.5578 | 81.03965 | 99.47188 | 101.867 | 85.82609 | 64.51419 | 91.32302 | 92.18368 | 107.6119 | 108.9599 | 122.3475 | 93.46673 | 98.38324 | 91.90153 | 117.2199 | 112.2549 |
| CUST_55291_PI429010886 | M109463 | 0.024415 | 15 | 3 | | | | | | 15000.31 | 6253.288 | 5080.996 | 27914.81 | 18694.18 | 36281.72 | 24777.72 | 6833.331 | 10117.01 | 286.997 | 13696.19 | 125.6499 | 11413.33 | 3551.684 | 9715.562 | 9919.546 | 23879.8 | 24777.72 | 15000.31 | 1447.664 | 15922.04 | 8783.054 | 12651.8 | 26902.5 | 1875.725 | 15199.11 | 118.1469 |
| CUST_39353_PI429010886 | M38183 | 0.024415 | 18 | 3 | | | | | | 11942.94 | 5201.099 | 4357.297 | 23085.06 | 15892.67 | 33351.13 | 20339.21 | 6091.908 | 8156.263 | 143.9734 | 11510.47 | 94.5989 | 7930.333 | 2797.965 | 7347.807 | 7909.769 | 19773.16 | 20138.4 | 11566.7 | 1170.02 | 14375.97 | 7930.333 | 10866.21 | 25976.81 | 1229.858 | 12253.96 | 79.78328 |
| CUST_42597_PI429010886 | M60678 | 0.024418 | 21 | 9 | | | | | | 16.94555 | 20.88392 | 24.36264 | 32.17021 | 25.74156 | 38.64078 | 26.98754 | 32.3288 | 25.87828 | 27.31503 | 29.09612 | 27.69147 | 24.74877 | 30.558 | 7.099269 | 24.51394 | 26.41719 | 24.4797 | 23.26561 | 40.45279 | 33.88263 | 20.86543 | 23.69356 | 24.46151 | 28.35396 | 22.74008 | 25.5267 |
| CUST_24677_PI429010886 | N51066 | 0.024439 | 18 | 0 | | | | | | 26.97522 | 16.66456 | 28.60372 | 23.6887 | 23.52324 | 18.61514 | 27.13598 | 9.744311 | 13.01989 | 25.38172 | 9.300708 | 16.49958 | 13.92846 | 18.27067 | 18.84012 | 27.27496 | 10.12954 | 35.88462 | 26.44421 | 25.25877 | 29.05092 | 17.69248 | 31.68203 | 23.82095 | 18.08051 | 17.03814 | 15.23056 |
| CUST_41793_PI429010886 | M58570 | 0.024458 | 21 | 12 | 443699865 | hypothetical protein CAPTEDRAFT_20554 [Capitella | | | 5.71E-17 | 53.96566 | 84.56429 | 54.6811 | 49.8518 | 42.11302 | 37.93423 | 38.64362 | 39.13986 | 45.62376 | 64.08758 | 53.17437 | 62.59195 | 81.48802 | 105.1159 | 74.97194 | 72.03576 | 43.48001 | 44.75093 | 45.2026 | 80.04179 | 65.49916 | 75.00155 | 69.216 | 37.49092 | 51.98858 | 55.26178 | 59.052 |
| CUST_38467_PI429010886 | M27044 | 0.024459 | 12 | 9 | | | | | | 361.8439 | 391.9101 | 366.4854 | 162.141 | 318.3844 | 212.9083 | 414.7856 | 329.2355 | 381.9407 | 621.5625 | 345.5828 | 426.2225 | 268.9268 | 294.6436 | 403.1472 | 265.8178 | 262.5059 | 276.4683 | 482.5207 | 308.0149 | 328.3197 | 317.1399 | 321.2041 | 332.753 | 205.2221 | 319.3199 | 384.4603 |
| CUST_32805_PI429010886 | M5150 | 0.024459 | 12 | 3 | | | | | | 85.61256 | 92.70294 | 64.61143 | 56.83575 | 91.31323 | 94.51496 | 83.08523 | 91.49463 | 77.87542 | 72.71237 | 49.10355 | 57.5416 | 52.7291 | 98.57526 | 77.05404 | 110.0483 | 95.18251 | 103.9198 | 67.50199 | 60.19028 | 58.73387 | 90.11953 | 47.79729 | 52.63886 | 145.9205 | 51.75281 | 95.63731 |
| CUST_4586_PI429010886 | N17083 | 0.02448 | 12 | 3 | | | | | | 23.20447 | 30.81693 | 23.77465 | 57.60056 | 97.282 | 210.9764 | 91.72554 | 30.74654 | 25.33989 | 34.46849 | 66.11527 | 24.27461 | 22.51683 | 41.02914 | 35.04798 | 40.45663 | 100.2916 | 56.46834 | 95.47811 | 25.70084 | 42.12986 | 45.90172 | 32.59686 | 44.95044 | 30.88328 | 31.8356 | 47.95255 |
| CUST_20143_PI429010886 | N42882 | 0.02448 | 12 | 3 | | | | | | 47.19565 | 49.46138 | 41.86651 | 49.45564 | 53.2143 | 61.14077 | 49.78435 | 52.31892 | 47.91134 | 56.82909 | 47.76946 | 41.97898 | 46.42845 | 43.37513 | 41.39181 | 69.90395 | 62.85713 | 57.94186 | 38.77187 | 46.23898 | 49.78435 | 65.19644 | 51.37925 | 46.96643 | 48.04331 | 42.70658 | 48.87456 |
| CUST_4185_PI429010886 | N16187 | 0.024487 | 15 | 9 | | | | | | 129.2002 | 99.73067 | 104.7208 | 97.89619 | 116.6845 | 72.62704 | 74.83385 | 119.1803 | 112.9419 | 111.8858 | 240.2928 | 259.439 | 164.0579 | 153.2734 | 156.539 | 127.5355 | 92.25373 | 99.31284 | 91.6735 | 273.7876 | 101.5112 | 100.651 | 143.4489 | 70.7113 | 153.4856 | 135.403 | 173.0953 |
| CUST_23728_PI429010886 | N49360 | 0.0245 | 21 | 0 | | | | | | 25.61197 | 21.60172 | 19.90164 | 14.66602 | 16.69997 | 15.79004 | 15.66371 | 9.877583 | 5.358734 | 17.40347 | 15.49433 | 13.3566 | 16.10864 | 6.102462 | 14.64036 | 24.33459 | 24.40654 | 20.80589 | 21.27634 | 12.53403 | 15.16412 | 43.82673 | 24.21253 | 9.970778 | 15.60949 | 15.69084 | 20.71154 |
| CUST_36057_PI429010886 | M14479 | 0.02452 | 12 | 0 | | | | | | 12.67743 | 15.72277 | 19.07106 | 7.831079 | 17.35771 | NA | 9.846676 | 7.525118 | NA | 9.467102 | 12.17117 | 10.33138 | 12.19042 | 11.27882 | 7.781741 | 12.96349 | 9.508624 | 11.34226 | 4.024049 | 6.603602 | 7.92158 | 14.10202 | 4.772717 | NA | 17.58162 | NA | 11.71206 |
| CUST_19709_PI429010886 | N42049 | 0.024532 | 24 | 0 | | | | | | 19.16951 | 15.14331 | 8.487802 | 15.83863 | 5.471124 | 12.73078 | 8.058299 | 12.24661 | 5.303282 | 12.79089 | 8.357942 | 13.83327 | 11.06582 | 7.952703 | 11.22442 | 10.41028 | 6.864974 | NA | 16.97579 | 7.718069 | 10.32292 | 8.466065 | 11.51778 | 11.46217 | 19.70026 | 21.00749 | 16.20777 |
| CUST_2997_PI429010886 | N13570 | 0.024545 | 24 | 0 | 56759584 | Parcxpwfx02 [Periplaneta americana] | | | 3.21E-30 | 86.4072 | 93.70583 | 111.1358 | 85.83424 | 92.73234 | 115.3926 | 107.1945 | 69.48443 | 80.76562 | 85.24568 | 103.6413 | 77.06228 | 53.79628 | 46.64901 | 40.26663 | 102.7422 | 90.42587 | 78.20249 | 117.5697 | 82.51706 | 91.43481 | 93.59991 | 78.19261 | 104.474 | 108.0902 | 97.47845 | 70.61376 |
| CUST_25159_PI429010886 | N51888 | 0.024547 | 21 | 0 | | | | | | 33.49263 | 16.28788 | 26.61439 | 15.00987 | 14.33088 | 24.02357 | 19.35261 | 16.99723 | 8.771173 | 14.83888 | 15.65479 | 11.84887 | 15.88543 | 17.01911 | 22.15217 | 11.18709 | 20.86179 | 18.16906 | 16.04975 | 15.35258 | 19.59409 | 18.34756 | 25.18232 | 23.85138 | 14.07874 | 18.554 | 27.56341 |
| CUST_42907_PI429010886 | M61565 | 0.024568 | 21 | 3 | 270014550 | hypothetical protein TcasGA2_TC004583 [Tribolium | | | 2.82E-58 | 41.75113 | 25.83997 | 47.14504 | 29.5273 | 34.58049 | 87.75721 | 30.46627 | 32.49365 | 26.69852 | 33.00843 | 27.44977 | 36.33335 | 19.76039 | 25.41068 | 29.73496 | 34.8398 | 28.47129 | 23.76325 | 28.98746 | 26.83617 | 36.89793 | 45.50116 | 40.883 | 24.84105 | 50.50708 | 39.25762 | 31.04579 |
| CUST_16087_PI429010886 | N35632 | 0.024579 | 24 | 18 | 336024847 | Mesorhizobium opportunistum WSM2075, complet | Metabolic process | Oxidation-reduction process | 3.1E-117 | 23.92174 | 33.10045 | 14.20045 | 17.38288 | 21.75394 | 15.82118 | 20.87033 | 12.29064 | 17.66287 | 31.79676 | 31.50111 | 16.20296 | 20.17771 | 24.15613 | 11.92505 | 26.48449 | 43.40546 | 19.45997 | 30.93804 | 42.81625 | 22.48589 | 28.31129 | 26.93871 | 31.10084 | 28.6303 | 22.14748 | 23.88015 |
| CUST_5897_PI429010886 | N19346 | 0.024612 | 24 | 15 | | | | | | 88.96346 | 60.57138 | 54.43209 | 41.85446 | 51.21188 | 42.73401 | 72.3461 | 59.31402 | 57.17501 | 56.18196 | 52.52687 | 62.3009 | 69.751 | 70.95494 | 61.8504 | 61.31684 | 68.1054 | 75.22711 | 64.8492 | 77.26285 | 54.17026 | 100.0169 | 64.34064 | 52.31892 | 70.47227 | 44.08227 | 59.40544 |
| CUST_35641_PI429010886 | M13089 | 0.024618 | 24 | 3 | | | | | | 156.8837 | 72.76448 | 9.828551 | 228.8706 | 50.93999 | 92.61954 | 101.4116 | 35.83044 | 86.63308 | 12.22487 | 19.90164 | 6.561972 | 90.29804 | 29.08151 | 92.8298 | 70.64745 | 32.36995 | 84.06167 | 6.924715 | 5.92867 | 4.478759 | 37.04426 | 9.583323 | 218.8861 | 14.88157 | 26.8901 | 60.49322 |
| CUST_48545_PI429010886 | M80928 | 0.024618 | 24 | 21 | | | | | | 163.6601 | 194.8105 | 197.9029 | 217.3065 | 198.3401 | 171.2071 | 150.434 | 149.4128 | 158.1732 | 198.2503 | 182.204 | 190.4443 | 180.3315 | 200.1336 | 223.6419 | 236.2498 | 156.4704 | 203.1495 | 175.6005 | 185.59 | 184.8426 | 230.3934 | 215.7123 | 209.6694 | 221.6861 | 212.9083 | 224.2596 |
| CUST_32162_PI429010886 | M3400 | 0.024638 | 15 | 9 | 321469343 | hypothetical protein DAPPUDRAFT_304059 [Daphn | Transport | Intracellular transport | 3.3E-41 | 121.6348 | 140.3976 | 105.2081 | 143.8657 | 131.4266 | 105.1478 | 82.91863 | 115.8195 | 147.4736 | 177.8716 | 140.1347 | 177.5136 | 128.0504 | 138.4518 | 134.7426 | 115.1051 | 90.09804 | 134.2642 | 106.2586 | 129.0466 | 135.2179 | 148.2643 | 112.609 | 55.16975 | 232.021 | 144.8631 | 179.5942 |
| CUST_12497_PI429010886 | N29558 | 0.02464 | 24 | 15 | | | | | | 659.0457 | 778.415 | 682.2742 | 527.8694 | 613.0465 | 611.7017 | 472.997 | 571.9057 | 528.1283 | 729.8175 | 612.793 | 703.6838 | 610.0761 | 769.8974 | 706.9904 | 892.3589 | 683.9239 | 728.235 | 646.0018 | 868.3571 | 613.6489 | 893.6919 | 625.9634 | 603.6169 | 689.4854 | 669.4372 | 902.7962 |
| CUST_21081_PI429010886 | N44596 | 0.024687 | 24 | 15 | 548340141 | PREDICTED: brefeldin A-inhibited guanine nucleotid | | | 1.47E-58 | 133.3139 | 159.1972 | 158.8254 | 126.539 | 148.2643 | 153.5797 | 119.3258 | 102.7727 | 119.6887 | 122.3027 | 145.861 | 141.8161 | 125.6996 | 162.8265 | 176.2631 | 187.5374 | 137.649 | 154.7047 | 133.9267 | 182.9909 | 127.9952 | 177.6974 | 135.6641 | 128.969 | 164.9397 | 128.2848 | 153.9392 |
| CUST_40970_PI429010886 | M56512 | 0.024695 | 24 | 0 | | | | | | 27.49771 | 25.33406 | 24.16065 | 30.45321 | 9.651747 | 24.96534 | 38.86489 | 8.434609 | 30.59854 | 21.15997 | 21.0901 | 22.8759 | 14.64247 | 15.32313 | 13.32018 | 30.11515 | 28.31689 | 16.70939 | 9.813406 | 18.44972 | 19.62227 | 20.2137 | 36.63495 | 16.82479 | 77.95844 | 22.90764 | 59.12378 |
| CUST_36453_PI429010886 | M15935 | 0.024708 | 21 | 12 | | | | | | 127.5355 | 210.5151 | 127.001 | 101.8542 | 115.6165 | 77.1951 | 77.98525 | 77.23965 | 85.37214 | 149.4288 | 142.2307 | 168.7503 | 135.0089 | 159.5233 | 153.0835 | 199.6541 | 102.5272 | 139.1034 | 106.1871 | 195.487 | 145.5994 | 195.487 | 150.2035 | 96.19258 | 153.4593 | 88.09249 | 117.9167 |
| CUST_18073_PI429010886 | N39091 | 0.024708 | 24 | 18 | | | | | | 55.52574 | 53.66581 | 63.98783 | 42.26619 | 41.06999 | 64.62304 | 56.07148 | 51.4111 | 51.50234 | 74.33243 | 39.0395 | 79.36014 | 59.50102 | 56.91101 | 54.14016 | 65.75053 | 58.33665 | 70.94855 | 71.83981 | 69.88371 | 73.0191 | 71.64239 | 57.57631 | 40.69465 | 62.0828 | 53.64846 | 56.27804 |
| CUST_56802_PI429010886 | M114677 | 0.024726 | 18 | 9 | | | | | | 970.3266 | 1173.656 | 1194.964 | 729.8175 | 909.1015 | 832.0351 | 797.7975 | 983.5467 | 1122.017 | 1292.668 | 1151.621 | 1344.053 | 1176.647 | 1333.433 | 1199.213 | 1286.564 | 1078.296 | 897.1177 | 1036.891 | 1417.195 | 1013.618 | 1320.152 | 1006.198 | 698.4337 | 1465.818 | 940.8909 | 1330.395 |
| CUST_43799_PI429010886 | M64007 | 0.024726 | 24 | 12 | 242013543 | conserved hypothetical protein [Pediculus humanus | | | 2.04E-15 | 28.37529 | 43.0609 | 35.22807 | 18.91365 | 26.23964 | 27.42267 | 29.49638 | 33.26462 | 34.6933 | 50.82151 | 21.12385 | 33.86551 | 43.69222 | 60.34181 | 53.37349 | 48.42271 | 28.78291 | 32.26938 | 49.65196 | 38.41593 | 34.44235 | 46.05816 | 35.65161 | 19.08808 | 51.00123 | 24.62356 | 42.29494 |
| CUST_2071_PI429010886 | N11496 | 0.024728 | 12 | 9 | 290908989 | myosin light chain [Marsupenaeus japonicus] | | | 3.27E-32 | 672.9863 | 957.5442 | 924.5282 | 734.477 | 530.042 | 595.4395 | 730.7862 | 790.7325 | 782.9369 | 792.4242 | 778.8569 | 920.3504 | 753.2014 | 780.6851 | 744.8667 | 914.3958 | 583.2176 | 604.9689 | 737.499 | 1496.572 | 629.802 | 987.5052 | 801.0442 | 747.636 | 630.9156 | 681.3848 | 843.5117 |
| CUST_7592_PI429010886 | N21777 | 0.024728 | 12 | 9 | | | | | | 12.94894 | 136.1666 | 104.0004 | 18.53102 | 10.90106 | 22.59328 | 43.42703 | 25.42315 | 41.76249 | 37.04826 | 53.07973 | 52.96595 | 39.19383 | 23.99863 | 29.78164 | 37.1484 | 24.23314 | 11.27882 | 50.57194 | 32.68774 | 14.16465 | 401.6619 | 61.5801 | 24.11784 | 79.47713 | 17.46064 | 41.32867 |
| CUST_10040_PI429010886 | N25604 | 0.024728 | 12 | 3 | | | | | | 29.29243 | 59.43662 | 25.19666 | 34.34564 | 36.55124 | 49.41638 | 42.53656 | 39.43541 | 33.7306 | 17.23883 | 25.1894 | 26.48449 | 30.04952 | 28.7462 | 45.75802 | 48.88625 | 31.73942 | 48.31638 | 33.7899 | 26.08978 | 27.28673 | 38.79122 | 21.01736 | 36.49315 | 32.05856 | 32.99167 | 30.10596 |
| CUST_19360_PI429010886 | N41448 | 0.024728 | 12 | 9 | | | | | | 25.98413 | 59.51435 | 47.87924 | 41.0817 | 46.50141 | 21.06491 | 32.7645 | 24.91029 | 56.41974 | 70.46014 | 46.57205 | 73.64901 | 37.9853 | 46.24589 | 48.6393 | 51.51243 | 9.476285 | 19.2789 | 36.77053 | 57.42962 | 59.09902 | 81.73916 | 64.18609 | 37.34484 | 76.2979 | 40.4329 | 63.55528 |
| CUST_9609_PI429010886 | N24917 | 0.024734 | 21 | 15 | 307180358 | Histone-lysine N-methyltransferase trithorax [Camp | Metabolic process | Other | 3.85E-54 | 262.0585 | 279.0812 | 236.3355 | 230.8944 | 240.3331 | 198.4542 | 196.157 | 188.1161 | 214.59 | 265.3779 | 243.2097 | 198.8279 | 249.9317 | 238.7409 | 246.3068 | 392.4795 | 272.1063 | 231.2637 | 262.6197 | 325.3686 | 204.4866 | 290.3758 | 211.5135 | 203.2288 | 277.5533 | 186.5287 | 239.571 |
| CUST_38675_PI429010886 | M29049 | 0.024766 | 21 | 12 | | | | | | 2423.127 | 897.9401 | 1778.896 | 693.5345 | 633.8371 | 1105.691 | 933.1038 | 617.029 | 559.6795 | 1463.065 | 931.7811 | 1670.5 | 1821.025 | 1966.099 | 2610.308 | 904.3304 | 1305.146 | 1045.15 | 477.7327 | 1500.831 | 1285.231 | 1932.967 | 1685.531 | 1731.857 | 1401.401 | 725.8016 | 1022.968 |
| CUST_22528_PI429010886 | N47189 | 0.024778 | 15 | 12 | | | | | | NA | 12.45838 | 10.23206 | 7.619757 | 4.711545 | 11.30957 | 7.970135 | NA | 7.577948 | 10.34644 | 13.46425 | 12.13695 | 9.672443 | NA | 13.52947 | 11.73735 | 7.635215 | 5.448479 | NA | 4.514 | 8.273204 | 9.526791 | NA | NA | 15.43638 | 12.21045 | 8.117917 |
| CUST_49980_PI429010886 | M88081 | 0.024786 | 15 | 12 | | | | | | 24.37229 | 53.53627 | 39.73253 | 16.34764 | 15.29619 | NA | 22.26808 | 21.18145 | 33.46044 | 23.194 | 18.87506 | 29.13595 | 36.82802 | 40.1303 | 31.59119 | 22.77856 | 23.22371 | 19.75734 | 21.78927 | 22.84227 | 28.99027 | 18.554 | 28.54572 | 19.55556 | 58.50555 | 24.03557 | 37.30131 |
| CUST_27293_PI429010886 | N55797 | 0.024786 | 12 | 0 | | | | | | 8.638278 | 14.02581 | 7.892117 | NA | 5.164789 | NA | 7.587626 | 4.614477 | 5.61067 | 4.850616 | 6.141389 | 6.36012 | NA | 18.69493 | 9.257427 | 18.02828 | 10.08948 | 7.06808 | 10.02077 | 6.059255 | 8.567471 | 8.026683 | NA | NA | 10.08113 | 13.40926 | 21.4174 |
| CUST_50323_PI429010886 | M89444 | 0.024793 | 18 | 12 | | | | | | 626.8612 | 726.9909 | 209.9313 | 163.1844 | 384.4603 | 224.5142 | 1530.336 | 287.8668 | 1661.324 | 942.3167 | 316.5936 | 1637.238 | 1535.786 | 1250.32 | 752.5982 | 807.1828 | 433.2734 | 464.1138 | 1439.715 | 120.6692 | 672.3398 | 334.6678 | 534.4051 | 385.2863 | 640.949 | 241.0332 | 1244.647 |
| CUST_3975_PI429010886 | N15703 | 0.024793 | 18 | 12 | | | | | | 1022.691 | 1154.447 | 304.1069 | 423.4903 | 566.4251 | 362.9082 | 1998.595 | 434.1796 | 2541.147 | 1428.149 | 571.4497 | 2811.816 | 1943.851 | 1751.202 | 1487.872 | 1356.986 | 739.0931 | 804.8286 | 2365.487 | 178.3086 | 884.4098 | 672.763 | 721.8143 | 595.8659 | 1077.802 | 340.4019 | 1631.709 |
| CUST_14407_PI429010886 | N32833 | 0.024793 | 15 | 0 | | | | | | 933.1038 | 983.8663 | 717.4734 | 1032.594 | 846.1561 | 867.4252 | 709.1206 | 540.5402 | 485.0905 | 817.7915 | 524.5768 | 611.8341 | 644.8914 | 813.1389 | 912.0221 | 1319.125 | 656.7764 | 1115.976 | 756.7863 | 760.7539 | 735.5143 | 1365.452 | 651.6164 | 899.2493 | 810.2943 | 583.9827 | 472.3127 |
| CUST_20370_PI429010886 | N43324 | 0.024794 | 15 | 9 | | | | | | 53.88876 | 26.08326 | 19.95634 | 41.18311 | 7.559884 | 30.27191 | 126.2324 | 21.84747 | 64.95763 | 246.4698 | 36.14137 | 40.15861 | 30.69155 | 23.48661 | 69.36484 | 39.25762 | 66.48237 | 18.65246 | 5.254253 | 13.7932 | 8.66034 | 70.06431 | 75.98951 | 80.81357 | 18.7688 | 78.40746 | 155.0892 |
| CUST_29282_PI429010886 | N59545 | 0.024794 | 15 | 9 | | | | | | 26.80447 | 61.64233 | 49.95028 | 51.273 | 41.83506 | 50.69847 | 56.54516 | 48.82597 | 92.34903 | 68.71451 | 54.01314 | 99.20651 | 49.66578 | 113.8462 | 56.65877 | 32.09117 | 38.42916 | 93.25437 | 22.89626 | 24.43037 | 59.60108 | 43.26504 | 91.88705 | 106.1516 | 92.52049 | 79.91242 | 107.1133 |
| CUST_31981_PI429010886 | M2902 | 0.024796 | 12 | 9 | 83318955 | hypothetical protein [Platynereis dumerilii] | Cellular component organization | Mitochondrion organization | 3.15E-41 | 37.67896 | 43.21123 | 50.78152 | 25.02448 | 36.60913 | 25.58851 | 23.67732 | 33.58065 | 32.47936 | 46.01632 | 35.11191 | 53.20475 | 30.6709 | 82.76433 | 44.11042 | 39.25017 | 24.16065 | 35.28945 | 43.7253 | 38.46426 | 42.36335 | 52.04647 | 78.97339 | 41.96975 | 92.77688 | 35.35004 | 50.56808 |
| CUST_52975_PI429010886 | M99651 | 0.024847 | 21 | 9 | 321473040 | hypothetical protein DAPPUDRAFT_239230 [Daphn | | | 4.23E-10 | 17.65481 | 21.36888 | 30.38324 | 30.48036 | 50.98064 | 25.99654 | 33.17041 | 57.8814 | 40.45663 | 30.33523 | 67.7616 | 62.70075 | 46.69563 | 33.35026 | 30.01154 | 31.13642 | 31.44747 | 25.62143 | 38.17748 | 134.4195 | 53.83209 | 18.92054 | 32.01033 | 24.4716 | 20.31401 | 52.8414 | 41.86651 |
| CUST_840_PI429010886 | N7492 | 0.024855 | 18 | 15 | | | | | | 808.2235 | 1264.096 | 768.5376 | 670.4666 | 825.7659 | 742.2219 | 644.7638 | 688.5897 | 659.1977 | 719.37 | 807.4639 | 956.4623 | 821.28 | 743.4007 | 776.3963 | 1178.599 | 744.2633 | 840.1524 | 808.4761 | 974.3808 | 705.7628 | 1181.87 | 690.9208 | 668.6149 | 1096.765 | 640.4514 | 693.0321 |
| CUST_36736_PI429010886 | M16697 | 0.024869 | 12 | 3 | 270161184 | i-type lysozyme-like protein 2 [Penaeus monodon] | Metabolic process | Carbohydrate metabolic process | 2.04E-12 | 68.24289 | 23.38781 | NA | 240.7867 | 118.7885 | 441.6462 | 173.1369 | 113.3117 | 92.52049 | 19.56685 | 66.67878 | 11.94919 | 139.7005 | 34.11202 | 91.86368 | 68.29075 | 188.2993 | 200.6444 | 197.2642 | 18.22302 | 22.97231 | 77.05404 | 57.27407 | 138.4302 | 48.17007 | 161.4354 | 32.26335 |
| CUST_35517_PI429010886 | M12768 | 0.024875 | 21 | 15 | 380017496 | PREDICTED: UBX domain-containing protein 4-like [ | | | 6.48E-19 | 28.45405 | 29.30762 | 35.48044 | 32.48228 | 27.76638 | 25.93179 | 28.84855 | 23.5585 | 29.02633 | 26.3358 | 34.03186 | 33.84879 | 25.22662 | 38.79996 | 32.87841 | 38.89725 | 31.35346 | 36.68252 | 34.45811 | 30.44396 | 29.92091 | 30.14577 | 33.63678 | 28.81252 | 33.1299 | 24.94778 | 31.49086 |
| CUST_48059_PI429010886 | M78726 | 0.024913 | 15 | 0 | 197940771 | Euphausia superba mitochondrion, partial genome | | | 1.6E-149 | 78081.9 | 72295.32 | 73854.47 | 79324.15 | 88276.1 | 58715.07 | 46511.8 | 56816.44 | 83322.86 | 34070.11 | 56627.52 | 52133.5 | 96660.96 | 51921.46 | 56054.85 | 86620.4 | 61640.76 | 79324.15 | 78891.09 | 69607.7 | 71869.37 | 83322.86 | 64277.46 | 73477.97 | 54505.68 | 63067.91 | 56816.44 |
| CUST_14014_PI429010886 | N32178 | 0.024913 | 18 | 0 | 241560240 | CDK5 regulatory subunit-associated protein, putativ | Nucleic acid metabolism | RNA metabolic process | 2.24E-29 | 40.79309 | 36.8021 | 45.2026 | 50.45103 | 33.2742 | 32.80342 | 30.38324 | 42.89937 | 31.64126 | 31.1463 | 22.46676 | 39.52923 | 29.43926 | 36.08462 | 51.32239 | 58.18474 | 40.68573 | 35.66685 | 21.64916 | 115.7889 | 102.0104 | 36.23123 | 43.12293 | 38.94325 | 31.35806 | 28.58408 | 20.69763 |
| CUST_31393_PI429010886 | M1423 | 0.024913 | 24 | 3 | | | | | | 134.5271 | 266.722 | 189.3905 | 247.7341 | 248.959 | 225.5174 | 142.821 | 163.2888 | 119.3665 | 208.3184 | 155.1093 | 94.75367 | 91.76014 | 176.601 | 16.11615 | 142.6758 | 161.9804 | 244.4939 | 156.8837 | 185.8187 | 201.2707 | 186.2827 | 158.3894 | 171.1038 | 248.8723 | 125.099 | 207.4667 |
| CUST_10595_PI429010886 | N26485 | 0.024927 | 18 | 9 | 321467979 | hypothetical protein DAPPUDRAFT_304984 [Daphn | Metabolic process | Other | 2.57E-32 | 60.24307 | 44.8896 | 77.74288 | 49.56937 | 53.96058 | 58.51806 | 60.53435 | 79.73386 | 90.91352 | 93.25437 | 81.16843 | 105.5819 | 82.51706 | 82.8752 | 115.5725 | 84.43869 | 75.97215 | 71.86244 | 70.61832 | 138.0091 | 96.8323 | 61.97713 | 90.91352 | 65.80832 | 87.35248 | 95.24605 | 114.9975 |

| Probe ID | ID | p-value | Period | Phase | EBI ID | Annotation | GO category | Go term | E-value | 0 | 0 | 0 | 3 | 3 | 3 | 6 | 6 | 6 | 9 | 9 | 9 | 12 | 12 | 12 | 15 | 15 | 15 | 18 | 18 | 18 | 21 | 21 | 21 | 24 | 24 | 24 |
|---|---|---|---|---|---|---|---|---|---|---|---|---|---|---|---|---|---|---|---|---|---|---|---|---|---|---|---|---|---|---|---|---|---|---|---|---|
| CUST_56552_PI429010886 | M114082 | 0.024928 | 18 | 12 | | | | | | 749.2477 | 737.7582 | 738.6637 | 680.3748 | 648.4582 | 618.5189 | 703.3407 | 674.0173 | 686.5061 | 842.0657 | 720.2695 | 909.4624 | 772.6877 | 848.6156 | 931.4066 | 1090.829 | 698.2239 | 761.273 | 807.9372 | 1025.618 | 610.2139 | 1011.687 | 602.6317 | 578.5402 | 1076.18 | 747.636 | 963.151 |
| CUST_28183_PI429010886 | N57448 | 0.024944 | 21 | 12 | | | | | | 44.84166 | 27.34334 | 31.03981 | 20.04708 | 24.89381 | 24.20353 | 26.65108 | 17.04303 | 17.41767 | 36.29774 | 32.46691 | 31.23744 | 34.86849 | 34.85305 | 37.63897 | 43.25052 | 27.36512 | 28.55239 | 34.76984 | 42.89021 | 33.09246 | 49.93988 | 23.94075 | 25.80322 | 45.62788 | 11.65987 | 34.24814 |
| CUST_39621_PI429010886 | M41892 | 0.024959 | 18 | 3 | 207298842 | Homarus americanus cytoplasmic type actin 1 mRN | Cellular Process | Cell growth | 5.78E-74 | 48465.49 | 60338.91 | 28694.35 | 56627.52 | 55443.98 | 52713.61 | 44409.28 | 35159.1 | 45970.08 | 42714.39 | 44409.28 | 45577.47 | 38680.42 | 30891.2 | 44277.38 | 45970.08 | 38079.24 | 49418.23 | 47564.51 | 44923.37 | 44666.31 | 47710.56 | 35264.41 | 49418.23 | 46148.42 | 42869.03 | 39067.89 |
| CUST_37258_PI429010886 | M18383 | 0.02496 | 24 | 3 | 326781695 | crustacyanin subunit A [Fenneropenaeus merguiens | | | 2.14E-12 | 792.4242 | 255.3013 | 140.1704 | 2270.606 | 730.6182 | 772.6877 | 2029.752 | 170.6697 | 1928.541 | 96.1439 | 19.97654 | 9.783545 | 679.9331 | 79.44802 | 1122.467 | 2089.376 | 73.46154 | 1315.839 | 35.89324 | 40.68988 | 8.978228 | 360.8661 | 40.56586 | 384.1514 | 169.9489 | 57.84964 | 296.5251 |
| CUST_31001_PI429010886 | M423 | 0.024962 | 24 | 15 | 260790040 | hypothetical protein BRAFLDRAFT_240825 [Branchi | Metabolic process | Other | 6.55E-36 | 298.7909 | 303.7719 | 338.1543 | 260.2651 | 291.9341 | 245.7815 | 217.4459 | 259.8913 | 303.0187 | 384.7174 | 278.0994 | 358.3575 | 306.5697 | 386.8238 | 336.3354 | 356.6641 | 311.2463 | 310.0283 | 285.7468 | 354.2035 | 349.8347 | 339.6011 | 324.8235 | 245.8233 | 305.4399 | 298.7909 | 367.6066 |
| CUST_19517_PI429010886 | N41703 | 0.024962 | 24 | 18 | | | | | | 5.767846 | 6.284506 | NA | NA | 5.601747 | 7.451482 | 26.23144 | 7.623642 | 6.110369 | 22.24779 | 11.87142 | NA | 5.016515 | 30.52136 | 9.631079 | 8.035951 | NA | 33.52107 | 24.34006 | 46.63569 | 57.72819 | 84.15456 | 37.63001 | NA | 8.956327 | 29.43141 | 44.84606 |
| CUST_46167_PI429010886 | M71624 | 0.024964 | 24 | 12 | | | | | | 20.84589 | 20.22094 | 32.36995 | 17.94343 | 26.22091 | 22.90466 | 22.15879 | 28.51585 | 26.13341 | 32.24939 | 27.4471 | 44.63407 | 35.88829 | 36.10574 | 39.72298 | 33.5432 | 25.33771 | 23.35223 | 30.41595 | 36.9176 | 41.75433 | 31.2144 | 35.66685 | 28.1626 | 30.01154 | 19.69401 | 37.30507 |
| CUST_53840_PI429010886 | M103385 | 0.024967 | 24 | 3 | | | | | | 56.05475 | 36.85933 | 78.92088 | 64.52054 | 54.40089 | 67.31728 | 68.49231 | 53.53627 | 65.65578 | 73.73837 | 44.93417 | 45.79191 | 39.66277 | 52.45258 | 41.77165 | 43.52847 | 49.79493 | 44.01861 | 38.92288 | 27.68618 | 65.58458 | 51.67859 | 83.16839 | 45.82893 | 54.6573 | 52.97067 | 55.39377 |
| CUST_32724_PI429010886 | M4937 | 0.024969 | 12 | 0 | 54400676 | ORM1-like protein 3 [Danio rerio] >gi\|81174971\|sp | Protein metabolism | Regulation of translational elongation | 3.53E-52 | 168.0101 | 182.0483 | 144.7304 | 125.751 | 121.4442 | 112.0103 | 113.2611 | 103.4856 | 113.3684 | 175.1085 | 122.4667 | 162.1787 | 108.6304 | 142.5643 | 154.4215 | 187.3568 | 101.9838 | 134.2991 | 117.9689 | 113.6011 | 101.7734 | 159.5684 | 107.5704 | 120.9718 | 182.965 | 125.027 | 129.6012 |
| CUST_9646_PI429010886 | N24981 | 0.024969 | 12 | 0 | | | | | | 48.59055 | 99.21099 | 65.11921 | 67.92832 | 16.80068 | 42.25718 | 46.39793 | 99.89328 | 13.32908 | 17.62133 | 66.87005 | 69.54952 | 63.33459 | 73.07849 | 120.3098 | 49.11669 | 24.86718 | 39.04491 | 49.04149 | 15.96673 | 43.12973 | 41.14845 | 47.75268 | 24.86718 | 51.09155 | 185.0177 | 146.3045 |
| CUST_30427_PI429010886 | N61872 | 0.024969 | 12 | 0 | | | | | | 89.37758 | 110.8879 | 98.43882 | 84.62693 | 82.27324 | 91.90962 | 78.35123 | 69.16053 | 71.43104 | 84.84663 | 78.2069 | 79.51299 | 96.01566 | 88.64024 | 75.94687 | 95.5926 | 84.1835 | 75.2095 | 75.10177 | 90.20594 | 73.55823 | 107.2874 | 84.42877 | 77.04659 | 101.8407 | 73.05511 | 94.65769 |
| CUST_34013_PI429010886 | M8501 | 0.02497 | 12 | 0 | | | | | | 140.5313 | 245.6075 | 182.2902 | 119.6777 | 100.0857 | 130.3469 | 137.0769 | 124.2716 | 118.04 | 149.5977 | 138.3413 | 167.3634 | 123.3713 | 152.424 | 147.0435 | 190.8326 | 145.1839 | 127.2574 | 125.8034 | 147.3848 | 114.2091 | 198.8522 | 137.2513 | 128.5685 | 203.8732 | 149.2677 | 156.562 |
| CUST_35073_PI429010886 | M11488 | 0.024979 | 21 | 9 | | | | | | 61.11029 | 71.46757 | 47.80852 | 76.54184 | 133.5939 | 70.51648 | 87.51279 | 158.3361 | 124.2868 | 59.9743 | 189.3668 | 181.6996 | 146.0878 | 51.50855 | 76.94992 | 82.14765 | 85.3343 | 55.65323 | 61.07679 | 307.7804 | 111.8286 | 63.16675 | 68.88215 | 53.80266 | 80.68886 | 93.44686 | 77.49116 |
| CUST_24969_PI429010886 | N51557 | 0.024994 | 18 | 0 | | | | | | 70.50836 | 29.76829 | 55.62798 | 31.95513 | 35.02904 | 62.71939 | 20.59992 | 23.92874 | 24.99806 | 28.63701 | 25.22662 | 51.43692 | 26.72669 | 36.58254 | 49.15395 | 32.24298 | 28.43068 | 48.72124 | 42.9046 | 42.53014 | 40.01887 | 35.84618 | 28.90658 | 27.10054 | 23.85138 | 40.46782 | 39.17807 |
| CUST_11331_PI429010886 | N27635 | 0.025011 | 18 | 6 | | | | | | 25.749 | 19.24807 | 21.09489 | 18.95813 | 32.67642 | 28.8964 | 54.05301 | 25.03856 | 35.13913 | 14.18613 | 20.72901 | 29.8401 | 52.34805 | 51.44191 | 34.92205 | 15.72851 | 13.96065 | 13.96779 | 28.97523 | 23.69356 | 21.27079 | 4.53627 | 31.85611 | 17.94144 | 41.11909 | 25.38492 | 37.65143 |
| CUST_15128_PI429010886 | N34052 | 0.025025 | 21 | 3 | | | | | | 127.4128 | 277.8925 | 255.1996 | 244.2771 | 254.3839 | 317.8203 | 233.5692 | 296.9284 | 253.9339 | 260.373 | 199.8275 | 314.7546 | 258.7852 | 232.8026 | 85.07901 | 281.692 | 212.1945 | 210.4274 | 163.8946 | 206.1326 | 228.7951 | 235.6934 | 309.5934 | 318.0852 | 325.1931 | 311.5276 | 271.4917 |
| CUST_43033_PI429010886 | M61914 | 0.025028 | 21 | 3 | | | | | | 113.5868 | 256.1518 | 127.3371 | 507.1661 | 203.9202 | 242.4336 | 126.539 | 116.6217 | 114.3896 | 128.0504 | 52.35985 | 176.8527 | 89.65503 | 99.94463 | 136.491 | 119.4557 | 38.57925 | 139.9485 | 64.87589 | 115.8195 | 216.4474 | 184.7859 | 208.552 | 123.4738 | 237.3703 | 235.6197 | 79.48728 |
| CUST_15456_PI429010886 | N34609 | 0.025028 | 15 | 12 | | | | | | 55.70806 | 69.00002 | 62.45524 | 52.29382 | 42.68003 | 42.5721 | 31.30264 | 27.50275 | 41.68626 | 71.03904 | 49.04708 | 73.94862 | 45.64715 | 69.47796 | 72.73041 | 78.04542 | 35.13338 | 57.97853 | 51.17489 | 58.0658 | 64.21464 | 47.42461 | 52.80587 | 50.22122 | 52.43379 | 47.34484 | 66.09773 |
| CUST_53814_PI429010886 | M103304 | 0.025052 | 24 | 18 | | | | | | 21.05993 | 25.4055 | 26.73153 | 15.28811 | 14.09025 | 19.66959 | 18.38425 | 16.32704 | 23.53335 | 20.15879 | 16.38885 | 29.71613 | 14.03191 | 15.25233 | 23.25736 | 28.08839 | 21.05098 | 26.03397 | 31.42645 | 19.40925 | 25.05128 | 24.52447 | 24.76468 | 24.7547 | 20.11768 | 23.72626 | 23.99379 |
| CUST_11164_PI429010886 | N27371 | 0.025055 | 18 | 15 | | | | | | 795.7542 | 1864.705 | 707.1584 | 874.1627 | 1696.945 | 1508.034 | NA | NA | 68.08199 | 166.0856 | 582.2842 | 839.5092 | 423.2024 | 2063.321 | 2006.856 | 2892.813 | NA | 2130.831 | 362.1966 | 3667.985 | 617.9947 | 984.6642 | 597.1763 | 542.3878 | 1501.641 | 106.3744 | 100.6291 |
| CUST_39209_PI429010886 | M36129 | 0.025084 | 15 | 3 | 543335553 | Na+/K+ ATPase [Penaeus monodon] | Transport | Ion transport | 5.28E-40 | 1777.044 | 1444.045 | 1226.144 | 1961.856 | 1933.842 | 2052.131 | 1769.215 | 1230.999 | 1847.968 | 1632.477 | 1574.342 | 1369.558 | 1631.709 | 1390.937 | 1098.078 | 2007.972 | 1597.443 | 1712.532 | 1999.641 | 1639.617 | 1433.821 | 1823.946 | 1129.308 | 1665.324 | 1580.29 | 1785.596 | 1382.151 |
| CUST_55296_PI429010886 | M109479 | 0.0251 | 15 | 9 | | | | | | 498.6758 | 555.454 | 687.2662 | 538.5366 | 554.9764 | 551.8489 | 515.0601 | 539.6207 | 595.5631 | 696.7816 | 623.9709 | 706.7803 | 557.4875 | 695.5556 | 804.2481 | 679.3339 | 503.4817 | 546.956 | 548.3934 | 603.9714 | 631.1117 | 605.6379 | 597.3714 | 492.7259 | 718.917 | 689.4854 | 772.946 |
| CUST_6415_PI429010886 | N20070 | 0.0251 | 15 | 0 | 332029157 | Bcl-2-like protein 1 [Acromyrmex echinatior] | Cellular Process | Cell death | 1.25E-24 | 97.3486 | 64.45839 | 100.566 | 62.45524 | 60.43419 | 64.03011 | 48.21189 | 64.95763 | 69.14635 | 72.00841 | 69.51764 | 60.77185 | 32.54445 | 65.30029 | 66.56405 | 70.92151 | 71.79355 | 63.71417 | 51.34112 | 69.53748 | 61.60388 | 52.38937 | 45.2026 | 39.88324 | 57.80004 | 47.02813 | 61.46953 |
| CUST_34289_PI429010886 | M9247 | 0.0251 | 15 | 12 | 270013885 | hypothetical protein TcasGA2_TC012551 [Tribolium | | | 2.4E-18 | 154.9491 | 162.1184 | 263.6263 | 111.8993 | 187.181 | 90.3035 | 229.178 | 317.9758 | 258.545 | 262.1655 | 239.3938 | 270.6659 | 452.9912 | 343.3378 | 283.6461 | 181.9242 | 410.8257 | 108.829 | 359.7386 | 114.3208 | 171.1038 | 176.9698 | 285.3755 | 181.0372 | 324.8963 | 138.6419 | 331.1696 |
| CUST_2272_PI429010886 | N12003 | 0.0251 | 15 | 12 | 443697440 | hypothetical protein CAPTEDRAFT_223046 [Capitell | Transport | Protein transport | 1.75E-23 | 25.03208 | 25.82153 | 21.81748 | 23.57221 | 21.73089 | 25.69538 | 26.34227 | 19.48105 | 22.14979 | 28.44308 | 25.14127 | 33.65406 | 33.23641 | 29.32561 | 39.36991 | 22.41474 | 23.79492 | 30.09768 | 20.18328 | 27.79833 | 12.37483 | 29.57087 | 20.58261 | 30.2284 | 32.69544 | 19.19079 | 27.60836 |
| CUST_7236_PI429010886 | N21236 | 0.025105 | 24 | 12 | | | | | | 15.59286 | 11.9176 | 25.31589 | 9.339132 | 11.34226 | 14.25137 | 16.28788 | 7.811739 | 11.93548 | 23.6887 | 13.28864 | 20.03907 | 17.44803 | 17.52979 | 22.44161 | 16.86014 | 19.48765 | 19.28798 | 15.73743 | 17.37315 | 6.811352 | 17.25469 | 24.86526 | NA | 14.99955 | 11.15573 | 16.97989 |
| CUST_1665_PI429010886 | N10390 | 0.025111 | 18 | 3 | | | | | | 724.0591 | 728.0084 | NA | 2117.402 | 1264.096 | 1057.689 | 989.1564 | 33.39553 | 828.1362 | 5.241968 | 36.58254 | 8.047455 | 481.7893 | 1004.727 | 1003.971 | 1472.72 | NA | 1174.011 | 6.172262 | 949.0578 | 149.0336 | 2972.831 | 99.48375 | 1096.765 | 416.7544 | 26.8663 | NA |
| CUST_33592_PI429010886 | M7365 | 0.025115 | 21 | 15 | | | | | | 431.3053 | 290.1265 | 395.4574 | 202.7615 | 340.057 | 281.8261 | 287.9369 | 279.9837 | 285.8071 | 580.8644 | 460.7596 | 465.3498 | 322.7948 | 384.4603 | 298.4347 | 622.9374 | 359.3716 | 556.7295 | 353.785 | 240.2928 | 205.2221 | 345.9993 | 395.6626 | 392.2917 | 291.6371 | 286.6682 | 274.7936 |
| CUST_51892_PI429010886 | M95567 | 0.025126 | 24 | 21 | 321456860 | hypothetical protein DAPPUDRAFT_330564 [Daphn | | | 9.16E-41 | 32.7645 | 29.55233 | 41.22879 | 21.80116 | 33.00505 | 27.37719 | 31.44965 | 18.39819 | 27.51414 | 24.94079 | 33.2742 | 9.54588 | 16.93718 | 34.87551 | 22.06655 | 29.96643 | 34.55194 | 19.25465 | 33.42247 | 31.0325 | 31.90127 | 37.32162 | 33.59157 | 33.35513 | 37.86103 | 26.60252 | 30.4923 |
| CUST_54840_PI429010886 | M107924 | 0.025145 | 12 | 3 | | | | | | 62.82655 | 23.82334 | NA | 89.37758 | 122.5827 | 192.8311 | 53.91424 | 91.23322 | 26.25499 | 7.61532 | 35.94919 | 6.790175 | 35.13913 | 25.53913 | 58.38951 | 13.64186 | 243.9591 | 219.715 | 82.78386 | 5.677532 | 13.21125 | 29.04226 | 5.460269 | 64.62929 | 5.346894 | 82.33655 | 10.2558 |
| CUST_44270_PI429010886 | M65456 | 0.025194 | 15 | 0 | | | | | | 213.275 | 246.5795 | 157.1495 | 204.2904 | 154.0002 | 161.0453 | 126.8312 | 137.351 | 120.9579 | 173.2729 | 146.7019 | 185.7639 | 188.427 | 193.773 | 203.4225 | 227.6752 | 153.5797 | 199.8756 | 192.324 | 173.7422 | 151.5005 | 243.3595 | 151.752 | 174.8053 | 189.7994 | 188.1623 | 154.4419 |
| CUST_22715_PI429010886 | N47523 | 0.025201 | 24 | 15 | 345489320 | PREDICTED: hypothetical protein LOC100677857 isc | | | 4.46E-23 | 14.11041 | 18.4372 | 26.76358 | 10.95916 | 13.18285 | 12.06791 | 14.72238 | 12.97975 | 23.43573 | 14.58718 | 21.35291 | 35.88829 | 15.83738 | 31.91513 | 25.91041 | 20.58435 | 31.89471 | 27.22941 | 18.6269 | 19.21235 | 21.15466 | 31.28165 | 16.84352 | 16.32267 | 25.60131 | 23.85638 | 31.56077 |
| CUST_31472_PI429010886 | M1613 | 0.025203 | 18 | 3 | 321474963 | hypothetical protein DAPPUDRAFT_309103 [Daphn | | | 3.65E-75 | 48.65088 | 67.30873 | 33.88658 | 88.6992 | 47.69004 | 52.68897 | 31.47618 | 19.09218 | 34.42302 | 37.10254 | 22.63247 | 64.63524 | 28.05565 | 28.43068 | 30.68445 | 49.88882 | 15.61522 | 34.74686 | 25.27723 | 39.06616 | 44.32414 | 37.74785 | 66.50022 | 40.20429 | 48.5802 | 46.48282 | 30.23517 |
| CUST_8093_PI429010886 | N22528 | 0.025203 | 24 | 18 | | | | | | 22.5314 | 19.89653 | 35.54408 | 21.44879 | 18.97288 | 16.92026 | 21.58196 | 26.42807 | 27.06117 | 31.94051 | 20.52907 | 38.4769 | 26.56221 | 44.41428 | 36.68252 | 38.74854 | 24.45953 | 24.03945 | 29.88417 | 33.01533 | 32.33975 | 31.51891 | 31.20174 | 23.43377 | 30.07667 | 27.37091 | 51.71837 |
| CUST_10936_PI429010886 | N27019 | 0.025203 | 24 | 21 | 291236785 | PREDICTED: origin recognition complex, subunit 1-li | Symbiosis, encompassing mutualism through parasitism | Other | 2.17E-09 | 14.45048 | 25.74156 | 19.0691 | 14.39019 | 15.63271 | 9.184475 | 14.33237 | 13.71667 | 9.488473 | 18.85554 | 14.1362 | 15.59485 | 16.3703 | 11.70566 | 19.99756 | 30.56491 | 15.21068 | 54.35965 | 17.62531 | 14.16669 | 16.54774 | 26.23528 | 15.9031 | 18.35292 | 15.26938 | 18.08454 | 19.39031 |
| CUST_33076_PI429010886 | M5910 | 0.025203 | 21 | 0 | | | | | | 122.3884 | 146.7445 | 112.1069 | 112.9234 | 104.474 | 120.6113 | 101.3914 | 94.36809 | 104.964 | 136.5329 | 102.8709 | 133.0951 | 99.2772 | 82.49999 | 100.3848 | 108.9284 | 93.13261 | 111.7621 | 117.3341 | 128.0902 | 116.8917 | 156.4704 | 118.55 | 107.6969 | 132.88 | 87.96508 | 109.3042 |
| CUST_16021_PI429010886 | N35525 | 0.025223 | 18 | 3 | | | | | | 67.40392 | 94.97923 | 93.71752 | 95.76592 | 76.35962 | 117.2072 | 67.98118 | 84.2393 | 67.18762 | 90.50136 | 57.36487 | 70.09922 | 64.3589 | 88.84142 | 82.07629 | 86.66049 | 85.81376 | 96.28021 | 81.92913 | 74.62941 | 104.363 | 96.86826 | 111.9958 | 97.73561 | 114.529 | 72.61909 | 104.205 |
| CUST_14196_PI429010886 | N32470 | 0.025232 | 24 | 3 | | | | | | 9.963882 | 7.116185 | 17.47454 | NA | 31.49741 | 45.55401 | 5.294876 | 4.37219 | 3.322443 | 20.76211 | 4.180218 | 8.741416 | 6.572155 | 3.410823 | 3.445255 | NA | 6.548124 | NA | 5.611283 | 3.816856 | 7.246865 | 8.402814 | 11.71876 | 5.397572 | NA | NA | 5.901635 |
| CUST_15270_PI429010886 | N34302 | 0.025253 | 12 | 0 | | | | | | 39.09942 | 34.2205 | 48.67019 | 26.92163 | 16.69384 | 20.26285 | 14.07538 | 30.66803 | 26.65108 | 36.68661 | 21.91785 | 10.01001 | 25.06956 | 47.01326 | 31.04579 | 53.65979 | 19.43524 | 40.76277 | 19.55939 | 17.16641 | 16.28372 | 22.3538 | 36.38283 | 23.24144 | 48.84271 | 22.49197 | 34.92634 |
| CUST_18877_PI429010886 | N40568 | 0.025253 | 12 | 0 | 242024511 | ankyrin-1, putative [Pediculus humanus corporis] >g | Metabolic process | Lipid metabolic process | 4.24E-74 | 53.12725 | 51.75281 | 58.69441 | 32.86117 | 55.92013 | 49.84204 | 46.69134 | 37.89768 | 40.45279 | 53.8998 | 51.6678 | 55.57729 | 52.95164 | 78.33945 | 57.07573 | 50.91028 | 61.43801 | 48.69326 | 59.81399 | 39.61101 | 50.8634 | 73.1185 | 53.07973 | 64.61143 | 83.48682 | 54.13488 | 64.90243 |
| CUST_31573_PI429010886 | M1878 | 0.025279 | 21 | 18 | 30584201 | Homo sapiens peroxisomal D3,D2-enoyl-CoA isome | Metabolic process | Lipid metabolic process | 6.23E-63 | 95.25723 | 72.11758 | 80.49518 | 61.1739 | 47.53841 | 66.44095 | 46.12938 | 79.5827 | 71.47658 | 114.1191 | 67.94324 | 94.97923 | 72.1095 | 79.61927 | 52.92115 | 63.50632 | 101.5014 | 80.26839 | 102.1823 | 93.1869 | 54.90496 | 72.78118 | 70.33128 | 68.56023 | 68.761 | 34.54793 | 61.09062 |
| CUST_46868_PI429010886 | M74002 | 0.025284 | 21 | 18 | | | | | | 39.30201 | 36.71638 | 37.10843 | 29.05729 | 33.58859 | 21.68228 | 48.87456 | 22.52113 | 34.48125 | 36.07548 | 31.96544 | 35.44546 | 35.32948 | 41.65038 | 27.53905 | 41.92389 | 31.30264 | 30.9722 | 41.93775 | 42.64305 | 36.83973 | 41.46823 | 39.52923 | 23.94075 | 34.28069 | 31.89471 | 30.06426 |
| CUST_14514_PI429010886 | N33003 | 0.025285 | 15 | 9 | | | | | | 52.78657 | 51.61085 | 183.091 | 59.10759 | 57.79515 | 65.39564 | 84.30944 | 118.378 | 126.9103 | 163.9694 | 72.83324 | 83.71896 | 135.9627 | 443.4781 | 76.23608 | 59.21305 | 113.8702 | 58.46928 | 51.7472 | 82.38056 | 74.60621 | 111.5352 | 330.882 | 115.284 | 236.449 | 88.87538 | 251.1828 |
| CUST_15603_PI429010886 | N34852 | 0.025288 | 24 | 15 | | | | | | 14.96828 | 18.95556 | 10.01115 | 18.54361 | 18.24494 | 17.14547 | 12.27296 | 5.502089 | 14.0442 | 13.26743 | 17.82897 | 16.61891 | 22.72881 | 12.68586 | 17.50362 | 49.52577 | 19.97113 | 21.17508 | 14.5578 | 21.71424 | 28.229 | 26.28688 | 16.9925 | 15.56753 | 22.1684 | 3.793388 | 18.28609 |
| CUST_45721_PI429010886 | M70056 | 0.02529 | 21 | 15 | 321464435 | hypothetical protein DAPPUDRAFT_306779 [Daphn | | | 1.04E-36 | 93.00061 | 82.97709 | 78.52355 | 86.23591 | 95.07199 | 100.0527 | 79.60953 | 78.0365 | 89.51747 | 104.5873 | 81.89181 | 83.77617 | 79.51299 | 103.3291 | 95.61724 | 154.0002 | 84.57831 | 105.711 | 95.689 | 105.1255 | 101.8114 | 103.4856 | 87.47537 | 106.4577 | 91.36505 | 78.68384 | 85.61256 |
| CUST_2383_PI429010886 | N12235 | 0.02529 | 21 | 15 | | | | | | 24.82512 | 67.98118 | 45.45196 | 16.38885 | 65.65578 | 30.21036 | 12.29346 | 35.88091 | 30.3471 | 38.57181 | 24.15872 | 63.67102 | 36.59066 | 50.17076 | 39.84998 | 102.1011 | 46.6 | 63.94078 | 42.86965 | 111.3415 | 49.94515 | 81.31167 | 23.94321 | 40.54346 | 53.76242 | 29.02633 | 47.98029 |
| CUST_54827_PI429010886 | M107854 | 0.025296 | 21 | 12 | | | | | | 27.63853 | 29.89381 | 69.29923 | 42.09877 | 27.14155 | 36.79155 | 24.41159 | 29.08714 | 38.91585 | 27.99749 | 33.07929 | 44.62585 | 36.18187 | 58.45028 | 64.50807 | 57.10423 | 50.30273 | 49.63154 | 42.18155 | 34.46151 | 43.20694 | 40.49183 | 35.4728 | 51.73011 | 26.28003 | 35.45118 | 51.63118 |
| CUST_3002_PI429010886 | N13577 | 0.025303 | 18 | 6 | 389615518 | mucin protein, partial [Papilio polytes] | | | 3.94E-17 | 71.26057 | 50.33907 | 127.0219 | 132.8952 | 150.434 | 292.8985 | 168.154 | 176.7462 | 187.5869 | 151.2216 | 156.4459 | 59.07548 | 118.5367 | 155.0892 | 97.65941 | 67.48761 | 311.3229 | 177.3959 | 131.2743 | 127.1944 | 129.5087 | 104.7208 | 207.2788 | 186.4927 | 247.4184 | 181.6707 | 271.3099 |
| CUST_17318_PI429010886 | N37766 | 0.025342 | 24 | 18 | | | | | | 59.58353 | 56.37885 | 47.10091 | 36.37581 | 33.69868 | 43.2321 | 39.87566 | 32.35642 | 39.71396 | 51.88107 | 51.73412 | 34.49254 | 40.49183 | 58.55654 | 41.96975 | 50.5231 | 44.60711 | 52.01848 | 49.08084 | 59.6055 | 52.01848 | 64.09512 | 48.89187 | 35.02904 | 77.73408 | 38.68077 | 52.8351 |
| CUST_46984_PI429010886 | M74401 | 0.025344 | 21 | 0 | | | | | | 16.5818 | 13.77454 | 18.24734 | 10.48808 | 10.98274 | NA | 10.87355 | 5.258608 | 12.6233 | 12.63519 | 5.790095 | 7.358125 | 8.813762 | 6.835739 | 6.888224 | 14.4448 | 19.24083 | 14.05126 | 9.501422 | 6.578887 | 7.344722 | 19.84447 | 43.41027 | 10.5119 | 6.852264 | 9.981072 | 8.601817 |
| CUST_33403_PI429010886 | M6802 | 0.025373 | 15 | 9 | | | | | | 156.5208 | 452.1056 | 398.8868 | 174.6176 | 128.6369 | 150.8963 | 140.364 | 219.1881 | 412.9301 | 322.593 | 492.1806 | 701.1457 | 289.4881 | 632.6214 | 383.4448 | 341.713 | 175.4567 | 198.5726 | 456.7992 | 312.7398 | 434.0155 | 718.4589 | 265.532 | 159.2366 | 796.983 | 528.1283 | 928.0578 |
| CUST_2385_PI429010886 | N12240 | 0.025406 | 15 | 3 | 380023984 | PREDICTED: LOW QUALITY PROTEIN: ATP-depender | | | 3.91E-29 | 1128.352 | 641.5416 | 1002.452 | 855.218 | 905.0641 | 977.7803 | 1319.125 | 992.543 | 693.7505 | 657.4366 | 638.2757 | 407.4423 | 703.3407 | 678.2021 | 613.6489 | 671.8789 | 1094.71 | 762.0199 | 1458.041 | 442.2873 | 702.9226 | 793.0472 | 880.2112 | 907.7978 | 638.1084 | 508.6284 | 566.4251 |
| CUST_15430_PI429010886 | N34569 | 0.025406 | 15 | 9 | 505844696 | PREDICTED: tyrosine-protein kinase Fer [Sorex aran | Protein metabolism | Cellular protein modification process | 1.55E-27 | 58.98538 | 58.53699 | 54.32801 | 69.89586 | 49.67787 | 57.05842 | 43.52847 | 45.6007 | 56.58921 | 85.493 | 85.1516 | 125.0459 | 68.47197 | 113.5137 | 100.4575 | 90.33731 | 64.3475 | 78.95217 | 66.52424 | 85.52397 | 55.26178 | 100.2315 | 89.49881 | 51.10924 | 98.92872 | 103.2289 | 135.8973 |
| CUST_51867_PI429010886 | M95462 | 0.025418 | 15 | 12 | | | | | | 22.32739 | 14.07257 | 23.63048 | 12.25145 | 12.80086 | 6.217406 | 9.432782 | 17.44175 | 14.09025 | 24.17334 | 23.83451 | 19.5463 | 13.65247 | 21.71424 | 21.31201 | 17.47076 | 13.20796 | 22.68223 | 6.004347 | 10.5119 | 12.49156 | 24.09915 | 17.07599 | 10.59324 | 16.5492 | 7.862605 | 13.58569 |
| CUST_5830_PI429010886 | N19258 | 0.025418 | 15 | 3 | 321460188 | hypothetical protein DAPPUDRAFT_309150 [Daphn | Metabolic process | Other | 1.1E-148 | 186.8185 | 191.145 | 169.6552 | 183.5489 | 256.9474 | 243.0834 | 196.7297 | 257.3603 | 154.3982 | 180.9607 | 175.4136 | 172.9267 | 168.154 | 140.974 | 151.3078 | 151.7333 | 208.7767 | 184.1431 | 234.8913 | 190.62 | 175.011 | 209.1953 | 150.5497 | 206.4165 | 175.5219 | 209.3879 | 134.2151 |
| CUST_23076_PI429010886 | N48219 | 0.025418 | 15 | 3 | | | | | | 21.28123 | 11.74047 | 12.83786 | 23.35223 | 25.40325 | 23.39071 | 10.48943 | 8.438285 | 12.65182 | 15.16412 | 8.609687 | 17.36312 | 10.32153 | 9.902997 | 10.48943 | 10.9225 | 18.7464 | 30.93394 | 15.34846 | 22.31737 | 18.59683 | 16.73391 | 5.6033 | 14.94458 | 18.88759 | 13.31647 | 9.989923 |
| CUST_26969_PI429010886 | N55200 | 0.025418 | 15 | 12 | | | | | | 12.89985 | 18.53904 | 22.29454 | 8.810922 | 19.34811 | 14.00731 | 11.60477 | 10.64168 | 10.38108 | 24.91507 | 16.66622 | 19.01762 | 19.37009 | 30.69467 | 15.34846 | 30.81693 | 15.557 | 19.29325 | 15.20537 | 27.23758 | 12.89788 | 22.32739 | 12.94894 | 8.802891 | 29.89732 | 15.1109 | 27.50907 |
| CUST_54355_PI429010886 | M105700 | 0.025437 | 21 | 15 | 537242084 | brevican core protein [Cricetulus griseus] | | | 1.82E-08 | 159.1135 | 251.7054 | 145.0813 | 225.2357 | 185.7639 | 85.26543 | 461.7538 | 202.301 | 340.6846 | 355.481 | 247.4599 | 273.4298 | 263.3865 | 413.4726 | 261.6417 | 462.0916 | 342.746 | 283.6942 | 713.8389 | 131.7341 | 368.0062 | 278.469 | 158.2183 | 113.9832 | 286.9075 | 127.112 | 335.8595 |
| CUST_12868_PI429010886 | N30176 | 0.025445 | 21 | 0 | | | | | | 36.69684 | 43.363 | 68.24733 | 27.74158 | 31.2636 | 16.87967 | 32.38846 | 30.33968 | 27.56643 | 29.92416 | 30.15316 | 12.58347 | 33.72458 | 38.36098 | 28.13722 | 45.85649 | 32.80956 | 22.14618 | 22.34365 | 25.04089 | 27.00493 | 45.02522 | 42.78187 | 33.14087 | 33.47284 | 29.40744 | 33.41208 |
| CUST_20164_PI429010886 | N42927 | 0.025447 | 18 | 0 | | | | | | 51.65858 | 38.1318 | 31.78038 | 33.34314 | 28.64267 | 27.085 | 26.73933 | 29.57087 | 12.35967 | 32.45773 | 28.89154 | 21.46638 | 25.33083 | 25.38784 | 37.68617 | 44.38619 | 19.50517 | 38.64362 | 37.27794 | 32.81991 | 25.28525 | 29.78445 | 18.92641 | 39.84647 | 19.72601 | 25.38172 | 20.94079 |
| CUST_34066_PI429010886 | M8647 | 0.025451 | 15 | 9 | 25229108 | chitinase [Litopenaeus vannamei] | Metabolic process | Carbohydrate metabolic process | 3.65E-58 | NA | 95.72441 | 39.61818 | 198.7421 | 272.4885 | 165.5416 | 121.284 | 484.1582 | 349.0566 | 76.02137 | 707.905 | 792.4242 | 154.3359 | 142.3214 | 114.5736 | 4.766047 | 99.25819 | NA | 93.62063 | 686.1452 | 298.7079 | NA | 182.5915 | 98.52867 | NA | 651.6164 | 299.1597 |
| CUST_22344_PI429010886 | N46859 | 0.025454 | 21 | 6 | 339235371 | putative glucosylceramidase [Trichinella spiralis] >g | Metabolic process | Lipid metabolic process | 1.7E-18 | 3.977236 | 19.17521 | 5.285615 | 1581.192 | 74.3064 | 38.28234 | 50.12098 | 219.5427 | 204.3548 | 37.5742 | 150.4655 | 159.5684 | 101.6113 | 31.01397 | 54.24943 | NA | 60.89252 | NA | 27.39567 | 249.0021 | 61.22888 | 4.798002 | 77.2245 | 39.79718 | NA | 165.0586 | 156.6593 |
| CUST_54077_PI429010886 | M104417 | 0.025462 | 18 | 3 | 157116023 | sulfotransferase (sult) [Aedes aegypti] >gi\|1088766 | | | 1.51E-11 | 10.22437 | 4.311251 | 11.41469 | 14.88541 | 11.82579 | 17.95452 | 8.359315 | 11.63036 | 9.945177 | 7.180488 | 9.774844 | NA | 3.650768 | NA | 3.894163 | 11.21025 | 12.75753 | 8.824107 | 10.65599 | 7.621463 | 6.18554 | 8.400369 | NA | NA | 11.10317 | 6.503335 | 4.632569 |
| CUST_8807_PI429010886 | N23621 | 0.025517 | 18 | 15 | 321468540 | hypothetical protein DAPPUDRAFT_304428 [Daphn | | | 7.04E-34 | 23.63855 | 23.73387 | 17.93968 | 18.4928 | 17.04303 | 9.591065 | 14.07874 | 19.6064 | 16.75298 | 28.7982 | 14.07257 | 13.65114 | 16.24662 | 15.81096 | 15.97633 | 26.54031 | 22.58851 | 26.15676 | 17.27797 | 33.76678 | 17.38798 | 19.92242 | 14.59396 | NA | 16.01505 | 16.42728 | 17.23467 |
| CUST_31983_PI429010886 | M2907 | 0.025521 | 15 | 0 | | | | | | 339.6011 | 317.1399 | 307.5089 | 280.0685 | 271.6132 | 290.1265 | 298.7909 | 266.0394 | 239.2718 | 241.7291 | 262.4225 | 235.9573 | 250.3151 | 302.1561 | 226.2023 | 384.9318 | 326.2957 | 286.8668 | 375.5434 | 271.6624 | 290.175 | 357.356 | 234.823 | 236.6906 | 405.6393 | 242.3388 | 236.9951 |
| CUST_49321_PI429010886 | M85184 | 0.025528 | 12 | 0 | 546675121 | hypothetical protein D910_03776 [Dendroctonus p | | | 1.26E-09 | 132.8034 | 193.3347 | 240.5344 | 120.9219 | 139.3127 | 91.21234 | 115.7448 | 123.6533 | 134.8934 | 252.0883 | 150.7034 | 204.8478 | 177.8158 | 250.3612 | 194.9477 | 194.777 | 150.914 | 136.2681 | 136.1838 | 202.4041 | 165.4521 | 221.0933 | 214.1332 | 137.8094 | 168.6291 | 227.4117 | 258.1697 |
| CUST_56794_PI429010886 | M114663 | 0.025536 | 12 | 0 | | | | | | 106.4186 | 96.80182 | 114.0935 | 92.42083 | 94.87745 | 134.8111 | 91.55434 | 88.85357 | 86.3602 | 100.2834 | 87.60236 | 178.5686 | 94.17604 | 125.9794 | 155.8809 | 102.6532 | 95.31428 | 111.1235 | 78.97339 | 96.42858 | 111.5216 | 111.7487 | 117.5843 | 107.8324 | 131.5284 | 133.1553 | 129.8789 |
| CUST_44899_PI429010886 | M67352 | 0.025536 | 12 | 6 | 330976894 | ubiquitin-conjugating enzyme E2 b [Eriocheir sinens | | | 2.33E-08 | 31.35346 | 25.58065 | 16.85432 | 36.93108 | 48.53244 | 23.86269 | 40.42007 | 45.74288 | 47.14504 | 34.23531 | 36.44258 | 27.93228 | 41.81244 | 30.80438 | 24.90512 | 37.0569 | 18.35726 | 32.56779 | 44.42212 | 45.55959 | 35.36774 | 25.21391 | 33.2742 | 16.74068 | 28.80255 | 25.44022 | 28.61965 |
| CUST_5383_PI429010886 | N18622 | 0.025536 | 12 | 0 | 443727713 | hypothetical protein CAPTEDRAFT_220586 [Capitell | | | 1.19E-84 | 72.91578 | 60.60207 | 55.58682 | 69.23488 | 59.18762 | 61.60388 | 52.69412 | 55.03461 | 58.7985 | 53.99181 | 58.27728 | 78.84468 | 65.45683 | 92.24128 | 70.56572 | 62.506 | 56.00276 | 55.57729 | 60.40681 | 59.6812 | 42.9717 | 74.35805 | 87.09496 | 72.84986 | 75.2095 | 81.45472 | 69.29923 |
| CUST_33239_PI429010886 | M6370 | 0.025544 | 15 | 12 | 260815341 | hypothetical protein BRAFLDRAFT_56774 [Branchio | | | 2.19E-24 | 1563.475 | 1857.991 | 1751.759 | 1372.611 | 1261.977 | 1284.605 | 1056.762 | 1114.056 | 1362.62 | 1735.328 | 1378.873 | 2235.234 | 1504.155 | 1911.091 | 2049.694 | 2302.386 | 1335.109 | 1567.155 | 1487.872 | 1751.202 | 1521.376 | 1860.994 | 1469.038 | 1371.865 | 2070.325 | 1575.017 | 2140.816 |
| CUST_20482_PI429010886 | N43537 | 0.025554 | 18 | 6 | 546684670 | hypothetical protein D910_11568 [Dendroctonus p | Protein metabolism | Proteolysis | 4.89E-38 | 34.17683 | 41.73826 | 38.95593 | 35.5525 | 36.80555 | 16.31556 | 50.68803 | 29.49638 | 60.21125 | 42.00978 | 42.46725 | 42.99343 | 36.91363 | 41.06137 | 24.59251 | 30.64068 | 17.47239 | 21.27347 | 58.4208 | 26.15331 | 31.24487 | 47.79729 | 34.35393 | 27.0727 | 37.53618 | 40.81311 | 63.43062 |
| CUST_25213_PI429010886 | N51985 | 0.025558 | 12 | 0 | | | | | | 24.87004 | NA | 32.21416 | 37.93755 | 12.69552 | 25.20056 | NA | 5.230054 | 14.43375 | 11.38323 | 8.168831 | 20.39383 | 15.46051 | 25.53642 | 11.17232 | 21.7647 | NA | 8.359315 | 9.439346 | 19.58619 | 6.016058 | 16.83375 | 23.24949 | 13.87032 | 50.15308 | 27.42777 | 23.14173 |
| CUST_55961_PI429010886 | M112331 | 0.025572 | 18 | 3 | 242020610 | protein kinase C and casein kinase substrate in neu | Metabolic process | Other | 2.64E-07 | 592.481 | 393.933 | 957.2333 | 2400.961 | 1134.542 | 1500.167 | 1553.02 | 947.8741 | 56.23475 | 31.77739 | 1292.668 | 15.09377 | 9.326745 | 19.18935 | 538.1956 | 5.931156 | 60.84139 | 21.48139 | 721.5575 | 4.961812 | 995.7921 | 11.14291 | 1497.212 | 1278.963 | 4.353703 | NA | 15.22654 |
| CUST_38399_PI429010886 | M26241 | 0.025593 | 24 | 21 | | | | | | 208.1801 | 214.5527 | 253.3806 | 247.9972 | 209.2252 | 199.8275 | 200.0776 | 239.3938 | 199.6316 | 209.0603 | 199.4521 | 222.578 | 225.3313 | 234.7465 | 237.3209 | 276.6492 | 225.6208 | 272.3366 | 221.425 | 176.1772 | 207.5948 | 261.0789 | 267.0284 | 308.0795 | 243.6339 | 250.1572 | 233.5227 |
| CUST_22789_PI429010886 | N47674 | 0.025598 | 18 | 3 | | | | | | 14.09213 | 11.21201 | 12.4383 | 17.1086 | 8.697209 | 21.27912 | 15.7789 | 8.256397 | 9.187257 | 6.037926 | 6.050323 | NA | 4.625348 | NA | 26.77723 | NA | 8.322322 | NA | 4.836773 | 9.452942 | 13.16726 | 9.768636 | 17.34013 | 14.18973 | 7.398161 | 6.276753 | 6.765406 |
| CUST_28379_PI429010886 | N57838 | 0.025612 | 24 | 9 | | | | | | 17.96838 | 10.41181 | 4.228707 | 21.18631 | 10.08948 | NA | NA | 18.20852 | 16.61891 | NA | 22.78329 | 24.5556 | 21.30243 | 12.31007 | 16.20611 | 14.70767 | 18.03617 | NA | 6.268183 | 23.5201 | 16.33477 | 8.613049 | 14.70218 | NA | 9.15807 | 13.14489 | 11.98424 |
| CUST_13666_PI429010886 | N31554 | 0.025632 | 24 | 18 | | | | | | 54.45016 | 39.07685 | 58.58633 | 43.5699 | 42.34931 | 44.83858 | 43.45754 | 45.2026 | 40.09922 | 65.76284 | 45.03591 | 49.94515 | 74.92071 | 54.09438 | 52.43827 | 51.43059 | 58.64138 | 51.59241 | 49.5373 | 53.26328 | 64.45055 | 88.5487 | 43.93404 | 41.5428 | 46.27475 | 46.54214 | 61.05832 |
| CUST_56056_PI429010886 | M112642 | 0.025661 | 15 | 12 | 321477208 | hypothetical protein DAPPUDRAFT_311768 [Daphn | Transport | Ion transport | 6.63E-24 | 214.8824 | 246.4302 | 311.1732 | 188.3767 | 158.8254 | 173.7422 | 200.4301 | 277.8488 | 270.3119 | 323.3503 | 214.4744 | 206.2372 | 224.7802 | 375.1955 | 240.6035 | 241.1795 | 184.2307 | 188.8504 | 209.7275 | 196.8321 | 186.2582 | 239.571 | 284.4719 | 172.3811 | 264.9674 | 180.3864 | 315.7204 |
| CUST_9040_PI429010886 | N23975 | 0.025661 | 15 | 12 | | | | | | 135.8061 | 147.6293 | 183.0597 | 97.99145 | 66.71625 | 42.7734 | 111.8739 | 205.0447 | 158.496 | 219.7388 | 100.9197 | 120.6692 | 163.6601 | 188.427 | 118.3504 | 147.1696 | 105.711 | 92.50008 | 116.73 | 100.1395 | 106.4846 | 174.4075 | 132.4207 | 71.04758 | 206.5724 | 105.1255 | 134.4036 |
| CUST_24760_PI429010886 | N51210 | 0.025684 | 21 | 18 | | | | | | 27.97666 | 20.75304 | 48.10487 | 25.76975 | 25.11853 | 24.84105 | 28.73136 | 12.77138 | 20.36175 | 32.28075 | 38.9709 | 38.81815 | 17.16495 | 31.13983 | 15.16642 | 34.16228 | 36.31145 | 18.45614 | 43.31294 | 38.34742 | 48.25178 | 42.44777 | 41.18849 | 29.12035 | 54.26471 | 22.1684 | 22.97006 |
| CUST_30050_PI429010886 | N61085 | 0.025684 | 21 | 0 | | | | | | 21.21967 | 17.70164 | 23.06016 | 22.38584 | 14.44097 | 17.03915 | 18.34116 | 14.75247 | 16.45644 | 17.61362 | 8.912458 | 7.560847 | 8.642479 | 24.79562 | 16.67559 | 14.30414 | 14.44266 | 24.42075 | 25.21391 | 14.9294 | 14.37562 | 16.89118 | 45.54848 | 29.30133 | 15.92609 | 13.16888 | 42.29018 |
| CUST_21340_PI429010886 | N45039 | 0.025685 | 18 | 0 | | | | | | 15.60562 | 20.76696 | 19.50089 | 16.03098 | 14.8732 | 18.65856 | 16.31556 | 10.27676 | 5.652126 | 21.00749 | 6.714373 | 9.591065 | 14.47174 | 11.61617 | 10.72419 | 21.49011 | 13.74631 | 12.14923 | 23.34144 | 16.27254 | 21.75697 | 11.8505 | 14.68799 | 11.09239 | 18.99317 | 13.01381 | 17.06586 |
| CUST_11842_PI429010886 | N28502 | 0.02569 | 15 | 3 | | | | | | 27.24907 | NA | 39.47357 | 138.2766 | 192.4758 | 254.9115 | 218.3262 | 112.5941 | 13.64415 | NA | 146.622 | NA | 31.34507 | 3.770056 | 14.5219 | 3.792944 | 253.1583 | 215.8293 | 194.0018 | NA | 283.5032 | 9.399234 | 228.3459 | 205.2921 | 4.501566 | 121.5472 | 9.60066 |
| CUST_41343_PI429010886 | M57502 | 0.025699 | 21 | 12 | | | | | | 5.685055 | 10.00617 | 5.199836 | NA | 7.279581 | NA | 4.894593 | 3.461391 | 4.785934 | 8.172783 | 5.72681 | 6.596635 | 37.73674 | NA | 38.62431 | 10.6935 | 4.69527 | NA | 12.72475 | 13.3617 | 8.416999 | 3.755326 | NA | 8.492959 | 7.92158 | 3.872507 | 11.85185 |
| CUST_48764_PI429010886 | M82085 | 0.025712 | 15 | 3 | 345496578 | PREDICTED: hypothetical protein LOC100678104 [N | | | 2.14E-16 | 10.65167 | 9.108247 | 21.14283 | 13.46425 | 9.56672 | 15.82858 | 11.22575 | 10.51343 | 10.09605 | 10.18199 | 8.512073 | 6.969672 | 9.375364 | 8.230173 | 11.74368 | 4.322357 | 19.77641 | 12.84068 | 17.17879 | 11.50923 | 15.97144 | 8.726312 | 8.601817 | 14.60539 | 10.45663 | 9.683196 | 7.179499 |
| CUST_1638_PI429010886 | N10306 | 0.025712 | 15 | 0 | | | | | | 227.3449 | 212.0939 | 177.0624 | 145.0813 | 197.9679 | 171.2801 | 111.1358 | 81.72278 | 86.31209 | 125.183 | 256.0552 | 166.9686 | 77.14278 | 134.8934 | 91.43481 | 244.3099 | 195.2684 | 198.9264 | 151.0368 | 235.3955 | 144.2537 | 222.5361 | 70.46616 | 83.31796 | 215.2456 | 91.52825 | 167.9037 |
| CUST_17992_PI429010886 | N38962 | 0.025712 | 15 | 9 | | | | | | 37.59821 | 44.91149 | 57.30764 | 34.49959 | 36.32136 | 33.16699 | 36.53489 | 38.06259 | 43.93802 | 74.81863 | 37.60789 | 71.2683 | 35.7636 | 65.84868 | 57.97363 | 65.43441 | 44.95044 | 48.17521 | 39.15692 | 53.02941 | 40.21556 | 55.83606 | 55.58103 | 45.54446 | 50.06107 | 51.09668 | 60.21705 |
| CUST_30512_PI429010886 | N62035 | 0.025722 | 21 | 0 | | | | | | 39.10739 | 38.07238 | 31.65825 | 37.70021 | 26.34227 | 43.00832 | 28.9272 | 29.44199 | 30.31727 | 27.08074 | 28.65357 | 30.24809 | 25.66555 | 37.26268 | 33.15216 | 27.20765 | 18.67864 | 28.62243 | 29.40744 | 28.98746 | 42.07479 | 30.76819 | 40.22531 | 32.95281 | 30.68445 | 23.75855 | 36.8491 |
| CUST_46980_PI429010886 | M74384 | 0.025735 | 15 | 12 | | | | | | 114.5149 | 88.00476 | 134.5033 | 113.1518 | 106.0522 | 108.9284 | 97.79572 | 86.57696 | 107.3899 | 119.3258 | 111.8858 | 114.8344 | 111.485 | 127.0219 | 134.6193 | 153.6882 | 117.7023 | 122.7034 | 111.812 | 122.8712 | 98.04327 | 132.7602 | 103.0243 | 91.48208 | 121.8439 | 116.6217 | 123.9078 |
| CUST_13104_PI429010886 | N30585 | 0.02574 | 21 | 0 | 321462933 | hypothetical protein DAPPUDRAFT_200344 [Daphn | Protein metabolism | Ribosome biogenesis | 2.64E-24 | 120.2505 | 113.1785 | 129.5889 | 73.1185 | 97.68675 | 100.955 | 85.65603 | 73.93015 | 90.31563 | 104.0557 | 86.97645 | 126.1607 | 82.84904 | 98.59007 | 112.9777 | 163.162 | 92.9186 | 185.5201 | 105.1778 | 103.7009 | 103.4079 | 123.9578 | 109.6371 | 104.5579 | 112.4311 | 98.62916 | 143.069 |
| CUST_19159_PI429010886 | N41074 | 0.025785 | 24 | 18 | 443696351 | hypothetical protein CAPTEDRAFT_181403 [Capitell | | | 2.48E-07 | 6.724594 | 13.32448 | 7.099269 | NA | 8.284158 | NA | 6.488863 | 5.036968 | 5.398604 | 6.502459 | 8.322322 | 7.029716 | 11.27882 | 12.50732 | 12.81434 | 16.9925 | 4.068602 | 17.24085 | 7.967454 | 21.46638 | 15.15404 | 5.890122 | 11.17512 | 8.004105 | 22.10871 | 7.009314 | 12.03892 |
| CUST_41727_PI429010886 | M58401 | 0.025791 | 12 | 9 | | | | | | 15.12966 | 17.47454 | NA | 10.94929 | 18.64838 | 11.72847 | 18.48046 | 13.17601 | 5.013897 | 29.97999 | 20.10533 | 32.46954 | 16.42376 | 8.813762 | 10.78323 | 9.243743 | 14.43577 | 16.1321 | 17.91813 | 20.67135 | 30.65659 | 28.02151 | 20.85646 | 10.95448 | 14.13431 | 10.91945 | 7.944424 |
| CUST_45421_PI429010886 | M69050 | 0.025797 | 21 | 12 | | | | | | 268.82 | 122.7515 | 102.5106 | 169.9973 | 168.2432 | 88.01513 | 285.5481 | 253.5864 | 299.6232 | 247.1436 | 117.2337 | 200.0776 | 216.2628 | 537.1757 | 318.0852 | 245.1154 | 133.9706 | 191.2063 | 73.52413 | 512.8422 | 327.2015 | 192.6463 | 170.1249 | 184.7859 | 163.7139 | 95.7753 | 134.4594 |
| CUST_56500_PI429010886 | M113946 | 0.02582 | 12 | 0 | | | | | | 637.7954 | 828.3608 | 822.5821 | 766.6399 | 644.1161 | 836.4238 | 557.1607 | 608.0744 | 686.6887 | 724.6164 | 593.331 | 858.7607 | 627.6184 | 885.3464 | 832.0351 | 824.9455 | 568.8782 | 808.9549 | 553.2164 | 709.528 | 796.2928 | 693.2829 | 728.0084 | 650.2354 | 834.3363 | 943.689 | 954.6687 |
| CUST_31544_PI429010886 | M1796 | 0.02582 | 12 | 0 | 156544361 | PREDICTED: putative methyltransferase NSUN4-like | | | 7.35E-79 | 31.46674 | 44.11518 | 35.91607 | 24.03557 | 23.99379 | 28.36368 | 24.12979 | 19.24611 | 27.42777 | 36.31145 | 26.92776 | 27.47752 | 23.17075 | 41.89757 | 35.01718 | 30.68045 | 23.06645 | 23.94925 | 11.74757 | 20.35771 | 24.2252 | 29.47907 | 30.89566 | 29.36585 | 24.64992 | 20.98763 | 36.66423 |
| CUST_32271_PI429010886 | M3680 | 0.02582 | 12 | 0 | | | | | | 367.921 | 429.9553 | 465.1151 | 424.2638 | 394.3244 | 363.6175 | 324.2442 | 320.693 | 386.8992 | 524.7212 | 375.0345 | 464.8665 | 346.3085 | 541.9679 | 412.3362 | 424.7309 | 376.1162 | 360.5617 | 366.8876 | 320.1726 | 384.4603 | 469.6397 | 431.842 | 342.6916 | 602.6317 | 429.7605 | 606.3721 |
| CUST_42091_PI429010886 | M59374 | 0.02582 | 12 | 0 | | | | | | 13.80995 | 7.20194 | 18.77767 | 11.06582 | 11.03659 | 10.23633 | 6.064337 | 12.8856 | 5.519534 | 14.00339 | 9.891852 | 14.1603 | 9.48088 | 11.73913 | 20.97642 | 17.85063 | 8.472888 | 17.78016 | 7.476065 | 11.02488 | 6.648806 | 13.51693 | 11.14181 | 15.1983 | 13.53466 | 19.45678 | 19.3208 |
| CUST_42602_PI429010886 | M60691 | 0.02582 | 12 | 0 | | | | | | 1042.392 | 2166.108 | 1874.82 | 1397.451 | 1726.714 | 1798.294 | 1405.495 | 1524.206 | 1542.262 | 1687.609 | 1515.268 | 1917.136 | 1601.013 | 2549.933 | 2109.682 | 1785.596 | 1281.734 | 1673.62 | 1530.336 | 1558.572 | 1389.409 | 2172.159 | 1621.23 | 1667.846 | 2296.235 | 1660.585 | 2454.105 |
| CUST_33824_PI429010886 | M7962 | 0.02582 | 12 | 0 | | | | | | 235.0098 | 219.9262 | 256.1116 | 292.4718 | 216.9356 | 226.151 | 153.4856 | 184.9585 | 209.7628 | 229.7926 | 212.2832 | 295.5186 | 214.361 | 342.0536 | 253.1078 | 294.596 | 175.4895 | 250.8554 | 225.1487 | 216.5751 | 166.2291 | 294.6436 | 204.7153 | 210.3141 | 300.4818 | 231.0331 | 227.3449 |
| CUST_9090_PI429010886 | N24050 | 0.02582 | 12 | 0 | 4996073 | U86 [Human herpesvirus 6B] | | | 8.94E-07 | 62.96663 | 77.90078 | 57.88958 | 63.04603 | 65.42078 | 53.52565 | 51.00626 | 43.87581 | 60.58205 | 66.82399 | 46.13849 | 73.33005 | 73.70231 | 73.38298 | 64.55964 | 82.55028 | 47.42461 | 68.22048 | 41.00395 | 66.4223 | 57.39031 | 68.08199 | 58.25188 | 59.07909 | 83.32632 | 62.4936 | 85.05317 |
| CUST_12669_PI429010886 | N29844 | 0.025821 | 12 | 6 | 321461967 | hypothetical protein DAPPUDRAFT_308028 [Daphn | Metabolic process | Generation of precursor metabolites and energy | 5.9E-46 | 2055.192 | 2457.891 | 2545.683 | 1409.58 | 2300.758 | 1903.519 | 2740.182 | 2556.905 | 2243.877 | 1896.247 | 2388.967 | 3082.77 | 2117.402 | 1577.266 | 1967.236 | 1986.907 | 2387.546 | 1979.892 | 2779.017 | 3618.732 | 2478.692 | 2106.309 | 1899.44 | 2147.931 | 1525.514 | 1751.759 | 1934.877 |
| CUST_31061_PI429010886 | M593 | 0.025824 | 18 | 15 | | | | | | 615.9204 | 751.2737 | 638.9314 | 818.6448 | 873.2696 | 989.1564 | 715.6525 | 607.7896 | 567.608 | 627.0644 | 796.5277 | 687.2662 | 576.6597 | 786.4352 | 758.8197 | 968.7311 | 1008.511 | 881.5993 | 896.8013 | 1000.153 | 654.7049 | 726.086 | 686.5061 | 749.2477 | 686.3511 | 659.4054 | 663.9332 |
| CUST_50615_PI429010886 | M90438 | 0.025824 | 18 | 15 | | | | | | 121.6208 | 135.78 | 136.023 | 124.5589 | 111.7362 | 136.3426 | 134.4388 | 131.1966 | 130.8664 | 160.9512 | 130.357 | 145.8124 | 142.4549 | 161.7779 | 120.1261 | 174.9558 | 145.5389 | 179.5033 | 140.7358 | 134.6919 | 134.1962 | 98.47983 | 128.1595 | 177.456 | 136.2319 | 137.9332 | 138.9365 |
| CUST_34274_PI429010886 | M9211 | 0.025824 | 18 | 15 | | | | | | 288.8149 | 424.4597 | 302.3576 | 265.7722 | 352.188 | 375.1189 | 252.0883 | 224.2244 | 296.4747 | 315.5852 | 294.9448 | 319.5171 | 272.1926 | 306.2123 | 394.8691 | 568.6876 | 293.3716 | 481.7893 | 294.6436 | 324.3863 | 312.5243 | 427.1137 | 255.5791 | 325.4382 | 290.3758 | 247.8273 | 292.1061 |
| CUST_55332_PI429010886 | M109596 | 0.025825 | 15 | 12 | | | | | | 135.9242 | 145.1303 | 173.6625 | 144.8631 | 138.0748 | 131.863 | 136.1838 | 164.7416 | 156.0301 | 196.9594 | 143.2947 | 154.542 | 150.3901 | 199.4211 | 182.7865 | 179.616 | 174.7413 | 144.5655 | 141.9197 | 155.0892 | 146.2634 | 195.4236 | 165.2021 | 120.9579 | 201.3287 | 169.6125 | 195.1585 |
| CUST_37979_PI429010886 | M22563 | 0.02583 | 15 | 0 | | | | | | 576.3102 | 540.3776 | 813.9324 | 674.0173 | 443.4781 | 867.1839 | 205.6642 | 297.4174 | 212.9083 | 495.5775 | 554.8262 | 518.3277 | 114.6578 | 583.9827 | 374.607 | 520.2836 | 512.6993 | 726.9909 | 416.025 | 1019.767 | 645.1219 | 513.5475 | 364.475 | 366.1623 | 489.1062 | 610.6415 | 410.1622 |
| CUST_44_PI429010886 | N187 | 0.025833 | 18 | 3 | | | | | | 12.19042 | 9.692914 | 8.00826 | 12.67037 | 15.1999 | 14.8651 | 11.64471 | NA | NA | NA | 6.224633 | 5.311014 | 11.38637 | NA | 8.269849 | 13.12545 | 15.13091 | 12.26155 | 11.0938 | 7.145089 | 8.743791 | 11.42268 | 18.60353 | 23.4652 | 8.044043 | 7.26746 | 4.482949 |
| CUST_26720_PI429010886 | N54762 | 0.025835 | 18 | 12 | | | | | | 12.65789 | 12.26689 | 17.12595 | 11.50042 | 14.83018 | NA | NA | 19.42496 | 56.93106 | 26.4784 | 17.16641 | 30.75644 | 34.11202 | 53.9397 | 37.66305 | 25.71973 | 12.49275 | 18.89688 | 6.874216 | 23.32 | 18.02634 | 25.76451 | 40.20429 | 3.671657 | 40.50125 | 18.04108 | 39.29501 |
| CUST_32707_PI429010886 | M4885 | 0.025853 | 21 | 3 | 321473145 | hypothetical protein DAPPUDRAFT_315291 [Daphn | | | 2.01E-09 | 1468.172 | 534.2439 | 19.40841 | 3046.724 | 632.2521 | 3230.715 | 1541.439 | 679.3339 | 1652.112 | NA | 52.14117 | 3.901966 | 1715.766 | 3.48828 | 1013.924 | 213.0864 | 534.5603 | 782.2398 | 65.59001 | 9.827509 | 81.45472 | 135.1252 | 45.80703 | 1983.417 | NA | 1648.471 | NA |
| CUST_42262_PI429010886 | M59828 | 0.025857 | 21 | 3 | 507645990 | PREDICTED: isoprenoid synthase domain-containing | Metabolic process | Lipid metabolic process | 1.86E-23 | 1616.675 | 4322.611 | 2085.292 | 6505.037 | 2774.636 | 2980.843 | 1748.457 | 1871.332 | 1584.97 | 2500.44 | 1035.582 | 2630.661 | 1045.476 | 2243.877 | 358.225 | 2206.497 | 900.1864 | 1811.1 | 1296.822 | 1801.38 | 2911.974 | 3034.646 | 2583.846 | 840.1524 | 3225.426 | 3582.089 | 1547.246 |
| CUST_17292_PI429010886 | N37723 | 0.025857 | 21 | 3 | | | | | | 108.3037 | 90.60771 | 126.727 | 110.0647 | 121.4237 | 126.539 | 106.4697 | 97.71155 | 121.0641 | 101.3443 | 119.841 | 97.63395 | 101.4644 | 107.1033 | 61.93566 | 94.84242 | 123.4881 | 100.0297 | 112.9639 | 81.29483 | 127.4262 | 111.5705 | 127.5671 | 114.0301 | 131.0829 | 113.1785 | 143.2327 |
| CUST_23798_PI429010886 | N49489 | 0.025869 | 24 | 6 | | | | | | 218.8861 | 126.1607 | NA | 302.3102 | 77.70756 | 136.023 | 328.4337 | 144.9791 | 283.5032 | NA | 10.82851 | NA | 325.6779 | NA | 113.1785 | 40.55137 | 53.48774 | 88.78823 | 32.83309 | 4.183366 | 5.922622 | 12.18164 | 26.72094 | 346.7902 | 5.219351 | 171.427 | NA |
| CUST_47440_PI429010886 | M76131 | 0.025884 | 21 | 0 | 307203790 | Choline/ethanolaminephosphotransferase 1 [Harpe | Metabolic process | Lipid metabolic process | 8.35E-21 | 19.31282 | 19.07608 | 22.28112 | 19.4114 | 11.51207 | 15.32925 | 20.4401 | 11.35064 | 14.06114 | 13.50266 | 15.1414 | 8.937423 | 16.77971 | 11.45279 | 11.93548 | 13.46088 | 14.05923 | 23.59085 | 10.52275 | 14.65658 | 28.66257 | 17.50878 | 21.38676 | 21.75964 | 13.1549 | 13.73069 | 19.82899 |
| CUST_39170_PI429010886 | M35556 | 0.02589 | 24 | 6 | | | | | | 177.5678 | 80.43582 | 10.85726 | 171.2071 | 60.38361 | 99.87407 | 182.3936 | 103.7577 | 162.5301 | 12.11676 | 20.51423 | 17.16495 | 149.6184 | 18.30638 | 57.96208 | 26.8663 | 43.75138 | 67.90618 | 28.24659 | 6.758426 | 7.308005 | 21.33598 | 10.70994 | 166.0384 | 11.21303 | 75.45183 | 39.36991 |
| CUST_55854_PI429010886 | M111893 | 0.025891 | 15 | 12 | | | | | | 1182.845 | 636.3168 | 1498.845 | 184.5783 | 1185.368 | 1365.452 | 961.8209 | 1113.695 | 1246.44 | 1606.601 | 1100.556 | 1623.972 | 1512.912 | 1991.454 | 1915.9 | 1992.587 | 1022.013 | 1241.135 | 1105.691 | 965.2192 | 1342.932 | 988.0968 | 1373.566 | 719.1308 | 1491.426 | 1155.687 | 1958.473 |
| CUST_20584_PI429010886 | N43701 | 0.025891 | 12 | 0 | | | | | | 104.2917 | 139.5841 | 152.0345 | 98.50813 | 115.6759 | 112.2136 | 101.0858 | 113.3918 | 106.6963 | 113.6641 | 110.5556 | 140.6429 | 130.7243 | 140.8976 | 146.585 | 143.1059 | 116.4584 | 119.0242 | 128.1445 | 116.9082 | 139.2832 | 132.2265 | 116.7957 | 113.8579 | 148.3869 | 144.1866 | 154.4215 |
| CUST_25113_PI429010886 | N51806 | 0.025904 | 15 | 3 | 121484216 | cuticle protein CB6 [Portunus pelagicus] | | | 3.08E-16 | 378.7197 | 44.37026 | 453.8761 | 648.7533 | 253.1914 | 1619.494 | 558.5528 | 386.4471 | 185.0177 | 44.34868 | 270.6659 | NA | 236.5291 | 80.34778 | 106.2399 | NA | 867.4252 | 553.2164 | 679.9331 | NA | 495.4416 | 8.164956 | 471.6311 | 801.3257 | 19.39824 | 547.117 | 8.328299 |
| CUST_27926_PI429010886 | N56955 | 0.02591 | 18 | 12 | | | | | | 106.1322 | 139.9664 | 115.6518 | 80.78349 | 105.9691 | 111.2474 | 109.3157 | 142.727 | 106.9318 | 144.6491 | 121.1456 | 111.685 | 110.7482 | 139.5098 | 164.6258 | 133.1717 | 188.4088 | 105.5375 | 116.6057 | 113.3258 | 96.91901 | 136.0755 | 94.89615 | 102.5272 | 114.3038 | 104.0321 | 120.7171 |
| CUST_47861_PI429010886 | M77892 | 0.025917 | 24 | 21 | 242332534 | uncharacterized protein LOC100041979 [Mus musc | | | 5.91E-13 | 12.2683 | 15.5539 | 17.31897 | 16.03532 | 14.9512 | 9.745966 | 6.847004 | 10.27406 | 14.26798 | 18.01173 | 15.23737 | 15.93948 | 17.78423 | 15.63419 | 13.19231 | 15.94807 | 12.71344 | 12.48145 | 15.36816 | 30.08532 | 23.17379 | 18.59923 | 16.44885 | NA | 18.02234 | 21.75179 | 24.28604 |
| CUST_41264_PI429010886 | M57279 | 0.025919 | 21 | 9 | 543364768 | PREDICTED: dehydrogenase/reductase SDR family n | Metabolic process | Oxidation-reduction process | 8.68E-55 | 166.8192 | 446.6411 | 435.7052 | 146.765 | 156.6286 | 112.4193 | 146.3889 | 265.9049 | 301.5058 | 474.1045 | 258.7852 | 414.6092 | 302.4916 | 243.7531 | 345.7017 | 215.4716 | 120.5518 | 203.7035 | 99.45401 | 296.3167 | 105.6505 | 192.4131 | 239.9392 | 178.0175 | 72.40334 | 177.896 | 238.455 |

| Probe ID | ID | p-value | Period | Phase | EBI ID | Annotation | GO category | Go term | E-value | 0 | 0 | 0 | 3 | 3 | 3 | 6 | 6 | 6 | 9 | 9 | 9 | 12 | 12 | 12 | 15 | 15 | 15 | 18 | 18 | 18 | 21 | 21 | 21 | 24 | 24 | 24 |
|---|---|---|---|---|---|---|---|---|---|---|---|---|---|---|---|---|---|---|---|---|---|---|---|---|---|---|---|---|---|---|---|---|---|---|---|---|
| CUST_4720_PI429010886 | N17339 | 0.025919 | 12 | 0 | | | | | | 494.3438 | 556.906 | 541.9679 | 435.1229 | 413.9155 | 428.865 | 375.1955 | 475.874 | 445.1412 | 523.2717 | 457.4348 | 769.0647 | 502.1624 | 717.2947 | 729.346 | 635.6512 | 465.7878 | 487.0643 | 481.1352 | 582.6377 | 536.8972 | 644.6096 | 574.2435 | 463.9991 | 569.2573 | 636.5467 | 661.6789 |
| CUST_55210_PI429010886 | M109129 | 0.025933 | 15 | 0 | | | | | | 13.23043 | 55.5206 | NA | 15.50146 | 9.111062 | 32.42745 | 7.975609 | 6.925886 | 11.94414 | 6.856722 | 9.479243 | 4.773638 | 4.255656 | 8.065131 | 20.35314 | 9.322057 | 10.68951 | 16.38286 | 11.58371 | 6.497327 | 11.40559 | NA | 7.493829 | 29.86056 | 6.456509 | 3.256875 | NA |
| CUST_56884_PI429010886 | M114833 | 0.025947 | 21 | 15 | | | | | | 308.5953 | 379.2779 | 377.4878 | 303.1495 | 294.2777 | 448.5246 | 236.449 | 277.4454 | 293.3227 | 314.5564 | 296.4747 | 439.5513 | 312.9412 | 335.1161 | 334.379 | 453.5801 | 303.1495 | 463.2589 | 364.7093 | 407.8386 | 324.6687 | 400.1681 | 328.2492 | 266.2431 | 375.8398 | 320.4556 | 369.6113 |
| CUST_13008_PI429010886 | N30431 | 0.025961 | 21 | 18 | 517080475 | hypothetical protein [Bradyrhizobium elkanii] | | | 1.65E-45 | 19.12122 | NA | 22.57752 | 7.641633 | 8.937423 | 9.823191 | 32.67642 | 33.37654 | 3.527959 | 10.33897 | 17.64058 | NA | 18.40021 | 27.04395 | 7.463527 | NA | 54.89949 | 10.51492 | 23.71704 | NA | 35.86299 | NA | 30.60709 | 19.56461 | 6.900525 | NA | 5.403396 |
| CUST_50138_PI429010886 | M88791 | 0.025967 | 21 | 18 | | | | | | 22.37068 | 27.17605 | 20.48097 | 17.42826 | 26.30221 | 23.97968 | 20.77886 | 22.19942 | 15.13462 | 14.19367 | 19.57848 | 16.66456 | 24.35938 | 10.92086 | 8.58357 | 18.90163 | 42.1732 | 28.74055 | 30.31987 | 40.80851 | 27.41751 | 25.97958 | 8.633134 | 17.47454 | 26.73374 | 10.64869 | 27.49771 |
| CUST_34826_PI429010886 | M10812 | 0.025976 | 18 | 3 | | | | | | 66.04832 | 69.39137 | 66.4223 | 105.4346 | 78.65203 | 76.49245 | 65.96571 | 69.27267 | 63.90408 | 56.56158 | 48.75681 | 54.17026 | 48.3882 | 84.09478 | 50.28834 | 86.82244 | 75.27932 | 77.55826 | 61.6776 | 56.02742 | 50.33907 | 121.284 | 67.92832 | 69.31912 | 103.5194 | 58.46928 | 69.46903 |
| CUST_51455_PI429010886 | M93607 | 0.025986 | 24 | 15 | | | | | | 247.6825 | 344.2985 | 319.007 | 284.3688 | 288.3422 | 302.6662 | 223.8084 | 273.0127 | 280.6238 | 290.4858 | 238.0845 | 302.0506 | 243.4729 | 356.8925 | 302.7176 | 384.6311 | 343.4753 | 405.2638 | 317.3299 | 303.4935 | 233.9335 | 400.1052 | 341.396 | 270.8273 | 347.9148 | 224.7458 | 315.0245 |
| CUST_49134_PI429010886 | M84288 | 0.025986 | 18 | 12 | | | | | | 4.361188 | 22.07787 | 8.474659 | 13.79507 | 22.49622 | NA | 390.973 | 79.13143 | 55.29319 | 37.06438 | 132.3396 | 10.57913 | 87.15435 | 382.7566 | 282.166 | 49.41092 | NA | NA | 13.74963 | 9.915982 | 225.8046 | 13.78438 | 64.64151 | 77.85613 | 52.97067 | 58.25188 | 70.64122 |
| CUST_10725_PI429010886 | N26689 | 0.026007 | 15 | 3 | 126340701 | PREDICTED: muskelin [Monodelphis domestica] | | | 6.97E-77 | 7.477595 | 16.08188 | 18.33154 | 10.97186 | 10.34912 | 14.51263 | 10.32521 | 15.91598 | 12.76773 | 9.148382 | 10.682 | 8.500792 | 12.53922 | 12.31301 | 16.16705 | 20.35314 | 17.5796 | 7.104442 | NA | 18.74202 | 20.65238 | 9.060793 | 10.7115 | 13.13833 | 10.60724 | 10.03982 | 5.419015 |
| CUST_43432_PI429010886 | M63030 | 0.026014 | 24 | 21 | | | | | | 12.40589 | 11.35432 | 9.830811 | 9.073537 | 14.6546 | 17.65061 | 11.69586 | 3.774436 | 5.917066 | 13.55604 | 5.917836 | 8.877949 | 8.612092 | 12.9382 | 13.56887 | 20.11049 | 9.505528 | 15.65479 | 12.60802 | 10.86137 | 11.57971 | 27.65968 | 14.5269 | 15.71416 | 8.798065 | NA | 9.106984 |
| CUST_47610_PI429010886 | M76864 | 0.026018 | 15 | 0 | | | | | | 147.2771 | 154.1347 | 121.7699 | 132.8611 | 98.41575 | 96.37601 | 87.80183 | 118.1621 | 89.20945 | 118.2155 | 102.4176 | 126.3583 | 111.4706 | 105.5208 | 106.4305 | 168.3796 | 123.2567 | 124.447 | 99.36845 | 122.7974 | 117.3039 | 144.7064 | 109.5408 | 79.14043 | 130.4682 | 96.06297 | 118.3632 |
| CUST_3246_PI429010886 | N14096 | 0.026018 | 15 | 0 | | | | | | 6065.211 | 8627.68 | 6863.631 | 4420.361 | 6059.267 | 8802.545 | 2009.101 | 2205.198 | 2576.961 | 4194.154 | 5585.575 | 7516.748 | 1332.781 | 5717.187 | 4357.297 | 5933.737 | 3991.112 | 7516.748 | 3432.751 | 10190.76 | 6577.842 | 4532.248 | 2984.905 | 3584.321 | 3805.814 | 6818.919 | 5065.56 |
| CUST_8697_PI429010886 | N23437 | 0.02602 | 15 | 0 | | | | | | 565.5557 | 472.997 | 440.6015 | 445.5129 | 463.7968 | 433.4824 | 456.1991 | 312.1828 | 301.7456 | 362.4934 | 433.2734 | 275.2679 | 327.7802 | 360.1121 | 464.198 | 471.7451 | 488.97 | 492.8667 | 530.9675 | 384.4603 | 396.0145 | 545.2224 | 303.0187 | 454.2849 | 468.9522 | 398.3325 | 374.1535 |
| CUST_17090_PI429010886 | N37343 | 0.026041 | 12 | 3 | | | | | | 11.84044 | 4.690123 | 8.73506 | NA | 10.93801 | 11.37412 | 9.188895 | 8.042965 | NA | 4.246572 | 7.131715 | 7.860635 | 6.02125 | 16.84886 | 6.522752 | 12.50339 | 17.39906 | 12.59547 | 11.95869 | 9.279943 | 9.388689 | 6.71361 | NA | 6.525733 | 13.93595 | 24.82512 | 5.841607 |
| CUST_18352_PI429010886 | N39634 | 0.026053 | 15 | 12 | | | | | | 363.7161 | 326.9766 | 511.8133 | 331.6154 | 425.2074 | 302.6146 | 295.1449 | 321.3108 | 244.0376 | 459.0733 | 376.1162 | 568.5491 | 302.0506 | 589.4448 | 471.0971 | 565.8837 | 338.1543 | 493.4379 | 434.7605 | 397.8439 | 394.7994 | 347.8665 | 363.0918 | 298.1495 | 493.7491 | 344.0991 | 540.6649 |
| CUST_25387_PI429010886 | N52329 | 0.026053 | 15 | 12 | | | | | | 24.52679 | 19.41782 | 27.49771 | 19.85636 | 19.08808 | 24.99293 | 19.62227 | 10.42653 | 26.44706 | 33.76983 | 21.37183 | 21.63352 | 27.39567 | 32.10959 | 40.85856 | 34.22484 | 21.39591 | 27.56643 | 18.19015 | 13.26879 | 13.97389 | 24.4797 | 33.37295 | 26.47171 | 16.64136 | 18.7509 | 25.70354 |
| CUST_30326_PI429010886 | N61640 | 0.026053 | 15 | 12 | | | | | | 230.4843 | 209.1953 | 322.671 | 172.9097 | 202.1435 | 232.8026 | 227.8652 | 282.166 | 268.6688 | 213.275 | 252.6253 | 308.7172 | 286.6682 | 362.3529 | 361.1547 | 318.8664 | 266.7622 | 281.599 | 231.3759 | 328.9809 | 180.6083 | 302.2127 | 253.8057 | 188.427 | 396.7445 | 238.7409 | 370.4877 |
| CUST_20127_PI429010886 | N42855 | 0.026056 | 18 | 15 | 405951390 | Kynureninase [Crassostrea gigas] | | | 6.25E-08 | 32.77063 | 24.54149 | 15.06711 | 27.30432 | 22.6112 | 10.48086 | 13.98699 | 11.98424 | 9.416182 | 26.46256 | 8.763789 | 17.83632 | 25.87828 | 16.80263 | 16.32267 | 50.50708 | NA | 18.10904 | 16.93603 | 15.99975 | 19.73096 | 37.22354 | NA | 29.65544 | 14.1362 | 8.718884 | 10.03383 |
| CUST_34686_PI429010886 | M10340 | 0.026061 | 24 | 6 | | | Metabolic process | Carbohydrate metabolic process | | 962.2957 | 4001.148 | 1827.911 | 2044.563 | 905.0641 | 775.2325 | 1161.62 | 2365.487 | 1026.418 | 661.6789 | 895.8021 | 1077.802 | 710.4011 | 669.2807 | 698.8251 | 497.3582 | 221.2065 | 302.2555 | 70.69633 | 1682.988 | 466.7074 | 107.4484 | 2136.057 | 649.3217 | 29.05729 | 810.0074 | 473.8216 |
| CUST_36045_PI429010886 | M14427 | 0.026081 | 21 | 15 | | | | | | 13.79996 | 12.44135 | 16.46586 | 34.89593 | 11.47955 | 11.77319 | 7.151549 | 7.751416 | 5.76332 | 13.12407 | 8.668476 | 13.33386 | 13.55355 | 7.690783 | 14.63109 | 14.69551 | 12.50951 | 46.15285 | 11.49641 | 18.40328 | 12.43735 | 18.71748 | 14.11161 | 8.402814 | 18.30885 | 9.665421 | 9.498899 |
| CUST_16163_PI429010886 | N35767 | 0.02609 | 24 | 21 | 380024475 | PREDICTED: rho-related BTB domain-containing pro | | | 2.78E-25 | 38.18832 | 39.79339 | 53.15786 | 37.61954 | 36.26174 | 29.11768 | 37.34183 | 29.85387 | 30.77101 | 37.53024 | 42.9046 | 36.41953 | 27.52267 | 48.90708 | 35.89648 | 38.35721 | 42.24487 | 44.75473 | 36.57353 | 42.34616 | 50.14442 | 56.34897 | 38.91585 | 38.85028 | 36.69997 | 48.21189 | 44.28544 |
| CUST_38825_PI429010886 | M30981 | 0.026122 | 12 | 3 | | | | | | 15872.37 | 6571.189 | 5528.982 | 27975.24 | 21011.69 | 45084.56 | 26858.78 | 7717.52 | 10819.14 | 370.4089 | 15369.79 | 179.1099 | 11289.07 | 4083.732 | 9855.517 | 11122.77 | 27577.88 | 33985.8 | 16676.56 | 1404.631 | 17865.21 | 9287.843 | 15743.72 | 33268.08 | 1703.869 | 17900.98 | 137.2146 |
| CUST_1796_PI429010886 | N10727 | 0.026122 | 12 | 3 | 187764371 | spatzle protein [Fenneropenaeus chinensis] | | | 6.2E-87 | 4274.83 | 1811.1 | 1186.311 | 16607.59 | 9305.385 | 20208.21 | 8188.092 | 4797.809 | 1452.711 | 253.8408 | 10649.47 | 249.8541 | 3062.827 | 517.8895 | 2104.851 | 1067.598 | 17482.08 | 14295.94 | 7479.639 | 505.3693 | 11143.54 | 1045.788 | 7950.432 | 10451.76 | 334.5946 | 6116.034 | 146.8141 |
| CUST_38075_PI429010886 | M23222 | 0.026143 | 18 | 0 | 321455420 | hypothetical protein DAPPUDRAFT_218978 [Daphn | Metabolic process | Carbohydrate metabolic process | 6.7E-81 | 125.4528 | 112.3919 | 156.1886 | 98.23281 | 42.30429 | 61.19321 | 17.02832 | 83.58732 | 24.11784 | 56.91101 | 43.75577 | 50.49286 | NA | 80.96373 | 36.39578 | 68.00621 | 48.87456 | 28.67208 | 59.71534 | 136.0526 | 102.1576 | 122.5319 | 40.10273 | 49.29932 | 30.18791 | 121.0216 | 73.76805 |
| CUST_36991_PI429010886 | M17476 | 0.026146 | 18 | 9 | | | Transport | Lipid transport | | 211.0494 | 300.7234 | 202.8118 | 178.8821 | 269.0643 | 160.7094 | 268.6359 | 272.8585 | 241.2255 | 290.5429 | 338.5409 | 241.3085 | 253.9339 | 270.9139 | 283.3904 | 338.9665 | 249.9907 | 218.5128 | 264.5866 | 380.8862 | 181.4625 | 225.8046 | 215.5744 | 149.974 | 278.3606 | 204.7153 | 304.437 |
| CUST_30466_PI429010886 | N61953 | 0.026146 | 18 | 15 | 170032728 | smad4 [Culex quinquefasciatus] >gi\|167873062\|gb | Signal transduction | Other | 2.05E-12 | 19.18101 | 19.20545 | 17.91398 | 15.36071 | 17.64932 | 7.65571 | 10.92713 | 27.9397 | 19.08147 | 145.9962 | 18.45819 | 20.82009 | 19.05569 | 23.32541 | 15.91213 | 24.68274 | 50.46214 | 21.54483 | 25.60312 | 15.74407 | 26.60013 | 11.80273 | 23.86861 | 11.55948 | 16.97989 | 15.3003 | 29.62034 |
| CUST_39425_PI429010886 | M39203 | 0.026157 | 21 | 12 | 16751924 | Euphausia superba large subunit ribosomal RNA ger | | | 4.95E-84 | 34.01746 | 960.3576 | 78.66048 | 774.7379 | 1233.738 | 153.1717 | 478.074 | 4847.521 | 3225.426 | 511.1819 | 4068.874 | 5717.187 | 8470.008 | 325.4847 | 5641.729 | 27.9866 | 2028.657 | 13.43047 | 70.70475 | 37163.2 | 1578.633 | 22.71614 | 318.8167 | 316.2685 | 28.23315 | 1701.136 | 1891.601 |
| CUST_54787_PI429010886 | M107681 | 0.026184 | 18 | 12 | | | | | | 18.48661 | 26.73933 | 30.53983 | 20.4513 | 20.33392 | 23.31758 | 16.82798 | 37.83723 | 30.83942 | 46.19529 | 21.3795 | 19.48765 | 32.70344 | 39.93998 | 38.88459 | 24.87004 | 36.92111 | 28.10538 | 31.92608 | 28.95462 | 32.858 | 26.32207 | 28.47733 | 13.33083 | 27.58634 | 24.56057 | 41.14845 |
| CUST_28055_PI429010886 | N57188 | 0.026185 | 21 | 12 | | | | | | 50.67859 | 66.938 | 12.5724 | 84.2989 | 7.112561 | 9.202277 | 19.10728 | 18.95062 | 14.1264 | 13.28314 | 14.98994 | 16.60048 | 39.38447 | 30.74148 | 44.7891 | 32.7645 | 16.23932 | 22.74317 | 14.69878 | 21.33841 | 17.65481 | 17.19687 | 27.9397 | 20.78572 | 9.709104 | 11.91576 | 11.79192 |
| CUST_24540_PI429010886 | N50806 | 0.026188 | 21 | 12 | | | | | | 114.1048 | 77.95158 | 111.5216 | 76.71565 | 95.02448 | 96.8234 | 93.66147 | 77.76831 | 75.32357 | 100.8857 | 92.61954 | 95.57811 | 118.0698 | 107.1617 | 115.5444 | 97.1202 | 115.1182 | 112.1069 | 89.59424 | 95.25723 | 98.94163 | 103.654 | 121.4562 | 101.2537 | 132.2831 | 87.13975 | 97.80592 |
| CUST_44146_PI429010886 | M65063 | 0.026196 | 15 | 3 | 391327707 | PREDICTED: uncharacterized protein LOC10090595( | | | 4.5E-23 | 105.3949 | 283.2818 | NA | 417.2965 | 179.1629 | 82.3571 | 318.2412 | 10.9956 | 162.5022 | 7.643809 | 3.790237 | NA | 69.24723 | 13.17601 | 64.52054 | 207.6805 | 13.61539 | 124.4126 | 14.13803 | NA | NA | 86.36905 | NA | 39.70495 | 35.7959 | NA | 11.21025 |
| CUST_47004_PI429010886 | M74467 | 0.026219 | 24 | 0 | | | | | | 30.17169 | 45.59444 | 34.91643 | 27.48106 | 27.79102 | 41.12787 | 43.94258 | 28.69117 | 33.24975 | 29.30133 | 32.73752 | 46.13849 | 23.44259 | 26.89938 | 24.95301 | 33.40072 | 28.34245 | 25.28773 | 31.39582 | 30.87561 | 33.66515 | 46.25868 | 23.78915 | 25.91676 | 51.51049 | 33.87876 | 37.60789 |
| CUST_1748_PI429010886 | N10594 | 0.026239 | 18 | 3 | 321470924 | hypothetical protein DAPPUDRAFT_302853 [Daphn | | | 7.71E-12 | 9.775801 | 19.86545 | 12.51404 | 28.74055 | 24.20666 | 15.44569 | 15.32781 | 15.92609 | 12.73533 | 6.824426 | 16.32095 | 14.95825 | 11.12554 | 12.97798 | 11.19433 | 11.37559 | 30.53018 | 10.42653 | 26.18577 | 22.15217 | 15.37012 | 15.37345 | 25.90815 | 25.42895 | 15.88827 | 26.13131 | 14.2617 |
| CUST_32529_PI429010886 | M4351 | 0.026242 | 18 | 3 | 328776107 | PREDICTED: anaphase-promoting complex subunit | | | 1.96E-34 | 20.92823 | 12.43272 | 15.11963 | 28.35396 | 18.42911 | 16.62399 | 20.56951 | 13.48586 | 9.138723 | 12.53064 | 14.00731 | 7.428537 | 12.20158 | 11.46694 | 11.95099 | 13.8859 | 17.47586 | 11.82733 | 25.37501 | 7.515463 | 19.07791 | 10.51492 | 26.67812 | 17.86001 | 12.66387 | 13.64284 | 16.03304 |
| CUST_48584_PI429010886 | M81131 | 0.026273 | 21 | 0 | | | | | | 34.14111 | 11.02334 | 43.54805 | 25.65833 | 19.52138 | 29.04798 | 15.73595 | 23.92584 | 6.939821 | 22.08937 | 12.34338 | 19.46833 | 5.097353 | 21.95843 | 9.87455 | 24.62608 | 31.61999 | 28.31399 | 37.19186 | 12.95466 | 32.25819 | 18.45401 | 39.3558 | 26.68081 | 27.90015 | 7.957708 | 30.0232 |
| CUST_10906_PI429010886 | N26972 | 0.02628 | 15 | 0 | 321459545 | hypothetical protein DAPPUDRAFT_309387 [Daphn | | | 2.23E-38 | 18.93037 | 15.09234 | NA | 18.86217 | 9.13082 | 14.64853 | 8.029282 | 5.738619 | 4.71542 | 8.689343 | 6.246892 | 3.8418 | 13.79851 | NA | 19.88848 | 16.59703 | 6.469129 | 28.84595 | NA | 7.162712 | 9.601564 | 9.048255 | NA | 12.44135 | 11.61617 | 4.16646 | 11.42367 |
| CUST_41048_PI429010886 | M56756 | 0.026311 | 24 | 15 | | | | | | 701.6345 | 837.729 | 613.6489 | 638.6578 | 684.5685 | 594.5741 | 511.4224 | 496.5233 | 557.82 | 808.7225 | 592.8448 | 790.1209 | 599.656 | 740.6945 | 813.6581 | 977.0262 | 678.7202 | 710.9044 | 640.949 | 822.5821 | 662.9564 | 954.6687 | 644.8914 | 656.3922 | 905.952 | 571.296 | 807.4639 |
| CUST_40700_PI429010886 | M55895 | 0.026324 | 15 | 9 | | | | | | 324.4624 | 276.4683 | 336.2744 | 259.6247 | 285.7004 | 212.501 | 328.3818 | 261.3191 | 288.5922 | 333.6383 | 322.3624 | 399.641 | 302.3576 | 371.054 | 328.9809 | 355.0658 | 319.8933 | 304.1627 | 303.0793 | 341.6507 | 302.2127 | 420.5887 | 313.9465 | 265.6039 | 411.3644 | 311.1732 | 423.0023 |
| CUST_2028_PI429010886 | N11361 | 0.026324 | 15 | 0 | | | | | | 362.7402 | 392.6279 | 269.3458 | 282.8614 | 308.7172 | 326.0904 | 237.799 | 215.9452 | 238.8964 | 371.1359 | 268.079 | 301.6859 | 271.6624 | 284.9694 | 286.8104 | 459.9274 | 307.6894 | 332.4166 | 332.6812 | 319.6424 | 266.4816 | 422.0878 | 261.0789 | 248.428 | 443.6521 | 263.9741 | 312.3654 |
| CUST_5945_PI429010886 | N19409 | 0.026324 | 15 | 0 | | | | | | 127.9775 | 277.5533 | 110.2326 | 105.4208 | 128.8934 | 138.7005 | 82.60943 | 73.60851 | 54.75352 | 102.022 | 65.64051 | 100.2082 | 69.69491 | 71.74241 | 86.35271 | 94.51496 | 142.7716 | 100.0986 | 114.787 | 86.96346 | 71.15715 | 84.41305 | 75.74491 | 70.76377 | 146.3889 | 85.80553 | 58.02431 |
| CUST_43491_PI429010886 | M63190 | 0.026326 | 21 | 18 | | | | | | 76.25978 | 73.37448 | 40.71208 | 101.6485 | 74.94569 | 44.58058 | 42.68895 | 36.48665 | 36.59066 | 60.66856 | 52.49227 | 43.94789 | 57.44502 | 68.3135 | 51.01709 | 85.86351 | 58.08045 | 71.64903 | 77.6254 | 71.09226 | 56.83575 | 91.22284 | 49.49452 | 53.1957 | 93.19487 | 48.71321 | 51.60182 |
| CUST_1071_PI429010886 | N8406 | 0.026364 | 24 | 6 | 478261422 | hypothetical protein YQE_02808, partial [Dendroctc | Protein metabolism | Proteolysis | 1.92E-13 | 586.9437 | 1099.822 | 819.682 | 1163.545 | 571.094 | 413.2538 | 702.0078 | 1203.954 | 416.363 | 633.8371 | 422.8357 | 366.8204 | 529.9042 | 623.5116 | 368.7924 | 152.5473 | 175.1085 | 21.25027 | 130.5299 | 545.9123 | 138.6787 | 9.013974 | 681.1329 | 148.65 | 11.73596 | 209.8626 | 125.9625 |
| CUST_54432_PI429010886 | M105998 | 0.026371 | 15 | 12 | 3913717 | RecName: Full=Guanine nucleotide-binding protein | Signal transduction | Other | 7.43E-10 | 34.04318 | 53.45236 | 50.29826 | 43.04145 | 23.62815 | 33.61732 | 34.54419 | 16.94759 | 29.5952 | 42.68003 | 16.98325 | 68.26569 | 55.31379 | 59.38984 | 73.21949 | 62.1341 | 42.44469 | 38.66452 | 38.76201 | 42.88135 | 33.61445 | 78.48034 | 33.45717 | 37.9894 | 33.3136 | 56.79499 | 46.23463 |
| CUST_1187_PI429010886 | N8777 | 0.026371 | 15 | 6 | | | | | | 49.30624 | 65.20986 | 30.25328 | 52.08646 | 69.95364 | 34.6933 | 78.74635 | 55.13187 | 40.34574 | 51.31053 | 46.74774 | 45.1372 | 26.869 | 16.86851 | 28.64267 | 60.9102 | 21.97161 | 49.36928 | 48.2481 | 52.13588 | 32.94969 | 65.89913 | 168.5282 | 30.74654 | 41.44167 | 35.33784 | 40.87212 |
| CUST_49746_PI429010886 | M86966 | 0.026375 | 18 | 0 | | | | | | 80.53149 | 55.95035 | 93.77397 | 60.03661 | 73.51179 | 55.52574 | 76.53451 | 56.25359 | 67.39787 | 47.81705 | 59.99556 | 38.63759 | 30.13575 | 33.80016 | 26.38163 | 58.00017 | 102.0348 | 50.97047 | 51.38396 | 76.33468 | 63.40813 | 54.13488 | 58.38163 | 49.10837 | 45.29693 | 87.99607 | 45.09375 |
| CUST_5562_PI429010886 | N18875 | 0.026395 | 15 | 3 | 383852356 | PREDICTED: myosin-IB-like [Megachile rotundata] | | | | 46.27475 | 28.21862 | 30.54779 | 39.1747 | 47.20161 | 102.9589 | 50.00676 | 50.45571 | 45.33809 | 25.96869 | 38.20162 | 23.63331 | 30.01523 | 30.37645 | 37.02581 | 42.92316 | 62.84333 | 49.28963 | 79.84889 | 50.81627 | 38.55322 | 18.62216 | 33.56673 | 50.80203 | 30.55308 | 58.59263 | 28.44009 |
| CUST_42157_PI429010886 | M59570 | 0.026403 | 18 | 6 | | | | | | 61.74634 | 33.32019 | 30.9722 | 64.50028 | 41.44605 | 72.3461 | 105.5819 | 45.87335 | 74.68625 | 41.71517 | 40.04237 | 47.23493 | 40.14953 | 21.54181 | 38.91166 | 37.79341 | 66.6859 | 32.8723 | 114.7111 | 24.58201 | 53.29849 | 34.2205 | 63.7264 | 97.17307 | 78.02809 | 50.9755 | 76.12074 |
| CUST_55839_PI429010886 | M111847 | 0.026419 | 15 | 9 | 293347793 | PREDICTED: zinc finger protein 709-like [Rattus norv | | | 3.52E-54 | 14.1603 | 10.68037 | 7.372745 | 8.669897 | 4.653863 | 12.50339 | 15.73055 | 11.68992 | 13.07347 | 22.39686 | 14.25137 | 16.62935 | 6.943814 | 19.13431 | 11.73596 | 33.86551 | 8.986987 | 6.412722 | 15.09075 | 7.610851 | 14.50091 | 19.14299 | 6.382168 | 16.9925 | 21.85231 | 19.24611 | 19.69856 |
| CUST_21450_PI429010886 | N45245 | 0.026424 | 18 | 0 | | | | | | 18.85135 | 26.54903 | 23.12065 | 23.67058 | 10.98742 | 15.25416 | 15.05054 | 15.96047 | 18.75909 | 19.1562 | 18.10445 | 21.62724 | 11.83887 | 10.08763 | 14.9864 | 21.84747 | 15.81697 | 24.57313 | 22.25705 | 22.63508 | 19.61371 | 18.13609 | 20.70543 | 12.96922 | 17.14756 | 11.90992 | 10.86638 |
| CUST_53131_PI429010886 | M100331 | 0.026431 | 18 | 6 | | | | | | NA | 59.67663 | 15.88699 | 43.38005 | 31.94483 | NA | NA | 415.0913 | 423.2024 | 24.34275 | 116.4744 | 122.452 | 110.4575 | 31.0625 | 107.174 | NA | 20.83003 | NA | 40.54674 | 1129.849 | 117.3985 | 29.23931 | 141.8752 | 113.1265 | NA | 179.3756 | 168.154 |
| CUST_18745_PI429010886 | N40339 | 0.026443 | 24 | 18 | | | | | | 43.59959 | 70.169 | 70.77093 | 49.49452 | 51.9493 | 59.2457 | 51.83037 | 50.33169 | 59.37489 | 118.0091 | 73.78486 | 103.6651 | 55.41559 | 84.81787 | 74.45078 | 73.3681 | 94.08412 | 64.99075 | 96.47975 | 90.11072 | 73.41537 | 76.68397 | 88.58239 | 32.86388 | 70.33128 | 68.07541 | 87.50342 |
| CUST_1373_PI429010886 | N9437 | 0.026446 | 21 | 18 | | | | | | 244.2771 | 397.7001 | 254.8434 | 225.5895 | 333.5081 | 273.9149 | 135.7577 | 111.8492 | 179.7697 | 295.0205 | 204.1442 | 384.1514 | 207.885 | 359.7386 | 417.6101 | 574.4912 | 111.4084 | 494.4534 | 229.13 | 505.2828 | 299.0341 | 720.0434 | 255.4386 | 266.5285 | 341.9433 | 224.9679 | 235.0098 |
| CUST_30472_PI429010886 | N61966 | 0.026486 | 24 | 6 | | | | | | 35.48457 | 42.46725 | 56.28307 | 49.59376 | 42.48584 | 62.54934 | 66.04832 | 44.85914 | 73.30133 | 45.67726 | 33.70252 | 40.95911 | 30.7925 | 49.51255 | 60.85469 | 48.36598 | 64.96286 | 47.65996 | 32.2878 | 35.24075 | 38.86489 | 44.01861 | 38.74344 | 32.55416 | 40.19376 | 34.90742 | 47.63767 |
| CUST_54789_PI429010886 | M107683 | 0.026489 | 12 | 0 | 197940771 | Euphausia superba mitochondrion, partial genome | Transport | Ion transport | 1.3E-104 | 3607.364 | 6189.048 | 4232.305 | 2218.572 | 2527.895 | 1983.417 | 3147.989 | 4232.305 | 4407.135 | 4348.32 | 3530.217 | 6100.209 | 3372.782 | 4738.823 | 5293.128 | 4201.227 | 2659.902 | 3352.836 | 3074.469 | 5980.233 | 3469.507 | 4031.594 | 4313.91 | 2531.781 | 4639.445 | 3569.653 | 5515.427 |
| CUST_39983_PI429010886 | M47802 | 0.026489 | 18 | 3 | | | | | | 6767.816 | 3175.174 | 2869.202 | 13907.98 | 9686 | 22281.96 | 13375.82 | 3711.262 | 5688.498 | 171.1038 | 6986.943 | 67.30873 | 5445.033 | 2139.684 | 4607.367 | 4782.509 | 12715.79 | 14805.59 | 7628.757 | 709.6744 | 8470.008 | 3972.597 | 7637.386 | 15537.42 | 889.3445 | 8783.054 | 83.93301 |
| CUST_39985_PI429010886 | M47887 | 0.026496 | 21 | 3 | | | | | | 20.79607 | 24.59022 | 16.07261 | 24.18418 | 24.9855 | 17.50878 | 12.95945 | 10.7968 | 15.15404 | 14.41386 | 12.71932 | 21.55583 | 10.30184 | 11.30247 | 13.04197 | 20.56778 | 14.66107 | 18.2356 | 10.87507 | 17.31232 | 24.21798 | 12.90641 | 33.64032 | 11.80446 | 18.46372 | 16.44566 | 23.18287 |
| CUST_10522_PI429010886 | N26368 | 0.026496 | 24 | 18 | | | | | | 320.3175 | 319.3199 | 298.53 | 264.1731 | 301.467 | 306.5332 | 261.7284 | 258.602 | 295.3248 | 369.6113 | 279.6762 | 306.8029 | 246.92 | 285.7468 | 272.1063 | 388.1247 | 311.9303 | 344.7288 | 329.9922 | 383.225 | 347.3329 | 325.6779 | 236.6906 | 302.6662 | 397.9322 | 295.9077 | 401.8543 |
| CUST_25302_PI429010886 | N52153 | 0.026496 | 24 | 15 | | | | | | 55.85751 | 50.68803 | 49.33073 | 40.81576 | 33.47045 | 40.95098 | 43.76564 | 38.43703 | 45.68644 | 56.58363 | 59.61145 | 52.55217 | 36.08462 | 64.1188 | 49.8819 | 58.11529 | 49.87667 | 58.64138 | 48.62311 | 54.98545 | 58.7985 | 67.71039 | 55.28288 | 55.79937 | 75.30403 | 42.40387 | 47.96622 |
| CUST_24205_PI429010886 | N50226 | 0.026512 | 21 | 0 | | | | | | 15.42553 | 10.67164 | 18.40643 | 4.958353 | 10.21711 | 11.84191 | 17.84645 | 12.82143 | 14.08649 | 8.438285 | 9.922626 | 8.906902 | 7.074973 | 3.941649 | 3.770663 | 4.544747 | 79.08585 | 7.916656 | 14.90751 | 5.769447 | 13.89131 | 11.3725 | 13.46088 | 24.85393 | 11.38323 | 21.58422 | 4.848883 |
| CUST_22382_PI429010886 | N46930 | 0.026512 | 21 | 12 | | | | | | 102.0693 | 159.9839 | 95.19444 | 86.6967 | 65.93703 | 26.93068 | 89.35703 | 78.54463 | 106.3073 | 121.0793 | 91.6409 | 134.5033 | 102.1933 | 155.5527 | 112.8577 | 103.5324 | 90.19499 | 119.8071 | 117.5843 | 83.50755 | 109.7061 | 144.5986 | 88.6992 | 99.84559 | 121.3759 | 45.7289 | 53.40548 |
| CUST_33841_PI429010886 | M8003 | 0.026524 | 24 | 0 | | | | | | 148.7285 | 164.8518 | 144.77 | 111.637 | 114.1048 | 157.8626 | 126.8079 | 113.0678 | 128.5525 | 138.0153 | 100.2724 | 142.821 | 95.13953 | 109.2258 | 128.7791 | 123.7772 | 123.2882 | 164.6258 | 125.5232 | 105.4346 | 121.4973 | 163.4134 | 102.2185 | 133.2798 | 165.9503 | 130.4682 | 143.8508 |
| CUST_1778_PI429010886 | N10679 | 0.026535 | 15 | 12 | 380503698 | sarcoplasmic calcium-binding protein [Marsupenae | | | 4.7E-66 | 2692.301 | 5278.442 | 4008.447 | 2688.606 | 3419.141 | 3126.195 | 2331.325 | 3130.59 | 3414.171 | 4561.712 | 3551.684 | 4787.683 | 3472.182 | 3968.239 | 5060.995 | 4847.521 | 3156.693 | 3509.529 | 4038.67 | 5176.163 | 3879.679 | 3271.146 | 3092.843 | 2184.967 | 4253.825 | 3886.072 | 4791.679 |
| CUST_42654_PI429010886 | M60870 | 0.026537 | 18 | 0 | | | | | | 43.0244 | 45.14009 | 65.68089 | 37.11592 | 38.74854 | 36.8462 | 41.6388 | 35.05162 | 21.74146 | 40.90818 | 36.55477 | 24.17916 | 33.88658 | 27.63304 | 39.10739 | 41.27495 | 28.63363 | 35.89648 | 37.83723 | 48.51868 | 32.41678 | 49.05418 | 30.79135 | 27.77642 | 32.74781 | 17.947 | 25.88089 |
| CUST_1828_PI429010886 | N10822 | 0.026539 | 12 | 0 | | | | | | NA | 14.93734 | 10.20734 | NA | 3.960392 | NA | NA | 3.673331 | 11.42007 | 19.29786 | 4.056221 | 13.13833 | 10.08636 | 21.72694 | 25.49244 | 12.53064 | NA | 9.530867 | 3.717105 | 4.494932 | 12.11037 | 31.86259 | 23.31758 | 12.45327 | 50.27036 | 23.77956 | 43.1379 |
| CUST_25191_PI429010886 | N51946 | 0.026554 | 12 | 9 | | | | | | 32.86117 | NA | 11.45774 | 73.55823 | 3.895848 | 7.39706 | NA | NA | 6.810134 | 61.36332 | 60.6558 | 157.283 | 114.2465 | 16.02003 | 48.08498 | NA | 5.579312 | 10.7921 | 6.574274 | 55.55289 | 60.61979 | 132.0478 | 10.76087 | 79.76051 | 29.90756 | 53.33947 | 41.59799 |
| CUST_40278_PI429010886 | M52406 | 0.026574 | 18 | 12 | | | | | | 766.6399 | 1184.785 | 1202.61 | 260.2212 | 717.1047 | 627.4273 | 818.6448 | 838.9983 | 617.5712 | 1013.924 | 1059.917 | 982.113 | 956.7714 | 1581.923 | 841.4264 | 1013.272 | 625.0062 | 1217.47 | 629.0535 | 1315.293 | 638.47 | 730.6182 | 785.705 | 709.8502 | 968.7311 | 659.1977 | 604.4329 |
| CUST_5958_PI429010886 | N19426 | 0.026574 | 15 | 0 | | | | | | 162.3056 | 119.6887 | 95.14966 | 70.32547 | 77.1366 | 80.11662 | 76.64595 | 72.71237 | 83.43282 | 109.1441 | 97.65941 | 109.9302 | 71.72159 | 99.75328 | 111.9496 | 126.1763 | 90.45788 | 101.0126 | 87.03787 | 124.1999 | 92.04256 | 93.73888 | 85.48477 | 67.27473 | 103.8804 | 105.2803 | 115.1051 |
| CUST_56160_PI429010886 | M112995 | 0.026585 | 24 | 15 | | | | | | 541.8489 | 696.9471 | 663.5689 | 580.161 | 618.8739 | 685.1065 | 585.2479 | 591.157 | 582.4599 | 664.1086 | 623.5116 | 741.9924 | 579.7012 | 789.9286 | 657.6448 | 781.4539 | 750.1475 | 737.2359 | 697.8167 | 643.5529 | 572.0401 | 759.8078 | 752.5982 | 715.6525 | 679.1732 | 597.5638 | 611.4557 |
| CUST_50025_PI429010886 | M88300 | 0.026591 | 12 | 3 | | | | | | 9.355583 | 9.343228 | 7.561772 | 23.62378 | 14.5497 | 48.24061 | 14.76465 | 12.69552 | 8.740277 | 7.532651 | 4.316396 | 17.19461 | 13.78911 | NA | 7.855725 | 10.15288 | 13.86795 | 12.53584 | 10.31716 | NA | 4.085334 | 9.300708 | NA | 7.193295 | 9.282132 | 12.25145 | 11.8786 |
| CUST_52687_PI429010886 | M98466 | 0.026592 | 12 | 0 | 260792312 | hypothetical protein BRAFLDRAFT_140040 [Branchi | | | 8.65E-19 | 39.71872 | 71.74926 | 36.09682 | 20.23721 | 4.527621 | NA | 14.2617 | 35.78629 | 43.50773 | 13.43349 | 16.87557 | 63.38531 | 45.63767 | 80.24894 | 59.05765 | 107.0039 | NA | 35.7959 | 5.999228 | 38.67688 | 31.46479 | 13.33763 | 67.08962 | 65.9853 | 122.1037 | 155.979 | 155.7397 |
| CUST_55505_PI429010886 | M110400 | 0.02663 | 15 | 9 | 365776023 | Penaeus monodon beta-actin mRNA, complete cds | | | 2.15E-74 | 15.71246 | 19.60466 | 18.58096 | 20.98935 | 15.07677 | 8.632122 | 17.47239 | 20.87638 | 24.97756 | 24.09666 | 17.11711 | 38.19514 | 15.3449 | 27.87681 | 22.89976 | 22.30489 | 20.78351 | 17.09174 | 16.92439 | 11.63591 | 23.22645 | 27.0144 | 22.86023 | 16.45078 | 20.74032 | 29.21516 | 35.82359 |
| CUST_56186_PI429010886 | M113051 | 0.02663 | 15 | 9 | 371591236 | Euphausia superba voucher 760M mitochondrion, p | Metabolic process | Generation of precursor metabolites and energy | | 561.4598 | 689.8687 | 1041.548 | 480.1775 | 648.6302 | 814.2205 | 707.1584 | 657.8288 | 862.42 | 552.8917 | 802.2989 | 1067.963 | 765.4435 | 1251.807 | 766.6399 | 741.4101 | 756.7863 | 853.9995 | 676.1626 | 751.781 | 679.1732 | 782.7144 | 769.8974 | 781.185 | 871.9238 | 1038.971 | 1129.849 |
| CUST_32108_PI429010886 | M3247 | 0.02663 | 15 | 9 | 321472283 | hypothetical protein DAPPUDRAFT_301838 [Daphn | Cellular Process | Cell cycle | 4E-142 | 166.5598 | 160.2968 | 144.8962 | 114.7111 | 121.4973 | 124.1467 | 128.3244 | 116.0813 | 145.4832 | 188.6253 | 106.8563 | 183.5489 | 146.8674 | 179.6526 | 176.7991 | 173.6625 | 131.4711 | 140.548 | 179.9875 | 128.1445 | 125.5811 | 167.4539 | 126.1331 | 113.1785 | 185.8187 | 150.1195 | 218.4011 |
| CUST_31044_PI429010886 | M545 | 0.02663 | 15 | 9 | 321463706 | hypothetical protein DAPPUDRAFT_56817 [Daphnia | Metabolic process | Cellular amino acid metabolic process | 1.07E-53 | 282.5343 | 546.5203 | 484.1582 | 289.2842 | 308.0795 | 234.853 | 262.6696 | 513.0951 | 640.2097 | 487.486 | 520.2836 | 667.7123 | 471.3393 | 903.1657 | 402.2817 | 620.3013 | 309.8242 | 390.973 | 269.0643 | 695.1115 | 275.4021 | 717.2947 | 386.0388 | 289.1774 | 1238.144 | 311.4551 | 783.1618 |
| CUST_41216_PI429010886 | M57153 | 0.02663 | 15 | 0 | | | | | | 101.5396 | 87.19276 | 73.91159 | 65.06017 | 81.78469 | 74.62941 | 64.33014 | 61.32673 | 67.25956 | 71.93323 | 74.41568 | 76.10603 | 63.56474 | 77.66626 | 80.62509 | 108.0902 | 67.32952 | 93.60773 | 91.73772 | 59.78571 | 60.86834 | 73.94862 | 49.65587 | 65.65578 | 92.25373 | 52.42324 | 42.02786 |
| CUST_42949_PI429010886 | M61694 | 0.02663 | 15 | 9 | 222543956 | nuclear autoantigenic sperm protein [Penaeus mon | | | 1.06E-25 | 52.4826 | 73.14043 | 48.28635 | 48.67019 | 46.98145 | 31.2144 | 37.75183 | 47.31501 | 60.79572 | 87.51279 | 61.43267 | 78.80354 | 63.72139 | 74.37782 | 56.47886 | 60.03661 | 32.02812 | 55.85751 | 42.11724 | 62.01223 | 59.00876 | 74.68625 | 52.40666 | 39.14912 | 74.52928 | 46.69134 | 82.58698 |
| CUST_2647_PI429010886 | N12831 | 0.02663 | 15 | 9 | | | | | | 56.67806 | 64.83329 | 62.99752 | 65.14977 | 57.58322 | 50.37741 | 32.06861 | 57.93571 | 53.25416 | 91.09799 | 75.33825 | 90.68288 | 71.76497 | 75.13415 | 53.8998 | 74.86136 | 36.29774 | 52.85191 | 86.97645 | 55.26922 | 51.79552 | 73.09859 | 49.40423 | 66.28856 | 91.12979 | 77.12763 | 67.52161 |
| CUST_42288_PI429010886 | M59912 | 0.026636 | 18 | 15 | | | | | | 163.051 | 160.6917 | 163.791 | 127.9641 | 118.275 | 161.0222 | 117.621 | 138.8758 | 98.75692 | 182.3936 | 160.0314 | 185.7109 | 140.3134 | 125.3729 | 157.17 | 155.3131 | 184.8973 | 198.2503 | 171.0319 | 161.861 | 135.2525 | 174.7759 | 137.687 | 139.8446 | 122.1728 | 185.2951 | 113.6906 |
| CUST_32495_PI429010886 | M4269 | 0.02664 | 15 | 9 | | | | | | 8.680488 | 11.07003 | 33.36548 | 18.47168 | 5.094187 | 26.03397 | 10.97186 | 80.96373 | 95.02448 | 54.43209 | 92.47211 | 103.7436 | 22.45136 | 108.8006 | 53.67582 | 25.86961 | 26.85111 | 27.52766 | 27.94887 | 108.0186 | 14.38683 | 110.0909 | 81.87922 | 8.52275 | 163.6107 | 44.36476 | 209.5944 |
| CUST_30973_PI429010886 | M345 | 0.026645 | 21 | 18 | | | | | | 1460.033 | 1638.031 | 1483.935 | 1362.62 | 1246.008 | 1345.526 | 1220.626 | 1080.655 | 1205.667 | 1561.567 | 1169.079 | 1399.299 | 981.8641 | 1678.528 | 1488.789 | 1730.823 | 1275.086 | 1383.78 | 1774.992 | 1351.741 | 1425.514 | 1576.033 | 1398.489 | 1367.805 | 1504.155 | 1249.348 | 1435.814 |
| CUST_3936_PI429010886 | N15614 | 0.026647 | 24 | 18 | | | | | | 16.28372 | 8.318727 | 11.43131 | 10.94786 | 7.179499 | 10.01823 | 14.83131 | 12.59071 | 3.359941 | 15.40925 | 22.82948 | 10.46847 | 17.23467 | 13.07005 | 8.055145 | 23.62155 | 58.14864 | 9.748744 | 24.33679 | 27.54224 | 11.31627 | 20.88122 | 16.5713 | 17.90806 | 17.88327 | 6.65508 | 23.194 |
| CUST_762_PI429010886 | N7201 | 0.026662 | 21 | 9 | 124245110 | tetraspanins-like protein-8 [Fenneropenaeus chiner | | | 4.97E-35 | 12.4022 | 20.13319 | 13.74525 | 47.12699 | 57.36487 | 23.94321 | 34.80955 | 175.2355 | 99.93216 | 24.69054 | 188.0209 | 236.0839 | 85.0717 | 14.28453 | 44.40996 | 7.609769 | 72.3461 | NA | 11.76966 | 350.0282 | 84.59116 | 23.4129 | 24.24769 | 30.91745 | 22.60935 | 98.71598 | 70.94036 |
| CUST_34276_PI429010886 | M9215 | 0.026677 | 15 | 9 | 371591197 | Euphausia superba voucher 841M mitochondrion, p | Transport | Ion transport | | 4.172653 | 7.304592 | NA | 37.29408 | 34.4458 | 26.77489 | 16.31911 | 91.84392 | 24.59907 | 11.31528 | 106.9501 | 146.9814 | 37.91131 | 17.40347 | 19.99051 | 4.187339 | 12.80404 | NA | 7.996886 | 175.3631 | 22.2321 | NA | 52.97579 | 28.94202 | NA | 33.41929 | 63.54061 |
| CUST_36279_PI429010886 | M15377 | 0.026689 | 15 | 12 | | | | | | 169.2006 | 111.5216 | 282.8614 | 176.1772 | 102.7622 | 155.8377 | 175.4136 | 223.7779 | 192.6124 | 238.3252 | 128.8206 | 182.5915 | 184.9236 | 278.9995 | 208.515 | 156.8208 | 187.9283 | 152.1158 | 164.9826 | 112.3805 | 162.719 | 189.0327 | 206.5724 | 153.0541 | 258.8229 | 199.8756 | 199.0448 |
| CUST_46496_PI429010886 | M72820 | 0.026689 | 15 | 3 | | | | | | 53.02941 | 69.41655 | 96.38791 | 84.64574 | 75.69546 | 68.70977 | 58.37612 | 60.00971 | 52.91094 | 57.60743 | 83.79573 | 55.58682 | 47.5011 | 58.56755 | 40.36651 | 47.48523 | 67.61178 | 45.73763 | 76.84997 | 85.10975 | 67.01506 | 47.43001 | 59.71534 | 47.88942 | 60.90428 | 57.97363 | 59.38223 |
| CUST_11684_PI429010886 | N28235 | 0.026691 | 21 | 12 | | | | | | 41.82918 | 42.98059 | 38.51022 | 22.88357 | 38.49435 | 35.78224 | 30.85363 | 36.35234 | 30.72063 | 55.45229 | 41.63072 | 48.93138 | 40.46037 | 50.12098 | 52.89103 | 48.34221 | 39.77522 | 48.79079 | 39.52248 | 48.32025 | 47.80852 | 51.95916 | 46.44351 | 36.29774 | 52.38415 | 36.20575 | 58.53699 |
| CUST_33459_PI429010886 | M6944 | 0.026715 | 15 | 12 | | | | | | 2063.321 | 2516.877 | 4345.199 | 2060.137 | 1684.016 | 2031.538 | 1968.333 | 2212.175 | 3411.47 | 2565.951 | 2474.132 | 5868.528 | 3339.741 | 3425.229 | 5239.377 | 4365.095 | 1287.748 | 3453.79 | 1644.909 | 2455.41 | 3590.082 | 5464.631 | 3016.912 | 2335.152 | 5188.094 | 3818.838 | 3792.107 |
| CUST_51384_PI429010886 | M93343 | 0.026717 | 24 | 18 | 524928295 | PREDICTED: apoptosis regulatory protein Siva isofor | Signal transduction | Other | 4.08E-09 | 48.39289 | 48.69863 | 56.09789 | 57.52971 | 56.04356 | 46.77465 | 39.57761 | 52.08136 | 35.91607 | 73.25256 | 46.23463 | 52.81394 | 49.84204 | 55.79477 | 55.35407 | 72.54647 | 51.30486 | 54.3948 | 59.35346 | 67.64264 | 61.00953 | 79.97877 | 57.16139 | 43.57816 | 68.75407 | 36.19462 | 54.61808 |
| CUST_46608_PI429010886 | M73149 | 0.026719 | 24 | 12 | 321458613 | hypothetical protein DAPPUDRAFT_300846 [Daphn | | | 2.09E-12 | 24.9855 | 42.68003 | 25.56608 | 30.10596 | 28.48875 | 24.67398 | 31.82347 | 31.59119 | 33.04717 | 66.57113 | 32.36995 | 52.78112 | 41.59799 | 65.89913 | 46.54874 | 49.98628 | 34.51949 | 28.46803 | 39.27986 | 64.28336 | 50.78152 | 35.56782 | 34.56663 | 40.97284 | 59.54485 | 22.81381 | 37.09799 |
| CUST_28058_PI429010886 | N57196 | 0.026719 | 24 | 0 | | | | | | 90.34874 | 123.1656 | 88.24844 | 72.04379 | 58.84482 | 51.82417 | 74.89819 | 68.09627 | 66.48727 | 81.84143 | 59.50688 | 60.7605 | 57.55495 | 82.57755 | 76.07191 | 120.6382 | 82.9851 | 75.81603 | 92.42083 | 101.5278 | 78.07189 | 90.79757 | 76.39741 | 60.71887 | 98.53734 | 90.33731 | 97.01852 |
| CUST_2161_PI429010886 | N11726 | 0.026725 | 15 | 9 | | | | | | 46.45766 | 4.283628 | 20.70392 | 65.9853 | 46.04576 | 27.07724 | 73.64901 | 54.51541 | 60.44797 | 91.05924 | 80.15079 | 75.4776 | 71.83386 | 32.65993 | 41.39686 | 79.18359 | 22.26173 | 27.91206 | 30.26197 | 105.3539 | 8.706462 | 78.33196 | 74.20243 | 54.38291 | 64.70488 | 19.52364 | 95.36046 |
| CUST_53417_PI429010886 | M101615 | 0.026726 | 21 | 18 | 62126070 | C-type lectin protein [Fenneropenaeus chinensis] | | | 2.84E-09 | 1105.302 | 840.3682 | 1195.55 | 619.2505 | 533.8243 | 171.0028 | 131.2193 | 181.6229 | 832.672 | 2128.599 | 359.0948 | 598.0415 | 710.9044 | 208.39 | 608.2249 | 661.8313 | 153.2103 | 1446.386 | 799.3451 | 1103.397 | 2398.993 | 356.5042 | 2117.402 | 1066.114 | 664.7021 | 127.723 | 628.3128 |
| CUST_40967_PI429010886 | M56507 | 0.026749 | 21 | 3 | | | | | | 11.16309 | 12.7744 | 19.95383 | 35.6179 | 16.94406 | 41.81244 | 7.747649 | 13.12407 | 12.98148 | 13.60386 | 7.279581 | 20.30647 | 10.33277 | 14.41386 | 17.58889 | 28.92395 | 6.695841 | 7.948839 | 4.370166 | 10.25442 | 13.22555 | 14.68459 | 10.68951 | 16.8955 | 18.71104 | 19.84447 | NA |
| CUST_33325_PI429010886 | M6602 | 0.02676 | 15 | 12 | 405974697 | C-terminal-binding protein [Crassostrea gigas] | | | 2.45E-29 | 12.73078 | 12.13161 | 28.93334 | 11.52352 | 14.62058 | 9.179429 | 10.16422 | 21.96556 | 20.5469 | 19.18935 | 15.32584 | 25.49499 | 27.73533 | 28.64039 | 20.5721 | 17.89445 | 25.02094 | NA | 19.26978 | 18.66977 | 12.67376 | 21.43485 | 17.99854 | 13.72174 | 31.50476 | 15.1999 | 31.15965 |
| CUST_1458_PI429010886 | N9705 | 0.026795 | 24 | 15 | 50513239 | calpain B [Gecarcinus lateralis] | Protein metabolism | Proteolysis | | 178.4456 | 191.7997 | 207.853 | 157.0455 | 185.2951 | 176.9135 | 134.5033 | 158.4788 | 157.4172 | 131.9258 | 130.6003 | 220.485 | 194.605 | 262.8518 | 123.0412 | 299.9058 | 205.1968 | 275.4021 | 206.9847 | 173.0247 | 221.0933 | 232.5572 | 197.1531 | 120.3899 | 165.3446 | 227.9039 | 211.4366 |
| CUST_31974_PI429010886 | M2881 | 0.026795 | 24 | 18 | | | | | | 171.5942 | 350.7295 | 191.4981 | 167.9521 | 154.9678 | 150.6483 | 159.3888 | 163.0297 | 169.275 | 193.556 | 175.9214 | 281.2623 | 231.3759 | 291.5581 | 166.9427 | 290.9496 | 181.0152 | 206.6326 | 188.5235 | 254.0209 | 234.4127 | 203.1221 | 214.4445 | 165.3668 | 301.2285 | 194.8606 | 232.8505 |
| CUST_56507_PI429010886 | M113962 | 0.026826 | 18 | 3 | | | | | | 4161.995 | 4019.029 | 2520.419 | 36543.07 | 35264.41 | 34870.47 | 17031.04 | 19923.01 | 1041.277 | 215.0865 | 22662.24 | 115.6165 | 4267.625 | 315.8463 | 3365.303 | 190.7327 | 14950.27 | 4004.804 | 10851.4 | 188.3253 | 23818.16 | 778.2204 | 17110.44 | 25496.93 | 311.8189 | 9715.562 | 130.2863 |
| CUST_13147_PI429010886 | N30657 | 0.026829 | 24 | 0 | | | | | | 235.3127 | 220.511 | 232.3859 | 98.53734 | 205.4212 | 193.2088 | 152.5866 | 166.7568 | 147.8113 | 192.7007 | 231.1582 | 152.7158 | 107.0039 | 146.9171 | 45.87664 | 222.5361 | 272.1441 | 176.8527 | 193.0075 | 186.6438 | 231.1023 | 148.6276 | 195.2684 | 198.5364 | 346.9119 | 130.1875 | 242.0784 |
| CUST_48486_PI429010886 | M80588 | 0.026833 | 24 | 0 | 195587425 | GD13338 [Drosophila simulans] >gi\|194195474\|gb | Cellular component organization | Cytoskeleton organization | 1.8E-08 | 78.90095 | 99.95963 | 118.1291 | 63.86613 | 90.65923 | 89.11931 | 52.48792 | 82.09306 | 102.0693 | 63.8461 | 75.63407 | 83.83973 | 73.37448 | 92.45305 | 28.18417 | 46.85704 | 59.96262 | 78.35123 | 49.48526 | 75.85953 | 151.2427 | 48.50508 | 104.363 | 148.3091 | 99.50846 | 106.2399 | 132.2265 |
| CUST_19050_PI429010886 | N40899 | 0.026837 | 12 | 3 | | | | | | 14.24812 | 26.66367 | 12.87351 | 30.46627 | 41.5428 | 40.6037 | 26.82709 | 11.9716 | 19.10864 | 17.06824 | 23.76908 | 23.89082 | 10.54628 | 23.00078 | 19.93091 | 27.88621 | 23.97713 | 37.67218 | 18.70704 | 35.43275 | 18.19015 | 25.77821 | 27.49426 | 39.0395 | 27.13002 | 10.06856 | 17.67128 |
| CUST_7810_PI429010886 | N22117 | 0.026852 | 21 | 12 | 459175521 | PREDICTED: beta-hexosaminidase subunit alpha-like | Metabolic process | Carbohydrate metabolic process | 2.5E-99 | 70.80146 | 86.86206 | 53.10218 | 141.5325 | 86.59518 | 62.61352 | 66.57113 | 202.262 | 167.9037 | 103.0871 | 179.2308 | 136.7912 | 204.9855 | 119.228 | 143.406 | 90.09804 | 84.1757 | 65.13839 | 75.7764 | 230.1517 | 154.1768 | 127.1944 | 69.80023 | 54.11265 | 64.41721 | 122.4345 | 65.20986 |
| CUST_1387_PI429010886 | N9476 | 0.026853 | 21 | 18 | 112420518 | Gasterosteus aculeatus clone CFW246-H10 mRNA s | Metabolic process | Carbohydrate metabolic process | 1.41E-98 | 282.1142 | 653.3522 | 273.733 | 386.5802 | 255.4957 | 280.12 | 187.9002 | 187.9811 | 234.5938 | 524.4761 | 258.4896 | 499.4614 | 337.0265 | 321.803 | 513.2283 | 531.6819 | 158.4097 | 424.6374 | 328.8667 | 478.074 | 420.1077 | 443.3821 | 274.1744 | 449.5149 | 308.9771 | 419.7481 | 242.1214 |
| CUST_4365_PI429010886 | N16604 | 0.026877 | 21 | 3 | | | | | | 98.43882 | 103.0771 | 111.2896 | 108.077 | 97.84604 | 114.3208 | 103.2145 | 80.97211 | 97.16739 | 90.48379 | 99.17539 | 99.90716 | 80.6942 | 103.3649 | 83.85047 | 89.6226 | 101.2666 | 84.33492 | 110.0025 | 100.5781 | 107.7388 | 73.62026 | 99.66003 | 97.01852 | 113.1785 | 104.9922 | 114.2465 |
| CUST_36523_PI429010886 | M16126 | 0.026879 | 18 | 12 | | | | | | 1058.786 | 1029.814 | 1166.392 | 816.6897 | 913.8189 | 927.1088 | 966.4783 | 988.77 | 1022.449 | 981.8641 | 1038.465 | 1307.703 | 1088.106 | 1403.935 | 1069.858 | 1576.033 | 979.7027 | 1012.95 | 1059.331 | 1377.193 | 812.5634 | 1382.151 | 927.1088 | 851.7359 | 1441.16 | 973.4615 | 1522.93 |
| CUST_36171_PI429010886 | M14869 | 0.026936 | 15 | 9 | | | | | | 203.2991 | 269.8655 | 270.707 | 186.9949 | 222.6303 | 222.8598 | 145.0486 | 214.5095 | 231.8541 | 324.6687 | 256.1926 | 331.3738 | 229.6885 | 303.7146 | 302.3102 | 337.9324 | 216.9071 | 241.7291 | 203.7035 | 289.423 | 222.1754 | 298.1495 | 265.0674 | 148.1555 | 307.2905 | 261.0269 | 394.6245 |
| CUST_36925_PI429010886 | M17242 | 0.026936 | 15 | 9 | | | | | | 114.165 | 147.911 | 163.1012 | 128.6916 | 125.3173 | 112.2413 | 111.8008 | 76.62236 | 125.408 | 156.7999 | 139.4507 | 163.5179 | 117.6303 | 137.687 | 112.304 | 141.0765 | 92.09827 | 127.2103 | 126.8764 | 115.1051 | 111.0786 | 129.4744 | 129.1685 | 91.81369 | 153.1511 | 141.3981 | 169.0201 |
| CUST_32612_PI429010886 | M4573 | 0.026936 | 15 | 0 | 321455420 | hypothetical protein DAPPUDRAFT_218978 [Daphn | Metabolic process | Carbohydrate metabolic process | 1.42E-86 | 1240.783 | 1639.617 | 1714.256 | 1181.87 | 1460.033 | 2002.228 | 518.9265 | 458.8168 | 786.706 | 981.4865 | 1633.35 | 1743.746 | 393.405 | 1435.197 | 888.7344 | 2113.685 | 1178.599 | 1948.38 | 1089.985 | 1855.858 | 1421.77 | 1248.106 | 975.9966 | 869.0196 | 1360.127 | 1332.781 | 1497.212 |
| CUST_46352_PI429010886 | M72339 | 0.026936 | 15 | 9 | | | | | | 554.8262 | 534.723 | 702.4802 | 593.331 | 580.3063 | 507.2776 | 529.4905 | 486.9644 | 593.5055 | 778.2204 | 553.6437 | 800.3484 | 584.5555 | 782.9369 | 607.2425 | 670.4666 | 460.7596 | 607.7896 | 484.2538 | 641.7483 | 505.0665 | 792.7128 | 707.905 | 538.8855 | 787.1975 | 645.5341 | 791.8244 |
| CUST_51030_PI429010886 | M91854 | 0.026936 | 15 | 9 | | | | | | 56.35545 | 56.62309 | 88.10949 | 37.33083 | 39.288 | 47.26147 | 61.89778 | 71.80124 | 96.84604 | 77.76127 | 64.26853 | 62.45524 | 59.84297 | 89.59424 | 56.40861 | 100.2406 | 69.22629 | 68.85144 | 53.88401 | 47.3594 | 67.55015 | 55.90718 | 62.75575 | 62.09543 | 134.5271 | 49.32644 | 180.1444 |
| CUST_9414_PI429010886 | N24584 | 0.026936 | 15 | 9 | | | | | | 76.24385 | 67.26789 | 69.39137 | 44.42567 | 47.05803 | 48.33175 | 41.67274 | 47.17722 | 54.33444 | 85.46155 | 58.0737 | 125.6357 | 58.59263 | 80.79066 | 73.53135 | 115.5511 | 58.36485 | 52.88574 | 58.79065 | 63.06953 | 56.95742 | 83.12577 | 98.42661 | 41.65544 | 82.55028 | 75.50768 | 113.976 |
| CUST_1342_PI429010886 | N9346 | 0.026936 | 15 | 9 | | | | | | 416.4515 | 543.6335 | 633.0392 | 511.4224 | 434.7605 | 429.6585 | 424.9332 | 435.0294 | 506.3576 | 806.919 | 469.1551 | 726.9909 | 480.0427 | 706.0046 | 579.7012 | 617.5712 | 357.7037 | 478.6306 | 485.2905 | 524.5768 | 469.4534 | 816.9007 | 589.4448 | 592.5758 | 1141.431 | 611.5405 | 1050.577 |
| CUST_35647_PI429010886 | M13132 | 0.026968 | 24 | 18 | | | | | | 51.73011 | 59.0152 | 62.56969 | 55.37064 | 53.88401 | 52.71293 | 58.60996 | 48.34221 | 54.4744 | 58.87771 | 26.48449 | 69.22629 | 60.37722 | 88.49666 | 71.98272 | 66.6859 | 65.80017 | 67.75526 | 77.14278 | 98.76997 | 50.84587 | 91.02196 | 64.07997 | 62.65132 | 74.92071 | 67.69277 | 79.59377 |
| CUST_15242_PI429010886 | N34255 | 0.026968 | 24 | 3 | | | | | | 42.86965 | 100.2579 | 44.07419 | 185.7909 | 54.04063 | 118.9329 | 101.6485 | 49.13393 | 93.55728 | 38.56543 | 17.08079 | 45.9505 | 64.21464 | 51.10924 | 135.7103 | 42.53957 | 22.61376 | 45.99048 | 26.16206 | 37.69724 | 49.06607 | 68.29796 | 31.32726 | 56.0501 | 50.57194 | 81.79478 | 30.10233 |
| CUST_28743_PI429010886 | N58534 | 0.026968 | 24 | 21 | | | | | | 183.3264 | 136.25 | 198.5726 | 125.3173 | 132.033 | 150.9664 | 139.5841 | 155.1312 | 160.4594 | 175.0831 | 173.9876 | 141.3582 | 149.0785 | 168.8237 | 181.7276 | 216.2249 | 189.5057 | 190.5242 | 125.1284 | 160.8926 | 140.8613 | 237.6429 | 210.2047 | 190.6724 | 194.1209 | 184.9236 | 185.3252 |
| CUST_37031_PI429010886 | M17615 | 0.026982 | 15 | 12 | 260784338 | hypothetical protein BRAFLDRAFT_241455 [Branchi | Metabolic process | Cellular amino acid metabolic process | 1.04E-76 | 71.40808 | 93.90071 | 69.17707 | 62.08961 | 66.72453 | 35.54408 | 34.3148 | 33.52107 | 42.20687 | 103.2892 | 68.35087 | 84.30944 | 61.56139 | 70.27178 | 74.88917 | 103.2145 | 49.07593 | 69.46903 | 42.5721 | 88.85357 | 67.41072 | 91.95171 | 39.32188 | 34.52588 | 72.3404 | 38.73964 | 55.75375 |
| CUST_31335_PI429010886 | M1274 | 0.027006 | 15 | 12 | 55742260 | nudix-type motif 2 [Xenopus (Silurana) tropicalis] >g | | | 2.73E-45 | 89.86196 | 161.1809 | 69.85356 | 107.1844 | 94.99099 | 84.85912 | 66.26094 | 49.8819 | 59.36711 | 119.2945 | 85.31136 | 115.0528 | 84.722 | 104.0193 | 112.3805 | 141.9566 | 64.04953 | 99.20203 | 85.42654 | 96.70714 | 89.70412 | 81.72278 | 84.44547 | 74.96433 | 105.2542 | 71.04758 | 97.56553 |
| CUST_44431_PI429010886 | M65969 | 0.027006 | 15 | 12 | | | | | | 17.8341 | 28.93678 | 20.71416 | 13.71806 | 14.53418 | 12.89178 | 22.00022 | 10.91384 | 15.5705 | 15.79004 | 25.78586 | 23.98273 | 11.89878 | 46.44979 | 27.16077 | 24.94581 | 23.10525 | 31.49741 | 21.87979 | 17.12595 | 14.8651 | 22.02831 | 27.32872 | 8.461505 | 29.44199 | 30.95577 | 30.80438 |
| CUST_44563_PI429010886 | M66327 | 0.027006 | 15 | 12 | | | | | | 58.36485 | 78.76718 | 101.2666 | 52.42324 | 38.59666 | 66.05732 | 76.19351 | 59.0899 | 78.97339 | 45.53002 | 28.5055 | 75.85108 | 88.28345 | 118.1621 | 92.08467 | 50.09589 | 30.61606 | 72.61909 | 54.37737 | 36.78858 | 32.43294 | 50.39753 | 38.54962 | 73.69227 | 72.59952 | 76.75659 | 58.87201 |
| CUST_1814_PI429010886 | N10772 | 0.027006 | 15 | 12 | | | | | | 26.54031 | 28.39147 | 32.29997 | 14.9321 | 23.5177 | 17.30092 | 16.68621 | 14.35201 | 14.13803 | 24.11521 | 14.38683 | 34.77329 | 27.64994 | 35.40201 | 23.31758 | 17.66889 | 26.07711 | 13.01989 | 21.89117 | 26.33847 | 14.9648 | 33.08815 | 16.85251 | 14.09213 | 23.22645 | 22.25913 | 18.70952 |
| CUST_15423_PI429010886 | N34555 | 0.027006 | 15 | 12 | | | | | | 293.5772 | 249.9317 | 364.9554 | 277.3932 | 275.3091 | 379.0729 | 214.2001 | 246.8808 | 242.2528 | 333.2348 | 304.5092 | 440.8097 | 299.1597 | 370.9223 | 400.6975 | 347.0333 | 301.1632 | 361.7087 | 305.1009 | 343.2786 | 377.1617 | 288.4469 | 311.1164 | 251.312 | 329.8367 | 371.903 | 363.7161 |

| Probe ID | ID | p-value | Period | Phase | EBI ID | Annotation | GO category | Go term | E-value | 0 | 0 | 0 | 3 | 3 | 3 | 6 | 6 | 6 | 9 | 9 | 9 | 12 | 12 | 12 | 15 | 15 | 15 | 18 | 18 | 18 | 21 | 21 | 21 | 24 | 24 | 24 |
|---|---|---|---|---|---|---|---|---|---|---|---|---|---|---|---|---|---|---|---|---|---|---|---|---|---|---|---|---|---|---|---|---|---|---|---|---|
| CUST_15620_PI429010886 | N34874 | 0.027006 | 15 | 12 | | | | | | 80.14138 | 50.82916 | 64.56424 | 55.83606 | 31.33759 | 22.26367 | 67.74837 | 120.8183 | 66.53186 | 55.64622 | 46.66161 | 38.26462 | 94.08412 | 81.87922 | 61.79243 | 77.28747 | 79.14043 | 40.33441 | 58.12189 | 35.23712 | 23.79718 | 72.61909 | 36.5186 | 62.46096 | 91.6409 | 46.52112 | 71.50258 |
| CUST_77_PI429010886 | N476 | 0.027006 | 15 | 12 | | | | | | 80.22855 | 84.12226 | 77.97597 | 67.72476 | 62.91706 | 74.89819 | 65.30643 | 64.125 | 70.33128 | 84.11167 | 60.61424 | 94.92777 | 74.11739 | 97.21485 | 105.9691 | 88.60515 | 63.93448 | 80.13478 | 73.04458 | 75.56946 | 68.82889 | 87.85163 | 71.82501 | 62.52529 | 74.84295 | 97.36237 | 87.78461 |
| CUST_23002_PI429010886 | N48089 | 0.027006 | 15 | 12 | | | | | | 41.26062 | 40.07867 | 62.67301 | 45.24787 | 49.51746 | 64.30394 | 40.79687 | 42.07479 | 47.49104 | 49.24687 | 36.42394 | 78.15144 | 50.85184 | 67.60441 | 82.41691 | 55.98343 | 62.94659 | 42.76199 | 44.46924 | 52.11124 | 62.78204 | 43.439 | 37.30131 | 34.49254 | 60.26272 | 73.96327 | 66.92364 |
| CUST_23583_PI429010886 | N49066 | 0.027006 | 15 | 3 | | | | | | 47.71565 | 83.36467 | 42.56702 | 60.33388 | 63.83994 | 95.27106 | 65.22254 | 81.52938 | 35.57579 | 69.18954 | 45.95551 | 46.51079 | 42.24809 | 41.93393 | 15.83311 | 67.12853 | 99.52485 | 49.03345 | 84.86918 | 74.61343 | 50.8348 | 78.63344 | 46.25868 | 76.11306 | 69.31912 | 37.67896 | 57.92964 |
| CUST_32955_PI429010886 | M5559 | 0.027008 | 21 | 15 | | | | | | 84.57304 | 77.69279 | 68.46628 | 62.38529 | 77.44837 | 68.0898 | 69.24723 | 61.13394 | 59.80641 | 88.4815 | 69.61944 | 77.45737 | 70.52611 | 80.61162 | 60.6558 | 91.04966 | 86.58356 | 87.11963 | 85.26543 | 71.9913 | 61.51764 | 83.31796 | 68.48249 | 58.62811 | 98.95253 | 58.11529 | 75.04971 |
| CUST_44584_PI429010886 | M66385 | 0.027031 | 24 | 18 | 260814680 | hypothetical protein BRAFLDRAFT_60702 [Branchio | | | 4.99E-11 | 15.81697 | 12.87174 | 12.30868 | 4.297852 | 17.34168 | 15.73743 | 5.500018 | 9.485366 | 12.38626 | 16.18436 | 17.69892 | 18.69291 | 13.21509 | 13.12545 | 7.213286 | 15.56439 | 15.29468 | 16.80437 | 16.86851 | 31.39871 | 20.1154 | 3.795256 | 21.95116 | 18.70704 | 13.67972 | 16.94046 | 10.41181 |
| CUST_35404_PI429010886 | M12497 | 0.027047 | 15 | 9 | | | | | | 160.2021 | 138.0091 | 154.3834 | 143.3153 | 126.6371 | 125.3899 | 183.091 | 154.2025 | 178.0431 | 203.006 | 162.5528 | 198.9264 | 149.9525 | 184.9585 | 170.4197 | 203.9202 | 240.682 | 158.2442 | 176.5188 | 176.4383 | 178.833 | 195.0326 | 153.0835 | 154.1562 | 258.9285 | 179.0604 | 201.3867 |
| CUST_11006_PI429010886 | N27120 | 0.027115 | 18 | 15 | | | | | | 27.48409 | 24.49848 | 24.8301 | 23.02707 | 19.59849 | 21.87774 | 21.80116 | 18.66441 | 18.58498 | 22.97663 | 18.73831 | 22.17757 | 12.98148 | 29.92091 | 27.32872 | 22.76882 | 25.62613 | 28.63363 | 20.8433 | 14.36186 | 14.53278 | 20.62307 | 26.50986 | 18.46537 | 13.95627 | 19.36095 | 7.499239 |
| CUST_14471_PI429010886 | N32941 | 0.027116 | 24 | 6 | | | | | | 153.961 | 57.78767 | 11.16007 | 165.8485 | 39.51034 | 74.04651 | 176.7754 | 75.27932 | 149.5758 | 7.779891 | 8.957551 | NA | 116.8917 | 10.41586 | 63.75313 | 17.99221 | 30.85363 | 37.26268 | 20.68548 | 3.990879 | 7.173073 | 9.479243 | 6.810134 | 128.673 | 5.171846 | 84.2393 | 17.24927 |
| CUST_11698_PI429010886 | N28258 | 0.027118 | 15 | 0 | | | | | | 23.41982 | 67.25349 | 28.54572 | 33.81529 | 43.9229 | 65.10535 | 33.8279 | 32.97999 | 21.10666 | 55.91435 | 59.22278 | 45.49623 | 49.20084 | 61.5801 | 34.47136 | 66.89269 | 74.06718 | 70.3928 | 43.00832 | 29.12321 | 25.59155 | 27.40178 | 39.09942 | 38.57925 | 40.94647 | 45.43527 | 40.01887 |
| CUST_3700_PI429010886 | N15078 | 0.027118 | 18 | 3 | | | | | | 48.84923 | 118.3064 | NA | 532.3882 | 272.5517 | 298.9754 | 137.7783 | 8.822611 | 105.242 | 6.337987 | 4.033641 | NA | 21.9208 | 69.03435 | 123.8729 | 420.1077 | 5.078333 | 292.0038 | 15.43825 | 44.11886 | 5.683749 | 364.7093 | NA | 290.0281 | 125.7351 | NA | NA |
| CUST_51376_PI429010886 | M93316 | 0.027134 | 24 | 0 | | | | | | 25.11484 | 32.57877 | 24.61213 | 18.27067 | 22.50429 | 24.50538 | 18.65246 | 17.33078 | 28.9598 | 28.81723 | 27.49118 | 24.08567 | 16.81632 | 23.40412 | 18.39223 | 28.93067 | 19.27159 | 24.64126 | 24.2252 | 14.35036 | 23.87245 | 24.86718 | 24.0618 | 25.83743 | 26.88741 | 27.13598 | 32.90555 |
| CUST_36349_PI429010886 | M15682 | 0.027137 | 24 | 12 | | | Metabolic process | Carbohydrate metabolic process | | 3.905813 | NA | 25.352 | 12.56761 | 13.35477 | 5.864902 | 9.42148 | 40.55137 | 25.04089 | 8.669897 | 22.4434 | 34.40827 | 40.81934 | 29.11372 | 27.1975 | 20.11281 | 27.89184 | NA | 24.25926 | 66.85557 | 30.32568 | 11.83103 | 31.05637 | NA | 17.14547 | 16.71521 | 8.260264 |
| CUST_45186_PI429010886 | M68268 | 0.027143 | 15 | 6 | | | | | | 10.90408 | 19.78239 | 15.64186 | 11.83012 | 17.43083 | 13.19079 | 23.16574 | 22.25206 | 16.63993 | 13.71042 | 14.54491 | 10.16134 | 10.95578 | 13.93866 | 4.847534 | 10.65864 | 19.94747 | 14.28453 | NA | 16.11003 | 12.38228 | 15.80527 | 19.05757 | NA | 22.58247 | 11.38749 | 16.43274 |
| CUST_10301_PI429010886 | N26028 | 0.027148 | 24 | 21 | | | | | | 84.1293 | 93.94852 | 95.7753 | 85.38257 | 79.20223 | 77.43118 | 77.21633 | 75.30403 | 78.75847 | 93.72794 | 66.62115 | 84.33492 | 79.74735 | 101.8251 | 102.6905 | 85.64499 | 85.03701 | 85.54854 | 91.1558 | 84.03407 | 91.40169 | 106.8104 | 113.9625 | 83.57768 | 67.82256 | 107.6827 | 94.85349 |
| CUST_3424_PI429010886 | N14468 | 0.027166 | 24 | 6 | | | | | | 73.5024 | 72.64262 | 85.83424 | 85.34367 | 61.72335 | 58.42676 | 91.4434 | 74.15763 | 84.20531 | 69.50944 | 55.30305 | 49.65587 | 62.08961 | 65.04228 | 57.46153 | 58.77193 | 53.04479 | 58.31834 | 66.33801 | 45.53479 | 50.61028 | 73.14833 | 56.37885 | 54.01898 | 74.20243 | 66.22546 | 44.39886 |
| CUST_34543_PI429010886 | M9973 | 0.027169 | 21 | 18 | | | | | | 82.20584 | 93.56818 | 49.10837 | 63.73904 | 76.02137 | 53.22735 | 46.92136 | 45.3426 | 49.53059 | 61.25018 | 63.12362 | 90.77809 | 61.89112 | 60.39123 | 63.03207 | 104.9497 | 54.80919 | 78.25555 | 76.11306 | 93.32222 | 66.25397 | 97.50342 | 85.41552 | 47.05803 | 77.04659 | 46.3492 | 65.70437 |
| CUST_20450_PI429010886 | N43466 | 0.02717 | 21 | 0 | | | | | | 81.63335 | 102.8432 | 93.49845 | 86.07302 | 80.38938 | 67.90618 | 43.03847 | 59.3369 | 65.34071 | 63.22526 | 75.32357 | 90.51231 | 57.69532 | 89.67555 | 50.42986 | 98.98439 | 58.01793 | 83.39175 | 95.72441 | 68.1364 | 84.2989 | 118.495 | 74.67944 | 85.493 | 63.95336 | 56.36185 | 87.85163 |
| CUST_32030_PI429010886 | M3052 | 0.027172 | 21 | 12 | | | | | | 15.13318 | 9.583323 | 14.85731 | NA | 9.517811 | 9.375364 | 4.231651 | 10.75755 | 14.01751 | 14.43734 | 16.32704 | 16.0016 | 17.47076 | 17.30489 | 14.42452 | 12.57708 | 13.60386 | 11.57836 | 16.63365 | 12.83923 | 7.613075 | 15.73743 | 15.32584 | 14.86246 | 10.67877 | 6.19599 | 12.02328 |
| CUST_32834_PI429010886 | M5220 | 0.027176 | 24 | 0 | | | | | | 179.5583 | 170.1249 | 117.7722 | 167.5472 | 164.8776 | 143.9244 | 144.1199 | 127.7733 | 113.6011 | 149.3076 | 117.0962 | 158.101 | 99.74324 | 119.1024 | 110.0194 | 140.8068 | 129.6714 | 126.6031 | 173.9876 | 143.0859 | 154.4215 | 131.0159 | 108.8172 | 148.9974 | 147.2113 | 170.3395 | 134.0378 |
| CUST_20515_PI429010886 | N43588 | 0.027178 | 12 | 0 | | | | | | 15.8263 | 9.213601 | 24.1047 | 15.33408 | 8.353787 | NA | 14.5414 | 10.70265 | 8.496964 | 10.9756 | 12.36577 | 13.18006 | 21.12654 | 21.17074 | 27.05448 | 19.12543 | 17.43453 | 12.73395 | 23.42234 | 5.614366 | 6.561972 | 11.44622 | 18.47842 | 17.12071 | 18.33154 | NA | NA |
| CUST_29406_PI429010886 | N59799 | 0.027199 | 15 | 0 | | | | | | 153.5542 | 127.6673 | 143.0445 | 136.9776 | 117.7161 | 93.95638 | 108.1985 | 106.3173 | 113.6504 | 159.9242 | 135.1566 | 137.2513 | 117.8738 | 172.082 | 109.5638 | 165.5033 | 137.9515 | 137.8443 | 125.2228 | 119.7503 | 139.9485 | 91.76014 | 128.195 | 120.6234 | 181.075 | 128.5685 | 157.4172 |
| CUST_33607_PI429010886 | M7412 | 0.027233 | 24 | 12 | 405960113 | Ubiquitin carboxyl-terminal hydrolase 15 [Crassostr | | | 3.47E-08 | 7.089313 | 14.62912 | 23.88653 | 8.07676 | 9.687487 | 5.652126 | 17.75139 | 17.29417 | 27.35532 | 13.06014 | 22.04537 | 16.56559 | 21.83253 | 25.40852 | 26.3141 | 18.41069 | 16.29894 | 12.30062 | 15.22485 | 29.20693 | 16.07923 | 24.13249 | 15.01997 | 14.1448 | 22.30073 | 13.39381 | 19.85963 |
| CUST_52944_PI429010886 | M99570 | 0.027233 | 24 | 15 | 260831558 | hypothetical protein BRAFLDRAFT_65954 [Branchio | | | 1.65E-10 | 85.15981 | 127.6283 | 147.7228 | 99.75328 | 88.96346 | 97.23707 | 85.56392 | 91.86368 | 97.85969 | 151.2427 | 99.68173 | 157.378 | 93.03197 | 143.069 | 118.04 | 124.009 | 158.8514 | 193.6603 | 108.3614 | 108.7837 | 112.3919 | 128.9813 | 144.2098 | 111.5107 | 150.1848 | 130.2384 | 107.3035 |
| CUST_49535_PI429010886 | M86007 | 0.027238 | 15 | 6 | | | | | | 258.7852 | 653.6532 | 596.8255 | 171.5473 | 1101.197 | 1043.343 | 549.9307 | 283.8348 | 1089.128 | 818.4343 | 392.2104 | 1201.959 | 422.2989 | 1097.209 | 982.113 | 496.5233 | 181.4858 | 445.5129 | 506.1361 | 729.346 | 1002.452 | 4058.299 | 1131.729 | 2163.813 | 1193.503 | 986.4774 | 1343.537 |
| CUST_39069_PI429010886 | M34188 | 0.027242 | 24 | 15 | 225875099 | Euphausia superba cry gene for cryptochrome, exor | | | | 998.2355 | 1270.943 | 1396.326 | 783.1618 | 1078.296 | 1034.145 | 754.7954 | 1037.639 | 1106.085 | 1310.544 | 1115.083 | 1483.935 | 1163.104 | 1548.599 | 1419.195 | 1765.654 | 1126.811 | 1328.52 | 1270.943 | 1450.058 | 1125.765 | 1472.13 | 1174.994 | 1019.767 | 1315.839 | 1246.965 | 1499.492 |
| CUST_39283_PI429010886 | M37201 | 0.027242 | 15 | 3 | 152949487 | calcification associated soluble matrix protein 2 [Prc | | | 7.3E-23 | 9937.704 | 9519.823 | 3739.652 | 27773.77 | 27270.14 | 40354.05 | 24132.63 | 9686 | 9261.186 | 452.4978 | 20443.66 | 505.6922 | 9715.562 | 771.0971 | 5319.238 | 1751.759 | 27773.77 | 14205.89 | 23030.7 | 442.9673 | 24647.46 | 1620.39 | 20865.58 | 29281.85 | 740.6945 | 12422.81 | 448.6531 |
| CUST_49936_PI429010886 | M87794 | 0.027242 | 15 | 9 | | | | | | 49.14895 | 50.45571 | 61.24287 | 35.35407 | 38.80762 | 54.79813 | 44.24444 | 48.92098 | 63.87286 | 97.70002 | 46.08794 | 57.59047 | 47.3594 | 49.99255 | 64.26853 | 59.1427 | 61.59565 | 53.59509 | 42.70658 | 60.47979 | 39.32188 | 50.11342 | 44.98106 | 33.73933 | 75.73714 | 77.49116 | 71.09226 |
| CUST_9162_PI429010886 | N24159 | 0.027242 | 15 | 9 | | | | | | 2636.548 | 9965.559 | 4733.383 | 2805.418 | 1234.796 | 315.8463 | 418.4668 | 1724.056 | 7479.639 | 7828.426 | 7197.042 | 15199.11 | 3572.519 | 8515.08 | 5197.227 | 14775.04 | 1041.277 | 3717.434 | 4827.614 | 8129.488 | 6621.858 | 10015.79 | 6750.846 | 891.3926 | 15221.8 | 10143.74 | 15458.08 |
| CUST_12036_PI429010886 | N28816 | 0.027242 | 15 | 9 | 37595925 | signal transducer and activator of transcription [Per | Signal transduction | Other | 1.14E-85 | 40.15861 | 34.0757 | 45.63167 | 26.01832 | 27.38976 | 18.46967 | 38.49903 | 34.7228 | 29.98387 | 78.16064 | 24.90512 | 45.05944 | 22.25206 | 45.81525 | 38.38764 | 52.91645 | 37.7275 | 40.66714 | 44.88618 | 31.77739 | 29.66792 | 45.24787 | 35.48044 | 31.81303 | 46.27883 | 44.70504 | 72.44248 |
| CUST_17240_PI429010886 | N37637 | 0.027242 | 15 | 9 | | | | | | 280.12 | 318.2985 | 338.4186 | 234.9258 | 256.7514 | 319.8266 | 272.5517 | 261.8083 | 270.0349 | 376.0037 | 280.5674 | 362.1966 | 277.4454 | 331.1092 | 325.6779 | 342.3066 | 251.5651 | 288.6485 | 267.38 | 285.421 | 300.4403 | 357.152 | 297.209 | 256.281 | 342.2472 | 349.8347 | 341.847 |
| CUST_422_PI429010886 | N5133 | 0.027242 | 15 | 9 | 220172371 | putative elastin a [Rimicaris exoculata] | | | 7.65E-13 | 6630.238 | 25196.95 | 13907.98 | 1748.457 | 3929.651 | 276.6492 | 698.4337 | 9341.534 | 24425.67 | 37739.19 | 31314.74 | 48876.8 | 21773.32 | 30443.79 | 25081.91 | 37972.43 | 5197.227 | 17482.08 | 31492.49 | 33148.92 | 30443.79 | 49258.66 | 23241.72 | 1564.24 | 54279.51 | 38947.83 | 56447.03 |
| CUST_4044_PI429010886 | N15871 | 0.027256 | 12 | 0 | | | | | | 55.08741 | 65.51399 | 104.6652 | 67.36225 | 66.51353 | 65.23612 | 64.58903 | 67.12853 | 85.59472 | 60.37722 | 62.23675 | 71.35987 | 98.62916 | 90.52141 | 83.53882 | 87.5621 | 93.87602 | 81.02279 | 57.59047 | 87.55497 | 62.12557 | 54.38291 | 70.32547 | 51.62562 | 83.45905 | 84.60749 | 112.0225 |
| CUST_13292_PI429010886 | N30922 | 0.027289 | 24 | 18 | | | | | | 316.2685 | 364.475 | 341.396 | 220.6402 | 265.9908 | 195.9052 | 281.0895 | 248.3342 | 301.6337 | 227.1802 | 321.8946 | 399.0919 | 287.7468 | 328.7379 | 322.593 | 420.025 | 335.4811 | 306.9203 | 401.3173 | 373.6828 | 248.428 | 478.4338 | 278.6632 | 227.6752 | 405.4301 | 227.225 | 316.9025 |
| CUST_46832_PI429010886 | M73866 | 0.027324 | 15 | 12 | | | | | | 28.32496 | 23.21071 | 26.9442 | 18.28609 | 20.75152 | 19.32948 | 23.91073 | 23.23227 | 17.74547 | 20.144 | 25.5782 | 21.99372 | 25.03208 | 21.82183 | 30.37957 | 21.36888 | 21.80317 | 22.87323 | 19.94747 | 17.21077 | 15.7161 | 27.12757 | 16.22684 | 20.55898 | 23.49823 | 7.525118 | 22.10198 |
| CUST_2305_PI429010886 | N12067 | 0.027324 | 15 | 12 | 241835601 | anti-silencing protein, putative [Ixodes scapularis] > | Cellular component organization | Chromosome organization | 1.3E-71 | 116.178 | 99.84559 | 93.36286 | 78.45985 | 96.1698 | 54.91034 | 90.68288 | 58.23374 | 65.14347 | 123.2747 | 87.59441 | 92.42083 | 95.74719 | 96.9916 | 91.17869 | 119.4425 | 89.21981 | 74.66117 | 78.92088 | 99.50846 | 93.28305 | 83.5291 | 84.68307 | 59.3369 | 126.8312 | 86.56723 | 90.76835 |
| CUST_6058_PI429010886 | N19572 | 0.027369 | 21 | 15 | | | | | | 390.2834 | 430.8818 | 253.9339 | 343.5515 | 328.7379 | 323.5707 | 259.3271 | 253.8833 | 241.5998 | 367.5439 | 291.1632 | 363.3814 | 286.7556 | 275.0029 | 277.6024 | 460.3656 | 358.225 | 389.6265 | 395.7641 | 347.0333 | 305.4399 | 421.2748 | 264.269 | 306.5697 | 363.8764 | 266.6447 | 313.9465 |
| CUST_11211_PI429010886 | N27450 | 0.027381 | 15 | 9 | | | | | | 11.55416 | 24.69322 | 11.63036 | 9.456396 | 6.974362 | 9.422886 | 13.85759 | 4.325683 | 15.07677 | 20.09831 | 10.17638 | 10.69036 | 15.76954 | 12.43574 | 14.47801 | 15.73231 | 9.501422 | 6.976589 | 7.378942 | 9.501422 | 6.140199 | 18.76711 | 17.22674 | 5.923247 | 20.04541 | 13.36717 | 17.40769 |
| CUST_33102_PI429010886 | M5972 | 0.027392 | 21 | 15 | 156938909 | thaumatin-like protein [Suidasia medanensis] | | | 4.09E-38 | 763.4512 | 800.3484 | 799.3451 | 268.588 | 287.0591 | 616.4707 | 501.1913 | 603.9714 | 196.032 | 811.5226 | 683.3484 | 675.0461 | 392.3971 | 546.6515 | 312.4689 | 887.1414 | 1182.845 | 883.1215 | 942.6383 | 234.5938 | 371.2619 | 822.5821 | 691.1905 | 443.5662 | 402.5662 | 680.741 | 435.6005 |
| CUST_34905_PI429010886 | M11040 | 0.027409 | 12 | 0 | | | | | | 421.2064 | 407.5311 | 412.4272 | 305.0519 | 262.1655 | 237.9979 | 285.3112 | 295.7576 | 317.5778 | 377.8878 | 259.5337 | 337.2385 | 340.7478 | 358.55 | 420.4304 | 501.4801 | 326.7925 | 336.8185 | 322.4251 | 375.1955 | 292.8985 | 476.8506 | 357.7037 | 298.7909 | 412.0745 | 361.9781 | 374.682 |
| CUST_38832_PI429010886 | M31027 | 0.027438 | 18 | 15 | | | | | | 2380.879 | 3748.628 | 1348.92 | 1147.595 | 4667.789 | 3902.514 | 437.4103 | 456.1991 | 1179.34 | 3264.73 | 1762.677 | 2071.989 | 854.2242 | 2543.989 | 1728.225 | 6240.976 | 1095.161 | 5004.678 | 1981.218 | 3680.553 | 1849.813 | 1428.874 | 1364.277 | 1176.647 | 1119.759 | 1647.631 | 1275.086 |
| CUST_32288_PI429010886 | M3704 | 0.027438 | 18 | 9 | | | | | | 1124.753 | 1213.834 | 829.8828 | 942.6383 | 1183.738 | 768.5376 | 981.0866 | 1354.655 | 997.0196 | 1138.766 | 1311.23 | 1686.366 | 1233.197 | 976.6512 | 1222.467 | 1373.036 | 1012.516 | 848.334 | 1276.669 | 2434.013 | 1231.516 | 980.7968 | 834.9559 | 721.8143 | 1226.144 | 1007.431 | 1141.431 |
| CUST_47387_PI429010886 | M75919 | 0.027453 | 24 | 15 | | | | | | 97.75958 | 102.6905 | 122.6448 | 90.54311 | 108.2468 | 93.55728 | 83.00511 | 88.77007 | 96.34482 | 123.3713 | 103.1898 | 119.3119 | 100.1573 | 126.7933 | 115.7889 | 132.652 | 100.4962 | 106.872 | 107.2382 | 112.8267 | 105.1478 | 132.473 | 102.1333 | 104.205 | 99.25819 | 95.98918 | 142.6996 |
| CUST_42398_PI429010886 | M60188 | 0.027479 | 21 | 18 | | | | | | 15.46051 | 10.2343 | 11.16751 | 3.791189 | 8.29527 | 9.204642 | 12.08287 | 18.08937 | 17.72667 | 13.37838 | 17.14547 | 13.67972 | 12.11941 | NA | 7.077971 | 14.18297 | 10.42781 | 8.982813 | 14.49923 | 19.50089 | 13.09346 | 8.6774 | 10.2415 | NA | 9.207048 | 7.447234 | 5.45314 |
| CUST_44948_PI429010886 | M67491 | 0.027491 | 15 | 9 | | | | | | 12.4643 | 12.92194 | 19.20978 | 12.01567 | 13.19382 | 17.63842 | 22.49442 | 13.32448 | 20.23489 | 20.47188 | 36.48332 | 37.25447 | 13.28314 | 8.855559 | 15.13318 | 11.51639 | 15.99082 | 19.04546 | 10.51996 | 13.86795 | 19.8522 | 17.54318 | 9.605462 | 13.20123 | 16.73161 | 32.13434 | 28.94785 |
| CUST_36079_PI429010886 | M14565 | 0.027494 | 21 | 0 | 328796226 | mitochondrial ATP synthase gamma subunit precur | Metabolic process | Generation of precursor metabolites and energy | 4.83E-98 | 2140.816 | 4270.599 | 2020.188 | 1342.338 | 1719.247 | 1172.595 | 912.4218 | 2100.151 | 1294.709 | 2139.684 | 1406.77 | 949.8478 | 949.0578 | 1715.766 | 646.7171 | 2476.731 | 1601.861 | 816.1069 | 1162.112 | 2807.026 | 849.0108 | 3676.785 | 1562.541 | 2669.53 | 1523.414 | 1277.111 | 1114.646 |
| CUST_5795_PI429010886 | N19207 | 0.027494 | 21 | 12 | 321473830 | hypothetical protein DAPPUDRAFT_99108 [Daphnia | Protein metabolism | Protein folding | 3.5E-70 | 81.75759 | 90.55235 | 76.674 | 65.60373 | 67.04779 | 47.67267 | 69.70264 | 44.62585 | 83.59685 | 83.95292 | 66.59221 | 113.976 | 159.3003 | 83.58732 | 147.8481 | 99.74324 | 49.93988 | 69.42462 | 71.14697 | 108.9165 | 61.77428 | 77.4993 | 100.5297 | 67.59014 | 70.99743 | 62.34386 | 66.3015 |
| CUST_31603_PI429010886 | M1947 | 0.0275 | 24 | 21 | 321468302 | hypothetical protein DAPPUDRAFT_104373 [Daphn | | | 8.53E-52 | 108.064 | 127.7879 | 79.64311 | 65.83024 | 96.44756 | 89.14278 | 66.86178 | 75.24399 | 69.88371 | 103.7132 | 83.87586 | 82.36794 | 72.71237 | 85.29111 | 92.31619 | 135.074 | 84.45921 | 71.72159 | 86.47473 | 113.5525 | 92.29442 | 118.0563 | 85.83424 | 101.5112 | 124.9549 | 93.10199 | 107.3899 |
| CUST_52256_PI429010886 | M96867 | 0.0275 | 24 | 21 | | | | | | 45.70165 | 43.8795 | 64.4873 | 40.45279 | 52.70102 | 32.63377 | 36.41953 | 16.47009 | 31.50694 | 40.59153 | 32.19898 | 42.54782 | 43.43615 | 45.80326 | 47.88942 | 69.17707 | 35.24786 | 60.90428 | 37.11863 | 47.1063 | 46.37163 | 68.06658 | 32.91963 | 48.30048 | 44.06361 | 61.1739 | 81.46986 |
| CUST_6893_PI429010886 | N20742 | 0.0275 | 24 | 18 | | | | | | 155.1509 | 223.9394 | 211.9082 | 163.7665 | 157.1144 | 174.3884 | 180.016 | 179.3955 | 181.4858 | 304.7525 | 193.556 | 249.8933 | 207.1975 | 220.5819 | 260.1385 | 210.8661 | 226.3675 | 186.3153 | 208.9685 | 272.638 | 209.4461 | 251.0973 | 219.1432 | 228.547 | 223.7478 | 199.7111 | 234.141 |
| CUST_874_PI429010886 | N7649 | 0.027527 | 21 | 6 | 255983833 | glutathione S-transferase [Eriocheir sinensis] | | | 2.23E-55 | 35.17499 | 79.09571 | 33.1299 | 167.2734 | 513.4234 | 181.8416 | 157.4775 | 1051.549 | 589.1626 | 97.20572 | 477.1155 | 668.0541 | 721.1961 | 78.66048 | 385.9721 | 150.5497 | 271.7719 | 126.6371 | 75.37057 | 2224.226 | 450.8925 | 66.4925 | 135.9627 | 196.5334 | 113.5681 | 653.8395 | 405.9943 |
| CUST_23922_PI429010886 | N49705 | 0.027529 | 21 | 15 | | | | | | 25.27217 | 34.52588 | 27.66265 | 9.530867 | 26.7682 | NA | 3.853843 | 16.11142 | 15.99247 | 15.68875 | 10.80018 | 26.61953 | 12.81312 | 22.58452 | 26.22286 | 50.99575 | 21.0901 | 29.68336 | 19.59595 | 29.72221 | 18.15465 | 25.69293 | 25.33083 | 16.43274 | 26.30532 | 19.70849 | 16.0166 |
| CUST_37421_PI429010886 | M18968 | 0.027548 | 15 | 0 | 223403273 | sarcoplasmic calcium-binding protein [Litopenaeus | | | 1.32E-86 | 5430.703 | 5991.557 | 3661.505 | 4584.946 | 5181.094 | 4979.355 | 2708.301 | 3152.492 | 2997.206 | 4854.251 | 3453.79 | 5933.737 | 2442.431 | 3809.024 | 3651.445 | 6744.801 | 5035.54 | 6307.408 | 4490.741 | 5023.801 | 3509.529 | 4432.801 | 4371.861 | 4058.299 | 5609.183 | 5430.703 | 4424.066 |
| CUST_37903_PI429010886 | M22186 | 0.027548 | 15 | 9 | 321475563 | hypothetical protein DAPPUDRAFT_307836 [Daphn | | | 3.88E-14 | 362.3529 | 442.0725 | 463.9991 | 357.7037 | 358.2812 | 376.4458 | 368.1562 | 391.5301 | 443.4781 | 435.2247 | 357.4809 | 507.5628 | 388.0401 | 500.2189 | 472.4043 | 565.3295 | 342.5512 | 413.2538 | 352.4518 | 389.0546 | 410.3497 | 602.798 | 453.0933 | 433.7721 | 506.8788 | 501.4801 | 543.5013 |
| CUST_42491_PI429010886 | M60404 | 0.027548 | 15 | 9 | 9186884 | BCS-1 [Amphibalanus amphitrite] | | | 1.1E-08 | 171.0777 | 537.9358 | 388.1247 | 59.58353 | 254.3377 | 44.98106 | 68.63903 | 359.57 | 452.4018 | 728.235 | 194.4818 | 617.029 | 233.6523 | 572.0401 | 277.2896 | 566.0346 | 107.8082 | 262.1655 | 166.6372 | 499.2279 | 246.0125 | 406.809 | 394.5293 | 14.0382 | 633.9622 | 362.7402 | 661.8313 |
| CUST_33542_PI429010886 | M7219 | 0.027548 | 15 | 0 | 242021988 | cAMP-dependent protein kinase catalytic subunit, p | Signal transduction | Intracellular signal transduction | 2.78E-22 | 42.89451 | 39.44549 | 42.9046 | 31.8793 | 38.52489 | 41.94819 | 33.98233 | 30.52691 | 24.1047 | 42.6624 | 37.51655 | 34.119 | 26.83276 | 25.55814 | 35.9063 | 48.48483 | 51.28805 | 44.07419 | 33.28088 | 37.1484 | 34.50513 | 41.11502 | 21.17943 | 37.07575 | 56.16292 | 32.87464 | 49.03345 |
| CUST_52329_PI429010886 | M97076 | 0.027548 | 15 | 9 | 334326889 | PREDICTED: zinc finger protein 287-like [Monodelph | | | 2.97E-26 | 50.21619 | 65.38596 | 57.18723 | 44.87166 | 45.88252 | 42.10422 | 59.6812 | 47.08024 | 67.30267 | 78.70239 | 64.3475 | 74.21285 | 47.51073 | 69.20807 | 47.29592 | 81.85243 | 48.34765 | 55.17531 | 62.71939 | 64.97549 | 67.85099 | 73.44541 | 67.7681 | 55.08741 | 85.30129 | 80.70446 | 71.05715 |
| CUST_903_PI429010886 | N7758 | 0.027551 | 24 | 12 | | | | | | 484.7143 | 569.2573 | 731.0066 | 499.859 | 506.0078 | 586.2213 | 605.6379 | 607.4385 | 614.0398 | 559.1604 | 524.4761 | 715.3982 | 652.3862 | 765.4435 | 759.8078 | 697.8167 | 653.1387 | 735.196 | 606.0546 | 614.0398 | 529.4905 | 676.3702 | 590.4824 | 569.9186 | 841.7695 | 541.7506 | 753.467 |
| CUST_40736_PI429010886 | M55967 | 0.027557 | 24 | 15 | 270009780 | hypothetical protein TcasGA2_TC009077 [Tribolium | | | 1.49E-30 | 237.3209 | 188.0209 | 210.2272 | 184.3061 | 156.4871 | 217.5163 | 165.7972 | 174.1985 | 203.482 | 250.8185 | 254.095 | 231.4586 | 187.0582 | 265.7201 | 241.2571 | 292.7654 | 225.7607 | 300.2063 | 227.1414 | 242.1214 | 181.7276 | 256.8548 | 273.6903 | 219.1432 | 296.8833 | 231.8541 | 242.1667 |
| CUST_43172_PI429010886 | M62271 | 0.027557 | 24 | 15 | 242019119 | protein phosphatase 1 regulatory subunit, putative | | | 3.9E-20 | 51.51243 | 52.09599 | 50.85762 | 62.93288 | 46.45766 | 36.69017 | 21.25435 | 32.81991 | 46.83381 | 56.67223 | 42.04788 | 53.96058 | 46.26909 | 55.09266 | 24.38309 | 66.23901 | 84.31618 | 62.87324 | 47.63767 | 63.52108 | 38.05769 | 57.70169 | 47.59295 | 105.5819 | 60.94428 | 44.07915 | 58.51806 |
| CUST_16177_PI429010886 | N35795 | 0.027562 | 15 | 0 | | | | | | 59.52293 | 123.5891 | 30.83099 | 37.78218 | 38.98332 | 47.64968 | 33.62105 | 34.31018 | 23.86861 | 39.56391 | 28.63701 | 24.66303 | 25.02916 | 20.34468 | 14.33423 | 48.68295 | 43.94789 | 40.48521 | 38.54256 | 25.6094 | 26.6361 | 28.89154 | 33.48083 | 29.28006 | 32.59686 | 31.27363 | 22.19942 |
| CUST_13035_PI429010886 | N30473 | 0.027574 | 18 | 15 | | | | | | 40.1699 | 66.36423 | 43.1896 | 41.35027 | 29.82235 | 37.10603 | 37.77438 | 27.78206 | 49.44344 | 59.67038 | 28.23631 | 58.23374 | 63.30844 | 35.59734 | 59.67038 | 60.78952 | 30.08888 | 81.91685 | 43.10871 | 50.19147 | 49.688 | 59.12378 | 37.87644 | 33.96909 | 50.89404 | 18.6314 | 28.22043 |
| CUST_32295_PI429010886 | M3720 | 0.02761 | 24 | 21 | | | | | | 48.11605 | 51.53321 | 48.88298 | 35.36774 | 44.68637 | 28.26641 | 32.17296 | 26.93627 | 36.94944 | 50.12589 | 61.5685 | 50.84587 | 45.46228 | 57.74713 | 26.13131 | 74.66117 | 33.04443 | 53.8551 | 46.13849 | 64.99075 | 50.59835 | 73.64901 | 59.23972 | 44.86385 | 59.8939 | 49.68963 | 56.64713 |
| CUST_6080_PI429010886 | N19603 | 0.02761 | 21 | 18 | | | | | | 281.2623 | 376.6382 | 256.8944 | 219.3834 | 258.3561 | 264.9246 | 213.5752 | 199.2344 | 225.8273 | 331.7667 | 254.8434 | 306.1523 | 254.9115 | 261.7284 | 234.4127 | 431.7288 | 204.7153 | 313.3535 | 263.6263 | 339.9729 | 283.8866 | 359.297 | 269.1187 | 239.1825 | 296.5712 | 261.4696 | 253.5058 |
| CUST_18778_PI429010886 | N40391 | 0.02761 | 21 | 15 | 321476640 | hypothetical protein DAPPUDRAFT_312099 [Daphn | Transport | Transmembrane transport | 2.3E-19 | 27.81087 | 18.06907 | 33.42247 | 4.550003 | 10.46694 | 8.902179 | 30.99832 | 20.34468 | 18.37554 | 17.19879 | 28.3396 | 21.43269 | 24.86526 | 23.34144 | 20.62529 | 26.32739 | 49.64767 | 22.63948 | 10.21233 | 26.04992 | 21.54181 | 14.51567 | 32.93338 | 24.96011 | 22.83945 | 15.70628 | 19.25276 |
| CUST_57041_PI429010886 | E108 | 0.027642 | 15 | 6 | | | | | | 8.58357 | 8.935164 | 13.80995 | 8.687252 | 12.97798 | 9.576049 | 17.96665 | 10.50509 | 14.10671 | 5.437546 | 7.228787 | 12.87701 | 4.56075 | 12.35967 | 8.167815 | 11.09833 | 12.6398 | 14.88937 | 8.637107 | 6.820714 | 13.87516 | 13.43349 | 15.03992 | 20.13696 | 10.2343 | 7.123404 | 8.883037 |
| CUST_52243_PI429010886 | M96830 | 0.027642 | 15 | 3 | | | | | | 68.01317 | 42.24487 | 65.15734 | 68.31995 | 78.70239 | 71.03479 | 78.24583 | 99.42759 | 40.42484 | 56.65877 | 47.73713 | 42.92316 | 57.16139 | 39.81638 | 27.53031 | 66.71625 | 66.45252 | 44.84989 | 56.83575 | 52.09002 | 68.46628 | 51.59735 | 51.18041 | 88.00476 | 66.03995 | 25.99016 | 50.99575 |
| CUST_24018_PI429010886 | N49871 | 0.027642 | 15 | 12 | | | | | | 29.38995 | 44.1761 | 36.09146 | 35.9589 | 30.23517 | 38.76201 | 22.41474 | 23.48661 | 24.18723 | 38.41183 | 26.03157 | 38.96658 | 36.62478 | 62.48029 | 38.50305 | 57.18723 | 24.67089 | 49.62512 | 40.3031 | 29.84652 | 28.50232 | 43.00468 | 18.4152 | 31.32056 | 43.2321 | 28.33335 | 33.86551 |
| CUST_1376_PI429010886 | N9440 | 0.027642 | 15 | 12 | | | | | | 40.64999 | 39.08137 | 39.84647 | 29.45107 | 31.93715 | 57.72307 | 22.90764 | 32.77838 | 31.47929 | 41.14049 | 48.86193 | 28.53623 | 20.21559 | 52.98961 | 50.80203 | 53.78486 | 48.30896 | 40.52064 | 37.52679 | 23.4104 | 33.22111 | 31.96138 | 21.97651 | 32.8525 | 44.23444 | 49.27153 | 63.25496 |
| CUST_6407_PI429010886 | N20061 | 0.027653 | 24 | 15 | | | | | | 1408.714 | 1841.426 | 1422.337 | 1313.707 | 1171.568 | 1102.647 | 843.5117 | 1120.861 | 1165.064 | 1591.245 | 1170.02 | 1773.273 | 1323.05 | 1902.402 | 1804.906 | 2358.256 | 1296.822 | 1456.136 | 1449.375 | 1734.303 | 1325.228 | 1998.595 | 1425.514 | 1022.013 | 1794.046 | 1347.073 | 1729.171 |
| CUST_19199_PI429010886 | N41154 | 0.027665 | 18 | 15 | | | | | | 9.958012 | 6.387554 | 9.448882 | NA | NA | NA | 6.418804 | NA | 3.748274 | 11.37699 | 4.098171 | 4.345516 | 11.78106 | 8.651114 | 13.13227 | NA | 38.65653 | 13.08345 | 9.43779 | 5.147446 | 9.468387 | 8.337419 | 7.015011 | NA | 7.359153 | 5.633982 | 10.27942 |
| CUST_12634_PI429010886 | N29781 | 0.027683 | 21 | 0 | | | | | | 493.7491 | NA | 242.4714 | 107.4719 | 45.94496 | 287.0591 | 814.5413 | 193.8001 | 29.65821 | 272.5517 | 105.8358 | 47.83293 | NA | 16.10308 | 67.83607 | 24.35681 | 142.4711 | 18.66797 | 225.0823 | 66.46786 | 32.62765 | 327.9176 | 413.4726 | 328.3818 | 12.88422 | 57.99026 | 90.50136 |
| CUST_36439_PI429010886 | M15896 | 0.027702 | 24 | 0 | 242015516 | Splicing factor, putative [Pediculus humanus corpor | | | 8.47E-18 | 19.29786 | 27.23134 | 21.56454 | 38.33878 | 20.45683 | 16.49199 | 11.79877 | 9.415283 | 15.73595 | 20.09233 | 13.92466 | 17.92595 | 13.69809 | 12.0248 | 5.258608 | 8.943033 | 10.53669 | 13.03814 | 21.87083 | 18.81492 | 25.62877 | 9.618599 | NA | 12.7372 | 21.89563 | 10.32292 | 24.57116 |
| CUST_40596_PI429010886 | M55564 | 0.027703 | 15 | 9 | | | | | | 30.9626 | 25.15397 | 85.95967 | NA | 5.565471 | NA | NA | 33.04443 | 189.684 | 91.85305 | 203.1495 | 221.2444 | 65.93142 | 272.7999 | 176.4102 | 95.39909 | NA | 37.06882 | 74.6525 | 377.8878 | 65.95253 | 196.5038 | 159.9075 | NA | 740.0272 | 103.8532 | 624.2921 |
| CUST_30167_PI429010886 | N61309 | 0.027738 | 18 | 15 | | | | | | 47.67821 | 48.87835 | 67.57577 | 47.8959 | 47.62717 | 56.50237 | 47.43001 | 52.08646 | 52.96132 | 60.80176 | 61.1739 | 62.84333 | 51.57463 | 74.68625 | 62.96663 | 58.72685 | 69.70264 | 65.60373 | 50.25535 | 57.04658 | 62.5561 | 51.73816 | 52.645 | 56.38454 | 63.81737 | 55.29319 | 68.80724 |
| CUST_3599_PI429010886 | N14852 | 0.02774 | 24 | 21 | | | | | | 72.21756 | 104.7823 | 108.2468 | 74.33243 | 98.6386 | 81.25016 | 71.38963 | 79.66167 | 78.54463 | 117.8738 | 120.1854 | 85.17905 | 89.57185 | 109.6904 | 106.002 | 93.24144 | 147.7426 | 80.39867 | 95.21856 | 136.8231 | 117.9497 | 178.255 | 126.0122 | 103.0871 | 123.4235 | 76.22636 | 45.32916 |
| CUST_35539_PI429010886 | M12823 | 0.027744 | 18 | 12 | | | | | | 378.1407 | 453.0933 | 434.9301 | 305.4399 | 382.2635 | 383.351 | 380.3886 | 357.152 | 489.4967 | 352.5288 | 425.8413 | 590.149 | 550.9319 | 663.1345 | 628.1675 | 655.2712 | 485.2041 | 479.5081 | 464.7526 | 561.1634 | 334.5323 | 596.0223 | 363.0024 | 315.2378 | 728.4044 | 306.5332 | 582.6377 |
| CUST_39251_PI429010886 | M36715 | 0.02776 | 18 | 3 | | | | | | 1119.759 | 702.9226 | 368.0783 | 4313.91 | 2001.122 | 5627.009 | 2019.046 | 1797.174 | 1468.172 | 229.4529 | 812.8751 | 194.4273 | 1338.117 | 658.1422 | 606.6133 | 523.5388 | 2393.921 | 3211.25 | 764.3328 | 359.4804 | 947.2744 | 1287.748 | 889.7966 | 3511.533 | 399.1635 | 1972.844 | 288.6485 |
| CUST_6799_PI429010886 | N20615 | 0.02776 | 18 | 9 | 221060551 | Plasmodium knowlesi strain H fibrillarin (PKH_1339 | Nucleic acid metabolism | RNA metabolic process | 9.07E-62 | 551.5652 | 912.0221 | 511.1819 | 378.4398 | 463.1518 | 269.3458 | 378.1899 | 521.1997 | 510.0071 | 704.3489 | 585.5822 | 644.8914 | 594.1197 | 555.5605 | 578.1088 | 863.4186 | 274.1119 | 457.7503 | 430.0642 | 689.0956 | 569.2573 | 549.2217 | 497.5175 | 423.0023 | 588.5777 | 488.4404 | 655.3909 |
| CUST_3595_PI429010886 | N14845 | 0.027821 | 18 | 12 | | | | | | 51.57463 | 42.47169 | 80.14138 | 30.42832 | 35.11191 | 59.90035 | 46.83015 | 47.59957 | 64.28764 | 39.40451 | 57.54706 | 71.81768 | 74.96433 | 93.15439 | 104.1327 | 88.43444 | 60.80817 | 68.82889 | 76.0951 | 80.18725 | 41.94521 | 89.36758 | 55.55289 | 38.95233 | 68.72963 | 61.02204 | 94.99099 |
| CUST_716_PI429010886 | N6899 | 0.027821 | 21 | 12 | | | | | | 139.8337 | 109.002 | 96.71335 | 56.32248 | 111.8634 | 41.82918 | 133.7417 | 97.90896 | 66.20577 | 143.6492 | 139.8784 | 102.5774 | 169.2316 | 135.1811 | 128.3894 | 68.58333 | 115.0228 | 82.12587 | 145.6105 | 126.6662 | 52.17878 | 99.31284 | 134.7571 | 171.6223 | 76.61613 | 13.56421 | 57.44502 |
| CUST_14776_PI429010886 | N33452 | 0.027825 | 18 | 0 | | | | | | 107.2744 | 113.0325 | 79.1048 | 57.05842 | 94.02768 | 116.6405 | 72.75459 | 75.45906 | 56.58363 | 74.12883 | 89.48953 | 58.49374 | 70.35427 | 56.36899 | 63.27271 | 76.71565 | 105.0612 | 91.59828 | 86.84519 | 108.6889 | 67.92832 | 84.28941 | 72.77354 | 92.15955 | 69.00612 | 60.55923 | 71.85634 |
| CUST_5963_PI429010886 | N19433 | 0.027828 | 21 | 15 | | | | | | 832.0351 | 782.9369 | 769.5294 | 678.9878 | 603.2889 | 680.741 | 719.1308 | 563.0019 | 604.2963 | 734.7434 | 616.6013 | 577.1878 | 635.2846 | 592.5758 | 830.2031 | 971.7833 | 740.9987 | 697.5569 | 887.8552 | 820.7972 | 606.4913 | 851.7359 | 643.7576 | 611.8341 | 778.2204 | 602.9273 | 653.9628 |
| CUST_39413_PI429010886 | M39064 | 0.027839 | 12 | 0 | | | | | | 9530.181 | 14205.89 | 9686 | 7175.165 | 6441.669 | 5125.683 | 7828.426 | 7221.522 | 8086.505 | 8675.932 | 7239.058 | 11882.91 | 8015.915 | 9541.284 | 9407.387 | 11619.8 | 6483.048 | 8733.657 | 7864.497 | 8304.282 | 7083.117 | 13322.16 | 7212.951 | 6571.189 | 11061.33 | 7876.866 | 10524.41 |
| CUST_41109_PI429010886 | M56894 | 0.027839 | 15 | 9 | | | | | | 31.63804 | 92.01375 | 84.58422 | 57.44502 | 56.60227 | 82.91863 | 67.59014 | 58.22987 | 81.25969 | 79.65072 | 78.67424 | 109.3881 | 69.9232 | 92.67278 | 158.9804 | 56.68336 | 70.02583 | 115.7238 | 52.97067 | 39.25762 | 74.93674 | 88.80055 | 99.93216 | 98.23281 | 101.7195 | 73.85269 | 125.3899 |
| CUST_916_PI429010886 | N7818 | 0.027839 | 15 | 9 | 543335593 | innexin 2 [Penaeus monodon] | | | 9.55E-22 | 570.6164 | 858.2338 | 676.5905 | 563.1994 | 504.5252 | 363.5385 | 380.2921 | 553.2164 | 678.3407 | 1124.753 | 551.1 | 1165.934 | 831.7121 | 924.5282 | 1066.79 | 1151.226 | 392.0959 | 640.4514 | 481.9422 | 781.4539 | 613.1363 | 1118.972 | 722.474 | 503.6617 | 1039.457 | 643.9207 | 1073.527 |
| CUST_53288_PI429010886 | M101108 | 0.027853 | 24 | 21 | | | | | | 30.81978 | 50.8107 | 54.32031 | 37.39567 | 31.12763 | 33.03437 | 23.04508 | 29.14509 | 39.56391 | 37.49557 | 33.68931 | 37.24189 | 29.6316 | 35.78629 | 34.31858 | 60.26272 | 43.80188 | 71.47658 | 42.97599 | 32.92673 | 42.33366 | 59.27376 | 51.91237 | 43.55173 | 64.36368 | 33.00843 | 46.79864 |
| CUST_3067_PI429010886 | N13726 | 0.027854 | 15 | 9 | | | | | | 10.8378 | 17.49601 | 48.08 | 5.941184 | NA | NA | 7.952703 | 90.99167 | 222.4725 | 157.4172 | 103.802 | 116.0676 | 88.29574 | 276.6094 | 96.5915 | 39.85765 | 53.17437 | 43.78734 | 18.01905 | 187.7825 | 13.41841 | 364.8884 | 135.6374 | NA | 361.2261 | 85.91746 | 325.4847 |
| CUST_54515_PI429010886 | M106316 | 0.027854 | 15 | 0 | | | | | | 83.43282 | 122.3193 | 132.0735 | 71.62646 | 123.2134 | 172.5221 | 63.96858 | 16.28372 | 71.70446 | 70.23629 | 127.0345 | 125.719 | 40.82323 | 73.26024 | 78.04542 | 140.6816 | 118.3504 | 134.79 | 82.8752 | 73.66521 | 108.5223 | 77.00574 | 69.17707 | 87.70762 | 133.3923 | 70.2629 | 113.1265 |
| CUST_55861_PI429010886 | M111910 | 0.027854 | 15 | 9 | | | | | | 200.5357 | 244.4939 | 261.8822 | 210.4575 | 214.0659 | 242.5125 | 209.6917 | 224.015 | 245.1154 | 323.6189 | 259.6731 | 311.3229 | 219.6484 | 292.1904 | 218.6003 | 232.3124 | 233.2859 | 304.6076 | 229.5755 | 251.9218 | 293.8285 | 281.3948 | 299.6797 | 261.2237 | 331.7667 | 307.9072 | 341.9848 |
| CUST_56788_PI429010886 | M114654 | 0.027854 | 15 | 9 | | | | | | 72.32108 | 66.46046 | 78.48723 | 68.85144 | 56.43628 | 61.1536 | 78.20249 | 79.22919 | 69.9701 | 91.46281 | 63.60606 | 78.72636 | 68.35087 | 108.7588 | 81.92913 | 99.52485 | 75.42436 | 98.4006 | 78.86687 | 73.84429 | 68.33792 | 94.36809 | 85.56392 | 72.13281 | 106.002 | 100.9909 | 142.1431 |
| CUST_3040_PI429010886 | N13661 | 0.027854 | 15 | 0 | | | | | | 1953.939 | 1955.85 | 1598.096 | 561.029 | 3595.062 | 1385.628 | 1487.266 | 1159.875 | 676.9355 | 3938.093 | 1090.456 | 2211.182 | 1190.624 | 1246.44 | 1223.726 | 2292.412 | 2253.027 | 4182.62 | 1003.971 | 1058.786 | 1332.781 | 1501.641 | 884.0735 | 1084.271 | 1200.709 | 1129.849 | 1598.927 |
| CUST_9469_PI429010886 | N24683 | 0.027854 | 15 | 9 | | | | | | 112.0225 | 441.5814 | 290.4858 | 104.4054 | 150.8963 | 104.5986 | 74.85405 | 242.8547 | 456.6758 | 468.3619 | 217.6167 | 779.5608 | 247.1825 | 578.8676 | 408.8989 | 608.0744 | 208.8382 | 686.6887 | 156.6593 | 214.9913 | 232.8505 | 1107.396 | 113.9031 | 620.6433 | 601.1869 | 378.0737 | 856.5552 |
| CUST_36549_PI429010886 | M16207 | 0.027875 | 21 | 0 | 291239983 | PREDICTED: fructosamine 3 kinase related protein-l | | | 2.18E-46 | 81.03057 | 96.47975 | 85.29111 | 40.97837 | 64.03635 | 63.36121 | 63.82351 | 40.46466 | 57.9197 | 59.40544 | 59.35924 | 49.95685 | 68.03939 | 62.86517 | 53.86751 | 113.9135 | 29.6615 | 86.22581 | 76.75659 | 48.98425 | 74.89819 | 75.86883 | 70.30533 | 56.26041 | 68.51154 | 54.64518 | 49.28963 |
| CUST_21491_PI429010886 | N45316 | 0.027882 | 24 | 15 | | | | | | 25.13071 | 16.03903 | 24.33679 | 25.68325 | 24.30245 | 29.24265 | 17.17252 | 14.48423 | 20.47619 | 33.18761 | 18.7895 | 26.26256 | 23.28379 | 31.82653 | 33.7306 | 44.53615 | 34.70757 | 37.00487 | 44.89448 | 20.88813 | 29.30762 | 33.51061 | 12.27788 | 23.13699 | 33.76678 | 36.69017 | 36.04752 |
| CUST_56070_PI429010886 | M112674 | 0.027905 | 12 | 9 | | | | | | 120.0167 | 131.4056 | 297.8606 | 135.6641 | 126.7933 | 162.2752 | 82.64391 | 180.4354 | 197.9328 | 352.3859 | 265.3147 | 339.6011 | 244.4297 | 278.7763 | 293.4659 | 278.397 | 237.166 | 160.6252 | 187.9811 | 247.6825 | 269.1618 | 250.2782 | 384.5478 | 263.6263 | 334.9967 | 173.7422 | 264.7036 |
| CUST_45249_PI429010886 | M68482 | 0.027905 | 12 | 9 | | | | | | 91927.09 | 82964.44 | 84866.33 | 87648.66 | 78081.9 | 78574.02 | 88276.1 | 71869.37 | 90253.54 | 88276.1 | 88771.33 | 90253.54 | 77729.71 | 86620.4 | 74829.64 | 65872.24 | 67921.27 | 64763.62 | 85436.14 | 81077.7 | 93467.7 | 76476.71 | 92843.62 | 92382.94 | 83322.86 | 91927.09 | 91360.38 |
| CUST_34746_PI429010886 | M10540 | 0.027905 | 12 | 0 | | | | | | 21.75179 | 130.1677 | 138.8211 | 15.03227 | 33.64659 | 14.1847 | 43.64762 | 29.72938 | 48.85705 | 64.26853 | 63.53447 | 78.92088 | 51.61085 | 187.805 | 90.70315 | 107.9692 | 44.78592 | 82.82848 | 46.65359 | 118.9935 | 48.28635 | 186.9949 | 125.183 | 107.8082 | 199.7685 | 49.40423 | 145.9616 |
| CUST_19309_PI429010886 | N41359 | 0.027909 | 15 | 9 | | | | | | 37.61954 | 43.08353 | 51.99458 | 59.9743 | 44.87442 | 47.09025 | 38.53689 | 49.58479 | 59.34275 | 59.96834 | 61.72335 | 53.53068 | 62.42674 | 69.97891 | 58.00017 | 59.30066 | 47.40881 | 50.4975 | 48.57108 | 59.07548 | 48.25771 | 77.6254 | 49.42792 | 47.24905 | 66.28161 | 63.12362 | 75.18156 |
| CUST_31029_PI429010886 | M513 | 0.027931 | 12 | 9 | | | | | | 34.21339 | 33.06129 | 25.31297 | 25.93979 | 29.57087 | 27.13598 | 25.88533 | 25.48335 | 24.05186 | 40.22531 | 41.01337 | 45.6007 | 28.96949 | 43.05396 | 35.1842 | 33.90631 | 25.8162 | 28.05565 | 33.77978 | 44.0999 | 38.01489 | 37.39567 | 35.4058 | 34.03541 | 37.25067 | 31.93333 | 41.56303 |
| CUST_44072_PI429010886 | M64852 | 0.027959 | 15 | 12 | 390368084 | PREDICTED: histone-lysine N-methyltransferase PRI | | | 1.1E-37 | 24.05603 | 35.23447 | 27.30762 | 15.84737 | 22.70524 | 15.54049 | 17.70702 | 13.22245 | 32.86616 | 34.72656 | 23.22645 | 36.23985 | 36.29351 | 39.61101 | 17.81453 | 30.37957 | 15.26731 | 31.28664 | 24.22757 | 21.98819 | 24.66033 | 34.57302 | 27.26909 | 29.78998 | 33.94434 | 24.49848 | 30.99084 |
| CUST_1861_PI429010886 | N10900 | 0.027959 | 15 | 12 | | | | | | 4381.739 | 116.8209 | 8643.087 | 191.886 | 161.4564 | 598.9907 | 2502.493 | 8504.473 | 7764.128 | 10866.21 | 402.5662 | 427.0325 | 10117.01 | 18410.6 | 9541.284 | 320.2376 | 5824.829 | 346.4727 | 2915.652 | 1309.194 | 1127.217 | 2982.876 | 5762.468 | 1074.839 | 8053.863 | 718.2136 | 9978.004 |
| CUST_26534_PI429010886 | N54410 | 0.027963 | 18 | 3 | | | | | | 120.9454 | 104.5873 | 144.4632 | 193.7403 | 145.308 | 82.82132 | 231.4996 | 52.36466 | 72.30449 | 125.3551 | 152.1653 | 92.41138 | 53.62183 | 51.24529 | 65.80832 | 117.7304 | 50.88285 | 81.76577 | 104.3742 | 41.40356 | 112.8702 | 195.4506 | 186.8185 | 102.5891 | 85.00815 | 147.073 | 180.7626 |
| CUST_27684_PI429010886 | N56510 | 0.027997 | 15 | 0 | | | | | | 17.69892 | 16.54774 | 15.15404 | 18.31307 | 14.4879 | 22.89626 | 14.53096 | 14.93945 | 16.71521 | 16.91861 | 19.84447 | 15.14528 | 11.23298 | 20.95946 | 20.42128 | 22.52377 | 18.57528 | 23.00815 | 17.23467 | 16.62548 | 19.26372 | 16.37181 | 12.45979 | 7.560847 | 20.38654 | 9.819588 | 15.81556 |
| CUST_41400_PI429010886 | M57648 | 0.028044 | 21 | 0 | | | | | | 11.46886 | NA | 9.956467 | 17.18399 | 5.321149 | 15.7271 | 8.778682 | 10.01115 | 7.157803 | 6.374853 | 6.391855 | NA | 5.304061 | 6.872039 | 8.309227 | 9.771392 | 8.031655 | 9.418869 | 7.733411 | 6.967135 | NA | NA | 8.228156 | 15.73231 | 10.48257 | 7.706127 | 9.769998 |
| CUST_532_PI429010886 | N5869 | 0.028048 | 24 | 0 | | | | | | 40.78834 | 39.38726 | 44.74623 | 38.79646 | 31.98439 | 28.3053 | 36.14573 | 29.49276 | 28.36368 | 46.22541 | 30.14229 | 25.75422 | 18.76417 | 21.82017 | 32.97611 | 47.18136 | 48.21613 | 31.70016 | 38.53317 | 43.43615 | 29.31162 | 39.02972 | 32.53358 | 45.54446 | 56.30906 | 46.33014 | [illegible] |
| CUST_24641_PI429010886 | N51000 | 0.028077 | 15 | 12 | | | | | | 17.79901 | 9.920366 | 21.83957 | 18.57232 | 9.847401 | 17.12281 | 15.1109 | 14.99273 | 14.24812 | 17.66078 | 16.33291 | 18.25337 | 20.87638 | 30.19994 | 20.43255 | 19.77419 | 19.37009 | 21.00534 | 19.47107 | 11.50698 | 7.191939 | 20.1457 | 23.91073 | 17.97978 | 30.31987 | 14.34274 | 20.6694 |
| CUST_14894_PI429010886 | N33649 | 0.028109 | 24 | 15 | 242016868 | ATP-dependent RNA helicase, putative [Pediculus h | | | 1.03E-18 | 55.96041 | 47.7103 | 107.533 | 38.94819 | 43.38758 | 42.06392 | 44.47869 | 37.06438 | 43.98243 | 76.55131 | 37.67218 | 58.15467 | 54.22335 | 80.41737 | 71.11204 | 84.60083 | 53.76242 | 85.06299 | 49.42389 | 55.85206 | 65.51399 | 92.63222 | 61.95208 | 67.67878 | 36.12687 | 58.76571 | 91.17869 |
| CUST_30349_PI429010886 | N61687 | 0.028144 | 24 | 3 | | | | | | 11.80809 | 11.37412 | 11.93548 | NA | 14.97499 | 9.473492 | 13.07843 | NA | 10.8378 | 13.07843 | 12.74489 | 9.085039 | 10.32292 | NA | 9.004162 | 10.06856 | 18.59923 | 9.136468 | 7.792392 | 6.236709 | 7.54037 | 10.77469 | NA | 4.208763 | 18.37346 | 12.18023 | 10.24027 |
| CUST_12330_PI429010886 | N29296 | 0.028149 | 21 | 3 | | | | | | 139.8784 | 118.8413 | 81.57207 | 141.4486 | 254.6874 | 203.3538 | 123.349 | 98.81579 | 42.65591 | 194.4273 | 50.66205 | 283.3904 | 12.35209 | 10.49198 | 89.19702 | 97.51669 | 144.8143 | 108.3885 | 250.9845 | 90.08902 | 376.8596 | 163.7139 | 145.3839 | 52.06984 | 247.3239 | 128.1757 | 425.2074 |
| CUST_35535_PI429010886 | M12818 | 0.028152 | 24 | 21 | | | | | | 36.29774 | 60.54854 | 15.557 | 68.82889 | 41.87571 | 29.45107 | 26.03903 | 19.83923 | 19.04367 | 33.53978 | 34.77329 | 47.327 | 37.36976 | 61.22888 | 51.62109 | 76.63992 | 36.41652 | 75.27932 | 20.6809 | 52.53463 | 41.2187 | 76.20212 | 43.97029 | 53.02384 | 45.00666 | 38.0304 | 26.24834 |
| CUST_46840_PI429010886 | M73895 | 0.028158 | 24 | 0 | | | | | | 32.41678 | 31.07173 | 32.96433 | 27.82792 | 15.9743 | 25.88326 | 20.90912 | 24.0618 | 15.79172 | 20.41547 | 7.053731 | 28.55239 | 12.23281 | 21.24048 | 17.8341 | 16.89973 | 13.20272 | 19.93792 | 27.50275 | 14.09963 | 23.69356 | 17.00366 | 27.52766 | 8.228156 | 21.27634 | 27.0727 | 18.10067 |
| CUST_48077_PI429010886 | M78787 | 0.028158 | 24 | 0 | | | | | | 48.92704 | 63.29578 | 95.42654 | 27.91558 | 46.67853 | 22.97663 | 41.65038 | 50.20569 | 40.76277 | 59.8939 | 35.84962 | 29.14509 | 50.65756 | 49.56937 | 48.67882 | 58.28332 | 30.59095 | 47.44495 | 56.84346 | 46.62471 | 73.75273 | 70.98423 | 63.4424 | 38.11743 | 85.82609 | 64.48135 | 81.31167 |
| CUST_19939_PI429010886 | N42484 | 0.028159 | 18 | 12 | | | | | | 397.0596 | 217.7055 | 360.9417 | 134.2991 | 173.0247 | 119.3927 | 314.4386 | 444.8946 | 207.0425 | 405.8466 | 213.9187 | 208.39 | 387.0723 | 348.906 | 216.4729 | 251.7054 | 292.0563 | 176.2005 | 245.1598 | 233.5227 | 153.734 | 290.5429 | 165.4521 | 136.0993 | 344.24 | 229.5755 | 260.373 |
| CUST_30733_PI429010886 | N62515 | 0.028159 | 12 | 0 | | | | | | 29.99408 | 30.68045 | 39.44972 | 16.67738 | 30.33968 | 17.91994 | 28.56717 | 21.77287 | 22.9097 | 33.05506 | 31.6776 | 30.12445 | 29.36585 | 39.01912 | 41.82009 | 36.93404 | 26.64032 | 19.79646 | 21.8729 | 39.46953 | 23.64694 | 40.38558 | 29.00316 | 27.3183 | 31.99053 | 33.07929 | 28.83204 |
| CUST_14572_PI429010886 | N33095 | 0.028161 | 15 | 9 | | | | | | 131.1566 | 183.9501 | 134.6396 | 121.0016 | 105.2542 | 150.2252 | 153.3563 | 153.6651 | 154.3563 | 193.3576 | 113.1518 | 247.7794 | 134.0969 | 194.5182 | 181.6229 | 155.4878 | 147.5068 | 224.9139 | 154.542 | 138.4518 | 114.0128 | 194.3549 | 185.7359 | 159.0669 | 213.5752 | 168.1988 | 184.5383 |
| CUST_6322_PI429010886 | N19946 | 0.02818 | 12 | 3 | 548531516 | PREDICTED: E3 ubiquitin-protein ligase HUWE1 isof | | | 2.7E-124 | 116.7605 | 161.827 | 126.8312 | 219.3055 | 115.6518 | 216.5438 | 87.17451 | 73.81016 | 59.35346 | 101.2537 | 75.58303 | 55.89482 | 83.6912 | 121.7341 | 90.44569 | 133.006 | 128.2524 | 135.3289 | 198.008 | 79.69567 | 59.55084 | 134.244 | 68.64401 | 113.6011 | 100.0527 | 115.5316 | 95.73603 |
| CUST_54083_PI429010886 | M104472 | 0.028195 | 24 | 18 | | | | | | 631.1117 | 639.8175 | 729.1026 | 515.4056 | 536.7924 | 530.3833 | 519.3194 | 494.4534 | 500.086 | 692.7803 | 542.234 | 818.9073 | 541.6023 | 685.3204 | 662.5447 | 832.3284 | 644.6096 | 594.7631 | 609.1465 | 670.6469 | 689.4854 | 719.1308 | 661.1589 | 640.6647 | 805.5548 | 581 | 851.7359 |
| CUST_35466_PI429010886 | M12642 | 0.028205 | 24 | 21 | | | | | | 123.669 | 105.1159 | 137.687 | 87.5621 | 85.6898 | 120.7327 | 85.77208 | 86.72657 | 60.71325 | 95.47811 | 105.7694 | 140.5657 | 70.14592 | 120.7487 | 102.0104 | 176.5494 | 127.4128 | 150.0554 | 126.1331 | 104.5131 | 122.0182 | 126.4216 | 113.525 | 149.7134 | 191.3655 | 203.8732 | 205.0143 |
| CUST_15435_PI429010886 | N34576 | 0.028205 | 24 | 3 | | | | | | 240.4265 | 146.882 | 170.6145 | 207.0153 | 155.4878 | 219.715 | 168.4127 | 95.65165 | 178.8821 | 115.1745 | 170.2922 | 128.9553 | 151.7333 | 145.2632 | 146.622 | 187.0582 | 117.8738 | 138.4086 | 103.8441 | 102.0461 | 107.3764 | 219.0379 | 145.3456 | 149.1265 | 247.0492 | 143.7068 | 144.2982 |

| Probe ID | ID | p-value | Period | Phase | EBI ID | Annotation | GO category | Go term | E-value | 0 | 0 | 0 | 3 | 3 | 3 | 6 | 6 | 6 | 9 | 9 | 9 | 12 | 12 | 12 | 15 | 15 | 15 | 18 | 18 | 18 | 21 | 21 | 21 | 24 | 24 | 24 |
|---|---|---|---|---|---|---|---|---|---|---|---|---|---|---|---|---|---|---|---|---|---|---|---|---|---|---|---|---|---|---|---|---|---|---|---|---|
| CUST_21245_PI429010886 | N44881 | 0.028206 | 24 | 15 | | | | | | 177.5884 | 220.6402 | 199.4211 | 217.37 | 145.9425 | 210.9062 | 203.4225 | 175.9719 | 159.2366 | 243.2786 | 186.1374 | 210.5891 | 179.0014 | 211.4366 | 271.9546 | 234.2305 | 224.1509 | 238.1723 | 237.5448 | 241.7686 | 183.8985 | 223.0044 | 208.7066 | 234.017 | 230.36 | 278.6632 | 185.6237 |
| CUST_8342_PI429010886 | N22906 | 0.028226 | 15 | 3 | | | | | | 546.956 | 509.8767 | 321.0768 | 554.8262 | 429.6585 | 601.962 | 514.6723 | 410.1622 | 428.333 | 359.0068 | 365.1838 | 427.1137 | 433.3733 | 426.6051 | 390.7246 | 465.6558 | 476.7276 | 468.7286 | 473.9584 | 391.0528 | 350.3639 | 454.8231 | 362.2892 | 525.2083 | 385.9721 | 313.8962 | 286.4677 |
| CUST_15477_PI429010886 | N34639 | 0.028228 | 15 | 9 | | | | | | 43.75577 | 101.2088 | 100.6181 | 66.90864 | 69.86983 | 103.4856 | 124.9854 | 109.2258 | 127.3036 | 161.2704 | 80.72822 | 119.7354 | 47.63767 | 150.7391 | 108.1857 | 43.05742 | 79.29506 | 133.2442 | 58.08697 | 53.94407 | 110.3002 | 85.72262 | 168.3078 | 204.8186 | 126.6474 | 137.351 | 159.741 |
| CUST_5269_PI429010886 | N18473 | 0.02823 | 12 | 0 | 291236218 | PREDICTED: NucleOLar protein family member (nol- | | | 5.48E-41 | 50.71291 | 55.55874 | 48.52333 | 34.86128 | 38.93982 | 13.47949 | 32.48228 | 27.9685 | 32.36622 | 55.78991 | 40.05813 | 60.5059 | 36.78203 | 66.30772 | 28.86203 | 49.81881 | 25.72813 | 30.2585 | 34.55538 | 51.61587 | 47.90107 | 47.71977 | 40.20059 | 30.81978 | 65.92191 | 55.80731 | 58.892 |
| CUST_42038_PI429010886 | M59219 | 0.028277 | 15 | 12 | 548531516 | PREDICTED: E3 ubiquitin-protein ligase HUWE1 isof | | | 2.04E-92 | 56.95742 | 44.67525 | 58.597 | 76.924 | 47.13629 | 48.92098 | 44.66322 | 47.14504 | 38.97838 | 55.86399 | 58.09597 | 64.45055 | 63.85949 | 58.36485 | 62.96106 | 53.36598 | 59.86951 | 58.60431 | 44.09424 | 56.73383 | 51.25067 | 74.71666 | 34.11542 | 26.86002 | 69.60585 | 64.77801 | 60.93069 |
| CUST_21563_PI429010886 | N45439 | 0.028313 | 18 | 15 | | | | | | 24.05186 | 16.77789 | 20.06061 | 24.30558 | 14.07075 | 18.57232 | 9.823191 | 12.46283 | 9.834889 | 30.54342 | 18.57026 | 22.4606 | 16.94914 | 14.06235 | 11.52352 | 22.93214 | 26.29349 | 23.63855 | 25.1894 | 74.15763 | 19.9835 | 13.67832 | 15.5215 | 8.963404 | 5.805366 | 25.36775 | 18.93037 |
| CUST_57337_PI429010886 | E6446 | 0.028335 | 15 | 9 | | | | | | 55.9701 | 67.50801 | 74.63865 | 57.99026 | 54.23167 | 61.05832 | 51.31578 | 52.96132 | 70.80885 | 70.32547 | 102.7959 | 77.64007 | 55.93124 | 77.89081 | 71.41994 | 65.1875 | 67.46884 | 65.43441 | 66.66314 | 66.25397 | 61.18601 | 61.49124 | 64.39273 | 64.81878 | 88.40608 | 58.15467 | 89.41173 |
| CUST_29000_PI429010886 | N58999 | 0.028366 | 18 | 9 | | | | | | 42.53656 | 35.63559 | 56.19324 | 19.82899 | 24.6471 | 22.79184 | 35.81535 | 43.30808 | 37.1484 | 67.04779 | 42.82585 | 51.91237 | 43.64196 | 44.54185 | 53.69064 | 48.21189 | 58.597 | 35.54827 | 58.25188 | 50.039 | 29.76829 | 48.61196 | 41.80062 | 34.10794 | 47.80852 | 46.55207 | 58.05303 |
| CUST_15304_PI429010886 | N34365 | 0.028378 | 24 | 15 | | | | | | 20.12103 | 28.3608 | 8.498279 | 20.71589 | 26.26779 | 13.2528 | 19.35495 | 17.0738 | 12.69324 | 26.52817 | 23.64095 | 29.64495 | 30.75309 | 28.1987 | 23.29568 | 40.3031 | 21.3795 | 30.53648 | 26.38163 | 37.33468 | 13.06491 | 32.8295 | NA | 24.87004 | 23.51171 | 20.88612 | 23.60076 |
| CUST_17542_PI429010886 | N38148 | 0.028378 | 24 | 0 | 170038740 | phospholipid scramblase 1 [Culex quinquefasciatus] | | | 2.48E-28 | 41.55626 | 37.4516 | 40.33441 | 37.53618 | 37.12271 | 33.36548 | 32.45773 | 34.53803 | 43.62743 | 33.51452 | 22.5314 | 27.38684 | 28.10538 | 27.89681 | 36.48332 | 29.82235 | 28.62456 | 15.5304 | 34.86849 | 48.17521 | 37.31704 | 31.95868 | 24.86718 | 42.27106 | 28.03356 | 56.8218 | 36.29774 |
| CUST_53481_PI429010886 | M101886 | 0.028382 | 21 | 15 | | | | | | 6.037096 | 8.387454 | 7.728763 | 9.464207 | 6.494902 | 7.842686 | 3.76154 | 8.419112 | NA | 10.43749 | 4.458762 | 10.21387 | 6.412722 | 10.99151 | 6.616037 | 12.80086 | 8.707775 | 11.32033 | 12.17117 | 9.274758 | 11.52569 | 6.793654 | NA | NA | 9.963882 | 5.900827 | 5.92639 |
| CUST_25163_PI429010886 | N51893 | 0.028411 | 12 | 0 | | | | | | 562.1725 | 580.6994 | 491.272 | 555.3104 | 485.7919 | 602.6317 | 468.822 | 469.6397 | 531.5557 | 702.2549 | 534.5603 | 739.8087 | 533.4173 | 642.8014 | 743.4007 | 477.8507 | 501.702 | 494.5663 | 491.0819 | 587.8154 | 529.9042 | 689.4854 | 557.1607 | 512.0216 | 748.7335 | 719.6282 | 628.8832 |
| CUST_43455_PI429010886 | M63104 | 0.028425 | 21 | 0 | | | | | | 52.80847 | 31.15588 | 54.5227 | 38.72684 | 37.54361 | 31.43817 | 39.7396 | 31.76384 | 53.29328 | 35.7636 | 23.56221 | 28.1163 | 30.96598 | 33.18183 | 18.65856 | 23.38605 | 37.17994 | 35.6576 | 30.87561 | 23.81358 | 39.44972 | 34.47823 | 48.21189 | 49.01378 | 52.9276 | 32.08471 | 35.86628 |
| CUST_42999_PI429010886 | M61815 | 0.028448 | 24 | 21 | | | | | | 14.91349 | 21.8123 | 20.19663 | 18.98962 | 14.64501 | 11.38971 | 11.36366 | 16.17384 | 20.71979 | 33.47045 | 20.28753 | 25.04089 | 20.36175 | 17.01511 | 8.727631 | 30.74351 | 18.29941 | 25.5267 | 16.18083 | 28.41298 | 68.06658 | 24.37698 | 59.11379 | 36.55801 | 33.56428 | 16.47211 | 23.96204 |
| CUST_48886_PI429010886 | M82714 | 0.028448 | 21 | 3 | 91088399 | PREDICTED: similar to Rapgap1 CG34374-PF [Triboli | Signal transduction | Other | 2.46E-24 | 49.67787 | 48.50963 | 46.33994 | 56.50947 | 47.27714 | 51.62109 | 61.64233 | 36.72937 | 47.5011 | 49.97285 | 37.88014 | 35.91866 | 42.26034 | 46.81158 | 39.94582 | 41.86651 | 56.65877 | 52.38415 | 36.53489 | 48.03764 | 55.15261 | 57.62155 | 60.81378 | 53.52565 | 82.72689 | 49.13393 | 63.34141 |
| CUST_47971_PI429010886 | M78333 | 0.028476 | 21 | 3 | | | | | | 111.1756 | 109.8291 | 107.8324 | 147.687 | 136.6153 | 118.7356 | 107.0174 | 90.28628 | 96.97877 | 132.033 | 116.1614 | 107.2382 | 89.93577 | 115.0393 | 93.55728 | 148.1409 | 107.533 | 109.002 | 120.5667 | 117.9689 | 99.58413 | 130.0885 | 197.3524 | 95.51753 | 142.821 | 137.4615 | 131.7044 |
| CUST_22172_PI429010886 | N46564 | 0.028478 | 18 | 6 | 260831342 | hypothetical protein BRAFLDRAFT_202728 [Branchi | Metabolic process | Oxidation-reduction process | 1.92E-34 | 15.18687 | 6.406079 | 14.50765 | 7.737261 | 5.533896 | 6.07716 | 18.12511 | 13.11969 | 15.42925 | 12.75297 | 7.84373 | 9.374459 | 7.733411 | 12.91624 | 7.811739 | 5.185762 | 8.613965 | 3.565422 | 9.073537 | 3.260163 | 6.580137 | 7.700308 | 20.91496 | 7.745791 | 14.06382 | NA | 11.16121 |
| CUST_143_PI429010886 | N1541 | 0.028479 | 12 | 0 | | | | | | 17110.44 | 9937.704 | 27412.06 | 2125.97 | 4854.251 | 2580.486 | 16297.18 | 26713.02 | 23137.01 | 26783.35 | 14054.76 | 13487.6 | 20646.45 | 29764.17 | 14529.33 | 8304.282 | 20372.97 | 8421.341 | 16571.88 | 11013.97 | 12175.63 | 17165.71 | 23998.85 | 8527.974 | 30360.4 | 8955.736 | 26713.02 |
| CUST_22211_PI429010886 | N46633 | 0.028479 | 12 | 0 | | | | | | 77.52659 | 88.92819 | 157.3603 | 50.00676 | 35.12261 | 25.49244 | 77.5823 | 134.5271 | 120.7327 | 158.101 | 62.03099 | 62.23219 | 109.3295 | 109.9853 | 108.9872 | 89.27715 | 59.35924 | 43.55575 | 56.65296 | 93.94852 | 66.09773 | 87.81308 | 93.10199 | 46.75809 | 109.6371 | 79.36014 | 135.7237 |
| CUST_1169_PI429010886 | N8723 | 0.028479 | 12 | 0 | | | | | | 1254.757 | 1574.342 | 1557.99 | 1344.532 | 1184.216 | 1275.591 | 1030.293 | 1162.112 | 1394.051 | 1550.902 | 1268.692 | 1708.455 | 1356.243 | 1895.191 | 1904.883 | 1712.532 | 1230.999 | 1447.664 | 1093.408 | 1516.22 | 1424.308 | 1734.303 | 1394.051 | 1324.078 | 1713.527 | 1451.905 | 1846.031 |
| CUST_2277_PI429010886 | N12011 | 0.028488 | 21 | 9 | 321466429 | hypothetical protein DAPPUDRAFT_225662 [Daphn | | | 2.12E-15 | 14.72906 | 80.87419 | 64.79479 | 187.6138 | 281.447 | 66.67032 | 140.4504 | 643.2335 | 359.3716 | 69.90395 | 638.47 | 673.3658 | 443.4781 | 94.40103 | 163.4951 | NA | 294.4013 | 13.74046 | 59.40092 | 1394.051 | 394.7279 | 8.546868 | 96.24145 | 83.51601 | 21.59935 | 307.5089 | 304.942 |
| CUST_7297_PI429010886 | N21337 | 0.028494 | 18 | 3 | | | | | | 39.05552 | 30.74654 | 41.28276 | 222.9038 | 96.61745 | 174.4788 | 103.8804 | 54.85716 | 44.38974 | 37.68617 | 87.81308 | 55.85206 | 44.06771 | 34.36064 | 26.56221 | 36.7129 | 53.83209 | 64.35394 | 41.46823 | 30.3471 | 122.5998 | 39.61101 | 103.0771 | 81.85243 | 61.911 | 105.1367 | 36.30688 |
| CUST_5467_PI429010886 | N18743 | 0.028499 | 21 | 15 | | | | | | 192.6772 | 227.3449 | 236.9573 | 202.6446 | 258.9285 | 217.0599 | 224.7094 | 176.0053 | 216.0765 | 181.3561 | 173.4253 | 189.9402 | 201.2454 | 225.0368 | 189.3905 | 252.9891 | 217.0982 | 307.9665 | 229.9564 | 221.9188 | 265.1519 | 279.1889 | 177.5136 | 198.0363 | 289.4881 | 192.8746 | 220.2522 |
| CUST_33900_PI429010886 | M8192 | 0.02851 | 18 | 9 | | | | | | 19.51092 | 12.59239 | 20.40858 | 15.52381 | 15.88403 | 10.35023 | 16.63804 | 13.34989 | 16.15585 | 17.65649 | 18.11915 | 25.61415 | 11.28356 | 25.03856 | 18.25998 | 16.71152 | 20.28225 | 20.14785 | 14.81603 | 21.67714 | 17.66078 | 12.61842 | 11.9014 | 11.49641 | 27.06658 | 13.92846 | 26.87814 |
| CUST_38877_PI429010886 | M31478 | 0.028518 | 21 | 6 | 321479210 | hypothetical protein DAPPUDRAFT_299971 [Daphn | | | 2.08E-14 | 6.541514 | 1310.544 | 510.1908 | 2134.91 | 2306.592 | 1051.932 | 1365.452 | 6448.481 | 5013.208 | 1396.326 | 4064.703 | 6026.937 | 3208.657 | 1038.033 | 2033.91 | NA | 1725.627 | NA | 624.47 | 13734.78 | 4461.585 | 8.351204 | 1827.911 | 1528.209 | NA | 4773.42 | 4847.521 |
| CUST_24558_PI429010886 | N50837 | 0.028521 | 21 | 18 | | | | | | 35.80705 | 27.26909 | 34.81682 | 40.71927 | 28.88408 | 36.01731 | 24.28972 | 24.88619 | 21.79421 | 22.82685 | 32.68104 | 29.72221 | 31.14945 | 25.82466 | 35.82003 | 42.20313 | 36.32136 | 28.24069 | 38.25818 | 43.96447 | 29.00747 | 34.05422 | 26.48449 | 31.5882 | 27.13842 | 36.77765 | 30.85708 |
| CUST_7635_PI429010886 | N21848 | 0.028523 | 24 | 15 | | | | | | 276.7912 | 299.4458 | 368.2489 | 214.1014 | 249.8541 | 247.6419 | 220.8734 | 209.5249 | 275.4605 | 344.0193 | 271.3533 | 389.6265 | 283.9501 | 346.965 | 373.896 | 360.9417 | 303.2741 | 341.4469 | 328.2492 | 348.9836 | 271.4065 | 372.5738 | 307.9072 | 248.7474 | 329.0332 | 280.0685 | 359.8163 |
| CUST_25784_PI429010886 | N53060 | 0.028533 | 18 | 0 | | | | | | 21.69636 | 18.7709 | 28.91721 | 13.00375 | 6.976589 | 17.67291 | 17.85063 | NA | 13.64087 | 14.33423 | 12.26155 | 11.57662 | 8.023445 | 27.29319 | 4.970591 | 8.652112 | 13.9884 | 10.87355 | 54.99254 | 19.89653 | 24.8275 | 10.86868 | 26.66367 | 18.2356 | 17.64058 | 12.70528 | 30.3611 |
| CUST_11111_PI429010886 | N27283 | 0.028535 | 24 | 0 | | | | | | 77.60864 | 171.5722 | 130.7837 | 89.33478 | 147.2999 | 102.3423 | 102.4176 | 99.7668 | 125.099 | 179.8168 | 101.9095 | 115.6759 | 75.50768 | 95.18251 | 88.31622 | 108.1674 | 98.73097 | 112.6894 | 115.1567 | 86.9224 | 130.357 | 114.1483 | 179.3955 | 131.7044 | 141.1131 | 127.3248 | 122.5181 |
| CUST_54262_PI429010886 | M105321 | 0.028536 | 15 | 3 | | | | | | 125.8598 | 45.48365 | 122.7332 | 238.6203 | 149.8301 | 221.0933 | 180.2584 | 217.8082 | 55.32634 | 11.90477 | 158.4788 | NA | 76.44833 | 29.18222 | 38.6903 | NA | 126.3312 | 17.61531 | 101.7238 | NA | 109.9853 | NA | 61.40092 | 211.0801 | 8.806922 | 13.44962 | NA |
| CUST_6326_PI429010886 | N19950 | 0.028554 | 12 | 0 | | | | | | 164.7626 | 182.7865 | 222.2031 | 99.47188 | 111.5705 | 109.0193 | 122.0534 | 156.9601 | 168.3796 | 228.0795 | 146.8674 | 203.5264 | 160.0546 | 210.7973 | 165.5033 | 190.645 | 139.7482 | 147.1318 | 161.4564 | 158.1524 | 166.8454 | 194.4538 | 193.6314 | 89.31811 | 210.6402 | 159.8607 | 209.2252 |
| CUST_33916_PI429010886 | M8231 | 0.028558 | 24 | 18 | | | | | | 38.88459 | 18.99531 | 17.96665 | 15.54567 | 19.50089 | 20.75304 | 28.38786 | 23.56736 | 25.99299 | 37.38785 | 16.70318 | 14.02745 | 19.06154 | 22.80672 | 29.54213 | 34.6933 | 40.83264 | 52.85888 | 53.53627 | 36.14912 | 35.23712 | 59.68907 | 17.61362 | 23.11751 | 34.62398 | 37.93423 | 32.47618 |
| CUST_40778_PI429010886 | M56059 | 0.028586 | 12 | 9 | 270003094 | hypothetical protein TcasGA2_TC000123 [Tribolium | Nucleic acid metabolism | Regulation of gene expression | 5.34E-13 | 80.23994 | 132.8409 | 79.84889 | 62.74739 | 69.19616 | 53.84406 | 44.07091 | 62.99128 | 78.08745 | 119.3119 | 88.40608 | 139.2667 | 87.74689 | 118.233 | 112.1531 | 103.202 | 72.81129 | 73.5908 | 71.66601 | 120.1692 | 93.60773 | 99.82512 | 120.1854 | 82.59837 | 80.63947 | 88.72989 | 152.6328 |
| CUST_2718_PI429010886 | N12972 | 0.028586 | 21 | 12 | 345496989 | PREDICTED: probable peroxisomal acyl-coenzyme A | Metabolic process | Lipid metabolic process | 4.5E-149 | NA | 5.649998 | NA | 10.27148 | 25.68325 | NA | 20.44923 | 18.92641 | 10.05594 | 22.68396 | 74.33243 | 65.10535 | 131.259 | NA | 69.14635 | 27.80089 | 24.76741 | 19.58405 | 14.24335 | 270.6283 | 30.14891 | 12.6973 | 12.40402 | 13.15672 | 4.66559 | 67.12853 | 115.7617 |
| CUST_37690_PI429010886 | M20696 | 0.028595 | 15 | 12 | 7320913 | masquerade-like protein [Pacifastacus leniusculus] | Protein metabolism | Proteolysis | 1.1E-100 | 1219.823 | 1010.922 | 1395.016 | 718.2136 | 498.8709 | 262.1655 | 1715.766 | 994.7785 | 1404.631 | 1663.806 | 518.8148 | 988.0968 | 2341.487 | 2351.436 | 1655.791 | 1717.641 | 1377.193 | 579.8544 | 1000.153 | 1085.565 | 764.0731 | 1920.006 | 1801.38 | 743.802 | 3138.529 | 656.3922 | 3219.948 |
| CUST_19042_PI429010886 | N40886 | 0.028595 | 15 | 12 | | | | | | 14.99431 | 20.02214 | 8.346516 | 4.26332 | 5.524891 | 5.05173 | 5.92558 | 9.79486 | 4.875781 | 17.72667 | 6.810134 | 11.84191 | 7.682987 | 21.5895 | 8.494473 | 13.49483 | 11.21679 | 8.854627 | 10.32292 | 5.063659 | 5.330047 | 16.12591 | 8.195858 | 10.9768 | 18.09887 | 9.524556 | 12.0863 |
| CUST_53535_PI429010886 | M102151 | 0.028629 | 21 | 3 | | | | | | 43.87581 | 33.92714 | 45.4417 | 33.42509 | 44.47869 | 49.3486 | 23.6887 | 35.95337 | 29.75085 | 30.45779 | 29.89381 | 34.1984 | 25.64937 | 35.26822 | 33.26462 | 32.54126 | 39.96875 | 41.75113 | 35.01361 | 20.65721 | 38.0304 | 33.41929 | 38.81815 | 46.00478 | 35.9369 | 41.76249 | 43.5925 |
| CUST_22486_PI429010886 | N47124 | 0.028634 | 24 | 21 | | | | | | 33.34314 | 39.08829 | 16.53843 | 27.06658 | 37.92303 | 32.18854 | 15.18687 | 15.64687 | 21.07965 | 37.8309 | 23.86522 | 15.37345 | 30.14891 | 28.51882 | 23.74624 | 32.00727 | 28.74973 | 34.57676 | 21.80535 | 27.78422 | 23.05184 | 38.12468 | 42.71532 | 29.25886 | 24.23621 | 37.34877 | 25.97297 |
| CUST_46127_PI429010886 | M71476 | 0.02871 | 15 | 12 | 321470910 | hypothetical protein DAPPUDRAFT_49564 [Daphnia | Signal transduction | Other | 3.75E-30 | 16.40268 | 22.5725 | 25.37243 | 4.894593 | 10.80337 | 12.09198 | 15.93573 | 13.98699 | 17.5674 | 20.99955 | 16.8955 | 14.67098 | 14.60212 | 23.47811 | 27.33369 | 23.5201 | 19.66959 | 17.81636 | 17.20064 | 13.57199 | 16.82166 | 21.99372 | 23.21852 | 13.99239 | 19.01271 | 19.51092 | 11.06447 |
| CUST_26740_PI429010886 | N54794 | 0.028726 | 21 | 3 | | | | | | 172.498 | 182.4397 | 224.8249 | 196.6343 | 194.0962 | 233.088 | 200.1336 | 188.7518 | 213.3037 | 220.3651 | 232.5962 | 207.7149 | 163.1012 | 185.7359 | 189.7693 | 169.6808 | 207.0153 | 151.6234 | 156.8624 | 175.011 | 239.7925 | 187.9002 | 227.0035 | 232.8026 | 253.6933 | 193.5749 | 239.7427 |
| CUST_38835_PI429010886 | M31062 | 0.028729 | 18 | 3 | | | | | | 4979.355 | 2756.174 | 848.6156 | 14950.27 | 6026.937 | 17482.08 | 7197.042 | 2977.308 | 6148.012 | 503.8676 | 1695.261 | 593.6703 | 4399.213 | 1955.85 | 4221.337 | 1883.709 | 7119.249 | 9770.164 | 3077.216 | 1728.225 | 2168.863 | 5349.606 | 2623.017 | 13124.49 | 1040.197 | 6863.631 | 444.5828 |
| CUST_43202_PI429010886 | M62393 | 0.028729 | 18 | 9 | | | | | | 16.06632 | 28.13335 | 29.68336 | 17.79901 | 18.68487 | 16.46383 | 11.73206 | 21.23846 | 19.78805 | 34.80653 | 25.62613 | 34.6052 | 14.80507 | 22.86472 | 26.80447 | 23.5068 | 17.19461 | 22.32946 | 19.9426 | 25.95428 | 26.3358 | 15.15061 | 7.863762 | 18.4152 | 19.58019 | 30.3731 | 17.42213 |
| CUST_53613_PI429010886 | M102478 | 0.028735 | 24 | 3 | | | | | | 326.4884 | 1569.266 | 434.4861 | 1907.989 | 638.47 | 780.6851 | 391.4581 | 540.3776 | 380.6318 | 499.6354 | 265.1181 | 880.8883 | 400.3298 | 517.0533 | 574.2435 | 499.2279 | 114.7999 | 398.993 | 238.8193 | 188.3473 | 578.2355 | 403.2396 | 619.0377 | 376.6382 | 658.1422 | 1009.233 | 340.2645 |
| CUST_16176_PI429010886 | N35794 | 0.028745 | 24 | 0 | | | | | | 33.77253 | 39.56391 | 44.76886 | 21.14025 | 31.61657 | 31.24487 | 33.34314 | 20.75304 | 26.34872 | 34.26964 | 21.9039 | 28.9598 | 26.97875 | 27.00762 | 34.34807 | 37.62291 | 32.84186 | 30.43159 | 22.71378 | 26.17206 | 34.73965 | 32.26619 | 32.47256 | 25.00635 | 39.25313 | 36.62478 | 35.93356 |
| CUST_41081_PI429010886 | M56836 | 0.028757 | 21 | 15 | | | | | | 436.3193 | 339.3078 | 496.4081 | 502.3399 | 475.6507 | 447.8279 | 436.7207 | 364.475 | 592.9907 | 684.7245 | 660.8688 | 918.9933 | 706.1971 | 686.1452 | 576.8367 | 731.4443 | 636.8404 | 769.8974 | 452.2817 | 586.9437 | 460.5023 | 397.0596 | 646.2272 | 638.6578 | 598.2934 | 447.1828 | 798.0318 |
| CUST_18515_PI429010886 | N39933 | 0.02876 | 15 | 0 | | | | | | 31.16623 | 25.36189 | 30.82234 | 17.54469 | 15.50269 | 22.74008 | 19.49887 | 16.46215 | 16.91691 | 17.92979 | 21.32406 | 22.8004 | 17.51958 | 21.89788 | 11.55683 | 30.32196 | 23.90612 | 26.63149 | 32.66548 | 30.0232 | 15.27365 | 25.32849 | 17.19113 | 13.95948 | 27.01753 | 17.67291 | 23.63855 |
| CUST_8745_PI429010886 | N23509 | 0.028761 | 21 | 15 | | | | | | 327.6399 | 377.0882 | 344.8512 | 314.8877 | 365.6765 | 507.983 | 359.57 | 289.8985 | 318.1453 | 374.5146 | 333.2881 | 303.9813 | 337.9324 | 319.9685 | 307.1797 | 430.7898 | 420.504 | 436.6018 | 432.8549 | 319.0748 | 358.6229 | 347.2399 | 400.1052 | 391.4581 | 335.8009 | 323.3503 | 308.2184 |
| CUST_32277_PI429010886 | M3688 | 0.028766 | 24 | 0 | 443721461 | hypothetical protein CAPTEDRAFT_17890 [Capitella | Symbiosis, encompassing mutualism through parasitism | Viral process | 7.48E-55 | 10.4406 | 9.456396 | 11.5275 | 8.352146 | 8.140928 | 19.2383 | 4.876287 | 10.94377 | 4.872328 | 11.1247 | 3.956825 | 6.818591 | 8.945536 | 6.320653 | 5.75088 | 5.84802 | NA | NA | NA | 12.82499 | 7.098337 | 11.4448 | NA | NA | 13.16888 | 11.32653 | 11.3725 |
| CUST_2343_PI429010886 | N12145 | 0.028799 | 12 | 3 | 214012051 | ADP/ATP translocase [Artemia franciscana] | | | 2.1E-39 | 1529.572 | 1177.081 | 1193.941 | 2475.35 | 2452.337 | 6372.368 | 1856.885 | 2027.433 | 1236.95 | 975.9966 | 2154.443 | 1302.095 | 1584.97 | 889.7966 | 561.7404 | 1110.444 | 3835.814 | 3902.514 | 2497.013 | 1433.182 | 1767.465 | 867.4252 | 1419.195 | 2400.961 | 1154.959 | 3787.685 | 1290.993 |
| CUST_13595_PI429010886 | N31439 | 0.028799 | 15 | 9 | | | | | | 87.09496 | 116.1614 | 73.22698 | 77.82271 | 72.82575 | 61.5685 | 59.53415 | 70.13143 | 115.5867 | 109.7371 | 70.88181 | 137.5279 | 125.3899 | 87.60236 | 103.5957 | 111.7039 | 29.5273 | 78.38473 | 87.98669 | 64.648 | 71.01876 | 109.4106 | 77.60864 | 90.72052 | 110.3421 | 92.75345 | 119.3665 |
| CUST_3097_PI429010886 | N13783 | 0.028803 | 24 | 6 | 27805448 | RecName: Full=Peptidyl-prolyl cis-trans isomerase E | Protein metabolism | Protein folding | 9.54E-45 | 707.3962 | 515.8865 | 224.0471 | 457.9897 | 170.8315 | 216.741 | 702.4802 | 352.0546 | 691.4084 | 310.4051 | 148.0647 | 122.1037 | 484.8203 | 238.0396 | 245.0768 | 290.0767 | 167.6977 | 109.3629 | 149.3496 | 198.9264 | 147.7682 | 206.6019 | 171.8979 | 268.3232 | 337.9324 | 190.3851 | 161.2028 |
| CUST_3659_PI429010886 | N14981 | 0.028808 | 18 | 12 | | | | | | 277.5533 | 274.9043 | 393.5988 | 230.6845 | 257.455 | 310.7615 | 274.6475 | 300.9857 | 332.5951 | 276.5189 | 333.4458 | 412.1707 | 335.5871 | 406.809 | 370.9949 | 400.5981 | 343.0578 | 354.2504 | 352.327 | 330.1788 | 240.8565 | 374.7559 | 316.8471 | 189.9733 | 403.0406 | 290.7849 | 419.3343 |
| CUST_10101_PI429010886 | N25695 | 0.028813 | 18 | 15 | 291238977 | PREDICTED: rCG56742-like, partial [Saccoglossus ko | | | 1.72E-45 | 70.61376 | 55.37064 | 87.29557 | 65.20347 | 66.65717 | 71.94962 | 71.07701 | 65.28197 | 69.07358 | 66.24577 | 87.69732 | 77.16796 | 88.4462 | 102.9589 | 83.14826 | 103.7718 | 100.3232 | 82.51706 | 89.47031 | 95.368 | 57.41889 | 79.36987 | 76.91503 | 41.10188 | 102.6107 | 78.12041 | 104.4971 |
| CUST_42712_PI429010886 | M61040 | 0.028818 | 18 | 12 | 307197899 | Transmembrane and TPR repeat-containing protein | | | 1.17E-31 | 7.535575 | 8.950802 | 22.95471 | 12.9342 | 8.252454 | 3.767545 | 25.45721 | 13.45283 | 14.23755 | NA | 27.47176 | 42.09877 | 22.50662 | 57.10862 | 30.96598 | 21.01435 | 6.49944 | 14.73365 | 8.15397 | 34.75205 | NA | 9.928945 | 22.66789 | NA | 22.54321 | 8.426447 | 15.33118 |
| CUST_39373_PI429010886 | M38509 | 0.028835 | 12 | 0 | | | | | | 9.031084 | 1461.877 | 710.6638 | 12.87509 | 16.62548 | 12.4831 | 8.792314 | 4.438084 | 12.0863 | 1001.62 | 283.0254 | 912.0221 | 75.51588 | 56.0501 | 20.89839 | 10.80445 | 11.81437 | 12.65789 | NA | 7.542588 | 5.269474 | NA | NA | 12.55296 | 1050.577 | 17.78423 | 6.702805 |
| CUST_56467_PI429010886 | M113871 | 0.028839 | 21 | 18 | | | | | | 85.77208 | 71.58792 | 91.82524 | 52.15768 | 96.84604 | 89.50659 | 75.62563 | 69.72006 | 51.37508 | 77.24703 | 68.39623 | 74.82753 | 56.80732 | 71.55597 | 49.41092 | 54.76027 | 100.651 | 97.282 | 116.8757 | 94.72175 | 98.03383 | 85.07901 | 101.6676 | 51.73412 | 87.65825 | 60.60207 | 70.05382 |
| CUST_4485_PI429010886 | N16869 | 0.028841 | 24 | 0 | | | | | | 137.8813 | 83.60593 | 117.7304 | 99.2772 | 53.86751 | 111.7487 | 71.77967 | 75.49081 | 77.21633 | 80.842 | 78.33196 | 54.85007 | 61.31684 | 91.14746 | 72.9595 | 55.01668 | 87.11006 | 73.49284 | 60.28242 | 111.637 | 84.19357 | 61.02614 | 40.62506 | 84.35593 | 114.6697 | 99.1645 | 119.9416 |
| CUST_33140_PI429010886 | M6071 | 0.028853 | 21 | 9 | 307207514 | Uncharacterized MFS-type transporter C19orf28-lik | | | 4.45E-07 | 13.62289 | 20.87033 | 9.691153 | 26.97522 | 21.61291 | 27.25419 | 23.65678 | 67.9638 | 50.99575 | 15.84016 | 106.3173 | 128.3615 | 46.51079 | 25.54793 | 29.71896 | 18.79291 | 9.877583 | 20.17126 | NA | 132.7894 | 75.48481 | 29.88134 | 7.982144 | 10.09227 | 13.67397 | 40.22531 | 51.1868 |
| CUST_55980_PI429010886 | M112406 | 0.028879 | 12 | 0 | | | | | | 386.0388 | 572.2834 | 462.321 | 294.7498 | 390.2131 | 416.4515 | 363.1719 | 412.4272 | 356.1302 | 439.2849 | 412.3362 | 635.6512 | 511.1819 | 478.5158 | 551.2455 | 598.4797 | 407.1868 | 352.2533 | 402.6962 | 472.773 | 435.0294 | 500.5021 | 465.4504 | 404.4491 | 500.9078 | 484.0144 | 518.0097 |
| CUST_57022_PI429010886 | M115140 | 0.028879 | 12 | 0 | | | | | | 525.1056 | 696.7816 | 746.6817 | 458.3652 | 650.0167 | 522.6581 | 465.4504 | 530.042 | 574.6243 | 707.3962 | 573.864 | 746.9886 | 596.0223 | 691.1905 | 607.116 | 534.9773 | 526.3482 | 634.9072 | 545.6531 | 679.1732 | 549.5559 | 638.9314 | 652.3862 | 568.3832 | 771.3694 | 605.4467 | 793.9994 |
| CUST_38676_PI429010886 | M29052 | 0.028879 | 12 | 3 | | | | | | 1509.413 | 667.0295 | 113.0821 | 7737.591 | 7960.065 | 14035.86 | 6448.481 | 3060.282 | 1533.433 | 11.85797 | 6426.682 | 13.25603 | 1563.475 | 6.445454 | 966.4783 | 149.0785 | 11673.13 | 11259.43 | 8696.127 | 3.698137 | 5055.748 | 57.8814 | 4015.965 | 7579.447 | 40.94065 | 7395.598 | 3.873572 |
| CUST_43093_PI429010886 | M62055 | 0.028879 | 12 | 0 | | | | | | 96.06297 | 91.98586 | 167.1839 | 91.11111 | 101.3567 | 111.0532 | 90.3266 | 96.91901 | 114.5736 | 128.1757 | 104.9253 | 156.9005 | 105.3539 | 151.2035 | 138.2244 | 100.7779 | 103.1588 | 93.84895 | 120.2042 | 104.9922 | 116.73 | 129.3352 | 141.3582 | 117.0097 | 125.5811 | 136.9448 | 166.2291 |
| CUST_6889_PI429010886 | N20737 | 0.028879 | 12 | 0 | 321478589 | hypothetical protein DAPPUDRAFT_303058 [Daphn | Nucleic acid metabolism | RNA metabolic process | 2E-111 | 130.1875 | 161.7305 | 92.52049 | 70.27178 | 72.28479 | 46.83381 | 70.2561 | 88.7086 | 102.7524 | 143.2735 | 80.94247 | 111.1756 | 112.5941 | 94.49669 | 95.23078 | 99.38154 | 97.43479 | 80.48768 | 87.38085 | 115.8059 | 108.9753 | 111.3772 | 108.3885 | 74.3064 | 122.2551 | 101.4752 | 171.8172 |
| CUST_50016_PI429010886 | M88273 | 0.028879 | 12 | 0 | 339721487 | proliferating cell nuclear antigen [Litopenaeus vann | Nucleic acid metabolism | DNA replication | 7.21E-26 | 248.8286 | 623.8166 | 456.2722 | 193.556 | 224.9139 | 170.317 | 220.511 | 305.2873 | 315.7204 | 477.2259 | 208.027 | 487.7611 | 374.9159 | 464.9946 | 455.2984 | 411.957 | 234.017 | 364.9554 | 232.9884 | 461.0025 | 350.5238 | 404.0517 | 420.025 | 175.4362 | 671.0656 | 354.5586 | 597.939 |
| CUST_22966_PI429010886 | N48017 | 0.028897 | 21 | 18 | | | | | | 30.62521 | 24.65512 | 14.48619 | 18.87315 | 22.43908 | 24.15363 | 15.87123 | 11.82115 | 13.87948 | 24.40654 | 18.61977 | 21.23397 | 20.208 | 21.23667 | 20.04961 | 28.85616 | 17.31232 | 28.05064 | 30.91745 | 26.61223 | 18.97043 | 27.76033 | 10.96904 | 23.4129 | 22.36658 | 24.11521 | 18.51985 |
| CUST_20313_PI429010886 | N43216 | 0.028898 | 24 | 21 | | | | | | 15.49062 | 10.32811 | 25.26607 | NA | 9.214884 | NA | 8.505871 | 5.547594 | 8.198298 | 14.24335 | 7.179499 | 6.194786 | 13.67397 | 11.96649 | 13.20796 | 15.7841 | 7.583588 | 23.69356 | 3.857393 | 11.92189 | NA | 19.50517 | 19.13867 | 12.52724 | 12.37856 | 10.93346 | 11.34133 |
| CUST_32331_PI429010886 | M3825 | 0.028906 | 18 | 12 | 260830290 | hypothetical protein BRAFLDRAFT_60087 [Branchio | Cellular component organization | Cytoskeleton organization | 1.44E-20 | 48.29446 | 62.00083 | 40.3065 | 33.49263 | 42.95918 | 45.66919 | 46.90121 | 60.66856 | 52.9276 | 59.18336 | 56.39846 | 62.07028 | 57.04658 | 58.4329 | 65.02562 | 71.48709 | 44.4501 | 44.73069 | 46.65817 | 57.33863 | 42.2531 | 63.42029 | 42.77108 | 29.61528 | 55.18575 | 30.27835 | 58.06032 |
| CUST_44592_PI429010886 | M66419 | 0.028911 | 24 | 18 | | | | | | 133.987 | 118.2001 | 135.5002 | 98.129 | 124.4264 | 94.87745 | 109.3881 | 134.7073 | 125.3551 | 125.1284 | 134.8934 | 160.598 | 160.3449 | 123.321 | 121.7165 | 157.8794 | 152.6328 | 114.6191 | 191.9229 | 134.2151 | 147.2999 | 146.8674 | 126.6211 | 160.5748 | 153.6441 | 122.5181 | 112.4193 |
| CUST_47036_PI429010886 | M74613 | 0.028911 | 24 | 21 | | | | | | 100.965 | 73.57494 | 61.72335 | 100.4575 | 53.92569 | 30.82801 | 92.67278 | 42.48986 | 83.50755 | 115.8949 | 102.5545 | 60.94428 | 105.2005 | 66.67032 | 84.73278 | 86.7497 | 49.60877 | 113.7893 | 96.91901 | 109.1768 | 109.0963 | 190.6724 | 106.2054 | 126.3583 | 99.63032 | 58.74569 | 91.32302 |
| CUST_2802_PI429010886 | N13139 | 0.028911 | 24 | 21 | | | Nucleic acid metabolism | RNA metabolic process | | 337.2385 | 479.0678 | 285.0395 | 239.3095 | 286.8104 | 245.3745 | 271.2223 | 278.6013 | 252.0389 | 462.321 | 264.9246 | 303.55 | 282.2712 | 290.995 | 275.0029 | 478.8398 | 243.8028 | 309.1008 | 297.6429 | 358.0335 | 287.1125 | 428.865 | 337.9324 | 382.9442 | 377.4878 | 251.4349 | 318.5558 |
| CUST_32042_PI429010886 | M3082 | 0.028913 | 18 | 0 | | | | | | 539.4382 | 466.4832 | 546.8107 | 352.327 | 574.3589 | 461.3593 | 528.618 | 284.7868 | 300.7234 | 356.4277 | 671.8789 | 396.481 | 335.9236 | 359.57 | 361.6235 | 405.164 | 840.1524 | 430.3425 | 617.6618 | 363.5385 | 573.9804 | 346.8405 | 791.0227 | 724.6164 | 372.0481 | 279.9837 | 300.3152 |
| CUST_25976_PI429010886 | N53420 | 0.028924 | 15 | 9 | | | | | | 146.8007 | 192.6772 | 198.7167 | 192.557 | 221.06 | 185.8695 | 162.0012 | 198.308 | 201.5302 | 220.2522 | 207.5722 | 213.0233 | 215.2013 | 231.8976 | 140.2437 | 204.2578 | 155.2181 | 172.8801 | 140.6816 | 185.2303 | 202.9774 | 181.5127 | 255.0746 | 242.8033 | 239.4694 | 244.0822 | 265.8178 |
| CUST_15072_PI429010886 | N33963 | 0.028926 | 12 | 9 | | | | | | 194.5783 | 141.2301 | 169.8261 | 142.216 | 155.4298 | 176.4808 | 173.1695 | 151.0154 | 140.0797 | 179.2925 | 178.5939 | 173.3481 | 63.00378 | 188.1161 | 106.8281 | 146.3194 | 164.2252 | 58.52324 | 174.4245 | 175.6726 | 76.06574 | 202.262 | 209.4461 | 206.8341 | 64.75716 | 173.3233 | 159.338 |
| CUST_22675_PI429010886 | N47437 | 0.028945 | 15 | 12 | | | | | | 7.555334 | 10.92516 | 9.371383 | 7.243822 | 15.18848 | 16.4753 | 8.373525 | 3.923061 | 8.635923 | 13.82413 | 9.77251 | 9.589321 | 83.83314 | 9.614111 | 17.60432 | 23.09604 | 13.96315 | 15.68172 | 12.05594 | 8.418027 | 9.412838 | 14.60951 | 8.152575 | 8.3032 | 10.93941 | 12.96349 | 7.030976 |
| CUST_22855_PI429010886 | N47803 | 0.028957 | 21 | 3 | | | | | | 21.73902 | 17.03463 | 27.23432 | 27.9397 | 28.04382 | 41.34285 | 29.50356 | 20.51687 | 16.90946 | 29.47649 | 10.17778 | 16.70939 | 22.13675 | 28.03123 | 13.65887 | 19.63631 | 23.23883 | 28.69117 | 27.69749 | 18.52484 | 12.37039 | 19.78441 | 21.33294 | 24.40411 | 28.15071 | 36.81255 | 29.9042 |
| CUST_17790_PI429010886 | N38594 | 0.028964 | 21 | 12 | | | | | | 4.99831 | 8.254327 | 7.074973 | NA | 5.914203 | 7.941557 | NA | 4.280152 | 4.622941 | NA | 6.403312 | 9.805454 | 11.50698 | NA | 10.99151 | 19.32322 | 8.313099 | 10.13183 | 9.444187 | 7.611898 | 4.625815 | 8.697209 | NA | NA | 11.51709 | 7.613075 | 5.697389 |
| CUST_29983_PI429010886 | N60946 | 0.028967 | 21 | 0 | | | | | | 88.02287 | 86.43501 | 81.55572 | 74.22931 | 92.34903 | 76.13932 | 77.2785 | 80.5848 | 64.60382 | 66.11527 | 74.06105 | 69.72451 | 79.91242 | 58.74569 | 71.44429 | 73.75273 | 83.6912 | 60.35512 | 70.80885 | 82.55028 | 86.60546 | 79.80104 | 78.07189 | 90.20594 | 98.129 | 74.62941 | 57.96208 |
| CUST_5699_PI429010886 | N19065 | 0.028979 | 15 | 3 | 321459462 | hypothetical protein DAPPUDRAFT_328087 [Daphn | | | 3.88E-36 | 66.07282 | 44.21249 | 57.95385 | 113.5525 | 67.31728 | 94.36809 | 96.72232 | 51.37925 | 41.92389 | 28.13108 | 50.36094 | 11.81437 | 49.46731 | 58.87201 | 56.01567 | 39.62708 | 105.8358 | 66.81883 | 67.18762 | 30.64068 | 48.99962 | 52.7291 | 53.63782 | 75.4776 | 54.45697 | 31.10084 | 26.1905 |
| CUST_35339_PI429010886 | M12315 | 0.028983 | 24 | 9 | | | | | | 9.808079 | 24.05405 | 8.250428 | 15.9743 | 13.26351 | 23.42758 | 58.36102 | 162.141 | 143.9734 | 28.19099 | 44.92995 | 39.93503 | 11.50042 | 129.6229 | 57.07016 | 16.65148 | 20.78747 | 10.71453 | 20.60646 | 76.99966 | 23.38781 | 18.10067 | 19.12122 | 20.89343 | 9.753321 | 21.68228 | 11.46886 |
| CUST_4159_PI429010886 | N16132 | 0.028984 | 15 | 12 | | | | | | 62.75575 | 52.86329 | 81.37626 | 58.00667 | 56.23475 | 57.48318 | 50.94926 | 45.52189 | 51.20753 | 69.52372 | 48.05138 | 62.73269 | 52.11928 | 88.55995 | 73.59863 | 59.87756 | 72.37554 | 199.7111 | 70.30533 | 50.63623 | 56.24668 | 54.73192 | 59.36711 | 58.47643 | 64.81878 | 73.39754 | 56.37263 |
| CUST_13280_PI429010886 | N30904 | 0.028993 | 18 | 15 | | | | | | 125.2677 | 73.91159 | 124.2579 | 60.68127 | 97.20572 | 108.4314 | 105.322 | 100.2082 | 91.32302 | 69.05841 | 107.8449 | 87.31709 | 117.5697 | 133.4052 | 120.6692 | 131.4711 | 170.9831 | 114.3617 | 147.523 | 125.5041 | 80.03068 | 112.8267 | 89.4301 | 61.38183 | 145.8455 | 68.55373 | 110.3002 |
| CUST_32865_PI429010886 | M5311 | 0.029079 | 15 | 12 | | | | | | 4909.967 | 4715.889 | 5188.094 | 3237.592 | 3921.02 | 4494.887 | 3520.151 | 3621.392 | 5283.671 | 3676.785 | 4451.275 | 6434.019 | 4643.325 | 6260.184 | 6253.288 | 8772.974 | 4098.525 | 6434.019 | 4004.804 | 3902.514 | 2910.582 | 8854.389 | 2766.721 | 3483.315 | 7950.432 | 5372.754 | 8942.257 |
| CUST_55843_PI429010886 | M111854 | 0.029088 | 21 | 6 | | | | | | 8.010366 | 14.64247 | 12.24024 | 19.43895 | 41.19265 | 23.99191 | 34.16228 | 117.2337 | 96.76761 | 17.55969 | 71.84934 | 76.6588 | 51.56072 | 7.52281 | 30.45321 | NA | 26.20596 | NA | 8.496964 | 138.8365 | 80.37038 | 7.310152 | 26.56923 | 22.69512 | NA | 52.12333 | 59.19392 |
| CUST_46513_PI429010886 | M72871 | 0.02911 | 15 | 3 | | | | | | 52.50431 | 42.89451 | 36.04028 | 91.54224 | 48.56555 | 26.98034 | 38.54575 | 65.06553 | 33.84879 | 71.54826 | 14.14304 | 35.74774 | 5.331416 | 23.13514 | 18.08454 | 27.05448 | 22.13675 | 18.76417 | 32.90861 | 83.57768 | 34.21609 | 21.03821 | 80.13478 | 44.08227 | 15.91598 | 9.208223 | NA |
| CUST_28582_PI429010886 | N58234 | 0.029119 | 12 | 0 | | | | | | 36.62775 | 74.6925 | 113.4502 | 6.586963 | 21.80116 | 14.92131 | 23.05184 | 36.23666 | 37.84899 | 47.20692 | 23.35433 | 18.40425 | 53.83209 | 77.26285 | 43.61983 | 60.47407 | 39.13986 | 32.12754 | 53.1338 | 29.69991 | 27.52478 | 57.32582 | 44.38974 | 35.58868 | 198.2869 | 30.88907 | 182.6771 |
| CUST_57028_PI429010886 | M115150 | 0.02913 | 21 | 6 | | | | | | 115.1745 | 68.00621 | 159.338 | 45.488 | 52.12912 | 147.895 | 127.3371 | 130.5635 | 102.0104 | 113.0325 | 111.4482 | 75.16329 | 102.5545 | 72.83324 | 69.07358 | 85.75328 | 96.90956 | 100.0169 | 45.58985 | 40.41641 | 57.76414 | 126.2074 | 57.53634 | 127.7733 | 104.1327 | 133.8595 | 131.3569 |
| CUST_31383_PI429010886 | M1407 | 0.029136 | 18 | 15 | | | | | | 107.1532 | 114.8783 | 77.1205 | 83.75206 | 87.67204 | 122.452 | 93.99876 | 59.98068 | 62.23675 | 70.35427 | 66.4925 | 91.22284 | 80.0595 | 109.19 | 115.5867 | 109.8741 | 107.898 | 108.6974 | 120.4034 | 103.7132 | 83.2292 | 89.28732 | 75.82559 | 115.1745 | 88.3275 | 78.69319 | 69.63319 |
| CUST_37588_PI429010886 | M19646 | 0.029137 | 21 | 15 | | | | | | 21.57749 | 7.324098 | 30.87561 | 6.064337 | 4.967969 | NA | 9.388689 | 9.41784 | 12.60293 | 10.22575 | 8.743791 | 6.130259 | 9.954929 | 10.58947 | 10.99426 | 12.30261 | 11.48488 | 21.61481 | 4.197202 | 11.31627 | 9.464207 | 14.67473 | 14.19691 | 10.94377 | 7.464698 | NA | 6.456509 |
| CUST_5396_PI429010886 | N18642 | 0.029146 | 18 | 12 | | | | | | 173.6414 | 182.2902 | 178.9173 | 112.3653 | 116.6217 | 102.1011 | 124.732 | 167.7564 | 131.7223 | 209.1953 | 142.0394 | 211.0801 | 154.7689 | 182.494 | 212.773 | 238.7756 | 134.6396 | 166.9427 | 141.7869 | 209.6917 | 151.1785 | 191.3918 | 158.6824 | 99.67314 | 176.1772 | 146.0474 | 199.0047 |
| CUST_1464_PI429010886 | N9719 | 0.029149 | 15 | 3 | | | | | | 1268.323 | 504.2482 | NA | 2280.777 | 926.3799 | 3543.323 | 1124.348 | 1968.333 | 740.9987 | 5.420008 | 418.3911 | 5.84802 | 1914.117 | 15.89011 | 832.3284 | 35.82698 | 2232.073 | 2379.147 | 3358.264 | NA | 119.1803 | 11.15858 | 68.1054 | 1577.693 | 11.24847 | 2441.116 | 4.912682 |
| CUST_8616_PI429010886 | N23308 | 0.02916 | 12 | 9 | 345484846 | PREDICTED: glycogen debranching enzyme-like [Nas | Metabolic process | Carbohydrate metabolic process | 9.47E-70 | 7.109363 | NA | 7.849959 | 7.597404 | NA | 7.483872 | 7.176452 | 8.967811 | 7.738172 | 11.68572 | 15.81822 | 9.902997 | 9.070174 | 6.348981 | 6.12441 | 4.758947 | 5.059565 | 4.827843 | 9.035702 | 8.297472 | NA | 3.723067 | NA | 10.6257 | 7.319054 | 5.230695 | 13.88119 |
| CUST_28258_PI429010886 | N57614 | 0.029169 | 21 | 0 | | | | | | 13.38957 | 21.27347 | 17.27797 | 10.53088 | 13.76785 | 12.16314 | NA | 8.274264 | 11.36366 | 13.75362 | 12.89985 | 6.092564 | 9.74748 | 11.07003 | 12.03276 | 18.29071 | 6.028359 | 16.53332 | 9.928945 | 16.30281 | 24.44489 | 22.53922 | 12.90802 | 13.33911 | 16.08188 | 8.352146 | 12.06791 |
| CUST_5975_PI429010886 | N19452 | 0.029188 | 15 | 0 | | | | | | 781.9696 | 678.9878 | 656.7764 | 496.5233 | 602.6317 | 705.2005 | 604.5341 | 532.3882 | 539.1803 | 495.7993 | 448.0031 | 599.494 | 618.6744 | 598.2934 | 782.4585 | 777.7644 | 568.0071 | 684.1373 | 607.116 | 640.0171 | 541.9679 | 745.6971 | 591.7087 | 695.332 | 551.2455 | 485.4409 | 647.0884 |
| CUST_13817_PI429010886 | N31827 | 0.029203 | 12 | 0 | 321479469 | hypothetical protein DAPPUDRAFT_300013 [Daphn | | | 2.27E-19 | 322.0219 | 345.8615 | 353.3222 | 300.0603 | 235.0591 | 243.2097 | 231.648 | 284.1504 | 300.9857 | 375.2702 | 315.1705 | 374.682 | 272.9055 | 329.6909 | 466.3395 | 427.2547 | 289.8985 | 261.3766 | 299.389 | 373.1186 | 305.5566 | 287.9369 | 368.0783 | 307.8404 | 420.4304 | 381.1947 | 427.0325 |
| CUST_20361_PI429010886 | N43299 | 0.029203 | 12 | 3 | 307169177 | Ecdysone-induced protein 74EF isoform B [Campon | Nucleic acid metabolism | Regulation of gene expression | 9.24E-13 | 27.56643 | 22.43297 | 29.89152 | 42.45161 | 93.91055 | 128.4766 | 118.5121 | 30.62163 | 36.88342 | 12.18324 | 141.6171 | 27.63853 | 37.04426 | 24.05405 | 24.67089 | 35.78629 | 235.838 | 87.40211 | 100.7905 | 31.9232 | 58.12189 | 21.28336 | 54.17626 | 60.76524 | 37.7275 | 28.6303 | 35.1434 |
| CUST_29262_PI429010886 | N59505 | 0.029219 | 15 | 3 | | | | | | 44.90621 | 46.93128 | 40.95384 | 43.23619 | 40.42007 | 86.35271 | 30.10596 | 56.76191 | 46.12938 | 39.88324 | 40.32605 | 30.80438 | 34.57676 | 36.5186 | 33.11438 | 40.42785 | 36.21438 | 44.96572 | 47.84187 | 41.2427 | 51.42712 | 38.17748 | 35.09563 | 61.40781 | 53.32175 | 37.88861 | 33.62817 |
| CUST_9461_PI429010886 | N24665 | 0.029231 | 12 | 3 | | | | | | 16328.69 | 6962.573 | 5699.333 | 30232.55 | 21318.49 | 43024.52 | 23297.76 | 7960.065 | 10348.94 | 352.8752 | 14829.9 | 131.4711 | 12070.48 | 4028.762 | 10831.29 | 11104 | 24647.46 | 31801.7 | 16762.52 | 1596.656 | 17165.71 | 10117.01 | 13858.59 | 31396.1 | 1961.856 | 17482.08 | 163.3744 |
| CUST_17431_PI429010886 | N37946 | 0.029231 | 12 | 3 | | | | | | 15604.09 | 5407.054 | 4864.618 | 27464.78 | 19037.21 | 39067.89 | 25595.75 | 7306.322 | 9501.999 | 308.9771 | 13438.24 | 112.9419 | 11030.7 | 3994.868 | 9320.998 | 10282.77 | 23934.78 | 28694.35 | 14559.32 | 1446.944 | 16791.57 | 8667.659 | 13226.16 | 26406.52 | 1584.97 | 16887.61 | 115.8594 |
| CUST_34632_PI429010886 | M10191 | 0.029237 | 15 | 6 | | | | | | 232.9884 | 339.2296 | 451.0636 | 574.8968 | 711.9048 | 1211.945 | 496.4081 | 1482.523 | 1227.773 | 1721.92 | 392.2917 | 469.4022 | NA | 529.3841 | 310.4773 | 2149.927 | 540.5402 | 1140.952 | 788.6009 | 455.5337 | 595.028 | 1737.368 | 789.4136 | 1608.297 | 542.8448 | 686.1452 | 351.5336 |
| CUST_32120_PI429010886 | M3279 | 0.029263 | 24 | 6 | 410509307 | Penaeus monodon MYH2 mRNA for myosin heavy c | | | 2E-129 | 84.13816 | 166.9686 | 149.6388 | 201.3287 | 126.0286 | 93.99876 | 179.2612 | 187.4537 | 153.4856 | 159.9839 | 172.9097 | 184.0938 | 101.637 | 119.5972 | 129.4038 | 49.74933 | 152.7363 | 44.22632 | 118.4142 | 174.4075 | 96.94706 | 73.53135 | 149.7694 | 82.46901 | 43.11192 | 143.3629 | 123.2283 |
| CUST_21384_PI429010886 | N45122 | 0.029266 | 12 | 3 | 321475473 | hypothetical protein DAPPUDRAFT_208250 [Daphn | | | 6.71E-56 | 119.9827 | 123.0876 | 90.09804 | 142.1431 | 97.3988 | 226.5808 | 136.7775 | 89.57185 | 89.26714 | 78.37536 | 81.52938 | 95.54961 | 103.7315 | 89.07632 | 181.4858 | 106.2586 | 174.0152 | 154.7492 | 262.8518 | 84.36786 | 111.8008 | 110.7787 | 85.24568 | 97.19605 | 91.77189 | 193.1764 | 104.474 |
| CUST_16538_PI429010886 | N36427 | 0.029273 | 24 | 21 | | | | | | 38.74344 | 272.6021 | 219.061 | 21.96294 | 49.46138 | 17.52653 | 13.97554 | 25.5782 | 40.9233 | 96.70093 | 42.78187 | 106.908 | 57.81384 | 107.6119 | 77.51881 | 125.027 | 5.241968 | 108.0008 | 61.0979 | 124.8573 | 82.90947 | 129.6421 | 109.0193 | 23.61475 | 56.43628 | 70.15478 | 88.53305 |
| CUST_41248_PI429010886 | M57237 | 0.029322 | 18 | 0 | | | | | | 7.990456 | 6.971491 | NA | 8.919059 | 4.215242 | NA | NA | 4.432136 | 3.591713 | 12.0248 | 4.401188 | 3.805775 | 5.631097 | 6.655856 | 4.435803 | 8.58357 | 6.348013 | NA | 6.766361 | 8.33181 | 7.840127 | NA | 7.82506 | 4.785934 | 6.202333 | NA | 4.104957 |
| CUST_29493_PI429010886 | N59975 | 0.029346 | 24 | 6 | | | | | | 112.7145 | 113.2611 | 171.3591 | 110.4128 | 99.67314 | 152.7363 | 145.2889 | 154.3834 | 119.9416 | 197.5074 | 101.3304 | 130.4535 | 118.9935 | 125.3899 | 93.44686 | 87.19276 | 98.91679 | 107.2874 | 111.0646 | 80.38938 | 91.82524 | 114.5149 | 159.8062 | 111.027 | 111.5216 | 108.8006 | 114.7256 |
| CUST_13473_PI429010886 | N31248 | 0.029353 | 24 | 15 | | | | | | 61.5 | 58.12612 | 54.79286 | 51.73816 | 63.39049 | 44.27177 | 54.99653 | 97.17307 | 66.78152 | 94.81013 | 79.95313 | 64.37974 | 73.04458 | 61.0979 | 101.5804 | 94.77604 | 68.72963 | 85.01801 | 79.86307 | 76.6588 | 101.9359 | 60.27464 | 58.46315 | 97.1202 | 58.84482 | 113.5368 | 55.30825 |
| CUST_31996_PI429010886 | M2944 | 0.029357 | 24 | 15 | | | | | | 29.80754 | 16.39729 | 19.79646 | 15.05287 | 11.71408 | 12.19762 | 11.16571 | 13.70338 | 13.66038 | 20.68784 | 26.17206 | 22.42981 | 8.683756 | 19.89057 | 10.20858 | 44.82294 | 26.01055 | 31.43539 | 13.3861 | 32.13794 | 19.09041 | 12.74804 | 19.36095 | NA | 21.32852 | 12.45001 | 21.84747 |
| CUST_49952_PI429010886 | M87857 | 0.029366 | 12 | 0 | | | | | | 92.19432 | 128.2673 | 90.03399 | 96.5167 | 87.2755 | 109.0854 | 78.16064 | 94.72175 | 74.99621 | 123.2423 | 82.57755 | 129.5554 | 124.2142 | 98.42661 | 126.7933 | 105.8252 | 67.70114 | 93.00061 | 72.26362 | 94.127 | 87.54624 | 104.4202 | 93.1869 | 67.9514 | 121.1638 | 113.5525 | 125.5232 |
| CUST_46262_PI429010886 | M71987 | 0.029388 | 24 | 15 | | | | | | 28.81723 | 27.90293 | 28.06937 | 24.01772 | 25.89927 | 26.91798 | 29.46496 | 17.18186 | 14.84209 | 26.70632 | 16.62775 | 25.02448 | 14.12299 | 19.78599 | 27.39302 | 32.15505 | 38.25121 | 39.84188 | 33.57067 | 30.48948 | 24.85393 | 29.99408 | 34.64172 | 25.79784 | 34.97152 | 30.11859 | 39.57456 |
| CUST_28368_PI429010886 | N57818 | 0.029421 | 21 | 3 | | | | | | 36.06929 | 31.08481 | 41.99643 | 67.60441 | 56.01567 | 59.62185 | 64.30394 | 43.99672 | 34.15181 | 57.29447 | 12.95466 | 59.6055 | 43.60346 | 44.80164 | 48.9688 | 37.97967 | 36.00037 | 45.7289 | 25.5782 | 37.04426 | 47.15656 | 51.69046 | 42.24809 | 47.63767 | 60.05679 | 41.20843 | 52.33357 |
| CUST_30951_PI429010886 | M292 | 0.029437 | 21 | 0 | 91087405 | PREDICTED: similar to structural maintenance of chı | Response to stress | DNA repair | 6.01E-53 | 251.4349 | 82.83897 | 486.6501 | 268.0379 | 99.59438 | 182.9016 | 167.1292 | 123.9789 | 93.55006 | 89.35703 | 188.9774 | 90.92371 | 73.44541 | 79.56143 | 32.12364 | 78.86687 | 228.1824 | 183.9787 | 153.2997 | 74.66117 | 315.8463 | 90.49352 | 211.1942 | 416.2434 | 70.6776 | 134.0969 | 79.95313 |
| CUST_2793_PI429010886 | N13119 | 0.029437 | 24 | 0 | | | Protein metabolism | Proteolysis | | 23.73387 | 25.77304 | 20.9065 | 15.94381 | 24.02357 | 16.81632 | 11.15122 | 15.08742 | 14.96269 | 14.5578 | 15.66502 | 17.49601 | 13.11618 | 16.59282 | 14.97314 | 23.49823 | 13.16396 | 21.89117 | 20.50468 | 27.87681 | 25.50065 | 16.64978 | 13.48403 | 11.73735 | 33.99233 | 23.57785 | 18.14702 |
| CUST_13230_PI429010886 | N30819 | 0.02944 | 15 | 12 | | | | | | 89.15191 | 69.25418 | 922.2736 | 13.11816 | 101.1596 | 112.8118 | 217.6167 | 1438.603 | 1555.197 | 416.8412 | 264.3268 | 215.1259 | 981.0866 | 1464.638 | 455.8444 | 137.5442 | 991.6341 | 37.1484 | 194.3207 | 178.833 | 133.1932 | 295.08 | 404.7043 | 90.84494 | 869.0196 | 242.7155 | 240.1049 |
| CUST_23770_PI429010886 | N49436 | 0.029444 | 12 | 3 | | | | | | 18.19818 | NA | NA | 25.51724 | 6.153881 | 53.2143 | 19.49159 | 25.70664 | 8.478155 | 4.816403 | 6.087274 | 7.97358 | 14.66408 | NA | 7.742712 | NA | 34.88888 | 53.92569 | 41.34656 | 5.92558 | 13.09531 | 5.247077 | 10.46847 | 20.20434 | 6.275836 | 22.50429 | 10.80725 |
| CUST_1824_PI429010886 | N10805 | 0.029469 | 24 | 3 | | | | | | 769.2478 | 1658.285 | 909.1015 | 3008.795 | 1485.965 | 1492.073 | 1581.923 | 1568.166 | 78.86687 | 66.90174 | 1600.359 | 8.972594 | 29.82743 | 156.0758 | 548.7959 | 18.3679 | 33.57067 | 14.42119 | 514.8406 | 12.50339 | 721.5575 | NA | 916.8157 | 828.8539 | NA | 12.39937 | 7.634015 |
| CUST_35185_PI429010886 | M11828 | 0.029471 | 21 | 0 | | | | | | 15.27765 | 17.85063 | 26.76116 | 23.21566 | 23.03433 | 15.62744 | 4.143348 | 5.389345 | 13.15835 | 13.08205 | 25.45721 | 6.515096 | NA | 12.58067 | 14.25474 | 19.07974 | 16.78585 | 30.08532 | 15.97633 | 20.50362 | 11.05702 | 35.82359 | NA | NA | 15.38675 | 6.637612 | 15.84016 |
| CUST_3969_PI429010886 | N15683 | 0.029473 | 18 | 9 | | | | | | 100.0727 | 96.63027 | 114.2883 | 109.4535 | 83.32632 | 111.625 | 118.7227 | 90.72781 | 105.7372 | 118.6098 | 110.8588 | 118.2899 | 105.711 | 103.7315 | 62.26361 | 85.31136 | 80.72119 | 87.14788 | 78.61543 | 90.06996 | 113.6641 | 94.5785 | 133.7601 | 108.9599 | 98.04327 | 112.5022 | 117.6032 |
| CUST_18410_PI429010886 | N39752 | 0.029478 | 21 | 9 | 190663314 | glutathione S-transferase theta 1 [Tigriopus japonic | | | 4.27E-10 | 14.36186 | 16.4683 | 18.10445 | 7.983639 | 22.35568 | 13.42458 | 20.67362 | 41.77517 | 42.79462 | 23.18287 | 25.9871 | 31.70409 | 16.95126 | 21.49202 | 26.02132 | 28.01496 | 11.9176 | 21.36888 | 25.80044 | 44.76277 | 28.81481 | 11.14031 | 17.9311 | 16.63804 | 18.73459 | 14.59908 | 18.2356 |
| CUST_49422_PI429010886 | M85589 | 0.029481 | 21 | 3 | | | | | | 20.35314 | 13.78911 | 21.44644 | 16.08973 | 17.75499 | 33.06846 | 20.62738 | 19.26978 | 13.14141 | 17.28228 | 11.95482 | 15.49226 | 7.358125 | 19.26103 | 7.935304 | 20.72678 | 18.04709 | 12.90988 | 21.15229 | 12.04909 | 16.68994 | 27.3183 | 25.87167 | 9.594729 | 21.49202 | 20.12103 | 25.82466 |
| CUST_16357_PI429010886 | N36101 | 0.029509 | 18 | 15 | | | | | | 39.22563 | 33.32679 | 25.95987 | 17.17661 | 28.22043 | 22.45136 | 19.51255 | 21.69885 | 24.5772 | 33.99968 | 33.08345 | 24.57313 | 26.16897 | 21.72163 | 24.15363 | 35.72924 | 32.9302 | 39.22918 | 33.96909 | 28.42135 | 12.70528 | 27.25206 | 24.44489 | 25.00056 | 33.3239 | 25.24454 | 15.35699 |
| CUST_9198_PI429010886 | N24218 | 0.029511 | 21 | 0 | | | | | | 579.0482 | NA | 710.9044 | 62.96663 | 137.3696 | 127.1338 | 647.6977 | 119.9123 | 43.49365 | 52.63381 | 166.7787 | NA | 56.30906 | 58.20859 | 20.81041 | NA | 284.3166 | 36.37581 | 255.9101 | NA | 477.9922 | NA | 739.338 | 677.7517 | 10.73106 | NA | 4.545299 |

| Probe ID | ID | p-value | Period | Phase | EBI ID | Annotation | GO category | Go term | E-value | 0 | 0 | 0 | 3 | 3 | 3 | 6 | 6 | 6 | 9 | 9 | 9 | 12 | 12 | 12 | 15 | 15 | 15 | 18 | 18 | 18 | 21 | 21 | 21 | 24 | 24 | 24 |
|---|---|---|---|---|---|---|---|---|---|---|---|---|---|---|---|---|---|---|---|---|---|---|---|---|---|---|---|---|---|---|---|---|---|---|---|---|
| CUST_41336_PI429010886 | M57477 | 0.029527 | 15 | 0 | | | | | | 653.9628 | 698.4337 | 519.6135 | 414.2851 | 478.2055 | 517.6852 | 457.3191 | 433.7721 | 383.7251 | 493.0904 | 505.5887 | 584.1415 | 339.1595 | 427.4471 | 508.5068 | 801.7718 | 560.8883 | 624.2921 | 536.4898 | 573.864 | 475.032 | 650.2354 | 399.1635 | 419.7481 | 942.3167 | 530.2702 | 597.0282 |
| CUST_52184_PI429010886 | M96678 | 0.029527 | 15 | 0 | | | | | | 54.76633 | 91.56459 | 77.47192 | 48.3123 | 58.87771 | 63.89787 | 48.59467 | 56.65877 | 43.27178 | 66.97079 | 55.25124 | 62.78204 | 60.3963 | 69.42462 | 63.35686 | 70.108 | 65.59001 | 75.04275 | 53.65979 | 65.42947 | 61.53942 | 54.07518 | 81.18707 | 55.59388 | 65.82296 | 49.3195 | 69.15357 |
| CUST_46502_PI429010886 | M72842 | 0.029528 | 12 | 0 | 307208663 | ER degradation-enhancing alpha-mannosidase-like | | | 7.4E-15 | 66.78979 | 20.10327 | 62.21051 | 15.94162 | 28.03992 | 18.42911 | 42.44034 | 48.13849 | 59.78571 | 51.51243 | 29.40344 | 35.21227 | 51.58072 | 80.13478 | 37.75935 | 32.71054 | 33.06433 | 17.99464 | 37.22874 | 32.02557 | 29.33547 | 58.00667 | 59.03159 | 23.16735 | 60.10166 | 19.96165 | 74.19557 |
| CUST_23965_PI429010886 | N49785 | 0.029528 | 12 | 3 | | | | | | 2212.175 | 2061.289 | 919.6285 | 11545.02 | 17234.2 | 18116.83 | 13147.15 | 5105.143 | 1918.015 | 746.9886 | 15482.55 | 1087.079 | 2884.034 | 797.7975 | 2112.767 | 1112.909 | 17837.74 | 13275.24 | 9530.181 | 581.5974 | 11816.95 | 1195.55 | 8493.602 | 11013.97 | 897.6332 | 7536.588 | 576.5142 |
| CUST_39857_PI429010886 | M45821 | 0.029554 | 12 | 3 | | | | | | 151.8403 | 18.21746 | 35.35004 | 1154.959 | 316.122 | 1904.883 | 568.6876 | 403.6614 | 75.64933 | 3.7334 | 340.8239 | NA | 139.6592 | 7.744827 | 56.77783 | NA | 1211.945 | 1086.056 | 243.0834 | NA | 139.9854 | 7.80639 | 132.7469 | 759.5836 | NA | 862.8974 | NA |
| CUST_14739_PI429010886 | N33384 | 0.029554 | 21 | 15 | | | | | | 339.7564 | 310.5573 | 258.0874 | 235.8718 | 281.2963 | 287.2312 | 241.9211 | 221.2763 | 200.3064 | 330.882 | 299.5774 | 264.3669 | 219.4136 | 261.767 | 217.5495 | 484.0144 | 334.1058 | 325.5645 | 363.8038 | 295.2655 | 279.1889 | 363.2975 | 223.1512 | 259.0619 | 475.032 | 198.8279 | 278.6013 |
| CUST_46036_PI429010886 | M71146 | 0.02958 | 21 | 18 | | | | | | 24.8275 | 46.60476 | 34.26311 | 15.27365 | 23.531 | 5.857243 | 15.62744 | 7.663615 | 10.42321 | 43.33729 | 19.54896 | 25.99897 | 24.06718 | 27.24092 | 29.90756 | 38.16824 | 18.57026 | 39.70836 | 17.49601 | 41.12468 | 31.78038 | 46.64901 | 23.59085 | 21.16222 | 22.91218 | 21.81525 | 35.19263 |
| CUST_31834_PI429010886 | M2521 | 0.0296 | 15 | 12 | | | | | | 791.2722 | 2179.192 | 1281.141 | 721.1961 | 465.2249 | 378.0737 | 393.3268 | 119.1299 | 790.5889 | 1504.846 | 898.9921 | 2504.679 | 905.952 | 901.9536 | 1657.221 | 1240.437 | 52.62093 | 358.0335 | 904.602 | 1660.585 | 1386.212 | 466.0044 | 225.693 | 226.0436 | 895.455 | 877.6784 | 1081.506 |
| CUST_12219_PI429010886 | N29121 | 0.029606 | 12 | 3 | 478250713 | hypothetical protein YQE_12134, partial [Dendroct | Metabolic process | Other | 1.82E-21 | 172.082 | 216.6058 | 90.38098 | 251.5259 | 186.9387 | 295.2206 | 225.7607 | 165.0319 | 228.3459 | 74.22931 | 65.63314 | 78.08745 | 171.4068 | 77.90078 | 115.9538 | 154.6378 | 160.259 | 231.8976 | 125.7869 | 73.26024 | 94.92777 | 101.6485 | 64.71901 | 318.4396 | 82.86721 | 179.7938 | 72.40334 |
| CUST_13568_PI429010886 | N31394 | 0.029606 | 12 | 3 | 262401023 | cytochrome c oxidase subunit VIIA putative [Scylla p | Metabolic process | Generation of precursor metabolites and energy | 4.62E-07 | 528.5021 | 503.4817 | 534.5603 | 675.7327 | 698.8251 | 825.7659 | 386.2115 | 648.6302 | 470.7834 | 463.903 | 441.3844 | 486.5202 | 492.3863 | 765.0242 | 592.481 | 599.7852 | 554.4782 | 742.6323 | 618.8739 | 902.7962 | 479.0678 | 750.8373 | 531.2482 | 369.6113 | 501.3373 | 591.7087 | 624.8531 |
| CUST_19310_PI429010886 | N41361 | 0.029607 | 18 | 9 | | | | | | 11.70041 | 7.967454 | NA | 5.415271 | 3.812859 | 5.254253 | NA | 5.405551 | NA | 15.7975 | 15.83559 | 14.13803 | 5.673212 | 7.57659 | 17.43627 | 7.138401 | 6.241278 | 17.5491 | 15.67634 | 4.425369 | 5.795169 | 6.782422 | NA | NA | 13.38001 | 9.695604 | 8.221669 |
| CUST_41537_PI429010886 | M57984 | 0.029616 | 24 | 18 | | | | | | 70.88996 | 72.1551 | 57.35641 | 62.54934 | 52.11537 | 48.09045 | 50.57194 | 50.43862 | 91.13848 | 84.41305 | 86.15616 | 115.3266 | 76.16727 | 84.05405 | 91.35555 | 88.58239 | 23.38375 | 71.38963 | 98.45237 | 93.34264 | 106.4305 | 87.75721 | 80.61916 | 36.35659 | 70.27178 | 88.17592 | 78.93037 |
| CUST_48862_PI429010886 | M82582 | 0.029616 | 24 | 21 | | | | | | 466.0826 | 503.9702 | 549.6818 | 424.5435 | 407.5311 | 480.2626 | 391.9101 | 415.3866 | 450.1209 | 486.1656 | 422.1729 | 523.8983 | 437.1883 | 538.1956 | 544.9594 | 618.5189 | 420.6788 | 471.0971 | 420.2091 | 624.13 | 570.6164 | 580.6994 | 570.1143 | 471.9637 | 594.1197 | 545.5317 | 654.7049 |
| CUST_30023_PI429010886 | N61025 | 0.029616 | 24 | 21 | | | | | | 72.97459 | 61.95712 | 64.36368 | 46.56491 | 61.3315 | 67.5589 | 50.71291 | 70.30533 | 63.61419 | 67.50801 | 49.76955 | 80.4086 | 58.96311 | 78.86687 | 51.83037 | 69.63319 | 69.87848 | 71.73214 | 65.71273 | 70.69633 | 73.63983 | 84.1757 | 78.84971 | 74.95386 | 78.93037 | 68.37004 | 69.56934 |
| CUST_35478_PI429010886 | M12680 | 0.029623 | 15 | 9 | | | | | | 763.8572 | 865.174 | 916.8157 | 727.4325 | 737.499 | 609.4542 | 836.7397 | 672.3398 | 793.9994 | 950.2628 | 970.3266 | 922.8399 | 702.0078 | 840.3682 | 757.8647 | 945.5514 | 701.1457 | 806.0846 | 835.782 | 911.24 | 694.5441 | 925.8412 | 605.4467 | 650.0167 | 1142.567 | 797.3133 | 971.7833 |
| CUST_53968_PI429010886 | M103917 | 0.029653 | 12 | 0 | | | | | | 13.19382 | 11.3957 | 16.33892 | 4.049919 | 5.364535 | 9.253041 | NA | 7.012133 | 5.601268 | 9.956467 | 6.083321 | 8.724159 | 4.642534 | NA | 9.02845 | 13.11969 | 15.87452 | 8.351204 | 8.209591 | 4.413551 | 5.282757 | 3.453675 | NA | 4.846612 | 14.67882 | 20.53586 | 18.96681 |
| CUST_4324_PI429010886 | N16515 | 0.029661 | 18 | 3 | | | | | | 58.62419 | 3.594225 | 60.26917 | 86.13317 | 113.3117 | 194.6574 | 145.9425 | 30.58011 | 4.4068 | 4.743067 | 92.4425 | 21.39305 | 23.79718 | NA | 9.104602 | 3.604472 | 191.2063 | 63.53447 | 193.7403 | NA | 42.6663 | NA | 201.1076 | 251.404 | NA | 55.67871 | 5.045546 |
| CUST_49543_PI429010886 | M86045 | 0.029672 | 21 | 0 | | | | | | 157.1144 | 168.7959 | 170.6936 | 161.1809 | 142.9849 | 166.0384 | 122.3193 | 130.5819 | 143.4947 | 147.1696 | 136.3782 | 189.7386 | 149.5758 | 197.6933 | 164.4415 | 156.9336 | 146.4472 | 165.2222 | 145.0637 | 162.3514 | 146.585 | 168.177 | 151.707 | 165.7787 | 144.7517 | 150.449 | 153.7764 |
| CUST_6348_PI429010886 | N19982 | 0.029673 | 18 | 6 | 321467616 | hypothetical protein DAPPUDRAFT_320238 [Daphn | | | 6.7E-30 | 118.6617 | 151.6013 | 117.746 | 170.7553 | 144.7517 | 116.0377 | 168.2847 | 150.286 | 161.8906 | 116.8091 | 167.4717 | 162.9163 | 154.6585 | 160.1816 | 112.517 | 145.0486 | 115.9131 | 92.96667 | 147.7228 | 145.4402 | 113.2498 | 124.2579 | 121.3759 | 102.2185 | 167.6742 | 136.7554 | 235.9095 |
| CUST_49478_PI429010886 | M85816 | 0.029686 | 24 | 21 | | | | | | 19.6024 | 118.1469 | 30.83339 | 57.16139 | 18.51256 | 22.20487 | 24.91328 | 12.29952 | 21.69445 | 27.73533 | 22.88862 | 24.135 | 29.94174 | 20.04708 | 19.58619 | 43.19614 | 27.92893 | 35.49419 | 26.87538 | 30.96855 | 49.79836 | 37.96514 | 23.565 | 59.0899 | 23.20447 | 15.59485 | 32.70344 |
| CUST_16119_PI429010886 | N35693 | 0.029687 | 15 | 3 | | | | | | 65.8929 | 46.88603 | 40.24548 | 62.94659 | 43.8795 | 50.48246 | 86.44204 | 50.53472 | 31.91513 | 58.00017 | 47.43001 | 13.50761 | 36.01332 | 26.37038 | 20.36175 | 90.55235 | 101.9359 | 28.44656 | 107.8556 | 117.3157 | 95.96344 | 55.16431 | 24.27461 | NA | 52.42987 | 42.62942 | 57.61472 |
| CUST_26867_PI429010886 | N55011 | 0.029688 | 12 | 9 | 390362121 | PREDICTED: uncharacterized protein LOC10089295 | | | 9.61E-10 | 5.298018 | NA | 29.62034 | 3.378824 | 4.322357 | 14.91349 | NA | 20.65925 | 8.026683 | 14.75394 | NA | 29.3424 | 7.613075 | 18.19818 | 23.15048 | 5.366146 | 3.667746 | NA | 5.268714 | 8.464949 | 6.508712 | 22.25705 | 17.57797 | NA | 9.972812 | 8.698364 | 3.60476 |
| CUST_50731_PI429010886 | M90812 | 0.029707 | 15 | 0 | | | | | | 11.17351 | 21.62181 | 9.927073 | 10.36474 | 14.13095 | 19.39824 | 9.033618 | 9.673986 | 7.272559 | 11.78596 | 8.630999 | 8.687252 | 9.384386 | 8.734424 | 14.9321 | 24.81549 | 9.666802 | 18.73049 | 12.54687 | 11.92305 | 7.388302 | 13.67397 | 15.2073 | 9.907267 | 9.272219 | NA | 6.989719 |
| CUST_41340_PI429010886 | M57496 | 0.02972 | 12 | 0 | | | | | | 83.06954 | 92.4425 | 72.19392 | 78.39762 | 70.81749 | 59.67663 | 47.59957 | 43.70884 | 64.61143 | 74.18777 | 71.72159 | 75.7673 | 77.2785 | 76.924 | 92.1688 | 121.5886 | 49.13393 | 72.52738 | 70.60594 | 101.1723 | 65.96571 | 117.8039 | 67.05664 | 59.65312 | 106.1754 | 71.89357 | 108.7588 |
| CUST_55493_PI429010886 | M110350 | 0.029748 | 12 | 0 | | | | | | 34.67799 | 34.76682 | 34.60947 | 18.43584 | 20.58696 | 35.41996 | 14.35419 | 22.40135 | 30.74148 | 37.52021 | 31.86259 | 45.15073 | 20.48821 | 43.96447 | 42.23347 | 33.54956 | 20.80857 | 28.68237 | 27.77246 | 24.51394 | 37.14508 | 34.52311 | 30.96855 | 18.20618 | 40.1276 | 37.77885 | 49.02873 |
| CUST_46503_PI429010886 | M72843 | 0.029824 | 15 | 0 | | | | | | 46.66745 | 56.41128 | 27.17883 | 59.75541 | 46.96117 | 42.09877 | 46.90544 | 38.97496 | 33.20236 | 36.06457 | 35.91866 | 30.23833 | 39.37435 | 26.04444 | 42.1461 | 44.0999 | 49.59839 | 47.93942 | 39.63454 | 44.98924 | 36.82429 | 59.82999 | 20.51687 | 36.73446 | 43.05742 | 32.88145 | 23.49823 |
| CUST_50552_PI429010886 | M90241 | 0.029824 | 15 | 9 | | | | | | 120.4315 | 73.59863 | 176.5007 | 48.71731 | 99.4422 | 86.20359 | 151.2427 | 92.47211 | 107.638 | 138.4789 | 122.7974 | 158.6127 | 115.1438 | 121.3307 | 118.5634 | 101.4752 | 142.4549 | 80.02167 | 98.51875 | 101.8542 | 118.0864 | 90.78956 | 158.4097 | 151.7333 | 137.3696 | 103.384 | 175.7472 |
| CUST_13767_PI429010886 | N31734 | 0.029836 | 24 | 6 | | | | | | 35.39935 | 12.51969 | 13.71806 | 41.8887 | 8.201884 | 35.76089 | 64.50807 | 49.15395 | 54.71071 | 6.49944 | 11.65277 | 16.96557 | 22.24073 | 11.23684 | 21.89378 | 8.291054 | 15.07677 | 19.54386 | 12.35607 | 8.353787 | 19.7328 | 5.377206 | 13.98077 | 12.76656 | 15.4684 | 48.63366 | 10.03982 |
| CUST_23699_PI429010886 | N49306 | 0.029836 | 24 | 21 | | | | | | 93.27103 | 141.0429 | 108.7711 | 101.92 | 97.97139 | 106.5708 | 99.30005 | 83.32632 | 85.95 | 135.9242 | 99.82512 | 138.9365 | 92.13028 | 139.0764 | 138.3597 | 156.3748 | 73.52413 | 140.147 | 95.12719 | 141.7869 | 125.2377 | 154.793 | 110.737 | 130.3743 | 132.109 | 116.2211 | 111.9132 |
| CUST_38112_PI429010886 | M23418 | 0.029843 | 15 | 12 | | | | | | 12673.02 | 301.849 | 29010.89 | 260.0114 | 707.1584 | 490.0839 | 16358.95 | 40491.1 | 34676.57 | 37163.2 | 5534.91 | 5029.59 | 35264.41 | 47564.51 | 23580.44 | 1273.05 | 17078.69 | 570.8436 | 9541.284 | 5732.178 | 4604.537 | 15704.56 | 30082.79 | 3055.899 | 32869.85 | 2727.828 | 31231.03 |
| CUST_9358_PI429010886 | N24486 | 0.029852 | 12 | 3 | 13898841 | CUB-serine protease [Panulirus argus] | Protein metabolism | Proteolysis | 2.59E-21 | 64.31677 | 29.25886 | 44.00542 | 159.9242 | 104.6153 | 295.5186 | 206.2668 | 134.02 | 62.00083 | 89.33478 | 264.4703 | 38.18132 | 72.11758 | 76.15742 | 60.5947 | 67.34873 | 346.7104 | 254.2335 | 114.3208 | 85.17215 | 119.245 | 76.18529 | 123.0174 | 161.7305 | 199.6541 | 84.11167 | 37.94932 |
| CUST_21616_PI429010886 | N45535 | 0.029852 | 12 | 3 | | | | | | 98.39109 | 62.03099 | 20.02214 | 182.709 | 79.84889 | 122.9865 | 169.8899 | 83.40091 | 111.3143 | 72.11758 | 114.5149 | 60.8738 | 81.61454 | 41.60369 | 64.39273 | 164.3174 | 125.8218 | 156.2584 | 122.404 | 54.61808 | 57.03895 | 133.9111 | 26.41485 | 74.51983 | 66.89269 | 121.4562 | 50.59331 |
| CUST_17734_PI429010886 | N38497 | 0.029865 | 21 | 3 | | | | | | 6.584593 | 8.959775 | 3.707704 | 13.17056 | 10.7039 | NA | 11.8016 | 5.820473 | 8.149809 | 13.50761 | 7.074973 | 5.010162 | 5.307944 | 8.723307 | 5.811802 | 5.515193 | 4.842579 | 6.779112 | 9.532078 | 10.86536 | 8.927589 | 8.086549 | 7.024562 | 10.50787 | 12.40589 | NA | 9.751886 |
| CUST_9770_PI429010886 | N25181 | 0.029871 | 21 | 3 | 242012890 | pre-mRNA-splicing factor ATP-dependent RNA helic | Nucleic acid metabolism | RNA metabolic process | 1.89E-58 | 542.234 | 1539.294 | 666.6962 | 1783.909 | 847.9888 | 1107.396 | 638.1084 | 582.4599 | 627.8304 | 558.5528 | 293.8285 | 452.1705 | 409.0918 | 685.9612 | 492.3863 | 716.0786 | 233.828 | 571.296 | 301.6859 | 599.656 | 1045.15 | 533.2467 | 1036.891 | 307.3807 | 1033.021 | 1139.145 | 436.2219 |
| CUST_46063_PI429010886 | M71237 | 0.029887 | 21 | 15 | | | | | | 128.9362 | 103.8532 | 124.4264 | 88.01513 | 106.2503 | 109.9853 | 101.5014 | 83.00511 | 84.44547 | 104.4433 | 118.4488 | 92.05581 | 88.42631 | 129.0306 | 84.61863 | 110.8154 | 151.0154 | 132.5328 | 164.2928 | 105.242 | 97.43479 | 138.3904 | 102.7268 | 104.1514 | 141.1886 | 94.71285 | 106.3603 |
| CUST_49989_PI429010886 | M88117 | 0.029893 | 21 | 0 | | | | | | 82.06433 | 43.90905 | 68.5761 | 60.82114 | 61.35068 | 73.44541 | 47.99403 | 61.40781 | 60.28242 | 73.02744 | 51.95466 | 63.53447 | 37.65478 | 46.55207 | 49.44854 | 55.18575 | 64.58903 | 74.42211 | 59.48755 | 52.04647 | 57.78122 | 74.64567 | 74.22065 | 60.4668 | 56.63828 | 73.09177 | 62.1341 |
| CUST_13994_PI429010886 | N32142 | 0.029903 | 15 | 12 | 290462133 | tRNA 2-thiocytidine biosynthesis protein ttcA [Lepe | Metabolic process | Other | 1.81E-26 | 57.06419 | 57.46891 | 60.93646 | 33.78634 | 45.55959 | 26.94977 | 44.61606 | 40.24548 | 44.331 | 77.26285 | 42.82196 | 63.11741 | 45.99048 | 62.89604 | 58.70799 | 64.68215 | 42.72999 | 60.17363 | 53.00673 | 54.56998 | 49.80346 | 55.27747 | 50.38211 | 25.92862 | 56.8218 | 41.86303 | 68.43089 |
| CUST_35387_PI429010886 | M12450 | 0.029907 | 24 | 15 | 242013849 | A kinase anchor protein, putative [Pediculus human | Metabolic process | Other | 1.67E-33 | 61.60941 | 87.42512 | 67.9514 | 46.55991 | 37.30507 | 52.32373 | 36.62478 | 37.50197 | 66.03172 | 65.19644 | 65.8929 | 73.74514 | 61.04516 | 73.16394 | 66.11527 | 82.1182 | 63.79444 | 85.83424 | 60.67368 | 80.51303 | 62.51938 | 65.08584 | 56.43061 | 83.21112 | 71.22702 | 66.34998 | 59.48755 |
| CUST_20851_PI429010886 | N44187 | 0.02991 | 15 | 6 | | | | | | 8.573429 | NA | 3.517415 | 58.20859 | 8.881606 | 92.01375 | 57.16139 | 58.81306 | 47.53397 | 8.33181 | NA | 60.66262 | 39.58582 | NA | 26.2903 | 19.97907 | 12.80802 | 19.17708 | 22.43724 | NA | 28.77687 | 27.25419 | NA | 46.29572 | 25.17921 | 55.92617 | 59.17148 |
| CUST_33798_PI429010886 | M7907 | 0.029911 | 15 | 9 | 345484011 | PREDICTED: Nasonia vitripennis replication factor C | Developmental process | Multicellular organismal development | 2.89E-56 | 72.2954 | 89.87648 | 98.55351 | 68.25442 | 72.76448 | 76.73952 | 69.18954 | 143.406 | 157.5293 | 127.3882 | 77.98525 | 108.8172 | 100.397 | 103.2411 | 113.0468 | 141.72 | 56.63828 | 87.6112 | 72.3461 | 70.41805 | 81.27628 | 118.0238 | 90.38098 | 51.05906 | 131.7223 | 100.6181 | 117.6882 |
| CUST_9156_PI429010886 | N24150 | 0.029925 | 24 | 15 | | | | | | 67.62921 | 86.64513 | 72.4658 | 73.07164 | 56.10477 | 58.33665 | 55.81208 | 65.38012 | 72.14746 | 81.9093 | 61.25018 | 70.94855 | 79.39521 | 82.07629 | 64.75716 | 87.05061 | 79.89836 | 91.91837 | 74.08523 | 65.22254 | 66.46046 | 117.8175 | 108.3285 | 65.80017 | 62.99752 | 70.05382 | 73.38298 |
| CUST_5472_PI429010886 | N18749 | 0.029929 | 21 | 9 | 85677254 | innexin inx2 [Homarus gammarus] | | | 2.53E-49 | 3.648462 | 8.658987 | NA | 10.95448 | 24.64992 | 5.123692 | 17.42611 | 50.43461 | 23.92874 | NA | 55.78991 | 46.27475 | 26.24303 | 46.53178 | 9.240522 | 5.880062 | 24.66033 | NA | 7.382145 | 75.66735 | 25.91379 | NA | 15.66989 | NA | 6.576173 | 19.82309 | 22.97231 |
| CUST_12159_PI429010886 | N29023 | 0.029933 | 21 | 15 | | | | | | 49.72048 | 51.32239 | 41.16431 | 48.76564 | 48.89685 | 37.00144 | 38.24749 | 40.04237 | 40.87555 | 70.7166 | 47.06657 | 47.61035 | 37.60789 | 53.28087 | 58.61502 | 52.11124 | 66.95495 | 47.20161 | 59.52293 | 48.9358 | 42.76199 | 51.75281 | 42.17788 | 53.15159 | 53.00119 | 40.86391 | 33.36125 |
| CUST_6875_PI429010886 | N20719 | 0.029944 | 15 | 0 | 262401075 | putative myosin regulatory light chain 2 smooth mu | | | 5.13E-88 | 1669.785 | 1819.925 | 1439.161 | 1594.166 | 1612.797 | 2027.433 | 2005.797 | 1233.738 | 1685.531 | 947.592 | 1331.49 | 1421.77 | 1557.483 | 1325.963 | 1718.489 | 1906.946 | 2003.234 | 1931.541 | 1989.387 | 1101.197 | 1330.395 | 1640.228 | 1097.209 | 1794.046 | 1388.938 | 1315.293 | 1042.025 |
| CUST_11257_PI429010886 | N27521 | 0.029944 | 15 | 9 | 260828245 | hypothetical protein BRAFLDRAFT_91042 [Branchio | Response to stress | DNA repair | 4.18E-63 | 34.05805 | 68.71451 | 44.23444 | 30.99084 | 47.94376 | 51.43059 | 57.78767 | 45.61861 | 71.50258 | 58.67101 | 66.01381 | 79.89836 | 44.50009 | 74.10963 | 53.58897 | 42.74237 | 45.02093 | 87.35248 | 38.26462 | 47.75268 | 75.7296 | 53.73015 | 94.07093 | 88.66033 | 73.17053 | 81.71205 | 128.3615 |
| CUST_12874_PI429010886 | N30187 | 0.029947 | 12 | 9 | | | | | | 123.056 | 75.64933 | 142.5643 | 65.63314 | 114.5891 | 92.50008 | 91.42252 | 120.3504 | 129.3708 | 141.1309 | 135.7577 | 145.9205 | 106.6375 | 140.0797 | 152.3983 | 96.25237 | 111.151 | 118.7227 | 115.9131 | 138.4302 | 121.4843 | 119.1161 | 145.749 | 116.9944 | 111.5529 | 135.6517 | 176.3364 |
| CUST_55180_PI429010886 | M109025 | 0.029955 | 12 | 0 | 91090962 | PREDICTED: similar to hypoxia associated factor [Tri | | | 2.09E-08 | 13.79507 | 8.844206 | 8.355196 | 5.910691 | 6.417851 | 10.44214 | 3.509222 | 7.768024 | 11.09741 | 6.391855 | 5.101628 | 8.756045 | 9.061963 | 9.898027 | NA | 5.037698 | NA | 15.93149 | 4.325683 | 4.554045 | 6.870124 | 4.629241 | 6.423011 | 9.696806 | 10.21823 | 15.01303 | 15.94563 |
| CUST_29497_PI429010886 | N59983 | 0.029957 | 15 | 9 | | | | | | 30.52691 | 47.27714 | 42.95145 | 33.33921 | 43.2321 | 35.6576 | 48.50963 | 35.10761 | 82.19075 | 33.48083 | 42.39566 | 91.59828 | 58.01793 | 67.49489 | 61.32192 | 29.02633 | 27.10054 | 24.51944 | 50.68803 | 25.12826 | 44.84166 | 28.56717 | 55.84573 | 40.55619 | 48.69863 | 51.50855 | 87.68051 |
| CUST_50182_PI429010886 | M88952 | 0.029968 | 24 | 21 | 291225045 | PREDICTED: zinc finger protein 45-like [Saccoglossu | | | 5.58E-23 | 34.71929 | 42.33801 | 43.82278 | 38.35427 | 45.04433 | 46.64002 | 38.62767 | 39.82861 | 45.78297 | 59.22654 | 47.08024 | 57.46891 | 42.57633 | 65.10535 | 60.10929 | 60.15877 | 47.16593 | 63.25496 | 66.72453 | 54.89215 | 57.00393 | 68.40354 | 59.82242 | 68.63241 | 73.33005 | 38.58794 | 66.04832 |
| CUST_21446_PI429010886 | N45238 | 0.029968 | 24 | 18 | 241622329 | ras-responsive element binding protein, putative [I | | | 7.24E-26 | 16.75535 | 21.08222 | 20.5469 | 12.96775 | 12.90145 | 11.43555 | 14.36868 | 20.25455 | 21.35291 | 28.47434 | 20.69556 | 21.17293 | 12.41093 | 38.42634 | 14.16669 | 23.74918 | 16.06632 | 22.87112 | 24.99293 | 22.1046 | 21.89117 | 21.83957 | 14.57533 | 23.31758 | 23.73141 | 16.20296 | 23.42758 |
| CUST_22714_PI429010886 | N47520 | 0.029968 | 24 | 3 | | | | | | 197.9029 | 239.6937 | 149.5538 | 193.4196 | 139.7302 | 198.1863 | 178.4972 | 130.3328 | 211.6792 | 132.7049 | 120.9334 | 248.652 | 137.5651 | 78.04542 | 116.8209 | 204.0863 | 74.48084 | 105.8984 | 102.4425 | 42.71853 | 87.80183 | 197.3275 | 143.6702 | 162.719 | 112.2413 | 91.47195 | 90.85633 |
| CUST_10765_PI429010886 | N26752 | 0.03003 | 21 | 0 | | | Developmental process | Multicellular organismal development | | 34.35393 | 49.51746 | 54.74747 | 46.93642 | 23.1605 | 35.57579 | 21.74369 | 14.66408 | 16.98127 | 23.63855 | 26.90876 | 42.18503 | 34.33169 | 40.41132 | 30.19101 | 32.66922 | 13.24864 | 30.63369 | 9.695604 | 70.08131 | 40.75734 | 46.20996 | 60.3963 | 23.43573 | 25.52881 | 37.20896 | 30.07667 |
| CUST_22876_PI429010886 | N47836 | 0.030074 | 12 | 0 | | | | | | 486.3734 | 435.9739 | 438.6413 | 363.6175 | 412.7593 | 415.2193 | 288.2901 | 239.4694 | 259.0023 | 518.627 | 413.3489 | 462.0916 | 296.0085 | 369.7785 | 469.1551 | 640.6647 | 375.1955 | 463.903 | 404.5388 | 402.6363 | 357.6255 | 498.3855 | 403.2396 | 438.1052 | 556.543 | 540.0536 | 493.9819 |
| CUST_15888_PI429010886 | N35300 | 0.03008 | 24 | 21 | | | | | | 50.91495 | 34.65808 | 22.82362 | 24.32948 | 14.38818 | 22.41298 | 23.33262 | 19.01762 | 23.17379 | 24.31817 | 19.37414 | 26.31122 | 32.83309 | 54.46899 | 35.13913 | 39.44223 | 26.37567 | 29.80754 | 31.1463 | 34.25879 | 23.67732 | 31.34507 | 27.50275 | 40.03501 | 39.90543 | 34.16228 | 27.39792 |
| CUST_1552_PI429010886 | N10041 | 0.030082 | 12 | 0 | | | | | | 66.41721 | 60.42655 | 73.97012 | 61.28936 | 51.07827 | 64.99854 | 49.34012 | 66.86178 | 58.85113 | 65.03693 | 52.00681 | 68.83612 | 59.34275 | 94.50596 | 88.46488 | 69.33625 | 54.77183 | 56.11599 | 70.61376 | 51.88107 | 64.86139 | 71.79355 | 69.40293 | 51.7472 | 89.67555 | 81.59575 | 74.62941 |
| CUST_436_PI429010886 | N5162 | 0.030082 | 15 | 9 | | | | | | 1271.51 | 1413.053 | 1357.763 | 1207.621 | 1155.687 | 1172.181 | 1054.214 | 1104.361 | 1139.145 | 1560.88 | 1312.967 | 1568.166 | 1191.932 | 1493.465 | 1447.664 | 1811.1 | 1251.807 | 1276.669 | 1100.556 | 1633.35 | 1080.128 | 1635.051 | 1245.533 | 1185.368 | 1968.333 | 1293.201 | 1467.586 |
| CUST_53994_PI429010886 | M104013 | 0.030085 | 15 | 12 | 321466304 | adenylyl cyclase [Daphnia pulex] | | | 8.99E-16 | 23.17801 | 16.88307 | 28.79209 | 48.44993 | 6.44346 | 21.34327 | 16.23047 | 30.15999 | 27.10661 | 43.28801 | 20.95699 | 38.43363 | 34.59885 | 92.12003 | 33.80016 | 24.64126 | 24.37063 | 33.35513 | 16.78585 | 16.25775 | 30.95933 | 42.34172 | 24.7547 | 18.33365 | 42.17788 | 32.8525 | 48.40257 |
| CUST_44207_PI429010886 | M65262 | 0.030115 | 24 | 18 | 465957132 | UDP-GalNAc:beta-1,3-N-acetylgalactosaminyltransf | Metabolic process | Carbohydrate metabolic process | 3.64E-09 | 16.42376 | 28.50866 | 15.17161 | 8.700812 | 15.79313 | 11.53559 | 13.7932 | 22.75684 | 26.10123 | 35.55848 | 21.61111 | 28.33698 | 19.14299 | 45.16608 | 16.29644 | 18.16713 | 22.69042 | 27.10385 | 36.72024 | 33.52107 | 24.26118 | 21.55583 | 20.46944 | NA | 25.74156 | 30.09468 | 22.02142 |
| CUST_10773_PI429010886 | N26767 | 0.030121 | 21 | 15 | 260806841 | hypothetical protein BRAFLDRAFT_69647 [Branchio | | | 8.05E-10 | 14.13262 | 23.29568 | 20.86339 | 16.84886 | 15.89427 | 10.24818 | 16.43851 | 15.97964 | 15.68573 | 35.04798 | 23.38375 | 18.76152 | 14.68167 | 16.11829 | 19.34576 | 20.75737 | 24.96736 | 31.11066 | 43.12293 | 19.3955 | 13.08345 | 20.50575 | 26.92502 | 20.91496 | 12.85718 | 13.41264 | 20.23937 |
| CUST_18982_PI429010886 | N40766 | 0.030121 | 21 | 0 | | | | | | 56.88497 | 46.33014 | 46.73511 | 37.00144 | 26.65472 | 60.82746 | 30.5127 | 40.1071 | 32.3787 | 32.95281 | 32.24298 | 28.99306 | 50.18617 | 46.26909 | 43.20694 | 37.52322 | 34.08686 | 37.18492 | 60.56579 | 28.67507 | 44.28544 | 54.85007 | 41.67823 | 49.88882 | 62.79742 | 37.85261 | 36.33605 |
| CUST_41411_PI429010886 | M57676 | 0.030155 | 12 | 3 | | | Metabolic process | Carbohydrate metabolic process | | 572.8694 | 132.2443 | 93.58403 | 802.0583 | 859.9982 | 1436.981 | 551.706 | 461.6522 | 266.1283 | 11.53748 | 389.9711 | 7.164995 | 365.0271 | 14.76465 | 113.1518 | 52.24124 | 1770.146 | 1280.117 | 821.7869 | 17.67291 | 390.5304 | 35.60156 | 335.4811 | 653.9628 | 11.47168 | 1013.924 | 5.180096 |
| CUST_4611_PI429010886 | N17129 | 0.030155 | 15 | 9 | | | | | | 222.5361 | 273.1684 | 248.1135 | 112.7975 | 248.652 | 180.634 | 283.8348 | 140.6816 | 201.5878 | 345.0421 | 225.9576 | 480.999 | 244.0822 | 306.093 | 322.0841 | 197.122 | 194.8105 | 210.7138 | 293.9979 | 164.8776 | 250.587 | 278.8959 | 229.7558 | 248.8286 | 314.246 | 247.961 | 323.8163 |
| CUST_3930_PI429010886 | N15602 | 0.030156 | 15 | 9 | | | | | | 851.0651 | 4064.703 | 3143.095 | 245.4886 | 360.037 | NA | 350.912 | 2273.444 | 4031.594 | 4025.068 | 2580.486 | 4738.823 | 1581.192 | 5819.009 | 4318.11 | 4270.599 | 968.7311 | 1560.88 | 2117.402 | 3972.597 | 1750.319 | 4724.809 | 3659.313 | 64.93326 | 6962.573 | 3842.983 | 8147.956 |
| CUST_4628_PI429010886 | N17163 | 0.030185 | 24 | 21 | | | | | | 18.32343 | 18.78533 | 20.05897 | 12.71046 | 10.33138 | 11.3438 | 18.11108 | 48.6393 | 16.33892 | 11.42482 | 4.695828 | 15.19248 | 10.58644 | 13.23183 | 15.40573 | 21.54483 | 23.57221 | 15.60331 | 8.167031 | 19.10174 | 16.4346 | 29.04798 | 22.19563 | 18.30186 | 16.23152 | 11.30247 | 20.09831 |
| CUST_1265_PI429010886 | N9074 | 0.030218 | 18 | 3 | | | | | | 1202.61 | 991.907 | 545.8082 | 1003.971 | 869.992 | 966.1688 | 1257.529 | 734.7434 | 836.7397 | 490.166 | 471.0971 | 438.5463 | 606.7586 | 417.1018 | 786.1873 | 851.4085 | 697.3779 | 550.7829 | 765.7162 | 474.1045 | 526.943 | 843.8131 | 497.8615 | 1080.128 | 506.7573 | 807.7825 | 373.5295 |
| CUST_29377_PI429010886 | N59742 | 0.030218 | 12 | 3 | | | | | | 92.20534 | 30.06738 | 16.02173 | 127.9469 | 86.45155 | 123.3865 | 117.7023 | 78.59764 | 21.44879 | 9.953883 | 65.33287 | 8.491672 | 73.33698 | 12.42474 | 18.35945 | 24.80133 | 192.1486 | 94.40103 | 151.7898 | 15.00335 | 43.14736 | 24.76116 | 17.18634 | 63.04603 | 9.326745 | 49.50242 | NA |
| CUST_42608_PI429010886 | M60719 | 0.03022 | 18 | 3 | 283854609 | DEAD(Asp-Glu-Ala-Asp) box polypeptide 5 [Macrob | | | 2.79E-49 | 35.4728 | 102.2299 | 40.80069 | 148.3091 | 51.7472 | 83.16029 | 39.41947 | 42.26034 | 28.65673 | 37.25844 | 19.69614 | 60.10166 | 27.07492 | 33.11438 | 42.5506 | 35.678 | 18.02634 | 45.54446 | 21.81525 | 37.08698 | 61.68471 | 52.70775 | 60.61979 | 41.50969 | 55.03461 | 88.78018 | 25.89264 |
| CUST_1858_PI429010886 | N10896 | 0.030243 | 18 | 3 | | | | | | 258.7295 | 806.3497 | 386.2115 | 1099.35 | 274.7936 | 909.4624 | 290.2221 | 510.2851 | 238.1723 | 256.3774 | 155.1933 | 573.39 | 331.4917 | 334.4771 | 178.4972 | 344.9738 | 141.7648 | 300.885 | 171.7375 | 365.1209 | 487.8771 | 424.4597 | 482.5207 | 586.8079 | 443.4781 | 726.329 | 280.4106 |
| CUST_11301_PI429010886 | N27588 | 0.030252 | 21 | 15 | 345490661 | PREDICTED: transmembrane protein 184B-like isofo | | | 2.71E-51 | 11.49641 | 5.620885 | 15.24741 | 9.896666 | 4.994508 | 9.143497 | 8.383502 | 4.82051 | NA | 3.778452 | 8.933611 | 5.296016 | 8.29257 | 9.016795 | 14.47943 | 16.07442 | 10.56079 | 13.82074 | 11.95622 | 14.30732 | 9.837542 | 12.12113 | 11.18163 | 10.65706 | 4.273874 | 30.25328 | 16.14981 |
| CUST_37244_PI429010886 | M18353 | 0.030282 | 21 | 9 | | | | | | 35.00516 | 37.86895 | 28.03701 | 28.55509 | 23.94659 | 19.17264 | 25.31589 | 28.73411 | 29.03947 | 52.8414 | 48.16535 | 51.35337 | 32.48902 | 37.46264 | 32.9302 | 44.87166 | 18.52264 | 34.91922 | 44.50009 | 37.20514 | 45.25329 | 50.71879 | 30.83099 | 22.76479 | 32.75472 | 30.2284 | 29.78164 |
| CUST_46142_PI429010886 | M71538 | 0.030285 | 15 | 0 | 53801334 | Crassostrea gigas beta-tubulin mRNA, partial cds | Cellular Process | Cell cycle | 5.61E-60 | 2564.647 | 3065.467 | 1484.716 | 3062.827 | 2604.753 | 2384.394 | 2306.592 | 2213.328 | 2016.816 | 2079.573 | 2223.051 | 1629.467 | 2573.258 | 1631.11 | 1909.916 | 2894.613 | 2658.303 | 2424.452 | 2343.419 | 2304.824 | 1678.528 | 2826.022 | 1254.757 | 2282.54 | 2211.182 | 1999.641 | 1519.583 |
| CUST_34827_PI429010886 | M10814 | 0.030287 | 12 | 3 | | | | | | 365.3412 | 196.2537 | 221.9975 | 295.7576 | 395.3575 | 620.3013 | 334.9255 | 330.4772 | 231.6114 | 113.3786 | 182.2638 | 62.96106 | 246.8318 | 159.3888 | 225.9576 | 210.0878 | 670.866 | 631.6256 | 441.8735 | 80.27864 | 185.1977 | 189.2062 | 176.3616 | 500.086 | 175.5769 | 351.5336 | 48.53244 |
| CUST_39883_PI429010886 | M46171 | 0.030287 | 12 | 3 | 321473230 | hypothetical protein DAPPUDRAFT_239294 [Daphn | | | 3.57E-15 | 915.6162 | 734.477 | 109.4106 | 2230.655 | 1617.488 | 3192.934 | 1731.857 | 709.1206 | 1326.638 | 298.4347 | 653.4911 | 278.6632 | 1198.309 | 628.3128 | 588.4339 | 877.6784 | 2382.34 | 2571.52 | 528.8856 | 398.6724 | 462.0916 | 966.4783 | 308.3369 | 2175.001 | 416.5528 | 1155.257 | 274.296 |
| CUST_26519_PI429010886 | N54386 | 0.030287 | 12 | 9 | | | | | | 211.1514 | 159.02 | 205.5955 | 183.4397 | 165.0586 | 165.5224 | 204.3871 | 191.9895 | 203.2288 | 211.9082 | 218.9824 | 209.9951 | 202.7268 | 248.7014 | 157.6456 | 211.85 | 187.2939 | 181.4625 | 193.8296 | 217.0054 | 224.8249 | 222.6053 | 214.9497 | 220.485 | 231.8131 | 197.9328 | 233.8998 |
| CUST_14916_PI429010886 | N33685 | 0.030317 | 21 | 0 | | | | | | 24.15613 | 20.3649 | 23.77956 | 11.13159 | 16.51889 | 15.99774 | 12.75753 | 18.72625 | 9.725914 | 15.87262 | 13.89481 | 18.47667 | 21.21658 | 15.35699 | 9.939882 | 17.08982 | 15.89674 | 10.67318 | 22.94293 | 17.26689 | 21.57953 | 15.71949 | 20.27043 | 19.40925 | 19.86296 | 9.846676 | 26.41485 |
| CUST_56942_PI429010886 | M114963 | 0.030321 | 24 | 21 | | | | | | 39.26949 | 60.21125 | 62.72678 | 42.47169 | 51.07289 | 54.94382 | 35.31167 | 50.99115 | 46.75809 | 42.44777 | 55.09266 | 44.12842 | 49.85667 | 69.46903 | 59.75541 | 62.44095 | 57.55495 | 51.52932 | 40.13905 | 67.58357 | 57.12722 | 69.08941 | 61.89112 | 58.12189 | 63.15525 | 62.21051 | 66.40412 |
| CUST_43925_PI429010886 | M64342 | 0.030321 | 24 | 18 | | | | | | 21.29353 | 28.82637 | 15.33595 | 18.37346 | 18.3198 | 4.110038 | 7.879494 | 10.03222 | 33.37654 | 33.41929 | 19.65043 | 16.9925 | 24.58762 | 23.38175 | 15.24921 | 25.18558 | 10.11533 | 26.80742 | 48.30896 | 23.53335 | 44.46924 | 41.74772 | 31.73044 | 9.942787 | 21.21967 | 17.33333 | 28.69415 |
| CUST_15654_PI429010886 | N34927 | 0.030321 | 24 | 21 | | | | | | 14.81806 | 7.180488 | 9.720983 | 11.3957 | 16.45078 | 8.965849 | 6.26744 | 8.781809 | 8.201884 | 16.37326 | 13.69985 | 4.07315 | 8.505871 | 15.99774 | 10.46311 | 14.71698 | 20.47876 | 16.5578 | 8.535901 | 12.7217 | 29.0776 | 20.63001 | 12.59239 | 16.74792 | 18.19204 | 17.34784 | 14.68592 |
| CUST_43230_PI429010886 | M62474 | 0.030335 | 24 | 18 | 290792623 | cytochrome P450 [Litopenaeus vannamei] | Metabolic process | Oxidation-reduction process | 2.84E-68 | 207.5377 | 228.1481 | 173.3024 | 178.0431 | 159.9242 | 100.5781 | 120.0037 | 150.7201 | 189.1194 | 223.1811 | 209.3591 | 269.2314 | 199.6541 | 239.0491 | 306.8029 | 281.0282 | 131.2414 | 169.6296 | 222.5361 | 306.6185 | 218.4381 | 223.4595 | 219.8055 | 141.6867 | 200.108 | 213.8921 | 200.9243 |
| CUST_27225_PI429010886 | N55670 | 0.030349 | 24 | 21 | | | | | | 10.17288 | 5.707042 | 6.523496 | 5.574158 | 6.445454 | 26.13969 | 8.870531 | 6.833163 | NA | 5.181728 | 5.934011 | NA | 4.309618 | NA | 3.683664 | 10.13899 | 21.24533 | 10.49518 | 8.255312 | 11.0059 | 8.769916 | 34.05076 | 10.87645 | 8.916592 | NA | NA | 7.846171 |
| CUST_46_PI429010886 | N191 | 0.03038 | 24 | 21 | | | | | | 197.2642 | 277.4454 | 270.3602 | 247.1825 | 177.1267 | 166.5412 | 160.4181 | 176.94 | 174.6884 | 455.0104 | 168.4747 | 312.6532 | 184.654 | 214.5095 | 147.9354 | 200.108 | 179.0604 | 203.5264 | 187.4158 | 210.6402 | 271.2785 | 278.1576 | 335.1917 | 259.4845 | 165.6637 | 382.8597 | 191.9229 |
| CUST_49621_PI429010886 | M86426 | 0.030384 | 15 | 12 | | | | | | 55.20237 | 47.76338 | 35.42633 | 32.26619 | 46.682 | 39.09942 | 38.59666 | 26.93342 | 33.9856 | 34.86394 | 28.20666 | 31.90818 | 51.42128 | 45.93985 | 55.54166 | 49.86955 | 29.69991 | 53.82575 | 30.82595 | 30.95933 | 26.42193 | 46.25026 | 30.16973 | 25.4055 | 41.46468 | 27.69749 | 37.79822 |
| CUST_41155_PI429010886 | M57009 | 0.030388 | 21 | 0 | | | | | | 45.09974 | 26.92776 | 65.24322 | 24.81825 | 31.88893 | 29.44853 | 46.23463 | 24.34759 | 23.20447 | 19.2217 | 30.02077 | 28.43336 | 33.22111 | 37.06061 | 35.53033 | 20.36028 | 40.1699 | 45.31583 | 73.21949 | 17.02082 | 48.54231 | 47.68335 | 44.73069 | 53.59509 | 22.54108 | 24.23014 | 21.036 |
| CUST_41153_PI429010886 | M57003 | 0.030396 | 21 | 9 | 66532480 | PREDICTED: androgen-induced gene 1 protein-like i | | | 9.81E-27 | 53.15786 | 38.84241 | 41.65038 | 37.56417 | 62.39657 | 48.98425 | 30.47404 | 105.607 | 81.54717 | 63.37918 | 97.77159 | 106.9663 | 49.02476 | 51.90291 | 57.22072 | 69.71071 | 52.9276 | 78.66644 | 50.11342 | 160.5552 | 81.40583 | 44.83858 | 46.42413 | 42.99343 | 40.95911 | 49.74933 | 57.256 |
| CUST_3935_PI429010886 | N15613 | 0.030417 | 21 | 0 | | | | | | 9.883505 | 9.388689 | 17.81187 | 10.87507 | 6.91834 | 6.423011 | 5.396719 | 6.438886 | 4.929201 | 13.38378 | 4.654443 | 6.800737 | NA | 4.162571 | 5.220847 | 22.59879 | 10.80725 | 13.45433 | NA | 8.29257 | NA | 4.903777 | 25.24216 | 17.40133 | 10.8676 | 6.903956 | 7.334339 |
| CUST_12614_PI429010886 | N29745 | 0.030417 | 18 | 12 | | | | | | 28.9598 | 28.67208 | 22.46676 | 17.98401 | 33.23976 | 17.76828 | 22.48019 | 21.7799 | 21.71687 | 24.86718 | 25.93469 | 23.48892 | 26.44706 | 26.1797 | 30.60937 | 37.65902 | 22.46339 | 23.14834 | 23.22371 | 48.49494 | 19.96619 | 22.24289 | 15.76357 | 17.84852 | 37.82706 | 14.78884 | 16.48255 |
| CUST_33629_PI429010886 | M7465 | 0.030418 | 24 | 3 | | | | | | 1260.686 | 3432.751 | 1823.946 | 4322.611 | 1978.873 | 2469.728 | 1494.92 | 1525.514 | 1730.048 | 1733.695 | 885.3464 | 2354.203 | 1022.968 | 1786.373 | 949.0578 | 1816.167 | 620.1455 | 1162.648 | 829.8828 | 1311.23 | 2556.905 | 865.9484 | 2672.36 | 1441.828 | 2264.822 | 2421.023 | 1356.986 |
| CUST_34156_PI429010886 | M8906 | 0.03042 | 21 | 18 | | | | | | 963.6143 | 1086.536 | 1264.445 | 1216.97 | 1154.959 | 921.275 | 1067.136 | 281.874 | 540.3776 | 1263.557 | 856.1081 | 1167.756 | 731.0066 | 1278.309 | 1363.618 | 1592.642 | 937.1457 | 1080.128 | 1149.925 | 1022.449 | 1460.496 | 1684.561 | 960.7453 | 1433.182 | 819.1664 | 599.927 | 869.29 |
| CUST_20565_PI429010886 | N43666 | 0.030447 | 15 | 9 | | | | | | 95.02448 | 21.55583 | 201.3287 | NA | 8.979148 | NA | 91.02902 | 103.6651 | 130.6774 | 355.6761 | 138.4789 | 285.8605 | 167.2066 | 164.8518 | 136.3625 | 142.7994 | 131.3745 | 48.6393 | 216.807 | 84.95079 | 243.1192 | 189.4251 | 226.3317 | 67.71736 | 207.0425 | 246.92 | 414.3494 |
| CUST_32899_PI429010886 | M5422 | 0.030451 | 24 | 15 | 13624645 | arrestin [Libelloides macaronius] | Signal transduction | Other | 1.13E-95 | 43.05396 | 173.6164 | 71.46149 | 86.15616 | 33.24975 | 42.81625 | 42.5506 | 107.1033 | 26.60787 | 79.35074 | 139.2667 | 53.05965 | 83.5291 | 97.10121 | 77.0714 | 161.1265 | 236.2907 | 137.4942 | 108.7711 | 144.4421 | 41.01337 | 115.7071 | 50.67294 | 47.3803 | 131.2414 | 124.6537 | 109.9447 |
| CUST_22969_PI429010886 | N48024 | 0.030485 | 24 | 21 | | | | | | 42.0037 | 32.19898 | 33.49853 | 50.34321 | 25.30759 | 29.34549 | 32.59218 | 32.90178 | 34.63858 | 33.7306 | 31.12763 | 41.79247 | 26.77939 | 33.92046 | 21.5295 | 41.70879 | 23.4832 | 30.65915 | 31.2636 | 39.00007 | 42.02786 | 44.48344 | 53.77384 | 42.54336 | 46.25026 | 33.61445 | 47.69448 |
| CUST_43906_PI429010886 | M64302 | 0.030491 | 15 | 9 | 260829621 | hypothetical protein BRAFLDRAFT_264920 [Branchi | Metabolic process | Cellular amino acid metabolic process | 3.28E-24 | 13.84485 | 9.733637 | 17.80913 | NA | 5.741678 | 6.020181 | 8.160107 | 11.4768 | 15.83863 | 18.84701 | 17.21816 | 23.92584 | 14.30234 | 16.14788 | 7.405027 | 23.24391 | 9.318343 | 8.000521 | 9.328195 | 21.6649 | 18.28004 | 8.432252 | 17.3861 | 9.913065 | 29.78164 | 28.65128 | 27.95797 |
| CUST_36844_PI429010886 | M17011 | 0.030505 | 21 | 15 | | | | | | 50.77711 | 43.45754 | 38.68077 | 33.43569 | 22.95471 | 26.57746 | 17.8869 | 22.74317 | 29.50649 | 39.59389 | 30.42483 | 58.92238 | 30.64068 | 49.20588 | 45.20819 | 65.50557 | 21.67212 | 50.56808 | 33.96553 | 47.23493 | 35.20549 | 55.00033 | 48.97519 | 25.78047 | 44.38974 | 24.48268 | 34.9676 |
| CUST_3893_PI429010886 | N15501 | 0.030508 | 24 | 6 | | | | | | 197.0135 | 321.0768 | 524.2243 | 210.0239 | 168.1988 | 202.0404 | 293.414 | 148.4609 | 224.884 | 43.38758 | 117.2199 | 157.6619 | 90.44569 | 171.427 | 94.36809 | 162.2752 | 188.9157 | 138.4789 | 96.07604 | 100.1275 | 77.26285 | 34.9503 | 55.27747 | 20.80086 | 139.6803 | 278.5705 | 158.6525 |
| CUST_36459_PI429010886 | M15949 | 0.030512 | 24 | 18 | | | | | | 12.9711 | 13.26351 | 10.31476 | 7.837273 | 17.37911 | NA | 5.631939 | 9.529604 | 13.19962 | 23.65146 | 10.35975 | 21.78927 | 16.64512 | 14.15378 | 16.62399 | 24.69054 | 14.05481 | 16.18253 | 22.45542 | 16.4888 | 18.15598 | 24.4716 | 14.71224 | 20.55693 | 16.69997 | 15.63345 | 15.1999 |
| CUST_55900_PI429010886 | M112053 | 0.030532 | 15 | 12 | | | | | | 1377.193 | 1216.535 | 2431.925 | 1193.094 | 995.7921 | 1026.845 | 1245.02 | 1332.781 | 1717.641 | 1653.841 | 1199.213 | 2621.227 | 1929.904 | 2514.893 | 2673.681 | 1936.604 | 1046.312 | 2264.822 | 1724.959 | 1451.905 | 2319.618 | 2538.071 | 1823.162 | 1372.611 | 2690.41 | 1973.742 | 2485.559 |
| CUST_34374_PI429010886 | M9470 | 0.030532 | 12 | 3 | | | | | | 212.6412 | 134.9903 | 40.40197 | 269.7651 | 233.1687 | 463.9991 | 257.8206 | 168.9486 | 183.266 | 63.53447 | 113.6011 | 52.82166 | 207.5377 | 90.94074 | 183.4677 | 192.7273 | 437.4848 | 536.7924 | 303.6686 | 81.33169 | 71.31512 | 220.1671 | 66.22546 | 267.177 | 105.5375 | 218.3262 | 24.34524 |
| CUST_6637_PI429010886 | N20382 | 0.030532 | 15 | 3 | | | | | | 220.5435 | 95.23078 | 81.49546 | 316.5285 | 422.2989 | 355.193 | 430.0642 | 235.838 | 94.02768 | 96.85587 | 326.6831 | 31.13642 | 173.6164 | 57.65633 | 88.85357 | 110.5649 | 556.2338 | 200.4301 | 548.077 | 68.37525 | 256.0146 | 110.4991 | 245.0961 | 332.8858 | 106.8828 | 152.565 | 69.35049 |
| CUST_36623_PI429010886 | M16376 | 0.030545 | 24 | 15 | | | | | | 8.413487 | 6.938783 | NA | 6.299452 | 4.657997 | 6.74367 | 6.262467 | 13.30523 | 14.19203 | 8.214888 | 4.789111 | 5.87006 | 11.60948 | 10.44442 | 18.11698 | 9.867365 | 41.92727 | NA | 9.126873 | 12.32189 | 9.493787 | 10.91708 | 14.48619 | NA | 12.58519 | 7.03993 | 4.272751 |
| CUST_46177_PI429010886 | M71684 | 0.030558 | 21 | 12 | 293629636 | hemocyanin HC4 [Limnoria quadripunctata] | | | 1.85E-20 | 5.210194 | 14.00927 | NA | 14.64247 | 32.90555 | NA | 21.61111 | 77.57474 | 19.98178 | 11.09833 | 64.49483 | 45.3118 | 291.3805 | NA | 100.3042 | 3.601988 | 74.66117 | NA | 9.5748 | 2663.095 | 84.64574 | NA | 5.019313 | NA | 5.66706 | 11.83012 | 70.85223 |
| CUST_26779_PI429010886 | N54860 | 0.030566 | 15 | 12 | | | | | | 19.57585 | 18.70291 | 18.16906 | 18.1574 | 17.60432 | 24.64126 | 10.47118 | 12.21189 | 14.40922 | 33.30259 | 19.48537 | 18.40839 | NA | 26.08326 | 27.20765 | 16.33068 | 14.87852 | 24.0618 | 9.060793 | 23.93394 | 20.11905 | 17.55503 | 18.50772 | 14.43213 | 34.50513 | 12.11329 | 25.27468 |
| CUST_2406_PI429010886 | N12285 | 0.030626 | 18 | 9 | 386376737 | hematopoietic prostaglandin D synthase [Penaeus r | | | 4.25E-61 | 537.9358 | 1154.959 | 974.9401 | 760.7539 | 355.3482 | 173.1695 | 833.7688 | 1335.109 | 1097.654 | 1551.584 | 802.5661 | 1576.839 | 791.5217 | 1323.05 | 908.1498 | 515.4981 | 603.6169 | 230.36 | 440.8097 | 1561.567 | 442.9673 | 428.4141 | 1011.26 | 266.9045 | 267.9382 | 521.3278 | 1111.525 |
| CUST_53751_PI429010886 | M103051 | 0.030627 | 12 | 3 | | | | | | 119.0071 | 39.33444 | 47.92719 | 92.96667 | 103.1223 | 93.5311 | 150.286 | 71.49639 | 135.2716 | 35.12625 | 32.82642 | 31.55452 | 102.7524 | 34.32332 | 73.81964 | 84.96672 | 39.4307 | 86.36905 | 19.56129 | 77.83266 | 68.91301 | 47.54347 | 30.63369 | 57.31513 | 46.98558 | 35.55553 | 36.54259 |
| CUST_51722_PI429010886 | M94727 | 0.030627 | 12 | 3 | 321477834 | hypothetical protein DAPPUDRAFT_311166 [Daphn | Protein metabolism | Proteolysis | 7.83E-19 | 209.4461 | 215.1259 | 30.91745 | 259.1574 | 322.7291 | 255.8665 | 137.2146 | 130.3926 | 74.23824 | 105.19 | 82.55028 | 67.12072 | 214.8393 | 112.4734 | 140.228 | 220.2228 | 114.1927 | 219.01 | 114.443 | 124.8107 | 78.9119 | 212.9538 | 48.069 | 161.8434 | 131.3745 | 119.7044 | 35.80323 |
| CUST_17322_PI429010886 | N37774 | 0.03065 | 15 | 12 | 242004984 | Plexin-A4 precursor, putative [Pediculus humanus c | Developmental process | Multicellular organismal development | 1.38E-52 | 12.95606 | 21.87979 | 11.36992 | 23.13337 | 18.0662 | 19.79173 | 18.81916 | 12.69121 | 13.73363 | 27.23134 | 16.71353 | 20.25455 | 24.21253 | 41.19265 | 35.35407 | 28.15798 | 22.13024 | 21.83957 | 44.1334 | 21.98126 | 16.06632 | 23.34607 | 10.73592 | 16.13381 | 28.54572 | 21.73658 | 25.13071 |
| CUST_39279_PI429010886 | M37155 | 0.030662 | 15 | 3 | | | | | | 392.0026 | 88.43444 | 418.2809 | 2915.652 | 12782.74 | 6727.843 | 15246.84 | 1226.638 | 182.0796 | 20.80086 | 13555.43 | NA | 97.93806 | 72.54647 | 53.87443 | 4.077197 | 15327.79 | 5861.755 | 19870.47 | NA | 17506.5 | 4.082996 | 15516.43 | 17110.44 | 49.42389 | 1271.998 | NA |
| CUST_53634_PI429010886 | M102583 | 0.030673 | 24 | 21 | | | | | | 419.9089 | 348.0871 | 406.06 | 246.1869 | 303.55 | 411.2669 | 299.7277 | 322.671 | 320.5083 | 416.9251 | 383.093 | 389.157 | 328.0426 | 317.3817 | 338.4186 | 359.8853 | 411.5645 | 391.1505 | 356.7855 | 405.5199 | 390.1389 | 377.3372 | 446.6411 | 444.1766 | 480.999 | 401.8543 | 476.8506 |
| CUST_53841_PI429010886 | M103386 | 0.030673 | 24 | 21 | | | | | | 113.8196 | 104.5462 | 118.9466 | 95.12719 | 110.6759 | 106.8563 | 94.79905 | 106.4305 | 105.0471 | 105.9305 | 102.3344 | 108.1857 | 104.8657 | 134.4036 | 121.9392 | 125.5232 | 126.2448 | 120.9454 | 98.41575 | 107.4857 | 110.5811 | 126.3438 | 117.8175 | 530.2702 | 135.6184 | 128.7649 | 139.7632 |
| CUST_35486_PI429010886 | M12692 | 0.030673 | 24 | 21 | | | | | | 49.03345 | 53.69064 | 46.37649 | 36.56569 | 61.25792 | 48.70779 | 31.96544 | 30.88587 | 33.67217 | 59.68907 | 32.31352 | 56.10477 | 32.93932 | 37.19463 | 40.54346 | 61.1802 | 36.3007 | 52.02853 | 38.91166 | 52.26577 | 49.93144 | 81.9929 | 49.98628 | 50.05524 | 54.14583 | 38.40419 | 51.4872 |
| CUST_48211_PI429010886 | M79359 | 0.030673 | 24 | 21 | 443711333 | hypothetical protein CAPTEDRAFT_140571 [Capitell | | | 6.12E-17 | 56.25359 | 59.13459 | 56.05994 | 45.71121 | 54.38291 | 42.33801 | 53.41869 | 46.38676 | 49.76383 | 75.50768 | 48.32558 | 67.40392 | 39.27214 | 58.56187 | 47.77962 | 66.85557 | 49.90548 | 58.03638 | 54.63011 | 50.78563 | 54.26471 | 66.12938 | 75.1394 | 85.27513 | 69.56167 | 62.35651 | 70.7166 |
| CUST_24359_PI429010886 | N50479 | 0.030673 | 24 | 21 | | | | | | 56.51605 | 78.52355 | 56.44254 | 59.06419 | 60.56579 | 39.07685 | 49.03725 | 54.67468 | 54.11265 | 84.13816 | 52.78112 | 87.57209 | 66.24577 | 60.80176 | 90.79757 | 93.80773 | 43.43615 | 61.25018 | 55.24489 | 82.01377 | 71.80124 | 98.65062 | 67.3892 | 104.964 | 80.38356 | 57.16139 | 86.3602 |
| CUST_8874_PI429010886 | N23733 | 0.030678 | 15 | 0 | | | | | | 231.1023 | 307.1296 | 189.0068 | 112.5022 | 246.0125 | 132.7469 | 146.2229 | 133.7926 | 71.93323 | 217.5163 | 161.0222 | 128.3894 | 140.974 | 108.5779 | 155.4485 | 202.7268 | 194.5182 | 164.3449 | 196.129 | 236.1903 | 168.99 | 140.8238 | 87.75721 | 117.1631 | 203.7634 | 140.5892 | 227.0558 |
| CUST_5089_PI429010886 | N18244 | 0.030699 | 21 | 0 | | | | | | 25.02916 | 13.66936 | 14.61919 | 5.662031 | 9.549172 | 10.89133 | 10.50364 | NA | 9.137413 | NA | 5.545681 | 3.744219 | 9.82119 | NA | 6.103455 | 7.561772 | 15.02569 | 9.910844 | 21.18827 | 6.184656 | 6.219397 | 11.98265 | 6.872975 | NA | 13.22714 | 7.14839 | 5.037698 |
| CUST_14451_PI429010886 | N32908 | 0.030729 | 12 | 0 | 507706013 | PREDICTED: probable E3 ubiquitin-protein ligase HE | | | 1.03E-34 | 118.3064 | 122.9865 | 123.1656 | 139.1034 | 146.3889 | 169.6125 | 91.78258 | 80.15845 | 125.4703 | 138.4649 | 86.35271 | 116.3059 | 90.52141 | 148.1409 | 149.0167 | 141.3142 | 93.10199 | 143.9014 | 95.39909 | 95.47811 | 115.7071 | 137.5279 | 100.8686 | 102.782 | 170.6936 | 162.8992 | 150.78 |
| CUST_25547_PI429010886 | N52614 | 0.030773 | 21 | 18 | 321464782 | hypothetical protein DAPPUDRAFT_214208 [Daphn | | | 7.87E-44 | 203.9899 | 348.4045 | 118.7738 | 203.7326 | 250.7705 | 77.09407 | 45.25874 | 52.35985 | 50.40874 | 278.2072 | 205.7316 | 294.1484 | 192.2628 | 345.7818 | 318.02 | 321.5473 | 46.22113 | 219.5762 | 101.4116 | 748.9581 | 325.619 | 543.3625 | 185.7909 | 143.9244 | 195.0054 | 119.885 | 95.39909 |
| CUST_43211_PI429010886 | M62413 | 0.030791 | 15 | 9 | | | | | | 217.0054 | 226.3675 | 205.392 | 105.1255 | 195.1881 | 155.7882 | 96.67753 | 72.22286 | 143.3153 | 248.7921 | 310.2077 | 345.7017 | 194.5783 | 204.4512 | 229.0599 | 323.9834 | 176.2225 | 254.2781 | 161.6619 | 177.4848 | 172.9267 | 172.0048 | 200.0082 | 244.4592 | 233.7145 | 196.9122 | 202.4642 |
| CUST_55024_PI429010886 | M108428 | 0.030814 | 24 | 21 | 321470471 | hypothetical protein DAPPUDRAFT_303500 [Daphn | Protein metabolism | Proteolysis | 1.96E-68 | 58.03044 | 54.56432 | 88.06388 | 69.80023 | 71.84934 | 76.57062 | 66.2145 | 75.08335 | 55.62798 | 80.13478 | 67.73286 | 70.36056 | 65.26348 | 77.68249 | 70.76377 | 85.42654 | 90.26625 | 74.67944 | 90.76835 | 81.66432 | 69.55824 | 82.06433 | 89.30785 | 81.61454 | 75.41714 | 66.62115 | 67.78689 |
| CUST_9351_PI429010886 | N24479 | 0.030834 | 24 | 18 | | | | | | 147.3218 | 131.6883 | 177.9272 | 117.2885 | 114.917 | 112.1531 | 132.652 | 135.061 | 133.2798 | 221.2763 | 151.8603 | 180.9607 | 143.9734 | 194.6782 | 342.1102 | 191.4276 | 145.9425 | 147.3848 | 169.3778 | 176.5494 | 150.0374 | 203.6685 | 168.8733 | 146.882 | 216.4474 | 136.3426 | 188.1161 |
| CUST_32332_PI429010886 | M3840 | 0.03084 | 18 | 0 | 148223481 | thioredoxin 2 [Xenopus laevis] >gi\|27694724\|gb\|A | Metabolic process | Oxidation-reduction process | 3.38E-36 | 406.3292 | 509.1564 | 317.6417 | 393.1602 | 341.0906 | 367.7937 | 310.2726 | 309.6337 | 317.2087 | 324.9748 | 343.6141 | 448.6531 | 321.6396 | 310.0812 | 358.7125 | 388.4984 | 322.0841 | 370.9949 | 339.1053 | 455.0936 | 386.5159 | 470.7834 | 261.511 | 337.8584 | 332.2552 | 300.4818 | 338.6596 |
| CUST_13884_PI429010886 | N31935 | 0.030846 | 18 | 3 | | | | | | 178.6287 | 177.4848 | 125.3551 | 209.4896 | 201.2454 | 174.5672 | 203.8966 | 103.7718 | 131.6163 | 78.21989 | 157.7661 | 117.863 | 97.67416 | 132.7469 | 188.7198 | 218.242 | 173.2522 | 190.0092 | 160.4181 | 136.4264 | 164.9826 | 217.37 | 165.3002 | 193.5271 | 134.9903 | 98.90127 | 82.46901 |

| Probe ID | ID | p-value | Period | Phase | EBI ID | Annotation | GO category | Go term | E-value | 0 | 0 | 0 | 3 | 3 | 3 | 6 | 6 | 6 | 9 | 9 | 9 | 12 | 12 | 12 | 15 | 15 | 15 | 18 | 18 | 18 | 21 | 21 | 21 | 24 | 24 | 24 |
|---|---|---|---|---|---|---|---|---|---|---|---|---|---|---|---|---|---|---|---|---|---|---|---|---|---|---|---|---|---|---|---|---|---|---|---|---|
| CUST_39676_PI429010886 | M42735 | 0.030852 | 18 | 6 | | | | | | NA | 36.63495 | 25.90815 | 39.51821 | 36.87579 | 30.43159 | 44.89448 | 60.9102 | 74.21285 | 21.31201 | 39.79718 | 58.5117 | 40.47688 | 30.0232 | 37.87276 | NA | 35.34065 | NA | 16.31911 | 55.50038 | 62.81643 | NA | 65.03148 | 23.78727 | NA | 75.98025 | 69.39137 |
| CUST_39578_PI429010886 | M41336 | 0.030879 | 12 | 0 | | | | | | 1889.582 | 2718.679 | 2397.429 | 2186.819 | 1993.696 | 1277.616 | 1478.956 | 1463.822 | 1704.986 | 2321.278 | 1924.188 | 3456.298 | 2159.598 | 2779.017 | 2863.818 | 2656.499 | 1630.188 | 2076.382 | 1679.237 | 2615.876 | 2542.502 | 2768.781 | 2142.216 | 1911.091 | 2773.22 | 3031.864 | 3098.049 |
| CUST_43615_PI429010886 | M63511 | 0.030885 | 21 | 15 | 524871421 | PREDICTED: phosphatidylinositol N-acetylglucosami | Protein metabolism | Cellular protein modification process | 9.9E-34 | 42.95145 | 40.81311 | 57.86841 | 35.89984 | 37.88014 | 38.77187 | 34.12708 | 44.21249 | 37.86895 | 54.4744 | 34.71929 | 69.01474 | 41.87987 | 60.0207 | 40.41641 | 49.64123 | 48.56555 | 45.77355 | 43.56595 | 56.67806 | 46.84472 | 45.74744 | 41.96975 | 25.27468 | 52.05083 | 30.81693 | 39.45879 |
| CUST_14764_PI429010886 | N33433 | 0.030895 | 21 | 0 | 91091108 | PREDICTED: similar to TFIIA [Tribolium castaneum] | Nucleic acid metabolism | RNA metabolic process | 5.4E-11 | 11.55275 | NA | 11.02206 | 9.03782 | 7.665956 | 4.05387 | 7.543719 | 3.839586 | NA | 7.886773 | 4.292685 | 24.60142 | 4.010865 | 8.960873 | 9.357193 | 7.956167 | 7.710119 | 10.68692 | NA | 4.637609 | NA | 14.06926 | NA | 29.62857 | 9.910844 | 4.317971 | 20.25021 |
| CUST_48873_PI429010886 | M82629 | 0.030896 | 24 | 21 | | | | | | 20.81041 | 12.06791 | 10.10137 | 5.191851 | 7.779891 | 15.90721 | NA | 8.668476 | 11.08478 | 8.333 | 6.259329 | 20.44316 | 7.611898 | 12.07646 | 14.37169 | 10.11891 | 18.20852 | 21.80116 | 24.12233 | 19.12737 | 9.308024 | 20.48821 | 31.33095 | 15.09945 | 19.52925 | 9.765189 | 12.74489 |
| CUST_38512_PI429010886 | M27567 | 0.030914 | 18 | 12 | | | | | | 632.0615 | 714.2963 | 200.6692 | 290.0767 | 370.9223 | 235.0591 | 1387.428 | 271.4065 | 1458.732 | 994.4459 | 330.1179 | 1601.861 | 1611.105 | 886.1313 | 750.3627 | 903.546 | 454.8231 | 408.5798 | 1390.937 | 121.1966 | 635.437 | 292.4718 | 514.6723 | 325.8898 | 639.5245 | 237.3703 | 1068.926 |
| CUST_56967_PI429010886 | M115019 | 0.030915 | 21 | 9 | | | | | | 419.5293 | 601.962 | 548.6331 | 631.1117 | 674.0173 | 602.4628 | 451.4433 | 1066.114 | 911.683 | 573.6965 | 1769.215 | 1797.174 | 1218.887 | 731.4443 | 638.1084 | 457.6266 | 860.5065 | 600.1331 | 494.6999 | 1179.987 | 478.074 | 542.5507 | 562.3412 | 594.5741 | 547.3353 | 537.701 | 874.5756 |
| CUST_26145_PI429010886 | N53748 | 0.030923 | 21 | 6 | | | | | | 36.76731 | 38.01489 | 36.47986 | 45.62376 | 56.31693 | 43.17605 | 86.54045 | 74.54819 | 62.5758 | 68.63903 | 80.05215 | 20.51687 | 32.02557 | 22.53424 | 28.82637 | 29.12589 | 75.5762 | 64.125 | 45.11398 | 19.55939 | 38.06259 | 61.98456 | 6.017128 | 35.54827 | 67.42469 | 39.42313 | 44.70504 |
| CUST_37587_PI429010886 | M19645 | 0.030952 | 24 | 9 | | | | | | 276.0613 | 265.0674 | 233.7917 | 291.6879 | 290.4297 | 182.5915 | 247.7794 | 301.291 | 259.3866 | 279.387 | 346.3085 | 364.5694 | 326.1708 | 239.9392 | 326.7477 | 214.0659 | 160.974 | 138.9538 | 244.775 | 676.5905 | 248.1506 | 233.4512 | 232.1489 | 211.85 | 226.2023 | 284.7376 | 241.2571 |
| CUST_28345_PI429010886 | N57768 | 0.030956 | 24 | 0 | | | | | | 48.00546 | 60.27464 | 50.55699 | 51.40578 | 46.95662 | 40.55619 | 41.04278 | 47.66523 | 43.65582 | 60.29484 | 37.85605 | 37.25844 | 50.9289 | 30.27835 | 21.77078 | 49.37977 | 37.67574 | 35.55553 | 47.65494 | 69.73102 | 59.57796 | 39.75659 | 39.25313 | 58.96311 | 70.88996 | 41.55123 | 66.09037 |
| CUST_37431_PI429010886 | M18998 | 0.030968 | 12 | 3 | | | | | | 11799.5 | 21636.27 | 706.4958 | 24841.44 | 8888.62 | 7637.386 | 17506.5 | 601.7703 | 18694.18 | 553.6437 | 442.4276 | 461.1208 | 6091.908 | 1276.133 | 8995.888 | 17268.09 | 881.5993 | 12374.53 | 935.7487 | 728.4044 | 472.8988 | 7154.842 | 366.6536 | 4808.982 | 4584.946 | 1126.811 | 2076.382 |
| CUST_2565_PI429010886 | N12642 | 0.030968 | 12 | 3 | | | | | | 1540.227 | 1075.686 | 1593.287 | 1492.759 | 2109.682 | 3914.042 | 1626.337 | 1123.855 | 143.002 | 618.6744 | 520.9934 | 220.4105 | 2491.849 | 680.1392 | 1674.38 | 1186.768 | 1617.488 | 1872.445 | 969.1321 | 696.5534 | 899.8161 | 382.4385 | 901.5294 | 2309.39 | 822.5821 | 3281.126 | 554.9764 |
| CUST_31857_PI429010886 | M2584 | 0.030974 | 24 | 21 | | | | | | 711.9048 | 625.7026 | 539.016 | 576.6597 | 515.8865 | 377.4127 | 657.1327 | 379.2779 | 465.6558 | 538.5366 | 650.8924 | 491.7148 | 440.22 | 423.3887 | 578.6895 | 681.7268 | 862.42 | 575.2228 | 623.0865 | 611.8341 | 717.957 | 751.2737 | 636.6917 | 834.3363 | 624.47 | 395.6626 | 377.2506 |
| CUST_52843_PI429010886 | M99179 | 0.030985 | 18 | 0 | | | | | | 88.34755 | 68.15284 | 61.46953 | 61.75222 | 56.61759 | 62.03099 | 58.25188 | 68.90417 | 47.55222 | 57.12722 | 50.18617 | 43.18074 | 50.23482 | 57.90171 | 44.58945 | 78.99159 | 63.38531 | 52.73715 | 85.95967 | 59.34275 | 58.91707 | 77.69906 | 45.24266 | 50.1618 | 73.62026 | 35.03861 | 56.03701 |
| CUST_29921_PI429010886 | N60821 | 0.030989 | 24 | 15 | | | | | | 424.2001 | 428.9575 | 573.9804 | 336.9449 | 481.9422 | 499.9431 | 369.0374 | 288.3979 | 282.166 | 453.4712 | 513.6722 | 375.1189 | 267.9382 | 340.1148 | 469.5046 | 563.6523 | 602.1279 | 765.4435 | 729.8175 | 569.7753 | 461.3593 | 401.1607 | 455.0104 | 305.6803 | 868.3571 | 401.0158 | 697.0634 |
| CUST_53033_PI429010886 | M99817 | 0.031012 | 24 | 0 | | | | | | 21.91188 | 35.90257 | 27.87681 | 16.44746 | 16.20429 | 26.4083 | 48.40695 | 6.701803 | 11.56577 | 13.53347 | 26.73153 | 26.6702 | 10.35813 | 14.61619 | NA | 12.81312 | 11.41581 | 25.30759 | 4.078497 | 12.09871 | 29.87357 | 29.55233 | 34.40003 | 31.47236 | 35.13017 | 46.0983 | 25.8552 |
| CUST_29096_PI429010886 | N59197 | 0.031015 | 21 | 6 | | | | | | 65.93142 | 35.82003 | 14.11808 | 61.47627 | 12.73395 | 57.86313 | 90.79757 | 134.9314 | 90.82199 | NA | 11.84337 | NA | 286.5169 | 7.967454 | 144.8798 | 22.41298 | 26.10463 | 28.64267 | 15.73595 | 6.15687 | 11.4448 | 10.8378 | 16.97579 | 114.649 | 25.01541 | 93.62063 | 19.49282 |
| CUST_23364_PI429010886 | N48713 | 0.031026 | 24 | 18 | 414151640 | prophenoloxidase activating factor [Litopenaeus va | Protein metabolism | Proteolysis | 1.55E-23 | 407.9548 | 187.5065 | 482.0705 | 103.5194 | 137.9515 | 128.1757 | 368.5464 | 302.8326 | 167.8576 | 411.751 | 455.6129 | 244.8254 | 260.055 | 342.6195 | 235.7566 | 267.8998 | 452.4978 | 224.9966 | 566.9632 | 402.0451 | 352.327 | 284.9694 | 404.3563 | 393.9841 | 386.5159 | 216.807 | 367.1896 |
| CUST_20765_PI429010886 | N44016 | 0.031034 | 24 | 18 | | | | | | 211.6272 | 479.9166 | 224.2596 | 148.3671 | 278.0994 | 101.1723 | 14.8118 | 5.717554 | 118.5367 | 479.3073 | 309.9578 | 661.1589 | 189.0849 | 338.2555 | 393.782 | 586.2213 | 15.51946 | 301.467 | 280.165 | 559.6795 | 457.038 | 658.0264 | 269.5259 | 195.2252 | 300.3777 | 259.3866 | 208.936 |
| CUST_56571_PI429010886 | M114137 | 0.031035 | 18 | 15 | | | | | | 34.0469 | 31.11356 | 9.289553 | 27.57506 | 26.73933 | 33.09246 | 28.01868 | 12.49275 | 26.71614 | 21.51397 | 28.38786 | 28.34529 | 28.78598 | 25.09128 | 19.4153 | 30.9722 | 48.72653 | 29.18222 | 38.86122 | 33.16699 | 27.14824 | 24.2954 | 27.60111 | 15.89244 | 26.82962 | 20.56111 | 22.13275 |
| CUST_56664_PI429010886 | M114372 | 0.031035 | 18 | 9 | | | | | | 51.90738 | 26.37038 | 33.86551 | 19.59595 | 68.39623 | 23.63583 | 29.14178 | 24.87004 | 36.34025 | 142.9167 | 59.84805 | 53.99181 | 81.72278 | 27.88257 | 15.91075 | 46.66161 | 55.63288 | 58.72685 | 44.71939 | 47.76946 | 70.32547 | 24.0728 | 23.23227 | 20.2329 | 13.85408 | 31.07173 | 47.56602 |
| CUST_3411_PI429010886 | N14451 | 0.031035 | 18 | 9 | 386376737 | hematopoietic prostaglandin D synthase [Penaeus r | Metabolic process | Cellular amino acid metabolic process | 1.12E-46 | 592.0389 | 702.7008 | 1307.703 | 390.8735 | 465.1151 | 320.9097 | 741.9924 | 1512.912 | 1689.395 | 1060.287 | 1117.033 | 1672.079 | 1032.594 | 1011.687 | 1040.197 | 1428.874 | 972.4433 | 442.1751 | 981.4865 | 1369.558 | 425.7405 | 1117.033 | 1025.99 | 295.3859 | 752.803 | 521.8521 | 1440.453 |
| CUST_14281_PI429010886 | N32627 | 0.031035 | 18 | 9 | | | | | | 219.9975 | 272.019 | 239.3938 | 245.5656 | 241.0846 | 226.4213 | 262.8518 | 273.6399 | 252.5773 | 350.5877 | 251.6265 | 358.2812 | 248.2496 | 282.4175 | 307.2905 | 354.1413 | 229.5365 | 249.0021 | 230.0814 | 261.6019 | 205.7973 | 335.9236 | 284.6531 | 251.1828 | 249.9907 | 248.3781 | 272.019 |
| CUST_55952_PI429010886 | M112280 | 0.031039 | 15 | 12 | | | | | | 136.5699 | 127.0638 | 164.6734 | 134.162 | 127.601 | 146.3743 | 104.6652 | 130.4101 | 135.3099 | 143.3153 | 136.25 | 167.0312 | 135.9242 | 169.0651 | 144.2346 | 171.1636 | 246.1531 | 157.1911 | 122.4782 | 135.4807 | 124.9854 | 137.196 | 142.6905 | 120.2795 | 157.9551 | 138.133 | 149.1265 |
| CUST_49223_PI429010886 | M84761 | 0.031047 | 24 | 3 | | | | | | 6.609639 | 9.801459 | 12.92626 | 40.65724 | 18.20414 | 25.15936 | 16.12591 | 13.76394 | 11.46096 | 13.19581 | 9.308024 | 9.140795 | 8.189309 | 7.186236 | 22.49197 | 5.341143 | 12.66387 | 13.82239 | 16.29756 | 6.088354 | 8.643568 | 9.47114 | 10.66241 | 17.48039 | 10.21387 | 14.64501 | 10.34523 |
| CUST_1023_PI429010886 | N8249 | 0.03106 | 21 | 15 | 123445462 | hypothetical protein [Trichomonas vaginalis G3] >gi | | | 9.28E-08 | 454.0927 | 9.458785 | 670.6469 | 42.16582 | 137.8443 | 137.604 | 545.1015 | 363.2398 | 310.9824 | 830.2031 | 321.0768 | NA | 385.1644 | 446.0696 | 169.5826 | 40.8947 | 928.0578 | 386.6669 | 1019.767 | NA | 217.6655 | 12.26536 | 129.1352 | 113.2085 | 7.013611 | 87.899 | 32.04673 |
| CUST_11573_PI429010886 | N28053 | 0.031084 | 15 | 12 | | | | | | 42.18909 | 45.31985 | 104.2505 | 7.15372 | 9.0794 | 12.41778 | 32.43294 | 68.16653 | 82.49299 | 53.59509 | 42.00978 | 86.27625 | 126.4216 | 151.6013 | 60.52796 | 6.448841 | 24.29286 | 25.42066 | 48.41716 | 49.31188 | 52.62093 | 29.94174 | 92.53187 | NA | 61.75898 | 87.29557 | 139.359 |
| CUST_13262_PI429010886 | N30869 | 0.031084 | 15 | 3 | | | | | | 29.23931 | 52.36466 | 37.77068 | 147.1318 | 55.44777 | 37.67574 | 22.68396 | 52.8414 | 17.31011 | 114.0128 | 17.28408 | 46.17281 | 4.429435 | 36.47059 | 25.1358 | 46.36142 | 23.27419 | 15.71416 | 57.68381 | 107.666 | 31.78356 | 29.11768 | 30.17589 | 28.44909 | 28.70795 | NA | 9.558027 |
| CUST_25687_PI429010886 | N52880 | 0.031111 | 24 | 15 | | | | | | 45.76846 | 50.56808 | 32.99848 | 29.86056 | 37.77068 | 31.19824 | 34.52311 | 27.47445 | 32.7645 | 39.68514 | 33.68076 | 47.69448 | 40.73202 | 41.4213 | 57.57631 | 53.29328 | 34.65808 | 55.50038 | 44.03947 | 50.40874 | 45.0701 | 51.32807 | 38.42916 | 43.8795 | 53.47899 | 47.40881 | 63.05647 |
| CUST_44432_PI429010886 | M65974 | 0.031113 | 21 | 15 | | | | | | 42.95918 | 84.06167 | 56.81475 | 61.67082 | 60.30436 | 51.53885 | 40.37133 | 38.55767 | 48.95474 | 68.24289 | 44.75473 | 30.17169 | 50.38632 | 67.25956 | 51.58072 | 91.51834 | 44.84989 | 116.2937 | 62.86517 | 63.15525 | 69.57456 | 65.9853 | 42.07479 | 57.05842 | 111.6728 | 39.37435 | 49.84719 |
| CUST_14253_PI429010886 | N32577 | 0.031115 | 21 | 3 | | | | | | 78.72636 | 47.82182 | 84.84663 | 101.7048 | 77.6254 | 71.84934 | 58.31834 | 52.02853 | 81.0784 | 78.66048 | 32.33975 | 80.54851 | 36.87107 | 55.02956 | 30.0795 | 50.95506 | 32.05062 | 56.60803 | 40.2115 | 31.34117 | 136.2095 | 45.09974 | 145.2889 | 55.20849 | 84.85912 | 65.36011 | 76.50308 |
| CUST_29937_PI429010886 | N60846 | 0.031124 | 18 | 3 | | | | | | 12.31301 | 28.44009 | NA | 80.63947 | 53.76242 | 68.50419 | 16.11274 | NA | 34.1956 | 3.222009 | 4.111374 | NA | 8.552148 | 76.353 | 59.35924 | 76.07191 | NA | 66.62115 | 9.253041 | 45.06422 | 9.064427 | 125.6499 | NA | 101.867 | 48.80606 | NA | 11.55822 |
| CUST_26818_PI429010886 | N54935 | 0.031135 | 12 | 0 | | | | | | 86.68692 | 72.7025 | 80.72119 | 68.5998 | 62.26361 | 61.96906 | 70.36056 | 51.0383 | 62.20164 | 43.5699 | 67.86664 | 54.85716 | 69.40293 | 109.2001 | 92.15955 | 76.69076 | 65.88576 | 70.20247 | 53.53068 | 59.90035 | 61.43267 | 67.11047 | 70.65347 | 81.44839 | 102.5272 | 44.91724 | 61.63016 |
| CUST_5495_PI429010886 | N18776 | 0.031154 | 24 | 21 | 241687507 | DNA replication factor/protein phosphatase inhibit | Cellular component organization | Chromosome organization | 5.14E-87 | 328.9267 | 454.3834 | 298.484 | 272.4885 | 312.7398 | 259.6731 | 216.4474 | 206.5428 | 255.6195 | 364.6302 | 275.6931 | 321.2502 | 271.5735 | 328.0426 | 309.1695 | 499.9431 | 231.3759 | 316.122 | 300.3777 | 382.7566 | 345.5828 | 404.7043 | 351.303 | 268.7143 | 438.9413 | 275.5962 | 371.6995 |
| CUST_11249_PI429010886 | N27511 | 0.031154 | 24 | 15 | | | | | | 254.095 | 331.1092 | 325.619 | 227.9427 | 250.0134 | 258.545 | 251.2548 | 219.7388 | 314.6224 | 301.3697 | 296.9284 | 447.6451 | 245.2785 | 504.1608 | 388.8828 | 444.3125 | 289.7475 | 442.5257 | 382.0118 | 338.7294 | 332.6812 | 376.27 | 377.56 | 292.0563 | 414.2198 | 305.9118 | 414.2851 |
| CUST_1205_PI429010886 | N8825 | 0.031154 | 24 | 18 | 321475487 | hypothetical protein DAPPUDRAFT_230482 [Daphn | | | 4.11E-18 | 32.99565 | 23.89355 | 20.69091 | 16.71152 | 19.2347 | 18.87721 | 14.44097 | 23.40583 | 19.38333 | 19.94541 | 20.79841 | 18.71748 | 19.4153 | 19.61727 | 21.57749 | 29.52168 | 16.22526 | 37.93755 | 30.5069 | 24.35938 | 26.44892 | 27.66265 | 18.85349 | 24.91723 | 27.35532 | 27.16077 | 23.77956 |
| CUST_36006_PI429010886 | M14267 | 0.031157 | 24 | 0 | | | | | | 25.25393 | 19.69614 | 35.12261 | 22.09437 | 17.20887 | 16.58665 | 14.99759 | 18.90357 | 16.75931 | 17.24279 | 17.80566 | 9.036719 | 13.79131 | 22.98483 | 18.81158 | 25.75422 | 18.77363 | 12.12113 | 22.95471 | 17.54469 | 29.23595 | 23.78457 | 25.12351 | 15.00987 | 22.13675 | 26.38163 | 18.71355 |
| CUST_39904_PI429010886 | M46616 | 0.031186 | 15 | 12 | | | | | | 1544.651 | 3225.426 | 3013.914 | 929.0194 | 1167.756 | 835.782 | 2003.234 | 2838.594 | 5152.319 | 4388.254 | 2022.724 | 4154.657 | 5596.154 | 6197.441 | 2539.516 | 5609.183 | 3110.134 | 3175.174 | 2478.692 | 3402.667 | 2054.295 | 5534.91 | 4444.002 | 1850.903 | 9647.722 | 2541.147 | 13044.56 |
| CUST_36049_PI429010886 | M14449 | 0.031188 | 21 | 0 | 524916152 | PREDICTED: serine/threonine-protein phosphatase | | | 2.2E-18 | 23.31493 | 23.98815 | 24.31347 | 24.48268 | 24.72926 | 18.80919 | 22.11953 | 20.46944 | 16.18253 | 21.13775 | 25.77541 | 18.08937 | 23.194 | 27.45261 | 19.49159 | 19.83251 | 17.14547 | 11.56735 | 27.19369 | 25.91941 | 19.88848 | 29.14178 | 29.70687 | 26.41485 | 30.95206 | 21.1196 | 23.30384 |
| CUST_29239_PI429010886 | N59453 | 0.031196 | 18 | 3 | | | | | | 30.61198 | 61.1802 | 40.65477 | 130.9454 | 36.59373 | 60.55923 | 24.31817 | 20.60841 | 27.58634 | 39.04491 | 11.99669 | 40.09244 | 20.72467 | 32.08763 | 12.25586 | 34.64172 | 9.142203 | 22.79581 | 20.3394 | 26.26527 | 59.91437 | 23.47003 | 71.62646 | 39.18602 | 38.57925 | 51.60744 | 20.14785 |
| CUST_35454_PI429010886 | M12614 | 0.03121 | 12 | 0 | 524871938 | PREDICTED: snurportin-1-like [Aplysia californica] | Nucleic acid metabolism | RNA metabolic process | 2.05E-50 | 32.29255 | 51.41614 | 34.74686 | 30.50923 | 27.9866 | 37.93755 | 21.56454 | 18.57026 | 27.15098 | 39.72298 | 23.72419 | 25.37501 | 24.30245 | 30.26846 | 28.23631 | 37.70458 | 15.9338 | 33.01166 | 25.66555 | 25.38172 | 23.09604 | 34.0026 | 30.80907 | 27.85193 | 45.95892 | 31.72455 | 30.94543 |
| CUST_50147_PI429010886 | M88813 | 0.031226 | 12 | 6 | | | | | | 91.02196 | 80.29877 | 103.4079 | 94.5785 | 128.037 | 113.9294 | 135.6641 | 105.1159 | 76.46353 | 130.6774 | 166.2053 | 64.04369 | 112.169 | 110.5098 | 75.63407 | 92.43125 | 94.97923 | 94.127 | 146.5997 | 117.0372 | 150.434 | 109.9175 | 118.6904 | 115.2533 | 86.35271 | 80.68026 | 55.57729 |
| CUST_51937_PI429010886 | M95741 | 0.031226 | 12 | 0 | | | | | | 65.04228 | 70.31729 | 60.87936 | 30.15999 | 53.68104 | 40.1276 | 46.37163 | 40.97284 | 48.67472 | 65.77588 | 43.52847 | 48.26954 | 60.71887 | 54.93851 | 48.87456 | 32.7846 | 55.02436 | 47.51659 | 39.35128 | 41.10579 | 44.90024 | 73.27672 | 45.87335 | 33.81235 | 66.30772 | 42.58108 | 62.79742 |
| CUST_52836_PI429010886 | M99161 | 0.031226 | 12 | 6 | 321462309 | hypothetical protein DAPPUDRAFT_307947 [Daphn | | | 2.17E-35 | 65.07248 | 86.27625 | 85.63673 | 116.0217 | 110.6854 | 86.86206 | 112.5793 | 85.94103 | 87.80183 | 106.6656 | 96.17817 | 90.11953 | 90.24634 | 79.96242 | 94.63457 | 121.4004 | 99.08673 | 103.654 | 122.5181 | 123.8085 | 84.30944 | 134.4388 | 97.23707 | 102.4301 | 84.96006 | 88.37144 | 77.79872 |
| CUST_7153_PI429010886 | N21115 | 0.031226 | 12 | 3 | | | | | | 158.6127 | 144.5043 | 94.40103 | 239.7925 | 204.2199 | 294.0478 | 190.0374 | 146.0878 | 110.3002 | 129.2905 | 150.4806 | 105.1609 | 144.5986 | 114.926 | 123.056 | 143.4947 | 236.449 | 190.2377 | 196.4719 | 114.8783 | 142.1088 | 138.7812 | 98.6386 | 184.3061 | 115.9278 | 180.0766 | 116.5533 |
| CUST_50971_PI429010886 | M91610 | 0.031253 | 15 | 0 | | | | | | 21.38369 | 27.88984 | 13.46781 | 19.36095 | 8.480876 | 4.766739 | 16.48255 | 6.576173 | 11.12639 | 14.06725 | 13.07663 | 8.957551 | 15.00335 | NA | 9.135099 | 22.76207 | 29.93503 | 14.41094 | 4.996795 | 15.01453 | 20.06809 | 11.09239 | 3.684245 | 10.04101 | 14.00188 | 14.09542 | 27.10661 |
| CUST_3345_PI429010886 | N14317 | 0.031253 | 15 | 9 | 321473526 | hypothetical protein DAPPUDRAFT_223002 [Daphn | Metabolic process | Carbohydrate metabolic process | 2.46E-20 | 125.3376 | 421.2064 | 540.5402 | 33.06129 | 186.1374 | 57.9941 | 68.86603 | 48.23121 | 400.3298 | 197.9029 | 480.7772 | 563.6523 | NA | 451.3065 | 189.144 | 420.8347 | 79.17523 | 434.7605 | 285.1566 | 135.1036 | 354.8939 | 482.5207 | 308.2627 | 12.68424 | 859.7051 | 310.1413 | 1285.231 |
| CUST_36384_PI429010886 | M15768 | 0.031266 | 24 | 18 | 345492871 | PREDICTED: NFX1-type zinc finger-containing protei | | | 1.49E-72 | 10.74554 | NA | 10.14304 | 11.50536 | 11.08171 | 8.527754 | 9.488473 | 11.14983 | 6.895161 | 15.48316 | 9.645798 | 20.17126 | 10.12547 | 11.59715 | 3.726808 | 16.53651 | 6.899472 | 8.257726 | 16.39244 | 19.76938 | 17.01911 | 16.00379 | 12.60615 | NA | 12.59903 | 13.86466 | 16.8263 |
| CUST_45166_PI429010886 | M68183 | 0.031305 | 15 | 0 | | | | | | 95.89707 | 76.77824 | 98.4006 | 70.99743 | 75.39347 | 78.63344 | 56.46018 | 78.32339 | 65.38012 | 72.00003 | 60.01583 | 40.25214 | 78.44378 | 70.24827 | 61.05172 | 61.95208 | 76.38976 | 134.577 | 44.69027 | 57.43585 | 44.87845 | 68.37004 | 69.81278 | 71.69416 | 67.9514 | 55.96041 | 55.82371 |
| CUST_43079_PI429010886 | M62019 | 0.031308 | 12 | 9 | | | | | | 116.8326 | 52.37245 | 32.16411 | 26.17436 | 206.0341 | 5.977954 | 77.42171 | 72.8194 | 51.69046 | 396.1095 | 56.46834 | 150.3479 | 67.05664 | 36.10137 | 29.14178 | 61.7839 | 34.1704 | 17.06407 | 85.35234 | 31.99517 | 43.62391 | 83.42195 | 112.8928 | 118.2001 | 8.608528 | 53.04479 | 155.4878 |
| CUST_43382_PI429010886 | M62890 | 0.031308 | 12 | 9 | | | | | | 319.3199 | 297.5373 | 573.0502 | 46.00056 | 146.5131 | 109.4916 | 318.2985 | 165.4521 | 71.46757 | 578.5402 | 82.39034 | 446.4087 | 222.1142 | 376.1162 | 241.3085 | 67.03346 | 143.0236 | 50.87205 | 494.0702 | 306.2716 | 372.2181 | 449.2825 | 226.7383 | 147.3848 | 297.3146 | 202.9175 | 413.4726 |
| CUST_49435_PI429010886 | M85635 | 0.031308 | 12 | 3 | | | | | | 69.43885 | 79.88802 | 84.19357 | 89.09879 | 86.70732 | 117.6882 | 77.63254 | 73.86349 | 88.47454 | 85.30129 | 73.21264 | 78.07189 | 63.35686 | 82.46901 | 72.52738 | 96.56866 | 108.7487 | 99.39132 | 135.5775 | 77.0714 | 74.64567 | 86.32314 | 71.33278 | 68.83612 | 106.4697 | 75.87721 | 81.94846 |
| CUST_14380_PI429010886 | N32785 | 0.031308 | 12 | 9 | | | | | | 337.3367 | 604.4329 | 520.4614 | 441.7468 | 391.6722 | 309.6337 | 354.6545 | 510.0071 | 410.06 | 580.436 | 544.2536 | 525.8609 | 403.0406 | 407.7342 | 388.1952 | 383.652 | 476.5945 | 323.4046 | 441.0416 | 574.3589 | 461.2479 | 429.1228 | 637.6126 | 443.7417 | 554.0852 | 390.2834 | 443.7417 |
| CUST_20216_PI429010886 | N43035 | 0.031308 | 12 | 9 | | | | | | 24.32155 | 199.3094 | 305.6076 | 21.66748 | 3.926744 | 27.83166 | 149.8536 | 342.0536 | 421.2064 | 484.7143 | 114.8562 | 228.5019 | 131.9084 | 239.0183 | 145.4402 | 47.11037 | 145.794 | 17.46274 | 59.00313 | 495.0933 | 71.09226 | 353.785 | 438.1052 | 10.59051 | 106.8983 | 216.0426 | 113.1785 |
| CUST_843_PI429010886 | N7514 | 0.031308 | 12 | 3 | | | | | | 2127.118 | 456.2722 | 82.04247 | 3699.019 | 1045.788 | 6894.743 | 1418.381 | 3960.835 | 881.5993 | 66.29488 | 196.0648 | 31.96946 | 2421.023 | 40.38893 | 692.7803 | 82.18107 | 3988.418 | 4797.809 | 2653.866 | 59.38984 | 216.0765 | 69.83541 | 37.77885 | 3440.367 | 56.56684 | 6516.822 | 50.79816 |
| CUST_19124_PI429010886 | N41014 | 0.031308 | 24 | 21 | | | | | | 33.0759 | 36.95565 | 33.71856 | 33.14087 | 41.01337 | 32.03583 | 53.37885 | 29.10242 | 35.82359 | 16.70546 | 41.99643 | 32.79731 | 28.77687 | 37.24189 | 7.831079 | 12.75753 | 27.72317 | 42.08627 | 26.01379 | 31.86259 | 54.93259 | 48.21189 | 52.72315 | 77.35795 | 38.93621 | 57.56078 | 38.29585 |
| CUST_3305_PI429010886 | N14225 | 0.031315 | 18 | 12 | | | | | | 44.23444 | 45.73763 | 39.80101 | 46.48282 | 47.18136 | 33.35513 | 62.70075 | 51.14708 | 58.8975 | 57.48318 | 61.5801 | 45.39483 | 62.406 | 60.72482 | 51.2779 | 55.30825 | 62.79742 | 56.55039 | 48.85705 | 43.49759 | 60.71325 | 55.45871 | 54.41833 | 53.04998 | 55.90028 | 55.66127 | 64.22812 |
| CUST_8435_PI429010886 | N23035 | 0.031331 | 18 | 15 | 91078418 | PREDICTED: similar to cytochrome c oxidase assemb | Protein metabolism | Macromolecular complex assembly | 1.44E-80 | 165.8485 | 204.9184 | 140.4504 | 129.8621 | 163.3073 | 177.6145 | 141.2095 | 159.6044 | 157.3097 | 207.6455 | 158.7737 | 190.7327 | 163.7665 | 153.734 | 150.6483 | 205.3646 | 165.2432 | 194.6975 | 188.6896 | 184.0038 | 133.3139 | 181.4858 | 141.0077 | 157.0047 | 154.2676 | 131.0829 | 131.2903 |
| CUST_6078_PI429010886 | N19599 | 0.031339 | 24 | 21 | | | | | | 49.97285 | 52.33357 | 70.24827 | 46.01155 | 43.52847 | 57.73367 | 37.11592 | 44.18564 | 70.169 | 42.30429 | 40.45279 | 55.45871 | 47.26147 | 52.48792 | 53.51251 | 52.09002 | 48.79699 | 45.42072 | 101.4215 | 42.45161 | 66.00564 | 65.3527 | 74.54008 | 66.50022 | 54.49444 | 71.09226 | 84.06167 |
| CUST_18016_PI429010886 | N39002 | 0.031339 | 24 | 15 | | | | | | 78.20249 | 56.57261 | 65.20986 | 41.7785 | 55.62798 | 62.42019 | 63.38531 | 59.30743 | 93.67647 | 81.42045 | 68.33006 | 63.90408 | 59.9279 | 80.0595 | 87.76889 | 65.54211 | 105.9574 | 79.45781 | 78.51483 | 78.67424 | 75.84454 | 70.58339 | 56.80303 | 71.03479 | 96.94706 | 65.18101 | 102.5637 |
| CUST_53248_PI429010886 | M100985 | 0.031355 | 24 | 0 | | | | | | 15.96275 | 14.57957 | 12.67037 | 7.683919 | 10.30348 | 11.57662 | 18.15993 | 5.086622 | NA | NA | 5.758268 | 19.68268 | 10.01115 | 10.72726 | 5.349898 | 7.071532 | NA | 3.727089 | 17.9819 | 8.180112 | 8.446645 | NA | NA | 16.5578 | 22.14748 | NA | 27.38056 |
| CUST_32412_PI429010886 | M4050 | 0.031366 | 15 | 3 | | | Metabolic process | Carbohydrate metabolic process | | 154.8716 | 107.0545 | 48.66243 | 458.0989 | 512.0216 | 715.8969 | 613.4417 | 185.7639 | 84.83474 | 6.21115 | 413.9155 | NA | 95.14966 | 10.5119 | 57.65114 | 36.44639 | 895.455 | 597.1763 | 422.7364 | NA | 333.6383 | 58.64138 | 265.3779 | 360.1121 | 5.611283 | 349.6083 | NA |
| CUST_11861_PI429010886 | N28529 | 0.031372 | 15 | 9 | | | | | | 58.19889 | 67.17456 | 78.12789 | 64.08758 | 60.12977 | 73.76113 | 49.28537 | 56.7576 | 68.6762 | 81.52938 | 69.81278 | 107.0816 | 51.37508 | 79.91242 | 66.46046 | 58.892 | 50.12098 | 66.84482 | 52.93772 | 64.75173 | 60.47979 | 83.06141 | 69.29923 | 60.58837 | 95.39206 | 72.90791 | 84.10524 |
| CUST_15177_PI429010886 | N34138 | 0.031372 | 21 | 3 | | | | | | 76.29064 | 154.8286 | 83.88709 | 120.2042 | 109.5408 | 88.13649 | 72.18474 | 91.68314 | 85.57408 | 123.1997 | 104.0557 | 109.9704 | 79.043 | 103.3151 | 85.81376 | 73.07849 | 68.33006 | 83.25716 | 85.18737 | 104.5462 | 94.48599 | 131.9258 | 94.99099 | 86.05551 | 154.7047 | 90.06088 | 112.6218 |
| CUST_53810_PI429010886 | M103291 | 0.031379 | 24 | 21 | | | | | | 14.17659 | 8.262678 | 23.88883 | 10.65864 | 4.032896 | 25.39336 | 11.75847 | 15.04347 | 17.59108 | 16.19477 | 18.16713 | 19.31538 | 22.30707 | 18.41912 | 21.00749 | 16.69534 | 22.52813 | 17.04861 | 19.68628 | 15.15645 | 28.19636 | 22.9227 | 24.99534 | 34.74686 | 20.38138 | 25.93469 | 17.16134 |
| CUST_12471_PI429010886 | N29517 | 0.031379 | 24 | 21 | 219815923 | IMD [Litopenaeus vannamei] | Signal transduction | Other | 6.61E-17 | 147.9921 | 202.005 | 164.0384 | 193.9282 | 140.3134 | 179.9875 | 98.10524 | 98.51344 | 149.0785 | 173.7422 | 130.1545 | 237.0939 | 188.9774 | 175.6552 | 212.9538 | 243.8028 | 86.84519 | 214.0659 | 158.5656 | 195.6184 | 169.4541 | 269.8655 | 213.4663 | 188.7518 | 208.4543 | 181.5403 | 196.0648 |
| CUST_26521_PI429010886 | N54390 | 0.031379 | 24 | 21 | | | | | | 137.2146 | 257.2108 | 164.3174 | 148.4035 | 168.8426 | 164.6529 | 147.0211 | 123.7589 | 152.4677 | 293.1844 | 158.3621 | 189.6625 | 154.222 | 171.9533 | 222.7411 | 219.8055 | 147.4899 | 149.1265 | 190.645 | 170.6324 | 176.6512 | 246.2665 | 185.2951 | 255.3452 | 171.4068 | 180.3315 | 176.2795 |
| CUST_34483_PI429010886 | M9802 | 0.031384 | 18 | 15 | | | | | | 179.4478 | 181.7953 | 209.2252 | 142.8582 | 175.2355 | 164.7416 | 161.8434 | 142.0014 | 155.5901 | 211.2278 | 154.793 | 181.4858 | 156.2584 | 207.4294 | 179.2925 | 192.586 | 185.4691 | 197.0135 | 206.6019 | 151.2719 | 191.5641 | 130.4682 | 198.4267 | 169.4295 | 181.6996 | 132.7602 | 169.3227 |
| CUST_1928_PI429010886 | N11098 | 0.031384 | 24 | 15 | | | | | | 2658.303 | 2567.686 | 2286.095 | 1851.755 | 2428.103 | 1838.739 | 1728.225 | 1827.911 | 2062.191 | 3010.148 | 1659.929 | 3748.628 | 2291.102 | 2278.385 | 2963.617 | 3296.897 | 2493.282 | 3616.168 | 2292.412 | 2371.653 | 2371.653 | 2706.735 | 2086.783 | 2091.598 | 2934.424 | 1963.68 | 2817.021 |
| CUST_13304_PI429010886 | N30953 | 0.031402 | 24 | 6 | | | | | | 10.84086 | NA | NA | NA | 7.783894 | 14.19523 | 93.80027 | 11.55545 | 9.143497 | 9.352687 | 10.93801 | 12.86855 | 4.470524 | 8.287779 | 5.023732 | 10.07591 | 8.291054 | NA | 6.510984 | 6.414725 | 6.453334 | 13.41264 | 6.830664 | 7.661152 | 7.513993 | NA | 5.422731 |
| CUST_48727_PI429010886 | M81876 | 0.031406 | 15 | 6 | | | | | | 115.7617 | 13.33386 | 15.91395 | 37.36668 | 6.630788 | 16.20777 | 83.44238 | 31.14323 | 54.17626 | 30.62818 | 11.22699 | 50.74837 | 20.03408 | NA | 16.59129 | 17.132 | 10.31716 | 33.89069 | 44.22142 | 21.0027 | 44.38619 | 73.86349 | 41.14494 | 58.52324 | 55.11221 | 23.73387 | 52.74988 |
| CUST_698_PI429010886 | N6799 | 0.031409 | 24 | 6 | 321469659 | hypothetical protein DAPPUDRAFT_318250 [Daphn | Metabolic process | Cellular amino acid metabolic process | 1.3E-51 | 69.53748 | 63.87286 | 112.9639 | 91.24943 | 51.97626 | 31.1463 | 63.15525 | 134.4739 | 83.97119 | 90.58155 | 53.18376 | 63.42473 | 46.83015 | 30.04719 | 11.20677 | 16.06458 | 42.61497 | 14.83503 | 33.69201 | 166.4452 | 25.17015 | 44.5467 | 58.58074 | 26.27705 | 26.4245 | 55.11221 | 105.1778 |
| CUST_37644_PI429010886 | M20185 | 0.03141 | 21 | 9 | | | | | | 7.928032 | 1828.604 | 583.9827 | 3680.553 | 9501.999 | 1633.35 | 2102.543 | 20865.58 | 16607.59 | 884.4098 | 24587.25 | 26713.02 | 16119.35 | 1920.945 | 10190.76 | 10.34165 | 5050.626 | NA | 990.2048 | 28378.02 | 21690.05 | 11.34865 | 1968.333 | 1478.956 | 8.403892 | 6461.792 | 14559.32 |
| CUST_36706_PI429010886 | M16598 | 0.03141 | 15 | 0 | | | | | | 270.2714 | 251.0973 | 252.4492 | 212.773 | 231.0649 | 245.7365 | 189.4251 | 184.4849 | 191.5641 | 250.9845 | 215.028 | 205.5955 | 156.5024 | 200.2396 | 218.2149 | 287.6352 | 231.0649 | 252.9368 | 245.9376 | 222.7411 | 229.3254 | 343.6779 | 195.4506 | 211.6792 | 317.032 | 221.165 | 253.6933 |
| CUST_55421_PI429010886 | M110019 | 0.031444 | 15 | 9 | | | | | | 119.9009 | 84.74127 | 176.0261 | 109.3542 | 86.43501 | 86.75819 | 149.1678 | 133.9999 | 168.177 | 210.7453 | 164.6734 | 135.6374 | 125.8034 | 143.4947 | 104.0321 | 153.6441 | 142.632 | 112.9639 | 126.8312 | 129.6714 | 134.9686 | 147.5068 | 113.2085 | 114.3333 | 190.8326 | 120.5518 | 211.85 |
| CUST_34224_PI429010886 | M9102 | 0.031444 | 15 | 0 | | | | | | 65.77588 | 55.48994 | 49.13393 | 47.11613 | 59.6416 | 44.04347 | 36.74157 | 46.33014 | 41.99643 | 59.34275 | 54.10071 | 47.47988 | 54.59618 | 47.36571 | 42.76605 | 72.3532 | 61.40092 | 60.42655 | 66.63641 | 54.15061 | 38.92558 | 64.67521 | 36.8491 | 40.50555 | 70.41805 | 34.78955 | 53.22735 |
| CUST_44867_PI429010886 | M67257 | 0.031457 | 15 | 12 | 220172363 | putative articulin p60 [Rimicaris exoculata] | | | 8.12E-12 | 15662.95 | 460.255 | 39731.64 | 625.0062 | 1393.263 | 1573.105 | 23632.71 | 67520.86 | 49654.41 | 48296.18 | 7686.447 | 8046.364 | 51007.02 | 80123.56 | 39918.27 | 1744.83 | 24777.72 | 1799.13 | 15246.84 | 8147.956 | 7034.347 | 27353.46 | 46148.42 | 7060.087 | 45084.56 | 4763.632 | 43573.57 |
| CUST_13704_PI429010886 | N31625 | 0.031468 | 21 | 0 | 195446372 | GK10855 [Drosophila willistoni] >gi\|194166835\|gb | | | 2.98E-62 | 13.11969 | 13.72673 | NA | 8.601817 | 18.32163 | NA | 13.22555 | 9.743013 | 5.333112 | 3.841155 | 14.84417 | 10.62424 | 5.120064 | 4.009968 | 6.229031 | 22.22394 | 13.54646 | 18.07887 | 11.63331 | 8.681407 | 8.585981 | 19.39824 | 11.0938 | 50.28232 | 13.47393 | 8.469456 | 11.0861 |
| CUST_29594_PI429010886 | N60164 | 0.031486 | 21 | 18 | 242023295 | guanine nucleotide exchange factor, putative [Pedic | | | 3.86E-07 | 197.44 | 253.025 | 266.2431 | 139.8015 | 230.6606 | 206.6326 | 176.3863 | 203.2288 | 175.8911 | 227.0976 | 245.2402 | 219.01 | 183.786 | 225.4439 | 229.4944 | 273.0635 | 206.6326 | 157.3996 | 250.6863 | 232.5216 | 259.5337 | 252.7105 | 246.5795 | 181.1004 | 222.1142 | 197.7545 | 202.0834 |
| CUST_35066_PI429010886 | M11470 | 0.031489 | 12 | 0 | | | | | | 83.48026 | 105.607 | 96.9668 | 84.38957 | 94.00857 | 83.41229 | 73.82839 | 74.57029 | 76.6588 | 81.93796 | 76.60738 | 94.49669 | 93.33448 | 101.4215 | 91.11943 | 99.7668 | 85.34367 | 64.73839 | 79.30391 | 94.16491 | 76.07766 | 102.2185 | 94.13978 | 89.70412 | 97.83625 | 90.29804 | 86.64513 |
| CUST_35440_PI429010886 | M12570 | 0.031489 | 12 | 6 | | | | | | 41.0381 | 43.43615 | 21.37183 | 38.09087 | 43.09035 | 31.21185 | 47.09025 | 49.76383 | 55.01668 | 46.92136 | 41.62677 | 29.97999 | 36.72937 | 28.66914 | 32.79731 | 39.0714 | 36.69684 | 28.6962 | 37.92604 | 50.08377 | 47.10091 | 32.10636 | 32.80705 | 29.99408 | 36.62028 | 38.14903 | 29.88865 |
| CUST_49629_PI429010886 | M86452 | 0.031489 | 12 | 0 | | | | | | 716.0786 | 861.4839 | 860.1543 | 795.9721 | 751.781 | 775.6671 | 735.5143 | 570.8436 | 700.208 | 897.4259 | 750.1475 | 865.174 | 797.3133 | 892.8593 | 814.5413 | 1021.127 | 783.8018 | 787.7599 | 775.4257 | 821.28 | 781.7106 | 959.2541 | 855.8158 | 790.1209 | 1040.9 | 875.926 | 970.3266 |
| CUST_15811_PI429010886 | N35184 | 0.031489 | 12 | 0 | | | | | | 128.0504 | 136.0993 | 111.9784 | 115.5186 | 90.70315 | 80.61916 | 74.86136 | 88.71715 | 96.05386 | 112.0385 | 113.6358 | 115.8883 | 97.49057 | 95.71563 | 116.5433 | 186.6799 | 102.0693 | 111.5352 | 94.63457 | 101.4018 | 83.39175 | 160.0995 | 107.3346 | 90.09804 | 137.1215 | 109.0449 | 127.6382 |
| CUST_17023_PI429010886 | N37247 | 0.031489 | 12 | 0 | | | | | | 216.304 | 219.0917 | 267.8414 | 196.5633 | 224.858 | 219.0917 | 202.4996 | 218.804 | 204.2578 | 237.8356 | 207.8011 | 279.8719 | 238.1723 | 283.0956 | 244.3511 | 205.2623 | 218.9512 | 280.7715 | 191.886 | 262.8116 | 210.7453 | 225.5174 | 255.2471 | 211.7142 | 263.2877 | 238.4211 | 265.532 |
| CUST_39434_PI429010886 | M39314 | 0.031494 | 18 | 12 | | | | | | 4965.834 | 8219.035 | 5654.855 | 4635.589 | 4803.756 | 5477.513 | 5262.203 | 5515.427 | 5515.427 | 9475.141 | 3526.312 | 8582.669 | 7853.842 | 7729.059 | 9937.704 | 10083.97 | 4782.509 | 5050.626 | 5119.45 | 8448.526 | 4847.521 | 7107.78 | 4392.265 | 3644.859 | 6454.955 | 5319.238 | 6794.71 |
| CUST_41788_PI429010886 | M58559 | 0.031494 | 18 | 12 | | | | | | 338.5409 | 322.593 | 176.9698 | 192.586 | 293.013 | 205.2623 | 347.7401 | 208.7483 | 295.0205 | 239.0183 | 236.4243 | 394.958 | 493.4379 | 401.4797 | 390.3686 | 489.4967 | 253.025 | 211.9082 | 403.4054 | 171.9533 | 222.8389 | 357.4809 | 138.0091 | 150.5251 | 397.8439 | 184.0938 | 406.9652 |
| CUST_40401_PI429010886 | M54983 | 0.031498 | 24 | 0 | | | | | | 19.50517 | 23.49374 | 27.24651 | 22.42598 | 20.55693 | 6.208299 | 17.53198 | 16.95965 | 23.41982 | 17.37911 | 21.92529 | 20.38984 | 17.92211 | 18.55589 | 18.6314 | 23.32 | 16.30451 | 14.46019 | 20.95946 | 18.92054 | 18.49479 | 23.59619 | 13.86613 | 231.0649 | 26.4059 | 22.83945 | 24.51087 |
| CUST_50460_PI429010886 | M89894 | 0.031501 | 24 | 21 | 66510919 | PREDICTED: dosage compensation regulator isoforn | | | 2.71E-44 | 7.594313 | 13.7877 | 11.78251 | 10.7321 | 13.05013 | 10.12547 | 8.953175 | 4.573813 | 7.490316 | 21.90983 | 4.202049 | 6.905105 | 6.327324 | 11.57436 | 17.42611 | 16.41995 | NA | 10.07219 | 5.338547 | 13.74631 | 19.40757 | 21.78927 | 16.68444 | NA | 11.94615 | 13.81689 | 18.20618 |
| CUST_42517_PI429010886 | M60486 | 0.031525 | 24 | 21 | | | | | | 34.76295 | 51.18041 | 71.45222 | 42.83757 | 55.00033 | 58.8975 | 33.09643 | 51.07827 | 48.26458 | 50.71291 | 41.4548 | 30.89913 | 56.2163 | 30.05351 | 38.153 | 44.67201 | 58.23374 | 57.664 | 48.07404 | 49.65587 | 63.46235 | 52.14117 | 59.57796 | 63.65227 | 64.9677 | 40.68573 | 72.55972 |
| CUST_43402_PI429010886 | M62946 | 0.031553 | 24 | 21 | | | | | | 14.39574 | 18.19818 | 17.15702 | 21.95116 | 15.66738 | 13.08554 | 13.21272 | 7.416629 | 14.54874 | 14.70767 | 13.9884 | 11.13307 | 13.03936 | 16.8955 | 11.11331 | 22.69042 | 15.80527 | 9.934601 | 19.06265 | 17.48685 | 18.0708 | 25.50611 | 20.71154 | 13.54494 | 32.18334 | 21.08658 | 15.39972 |
| CUST_36567_PI429010886 | M16251 | 0.031568 | 24 | 18 | | | | | | 72.22304 | 43.41776 | 43.01496 | 36.69997 | 37.39567 | 53.94407 | 44.18564 | 35.27392 | 42.86103 | 52.79712 | 42.242 | 47.94376 | 32.88145 | 41.59799 | 46.55609 | 46.61273 | 63.75313 | 41.37701 | 63.82351 | 58.75213 | 48.99561 | 54.73744 | 49.63764 | 48.85338 | 49.86955 | 47.95704 | 49.16869 |
| CUST_17744_PI429010886 | N38518 | 0.03157 | 24 | 21 | | | | | | 147.0211 | 163.6892 | 153.5041 | 185.6524 | 126.3708 | 165.7787 | 99.26813 | 113.4502 | 111.5705 | 136.2681 | 103.5957 | 136.8231 | 107.4719 | 162.9163 | 134.5392 | 208.8792 | 151.7723 | 183.2331 | 141.5789 | 148.1205 | 139.1034 | 186.1374 | 118.905 | 175.6005 | 167.6463 | 142.3214 | 158.4788 |
| CUST_40546_PI429010886 | M55421 | 0.031573 | 12 | 0 | | | | | | 51.87547 | 77.23965 | 50.63623 | 52.8414 | 47.33315 | 32.13072 | 50.58779 | 55.65323 | 33.95306 | 50.91028 | 45.39895 | 47.75744 | 49.8518 | 58.2166 | 52.97067 | 52.96132 | 41.55626 | 45.38512 | 49.73229 | 45.10479 | 38.61391 | 56.65877 | 48.39289 | 45.11398 | 68.61799 | 48.61196 | 54.12178 |
| CUST_41049_PI429010886 | M56757 | 0.031598 | 21 | 6 | | | | | | 50.41901 | 74.14418 | 69.20227 | 50.48761 | 67.9344 | 54.81682 | 84.07185 | 88.45598 | 84.75109 | 77.2785 | 57.70169 | 78.79452 | 66.48727 | 91.87634 | 74.80865 | 65.48233 | 48.65088 | 33.50669 | 52.02853 | 70.7113 | 42.06008 | 52.80587 | 96.06297 | 58.10187 | 29.53988 | 79.01755 | 83.07834 |
| CUST_5815_PI429010886 | N19234 | 0.031598 | 21 | 6 | 505841261 | PREDICTED: origin recognition complex subunit 4 [S | Cellular Process | Cell cycle | 1.6E-104 | 61.14806 | 56.89861 | 64.77801 | 42.6726 | 52.10062 | 81.31771 | 69.45519 | 72.24742 | 67.73286 | 77.44015 | 63.99533 | 49.86955 | 60.85469 | 59.8939 | 61.2655 | 59.03159 | 64.09512 | 37.40747 | 42.00978 | 55.67313 | 55.09266 | 72.05058 | 70.17729 | 58.59263 | 78.54463 | 55.90028 | 82.71117 |
| CUST_56866_PI429010886 | M114794 | 0.031601 | 24 | 21 | | | | | | 10.10937 | 8.366386 | 11.06786 | 5.637553 | 6.066547 | 11.9534 | 13.93254 | NA | 6.739431 | 5.058833 | 5.569488 | NA | 8.992487 | 10.48357 | 6.11955 | 8.348712 | 8.760123 | 9.105658 | 9.142203 | 5.710989 | 9.177025 | 11.25284 | NA | 13.16535 | 14.57268 | 9.138723 | 6.910269 |
| CUST_47153_PI429010886 | M74972 | 0.031609 | 18 | 12 | | | | | | 16.0444 | 20.29574 | 19.30172 | 11.05263 | 18.25166 | 8.748535 | 12.4451 | 7.884889 | 20.81295 | 20.84589 | 20.16285 | 26.4818 | 28.5643 | 25.69538 | 25.68589 | 14.32411 | 9.811903 | 22.19563 | 14.47801 | 23.80171 | 14.94965 | 26.869 | 12.41778 | 9.982619 | 23.79915 | 12.32915 | 24.53437 |
| CUST_35739_PI429010886 | M13356 | 0.031611 | 18 | 0 | | | | | | 42.50976 | 46.28376 | 44.84166 | 65.25047 | 45.69115 | 37.69372 | 30.56491 | 19.25911 | 31.32726 | 40.44483 | 39.27214 | 51.28378 | 32.55113 | 39.22918 | 42.06993 | 48.5802 | 36.35659 | 42.2764 | 47.0949 | 34.98674 | 50.04478 | 50.02767 | 37.61167 | 41.50205 | 43.7253 | 25.1646 | 34.1956 |
| CUST_12003_PI429010886 | N28761 | 0.031614 | 18 | 15 | 443716148 | hypothetical protein CAPTEDRAFT_193197 [Capitell | | | 5.99E-10 | 11.25926 | 9.354027 | 8.147639 | 8.960873 | 7.444212 | NA | 10.68832 | 6.881575 | 15.7177 | NA | 14.30544 | 12.59547 | 10.40388 | 12.43401 | 13.70505 | 15.6362 | 17.4379 | 18.93037 | 11.07843 | 8.212232 | NA | 17.78016 | 8.914093 | 6.95796 | 10.82077 | 7.934263 | 10.90827 |
| CUST_1729_PI429010886 | N10556 | 0.031681 | 21 | 9 | 75022291 | RecName: Full=Oplophorus-luciferin 2-monooxyger | | | 1.31E-24 | NA | 93.96395 | 52.50431 | 376.8596 | 607.7896 | 104.4971 | 104.6277 | 861.1597 | 833.7688 | NA | 844.11 | 1021.127 | 1390.937 | 227.1414 | 790.1209 | NA | 368.5464 | NA | 144.7304 | 1413.053 | 542.234 | 6.675568 | 510.619 | 311.2463 | NA | 1854.457 | 148.7446 |
| CUST_27383_PI429010886 | N55954 | 0.031694 | 24 | 21 | | | | | | 49.44854 | 66.10733 | 64.8125 | 58.59263 | 45.22744 | 57.32094 | 49.93144 | 54.74214 | 53.55772 | 68.56023 | 49.25815 | 47.37598 | 52.56534 | 51.75764 | 50.77029 | 60.20751 | 42.242 | 61.00953 | 58.37612 | 52.53115 | 58.78466 | 61.7839 | 68.49231 | 68.38918 | 73.54244 | 50.37311 | 58.08697 |
| CUST_31632_PI429010886 | M2022 | 0.031717 | 15 | 3 | 18916919 | cyclic AMP-regulated protein like protein [Marsupe | | | 3.73E-51 | 600.2864 | 820.2371 | 314.8877 | 752.0253 | 632.4528 | 847.0661 | 670.3311 | 414.4964 | 510.1908 | 352.327 | 375.1189 | 458.3652 | 581.4141 | 537.5555 | 754.5887 | 729.8175 | 562.0583 | 820.2371 | 777.3766 | 591.3951 | 561.4598 | 894.0864 | 362.6784 | 791.0227 | 416.1299 | 540.8491 | 389.3563 |
| CUST_38394_PI429010886 | M26191 | 0.031722 | 18 | 3 | 460838092 | strongly chitin-binding protein-1 [Procambarus clarl | | | 8.9E-24 | 246.1531 | 113.4637 | 11.33971 | 792.1417 | 1465.818 | 1040.536 | 660.5155 | 353.0021 | 77.97597 | 7.070433 | 1027.698 | 6.324533 | 255.4386 | 37.90698 | 109.19 | 109.5894 | 943.3673 | 327.9867 | 1041.548 | 23.63331 | 592.0389 | 156.9601 | 905.6425 | 1070.179 | 62.67301 | 416.9251 | NA |
| CUST_40228_PI429010886 | M51913 | 0.031723 | 12 | 0 | | | | | | 382.1801 | 296.3167 | 387.6806 | 276.0613 | 284.8452 | 221.425 | 257.0386 | 302.6662 | 302.9535 | 333.8649 | 407.4423 | 357.862 | 385.3789 | 519.4558 | 287.3115 | 344.6643 | 313.5825 | 337.3994 | 296.4747 | 400.5132 | 443.9779 | 445.9535 | 454.9277 | 397.4231 | 512.9488 | 467.8704 | 609.308 |
| CUST_43606_PI429010886 | M63489 | 0.031723 | 15 | 12 | | | | | | 39067.89 | 18556.73 | 62855.9 | 3991.112 | 9448.983 | 5712.144 | 34870.47 | 60338.91 | 64763.62 | 62522.62 | 32800.86 | 31148.55 | 51921.46 | 73076.54 | 41266.06 | 17402.33 | 41577.33 | 20248.42 | 38580.75 | 30891.2 | 32962.39 | 45970.08 | 63067.91 | 23420.74 | 66128 | 26474.55 | 65192.57 |
| CUST_19808_PI429010886 | N42231 | 0.031726 | 24 | 9 | | | | | | 65.61755 | 85.96929 | 67.52947 | 93.58403 | 81.97482 | 100.3587 | 73.26779 | 46.61273 | 75.10926 | 87.44346 | 80.22855 | 145.6105 | 66.78979 | 75.01889 | 94.88698 | 75.91487 | 72.6348 | 90.20594 | 64.52054 | 70.75343 | 80.88726 | 60.0207 | 62.48029 | 60.80176 | 78.50617 | 90.76835 | 81.2413 |
| CUST_9072_PI429010886 | N24018 | 0.031728 | 24 | 3 | 321458954 | hypothetical protein DAPPUDRAFT_328542 [Daphn | | | 5.24E-31 | 34.89251 | 23.92396 | 34.55194 | 33.86813 | 42.02786 | 43.72859 | 39.03429 | 47.88363 | 35.91866 | 22.29671 | 13.8828 | 26.51493 | 42.1461 | 28.5492 | 40.98703 | 21.98126 | 30.6513 | 31.07494 | 30.16185 | 21.14283 | 23.14492 | 30.89566 | 25.19666 | 47.55714 | 29.03344 | 52.79712 | 33.46044 |
| CUST_8976_PI429010886 | N23880 | 0.03173 | 24 | 0 | | | | | | 453.7002 | 533.6685 | 490.4258 | 457.1179 | 411.5645 | 532.3882 | 381.737 | 335.677 | 401.3938 | 411.4634 | 432.5053 | 439.0329 | 348.8261 | 446.4087 | 406.3292 | 600.7483 | 412.3362 | 429.0337 | 455.9675 | 428.865 | 393.5988 | 461.9687 | 420.3153 | 403.0406 | 579.2037 | 488.3379 | 456.9093 |
| CUST_37263_PI429010886 | M18425 | 0.031731 | 24 | 21 | | | | | | 24.97464 | 40.62506 | 17.60432 | 26.14965 | 23.61053 | 13.88977 | 15.49062 | 19.15845 | 14.62416 | 34.7029 | 18.1616 | 29.2624 | 25.75668 | 29.53645 | 31.21185 | 36.39578 | 5.311014 | 30.0571 | 17.39698 | 35.18679 | 22.40881 | 44.12842 | 28.16847 | 57.03251 | 37.64162 | 18.95556 | 26.78992 |
| CUST_48017_PI429010886 | M78548 | 0.031747 | 21 | 0 | 395517443 | PREDICTED: zinc finger protein 252-like, partial [Sar | | | 6.87E-24 | 12.68586 | 13.1549 | 16.10864 | 10.73752 | 7.719905 | 10.00617 | 8.060022 | 5.275871 | 11.81981 | 9.122458 | 8.285257 | 10.14742 | 5.327653 | 11.8448 | 10.91844 | 10.43918 | 6.898311 | 13.84136 | 17.22867 | 11.36478 | 6.391082 | 21.05993 | 10.5119 | 16.25197 | 17.31897 | 4.083444 | 18.69874 |
| CUST_26357_PI429010886 | N54107 | 0.031757 | 12 | 3 | | | | | | 19.50736 | 6.760169 | NA | 13.93436 | 12.67185 | 20.23079 | 7.840127 | 10.6528 | 10.52781 | 7.638107 | 4.83618 | 4.511976 | 10.09872 | NA | 9.154209 | 5.346894 | 20.00381 | 19.74119 | 5.805366 | 6.920375 | 6.752371 | 7.298488 | 7.042021 | 7.871973 | 6.592569 | 8.815034 | 7.091579 |
| CUST_10914_PI429010886 | N26983 | 0.031811 | 24 | 0 | | | | | | 38.05146 | 43.99672 | 26.49155 | 32.46954 | 23.65401 | 19.78239 | 45.68153 | 28.74055 | 22.5025 | 34.99807 | 24.35938 | 22.40135 | 27.53274 | 16.26898 | 19.14778 | 25.11237 | 18.10067 | 26.07711 | 15.46253 | 23.37953 | 26.8663 | 37.49557 | 28.9143 | 10.68521 | 38.09087 | 25.70084 | 37.87276 |
| CUST_53977_PI429010886 | M103947 | 0.031817 | 21 | 6 | | | | | | 29.11768 | 31.46674 | 36.15703 | 33.26161 | 78.80354 | 60.53435 | 54.34169 | 158.4583 | 159.9839 | 31.56943 | 143.2572 | 140.4751 | 72.6524 | 49.57451 | 35.83833 | 37.20896 | 60.5193 | 40.42484 | 66.40412 | 196.129 | 90.37187 | 33.46044 | 60.01583 | 24.2722 | 49.40423 | 87.06371 | 46.77892 |
| CUST_25097_PI429010886 | N51778 | 0.031822 | 18 | 15 | | | | | | 52.22902 | 82.41691 | 75.72117 | 50.28232 | 84.30944 | 73.23407 | 65.99744 | 64.26189 | 63.32035 | 81.95659 | 86.05551 | 97.97139 | 77.37108 | 74.17182 | 90.74799 | 91.69639 | 93.1664 | 74.68625 | 76.25978 | 77.43118 | 66.37927 | 52.36466 | 88.6126 | 61.59565 | 67.46884 | 69.70264 | 68.05219 |
| CUST_7625_PI429010886 | N21827 | 0.031828 | 15 | 9 | 391328182 | PREDICTED: uncharacterized protein LOC10090848 | | | 1.42E-17 | 83.41229 | 132.3882 | 127.2574 | 103.2771 | 105.2326 | 114.2745 | 102.782 | 175.6284 | 166.0617 | 177.896 | 110.1036 | 160.5748 | 131.8093 | 107.0931 | 161.0687 | 151.4764 | 108.1306 | 120.0446 | 79.79104 | 128.2524 | 130.2006 | 143.9489 | 140.2989 | 151.9116 | 155.8094 | 159.338 | 134.8111 |
| CUST_13125_PI429010886 | N30623 | 0.031828 | 15 | 0 | | | | | | 639.5245 | 611.2062 | 577.0235 | 483.6862 | 501.702 | 622.7518 | 421.0101 | 450.649 | 461.1208 | 560.0158 | 465.5606 | 586.9437 | 392.2917 | 524.2988 | 550.2491 | 755.2577 | 579.0482 | 673.1948 | 496.5233 | 607.5979 | 462.9458 | 656.7764 | 386.742 | 525.7241 | 733.3828 | 509.0016 | 601.5918 |
| CUST_33874_PI429010886 | M8106 | 0.031829 | 21 | 9 | 291235674 | PREDICTED: serine carboxypeptidase 1-like [Saccogl | Protein metabolism | Proteolysis | 4.32E-86 | NA | 25.42619 | 7.911129 | 162.1787 | 414.9669 | 57.97363 | 115.7331 | 540.7565 | 107.4719 | 84.90321 | 1090.829 | 1091.244 | 287.9804 | 55.14628 | 80.06962 | NA | 186.0312 | NA | 31.56711 | 1209.913 | 500.7784 | NA | 38.60384 | 36.21632 | NA | 197.44 | 230.7168 |
| CUST_8974_PI429010886 | N23878 | 0.031843 | 12 | 0 | | | | | | 109.3629 | 99.19224 | 124.9715 | 56.52083 | 83.46951 | 72.03066 | 73.25256 | 72.66838 | 54.16396 | 110.765 | 79.99032 | 87.44346 | 60.39123 | 71.10646 | 87.71808 | 106.4305 | 89.84183 | 97.95028 | 65.16761 | 86.49079 | 60.58205 | 81.02279 | 75.22006 | 65.33287 | 162.5022 | 88.33792 | 121.7504 |
| CUST_29916_PI429010886 | N60815 | 0.031843 | 12 | 0 | | | | | | 637.6126 | 782.7144 | 805.2979 | 710.9044 | 604.5341 | 768.0399 | 538.0354 | 536.7924 | 598.0415 | 787.1975 | 502.9268 | 866.5099 | 587.1049 | 854.7156 | 953.9498 | 687.0726 | 609.869 | 733.1697 | 614.8997 | 676.7937 | 815.0535 | 771.0971 | 689.3131 | 584.697 | 791.5217 | 900.4832 | 900.857 |
| CUST_40880_PI429010886 | M56288 | 0.031851 | 18 | 3 | 242010783 | conserved hypothetical protein [Pediculus humanus | Response to stress | Response to oxidative stress | 7.67E-34 | 29.86645 | 15.13796 | 7.398161 | 64.23456 | 45.61086 | 92.79213 | 60.29484 | 41.25188 | 43.5699 | 4.956681 | 11.23137 | 3.697853 | 21.80952 | 20.28929 | 36.41652 | 31.33456 | 40.83694 | 52.29382 | 43.75138 | 7.897946 | 5.304061 | 43.65882 | 17.49952 | 54.5396 | 24.54709 | 57.26217 | 9.462739 |

| Probe ID | ID | p-value | Period | Phase | EBI ID | Annotation | GO category | Go term | E-value | 0 | 0 | 0 | 3 | 3 | 3 | 6 | 6 | 6 | 9 | 9 | 9 | 12 | 12 | 12 | 15 | 15 | 15 | 18 | 18 | 18 | 21 | 21 | 21 | 24 | 24 | 24 |
|---|---|---|---|---|---|---|---|---|---|---|---|---|---|---|---|---|---|---|---|---|---|---|---|---|---|---|---|---|---|---|---|---|---|---|---|---|
| CUST_4606_PI429010886 | N17117 | 0.031851 | 18 | 9 | | | | | | 642.1649 | 801.3257 | 1434.403 | 112.0774 | 82.90011 | 19.88004 | 403.8464 | 2081.941 | 3296.897 | 1547.246 | 833.259 | 2178.033 | 1670.981 | 1782.524 | 1276.133 | 397.2225 | 1049.194 | 186.2582 | 613.8279 | 2786.84 | 619.2505 | 600.7483 | 1946.546 | 95.25723 | 1498.845 | 863.1365 | 1464.638 |
| CUST_17106_PI429010886 | N37370 | 0.031851 | 18 | 3 | | | | | | 747.636 | 796.983 | 674.6226 | 851.7359 | 798.3051 | 1038.971 | 763.8572 | 606.0546 | 573.5264 | 628.02 | 747.806 | 596.3171 | 502.9268 | 726.6345 | 691.1905 | 744.2633 | 984.9879 | 975.7147 | 719.37 | 748.4841 | 621.3573 | 845.2412 | 746.9886 | 771.3694 | 637.7954 | 770.7121 | 626.6888 |
| CUST_51232_PI429010886 | M92717 | 0.031852 | 15 | 0 | | | | | | 844.11 | 1011.687 | 815.8741 | 601.3413 | 1005.284 | 510.9063 | 726.086 | 420.5887 | 495.5775 | 552.5713 | 498.9877 | 535.6554 | 493.7491 | 815.5774 | 284.9062 | 1073.527 | 457.3191 | 618.1584 | 535.7875 | 672.3398 | 719.6282 | 505.8775 | 411.1682 | 497.8615 | 358.4672 | 582.2842 | 437.4848 |
| CUST_32113_PI429010886 | M3254 | 0.03186 | 15 | 3 | 387165442 | C-type lectin 1 [Marsupenaeus japonicus] | | | 1.04E-16 | 68.79393 | 32.82252 | 67.37108 | 84.1647 | 46.9109 | 141.2568 | 69.28034 | 34.03186 | 56.18196 | 23.8404 | 40.69465 | 31.71799 | 27.85193 | 28.93678 | 24.09426 | 95.99909 | 38.07688 | 102.7959 | 102.4647 | 35.78629 | 29.66445 | 39.00381 | 33.05172 | 46.73511 | 37.50442 | 60.08957 | 28.53623 |
| CUST_27311_PI429010886 | N55826 | 0.03186 | 15 | 12 | | | | | | 9.313914 | 13.25045 | 19.25031 | 14.01751 | 14.89947 | 18.12837 | 12.88874 | 13.58792 | 14.91902 | 18.35292 | 17.15538 | 18.93949 | 24.36596 | 20.46944 | 30.66153 | 34.35136 | 20.98763 | 8.219245 | 10.18097 | 18.53665 | 15.93774 | 16.0587 | 17.54073 | 10.24932 | 24.35068 | 25.01145 | 28.55239 |
| CUST_55969_PI429010886 | M112375 | 0.031869 | 18 | 12 | | | | | | 718.4589 | 855.518 | 933.1038 | 720.9482 | 733.7989 | 756.7863 | 630.9156 | 783.1618 | 845.9343 | 836.4238 | 808.2235 | 1179.34 | 845.457 | 1130.431 | 937.6859 | 961.1254 | 871.9238 | 869.29 | 798.5706 | 962.6096 | 792.4242 | 986.0892 | 683.1376 | 686.896 | 1144.142 | 780.6851 | 1078.601 |
| CUST_16608_PI429010886 | N36560 | 0.031876 | 15 | 3 | | | | | | 114.9742 | 65.14347 | 116.516 | 140.4217 | 117.7023 | 299.9648 | 145.4633 | 94.85349 | 80.44402 | 73.18236 | 135.0089 | 56.70249 | 63.09759 | 81.44042 | 54.15061 | 68.46628 | 239.3095 | 191.2388 | 152.7363 | 62.583 | 155.0084 | 89.66356 | 165.2222 | 187.1216 | 86.06426 | 132.0735 | 60.89775 |
| CUST_34807_PI429010886 | M10745 | 0.031883 | 21 | 3 | | | | | | 326.1708 | 245.408 | 213.4066 | 174.3884 | 272.9711 | 326.6284 | 253.025 | 252.1679 | 218.636 | 317.0842 | 217.2265 | 263.6263 | 127.8287 | 197.4111 | 235.5325 | 263.5218 | 218.6882 | 295.0205 | 244.9337 | 187.5374 | 253.2546 | 359.3716 | 228.9547 | 299.389 | 325.4382 | 407.8386 | 458.0989 |
| CUST_25817_PI429010886 | N53108 | 0.031901 | 24 | 21 | | | | | | 9.180913 | 5.424341 | 13.13514 | NA | 9.745966 | 5.908014 | 5.392196 | 6.39392 | 7.3856 | 3.709813 | 8.964575 | 8.984223 | 6.407772 | 11.11919 | 10.27034 | 11.65798 | 13.01679 | NA | 9.343228 | 11.21427 | 5.071599 | 9.371383 | 13.67691 | 14.19523 | 9.744311 | 7.901749 | 5.345394 |
| CUST_1842_PI429010886 | N10862 | 0.03192 | 12 | 0 | | | | | | 214.7985 | 634.9072 | 739.6144 | 22.14395 | 77.49116 | 49.83701 | 114.5611 | 176.2631 | 662.2599 | 600.7483 | 327.598 | 798.8942 | 634.7435 | 650.7381 | 583.9827 | 544.9594 | 199.9465 | 235.5325 | 397.8439 | 1429.583 | 396.6524 | 934.5759 | 617.3843 | 43.9229 | 790.1209 | 793.0472 | 1507.459 |
| CUST_3586_PI429010886 | N14826 | 0.031931 | 18 | 3 | 157123293 | hypothetical protein AaeL_AAEL009478 [Aedes aeg | | | 5.04E-07 | 599.927 | 477.7327 | 194.6782 | 6920.477 | 8352.868 | 7579.447 | 2796.121 | 4873.025 | 286.5169 | 46.93642 | 4557.188 | 35.69567 | 1210.418 | 50.90018 | 309.517 | 53.22735 | 1600.359 | 220.485 | 2786.84 | 36.82802 | 3644.859 | 51.52932 | 2256.115 | 4098.525 | 48.69326 | 1294.143 | 59.94958 |
| CUST_3656_PI429010886 | N14974 | 0.031997 | 18 | 6 | 532759720 | antimicrobial peptide type 1 precursor Ic [Pandalop | | | 1.16E-18 | 87.2868 | 117.2428 | 50.74837 | 56.56684 | 241.1275 | 39.65367 | 1669.785 | 55.71375 | 2249.222 | 202.4642 | 70.86123 | 607.5979 | 897.4259 | 663.4104 | 595.7445 | 346.2493 | 741.2403 | 87.01627 | 121.2986 | 31.91824 | 142.963 | 293.4659 | 65.36011 | 754.1104 | 1169.504 | 399.5497 | 1467.042 |
| CUST_18429_PI429010886 | N39783 | 0.031997 | 18 | 6 | | | | | | 24.55823 | 160.3449 | 15.99082 | 75.58983 | 74.51037 | 32.45148 | 946.4318 | 114.9975 | 1024.31 | 157.2555 | 28.58102 | 309.7678 | 301.849 | 348.8261 | 299.9058 | 198.8522 | 365.3412 | 16.08973 | 154.9129 | 14.34646 | 190.2685 | 312.7841 | 27.60836 | 354.5586 | 918.9933 | 75.45906 | 449.3849 |
| CUST_46406_PI429010886 | M72565 | 0.032008 | 24 | 0 | 321470123 | hypothetical protein DAPPUDRAFT_303592 [Daphn | | | 7.91E-37 | 15.29079 | 15.37649 | 18.35726 | 12.07791 | 12.46866 | 7.840127 | 14.31366 | 6.897073 | 24.79562 | 17.30092 | 10.52396 | 11.74252 | 6.712633 | 11.05263 | 11.28199 | 21.25262 | 7.434035 | 13.90384 | 12.26356 | 28.81723 | 12.97975 | 14.71101 | 20.51967 | 15.05054 | 16.67738 | 16.55564 | 17.62531 |
| CUST_19710_PI429010886 | N42050 | 0.032009 | 24 | 21 | | | | | | 297.4787 | 1094.409 | 721.1961 | 404.8823 | 558.5528 | 265.6039 | 275.6487 | 341.9848 | 546.956 | 956.4623 | 754.3579 | 971.7833 | 1233.738 | 2089.376 | 1839.737 | 1616.675 | 511.3259 | 1080.655 | 377.1617 | 1402.469 | 530.8519 | 2766.721 | 2195.833 | 999.4148 | 2292.412 | 785.0368 | 3627.549 |
| CUST_15158_PI429010886 | N34099 | 0.032014 | 18 | 9 | | | | | | 68.63903 | 188.8906 | 368.9642 | 252.0389 | 39.3558 | 22.77856 | 251.8524 | 303.854 | 287.6352 | 507.983 | 661.523 | 1000.816 | 25.40127 | 441.7468 | 220.5435 | 326.7925 | 124.0489 | 169.8718 | 49.28537 | 375.5434 | 195.4236 | 264.7951 | 269.0306 | 40.44483 | 56.60227 | 135.7237 | 280.165 |
| CUST_49021_PI429010886 | M83649 | 0.032028 | 21 | 15 | | | | | | 107.6252 | 38.14082 | 40.03501 | 32.23977 | 58.28332 | 24.73576 | 82.50766 | 58.88281 | 81.73063 | 74.75705 | 106.3073 | 65.01299 | 46.35165 | 109.167 | 5.365255 | 104.8092 | 95.78605 | 173.944 | 47.20692 | 96.80182 | 33.35513 | 70.41805 | 55.33057 | 44.82294 | 73.83558 | 35.9416 | 25.82739 |
| CUST_54641_PI429010886 | M106998 | 0.032039 | 12 | 3 | | | | | | 48.44291 | 30.04719 | 7.070433 | 57.80685 | 49.24687 | 78.78492 | 26.38163 | 28.99306 | 18.49721 | 11.10065 | 21.74923 | NA | 37.68617 | NA | 27.03336 | 27.93228 | 68.2359 | 77.25415 | 52.92115 | 7.496731 | 12.89985 | 27.77246 | 6.55575 | 50.67294 | 19.11241 | 52.80847 | 5.51219 |
| CUST_38504_PI429010886 | M27503 | 0.03204 | 21 | 3 | | | | | | 411.957 | 494.8206 | 602.4628 | 553.514 | 694.7611 | 539.016 | 524.852 | 615.7661 | 647.6977 | 629.6206 | 538.5366 | 550.4344 | 347.4375 | 462.0916 | 452.1705 | 678.5082 | 571.094 | 659.0457 | 482.287 | 432.1375 | 420.8347 | 925.4363 | 490.166 | 479.9166 | 824.9455 | 580.436 | 881.8414 |
| CUST_27894_PI429010886 | N56896 | 0.032045 | 21 | 18 | | | | | | 26.23144 | 21.2243 | 27.35532 | 24.23621 | 27.01961 | 21.74369 | 25.43424 | 16.43668 | 26.5649 | 32.34301 | 19.42864 | 18.49894 | 14.97 | 9.82119 | 9.108247 | 25.66555 | 30.68045 | 20.47876 | 31.71523 | 23.33049 | 35.97533 | 29.41046 | 19.66479 | 37.44113 | 49.02873 | 17.42054 | 23.99602 |
| CUST_43411_PI429010886 | M62977 | 0.032048 | 15 | 3 | 241598567 | plexin A, putative [Ixodes scapularis] >gi\|21550047! | Developmental process | Multicellular organismal development | 1.06E-27 | 137.9678 | 144.3925 | 151.8603 | 132.1519 | 252.8575 | 120.2323 | 157.3097 | 152.7823 | 83.63981 | 92.41138 | 143.9014 | 121.6679 | 53.28731 | 78.59764 | 90.77809 | 373.4486 | 146.5997 | 276.1029 | 148.6709 | 178.3785 | 161.861 | 233.5227 | 82.70078 | 191.3918 | 127.5355 | 70.94855 | 100.955 |
| CUST_40792_PI429010886 | M56091 | 0.032058 | 24 | 0 | | | | | | 562.0583 | 653.9628 | 652.3862 | 571.9057 | 558.0588 | 584.1415 | 482.1854 | 534.0061 | 573.864 | 534.8364 | 550.0892 | 568.6876 | 482.0705 | 751.4303 | 471.7451 | 703.3407 | 576.5142 | 614.5524 | 560.4476 | 601.3413 | 580.6994 | 597.0282 | 644.4395 | 601.962 | 757.419 | 565.6978 | 754.5887 |
| CUST_55412_PI429010886 | M109991 | 0.032071 | 15 | 3 | | | | | | 435.6005 | 132.6083 | 131.1802 | 821.4933 | 824.3794 | 1673.62 | 700.208 | 352.7559 | 238.0396 | 8.440373 | 547.8035 | NA | 292.8985 | 6.7534 | 158.2669 | 57.90171 | 2232.866 | 1247.588 | 847.8005 | NA | 427.3564 | 18.33154 | 334.1058 | 845.6802 | 9.60066 | 641.17 | NA |
| CUST_43588_PI429010886 | M63424 | 0.032084 | 24 | 21 | | | | | | 46.47367 | 42.14262 | 50.82916 | 38.98692 | 38.53689 | 61.38183 | 37.41832 | 41.48865 | 50.4975 | 55.08741 | 42.64756 | 53.61735 | 37.45905 | 61.2083 | 60.26272 | 42.52046 | 43.71284 | 45.98092 | 48.65694 | 44.2637 | 55.38718 | 57.97853 | 61.2655 | 72.60868 | 51.72418 | 55.17531 | 69.87848 |
| CUST_16512_PI429010886 | N36361 | 0.032084 | 24 | 21 | 511919090 | PREDICTED: zinc finger protein 391 isoform X1 [Mus | Nucleic acid metabolism | Regulation of gene expression | 1.71E-08 | 12.28913 | 11.91905 | 15.15241 | 10.85446 | 15.85477 | 19.31538 | 4.793918 | 13.34705 | 7.86472 | 17.25337 | 13.50761 | 11.50211 | 11.00785 | 22.35102 | 13.90831 | 16.56737 | 13.30348 | 16.1679 | 19.95159 | 13.32018 | 11.11786 | 30.19994 | 17.86172 | 22.65868 | 15.03778 | 22.76882 | 9.867365 |
| CUST_28587_PI429010886 | N58244 | 0.032084 | 24 | 21 | | | | | | 129.4353 | 131.1004 | 136.9448 | 101.0858 | 139.8784 | 148.3243 | 128.7232 | 81.44839 | 108.7243 | 154.6378 | 104.2347 | 180.016 | 127.601 | 169.0201 | 162.775 | 181.6229 | 102.9324 | 176.9135 | 187.276 | 118.2001 | 154.2483 | 183.3903 | 145.7727 | 326.0308 | 162.6071 | 138.4789 | 158.6127 |
| CUST_7295_PI429010886 | N21335 | 0.032091 | 21 | 15 | | | | | | 471.7451 | 509.603 | 537.4529 | 293.704 | 482.4063 | 446.7333 | 381.8763 | 343.8588 | 297.14 | 495.3471 | 563.1994 | 400.3298 | 268.6688 | 356.7158 | 400.5132 | 833.259 | 541.8489 | 575.8176 | 541.4466 | 499.1257 | 400.5981 | 493.0904 | 371.5506 | 375.6905 | 676.1626 | 334.9967 | 651.9918 |
| CUST_20387_PI429010886 | N43349 | 0.032094 | 15 | 12 | 332029856 | Tyrosine-protein phosphatase non-receptor type 23 | | | 9.98E-38 | 30.89566 | 36.55477 | 30.90791 | 26.27126 | 34.36554 | 27.79102 | 26.30031 | 27.77642 | 26.78191 | 40.66388 | 31.34763 | 24.8962 | 29.75085 | 44.16686 | 40.48796 | 39.98138 | 32.63646 | 36.29774 | 42.5506 | 38.6481 | 30.01967 | 35.19889 | 13.19779 | 25.60312 | 35.99615 | 24.03363 | 34.1984 |
| CUST_13006_PI429010886 | N30429 | 0.032117 | 24 | 0 | | | | | | 16.78445 | 16.21134 | 17.21816 | 14.926 | 13.4766 | 10.76236 | 6.805373 | 10.88451 | 5.553832 | 13.01876 | 7.818797 | 20.18328 | 10.62249 | 10.08948 | 11.22009 | 12.90269 | 10.24932 | 15.17526 | 10.57646 | 12.91897 | 12.63519 | 8.830666 | 13.28864 | 19.42496 | 17.11368 | 12.47348 | 30.37057 |
| CUST_13810_PI429010886 | N31814 | 0.032117 | 24 | 0 | | | | | | 220.7874 | 208.027 | 241.4805 | 179.3477 | 176.8527 | 210.3335 | 156.8208 | 174.5449 | 174.3026 | 210.3335 | 180.7887 | 170.9183 | 133.668 | 164.4185 | 108.064 | 225.4062 | 220.5435 | 191.1723 | 191.3082 | 146.9171 | 182.494 | 189.8565 | 166.8689 | 176.1138 | 230.6606 | 165.5714 | 197.6731 |
| CUST_17897_PI429010886 | N38793 | 0.032117 | 24 | 0 | | | | | | 17.65858 | 21.05098 | 17.6966 | 13.66784 | 10.338 | 23.25478 | 9.02845 | 20.71589 | 16.79088 | 15.72277 | 16.16875 | 16.80068 | 7.617624 | 7.507529 | 4.189741 | 18.97898 | 14.42793 | 10.32153 | 6.030949 | 21.19291 | 9.56672 | 20.95495 | 16.17624 | 13.21125 | 19.09764 | 16.56922 | 15.10512 |
| CUST_29157_PI429010886 | N59306 | 0.032117 | 24 | 0 | | | | | | 110.9853 | 114.6323 | 108.1041 | 73.91159 | 87.32671 | 96.1698 | 56.69148 | 52.46763 | 68.9769 | 120.9579 | 100.9909 | 93.84895 | 70.04597 | 81.95659 | 63.90408 | 102.639 | 84.05405 | 83.05308 | 72.57482 | 126.9474 | 104.8392 | 149.3496 | 88.92819 | 52.11928 | 143.2735 | 90.25676 | 138.8211 |
| CUST_30790_PI429010886 | N62705 | 0.032117 | 24 | 0 | | | | | | 125.4314 | 137.2699 | 155.8611 | 137.1056 | 91.40169 | 112.7046 | 64.99854 | 103.3491 | 117.7722 | 128.673 | 97.85969 | 117.0501 | 87.96508 | 88.94052 | 128.037 | 124.3983 | 77.35795 | 122.404 | 62.69277 | 118.4142 | 92.53187 | 149.2244 | 132.8034 | 71.95612 | 139.419 | 124.3644 | 153.8729 |
| CUST_25154_PI429010886 | N51880 | 0.032136 | 24 | 3 | | | | | | 18.78533 | 15.444 | 13.49282 | 16.17213 | 19.57173 | NA | 19.11241 | 8.86752 | 8.956327 | 17.66287 | 8.573429 | 9.493787 | 9.008803 | 10.62249 | 13.41264 | 8.148866 | 6.823408 | NA | 7.697859 | 9.216332 | 5.497955 | 20.92823 | NA | NA | 16.26432 | 9.917243 | 7.941557 |
| CUST_37994_PI429010886 | M22660 | 0.032154 | 12 | 3 | | | | | | 1208.126 | 1272.526 | 33.24397 | 2325.953 | 1031.453 | 2315.282 | 1245.533 | 1095.532 | 930.8848 | 51.85122 | 300.2063 | 43.37106 | 1047.078 | 326.9766 | 642.1649 | 1366.025 | 1819.925 | 2292.412 | 1716.583 | 210.2559 | 125.4876 | 1276.669 | NA | 1544.651 | 801.0442 | 1243.416 | NA |
| CUST_35999_PI429010886 | M14249 | 0.032157 | 24 | 12 | | | | | | 69.16983 | 60.77185 | 53.88401 | 59.59032 | 54.61808 | 78.07189 | 76.12074 | 55.99321 | 57.256 | 82.67004 | 56.36899 | 87.87096 | 65.97871 | 70.68641 | 95.57811 | 85.38257 | 61.64915 | 70.48615 | 60.83438 | 69.36484 | 64.00351 | 83.11485 | 71.37464 | 60.03661 | 51.73816 | 49.11669 | 69.06525 |
| CUST_52216_PI429010886 | M96747 | 0.032186 | 21 | 3 | | | | | | 204.1442 | 279.5177 | 245.5292 | 445.4133 | 258.0874 | 270.4372 | 226.8032 | 187.7096 | 166.8454 | 214.6269 | 167.1087 | 214.7331 | 158.0023 | 230.3934 | 215.0582 | 207.6455 | 176.2631 | 228.9547 | 170.3395 | 188.9157 | 242.8033 | 281.7845 | 257.9077 | 141.4697 | 336.4536 | 277.125 | 191.1893 |
| CUST_1081_PI429010886 | N8431 | 0.032197 | 12 | 0 | | | | | | 68.43089 | 91.4434 | 64.55964 | 63.06953 | 56.57261 | 38.32192 | 49.56937 | 55.45229 | 50.56281 | 67.10317 | 43.93404 | 74.316 | 61.86952 | 68.33006 | 69.44924 | 59.68907 | 45.18835 | 60.82746 | 50.64584 | 61.44485 | 59.1427 | 76.95909 | 55.76383 | 52.2983 | 79.4217 | 48.33618 | 81.9929 |
| CUST_35851_PI429010886 | M13702 | 0.032199 | 24 | 6 | | | | | | 86.51241 | 68.91937 | 18.689 | 87.71808 | 29.00747 | 94.99099 | 162.2159 | 105.1 | 123.8281 | 19.64381 | 17.64932 | 10.87507 | 120.5667 | 10.24258 | 62.75575 | 18.69291 | 31.37421 | 20.33392 | 32.28425 | 13.72321 | 24.20353 | 50.61812 | 12.80617 | 165.0319 | 43.59595 | 58.03044 | 41.29432 |
| CUST_32072_PI429010886 | M3158 | 0.032199 | 24 | 15 | 38232236 | Armadillidium vulgare clone Avu2Apol RNA polyme | Metabolic process | Nucleobase-containing compound metabolic process | | 231.5437 | 356.2218 | 263.7458 | 193.0913 | 239.6059 | 247.4184 | 180.3041 | 218.6882 | 189.8916 | 339.8212 | 206.0656 | 317.2703 | 225.1487 | 295.0205 | 296.6828 | 413.4726 | 248.5464 | 372.0481 | 274.9598 | 274.0681 | 201.5878 | 266.851 | 292.3195 | 208.7767 | 230.9992 | 300.2764 | 290.3758 |
| CUST_9240_PI429010886 | N24285 | 0.032211 | 15 | 0 | | | | | | 29.05431 | 76.07191 | 46.75333 | 18.88563 | 51.87547 | 16.31911 | 37.96113 | 10.58644 | 17.95452 | 51.12395 | 26.19976 | 50.85762 | 13.38764 | 37.56088 | 31.19161 | 47.1063 | 30.90181 | 74.63865 | 24.18418 | 26.77723 | 19.81178 | 13.77941 | 26.39959 | 11.78768 | 9.72827 | 19.48537 | 32.56779 |
| CUST_20363_PI429010886 | N43306 | 0.032211 | 15 | 0 | | | | | | 145.5389 | 98.07031 | 149.9525 | 98.34685 | 106.7511 | 96.92806 | 77.16796 | 67.77286 | 103.1898 | 150.242 | 93.49845 | 79.043 | 84.74127 | 126.1607 | 110.3777 | 114.6697 | 145.3984 | 171.2587 | 109.9853 | 93.90071 | 126.539 | 93.34264 | 91.62991 | 124.0862 | 174.0963 | 113.4366 | 139.8784 |
| CUST_31020_PI429010886 | M480 | 0.032216 | 21 | 18 | 321461570 | hypothetical protein DAPPUDRAFT_254259 [Daphn | | | 2.59E-23 | 13.46781 | 18.7895 | NA | 24.71119 | 15.69467 | 6.328546 | 3.717408 | 65.46343 | 8.764694 | NA | 6.299452 | 3.783207 | 29.73496 | 14.07729 | 14.93734 | 23.51614 | 8.912458 | 12.11785 | 16.75298 | 60.63941 | 37.66305 | 38.02365 | NA | 15.12292 | 11.42142 | 24.23967 | 9.754482 |
| CUST_1950_PI429010886 | N11150 | 0.032242 | 21 | 15 | | | | | | 44.94077 | 38.79122 | 30.86904 | 28.99536 | 46.07297 | 44.45342 | 51.15902 | 53.37349 | 41.68251 | 30.03474 | 38.09087 | 64.33014 | 63.01798 | 60.24939 | 40.69465 | 69.72451 | 39.91731 | 55.24489 | 72.9477 | 54.20454 | 60.03661 | 40.33441 | 37.63465 | 27.75224 | 42.35962 | 39.85359 | 27.43011 |
| CUST_7939_PI429010886 | N22300 | 0.032243 | 18 | 12 | 195436302 | GK22112 [Drosophila willistoni] >gi\|300681124\|sp\| | Transport | Transmembrane transport | 2.97E-48 | 86.12374 | 73.76113 | 161.7557 | 38.11258 | 27.95174 | 37.21299 | 63.3148 | 187.4758 | 123.0412 | 134.1419 | 55.5381 | 92.37125 | 110.7189 | 146.9814 | 93.09068 | 68.2359 | 100.4445 | 54.80919 | 78.07189 | 98.31044 | 55.33618 | 56.41396 | 100.5781 | 45.18256 | 96.72232 | 66.2145 | 64.03635 |
| CUST_35710_PI429010886 | M13276 | 0.03226 | 18 | 15 | | | | | | 164.1721 | 164.3449 | 128.0504 | 125.6357 | 152.4677 | 156.5024 | 138.6787 | 128.8389 | 106.1516 | 145.5578 | 123.0707 | 202.0834 | 121.8291 | 118.675 | 150.286 | 202.5331 | 170.8572 | 191.6398 | 152.0093 | 157.4409 | 122.1524 | 223.7478 | 145.899 | 139.4961 | 148.6917 | 132.1335 | 93.03197 |
| CUST_7302_PI429010886 | N21342 | 0.032283 | 21 | 12 | | | | | | 81.94846 | 88.24844 | 99.95963 | 98.98439 | 93.00061 | 63.40813 | 79.08585 | 144.7064 | 120.5667 | 121.7504 | 96.11914 | 147.5068 | 147.3848 | 114.6697 | 138.2104 | 96.46972 | 90.57431 | 98.42661 | 91.35555 | 242.9704 | 75.92185 | 129.1009 | 90.95085 | 60.80817 | 102.3532 | 95.45887 | 114.0787 |
| CUST_43030_PI429010886 | M61904 | 0.032286 | 12 | 3 | | | | | | 332.0505 | 378.6086 | 246.7846 | 347.2399 | 297.209 | 361.5394 | 315.4574 | 281.447 | 254.2781 | 287.2312 | 285.8071 | 299.389 | 273.6399 | 324.4624 | 374.9159 | 437.4103 | 343.9276 | 332.0008 | 342.5053 | 263.7458 | 306.2716 | 281.2623 | 245.6875 | 307.1296 | 299.285 | 308.8946 | 295.8079 |
| CUST_47182_PI429010886 | M75089 | 0.032286 | 12 | 9 | | | | | | 100.2724 | 132.0735 | 1479.619 | 18.20618 | 34.59277 | 18.37346 | 276.2557 | 441.7468 | 1328.909 | 1308.744 | 195.8687 | 824.5491 | 597.1763 | 921.275 | 46.3344 | 5.447638 | 484.607 | 32.50599 | 118.1115 | 337.3367 | 204.0863 | 580.0174 | 1859.87 | 66.41721 | 2219.95 | 75.40046 | 2296.235 |
| CUST_47352_PI429010886 | M75744 | 0.032286 | 12 | 0 | 328899629 | ribosomal protein L36 [Procambarus clarkii] | Protein metabolism | Ribosome biogenesis | 1.7E-14 | 1851.755 | 3288.575 | 2016.816 | 1742.655 | 1747.319 | 1809.996 | 1776.211 | 1909.003 | 1942.982 | 2704.165 | 1981.218 | 3029.514 | 2125.97 | 1973.742 | 2062.191 | 2725.555 | 1497.781 | 1676.557 | 1613.342 | 2060.137 | 2040.559 | 2500.44 | 1974.817 | 1946.546 | 2299.417 | 2138.481 | 1983.417 |
| CUST_38204_PI429010886 | M24132 | 0.032322 | 21 | 0 | | | | | | 10.4869 | 15.54357 | 10.93036 | 9.945177 | 7.933391 | NA | 4.66275 | 6.527295 | 4.813825 | 8.207161 | 4.734116 | 9.275747 | 6.738368 | 8.972594 | 7.680335 | 5.558712 | NA | NA | 7.350712 | 14.66496 | 8.5134 | 6.747408 | NA | NA | 8.613049 | 7.585958 | 10.61167 |
| CUST_6605_PI429010886 | N20338 | 0.032336 | 18 | 3 | | | | | | 15.35034 | 15.21068 | 11.63852 | 12.47694 | 20.24671 | 14.17029 | 14.4689 | 18.67218 | 14.89947 | 6.407772 | 8.10577 | 9.786144 | 10.04812 | 15.09377 | 11.89545 | 12.92033 | 11.91408 | 10.56393 | 15.7975 | 16.91321 | 12.73533 | 30.18791 | 10.00867 | NA | 22.41298 | 11.38637 | 3.770056 |
| CUST_48055_PI429010886 | M78714 | 0.032364 | 24 | 18 | | | | | | 10.87507 | 5.891455 | 13.11618 | 9.06304 | 9.69944 | 5.844471 | 6.876408 | 7.901749 | 11.37788 | 8.353787 | 4.659099 | 15.69237 | 13.03254 | 21.68921 | 14.20342 | 11.98928 | 7.08513 | 7.91474 | 17.54318 | 12.27925 | 24.30007 | 16.89973 | 19.98178 | 9.84998 | 10.02441 | 19.45151 | 11.90309 |
| CUST_8441_PI429010886 | N23042 | 0.032364 | 21 | 0 | 405962076 | Agmatinase, mitochondrial [Crassostrea gigas] | Metabolic process | Nitrogen compound metabolic process | 6.64E-94 | 25.97958 | 38.68077 | 25.76223 | 21.81748 | 23.93111 | 18.0662 | 12.34688 | 11.21427 | 14.84417 | 24.2252 | 35.01361 | 24.6471 | 20.47876 | 24.8962 | 25.46966 | 28.44009 | 48.09585 | 19.86843 | 25.25647 | 24.51944 | 22.63022 | 32.17021 | 24.07489 | 30.6438 | 41.63417 | 19.11453 | 39.97721 |
| CUST_45223_PI429010886 | M68391 | 0.032409 | 24 | 12 | | | | | | 202.301 | 143.2735 | 213.7802 | 167.982 | 198.0363 | 149.7694 | 209.3591 | 228.3136 | 231.7343 | 203.7326 | 200.0776 | 332.1138 | 226.0846 | 283.788 | 251.0607 | 225.8046 | 204.3871 | 240.9894 | 189.8316 | 213.366 | 206.8026 | 242.9037 | 234.3391 | 212.8309 | 209.4461 | 169.2006 | 286.9542 |
| CUST_43426_PI429010886 | M63019 | 0.032436 | 24 | 3 | | | | | | 16.16294 | 7.294658 | 14.79665 | 16.77789 | 16.22239 | 46.41206 | 8.042965 | 16.3435 | 6.152294 | 11.56735 | 9.316937 | 12.85382 | 7.997831 | 7.250623 | 17.63614 | 16.31198 | 7.131715 | 17.66603 | 6.630788 | 7.940312 | 3.686535 | 8.610816 | 4.636066 | 8.639749 | 15.3003 | 18.0708 | 6.482775 |
| CUST_227_PI429010886 | N2855 | 0.032436 | 24 | 21 | | | | | | 20.09671 | 18.48442 | 18.85135 | 23.34418 | 17.27797 | 30.18124 | 11.51911 | 15.79004 | 12.87351 | 24.6471 | 16.78585 | 28.81723 | 15.25038 | 31.25182 | 21.37183 | 19.66703 | 18.70704 | 17.69892 | 16.80616 | 28.06711 | 26.20852 | 30.24809 | 26.79789 | 23.75572 | 25.50611 | 20.92648 | 26.08978 |
| CUST_6316_PI429010886 | N19939 | 0.03246 | 24 | 15 | | | | | | 948.388 | 1038.971 | 837.3907 | 920.0256 | 883.6545 | 1027.258 | 724.3304 | 793.0472 | 696.9471 | 849.9143 | 953.5865 | 1019.044 | 627.0644 | 900.1864 | 829.1909 | 1199.593 | 1036.891 | 1165.934 | 907.1459 | 1294.143 | 978.4784 | 950.5113 | 652.7665 | 885.7556 | 1115.083 | 1020.365 | 932.1915 |
| CUST_1363_PI429010886 | N9408 | 0.03246 | 18 | 9 | | | | | | 54.56432 | 58.66467 | 79.26193 | 49.14895 | 58.20859 | 49.16869 | 71.14036 | 51.24109 | 68.56023 | 72.32108 | 60.78457 | 107.9401 | 66.27543 | 89.00024 | 73.1185 | 77.72793 | 55.68518 | 70.23629 | 60.73689 | 78.0365 | 59.41199 | 78.60763 | 56.07774 | 42.33801 | 102.0348 | 66.20577 | 93.33448 |
| CUST_40303_PI429010886 | M52975 | 0.032464 | 15 | 9 | | | | | | 21.93936 | 35.37802 | 45.77355 | 27.41751 | 19.3883 | 27.4871 | 29.53468 | 21.75964 | 46.79864 | 45.53479 | 28.58408 | 43.26125 | 24.14057 | 62.21051 | NA | 23.3977 | 21.53946 | 78.84971 | 19.51952 | 19.04546 | 24.92176 | 8.633134 | 44.75093 | 30.49715 | 29.41046 | 54.96916 | 53.1338 |
| CUST_6578_PI429010886 | N20295 | 0.032482 | 21 | 15 | | | | | | 36.45046 | 37.01331 | 34.21609 | 35.71333 | 30.55308 | 48.6393 | 26.72979 | 25.41871 | 20.32379 | 39.13986 | 30.49715 | 39.57145 | 25.40325 | 24.30007 | 28.29864 | 40.20059 | 47.29592 | 41.53959 | 34.14111 | 42.35334 | 28.24069 | 39.63454 | 27.65747 | 39.04491 | 36.80914 | 31.36464 | 29.13595 |
| CUST_4261_PI429010886 | N16349 | 0.0325 | 24 | 18 | | | | | | 146.1343 | 103.2145 | 151.7333 | 90.04123 | 98.74368 | 172.1318 | 113.0325 | 97.37669 | 90.36196 | 73.95574 | 141.0607 | 149.7134 | 79.84151 | 128.2848 | 138.5786 | 130.8991 | 171.3142 | 229.2068 | 147.7682 | 165.3446 | 178.9173 | 146.4472 | 136.2681 | 136.25 | 217.2745 | 134.8111 | 175.7782 |
| CUST_53217_PI429010886 | M100772 | 0.032504 | 24 | 0 | | | | | | 13.3861 | 17.97978 | 14.66789 | 9.242542 | 14.21999 | 8.892334 | 11.43831 | 7.245575 | 6.402614 | 20.72901 | 6.542661 | 15.40175 | 7.774382 | 8.672182 | 6.822524 | 11.55948 | 9.137413 | 8.032568 | 10.88348 | 11.30106 | 6.481784 | 12.35209 | 14.44838 | 13.06014 | 15.73743 | 3.491736 | 26.16897 |
| CUST_1380_PI429010886 | N9450 | 0.032504 | 24 | 0 | 443692836 | hypothetical protein CAPTEDRAFT_152167 [Capitell | | | 6.3E-136 | 210.8247 | 216.807 | 214.1332 | 194.762 | 214.9913 | 185.9766 | 186.006 | 160.9168 | 176.4383 | 223.9394 | 177.6145 | 198.4267 | 163.6892 | 200.21 | 182.5915 | 237.9578 | 165.1872 | 210.1212 | 218.3592 | 185.5593 | 197.0135 | 281.447 | 199.1974 | 190.5009 | 329.5353 | 258.8229 | 219.6129 |
| CUST_8396_PI429010886 | N22980 | 0.032514 | 15 | 12 | 546684238 | hypothetical protein D910_11229 [Dendroctonus po | | | 3.54E-43 | 11.88893 | NA | 13.54122 | NA | 5.88693 | 3.818526 | 7.861738 | 5.160214 | 6.543554 | 13.77798 | 5.542516 | 9.87019 | NA | 14.32411 | NA | 6.187658 | 13.15317 | NA | 9.774844 | 5.81075 | 5.819588 | 8.047455 | 7.133972 | 3.801419 | 11.56577 | 10.91708 | 6.730364 |
| CUST_7926_PI429010886 | N22283 | 0.03252 | 18 | 3 | 195426970 | GK20959 [Drosophila willistoni] >gi\|194157639\|gb | | | 4.77E-08 | 146.0878 | 153.734 | 30.12812 | 171.1038 | 313.2862 | 357.2284 | 83.77617 | 67.99795 | 42.45161 | 76.00655 | 56.63828 | 27.17605 | 53.22735 | 55.73505 | 49.06607 | 121.2116 | 136.8618 | 117.1353 | 198.5143 | 27.25206 | 42.39566 | 121.4004 | 33.86006 | 166.8689 | 94.55772 | 99.87407 | 22.58452 |
| CUST_47053_PI429010886 | M74663 | 0.032546 | 18 | 3 | 9186884 | BCS-1 [Amphibalanus amphitrite] | | | 1.48E-08 | 388.6405 | 17.46532 | 424.2638 | 652.581 | 1180.515 | 2189.348 | 1617.488 | 643.0031 | 177.4241 | 7.304592 | 1616.675 | 10.83953 | 270.7613 | 84.015 | 134.0378 | 8.622966 | 2483.895 | 2058.855 | 2197.498 | NA | 1150.482 | NA | 2863.818 | 3569.653 | NA | 818.0738 | 8.830666 |
| CUST_21432_PI429010886 | N45207 | 0.032547 | 12 | 0 | | | | | | 10.85866 | 10.54361 | NA | 6.180449 | 8.953175 | 7.278197 | 5.322036 | 8.121387 | 7.493829 | 19.64813 | 4.853908 | 6.139615 | 23.45456 | 6.814705 | 9.057647 | 10.69036 | 8.730965 | 13.17448 | 6.775327 | NA | 8.128829 | 8.225765 | 13.42229 | 10.14162 | 13.76206 | 12.93141 | 21.8588 |
| CUST_52668_PI429010886 | M98389 | 0.032549 | 12 | 3 | 478253126 | hypothetical protein YQE_09748, partial [Dendroctc | | | 1.1E-12 | 21.7778 | 14.96828 | 16.80616 | 18.30388 | 18.06411 | 21.17508 | 14.99598 | 10.61167 | 19.7129 | 10.47807 | 15.10512 | 10.91945 | 11.99376 | 11.27392 | 21.93936 | 17.99464 | 22.51909 | 16.93603 | 18.17304 | 12.49458 | 16.9221 | 9.122458 | 11.56124 | 19.88374 | 20.49506 | NA | 13.36038 |
| CUST_6411_PI429010886 | N20066 | 0.032549 | 12 | 3 | | | Metabolic process | Oxidation-reduction process | | 23.13337 | 16.65858 | 16.46028 | 44.38038 | 19.87307 | 26.90259 | 38.68605 | 13.09531 | 18.56257 | 16.29403 | 4.236766 | 19.02231 | 20.20612 | 22.6017 | 26.18825 | 30.78534 | 6.782422 | 33.24682 | 8.47166 | 25.92862 | 9.560965 | 10.41181 | 12.21701 | 15.15061 | 16.93029 | 19.94747 | NA |
| CUST_4129_PI429010886 | N16081 | 0.032551 | 12 | 0 | | | | | | 219.5762 | 215.4042 | 205.3314 | 193.8001 | 201.5551 | 284.093 | 179.3477 | 161.1033 | 178.833 | 186.9659 | 215.7896 | 301.5634 | 161.7557 | 230.7819 | 316.8471 | 255.7893 | 180.016 | 220.7134 | 164.2756 | 179.3477 | 222.2031 | 213.51 | 192.1754 | 192.9815 | 229.2792 | 272.9711 | 234.823 |
| CUST_20656_PI429010886 | N43829 | 0.032557 | 12 | 9 | | | | | | 101.4116 | 247.8745 | 146.2438 | 26.45407 | 32.4969 | 17.24279 | 37.57841 | 106.5597 | 189.4567 | 193.4196 | 122.8182 | 238.6203 | 161.355 | 368.5464 | 179.5282 | 212.1657 | 59.07909 | 78.44378 | 184.2307 | 282.651 | 126.3916 | 473.1073 | 206.8694 | NA | 990.2048 | 192.586 | 404.0517 |
| CUST_34129_PI429010886 | M8843 | 0.032581 | 21 | 18 | | | | | | 667.5342 | 1504.155 | 468.7286 | 386.5159 | 901.1709 | 299.389 | 30.24099 | 21.09489 | 217.6167 | 901.9536 | 562.6249 | 1122.017 | 564.8923 | 526.5682 | 605.4467 | 1405.495 | 25.87828 | 722.796 | 518.4033 | 1174.994 | 761.273 | 1184.785 | 615.3731 | 374.5146 | 574.6243 | 468.3619 | 437.1883 |
| CUST_46784_PI429010886 | M73709 | 0.032595 | 15 | 6 | | | | | | 13.00011 | 34.84639 | 42.19441 | 28.93678 | 26.88229 | 28.00512 | 54.35449 | 28.1812 | 40.84912 | 19.44494 | 31.13983 | 53.11468 | 33.01166 | 42.15075 | 26.71164 | 15.67417 | 16.2854 | 34.58379 | 26.12256 | 25.05128 | 67.64264 | 80.32797 | 73.33698 | 55.2283 | 38.99636 | 47.93942 | 68.66774 |
| CUST_5013_PI429010886 | N18150 | 0.032595 | 15 | 3 | 261289447 | hypothetical protein BRAFLDRAFT_63199 [Branchio | Cellular Process | Other | 1.93E-14 | 319.1396 | 129.735 | 267.2122 | 351.4461 | 414.2198 | 764.7939 | 392.7909 | 272.5517 | 149.2467 | 113.0821 | 383.2896 | 110.4991 | 173.881 | 120.5952 | 197.3524 | 132.3882 | 655.0692 | 415.3047 | 693.2829 | 112.3919 | 414.2198 | 130.6774 | 410.7156 | 524.4761 | 127.3563 | 327.7802 | 118.5941 |
| CUST_7134_PI429010886 | N21087 | 0.032595 | 15 | 0 | | | | | | 1941.576 | 2356.592 | 2132.178 | 942.9477 | 1659.929 | 1937.537 | 1334.604 | 1503.323 | 1407.859 | 1901.328 | 1614.966 | 2221.45 | 1053.349 | 1915.9 | 1891.601 | 2626.728 | 1619.494 | 2669.53 | 1868.48 | 2300.758 | 1642.187 | 1875.725 | 1487.872 | 1522.93 | 2851.724 | 1834.08 | 2693.745 |
| CUST_3680_PI429010886 | N15033 | 0.032635 | 21 | 12 | 321462196 | hypothetical protein DAPPUDRAFT_325511 [Daphn | | | 8.27E-20 | 55.38216 | 46.47908 | 55.76383 | 65.43441 | 47.34484 | 52.61483 | 58.40782 | 64.33014 | 50.93999 | 73.45438 | 52.27055 | 67.03346 | 51.92326 | 80.4611 | 91.70502 | 64.87589 | 56.56158 | 53.73015 | 75.55792 | 77.08541 | 48.53244 | 34.0946 | 49.25815 | 53.78486 | 62.27945 | 66.24577 | 63.07552 |
| CUST_15762_PI429010886 | N35097 | 0.032635 | 12 | 3 | | | | | | 38.39957 | 25.27977 | 28.63701 | 63.5725 | 52.8747 | 79.34273 | 45.22377 | 39.55173 | 32.29255 | 32.67128 | 41.37211 | 19.27159 | 35.65161 | 27.91811 | 31.88231 | 45.04433 | 81.82955 | 59.82999 | 39.98138 | 34.00581 | 30.13575 | 45.75802 | 36.69684 | 48.09045 | 44.07419 | 48.50963 | 36.98139 |
| CUST_30929_PI429010886 | M241 | 0.032653 | 15 | 0 | 270007066 | hypothetical protein TcasGA2_TC013516 [Tribolium | Protein metabolism | Proteolysis | 5.84E-74 | 95.19444 | 71.2117 | 59.72927 | 86.99215 | 62.10992 | 86.39769 | 79.96242 | 65.71273 | 72.65998 | 46.8066 | 42.09528 | 43.45323 | 65.73281 | 62.67964 | 67.54243 | 89.77998 | 61.8328 | 65.64051 | 86.67105 | 65.26948 | 51.28805 | 70.63158 | 51.33619 | 77.72793 | 64.94243 | 56.74919 | 42.01359 |
| CUST_51431_PI429010886 | M93520 | 0.032654 | 15 | 9 | 546686167 | hypothetical protein D910_12814, partial [Dendroct | | | 3.71E-09 | 5.360311 | 11.6401 | 9.959454 | 13.41075 | 6.287022 | 8.496964 | NA | 10.35144 | 18.28609 | 19.91373 | 7.398161 | 17.75695 | 11.42007 | 10.75755 | 8.333 | 10.37139 | 5.49933 | 12.39257 | 8.943033 | 12.77316 | 18.80396 | 11.16751 | 22.6112 | 14.31922 | 24.77587 | 17.38798 | 19.69614 |
| CUST_43504_PI429010886 | M63222 | 0.032666 | 21 | 15 | 321475219 | hypothetical protein DAPPUDRAFT_308482 [Daphn | Metabolic process | Cellular amino acid metabolic process | 7.24E-79 | 9.578663 | 11.18163 | 19.23025 | NA | 12.60615 | 10.21387 | 14.4735 | 14.53418 | 15.58918 | 10.10137 | 10.28563 | 11.08171 | 20.28061 | 16.51607 | 27.88621 | 13.73574 | 42.06008 | 24.62111 | 12.24951 | 14.01595 | 18.65246 | 16.71521 | 20.99763 | 42.1391 | 7.835676 | 13.1549 | 11.52352 |
| CUST_36425_PI429010886 | M15869 | 0.032666 | 15 | 12 | | | | | | 232.1489 | 280.8226 | 217.0982 | 219.4136 | 204.4866 | 171.8712 | 162.5528 | 169.5063 | 184.3916 | 261.6019 | 204.658 | 236.9573 | 245.1598 | 234.6626 | 244.904 | 340.4766 | 170.9183 | 208.7767 | 218.7669 | 274.6972 | 194.4818 | 279.7821 | 190.4729 | 146.1108 | 259.8913 | 194.8827 | 216.5438 |
| CUST_25117_PI429010886 | N51811 | 0.032666 | 15 | 12 | | | | | | 62.67301 | 62.89604 | 60.12109 | 55.84573 | 51.4 | 57.52971 | 35.38465 | 38.29927 | 44.26787 | 73.88403 | 59.86271 | 62.0828 | 51.53321 | 68.52849 | 63.1995 | 72.72106 | 52.01256 | 64.59746 | 37.34183 | 64.17425 | 76.31563 | 56.49623 | 54.23167 | 31.16262 | 71.05715 | 53.3996 | 63.78913 |
| CUST_40838_PI429010886 | M56202 | 0.032668 | 18 | 3 | 195445278 | GK11959 [Drosophila willistoni] >gi\|194166340\|gb | Protein metabolism | Protein folding | 7.69E-15 | 170.3395 | 205.8446 | 230.6845 | 277.1919 | 197.6076 | 200.184 | 183.2331 | 145.9801 | 162.2159 | 202.5771 | 145.9801 | 145.5389 | 169.5547 | 190.9462 | 168.9219 | 181.5662 | 173.5211 | 182.4169 | 130.8168 | 186.9949 | 193.2364 | 234.7048 | 310.7127 | 154.738 | 194.7223 | 179.7058 | 189.4794 |
| CUST_20918_PI429010886 | N44311 | 0.032668 | 18 | 9 | | | | | | 132.4401 | 98.38324 | 120.9454 | 134.5558 | 141.8161 | 133.0251 | 121.9054 | 132.652 | 87.26148 | 173.5738 | 136.3926 | 222.4378 | 111.0786 | 157.9764 | 153.0166 | 159.5474 | 121.6835 | 143.2947 | 111.8858 | 143.2947 | 109.2372 | 152.3983 | 115.9278 | 115.7889 | 219.061 | 156.8208 | 144.77 |
| CUST_45497_PI429010886 | M69299 | 0.032692 | 15 | 9 | | | | | | 447.4667 | 451.4433 | 583.4081 | 399.641 | 376.7825 | 387.589 | 329.2355 | 392.5464 | 468.4803 | 551.1 | 443.3821 | 672.763 | 433.7019 | 651.2944 | 603.0908 | 541.31 | 433.8707 | 467.7939 | 456.5493 | 494.3438 | 426.5023 | 465.7878 | 466.4832 | 376.7168 | 615.7661 | 452.4978 | 665.3114 |
| CUST_12012_PI429010886 | N28773 | 0.03273 | 15 | 3 | 163868840 | autotransporter [Bartonella tribocorum CIP 105476 | | | 6.12E-07 | 755.0608 | 1634.318 | 467.4407 | 3130.59 | 3946.907 | 3320.318 | 1167.349 | 2659.902 | 146.2229 | 38.96253 | 976.6512 | 34.66525 | 2032.79 | 47.49605 | 1464.638 | 174.4788 | 1001.62 | 154.5663 | 2009.101 | 31.00211 | 752.803 | NA | 216.3677 | 2361.621 | 4.445946 | 466.9714 | 37.56417 |
| CUST_2456_PI429010886 | N12401 | 0.03274 | 21 | 0 | | | | | | 381.1947 | 497.7449 | 472.1948 | 275.8443 | 369.6879 | 326.4884 | 335.333 | 312.4689 | 256.9966 | 457.1179 | 360.5617 | 338.7848 | 276.3669 | 402.3792 | 299.0341 | 508.8245 | 309.7028 | 377.4127 | 392.2104 | 294.0478 | 395.5442 | 427.3564 | 518.0097 | 425.9278 | 564.2409 | 292.3676 | 435.5217 |
| CUST_25515_PI429010886 | N52554 | 0.03274 | 24 | 18 | | | | | | 316.5285 | 332.8858 | 277.7994 | 286.423 | 272.1441 | 229.8288 | 168.9693 | 178.0648 | 227.704 | 332.6812 | 297.0259 | 309.5934 | 222.4378 | 360.4157 | 256.1518 | 441.9667 | 235.5779 | 319.5751 | 254.6455 | 423.5928 | 320.9097 | 348.0084 | 305.8476 | 292.9566 | 360.1952 | 278.9452 | 417.2965 |
| CUST_35197_PI429010886 | M11868 | 0.032753 | 21 | 6 | 321478381 | hypothetical protein DAPPUDRAFT_303174 [Daphn | | | 1.97E-17 | 89.31811 | 46.70127 | 39.67691 | 85.36118 | 57.00393 | 87.2755 | 80.63947 | 77.98525 | 107.533 | 50.65259 | 48.07404 | 59.54039 | 68.45737 | 49.96713 | 93.34264 | 37.68617 | 45.08069 | 55.78991 | 38.92892 | 68.69981 | 78.52355 | 55.62148 | 63.70797 | 57.32094 | 62.64364 | 65.83727 | 62.83869 |
| CUST_36177_PI429010886 | M14897 | 0.032753 | 24 | 21 | 241595281 | NudC domain-containing protein, putative [Ixodes s | | | 6.12E-30 | 122.3027 | 62.5561 | 74.58958 | 46.74005 | 59.52293 | 47.92719 | 59.3952 | 54.60894 | 52.26081 | 106.8281 | 66.13621 | 57.00393 | 42.10755 | 59.22654 | 52.34171 | 111.7908 | 61.2083 | 167.8299 | 84.58422 | 114.7999 | 49.81881 | 120.6382 | 83.4507 | 86.12374 | 102.3996 | 66.40412 | 83.04083 |
| CUST_45257_PI429010886 | M68503 | 0.032753 | 18 | 12 | | | | | | 103.9314 | 80.54851 | 107.1033 | 80.52163 | 85.02582 | 75.22711 | 117.9903 | 118.3239 | 95.87365 | 109.7731 | 87.90722 | 109.1768 | 115.1327 | 107.7642 | 111.9784 | 106.002 | 116.178 | 86.3406 | 111.685 | 111.1116 | 97.74764 | 109.2001 | 90.85633 | 86.97645 | 107.0174 | 84.25035 | 121.9257 |
| CUST_13544_PI429010886 | N31361 | 0.032753 | 21 | 0 | | | | | | 67.28983 | 27.21328 | 78.62359 | 43.9135 | 32.51998 | 52.76183 | 64.65683 | 36.18187 | 43.78734 | 48.50046 | 51.03287 | 23.31493 | 26.69312 | 41.33633 | 18.18378 | 49.92642 | 78.30025 | 39.78497 | 47.60452 | 32.67642 | 50.74126 | 53.6056 | 58.11529 | 53.51858 | 56.62309 | 39.40978 | 57.91335 |
| CUST_23416_PI429010886 | N48803 | 0.032753 | 21 | 3 | | | | | | 94.04891 | 90.9816 | 52.37245 | 104.0821 | 154.5247 | 80.70446 | 84.20531 | 64.31019 | 59.96834 | 91.70502 | 79.82568 | 75.31286 | 67.74201 | 62.18234 | 68.80103 | 75.45183 | 61.45036 | 74.86136 | 63.06953 | 97.82533 | 81.92166 | 107.6537 | 75.96227 | 68.77778 | 96.58099 | 77.00574 | 77.86556 |
| CUST_40964_PI429010886 | M56499 | 0.03279 | 21 | 15 | | | | | | 50.92195 | 91.46281 | 43.06915 | 38.24749 | 48.36598 | 38.94325 | 61.98456 | 56.26704 | 47.94376 | 59.48755 | 49.37485 | 50.02348 | 48.70302 | 40.89152 | 35.99615 | 64.31677 | 62.16793 | 55.5381 | 52.91094 | 57.96698 | 44.58572 | 56.13335 | 42.98911 | 47.83781 | 48.35331 | 32.8503 | 43.80561 |
| CUST_46744_PI429010886 | M73575 | 0.032794 | 24 | 18 | | | | | | 12.56262 | 9.396038 | 19.81449 | 8.315583 | 9.853418 | 16.63144 | 20.8155 | 14.33928 | 10.50364 | 23.81072 | 9.418869 | 18.04537 | 18.42911 | 28.48875 | 15.76192 | 21.58196 | 24.59022 | 17.74325 | 27.25682 | 27.94276 | 17.13473 | 12.51689 | 18.46027 | 10.67575 | 18.80651 | 13.38001 | 21.0745 |
| CUST_9174_PI429010886 | N24180 | 0.032794 | 21 | 18 | 321477120 | hypothetical protein DAPPUDRAFT_305695 [Daphn | Metabolic process | Nucleobase-containing compound metabolic process | 3.1E-103 | 53.26328 | 96.20672 | 81.82955 | 70.46616 | 50.403 | 37.5742 | 34.91643 | 16.1391 | 41.27063 | 99.09557 | 57.24358 | 154.2483 | 57.40815 | 70.13886 | 91.32302 | 83.23552 | 26.97315 | 64.50028 | 65.06017 | 119.508 | 74.63865 | 98.2447 | 60.63941 | 47.79729 | 70.75343 | 45.74744 | 55.87064 |
| CUST_47464_PI429010886 | M76227 | 0.032809 | 24 | 15 | | | | | | 1115.083 | 1224.871 | 1211.945 | 1240.437 | 1126.811 | 1126.187 | 1003.547 | 965.2192 | 1040.197 | 1433.821 | 1081.105 | 1154.447 | 1112.909 | 1462.44 | 1309.784 | 1654.803 | 1297.484 | 1341.448 | 1134.542 | 1235.847 | 1157.228 | 1526.305 | 1064.697 | 1097.209 | 1556.767 | 1275.591 | 1525.167 |
| CUST_26850_PI429010886 | N54984 | 0.032809 | 18 | 3 | | | | | | 181.7533 | 68.55373 | 202.1751 | 212.6412 | 1219.823 | 594.2743 | 1249.958 | 211.1514 | 99.2772 | 98.02439 | 1542.825 | 94.84242 | 88.66977 | 93.48735 | 76.39741 | 73.47743 | 2033.91 | 339.7564 | 526.8072 | 100.542 | 1855.858 | 90.40629 | 1933.842 | 1918.015 | 131.9438 | 174.2228 | 104.0193 |
| CUST_21191_PI429010886 | N44794 | 0.032815 | 18 | 3 | | | | | | 14.8801 | 13.40566 | 14.63709 | 18.84163 | 12.05594 | 41.84554 | 23.5068 | 15.06396 | 16.42915 | 11.21427 | 12.08102 | 5.815262 | 12.73533 | 3.667917 | 10.59486 | 32.49365 | 44.40996 | 28.37529 | 33.30259 | 10.53799 | 17.13072 | 16.90667 | 33.89069 | 25.86961 | 20.23079 | 16.18695 | 13.77293 |
| CUST_46725_PI429010886 | M73528 | 0.032832 | 15 | 9 | | | | | | 10.95448 | 10.05721 | 8.858469 | 6.477097 | 8.074741 | 4.163533 | NA | 4.637252 | 10.81881 | 26.03036 | 7.268707 | 12.64236 | 6.122347 | 12.75297 | 4.292685 | 7.122745 | NA | 9.562437 | 6.109539 | 7.876336 | 5.92558 | 61.5801 | NA | 9.320692 | 11.50042 | 10.99683 | 11.66976 |
| CUST_8820_PI429010886 | N23647 | 0.032836 | 21 | 0 | | | | | | 39.16663 | 20.82009 | 39.31065 | 26.81013 | 24.73576 | 23.67058 | 34.50298 | NA | NA | 8.563205 | 13.22412 | NA | 24.2001 | 30.99084 | 12.33347 | 26.85111 | 10.13526 | 18.62971 | 31.49086 | 190.8098 | 20.38809 | 33.09246 | 24.16344 | 36.28401 | 25.34187 | 15.75973 | 11.48488 |
| CUST_20417_PI429010886 | N43404 | 0.032851 | 21 | 9 | | | | | | 56.92511 | 36.00421 | 54.19041 | 178.4123 | 79.30391 | 128.3042 | 70.75343 | 63.79444 | 47.56602 | 69.63319 | 79.63437 | 102.8187 | 73.1075 | 64.16127 | 110.737 | 84.81787 | 59.67663 | 100.1974 | 49.31188 | 63.13991 | 52.99425 | 58.91707 | 55.0673 | 69.19616 | 63.54619 | 93.86099 | 43.21123 |
| CUST_14827_PI429010886 | N33535 | 0.032859 | 18 | 15 | | | | | | 667.0295 | 806.58 | 528.5021 | 522.6581 | 480.1775 | 473.6842 | 607.5979 | 611.3709 | 572.4248 | 768.5376 | 503.2472 | 608.5272 | 740.9987 | 568.9937 | 519.0655 | 704.3489 | 736.5339 | 580.8644 | 653.8395 | 606.6133 | 388.4161 | 604.157 | 498.9877 | 497.0198 | 532.1598 | 329.8367 | 558.0588 |
| CUST_9967_PI429010886 | N25482 | 0.032887 | 24 | 6 | | | | | | 62.04982 | 38.16452 | 10.52396 | 59.84297 | 20.46617 | 37.61954 | 71.11204 | 84.03407 | 52.59799 | 18.01506 | 10.02441 | 10.52136 | 114.4714 | 23.60076 | 37.76259 | 17.35915 | 21.79421 | 16.14566 | 34.77329 | 6.229675 | 6.952548 | 22.00833 | 9.305534 | 52.31329 | 22.68707 | 58.16774 | 10.87645 |
| CUST_11761_PI429010886 | N28356 | 0.032961 | 15 | 0 | | | | | | 158.8514 | 131.9438 | 124.1467 | 110.6854 | 115.3266 | 109.7371 | 100.1156 | 115.2365 | 81.1623 | 105.4346 | 89.68808 | 95.86282 | 71.30124 | 126.1505 | 122.84 | 143.3153 | 120.9454 | 142.4863 | 121.4004 | 123.0412 | 91.58478 | 149.507 | 94.26766 | 104.0648 | 143.3418 | 93.48735 | 115.9538 |
| CUST_34193_PI429010886 | M9014 | 0.032978 | 15 | 0 | | | | | | 1052.364 | 1335.109 | 846.752 | 585.8783 | 959.2541 | 822.0802 | 596.3171 | 671.7237 | 643.0031 | 1112.064 | 866.5099 | 1034.677 | 671.3234 | 850.7537 | 890.1484 | 1234.248 | 1063.11 | 965.4945 | 898.9921 | 872.488 | 861.1597 | 1009.233 | 790.1209 | 779.1452 | 1267.407 | 794.1984 | 980.1159 |
| CUST_18882_PI429010886 | N40575 | 0.032978 | 15 | 6 | 334326753 | PREDICTED: LOW QUALITY PROTEIN: transmembrar | Protein metabolism | Proteolysis | 9.26E-13 | 42.31884 | 48.1448 | 51.59735 | 44.87845 | 55.6932 | 39.42673 | 104.2917 | 88.06388 | 81.70156 | 100.4818 | 59.16475 | 46.45397 | 75.94687 | 63.80077 | 48.79699 | 41.87987 | 42.24809 | 46.96643 | 97.62464 | 50.10243 | 61.6776 | 51.1868 | 72.00003 | 50.9289 | 44.74185 | 47.90107 | 74.40209 |
| CUST_623_PI429010886 | N6382 | 0.032978 | 15 | 3 | | | Developmental process | Muscle structure development | | 10006.61 | 9371.785 | 5726.098 | 15000.31 | 11289.07 | 10610.68 | 11983.26 | 6571.189 | 6307.408 | 4842.442 | 8129.488 | 3783.178 | 6871.867 | 3908.116 | 5349.606 | 6267.492 | 9261.186 | 9271.524 | 11908.36 | 3842.983 | 6240.976 | 7071.027 | 5035.54 | 9271.524 | 4169.34 | 7373.59 | 2638.062 |
| CUST_671_PI429010886 | N6674 | 0.032978 | 15 | 9 | | | | | | 281.874 | 546.6515 | 921.954 | 198.1233 | 142.3547 | 210.7973 | 277.4972 | 1461.256 | 3318.146 | 1379.405 | 1738.403 | 3259.251 | 825.7659 | 4080.667 | 2293.562 | 1664.492 | 961.8209 | 532.1598 | 1219.272 | 3911.313 | 752.1706 | 4557.188 | 2096.527 | 135.0881 | 6794.71 | 1534.021 | 6059.267 |
| CUST_21339_PI429010886 | N45038 | 0.03299 | 24 | 0 | | | | | | 14.48423 | 12.37483 | 16.30792 | 6.604961 | 18.02234 | 18.21393 | 12.77633 | 9.140795 | 6.473366 | 11.99536 | 8.463865 | 12.34688 | 9.07643 | 13.7877 | 6.107648 | 14.24812 | 9.343228 | 12.10746 | 24.28 | 13.20796 | 13.10037 | 11.55822 | 19.55939 | 14.47659 | 18.02985 | 9.035702 | 19.87086 |
| CUST_41817_PI429010886 | M58617 | 0.032993 | 21 | 12 | | | | | | 223.0503 | 292.0563 | 272.8585 | 215.2013 | 227.3449 | 207.8011 | 155.0302 | 231.5694 | 201.7309 | 282.0094 | 221.8177 | 297.6952 | 247.7794 | 339.9729 | 308.5953 | 328.6838 | 225.2614 | 252.0389 | 233.9751 | 293.9351 | 241.5289 | 335.333 | 242.6123 | 218.173 | 284.7868 | 233.7145 | 285.421 |
| CUST_36943_PI429010886 | M17285 | 0.032993 | 18 | 3 | | | | | | 708.9187 | 681.5488 | 738.2515 | 1591.999 | 1457.169 | 3132.563 | 2160.998 | 897.4259 | 812.8751 | 590.149 | 2017.499 | 692.5393 | 762.7979 | 750.5959 | 437.8204 | 646.0018 | 3113.095 | 2060.137 | 1163.104 | 612.793 | 1995.819 | 885.7556 | 2351.436 | 2833.332 | 862.6591 | 1490.148 | 815.5774 |
| CUST_44556_PI429010886 | M66311 | 0.032993 | 18 | 12 | | | | | | 19.03069 | 20.34203 | 22.39477 | 6.468226 | 17.01109 | 13.5897 | 22.52813 | 24.1098 | 24.96359 | 21.35972 | 18.44972 | 33.33921 | 23.5415 | 38.46426 | 28.60108 | 24.04991 | 17.62325 | 21.03821 | 20.25021 | 35.73798 | 24.93429 | 23.31493 | 18.62216 | 20.3261 | 37.30131 | 22.9227 | 35.992 |
| CUST_39844_PI429010886 | M45613 | 0.032998 | 12 | 0 | | | | | | 12.83327 | 11.07141 | 7.897325 | 9.013974 | 6.520113 | NA | 5.394188 | 3.673461 | 13.07005 | 12.39937 | 6.74367 | 12.00862 | 59.42335 | 13.71506 | 10.88684 | 9.987338 | 4.885315 | 15.1999 | 5.522656 | 6.66543 | 12.25902 | 4.301678 | 9.203374 | 11.11786 | 13.14971 | 15.38835 | 20.26285 |
| CUST_6482_PI429010886 | N20159 | 0.033 | 15 | 3 | | | | | | 2332.981 | 1837.082 | 1573.105 | 2457.891 | 2130.831 | 2527.895 | 2175.001 | 1853.071 | 1532.545 | 1408.714 | 2085.292 | 1344.532 | 1809.996 | 1159.875 | 1346.591 | 1895.191 | 3046.724 | 1742.655 | 2162.684 | 1324.078 | 1909.916 | 1642.187 | 1889.582 | 2444.229 | 1688.453 | 1826.751 | 1227.15 |
| CUST_27744_PI429010886 | N56621 | 0.033014 | 24 | 15 | | | | | | 249.3375 | 313.7524 | 245.408 | 240.1418 | 186.883 | 225.1125 | 156.4118 | 177.5136 | 179.5583 | 302.7176 | 208.4543 | 309.1695 | 212.4692 | 249.8117 | 347.3329 | 317.6417 | 264.1731 | 293.0543 | 191.5641 | 298.5904 | 205.078 | 422.7364 | 233.9751 | 235.2753 | 242.1667 | 254.7626 | 272.7482 |
| CUST_17509_PI429010886 | N38092 | 0.03302 | 15 | 0 | | | | | | 123.2423 | 173.0713 | 84.4182 | 141.2731 | 160.2794 | 127.3563 | 190.1658 | 79.0243 | 147.4281 | 95.98918 | 79.61927 | 82.6196 | 104.71 | 89.48953 | 142.4863 | 181.3561 | 89.03619 | 175.4895 | 117.3985 | 127.4851 | 93.6298 | 132.3017 | 67.20205 | 96.75892 | 74.51037 | 62.70075 | 42.15632 |
| CUST_37917_PI429010886 | M22229 | 0.033027 | 24 | 3 | 155966352 | putative cuticle protein [Lepeophtheirus salmonis] | | | 1.7E-24 | 1098.078 | 479.189 | NA | 2454.105 | 218.3262 | 1128.352 | 705.7628 | 1316.22 | 632.6214 | NA | 5.354146 | 20.6419 | 869.0196 | 194.5472 | 338.5891 | 169.1204 | 164.3449 | 305.3843 | 17.53198 | 53.86098 | 12.50047 | 179.5282 | NA | 2676.76 | 19.30884 | 1545.528 | NA |
| CUST_56982_PI429010886 | M115048 | 0.03307 | 15 | 12 | | | | | | 1884.761 | 2636.548 | 1746.637 | 1696.945 | 1727.428 | 1344.532 | 1315.293 | 1423.398 | 1565.179 | 2430.624 | 1506.006 | 2139.684 | 1760.95 | 2081.036 | 2509.921 | 2953.556 | 1478.287 | 2069.143 | 1650.111 | 2447.295 | 1895.191 | 2346.537 | 1631.709 | 1044.56 | 2529.511 | 1945.509 | 2361.621 |

| Probe ID | ID | p-value | Period | Phase | EBI ID | Annotation | GO category | Go term | E-value | 0 | 0 | 0 | 3 | 3 | 3 | 6 | 6 | 6 | 9 | 9 | 9 | 12 | 12 | 12 | 15 | 15 | 15 | 18 | 18 | 18 | 21 | 21 | 21 | 24 | 24 | 24 |
|---|---|---|---|---|---|---|---|---|---|---|---|---|---|---|---|---|---|---|---|---|---|---|---|---|---|---|---|---|---|---|---|---|---|---|---|---|
| CUST_31773_PI429010886 | M2367 | 0.03307 | 15 | 12 | | | | | | 128.4938 | 174.4075 | 128.195 | 124.6537 | 113.1785 | 131.2903 | 118.1469 | 100.1395 | 116.1174 | 139.0161 | 142.5067 | 132.652 | 109.8428 | 147.3848 | 190.2685 | 158.0023 | 104.6277 | 157.9028 | 147.1104 | 123.7589 | 136.7554 | 136.6377 | 104.9922 | 121.6679 | 144.9326 | 140.8407 | 136.5503 |
| CUST_39341_PI429010886 | M38099 | 0.03307 | 15 | 12 | | | | | | 4190.35 | 3561.268 | 8827.561 | 1923.113 | 1642.855 | 1166.811 | 4827.614 | 10259.45 | 8866.604 | 7318.038 | 1833.381 | 3496.725 | 8404.925 | 7802.451 | 4941.562 | 6168.628 | 3811.495 | 2513.183 | 2826.022 | 4322.611 | 1462.44 | 4647.339 | 3295.06 | 999.7477 | 7457.426 | 2221.45 | 9686 |
| CUST_45578_PI429010886 | M69557 | 0.03307 | 15 | 12 | 383855680 | PREDICTED: uncharacterized protein LOC10087672 | | | 4.35E-31 | 23.52324 | 27.79833 | 25.29278 | 23.95916 | 18.36981 | 28.97523 | 18.4326 | 28.4998 | 19.27396 | 31.23744 | 24.72309 | 39.25313 | 24.15101 | 63.83268 | 46.67338 | 38.73964 | 38.20449 | 40.02682 | 24.40654 | 28.16499 | 25.25069 | 36.93108 | 29.11768 | 36.85539 | 35.69567 | 42.82585 | 34.48913 |
| CUST_51702_PI429010886 | M94637 | 0.033075 | 15 | 9 | 390352602 | PREDICTED: zinc finger protein 84-like [Strongyloce | | | 1.39E-17 | 7.995482 | 5.996868 | 7.575226 | 4.388734 | 4.451742 | NA | 3.942482 | 10.87917 | 5.706029 | 17.3861 | 12.21189 | 6.821529 | 6.852264 | 9.76387 | 7.608505 | 15.62361 | 4.930335 | NA | 5.792463 | 5.444796 | NA | 13.21926 | 9.07643 | NA | 14.88378 | 7.409357 | 9.208223 |
| CUST_49886_PI429010886 | M87594 | 0.0331 | 24 | 6 | 332217572 | PREDICTED: tryptophan 2,3-dioxygenase [Nomascu | Metabolic process | Cellular amino acid metabolic process | 2.97E-11 | 22.21414 | 11.06447 | 18.68272 | NA | 7.506584 | 28.54572 | 21.50683 | 28.83526 | 25.47915 | 18.90163 | 6.004347 | 10.01823 | 15.97633 | 15.73595 | 14.71101 | 15.54715 | 12.3071 | NA | 6.159956 | 12.89444 | 7.164995 | 20.53586 | 8.130029 | NA | 18.06411 | 6.11133 | 11.01728 |
| CUST_25217_PI429010886 | N52001 | 0.033116 | 15 | 0 | | | | | | 92.95468 | 53.99181 | 52.39551 | 93.69637 | 51.6678 | 107.5904 | 78.64329 | 51.36009 | 78.60763 | 28.86885 | 28.9272 | 26.8663 | 46.64388 | 48.56555 | 57.64388 | 44.89448 | 74.21285 | 74.03663 | 35.6284 | 19.63409 | 36.61393 | 41.04712 | 36.73877 | 90.60771 | 27.68618 | 44.78041 | 20.87254 |
| CUST_45232_PI429010886 | M68415 | 0.033131 | 24 | 9 | | | | | | 11.53905 | 10.51766 | 13.06491 | 9.833527 | 10.71609 | 11.87142 | 14.24578 | 19.04932 | 24.2228 | 10.98618 | 22.90466 | 17.3497 | 10.94511 | 3.668771 | 17.83843 | 15.82858 | 9.949298 | 13.18285 | 14.82365 | 29.65277 | 26.50017 | 4.175243 | 5.327653 | 8.420369 | 6.782422 | 9.704431 | 13.6193 |
| CUST_48116_PI429010886 | M78933 | 0.033172 | 21 | 0 | | | | | | 86.05551 | 79.19112 | 88.5487 | 81.52938 | 58.66467 | 54.64518 | 95.32771 | 62.5636 | 57.26217 | 46.78356 | 53.44251 | 50.41901 | 51.7675 | 64.33014 | 71.48709 | 79.80797 | 50.546 | 65.91381 | 60.82746 | 49.09733 | 32.13794 | 76.353 | 69.9232 | 88.7086 | 61.75222 | 32.16411 | 53.63219 |
| CUST_37049_PI429010886 | M17683 | 0.033196 | 21 | 18 | 157107416 | n-acetylgalactosaminyltransferase [Aedes aegypti] | Metabolic process | Carbohydrate metabolic process | 4.13E-48 | 107.3414 | 83.0149 | 153.6022 | 40.38558 | 71.43726 | 60.24939 | 68.12178 | 60.24307 | 52.65189 | 86.00719 | 67.08962 | 137.9332 | 81.13095 | 103.4451 | 94.41213 | 71.5379 | 73.33698 | 111.7362 | 59.59032 | 118.6617 | 109.1565 | 92.73234 | 98.2447 | 93.76216 | 56.69692 | 103.2771 | 77.3067 |
| CUST_33124_PI429010886 | M6029 | 0.033204 | 21 | 18 | | | | | | 178.255 | 344.803 | 263.9741 | 203.324 | 251.2251 | 190.588 | 120.2795 | 136.3426 | 175.5504 | 269.9577 | 228.095 | 480.7772 | 204.2199 | 237.4261 | 300.2764 | 344.8512 | 144.8962 | 275.2295 | 224.3549 | 344.4277 | 329.9108 | 289.2842 | 233.8998 | 199.6316 | 236.2907 | 222.818 | 235.9573 |
| CUST_17139_PI429010886 | N37418 | 0.033209 | 15 | 9 | | | | | | 51.91715 | 59.05765 | 62.60675 | 49.98628 | 31.721 | 43.94258 | 39.63454 | 53.40548 | 64.45055 | 69.87848 | 65.60373 | 59.46444 | 56.07148 | 75.1888 | 66.26094 | 77.57474 | 49.36928 | 59.43076 | 45.15073 | 61.911 | 57.42962 | 65.85741 | 79.07032 | 41.57514 | 96.15886 | 53.02941 | 71.87825 |
| CUST_18052_PI429010886 | N39059 | 0.033228 | 15 | 12 | | | | | | 19.74282 | 99.42759 | 151.4536 | 55.01668 | 80.21197 | 145.5994 | 598.2934 | 58.58633 | 107.9401 | NA | NA | 54.84373 | 1165.934 | 1242.81 | 562.4407 | 113.5681 | 5.044094 | 94.51496 | 307.7422 | 5.868354 | 11.98753 | 83.19226 | 224.9679 | 123.0876 | 520.7262 | 249.5102 | 277.0736 |
| CUST_56480_PI429010886 | M113905 | 0.033236 | 18 | 15 | 77743875 | Euphausia superba isolate DGGE band AntEuk13-5 | | | 1.7E-111 | 64.19796 | 51.10313 | 39.38447 | 40.14953 | 44.014 | 55.5206 | 40.14648 | 37.13804 | 34.78474 | 43.5437 | 45.35819 | 44.86761 | 42.71137 | 50.61028 | 62.43324 | 74.27647 | 51.49206 | 73.87582 | 40.36236 | 59.41826 | 25.19237 | 54.1974 | 52.88081 | 38.93982 | 69.74095 | 44.34868 | 35.13913 |
| CUST_18614_PI429010886 | N40098 | 0.03324 | 18 | 3 | | | | | | 77.24703 | 91.24943 | 79.80797 | 91.7472 | 100.2191 | 101.0493 | 119.8071 | 95.39909 | 70.23023 | 77.95844 | 81.49546 | 66.2145 | 75.13415 | 69.19616 | 64.1319 | 79.0521 | 137.1423 | 88.38108 | 87.88231 | 66.36423 | 99.99373 | 92.18368 | 97.92701 | 84.13816 | 102.0104 | 66.64273 | 74.33243 |
| CUST_7923_PI429010886 | N22279 | 0.033242 | 18 | 9 | 213515230 | Dehydrogenase/reductase SDR family member 11 [ | Metabolic process | Oxidation-reduction process | 1.24E-68 | 103.6413 | 103.0626 | 73.71157 | 85.74608 | 142.2559 | 50.81627 | 53.46776 | 109.1441 | 153.8729 | 198.4542 | 236.0839 | 266.2859 | 117.2072 | 101.2912 | 89.30785 | 175.6284 | 117.6032 | 94.95262 | 103.5442 | 225.8273 | 113.1427 | 115.0086 | 71.86244 | 55.04289 | 115.9538 | 73.1185 | 105.6225 |
| CUST_32634_PI429010886 | M4642 | 0.033252 | 18 | 12 | 383849433 | PREDICTED: mediator of RNA polymerase II transcri | Nucleic acid metabolism | Regulation of gene expression | 8.12E-24 | 14.85125 | 11.53559 | 26.61953 | 16.15147 | 12.27788 | NA | 9.675354 | 38.84241 | 20.208 | 20.08697 | 26.45134 | 22.64731 | 20.30647 | 24.77587 | 162.6703 | 27.02172 | 16.16875 | 14.1623 | 24.11276 | 19.32542 | 17.89088 | 14.94106 | 15.69084 | 14.69352 | 21.5495 | 3.208088 | 18.24494 |
| CUST_45678_PI429010886 | M69880 | 0.033267 | 24 | 21 | | | | | | 91.72554 | 72.97459 | 98.26203 | 64.76438 | 77.20798 | 88.09249 | 62.36156 | 63.43828 | 79.16747 | 113.6906 | 66.19578 | 73.00251 | 64.52054 | 67.62103 | 77.59246 | 82.65719 | 79.64311 | 76.06574 | 81.59575 | 95.23078 | 87.50342 | 74.48084 | 89.31811 | 82.49299 | 84.00641 | 85.63673 | 106.7139 |
| CUST_44389_PI429010886 | M65800 | 0.033269 | 18 | 6 | | | | | | 434.7605 | 441.7468 | 631.6256 | 309.2864 | 515.0601 | 582.6377 | 582.2842 | 483.2456 | 529.7833 | 569.6586 | 487.6477 | 538.1956 | 299.7832 | 462.6916 | 157.0047 | 440.22 | 477.2259 | 436.7207 | 397.5988 | 428.5138 | 661.3119 | 453.0933 | 606.7586 | 605.4467 | 661.3119 | 574.3589 | 668.4719 |
| CUST_15978_PI429010886 | N35444 | 0.033278 | 24 | 0 | 443689970 | hypothetical protein CAPTEDRAFT_194244 [Capitell | | | 5.28E-08 | 42.35334 | 46.0364 | 61.3315 | 24.12979 | 26.04992 | 36.26694 | 26.1797 | 18.92054 | 23.55239 | 57.63741 | 47.47127 | 44.99497 | 15.91395 | 22.92066 | 22.35984 | 38.16452 | 39.72839 | 49.74933 | 25.24454 | 19.08808 | 30.86904 | 28.64039 | 53.18376 | 48.25178 | 51.69686 | 38.59666 | 48.28142 |
| CUST_28454_PI429010886 | N57979 | 0.033278 | 24 | 0 | 308220078 | LIM class homeobox transcription factor Lmx [Mner | | | 1.46E-11 | 43.32695 | 46.74367 | 37.01331 | 21.15229 | 43.04145 | 46.23463 | 25.64332 | 40.31691 | 21.0027 | 23.33262 | 28.01496 | 38.36859 | 21.71424 | 41.8887 | 20.98522 | 44.26787 | 25.98413 | 27.05448 | 22.36658 | 25.03856 | 27.77944 | 35.7636 | 25.888 | 40.37133 | 43.99672 | 47.91134 | 27.17008 |
| CUST_24753_PI429010886 | N51189 | 0.033297 | 18 | 3 | | | | | | 9.87019 | 21.22218 | NA | 22.17314 | 50.12098 | 48.8169 | 23.4129 | 5.874241 | 22.19735 | NA | 4.035125 | NA | 5.315305 | 29.83449 | 19.9741 | 37.73674 | 8.130029 | 29.50356 | 16.43668 | 12.43137 | 8.582555 | 61.3758 | NA | 38.05146 | 23.68051 | 8.045043 | NA |
| CUST_6767_PI429010886 | N20574 | 0.033309 | 15 | 9 | | | | | | 25.63785 | 23.91073 | 22.13275 | 15.06062 | 34.84989 | 24.01772 | 26.03397 | 24.37441 | 22.47267 | 36.58662 | 28.54216 | 39.48748 | 19.15208 | 34.31858 | 23.00078 | 26.83996 | 24.45653 | 21.4644 | 40.58787 | 18.81645 | 22.01177 | 32.99167 | 28.96358 | 22.31012 | 33.27763 | 41.69983 | 40.13494 |
| CUST_42997_PI429010886 | M61813 | 0.03331 | 18 | 12 | | | | | | 52.32373 | 93.87602 | 84.83474 | 73.73041 | 75.79683 | 97.19605 | 101.9095 | 82.17099 | 102.1333 | 113.8462 | 90.79757 | 150.9847 | 77.34 | 152.1653 | 166.6372 | 65.82296 | 104.4333 | 192.1174 | 64.72483 | 62.77579 | 102.0461 | 91.02902 | 155.1735 | 139.7174 | 109.7371 | 105.2081 | 124.8931 |
| CUST_39038_PI429010886 | M33821 | 0.033319 | 24 | 0 | 528314838 | ubiquitin [Schizosaccharomyces cryophilus OY26] | Metabolic process | Oxidation-reduction process | 1.77E-22 | 78.24583 | 64.35394 | 91.51834 | 50.61028 | 49.63764 | 70.58805 | 49.65196 | 73.62986 | 57.54706 | 40.22053 | 48.85338 | 72.18474 | 45.45728 | 56.95742 | 50.65756 | 49.0094 | 48.78617 | 41.52389 | 60.22941 | 65.36594 | 84.46837 | 48.62912 | 75.37057 | 55.96041 | 55.43705 | 50.92195 | 101.891 |
| CUST_13092_PI429010886 | N30559 | 0.033323 | 21 | 3 | 189240910 | PREDICTED: similar to glucosyl/glucuronosyl transfe | Metabolic process | Other | 1.22E-37 | 200.6444 | 415.7405 | 249.4607 | 767.811 | 316.7444 | 400.3298 | 230.9612 | 203.482 | 268.2743 | 271.6624 | 121.9392 | 329.9922 | 149.811 | 289.9742 | 171.5147 | 257.6478 | 121.562 | 142.2748 | 103.654 | 236.0839 | 427.6746 | 228.3136 | 446.7333 | 240.474 | 383.8497 | 415.7405 | 207.3277 |
| CUST_44074_PI429010886 | M64858 | 0.033351 | 21 | 9 | | | | | | 143.0236 | 122.5622 | 158.7737 | 75.43644 | 106.7999 | 77.02903 | 96.689 | 147.8312 | 113.9999 | 203.9899 | 149.9119 | 115.5186 | 96.30815 | 132.8611 | 104.5986 | 116.321 | 133.2798 | 108.1406 | 93.07732 | 96.28021 | 108.2633 | 95.73603 | 166.519 | 61.32192 | 79.80104 | 90.44569 | 70.92151 |
| CUST_41871_PI429010886 | M58750 | 0.033352 | 21 | 18 | | | | | | 20.37385 | 43.81829 | 19.26616 | 18.36981 | 34.71088 | 20.58696 | 18.19015 | 16.12591 | 8.440373 | 33.0759 | 19.47107 | 18.85808 | 25.86296 | 12.57863 | 19.15374 | 22.97943 | 19.32542 | 19.08808 | 47.75744 | 32.86616 | 35.47721 | 22.91473 | 16.21551 | 10.19353 | 23.6747 | 10.79319 | 26.60787 |
| CUST_53386_PI429010886 | M101503 | 0.033362 | 15 | 9 | | | | | | 57.63741 | 62.5636 | 74.60621 | 53.20475 | 53.07285 | 63.48536 | 47.98029 | 66.94583 | 68.77778 | 80.54851 | 65.25641 | 101.7152 | 64.92708 | 91.00006 | 82.49299 | 77.55826 | 53.48278 | 81.93796 | 63.52784 | 69.9701 | 77.48229 | 69.51764 | 90.59403 | 68.45737 | 74.29008 | 85.20601 | 107.354 |
| CUST_31631_PI429010886 | M2016 | 0.033362 | 15 | 9 | 262401207 | ubiquitin associated protein 2-like protein [Scylla pa | | | 7.71E-12 | 233.8649 | 278.8318 | 269.9974 | 231.8976 | 228.3928 | 221.9975 | 182.0796 | 235.8014 | 271.4917 | 338.5409 | 243.2786 | 315.9066 | 256.8548 | 316.8071 | 267.177 | 301.467 | 254.0567 | 233.124 | 252.5773 | 292.5508 | 282.651 | 300.1314 | 290.2221 | 187.251 | 355.116 | 295.08 | 379.8142 |
| CUST_26806_PI429010886 | N54916 | 0.033362 | 15 | 9 | | | | | | 55.45871 | 50.88842 | 43.44584 | 42.82196 | 41.06137 | 41.06525 | 48.35331 | 54.5396 | 48.25178 | 55.61005 | 49.03725 | 57.33863 | 58.82472 | 50.87205 | 54.61808 | 71.08447 | 48.16109 | 47.19565 | 51.83879 | 63.21301 | 40.52511 | 68.69041 | 54.56432 | 26.82401 | 69.53162 | 61.45704 | 58.85973 |
| CUST_55002_PI429010886 | M108370 | 0.033381 | 21 | 6 | 260787569 | hypothetical protein BRAFLDRAFT_89744 [Branchio | Metabolic process | Other | 2.57E-16 | 3.624389 | 30.0571 | 17.22225 | 108.6177 | 101.7598 | 68.66774 | 62.33854 | 300.7234 | 301.1198 | 22.20774 | 189.2302 | 257.7314 | 178.3785 | 50.10697 | 126.1505 | 7.979138 | 86.63308 | NA | 50.87205 | 440.3254 | 205.4212 | 5.271736 | 71.57464 | 55.10664 | NA | 142.4348 | 119.4425 |
| CUST_38668_PI429010886 | M28969 | 0.033387 | 15 | 12 | | | | | | 27.18226 | 19.05569 | 26.66367 | 16.83181 | 14.27753 | 14.66496 | 24.70162 | 20.16612 | 38.04406 | 29.72938 | 33.62817 | 21.61979 | 30.18124 | 19.45997 | 24.93429 | 29.79322 | 8.676162 | 37.44113 | 15.41864 | 20.49506 | 8.545589 | 18.17921 | 55.6145 | 17.19879 | 22.80223 | 38.89725 | 17.74945 |
| CUST_4360_PI429010886 | N16593 | 0.033387 | 12 | 0 | | | | | | 23.16574 | 25.64589 | 32.04305 | 23.00815 | 22.83945 | 9.829719 | 15.65642 | 22.91218 | 17.37315 | 28.71019 | 20.01979 | 26.49443 | 12.98669 | 44.72593 | 19.93306 | 23.98815 | 24.75921 | 28.53148 | 13.87948 | 17.73598 | 18.40425 | 26.70421 | 30.54779 | 15.2636 | 28.84183 | 36.8424 | 27.08746 |
| CUST_3414_PI429010886 | N14456 | 0.033396 | 21 | 3 | 158295014 | AGAP005928-PA [Anopheles gambiae str. PEST] >gi | | | 4.12E-07 | 646.5567 | 482.6309 | 912.7145 | 1929.904 | 945.2395 | 1067.963 | 714.9465 | 723.6629 | 737.2359 | 816.1069 | 406.244 | 858.2338 | 457.1179 | 997.8681 | 316.3545 | 535.2858 | 363.3814 | 682.2742 | 445.2354 | 333.2348 | 1136.737 | 420.025 | 1135.995 | 604.5341 | 1117.627 | 1004.727 | 590.6242 |
| CUST_4454_PI429010886 | N16791 | 0.033439 | 24 | 0 | | | | | | 37.49557 | 33.11671 | 42.70151 | 35.96223 | 40.81576 | 33.43214 | 37.91438 | 24.8201 | 35.06879 | 36.17076 | 27.62424 | 28.34801 | 17.06824 | 34.82515 | 25.52353 | 37.48336 | 29.25886 | 20.23079 | 37.30131 | 27.3183 | 33.63387 | 34.03924 | 46.20996 | 34.67799 | 34.04318 | 36.83629 | 43.60712 |
| CUST_13854_PI429010886 | N31892 | 0.033468 | 12 | 0 | | | | | | 21.28336 | 18.03887 | 18.49721 | 5.059565 | 21.03394 | NA | 7.34732 | 9.748744 | 13.33577 | 17.48685 | 10.5422 | 18.98793 | 11.55948 | 17.43453 | 14.94259 | 23.88653 | 11.92804 | 17.32475 | 10.13647 | 7.622506 | 14.31543 | 13.97389 | 16.26691 | 16.16705 | 32.42745 | 11.98592 | 24.38309 |
| CUST_7844_PI429010886 | N22167 | 0.033473 | 15 | 12 | 291237036 | PREDICTED: Saccoglossus kowalevskii mps one bind | Cellular Process | Cell cycle | 1.49E-73 | 505.1623 | 733.7989 | 525.5752 | 544.2536 | 533.5457 | 465.7878 | 383.017 | 388.1247 | 426.5023 | 531.1131 | 478.7395 | 478.9465 | 541.4466 | 599.927 | 674.891 | 908.5337 | 426.2225 | 598.0415 | 449.7775 | 612.9216 | 548.2244 | 795.1421 | 449.7775 | 405.7694 | 576.1328 | 511.4224 | 659.0457 |
| CUST_16640_PI429010886 | N36612 | 0.033473 | 15 | 12 | 345488448 | PREDICTED: hypothetical protein LOC100114737 [N | | | 8.79E-11 | 37.13071 | 29.69394 | 37.25447 | 29.91175 | 39.51821 | 33.29134 | 27.95797 | 34.08252 | 28.18417 | 35.28275 | 34.64172 | 63.73239 | 32.87464 | 53.82575 | 52.47261 | 35.40201 | 38.91955 | 38.33878 | 24.35681 | 40.09922 | 39.7396 | 32.37576 | 27.06429 | 30.73213 | 45.50557 | 41.36078 | 33.56673 |
| CUST_3776_PI429010886 | N15245 | 0.033516 | 24 | 6 | | | | | | 7560.283 | 3.818452 | 30145.84 | NA | NA | NA | 21011.69 | 25830.1 | 21773.32 | 16515.38 | 52.25211 | 42.40805 | 14135.18 | 15221.8 | 8461.907 | 11.32516 | 10542.53 | 17.04303 | 4510.96 | 55.20849 | 61.69829 | 157.4606 | 4873.025 | 88.63339 | 4959.867 | 34.53803 | 3350.556 |
| CUST_51894_PI429010886 | M95577 | 0.033523 | 15 | 3 | | | | | | 35.31845 | 20.97642 | NA | 30.81693 | 12.33686 | 33.03817 | 37.87644 | 19.78805 | 34.10794 | 13.71331 | 13.42609 | 8.790994 | 13.5705 | 6.697246 | 8.600646 | 10.12007 | 21.55583 | 28.59732 | 40.56158 | NA | 14.91181 | NA | 8.721751 | 19.69856 | 14.22183 | 13.34528 | 7.410795 |
| CUST_38928_PI429010886 | M32160 | 0.033567 | 15 | 12 | | | | | | 76927.64 | 44277.38 | 107232.5 | 12422.81 | 25360.97 | 9552.668 | 80636.04 | 101693.2 | 99042.74 | 101136 | 61640.76 | 49052.57 | 76927.64 | 110974.5 | 73076.54 | 33895.99 | 90794.74 | 44666.31 | 68571.16 | 43935.4 | 57198.62 | 75608.53 | 102912.5 | 33895.99 | 102912.5 | 51514.17 | 109583.6 |
| CUST_31730_PI429010886 | M2257 | 0.033574 | 21 | 18 | 321458840 | hypothetical protein DAPPUDRAFT_228516 [Daphni | | | 9.6E-177 | 107.174 | 107.4484 | 78.01256 | 105.0313 | 139.1997 | 90.43345 | 73.09177 | 77.70756 | 87.65825 | 105.8816 | 107.9692 | 109.2818 | 89.80969 | 128.0141 | 129.8259 | 121.2504 | 101.3168 | 122.4667 | 102.6636 | 173.9115 | 110.737 | 147.4736 | 79.72112 | 97.82533 | 109.2818 | 91.43481 | 102.3756 |
| CUST_4024_PI429010886 | N15817 | 0.033601 | 15 | 12 | 339232928 | putative zinc finger protein [Trichinella spiralis] >gi\| | | | 5.52E-16 | 1361.387 | 1576.033 | 1866.472 | 1548.023 | 1306.431 | 1207.044 | 1284.605 | 1321.214 | 1392.18 | 1589.601 | 1352.942 | 1890.591 | 1438.603 | 1954.96 | 1793.14 | 1849.813 | 1385.14 | 1617.488 | 1400.033 | 1752.574 | 1427.503 | 1749.353 | 1407.359 | 1332.781 | 1933.842 | 1811.851 | 1893.755 |
| CUST_11295_PI429010886 | N27577 | 0.03361 | 18 | 12 | 321466080 | hypothetical protein DAPPUDRAFT_305861 [Daphni | | | 5.22E-52 | 11.71876 | 19.48537 | 21.5495 | 7.617624 | 18.7049 | 11.07586 | 10.55308 | 14.38334 | 17.27797 | 23.91343 | 11.66349 | 23.24949 | 18.0732 | 25.74383 | 30.79365 | 12.7848 | 19.9426 | 15.84353 | 19.68008 | 18.58833 | 12.71803 | 13.6354 | 19.23025 | 10.23633 | 28.44656 | 17.52814 | 15.79172 |
| CUST_49056_PI429010886 | M83834 | 0.033614 | 24 | 18 | | | | | | 10.65025 | 11.75847 | 24.69848 | 13.92846 | 10.75121 | 7.731153 | 14.14304 | 12.36888 | 17.68838 | 7.442152 | 11.99992 | 13.94276 | 12.58824 | 15.86747 | 16.67738 | 17.78423 | 16.45281 | 31.2144 | 25.93469 | 13.75107 | 18.49081 | 18.92641 | 12.57863 | 24.08827 | 14.59396 | 14.08315 | 20.28484 |
| CUST_22004_PI429010886 | N46243 | 0.03365 | 15 | 0 | | | | | | 210.4017 | 119.6528 | 172.7406 | 91.88705 | 154.8286 | 168.8237 | 192.9815 | 106.8828 | 104.7208 | 95.54049 | 197.4111 | 80.85722 | 133.987 | 138.4956 | 125.5654 | 148.3475 | 282.3655 | 148.0082 | 273.965 | 101.4215 | 201.085 | 132.0609 | 133.1932 | 169.0651 | 100.734 | 108.4515 | 81.16843 |
| CUST_38466_PI429010886 | M27031 | 0.033678 | 21 | 3 | | | | | | 197.4111 | 807.1828 | 158.3361 | 447.6451 | 956.1852 | 777.3766 | 595.305 | 275.1358 | 604.157 | 653.8395 | 455.0104 | 965.2192 | 281.2963 | 368.614 | 309.1008 | 902.1996 | 143.517 | 433.4824 | 145.3456 | 177.1551 | 221.873 | 787.7599 | 538.667 | 450.5804 | 1324.078 | 267.9382 | 914.9994 |
| CUST_3014_PI429010886 | N13604 | 0.03371 | 15 | 12 | 241999376 | ubiquitin protein ligase [Ixodes scapularis] >gi\|2154 | Protein metabolism | Cellular protein modification process | 6.53E-34 | 221.5021 | 134.2991 | 534.723 | 149.9525 | 149.1678 | 166.8454 | 325.0806 | 801.3257 | 471.3393 | 473.1073 | 229.6885 | 195.487 | 512.5692 | 1076.531 | 349.9147 | 129.8621 | 457.1179 | 124.7213 | 184.8973 | 163.3744 | 180.4095 | 253.1914 | 790.7325 | 173.3024 | 585.2479 | 189.4794 | 660.5155 |
| CUST_46703_PI429010886 | M73456 | 0.03372 | 18 | 12 | 321457856 | hypothetical protein DAPPUDRAFT_62792 [Daphnia | | | 1.98E-10 | 42.02786 | 37.04426 | 50.06979 | 41.22377 | 33.52618 | 21.56454 | 39.39544 | 44.20781 | 39.64114 | 55.75375 | 37.57841 | 44.04837 | 56.20414 | 47.93244 | 56.18196 | 53.03978 | 56.80732 | 41.13711 | 44.52812 | 49.02131 | 45.87335 | 43.17145 | 52.46763 | 35.4058 | 58.13824 | 36.82429 | 70.48615 |
| CUST_56124_PI429010886 | M112857 | 0.033745 | 15 | 9 | | | | | | 480.665 | 558.2631 | 607.9318 | 579.2037 | 384.7174 | 509.1564 | 411.751 | 507.1661 | 484.3691 | 728.8915 | 514.8406 | 764.3328 | 517.6852 | 611.5405 | 638.7779 | 693.2829 | 499.2279 | 562.6249 | 479.9166 | 659.8695 | 521.7265 | 709.528 | 647.9128 | 627.6184 | 697.8167 | 687.964 | 681.8773 |
| CUST_39453_PI429010886 | M39628 | 0.033745 | 15 | 9 | | | | | | 405.9943 | 383.7251 | 515.1721 | 412.3362 | 372.4888 | 355.6114 | 318.5103 | 365.5076 | 410.7156 | 552.4266 | 425.1252 | 647.3023 | 390.7936 | 666.8781 | 471.6311 | 450.4975 | 406.5948 | 407.0568 | 431.7288 | 470.9976 | 406.244 | 481.413 | 484.3691 | 432.0274 | 575.3599 | 472.997 | 621.7238 |
| CUST_115_PI429010886 | N1114 | 0.033745 | 15 | 9 | | | | | | 49.09733 | 83.86935 | 84.91262 | 43.14736 | 83.02241 | 76.16727 | 99.94463 | 84.62693 | 116.7432 | 85.22504 | 82.38056 | 156.3501 | 55.14182 | 115.284 | 128.4068 | 45.28288 | 87.90722 | 140.3479 | 62.34833 | 49.76955 | 107.3346 | 78.82383 | 104.7823 | 110.8992 | 129.0466 | 111.2819 | 117.3776 |
| CUST_11233_PI429010886 | N27485 | 0.033745 | 15 | 9 | | | | | | 29.50356 | 43.62743 | 50.2407 | 36.94269 | 31.87279 | 24.28324 | 25.07401 | 41.86303 | 35.56782 | 48.38349 | 33.15625 | 102.0348 | 36.73446 | 44.51031 | 48.10487 | 51.38396 | 29.96087 | 25.87358 | 33.14786 | 42.58108 | 33.76323 | 43.05396 | 33.8457 | 25.75668 | 46.88603 | 57.16741 | 61.08538 |
| CUST_12449_PI429010886 | N29475 | 0.033745 | 15 | 9 | | | | | | 113.976 | 225.8611 | 230.5203 | 111.625 | 99.32217 | 25.36775 | 74.34861 | 173.0713 | 300.8257 | 412.4272 | 174.0152 | 392.2104 | 248.428 | 254.3839 | 198.5143 | 299.4458 | 91.90962 | 102.2185 | 195.3573 | 285.8605 | 188.8504 | 244.8705 | 327.598 | 63.12362 | 496.875 | 212.6792 | 556.0784 |
| CUST_14414_PI429010886 | N32843 | 0.033745 | 15 | 0 | | | | | | 4041.003 | 6468.264 | 6142.292 | 3429.061 | 5029.59 | 6941.938 | 1931.541 | 1346.591 | 3055.899 | 3240.576 | 5482.481 | 7002.188 | 1378.434 | 4647.339 | 4098.525 | 7960.065 | 3704.973 | 7282.171 | 4484.875 | 5868.528 | 4696.717 | 4988.507 | 2949.668 | 3011.822 | 4973.61 | 3739.652 | 4773.42 |
| CUST_28351_PI429010886 | N57782 | 0.033745 | 15 | 9 | 357612120 | hypothetical protein KGM_08998 [Danaus plexippu | | | 3.49E-12 | 28.48875 | 38.7854 | 33.77253 | 25.97694 | 34.82515 | 40.25214 | 33.49626 | 21.18978 | 25.2086 | 43.48001 | 37.77068 | 43.34277 | 28.64539 | 40.51578 | 41.91362 | 37.86103 | 41.77165 | 38.94325 | 31.12763 | 38.08646 | 31.42286 | 38.25818 | 33.86312 | 32.08077 | 44.55249 | 42.89021 | 40.40681 |
| CUST_46154_PI429010886 | M71582 | 0.033751 | 15 | 0 | | | | | | 28.31399 | 27.60309 | 17.02546 | 15.20537 | 28.61737 | 25.10042 | NA | 8.318727 | NA | 10.19353 | 9.135099 | 3.793248 | 27.20765 | 14.05126 | 8.669897 | 27.48106 | 8.520664 | 23.43097 | NA | 8.540006 | NA | 6.006114 | NA | 11.14433 | 19.21235 | 8.142064 | 5.781367 |
| CUST_34161_PI429010886 | M8914 | 0.033753 | 24 | 18 | | | | | | 766.3346 | 902.7962 | 676.9355 | 611.5405 | 606.0546 | 495.4416 | 558.7985 | 551.1 | 652.3862 | 707.905 | 665.8633 | 549.5559 | 620.3013 | 774.7379 | 861.1597 | 806.919 | 660.0703 | 638.47 | 736.1144 | 853.9995 | 668.9099 | 893.461 | 711.432 | 568.8782 | 772.6877 | 622.9374 | 718.6829 |
| CUST_1822_PI429010886 | N10799 | 0.033763 | 18 | 3 | 321464620 | hypothetical protein DAPPUDRAFT_306723 [Daphni | | | 1.05E-24 | 457.3191 | 660.0703 | NA | 2529.511 | 1512.011 | 932.818 | 825.1657 | 6.193263 | 599.2901 | NA | 8.289001 | NA | 236.4243 | 612.793 | 693.8801 | 1638.031 | 11.34226 | 1011.687 | NA | 517.8895 | 62.45524 | 2295.055 | NA | 893.1457 | 188.9774 | 13.27508 | NA |
| CUST_36670_PI429010886 | M16500 | 0.033763 | 21 | 3 | 321457271 | hypothetical protein DAPPUDRAFT_301481 [Daphni | | | 7.11E-39 | 108.5223 | 90.21463 | 93.55728 | 129.1185 | 94.50596 | 133.1101 | 73.45438 | 90.31563 | 80.35671 | 62.71939 | 69.56934 | 116.5651 | 66.09037 | 129.3867 | 119.1955 | 85.95 | 70.00254 | 122.0311 | 86.78972 | 69.86983 | 76.38127 | 128.7649 | 86.51241 | 88.40608 | 95.17141 | 125.4528 | 120.5667 |
| CUST_47015_PI429010886 | M74507 | 0.03378 | 24 | 15 | 260786469 | hypothetical protein BRAFLDRAFT_124711 [Branchi | Cellular component organization | Cytoskeleton organization | 1.51E-39 | 38.121 | 38.10393 | 43.48893 | 31.46479 | 39.0714 | 50.26445 | 21.57749 | 32.52687 | 39.36593 | 35.77142 | 38.18452 | 57.03895 | 43.07866 | 41.53959 | 46.00056 | 55.83606 | 55.4319 | 47.0488 | 50.31908 | 44.25982 | 43.17605 | 52.49852 | 36.50859 | 34.06071 | 48.86193 | 42.56702 | 55.79937 |
| CUST_55582_PI429010886 | M110835 | 0.033825 | 24 | 0 | 291220976 | PREDICTED: hypothetical protein [Saccoglossus kow | | | 6.85E-08 | 18.21241 | 23.4957 | 26.60537 | 22.07122 | 21.31694 | 13.92025 | 16.04069 | 8.053924 | 16.34959 | 20.39383 | 13.37205 | 14.73176 | 16.02367 | 8.29527 | 21.44476 | 33.68931 | 9.776853 | 11.29494 | 7.516724 | 16.85059 | 16.87735 | 29.50649 | 11.98928 | 14.73565 | 24.56849 | 31.42286 | 21.35775 |
| CUST_31708_PI429010886 | M2214 | 0.033829 | 18 | 15 | | | Protein metabolism | Proteolysis | | 130.6215 | 141.4128 | 112.4033 | 130.7466 | 207.2391 | 119.1803 | 78.30552 | 47.62012 | 64.50028 | 163.4134 | 248.2061 | 106.1871 | 69.13832 | 78.38473 | 55.90028 | 189.3158 | 217.3065 | 157.4409 | 144.4632 | 183.1759 | 125.6016 | 170.3963 | 87.41325 | 90.65923 | 199.2344 | 57.63741 | 73.15736 |
| CUST_36683_PI429010886 | M16535 | 0.033876 | 15 | 12 | 431908898 | EF-hand calcium-binding domain-containing protei | | | 2.17E-62 | 169.4763 | 197.5737 | 171.5473 | 117.1499 | 139.1588 | 144.8456 | 135.9831 | 140.8068 | 140.8068 | 192.7273 | 130.4407 | 186.3653 | 180.9857 | 170.6145 | 215.9695 | 222.4127 | 148.1409 | 150.0225 | 140.6597 | 176.7754 | 132.0113 | 195.5222 | 159.741 | 137.2513 | 225.2614 | 142.5887 | 170.2053 |
| CUST_31051_PI429010886 | M561 | 0.033876 | 15 | 3 | 83628250 | arthrodial cuticle protein AMP16.3 [Callinectes sapi | | | 6.95E-08 | 415.0913 | 112.3183 | 681.7268 | 644.7638 | 447.0926 | 1065.852 | 578.5402 | 499.7486 | 257.3277 | 205.2623 | 320.3175 | 204.0536 | 118.6343 | 164.9622 | 117.8039 | 147.5789 | 822.9643 | 607.4385 | 929.2639 | 72.41676 | 562.4407 | 145.9962 | 714.7333 | 700.9759 | 150.6133 | 260.4633 | 184.0938 |
| CUST_4222_PI429010886 | N16266 | 0.033876 | 15 | 3 | 241627388 | conserved hypothetical protein [Ixodes scapularis] | | | 3.89E-27 | 20.8155 | 6.161897 | 14.18297 | 17.65061 | 16.63564 | 23.16293 | 7.762745 | 9.343228 | 16.41772 | 15.65293 | 4.674911 | 13.74799 | 8.377586 | 11.55275 | 7.513993 | 24.25592 | 9.979521 | 15.89427 | 15.07523 | 18.34346 | 12.65934 | 14.5219 | 11.78596 | 11.27249 | 12.30868 | 13.82753 | 6.630038 |
| CUST_4903_PI429010886 | N17747 | 0.033876 | 15 | 12 | | | | | | 974.6113 | 1057.689 | 1238.144 | 1038.465 | 929.5412 | 1102.275 | 941.2341 | 891.7466 | 981.8641 | 1146.628 | 882.5331 | 1178.599 | 1020.365 | 6253.288 | 1668.794 | 1453.376 | 999.0406 | 1139.583 | 1009.897 | 1246.965 | 989.1564 | 1329.963 | 957.2333 | 934.9001 | 1310.544 | 1134.542 | 1444.613 |
| CUST_10598_PI429010886 | N26489 | 0.033876 | 15 | 12 | | | | | | 45.05944 | 47.03677 | 43.77809 | 21.51773 | 61.56139 | 40.54346 | 37.26633 | 26.23528 | 37.96113 | 45.62788 | 29.69737 | 62.1467 | 49.09733 | 47.53397 | 59.52293 | 47.61035 | 31.0625 | 61.12874 | 50.11711 | 44.94077 | 59.42335 | 34.45811 | 30.48607 | 42.09528 | 44.7891 | 46.08794 | 60.6558 |
| CUST_16433_PI429010886 | N36227 | 0.033876 | 15 | 12 | | | | | | 71.15715 | 64.88975 | 83.44238 | 49.18419 | 67.98118 | 52.31892 | 35.00866 | 44.331 | 54.46899 | 75.37057 | 57.99026 | 69.48443 | 66.3431 | 55.18575 | 74.36967 | 71.03479 | 53.33275 | 47.28217 | 56.38454 | 73.44541 | 75.60669 | 47.82182 | 53.9397 | 45.20819 | 67.28983 | 55.30305 | 64.97549 |
| CUST_51115_PI429010886 | M92261 | 0.033893 | 18 | 15 | | | | | | 529.4905 | 919.6285 | 530.042 | 524.0613 | 680.5523 | 492.0126 | 429.7605 | 523.7505 | 426.3841 | 702.4802 | 659.4054 | 816.1069 | 513.6722 | 626.4662 | 679.3339 | 922.8399 | 643.5529 | 520.9934 | 548.7959 | 910.5801 | 617.8466 | 678.3407 | 678.2021 | 542.7169 | 484.607 | 423.2024 | 540.1617 |
| CUST_49605_PI429010886 | M86368 | 0.033902 | 24 | 12 | | | | | | 8.543285 | 5.78191 | 16.75121 | 8.508012 | 6.081579 | 7.39029 | 19.30622 | 10.75291 | 10.2415 | 30.47404 | 17.68083 | 15.33784 | 21.92688 | 14.34446 | 21.65997 | 14.75649 | 17.03463 | 15.79004 | 3.519562 | 22.98966 | 47.67267 | 12.10746 | 15.17161 | 11.58541 | 15.35034 | 20.56296 | 4.785934 |
| CUST_54916_PI429010886 | M108155 | 0.033914 | 12 | 3 | 157120354 | hypothetical protein AaeL_AAEL008947 [Aedes aeg | Cellular component organization | Cytoskeleton organization | 3.07E-33 | 85.89616 | 19.2217 | 14.14994 | 185.59 | 121.5117 | 409.6774 | 120.1521 | 147.5581 | 40.89152 | 26.28688 | 134.6919 | 21.18827 | 54.17026 | 7.625766 | 22.84721 | 24.135 | 212.773 | 244.8705 | 227.3449 | 32.10959 | 95.86282 | 39.05895 | 48.41716 | 100.5781 | 47.53397 | 270.0349 | 26.75249 |
| CUST_17396_PI429010886 | N37895 | 0.033916 | 21 | 3 | | | | | | 40.16219 | 9.082334 | NA | 33.5333 | 32.13072 | 62.39657 | 36.89793 | 20.75737 | 31.00505 | 57.87372 | 7.594313 | 9.516775 | 10.32671 | 13.58792 | 19.2011 | 10.23633 | 15.36478 | 31.43005 | 6.893139 | 100.7517 | 68.44169 | 70.23629 | 25.19447 | 16.62548 | 121.5738 | 57.01626 | 96.95634 |
| CUST_5822_PI429010886 | N19248 | 0.033924 | 18 | 6 | 328705463 | PREDICTED: hypothetical protein LOC100570122 [A | | | 7.6E-62 | 118.5239 | 138.0366 | 143.1059 | 213.6068 | 166.0384 | 170.3686 | 148.0995 | 212.8755 | 193.0602 | 238.8599 | 105.6941 | 206.1326 | 172.5998 | 242.0042 | 193.108 | 212.5355 | 138.4789 | 248.5464 | 143.4489 | 177.2677 | 130.1677 | 325.1931 | 141.6752 | 172.9097 | 273.965 | 298.9754 | 267.2735 |
| CUST_10988_PI429010886 | N27095 | 0.033928 | 24 | 18 | 530607984 | PREDICTED: DNA polymerase epsilon subunit 3 isof | | | 2.45E-21 | 13.48403 | 5.990926 | 11.28834 | 4.356707 | 4.095655 | 5.128467 | 12.05879 | NA | 7.411834 | 8.745116 | 10.04728 | 15.19101 | 11.24691 | 22.60724 | 9.766658 | 20.17771 | NA | 8.98024 | 78.19261 | 10.83953 | NA | 9.152945 | 8.881606 | NA | 15.29079 | 12.66733 | 10.59486 |
| CUST_8383_PI429010886 | N22965 | 0.033939 | 12 | 0 | | | | | | 198.6688 | 267.983 | 234.853 | 184.5131 | 186.0312 | 179.8447 | 141.8325 | 145.1427 | 162.251 | 246.5093 | 180.3041 | 214.5304 | 189.7386 | 249.0021 | 225.3313 | 249.7124 | 140.7358 | 200.6114 | 174.6367 | 227.6752 | 223.7478 | 272.3366 | 209.6917 | 199.0699 | 257.6826 | 221.6459 | 280.2255 |
| CUST_29164_PI429010886 | N59314 | 0.033981 | 18 | 6 | | | | | | 39.27655 | 15.7022 | 34.40352 | 22.79581 | 38.95593 | 32.61875 | 42.59897 | 11.3595 | 91.04016 | 27.16436 | 32.74461 | 23.6887 | 19.30622 | 33.40373 | 15.51056 | 18.98962 | 23.2298 | 15.63777 | 30.77501 | 23.60773 | 28.64039 | 21.96556 | 47.17722 | 16.53843 | 40.71208 | 39.78059 | 46.02004 |
| CUST_38851_PI429010886 | M31227 | 0.034 | 21 | 9 | 328899290 | receptor accessory protein 5 [Procambarus clarkii] | | | 3.12E-53 | 4.496036 | 13.06127 | 3.502463 | 10.70732 | 38.53317 | 6.843848 | 15.42332 | 105.5931 | 63.85255 | 11.10317 | 100.955 | 116.2535 | 76.25978 | 7.729578 | 42.48584 | NA | 37.61954 | NA | 8.925496 | 151.7333 | 45.38512 | NA | 15.29619 | 9.105658 | NA | 46.4361 | 38.41183 |
| CUST_55377_PI429010886 | M109833 | 0.034007 | 15 | 9 | | | | | | 360.9417 | 333.708 | 391.0528 | 319.448 | 321.4904 | 309.2864 | 315.1705 | 252.9891 | 323.2059 | 402.5662 | 314.4386 | 388.6405 | 347.1114 | 421.7857 | 304.3497 | 368.9642 | 305.9118 | 327.535 | 263.3865 | 310.0812 | 398.8868 | 378.7944 | 391.6722 | 342.3066 | 457.8737 | 400.0206 | 424.0774 |
| CUST_43073_PI429010886 | M62006 | 0.03401 | 18 | 12 | | | | | | 20.61883 | 13.2544 | 15.4721 | 20.55898 | 10.70821 | 19.54896 | 17.43083 | 20.91693 | 20.19405 | 20.9954 | 11.43263 | 28.86885 | 24.73262 | 27.36205 | 29.16341 | 22.53424 | 16.29756 | 15.93573 | 15.7271 | 19.22009 | 24.08827 | 26.26779 | 17.84395 | 17.21299 | 24.41159 | 23.42495 | 27.06117 |
| CUST_53037_PI429010886 | M99839 | 0.034024 | 24 | 6 | | | | | | 509.603 | 474.7169 | 344.6643 | 640.2097 | 578.8676 | 573.9804 | 484.2538 | 783.5804 | 585.2479 | 341.0906 | 699.2311 | 760.5296 | 679.5311 | 321.0768 | 622.024 | 462.6916 | 519.7383 | 366.8204 | 480.7772 | 1069.225 | 531.9567 | 293.5772 | 397.7744 | 452.2817 | 297.0813 | 560.1437 | 453.0933 |
| CUST_18675_PI429010886 | N40206 | 0.03403 | 21 | 12 | | | | | | 17.19687 | 21.7799 | 20.73682 | 8.764694 | 31.8356 | 10.49992 | 17.56531 | 13.31647 | 15.14331 | 27.43011 | 18.53904 | 19.99534 | 30.98507 | 25.71698 | 20.66296 | 26.73668 | 17.90553 | 25.60131 | 23.48661 | 24.48268 | 12.15775 | 17.86172 | 21.61111 | 13.48767 | 12.1506 | 14.09753 | 34.0946 |
| CUST_49345_PI429010886 | M85289 | 0.034045 | 24 | 21 | | | | | | 14.43921 | 9.924045 | NA | NA | 8.247776 | NA | 14.84209 | 8.494473 | 17.86001 | 4.90426 | 4.337085 | 6.852264 | 13.22555 | 33.67567 | 11.73379 | NA | 23.81911 | NA | 16.50813 | 19.49659 | 5.711785 | 22.04791 | 29.33184 | 18.52264 | 27.40469 | 5.616236 | 27.87925 |
| CUST_41123_PI429010886 | M56919 | 0.034047 | 24 | 21 | 291241993 | PREDICTED: Saccoglossus kowalevskii cytoplasmic d | Cellular component organization | Cytoskeleton organization | 1.55E-78 | 16.3703 | 17.05906 | 12.87509 | 18.01506 | 15.5488 | 14.67098 | 23.76056 | 21.09286 | 16.68074 | 19.18311 | 11.13919 | 13.07843 | 18.91365 | 21.89247 | 19.70298 | 28.16847 | 13.72174 | 25.43742 | 22.76207 | 20.78747 | 25.76654 | 27.8177 | 89.80198 | 23.01108 | 23.35894 | 28.32496 | 18.13226 |
| CUST_3272_PI429010886 | N14157 | 0.034047 | 24 | 18 | 260828941 | hypothetical protein BRAFLDRAFT_114977 [Branchi | | | 1.18E-88 | 35.00866 | 41.72002 | 38.37992 | 25.57234 | 39.7396 | 31.40893 | 24.86718 | 25.14839 | 44.95443 | 55.42181 | 30.09468 | 63.49666 | 37.44766 | 47.38756 | 30.90791 | 68.66167 | 31.07173 | 43.32695 | 71.64239 | 46.87213 | 50.50708 | 59.41826 | 46.84472 | 25.17616 | 66.18886 | 41.93393 | 47.19112 |
| CUST_20211_PI429010886 | N43028 | 0.034047 | 24 | 18 | 29134779 | projectin [Procambarus clarkii] | | | 3.08E-24 | 211.3121 | 172.408 | 214.7331 | 193.773 | 192.5322 | 200.9243 | 160.7305 | 182.4169 | 184.3061 | 251.662 | 242.4336 | 245.4514 | 196.9594 | 213.9187 | 188.0537 | 218.804 | 246.8808 | 175.6284 | 272.7482 | 233.6069 | 209.0603 | 251.8789 | 214.9913 | 198.5726 | 220.1334 | 208.2838 | 248.3342 |
| CUST_47964_PI429010886 | M78308 | 0.034052 | 24 | 0 | | | | | | 68.26569 | 38.35721 | 55.18575 | 33.58412 | 37.80277 | 47.13629 | 43.5925 | 32.80342 | 30.16372 | 44.64106 | 36.64169 | 49.96135 | 23.89355 | 27.80573 | 25.24216 | 48.40257 | 45.77355 | 58.35369 | 44.10538 | 42.242 | 44.34401 | 30.29627 | 50.5231 | 40.80069 | 61.2083 | 43.88802 | 53.36025 |
| CUST_3662_PI429010886 | N14987 | 0.034052 | 24 | 0 | | | | | | 366.4854 | 185.7359 | 120.114 | 76.96996 | 191.886 | 168.177 | 152.9488 | 96.33377 | 102.0573 | 155.5733 | 258.9285 | 179.8168 | 41.63417 | 109.2372 | 74.21285 | 159.5837 | 122.5622 | 167.9037 | 123.444 | 101.2088 | 63.20604 | 159.5474 | 90.11072 | 129.5889 | 319.8266 | 184.0607 | 174.4075 |
| CUST_11375_PI429010886 | N27712 | 0.034052 | 24 | 12 | 123977013 | hypothetical protein [Trichomonas vaginalis G3] >gi | | | 1.41E-07 | 11.26822 | 13.11969 | 13.97389 | 20.10327 | 15.73939 | 11.27734 | 17.15146 | 59.63236 | 12.7744 | 17.24751 | 18.14179 | 14.42559 | 17.34784 | 26.35743 | 23.49823 | 18.49721 | 16.83941 | 15.20537 | 16.42197 | 24.17916 | 25.04386 | 41.2427 | 13.54494 | 14.71101 | 22.27554 | 11.57286 | 14.24976 |
| CUST_4431_PI429010886 | N16740 | 0.034052 | 15 | 9 | 310693525 | truncated cathepsin A [Eriocheir sinensis] | Protein metabolism | Proteolysis | 9.57E-10 | 3.824188 | 498.3855 | 235.7566 | 1188.466 | 845.2412 | 590.6242 | 516.2214 | 2095.03 | 1934.877 | 210.1212 | 1896.247 | 3003.506 | 763.4512 | 665.511 | 651.5039 | 4.542912 | 517.0533 | NA | 250.6384 | 3365.303 | 1801.38 | NA | 700.4893 | 525.5752 | NA | 1952.727 | 1601.861 |
| CUST_46536_PI429010886 | M72933 | 0.034084 | 15 | 0 | | | | | | 142.7507 | 143.7253 | 92.10717 | 138.8045 | 121.6348 | 104.2766 | 95.54049 | 98.62916 | 82.05363 | 107.638 | 91.78258 | 93.80773 | 95.86282 | 103.1666 | 114.0301 | 198.7745 | 120.1371 | 128.6916 | 100.2495 | 121.3613 | 88.37144 | 195.2959 | 72.4241 | 89.07632 | 137.2513 | 88.34755 | 76.46353 |
| CUST_26121_PI429010886 | N53707 | 0.0341 | 21 | 15 | 291242073 | PREDICTED: predicted protein-like [Saccoglossus ko | | | 1.61E-09 | 21.84275 | 6.823408 | NA | 10.12151 | 16.66793 | 14.04228 | 9.347162 | 6.034486 | 7.640309 | 6.429353 | 16.39504 | 14.24335 | 6.670159 | 35.9589 | 5.574158 | 16.2854 | 17.63614 | 69.08039 | 14.99273 | 16.16705 | NA | 10.24932 | 9.091001 | NA | 14.66262 | NA | 8.47166 |
| CUST_37165_PI429010886 | M18061 | 0.034112 | 18 | 0 | 40809702 | crustocalcin-a [Marsupenaeus japonicus] | | | 2.33E-07 | 170.4767 | NA | 219.4603 | 14.64853 | 63.40813 | 125.0127 | 261.6417 | 50.84005 | 27.74158 | 21.6649 | 165.7579 | 7.880481 | 28.16499 | 23.06972 | 15.64945 | NA | 207.0425 | 65.92191 | 231.4586 | NA | 143.5804 | NA | 168.9486 | 136.2923 | 5.105862 | NA | NA |
| CUST_11138_PI429010886 | N27328 | 0.034127 | 21 | 6 | | | | | | 45.64241 | 25.54204 | 42.79014 | 44.24444 | 37.07935 | 50.42448 | 53.03364 | 117.3985 | 92.20534 | 109.0299 | 42.81164 | 56.23475 | 67.52947 | 82.6351 | 30.32196 | 49.49037 | 33.25728 | 31.1168 | 50.84587 | 68.52038 | 33.87601 | 40.97284 | 49.10355 | 13.1549 | 87.44346 | 30.33968 | 86.4151 |
| CUST_54509_PI429010886 | M106292 | 0.034129 | 15 | 9 | | | | | | 68.66774 | 33.48853 | 80.842 | 20.61498 | 35.72502 | 26.69563 | 58.58633 | 48.86699 | 26.50986 | 76.50308 | 61.35668 | 50.46685 | 40.59153 | 38.84241 | 7.340015 | 37.36976 | 43.62391 | 21.58196 | 16.50096 | 28.15466 | 50.38211 | 36.91363 | 61.11567 | 60.16545 | 77.2785 | 29.9042 | 92.13928 |
| CUST_38521_PI429010886 | M27648 | 0.034129 | 15 | 3 | | | | | | 1066.553 | 518.3277 | 51.54875 | 2370.125 | 2385.821 | 2988.348 | 1984.848 | 2082.977 | 741.4101 | 19.72298 | 913.8189 | 21.69169 | 1374.989 | 51.34112 | 553.514 | 341.9433 | 1895.191 | 1295.233 | 2545.683 | 40.11789 | 379.8142 | 106.002 | 217.8082 | 1351.741 | 54.94382 | 1260.686 | 7.63023 |
| CUST_40543_PI429010886 | M55418 | 0.034129 | 15 | 3 | | | | | | 921.6949 | 968.3869 | 370.3508 | 2114.712 | 1406.77 | 1469.899 | 1082.038 | 446.6411 | 1109.944 | 396.481 | 379.2779 | 377.4127 | 796.2928 | 1020.69 | 1403.935 | 1397.451 | 517.5225 | 1054.214 | 464.4064 | 903.1657 | 541.31 | 1596.656 | 514.5197 | 979.7027 | 746.9886 | 224.1286 | 294.8656 |
| CUST_43053_PI429010886 | M61951 | 0.034129 | 15 | 3 | | | | | | 17.96396 | 6.374853 | 16.23324 | 15.62557 | 16.35532 | 26.78447 | 27.05759 | 24.0422 | 26.03903 | 9.001207 | 11.72403 | 10.11773 | 26.2515 | 16.06241 | 16.23152 | 5.525809 | 16.39504 | 19.44365 | 30.97619 | 44.18048 | 25.68021 | 21.30243 | 13.42877 | 17.81842 | 9.41784 | 13.68318 | 18.88981 |
| CUST_46257_PI429010886 | M71966 | 0.034129 | 15 | 3 | 118766718 | beta-tubulin [Oxymonadida environmental sample] | Cellular component organization | Cytoskeleton organization | 7.72E-20 | 22.20953 | 17.34629 | 10.96329 | 29.53645 | 26.24303 | 24.78858 | 29.61833 | 13.34357 | 14.82674 | 16.80832 | 14.77313 | 4.862972 | 42.02786 | 10.82471 | 14.8977 | 18.57693 | 31.21749 | 17.13473 | 20.27214 | 18.32541 | 22.21172 | 19.54386 | 6.490205 | 27.2017 | 15.64186 | 15.97793 | 9.136468 |
| CUST_48849_PI429010886 | M82488 | 0.034129 | 15 | 9 | | | | | | 1046.312 | 1292.191 | 1556.767 | 1162.112 | 1152.374 | 1240.783 | 1079.252 | 1224.871 | 1475.875 | 1641.174 | 1248.552 | 1763.663 | 1168.111 | 2219.95 | 1433.821 | 1419.195 | 1266.139 | 1320.618 | 1280.117 | 1395.016 | 1423.398 | 1445.794 | 1434.403 | 1174.454 | 1653.841 | 1544.651 | 2013.592 |
| CUST_34152_PI429010886 | M8895 | 0.034129 | 15 | 9 | | | | | | 154.9299 | 107.533 | 146.882 | 117.9689 | 120.8183 | 78.77563 | 57.37114 | 78.66644 | 125.9958 | 407.7342 | 392.3971 | 226.1214 | 32.46954 | 201.0518 | 264.026 | 529.0811 | 12.63007 | 173.4432 | 149.6657 | 24.81549 | 123.444 | 213.7556 | 204.0536 | 70.01711 | 679.7235 | 133.006 | 185.104 |
| CUST_52686_PI429010886 | M98464 | 0.034129 | 15 | 9 | | | | | | 34.6609 | 42.11302 | 42.98324 | 37.38397 | 32.18039 | 34.11542 | 37.3381 | 35.21995 | 32.17021 | 39.87225 | 43.32695 | 48.68295 | 31.90512 | 42.93894 | 50.4975 | 53.33947 | 38.80762 | 35.29256 | 45.23797 | 41.73275 | 27.8344 | 45.78297 | 41.41188 | 43.06915 | 60.09613 | 50.63623 | 50.92195 |
| CUST_6335_PI429010886 | N19969 | 0.034129 | 15 | 0 | 416656 | RecName: Full=Arrestin homolog >gi\|298756\|gb\|A | Signal transduction | Other | 9.1E-87 | 1784.695 | 1835.374 | 1744.83 | 1012.516 | 1585.767 | 1664.492 | 1117.033 | 1124.753 | 941.2341 | 1882.712 | 1467.586 | 1491.426 | 822.9643 | 1129.849 | 1240.783 | 2497.013 | 1816.167 | 1939.579 | 1378.873 | 1816.167 | 1372.611 | 1525.167 | 1175.49 | 1287.066 | 2624.92 | 1432.157 | 2218.572 |
| CUST_9715_PI429010886 | N25085 | 0.034129 | 15 | 9 | | | | | | 39.79339 | 46.6 | 41.47521 | 37.24189 | 28.51585 | 31.80677 | 28.76281 | 42.00009 | 47.97063 | 55.77394 | 34.53803 | 52.10631 | 35.70225 | 45.84492 | 57.72819 | 48.98952 | 37.96902 | 29.57087 | 37.68617 | 44.77456 | 30.705 | 44.85914 | 41.909 | 28.3608 | 60.70574 | 41.71517 | 60.57138 |
| CUST_272_PI429010886 | N3246 | 0.034129 | 15 | 9 | 260793094 | hypothetical protein BRAFLDRAFT_92778 [Branchio | | | 1.98E-07 | 1348.92 | 1106.942 | 1103.397 | 840.3682 | 828.5874 | 889.0555 | 874.1627 | 582.6377 | 1336.322 | 1342.338 | 608.0744 | 1554.539 | 840.6106 | 1451.1 | 1412.634 | 1435.197 | 627.2172 | 1034.677 | 615.9204 | 865.4579 | 863.9077 | 1838.739 | 1436.981 | 1146 | 1227.15 | 1569.266 | 1660.585 |
| CUST_21620_PI429010886 | N45541 | 0.034129 | 15 | 0 | | | | | | 1315.839 | 1496.572 | 1098.749 | 692.5393 | 1216.535 | 1110.941 | 690.9208 | 1047.491 | 628.4644 | 1250.839 | 1043.787 | 998.2355 | 699.2311 | 809.6878 | 799.5941 | 1609.005 | 1484.716 | 1663.063 | 955.4895 | 1454.733 | 972.1119 | 1265.474 | 808.2235 | 795.1421 | 2009.101 | 767.5196 | 1303.875 |
| CUST_23799_PI429010886 | N49492 | 0.034129 | 15 | 9 | | | | | | 31.1168 | 35.19595 | 33.69477 | 11.27392 | 25.21717 | 14.73825 | 16.75121 | 22.64731 | 28.10839 | 32.61498 | 23.52604 | 42.02191 | 27.55645 | 25.06406 | 31.79024 | 26.52817 | 29.38995 | 17.75139 | 19.01531 | 17.18917 | 24.51087 | 38.78884 | 40.90818 | 16.66456 | 39.16663 | 23.3229 | 40.74811 |
| CUST_755_PI429010886 | N7172 | 0.034129 | 15 | 6 | | | | | | 74.00809 | 66.20577 | 66.06376 | 80.47827 | 84.28941 | 79.39521 | 112.8438 | 177.9987 | 94.06166 | 79.30391 | 99.08673 | 54.0716 | 86.87439 | 101.2088 | 79.91242 | 57.27407 | 123.6053 | 75.2717 | 82.08362 | 92.05581 | 74.7826 | 112.169 | 86.14614 | 83.83973 | 106.1639 | 58.0658 | 78.06614 |
| CUST_2749_PI429010886 | N13033 | 0.034135 | 18 | 9 | 321453216 | hypothetical protein DAPPUDRAFT_304899 [Daphn | Transport | Protein transport | 9.5E-114 | 6.238311 | 10.99994 | 3.793848 | 20.76905 | 11.0861 | 11.65447 | 11.27574 | 11.03769 | 14.5219 | 16.72128 | 9.819588 | 14.83746 | 21.49928 | 12.30261 | 7.491442 | 13.84341 | 10.13183 | 7.970984 | 9.739447 | 7.178303 | 5.793257 | 6.740179 | NA | 19.07331 | 8.638278 | 8.281111 | 14.24194 |
| CUST_11782_PI429010886 | N28395 | 0.034144 | 18 | 0 | | | | | | 226.6782 | 239.571 | 223.2901 | 189.0327 | 221.5021 | 213.9538 | 219.4603 | 175.7224 | 178.1983 | 273.6024 | 183.4677 | 177.6428 | 144.9989 | 196.2921 | 186.3653 | 264.2251 | 230.9992 | 202.9175 | 254.0567 | 207.3277 | 216.9071 | 222.818 | 222.9397 | 194.5182 | 253.8057 | 189.5057 | 201.1422 |
| CUST_19257_PI429010886 | N41263 | 0.03416 | 15 | 3 | | | | | | 32.48228 | 124.3644 | 11.91905 | 498.1212 | 149.7134 | 170.0196 | 79.29506 | 7.672382 | 81.82047 | 5.158093 | 8.692145 | NA | 10.01285 | 11.63591 | 105.4997 | 132.8222 | 8.396527 | 137.2358 | NA | 3.831335 | NA | 71.29059 | 9.474956 | 55.20237 | 8.267442 | 12.52538 | NA |
| CUST_32071_PI429010886 | M3157 | 0.03418 | 24 | 21 | | | | | | 539.1803 | 713.8389 | 610.9693 | 417.2023 | 533.6685 | 542.7169 | 373.3663 | 458.9977 | 433.0579 | 582.8028 | 557.3105 | 720.0434 | 426.1051 | 609.308 | 35.4902 | 655.3909 | 600.2864 | 547.465 | 740.2282 | 557.0435 | 547.117 | 580.436 | 674.6226 | 597.0282 | 610.8058 | 467.0909 | 588.1169 |
| CUST_31816_PI429010886 | M2478 | 0.034187 | 24 | 0 | | | | | | 185.2951 | 77.21633 | 127.8639 | 194.5182 | 43.50773 | 92.15955 | 52.72315 | 107.5704 | 26.07451 | 78.06614 | 53.62183 | 68.6762 | 42.52496 | 101.3667 | 61.41511 | 116.1174 | 85.54854 | 75.7868 | 49.25162 | 110.8154 | 66.50022 | 164.4877 | 47.72724 | 88.47454 | 138.9365 | 185.59 | 190.5242 |
| CUST_46514_PI429010886 | M72873 | 0.034237 | 21 | 18 | | | | | | 49.39411 | 61.88462 | 38.09087 | 49.7894 | 46.88603 | 30.28193 | 21.77078 | 31.86259 | 26.13604 | 58.5117 | 40.64728 | 57.27407 | 37.86509 | 50.49286 | 42.03093 | 51.02123 | 32.70652 | 55.67871 | 50.39753 | 51.48154 | 50.38632 | 54.05301 | 53.96058 | 36.46183 | 45.66919 | 34.20754 | 45.22744 |
| CUST_31191_PI429010886 | M940 | 0.034237 | 15 | 0 | | | | | | 3098.049 | 3821.032 | 2659.902 | 1796.352 | 2706.735 | 2280.777 | 1750.319 | 1868.48 | 1642.855 | 3624.616 | 2794.005 | 3181.861 | 1864.705 | 2179.192 | 2502.493 | 4528.171 | 2800.799 | 3276.015 | 2738.341 | 3466.9 | 2242.56 | 3610.458 | 1977.124 | 2014.561 | 3748.628 | 2030.719 | 2999.441 |
| CUST_27442_PI429010886 | N56064 | 0.034245 | 24 | 6 | | | | | | 227.0266 | 234.6243 | 31.82653 | 391.5964 | 91.07591 | 137.8443 | 175.6005 | 298.2497 | 158.7119 | 15.84192 | 23.42234 | 7.568679 | 274.49 | 27.58029 | 144.7064 | 62.10992 | 101.6676 | 61.63016 | 21.24309 | 11.30787 | 9.802984 | 26.26256 | 24.11521 | 270.8602 | 22.49442 | 64.49483 | 15.75243 |
| CUST_19024_PI429010886 | N40848 | 0.034251 | 24 | 21 | | | | | | 21.94249 | 13.89928 | 12.12283 | 17.7958 | 12.04602 | 13.29711 | 10.682 | 8.588693 | 8.711267 | 13.91626 | 5.821706 | 18.0708 | 17.6966 | 15.60562 | 6.844642 | 11.22575 | 8.548078 | 13.71506 | 13.43228 | 14.22674 | 17.55275 | 20.88612 | 14.65028 | 19.14111 | 17.52814 | 15.19655 | 23.78727 |
| CUST_7854_PI429010886 | N22179 | 0.034258 | 21 | 3 | | | | | | 302.092 | 222.6053 | 196.8724 | 242.4336 | 339.3609 | 296.4182 | 237.0425 | 256.5714 | 210.6087 | 299.502 | 251.9665 | 274.6972 | 185.6524 | 191.2652 | 169.7349 | 243.4369 | 288.0337 | 205.2921 | 268.2351 | 268.3232 | 171.6223 | 279.4425 | 218.4011 | 244.5361 | 357.7936 | 219.7749 | 288.7609 |
| CUST_8252_PI429010886 | N22774 | 0.034258 | 15 | 3 | | | | | | 159.6853 | 12.31301 | 200.3982 | 276.7604 | 286.997 | 396.1913 | 409.8763 | 167.9037 | 54.89215 | NA | 408.3975 | 3.517971 | 63.80913 | 7.053731 | 31.65107 | 6.289108 | 1020.365 | 150.9664 | 933.7706 | NA | 273.6399 | NA | 278.0994 | 414.4964 | 9.192825 | 91.49463 | NA |
| CUST_821_PI429010886 | N7415 | 0.034272 | 24 | 21 | | | | | | 16.97989 | 17.61748 | 24.69054 | 9.840112 | 15.39972 | 15.92196 | NA | NA | 15.08227 | 14.4635 | 6.104204 | 17.80075 | 7.744827 | 18.20414 | 16.43851 | 23.36932 | 8.509556 | 23.2774 | 15.77707 | 17.65649 | 11.86129 | 26.4083 | 22.07787 | NA | 18.41753 | 15.32925 | 18.27526 |

| Probe ID | ID | p-value | Period | Phase | EBI ID | Annotation | GO category | Go term | E-value | 0 | 0 | 0 | 3 | 3 | 3 | 6 | 6 | 6 | 9 | 9 | 9 | 12 | 12 | 12 | 15 | 15 | 15 | 18 | 18 | 18 | 21 | 21 | 21 | 24 | 24 | 24 |
|---|---|---|---|---|---|---|---|---|---|---|---|---|---|---|---|---|---|---|---|---|---|---|---|---|---|---|---|---|---|---|---|---|---|---|---|---|
| CUST_56798_PI429010886 | M114668 | 0.03428 | 15 | 12 | | | | | | 567.0781 | 629.6206 | 771.3694 | 495.0933 | 549.794 | 487.7611 | 506.9726 | 557.4875 | 585.4036 | 683.1376 | 539.016 | 740.6945 | 603.6169 | 783.8018 | 731.4443 | 824.1385 | 593.331 | 559.1604 | 566.4251 | 729.1026 | 548.6331 | 769.2478 | 595.8659 | 382.2635 | 798.5706 | 580.0174 | 838.3296 |
| CUST_39172_PI429010886 | M35591 | 0.03428 | 15 | 12 | | | | | | 10348.94 | 216.5095 | 22662.24 | 373.0312 | 390.1389 | 1006.198 | 9670.846 | 25748.12 | 23368.13 | 24915.2 | 1548.599 | 1499.492 | 24478.23 | 43151.77 | 23469.53 | 670.112 | 12334.51 | 1050.577 | 8627.68 | 2316.991 | 2089.376 | 9475.141 | 19491.76 | 3138.529 | 17865.21 | 1542.825 | 18694.18 |
| CUST_31060_PI429010886 | M586 | 0.03428 | 15 | 12 | 405970980 | Cytosolic Fe-S cluster assembly factor NUBP1-like p | Metabolic process | Other | 1.7E-122 | 141.4697 | 158.8254 | 164.6012 | 96.78797 | 101.6113 | 128.9944 | 118.6904 | 141.4904 | 148.3869 | 196.157 | 125.6844 | 180.9857 | 141.2568 | 191.4981 | 162.141 | 180.3315 | 129.5554 | 124.7724 | 103.0626 | 155.649 | 139.4033 | 165.0799 | 121.9392 | 130.6774 | 141.6867 | 124.009 | 170.2454 |
| CUST_15545_PI429010886 | N34760 | 0.03428 | 24 | 18 | | | | | | 1027.258 | 993.3629 | 1081.506 | 911.683 | 917.7643 | 724.3304 | 1025.258 | 767.811 | 734.477 | 1104.958 | 794.1984 | 890.6855 | 884.4098 | 1313.707 | 953.2915 | 1739.21 | 917.4556 | 924.2614 | 1098.078 | 1127.801 | 961.1254 | 1534.021 | 1056.312 | 870.876 | 1274.525 | 794.4801 | 1030.678 |
| CUST_46857_PI429010886 | M73965 | 0.03429 | 21 | 15 | | | | | | 41.91362 | 38.73495 | 39.06311 | 35.39157 | 35.992 | 53.33275 | 32.68774 | 45.488 | 49.48526 | 47.95255 | 34.57302 | 29.06792 | 40.67008 | 46.42845 | 43.67532 | 42.92761 | 67.45213 | 70.169 | 54.79813 | 78.72636 | 62.36898 | 46.02417 | 42.36335 | 35.44546 | 55.92013 | 30.88328 | 41.30417 |
| CUST_30906_PI429010886 | M182 | 0.034301 | 18 | 9 | 332021445 | Putative protein phosphatase [Acromyrmex echinat | Protein metabolism | Cellular protein modification process | 4.38E-48 | 129.6421 | 144.77 | 128.7232 | 73.97012 | 118.3919 | 72.78887 | 82.82132 | 113.0821 | 148.9405 | 233.1687 | 135.8061 | 193.556 | 155.8927 | 195.0326 | 125.6499 | 242.1214 | 119.3927 | 112.3805 | 148.7285 | 210.4017 | 108.5779 | 114.8783 | 140.036 | 91.98586 | 189.3407 | 103.7863 | 247.6419 |
| CUST_37359_PI429010886 | M18783 | 0.034308 | 12 | 0 | | | | | | 188.658 | 473.327 | 429.3715 | 293.1271 | 182.1441 | 27.53031 | 60.81378 | 224.0801 | 382.5164 | 439.9985 | 273.393 | 543.3625 | 357.152 | 528.7455 | 470.5429 | 333.3682 | 137.8924 | 342.0536 | 281.2623 | 394.7994 | 407.6622 | 409.7595 | 459.5005 | 81.95659 | 310.8131 | 433.0579 | 496.6049 |
| CUST_52742_PI429010886 | M98736 | 0.034308 | 12 | 3 | 2660868 | fast tropomyosin isoform [Homarus americanus] | | | 2.05E-12 | 103.0626 | 103.5822 | 70.11707 | 129.1538 | 105.8984 | 139.9078 | 139.7302 | 95.7753 | 67.41072 | 88.07214 | 76.72395 | 73.45438 | 88.45598 | 78.16064 | 64.46821 | 87.70762 | 139.419 | 118.9935 | 113.1785 | 82.95644 | 87.34049 | 104.474 | 85.19825 | 104.2212 | 72.00841 | 95.82559 | 69.17707 |
| CUST_8170_PI429010886 | N22640 | 0.034308 | 12 | 0 | | | Nucleic acid metabolism | DNA replication | | 13.25777 | 21.28336 | 17.80745 | 10.69036 | 14.62285 | 16.11003 | 14.91349 | 14.35036 | 20.67573 | 19.27695 | 17.63322 | 28.78903 | 18.18793 | 21.26038 | 24.91507 | 17.80424 | 9.86011 | 10.59893 | 23.87245 | 17.13668 | 15.67417 | 20.17126 | 13.31366 | 17.09735 | 25.02916 | 19.79646 | 19.96165 |
| CUST_11149_PI429010886 | N27348 | 0.034308 | 12 | 3 | | | | | | 26.89586 | 6.454629 | 8.930028 | 102.5774 | 48.50963 | 227.9039 | 29.96087 | 52.42987 | 16.90254 | 17.76311 | 40.28418 | 9.631079 | 28.28611 | 16.20777 | 14.90554 | 30.06426 | 173.3481 | 95.86282 | 37.1484 | 14.33789 | 25.97958 | 26.67547 | 32.22652 | 47.36571 | 16.28372 | 78.78492 | 37.818 |
| CUST_31108_PI429010886 | M720 | 0.034343 | 21 | 6 | 242022061 | eukaryotic translation initiation factor 4E type, puta | Protein metabolism | Regulation of translational initiation | 3.67E-67 | 41.26402 | 41.02914 | 44.25018 | 60.05679 | 50.46685 | 68.45737 | 58.55654 | 67.73286 | 58.47643 | 30.70184 | 41.35027 | 45.27452 | 51.9493 | 57.75281 | 38.4769 | 42.0436 | 58.77193 | 51.54393 | 49.91173 | 39.288 | 26.69563 | 44.03128 | 32.75472 | 60.30436 | 46.52112 | 46.85243 | 41.71517 |
| CUST_5307_PI429010886 | N18522 | 0.034343 | 24 | 18 | | | Protein metabolism | Cellular protein modification process | | 643.0031 | 805.8232 | 535.7875 | 573.9804 | 583.8403 | 559.8475 | 594.8858 | 538.0354 | 471.9637 | 643.0031 | 603.7585 | 721.1961 | 602.272 | 769.5294 | 651.2944 | 919.3319 | 642.3044 | 545.6531 | 659.1977 | 827.8275 | 608.3741 | 773.4201 | 742.9123 | 593.8074 | 702.4802 | 610.0761 | 736.3462 |
| CUST_29371_PI429010886 | N59731 | 0.034357 | 24 | 18 | | | | | | 21.77287 | 9.609115 | 7.511797 | 8.316685 | 14.31366 | 8.435831 | 20.25455 | 10.94511 | 4.956681 | 24.15363 | 22.72662 | 6.408788 | 20.35314 | 12.20709 | NA | 21.1658 | 28.51585 | 14.54874 | 25.46475 | 21.97852 | 29.42444 | 20.38809 | 6.257639 | 15.33921 | 23.75128 | NA | 23.61053 |
| CUST_8901_PI429010886 | N23769 | 0.03439 | 21 | 3 | | | | | | 41.44167 | 39.39544 | 51.25727 | 69.36484 | 42.48986 | 55.25124 | 30.12812 | 54.88431 | 35.69319 | 35.57579 | 40.53866 | 42.57633 | 32.51731 | 51.60182 | 27.19088 | 39.33849 | 37.63897 | 36.95956 | 33.92714 | 28.55766 | 55.90718 | 48.43649 | 42.13297 | 34.56933 | 37.66305 | 50.19147 | 53.35562 |
| CUST_11368_PI429010886 | N27700 | 0.034401 | 24 | 0 | | | | | | 320.0372 | 418.1872 | 344.9738 | 300.2063 | 358.7967 | 366.3446 | 279.7821 | 312.2468 | 294.3403 | 341.9433 | 303.1495 | 337.1609 | 368.1562 | 299.5774 | 235.0098 | 319.3199 | 305.2258 | 300.8257 | 180.3556 | 288.2901 | 337.0265 | 399.475 | 372.4888 | 363.8038 | 478.4338 | 306.5697 | 408.7569 |
| CUST_53689_PI429010886 | M102814 | 0.034422 | 24 | 0 | 260785109 | hypothetical protein BRAFLDRAFT_194496 [Branchi | | | 4.76E-26 | 27.85864 | 32.99848 | 37.34877 | 20.54044 | 29.12035 | 22.38112 | 16.69997 | 13.33386 | 22.62556 | 27.58634 | 22.01914 | 20.22864 | 12.80948 | 12.94569 | NA | 30.45027 | 19.06154 | 20.04102 | 17.78988 | 15.96275 | 13.72842 | 19.52925 | 19.60862 | 12.07646 | 25.72198 | 27.56341 | 18.68272 |
| CUST_34081_PI429010886 | M8715 | 0.034427 | 21 | 0 | | | | | | 17.67709 | 11.93121 | NA | 7.173073 | 14.2617 | 5.132987 | 9.485366 | 9.064427 | 6.05735 | 13.59135 | 7.13523 | 4.250522 | 6.46725 | 12.49757 | 6.344872 | 13.55604 | 9.24163 | 8.701981 | 6.485495 | 7.458433 | NA | 17.47239 | 24.96011 | 19.10453 | 16.09421 | NA | 4.164288 |
| CUST_32954_PI429010886 | M5558 | 0.034427 | 21 | 18 | 321477672 | zinc finger protein [Daphnia pulex] | | | 1.34E-39 | 135.6374 | 117.1631 | 105.3949 | 71.11942 | 106.2741 | 48.69863 | 77.24703 | 54.73192 | 70.29196 | 142.7994 | 105.2803 | 80.1769 | 79.36987 | 90.8776 | 74.70996 | 133.8595 | 95.63731 | 108.7487 | 107.7242 | 108.4917 | 95.87365 | 131.513 | 77.5423 | 94.5785 | 157.9764 | 94.17604 | 93.5311 |
| CUST_49261_PI429010886 | M84937 | 0.034431 | 15 | 9 | | | | | | 49.02131 | 45.07578 | 69.83541 | 34.51534 | 45.30656 | 50.88842 | 41.86651 | 50.77711 | 49.36928 | 62.1958 | 53.12725 | 55.76383 | 50.75355 | 44.98106 | 48.10026 | 37.96514 | 42.08627 | 47.57087 | 45.38957 | 44.00078 | 50.02348 | 44.51031 | 45.26792 | 38.93621 | 69.65899 | 53.15159 | 83.383 |
| CUST_42028_PI429010886 | M59192 | 0.034434 | 24 | 18 | 350417077 | PREDICTED: protein lap4-like isoform 3 [Bombus im | | | 3.5E-36 | 14.99598 | 24.77587 | 18.12837 | 11.14656 | 15.09377 | 14.55595 | 12.96451 | 16.20777 | 12.2919 | 20.859 | 17.17252 | 35.84618 | 19.50301 | 19.87757 | 20.08306 | 24.8275 | 7.832427 | 20.55089 | 23.38781 | 25.63163 | 17.64058 | 28.08839 | 15.27765 | 18.34756 | 20.11905 | 21.38108 | 23.2348 |
| CUST_9290_PI429010886 | N24380 | 0.034434 | 21 | 3 | 383852228 | PREDICTED: major antigen-like [Megachile rotundat | | | 2.83E-10 | 189.6625 | 691.1905 | 261.2237 | 861.4839 | 330.6123 | 509.8767 | 240.4265 | 319.7057 | 218.4835 | 216.1505 | 147.7228 | 367.1141 | 210.1562 | 289.423 | 203.324 | 284.093 | 114.2745 | 213.9187 | 122.8973 | 270.4372 | 343.6779 | 224.7094 | 259.8087 | 262.9561 | 413.7293 | 554.3622 | 201.1709 |
| CUST_8834_PI429010886 | N23668 | 0.034434 | 24 | 18 | 270013578 | hypothetical protein TcasGA2_TC012198 [Tribolium | Developmental process | Multicellular organismal development | 1.25E-19 | 23.97713 | 22.13675 | 21.03394 | 10.85866 | 30.72063 | 15.04347 | 15.90511 | 12.19407 | 19.4114 | 38.97496 | 20.40333 | 34.2205 | 20.58435 | 31.15277 | 24.58201 | 25.73562 | 13.18143 | 18.98793 | 31.6776 | 28.80614 | 32.56046 | 27.67304 | 30.90181 | 27.01225 | 29.75783 | 27.0513 | 30.82595 |
| CUST_50406_PI429010886 | M89711 | 0.034439 | 24 | 0 | 443706934 | hypothetical protein CAPTEDRAFT_164592 [Capitell | | | 1.11E-17 | 65.84223 | 65.28197 | 80.18725 | 56.05994 | 48.66243 | 41.79247 | 61.5685 | 57.61472 | 65.17572 | 68.01317 | 50.8686 | 61.74032 | 53.9799 | 60.77736 | 53.01337 | 67.28983 | 48.10026 | 62.32467 | 61.95712 | 59.83655 | 59.9058 | 72.2954 | 73.75273 | 45.77355 | 88.15874 | 77.45737 | 81.29483 |
| CUST_5455_PI429010886 | N18727 | 0.034494 | 21 | 0 | 321472375 | hypothetical protein DAPPUDRAFT_301948 [Daphn | Signal transduction | Cell-cell signaling | 6.11E-19 | 78.9119 | 64.04953 | 101.9838 | 70.90194 | 73.62026 | 48.53244 | 100.0857 | 49.16013 | 56.11599 | 51.21188 | 73.53135 | 39.58144 | 33.76983 | 58.62811 | 40.39432 | 55.04289 | 73.09859 | 51.5653 | 120.2652 | 30.2284 | 119.6777 | 77.76831 | 98.34685 | 80.5848 | 80.71386 | 32.01033 | 57.09554 |
| CUST_38591_PI429010886 | M28372 | 0.034512 | 15 | 9 | | | | | | 642.3044 | 979.2899 | 1007.032 | 726.7504 | 830.4425 | 918.9933 | 761.5045 | 756.3176 | 831.0172 | 1166.392 | 764.5382 | 1135.012 | 843.5117 | 1099.35 | 946.7835 | 963.6143 | 742.6323 | 832.9715 | 769.8974 | 849.0108 | 810.6402 | 1071.523 | 919.6285 | 798.0318 | 1150.815 | 1010.24 | 1070.179 |
| CUST_32873_PI429010886 | M5330 | 0.034512 | 15 | 9 | 2290213 | Lian-Aa1 retrotransposon protein [Aedes aegypti] | | | 1.23E-57 | 629.4397 | 737.499 | 775.9372 | 472.0832 | 564.1196 | 723.4421 | 540.1617 | 638.1084 | 770.9286 | 966.1688 | 639.6792 | 930.2399 | 638.7779 | 897.4259 | 694.3101 | 917.4556 | 672.3398 | 716.9224 | 687.964 | 867.1839 | 642.1649 | 747.806 | 741.2403 | 970.6979 | 929.9665 | 991.6341 | 1002.452 |
| CUST_42380_PI429010886 | M60142 | 0.034512 | 15 | 3 | | | | | | 6336.037 | 1623.132 | 7266.955 | 10478.08 | 8955.736 | 9795.872 | 9697.645 | 6073.086 | 764.7939 | 543.6335 | 9305.385 | 19.01959 | 955.8025 | 577.6505 | 944.3898 | 110.4435 | 7435.628 | 1777.79 | 6962.573 | 30.01523 | 11983.26 | 31.93715 | 10143.74 | 9770.164 | 55.53245 | 1568.166 | 163.2245 |
| CUST_45618_PI429010886 | M69686 | 0.034512 | 15 | 3 | | | | | | 28.57032 | 15.57429 | 14.87518 | 26.78191 | 28.20406 | 30.73521 | 22.37306 | 22.20487 | 14.27406 | 15.00181 | 18.44132 | 11.51496 | 18.88759 | 11.83225 | 29.05988 | 13.93436 | 26.58331 | 19.26616 | 17.46064 | 31.79024 | 17.96838 | 21.7849 | 20.72901 | 21.51397 | 15.98525 | 13.05013 | 18.48442 |
| CUST_46368_PI429010886 | M72411 | 0.034512 | 15 | 0 | | | | | | 156.7533 | 148.65 | 142.6758 | 109.0596 | 165.2021 | 223.8787 | 131.3416 | 56.03701 | 103.7315 | 108.5779 | 205.4212 | 161.2704 | 89.87648 | 98.50813 | 131.4711 | 191.5641 | 270.1288 | 221.852 | 181.7853 | 140.4751 | 132.8611 | 143.4699 | 87.18471 | 97.91604 | 200.9848 | 128.623 | 153.5542 |
| CUST_6903_PI429010886 | N20755 | 0.034512 | 15 | 0 | 328779109 | PREDICTED: Apis mellifera vinculin (Vinc), mRNA | Cellular Process | Cell adhesion | 1.43E-84 | 29.13814 | 27.71267 | 30.6709 | 20.0846 | 26.19664 | 17.10987 | 19.20292 | 20.74032 | 28.5492 | 23.2298 | 26.93627 | 24.0705 | 27.00978 | 21.93409 | 20.62738 | 23.39071 | 29.29583 | 33.97352 | 37.24643 | 23.45771 | 12.78166 | 16.46122 | 17.34168 | 15.33408 | 23.76609 | 41.0817 | 26.31693 |
| CUST_25245_PI429010886 | N52057 | 0.034512 | 15 | 9 | | | | | | 73.91931 | 81.57207 | 89.15191 | 11.92804 | 71.78694 | 73.90307 | 83.28321 | 96.75892 | 75.93064 | 71.37464 | 98.42661 | 55.55874 | 71.56769 | 110.765 | 74.71666 | 102.9429 | 95.17141 | 80.43582 | 44.78592 | 70.55676 | 51.14292 | 143.5589 | 69.04997 | 98.14502 | 161.2028 | 100.1493 | 140.2821 |
| CUST_32228_PI429010886 | M3582 | 0.034527 | 21 | 9 | | | | | | 271.6132 | 325.8124 | 343.8588 | 199.1321 | 224.0471 | 172.7143 | 221.6212 | 333.2348 | 303.1495 | 375.1955 | 238.2063 | 381.6649 | 252.6253 | 282.5938 | 256.1926 | 244.3511 | 230.2231 | 271.918 | 233.7917 | 305.2873 | 231.6859 | 215.7523 | 299.0972 | 205.392 | 260.3136 | 183.5489 | 206.3065 |
| CUST_20791_PI429010886 | N44060 | 0.034528 | 15 | 3 | | | | | | 2756.174 | 44.00542 | 4471.709 | 1275.591 | 2215.807 | 3092.843 | 4887.374 | 955.8025 | 267.2735 | 137.1593 | 2587.199 | 6.980107 | 325.0318 | 302.8326 | 212.6792 | 13.06491 | 4979.355 | 2529.511 | 5013.208 | NA | 5009.146 | 6.282328 | 2975.526 | 3421.62 | 34.52588 | 1239.225 | 43.49365 |
| CUST_15114_PI429010886 | N34032 | 0.034576 | 21 | 0 | | | | | | 72.8194 | 63.73239 | 47.43001 | 42.08627 | 46.16276 | 46.1038 | 35.18059 | 40.10273 | 28.35783 | 65.20986 | 52.41832 | 36.46662 | 39.70082 | 36.85539 | 35.6143 | 57.01626 | 60.83438 | 66.19578 | 36.73446 | 57.50407 | 44.19983 | 82.67802 | 43.15798 | 47.82182 | 62.65132 | 39.55173 | 53.36598 |
| CUST_28842_PI429010886 | N58710 | 0.034576 | 21 | 0 | | | | | | 49.65587 | 43.06591 | 68.14294 | 27.70089 | 16.79285 | 18.52719 | 20.44316 | 12.6884 | 17.93802 | 49.31188 | 12.65789 | 33.89428 | 28.3838 | 22.7759 | 34.13077 | 49.51255 | 8.835496 | 47.29592 | 27.40752 | 46.85243 | 17.73942 | 67.99795 | 40.2964 | 38.96658 | 6.822524 | 14.48109 | 45.61086 |
| CUST_55534_PI429010886 | M110554 | 0.034605 | 24 | 12 | | | | | | 55.93797 | 66.15751 | 122.5481 | 104.302 | 61.64915 | 88.72989 | 74.64909 | 75.86883 | 128.8011 | 76.30835 | 94.44207 | 153.6441 | 121.562 | 244.4297 | 106.3929 | 91.80446 | 136.1436 | 110.0483 | 61.88462 | 66.71625 | 70.92151 | 113.0325 | 87.36382 | 84.41305 | 60.63941 | 91.00006 | 79.92252 |
| CUST_42508_PI429010886 | M60461 | 0.034627 | 18 | 15 | | | | | | 79.46381 | 91.60957 | 83.87586 | 89.64325 | 82.62874 | 53.77384 | 61.58977 | 41.35027 | 48.70779 | 104.976 | 91.18913 | 118.0864 | 92.52049 | 80.22855 | 89.90347 | 97.4551 | 60.67368 | 123.1229 | 80.1659 | 113.4075 | 86.70732 | 121.4843 | 60.16545 | 81.08583 | 61.76631 | 66.57113 | 82.30578 |
| CUST_31440_PI429010886 | M1533 | 0.034644 | 24 | 15 | | | | | | 2665.55 | 2415.728 | 2156.875 | 1953.939 | 2690.41 | 2400.961 | 1525.514 | 1738.403 | 1647.631 | 2735.202 | 2439.013 | 2461.297 | 1539.294 | 1913.282 | 2092.578 | 4253.825 | 2606.005 | 2937.772 | 2688.606 | 3143.095 | 2404.795 | 3590.082 | 1779.751 | 2046.644 | 3564.266 | 1923.113 | 3077.216 |
| CUST_19000_PI429010886 | N40808 | 0.03467 | 21 | 3 | | | | | | 6454.955 | 12034.22 | 8015.915 | 17966.78 | 11601.88 | 10190.76 | 4150.569 | 4041.003 | 7717.52 | 8582.669 | 4424.066 | 11779.89 | 5674.79 | 7128.301 | 3917.614 | 10387.85 | 3276.015 | 8391.959 | 2621.227 | 4808.982 | 8504.473 | 8611.654 | 9670.846 | 6078.985 | 11762.42 | 14600.6 | 6426.682 |
| CUST_36073_PI429010886 | M14542 | 0.034683 | 15 | 12 | 242005387 | cAMP-dependent protein kinase catalytic subunit, p | Signal transduction | Other | 1.41E-54 | 109.6567 | 116.9761 | 146.0723 | 104.0978 | 95.91036 | 122.7974 | 98.7787 | 88.05483 | 108.6889 | 116.5651 | 111.5705 | 171.1636 | 108.7377 | 139.7302 | 152.3775 | 127.7879 | 106.9908 | 108.3734 | 131.8938 | 132.5094 | 93.69637 | 117.6603 | 105.5375 | 93.37534 | 150.2252 | 135.0881 | 149.8707 |
| CUST_44963_PI429010886 | M67545 | 0.034683 | 15 | 12 | | | | | | 33.99576 | 36.29122 | 37.94571 | 27.32084 | 21.17293 | 22.98483 | 28.1163 | 39.88324 | 31.1045 | 39.85359 | 27.05448 | 41.47082 | 31.68997 | 38.32695 | 38.86122 | 45.30656 | 35.07318 | 40.07222 | 28.51242 | 31.74172 | 20.92107 | 44.08735 | 32.42452 | 26.06598 | 36.67205 | 30.04394 | 38.13489 |
| CUST_6334_PI429010886 | N19964 | 0.034683 | 15 | 12 | 227122178 | masquerade-like serine proteinase-like protein 2 [P | Protein metabolism | Proteolysis | 6.63E-79 | 1270.943 | 1606.601 | 1467.042 | 1009.233 | 916.49 | 575.3599 | 1501.641 | 1617.488 | 1739.21 | 1664.492 | 1006.198 | 1106.54 | 1386.212 | 1860.994 | 1681.036 | 1451.905 | 1131.289 | 933.4785 | 1320.618 | 1073.06 | 797.7975 | 1602.617 | 1240.783 | 1076.18 | 1466.368 | 914.3958 | 1462.44 |
| CUST_11990_PI429010886 | N28737 | 0.034683 | 15 | 12 | 395529788 | PREDICTED: uncharacterized protein LOC10092264 | | | 3.44E-10 | 48.45297 | 55.25615 | 81.44839 | 40.86391 | 58.74569 | 30.37645 | 46.96117 | 62.38529 | 50.09589 | 94.95262 | 54.38291 | 63.08907 | 66.72453 | 84.34439 | 68.44956 | 76.06574 | 42.77863 | 62.406 | 46.30373 | 57.26217 | 62.77579 | 80.9345 | 78.43466 | 44.97088 | 83.26471 | 49.59376 | 63.01798 |
| CUST_13216_PI429010886 | N30794 | 0.034683 | 15 | 12 | | | | | | 137.1215 | 229.7558 | 155.2537 | 120.3232 | 129.065 | 162.5528 | 120.6514 | 92.74338 | 135.1036 | 182.6137 | 136.3233 | 186.9387 | 137.0179 | 193.0385 | 152.3321 | 174.1468 | 140.8407 | 137.9867 | 150.434 | 100.3487 | 133.5047 | 180.9857 | 86.44204 | 99.8601 | 208.1099 | 130.5819 | 109.8428 |
| CUST_25036_PI429010886 | N51672 | 0.034683 | 15 | 12 | 321461624 | hypothetical protein DAPPUDRAFT_326012 [Daphn | | | 8.49E-14 | 34.43474 | 33.84879 | 49.23486 | 36.96378 | 37.08279 | 32.93338 | 25.49499 | 30.33165 | 36.7953 | 49.81881 | 36.65626 | 48.39849 | 41.87571 | 53.20475 | 49.03345 | 69.83541 | 42.89451 | 39.58944 | 46.29899 | 45.7065 | 28.20146 | 32.10376 | 34.91643 | 43.28403 | 54.92635 | 42.98911 | 54.56432 |
| CUST_32069_PI429010886 | M3152 | 0.034697 | 24 | 6 | | | | | | 1719.247 | 1253.921 | NA | 3653.581 | 273.2835 | 4161.995 | 3124.162 | 3249.343 | 3266.951 | NA | 71.83981 | NA | 1952.727 | NA | 1340.855 | 272.3366 | 479.9166 | 717.1047 | 1040.536 | NA | 53.08483 | 17.23726 | 41.62677 | 2219.95 | NA | 2299.417 | 31.31664 |
| CUST_6825_PI429010886 | N20649 | 0.034703 | 12 | 0 | | | | | | 73.28621 | 60.63941 | 78.59764 | 33.95306 | 49.08084 | 45.50116 | 55.29787 | 63.78913 | 65.30643 | 110.9478 | 63.8461 | 66.28161 | 62.76241 | 86.64513 | 71.84934 | 53.12725 | 63.05647 | 47.46581 | 75.61377 | 38.06259 | 70.64745 | 56.33375 | 70.63158 | 66.79779 | 61.45036 | 62.46096 | 106.9318 |
| CUST_17679_PI429010886 | N38399 | 0.034703 | 12 | 0 | | | | | | 53.01337 | 89.24944 | 201.5878 | 27.88984 | 20.09233 | 12.22036 | 41.06999 | 160.5748 | 175.4136 | 105.6941 | 77.95844 | 119.6384 | 151.582 | 387.8661 | 234.853 | 60.54253 | 120.3338 | 53.86098 | 61.09062 | 221.8177 | 142.418 | 267.177 | 299.837 | 71.97535 | 191.2388 | 152.4677 | 228.2456 |
| CUST_24603_PI429010886 | N50919 | 0.034708 | 24 | 21 | | | | | | 52.20745 | 32.43577 | 49.37977 | 34.88582 | 29.75783 | 32.20795 | 32.15263 | 42.16986 | 31.22335 | 35.21227 | 43.28403 | 36.80914 | 30.27835 | 39.8885 | 36.46662 | 50.14002 | 38.93982 | 50.80674 | 44.92995 | 40.97284 | 37.40452 | 48.61666 | 58.96932 | 38.88459 | 44.97088 | 33.75702 | 48.22079 |
| CUST_48381_PI429010886 | M80085 | 0.03471 | 18 | 9 | 512922551 | PREDICTED: poly(U)-specific endoribonuclease hom | | | 7.83E-12 | 22.74008 | 16.34592 | 24.09666 | 13.43228 | 11.92991 | 20.39615 | 6.74367 | 22.42981 | 28.27144 | 28.32791 | 38.68077 | 26.64032 | 17.73598 | 8.86752 | 24.93734 | 27.91206 | 9.880246 | 20.6935 | 20.2137 | 16.45281 | 14.64501 | 10.46038 | 10.85593 | 18.87506 | 22.04537 | 12.94311 | 20.35525 |
| CUST_21173_PI429010886 | N44764 | 0.03471 | 18 | 15 | | | | | | 42.98911 | 35.6179 | 46.00876 | 37.25844 | 34.41491 | 37.49092 | 55.02956 | 48.94831 | 53.70396 | 34.42762 | 37.22562 | 39.38726 | 45.63167 | 51.43692 | 44.331 | 58.96311 | 55.57175 | 45.3118 | 62.83219 | 42.72999 | 42.32326 | 36.58254 | 32.54901 | 27.70733 | 81.39522 | 35.95337 | 76.33468 |
| CUST_13920_PI429010886 | N32015 | 0.034722 | 12 | 9 | | | | | | 205.5305 | 229.6175 | 319.8266 | 203.4225 | 220.1334 | 230.8944 | 214.9913 | 244.0376 | 231.1582 | 284.0133 | 272.4885 | 282.2712 | 301.5058 | 330.1179 | 282.7521 | 240.8224 | 233.6069 | 211.6967 | 272.9055 | 370.2985 | 225.9175 | 460.6363 | 252.2065 | 308.5953 | 227.642 | 215.4042 | 278.9452 |
| CUST_28383_PI429010886 | N57842 | 0.034728 | 15 | 12 | | | | | | 6.423606 | 10.38786 | 9.216332 | NA | 11.67245 | 5.950176 | 10.10842 | 6.275836 | 6.257639 | 13.7972 | 19.25031 | 15.72131 | 20.01489 | 13.48403 | 27.45566 | 10.1142 | 6.252271 | 15.70756 | 8.3032 | 9.664283 | 9.886641 | 26.71388 | 11.48488 | NA | 7.316619 | 12.28413 | 14.25774 |
| CUST_53548_PI429010886 | M102208 | 0.034757 | 24 | 21 | | | | | | 21.02638 | 8.464949 | 10.02441 | 3.909489 | 7.643809 | 8.167815 | NA | 10.94786 | 3.802865 | 7.993052 | 8.949545 | 3.516817 | 8.442875 | 8.557801 | 4.89178 | 6.824426 | 12.39257 | 12.29064 | 8.317554 | NA | 15.4125 | 19.47912 | 12.88422 | NA | 17.28828 | 3.427839 | 20.08306 |
| CUST_55424_PI429010886 | M110029 | 0.034774 | 24 | 12 | | | | | | 255.0348 | 248.959 | 282.8059 | 211.1137 | 222.5078 | 248.1506 | 213.8921 | 222.2769 | 243.5502 | 269.7173 | 243.8404 | 359.4272 | 261.2742 | 345.3911 | 374.3698 | 315.0568 | 237.5448 | 296.3588 | 264.8381 | 290.8448 | 272.4368 | 311.8189 | 299.9058 | 228.3459 | 285.9079 | 285.0395 | 351.983 |
| CUST_33430_PI429010886 | M6863 | 0.034779 | 24 | 3 | | | | | | 49.81358 | 32.57176 | NA | 96.97877 | 39.48403 | 98.50813 | 54.95718 | 33.81876 | 30.58787 | NA | 29.32288 | NA | 19.20978 | 7.083214 | 21.74923 | 13.60023 | 40.53465 | 35.41597 | 50.3484 | NA | 4.89178 | 25.8552 | NA | 37.65143 | 6.626935 | 68.46628 | NA |
| CUST_55896_PI429010886 | M112043 | 0.034798 | 15 | 9 | | | | | | 48.37153 | 29.42815 | 77.77973 | 26.60013 | 27.80089 | 41.76249 | 42.09877 | 73.09177 | 53.04479 | 73.07849 | 35.74774 | 56.37263 | 49.43857 | 189.8916 | 46.66745 | 23.02991 | 43.60346 | 43.41776 | 65.09833 | 25.2086 | 36.95956 | 54.81682 | 80.63947 | 61.02614 | 61.00953 | 67.74201 | 75.91487 |
| CUST_34282_PI429010886 | M9237 | 0.0348 | 21 | 15 | | | | | | 32.68774 | 39.67691 | 44.85914 | 33.52987 | 25.51724 | 38.93621 | 26.96892 | 30.91745 | 34.14561 | 36.51599 | 33.49853 | 48.2481 | 25.13071 | 32.23977 | 41.74772 | 44.63016 | 40.98319 | 74.38643 | 35.49911 | 58.8975 | 32.51286 | 37.4709 | 40.45663 | 39.37758 | 43.12293 | 32.3494 | 35.57881 |
| CUST_55701_PI429010886 | M111370 | 0.034827 | 24 | 21 | | | | | | 16.51009 | 29.39664 | 14.63889 | 24.41426 | 14.92131 | 16.85059 | 9.336649 | 12.65013 | 12.58347 | 12.2919 | 5.137251 | 17.08729 | 11.63703 | 18.39223 | 7.359153 | 28.07627 | 11.66976 | 24.2228 | 9.091001 | 16.23717 | 19.25722 | 22.76207 | 15.91598 | 23.14492 | 19.07331 | 15.32584 | 19.06154 |
| CUST_47526_PI429010886 | M76470 | 0.034831 | 15 | 12 | | | | | | 73.84429 | 58.79065 | 82.36794 | 50.19562 | 57.28882 | 73.39754 | 66.16504 | 62.07681 | 56.40373 | 70.33128 | 55.31379 | 81.52938 | 78.16064 | 100.8047 | 84.1293 | 85.37214 | 65.76942 | 70.52611 | 64.09512 | 70.39937 | 67.28983 | 93.45456 | 71.91814 | 67.61178 | 80.14138 | 67.64264 | 83.87586 |
| CUST_34814_PI429010886 | M10776 | 0.034844 | 24 | 3 | | | | | | 275.7295 | 155.4878 | 113.1427 | 415.5762 | 145.4981 | 318.2412 | 383.351 | 248.4954 | 301.467 | 67.5589 | 55.52574 | 58.31001 | 279.7821 | 74.10143 | 166.9868 | 96.63027 | 141.8325 | 161.0222 | 132.88 | 68.06658 | 49.63154 | 130.3743 | 102.1576 | 214.2583 | 84.19357 | 297.5859 | 94.46416 |
| CUST_9492_PI429010886 | N24728 | 0.034844 | 21 | 3 | | | | | | 80.50367 | 64.17425 | 49.1644 | 77.08541 | 61.34598 | 66.39266 | 46.47367 | 76.28297 | 44.52812 | 76.353 | 33.1299 | 48.81237 | 55.6145 | 63.45518 | 61.12874 | 52.98238 | 40.97837 | 41.7785 | 49.62512 | 55.15882 | 36.606 | 51.42712 | 56.99104 | 23.47811 | 79.22919 | 53.38747 | 64.72483 |
| CUST_42295_PI429010886 | M59936 | 0.034895 | 15 | 9 | 371591236 | Euphausia superba voucher 760M mitochondrion, p | | | | 82964.44 | 70048.09 | 76927.64 | 95621.16 | 97928.01 | 80123.56 | 82964.44 | 42140.05 | 79324.15 | 103589.1 | 91360.38 | 85436.14 | 89222.73 | 71869.37 | 81077.7 | 66128 | 74829.64 | 77729.71 | 76141.25 | 73477.97 | 94450 | 84866.33 | 86620.4 | 97928.01 | 92843.62 | 90253.54 | 88771.33 |
| CUST_36860_PI429010886 | M17055 | 0.034896 | 15 | 0 | | | | | | 12.10209 | 32.62278 | 32.52286 | 26.90876 | 31.56943 | 10.77751 | 11.17643 | 22.21962 | 9.810562 | 25.68325 | 20.70392 | 24.08063 | 20.30807 | 49.28963 | 19.39824 | 28.28611 | 22.18811 | 29.20057 | 26.34508 | 23.06645 | 17.43225 | 19.35656 | 11.77781 | 12.59383 | 28.5492 | 20.0133 | 28.8656 |
| CUST_45661_PI429010886 | M69844 | 0.034896 | 15 | 9 | | | | | | 16.96167 | 21.75697 | 21.24309 | 12.41217 | 14.79848 | 16.37181 | 16.61727 | 21.13287 | 22.6112 | 20.70392 | 19.43895 | 27.25419 | 26.50391 | 18.08249 | 14.30732 | 22.43724 | 19.11241 | 19.98856 | 15.11263 | 16.50592 | 25.27468 | 17.09174 | 19.38108 | 20.43418 | 21.15229 | 22.40634 | 25.19447 |
| CUST_7699_PI429010886 | N21943 | 0.034896 | 15 | 9 | | | | | | 40.05116 | 56.05994 | 46.87213 | 57.70169 | 42.88574 | 28.94785 | 32.56779 | 47.21974 | 37.49843 | 61.84039 | 45.71121 | 63.02348 | 36.67205 | 55.87623 | 49.3486 | 53.41869 | 30.28541 | 52.83011 | 41.8887 | 44.1761 | 40.99957 | 59.56411 | 52.34805 | 36.27978 | 64.62304 | 65.99744 | 63.34887 |
| CUST_1254_PI429010886 | N9039 | 0.034896 | 15 | 9 | 242014887 | conserved hypothetical protein [Pediculus humanus | Nucleic acid metabolism | Regulation of gene expression | 2.33E-81 | 215.5318 | 182.6397 | 232.1818 | 181.9887 | 182.204 | 203.1495 | 150.3647 | 181.7752 | 220.1334 | 221.9188 | 192.442 | 266.7622 | 171.7375 | 266.7622 | 216.6952 | 241.2571 | 237.1988 | 237.7577 | 175.8238 | 220.1334 | 177.8716 | 214.4744 | 238.8193 | 202.6087 | 246.2665 | 243.0834 | 244.9911 |
| CUST_6772_PI429010886 | N20580 | 0.034919 | 24 | 0 | 260790163 | hypothetical protein BRAFLDRAFT_83391 [Branchio | | | 5.43E-40 | 15.32781 | 20.20434 | 16.08792 | 21.57749 | 16.04975 | 14.33423 | 9.865884 | 9.5748 | 14.8732 | 18.88981 | 13.87668 | 23.04991 | 7.614141 | 10.39893 | 12.88026 | 25.17015 | 10.63992 | 15.43461 | 12.45682 | 12.18711 | 20.85111 | 12.17439 | 27.33167 | 22.52377 | 23.90612 | 39.47942 | 19.70026 |
| CUST_16579_PI429010886 | N36495 | 0.034919 | 24 | 18 | | | | | | 24.09426 | 17.71924 | 27.78206 | 20.22452 | 23.22056 | 22.95261 | 22.1046 | 18.21042 | 21.42649 | 35.20829 | 18.79291 | 20.15665 | 23.5068 | 20.89343 | 26.39959 | 26.16386 | 28.58747 | 24.09666 | 27.44439 | 21.94249 | 34.75205 | 24.75921 | 32.816 | 23.73784 | 26.81013 | 21.89788 | 29.15809 |
| CUST_12144_PI429010886 | N28998 | 0.034943 | 21 | 15 | | | | | | 499.1257 | 467.2192 | 571.5932 | 372.0481 | 436.1525 | 471.8606 | 476.1852 | 334.379 | 435.8026 | 292.3195 | 458.9977 | 568.2975 | 456.3557 | 506.5013 | 480.7772 | 836.7397 | 500.7784 | 572.5618 | 642.6469 | 574.2435 | 454.6218 | 715.1399 | 406.5096 | 390.2834 | 675.3956 | 375.3397 | 457.7503 |
| CUST_11541_PI429010886 | N27992 | 0.034958 | 15 | 3 | | | | | | 63.5725 | 85.69951 | 77.83266 | 57.10423 | 103.5565 | 99.32217 | 91.52825 | 74.38643 | 51.52932 | 100.8047 | 94.26766 | 60.3963 | 58.19173 | 50.14442 | 49.71501 | 59.75541 | 93.98995 | 66.32627 | 114.8221 | 77.52659 | 80.75638 | 87.12746 | 86.4248 | 93.06352 | 59.9279 | 54.10071 | 40.56158 |
| CUST_2088_PI429010886 | N11540 | 0.03496 | 15 | 9 | 66276651 | chitinase [Fenneropenaeus chinensis] | Metabolic process | Carbohydrate metabolic process | 1.3E-172 | 4.852014 | 569.1252 | 231.4996 | 1320.152 | 1442.354 | 756.1137 | 610.5161 | 3271.146 | 2282.54 | 766.9049 | 2534.877 | 3213.851 | 1428.874 | 444.4951 | 871.2706 | NA | 561.1634 | NA | 443.9779 | 5762.468 | 2420.027 | NA | 876.7586 | 612.4623 | NA | 2533.526 | 1770.146 |
| CUST_47782_PI429010886 | M77604 | 0.034961 | 24 | 18 | | | | | | 30.112 | 34.34564 | 34.28069 | 27.71788 | 20.71416 | 24.0705 | 23.84605 | 22.92991 | 29.97999 | 37.60789 | 23.03433 | 26.59238 | 32.00329 | 31.93333 | 33.10045 | 34.8364 | 30.10928 | 29.81522 | 23.08262 | 38.25818 | 38.50578 | 33.14786 | 35.49419 | 25.62877 | 31.88893 | 35.81064 | 35.37131 |
| CUST_6331_PI429010886 | N19960 | 0.034962 | 24 | 15 | | | | | | 1012.109 | 1048.763 | 1112.064 | 635.437 | 811.2478 | 933.4785 | 612.1213 | 644.6096 | 527.0813 | 1036.073 | 929.2639 | 1083.296 | 566.9632 | 715.1399 | 899.5738 | 1242.81 | 1082.904 | 1263.557 | 923.7156 | 1061.893 | 883.6545 | 1253.307 | 650.3692 | 817.1902 | 1620.39 | 867.8685 | 1320.152 |
| CUST_371_PI429010886 | N4645 | 0.034965 | 21 | 3 | 363540724 | mg274 gene product [Megavirus chiliensis] >gi\|350 | Protein metabolism | Proteolysis | 1.09E-08 | 456.6758 | 44.04347 | NA | 862.6591 | 178.3541 | 882.153 | 347.6562 | 486.3734 | 193.0385 | NA | 28.32496 | NA | 292.5508 | 6.956638 | 103.8673 | 26.8214 | 607.4385 | 481.5308 | 52.9276 | 9.548233 | 10.86137 | 39.41947 | 16.35722 | 683.3484 | NA | 534.5603 | NA |
| CUST_39660_PI429010886 | M42375 | 0.034967 | 21 | 3 | | | | | | 31.15965 | 26.43248 | 33.49263 | 36.64631 | 24.74137 | 34.0946 | 20.36028 | 19.31282 | 30.89566 | 30.17815 | 25.27723 | 14.55221 | 26.85111 | 29.75783 | 20.31401 | 20.17559 | 24.54709 | 31.37421 | 29.25886 | 20.12366 | 43.19614 | 41.7785 | 20.46051 | 39.92228 | 32.21966 | 43.17145 | 47.07665 |
| CUST_4836_PI429010886 | N17587 | 0.035042 | 15 | 0 | | | | | | 46.53779 | 25.22098 | 56.29594 | 21.70738 | 21.09489 | 7.113361 | 52.66271 | 19.92665 | 43.00099 | 8.334073 | 11.81437 | 10.01939 | 35.03522 | 42.18909 | 30.18124 | 80.8031 | 78.30552 | 22.82685 | 34.97912 | NA | 4.908172 | 14.49351 | 7.061176 | 9.416182 | 25.47199 | 18.24055 | 17.03463 |
| CUST_34546_PI429010886 | M9982 | 0.035046 | 21 | 18 | 321469961 | hypothetical protein DAPPUDRAFT_318167 [Daphn | Metabolic process | Cellular amino acid metabolic process | 5.26E-54 | 75.27932 | 107.6537 | 59.80108 | 51.16457 | 107.2518 | 56.82909 | 58.80718 | 65.32582 | 61.19321 | 91.1558 | 87.10078 | 93.01036 | 78.06614 | 51.45306 | 77.84223 | 103.3151 | 87.42512 | 72.45575 | 98.22103 | 106.0325 | 95.91036 | 101.7859 | 65.82296 | 62.6571 | 70.58339 | 58.12612 | 65.04995 |
| CUST_36530_PI429010886 | M16159 | 0.035052 | 15 | 0 | | | | | | 308.4843 | 369.8403 | 273.9149 | 238.1723 | 287.4102 | 261.8822 | 165.4234 | 172.831 | 192.9815 | 333.2881 | 282.651 | 282.1142 | 193.1525 | 269.8655 | 274.0115 | 336.2223 | 309.517 | 290.8448 | 350.4435 | 278.7763 | 251.4731 | 334.1776 | 229.5755 | 242.0784 | 384.1514 | 220.485 | 293.704 |
| CUST_41244_PI429010886 | M57230 | 0.035086 | 15 | 12 | | | | | | 66.36423 | 51.89822 | 69.2413 | 51.44191 | 54.79286 | 79.12414 | 59.38984 | 63.41464 | 64.83984 | 53.20866 | 53.08483 | 63.17657 | 221.9975 | 81.20466 | 62.12557 | 64.55134 | 59.68907 | 66.43118 | 47.90583 | 48.57594 | 48.05852 | 56.62309 | 65.87593 | 59.31402 | 58.54952 | 67.40392 | 76.73952 |
| CUST_45869_PI429010886 | M70576 | 0.035086 | 15 | 12 | | | | | | 244.8705 | 197.0393 | 582.2842 | 190.588 | 207.4667 | 251.404 | 302.4916 | 335.333 | 421.6972 | 436.6018 | 202.1175 | 290.3277 | 345.3911 | 856.1081 | 343.3378 | 254.8434 | 466.0826 | 236.9243 | 247.7341 | 247.0952 | 219.4848 | 386.8992 | 602.798 | 220.4437 | 617.3843 | 254.189 | 669.0767 |
| CUST_8615_PI429010886 | N23305 | 0.035086 | 15 | 6 | | | | | | 91.62991 | 99.52485 | 272.4368 | 112.6761 | 50.78563 | 105.8112 | 130.9642 | 116.1614 | 147.687 | 118.4142 | 109.8598 | 88.52351 | 46.49334 | 30.5127 | 27.34913 | 60.05019 | 121.8904 | 80.57644 | 67.90618 | 55.96041 | 143.069 | 28.53867 | 171.2801 | 173.1695 | 37.38785 | 74.06718 | 93.84895 |
| CUST_17817_PI429010886 | N38643 | 0.035086 | 15 | 12 | | | | | | 138.0748 | 318.6961 | 154.0684 | 51.32239 | 155.0084 | 46.05345 | 2158.318 | 121.7341 | 2608.992 | 398.0093 | 25.41068 | 849.3126 | 1761.883 | 1010.922 | 1058.251 | 988.4432 | 1035.582 | 136.3625 | 222.3674 | 99.87407 | 215.4716 | 543.9462 | 235.4286 | 1361.981 | 1048.248 | 314.7546 | 1475.875 |
| CUST_51144_PI429010886 | M92357 | 0.035093 | 21 | 15 | | | | | | 21.50916 | 11.92189 | 17.97541 | 13.3566 | 14.71224 | 16.62548 | 15.56605 | 16.48691 | 16.71152 | 14.72748 | 9.194369 | 13.26879 | 11.99256 | 19.82309 | 16.59282 | 19.56278 | 17.94343 | 24.52204 | 22.42981 | 18.83592 | 19.19934 | 22.98966 | 12.05188 | 24.48268 | 14.04854 | 4.870293 | 18.53102 |
| CUST_47419_PI429010886 | M76033 | 0.035097 | 12 | 3 | | | | | | 155.7397 | 60.82114 | 43.26125 | 350.2055 | 261.6019 | 755.0608 | 229.3656 | 263.4332 | 80.92356 | 52.63886 | 229.2068 | 44.62034 | 121.5738 | 54.6573 | 68.26569 | 79.36014 | 367.921 | 308.7717 | 473.6842 | 52.59262 | 129.2719 | 77.39037 | 126.2324 | 206.2132 | 59.50102 | 354.4714 | 60.68591 |
| CUST_47810_PI429010886 | M77692 | 0.035097 | 12 | 9 | | | | | | 25.00056 | 33.23641 | 19.85963 | 37.22874 | 19.58019 | 24.48509 | 41.28276 | 19.59849 | 56.47376 | 32.87464 | 27.39792 | 42.14262 | 25.5782 | 37.87644 | 23.53335 | 30.88019 | 19.02839 | 29.95175 | 36.53812 | 39.93998 | 34.29142 | 47.05803 | 32.53358 | 56.32832 | 8.756045 | 11.53748 | 24.40654 |
| CUST_50261_PI429010886 | M89220 | 0.035097 | 12 | 3 | | | | | | 459.5005 | 441.3844 | 194.5182 | 1627.141 | 979.2899 | 3200.017 | 1260.686 | 633.8371 | 608.3741 | 44.80736 | 721.1961 | 34.32725 | 570.4962 | 171.9322 | 594.8858 | 375.4995 | 1658.285 | 2542.502 | 705.2005 | 99.10652 | 608.8662 | 369.6113 | 584.1415 | 1531.156 | 83.53882 | 1476.818 | 48.15042 |
| CUST_32733_PI429010886 | M4956 | 0.035107 | 21 | 18 | | | | | | 25.9871 | 231.931 | 127.9952 | NA | 25.44437 | 33.93687 | 21.53411 | 9.937128 | 39.79339 | 186.006 | 61.71609 | 112.457 | 138.7812 | 73.82839 | 44.1334 | 146.7819 | 70.99743 | 67.71039 | 158.1524 | 83.21855 | 46.99705 | 95.21856 | 110.8154 | 76.76597 | 86.4072 | 36.33605 | 30.83662 |
| CUST_48576_PI429010886 | M81118 | 0.035111 | 24 | 3 | | | | | | NA | 22.23809 | NA | 45.47327 | 21.10951 | 48.30459 | 7.533977 | 37.36976 | 15.97964 | 47.327 | 11.60175 | 62.12557 | 3.832676 | 6.884007 | 6.346476 | 10.90695 | 29.0899 | 7.579956 | 10.15754 | 30.92026 | NA | 102.8187 | 12.61842 | 6.697246 | 22.62792 | 18.80651 | NA |
| CUST_31636_PI429010886 | M2029 | 0.035118 | 18 | 9 | 321463221 | putative glycogen synthase [Daphnia pulex] | Metabolic process | Generation of precursor metabolites and energy | 2.54E-80 | 253.7351 | 258.3561 | 287.2312 | 110.2887 | 171.8979 | 185.0177 | 201.9396 | 372.8945 | 219.9975 | 286.3672 | 273.5534 | 364.4089 | 309.5934 | 232.9495 | 219.4848 | 168.1071 | 230.7517 | 146.1758 | 181.5403 | 319.7057 | 182.5658 | 212.3302 | 227.3812 | 125.6673 | 252.0883 | 206.0933 | 224.6063 |
| CUST_44179_PI429010886 | M65183 | 0.035118 | 18 | 15 | 157114890 | UDP-glucose 4-epimerase [Aedes aegypti] >gi\|1088 | Metabolic process | Nucleobase-containing compound metabolic process | 1.03E-27 | 151.8851 | 169.7046 | 135.8661 | 90.36196 | 207.9906 | 179.4697 | 99.42759 | 89.19702 | 94.7426 | 218.173 | 113.3684 | 225.1125 | 127.6888 | 142.4863 | 121.8737 | 232.484 | 239.3501 | 252.2065 | 113.2085 | 85.90922 | 98.2447 | 233.8649 | 138.9124 | 162.1184 | 117.7597 | 128.4938 | 110.8154 |
| CUST_10352_PI429010886 | N26105 | 0.035118 | 18 | 15 | | | | | | 116.7432 | 102.3183 | 157.7092 | 78.35921 | 83.05308 | 72.8194 | 95.86282 | 95.86282 | 84.90321 | 103.3649 | 95.54961 | 90.01113 | 87.69732 | 89.4301 | 86.36905 | 126.1331 | 124.8931 | 90.72781 | 104.8657 | 98.00231 | 95.98918 | 94.127 | 83.78522 | 56.18729 | 129.6012 | 74.92755 | 93.28305 |
| CUST_12544_PI429010886 | N29631 | 0.035118 | 18 | 15 | | | | | | 32.01382 | 23.69143 | 29.76053 | 17.45526 | 22.82685 | 18.67218 | 32.31352 | 24.11276 | 11.60175 | 21.40056 | 22.65868 | 24.62608 | 27.39792 | 17.57463 | 24.77058 | 28.69415 | 32.55631 | 34.23531 | 24.54416 | 23.98815 | 28.54572 | 19.03935 | 22.59879 | 25.43742 | 31.03692 | 28.42752 | 24.80361 |
| CUST_4444_PI429010886 | N16773 | 0.035124 | 18 | 15 | | | | | | 12.4096 | 25.75668 | 16.0606 | 8.914093 | 13.3861 | 6.578887 | 8.269849 | 7.647212 | 9.116313 | 10.65025 | 18.22302 | 19.75734 | 14.38683 | 16.54329 | 12.83461 | 21.77078 | 12.05188 | 19.70849 | 11.41581 | 27.49986 | 18.00026 | 20.4413 | 12.16205 | 5.592987 | 15.90941 | 5.916179 | 12.97303 |
| CUST_10499_PI429010886 | N26331 | 0.035127 | 12 | 6 | | | | | | 19.03935 | 7.936545 | 10.71844 | 21.59935 | 17.70313 | NA | 19.02605 | 18.10445 | NA | 16.57948 | 23.77465 | 8.351204 | 15.87666 | 17.16134 | 7.727602 | 11.14656 | 24.6471 | 7.943105 | 25.68589 | 21.68419 | 17.37163 | 17.14295 | 11.35064 | 11.86943 | 16.23717 | 11.27392 | 8.960873 |
| CUST_44757_PI429010886 | M66893 | 0.035134 | 24 | 3 | | | | | | 345.9993 | 176.2423 | 938.9786 | 871.2706 | 770.1948 | 1066.553 | 785.0368 | 847.5292 | 307.7804 | 117.1499 | 483.597 | 56.67223 | 77.3067 | 112.6306 | 217.9121 | 48.32025 | 221.5021 | 106.4305 | 369.6879 | 38.30389 | 636.5467 | 47.79169 | 658.6556 | 601.0454 | 56.11599 | 120.3747 | 44.40267 |
| CUST_5238_PI429010886 | N18437 | 0.035148 | 21 | 15 | | | | | | 327.7802 | 447.4667 | 267.7928 | 267.7928 | 379.3496 | 241.3085 | 222.9689 | 202.2232 | 249.5102 | 347.5866 | 253.5447 | 388.0401 | 253.8833 | 302.566 | 315.7204 | 455.9675 | 269.5259 | 357.7037 | 278.469 | 399.641 | 270.7613 | 394.7279 | 265.4892 | 268.6359 | 358.2812 | 265.7722 | 330.6123 |
| CUST_21456_PI429010886 | N45258 | 0.035148 | 24 | 18 | | | | | | 12.74489 | 28.3396 | 22.70524 | 16.1429 | 9.218873 | 20.67135 | 8.774888 | 15.5705 | 18.7049 | 18.79959 | 11.22575 | 24.65512 | 15.37925 | 22.25705 | 13.0885 | 28.4998 | 17.09347 | 24.26979 | 22.22394 | 22.28394 | 28.5492 | 19.59849 | 29.07242 | 19.58019 | 49.09421 | 21.86589 | 17.81453 |
| CUST_40024_PI429010886 | M48768 | 0.035164 | 24 | 18 | | | | | | 227.3812 | 198.1863 | 75.25456 | 41.76704 | 20.22094 | 40.36236 | 168.2221 | 58.25188 | 104.0193 | 165.0586 | 68.52849 | 41.09904 | 54.15061 | 51.99458 | 106.6808 | 494.4534 | 66.59221 | 340.8239 | 742.6323 | 104.363 | 330.8112 | 50.90018 | 14.14304 | 370.9949 | 11.16458 | 152.2232 | 262.997 |
| CUST_4249_PI429010886 | N16321 | 0.035164 | 24 | 21 | | | | | | 76.00655 | 86.95691 | 100.944 | 86.49959 | 106.1954 | 142.34 | 71.3067 | 98.29921 | 79.57182 | 70.15478 | 108.4414 | 90.84494 | 100.1974 | 76.40353 | 83.7299 | 102.5891 | 154.9299 | 105.4471 | 109.9562 | 96.95634 | 115.2365 | 102.4945 | 112.1531 | 156.4871 | 97.46537 | 111.3513 | 83.56858 |
| CUST_23361_PI429010886 | N48704 | 0.035167 | 24 | 18 | | | | | | 47.0845 | 50.90018 | 55.64622 | 34.43912 | 36.09309 | 55.42181 | 31.74877 | 35.58868 | 40.76795 | 47.97564 | 37.86103 | 38.18832 | 33.52107 | 39.38726 | 39.53295 | 61.54664 | 36.78596 | 46.58823 | 73.28621 | 58.46928 | 51.69686 | 42.37752 | 66.11527 | 37.84521 | 86.7972 | 45.69115 | 74.6525 |
| CUST_37934_PI429010886 | M22298 | 0.035208 | 24 | 18 | 507697569 | PREDICTED: 14 kDa phosphohistidine phosphatase [ | | | 7.29E-34 | 117.0641 | 196.9122 | 145.0637 | 277.3264 | 158.4321 | 103.654 | 91.59828 | 93.59388 | 116.2092 | 180.8735 | 132.3201 | 261.7284 | 178.0175 | 143.9014 | 176.6512 | 189.5687 | 83.87586 | 136.3105 | 181.6229 | 317.6417 | 192.442 | 202.005 | 141.0947 | 163.8459 | 168.2847 | 160.9512 | 188.427 |
| CUST_18456_PI429010886 | N39827 | 0.03521 | 12 | 3 | | | | | | 35.95633 | 51.04982 | 50.08673 | 53.6538 | 102.6636 | 85.95967 | 67.37108 | 33.88658 | 30.16973 | 63.12362 | 60.20751 | 44.52812 | 39.97721 | 43.72859 | 37.12271 | 45.35228 | 113.7469 | 60.62589 | 103.6887 | 52.56534 | 65.08 | 52.09002 | 62.24459 | 57.48318 | 62.94032 | 36.12242 | 38.96658 |
| CUST_50768_PI429010886 | M90936 | 0.035222 | 15 | 0 | | | | | | 20.90912 | 19.36347 | 15.42734 | 11.10641 | 10.76087 | 9.432782 | NA | NA | 10.52513 | 8.840456 | 6.913911 | 14.68311 | 14.15156 | 17.7146 | 4.808805 | 14.64036 | NA | 26.24303 | 13.04417 | 10.46578 | 21.19713 | 17.3695 | NA | 3.336108 | 14.49351 | 6.748738 | 8.553452 |
| CUST_49851_PI429010886 | M87431 | 0.035254 | 24 | 18 | | | | | | 49.26562 | 45.91808 | 62.43324 | 47.51073 | 47.02203 | 30.55308 | 35.91157 | 48.08 | 35.69903 | 57.76414 | 61.87735 | 58.59263 | 44.40636 | 56.45424 | 54.64518 | 67.75526 | 50.18155 | 50.9653 | 59.86951 | 69.96115 | 53.77384 | 68.22752 | 55.05355 | 52.98961 | 59.96834 | 46.6 | 69.86983 |
| CUST_50865_PI429010886 | M91275 | 0.035254 | 15 | 12 | | | | | | 39.21347 | 60.57719 | 38.74854 | 25.22969 | 28.04755 | 25.86296 | 49.81001 | 28.31921 | 86.14614 | 39.26587 | 32.20521 | 79.60953 | 59.38223 | 45.7065 | 57.78122 | 91.85305 | 44.17135 | 102.8187 | 42.34931 | 39.76167 | 22.5725 | 59.42335 | 31.1168 | 56.72941 | 55.65323 | 42.49968 | 53.51251 |
| CUST_8386_PI429010886 | N22968 | 0.035254 | 21 | 18 | | | Metabolic process | Cellular amino acid metabolic process | | 105.6941 | 155.9427 | 118.4789 | 101.4644 | 85.77208 | 75.43644 | 21.26332 | 37.35904 | 94.5785 | 97.37669 | 145.5578 | 121.5738 | 91.12979 | 107.0174 | 85.03701 | 92.93954 | 56.64713 | 55.72398 | 132.7469 | 153.3563 | 146.4231 | 175.7224 | 53.78964 | 111.0786 | 82.01377 | 126.6371 | 115.0393 |
| CUST_12314_PI429010886 | N29276 | 0.035254 | 21 | 6 | 380029118 | PREDICTED: uncharacterized protein LOC10087286 | | | 1.3E-30 | 59.6812 | 30.42483 | 80.05215 | 60.97252 | 50.78152 | 84.87934 | 125.183 | 92.50739 | 72.55972 | 68.95801 | 47.02813 | 39.02375 | 63.03207 | 56.82909 | 59.74927 | 45.67726 | 76.88221 | 51.53885 | 62.08961 | 37.65143 | 42.62004 | 41.77165 | 60.12109 | 66.3431 | 53.31549 | 55.85751 | 67.11047 |
| CUST_30953_PI429010886 | M297 | 0.035269 | 21 | 9 | 260835118 | 3-hydroxybutyrate dehydrogenase [Branchiostoma | Metabolic process | Other | 2.76E-68 | 6.718366 | 61.14077 | 12.40735 | 51.73412 | 110.4288 | 31.88231 | 60.41389 | 324.2442 | 270.9645 | 26.9466 | 389.0546 | 544.0717 | 233.7572 | 46.31902 | 129.6012 | 12.17117 | 114.3743 | 9.720983 | 32.35941 | 494.4534 | 316.122 | 3.733452 | 48.16109 | 42.15632 | 14.13262 | 213.8072 | 202.1751 |
| CUST_42006_PI429010886 | M59133 | 0.035279 | 15 | 9 | | | | | | 96.24145 | 92.24128 | 137.4373 | 75.33825 | 89.86196 | 97.87109 | 85.13186 | 99.07551 | 126.5253 | 132.4059 | 108.6016 | 117.8039 | 90.43345 | 107.6827 | 78.50617 | 106.9755 | 83.89746 | 113.6118 | 99.07551 | 84.93968 | 104.1211 | 92.43125 | 115.1567 | 85.51349 | 139.1034 | 112.0225 | 132.7894 |
| CUST_44183_PI429010886 | M65195 | 0.035279 | 15 | 0 | | | | | | 405.2638 | 370.0362 | 429.6585 | 283.6942 | 559.2711 | 579.7012 | 234.555 | 111.5107 | 231.235 | 360.4157 | 376.4458 | 521.397 | 115.6518 | 264.6685 | 187.869 | 471.4881 | 441.149 | 406.8847 | 375.2702 | 278.7196 | 290.8448 | 313.2862 | 227.225 | 235.6197 | 414.4964 | 291.2128 | 380.3886 |
| CUST_49102_PI429010886 | M84098 | 0.035279 | 15 | 9 | 341650460 | IKKepsilon2 [Litopenaeus vannamei] | | | 2.5E-24 | 44.62034 | 34.96354 | 47.20692 | 18.59923 | 21.81748 | 34.73538 | 37.36325 | 35.57263 | 29.13814 | 120.1854 | 37.04826 | 41.84077 | 30.85708 | 23.14834 | 38.39166 | 43.39642 | 39.99252 | 36.69997 | 35.21227 | 32.51998 | 30.69155 | 39.40002 | 42.96262 | 39.01912 | 47.19112 | 63.23408 | 30.98188 |
| CUST_13175_PI429010886 | N30714 | 0.035279 | 15 | 0 | | | | | | 174.7759 | 164.3174 | 135.061 | 84.86918 | 167.5217 | 151.5629 | 126.6828 | 107.4719 | 97.61331 | 150.9399 | 155.2181 | 101.8776 | 95.66411 | 98.04327 | 122.5181 | 194.605 | 176.4634 | 162.9606 | 155.9176 | 136.5699 | 116.1332 | 142.8727 | 103.0015 | 91.87634 | 192.7945 | 122.4782 | 154.3834 |
| CUST_21102_PI429010886 | N44633 | 0.035279 | 15 | 9 | | | | | | 46.64002 | 73.3681 | 46.34675 | 45.82436 | 48.05852 | 49.13878 | 40.67008 | 60.17363 | 33.90175 | 50.66205 | 55.70806 | 66.87846 | 53.81515 | 46.87213 | 21.32406 | 54.08762 | 57.70169 | 60.91532 | 43.00099 | 33.62524 | 44.25018 | 49.84719 | 43.87581 | 61.3315 | 60.7605 | 64.51419 | 81.03965 |
| CUST_34706_PI429010886 | M10412 | 0.03528 | 18 | 12 | | | | | | 56.83575 | 63.42029 | 71.39836 | 47.24387 | 54.83325 | 25.35443 | 50.70831 | 105.9305 | 86.39769 | 82.90947 | 42.43356 | 58.77783 | 84.36786 | 77.21633 | 68.72308 | 50.82151 | 42.53339 | 52.93772 | 39.32188 | 75.50768 | 44.85914 | 38.55767 | 47.07665 | 41.99643 | 56.13335 | 43.28403 | 75.51588 |
| CUST_36173_PI429010886 | M14876 | 0.035292 | 18 | 0 | | | | | | 147.0435 | 144.1009 | 110.7887 | 123.6231 | 115.3546 | 100.7109 | 91.71625 | 95.87365 | 90.08902 | 110.7887 | 113.7469 | 110.0909 | 122.1188 | 108.6696 | 102.8784 | 137.5279 | 97.91604 | 116.4203 | 102.4535 | 126.7437 | 100.64 | 148.8486 | 92.8298 | 128.5363 | 113.7364 | 82.62874 | 81.19526 |
| CUST_33687_PI429010886 | M7611 | 0.035309 | 21 | 18 | 321463478 | hypothetical protein DAPPUDRAFT_226671 [Daphn | Signal transduction | Other | 9.42E-19 | 68.50419 | 72.5683 | 88.01513 | 62.406 | 67.79763 | 49.1644 | 60.05019 | 57.50407 | 66.37927 | 92.71397 | 59.56411 | 67.26789 | 57.97853 | 79.13143 | 81.11384 | 96.05386 | 52.74471 | 87.12746 | 76.21064 | 91.72554 | 94.30937 | 126.0122 | 58.75806 | 64.16931 | 64.54153 | 53.73015 | 71.2192 |
| CUST_42603_PI429010886 | M60692 | 0.035315 | 15 | 9 | | | | | | 288.5922 | 308.7717 | 392.4795 | 318.2985 | 379.2779 | 434.3733 | 394.8691 | 351.7659 | 322.9274 | 406.6708 | 387.8661 | 413.1358 | 319.7057 | 414.8654 | 275.3515 | 297.7533 | 394.3244 | 334.7375 | 312.1179 | 357.5465 | 369.8842 | 292.1061 | 386.3188 | 390.3686 | 423.767 | 471.9637 | 419.5293 |
| CUST_8658_PI429010886 | N23368 | 0.035315 | 15 | 9 | 321473349 | hypothetical protein DAPPUDRAFT_194528 [Daphn | Protein metabolism | Cellular protein modification process | 3.46E-60 | 20.30647 | 17.81187 | 24.34524 | 22.54583 | 29.28006 | 23.1075 | 31.95513 | 22.41474 | 22.99283 | 31.89471 | 31.45667 | 35.30492 | 25.19666 | 27.39792 | 24.45953 | 26.58056 | 26.4083 | 17.84852 | 40.81311 | 20.70392 | 27.25206 | 24.00128 | 28.78903 | 18.88759 | 31.71523 | 35.37524 | 24.54709 |
| CUST_52298_PI429010886 | M96989 | 0.035352 | 15 | 0 | | | | | | 57.80004 | 46.60859 | 66.76607 | 53.88401 | 36.99055 | 40.17935 | 45.53479 | 42.61032 | 45.62788 | 46.68828 | 39.8966 | 43.44981 | 54.1859 | 82.41691 | 53.48278 | 58.94983 | 59.79684 | 52.10062 | 52.58293 | 63.40813 | 35.5525 | 51.29496 | 51.58072 | 37.61167 | 55.16431 | 47.64968 | 46.67853 |

| Probe ID | ID | p-value | Period | Phase | EBI ID | Annotation | GO category | Go term | E-value | 0 | 0 | 0 | 3 | 3 | 3 | 6 | 6 | 6 | 9 | 9 | 9 | 12 | 12 | 12 | 15 | 15 | 15 | 18 | 18 | 18 | 21 | 21 | 21 | 24 | 24 | 24 |
|---|---|---|---|---|---|---|---|---|---|---|---|---|---|---|---|---|---|---|---|---|---|---|---|---|---|---|---|---|---|---|---|---|---|---|---|---|
| CUST_9667_PI429010886 | N25018 | 0.035357 | 15 | 0 | 66499810 | PREDICTED: cleavage and polyadenylation specificit | Nucleic acid metabolism | RNA metabolic process | 1.2E-99 | 52.91094 | 68.22752 | 37.48336 | 46.02927 | 41.74272 | 45.39483 | 28.73411 | 18.98392 | 41.22879 | 47.63767 | 42.49492 | 45.79823 | 49.57451 | 34.98328 | 67.01506 | 68.01317 | 19.52364 | 60.3963 | 42.69298 | 49.1644 | 42.84657 | 42.82585 | 40.1699 | 35.97533 | 49.71501 | 34.36888 | 39.64527 |
| CUST_52266_PI429010886 | M96910 | 0.035377 | 18 | 3 | | | | | | 166.8948 | 423.0023 | 235.8718 | 675.9235 | 220.8472 | 360.1121 | 175.7224 | 192.324 | 167.0167 | 166.2981 | 84.43869 | 267.4223 | 138.7624 | 208.2116 | 181.0152 | 201.7725 | 69.16983 | 188.591 | 107.6827 | 171.9761 | 309.1008 | 237.0939 | 285.7468 | 194.3859 | 252.0041 | 362.0452 | 139.0616 |
| CUST_46260_PI429010886 | M71975 | 0.035405 | 18 | 3 | | | | | | 316.6467 | 176.6512 | 211.2278 | 159.3888 | 212.3072 | 247.7341 | 158.2183 | 100.1573 | 99.2894 | 228.2456 | 170.8315 | 220.8734 | 71.65614 | 130.8512 | 79.21035 | 46.69134 | 172.2992 | 146.882 | 98.46582 | 120.5518 | 126.1032 | 242.302 | 171.3142 | 228.8706 | 117.7722 | 66.86178 | 99.69352 |
| CUST_1366_PI429010886 | N9420 | 0.03541 | 21 | 9 | 321470487 | hypothetical protein DAPPUDRAFT_303430 [Daphn | | | 5.7E-108 | 296.6312 | 376.1162 | 220.8472 | 225.6208 | 268.0379 | 186.5931 | 276.9079 | 364.4089 | 208.4543 | 369.0374 | 482.753 | 527.4806 | 491.272 | 342.8578 | 270.4039 | 277.7171 | 301.6859 | 190.0964 | 504.8164 | 270.9139 | 363.0918 | 338.0611 | 286.0114 | 229.9104 | 462.7955 | 191.6398 | 184.2548 |
| CUST_54781_PI429010886 | M107652 | 0.035419 | 21 | 3 | | | | | | 17.55275 | 10.88915 | 9.301993 | 13.44726 | 18.65856 | 9.105658 | 11.29639 | 4.488345 | 9.673986 | 12.75496 | 6.796716 | 12.59383 | 8.004105 | 7.010504 | 5.601747 | 8.003112 | 12.60946 | 10.64535 | 19.46131 | 7.786537 | 7.327597 | 15.28275 | 8.197045 | 58.88281 | 16.75298 | 12.22036 | 17.30092 |
| CUST_22742_PI429010886 | N47591 | 0.035436 | 21 | 0 | | | | | | 60.24939 | 57.32094 | 70.61832 | 52.38415 | 39.667 | 40.59908 | 40.35311 | 45.63767 | 38.89335 | 53.49548 | 34.45395 | 62.67301 | 50.45103 | 54.79286 | 53.51858 | 65.75656 | 43.9229 | 54.37221 | 68.25442 | 52.08136 | 52.79221 | 77.63254 | 52.70102 | 51.58632 | 62.48649 | 46.55991 | 63.60606 |
| CUST_47247_PI429010886 | M75335 | 0.03544 | 18 | 9 | | | | | | 2923.398 | 4345.199 | 4201.227 | 3687.08 | 3899.401 | 3312.37 | 2454.105 | 5477.513 | 5105.143 | 3842.983 | 6925.74 | 11327.23 | 5717.187 | 5411.773 | 5372.754 | 4083.732 | 3243.287 | 3673.827 | 3320.318 | 6794.71 | 3534.122 | 4461.585 | 3466.9 | 2723.994 | 4544.383 | 4528.171 | 6225.291 |
| CUST_55098_PI429010886 | M108722 | 0.035489 | 15 | 12 | | | | | | 680.1392 | 714.7333 | 776.1766 | 765.4435 | 615.211 | 704.3489 | 561.5311 | 669.8776 | 709.6744 | 715.8969 | 637.2498 | 813.1389 | 697.0634 | 926.1166 | 916.49 | 922.2736 | 689.4854 | 791.0227 | 698.6187 | 693.2829 | 732.5018 | 676.9355 | 755.0608 | 518.9265 | 745.5335 | 764.3328 | 964.9461 |
| CUST_36230_PI429010886 | M15101 | 0.035489 | 15 | 12 | | | | | | 18.68272 | 35.37131 | 28.85946 | 21.77078 | 18.66111 | 38.57925 | 19.25031 | 24.61812 | 20.06061 | 36.06105 | 30.0795 | 30.74654 | 29.1329 | 37.33468 | 40.75297 | 34.82944 | 24.19734 | 33.23641 | 25.95428 | 22.61839 | 24.75921 | 46.09323 | 26.45722 | 27.13598 | 28.74055 | 31.89828 | 33.90948 |
| CUST_47025_PI429010886 | M74563 | 0.035489 | 15 | 12 | | | | | | 33.88658 | 26.14767 | 41.59029 | 13.56063 | 16.91106 | 22.6653 | 24.3983 | 42.91318 | 33.14406 | 72.26362 | 21.91188 | 35.60156 | 33.38385 | 46.65359 | 36.12687 | 46.37163 | 29.66792 | 25.01145 | 28.19636 | 30.9722 | 25.46747 | 31.267 | 42.81625 | 14.57688 | 42.30429 | 23.91586 | 46.61752 |
| CUST_47642_PI429010886 | M77016 | 0.035489 | 15 | 3 | | | | | | 45.46745 | 55.87623 | 18.0708 | 61.01573 | 48.92098 | 43.5699 | 39.79718 | 36.77308 | 25.68978 | 34.39152 | 41.46468 | 23.93813 | 30.29871 | 27.71495 | 15.22485 | 35.33784 | 50.45571 | 39.23735 | 56.67223 | 48.92704 | 25.97297 | 38.19514 | 25.80604 | 30.21389 | 38.11743 | 26.43455 | 25.888 |
| CUST_48906_PI429010886 | M82834 | 0.035489 | 15 | 12 | | | | | | 74.54819 | 89.80198 | 123.4578 | 63.37918 | 60.44797 | 75.4087 | 65.36594 | 94.92777 | 127.5818 | 108.9284 | 74.76616 | 105.1159 | 92.81476 | 114.743 | 111.7908 | 117.621 | 64.29604 | 95.36046 | 76.96996 | 96.8323 | 70.74696 | 85.06299 | 86.38663 | 95.86282 | 78.06614 | 104.1514 | 107.6827 |
| CUST_6959_PI429010886 | N20836 | 0.035489 | 15 | 3 | | | | | | 62.32467 | 57.70169 | 61.2231 | 65.46343 | 97.3486 | 93.21704 | 81.1623 | 56.37263 | 36.58662 | 55.45871 | 74.82753 | 42.72555 | 46.9717 | 51.49206 | 31.68743 | 42.85091 | 114.1048 | 85.78724 | 123.9078 | 44.38974 | 75.4776 | 49.37485 | 71.02655 | 103.5678 | 45.06422 | 59.84297 | 31.74511 |
| CUST_19521_PI429010886 | N41709 | 0.035489 | 15 | 12 | | | | | | 743.4007 | 915.8521 | 723.9047 | 855.518 | 753.2014 | 947.8741 | 551.2455 | 592.1986 | 630.6794 | 834.6224 | 842.0657 | 1078.601 | 633.0392 | 865.6927 | 1129.849 | 938.6625 | 910.5801 | 772.4833 | 748.9581 | 850.4702 | 728.8915 | 840.6106 | 716.5268 | 604.4329 | 633.0392 | 863.4186 | 769.0647 |
| CUST_24449_PI429010886 | N50647 | 0.035489 | 15 | 12 | | | | | | 70.47227 | 55.45229 | 64.90243 | 50.88285 | 40.19775 | 61.89112 | 33.80408 | 32.10055 | 41.20095 | 65.09219 | 60.83438 | 66.53186 | 66.37927 | 43.84173 | 62.31885 | 52.26577 | 51.95466 | 50.59835 | 57.45413 | 43.97896 | 47.41918 | 52.96595 | 54.89949 | 40.00557 | 43.57816 | 55.20237 | 44.37026 |
| CUST_44936_PI429010886 | M67460 | 0.035491 | 12 | 0 | | | | | | 886.7923 | 1019.446 | 921.6949 | 680.1392 | 736.1144 | 678.3407 | 744.0064 | 738.0296 | 830.6821 | 1076.531 | 768.2695 | 873.2696 | 810.0074 | 1114.056 | 955.4895 | 1156.728 | 768.2695 | 723.6629 | 866.8694 | 960.3576 | 677.5621 | 1182.29 | 959.6386 | 744.5402 | 1183.738 | 769.8974 | 1043.787 |
| CUST_48031_PI429010886 | M78612 | 0.035491 | 12 | 3 | 242019220 | conserved hypothetical protein [Pediculus humanus | | | 7.42E-56 | 258.7017 | 190.6724 | 154.9827 | 463.5935 | 282.9535 | 469.9116 | 434.5491 | 188.4088 | 228.4344 | 63.73904 | 216.0426 | 87.35248 | 187.6138 | 48.91209 | 187.1216 | 94.34866 | 391.6722 | 284.7376 | 282.9109 | 68.9769 | 207.853 | 60.43419 | 181.9024 | 311.1164 | 61.02614 | 208.9109 | 62.63151 |
| CUST_9143_PI429010886 | N24129 | 0.035491 | 12 | 3 | | | | | | 31.81697 | 10.57913 | 22.64731 | 30.42483 | 56.55685 | 58.23374 | 29.26565 | 31.73044 | 25.23241 | 33.39553 | 45.70165 | 20.80307 | 21.68419 | 34.58756 | 22.59879 | 23.83766 | 52.40666 | 39.91731 | 34.90742 | 31.75251 | 38.36474 | 25.5267 | 27.78747 | 30.90472 | 22.88862 | 23.36677 | 31.00505 |
| CUST_35129_PI429010886 | M11671 | 0.035516 | 18 | 9 | | | | | | 55.76859 | 85.141 | 82.02402 | 62.4741 | 44.45813 | 55.47209 | 53.15786 | 60.31958 | 43.44238 | 94.13978 | 111.7908 | 78.27179 | 84.27965 | 86.78972 | 71.38963 | 89.35703 | 59.7424 | 69.08941 | 86.81547 | 82.64391 | 63.28709 | 86.33154 | 64.74408 | 50.71291 | 77.56897 | 81.09417 | 63.1832 |
| CUST_54021_PI429010886 | M104129 | 0.035534 | 18 | 0 | | | | | | 198.8279 | NA | 254.9115 | 27.8344 | 66.50645 | 90.37187 | 364.4089 | 98.09433 | 57.41489 | 7.485838 | 143.1678 | NA | 39.67041 | 42.42067 | 22.72881 | NA | 243.0834 | 99.34504 | 360.9417 | NA | 166.5412 | NA | 139.7302 | 169.2493 | NA | 50.91495 | 5.502777 |
| CUST_16577_PI429010886 | N36493 | 0.035544 | 12 | 3 | | | | | | 44.65427 | 110.9726 | 109.8741 | 26.72384 | 76.668 | 97.50342 | 74.58008 | 73.74514 | 77.1122 | 6.971491 | 27.34621 | 15.29798 | 3.902692 | 15.64506 | 60.47979 | 89.82702 | 131.1966 | 170.2922 | 13.08554 | 6.641946 | 21.81748 | NA | NA | NA | 97.5983 | 77.38462 | 65.60373 |
| CUST_13510_PI429010886 | N31306 | 0.035556 | 24 | 21 | 345487070 | PREDICTED: microtubule-actin cross-linking factor 1 | | | 6.95E-19 | 38.29585 | 51.4 | 46.16699 | 50.84587 | 39.45879 | 43.07403 | 40.44128 | 40.78513 | 48.8169 | 61.99224 | 43.74674 | 43.71284 | 48.11605 | 51.63118 | 46.74774 | 51.98058 | 41.50677 | 54.5227 | 56.88203 | 53.57478 | 51.66322 | 55.54166 | 70.12587 | 45.54848 | 66.2145 | 54.93259 | 55.93124 |
| CUST_23362_PI429010886 | N48711 | 0.0356 | 24 | 0 | | | | | | 725.0021 | 2413.596 | 2485.559 | 427.8624 | 1359.544 | 847.8005 | 370.13 | 194.4818 | 1640.228 | 582.9606 | 971.1081 | 2408.505 | 58.22987 | 167.4333 | 272.3924 | 2536.388 | 832.3284 | 1670.5 | 1351.741 | 716.2921 | 768.2695 | 1649.441 | 1071.523 | 143.2572 | 2237.5 | 1211.313 | 3503.673 |
| CUST_53221_PI429010886 | M100778 | 0.03561 | 15 | 9 | | | | | | 1134.016 | 1256.525 | 2352.559 | 1183.341 | 884.6458 | 1015.729 | 997.4048 | 1214.782 | 1547.246 | 1537.216 | 1282.355 | 2923.398 | 1520.67 | 2151.64 | 2370.125 | 2147.931 | 731.0066 | 1848.861 | 1463.822 | 1173.656 | 1724.959 | 2980.843 | 1771.34 | 1462.44 | 2695.193 | 2007.972 | 1968.333 |
| CUST_50872_PI429010886 | M91294 | 0.035626 | 21 | 0 | | | | | | 22.87323 | 15.75243 | 34.47396 | 8.345317 | 20.62099 | 16.44239 | 11.28356 | 12.53922 | 7.920199 | 19.46833 | 9.102009 | 11.69762 | 4.387413 | 22.78742 | 3.849709 | 16.74929 | 12.2288 | 12.2288 | 17.10987 | 12.79089 | 12.60453 | 20.17321 | NA | 15.37785 | 21.83532 | 7.986139 | 16.77607 |
| CUST_42957_PI429010886 | M61725 | 0.035646 | 12 | 9 | | | | | | 56.40373 | 34.9503 | 34.45811 | NA | 14.73565 | 11.93292 | 3.69797 | 30.10233 | 24.6038 | 78.14128 | 30.78534 | 61.72981 | NA | 11.97713 | NA | 10.46578 | NA | 16.39504 | 24.96534 | 18.25998 | 11.85674 | 29.65544 | 30.53018 | NA | 37.65478 | NA | 10.97825 |
| CUST_50277_PI429010886 | M89290 | 0.035655 | 24 | 9 | | | | | | 4.705963 | 11.15682 | NA | 31.87606 | 6.800737 | NA | 22.73782 | 19.13027 | 11.98753 | 11.41581 | 20.95213 | 27.55378 | 21.56963 | 11.5275 | 27.49118 | 21.96744 | 17.38798 | 17.71924 | 7.141791 | 46.66745 | 7.232248 | 10.06452 | 19.95159 | 8.503639 | 5.78592 | 15.53244 | 5.746337 |
| CUST_56360_PI429010886 | M113594 | 0.035663 | 21 | 18 | | | | | | 15.02147 | 12.32604 | 9.409299 | 12.23011 | 6.801891 | 16.13004 | NA | 15.24047 | 5.107128 | 8.662743 | 6.26744 | 11.02488 | 5.554616 | 6.287022 | 5.204531 | 14.56343 | 12.28125 | 22.28917 | 18.93037 | 9.211504 | 15.42005 | 18.04709 | 12.79969 | 10.24643 | 13.75465 | 9.927073 | 10.23206 |
| CUST_26965_PI429010886 | N55195 | 0.035664 | 24 | 21 | | | | | | 18.35726 | 10.11891 | 12.16044 | 21.74146 | 9.136468 | 14.73662 | 9.558027 | 8.972594 | 10.99269 | 16.46586 | 13.12868 | 12.0863 | 10.06555 | 24.54416 | 14.68043 | 18.4246 | 12.6973 | 13.95459 | 15.29079 | 13.04571 | 43.75138 | 20.04708 | 23.63048 | 12.36728 | 16.16705 | 14.13095 | 18.94519 |
| CUST_52442_PI429010886 | M97463 | 0.035702 | 24 | 15 | | | | | | 41.17264 | 46.25026 | 50.4975 | 28.33335 | 33.39553 | 29.2332 | 38.30158 | 34.74686 | 41.15689 | 42.10755 | 30.6513 | 41.65923 | 32.84559 | 32.3288 | 52.65189 | 49.54106 | 52.91094 | 53.04998 | 36.4354 | 38.93982 | 21.97651 | 43.9135 | 42.18909 | 27.55645 | 35.97533 | 36.64631 | 40.5109 |
| CUST_42724_PI429010886 | M61077 | 0.035727 | 15 | 0 | | | | | | 59.07909 | 186.5287 | 106.5468 | 51.33151 | 48.9688 | 30.85708 | 98.49703 | 51.73816 | 72.32108 | 43.69222 | 37.68617 | 27.4118 | 43.87014 | 93.78702 | 72.23286 | 137.687 | 183.8412 | 44.36476 | 83.66854 | NA | 22.22168 | 6.86152 | NA | NA | 16.20611 | 55.87064 | 46.60859 |
| CUST_47800_PI429010886 | M77654 | 0.035731 | 15 | 9 | | | | | | 245.8233 | 230.1517 | 296.0927 | 217.2745 | 238.8193 | 207.3956 | 245.5656 | 242.9704 | 282.7521 | 297.5373 | 268.82 | 261.123 | 229.2792 | 363.5385 | 281.2019 | 304.0369 | 224.5779 | 280.12 | 254.1554 | 242.7155 | 249.3375 | 284.8452 | 237.3703 | 209.83 | 385.0122 | 278.8318 | 289.679 |
| CUST_5683_PI429010886 | N19037 | 0.035731 | 15 | 0 | | | | | | 207.3956 | 248.2061 | 249.7604 | 187.9283 | 248.8723 | 248.3781 | 179.616 | 165.6162 | 180.0766 | 227.312 | 273.7876 | 274.0115 | 190.9766 | 209.3591 | 198.4542 | 304.2121 | 220.6157 | 290.2748 | 189.3158 | 269.2795 | 189.5057 | 222.578 | 184.1183 | 200.7582 | 253.3366 | 174.7413 | 242.9037 |
| CUST_18227_PI429010886 | N39388 | 0.035731 | 12 | 3 | 478261422 | hypothetical protein YQE_02808, partial [Dendroctc | Protein metabolism | Proteolysis | 1.31E-22 | 57.34886 | 144.9545 | 22.0325 | 521.963 | 470.6652 | 536.1688 | 242.5645 | 27.07724 | 223.0851 | 29.32288 | 4.948921 | 11.4747 | 36.50003 | 161.3901 | 309.2864 | 722.796 | 45.21982 | 562.1725 | 60.29484 | 53.33947 | 19.74451 | 532.0502 | 28.24369 | 319.745 | 145.749 | 37.06882 | 23.55239 |
| CUST_43440_PI429010886 | M63052 | 0.035776 | 15 | 9 | | | | | | 104.4835 | 101.3168 | 122.0311 | 84.32383 | 101.5527 | 125.9958 | 107.7242 | 111.8286 | 130.8825 | 139.1812 | 108.4109 | 121.0512 | 97.4551 | 127.2256 | 131.1671 | 106.5216 | 115.3001 | 101.7238 | 107.4359 | 108.9872 | 114.7609 | 136.1838 | 118.7528 | 131.2743 | 119.0883 | 134.244 | 127.0971 |
| CUST_3609_PI429010886 | N14873 | 0.035776 | 15 | 9 | | | | | | 129.7547 | 284.8452 | 706.0046 | 12.07272 | 75.93064 | 9.013974 | 220.6402 | 776.8406 | 1619.494 | 926.1166 | 720.48 | 1185.878 | 332.8858 | 1259.651 | 595.305 | 229.4054 | 444.7159 | 210.0239 | 302.1561 | 1316.22 | 294.2267 | 1135.995 | 796.2928 | 26.45722 | 1923.113 | 250.8965 | 1018.327 |
| CUST_56496_PI429010886 | M113933 | 0.035799 | 12 | 0 | | | | | | 1170.02 | 1560.88 | 1703.115 | 1049.194 | 1043.343 | 1058.786 | 1143.075 | 1148.187 | 1419.195 | 1492.759 | 1259.651 | 1485.965 | 1232.59 | 1734.303 | 1773.273 | 1718.489 | 1127.801 | 1316.22 | 1366.468 | 1730.823 | 1287.748 | 1662.371 | 1432.157 | 1236.95 | 1565.179 | 1278.309 | 1696.249 |
| CUST_33287_PI429010886 | M6504 | 0.035805 | 24 | 15 | 459937336 | putative TRP channel protein [Periplaneta american | Transport | Ion transport | 3.5E-159 | 1085.118 | 1136.737 | 1074.839 | 651.2944 | 1079.252 | 1113.695 | 754.5887 | 810.6402 | 709.1206 | 1028.799 | 1036.073 | 1099.653 | 625.8341 | 1050.577 | 991.3315 | 1327.226 | 1163.545 | 1302.682 | 1103.397 | 1126.811 | 942.9477 | 1301.471 | 892.3589 | 790.5889 | 1858.933 | 1035.582 | 1205.667 |
| CUST_33730_PI429010886 | M7734 | 0.035826 | 15 | 9 | 321469223 | hypothetical protein DAPPUDRAFT_243998 [Daphn | Metabolic process | Carbohydrate metabolic process | 7.7E-138 | 31.24843 | 203.2739 | 123.6053 | 278.8318 | 317.8203 | 114.6697 | 267.6598 | 712.1447 | 663.1345 | 264.2251 | 765.9632 | 1041.277 | 451.5463 | 132.4515 | 282.2114 | 5.893349 | 170.0428 | NA | 95.17141 | 1680.099 | 328.6838 | NA | 311.5276 | 189.3668 | NA | 241.4805 | 641.7483 |
| CUST_15747_PI429010886 | N35075 | 0.035866 | 15 | 0 | | | | | | 300.5238 | 309.1008 | 251.5651 | 205.078 | 245.2402 | 275.0389 | 223.6419 | 216.6952 | 192.4758 | 256.9474 | 232.3526 | 246.1869 | 190.7772 | 276.5696 | 251.6265 | 314.9825 | 262.6696 | 284.1504 | 289.4881 | 267.7292 | 238.6573 | 312.6532 | 213.1567 | 204.9184 | 397.0596 | 244.2379 | 289.3731 |
| CUST_26083_PI429010886 | N53628 | 0.03587 | 24 | 15 | | | | | | 33.11438 | 30.76441 | 35.87751 | 27.4471 | 27.13299 | 20.75737 | 23.81679 | 28.70508 | 29.71386 | 40.14648 | 20.4859 | 44.91149 | 32.27766 | 42.29841 | 30.60425 | 55.11221 | 25.25393 | 44.99497 | 36.02402 | 37.86509 | 42.88135 | 34.73965 | 42.06008 | 34.40003 | 49.67248 | 43.80561 | 46.1038 |
| CUST_33956_PI429010886 | M8360 | 0.035878 | 21 | 15 | | | | | | 357.152 | 445.5129 | 275.8443 | 374.4434 | 410.06 | 378.5438 | 263.6263 | 292.3676 | 234.1059 | 264.7951 | 338.3238 | 344.803 | 285.4569 | 332.4804 | 366.8204 | 479.585 | 352.8752 | 409.1687 | 395.3575 | 406.809 | 307.1797 | 467.665 | 296.3167 | 309.1695 | 353.6127 | 352.3859 | 289.9742 |
| CUST_48279_PI429010886 | M79638 | 0.035886 | 12 | 0 | | | | | | 18.84309 | 18.30186 | 40.42785 | 9.82119 | 8.788925 | 14.39019 | 6.379564 | 8.110471 | 23.21318 | 11.32143 | 36.5984 | 16.53651 | 17.23883 | 25.00865 | 17.86554 | 13.36577 | 11.01728 | 10.46311 | 10.78602 | 19.19079 | 11.55683 | 15.56605 | 22.11953 | 14.70218 | 20.88813 | 9.913065 | 16.72128 |
| CUST_50546_PI429010886 | M90221 | 0.035886 | 12 | 3 | | | | | | 33.66143 | 26.07711 | 34.17378 | 43.28157 | 53.05965 | 43.961 | 64.26853 | 49.57895 | 34.88582 | 33.94751 | 58.12189 | 41.15368 | 53.05965 | 23.70484 | 20.05897 | 84.62693 | 63.41464 | 53.76242 | 48.23603 | 38.56828 | 54.77183 | 40.40681 | 36.52603 | 88.3593 | 28.28914 | 51.31578 | 41.43272 |
| CUST_23777_PI429010886 | N49446 | 0.035886 | 21 | 15 | | | | | | 432.1375 | 513.4234 | 534.0061 | 395.9375 | 472.8988 | 494.6999 | 463.6969 | 463.4816 | 458.3652 | 350.0802 | 473.1073 | 359.57 | 482.753 | 394.7279 | 455.0936 | 601.0454 | 514.0171 | 501.702 | 535.1571 | 530.8519 | 488.8934 | 512.5692 | 483.597 | 351.983 | 688.7233 | 420.5887 | 595.028 |
| CUST_44242_PI429010886 | M65366 | 0.035893 | 15 | 12 | 321469964 | hypothetical protein DAPPUDRAFT_303780 [Daphn | | | 2.15E-33 | 52.29382 | 59.9559 | 61.93065 | 38.56828 | 43.90259 | 57.62155 | 25.352 | 35.96518 | 55.09266 | 51.60182 | 40.28049 | 121.4703 | 46.84472 | 80.38356 | 73.9797 | 59.79177 | 34.10794 | 58.99785 | 47.99919 | 59.79684 | 57.52971 | 52.18439 | 51.1868 | 33.2742 | 71.51375 | 48.3882 | 64.5831 |
| CUST_17905_PI429010886 | N38807 | 0.035894 | 24 | 18 | 350399191 | PREDICTED: atlastin-like isoform 1 [Bombus impatie | | | 8.8E-19 | 74.32582 | 66.50022 | 45.11398 | 47.12249 | 47.09025 | 36.48665 | 74.03663 | 32.80705 | 46.92598 | 67.02702 | 79.20223 | 63.08199 | 58.69441 | 56.19981 | 50.45571 | 74.55697 | 133.7749 | 39.79339 | 85.52397 | 138.0748 | 64.62304 | 68.38203 | 48.47467 | 53.36025 | 65.28922 | 33.30987 | 61.24287 |
| CUST_27504_PI429010886 | N56176 | 0.035915 | 21 | 9 | 260806438 | hypothetical protein BRAFLDRAFT_85692 [Branchio | Metabolic process | Other | 7.4E-18 | 17.90404 | 8.630999 | 7.842686 | 27.04148 | 14.63889 | 14.9864 | 14.68592 | 21.66748 | 18.72053 | 16.75931 | 31.01112 | 22.27554 | 38.05769 | 20.96144 | 9.959454 | 8.974986 | 12.10746 | 11.39853 | 14.05599 | 36.2246 | 32.58037 | 8.401293 | 7.421979 | NA | 12.50732 | 29.24532 | 15.07014 |
| CUST_14702_PI429010886 | N33323 | 0.035923 | 15 | 12 | | | | | | 14.18775 | 31.61657 | 14.39423 | 22.23059 | 18.10445 | 20.66537 | 15.60693 | 20.65238 | 10.90827 | 25.24454 | 18.85349 | 31.47929 | 25.93179 | 24.43037 | 15.70408 | 26.55213 | 23.84605 | 26.66659 | 23.13699 | 20.77685 | 22.11226 | 19.63409 | 18.80396 | 14.82167 | 24.41426 | 21.07244 | 19.60862 |
| CUST_55870_PI429010886 | M111947 | 0.035959 | 21 | 12 | | | | | | 36.69017 | 31.68444 | 36.72937 | 27.14607 | 34.05076 | 42.75674 | 28.1987 | 35.16453 | 28.16499 | 37.06882 | 31.82901 | 31.04579 | 37.11196 | 40.52511 | 47.59957 | 31.8 | 38.71497 | 38.63759 | 39.57761 | 39.86606 | 37.50798 | 35.17156 | 37.91955 | 22.19942 | 37.67218 | 33.84879 | 32.61498 |
| CUST_10004_PI429010886 | N25551 | 0.036003 | 24 | 15 | | | | | | 18.01314 | 16.68994 | 25.02916 | 13.05889 | 10.94103 | 19.52925 | 8.588693 | 10.23894 | 12.36405 | 9.824879 | 14.76986 | 17.53908 | 10.3005 | 30.18478 | 17.33858 | 22.95261 | 15.52381 | 23.1013 | 16.46028 | 20.99763 | 12.43272 | 18.88215 | 22.78989 | 14.56714 | 13.55355 | 21.90202 | 19.01531 |
| CUST_35642_PI429010886 | M13093 | 0.036015 | 21 | 15 | | | | | | 515.4981 | 523.5388 | 582.1613 | 537.0505 | 472.5516 | 561.2769 | 379.8142 | 445.9535 | 460.0363 | 553.514 | 477.9922 | 604.5341 | 400.1052 | 548.077 | 484.607 | 719.1308 | 610.8058 | 541.31 | 613.6489 | 625.7026 | 493.4379 | 690.9208 | 500.7784 | 438.6413 | 569.6586 | 440.5078 | 558.4057 |
| CUST_31067_PI429010886 | M604 | 0.036015 | 18 | 0 | 242003046 | WD-repeatcontaining protein, putative [Pediculus h | | | | 70.94855 | 66.81139 | 58.96311 | 49.58479 | 86.36905 | 40.81576 | 53.81515 | 41.70336 | 56.80303 | 64.40125 | 64.04369 | 72.05819 | 66.7414 | 48.62311 | 57.09018 | 60.98516 | 55.78991 | 79.17523 | 71.28268 | 63.88437 | 65.46343 | 57.78767 | 63.77772 | 57.83783 | 58.67101 | 51.7068 | 48.88298 |
| CUST_53650_PI429010886 | M102649 | 0.036021 | 15 | 3 | | | | | | 20.96144 | 7.50435 | 9.661698 | 30.48607 | 20.9065 | 24.3684 | NA | 16.38286 | NA | 28.46316 | 22.33967 | 10.40896 | 9.823191 | 15.53244 | 6.779112 | 13.17601 | 21.74923 | 17.04092 | 29.46873 | NA | 20.90047 | 9.905555 | NA | NA | 19.28584 | 6.849615 | 23.7918 |
| CUST_35349_PI429010886 | M12337 | 0.036023 | 12 | 0 | 390345459 | PREDICTED: zinc finger protein 84-like [Strongylocer | | | 2.31E-55 | 7.54037 | 15.02569 | 9.336649 | 5.56425 | 9.02845 | 13.05441 | NA | 16.1321 | 8.488945 | 6.712633 | 7.756103 | 10.93346 | 30.11515 | 13.71994 | NA | 9.104602 | 8.486348 | 8.406805 | 5.986055 | 4.622941 | NA | 8.468531 | 11.15573 | NA | 13.58403 | 19.96449 | 18.90357 |
| CUST_35810_PI429010886 | M13573 | 0.036028 | 21 | 15 | | | | | | 207.0153 | 272.3924 | 210.5151 | 226.8346 | 208.7767 | 228.1106 | 159.6292 | 174.4245 | 180.162 | 256.6914 | 213.6947 | 231.3422 | 160.974 | 205.8829 | 246.0125 | 304.1069 | 214.5095 | 323.9834 | 224.3204 | 273.6399 | 222.2406 | 288.4954 | 204.658 | 191.9895 | 262.1655 | 215.2857 | 213.0864 |
| CUST_52104_PI429010886 | M96417 | 0.036048 | 24 | 6 | | | | | | 200.9848 | 167.6229 | 11.13485 | 241.7291 | 53.57981 | 166.9427 | 207.0153 | 232.6257 | 183.2048 | NA | 19.28099 | 13.98077 | 267.6598 | 12.45838 | 150.6325 | 40.32087 | 49.7894 | 74.6525 | 16.06458 | 9.714422 | 14.87518 | 26.43248 | 10.57804 | 297.8198 | 23.60773 | 84.05405 | 16.97785 |
| CUST_10621_PI429010886 | N26524 | 0.036074 | 24 | 21 | | | | | | 635.0709 | 754.5887 | 726.7504 | 563.9632 | 658.1422 | 585.2479 | 542.3878 | 570.3497 | 653.9628 | 859.0461 | 628.4644 | 680.3748 | 592.5758 | 866.8694 | 679.9331 | 875.295 | 631.2945 | 671.5411 | 687.7759 | 764.0731 | 706.1971 | 927.1088 | 762.7979 | 632.2521 | 891.7466 | 672.0887 | 826.4016 |
| CUST_30849_PI429010886 | N63031 | 0.036074 | 15 | 12 | | | | | | 5.92867 | 8.774888 | 15.63054 | 4.489555 | 5.98865 | 6.784674 | 12.38076 | 13.32777 | 12.17439 | 11.38749 | 4.66886 | 14.2605 | 14.73825 | 17.13668 | 9.530867 | 19.40005 | 13.50761 | 15.69662 | 9.658216 | 6.648806 | 8.328299 | 14.23976 | 9.422886 | 17.33696 | 8.800802 | 17.02082 | 16.62775 |
| CUST_51494_PI429010886 | M93725 | 0.036078 | 24 | 18 | | | | | | 106.4305 | 80.36337 | 148.886 | 90.90134 | 79.81539 | 64.46821 | 84.76204 | 118.5634 | 121.8439 | 85.29111 | 108.3189 | 124.8411 | 109.19 | 148.6917 | 166.6587 | 121.0016 | 146.6632 | 139.4033 | 202.6837 | 86.38663 | 133.9267 | 201.7937 | 144.2774 | 104.4054 | 175.7224 | 140.2062 | 127.916 |
| CUST_23447_PI429010886 | N48854 | 0.036103 | 24 | 21 | | | | | | 12.50339 | 34.15181 | 28.2689 | 11.47303 | 14.56714 | 15.43128 | 12.45001 | 12.79813 | 11.4747 | 10.5667 | 13.99028 | 25.352 | 14.36708 | 26.79789 | 15.5488 | 25.39336 | 14.42276 | 18.04108 | 17.73942 | 11.38442 | 9.469496 | 25.15397 | 22.89148 | 35.56782 | 17.40769 | 21.8123 | 23.29073 |
| CUST_36023_PI429010886 | M14315 | 0.03611 | 15 | 6 | 321467326 | GST-N-Metaxin-like protein [Daphnia pulex] | | | 2.68E-15 | 12.13343 | 18.12277 | 9.751886 | 31.40553 | 25.61779 | 27.7492 | 53.28087 | NA | NA | 14.92766 | 28.7018 | 6.593457 | 6.850987 | NA | 3.96015 | 10.68521 | 29.59116 | 21.57749 | 34.76295 | 8.367475 | 26.08713 | 10.94248 | 24.48268 | 40.24548 | 14.9472 | 12.04766 | NA |
| CUST_6764_PI429010886 | N20569 | 0.036112 | 18 | 6 | 46849491 | glycinamide ribonucleotide synthetase-aminoimida | Metabolic process | Nucleobase-containing compound metabolic process | 7E-112 | 98.0082 | 158.7737 | 147.1696 | 125.2677 | 124.672 | 148.9557 | 241.2255 | 163.791 | 235.8014 | 186.3956 | 189.4567 | 228.2235 | 146.3399 | 212.0119 | 206.7552 | 235.7212 | 281.652 | 231.6114 | 149.5977 | 134.0378 | 111.4345 | 209.6694 | 226.8032 | 151.6449 | 314.5564 | 142.2559 | 257.7314 |
| CUST_56944_PI429010886 | M114968 | 0.036118 | 21 | 3 | | | | | | 11.63591 | 9.262293 | 13.53466 | 16.76894 | 14.4005 | 14.84209 | 17.37911 | 12.43952 | 13.99622 | 5.169974 | 11.42268 | 7.768024 | 10.73339 | NA | 11.71083 | 11.03659 | 7.743875 | 8.821462 | 19.08808 | 9.476285 | 19.93091 | 7.982144 | 11.33312 | 8.664964 | 10.16422 | 37.79822 | 26.0521 |
| CUST_50278_PI429010886 | M89294 | 0.036125 | 21 | 3 | 410950364 | PREDICTED: zinc finger protein 558 [Felis catus] | Nucleic acid metabolism | Regulation of gene expression | 2.8E-14 | 1050.577 | 2223.051 | 1321.79 | 3416.788 | 1591.245 | 1710.023 | 1140.062 | 1082.445 | 1133.519 | 1419.795 | 774.5083 | 1613.342 | 793.8063 | 1502.524 | 920.0256 | 1359.544 | 588.9896 | 1266.654 | 677.9305 | 973.4615 | 2026.043 | 1797.174 | 2049.694 | 1130.917 | 2133.627 | 1911.091 | 1192.165 |
| CUST_12155_PI429010886 | N29016 | 0.036126 | 18 | 12 | | | | | | 9.106984 | 24.84355 | 11.00169 | 8.120251 | 4.927606 | 6.295535 | 21.44879 | 68.5913 | 5.105169 | 21.23667 | 4.346071 | 17.7534 | 21.8835 | 27.96355 | 23.03433 | 29.38047 | 14.91181 | 24.42797 | 21.76863 | 10.97938 | 11.32653 | 13.73772 | 10.07966 | NA | 39.78497 | 15.08419 | 38.77187 |
| CUST_482_PI429010886 | N5602 | 0.036133 | 15 | 3 | | | | | | 30.2284 | 16.51743 | 8.855559 | 132.4059 | 45.09974 | 163.9899 | 61.66407 | 29.02633 | 33.23641 | 13.12705 | 11.93548 | 5.746337 | 38.0368 | 31.28664 | 39.64527 | 15.93573 | 71.43104 | 64.00351 | 36.5984 | 14.03191 | 23.59085 | 41.35027 | 14.88541 | 95.17141 | 12.0315 | 33.23109 | 7.656642 |
| CUST_6217_PI429010886 | N19790 | 0.036156 | 15 | 0 | 119117126 | Cryptocercus punctulatus alpha-tubulin (Atub) mRN | Cellular component organization | Cytoskeleton organization | | 2195.833 | 2201.928 | 1987.549 | 2298.025 | 1331.49 | 1924.978 | 1823.162 | 1955.85 | 1680.099 | 1560.159 | 1091.604 | 1112.064 | 1229.288 | 1421.77 | 1390.385 | 2092.578 | 1863.078 | 2142.216 | 1959.594 | 1243.416 | 1185.878 | 1367.805 | 1327.226 | 2090.413 | 1320.618 | 1966.099 | 1430.089 |
| CUST_4353_PI429010886 | N16577 | 0.036165 | 18 | 3 | | | | | | 7.384155 | 35.89984 | 14.29372 | 50.22468 | NA | 31.52203 | 20.1457 | 5.497161 | 3.476526 | 8.00826 | 3.861406 | 7.946536 | 6.823408 | 3.828839 | 4.720527 | 52.21271 | 29.92091 | 75.86883 | 5.131288 | 35.01361 | 13.31508 | 44.31783 | 21.7799 | 17.90553 | 17.4398 | 16.30792 | 31.65825 |
| CUST_33073_PI429010886 | M5901 | 0.036174 | 18 | 6 | 256033192 | triacylglycerol lipase [Litopenaeus vannamei] | | | 1.27E-90 | 10.5296 | 246.92 | 87.37332 | 379.2779 | 440.9199 | 198.4267 | 224.7458 | 1102.647 | 743.5954 | 213.0233 | 612.1213 | 674.0173 | 587.8154 | 154.2025 | 413.9155 | 5.177142 | 374.9864 | 10.05309 | 104.1327 | 2362.762 | 837.729 | NA | 306.5332 | 222.9397 | NA | 561.9199 | 535.4947 |
| CUST_4380_PI429010886 | N16633 | 0.036179 | 12 | 0 | 89520371 | transposase [Heliothis virescens] | | | 5.06E-07 | 37.51655 | 32.87841 | 53.02941 | 39.44549 | 30.13914 | 10.87219 | 45.25329 | 27.75224 | 21.80739 | 31.99517 | 28.82923 | 19.8522 | 47.20692 | 53.01337 | 39.80783 | 39.64527 | 27.68104 | 40.78034 | 38.08235 | 26.41719 | 28.77383 | 34.53064 | 31.56943 | 27.18775 | 42.82585 | 35.43589 | 31.71799 |
| CUST_21605_PI429010886 | N45508 | 0.036207 | 15 | 12 | 91084129 | PREDICTED: similar to ATP-binding cassette transpo | | | 2.57E-31 | 88.33792 | 52.13588 | 116.5651 | 37.94143 | 46.12938 | 45.40667 | 71.68684 | 53.29328 | 58.53699 | 84.2393 | 65.59604 | 64.42438 | 141.6752 | 81.23395 | 52.66699 | 55.88131 | 60.96666 | 41.25578 | 62.94659 | 40.80459 | 61.2083 | 59.57181 | 52.01256 | 61.24287 | 60.39123 | 45.60489 | 77.0714 |
| CUST_56306_PI429010886 | M113460 | 0.036238 | 24 | 12 | | | | | | 1730.048 | 953.5865 | 507.983 | 786.1873 | 423.2024 | 124.3489 | 877.4226 | 837.9656 | 489.8149 | 722.474 | 670.3311 | 1213.318 | 1862.262 | 1695.261 | 1993.696 | 1615.873 | 494.4534 | 927.1088 | 2104.851 | 1338.117 | 912.0221 | 829.8828 | 706.9904 | 1342.932 | 146.5685 | 926.7413 | 2356.592 |
| CUST_32279_PI429010886 | M3691 | 0.036252 | 24 | 9 | 238625733 | mannose receptor [Rimicaris exoculata] | | | 1.11E-23 | 603.6169 | 1015.729 | 956.4623 | 910.8643 | 1016.851 | 251.3539 | 713.1739 | 1127.801 | 689.6848 | 1062.278 | 909.1015 | 1400.033 | 345.2409 | 277.7527 | 325.1246 | 1059.917 | 363.7161 | 303.6176 | 187.2169 | 483.0972 | 208.8382 | 133.8101 | 805.0049 | 478.7395 | 46.69134 | 426.2225 | 270.9645 |
| CUST_32221_PI429010886 | M3560 | 0.03626 | 15 | 3 | | | | | | 7.809814 | 12.71803 | 24.96011 | NA | 14.6922 | 23.70291 | NA | 5.37195 | 11.6615 | 8.508012 | 9.248628 | 12.03567 | 9.150748 | 15.9244 | 10.42781 | 10.77921 | 17.47076 | 15.29619 | 17.19687 | 12.76533 | 13.67397 | 16.84886 | 12.54065 | 19.63409 | 9.334862 | 10.7807 | 8.858469 |
| CUST_10897_PI429010886 | N26961 | 0.036262 | 18 | 6 | 321479210 | hypothetical protein DAPPUDRAFT_299971 [Daphn | | | 1.32E-14 | NA | 145.5155 | 66.64899 | 232.9884 | 392.0026 | 87.22914 | 112.9069 | 1248.106 | 1065.113 | 185.8972 | 405.9943 | 596.6167 | 159.7611 | 71.95612 | 71.87199 | 4.581602 | 120.3899 | NA | 50.77029 | 1709.2 | 245.6501 | 8.479761 | 163.8724 | 145.2433 | NA | 651.0924 | 972.1119 |
| CUST_26614_PI429010886 | N54541 | 0.036273 | 24 | 18 | 471417319 | PREDICTED: unconventional myosin-VI [Trichechus | | | 8.28E-38 | 106.5708 | 105.2081 | 89.35703 | 94.09795 | 97.58263 | 89.09879 | 78.76718 | 58.4329 | 66.79779 | 112.6761 | 91.24943 | 100.4962 | 65.28197 | 98.82662 | 124.6537 | 92.19432 | 82.6196 | 92.20534 | 101.6806 | 152.4677 | 93.19487 | 128.969 | 91.27166 | 98.42661 | 102.6011 | 85.10975 | 111.5352 |
| CUST_37466_PI429010886 | M19128 | 0.03628 | 21 | 0 | 405969873 | hypothetical protein CGI_10022520 [Crassostrea gig | | | 8.23E-29 | 50.9755 | 77.38027 | NA | 118.55 | 45.64715 | 46.53779 | 4.87499 | NA | 14.69744 | 4.93182 | 9.055842 | 8.394638 | 4.275158 | 44.60711 | 45.01588 | 55.29787 | NA | 55.20237 | NA | 25.08853 | 8.709062 | 114.1927 | 7.859342 | 111.6499 | 49.41638 | 5.241968 | NA |
| CUST_6885_PI429010886 | N20732 | 0.03628 | 12 | 9 | 242025142 | myosin-18A, putative [Pediculus humanus corporis] | | | 9.82E-60 | 7.358125 | 10.8378 | 16.54774 | 11.65277 | 15.49062 | 21.50916 | 16.42728 | 11.68016 | 12.25902 | 18.56257 | 24.92567 | 17.33078 | 10.37845 | 7.997831 | 10.91573 | 13.21776 | 23.60284 | 13.26048 | 14.51697 | 13.05013 | 17.59108 | 14.98837 | 20.48326 | 26.869 | 14.12148 | 15.81986 | 15.33279 |
| CUST_15290_PI429010886 | N34336 | 0.03628 | 12 | 0 | | | | | | 55.81794 | 141.8494 | 180.3315 | 81.31167 | 66.67878 | 33.77621 | 37.66305 | 56.79499 | 69.72006 | 93.80027 | 29.16954 | 233.828 | 128.3244 | 130.1396 | 114.2465 | 96.8234 | 27.90015 | 25.51724 | 41.25578 | 92.5954 | 83.43282 | 61.28097 | 103.0974 | 36.39953 | 142.8394 | 60.45419 | 185.5593 |
| CUST_16888_PI429010886 | N37031 | 0.03628 | 12 | 0 | | | | | | 89.27715 | 121.3454 | 127.5485 | 82.55028 | 86.28429 | 73.55011 | 72.61909 | 58.69441 | 88.86362 | 96.74817 | 89.18534 | 114.443 | 75.70522 | 113.2721 | 120.2042 | 149.2677 | 42.36335 | 85.29111 | 102.6772 | 106.1066 | 96.05386 | 112.5615 | 96.6566 | 83.66854 | 117.4728 | 97.5983 | 123.0412 |
| CUST_25629_PI429010886 | N52771 | 0.03628 | 12 | 0 | | | | | | 67.15618 | 29.86056 | 55.61005 | 17.34168 | 25.06406 | 43.98243 | 36.27265 | 44.70035 | 37.72421 | 40.73202 | 40.07222 | 33.63678 | 44.7891 | 45.488 | 32.50993 | 23.94075 | 34.71503 | 33.24975 | 39.17807 | 29.0899 | 38.60692 | 37.95647 | 45.04433 | 26.83276 | 62.36898 | 39.98991 | 40.16678 |
| CUST_41010_PI429010886 | M56613 | 0.036296 | 15 | 12 | 546674638 | hypothetical protein D910_03395 [Dendroctonus p | Metabolic process | Nucleobase-containing compound metabolic process | 1.78E-22 | 154.7269 | 184.9825 | 153.4593 | 136.8231 | 170.6499 | 175.8238 | 128.2056 | 133.7064 | 136.3233 | 164.8518 | 161.9364 | 201.7309 | 254.1554 | 157.9028 | 254.6874 | 190.2958 | 162.0012 | 157.5747 | 149.2083 | 190.7772 | 150.5005 | 167.7564 | 154.0296 | 166.8948 | 173.9653 | 165.8485 | 181.7276 |
| CUST_6268_PI429010886 | N19875 | 0.036296 | 15 | 12 | | | | | | 1558.572 | 2564.647 | 1251.3 | 1523.414 | 1614.376 | 1358.264 | 1115.54 | 1198.73 | 1202.334 | 1794.046 | 1354.655 | 1995.819 | 1692.067 | 1847.968 | 1849.813 | 2818.479 | 1129.849 | 1370.685 | 1453.376 | 1821.885 | 1410.909 | 2318.551 | 1194.964 | 990.5143 | 1663.806 | 1318.564 | 1602.617 |
| CUST_7300_PI429010886 | N21340 | 0.036296 | 15 | 12 | | | | | | 1584.97 | 1771.34 | 1485.346 | 1521.376 | 1186.768 | 1083.765 | 1197.011 | 1459.126 | 1492.073 | 1987.549 | 1329.318 | 1542.825 | 1746.637 | 1823.162 | 1669.785 | 2261.77 | 1276.669 | 1028.799 | 1451.905 | 1371.349 | 867.8685 | 1881.402 | 1568.166 | 996.2576 | 1973.742 | 1148.187 | 1620.39 |
| CUST_37601_PI429010886 | M19739 | 0.036343 | 18 | 15 | 13898841 | CUB-serine protease [Panulirus argus] | Protein metabolism | Proteolysis | 1.55E-48 | 5188.094 | 4864.618 | 4444.002 | 1186.768 | 5757.178 | 3372.782 | 4584.946 | 3676.785 | 4859.11 | 5596.154 | 3673.827 | 6085.653 | 5627.009 | 5780.138 | 3453.79 | 6247.817 | 4537.099 | 6528.23 | 2906.073 | 5774.702 | 4213.146 | 3739.652 | 3136.621 | 2595.092 | 3692.777 | 4630.476 | 6738.231 |
| CUST_7449_PI429010886 | N21568 | 0.036343 | 18 | 3 | 157119701 | cuticle protein, putative [Aedes aegypti] >gi\|10887 | | | 1.26E-23 | 3230.715 | 1475.875 | 652.7665 | 9271.524 | 4058.299 | 12445.58 | 5004.678 | 2553.637 | 4289.879 | 344.6643 | 1228.741 | 325.619 | 2918.106 | 1316.22 | 2894.613 | 1246.008 | 4773.42 | 5836.719 | 1725.627 | 1148.695 | 1680.099 | 2879.829 | 1802.411 | 9261.186 | 811.2478 | 4146.864 | 339.3609 |
| CUST_15123_PI429010886 | N34045 | 0.036344 | 21 | 0 | 113205550 | nth endonuclease III-like 1 [Xenopus (Silurana) trop | Response to stress | DNA repair | 1.11E-37 | 59.04082 | 44.62034 | 91.58478 | 67.80419 | 43.83694 | 23.67732 | 37.71676 | 48.61666 | 52.36466 | 47.42461 | 33.9856 | 16.77093 | 19.92665 | 21.11131 | 24.94581 | 16.32095 | 40.5109 | 41.56705 | 35.78629 | 23.89082 | 37.47945 | 26.66065 | 60.07749 | 43.05742 | 67.17456 | 22.00022 | 26.69852 |
| CUST_48648_PI429010886 | M81469 | 0.036351 | 15 | 12 | | | | | | 78.77563 | 64.68958 | 81.46299 | 59.00313 | 64.45839 | 78.08745 | 57.42327 | 59.88574 | 65.32582 | 92.50008 | 73.42232 | 108.7243 | 71.00916 | 103.0626 | 89.58206 | 109.7501 | 79.88008 | 82.14765 | 75.71395 | 98.4006 | 66.91698 | 77.73408 | 90.93451 | 55.82952 | 96.91901 | 81.49546 | 89.50659 |
| CUST_29638_PI429010886 | N60274 | 0.036391 | 15 | 3 | | | | | | 9.312357 | 25.65833 | 11.74368 | 21.05341 | 19.9835 | 13.2165 | 11.11044 | 9.787782 | 7.44877 | 7.629199 | 6.988725 | 15.49062 | 7.751416 | NA | 20.7312 | 8.581401 | 16.0444 | 15.5721 | NA | 20.34669 | 30.21036 | 26.64821 | 7.516724 | 11.89545 | 16.74929 | 9.217559 | 4.657997 |
| CUST_20486_PI429010886 | N43542 | 0.036459 | 12 | 0 | 91087807 | PREDICTED: similar to vacuolar protein sorting 35 [T | | | 5.02E-19 | 72.93993 | 68.89697 | 65.38596 | 66.15125 | 74.06105 | 75.12567 | 48.41716 | 54.79813 | 76.10603 | 76.7303 | 71.9913 | 70.92151 | 78.41704 | 75.7673 | 79.41411 | 97.10121 | 55.9772 | 69.89082 | 66.26607 | 70.58339 | 55.57729 | 62.39657 | 65.64051 | 75.26431 | 88.28345 | 76.73952 | 79.33173 |
| CUST_30227_PI429010886 | N61440 | 0.036461 | 15 | 0 | | | | | | 76.79471 | 78.53416 | 74.64567 | 47.5011 | 48.20555 | 69.07358 | 39.31065 | 51.25067 | 56.85652 | 63.27271 | 53.77942 | 48.67882 | 58.597 | 58.22987 | 74.3064 | 77.08541 | 68.1165 | 63.68912 | 50.9755 | 78.68384 | 53.79628 | 71.52214 | 57.34357 | 55.74249 | 71.97535 | 40.51578 | 69.05841 |
| CUST_27532_PI429010886 | N56226 | 0.036517 | 21 | 18 | | | | | | 9.816503 | 13.89365 | 29.87699 | 7.478506 | NA | 8.201884 | 4.059514 | NA | 9.78139 | 11.6078 | 4.024049 | 32.29627 | 10.57353 | 14.11808 | 10.2112 | 23.04508 | 9.267153 | 13.69402 | 13.76785 | 22.84721 | 15.02993 | 13.65114 | 19.26978 | NA | 15.53324 | 5.983777 | 11.93851 |
| CUST_22728_PI429010886 | N47548 | 0.036521 | 12 | 0 | | | | | | 10.17638 | 8.48246 | 14.92131 | 5.010923 | 4.776802 | 15.62884 | NA | 11.00948 | 4.634681 | 6.098401 | 7.631181 | 5.698164 | 131.7341 | 17.17022 | 14.09213 | 11.18584 | NA | 9.202277 | 5.82746 | 9.73488 | 7.20194 | 7.210827 | 11.54084 | NA | 13.60767 | 10.60724 | 10.9288 |
| CUST_10759_PI429010886 | N26741 | 0.036523 | 21 | 15 | | | | | | 233.2127 | 194.5783 | 159.4135 | 159.4333 | 200.4624 | 147.8481 | 161.5676 | 153.1511 | 149.3076 | 250.2094 | 180.6894 | 164.1481 | 159.1475 | 170.2284 | 142.3777 | 289.8985 | 262.1121 | 216.1856 | 247.9064 | 181.8837 | 139.7005 | 224.3925 | 195.7607 | 175.5504 | 167.1839 | 145.8124 | 183.6281 |
| CUST_2548_PI429010886 | N12592 | 0.036524 | 21 | 0 | | | | | | 43.81006 | 54.49444 | 84.65687 | 51.5559 | 40.26187 | 37.65902 | 21.39591 | 18.01905 | 17.29878 | 34.1984 | 38.51373 | 38.52489 | 38.58794 | 53.83209 | 50.92195 | 67.81299 | 34.27813 | 17.0365 | 59.92145 | 19.74119 | 43.76068 | 41.62677 | 52.56534 | 37.85605 | 53.26328 | 31.28349 | 55.88861 |
| CUST_5291_PI429010886 | N18501 | 0.036524 | 21 | 0 | 321461881 | hypothetical protein DAPPUDRAFT_200622 [Daphn | Cellular Process | Cell cycle | 2.47E-53 | 115.837 | 126.2208 | 117.3157 | 79.86307 | 87.26858 | 79.32298 | 70.57215 | 76.78516 | 89.48953 | 116.7026 | 77.44015 | 83.69759 | 91.70502 | 95.47811 | 106.5597 | 114.845 | 82.74334 | 105.9305 | 86.58356 | 88.97552 | 92.07382 | 114.1768 | 86.09876 | 109.0854 | 128.8934 | 83.42195 | 102.9324 |
| CUST_30257_PI429010886 | N61502 | 0.036524 | 12 | 0 | | | | | | 49.06607 | 69.28978 | 91.50805 | 41.47981 | 30.73521 | 33.79348 | 40.63456 | 33.00184 | 42.92761 | 45.65948 | 34.10344 | 34.92205 | 54.61808 | 82.52826 | 54.1859 | 87.80183 | 36.83629 | 53.75298 | 38.2551 | 50.69847 | 61.09062 | 71.11942 | 36.90619 | 54.27879 | 68.44169 | 56.05994 | 82.78386 |
| CUST_4137_PI429010886 | N16094 | 0.036534 | 24 | 21 | 189241054 | PREDICTED: similar to CG32169 CG32169-PA [Tribol | | | 2.18E-33 | 149.0992 | 131.0636 | 187.5065 | 106.8104 | 137.7352 | 134.9903 | 107.3899 | 121.3454 | 114.6697 | 179.0604 | 131.6679 | 115.7331 | 107.4983 | 147.8481 | 178.1983 | 196.9798 | 137.0908 | 164.2756 | 138.5572 | 164.6529 | 136.51 | 197.9679 | 143.2115 | 148.8058 | 148.6276 | 133.987 | 204.946 |
| CUST_40365_PI429010886 | M54459 | 0.036577 | 15 | 9 | | | | | | 14.95429 | 41.78277 | 41.47981 | 37.11196 | 31.46479 | 47.81705 | 48.28142 | 31.09423 | 46.629 | 44.53184 | 47.59957 | 86.72657 | 40.07867 | 58.94983 | 44.50661 | 10.76813 | 31.65427 | 70.15478 | 27.36205 | 37.76644 | 59.9559 | 37.9894 | 87.80183 | 84.58422 | 60.08957 | 75.83538 | 64.41009 |
| CUST_43867_PI429010886 | M64199 | 0.03658 | 18 | 0 | | | | | | 656.5643 | 34.7772 | 550.6217 | 326.0308 | 432.3349 | 600.4305 | 703.4699 | 221.9188 | 35.60156 | 20.63783 | 356.8925 | NA | 93.75665 | 11.83405 | 27.0513 | 5.550742 | 730.6182 | 243.6339 | 770.1948 | NA | 343.2786 | NA | 517.5225 | 621.5625 | NA | 181.3281 | NA |
| CUST_31339_PI429010886 | M1296 | 0.03659 | 15 | 3 | 332859533 | PREDICTED: neurofilament heavy polypeptide isofo | | | 4.28E-10 | 225.4062 | 78.02809 | 223.676 | 321.8946 | 341.396 | 532.9396 | 240.0651 | 200.8635 | 106.7633 | 71.30124 | 257.4087 | 95.20545 | 134.0744 | 95.62622 | 121.1298 | 82.71117 | 479.4117 | 333.1118 | 516.8489 | 87.50342 | 261.767 | 108.4109 | 275.2679 | 382.4385 | 94.17604 | 268.82 | 67.237 |
| CUST_39689_PI429010886 | M42938 | 0.03659 | 15 | 9 | | | | | | 1614.376 | 3469.507 | 3558.109 | 1158.693 | 1510.415 | 751.1067 | 1157.228 | 1569.952 | 2296.235 | 3456.298 | 2118.286 | 5188.094 | 3448.497 | 3493.781 | 3278.647 | 5356.619 | 1007.431 | 3098.049 | 1731.857 | 3006.75 | 2722.22 | 3896.561 | 3764.548 | 2026.043 | 5663.977 | 2286.095 | 5903.55 |
| CUST_5732_PI429010886 | N19117 | 0.03659 | 18 | 12 | | | Nucleic acid metabolism | Regulation of gene expression | | 128.4226 | 117.4158 | 172.1318 | 107.4857 | 94.81013 | 93.96395 | 135.8274 | 157.9551 | 137.1056 | 175.5504 | 132.0735 | 113.7469 | 125.6016 | 160.9995 | 144.5043 | 127.8994 | 154.2483 | 136.9448 | 148.6276 | 120.0646 | 79.32298 | 107.4983 | 141.2731 | 87.899 | 161.7779 | 115.7448 | 161.1809 |
| CUST_20161_PI429010886 | N42921 | 0.036614 | 18 | 12 | | | | | | 29.60942 | 20.3487 | 30.48036 | 9.72694 | 20.91496 | 17.86554 | 26.44421 | 17.80246 | 19.37414 | 26.4245 | 18.94519 | 26.25702 | 21.81525 | 24.98792 | 24.83589 | 30.2284 | 11.39415 | 20.77162 | 18.34511 | 26.18825 | 20.62099 | 18.70704 | 10.65706 | 10.27406 | 14.49923 | 12.67743 | 11.30336 |
| CUST_23671_PI429010886 | N49247 | 0.036614 | 24 | 0 | | | | | | 52.034 | 22.95261 | 51.10924 | 21.7647 | 32.83588 | 38.41183 | 44.60711 | 25.97092 | 20.43695 | 13.04046 | 32.71805 | 16.78445 | 19.42074 | 17.33525 | 23.35652 | 33.8279 | 44.06771 | 29.42101 | 51.01157 | 14.31728 | 52.22471 | 21.8588 | 32.55416 | 41.87571 | 33.14786 | 35.33485 | 40.11789 |
| CUST_47413_PI429010886 | M76012 | 0.036623 | 15 | 3 | 321471596 | hypothetical protein DAPPUDRAFT_302342 [Daphn | | | 5.38E-13 | 1184.216 | 441.5046 | 301.5634 | 10649.47 | 7669.986 | 21478.53 | 7647.424 | 2957.256 | 663.5689 | 32.33975 | 7628.757 | 14.5958 | 1503.323 | 69.71071 | 816.1069 | 119.1637 | 11222.08 | 9371.785 | 3692.777 | 98.22103 | 9638.897 | 207.5948 | 5769.917 | 9898.02 | 20.39383 | 8036.745 | 10.21823 |
| CUST_17013_PI429010886 | N37228 | 0.036625 | 15 | 0 | | | | | | 22.41939 | 17.35357 | 21.10951 | 42.58108 | 17.21639 | 15.80722 | 18.68705 | 15.33118 | 42.51309 | 9.375364 | 7.808479 | 19.44494 | 17.45875 | 17.63842 | 21.35072 | 30.9626 | NA | 30.27449 | 7.002324 | 29.18761 | 14.4666 | 26.43937 | 13.17448 | 20.92823 | 13.98209 | 6.953549 | 11.73379 |
| CUST_31386_PI429010886 | M1413 | 0.036651 | 24 | 0 | 156543086 | PREDICTED: trafficking protein particle complex sub | | | 5E-70 | 36.91363 | 51.50855 | 33.51766 | 24.67398 | 15.5279 | 18.27526 | 32.80342 | 15.58064 | 31.74172 | 18.28397 | 23.93544 | 23.00815 | 23.39301 | 15.23856 | 23.58263 | 20.39615 | 20.23721 | 17.80745 | 15.29306 | 22.63022 | 17.77803 | 27.2017 | 16.76285 | 27.47752 | 20.00381 | 28.97849 | 37.61167 |
| CUST_10609_PI429010886 | N26503 | 0.036669 | 15 | 3 | | | | | | 144.2537 | 19.01018 | 174.4075 | 41.12787 | 112.5941 | 91.42252 | 199.344 | 30.64706 | 23.07793 | 30.93077 | 129.5889 | 20.34336 | 19.98178 | 18.17304 | 26.65108 | 32.10376 | 184.3384 | 62.2514 | 208.1099 | 33.4065 | 139.376 | 29.32561 | 158.8004 | 200.6692 | 24.2514 | 29.46496 | 16.27803 |
| CUST_3996_PI429010886 | N15747 | 0.036699 | 15 | 12 | | | | | | 16.86851 | 15.08925 | 26.92163 | 16.26691 | 11.62058 | 13.28477 | 14.39924 | 18.80192 | 16.73391 | 26.21698 | 18.91365 | 13.05441 | 15.94162 | 26.73374 | 22.30073 | 19.19726 | 22.27554 | 20.66537 | 17.80424 | 15.6362 | 12.91624 | 21.80952 | 22.55762 | 16.17624 | 25.25393 | 14.79157 | 26.72979 |
| CUST_56634_PI429010886 | M114258 | 0.036725 | 21 | 12 | | | | | | 1901.328 | 1257.974 | 1507.459 | 552.6602 | 2237.5 | 1398.489 | 2001.122 | 1289.943 | 1364.868 | 2850.368 | 1228.27 | 1705.674 | 2644.158 | 1802.411 | 2093.981 | 1823.162 | 1834.08 | 2601.307 | 1684.016 | 1850.903 | 1393.263 | 1999.641 | 744.0064 | 657.2757 | 843.8131 | 1189.668 | 1451.905 |
| CUST_33676_PI429010886 | M7582 | 0.036734 | 21 | 3 | 338224394 | ADP-ribosylation factor GTPase-activating protein [S | | | 1.45E-13 | 20.87462 | 19.3376 | 13.55063 | 19.12864 | 61.32673 | 25.45947 | 10.07591 | 10.54822 | 10.2558 | 19.87307 | 22.48304 | 30.25192 | 7.943105 | 12.28582 | 9.08845 | 19.97113 | 20.75152 | 16.31911 | 19.81178 | 13.70684 | 17.42826 | 33.71073 | 12.82499 | 25.31297 | 34.0026 | 13.13981 | 42.46136 |
| CUST_8793_PI429010886 | N23598 | 0.036735 | 18 | 12 | | | | | | 44.92995 | 65.70437 | 52.27055 | 74.97848 | 39.99701 | 35.08626 | 52.33357 | 52.35268 | 74.54819 | 66.72453 | 82.9851 | 82.43374 | 101.6948 | 112.9234 | 89.47031 | 65.96017 | 39.28519 | 78.48034 | 69.20807 | 92.13028 | 51.9493 | 86.37756 | 32.77063 | 62.94659 | 62.74069 | 115.3546 | 83.33435 |
| CUST_30176_PI429010886 | N61330 | 0.036735 | 24 | 0 | | | | | | 17.12281 | 19.03935 | 29.49638 | 19.12737 | 17.21299 | 18.71355 | 13.25918 | 17.19879 | 15.93774 | 20.19405 | 16.75738 | 12.46997 | 8.869182 | 22.65868 | 8.883037 | 16.13535 | 22.88862 | 8.745116 | 21.4644 | 16.53144 | 22.66107 | 25.01145 | 24.36264 | 17.02832 | 26.75249 | 44.20422 | 15.80798 |
| CUST_19237_PI429010886 | N41228 | 0.03674 | 21 | 15 | | | | | | 157.8626 | 351.1934 | 198.8955 | 144.3614 | 93.50952 | 127.3882 | 150.2666 | 123.9463 | 100.2082 | 126.3583 | 74.64567 | 170.6145 | 108.8006 | 274.2312 | 236.2204 | 401.9872 | 169.3227 | 248.3342 | 219.7388 | 194.9182 | 210.5151 | 153.6882 | 111.4084 | 173.7221 | 338.4186 | 225.4439 | 258.6304 |
| CUST_54381_PI429010886 | M105806 | 0.036749 | 24 | 15 | | | | | | 202.7794 | 241.6881 | 247.6419 | 182.318 | 201.8347 | 234.4818 | 196.9122 | 177.2677 | 190.5242 | 214.921 | 205.6287 | 212.2832 | 189.8916 | 224.3204 | 228.6997 | 251.312 | 215.9452 | 245.6501 | 216 | 228.4677 | 211.6272 | 240.7867 | 223.8346 | 197.7545 | 246.2258 | 222.7049 | 237.3703 |
| CUST_2185_PI429010886 | N11816 | 0.036756 | 18 | 6 | | | | | | 26.50391 | 29.9042 | 36.72537 | 33.81235 | 51.00626 | 52.49852 | 30.77501 | 52.42324 | 48.49494 | 39.46416 | 48.49058 | 68.6262 | 26.28688 | 27.84094 | 40.09244 | 30.05351 | 44.4293 | 26.50702 | 33.49263 | 56.94451 | 43.55575 | 46.60859 | 36.41327 | 45.85232 | 52.78657 | 39.73253 | 52.57619 |
| CUST_52839_PI429010886 | M99169 | 0.036784 | 24 | 15 | 321474793 | hypothetical protein DAPPUDRAFT_237230 [Daphn | | | 1.64E-09 | 12.18711 | 7.683919 | 8.504674 | 7.391703 | 5.239566 | NA | 6.529408 | 5.231414 | 14.35732 | 9.960996 | 9.76387 | 11.28199 | 10.04101 | 20.66741 | 13.73069 | 10.97439 | 17.66889 | 24.0044 | 12.0262 | 7.447234 | 13.00375 | 14.38334 | 16.5954 | 6.249438 | 23.92174 | 9.656691 | 7.592295 |
| CUST_12869_PI429010886 | N30178 | 0.036789 | 15 | 3 | | | | | | 32.51731 | 58.42676 | 10.69461 | 313.8184 | 109.8902 | 95.84863 | 69.00612 | 8.103168 | 34.23165 | 12.14923 | 12.03276 | 5.961815 | 24.61213 | 57.76074 | 61.86952 | 125.183 | NA | 70.24276 | 7.975609 | 87.68861 | 20.0846 | 223.1811 | 10.30492 | 84.15456 | 40.09244 | 7.43529 | 9.277187 |

| Probe ID | ID | p-value | Period | Phase | EBI ID | Annotation | GO category | Go term | E-value | 0 | 0 | 0 | 3 | 3 | 3 | 6 | 6 | 6 | 9 | 9 | 9 | 12 | 12 | 12 | 15 | 15 | 15 | 18 | 18 | 18 | 21 | 21 | 21 | 24 | 24 | 24 |
|---|---|---|---|---|---|---|---|---|---|---|---|---|---|---|---|---|---|---|---|---|---|---|---|---|---|---|---|---|---|---|---|---|---|---|---|---|
| CUST_33912_PI429010886 | M8225 | 0.036817 | 21 | 18 | 321474382 | hypothetical protein DAPPUDRAFT_230544 [Daphn | | | 6.8E-32 | 53.47899 | 63.26276 | 42.53339 | 30.705 | 222.2769 | 18.40232 | 51.47755 | 29.28006 | 20.4413 | 176.0861 | 170.6324 | 186.167 | 22.21172 | 67.78689 | 65.93703 | 60.16545 | 72.3404 | 100.2495 | 62.28748 | 360.7127 | 146.3045 | 37.94932 | 106.3333 | 64.55134 | 32.86616 | 29.66792 | 57.32094 |
| CUST_52089_PI429010886 | M96382 | 0.036842 | 15 | 9 | | | | | | 15.44955 | 25.09429 | 7.378942 | 7.916656 | 7.053731 | 7.002324 | 21.17074 | 76.38976 | 5.147446 | 16.0073 | 13.58792 | 17.74139 | 12.2697 | 13.97554 | 13.79851 | 14.85271 | 9.200881 | 7.646317 | 10.0911 | 6.538282 | 12.65638 | 14.00927 | NA | NA | 22.69834 | NA | 15.46627 |
| CUST_53363_PI429010886 | M101413 | 0.036863 | 12 | 9 | | | | | | 14.70218 | 8.479761 | 15.53405 | 13.75707 | 12.73533 | 17.58365 | 3.550382 | 14.0658 | 5.302715 | 40.1229 | 22.61376 | 30.9788 | 18.88215 | 28.23631 | 13.76785 | 11.26192 | 16.25929 | 12.25717 | 21.68419 | 17.59799 | 24.91723 | 16.18436 | 16.52754 | 22.54321 | 12.09063 | 13.2704 | 13.53466 |
| CUST_16218_PI429010886 | N35866 | 0.036863 | 21 | 0 | | | | | | 65.45683 | 52.73287 | 76.03863 | 41.70336 | 40.84069 | 58.01161 | 52.22902 | 49.33073 | 30.68445 | 40.68218 | 51.32807 | 25.63785 | 56.77783 | 22.25418 | 42.55742 | 39.05552 | 52.29382 | 36.55477 | 41.29432 | 38.01489 | 36.10574 | 49.29932 | 54.08762 | 54.88431 | 41.0381 | 52.97579 | 36.69997 |
| CUST_39199_PI429010886 | M36036 | 0.036864 | 18 | 15 | | | | | | 3630.71 | 5256.29 | 3847.06 | 3701.465 | 3303.552 | 3197.843 | 2491.849 | 3144.897 | 2727.828 | 5060.995 | 3717.434 | 4763.632 | 3787.685 | 3872.65 | 2176.36 | 5705.785 | 3380.982 | 3500.164 | 3956.693 | 4579.426 | 3634.672 | 4827.614 | 3474.89 | 2756.174 | 3772.58 | 2704.165 | 3095.586 |
| CUST_13682_PI429010886 | N31583 | 0.036864 | 18 | 15 | | | | | | 98.04327 | 124.8931 | 131.3569 | 99.23417 | 97.88296 | 100.7206 | 87.40211 | 73.77697 | 101.0493 | 75.80349 | 131.838 | 126.8628 | 87.51279 | 121.0216 | 120.8052 | 127.001 | 80.70446 | 108.2888 | 101.7152 | 99.40267 | 124.3644 | 110.652 | 91.69639 | 93.80773 | 91.35555 | 79.43105 | 84.1293 |
| CUST_32254_PI429010886 | M3641 | 0.036866 | 12 | 9 | | | | | | 45.79191 | 36.02402 | 49.1739 | 40.58787 | 36.3679 | 45.66434 | 58.597 | 43.33449 | 51.05482 | 69.54952 | 52.15768 | 60.95703 | 47.82652 | 40.94065 | 37.99335 | 30.99084 | 64.23456 | 49.67248 | 42.98059 | 25.65833 | 44.40267 | 52.21271 | 64.49483 | 59.84805 | 46.45766 | 52.81394 | 37.22874 |
| CUST_22485_PI429010886 | N47123 | 0.036898 | 12 | 0 | 242004048 | glycerol-3-phosphate acyltransferase, putative [Ped | Metabolic process | Other | 6.88E-24 | 32.77838 | 21.76618 | 13.22835 | 8.165976 | 16.87203 | 15.12966 | 10.2343 | 12.38626 | 7.305705 | 19.07974 | 14.55595 | 14.36708 | 17.42611 | 13.12868 | 26.82401 | 21.43485 | 14.1264 | 15.7577 | 14.59396 | 17.69459 | 11.56997 | 26.16563 | 10.36359 | 19.37234 | 21.05341 | 17.85771 | 20.31875 |
| CUST_8548_PI429010886 | N23206 | 0.036942 | 12 | 3 | | | | | | 21.19713 | 7.792392 | 24.84105 | 26.68081 | 29.72938 | 26.62548 | 23.28108 | 13.36717 | 13.89481 | 5.101628 | 8.548078 | NA | 9.934601 | 14.94259 | 17.56531 | 28.04755 | 24.51944 | 38.09997 | 8.957551 | 7.573982 | NA | 20.40111 | 11.54827 | 29.08411 | 15.73939 | 3.780328 | NA |
| CUST_14906_PI429010886 | N33671 | 0.036961 | 15 | 9 | | | | | | 9.23209 | 10.55447 | 36.07274 | 12.51827 | 12.68973 | NA | 15.50857 | 25.89483 | 27.84917 | 40.71208 | 23.89355 | 25.54596 | 23.33049 | 37.46672 | 24.0077 | 29.92416 | 14.31922 | 15.24223 | 19.41782 | 23.04991 | 15.5279 | 33.93687 | 37.42124 | 11.30787 | 46.991 | 21.14577 | 45.27903 |
| CUST_15051_PI429010886 | N33931 | 0.036991 | 24 | 18 | 195576256 | GD22775 [Drosophila simulans] >gi\|194190001\|gb | Transport | Ion transport | 5.14E-19 | 1241.637 | 2182.975 | 1065.529 | 932.5265 | 1142.567 | 1099.991 | 1120.861 | 1157.228 | 1294.143 | 1200.709 | 1161.62 | 1695.261 | 1352.582 | 1454.733 | 1689.395 | 1492.073 | 1284.605 | 1157.816 | 1390.385 | 1868.48 | 1178.599 | 1530.336 | 1152.92 | 1437.865 | 1163.104 | 1332.224 | 1500.167 |
| CUST_6579_PI429010886 | N20298 | 0.036997 | 15 | 9 | 260833404 | hypothetical protein BRAFLDRAFT_117120 [Branchi | | | 1.76E-81 | 75.22711 | 84.35593 | 108.6304 | 90.12961 | 105.86 | 81.94846 | 73.13314 | 87.31709 | 115.1886 | 100.3711 | 129.735 | 134.0609 | 82.46049 | 114.7719 | 91.02196 | 98.51875 | 76.13932 | 82.25514 | 66.70898 | 137.33 | 90.06996 | 102.0573 | 117.6603 | 103.654 | 102.3756 | 117.8738 | 130.9454 |
| CUST_17507_PI429010886 | N38088 | 0.037015 | 15 | 12 | | | | | | 11.65145 | NA | 4.33418 | 6.482775 | 4.62769 | NA | 3.81618 | 4.822002 | 6.205172 | 11.4538 | 13.18006 | 12.79677 | 13.57654 | 15.7161 | 16.81213 | 8.991035 | NA | 10.23296 | 10.70092 | 10.32153 | 9.864571 | 13.80154 | NA | 11.33598 | 10.96329 | 35.39157 | 8.381146 |
| CUST_2792_PI429010886 | N13117 | 0.037024 | 15 | 3 | 91092632 | PREDICTED: similar to AGAP007416-PA [Tribolium c | Nucleic acid metabolism | Regulation of gene expression | 2.4E-106 | 350.7295 | 214.4171 | 117.2428 | 636.8404 | 385.1644 | 671.7237 | 447.1828 | 337.4667 | 193.267 | 108.4314 | 290.995 | 81.5056 | 291.8868 | 161.8008 | 203.1221 | 162.9163 | 597.939 | 361.4417 | 397.7744 | 132.3201 | 177.456 | 232.1034 | 143.9489 | 360.4157 | 147.1696 | 334.0181 | 65.68089 |
| CUST_4320_PI429010886 | N16505 | 0.037031 | 21 | 18 | 524914725 | PREDICTED: zinc transporter ZIP10-like [Aplysia calif | Transport | Ion transport | 1.02E-07 | 18.18555 | 19.03069 | 20.43255 | 11.09239 | 12.53746 | 19.12737 | 7.138401 | 6.62402 | 6.684024 | 17.61145 | 24.3983 | 13.51055 | 14.89135 | 16.81213 | 11.70041 | 23.51171 | 13.02142 | 16.21134 | 20.93844 | 16.8622 | 19.89057 | 18.13226 | 18.86017 | 9.139595 | 22.96785 | 16.74068 | 16.90667 |
| CUST_44806_PI429010886 | M67052 | 0.037039 | 18 | 3 | | | | | | 3087.397 | 1104.958 | 598.6499 | 16707.04 | 6350.776 | 21810.18 | 12697.77 | 4144.215 | 2684.729 | 52.78657 | 9855.517 | 7.757233 | 2101.303 | 1106.54 | 1837.915 | 1198.309 | 14583.52 | 13673.74 | 5113.292 | 241.4042 | 10200.91 | 2695.193 | 13030.53 | 19357.18 | 338.8337 | 6563.249 | 34.11542 |
| CUST_16084_PI429010886 | N35629 | 0.037049 | 21 | 0 | | | | | | 107.0174 | 113.3559 | 110.5556 | 85.66826 | 90.84494 | 84.34439 | 78.21989 | 90.6343 | 71.35403 | 105.7587 | 87.44346 | 105.19 | 66.22546 | 85.58471 | 73.00251 | 100.2834 | 104.1782 | 79.71189 | 122.5481 | 81.69291 | 85.6898 | 85.82609 | 115.6651 | 94.72175 | 78.83809 | 69.04216 | 90.47199 |
| CUST_19892_PI429010886 | N42398 | 0.03705 | 18 | 3 | | | | | | 9.228812 | 11.08009 | 10.25265 | 11.69128 | 33.80907 | 19.84931 | NA | NA | 14.61919 | 7.738172 | 8.768772 | NA | 3.977959 | 20.25021 | 4.128195 | 4.791154 | 7.155832 | 8.723307 | 15.13462 | 4.622331 | 11.47799 | NA | 7.88778 | 32.26103 | 4.378214 | 48.49058 | 15.93774 |
| CUST_11798_PI429010886 | N28419 | 0.037064 | 18 | 3 | | | | | | 7.808479 | 23.78457 | NA | 61.27293 | 10.40896 | 19.8522 | 8.315583 | 13.63796 | 10.03833 | 4.06003 | 3.952125 | 7.865975 | 4.812279 | 7.319807 | 16.50592 | 31.47618 | NA | 8.236094 | 3.615981 | NA | 13.75242 | 12.97654 | 9.633666 | 211.1514 | 10.09605 | 36.70412 | 6.13761 |
| CUST_9283_PI429010886 | N24369 | 0.037068 | 24 | 15 | | | | | | 552.7878 | 677.3854 | 773.1832 | 444.9718 | 488.4404 | 613.4417 | 439.2849 | 516.8489 | 440.5078 | 711.432 | 606.4913 | 762.5883 | 278.5157 | 624.8531 | 588.293 | 803.3312 | 784.2356 | 862.1393 | 741.9924 | 597.3714 | 540.7565 | 827.8275 | 397.8439 | 475.7608 | 900.1864 | 499.3294 | 731.0066 |
| CUST_2381_PI429010886 | N12231 | 0.037075 | 18 | 15 | | | | | | 1599.783 | 4353.113 | 994.7785 | 2693.745 | 2247.598 | 1409.58 | 987.1593 | 87.50342 | 909.4624 | 1950.603 | 1263.014 | 2516.877 | 1877.805 | 2370.125 | 2465.06 | 4676.187 | NA | 2567.686 | 641.5416 | 4058.299 | 1428.149 | 3309.537 | 880.6305 | 1380.113 | 2750.47 | 902.7962 | 487.6477 |
| CUST_29448_PI429010886 | N59876 | 0.037097 | 12 | 3 | 242016983 | hypothetical protein Phum_PHUM412790 [Pediculu | | | 8.17E-17 | 61.19321 | 22.63022 | NA | 53.76881 | 30.48607 | 61.25018 | 48.3768 | 43.53241 | 36.78858 | 17.17494 | 9.751886 | 6.900525 | 44.1334 | 14.63709 | 32.83834 | 26.60013 | 47.24387 | 29.22246 | 34.49959 | 19.06722 | 18.96046 | 24.11521 | 11.65145 | 43.14736 | 15.35498 | 23.99602 | 12.31007 |
| CUST_16798_PI429010886 | N36888 | 0.037103 | 15 | 12 | | | | | | 32.71054 | 37.818 | 38.16452 | 36.98363 | 33.42509 | 44.03947 | 29.58843 | 33.01824 | 32.03971 | 40.77403 | 31.64376 | 37.95647 | 30.88587 | 50.18617 | 40.24278 | 38.22991 | 36.06457 | 58.20366 | 34.2549 | 38.33878 | 35.45648 | 25.75422 | 36.63133 | 28.4998 | 42.16582 | 32.99565 | 38.88459 |
| CUST_17727_PI429010886 | N38486 | 0.037158 | 24 | 0 | | | | | | 20.28864 | 19.76676 | 20.55453 | 20.2329 | 18.84163 | 20.56557 | 17.0998 | 19.6916 | 13.69236 | 16.83181 | 15.06859 | 15.93149 | 15.06535 | 16.03903 | 8.98024 | 22.73086 | 19.83086 | 23.26561 | 17.83033 | 18.26586 | 20.36313 | 21.19536 | 15.56605 | 16.97579 | 22.32278 | 16.43932 | 27.41751 |
| CUST_40556_PI429010886 | M55451 | 0.03716 | 18 | 15 | 327259240 | PREDICTED: ATP-dependent RNA helicase DDX24-lik | | | 7.42E-22 | 13.69402 | 22.64182 | 19.6594 | 11.70937 | 13.532 | 7.254393 | 16.57948 | 18.689 | 17.76635 | 35.59734 | 17.02546 | 10.32521 | 18.90163 | 17.37315 | 16.45281 | 26.61953 | 18.76417 | 17.16641 | 13.30289 | 22.9227 | 21.08658 | 13.19382 | 15.20148 | 9.654274 | 22.16225 | 17.26212 | 33.19093 |
| CUST_13044_PI429010886 | N30487 | 0.037179 | 15 | 12 | | | | | | 90.17193 | 117.863 | 74.71666 | 89.48953 | 89.59424 | 86.94051 | 69.4087 | 73.18236 | 80.72822 | 98.00231 | 97.6483 | 152.3559 | 120.7171 | 122.3609 | 112.6894 | 127.0971 | 67.5589 | 139.1997 | 99.22324 | 134.3348 | 78.12789 | 90.6343 | 97.38686 | 87.45287 | 110.4846 | 90.72052 | 98.88783 |
| CUST_30752_PI429010886 | N62561 | 0.037187 | 18 | 12 | | | | | | 127.6888 | 136.5699 | 155.0302 | 117.9497 | 128.7368 | 131.838 | 126.5542 | 123.5891 | 142.4711 | 158.2442 | 116.3422 | 165.1224 | 166.5598 | 173.5211 | 161.9593 | 166.8022 | 153.3324 | 140.5313 | 138.4956 | 166.6587 | 169.847 | 123.321 | 145.8314 | 125.408 | 188.4563 | 120.4034 | 168.0579 |
| CUST_26342_PI429010886 | N54078 | 0.037211 | 18 | 15 | | | | | | 9.947764 | 6.606849 | 10.18199 | 8.32722 | 8.055935 | NA | NA | 3.727813 | 5.11464 | 8.137781 | 8.046291 | 11.68572 | NA | 11.9678 | 7.299608 | 13.36038 | 10.07859 | 36.82429 | 8.338462 | NA | 5.162518 | 10.644 | NA | 3.383341 | 6.46725 | NA | 10.16697 |
| CUST_5465_PI429010886 | N18741 | 0.037224 | 18 | 15 | 345491979 | PREDICTED: hypothetical protein LOC100678008 [N | Signal transduction | Intracellular signal transduction | 3.94E-26 | 1646.767 | 1719.247 | 1604.147 | 1066.79 | 1267.407 | 1204.562 | 1212.358 | 1331.49 | 1067.598 | 1964.934 | 1610.651 | 1572.497 | 887.5191 | 1497.781 | 1366.025 | 2093.981 | 1795.627 | 1998.595 | 1494.238 | 1774.043 | 1368.295 | 1990.565 | 1164.641 | 1321.214 | 2326.832 | 1408.714 | 2027.433 |
| CUST_4084_PI429010886 | N15970 | 0.037246 | 18 | 3 | 443695706 | hypothetical protein CAPTEDRAFT_227200, partial [ | | | 1.31E-22 | 114.6697 | 72.17688 | 164.5164 | 106.1066 | 159.2768 | 130.3926 | 163.569 | 72.18474 | 87.60236 | 71.27533 | 174.9558 | 56.24668 | 70.20247 | 62.85713 | 84.55489 | 96.75892 | 194.3549 | 104.4835 | 146.902 | 96.23175 | 152.1844 | 97.79572 | 163.2888 | 199.0448 | 73.27672 | 52.11124 | 79.93349 |
| CUST_14912_PI429010886 | N33677 | 0.037246 | 18 | 6 | | | | | | 231.4164 | 125.751 | 589.9381 | 187.5065 | 182.0483 | 173.2729 | 534.2439 | 918.3868 | 509.3966 | 579.7012 | 313.4993 | 259.3271 | 663.4104 | 924.8591 | 257.9077 | 166.9427 | 792.7128 | 132.4059 | 180.0476 | 139.8015 | 245.2785 | 333.1118 | 520.7262 | 115.7777 | 736.5339 | 294.8062 | 666.8781 |
| CUST_34797_PI429010886 | M10701 | 0.037294 | 12 | 3 | | | | | | 131.7341 | 83.5291 | 18.75909 | 235.9095 | 191.6952 | 286.8668 | 154.0476 | 122.7816 | 56.12021 | 14.32775 | 61.40781 | 6.601733 | 152.1158 | 13.14141 | 87.6188 | 61.40092 | 205.9692 | 147.7053 | 137.687 | 16.34119 | 39.35128 | 62.00083 | 27.14357 | 187.6821 | 26.71388 | 144.9791 | 7.553772 |
| CUST_4453_PI429010886 | N16787 | 0.037297 | 21 | 0 | 321470214 | hypothetical protein DAPPUDRAFT_317955 [Daphn | Developmental process | Multicellular organismal development | 4.46E-15 | 260.5944 | 301.5058 | 281.3948 | 170.2284 | 288.1636 | 238.5792 | 195.9585 | 136.7554 | 192.1486 | 214.2583 | 129.5087 | 214.6655 | 126.6662 | 199.7685 | 168.58 | 402.0451 | 172.7709 | 311.2463 | 180.5686 | 274.7936 | 210.6087 | 406.8847 | 190.0645 | 221.873 | 265.0157 | 178.862 | 211.1514 |
| CUST_246_PI429010886 | N2999 | 0.0373 | 15 | 9 | | | | | | 27.33167 | 60.67368 | 47.71977 | 27.42267 | 64.66231 | 24.76741 | 28.88408 | 34.57302 | 68.41788 | 117.3341 | 44.34868 | 126.8447 | 65.22887 | 116.4869 | 53.53627 | 52.62093 | 18.28861 | 49.11313 | 44.44423 | 40.59153 | 57.51717 | 96.44756 | 39.37435 | 24.62928 | 97.5299 | 59.46938 | 130.4973 |
| CUST_8636_PI429010886 | N23335 | 0.037303 | 24 | 18 | | | | | | 46.52715 | 44.09799 | 61.5801 | 43.53241 | 40.42785 | 43.41406 | 43.06591 | 41.46823 | 47.96622 | 59.46938 | 54.16396 | 58.14321 | 43.03847 | 63.91001 | 57.31513 | 48.12746 | 40.15861 | 59.06419 | 64.03011 | 63.83268 | 59.52293 | 37.87644 | 63.37918 | 52.32373 | 59.9743 | 54.66133 | 72.08475 |
| CUST_1198_PI429010886 | N8809 | 0.037321 | 12 | 0 | | | | | | 37.87644 | 45.60489 | 57.24358 | 28.51242 | 38.11258 | 34.41851 | 24.23314 | 27.23432 | 28.46316 | 41.44605 | 48.8169 | 66.64273 | 39.23286 | 52.86329 | 60.14737 | 71.2117 | 37.92303 | 41.00705 | 39.49159 | 36.10137 | 45.25874 | 54.71071 | 43.79745 | 39.35128 | 56.87504 | 43.84173 | 61.77428 |
| CUST_9737_PI429010886 | N25120 | 0.037326 | 21 | 12 | | | | | | 17.71924 | 10.88684 | 23.1311 | 17.87663 | 18.72625 | 14.32267 | 32.90861 | 23.51373 | 22.74587 | 11.79192 | 30.95577 | 18.82741 | 26.09533 | 26.06265 | 27.51937 | 28.20146 | 20.36175 | 17.22225 | 32.75472 | 33.77253 | 18.17736 | 21.64092 | 8.752945 | 11.14983 | 21.75179 | 16.71936 | 18.13841 |
| CUST_32928_PI429010886 | M5500 | 0.03733 | 21 | 18 | 321464782 | hypothetical protein DAPPUDRAFT_214208 [Daphn | | | 8.69E-48 | 52.45258 | 76.03079 | 33.61445 | 55.64622 | 70.62647 | 10.34781 | 6.62402 | 4.145387 | 8.616285 | 70.85223 | 47.16106 | 69.36484 | 44.22142 | 60.52796 | 57.42962 | 68.81509 | NA | 29.93503 | 23.69577 | 176.3124 | 93.33448 | 161.6167 | 44.21756 | 31.87606 | 45.42508 | 24.16344 | 18.65035 |
| CUST_4564_PI429010886 | N17037 | 0.037337 | 24 | 15 | | | | | | 27.33674 | 32.00727 | 37.93423 | 26.81787 | 36.115 | 33.1987 | 29.46873 | 32.45773 | 30.21389 | 30.63669 | 40.103 | 40.37974 | 38.65189 | 51.04982 | 35.48686 | 44.80164 | 48.3768 | 32.64929 | 17.42213 | 49.07171 | 36.4354 | 102.6772 | 42.6663 | 34.84639 | 28.93983 | 33.37295 | 29.76585 |
| CUST_33094_PI429010886 | M5956 | 0.037369 | 15 | 6 | | | | | | 23.66154 | 78.00387 | 18.88563 | 46.63569 | 19.96165 | 24.21798 | 40.82323 | 28.1226 | 80.73902 | 8.238355 | 8.995799 | 6.366804 | 9.345594 | 6.282328 | 23.43573 | 44.03947 | 12.26155 | 28.93067 | 24.27461 | 34.17378 | 31.35346 | 45.30656 | 36.4309 | 20.54425 | 22.55627 | 32.7846 | 31.37421 |
| CUST_54740_PI429010886 | M107419 | 0.037371 | 24 | 12 | | | | | | 50.85762 | 38.05463 | 30.89566 | 44.11518 | 38.16452 | 28.27144 | 82.16288 | 65.19644 | 80.75638 | 50.03352 | 78.50617 | 70.80146 | 75.16329 | 65.96017 | 94.46416 | 70.94036 | 87.6112 | 46.78356 | 81.55572 | 101.1328 | 46.01155 | 49.55005 | 64.41009 | 51.87547 | 78.32339 | 47.04186 | 78.02809 |
| CUST_22469_PI429010886 | N47086 | 0.037374 | 18 | 6 | | | | | | 15.66371 | 44.90621 | 38.33525 | 28.39147 | 32.816 | 42.64305 | 52.11928 | 16.21134 | 65.18101 | 20.3261 | 56.30906 | 56.53393 | 34.66525 | 39.94945 | 12.45682 | 14.4465 | 21.23397 | 44.48344 | 14.70379 | 21.26038 | 62.25774 | 23.13337 | 50.06107 | 78.35921 | 56.76151 | 66.84482 | 75.44459 |
| CUST_36270_PI429010886 | M15270 | 0.037443 | 21 | 0 | 242015484 | tyrosine-protein kinase Src42A, putative [Pediculus | Protein metabolism | Cellular protein modification process | 8.6E-118 | 15.79172 | 11.55822 | 17.24279 | 9.262293 | 14.87074 | 17.77043 | 10.88451 | 4.793305 | 7.07379 | 13.20951 | 5.81607 | 10.47683 | 13.43914 | 8.480876 | 8.116528 | 7.596526 | 5.111572 | 21.02215 | 4.854945 | 11.65616 | 7.161801 | 15.51399 | 15.63968 | 15.8658 | 13.85312 | 7.661152 | 11.4448 |
| CUST_10740_PI429010886 | N26712 | 0.037448 | 24 | 21 | | | | | | 57.83187 | 58.31834 | 65.70437 | 57.78122 | 46.19013 | 56.70973 | 57.8814 | 53.17437 | 46.26383 | 58.28332 | 49.29515 | 62.54347 | 56.52669 | 54.86156 | 59.31402 | 63.75313 | 63.06213 | 57.80004 | 62.18234 | 40.90354 | 62.96106 | 82.91863 | 60.09613 | 61.46235 | 68.41788 | 58.82472 | 55.44212 |
| CUST_23136_PI429010886 | N48315 | 0.037448 | 24 | 21 | | | | | | 54.07518 | 49.86266 | 91.0847 | 32.59686 | 50.84587 | 54.36604 | 52.18439 | 46.25868 | 44.4356 | 50.46214 | 62.92532 | 76.07766 | 58.23835 | 54.80919 | 55.54708 | 50.31204 | 65.79499 | 61.88462 | 70.83788 | 48.60566 | 70.41805 | 100.5554 | 56.40373 | 77.94557 | 41.73826 | 52.47732 | 56.60227 |
| CUST_47194_PI429010886 | M75143 | 0.037476 | 18 | 15 | | | | | | 186.6438 | 241.9211 | 162.2752 | 185.6828 | 168.58 | 137.1215 | 154.8105 | 160.6917 | 144.6167 | 226.4213 | 191.2063 | 164.9098 | 181.9242 | 163.4482 | 155.7012 | 278.5157 | 204.658 | 196.157 | 194.4273 | 165.3955 | 163.791 | 246.8318 | 129.065 | 134.4388 | 219.0379 | 125.8442 | 158.91 |
| CUST_54131_PI429010886 | M104717 | 0.037478 | 24 | 15 | | | | | | 61.91569 | 51.86061 | 72.2558 | 44.95044 | 50.92195 | 54.87191 | 43.5437 | 53.12725 | 40.59586 | 69.88371 | 38.50578 | 49.36928 | 60.15341 | 69.39137 | 67.7616 | 73.87582 | 57.36487 | 68.2813 | 61.93065 | 70.46616 | 56.17296 | 51.00123 | 68.50419 | 45.22377 | 85.74608 | 54.1974 | 65.93703 |
| CUST_48982_PI429010886 | M83352 | 0.037539 | 15 | 12 | | | Cellular Process | Cell adhesion | | 170.9183 | 111.3415 | 153.4856 | 100.1395 | 127.1944 | 137.33 | 189.8916 | 134.6193 | 159.3655 | 146.1108 | 110.4128 | 199.8756 | 210.2883 | 224.7458 | 173.4253 | 163.3967 | 222.0782 | 153.6022 | 203.4225 | 109.002 | 123.6533 | 167.0312 | 123.9578 | 158.7513 | 170.2702 | 101.565 | 217.9939 |
| CUST_32587_PI429010886 | M4497 | 0.037555 | 21 | 6 | 321474954 | hypothetical protein DAPPUDRAFT_309106 [Daphn | | | 8.7E-180 | 4.900232 | 176.0509 | 84.67784 | 340.5982 | 418.1195 | 176.2005 | 215.7896 | 1128.807 | 718.917 | 153.5334 | 830.4425 | 988.77 | 622.024 | 139.5229 | 408.2171 | NA | 308.0149 | 9.379442 | 122.4667 | 1856.885 | 697.5569 | 7.386661 | 278.9452 | 244.775 | NA | 929.0194 | 633.6093 |
| CUST_9442_PI429010886 | N24630 | 0.037627 | 24 | 15 | | | | | | 35.4058 | 27.39302 | 38.62431 | 29.30133 | 25.17336 | 21.2067 | 41.07674 | 20.23489 | 17.17879 | 36.20575 | 29.29243 | 26.64522 | 28.49553 | 40.24548 | 41.8249 | 33.99233 | 36.68661 | 48.17972 | 43.01496 | 34.96354 | 32.01033 | 37.28683 | 39.82447 | 28.22595 | 33.56673 | 32.64658 | 38.92288 |
| CUST_20876_PI429010886 | N44229 | 0.037684 | 24 | 3 | 77743875 | Euphausia superba isolate DGGE band AntEuk13-5 : | | | 1.64E-74 | 37461.84 | 67084.62 | 22307.76 | 45084.56 | 50017.11 | 47369.29 | 36046.8 | 42714.39 | 43024.52 | 37040.41 | 60957.53 | 39606.34 | 58446.35 | 29992.45 | 8094.902 | 24478.23 | 36912.34 | 23420.74 | 35818.08 | 35362.62 | 41407.04 | 44409.28 | 24478.23 | 11313.76 | 40846.53 | 41266.06 | 54279.51 |
| CUST_16655_PI429010886 | N36642 | 0.037688 | 15 | 12 | | | | | | 48.24061 | 50.40874 | 55.21295 | 46.40223 | 49.49452 | 47.7103 | 32.27198 | 48.40695 | 50.0808 | 45.02522 | 67.32283 | 57.17501 | 48.45297 | 66.65717 | 59.33007 | 52.63886 | 54.3948 | 56.73383 | 41.59399 | 46.07745 | 53.63219 | 47.54787 | 53.90994 | 48.33175 | 56.29594 | 43.71768 | 57.30764 |
| CUST_54611_PI429010886 | M106849 | 0.037723 | 12 | 9 | | | | | | 184.0607 | 141.2095 | 262.1655 | 67.10317 | 144.3428 | 113.6906 | 98.75692 | 68.91937 | 115.0393 | 183.5489 | 218.0605 | 248.8723 | 174.8815 | 104.8392 | 103.1666 | 64.73185 | 153.5041 | 84.62693 | 113.9294 | 134.7752 | 118.3356 | 184.9585 | 141.7485 | 111.685 | 168.1071 | 160.531 | 165.0799 |
| CUST_50666_PI429010886 | M90597 | 0.037723 | 12 | 9 | | | | | | 28.82923 | 29.13814 | 79.65072 | 30.98188 | 30.36422 | 31.72455 | 44.90621 | 51.32239 | 49.43277 | 55.0673 | 65.15734 | 74.18015 | 53.8551 | 43.24561 | 39.33067 | 53.56751 | 54.28784 | 35.51693 | 38.18783 | 54.58347 | 51.86061 | 42.87529 | 65.91381 | 56.59481 | 51.64328 | 35.68582 | 64.26189 |
| CUST_22570_PI429010886 | N47253 | 0.037723 | 12 | 9 | | | | | | 285.8605 | 124.6404 | 248.7014 | 137.1215 | 179.3477 | 123.9578 | 112.0774 | 139.8337 | 108.8637 | 213.4377 | 232.8505 | 260.9034 | 210.3335 | 199.0699 | 184.8973 | 202.6837 | 161.54 | 126.0286 | 140.036 | 146.4231 | 150.7034 | 281.8261 | 241.0332 | 214.3213 | 444.3979 | 170.2702 | 195.6579 |
| CUST_30802_PI429010886 | N62761 | 0.037735 | 12 | 0 | | | | | | 61.59565 | 62.01844 | 64.88975 | 39.18988 | 43.24186 | 38.81418 | 45.78676 | 45.08487 | 40.08304 | 60.34181 | 42.68463 | 43.49365 | 49.79493 | 55.20849 | 62.28748 | 57.07016 | 42.95918 | 81.58984 | 45.08487 | 45.47762 | 50.69847 | 63.08907 | 45.75802 | 44.56286 | 59.18336 | 50.28834 | 69.40293 |
| CUST_38516_PI429010886 | M27604 | 0.037751 | 24 | 18 | | | | | | 81.73063 | 108.3189 | 74.16318 | 41.15368 | 86.71532 | 63.01798 | 23.47569 | 23.1311 | 38.46426 | 78.48723 | 57.96208 | 141.4128 | 62.39657 | 104.1211 | 112.4734 | 109.5748 | 27.43011 | 134.5271 | 52.89696 | 155.7184 | 133.4505 | 165.9754 | 71.58792 | 73.31065 | 75.3452 | 121.7504 | 101.5278 |
| CUST_38355_PI429010886 | M25807 | 0.037753 | 15 | 12 | | | | | | 13.14489 | 14.74437 | 26.07711 | 16.74792 | 7.965022 | NA | 5.08183 | 13.05601 | 11.17351 | 15.84353 | 10.01379 | 18.89922 | 14.99955 | 24.85393 | 37.53618 | 64.11406 | 11.59303 | 23.06645 | 19.88268 | 18.87721 | 13.13514 | 15.3592 | 16.40095 | 10.58033 | 18.35021 | 16.85432 | 16.2897 |
| CUST_30871_PI429010886 | M61 | 0.037763 | 21 | 18 | | | Transport | Intracellular transport | | 574.2435 | 714.0634 | 484.9716 | 505.3693 | 500.086 | 429.3715 | 419.9089 | 422.0878 | 456.9093 | 706.0046 | 471.4881 | 621.2292 | 501.3373 | 481.7893 | 528.7455 | 797.54 | 414.7856 | 497.0198 | 527.3498 | 661.3119 | 584.3722 | 674.4353 | 504.2482 | 480.999 | 643.3515 | 412.7593 | 438.1052 |
| CUST_51834_PI429010886 | M95322 | 0.037763 | 18 | 6 | | | | | | 3879.679 | 4729.674 | 4876.999 | 4110.832 | 4839.291 | 6679.096 | 6018.766 | 6128.181 | 6273.384 | 4941.562 | 3699.019 | 4959.867 | 3327.699 | 4803.756 | 4182.62 | 3683.38 | 6116.034 | 5188.094 | 5545.717 | 2892.813 | 4091.647 | 4309.606 | 3879.679 | 7188.247 | 6128.181 | 8745.677 | 5098.883 |
| CUST_32617_PI429010886 | M4594 | 0.03777 | 15 | 9 | 321455842 | hypothetical protein DAPPUDRAFT_302246 [Daphn | | | 4.9E-31 | 185.5006 | 121.0016 | 102.9919 | 133.7242 | 176.7991 | 103.3023 | 198.5143 | 311.7466 | 241.1275 | 237.7577 | 348.5888 | 370.4877 | 429.1228 | 187.5374 | 235.2753 | 98.32258 | 210.3335 | 109.7371 | 113.976 | 612.5844 | 297.3146 | 145.5578 | 223.8084 | 107.9121 | 105.6225 | 364.6302 | 418.2809 |
| CUST_12104_PI429010886 | N28930 | 0.037777 | 12 | 0 | | | | | | 44.09799 | 58.41483 | 69.38283 | 42.09086 | 22.64961 | 43.50773 | 38.49034 | 43.44584 | 37.84181 | 43.68024 | 27.80573 | 55.67313 | 44.30287 | 77.10171 | 59.50688 | 61.43267 | 46.75809 | 43.33061 | 35.60906 | 48.16109 | 46.83381 | 41.12787 | 69.9145 | 39.63814 | 48.91662 | 52.71293 | 50.48246 |
| CUST_17302_PI429010886 | N37739 | 0.037777 | 12 | 0 | | | | | | 30.86904 | 23.2088 | 16.74268 | 14.31543 | 17.36105 | 21.99372 | 18.6374 | 7.838281 | 7.626632 | 31.66159 | 11.8786 | 30.42483 | 18.27296 | 16.49701 | 16.81632 | 16.30792 | 8.5134 | 14.32589 | 7.176452 | 10.51343 | 7.681885 | 32.93932 | 11.69762 | 11.96496 | 12.01996 | 17.19879 | 21.28123 |
| CUST_10964_PI429010886 | N27059 | 0.037778 | 15 | 0 | | | | | | 86.63308 | 120.3338 | 75.56946 | 68.72308 | 90.75806 | 78.49411 | 62.28748 | 71.87199 | 69.98559 | 83.53882 | 72.85867 | 71.03479 | 52.47261 | 93.86099 | 83.95292 | 82.83897 | 88.25864 | 79.62582 | 96.9916 | 78.35123 | 78.26326 | 87.70762 | 63.59288 | 68.38203 | 78.72104 | 63.92583 | 73.21949 |
| CUST_27394_PI429010886 | N55968 | 0.037816 | 21 | 9 | | | | | | 94.83175 | 111.8492 | 79.30391 | 93.28305 | 148.3475 | 71.86244 | 108.7377 | 134.7426 | 126.0286 | 124.8411 | 169.3227 | 159.0362 | 113.2611 | 98.75692 | 51.75281 | 93.77397 | 70.28439 | 73.88403 | 82.86721 | 194.1209 | 107.666 | 124.7587 | 46.40223 | 94.13978 | 95.81155 | 107.7642 | 106.9212 |
| CUST_48479_PI429010886 | M80552 | 0.037835 | 18 | 6 | | | | | | 39.12009 | 33.69477 | 29.02946 | 47.36571 | 46.629 | 50.04999 | 73.94862 | 48.44993 | 49.02476 | 50.8686 | 35.57263 | 51.39386 | 59.74927 | 63.96144 | 68.25442 | 44.24001 | 51.11368 | 30.65462 | 85.65603 | 18.11507 | 34.51534 | 67.42469 | 33.6052 | 30.83942 | 84.45921 | 51.57764 | 80.20389 |
| CUST_46705_PI429010886 | M73459 | 0.037836 | 15 | 9 | | | | | | 8.006014 | 9.181579 | 8.5134 | 7.112561 | 14.90554 | 9.194369 | 10.62249 | NA | 11.48964 | 16.61727 | 10.90695 | 11.28356 | 9.534612 | 14.19901 | 14.18613 | 11.78437 | 5.893349 | 13.43228 | 18.76417 | 7.155832 | 15.2636 | 18.98392 | 16.22392 | NA | 15.00181 | 18.71748 | 23.9514 |
| CUST_6056_PI429010886 | N19570 | 0.037852 | 15 | 9 | 242006280 | NADPH--cytochrome P450, putative [Pediculus hum | Metabolic process | Oxidation-reduction process | 2.3E-163 | 88.10192 | 94.90628 | 104.5344 | 100.566 | 114.1483 | 72.05058 | 107.8556 | 126.0286 | 122.1353 | 134.6768 | 115.4049 | 102.5545 | 121.9844 | 132.9869 | 94.61126 | 98.61381 | 87.91683 | 86.02859 | 96.29479 | 175.7224 | 106.2399 | 111.7362 | 100.5915 | 68.1923 | 121.4562 | 100.965 | 168.9693 |
| CUST_5676_PI429010886 | N19025 | 0.037854 | 21 | 15 | | | | | | 1393.263 | 1722.719 | 1270.234 | 1056.762 | 1194.442 | 1217.974 | 1082.445 | 1056.312 | 1062.278 | 1453.376 | 1265.474 | 1651.385 | 1170.459 | 1414.097 | 1525.167 | 2101.303 | 1255.497 | 1607.248 | 1513.56 | 1460.033 | 1166.811 | 1570.987 | 1195.55 | 1074.496 | 1615.873 | 1034.145 | 1461.256 |
| CUST_44469_PI429010886 | M66074 | 0.037856 | 12 | 9 | | | | | | 26.4245 | 16.43063 | 17.52653 | 53.1957 | 9.823191 | 20.78959 | 26.57746 | 28.6271 | 22.30272 | 53.78964 | 47.13105 | 118.7356 | 35.07942 | 26.00383 | 14.23571 | 22.54583 | 50.29336 | 16.10718 | 30.78534 | 17.25337 | 32.83309 | 44.75473 | 29.31162 | 62.00733 | 25.88326 | 15.17526 | 43.01496 |
| CUST_16472_PI429010886 | N36294 | 0.037856 | 15 | 9 | | | | | | 159.8062 | 114.7719 | 115.6651 | 103.4972 | 89.54002 | 120.1261 | 84.92511 | 119.3119 | 83.16029 | 149.4702 | 184.5383 | 168.032 | 132.2661 | 101.92 | 139.8015 | 136.6832 | 116.9508 | 120.3899 | 121.6208 | 166.4887 | 135.8061 | 137.3083 | 145.861 | 142.3547 | 160.531 | 158.5656 | 177.4241 |
| CUST_28622_PI429010886 | N58311 | 0.037856 | 15 | 0 | | | | | | 190.0092 | 114.9376 | 113.9625 | 161.1265 | 109.4643 | 135.7103 | 148.9832 | 124.513 | 108.4109 | 105.3648 | 83.19226 | 55.48994 | 105.8252 | 123.2134 | 114.2883 | 108.8637 | 121.2134 | 117.3985 | 140.0198 | 84.04395 | 98.7787 | 104.8909 | 77.55826 | 114.743 | 110.6458 | 89.05105 | 72.52738 |
| CUST_41799_PI429010886 | M58579 | 0.037868 | 21 | 9 | | | | | | 36.92762 | 42.91318 | 46.05345 | 38.95593 | 44.80164 | 25.03856 | 53.53068 | 63.73239 | 64.87589 | 76.26718 | 97.29292 | 90.54311 | 79.29506 | 43.90259 | 37.96113 | 35.52696 | 76.43974 | 30.00667 | 59.98757 | 94.50596 | 99.32217 | 31.45667 | 52.98238 | 40.86821 | 40.77106 | 48.42693 | 62.51938 |
| CUST_22046_PI429010886 | N46327 | 0.037868 | 21 | 9 | | | | | | 34.53375 | 41.67823 | 37.49557 | 37.45529 | 39.03429 | 41.8722 | 49.77895 | 62.09543 | 33.86006 | 55.23983 | 54.03548 | 48.20555 | 47.77962 | 57.028 | 48.41112 | 48.50046 | 51.37093 | 38.59321 | 56.16292 | 44.32754 | 36.16806 | 43.79306 | 36.85539 | 41.05122 | 58.41483 | 38.8107 | 52.76535 |
| CUST_43898_PI429010886 | M64286 | 0.037877 | 15 | 12 | | | | | | 57.93571 | 25.26918 | 48.33618 | 35.48457 | 19.01531 | 36.14912 | 28.74055 | 33.54623 | 44.47869 | 32.15824 | 29.36585 | 49.26562 | 37.08279 | 59.72331 | 62.06295 | 48.19648 | 47.40164 | 48.10487 | 39.12985 | 35.678 | 39.14912 | 35.9589 | 42.42487 | 33.38032 | 44.22939 | 29.94487 | 40.85343 |
| CUST_41525_PI429010886 | M57959 | 0.037914 | 21 | 9 | | | | | | 388.8828 | 329.648 | 199.8275 | 524.852 | 122.2181 | 77.45737 | 294.0478 | 146.5035 | 163.9694 | 761.5045 | 306.5697 | 818.0738 | 494.5663 | 400.26 | 288.1636 | 299.6797 | 477.7327 | 353.1722 | 686.1452 | 78.01974 | 499.6354 | 226.5808 | 206.4448 | 179.0014 | 114.4264 | 99.95963 | 351.303 |
| CUST_6721_PI429010886 | N20512 | 0.037927 | 18 | 3 | 242018020 | chitin binding peritrophin-A, putative [Pediculus hu | Metabolic process | Carbohydrate metabolic process | 8.57E-53 | 921.954 | 878.7545 | 310.7615 | 1413.603 | 855.518 | 1622.403 | 910.8643 | 800.6759 | 709.8502 | 215.4042 | 370.5671 | 191.6398 | 816.9007 | 375.0345 | 626.6888 | 210.4862 | 836.4238 | 697.8167 | 821.28 | 177.6724 | 403.6614 | 319.5751 | 390.2131 | 1474.289 | 237.9203 | 765.4435 | 162.3309 |
| CUST_30806_PI429010886 | N62766 | 0.037945 | 12 | 9 | 291225229 | PREDICTED: Flavin reductase-like [Saccoglossus kow | | | 2.8E-09 | 39.63454 | 37.17503 | 56.49623 | 27.14824 | 39.31816 | 9.029764 | 40.00171 | 113.9999 | 77.5823 | 63.90408 | 102.1933 | 95.08289 | 52.61483 | 52.43827 | 40.23874 | 36.27265 | 53.04998 | 28.23631 | 54.6863 | 130.1208 | 79.69567 | 58.14864 | 49.49896 | 24.44489 | 46.45397 | 34.58756 | 49.38456 |
| CUST_33334_PI429010886 | M6631 | 0.037961 | 24 | 3 | 546675233 | hypothetical protein D910_03875, partial [Dendroc | | | 2.98E-07 | 20.31401 | 10.56079 | 13.2528 | 38.14903 | 49.50242 | 34.05422 | 68.15284 | 12.94753 | 31.19824 | 14.28302 | 43.93042 | NA | 14.24194 | 17.05374 | 27.51414 | 28.62243 | 15.37156 | 25.46248 | 4.113524 | 6.536487 | 22.28112 | 32.90555 | 9.800176 | 30.29871 | 23.40412 | 17.56349 | 9.974505 |
| CUST_29219_PI429010886 | N59423 | 0.037995 | 15 | 12 | | | | | | 19.48105 | 34.29142 | NA | 26.73933 | 18.39671 | 10.57353 | 16.5596 | 10.82989 | 11.72586 | 25.75169 | 13.02142 | 26.75604 | 27.69749 | 42.18155 | 24.43342 | 44.82913 | 33.56101 | 18.43075 | 7.648203 | 13.51055 | 8.965849 | 13.02465 | 25.37889 | 19.74451 | 20.97422 | 4.106801 | 17.37482 |
| CUST_5881_PI429010886 | N19328 | 0.037996 | 15 | 0 | 321471618 | hypothetical protein DAPPUDRAFT_316364 [Daphn | | | 1.56E-51 | 23.69356 | 17.23467 | 15.03778 | 16.93029 | 11.20539 | 22.61376 | 5.762314 | 6.766361 | 9.613143 | 14.74805 | 9.807043 | 10.6327 | 7.807289 | 15.65479 | 16.10308 | 16.27412 | 11.52569 | 13.81321 | 8.518007 | 23.2348 | NA | 11.3111 | NA | 10.46311 | 14.92301 | 17.53908 | 12.55922 |
| CUST_8133_PI429010886 | N22583 | 0.038014 | 15 | 12 | 380023234 | PREDICTED: uncharacterized protein LOC10086930 | | | 1.94E-35 | 21.68419 | 20.10727 | 20.08095 | 8.599447 | 8.669897 | 11.57971 | 15.2073 | 11.58688 | 19.43095 | 25.15661 | 9.138723 | 19.77419 | 21.00917 | 26.61953 | 16.79729 | 20.48326 | 9.958012 | 18.96468 | 12.38785 | 16.28143 | 17.15927 | 16.10864 | 22.87323 | 8.883037 | 18.50615 | 10.79562 | 16.87055 |
| CUST_43245_PI429010886 | M62518 | 0.038031 | 18 | 9 | | | | | | 1677.906 | 1464.638 | 963.6143 | 600.7483 | 393.4936 | 303.2078 | 1051.549 | 1135.012 | 1170.02 | 3143.095 | 1050.96 | 1933.842 | 1312.47 | 1113.695 | 2366.854 | 1461.256 | 654.1515 | 854.951 | 2303.489 | 997.8681 | 1145.481 | 1741.833 | 942.6383 | 772.946 | 785.705 | 925.8412 | 1527.118 |
| CUST_56948_PI429010886 | M114975 | 0.038039 | 18 | 9 | | | | | | 590.3441 | 577.1878 | 808.7225 | 514.6723 | 654.3393 | 699.0502 | 682.478 | 722.0011 | 713.8389 | 808.2235 | 783.3097 | 810.0074 | 747.806 | 830.2031 | 683.7716 | 736.5339 | 774.5083 | 604.4329 | 719.6282 | 762.3115 | 660.7381 | 743.5954 | 707.6225 | 663.7859 | 752.1706 | 595.028 | 905.952 |
| CUST_12759_PI429010886 | N29991 | 0.038071 | 18 | 3 | | | | | | 124.2868 | 115.1182 | 108.1985 | 115.5 | 105.7255 | 104.1211 | 138.5572 | 61.1739 | 92.57501 | 68.56896 | 81.87168 | 69.46349 | 49.30624 | 74.29008 | 88.25864 | 98.05949 | 86.04961 | 90.58155 | 90.38098 | 71.98272 | 62.18234 | 106.2285 | 92.65529 | 76.71565 | 95.12719 | 50.40874 | 63.12362 |
| CUST_31771_PI429010886 | M2362 | 0.038152 | 12 | 9 | | | | | | 136.3105 | 186.6438 | 191.886 | 158.8804 | 161.54 | 92.1688 | 141.7648 | 136.4739 | 151.6234 | 251.5259 | 199.6316 | 213.3037 | 165.6398 | 193.3866 | 180.634 | 183.9787 | 177.2358 | 126.9474 | 210.0511 | 184.6172 | 175.6552 | 256.8944 | 194.2624 | 131.8938 | 243.9078 | 173.4432 | 219.3496 |
| CUST_7943_PI429010886 | N22306 | 0.038152 | 12 | 3 | | | | | | 90.53385 | 89.4807 | 33.53978 | 152.7576 | 102.7422 | 127.2704 | 90.9816 | 60.5947 | 45.91808 | 58.70044 | 57.72819 | 61.53357 | 79.19112 | 68.58333 | 52.69412 | 105.2542 | 110.192 | 129.8119 | 123.7589 | 79.92252 | 54.07518 | 160.2968 | 35.44816 | 70.05382 | 108.1857 | 76.2979 | 41.72002 |
| CUST_53097_PI429010886 | M100132 | 0.038157 | 24 | 15 | | | | | | 157.3996 | 130.4535 | 143.124 | 105.3539 | 120.2323 | 146.0295 | 122.6286 | 109.5988 | 129.8445 | 134.3159 | 140.4954 | 126.1032 | 107.9913 | 145.0637 | 115.6002 | 167.5706 | 148.8262 | 165.7579 | 136.6618 | 181.2412 | 106.7278 | 142.9167 | 147.341 | 138.8541 | 138.0216 | 123.2882 | 157.6076 |
| CUST_7618_PI429010886 | N21816 | 0.038167 | 21 | 15 | 158301687 | AGAP001738-PA [Anopheles gambiae str. PEST] >gi | Protein metabolism | Ribosome biogenesis | 2.46E-45 | 207.8011 | 259.6731 | 196.8321 | 171.2801 | 196.2537 | 138.0748 | 144.2774 | 158.5869 | 153.5542 | 249.5102 | 207.0425 | 221.873 | 198.0363 | 145.9425 | 193.8995 | 272.9055 | 156.6593 | 228.274 | 200.9492 | 234.7465 | 199.9828 | 207.9222 | 201.0518 | 174.6884 | 183.574 | 151.9691 | 211.3121 |
| CUST_44812_PI429010886 | M67075 | 0.038201 | 18 | 15 | | | | | | 1064.697 | 539.6207 | 883.3994 | 309.7678 | 1282.889 | 774.2499 | 1089.985 | 745.8794 | 818.9073 | 1328.52 | 633.3531 | 843.5117 | 1284.605 | 893.1457 | 1234.796 | 1091.244 | 1038.033 | 1528.209 | 916.176 | 936.7815 | 848.6156 | 1102.979 | 472.3127 | 346.3085 | 393.8505 | 820.5141 | 902.1996 |
| CUST_36298_PI429010886 | M15493 | 0.038216 | 12 | 0 | 321468144 | hypothetical protein DAPPUDRAFT_304936 [Daphn | Metabolic process | Lipid metabolic process | 2.7E-49 | 240.2315 | 286.3672 | 273.6903 | 246.3941 | 252.3062 | 239.9082 | 171.2286 | 198.8279 | 200.3982 | 206.4448 | 209.5944 | 281.652 | 216.807 | 266.851 | 253.6933 | 253.3806 | 196.3862 | 230.6259 | 166.1109 | 245.408 | 333.5908 | 287.4102 | 229.7926 | 222.6053 | 251.1828 | 379.0729 | 291.4361 |
| CUST_46742_PI429010886 | M73571 | 0.038216 | 12 | 0 | | | | | | 84.87934 | 91.78258 | 97.32524 | 87.09496 | 73.15736 | 62.29412 | 69.56934 | 70.89744 | 84.47788 | 92.1688 | 73.57494 | 73.3681 | 76.81043 | 79.95313 | 68.78674 | 79.12414 | 70.82822 | 86.00719 | 72.64262 | 67.9344 | 68.77033 | 95.70219 | 74.71666 | 84.53623 | 108.1565 | 82.10096 | 88.14739 |
| CUST_48175_PI429010886 | M79234 | 0.038216 | 12 | 0 | | | | | | 5417.146 | 6168.628 | 4965.834 | 2876.173 | 3802.906 | 4965.834 | 2506.708 | 3414.171 | 1782.524 | 5609.183 | 3792.107 | 5018.825 | 5176.163 | 6217.899 | 6614.331 | 8643.087 | 2329.908 | 2361.621 | 2269.213 | 5197.227 | 4293.225 | 8391.959 | 4864.618 | 4225.122 | 4365.095 | 4617.753 | 5367.717 |
| CUST_31973_PI429010886 | M2879 | 0.038227 | 15 | 12 | | | | | | 71.57464 | 81.62354 | 71.19165 | 47.10091 | 65.36011 | 52.67412 | 54.3134 | 43.21618 | 64.8492 | 80.20389 | 68.8749 | 89.27715 | 69.00612 | 81.84143 | 76.50308 | 120.9878 | 45.57 | 79.32298 | 53.37885 | 71.22702 | 74.54819 | 72.03576 | 53.73593 | 59.1427 | 93.47603 | 70.46616 | 79.96242 |
| CUST_3479_PI429010886 | N14587 | 0.038229 | 18 | 3 | | | | | | 24.17719 | NA | 7.104442 | 24.65512 | 4.672014 | 12.71803 | 18.68487 | 5.751851 | 22.63508 | 8.427427 | 6.616037 | NA | 9.461053 | NA | 3.74627 | 6.474974 | 7.063727 | 3.655859 | 6.786633 | NA | 6.512863 | 42.48584 | NA | 34.72656 | 12.95945 | 15.68172 | 4.346382 |
| CUST_8269_PI429010886 | N22799 | 0.038232 | 18 | 15 | | | | | | 110.0373 | 119.228 | 106.1812 | 107.512 | 101.3798 | 78.73486 | 83.42195 | 96.42858 | 82.21549 | 131.2193 | 95.47811 | 108.5891 | 95.25723 | 118.9329 | 124.1661 | 148.5237 | 115.9402 | 87.97637 | 113.7469 | 125.027 | 88.43444 | 121.562 | 112.4311 | 77.27026 | 103.9314 | 57.52971 | 104.1211 |
| CUST_36554_PI429010886 | M16216 | 0.038257 | 15 | 3 | | | | | | 81.16843 | 30.92489 | 79.49534 | 117.7722 | 171.7601 | 132.3781 | 189.3668 | 41.16894 | 39.40451 | 12.99442 | 117.7722 | 18.89448 | 33.92714 | 34.49959 | 45.09375 | 50.96015 | 262.8518 | 130.9135 | 174.0152 | 16.58905 | 158.635 | 33.50669 | 147.0019 | 220.3236 | 28.48875 | 46.36142 | 14.81603 |
| CUST_23418_PI429010886 | N48805 | 0.038314 | 24 | 15 | | | | | | 174.6367 | 237.1988 | 246.5795 | 169.802 | 178.0175 | 189.2302 | 213.6068 | 265.6599 | 250.587 | 251.8789 | 192.7945 | 314.6224 | 262.76 | 323.5707 | 243.595 | 387.779 | 224.2244 | 269.9577 | 276.9079 | 267.2735 | 247.587 | 327.3251 | 185.8424 | 190.0964 | 282.7521 | 174.8316 | 277.7994 |
| CUST_39558_PI429010886 | M41211 | 0.038316 | 21 | 0 | | | | | | 193.0913 | 184.4394 | 197.2996 | 159.4512 | 183.021 | 173.7958 | 158.6824 | 166.4887 | 160.9334 | 168.9693 | 180.8415 | 155.7184 | 165.0586 | 196.9798 | 131.8093 | 213.2126 | 161.0222 | 188.3767 | 206.9171 | 182.9432 | 167.5472 | 194.9822 | 191.2388 | 168.4961 | 217.4803 | 176.601 | 170.7553 |
| CUST_33917_PI429010886 | M8234 | 0.038361 | 24 | 21 | 321474219 | hypothetical protein DAPPUDRAFT_300212 [Daphn | Protein metabolism | Ribosome biogenesis | 2.38E-63 | 1374.989 | 1451.1 | 1642.187 | 1201.319 | 1195.55 | 1111.525 | 1149.925 | 1186.311 | 1172.181 | 1242.81 | 1453.962 | 1938.653 | 966.7908 | 1535.786 | 1239.798 | 2062.191 | 1135.012 | 1443.25 | 1338.953 | 1506.739 | 1328.52 | 1849.813 | 1572.497 | 1344.053 | 1266.139 | 1256.084 | 1689.395 |
| CUST_8741_PI429010886 | N23504 | 0.038361 | 24 | 21 | 321471077 | hypothetical protein DAPPUDRAFT_302835 [Daphn | | | 1.34E-07 | 115.1567 | 105.4884 | 95.32771 | 81.45472 | 85.35234 | 99.8601 | 97.85969 | 102.9808 | 94.40103 | 106.7139 | 88.66977 | 91.25978 | 91.91837 | 112.9528 | 113.2396 | 117.0641 | 110.5098 | 106.8104 | 106.8983 | 109.6904 | 79.19112 | 140.5991 | 113.9832 | 158.101 | 114.2091 | 110.5811 | 81.05888 |
| CUST_46394_PI429010886 | M72505 | 0.038365 | 15 | 9 | | | | | | 427.2547 | 1160.295 | 1040.536 | 168.0847 | 236.5825 | NA | 264.9674 | 1025.258 | 1463.822 | 1814.412 | 854.951 | 1747.319 | 2010.691 | 2614.485 | 1555.197 | 541.0041 | 460.0363 | 1601.861 | 741.4101 | 2002.228 | 944.6524 | 2780.239 | 2890.576 | 755.8427 | 3034.646 | 1273.05 | 4763.632 |
| CUST_34203_PI429010886 | M9051 | 0.038365 | 15 | 9 | 321466121 | hypothetical protein DAPPUDRAFT_305875 [Daphn | | | 1.96E-10 | 105.7587 | 264.8881 | 235.3617 | 60.47979 | 56.59481 | NA | 10.31615 | 55.74249 | 248.4954 | 354.2504 | 218.6003 | 807.1828 | 146.3889 | 383.9376 | 352.327 | 322.593 | 54.16396 | 310.2726 | 206.9547 | 363.8764 | 220.8472 | 282.7521 | 342.6916 | 12.64489 | 401.7586 | 417.6101 | 709.528 |
| CUST_53686_PI429010886 | M102803 | 0.038365 | 21 | 0 | 321471954 | hypothetical protein DAPPUDRAFT_315933 [Daphn | Metabolic process | Lipid metabolic process | 7.84E-18 | 16.95326 | 30.18791 | 15.32313 | 25.33406 | 17.13668 | 18.29941 | 7.795868 | 5.166249 | 6.195387 | 9.864571 | 11.47955 | 30.17589 | 9.996492 | 6.49944 | 12.47694 | 21.07244 | 11.01558 | 8.673687 | 9.407802 | 11.52917 | 7.807289 | 27.9603 | NA | NA | 9.519535 | 8.103168 | 7.788223 |
| CUST_45316_PI429010886 | M68712 | 0.038372 | 18 | 15 | 345487422 | PREDICTED: transient receptor potential cation char | | | 2.22E-13 | 20.75304 | 22.68396 | 15.65092 | 19.01959 | 17.45713 | 11.40403 | 16.76094 | 14.4689 | 17.35176 | 14.05126 | 13.32908 | 87.26858 | 11.32307 | 14.45048 | 24.0648 | 30.98188 | 25.74156 | 17.17022 | 21.7799 | 25.76654 | 18.39223 | 20.22452 | 13.86905 | 25.42066 | 17.72933 | 7.873199 | 10.8708 |
| CUST_32236_PI429010886 | M3601 | 0.038378 | 15 | 12 | | | | | | 73.69227 | 65.34609 | 64.75716 | 59.34665 | 53.84859 | 69.04997 | 51.97066 | 55.32113 | 44.16686 | 76.69076 | 67.22077 | 70.52611 | 57.00393 | 67.97289 | 86.01753 | 89.24944 | 58.91707 | 74.3064 | 86.94051 | 74.3942 | 61.74634 | 72.83324 | 49.12739 | 54.72536 | 76.24385 | 56.2163 | 74.58008 |
| CUST_57143_PI429010886 | E1475 | 0.038379 | 18 | 9 | 241725974 | guanine nucleotide-binding protein, putative [Ixode | Metabolic process | Other | 1.44E-14 | 288.3979 | 355.3996 | 324.6687 | 207.7747 | 217.7055 | 209.7628 | 176.9698 | 255.5333 | 227.6752 | 339.0413 | 299.3397 | 373.826 | 265.8178 | 246.1025 | 253.7689 | 370.6555 | 209.83 | 260.5944 | 251.3539 | 229.2068 | 240.9019 | 251.7868 | 280.971 | 210.6402 | 338.5409 | 213.0626 | 261.922 |
| CUST_16443_PI429010886 | N36248 | 0.038499 | 18 | 12 | | | | | | 18.57693 | 17.65481 | 13.76206 | 7.935304 | 7.079231 | 9.568409 | NA | 5.691879 | 11.49926 | NA | 4.108112 | 12.81312 | 22.08177 | NA | 141.0947 | 22.74317 | 14.37169 | 12.67376 | 7.368786 | 35.58868 | 8.343945 | 16.36148 | 7.837273 | 8.889261 | 12.5052 | 7.90501 | 8.291054 |
| CUST_1708_PI429010886 | N10497 | 0.038499 | 24 | 12 | | | | | | 5.695981 | 5.509418 | NA | 9.177025 | 8.564683 | 6.052528 | 6.825412 | 13.04696 | 7.180488 | NA | 12.14661 | 18.6166 | 28.9666 | 33.1071 | 46.40223 | 3.967645 | 4.980983 | NA | 42.83336 | 8.232782 | 21.2243 | NA | NA | 9.112459 | NA | 4.642534 | 21.97852 |
| CUST_20232_PI429010886 | N43061 | 0.03855 | 18 | 12 | | | | | | 342.1102 | NA | 1041.277 | 7.007076 | 7.14839 | 11.04457 | 639.8175 | 1267.018 | 844.11 | 485.9211 | 7.202889 | NA | 639.3223 | 792.7128 | 327.535 | NA | 698.4337 | 11.94919 | 321.753 | 6.895161 | 12.3974 | 9.943996 | 69.07358 | 6.250301 | 65.21686 | 12.75137 | 147.4281 |
| CUST_9650_PI429010886 | N24996 | 0.03857 | 18 | 9 | | | | | | 7.184973 | NA | 9.642662 | 9.043293 | 12.83461 | NA | NA | 8.505871 | NA | 14.27944 | 11.94615 | 36.88342 | 9.451067 | 16.25623 | 9.591065 | 15.61765 | 7.571579 | 9.901573 | 9.221505 | 3.322515 | 7.13523 | 6.351896 | 16.80921 | NA | 18.39397 | 8.732125 | 12.83593 |
| CUST_14957_PI429010886 | N33759 | 0.03858 | 12 | 9 | | | | | | 1580.29 | 1626.337 | 1533.433 | 955.4895 | 1271.998 | 763.2137 | 1550.902 | 1416.05 | 1587.608 | 4062.216 | 1409.133 | 2624.92 | 1265.003 | 1687.609 | 2747.282 | 1950.603 | 1003.971 | 1156.728 | 2370.125 | 857.8725 | 1573.833 | 1685.531 | 1843.043 | 1883.709 | 746.4019 | 1841.426 | 2456.459 |
| CUST_29781_PI429010886 | N60531 | 0.038581 | 24 | 15 | | | | | | 6.969672 | 6.749952 | 8.462821 | 7.518909 | 5.404635 | NA | 8.414372 | 9.798163 | 9.162149 | 24.22062 | 14.0442 | 9.430417 | NA | 7.710119 | NA | 11.13919 | 12.67545 | 13.7972 | 5.906131 | 12.36224 | 17.24517 | NA | 6.796716 | 9.827509 | 9.514057 | 9.603713 | 10.84809 |
| CUST_6354_PI429010886 | N19992 | 0.038607 | 21 | 9 | 344264210 | PREDICTED: arginase-1-like [Loxodonta africana] | Metabolic process | Cellular amino acid metabolic process | 4.43E-53 | 43.70884 | 59.59632 | 24.08063 | 16.93603 | 71.20471 | 22.8042 | 48.99962 | 52.51409 | 42.89021 | 189.2921 | 73.95574 | 70.50836 | 42.68895 | 49.23067 | 85.20601 | 40.13494 | 48.25178 | 40.23032 | 69.98559 | 24.99806 | 12.1266 | 40.44483 | 69.55824 | 21.89378 | 55.47668 | 7.800886 | 36.94269 |
| CUST_31290_PI429010886 | M1152 | 0.038617 | 18 | 12 | | | | | | 413.616 | 649.3217 | 605.8766 | 383.093 | 435.5217 | 259.439 | 490.4258 | 611.2062 | 499.7486 | 606.4913 | 574.7836 | 688.5897 | 593.5055 | 740.0272 | 634.4094 | 689.0956 | 553.2164 | 491.0083 | 559.3835 | 991.6341 | 574.7836 | 674.0173 | 552.1675 | 380.2921 | 532.6708 | 682.478 | 677.9305 |
| CUST_2536_PI429010886 | N12576 | 0.038617 | 18 | 3 | | | | | | 326.7477 | 62.94032 | 418.1872 | 398.6724 | 558.2631 | 417.8136 | 651.2944 | 304.5092 | 58.15995 | 43.62391 | 444.5828 | 24.40888 | 28.75932 | 22.91836 | 73.26024 | 11.71536 | 227.8356 | 74.40778 | 354.8939 | 20.52466 | 477.8507 | 35.6894 | 723.9047 | 787.7599 | 11.16309 | 55.02956 | 14.03086 |
| CUST_23334_PI429010886 | N48656 | 0.03864 | 21 | 3 | 446987672 | membrane protein [Proteobacteria] >gi\|229336051 | | | 6.97E-37 | 33.43942 | 44.46924 | 30.11515 | 54.23167 | 54.96916 | 58.45028 | 33.23404 | 42.7734 | 30.93804 | 33.76025 | 25.66555 | 48.64598 | 33.03437 | 34.51534 | 40.80851 | 44.25502 | 33.30691 | 54.62458 | 35.2168 | 14.98523 | 39.52923 | 39.27986 | 37.02134 | 34.51101 | 47.62012 | 47.29164 | 56.31693 |
| CUST_49024_PI429010886 | M83657 | 0.038641 | 18 | 6 | | | | | | 18.43075 | 25.27468 | 22.44547 | 19.11241 | 29.67419 | 35.39935 | 26.22286 | 22.94537 | 41.2187 | 26.56923 | 29.70687 | 58.85973 | 22.9275 | 37.04031 | 11.12791 | 10.11533 | 19.41296 | 50.55173 | 10.75291 | 6.299452 | 26.0691 | 15.24047 | 53.76242 | 70.47896 | 32.73752 | 46.17776 | 68.55373 |
| CUST_55914_PI429010886 | M112119 | 0.038644 | 21 | 3 | | | | | | 590.6242 | 498.6758 | 516.7166 | 643.2335 | 408.32 | 667.1794 | 521.7265 | 631.8289 | 607.9318 | 619.0377 | 491.0083 | 373.7568 | 355.0658 | 451.4433 | 352.1132 | 868.721 | 322.9274 | 344.6094 | 799.8512 | 275.8944 | 767.5196 | 1044.56 | 517.0533 | 618.5189 | 828.5874 | 780.4444 | 687.7759 |
| CUST_56739_PI429010886 | M114550 | 0.038655 | 12 | 0 | | | | | | 3243.287 | 3960.835 | 3200.017 | 2774.636 | 2047.628 | 2854.292 | 2147.931 | 2318.551 | 2814.544 | 3195.434 | 2393.921 | 3893.391 | 2851.724 | 3143.095 | 3661.505 | 4506.751 | 2278.385 | 3029.514 | 1814.412 | 3105.643 | 3551.684 | 4221.337 | 3031.864 | 2083.983 | 4451.275 | 4660.817 | 4250.591 |

| Probe ID | ID | p-value | Period | Phase | EBI ID | Annotation | GO category | Go term | E-value | 0 | 0 | 0 | 3 | 3 | 3 | 6 | 6 | 6 | 9 | 9 | 9 | 12 | 12 | 12 | 15 | 15 | 15 | 18 | 18 | 18 | 21 | 21 | 21 | 24 | 24 | 24 |
|---|---|---|---|---|---|---|---|---|---|---|---|---|---|---|---|---|---|---|---|---|---|---|---|---|---|---|---|---|---|---|---|---|---|---|---|---|
| CUST_15605_PI429010886 | N34854 | 0.038655 | 12 | 0 | | | | | | 136.2681 | 154.8105 | 162.251 | 130.3328 | 109.8741 | 126.7437 | 95.97714 | 95.29429 | 93.19487 | 159.6044 | 129.3158 | 183.4677 | 125.0791 | 150.3171 | 160.9995 | 168.3529 | 99.79034 | 157.17 | 106.7511 | 147.1503 | 139.1216 | 163.0075 | 132.9126 | 111.6628 | 173.4736 | 150.6483 | 169.5826 |
| CUST_28080_PI429010886 | N57252 | 0.038655 | 12 | 0 | | | | | | 1715.766 | 2701.759 | 1958.473 | 1948.38 | 1644.909 | 1773.273 | 1335.109 | 1403.267 | 1672.079 | 2142.216 | 1551.584 | 2316.991 | 1687.609 | 2260.474 | 2491.849 | 2456.459 | 1469.038 | 1911.091 | 1594.841 | 2280.777 | 1880.44 | 2547.522 | 1807.03 | 1665.986 | 2704.165 | 2184.967 | 2462.824 |
| CUST_46867_PI429010886 | M73998 | 0.038664 | 18 | 9 | | | | | | 195.3573 | 230.2846 | 239.6393 | 224.7802 | 124.7036 | 221.6459 | 211.85 | 249.0492 | 229.4054 | 428.9575 | 147.4899 | 314.246 | 149.1678 | 339.5005 | 260.9414 | 385.8989 | 264.6685 | 226.3675 | 217.9412 | 101.0393 | 206.8341 | 149.7694 | 214.7331 | 114.9009 | 229.7558 | 160.259 | 275.0769 |
| CUST_19106_PI429010886 | N40990 | 0.038677 | 24 | 21 | | | | | | 16.40616 | 11.61773 | 8.593635 | 12.74489 | 8.930028 | 5.841607 | 13.08017 | 8.019973 | 6.36012 | 14.96828 | 10.19195 | 9.070174 | 20.59519 | 15.92833 | 14.31922 | 19.38333 | 12.60453 | 17.00167 | NA | 6.814705 | 14.12974 | 16.13535 | 34.1704 | 16.5954 | 18.85349 | 8.111614 | 10.79019 |
| CUST_54873_PI429010886 | M108063 | 0.038679 | 15 | 9 | | | | | | 192.2319 | 147.2113 | 207.5377 | 159.338 | 355.5348 | 228.5019 | 224.6396 | 191.6952 | 205.4212 | 262.4225 | 242.7155 | 262.997 | 191.886 | 225.8926 | 204.0863 | 227.1802 | 218.9157 | 168.1071 | 251.7868 | 213.4377 | 223.0044 | 341.713 | 203.8732 | 236.856 | 276.6094 | 237.8874 | 235.6566 |
| CUST_38749_PI429010886 | M29975 | 0.038707 | 15 | 12 | | | | | | 7960.065 | 164.7626 | 17865.21 | 129.5554 | 420.7782 | 341.7763 | 11650.8 | 26858.78 | 22360.77 | 25017.78 | 3440.367 | 3240.576 | 20248.42 | 29378.84 | 16394.35 | 684.1373 | 11864.38 | 404.8823 | 7017.655 | 3610.458 | 3057.78 | 9726.619 | 19224.7 | 2441.116 | 20102.84 | 1522.087 | 23297.76 |
| CUST_29039_PI429010886 | N59083 | 0.038711 | 15 | 12 | | | | | | 67.19434 | 70.32547 | 66.81883 | 64.22812 | 52.38415 | 53.76881 | 46.17281 | 59.22654 | 66.62755 | 73.90307 | 67.69277 | 82.65719 | 59.70983 | 85.38257 | 78.75847 | 73.24512 | 55.45229 | 55.11221 | 57.09554 | 72.3636 | 57.6895 | 74.7945 | 55.98779 | 67.92832 | 69.82734 | 63.64812 | 73.14833 |
| CUST_9303_PI429010886 | N24406 | 0.038717 | 21 | 18 | | | | | | 157.9764 | 167.2066 | 107.409 | 148.7446 | 155.4149 | 126.1505 | 104.3886 | 105.5819 | 102.2534 | 121.9257 | 132.7894 | 146.6794 | 130.6959 | 99.11857 | 133.2798 | 164.6734 | 117.8888 | 174.2228 | 136.491 | 193.556 | 121.3613 | 169.8718 | 107.3035 | 155.1735 | 134.2991 | 110.2442 | 130.2691 |
| CUST_38464_PI429010886 | M27020 | 0.038726 | 21 | 12 | | | | | | 15.86933 | 20.208 | 16.58665 | 8.836706 | 17.79901 | 12.33347 | 11.35432 | 16.7373 | 17.76828 | 23.00521 | 15.78045 | 33.67567 | 24.21798 | 24.07736 | 18.84499 | 20.59519 | 9.801459 | 26.8901 | 13.39222 | 18.67439 | 22.9275 | 16.23539 | 20.5469 | 19.92471 | 13.00011 | 14.84417 | 22.77856 |
| CUST_34556_PI429010886 | M10009 | 0.038735 | 15 | 3 | | | | | | 186.167 | 102.5272 | 214.6269 | 132.3396 | 149.8707 | 190.62 | 315.6417 | 117.7597 | 125.0791 | 56.40861 | 230.2846 | 55.21772 | 78.01974 | 115.5444 | 74.16318 | 81.71205 | 264.026 | 145.1839 | 241.2571 | 78.19261 | 208.936 | 86.25852 | 143.7672 | 192.7647 | 62.39032 | 84.79763 | 41.01337 |
| CUST_23578_PI429010886 | N49058 | 0.038735 | 15 | 0 | | | | | | 182.4169 | 155.2537 | 208.0738 | 135.2716 | 137.3083 | 157.6076 | 159.2768 | 125.3551 | 133.891 | 190.62 | 150.449 | 171.2286 | 157.5 | 162.7472 | 167.776 | 175.2355 | 181.9242 | 164.6926 | 168.7959 | 153.3961 | 137.0338 | 164.2928 | 153.0541 | 150.9664 | 172.9946 | 144.1452 | 164.7626 |
| CUST_14737_PI429010886 | N33379 | 0.038742 | 18 | 15 | 189239442 | PREDICTED: similar to Nedd4 CG7555-PC [Tribolium | Protein metabolism | Cellular protein modification process | 9.96E-53 | 17.25706 | 21.82183 | 24.17061 | 24.09176 | 20.20177 | 17.47239 | 18.21981 | 19.76039 | 16.25775 | 7.810859 | 17.19336 | 20.17321 | 26.34872 | 20.3261 | 32.23691 | 21.43971 | 28.98746 | 29.97999 | 24.05186 | 46.28376 | 17.31462 | 18.40425 | 17.20887 | 21.23397 | 21.99572 | 8.136532 | 27.76301 |
| CUST_8353_PI429010886 | N22917 | 0.038805 | 15 | 9 | | | | | | 76.50308 | 52.95164 | 38.73055 | 75.56946 | 79.35074 | 38.29927 | 78.36561 | 169.2493 | 120.6382 | 78.81351 | 116.962 | 155.8377 | 108.3734 | 52.99425 | 76.00655 | 56.0501 | 84.65687 | 31.36464 | 67.95716 | 147.2361 | 98.09433 | 88.77007 | 63.105 | 64.72483 | 73.18236 | 117.1213 | 127.2256 |
| CUST_42139_PI429010886 | M59511 | 0.038811 | 21 | 6 | 75022291 | RecName: Full=Oplophorus-luciferin 2-monooxyger | | | 1.95E-10 | NA | 12.41521 | NA | 25.80044 | 29.79322 | 15.36262 | 16.16487 | 90.90134 | 50.61028 | 18.66441 | 37.16027 | 24.43879 | 22.41939 | 7.374786 | 13.6708 | NA | 10.03833 | NA | 8.995799 | 76.24971 | 18.2774 | NA | 9.427695 | 13.51693 | NA | 18.56257 | 27.4118 |
| CUST_36080_PI429010886 | M14569 | 0.038811 | 15 | 0 | | | | | | 240.2928 | 371.1996 | 225.4439 | 217.1338 | 216.304 | 163.8946 | 143.8657 | 113.9031 | 143.9903 | 220.4105 | 173.5554 | 211.1137 | 204.2904 | 164.0893 | 225.5174 | 305.0243 | 146.3045 | 224.3925 | 190.7563 | 252.9891 | 209.83 | 252.4045 | 181.1348 | 163.8946 | 187.0582 | 226.3675 | 236.9243 |
| CUST_40542_PI429010886 | M55413 | 0.038818 | 24 | 21 | 443699690 | hypothetical protein CAPTEDRAFT_212468 [Capitell | Nucleic acid metabolism | DNA replication | 4.99E-21 | 80.49518 | 90.69277 | 111.7257 | 80.1769 | 72.11758 | 94.5785 | 75.16329 | 82.4046 | 72.6524 | 101.8407 | 80.54851 | 84.77843 | 82.07629 | 112.2705 | 94.27998 | 112.4734 | 98.56528 | 92.89875 | 92.41138 | 85.26543 | 105.6225 | 104.1327 | 96.58099 | 100.0408 | 110.605 | 98.41575 | 138.5342 |
| CUST_31348_PI429010886 | M1316 | 0.03883 | 12 | 3 | | | | | | 676.1626 | 400.0206 | 609.1465 | 1117.627 | 722.2644 | 1105.302 | 904.602 | 395.3575 | 443.0418 | 409.277 | 495.7193 | 218.9512 | 561.9199 | 699.845 | 778.015 | 684.3658 | 1180.515 | 956.1852 | 1029.814 | 350.2692 | 470.0153 | 665.511 | 602.1279 | 862.8974 | 565.3295 | 429.8795 | 341.2566 |
| CUST_33135_PI429010886 | M6056 | 0.03883 | 15 | 9 | 521028669 | Zinc finger protein 2 like protein [Myotis brandtii] | | | 1.78E-09 | 11.73379 | 14.53096 | 20.87638 | 15.06859 | 4.761352 | 21.47183 | 19.58231 | 17.25706 | 17.42054 | 27.69147 | 33.76983 | 16.68994 | 14.14176 | 22.85699 | 11.99992 | 17.73394 | 18.93518 | 14.31543 | 13.64087 | 18.62971 | 14.22529 | 19.97907 | 28.00895 | 39.91268 | 22.20487 | 35.16159 | 24.61812 |
| CUST_35908_PI429010886 | M13874 | 0.03885 | 15 | 12 | 321476759 | hypothetical protein DAPPUDRAFT_207426 [Daphn | | | 2.6E-167 | 16.04256 | 6.78591 | NA | 7.993052 | 6.102462 | 10.54361 | 6.534722 | 11.31757 | 8.048476 | 8.090428 | 6.161897 | 11.64471 | 13.60227 | 13.84341 | 12.73533 | 14.52959 | 17.42054 | 23.01108 | 3.983369 | 7.915611 | 5.015188 | 15.50513 | NA | 13.84831 | 19.5508 | 14.81438 | 12.41521 |
| CUST_9363_PI429010886 | N24494 | 0.038874 | 15 | 9 | 512863611 | PREDICTED: LOW QUALITY PROTEIN: YLP motif-cont | | | 1.01E-36 | 4.666535 | NA | 11.59883 | NA | NA | 7.696778 | 4.342682 | 8.127017 | 20.52466 | 22.0997 | 37.93047 | 14.92766 | 4.389591 | 17.90553 | 10.8676 | 12.85526 | 5.05047 | 10.74149 | 9.562437 | 10.44442 | 16.43668 | 13.19581 | 26.30031 | NA | 23.41982 | 11.77935 | 20.98522 |
| CUST_40905_PI429010886 | M56352 | 0.038875 | 15 | 3 | | | | | | 217.5886 | 59.92145 | 29.69991 | 1104.361 | 929.0194 | 1595.711 | 1071.809 | 274.404 | 137.6307 | 25.62613 | 1242.051 | 16.10523 | 71.46757 | 25.61197 | 98.27597 | 40.78834 | 890.3835 | 409.5769 | 453.8761 | 28.11334 | 1048.763 | 39.73588 | 853.9995 | 987.1593 | 27.51414 | 144.0774 | 95.76592 |
| CUST_39967_PI429010886 | M47590 | 0.038891 | 15 | 12 | | | | | | 2538.071 | 3384.084 | 2978.783 | 2773.22 | 2516.877 | 2712.01 | 2125.97 | 2478.692 | 2507.968 | 2910.582 | 2349.941 | 3327.699 | 2921.018 | 3861.408 | 4080.667 | 4186.218 | 2551.399 | 3158.604 | 2784.905 | 3847.06 | 2563.253 | 3911.313 | 2612.527 | 2612.527 | 3213.851 | 2879.829 | 3534.122 |
| CUST_4267_PI429010886 | N16367 | 0.038895 | 15 | 9 | | | | | | 164.2928 | 12.79969 | 262.997 | 13.70338 | 10.90286 | 7.303907 | 95.46826 | 266.2431 | 106.908 | 134.486 | 111.8634 | 20.95213 | 74.80865 | 119.585 | 48.35952 | 11.84727 | 82.929 | NA | 15.8511 | 19.20292 | 50.30627 | 51.36009 | 40.01887 | 44.62585 | 94.51496 | NA | 174.0729 |
| CUST_37955_PI429010886 | M22454 | 0.038903 | 18 | 3 | 355344172 | Collinia beringensis isolate 12 18S ribosomal RNA ge | | | 8.8E-152 | 5.747087 | NA | NA | 46.55207 | 19.83086 | 43.88387 | 5.459208 | 6.294694 | NA | NA | 23.39071 | 6.068824 | 8.035951 | 8.706462 | 12.75297 | 5.503527 | 18.06411 | NA | 14.93582 | 7.934263 | 17.16641 | 27.99312 | 18.22767 | 48.09585 | NA | 19.28281 | 5.102784 |
| CUST_38966_PI429010886 | M32708 | 0.038917 | 21 | 6 | 321463770 | putative triacylglycerol lipase [Daphnia pulex] | Metabolic process | Lipid metabolic process | 2.69E-50 | 7.934263 | 303.854 | 122.3609 | 550.0892 | 652.2033 | 264.6314 | 335.9236 | 1741.833 | 1154.447 | 247.6419 | 1020.69 | 1168.111 | 849.9143 | 235.9095 | 521.1997 | 7.974486 | 411.6609 | NA | 200.3686 | 3003.506 | 850.7537 | 5.764682 | 418.1872 | 320.5083 | 21.13287 | 795.1421 | 833.5719 |
| CUST_8831_PI429010886 | N23663 | 0.038928 | 18 | 0 | | | | | | 34.4458 | 23.28108 | NA | 21.84275 | 19.96619 | 9.078323 | 14.69744 | 10.99038 | 4.454763 | 20.31875 | 16.20103 | 12.03392 | 12.32915 | 19.85432 | 12.36577 | 17.61017 | 34.0026 | 19.78961 | 23.30079 | 10.37992 | NA | 34.03541 | 13.80323 | 16.8197 | 22.07122 | 5.837543 | 6.037926 |
| CUST_16013_PI429010886 | N35510 | 0.038935 | 21 | 12 | 345481110 | PREDICTED: spectrin beta chain, brain 1-like [Nason | | | 8.57E-51 | 46.59363 | 43.14351 | 47.47127 | 53.16284 | 43.58778 | 52.04012 | 38.37256 | 45.63167 | 36.25388 | 42.07991 | 45.64241 | 36.92111 | 52.24124 | 57.30764 | 58.36968 | 50.94392 | 51.06646 | 60.00077 | 58.22987 | 44.4501 | 52.48792 | 55.39377 | 45.12848 | 42.77863 | 48.62912 | 55.13187 | 37.48336 |
| CUST_51351_PI429010886 | M93219 | 0.038964 | 15 | 12 | | | | | | 43.89224 | 66.95495 | 42.53957 | 30.98507 | 40.73202 | 27.80573 | 32.55631 | 37.71676 | 37.23147 | 58.36485 | 38.84241 | 58.97911 | 66.92953 | 48.71321 | 47.16593 | 45.66434 | 52.70102 | 32.48585 | 32.13794 | 50.34321 | 34.56663 | 53.29328 | 38.94819 | 23.45456 | 44.99497 | 48.03764 | 26.87538 |
| CUST_32847_PI429010886 | M5258 | 0.038976 | 21 | 15 | 356651206 | caspase 3C [Macrobrachium rosenbergii] | | | 6.76E-21 | 27.65371 | 40.56586 | 32.81991 | 21.01979 | 29.9579 | 15.24921 | 35.32612 | 29.97696 | 27.36512 | 31.24487 | 35.9369 | 35.9369 | 35.61182 | 33.21431 | 19.58231 | 52.40666 | 53.08483 | 40.08304 | 60.57719 | 39.16663 | 17.3497 | 72.88198 | 24.55066 | 16.96853 | 60.87936 | 15.16412 | 25.99897 |
| CUST_34062_PI429010886 | M8639 | 0.03898 | 21 | 18 | | | | | | 903.9804 | 1328.52 | 1092.486 | 810.0074 | 1119.369 | 842.3459 | 644.1161 | 740.6945 | 746.6817 | 1271.51 | 710.6638 | 1722.719 | 824.9455 | 806.0846 | 1092.486 | 1210.833 | 715.3982 | 906.5131 | 1082.904 | 1264.445 | 1141.431 | 1506.739 | 921.954 | 784.2356 | 964.5529 | 694.3101 | 890.3835 |
| CUST_4116_PI429010886 | N16052 | 0.039009 | 12 | 9 | 166952321 | ribosomal protein rpl35a [Lineus viridis] | Protein metabolism | Ribosome biogenesis | 1.22E-40 | 3832.303 | 5705.785 | 4083.732 | 3858.586 | 3345.256 | 3306.501 | 3530.217 | 3934.354 | 3929.651 | 4008.447 | 3483.315 | 6483.048 | 3997.898 | 3960.835 | 3517.336 | 3048.703 | 2671.216 | 3543.323 | 2393.921 | 5596.154 | 3345.256 | 5492.691 | 3968.239 | 4041.003 | 3169.636 | 5035.54 | 5349.606 |
| CUST_9524_PI429010886 | N24779 | 0.039065 | 15 | 0 | 307197722 | hypothetical protein EAI_02279 [Harpegnathos salt | | | 8.81E-41 | 2511.522 | 3113.095 | 1998.595 | 3116.138 | 2509.921 | 2814.544 | 1897.37 | 1679.237 | 1598.096 | 2163.813 | 2092.578 | 1862.262 | 2085.292 | 2270.606 | 2663.095 | 2731.57 | 2169.749 | 3240.576 | 3208.657 | 1886.936 | 2280.777 | 2263.289 | 1839.737 | 2238.739 | 2324.619 | 3154.98 | 2119.433 |
| CUST_37305_PI429010886 | M18552 | 0.039067 | 18 | 9 | 345479078 | PREDICTED: Nasonia vitripennis ribonucleoside-dipl | Nucleic acid metabolism | DNA replication | 4.6E-73 | 115.1051 | 171.8712 | 104.3393 | 121.8904 | 121.0512 | 66.36423 | 123.9463 | 156.6286 | 140.5892 | 179.8682 | 155.3131 | 175.6284 | 208.7066 | 156.3918 | 145.3261 | 238.7409 | 139.1997 | 120.2795 | 162.7472 | 139.4033 | 118.8413 | 181.3801 | 114.2624 | 81.49546 | 305.4951 | 109.1135 | 165.6901 |
| CUST_4307_PI429010886 | N16464 | 0.039069 | 15 | 3 | | | | | | 3934.354 | 920.7129 | 47.69448 | 14710.21 | 5009.146 | 28551.51 | 5688.498 | 8046.364 | 2123.129 | 5.529046 | 1430.089 | NA | 4876.999 | 15.98124 | 2562.095 | 48.62311 | 10348.94 | 11524.4 | 12983.85 | NA | 1571.524 | 8.396527 | 452.0017 | 8436.312 | 7.311535 | 13104.17 | 7.088352 |
| CUST_23163_PI429010886 | N48362 | 0.039094 | 18 | 12 | | | Transport | Ion transport | | 5769.917 | 6794.71 | 7347.807 | 4613.187 | 5336.786 | 7273.892 | 5681.469 | 7026.307 | 8414.501 | 8086.505 | 5520.509 | 8278.281 | 7890.624 | 8504.473 | 10387.85 | 8461.907 | 4876.999 | 7119.249 | 6621.858 | 7536.588 | 6359.374 | 6925.74 | 6336.037 | 5774.702 | 6407.392 | 10899.12 | 8196.001 |
| CUST_29149_PI429010886 | N59289 | 0.039095 | 12 | 0 | | | | | | 167.4333 | 151.0368 | 170.3395 | 147.341 | 153.1511 | 241.4042 | 128.8389 | 140.4954 | 160.9995 | 164.8776 | 134.8756 | 211.9851 | 162.4876 | 198.2178 | 153.5542 | 122.3609 | 159.948 | 193.3347 | 141.6349 | 127.6161 | 160.6252 | 171.3142 | 161.3901 | 165.6637 | 217.5163 | 242.302 | 196.9122 |
| CUST_34644_PI429010886 | M10222 | 0.039096 | 24 | 0 | | | | | | 175.1768 | 232.9174 | 131.3133 | 119.3927 | 145.0637 | 133.2626 | 150.4117 | 113.9294 | 156.5024 | 173.9115 | 138.5194 | 145.794 | 135.7103 | 137.4175 | 129.6421 | 185.2951 | 109.0596 | 149.5977 | 109.399 | 178.8016 | 138.5342 | 185.6237 | 176.2631 | 149.6919 | 196.9351 | 212.7445 | 259.0023 |
| CUST_8478_PI429010886 | N23100 | 0.039109 | 15 | 3 | 405965651 | Mitochondrial sodium/hydrogen exchanger NHA2 [ | Transport | Ion transport | 1.07E-27 | 86.65167 | 22.45787 | 92.30386 | 44.62585 | 84.36786 | 102.477 | 147.6693 | 37.56967 | 21.18145 | 18.14003 | 107.409 | NA | 20.09831 | 16.19147 | 21.38676 | 22.44161 | 159.5474 | 37.9742 | 176.3124 | 30.66803 | 78.51483 | 18.15598 | 100.6291 | 87.49111 | 15.12815 | 27.67518 | 11.93121 |
| CUST_19406_PI429010886 | N41524 | 0.039112 | 15 | 9 | 443732030 | hypothetical protein CAPTEDRAFT_222010 [Capitell | Transport | Other | 3.45E-28 | 60.10166 | 60.35512 | 53.78964 | 55.81794 | 58.0658 | 94.84242 | 80.24894 | 76.84997 | 76.17525 | 70.61376 | 72.3404 | 107.6007 | 65.31793 | 79.23712 | 57.86841 | 93.65476 | 56.80303 | 79.21992 | 71.2192 | 69.50944 | 69.34114 | 90.21463 | 67.71736 | 67.48171 | 90.92371 | 93.67647 | 91.94088 |
| CUST_2764_PI429010886 | N13057 | 0.039121 | 24 | 21 | 383862764 | PREDICTED: uncharacterized protein LOC10087882 | Nucleic acid metabolism | Regulation of gene expression | 8.99E-61 | 9.048255 | 12.0262 | 15.83559 | 8.057073 | 5.622844 | 9.315276 | 11.49641 | 5.958556 | 12.66068 | 4.805296 | 6.606034 | 12.66619 | 10.44442 | 22.08663 | 11.89031 | 28.04382 | 6.870124 | 16.71353 | 12.84177 | 19.11241 | 10.59996 | 27.75759 | 16.47211 | 12.17805 | 20.90506 | 11.73206 | 15.47775 |
| CUST_47624_PI429010886 | M76943 | 0.039136 | 12 | 3 | | | | | | 349.978 | 380.2117 | 95.46826 | 780.9267 | 292.3676 | 1091.604 | 695.1115 | 385.6213 | 499.2279 | 277.7171 | 388.1247 | 53.50545 | 289.0715 | 276.2073 | 274.0681 | 218.3262 | 517.1879 | 408.6642 | 503.4817 | 344.9738 | 493.9819 | 655.7469 | 145.6855 | 359.57 | 204.8186 | 458.895 | 51.95466 |
| CUST_54359_PI429010886 | M105717 | 0.039138 | 15 | 6 | | | | | | 275.2679 | 42.80091 | 541.7506 | 345.5168 | 370.8487 | 458.9977 | 493.0904 | 558.0588 | 141.3981 | 45.88252 | 189.684 | 6.594234 | 59.26817 | 27.14607 | 39.30599 | NA | 205.4682 | NA | 242.6521 | 3.972829 | 377.6826 | NA | 380.2117 | 495.7193 | NA | 3.774135 | 4.675508 |
| CUST_30773_PI429010886 | N62640 | 0.039143 | 24 | 21 | | | | | | 34.29722 | 28.57866 | 31.8793 | 27.09386 | 23.31758 | 17.08222 | 22.0851 | 17.58365 | 24.75742 | 23.60773 | 20.91693 | 17.15304 | 21.5046 | 44.66782 | 33.49263 | 31.80677 | 23.35894 | 17.61934 | 33.15216 | 21.7968 | 33.7899 | 28.15071 | 36.78203 | 27.4471 | 33.45456 | 19.15208 | 33.26462 |
| CUST_40955_PI429010886 | M56475 | 0.039148 | 18 | 3 | 345497413 | PREDICTED: hypothetical protein LOC100114875 [N | | | 9.43E-49 | 328.5456 | 531.9567 | 417.1018 | 728.8915 | 543.0155 | 637.0757 | 358.225 | 272.4368 | 342.4231 | 424.2638 | 222.6303 | 546.5203 | 256.7514 | 459.5005 | 269.1618 | 447.2563 | 158.8254 | 380.0505 | 201.3867 | 212.1377 | 613.0465 | 523.093 | 600.1331 | 374.3698 | 512.8422 | 661.0472 | 269.0306 |
| CUST_42893_PI429010886 | M61533 | 0.039162 | 12 | 3 | | | | | | 239.6059 | 80.25744 | 145.749 | 339.5005 | 320.2376 | 600.1331 | 346.7104 | 154.222 | 79.35074 | 44.4501 | 284.5979 | 75.10926 | 118.9193 | 40.40681 | 112.3183 | 74.29802 | 602.9273 | 364.0324 | 491.9013 | 51.36601 | 309.3892 | 48.44993 | 334.2433 | 364.1216 | 50.7592 | 253.4186 | 42.08627 |
| CUST_8017_PI429010886 | N22425 | 0.039162 | 15 | 3 | | | | | | 59.99556 | 27.16781 | 76.40353 | 63.85255 | 72.6348 | 140.5991 | 113.4366 | 41.84077 | 23.06972 | 20.65041 | 88.95458 | 35.81535 | 27.4471 | 26.06598 | 19.6198 | 53.63782 | 108.1041 | 71.97535 | 180.2796 | 21.28517 | 56.32248 | 36.19875 | 65.18101 | 93.19487 | 21.84275 | 32.95758 | 8.246449 |
| CUST_51196_PI429010886 | M92556 | 0.039165 | 21 | 0 | | | | | | 13.28156 | 19.16559 | 14.02745 | 8.648968 | 10.83953 | 10.22956 | 11.33051 | 14.56893 | 10.99038 | 16.8622 | 4.035717 | 8.688469 | 10.12007 | 8.863586 | 9.985243 | 14.68167 | 8.877949 | NA | 15.41448 | 13.13833 | 13.20796 | 13.27508 | 22.03029 | 16.36632 | 13.79851 | 7.240943 | 9.275747 |
| CUST_17521_PI429010886 | N38110 | 0.039213 | 15 | 0 | | | | | | 68.03939 | 62.86517 | 64.28764 | 33.17446 | 43.37513 | 58.08045 | 33.52618 | 32.17709 | 50.8107 | 56.72265 | 42.02786 | 66.37222 | 40.03501 | 47.80852 | 39.67377 | 53.17877 | 52.73715 | 60.12109 | 44.70035 | 52.88574 | 48.67019 | 24.05405 | 44.67201 | 53.23845 | 48.27575 | 40.32087 | 41.69983 |
| CUST_39614_PI429010886 | M41775 | 0.039264 | 24 | 15 | | | | | | 384.6311 | 400.5981 | 436.0745 | 339.6011 | 364.8104 | 366.089 | 357.4809 | 360.4943 | 380.9707 | 455.5337 | 347.2399 | 416.2434 | 382.0118 | 492.3863 | 434.1796 | 469.7466 | 426.1051 | 408.8064 | 418.4668 | 497.1091 | 373.2835 | 421.5075 | 390.7936 | 369.3306 | 486.0261 | 395.4574 | 512.2788 |
| CUST_51705_PI429010886 | M94646 | 0.039272 | 12 | 9 | 260823076 | hypothetical protein BRAFLDRAFT_119786 [Branchi | | | 1.76E-29 | 23.60773 | 16.15388 | 18.19988 | 11.99669 | 11.94615 | 11.03039 | 25.15661 | 9.308024 | 23.56736 | 18.54155 | 13.37838 | 33.34314 | 21.90983 | 13.8146 | 10.31132 | 23.26041 | 7.64912 | 23.96961 | 15.39724 | 25.45947 | 29.63396 | 200.4301 | 31.86956 | 38.56828 | 14.1264 | 21.036 | 31.97801 |
| CUST_5123_PI429010886 | N18288 | 0.039272 | 21 | 18 | 541906833 | casein kinase 1 epsilon [Eurydice pulchra] | Protein metabolism | Cellular protein modification process | | 781.4539 | 538.8855 | 841.0533 | 460.8905 | 513.6722 | 490.2884 | 625.9634 | 512.6993 | 586.0335 | 614.705 | 664.7021 | 655.5465 | 494.3438 | 648.7533 | 557.1607 | 635.437 | 790.7325 | 566.7662 | 683.5585 | 556.7295 | 700.4893 | 622.3874 | 755.8427 | 803.5259 | 588.293 | 521.963 | 555.7341 |
| CUST_56264_PI429010886 | M113307 | 0.039275 | 24 | 21 | 91079024 | PREDICTED: similar to predicted protein [Tribolium | | | 8.58E-35 | 71.45222 | 84.19357 | 82.32602 | 70.169 | 79.18359 | 65.30029 | 42.69298 | 65.14347 | 72.91578 | 73.97012 | 69.20227 | 90.8645 | 55.82371 | 73.63983 | 69.37437 | 97.74764 | 58.05303 | 76.30835 | 77.69279 | 83.20205 | 68.52038 | 89.33478 | 110.6986 | 71.54826 | 88.4815 | 67.71039 | 84.09478 |
| CUST_35427_PI429010886 | M12543 | 0.039275 | 24 | 21 | | | | | | 229.4944 | 267.8998 | 322.9274 | 261.2742 | 207.7149 | 289.7899 | 172.5457 | 178.6581 | 224.0471 | 244.775 | 153.7558 | 319.0748 | 198.7745 | 265.0157 | 280.2255 | 358.6229 | 218.8408 | 252.3543 | 271.8242 | 224.0801 | 250.1141 | 295.8079 | 304.9967 | 270.3602 | 287.6725 | 336.682 | 279.5177 |
| CUST_47734_PI429010886 | M77369 | 0.039275 | 24 | 21 | | | | | | 331.6154 | 274.7936 | 342.3707 | 278.0457 | 293.6394 | 310.6611 | 291.1029 | 252.8082 | 257.8206 | 301.467 | 351.1194 | 367.4512 | 246.2258 | 303.6686 | 210.2047 | 309.8951 | 285.4947 | 292.7227 | 284.6976 | 369.6879 | 344.0991 | 357.7037 | 366.3446 | 363.3814 | 354.5586 | 270.6283 | 428.1275 |
| CUST_32569_PI429010886 | M4455 | 0.039282 | 18 | 9 | | | | | | 922.2736 | 1149.925 | 2012.591 | 154.8286 | 125.6673 | 31.63804 | 787.7599 | 3549.404 | 4571.032 | 2370.125 | 1236.256 | 2953.556 | 2112.767 | 1937.537 | 2041.736 | 581.8912 | 1472.72 | 236.0009 | 864.2169 | 4738.823 | 983.5467 | 1087.079 | 2774.636 | 126.8312 | 2500.44 | 1075.314 | 2288.66 |
| CUST_48686_PI429010886 | M81626 | 0.039282 | 15 | 12 | | | | | | 368.3133 | 1059.331 | 549.0001 | 274.9598 | 348.6809 | 168.8733 | 864.8993 | 583.066 | 1269.191 | 1027.997 | 526.8072 | 953.2915 | 1481.812 | 1487.266 | 1102.647 | 2022.724 | 612.1213 | 873.8827 | 463.9991 | 1914.117 | 517.4057 | 2012.591 | 1739.21 | 1470.792 | 5744.456 | 1119.369 | 5824.829 |
| CUST_703_PI429010886 | N6835 | 0.039282 | 18 | 9 | | | | | | 960.3576 | 1277.616 | 2066.68 | 160.8449 | 118.1621 | 37.74785 | 770.9286 | 3855.839 | 4711.518 | 2520.419 | 1294.709 | 2890.576 | 2276.234 | 2374.602 | 2189.348 | 640.0171 | 1552.183 | 189.3158 | 924.5282 | 4186.218 | 1021.127 | 1058.251 | 2945.87 | 123.2134 | 2645.933 | 1317.659 | 2112.767 |
| CUST_21718_PI429010886 | N45730 | 0.039378 | 21 | 15 | | | | | | 9.773611 | 7.527497 | 8.542736 | 10.91573 | 9.118641 | 13.72673 | 10.83953 | 9.14409 | 9.166269 | 6.049495 | 12.39561 | 17.31011 | 13.05601 | 13.96779 | 10.07219 | 14.81603 | 12.0315 | 23.84861 | 15.51056 | 13.99028 | 19.00448 | 10.22712 | 8.591341 | 11.40101 | 11.82115 | 12.23618 | 12.71188 |
| CUST_53636_PI429010886 | M102588 | 0.039387 | 18 | 3 | | | | | | 5.462213 | 18.56257 | 12.91769 | 11.00169 | 6.86682 | 15.21068 | 7.509358 | 13.41075 | 10.95668 | 9.48088 | 11.1247 | 10.99269 | 4.405905 | 3.707188 | NA | 6.461537 | NA | 4.080756 | 8.510732 | 7.70702 | 17.74139 | 13.92466 | 30.3611 | 10.64869 | 12.8699 | 8.840456 | 5.563346 |
| CUST_31646_PI429010886 | M2047 | 0.039395 | 24 | 9 | 189240635 | PREDICTED: similar to AGAP009835-PA [Tribolium c | Transport | Transmembrane transport | 9.8E-76 | 33.93687 | 39.40978 | 28.23857 | 50.8634 | 49.37977 | 25.14127 | 42.11302 | 106.3333 | 56.99751 | 35.12261 | 117.4327 | 105.8112 | 94.83175 | 28.13108 | 49.14494 | 39.0395 | 17.68083 | 17.67291 | 23.27942 | 163.791 | 37.30131 | 33.95071 | 21.53219 | 29.8311 | 15.24047 | 51.28805 | 42.74724 |
| CUST_29941_PI429010886 | N60855 | 0.039409 | 24 | 9 | | | | | | 19.62227 | 21.30938 | 20.1457 | 17.99464 | 11.75215 | 22.91218 | 23.51373 | 15.97793 | 23.40583 | 28.44656 | 24.62356 | 21.75179 | 49.89799 | 18.11507 | 23.41598 | 14.07874 | 14.67473 | 18.96864 | 5.51219 | 33.37295 | 6.965876 | 12.34018 | 29.78998 | 7.074973 | 15.02993 | 8.663836 | 14.46019 |
| CUST_48474_PI429010886 | M80505 | 0.039425 | 24 | 21 | | | | | | 134.0378 | 181.6996 | 225.8046 | 127.0345 | 171.427 | 186.3412 | 142.0255 | 162.9163 | 134.4036 | 138.6787 | 129.2905 | 98.46582 | 160.0314 | 207.6805 | 118.2155 | 173.9653 | 140.5991 | 181.7533 | 157.0892 | 163.0075 | 196.5633 | 197.7844 | 186.5931 | 171.8712 | 218.3262 | 116.7026 | 238.5428 |
| CUST_31621_PI429010886 | M1985 | 0.039438 | 12 | 9 | | | | | | 140.2611 | 170.2702 | 115.0699 | 79.0521 | 70.53195 | 83.25716 | 79.94364 | 83.56086 | 97.07573 | 117.2428 | 97.20572 | 130.7948 | 123.669 | 92.31619 | 82.26332 | 70.44162 | 81.87168 | 83.64909 | 53.26328 | 239.9876 | 73.30133 | 118.7738 | 135.1423 | 97.78298 | 107.2622 | 98.6386 | 69.54952 |
| CUST_14031_PI429010886 | N32208 | 0.039438 | 12 | 9 | 242003995 | Sorting nexin-9, putative [Pediculus humanus corpo | Signal transduction | Cell communication | 5.19E-29 | 38.49435 | 34.41169 | 32.62278 | 27.32583 | 31.39582 | 28.59435 | 35.84962 | 25.56096 | 37.43755 | 49.18419 | 38.29585 | 33.19476 | 30.33523 | 31.78038 | 34.35393 | 37.04426 | 30.41187 | 28.55509 | 22.57964 | 36.93108 | 36.77308 | 41.9571 | 43.48424 | 31.40253 | 40.14648 | 39.99252 | 29.81183 |
| CUST_20502_PI429010886 | N43568 | 0.039438 | 12 | 9 | | | | | | 5.642111 | 22.54108 | 11.45279 | 18.11915 | 13.98352 | 9.800176 | 17.92979 | 15.18207 | 13.82413 | 28.26641 | 19.20978 | 19.7328 | 19.90878 | 13.77454 | 19.46511 | 16.36491 | 9.385032 | 9.472519 | 20.82676 | 17.55503 | 15.04503 | 15.036 | 23.37181 | 18.71355 | 13.95252 | 18.18793 | 19.95383 |
| CUST_16077_PI429010886 | N35615 | 0.039439 | 15 | 12 | | | | | | 27.68104 | 24.50817 | 36.75997 | 14.31366 | 14.05599 | 14.49726 | 22.27756 | 26.38163 | 22.53631 | 36.10883 | 10.89307 | 22.42169 | 20.53586 | 40.08825 | 33.29463 | 25.42066 | 26.04145 | 35.45648 | 32.54445 | 22.0997 | 17.76083 | 21.71104 | 23.00326 | 22.91619 | 30.48251 | 25.87632 | 26.49155 |
| CUST_26147_PI429010886 | N53750 | 0.039451 | 21 | 0 | | | | | | 585.2479 | 708.9187 | 417.4973 | 530.042 | 536.6546 | 594.7631 | 465.5606 | 378.7944 | 429.6585 | 492.3863 | 368.6847 | 395.2528 | 477.6391 | 373.6828 | 562.1725 | 936.7815 | 414.6092 | 527.4806 | 528.5021 | 600.4305 | 398.2452 | 708.2954 | 429.9553 | 568.2036 | 520.9934 | 465.9125 | 479.0678 |
| CUST_17403_PI429010886 | N37908 | 0.039471 | 24 | 21 | | | | | | 8.714119 | 10.24518 | 11.55416 | 6.233261 | 8.180112 | 21.45154 | 7.565304 | 6.030448 | 6.960178 | 28.88138 | 4.079716 | 4.610203 | 7.573982 | 7.666723 | NA | 9.428784 | 15.52639 | 4.42078 | 9.102009 | 10.00209 | 7.851792 | 13.11481 | NA | NA | 9.779594 | 9.22307 | 9.508624 |
| CUST_35729_PI429010886 | M13329 | 0.039489 | 21 | 12 | | | | | | 326.7925 | 323.4981 | 261.6861 | 352.188 | 222.2031 | 332.0008 | 248.1135 | 374.4434 | 376.9333 | 343.3863 | 248.7474 | 385.6213 | 371.6995 | 346.7104 | 366.8876 | 312.5243 | 330.9233 | 280.3633 | 307.2464 | 300.9186 | 216.807 | 271.0795 | 297.8606 | 306.5332 | 302.092 | 313.6383 | 379.9539 |
| CUST_4857_PI429010886 | N17625 | 0.039495 | 24 | 3 | | | | | | 30.81693 | 24.27705 | 35.45648 | 33.81876 | 57.72819 | 36.39578 | 50.85184 | 32.45148 | 31.79024 | 31.5882 | 43.85634 | 31.60336 | 22.89626 | 34.62398 | 32.65296 | 26.75848 | 29.27092 | 30.35236 | 29.35151 | 29.96972 | 40.70276 | 29.55843 | 23.78208 | 37.19805 | 30.72063 | 33.00505 | 33.61732 |
| CUST_55403_PI429010886 | M109956 | 0.039497 | 15 | 12 | | | | | | 286.7058 | 265.532 | 335.74 | 268.3232 | 235.0953 | 272.4885 | 254.8048 | 253.3806 | 276.4683 | 314.3652 | 277.8925 | 335.8009 | 286.9542 | 364.8104 | 375.6905 | 358.7967 | 297.9086 | 295.8079 | 280.3132 | 308.3369 | 309.2864 | 390.4272 | 293.9351 | 269.6735 | 340.9466 | 313.2862 | 378.6086 |
| CUST_32644_PI429010886 | M4676 | 0.039534 | 12 | 0 | 241846314 | SEL-1, putative [Ixodes scapularis] >gi\|215509773\| | Metabolic process | Other | 1.1E-122 | 53.70396 | 76.79471 | 58.22303 | 93.40468 | 63.82351 | 38.36474 | 33.37295 | 35.59331 | 47.62717 | 82.93626 | 54.58961 | 77.09407 | 35.92246 | 59.75541 | 91.13848 | 73.62986 | 39.96079 | 58.27233 | 51.90738 | 27.66624 | 38.18452 | 58.26543 | 55.21772 | 48.9358 | 70.99058 | 76.20212 | 68.44956 |
| CUST_13042_PI429010886 | N30481 | 0.039534 | 12 | 0 | | | | | | 54.95718 | 72.85867 | 41.72747 | 46.37163 | 55.14628 | 47.327 | 34.47136 | 44.27177 | 40.78513 | 66.01381 | 42.21564 | 48.10026 | 50.99115 | 57.20678 | 53.49548 | 61.96298 | 51.97626 | 50.05524 | 40.17935 | 52.60357 | 36.44258 | 58.7202 | 47.73188 | 40.64326 | 69.48443 | 46.66745 | 52.50431 |
| CUST_21977_PI429010886 | N46194 | 0.039534 | 12 | 0 | | | | | | 37.89768 | 23.69772 | 31.90127 | 23.82568 | 25.14127 | 27.88984 | 18.74861 | 29.11133 | 23.31281 | 33.84879 | 20.52725 | 21.89117 | 34.68251 | 28.66257 | 39.12009 | 30.23833 | 26.38969 | 28.07255 | 19.52138 | 33.06129 | 26.79307 | 40.31691 | 22.15408 | 26.40308 | 40.90818 | 29.15459 | 46.23898 |
| CUST_22516_PI429010886 | N47174 | 0.039534 | 12 | 0 | | | | | | 181.6996 | 213.0626 | 238.6203 | 156.6119 | 150.3171 | 185.2639 | 133.7926 | 131.513 | 286.6682 | 163.4728 | 137.478 | 200.1336 | 156.6593 | 195.1137 | 229.6885 | 164.3802 | 151.1369 | 200.21 | 179.1322 | 146.0101 | 157.3603 | 188.3767 | 168.032 | 158.7119 | 169.6552 | 327.3251 | 191.0756 |
| CUST_27307_PI429010886 | N55820 | 0.039534 | 12 | 0 | | | | | | 183.6909 | 302.4916 | 278.9995 | 229.6175 | 193.9691 | 242.0042 | 185.7109 | 189.4794 | 224.5779 | 244.0376 | 176.4634 | 285.2578 | 204.7693 | 288.2204 | 342.6916 | 246.3068 | 198.9664 | 268.1153 | 200.9243 | 228.3136 | 212.8309 | 307.9072 | 234.1955 | 185.414 | 252.8872 | 292.1061 | 323.3503 |
| CUST_28613_PI429010886 | N58293 | 0.039534 | 12 | 0 | | | | | | 96.56866 | 166.8022 | 139.8446 | 119.585 | 102.2413 | 100.1493 | 77.35795 | 69.90395 | 82.1182 | 155.3616 | 65.70437 | 236.8954 | 140.2062 | 181.1767 | 138.4518 | 110.6986 | 99.93216 | 146.6394 | 97.87109 | 123.1997 | 152.5866 | 105.0723 | 132.0957 | 136.0004 | 153.0835 | 139.6803 | 183.3031 |
| CUST_841_PI429010886 | N7499 | 0.039534 | 12 | 6 | 241096187 | Mid1-interacting protein, putative [Ixodes scapulari | | | 6.64E-13 | 61.25018 | 100.4062 | 80.5678 | 84.27965 | 142.8727 | 51.02755 | 150.6728 | 112.0774 | 65.20347 | 109.5748 | 100.0727 | 85.62903 | 83.16029 | 68.50419 | 45.03054 | 123.4117 | 161.1553 | 58.85113 | 126.3312 | 87.91683 | 111.2474 | 105.5819 | 115.6165 | 88.80055 | 79.19112 | 61.98456 | 66.3431 |
| CUST_17555_PI429010886 | N38170 | 0.03955 | 21 | 18 | | | | | | 24.42797 | 30.86285 | 33.50292 | 17.96665 | 21.52056 | 30.81693 | 13.81863 | 20.84115 | 24.53437 | 28.28611 | 22.85266 | 89.20945 | 21.21967 | 31.68444 | 32.36995 | 39.02375 | 25.84583 | 27.42544 | 29.8328 | 31.73044 | 33.50669 | 21.61669 | 31.2907 | 26.21095 | 29.43926 | 34.90297 | 22.06407 |
| CUST_20538_PI429010886 | N43628 | 0.039571 | 18 | 3 | 546680770 | hypothetical protein D910_08318 [Dendroctonus p | | | 1.22E-20 | 176.1434 | 131.5284 | 111.1116 | 155.5733 | 215.3368 | 194.8405 | 199.7913 | 161.2431 | 87.13975 | 97.05352 | 235.6934 | 81.41269 | 114.3485 | 66.40981 | 81.03057 | 122.2551 | 336.5091 | 153.3961 | 290.175 | 92.73234 | 126.5833 | 165.4769 | 189.4251 | 202.9175 | 126.3312 | 122.7034 | 56.92511 |
| CUST_18307_PI429010886 | N39545 | 0.039575 | 24 | 0 | | | | | | 12.98486 | 7.431826 | 57.02142 | 6.610857 | 6.510984 | 16.8197 | NA | 4.698051 | 5.040368 | 6.39392 | 8.747631 | 8.057073 | 6.00986 | 6.301983 | 6.658652 | 5.56157 | 11.86309 | 7.727602 | 6.736876 | 18.18378 | 5.257423 | 11.61617 | 10.50364 | NA | 15.97964 | 10.69036 | 14.74259 |
| CUST_52179_PI429010886 | M96658 | 0.039576 | 18 | 12 | | | | | | 23.94321 | 29.82235 | 36.58254 | 32.45148 | 39.28519 | 45.95892 | 36.55124 | 41.65923 | 53.9331 | 25.21391 | 35.78629 | 83.36467 | 43.39642 | 53.07973 | 89.25924 | 132.2265 | 29.19475 | 56.57948 | 40.52064 | 12.14051 | 32.74092 | 34.60947 | 29.62583 | 28.14284 | 56.95742 | 60.17363 | 51.50234 |
| CUST_41144_PI429010886 | M56981 | 0.039581 | 18 | 3 | | | | | | 38.16824 | 17.39906 | 21.58663 | 34.82944 | 30.81693 | 41.30417 | 19.95383 | 26.28003 | 21.14577 | 26.26779 | 23.26869 | 22.43908 | 24.42075 | 28.23857 | 11.50698 | 23.61475 | 24.54416 | 26.58995 | 24.21006 | 30.41187 | 28.45711 | 32.13794 | 24.17061 | 37.53618 | 24.39107 | 27.28895 | 20.47188 |
| CUST_45945_PI429010886 | M70837 | 0.039582 | 15 | 12 | 390367765 | PREDICTED: uncharacterized protein LOC10089241 | | | 9.53E-13 | 6.406079 | 8.319795 | NA | 7.616402 | 12.04261 | 14.83131 | 4.852014 | 6.115825 | 4.767403 | 15.38675 | 8.401293 | 8.680488 | NA | 13.43914 | NA | 11.36478 | 10.93186 | NA | 8.717232 | 9.016795 | 6.999938 | 8.240312 | NA | NA | 12.54687 | 13.00375 | 10.72561 |
| CUST_16806_PI429010886 | N36900 | 0.039583 | 15 | 9 | | | | | | 208.5959 | 184.5383 | 253.6933 | 204.3548 | 200.041 | 234.1955 | 211.951 | 227.9039 | 269.8218 | 302.1561 | 201.008 | 327.9867 | 244.2379 | 391.346 | 276.8504 | 213.6947 | 194.2325 | 258.3105 | 236.6472 | 205.392 | 267.7292 | 243.2097 | 255.6195 | 205.1195 | 260.5389 | 293.704 | 332.54 |
| CUST_42052_PI429010886 | M59269 | 0.039585 | 24 | 0 | | | | | | 30.04719 | 28.78291 | 16.8666 | 21.61291 | 19.55939 | 21.43971 | 15.94381 | 24.23014 | 23.83451 | 22.38584 | 18.91531 | 23.01108 | 17.15146 | 14.12148 | 10.7174 | 25.10042 | 15.88403 | 14.34646 | 20.06405 | 20.26123 | 25.33406 | 24.77801 | 28.77061 | 14.62779 | 28.46598 | 26.47532 | 15.48139 |
| CUST_4847_PI429010886 | N17605 | 0.039592 | 24 | 9 | | | | | | 48.35331 | 57.41889 | 48.78129 | 41.66498 | 71.57902 | 51.98858 | 50.90018 | 68.61799 | 51.86583 | 76.71565 | 70.70475 | 63.27865 | 32.56779 | 54.01898 | 45.29251 | 61.30991 | 82.21549 | 54.71071 | 75.98951 | 58.57388 | 63.41464 | 44.76886 | 46.95122 | 35.38107 | 50.01173 | 50.67859 | 61.03108 |
| CUST_41426_PI429010886 | M57702 | 0.039612 | 18 | 15 | | | | | | 48.21613 | 152.8184 | 167.9037 | 3.795987 | 91.49463 | 87.62612 | 6.337987 | 120.5518 | 67.31728 | 224.3204 | 71.79355 | 104.0821 | 86.67105 | 155.1933 | 131.863 | 207.9222 | 115.4184 | 175.2604 | 140.7073 | 80.60147 | 41.55626 | 85.9812 | 31.53429 | 82.25514 | 142.7507 | 84.31618 | 182.5193 |
| CUST_2266_PI429010886 | N11990 | 0.039618 | 21 | 0 | 91087225 | PREDICTED: similar to CG1973 CG1973-PA [Triboliu | Protein metabolism | Cellular protein modification process | 8.32E-90 | 30.44094 | 22.5314 | 39.61101 | 18.48661 | 19.83923 | 18.13841 | 21.80535 | 20.12835 | 23.59619 | 18.05541 | 14.13977 | 14.30732 | 18.24734 | 19.73096 | 19.04546 | 31.6082 | 11.93121 | 16.91861 | 32.39452 | 14.36537 | 24.11521 | 19.44155 | 22.54321 | 28.20666 | 25.6517 | 11.41745 | 23.73387 |
| CUST_35977_PI429010886 | M14172 | 0.039661 | 24 | 18 | | | | | | 363.3814 | 346.0687 | 425.5183 | 350.314 | 331.6846 | 339.8879 | 300.9186 | 318.1453 | 336.8758 | 410.0103 | 331.8685 | 374.2323 | 287.6352 | 315.9925 | 354.9858 | 339.7564 | 366.7345 | 387.3252 | 414.8654 | 386.3769 | 379.2779 | 390.5304 | 396.9865 | 316.971 | 381.0616 | 332.4166 | 561.4598 |
| CUST_55723_PI429010886 | M111475 | 0.039691 | 15 | 9 | | | | | | 99.6429 | 89.30785 | 113.4221 | 79.59942 | 98.59007 | 75.75933 | 62.31885 | 102.4535 | 86.08273 | 127.6161 | 99.40267 | 136.6153 | 97.55406 | 114.649 | 107.6007 | 99.88938 | 103.9666 | 108.0902 | 128.5525 | 96.44756 | 101.7048 | 126.8312 | 99.06334 | 85.42654 | 125.5467 | 114.1326 | 149.8948 |
| CUST_31939_PI429010886 | M2778 | 0.039691 | 15 | 9 | 241560357 | glyoxylate/hydroxypyruvate reductase, putative [Ix | Metabolic process | Cellular amino acid metabolic process | 3.4E-147 | 21.5895 | 21.01435 | 46.30865 | 31.33456 | 23.38175 | 26.8539 | 21.22623 | 41.09065 | 34.29722 | 43.13359 | 33.98937 | 50.51683 | 33.76678 | 49.79836 | 39.86189 | 36.10883 | 43.38758 | 41.93393 | 26.92163 | 27.59937 | 38.80762 | 42.53957 | 32.56046 | 23.40412 | 49.91173 | 34.29722 | 48.30896 |
| CUST_6148_PI429010886 | N19691 | 0.039691 | 15 | 9 | | | | | | 16.08632 | 41.35575 | 30.74938 | 25.83289 | 24.98792 | 14.50223 | 16.41995 | 31.86571 | 19.57848 | 44.06771 | 98.45237 | 34.73965 | 23.52815 | 44.71419 | 26.01379 | 30.35559 | 8.230173 | 31.32726 | 19.52925 | 14.77313 | 19.14299 | 31.58406 | 25.58851 | 24.26504 | 53.62183 | 20.49506 | 21.17293 |
| CUST_11582_PI429010886 | N28069 | 0.039691 | 15 | 3 | 225713136 | Cuticle protein 7 [Lepeophtheirus salmonis] | | | 1.45E-22 | 812.8751 | 82.28242 | 1232.59 | 1289.132 | 1291.558 | 2278.385 | 1977.124 | 324.8235 | 189.8316 | 31.18147 | 1395.696 | 12.49156 | 219.8889 | 118.905 | 118.495 | 39.82044 | 3421.62 | 1895.191 | 2725.555 | 16.77607 | 2346.537 | 46.11982 | 2041.736 | 2159.598 | 21.9039 | 621.8584 | 12.31182 |
| CUST_30888_PI429010886 | M118 | 0.039692 | 24 | 9 | | | Protein metabolism | Ribosome biogenesis | | 583.5785 | 570.2153 | 492.3863 | 592.7227 | 623.9709 | 689.3131 | 549.0001 | 656.1679 | 664.3088 | 522.9557 | 725.8016 | 995.2843 | 759.2915 | 512.6993 | 707.905 | 654.4842 | 611.2062 | 622.024 | 712.3784 | 905.268 | 656.5643 | 642.3044 | 447.0926 | 635.9764 | 460.8905 | 610.2139 | 541.31 |
| CUST_24199_PI429010886 | N50214 | 0.039725 | 15 | 12 | | | | | | 1203.954 | 1191.699 | 1216.97 | 998.694 | 1273.05 | 1169.504 | 1078.296 | 1143.569 | 1247.588 | 1303.875 | 1180.515 | 1567.155 | 1607.248 | 1791.281 | 1462.44 | 1508.69 | 1650.111 | 1387.967 | 1301.471 | 1502.524 | 955.4895 | 1525.514 | 1521.376 | 1158.693 | 2137.444 | 1228.741 | 2227.948 |
| CUST_48083_PI429010886 | M78810 | 0.039731 | 24 | 21 | | | | | | 198.308 | 222.5078 | 200.4624 | 187.387 | 214.4171 | 179.0874 | 174.4788 | 206.1326 | 174.0729 | 259.2819 | 227.1414 | 224.3204 | 181.075 | 252.0389 | 113.525 | 276.4162 | 219.0379 | 166.7568 | 267.619 | 247.587 | 216.5095 | 273.4925 | 292.7227 | 219.1881 | 332.4166 | 251.7054 | 247.7794 |
| CUST_10830_PI429010886 | N26856 | 0.039731 | 24 | 21 | | | | | | 563.0019 | 634.7435 | 686.896 | 587.4356 | 631.1117 | 662.7679 | 527.3498 | 568.0071 | 583.3128 | 669.4372 | 597.1763 | 596.8255 | 602.4628 | 688.9088 | 463.903 | 705.3837 | 670.3311 | 568.2036 | 545.2224 | 658.8544 | 670.866 | 707.3962 | 711.9048 | 614.0398 | 782.7144 | 663.9332 | 734.9802 |
| CUST_2665_PI429010886 | N12863 | 0.039733 | 18 | 15 | | | | | | 640.0171 | 573.5264 | 604.157 | 571.094 | 570.9837 | 601.5918 | 554.2309 | 456.5493 | 382.1801 | 579.5177 | 549.0001 | 570.6164 | 548.6331 | 552.3175 | 524.5768 | 644.8914 | 734.477 | 538.4051 | 639.3223 | 532.5435 | 544.2536 | 624.2921 | 537.9358 | 537.4529 | 603.0908 | 519.8548 | 479.585 |
| CUST_50467_PI429010886 | M89931 | 0.039833 | 24 | 3 | | | | | | 76.30835 | 63.36658 | 85.91746 | 74.06718 | 117.0097 | 192.4131 | 114.1326 | 61.75222 | 63.6369 | 54.3948 | 99.91859 | 71.83981 | 45.19753 | 34.18144 | 47.44495 | 46.16276 | 108.7837 | 68.5761 | 61.51764 | 54.72113 | 121.9257 | 74.50049 | 46.71536 | 71.94962 | 33.28766 | 117.6032 | 55.3496 |
| CUST_23175_PI429010886 | N48387 | 0.039833 | 18 | 12 | | | | | | 22.79581 | 15.89427 | 35.4728 | 23.71704 | 19.4114 | 24.21006 | 22.28394 | 21.02215 | 27.91558 | 17.58731 | 31.24487 | 36.55124 | 28.01496 | 33.77253 | 38.33525 | 34.48473 | 24.59542 | 30.77101 | 23.53859 | 34.51101 | 12.88422 | 22.2285 | 25.77541 | 12.44781 | 35.6894 | 32.61498 | 28.2496 |
| CUST_33044_PI429010886 | M5818 | 0.03984 | 15 | 9 | | | | | | 163.1486 | 236.1246 | 215.9006 | 224.1509 | 274.5343 | 206.3446 | 216.5095 | 397.8439 | 277.4454 | 286.7058 | 426.3129 | 421.3888 | 324.7416 | 242.5645 | 317.9758 | 225.3313 | 241.7686 | 241.9211 | 278.0457 | 461.3593 | 365.6765 | 292.0563 | 253.9783 | 240.9894 | 299.837 | 236.2907 | 321.753 |
| CUST_41208_PI429010886 | M57132 | 0.039847 | 24 | 15 | | | | | | 303.4935 | 326.7925 | 219.6868 | 201.1422 | 246.3705 | 315.2378 | 211.7804 | 237.3703 | 236.4243 | 409.4886 | 258.8229 | 330.8112 | 223.9141 | 255.0746 | 329.0332 | 495.8952 | 342.6916 | 352.327 | 311.6936 | 496.7325 | 393.6847 | 410.239 | 196.2921 | 275.1358 | 524.852 | 318.9508 | 308.2627 |
| CUST_32472_PI429010886 | M4201 | 0.039861 | 21 | 9 | | | | | | 242.1214 | 245.6501 | 333.7959 | 181.2698 | 163.329 | 204.4512 | 229.4944 | 257.9388 | 244.904 | 343.8588 | 258.2202 | 297.5859 | 242.9704 | 330.1788 | 285.2001 | 271.918 | 247.9064 | 254.8434 | 279.0812 | 270.8602 | 252.2638 | 291.1029 | 284.1504 | 194.9477 | 322.0841 | 236.8108 | 214.0659 |
| CUST_46080_PI429010886 | M71304 | 0.03987 | 21 | 9 | | | | | | 11.21427 | 295.4403 | 76.89998 | 601.0454 | 1580.29 | 222.3148 | 469.4022 | 4396.276 | 2388.967 | 122.3884 | 4528.171 | 4524.584 | 3739.652 | 286.3672 | 1213.318 | 11.55275 | 1102.275 | NA | 169.5063 | 6863.631 | 2788.353 | 8.850436 | 291.4361 | 237.166 | 6.334658 | 1000.153 | 2144.378 |
| CUST_14953_PI429010886 | N33750 | 0.039871 | 21 | 15 | | | | | | 132.4891 | 133.2798 | 120.0446 | 112.1069 | 113.7364 | 104.976 | 113.7623 | 77.34 | 119.4425 | 165.2021 | 139.8015 | 147.4736 | 110.5556 | 126.4216 | 126.727 | 197.7278 | 115.4907 | 186.0597 | 109.3157 | 139.5229 | 99.13646 | 145.8124 | 118.7885 | 128.6369 | 107.7787 | 78.64329 | 121.0793 |
| CUST_29075_PI429010886 | N59150 | 0.03993 | 21 | 0 | | | | | | 11.30106 | 11.80809 | 28.6303 | 14.77643 | NA | NA | NA | NA | 9.273731 | 11.90309 | 9.204642 | 9.908813 | 8.724159 | NA | 11.38637 | 5.315305 | 8.07058 | 9.42148 | 4.121406 | 12.72627 | 5.596499 | 12.24457 | 9.380587 | 12.59239 | 9.591065 | 19.28099 | 10.28851 |
| CUST_38205_PI429010886 | M24136 | 0.039936 | 21 | 9 | | | | | | 897.9401 | 618.5189 | 415.2193 | 460.255 | 312.1828 | 259.5794 | 816.1069 | 166.8948 | 484.5005 | 1329.318 | 532.8085 | 2555.115 | 690.9208 | 603.4589 | 503.2472 | 399.93 | 591.0201 | 448.2914 | 1601.013 | 264.8381 | 1174.994 | 274.9598 | 497.7449 | 280.971 | 124.672 | 424.3711 | 495.0933 |
| CUST_14557_PI429010886 | N33073 | 0.039961 | 24 | 0 | | | | | | 180.3041 | 159.4512 | 133.064 | 140.3134 | 151.0826 | 139.6803 | 125.1102 | 132.3624 | 119.5708 | 154.1347 | 114.0624 | 144.9176 | 118.7738 | 128.7123 | 110.3957 | 132.5328 | 116.5927 | 114.3743 | 136.9633 | 106.3847 | 120.0167 | 152.263 | 113.3621 | 126.3312 | 164.7204 | 129.419 | 118.8883 |
| CUST_52899_PI429010886 | M99363 | 0.039968 | 21 | 18 | | | | | | 10.86137 | NA | NA | 7.111485 | 4.161879 | 6.563098 | 4.79181 | 16.02003 | 5.497161 | NA | 17.87663 | 20.83504 | 10.31476 | NA | 8.783511 | 4.251678 | 9.895974 | NA | NA | 24.80615 | 14.23639 | 13.21776 | 8.12515 | 6.436681 | 5.029969 | 5.374335 | 4.916968 |
| CUST_36311_PI429010886 | M15537 | 0.039973 | 21 | 9 | 507143694 | family 7 cellobiohydrolase [Chelura terebrans] | Metabolic process | Carbohydrate metabolic process | 3.44E-52 | NA | 25.96531 | 14.77827 | 77.56897 | 65.40248 | 40.70657 | 74.40778 | 194.8827 | 147.5412 | 21.15229 | 202.4041 | 215.4319 | 169.8261 | 38.74344 | 83.36467 | NA | 88.96346 | NA | 15.21532 | 373.2026 | 103.2892 | 5.820473 | 58.28332 | 49.91574 | NA | 164.8298 | 129.8119 |
| CUST_22733_PI429010886 | N47564 | 0.040002 | 15 | 9 | | | | | | 16.90108 | 12.71188 | 14.68459 | 5.071599 | 8.552148 | NA | 9.031084 | 18.35021 | 25.51149 | 20.69763 | 15.76954 | 30.26846 | 30.4033 | 18.39819 | 19.93792 | 20.05683 | 17.87837 | 14.57688 | 13.85759 | 24.5371 | 8.787338 | 18.36323 | 23.99191 | 11.0048 | 35.98798 | 10.08113 | 20.87902 |
| CUST_22750_PI429010886 | N47609 | 0.040016 | 21 | 0 | | | | | | 141.7485 | 47.37598 | 153.8239 | 50.73538 | 92.24128 | 51.5698 | 140.4954 | 55.79937 | 37.07575 | 39.45879 | 78.13459 | 41.52389 | 43.44981 | 41.13272 | 39.30201 | 48.65694 | 156.2332 | 62.89604 | 92.65529 | 41.8722 | 122.2373 | 60.84139 | 136.6832 | 127.0833 | 58.64723 | 42.9046 | 36.16119 |
| CUST_55189_PI429010886 | M109047 | 0.040062 | 12 | 3 | 290560934 | cuticular protein RR-1 motif 43 precursor [Bombyx | | | 5.42E-07 | 657.6448 | 285.8605 | 56.41396 | 1952.727 | 1495.765 | 3175.174 | 2004.816 | 694.099 | 677.7517 | 7.179499 | 1565.179 | 16.97291 | 601.1869 | 86.39769 | 278.2072 | 140.8807 | 2888.695 | 2057.769 | 818.9073 | 60.05679 | 2143.344 | 507.5628 | 1591.245 | 2923.398 | 65.77588 | 1344.532 | 28.67208 |
| CUST_5497_PI429010886 | N18780 | 0.04007 | 24 | 15 | 157106752 | nuclear pore complex protein nup214 [Aedes aegyp | | | 1.95E-22 | 46.36775 | 46.14411 | 65.84223 | 39.20847 | 58.29649 | 46.57205 | 42.7734 | 45.52612 | 58.66467 | 73.70231 | 54.92234 | 123.0876 | 62.03099 | 74.89819 | 76.69076 | 87.16389 | 62.67964 | 74.18777 | 51.4111 | 84.19357 | 63.17657 | 78.39028 | 62.53094 | 52.45258 | 77.60864 | 67.50801 | 81.31771 |
| CUST_38141_PI429010886 | M23617 | 0.040074 | 24 | 0 | 197940771 | Euphausia superba mitochondrion, partial genome | | | 4.4E-107 | 4455.764 | 6738.231 | 3805.814 | 3281.126 | 5211.698 | 3219.948 | 3067.321 | 3988.418 | 3453.79 | 4190.35 | 4506.751 | 4197.681 | 3756.632 | 3561.268 | 2838.594 | 3051.185 | 3783.178 | 3670.658 | 4058.299 | 6426.682 | 4257.291 | 4759.605 | 4197.681 | 3429.061 | 5454.8 | 4257.291 | 5039.929 |
| CUST_32443_PI429010886 | M4132 | 0.040079 | 24 | 0 | 443685184 | hypothetical protein CAPTEDRAFT_179128 [Capitell | Transport | Other | 1.69E-51 | 7.338708 | NA | 9.057109 | 5.75088 | 4.353182 | NA | 5.975218 | 5.309356 | 3.423505 | 10.70604 | 4.655316 | 4.550924 | NA | NA | 3.776514 | 5.777163 | 5.920001 | 9.312357 | 4.707461 | 6.865815 | 5.997689 | 12.2683 | NA | NA | 9.387006 | 16.59129 | 6.166942 |
| CUST_41968_PI429010886 | M59025 | 0.040081 | 24 | 21 | | | | | | 185.9766 | 199.2344 | 212.2276 | 249.6087 | 235.7212 | 302.7759 | 172.436 | 155.4485 | 173.7706 | 192.922 | 195.0054 | 218.4835 | 126.6474 | 223.1512 | 214.5713 | 234.141 | 243.9078 | 297.14 | 225.5174 | 242.3388 | 214.59 | 281.5398 | 211.6272 | 239.521 | 241.3477 | 184.7518 | 221.1299 |
| CUST_49474_PI429010886 | M85795 | 0.040101 | 21 | 9 | | | | | | 45.81525 | 37.54075 | 25.86961 | 42.39566 | 55.3496 | 30.95206 | 38.86122 | 78.17526 | 36.27265 | 44.87166 | 108.9403 | 83.89746 | 75.82559 | 32.89038 | 45.60489 | 49.38887 | 50.67859 | 37.54361 | 41.43272 | 124.2579 | 68.58333 | 34.25171 | 28.51585 | 36.5984 | 41.78277 | 41.31249 | 40.10273 |
| CUST_41744_PI429010886 | M58440 | 0.040151 | 24 | 6 | 516674472 | hypothetical protein [euryarchaeote SCGC AAA261- | Metabolic process | Carbohydrate metabolic process | 4.24E-23 | 80.47827 | 90.25676 | 77.27026 | 108.1195 | 91.54224 | 124.1861 | 106.4489 | 110.5233 | 100.1156 | 115.6165 | 90.49352 | 104.5131 | 82.58698 | 94.5785 | 71.02655 | 102.9324 | 75.64933 | 87.77632 | 59.12871 | 132.1335 | 76.40353 | 39.46416 | 119.3258 | 60.00077 | 79.38753 | 91.32302 | 77.91084 |

| Probe ID | ID | p-value | Period | Phase | EBI ID | Annotation | GO category | Go term | E-value | 0 | 0 | 0 | 3 | 3 | 3 | 6 | 6 | 6 | 9 | 9 | 9 | 12 | 12 | 12 | 15 | 15 | 15 | 18 | 18 | 18 | 21 | 21 | 21 | 24 | 24 | 24 |
|---|---|---|---|---|---|---|---|---|---|---|---|---|---|---|---|---|---|---|---|---|---|---|---|---|---|---|---|---|---|---|---|---|---|---|---|---|
| CUST_11153_PI429010886 | N27355 | 0.040151 | 21 | 0 | | | | | | 638.47 | 659.6239 | 496.5233 | 456.0552 | 432.6248 | 348.2614 | 326.6284 | 342.1732 | 334.9967 | 568.0071 | 470.4395 | 540.3776 | 482.287 | 525.3464 | 420.8347 | 678.9878 | 412.2515 | 407.3532 | 433.2734 | 583.2176 | 531.5557 | 596.4585 | 528.248 | 433.0579 | 554.6572 | 556.906 | 500.086 |
| CUST_15470_PI429010886 | N34629 | 0.040151 | 21 | 0 | | | | | | 548.7959 | 901.5294 | 722.796 | 622.3874 | 596.3171 | 618.1584 | 498.3855 | 568.9937 | 601.5918 | 637.2498 | 546.956 | 525.2083 | 643.2335 | 601.0454 | 557.82 | 559.0577 | 550.9319 | 666.6962 | 597.939 | 708.7623 | 627.8304 | 780.6851 | 618.6744 | 636.6917 | 771.6454 | 365.1838 | 762.0199 |
| CUST_19767_PI429010886 | N42154 | 0.040151 | 24 | 18 | | | | | | 18.59683 | 32.38846 | 16.02537 | 11.13485 | 20.66296 | 13.57374 | 11.30106 | 15.51771 | 20.45683 | 32.99167 | 26.24834 | 29.03947 | 23.85638 | 26.72979 | 31.97801 | 32.26619 | 14.75825 | 39.48403 | 27.98319 | 22.31012 | 36.89165 | 32.3288 | 23.40827 | 23.77247 | 30.19101 | 16.82979 | 44.2637 |
| CUST_28507_PI429010886 | N58083 | 0.040151 | 21 | 0 | | | | | | 87.57209 | 76.88221 | 71.18669 | 83.16839 | 77.55826 | 56.37885 | 59.30066 | 61.91569 | 61.54664 | 53.55772 | 70.72361 | 65.38596 | 55.6932 | 103.0385 | 68.90417 | 97.80592 | 57.32582 | 73.93015 | 57.37114 | 86.9841 | 75.45183 | 79.21992 | 83.41229 | 83.07834 | 63.46235 | 86.14614 | 62.82655 |
| CUST_41416_PI429010886 | M57682 | 0.040169 | 15 | 0 | | | | | | 187.4537 | 164.0893 | 161.1265 | 115.9016 | 151.5201 | 143.9734 | 148.886 | 143.9244 | 124.6867 | 178.9487 | 157.9764 | 145.6295 | 108.3189 | 160.2216 | 121.5472 | 203.453 | 199.1974 | 172.213 | 184.2011 | 142.6158 | 152.8002 | 146.2229 | 146.902 | 133.5211 | 231.1582 | 141.0258 | 154.1139 |
| CUST_45880_PI429010886 | M70604 | 0.040169 | 15 | 9 | | | | | | 659.1977 | 805.0049 | 810.6402 | 656.7764 | 572.181 | 624.2921 | 550.0892 | 691.5947 | 731.0066 | 886.3986 | 688.5897 | 1003.547 | 704.1186 | 837.1072 | 822.5821 | 953.5865 | 661.9537 | 724.3304 | 722.474 | 776.8406 | 659.8695 | 929.2639 | 715.8969 | 600.7483 | 975.9966 | 701.8561 | 939.4252 |
| CUST_34177_PI429010886 | M8958 | 0.040169 | 15 | 0 | | | | | | 182.8782 | 201.1997 | 187.4537 | 169.9162 | 193.8001 | 147.2543 | 166.3173 | 159.2586 | 133.3139 | 189.0589 | 174.9214 | 176.885 | 137.8443 | 161.1033 | 146.6394 | 194.2624 | 212.4411 | 178.2181 | 183.5489 | 197.122 | 163.3967 | 181.7276 | 157.5503 | 136.7329 | 205.1599 | 140.1157 | 160.2021 |
| CUST_2574_PI429010886 | N12675 | 0.040169 | 15 | 9 | | | | | | 896.5197 | 650.8924 | 640.4514 | 717.1047 | 800.6759 | 629.6206 | 602.9273 | 581.5974 | 926.7413 | 988.4432 | 496.1582 | 1214.293 | 874.9124 | 781.7106 | 678.3407 | 994.0668 | 455.0936 | 669.4372 | 501.0805 | 637.7954 | 630.6794 | 1197.469 | 704.3489 | 681.1329 | 1091.244 | 1373.036 | 1256.084 |
| CUST_3038_PI429010886 | N13655 | 0.040169 | 15 | 0 | 242021782 | conserved hypothetical protein [Pediculus humanus | Metabolic process | Other | 1.92E-40 | 227.7485 | 224.668 | 192.0579 | 186.2827 | 192.557 | 193.8995 | 170.2702 | 184.4143 | 168.0847 | 225.2988 | 163.2245 | 193.8001 | 146.9171 | 153.3961 | 194.0962 | 262.4225 | 205.078 | 200.7871 | 202.3386 | 224.9139 | 187.6821 | 200.2689 | 155.0892 | 145.794 | 223.1189 | 168.177 | 168.329 |
| CUST_3245_PI429010886 | N14095 | 0.040169 | 15 | 0 | | | | | | 5800.891 | 8147.956 | 7568.915 | 4427.545 | 5396.825 | 8391.959 | 2363.842 | 2230.655 | 2975.526 | 2829.871 | 5498.252 | 8265.934 | 1421.097 | 5613.6 | 4253.825 | 6879.978 | 4114.35 | 7347.807 | 3921.02 | 9882.018 | 6033.954 | 4859.11 | 2911.974 | 2988.348 | 4696.717 | 6247.817 | 4427.545 |
| CUST_42219_PI429010886 | M59713 | 0.04017 | 24 | 0 | | | | | | 41.92727 | 71.93323 | 95.368 | 42.69742 | 50.17569 | 64.79479 | 37.65902 | 46.78356 | 43.44584 | 43.82278 | 54.84373 | 46.93642 | 42.98911 | 35.73252 | 52.05083 | 42.242 | 35.8423 | 51.48154 | 46.83381 | 48.69863 | 43.52397 | 48.59055 | 41.01917 | 53.89507 | 58.05303 | 53.31549 | 68.25442 |
| CUST_47501_PI429010886 | M76361 | 0.040185 | 12 | 3 | 321469585 | hypothetical protein DAPPUDRAFT_318494 [Daphn | | | 1.29E-14 | 94.48599 | 60.5059 | 48.22578 | 146.3509 | 119.7932 | 114.3743 | 109.9853 | 83.95292 | 46.55609 | 53.26328 | 70.15478 | 29.40744 | 84.25035 | 72.82575 | 41.28967 | 90.49352 | 105.9897 | 92.13028 | 121.8737 | 86.95691 | 40.96307 | 145.9616 | 25.99016 | 61.96298 | 108.9599 | 51.36601 | 29.83725 |
| CUST_48671_PI429010886 | M81571 | 0.040185 | 15 | 12 | | | | | | 59.00876 | 75.79683 | 51.44191 | 62.53094 | 63.94078 | 47.51659 | 41.18311 | 52.24698 | 44.13738 | 59.84297 | 31.5882 | 62.22607 | 66.12938 | 56.54516 | 65.83024 | 82.55661 | 52.63381 | 70.7113 | 48.10026 | 55.72398 | 31.45667 | 47.8959 | 60.41993 | 40.89955 | 67.04072 | 44.7891 | 56.93106 |
| CUST_46856_PI429010886 | M73959 | 0.040188 | 24 | 21 | | | | | | 44.91149 | 46.36775 | 45.3644 | 41.27063 | 39.97721 | 33.22111 | 25.80604 | 28.93334 | 26.54351 | 52.17339 | 32.2878 | 41.8585 | 33.91698 | 43.80188 | 46.80174 | 55.26178 | 28.22043 | 41.89757 | 51.23628 | 27.93572 | 39.8936 | 55.84573 | 55.38718 | 32.09117 | 49.93988 | 33.89729 | 51.14708 |
| CUST_3952_PI429010886 | N15649 | 0.040188 | 24 | 6 | | | | | | 117.2732 | 82.9851 | 159.5474 | 40.47688 | 73.34493 | 86.59518 | 123.7264 | 173.3717 | 99.30005 | 171.0565 | 91.20024 | 71.02655 | 93.36286 | 58.87771 | 65.57569 | 71.18045 | 81.84143 | 66.14351 | 113.3684 | 50.18155 | 45.68153 | 37.60789 | 78.40746 | 56.42615 | 95.67336 | 49.61333 | 91.87634 |
| CUST_11247_PI429010886 | N27507 | 0.040188 | 24 | 21 | | | | | | 191.4774 | 212.1377 | 193.4709 | 207.4667 | 176.6255 | 97.68675 | 152.263 | 142.7716 | 152.8609 | 234.853 | 179.9528 | 234.5938 | 161.9364 | 220.2915 | 166.9686 | 306.2123 | 165.1538 | 202.1751 | 193.4964 | 167.2954 | 210.4274 | 241.1275 | 214.3213 | 205.909 | 226.242 | 164.4185 | 184.4394 |
| CUST_42384_PI429010886 | M60150 | 0.040192 | 18 | 15 | | | | | | 252.0883 | 266.1879 | 190.8837 | 172.1642 | 214.6655 | 206.0341 | 178.5202 | 143.124 | 177.8158 | 259.8087 | 208.8382 | 279.5177 | 190.7327 | 241.8211 | 221.7682 | 296.1446 | 197.7278 | 260.7005 | 208.39 | 239.1825 | 200.041 | 287.1125 | 206.469 | 195.9052 | 221.165 | 174.5081 | 206.0341 |
| CUST_34040_PI429010886 | M8570 | 0.04021 | 18 | 9 | | | | | | 31.20492 | 73.51179 | 64.00351 | 26.07952 | 38.1318 | 32.858 | 24.5797 | 42.00978 | 29.50356 | 68.61799 | 35.91157 | 66.07282 | 42.74724 | 48.05852 | 46.12938 | 55.75917 | 29.28851 | 33.51452 | 36.05492 | 37.58456 | 35.19595 | 38.22525 | 28.68856 | 28.06711 | 56.34897 | 38.24304 | 46.32451 |
| CUST_35175_PI429010886 | M11808 | 0.040226 | 18 | 12 | | | | | | 160.8227 | 166.1651 | 172.0048 | 184.0607 | 136.3926 | 140.2437 | 155.7882 | 172.7709 | 165.4769 | 202.7268 | 168.032 | 202.438 | 168.5282 | 240.9894 | 192.7945 | 204.9855 | 147.3848 | 179.1629 | 159.5684 | 203.482 | 155.8809 | 182.318 | 158.2045 | 149.8948 | 186.7272 | 274.6475 | 169.1739 |
| CUST_1854_PI429010886 | N10891 | 0.040229 | 24 | 18 | | | | | | 72.90791 | 83.83314 | 97.4551 | 89.38803 | 81.34562 | 50.99115 | 71.80124 | 72.88198 | 64.26189 | 86.93205 | 68.71451 | 84.43869 | 80.26839 | 81.73916 | 77.80891 | 89.70412 | 70.47896 | 76.70634 | 88.55995 | 89.70412 | 95.54049 | 101.6948 | 87.51279 | 75.74491 | 110.8879 | 82.22696 | 107.2382 |
| CUST_49644_PI429010886 | M86524 | 0.040229 | 12 | 9 | | | | | | 8.724159 | 7.161801 | 7.625766 | 8.572306 | 9.995172 | NA | 6.238311 | 18.82741 | 17.25469 | 10.47683 | 27.76033 | 34.71503 | 11.82733 | 11.4747 | 7.07379 | 9.691153 | 5.546661 | 3.737061 | 4.390741 | 34.88082 | 10.03121 | NA | 21.82017 | NA | 4.664693 | 14.85468 | 17.01358 |
| CUST_29182_PI429010886 | N59348 | 0.040235 | 15 | 12 | 321473698 | hypothetical protein DAPPUDRAFT_194445 [Daphn | Protein metabolism | Ribosome biogenesis | 2.23E-07 | 83.57768 | 108.0008 | 74.08523 | 83.21112 | 88.78823 | 58.40782 | 55.87623 | 87.42512 | 86.25852 | 93.00061 | 79.80104 | 99.52485 | 108.1195 | 96.07604 | 92.70294 | 113.9294 | 92.12003 | 109.8902 | 70.82822 | 76.25978 | 77.28747 | 101.3798 | 91.94088 | 77.2785 | 100.336 | 57.91335 | 90.11072 |
| CUST_25751_PI429010886 | N52991 | 0.040319 | 21 | 3 | 283854609 | DEAD(Asp-Glu-Ala-Asp) box polypeptide 5 [Macrobı | | | 3.44E-44 | 8.368824 | 23.531 | 27.58334 | 55.35407 | 15.65986 | 27.99024 | 12.0315 | 11.55822 | 16.11829 | 14.39423 | 13.13021 | NA | 14.07874 | 12.82841 | 20.70392 | 18.36165 | 8.991035 | 11.15284 | 10.14024 | 16.22239 | 13.25045 | 18.55819 | 24.14057 | 19.93792 | 34.16228 | 30.64706 | 13.12172 |
| CUST_1500_PI429010886 | N9832 | 0.040339 | 21 | 15 | 158830131 | tetraspanins-like protein CD63 [Fenneropenaeus ch | | | 1.62E-51 | 25.11237 | 6.848027 | 20.13696 | 14.11161 | 8.777159 | 11.56285 | 23.96699 | 16.11829 | 12.502 | 9.977459 | 17.31897 | 12.34338 | 68.52849 | 14.12974 | 17.9819 | 63.96144 | 13.13021 | 23.39071 | 20.6453 | 4.587142 | NA | 14.8651 | 23.4129 | 14.57957 | 7.902818 | 7.451482 | 9.230112 |
| CUST_39281_PI429010886 | M37163 | 0.040341 | 21 | 15 | | | | | | 952.3475 | 1290.993 | 1656.441 | 609.0182 | 879.939 | 901.1709 | 1034.677 | 907.7978 | 1076.18 | 1010.552 | 979.7027 | 1039.457 | 1193.503 | 1061.893 | 877.0953 | 1162.648 | 972.7549 | 1060.588 | 728.657 | 1387.967 | 869.5437 | 1085.118 | 861.4839 | 730.2123 | 732.8997 | 696.3149 | 775.9372 |
| CUST_17930_PI429010886 | N38855 | 0.040341 | 21 | 9 | | | | | | 29.19475 | 41.39686 | 51.96426 | 89.6226 | 180.1028 | 56.48454 | 87.899 | 143.3629 | 117.8313 | 45.48365 | 261.8822 | 265.3779 | 268.7796 | 59.1427 | 145.7071 | 41.95242 | 134.9314 | 43.86454 | 37.54075 | 731.4443 | 175.7782 | 32.36995 | 98.51875 | 86.82244 | 46.0364 | 110.2618 | 146.8007 |
| CUST_42510_PI429010886 | M60467 | 0.040376 | 21 | 6 | | | | | | 81.17779 | 82.91863 | 124.1661 | 129.4503 | 149.7905 | 43.57816 | 189.3158 | 408.9972 | 338.1543 | 120.1371 | 343.9276 | 258.2663 | 308.8946 | 110.6986 | 164.7416 | 87.65825 | 85.10975 | 42.97599 | 117.3497 | 585.2479 | 144.1866 | 180.5428 | 108.9026 | 57.42327 | 71.67319 | 260.0114 | 272.9055 |
| CUST_42259_PI429010886 | M59821 | 0.04039 | 18 | 9 | 321452138 | hypothetical protein DAPPUDRAFT_306997 [Daphn | | | 1.87E-57 | 286.6682 | 381.5724 | 353.5438 | 257.2859 | 323.1264 | 235.9573 | 267.619 | 258.6664 | 292.3195 | 456.7992 | 460.5023 | 307.3807 | 378.7197 | 319.2197 | 361.0091 | 350.4435 | 206.4448 | 250.9845 | 429.1228 | 297.4787 | 344.9163 | 303.9813 | 186.4327 | 130.8825 | 219.9975 | 264.7036 | 327.9176 |
| CUST_21065_PI429010886 | N44559 | 0.040395 | 24 | 3 | | | | | | 41.27857 | 16.40268 | NA | 46.33014 | 15.89011 | 56.1387 | 42.23748 | 31.95513 | 28.7982 | 7.860635 | 13.68168 | 7.059957 | 29.36926 | NA | 21.99145 | 15.87262 | 7.915611 | 11.03659 | 8.003112 | 8.697209 | 14.27082 | 14.65365 | NA | 31.42286 | 4.654918 | 22.78989 | 8.979148 |
| CUST_13004_PI429010886 | N30426 | 0.040413 | 12 | 0 | | | | | | 13.90831 | 20.72239 | 19.21235 | 20.92107 | 10.02077 | 10.78602 | 9.457848 | 7.297269 | 13.00011 | 14.43577 | 12.42241 | 17.32922 | 11.75341 | 20.65497 | 18.55819 | 18.60572 | 18.13433 | 14.26332 | 14.37358 | 9.13082 | 10.95448 | 21.74647 | 13.56226 | 13.8397 | 13.76394 | 14.27562 | 23.82568 |
| CUST_20000_PI429010886 | N42619 | 0.040413 | 12 | 0 | | | | | | 1274.119 | 1721.92 | 1445.235 | 1208.768 | 1137.521 | 1256.992 | 949.8478 | 959.2541 | 1274.525 | 1426.122 | 1039.842 | 1524.773 | 1131.289 | 1533.433 | 1569.266 | 1703.869 | 939.7655 | 1149.925 | 1052.869 | 1517.877 | 1187.094 | 1513.56 | 1197.011 | 1090.456 | 1458.041 | 1594.841 | 2016.816 |
| CUST_23053_PI429010886 | N48175 | 0.040413 | 12 | 0 | | | | | | 1687.609 | 2110.744 | 1480.417 | 1565.628 | 1813.091 | 1964.934 | 1338.117 | 1101.728 | 1542.825 | 1639.617 | 1461.877 | 2563.253 | 1567.155 | 1778.896 | 2579.022 | 2761.396 | 1512.912 | 1935.871 | 1194.964 | 1832.42 | 1474.289 | 1882.712 | 1576.033 | 1513.56 | 2123.129 | 2456.459 | 2324.619 |
| CUST_29389_PI429010886 | N59767 | 0.040413 | 12 | 6 | | | | | | 18.88046 | 36.40882 | 14.26332 | 33.21431 | 19.46629 | 36.16806 | 59.30743 | 41.18311 | 36.52603 | 39.17072 | 29.17928 | 24.58529 | 22.4434 | 29.98387 | 20.29145 | 43.09498 | 39.90155 | 23.12649 | 55.88131 | 10.99994 | 63.52108 | 48.73587 | 20.74695 | 17.7744 | 23.32541 | 19.91592 | 21.05341 |
| CUST_1912_PI429010886 | N11042 | 0.040423 | 24 | 21 | | | | | | 59.56411 | 67.84362 | 80.97211 | 23.19661 | 44.04347 | 51.88107 | 44.4641 | 54.38941 | 63.29578 | 64.49483 | 33.22506 | 24.35681 | 42.29018 | 59.18336 | 37.78218 | 58.78466 | 74.86726 | 62.98504 | 62.54934 | 53.21879 | 67.10317 | 126.5833 | 92.09827 | 30.69887 | 56.33375 | 77.03965 | 72.12484 |
| CUST_30336_PI429010886 | N61655 | 0.040446 | 24 | 18 | | | | | | 350.8381 | 392.0959 | 416.7544 | 321.2041 | 349.3181 | 373.6035 | 324.8235 | 303.4935 | 324.8235 | 420.2091 | 329.2875 | 447.2563 | 380.0505 | 370.213 | 478.8398 | 454.0927 | 385.7092 | 382.663 | 363.2398 | 467.8704 | 394.2069 | 507.2776 | 406.809 | 313.8184 | 418.4668 | 375.4169 | 491.7148 |
| CUST_46440_PI429010886 | M72658 | 0.040474 | 12 | 3 | 321449650 | hypothetical protein DAPPUDRAFT_120652 [Daphn | | | 4.34E-30 | 39.11234 | 18.6269 | 40.70657 | 55.74249 | 45.19753 | 39.67691 | 46.40711 | 19.1562 | 38.44451 | 10.90286 | 28.99306 | 8.134417 | 31.24239 | 26.25499 | 33.01824 | 39.48748 | 58.58633 | 54.60219 | 29.45836 | 13.22412 | 24.2954 | 41.06525 | 26.04992 | 46.71096 | 31.19434 | 18.81492 | 21.3795 |
| CUST_50604_PI429010886 | M90411 | 0.040488 | 21 | 18 | | | | | | 30.27835 | 11.10882 | 26.60252 | 7.414036 | 11.08478 | 16.10718 | 15.91213 | 23.30384 | 18.10445 | 16.63993 | 18.75416 | 21.97852 | 24.03557 | 38.45723 | 21.80116 | 50.42448 | 6.616835 | 23.75855 | 31.58406 | 29.9042 | 13.53636 | 28.44656 | 22.1046 | 15.28371 | 29.02323 | 7.084189 | 12.13602 |
| CUST_47715_PI429010886 | M77293 | 0.040498 | 21 | 6 | | | | | | NA | 48.76067 | 20.3487 | 91.84392 | 120.0446 | 11.97713 | 63.98224 | 308.0795 | 215.6089 | 28.41713 | 213.5411 | 217.0054 | 304.9967 | 63.64812 | 117.7023 | NA | 87.6188 | NA | 18.45614 | 594.8858 | 187.869 | 5.551541 | 16.53993 | 16.51414 | 9.564156 | 93.6298 | 279.1889 |
| CUST_28322_PI429010886 | N57723 | 0.040512 | 15 | 12 | | | | | | 96.01566 | 18.84309 | 51.0829 | 36.08743 | 69.18954 | 75.7296 | 28.57866 | 16.68994 | 32.94969 | 29.38623 | 9.482226 | 98.21029 | 110.3421 | 47.07001 | 26.50017 | 20.49506 | 102.4945 | 87.5621 | 43.58778 | 22.79184 | 26.04444 | 15.557 | 12.66387 | 18.25337 | 46.56491 | 23.14292 | 68.09627 |
| CUST_28085_PI429010886 | N57270 | 0.040523 | 24 | 21 | | | | | | 9.717046 | 8.616285 | 11.39853 | 12.97507 | 8.469456 | 21.80952 | 10.17288 | 9.413901 | 5.567101 | 10.00867 | 12.74006 | 8.250428 | 10.60899 | NA | 7.20793 | 12.51404 | 19.16281 | 12.99803 | 14.28677 | 11.33051 | 14.63523 | 15.75973 | 12.90269 | NA | 14.62779 | 12.4096 | 12.16044 |
| CUST_52652_PI429010886 | M98325 | 0.040542 | 21 | 0 | | | | | | 44.4641 | 20.82676 | 15.30761 | 26.30532 | 18.6803 | 21.8498 | 22.9575 | 16.65148 | 12.84641 | 15.13796 | 23.12297 | 7.298488 | 6.395897 | 17.2249 | 11.0048 | 24.43037 | 28.82637 | 30.24809 | 30.76441 | 8.886857 | 20.80589 | 27.87027 | 61.2083 | 29.14874 | 20.63276 | 8.784908 | 5.116935 |
| CUST_19799_PI429010886 | N42212 | 0.040546 | 24 | 0 | | | | | | 106.5084 | 129.5889 | 129.4038 | 135.0881 | 100.9074 | 133.6452 | 89.25924 | 111.5529 | 90.12961 | 127.8042 | 103.7436 | 99.03782 | 121.2697 | 97.24592 | 122.8574 | 201.3287 | 80.1769 | 85.71149 | 99.48375 | 102.7268 | 122.8712 | 113.1518 | 92.25373 | 78.48034 | 282.166 | 319.6424 | 292.3676 |
| CUST_33385_PI429010886 | M6755 | 0.040557 | 21 | 3 | 281337645 | hypothetical protein PANDA_009536 [Ailuropoda m | | | 4.26E-07 | 149.147 | 128.673 | 137.196 | 144.8798 | 103.3491 | 181.649 | 107.0416 | 163.569 | 133.4278 | 166.0384 | 128.7498 | 127.8287 | 172.408 | 137.604 | 142.1826 | 143.0445 | 122.1911 | 102.8545 | 131.327 | 125.0791 | 77.46325 | 133.7601 | 137.6307 | 118.3632 | 178.0431 | 200.8635 | 244.5361 |
| CUST_31352_PI429010886 | M1331 | 0.040562 | 24 | 0 | | | | | | 1481.812 | 1995.819 | 1460.496 | 1376.074 | 1145.003 | 1311.23 | 1140.432 | 1246.965 | 1163.104 | 1441.16 | 1428.149 | 1805.829 | 950.5113 | 1211.313 | 1248.908 | 1684.016 | 1448.061 | 1344.532 | 1403.935 | 1849.813 | 1390.937 | 1611.105 | 1335.768 | 1302.095 | 1695.261 | 1491.426 | 1621.924 |
| CUST_7000_PI429010886 | N20895 | 0.040562 | 24 | 0 | 194900772 | GG21285 [Drosophila erecta] >gi\|257096534\|sp\|B3 | Protein metabolism | Proteolysis | 4.18E-63 | 73.17053 | 58.32457 | 53.36598 | 49.04149 | 48.85338 | 50.65756 | 41.7785 | 43.20136 | 47.80199 | 66.59221 | 40.80459 | 33.38032 | 48.85949 | 41.12787 | 29.23595 | 58.42676 | 44.38038 | 44.52089 | 44.16686 | 40.03126 | 31.54083 | 55.29319 | 21.73089 | 42.1461 | 44.02245 | 70.77767 | 62.84333 |
| CUST_34254_PI429010886 | M9176 | 0.040584 | 21 | 9 | | | | | | 6.787758 | 9.017882 | 7.573982 | 11.77184 | 26.79563 | 22.45787 | 4.893462 | 62.70814 | 29.06247 | 12.44359 | 97.22671 | 85.69951 | 43.31707 | 12.70382 | 24.32573 | 4.451004 | 21.2243 | 3.926918 | 9.290785 | 83.99654 | 54.01898 | 5.756028 | 13.66257 | 7.17066 | 8.437018 | 35.96223 | 25.69829 |
| CUST_15687_PI429010886 | N34976 | 0.04059 | 21 | 18 | | | | | | 1295.233 | 1659.929 | 1581.923 | 761.7734 | 1189.103 | 1001.62 | 811.2478 | 920.7129 | 894.9707 | 1238.144 | 1146.628 | 1329.963 | 792.1417 | 927.8138 | 981.0866 | 1333.433 | 1035.008 | 1016.492 | 1246.44 | 1789.616 | 1337.58 | 1127.217 | 1158.693 | 755.0608 | 1684.016 | 1104.361 | 1323.05 |
| CUST_8610_PI429010886 | N23300 | 0.040601 | 12 | 0 | | | | | | 48.65694 | 36.36466 | 36.69017 | 19.64586 | 33.41208 | 31.93333 | 26.35743 | 31.23112 | 26.98754 | 35.78945 | 37.74031 | 35.72502 | 35.9416 | 34.55194 | 34.75205 | 23.25736 | 20.80086 | 17.19687 | 34.74405 | 32.97611 | 16.25775 | 31.18147 | 22.91473 | 27.29319 | 34.91643 | 25.3441 | 24.60995 |
| CUST_12203_PI429010886 | N29098 | 0.040601 | 12 | 0 | | | | | | 320.1726 | 351.6868 | 432.6248 | 232.021 | 247.7794 | 225.2614 | 304.1627 | 308.0149 | 328.9809 | 414.0246 | 280.4106 | 388.4161 | 317.4719 | 426.0225 | 393.405 | 409.277 | 315.9925 | 328.5456 | 334.9967 | 343.3863 | 351.1934 | 354.7905 | 376.7168 | 296.8833 | 418.6867 | 325.2563 | 429.5807 |
| CUST_28100_PI429010886 | N57300 | 0.040601 | 24 | 18 | | | | | | 184.2307 | 84.88556 | 338.0611 | 101.7734 | 123.2567 | 72.83324 | 123.7589 | 196.4155 | 60.75286 | 138.5572 | 69.93057 | 60.93646 | 89.15191 | 205.3646 | 127.8836 | 157.0455 | 219.9975 | 112.8928 | 556.906 | 115.3926 | 460.7596 | 257.9388 | 189.144 | 73.21949 | 109.8598 | 132.2924 | 231.7766 |
| CUST_41170_PI429010886 | M57047 | 0.04061 | 18 | 15 | | | | | | 41.86303 | 44.98924 | 26.26256 | 23.31281 | 27.69389 | 21.4644 | 22.10198 | 29.11768 | 18.84309 | 31.60525 | 25.58065 | 19.99274 | 25.54793 | 29.9042 | 40.35311 | 96.9916 | 29.00481 | 30.21389 | 26.67812 | 28.35396 | 21.51397 | 44.75093 | 13.69402 | 41.7589 | 24.65764 | 20.93627 | 21.91785 |
| CUST_709_PI429010886 | N6878 | 0.04061 | 24 | 0 | | | | | | 60.47979 | 55.74802 | 40.78034 | 37.23914 | 46.04195 | 27.59937 | 40.19775 | 32.19156 | 31.12088 | 52.53115 | 27.25206 | 28.97849 | 29.62034 | 30.27449 | 27.92484 | 52.08136 | 22.14748 | 29.93088 | 72.74687 | 37.62291 | 32.6566 | 64.55134 | 39.22239 | 11.73596 | 58.91203 | 34.45395 | 36.01332 |
| CUST_45208_PI429010886 | M68323 | 0.040614 | 21 | 12 | | | | | | 130.1677 | 172.5457 | 194.4273 | 169.5063 | 139.7854 | 162.2159 | 141.4486 | 147.0019 | 126.8312 | 197.0393 | 163.8459 | 159.4333 | 168.2678 | 215.4716 | 233.124 | 232.5216 | 189.8916 | 148.4609 | 164.6012 | 187.2939 | 160.259 | 206.1701 | 159.4972 | 179.4697 | 181.0372 | 149.4495 | 182.9016 |
| CUST_27302_PI429010886 | N55808 | 0.040621 | 15 | 12 | | | | | | 365.0271 | 359.6577 | 493.4379 | 303.0187 | 335.8595 | 451.0636 | 362.8276 | 304.6076 | 429.8795 | 444.8018 | 333.1788 | 610.2139 | 420.7782 | 606.0546 | 686.5061 | 528.8856 | 404.5388 | 435.3919 | 331.7667 | 414.0246 | 460.8905 | 373.6828 | 400.26 | 383.7251 | 506.3576 | 441.5046 | 613.1363 |
| CUST_45876_PI429010886 | M70591 | 0.040645 | 24 | 21 | 270005635 | hypothetical protein TcasGA2_TC007718 [Tribolium | | | 8.59E-19 | 38.54256 | 38.6481 | 26.81554 | 34.22484 | 37.96902 | 35.19889 | 26.26779 | 29.2624 | 24.19522 | 38.82938 | 41.05122 | 28.93983 | 30.0571 | 36.25025 | 39.36079 | 34.27356 | 42.0037 | 41.83506 | 37.12735 | 48.1847 | 35.50744 | 51.85122 | 37.87276 | 34.46151 | 48.78129 | 37.4709 | 39.70495 |
| CUST_3528_PI429010886 | N14692 | 0.040645 | 24 | 6 | | | | | | 16358.95 | 989.5639 | 21318.49 | 10575.43 | 18116.83 | 14493.88 | 24647.46 | 23934.78 | 14928.07 | 15109.1 | 17576.39 | 185.1977 | 12782.74 | 10341.13 | 8550.193 | 133.5939 | 27642.69 | 8470.008 | 25496.93 | 196.7621 | 8327.109 | 388.7869 | 2457.891 | 4973.61 | 1140.432 | 6495.601 | 1634.318 |
| CUST_3816_PI429010886 | N15339 | 0.040645 | 24 | 21 | 2636680 | pol [Schistosoma mansoni] | Nucleic acid metabolism | DNA replication | 1.82E-16 | 14.06725 | 16.39244 | 25.17616 | 15.78638 | 14.48619 | 19.43095 | 11.91129 | 8.841942 | 18.89221 | 16.30067 | 15.38497 | 22.44818 | 11.34711 | 23.90824 | 13.11125 | 19.49887 | 14.03531 | 28.15071 | 12.81434 | 18.73459 | 16.1997 | 21.24048 | 35.11564 | 48.00964 | 20.51216 | 17.23467 | 24.76741 |
| CUST_25634_PI429010886 | N52779 | 0.040645 | 24 | 18 | | | | | | 44.50009 | 34.65148 | 43.56595 | 29.72539 | 30.14891 | 33.54623 | 42.91838 | 30.77501 | 38.46101 | 42.79014 | 35.53745 | 40.54674 | 39.42313 | 43.99167 | 40.55137 | 52.43379 | 39.41947 | 40.05813 | 41.67274 | 41.909 | 57.65114 | 46.00478 | 43.7836 | 36.53021 | 48.59055 | 27.98319 | 52.63886 |
| CUST_55011_PI429010886 | M108402 | 0.040645 | 21 | 0 | | | | | | 17.35771 | 27.53274 | 10.23894 | 12.95466 | 16.72612 | NA | 16.04256 | 15.11963 | 11.56409 | 26.08326 | 12.78761 | 16.36343 | 12.23618 | 13.92466 | 11.07141 | 9.930283 | 6.60261 | 9.661698 | 15.40175 | 17.73774 | 13.4001 | 22.13024 | 19.56685 | 11.71707 | 21.71687 | 10.40388 | 19.45151 |
| CUST_47065_PI429010886 | M74699 | 0.040648 | 15 | 0 | | | | | | 53.27432 | 58.04233 | 73.60851 | 72.79951 | 56.91814 | 68.17959 | 36.02402 | 46.25392 | 40.68988 | 44.0607 | 51.04482 | 49.8924 | 44.59831 | 49.51746 | 58.78466 | 64.42438 | 50.77711 | 57.96698 | 46.22951 | 48.5802 | 54.72536 | 59.61145 | 40.17935 | 44.63407 | 59.93656 | 63.80913 | 49.21663 |
| CUST_33835_PI429010886 | M7984 | 0.040648 | 15 | 0 | | | | | | 387.8661 | 376.8596 | 336.5695 | 299.0972 | 379.6269 | 334.4771 | 280.12 | 280.2656 | 288.6485 | 334.4771 | 281.652 | 357.2284 | 244.775 | 395.7641 | 305.0519 | 452.9912 | 387.5117 | 422.2989 | 359.2285 | 345.5828 | 288.1296 | 402.3792 | 307.2464 | 298.3902 | 405.8466 | 345.8615 | 363.2975 |
| CUST_7882_PI429010886 | N22214 | 0.040648 | 15 | 0 | 321463476 | hypothetical protein DAPPUDRAFT_307279 [Daphn | | | 1.37E-35 | 104.5244 | 115.8883 | 136.6977 | 94.10691 | 119.1476 | 103.0015 | 103.202 | 84.30944 | 113.2498 | 115.4344 | 115.1327 | 111.3283 | 116.1332 | 119.7932 | 90.99167 | 126.5103 | 131.3133 | 113.0933 | 135.3886 | 110.5384 | 117.8313 | 98.02439 | 102.639 | 98.79644 | 133.7417 | 104.9421 | 117.2072 |
| CUST_8660_PI429010886 | N23374 | 0.040648 | 15 | 0 | 260793621 | hypothetical protein BRAFLDRAFT_123545 [Branchi | Metabolic process | Carbohydrate metabolic process | 5.94E-66 | 121.4843 | 151.2427 | 194.5783 | 285.6058 | 154.8485 | 193.5271 | 78.17526 | 42.8661 | 75.30403 | 108.4109 | 215.4716 | 135.3289 | 59.9058 | 79.5425 | 79.5827 | 154.6176 | 123.6359 | 209.9313 | 119.1299 | 142.4348 | 153.2997 | 98.16979 | 81.82955 | 77.08541 | 149.1265 | 96.26654 | 96.25237 |
| CUST_4083_PI429010886 | N15969 | 0.04066 | 21 | 18 | 402589316 | actin [Wuchereria bancrofti] | | | 9.88E-15 | 58.49374 | 33.3239 | 57.58322 | 54.27461 | 70.44162 | 47.36571 | 78.39762 | 37.36325 | 27.13299 | 34.0788 | 79.84889 | 26.03397 | 27.71788 | 31.11356 | 44.52812 | 44.97088 | 94.91653 | 60.18184 | 82.51706 | 40.52064 | 79.95313 | 55.64028 | 73.40872 | 95.368 | 31.91513 | 38.82258 | 32.09448 |
| CUST_14256_PI429010886 | N32583 | 0.040666 | 12 | 9 | | | | | | 11.47799 | 12.68722 | 19.88268 | 8.967811 | 10.20122 | 5.026974 | 9.761566 | 13.26521 | 10.87917 | 11.93548 | 15.11263 | 17.50149 | 12.31007 | 14.09963 | 16.79285 | 13.28314 | 10.56393 | 22.72441 | 16.39936 | 11.13027 | 12.75871 | 24.78008 | 16.05605 | 19.78805 | 15.51771 | 9.871686 | 14.55066 |
| CUST_9544_PI429010886 | N24811 | 0.040681 | 18 | 3 | 321460073 | eIF2B-alpha protein [Daphnia pulex] | Protein metabolism | Regulation of translational initiation | 2.13E-15 | 38.86122 | 70.94855 | 52.063 | 37.18815 | 53.14405 | 117.8039 | 74.27647 | 37.19463 | 45.87335 | 50.28834 | 55.24489 | 56.71651 | 50.24503 | 46.02927 | 36.51599 | 34.34564 | 49.74292 | 54.66133 | 30.62521 | 50.31908 | 57.08439 | 61.66407 | 65.29553 | 100.4445 | 60.23492 | 63.88437 | 69.25418 |
| CUST_2209_PI429010886 | N11876 | 0.040708 | 24 | 15 | 74095343 | aurora/Ipl1p-related kinase [Marthasterias glacialis | Developmental process | Multicellular organismal development | 5E-119 | 46.57707 | 70.06431 | 53.47338 | 20.56557 | 36.33605 | 16.73391 | 49.02476 | 36.03165 | 50.3484 | 53.22735 | 50.71291 | 74.66823 | 46.85704 | 45.3426 | 56.17296 | 75.99857 | 30.16839 | 70.92151 | 46.00478 | 70.98423 | 65.20986 | 61.60941 | 56.23475 | 42.01359 | 61.05832 | 58.20859 | 50.32404 |
| CUST_898_PI429010886 | N7746 | 0.040744 | 15 | 12 | | | | | | 978.4784 | 1200.158 | 1151.621 | 920.9532 | 911.683 | 991.907 | 801.7718 | 922.2736 | 1037.274 | 1284.158 | 943.689 | 1670.981 | 926.1166 | 1455.397 | 1435.197 | 86045.29 | 1149.925 | 1122.959 | 1036.441 | 1116.604 | 996.654 | 1290.488 | 1270.943 | 1055.576 | 1269.774 | 1166.392 | 1351.741 |
| CUST_46963_PI429010886 | M74320 | 0.040789 | 21 | 18 | | | | | | 56.1387 | 51.93952 | 30.26197 | 45.51068 | 36.81255 | 36.85539 | 42.20687 | 18.76152 | 36.0581 | 43.27178 | 38.56175 | 23.55239 | 22.64731 | 45.33809 | 29.85007 | 46.47367 | 43.46213 | 29.88417 | 54.09438 | 50.19562 | 49.41638 | 58.60996 | 34.47136 | 25.72198 | 47.21974 | 31.9127 | 37.7275 |
| CUST_12827_PI429010886 | N30114 | 0.040829 | 12 | 0 | | | | | | 78.62359 | 115.3764 | 114.3208 | 61.2655 | 68.53497 | 51.97066 | 58.36485 | 65.27575 | 86.64513 | 106.002 | 74.35805 | 110.9326 | 94.33627 | 99.59438 | 96.30815 | 134.8111 | 50.85184 | 88.53305 | 73.803 | 93.67647 | 66.16504 | 108.829 | 86.94051 | 62.70075 | 106.7278 | 87.25084 | 85.62903 |
| CUST_55121_PI429010886 | M108794 | 0.040862 | 21 | 12 | | | | | | 677.9305 | 514.0171 | 638.7779 | 244.0822 | 933.7706 | 635.9764 | 696.9471 | 595.8659 | 530.042 | 1210.626 | 506.6135 | 677.3854 | 953.9498 | 802.0583 | 634.2891 | 661.3119 | 739.6144 | 1199.593 | 595.7445 | 573.39 | 641.3583 | 772.1398 | 348.6809 | 298.4347 | 286.7058 | 529.2101 | 566.2341 |
| CUST_33470_PI429010886 | M6970 | 0.040875 | 15 | 0 | | | | | | 307.4267 | 281.9234 | 291.9341 | 262.5059 | 261.4696 | 377.0163 | 344.7288 | 227.7485 | 272.1926 | 136.5811 | 319.745 | 254.5401 | 234.9764 | 288.3979 | 284.2107 | 466.0826 | 330.4301 | 332.0505 | 340.4766 | 282.2712 | 255.3452 | 425.3087 | 239.0792 | 277.8488 | 280.971 | 198.4542 | 205.8829 |
| CUST_39045_PI429010886 | M33891 | 0.040881 | 18 | 3 | 9186884 | BCS-1 [Amphibalanus amphitrite] | | | 6.6E-11 | 1735.328 | 821.0172 | 310.6611 | 3772.58 | 1501.641 | 3890.087 | 2042.507 | 785.4766 | 1467.586 | 71.72159 | 731.2482 | 63.21301 | 853.1958 | 513.5475 | 677.3854 | 1115.54 | 2096.527 | 2661.313 | 793.9994 | 288.7076 | 921.6949 | 1627.802 | 622.024 | 3156.693 | 251.8789 | 2170.679 | 27.29319 |
| CUST_47890_PI429010886 | M78039 | 0.040892 | 24 | 15 | | | | | | 14.9648 | 9.760194 | NA | NA | 8.541205 | 10.31476 | 11.8505 | 5.614366 | 8.698364 | 15.54357 | 8.965849 | 17.39038 | 10.83635 | 17.9819 | 9.993532 | 14.38818 | 13.91228 | 19.73697 | 13.36864 | 14.70218 | 15.11766 | 10.32397 | 16.38885 | 11.58977 | 12.17117 | 14.71926 | 14.5219 |
| CUST_49745_PI429010886 | M86964 | 0.040904 | 21 | 9 | | | | | | 96.52778 | 61.30991 | 179.7938 | 112.4734 | 149.147 | 108.4917 | 149.2835 | 366.089 | 273.0635 | 119.9288 | 593.1266 | 454.9277 | 346.9119 | 168.4504 | 127.6673 | 77.80891 | 234.5938 | 135.2179 | 133.561 | 296.0384 | 152.2232 | 85.83424 | 135.0089 | 136.0755 | 118.3919 | 111.9496 | 192.7945 |
| CUST_40649_PI429010886 | M55740 | 0.040908 | 21 | 18 | 443690382 | hypothetical protein CAPTEDRAFT_181480 [Capitell | | | 1.53E-26 | 11.69128 | 7.081402 | 17.73142 | NA | 7.907569 | 12.20042 | 10.11181 | 5.349898 | NA | 10.32292 | 4.388362 | 6.756825 | 8.357942 | 9.867365 | 9.11334 | 10.99803 | 10.55953 | 19.31761 | 10.20858 | 11.84044 | 11.79877 | 7.732289 | 12.93967 | NA | 11.00334 | 7.994315 | NA |
| CUST_33950_PI429010886 | M8337 | 0.04091 | 24 | 15 | | | | | | 5.421444 | 11.12035 | 14.38334 | 11.46533 | 13.12172 | 8.326131 | 6.375755 | 6.296703 | 5.447638 | 8.699683 | 8.93642 | 6.759208 | 5.548767 | 15.1027 | NA | 19.58019 | 34.2288 | 15.86933 | 5.107711 | 9.104602 | 17.14547 | 11.88135 | 11.82733 | NA | 11.04742 | 9.5748 | 18.97492 |
| CUST_17966_PI429010886 | N38917 | 0.040922 | 21 | 9 | 460027843 | prostaglandin F synthase [Penaeus monodon] | Metabolic process | Oxidation-reduction process | 3.58E-41 | 32.80705 | 23.41598 | 34.55538 | 26.4784 | 36.91363 | 38.53689 | 55.20849 | 94.42153 | 70.55676 | 35.98369 | 80.74596 | 103.1342 | 68.58333 | 41.8585 | 39.13986 | 37.41832 | 37.64513 | 14.10338 | 38.15596 | 118.8596 | 56.57948 | 28.74973 | 47.84739 | 24.29748 | 16.99938 | 28.22043 | 85.75328 |
| CUST_51082_PI429010886 | M92093 | 0.040964 | 15 | 0 | | | | | | 38.12468 | 30.58787 | 46.78872 | 33.47284 | 15.1983 | 19.49887 | 14.47801 | 21.70044 | 22.7759 | 22.46676 | 31.92608 | 19.19334 | 24.2252 | 33.84879 | 22.12824 | 32.61136 | 33.99968 | 29.41046 | 21.60172 | 37.9853 | 35.91157 | 21.9058 | 19.26616 | 33.54623 | 22.73323 | 24.02788 | 34.05076 |
| CUST_18462_PI429010886 | N39836 | 0.040977 | 21 | 18 | | | Signal transduction | Other | | 44.92544 | 35.1468 | 24.9855 | 30.66462 | 34.48125 | 20.31618 | 35.09944 | 23.99863 | 35.992 | 28.48104 | 33.92046 | 32.03583 | 36.01731 | 30.34969 | 25.9573 | 47.8728 | 32.10959 | 31.14945 | 29.04226 | 36.87107 | 53.29328 | 49.73773 | 32.53107 | 34.35136 | 24.0044 | 20.41292 | 16.67648 |
| CUST_45064_PI429010886 | M67890 | 0.040994 | 24 | 18 | | | | | | 111.1235 | 114.3617 | 120.5115 | 105.4104 | 102.477 | 109.0963 | 104.3119 | 105.3539 | 99.4135 | 127.1338 | 93.83775 | 111.3415 | 100.0297 | 159.741 | 133.9111 | 119.1161 | 97.95028 | 112.064 | 136.2319 | 120.5234 | 123.5891 | 138.0366 | 114.4563 | 110.7787 | 149.7694 | 95.61724 | 132.4059 |
| CUST_19086_PI429010886 | N40955 | 0.040997 | 18 | 9 | | | | | | 58.597 | 75.54672 | 58.94983 | 47.17722 | 88.57155 | 72.71237 | 76.77824 | 65.46937 | 82.00472 | 93.37534 | 143.0236 | 100.2406 | 76.74889 | 50.0808 | 64.5831 | 80.73902 | 90.55235 | 64.56992 | 61.34598 | 80.4086 | 77.50869 | 71.97535 | 99.19224 | 65.26348 | 106.4989 | 73.14043 | 103.0974 |
| CUST_19885_PI429010886 | N42385 | 0.041027 | 15 | 12 | | | | | | 472.651 | 461.4541 | 485.4409 | 303.2741 | 374.2323 | 429.2473 | 271.4065 | 372.8033 | 331.2853 | 480.5601 | 436.3193 | 552.3175 | 426.8105 | 469.1551 | 510.3855 | 518.0939 | 466.4129 | 406.6708 | 465.2249 | 461.7538 | 374.607 | 410.9902 | 478.2055 | 391.0528 | 550.7829 | 385.0916 | 544.2536 |
| CUST_38491_PI429010886 | M27324 | 0.041049 | 18 | 12 | | | | | | 365.7398 | 1584.97 | 1145.481 | 566.0346 | 133.8595 | 26.23793 | 393.4936 | 796.5277 | 876.1774 | 2046.644 | 497.2573 | 1391.685 | 1228.741 | 2041.736 | 1636.442 | 612.1213 | 789.0995 | 172.082 | 889.0555 | 2303.489 | 1621.924 | 528.1283 | 1792.391 | 134.4036 | 184.3916 | 790.5889 | 1300.946 |
| CUST_16291_PI429010886 | N35985 | 0.041051 | 24 | 0 | 543341861 | PREDICTED: zinc finger protein 236 [Pseudopodoces | | | 6.67E-11 | 46.507 | 42.31884 | 39.0714 | 39.46953 | 32.7645 | 18.11915 | 20.6694 | 26.14633 | 29.30417 | 37.2354 | 43.44238 | 31.65107 | 19.59849 | 39.76167 | 17.5765 | 37.18815 | 29.05092 | 32.31627 | 35.30842 | 33.12364 | 32.30335 | 47.83293 | 43.61983 | 20.98222 | 59.02601 | 34.57302 | 44.90621 |
| CUST_5986_PI429010886 | N19470 | 0.041053 | 15 | 9 | | | Metabolic process | Lipid metabolic process | | 172.6317 | 263.2877 | 252.9891 | 114.0128 | 255.6621 | 192.6124 | 136.2681 | 193.108 | 151.4326 | 204.4512 | 311.0329 | 338.2555 | 376.9333 | 195.9288 | 325.2563 | 177.7855 | 226.2023 | 159.5684 | 122.0534 | 444.8946 | 186.0597 | 195.6579 | 194.8405 | 202.4996 | 201.1076 | 267.4607 | 421.5075 |
| CUST_34980_PI429010886 | M11229 | 0.041053 | 21 | 9 | 302887532 | hypothetical protein NECHADRAFT_24316 [Nectria | | | 2.52E-09 | 15.67417 | 30.96598 | 11.63165 | 49.52106 | 83.41229 | 21.90202 | 33.76323 | 160.7305 | 116.9236 | 27.51169 | 138.7624 | 159.2366 | 50.79816 | 26.50195 | 36.01332 | 36.40199 | 40.08304 | 7.794721 | 17.79901 | 219.3834 | 108.3885 | 13.41841 | 35.71639 | 26.10463 | 13.30229 | 69.68123 | 66.32011 |
| CUST_55581_PI429010886 | M110823 | 0.041065 | 15 | 12 | | | | | | 61.81309 | 52.4826 | 61.67082 | 40.73202 | 40.05813 | 52.19516 | 31.41951 | 39.15692 | 41.28967 | 67.41874 | 42.96811 | 59.98068 | 53.39323 | 59.30743 | 59.61663 | 61.911 | 40.09922 | 57.2685 | 57.67685 | 49.7894 | 38.0269 | 74.88917 | 38.51713 | 39.8885 | 42.8661 | 32.98902 | 50.74126 |
| CUST_28389_PI429010886 | N57850 | 0.041065 | 15 | 12 | | | | | | 10.2754 | 7.362688 | 5.503527 | 7.970135 | 6.071276 | 11.58977 | 6.889529 | 8.298481 | 14.85468 | 20.41758 | 9.920366 | 5.722118 | 15.68356 | 17.80566 | 11.85349 | 11.83405 | 11.44906 | 22.88601 | 8.089072 | 7.706127 | 8.091683 | 10.43583 | 12.89633 | 15.10731 | 12.49156 | 18.13609 | 15.88543 |
| CUST_20365_PI429010886 | N43311 | 0.041078 | 12 | 0 | | | | | | 21.21095 | 20.29145 | 19.2789 | 13.80666 | 13.92993 | 14.96828 | 15.90721 | 14.70767 | 12.45327 | 11.49531 | 14.85609 | 26.93627 | 14.25304 | 22.94743 | 19.61727 | 13.8928 | 10.39792 | 19.26978 | 11.412 | 15.76549 | 27.61834 | 18.56806 | 19.72906 | 24.44247 | 24.94331 | 23.75128 | 27.09386 |
| CUST_9190_PI429010886 | N24206 | 0.041111 | 24 | 15 | | | | | | 521.7265 | 721.5575 | 667.8908 | 575.3599 | 525.5752 | 581.1144 | 447.7446 | 447.5499 | 523.5388 | 623.6818 | 512.5692 | 801.3257 | 545.6531 | 772.6877 | 765.0242 | 878.2313 | 552.5713 | 693.2829 | 634.2891 | 677.3854 | 683.7716 | 799.3451 | 593.5055 | 541.117 | 699.0502 | 599.2901 | 770.9286 |
| CUST_31363_PI429010886 | M1354 | 0.041124 | 18 | 3 | | | | | | 955.8025 | 721.8143 | 497.1091 | 760.5296 | 786.4352 | 755.8427 | 816.3533 | 512.8422 | 521.397 | 478.4338 | 545.8082 | 391.6722 | 660.8688 | 399.93 | 505.8775 | 673.1948 | 773.6682 | 653.6532 | 790.5889 | 470.5429 | 495.7993 | 716.6989 | 543.3625 | 834.9559 | 555.3104 | 454.2849 | 470.2955 |
| CUST_4631_PI429010886 | N17169 | 0.041124 | 24 | 15 | 431808832 | ankyrin repeat-containing protein [Brachyspira pilos | | | 9.99E-07 | 11.4368 | NA | 10.56393 | 13.18579 | 19.81449 | 26.06265 | 48.67019 | 54.04063 | NA | 9.711718 | 23.35652 | NA | 43.35936 | 35.05162 | 23.42758 | NA | 78.60763 | 35.07942 | 96.41487 | 4.476001 | 13.04696 | 13.68648 | 22.9575 | 14.68592 | 13.50586 | NA | 6.698064 |
| CUST_33894_PI429010886 | M8171 | 0.041126 | 15 | 9 | | | | | | 298.484 | 194.605 | 339.1053 | 234.6626 | 259.7236 | 371.2619 | 309.3403 | 274.6972 | 302.7759 | 307.0541 | 273.4298 | 398.7713 | 241.3085 | 343.1257 | 334.1776 | 327.7237 | 242.8033 | 302.7176 | 239.6937 | 290.7849 | 291.8279 | 325.3231 | 328.6838 | 305.9676 | 361.8439 | 465.5606 | 436.0745 |
| CUST_5210_PI429010886 | N18403 | 0.041126 | 15 | 3 | 332023022 | Ornithine aminotransferase, mitochondrial [Acromy | | | 4.8E-176 | 115.1327 | 82.76433 | 127.0971 | 140.6429 | 106.908 | 204.8186 | 204.2904 | 58.72685 | 106.2171 | 62.12557 | 102.9204 | 78.83085 | 100.4336 | 79.37795 | 89.419 | 100.5554 | 175.6726 | 112.7849 | 157.231 | 76.46353 | 133.7417 | 68.09627 | 93.75113 | 151.6234 | 67.55015 | 104.6277 | 89.75292 |
| CUST_11062_PI429010886 | N27206 | 0.041126 | 15 | 3 | | | | | | 62.04074 | 53.16758 | 47.19112 | 77.83266 | 202.7268 | 195.392 | 188.9473 | 46.33994 | 32.70652 | 36.60913 | 119.6777 | 44.01861 | 52.34171 | 58.10187 | 48.53244 | 67.99795 | 156.7272 | 119.1161 | 126.4569 | 42.63354 | 66.10733 | 62.45524 | 52.96595 | 82.24759 | 51.57463 | 46.95122 | 45.24266 |
| CUST_19770_PI429010886 | N42160 | 0.041172 | 18 | 12 | | | | | | 32.19156 | 36.51599 | 29.36926 | 20.76211 | 28.93067 | 13.42725 | 60.20751 | 29.12856 | 49.40038 | 40.80851 | 21.82911 | 54.37221 | 49.97285 | 52.29382 | 63.17657 | 45.37412 | 20.16106 | 39.76167 | 63.42029 | 19.77641 | 38.94325 | 64.31019 | 29.19475 | 21.32406 | 32.39452 | 38.56175 | 30.53018 |
| CUST_54127_PI429010886 | M104702 | 0.041186 | 24 | 12 | | | | | | 117.2582 | 129.4353 | 190.6989 | 113.525 | 139.7632 | 128.9362 | 117.0813 | 142.5643 | 106.6375 | 176.2631 | 134.659 | 161.0453 | 188.7518 | 293.8285 | 220.0942 | 173.2522 | 140.8613 | 116.1033 | 151.582 | 199.1618 | 191.145 | 219.4603 | 161.355 | 95.09391 | 156.4118 | 145.578 | 185.5006 |
| CUST_56390_PI429010886 | M113659 | 0.041186 | 24 | 6 | | | | | | 35.51693 | 51.69046 | 46.76971 | 76.94992 | 52.14738 | 18.75909 | 60.54854 | 87.09496 | 55.20237 | 69.76759 | 52.11537 | 58.42676 | 19.78441 | 29.99986 | 18.7049 | 58.70799 | 24.0618 | 21.56682 | 19.1106 | 37.26268 | 32.85525 | 12.28125 | 40.01136 | 44.05203 | 5.934011 | 25.12351 | 34.82944 |
| CUST_57336_PI429010886 | E6444 | 0.041187 | 24 | 18 | 374095290 | voltage-dependent non-L-type calcium channel alpł | Transport | Ion transport | 5.32E-64 | 38.24749 | 29.6775 | 38.121 | 26.45984 | 38.17748 | 40.10273 | 43.08722 | 30.0232 | 29.35151 | 38.39579 | 44.97088 | 36.25859 | 19.58231 | 33.41208 | 38.97496 | 44.4501 | 49.24106 | 41.6172 | 69.33625 | 43.93042 | 44.58058 | 45.62788 | 39.24656 | 34.05076 | 69.27267 | 33.51766 | 43.91811 |
| CUST_17531_PI429010886 | N38129 | 0.041187 | 21 | 0 | | | | | | 128.0902 | 133.7417 | 186.9387 | 124.8716 | 115.1745 | 114.8783 | 98.80707 | 105.3648 | 86.94051 | 194.6574 | 128.9944 | 124.5285 | 116.0676 | 114.0443 | 107.2382 | 190.4443 | 155.8611 | 89.6226 | 125.6673 | 123.8447 | 140.6429 | 154.595 | 134.6919 | 138.7624 | 164.4613 | 91.4434 | 107.0545 |
| CUST_48789_PI429010886 | M82214 | 0.041236 | 18 | 0 | | | | | | 158.9804 | 123.1846 | 164.5463 | 11.9176 | 151.0368 | 69.78353 | 73.07164 | 50.06979 | 82.47663 | 121.6348 | 130.4535 | 87.34049 | 40.95098 | 79.94364 | 56.88203 | 104.0557 | 114.6191 | 91.99262 | 138.4518 | 69.18246 | 131.0289 | 69.04997 | 73.22698 | 77.29645 | 98.02439 | 37.04826 | 67.07557 |
| CUST_12211_PI429010886 | N29108 | 0.041236 | 18 | 0 | 34576191 | PMAV [Penaeus monodon] >gi\|110264573\|gb\|ABG | | | 2.91E-09 | 254.4554 | 593.1266 | 384.2391 | 252.6253 | 503.9702 | 223.2901 | 32.78743 | 120.8733 | 28.02833 | 305.1659 | 253.7351 | 242.0042 | 36.09682 | 267.983 | 100.1573 | 247.3742 | 33.1843 | 88.87538 | 294.8062 | 335.1161 | 289.2842 | 238.941 | 59.6416 | 38.1599 | 165.7579 | 164.3449 | 271.6132 |
| CUST_19051_PI429010886 | N40901 | 0.041236 | 18 | 6 | | | | | | 12.66733 | 6.31804 | 9.09473 | 28.70795 | 61.46953 | 9.394559 | 31.36464 | 9.217559 | 32.58197 | 11.22853 | 14.15536 | 18.97288 | 20.42712 | 45.66434 | 29.71386 | 29.43648 | 16.84704 | 16.49199 | 14.00504 | 5.210194 | 16.66622 | 22.03473 | 6.337987 | 21.82017 | 25.58242 | 24.37229 | 36.40505 |
| CUST_54651_PI429010886 | M107043 | 0.041264 | 24 | 0 | | | | | | 19.2011 | 8.743791 | 7.460722 | 9.642662 | 11.72403 | 7.653039 | 11.24123 | 7.026744 | 4.890483 | 9.061963 | 34.35393 | NA | 7.02756 | NA | 5.61067 | 7.05569 | 9.124 | 4.31702 | 6.552993 | 10.34523 | 8.937423 | 4.849769 | 11.93548 | 7.334339 | 12.63126 | 14.13977 | 21.13597 |
| CUST_27087_PI429010886 | N55426 | 0.041302 | 15 | 9 | 390362922 | PREDICTED: zinc finger protein Xfin-like [Strongyloc | | | 3.74E-26 | 60.72482 | 62.62665 | 84.11167 | 40.18446 | 59.47675 | 50.21157 | 32.00329 | 57.70169 | 57.70169 | 62.74739 | 52.06984 | 91.94088 | 49.998 | 68.03939 | 56.12021 | 65.06553 | 40.90354 | 52.67412 | 52.1905 | 53.75298 | 58.25978 | 66.67032 | 70.57215 | 62.83869 | 86.7497 | 66.61406 | 86.32314 |
| CUST_6245_PI429010886 | N19844 | 0.041326 | 18 | 3 | 321478487 | cyclin G-like protein [Daphnia pulex] | | | 9.29E-58 | 110.7189 | 91.55434 | 98.70636 | 102.2185 | 146.765 | 127.1757 | 159.4512 | 116.8603 | 82.01377 | 109.3542 | 159.2127 | 78.44959 | 82.49999 | 75.75933 | 69.20227 | 92.8298 | 196.9594 | 111.7908 | 136.4264 | 97.15481 | 108.6787 | 117.4158 | 135.6803 | 125.9322 | 145.861 | 94.24344 | 84.04395 |
| CUST_35853_PI429010886 | M13711 | 0.041351 | 15 | 9 | 4585366 | extracellular superoxide dismutase precursor [Pacif | Metabolic process | Oxidation-reduction process | 1.5E-43 | 823.2501 | 393.1602 | 2434.013 | 264.7951 | 329.9108 | 433.59 | 929.5412 | 2779.017 | 2740.182 | 3411.47 | 593.6703 | 728.8915 | 1623.972 | 4381.739 | 2155.605 | 580.6994 | 2163.813 | 491.7148 | 613.2651 | 2534.877 | 474.1045 | 3667.985 | 3661.505 | 537.8198 | 4955.555 | 1336.854 | 4769.116 |
| CUST_1142_PI429010886 | N8625 | 0.041379 | 24 | 18 | | | | | | 39.0395 | 45.58535 | 55.17531 | 36.0581 | 39.36991 | 16.63365 | 31.79024 | 38.00715 | 36.24375 | 48.53823 | 35.60906 | 44.27668 | 37.20514 | 49.42792 | 40.86391 | 57.21451 | 40.95911 | 45.49238 | 37.65143 | 46.69134 | 54.46306 | 63.00378 | 50.21619 | 36.31618 | 51.05482 | 26.66367 | 46.83015 |
| CUST_31665_PI429010886 | M2115 | 0.041423 | 21 | 15 | 322791260 | hypothetical protein SINV_02753 [Solenopsis invictı | | | 1.5E-104 | 75.2875 | 112.4452 | 81.5806 | 96.24145 | 70.24827 | 69.88371 | 64.71901 | 77.28747 | 82.55028 | 136.6377 | 76.353 | 91.66231 | 92.70294 | 78.41704 | 71.46149 | 128.8206 | 107.3346 | 86.78972 | 116.178 | 110.5811 | 79.0243 | 83.74259 | 90.36196 | 78.54463 | 79.06168 | 56.00982 | 87.67204 |
| CUST_52_PI429010886 | N229 | 0.041426 | 15 | 0 | | | | | | 404.1368 | 319.448 | 292.3195 | 306.4375 | 356.2894 | 385.8062 | 369.4169 | 254.0567 | 303.4935 | 288.5922 | 286.5693 | 310.6611 | 287.2755 | 280.7168 | 280.3633 | 372.8033 | 348.6809 | 439.0329 | 392.6279 | 306.8772 | 311.0329 | 304.5092 | 293.5299 | 323.6761 | 357.4809 | 269.5764 | 300.0603 |
| CUST_41230_PI429010886 | M57188 | 0.041478 | 18 | 12 | | | | | | 15.12127 | 26.81319 | 16.96167 | 16.5713 | 23.36932 | 11.18927 | 20.34468 | 18.03617 | 17.02082 | 23.15281 | 16.87735 | 20.76905 | 22.91473 | 24.94581 | 26.45407 | 20.47619 | 12.0262 | 17.14756 | 18.72625 | 37.77885 | 12.94169 | 16.83941 | 14.94965 | 7.05569 | 21.20179 | 12.20426 | 23.12912 |
| CUST_19571_PI429010886 | N41796 | 0.041478 | 18 | 12 | | | | | | 97.70002 | 135.2716 | 145.578 | 74.7945 | 69.34114 | 60.00971 | 109.5513 | 103.0243 | 94.55772 | 133.1101 | 78.24583 | 105.4208 | 102.5891 | 117.487 | 118.3632 | 124.8411 | 102.1011 | 88.15874 | 124.2426 | 84.45921 | 83.99654 | 70.5402 | 129.896 | 56.82909 | 100.1573 | 101.1982 | 111.1756 |
| CUST_48052_PI429010886 | M78704 | 0.041483 | 12 | 9 | 307715368 | arginine kinase 1 [Macrobrachium rosenbergii] | | | 5.32E-53 | 30.10233 | 10.74664 | 32.01033 | 35.06647 | 23.01368 | 27.27727 | 23.25228 | 27.26616 | 16.50096 | 37.99335 | 32.55631 | 45.12848 | 18.35945 | 32.41392 | 13.77121 | 28.81481 | 66.56405 | 46.83381 | 34.01997 | 31.68203 | 36.71638 | 32.47618 | 73.0191 | 33.83173 | 26.4818 | 18.80396 | 20.97008 |
| CUST_2907_PI429010886 | N13363 | 0.041494 | 24 | 18 | | | | | | 979.7027 | 1080.655 | 955.8025 | 928.0578 | 946.4318 | 959.9717 | 718.4589 | 752.9771 | 877.4226 | 996.654 | 1004.987 | 1066.553 | 852.9478 | 1165.934 | 1144.662 | 1468.172 | 949.8478 | 1097.209 | 1045.788 | 1366.468 | 1076.944 | 1325.963 | 831.3875 | 955.4895 | 1405.495 | 1112.549 | 1423.398 |
| CUST_238_PI429010886 | N2929 | 0.041494 | 24 | 18 | | | | | | 1708.455 | 17234.2 | 5888.291 | 4696.717 | 11942.94 | 2756.174 | 655.2712 | 951.6021 | 7560.283 | 12404.67 | 4467.857 | 18023.86 | 12502.58 | 9371.785 | 13226.16 | 15271.23 | 903.1657 | 11400.82 | 10200.91 | 18694.18 | 13907.98 | 24425.67 | 9193.951 | 10387.85 | 11601.88 | 7248.432 | 9271.524 |
| CUST_17398_PI429010886 | N37899 | 0.041494 | 24 | 15 | | | | | | 106.023 | 86.59518 | 125.7351 | 85.59472 | 88.39516 | 106.7278 | 90.13978 | 118.1115 | 111.2474 | 105.242 | 106.2171 | 99.74324 | 120.7928 | 136.6618 | 133.891 | 137.3935 | 138.7624 | 110.4288 | 121.7341 | 102.5401 | 108.1041 | 144.6688 | 93.27103 | 106.8828 | 132.2049 | 112.4908 | 121.7341 |
| CUST_22303_PI429010886 | N46780 | 0.041515 | 12 | 0 | | | | | | 37.6674 | 35.15108 | 52.40666 | 11.34588 | 9.054022 | 36.95956 | 16.11829 | 10.98742 | 16.43851 | 7.400394 | 15.7841 | 18.72053 | 22.77039 | 36.42394 | 11.92638 | 3.855158 | 47.23018 | 29.17612 | 10.68692 | 5.316105 | 8.5745 | 22.66107 | 51.21188 | NA | 30.07342 | 15.86933 | 47.83293 |
| CUST_31356_PI429010886 | M1338 | 0.041527 | 24 | 18 | 189234415 | PREDICTED: similar to AGAP004605-PA [Tribolium c | | | 1.38E-25 | 134.486 | 175.4567 | 109.0596 | 114.5611 | 113.1645 | 113.7768 | 78.00387 | 103.6762 | 109.0596 | 155.3371 | 118.9612 | 161.711 | 110.2887 | 129.5554 | 143.6875 | 180.8415 | 113.0678 | 135.7237 | 120.3232 | 163.5397 | 137.687 | 147.3218 | 130.4101 | 124.2142 | 144.9791 | 122.2181 | 151.6013 |
| CUST_33337_PI429010886 | M6642 | 0.04154 | 24 | 0 | | | | | | 84.58422 | 97.3486 | 85.72262 | 67.84362 | 69.77447 | 84.57304 | 66.67878 | 95.57811 | 74.51037 | 88.18553 | 62.1958 | 99.42759 | 44.39447 | 64.18045 | 58.51806 | 48.90211 | 86.02859 | 70.45058 | 81.52938 | 62.27945 | 57.77749 | 77.38462 | 80.12587 | 53.29328 | 82.78386 | 93.36286 | 76.42108 |

| Probe ID | ID | p-value | Period | Phase | EBI ID | Annotation | GO category | Go term | E-value | 0 | 0 | 0 | 3 | 3 | 3 | 6 | 6 | 6 | 9 | 9 | 9 | 12 | 12 | 12 | 15 | 15 | 15 | 18 | 18 | 18 | 21 | 21 | 21 | 24 | 24 | 24 |
|---|---|---|---|---|---|---|---|---|---|---|---|---|---|---|---|---|---|---|---|---|---|---|---|---|---|---|---|---|---|---|---|---|---|---|---|---|
| CUST_48351_PI429010886 | M79962 | 0.041552 | 21 | 12 | 405961122 | Inversin [Crassostrea gigas] | Developmental process | Embryo development | 1.44E-11 | 6.580137 | 4.487534 | 6.616037 | NA | 10.59996 | 3.884204 | NA | 4.253349 | 9.208223 | 16.43274 | 3.905122 | 12.73533 | 8.389601 | 30.34164 | 39.11677 | 6.43023 | 7.837273 | 11.63703 | 10.22197 | 7.541361 | 4.942301 | 8.219245 | NA | 3.200219 | 12.58978 | 3.924538 | 9.402874 |
| CUST_2736_PI429010886 | N13007 | 0.041556 | 12 | 0 | 512127157 | checkpoint kinase 1 [Daphnia pulex] | Protein metabolism | Cellular protein modification process | 1.3E-142 | 420.9194 | 634.2891 | 475.7608 | 320.3175 | 385.3789 | 217.159 | 370.5671 | 355.5079 | 412.4272 | 667.7123 | 413.4726 | 601.5918 | 449.7775 | 431.1968 | 451.7787 | 764.3328 | 335.1161 | 340.2645 | 382.5164 | 591.5595 | 374.1535 | 558.6606 | 402.9446 | 320.4556 | 688.1653 | 388.5645 | 598.2934 |
| CUST_21689_PI429010886 | N45669 | 0.041558 | 24 | 3 | | | | | | 64.66893 | 53.35103 | 80.32797 | 127.5818 | 49.48526 | 72.39117 | 62.08961 | 54.77183 | 43.34277 | 48.86699 | 48.83879 | 38.0269 | 50.31908 | 53.78964 | 82.24759 | 40.93552 | 51.25727 | 42.62942 | 36.72937 | 41.41686 | 44.67525 | 42.39566 | 48.5802 | 68.58333 | 48.29446 | 47.5011 | 38.1599 |
| CUST_7381_PI429010886 | N21458 | 0.041561 | 15 | 12 | | | | | | 61.3315 | 54.03548 | 66.12218 | 59.36711 | 65.54211 | 69.64081 | 48.13345 | 52.52687 | 64.68215 | 76.41163 | 65.56878 | 78.97339 | 81.09417 | 88.24844 | 67.21241 | 65.04228 | 68.29075 | 82.93626 | 61.1536 | 80.1769 | 58.6875 | 70.31729 | 67.12072 | 65.29553 | 74.22065 | 72.88198 | 74.25035 |
| CUST_8742_PI429010886 | N23505 | 0.041586 | 21 | 15 | 321470898 | hypothetical protein DAPPUDRAFT_49503 [Daphnia | Metabolic process | Oxidation-reduction process | 9.61E-63 | 648.0827 | 610.0761 | 589.2877 | 269.0643 | 358.3575 | 377.2506 | 479.9166 | 449.8886 | 500.7784 | 648.9401 | 453.0933 | 496.4081 | 722.2644 | 513.2283 | 498.3855 | 949.0578 | 215.6344 | 558.4057 | 701.3851 | 419.1452 | 555.136 | 579.5177 | 611.9605 | 406.6708 | 316.4275 | 371.9875 | 596.8255 |
| CUST_28265_PI429010886 | N57626 | 0.041594 | 24 | 15 | | | | | | 245.0284 | 221.06 | 233.2471 | 217.7483 | 224.6063 | 183.786 | 191.0756 | 202.5331 | 224.2244 | 229.1012 | 205.6642 | 264.8381 | 252.4954 | 284.6976 | 227.3812 | 267.4607 | 238.7009 | 251.6265 | 242.1214 | 298.1495 | 216.3391 | 286.8668 | 221.4718 | 211.6272 | 321.696 | 230.2231 | 231.3422 |
| CUST_54701_PI429010886 | M107218 | 0.041604 | 15 | 9 | 321477444 | hypothetical protein DAPPUDRAFT_305606 [Daphn | Signal transduction | Other | 2.4E-21 | 28.84183 | 45.84492 | 40.22053 | 36.78858 | 35.09563 | 28.49366 | 43.90259 | 34.40003 | 46.1565 | 51.15134 | 40.39881 | 50.37741 | 42.68003 | 39.74876 | 40.56586 | 47.47594 | 48.24061 | 37.79341 | 35.55553 | 35.64343 | 28.19099 | 47.59957 | 44.07419 | 36.85229 | 45.12848 | 34.18647 | 41.8585 |
| CUST_43559_PI429010886 | M63351 | 0.041604 | 15 | 0 | | | | | | 368.494 | 396.1913 | 300.9857 | 264.269 | 327.0942 | 272.638 | 204.4226 | 240.682 | 244.4939 | 340.057 | 328.5456 | 382.4385 | 251.4349 | 338.7294 | 256.52 | 414.4964 | 315.4574 | 277.0736 | 326.6284 | 350.793 | 265.4463 | 302.7759 | 287.6352 | 228.2235 | 400.1052 | 240.7867 | 310.6611 |
| CUST_34191_PI429010886 | M9012 | 0.041604 | 15 | 0 | | | | | | 626.2648 | 661.8313 | 561.9199 | 417.9411 | 532.0502 | 437.5902 | 318.6961 | 396.3895 | 420.025 | 591.157 | 476.472 | 584.5557 | 373.5295 | 539.4382 | 583.066 | 704.9925 | 471.0971 | 633.8371 | 423.5928 | 510.2851 | 529.3841 | 563.9632 | 430.1259 | 545.3681 | 627.6184 | 465.3498 | 577.9827 |
| CUST_26233_PI429010886 | N53889 | 0.041604 | 15 | 9 | | | | | | 13.39116 | 14.82167 | 28.09705 | 12.43401 | 18.87721 | 15.58064 | 10.01939 | 17.64058 | 18.02985 | 26.37834 | 16.0514 | 39.38447 | 18.8495 | 16.04256 | 44.20781 | 19.01271 | 13.99239 | 14.89947 | 12.72627 | 15.5384 | 18.61977 | 17.83632 | 10.14992 | 21.99795 | 19.84931 | 252.3062 | 41.6172 |
| CUST_17291_PI429010886 | N37721 | 0.041605 | 24 | 0 | | | | | | 140.7563 | 111.6499 | 124.2142 | 117.847 | 104.3119 | 131.3133 | 93.13261 | 70.32547 | 97.1202 | 114.7719 | 83.74259 | 98.69728 | 71.16635 | 112.7504 | 109.5408 | 123.7165 | 120.4315 | 110.192 | 153.1717 | 91.6409 | 113.4862 | 117.1213 | 66.48727 | 83.87586 | 123.2283 | 148.5237 | 160.804 |
| CUST_7930_PI429010886 | N22287 | 0.041609 | 24 | 18 | | | | | | 131.2193 | 117.5843 | 149.4128 | 129.3158 | 129.1009 | 144.7965 | 123.0707 | 99.68173 | 79.77146 | 136.3926 | 136.9776 | 135.3575 | 74.59866 | 172.0048 | 126.1763 | 133.7242 | 148.0447 | 195.9288 | 181.4093 | 177.456 | 138.0366 | 141.6349 | 117.0641 | 129.0136 | 187.6138 | 102.6107 | 152.9488 |
| CUST_44542_PI429010886 | M66283 | 0.041631 | 21 | 9 | | | | | | 21.30503 | 94.75367 | 51.75281 | 3756.632 | 1110.941 | 3156.693 | 156.2928 | 40.79309 | 150.0374 | 837.729 | 191.6952 | 732.5018 | 778.415 | 45.52189 | 330.9853 | 59.35924 | 781.4539 | 588.4339 | 27.86411 | 62.66235 | 2270.606 | 29.21516 | 58.55654 | 25.18232 | 99.26813 | 53.23845 | 50.16596 |
| CUST_26219_PI429010886 | N53867 | 0.041631 | 24 | 21 | | | | | | 72.58192 | 68.02926 | 63.37918 | 51.70242 | 59.43662 | 47.74853 | 51.61587 | 59.0851 | 60.18184 | 58.95549 | 51.10313 | 68.85742 | 69.35049 | 50.41387 | 71.2192 | 80.02167 | 59.64714 | 66.10733 | 58.66467 | 56.62309 | 75.1394 | 71.77101 | 74.84295 | 72.89846 | 93.92221 | 62.52529 | 81.7755 |
| CUST_54296_PI429010886 | M105473 | 0.041633 | 18 | 0 | | | | | | 98.37238 | NA | 191.7619 | 45.61505 | 96.28021 | 157.2097 | 155.2181 | 129.5889 | 43.76068 | 64.4873 | 68.19793 | NA | 42.36335 | 52.14117 | 35.32612 | NA | 77.82271 | 41.65038 | 115.8195 | NA | 186.1103 | NA | 69.68123 | 84.73278 | 5.704319 | 126.8628 | 24.87256 |
| CUST_41250_PI429010886 | M57245 | 0.041648 | 24 | 21 | 118136261 | beta-actin [Penaeus monodon] | | | 8.86E-30 | 23.17992 | 18.18164 | 12.70382 | 12.6398 | 26.32456 | 13.75242 | 11.38971 | 8.706462 | 5.142003 | 19.00448 | 16.39244 | 15.79313 | 11.48964 | 17.61145 | 6.927136 | 13.74381 | 24.10237 | 10.66902 | 12.20297 | 17.12281 | 7.396068 | 22.12202 | 25.28773 | 20.25895 | 13.31508 | NA | 14.75975 |
| CUST_40103_PI429010886 | M50240 | 0.041719 | 24 | 15 | | | | | | 139.545 | 162.0779 | 145.5389 | 115.5725 | 128.2363 | 143.4699 | 108.4515 | 108.4314 | 97.3486 | 162.8741 | 155.7184 | 138.969 | 100.2315 | 164.6258 | 143.2947 | 195.9916 | 143.4947 | 180.4919 | 143.406 | 166.1459 | 115.1182 | 163.791 | 132.8952 | 120.2323 | 209.83 | 118.233 | 197.2642 |
| CUST_41734_PI429010886 | M58421 | 0.041762 | 24 | 18 | 543722690 | PREDICTED: serine/threonine-protein phosphatase | | | 2.48E-85 | 8.612092 | 8.045043 | 23.42758 | NA | 10.91708 | 3.628431 | NA | NA | 6.067975 | 7.711168 | 8.031655 | 8.259244 | 4.819923 | 14.58718 | 7.850591 | 23.57785 | NA | NA | 27.17883 | 22.02561 | 13.06014 | 17.22674 | 18.22532 | NA | 5.446361 | 8.917899 | 32.73103 |
| CUST_11502_PI429010886 | N27918 | 0.041783 | 24 | 15 | | | | | | 18.46255 | 27.384 | 25.93179 | 11.16751 | 27.35753 | 9.400307 | 23.33499 | 19.11878 | 15.26556 | 34.67355 | 29.88865 | 29.21265 | 15.01997 | 11.05595 | 24.64992 | 36.45455 | 26.10687 | 62.07681 | 19.48765 | 19.3376 | 43.39642 | 34.46849 | 34.73965 | 38.6196 | 26.13131 | 25.352 | 16.67239 |
| CUST_46607_PI429010886 | M73145 | 0.041819 | 24 | 0 | | | | | | 6.689971 | 10.38291 | 18.87895 | NA | 8.24404 | 7.03873 | NA | 8.576964 | 5.137936 | 10.28962 | 6.188539 | NA | 6.975434 | 8.286303 | 6.531816 | NA | 7.091579 | 8.953175 | 10.22575 | 12.57101 | 7.313769 | 10.92086 | 6.563098 | NA | 13.18143 | 14.42559 | 17.85258 |
| CUST_15391_PI429010886 | N34506 | 0.041824 | 18 | 3 | 321474731 | hypothetical protein DAPPUDRAFT_309035 [Daphn | | | 5.99E-22 | 19.09504 | 25.52881 | 15.66989 | 43.74083 | 106.3173 | 65.4438 | 82.75397 | 31.25517 | 18.80396 | 15.62744 | 148.1764 | 17.59799 | 18.94364 | 11.99992 | 7.968722 | 18.04537 | 74.82753 | 33.12696 | 50.04478 | 14.14808 | 44.50661 | 15.99975 | 56.37263 | 64.22812 | 27.67304 | 14.49726 | 23.32 |
| CUST_23502_PI429010886 | N48941 | 0.041824 | 15 | 9 | 242007895 | Huntingtin-associated protein-interacting protein, p | Metabolic process | Cellular amino acid metabolic process | 3.57E-33 | 336.5091 | 324.6687 | 326.4884 | 273.0635 | 313.3535 | 280.3132 | 317.7577 | 305.3843 | 342.746 | 409.385 | 319.3199 | 397.2225 | 320.6371 | 487.486 | 382.3424 | 406.3292 | 372.8945 | 340.4766 | 368.614 | 391.6722 | 302.1561 | 395.3575 | 386.2115 | 270.6659 | 431.462 | 350.793 | 471.8606 |
| CUST_11017_PI429010886 | N27133 | 0.041835 | 21 | 3 | | | | | | 154.4215 | 321.696 | 147.5068 | 344.0991 | 193.0913 | 206.3839 | 138.2244 | 132.1335 | 153.7558 | 154.8889 | 99.32217 | 201.1997 | 107.7242 | 186.4327 | 179.9875 | 167.4333 | 78.94301 | 156.7999 | 111.625 | 135.5426 | 203.2288 | 193.8618 | 186.7835 | 137.5108 | 257.6478 | 300.3777 | 159.4333 |
| CUST_21259_PI429010886 | N44905 | 0.041844 | 18 | 12 | | | | | | 23.44768 | 32.79034 | 31.69618 | 17.55108 | 15.55047 | 16.36632 | 35.58457 | 20.88813 | 14.61247 | 21.55161 | 13.24259 | 32.14855 | 23.62378 | 32.47256 | 33.26161 | 18.08249 | 14.16465 | NA | 13.64945 | 33.24397 | 9.530867 | 10.03982 | 6.172262 | 16.77093 | 25.81867 | 23.4832 | 31.47236 |
| CUST_21768_PI429010886 | N45817 | 0.041853 | 18 | 9 | | | | | | 14.98214 | 23.16574 | 9.425811 | 16.04554 | 16.89973 | 6.176883 | 6.799702 | 21.07701 | 21.68681 | 22.02378 | 29.15169 | 22.69834 | 20.50947 | 18.29071 | 22.04079 | 16.96368 | 17.47741 | 15.18207 | 12.82706 | 36.41327 | 24.42322 | 21.69445 | 5.82395 | 19.12864 | 22.72441 | 22.64343 | 27.16436 |
| CUST_27634_PI429010886 | N56412 | 0.041867 | 21 | 18 | | | | | | 13.32777 | 18.22141 | 9.72432 | 17.76828 | 7.255725 | 7.234602 | 5.951528 | 4.952477 | NA | 15.0972 | 11.2994 | 3.703251 | 12.50339 | 3.957334 | 8.078376 | NA | 10.42464 | NA | 10.68382 | 20.63988 | NA | 10.71996 | 17.44175 | NA | 7.641633 | 8.978228 | NA |
| CUST_54062_PI429010886 | M104332 | 0.041881 | 21 | 0 | | | | | | 19.02839 | 13.2528 | 18.54361 | 9.848126 | 5.288214 | 12.49458 | NA | 8.371508 | 11.07586 | 6.675568 | 4.231651 | 42.5506 | 5.986055 | 5.571287 | 12.0262 | 11.74368 | 7.374786 | 15.5705 | 7.609769 | 5.76332 | 5.277704 | 14.20484 | 45.38957 | 10.6528 | 13.57374 | 11.65616 | 16.80263 |
| CUST_9924_PI429010886 | N25417 | 0.04189 | 15 | 12 | | | | | | 149.7694 | 176.3863 | 187.6525 | 144.0112 | 130.0042 | 134.2151 | 131.8938 | 143.0859 | 167.8836 | 243.166 | 145.578 | 448.7865 | 150.242 | 202.4041 | 207.2391 | 175.6726 | 128.8708 | 198.2178 | 122.1728 | 177.2148 | 164.0384 | 159.1708 | 168.8426 | 154.0684 | 166.6809 | 167.5472 | 192.7647 |
| CUST_39420_PI429010886 | M39149 | 0.041927 | 15 | 3 | 321457991 | hypothetical protein DAPPUDRAFT_301173 [Daphn | | | 1.98E-18 | 122.2181 | 97.29292 | 73.84429 | 361.6235 | 244.775 | 1050.96 | 510.0071 | 121.8737 | 144.3781 | NA | 334.4771 | NA | 116.178 | 19.85636 | 90.3266 | 34.17683 | 764.5382 | 625.9634 | 309.5934 | NA | 726.7504 | 56.42615 | 500.2189 | 775.4257 | 22.62086 | 302.8941 | 4.709883 |
| CUST_9949_PI429010886 | N25455 | 0.041954 | 18 | 3 | | | | | | 34.24386 | 25.24454 | 15.93573 | 45.39483 | 34.39152 | 35.53394 | 35.35004 | 16.31198 | 17.78619 | 35.6179 | 26.18577 | 22.5025 | 24.8275 | 18.36555 | 19.65043 | 32.41392 | 29.05729 | 31.59119 | 33.56673 | 26.80447 | 33.48083 | 41.27857 | 22.98207 | 33.86813 | 33.58412 | 22.68707 | 19.59409 |
| CUST_35778_PI429010886 | M13490 | 0.041959 | 21 | 0 | | | | | | 202.8821 | 187.2169 | 154.1768 | 150.6325 | 192.7007 | 140.7156 | 146.9588 | 140.2062 | 158.2045 | 161.355 | 130.0221 | 153.8044 | 110.5098 | 124.7724 | 136.8476 | 229.0599 | 129.5373 | 163.7445 | 165.5033 | 171.451 | 120.3338 | 235.6197 | 171.427 | 134.2642 | 183.7672 | 189.9733 | 128.7498 |
| CUST_9649_PI429010886 | N24995 | 0.041959 | 21 | 12 | | | | | | 47.95704 | 71.41994 | 56.33375 | 81.03965 | 118.6493 | 91.41127 | 138.8541 | 91.85305 | 84.1647 | 228.7451 | 28.61965 | 108.6974 | 117.8313 | 201.7309 | 134.7073 | 90.13978 | 87.35248 | 94.2195 | 177.0624 | 7.182647 | 112.0225 | 109.9447 | 56.91814 | 55.01668 | 105.0313 | 38.07238 | 126.8192 |
| CUST_11186_PI429010886 | N27405 | 0.041977 | 21 | 0 | 156544105 | PREDICTED: uracil phosphoribosyltransferase homo | | | 3.88E-52 | 81.45472 | 77.74288 | 63.01798 | 54.80919 | 58.73387 | 70.53195 | 101.8998 | 52.063 | 53.78486 | 53.18376 | 69.17707 | 59.19994 | 49.61333 | 59.03159 | 70.99743 | 68.1054 | 78.00387 | 65.39564 | 65.43441 | 68.25442 | 59.30743 | 77.64007 | 79.09571 | 101.0858 | 56.24014 | 68.39623 | 45.51068 |
| CUST_761_PI429010886 | N7195 | 0.042002 | 24 | 3 | | | | | | 301.4214 | 98.49703 | 42.80091 | 271.2223 | 48.48483 | 70.03699 | 244.4297 | 57.23179 | 237.166 | 34.28772 | 35.51693 | 38.153 | 191.5641 | 46.54214 | 113.9135 | 60.02433 | 35.98369 | 42.22821 | 35.15442 | 39.52535 | 37.11196 | 46.22113 | 47.65494 | 115.9694 | 54.83755 | 63.95336 | 42.88135 |
| CUST_53812_PI429010886 | M103299 | 0.042011 | 15 | 0 | | | | | | 34.25171 | 37.88014 | 27.33167 | 31.49086 | 24.65512 | 35.88462 | 25.75668 | 24.19734 | 27.39567 | 18.88215 | 13.2803 | 24.92567 | 19.80974 | 13.36717 | 23.04237 | 33.71856 | 24.52447 | 38.44451 | 24.64442 | 16.65858 | 28.58102 | 20.53839 | 30.27835 | 22.24561 | 29.21058 | 22.14979 | 26.96538 |
| CUST_48646_PI429010886 | M81448 | 0.042015 | 24 | 3 | | | | | | 16.17624 | 33.51452 | 25.29581 | 24.91507 | 31.62724 | 27.95174 | 15.89674 | 19.32542 | 8.238355 | 43.04692 | 18.05758 | 21.38369 | 19.98576 | 12.18876 | 29.27092 | 20.89343 | 15.53405 | 21.94699 | 10.35023 | 6.760169 | 6.708563 | 22.43297 | 23.28835 | 17.16134 | 21.44879 | 19.19726 | 11.87577 |
| CUST_47177_PI429010886 | M75053 | 0.042018 | 18 | 12 | | | | | | 66.25397 | 72.3404 | 71.81025 | 76.52417 | 71.87825 | 75.06598 | 85.31136 | 73.68557 | 75.1888 | 95.86282 | 61.11567 | 61.03108 | 80.21909 | 94.37733 | 125.9169 | 93.17664 | 108.7377 | 119.8572 | 81.42045 | 57.64388 | 52.79712 | 78.61543 | 50.33169 | 72.92837 | 147.9921 | 74.4613 | 90.92371 |
| CUST_11834_PI429010886 | N28489 | 0.042028 | 24 | 0 | 291226812 | PREDICTED: F-box protein 31-like [Saccoglossus kow | Cellular Process | Cell cycle | 4.25E-19 | 35.13017 | 41.68991 | 52.0001 | 38.51713 | 30.30557 | 32.83588 | 18.84309 | 45.7065 | 41.65544 | 43.14736 | 22.11463 | 37.59059 | 20.55297 | 25.93469 | 35.96223 | 30.45321 | 33.66812 | 26.92163 | 46.11558 | 26.74645 | 36.42394 | 32.88602 | 35.17156 | 50.65756 | 37.19805 | 96.29479 | 38.99636 |
| CUST_35709_PI429010886 | M13274 | 0.042034 | 12 | 0 | | | | | | 311.6128 | 442.2873 | 425.4184 | 110.4575 | 146.1343 | 70.68641 | 158.6127 | 188.3767 | 232.5216 | 519.4558 | 245.6075 | 333.1788 | 293.1844 | 302.7759 | 245.2785 | 489.1062 | 169.0201 | 234.3391 | 257.3603 | 308.2184 | 217.7483 | 377.8878 | 309.2864 | 125.2116 | 443.8496 | 251.0141 | 331.4917 |
| CUST_38111_PI429010886 | M23412 | 0.042034 | 12 | 3 | | | | | | 171.7601 | 91.42252 | 31.71523 | 671.3234 | 550.0892 | 1351.741 | 720.7013 | 302.566 | 162.9163 | 25.04907 | 378.7944 | 20.74695 | 163.7445 | 50.9755 | 111.7621 | 83.83314 | 1166.392 | 945.8187 | 194.777 | 31.68743 | 689.3131 | 93.92221 | 539.2831 | 1013.272 | 27.09043 | 352.2533 | 16.3681 |
| CUST_32974_PI429010886 | M5604 | 0.042034 | 12 | 3 | | | Metabolic process | Carbohydrate metabolic process | | 1740.855 | 724.0591 | 179.678 | 3934.354 | 4719.422 | 6903.073 | 4944.792 | 2041.736 | 1264.445 | 95.20545 | 5903.55 | 111.637 | 1690.584 | 142.6158 | 1070.988 | 456.7992 | 8378.604 | 5663.977 | 3624.616 | 228.3928 | 4135.156 | 338.2007 | 2794.005 | 5080.996 | 114.9376 | 3213.851 | 85.54854 |
| CUST_17541_PI429010886 | N38147 | 0.042034 | 12 | 3 | 345495435 | PREDICTED: protein O-mannosyl-transferase 2-like [ | Metabolic process | Carbohydrate metabolic process | 6.52E-32 | 21.22623 | 15.00845 | 11.01084 | 40.62506 | 17.83632 | 68.81509 | 22.23809 | 35.25245 | 12.41093 | 12.26032 | 24.68274 | 9.886641 | 11.74904 | 8.765923 | 14.54005 | 12.72475 | 60.78952 | 51.4111 | 18.58338 | 15.60562 | 16.13381 | 13.2704 | 34.69687 | 75.22006 | 8.357942 | 34.65808 | 8.585981 |
| CUST_48444_PI429010886 | M80356 | 0.042045 | 24 | 0 | 270015064 | hypothetical protein TcasGA2_TC014226 [Tribolium | | | 1.03E-19 | 21.14577 | 13.60767 | 17.4527 | 11.80651 | 22.28394 | 11.64935 | 20.93627 | 15.60189 | 14.61619 | 11.62851 | 13.56887 | 8.690781 | 13.80995 | 9.952171 | 10.76549 | 12.21534 | 21.2878 | 12.88708 | 26.46256 | 12.89633 | 16.08792 | 20.55453 | 14.09025 | 18.66273 | 33.20597 | 9.299202 | 21.55161 |
| CUST_56828_PI429010886 | M114725 | 0.042082 | 15 | 9 | | | | | | 118.6228 | 149.8707 | 179.2308 | 104.7962 | 135.8973 | 104.302 | 112.8702 | 108.7588 | 173.4432 | 153.961 | 146.4231 | 183.3264 | 150.78 | 225.6208 | 145.5389 | 146.882 | 118.6617 | 145.1427 | 151.1026 | 149.0167 | 117.7597 | 176.8268 | 125.0127 | 92.37125 | 234.3391 | 147.2999 | 187.7096 |
| CUST_4384_PI429010886 | N16643 | 0.042082 | 15 | 9 | | | | | | 100.2916 | 98.21029 | 111.3415 | 44.92995 | 56.00276 | 57.10862 | 73.48596 | 81.42045 | 46.27475 | 208.0738 | 99.09557 | 65.39564 | 86.37756 | 65.21686 | 58.12612 | 57.48318 | 129.4911 | 50.58305 | 56.44254 | 64.57467 | 48.10487 | 106.7633 | 65.01299 | 63.43062 | 65.32582 | 70.61832 | 82.64391 |
| CUST_20401_PI429010886 | N43376 | 0.042082 | 15 | 9 | | | | | | 94.06166 | 78.93037 | 69.78353 | 74.11739 | 54.87756 | 53.57981 | 44.1334 | 98.97177 | 84.15456 | 123.6359 | 88.34755 | 92.13028 | 71.74241 | 100.4445 | 89.16253 | 86.4151 | 70.4935 | 72.93993 | 54.3948 | 74.86136 | 73.96327 | 120.5371 | 94.76564 | 50.2407 | 163.4728 | 74.58008 | 80.34778 |
| CUST_16773_PI429010886 | N36846 | 0.042112 | 24 | 21 | | | | | | 46.19956 | 46.20996 | 50.88285 | 36.47986 | 31.47929 | 39.414 | 30.08229 | 29.73209 | 41.72002 | 52.20238 | 32.84559 | 39.83487 | 35.69567 | 52.58293 | 45.24787 | 53.55422 | 39.99701 | 41.28276 | 49.64767 | 43.84173 | 48.40257 | 46.12593 | 53.16758 | 42.69298 | 59.7424 | 34.79371 | 63.83268 |
| CUST_42664_PI429010886 | M60899 | 0.042116 | 21 | 6 | 328899769 | ribosomal protein S19 [Procambarus clarkii] | Protein metabolism | Ribosome biogenesis | 3.37E-31 | 25.73562 | 21.56963 | 19.75556 | 32.58533 | 23.19911 | 25.04089 | 44.91724 | 52.48792 | 38.37992 | 37.02998 | 20.99955 | 41.23729 | 26.29806 | 25.85184 | 25.38172 | 5.400362 | 27.85465 | 20.52725 | 41.51371 | 42.97599 | 20.23937 | 21.08444 | 34.90742 | 24.33679 | 35.15805 | 25.8104 | 22.89389 |
| CUST_28267_PI429010886 | N57634 | 0.042135 | 18 | 9 | | | | | | 10.00617 | 4.39723 | 9.384386 | 6.455442 | 10.48086 | NA | 10.0999 | 6.781376 | 6.376695 | 16.1997 | 12.6398 | 12.39128 | 11.4448 | 16.67559 | 8.421448 | 16.63365 | 9.939882 | NA | 12.15775 | 7.620705 | NA | 7.610851 | 7.513993 | NA | 10.05076 | 11.46694 | 7.426569 |
| CUST_14113_PI429010886 | N32346 | 0.042145 | 21 | 18 | | | | | | 199.7111 | 175.0831 | 239.6059 | 137.649 | 170.0428 | 155.5901 | 165.3668 | 206.3065 | 167.321 | 272.1441 | 194.4273 | 159.878 | 179.6526 | 189.7693 | 184.4849 | 232.766 | 192.442 | 158.6525 | 235.2753 | 218.5606 | 198.9264 | 236.0508 | 191.4276 | 156.4282 | 193.2364 | 135.6641 | 176.5718 |
| CUST_4155_PI429010886 | N16123 | 0.042152 | 24 | 15 | 390362924 | PREDICTED: zinc finger protein 84-like [Strongylocer | | | 3.5E-15 | 286.0749 | 397.0596 | 306.479 | 257.8206 | 290.8448 | 234.9764 | 246.8808 | 208.138 | 339.4134 | 511.5434 | 352.327 | 535.2858 | 247.8273 | 343.7569 | 358.7967 | 684.8725 | 341.3294 | 376.5573 | 312.7398 | 417.7156 | 313.7524 | 502.4781 | 320.8355 | 264.5273 | 492.9704 | 280.5129 | 360.4943 |
| CUST_6395_PI429010886 | N20047 | 0.042152 | 24 | 18 | 154416763 | viral A-type inclusion protein [Trichomonas vaginali | | | 2.89E-13 | 9.855895 | 14.80676 | 13.52681 | 15.93149 | 10.18458 | 6.031977 | 8.652112 | 6.930953 | 10.46847 | 16.10523 | 12.22036 | 19.08628 | 21.74923 | 17.93968 | 20.52725 | 23.1883 | 12.71046 | 13.68168 | 14.25924 | 17.83632 | 25.45721 | 8.446645 | 19.42289 | 23.49823 | 32.3787 | 7.860635 | 16.39244 |
| CUST_30286_PI429010886 | N61555 | 0.042152 | 21 | 9 | | | | | | 19.61371 | 16.15147 | 19.09504 | 15.24413 | 17.62133 | 24.32155 | 31.33456 | 23.72867 | 22.69285 | 22.64587 | 25.31087 | 32.26938 | 18.73831 | 27.6707 | 11.32033 | 17.13072 | 34.52311 | 28.36368 | 24.71374 | 13.99388 | 24.56345 | 13.50761 | 22.13451 | 9.490926 | 29.84652 | 20.53839 | 14.62285 |
| CUST_8895_PI429010886 | N23761 | 0.042166 | 24 | 0 | 91082923 | PREDICTED: similar to transglutaminase [Tribolium | | | 2.72E-83 | 20.25455 | NA | 21.81525 | 17.61145 | 9.001207 | 10.99683 | 8.633134 | 10.21387 | 12.27121 | 9.928249 | 10.6935 | 10.41586 | 11.78894 | 25.51449 | 12.92974 | 5.436916 | 8.663836 | 3.646152 | 7.162712 | 9.722947 | 12.84068 | 8.958591 | NA | 9.659531 | 27.53501 | 14.41764 | 9.808079 |
| CUST_42010_PI429010886 | M59143 | 0.042181 | 18 | 9 | | | | | | 137.5651 | 161.6619 | 161.9364 | 118.2155 | 134.244 | 128.3615 | 116.7432 | 112.3183 | 150.5497 | 218.6882 | 141.5508 | 189.3905 | 133.5211 | 178.3785 | 166.7787 | 212.773 | 146.622 | 147.0019 | 129.6855 | 178.0648 | 124.2579 | 153.5542 | 137.8924 | 107.543 | 188.7518 | 132.9126 | 163.2888 |
| CUST_44499_PI429010886 | M66146 | 0.042184 | 21 | 3 | | | | | | 7.100449 | 5.241968 | 8.949545 | 11.30649 | 23.54965 | 16.19147 | 9.751886 | 45.9505 | 10.5712 | 6.451538 | 3.916691 | 8.064111 | NA | 6.829705 | NA | 6.604961 | 5.243296 | NA | NA | 7.357022 | 21.2878 | NA | 20.3649 | 5.142003 | 13.97389 | 16.08792 | NA |
| CUST_48687_PI429010886 | M81637 | 0.042195 | 18 | 6 | | | | | | 121.2986 | 137.6985 | 181.5403 | 193.3347 | 179.4697 | 274.1744 | 194.6574 | 142.4711 | 231.5437 | 139.2832 | 232.9174 | 334.8266 | 144.1199 | 248.9182 | 209.0873 | 195.2684 | 163.2245 | 298.3902 | 147.7053 | 140.2062 | 179.3477 | 172.6882 | 211.0236 | 255.7006 | 239.3501 | 425.0392 | 352.4518 |
| CUST_40583_PI429010886 | M55538 | 0.0422 | 24 | 3 | | | | | | 5.550178 | 19.67357 | 14.36186 | 22.07292 | 23.48892 | 18.49894 | 20.22452 | 25.9425 | 8.884013 | 9.373204 | 22.79184 | 11.93292 | 6.591511 | 14.88541 | 12.39128 | 8.788925 | 14.06382 | 11.36478 | 10.5296 | 13.96931 | 19.11453 | 18.48661 | 14.95261 | 11.86309 | 16.98127 | 12.68111 | 16.24458 |
| CUST_45396_PI429010886 | M68982 | 0.042201 | 15 | 9 | 241678636 | conserved hypothetical protein [Ixodes scapularis] > | | | 1.76E-48 | 12.99648 | 24.44247 | 13.70505 | 14.90147 | 16.11943 | NA | 11.5275 | 20.60423 | 22.29224 | 26.64522 | 26.3191 | 31.51141 | 18.50987 | 27.36801 | 17.27797 | 38.12809 | 9.071169 | 10.70821 | 15.7734 | 23.89866 | 30.78076 | 20.15665 | 22.54108 | 16.03304 | 28.9143 | 22.85484 | 36.8462 |
| CUST_52328_PI429010886 | M97075 | 0.042213 | 21 | 0 | | | | | | 30.54342 | 35.31845 | 19.56884 | 15.48667 | 18.20163 | 5.558018 | 25.5315 | 58.71363 | 14.66408 | 20.42397 | 12.04602 | NA | 22.92529 | 6.378621 | 14.43213 | 24.80361 | 36.61393 | 14.64247 | 27.14824 | 23.91586 | 18.12277 | 31.37421 | 52.95164 | 25.02448 | 25.83997 | 8.727631 | 26.30031 |
| CUST_27923_PI429010886 | N56949 | 0.042216 | 21 | 9 | 410509308 | myosin heavy chain type 2 [Penaeus monodon] | | | 9.41E-21 | 21.99795 | 32.32519 | 34.26964 | 25.63785 | 26.35743 | 32.78743 | 25.17015 | 54.01314 | 56.04356 | 35.36241 | 48.94831 | 73.62026 | 27.30432 | 25.26092 | 41.94521 | 26.23144 | 13.93866 | 23.18582 | 15.08925 | 56.56684 | 37.41038 | 18.2618 | 23.82095 | 28.5055 | 28.33335 | 47.94376 | 46.84888 |
| CUST_54909_PI429010886 | M108140 | 0.042218 | 24 | 0 | | | | | | 841.0533 | 889.7966 | 889.0555 | 887.1414 | 838.6949 | 784.4175 | 834.3363 | 744.5402 | 781.185 | 1003.971 | 730.2123 | 754.3579 | 760.7539 | 877.6784 | 854.2242 | 1068.926 | 796.2928 | 873.1071 | 847.0661 | 841.7695 | 837.1072 | 1064.697 | 894.0864 | 820.2371 | 1181.87 | 949.8478 | 1038.033 |
| CUST_8080_PI429010886 | N22512 | 0.042222 | 15 | 3 | | | | | | 22.53424 | 10.89022 | 9.350056 | 18.78315 | 20.16612 | 41.33159 | 18.56074 | 12.63126 | 7.300647 | 12.72931 | 12.92033 | NA | 5.826839 | 6.806239 | 10.60724 | 7.666723 | 28.3805 | 34.10794 | 8.733788 | 14.61247 | 21.93061 | 9.119784 | 26.44892 | 37.54698 | 7.284707 | 7.906354 | 10.36359 |
| CUST_33908_PI429010886 | M8210 | 0.042272 | 15 | 12 | | | | | | 213.1848 | 237.679 | 232.5572 | 216.807 | 187.7825 | 201.2707 | 174.2465 | 187.4158 | 197.0951 | 268.2743 | 189.5057 | 234.0614 | 183.1526 | 249.8541 | 257.2859 | 238.1333 | 226.4554 | 267.7928 | 205.6958 | 207.7149 | 204.3252 | 266.346 | 206.6019 | 181.2996 | 300.5238 | 219.8055 | 242.9704 |
| CUST_444_PI429010886 | N5213 | 0.042272 | 15 | 12 | | | Protein metabolism | Ribosome biogenesis | | 224.8249 | 195.8687 | 202.7268 | 168.8426 | 132.7894 | 152.675 | 198.3971 | 172.0048 | 209.9313 | 225.3596 | 148.65 | 208.7066 | 222.1754 | 157.0244 | 185.8695 | 192.6772 | 211.951 | 187.4158 | 151.5629 | 123.9272 | 123.9463 | 198.6919 | 157.1805 | 137.5853 | 227.3812 | 160.4594 | 184.2011 |
| CUST_55414_PI429010886 | M109998 | 0.042292 | 24 | 21 | | | | | | 49.72682 | 53.22735 | 71.46757 | 51.53321 | 59.50688 | 85.81376 | 49.24106 | 56.96512 | 46.64901 | 73.73041 | 63.83268 | 62.6571 | 40.33441 | 53.12043 | 39.57761 | 50.8686 | 87.49111 | 65.36594 | 70.45058 | 62.39032 | 67.97289 | 67.39787 | 73.71157 | 85.73944 | 66.99196 | 64.0654 | 57.90878 |
| CUST_14193_PI429010886 | N32465 | 0.042292 | 24 | 0 | 196006105 | hypothetical protein TRIADDRAFT_56539 [Trichopla | Metabolic process | Carbohydrate metabolic process | 8.34E-16 | 74.43452 | 57.59047 | 56.95101 | 42.11724 | 60.10166 | 54.66133 | 59.02601 | 48.76564 | 52.72315 | 70.83788 | 38.91166 | 66.18886 | 50.8634 | 43.24186 | 43.59595 | 62.54347 | 52.24124 | 52.35268 | 75.51588 | 45.05092 | 51.38396 | 67.09564 | 60.10166 | 67.3827 | 67.31728 | 58.75806 | 82.929 |
| CUST_10869_PI429010886 | N26919 | 0.04239 | 21 | 18 | | | | | | 19.58619 | 19.3883 | 19.02231 | 11.20164 | 14.57533 | 18.14494 | 10.20401 | 9.098614 | 10.02837 | 21.86356 | 23.24391 | 17.05906 | 17.52814 | 11.38971 | 16.35532 | 13.8468 | 11.17512 | 23.06016 | 13.77293 | 27.29319 | 19.91992 | 18.58498 | 24.40411 | 12.72782 | 32.64929 | 10.58768 | 12.00275 |
| CUST_13507_PI429010886 | N31303 | 0.042394 | 15 | 0 | | | | | | 59.57796 | 60.91532 | 61.59565 | 43.65248 | 69.17707 | 43.40065 | 71.87199 | 40.79687 | 44.97088 | 45.55959 | 64.36368 | 45.02093 | 55.66641 | 60.97776 | 36.75622 | 77.32264 | 60.73689 | 73.26779 | 80.46995 | 40.95098 | 58.15995 | 7.759101 | 46.70127 | 29.2332 | 42.82196 | 13.99239 | 46.31285 |
| CUST_2608_PI429010886 | N12749 | 0.042427 | 15 | 0 | | | Cellular component organization | Cytoskeleton organization | | 7282.171 | 5712.144 | 6717.481 | 4553.274 | 5035.54 | 5023.801 | 6100.209 | 6441.669 | 5609.183 | 6108.092 | 3925.917 | 3161.551 | 6217.899 | 6065.211 | 5613.6 | 6587.101 | 6992.837 | 5119.45 | 5498.252 | 3435.959 | 3352.836 | 4941.562 | 4128.792 | 4361.48 | 5060.995 | 2747.282 | 4451.275 |
| CUST_50537_PI429010886 | M90174 | 0.042431 | 18 | 12 | | | | | | 1290.488 | 1895.191 | 1564.24 | 852.6077 | 871.62 | 767.811 | 1153.433 | 1570.987 | 1733.695 | 1187.991 | 1284.158 | 1557.99 | 1052.869 | 1977.87 | 1627.141 | 1649.441 | 947.8741 | 1185.878 | 1058.251 | 1099.991 | 1484.716 | 1439.161 | 1070.582 | 741.589 | 1187.094 | 1127.217 | 1609.757 |
| CUST_14271_PI429010886 | N32608 | 0.042477 | 21 | 0 | | | | | | 45.29251 | 21.00917 | 33.95872 | 8.442875 | 11.58812 | 27.51414 | 25.96869 | 23.01868 | 13.37838 | 14.54325 | 26.19371 | 23.38605 | 12.86372 | 20.79384 | 9.633666 | 15.80258 | 13.12705 | 38.56543 | 15.75973 | 35.22469 | 23.94321 | 28.99306 | 32.13072 | 33.22111 | 22.06161 | 22.44818 | 24.68775 |
| CUST_33776_PI429010886 | M7862 | 0.042479 | 18 | 9 | | | | | | 14.13431 | 7.772166 | 8.165976 | 15.28275 | 17.24279 | 9.811903 | 10.87507 | 14.47174 | 10.78913 | 13.75845 | 15.28275 | 31.37091 | 22.06655 | 12.4096 | 16.72767 | 15.16642 | 12.89788 | 12.35967 | 21.13287 | 8.801809 | 7.3856 | 14.07399 | 12.39561 | 8.941099 | 21.21095 | 14.43577 | 16.56559 |
| CUST_5161_PI429010886 | N18339 | 0.04248 | 21 | 15 | 156544470 | PREDICTED: hsp90 co-chaperone Cdc37-like [Nason | | | 7.2E-106 | 404.3563 | 521.8521 | 404.2573 | 394.7279 | 353.8817 | 308.6668 | 348.6809 | 321.374 | 328.6116 | 527.2284 | 358.9421 | 432.6248 | 393.1602 | 365.5076 | 349.7536 | 601.1869 | 408.7569 | 423.1161 | 431.462 | 478.4338 | 369.1054 | 479.5081 | 340.057 | 321.753 | 466.4129 | 301.7927 | 434.4861 |
| CUST_49338_PI429010886 | M85247 | 0.042487 | 18 | 12 | | | | | | 84.1757 | 112.6604 | 115.4344 | 60.95084 | 69.11088 | 89.10973 | 74.41568 | 68.25944 | 106.9755 | 58.34154 | 112.7651 | 162.5301 | 92.46177 | 160.7094 | 128.3615 | 143.2947 | 93.35337 | 149.2835 | 91.6409 | 122.7332 | 92.65529 | 112.6761 | 82.55028 | 102.9667 | 117.0813 | 79.97123 | 154.8889 |
| CUST_31269_PI429010886 | M1109 | 0.042511 | 12 | 3 | 242014826 | transient receptor potential locus C protein precurs | Metabolic process | Other | 2.87E-54 | 204.0225 | 168.3529 | 215.7523 | 274.1744 | 227.4117 | 300.885 | 204.629 | 216.1505 | 198.0625 | 218.9824 | 222.4127 | 211.951 | 227.6752 | 173.4981 | 190.3154 | 275.2679 | 215.0865 | 238.3762 | 276.0613 | 299.5774 | 242.3388 | 174.5672 | 194.3207 | 184.7518 | 133.668 | 215.6784 | 246.92 |
| CUST_32198_PI429010886 | M3501 | 0.042511 | 12 | 3 | | | Signal transduction | Cell-cell signaling | | 114.9009 | 189.3905 | 78.97339 | 137.9332 | 181.5127 | 201.6148 | 171.4799 | 115.7071 | 113.1942 | 124.0489 | 146.8141 | 83.70947 | 122.2713 | 98.71598 | 133.5047 | 142.3547 | 225.5895 | 205.6642 | 171.2286 | 116.4584 | 110.6759 | 210.2559 | 105.9574 | 135.3575 | 93.34264 | 122.6882 | 82.26332 |
| CUST_6494_PI429010886 | N20175 | 0.042511 | 12 | 0 | | | | | | 318.2412 | 409.7595 | 348.906 | 311.1732 | 305.3469 | 272.7482 | 294.8062 | 313.3535 | 321.803 | 444.3125 | 278.2553 | 444.8946 | 316.5285 | 407.5311 | 466.1947 | 384.1514 | 271.5735 | 278.8959 | 372.5738 | 361.9781 | 309.8242 | 391.819 | 349.6893 | 287.2312 | 444.4951 | 329.9922 | 430.6917 |
| CUST_18224_PI429010886 | N39382 | 0.042511 | 12 | 3 | | | | | | 67.82991 | 40.34341 | 48.33175 | 106.8412 | 118.7113 | 146.3194 | 159.7611 | 66.66314 | 52.70102 | 39.24656 | 104.0978 | 42.01359 | 70.33128 | 42.5721 | 86.9841 | 83.32632 | 182.4169 | 95.23078 | 89.38803 | 38.44451 | 93.06352 | 66.99867 | 84.00641 | 121.284 | 64.35394 | 66.48237 | 36.77053 |
| CUST_36978_PI429010886 | M17397 | 0.04252 | 24 | 21 | | | | | | 53.74643 | 76.98006 | 53.39323 | 51.58632 | 41.49369 | 23.1883 | 27.14357 | 51.37508 | 44.38619 | 69.03435 | 44.19983 | 57.73367 | 57.52971 | 65.64051 | 54.30012 | 78.35921 | 45.68153 | 72.09388 | 67.3892 | 67.12853 | 24.19522 | 83.36467 | 66.15125 | 58.17945 | 81.55572 | 55.75917 | 41.47521 |
| CUST_55348_PI429010886 | M109696 | 0.04256 | 15 | 3 | | | | | | 165.1872 | 66.022 | 65.16761 | 187.0582 | 111.8993 | 244.8254 | 171.9533 | 102.477 | 81.46986 | 28.14805 | 101.218 | 19.73888 | 116.516 | 21.12887 | 78.06614 | 34.32725 | 170.1475 | 119.0071 | 115.0699 | 22.85123 | 80.49518 | 34.86128 | 56.19981 | 239.9392 | 19.24305 | 64.19796 | 6.649752 |
| CUST_56762_PI429010886 | M114601 | 0.04256 | 15 | 9 | | | | | | 620.4611 | 609.1465 | 794.1984 | 329.2355 | 616.7782 | 396.3895 | 585.4036 | 627.8304 | 637.6126 | 682.9708 | 643.7576 | 803.5259 | 627.2172 | 696.015 | 772.1398 | 569.2573 | 725.0021 | 643.2335 | 700.6808 | 656.1679 | 598.2934 | 592.8448 | 775.9372 | 603.4589 | 750.3627 | 644.7638 | 835.4969 |
| CUST_37405_PI429010886 | M18915 | 0.04256 | 15 | 9 | | | | | | 1865.638 | 7737.591 | 2312.077 | 2549.933 | 3644.859 | 3179.631 | 2992.796 | 1952.727 | 5181.094 | 7042.885 | 3879.679 | 11510.47 | 4901.718 | 5004.678 | 5023.801 | 8024.987 | 1376.074 | 3921.02 | 2980.843 | 2908.57 | 3292.379 | 8733.657 | 3699.019 | 3095.586 | 9207.498 | 3285.957 | 10321.81 |
| CUST_40220_PI429010886 | M51849 | 0.04256 | 15 | 9 | | | | | | 135.0409 | 173.0247 | 206.6657 | 167.1583 | 169.9973 | 180.9317 | 161.7305 | 169.5826 | 167.6742 | 208.4275 | 179.8168 | 288.3979 | 192.7273 | 198.8522 | 173.2522 | 191.0756 | 219.4136 | 184.4394 | 167.4943 | 199.281 | 127.5355 | 208.138 | 184.7518 | 212.9538 | 212.0939 | 183.2048 | 273.0127 |
| CUST_41994_PI429010886 | M59111 | 0.04256 | 15 | 9 | 170037782 | bifunctional protein folD [Culex quinquefasciatus] > | Metabolic process | Other | 8.08E-25 | 146.5035 | 148.3091 | 263.345 | 144.8456 | 143.7672 | 152.1653 | 146.8141 | 177.3442 | 211.5135 | 170.0663 | 180.1966 | 181.4398 | 140.5657 | 163.9899 | 112.3515 | 157.17 | 203.1953 | 165.9249 | 104.0821 | 134.8244 | 127.0638 | 180.8997 | 183.528 | 157.3097 | 241.5575 | 163.5913 | 161.0453 |
| CUST_42105_PI429010886 | M59408 | 0.04256 | 15 | 9 | | | | | | 120.7611 | 129.718 | 116.6405 | 108.8172 | 114.443 | 100.2724 | 101.1196 | 96.09446 | 151.4063 | 160.2216 | 123.0876 | 166.2291 | 119.4425 | 131.9258 | 118.802 | 146.9588 | 114.9376 | 158.2183 | 106.3173 | 153.6236 | 131.4929 | 157.5293 | 145.0637 | 115.7617 | 137.759 | 154.1347 | 155.1735 |
| CUST_44227_PI429010886 | M65323 | 0.04256 | 15 | 9 | 291227245 | PREDICTED: hypothetical protein, partial [Saccoglos | | | 9.13E-08 | 22.89389 | 20.58435 | 41.60845 | 23.33698 | 20.74944 | 21.07029 | 17.14756 | 23.9514 | 23.32541 | 32.30669 | 34.67799 | 33.29815 | 21.20384 | 42.67635 | 23.09604 | 27.80787 | 26.50702 | 20.90047 | 21.64092 | 27.4471 | 22.75236 | 19.73888 | 32.32519 | 23.74624 | 26.0691 | 179.5033 | 34.29722 |
| CUST_3735_PI429010886 | N15159 | 0.04256 | 15 | 6 | | | | | | 18.45187 | 26.55907 | 44.56286 | 33.63387 | 19.86545 | 61.20199 | 46.55207 | 56.43628 | 78.56402 | 17.13072 | 25.62877 | 38.55322 | 35.08291 | 37.818 | 36.6668 | 33.2742 | 32.47936 | 28.2496 | 39.48403 | 48.75681 | 46.49334 | 39.60163 | 46.08198 | 36.48332 | 44.78041 | 32.26619 | 51.2779 |
| CUST_6389_PI429010886 | N20038 | 0.04256 | 15 | 9 | 321464750 | hypothetical protein DAPPUDRAFT_323067 [Daphn | | | 6.96E-07 | 367.3426 | 519.0655 | 459.9274 | 319.8933 | 306.5332 | 250.8185 | 288.9328 | 387.3252 | 436.0745 | 570.4962 | 409.277 | 642.1649 | 420.9194 | 467.9877 | 489.6976 | 577.33 | 318.9508 | 405.9943 | 357.7936 | 640.6647 | 456.2722 | 549.5559 | 488.4404 | 322.2737 | 518.9265 | 503.3426 | 629.4397 |
| CUST_12289_PI429010886 | N29237 | 0.04256 | 15 | 9 | | | | | | 33.72715 | 29.10476 | 13.05013 | 26.33096 | 20.49048 | 33.18761 | 20.77394 | 18.25337 | 17.98995 | 28.21862 | 38.723 | 41.94819 | 22.49442 | 30.6513 | 36.57035 | 28.09437 | 21.98421 | 32.36622 | 30.25566 | 23.48661 | 22.90261 | 35.96862 | 25.41068 | 35.57579 | 65.46937 | 44.13738 | 36.39953 |
| CUST_14446_PI429010886 | N32898 | 0.04256 | 15 | 9 | 328899290 | receptor accessory protein 5 [Procambarus clarkii] | | | 1.54E-08 | 181.8579 | 160.3693 | 151.4764 | 114.3617 | 196.2921 | 154.5009 | 158.4583 | 130.2535 | 151.2877 | 207.5377 | 204.5915 | 278.5705 | 220.8472 | 197.5737 | 135.8061 | 176.3124 | 257.6826 | 215.6344 | 174.2722 | 129.7027 | 168.7755 | 183.4917 | 157.1805 | 224.5142 | 231.6114 | 191.3918 | 188.1623 |
| CUST_17002_PI429010886 | N37212 | 0.04256 | 15 | 3 | | | | | | 8562.999 | 2995.003 | 5681.469 | 10130.67 | 12898.01 | 12273.46 | 12502.58 | 4213.146 | 4205.626 | 1342.932 | 10451.76 | 1087.534 | 3181.861 | 1497.212 | 2842.143 | 2215.807 | 13937.5 | 8942.257 | 8675.932 | 1230.543 | 13275.24 | 1983.417 | 10964.51 | 16119.35 | 924.2614 | 4436.798 | 817.4781 |
| CUST_20994_PI429010886 | N44434 | 0.04256 | 15 | 3 | | | | | | 625.8341 | 203.1953 | 432.5053 | 1345.526 | 1323.05 | 2536.388 | 1308.281 | 606.4913 | 276.5696 | 38.24749 | 1335.768 | 26.45134 | 408.2171 | 83.89746 | 228.6362 | 110.765 | 2431.925 | 1312.47 | 1603.184 | 28.68501 | 994.7785 | 128.1595 | 1070.988 | 1530.336 | 45.58985 | 849.9143 | 10.94634 |
| CUST_5782_PI429010886 | N19190 | 0.042563 | 21 | 9 | 321474141 | salivary alkaline phosphatase [Daphnia pulex] | Metabolic process | Lipid metabolic process | 3.1E-114 | NA | 36.80555 | 17.50524 | 55.13187 | 121.1638 | 21.35972 | 29.3424 | 355.7822 | 221.7682 | NA | 310.1413 | 373.2026 | 237.5773 | 19.82458 | 88.66977 | NA | 96.50257 | NA | 12.56761 | 582.1613 | 304.6076 | NA | 30.14229 | 20.38984 | NA | 127.2704 | 169.802 |
| CUST_32800_PI429010886 | M5140 | 0.042571 | 24 | 21 | | | | | | 140.1157 | 140.4217 | 134.7426 | 143.6875 | 156.6119 | 159.5684 | 106.2171 | 112.7651 | 111.0646 | 148.1555 | 110.7081 | 127.0114 | 94.11857 | 139.3127 | 136.2681 | 157.231 | 126.1331 | 160.4594 | 136.25 | 149.9119 | 145.3456 | 225.5499 | 133.4937 | 159.0362 | 189.8916 | 154.9491 | 162.5022 |
| CUST_7796_PI429010886 | N22088 | 0.04261 | 21 | 6 | | | | | | 15.69662 | 72.3532 | 9.199507 | 195.2252 | 10.40557 | NA | 172.213 | 5.024342 | 169.1739 | 83.30915 | 11.26345 | 24.81825 | 93.20644 | 16.85251 | 30.43732 | 4.923415 | NA | 7.608505 | NA | 12.48145 | 13.68935 | 20.26285 | 22.52377 | 18.18164 | 25.15661 | 91.0847 | 30.83099 |
| CUST_49120_PI429010886 | M84192 | 0.042619 | 18 | 9 | 302320972 | Taku spinosocarinatus voucher LIRS07-26 28S ribos | | | 2.4E-148 | 104856.2 | 86620.4 | 87164.76 | 109583.6 | 105375.3 | 96660.96 | 118088.6 | 105375.3 | 93467.7 | 119417.1 | 111801.3 | 106276.7 | 113412.4 | 91927.09 | 110974.5 | 69607.7 | 110259.4 | 99042.74 | 104141 | 104856.2 | 99042.74 | 108910.4 | 89699.25 | 102282.3 | 115564.1 | 100388.2 | 106276.7 |
| CUST_30005_PI429010886 | N60983 | 0.042621 | 24 | 21 | | | | | | 8.857132 | 8.596197 | NA | 8.839067 | 10.58195 | 11.24691 | 20.06593 | 15.79548 | 8.542736 | 17.83843 | 8.773525 | 8.019973 | 11.18163 | 7.834552 | 10.7115 | 20.3394 | 17.37163 | 10.7968 | 15.30179 | 7.838281 | 21.40881 | 17.51574 | 30.41187 | 53.13993 | 23.97968 | 16.83754 | 18.3679 |
| CUST_2583_PI429010886 | N12697 | 0.042624 | 24 | 18 | | | | | | 17.47454 | 23.34418 | 20.67362 | 21.7799 | 12.78901 | 17.90553 | 8.386399 | 10.81563 | 11.45774 | 23.73784 | 22.07122 | 16.22526 | 14.94106 | 25.5267 | 16.91528 | 23.08011 | 18.7509 | 18.65035 | 20.79384 | 20.32379 | 27.07724 | 17.64932 | 23.29073 | 21.73089 | 34.05805 | 24.68775 | 21.33841 |
| CUST_38506_PI429010886 | M27519 | 0.042628 | 18 | 15 | | | | | | 403.4755 | 279.387 | 148.2477 | 50.15308 | 382.5164 | 24.49848 | 286.1419 | 305.4399 | 258.6304 | 298.0242 | 1009.233 | 341.713 | 495.2283 | 477.5078 | 379.0179 | 672.5182 | 302.3576 | 667.377 | 532.9396 | 41.18849 | 269.2555 | 63.85949 | 54.5396 | 4544.383 | 78.04542 | 211.951 | 840.6106 |
| CUST_35940_PI429010886 | M14034 | 0.042649 | 18 | 15 | | | | | | 308.2184 | 538.5366 | 495.0933 | 227.5328 | 441.149 | 370.4877 | 242.3388 | 244.4939 | 186.7272 | 233.9751 | 257.0697 | 457.9897 | 194.4273 | 354.8858 | 308.9771 | 390.7246 | 582.6377 | 441.8735 | 458.8168 | 239.2282 | 297.4787 | 401.9872 | 359.7386 | 358.1231 | 403.3076 | 233.9751 | 282.8614 |
| CUST_5820_PI429010886 | N19243 | 0.042666 | 18 | 9 | | | | | | 423.9576 | 393.5988 | 453.9938 | 132.109 | 366.4064 | 374.0543 | 388.3479 | 480.2626 | 498.7637 | 668.4719 | 379.2136 | 543.5013 | 485.0905 | 515.8865 | 501.3373 | 508.3277 | 446.3108 | 509.2578 | 538.8855 | 469.28 | 276.6094 | 341.2566 | 712.5461 | 266.4363 | 343.0578 | 674.6226 | 647.5069 |
| CUST_14212_PI429010886 | N32494 | 0.042694 | 15 | 0 | | | | | | 457.1179 | 570.1143 | 403.1472 | 288.5922 | 433.4824 | 370.5671 | 322.593 | 250.1572 | 272.9383 | 207.4294 | 319.4017 | 430.0642 | 270.0349 | 360.9417 | 319.8933 | 441.5814 | 479.189 | 433.8707 | 391.346 | 304.5545 | 272.638 | 497.5175 | 348.3321 | 351.1194 | 533.8243 | 248.7014 | 319.5171 |
| CUST_19516_PI429010886 | N41702 | 0.04273 | 18 | 12 | | | | | | 30.91161 | 52.1905 | 37.18492 | 41.89305 | 30.26479 | 27.1975 | 31.80677 | 13.11816 | 21.37656 | 35.76089 | 27.72962 | 43.79306 | 32.90861 | 52.16399 | 49.69905 | 55.9701 | 26.66659 | 49.59839 | 26.64522 | 44.61606 | 38.04057 | 56.93106 | 28.35396 | 39.25762 | 33.48474 | 30.34969 | 24.69054 |
| CUST_53820_PI429010886 | M103322 | 0.042746 | 18 | 9 | 270002715 | hypothetical protein TcasGA2_TC016161 [Tribolium | | | 3.03E-32 | 42.16582 | 43.51975 | 36.95956 | 35.20549 | 24.58201 | 14.84953 | 25.27977 | 25.11237 | 34.91043 | 57.65633 | 40.03126 | 49.01378 | 36.115 | 50.85762 | 42.08627 | 57.33863 | 38.54962 | 39.09341 | 35.18679 | 48.30896 | 40.14284 | 42.79014 | 35.59331 | 21.11131 | 68.07541 | 41.26062 | 24.44087 |
| CUST_17025_PI429010886 | N37250 | 0.042747 | 18 | 0 | 495400722 | nucleoside-diphosphate-sugar epimerase [Phylloba | Metabolic process | Other | 4.23E-42 | 4524.584 | 121.7504 | 5055.748 | 769.5294 | 2005.797 | 1778.896 | 6240.976 | 541.7506 | 132.7469 | 106.3446 | 2856.456 | 69.39137 | 150.1848 | 103.1477 | 78.63344 | 85.10975 | 4733.383 | 952.3475 | 5382.99 | 122.2713 | 3946.907 | 136.2095 | 4868.886 | 4617.753 | 115.4469 | 500.086 | 86.30175 |
| CUST_28958_PI429010886 | N58915 | 0.042747 | 21 | 0 | | | | | | 10.26743 | 6.745411 | 14.40922 | NA | 7.90501 | 8.447696 | 9.278391 | 4.442076 | 9.526791 | 8.083245 | 17.65271 | 3.886854 | 3.755761 | NA | 3.894967 | 6.19695 | 14.55595 | NA | 4.97539 | 12.28249 | 9.349048 | 11.00785 | 36.58662 | 27.24397 | 5.087451 | 5.72427 | NA |
| CUST_40197_PI429010886 | M51617 | 0.042762 | 15 | 12 | | | | | | 66.35653 | 130.9315 | 123.0016 | 88.72989 | 105.3783 | 143.8832 | 135.1423 | 83.74259 | 148.544 | 122.3475 | 145.0813 | 213.366 | 100.64 | 217.0054 | 162.7472 | 118.1469 | 105.2803 | 229.9104 | 78.48034 | 93.49845 | 88.74926 | 102.3996 | 174.7759 | 153.0835 | 144.6868 | 122.5622 | 211.9082 |
| CUST_26545_PI429010886 | N54427 | 0.042792 | 21 | 15 | | | | | | 27.97387 | 14.38028 | 52.33357 | 15.42925 | 23.30725 | 29.70337 | 12.29647 | 15.56605 | 26.69053 | NA | 14.67882 | 22.96015 | 14.4735 | 25.67741 | 19.06154 | 26.85728 | 40.20059 | 34.54086 | 30.48948 | 28.77687 | 35.77142 | 22.83945 | 17.60822 | 22.33967 | 31.4416 | 19.09218 | 22.74587 |
| CUST_25813_PI429010886 | N53104 | 0.042794 | 15 | 0 | | | | | | 52.8414 | 40.1276 | 53.35103 | 18.69696 | 32.23691 | 49.65196 | 35.05162 | 39.22918 | 35.25245 | 37.67574 | 24.97464 | 36.28401 | 31.06527 | 32.62278 | 41.44167 | 46.20496 | 45.00065 | 56.72941 | 34.01329 | 43.83694 | 40.62855 | 50.45103 | 39.90906 | 31.56711 | 41.28276 | 38.13489 | 32.58197 |
| CUST_35473_PI429010886 | M12658 | 0.042795 | 15 | 3 | | | | | | 96.92806 | 59.50688 | 63.50632 | 140.0997 | 180.0766 | 238.455 | 121.7165 | 134.6396 | 53.88876 | 78.32339 | 123.6533 | 36.52268 | 75.98951 | 43.5925 | 51.32807 | 66.09037 | 196.3252 | 174.2465 | 217.0054 | 81.04828 | 73.99757 | 93.37534 | 77.59246 | 105.242 | 100.3848 | 223.3365 | 52.08136 |
| CUST_1617_PI429010886 | N10246 | 0.042795 | 15 | 12 | | | | | | 652.581 | 867.8685 | 729.8175 | 453.5801 | 489.2052 | 642.1649 | 458.217 | 639.5245 | 639.8175 | 783.9719 | 623.3295 | 1195.978 | 690.2519 | 927.5266 | 1029.372 | 813.1389 | 524.5768 | 649.7314 | 580.3063 | 714.2963 | 730.2123 | 784.2356 | 771.9192 | 616.9292 | 950.2628 | 546.0726 | 870.3572 |
| CUST_28290_PI429010886 | N57675 | 0.042795 | 24 | 21 | | | | | | 281.7845 | 281.5398 | 280.8839 | 234.3391 | 251.7054 | 214.6655 | 267.8414 | 228.4344 | 226.9563 | 307.7804 | 243.3928 | 282.7521 | 250.1141 | 316.5936 | 280.6713 | 289.423 | 245.2402 | 269.2314 | 271.3099 | 286.4677 | 279.2899 | 294.2267 | 301.1198 | 277.9987 | 358.9421 | 277.8488 | 349.9147 |
| CUST_20968_PI429010886 | N44388 | 0.042816 | 18 | 3 | 196014793 | hypothetical protein TRIADDRAFT_61245 [Trichopla | Protein metabolism | Proteolysis | 1.4E-16 | 13.99779 | 21.52056 | 19.42074 | 50.33169 | 14.32589 | 30.72063 | 16.67423 | 5.276803 | 18.79689 | 10.02549 | 4.524578 | 10.74821 | 4.149199 | 18.61046 | 5.570616 | 15.37508 | 9.592481 | 11.85797 | NA | 8.39917 | 32.76173 | 12.45527 | 43.94789 | 10.6935 | 14.76626 | 19.50736 | 5.904393 |
| CUST_51165_PI429010886 | M92437 | 0.042817 | 21 | 15 | 321466578 | hypothetical protein DAPPUDRAFT_54175 [Daphnia | | | 1.78E-57 | 40.14953 | 52.66699 | 47.12249 | 59.1427 | 47.14504 | 46.56491 | 41.99643 | 68.02073 | 38.153 | 77.23965 | 47.21351 | 68.5444 | 72.85867 | 84.22907 | 70.02583 | 125.6499 | 47.02203 | 144.2537 | 54.66707 | 56.09789 | 46.04576 | 71.23473 | 46.34675 | 45.55959 | 75.17352 | 49.48526 | 72.43161 |
| CUST_32548_PI429010886 | M4396 | 0.042831 | 24 | 0 | 478250384 | hypothetical protein YQE_12284, partial [Dendroct | | | 4.73E-33 | 64.11406 | 52.91094 | 83.07834 | 67.14861 | 62.63918 | 71.40808 | 50.48246 | 50.57708 | 48.94359 | 67.59725 | 41.20481 | 71.91218 | 31.59119 | 57.20099 | 52.97579 | 55.92013 | 61.5801 | 78.15144 | 54.19041 | 38.60175 | 60.43972 | 64.58903 | 39.39152 | 59.33007 | 73.78486 | 73.02744 | 76.23608 |
| CUST_49457_PI429010886 | M85721 | 0.042831 | 24 | 0 | | | | | | 52.645 | 28.93334 | 53.26328 | 97.72266 | 46.58823 | 43.67133 | 20.83504 | 23.44093 | 27.10385 | 34.58379 | 34.79732 | 46.91616 | 15.49062 | 23.12065 | 28.48104 | 54.95061 | 29.12856 | 51.26857 | 32.11242 | 25.70354 | 40.05116 | 31.12392 | 40.63191 | 50.92195 | 47.74853 | 47.0845 | 45.76846 |
| CUST_11428_PI429010886 | N27788 | 0.042837 | 24 | 15 | | | | | | 7.986139 | 10.20401 | 9.034713 | 6.326533 | 13.32189 | 11.53905 | 4.097744 | 8.486348 | 8.164956 | 8.768772 | 7.894781 | 15.72277 | 10.90408 | 12.55563 | 18.92641 | 15.99431 | 15.0068 | NA | 8.504674 | 14.57957 | 9.301993 | 19.74451 | 12.05594 | 11.56409 | 15.5304 | 8.10145 | 11.3595 |
| CUST_56151_PI429010886 | M112976 | 0.042846 | 24 | 12 | | | | | | 188.2993 | 237.3209 | 210.2559 | 187.5869 | 189.5968 | 186.2582 | 162.7472 | 189.9733 | 200.2396 | 235.7566 | 203.3901 | 258.9692 | 203.324 | 276.0613 | 273.4298 | 267.7928 | 193.4709 | 212.4411 | 201.9074 | 250.9845 | 212.1945 | 299.2346 | 259.8913 | 190.8098 | 228.547 | 233.3626 | 261.3191 |
| CUST_43262_PI429010886 | M62564 | 0.04285 | 15 | 0 | | | | | | 217.4042 | 271.6624 | 230.5922 | 227.9039 | 193.3073 | 202.301 | 139.545 | 142.5887 | 181.6707 | 252.4492 | 195.2684 | 289.2197 | 177.4848 | 190.62 | 280.2656 | 293.6394 | 179.3756 | 256.281 | 180.4095 | 236.2204 | 223.1811 | 245.8985 | 177.0904 | 174.9779 | 241.0846 | 146.3889 | 191.3655 |

| Probe ID | ID | p-value | Period | Phase | EBI ID | Annotation | GO category | Go term | E-value | 0 | 0 | 0 | 3 | 3 | 3 | 6 | 6 | 6 | 9 | 9 | 9 | 12 | 12 | 12 | 15 | 15 | 15 | 18 | 18 | 18 | 21 | 21 | 21 | 24 | 24 | 24 |
|---|---|---|---|---|---|---|---|---|---|---|---|---|---|---|---|---|---|---|---|---|---|---|---|---|---|---|---|---|---|---|---|---|---|---|---|---|
| CUST_37651_PI429010886 | M20253 | 0.042879 | 21 | 15 | 51104825 | Euphausia superba small subunit ribosomal RNA ge | | | | 746.6817 | 380.9707 | 725.1936 | 217.5886 | 855.218 | 594.1197 | 865.4579 | 575.3599 | 739.6144 | 1192.582 | 471.9637 | 516.2214 | 1353.303 | 853.6849 | 716.6989 | 1006.198 | 1028.799 | 888.1875 | 663.5689 | 800.6759 | 882.8865 | 1131.289 | 471.9637 | 505.3693 | 388.966 | 466.1947 | 987.1593 |
| CUST_7440_PI429010886 | N21555 | 0.042879 | 24 | 15 | 327261663 | PREDICTED: Anolis carolinensis karyopherin alpha 5 | | | 9.93E-71 | 221.425 | 170.5103 | 163.9462 | 174.1985 | 186.006 | 156.6119 | 170.4197 | 153.5797 | 175.4895 | 170.8864 | 196.5334 | 208.2116 | 167.4717 | 203.6685 | 193.8618 | 205.9692 | 213.6068 | 239.0792 | 219.715 | 186.0881 | 143.4489 | 193.4709 | 177.7855 | 187.7825 | 197.2642 | 142.4549 | 193.6848 |
| CUST_26540_PI429010886 | N54419 | 0.042881 | 18 | 15 | | | | | | 73.82839 | 120.9878 | 86.00719 | 83.07834 | 64.92708 | 82.07629 | 81.22317 | 81.45472 | 67.69277 | 88.31622 | 96.25237 | 138.7005 | 85.8526 | 120.1854 | 107.3764 | 123.1846 | 82.80093 | 97.22671 | 89.91874 | 94.36809 | 76.72395 | 109.6904 | 81.42045 | 62.46895 | 95.83965 | 67.61178 | 97.16739 |
| CUST_46611_PI429010886 | M73161 | 0.0429 | 12 | 0 | | | | | | 52.98961 | 58.63339 | 54.57781 | 42.44034 | 43.03291 | 44.1761 | 34.30707 | 38.18452 | 40.50125 | 55.48383 | 52.27598 | 45.66434 | 64.30394 | 46.91616 | 56.30906 | 69.70264 | 38.99636 | 45.64241 | 45.05595 | 50.71879 | 41.70879 | 63.37317 | 46.991 | 44.11518 | 55.63288 | 30.17169 | 48.74628 |
| CUST_49490_PI429010886 | M85847 | 0.0429 | 12 | 0 | | | | | | 111.6728 | 96.90956 | 92.34903 | 76.42859 | 70.28439 | 96.81237 | 81.73063 | 55.66641 | 81.68427 | 67.45213 | 87.71808 | 94.5785 | 77.1205 | 95.16133 | 98.36103 | 112.5022 | 88.3275 | 115.9694 | 76.87346 | 85.46155 | 77.38027 | 93.38696 | 84.51009 | 99.17539 | 93.05164 | 102.9589 | 101.565 |
| CUST_57146_PI429010886 | E1547 | 0.042905 | 12 | 3 | 321472355 | hypothetical protein DAPPUDRAFT_100611 [Daphn | Cellular component organization | Cytoskeleton organization | 6.45E-30 | 6136.091 | 7662.198 | 5367.717 | 8138.075 | 8448.526 | 10366.97 | 6679.096 | 5176.163 | 3917.614 | 4064.703 | 6267.492 | 3934.354 | 5130.907 | 3082.77 | 6078.985 | 9670.846 | 5663.977 | 7864.497 | 7207.278 | 4598.774 | 4833.67 | 6366.193 | 3572.519 | 7536.588 | 4676.187 | 6180.646 | 2587.199 |
| CUST_49154_PI429010886 | M84429 | 0.042908 | 18 | 12 | 321471830 | hypothetical protein DAPPUDRAFT_302359 [Daphn | Transport | Ion transport | 1.17E-39 | 439.7553 | 262.4225 | 653.1387 | 179.5583 | 264.1236 | 260.5944 | 588.9896 | 531.8244 | 398.993 | 492.0126 | 276.6492 | 385.5507 | 471.2464 | 726.329 | 409.6774 | 326.8661 | 408.6642 | 262.997 | 414.6925 | 314.5564 | 313.2163 | 413.0515 | 319.5171 | 257.5442 | 413.3489 | 353.0802 | 419.6576 |
| CUST_49138_PI429010886 | M84309 | 0.042924 | 24 | 18 | | | | | | 35.74774 | 34.80311 | 42.68895 | 24.55823 | 32.91264 | 33.53684 | 27.14155 | 38.55322 | 37.83723 | 55.70082 | 42.72555 | 73.47109 | 42.17788 | 62.04074 | 53.64846 | 64.70488 | 31.55142 | 45.75251 | 47.35331 | 60.42655 | 51.04982 | 63.68912 | 35.6576 | 41.0817 | 52.79221 | 63.96858 | 45.86806 |
| CUST_32730_PI429010886 | M4946 | 0.042943 | 18 | 6 | | | | | | 22.00833 | 29.96361 | 18.20163 | 39.99701 | 12.15775 | 20.66741 | 20.34203 | 77.51881 | 33.54956 | 37.77885 | 26.27126 | 33.46666 | 19.3658 | 45.7289 | 12.56568 | 20.50772 | 12.61647 | 13.65247 | 7.898568 | 29.89381 | 11.55822 | 20.22094 | 42.57633 | 29.60942 | 45.215 | 25.60611 | 37.66305 |
| CUST_10629_PI429010886 | N26541 | 0.042944 | 15 | 9 | | | | | | 1342.338 | 1936.604 | 1748.457 | 1284.158 | 1832.42 | 1902.402 | 1761.883 | 1137.917 | 1954.96 | 2111.683 | 1851.755 | 2314.02 | 1623.132 | 2610.308 | 1663.806 | 1201.319 | 1715.766 | 1586.719 | 1576.033 | 1622.403 | 1971.63 | 1272.526 | 1837.915 | 1796.352 | 2466.842 | 2356.592 | 2531.781 |
| CUST_43953_PI429010886 | M64453 | 0.042951 | 24 | 18 | | | | | | 48.17972 | 55.63288 | 44.53184 | 25.04089 | 36.72024 | 22.30707 | 27.27496 | 25.69829 | 25.10262 | 50.22122 | 32.35642 | 41.96975 | 37.25447 | 38.37639 | 30.3611 | 50.25535 | 46.22951 | 42.91838 | 55.38216 | 45.28763 | 32.20238 | 59.30743 | 24.79214 | 40.20735 | 54.76633 | 31.83203 | 55.03461 |
| CUST_55118_PI429010886 | M108788 | 0.042989 | 12 | 0 | | | | | | 482.5207 | 241.2255 | 144.2982 | 16.1574 | 51.7068 | 27.36512 | 142.7507 | 182.3659 | 123.5563 | 482.976 | 130.9315 | 139.0764 | 239.521 | 136.6977 | 100.1493 | 101.6485 | 77.29645 | 76.87346 | 200.8366 | 86.46205 | 101.7598 | 234.3704 | 47.57087 | 78.95924 | 17.24279 | 183.1526 | 407.1868 |
| CUST_41714_PI429010886 | M58373 | 0.042989 | 12 | 0 | | | | | | 564.1196 | 261.4208 | 737.0066 | 496.4081 | 142.0582 | 51.15134 | 827.2898 | 376.7825 | 275.8443 | 854.7156 | 426.1051 | 384.0577 | 584.8769 | 877.4226 | 870.5556 | 145.3839 | 182.2638 | 80.99667 | 1263.014 | 244.4592 | 534.8364 | 302.092 | 800.3484 | 241.9617 | 868.721 | 304.5545 | 384.9318 |
| CUST_1685_PI429010886 | N10435 | 0.042989 | 12 | 3 | 242016965 | Aminopeptidase N precursor, putative [Pediculus h | Protein metabolism | Proteolysis | 1.1E-172 | 378.5438 | 276.4162 | 464.198 | 805.0049 | 801.7718 | 1370.685 | 767.2232 | 578.2355 | 405.3248 | 323.0511 | 885.0858 | 410.06 | 384.5478 | 422.1729 | 359.0948 | 329.3607 | 968.3869 | 883.3994 | 1137.917 | 316.122 | 931.4066 | 404.9556 | 771.0971 | 794.9156 | 317.0842 | 748.4841 | 356.2894 |
| CUST_338_PI429010886 | N4062 | 0.042989 | 12 | 0 | 321460896 | hypothetical protein DAPPUDRAFT_216354 [Daphn | Protein metabolism | Proteolysis | 8.6E-36 | 4064.703 | 4036.57 | 3792.107 | 2574.868 | 2851.724 | 2104.851 | 2704.165 | 3098.049 | 3074.469 | 2957.256 | 3130.59 | 4205.626 | 4205.626 | 3756.632 | 3271.146 | 3561.268 | 2254.33 | 2856.456 | 2019.046 | 5836.719 | 2995.003 | 4353.113 | 3116.138 | 2332.981 | 3485.611 | 2963.617 | 3208.657 |
| CUST_20935_PI429010886 | N44332 | 0.042989 | 12 | 0 | | | | | | 24.59907 | 19.64813 | 39.81638 | 19.97654 | 13.80154 | 3.426123 | 23.87529 | 35.56458 | 18.54906 | 30.65915 | 18.689 | 23.66154 | 24.36264 | 30.9626 | 21.53411 | 16.68444 | 17.07869 | 24.73576 | 18.48864 | 20.72678 | 18.41069 | 28.5055 | 26.02411 | 23.85972 | 40.81576 | 15.12815 | 31.62724 |
| CUST_37112_PI429010886 | M17877 | 0.042994 | 24 | 18 | | | | | | 144.5655 | 152.565 | 154.595 | 144.5043 | 138.8365 | 152.0093 | 124.0862 | 118.275 | 140.7156 | 165.8485 | 133.4937 | 181.0372 | 150.6133 | 176.1434 | 162.1587 | 165.1224 | 152.2451 | 171.784 | 178.8821 | 171.0319 | 151.8851 | 177.896 | 165.716 | 143.1896 | 186.883 | 146.7819 | 196.1855 |
| CUST_29745_PI429010886 | N60463 | 0.042994 | 24 | 18 | | | | | | 68.46628 | 61.23542 | 75.11707 | 61.93065 | 60.72981 | 62.51292 | 35.51693 | 66.83042 | 52.74988 | 73.26779 | 67.68545 | 59.63236 | 78.17016 | 65.0047 | 78.70239 | 62.04074 | 71.34779 | 53.26328 | 68.07541 | 88.43444 | 78.67424 | 84.04395 | 53.52565 | 81.31771 | 86.65167 | 65.72077 | 75.36334 |
| CUST_17560_PI429010886 | N38184 | 0.042995 | 21 | 3 | | | | | | 23.61236 | 20.74944 | 9.456396 | 47.47127 | 34.65808 | 35.34739 | 26.43248 | 18.25747 | 18.60572 | 19.7715 | 23.54424 | 18.90163 | 17.0738 | 17.62699 | 18.49081 | 21.86875 | 13.11816 | 29.14178 | 24.35448 | 82.97709 | 15.68875 | 31.07874 | 7.113361 | 12.0052 | 26.41719 | 51.81515 | 29.37298 |
| CUST_56307_PI429010886 | M113465 | 0.043028 | 15 | 12 | | | | | | 3022.502 | 4228.899 | 3318.146 | 2845.968 | 2585.542 | 2932.434 | 2061.289 | 2626.728 | 2850.368 | 3384.084 | 2454.105 | 4381.739 | 3161.551 | 3511.533 | 4532.248 | 5233.779 | 2359.813 | 3048.703 | 2788.353 | 4038.67 | 2858.942 | 3965.108 | 2892.813 | 2247.598 | 3981.392 | 3407.322 | 4225.122 |
| CUST_42089_PI429010886 | M59370 | 0.043028 | 24 | 21 | | | | | | 35.9369 | 38.53966 | 41.73826 | 33.69868 | 32.97611 | 39.94945 | 32.64658 | 25.35696 | 29.60276 | 33.82224 | 39.51821 | 58.20859 | 34.92634 | 44.70504 | 49.76383 | 49.37485 | 32.73413 | 51.21719 | 32.02557 | 34.7228 | 43.29309 | 40.03501 | 61.51764 | 43.96447 | 38.64362 | 82.60943 | 63.97505 |
| CUST_37091_PI429010886 | M17828 | 0.043039 | 15 | 3 | | | | | | 340.5239 | 290.2748 | 166.2726 | 417.8136 | 260.7973 | 390.7936 | 292.4718 | 259.3271 | 309.4606 | 222.9689 | 184.7518 | 220.9933 | 318.4396 | 231.6114 | 271.5735 | 303.3749 | 240.8224 | 355.3482 | 340.5239 | 255.7893 | 216.5751 | 306.5332 | 211.1137 | 418.4668 | 186.7544 | 227.0266 | 191.1893 |
| CUST_45206_PI429010886 | M68315 | 0.043039 | 15 | 9 | | | | | | 53.71399 | 61.2083 | 47.5011 | 42.45744 | 41.96975 | 32.15505 | 38.89725 | 34.33169 | 47.19565 | 68.86603 | 35.95337 | 66.92953 | 43.79745 | 52.65189 | 65.03148 | 61.81926 | 31.96138 | 60.70574 | 47.05374 | 60.28718 | 41.49369 | 65.08 | 52.4135 | 44.62034 | 65.49916 | 60.99798 | 64.18045 |
| CUST_48069_PI429010886 | M78758 | 0.043039 | 15 | 9 | | | | | | 40.09244 | 20.98522 | 41.95242 | 29.85793 | 34.2205 | 35.94612 | 40.03759 | 44.00078 | 43.06591 | 44.69546 | 42.38165 | 52.70102 | 33.75052 | 44.50009 | 19.19007 | 41.47981 | 47.14099 | 35.94919 | 33.17041 | 34.70757 | 44.15212 | 43.69222 | 44.09799 | 41.12787 | 63.78205 | 38.09477 | 48.05852 |
| CUST_26433_PI429010886 | N54236 | 0.043039 | 15 | 9 | | | | | | 811.2478 | 1003.294 | 1178.063 | 912.7145 | 773.8104 | 933.7706 | 801.0442 | 867.1839 | 1023.799 | 1064.349 | 930.2399 | 1358.264 | 962.2957 | 1262.411 | 1064.349 | 1051.549 | 1044.56 | 981.8641 | 986.7668 | 1102.979 | 956.4623 | 1248.908 | 1054.214 | 877.9874 | 1270.943 | 1181.488 | 1309.194 |
| CUST_28470_PI429010886 | N58007 | 0.043039 | 15 | 3 | | | | | | 24.90221 | 19.95383 | 19.48105 | 34.7228 | 26.21698 | 31.49086 | 25.56096 | 12.85919 | 25.94523 | 22.34831 | 15.93573 | 23.51614 | 27.60111 | 32.80705 | 12.14502 | 21.80535 | 33.06129 | 26.03157 | 26.90259 | 24.96736 | 27.79833 | 20.41951 | 28.44656 | 36.17076 | 18.74405 | 17.49952 | 20.3487 |
| CUST_11589_PI429010886 | N28081 | 0.043039 | 18 | 9 | 380013858 | PREDICTED: Apis florea serine/threonine-protein ki | Protein metabolism | Cellular protein modification process | 3.3E-103 | 40.80459 | 38.98332 | 63.90408 | 52.78112 | 90.34874 | 78.95217 | 32.76173 | 42.70658 | 69.81278 | 89.38803 | 96.19258 | 79.88802 | 38.6903 | 85.99449 | 60.53815 | 58.03044 | 51.54393 | 56.96512 | 45.4027 | 50.65259 | 44.22939 | 65.39564 | 59.0152 | 33.66515 | 60.04192 | 48.5802 | 75.92185 |
| CUST_20757_PI429010886 | N44005 | 0.043076 | 21 | 3 | | | | | | 132.0957 | 124.447 | 135.7577 | 140.7563 | 134.8409 | 118.9466 | 161.4564 | 109.0193 | 142.5512 | 106.2503 | 149.3929 | 148.5237 | 91.09799 | 109.4262 | 83.11485 | 137.9332 | 130.2384 | 134.4388 | 117.847 | 73.95574 | 99.75328 | 100.2082 | 117.3039 | 153.7764 | 206.0933 | 125.5041 | 174.0152 |
| CUST_50764_PI429010886 | M90922 | 0.043114 | 15 | 12 | | | | | | 16.10308 | 18.55819 | 15.86385 | 10.67164 | 7.249166 | 7.708974 | 13.66936 | 5.949232 | 21.76618 | 10.41346 | 14.24501 | 12.63126 | 11.01284 | NA | 18.70053 | 29.45107 | 12.68586 | 3.350493 | 3.828115 | 4.773638 | 8.664964 | 11.41469 | 13.26174 | NA | 15.84353 | 12.77633 | 18.93949 |
| CUST_2699_PI429010886 | N12933 | 0.043177 | 12 | 0 | | | | | | 873.2696 | 836.1144 | 1351.741 | 479.7862 | 276.2557 | 157.1495 | 1102.979 | 1670.981 | 1141.431 | 1787.33 | 654.7049 | 932.818 | 1354.655 | 1592.642 | 961.8209 | 246.7296 | 518.3277 | 154.4419 | 455.0936 | 1248.908 | 415.5762 | 854.951 | 1777.79 | 338.8337 | 1045.476 | 641.887 | 1271.998 |
| CUST_8403_PI429010886 | N22987 | 0.043192 | 21 | 18 | 321469674 | hypothetical protein DAPPUDRAFT_303843 [Daphni | | | 6.02E-34 | 170.7231 | 274.0115 | 114.5611 | 173.7958 | 134.1419 | 139.4507 | 97.62464 | 103.5822 | 110.7189 | 125.9322 | 143.0445 | 186.1933 | 103.202 | 137.5108 | 174.8583 | 266.4363 | 97.77159 | 145.308 | 146.2438 | 205.5955 | 185.0427 | 232.3859 | 126.8079 | 138.8211 | 176.2225 | 114.4015 | 171.6689 |
| CUST_46864_PI429010886 | M73995 | 0.043215 | 24 | 21 | | | | | | 16.03304 | 15.1736 | 12.30553 | 22.65536 | 13.2165 | 15.28275 | 21.31201 | 10.07219 | 13.11271 | 22.4434 | 13.89365 | 10.037 | 16.15388 | 7.543719 | 6.466225 | 19.52762 | 18.28397 | 4.187339 | 22.50662 | 21.4691 | 13.732 | 28.27702 | 23.13514 | 22.4434 | 27.19369 | 7.977931 | 28.57032 |
| CUST_34108_PI429010886 | M8793 | 0.043215 | 24 | 21 | 1562538 | Homarus americanus alpha-III tubulin mRNA, comp | Cellular component organization | Cytoskeleton organization | | 751.4303 | 1055.576 | 717.957 | 776.8406 | 586.6898 | 682.9708 | 742.4096 | 908.82 | 629.2275 | 1256.525 | 575.3599 | 865.9484 | 663.9332 | 635.813 | 532.5435 | 1536.448 | 927.5266 | 909.4624 | 888.1875 | 683.9239 | 810.0074 | 1109.944 | 973.4615 | 1115.54 | 1022.013 | 944.3898 | 731.2482 |
| CUST_15504_PI429010886 | N34688 | 0.043215 | 24 | 15 | | | | | | 248.3781 | 267.8414 | 291.8279 | 278.0994 | 275.9866 | 385.5507 | 251.7054 | 237.2477 | 277.3932 | 316.8471 | 268.1467 | 290.7302 | 290.1265 | 333.2348 | 406.809 | 345.7818 | 324.1237 | 361.5394 | 285.7004 | 312.1179 | 303.0793 | 376.0453 | 297.0259 | 290.2748 | 347.2399 | 274.6972 | 350.2692 |
| CUST_18804_PI429010886 | N40441 | 0.043217 | 18 | 15 | | | | | | 101.9838 | 98.84179 | 81.82047 | 119.9416 | 101.6252 | 128.5235 | 95.04998 | 78.62359 | 76.16727 | 118.9612 | 98.27597 | 99.07551 | 89.71539 | 105.2803 | 98.37238 | 160.0108 | 134.5558 | 129.7943 | 113.7623 | 136.8846 | 93.38696 | 116.5788 | 72.2558 | 84.07185 | 134.7225 | 93.83775 | 80.78349 |
| CUST_43417_PI429010886 | M62987 | 0.04324 | 21 | 18 | 242018035 | tpr repeat nuclear phosphoprotein, putative [Pedicu | | | 2.42E-82 | 31.721 | 29.43141 | 25.87828 | 27.80787 | 36.11849 | 18.47667 | 16.8955 | 15.93573 | 13.5398 | 34.54793 | 20.83932 | 30.66153 | 25.96869 | 31.80323 | 28.46598 | 34.86849 | 23.34607 | 34.1956 | 35.27881 | 57.28882 | 20.61498 | 33.11438 | 22.78539 | 13.90672 | 31.39141 | 22.41939 | 26.76517 |
| CUST_45275_PI429010886 | M68558 | 0.043242 | 24 | 15 | | | | | | 11.63852 | 8.181079 | 10.39055 | 15.73405 | 11.01084 | 14.21003 | 9.814804 | 12.36089 | 15.49638 | 14.70379 | 11.36093 | 115.1182 | 18.61312 | 12.24661 | 7.222005 | 21.4035 | 15.37012 | 24.43037 | 10.40557 | 10.1832 | 12.77138 | 16.1391 | 16.49199 | 13.55467 | 9.977459 | 8.951802 | NA |
| CUST_55242_PI429010886 | M109268 | 0.043243 | 24 | 0 | | | | | | 164.6734 | 127.8836 | 188.6253 | 80.15845 | 126.5686 | 130.1677 | 128.0902 | 112.8118 | 171.3364 | 115.8949 | 89.89615 | 97.80592 | 131.1249 | 160.7305 | 104.5579 | 130.5819 | 110.0751 | 124.1999 | 111.9784 | 89.56339 | 111.9633 | 135.8479 | 91.58478 | 126.0286 | 132.6083 | 148.572 | 193.3073 |
| CUST_6919_PI429010886 | N20779 | 0.043275 | 24 | 0 | 156399889 | predicted protein [Nematostella vectensis] >gi\|156 | | | 2.51E-63 | 9.489751 | 25.38172 | 26.58331 | 13.76785 | 10.49349 | 13.35477 | 5.673834 | NA | 14.45048 | 6.62402 | 8.502113 | 14.12465 | 5.716003 | 6.701002 | 14.28863 | 20.02647 | 5.046239 | 16.74929 | 8.257726 | 21.87083 | 35.0422 | 16.02878 | 10.21924 | 20.9065 | 17.59108 | 18.33365 | 20.44923 |
| CUST_38868_PI429010886 | M31399 | 0.04329 | 15 | 12 | 546676950 | hypothetical protein D910_05262 [Dendroctonus p | Protein metabolism | Ribosome biogenesis | 6.73E-22 | 178.5939 | 237.5773 | 195.7228 | 151.2216 | 161.6849 | 157.5 | 178.7072 | 191.6146 | 214.2583 | 217.5495 | 171.8504 | 235.0953 | 189.6625 | 204.2904 | 229.0179 | 261.8083 | 139.1812 | 187.276 | 157.0455 | 202.0834 | 157.7478 | 227.8652 | 171.5473 | 182.6771 | 167.8299 | 196.9351 | 211.7401 |
| CUST_49126_PI429010886 | M84241 | 0.04329 | 15 | 12 | | | | | | 67.16912 | 49.7894 | 44.83381 | 51.17489 | 47.24387 | 44.34401 | 42.73401 | 39.9644 | 43.39642 | 56.76781 | 39.59389 | 49.44344 | 53.43124 | 54.01314 | 53.97066 | 52.0001 | 52.02242 | 46.26909 | 72.49991 | 63.64812 | 41.78704 | 50.76541 | 47.20692 | 36.37581 | 64.3699 | 44.63016 | 45.59444 |
| CUST_27798_PI429010886 | N56712 | 0.04329 | 15 | 12 | | | | | | 48.33175 | 62.41453 | 73.14043 | 80.18725 | 48.67019 | 54.37221 | 57.32582 | 79.78328 | 72.22304 | 45.65482 | 61.74634 | 68.47784 | 89.64325 | 67.87327 | 98.02439 | 92.60851 | 33.77978 | 73.04458 | 45.44604 | 55.57729 | 42.21202 | 61.0979 | 51.58632 | 54.61808 | 61.70387 | 83.14826 | 73.98788 |
| CUST_56941_PI429010886 | M114961 | 0.043292 | 15 | 9 | | | | | | 988.4432 | 1040.536 | 1250.32 | 888.436 | 1072.593 | 1044.56 | 919.6285 | 972.7549 | 1199.213 | 1338.117 | 1135.445 | 1270.234 | 1043.343 | 1578.633 | 1027.258 | 818.4343 | 931.1392 | 1141.431 | 976.3736 | 1172.181 | 1240.783 | 1376.551 | 1120.861 | 980.4603 | 1576.033 | 1362.62 | 1613.342 |
| CUST_25711_PI429010886 | N52926 | 0.043318 | 12 | 0 | | | | | | 54.35965 | 78.08745 | 79.14778 | 55.98343 | 58.46928 | 42.46725 | 45.4417 | 69.61278 | 50.87772 | 109.1565 | 59.25275 | 74.02683 | 99.52485 | 94.88698 | 83.6912 | 101.637 | 42.53957 | 73.1075 | 104.5873 | 66.63641 | 52.72315 | 77.45737 | 69.28978 | 71.66601 | 111.9364 | 60.34766 | 119.7198 |
| CUST_48197_PI429010886 | M79298 | 0.04332 | 21 | 18 | | | | | | 19.99534 | 16.70757 | 17.59292 | 16.12785 | 10.08763 | 7.243822 | 8.897078 | 4.524037 | 14.94259 | 19.73697 | 8.039625 | 18.03173 | NA | 10.97339 | 5.029486 | 20.48821 | 14.20342 | 8.082183 | 27.34621 | 11.8227 | 15.51771 | 23.34857 | 16.25929 | 10.61682 | 14.1603 | 9.941638 | 8.490289 |
| CUST_22580_PI429010886 | N47272 | 0.043329 | 21 | 3 | | | | | | 19.38567 | 28.23857 | 19.16951 | 35.53745 | 39.09942 | 31.92608 | 7.046406 | 4.67588 | 10.9847 | 55.82371 | 5.330779 | 17.71709 | 20.35771 | 14.72748 | 21.7799 | 38.93982 | 4.458351 | 25.49244 | 25.08131 | 18.24494 | 27.4201 | 27.00004 | 30.09768 | 11.22575 | 44.66782 | 38.04406 | 52.59262 |
| CUST_39667_PI429010886 | M42506 | 0.04333 | 18 | 3 | | | | | | 13.85986 | 15.08742 | 20.16285 | 55.91435 | 63.06953 | 89.71539 | 44.13738 | 30.08888 | 13.39856 | 3.525109 | 40.44714 | 5.731619 | 16.04975 | NA | 12.78311 | 7.501961 | 75.82559 | 67.43306 | 21.02441 | 8.149809 | 77.12763 | 16.2854 | 37.33083 | 92.63222 | 5.382165 | 28.9143 | 18.21746 |
| CUST_7814_PI429010886 | N22121 | 0.043349 | 15 | 12 | | | | | | 84.69362 | 67.50199 | 112.7651 | 60.02795 | 62.97162 | 71.2117 | 80.41737 | 84.86918 | 72.62704 | 81.81059 | 76.20212 | 79.40489 | 78.36561 | 91.6512 | 87.96508 | 94.43038 | 98.59007 | 77.18047 | 77.69906 | 65.76284 | 88.3275 | 72.686 | 83.71896 | 75.82559 | 93.70583 | 78.83085 | 104.9178 |
| CUST_46619_PI429010886 | M73190 | 0.043358 | 24 | 21 | | | | | | 6.492705 | 12.28125 | 12.63616 | 7.990456 | 8.321397 | 6.157906 | 5.899189 | 7.411834 | 5.184488 | 7.891053 | 9.279943 | 4.076501 | 8.279758 | 10.23894 | 6.513918 | 6.94077 | 7.059957 | 15.33921 | NA | 9.230964 | NA | 10.62424 | NA | 9.177025 | 11.65145 | 7.983639 | NA |
| CUST_46897_PI429010886 | M74108 | 0.043412 | 21 | 9 | | | | | | 25.7937 | 27.71495 | 30.59469 | 28.97255 | 16.3435 | 30.74938 | 30.75954 | 54.40561 | 40.33441 | 36.05134 | 76.44833 | 73.78486 | 46.00056 | 42.42487 | 27.9397 | 25.82153 | 17.91014 | 23.17075 | 30.80046 | 70.5402 | 48.16535 | 16.40969 | 28.29582 | 35.55553 | 15.21915 | 34.27356 | 52.58293 |
| CUST_45736_PI429010886 | M70093 | 0.043424 | 21 | 18 | 321455020 | hypothetical protein DAPPUDRAFT_3691 [Daphnia | Signal transduction | Other | 3.27E-26 | 26.4784 | 20.5469 | 17.54669 | 20.09233 | 24.30839 | 13.23625 | 29.2332 | 13.8397 | 8.3032 | 15.09945 | 19.22658 | 10.89133 | 22.94293 | 17.15702 | 30.57199 | 28.53623 | 25.04386 | 21.02638 | 34.98674 | 35.07942 | 18.33154 | 18.05541 | 16.16487 | 22.62556 | 28.64039 | 5.673212 | 16.71789 |
| CUST_35252_PI429010886 | M12048 | 0.043456 | 24 | 3 | | | | | | 72.48039 | 68.82274 | NA | 78.07189 | 45.2026 | 84.52872 | 59.11379 | 9.021728 | 58.892 | 3.98105 | 4.991219 | NA | 35.40201 | 12.98781 | 47.03677 | 39.01912 | 9.492295 | 44.56286 | 5.194327 | 14.1847 | 8.044043 | 35.11564 | NA | 37.09496 | 16.08378 | 12.29489 | 15.50269 |
| CUST_53319_PI429010886 | M101263 | 0.043467 | 12 | 0 | | | | | | 218.9512 | 264.5273 | 302.8941 | 244.1166 | 253.9783 | 272.8585 | 258.1237 | 225.2988 | 260.0898 | 254.4207 | 236.8954 | 330.1179 | 254.9978 | 330.3612 | 339.4134 | 237.5448 | 228.0216 | 233.6069 | 177.8716 | 280.0237 | 266.4363 | 291.5938 | 280.9299 | 260.9776 | 315.3064 | 302.3576 | 283.788 |
| CUST_54572_PI429010886 | M106601 | 0.043467 | 12 | 0 | | | | | | 65.81652 | 63.66464 | 41.41686 | 53.99181 | 29.34549 | 40.99957 | 35.39506 | 34.78474 | 47.99919 | 79.76051 | 43.88802 | 46.38212 | 56.11071 | 53.66581 | 60.36203 | 56.11599 | 27.35532 | 42.01359 | 39.68132 | 54.83325 | 53.11468 | 64.52054 | 46.88042 | 31.02312 | 87.57209 | 64.29604 | 96.42858 |
| CUST_31868_PI429010886 | M2617 | 0.043467 | 12 | 3 | 443734794 | hypothetical protein CAPTEDRAFT_73288, partial [C | Nucleic acid metabolism | RNA metabolic process | 3.69E-42 | 450.0163 | 208.1099 | 156.6764 | 1656.441 | 2679.021 | 3390.638 | 2718.679 | 1070.179 | 301.0804 | 96.72232 | 2555.115 | 104.0321 | 452.8393 | 96.92806 | 299.2346 | 156.0758 | 4051.321 | 1856.885 | 1828.604 | 92.48444 | 1652.112 | 176.4808 | 1370.685 | 1782.524 | 153.376 | 1022.449 | 82.72689 |
| CUST_48602_PI429010886 | M81219 | 0.043467 | 12 | 0 | | | | | | 41.41188 | 36.91363 | 67.13373 | 46.77892 | 32.32519 | 44.17135 | 37.19186 | 32.19898 | 43.66151 | 38.64362 | 29.22246 | 32.57877 | 32.47618 | 48.85338 | 49.01735 | 51.34688 | 36.77308 | 41.65923 | 33.69477 | 36.39578 | 37.20061 | 48.00546 | 32.70344 | 31.92933 | 62.97836 | 40.90354 | 51.62562 |
| CUST_2970_PI429010886 | N13505 | 0.043494 | 24 | 18 | 242009661 | Bromodomain-containing protein, putative [Pedicul | Metabolic process | Other | 1.65E-50 | 7.413154 | 10.91708 | 10.6154 | 10.82657 | 11.0048 | 12.56262 | 7.959892 | 5.063659 | 10.53948 | 15.66371 | 15.28371 | 12.20709 | 6.868272 | 8.212232 | 8.316685 | 14.56009 | 8.815034 | 12.95753 | 16.91106 | 16.2897 | 15.63419 | 17.01511 | 9.199507 | 11.98424 | 15.63419 | 7.396068 | 16.19147 |
| CUST_20141_PI429010886 | N42875 | 0.043495 | 24 | 0 | | | | | | 30.69887 | 30.93077 | 25.20311 | 18.87168 | 19.16951 | 16.8263 | 23.26561 | 16.0016 | 19.46629 | 29.17928 | 28.53359 | 24.64992 | 14.20826 | 16.91106 | 9.684471 | 17.29878 | 18.22532 | 25.36775 | 24.38309 | 22.52113 | 37.4516 | 17.99464 | 32.45148 | 10.36736 | 42.7734 | 21.53652 | 31.45667 |
| CUST_55201_PI429010886 | M109100 | 0.043508 | 18 | 6 | | | | | | 21.11367 | 24.37441 | 16.65858 | 24.53437 | 6.952548 | 24.42522 | 13.17448 | 45.22744 | 14.64853 | 13.32908 | 28.02833 | 25.52881 | 20.18529 | 7.757233 | 5.7379 | 10.24643 | 16.92439 | 12.65934 | 19.82899 | 16.76285 | 32.97611 | 39.33067 | 29.74088 | 15.5488 | 33.62105 | 33.56101 | 32.7645 |
| CUST_28235_PI429010886 | N57564 | 0.043508 | 24 | 18 | | | | | | 136.3426 | 137.8094 | 146.0101 | 84.40117 | 94.26766 | 86.56723 | 102.2413 | 88.34755 | 101.2537 | 196.129 | 119.4767 | 132.8952 | 102.205 | 168.3078 | 152.988 | 212.2276 | 96.56866 | 122.9325 | 149.2677 | 258.7295 | 116.8326 | 142.5243 | 99.73067 | 91.90962 | 232.8026 | 112.3919 | 193.1764 |
| CUST_55561_PI429010886 | M110723 | 0.043517 | 15 | 0 | | | | | | 3446.227 | 5035.54 | 2509.921 | 2682.549 | 4073.081 | 3303.552 | 1506.739 | 834.6224 | 1561.567 | 3342.501 | 3991.112 | 3278.647 | 1244.117 | 2062.191 | 1937.537 | 5688.498 | 3296.897 | 3216.436 | 2645.933 | 4374.715 | 2794.005 | 3249.343 | 1728.225 | 1442.354 | 3908.116 | 1902.402 | 2850.368 |
| CUST_52774_PI429010886 | M98857 | 0.043517 | 15 | 9 | | | | | | 205.1599 | 227.6752 | 276.8504 | 169.847 | 189.0849 | 199.1036 | 190.0645 | 201.5551 | 230.3934 | 271.8631 | 209.0287 | 263.6735 | 201.4342 | 239.3938 | 190.3637 | 254.9978 | 208.3184 | 190.3154 | 195.9585 | 229.245 | 216.9356 | 215.8293 | 211.9851 | 188.1392 | 345.9305 | 230.1517 | 260.5389 |
| CUST_6230_PI429010886 | N19810 | 0.043517 | 15 | 9 | | | | | | 23.565 | 28.39426 | 31.05901 | 17.18028 | 17.3028 | 12.08465 | 19.93306 | 15.24413 | 25.5511 | 27.50644 | 30.90791 | 32.75095 | 20.32835 | 26.30532 | 27.62987 | 25.33083 | 23.64437 | 24.13249 | 21.93695 | 25.37889 | 35.91866 | 30.112 | 32.10959 | 16.40766 | 36.23362 | 26.86002 | 34.35136 |
| CUST_6261_PI429010886 | N19863 | 0.043517 | 15 | 6 | | | | | | 32.50993 | 44.4501 | 34.69687 | 49.95028 | 36.16119 | 27.24397 | 67.9344 | 37.59821 | 61.31684 | 47.90107 | 32.96805 | 30.88328 | 28.5055 | 59.46938 | 57.16139 | 56.93106 | 36.01332 | 33.29134 | 55.57175 | 48.5148 | 54.58961 | 196.8321 | 92.1489 | 102.9324 | 44.40636 | 54.96227 | 49.89799 |
| CUST_10605_PI429010886 | N26499 | 0.043517 | 15 | 9 | | | | | | 188.2724 | 194.9477 | 216.6058 | 157.283 | 203.324 | 215.8293 | 170.0663 | 206.6992 | 195.555 | 299.5774 | 194.9477 | 256.8125 | 190.5242 | 235.2181 | 211.1942 | 250.7705 | 214.6655 | 248.5974 | 194.3859 | 190.0645 | 174.5081 | 192.5048 | 200.6692 | 223.2546 | 306.2716 | 203.1221 | 275.9866 |
| CUST_26123_PI429010886 | N53710 | 0.043517 | 15 | 9 | | | | | | 65.85741 | 96.38791 | 78.24583 | 62.44513 | 63.83268 | 76.52417 | 46.48282 | 68.78674 | 65.8929 | 97.99145 | 59.42335 | 102.9808 | 65.99023 | 83.1842 | 85.23443 | 73.61433 | 70.13143 | 73.35296 | 49.59839 | 62.79742 | 67.07557 | 80.00094 | 96.25237 | 74.74125 | 90.96114 | 70.3766 | 84.64574 |
| CUST_749_PI429010886 | N7131 | 0.043517 | 15 | 9 | | | | | | 1500.167 | 3272.814 | 946.2082 | 1237.49 | 2397.429 | 925.1976 | 2251.803 | 1075.686 | 3644.859 | 3821.032 | 2101.303 | 6505.037 | 1892.721 | 1827.911 | 2551.399 | 4950.976 | 451.6758 | 2807.026 | 1318.096 | 3204.304 | 1646.767 | 5086.964 | 1786.373 | 2624.92 | 3744.536 | 3493.781 | 4617.753 |
| CUST_1445_PI429010886 | N9663 | 0.043517 | 15 | 9 | | | | | | 1948.38 | 4544.383 | 953.5865 | 1777.044 | 976.6512 | 250.6863 | 128.1244 | 658.6556 | 4992.636 | 4091.647 | 3534.122 | 9660.218 | 2354.203 | 5430.703 | 3342.501 | 10044.17 | 442.6624 | 3459.777 | 491.272 | 6767.816 | 1346.091 | 2457.891 | 3972.597 | 822.3869 | 4797.809 | 6630.238 | 8414.501 |
| CUST_56045_PI429010886 | M112614 | 0.043523 | 18 | 12 | | | | | | 13.85408 | 17.22867 | 21.06491 | 15.27765 | 23.62378 | 24.93189 | 28.17746 | 28.48354 | 21.58663 | 21.99145 | 24.38855 | 33.76983 | 38.22991 | 27.78747 | 25.15661 | 24.96359 | 16.49958 | 20.17559 | 11.24691 | 18.11108 | 34.71503 | 26.68081 | 20.12612 | 20.76211 | 13.95091 | 24.39107 | 34.74686 |
| CUST_11397_PI429010886 | N27746 | 0.043529 | 24 | 21 | | | | | | 20.83932 | 10.43455 | 27.88984 | 16.0073 | 9.925474 | 13.80666 | 14.99955 | 11.33171 | 9.959454 | 15.37925 | 13.54322 | 17.48945 | 13.89365 | 18.42911 | 24.5772 | 29.0109 | 18.46372 | 24.19734 | 12.69121 | 13.87348 | 12.53403 | 33.14087 | 19.75734 | 21.53411 | 22.17314 | 25.12948 | 18.73257 |
| CUST_28466_PI429010886 | N58003 | 0.043529 | 15 | 12 | | | | | | 1675.386 | 2136.057 | 1627.141 | 1418.381 | 1493.465 | 1453.376 | 1136.737 | 1387.967 | 1416.05 | 1902.402 | 1398.489 | 2688.606 | 1773.273 | 1862.262 | 2877.641 | 2645.933 | 1212.358 | 1583.066 | 1355.174 | 2513.183 | 1632.477 | 2070.325 | 1550.902 | 1227.773 | 2151.64 | 1858.933 | 2316.991 |
| CUST_40804_PI429010886 | M56125 | 0.043558 | 15 | 0 | | | | | | 1298.64 | 2288.66 | 1651.385 | 978.9007 | 1241.637 | 1199.593 | 1010.552 | 765.2983 | 1173.656 | 1178.991 | 1332.224 | 1837.915 | 1073.06 | 1414.763 | 1553.73 | 2186.819 | 1415.482 | 1687.609 | 1193.503 | 1332.781 | 1119.759 | 1725.627 | 1388.367 | 1031.453 | 2140.816 | 1286.564 | 1651.385 |
| CUST_36055_PI429010886 | M14473 | 0.043566 | 15 | 9 | | | | | | 11.85674 | 11.71206 | 24.58762 | NA | 18.31489 | 18.63959 | 26.25499 | 31.24487 | 34.36888 | 25.60131 | 32.83834 | 30.62818 | 18.6166 | 37.25844 | 15.46627 | 26.43455 | 17.37482 | 12.92974 | 13.33386 | 31.65107 | 15.24413 | 37.24189 | 25.17921 | NA | 44.31444 | 6.699155 | 32.16644 |
| CUST_51726_PI429010886 | M94741 | 0.043588 | 18 | 15 | | | | | | 9.442336 | 12.55 | 11.07141 | 10.48943 | 10.32521 | 3.561028 | 12.80086 | 10.36994 | 6.597762 | 13.18773 | 13.34989 | NA | 7.80639 | 13.73363 | 18.74861 | 28.64039 | 6.281409 | 16.32267 | 13.24553 | 15.07352 | 16.11406 | 6.369665 | NA | NA | 17.90806 | 4.808163 | 13.34357 |
| CUST_55607_PI429010886 | M110932 | 0.043635 | 18 | 12 | | | | | | 14.37562 | 23.83451 | 28.60828 | 65.56207 | 24.08827 | 18.96468 | 19.14299 | 17.85063 | 16.94555 | 10.06452 | 23.90131 | 30.40511 | 48.1847 | 49.08991 | 32.88602 | 35.32273 | 31.52203 | 22.00276 | 18.2155 | 38.71068 | 23.46287 | 31.54083 | 23.95916 | 19.5413 | 16.78585 | 9.650569 | 31.17481 |
| CUST_22977_PI429010886 | N48040 | 0.043664 | 24 | 6 | 380030133 | PREDICTED: F-box only protein 21-like [Apis florea] | | | 5.83E-15 | 33.24397 | 29.40744 | 25.63163 | 11.84611 | 26.73153 | 31.5798 | 38.14903 | 43.66792 | 32.87464 | 23.68051 | 24.70497 | 43.961 | 35.46793 | 21.17737 | 32.06361 | 31.00851 | 25.03856 | 23.00326 | 23.97462 | 19.54386 | 19.1264 | 29.88417 | 39.95554 | 16.78585 | 29.33184 | 25.47915 | 27.36205 |
| CUST_1669_PI429010886 | N10400 | 0.043685 | 21 | 9 | 291222242 | PREDICTED: predicted protein-like [Saccoglossus ko | | | 1.2E-107 | NA | 12.94311 | 10.21556 | 19.29325 | 32.86388 | 5.60979 | 13.59135 | 76.22636 | 53.22359 | 21.04949 | 107.955 | 92.96667 | 72.28479 | 9.48088 | 25.39002 | 8.299606 | 29.47649 | NA | 13.7877 | 188.1161 | 86.63308 | 4.861634 | NA | 5.930374 | NA | 42.9717 | 56.89861 |
| CUST_55495_PI429010886 | M110357 | 0.043694 | 18 | 15 | | | | | | 30.38917 | 36.76731 | 21.00534 | 23.15281 | 24.69594 | 9.269541 | 23.2348 | 19.74682 | 27.31066 | 34.32332 | 20.44923 | 26.92776 | 43.05396 | 23.74079 | 25.75169 | 31.40553 | 31.53112 | 29.38623 | 24.89184 | 29.93088 | 12.79367 | 26.50986 | 20.73391 | 14.57268 | 33.87601 | 25.83743 | 16.36632 |
| CUST_52690_PI429010886 | M98470 | 0.043694 | 18 | 15 | | | | | | 130.8991 | 139.419 | 97.40947 | 93.11072 | 151.8851 | 134.8756 | 98.57526 | 109.5408 | 119.2623 | 150.78 | 124.6404 | 109.3157 | 89.24944 | 121.9257 | 96.23175 | 161.4564 | 169.7349 | 155.9586 | 155.5901 | 152.1844 | 92.97872 | 158.5869 | 134.1419 | 108.9403 | 132.9281 | 72.50841 | 83.69759 |
| CUST_29345_PI429010886 | N59681 | 0.043694 | 18 | 15 | | | | | | 26.33847 | 20.4401 | 15.96275 | 12.0248 | 16.78198 | 15.15808 | 15.7161 | 19.12346 | 16.56737 | 24.66303 | 23.71704 | 26.33096 | 20.83003 | 18.21042 | 24.0728 | 30.65462 | 25.52353 | 25.80604 | 22.23809 | 34.29372 | 9.159424 | 22.7667 | 13.36577 | 12.42997 | 27.17268 | 27.59686 | 28.38786 |
| CUST_8333_PI429010886 | N22888 | 0.043702 | 21 | 3 | 260805919 | hypothetical protein BRAFLDRAFT_286073 [Branchi | | | 1.59E-34 | 8.783511 | 6.397942 | 12.33347 | 13.70505 | 8.248819 | NA | 4.767964 | 5.611283 | NA | 9.946499 | 8.47166 | 11.81299 | 9.485366 | 11.6078 | 4.773638 | 4.066856 | 6.201123 | 7.885901 | NA | 6.731135 | 15.61967 | 11.51911 | NA | NA | 27.83166 | 24.1375 | 20.75541 |
| CUST_36939_PI429010886 | M17271 | 0.043728 | 12 | 3 | | | | | | 23.90612 | 16.61483 | 18.02985 | 25.31589 | 29.15169 | 46.40223 | 57.58322 | 32.07598 | 23.70999 | 23.85138 | 19.98856 | 25.95428 | 27.75759 | 23.52604 | 33.03437 | 25.83289 | 35.58868 | 50.85184 | 44.38974 | 20.8433 | 15.36071 | 23.62598 | 19.86296 | 16.92439 | 23.75855 | 29.26863 | 25.33406 |
| CUST_38743_PI429010886 | M29892 | 0.043734 | 15 | 9 | | | | | | 993.7035 | 1109.944 | 1146 | 909.757 | 827.2898 | 920.7129 | 682.7483 | 826.4016 | 1016.851 | 1285.806 | 888.1875 | 1281.734 | 960.3576 | 1391.685 | 1202.334 | 1271.51 | 913.0418 | 1110.444 | 912.4218 | 1047.894 | 1063.638 | 1269.191 | 1238.144 | 897.6332 | 1330.395 | 1109.944 | 1353.303 |
| CUST_42483_PI429010886 | M60390 | 0.043735 | 24 | 15 | 488518269 | PREDICTED: la-related protein 6 [Dasypus novemci | | | 1.66E-08 | 63.02348 | 40.82323 | 39.93998 | 37.72041 | 32.88602 | 31.13379 | 32.94683 | 32.88145 | 28.97849 | 73.22698 | 47.80852 | 33.71856 | 29.23595 | 42.31429 | 38.6196 | 64.26853 | 47.64968 | 65.83024 | 58.23835 | 55.37764 | 37.42448 | 51.0864 | 32.3787 | 35.16453 | 85.79457 | 45.16608 | 55.81208 |
| CUST_43718_PI429010886 | M63785 | 0.043735 | 24 | 15 | | | | | | 42.24487 | 49.02873 | 21.52056 | 35.07318 | 31.56077 | 15.91213 | 24.31121 | 24.94079 | 26.65472 | 38.80417 | 27.01753 | 35.33485 | 25.06406 | 32.45453 | 30.3471 | 48.17521 | 59.70983 | 40.4329 | 31.75251 | 38.93621 | 42.86965 | 44.55249 | 30.70822 | 28.3805 | 50.4975 | 38.55322 | 47.20161 |
| CUST_17582_PI429010886 | N38222 | 0.043735 | 24 | 15 | | | | | | 59.51435 | 54.62458 | 53.50062 | 53.79628 | 45.36871 | 77.59246 | 41.68991 | 44.31444 | 42.23347 | 59.29234 | 43.42171 | 84.47788 | 59.12378 | 57.48318 | 74.01735 | 75.13415 | 61.71609 | 71.2117 | 57.86841 | 76.03863 | 56.70973 | 64.19796 | 59.8939 | 45.04433 | 62.45053 | 52.33717 | 96.91901 |
| CUST_3501_PI429010886 | N14631 | 0.043754 | 18 | 6 | 504163658 | PREDICTED: D-dopachrome decarboxylase-like [Och | | | 1.07E-13 | 146.9814 | 133.7242 | 253.8408 | 143.1502 | 192.1973 | 171.7093 | 147.5412 | 337.0265 | 391.1505 | 116.6217 | 255.3857 | 297.361 | 221.5886 | 187.6138 | 253.8833 | 90.62378 | 78.55433 | 110.9478 | 149.9525 | 285.3755 | 157.6874 | 164.7862 | 151.9294 | 146.1555 | 83.71896 | 336.0629 | 232.5572 |
| CUST_39034_PI429010886 | M33746 | 0.04379 | 18 | 3 | | | | | | 612.1213 | 241.0332 | 270.1288 | 903.1657 | 965.4945 | 1785.596 | 1577.693 | 504.7239 | 692.0661 | 4.992877 | 748.2524 | 5.92639 | 410.9902 | 227.0558 | 383.9376 | 414.0246 | 1501.641 | 1419.195 | 803.077 | 27.64994 | 748.9581 | 377.6826 | 834.6224 | 2049.694 | 63.1995 | 953.5865 | 9.204642 |
| CUST_56322_PI429010886 | M113494 | 0.0438 | 15 | 12 | | | | | | 613.2651 | 749.7018 | 812.5634 | 714.9465 | 628.1675 | 837.1072 | 541.6023 | 606.6133 | 629.9728 | 888.436 | 655.7469 | 1146.628 | 721.3883 | 980.1159 | 1183.738 | 1092.036 | 651.5039 | 766.6399 | 764.5382 | 847.9888 | 801.3257 | 832.3284 | 720.7013 | 599.494 | 923.2313 | 840.6106 | 949.8478 |
| CUST_9976_PI429010886 | N25496 | 0.0438 | 15 | 12 | 328788969 | PREDICTED: leucine-rich repeat-containing protein | | | 1.46E-84 | 21.33598 | 17.44361 | 23.73387 | 24.26979 | 14.8801 | 11.51496 | 17.58889 | 12.91295 | 15.98363 | 15.88699 | 14.55066 | 25.39589 | 22.50936 | 23.88015 | 25.19237 | 33.62524 | 17.19687 | 8.667042 | 21.28336 | 19.66479 | 9.309107 | 13.74195 | 13.06649 | 10.84393 | 19.67357 | 11.96496 | 20.49973 |
| CUST_15396_PI429010886 | N34512 | 0.043801 | 21 | 0 | 242019615 | conserved hypothetical protein [Pediculus humanus | | | 6.17E-57 | 50.31204 | 29.94885 | 71.59535 | 32.77524 | 42.82585 | 46.88042 | 51.24109 | 34.91043 | 26.56923 | 34.1704 | 55.92013 | 33.42828 | 20.94349 | 20.04541 | 21.5046 | 29.23595 | 79.71189 | 27.92065 | 62.89021 | 28.69117 | 43.59959 | 31.19161 | 62.99752 | 66.40981 | 35.80323 | 28.38786 | 21.77078 |
| CUST_13738_PI429010886 | N31689 | 0.043807 | 15 | 9 | | | | | | 13.0885 | 487.5504 | 261.6019 | 422.5155 | 602.1279 | 259.2052 | 358.1231 | 1460.496 | 1075.314 | 311.7466 | 1132.32 | 1332.224 | 799.8512 | 259.9699 | 547.6349 | 7.279581 | 330.6772 | 7.138401 | 167.4333 | 3269.374 | 1049.194 | 7.952703 | 558.7985 | 426.8105 | NA | 1021.64 | 793.0472 |
| CUST_21715_PI429010886 | N45724 | 0.043815 | 15 | 3 | | | | | | 125.2532 | 110.4846 | 94.75367 | 120.4142 | 119.5328 | 144.5043 | 111.7039 | 66.46046 | 78.63344 | 74.93674 | 95.91036 | 74.52928 | 70.80885 | 72.85867 | 104.8214 | 108.6696 | 110.8154 | 103.7315 | 106.7397 | 89.45826 | 71.75603 | 81.45472 | 91.13848 | 114.5611 | 77.1951 | 73.07164 | 74.13818 |
| CUST_46444_PI429010886 | M72663 | 0.043821 | 21 | 15 | | | | | | 196.8321 | 172.7406 | 166.7568 | 130.6431 | 208.3184 | 167.6463 | 157.2555 | 161.3046 | 136.1838 | 170.4197 | 198.6003 | 172.9097 | 179.0372 | 149.3697 | 160.7094 | 242.302 | 226.768 | 184.4143 | 232.3526 | 147.5581 | 174.5925 | 176.4634 | 172.4652 | 156.3918 | 208.9955 | 141.9566 | 170.5765 |
| CUST_14411_PI429010886 | N32840 | 0.043821 | 18 | 6 | | | | | | 8.995799 | 6.858047 | 11.64812 | 7.278197 | NA | 7.774382 | 13.18436 | 16.75121 | 12.92801 | 17.0365 | 7.216307 | 9.605462 | 12.98903 | 21.16443 | 6.681729 | 4.954854 | 12.80617 | 7.774382 | 11.51778 | 4.279937 | 6.433191 | 10.46975 | 26.49443 | NA | 21.96294 | NA | 18.26372 |
| CUST_6471_PI429010886 | N20142 | 0.043826 | 24 | 15 | 241833897 | secreted protein, putative [Ixodes scapularis] >gi\|2 | | | 4.67E-16 | 167.6463 | 129.6012 | 130.1016 | 143.3418 | 151.752 | 187.869 | 142.4863 | 125.1948 | 149.6657 | 159.0029 | 137.2513 | 181.1767 | 123.2747 | 227.9427 | 230.3934 | 189.5057 | 175.4136 | 189.4794 | 187.1216 | 166.9208 | 147.5934 | 205.1599 | 156.8624 | 165.9249 | 149.3496 | 155.7397 | 137.4615 |
| CUST_36398_PI429010886 | M15800 | 0.043847 | 12 | 3 | | | | | | 171.1195 | 376.0453 | 14.18148 | 893.461 | 428.865 | 410.9035 | 237.3209 | 12.56927 | 187.4158 | 12.4672 | 20.40507 | 17.28828 | 74.86726 | 393.8505 | 302.2127 | 566.2341 | 26.55213 | 484.3691 | 24.6789 | 425.5183 | 87.40211 | 838.9983 | 11.99825 | 441.9667 | 216.9071 | 23.72179 | 21.20862 |
| CUST_33244_PI429010886 | M6382 | 0.043847 | 15 | 9 | | | | | | 90.05554 | 177.8716 | 104.474 | 67.64264 | 86.87439 | 47.86055 | 52.62699 | 68.17959 | 108.1985 | 175.6005 | 147.1881 | 228.4344 | 95.02448 | 134.1249 | 137.058 | 186.6438 | 75.62563 | 95.80114 | 147.6693 | 114.529 | 112.609 | 155.7397 | 110.6199 | 43.12293 | 175.9447 | 145.8455 | 204.9855 |
| CUST_56669_PI429010886 | M114385 | 0.043876 | 21 | 15 | | | | | | 143.8832 | 71.95612 | 123.8085 | 16.44239 | 141.0077 | 115.9278 | 108.6016 | 146.6632 | 70.29878 | 140.5892 | 89.80969 | 102.9429 | 158.3894 | 173.4253 | 68.1165 | 132.6337 | 162.7472 | 187.6525 | 172.3811 | 144.3925 | 122.6286 | 120.2042 | 107.1945 | 63.98224 | 42.16986 | 107.2229 | 129.0136 |
| CUST_40966_PI429010886 | M56505 | 0.043892 | 12 | 6 | | | | | | 63.03207 | 120.114 | 81.68427 | 52.60357 | 339.5005 | 90.50136 | 85.69951 | 99.36845 | 180.5686 | 100.1573 | 109.0752 | 128.2363 | 59.16475 | 36.74536 | 45.54848 | 100.9764 | 147.7682 | 76.88757 | 174.0405 | 79.1048 | 249.8933 | 131.6679 | 167.9521 | 84.93968 | 84.55489 | 101.3443 | 57.83187 |
| CUST_38380_PI429010886 | M26026 | 0.043904 | 18 | 9 | | | | | | 17.51958 | 9.645798 | 14.85125 | NA | 9.071169 | 8.208279 | 11.73379 | 10.69461 | 14.34788 | 20.58016 | 17.89088 | 35.51693 | 10.94929 | NA | NA | 7.359153 | 22.86232 | 28.08233 | 28.23631 | 6.13761 | 24.07736 | 9.149574 | NA | 7.746908 | 5.560888 | 14.98214 | 13.19779 |
| CUST_26287_PI429010886 | N53985 | 0.043917 | 12 | 0 | | | | | | 36.39953 | 43.31294 | 43.75138 | 28.82637 | 19.0844 | 18.0732 | 18.84012 | 32.35284 | 23.61845 | 35.57263 | 26.08326 | 26.85111 | 27.384 | 28.6962 | 35.37802 | 40.23874 | 24.94331 | 32.19156 | 22.91218 | 41.04712 | 27.18447 | 41.49836 | 27.97387 | 29.53645 | 52.8414 | 32.63377 | 33.11671 |
| CUST_43487_PI429010886 | M63179 | 0.043919 | 21 | 15 | | | | | | 370.2985 | 366.4854 | 277.7527 | 335.8595 | 286.2536 | 283.0956 | 265.2111 | 300.7661 | 297.361 | 316.971 | 274.7936 | 268.6688 | 269.0643 | 321.4467 | 344.0991 | 416.8412 | 316.7444 | 330.4772 | 310.5967 | 354.8939 | 327.2675 | 406.7395 | 276.2073 | 322.0219 | 350.0802 | 305.6803 | 252.0389 |
| CUST_38120_PI429010886 | M23440 | 0.043944 | 12 | 0 | | | | | | 3799.249 | 5080.996 | 2915.652 | 756.9801 | 738.2515 | 306.6185 | 5726.098 | 938.6625 | 1158.222 | 1704.986 | 1512.912 | 2168.863 | 1477.505 | 1317.338 | 5498.252 | 995.2843 | 668.0541 | 1011.26 | 3818.838 | 1478.287 | 2617.426 | 477.7327 | 1115.976 | 2448.925 | 926.1166 | 3276.015 | 4598.774 |
| CUST_39951_PI429010886 | M47399 | 0.043944 | 12 | 0 | | | | | | 3692.777 | 6247.817 | 3092.843 | 953.2915 | 857.8725 | 372.4888 | 6247.817 | 1096.334 | 1245.02 | 1807.03 | 1646.767 | 2700.337 | 1735.328 | 1413.603 | 6687.729 | 1264.096 | 696.7816 | 1184.216 | 4120.1 | 1756.162 | 2886.71 | 540.7565 | 1224.871 | 3001.59 | 1071.523 | 3687.08 | 4937.213 |
| CUST_5430_PI429010886 | N18690 | 0.043944 | 12 | 3 | 239812361 | Colias eurytheme clone PGI3-31_10 phosphoglucos | Metabolic process | Carbohydrate metabolic process | 8.9E-119 | 410.3497 | 593.8074 | 322.7291 | 581.8912 | 562.1725 | 568.2975 | 603.0098 | 350.0282 | 476.0617 | 365.1838 | 463.0515 | 538.667 | 404.8094 | 342.1732 | 427.4471 | 541.9679 | 533.5457 | 571.9057 | 537.701 | 463.5935 | 353.0021 | 561.7404 | 334.4771 | 540.3776 | 394.8691 | 365.8372 | 312.5243 |
| CUST_11791_PI429010886 | N28408 | 0.043973 | 18 | 6 | | | | | | 15.30361 | 4.719827 | NA | 6.448841 | 12.51404 | 20.95213 | 15.29619 | 28.19099 | 24.97091 | 24.67089 | 8.863586 | 14.47659 | 20.28753 | 7.072564 | 11.48274 | 16.39936 | 10.26589 | NA | 18.75416 | 11.22575 | 23.95732 | NA | 28.58102 | 18.02828 | 21.33093 | 33.91251 | 31.09423 |
| CUST_20605_PI429010886 | N43740 | 0.04398 | 21 | 9 | | | | | | 35.93002 | 24.9077 | 28.25151 | 20.37942 | 20.10533 | 8.56209 | 26.41986 | 24.52447 | 23.53859 | 27.52766 | 28.9598 | 27.2271 | 22.45542 | 25.0272 | 25.82998 | 20.20612 | 24.95301 | 25.91379 | 28.83526 | 19.32322 | 21.60941 | 24.37441 | 26.80742 | 23.75346 | 24.21253 | 18.03173 | 23.26561 |
| CUST_54632_PI429010886 | M106936 | 0.043983 | 24 | 0 | | | | | | 517.6852 | 720.2695 | 684.7245 | 612.4623 | 507.0685 | 665.6609 | 413.4726 | 531.3865 | 576.6597 | 802.0583 | 589.2877 | 695.332 | 518.0097 | 491.7148 | 473.1073 | 643.9207 | 426.6051 | 545.1015 | 645.4311 | 481.413 | 699.2311 | 555.7341 | 719.6282 | 306.3807 | 734.7434 | 806.3497 | 729.8175 |
| CUST_39044_PI429010886 | M33890 | 0.043994 | 24 | 18 | 237847769 | beta-N-acetylglucosaminidase [Litopenaeus vannan | Metabolic process | Carbohydrate metabolic process | 8.64E-28 | 129062.7 | 145034.3 | 111801.3 | 118691.9 | 110974.5 | 51921.46 | 19571.84 | 32430.96 | 133113.8 | 143243.9 | 123526 | 157589.4 | 105375.3 | 101693.2 | 135996.9 | 153607.2 | 20761.9 | 169441.2 | 145034.3 | 152308.4 | 150249.1 | 157589.4 | 133987.5 | 54992.02 | 148780.5 | 143960.1 | 130986.3 |
| CUST_20052_PI429010886 | N42719 | 0.043994 | 24 | 6 | | | | | | 312.1828 | 490.2884 | 225.3313 | 291.5581 | 198.7421 | 292.1061 | 414.8654 | 339.1053 | 299.6797 | 128.2179 | 333.2348 | 121.6835 | 231.2637 | 142.963 | 197.9328 | 208.3478 | 283.788 | 136.3233 | 168.9219 | 189.1715 | 107.0039 | 180.6083 | 244.775 | 232.5216 | 299.4458 | 162.3309 | 187.6138 |
| CUST_21990_PI429010886 | N46222 | 0.043994 | 24 | 15 | | | | | | 72.7025 | 12.00128 | 85.17905 | 23.86269 | 61.62124 | 47.55714 | 114.917 | 62.5561 | 107.8324 | 116.5044 | 55.67871 | 35.87751 | 168.6715 | 66.1724 | 48.11605 | 99.91859 | 143.3153 | 88.72989 | 78.76718 | 61.19321 | 137.0908 | 118.8596 | 64.90973 | 79.62582 | 63.38531 | 50.33169 | 98.22103 |
| CUST_42252_PI429010886 | M59804 | 0.043995 | 15 | 0 | | | | | | 74.88 | 104.964 | 131.2414 | 96.21869 | 101.7152 | 178.5686 | 38.88802 | 18.90043 | 66.00564 | 75.27932 | 107.1617 | 104.351 | 35.85352 | 78.85902 | 75.3452 | 131.7341 | 92.92758 | 140.3134 | 79.09571 | 101.5112 | 108.1674 | 81.67574 | 54.04881 | 49.80346 | 99.05161 | 113.3684 | 114.6323 |
| CUST_52779_PI429010886 | M98876 | 0.043995 | 15 | 9 | 345489996 | PREDICTED: hypothetical protein LOC100678781 [N | | | 2.91E-22 | 11.35551 | 23.38175 | 26.33364 | 19.13027 | 10.84227 | 20.84836 | 24.58762 | 20.66296 | 12.76656 | 21.4035 | 24.17334 | 31.73369 | 15.70628 | 26.37273 | 18.20414 | 11.95734 | 23.46033 | 25.09429 | 11.23137 | 20.73834 | 26.34872 | 25.01801 | 30.6195 | 16.01307 | 33.76025 | 29.86645 | 33.76678 |
| CUST_10039_PI429010886 | N25603 | 0.043995 | 15 | 9 | | | | | | 54.85716 | 66.76607 | 67.71039 | 53.9331 | 58.544 | 74.40209 | 44.92544 | 53.61134 | 70.66973 | 81.36657 | 41.06525 | 77.20798 | 67.41072 | 67.02702 | 75.99857 | 69.52372 | 53.50062 | 69.80469 | 58.87201 | 55.01225 | 64.54153 | 75.17352 | 75.93064 | 68.06012 | 79.88802 | 76.13058 | 79.97877 |
| CUST_13085_PI429010886 | N30549 | 0.043995 | 15 | 9 | | | | | | 59.1427 | 59.62185 | 64.4321 | 50.18155 | 50.33907 | 50.60456 | 53.07973 | 64.8815 | 69.39137 | 61.98456 | 65.10535 | 71.27533 | 55.55874 | 57.93571 | 56.99104 | 75.82559 | 59.74927 | 62.04074 | 44.95044 | 69.04216 | 45.46228 | 71.43726 | 62.23219 | 53.49548 | 75.48481 | 57.00393 | 74.41568 |
| CUST_19960_PI429010886 | N42519 | 0.043995 | 15 | 9 | | | | | | 80.31713 | 105.2542 | 120.7327 | 101.3914 | 100.2834 | 102.639 | 88.98374 | 90.44569 | 113.7893 | 125.9958 | 114.2883 | 161.2028 | 107.1532 | 144.7304 | 102.3996 | 129.4353 | 108.5223 | 102.8937 | 93.99876 | 112.7651 | 115.8059 | 146.2634 | 128.4938 | 92.01375 | 142.124 | 153.3563 | 141.1734 |
| CUST_23871_PI429010886 | N49622 | 0.043995 | 15 | 9 | | | | | | 212.3072 | 238.1333 | 279.0812 | 215.167 | 201.2707 | 180.0476 | 225.3313 | 277.0077 | 239.9392 | 343.9276 | 236.1903 | 257.5442 | 193.3866 | 281.9612 | 223.8787 | 227.4444 | 229.13 | 259.2052 | 241.0846 | 182.204 | 222.9689 | 300.7661 | 289.2842 | 214.0415 | 279.1889 | 227.4444 | 283.8348 |
| CUST_56019_PI429010886 | M112553 | 0.044029 | 15 | 12 | | | | | | 1332.781 | 1430.775 | 1441.828 | 1235.847 | 1274.119 | 1109.944 | 1120.205 | 1161.62 | 1167.349 | 1550.226 | 1330.395 | 1610.651 | 1329.318 | 1524.773 | 1622.403 | 1815.297 | 1187.094 | 1186.768 | 1281.734 | 1515.268 | 1227.15 | 1621.924 | 1187.991 | 1105.691 | 1735.328 | 1289.132 | 1789.616 |
| CUST_52850_PI429010886 | M99200 | 0.044035 | 18 | 6 | | | | | | 10.29815 | 9.391276 | 25.77541 | NA | 11.03355 | NA | 19.33417 | 17.09566 | 19.40236 | 23.54965 | 12.32751 | 13.08554 | 10.63659 | 16.11406 | 18.00776 | 12.04133 | 9.758601 | 14.51392 | 15.84353 | 15.41663 | 10.00344 | 13.61134 | 9.198308 | NA | 16.36148 | 16.67648 | 29.43648 |

| Probe ID | ID | p-value | Period | Phase | EBI ID | Annotation | GO category | Go term | E-value | 0 | 0 | 0 | 3 | 3 | 3 | 6 | 6 | 6 | 9 | 9 | 9 | 12 | 12 | 12 | 15 | 15 | 15 | 18 | 18 | 18 | 21 | 21 | 21 | 24 | 24 | 24 |
|---|---|---|---|---|---|---|---|---|---|---|---|---|---|---|---|---|---|---|---|---|---|---|---|---|---|---|---|---|---|---|---|---|---|---|---|---|
| CUST_51680_PI429010886 | M94549 | 0.044065 | 15 | 12 | | | | | | 9.684471 | 17.12411 | 19.31282 | 35.1893 | 10.86396 | 15.14331 | 10.13043 | 8.386399 | 12.91624 | 18.63959 | 5.868354 | 8.1716 | 20.79184 | NA | 61.69829 | 26.28003 | 12.60126 | 42.40387 | 17.71924 | 13.70338 | 10.50509 | NA | NA | 7.407239 | 10.6028 | 23.09604 | 10.31819 |
| CUST_1991_PI429010886 | N11252 | 0.044066 | 24 | 15 | | | | | | 40.23032 | 46.72383 | 59.07909 | 30.41942 | 49.79836 | 82.44448 | 41.28609 | 42.33366 | 52.15419 | 40.50555 | 48.26954 | 63.11008 | 54.45697 | 64.53642 | 57.92964 | 54.79813 | 90.82199 | 70.33751 | 74.7726 | 37.54361 | 64.03011 | 43.91811 | 59.2457 | 58.92815 | 50.77029 | 43.82278 | 46.56491 |
| CUST_53670_PI429010886 | M102717 | 0.044076 | 21 | 15 | | | | | | 34.47136 | 29.17357 | 19.30381 | 29.81183 | 23.95482 | 24.80361 | 31.10257 | 16.85251 | 23.43097 | 28.57593 | 22.55376 | 30.63369 | 28.70795 | 22.16055 | 38.73055 | 35.66432 | 36.9176 | 31.55142 | 29.31162 | 27.38056 | 30.2284 | 34.38336 | 32.70652 | 26.90259 | 30.9626 | 20.88392 | 23.90612 |
| CUST_6428_PI429010886 | N20089 | 0.044089 | 24 | 21 | 321477877 | hypothetical protein DAPPUDRAFT_206599 [Daphn | | | 3.12E-60 | 78.02809 | 75.75933 | 62.08961 | 83.63981 | 89.67555 | 90.01113 | 73.84429 | 72.62704 | 84.56429 | 95.63731 | 70.87083 | 83.40091 | 66.91698 | 74.07672 | 76.11306 | 96.81237 | 115.6902 | 91.40169 | 97.17307 | 86.48234 | 84.56429 | 100.9764 | 112.7504 | 98.22103 | 72.45575 | 82.30578 | 84.82591 |
| CUST_52511_PI429010886 | M97717 | 0.044096 | 15 | 9 | | | | | | 50.27036 | 49.3486 | 63.27271 | 42.02786 | 41.4213 | 36.4309 | 39.99252 | 41.7241 | 49.81881 | 58.45028 | 45.85649 | 127.001 | 48.85338 | 60.70574 | 57.37114 | 79.59942 | 40.48072 | 47.58792 | 47.5835 | 52.69412 | 44.63765 | 64.88975 | 39.46416 | 51.67859 | 57.62155 | 57.93571 | 76.353 |
| CUST_44841_PI429010886 | M67165 | 0.044106 | 15 | 9 | 260834499 | hypothetical protein BRAFLDRAFT_100078 [Branchi | | | 1.53E-27 | 7.941557 | 8.773525 | NA | NA | 11.09833 | 7.066118 | 6.221163 | 61.63016 | 12.41778 | 18.52264 | NA | 14.59396 | 5.012567 | NA | 8.255312 | 7.520119 | 5.469892 | 8.237262 | 16.91528 | NA | 7.310152 | 9.69944 | 10.85271 | 17.35915 | 13.72174 | 19.24305 | 11.7681 |
| CUST_26929_PI429010886 | N55143 | 0.044143 | 18 | 12 | | | | | | 119.1161 | 162.0946 | 228.0795 | 269.4831 | 167.5706 | 207.9222 | 181.1767 | 268.7143 | 197.6933 | 230.6845 | 173.5738 | 301.467 | 264.7596 | 280.0685 | 305.9676 | 193.3866 | 197.6076 | 224.4296 | 183.266 | 188.0209 | 150.8706 | 310.4051 | 194.4273 | 182.5915 | 186.6438 | 303.0187 | 241.638 |
| CUST_3357_PI429010886 | N14353 | 0.044186 | 21 | 15 | 242021864 | Amphiphysin, putative [Pediculus humanus corporis | | | 4.98E-49 | 25.43424 | 51.32807 | 31.13642 | 33.67567 | 29.6615 | 34.79371 | 21.59499 | 29.51147 | 23.53568 | 28.58102 | 26.07952 | 29.43141 | 31.2907 | 52.08136 | 18.66977 | 34.60947 | 35.19595 | 47.14099 | 36.43958 | 33.23976 | 31.18733 | 32.50993 | 25.88533 | 39.09341 | 27.48106 | 22.49442 | 45.42508 |
| CUST_3463_PI429010886 | N14553 | 0.044186 | 24 | 18 | | | | | | 14.36708 | 40.53866 | 18.65856 | 13.89803 | 12.74489 | 15.53405 | 15.70076 | 12.81601 | 18.35021 | 21.49202 | 15.34644 | 20.93844 | 17.45526 | 26.78191 | 32.42452 | 23.21852 | 13.21272 | 43.11192 | 15.89244 | 30.83942 | 25.95987 | 15.77573 | 18.03368 | 15.56261 | 24.69322 | 31.17481 | 15.02569 |
| CUST_45837_PI429010886 | M70452 | 0.044204 | 24 | 0 | | | | | | 94.42153 | 103.5678 | 68.44169 | 44.39447 | 53.36598 | 79.88802 | 66.29488 | 54.44415 | 43.26504 | 40.98995 | 42.45744 | 37.21745 | 71.41994 | 40.91962 | 40.59586 | 52.32373 | 78.77563 | 98.59007 | 81.36657 | 35.23219 | 37.62291 | 50.12589 | 45.83381 | 74.54008 | 57.49062 | 62.82655 | 53.95069 |
| CUST_38457_PI429010886 | M26896 | 0.044208 | 18 | 9 | 321478301 | hypothetical protein DAPPUDRAFT_303131 [Daphn | Metabolic process | Carbohydrate metabolic process | 5.17E-24 | 651.2944 | 1115.976 | 816.6897 | 829.4016 | 903.9804 | 401.7586 | 641.3583 | 1009.233 | 1039.842 | 1507.459 | 1188.466 | 1295.841 | 1168.631 | 1075.314 | 909.4624 | 1233.738 | 519.4558 | 670.112 | 985.3973 | 1599.783 | 1027.698 | 1355.174 | 631.4343 | 439.9985 | 996.654 | 1023.389 | 1437.865 |
| CUST_44087_PI429010886 | M64888 | 0.044208 | 18 | 9 | | | | | | 63.60606 | 50.28232 | 40.04237 | 76.06574 | 50.61028 | 50.85184 | 56.59481 | 59.64714 | 63.54619 | 96.92806 | 97.42055 | 61.58977 | 64.0654 | 46.02417 | 55.23417 | 60.29973 | 78.83809 | 59.20737 | 59.31402 | 51.17489 | 43.15798 | 58.53061 | 70.05382 | 64.29604 | 70.08934 | 61.00433 | 57.76414 |
| CUST_46457_PI429010886 | M72709 | 0.044208 | 18 | 15 | | | | | | 101.1196 | 126.8192 | 95.36046 | 98.82662 | 86.03759 | 105.6225 | 75.85953 | 76.25978 | 93.83775 | 116.9236 | 83.91514 | 123.669 | 84.77843 | 99.23417 | 109.2144 | 110.1036 | 103.3649 | 141.0077 | 73.84429 | 107.7922 | 58.74124 | 107.0314 | 93.80773 | 94.90628 | 97.47845 | 61.2231 | 87.92471 |
| CUST_12204_PI429010886 | N29099 | 0.044232 | 18 | 9 | 260794975 | hypothetical protein BRAFLDRAFT_68970 [Branchio | | | 5.62E-37 | 11.21854 | 10.35656 | 15.98525 | 9.442336 | 8.853279 | 4.789688 | 6.762779 | 13.14309 | 22.5259 | NA | 21.16443 | 13.49282 | 12.3155 | 26.84868 | 16.31327 | 16.45644 | 12.78311 | 15.2073 | 14.77827 | 20.61498 | 7.348282 | 19.51952 | 10.09872 | 5.742324 | 17.20887 | 17.79901 | 13.99622 |
| CUST_34940_PI429010886 | M11130 | 0.044233 | 24 | 6 | | | Protein metabolism | Proteolysis | | 386.3769 | 1135.445 | 893.1457 | 929.9665 | 247.1825 | 348.906 | 565.8837 | 976.6512 | 389.4334 | 622.9374 | 358.8712 | 402.6363 | 230.7819 | 461.7538 | 290.4858 | 191.0009 | 132.88 | 28.85616 | 74.19557 | 474.2296 | 172.8115 | 15.53244 | 803.077 | 186.2287 | 4.443923 | 239.0491 | 86.37756 |
| CUST_39212_PI429010886 | M36171 | 0.044233 | 21 | 9 | 77743875 | Euphausia superba isolate DGGE band AntEuk13-5 1 | | | | 25.52007 | 46.53178 | 50.8348 | 97.40947 | 90.91352 | 42.44034 | 71.83981 | 346.6366 | 96.95634 | 64.66231 | 367.6066 | 364.2104 | 844.11 | 32.6735 | 298.0928 | 19.83493 | 171.1038 | 21.89378 | 43.14736 | 7221.522 | 153.44 | 34.97912 | 39.44223 | 22.33694 | 38.91585 | 190.1658 | 360.2672 |
| CUST_44568_PI429010886 | M66337 | 0.044233 | 24 | 18 | | | | | | 170.6145 | 202.6446 | 129.4503 | 87.85163 | 141.9837 | 110.9478 | 116.4037 | 97.62464 | 138.5194 | 172.1829 | 158.9804 | 170.2053 | 148.6917 | 127.3563 | 154.7492 | 240.0651 | 93.09068 | 154.463 | 205.7316 | 163.3073 | 191.3918 | 225.693 | 180.6083 | 115.8704 | 160.5552 | 123.4117 | 182.494 |
| CUST_42961_PI429010886 | M61734 | 0.044242 | 21 | 0 | | | | | | 127.112 | 127.6888 | 138.5998 | 114.5736 | 118.495 | 111.5705 | 121.3613 | 103.802 | 101.0393 | 137.4373 | 122.9126 | 99.57087 | 125.408 | 133.5801 | 116.8091 | 127.3882 | 145.2632 | 121.4442 | 125.9958 | 111.1643 | 129.0136 | 130.5118 | 134.5558 | 127.0219 | 171.8172 | 96.74817 | 121.6835 |
| CUST_3800_PI429010886 | N15301 | 0.044252 | 24 | 6 | | | | | | 17.03915 | 19.53272 | 14.87074 | 18.72449 | 23.45771 | 3.614894 | 19.29593 | 21.86136 | 26.77181 | 36.89793 | 20.24475 | 17.93968 | 19.96619 | 16.09421 | 18.10067 | 13.45079 | 12.90145 | 14.12148 | 20.24475 | 25.10875 | 21.01736 | 10.95786 | 8.491672 | 9.124 | 19.4153 | 21.32603 | 19.37939 |
| CUST_18702_PI429010886 | N40252 | 0.044256 | 24 | 15 | 332023058 | Protein lap4 [Acromyrmex echinatior] | | | 9.97E-44 | 42.06392 | 50.46685 | 42.80624 | 38.84613 | 39.68929 | 60.19936 | 33.28531 | 44.28069 | 34.95466 | 52.09002 | 40.75734 | 38.95233 | 44.31012 | 53.77942 | 35.8731 | 67.44354 | 84.93221 | 60.12977 | 50.88285 | 55.59388 | 38.73495 | 68.61072 | 29.85793 | 43.21123 | 48.71321 | 56.07774 | 51.54875 |
| CUST_41126_PI429010886 | M56931 | 0.044264 | 18 | 9 | | | | | | 29.52396 | 45.22744 | 45.07578 | 24.86204 | 30.59095 | 18.01173 | 36.98717 | 55.35407 | 59.41199 | 66.37222 | 40.54346 | 47.48523 | 36.29774 | 37.31224 | 29.33184 | 60.41993 | 23.1151 | 39.90876 | 48.25771 | 21.40624 | 27.49771 | 44.64991 | 27.30026 | 21.52428 | 56.94451 | 30.27191 | 57.08439 |
| CUST_2760_PI429010886 | N13048 | 0.044283 | 21 | 0 | 110671859 | ALG-2 interacting protein x [Penaeus monodon] | | | 2.58E-62 | 15.29798 | 11.39853 | 33.21431 | 16.53144 | 6.62402 | 11.11646 | 12.14051 | 7.213286 | 8.845266 | 5.311014 | 9.313914 | NA | 23.22371 | 12.01567 | 13.1549 | 10.74056 | 7.427442 | 14.47174 | 9.345594 | 18.6803 | 6.383068 | 16.02003 | 24.10675 | 19.12737 | 5.942845 | 9.830811 | 7.482892 |
| CUST_33477_PI429010886 | M6983 | 0.044287 | 15 | 9 | | | | | | 1258.369 | 1140.062 | 1230.543 | 1030.678 | 1066.79 | 917.4556 | 999.7477 | 1013.924 | 1119.759 | 1245.02 | 1069.858 | 1724.056 | 1166.811 | 1271.51 | 1223.156 | 1642.187 | 1123.855 | 963.151 | 1050.577 | 1444.045 | 1132.32 | 1446.944 | 1181.018 | 637.4283 | 1747.319 | 1401.401 | 1690.584 |
| CUST_44418_PI429010886 | M65900 | 0.044298 | 24 | 18 | 242005302 | ankyrin repeat domain-containing protein, putative | | | 1.04E-24 | 11.412 | 9.625872 | 5.666175 | 12.10865 | 12.6532 | 9.601564 | 5.973441 | 7.995482 | 3.62823 | 16.44566 | 5.526487 | 8.908142 | 5.841607 | 7.93225 | 6.839858 | 18.87168 | 10.01939 | NA | 15.64506 | 15.02374 | NA | 15.26731 | 4.424481 | 14.39261 | 9.87605 | NA | NA |
| CUST_30855_PI429010886 | M14 | 0.044309 | 15 | 12 | | | | | | 106.5844 | 107.666 | 120.8319 | 90.74799 | 95.74719 | 102.0907 | 116.2211 | 74.36967 | 120.7015 | 103.9198 | 70.80146 | 154.9678 | 100.1395 | 176.2631 | 127.601 | 144.5655 | 92.42083 | 116.8326 | 88.31622 | 83.32632 | 99.52485 | 104.9922 | 70.80885 | 97.93806 | 140.4217 | 103.6762 | 141.1131 |
| CUST_31516_PI429010886 | M1703 | 0.044309 | 15 | 12 | | | | | | 84.96672 | 105.2698 | 67.22918 | 75.5762 | 83.85047 | 85.493 | 67.55015 | 60.14737 | 70.13886 | 79.28399 | 80.6942 | 134.595 | 90.76835 | 82.48421 | 123.0876 | 111.4235 | 69.00002 | 104.164 | 64.45055 | 119.4557 | 85.65603 | 81.22317 | 80.79066 | 57.63741 | 84.61863 | 68.25944 | 89.88488 |
| CUST_38041_PI429010886 | M23000 | 0.044309 | 15 | 12 | | | | | | 4154.657 | 5726.098 | 5157.962 | 4701.722 | 3827.342 | 4447.018 | 3511.533 | 3772.58 | 4782.509 | 5372.754 | 3960.835 | 8304.282 | 5039.929 | 6920.477 | 7330.797 | 6416.964 | 3384.084 | 5757.178 | 3981.392 | 5991.557 | 4594.669 | 5407.054 | 4420.361 | 3886.072 | 6329.544 | 5751.466 | 6621.858 |
| CUST_43760_PI429010886 | M63883 | 0.044309 | 15 | 3 | 189236380 | PREDICTED: similar to IP3K2 CG34359-PC [Tribolium | | | 1.65E-52 | 68.85742 | 45.78676 | 63.34887 | 74.74125 | 97.70002 | 70.05382 | 99.45401 | 71.9016 | 42.05465 | 45.84492 | 67.60441 | 39.82447 | 56.76191 | 37.14124 | 44.09424 | 51.41614 | 87.26858 | 65.42947 | 87.87096 | 65.28922 | 45.98092 | 54.5276 | 57.59651 | 52.56534 | 66.45252 | 59.72331 | 32.3494 |
| CUST_5141_PI429010886 | N18309 | 0.044309 | 15 | 12 | 321468460 | hypothetical protein DAPPUDRAFT_52407 [Daphnia | Protein metabolism | Cellular protein modification process | 1.1E-107 | 112.9946 | 151.5629 | 122.4782 | 86.7497 | 87.07504 | 53.00119 | 80.05215 | 125.4876 | 121.7504 | 161.7779 | 92.38118 | 187.8401 | 116.5433 | 174.5449 | 143.2115 | 175.011 | 95.32771 | 117.4158 | 106.4056 | 147.0435 | 85.8526 | 150.2666 | 141.4904 | 79.55126 | 160.3449 | 104.2505 | 171.2071 |
| CUST_8758_PI429010886 | N23543 | 0.044309 | 15 | 12 | 321474804 | hypothetical protein DAPPUDRAFT_309070 [Daphn | Metabolic process | Other | 2.04E-97 | 185.8187 | 300.5825 | 257.6478 | 257.2108 | 208.6342 | 264.1236 | 225.5174 | 199.0699 | 266.0871 | 378.5438 | 194.6296 | 229.3254 | 295.9077 | 372.2181 | 312.5243 | 295.9544 | 287.9369 | 249.9907 | 237.9203 | 200.4624 | 228.3136 | 311.4551 | 272.4885 | 203.482 | 267.1348 | 298.8597 | 275.547 |
| CUST_8765_PI429010886 | N23555 | 0.044309 | 15 | 12 | | | | | | 87.20085 | 85.90922 | 96.55629 | 80.842 | 76.38976 | 78.53416 | 48.48483 | 63.1832 | 69.58984 | 78.27999 | 52.47261 | 103.3725 | 92.8298 | 87.03787 | 87.6112 | 98.46582 | 48.55278 | 56.27319 | 76.82089 | 76.34394 | 79.66167 | 75.22006 | 87.20085 | 58.81878 | 76.81043 | 71.38156 | 67.27473 |
| CUST_9053_PI429010886 | N23992 | 0.044309 | 15 | 12 | | | | | | 83.56858 | 120.2505 | 107.7093 | 86.01753 | 74.89819 | 72.30449 | 50.039 | 62.5758 | 69.18246 | 106.4056 | 71.74926 | 110.9624 | 87.54624 | 103.0771 | 64.57467 | 90.8645 | 59.86271 | 81.94846 | 81.47819 | 59.6416 | 89.32488 | 58.74124 | 75.25456 | 55.48994 | 87.6112 | 45.1918 | 93.83775 |
| CUST_12162_PI429010886 | N29027 | 0.044309 | 15 | 6 | 260820407 | hypothetical protein BRAFLDRAFT_116233 [Branchi | Metabolic process | Nucleobase-containing compound metabolic process | 8.64E-38 | 97.47845 | 96.34482 | 134.0609 | 87.83251 | 105.1 | 90.53385 | 106.6808 | 162.4501 | 108.6696 | 134.6396 | 85.45263 | 139.419 | 80.9845 | 82.09306 | 88.22213 | 88.55995 | 71.61792 | 99.32217 | 98.37238 | 120.6234 | 103.7577 | 102.4535 | 131.6163 | 97.56553 | 87.41325 | 111.8286 | 112.2705 |
| CUST_23841_PI429010886 | N49569 | 0.044309 | 15 | 12 | | | | | | 105.2005 | 106.4989 | 91.36505 | 112.8267 | 68.77033 | 74.66117 | 70.7166 | 85.05317 | 105.5089 | 125.4528 | 77.03965 | 113.1785 | 111.8858 | 132.5094 | 121.4703 | 131.1004 | 70.84411 | 103.1898 | 79.38753 | 113.8462 | 82.44448 | 106.4056 | 109.5287 | 61.16623 | 124.0489 | 117.847 | 132.4401 |
| CUST_37230_PI429010886 | M18282 | 0.044314 | 12 | 0 | 328781555 | PREDICTED: tRNA-splicing endonuclease subunit Se | Nucleic acid metabolism | RNA metabolic process | 6.49E-43 | 41.65923 | 34.82515 | 53.61134 | 28.77687 | 20.09671 | 24.35068 | 20.42712 | 28.66586 | 32.73752 | 41.73826 | 23.78915 | 416.363 | 36.39221 | 53.11468 | 27.7492 | 32.94969 | 18.9533 | 30.27835 | 31.32413 | 19.58405 | 19.12864 | 38.37992 | 42.29494 | 30.04146 | 34.33884 | 31.56711 | 30.89566 |
| CUST_30735_PI429010886 | N62519 | 0.044314 | 12 | 3 | | | | | | 104.6277 | 100.9304 | 66.75988 | 143.2572 | 237.1286 | 263.8365 | 269.7173 | 91.42252 | 68.52038 | 80.85722 | 181.5127 | 77.34848 | 87.45287 | 68.95801 | 68.02926 | 83.10449 | 298.3253 | 131.9783 | 241.7686 | 66.83042 | 141.9197 | 92.34903 | 134.7073 | 160.531 | 71.76497 | 99.30005 | 58.41483 |
| CUST_16046_PI429010886 | N35560 | 0.04432 | 15 | 0 | | | | | | 235.3955 | 202.6087 | 204.1442 | 179.5282 | 213.8072 | 201.6936 | 194.2907 | 130.3743 | 160.531 | 147.5934 | 174.8583 | 141.1886 | 142.2988 | 189.5057 | 175.8911 | 159.741 | 230.1871 | 322.5056 | 170.9469 | 193.5271 | 204.2904 | 211.1942 | 151.5005 | 164.3174 | 160.1083 | 221.2444 | 183.266 |
| CUST_24208_PI429010886 | N50229 | 0.04432 | 18 | 12 | | | | | | 488.5796 | 430.7898 | 358.6229 | 364.5694 | 544.2536 | 296.0384 | 369.1054 | 365.2576 | 336.6312 | 467.4407 | 636.6917 | 734.2085 | 649.8854 | 549.0001 | 510.9063 | 623.8166 | 379.0729 | 461.882 | 312.4226 | 1122.017 | 545.1015 | 593.968 | 303.4935 | 356.4277 | 505.1623 | 452.9164 | 541.9679 |
| CUST_14362_PI429010886 | N32761 | 0.044345 | 15 | 9 | 465980330 | Nucleolar protein 14 [Chelonia mydas] | Nucleic acid metabolism | RNA metabolic process | 1.07E-20 | 125.9453 | 197.2642 | 161.6167 | 105.5375 | 119.3258 | 99.67314 | 117.6403 | 120.7762 | 147.5068 | 211.4366 | 137.9332 | 204.1086 | 162.3872 | 185.8187 | 137.8094 | 197.6933 | 100.5554 | 145.794 | 143.7068 | 180.2796 | 158.496 | 179.616 | 143.8508 | 88.75833 | 205.4682 | 151.7723 | 209.83 |
| CUST_20425_PI429010886 | N43413 | 0.044378 | 24 | 3 | | | | | | 810.6402 | 530.8519 | NA | 793.9994 | 289.8469 | 333.2881 | 698.6187 | 48.96007 | 601.7703 | NA | NA | NA | 484.607 | 34.84989 | 321.696 | 266.722 | NA | 298.3253 | 3.635833 | 26.6361 | 3.527616 | 212.2526 | NA | 506.1361 | 28.47129 | 32.89748 | 3.545379 |
| CUST_46087_PI429010886 | M71333 | 0.044396 | 21 | 18 | 443696486 | hypothetical protein CAPTEDRAFT_172435 [Capitell | | | 2.55E-18 | 75.2717 | 82.78386 | 68.64401 | 56.36899 | 87.87096 | 56.13335 | 43.48001 | 49.02476 | 46.991 | 111.7257 | 64.20475 | 69.59584 | 66.13621 | 64.18609 | 68.45737 | 81.82955 | 42.84657 | 86.31209 | 60.97252 | 79.24559 | 80.15845 | 84.722 | 71.85634 | 66.75988 | 68.52849 | 61.86332 | 57.59651 |
| CUST_17353_PI429010886 | N37821 | 0.044413 | 18 | 9 | | | | | | 21.99372 | 32.11242 | 30.97619 | 22.79797 | 18.19413 | 23.00326 | 24.45953 | 21.61291 | 23.88883 | 37.28203 | 23.92874 | 48.96007 | 25.70952 | 36.23362 | 28.59095 | 37.71174 | 18.93228 | 42.34616 | 29.00481 | 26.63383 | 20.69091 | 27.60111 | 22.29454 | 20.4413 | 26.44212 | 27.17605 | 44.68023 |
| CUST_24542_PI429010886 | N50810 | 0.044413 | 12 | 3 | | | | | | 1970.653 | 793.0472 | 894.6121 | 4480.043 | 3132.563 | 7811.413 | 3517.336 | 810.2943 | 1444.613 | 120.6382 | 1992.587 | 131.3416 | 1263.557 | 852.6077 | 954.3357 | 934.5759 | 4579.426 | 5133.687 | 2424.452 | 191.0009 | 3489.908 | 1237.49 | 2387.546 | 5152.319 | 320.2376 | 3237.592 | 113.475 |
| CUST_5656_PI429010886 | N18999 | 0.044447 | 18 | 0 | | | | | | 1929.904 | 575.3599 | 1729.171 | 535.7875 | 1060.287 | 691.4084 | 1793.14 | 627.2172 | 606.7586 | 454.184 | 1409.58 | 492.7259 | 581.5974 | 587.4356 | 511.9204 | 474.2296 | 1634.318 | 473.8216 | 1746.637 | 670.112 | 963.6143 | 721.5575 | 1704.986 | 1498.845 | 749.7018 | 525.7241 | 599.1678 |
| CUST_813_PI429010886 | N7400 | 0.044451 | 21 | 6 | | | | | | 55.55289 | 222.7049 | 125.0127 | 459.0733 | 211.3962 | 379.0179 | 146.0723 | 514.1495 | 531.3865 | 190.5242 | 437.7044 | 685.9612 | 327.0942 | 463.2589 | 356.6641 | 85.19825 | 165.9503 | 114.443 | 123.6231 | 1215.238 | 434.2638 | 76.98006 | 181.4858 | 121.5738 | 81.86063 | 549.2217 | 519.8548 |
| CUST_48027_PI429010886 | M78587 | 0.044529 | 18 | 3 | | | | | | 5.510093 | 14.35732 | 13.61134 | NA | 20.31401 | 9.166269 | 4.541579 | 10.47429 | NA | 5.777163 | 11.76447 | 11.49808 | 3.992492 | 3.337384 | NA | 7.452286 | 7.544684 | 9.044875 | 10.7968 | 4.716459 | 7.106027 | 20.4513 | NA | NA | 7.891053 | 7.785373 | 8.564683 |
| CUST_48531_PI429010886 | M80827 | 0.044529 | 15 | 12 | | | | | | 692.3236 | 823.8136 | 869.29 | 653.8395 | 681.5488 | 859.4575 | 526.4672 | 616.7782 | 710.0284 | 804.2481 | 599.2901 | 1171.568 | 712.5461 | 1027.997 | 1048.248 | 1022.449 | 612.9216 | 824.1385 | 675.3956 | 859.0461 | 740.4281 | 816.1069 | 778.015 | 607.4385 | 894.6121 | 881.5993 | 912.0221 |
| CUST_25744_PI429010886 | N52976 | 0.04455 | 24 | 15 | | | | | | 86.07302 | 97.24592 | 112.5022 | 108.6016 | 74.84295 | 79.08585 | 77.69906 | 67.36225 | 80.19532 | 132.5094 | 99.99373 | 132.9657 | 96.37601 | 131.4711 | 131.1403 | 154.1768 | 97.42055 | 104.192 | 111.1358 | 92.46177 | 108.3614 | 110.9974 | 105.3949 | 118.5121 | 98.86383 | 89.19702 | 110.273 |
| CUST_17412_PI429010886 | N37921 | 0.044565 | 18 | 12 | | | | | | 16.46586 | 9.464207 | 38.84241 | NA | NA | 4.852706 | 25.88998 | 25.6517 | 20.63783 | 17.75939 | 20.14149 | 19.13785 | 41.89305 | 36.67205 | 27.47752 | 13.27837 | 18.84012 | NA | 49.688 | 16.29644 | 26.77489 | 10.45417 | 20.01979 | 13.4823 | 13.37205 | NA | 16.53144 |
| CUST_34595_PI429010886 | M10110 | 0.044628 | 18 | 3 | 48094341 | PREDICTED: myophilin [Apis mellifera] | | | 2.3E-68 | 144.6868 | 48.66621 | 166.2545 | 75.2875 | 455.1929 | 187.5572 | 467.2192 | 46.20496 | 42.40805 | 40.2115 | 416.7544 | 51.32807 | 36.99055 | 52.25652 | 44.97678 | 72.17688 | 714.2963 | 165.4521 | 455.4031 | 42.21202 | 585.0822 | 64.03011 | 812.8751 | 791.5217 | 52.61483 | 49.49037 | 54.10775 |
| CUST_5045_PI429010886 | N18190 | 0.044688 | 24 | 18 | 524911143 | PREDICTED: DNA ligase 1-like [Aplysia californica] | Response to stress | DNA repair | | 103.0871 | 139.2667 | 85.15981 | 73.41537 | 80.5848 | 75.31286 | 53.07285 | 94.06166 | 100.0068 | 92.8298 | 88.71715 | 136.4498 | 89.30785 | 109.0193 | 134.595 | 165.6901 | 94.5989 | 95.37902 | 100.7517 | 128.0141 | 118.1013 | 111.4706 | 109.4916 | 75.43095 | 126.1763 | 97.46537 | 121.4237 |
| CUST_29287_PI429010886 | N59554 | 0.044691 | 24 | 18 | | | | | | 103.6887 | 130.7243 | 113.3117 | 87.54624 | 89.00024 | 73.69227 | 71.16635 | 53.03364 | 92.31619 | 122.5998 | 93.82886 | 118.2155 | 79.16747 | 106.7633 | 87.57209 | 125.1451 | 68.95801 | 107.3346 | 97.03372 | 126.8764 | 152.4677 | 95.52923 | 120.4628 | 76.75659 | 136.9448 | 106.7139 | 112.7046 |
| CUST_29407_PI429010886 | N59800 | 0.044714 | 15 | 6 | | | | | | 85.77984 | 78.49411 | 94.07093 | 106.5597 | 95.12719 | 74.14418 | 110.2048 | 85.18737 | 93.82886 | 136.5503 | 82.29118 | 114.8668 | 83.42195 | 81.18707 | 72.59038 | 85.94103 | 78.13459 | 89.16253 | 91.36505 | 93.10199 | 115.1327 | 100.0986 | 122.0182 | 99.25819 | 88.63339 | 243.718 | 100.336 |
| CUST_44724_PI429010886 | M66784 | 0.044715 | 15 | 3 | | | | | | 20.6935 | 17.88508 | 9.218873 | 76.61613 | 71.47658 | 138.0091 | 86.24828 | 51.5653 | 35.91157 | 3.804265 | 43.10871 | NA | 37.32614 | 11.7971 | 22.38584 | 7.885901 | 129.5236 | 70.02583 | 52.801 | NA | 53.44251 | 7.844929 | 64.42438 | 89.43652 | 9.183116 | 32.51286 | 11.20539 |
| CUST_16495_PI429010886 | N36332 | 0.044719 | 12 | 0 | | | | | | 83.75206 | 75.12567 | 94.34866 | 67.65513 | 57.61472 | 50.22122 | 61.05172 | 52.33717 | 89.10973 | 90.65923 | 64.14878 | 73.29485 | 70.03699 | 106.7139 | 68.44169 | 53.1957 | 58.00017 | 79.18359 | 67.65513 | 64.33014 | 79.73386 | 122.0534 | 82.62874 | 79.07776 | 70.76377 | 84.63515 | 73.68557 |
| CUST_20432_PI429010886 | N43426 | 0.044719 | 12 | 0 | | | | | | 685.806 | 588.5777 | 788.3593 | 461.6522 | 569.4459 | 671.3234 | 532.0502 | 537.4529 | 453.3672 | 650.5246 | 556.0784 | 838.3296 | 619.0377 | 606.3721 | 715.8969 | 556.2338 | 544.2536 | 529.6585 | 525.4442 | 631.4343 | 500.6766 | 839.8931 | 742.4096 | 667.8908 | 649.1033 | 607.2425 | 728.657 |
| CUST_37848_PI429010886 | M21859 | 0.044722 | 18 | 9 | 260801594 | Branchiostoma floridae hypothetical protein, mRNA | Cellular component organization | Chromosome organization | 2.79E-93 | 425.5183 | 1063.11 | 615.9204 | 593.5055 | 676.7937 | 511.8133 | 472.651 | 576.6597 | 803.077 | 899.5738 | 620.1455 | 1299.658 | 811.2478 | 859.4575 | 844.933 | 1066.553 | 496.4081 | 724.6164 | 806.0846 | 793.0472 | 737.2359 | 573.6965 | 633.0392 | 534.0061 | 1186.768 | 747.2269 | 952.3475 |
| CUST_45511_PI429010886 | M69334 | 0.044722 | 18 | 15 | 321473002 | hypothetical protein DAPPUDRAFT_223051 [Daphn | | | 2.84E-14 | 17.02546 | 14.72582 | 11.49258 | 15.51227 | 12.67545 | 11.06582 | 7.989468 | 11.37935 | 8.3117 | 17.00167 | 17.60068 | 24.21006 | 8.827909 | 13.47795 | 7.635215 | 22.16055 | 15.82481 | 7.703897 | 11.92046 | 14.59759 | 15.60189 | 14.24976 | 6.604961 | 10.62697 | 7.659037 | 7.323042 | 9.833527 |
| CUST_14493_PI429010886 | N32975 | 0.044723 | 24 | 15 | | | | | | 8.769916 | 13.18436 | 10.20522 | NA | 10.7807 | 7.368786 | 6.910269 | 3.747255 | 6.895828 | 5.640027 | 14.72238 | 7.102715 | 18.82741 | 16.23932 | 7.408115 | NA | 14.73565 | NA | 12.84324 | NA | 4.988225 | 15.5304 | 10.37567 | NA | 12.38947 | 12.88874 | NA |
| CUST_17496_PI429010886 | N38069 | 0.044747 | 21 | 15 | | | | | | 140.8613 | 120.5115 | 147.2771 | 99.84559 | 116.6557 | 116.73 | 108.6974 | 94.54715 | 106.1066 | 133.9495 | 102.782 | 119.841 | 115.9538 | 132.88 | 83.24628 | 145.9205 | 133.2264 | 144.3428 | 99.11857 | 150.8429 | 142.194 | 144.9791 | 117.9167 | 114.9975 | 129.6855 | 102.2185 | 159.32 |
| CUST_36588_PI429010886 | M16302 | 0.044748 | 24 | 21 | 241632816 | ETS domain-containing protein Elk-4, putative [Ixod | Nucleic acid metabolism | Regulation of gene expression | 5.95E-48 | 14.94965 | NA | 10.3727 | 9.008803 | 16.4962 | 6.047731 | 8.578901 | 5.144335 | 9.581554 | 9.403927 | 6.566142 | 8.190659 | 10.33897 | 12.8856 | 14.68167 | 17.76083 | 14.33088 | 7.710119 | NA | 8.73506 | 14.51697 | 15.88213 | 14.70984 | 10.73459 | 7.242163 | 49.69406 | 13.44859 |
| CUST_36114_PI429010886 | M14674 | 0.044753 | 24 | 0 | | | | | | 18.08454 | 26.80106 | 28.55766 | 10.53251 | 7.579956 | 14.27753 | 19.99534 | 21.22218 | 15.78237 | 17.93968 | 16.54538 | 17.66078 | 14.3364 | 17.1412 | 16.99938 | 25.00056 | 8.128829 | 11.68857 | 23.52815 | 19.24305 | 22.55627 | 19.60466 | 21.94448 | 13.03387 | 23.53859 | 29.15169 | 30.61198 |
| CUST_35930_PI429010886 | M14001 | 0.04476 | 21 | 18 | | | | | | 268.1153 | 354.9858 | 224.6063 | 192.7945 | 178.7372 | 211.1137 | 176.4634 | 212.4692 | 159.0029 | 291.7598 | 204.4866 | 179.7938 | 184.0607 | 222.2406 | 250.8965 | 333.6383 | 223.1189 | 217.6167 | 230.3934 | 313.5825 | 218.1405 | 346.5286 | 188.427 | 207.6805 | 349.6083 | 219.3834 | 229.0179 |
| CUST_12559_PI429010886 | N29651 | 0.044783 | 15 | 12 | | | | | | 904.602 | 1002.084 | 731.6805 | 677.1285 | 606.4913 | 644.3019 | 604.7306 | 648.9401 | 666.3149 | 914.3958 | 660.5155 | 831.3875 | 769.0647 | 878.7545 | 911.24 | 1013.924 | 696.3149 | 738.2515 | 934.2348 | 846.1561 | 790.5889 | 958.279 | 672.9863 | 618.8739 | 956.1852 | 707.3962 | 894.0864 |
| CUST_45070_PI429010886 | M67909 | 0.044812 | 12 | 0 | 242011956 | ubiquitin conjugation factor E4 B, putative [Pediculu | Protein metabolism | Proteolysis | 3.22E-29 | 40.97837 | 60.02795 | 66.52424 | 35.48044 | 39.14518 | 44.98472 | 45.83957 | 41.67274 | 43.68839 | 54.51541 | 40.03759 | 50.00256 | 43.35404 | 54.87191 | 53.25416 | 58.01793 | 48.81237 | 43.80188 | 47.80199 | 36.5186 | 31.54852 | 73.88403 | 49.84719 | 32.65296 | 60.0207 | 53.48774 | 58.0737 |
| CUST_31419_PI429010886 | M1483 | 0.044838 | 24 | 18 | 391331678 | PREDICTED: transcription elongation factor S-II-like | Protein metabolism | Regulation of translational elongation | 4.73E-64 | 6.433191 | 15.5721 | 8.799195 | 12.57101 | 7.487023 | 3.689388 | NA | 5.630584 | 8.602798 | 11.88135 | 9.647019 | 15.83559 | 19.86545 | 10.19086 | 10.54822 | 18.07887 | 8.152575 | NA | 16.95126 | 35.31167 | 12.95283 | 12.01996 | 4.86085 | 35.18059 | 15.37012 | 11.86943 | 14.53418 |
| CUST_46759_PI429010886 | M73636 | 0.044868 | 15 | 12 | | | | | | 24.28972 | 19.676 | 15.59485 | 16.93182 | 25.53352 | 15.77707 | 21.92246 | 18.61312 | 12.60453 | 22.69042 | 21.27347 | 18.1208 | 32.53107 | 26.51493 | 28.67208 | 20.26123 | 34.58049 | 24.39614 | 30.07667 | 22.90261 | 19.38567 | 30.98764 | 16.56559 | 15.2034 | 28.55766 | 15.7841 | 21.7647 |
| CUST_37825_PI429010886 | M21773 | 0.044889 | 18 | 3 | 256539086 | fatty acids binding protein [Fenneropenaeus chinen | Transport | Other | 4.11E-25 | 8.3032 | 5.105169 | 7.550196 | NA | 1640.228 | 15.03992 | 5.769447 | 4.723754 | 3.787986 | 5.494496 | 4.207721 | 4.049112 | NA | NA | 3.52009 | 5.526487 | 1529.572 | NA | 1266.139 | 1201.319 | 5.970417 | 7.967454 | 12.32915 | NA | 15.5279 | 2123.129 | 4.980145 |
| CUST_32643_PI429010886 | M4674 | 0.044899 | 24 | 0 | 528469211 | PREDICTED: mucin-22-like [Danio rerio] | | | 2.09E-07 | 91.12979 | 120.579 | 125.8769 | 65.45683 | 76.64595 | 95.88672 | 83.48026 | 79.23712 | 81.41269 | 135.2716 | 130.7948 | 110.8992 | 78.44959 | 90.41498 | 71.91814 | 113.5017 | 104.6828 | 102.6107 | 102.3344 | 101.2912 | 116.3867 | 113.6118 | 123.7589 | 124.8411 | 165.3668 | 130.6431 | 115.0699 |
| CUST_3269_PI429010886 | N14145 | 0.044899 | 24 | 3 | | | | | | 48.1847 | 62.65132 | 55.79937 | 62.72678 | 67.78689 | 68.1923 | 60.3241 | 63.02348 | 56.29594 | 49.48526 | 37.73221 | 54.21273 | 56.59481 | 65.25641 | 58.81878 | 57.56078 | 41.74272 | 51.35337 | 78.39028 | 40.70276 | 39.43891 | 48.05852 | 35.15805 | 43.32293 | 24.63631 | 55.88861 | 51.92326 |
| CUST_11550_PI429010886 | N28006 | 0.044899 | 15 | 3 | | | | | | 129.8621 | 92.38705 | 39.20036 | 155.649 | 125.6499 | 163.3967 | 115.8059 | 112.8928 | 52.62093 | 70.66226 | 63.21301 | 30.59854 | 99.79838 | 48.82147 | 71.9913 | 85.77984 | 151.3617 | 101.6676 | 131.1249 | 56.95101 | 59.48755 | 77.67565 | 72.07584 | 113.3684 | 71.38156 | 104.0821 | 38.0304 |
| CUST_12180_PI429010886 | N29057 | 0.044911 | 15 | 9 | | | | | | 143.9244 | 140.364 | 104.6153 | 43.1379 | 52.68156 | 70.94036 | 52.54728 | 72.27253 | 65.1875 | 479.6761 | 140.2437 | 394.8691 | NA | 168.6014 | 155.0724 | 290.4297 | NA | 136.5699 | 63.92583 | 154.3359 | 182.5193 | 53.69064 | 221.0323 | 271.5235 | 245.6875 | 107.8082 | 174.4788 |
| CUST_45988_PI429010886 | M71012 | 0.044924 | 15 | 9 | | | | | | 30.24466 | 32.17296 | 29.9579 | 10.46161 | 26.09533 | 22.43297 | 30.30557 | 21.43485 | 23.2298 | 51.70242 | 29.02633 | 38.35721 | 35.992 | 38.88089 | 47.05374 | 38.4769 | 17.96838 | 38.11743 | 36.41953 | 38.54962 | 33.80016 | 38.43703 | 34.2288 | 21.59129 | 51.00626 | 44.57334 | 55.23417 |
| CUST_44644_PI429010886 | M66559 | 0.044959 | 21 | 12 | | | | | | NA | 26.72669 | 9.524556 | 85.10975 | 50.8634 | 12.43401 | 26.66367 | 297.7533 | 57.06419 | 25.90485 | 234.2305 | 215.7123 | 499.6354 | 9.105658 | 235.0953 | 8.818215 | 113.2085 | NA | 16.0073 | 4672.866 | 83.67885 | 6.209359 | 5.594409 | 13.36717 | 8.654424 | 105.7694 | 169.7841 |
| CUST_41328_PI429010886 | M57456 | 0.044961 | 24 | 0 | 321463376 | hypothetical protein DAPPUDRAFT_226732 [Daphn | Transport | Ion transport | 1.21E-10 | 60.87936 | 85.39372 | 97.81711 | 37.95272 | 59.70379 | 76.25978 | 65.49092 | 50.02348 | 62.07681 | 77.41461 | 65.06553 | 59.47675 | 39.414 | 43.20694 | 44.70504 | 69.41655 | 78.56402 | 75.43095 | 81.21391 | 52.14738 | 53.38747 | 46.58164 | 51.80467 | 52.74471 | 72.83324 | 74.02683 | 105.57 |
| CUST_51397_PI429010886 | M93390 | 0.045033 | 21 | 9 | | | | | | NA | 240.9417 | 49.27786 | 306.9757 | 455.6129 | 108.3189 | 326.6831 | 3192.934 | 2030.719 | 90.64646 | 2109.682 | 2457.891 | 3110.134 | 102.5401 | 1259.079 | NA | 857.1846 | NA | 83.26471 | 12744.97 | 832.0351 | 4.087188 | 206.7246 | 178.4123 | 6.563098 | 741.4101 | 1466.368 |
| CUST_6433_PI429010886 | N20095 | 0.045037 | 24 | 0 | | | | | | 33.52107 | 43.66792 | 35.7636 | 29.27728 | 32.07184 | 25.37889 | 22.10673 | 34.89251 | 21.77078 | 21.12385 | 23.20447 | 20.82459 | 23.73784 | 16.44239 | 24.59022 | 184810.5 | 24.32155 | 23.85453 | 32.42452 | 21.9039 | 18.69874 | 34.91364 | 24.28604 | 13.13514 | 43.15798 | 24.64442 | 23.75346 |
| CUST_37249_PI429010886 | M18363 | 0.045066 | 18 | 3 | | | | | | 1898.136 | 7091.211 | 2888.695 | 7686.447 | 3163.856 | 5265.647 | 1905.993 | 2531.781 | 2948.044 | 2890.576 | 1701.136 | 4680.081 | 2142.216 | 3375.684 | 2276.234 | 3355.686 | 1371.865 | 2203.176 | 1604.147 | 3034.646 | 4031.594 | 3929.651 | 5262.203 | 3074.469 | 4371.861 | 5356.619 | 2504.679 |
| CUST_44941_PI429010886 | M67470 | 0.045078 | 24 | 18 | | | | | | 64.48135 | 152.8418 | 143.8832 | 90.47199 | 120.3232 | 69.98559 | 112.8267 | 86.38663 | 78.76718 | 164.0384 | 139.5841 | 145.1035 | 144.6491 | 173.1695 | 172.6882 | 268.9771 | 192.2865 | 86.54824 | 139.8015 | 286.0114 | 156.4459 | 156.1682 | 192.5322 | 55.85751 | 285.6495 | 104.2639 | 206.3839 |
| CUST_54159_PI429010886 | M104867 | 0.04508 | 21 | 18 | | | | | | 74.84295 | 107.354 | 80.81357 | 74.85405 | 75.98025 | 59.87756 | 45.77355 | 45.77928 | 63.49666 | 97.18779 | 60.33388 | 97.37669 | 62.11683 | 91.30112 | 94.42153 | 92.48444 | 68.24289 | 87.32671 | 74.75705 | 99.91859 | 96.38791 | 152.2451 | 66.94583 | 67.30873 | 72.92837 | 58.85973 | 78.70239 |
| CUST_27310_PI429010886 | N55825 | 0.045092 | 15 | 9 | | | | | | 518.3277 | 601.0454 | 575.6787 | 393.2523 | 455.4031 | 451.4433 | 408.6642 | 448.6531 | 524.4761 | 724.0591 | 491.9013 | 628.02 | 526.3482 | 652.581 | 587.1049 | 737.2359 | 470.4395 | 523.7505 | 480.665 | 614.8997 | 544.3999 | 624.13 | 634.1523 | 456.9093 | 744.0064 | 553.0837 | 644.3019 |
| CUST_36672_PI429010886 | M16504 | 0.045103 | 21 | 6 | | | | | | 51.02123 | NA | 8.495784 | 45.44604 | 5.244064 | 37.61167 | 936.0363 | NA | 899.2493 | 33.92714 | 6.644648 | 20.33108 | 288.1296 | 21.94448 | 158.8254 | 23.67253 | NA | 24.32573 | 8.305626 | 8.313099 | 8.80889 | 18.61046 | 20.18529 | 18.85135 | 11.65447 | 41.05122 | 13.43228 |
| CUST_47185_PI429010886 | M75100 | 0.045203 | 15 | 3 | | | | | | 46.7059 | 41.26402 | 47.11613 | 68.69041 | 37.71676 | 44.18564 | 47.65494 | 38.87769 | 35.10359 | 32.79034 | 19.98178 | 31.32413 | 49.68201 | 28.56717 | 26.0691 | 59.32225 | 38.64078 | 37.88493 | 44.15772 | 27.87027 | 67.08962 | 73.20424 | 30.64068 | 41.7241 | 23.44768 | 35.96518 | 31.77138 |
| CUST_50148_PI429010886 | M88815 | 0.045203 | 24 | 0 | 270009743 | hypothetical protein TcasGA2_TC009040 [Tribolium | Nucleic acid metabolism | RNA metabolic process | 6.33E-14 | 486.9644 | 781.7106 | 789.7368 | 427.0325 | 694.099 | 955.8025 | 522.6581 | 542.8448 | 641.7483 | 659.6239 | 357.9328 | 850.7537 | 379.3496 | 647.3023 | 250.2782 | 508.5068 | 227.225 | 423.2024 | 329.0813 | 177.8416 | 985.3973 | 678.9878 | 1068.926 | 610.2139 | 839.5092 | 970.0099 | 555.454 |
| CUST_19711_PI429010886 | N42052 | 0.045203 | 15 | 3 | | | | | | 10.11094 | 18.44132 | 19.90626 | 12.85382 | 13.82413 | 24.08567 | 14.52959 | 10.4013 | 11.67245 | 13.82537 | 8.462821 | 11.76608 | 13.30045 | 8.587225 | 10.99269 | 16.39244 | 11.99992 | 9.13082 | 13.74381 | 20.79841 | 19.04367 | 17.17022 | 10.11181 | 20.41758 | 13.97697 | 12.79498 | 5.653542 |
| CUST_438_PI429010886 | N5182 | 0.045205 | 18 | 6 | | | | | | 1868.48 | 1477.505 | 2651.764 | 738.4538 | 1937.537 | 1653.841 | 2232.073 | 2770.165 | 2935.657 | 2603.161 | 1631.11 | 1926.251 | 2474.132 | 2229.117 | 1745.549 | 4510.96 | 1909.916 | 2031.538 | 1113.282 | 1621.23 | 1510.415 | 2698.741 | 2167.205 | 1718.489 | 2756.174 | 1503.323 | 2833.332 |
| CUST_35198_PI429010886 | M11873 | 0.045211 | 15 | 3 | | | | | | 200.2396 | 28.25834 | 168.032 | 519.6135 | 484.2538 | 1178.599 | 611.7017 | 287.5478 | 165.8485 | 6.312211 | 714.7333 | NA | 147.3674 | 26.29189 | 85.00815 | 17.0738 | 1028.397 | 674.4353 | 625.7026 | NA | 666.1474 | 16.88691 | 431.3053 | 626.8612 | 7.860635 | 507.4019 | 4.747131 |
| CUST_46604_PI429010886 | M73135 | 0.045233 | 15 | 9 | | | | | | 77.45737 | 84.03407 | 75.66735 | 78.56402 | 95.54049 | 72.93993 | 77.94557 | 85.69951 | 80.66119 | 96.90956 | 90.95085 | 92.35924 | 66.5854 | 75.39347 | 86.20359 | 93.1869 | 72.98413 | 72.67632 | 79.51299 | 89.4755 | 81.57207 | 106.1754 | 81.73916 | 107.5904 | 92.5954 | 84.10524 | 103.384 |
| CUST_11758_PI429010886 | N28353 | 0.045236 | 18 | 9 | 390343325 | PREDICTED: RNA-directed DNA polymerase from m | Nucleic acid metabolism | DNA replication | 3.35E-17 | 26.0056 | 21.08444 | 32.83309 | 28.16035 | 23.83176 | 21.64092 | 28.3838 | 38.1318 | 29.75783 | 38.41593 | 42.03557 | 24.2228 | 32.38272 | 38.02365 | 26.16897 | 27.97666 | 46.25026 | 34.37176 | 32.39752 | 38.723 | 31.94483 | 32.52687 | 20.1154 | 20.859 | 39.22918 | 36.67205 | 33.3136 |
| CUST_14128_PI429010886 | N32370 | 0.045236 | 18 | 3 | | | | | | 242.0042 | 202.8118 | 20.76211 | 344.7288 | 368.8573 | 364.2104 | 156.2777 | 74.74125 | 168.4127 | 31.74511 | 29.62034 | 41.61311 | 107.1617 | 273.7876 | 258.3561 | 344.2985 | 39.03429 | 366.3446 | 26.75249 | 194.9822 | 58.46315 | 514.6723 | 41.13711 | 489.8149 | 219.715 | 71.34779 | 38.43988 |
| CUST_4797_PI429010886 | N17501 | 0.045274 | 24 | 18 | | | | | | 54.86684 | 16.51187 | 58.94211 | 37.92303 | 40.40681 | 38.19514 | 12.50047 | 38.48636 | 40.63191 | 39.50189 | 60.04192 | 46.09323 | 34.65468 | 56.93106 | 47.20692 | 53.37349 | 49.64123 | 40.48796 | 77.18047 | 40.04237 | 76.00655 | 30.04952 | 52.3781 | 59.68907 | 54.74214 | 47.06657 | 50.57708 |
| CUST_46504_PI429010886 | M72852 | 0.045276 | 24 | 21 | | | | | | 1160.295 | 1367.805 | 1359.544 | 908.82 | 937.6859 | 1200.709 | 940.8909 | 1076.18 | 1041.548 | 1108.664 | 980.7968 | 1590.414 | 1008.511 | 1529.041 | 1349.576 | 1544.651 | 1167.349 | 1269.191 | 1164.105 | 1283.349 | 1027.258 | 1430.775 | 1532.545 | 1288.361 | 1252.158 | 1070.582 | 1241.135 |
| CUST_31661_PI429010886 | M2101 | 0.04528 | 24 | 15 | | | | | | 604.2963 | 686.6887 | 564.5345 | 601.5918 | 698.2239 | 686.5061 | 544.9594 | 595.1496 | 489.338 | 751.1067 | 634.9072 | 609.4542 | 536.0428 | 653.8395 | 580.436 | 800.0152 | 706.7803 | 665.511 | 742.4096 | 611.2062 | 634.9072 | 850.7537 | 582.4599 | 617.3843 | 702.0078 | 608.0744 | 596.0223 |
| CUST_24031_PI429010886 | N49901 | 0.04528 | 24 | 15 | | | | | | 257.9077 | 218.4011 | 248.1506 | 200.3982 | 224.668 | 248.652 | 233.828 | 186.8543 | 146.0295 | 282.6926 | 314.246 | 231.4996 | 160.1366 | 179.3756 | 217.159 | 286.3672 | 330.4772 | 279.0812 | 279.6762 | 281.692 | 214.1332 | 228.1481 | 240.6035 | 237.506 | 300.885 | 234.521 | 235.7566 |
| CUST_46291_PI429010886 | M72085 | 0.045283 | 12 | 6 | | | | | | 54.04063 | 51.02755 | 61.84039 | 55.50473 | 50.11342 | 42.02191 | 57.73367 | 58.7202 | 69.1026 | 48.03764 | 62.583 | 60.71887 | 29.58843 | 17.75499 | 40.88839 | 61.40781 | 79.32298 | 62.18234 | 72.78118 | 54.3948 | 38.09477 | 52.645 | 46.27475 | 52.02853 | 34.27813 | 44.74185 | 29.08411 |
| CUST_29516_PI429010886 | N60023 | 0.045283 | 12 | 9 | | | | | | 18.33589 | 13.97389 | 20.85111 | 10.45663 | 12.50339 | 12.48145 | 11.28834 | 12.57863 | 14.20826 | 16.76285 | 12.07791 | 15.41864 | 10.53088 | 35.48457 | 18.29071 | 9.255767 | 8.697209 | 11.70041 | 8.29257 | 19.35495 | 17.87442 | 28.19347 | 19.17708 | 14.30414 | 18.60353 | 11.84044 | 14.81994 |
| CUST_57301_PI429010886 | E4652 | 0.045297 | 24 | 18 | 345316531 | PREDICTED: beta-1,4-mannosyl-glycoprotein 4-beta | Metabolic process | Carbohydrate metabolic process | 2.61E-15 | 20.2137 | 16.54136 | 9.889085 | 22.74317 | 7.061176 | 18.0096 | 16.74929 | 10.79886 | 15.89011 | 14.65365 | 10.44576 | 14.55387 | 22.41474 | 25.11237 | 27.65968 | 17.53908 | 13.55355 | 23.31758 | 20.07409 | 27.95797 | 26.64032 | 16.81632 | 33.89428 | 18.83139 | 25.29581 | 14.8771 | 30.64068 |
| CUST_7517_PI429010886 | N21664 | 0.045299 | 21 | 15 | 322795781 | hypothetical protein SINV_10943 [Solenopsis invicta | | | 7.09E-53 | 36.18187 | 42.98911 | 31.39582 | 38.76201 | 41.13711 | 27.89551 | 17.51958 | 14.91349 | 21.7873 | 48.25771 | 33.93351 | 34.95753 | 30.3584 | 40.29977 | 26.56923 | 55.6145 | 20.48821 | 51.46072 | 34.17378 | 46.53779 | 45.37412 | 47.32005 | 31.06856 | 23.38605 | 42.16099 | 32.59218 | 36.10883 |
| CUST_26181_PI429010886 | N53807 | 0.045304 | 15 | 12 | 339234267 | ETS factor [Trichinella spiralis] >gi\|316978761\|gb\|E | Nucleic acid metabolism | Regulation of gene expression | 2.58E-15 | 21.81525 | 58.54952 | 77.04659 | 31.97403 | 24.79214 | 6.711326 | NA | 97.81711 | 85.18737 | 159.8062 | 34.27356 | 48.12746 | 77.71996 | 131.2743 | 56.00276 | 71.17303 | 62.42019 | 26.84327 | 29.11372 | 58.00017 | 44.75093 | 68.84477 | 107.6537 | NA | 130.3743 | 47.36571 | 107.9692 |
| CUST_10365_PI429010886 | N26125 | 0.045313 | 12 | 0 | | | | | | NA | 13.54808 | 9.376909 | 12.81122 | 7.483872 | NA | 5.302715 | 12.03892 | 4.41284 | 18.19605 | 7.277507 | 5.293256 | 6.173008 | 23.88883 | 14.1684 | 11.91263 | 6.925886 | NA | NA | 11.86943 | 7.251886 | 5.124238 | 5.349898 | NA | 22.18561 | 13.34357 | 12.26536 |
| CUST_5205_PI429010886 | N18397 | 0.045318 | 24 | 21 | 321477675 | hypothetical protein DAPPUDRAFT_304710 [Daphn | | | 1.5E-84 | 92.32556 | 148.9974 | 87.21952 | 106.908 | 81.28485 | 72.4241 | 70.63158 | 53.99181 | 60.05019 | 121.0512 | 73.28621 | 97.14315 | 75.68737 | 93.82886 | 100.734 | 125.1948 | 41.10188 | 108.3614 | 80.74596 | 129.5236 | 72.65998 | 118.802 | 99.10652 | 93.15439 | 80.20389 | 88.04251 | 78.78492 |
| CUST_7556_PI429010886 | N21724 | 0.045327 | 15 | 0 | | | | | | 375.8398 | 553.0837 | 381.9407 | 348.4045 | 330.2472 | 311.7466 | 231.8541 | 244.6362 | 241.3085 | 442.5257 | 327.6399 | 535.7875 | 295.0205 | 350.0802 | 464.6636 | 527.3498 | 263.7999 | 448.0031 | 333.6383 | 467.0909 | 410.4134 | 471.2464 | 302.4517 | 257.3603 | 401.4797 | 344.2985 | 374.9159 |
| CUST_34671_PI429010886 | M10301 | 0.045328 | 15 | 3 | | | | | | 79.99032 | 57.10862 | 31.64126 | 106.1871 | 60.34181 | 67.57577 | 67.34293 | 27.41533 | 60.56579 | 63.17657 | 65.09833 | 30.82801 | 35.30492 | 36.10883 | 19.98856 | 71.09841 | 56.48952 | 37.45529 | 59.37489 | 65.6996 | 64.30394 | 89.4807 | 49.8924 | 20.27043 | 58.93488 | 59.05765 | 51.98058 |
| CUST_55212_PI429010886 | M109134 | 0.045328 | 15 | 12 | | | | | | 3295.06 | 6536.631 | 3398.882 | 3147.989 | 3136.621 | 3394.033 | 2246.519 | 2911.974 | 3285.957 | 4098.525 | 2692.301 | 5927.379 | 4427.545 | 4357.297 | 4915.943 | 4729.674 | 2140.816 | 3783.178 | 2963.617 | 4427.545 | 2811.816 | 4519.983 | 2773.22 | 2874.609 | 3783.178 | 3832.303 | 4368.248 |
| CUST_39895_PI429010886 | M46329 | 0.045328 | 15 | 3 | 121484216 | cuticle protein CB6 [Portunus pelagicus] | | | 9.64E-11 | 18.77767 | 9.624456 | 30.68719 | 877.9874 | 1386.924 | 2565.951 | 854.2242 | 174.1781 | 3.741016 | 7.91389 | 905.268 | 4.769711 | 5.544303 | 3.548035 | 34.05422 | 7.010504 | 1897.37 | 823.2501 | 2472.114 | 4.770186 | 386.0388 | 9.103248 | 396.1095 | 447.9038 | 9.996492 | 206.5059 | 9.008803 |
| CUST_165_PI429010886 | N1995 | 0.045328 | 15 | 12 | | | | | | 85436.14 | 1846.031 | 101693.2 | 9950.402 | 16062.45 | 26037.41 | 86620.4 | 74829.64 | 95621.16 | 86045.29 | 45084.56 | 27018.31 | 102912.5 | 103589.1 | 104856.2 | 7930.333 | 96143.7 | 21119.13 | 67520.86 | 29210.68 | 47564.51 | 56816.44 | 97928.01 | 45272.91 | 88771.33 | 16734.53 | 95621.16 |
| CUST_16762_PI429010886 | N36824 | 0.045328 | 15 | 12 | | | | | | 29.17357 | 32.73103 | 40.79687 | 15.11263 | 20.13562 | 22.78742 | 31.41586 | 37.77068 | 33.92714 | 31.98439 | 32.11242 | 31.47236 | 32.51286 | 50.71879 | 28.9143 | 30.20292 | 34.32725 | 25.37243 | 26.76517 | 15.23737 | 30.01154 | 19.96449 | 45.95551 | 23.88653 | 37.83427 | 19.37234 | 39.0395 |
| CUST_17706_PI429010886 | N38442 | 0.045328 | 15 | 12 | | | | | | 248.7921 | 201.3287 | 197.3275 | 130.1545 | 147.7053 | 149.4495 | 108.4314 | 249.6664 | 182.4397 | 210.4017 | 137.5279 | 151.582 | 236.1246 | 303.7719 | 277.0077 | 231.235 | 135.3575 | 179.7342 | 158.2913 | 156.2584 | 163.0297 | 296.7865 | 211.9082 | 156.3082 | 227.7263 | 145.5578 | 248.2061 |
| CUST_27876_PI429010886 | N56856 | 0.045328 | 15 | 12 | | | | | | 92.93954 | 98.45237 | 126.2074 | 68.95198 | 104.2766 | 98.65062 | 70.44162 | 113.5017 | 110.7081 | 99.21099 | 73.83558 | 158.9341 | 134.7426 | 120.7928 | 126.0646 | 132.551 | 85.50355 | 128.5363 | 74.62941 | 99.89328 | 80.77462 | 102.477 | 108.6177 | 112.9777 | 111.8286 | 96.9668 | 134.6919 |
| CUST_8607_PI429010886 | N23297 | 0.045342 | 15 | 3 | | | | | | 607.4385 | 301.6337 | 350.314 | 389.2749 | 431.842 | 475.2865 | 728.8915 | 331.6846 | 401.8543 | 212.4051 | 233.6523 | 63.70797 | 313.9465 | 335.677 | 346.1184 | 322.2147 | 454.9277 | 338.4841 | 315.8463 | 173.2308 | 325.3231 | 299.9648 | 450.4975 | 649.5445 | 157.2097 | 170.9183 | 89.14278 |
| CUST_34389_PI429010886 | M9522 | 0.045371 | 15 | 12 | 193591726 | PREDICTED: zinc finger protein 271-like [Acyrthosipl | | | 1.92E-29 | 75.50768 | 150.3479 | 86.3602 | 82.95644 | 92.12003 | 92.18368 | 90.22371 | 114.6015 | 92.21711 | 166.9686 | 110.2442 | 126.1892 | 109.3629 | 137.5279 | 132.88 | 129.6012 | 95.60632 | 125.2532 | 105.4346 | 101.1532 | 101.9095 | 136.6832 | 105.9691 | 131.1004 | 110.0194 | 142.1431 | 101.3304 |
| CUST_32057_PI429010886 | M3130 | 0.045451 | 15 | 12 | 405960295 | Fumarylacetoacetase [Crassostrea gigas] | Metabolic process | Cellular amino acid metabolic process | 7.9E-177 | 849.0108 | 3008.795 | 1189.668 | 1102.979 | 869.29 | 894.6121 | 455.2984 | 714.7333 | 887.8552 | 2151.64 | 1071.523 | 2032.79 | 1486.604 | 1197.916 | 2163.813 | 3029.514 | 647.5069 | 1409.58 | 1380.785 | 2244.992 | 1361.981 | 1114.056 | 1095.532 | 978.4784 | 1824.738 | 1445.235 | 1340.303 |
| CUST_16706_PI429010886 | N36726 | 0.045454 | 15 | 9 | | | | | | 69.13041 | 4.286001 | 10.5296 | NA | 58.77783 | 19.80135 | NA | 522.9557 | 511.4224 | 716.2921 | 11.00334 | 697.5569 | 4.536687 | 124.009 | 60.04192 | 340.4019 | 67.17456 | 8.761286 | NA | 108.3189 | 19.02605 | NA | 40.39432 | NA | 151.2877 | 601.7703 | 390.973 |
| CUST_37800_PI429010886 | M21652 | 0.045482 | 12 | 3 | | | | | | 2192.843 | 487.6477 | 262.5661 | 12388.76 | 820.2371 | 12538.73 | 3036.431 | 3590.082 | 2312.077 | 29.47907 | 430.1259 | NA | 3262.349 | 48.10487 | 1354.655 | NA | 4655.618 | 4787.683 | 1128.807 | NA | 500.9078 | 9.987338 | 513.4234 | 8627.68 | NA | 11259.43 | NA |
| CUST_26699_PI429010886 | N54725 | 0.045494 | 24 | 6 | | | | | | 38.05769 | 42.88574 | 51.97626 | 40.29977 | 42.42487 | 107.4359 | 86.60546 | 81.63335 | 55.66641 | 44.92099 | 32.18557 | 31.92079 | 43.07403 | 48.85705 | 38.22991 | 37.77885 | 88.78823 | 60.43419 | 33.20236 | 26.38969 | 30.76819 | 50.22974 | 34.39152 | 51.37925 | 40.08304 | 30.60937 | 32.06599 |
| CUST_26777_PI429010886 | N54858 | 0.045503 | 21 | 15 | | | | | | 39.22918 | 30.93804 | 34.05076 | 16.9342 | 25.46475 | 19.72906 | 16.88939 | 22.63948 | 18.25337 | 33.26796 | 27.47752 | 27.13842 | 23.99602 | 26.03629 | 20.80589 | 34.38812 | 23.20138 | 42.38554 | 32.96805 | 20.54044 | 28.59095 | 35.64771 | 29.22246 | 19.60466 | 24.79214 | 21.95358 | 30.33523 |
| CUST_39595_PI429010886 | M41570 | 0.045525 | 21 | 9 | | | | | | 7.355758 | 8.758494 | 7.018038 | 21.74923 | 26.67547 | 3.627518 | 18.27526 | 51.17489 | 15.84016 | 19.89653 | 55.89482 | 41.74772 | 61.1802 | 13.93436 | 18.10904 | NA | 13.37557 | 11.23298 | 3.427938 | 111.5879 | 47.20161 | 7.602225 | 20.02647 | 7.563005 | 6.769475 | 14.98523 | 25.87358 |
| CUST_46051_PI429010886 | M71199 | 0.04553 | 12 | 0 | | | | | | 36.65319 | 30.60709 | 23.64095 | 28.06449 | 30.65659 | 31.36762 | 33.42828 | 21.17943 | 26.72979 | 19.41782 | 20.36175 | 30.73213 | 27.99749 | 32.11685 | 38.25121 | 25.97694 | 28.75592 | 28.48875 | 24.51944 | 18.65246 | 23.50904 | 25.90485 | 21.55583 | 30.97619 | 35.34739 | 26.869 | 31.53803 |
| CUST_16337_PI429010886 | N36065 | 0.04553 | 12 | 0 | | | | | | 68.39623 | 64.3475 | 55.59887 | 63.58566 | 45.69586 | 75.54672 | 56.62309 | 48.66621 | 47.3028 | 51.7472 | 36.9383 | 47.26675 | 38.27387 | 77.23129 | 72.14746 | 69.35049 | 52.43379 | 61.15658 | 42.40805 | 41.13711 | 55.08741 | 70.3852 | 36.87107 | 57.65114 | 84.2614 | 67.42469 | 65.06553 |
| CUST_46596_PI429010886 | M73109 | 0.045531 | 15 | 0 | | | | | | 79.6699 | 71.00916 | 72.59952 | 48.66243 | 62.13897 | 80.6942 | 71.9913 | 97.91604 | 48.00964 | 66.48727 | 63.34887 | 48.88298 | 50.56808 | 47.58792 | 52.54728 | 67.55015 | 87.9523 | 88.59096 | 83.76494 | 47.9222 | 56.76191 | 49.62512 | 62.34386 | 80.04179 | 34.18999 | 46.72755 | 32.97611 |

| Probe ID | ID | p-value | Period | Phase | EBI ID | Annotation | GO category | Go term | E-value | 0 | 0 | 0 | 3 | 3 | 3 | 6 | 6 | 6 | 9 | 9 | 9 | 12 | 12 | 12 | 15 | 15 | 15 | 18 | 18 | 18 | 21 | 21 | 21 | 24 | 24 | 24 |
|---|---|---|---|---|---|---|---|---|---|---|---|---|---|---|---|---|---|---|---|---|---|---|---|---|---|---|---|---|---|---|---|---|---|---|---|---|
| CUST_17311_PI429010886 | N37755 | 0.045544 | 12 | 0 | | | | | | 81.49546 | 85.47277 | 91.47195 | 48.84271 | 52.034 | 44.62585 | 46.42413 | 65.31258 | 64.94243 | 78.101 | 56.52083 | 71.48709 | 76.19351 | 64.5831 | 81.31771 | 109.5638 | 69.0257 | 82.67004 | 58.40782 | 75.2095 | 60.13681 | 89.419 | 71.43726 | 37.52679 | 85.60322 | 75.29438 | 94.7426 |
| CUST_37650_PI429010886 | M20251 | 0.045545 | 21 | 15 | | | | | | 16.5713 | 12.53208 | 28.66257 | NA | 6.606034 | 8.346516 | 12.83173 | 23.05797 | 11.02819 | 14.17862 | 4.611346 | 6.74367 | 15.45873 | 14.15692 | 12.63269 | NA | 27.38976 | 15.22088 | 17.25976 | NA | 11.61773 | NA | 19.91373 | 10.87219 | 9.934601 | 12.61292 | 10.79319 |
| CUST_2271_PI429010886 | N12001 | 0.045556 | 12 | 9 | 156541526 | PREDICTED: cullin-4B-like [Nasonia vitripennis] | Protein metabolism | Proteolysis | 1.6E-122 | 88.65038 | 106.023 | 82.76433 | 63.76139 | 86.32314 | 89.74363 | 109.7731 | 80.03068 | 64.00351 | 140.0198 | 80.87419 | 107.512 | 81.69291 | 54.0232 | 72.61909 | 92.53187 | 84.88556 | 77.02903 | 118.8596 | 89.03619 | 104.2212 | 89.93577 | 106.023 | 127.7418 | 81.31771 | 78.29209 | 75.45183 |
| CUST_56046_PI429010886 | M112617 | 0.045562 | 18 | 3 | | | | | | 74.96433 | 57.02142 | 27.43923 | 72.66838 | 32.66922 | 45.63167 | 51.87045 | 32.62278 | 35.57263 | 32.11685 | 17.17879 | 54.99653 | 32.60067 | 25.46475 | 28.54216 | 38.75769 | 17.64732 | 34.17378 | 19.24807 | 15.69237 | 21.22623 | 50.43862 | 65.4438 | 79.1048 | 26.49815 | 22.8004 | 33.92714 |
| CUST_13157_PI429010886 | N30681 | 0.045585 | 21 | 0 | | | | | | 75.04275 | 69.69491 | 68.77033 | 42.54782 | 52.93292 | 31.90818 | 46.29572 | 248.3781 | 41.87987 | 81.86063 | 43.961 | 46.74367 | 32.55631 | 30.37057 | 28.19347 | 93.59991 | 57.02142 | 56.28989 | 56.99751 | 57.73367 | 58.85973 | 79.43105 | 64.99075 | 36.6789 | 49.21104 | 61.63016 | 49.02476 |
| CUST_5950_PI429010886 | N19417 | 0.045599 | 24 | 6 | | | | | | 542.3878 | 355.193 | NA | 704.9925 | 145.1631 | 555.3104 | 675.7327 | 432.4326 | 592.7227 | NA | 33.41929 | NA | 501.1913 | 3.536967 | 281.7845 | 60.22346 | 171.7375 | 220.8734 | 214.2001 | NA | 16.1574 | 12.55922 | 19.12346 | 545.5317 | NA | 411.2669 | NA |
| CUST_34655_PI429010886 | M10251 | 0.045615 | 12 | 0 | 270013418 | hypothetical protein TcasGA2_TC012014 [Tribolium | Nucleic acid metabolism | RNA metabolic process | 6.36E-22 | 50.75355 | 57.98645 | 42.71853 | 37.5119 | 43.66792 | 34.08252 | 21.32406 | 31.35806 | 30.66153 | 40.2482 | 46.26909 | 65.74131 | 86.64513 | 52.2983 | 35.58868 | 55.93124 | 32.98635 | 54.87191 | 32.53107 | 38.44815 | 33.45023 | 55.44212 | 44.18564 | 48.43308 | 50.8686 | 31.39141 | 45.29693 |
| CUST_4037_PI429010886 | N15861 | 0.045628 | 24 | 15 | 383855622 | PREDICTED: neurobeachin-like [Megachile rotundat | Developmental process | Multicellular organismal development | 1.28E-76 | 41.06137 | 41.23292 | 60.35512 | 32.35642 | 33.59506 | 26.50986 | 31.08481 | 29.00151 | 37.99673 | 55.6042 | 39.1027 | 29.51436 | 33.16699 | 50.2602 | 43.57816 | 50.27036 | 51.92939 | 44.90621 | 48.84923 | 44.014 | 33.55523 | 71.83981 | 39.12009 | 41.43739 | 68.84477 | 29.17357 | 60.17363 |
| CUST_14779_PI429010886 | N33457 | 0.045628 | 21 | 3 | | | | | | 75.52934 | 253.4728 | 82.82132 | 350.0282 | 128.0504 | 178.833 | 95.18251 | 78.72104 | 68.03939 | 84.28941 | 41.0381 | 120.8319 | 68.26569 | 91.91837 | 86.54824 | 88.87538 | 32.89748 | 77.89081 | 48.67019 | 68.73669 | 164.6734 | 86.2674 | 138.8365 | 98.56528 | 158.5656 | 229.7242 | 67.50801 |
| CUST_6480_PI429010886 | N20156 | 0.04563 | 18 | 0 | 321461563 | hypothetical protein DAPPUDRAFT_308176 [Daphn | Protein metabolism | Ribosome biogenesis | 1.31E-95 | 120.9334 | 152.5355 | 140.7358 | 68.18607 | 114.7111 | 116.5433 | 100.8309 | 95.10396 | 103.384 | 100.955 | 91.04016 | 122.8712 | 93.44686 | 77.37108 | 86.78972 | 108.0186 | 83.09489 | 151.4536 | 111.5529 | 145.0486 | 114.0443 | 96.61745 | 117.4728 | 70.7847 | 97.74764 | 79.55126 | 83.0149 |
| CUST_38918_PI429010886 | M31949 | 0.045671 | 12 | 3 | | | | | | 892.629 | 710.4011 | 713.6188 | 982.4374 | 1122.467 | 1159.33 | 1102.275 | 694.7611 | 670.3311 | 685.5063 | 818.0738 | 543.0155 | 618.3611 | 557.6662 | 882.8865 | 1064.697 | 1291.558 | 1079.829 | 1127.217 | 634.6129 | 816.3533 | 938.0188 | 763.6837 | 844.3643 | 822.9643 | 771.3694 | 823.8136 |
| CUST_54667_PI429010886 | M107092 | 0.04569 | 18 | 3 | | | | | | 354.8939 | 329.7729 | 303.7719 | 386.0388 | 371.1359 | 357.862 | 359.297 | 268.82 | 248.3781 | 328.5456 | 380.2921 | 321.803 | 200.7582 | 274.4437 | 228.1481 | 329.2355 | 442.5257 | 287.2312 | 356.9822 | 224.2596 | 318.8167 | 379.5119 | 343.0578 | 302.4916 | 469.1551 | 317.2087 | 295.5774 |
| CUST_54549_PI429010886 | M106471 | 0.045714 | 21 | 0 | | | | | | 80.37733 | 105.6225 | 89.34718 | 87.76889 | 86.46205 | 81.70156 | 64.57467 | 60.63321 | 64.24619 | 64.48135 | 70.94036 | 36.41327 | 55.36449 | 59.10759 | 68.77033 | 118.9466 | 51.82417 | 89.80969 | 54.91632 | 84.59116 | 58.24456 | 132.5094 | 68.35087 | 80.06962 | 79.76051 | 55.62798 | 52.05083 |
| CUST_51253_PI429010886 | M92816 | 0.045719 | 24 | 18 | | | | | | 10.4013 | 43.28801 | 19.63157 | 38.02052 | 22.28718 | 6.874216 | 15.2714 | 13.75242 | 7.022738 | 57.06419 | 22.20218 | 35.67075 | 39.74462 | 13.16726 | 25.29278 | 47.00702 | 14.85125 | 36.7953 | 25.84583 | 44.07915 | 47.53841 | 40.23032 | 24.15613 | 14.15378 | 43.01496 | 17.00952 | 23.91945 |
| CUST_32211_PI429010886 | M3534 | 0.04575 | 24 | 15 | | | | | | 773.1832 | 954.6687 | 643.5529 | 722.955 | 865.6927 | 824.3794 | 719.8537 | 608.3741 | 540.3051 | 862.1393 | 783.8018 | 739.338 | 604.157 | 664.7021 | 761.273 | 1027.997 | 940.8909 | 911.683 | 788.0347 | 785.9549 | 571.7132 | 857.8725 | 641.5416 | 672.5182 | 935.7487 | 596.4585 | 517.1879 |
| CUST_54179_PI429010886 | M104957 | 0.045757 | 24 | 21 | | | | | | 41.94521 | 58.4329 | 57.40224 | 49.96713 | 59.35924 | 57.69532 | 41.91362 | 53.15786 | 53.69064 | 54.63011 | 63.66464 | 68.18607 | 53.54974 | 42.92316 | 46.74367 | 41.50677 | 54.6863 | 49.66096 | 45.71627 | 77.60864 | 59.23972 | 73.93957 | 63.82351 | 70.23023 | 71.54826 | 65.81652 | 69.94604 |
| CUST_10233_PI429010886 | N25921 | 0.04578 | 18 | 3 | | | | | | 23.5201 | 20.11049 | 20.54425 | 37.9853 | 33.75702 | 37.94571 | 24.44701 | 24.54709 | 30.99084 | 33.26161 | 27.06954 | 26.70632 | 18.70952 | 24.99108 | 25.5267 | 33.93351 | 25.47199 | 51.56072 | 28.42752 | 22.75236 | 32.03181 | 41.32867 | 30.34438 | 35.02024 | 18.05541 | 36.47059 | 30.08532 |
| CUST_53926_PI429010886 | M103734 | 0.045794 | 24 | 15 | | | | | | 151.2877 | 125.4314 | 167.0802 | 128.9362 | 129.8621 | 170.8572 | 156.8624 | 120.114 | 109.9562 | 168.7266 | 252.0389 | 191.1723 | 140.364 | 167.4108 | 166.4042 | 260.373 | 148.3869 | 198.0363 | 159.3888 | 245.1598 | 270.2125 | 188.7518 | 143.8341 | 148.3869 | 146.0723 | 168.58 | 174.3314 |
| CUST_40954_PI429010886 | M56473 | 0.045794 | 24 | 15 | 391336842 | PREDICTED: long-chain-fatty-acid--CoA ligase 4-like | Metabolic process | Other | 8.05E-67 | 136.9094 | 124.7036 | 135.3886 | 85.31136 | 137.2513 | 134.3489 | 112.7335 | 107.1844 | 111.0395 | 171.8504 | 137.865 | 147.7426 | 60.52796 | 120.496 | 117.5843 | 164.2756 | 151.4536 | 189.4251 | 150.3479 | 152.8418 | 158.8254 | 169.2316 | 125.5811 | 142.3214 | 205.2921 | 172.498 | 204.885 |
| CUST_44612_PI429010886 | M66475 | 0.045804 | 15 | 0 | 443690866 | hypothetical protein CAPTEDRAFT_204931 [Capitell | | | 2.66E-20 | 12.79677 | 14.00188 | 14.37562 | 16.65858 | 12.20868 | 6.931825 | 5.286981 | 5.830969 | 10.10842 | 12.34018 | 4.811712 | 14.70218 | 8.007045 | 12.48018 | 13.97554 | 10.70475 | 14.90554 | 13.01044 | NA | 6.825412 | 8.253508 | 12.76533 | 5.72427 | NA | 11.43831 | 4.879881 | 10.49349 |
| CUST_57125_PI429010886 | E1235 | 0.045821 | 12 | 3 | 156553659 | PREDICTED: nuclear receptor 2C2-associated protei | Cellular Process | Cell adhesion | 1.85E-21 | 8.180112 | 11.01728 | 12.14326 | NA | 12.33539 | 16.91691 | 10.04812 | 8.353787 | NA | 10.66414 | 9.673986 | 5.369319 | 5.458239 | 12.84177 | 8.259244 | 20.63988 | NA | 20.65721 | 11.17512 | 13.58792 | NA | 16.75298 | 7.423086 | 3.678115 | 11.12353 | 8.908142 | 22.16055 |
| CUST_50890_PI429010886 | M91360 | 0.045832 | 18 | 15 | | | | | | 40.1147 | 39.86189 | 26.05952 | 74.50049 | 54.69269 | 50.2407 | 32.54126 | 15.37925 | 25.37501 | 36.08065 | 21.07244 | 43.71768 | 61.02204 | 55.17531 | 64.00351 | 93.59388 | 43.85634 | 100.468 | 30.92026 | 62.46895 | 28.23857 | 56.86246 | 25.29581 | 44.07419 | 43.69907 | 28.48354 | 35.56782 |
| CUST_53571_PI429010886 | M102302 | 0.045837 | 15 | 12 | 444516848 | Tankyrase-1 [Tupaia chinensis] | | | 2.59E-14 | 22.52813 | 27.49986 | 31.02643 | 30.45027 | 27.8201 | 20.62529 | 32.50043 | 27.0144 | 23.4104 | 22.14979 | 38.09477 | 29.24887 | 34.69687 | 31.7359 | 40.98995 | 45.64715 | 29.74822 | 22.00022 | 20.86179 | 21.60382 | 28.06711 | 34.94529 | 32.02812 | 23.8296 | 32.60782 | 24.83878 | 41.66889 |
| CUST_37012_PI429010886 | M17537 | 0.045837 | 15 | 3 | | | | | | 138.3413 | 106.1197 | 51.73412 | 68.49231 | 26.75249 | 12.10209 | 71.19165 | 15.70949 | 49.18958 | 59.12378 | 77.74288 | 57.36487 | 52.3781 | 112.9419 | 72.71237 | 94.06166 | 36.62775 | 66.20577 | 42.18503 | 29.24265 | 28.28914 | 95.84863 | 53.03978 | 76.07766 | 129.3158 | 66.01381 | 46.47367 |
| CUST_47075_PI429010886 | M74730 | 0.045837 | 15 | 3 | 383848471 | PREDICTED: uncharacterized protein LOC10087850 | | | 9.04E-32 | 28.05064 | 19.87086 | 30.03474 | 41.55928 | 36.89165 | 54.58347 | 42.84657 | 19.06722 | 19.74119 | 26.44706 | 39.16663 | 21.07244 | 20.38809 | 27.43011 | 26.62182 | 28.61737 | 53.98541 | 35.74774 | 48.31638 | 46.95889 | 37.29054 | 39.45879 | 21.22872 | 25.1646 | 38.12809 | 32.45453 | 28.26127 |
| CUST_50276_PI429010886 | M89282 | 0.045837 | 15 | 12 | | | | | | 476.2802 | 620.7934 | 611.7017 | 513.9071 | 432.4326 | 638.2757 | 428.865 | 463.6969 | 478.074 | 570.1143 | 494.9533 | 822.9643 | 553.9482 | 724.7977 | 966.7908 | 683.3484 | 438.1052 | 559.2711 | 524.2243 | 595.305 | 518.2235 | 636.6917 | 521.8521 | 465.6558 | 573.39 | 676.9355 | 703.0939 |
| CUST_4286_PI429010886 | N16406 | 0.045847 | 15 | 12 | | | | | | 169.9162 | 213.3434 | 212.9853 | 180.4095 | 197.1816 | 196.4719 | 154.8889 | 171.5942 | 154.9491 | 192.2628 | 190.3369 | 179.5282 | 195.1331 | 224.5142 | 188.2417 | 172.8801 | 196.6343 | 158.9804 | 173.8566 | 227.0035 | 219.5762 | 141.7485 | 157.9269 | 178.6841 | 192.7007 | 194.3859 | 255.7006 |
| CUST_52320_PI429010886 | M97048 | 0.045855 | 24 | 0 | | | | | | 73.73837 | 64.23886 | 50.80203 | 38.95233 | 54.17026 | 57.64388 | 49.04708 | 42.05204 | 48.09585 | 50.99115 | 43.75138 | 42.42487 | 44.07419 | 38.8107 | 41.92389 | 63.24801 | 57.97853 | 57.92964 | 68.26569 | 52.30747 | 53.74643 | 65.49916 | 46.77892 | 43.46213 | 100.651 | 42.59897 | 74.00809 |
| CUST_28864_PI429010886 | N58750 | 0.045867 | 24 | 0 | | | | | | 46.12938 | 32.66922 | 45.86308 | 28.75284 | 44.27177 | 37.96902 | 20.82459 | 29.82743 | 33.87876 | 43.57816 | 26.37834 | 42.10422 | 37.61954 | 38.25818 | 23.70999 | 33.81876 | 21.19291 | 27.13598 | 33.12364 | 30.00329 | 49.57895 | 37.40452 | 35.05162 | 21.72163 | 41.50677 | 38.66042 | 55.784 |
| CUST_27431_PI429010886 | N56048 | 0.045879 | 24 | 0 | | | | | | 57.73367 | 56.76191 | 80.90583 | 57.63741 | 62.32467 | 79.4217 | 43.81829 | 45.24266 | 52.93772 | 47.26675 | 58.85113 | 34.59885 | 45.65948 | 53.61134 | 43.04692 | 91.22284 | 73.38298 | 83.21112 | 66.60678 | 70.72361 | 55.16975 | 69.93933 | 49.71058 | 60.54253 | 77.01708 | 68.02926 | 117.2732 |
| CUST_12058_PI429010886 | N28855 | 0.045904 | 15 | 0 | | | | | | 48.62311 | 58.47643 | 63.61419 | 32.44657 | 15.96047 | 18.11602 | 22.29454 | 21.59499 | 21.82677 | 11.84887 | 24.983 | 22.18079 | 13.65469 | 9.180913 | NA | 28.22332 | NA | NA | NA | 34.69687 | 14.52536 | NA | 43.45754 | 18.61514 | 12.56568 | 8.723307 | 11.97085 |
| CUST_45152_PI429010886 | M68143 | 0.045932 | 21 | 9 | | | | | | 44.63765 | 87.05061 | 71.05715 | 67.64754 | 21.82183 | 30.78076 | 115.3125 | 138.2381 | 51.72418 | 65.46343 | 134.1419 | 105.4346 | 122.0311 | 131.4929 | 71.46757 | 50.58305 | 44.02718 | 65.77588 | 48.9358 | 145.1839 | 60.75286 | 79.22919 | 132.473 | 59.27376 | 36.15703 | 55.71823 | 36.47059 |
| CUST_21873_PI429010886 | N46016 | 0.045941 | 15 | 9 | | | | | | 6.820714 | 14.73825 | 14.13262 | 7.182647 | 9.316937 | NA | 16.96853 | 14.85731 | 12.20426 | 17.11553 | 20.61255 | 18.99123 | 10.53483 | 17.05513 | 12.05708 | 12.12459 | 10.71609 | NA | 8.487802 | 17.61748 | 8.098026 | 20.10327 | 7.286814 | NA | 23.35433 | NA | 11.12353 |
| CUST_21833_PI429010886 | N45939 | 0.045964 | 12 | 0 | | | | | | 25.02094 | 47.67267 | 33.63068 | 49.91173 | 23.71956 | 10.62697 | 19.16725 | 17.21816 | 24.33459 | 30.9788 | 21.8588 | 30.558 | 48.53244 | 58.93488 | 34.24386 | 33.25728 | 17.10186 | 33.18183 | 30.44396 | 20.859 | 26.26527 | 31.34763 | 28.82637 | 29.50649 | 39.31065 | 20.78572 | 23.80497 |
| CUST_53388_PI429010886 | M101513 | 0.046029 | 15 | 12 | 260832820 | hypothetical protein BRAFLDRAFT_120334 [Branchi | Protein metabolism | Cellular protein modification process | 1.5E-47 | 45.42072 | 58.83776 | 69.63319 | 49.3358 | 49.1739 | 56.08637 | 59.10759 | 44.41825 | 45.89685 | 94.03801 | 47.40597 | 71.93323 | 61.64915 | 77.38027 | 70.33128 | 64.25402 | 80.34778 | 52.28302 | 57.00887 | 60.29484 | 44.37542 | 56.9379 | 64.58903 | 28.47733 | 63.06953 | 45.58039 | 60.86834 |
| CUST_15343_PI429010886 | N34433 | 0.04603 | 15 | 12 | | | | | | 36.86394 | 50.4763 | 39.54839 | 46.35772 | 34.27813 | 41.99242 | 27.50275 | 30.9788 | 30.22418 | 42.8661 | 40.7292 | 55.33618 | 21.02441 | 51.38396 | 49.47471 | 47.71565 | 36.5619 | 45.2318 | 42.21202 | 34.30407 | 40.58399 | 42.36861 | 23.98815 | 34.02396 | 56.39846 | 51.62562 | 46.82282 |
| CUST_32824_PI429010886 | M5193 | 0.046049 | 15 | 0 | | | | | | 161.1033 | 250.5389 | 56.64713 | 201.6148 | 137.8813 | 144.9176 | 124.1661 | 30.42832 | 110.3777 | 65.84223 | 34.73538 | 46.63569 | 98.55351 | 72.33072 | 145.9962 | 155.649 | 23.21852 | 149.9525 | 46.87213 | 70.53195 | 58.62811 | 117.3039 | 52.42987 | 114.6015 | 57.60743 | 57.29447 | 36.22069 |
| CUST_22637_PI429010886 | N47377 | 0.046078 | 18 | 9 | | | | | | 6.943814 | 7.354715 | 10.41346 | NA | 7.092466 | 9.883505 | 10.91146 | 7.807289 | 11.10196 | NA | 22.13675 | 25.34187 | 19.32948 | 13.56887 | 14.98523 | NA | 10.36994 | 9.34052 | NA | 27.42544 | 14.59087 | 11.87443 | NA | 3.808743 | 33.30987 | 8.042965 | 11.82579 |
| CUST_52331_PI429010886 | M97084 | 0.046101 | 18 | 3 | | | | | | 73.61433 | 54.7161 | 102.4647 | 74.18777 | 77.32264 | 161.1553 | 87.81308 | 70.47896 | 86.2674 | 97.10121 | 78.54463 | 99.26813 | 94.03801 | 51.40578 | 50.18617 | 60.28718 | 51.37925 | 70.98423 | 65.26948 | 77.34 | 70.14592 | 86.94867 | 113.1045 | 105.6505 | 76.98006 | 425.1252 | 63.00905 |
| CUST_9937_PI429010886 | N25439 | 0.046123 | 21 | 3 | | | | | | 22.78329 | 22.19942 | 9.064427 | 23.97713 | 24.22757 | 25.77821 | 11.44906 | 15.37345 | 12.37483 | 19.2011 | 16.62053 | 15.47412 | 9.893302 | 15.66738 | 11.17351 | 19.30028 | NA | 24.61213 | 8.44134 | 9.103248 | 12.92501 | 14.95429 | 8.210685 | 10.97825 | 35.92246 | 26.08326 | 12.59547 |
| CUST_30020_PI429010886 | N61018 | 0.04618 | 21 | 3 | | | | | | 64.00351 | 42.02191 | 34.2288 | 56.0501 | 53.70396 | 72.9942 | 41.45965 | 52.88574 | 34.28772 | 51.7896 | 45.53002 | 38.31256 | 33.69477 | 41.61311 | 41.50205 | 39.52535 | 49.71501 | 67.73286 | 57.52971 | 50.44452 | 39.33849 | 62.08961 | 40.52511 | 59.92145 | 60.00971 | 57.27407 | 53.27432 |
| CUST_16536_PI429010886 | N36420 | 0.046208 | 15 | 12 | | | | | | NA | 95.06463 | 68.81509 | 32.18854 | 32.8295 | 47.11613 | 4.458762 | 9.739447 | 16.83531 | 220.8472 | 65.57569 | 214.59 | 122.2373 | 30.77807 | 68.38203 | 93.66147 | NA | NA | 43.5925 | 54.94382 | 113.8304 | 41.23729 | 60.20751 | 23.58263 | 302.0506 | 78.46976 | 385.5507 |
| CUST_39812_PI429010886 | M45029 | 0.046264 | 18 | 0 | | | | | | 24.52204 | 662.9564 | 553.7439 | 10.04101 | 11.74612 | 8.387454 | 20.3477 | 26.29583 | 19.57173 | 6.43023 | 12.7848 | 6.831546 | 12.61125 | 29.91744 | 8.233831 | 7.624707 | 20.93627 | 11.80446 | 20.3261 | 211.0494 | 11.59303 | 60.5947 | 19.76282 | 9.45038 | 12.94169 | 7.393906 | 10.10703 |
| CUST_12535_PI429010886 | N29615 | 0.046264 | 18 | 3 | | | | | | 56.41396 | 48.36598 | 63.6301 | 64.66893 | 83.43282 | 88.77007 | 84.58422 | 43.83106 | 38.81418 | 41.53296 | 60.93069 | 41.45045 | 39.126 | 51.07827 | 58.482 | 51.0383 | 99.57087 | 55.28288 | 78.01974 | 40.15861 | 64.5831 | 63.56474 | 59.57181 | 72.19392 | 55.84573 | 44.76886 | 55.43705 |
| CUST_22996_PI429010886 | N48080 | 0.046264 | 18 | 15 | | | | | | 33.01824 | 29.56124 | 14.31222 | 53.86098 | 38.36859 | 44.03128 | 24.36264 | 18.92468 | 23.25228 | 34.43117 | 26.64522 | 36.07548 | 30.77101 | 29.61528 | 27.70404 | 73.54244 | 47.03677 | 40.46037 | 13.46279 | 69.13832 | 25.36189 | 35.1842 | 30.26479 | 31.76384 | 16.26898 | 32.52286 | 9.687487 |
| CUST_33197_PI429010886 | M6249 | 0.046278 | 18 | 6 | | | | | | 5.460269 | 1500.167 | 1161.62 | NA | 16.5954 | 6.804229 | 385.0916 | 379.0729 | 323.6189 | 164.7626 | 226.3675 | 393.8505 | 191.2652 | 137.2146 | 111.6728 | 86.35271 | 14.70096 | 80.92356 | 203.324 | 58.26543 | 270.1737 | 112.5941 | 108.555 | 87.09496 | 280.3132 | 184.2793 | 337.0926 |
| CUST_4809_PI429010886 | N17525 | 0.046294 | 21 | 15 | | | | | | 29.01472 | 27.25206 | 32.10376 | 39.13386 | 28.57291 | 17.83264 | 53.20475 | 27.63541 | 43.40546 | 15.54567 | 48.21189 | 10.12411 | 27.90015 | 31.02908 | 30.88907 | 36.115 | 57.96208 | 58.83154 | 50.27036 | 27.52766 | 50.39753 | 28.04755 | 45.66434 | 61.82662 | 24.26504 | 24.61213 | 24.16344 |
| CUST_45310_PI429010886 | M68669 | 0.046298 | 18 | 9 | 242015135 | Thrombospondin-3 precursor, putative [Pediculus h | Cellular Process | Cell adhesion | 2.45E-23 | 389.3563 | 297.6952 | 212.2526 | 352.7559 | 575.2228 | 452.1056 | 545.5317 | 226.6106 | 294.6992 | 618.8739 | 554.3622 | 459.3991 | 341.9433 | 314.4386 | 231.6859 | 523.7505 | 616.4707 | 644.4395 | 139.1382 | 167.1583 | 235.2181 | 589.6619 | 316.8071 | 338.7294 | 436.3193 | 319.1396 | 415.7405 |
| CUST_23774_PI429010886 | N49442 | 0.0463 | 12 | 15 | | | | | | 8.956327 | NA | 7.480574 | 18.72053 | 5.24251 | NA | NA | 6.893139 | 7.137459 | 11.59303 | 8.479761 | 15.90074 | 11.41581 | 27.58334 | 19.57848 | 13.69592 | 7.478506 | 14.37562 | 9.53977 | 4.06544 | 8.176012 | 12.0248 | 5.502777 | 17.67558 | 15.48818 | 24.55066 | 15.95534 |
| CUST_5531_PI429010886 | N18825 | 0.046315 | [illegible] | 15 | | | | | | 583.066 | 761.7734 | 545.9123 | 615.3731 | 722.0011 | 526.8072 | 452.4978 | 417.4973 | 525.2083 | 678.2021 | 602.272 | 977.0262 | 536.1688 | 646.7171 | 672.3398 | 1318.564 | 591.7087 | 732.5018 | 634.9072 | 942.9477 | 707.1584 | 1128.352 | 585.6944 | 635.813 | 988.77 | 412.4272 | 755.5617 |
| CUST_7553_PI429010886 | N21719 | 0.046332 | 21 | 9 | 321457121 | hypothetical protein DAPPUDRAFT_330318 [Daphn | Protein metabolism | Proteolysis | 9.16E-25 | 5.69423 | 47.19112 | 24.135 | 73.47109 | 150.4806 | 77.95158 | 58.75213 | 275.0769 | 179.6526 | 38.90216 | 323.5707 | 384.3454 | 171.2587 | 45.44604 | 110.4435 | NA | 88.14739 | NA | 25.46966 | 382.0118 | 154.3834 | NA | 84.1757 | 49.59839 | NA | 176.9698 | 166.7311 |
| CUST_47754_PI429010886 | M77475 | 0.046333 | 18 | 12 | 291228352 | PREDICTED: CDC42 binding protein kinase alpha-like | Signal transduction | Intracellular signal transduction | 3.2E-21 | 27.17605 | 31.43817 | 23.62378 | 19.19079 | 23.12912 | 6.654035 | 20.25895 | 9.262293 | 23.38375 | 25.76654 | 22.14395 | 47.77962 | 33.2742 | 22.9575 | 32.62278 | 38.13489 | 10.23206 | 27.04148 | 20.14785 | 28.7982 | 19.62649 | 22.22394 | 14.84209 | 19.30622 | 40.04237 | 9.895282 | 18.49479 |
| CUST_10529_PI429010886 | N26376 | 0.046333 | 18 | 6 | | | | | | 64.4873 | 68.12178 | 59.12871 | 57.6895 | 83.39175 | 98.47983 | 187.276 | 71.79355 | 202.3705 | 84.015 | 75.69546 | 86.38663 | 96.89497 | 85.10115 | 65.0047 | 92.46177 | 139.562 | 103.5442 | 77.39037 | 40.52511 | 54.21273 | 46.79377 | 71.98272 | 89.14278 | 139.4961 | 82.53975 | 95.29429 |
| CUST_19176_PI429010886 | N41116 | 0.046339 | 24 | 9 | | | | | | 31.60035 | 38.82583 | 57.92964 | 41.36078 | 36.02623 | 26.23144 | 30.81693 | 28.23857 | 37.60789 | 34.92205 | 37.20061 | 49.82473 | 23.51614 | 31.61657 | 31.22768 | 32.11242 | 37.77068 | 25.58851 | 21.59935 | 22.38112 | 19.57173 | 17.39038 | 33.91251 | 27.44311 | 19.91752 | 27.0028 | 31.4416 |
| CUST_38172_PI429010886 | M23838 | 0.046346 | 15 | 12 | 227122184 | masquerade-like serine proteinase-like protein 3 [P | Protein metabolism | Proteolysis | 4.98E-17 | 2522.174 | 3128.702 | 2779.017 | 1571.524 | 886.3986 | 325.3231 | 3184.108 | 2461.297 | 3020.819 | 3673.827 | 1064.349 | 1998.595 | 5004.678 | 4480.043 | 2722.22 | 4021.809 | 2906.073 | 1089.985 | 1917.136 | 2777.472 | 1492.759 | 4135.156 | 3312.37 | 1265.474 | 7099.277 | 1165.381 | 7662.198 |
| CUST_17238_PI429010886 | N37631 | 0.046346 | 15 | 15 | 512817511 | PREDICTED: uncharacterized protein LOC10173106 | | | 8.96E-16 | 7.924854 | 13.06649 | 14.02581 | 4.859225 | 18.33968 | 9.836031 | 11.11786 | 19.88004 | 7.040982 | 8.97147 | 14.40423 | 12.88026 | 14.9294 | 28.71965 | 17.34415 | 10.33682 | 10.99683 | 15.95324 | 8.330776 | 5.637553 | 9.830811 | 16.34959 | 8.884013 | 11.83405 | 14.20826 | 7.260457 | 13.94454 |
| CUST_36231_PI429010886 | M15102 | 0.046348 | 18 | 12 | 380022121 | PREDICTED: LOW QUALITY PROTEIN: dystrophin, isc | | | 7.08E-29 | 191.0009 | 202.7615 | 220.0942 | 139.2832 | 184.9825 | 169.5547 | 144.2346 | 164.7862 | 189.3668 | 238.941 | 186.6214 | 332.0008 | 188.1392 | 245.2402 | 289.5961 | 292.8985 | 167.3467 | 230.7819 | 198.0625 | 276.7912 | 200.4301 | 239.3095 | 170.4197 | 179.5033 | 216.3677 | 222.3148 | 244.4297 |
| CUST_21958_PI429010886 | N46157 | 0.046367 | 18 | 3 | | | | | | 56.69692 | 42.73401 | 80.36337 | 118.4789 | 74.90972 | 136.6977 | 63.40813 | 41.32867 | 33.50292 | 36.72024 | 64.58903 | 37.31224 | 34.73053 | 48.35331 | 72.65998 | 91.83515 | 73.40872 | 108.2155 | 86.13317 | 58.04233 | 53.65979 | 83.7299 | 57.45413 | 96.689 | 52.24698 | 85.13186 | 31.42645 |
| CUST_55593_PI429010886 | M110884 | 0.046388 | 24 | 0 | | | | | | 521.963 | 591.8445 | 629.9728 | 586.2213 | 541.9679 | 578.1088 | 543.5013 | 457.9897 | 584.8769 | 587.2428 | 434.6643 | 422.7364 | 456.5493 | 670.112 | 297.0259 | 722.474 | 487.3364 | 608.2249 | 470.9976 | 613.6489 | 528.026 | 596.6167 | 506.8788 | 485.9211 | 864.8993 | 653.3522 | 787.4832 |
| CUST_49938_PI429010886 | M87797 | 0.046388 | 24 | 0 | | | | | | 37.14124 | 24.11521 | 46.25026 | 14.00731 | 23.04508 | 45.17801 | 19.02231 | 31.96544 | 34.74686 | 30.9722 | 22.38584 | 21.27634 | 25.10042 | 28.92063 | 33.27763 | 30.0795 | 28.90976 | 31.09423 | 20.47188 | 28.87402 | 30.58787 | 35.16159 | 32.68431 | 25.57234 | 40.73672 | 48.49494 | 43.40065 |
| CUST_18002_PI429010886 | N38977 | 0.046429 | 21 | 9 | | | | | | 5.798248 | 11.04742 | NA | 14.44266 | 5.327 | 5.390817 | NA | NA | 4.898275 | 20.41951 | 11.82406 | 28.12721 | 9.263386 | NA | 10.75903 | 17.44175 | 8.765923 | 11.58812 | 6.058176 | 8.624589 | 9.731006 | 13.47393 | 8.86752 | NA | 23.04991 | 9.581554 | 29.47907 |
| CUST_15298_PI429010886 | N34354 | 0.046436 | 21 | 15 | 291231297 | PREDICTED: hypothetical protein [Saccoglossus kow | | | 1.16E-23 | 108.7243 | 138.6217 | 150.3647 | 110.4846 | 85.83424 | 128.6369 | 60.93646 | 76.52417 | 120.8052 | 113.2243 | 98.47983 | 161.5046 | 90.15151 | 106.0819 | 113.1942 | 145.2632 | 120.7611 | 168.9693 | 137.649 | 103.4319 | 128.8934 | 123.4578 | 153.2734 | 100.999 | 114.6944 | 97.83625 | 116.5044 |
| CUST_26150_PI429010886 | M14810 | 0.046439 | 18 | 9 | 348563737 | PREDICTED: collagen alpha-5(IV) chain [Cavia porce | | | 1.2E-25 | 5.487456 | 6.844642 | 24.19522 | 13.0288 | 7.108049 | 21.42376 | 13.30523 | 15.32478 | 4.965994 | 20.5721 | 16.34764 | 13.88119 | NA | 11.51911 | 17.62133 | 7.804985 | 12.03567 | 13.07517 | 4.552819 | 9.04725 | 10.06856 | 10.3075 | 13.04197 | NA | 14.70379 | 26.29583 | 12.52724 |
| CUST_21061_PI429010886 | N44549 | 0.046448 | 15 | 9 | | | | | | 718.917 | 715.3982 | 643.2335 | 651.0924 | 645.7634 | 497.6528 | 726.6345 | 656.946 | 896.8013 | 1135.995 | 772.946 | 743.1041 | 525.5752 | 915.3775 | 576.3102 | 874.5756 | 542.7169 | 511.9204 | 805.2979 | 679.3339 | 672.0887 | 1217.47 | 648.9401 | 687.2662 | 1361.387 | 722.2644 | 1133.023 |
| CUST_45632_PI429010886 | M69728 | 0.046452 | 24 | 21 | 507983255 | PREDICTED: zinc finger protein 184 [Condylura crist | | | 1.61E-47 | 86.04961 | 89.00024 | 116.2937 | 82.6196 | 78.38473 | 84.22907 | 84.62693 | 79.30391 | 81.95659 | 98.92872 | 79.44802 | 85.81376 | 83.11485 | 99.89328 | 110.4288 | 91.6203 | 76.52417 | 93.86099 | 105.8358 | 86.63308 | 91.27166 | 96.50257 | 104.2917 | 98.00231 | 119.2791 | 82.86721 | 92.1489 |
| CUST_11470_PI429010886 | N27868 | 0.046527 | 15 | 12 | | | Metabolic process | Nucleobase-containing compound metabolic process | | 15.00335 | 24.68274 | 16.77475 | 10.64689 | 17.4527 | 13.7828 | 13.12705 | 12.78607 | 16.59129 | 19.6916 | 14.52793 | 18.81343 | 24.8123 | 23.25736 | 24.03757 | 19.63157 | NA | 27.35173 | 12.09545 | 21.2878 | 16.37181 | 40.26663 | 18.48046 | 9.711718 | 14.55387 | 18.54724 | 32.50331 |
| CUST_20051_PI429010886 | N42716 | 0.046553 | 12 | 9 | | | | | | 14.21164 | 3.79765 | 12.81601 | 11.38637 | 13.99028 | 21.90983 | 43.97029 | 7.157803 | 13.85139 | NA | 21.7968 | NA | 4.992877 | NA | 6.451538 | 6.837856 | 27.53274 | 26.22564 | 14.3364 | NA | 37.56088 | 10.47807 | 34.86849 | 61.15955 | NA | 4.696595 | 8.495784 |
| CUST_20550_PI429010886 | N43647 | 0.046629 | 18 | 3 | | | | | | 29.9042 | 72.67632 | 37.67574 | 102.0816 | 27.88621 | 72.13281 | 38.36474 | 39.54839 | 29.69394 | 17.74547 | 15.66989 | 39.5035 | 25.01801 | 17.47239 | NA | 23.27419 | 5.649998 | 31.92608 | 7.776362 | 24.86204 | 54.14016 | 18.81492 | 61.28097 | 44.0607 | 21.7761 | 60.0207 | 15.59665 |
| CUST_32835_PI429010886 | M5222 | 0.046653 | 24 | 9 | | | | | | 156.8208 | 257.772 | 19.58231 | 582.4599 | 67.67314 | 63.34887 | NA | 22.65868 | 19.61118 | 744.5402 | 492.0126 | 1473.383 | 654.914 | 625.332 | 618.8739 | 426.5023 | 11.3912 | 503.4817 | 80.78349 | 558.6606 | 93.97578 | 158.101 | 433.8707 | 31.64376 | 257.9765 | 27.4871 | 117.6403 |
| CUST_28211_PI429010886 | N57517 | 0.046669 | 15 | 6 | | | | | | 176.4808 | 101.1468 | 263.5785 | 40.61781 | 17.43225 | NA | 211.0801 | 480.0427 | 503.2472 | 413.8447 | 71.9016 | 64.41009 | 15.17161 | 28.71325 | 18.74035 | 8.140928 | 155.0892 | NA | 82.26332 | 84.27092 | 38.82938 | NA | 268.9771 | 68.34468 | 17.92595 | 52.95164 | 44.27668 |
| CUST_20889_PI429010886 | N44260 | 0.04668 | 21 | 3 | 193591726 | PREDICTED: zinc finger protein 271-like [Acyrthosipl | | | 7.36E-34 | 16.59282 | 16.69997 | 23.94075 | 20.94079 | 18.55819 | 21.5295 | 22.97943 | 10.66756 | 19.82066 | 16.97468 | 11.45279 | 19.19934 | 14.5269 | 13.95252 | 8.919059 | 18.72449 | 19.48977 | 22.25206 | 23.95732 | 19.03069 | 17.88508 | 14.09377 | 20.31071 | 85.01801 | 19.88848 | 23.11751 | 29.49638 |
| CUST_56401_PI429010886 | M115803 | 0.046701 | 12 | 12 | | | | | | 63.43828 | 64.16931 | 71.64239 | 55.07381 | 46.90121 | 61.53357 | 39.94582 | 48.38349 | 52.22902 | 78.53416 | 53.26961 | 52.50431 | 35.68582 | 76.24385 | 95.50344 | 64.96286 | 80.37038 | 70.88181 | 54.69269 | 54.60219 | 38.44815 | 65.79499 | 50.82916 | 73.54244 | 103.7132 | 68.15284 | 61.39492 |
| CUST_45617_PI429010886 | M69674 | 0.046701 | 12 | 0 | 91087755 | PREDICTED: similar to GA11514-PA [Tribolium casta | | | 1.82E-76 | 12.86372 | 14.11041 | 12.45001 | 13.08205 | 9.396038 | 10.86396 | 6.319033 | 5.407886 | 10.65431 | 13.72842 | 9.458785 | 5.099536 | 10.87219 | 12.75631 | 11.18584 | 14.40423 | 4.102467 | 10.69771 | 7.94792 | 6.823408 | 8.926798 | 13.43047 | 11.21554 | 14.38334 | 16.22818 | 10.55953 | 11.3912 |
| CUST_48595_PI429010886 | M81187 | 0.046701 | 12 | 0 | | | | | | 50.88285 | 38.31739 | 51.5653 | 43.06591 | 48.45867 | 56.80303 | 36.64988 | 42.09528 | 33.66812 | 48.87835 | 50.62301 | 42.69298 | 30.71736 | 50.4763 | 44.52089 | 52.034 | 44.59476 | 40.93552 | 44.47869 | 25.80322 | 28.96949 | 55.23417 | 40.83264 | 50.61028 | 66.67032 | 54.28784 | 61.54664 |
| CUST_38629_PI429010886 | M28683 | 0.046709 | 12 | 0 | | | | | | 8036.745 | 8265.934 | 11327.23 | 6042.693 | 5794.422 | 6148.012 | 6388.757 | 7435.628 | 8904.357 | 7974.043 | 7647.424 | 14629.47 | 9072.043 | 10819.14 | 17865.21 | 11327.23 | 4973.61 | 6879.978 | 9154.315 | 9448.983 | 9937.704 | 16000.49 | 8245.915 | 7002.188 | 12563.99 | 7457.426 | 11566.7 |
| CUST_55346_PI429010886 | M109694 | 0.04673 | 12 | 3 | | | | | | 24.50255 | 33.7899 | 8.496964 | 41.51371 | 46.507 | 63.73239 | 5.856518 | 31.13983 | 10.09344 | 12.61842 | 26.02132 | 7.001213 | 4.235405 | 14.90147 | 6.139615 | 58.40782 | 17.64932 | 30.3942 | 10.65167 | 5.837543 | 9.387006 | 7.178303 | NA | NA | 14.24976 | 43.83106 | 8.035951 |
| CUST_30909_PI429010886 | M197 | 0.046743 | 15 | 0 | | | | | | 79.97877 | 78.75847 | 83.85047 | 73.94862 | 49.92232 | 65.42947 | 47.83781 | 61.6776 | 67.08962 | 59.74927 | 60.92331 | 88.47454 | 70.108 | 79.45781 | 77.77973 | 97.04503 | 69.26327 | 76.49245 | 61.63578 | 102.6532 | 76.94992 | 76.41163 | 72.98413 | 57.36487 | 78.60763 | 77.67565 | 100.0857 |
| CUST_54503_PI429010886 | M106275 | 0.046772 | 15 | 12 | 195326584 | Drosophila sechellia GM24804 (Dsec\GM24804), m | Metabolic process | Carbohydrate metabolic process | 5.38E-55 | 10.76087 | 31.97801 | 9.576049 | 12.65789 | 11.53244 | 10.19086 | 10.36072 | 15.73405 | 8.881606 | 21.0745 | 8.218065 | 16.53332 | 23.34418 | 35.40982 | 14.38683 | 20.29845 | 16.25775 | 10.41894 | 17.96665 | 11.14656 | 16.21643 | 16.03098 | 7.408115 | 7.368786 | 30.0571 | 7.431826 | 16.96557 |
| CUST_57292_PI429010886 | E4472 | 0.046778 | 18 | 9 | | | | | | 191.0756 | 198.9664 | 280.7168 | 140.5892 | 185.7909 | 158.0759 | 154.2483 | 343.6779 | 298.1495 | 249.5597 | 243.9078 | 319.448 | 231.235 | 271.918 | 263.8863 | 232.6257 | 207.2391 | 191.9895 | 212.4692 | 375.1189 | 261.123 | 217.9939 | 234.7048 | 135.2179 | 184.7518 | 242.2528 | 277.125 |
| CUST_34392_PI429010886 | M9529 | 0.046778 | 18 | 9 | 170576227 | unspecific monooxygenase [Brugia malayi] >gi\|158 | | | 7.58E-07 | 37.30131 | 54.81682 | 55.74802 | 33.57067 | 36.75997 | 35.53745 | 40.11789 | 64.83329 | 51.46537 | 88.80055 | 47.3028 | 81.58984 | 34.0757 | 51.48154 | 41.8722 | 49.84204 | 39.06311 | 53.63219 | 46.66161 | 64.30394 | 52.04012 | 43.58778 | 41.05593 | 31.11356 | 63.47762 | 39.15253 | 63.89005 |
| CUST_49697_PI429010886 | M86750 | 0.046817 | 18 | 15 | | | | | | 82.3472 | 80.9845 | 67.54243 | 62.12557 | 60.68591 | 65.84868 | 68.1364 | 58.81878 | 76.21767 | 66.03995 | 63.105 | 82.31536 | 78.39028 | 77.1122 | 92.24128 | 95.23078 | 70.96812 | 75.91487 | 77.2245 | 50.93999 | 80.92356 | 63.36121 | 73.09859 | 76.29064 | 78.38473 | 67.28983 | 69.28034 |
| CUST_9212_PI429010886 | N24240 | 0.046822 | 21 | 18 | | | | | | 34.75551 | 23.12065 | 28.06449 | 6.942833 | 18.08701 | 32.82252 | 29.04567 | 28.09705 | 30.09221 | 37.00144 | 19.57413 | 42.1461 | 20.56111 | 45.4027 | 29.95175 | 41.25188 | 22.498 | 29.9579 | 28.36576 | 30.24099 | 41.31796 | 33.25306 | 25.55814 | 38.41593 | 20.76696 | 22.0325 | 19.4114 |
| CUST_19273_PI429010886 | N31743 | 0.046853 | 18 | 9 | 471221791 | hypothetical protein IMG5_174580 [Ichthyophthiriu | | | 6.87E-07 | 199.8275 | 186.6214 | 205.2623 | 203.0622 | 154.3172 | 172.8115 | 129.2905 | 170.9469 | 168.9486 | 207.3573 | 166.4251 | 170.4477 | 171.5942 | 180.1444 | 185.0177 | 204.946 | 197.2642 | 188.2724 | 177.2908 | 198.0922 | 171.1636 | 204.0225 | 176.2795 | 188.7857 | 232.5572 | 179.7697 | 229.8288 |
| CUST_16349_PI429010886 | M113561 | 0.046855 | 15 | 12 | | | | | | 5336.786 | 7355.509 | 5439.997 | 7017.655 | 6018.766 | 5330.558 | 3994.868 | 4763.632 | 4817.775 | 5940.317 | 4575.004 | 7701.667 | 5800.891 | 6802.929 | 8156.263 | 8876.599 | 4528.171 | 5424.543 | 5197.227 | 6528.23 | 5080.996 | 7188.247 | 5092.903 | 4293.225 | 7347.807 | 5726.098 | 6454.955 |
| CUST_38266_PI429010886 | M24832 | 0.046855 | 15 | 12 | | | | | | 11.42997 | 10.04937 | 28.74973 | 8.355196 | 6.205172 | 6.656764 | 6.755699 | 13.53636 | 21.32116 | 11.84727 | 8.484781 | 14.23976 | 8.07676 | 13.90672 | 17.41182 | 13.46995 | 13.04874 | 11.86639 | 9.86011 | 6.186749 | 9.227326 | 7.797143 | 11.95978 | 23.95916 | 10.08948 | 13.8146 | 14.79313 |
| CUST_32272_PI429010886 | M3681 | 0.046859 | 24 | 15 | 321475415 | hypothetical protein DAPPUDRAFT_313219 [Daphn | | | 7.74E-86 | 22.15879 | 13.50586 | 19.3883 | 17.50878 | 19.6594 | 17.36105 | 16.06458 | 15.23737 | 13.50139 | 13.68485 | 21.30938 | 23.72867 | 16.10523 | 27.67518 | 18.50987 | 23.49179 | 36.12687 | 28.89154 | 19.38567 | 23.07793 | 7.891053 | 26.79307 | 24.0077 | 15.85477 | 24.03945 | 17.7958 | 27.84642 |
| CUST_5731_PI429010886 | N19116 | 0.046909 | 24 | 9 | | | | | | 87.47537 | 157.8347 | 125.0791 | 194.0018 | 132.3017 | 127.8146 | 111.3637 | 145.8124 | 116.2535 | 184.0038 | 173.4432 | 178.9487 | 122.3193 | 160.3693 | 131.0829 | 115.7238 | 93.34264 | 103.3151 | 142.8394 | 178.0431 | 134.6193 | 139.216 | 163.6892 | 83.0149 | 91.71625 | 73.89571 | 83.88709 |
| CUST_40953_PI429010886 | M56472 | 0.046964 | 12 | 0 | | | | | | 59.17148 | 63.37317 | 80.82297 | 40.68218 | 47.73188 | 30.93394 | 44.07915 | 46.05816 | 65.04228 | 77.67565 | 54.79813 | 78.66644 | 52.29382 | 76.13058 | 72.24742 | 62.08961 | 51.22479 | 62.95412 | 55.44777 | 65.95253 | 73.13314 | 80.14138 | 92.32556 | 41.69983 | 81.26685 | 69.46903 | 85.84413 |
| CUST_42486_PI429010886 | M60396 | 0.046994 | 21 | 0 | | | | | | 61.3758 | 49.04708 | 55.90718 | 18.06411 | 30.75309 | 30.45027 | 36.16119 | 26.02914 | 35.43589 | 31.12088 | 25.20311 | 31.81303 | 26.75249 | 29.26565 | 30.37645 | 47.03677 | 33.63678 | 36.63495 | 43.80188 | 35.48686 | 17.91166 | 52.20745 | 28.33335 | 38.59967 | 44.39447 | 25.37 | 17.35771 |
| CUST_24377_PI429010886 | N50509 | 0.04702 | 21 | 3 | | | | | | 69.96115 | 121.4004 | 84.63515 | 198.8279 | 130.6774 | 161.8906 | 94.72175 | 85.63673 | 67.61178 | 81.62354 | 61.65538 | 78.31562 | 63.06213 | 78.76718 | 123.3865 | 105.0612 | 76.31563 | 94.23139 | 66.97079 | 62.07681 | 110.4128 | 92.84239 | 93.29834 | 84.10524 | 116.8091 | 143.5804 | 73.96327 |
| CUST_42324_PI429010886 | M60008 | 0.047026 | 21 | 0 | | | | | | 17.22867 | 17.43627 | 13.54646 | 7.768024 | 18.54361 | 12.02328 | 10.84393 | 13.80323 | 7.993052 | 7.646317 | 13.26879 | 5.940285 | 13.30229 | 8.055145 | 6.425284 | 18.81158 | 18.79454 | 13.94176 | 8.188306 | 9.725914 | 7.710119 | 18.58096 | 14.87518 | NA | 15.80258 | 4.140048 | 9.203374 |
| CUST_55963_PI429010886 | M112337 | 0.047031 | 12 | 0 | | | | | | 733.7989 | 716.5268 | 694.5441 | 634.1523 | 568.2036 | 560.4476 | 576.3102 | 599.2901 | 616.0789 | 846.4177 | 644.6096 | 820.0337 | 668.4719 | 736.5339 | 721.5575 | 809.3886 | 626.2648 | 621.0146 | 623.5116 | 806.919 | 713.6188 | 769.5294 | 655.3909 | 625.332 | 875.295 | 652.2033 | 857.5648 |
| CUST_822_PI429010886 | N7420 | 0.047031 | 18 | 0 | | | | | | 4201.227 | 5239.377 | 6049.937 | 6536.631 | 2475.35 | 7107.78 | 1055.909 | 1832.42 | 1385.628 | 3869.78 | 3100.565 | 5330.558 | 400.0206 | 6033.954 | 3893.391 | 4077.008 | 2648.384 | 4667.789 | 3152.492 | 6871.867 | 4062.216 | 4028.762 | 2250.529 | 1999.641 | 2147.931 | 5349.606 | 2232.866 |
| CUST_40026_PI429010886 | M48794 | 0.047034 | 24 | 18 | 345485280 | PREDICTED: mitochondrial import inner membrane | Transport | Protein transport | 9.4E-65 | 32.63054 | 24.55823 | 21.21095 | 20.65041 | 25.0272 | 19.87086 | 18.11698 | 20.27043 | 29.09978 | 32.38846 | 28.05565 | 34.41169 | 25.07677 | 34.0469 | 41.99643 | 36.89165 | 21.76863 | 26.75604 | 40.95911 | 40.86391 | 27.46314 | 23.81911 | 33.57067 | 21.91785 | 32.6566 | 22.26367 | 39.87971 |
| CUST_9248_PI429010886 | N24306 | 0.04704 | 18 | 6 | | | | | | 3.747172 | NA | NA | 18.25998 | 8.403892 | 7.879494 | 11.78251 | 17.05056 | 10.67318 | 10.27804 | 14.50091 | 17.36105 | 8.727631 | NA | NA | NA | 5.640027 | 11.16571 | 6.261173 | 6.613886 | 10.78913 | NA | 7.719905 | 16.29239 | 8.529362 | 17.3497 | 17.25706 |
| CUST_5171_PI429010886 | N18354 | 0.047057 | 24 | 21 | 357612299 | hypothetical protein KGM_05346 [Danaus plexippu | Protein metabolism | Cellular protein modification process | 9.86E-59 | 6.071276 | 16.09421 | 11.40403 | 8.519462 | 4.758391 | 5.324458 | 6.75468 | 8.036889 | 3.688492 | 16.40453 | 8.472888 | 15.88543 | 7.283318 | 9.3513 | 11.73054 | 15.62557 | NA | 8.361603 | 22.3581 | 5.420008 | 14.83018 | 14.72748 | 17.09347 | NA | 10.35813 | 9.891852 | 8.44134 |
| CUST_19046_PI429010886 | N40893 | 0.047081 | 12 | 3 | | | | | | 14.56893 | 11.76157 | 7.795868 | 29.75464 | 32.35284 | 67.85743 | 37.13071 | 22.18079 | 14.89599 | 8.257726 | 24.08063 | 6.552993 | 25.91379 | NA | 9.827509 | 15.81194 | 77.67565 | 34.4458 | 46.74005 | 6.824426 | 23.97181 | 5.759897 | 26.46581 | 56.71651 | 4.413149 | 11.56577 | 10.89944 |
| CUST_19121_PI429010886 | N41011 | 0.0471 | 21 | 0 | | | | | | 35.87751 | 52.90229 | 46.81599 | 36.40505 | 55.80731 | 43.77433 | 38.45312 | 34.84252 | 42.54336 | 36.48665 | 44.37542 | 44.34868 | 42.12986 | 34.45811 | 44.63407 | 33.48474 | 34.63494 | 47.47127 | 42.07991 | 48.50046 | 37.13409 | 77.59246 | 46.95662 | 66.87846 | 50.06979 | 42.22821 | 51.52932 |
| CUST_52654_PI429010886 | M98332 | 0.047122 | 24 | 15 | | | | | | 64.99854 | 33.58859 | NA | 32.83834 | 37.25447 | 39.32573 | 37.69372 | 60.53435 | 44.77456 | 512.8422 | 90.55235 | 113.0059 | NA | 105.8112 | 59.32225 | 163.0297 | NA | 89.61164 | 70.97584 | 95.18251 | 138.2562 | 39.0152 | 56.46834 | 176.5494 | 150.4655 | 95.47811 | 146.8674 |
| CUST_16421_PI429010886 | N36208 | 0.047143 | 24 | 0 | | | | | | 4.822002 | 28.43068 | 16.36343 | 47.75268 | 115.3926 | 55.30305 | 53.73015 | 164.1178 | 94.62564 | 14.45805 | 37.76644 | 24.28324 | 46.59363 | 30.90791 | 29.40344 | 8.813762 | 21.38839 | NA | 30.81693 | 100.7779 | 185.7909 | 12.39257 | 64.31019 | 67.25956 | 13.79996 | 58.18474 | 16.84352 |
| CUST_43503_PI429010886 | M63221 | 0.047187 | 21 | 18 | | | | | | 83.87586 | 146.3194 | 131.9637 | 69.72451 | 124.9715 | 78.27999 | 78.51483 | 71.58792 | 69.55824 | 103.6255 | 144.0441 | 128.5885 | 68.95801 | 130.0555 | 104.4054 | 135.7577 | 95.12719 | 129.4353 | 111.0786 | 105.1367 | 132.9489 | 155.7882 | 92.55609 | 95.84863 | 129.5236 | 77.82271 | 103.4225 |
| CUST_21273_PI429010886 | N44929 | 0.047244 | 12 | 0 | 328711363 | PREDICTED: zinc finger protein 845-like [Acyrthosipl | | | 2.16E-09 | 200.337 | 260.6408 | 229.5365 | 151.9486 | 195.487 | 131.1403 | 153.0373 | 150.9847 | 175.5769 | 203.0375 | 155.9986 | 191.528 | 183.6281 | 418.5656 | 181.1767 | 260.4633 | 182.5193 | 186.3956 | 142.5243 | 218.037 | 194.2624 | 215.6784 | 156.8398 | 128.7791 | 277.6024 | 208.8382 | 259.1574 |
| CUST_52383_PI429010886 | M97245 | 0.047269 | 15 | 0 | | | | | | 188.4563 | 200.7582 | 282.7521 | 179.1322 | 166.9208 | 193.3073 | 163.1553 | 198.9264 | 199.4521 | 239.4245 | 197.3524 | 249.6664 | 206.8341 | 297.9086 | 246.3468 | 233.7917 | 201.5551 | 266.3801 | 238.8599 | 193.2088 | 207.128 | 260.055 | 205.9317 | 188.3253 | 328.6838 | 230.36 | 237.2477 |
| CUST_43620_PI429010886 | M63520 | 0.047273 | 21 | 0 | | | | | | 36.93404 | 41.31796 | 43.5925 | 40.17403 | 35.65407 | 24.11784 | 28.65923 | 20.69091 | 30.00329 | 43.05396 | 26.04992 | 31.25517 | 17.92595 | 36.23123 | 32.59686 | 40.66087 | 33.54956 | 42.14262 | 35.07318 | 29.5952 | 43.70451 | 61.28936 | 45.47327 | 20.46617 | 45.89316 | 28.92395 | 39.52248 |
| CUST_7546_PI429010886 | N21708 | 0.047273 | 24 | 21 | 123502501 | hypothetical protein [Trichomonas vaginalis G3] >gi | | | 6.07E-07 | 174.1781 | 191.7619 | 212.8057 | 151.3617 | 139.0161 | 150.0374 | 150.1195 | 159.948 | 165.9754 | 182.3416 | 138.1473 | 186.0881 | 145.7727 | 257.6826 | 212.4692 | 201.6936 | 163.6601 | 177.6145 | 203.482 | 183.4677 | 175.2355 | 219.4136 | 224.7094 | 168.032 | 188.1904 | 185.0712 | 166.8022 |
| CUST_23759_PI429010886 | N49414 | 0.047273 | 12 | 0 | | | | | | 434.8523 | 407.4423 | 165.7972 | 646.4459 | 405.4301 | 472.5516 | 349.978 | 316.122 | 289.423 | 268.9771 | 289.679 | 222.6303 | 361.7651 | 279.2335 | 350.0802 | 521.963 | 378.3563 | 420.4304 | 380.9707 | 329.2355 | 231.8541 | 456.9093 | 190.5242 | 402.9446 | 398.993 | 316.6467 | 172.0048 |
| CUST_38012_PI429010886 | M22771 | 0.047283 | 12 | 9 | | | | | | 129.6855 | 663.5689 | 1028.799 | 24.91723 | 11.04906 | 27.86689 | 394.2069 | 896.5197 | 953.5865 | 1444.613 | 305.2873 | 793.9994 | 387.9318 | 823.5514 | 415.4709 | 126.405 | 275.9866 | 80.42722 | 128.4938 | 1446.386 | 107.1617 | 797.54 | 724.6164 | 25.36437 | 262.0585 | 634.9072 | 356.0402 |
| CUST_48730_PI429010886 | M81893 | 0.047286 | 18 | 15 | | | | | | 12.92383 | 10.23633 | 20.15665 | 15.07833 | 13.18579 | 22.58851 | 10.71325 | 8.654424 | 4.520354 | 9.611855 | 16.85623 | 3.898362 | 13.14806 | 19.02231 | 16.08792 | 21.49928 | 21.68419 | 16.90946 | 11.6794 | 7.474432 | 17.3946 | 9.712939 | 19.19334 | 10.4406 | 11.69456 | 5.285615 | 16.32095 |
| CUST_9830_PI429010886 | N25269 | 0.047299 | 18 | 6 | 405977152 | Putative ferric-chelate reductase 1 [Crassostrea gigas | Response to stress | Defence responce | 2.64E-09 | 255.3857 | 221.5886 | 725.6338 | 130.6215 | 69.37437 | 69.70264 | 334.3349 | 778.8569 | 597.939 | 453.3672 | 165.3002 | 209.7275 | 214.0415 | 320.7508 | 165.2222 | 137.5651 | 212.046 | 69.39137 | 153.6882 | 268.82 | 73.65478 | 212.9083 | 380.2921 | 84.14675 | 317.2703 | 293.3227 | 469.6397 |
| CUST_53966_PI429010886 | M103909 | 0.047303 | 24 | 0 | | | | | | 47.90107 | 41.71124 | 48.32558 | 32.91963 | 38.2326 | 43.33061 | 32.12754 | 14.80676 | 41.87571 | 38.09087 | 34.55194 | 38.77656 | 42.99716 | 45.65948 | 12.23461 | 31.31664 | 25.44661 | 42.12575 | 22.99548 | 38.89725 | 46.9109 | 32.46164 | 47.2395 | 50.14442 | 46.15285 | 38.26462 | 51.43059 |
| CUST_10272_PI429010886 | N25983 | 0.04733 | 21 | 0 | | | | | | 81.64235 | 154.0296 | 93.45456 | 73.89571 | 95.04998 | 72.40904 | 63.66464 | 63.68458 | 69.54952 | 133.4752 | 80.39867 | 214.2881 | 98.80707 | 110.737 | 127.4645 | 187.0261 | 53.80266 | 128.6369 | 92.34903 | 112.226 | 95.46826 | 105.3783 | 77.08541 | 99.10652 | 97.72266 | 75.59869 | 114.6944 |
| CUST_18488_PI429010886 | N39887 | 0.047333 | 12 | 3 | | | | | | 18.24285 | 23.33049 | 23.98555 | 34.20754 | 33.58412 | 34.91043 | 27.06117 | 6.177678 | 24.7547 | 8.753886 | 4.987261 | 15.90831 | 12.01567 | 16.67738 | 33.24682 | 229.2068 | 6.977617 | 57.1153 | NA | 13.36038 | 11.0581 | 32.02169 | 11.33779 | 20.58435 | 16.29078 | 4.599604 | NA |
| CUST_30935_PI429010886 | M261 | 0.047342 | 24 | 18 | 290562199 | Probable 39S ribosomal protein L49, mitochondrial | Protein metabolism | Ribosome biogenesis | 5.32E-17 | 94.54715 | 169.2006 | 109.6567 | 84.50068 | 99.09557 | 89.4807 | 89.77998 | 80.46995 | 89.4807 | 119.1476 | 92.30386 | 139.8628 | 107.1133 | 126.7437 | 167.9263 | 160.8449 | 71.31512 | 121.3759 | 135.9242 | 127.2256 | 106.5352 | 134.3348 | 96.1698 | 107.9913 | 110.0373 | 109.3451 | 118.1855 |
| CUST_42758_PI429010886 | M61171 | 0.047344 | 15 | 12 | 321471759 | hypothetical protein DAPPUDRAFT_302396 [Daphn | | | 1.36E-08 | 63.11741 | 63.33459 | 115.5 | 30.76819 | 45.76846 | 37.43361 | 61.59565 | 100.0727 | 79.56143 | 91.79308 | 59.68907 | 84.80692 | 80.44402 | 97.19605 | 74.98811 | 55.19772 | 71.39836 | 61.62124 | 66.27543 | 63.36121 | 63.34141 | 64.56992 | 82.06433 | 48.46285 | 103.6887 | 55.25615 | 88.10949 |
| CUST_46272_PI429010886 | M72033 | 0.047365 | 18 | 9 | | | | | | 13.63796 | 13.26174 | 28.93983 | 8.346516 | 16.11615 | 14.77643 | 12.55445 | 9.648135 | 16.35941 | 23.1151 | 19.60862 | 23.73141 | 18.4372 | 25.8162 | 22.05844 | 16.25775 | 13.53347 | 15.71146 | 11.36093 | 20.25261 | 23.25228 | 15.16191 | 15.56031 | 9.332574 | 13.54494 | 24.54709 | 22.75236 |
| CUST_469_PI429010886 | N5485 | 0.047365 | 21 | 3 | 256002943 | ribosomal protein L10a [Fenneropenaeus merguien | Protein metabolism | Ribosome biogenesis | 1.55E-28 | 18962.11 | 39606.34 | 21067.41 | 46851.42 | 32430.96 | 33148.92 | 18116.83 | 18927.17 | 21267.61 | 27353.46 | 15567.33 | 30232.55 | 18410.6 | 23759.64 | 12563.99 | 26343.04 | 10721.33 | 25830.1 | 14629.47 | 21431.7 | 34767.97 | 32610.41 | 25871.73 | 23706.31 | 26294.44 | 35468.64 | 19004.47 |
| CUST_23759_PI429010886 | N49414 | 0.047391 | 18 | 3 | | | | | | 37.33083 | 53.79628 | 70.94036 | 62.90335 | 55.07381 | 74.92071 | 16.69775 | 34.95753 | 35.1685 | 34.0946 | 40.93552 | 40.97837 | 36.9383 | 72.81129 | 26.73668 | 27.03336 | 19.6594 | 55.02956 | 26.48449 | 109.6802 | 79.76051 | 41.7589 | 64.83329 | 57.62155 | 42.5721 | 41.0381 | 41.41188 |
| CUST_36648_PI429010886 | M16451 | 0.047415 | 24 | 3 | | | | | | 450.2218 | 1204.562 | 493.331 | 1557.99 | 604.4329 | 641.7483 | 397.2225 | 474.8115 | 477.8507 | 526.019 | 265.8546 | 865.4579 | 272.1063 | 577.5152 | 414.4964 | 569.1252 | 155.1933 | 452.1705 | 231.1582 | 536.6546 | 715.8969 | 740.0272 | 657.1327 | 427.0325 | 940.5709 | 1003.971 | 491.272 |
| CUST_6205_PI429010886 | N19768 | 0.047415 | 21 | 12 | 443695138 | hypothetical protein CAPTEDRAFT_195081 [Capitell | | | 3.33E-09 | 16.24253 | 25.9573 | 19.89848 | 30.73213 | 18.87168 | 22.60935 | 11.20677 | 44.4501 | 20.25455 | 56.26704 | 26.44421 | 38.65189 | 30.61606 | 37.47537 | 38.92288 | 23.57221 | 21.29823 | 21.51773 | 33.22111 | 42.68003 | 29.49276 | 26.22091 | 23.86522 | 8.779513 | 32.87841 | 29.85793 | 28.00703 |
| CUST_22177_PI429010886 | N46573 | 0.047416 | 24 | 3 | 321471535 | hypothetical protein DAPPUDRAFT_316225 [Daphn | Protein metabolism | Proteolysis | 5.09E-27 | 94.75367 | 95.63731 | 104.7461 | 99.73067 | 108.9026 | 144.7517 | 92.58405 | 97.61331 | 135.1566 | 112.7335 | 94.56836 | 116.9236 | 130.6215 | 108.9599 | 93.1869 | 124.3822 | 91.42867 | 96.64522 | 104.1211 | 68.66167 | 62.29412 | 92.20534 | 76.78516 | 59.16475 | 83.88709 | 134.3075 | 139.419 |

| Probe ID | ID | p-value | Period | Phase | EBI ID | Annotation | GO category | Go term | E-value | 0 | 0 | 0 | 3 | 3 | 3 | 6 | 6 | 6 | 9 | 9 | 9 | 12 | 12 | 12 | 15 | 15 | 15 | 18 | 18 | 18 | 21 | 21 | 21 | 24 | 24 | 24 |
|---|---|---|---|---|---|---|---|---|---|---|---|---|---|---|---|---|---|---|---|---|---|---|---|---|---|---|---|---|---|---|---|---|---|---|---|---|
| CUST_31900_PI429010886 | M2696 | 0.047485 | 12 | 0 | 322792405 | hypothetical protein SINV_11858 [Solenopsis invicta | | | 1.23E-29 | 206.1701 | 192.7007 | 163.3967 | 155.3131 | 159.7825 | 152.5355 | 126.7035 | 138.5194 | 155.3897 | 161.4133 | 147.402 | 198.5364 | 194.747 | 207.0705 | 184.4394 | 245.6075 | 155.2361 | 173.1201 | 134.8627 | 177.2908 | 163.4482 | 213.9895 | 179.7938 | 144.0774 | 177.6724 | 192.4131 | 155.1312 |
| CUST_32245_PI429010886 | M3619 | 0.047512 | 18 | 0 | 13898841 | CUB-serine protease [Panulirus argus] | Protein metabolism | Proteolysis | 7.54E-55 | 406.244 | 613.2651 | 660.5155 | 239.9392 | 172.8801 | 56.31266 | 79.07032 | 226.5039 | 122.0311 | 129.8789 | 246.3068 | 227.0035 | 594.5741 | 590.8179 | 490.5451 | 24.33153 | NA | 10.43749 | 159.9648 | 1670.981 | 396.2899 | 7.591069 | 600.4305 | 351.3648 | 7.111485 | 203.3538 | NA |
| CUST_50551_PI429010886 | M90239 | 0.047546 | 21 | 3 | | | | | | 5.007287 | 6.831546 | NA | 6.572882 | 11.52078 | 13.99388 | NA | 5.684217 | NA | 7.410795 | 4.25424 | 6.559984 | 5.814516 | 3.444924 | NA | 10.31018 | 3.353899 | 8.810922 | 8.134417 | 8.342648 | 4.723031 | 10.13327 | 7.986139 | 7.411834 | 15.67417 | 15.5488 | 12.82338 |
| CUST_9843_PI429010886 | N25287 | 0.04758 | 18 | 12 | | | | | | 46.01632 | 108.4222 | 87.87096 | 49.95685 | 47.54787 | 34.48913 | 75.89665 | 84.92511 | 92.35924 | 171.7093 | 50.1618 | 95.72441 | 127.7612 | 91.42252 | 78.36561 | 82.73488 | 80.97211 | 73.59863 | 71.57902 | 106.788 | 57.56812 | 102.4301 | 55.85206 | 61.09062 | 88.85357 | 55.5381 | 83.51601 |
| CUST_56714_PI429010886 | M114497 | 0.047584 | 15 | 12 | | | | | | 85.30129 | 66.90174 | 111.027 | 66.40412 | 84.58422 | 83.50755 | 81.96733 | 88.18553 | 73.61433 | 105.9574 | 86.89271 | 107.2518 | 87.80183 | 123.9272 | 105.607 | 118.5121 | 82.67004 | 104.0004 | 64.47443 | 94.17604 | 89.93577 | 130.9454 | 98.18381 | 81.34562 | 107.9401 | 101.7342 | 121.2398 |
| CUST_37161_PI429010886 | M18048 | 0.047584 | 18 | 3 | | | | | | 124.1861 | 126.2814 | 16.63804 | 150.1517 | 146.3194 | 128.2848 | 132.8222 | 84.73278 | 63.39049 | 60.93069 | 47.54787 | 39.05895 | 116.7797 | 39.73253 | 67.08962 | 103.9537 | 95.9293 | 100.8309 | 93.19487 | 71.41994 | 45.20819 | 136.1246 | 35.0422 | 101.9474 | 74.27647 | 54.02913 | 36.387 |
| CUST_31829_PI429010886 | M2512 | 0.047584 | 15 | 3 | 91080075 | PREDICTED: similar to arf gtpase-activating protein | Signal transduction | Other | 5.75E-87 | 180.661 | 118.4632 | 94.11857 | 179.1099 | 184.3061 | 151.8851 | 144.7517 | 104.9053 | 89.49881 | 116.0676 | 129.6229 | 63.13278 | 113.2835 | 73.42972 | 88.77007 | 160.9512 | 153.132 | 89.70412 | 157.8347 | 128.8011 | 85.90922 | 123.3865 | 85.86351 | 123.349 | 131.0159 | 78.01256 | 74.48084 |
| CUST_27505_PI429010886 | N56179 | 0.047584 | 15 | 12 | | | | | | 205.7316 | 92.20534 | 486.8632 | 78.17016 | 59.72927 | 186.0881 | 590.149 | 492.5912 | 558.2631 | 551.3995 | 239.0491 | 277.4454 | 597.939 | 1364.868 | 301.9157 | 78.63344 | 473.8216 | 178.3086 | 344.24 | 178.4123 | 251.1408 | 722.0011 | 525.2083 | 103.0505 | 735.8213 | 275.6931 | 1100.556 |
| CUST_42789_PI429010886 | M61261 | 0.047593 | 18 | 3 | | | | | | 103.384 | 82.3571 | 87.15435 | 106.9501 | 109.7061 | 149.6184 | 211.9082 | 78.30025 | 78.53416 | 39.33849 | 93.37534 | 31.95131 | 70.11707 | 51.65313 | 56.21187 | 50.77029 | 131.39 | 77.10171 | 90.12961 | 20.92648 | 112.2413 | 39.06616 | 133.8722 | 145.6462 | 38.53689 | 82.67004 | 46.78872 |
| CUST_3535_PI429010886 | N14709 | 0.047593 | 12 | 0 | | | | | | 158.5157 | 176.8527 | 258.3105 | 169.0651 | 168.6014 | 175.8509 | 178.3086 | 165.1146 | 195.1881 | 207.0705 | 181.3801 | 218.1069 | 185.2303 | 233.7917 | 187.2169 | 182.6137 | 140.3134 | 163.4482 | 166.4042 | 166.2053 | 228.274 | 218.7669 | 239.6059 | 172.854 | 261.1858 | 194.0962 | 230.5922 |
| CUST_31956_PI429010886 | M2821 | 0.047599 | 21 | 18 | | | | | | 1362.62 | 1234.248 | 982.113 | 804.2481 | 1081.506 | 949.3416 | 978.1392 | 773.8104 | 817.4781 | 1083.296 | 895.455 | 1015.362 | 758.445 | 784.2356 | 963.9292 | 1340.855 | 1093.408 | 1077.327 | 1117.033 | 1135.445 | 1020.365 | 1373.036 | 872.488 | 933.1038 | 1170.459 | 787.7599 | 1082.445 |
| CUST_53714_PI429010886 | M102891 | 0.047601 | 15 | 0 | | | | | | 128.1595 | 133.3923 | 106.4846 | 141.7029 | 113.6011 | 126.1331 | 102.7028 | 96.71335 | 108.4314 | 105.9129 | 100.0408 | 94.26766 | 129.8259 | 89.5493 | 111.9132 | 116.4869 | 107.6827 | 148.8486 | 94.79006 | 92.10717 | 89.6226 | 117.7304 | 94.71285 | 142.963 | 68.45737 | 86.20359 | 89.74363 |
| CUST_36524_PI429010886 | M16133 | 0.047628 | 24 | 18 | | | | | | 25.53352 | 29.12321 | 11.9176 | 9.355583 | 11.83012 | 11.31528 | 10.35316 | 14.62779 | 16.46383 | 13.04696 | 12.12977 | 11.01728 | 10.58947 | 22.07292 | 14.59759 | 19.00199 | 13.35309 | 12.69121 | 38.45723 | 15.33595 | 19.31761 | 13.19231 | 14.64036 | 15.59858 | 21.91188 | 11.53905 | 18.28186 |
| CUST_37479_PI429010886 | M19177 | 0.047628 | 24 | 21 | 9367086 | Euphausia superba large subunit ribosomal RNA ger | Protein metabolism | Regulation of translational initiation | | 56.41974 | 89.95558 | 86.3406 | 78.00387 | 45.3248 | 66.4223 | 48.45867 | 49.0094 | 62.96663 | 86.85226 | 55.38718 | 122.6286 | 83.46951 | 88.12787 | 124.1861 | 76.59109 | 61.39492 | 102.4945 | 55.95565 | 94.99099 | 83.59685 | 96.21869 | 88.65038 | 122.84 | 70.90194 | 113.2976 | 65.61755 |
| CUST_51371_PI429010886 | M93301 | 0.047629 | 24 | 3 | | | | | | NA | 6.293928 | NA | 18.554 | 10.08113 | NA | NA | 10.09463 | 9.352687 | 15.036 | 15.72953 | 9.963882 | 12.06387 | 7.110255 | 7.259344 | 9.881836 | 4.36971 | NA | 7.52281 | 6.977617 | 6.955954 | 7.180488 | 10.23296 | NA | 12.63269 | 5.081264 | 17.3497 |
| CUST_7662_PI429010886 | N21885 | 0.047643 | 18 | 12 | | | | | | 805.0049 | 769.0647 | 647.6977 | 664.8684 | 498.1212 | 313.2163 | 755.2577 | 538.762 | 802.5661 | 949.0578 | 628.3128 | 919.6285 | 976.3736 | 1056.312 | 737.7582 | 1100.556 | 580.6994 | 562.3412 | 1201.959 | 592.8448 | 820.2371 | 877.4226 | 476.0617 | 511.7062 | 887.8552 | 422.9234 | 668.1948 |
| CUST_44609_PI429010886 | M66467 | 0.047655 | 18 | 9 | | | | | | 43.56037 | 102.0573 | 78.06614 | 72.40904 | 86.47473 | 75.90597 | 39.20847 | 143.8832 | 88.07214 | 145.4981 | 249.3051 | 118.1013 | 167.3841 | 130.6774 | 110.8479 | 72.52738 | 150.3901 | 65.93142 | 94.84242 | 67.99795 | 128.1445 | 67.89962 | 90.31563 | 85.56392 | 189.5687 | 41.78277 | 99.98303 |
| CUST_18340_PI429010886 | N39611 | 0.047697 | 18 | 12 | | | | | | 104.5873 | 128.623 | 132.5751 | 80.55811 | 86.51241 | 95.4903 | 99.60857 | 104.8753 | 93.47603 | 93.3114 | 84.73278 | 138.323 | 103.5442 | 135.9831 | 119.6528 | 128.9813 | 94.3579 | 133.0452 | 97.22671 | 108.8877 | 94.16491 | 87.10078 | 110.1333 | 74.44282 | 123.8281 | 99.8601 | 158.0023 |
| CUST_13118_PI429010886 | N30603 | 0.047708 | 15 | 0 | | | | | | 14.01249 | 13.55604 | 26.30031 | 10.88451 | 10.93186 | 9.79911 | NA | 9.354027 | 5.284088 | 12.54217 | 13.87948 | 12.88422 | 11.13307 | 33.72145 | 11.82579 | 20.96593 | 16.16705 | 11.25121 | 15.35034 | 7.517908 | 6.959325 | 15.34115 | 4.408739 | NA | 14.71698 | NA | 11.07279 |
| CUST_19465_PI429010886 | N41627 | 0.047713 | 24 | 3 | | | | | | 4427.545 | 13555.43 | 5807.129 | 6091.908 | 7819.87 | 9541.284 | 4701.722 | 5991.557 | 5565.727 | 6128.181 | 3071.798 | 9182.945 | 5171.854 | 2959.951 | 713.1739 | 7686.447 | 2081.941 | 5065.56 | 2629.007 | 3138.529 | 7579.447 | 6702.515 | 6744.801 | 5171.854 | 9686 | 5717.187 | 4537.099 |
| CUST_42330_PI429010886 | M60021 | 0.047728 | 18 | 3 | | | | | | 23.194 | 12.6619 | 9.928945 | 29.89732 | 19.30622 | 15.49062 | 20.24846 | 7.662248 | 14.19367 | NA | 4.346382 | NA | 15.51619 | 18.55819 | 11.73735 | 8.940041 | 5.354757 | 10.50509 | NA | 9.405002 | 4.927102 | 24.10675 | NA | 24.72926 | 4.632018 | 6.423606 | 4.527621 |
| CUST_49768_PI429010886 | M87041 | 0.047755 | 24 | 15 | | | | | | 34.94119 | 37.17994 | 29.44539 | 34.72656 | 30.74148 | 32.98295 | 24.71814 | 35.91157 | 29.77206 | 31.19824 | 53.63782 | 65.96017 | 34.54086 | 53.8998 | 53.42416 | 74.10143 | 31.2636 | 43.41027 | 36.37581 | 58.52324 | 35.88091 | 58.49374 | 53.51858 | 37.12735 | 31.73044 | 32.73103 | 39.68132 |
| CUST_16815_PI429010886 | N36911 | 0.047769 | 24 | 12 | 393905834 | CMGC/DYRK/PRP4 protein kinase [Loa loa] | Protein metabolism | Cellular protein modification process | 1.34E-49 | 16.11003 | 22.29224 | 17.58731 | 21.98642 | 22.32278 | 21.32852 | 15.62192 | 23.39301 | 23.91343 | 23.01868 | 25.48335 | 18.76417 | 23.03149 | 33.97352 | 30.08888 | 19.27695 | 25.82153 | 28.35783 | 23.34418 | 32.20238 | 26.86002 | 29.51436 | 26.03397 | 24.0648 | 20.20434 | 14.48282 | 28.80979 |
| CUST_43600_PI429010886 | M63470 | 0.047787 | 12 | 0 | | | | | | 1579.682 | 2189.348 | 2100.151 | 1718.489 | 1401.401 | 1684.016 | 1118.208 | 1427.503 | 1694.466 | 2091.598 | 1402.469 | 2292.412 | 1751.202 | 2237.5 | 2325.953 | 2134.91 | 1132.32 | 1659.051 | 1574.342 | 1875.725 | 1426.735 | 2269.213 | 1535.786 | 1348.276 | 2159.598 | 1953.939 | 2197.498 |
| CUST_26558_PI429010886 | N54446 | 0.047787 | 12 | 0 | | | | | | 39.54839 | 59.06419 | 54.16396 | 34.16739 | 35.26822 | 44.39447 | 39.97268 | 38.76201 | 32.15263 | 40.02231 | 34.29142 | 60.03661 | 39.99252 | 56.40373 | 36.44258 | 50.42986 | 43.91811 | 37.90698 | 28.49366 | 40.96307 | 35.92679 | 52.93772 | 33.75052 | 29.18222 | 53.22735 | 44.4293 | 68.61799 |
| CUST_38784_PI429010886 | M30337 | 0.04782 | 12 | 3 | 357618028 | hypothetical protein KGM_08122 [Danaus plexippu | | | 6.09E-10 | 174.0405 | 290.8954 | 213.0626 | 284.7376 | 431.5778 | 507.5628 | 246.3468 | 207.853 | 156.8208 | 200.5697 | 247.2726 | 135.3099 | 257.7314 | 167.2954 | 224.4296 | 308.528 | 254.4916 | 342.9573 | 320.2376 | 218.0605 | 163.9899 | 232.0593 | 168.2678 | 259.1574 | 179.8447 | 319.0748 | 203.2288 |
| CUST_8649_PI429010886 | N23357 | 0.04782 | 12 | 9 | | | | | | 75.13415 | 56.77264 | 85.45263 | 58.52324 | 48.98001 | 68.29796 | 74.36967 | 68.22048 | 69.34114 | 95.36046 | 52.01848 | 81.5369 | 63.3033 | 105.1367 | 78.20249 | 67.08317 | 72.2954 | 73.20424 | 81.48802 | 81.05888 | 80.76562 | 85.72992 | 87.88231 | 79.41411 | 90.50136 | 75.08335 | 86.36905 |
| CUST_12628_PI429010886 | N29770 | 0.04782 | 12 | 3 | | | | | | 53.53627 | 36.387 | 46.45397 | 70.86123 | 69.0257 | 176.5007 | 55.66127 | 72.00003 | 16.49783 | 29.52168 | 38.01489 | 10.51872 | 57.33863 | 43.76947 | 44.63765 | 48.81237 | 143.7068 | 137.4373 | 121.6056 | 9.301993 | 35.89324 | 50.79816 | 39.51034 | 62.5561 | 22.6112 | 78.93556 | 31.6776 |
| CUST_24048_PI429010886 | N49939 | 0.047827 | 24 | 21 | 443705981 | hypothetical protein CAPTEDRAFT_139004, partial [ | Cellular component organization | Cytoskeleton organization | 7.03E-21 | 332.1642 | 242.7679 | 401.7586 | 337.7668 | 352.8752 | 372.2181 | 374.9159 | 334.6678 | 323.4046 | 418.9595 | 452.1056 | 381.1947 | 317.6417 | 412.0745 | 235.3127 | 376.0453 | 579.8544 | 421.8908 | 538.762 | 381.5724 | 392.3971 | 433.7721 | 441.5046 | 473.5692 | 361.9781 | 380.2921 | 472.5516 |
| CUST_38630_PI429010886 | M28687 | 0.047849 | 21 | 3 | | | | | | 1410.909 | 1783.909 | 1709.2 | 1730.823 | 1731.857 | 1886.936 | 1638.912 | 1607.248 | 1643.929 | 2064.29 | 1715.766 | 2007.972 | 1515.268 | 2073.005 | 1168.111 | 1205.12 | 1651.385 | 1566.438 | 1178.991 | 1665.324 | 1804.906 | 1504.846 | 2514.893 | 1784.695 | 2346.537 | 2021.454 | 2271.87 |
| CUST_3888_PI429010886 | N15491 | 0.047895 | 18 | 6 | | | | | | 18.9048 | 16.96368 | 17.11883 | 18.36165 | 17.46532 | 16.87557 | 44.4501 | 17.55275 | 13.51834 | 26.1596 | 13.59657 | 27.49118 | 12.91897 | 10.11651 | 16.29239 | 16.78585 | 13.63953 | 15.99975 | 16.68074 | 14.08168 | 41.8249 | 23.72867 | 21.63352 | 20.99142 | 23.34144 | 14.97143 | 32.38525 |
| CUST_52837_PI429010886 | M99167 | 0.047908 | 24 | 12 | | | | | | 23.4957 | 46.46836 | 33.3239 | 22.94051 | 32.4042 | 81.74728 | 80.72119 | 35.57881 | 44.53184 | 72.48039 | 52.46279 | 56.77264 | 82.1182 | 84.14675 | 46.82282 | 24.85691 | 54.73192 | 40.68573 | 90.64646 | 70.08934 | 68.41788 | 63.14802 | 38.34336 | 42.86965 | 26.00383 | 46.87582 | 38.02365 |
| CUST_15370_PI429010886 | N34470 | 0.047943 | 21 | 0 | | | | | | 440.6015 | 334.9967 | 589.6619 | 328.0964 | 351.6009 | 329.2875 | 402.8509 | 317.1399 | 330.9233 | 374.4434 | 253.9783 | 379.9539 | 301.9881 | 368.397 | 464.3001 | 344.24 | 319.1396 | 360.7127 | 450.3936 | 404.4491 | 417.8136 | 443.0418 | 440.5078 | 377.0882 | 280.2255 | 364.4089 | 336.5695 |
| CUST_12358_PI429010886 | N29343 | 0.047951 | 24 | 0 | | | | | | 77.93692 | 69.34114 | 115.8195 | 44.58945 | 71.09841 | 65.78559 | 34.75205 | 55.58103 | 53.30954 | 107.666 | 68.95801 | 56.58363 | 40.99424 | 51.1689 | 70.00254 | 35.36774 | 42.50508 | 39.81145 | 49.3486 | 124.1092 | 55.88131 | 42.2806 | 44.44423 | 29.49638 | 58.23835 | 74.97848 | 61.28097 |
| CUST_48241_PI429010886 | M79485 | 0.047953 | 24 | 18 | | | | | | 28.93983 | 23.70999 | 3.731986 | 17.08079 | 22.06923 | 29.71613 | 33.17839 | 23.84339 | 18.33365 | 11.66655 | 20.75152 | 13.95744 | 14.86246 | 11.68857 | 25.18558 | 18.17304 | 13.48586 | 30.33968 | 32.71805 | 32.43294 | 31.69618 | 20.02214 | 37.31704 | 30.47678 | 23.16574 | 43.73764 | 66.08238 |
| CUST_34539_PI429010886 | M9956 | 0.047961 | 18 | 3 | | | | | | 57.32582 | 72.38234 | 55.81794 | 70.70475 | 74.41568 | 54.72536 | 58.39472 | 77.6254 | 44.28069 | 55.16975 | 59.33007 | 45.54024 | 48.81237 | 45.7637 | 33.56673 | 38.99636 | 57.11196 | 46.16699 | 66.43118 | 41.96975 | 47.53397 | 60.36203 | 47.61652 | 59.73525 | 44.06771 | 55.57175 | 54.60894 |
| CUST_14072_PI429010886 | N32283 | 0.047961 | 15 | 12 | | | | | | 116.5044 | 61.30366 | 86.5251 | 106.6154 | 79.21035 | 63.747 | 39.17072 | 78.35921 | 47.16593 | 92.74338 | 48.78129 | 73.51179 | 80.02167 | 118.5239 | 126.045 | 156.6593 | 92.13028 | 111.0646 | 74.03663 | 110.3002 | 90.40629 | 141.5625 | 66.53186 | 50.6408 | 141.0947 | 91.54224 | 95.44 |
| CUST_10494_PI429010886 | N26323 | 0.048003 | 18 | 6 | | | | | | 3809.024 | 1422.337 | 5167.16 | 8772.974 | 1722.719 | 8866.604 | 4503.558 | 8363.4 | 5843.06 | 7509.168 | 2036.617 | 1548.599 | 8336.973 | 10964.51 | 6563.249 | 973.9722 | 5787.347 | 4306.703 | 5927.379 | 1918.897 | 1679.237 | 5602.191 | 6350.776 | 5211.698 | 12034.22 | 6160.933 | 10451.76 |
| CUST_35127_PI429010886 | M11664 | 0.048008 | 15 | 0 | | | | | | 310.0283 | 165.8485 | 207.6805 | 215.8293 | 160.4822 | 227.9715 | 327.535 | 208.4543 | 181.4093 | 86.67899 | 169.8899 | 232.8505 | 237.3209 | 188.6253 | 246.1531 | 277.0077 | 254.6455 | 285.7468 | 266.1283 | 193.6314 | 156.3082 | 229.9936 | 162.1587 | 191.6146 | 174.1985 | 157.9028 | 160.9334 |
| CUST_36397_PI429010886 | M15798 | 0.048009 | 15 | 12 | | | | | | 50.53472 | 41.27857 | 43.99167 | 35.63559 | 44.05675 | 34.96354 | 38.96253 | 40.90818 | 31.98148 | 28.98446 | 48.28635 | 39.12985 | 51.11982 | 57.00887 | 51.52932 | 62.42019 | 54.1974 | 49.16869 | 63.55528 | 43.07403 | 30.04146 | 62.23219 | 37.82192 | 24.49005 | 61.50815 | 30.28193 | 43.41027 |
| CUST_15460_PI429010886 | N34615 | 0.048009 | 15 | 9 | | | | | | 8.847678 | 11.66655 | 11.80446 | 7.44339 | 6.436681 | NA | 7.444212 | 125.1284 | 8.123884 | 21.08222 | 9.706284 | 25.8352 | 10.43749 | 21.2878 | 17.17661 | 28.05304 | 6.626516 | 11.17946 | 4.945317 | 10.01823 | 12.6412 | 30.46421 | 13.87032 | 13.65469 | 14.25774 | 16.22818 | 26.14333 |
| CUST_40418_PI429010886 | M55024 | 0.048013 | 15 | 9 | | | | | | 35.96862 | 37.50798 | 44.98924 | 31.16892 | 43.11192 | 54.1974 | 49.07171 | 38.77187 | 74.74125 | 41.68626 | 59.96834 | 55.57175 | 37.67896 | 44.58058 | 30.24466 | 15.01118 | 37.12735 | 50.8348 | 25.71698 | 38.04684 | 54.77637 | 38.45723 | 44.02245 | 71.24301 | 39.12009 | 58.37612 | 71.17303 |
| CUST_16057_PI429010886 | N35578 | 0.048013 | 21 | 15 | | | | | | 124.4126 | 94.23139 | 99.4135 | 68.51154 | 65.52616 | 88.40608 | 127.8146 | 112.4033 | 80.30929 | 56.73383 | 104.2766 | 114.2091 | 109.7501 | 116.9944 | 121.7504 | 192.1754 | 126.6828 | 101.0607 | 114.0787 | 126.8447 | 84.45921 | 131.2743 | 47.67821 | 71.67319 | 88.97552 | 61.28936 | 117.1913 |
| CUST_7172_PI429010886 | N21141 | 0.048022 | 21 | 3 | | | | | | 121.2116 | 94.79905 | 122.4667 | 104.0193 | 107.543 | 140.4217 | 136.3233 | 87.58299 | 107.1033 | 126.5103 | 78.23845 | 83.1842 | 74.27647 | 125.1451 | 108.3037 | 105.7372 | 81.96733 | 92.50739 | 98.02439 | 87.92471 | 103.1477 | 121.0016 | 161.7305 | 119.7777 | 111.6628 | 149.3076 | 121.4562 |
| CUST_51560_PI429010886 | M93980 | 0.048035 | 21 | 15 | | | | | | 11.57662 | 7.953838 | 11.61617 | 6.582515 | 12.62537 | 10.9288 | 11.10785 | 6.534722 | 4.828419 | 3.744336 | 8.235102 | 13.3483 | 14.72582 | 7.304592 | 14.54874 | 14.05126 | 14.98994 | NA | 13.5897 | 11.68238 | 8.829376 | 12.73078 | NA | 9.125404 | 9.751886 | 5.721438 | 16.51889 |
| CUST_51972_PI429010886 | M95877 | 0.048063 | 21 | 6 | | | | | | 31.38208 | 23.28379 | 36.29774 | 64.64151 | 35.77964 | 102.8077 | 63.79444 | 48.77592 | 49.40423 | 25.20311 | 27.91811 | 39.91268 | 49.14494 | 149.1909 | 18.90357 | 24.21798 | 34.5638 | 34.75551 | 35.6576 | 32.89329 | 20.66296 | 25.83997 | 40.34341 | 53.58897 | 39.15692 | 64.34064 | 26.96892 |
| CUST_12376_PI429010886 | N29372 | 0.04807 | 24 | 18 | | | | | | 56.42615 | 100.9197 | 55.95035 | 60.81378 | 75.32357 | 61.43267 | 65.76942 | 68.36198 | 61.72981 | 55.3496 | 89.45826 | 75.72117 | 91.77189 | 93.01036 | 74.86136 | 96.07604 | 67.22918 | 82.69106 | 95.34993 | 94.97923 | 73.89571 | 93.07732 | 70.66973 | 57.84331 | 91.066 | 56.13335 | 57.51717 |
| CUST_40464_PI429010886 | M55190 | 0.048088 | 15 | 9 | | | | | | 20.63496 | 26.30221 | 24.57116 | 15.93948 | 23.04785 | 24.61213 | 21.67212 | 38.89725 | 24.52679 | 35.53033 | 32.48228 | 25.07539 | 25.78047 | 28.40654 | 26.97315 | 26.72669 | 27.92219 | 25.6517 | 25.51449 | 25.22662 | 22.93439 | 28.77061 | 30.68445 | 16.19147 | 32.02812 | 18.85349 | 42.6726 |
| CUST_16081_PI429010886 | N35623 | 0.048088 | 15 | 9 | | | | | | 38.99636 | 54.48084 | 50.11711 | 42.98911 | 49.67248 | 49.34012 | 29.14509 | 46.81599 | 50.43461 | 61.04516 | 51.96426 | 87.81308 | 55.93797 | 69.12024 | 70.6776 | 91.52825 | 45.37412 | 64.02386 | 54.49444 | 45.90172 | 57.07016 | 77.51881 | 45.65948 | 49.69406 | 63.52784 | 98.4006 | 97.93806 |
| CUST_3796_PI429010886 | N15280 | 0.048092 | 12 | 0 | | | | | | 12.08465 | 9.581554 | 8.265097 | 6.95796 | NA | NA | NA | 4.510154 | NA | 4.965994 | 4.07315 | 8.039625 | 7.097098 | 15.04174 | 6.214637 | 11.37147 | 5.217577 | 9.651747 | 6.668324 | 5.263094 | 5.180096 | 6.779112 | 6.219397 | NA | 6.911676 | NA | 7.886773 |
| CUST_36327_PI429010886 | M15608 | 0.048102 | 12 | 0 | | | | | | 337.8584 | 373.5295 | 392.0026 | 325.0318 | 320.8355 | 353.4537 | 317.5249 | 298.6335 | 311.0329 | 388.3479 | 321.1502 | 431.842 | 353.785 | 508.7091 | 355.8536 | 388.2685 | 410.06 | 372.2873 | 373.3663 | 389.6265 | 313.8962 | 438.1052 | 335.8009 | 323.0511 | 461.1208 | 354.0483 | 429.6585 |
| CUST_48303_PI429010886 | M79729 | 0.048117 | 15 | 12 | | | | | | 94.7334 | 85.52397 | 92.63222 | 91.49463 | 96.19258 | 115.0894 | 63.92583 | 90.06996 | 91.5749 | 96.41487 | 81.68427 | 118.2001 | 118.8596 | 116.8326 | 137.7783 | 109.3295 | 86.3602 | 120.6877 | 88.63339 | 106.3073 | 83.96253 | 105.19 | 93.76216 | 97.06276 | 105.2081 | 115.3001 | 107.3626 |
| CUST_1305_PI429010886 | N9218 | 0.048131 | 21 | 3 | 270161184 | i-type lysozyme-like protein 2 [Penaeus monodon] | Metabolic process | Carbohydrate metabolic process | 3.78E-37 | 22.41298 | 8.480876 | 25.77304 | 30.67432 | 8.221669 | 76.06574 | 24.00128 | 42.16099 | 11.58812 | 19.15208 | 12.91624 | 23.47811 | 18.2774 | 13.01498 | 19.26616 | 8.599447 | 39.93142 | 12.00275 | 24.52679 | 31.50111 | 23.53568 | 36.2246 | 56.35545 | 29.16341 | 54.71071 | 44.92995 | 35.93002 |
| CUST_55318_PI429010886 | M109537 | 0.048137 | 12 | 3 | | | | | | 1672.661 | 558.0588 | 489.6976 | 3082.77 | 2761.396 | 3835.814 | 2379.147 | 935.3105 | 705.3837 | 47.02203 | 1932.967 | 18.29194 | 963.6143 | 27.50644 | 461.6522 | 228.4344 | 4823.273 | 3027.574 | 2444.229 | 26.08713 | 1127.801 | 85.77208 | 1200.158 | 2582.117 | 28.888 | 2237.5 | 4.65851 |
| CUST_37172_PI429010886 | M18089 | 0.048137 | 12 | 0 | | | | | | 495.8952 | 1613.342 | 1078.296 | 252.9368 | 621.3573 | 115.8883 | 103.1666 | 610.9693 | 983.5467 | 1529.041 | 744.2633 | 1789.616 | 1040.9 | 1564.24 | 1482.523 | 1364.277 | 370.5671 | 688.1653 | 629.4397 | 1652.112 | 1056.312 | 1632.477 | 1060.588 | 220.4105 | 1954.96 | 1355.659 | 2073.005 |
| CUST_38519_PI429010886 | M27639 | 0.048137 | 18 | 9 | | | | | | 273.0127 | 275.0769 | 326.8661 | 296.3588 | 297.2502 | 319.3199 | 316.971 | 289.5961 | 312.3654 | 354.7905 | 296.6312 | 397.7744 | 285.2578 | 354.5586 | 357.7037 | 338.0611 | 326.9265 | 296.208 | 308.0149 | 318.2985 | 296.3167 | 326.7925 | 328.0964 | 277.0736 | 373.0312 | 353.689 | 350.5877 |
| CUST_32518_PI429010886 | M4332 | 0.048137 | 15 | 12 | 91094771 | PREDICTED: similar to AGAP000117-PA [Tribolium c | | | 9.81E-16 | 1642.187 | 4381.739 | 2513.183 | 473.2302 | 2213.328 | 154.7269 | 629.2275 | 906.266 | 1594.166 | 4604.537 | 2049.694 | 4274.83 | 1782.524 | 3013.914 | 2204.613 | 3988.418 | 907.4672 | 1332.781 | 945.8187 | 1370.685 | 1232.59 | 3567.184 | 1624.747 | 336.8185 | 2809.847 | 1235.847 | 2468.346 |
| CUST_41087_PI429010886 | M56847 | 0.048137 | 15 | 12 | 328786874 | PREDICTED: putative DNA helicase Ino80-like [Apis | | | 2.42E-13 | 121.4237 | 107.7492 | 151.2216 | 93.04202 | 109.8598 | 106.7139 | 86.7788 | 111.1235 | 147.0211 | 170.317 | 100.1573 | 148.544 | 136.0755 | 148.4479 | 149.3076 | 148.1555 | 109.7371 | 127.1757 | 117.3157 | 123.7403 | 114.926 | 128.3451 | 164.4415 | 110.1779 | 126.6031 | 137.9515 | 200.2689 |
| CUST_28232_PI429010886 | N57555 | 0.048137 | 15 | 3 | | | | | | 204.7885 | 300.5238 | 286.1419 | 256.1926 | 319.448 | 287.4768 | 311.8796 | 217.9412 | 178.0648 | 279.4425 | 274.5343 | 201.9074 | 187.869 | 268.2743 | 206.6019 | 236.1561 | 274.9598 | 241.3477 | 317.2087 | 236.9573 | 300.3152 | 247.7341 | 309.7028 | 211.1137 | 245.5292 | 175.2864 | 193.4435 |
| CUST_48575_PI429010886 | M81109 | 0.048137 | 24 | 15 | 332030460 | Polycomb protein Scm [Acromyrmex echinatior] | | | 5.99E-09 | 14.82853 | 10.73106 | 16.74792 | 17.50362 | 14.51104 | 19.99756 | 9.914777 | 16.48468 | 16.45281 | 19.02839 | 19.34225 | 20.54194 | 17.49801 | 25.10042 | 20.1457 | 28.36898 | 25.68589 | 16.76285 | 18.02828 | 12.01073 | 12.26356 | 27.84371 | NA | 13.67211 | 17.72399 | 12.91624 | 15.80258 |
| CUST_42803_PI429010886 | M61299 | 0.048158 | 24 | 15 | | | | | | 81.92166 | 74.87405 | 83.8218 | 67.28983 | 60.92331 | 69.69491 | 78.96798 | 71.77967 | 62.70075 | 80.91461 | 81.81059 | 112.0535 | 83.21855 | 86.93205 | 76.353 | 84.64574 | 83.07834 | 85.63673 | 92.80312 | 78.36561 | 66.15125 | 87.62612 | 88.18553 | 72.05058 | 97.2708 | 80.30929 | 74.47085 |
| CUST_35260_PI429010886 | M12068 | 0.048202 | 15 | 12 | 525026447 | PREDICTED: molybdopterin synthase catalytic subu | Metabolic process | Other | 8.74E-39 | 58.97911 | 77.1205 | 43.00832 | 40.00171 | 56.07148 | 31.86956 | 28.80614 | 45.94496 | 44.78592 | 88.22213 | 56.78823 | 64.03011 | 48.65694 | 75.10177 | 86.46205 | 82.8752 | 37.48716 | 71.9913 | 46.41206 | 61.33812 | 56.48952 | 57.29447 | 53.51251 | 38.21245 | 85.21423 | 50.53472 | 51.18041 |
| CUST_44156_PI429010886 | M65111 | 0.048216 | 24 | 21 | | | | | | 83.80399 | 69.55824 | 85.51349 | 75.2095 | 62.75575 | 103.0243 | 72.6524 | 63.08199 | 77.86556 | 63.40813 | 63.69473 | 84.00641 | 78.66644 | 89.66356 | 72.48039 | 93.07732 | 89.24944 | 96.49062 | 74.87405 | 85.40569 | 84.61863 | 94.61126 | 83.98715 | 89.69655 | 88.38108 | 106.011 | 82.38056 |
| CUST_21078_PI429010886 | N44591 | 0.048223 | 15 | 3 | | | | | | 42.64756 | 19.35495 | 54.28784 | 145.0637 | 196.032 | 252.0883 | 282.7521 | 76.88221 | 17.5216 | 9.510155 | 269.6735 | NA | 21.71104 | 9.745966 | 16.34764 | 15.70076 | 356.5583 | 93.80773 | 254.2335 | 18.50256 | 231.0331 | 14.08315 | 166.4668 | 193.3866 | 7.626632 | 59.41199 | 10.46161 |
| CUST_13875_PI429010886 | N31922 | 0.048246 | 18 | 3 | 307195519 | Seipin [Harpegnathos saltator] | | | 3.37E-20 | 42.61032 | 44.64991 | 29.74088 | 49.12739 | 51.00123 | 27.58334 | 37.06882 | 37.07575 | 26.69563 | 42.83336 | 39.85359 | 27.34621 | 17.61017 | 34.53375 | 20.45436 | 37.02581 | 40.24548 | 25.09705 | 22.84721 | 31.15277 | 27.25682 | 43.66792 | 37.72421 | 40.70657 | 46.37163 | 21.48139 | 34.16739 |
| CUST_31196_PI429010886 | M953 | 0.048305 | 12 | 3 | | | | | | 3.343563 | 1085.118 | 144.5043 | 1497.212 | 405.7694 | 835.4969 | 187.2169 | 1227.773 | 177.6428 | 131.9783 | NA | 63.43828 | 1053.785 | 301.6337 | 829.599 | 1180.515 | 1033.403 | 1643.929 | 1404.631 | 389.9711 | 232.766 | 276.7086 | NA | 463.0515 | 700.208 | 1219.272 | 71.9016 |
| CUST_44671_PI429010886 | M66627 | 0.048317 | 15 | 12 | | | | | | 4150.569 | 2908.57 | 3204.304 | 1453.962 | 2374.602 | 1948.38 | 2932.434 | 2497.013 | 2385.821 | 2900.662 | 2634.615 | 3006.75 | 3165.648 | 3921.02 | 2695.193 | 2972.831 | 2437.103 | 2925.159 | 2264.822 | 3673.827 | 2430.624 | 4484.875 | 2621.227 | 2373.328 | 2743.533 | 1987.549 | 2796.121 |
| CUST_4053_PI429010886 | N15892 | 0.04833 | 12 | 0 | | | | | | 8876.599 | 11413.33 | 10348.94 | 10451.76 | 9475.141 | 6654.81 | 8582.669 | 8574.409 | 9169.5 | 8995.888 | 7920.23 | 12983.85 | 8391.959 | 11089.86 | 10178.43 | 9046.466 | 8024.987 | 9485.91 | 7717.52 | 5188.094 | 9756.741 | 5009.146 | 9235.263 | 9670.846 | 10597.5 | 14659.71 | 11762.42 |
| CUST_8957_PI429010886 | N23853 | 0.04833 | 12 | 0 | | | | | | 157.7938 | 177.2677 | 148.8486 | 209.5249 | 159.0669 | 220.0942 | 105.4997 | 139.3127 | 135.7577 | 154.6585 | 159.4718 | 152.8418 | 129.2244 | 180.8997 | 168.8426 | 185.3559 | 142.6543 | 166.4668 | 136.2095 | 151.707 | 119.7932 | 201.3287 | 150.7034 | 144.8962 | 190.7772 | 216.4729 | 176.5188 |
| CUST_10616_PI429010886 | N26511 | 0.04833 | 12 | 0 | | | | | | 52.20238 | 56.00276 | 50.12098 | 40.63456 | 44.12842 | 55.80731 | 29.93088 | 36.65626 | 50.13498 | 51.0829 | 35.4902 | 62.44513 | 44.23444 | 50.22974 | 57.69532 | 56.63828 | 49.03725 | 51.00123 | 27.73257 | 58.12189 | 46.1038 | 50.87205 | 47.1063 | 38.81418 | 63.39728 | 69.42462 | 65.64906 |
| CUST_1532_PI429010886 | N9957 | 0.04833 | 12 | 0 | | | | | | 59.06419 | 65.76942 | 65.46343 | 49.03345 | 52.1682 | 47.3028 | 52.801 | 41.34285 | 40.06518 | 70.169 | 55.26922 | 61.36332 | 59.63236 | 56.55685 | 57.84964 | 67.52161 | 50.10243 | 55.09927 | 43.43615 | 65.9853 | 50.93999 | 68.39623 | 50.29336 | 41.54682 | 70.57215 | 55.95035 | 66.15125 |
| CUST_18017_PI429010886 | N39004 | 0.04833 | 12 | 3 | | | | | | 26.61953 | 44.27668 | 11.61125 | 83.86935 | 37.32614 | 42.98911 | 66.40412 | 12.16991 | 32.9302 | 10.08948 | 26.98494 | 6.981775 | 15.13796 | 6.718366 | 20.26123 | 31.66766 | 45.95551 | 25.69829 | 18.78315 | 14.56009 | 21.43032 | 33.74722 | 14.40546 | 30.30557 | 15.72585 | 12.7848 | 13.5281 |
| CUST_39494_PI429010886 | M40390 | 0.048358 | 12 | 9 | | | | | | 59.96262 | 180.8735 | 210.0239 | 45.23797 | 181.6707 | 195.689 | 86.49959 | 78.75847 | 257.0697 | 203.453 | 106.4697 | 323.3503 | 122.6119 | 297.6429 | 316.8071 | 148.6082 | 48.00964 | 142.6758 | 123.4881 | 196.9122 | 272.7999 | 560.1437 | 288.5334 | 365.8372 | 290.6552 | 287.3115 | 347.3822 |
| CUST_45708_PI429010886 | M70004 | 0.048358 | 12 | 3 | | | | | | 744.1349 | 813.1389 | 53.28731 | 1347.073 | 692.3236 | 556.2338 | 1400.624 | 151.0563 | 1113.282 | 34.9939 | 90.12961 | 22.24289 | 635.0709 | 100.3487 | 572.181 | 1137.521 | 210.5448 | 925.4363 | 166.3173 | 89.57185 | 85.06299 | 584.5557 | 94.63457 | 607.7896 | 258.7852 | 153.6441 | 100.3487 |
| CUST_4919_PI429010886 | N17787 | 0.048377 | 24 | 21 | | | Protein metabolism | Proteolysis | | 33.84232 | 44.014 | 38.18832 | 37.17503 | 31.6343 | 35.46061 | 25.37889 | 37.2354 | 32.15824 | 43.22077 | 30.39754 | 110.3616 | 31.17167 | 40.93552 | 44.90024 | 35.04798 | 28.7672 | 32.72316 | 52.47732 | 47.45553 | 40.39881 | 54.42343 | 59.67038 | 39.06311 | 47.24387 | 28.03123 | 47.52868 |
| CUST_1585_PI429010886 | N10149 | 0.048422 | 21 | 3 | | | | | | 372.1299 | 370.6555 | 726.9909 | 1217.974 | 648.7533 | 801.0442 | 680.1392 | 378.1407 | 501.1913 | 776.3963 | 341.0906 | 736.8107 | 307.6262 | 593.8074 | 217.7746 | 595.4395 | 258.4027 | 482.4063 | 339.4134 | 232.8026 | 1082.904 | 374.2323 | 1229.858 | 546.2052 | 615.5507 | 776.1766 | 380.5555 |
| CUST_8778_PI429010886 | N23573 | 0.048485 | 12 | 9 | | | | | | 64.41721 | 86.56723 | NA | 131.771 | 79.41411 | 104.363 | 30.83662 | 276.7086 | 244.0822 | 1595.711 | 873.6327 | 742.4096 | 563.7899 | 178.7072 | 249.0492 | 543.3625 | 71.94024 | 447.7446 | 463.903 | 490.2884 | 587.8154 | 372.7154 | 1612.177 | 253.7689 | 156.8624 | 751.2737 | 884.6458 |
| CUST_38981_PI429010886 | M32956 | 0.04852 | 21 | 9 | | | | | | 9.592481 | 71.07061 | 23.23227 | 131.3133 | 217.7746 | 35.91607 | 69.04216 | 419.7481 | 365.2576 | 63.05147 | 829.599 | 1073.921 | 268.079 | 25.22384 | 150.0554 | NA | 145.1035 | 13.84831 | 26.21698 | 1076.531 | 416.1299 | 22.04079 | 83.13765 | 43.29738 | 15.84934 | 228.3928 | 255.9554 |
| CUST_13159_PI429010886 | N30685 | 0.048549 | 15 | 12 | | | | | | 31.33456 | 38.86489 | 45.45728 | 27.00978 | 33.47616 | 29.43926 | 36.01332 | 25.53913 | 22.73323 | 53.8203 | 30.11515 | 41.11091 | 32.36995 | 42.69742 | 52.24698 | 43.98243 | 20.6419 | 42.26619 | 32.91264 | 42.4817 | 33.77978 | 17.48039 | 39.98138 | 22.32739 | 36.78858 | 38.59967 | 25.56884 |
| CUST_10856_PI429010886 | N26900 | 0.048549 | 15 | 0 | 321479277 | hypothetical protein DAPPUDRAFT_300153 [Daphn | | | 1.41E-32 | 10.71996 | 13.44588 | 15.12815 | 12.15878 | 7.515463 | 20.03907 | 4.488345 | 9.396038 | 5.219351 | 8.506994 | 7.430567 | 12.16044 | 8.559956 | 9.846676 | 12.42024 | 79.84889 | 16.97048 | 4.153912 | 7.22526 | 11.36366 | 7.058007 | 8.764694 | 6.120324 | NA | 11.24998 | 12.26356 | 13.33763 |
| CUST_28246_PI429010886 | N57588 | 0.048568 | 21 | 6 | | | | | | 32.10376 | 26.01832 | 34.53064 | 39.96875 | 75.49979 | 33.68381 | 49.95028 | 41.26062 | 33.98233 | 51.94426 | 28.6962 | 40.07222 | 28.75284 | 22.03473 | 18.15598 | 45.33363 | 27.40752 | 23.63048 | 40.51578 | 31.43539 | 37.04031 | 43.32695 | 33.16699 | 25.70952 | 42.81164 | 33.35513 | 34.24386 |
| CUST_44913_PI429010886 | M67395 | 0.048575 | 15 | 9 | | | | | | NA | 79.53245 | 45.71121 | 126.3312 | 119.0883 | 79.09571 | 157.0047 | 327.144 | 222.6599 | 81.87922 | 775.4257 | 903.546 | 252.0883 | 51.4872 | 150.3647 | 6.140199 | 107.0174 | NA | 65.6728 | 795.358 | 417.9411 | 15.4643 | 113.9135 | 75.81603 | NA | 370.4877 | 335.9972 |
| CUST_6712_PI429010886 | N20497 | 0.048624 | 15 | 12 | | | | | | 515.0601 | 652.7665 | 617.8466 | 496.0263 | 513.344 | 644.7638 | 413.2538 | 435.6005 | 469.6397 | 558.0588 | 510.76 | 783.8018 | 586.6898 | 773.8104 | 778.5698 | 690.6945 | 484.8203 | 627.6184 | 542.3878 | 637.0757 | 565.3295 | 702.2549 | 553.6437 | 536.0428 | 627.0644 | 584.5557 | 665.511 |
| CUST_25372_PI429010886 | N52293 | 0.048629 | 21 | 0 | | | | | | 14.86089 | NA | 13.42877 | 10.51343 | 13.83327 | 7.033695 | 5.059565 | 4.920856 | 4.948004 | 12.71344 | 6.324533 | 6.735195 | NA | 8.07676 | 8.724159 | 17.92364 | 7.812822 | 11.63036 | 15.08742 | 8.981694 | 7.727602 | 8.629296 | 10.00209 | 11.31528 | 22.76207 | 12.41645 | 8.746322 |
| CUST_44414_PI429010886 | M65887 | 0.048632 | 24 | 0 | 242007487 | conserved hypothetical protein [Pediculus humanus | Transport | Ion transport | 1.43E-29 | 12.78047 | 6.253831 | 14.04854 | NA | 8.413487 | 13.28864 | 8.526397 | 11.56735 | 8.130029 | 12.16799 | 4.647861 | 9.983821 | 7.430567 | NA | 6.610857 | 10.29398 | 8.329617 | 6.889529 | 8.700812 | 7.23801 | 6.21115 | 10.45509 | 7.354715 | NA | 12.4096 | NA | 14.77827 |
| CUST_43857_PI429010886 | M64168 | 0.048637 | 15 | 12 | | | | | | 1170.459 | 1261.977 | 1259.651 | 989.5639 | 1019.767 | 1013.924 | 947.592 | 911.24 | 998.694 | 1189.668 | 928.6222 | 1329.318 | 981.4865 | 1485.346 | 1370.195 | 1367.805 | 1043.787 | 1345.526 | 1063.11 | 1365.452 | 1097.654 | 1347.073 | 1099.991 | 943.689 | 1343.537 | 1460.496 | 1456.136 |
| CUST_5543_PI429010886 | N18838 | 0.048637 | 15 | 3 | | | | | | 57.46891 | 44.44423 | 44.1892 | 62.3009 | 35.43589 | 79.82568 | 53.77942 | 50.29336 | 43.11747 | 36.67205 | 32.30335 | 27.4692 | 31.60035 | 27.06117 | 34.8131 | 45.74288 | 49.57451 | 43.33449 | 44.63407 | 45.58039 | 41.25578 | 30.11859 | 35.41295 | 52.57619 | 31.74877 | 53.51858 | 33.50292 |
| CUST_13084_PI429010886 | N30548 | 0.048645 | 24 | 21 | | | | | | 42.25718 | 26.24303 | 40.74077 | 29.92091 | 37.69724 | 37.58201 | 34.82515 | 30.22418 | 27.05759 | 39.68929 | 29.26863 | 36.69017 | 28.93067 | 31.83203 | 186.006 | 45.80326 | 46.23898 | 37.62657 | 49.08991 | 41.26402 | 32.14525 | 47.75744 | 49.53059 | 33.83579 | 32.26335 | 32.78199 | 30.13158 |
| CUST_34860_PI429010886 | M10919 | 0.048682 | 15 | 0 | | | | | | 405.7694 | 395.9375 | 247.8273 | 344.2985 | 313.5825 | 315.5114 | 286.0749 | 263.2372 | 268.7796 | 310.1413 | 273.6903 | 272.4368 | 307.7422 | 325.4382 | 351.6439 | 455.0936 | 321.2502 | 359.2285 | 297.0813 | 418.8645 | 269.1187 | 346.5745 | 254.9978 | 280.0685 | 406.1396 | 264.5866 | 308.7717 |
| CUST_37971_PI429010886 | M22522 | 0.04869 | 12 | 3 | 321475115 | hypothetical protein DAPPUDRAFT_313056 [Daphn | | | 1.02E-24 | 223.4595 | 22.22394 | 259.0023 | 615.5507 | 554.4782 | 1167.349 | 939.7655 | 141.2095 | 126.5542 | 21.69885 | 959.9717 | 15.12966 | 71.19859 | 50.99115 | 41.43272 | 55.66641 | 1214.782 | 745.5335 | 311.6128 | 25.68325 | 791.2722 | 45.74288 | 357.862 | 655.3909 | 8.576964 | 150.1517 | 14.18297 |
| CUST_30966_PI429010886 | M335 | 0.04869 | 12 | 0 | 242019954 | DEAD box ATP-dependent RNA helicase, putative [P | | | 2.64E-19 | 116.2937 | 154.3834 | 135.9831 | 66.88544 | 71.38156 | 38.7854 | 60.71887 | 97.31477 | 120.0037 | 192.586 | 82.8076 | 168.4747 | 129.9942 | 163.051 | 119.2791 | 146.1977 | 40.31691 | 68.50419 | 63.34141 | 183.4917 | 92.80312 | 154.3172 | 102.1471 | 55.84117 | 159.1135 | 108.1041 | 170.2702 |
| CUST_3381_PI429010886 | N14396 | 0.04869 | 12 | 0 | | | | | | 47.03357 | 53.55422 | 65.19644 | 51.21188 | 55.49391 | 46.08198 | 52.08646 | 50.9289 | 49.68201 | 59.3369 | 62.07028 | 56.91101 | 46.57707 | 74.19557 | 72.49088 | 65.24322 | 48.86699 | 36.53812 | 40.84477 | 57.53634 | 57.14083 | 80.45236 | 62.6571 | 55.91435 | 98.71598 | 119.8071 | 67.7681 |
| CUST_6504_PI429010886 | N20189 | 0.04869 | 12 | 3 | | | | | | 324.8963 | 122.949 | 66.55641 | 2274.622 | 2421.023 | 4584.946 | 2874.609 | 653.6532 | 184.5383 | 10.93666 | 3068.87 | 11.36635 | 207.6455 | 25.56884 | 136.8075 | 115.8059 | 3564.266 | 2063.321 | 1840.589 | 43.13359 | 2083.983 | 87.98669 | 1482.523 | 2181.474 | 37.59059 | 1335.109 | 15.83863 |
| CUST_6544_PI429010886 | N20243 | 0.04869 | 18 | 3 | | | | | | 642.4467 | 343.9276 | 617.1846 | 479.585 | 468.6028 | 633.0392 | 617.9947 | 376.5573 | 468.0946 | 333.5081 | 373.1186 | 297.2502 | 361.7087 | 446.3108 | 485.0905 | 437.9769 | 456.3557 | 471.4881 | 549.0001 | 268.8761 | 402.1311 | 437.9165 | 460.1631 | 649.7314 | 328.2492 | 397.0596 | 309.7028 |
| CUST_19575_PI429010886 | N41801 | 0.04869 | 15 | 12 | | | | | | 135.4807 | 179.678 | 216.9356 | 169.275 | 146.5292 | 148.9131 | 177.8716 | 151.8851 | 134.486 | 210.676 | 153.8044 | 223.2254 | 184.7859 | 266.5934 | 201.8776 | 174.4245 | 185.8695 | 155.1735 | 145.578 | 199.7685 | 170.531 | 214.5527 | 187.5374 | 136.8618 | 189.4251 | 189.0849 | 237.6429 |
| CUST_29986_PI429010886 | N60950 | 0.048692 | 18 | 6 | | | | | | 35.5525 | 15.11263 | 16.00911 | 31.32413 | 24.83327 | 26.66065 | 27.75224 | 24.43342 | 30.69467 | 28.20989 | 29.83725 | 26.08713 | 28.15071 | 37.78545 | 20.83234 | 22.66107 | 31.07494 | 22.97663 | 22.68707 | 18.49479 | 22.81875 | 15.13091 | 35.01718 | 26.37038 | 36.09682 | 34.39507 | 37.35631 |
| CUST_25259_PI429010886 | N52078 | 0.048699 | 24 | 0 | | | | | | 148.5892 | 174.7134 | 126.8447 | 152.3559 | 145.6105 | 161.3046 | 120.2964 | 117.8738 | 133.3318 | 151.582 | 105.8718 | 145.8455 | 110.6986 | 102.5891 | 120.0646 | 162.3514 | 127.0483 | 164.3174 | 128.5235 | 116.0217 | 141.72 | 216.0765 | 103.8532 | 158.4788 | 167.4333 | 132.3882 | 156.0301 |
| CUST_52439_PI429010886 | M97449 | 0.0487 | 24 | 0 | | | | | | 30.66462 | 23.87788 | 33.86006 | 33.30259 | 21.19291 | 17.9021 | 23.22371 | 13.64087 | 18.73257 | 15.22088 | 11.17818 | 18.85349 | 13.11969 | 30.15316 | 15.66738 | 19.32948 | 47.80852 | 17.88028 | 15.85862 | 21.44644 | 31.62413 | 17.44361 | 37.84521 | 13.23183 | 22.14618 | 23.97181 | 24.14057 |
| CUST_17009_PI429010886 | N37221 | 0.048718 | 12 | 0 | | | | | | 13.8928 | 15.67417 | 26.50391 | 8.730965 | 8.188306 | 10.83121 | 7.678477 | 13.09856 | 8.618402 | 15.64506 | 10.76236 | 12.35607 | 14.69352 | 18.31489 | 18.64838 | 17.04499 | 8.205981 | 17.50362 | 8.455318 | 5.750105 | 16.09243 | 25.55609 | 8.741416 | 12.73246 | 12.29647 | 8.357942 | 24.32948 |
| CUST_955_PI429010886 | N7968 | 0.048721 | 24 | 9 | 291241039 | PREDICTED: Caenorhabditis EpoxidE Hydrolase fami | Metabolic process | Other | 4.8E-56 | 70.73325 | 80.95222 | 109.4779 | 69.31912 | 83.1842 | 33.86551 | 71.9016 | 202.8541 | 130.9315 | 111.6499 | 186.5659 | 212.9083 | 176.4102 | 58.20366 | 92.12003 | 66.18105 | 55.13187 | 23.36338 | 82.32602 | 342.1732 | 113.2611 | 39.32188 | 63.91001 | 29.82743 | 36.19062 | 121.8599 | 116.6057 |
| CUST_24122_PI429010886 | N50070 | 0.04873 | 21 | 18 | | | | | | 253.6933 | 106.872 | 194.9477 | 141.3142 | 140.2821 | 120.3232 | 315.7757 | 106.5844 | 81.96733 | 148.3475 | 349.6083 | 53.8203 | 149.0574 | 98.90127 | 43.44238 | 162.5301 | 709.6744 | 93.68609 | 306.9203 | 386.6669 | 242.302 | 150.5005 | 225.3313 | 268.82 | 119.885 | 67.46087 | 169.4763 |
| CUST_5411_PI429010886 | N18664 | 0.04873 | 21 | 0 | 544436677 | PREDICTED: dnaJ homolog subfamily C member 21 | | | 1.8E-61 | 62.17487 | 68.81509 | 55.20849 | 65.54862 | 47.78664 | 62.27229 | 54.58961 | 57.028 | 43.67532 | 40.3065 | 43.13359 | 38.54962 | 53.33275 | 49.91574 | 53.25946 | 69.82734 | 39.95554 | 67.58357 | 43.55575 | 46.97546 | 45.12848 | 64.70488 | 41.00395 | 838.9983 | 55.31379 | 45.85649 | 47.88363 |
| CUST_54472_PI429010886 | M106161 | 0.048804 | 24 | 18 | | | | | | 13.30863 | 9.387006 | 18.64164 | 6.748738 | 10.55729 | 10.08948 | 14.1847 | 10.15288 | 10.94248 | 8.950802 | 13.10998 | 12.70194 | 19.73888 | 13.7932 | 9.73225 | 18.29426 | 15.49998 | 14.48423 | 16.35532 | 16.00542 | 14.10338 | 18.2774 | 11.61125 | 21.19536 | 17.96131 | 14.21644 | 23.88653 |
| CUST_55911_PI429010886 | M112094 | 0.048804 | 24 | 21 | 390345457 | PREDICTED: zinc finger protein 84-like [Strongyloce | | | 1.7E-22 | 182.2638 | 226.6353 | 285.8071 | 164.0579 | 159.7175 | 204.8478 | 174.8583 | 177.758 | 177.5136 | 217.9412 | 198.9264 | 284.5979 | 189.7994 | 312.5243 | 203.1014 | 248.5464 | 192.898 | 227.9715 | 209.9313 | 232.7383 | 218.636 | 208.7066 | 262.8518 | 251.0607 | 248.2496 | 226.6782 | 285.1566 |
| CUST_3797_PI429010886 | N15281 | 0.048804 | 24 | 21 | | | | | | 346.1873 | 392.5464 | 471.2464 | 342.1102 | 340.4019 | 383.225 | 337.7668 | 366.6536 | 352.0546 | 360.8661 | 350.5877 | 448.5246 | 365.1209 | 410.594 | 399.1635 | 411.2669 | 391.819 | 390.4272 | 406.5096 | 374.2323 | 393.8505 | 458.895 | 444.8946 | 368.0783 | 466.0826 | 408.0489 | 442.9673 |
| CUST_38726_PI429010886 | M29673 | 0.04884 | 15 | 9 | 220172369 | cuticle protein [Rimicaris exoculata] | | | 3.2E-13 | 37.96514 | 74.08523 | 59.07548 | 68.63241 | 63.00378 | 77.92872 | 80.33892 | 58.892 | 64.8815 | 66.022 | 66.29488 | 111.5216 | 60.16545 | 78.48723 | 47.37022 | 39.18988 | 54.05301 | 64.22812 | 32.38272 | 41.28609 | 86.71532 | 58.58074 | 88.74926 | 108.8637 | 74.87405 | 75.33825 | 128.0667 |
| CUST_50247_PI429010886 | M89154 | 0.048841 | 12 | 0 | | | | | | 99.58413 | 90.70315 | 108.4222 | 43.7836 | 88.02287 | 81.34562 | 70.52611 | 73.26779 | 87.64594 | 103.0135 | 94.16491 | 98.2447 | 88.34755 | 83.90478 | 80.53946 | 83.80399 | 76.41163 | 82.07629 | 71.10646 | 89.92723 | 100.542 | 96.09446 | 93.91055 | 41.70336 | 107.898 | 99.36845 | 103.1223 |
| CUST_44539_PI429010886 | M66266 | 0.048873 | 12 | 0 | | | | | | 18.57367 | 46.48737 | 34.13077 | 35.16159 | 20.78572 | 21.49928 | 13.4001 | 17.19687 | 25.70354 | 30.95577 | 28.37201 | 29.32561 | 23.89642 | 35.6894 | 34.60204 | 54.23711 | 21.05341 | 38.96658 | 24.8597 | 24.19734 | 17.72933 | 43.67133 | 33.67217 | 25.21131 | 46.64388 | 32.48902 | 41.2187 |
| CUST_3494_PI429010886 | N14617 | 0.048873 | 12 | 0 | | | | | | 88.20739 | 124.7587 | 139.376 | 81.39522 | 87.6188 | 122.4191 | 75.66735 | 82.55028 | 85.3343 | 93.87602 | 82.47663 | 156.3748 | 99.71807 | 116.0529 | 117.2199 | 93.56818 | 89.19702 | 88.10949 | 86.64513 | 105.6617 | 96.47975 | 134.8627 | 109.7208 | 90.82199 | 100.5297 | 108.1306 | 133.4278 |
| CUST_22138_PI429010886 | N46501 | 0.048873 | 12 | 0 | | | | | | 173.9115 | 224.1873 | 191.6673 | 158.5869 | 153.1717 | 202.3386 | 135.6803 | 145.3659 | 134.1249 | 166.4668 | 134.7225 | 236.613 | 143.7672 | 171.6689 | 251.7054 | 186.8543 | 149.4128 | 193.3073 | 153.6236 | 187.5065 | 157.5747 | 207.1975 | 155.8809 | 140.8407 | 187.9811 | 222.578 | 227.4117 |
| CUST_25025_PI429010886 | N51655 | 0.048873 | 12 | 0 | | | | | | 40.76795 | 66.48727 | 51.88614 | 44.55872 | 39.38447 | 34.98674 | 24.20353 | 30.53983 | 48.98952 | 49.32644 | 38.96658 | 66.36423 | 46.08198 | 66.78152 | 65.68694 | 54.04063 | 21.27079 | 53.8203 | 20.14149 | 47.15656 | 44.25982 | 75.58303 | 58.09597 | 28.49131 | 63.98783 | 48.1448 | 69.39648 |
| CUST_25898_PI429010886 | N53262 | 0.048873 | 12 | 0 | | | | | | 112.7504 | 120.0318 | 99.57087 | 99.01075 | 103.5324 | 111.7621 | 93.72794 | 82.41691 | 89.6226 | 129.9323 | 98.86383 | 111.625 | 97.75958 | 108.8513 | 108.9599 | 141.7485 | 82.97709 | 113.475 | 91.56459 | 92.85168 | 100.3128 | 110.2048 | 79.52449 | 92.31619 | 117.0501 | 117.5516 | 119.2623 |
| CUST_1186_PI429010886 | N8773 | 0.048873 | 12 | 0 | | | | | | 65.79499 | 92.54562 | 111.7908 | 84.07185 | 61.85594 | 83.92582 | 56.97163 | 86.43501 | 65.6996 | 94.99099 | 76.28297 | 93.9354 | 93.10199 | 100.8857 | 66.25397 | 93.62063 | 73.1185 | 98.05949 | 72.90791 | 82.22696 | 84.02515 | 95.01446 | 92.06564 | 79.85552 | 103.9452 | 105.4619 | 118.9193 |
| CUST_38913_PI429010886 | M31924 | 0.048895 | 12 | 3 | 33468738 | calcification-associated peptide-1 [Procambarus cla | | | 3.02E-11 | 31148.55 | 20401.48 | 149.4288 | 73477.97 | 27642.69 | 99779.5 | 37461.84 | 31314.74 | 43369.66 | 65.88576 | 5119.45 | 77.60864 | 41887.92 | 1705.674 | 29210.68 | 8414.501 | 46851.42 | 72295.32 | 40354.05 | 352.3859 | 2232.866 | 2634.615 | 965.2192 | 43755.93 | 972.1119 | 53582.06 | 20.717 |
| CUST_7538_PI429010886 | N21697 | 0.048895 | 12 | 9 | | | Metabolic process | Nucleobase-containing compound metabolic process | | 43.41776 | 68.07541 | 51.21188 | 37.96113 | 30.47678 | 30.33968 | 40.25661 | 29.45836 | 31.65427 | 45.56467 | 52.63381 | 59.234 | 33.99968 | 35.6143 | 29.41419 | 55.36009 | 31.39871 | 28.90976 | 31.30264 | 65.65578 | 38.55767 | 49.74292 | 56.26704 | 39.02972 | 34.37952 | 42.77108 | 30.56491 |
| CUST_15642_PI429010886 | N34908 | 0.048895 | 12 | 9 | | | | | | 57.97363 | 55.96041 | 50.45103 | 31.07173 | 36.87107 | 48.34221 | 31.56943 | 36.79834 | 44.82294 | 62.13897 | 48.46881 | 81.10241 | 42.98324 | 54.96227 | 43.52397 | 45.84834 | 39.50189 | 47.03357 | 43.06591 | 51.77357 | 38.11258 | 59.62185 | 60.63321 | 37.65478 | 58.44181 | 51.21188 | 48.17521 |
| CUST_32392_PI429010886 | M3993 | 0.048912 | 24 | 0 | 270010258 | hypothetical protein TcasGA2_TC009637 [Tribolium | | | 1.43E-29 | 113.2396 | 79.48728 | 109.2372 | 63.88437 | 100.3128 | 50.19147 | 103.1763 | 63.95336 | 103.3151 | 107.2874 | 89.66356 | 84.19357 | 60.51107 | 86.91384 | 56.37263 | 75.84454 | 84.015 | 63.70797 | 83.51601 | 79.31367 | 81.74728 | 67.34293 | 95.689 | 64.83984 | 88.74926 | 145.2889 | 149.6388 |
| CUST_32051_PI429010886 | M3109 | 0.048989 | 15 | 3 | | | | | | 149.4702 | 134.7225 | 84.25035 | 190.8098 | 183.091 | 264.2251 | 133.2264 | 114.926 | 128.4938 | 112.6218 | 95.27106 | 151.5005 | 107.543 | 152.8803 | 120.7928 | 129.8119 | 162.5301 | 184.9585 | 141.5625 | 101.4497 | 142.9849 | 162.7985 | 119.9711 | 167.1839 | 113.1265 | 121.8132 | 111.9276 |
| CUST_24972_PI429010886 | N51564 | 0.048989 | 12 | 0 | | | | | | 21.41385 | 22.65536 | 19.64187 | 21.87774 | 12.16648 | 19.95854 | 6.736876 | 14.99759 | 11.8646 | 18.93949 | 14.04228 | 12.47217 | 11.58812 | 18.66627 | 21.89563 | 29.35511 | 15.08577 | 20.80086 | 10.06713 | 19.99274 | 20.13696 | 17.94144 | 8.06826 | 21.16443 | 23.11751 | 25.66555 | 28.75592 |
| CUST_4796_PI429010886 | N17497 | 0.04901 | 24 | 12 | | | | | | 8.887892 | NA | 11.14433 | 23.4652 | 9.922626 | 10.71844 | 10.90408 | 52.45794 | 12.31427 | 27.52267 | 16.49199 | 18.7049 | 16.60892 | 22.45787 | 23.45456 | 8.978228 | 9.089436 | NA | 22.14979 | 10.54982 | 19.47107 | 17.44803 | 15.45689 | 27.20765 | 7.227483 | 13.84485 | 4.946841 |

| Probe ID | ID | p-value | Period | Phase | EBI ID | Annotation | GO category | Go term | E-value | 0 | 0 | 0 | 3 | 3 | 3 | 6 | 6 | 6 | 9 | 9 | 9 | 12 | 12 | 12 | 15 | 15 | 15 | 18 | 18 | 18 | 21 | 21 | 21 | 24 | 24 | 24 |
|---|---|---|---|---|---|---|---|---|---|---|---|---|---|---|---|---|---|---|---|---|---|---|---|---|---|---|---|---|---|---|---|---|---|---|---|---|
| CUST_35569_PI429010886 | M12906 | 0.049013 | 15 | 3 | 321472396 | hypothetical protein DAPPUDRAFT_315872 [Daphn | Protein metabolism | Proteolysis | 3.4E-89 | 26.26527 | 28.69415 | NA | 35.86299 | 21.05993 | 49.2211 | 30.42832 | 31.15965 | 24.02555 | 15.20537 | 12.99168 | 13.6134 | 22.31012 | 17.8956 | 12.55296 | 13.86905 | 29.65821 | 30.92489 | 39.98991 | 9.811903 | 18.92054 | 20.77886 | NA | 28.99027 | 5.608929 | 25.56371 | 5.489718 |
| CUST_1958_PI429010886 | N11166 | 0.049016 | 18 | 3 | | | | | | 2434.013 | 2708.301 | 1463.065 | 29816.7 | 26474.55 | 29010.89 | 10217.96 | 12214.41 | 425.3087 | 25.83997 | 12610.19 | 17.5674 | 2716.899 | 142.3777 | 2434.013 | 19.44155 | 7490.614 | 2782.19 | 6042.693 | 7.53723 | 15000.31 | 324.8235 | 10949.63 | 15872.37 | 44.83858 | 6932.514 | 14.50091 |
| CUST_36557_PI429010886 | M16227 | 0.049034 | 21 | 18 | | | | | | 73.78486 | 97.10121 | 71.44429 | 70.98423 | 64.57467 | 50.25117 | 49.50242 | 55.81794 | 60.61979 | 82.97709 | 59.70983 | 102.6107 | 68.96573 | 49.84204 | 85.09002 | 99.33533 | 55.24489 | 69.05841 | 76.15742 | 87.83251 | 76.20212 | 84.55489 | 50.46685 | 71.30124 | 59.86271 | 68.85742 | 82.02402 |
| CUST_23758_PI429010886 | N49413 | 0.049055 | 21 | 15 | 321451124 | hypothetical protein DAPPUDRAFT_336270 [Daphn | | | 6.93E-17 | 11.69261 | 22.26173 | NA | 16.27803 | 33.31614 | 26.53218 | 18.83139 | NA | 18.4326 | 13.21272 | 4.86774 | 9.405002 | 12.27296 | 26.13131 | 27.00004 | 44.39886 | NA | 22.75029 | NA | 37.84899 | 21.64092 | 40.1699 | 16.95326 | 30.30845 | 6.853489 | NA | NA |
| CUST_41550_PI429010886 | M58010 | 0.04906 | 21 | 18 | | | | | | 17.62531 | 37.6475 | NA | 111.5107 | 118.5941 | 115.8594 | 25.34926 | 8.0157 | 12.32751 | 11.70566 | 12.29064 | NA | 11.00948 | 278.8959 | 175.6726 | 162.6071 | 13.45887 | 176.4102 | NA | 248.2496 | 80.21909 | 383.093 | 22.13275 | 227.4117 | 91.32302 | 14.30732 | 16.44746 |
| CUST_25764_PI429010886 | N53016 | 0.049085 | 21 | 12 | 37780426 | hemocyanin 2 [Pacifastacus leniusculus] | | | 2.32E-16 | NA | 88.81258 | 40.94647 | 265.4463 | 174.5081 | 40.00557 | 102.2773 | 1110.941 | 207.7427 | 113.3918 | 971.3866 | 1088.645 | 2656.499 | 37.56417 | 1224.331 | NA | 422.9234 | NA | 37.86509 | 19739.66 | 320.4556 | NA | 24.52204 | 18.1616 | NA | 452.9912 | 744.5402 |
| CUST_9355_PI429010886 | N24483 | 0.0491 | 21 | 0 | | | | | | 104.2505 | 100.1573 | 68.1923 | 51.75281 | 68.97204 | 45.33809 | 69.74095 | 47.47127 | 37.70765 | 57.85675 | 51.67197 | 60.43419 | 58.6875 | 48.21189 | 50.41387 | 94.16491 | 63.89787 | 63.06213 | 52.99425 | 90.68288 | 66.99196 | 50.24503 | 65.38596 | 69.30744 | 68.97204 | 61.28097 | 57.39701 |
| CUST_50783_PI429010886 | M90989 | 0.049107 | 12 | 0 | | | | | | 12.55296 | 8.834126 | NA | 9.09768 | 5.683749 | 9.666802 | 6.956638 | 6.468226 | NA | 9.320057 | 4.040067 | 7.035948 | 6.406079 | 9.939882 | 10.15583 | 6.487746 | NA | 8.431174 | 6.94159 | 6.597762 | 5.452395 | NA | NA | 7.822452 | 7.857013 | 10.96784 | NA |
| CUST_39087_PI429010886 | M34399 | 0.049125 | 15 | 9 | | | | | | 25.64937 | 66.32627 | 52.25652 | 24.86526 | 52.4826 | 48.3123 | 56.68336 | 28.58102 | 69.45519 | 39.18602 | 75.12567 | 77.46325 | 34.92205 | 78.08745 | 41.76249 | 18.22767 | 38.81418 | 73.28621 | 27.42777 | 28.2496 | 57.94186 | 41.10579 | 55.66641 | 96.41487 | 86.82244 | 67.29642 | 105.005 |
| CUST_35328_PI429010886 | M12274 | 0.049158 | 15 | 12 | | | | | | 6833.331 | 4524.584 | 2604.753 | 4374.715 | 1321.214 | 1483.935 | 1026.845 | 2747.282 | 3333.763 | 2725.555 | 1587.608 | 3067.321 | 3184.108 | 5407.054 | 4161.995 | 2638.062 | 3416.788 | 4015.963 | 2596.051 | 4748.861 | 1485.346 | 1893.755 | 2166.108 | 2108.426 | 2180.377 | 2434.013 | 2826.022 |
| CUST_49869_PI429010886 | M87511 | 0.049221 | 12 | 0 | 9367086 | Euphausia superba large subunit ribosomal RNA ger | | | 7.69E-69 | 121.9054 | NA | 354.2035 | 7.878584 | 18.17304 | 34.85305 | 155.0084 | 269.4831 | 164.4185 | 207.7747 | 43.52397 | 23.33499 | 178.4972 | 221.6212 | 183.021 | 9.486543 | 133.6264 | 25.68457 | 109.7371 | 20.05897 | 88.24844 | 106.8828 | 270.6659 | 90.26625 | 198.8955 | 23.61053 | 181.5662 |
| CUST_12037_PI429010886 | N28818 | 0.049223 | 24 | 21 | | | | | | 112.7335 | 116.6557 | 158.4583 | 104.1514 | 97.05352 | 128.9553 | 116.1911 | 128.1595 | 114.4136 | 135.3397 | 103.9891 | 91.24255 | 95.30462 | 124.8269 | 108.6304 | 140.3134 | 88.6992 | 139.545 | 146.8499 | 121.7504 | 135.1423 | 152.8418 | 168.329 | 118.3919 | 119.245 | 139.9485 | 135.2179 |
| CUST_2700_PI429010886 | N12935 | 0.049231 | 15 | 12 | | | | | | 472.3127 | 469.28 | 517.1879 | 381.1294 | 415.6575 | 408.32 | 390.7246 | 421.8908 | 464.3001 | 536.6546 | 432.7317 | 541.0041 | 471.9637 | 559.6795 | 552.1675 | 893.1457 | 419.4237 | 452.8393 | 463.3861 | 534.0061 | 428.333 | 567.7179 | 493.331 | 367.3426 | 648.0827 | 452.1056 | 562.8166 |
| CUST_57262_PI429010886 | E3125 | 0.049244 | 12 | 3 | 152949487 | calcification associated soluble matrix protein 2 [Prc | | | 2.04E-12 | 751.781 | 205.1599 | 662.2599 | 12523.21 | 18023.86 | 25871.73 | 12999.66 | 4901.718 | 545.8082 | 14.33789 | 18463.84 | 11.16911 | 613.2651 | 28.87669 | 463.3861 | 25.89927 | 15872.37 | 15221.8 | 10746.13 | 19.10728 | 14205.89 | 60.21125 | 8448.525 | 14295.94 | 11.27249 | 7239.058 | 5.346894 |
| CUST_3203_PI429010886 | N14013 | 0.049244 | 12 | 0 | | | | | | 642.6469 | 970.3266 | 801.5312 | 589.8071 | 524.852 | 737.7582 | 610.9693 | 523.5388 | 598.9907 | 855.518 | 753.467 | 915.3775 | 739.6144 | 779.7877 | 885.3464 | 1092.918 | 657.4366 | 661.1589 | 603.4589 | 753.7243 | 664.8684 | 1140.062 | 678.5082 | 608.5272 | 1135.995 | 687.2662 | 1003.294 |
| CUST_8272_PI429010886 | N22802 | 0.049244 | 18 | 9 | | | | | | 328.4958 | 437.8204 | 381.8035 | 265.2639 | 331.4379 | 304.8063 | 261.123 | 370.213 | 331.8125 | 412.2515 | 401.7586 | 392.7909 | 352.6063 | 384.5478 | 363.8038 | 472.651 | 404.4491 | 330.9233 | 321.6396 | 362.6784 | 304.1069 | 408.3975 | 306.5697 | 254.4207 | 521.8521 | 287.7468 | 409.0918 |
| CUST_489_PI429010886 | N5621 | 0.049244 | 15 | 12 | 298370749 | cathepsin B [Litopenaeus vannamei] | Protein metabolism | Proteolysis | 3.9E-150 | 444.7159 | 1069.514 | 650.5246 | 505.1623 | 401.3173 | 625.8341 | 441.1682 | 583.7272 | 496.4081 | 896.1269 | 634.1523 | 1115.976 | 686.1452 | 769.2478 | 768.7795 | 715.8969 | 602.4628 | 495.0933 | 456.6758 | 560.8883 | 698.0293 | 1132.32 | 646.4459 | 643.2335 | 793.8063 | 655.5465 | 638.7779 |
| CUST_12179_PI429010886 | N29056 | 0.049273 | 21 | 0 | 260799997 | hypothetical protein BRAFLDRAFT_72305 [Branchio | | | 7.53E-20 | 13.37838 | 16.2627 | 10.52781 | 7.434035 | 7.744827 | 20.60205 | 5.410089 | 11.49641 | 4.391667 | 8.991035 | 4.837303 | 8.510732 | 3.885096 | 9.987338 | 12.5724 | 19.42289 | 9.102009 | 25.36189 | 26.68081 | 11.13632 | 9.953883 | 14.49175 | 13.14971 | NA | 16.06632 | 4.962271 | 18.08249 |
| CUST_41808_PI429010886 | M58599 | 0.049339 | 21 | 15 | 29134779 | projectin [Procambarus clarkii] | Protein metabolism | Cellular protein modification process | 1.32E-87 | 145.2433 | 92.03214 | 108.4109 | 90.11072 | 97.75958 | 98.26203 | 84.79763 | 98.92872 | 100.542 | 111.685 | 131.3569 | 132.8034 | 100.3042 | 104.2766 | 91.90153 | 115.6759 | 151.5201 | 107.9692 | 119.1161 | 133.064 | 77.33177 | 105.711 | 128.9944 | 98.36103 | 102.2659 | 85.25376 | 95.39206 |
| CUST_54717_PI429010886 | M107298 | 0.049368 | 24 | 0 | | | | | | 651.6164 | 671.8789 | 790.7325 | 581.1144 | 579.2037 | 672.0887 | 543.0155 | 557.1607 | 643.9207 | 695.1115 | 622.3874 | 685.3204 | 537.9358 | 804.5642 | 558.4057 | 674.0173 | 510.619 | 611.3709 | 558.6606 | 650.8924 | 607.116 | 681.8773 | 731.6805 | 591.3951 | 833.5719 | 702.9226 | 846.752 |
| CUST_13670_PI429010886 | N31564 | 0.049392 | 24 | 21 | | | | | | 66.86178 | 99.22324 | 63.46985 | 77.12763 | 62.506 | 47.76946 | 45.47762 | 44.38619 | 65.08584 | 70.90857 | 73.23407 | 103.9314 | 95.29429 | 79.88802 | 79.0243 | 91.5749 | 59.25893 | 88.29574 | 75.48481 | 76.94138 | 77.15161 | 101.0126 | 104.1782 | 74.10143 | 94.40103 | 60.23492 | 80.71386 |
| CUST_14838_PI429010886 | N33553 | 0.049392 | 24 | 21 | | | | | | 125.7351 | 153.3961 | 160.8449 | 119.1024 | 124.1467 | 164.0579 | 128.9362 | 120.0167 | 129.2382 | 157.9764 | 117.2732 | 145.0185 | 133.7242 | 152.0579 | 158.0318 | 155.2361 | 152.1653 | 157.0455 | 132.3882 | 176.2005 | 147.402 | 160.8449 | 154.8889 | 164.6529 | 166.0195 | 153.132 | 186.7835 |
| CUST_18072_PI429010886 | N39090 | 0.049392 | 24 | 21 | 465954970 | Beta-1,4-galactosyltransferase 3 [Chelonia mydas] | Metabolic process | Carbohydrate metabolic process | 3.48E-20 | 66.60678 | 65.13281 | 62.63918 | 75.59869 | 120.7015 | 72.16939 | 41.909 | 33.62817 | 46.13849 | 53.79628 | 56.56684 | 72.3461 | 54.4284 | 89.91874 | 68.81509 | 107.5586 | 52.70775 | 87.22914 | 40.10273 | 142.1088 | 70.2561 | 110.1494 | 87.21952 | 83.06141 | 83.14826 | 49.30624 | 49.49023 |
| CUST_23503_PI429010886 | N48942 | 0.049397 | 21 | 0 | | | | | | 46.26383 | 39.97268 | 34.10344 | 28.58102 | 28.6962 | 18.05541 | 33.69201 | 14.44838 | 14.91728 | 21.84275 | 22.94962 | 32.46691 | 21.92896 | 39.13386 | 31.79359 | 37.39567 | 36.55801 | 25.72523 | 21.82183 | 33.37016 | 19.36786 | 46.95662 | 26.94089 | 41.23292 | 42.36335 | 34.77329 | 20.30155 |
| CUST_46096_PI429010886 | M71353 | 0.049416 | 12 | 0 | | | | | | 201.4981 | 165.6162 | 227.0266 | 144.9989 | 194.7223 | 207.4294 | 163.2552 | 189.6264 | 201.7725 | 186.9145 | 154.7492 | 188.658 | 186.2582 | 246.6015 | 220.4437 | 203.7326 | 175.1291 | 190.201 | 158.6127 | 179.7938 | 161.9153 | 220.9369 | 189.9402 | 181.2412 | 196.2921 | 228.9547 | 216.9753 |
| CUST_3524_PI429010886 | N14684 | 0.049416 | 12 | 0 | | | | | | 69.03435 | 88.14739 | 72.6524 | 56.05475 | 74.79904 | 61.80458 | 40.1071 | 47.17274 | 46.95662 | 82.29861 | 54.3948 | 85.77208 | 60.27464 | 64.59746 | 83.00511 | 69.34114 | 49.11669 | 68.21242 | 47.15148 | 94.1523 | 60.79572 | 66.60001 | 63.93448 | 44.1761 | 65.31258 | 90.21463 | 92.90826 |
| CUST_6985_PI429010886 | N20870 | 0.049416 | 12 | 0 | | | | | | 103.1477 | 79.68658 | 103.0243 | 97.23707 | 81.63335 | 129.3708 | 84.49247 | 85.02582 | 77.24703 | 85.0717 | 78.27179 | 85.67865 | 85.67865 | 89.26714 | 87.03787 | 101.9601 | 89.72544 | 73.47743 | 83.383 | 79.56143 | 71.13139 | 86.04961 | 97.25472 | 100.2724 | 85.25376 | 111.7487 | 104.8657 |
| CUST_11696_PI429010886 | N28256 | 0.049416 | 12 | 0 | 383855186 | PREDICTED: uncharacterized protein LOC10087841( | | | 1.68E-10 | 17.65061 | 30.25328 | 15.19101 | 19.07974 | 18.3679 | 12.46283 | 10.23296 | 10.76376 | 10.28467 | 32.8503 | 15.95534 | 17.21299 | 12.89788 | 21.62724 | 22.22394 | 41.56705 | 9.08762 | 22.56226 | 12.63783 | 13.60023 | 14.21003 | 20.16106 | 12.28413 | 16.4888 | 18.84499 | 20.14149 | 23.81358 |
| CUST_22070_PI429010886 | N46367 | 0.049416 | 12 | 0 | 321478103 | hypothetical protein DAPPUDRAFT_304684 [Daphn | | | 2.95E-35 | 53.36598 | 20.48821 | 35.12625 | 36.9176 | 11.69762 | 21.88139 | 27.82792 | 17.45526 | 14.43921 | 29.41742 | 20.91887 | 35.02438 | 26.66659 | 47.5229 | 16.44885 | 29.27092 | 19.3883 | 25.64332 | 14.48109 | 22.74587 | 16.83375 | 31.57401 | 39.20847 | 24.88398 | 31.20492 | 32.77063 | 41.83506 |
| CUST_28954_PI429010886 | N58911 | 0.049416 | 12 | 0 | | | | | | 25.88998 | 36.46183 | 21.05098 | 21.21658 | 24.04991 | 27.78422 | 19.9426 | 19.2789 | 26.58817 | 29.01971 | 32.74092 | 30.77501 | 19.05757 | 39.83487 | 41.8496 | 32.75095 | 23.35223 | 36.63834 | 27.68104 | 20.18733 | 19.22009 | 33.24975 | 34.61952 | 20.92107 | 50.54005 | 34.65468 | 29.38623 |
| CUST_5888_PI429010886 | N19335 | 0.049432 | 12 | 3 | | | | | | 2280.777 | 1975.879 | 1487.266 | 2892.813 | 1901.328 | 3952.869 | 1870.503 | 2465.06 | 1811.1 | 1624.747 | 1555.197 | 1692.067 | 1939.579 | 1471.445 | 1952.727 | 2070.325 | 2555.115 | 3084.768 | 2814.544 | 1389.409 | 1263.557 | 1876.716 | 1277.616 | 2459.721 | 1889.582 | 3315.042 | 1566.438 |
| CUST_6298_PI429010886 | N19918 | 0.049432 | 12 | 3 | | | | | | 90.36196 | 137.9515 | 72.91578 | 132.7287 | 131.1249 | 139.4961 | 116.0676 | 95.368 | 88.30586 | 102.8432 | 101.3443 | 74.34861 | 106.5981 | 145.2433 | 102.9429 | 97.24592 | 136.2681 | 116.8917 | 123.1656 | 130.6959 | 90.43345 | 96.8323 | 103.384 | 96.28021 | 107.3764 | 107.174 | 111.1891 |
| CUST_7248_PI429010886 | N21260 | 0.049432 | 12 | 3 | 546686148 | hypothetical protein D910_12796 [Dendroctonus pc | | | 1.9E-166 | 68.99159 | 64.66893 | 60.64562 | 82.08362 | 84.46837 | 90.34874 | 85.04454 | 62.75575 | 49.0094 | 56.34897 | 59.45067 | 48.01455 | 78.57748 | 81.39522 | 71.79355 | 64.4873 | 114.7609 | 55.90028 | 87.58299 | 48.09806 | 68.98284 | 66.90174 | 57.08439 | 59.6278 | 43.1896 | 61.07112 | 70.84411 |
| CUST_23222_PI429010886 | N48464 | 0.049432 | 12 | 3 | | | | | | 24.36264 | 26.86002 | 26.54903 | 28.11972 | 28.02151 | 28.93678 | 23.80754 | 22.47267 | 16.25929 | 27.02172 | 20.07855 | 16.26691 | 16.97048 | 27.03609 | 27.7711 | 34.92634 | 38.95233 | 37.79822 | 21.38108 | 19.70553 | 7.274775 | 33.45717 | 26.23144 | 14.28677 | 32.74092 | 24.97091 | 30.08229 |
| CUST_30231_PI429010886 | N61445 | 0.049432 | 12 | 9 | | | | | | 37.47945 | 52.02242 | 46.02417 | 31.74511 | 45.06422 | 31.95513 | 33.28766 | 78.14128 | 42.4817 | 58.14864 | 60.22346 | 35.57263 | 48.05138 | 33.95306 | 30.70184 | 40.09244 | 36.8424 | 19.96449 | 68.79393 | 36.52268 | 40.42007 | 45.87335 | 85.40569 | 74.55697 | 57.43992 | 24.65264 | 35.9589 |
| CUST_25752_PI429010886 | N52993 | 0.049452 | 18 | 12 | 395484809 | glutamine synthetase [Fenneropenaeus chinensis] > | | | 1.77E-16 | 344.4708 | 87.77632 | 638.6578 | 62.34667 | 189.7386 | 115.1327 | 300.3777 | 344.24 | 279.8319 | 318.1453 | 159.4135 | 132.2443 | 251.0973 | 351.2576 | 225.2614 | 160.2216 | 249.2154 | 140.6597 | 210.7453 | 95.4903 | 195.3573 | 87.84073 | 166.9208 | 116.0813 | 180.3864 | 130.357 | 219.8055 |
| CUST_38145_PI429010886 | M23646 | 0.049463 | 12 | 9 | 54042615 | arthrodial cuticle protein AMP6.0 [Callinectes sapid | | | 4.55E-16 | 577.33 | 642.8014 | 1091.604 | 292.1395 | 510.5227 | 270.9139 | 882.8865 | 622.7518 | 794.4801 | 903.9804 | 362.4934 | 610.3506 | 497.6538 | 827.8275 | 344.2985 | 264.3669 | 492.7259 | 227.604 | 487.486 | 502.1624 | 502.0515 | 917.7643 | 1320.152 | 457.1179 | 1307.703 | 364.5694 | 1377.953 |
| CUST_32377_PI429010886 | M3963 | 0.049464 | 21 | 6 | 321468506 | hypothetical protein DAPPUDRAFT_52203 [Daphnia | Metabolic process | Other | 3.03E-47 | 25.33771 | 33.14087 | 30.46627 | 32.65296 | 21.61481 | 22.43724 | 22.9868 | 34.93733 | 29.9146 | 26.04992 | 15.02774 | 33.52618 | 23.38175 | 15.30965 | 11.71408 | 15.81986 | 14.97499 | 20.48097 | 17.87105 | 22.62556 | 28.57291 | 41.40356 | 18.73257 | 25.24216 | 22.07787 | 23.13968 | 29.30417 |
| CUST_35068_PI429010886 | M11472 | 0.049469 | 15 | 12 | | | | | | 944.9878 | 910.5801 | 1056.762 | 625.332 | 778.415 | 877.6784 | 760.7539 | 766.9049 | 790.3406 | 937.1457 | 1189.668 | 1138.397 | 959.9717 | 1134.016 | 1259.651 | 1394.642 | 1047.078 | 886.7923 | 1046.312 | 1341.448 | 732.691 | 1210.833 | 842.3459 | 582.4599 | 1184.785 | 716.0786 | 1207.044 |
| CUST_39188_PI429010886 | M35849 | 0.04949 | 24 | 21 | | | | | | 8.496964 | 34.97152 | 27.30026 | 31.34507 | 22.82168 | 20.65925 | 16.17384 | 7.501961 | 23.27419 | 22.66333 | 23.07266 | 22.13451 | 21.96895 | 19.99274 | 12.94469 | 17.50524 | 10.85726 | 25.26334 | 12.93205 | 19.68474 | 28.55509 | 34.12708 | 58.85113 | 25.63451 | 48.31638 | 36.47682 | 20.01979 |
| CUST_4876_PI429010886 | N17673 | 0.04949 | 21 | 0 | | | | | | 31.04302 | 22.91836 | 17.00562 | 21.65997 | 18.5156 | 26.98251 | 12.69552 | 17.12411 | 12.79225 | 15.81194 | 20.37597 | 13.06127 | 11.29004 | 23.59869 | 9.535711 | 8.789967 | 20.27847 | 18.26372 | 13.58569 | 68.30614 | 20.94079 | 18.47407 | 25.71973 | 21.28336 | 23.36677 | 13.72673 | 20.24846 |
| CUST_41922_PI429010886 | M58886 | 0.049496 | 18 | 0 | 512932133 | PREDICTED: uncharacterized protein LOC10173755( | | | 5.61E-08 | 18.58833 | 13.27381 | 20.74695 | 16.30067 | 18.21746 | 8.582555 | 17.43453 | 10.86011 | 12.56761 | 14.16465 | 13.08017 | 10.53371 | 6.849615 | 14.60042 | 6.633519 | 20.57784 | 14.83503 | 21.15997 | 20.66537 | 20.04961 | 17.27797 | 15.25738 | 11.17818 | 6.417851 | 15.81556 | 12.29064 | 17.47586 |
| CUST_38460_PI429010886 | M26958 | 0.049524 | 15 | 9 | | | | | | 332.1138 | 340.1906 | 463.0515 | 331.5388 | 396.6524 | 223.9394 | 402.2817 | 184.7518 | 400.1681 | 346.3085 | 398.4336 | 404.8094 | 292.6086 | 469.825 | 172.213 | 291.4361 | 312.3161 | 280.7168 | 114.0624 | 242.302 | 447.8279 | 305.3843 | 435.3009 | 324.7416 | 471.0971 | 510.0071 | 495.2283 |
| CUST_20290_PI429010886 | N43176 | 0.049526 | 15 | 0 | | | | | | 68.35087 | 62.88366 | 63.48536 | 50.55173 | 49.65587 | 65.48233 | 49.83081 | 39.98138 | 48.63366 | 59.00313 | 39.01912 | 51.33619 | 43.80188 | 81.01356 | 63.28709 | 59.67663 | 60.6558 | 64.68215 | 60.07014 | 47.40881 | 56.68336 | 53.27432 | 53.26961 | 53.21879 | 74.26685 | 51.86583 | 41.89305 |
| CUST_49386_PI429010886 | M85461 | 0.049545 | 21 | 3 | | | | | | 76.96996 | 152.5866 | 84.93968 | 289.0715 | 98.02439 | 110.2048 | 78.27179 | 41.69502 | 71.67319 | 76.73952 | 39.60553 | 104.9421 | 58.94983 | 67.58357 | 50.19147 | 305.9676 | 21.95116 | 35.97953 | 34.25171 | 39.73588 | 140.3818 | 64.24619 | 123.2882 | 86.45155 | 111.9633 | 133.6865 | 59.20737 |
| CUST_5094_PI429010886 | N18249 | 0.049546 | 12 | 0 | 321460483 | hypothetical protein DAPPUDRAFT_308857 [Daphn | Developmental process | Muscle structure development | | 25.48959 | 43.23619 | 32.79034 | 28.57032 | 24.37229 | 22.53922 | 11.83225 | 22.61376 | 21.09976 | 32.65296 | 39.57145 | 40.02231 | 66.20577 | 25.30072 | 29.88417 | 57.7399 | 18.33154 | 31.95691 | 25.09705 | 31.05901 | 32.55631 | 30.26846 | 31.06527 | 26.64257 | 54.04881 | 39.77048 | 45.57 |
| CUST_40040_PI429010886 | M49130 | 0.049568 | 21 | 12 | 37780426 | hemocyanin 2 [Pacifastacus leniusculus] | | | 5.28E-65 | 4.853908 | 67.29642 | 21.8588 | 208.4543 | 153.9132 | 28.40282 | 105.5539 | 900.857 | 172.7143 | 97.73561 | 768.5376 | 878.6172 | 2064.29 | 37.11592 | 983.8663 | 4.56456 | 395.7641 | NA | 43.38758 | 18586.47 | 413.3489 | NA | 43.03291 | 12.40735 | 10.57913 | 312.052 | 595.1496 |
| CUST_49634_PI429010886 | M86468 | 0.049585 | 18 | 0 | | | | | | 40.2482 | NA | 57.80685 | 13.05013 | 45.16011 | 48.74628 | 51.192 | 11.05702 | 8.370071 | 7.744827 | 23.39301 | 7.215021 | 3.890093 | NA | 7.035948 | 8.621822 | 70.94855 | 39.58144 | 58.892 | 8.089072 | 56.69692 | 6.115825 | 97.61331 | 74.89819 | 6.112624 | 5.342715 | 9.173022 |
| CUST_23890_PI429010886 | N49651 | 0.049588 | 21 | 0 | | | | | | 43.62743 | 27.82378 | 60.85469 | 41.09417 | 39.29501 | 34.93063 | 34.96061 | 21.13597 | 23.58866 | 28.90402 | 33.65406 | 22.89626 | 26.38499 | 24.86526 | 33.2742 | 35.55848 | 27.10661 | 17.49952 | 32.84186 | 18.01173 | 18.86017 | 31.92933 | 31.49741 | 37.59059 | 45.01588 | 29.5179 | 28.03356 |
| CUST_38703_PI429010886 | M29239 | 0.049603 | 21 | 9 | | | | | | 5.40874 | 249.2154 | 68.49231 | 631.6256 | 1233.197 | 243.718 | 248.0682 | 4051.321 | 2612.527 | 180.661 | 3653.581 | 3730.34 | 3090.22 | 210.4862 | 1249.958 | NA | 854.2242 | NA | 133.4937 | 5080.996 | 3074.469 | NA | 246.3468 | 165.5714 | NA | 1245.02 | 1962.83 |
| CUST_51481_PI429010886 | M93684 | 0.049626 | 12 | 3 | | | | | | 743.1041 | 290.7849 | 22.14618 | 987.1593 | 635.9764 | 1409.133 | 948.7418 | 369.8403 | 523.7505 | 7.787579 | 355.0658 | 9.324813 | 513.5475 | 153.8044 | 310.7127 | 348.906 | 1227.15 | 1100.556 | 207.0705 | 117.5843 | 360.7127 | 510.5227 | 231.5694 | 951.6021 | 44.49519 | 513.6722 | NA |
| CUST_39284_PI429010886 | M37208 | 0.049638 | 21 | 9 | 349502991 | O-methyltransferase [Penaeus monodon] | | | 1.03E-30 | 66.99867 | 152.2767 | 77.02903 | 96.73765 | 82.69106 | 120.6877 | 178.2082 | 453.79 | 349.238 | 148.7446 | 353.25 | 370.4089 | 215.5744 | 138.5998 | 185.6237 | 65.60373 | 145.0185 | 77.14278 | 133.9111 | 576.5142 | 198.3971 | 80.61162 | 147.4736 | 166.3173 | 72.14089 | 234.6626 | 134.79 |
| CUST_796_PI429010886 | N7348 | 0.049671 | 21 | 9 | | | | | | 5.861533 | 1187.536 | 561.029 | 1667.846 | 1445.794 | 743.5954 | 853.1958 | 3832.303 | 2777.472 | 998.694 | 3243.287 | 5113.292 | 2489.582 | 690.4518 | 1589.601 | NA | 1042.897 | NA | 543.3625 | 7509.168 | 2247.598 | 17.28408 | 1309.784 | 861.4839 | 4.183645 | 2296.235 | 2050.871 |
| CUST_32970_PI429010886 | M5597 | 0.049694 | 18 | 15 | | | | | | 342.8578 | 400.26 | 355.7822 | 359.4804 | 440.22 | 479.7862 | 301.6859 | 328.4337 | 266.6447 | 356.8925 | 404.1368 | 343.3378 | 283.8866 | 306.5697 | 335.4811 | 438.8255 | 473.6842 | 461.2479 | 411.2669 | 373.3663 | 349.6083 | 439.1396 | 308.8946 | 291.2128 | 395.9375 | 304.7525 | 329.8367 |
| CUST_3176_PI429010886 | N13957 | 0.049697 | 24 | 18 | | | | | | 352.327 | 386.6669 | 404.8823 | 328.3818 | 316.4852 | 310.4051 | 296.9284 | 332.4804 | 347.1114 | 429.7605 | 339.8212 | 330.6123 | 342.3707 | 429.4606 | 315.9925 | 421.5075 | 341.4669 | 349.3181 | 383.2896 | 417.2023 | 361.9091 | 439.8846 | 428.333 | 334.2433 | 472.773 | 328.0426 | 395.8483 |
| CUST_51875_PI429010886 | M95501 | 0.049716 | 21 | 3 | | | | | | 38.51373 | 42.83336 | 43.98243 | 52.15419 | 42.25718 | 52.15768 | 39.37435 | 48.11605 | 23.12297 | 36.85229 | 28.66257 | 52.9063 | 29.32849 | 45.09026 | 41.70879 | 50.30273 | 46.55991 | 44.06771 | 29.96087 | 35.67075 | 43.20136 | 54.3134 | 46.12593 | 53.67582 | 80.34778 | 80.66119 | 60.24307 |
| CUST_47136_PI429010886 | M74923 | 0.049733 | 15 | 12 | | | | | | 233.3626 | 212.1657 | 190.4729 | 157.9269 | 205.2921 | 148.0276 | 392.2104 | 234.017 | 321.374 | 224.9966 | 145.7071 | 301.1632 | 433.8707 | 418.4668 | 336.8758 | 386.3769 | 212.3649 | 171.7601 | 365.3992 | 190.2377 | 184.3916 | 286.0749 | 166.5598 | 227.3812 | 303.0793 | 192.7273 | 474.4772 |
| CUST_39159_PI429010886 | M35402 | 0.049751 | 24 | 0 | | | | | | 122.5998 | 124.2142 | 112.0774 | 119.508 | 95.99909 | 108.9403 | 97.23707 | 100.0169 | 88.78823 | 99.55807 | 107.3346 | 118.04 | 91.04016 | 72.71237 | 106.5981 | 122.5622 | 103.109 | 110.0194 | 94.95262 | 138.1676 | 111.7143 | 106.5844 | 136.1838 | 91.55434 | 118.6343 | 105.4471 | 141.5325 |
| CUST_18369_PI429010886 | M39664 | 0.049761 | 24 | 12 | 332028268 | Neurogenic locus Notch protein [Acromyrmex echir | | | 4.1E-29 | 76.52417 | 65.06017 | 145.8825 | 74.7266 | 55.18575 | 93.98995 | 117.2582 | 113.8871 | 88.72989 | 108.6889 | 91.97543 | 88.19572 | 133.4505 | 156.9005 | 133.8101 | 127.8459 | 174.126 | 77.6254 | 117.9167 | 88.14739 | 75.7673 | 71.63476 | 150.434 | 93.12227 | 99.35803 | 78.77563 | 121.0946 |
| CUST_15058_PI429010886 | N33941 | 0.049782 | 12 | 0 | | | | | | 41.36452 | 34.20754 | 42.54782 | 23.3977 | 31.20174 | 18.29753 | 21.82017 | 50.68803 | 36.67205 | 50.18617 | 31.17772 | 38.12809 | 36.23985 | 52.56971 | 48.82597 | 36.76731 | 26.66065 | 35.56458 | 32.51286 | 31.09756 | 46.41206 | 45.03054 | 42.68003 | 22.85123 | 69.62631 | 33.40939 | 55.29787 |
| CUST_26249_PI429010886 | N53915 | 0.049794 | 18 | 0 | 321476738 | hypothetical protein DAPPUDRAFT_306430 [Daphn | Metabolic process | Other | 3.6E-17 | 43.70451 | 54.64196 | 50.2602 | 24.02357 | 24.0648 | 16.74268 | 26.84868 | 23.79492 | 31.04302 | 42.81164 | 29.5457 | 36.38283 | 39.67691 | 24.316 | 42.07479 | 47.69004 | 28.44009 | 38.82258 | 37.77524 | 44.63765 | 34.07208 | 48.43308 | 28.6271 | 31.1463 | 45.11939 | 22.85699 | 31.59119 |
| CUST_53672_PI429010886 | M102742 | 0.049798 | 24 | 15 | 328786053 | PREDICTED: CLIP-associating protein 1-A isoform 1 [ | | | 6.11E-26 | 43.90259 | 37.07935 | 44.60711 | 38.91585 | 40.83694 | 49.52577 | 46.11982 | 38.67242 | 42.68895 | 44.3588 | 45.66919 | 31.13379 | 46.97546 | 57.3007 | 44.87845 | 61.65538 | 59.16475 | 46.17281 | 67.06743 | 52.42987 | 33.10392 | 51.35337 | 43.95205 | 51.77357 | 57.75281 | 34.8398 | 42.30429 |
| CUST_35675_PI429010886 | M13208 | 0.049826 | 21 | 0 | | | | | | 29.94487 | 35.65852 | 31.56711 | 24.49305 | 28.7018 | 31.79359 | 23.62815 | 25.37501 | 22.60724 | 28.14021 | 27.3183 | 20.21498 | 24.84105 | 21.93936 | 17.3861 | 33.60101 | 38.45723 | 23.4129 | 26.60537 | 17.84197 | 25.79862 | 27.86411 | 32.39121 | 27.84917 | 20.37797 | 13.63459 | 25.59587 |
| CUST_18375_PI429010886 | N39674 | 0.04986 | 18 | 3 | | | | | | 87.14788 | 166.1287 | 80.8031 | 221.873 | 92.13028 | 133.6865 | 71.69416 | 65.76284 | 93.51914 | 84.91262 | 55.73505 | 125.2377 | 91.90557 | 33.11951 | 83.59685 | 91.12979 | 41.27063 | 81.86063 | 46.34675 | 102.3532 | 112.7849 | 113.2396 | 101.2088 | 90.85633 | 92.54562 | 144.9176 | 75.2095 |
| CUST_33062_PI429010886 | M5872 | 0.049861 | 24 | 15 | | | | | | 114.2465 | 122.3027 | 135.0881 | 120.9718 | 119.4557 | 112.5022 | 92.68449 | 91.39106 | 111.0142 | 137.8094 | 100.0527 | 114.0128 | 118.233 | 137.1056 | 136.9989 | 154.463 | 104.2347 | 138.4302 | 94.81726 | 139.9078 | 129.6421 | 147.4496 | 115.9538 | 121.2986 | 141.4128 | 102.3996 | 134.5392 |
| CUST_7164_PI429010886 | N21130 | 0.049869 | 15 | 0 | 443733197 | hypothetical protein CAPTEDRAFT_199760 [Capitell | | | 5.27E-23 | 435.3009 | 442.5257 | 407.1868 | 312.8606 | 402.7742 | 358.7125 | 322.8571 | 286.5693 | 343.3378 | 455.9675 | 415.3047 | 425.1252 | 333.1118 | 362.4143 | 342.746 | 448.0873 | 458.895 | 390.1389 | 412.1707 | 470.2955 | 353.3873 | 341.6507 | 381.8763 | 307.7422 | 436.9493 | 239.3501 | 455.2984 |
| CUST_18535_PI429010886 | N39975 | 0.049877 | 15 | 12 | | | | | | 9.457848 | 8.958591 | 15.1999 | 8.230173 | 6.166942 | 6.274269 | 12.84883 | 13.3861 | 6.221936 | 9.108247 | 4.175552 | 14.07538 | 11.97877 | 23.03149 | 13.13632 | 10.00064 | 9.911889 | 15.23737 | 13.56559 | 5.538092 | 8.491672 | 12.11493 | 9.213601 | NA | 16.11142 | 9.464207 | 13.50266 |
| CUST_45077_PI429010886 | M67926 | 0.049887 | 18 | 3 | | | | | | 9.181579 | 8.381146 | NA | 13.7661 | 10.96162 | 19.73455 | 7.223501 | 12.11037 | NA | 13.11125 | 6.678574 | 8.089072 | NA | 6.311063 | 7.195825 | 20.20434 | 7.912744 | 4.932666 | 4.597228 | 6.922333 | 10.46038 | 10.7321 | NA | 9.034713 | 9.632488 | 8.069484 | NA |
| CUST_32510_PI429010886 | M4306 | 0.049907 | 24 | 0 | | | | | | 53.46776 | 34.73965 | 49.61919 | 41.55626 | 23.39495 | 42.84203 | 39.80101 | 45.28763 | 31.99333 | 20.57577 | 25.01145 | 23.1311 | 22.92066 | 34.67355 | 17.78837 | 171.1038 | 28.9272 | 27.94605 | 37.0175 | 27.47752 | 32.81302 | 29.0776 | 26.49155 | 40.38558 | 37.27794 | 29.44199 | 29.39991 |
| CUST_13937_PI429010886 | N32048 | 0.049907 | 24 | 0 | | | | | | 2464.001 | 2863.818 | 3163.856 | 2564.647 | 2858.942 | 2704.165 | 3031.864 | 2441.116 | 2750.47 | 2595.092 | 2984.905 | 3264.73 | 2416.782 | 3673.827 | 1315.839 | 2842.143 | 2497.013 | 2336.801 | 2243.877 | 1869.455 | 3027.574 | 3024.762 | 3144.897 | 2442.431 | 3775.991 | 3711.262 | 3734.025 |
| CUST_16061_PI429010886 | N35583 | 0.049916 | 24 | 6 | 238625719 | SpAN-like protein [Rimicaris exoculata] | | | 5.64E-10 | 37.02134 | 68.46628 | 40.37133 | 115.0806 | 10.71453 | 42.16986 | 96.61745 | 149.8301 | 78.83809 | 27.84642 | 125.4528 | 117.5373 | 107.5586 | 42.25718 | 46.18431 | 19.40236 | 29.31162 | 15.97633 | 23.565 | 159.8062 | 35.678 | 27.20523 | 105.4471 | 24.1047 | 38.66042 | 49.69905 | 34.91922 |
| CUST_33318_PI429010886 | M6581 | 0.049917 | 15 | 3 | | | | | | 16.62231 | 12.88422 | 18.10692 | 10.66756 | 15.16191 | 29.52396 | 17.06111 | 10.37139 | 14.51263 | 9.282132 | 12.6619 | 42.9046 | 9.72694 | 8.253508 | 10.32671 | 11.79386 | 24.58529 | 23.57785 | 13.07843 | 26.61723 | 27.74359 | 10.03543 | 20.39615 | 19.25722 | 8.73928 | 10.20401 | 18.94364 |
| CUST_28746_PI429010886 | N58542 | 0.049917 | 15 | 6 | | | | | | 31.90127 | 16.32704 | 12.80948 | 30.82801 | 30.38389 | 26.60537 | 29.72938 | 11.61617 | 16.20429 | 28.02151 | 16.6126 | 11.72586 | 15.55244 | 18.54724 | 9.363982 | 39.31065 | 29.95513 | 21.69445 | 36.31145 | 26.81013 | 36.85229 | 31.78038 | 19.30028 | 9.99191 | 25.44661 | 12.52222 | 31.70016 |
| CUST_23234_PI429010886 | N48486 | 0.049924 | 21 | 0 | | | | | | 19.72298 | 40.28418 | 37.38785 | 19.73096 | 27.7391 | 8.610816 | 18.69088 | 23.24689 | 15.26731 | 17.18186 | 14.60716 | 16.44566 | 11.16309 | 43.43615 | 20.79284 | 34.9939 | 27.14824 | 30.3731 | 23.74624 | 7.891053 | 33.54623 | 29.0109 | 39.87566 | 26.56771 | 35.31845 | 18.07527 | 37.40452 |
| CUST_34377_PI429010886 | M9484 | 0.049952 | 12 | 0 | | | | | | 274.2312 | 259.4845 | 261.8083 | 161.711 | 232.1034 | 242.1667 | 235.1413 | 205.4682 | 228.547 | 280.6238 | 212.0939 | 235.4286 | 245.9792 | 271.7176 | 296.5251 | 381.5724 | 242.041 | 273.1122 | 244.0376 | 273.6903 | 231.1968 | 319.9685 | 217.4459 | 219.4848 | 382.1161 | 328.2844 | 319.1396 |
| CUST_9556_PI429010886 | N24831 | 0.049952 | 12 | 0 | | | | | | 241.638 | 260.7556 | 229.1012 | 208.552 | 198.5364 | 198.6003 | 201.1709 | 191.5641 | 226.6782 | 257.2859 | 227.3449 | 261.2742 | 207.5948 | 295.3859 | 262.2092 | 286.0749 | 155.3616 | 212.1159 | 213.0233 | 231.235 | 239.0491 | 199.1036 | 255.3013 | 216.4729 | 224.4715 | 250.5389 | 242.5645 |
| CUST_7157_PI429010886 | N21122 | 0.049959 | 12 | 6 | | | | | | 38.08646 | 62.02406 | 45.55401 | 58.18474 | 31.1045 | 68.46628 | 67.12072 | 50.71879 | 59.54039 | 65.04228 | 51.192 | 86.81547 | 40.02231 | 47.59295 | 48.22578 | 83.96253 | 31.57401 | 51.7068 | 90.83511 | 67.19434 | 94.97923 | 60.83438 | 41.55928 | 57.70597 | 54.5454 | 44.87166 | 51.69686 |
| CUST_9052_PI429010886 | N23991 | 0.049959 | 12 | 0 | | | | | | 171.6223 | 227.2732 | 170.4477 | 166.0856 | 145.794 | 135.6641 | 112.9639 | 118.7528 | 148.8262 | 198.6003 | 130.5455 | 159.6585 | 146.902 | 169.5063 | 175.2604 | 224.3204 | 110.0483 | 179.7938 | 113.5368 | 124.6537 | 174.2228 | 208.9109 | 157.3996 | 141.6752 | 215.3103 | 154.0002 | 184.1431 |
| CUST_29797_PI429010886 | N60572 | 0.049959 | 12 | 0 | | | | | | 9034.665 | 13322.16 | 11347.09 | 11762.42 | 10165.13 | 12253.96 | 6281.928 | 9249.246 | 9020.979 | 12651.8 | 11413.33 | 16638.44 | 10760.71 | 11864.38 | 16328.69 | 13468.67 | 9552.668 | 10348.94 | 9382.703 | 12175.63 | 10387.85 | 12762.11 | 10130.67 | 12673.02 | 15662.95 | 14471.07 | 14710.21 |
| CUST_1499_PI429010886 | N9825 | 0.049959 | 12 | 0 | | | | | | 137.759 | 116.8603 | 131.863 | 145.4981 | 98.98439 | 114.2341 | 85.71149 | 105.2954 | 85.25376 | 115.3063 | 85.51349 | 181.5403 | 170.0428 | 212.0939 | 116.6759 | 115.6002 | 108.2888 | 139.2667 | 118.6357 | 108.9753 | 87.62612 | 206.0341 | 101.0126 | 115.1327 | 84.22055 | 183.9247 | 150.1848 |
| CUST_41776_PI429010886 | M58535 | 0.049965 | 21 | 12 | | | | | | 152.9004 | 183.3645 | 184.8426 | 145.6647 | 132.7741 | 140.777 | 141.2301 | 162.9535 | 127.0971 | 178.0175 | 148.9405 | 181.7276 | 168.1346 | 158.1524 | 167.3467 | 159.0362 | 162.6527 | 143.124 | 118.1739 | 157.6874 | 124.672 | 171.9322 | 139.1812 | 154.0002 | 140.4504 | 108.5891 | 126.9651 |
| CUST_34590_PI429010886 | M10092 | 0.04998 | 24 | 21 | | | | | | 89.80198 | 117.3039 | 94.127 | 54.35965 | 98.73097 | 73.41537 | 70.87083 | 82.91863 | 69.25418 | 98.37238 | 73.00251 | 87.55497 | 68.77033 | 91.24255 | 107.4484 | 101.9474 | 86.95691 | 98.37238 | 79.14778 | 134.1962 | 75.55792 | 142.2988 | 106.8281 | 91.46281 | 142.632 | 104.1327 | 122.3884 |
| CUST_7889_PI429010886 | N22228 | 0.04998 | 24 | 18 | 321477199 | hypothetical protein DAPPUDRAFT_305543 [Daphn | Cellular Process | Cell growth | 1.52E-73 | 120.7762 | 144.4075 | 121.9844 | 99.81229 | 114.2624 | 79.97123 | 95.31428 | 108.6787 | 122.2373 | 137.5651 | 120.1261 | 154.5009 | 126.7767 | 163.0758 | 135.5608 | 165.0799 | 81.66432 | 122.84 | 136.6832 | 174.2228 | 134.4388 | 191.4276 | 145.9962 | 98.39109 | 156.6119 | 120.5667 | 157.3603 |
| CUST_26679_PI429010886 | N54679 | 0.04998 | 24 | 21 | | | | | | 12.0248 | 16.22526 | 17.87442 | 12.2697 | 14.21644 | 14.99759 | 6.219397 | 11.35686 | 13.18932 | 13.72174 | 12.96166 | 15.30179 | 14.82167 | 17.00952 | 8.989758 | 15.37649 | 14.65028 | 8.256397 | 16.23717 | 11.69261 | 9.519535 | 19.34025 | 15.69467 | 16.31911 | 14.21164 | 18.93037 | 16.77252 |
| CUST_7679_PI429010886 | N21906 | 0.049982 | 21 | 18 | | | | | | 714.2963 | 959.2541 | 713.1739 | 459.2637 | 486.9644 | 464.1138 | 399.5497 | 469.825 | 626.6888 | 760.2724 | 503.4817 | 760.0455 | 662.9564 | 659.8695 | 616.2727 | 1084.753 | 380.9707 | 759.5836 | 517.5225 | 1011.26 | 686.896 | 900.4832 | 712.1447 | 516.7166 | 635.0709 | 621.7238 | 621.5625 |
