# Supplementary material for: Analysis of the circadian transcriptome of the Antarctic krill Euphausia superba
Source: Sci Rep. 2019 Sep 25;9:13894. doi: 10.1038/s41598-019-50282-1 (PMC6761102; doi:10.1038/s41598-019-50282-1)
Supplement: Supplementary file 4 — Supplementary Table 3 [file 41598_2019_50282_MOESM4_ESM.pdf]

Analysis of the circadian transcriptome of the Antarctic krill *Euphausia superba*

Alberto Biscontin[1,2,*], Paolo Martini[1], Rodolfo Costa[1], Achim Kramer[2], Bettina Meyer[3,4,5], So Kawaguchi[6], Mathias Teschke[3], Cristiano De Pittà[1,*]

[1]Dipartimento di Biologia, Università degli Studi di Padova, Padova, Italy

[2]Laboratory of Chronobiology, Charité Universitätsmedizin Berlin, Berlin, Germany

[3]Section Polar Biological Oceanography, Alfred Wegener Institute Helmholtz Centre for Polar and Marine Research, Bremerhaven, Germany

[4]Institute for Chemistry and Biology of the Marine Environment, Carl von Ossietzky University of Oldenburg, Oldenburg, Germany

[5]Helmholtz Institute for Functional Marine Biodiversity (HIFMB) at the University of Oldenburg, 26111 Oldenburg, Germany

[6]Department of Environment and Heritage, Australian Antarctic Division, Kingston, Tasmania, Australia

[*]Corresponding authors:

Cristiano De Pittà, Dipartimento di Biologia, Università degli Studi di Padova, via U. Bassi 58/B 35131 Padova, Italy; Phone: +39-049-8276210; Fax: +39-049-8276209; e-mail address: cristiano.depitta@unipd.it

Alberto Biscontin, Dipartimento di Biologia, Università degli Studi di Padova, via U. Bassi 58/B 35131 Padova, Italy; Phone: +39-049-8276228; Fax: +39-049-8276209; e-mail address: alberto.biscontin@unipd.it

Supplementary Table 3. List of 1,564 circadian genes with a sinusoidal expression pattern both in LD and DD.

Expression levels of genes, identified by RAIN (adjusted p-value < 0.05) throughout the 24-hour cycle both in LD and DD, are shown. [a] Probe ID: ID of probe sequence in "Krill 2.0" Agilent microarray platform; [b]ID: accession number of krill master transcriptome [23]; [c]EBI ID: accession number of EBI; [d]Annotation = description of the gene; [e]e-value: score of GO functional annotation with BLAST2GO [f]GO term: gene ontology biological process; [g]GO child term: most fitting gene ontology child term to further refine the functional annotation; [h]p-value LD: RAIN Benjamini-Hochberg adjusted p-value in LD; [i]Period LD: period of oscillation in LD; [j]Phase LD: maximum peak of expression throughout the 24 hours cycle in LD; [k]p-value DD: RAIN Benjamini-Hochberg adjusted p-value in DD; [l]Period DD: period of oscillation in DD; [m]Phase DD: maximum peak of expression throughout the 24 hours cycle in DD; [n-an]mRNA expression levels in LD; [ao-bo]mRNA expression levels in DD.

Probe ID	ID	EBI ID	Annotation	E-value	GO categor	Go term	p-value LD	Period LD	Phase LD	p-value DD	Period DD	Phase DD	LD / DD expression levels (0–24 h)
CUST_5203_PI429010886	N18394				Signal tran	Other	2.474E-11	24	18	1.904E-05	24	21	[illegible]
CUST_39293_PI429010886	M37357						7.702E-08	18	9	9.606E-06	21	18	[illegible]
CUST_3609_PI429010886	N14873						8.615E-08	12	9	0.0357755	15	9	[illegible]
CUST_33205_PI429010886	M6266						4.689E-07	12	3	0.0011619	21	3	[illegible]
CUST_26818_PI429010886	N54935						1.012E-06	21	12	0.0311353	12	0	[illegible]
CUST_31297_PI429010886	M1163	156543561	PREDICTED: ubiquitin-conjugating	1.28E-60	Cellular Pr	Cell cycle	1.283E-06	24	21	0.0077325	12	0	[illegible]
CUST_3012_PI429010886	N13602						1.48E-06	12	9	0.0108866	15	9	[illegible]
CUST_49535_PI429010886	M86007						1.702E-06	24	0	0.0272382	15	6	[illegible]
CUST_2826_PI429010886	N13190						1.877E-06	24	21	0.0011047	15	0	[illegible]
CUST_12202_PI429010886	N29097	307177147	Acetyl-CoA carboxylase [Camponc	6.5E-21			2.017E-06	18	12	0.003202	21	9	[illegible]
CUST_9969_PI429010886	N25485				Signal tran	Other	2.138E-06	21	0	0.0014287	15	9	[illegible]
CUST_6952_PI429010886	N20825						2.149E-06	24	21	0.0131645	15	0	[illegible]
CUST_8587_PI429010886	N23265	291230131	PREDICTED: hypothetical protein |	2.81E-09	Metabolic	Other	2.262E-06	24	21	0.0050734	15	0	[illegible]
CUST_43975_PI429010886	M64509						3.754E-06	24	9	0.000323	15	3	[illegible]
CUST_15158_PI429010886	N34099						4.23E-06	24	18	0.0320137	18	9	[illegible]
CUST_38028_PI429010886	M22894						4.746E-06	12	9	0.0042147	15	12	[illegible]
CUST_54935_PI429010886	M108205				Metabolic	Carbohydr	5.559E-06	24	21	0.0176913	15	0	[illegible]
CUST_48438_PI429010886	M80321	197940771	Euphausia superba mitochondrior	2.34E-57			5.559E-06	24	21	0.0046874	15	0	[illegible]
CUST_14414_PI429010886	N32843						5.634E-06	24	21	0.0337453	15	0	[illegible]
CUST_16832_PI429010886	N36935						5.862E-06	24	15	0.0154703	24	0	[illegible]
CUST_51711_PI429010886	M94674						6.336E-06	12	0	0.0223736	15	6	[illegible]
CUST_7015_PI429010886	N20917						6.689E-06	24	18	0.0228863	21	6	[illegible]
CUST_15933_PI429010886	N35371						7.383E-06	18	3	0.0030172	21	3	[illegible]
CUST_7688_PI429010886	N21924						7.589E-06	24	18	0.0097641	24	21	[illegible]
CUST_33676_PI429010886	M7582	338224394	ADP-ribosylation factor GTPase-ac	1.45E-13			7.797E-06	12	0	0.0367341	21	3	[illegible]
CUST_11408_PI429010886	N27759						9.064E-06	24	18	0.0239216	12	9	[illegible]
CUST_32981_PI429010886	M5626						9.3E-06	24	21	0.0095968	15	0	[illegible]
CUST_42252_PI429010886	M59804						9.3E-06	24	21	0.0439951	15	0	[illegible]
CUST_37962_PI429010886	M22486						1.027E-05	15	9	0.0054264	15	12	[illegible]
CUST_56944_PI429010886	M114968						1.145E-05	18	9	0.0361175	21	3	[illegible]
CUST_41879_PI429010886	M58780						1.21E-05	24	12	0.0001631	24	12	[illegible]
CUST_31663_PI429010886	M2104						1.293E-05	24	21	0.0136531	15	0	[illegible]
CUST_38075_PI429010886	M23222	321455420	hypothetical protein DAPPUDRAF	6.7E-81	Metabolic	Carbohydr	1.294E-05	18	3	0.0261427	18	0	[illegible]
CUST_49139_PI429010886	M84314						1.311E-05	24	21	0.0026438	15	0	[illegible]
CUST_38504_PI429010886	M27503						1.355E-05	15	9	0.0320396	21	3	[illegible]
CUST_44183_PI429010886	M65195						1.441E-05	24	21	0.0352791	15	0	[illegible]
CUST_41715_PI429010886	M58374						1.46E-05	21	15	0.0048874	21	6	[illegible]
CUST_4608_PI429010886	N17124						1.611E-05	24	6	0.0011048	21	15	[illegible]
CUST_32899_PI429010886	M5422	13624645	arrestin [Libelloides macaronius]	1.13E-95	Signal tran	Other	1.656E-05	12	9	0.030451	24	15	[illegible]
CUST_11117_PI429010886	N27293						1.671E-05	24	0	0.024394	24	15	[illegible]
CUST_47182_PI429010886	M75089						1.86E-05	15	6	0.0322865	12	9	[illegible]
CUST_37979_PI429010886	M22563						2.005E-05	24	21	0.0258304	15	0	[illegible]
CUST_48484_PI429010886	M80570						2.091E-05	24	21	0.0025793	15	0	[illegible]
CUST_13764_PI429010886	N31731						2.165E-05	24	9	0.0048626	12	3	[illegible]
CUST_1638_PI429010886	N10306						2.205E-05	24	21	0.0257118	15	0	[illegible]
CUST_52479_PI429010886	M97580						2.291E-05	24	3	0.0210728	18	6	[illegible]
CUST_42396_PI429010886	M60180						2.304E-05	12	3	0.0039756	24	12	[illegible]
CUST_955_PI429010886	N7968	291241039	PREDICTED: Caenorhabditis Epoxi	4.8E-56	Metabolic	Other	2.31E-05	21	18	0.048721	24	9	[illegible]
CUST_57300_PI429010886	E4642	548856974	hypothetical protein AMTR_s0003	9.02E-08	Cellular co	Cytoskelet	2.389E-05	24	9	0.0112152	21	15	[illegible]
CUST_12694_PI429010886	N29883						2.401E-05	24	18	0.0012569	12	9	[illegible]
CUST_460_PI429010886	N5425						2.506E-05	12	9	0.0024048	15	12	[illegible]
CUST_2275_PI429010886	N12009						2.537E-05	21	6	0.0201113	24	6	[illegible]
CUST_38411_PI429010886	M26337						2.592E-05	24	15	0.0166886	18	9	[illegible]
CUST_6937_PI429010886	N20804						2.774E-05	24	18	0.0176269	21	9	[illegible]
CUST_31621_PI429010886	M1985						3.169E-05	24	18	0.0394378	12	9	[illegible]
CUST_34193_PI429010886	M9014						3.425E-05	24	21	0.0329783	15	0	[illegible]
CUST_31834_PI429010886	M2521						3.637E-05	24	15	0.0295995	15	12	[illegible]
CUST_14711_PI429010886	N33335						4.155E-05	24	3	0.0175514	18	6	[illegible]
CUST_542_PI429010886	N5908						4.22E-05	15	9	0.0058334	15	12	[illegible]
CUST_543_PI429010886	N5909						4.272E-05	15	9	0.0214008	15	9	[illegible]
CUST_2996_PI429010886	N13567	329565704	ribosomal protein L24 [Azumapec	4.27E-40	Protein me	Ribosome	4.307E-05	18	0	0.0047977	15	9	[illegible]
CUST_33058_PI429010886	M5855						5.026E-05	24	18	0.0011784	24	0	[illegible]
CUST_19676_PI429010886	N41996						5.034E-05	24	18	0.0001259	18	12	[illegible]
CUST_29745_PI429010886	N60463						5.036E-05	21	0	0.0429941	24	18	[illegible]
CUST_1672_PI429010886	N10407						5.094E-05	18	3	0.00671	24	0	[illegible]
CUST_29232_PI429010886	N59440						5.179E-05	24	18	0.0081064	24	18	[illegible]
CUST_31264_PI429010886	M1104						5.196E-05	24	21	0.0223736	15	0	[illegible]
CUST_39504_PI429010886	M40586						5.301E-05	12	9	0.0209168	15	12	[illegible]
CUST_36411_PI429010886	M15834						5.379E-05	24	6	0.0025656	24	15	[illegible]
CUST_38986_PI429010886	M33013						5.426E-05	24	12	0.0236455	21	[illegible]	[illegible]
CUST_38378_PI429010886	M26020						5.432E-05	12	9	0.0132454	15	[illegible]	[illegible]
CUST_34331_PI429010886	M9345	321468460	hypothetical protein DAPPUDRAF	8E-83	Protein me	Cellular pr	5.463E-05	24	21	0.0073884	24	[illegible]	[illegible]
CUST_56241_PI429010886	M113241						5.54E-05	24	12	0.0188615	12	[illegible]	[illegible]
CUST_38616_PI429010886	M28570						5.628E-05	12	9	0.0081726	15	[illegible]	[illegible]
CUST_33959_PI429010886	M8365						5.886E-05	15	9	0.0062464	15	[illegible]	[illegible]
CUST_54572_PI429010886	M106601						5.892E-05	24	0	0.0434665	12	[illegible]	[illegible]
CUST_16726_PI429010886	N36759						5.955E-05	12	9	0.0049698	15	[illegible]	[illegible]
CUST_13367_PI429010886	N31065	321478280	hypothetical protein DAPPUDRAF	1.5E-24	Transport	Ion transp	5.978E-05	18	15	0.0100708	15	[illegible]	[illegible]
CUST_30765_PI429010886	N62607						6.065E-05	18	3	4.132E-05	18	[illegible]	[illegible]
CUST_55495_PI429010886	M110357						6.182E-05	12	9	0.0436938	18	[illegible]	[illegible]
CUST_20422_PI429010886	N43410						6.236E-05	18	9	0.0092414	15	[illegible]	[illegible]
CUST_30543_PI429010886	N62093	442630141	masquerade, isoform C [Drosophi	6.41E-14	Protein me	Proteolysis	7.142E-05	12	3	0.0164827	18	[illegible]	[illegible]
CUST_3245_PI429010886	N14095						7.501E-05	24	21	0.0401695	15	[illegible]	[illegible]
CUST_37912_PI429010886	M22216						7.693E-05	12	9	0.0055621	15	[illegible]	[illegible]
CUST_14514_PI429010886	N33003						7.693E-05	12	9	0.0252849	15	[illegible]	[illegible]
CUST_16237_PI429010886	N35893						8.073E-05	12	9	0.0209168	15	[illegible]	[illegible]
CUST_43503_PI429010886	M63221						8.086E-05	12	0	0.0471868	21	[illegible]	[illegible]
CUST_6772_PI429010886	N20580	260790163	hypothetical protein BRAFLDRAFT	5.43E-40			8.162E-05	15	12	0.034919	24	[illegible]	[illegible]
CUST_39172_PI429010886	M35591						8.168E-05	12	9	0.0342796	15	[illegible]	[illegible]
CUST_33465_PI429010886	M6960						8.168E-05	12	9	0.0048745	15	[illegible]	[illegible]
CUST_39494_PI429010886	M40390						8.698E-05	24	0	0.0483576	12	[illegible]	[illegible]
CUST_11311_PI429010886	N27605						8.765E-05	24	3	0.0095168	15	[illegible]	[illegible]
CUST_36520_PI429010886	M16117						8.802E-05	15	9	0.0149537	15	[illegible]	[illegible]
CUST_43079_PI429010886	M62019						8.962E-05	21	6	0.0313079	12	[illegible]	[illegible]
CUST_7679_PI429010886	N21906						9.106E-05	24	15	0.0499818	21	[illegible]	[illegible]
CUST_7485_PI429010886	N21614	321463650	hypothetical protein DAPPUDRAF	5.37E-59			9.533E-05	24	15	0.0190314	24	[illegible]	[illegible]
CUST_8492_PI429010886	N23121	154416763	viral A-type inclusion protein [Tric	5.32E-11			9.59E-05	24	15	0.0164023	24	[illegible]	[illegible]
CUST_26697_PI429010886	N54721						9.617E-05	18	0	0.011516	18	[illegible]	[illegible]
CUST_29585_PI429010886	N60149						0.0001038	12	9	3.259E-06	15	[illegible]	[illegible]
CUST_42534_PI429010886	M60524	321457450	hypothetical protein DAPPUDRAF	1.45E-29			0.0001043	21	15	0.0224044	21	[illegible]	[illegible]
CUST_35902_PI429010886	M13860						0.00011	24	18	0.0181747	18	[illegible]	[illegible]
CUST_43228_PI429010886	M62469	512883568	PREDICTED: putative ferric-chelat	3.05E-08			0.0001106	12	9	8.38E-05	15	[illegible]	[illegible]
CUST_7287_PI429010886	N21321						0.0001116	24	15	0.0055786	21	[illegible]	[illegible]
CUST_84_PI429010886	N558						0.000112	12	9	0.0161057	15	[illegible]	[illegible]
CUST_461_PI429010886	N5429						0.0001134	12	9	0.0013054	15	[illegible]	[illegible]
CUST_38087_PI429010886	M23281						0.0001152	15	9	0.0049761	15	12	[illegible]
CUST_32393_PI429010886	M3994	321465430	hypothetical protein DAPPUDRAF	2.59E-32			0.0001161	12	9	0.0068603	12	9	[illegible]
CUST_1271_PI429010886	N9090	307169370	Chorion peroxidase [Camponotus	2.5E-104	Response t	Response t	0.0001174	24	6	0.0012707	12	9	[illegible]
CUST_48653_PI429010886	M81489						0.0001233	24	15	0.0200342	21	18	[illegible]
CUST_44563_PI429010886	M66327						0.0001283	15	6	0.0270063	15	12	[illegible]
CUST_19724_PI429010886	N42071						0.0001284	15	9	0.0223343	24	21	[illegible]
CUST_43840_PI429010886	M64131	242006829	conserved hypothetical protein [P	9.9E-07	Transport	Transmem	0.0001304	24	9	0.0240683	18	6	[illegible]
CUST_3900_PI429010886	N15523	443721341	hypothetical protein CAPTEDRAFT	1.06E-13			0.000135	24	0	0.0022451	24	15	[illegible]
CUST_3800_PI429010886	N15301						0.0001356	24	21	0.044252	24	6	[illegible]
CUST_1994_PI429010886	N11264						0.0001426	24	15	0.0045125	18	0	[illegible]
CUST_2063_PI429010886	N11469						0.0001456	24	21	0.0096443	24	9	[illegible]
CUST_22260_PI429010886	N46708						0.0001499	15	9	0.0043175	15	12	[illegible]
CUST_38971_PI429010886	M32801						0.0001557	15	9	0.0057655	15	12	[illegible]
CUST_47865_PI429010886	M77925						0.0001561	21	15	0.0201914	15	3	[illegible]
CUST_41927_PI429010886	M58897						0.0001619	24	18	0.0070728	18	9	[illegible]
CUST_40024_PI429010886	M48768						0.0001633	21	9	0.0351644	24	18	[illegible]
CUST_38112_PI429010886	M23418						0.0001655	12	9	0.0298434	15	12	[illegible]
CUST_26083_PI429010886	N53628						0.0001663	24	21	0.0358703	24	15	[illegible]
CUST_38749_PI429010886	M29975						0.0001694	12	9	0.0387067	15	12	[illegible]
CUST_143_PI429010886	N1541						0.0001713	12	9	0.0284785	12	0	[illegible]
CUST_51355_PI429010886	M93237						0.0001729	24	21	0.0035518	15	0	[illegible]
CUST_23503_PI429010886	N48942						0.000174	15	12	0.0493967	21	0	[illegible]
CUST_3251_PI429010886	N14108						0.0001758	15	0	0.0057574	15	0	[illegible]
CUST_5455_PI429010886	N18727	321472375	hypothetical protein DAPPUDRAF	6.11E-19	Signal tran	Cell-cell sig	0.0001773	24	3	0.0344944	21	0	[illegible]
CUST_12314_PI429010886	N29276	380029118	PREDICTED: uncharacterized prot	1.3E-30			0.0001773	24	3	0.0352537	21	6	[illegible]
CUST_37467_PI429010886	M19131	321455420	hypothetical protein DAPPUDRAF	9.82E-92	Metabolic	Carbohydr	0.0001788	24	21	0.0100808	15	0	[illegible]
CUST_8968_PI429010886	N23867						0.0001799	24	12	0.0001116	24	15	[illegible]
CUST_16337_PI429010886	N36065						0.000195	15	0	0.04553	12	0	[illegible]
CUST_11207_PI429010886	N27444						0.0001974	18	6	0.0080301	15	9	[illegible]
CUST_4345_PI429010886	N16556						0.0001997	15	6	0.0063553	15	12	[illegible]
CUST_38650_PI429010886	M28840						0.0002226	24	12	0.0112484	15	3	[illegible]
CUST_12012_PI429010886	N28773	163868840	autotransporter [Bartonella triboc	6.12E-07			0.0002276	15	3	0.0327298	15	3	[illegible]
CUST_40002_PI429010886	M48221						0.0002284	12	9	0.0056299	15	12	[illegible]
CUST_21563_PI429010886	N45439						0.0002287	21	0	0.0283134	18	15	[illegible]
CUST_40907_PI429010886	M56361	443696893	hypothetical protein CAPTEDRAFT	1.1E-09			0.0002318	21	18	0.0128185	12	0	[illegible]
CUST_2903_PI429010886	N13357	321455420	hypothetical protein DAPPUDRAF	4.73E-96	Metabolic	Carbohydr	0.000233	24	21	0.0104024	18	0	[illegible]
CUST_54359_PI429010886	M105717						0.0002338	24	3	0.0391376	15	6	[illegible]
CUST_18019_PI429010886	N39007						0.0002353	24	3	0.0243488	15	3	[illegible]
CUST_7796_PI429010886	N22088						0.0002381	18	15	0.0426097	21	6	[illegible]
CUST_17764_PI429010886	N38551	325303532	TPA_inf: hypothetical secreted pr	1.22E-12			0.0002394	24	18	0.0139862	24	12	[illegible]
CUST_51751_PI429010886	M94865						0.0002403	15	9	0.0042147	15	12	[illegible]
CUST_52473_PI429010886	M97567						0.0002422	12	9	0.0042661	15	12	[illegible]
CUST_20017_PI429010886	N42655						0.0002457	24	21	0.0136185	15	0	[illegible]
CUST_3735_PI429010886	N15159						0.0002477	12	3	0.0425605	15	6	[illegible]
CUST_36230_PI429010886	M15101						0.0002544	24	15	0.0354894	15	12	[illegible]
CUST_46341_PI429010886	M72300						0.0002544	24	21	0.0015383	24	12	[illegible]
CUST_49321_PI429010886	M85184	546675121	hypothetical protein D910_03776	1.26E-09			0.0002639	24	15	0.0255281	12	0	[illegible]
CUST_37011_PI429010886	M17535						0.0002675	24	21	0.0004646	15	9	[illegible]
CUST_49532_PI429010886	M85988						0.0002748	24	18	0.006438	15	9	[illegible]
CUST_19237_PI429010886	N41228						0.0002892	24	18	0.0367402	21	15	[illegible]
CUST_47352_PI429010886	M75744	328899629	ribosomal protein L36 [Procamba	1.7E-14	Protein me	Ribosome	0.0002964	24	15	0.0322865	12	0	[illegible]
CUST_24820_PI429010886	N51300	383856504	PREDICTED: actin-related protein	4.57E-52	Cellular co	Cytoskelet	0.0003022	21	0	0.0054173	21	6	[illegible]
CUST_2276_PI429010886	N12010						0.0003197	18	6	0.0230393	24	3	[illegible]
CUST_14380_PI429010886	N32785						0.0003233	24	18	0.0313079	12	9	[illegible]
CUST_83_PI429010886	N536						0.0003241	12	9	0.0013531	15	12	[illegible]
CUST_4822_PI429010886	N17554						0.0003274	15	6	3.151E-05	15	9	[illegible]
CUST_38016_PI429010886	M22785						0.000329	24	15	0.002176	18	9	[illegible]
CUST_15762_PI429010886	N35097						0.0003367	24	3	0.0326354	12	3	[illegible]
CUST_45180_PI429010886	M68247						0.0003412	18	9	0.0018062	24	12	[illegible]
CUST_39951_PI429010886	M47399						0.0003461	24	12	0.0439442	12	0	[illegible]

Probe ID	ID	EBI ID	Annotation	E-value	GO category	Go term	p-value LD	Period LD	Phase LD	p-value DD	Period DD	Phase DD	LD 0	LD 0–24 / DD 0–24 (remaining time points)
CUST_35451_PI429010886	M12607	295639972	chitinase 2 [Penaeus monodon]	5.4E-143	Metabolic	Carbohydr	0.000353	21	15	0.0124989	21	18	26.77594	[illegible]
CUST_37800_PI429010886	M21652						0.0003551	21	3	0.0454818	12	3	528.6354	[illegible]
CUST_39480_PI429010886	M39996						0.0003552	12	9	0.0075636	15	12	3331.869	[illegible]
CUST_2191_PI429010886	N11830				Protein me	Cellular pr	0.0003598	24	21	0.0001667	15	0	818.3565	[illegible]
CUST_50165_PI429010886	M88895						0.0003635	24	12	0.0236528	12	3	48.43357	[illegible]
CUST_10494_PI429010886	N26323						0.0003704	18	3	0.0480034	18	6	3039.361	[illegible]
CUST_41116_PI429010886	M56909	546685137	hypothetical protein D910_11939	4.37E-83			0.000386	24	21	0.0006698	15	9	356.7674	[illegible]
CUST_5731_PI429010886	N19116						0.0004016	24	15	0.0469089	24	9	96.36643	[illegible]
CUST_17339_PI429010886	N37797	270012294	hypothetical protein TcasGA2_TC(	2.36E-08			0.0004065	24	21	0.0010577	24	21	43.16883	[illegible]
CUST_42261_PI429010886	M59827						0.0004091	21	15	0.0166368	21	18	82.52242	[illegible]
CUST_22714_PI429010886	N47520						0.0004094	21	9	0.0299682	24	3	49.0091	[illegible]
CUST_51499_PI429010886	M93736						0.0004103	21	0	0.0090387	24	0	125.3559	[illegible]
CUST_36075_PI429010886	M14547						0.0004107	24	0	0.0181856	24	0	152.9126	[illegible]
CUST_34156_PI429010886	M8906						0.0004205	21	15	0.0304198	21	18	911.7924	[illegible]
CUST_462_PI429010886	N5434						0.0004294	12	9	0.0094008	15	12	427.8043	[illegible]
CUST_10300_PI429010886	N26026	322791130	hypothetical protein SINV_06374	2.33E-28	Metabolic	Oxidation-	0.0004299	21	15	0.0221832	24	6	32.73765	[illegible]
CUST_1191_PI429010886	N8789	187610687	small nuclear ribonucleoprotein p	3.39E-20			0.0004315	21	15	0.0006698	15	9	26.99962	[illegible]
CUST_31857_PI429010886	M2584						0.0004391	21	15	0.0309741	24	21	265.5849	[illegible]
CUST_53940_PI429010886	M103791						0.0004429	21	0	0.0070977	24	3	64.42993	[illegible]
CUST_16803_PI429010886	N36896						0.0004454	24	0	0.0010635	24	0	263.6152	[illegible]
CUST_51950_PI429010886	M95795						0.0004484	24	0	0.0079196	15	12	25.02228	[illegible]
CUST_7844_PI429010886	N22167	291237036	PREDICTED: Saccoglossus kowalev	1.49E-73	Cellular Pr	Cell cycle	0.0004485	24	15	0.033473	15	12	314.367	[illegible]
CUST_38120_PI429010886	M23440						0.0004856	24	12	0.0439442	12	0	272.3474	[illegible]
CUST_41426_PI429010886	M57702						0.0004905	18	0	0.0396117	18	15	25.37685	[illegible]
CUST_3919_PI429010886	N15567						0.0004925	15	0	0.0066175	18	0	92.36671	[illegible]
CUST_17818_PI429010886	N38644	321469866	hypothetical protein DAPPUDRAF	2.65E-07			0.0004925	15	6	0.0029686	15	9	770.3832	[illegible]
CUST_44267_PI429010886	M65450						0.0004958	21	0	0.0087013	24	15	27.11917	[illegible]
CUST_31718_PI429010886	M2239	291239931	PREDICTED: GK11735-like [Saccog	1.65E-13			0.0004981	12	6	0.021644	15	9	216.5705	[illegible]
CUST_14271_PI429010886	N32608						0.000499	21	15	0.0424767	21	0	14.94152	[illegible]
CUST_41563_PI429010886	M58041	260833120	hypothetical protein BRAFLDRAFT	2.86E-24			0.0005022	24	21	0.0029017	15	9	26.18151	[illegible]
CUST_21173_PI429010886	N44764						0.0005037	21	0	0.0347097	18	15	42.62368	[illegible]
CUST_13571_PI429010886	N31397						0.0005052	24	15	0.0001183	24	15	104.4781	[illegible]
CUST_44115_PI429010886	M64986						0.0005066	24	18	0.0022606	24	21	NA	[illegible]
CUST_31816_PI429010886	M2478						0.0005116	21	3	0.0341874	24	0	115.5105	[illegible]
CUST_32612_PI429010886	M4573	321455420	hypothetical protein DAPPUDRAF	1.42E-86	Metabolic	Carbohydr	0.0005154	24	21	0.0269362	15	0	1365.442	[illegible]
CUST_33321_PI429010886	M6590	156550289	PREDICTED: ileal sodium/bile acid	9.59E-33	Transport	Ion transp	0.0005173	18	3	0.0066281	21	3	30.89131	[illegible]
CUST_31636_PI429010886	M2029	321463221	putative glycogen synthase [Daph	2.54E-80	Metabolic	Generation	0.0005193	12	9	0.035118	18	9	125.4485	[illegible]
CUST_42756_PI429010886	M61169						0.0005196	12	3	0.0153474	18	6	18.48914	[illegible]
CUST_52593_PI429010886	M98110						0.0005286	24	3	0.0071489	21	0	26.39512	[illegible]
CUST_36376_PI429010886	M15752						0.0005352	24	21	0.0029328	15	12	132.0358	[illegible]
CUST_9016_PI429010886	N23938						0.0005519	24	9	0.0049292	21	18	24.65528	[illegible]
CUST_11340_PI429010886	N27650						0.0005748	24	18	0.0026061	12	0	543.5625	[illegible]
CUST_31189_PI429010886	M931	60652230	protease [Homarus americanus]	1.11E-24	Protein me	Proteolysis	0.0005791	18	15	0.0212319	21	15	1085.159	[illegible]
CUST_41906_PI429010886	M58852						0.000581	24	18	0.001139	15	9	75.81657	[illegible]
CUST_39982_PI429010886	M47801						0.0005853	12	9	0.01983	21	15	15.20248	[illegible]
CUST_11019_PI429010886	N27135	299782457	kinesin 2B [Nasonia vitripennis] >g	4.87E-30	Cellular co	Cytoskelet	0.0005931	21	0	0.011296	18	0	29.55798	[illegible]
CUST_44967_PI429010886	M67556						0.0005934	12	9	0.0016161	18	0	23.63327	[illegible]
CUST_34980_PI429010886	M11229	302887532	hypothetical protein NECHADRAF	2.52E-09			0.0005983	24	18	0.041053	21	9	4.285677	[illegible]
CUST_50152_PI429010886	M88841	512816354	PREDICTED: protocadherin Fat 1 is	6.87E-16	Cellular Pr	Cell adhesi	0.0006131	21	6	0.0065906	21	0	17.18131	[illegible]
CUST_7145_PI429010886	N21105						0.0006136	21	15	0.0231075	18	0	903.1163	[illegible]
CUST_716_PI429010886	N6899						0.0006181	24	0	0.0278213	21	12	73.95531	[illegible]
CUST_57285_PI429010886	E4142						0.0006223	12	9	0.0201849	15	9	6897.446	[illegible]
CUST_33314_PI429010886	M6573						0.0006258	15	9	0.0013679	12	9	13.66845	[illegible]
CUST_27024_PI429010886	N55314						0.0006282	24	21	0.0139211	15	9	54.1566	[illegible]
CUST_44867_PI429010886	M67257	220172363	putative articulin p60 [Rimicaris e:	8.12E-12			0.0006446	12	9	0.0314565	15	12	16622.66	[illegible]
CUST_46502_PI429010886	M72842	307208663	ER degradation-enhancing alpha-r	7.4E-15			0.0006594	12	9	0.0295276	12	0	24.5939	[illegible]
CUST_40731_PI429010886	M55957						0.0006668	12	9	0.0015337	12	0	113.1214	[illegible]
CUST_52088_PI429010886	M96376						0.0006742	12	9	0.0046979	24	18	24.93768	[illegible]
CUST_19688_PI429010886	N42013						0.0006757	24	21	0.0220883	18	15	361.0483	[illegible]
CUST_52730_PI429010886	M98691						0.0006766	15	9	0.018997	15	9	422.3982	[illegible]
CUST_2272_PI429010886	N12003	443697440	hypothetical protein CAPTEDRAFT	1.75E-23	Transport	Protein tra	0.0006861	24	15	0.0250999	15	12	36.615	[illegible]
CUST_35309_PI429010886	M12199						0.0006945	24	3	0.010455	12	3	39.25338	[illegible]
CUST_44242_PI429010886	M65366	321469964	hypothetical protein DAPPUDRAF	2.15E-33			0.0006945	24	21	0.0358927	15	12	41.84178	[illegible]
CUST_31624_PI429010886	M1994	380026333	PREDICTED: uncharacterized prot	1.75E-35			0.0007039	24	21	0.0129444	15	12	32.25164	[illegible]
CUST_53417_PI429010886	M101615	62126070	C-type lectin protein [Fenneropen	2.84E-09			0.0007133	18	0	0.0267262	21	18	1207.663	[illegible]
CUST_3246_PI429010886	N14096						0.0007133	24	21	0.0260179	15	0	6559.55	[illegible]
CUST_29188_PI429010886	N59357						0.0007227	24	21	0.0092935	24	21	103.3894	[illegible]
CUST_48872_PI429010886	M82628						0.0007273	15	0	0.0029458	21	0	157.2786	[illegible]
CUST_49980_PI429010886	M88081						0.000747	21	0	0.0247858	15	12	28.57269	[illegible]
CUST_13544_PI429010886	N31361						0.0007504	12	0	0.0327528	21	0	27.07259	[illegible]
CUST_9711_PI429010886	N25080	357619676	hypothetical protein KGM_21935	5.3E-16			0.0007552	21	0	0.0015898	24	15	15.81757	[illegible]
CUST_37133_PI429010886	M17951	345484846	PREDICTED: glycogen debranching	2.19E-65	Metabolic	Generation	0.0007602	24	21	0.0224021	18	15	10.11558	[illegible]
CUST_37616_PI429010886	M19862						0.0007687	24	15	0.0213247	21	9	80.82353	[illegible]
CUST_56331_PI429010886	M113518	546682942	hypothetical protein D910_10129	6.83E-08			0.0007696	24	6	0.0166851	18	3	329.7868	[illegible]
CUST_39701_PI429010886	M43090						0.000772	24	15	0.0011033	18	12	5157.305	[illegible]
CUST_4538_PI429010886	N16987						0.0007871	12	3	0.0012961	18	3	29.53009	[illegible]
CUST_45511_PI429010886	M69334	321473002	hypothetical protein DAPPUDRAF	2.84E-14			0.0007919	15	6	0.0447219	18	15	7.114823	[illegible]
CUST_16536_PI429010886	N36420						0.0007958	18	0	0.0462077	15	12	105.5064	[illegible]
CUST_37582_PI429010886	M19596						0.0008005	24	12	0.0185002	15	3	28.0791	[illegible]
CUST_29077_PI429010886	N59152						0.0008007	24	0	0.0091647	24	0	43.67283	[illegible]
CUST_21887_PI429010886	N46043						0.0008019	12	3	0.0005806	18	3	20.11731	[illegible]
CUST_12501_PI429010886	N29563						0.0008039	21	15	0.0069401	12	3	102.222	[illegible]
CUST_6331_PI429010886	N19960						0.0008071	24	21	0.0349621	24	15	935.4596	[illegible]
CUST_12475_PI429010886	N29524						0.0008071	24	21	0.0007439	12	0	1119.38	[illegible]
CUST_8548_PI429010886	N23206						0.000811	12	3	0.0369415	12	3	13.33462	[illegible]
CUST_38872_PI429010886	M31412	195447950	GK25804 [Drosophila willistoni] >g	5.01E-07			0.0008121	12	9	0.0042661	15	12	580.6289	[illegible]
CUST_12066_PI429010886	N28871						0.0008191	24	21	0.0006546	15	0	621.285	[illegible]
CUST_36322_PI429010886	M15588						0.0008223	12	9	0.0033474	24	18	28.47679	[illegible]
CUST_25344_PI429010886	N52236						0.0008294	15	12	1.687E-05	12	0	20.38623	[illegible]
CUST_45500_PI429010886	M69310						0.0008435	18	0	0.0138529	24	3	50.75197	[illegible]
CUST_49633_PI429010886	M86465	291235783	PREDICTED: galactose-3-O-sulfotr	8.07E-13	Metabolic	Lipid meta	0.0008474	15	0	0.0179656	15	3	57.36964	[illegible]
CUST_57336_PI429010886	E6444	374095290	voltage-dependent non-L-type cal	5.32E-64	Transport	Ion transp	0.0008485	12	0	0.0411873	24	18	46.17368	[illegible]
CUST_53670_PI429010886	M102717						0.0008528	15	12	0.0440761	21	15	36.02994	[illegible]
CUST_21856_PI429010886	N45981						0.0008595	24	9	0.0178952	15	3	135.4737	[illegible]
CUST_9352_PI429010886	N24480				Transport	Protein tra	0.0008645	15	3	0.0011266	18	12	106.1034	[illegible]
CUST_38878_PI429010886	M31485						0.0008907	24	12	0.0162907	12	0	163.4636	[illegible]
CUST_31592_PI429010886	M1920						0.0008934	12	9	0.0006725	15	12	203.9603	[illegible]
CUST_30297_PI429010886	N61574						0.0008934	12	9	0.0092212	18	15	19.39036	[illegible]
CUST_34717_PI429010886	M10447						0.0009035	12	3	0.047283	12	3	206.781	[illegible]
CUST_56458_PI429010886	M113843						0.0009061	12	3	0.018148	12	3	36.11568	[illegible]
CUST_43942_PI429010886	M64411	308220078	LIM class homeobox transcription	1.63E-19			0.0009479	18	12	0.0101636	24	21	18.69191	[illegible]
CUST_42778_PI429010886	M61230						0.000951	15	9	0.0218872	15	9	17.47661	[illegible]
CUST_28861_PI429010886	N58744	443715571	hypothetical protein CAPTEDRAFT	9.05E-08			0.0009568	18	15	0.010048	24	21	26.4274	[illegible]
CUST_5708_PI429010886	N19078						0.0009594	24	21	0.0029044	21	15	200.7515	[illegible]
CUST_28125_PI429010886	N57342						0.0009916	24	21	0.0202472	24	12	42.59404	[illegible]
CUST_18543_PI429010886	N39985						0.0010107	21	3	0.016228	21	0	26.62694	[illegible]
CUST_641_PI429010886	N6502						0.0010113	21	18	0.0001942	24	6	300.7552	[illegible]
CUST_6906_PI429010886	N20761	321470670	hypothetical protein DAPPUDRAF	7.76E-97	Metabolic	Cellular am	0.0010181	24	18	0.0015878	12	0	326.0295	[illegible]
CUST_8660_PI429010886	N23374	260793621	hypothetical protein BRAFLDRAFT	5.94E-66	Metabolic	Carbohydr	0.0010181	24	21	0.0406477	15	0	137.9345	[illegible]
CUST_4050_PI429010886	N15882						0.0010197	18	15	0.0058657	15	9	37.48759	[illegible]
CUST_38924_PI429010886	M32005						0.0010528	24	9	0.0228465	12	9	38.21258	[illegible]
CUST_16218_PI429010886	N35866						0.0010547	18	3	0.0368632	21	0	27.58739	[illegible]
CUST_39355_PI429010886	M38223						0.0010548	12	9	0.009749	15	12	6226.747	[illegible]
CUST_9156_PI429010886	N24150						0.0010577	24	18	0.0299246	24	15	49.85973	[illegible]
CUST_24512_PI429010886	N50750						0.0010751	12	3	0.0024917	15	12	NA	[illegible]
CUST_5622_PI429010886	N18959	351542215	exonuclease 3'-5' domain containi	7.41E-77	Metabolic	Nucleobas	0.0010874	21	18	0.0074164	12	0	6.010955	[illegible]
CUST_32198_PI429010886	M3501				Signal tran	Cell-cell sig	0.0010905	24	9	0.0425112	12	3	137.5078	[illegible]
CUST_5210_PI429010886	N18403	332023022	Ornithine aminotransferase, mito	4.8E-176			0.0010974	24	3	0.0411259	15	3	165.9265	[illegible]
CUST_7580_PI429010886	N21757						0.0011011	15	9	0.0058089	24	21	18.51954	[illegible]
CUST_46608_PI429010886	M73149	321458613	hypothetical protein DAPPUDRAF	2.09E-12			0.0011106	24	21	0.0267187	24	12	42.28326	[illegible]
CUST_56306_PI429010886	M113460						0.001115	24	12	0.0362375	24	12	214.3618	[illegible]
CUST_25271_PI429010886	N52096	543335593	innexin 2 [Penaeus monodon]	2.82E-09			0.0011169	24	3	0.0028061	18	3	6.36732	[illegible]
CUST_49615_PI429010886	M86406						0.0011291	18	0	0.0003204	15	9	146.658	[illegible]
CUST_32019_PI429010886	M3013						0.00113	24	21	0.0144301	15	9	1034.116	[illegible]
CUST_5668_PI429010886	N19015	405966738	Eukaryotic translation initiation fa	3.87E-14			0.0011304	21	15	0.0067439	24	15	54.08928	[illegible]
CUST_14344_PI429010886	N32731						0.0011325	12	3	0.0100708	15	3	341.84	[illegible]
CUST_13450_PI429010886	N31211						0.0011331	24	3	0.0206143	12	6	34.13078	[illegible]
CUST_31620_PI429010886	M1983						0.0011432	21	3	0.0084454	15	9	56.12288	[illegible]
CUST_7075_PI429010886	N21003						0.0011503	24	21	0.0010908	15	0	88.92336	[illegible]
CUST_17655_PI429010886	N38355						0.0011602	12	9	0.0063553	15	12	703.1577	[illegible]
CUST_30966_PI429010886	M335	242019954	DEAD box ATP-dependent RNA he	2.64E-19			0.0011621	24	18	0.0486904	12	0	68.82621	[illegible]
CUST_40928_PI429010886	M56407						0.0011939	15	6	0.0070297	15	12	73.9144	[illegible]
CUST_48479_PI429010886	M80552						0.0012016	12	9	0.0378352	18	6	53.21446	[illegible]
CUST_42507_PI429010886	M60457						0.0012073	15	0	0.0116514	15	3	289.5149	[illegible]
CUST_49189_PI429010886	M84589						0.0012083	18	6	0.0123912	12	0	179.5567	[illegible]
CUST_144_PI429010886	N1544						0.0012154	12	9	0.0045745	15	12	8107.941	[illegible]
CUST_2336_PI429010886	N12130						0.0012341	24	3	0.018796	12	3	102.3111	[illegible]
CUST_52439_PI429010886	M97449						0.0012354	21	15	0.0486996	24	0	10.46073	[illegible]
CUST_9703_PI429010886	N25070						0.0012563	24	15	0.0236198	21	0	22.58052	[illegible]
CUST_7939_PI429010886	N22300	195436302	GK22112 [Drosophila willistoni] >g	2.97E-48	Transport	Transmem	0.0012707	12	9	0.0322434	18	12	36.41438	[illegible]
CUST_51351_PI429010886	M93219						0.0012713	21	15	0.0389641	15	12	26.38647	[illegible]
CUST_31044_PI429010886	M545	321463706	hypothetical protein DAPPUDRAF	1.07E-53	Metabolic	Cellular am	0.0012787	24	18	0.0266301	15	9	197.3862	[illegible]
CUST_38919_PI429010886	M31957						0.0012897	15	9	0.0077532	15	9	2881.653	[illegible]
CUST_7343_PI429010886	N21406	339896293	cytochrome P450 [Bemisia tabaci]	2.79E-48	Metabolic	Oxidation-	0.0012953	18	15	0.0204009	15	12	404.7001	[illegible]
CUST_42494_PI429010886	M60414						0.0013077	24	0	0.0084476	15	0	93.01673	[illegible]
CUST_42491_PI429010886	M60404	9186884	BCS-1 [Amphibalanus amphitrite]	1.1E-08			0.0013121	18	0	0.0275484	15	9	242.7012	[illegible]
CUST_1219_PI429010886	N8897	321469994	hypothetical protein DAPPUDRAF	2.09E-20			0.0013302	24	9	8.086E-05	12	3	36.8117	[illegible]
CUST_29357_PI429010886	N59703						0.0013476	24	15	0.0057387	18	0	58.95577	[illegible]
CUST_17817_PI429010886	N38643						0.0013596	24	9	0.0350861	15	12	42.84965	[illegible]
CUST_11372_PI429010886	N27709						0.0013608	24	21	0.0214008	15	0	208.4776	[illegible]
CUST_32690_PI429010886	M4820	321472416	hypothetical protein DAPPUDRAF	9.99E-25			0.0013775	21	18	0.0113881	24	9	30.445	[illegible]
CUST_53318_PI429010886	M101251						0.0014038	24	9	8.902E-05	24	9	11.81411	[illegible]
CUST_6322_PI429010886	N19946	548531516	PREDICTED: E3 ubiquitin-protein l	2.7E-124			0.0014049	21	6	0.02818	12	3	82.53807	[illegible]
CUST_10067_PI429010886	N25641						0.0014121	18	6	0.0171213	24	6	20.92431	[illegible]
CUST_45215_PI429010886	M68354						0.0014227	24	21	0.0229171	24	15	207.8099	[illegible]
CUST_43015_PI429010886	M61864						0.0014315	24	12	0.0006282	18	9	22.32375	[illegible]
CUST_43551_PI429010886	M63338						0.0014404	21	12	0.0034129	21	9	7.342734	[illegible]
CUST_34525_PI429010886	M9914						0.0014412	24	3	0.0012713	21	18	125.5101	[illegible]
CUST_43143_PI429010886	M62188						0.0014424	12	0	0.0023536	15	0	206.1814	[illegible]
CUST_30227_PI429010886	N61440						0.0014458	15	9	0.0364609	15	0	47.67532	[illegible]
CUST_54026_PI429010886	M104151	321458008	hypothetical protein DAPPUDRAF	1.34E-46	Transport	Transmem	0.0014615	24	15	0.0141893	15	3	25.39051	[illegible]
CUST_53827_PI429010886	M103348						0.001465	21	9	0.0046886	18	3	3.592956	[illegible]
CUST_52801_PI429010886	M98971						0.0014759	24	21	0.0095168	15	12	67.79476	[illegible]
CUST_12144_PI429010886	N28998						0.0014778	24	18	0.0349435	21	15	370.3281	[illegible]
CUST_21460_PI429010886	N45262						0.0014898	24	21	0.015967	21	18	6.126911	[illegible]
CUST_1841_PI429010886	N10861	321466121	hypothetical protein DAPPUDRAF	9.61E-09			0.0014956	24	18	0.0062949	15	9	365.0376	[illegible]
CUST_24247_PI429010886	N50294						0.0014966	24	18	0.0050993	18	12	26.87774	[illegible]
CUST_10093_PI429010886	N25681	54042615	arthrodial cuticle protein AMP6.0	4.12E-15			0.0014975	24	18	0.0015479	15	9	1183.356	[illegible]
CUST_43401_PI429010886	M62945						0.0015122	18	6	0.0086765	18	12	58.28361	[illegible]
CUST_7302_PI429010886	N21342						0.0015151	24	21	0.0322832	21	12	118.9614	[illegible]

Probe ID	ID	EBI ID	Annotation	E-value	GO category	Go term	p-value LD	Period LD	Phase LD	p-value DD	Period DD	Phase DD
CUST_18535_PI429010886	N39975						0.0015296	24	21	0.0498768	15	12
CUST_23038_PI429010886	N48149						0.0015336	24	21	0.0025438	18	9
CUST_47128_PI429010886	M74889						0.0015521	24	21	0.0020841	18	15
CUST_25454_PI429010886	N52447						0.0015553	24	21	0.016038	24	15
CUST_33385_PI429010886	M6755	281337645	hypothetical protein PANDA_009	4.26E-07			0.001559	18	3	0.0405568	21	3
CUST_359_PI429010886	N4421	220172369	cuticle protein [Rimicaris exoculat	2.35E-29			0.0015871	15	3	0.0001873	15	12
CUST_27420_PI429010886	N56030	242002324	sugar transporter, putative [Ixode	2.1E-20	Transport	Carbohydr	0.0016151	24	15	0.0192926	21	15
CUST_3040_PI429010886	N13661						0.0016161	15	6	0.0278545	15	0
CUST_19182_PI429010886	N41125						0.0016176	24	15	0.019199	12	0
CUST_1861_PI429010886	N10900						0.0016235	12	9	0.0279594	15	12
CUST_38832_PI429010886	M31027						0.0016238	24	15	0.0274375	18	15
CUST_10604_PI429010886	N26497						0.0016272	24	0	0.0147418	24	0
CUST_38595_PI429010886	M28404						0.0016376	15	12	0.0124989	21	9
CUST_21360_PI429010886	N45078						0.00164	15	9	0.0054365	15	9
CUST_45000_PI429010886	M67675						0.0016421	12	3	0.0003321	24	21
CUST_32805_PI429010886	M5150						0.0016432	12	0	0.0244592	12	3
CUST_20425_PI429010886	N43413						0.0016437	21	0	0.0443775	24	3
CUST_33403_PI429010886	M6802						0.0016451	24	18	0.0253727	15	9
CUST_13466_PI429010886	N31238						0.0016663	21	15	0.0200048	12	9
CUST_54301_PI429010886	M105482						0.0016713	12	0	0.0211109	21	6
CUST_32126_PI429010886	M3294	321471368	hypothetical protein DAPPUDRAF	1.08E-28	Protein me	Proteolysis	0.0016736	18	9	0.0086088	12	3
CUST_28613_PI429010886	N58293						0.00169	15	0	0.039534	12	0
CUST_33073_PI429010886	M5901	256033192	triacylglycerol lipase [Litopenaeus	1.27E-90			0.0016916	15	6	0.0361743	18	6
CUST_13432_PI429010886	N31179						0.0016964	15	0	0.0040221	12	0
CUST_41264_PI429010886	M57279	543364768	PREDICTED: dehydrogenase/redu	8.68E-55	Metabolic	Oxidation-	0.0017187	24	18	0.0259187	21	9
CUST_39399_PI429010886	M38859						0.001742	24	15	0.0091821	21	18
CUST_48241_PI429010886	M79485						0.0017453	15	12	0.047953	24	18
CUST_6718_PI429010886	N20506	321471179	hypothetical protein DAPPUDRAF	2.14E-68			0.0017635	24	18	0.0016538	18	9
CUST_46051_PI429010886	M71199						0.0017883	18	15	0.04553	12	0
CUST_7274_PI429010886	N21303						0.0017944	21	15	0.0144397	24	21
CUST_43479_PI429010886	M63155						0.0018203	12	9	0.0009327	24	12
CUST_45132_PI429010886	M68092						0.0018315	15	12	0.0012184	21	15
CUST_20216_PI429010886	N43035						0.0018588	24	18	0.0313079	12	9
CUST_11942_PI429010886	N28667						0.0018655	24	12	1.424E-06	24	12
CUST_21916_PI429010886	N46093						0.0018816	15	12	0.0056694	24	21
CUST_13994_PI429010886	N32142	290462133	tRNA 2-thiocytidine biosynthesis p	1.81E-26	Metabolic	Other	0.0019093	24	18	0.0299032	15	12
CUST_20370_PI429010886	N43324						0.0019289	21	18	0.0247935	15	9
CUST_7158_PI429010886	N21123	345484364	PREDICTED: DNA-directed RNA po	5.43E-20	Nucleic aci	Transcripti	0.0019354	24	21	0.0175431	15	9
CUST_39038_PI429010886	M33821	528314838	ubiquitin [Schizosaccharomyces c	1.77E-22	Metabolic	Oxidation-	0.0019363	21	15	0.0333188	24	0
CUST_1415_PI429010886	N9562						0.001946	24	3	0.0239085	12	3
CUST_27140_PI429010886	N55515						0.0019606	24	21	0.0151915	15	9
CUST_27926_PI429010886	N56955						0.0019728	24	21	0.0259095	18	12
CUST_56697_PI429010886	M114441						0.0019984	24	21	0.0015383	24	12
CUST_37383_PI429010886	M18847	321455420	hypothetical protein DAPPUDRAF	5.49E-92	Metabolic	Carbohydr	0.0019984	24	21	0.0136185	15	0
CUST_2729_PI429010886	N12995	321455420	hypothetical protein DAPPUDRAF	4.97E-96	Metabolic	Carbohydr	0.0019984	24	21	0.0008638	15	0
CUST_53840_PI429010886	M103385						0.0020499	21	12	0.0249666	24	3
CUST_13649_PI429010886	N31528						0.0020571	15	12	0.0082575	24	0
CUST_5008_PI429010886	N18125						0.0020593	15	9	0.0021537	12	9
CUST_35296_PI429010886	M12167						0.0020662	24	21	0.0001223	15	9
CUST_2161_PI429010886	N11726						0.0020738	12	9	0.0267253	15	9
CUST_41816_PI429010886	M58616						0.0020994	12	0	0.0039705	15	0
CUST_25941_PI429010886	N53342	528522579	PREDICTED: gastrula zinc finger pr	4.98E-27			0.0021036	12	9	0.0008188	15	9
CUST_42577_PI429010886	M60630						0.0021182	18	15	0.0173293	15	0
CUST_41374_PI429010886	M57581						0.0021236	24	18	0.0006877	24	9
CUST_35500_PI429010886	M12728	260813390	hypothetical protein BRAFLDRAFT	1.78E-38			0.0021265	24	18	0.018932	15	12
CUST_21384_PI429010886	N45122	321475473	hypothetical protein DAPPUDRAF	6.71E-56			0.0021265	24	3	0.0292661	12	3
CUST_36426_PI429010886	M15871						0.0021287	12	3	0.0052419	12	9
CUST_7659_PI429010886	N21882						0.0021512	15	6	0.0154062	18	6
CUST_30937_PI429010886	M264						0.0021523	24	21	0.0101747	24	15
CUST_15455_PI429010886	N34608						0.0021777	24	21	0.0150512	12	0
CUST_17412_PI429010886	N37921						0.0021812	21	0	0.0445647	18	12
CUST_93_PI429010886	N737	532759730	antimicrobial peptide type 2 prec	1.31E-26			0.0021849	15	0	0.0113756	24	21
CUST_52872_PI429010886	M99276						0.0021897	18	12	0.0109338	18	12
CUST_7295_PI429010886	N21335						0.0021967	24	21	0.0320914	21	15
CUST_49967_PI429010886	M87978						0.0022038	12	9	0.0170829	15	12
CUST_45631_PI429010886	M69725	209418466	serpin 8 [Branchiostoma lanceolat	5.77E-31			0.0022243	18	15	0.0128065	21	12
CUST_11258_PI429010886	N27522						0.0022384	24	21	0.0099811	15	12
CUST_54667_PI429010886	M107092						0.0022539	12	3	0.0456903	18	3
CUST_13035_PI429010886	N30473						0.0022671	24	15	0.0275738	18	15
CUST_46101_PI429010886	M71369	332026908	hypothetical protein G5I_04441 [	5.79E-07			0.0022889	15	6	0.0096418	12	0
CUST_11377_PI429010886	N27715						0.0022986	18	15	0.0235969	21	0
CUST_26779_PI429010886	N54860						0.0023022	24	0	0.0305657	15	12
CUST_33927_PI429010886	M8267						0.0023129	18	9	0.0082345	12	0
CUST_8558_PI429010886	N23221						0.0023464	15	9	0.0083763	15	12
CUST_26147_PI429010886	N53750						0.0023599	18	15	0.0394506	21	0
CUST_37732_PI429010886	M21285	6831612	RecName: Full=60S ribosomal pro	8.7E-82	Protein me	Ribosome	0.0023775	24	15	1.622E-06	24	0
CUST_3763_PI429010886	N15223						0.0023818	24	15	0.0159607	15	0
CUST_35197_PI429010886	M11868	321478381	hypothetical protein DAPPUDRAF	1.97E-17			0.0023886	12	3	0.0327528	21	6
CUST_16066_PI429010886	N35591						0.0023918	15	6	0.0046259	15	12
CUST_18472_PI429010886	N39862						0.0024105	24	3	0.0216638	15	3
CUST_18654_PI429010886	N40175						0.0024155	21	6	0.004908	15	9
CUST_38012_PI429010886	M22771						0.0024245	24	18	0.047283	12	9
CUST_17684_PI429010886	N38408	193705824	PREDICTED: ankyrin repeat domai	1.25E-19			0.0024338	15	12	0.0006593	18	12
CUST_20012_PI429010886	N42645	242023449	conserved hypothetical protein [P	1.99E-07			0.0024392	24	21	0.0222888	18	12
CUST_55735_PI429010886	M111521						0.0024499	15	3	0.0090754	18	9
CUST_40596_PI429010886	M55564						0.002459	21	3	0.0277025	15	9
CUST_29139_PI429010886	N59269						0.0024619	15	9	0.0011257	24	18
CUST_37601_PI429010886	M19739	13898841	CUB-serine protease [Panulirus ar	1.55E-48	Protein me	Proteolysis	0.0024917	15	6	0.0363431	18	15
CUST_46036_PI429010886	M71146						0.002512	21	15	0.0295805	21	18
CUST_47881_PI429010886	M77998	170819724	reverse transcriptase [Daphnia pu	3.93E-11			0.0025146	24	18	0.0228158	24	0
CUST_56667_PI429010886	M114381						0.0025175	24	6	0.0050522	21	12
CUST_12037_PI429010886	N28818						0.0025221	21	0	0.0492232	24	21
CUST_14737_PI429010886	N33379	189239442	PREDICTED: similar to Nedd4 CG7	9.96E-53	Protein me	Cellular pr	0.0025399	15	12	0.0387419	18	15
CUST_19976_PI429010886	N42546						0.0025551	24	15	0.0083733	18	0
CUST_14953_PI429010886	N33750						0.0026009	24	15	0.039871	21	15
CUST_37376_PI429010886	M18825	256539086	fatty acids binding protein [Fenne	1.79E-31	Symbiosis,	Viral proce	0.0026061	24	0	0.0222151	21	9
CUST_55561_PI429010886	M110723						0.0026452	24	18	0.0435169	15	0
CUST_9151_PI429010886	N24141						0.0026679	21	15	0.012054	21	0
CUST_42887_PI429010886	M61517						0.0026795	24	21	0.0035979	15	12
CUST_3014_PI429010886	N13604	241999376	ubiquitin protein ligase [Ixodes sc	6.53E-34	Protein me	Cellular pr	0.0026891	12	9	0.0337096	15	12
CUST_36706_PI429010886	M16598						0.0027062	24	21	0.0314104	15	0
CUST_51834_PI429010886	M95322						0.002708	24	9	0.0377632	18	6
CUST_25524_PI429010886	N52570						0.0027148	24	21	0.0070297	15	12
CUST_20147_PI429010886	N42888						0.0027362	24	0	0.0078046	24	18
CUST_43606_PI429010886	M63489						0.0027379	12	9	0.0317226	15	12
CUST_41746_PI429010886	M58445						0.0027415	24	15	0.0040394	12	0
CUST_43004_PI429010886	M61827						0.0027415	24	0	0.0098338	15	0
CUST_35597_PI429010886	M12985						0.0027471	18	12	0.0068518	15	12
CUST_931_PI429010886	N7861						0.0028003	21	0	0.0213152	24	21
CUST_17351_PI429010886	N37819						0.0028085	24	18	0.0193525	12	0
CUST_14453_PI429010886	N32910						0.0028206	24	6	0.0050354	18	15
CUST_12628_PI429010886	N29770						0.0028225	21	9	0.0478203	12	3
CUST_874_PI429010886	N7649	255983833	glutathione S-transferase [Erioche	2.23E-55			0.0028354	21	18	0.0275274	21	6
CUST_37087_PI429010886	M17810						0.0028412	24	18	0.0174711	15	12
CUST_45876_PI429010886	M70591	270005635	hypothetical protein TcasGA2_TC	8.59E-19			0.0028447	24	0	0.0406446	24	21
CUST_33542_PI429010886	M7219	242021988	cAMP-dependent protein kinase c	2.78E-22	Signal tran	Intracellula	0.0028558	24	21	0.0275484	15	0
CUST_1366_PI429010886	N9420	321470487	hypothetical protein DAPPUDRAF	5.7E-108			0.0028646	15	12	0.0354102	21	9
CUST_23949_PI429010886	N49752						0.0028656	24	15	0.0045221	21	0
CUST_50566_PI429010886	M90295						0.0028695	15	9	0.0105491	24	15
CUST_9442_PI429010886	N24630						0.0028724	21	0	0.0376271	24	15
CUST_20529_PI429010886	N43614						0.0029017	15	9	0.000275	24	0
CUST_53520_PI429010886	M102096	321466278	notch 2 [Daphnia pulex]	1.17E-41			0.0029047	21	15	0.0009954	18	15
CUST_3272_PI429010886	N14157	260828941	hypothetical protein BRAFLDRAFT	1.18E-88			0.0029162	24	21	0.0340474	24	18
CUST_9869_PI429010886	N25327						0.0029263	24	21	0.0071689	24	15
CUST_1499_PI429010886	N9825						0.0029263	24	21	0.0499589	12	0
CUST_18778_PI429010886	N40391	321476640	hypothetical protein DAPPUDRAF	2.3E-19	Transport	Transmem	0.0029572	18	3	0.0276104	21	15
CUST_36530_PI429010886	M16159						0.0029616	24	21	0.0350519	15	0
CUST_14585_PI429010886	N33118						0.0029616	24	21	0.0104187	18	9
CUST_34632_PI429010886	M10191						0.002975	21	9	0.0292367	15	6
CUST_39595_PI429010886	M41570						0.0029779	15	12	0.0455248	21	9
CUST_18752_PI429010886	N40352						0.0030051	12	9	0.0047977	15	9
CUST_11871_PI429010886	N28544						0.0030344	24	18	0.0163836	12	0
CUST_12325_PI429010886	N29289						0.0030542	18	15	0.0095157	21	15
CUST_3411_PI429010886	N14451	386376737	hematopoietic prostaglandin D sy	1.12E-46	Metabolic	Cellular an	0.0031379	24	18	0.0310345	18	9
CUST_5139_PI429010886	N18307						0.0031493	21	15	0.0050855	18	15
CUST_50435_PI429010886	M89803						0.0031613	24	15	0.0212221	18	3
CUST_52654_PI429010886	M98332						0.0031742	15	0	0.0471223	24	15
CUST_22060_PI429010886	N46350						0.0031764	18	15	0.0117403	15	12
CUST_16706_PI429010886	N36726						0.0032005	15	6	0.0454539	15	9
CUST_822_PI429010886	N7420						0.0032031	24	21	0.0470305	18	0
CUST_13755_PI429010886	N31716	291235351	PREDICTED: zinc finger protein 34	2.25E-22			0.0032058	24	0	0.0063329	24	21
CUST_22647_PI429010886	N47395						0.0032384	12	9	0.0053558	24	21
CUST_33654_PI429010886	M7523						0.0032777	15	9	0.0008419	18	6
CUST_42588_PI429010886	M60658						0.0032816	12	6	0.0021757	15	9
CUST_13216_PI429010886	N30794						0.0032816	12	0	0.0346828	15	12
CUST_1842_PI429010886	N10862						0.0032821	21	18	0.0319196	12	0
CUST_6429_PI429010886	N20090						0.0033017	24	18	0.006438	15	9
CUST_36652_PI429010886	M16457						0.0033041	15	9	0.0011047	15	0
CUST_39596_PI429010886	M41608						0.0033132	15	9	0.002029	15	9
CUST_5994_PI429010886	N19479						0.0033393	18	15	0.003612	24	21
CUST_13736_PI429010886	N31686						0.0033679	15	12	0.0233913	18	0
CUST_47861_PI429010886	M77892	242332534	uncharacterized protein LOC1000	5.91E-13			0.0033703	21	3	0.0259172	24	21
CUST_2185_PI429010886	N11816						0.0033703	21	3	0.0367561	18	6
CUST_4329_PI429010886	N16524						0.0033703	21	12	0.000214	21	15
CUST_8901_PI429010886	N23769						0.0034169	24	3	0.0343904	21	3
CUST_27230_PI429010886	N55676						0.0034169	21	9	0.0083695	21	0
CUST_3714_PI429010886	N15115						0.0034456	12	6	0.0200368	24	0
CUST_12443_PI429010886	N29469						0.0034557	21	3	0.0204188	15	12
CUST_26181_PI429010886	N53807	339234267	ETS factor [Trichinella spiralis] >gi	2.58E-15	Nucleic aci	Regulation	0.0034619	12	9	0.0453035	15	12
CUST_20791_PI429010886	N44060						0.0034633	24	3	0.0345284	15	3
CUST_7134_PI429010886	N21087						0.0034708	24	21	0.0325948	15	0
CUST_37794_PI429010886	M21622	242007350	15 kDa selenoprotein precursor, p	4.84E-43			0.0035467	24	15	0.0094008	15	12
CUST_6232_PI429010886	N19820	443719661	hypothetical protein CAPTEDRAFT	3.3E-114	Metabolic	Lipid meta	0.0035831	12	9	0.0019088	18	12
CUST_6903_PI429010886	N20755	328779109	PREDICTED: Apis mellifera vinculir	1.43E-84	Cellular Pr	Cell adhesi	0.0036066	24	0	0.0345122	15	0
CUST_32412_PI429010886	M4050				Metabolic	Carbohydr	0.0036451	24	9	0.0313664	15	3
CUST_2450_PI429010886	N12377						0.0036602	24	21	0.0001829	15	12
CUST_43245_PI429010886	M62518						0.0036709	24	12	0.0380313	18	9
CUST_22270_PI429010886	N46722						0.0037084	24	21	0.0108649	21	15
CUST_4633_PI429010886	N17176						0.0037158	12	9	0.0116438	15	12
CUST_337_PI429010886	N4053						0.0037158	12	9	0.0002554	15	12

Probe ID	ID	EBI ID	Annotation	E-value	GO categor	Go term	p-value LD	Period LD	Phase LD	p-value DD	Period DD	Phase DD	LD 0
CUST_28842_PI429010886	N58710						0.0037192	18	12	0.0345763	21	0	22.99386
CUST_8432_PI429010886	N23030	405960555	Fanconi anemia group J protein [C	1.35E-21	Response t	DNA repai	0.0037565	24	18	0.0170829	15	12	30.85244
CUST_10764_PI429010886	N26750						0.0037565	24	18	0.0008088	21	9	26.37129
CUST_53910_PI429010886	M103670	260830643	hypothetical protein BRAFLDRAFT	1.68E-21	Metabolic	Other	0.0037602	24	18	0.0106628	24	15	18.08256
CUST_42258_PI429010886	M59819						0.0037602	24	18	0.0142513	21	6	23.78991
CUST_11500_PI429010886	N27915						0.0037721	24	15	0.0173067	15	0	596.2588
CUST_50872_PI429010886	M91294						0.0037873	15	12	0.0356256	21	0	6.203165
CUST_51082_PI429010886	M92093						0.0037938	21	6	0.0409638	15	0	11.34354
CUST_12158_PI429010886	N29022						0.0037966	18	12	0.0001631	24	0	21.3257
CUST_11864_PI429010886	N28533						0.0038528	24	21	0.0010313	24	21	77.49407
CUST_15430_PI429010886	N34569	505844696	PREDICTED: tyrosine-protein kina	1.55E-27	Protein me	Cellular pr	0.0038675	24	21	0.0254057	15	9	49.9052
CUST_53620_PI429010886	M102512	443716906	hypothetical protein CAPTEDRAFT	3.34E-35	Transport	Protein tra	0.0038694	24	12	0.0195699	12	0	199.588
CUST_16595_PI429010886	N36525						0.0039224	24	12	0.0125004	12	3	57.76704
CUST_51716_PI429010886	M94702						0.0039369	12	9	0.0006845	12	0	413.9686
CUST_19075_PI429010886	N40938	157127444	amino acid transporter [Aedes ae	5.23E-40			0.0039492	24	3	0.0044507	18	6	74.93498
CUST_53994_PI429010886	M104013	321466304	adenylyl cyclase [Daphnia pulex]	8.99E-16			0.0039678	12	0	0.0300851	15	12	26.68521
CUST_46368_PI429010886	M72411						0.0039741	24	15	0.0345122	15	0	126.9105
CUST_11137_PI429010886	N27327						0.0039973	24	21	0.0219474	21	15	51.66124
CUST_47462_PI429010886	M76220						0.0040114	24	21	0.015263	24	18	121.6909
CUST_50200_PI429010886	M89007						0.0040254	12	9	0.0072967	15	12	38.85553
CUST_14740_PI429010886	N33385						0.0040612	18	6	0.0183268	15	9	948.407
CUST_3642_PI429010886	N14949						0.0040697	12	3	0.00867	18	3	487.1319
CUST_34606_PI429010886	M10133						0.004074	24	15	0.0150512	12	0	194.5138
CUST_35610_PI429010886	M13016						0.0040936	24	18	0.0218872	15	9	156.0931
CUST_51253_PI429010886	M92816						0.0041292	24	18	0.0457188	24	18	12.96468
CUST_53363_PI429010886	M101413						0.0041316	15	0	0.0368632	12	9	19.20962
CUST_13568_PI429010886	N31394	262401023	cytochrome c oxidase subunit VII	4.62E-07	Metabolic	Generatio	0.0041418	24	6	0.0296064	12	3	406.4569
CUST_29457_PI429010886	N59901						0.0041418	24	21	0.0083281	12	0	97.10574
CUST_45894_PI429010886	M70652	256079279	procollagen-lysine2-oxoglutarate	2.36E-12	Protein me	Other	0.0041509	18	15	0.0048838	24	15	49.04743
CUST_34488_PI429010886	M9814						0.0041509	24	21	0.0038722	12	0	51.39588
CUST_27505_PI429010886	N56179						0.0041954	18	3	0.0475838	15	12	399.8074
CUST_31623_PI429010886	M1992						0.004208	24	15	0.0077548	18	0	73.02048
CUST_35101_PI429010886	M11577						0.004235	21	0	0.0026116	15	6	56.00071
CUST_45045_PI429010886	M67833						0.0042614	15	9	0.0066082	12	0	22.16637
CUST_50385_PI429010886	M89655						0.0042639	18	3	0.0026446	12	9	39.05628
CUST_24208_PI429010886	N50229						0.0042849	21	15	0.0443197	18	12	1242.744
CUST_20106_PI429010886	N42821	242023524	Heterogeneous nuclear ribonucle	6.17E-25			0.0042972	24	15	0.0148628	24	15	57.31291
CUST_8664_PI429010886	N23381						0.0043344	24	18	0.0135465	15	12	115.9671
CUST_17311_PI429010886	N37755						0.0043541	24	12	0.0455445	12	0	53.98068
CUST_18636_PI429010886	N40146						0.0043553	12	0	0.0067628	15	6	21.31707
CUST_38284_PI429010886	M25016	2414483	Crithidia fasciculata ribosomal DN				0.0043671	24	9	0.0015324	12	3	67.60011
CUST_28080_PI429010886	N57252						0.0044047	24	15	0.0386555	12	0	1692.044
CUST_37152_PI429010886	M18015						0.0044106	18	0	7.411E-05	21	0	549.679
CUST_2252_PI429010886	N11966	225714106	Cytosolic non-specific dipeptidase	5.3E-177	Protein me	Proteolysis	0.0044106	18	15	0.017359	24	9	49.08263
CUST_5454_PI429010886	N18726						0.0044232	15	0	0.001625	12	0	405.9933
CUST_6217_PI429010886	N19790	119117126	Cryptocercus punctulatus alpha-tu		Cellular co	Cytoskelet	0.0044584	24	3	0.0361562	15	0	1354.492
CUST_41839_PI429010886	M58675						0.0044686	21	9	0.0049507	12	3	269.5843
CUST_12721_PI429010886	N29935						0.004481	24	15	0.0017883	18	9	366.7062
CUST_6480_PI429010886	N20156	321461563	hypothetical protein DAPPUDRAF	1.31E-95	Protein me	Ribosome	0.0045121	24	21	0.0456297	18	0	57.06084
CUST_8475_PI429010886	N23096						0.0045121	24	15	0.0101747	24	15	342.0443
CUST_13810_PI429010886	N31814						0.004522	15	9	0.0321171	24	0	161.1223
CUST_43941_PI429010886	M64408						0.0045231	15	6	0.0060822	24	15	16.38359
CUST_72_PI429010886	N395						0.004527	24	21	0.0131033	12	0	70.44138
CUST_8563_PI429010886	N23226	307184380	Carbohydrate sulfotransferase 14	1.37E-19	Metabolic	Carbohydr	0.0045355	21	18	0.0132685	15	0	273.7185
CUST_53699_PI429010886	M102832						0.0045377	24	15	0.0113487	15	0	156.6513
CUST_34948_PI429010886	M11141						0.0045658	24	21	0.0237551	18	12	167.7764
CUST_30609_PI429010886	N62219						0.0045709	12	0	0.0002971	15	9	527.7959
CUST_31456_PI429010886	M1564						0.0046732	24	15	0.0070092	18	15	47.51044
CUST_18595_PI429010886	N40075						0.0046918	24	21	0.017753	15	0	38.71226
CUST_4127_PI429010886	N16077						0.0046924	24	9	0.0041376	15	3	25.82213
CUST_50625_PI429010886	M90466						0.0047303	12	0	0.0076932	24	15	119.6589
CUST_39563_PI429010886	M41234	341650466	Spz1 [Litopenaeus vannamei]	8.36E-88			0.0047644	24	3	0.0044047	24	6	211.7618
CUST_13817_PI429010886	N31827	321479469	hypothetical protein DAPPUDRAF	2.27E-19			0.0047644	24	15	0.0292031	12	0	291.4994
CUST_39473_PI429010886	M39930						0.0047715	24	15	5.062E-06	18	12	4415.56
CUST_28280_PI429010886	N57660						0.0047787	24	21	0.0011599	15	9	38.15601
CUST_36813_PI429010886	M16931						0.0048232	12	0	0.0130165	21	0	65.13726
CUST_15818_PI429010886	N35198						0.0048332	24	21	0.008307	15	0	125.4319
CUST_6133_PI429010886	N19672	242016587	hypothetical protein Phum_PHUN	5.63E-13			0.0048342	12	3	0.0036715	12	3	179.1302
CUST_39423_PI429010886	M39190	255653868	crustin 1 [Panulirus japonicus]	1.4E-08			0.0048417	18	3	0.015288	24	15	41.90666
CUST_13181_PI429010886	N30729				Metabolic	Carbohydr	0.0049136	12	6	0.0243267	15	9	4.035824
CUST_38905_PI429010886	M31812						0.0050187	24	6	0.0190902	24	3	136.6874
CUST_49817_PI429010886	M87287						0.0050896	24	15	0.0086911	24	15	54.05823
CUST_4606_PI429010886	N17117						0.0051173	12	9	0.0318512	18	9	616.5627
CUST_9840_PI429010886	N25282						0.0051312	21	0	0.0026787	21	0	197.5603
CUST_31191_PI429010886	M940						0.0051598	24	21	0.0342367	15	0	1996.997
CUST_36177_PI429010886	M14897	241595281	NudC domain-containing protein,	6.12E-30			0.0052131	12	0	0.0327528	24	21	76.38149
CUST_15351_PI429010886	N34443						0.0052275	24	0	0.02393	21	3	44.7999
CUST_11211_PI429010886	N27450						0.005272	24	3	0.0273812	15	9	9.392936
CUST_5196_PI429010886	N18387	383860504	PREDICTED: 7SK snRNA methylph	3.18E-82			0.0052783	24	21	0.0092139	15	9	133.8434
CUST_12303_PI429010886	N29258						0.0052783	24	21	0.0223014	24	6	19.98159
CUST_22738_PI429010886	N47579						0.0052904	24	21	0.0083029	21	9	21.23274
CUST_22986_PI429010886	N48057						0.0053001	12	3	0.0239216	12	3	133.1101
CUST_28288_PI429010886	N57673						0.0053305	24	12	0.0122437	18	15	28.83489
CUST_24177_PI429010886	N50173	219809644	Kazal-type serine proteinase inhib	1.33E-11	Protein me	Proteolysis	0.0053431	15	0	0.0136983	18	3	73.50942
CUST_24199_PI429010886	N50214						0.0053558	24	21	0.0397253	15	12	1286.33
CUST_45166_PI429010886	M68183						0.005368	18	3	0.0313055	15	0	45.1102
CUST_53709_PI429010886	M102862						0.0054211	24	21	0.0013517	24	15	414.4128
CUST_30391_PI429010886	N61781	157129622	hypothetical protein AaeL_AAEL0	1.12E-18			0.0054353	21	0	0.0175339	24	21	18.77458
CUST_31136_PI429010886	M798						0.0054864	24	18	0.0038485	12	9	98.67888
CUST_16611_PI429010886	N36566						0.0055146	21	9	0.0066316	12	9	13.35908
CUST_24073_PI429010886	N49983						0.0055161	15	0	0.0025078	18	12	10127.21
CUST_32569_PI429010886	M4455						0.005517	24	18	0.039282	18	9	793.898
CUST_21729_PI429010886	N45746						0.0055222	12	3	0.0165954	15	12	15.61685
CUST_24413_PI429010886	N50580						0.0055243	24	6	0.021409	18	12	106.2214
CUST_53953_PI429010886	M103850						0.0055969	24	9	0.0035094	15	3	478.2442
CUST_37841_PI429010886	M21826						0.005617	24	18	0.0062949	15	9	1271.126
CUST_15495_PI429010886	N34674	156552355	PREDICTED: hypothetical protein I	3.69E-09	Metabolic	Other	0.0056214	24	0	0.000296	18	15	25.39283
CUST_18882_PI429010886	N40575	334326753	PREDICTED: LOW QUALITY PROTE	9.26E-13	Protein me	Proteolysis	0.0056214	24	12	0.0329783	15	6	34.81137
CUST_19417_PI429010886	N41544						0.0056352	18	3	0.0236771	18	12	25.5566
CUST_4752_PI429010886	N17406						0.0056396	24	0	0.0111597	21	18	428.6927
CUST_6326_PI429010886	N19950						0.0056696	24	21	0.0285542	12	0	124.2797
CUST_27923_PI429010886	N56949	410509308	myosin heavy chain type 2 [Penae	9.41E-21			0.0056865	12	6	0.0422156	21	9	28.72644
CUST_31547_PI429010886	M1802						0.0057224	15	6	0.0061036	15	9	3428.918
CUST_54362_PI429010886	M105728						0.0057423	24	9	0.0144691	15	3	84.16166
CUST_38843_PI429010886	M31132	532759728	antimicrobial peptide type 2 precu	2.51E-30			0.0057423	24	6	8.25E-05	15	9	1433.817
CUST_33666_PI429010886	M7550						0.0057476	24	18	0.0077051	15	9	62.40045
CUST_56664_PI429010886	M114372						0.0057494	24	6	0.0310345	18	9	33.97232
CUST_16472_PI429010886	N36294						0.0057623	24	0	0.0378555	15	9	130.7332
CUST_45869_PI429010886	M70576						0.005877	15	9	0.0350861	15	12	354.5733
CUST_1436_PI429010886	N9625	238476875	crustin type I [Macrobrachium ros	2.87E-10			0.0058877	24	6	0.0015894	18	6	148.5291
CUST_9218_PI429010886	N24253						0.0059375	24	15	0.0128619	15	9	98.68778
CUST_15118_PI429010886	N34038						0.0059604	24	15	0.0068606	24	21	159.4235
CUST_46024_PI429010886	M71100						0.0060212	21	0	0.0082147	15	9	636.9918
CUST_51115_PI429010886	M92261						0.0060331	24	15	0.033893	18	15	287.4579
CUST_45518_PI429010886	M69362						0.0061058	24	15	0.000583	21	15	24.25396
CUST_34191_PI429010886	M9012						0.0061058	24	15	0.0416041	15	0	449.9406
CUST_5569_PI429010886	N18886						0.0061058	24	15	0.0014975	21	18	97.64083
CUST_9228_PI429010886	N24269						0.0061058	24	21	0.0003417	12	0	17.63035
CUST_34203_PI429010886	M9051	321466121	hypothetical protein DAPPUDRAF	1.96E-10			0.0061136	24	18	0.0383649	15	9	165.0428
CUST_24678_PI429010886	N51068						0.0061223	21	18	0.024373	21	3	17.0552
CUST_29594_PI429010886	N60164	242023295	guanine nucleotide exchange fact	3.86E-07			0.0061734	24	18	0.0314857	21	18	210.039
CUST_23597_PI429010886	N49090						0.0061991	15	3	0.0122013	24	6	42.48778
CUST_22580_PI429010886	N47272						0.0062149	21	15	0.0433288	21	3	3.933996
CUST_15620_PI429010886	N34874						0.0062505	12	9	0.0270063	15	12	47.90004
CUST_51441_PI429010886	M93544						0.0062664	24	15	0.000996	21	0	15.63697
CUST_39264_PI429010886	M36900	225712016	Cuticle protein 6 [Lepeophtheirus	2.62E-19			0.0062862	18	6	0.0046874	15	9	16348.66
CUST_38506_PI429010886	M27519						0.0063052	24	6	0.0426281	18	15	87.74995
CUST_32724_PI429010886	M4937	54400676	ORM1-like protein 3 [Danio rerio]	3.53E-52	Protein me	Regulation	0.0063238	24	15	0.0249688	12	0	120.0673
CUST_45038_PI429010886	M67802	123316129	Portunus pelagicus actin mRNA, p	3.53E-56			0.0063596	18	0	6.368E-05	18	0	47.80868
CUST_27532_PI429010886	N56226						0.0063965	24	21	0.0365169	21	18	11.81016
CUST_1532_PI429010886	N9957						0.0064008	24	0	0.0483298	12	0	61.86512
CUST_39150_PI429010886	M35302	493789484	hypothetical protein, partial [Lact	9.09E-08			0.006403	12	9	0.0001283	15	12	6276.978
CUST_11164_PI429010886	N27371						0.006417	21	6	0.0250553	18	15	1505.065
CUST_27857_PI429010886	N56824						0.0064183	21	18	0.0027876	18	0	133.4406
CUST_34254_PI429010886	M9176						0.0064347	21	15	0.0405839	21	9	4.846566
CUST_165_PI429010886	N1995						0.0064792	12	9	0.0453276	15	12	66946.06
CUST_39532_PI429010886	M40884						0.0065088	24	18	0.0008712	18	9	836.1158
CUST_11676_PI429010886	N28222						0.0065088	24	21	0.0019974	21	0	752.85
CUST_44659_PI429010886	M66592						0.0065127	21	0	0.0081224	15	9	40.17351
CUST_7728_PI429010886	N21981	307197312	Sodium-dependent neutral amino	9.9E-47	Transport	Neurotran	0.0065515	21	0	0.0040138	15	9	125.1074
CUST_36673_PI429010886	M16505						0.0065643	24	21	0.0014189	15	9	575.9481
CUST_1387_PI429010886	N9476	112420518	Gasterosteus aculeatus clone CFW	1.41E-98	Metabolic	Carbohydr	0.006573	24	15	0.0268534	21	18	185.9927
CUST_31966_PI429010886	M2853	321479500	hypothetical protein DAPPUDRAF	6.6E-28			0.0065848	15	6	0.0079378	15	9	373.6591
CUST_11886_PI429010886	N28574	46240812	crustin-like peptide type 2 [Marsu	1.52E-08			0.0066199	18	6	0.013009	15	12	322.1601
CUST_47810_PI429010886	M77692						0.0066878	24	0	0.0350969	12	9	33.51959
CUST_9937_PI429010886	N25439						0.0067047	24	15	0.0461231	21	3	12.99289
CUST_4185_PI429010886	N16187						0.006737	24	21	0.0244874	15	9	119.1547
CUST_20424_PI429010886	N43412						0.006737	21	9	0.0096865	21	0	11.51019
CUST_33135_PI429010886	M6056	521028669	Zinc finger protein 2 like protein [I	1.78E-09			0.006746	12	0	0.0388304	15	9	21.41005
CUST_36319_PI429010886	M15574						0.0067554	21	15	0.0018374	21	18	230.1554
CUST_31120_PI429010886	M739	291231880	PREDICTED: predicted protein-like	1.48E-41			0.0067632	12	0	0.0124831	24	15	189.9176
CUST_16275_PI429010886	N35954						0.00679	18	15	5.411E-06	24	21	126.6732
CUST_422_PI429010886	N5133	220172371	putative elastin a [Rimicaris exocu	7.65E-13			0.0068191	21	18	0.0272423	15	9	15900.37
CUST_20377_PI429010886	N43336						0.0068234	21	18	0.0101792	18	9	19.29052
CUST_22172_PI429010886	N46564	260831342	hypothetical protein BRAFLDRAFT	1.92E-34	Metabolic	Oxidation-	0.0068238	12	9	0.0284783	18	6	7.684798
CUST_3656_PI429010886	N14974	532759720	antimicrobial peptide type 1 precu	1.16E-18			0.0068277	24	9	0.0319969	18	6	51.49597
CUST_37049_PI429010886	M17683	157107416	n-acetylgalactosaminyltransferase	4.13E-48	Metabolic	Carbohydr	0.0068418	18	9	0.033196	21	18	44.58117
CUST_2676_PI429010886	N12887						0.0068606	24	21	0.0240809	24	15	525.5146
CUST_17318_PI429010886	N37766						0.0068606	24	21	0.0253423	24	18	48.92244
CUST_3501_PI429010886	N14631	504163658	PREDICTED: D-dopachrome decar	1.07E-13			0.0068753	18	0	0.0437538	18	6	138.0571
CUST_34546_PI429010886	M9982	321469961	hypothetical protein DAPPUDRAF	5.26E-54	Metabolic	Cellular am	0.0069125	24	15	0.0350457	21	18	43.69757
CUST_9646_PI429010886	N24981						0.006917	18	15	0.0249688	12	0	55.20546
CUST_42360_PI429010886	M60095	260811974	hypothetical protein BRAFLDRAFT	3.95E-17			0.0069365	12	3	0.0195699	12	3	121.0405
CUST_17772_PI429010886	N38565						0.0069435	24	18	0.0020816	15	9	98.478
CUST_4120_PI429010886	N16059						0.0069486	24	21	0.0026761	15	9	151.5229
CUST_51380_PI429010886	M93328				Metabolic	Cellular am	0.006993	24	21	0.0022986	18	9	39.82958
CUST_30542_PI429010886	N62090						0.0069961	21	18	0.012972	24	3	74.24613
CUST_42909_PI429010886	M61577	242006996	conserved hypothetical protein [P	1.45E-18			0.0070041	24	21	0.0010356	15	0	38.08216
CUST_48171_PI429010886	M79221						0.0070128	12	3	0.0012946	15	6	109.8644
CUST_50956_PI429010886	M91568						0.007032	24	15	0.012972	24	21	26.76803

Probe ID	ID	EBI ID	Annotation	E-value	GO categor	Go term	p-value LD	Period LD	Phase LD	p-value DD	Period DD	Phase DD	LD 0	LD/DD 0–24 (remaining columns)
CUST_44941_PI429010886	M67470						0.0070365	24	18	0.0450784	24	18	197.961	[illegible]
CUST_2599_PI429010886	N12731	345483293	PREDICTED: LOW QUALITY PROTE				0.0070365	24	21	0.0190025	24	18	1554.179	[illegible]
CUST_22211_PI429010886	N46633						0.0070825	24	21	0.0284785	12	0	105.2701	[illegible]
CUST_523_PI429010886	N5820						0.00711	21	15	0.001987	24	21	2085.588	[illegible]
CUST_32645_PI429010886	M4677	321462910	hypothetical protein DAPPUDRAF	6.83E-62	Protein me	Proteolysis	0.0071256	18	6	0.0001257	12	9	10.10472	[illegible]
CUST_54781_PI429010886	M107652						0.0071417	24	12	0.0354192	21	3	4.659382	[illegible]
CUST_36722_PI429010886	M16660	85677399	pigment-dispersing hormone 3 [M	3.45E-07	Signal tran	Other	0.0071465	21	12	0.0037162	24	21	142.0207	[illegible]
CUST_53098_PI429010886	M100133						0.0071936	18	3	0.016706	24	18	40.27654	[illegible]
CUST_52727_PI429010886	M98669						0.0072077	15	12	0.0217107	15	3	32.06294	[illegible]
CUST_2556_PI429010886	N12615						0.0072169	15	6	1.408E-06	21	9	20.91626	[illegible]
CUST_9786_PI429010886	N25204						0.0072553	21	15	0.0223736	15	0	29.31643	[illegible]
CUST_45089_PI429010886	M67974						0.0072692	24	9	0.004209	21	12	149.533	[illegible]
CUST_36556_PI429010886	M16224						0.0072932	12	0	0.0122913	24	0	105.0258	[illegible]
CUST_54916_PI429010886	M108155	157120354	hypothetical protein AaeL_AAEL0	3.07E-33	Cellular co	Cytoskelet	0.007325	24	9	0.0339138	12	3	24.21438	[illegible]
CUST_7800_PI429010886	N22098						0.0074227	24	21	0.0066833	12	0	92.17501	[illegible]
CUST_703_PI429010886	N6835						0.007428	24	18	0.039282	18	9	868.2894	[illegible]
CUST_16581_PI429010886	N36501				Metabolic	Lipid meta	0.0074711	15	12	3.356E-05	21	15	30.3574	[illegible]
CUST_6182_PI429010886	N19737						0.0074763	24	21	0.0037158	12	9	21.54756	[illegible]
CUST_53149_PI429010886	M100409						0.007486	15	0	0.0066957	24	3	141.539	[illegible]
CUST_5096_PI429010886	N18251						0.0075204	24	21	0.0044151	12	0	1931.709	[illegible]
CUST_15395_PI429010886	N34511						0.0075277	21	0	0.0205486	18	15	2509.266	[illegible]
CUST_28339_PI429010886	N57757						0.0075503	15	12	0.0046477	24	18	36.29093	[illegible]
CUST_37265_PI429010886	M18434	13898841	CUB-serine protease [Panulirus ar	8.86E-31	Protein me	Proteolysis	0.0075686	15	9	0.0060812	15	9	174.4941	[illegible]
CUST_13854_PI429010886	N31892						0.007617	15	6	0.0334676	12	0	9.721747	[illegible]
CUST_7889_PI429010886	N22228	321477199	hypothetical protein DAPPUDRAF	1.52E-73	Cellular Pr	Cell growth	0.0076324	24	18	0.04998	24	18	77.34627	[illegible]
CUST_1373_PI429010886	N9437						0.0076548	18	15	0.0264456	21	18	178.3394	[illegible]
CUST_38027_PI429010886	M22886						0.0076567	24	0	0.0104262	15	9	8430.296	[illegible]
CUST_43559_PI429010886	M63351						0.0077157	24	21	0.0416041	15	0	168.8172	[illegible]
CUST_43311_PI429010886	M62710						0.0077211	15	0	0.0145769	24	3	22.86698	[illegible]
CUST_16503_PI429010886	N36346	321473698	hypothetical protein DAPPUDRAF	2.89E-22			0.0077389	21	15	0.0152087	24	15	59.59396	[illegible]
CUST_25463_PI429010886	N52462	296245397	inhibitor of apoptosis protein [Lit	5.03E-27			0.0077794	18	0	0.0027076	24	0	77.9006	[illegible]
CUST_17893_PI429010886	N38786						0.0078075	24	6	0.0015749	21	12	17.40595	[illegible]
CUST_31060_PI429010886	M586	405970980	Cytosolic Fe-S cluster assembly fa	1.7E-122	Metabolic	Other	0.0078134	24	15	0.0342796	15	12	82.72734	[illegible]
CUST_36214_PI429010886	M15031						0.0078348	12	9	0.0165375	15	0	7.301642	[illegible]
CUST_49599_PI429010886	M86355						0.007835	12	9	0.017277	15	12	233.5329	[illegible]
CUST_48681_PI429010886	M81606						0.0078455	15	6	0.0016591	15	12	663.421	[illegible]
CUST_28259_PI429010886	N57617						0.007862	15	3	0.0166088	21	18	161.5143	[illegible]
CUST_29999_PI429010886	N60975						0.0078875	18	15	0.0042593	21	12	144.2677	[illegible]
CUST_43142_PI429010886	M62184						0.0078928	18	12	0.0044106	18	15	36.72221	[illegible]
CUST_25159_PI429010886	N51888						0.0079082	12	0	0.0245473	21	0	22.05957	[illegible]
CUST_37113_PI429010886	M17882	321472705	hypothetical protein DAPPUDRAF	5.81E-31			0.0079358	21	9	0.0039812	12	3	1123.543	[illegible]
CUST_14284_PI429010886	N32632						0.0079478	24	18	0.0005583	21	15	42.57027	[illegible]
CUST_9492_PI429010886	N24728						0.0079623	15	6	0.0348437	21	3	50.47426	[illegible]
CUST_4563_PI429010886	N17035						0.0080041	24	18	0.0082336	24	9	41.27805	[illegible]
CUST_33636_PI429010886	M7482						0.0080087	24	21	0.0009844	12	0	1547.502	[illegible]
CUST_21794_PI429010886	N45867						0.0080087	24	18	0.001987	21	0	41.62674	[illegible]
CUST_24707_PI429010886	N51124						0.0080126	21	0	0.0085892	18	0	108.1041	[illegible]
CUST_33009_PI429010886	M5713	291220823	PREDICTED: Saccoglossus kowalev				0.0080338	24	18	0.0066034	15	9	39.11041	[illegible]
CUST_18666_PI429010886	N40192	133754273	inhibitor of apoptosis protein [Per	5.25E-16			0.0080611	18	0	0.0010248	24	0	346.4474	[illegible]
CUST_23416_PI429010886	N48803						0.0080699	24	15	0.0327528	21	3	38.01913	[illegible]
CUST_55002_PI429010886	M108370	260787569	hypothetical protein BRAFLDRAFT	2.57E-16	Metabolic	Other	0.0080768	12	6	0.0333807	21	6	4.396195	[illegible]
CUST_7699_PI429010886	N21943						0.0081064	24	15	0.0348957	15	9	41.57926	[illegible]
CUST_37987_PI429010886	M22615	225680773	predicted protein [Paracoccidioide	8.48E-07			0.008147	21	6	0.0231015	15	12	3.97901	[illegible]
CUST_46514_PI429010886	M72873						0.008204	24	15	0.0342367	21	18	27.32487	[illegible]
CUST_11295_PI429010886	N27577	321466080	hypothetical protein DAPPUDRAF	5.22E-52			0.008204	24	21	0.0336096	18	12	19.92192	[illegible]
CUST_41048_PI429010886	M56756						0.0083017	24	15	0.0263107	24	15	491.1439	[illegible]
CUST_23075_PI429010886	N48215						0.0083017	24	21	0.0126434	15	12	50.50174	[illegible]
CUST_44074_PI429010886	M64858						0.0083344	24	15	0.0333515	21	9	104.9484	[illegible]
CUST_27164_PI429010886	N55561						0.0083857	21	6	0.0092734	24	0	14.65904	[illegible]
CUST_53571_PI429010886	M102302	444516848	Tankyrase-1 [Tupaia chinensis]	2.59E-14			0.0083938	24	6	0.0458369	15	12	23.67748	[illegible]
CUST_8632_PI429010886	N23331	321456752	hypothetical protein DAPPUDRAF	5.76E-31			0.0083994	24	15	0.0072917	15	0	95.28229	[illegible]
CUST_50888_PI429010886	M91350						0.0084668	12	9	0.0212765	15	12	7.994626	[illegible]
CUST_37172_PI429010886	M18089						0.0085607	24	15	0.0481371	12	0	799.0901	[illegible]
CUST_46130_PI429010886	M71487						0.0085621	24	3	0.003999	15	3	27.94531	[illegible]
CUST_3256_PI429010886	N14119	9186886	BCS-2 [Amphibalanus amphitrite]	1.79E-28			0.0085692	21	0	0.0236814	12	0	1416.053	[illegible]
CUST_8974_PI429010886	N23878						0.0086284	12	9	0.0318429	12	0	78.85311	[illegible]
CUST_4261_PI429010886	N16349						0.0086349	12	0	0.0324998	24	18	188.4673	[illegible]
CUST_49989_PI429010886	M88117						0.0086385	15	9	0.0298934	21	0	61.31201	[illegible]
CUST_15548_PI429010886	N34767						0.008653	24	3	0.0079512	24	15	23.4647	[illegible]
CUST_51229_PI429010886	M92702						0.0087053	21	6	0.0014284	21	3	10.66525	[illegible]
CUST_218_PI429010886	N2768						0.0087301	21	15	0.0052131	24	6	692.1008	[illegible]
CUST_19745_PI429010886	N42115						0.008747	12	0	0.0013517	18	15	29.24359	[illegible]
CUST_2160_PI429010886	N11724						0.0088095	18	0	0.0059787	24	21	27.77493	[illegible]
CUST_15942_PI429010886	N35384						0.008819	24	3	0.0015302	12	3	129.8849	[illegible]
CUST_15396_PI429010886	N34512	242019615	conserved hypothetical protein [P	6.17E-57			0.0088205	15	12	0.043801	21	0	26.28725	[illegible]
CUST_29427_PI429010886	N59841						0.0088421	21	0	0.0002458	24	3	3185.221	[illegible]
CUST_3235_PI429010886	N14075						0.008926	12	3	0.0103077	15	3	1442.661	[illegible]
CUST_45781_PI429010886	M70273						0.0089306	24	6	0.0053428	12	3	118.8089	[illegible]
CUST_12016_PI429010886	N28781						0.0089365	24	21	0.0165375	15	0	67.23484	[illegible]
CUST_1880_PI429010886	N10942						0.0089365	15	12	0.0002603	24	21	20.55824	[illegible]
CUST_11000_PI429010886	N27109						0.0089406	24	21	0.008823	24	18	159.4491	[illegible]
CUST_23163_PI429010886	N48362				Transport	Ion transp	0.0089406	24	18	0.0390938	18	12	4875.223	[illegible]
CUST_32582_PI429010886	M4478						0.0089437	24	15	0.0150238	18	3	8.391548	[illegible]
CUST_32518_PI429010886	M4332	91094771	PREDICTED: similar to AGAP00011	9.81E-16			0.0089696	24	0	0.0481371	15	12	1129.142	[illegible]
CUST_50998_PI429010886	M91728						0.0090251	12	9	0.006993	21	12	575.3242	[illegible]
CUST_51758_PI429010886	M94914	442769035	glycosyl-phosphatidylinositol-linke	3.23E-13	Metabolic	Nitrogen c	0.009061	12	0	0.0008638	15	9	206.2187	[illegible]
CUST_1085_PI429010886	N8443						0.0090718	18	0	0.0083994	24	18	542.7794	[illegible]
CUST_8820_PI429010886	N23647						0.0090737	15	12	0.0328355	21	0	23.82334	[illegible]
CUST_9544_PI429010886	N24811	321460073	eIF2B-alpha protein [Daphnia pule	2.13E-15	Protein me	Regulation	0.0090985	24	3	0.0406805	18	3	85.0591	[illegible]
CUST_42758_PI429010886	M61171	321471759	hypothetical protein DAPPUDRAF	1.36E-08			0.0091243	12	9	0.0473443	15	12	105.3673	[illegible]
CUST_3700_PI429010886	N15078						0.0091628	15	0	0.027118	18	3	317.4305	[illegible]
CUST_11650_PI429010886	N28179						0.0091759	24	18	0.0002718	12	0	62.03291	[illegible]
CUST_6812_PI429010886	N20632	291243678	PREDICTED: La autoantigen-like [S	1.2E-12			0.0092122	24	21	0.0220775	24	18	74.49329	[illegible]
CUST_45141_PI429010886	M68114	91090522	PREDICTED: similar to mitochondr	6.79E-10			0.0092608	18	0	0.0236814	12	9	284.3354	[illegible]
CUST_23063_PI429010886	N48192						0.0093259	24	18	0.0039134	15	12	68.27308	[illegible]
CUST_16479_PI429010886	N36305	211922663	opsin [Triops granarius]	5.3E-21			0.0093649	21	3	0.0044603	24	18	36.63747	[illegible]
CUST_13688_PI429010886	N31597						0.0093888	21	18	0.003129	15	0	399.0134	[illegible]
CUST_13474_PI429010886	N31252						0.0094112	24	21	0.02159	15	0	156.2876	[illegible]
CUST_52916_PI429010886	M99417						0.0094607	18	15	0.0074846	24	18	94.10473	[illegible]
CUST_57301_PI429010886	E4652	345316531	PREDICTED: beta-1,4-mannosyl-gl	2.61E-15	Metabolic	Carbohydr	0.0096242	24	21	0.0452973	24	18	29.58887	[illegible]
CUST_36079_PI429010886	M14565	328796226	mitochondrial ATP synthase gamn	4.83E-98	Metabolic	Generatior	0.0096592	18	15	0.027494	21	0	1091.098	[illegible]
CUST_13927_PI429010886	N32029						0.0096671	24	15	0.0011371	24	21	32.37762	[illegible]
CUST_52562_PI429010886	M97957						0.0096851	24	15	0.0128879	12	9	42.98408	[illegible]
CUST_32560_PI429010886	M4435	443717635	hypothetical protein CAPTEDRAFT	2.15E-50	Nucleic aci	RNA metal	0.0097355	18	12	0.0120968	21	18	16.11644	[illegible]
CUST_37413_PI429010886	M18944						0.0097546	12	9	0.0208812	15	9	7225.192	[illegible]
CUST_1194_PI429010886	N8797						0.0097641	24	18	0.0121918	15	12	3137.141	[illegible]
CUST_3565_PI429010886	N14765						0.0097746	24	21	0.0236814	12	0	64.99766	[illegible]
CUST_2594_PI429010886	N12719	91082911	PREDICTED: similar to dre4 CG182		Cellular Pr	Other	0.0097809	24	18	0.0194227	15	12	197.2544	[illegible]
CUST_48543_PI429010886	M80903						0.0098157	21	3	0.0227242	24	21	80.16082	[illegible]
CUST_52918_PI429010886	M99429	168824627	Euphausia superba isolate I241 cy	5.71E-93			0.0098719	12	0	1.983E-06	15	9	304.58	[illegible]
CUST_55723_PI429010886	M111475						0.0098817	24	21	0.0396913	15	9	100.7083	[illegible]
CUST_45946_PI429010886	M70838						0.0098817	24	3	0.006805	15	9	115.5968	[illegible]
CUST_27133_PI429010886	N55506	157132998	4-aminobutyrate aminotransferas	2.06E-36	Metabolic	Cellular an	0.0098817	24	18	8.359E-05	15	9	119.3101	[illegible]
CUST_1501_PI429010886	N9836						0.009905	24	21	0.0106325	12	0	3426.429	[illegible]
CUST_56031_PI429010886	M112588						0.0099305	21	15	0.0100232	24	15	57.49967	[illegible]
CUST_20135_PI429010886	N42869						0.0099994	24	18	0.0011078	18	9	152.096	[illegible]
CUST_29351_PI429010886	N59693						0.0099994	24	18	6.41E-06	18	0	26.14707	[illegible]
CUST_33124_PI429010886	M6029						0.0100594	18	15	0.033204	21	18	150.7719	[illegible]
CUST_7088_PI429010886	N21023	518897355	hypothetical protein [alpha prote	1.23E-27	Metabolic	Other	0.0101095	21	18	1.146E-05	18	12	74.62293	[illegible]
CUST_54515_PI429010886	M106316						0.010117	24	21	0.0278545	15	0	160.3187	[illegible]
CUST_26490_PI429010886	N54335						0.010117	24	18	0.016153	15	9	19.23397	[illegible]
CUST_25213_PI429010886	N51985						0.0101962	12	6	0.0255581	12	0	8.689291	[illegible]
CUST_41776_PI429010886	M58535						0.0102464	21	15	0.0499652	21	12	94.33013	[illegible]
CUST_6245_PI429010886	N19844	321478487	cyclin G-like protein [Daphnia pule	9.29E-58			0.0103293	15	12	0.0413265	18	3	84.63533	[illegible]
CUST_4044_PI429010886	N15871						0.0103849	21	18	0.0272563	12	0	108.2696	[illegible]
CUST_1680_PI429010886	N10420	195425743	GK10316 [Drosophila willistoni] >g	9.74E-16			0.0105385	18	15	0.0079082	12	0	33.85933	[illegible]
CUST_30687_PI429010886	N62401						0.0105505	21	0	0.0221474	21	12	98.66873	[illegible]
CUST_12036_PI429010886	N28816	37595925	signal transducer and activator of	1.14E-85	Signal tran	Other	0.0105735	24	15	0.0272423	15	9	23.8982	[illegible]
CUST_32674_PI429010886	M4771						0.0106408	24	9	0.0070092	18	3	8.131518	[illegible]
CUST_23710_PI429010886	N49332						0.0106858	24	6	0.0156612	18	15	13.77957	[illegible]
CUST_20161_PI429010886	N42921						0.0107507	15	0	0.0366144	18	12	13.28376	[illegible]
CUST_3806_PI429010886	N15313	255985514	Kazal-type proteinase inhibitor [Pa	1.02E-24			0.0108319	21	18	0.0025358	15	12	61.0126	[illegible]
CUST_32563_PI429010886	M4439						0.0108552	18	6	0.0230067	15	9	38.08917	[illegible]
CUST_246_PI429010886	N2999						0.0108977	18	15	0.0373002	15	9	35.33768	[illegible]
CUST_16421_PI429010886	N36208						0.0109436	12	6	0.0471629	12	6	11.54107	[illegible]
CUST_3138_PI429010886	N13881						0.0109725	12	9	0.0063912	18	6	75.3636	[illegible]
CUST_44584_PI429010886	M66385	260814680	hypothetical protein BRAFLDRAFT	4.99E-11			0.0110381	24	15	0.0270313	24	18	5.06766	[illegible]
CUST_34940_PI429010886	M11130				Protein me	Proteolysis	0.0110508	24	15	0.0442332	24	6	412.4307	[illegible]
CUST_35063_PI429010886	M11462	123507472	ankyrin repeat protein [Trichomo	1.42E-12			0.0110951	21	12	0.007618	24	9	293.568	[illegible]
CUST_15747_PI429010886	N35075						0.0111063	12	0	0.0358656	15	0	286.2354	[illegible]
CUST_5822_PI429010886	N19248	328705463	PREDICTED: hypothetical protein I	7.6E-62			0.0112004	24	18	0.0339244	18	6	185.937	[illegible]
CUST_47025_PI429010886	M74563						0.0112269	12	9	0.0354894	15	12	43.19018	[illegible]
CUST_2406_PI429010886	N12285	386376737	hematopoietic prostaglandin D sy	4.25E-61			0.0112269	24	18	0.0306262	18	9	432.3981	[illegible]
CUST_52403_PI429010886	M97333	321461236	hypothetical protein DAPPUDRAF	2.12E-09			0.0112887	15	6	0.0027083	15	9	459.3044	[illegible]
CUST_18072_PI429010886	N39090	465954970	Beta-1,4-galactosyltransferase 3 [	3.48E-20	Metabolic	Carbohydr	0.0113386	24	15	0.049392	24	21	51.87516	[illegible]
CUST_54330_PI429010886	M105618						0.011419	12	3	0.0146169	18	0	30.98378	[illegible]
CUST_47272_PI429010886	M75421	321476689	hypothetical protein DAPPUDRAF	2.8E-11			0.011419	12	3	0.0068714	24	9	25.52339	[illegible]
CUST_40966_PI429010886	M56505						0.0114392	15	6	0.0438918	12	6	158.9852	[illegible]
CUST_32120_PI429010886	M3279	410509307	Penaeus monodon MYH2 mRNA f	2E-129			0.0114557	18	15	0.0292631	24	6	87.48976	[illegible]
CUST_44757_PI429010886	M66893						0.0114689	24	3	0.0351335	24	3	242.1802	[illegible]
CUST_25971_PI429010886	N53411						0.0115052	24	0	0.0035687	12	0	30.21523	[illegible]
CUST_41709_PI429010886	M58360						0.0115473	12	9	0.0013067	21	12	15.7393	[illegible]
CUST_392_PI429010886	N4801	321466528	hypothetical protein DAPPUDRAF	2.58E-19			0.0116315	18	6	0.000459	15	9	2759.556	[illegible]
CUST_32685_PI429010886	M4810	149675211	cuticle proprotein proCP5.2 [Callir	5.32E-71			0.0116514	15	6	0.0124079	15	9	6758.872	[illegible]
CUST_245_PI429010886	N2986						0.0116749	18	15	0.0238868	18	9	10.61538	[illegible]
CUST_5161_PI429010886	N18339	156544470	PREDICTED: hsp90 co-chaperone (	7.2E-106			0.0116936	24	15	0.0424795	21	15	285.6824	[illegible]
CUST_13473_PI429010886	N31248						0.0116954	15	3	0.029353	24	15	35.98686	[illegible]
CUST_34598_PI429010886	M10120						0.0117184	24	12	0.0218872	15	0	47.30405	[illegible]
CUST_49842_PI429010886	M87391	528746119	C-type lectin 4 [Fenneropenaeus r	3.99E-08			0.0117296	24	15	0.0224749	21	18	44.63889	[illegible]
CUST_44434_PI429010886	M65978						0.0117549	24	9	0.0222192	15	0	335.6891	[illegible]
CUST_48873_PI429010886	M82629						0.0118027	15	9	0.030896	24	21	20.19825	[illegible]
CUST_46757_PI429010886	M73623						0.0118039	12	9	0.0205391	15	12	77.05387	[illegible]
CUST_8328_PI429010886	N22878	66499523	PREDICTED: peroxisomal membra	5.73E-85	Transport	Mitochonc	0.0118134	21	15	0.0113621	24	15	33.3276	[illegible]
CUST_34644_PI429010886	M10222						0.0118779	24	18	0.0390962	24	0	108.9127	[illegible]
CUST_31061_PI429010886	M593						0.0119015	21	12	0.0258236	18	15	454.3052	[illegible]
CUST_34392_PI429010886	M9529	170576227	unspecific monooxygenase [Brugi	7.58E-07			0.0119037	21	0	0.0467781	18	9	33.72001	[illegible]
CUST_8252_PI429010886	N22774						0.0119236	24	3	0.0342582	15	3	147.5974	[illegible]
CUST_32403_PI429010886	M4015				Metabolic	Cellular an	0.0119467	21	15	6.87E-05	24	15	107.2361	[illegible]
CUST_3853_PI429010886	N15407						0.0120743	24	21	0.0175431	15	9	254.9746	[illegible]
CUST_3116_PI429010886	N13829						0.0121063	12	6	0.0033814	12	0	2167.915	[illegible]

Probe ID	ID	EBI ID	Annotation	E-value	GO categor	Go term	p-value LD	Period LD	Phase LD	p-value DD	Period DD	Phase DD
CUST_2736_PI429010886	N13007	512127157	checkpoint kinase 1 [Daphnia pule	1.3E-142	Protein me	Cellular pr	0.0121905	24	18	0.0415559	12	0
CUST_20775_PI429010886	N44037						0.0122013	24	21	0.0240439	15	12
CUST_32644_PI429010886	M4676	241846314	SEL-1, putative [Ixodes scapularis]	1.1E-122	Metabolic	Other	0.012218	18	9	0.039534	12	0
CUST_52256_PI429010886	M96867						0.012218	18	15	0.0275003	24	21
CUST_16529_PI429010886	N36400	195162205	GL14252 [Drosophila persimilis] >	5.61E-10	Cellular Pr	Cell death	0.0122307	21	0	0.0242612	21	0
CUST_31969_PI429010886	M2866						0.0123468	24	18	0.0168017	24	18
CUST_50020_PI429010886	M88284						0.0123912	12	0	0.0132387	18	12
CUST_17978_PI429010886	N38938						0.0124229	24	3	0.0010147	18	3
CUST_46984_PI429010886	M74401						0.012464	12	9	0.0253437	21	0
CUST_25355_PI429010886	N52253						0.0124844	15	12	0.0156576	18	3
CUST_41799_PI429010886	M58579						0.0124928	15	3	0.0378677	21	9
CUST_39906_PI429010886	M46634						0.0125004	12	0	0.0019572	12	9
CUST_43526_PI429010886	M63277						0.0125593	15	0	0.0048528	15	0
CUST_31440_PI429010886	M1533						0.0126594	24	21	0.0346443	24	15
CUST_13698_PI429010886	N31614						0.0126594	24	21	0.0077532	15	0
CUST_14281_PI429010886	N32627						0.0127187	24	15	0.0310345	18	9
CUST_44725_PI429010886	M66795						0.0127504	24	3	0.0070259	12	3
CUST_29937_PI429010886	N60846						0.0127557	12	3	0.0311242	18	3
CUST_21397_PI429010886	N45146	260813040	hypothetical protein BRAFLDRAFT	2.35E-07	Metabolic	Nitrogen c	0.0127831	24	3	0.0126441	12	3
CUST_57276_PI429010886	E3651	4585366	extracellular superoxide dismutas	2.4E-21	Metabolic	Oxidation-	0.0127871	12	9	0.0105447	15	9
CUST_40077_PI429010886	M49819						0.0127964	18	15	0.0009676	24	21
CUST_4734_PI429010886	N17371						0.0128157	24	18	0.0027306	21	9
CUST_13415_PI429010886	N31139						0.0128157	24	18	0.0210597	21	12
CUST_761_PI429010886	N7195						0.0128185	12	6	0.0420019	24	3
CUST_52329_PI429010886	M97076	334326889	PREDICTED: zinc finger protein 28	2.97E-26			0.0128386	21	0	0.0275484	15	9
CUST_42105_PI429010886	M59408						0.0129913	18	15	0.0425605	15	9
CUST_1872_PI429010886	N10927						0.013094	24	3	0.0119376	18	3
CUST_42692_PI429010886	M60985						0.0131256	24	15	0.0057045	21	0
CUST_37125_PI429010886	M17932						0.0131283	24	18	0.0032594	21	0
CUST_25911_PI429010886	N53285	242022418	heat containing protein, putative	1.01E-11			0.0131286	24	15	0.0028646	15	12
CUST_31677_PI429010886	M2148						0.0131442	24	18	0.0023532	24	0
CUST_34544_PI429010886	M9977						0.0131442	24	18	0.0044811	21	18
CUST_21376_PI429010886	N45105						0.0131645	15	9	0.0015663	15	0
CUST_7810_PI429010886	N22117	459175521	PREDICTED: beta-hexosaminidase	2.5E-99	Metabolic	Carbohydr	0.0131854	12	3	0.0268515	21	12
CUST_50260_PI429010886	M89214						0.0132845	24	3	0.0079378	15	3
CUST_14076_PI429010886	N32287						0.0132845	24	18	0.0070297	15	12
CUST_35224_PI429010886	M11957						0.0133006	18	9	0.0172981	24	18
CUST_34184_PI429010886	M8985	383865231	PREDICTED: polypeptide N-acetylg	9.07E-16			0.0133006	18	15	0.0044106	18	15
CUST_31516_PI429010886	M1703						0.013396	24	15	0.044309	15	12
CUST_12905_PI429010886	N30244	391325031	PREDICTED: zinc finger protein 48	2.3E-11			0.0133976	24	18	0.0169465	18	12
CUST_10536_PI429010886	N26386						0.0134227	15	9	0.0202069	24	21
CUST_9613_PI429010886	N24924						0.0134408	24	18	0.0081224	15	9
CUST_52039_PI429010886	M96174	345486208	PREDICTED: neither inactivation n	6.94E-08			0.0134497	24	0	0.0151937	21	3
CUST_21808_PI429010886	N45889						0.0134672	15	9	0.0078204	12	0
CUST_4560_PI429010886	N17029						0.0134682	24	21	0.0074689	12	0
CUST_5185_PI429010886	N18371	345494023	PREDICTED: Nasonia vitripennis hy	3.3E-104	Transport	Protein tra	0.0135574	24	15	0.0110809	15	0
CUST_16538_PI429010886	N36427						0.0135971	24	18	0.0292734	24	21
CUST_29553_PI429010886	N60094	549834	RecName: Full=Zinc finger Y-chror	2.16E-09	Nucleic aci	Regulation	0.0135971	24	21	0.0120413	15	12
CUST_42545_PI429010886	M60556	321463530	hypothetical protein DAPPUDRAF	1.35E-16	Metabolic	Carbohydr	0.0136353	12	3	0.0122941	12	9
CUST_51759_PI429010886	M94916						0.0137534	24	18	0.0099523	15	9
CUST_32730_PI429010886	M4946						0.0137698	15	3	0.042943	18	6
CUST_335_PI429010886	N4034	170040829	predicted protein [Culex quinquef	9.59E-10			0.0137823	15	6	0.0006896	15	12
CUST_3936_PI429010886	N15614						0.0138392	21	12	0.0266472	24	18
CUST_25406_PI429010886	N52357						0.0138791	21	9	0.0121436	15	0
CUST_18488_PI429010886	N39887						0.0138904	15	0	0.0473326	12	3
CUST_5656_PI429010886	N18999						0.0139072	18	0	0.044447	18	0
CUST_37641_PI429010886	M20124						0.0139193	18	15	0.0017022	15	12
CUST_10522_PI429010886	N26368						0.0139792	24	0	0.0264964	24	18
CUST_50849_PI429010886	M91234						0.0139923	24	18	0.0033393	18	9
CUST_32221_PI429010886	M3560						0.014043	12	0	0.0362605	15	3
CUST_16460_PI429010886	N36270						0.014088	21	18	0.0181324	12	0
CUST_46414_PI429010886	M72608	405959130	Elongator complex protein 1 [Cras	1.77E-16			0.0142219	18	9	5.337E-06	24	18
CUST_38514_PI429010886	M27586						0.0142599	24	9	0.0104187	18	3
CUST_41687_PI429010886	M58310						0.0142599	24	0	0.0091716	24	15
CUST_25004_PI429010886	N51619						0.0142771	12	6	0.0041911	15	0
CUST_36450_PI429010886	M15926						0.0143378	24	15	0.001625	12	0
CUST_8969_PI429010886	N23868						0.0143378	24	15	0.0072274	24	15
CUST_34435_PI429010886	M9671						0.0143601	12	9	0.0044717	15	12
CUST_40769_PI429010886	M56047						0.0143843	24	15	0.0010789	18	3
CUST_2307_PI429010886	N12070						0.0144553	12	0	0.0210463	21	0
CUST_53194_PI429010886	M100662						0.0144563	12	6	0.024034	21	9
CUST_45680_PI429010886	M69891	321474894	hypothetical protein DAPPUDRAF	1.24E-17			0.0145252	12	3	0.0103448	15	0
CUST_40189_PI429010886	M51523						0.0145309	21	15	0.0219955	18	12
CUST_49201_PI429010886	M84656						0.0145384	24	0	0.0096443	24	18
CUST_15651_PI429010886	N34921						0.0145386	12	6	0.0071402	21	9
CUST_42993_PI429010886	M61807						0.0145832	12	0	0.021644	15	0
CUST_238_PI429010886	N2929						0.0146832	24	15	0.0414943	24	18
CUST_7550_PI429010886	N21714						0.0146836	18	6	0.0235358	24	18
CUST_56726_PI429010886	M114523						0.0146903	12	9	0.0035941	15	9
CUST_1140_PI429010886	N8622						0.0146903	12	9	0.0141868	15	12
CUST_20128_PI429010886	N42856						0.0147023	24	18	0.0209168	15	12
CUST_26614_PI429010886	N54541	471417319	PREDICTED: unconventional myos	8.28E-38			0.0147023	24	15	0.0362734	24	18
CUST_35751_PI429010886	M13413						0.014856	24	15	0.0110779	24	18
CUST_51941_PI429010886	M95754						0.0148745	24	6	0.0229898	12	9
CUST_50615_PI429010886	M90438						0.0148958	21	3	0.0258236	18	15
CUST_23265_PI429010886	N48538						0.0148958	24	0	3.625E-06	24	0
CUST_38741_PI429010886	M29865						0.0149112	24	12	0.0058429	18	12
CUST_31595_PI429010886	M1924	270007800	hypothetical protein TcasGA2_TC(	5.11E-07			0.0150204	12	9	0.0204281	15	9
CUST_19521_PI429010886	N41709						0.0150287	24	21	0.0354894	15	12
CUST_32057_PI429010886	M3130	405960295	Fumarylacetoacetase [Crassostrea	7.9E-177	Metabolic	Cellular an	0.0150585	24	18	0.0454507	15	12
CUST_49021_PI429010886	M83649						0.0150758	21	15	0.0320278	21	15
CUST_38837_PI429010886	M31074	156938905	profilin [Penaeus monodon]	2.42E-30	Cellular co	Cytoskelet	0.0150892	24	6	0.0207691	21	3
CUST_12362_PI429010886	N29348						0.0150892	24	18	0.0077532	15	0
CUST_17137_PI429010886	N37415						0.0150892	24	21	0.0131645	15	6
CUST_32608_PI429010886	M4562	443728134	hypothetical protein CAPTEDRAFT	8.25E-97			0.0150992	18	15	0.0018542	24	15
CUST_14128_PI429010886	N32370						0.0150992	18	15	0.045236	18	3
CUST_48059_PI429010886	M78726	197940771	Euphausia superba mitochondrior	1.6E-149			0.0151435	24	6	0.024913	15	0
CUST_16717_PI429010886	N36746						0.0152015	24	15	0.0212471	24	15
CUST_1398_PI429010886	N9515	321459536	hypothetical protein DAPPUDRAF	4.55E-37			0.0152015	24	15	0.000376	15	12
CUST_20565_PI429010886	N43666						0.0152058	12	9	0.0304467	15	9
CUST_55416_PI429010886	M110005						0.0152574	21	15	0.0013673	24	18
CUST_7538_PI429010886	N21697				Metabolic	Nucleobas	0.0152827	24	18	0.0488949	12	9
CUST_37246_PI429010886	M18356						0.015288	12	0	0.0241723	24	21
CUST_53975_PI429010886	M103943						0.0153529	21	9	0.0065554	12	3
CUST_33244_PI429010886	M6382						0.0153552	24	0	0.0438468	15	9
CUST_39199_PI429010886	M36036						0.0153682	15	0	0.0368641	18	15
CUST_391_PI429010886	N4800	225712386	Cuticle protein 6 [Lepeophtheirus	4.51E-21			0.0153838	15	6	0.0130132	15	9
CUST_22687_PI429010886	N47460	543171838	CD209 antigen-like protein D [Onc	9.1E-08			0.0154053	12	0	0.0005174	24	21
CUST_19138_PI429010886	N41036						0.0155386	15	12	0.005755	12	0
CUST_3680_PI429010886	N15033	321462196	hypothetical protein DAPPUDRAF	8.27E-20			0.0156567	24	12	0.0326354	21	12
CUST_48584_PI429010886	M81131						0.01568	12	0	0.0262733	21	0
CUST_56199_PI429010886	M113086						0.0157057	24	0	0.0050194	15	12
CUST_2852_PI429010886	N13248	85057633	hypothetical protein AYWB_353 [	6.11E-08			0.0157057	24	15	0.0221304	15	0
CUST_54789_PI429010886	M107683	197940771	Euphausia superba mitochondrior	1.3E-104	Transport	Ion transp	0.0157107	24	18	0.026489	12	0
CUST_36978_PI429010886	M17397						0.0157446	15	12	0.0425204	24	21
CUST_14957_PI429010886	N33759						0.015746	24	12	0.0385804	12	9
CUST_43432_PI429010886	M63030						0.0158233	24	15	0.0260141	24	21
CUST_38928_PI429010886	M32160						0.0158608	18	3	0.0335669	15	12
CUST_53842_PI429010886	M103387						0.015985	24	21	0.0038176	15	12
CUST_21024_PI429010886	N44494	351699829	Glucose-dependent insulinotropic	2.77E-09			0.0159976	24	18	0.006314	15	9
CUST_6335_PI429010886	N19969	416656	RecName: Full=Arrestin homolog	9.1E-87	Signal tran	Other	0.0160225	12	0	0.0341287	15	0
CUST_9031_PI429010886	N23963	115345804	hemolymph clottable protein [Litc	3.11E-15			0.0160584	12	6	0.0218917	21	9
CUST_26634_PI429010886	N54586						0.0160586	24	0	0.0216978	21	3
CUST_17595_PI429010886	N38245						0.0160842	18	9	0.0103293	15	3
CUST_49525_PI429010886	M85975	195999790	hypothetical protein TRIADDRAFT	1.06E-08	Transport	Ion transp	0.0161007	21	12	0.0080649	15	6
CUST_19121_PI429010886	N41011						0.0161316	18	12	0.0471003	21	0
CUST_6767_PI429010886	N20574						0.0162072	18	12	0.0333089	15	9
CUST_54269_PI429010886	M105340						0.0162666	24	18	0.0084444	15	9
CUST_37885_PI429010886	M22046	149675211	cuticle proprotein proCP5.2 [Callir	8.11E-41			0.0163143	15	6	0.0097153	15	9
CUST_36877_PI429010886	M17119						0.0163207	24	18	0.012676	12	0
CUST_37644_PI429010886	M20185						0.0163345	21	18	0.0314099	21	9
CUST_13281_PI429010886	N30906	443693165	hypothetical protein CAPTEDRAFT	4.16E-14			0.0166047	15	12	0.0009225	21	0
CUST_42938_PI429010886	M61668						0.0166066	24	18	0.0177642	24	18
CUST_10743_PI429010886	N26716						0.0166886	18	15	0.0024075	18	3
CUST_41673_PI429010886	M58284						0.016701	21	15	0.0050427	12	0
CUST_32277_PI429010886	M3688	443721461	hypothetical protein CAPTEDRAFT	7.48E-55	Symbiosis, Viral proce		0.0167423	15	12	0.0287657	24	0
CUST_390_PI429010886	N4792						0.0167423	15	6	0.0007066	15	12
CUST_43587_PI429010886	M63421	54042615	arthrodial cuticle protein AMP6.0	3.27E-15			0.0167876	24	18	0.0001616	12	9
CUST_11719_PI429010886	N28294	545182363	PREDICTED: LOW QUALITY PROTE	1.65E-13			0.0168701	21	3	0.0170961	21	0
CUST_31594_PI429010886	M1922						0.0168873	18	3	0.0074463	18	12
CUST_49282_PI429010886	M85009						0.0168887	15	12	0.0126912	18	0
CUST_30020_PI429010886	N61018						0.0168972	21	3	0.0461804	21	3
CUST_52687_PI429010886	M98466	260792312	hypothetical protein BRAFLDRAFT	8.65E-19			0.0168976	21	15	0.0265918	12	0
CUST_19050_PI429010886	N40899						0.0169087	21	9	0.0268369	12	3
CUST_42385_PI429010886	M60156						0.016915	24	6	0.0079794	21	15
CUST_21432_PI429010886	N45207						0.0169218	21	18	0.0325469	12	0
CUST_14072_PI429010886	N32283						0.0169444	21	15	0.0479611	15	12
CUST_1543_PI429010886	N10001						0.016965	12	3	0.0115869	12	9
CUST_32473_PI429010886	M4202						0.0170644	12	9	0.0007066	15	12
CUST_4267_PI429010886	N16367						0.017086	18	3	0.0388954	15	9
CUST_6354_PI429010886	N19992	344264210	PREDICTED: arginase-1-like [Loxoc	4.43E-53	Metabolic	Cellular an	0.0171044	24	6	0.0386068	21	9
CUST_37753_PI429010886	M21399	506968295	60S ribosomal protein L6 [Coptote	4.65E-61	Protein me	Ribosome	0.0171213	24	18	0.0118335	15	12
CUST_48263_PI429010886	M79590						0.0171213	24	21	0.0223448	15	0
CUST_29182_PI429010886	N59348	321473698	hypothetical protein DAPPUDRAF	2.23E-07	Protein me	Ribosome	0.0171247	21	15	0.0402346	15	12
CUST_3929_PI429010886	N15593	442633892	CG7632, isoform B [Drosophila me	5.67E-17			0.017126	21	15	0.0008277	21	6
CUST_3930_PI429010886	N15602						0.0172354	15	6	0.0301559	15	9
CUST_886_PI429010886	N7700						0.0172701	24	12	0.021069	18	15
CUST_57143_PI429010886	E1475	241725974	guanine nucleotide-binding protei	1.44E-14	Metabolic	Other	0.0173067	15	9	0.0383789	18	9
CUST_19858_PI429010886	N42331						0.0173276	24	21	0.0004813	15	9
CUST_23773_PI429010886	N49441	321461754	ABC transporter [Daphnia pulex]	2.43E-21			0.0173276	24	18	0.0042547	12	0
CUST_34013_PI429010886	M8501						0.01734	18	12	0.0249701	12	0
CUST_32384_PI429010886	M3981	91086121	PREDICTED: similar to adenine ph	5.42E-47	Metabolic	Nucleobas	0.0175037	18	12	0.0008941	21	9
CUST_11681_PI429010886	N28230						0.0175146	24	15	0.0044117	15	0
CUST_12001_PI429010886	N28759						0.0175339	24	21	0.0232595	24	18
CUST_43504_PI429010886	M63222	321475219	hypothetical protein DAPPUDRAF	7.24E-79	Metabolic	Cellular an	0.0176771	21	15	0.032666	21	15
CUST_43956_PI429010886	M64457						0.0177401	24	21	0.0034249	15	9
CUST_49621_PI429010886	M86426						0.017742	24	15	0.030384	15	12
CUST_28714_PI429010886	N58477						0.0177456	12	9	0.0179543	24	0
CUST_45351_PI429010886	M68819	260841562	hypothetical protein BRAFLDRAFT	3.1E-08			0.0177648	21	0	0.000555	24	18
CUST_36736_PI429010886	M16697	270161184	i-type lysozyme-like protein 2 [Per	2.04E-12	Metabolic	Carbohydr	0.0178415	21	9	0.0248685	12	3

Probe ID	ID	EBI ID	Annotation	E-value	GO categor	Go term	p-value LD	Period LD	Phase LD	p-value DD	Period DD	Phase DD	LD 0	Remaining LD/DD time points
CUST_9174_PI429010886	N24180	321477120	hypothetical protein DAPPUDRAF	3.1E-103	Metabolic	Nucleobas	0.0178807	18	15	0.0327941	21	18	55.19052	[illegible]
CUST_56229_PI429010886	M113201						0.0179464	24	21	0.0221304	15	9	37.77609	[illegible]
CUST_3308_PI429010886	N14229						0.0179695	24	21	0.0092413	15	12	109.9215	[illegible]
CUST_23793_PI429010886	N49480						0.0180344	24	18	0.011765	12	0	43.62652	[illegible]
CUST_9283_PI429010886	N24369						0.0180493	24	21	0.0370682	24	15	597.7182	[illegible]
CUST_46352_PI429010886	M72339						0.018126	24	15	0.0269362	15	9	558.6463	[illegible]
CUST_54447_PI429010886	M106057						0.0181527	24	21	0.0241904	12	3	23.20911	[illegible]
CUST_27254_PI429010886	N55716						0.018197	24	15	0.0007919	15	0	183.4751	[illegible]
CUST_43270_PI429010886	M62583						0.0182475	24	18	0.0236814	12	0	28.63092	[illegible]
CUST_41268_PI429010886	M57293						0.0182702	24	18	0.0046924	24	3	18.35004	[illegible]
CUST_16603_PI429010886	N36549						0.01828	18	3	0.0038727	18	9	30.2584	[illegible]
CUST_20653_PI429010886	N43824	321458840	hypothetical protein DAPPUDRAF	1.2E-15			0.0183231	15	12	0.0127013	18	12	5.98788	[illegible]
CUST_17930_PI429010886	N38855						0.0183736	12	9	0.0403406	21	9	40.0019	[illegible]
CUST_46856_PI429010886	M73959						0.0184244	24	21	0.0401879	24	21	32.68841	[illegible]
CUST_24031_PI429010886	N49901						0.0184492	24	21	0.0452801	24	15	329.8991	[illegible]
CUST_46291_PI429010886	M72085						0.0185842	18	0	0.0452827	12	6	66.18402	[illegible]
CUST_2415_PI429010886	N12299	303277513	predicted protein [Micromonas pu	9.84E-13			0.0185985	21	0	0.02203	15	9	241.54	[illegible]
CUST_39413_PI429010886	M39064						0.0186519	24	15	0.0278386	12	0	5540.01	[illegible]
CUST_10197_PI429010886	N25855	431838964	Lymphoid-specific helicase [Ptero	6.84E-07			0.0186519	24	21	0.0017238	15	12	44.35011	[illegible]
CUST_32643_PI429010886	M4674	528469211	PREDICTED: mucin-22-like [Danio	2.09E-07			0.0186779	24	18	0.0448994	24	0	84.24007	[illegible]
CUST_28506_PI429010886	N58079						0.0187441	15	0	0.0064013	21	9	17.14735	[illegible]
CUST_26542_PI429010886	N54422						0.018796	12	9	0.0008709	24	15	36.56104	[illegible]
CUST_15435_PI429010886	N34576						0.0188425	24	15	0.0282054	24	3	114.0434	[illegible]
CUST_12162_PI429010886	N29027	260820407	hypothetical protein BRAFLDRAFT	8.64E-38	Metabolic	Nucleobas	0.0189884	18	9	0.044309	15	6	109.1979	[illegible]
CUST_393_PI429010886	N4805	225712016	Cuticle protein 6 [Lepeophtheirus	1.35E-19			0.0191283	18	6	0.0008536	15	9	2889.672	[illegible]
CUST_20052_PI429010886	N42719						0.0191721	21	15	0.043994	24	6	137.2351	[illegible]
CUST_29939_PI429010886	N60852						0.0192544	24	15	0.0017238	15	12	49.3434	[illegible]
CUST_36091_PI429010886	M14594	330434836	hypothetical protein [Macrobrach	1.44E-12			0.0192956	18	15	0.0109823	21	9	72.57836	[illegible]
CUST_30562_PI429010886	N62125						0.0193203	24	3	0.0159203	15	0	18.66401	[illegible]
CUST_41186_PI429010886	M57088						0.0193257	24	9	0.0049371	15	3	120.3932	[illegible]
CUST_47512_PI429010886	M76418						0.0193257	24	15	0.0109996	18	0	155.2317	[illegible]
CUST_9363_PI429010886	N24494	512863611	PREDICTED: LOW QUALITY PROTE	1.01E-36			0.0194073	18	3	0.038874	15	9	11.68791	[illegible]
CUST_24638_PI429010886	N50996						0.0194296	12	3	0.0031256	21	18	55.84882	[illegible]
CUST_22630_PI429010886	N47363						0.0195337	18	9	0.0025087	18	3	15.46338	[illegible]
CUST_33665_PI429010886	M7549				Cellular co	Cytoskelet	0.0195617	24	3	0.0031049	21	15	20.81726	[illegible]
CUST_52568_PI429010886	M97992						0.0195617	24	3	0.0061518	15	3	571.5137	[illegible]
CUST_35398_PI429010886	M12477						0.0196065	15	3	0.0056651	18	0	16.76758	[illegible]
CUST_30871_PI429010886	M61				Transport	Intracellul	0.0197473	18	15	0.0377632	21	18	361.4106	[illegible]
CUST_36296_PI429010886	M15481	321462126	hypothetical protein DAPPUDRAF	4.57E-16			0.0198931	24	6	0.0046698	24	3	74.55481	[illegible]
CUST_45346_PI429010886	M68809						0.0199934	24	18	0.0004899	12	9	54.19504	[illegible]
CUST_41969_PI429010886	M59026						0.0200048	12	3	0.0003717	18	3	70.52075	[illegible]
CUST_42292_PI429010886	M59923	260833078	hypothetical protein BRAFLDRAFT	5.8E-07	Protein me	Cellular pr	0.0200069	15	0	0.0119536	24	21	37.14266	[illegible]
CUST_795_PI429010886	N7343						0.020035	15	6	0.0045771	15	9	5451.324	[illegible]
CUST_34377_PI429010886	M9484						0.0200704	12	0	0.0499518	12	0	224.2026	[illegible]
CUST_8572_PI429010886	N23238	405965499	Solute carrier family 35 member F	1.28E-30			0.0200755	24	12	0.0035687	12	6	18.2488	[illegible]
CUST_36150_PI429010886	M14810	348563737	PREDICTED: collagen alpha-5(IV) c	1.2E-25			0.0200894	21	3	0.0464388	18	9	10.25642	[illegible]
CUST_51772_PI429010886	M95000						0.0201271	21	18	0.0066579	12	9	190.1223	[illegible]
CUST_47341_PI429010886	M75696						0.0201849	15	6	0.0032904	15	12	853.4119	[illegible]
CUST_28668_PI429010886	N58395						0.0202231	24	21	0.0137742	18	9	48.98753	[illegible]
CUST_55227_PI429010886	M109198						0.0202441	24	6	0.0001799	18	15	654.3402	[illegible]
CUST_11382_PI429010886	N27723						0.0202472	18	15	0.0127964	21	18	199.2769	[illegible]
CUST_43136_PI429010886	M62161						0.0203878	12	0	0.0002303	21	3	34.25718	[illegible]
CUST_913_PI429010886	N7810	255653872	crustin 3 [Panulirus japonicus]	4.79E-08			0.0204022	15	9	0.0205629	15	12	68.38347	[illegible]
CUST_10794_PI429010886	N26801						0.0204572	24	0	0.0148741	12	0	299.8024	[illegible]
CUST_9570_PI429010886	N24855						0.0205554	21	0	0.0220327	24	18	50.48397	[illegible]
CUST_16443_PI429010886	N36248						0.0205554	21	9	0.0384987	18	12	3.634937	[illegible]
CUST_25752_PI429010886	N52993	395484809	glutamine synthetase [Fennerope	1.77E-16			0.020565	18	3	0.0494522	18	12	428.4643	[illegible]
CUST_51335_PI429010886	M93133	443695706	hypothetical protein CAPTEDRAFT	2.48E-18			0.0206961	12	3	0.0220387	15	3	124.0147	[illegible]
CUST_40969_PI429010886	M56511	321479500	hypothetical protein DAPPUDRAF	1.99E-27			0.0207208	12	9	0.0011185	15	9	105.3222	[illegible]
CUST_24612_PI429010886	N50942						0.0207264	12	3	0.0205722	24	6	228.0728	[illegible]
CUST_4083_PI429010886	N15969	402589316	actin [Wuchereria bancrofti]	9.88E-15			0.0207905	15	12	0.0406596	21	18	55.69808	[illegible]
CUST_10247_PI429010886	N25942						0.0209144	15	9	0.0130751	12	9	62.2112	[illegible]
CUST_35345_PI429010886	M12331	541906825	6-4 photolyase [Eurydice pulchra]	2.3E-124	Response t	DNA repai	0.0210565	21	18	0.0035079	18	0	38.70148	[illegible]
CUST_536_PI429010886	N5882	270013365	hypothetical protein TcasGA2_TC(	1.63E-08			0.0211668	21	12	0.0060306	21	3	15.5828	[illegible]
CUST_6636_PI429010886	N20381						0.0212135	12	3	0.0085339	21	18	97.34674	[illegible]
CUST_48601_PI429010886	M81218						0.0212927	12	0	0.0131696	24	15	6.842059	[illegible]
CUST_47847_PI429010886	M77826						0.0213329	15	0	0.006322	24	21	110.2483	[illegible]
CUST_50839_PI429010886	M91195						0.0213716	24	18	0.0104572	12	0	102.4622	[illegible]
CUST_3672_PI429010886	N15015						0.021562	21	0	0.0012539	15	12	57.67493	[illegible]
CUST_13471_PI429010886	N31246	241695265	integral membrane protein GPR17	1.97E-42	Signal tran	Other	0.0216278	15	12	0.0187045	21	9	21.80682	[illegible]
CUST_46867_PI429010886	M73998						0.0216638	15	6	0.0386636	18	9	334.4355	[illegible]
CUST_3798_PI429010886	N15293						0.0216692	18	6	0.0046239	15	9	129.5849	[illegible]
CUST_336_PI429010886	N4036	189242004	PREDICTED: hypothetical protein,	1.98E-08			0.0216935	18	9	0.0023759	15	12	202.5102	[illegible]
CUST_31670_PI429010886	M2133						0.0217685	24	0	0.0159607	15	9	161.1965	[illegible]
CUST_52313_PI429010886	M97028	309356629	Protein CBR-NAS-4 [Caenorhabdit	2.29E-19	Protein me	Proteolysis	0.0218048	24	9	0.0120413	15	12	NA	[illegible]
CUST_50322_PI429010886	M89442						0.0218469	18	3	0.0025213	12	0	22.9157	[illegible]
CUST_30983_PI429010886	M378	242021897	conserved hypothetical protein [P	4.1E-150	Metabolic	Cellular an	0.0218872	15	9	0.0208408	12	0	610.0117	[illegible]
CUST_56307_PI429010886	M113465						0.0219127	24	18	0.0430284	15	12	3633.442	[illegible]
CUST_2798_PI429010886	N13131	443725752	hypothetical protein CAPTEDRAFT	5.5E-179	Metabolic	Carbohydr	0.0219127	24	18	0.0054386	18	9	403.1106	[illegible]
CUST_6788_PI429010886	N20603						0.0219127	24	21	0.0223736	15	0	1091.584	[illegible]
CUST_31633_PI429010886	M2024						0.0219128	15	6	0.010455	12	0	174.0791	[illegible]
CUST_5242_PI429010886	N18441	345490321	PREDICTED: LOW QUALITY PROTE	3.02E-50	Nucleic aci	Regulation	0.0219379	21	12	0.0242849	24	21	205.4884	[illegible]
CUST_25191_PI429010886	N51946						0.0220794	24	18	0.0265545	12	9	5.136576	[illegible]
CUST_38877_PI429010886	M31478	321479210	hypothetical protein DAPPUDRAF	2.08E-14			0.0221131	12	6	0.0285176	21	6	NA	[illegible]
CUST_50387_PI429010886	M89664						0.0221618	15	6	0.0073397	24	21	51.09282	[illegible]
CUST_52516_PI429010886	M97743						0.0221832	24	21	0.0018962	15	12	233.0433	[illegible]
CUST_46257_PI429010886	M71966	118766718	beta-tubulin [Oxymonadida envirc	7.72E-20	Cellular co	Cytoskelet	0.0221977	24	3	0.0341287	15	3	12.46071	[illegible]
CUST_37774_PI429010886	M21508	170040829	predicted protein [Culex quinquef	2.72E-14			0.022206	15	6	0.0004989	15	12	366.556	[illegible]
CUST_331_PI429010886	N3958	117067248	ribosomal protein S26 [Branchiost	2.62E-30	Protein me	Ribosome	0.0222557	24	12	0.0204853	24	21	39.05992	[illegible]
CUST_15904_PI429010886	N35322						0.0222752	18	15	0.0242096	18	3	46.69101	[illegible]
CUST_17897_PI429010886	N38793						0.0222906	24	12	0.0321171	24	0	7.845494	[illegible]
CUST_32865_PI429010886	M5311						0.0223422	18	15	0.0290794	15	12	3660.946	[illegible]
CUST_33287_PI429010886	M6504	459937336	putative TRP channel protein [Per	3.5E-159	Transport	Ion transp	0.022412	24	21	0.0358045	24	15	801.0558	[illegible]
CUST_49339_PI429010886	M85248	321473453	hypothetical protein DAPPUDRAF	2.05E-20	Protein me	Proteolysis	0.022412	24	12	0.0005261	18	3	9.436321	[illegible]
CUST_1180_PI429010886	N8763						0.0224537	24	18	0.0069486	24	21	133.8226	[illegible]
CUST_25618_PI429010886	N52753						0.0225528	24	15	0.0044974	24	3	67.01762	[illegible]
CUST_55284_PI429010886	M109440	19921528	Asparaginyl-tRNA synthetase, isof	7.71E-13	Metabolic	Cellular an	0.022582	18	3	0.0210728	18	9	17.311	[illegible]
CUST_28215_PI429010886	N57529						0.0226099	18	12	0.0008954	21	0	9.325616	[illegible]
CUST_25898_PI429010886	N53262						0.022617	24	15	0.0488729	12	0	75.91716	[illegible]
CUST_4116_PI429010886	N16052	166952321	ribosomal protein rpl35a [Lineus v	1.22E-40	Protein me	Ribosome	0.022618	21	15	0.0390091	12	9	3043.672	[illegible]
CUST_56507_PI429010886	M113962						0.0227242	24	6	0.026826	18	3	1355.75	[illegible]
CUST_56540_PI429010886	M114046						0.0227242	24	6	0.0109564	15	3	189.6824	[illegible]
CUST_18227_PI429010886	N39388	478261422	hypothetical protein YQE_02808,	1.31E-22	Protein me	Proteolysis	0.0228465	12	3	0.0357315	12	3	210.5427	[illegible]
CUST_2381_PI429010886	N12231						0.02285	18	15	0.037075	18	15	1410.73	[illegible]
CUST_23841_PI429010886	N49569						0.0229948	24	18	0.044309	15	12	83.13651	[illegible]
CUST_48122_PI429010886	M78981						0.0230306	24	0	0.0187899	24	21	5.596537	[illegible]
CUST_6158_PI429010886	N19703	242010409	conserved hypothetical protein [P	2.75E-34			0.0230625	18	0	1.44E-05	21	0	536.9014	[illegible]
CUST_54262_PI429010886	M105321						0.0231286	24	3	0.0285356	15	3	50.63563	[illegible]
CUST_53476_PI429010886	M101877						0.0232653	24	6	0.018355	18	15	595.2796	[illegible]
CUST_35770_PI429010886	M13463						0.0232653	24	3	0.0180042	15	3	584.7373	[illegible]
CUST_44438_PI429010886	M65988	156539569	PREDICTED: SET and MYND domai	5.08E-09			0.0232653	24	21	0.0155677	24	0	58.67768	[illegible]
CUST_24092_PI429010886	N50011						0.0232653	24	21	0.0050985	24	0	91.27426	[illegible]
CUST_26699_PI429010886	N54725						0.0232653	24	3	0.0454938	24	6	22.98107	[illegible]
CUST_41550_PI429010886	M58010						0.0234975	21	15	0.0490602	21	18	77.41498	[illegible]
CUST_29287_PI429010886	N59554						0.0235098	24	15	0.0446915	24	18	62.65711	[illegible]
CUST_34609_PI429010886	M10141						0.0235325	21	0	0.0070985	18	15	17.25337	[illegible]
CUST_12073_PI429010886	N28887						0.0235358	24	21	0.0125084	18	0	349.9023	[illegible]
CUST_5950_PI429010886	N19417						0.0235922	18	6	0.045599	24	6	202.3368	[illegible]
CUST_22855_PI429010886	N47803						0.0237551	18	3	0.0289574	21	3	12.82511	[illegible]
CUST_3734_PI429010886	N15157						0.0238063	24	21	0.0133006	18	15	542.0349	[illegible]
CUST_5975_PI429010886	N19452						0.0239486	21	15	0.0291881	15	0	470.5066	[illegible]
CUST_29923_PI429010886	N60825						0.023988	12	9	0.0058461	24	6	2958.733	[illegible]
CUST_45403_PI429010886	M69002						0.0240788	24	6	0.0161217	18	9	31.91889	[illegible]
CUST_5478_PI429010886	N18757	321476119	hypothetical protein DAPPUDRAF	1.02E-79			0.024108	24	15	0.0162974	18	9	96.16362	[illegible]
CUST_28582_PI429010886	N58234						0.0243052	21	15	0.0291185	12	0	74.89382	[illegible]
CUST_39188_PI429010886	M35849						0.0243168	21	15	0.0494896	24	21	6.755262	[illegible]
CUST_39500_PI429010886	M40528						0.0243768	18	6	0.0078842	15	12	562.0064	[illegible]
CUST_1195_PI429010886	N8800						0.024402	15	6	0.0162974	15	12	2372.615	[illegible]
CUST_20981_PI429010886	N44415						0.0244025	24	15	0.0045771	15	9	36.07006	[illegible]
CUST_13222_PI429010886	N30807						0.0244561	24	9	1.234E-05	12	3	127.4702	[illegible]
CUST_25026_PI429010886	N51656						0.0244645	15	12	0.016463	12	3	11.77966	[illegible]
CUST_43907_PI429010886	M64303						0.0245324	18	6	0.0011599	15	9	176.429	[illegible]
CUST_11501_PI429010886	N27917						0.0246334	18	3	0.0158001	24	6	108.5148	[illegible]
CUST_39026_PI429010886	M33579						0.0246376	18	6	0.0015479	15	9	28561.19	[illegible]
CUST_1305_PI429010886	N9218	270161184	i-type lysozyme-like protein 2 [Per	3.78E-37	Metabolic	Carbohydr	0.0246428	24	3	0.0481312	21	3	25.91869	[illegible]
CUST_30923_PI429010886	M229				Protein me	Proteolysis	0.0246851	12	0	0.0008248	24	15	1321.031	[illegible]
CUST_36524_PI429010886	M16133						0.0247001	24	15	0.047628	24	18	14.10469	[illegible]
CUST_9556_PI429010886	N24831						0.0247001	24	15	0.0499518	12	0	148.892	[illegible]
CUST_41040_PI429010886	M56721	225718978	L-aspartate dehydrogenase [Calig	2.14E-36	Metabolic	Cellular an	0.0247279	12	9	0.0049631	15	9	158.7144	[illegible]
CUST_2791_PI429010886	N13115	242008167	vacuolar amino acid transporter, p	6.5E-99			0.0247279	12	3	0.0025286	21	6	54.71169	[illegible]
CUST_47882_PI429010886	M78000						0.0249916	18	15	0.0028269	24	9	107.7706	[illegible]
CUST_1264_PI429010886	N9070	156349174	hypothetical protein NEMVEDRAF	8.25E-11			0.0249936	21	9	0.01237	12	3	355.841	[illegible]
CUST_11257_PI429010886	N27521	260828245	hypothetical protein BRAFLDRAFT	4.18E-63	Response t	DNA repai	0.0249977	24	9	0.0299444	15	9	160.217	[illegible]
CUST_672_PI429010886	N6680	290462391	Cuticle protein 6 [Lepeophtheirus	4.4E-19			0.0249977	24	3	0.0113606	15	0	3688.899	[illegible]
CUST_10099_PI429010886	N25691	152013699	putative cuticle protein [Artemia f	1.19E-09			0.0249996	24	12	0.0016608	12	3	75.32925	[illegible]
CUST_49120_PI429010886	M84192	302320972	Taku spinosocarinatus voucher LIF	2.4E-148			0.025177	24	15	0.0426187	18	9	94790.65	[illegible]
CUST_32460_PI429010886	M4180						0.0252427	24	0	0.0172617	15	0	66.43371	[illegible]
CUST_45323_PI429010886	M68735						0.0252526	12	0	0.0042837	24	21	70.73344	[illegible]
CUST_52194_PI429010886	M96702						0.0252526	12	0	0.0118335	21	15	79.98506	[illegible]
CUST_6433_PI429010886	N20095						0.0252832	21	15	0.0450373	24	0	22.58797	[illegible]
CUST_15596_PI429010886	N34844						0.02532	18	12	0.0030458	24	15	15.73415	[illegible]
CUST_51443_PI429010886	M93553						0.0253238	21	3	0.0123468	24	21	65.34281	[illegible]
CUST_21450_PI429010886	N45245						0.0253485	18	3	0.0264244	18	0	13.27388	[illegible]
CUST_36842_PI429010886	M17008						0.0254597	24	6	0.001655	18	15	999.5752	[illegible]
CUST_12376_PI429010886	N29372						0.0255299	21	15	0.04807	24	18	47.60538	[illegible]
CUST_35156_PI429010886	M11751						0.0255425	21	6	0.0094347	18	0	15.71978	[illegible]
CUST_4010_PI429010886	N15779						0.0255929	24	3	0.0050874	21	3	73.52981	[illegible]
CUST_12835_PI429010886	N30128						0.0255929	24	15	0.0051598	24	21	33.48088	[illegible]
CUST_39087_PI429010886	M34399						0.0257268	24	9	0.0491245	15	9	170.7219	[illegible]
CUST_34187_PI429010886	M8997						0.0258572	24	12	0.0157594	12	0	87.99932	[illegible]
CUST_38788_PI429010886	M30395						0.025872	12	9	0.0059012	15	12	678.3225	[illegible]
CUST_6808_PI429010886	N20627	546683481	hypothetical protein D910_10583	7.31E-48			0.0258737	24	18	0.0099397	18	15	58.91249	[illegible]
CUST_3249_PI429010886	N14105	255732843	Candida tropicalis MYA-3404 NAD	6.53E-62	Transport	Ion transp	0.0258871	21	15	0.0036273	12	9	542.9066	[illegible]
CUST_21958_PI429010886	N46157						0.0259952	15	12	0.0463668	18	3	68.35388	[illegible]
CUST_17891_PI429010886	N38779	157126968	hypothetical protein AaeL_AAEL0	5.86E-15			0.0261038	12	0	0.0031067	24	0	30.52605	[illegible]
CUST_22810_PI429010886	N47713						0.0261038	12	0	0.0013183	24	0	178.1189	[illegible]
CUST_17398_PI429010886	N37899						0.0261464	18	3	0.0414943	24	15	113.636	[illegible]
CUST_8783_PI429010886	N23581	321459261	hypothetical protein DAPPUDRAF	5.62E-07			0.0261708	24	0	0.0097809	24	3	397.9972	[illegible]
CUST_4941_PI429010886	N17853						0.0262377	12	0	0.005617	24	3	46.91122	[illegible]
CUST_36554_PI429010886	M16216						0.0262978	24	12	0.0382566	15	3	24.05325	[illegible]

Probe ID	ID	EBI ID	Annotation	E-value	GO categor	Go term	p-value LD	Period LD	Phase LD	p-value DD	Period DD	Phase DD	LD 0
CUST_36080_PI429010886	M14569						0.0263262	24	18	0.0388113	15	0	187.7082
CUST_7181_PI429010886	N21156	321456676	hypothetical protein DAPPUDRAF	6.87E-63	Metabolic	Oxidation-	0.0264692	18	15	0.0157962	18	0	210.8385
CUST_40612_PI429010886	M55641						0.0265003	24	15	0.0021273	12	0	491.5182
CUST_30168_PI429010886	N61313						0.026524	24	0	0.0070365	24	21	36.81564
CUST_1201_PI429010886	N8817						0.0265311	21	0	0.0148614	24	12	27.63116
CUST_28040_PI429010886	N57161						0.0265847	24	18	0.0004699	15	9	129.5035
CUST_711_PI429010886	N6883	321472705	hypothetical protein DAPPUDRAF	1.27E-26			0.0266224	21	9	0.0190072	12	3	2920.075
CUST_34605_PI429010886	M10132						0.0266402	18	9	0.0032298	18	3	11.44626
CUST_50150_PI429010886	M88826						0.026754	24	6	0.0068019	18	3	102.5682
CUST_34905_PI429010886	M11040						0.0267833	24	15	0.0274094	12	0	235.9714
CUST_44701_PI429010886	M66722						0.0268871	24	9	0.0193525	12	3	46.56066
CUST_2993_PI429010886	N13563						0.026906	21	15	0.0001841	12	6	31.36919
CUST_44130_PI429010886	M65029	307202295	Segmentation protein cap'n'collar	3.43E-07			0.0269683	24	18	0.0100726	18	12	232.4494
CUST_25097_PI429010886	N51778						0.0269683	24	15	0.0318219	18	15	79.15396
CUST_34129_PI429010886	M8843						0.0270809	24	15	0.0325813	21	18	229.8267
CUST_34352_PI429010886	M9399						0.0270809	24	15	0.007597	24	15	332.5739
CUST_20515_PI429010886	N43588						0.0271477	24	6	0.0271779	12	0	20.87993
CUST_37222_PI429010886	M18256						0.0271664	24	18	0.0234733	12	0	161.9945
CUST_17403_PI429010886	N37908						0.027229	21	3	0.0394712	24	21	10.04856
CUST_31818_PI429010886	M2485						0.0272423	15	9	4.904E-05	24	18	314.5002
CUST_52686_PI429010886	M98464						0.0272893	24	21	0.0341287	15	9	33.98645
CUST_5465_PI429010886	N18741	345491979	PREDICTED: hypothetical protein l	3.94E-26	Signal tran	Intracellula	0.0272893	24	0	0.0372238	18	15	960.5506
CUST_24641_PI429010886	N51000						0.0273513	18	3	0.0280766	15	12	22.74717
CUST_50345_PI429010886	M89523						0.0274281	21	3	0.0170829	15	12	24.37029
CUST_47532_PI429010886	M76496						0.027474	18	3	0.0004501	18	9	19.44173
CUST_13094_PI429010886	N30563						0.0275155	24	12	0.0101065	12	3	123.2322
CUST_17510_PI429010886	N38094						0.0275808	12	3	0.0016608	12	3	385.121
CUST_423_PI429010886	N5141	220172371	putative elastin a [Rimicaris exocu	6.1E-13			0.027649	24	0	0.0101892	15	9	14071.14
CUST_23745_PI429010886	N49388						0.0278545	15	9	0.0193904	15	0	15.4109
CUST_33456_PI429010886	M6935	470380336	ubiquitin thiolesterase [Acantham	1.79E-33			0.0281551	18	9	0.0004568	24	0	14.95298
CUST_16582_PI429010886	N36503	47219760	unnamed protein product [Tetrao	6.93E-08			0.028158	24	15	0.0169682	21	0	9.653093
CUST_39211_PI429010886	M36163						0.02818	15	6	0.0206266	15	9	2275.002
CUST_42404_PI429010886	M60197	371591197	Euphausia superba voucher 841M	2E-118	Transport	Ion transp	0.0281949	21	18	0.0010922	24	0	329.5816
CUST_37012_PI429010886	M17537						0.0282054	24	3	0.0458369	15	12	28.10459
CUST_12203_PI429010886	N29098						0.0282054	24	21	0.0406006	12	0	254.6677
CUST_38467_PI429010886	M27044						0.028224	24	12	0.0244592	12	9	147.0849
CUST_44609_PI429010886	M66467						0.0282297	12	3	0.0476548	18	9	33.25164
CUST_54740_PI429010886	M107419						0.0282457	24	12	0.0373714	24	12	42.9709
CUST_32954_PI429010886	M5558	321477672	zinc finger protein [Daphnia pulex	1.34E-39			0.0282876	18	3	0.0344272	21	18	71.65566
CUST_5758_PI429010886	N19159						0.0283353	18	9	0.0092133	18	12	39.44924
CUST_49634_PI429010886	M86468						0.0283638	24	3	0.0495855	18	0	38.65654
CUST_36391_PI429010886	M15789						0.0284059	18	6	0.0008536	15	9	24984.55
CUST_33243_PI429010886	M6380	403182806	AAEL006520-PB [Aedes aegypti]	1.18E-11			0.0285669	15	9	0.0014522	21	0	36.44966
CUST_48197_PI429010886	M79298						0.0286374	15	12	0.0433197	21	18	4.257043
CUST_35583_PI429010886	M12949						0.0287393	24	6	0.0076714	18	6	180.3689
CUST_31773_PI429010886	M2367						0.028821	24	15	0.0330697	15	12	81.42279
CUST_22138_PI429010886	N46501						0.0289105	24	18	0.0488729	12	0	252.7743
CUST_38966_PI429010886	M32708	321463770	putative triacylglycerol lipase [Dap	2.69E-50	Metabolic	Lipid meta	0.0289785	12	6	0.038917	21	6	NA
CUST_27431_PI429010886	N56048						0.0293663	12	0	0.0458792	24	0	57.08138
CUST_4611_PI429010886	N17129						0.0293802	18	12	0.0301552	15	9	219.4299
CUST_2583_PI429010886	N12697						0.0293895	18	9	0.042624	24	18	12.32049
CUST_23261_PI429010886	N48529						0.0294861	21	9	0.01532	15	9	127.1629
CUST_41817_PI429010886	M58617						0.0295367	24	18	0.0329931	21	12	232.5186
CUST_38085_PI429010886	M23271						0.0295569	24	21	0.014752	21	3	441.1218
CUST_8104_PI429010886	N22542						0.0296018	21	15	0.0014778	24	21	92.0968
CUST_3392_PI429010886	N14417						0.0296157	24	6	0.0149394	15	3	920.7381
CUST_25411_PI429010886	N52364						0.0297022	24	3	0.0080085	15	3	11.65887
CUST_34282_PI429010886	M9237						0.0297093	15	9	0.0347999	21	15	27.71183
CUST_5948_PI429010886	N19415						0.0298357	18	15	0.0102814	18	0	442.5726
CUST_52430_PI429010886	M97422						0.0298521	12	0	0.0001378	12	0	132.7509
CUST_57053_PI429010886	E254						0.0299682	24	6	0.0166398	15	0	3274.995
CUST_45454_PI429010886	M69161						0.0299682	24	3	0.018997	15	3	540.0952
CUST_3169_PI429010886	N13946	326781695	crustacyanin subunit A [Fennerop	4.41E-16			0.0299682	24	21	0.0003008	15	3	24.16822
CUST_28864_PI429010886	N58750						0.0300871	21	0	0.0458665	24	0	55.37116
CUST_36731_PI429010886	M16687						0.0301637	18	3	0.0080623	18	6	6.772005
CUST_34062_PI429010886	M8639						0.0302817	24	15	0.0389796	21	18	614.5401
CUST_11957_PI429010886	N28689						0.0303208	24	3	0.0075625	18	3	101.5466
CUST_35473_PI429010886	M12658						0.0304041	24	3	0.0427946	15	3	59.67994
CUST_26521_PI429010886	N54390						0.0305027	21	15	0.0313785	24	21	106.6009
CUST_55896_PI429010886	M112043						0.0306249	15	9	0.0347977	15	9	73.70166
CUST_33904_PI429010886	M8197						0.0306261	15	6	0.0011461	15	9	222.3734
CUST_32477_PI429010886	M4220						0.0306273	12	9	0.001471	15	9	68.55444
CUST_2976_PI429010886	N13523						0.0306734	24	18	0.0239724	18	12	45.40562
CUST_8873_PI429010886	N23732	260799069	hypothetical protein BRAFLDRAFT	1.37E-45			0.0306734	24	21	0.0001731	15	9	16.49523
CUST_33268_PI429010886	M6449						0.030702	24	9	0.0197874	12	3	99.71725
CUST_671_PI429010886	N6674						0.0307119	24	21	0.0329783	15	9	1154.885
CUST_7413_PI429010886	N21510	242020240	senecionine N-oxygenase, putativ	5.47E-59	Metabolic	Oxidation-	0.0307449	21	0	0.0001655	12	9	95.69298
CUST_42345_PI429010886	M60055						0.0307869	18	3	0.0101892	15	9	141.6695
CUST_33986_PI429010886	M8431	124245110	tetraspanins-like protein-8 [Fenne	5.03E-08			0.030815	24	0	0.0120576	15	9	81.01838
CUST_25431_PI429010886	N52406						0.0308268	18	3	0.0166365	24	0	14.25928
CUST_37212_PI429010886	M18220						0.0308297	24	0	0.0032248	12	3	17.53638
CUST_32835_PI429010886	M5222						0.0308775	12	3	0.0466532	24	9	74.56881
CUST_10233_PI429010886	N25921						0.0309676	12	3	0.0457803	18	3	15.99496
CUST_30432_PI429010886	N61884						0.0309876	15	9	0.0157855	15	6	201.6125
CUST_45956_PI429010886	M70869	77743875	Euphausia superba isolate DGGE b	4.5E-176			0.0310059	24	0	0.0150038	18	3	98.36388
CUST_8976_PI429010886	N23880						0.0311125	24	0	0.0317302	24	0	370.4445
CUST_22316_PI429010886	N46801	395751682	PREDICTED: zinc finger protein 52	8.2E-09			0.0311326	18	9	0.0096883	24	21	12.44003
CUST_9915_PI429010886	N25398						0.0313079	12	9	0.0167298	15	9	67.36564
CUST_23040_PI429010886	N48151						0.0313079	12	9	0.0030794	24	12	29.80233
CUST_2404_PI429010886	N12279						0.0313085	24	21	0.0185653	24	21	61.18015
CUST_11821_PI429010886	N28458	198435252	PREDICTED: costars family protein	8.84E-25			0.0313391	24	15	0.0124928	15	12	47.10201
CUST_47075_PI429010886	M74730	383848471	PREDICTED: uncharacterized prote	9.04E-32			0.0315074	21	9	0.0458369	15	3	31.43117
CUST_40859_PI429010886	M56242	298162780	early cuticle protein 6 [Callinectes	1.62E-14			0.031517	24	12	0.023821	12	3	1627.485
CUST_2484_PI429010886	N12482						0.0315643	18	15	7.382E-05	15	0	1496.297
CUST_8796_PI429010886	N23604	471414688	PREDICTED: zinc finger protein 23	3.13E-38			0.0317081	18	9	0.0192499	21	3	3.945248
CUST_32246_PI429010886	M3621	380015144	PREDICTED: uncharacterized FAM	1.26E-61			0.031726	24	15	0.0005387	18	15	216.014
CUST_45093_PI429010886	M67982						0.0317837	21	15	0.0020738	12	3	183.8806
CUST_20164_PI429010886	N42927						0.0318512	18	15	0.0254465	18	0	31.11715
CUST_9497_PI429010886	N24736						0.031892	12	9	0.0012417	15	12	323.5302
CUST_45309_PI429010886	M68663						0.0319687	21	15	0.0016764	21	18	35.04281
CUST_19445_PI429010886	N41594	196002303	hypothetical protein TRIADDRAFT	6.71E-19	Metabolic	Other	0.0321171	24	0	0.0160043	18	12	71.83398
CUST_4519_PI429010886	N16955						0.0322572	18	15	0.0218872	15	9	269.2665
CUST_35427_PI429010886	M12543						0.0323842	24	21	0.0392746	24	21	289.2838
CUST_35729_PI429010886	M13329						0.0324092	15	6	0.0394885	21	12	195.6118
CUST_54325_PI429010886	M105588						0.0325505	12	0	0.0002255	21	6	345.674
CUST_51875_PI429010886	M95501						0.0326679	18	9	0.0497162	21	3	25.99138
CUST_842_PI429010886	N7506	442769035	glycosyl-phosphatidylinositol-linke	5.7E-62			0.0327953	21	18	0.007747	18	15	787.2783
CUST_43935_PI429010886	M64384						0.0328437	24	15	0.0022855	21	12	26.65836
CUST_56636_PI429010886	M114267						0.0328867	24	6	0.0195495	18	15	407.0205
CUST_49469_PI429010886	M85776	197361188	signal transducer and activator of	6.46E-22	Signal tran	Other	0.0328867	24	15	0.002805	15	9	130.9524
CUST_27088_PI429010886	N55427						0.0328867	24	9	0.023756	24	9	414.6538
CUST_28622_PI429010886	N58311						0.0328867	24	3	0.0378555	15	0	88.21411
CUST_13701_PI429010886	N31619						0.0329211	15	6	0.0006317	15	9	1648.551
CUST_9830_PI429010886	N25269	405977152	Putative ferric-chelate reductase 1	2.64E-09	Response t	Defence re	0.0329842	24	21	0.0472988	18	6	255.9552
CUST_18305_PI429010886	N39543						0.033014	18	9	0.0050945	24	21	218.889
CUST_38414_PI429010886	M26371						0.0331838	24	0	0.0047977	15	9	4349.21
CUST_32908_PI429010886	M5443	242007544	Pediculus humanus corporis prote	2.03E-63			0.0332736	24	15	0.009865	15	12	135.258
CUST_45721_PI429010886	M70056	321464435	hypothetical protein DAPPUDRAF	1.04E-36			0.0332736	24	15	0.0252903	21	15	91.82394
CUST_39159_PI429010886	M35402						0.033278	24	0	0.0497508	24	0	86.94104
CUST_4980_PI429010886	N18069	29134779	projectin [Procambarus clarkii]	7.8E-130			0.0334216	24	15	0.0014781	24	21	51.05459
CUST_11573_PI429010886	N28053						0.0334521	24	18	0.0310836	15	12	62.66935
CUST_37584_PI429010886	M19621						0.0336859	24	3	0.0181842	18	0	3851.521
CUST_49886_PI429010886	M87594	332217572	PREDICTED: tryptophan 2,3-dioxy	2.97E-11	Metabolic	Cellular am	0.0337632	21	0	0.0331001	24	6	24.82357
CUST_44064_PI429010886	M64830	91076314	PREDICTED: similar to spliceosom	3.6E-60	Nucleic aci	RNA metal	0.0337802	24	9	0.0112922	18	0	13.88764
CUST_16663_PI429010886	N36651						0.0338309	24	3	0.0216638	15	3	26.38902
CUST_9980_PI429010886	N25505	395856453	PREDICTED: E3 ubiquitin-protein l	5.12E-21			0.033893	18	9	0.0140224	24	0	119.8257
CUST_36029_PI429010886	M14333	123471670	ankyrin repeat protein [Trichomo	3.89E-30			0.0339343	21	0	0.0040936	24	18	20.228
CUST_4289_PI429010886	N16411						0.0339343	24	0	0.0007473	24	18	14.94729
CUST_15321_PI429010886	N34396						0.0340239	21	15	0.0139797	15	9	16.97814
CUST_20765_PI429010886	N44016						0.0340474	24	15	0.0310339	24	18	124.2633
CUST_29157_PI429010886	N59306						0.0340474	24	15	0.0321171	24	0	64.65354
CUST_19887_PI429010886	N42390	345498241	PREDICTED: transportin-1 [Nasoni	1.51E-54	Transport	Protein tra	0.0340605	21	6	0.0132845	24	21	20.21346
CUST_50794_PI429010886	M91031						0.0340794	24	6	0.022834	18	3	5.362097
CUST_49970_PI429010886	M87987	322791059	hypothetical protein SINV_09638	6.24E-09			0.0340836	12	9	0.0059012	15	12	30.6586
CUST_12173_PI429010886	N29044						0.0341123	21	0	0.0048528	15	9	58.16145
CUST_17326_PI429010886	N37780						0.0341287	15	9	4.289E-05	18	12	23.23151
CUST_35535_PI429010886	M12818						0.0343013	18	15	0.0281517	24	21	30.72933
CUST_37852_PI429010886	M21875						0.0343099	18	6	0.0115609	12	0	361.2613
CUST_15299_PI429010886	N34355	328709829	PREDICTED: heterogeneous nucle	5.05E-27			0.0343758	21	0	0.0103326	24	15	23.09017
CUST_14565_PI429010886	N33085						0.0344338	21	0	0.0059605	12	0	186.302
CUST_50265_PI429010886	M89232						0.0344343	24	15	0.024049	24	15	105.4679
CUST_22528_PI429010886	N47189						0.0344388	24	0	0.024778	15	12	7.801944
CUST_5123_PI429010886	N18288	541906833	casein kinase 1 epsilon [Eurydice p		Protein me	Cellular pr	0.0344745	15	0	0.0392716	21	18	579.8505
CUST_45837_PI429010886	M70452						0.0344983	24	6	0.0442041	24	0	29.28939
CUST_50117_PI429010886	M88687						0.0345533	18	9	0.0129294	24	3	NA
CUST_22570_PI429010886	N47253						0.0346828	15	6	0.0377231	12	9	195.1305
CUST_32122_PI429010886	M3286	260824760	hypothetical protein BRAFLDRAFT	1E-48			0.0348212	24	15	0.0105566	24	15	56.18215
CUST_47098_PI429010886	M74785	270010451	hypothetical protein TcasGA2_TC(	1.76E-25			0.0348212	24	15	0.0188793	24	18	22.03729
CUST_56186_PI429010886	M113051	371591236	Euphausia superba voucher 760M		Metabolic	Generatior	0.0348957	15	0	0.0266301	15	9	610.334
CUST_11747_PI429010886	N28340						0.0348957	15	9	3.201E-05	21	12	46.11055
CUST_14847_PI429010886	N33568						0.0349634	12	0	0.0076924	15	12	26.79608
CUST_44237_PI429010886	M65353	388855385	related to FPR2-FK506/rapamycin	4.63E-15	Protein me	Protein fol	0.0349879	24	21	1.958E-05	18	3	107.4112
CUST_8012_PI429010886	N22415	383859710	PREDICTED: transcription elongati	5.52E-40	Nucleic aci	Regulation	0.0350161	24	21	0.0131645	15	9	31.12893
CUST_32141_PI429010886	M3340	383852569	PREDICTED: uncharacterized prote	2.46E-37			0.035061	18	9	0.015805	21	9	25.79038
CUST_51261_PI429010886	M92858	260828125	beta-transducin repeat containing	1.23E-33			0.0350969	12	0	0.0146508	18	3	18.69988
CUST_56242_PI429010886	M113245						0.0351813	15	9	0.0212556	15	0	28.64244
CUST_15666_PI429010886	N34949	443697703	hypothetical protein CAPTEDRAFT	3.45E-27			0.0352791	15	9	0.000323	15	9	31.98442
CUST_41884_PI429010886	M58788	449471748	PREDICTED: cartilage intermediate	3.8E-12			0.0354046	24	21	0.0112364	24	0	1002.076
CUST_46513_PI429010886	M72871						0.0354552	12	0	0.0291098	15	3	4.851563
CUST_38491_PI429010886	M27324						0.0356521	12	3	0.0410495	18	12	264.0266
CUST_55573_PI429010886	M110788						0.03574	15	6	0.0012396	15	12	7.725643
CUST_31989_PI429010886	M2931	117959988	clottable protein [Marsupenaeus j	7.44E-30	Transport	Lipid trans	0.0357565	18	3	0.0143045	18	15	3.633402
CUST_41888_PI429010886	M58803						0.0358869	24	12	0.0004285	12	3	46.9383
CUST_37934_PI429010886	M22298	507697569	PREDICTED: 14 kDa phosphohistid	7.29E-34			0.0359347	18	15	0.0352081	24	18	118.8436
CUST_54087_PI429010886	M104483						0.0360029	18	15	0.0001774	21	3	22.74435
CUST_2266_PI429010886	N11990	91087225	PREDICTED: similar to CG1973 CG	8.32E-90	Protein me	Cellular pr	0.0362017	24	12	0.0396176	21	0	13.67194
CUST_12613_PI429010886	N29744						0.0363479	24	18	0.0006166	21	0	179.7279
CUST_37405_PI429010886	M18915						0.0365344	24	15	0.0425605	15	9	3480.931
CUST_23966_PI429010886	N49786						0.0365408	21	15	0.0122487	12	0	181.4867
CUST_31765_PI429010886	M2349						0.0367222	18	6	0.0010115	21	0	5.033666

Probe ID	ID	EBI ID	Annotation	E-value	GO categor	Go term	p-value LD	Period LD	Phase LD	p-value DD	Period DD	Phase DD	LD 0	
CUST_6463_PI429010886	N20132	345491105	PREDICTED: DOMON domain-cont	3.52E-40			0.0368215	21	3	0.0005196	21	18	48.95729	[illegible]
CUST_16733_PI429010886	N36772						0.0368934	18	15	0.0142313	24	18	30.31994	[illegible]
CUST_51384_PI429010886	M93343	524928295	PREDICTED: apoptosis regulatory	4.08E-09	Signal tran	Other	0.0369912	24	18	0.026717	24	18	29.06579	[illegible]
CUST_52067_PI429010886	M96303						0.0370672	24	9	0.0080301	15	3	219.3902	[illegible]
CUST_14600_PI429010886	N33152	242006716	conserved hypothetical protein [P	8.17E-49	Transport	Protein tra	0.0371598	18	9	0.0009589	24	18	24.0921	[illegible]
CUST_13940_PI429010886	N32052						0.0371685	21	15	0.0160926	18	15	30.58701	[illegible]
CUST_40392_PI429010886	M54968						0.0372273	15	6	0.0044115	24	18	137.1425	[illegible]
CUST_12449_PI429010886	N29475						0.0373007	24	0	0.0337453	15	9	190.9992	[illegible]
CUST_821_PI429010886	N7415						0.0373738	18	6	0.034272	24	21	19.47777	[illegible]
CUST_54753_PI429010886	M107465	317383196	myosin light chain [Penaeus monc	2.12E-15			0.0374395	12	0	0.0046323	15	6	17.8703	[illegible]
CUST_5840_PI429010886	N19275	321468789	hypothetical protein DAPPUDRAF	1.37E-87	Metabolic	Oxidation-	0.0374478	24	18	0.0210728	18	9	169.2749	[illegible]
CUST_9378_PI429010886	N24517						0.0374478	24	21	0.0167876	24	15	102.7974	[illegible]
CUST_1186_PI429010886	N8773						0.0374478	24	18	0.0488729	12	0	83.32737	[illegible]
CUST_6599_PI429010886	N20330	242011385	conserved hypothetical protein [P	5.14E-37			0.0374837	24	0	0.0177632	18	15	7.392041	[illegible]
CUST_44671_PI429010886	M66627						0.0375681	18	15	0.0483167	15	12	2571.491	[illegible]
CUST_26441_PI429010886	N54249						0.0379045	24	18	0.0214148	15	12	41.46154	[illegible]
CUST_23798_PI429010886	N49489						0.0381298	24	0	0.0258693	24	6	92.42138	[illegible]
CUST_10688_PI429010886	N26631						0.0381908	24	15	0.0218677	24	18	99.42287	[illegible]
CUST_18440_PI429010886	N39804						0.0381974	15	12	0.0091184	21	0	12.04588	[illegible]
CUST_326_PI429010886	N3906						0.0382133	24	3	0.0228097	15	0	142.6928	[illegible]
CUST_15687_PI429010886	N34976						0.0382948	21	15	0.0405904	21	18	1348.208	[illegible]
CUST_17857_PI429010886	N38727						0.0383133	18	9	0.001602	12	3	3.856309	[illegible]
CUST_613_PI429010886	N6328						0.0383569	18	0	0.0025148	15	9	51.66717	[illegible]
CUST_56788_PI429010886	M114654						0.0383612	24	21	0.0278545	15	9	83.93495	[illegible]
CUST_3865_PI429010886	N15440						0.0383612	24	21	0.0027898	15	12	40.5192	[illegible]
CUST_5452_PI429010886	N18724						0.0383612	24	18	0.0228832	12	0	1457.293	[illegible]
CUST_39940_PI429010886	M47110	18266071	collagenolytic serine protease [Pa	4.48E-29	Protein me	Proteolysis	0.0383953	24	21	0.0019044	21	9	55.80638	[illegible]
CUST_32617_PI429010886	M4594	321455842	hypothetical protein DAPPUDRAF	4.9E-31			0.0384017	18	0	0.0377703	15	9	131.8934	[illegible]
CUST_35608_PI429010886	M13011	291232275	PREDICTED: eukaryotic translation	1.4E-16			0.0384686	18	0	0.0103523	24	18	52.45861	[illegible]
CUST_21273_PI429010886	N44929	328711363	PREDICTED: zinc finger protein 84	2.16E-09			0.0385089	21	0	0.0472438	12	0	175.3842	[illegible]
CUST_15424_PI429010886	N34558						0.038513	21	18	0.0153357	15	6	504.0705	[illegible]
CUST_46080_PI429010886	M71304						0.0385531	24	18	0.03987	21	9	6.532525	[illegible]
CUST_35339_PI429010886	M12315						0.0387003	18	0	0.0289832	24	9	21.52625	[illegible]
CUST_31393_PI429010886	M1423						0.0387353	21	3	0.0249131	24	3	223.7206	[illegible]
CUST_9600_PI429010886	N24902						0.0387597	15	12	0.0073254	15	6	107.7409	[illegible]
CUST_41461_PI429010886	M57788						0.0388179	24	18	0.0079342	12	9	553.413	[illegible]
CUST_361_PI429010886	N4431						0.0388304	24	3	0.0152947	18	3	699.596	[illegible]
CUST_14191_PI429010886	N32460						0.0388487	15	9	0.0006831	21	18	6.652007	[illegible]
CUST_30023_PI429010886	N61025						0.0389204	21	6	0.0296157	24	21	75.48453	[illegible]
CUST_4454_PI429010886	N16791						0.038932	15	6	0.0334389	24	0	32.23946	[illegible]
CUST_28322_PI429010886	N57723						0.0389713	15	9	0.040512	15	12	141.0115	[illegible]
CUST_51150_PI429010886	M92373						0.0390091	12	9	0.0218872	15	9	18.96877	[illegible]
CUST_33470_PI429010886	M6970						0.0390293	21	15	0.0408747	15	0	207.5504	[illegible]
CUST_52280_PI429010886	M96945						0.0390408	18	6	0.0174806	15	12	6.409298	[illegible]
CUST_31563_PI429010886	M1844	242021017	conserved hypothetical protein [P	1.74E-52	Nucleic aci	Regulation	0.0393625	21	15	0.0041954	18	15	43.88384	[illegible]
CUST_50873_PI429010886	M91295						0.0394378	12	3	0.0021708	24	18	18.35824	[illegible]
CUST_4978_PI429010886	N18011						0.0395157	21	0	0.0128185	12	0	27.15396	[illegible]
CUST_41098_PI429010886	M56865	66508366	PREDICTED: inosine-5'-monophos	5.73E-93	Metabolic	Nucleobas	0.0396176	21	3	0.0041119	15	12	46.76121	[illegible]
CUST_17203_PI429010886	N37566						0.039859	21	15	0.0223768	24	6	108.5389	[illegible]
CUST_53548_PI429010886	M102208						0.0399733	12	0	0.0347566	24	21	17.78857	[illegible]
CUST_38598_PI429010886	M28437						0.0399733	12	0	0.0107769	18	0	46.09707	[illegible]
CUST_28211_PI429010886	N57517						0.0399821	21	18	0.0466695	15	6	106.7298	[illegible]
CUST_54611_PI429010886	M106849						0.0399945	24	15	0.0377231	12	9	125.025	[illegible]
CUST_33100_PI429010886	M5970						0.0399945	24	3	0.0236365	21	18	73.50137	[illegible]
CUST_48350_PI429010886	M79956						0.0401051	21	0	0.0038908	24	21	19.3323	[illegible]
CUST_2271_PI429010886	N12001	156541526	PREDICTED: cullin-4B-like [Nasoni	1.6E-122	Protein me	Proteolysis	0.0402542	21	15	0.0455556	12	9	122.4178	[illegible]
CUST_7913_PI429010886	N22265	241999648	src tyrosine kinase, putative [Ixod	4.6E-104	Developme	Other	0.0402773	24	0	0.011679	12	0	37.07884	[illegible]
CUST_21004_PI429010886	N44459						0.0404042	18	0	0.0048115	21	18	226.2405	[illegible]
CUST_31573_PI429010886	M1878	30584201	Homo sapiens peroxisomal D3,D2	6.23E-63	Metabolic	Lipid meta	0.0404046	21	12	0.0252788	21	18	32.13644	[illegible]
CUST_35709_PI429010886	M13274						0.0404463	21	18	0.0420336	12	0	227.1397	[illegible]
CUST_42776_PI429010886	M61225						0.0404846	21	18	0.020035	21	15	11.25219	[illegible]
CUST_5307_PI429010886	N18522				Protein me	Cellular pr	0.0404944	24	15	0.0343435	24	18	514.0806	[illegible]
CUST_17743_PI429010886	N38517	321468531	hypothetical protein DAPPUDRAF	2.41E-21	Nucleic aci	Regulation	0.0404944	24	15	0.0162035	12	0	18.57573	[illegible]
CUST_51416_PI429010886	M93434						0.0405623	24	0	0.0002548	12	0	267.0346	[illegible]
CUST_17286_PI429010886	N37713						0.0405722	21	18	0.0003704	18	9	219.8411	[illegible]
CUST_50147_PI429010886	M88813						0.0406114	24	12	0.0312259	12	6	71.88037	[illegible]
CUST_46265_PI429010886	M71998						0.0406446	24	18	0.0124079	15	9	190.613	[illegible]
CUST_47053_PI429010886	M74663	9186884	BCS-1 [Amphibalanus amphitrite]	1.48E-08			0.0406491	24	3	0.0325457	18	3	361.9725	[illegible]
CUST_50777_PI429010886	M90967						0.0406544	24	0	0.0067732	15	0	208.0709	[illegible]
CUST_23444_PI429010886	N48851						0.0407746	24	0	0.0026166	21	18	35.01298	[illegible]
CUST_11709_PI429010886	N28278						0.0407874	15	6	9.383E-06	15	9	17589.58	[illegible]
CUST_16061_PI429010886	N35583	238625719	SpAN-like protein [Rimicaris exocu	5.64E-10			0.0408353	12	3	0.0499157	24	6	25.65087	[illegible]
CUST_54900_PI429010886	M108126						0.0409091	21	12	0.0116149	21	3	NA	[illegible]
CUST_1694_PI429010886	N10456	380017443	PREDICTED: carboxypeptidase B-li	7.36E-65	Protein me	Proteolysis	0.041065	18	0	6.769E-05	24	12	525.9937	[illegible]
CUST_14480_PI429010886	N32957						0.0411013	24	21	0.0239475	12	0	91.18387	[illegible]
CUST_11321_PI429010886	N27619						0.0411321	24	18	0.0001795	15	9	37427.64	[illegible]
CUST_33447_PI429010886	M6907						0.0411403	24	21	0.0222144	21	12	13.98312	[illegible]
CUST_32245_PI429010886	M3619	13898841	CUB-serine protease [Panulirus ar	7.54E-55	Protein me	Proteolysis	0.0414766	24	15	0.0475123	18	0	69.71083	[illegible]
CUST_34491_PI429010886	M9826	537079664	zinc finger protein, partial [Cricetu	4.33E-45			0.0414834	24	18	0.0177401	24	18	43.55336	[illegible]
CUST_46087_PI429010886	M71333	443696486	hypothetical protein CAPTEDRAFT	2.55E-18			0.0414943	24	15	0.0443957	21	18	37.46702	[illegible]
CUST_47585_PI429010886	M76734						0.0414943	24	21	0.0012269	12	0	1592.484	[illegible]
CUST_21833_PI429010886	N45939						0.0414943	24	21	0.045964	12	0	9.71776	[illegible]
CUST_56390_PI429010886	M113659						0.0415949	24	15	0.0411864	24	6	26.99645	[illegible]
CUST_4950_PI429010886	N17877						0.0416439	18	15	0.0099651	21	0	18.19475	[illegible]
CUST_9508_PI429010886	N24758	240972665	chloride channel, putative [Ixodes	1.06E-51	Transport	Ion transp	0.0416527	24	15	0.0005252	15	0	53.96936	[illegible]
CUST_10763_PI429010886	N26749	260824441	hypothetical protein BRAFLDRAFT	2.82E-78			0.0416603	15	9	0.0018124	21	18	7.873998	[illegible]
CUST_34167_PI429010886	M8933						0.0416662	24	21	0.0178927	21	15	350.791	[illegible]
CUST_35208_PI429010886	M11904	328784017	PREDICTED: LOW QUALITY PROTE	9.07E-66	Signal tran	Other	0.0417625	15	12	0.0135031	12	3	26.61351	[illegible]
CUST_37636_PI429010886	M20050	77743875	Euphausia superba isolate DGGE b				0.0417993	21	18	0.0158566	24	15	172120.5	[illegible]
CUST_52858_PI429010886	M99237	533148364	PREDICTED: mitotic spindle-associ	1.81E-25			0.0419655	21	15	0.013159	24	0	14.61885	[illegible]
CUST_4030_PI429010886	N15847						0.0420146	24	18	0.0054938	18	15	28.84569	[illegible]
CUST_16033_PI429010886	N35540						0.0420146	24	3	0.0133158	15	3	62.40732	[illegible]
CUST_56465_PI429010886	M113865						0.0420173	21	0	0.00147	21	3	47.79939	[illegible]
CUST_46262_PI429010886	M71987						0.0420823	15	9	0.0293884	24	15	41.54672	[illegible]
CUST_26289_PI429010886	N53992						0.0420823	15	9	0.0040225	24	0	63.92981	[illegible]
CUST_48881_PI429010886	M82686						0.0422598	24	0	0.0001717	15	12	59.61189	[illegible]
CUST_7899_PI429010886	N22242	71159516	C-type lectin [Penaeus monodon]	9.67E-12			0.0422906	15	12	0.0160899	24	21	1128.19	[illegible]
CUST_20656_PI429010886	N43829						0.0425571	24	18	0.0325574	12	9	60.09389	[illegible]
CUST_44526_PI429010886	M66217	317419745	Diphthamide biosynthesis protein	4.23E-19	Protein me	Cellular pr	0.0426525	24	21	0.0037576	18	15	34.92175	[illegible]
CUST_39757_PI429010886	M44093	220172371	putative elastin a [Rimicaris exocu	1.56E-07			0.0426699	18	6	0.0004646	15	9	858.51	[illegible]
CUST_34320_PI429010886	M9318	242017663	anaphase-promoting complex sub	3.5E-41	Protein me	Proteolysis	0.0427118	24	18	0.017682	18	15	21.26556	[illegible]
CUST_11921_PI429010886	N28631						0.0427636	21	3	0.0153388	12	3	240.5773	[illegible]
CUST_1503_PI429010886	N9845				Metabolic	Carbohydr	0.0427636	24	15	0.016856	24	6	325.2782	[illegible]
CUST_38040_PI429010886	M22993						0.0429941	24	21	0.0219128	15	12	192.4063	[illegible]
CUST_47242_PI429010886	M75323	321469983	hypothetical protein DAPPUDRAF	5.75E-61	Protein me	Proteolysis	0.0429947	21	0	0.0151416	15	12	249.928	[illegible]
CUST_10988_PI429010886	N27095	530607984	PREDICTED: DNA polymerase epsi	2.45E-21			0.0431293	21	9	0.0339276	24	18	5.13998	[illegible]
CUST_56644_PI429010886	M114291	56968003	Anaspides tasmaniae internal trar	6.6E-100			0.0431504	24	6	0.0078205	18	15	277.8246	[illegible]
CUST_17297_PI429010886	N37731	345482168	PREDICTED: kinesin-like protein ui	1.95E-38			0.0432496	21	3	0.0158001	24	15	47.03106	[illegible]
CUST_2647_PI429010886	N12831						0.0432532	15	6	0.0266301	15	9	40.59456	[illegible]
CUST_14007_PI429010886	N32164						0.0432779	24	6	0.0017347	24	0	37.64453	[illegible]
CUST_9040_PI429010886	N23975						0.04338	24	21	0.0256611	15	12	64.09325	[illegible]
CUST_36279_PI429010886	M15377						0.0434038	24	21	0.0266886	15	12	162.7145	[illegible]
CUST_49538_PI429010886	M86024						0.043494	24	15	0.014279	24	18	87.03898	[illegible]
CUST_15051_PI429010886	N33931	195576256	GD22775 [Drosophila simulans] >g	5.14E-19	Transport	Ion transp	0.043494	24	15	0.0369912	24	18	773.0893	[illegible]
CUST_33992_PI429010886	M8448						0.0435298	24	0	0.0219128	15	6	54.66763	[illegible]
CUST_50793_PI429010886	M91027						0.043539	21	3	0.0222993	24	18	4.174325	[illegible]
CUST_6389_PI429010886	N20038	321464750	hypothetical protein DAPPUDRAF	6.96E-07			0.0435819	24	15	0.0425605	15	9	263.9286	[illegible]
CUST_3066_PI429010886	N13725						0.0437354	24	18	0.0002462	15	9	252.989	[illegible]
CUST_46465_PI429010886	M72731						0.043821	24	21	0.0209772	18	9	30.53594	[illegible]
CUST_33493_PI429010886	M7047	321463241	hypothetical protein DAPPUDRAF	4.97E-33	Metabolic	Cellular an	0.0438588	24	9	0.0238284	24	12	44.05907	[illegible]
CUST_52756_PI429010886	M98803						0.0438758	12	6	0.0005546	21	9	6.688662	[illegible]
CUST_22469_PI429010886	N47086						0.0439292	24	9	0.0373738	18	6	146.0664	[illegible]
CUST_34686_PI429010886	M10340				Metabolic	Carbohydr	0.043994	24	15	0.0260606	24	6	198.2176	[illegible]
CUST_16367_PI429010886	N36116						0.043994	24	15	0.0020213	18	12	14.69279	[illegible]
CUST_21448_PI429010886	N45241						0.0439957	24	0	0.0097563	12	0	114.4277	[illegible]
CUST_25113_PI429010886	N51806	121484216	cuticle protein CB6 [Portunus pela	3.08E-16			0.0442389	24	3	0.0259045	15	3	325.7046	[illegible]
CUST_47983_PI429010886	M78387	148226561	coiled-coil domain-containing pro	6.33E-30			0.0442965	21	0	0.0169906	15	12	9.328141	[illegible]
CUST_15811_PI429010886	N35184						0.044309	15	12	0.0314891	12	0	69.91553	[illegible]
CUST_55247_PI429010886	M109282						0.0444939	24	15	0.0140979	24	0	1409.395	[illegible]
CUST_4431_PI429010886	N16740	310693525	truncated cathepsin A [Eriocheir s	9.57E-10	Protein me	Proteolysis	0.0446671	12	6	0.0340525	15	9	11.2206	[illegible]
CUST_47754_PI429010886	M77475	291228352	PREDICTED: CDC42 binding protei	3.2E-21	Signal tran	Intracellula	0.0447219	18	15	0.046333	18	12	16.65157	[illegible]
CUST_27118_PI429010886	N55473						0.0447536	24	12	0.0046772	12	0	65.08782	[illegible]
CUST_6764_PI429010886	N20569	46849491	glycinamide ribonucleotide synthe	7E-112	Metabolic	Nucleobas	0.0447656	24	18	0.0361123	18	6	95.09356	[illegible]
CUST_444_PI429010886	N5213				Protein me	Ribosome	0.0448119	15	9	0.0422718	15	12	216.0939	[illegible]
CUST_3067_PI429010886	N13726						0.0448173	24	18	0.0278537	15	9	132.1609	[illegible]
CUST_4919_PI429010886	N17787				Protein me	Proteolysis	0.0449938	24	15	0.0483768	24	21	27.31402	[illegible]
CUST_47908_PI429010886	M78121	270007545	hypothetical protein TcasGA2_TC(	1.87E-21	Metabolic	Nucleobas	0.0451608	24	21	0.0076861	21	12	18.20871	[illegible]
CUST_6825_PI429010886	N20649						0.0452739	24	6	0.0347025	12	0	33.59475	[illegible]
CUST_12704_PI429010886	N29902						0.0453706	12	9	0.0002554	15	12	408.9306	[illegible]
CUST_13084_PI429010886	N30548						0.0453709	21	9	0.0486448	24	21	47.14137	[illegible]
CUST_42382_PI429010886	M60146						0.0454938	24	21	0.0013975	18	9	23.61225	[illegible]
CUST_5815_PI429010886	N19234	505841261	PREDICTED: origin recognition cor	1.6E-104	Cellular Pr	Cell cycle	0.0455048	21	9	0.0315983	21	6	54.86877	[illegible]
CUST_31632_PI429010886	M2022	18916919	cyclic AMP-regulated protein like	3.73E-51			0.0455508	21	15	0.0317167	15	3	449.0619	[illegible]
CUST_43230_PI429010886	M62474	290792623	cytochrome P450 [Litopenaeus va	2.84E-68	Metabolic	Oxidation-	0.04575	18	15	0.0303346	24	18	97.06787	[illegible]
CUST_3799_PI429010886	N15294						0.0458399	15	6	0.0140089	15	9	115.0038	[illegible]
CUST_859_PI429010886	N7593						0.0459302	18	0	0.002278	15	0	877.7497	[illegible]
CUST_11995_PI429010886	N28748						0.0459937	24	15	0.0204281	15	9	1275.515	[illegible]
CUST_14912_PI429010886	N33677						0.0462084	12	9	0.037246	18	6	293.934	[illegible]
CUST_48817_PI429010886	M82320						0.0463513	24	21	0.0072093	21	0	18.42453	[illegible]
CUST_38836_PI429010886	M31066						0.0464774	24	18	0.0001753	15	9	35607.66	[illegible]
CUST_30345_PI429010886	N61677						0.0467623	18	3	0.0191737	15	12	92.91315	[illegible]
CUST_39950_PI429010886	M47341	121484216	cuticle protein CB6 [Portunus pela	2.69E-12			0.046822	24	6	0.0039059	18	3	287.7668	[illegible]
CUST_35853_PI429010886	M13711	4585366	extracellular superoxide dismutas	1.5E-43	Metabolic	Oxidation-	0.0470466	12	9	0.0413508	15	9	1090.482	[illegible]
CUST_39281_PI429010886	M37163						0.0472922	18	15	0.0403406	21	15	997.9064	[illegible]
CUST_45539_PI429010886	M69422				Metabolic	Other	0.0473098	21	15	0.010605	15	0	5.126214	[illegible]
CUST_1464_PI429010886	N9719						0.0473357	24	9	0.029149	15	3	244.999	[illegible]
CUST_6693_PI429010886	N20470	156546442	PREDICTED: 1-acyl-sn-glycerol-3-p	8.34E-75	Metabolic	Other	0.0473883	24	15	0.0160525	21	9	51.23629	[illegible]
CUST_53451_PI429010886	M101760	328899820	ribosomal protein S7 [Procambaru	3.27E-35	Protein me	Ribosome	0.047512	21	15	0.0115473	12	9	310.8187	[illegible]
CUST_38478_PI429010886	M27180	242009985	zinc finger protein CCCH domain-c	3.07E-48			0.047628	24	21	7.909E-05	15	12	58.88834	[illegible]
CUST_21760_PI429010886	N45800						0.0479177	12	9	0.0051542	15	6	NA	[illegible]
CUST_6223_PI429010886	N19798	321477834	hypothetical protein DAPPUDRAF	6.48E-35	Protein me	Proteolysis	0.0479821	18	0	0.01532	15	0	143.92	[illegible]
CUST_11761_PI429010886	N28356						0.0480446	24	21	0.0329607	15	0	83.91692	[illegible]
CUST_14848_PI429010886	N33569						0.0483576	12	9	0.0057655	15	12	51.37499	[illegible]
CUST_53547_PI429010886	M102205	260808985	hypothetical protein BRAFLDRAFT	2.04E-12			0.0484912	18	9	0.0225042	21	9	NA	[illegible]
CUST_22030_PI429010886	N46299						0.0484914	24	12	0.0088846	21	0	25.7544	[illegible]
CUST_55419_PI429010886	M110013	270009780	hypothetical protein TcasGA2_TC(	5.59E-10			0.048652	18	6	0.0104815	18	6	1337.377	[illegible]
CUST_1685_PI429010886	N10435	242016965	Aminopeptidase N precursor, puta	1.1E-172	Protein me	Proteolysis	0.0486785	24	9	0.0429889	12	3	415.2055	[illegible]
CUST_37961_PI429010886	M22481						0.0486816	15	0	0.0157185	21	12	3101.358	[illegible]
CUST_6886_PI429010886	N20733						0.0487299	18	0	0.0019967	18	0	30.3261	[illegible]
CUST_43072_PI429010886	M61997	345486690	PREDICTED: phosphatidylinositol 3	5.18E-37			0.048804	24	21	0.0071936	18	15	132.2851	[illegible]

Probe ID	ID	EBI ID	Annotation	E-value	GO categor	Go term	p-value LD	Period LD	Phase LD	p-value DD	Period DD	Phase DD
CUST_42364_PI429010886	M60103						0.0488707	21	15	0.0183736	12	3
CUST_10906_PI429010886	N26972	321459545	hypothetical protein DAPPUDRAF	2.23E-38			0.0488949	12	3	0.0262796	15	0
CUST_33562_PI429010886	M7282						0.0489428	18	15	0.0058235	12	6
CUST_10936_PI429010886	N27019	291236785	PREDICTED: origin recognition cor	2.17E-09	Symbiosis, Other		0.0490959	24	21	0.0252026	24	21
CUST_19856_PI429010886	N42326	149409130	PREDICTED: COP9 signalosome co	5.39E-45	Developme	Embryo de	0.0491608	12	9	0.0145038	24	9
CUST_39434_PI429010886	M39314						0.049193	24	15	0.0314936	18	12
CUST_38820_PI429010886	M30943						0.0492606	24	15	0.0217022	18	12
CUST_14391_PI429010886	N32805						0.0492861	15	12	0.0184025	24	0
CUST_19229_PI429010886	N41219						0.0492883	21	18	0.0115083	18	12
CUST_41400_PI429010886	M57648						0.0492933	12	3	0.0280438	21	0
CUST_18352_PI429010886	N39634						0.049392	24	18	0.0260531	15	12
CUST_45580_PI429010886	M69570						0.0494159	12	0	0.0136311	21	3
CUST_35407_PI429010886	M12509						0.0494418	24	21	0.0055677	18	12
CUST_11186_PI429010886	N27405	156544105	PREDICTED: uracil phosphoribosyl	3.88E-52			0.0498263	21	12	0.0419773	21	0
CUST_1064_PI429010886	N8384						0.0498543	24	9	0.0150347	15	9
CUST_39663_PI429010886	M42411	493789484	hypothetical protein, partial [Lact	6.55E-09			0.0498689	15	6	0.0072967	15	12
CUST_56045_PI429010886	M112614						0.0499167	21	12	0.0435226	18	12
CUST_50250_PI429010886	M89164						0.049924	18	15	0.0080301	15	3
CUST_25270_PI429010886	N52095						0.0499546	24	6	0.0193159	21	3
CUST_56893_PI429010886	M114850						0.0499589	12	0	0.0048225	21	0
CUST_4155_PI429010886	N16123	390362924	PREDICTED: zinc finger protein 84	3.5E-15			0.04998	24	18	0.0421516	24	15

LD

Probe ID	0	0	0	3	3	3	6	6	6	9	9	9	12	12	12	15	15	15	18	18	18	21	21	21	24	24	24
CUST_42364_PI429010886	179.2149	172.748	163.8102	190.4954	210.3322	209.5237	188.991	176.5842	204.8848	239.4012	156.8401	205.2408	201.5828	202.4552	229.203	268.9316	196.1132	217.5068	200.1541	149.129	174.5649	193.4888	139.3207	165.2945	216.2603	164.7136	161.175
CUST_10906_PI429010886	23.14891	13.91642	10.49199	7.958428	14.28701	13.58386	21.44313	18.60134	20.33393	5.162816	5.848596	8.809458	9.676757	8.79216	7.55889	18.01447	20.21086	11.07276	8.825422	5.509501	12.89468	3.555623	7.947466	5.679021	13.5943	11.76708	3.369406
CUST_33562_PI429010886	146.5888	648.4382	454.7075	252.3498	150.5356	199.116	522.1886	821.966	537.7378	635.4957	329.4331	180.2441	342.9247	395.8718	408.0121	590.1632	393.358	771.2654	267.5084	372.8037	121.3291	199.588	167.9984	293.4576	364.8028	264.4099	187.0768
CUST_10936_PI429010886	9.358571	12.8289	16.00437	15.2353	13.13552	12.15876	15.52247	12.83426	7.856768	12.17645	11.43085	13.41447	12.98751	8.296655	10.40441	10.07945	12.59391	13.74572	17.73313	12.07005	12.939	13.95642	16.63046	21.63236	15.01966	12.95843	23.87086
CUST_19856_PI429010886	10.38036	6.093975	9.227637	4.395918	7.288803	4.866146	NA	4.081102	4.696988	8.068353	12.83829	7.340202	6.848153	4.703264	4.186342	6.429816	5.483339	3.118923	9.832908	7.751317	6.644311	8.974877	8.143822	8.441492	7.235998	NA	10.43378
CUST_39434_PI429010886	5271.495	9653.786	6837.265	6116.36	3321.698	3067.239	6956.592	6592.219	8522.605	5342.932	5907.39	4193.691	4531.71	3629.795	4334.63	8698.04	10337.3	10193.49	7232.62	6000.152	7872.926	5209.792	4829.887	6710.162	5496.275	4517.506	6125.57
CUST_38820_PI429010886	7912.634	14642.96	9226.663	8644.801	4234.05	5357.194	9096.626	10408.52	11206.93	7844.789	6929.595	5502.319	9516.402	5571.385	6393.352	12860.58	14470.28	14071.14	9129.704	9872.039	7750.118	8256.789	7316.568	9669	8238.918	7348.003	7993.785
CUST_14391_PI429010886	35.07134	40.70597	49.85348	26.69299	36.28088	34.03486	21.36854	46.57555	20.97815	31.70434	36.04692	28.47101	46.40366	47.41757	41.06053	40.56201	56.30846	46.40366	24.22486	45.43179	35.44329	38.97889	37.33345	28.5989	43.73952	45.29434	41.85499
CUST_19229_PI429010886	16.80772	11.52408	21.08201	14.17513	13.31268	3.736826	11.33397	15.63254	21.04316	5.039424	9.573755	15.0561	13.44807	15.44997	5.856234	12.31255	17.22544	19.20962	14.07152	25.6877	22.48894	9.01805	21.1337	17.42012	11.46447	9.398999	11.93346
CUST_41400_PI429010886	10.15678	5.692618	14.23051	16.66622	10.5599	17.0552	10.3429	NA	21.00252	8.04855	5.651239	10.07137	8.571918	6.517364	14.53618	11.7024	12.09079	17.69677	12.59391	6.247573	8.817048	13.2031	5.740467	5.868192	14.43423	15.2772	17.28788
CUST_18352_PI429010886	273.0279	195.1305	310.9804	298.5479	310.4343	254.7629	340.3327	332.8862	423.3824	609.0112	399.8777	447.5258	425.8369	386.3361	439.5135	490.4622	277.3623	503.9395	474.0818	422.6098	500.8894	393.0106	444.3539	494.3291	494.606	411.5622	609.2645
CUST_45580_PI429010886	71.14732	65.53387	101.2279	76.90215	61.68316	66.60981	56.55991	59.59396	63.24693	73.24973	59.99359	64.07605	106.3228	45.64468	97.30769	75.75538	80.79724	65.11364	69.28931	69.15316	62.07591	91.52744	58.42497	71.9236	86.41587	99.20976	75.06882
CUST_35407_PI429010886	75.0185	155.8206	122.1097	90.65467	108.1041	101.6228	87.83185	136.5677	124.3455	95.03516	123.7628	73.39156	96.37995	98.86808	103.914	118.8436	149.2467	143.7425	101.4235	138.4144	143.144	146.7486	134.2508	152.7137	120.0673	123.6152	155.885
CUST_11186_PI429010886	68.30294	53.25197	35.98686	42.32912	38.69332	55.19052	80.16082	57.57578	62.55614	36.00722	37.41195	59.72208	66.32665	70.43485	85.00598	73.94525	65.86788	62.62916	49.50343	30.2752	40.11852	42.84582	43.64463	56.72705	40.85968	47.34933	36.76966
CUST_1064_PI429010886	276.8158	36.49885	24.13409	339.8451	59.39503	82.3809	295.8529	54.73135	10.09623	349.6396	80.15283	96.10147	253.1631	65.67573	73.11579	72.75695	47.60538	169.34	14.64829	65.97782	11.81288	26.73097	36.05612	56.80856	5.148525	94.20496	43.76293
CUST_39663_PI429010886	1197.753	2242.146	1956.496	2350.317	2340.704	1842.758	2522.496	2711.745	3319.225	2146.15	2661.468	1944.792	3179.907	2835.943	2725.344	909.1828	1529.136	1016.824	1947.583	2430.729	2041.036	1734.409	2698.998	2956.694	1529.136	1961.653	3043.672
CUST_56045_PI429010886	11.57188	20.97815	9.534814	18.8606	11.68418	22.49316	12.3345	25.53705	24.40844	12.46202	15.97033	16.93597	16.64399	22.29988	23.65067	16.43359	21.36854	14.85649	15.86706	10.6362	25.44962	14.75102	7.07223	12.08533	11.31144	15.4677	19.71392
CUST_50250_PI429010886	27.26144	46.81937	11.69612	17.61125	20.01679	26.29331	22.37507	50.79258	22.80251	32.23579	22.01591	36.79887	29.41053	29.83399	20.16722	38.17039	53.99735	44.26795	33.92477	21.70341	38.49334	19.75896	47.05022	19.01772	29.17859	26.24865	20.32943
CUST_25270_PI429010886	32.08859	35.61115	NA	12.24986	69.08897	54.69951	52.97639	45.16295	62.71091	10.26423	24.94507	31.77263	19.36009	18.45414	27.1944	44.00493	27.49895	15.1083	9.34077	35.32719	7.219437	5.721366	36.15638	8.185146	8.924839	73.80869	NA
CUST_56893_PI429010886	103.9866	82.44777	85.63473	101.522	60.43161	70.49708	77.73937	55.85351	61.3418	99.1481	62.19095	75.37573	105.87	57.70575	89.26114	102.8149	103.5395	101.2701	84.4188	66.296	49.51848	102.4622	84.17542	87.55235	107.4885	86.60823	106.6432
CUST_4155_PI429010886	218.9572	382.6455	359.4486	261.3674	211.803	214.3618	263.251	335.5615	356.1688	230.8967	279.1333	263.0994	284.4662	261.7118	231.8895	314.3155	333.9088	376.8731	324.5457	345.8925	400.0672	230.0694	306.2147	361.5772	309.4164	299.9228	531.9945

DD

Probe ID	0	0	0	3	3	3	6	6	6	9	9	9	12	12	12	15	15	15	18	18	18	21	21	21	24	24	24
CUST_42364_PI429010886	182.0483	218.3592	172.9784	278.7763	286.5169	219.2135	193.5749	159.4512	185.6237	177.1267	183.6687	165.7374	152.2767	177.8158	165.2714	229.8746	178.7689	240.9417	190.2958	175.9719	190.0964	191.3655	164.6734	164.6012	274.296	179.678	166.519
CUST_10906_PI429010886	18.93037	15.09234	NA	18.86217	9.13082	14.64853	8.029282	5.738619	4.71542	8.689343	6.246892	3.8418	13.79851	NA	19.88848	16.59703	6.469129	28.84595	NA	7.162712	9.601564	9.048255	NA	12.44135	11.61617	4.16646	11.42367
CUST_33562_PI429010886	142.4348	1114.056	507.5628	308.3369	706.7803	1022.449	566.4251	477.9922	832.3284	535.1571	321.2041	963.151	172.498	634.6129	286.423	635.0709	276.5696	792.7128	1242.051	1068.447	1023.799	822.9643	377.1617	424.6374	127.723	279.1338	76.71565
CUST_10936_PI429010886	14.45048	25.74156	19.0691	14.39019	15.63271	9.184475	14.33237	13.71667	9.488473	18.85554	14.1362	15.59485	16.3703	11.70566	19.99756	30.56491	15.21068	54.35965	17.62531	14.16669	16.54774	26.23528	15.9031	18.35292	15.26938	18.08454	19.39031
CUST_19856_PI429010886	11.22575	6.522752	NA	9.748744	7.488246	NA	9.413901	NA	10.87507	21.45934	9.858236	12.64557	9.441058	10.86536	9.049354	12.04909	3.961673	7.490316	10.23894	4.495511	7.753636	6.143836	NA	NA	5.049338	5.888357	5.710989
CUST_39434_PI429010886	4965.834	8219.035	5654.855	4635.589	4803.756	5477.513	5262.203	5515.427	5515.427	9475.141	3526.312	8582.669	7853.842	7729.059	9937.704	10083.97	4782.509	5050.626	5119.45	8448.526	4847.521	7107.78	4392.265	3644.859	6454.955	5319.238	6794.71
CUST_38820_PI429010886	6350.776	10178.43	8176.608	4782.509	6600.16	6910.632	6654.81	5717.187	8597.6	12610.19	6461.792	10296.63	10247.75	11122.77	11864.38	12053.21	6234.905	10469.53	7930.333	10247.75	7794.866	11545.02	5447.86	5819.009	10685.37	7071.027	10366.97
CUST_14391_PI429010886	61.72981	64.48135	55.36009	53.30382	51.0829	58.04233	52.71293	53.01853	42.89451	47.83781	40.99957	46.76098	43.53241	47.43502	29.43926	42.35962	56.79499	51.9493	61.44485	50.88285	30.89566	59.07909	33.26161	37.93755	63.37918	47.39715	49.2211
CUST_19229_PI429010886	11.71083	14.5219	NA	11.88603	16.04761	5.867579	15.19655	14.51697	20.87033	15.17932	16.11406	20.02885	22.92991	43.39175	23.26041	20.6694	14.40694	9.365159	19.3587	22.47719	19.64813	6.911676	NA	13.35309	23.72626	30.52691	29.5483
CUST_41400_PI429010886	11.46886	NA	9.956467	17.18399	5.321149	15.7271	8.778682	10.01115	7.157803	6.374853	6.391855	NA	5.304061	6.872039	8.309227	9.771392	8.031655	9.418869	7.733411	6.967135	NA	NA	8.228156	15.73231	10.48257	7.706127	9.769998
CUST_18352_PI429010886	363.7161	326.9766	511.8133	331.6154	425.2074	302.6146	295.1449	321.3108	244.0376	459.0733	376.1162	568.5491	302.0506	589.4448	471.0971	565.8837	338.1543	493.4379	434.7605	397.8439	394.7994	347.8665	363.0918	298.1495	493.7491	344.0991	540.6649
CUST_45580_PI429010886	89.07632	57.14083	69.47796	82.89486	84.53623	77.1366	77.48229	51.13109	55.27747	86.64513	55.37764	79.30391	56.24668	53.15786	57.84331	79.71189	73.08468	76.31563	59.49267	67.15618	70.01711	73.28621	40.70657	79.99032	100.397	119.2945	115.7331
CUST_35407_PI429010886	146.8141	148.08	157.5747	115.6518	137.1056	124.4818	134.577	116.8442	144.7064	140.6181	150.9847	173.7958	145.1035	170.6697	181.0372	227.8652	143.5925	163.6601	144.8143	158.8004	102.7622	178.9173	84.1293	108.3734	165.5902	123.0876	134.282
CUST_11186_PI429010886	81.45472	77.74288	63.01798	54.80919	58.73387	70.53195	101.8998	52.063	53.78486	53.18376	69.17707	59.19994	49.61333	59.03159	70.99743	68.1054	78.00387	65.39564	65.43441	68.25442	59.30743	77.64007	79.09571	101.0858	56.24014	68.39623	45.51068
CUST_1064_PI429010886	33.82224	77.82271	64.8815	55.93124	68.01317	83.7299	83.99654	61.5685	114.2228	80.01037	83.83973	149.3268	48.10487	95.50344	57.72307	35.41295	56.0662	104.3119	40.98319	55.03461	70.72361	62.62665	92.18368	103.6255	102.5545	83.02241	139.9854
CUST_39663_PI429010886	2033.91	2854.292	3644.859	857.8725	1520.67	1162.112	6073.086	4594.669	6779.053	3653.581	2291.102	3120.564	5402.577	5807.129	3965.108	5239.377	4068.874	3060.282	3048.703	2235.234	2529.511	3952.869	5515.427	2877.641	8245.915	2970.275	11545.02
CUST_56045_PI429010886	13.85408	17.22867	21.06491	15.27765	23.62378	24.93189	28.17746	28.48354	21.58663	21.99145	24.38855	33.76983	38.22991	27.78747	25.15661	24.96359	16.49958	20.17559	11.24691	18.11108	34.71503	26.68081	20.12612	20.76211	13.95091	24.39107	34.74686
CUST_50250_PI429010886	52.75651	42.79014	22.55376	69.35049	43.20136	62.84333	34.95753	42.92316	30.02913	24.69322	24.76116	30.13158	28.87402	41.01917	42.84657	70.18575	28.53623	50.56281	38.09997	33.95306	40.06113	55.65323	32.73752	35.98369	33.75052	27.25419	16.46586
CUST_25270_PI429010886	22.91836	22.9575	9.181579	58.22303	12.01996	83.80399	56.15718	17.50878	28.18833	NA	7.02756	NA	42.11302	NA	32.37332	16.10308	18.44318	27.28895	11.99825	4.943292	7.638915	13.29711	NA	61.56139	NA	47.19112	NA
CUST_56893_PI429010886	89.77998	85.24568	102.3996	74.48926	83.10449	71.57902	79.9054	80.10966	92.38118	77.29645	71.98272	81.04828	72.62704	79.31367	74.75705	84.57831	83.48682	66.4223	65.86348	84.64574	78.05922	93.01036	98.55351	89.17313	98.23281	80.03068	72.8924
CUST_4155_PI429010886	286.0749	397.0596	306.479	257.8206	290.8448	234.9764	246.8808	208.138	339.4134	511.5434	352.327	535.2858	247.8273	343.7569	358.7967	684.8725	341.3294	376.5573	312.7398	417.7156	313.7524	502.4781	320.8355	264.5273	492.9704	280.5129	360.4943
