# Supplementary material for: Analysis of the circadian transcriptome of the Antarctic krill Euphausia superba
Source: Sci Rep. 2019 Sep 25;9:13894. doi: 10.1038/s41598-019-50282-1 (PMC6761102; doi:10.1038/s41598-019-50282-1)
Supplement: Supplementary file 5 — Supplementary Table 4 [file 41598_2019_50282_MOESM5_ESM.pdf]

Analysis of the circadian transcriptome of the Antarctic krill *Euphausia superba*

Alberto Biscontin[1,2,*], Paolo Martini[1], Rodolfo Costa[1], Achim Kramer[2], Bettina Meyer[3,4,5], So Kawaguchi[6], Mathias Teschke[3], Cristiano De Pittà[1,*]

[1]Dipartimento di Biologia, Università degli Studi di Padova, Padova, Italy

[2]Laboratory of Chronobiology, Charité Universitätsmedizin Berlin, Berlin, Germany

[3]Section Polar Biological Oceanography, Alfred Wegener Institute Helmholtz Centre for Polar and Marine Research, Bremerhaven, Germany

[4]Institute for Chemistry and Biology of the Marine Environment, Carl von Ossietzky University of Oldenburg, Oldenburg, Germany

[5]Helmholtz Institute for Functional Marine Biodiversity (HIFMB) at the University of Oldenburg, 26111 Oldenburg, Germany

[6]Department of Environment and Heritage, Australian Antarctic Division, Kingston, Tasmania, Australia

[*]Corresponding authors:

Cristiano De Pittà, Dipartimento di Biologia, Università degli Studi di Padova, via U. Bassi 58/B 35131 Padova, Italy; Phone: +39-049-8276210; Fax: +39-049-8276209; e-mail address: cristiano.depitta@unipd.it

Alberto Biscontin, Dipartimento di Biologia, Università degli Studi di Padova, via U. Bassi 58/B 35131 Padova, Italy; Phone: +39-049-8276228; Fax: +39-049-8276209; e-mail address: alberto.biscontin@unipd.it

Supplementary Table 4. List of 428 annotated circadian genes with a sinusoidal expression pattern both in LD and DD.

Functional annotation with BLAST2GO of circadian genes, identified by RAIN (adjusted *p*-value < 0.05) throughout the 24-hour cycle both in LD and DD, are shown. [a]ID Krill2: accession number of krill master transcriptome
[b]ID Krill1: accession number of krill diurnal transcriptome 14; [c]Annotation = description of the gene; [d]GO term: gene ontology biological process;
[e]GO child term: most fitting gene ontology child term to further refine the functional annotation.

ID krill2	ID krill1	Annotation	GO Term	GO Child Term
**Cellular component organization**				
M93133	isotig03139	actin [Crassostrea gigas]	Cellular component organization	Cytoskeleton organization
N15969		actin [Wuchereria bancrofti]	Cellular component organization	Cytoskeleton organization
E4642		beta-1 tubulin [Spodoptera frugiperda]	Cellular component organization	Cytoskeleton organization
M71966	isotig02534	beta-tubulin [Oxymonadida environmental sample]	Cellular component organization	Cytoskeleton organization
N19790		alpha-tubulin (Atub) mRNA, partial cds	Cellular component organization	Cytoskeleton organization
M2022		cyclic AMP-regulated protein like protein [Marsupenaeus japonicus]	Cellular component organization	Cytoskeleton organization
M7549		Katanin p80 WD40-containing subunit B1 [Camponotus floridanus]	Cellular component organization	Cytoskeleton organization
N27135		kinesin 2B [Nasonia vitripennis]	Cellular component organization	Cytoskeleton organization
N56949	isotig04195	myosin heavy chain type 2 [Penaeus monodon]	Cellular component organization	Cytoskeleton organization
M107465		myosin light chain [Penaeus monodon]	Cellular component organization	Cytoskeleton organization
M3279		Penaeus monodon MYH2 mRNA for myosin heavy chain type 2, complete cds	Cellular component organization	Cytoskeleton organization
M67802		Portunus pelagicus actin mRNA, partial cds	Cellular component organization	Cytoskeleton organization
N51300		PREDICTED: actin-related protein 2-like isoform 1 [Megachile rotundata]	Cellular component organization	Cytoskeleton organization
N28458		PREDICTED: costars family protein ABRACL-like [Ciona intestinalis]	Cellular component organization	Cytoskeleton organization
N45122		PREDICTED: filamin-A isoform X3 [Tribolium castaneum]	Cellular component organization	Cytoskeleton organization
N37731		PREDICTED: kinesin-like protein unc-104 [Nasonia vitripennis]	Cellular component organization	Cytoskeleton organization
N12731	GTJW03E08JUW9R	PREDICTED: LOW QUALITY PROTEIN: myosin-IIIa [Nasonia vitripennis]	Cellular component organization	Cytoskeleton organization
M739		PREDICTED: microtubule-associated proteins 1A/1B light chain 3C [Chelonia mydas]	Cellular component organization	Cytoskeleton organization
M110013		PREDICTED: similar to kakapo [Tribolium castaneum]	Cellular component organization	Cytoskeleton organization
N54541		PREDICTED: unconventional myosin-VI [Trichechus manatus latirostris]	Cellular component organization	Cytoskeleton organization
N43824		PREDICTED: unconventional myosin-VI isoform X1 [Poecilia formosa]	Cellular component organization	Cytoskeleton organization
M31074		profilin [Penaeus monodon]	Cellular component organization	Cytoskeleton organization
M67257		putative articulin p60 [Rimicaris exoculata]	Cellular component organization	Cytoskeleton organization
N23331		serine/threonine protein phosphatase 4 regulatory subunit [Ixodes scapularis]	Cellular component organization	Cytoskeleton organization
M42411		collagen-binding protein [Bacteroides cellulosilyticus]	Cellular component organization	Extracellular matrix organization
M98466		EGF-like domain-containing protein [Polysphondylium pallidum PN500]	Cellular component organization	Extracellular matrix organization
N25080		PREDICTED: serine-rich adhesin for platelets-like isoform X2 [Ceratitis capitata]	Cellular component organization	Extracellular matrix organization
M44093		putative elastin a [Rimicaris exoculata]	Cellular component organization	Extracellular matrix organization
N5133		putative elastin a [Rimicaris exoculata]	Cellular component organization	Extracellular matrix organization
N5141		putative elastin a [Rimicaris exoculata]	Cellular component organization	Extracellular matrix organization
N20132		DOMON domain-containing protein CG14681-like [Nasonia vitripennis]	Cellular component organization	
M14810		PREDICTED: collagen alpha-5(IV) chain [Cavia porcellus]	Cellular component organization	
**Cellular Process**				
N20755		PREDICTED: Apis mellifera vinculin (Vinc), mRNA	Cellular Process	Cell adhesion
M88841		PREDICTED: protocadherin Fat 1 isoform X5 [Xenopus (Silurana) tropicalis]	Cellular Process	Cell adhesion
N14119	isotig07477	BCS-2 [Amphibalanus amphitrite]	Cellular Process	Cell cycle
N19844		cyclin G-like protein [Daphnia pulex]	Cellular Process	Cell cycle
M99237		mitotic spindle-associated MMXD complex subunit MIP18 [Chinchilla lanigera]	Cellular Process	Cell cycle
N19234		PREDICTED: origin recognition complex subunit 4 [Sorex araneus]	Cellular Process	Cell cycle
N22167		PREDICTED: Saccoglossus kowalevskii mps one binder kinase activator-like 3-like	Cellular Process	Cell cycle
N18757		PREDICTED: dnaJ homolog subfamily C member 2-like [Pundamilia nyererei]	Cellular Process	Cell cycle regulation
M70056		Inhibitor of growth protein 1 [Camponotus floridanus]	Cellular Process	Cell death
M61577		PREDICTED: programmed cell death protein 2-like [Nasonia vitripennis]	Cellular Process	Cell death
N24494		PREDICTED: YLP motif-containing protein 1 [Xenopus (Silurana) tropicalis]	Cellular Process	Cell differentiation
N52462		inhibitor of apoptosis protein [Litopenaeus vannamei]	Cellular Process	Cell proliferation
N40192		inhibitor of apoptosis protein [Penaeus monodon]	Cellular Process	Cell proliferation
N12719		PREDICTED: similar to dre4 CG1828-PA [Tribolium castaneum]	Cellular Process	
**Developmental process**				
M85184		PREDICTED: titin-like isoform X1 [Apis mellifera]	Developmental process	Muscle structure development
N18069		projectin [Procambarus clarkii]	Developmental process	Muscle structure development
M11868		PREDICTED: alpha/beta hydrolase domain-containing protein 14A [Gallus gallus]	Developmental process	Nervous system development
M378		similar to receptor-type tyrosine-protein phosphatase N2 [Tribolium castaneum]	Developmental process	Nervous system development
M11462		ankyrin repeat protein [Trichomonas vaginalis G3]	Developmental process	
M14333		ankyrin repeat protein [Trichomonas vaginalis G3]	Developmental process	
N52096		innexin 2 [Penaeus monodon]	Developmental process	
M3501		innexin inx1 [Homarus gammarus]	Developmental process	
M64411		LIM class homeobox transcription factor Lmx [Mnemiopsis leidyi]	Developmental process	
N36400		Microtubule-associated protein futsch [Cerapachys biroi]	Developmental process	
M5443		Pediculus humanus corporis protein Mo25, putative, mRNA	Developmental process	
M65366		Pogo transposable element with KRAB domain [Crassostrea gigas]	Developmental process	
N4801	isotig00687	PREDICTED: calphotin-like [Acyrthosiphon pisum]	Developmental process	
N42326		PREDICTED: COP9 signalosome complex subunit 3 [Ornithorhynchus anatinus]	Developmental process	
N39388		PREDICTED: similar to serine proteinase stubble [Tribolium castaneum]	Developmental process	
M65029		Segmentation protein cap'n'collar [Harpegnathos saltator]	Developmental process	
N35583		SpAN-like protein [Rimicaris exoculata]	Developmental process	
N22265		src tyrosine kinase, putative [Ixodes scapularis]	Developmental process	
**Metabolic process**				
M108370		Oplophorus-luciferin 2-monooxygenase non-catalytic subunit [Oplophorus gracilirostris]	Metabolic process	Bioluminescence
M73149		Acid sphingomyelinase-like phosphodiesterase 3b, partial [Ophiophagus hannah]	Metabolic process	Carbohydrate metabolic process
N39090		Beta-1,4-galactosyltransferase 3 [Chelonia mydas]	Metabolic process	Carbohydrate metabolic process
N23226		Carbohydrate sulfotransferase 14 [Camponotus floridanus]	Metabolic process	Carbohydrate metabolic process
M12607		chitinase 2 [Penaeus monodon]	Metabolic process	Carbohydrate metabolic process (
M60556		galactoside 2-alpha-L-fucosyltransferase 2-like [Odobenus rosmarus divergens]	Metabolic process	Carbohydrate metabolic process
M18847	isotig06097	sp|Q7C3R3|XYLA_ENTFA Xylose isomerase OS=Enterococcus faecalis GN=xylA PE=3 SV=1	Metabolic process	Carbohydrate metabolic process
M16697		i-type lysozyme-like protein 2 [Penaeus monodon]	Metabolic process	Carbohydrate metabolic process
N9218		i-type lysozyme-like protein 2 [Penaeus monodon]	Metabolic process	Carbohydrate metabolic process
M17683		n-acetylgalactosaminyltransferase [Aedes aegypti]	Metabolic process	Carbohydrate metabolic process
E4652		PREDICTED: beta-1,4-mannosyl-glycoprotein 4-beta-N-acetylglucosaminyltransferase [Ornithorhynchus anatinus]	Metabolic process	Carbohydrate metabolic process
N22117		PREDICTED: xylose isomerase-like [Strongylocentrotus purpuratus]	Metabolic process	Carbohydrate metabolic process
M17951	GTJW03E08JCXIH	PREDICTED: glycogen debranching enzyme-like [Nasonia vitripennis]	Metabolic process	Carbohydrate metabolic process
N23374	isotig06708	PREDICTED: xylose isomerase [Ciona intestinalis]	Metabolic process	Carbohydrate metabolic process

ID krill2	ID krill1	Annotation	GO Term	GO Child Term
N13131		PREDICTED: xylose isomerase-like [Amphimedon queenslandica]	Metabolic process	Carbohydrate metabolic process
M108205		PREDICTED: xylose isomerase-like [Aplysia californica]	Metabolic process	Carbohydrate metabolic process
M19131	isotig07272	PREDICTED: xylose isomerase-like [Takifugu rubripes]	Metabolic process	Carbohydrate metabolic process
M23222	isotig07191	PREDICTED: xylose isomerase-like [Takifugu rubripes]	Metabolic process	Carbohydrate metabolic process
M4573	isotig08459	PREDICTED: xylose isomerase-like [Takifugu rubripes]	Metabolic process	Carbohydrate metabolic process
N13357		PREDICTED: xylose isomerase-like [Takifugu rubripes]	Metabolic process	Carbohydrate metabolic process
N12995		PREDICTED: xylose isomerase-like isoform X1 [Oreochromis niloticus]	Metabolic process	Carbohydrate metabolic process
M2029	FW09YNP01CT3PY	putative glycogen synthase [Daphnia pulex]	Metabolic process	Carbohydrate metabolic process
N55506		4-aminobutyrate aminotransferase [Aedes aegypti]	Metabolic process	Cellular amino acid metabolic pro
M109440		Asparaginyl-tRNA synthetase, isoform A [Drosophila melanogaster]	Metabolic process	Cellular amino acid metabolic pro
M3130		Fumarylacetoacetase [Crassostrea gigas]	Metabolic process	Cellular amino acid metabolic pro
N52993	isotig10007	glutamine synthetase [Fenneropenaeus chinensis]	Metabolic process	Cellular amino acid metabolic pro
M93328		glycine decarboxylase (predicted), isoform CRA_d [Rattus norvegicus]	Metabolic process	Cellular amino acid metabolic pro
N14451		hematopoietic prostaglandin D synthase [Penaeus monodon]	Metabolic process	Cellular amino acid metabolic pro
M545		kynurenine 3-monooxygenase [Capsaspora owczarzaki ATCC 30864]	Metabolic process	Cellular amino acid metabolic pro
M56721		L-aspartate dehydrogenase [Caligus clemensi]	Metabolic process	Cellular amino acid metabolic pro
M4562		methylcrotonoyl-CoA carboxylase beta chain, mitochondrial [Oreochromis niloticus]	Metabolic process	Cellular amino acid metabolic pro
N18403		Ornithine aminotransferase, mitochondrial [Acromyrmex echinatior]	Metabolic process	Cellular amino acid metabolic pro
M4015		PREDICTED: adenosylhomocysteinase A-like [Strongylocentrotus purpuratus]	Metabolic process	Cellular amino acid metabolic pro
N19992		PREDICTED: arginase-1-like [Loxodonta africana]	Metabolic process	Cellular amino acid metabolic pro
N19820		PREDICTED: threonine synthase-like 2 [Callorhinchus milii]	Metabolic process	Cellular amino acid metabolic pro
M87594		PREDICTED: tryptophan 2,3-dioxygenase [Nomascus leucogenys]	Metabolic process	Cellular amino acid metabolic pro
M7047		Prolyl 4-hydroxylase subunit alpha-1 [Crassostrea gigas]	Metabolic process	Cellular amino acid metabolic pro
M63222		sarcosine dehydrogenase, mitochondrial-like [Strongylocentrotus purpuratus]	Metabolic process	Cellular amino acid metabolic pro
M4050		putative Collagen alpha-1V chain [Danaus plexippus]	Metabolic process	Chitin metabolic process
M78726	contig03755	cytochrome c oxidase subunit I, partial (mitochondrion) [Neopetrolisthes maculatus]	Metabolic process	Generation of precursor metaboli
N31394		cytochrome c oxidase subunit VIIA putative [Scylla paramamosain]	Metabolic process	Generation of precursor metaboli
M99429	contig03831	Euphausia superba isolate I241 cytochrome c oxidase subunit I (COI) and NADH dehydrogenase subunit 1 (ND1) genes, partial cds; mitochondrial	Metabolic process	Generation of precursor metaboli
M113051		Euphausia superba voucher 760M mitochondrion, partial genome	Metabolic process	Generation of precursor metaboli
M14565		mitochondrial ATP synthase gamma subunit precursor [Litopenaeus vannamei]	Metabolic process	Generation of precursor metaboli
N33931		NADH dehydrogenase [ubiquinone] 1 subunit C2-like [Musca domestica]	Metabolic process	Generation of precursor metaboli
N14105		NADH-ubiquinone oxidoreductase 20 kDa subunit, mitochondrial precursor, mRNA [Candida tropicalis]	Metabolic process	Generation of precursor metaboli
M68114		PREDICTED: similar to mitochondrial NADH:ubiquinone oxidoreductase ESSS subunit, putative [Tribolium castaneum]	Metabolic process	Generation of precursor metaboli
N29097	FW09YNP02I2TOO	Acetyl-CoA carboxylase [Camponotus floridanus]	Metabolic process	Lipid metabolic process
N12285		hematopoietic prostaglandin D synthase [Penaeus monodon]	Metabolic process	Lipid metabolic process
M1878		Homo sapiens peroxisomal D3,D2-enoyl-CoA isomerase [synthetic construct]	Metabolic process	Lipid metabolic process
M86465		PREDICTED: galactose-3-O-sulfotransferase 1-like [Saccoglossus kowalevskii]	Metabolic process	Lipid metabolic process
N9420		PREDICTED: probable serine incorporator-like [Nasonia vitripennis]	Metabolic process	Lipid metabolic process
M32708		putative triacylglycerol lipase [Daphnia pulex]	Metabolic process	Lipid metabolic process
M5901		triacylglycerol lipase [Litopenaeus vannamei]	Metabolic process	Lipid metabolic process
N36501		triacylglycerol lipase [Litopenaeus vannamei]	Metabolic process	Lipid metabolic process
M94914		glycosyl-phosphatidylinositol-linked carbonic anhydrase [Litopenaeus vannamei]	Metabolic process	Nitrogen compound metabolic pr
N18959		exonuclease 3'-5' domain containing 2 [Xenopus laevis]	Metabolic process	Nucleobase-containing compound
N20569		glycinamide ribonucleotide synthetase-aminoimidazole ribonucleotide synthetase-glycinamide ribonucleotide transformylase [Branchiostoma belcheri]	Metabolic process	Nucleobase-containing compound
N29027		PREDICTED: guanine deaminase [Ficedula albicollis]	Metabolic process	Nucleobase-containing compound
M56865		PREDICTED: inosine-5'-monophosphate dehydrogenase isoform 1 [Apis mellifera]	Metabolic process	Nucleobase-containing compound
N21697		PREDICTED: probable queuine tRNA-ribosyltransferase [Acyrthosiphon pisum]	Metabolic process	Nucleobase-containing compound
M3981		PREDICTED: similar to adenine phosphoribosyltransferase [Tribolium castaneum]	Metabolic process	Nucleobase-containing compound
M78121		PREDICTED: similar to ribosomal pseudouridine synthase [Tribolium castaneum]	Metabolic process	Nucleobase-containing compound
N46564		PREDICTED: xanthine dehydrogenase/oxidase [Danio rerio]	Metabolic process	Nucleobase-containing compound
N24180		UDP-glucose 4-epimerase [Zootermopsis nevadensis]	Metabolic process	Nucleobase-containing compound
N21406		cytochrome P450 [Bemisia tabaci]	Metabolic process	Oxidation-reduction process
M62474		cytochrome P450 [Litopenaeus vannamei]	Metabolic process	Oxidation-reduction process
M57279		dehydrogenase/reductase SDR family member 11 [Pseudopodoces humilis]	Metabolic process	Oxidation-reduction process
E3651		extracellular superoxide dismutase precursor [Pacifastacus leniusculus]	Metabolic process	Oxidation-reduction process
M13711		extracellular superoxide dismutase precursor [Pacifastacus leniusculus]	Metabolic process	Oxidation-reduction process
N26026		Laccase-4 [Acromyrmex echinatior]	Metabolic process	Oxidation-reduction process
N21156		peptidylglycine alpha-amidating monooxygenase [Conus bullatus]	Metabolic process	Oxidation-reduction process
N19275		saccharopine dehydrogenase-like oxidoreductase-like [Saccoglossus kowalevskii]	Metabolic process	Oxidation-reduction process
N21510		senecionine N-oxygenase, putative [Pediculus humanus corporis]	Metabolic process	Oxidation-reduction process
M4594		thioredoxin [Ruditapes philippinarum]	Metabolic process	Oxidation-reduction process
M9529		unspecific monooxygenase [Brugia malayi]	Metabolic process	Oxidation-reduction process
N21023		dihydroflavonol 4-reductase [alpha proteobacterium SCGC AAA536-K22]	Metabolic process	Pigment
M75421		PREDICTED: LYR motif-containing protein 4B-like [Oreochromis niloticus]	Metabolic process	Small molecule metabolic process
N20470		1-acyl-sn-glycerol-3-phosphate acyltransferase gamma-like [Nasonia vitripennis]	Metabolic process	
M104013		adenylyl cyclase [Daphnia pulex]	Metabolic process	
M4820		carboxylesterase [Laodelphax striatella]	Metabolic process	
M586		Cytosolic Fe-S cluster assembly factor NUBP1-like protein [Crassostrea gigas]	Metabolic process	
E1475		guanine nucleotide-binding protein, putative [Ixodes scapularis]	Metabolic process	
N38779		PREDICTED: abhydrolase domain-containing protein 3-like [Bombus impatiens]	Metabolic process	
N45146		PREDICTED: allantoinase, mitochondrial-like [Ornithorhynchus anatinus]	Metabolic process	
N23265		PREDICTED: arylamine N-acetyltransferase 3-like [Microtus ochrogaster]	Metabolic process	
N7968		PREDICTED: Caenorhabditis EpoxidE Hydrolase family member (ceeh-1)-like [Saccoglossus kowalevskii]	Metabolic process	
N20506		PREDICTED: estrogen sulfotransferase-like [Apis mellifera]	Metabolic process	
N41594		PREDICTED: GDP-L-fucose synthase-like [Aplysia californica]	Metabolic process	
M65988		PREDICTED: SET and MYND domain-containing protein 4-like [Nasonia vitripennis]	Metabolic process	
M4676		SEL-1, putative [Ixodes scapularis]	Metabolic process	
N32142		tRNA 2-thiocytidine biosynthesis protein ttcA [Lepeophtheirus salmonis]	Metabolic process	
**Nucleic acid metabolism**				
M102205		DNA topoisomerase 3-alpha [Crassostrea gigas]	Nucleic acid metabolism	DNA metabolism
N27019		PREDICTED: origin recognition complex, subunit 1-like [Saccoglossus kowalevskii]	Nucleic acid metabolism	DNA replication
N27095		PREDICTED: DNA polymerase epsilon subunit 3 isoform X1 [Chrysemys picta bellii]	Nucleic acid metabolism	DNA replication
M108155		PREDICTED: protein misato-like isoform X1 [Musca domestica]	Nucleic acid metabolism	DNA replication
N38408		ankyrin repeat domain-containing protein 49-like [Acyrthosiphon pisum]	Nucleic acid metabolism	Regulation of gene expression

ID krill2	ID krill1	Annotation	GO Term	GO Child Term
M335		DEAD box ATP-dependent RNA helicase, putative [Pediculus humanus corporis]	Nucleic acid metabolism	Regulation of gene expression
M72608		Elongator complex protein 1 [Crassostrea gigas]	Nucleic acid metabolism	Regulation of gene expression
N53807		ETS factor [Trichinella spiralis]	Nucleic acid metabolism	Regulation of gene expression
M66385		GDNF-inducible zinc finger protein 1-like isoform X1 [Callorhinchus milii]	Nucleic acid metabolism	Regulation of gene expression
N18441		PREDICTED: ets DNA-binding protein pokkuri-like [Nasonia vitripennis]	Nucleic acid metabolism	Regulation of gene expression
N53342		PREDICTED: gastrula zinc finger protein XlCGF57.1-like, partial [Danio rerio]	Nucleic acid metabolism	Regulation of gene expression
M1844		PREDICTED: mediator of RNA polymerase II transcription subunit 8 isoform 2 [Nasonia vitripennis]	Nucleic acid metabolism	Regulation of gene expression
N61781		PREDICTED: protein charlatan-like isoform X1 [Ceratitis capitata]	Nucleic acid metabolism	Regulation of gene expression
N22415		PREDICTED: transcription elongation factor SPT5-like [Megachile rotundata]	Nucleic acid metabolism	Regulation of gene expression
N23604		PREDICTED: zinc finger protein 235 [Trichechus manatus latirostris]	Nucleic acid metabolism	Regulation of gene expression
M97076		PREDICTED: zinc finger protein 287-like [Monodelphis domestica]	Nucleic acid metabolism	Regulation of gene expression
N31716		PREDICTED: zinc finger protein 347-like [Saccoglossus kowalevskii]	Nucleic acid metabolism	Regulation of gene expression
N30244		PREDICTED: zinc finger protein 484-like [Metaseiulus occidentalis]	Nucleic acid metabolism	Regulation of gene expression
N46801		PREDICTED: zinc finger protein 528-like [Pongo abelii]	Nucleic acid metabolism	Regulation of gene expression
M77892	FW09YNP02IAVCY	PREDICTED: zinc finger protein 570-like [Elephantulus edwardii]	Nucleic acid metabolism	Regulation of gene expression
M12728		PREDICTED: zinc finger protein 596-like [Chrysochloris asiatica]	Nucleic acid metabolism	Regulation of gene expression
M58041		PREDICTED: zinc finger protein 658-like isoform X2 [Neolamprologus brichardi]	Nucleic acid metabolism	Regulation of gene expression
N44929		PREDICTED: zinc finger protein 845-like [Acyrthosiphon pisum]	Nucleic acid metabolism	Regulation of gene expression
N16123		PREDICTED: zinc finger protein 84-like [Strongylocentrotus purpuratus]	Nucleic acid metabolism	Regulation of gene expression
N15523		putative general transcription factor IIIC polypeptide 5 [Platynereis dumerilii]	Nucleic acid metabolism	Regulation of gene expression
M41234	isotig00290	Spz1 [Litopenaeus vannamei]	Nucleic acid metabolism	Regulation of gene expression
N38517		transcription initiation factor TFIID subunit 4-like [Metaseiulus occidentalis]	Nucleic acid metabolism	Regulation of gene expression
M5558		zinc finger protein [Daphnia pulex]	Nucleic acid metabolism	Regulation of gene expression
M6056		Zinc finger protein 2 like protein [Myotis brandtii]	Nucleic acid metabolism	Regulation of gene expression
N37797		zinc finger protein 516 [Danio rerio]	Nucleic acid metabolism	Regulation of gene expression
M27180		zinc finger protein CCCH domain-containing protein [Pediculus humanus corporis]	Nucleic acid metabolism	Regulation of gene expression
M9826		zinc finger protein, partial [Cricetulus griseus]	Nucleic acid metabolism	Regulation of gene expression
N60094		Zinc finger Y-chromosomal protein 1 [Xenopus laevis]	Nucleic acid metabolism	Regulation of gene expression
M114291		Anaspides tasmaniae internal transcribed spacer 2 and 28S ribosomal RNA gene	Nucleic acid metabolism	Regulation of gene expression, ep
N10420		Chromatin accessibility complex protein 1 [Acromyrmex echinatior]	Nucleic acid metabolism	Regulation of gene expression, ep
N20627		histone-lysine N-methyltransferase E(z) isoform 2 [Nasonia vitripennis]	Nucleic acid metabolism	Regulation of gene expression, ep
N31827		PREDICTED: protein Gawky isoform X5 [Tribolium castaneum]	Nucleic acid metabolism	Regulation of gene expression, ep
N19248	FW09YNP01EBAIE	putative DNA-mediated transposase [Helicoverpa zea]	Nucleic acid metabolism	Regulation of gene expression, ep
N22228		Exosome complex exonuclease RRP4, partial [Anas platyrhynchos]	Nucleic acid metabolism	Ribosome biogenesis
N34355		heterogeneous nuclear ribonucleoprotein 87F-like [Acyrthosiphon pisum]	Nucleic acid metabolism	RNA metabolic process
N18387		7SK snRNA methylphosphate capping enzyme-like [Megachile rotundata]	Nucleic acid metabolism	RNA metabolic process
N53285		heat containing protein, putative [Pediculus humanus corporis]	Nucleic acid metabolism	RNA metabolic process
N42821		Heterogeneous nuclear ribonucleoprotein A1 [Pediculus humanus corporis]	Nucleic acid metabolism	RNA metabolic process
M60095		PAB-dependent poly(A)-specific ribonuclease subunit 2-like [Meleagris gallopavo]	Nucleic acid metabolism	RNA metabolic process
M74785		PREDICTED: 5'-3' exoribonuclease 2 homolog [Ceratitis capitata]	Nucleic acid metabolism	RNA metabolic process
N28294		PREDICTED: LOW QUALITY PROTEIN: integrator complex subunit 1 [Equus caballus]	Nucleic acid metabolism	RNA metabolic process
M4435		PREDICTED: probable methyltransferase TARBP1-like [Aplysia californica]	Nucleic acid metabolism	RNA metabolic process
N19703		PREDICTED: protein SMG9-like [Astyanax mexicanus]	Nucleic acid metabolism	RNA metabolic process
N23732		PREDICTED: RNA polymerase II-associated protein 3-like [Ciona intestinalis]	Nucleic acid metabolism	RNA metabolic process
N26749		PREDICTED: serine/threonine-protein kinase SMG1-like [Astyanax mexicanus]	Nucleic acid metabolism	RNA metabolic process
M64830		PREDICTED: similar to spliceosome associated protein [Tribolium castaneum]	Nucleic acid metabolism	RNA metabolic process
N27405		PREDICTED: uracil phosphoribosyltransferase homolog [Nasonia vitripennis]	Nucleic acid metabolism	RNA metabolic process
M77998		reverse transcriptase [Daphnia pulex]	Nucleic acid metabolism	RNA metabolic process
N23581		serine/arginine repetitive matrix protein 2-like isoform X1 [Apis dorsata]	Nucleic acid metabolism	RNA metabolic process
N8789		small nuclear ribonucleoprotein polypeptide G [Penaeus monodon]	Nucleic acid metabolism	RNA metabolic process
M5713		splicing factor 3B subunit 1 [Trichinella spiralis]	Nucleic acid metabolism	RNA metabolic process
N21123		DNA-directed RNA polymerase I subunit RPA43-like [Nasonia vitripennis]	Nucleic acid metabolism	Transcription, DNA-dependent
N25855		Lymphoid-specific helicase [Pteropus alecto]	Nucleic acid metabolism	

Protein metabolism

N15593		valacyclovir hydrolase [Anopheles darlingi]	Protein metabolism	Cellular amino acid metabolic pro
N18288		casein kinase 1 epsilon [Eurydice pulchra]	Protein metabolism	Cellular protein modification proc
N13007		checkpoint kinase 1 [Daphnia pulex]	Protein metabolism	Cellular protein modification proc
M66217		Diphthamide biosynthesis protein 2 [Dicentrarchus labrax]	Protein metabolism	Cellular protein modification proc
N9476		dolichyl-diphosphooligosaccharide--protein glycosyltransferase subunit STT3A [Poecilia formosa]	Protein metabolism	Cellular protein modification proc
N11830	GTJW03E08JXILJ	hemocyte protein-glutamine gamma-glutamyltransferase-like [Bombyx mori]	Protein metabolism	Cellular protein modification proc
M8985		polypeptide N-acetylgalactosaminyltransferase 5-like [Megachile rotundata]	Protein metabolism	Cellular protein modification proc
M22298		PREDICTED: 14 kDa phosphohistidine phosphatase [Octodon degus]	Protein metabolism	Cellular protein modification proc
M85248		PREDICTED: glutaminyl-peptide cyclotransferase-like [Bombyx mori]	Protein metabolism	Cellular protein modification proc
M9982		PREDICTED: probable leucine--tRNA ligase, mitochondrial-like [Musca domestica]	Protein metabolism	Cellular protein modification proc
N33379		PREDICTED: similar to Nedd4 CG7555-PC [Tribolium castaneum]	Protein metabolism	Cellular protein modification proc
N34569		PREDICTED: tyrosine-protein kinase Fer [Sorex araneus]	Protein metabolism	Cellular protein modification proc
M61171		Protein FAM76A [Crassostrea gigas]	Protein metabolism	Cellular protein modification proc
M9345		protein kinase, putative [Ixodes scapularis]	Protein metabolism	Cellular protein modification proc
M59923		RpeA [Escherichia coli]	Protein metabolism	Cellular protein modification proc
N20761		serine/threonine protein kinase rio3, putative [Ixodes scapularis]	Protein metabolism	Cellular protein modification proc
N18522		upstream regulatory element binding protein 1 [Homo sapiens]	Protein metabolism	Cellular protein modification proc
M21622		15 kDa selenoprotein precursor, putative [Pediculus humanus corporis]	Protein metabolism	Protein folding
M60404		BCS-1 [Amphibalanus amphitrite]	Protein metabolism	Protein folding
M74663		BCS-1 [Amphibalanus amphitrite]	Protein metabolism	Protein folding
M65353	isotig05545	related to FPR2-FK506/rapamycin-binding protein of the ER [Ustilago hordei]	Protein metabolism	Protein folding
N10435		Aminopeptidase N precursor, putative [Pediculus humanus corporis]	Protein metabolism	Proteolysis
M9318		anaphase-promoting complex subunit, putative [Pediculus humanus corporis]	Protein metabolism	Proteolysis
N17787		caspase-3 [Culex quinquefasciatus]	Protein metabolism	Proteolysis
M4677		CLIP-domain serine protease subfamily A [Anopheles darlingi]	Protein metabolism	Proteolysis
M47110		collagenolytic serine protease [Paralithodes camtschaticus]	Protein metabolism	Proteolysis
M18434		CUB-serine protease [Panulirus argus]	Protein metabolism	Proteolysis
M19739		CUB-serine protease [Panulirus argus]	Protein metabolism	Proteolysis
M3619		CUB-serine protease [Panulirus argus]	Protein metabolism	Proteolysis
N11966		Cytosolic non-specific dipeptidase [Lepeophtheirus salmonis]	Protein metabolism	Proteolysis

ID krill2	ID krill1	Annotation	GO Term	GO Child Term
M72842		ER degradation-enhancing alpha-mannosidase-like 3 [Harpegnathos saltator]	Protein metabolism	Proteolysis
M92858		F-box/WD repeat-containing protein 11 [Xiphophorus maculatus]	Protein metabolism	Proteolysis
N50173		Kazal-type serine proteinase inhibitor 1 [Fenneropenaeus chinensis]	Protein metabolism	Proteolysis
M75323		matrix metalloproteinase 1, isoform C [Drosophila melanogaster]	Protein metabolism	Proteolysis
N40575		PREDICTED: transmembrane protease serine 9-like [Monodelphis domestica]	Protein metabolism	Proteolysis
N30906		PREDICTED: arginyl-tRNA--protein transferase 1-like [Neolamprologus brichardi]	Protein metabolism	Proteolysis
N10456		PREDICTED: carboxypeptidase B-like [Apis florea]	Protein metabolism	Proteolysis
N12001		PREDICTED: cullin-4B-like [Nasonia vitripennis]	Protein metabolism	Proteolysis
N19946		PREDICTED: E3 ubiquitin-protein ligase HUWE1 isoform X1 [Capra hircus]	Protein metabolism	Proteolysis
N25505		PREDICTED: E3 ubiquitin-protein ligase RNF123 [Otolemur garnettii]	Protein metabolism	Proteolysis
M70591		PREDICTED: E3 ubiquitin-protein ligase TRIM33-like isoform X4 [Ceratitis capitata]	Protein metabolism	Proteolysis
M1163		PREDICTED: ubiquitin-conjugating enzyme E2 C-like [Nasonia vitripennis]	Protein metabolism	Proteolysis
M56909		PREDICTED: ubiquitin-conjugating enzyme E2 H-like [Bombus terrestris]	Protein metabolism	Proteolysis
M931		protease [Homarus americanus]	Protein metabolism	Proteolysis
M97028		Protein CBR-NAS-4 [Caenorhabditis briggsae]	Protein metabolism	Proteolysis
M3294		putative chymotrypsin precursor-1 protein [Daphnia pulex]	Protein metabolism	Proteolysis
N15033		RING finger protein 180 [Danio rerio]	Protein metabolism	Proteolysis
M229		serine protease [Anopheles darlingi]	Protein metabolism	Proteolysis
M69725		serpin 8 [Branchiostoma lanceolatum]	Protein metabolism	Proteolysis
N16740		truncated cathepsin A [Eriocheir sinensis]	Protein metabolism	Proteolysis
M33821		ubiquitin [Schizosaccharomyces cryophilus OY26]	Protein metabolism	Proteolysis
N13604		ubiquitin protein ligase [Ixodes scapularis]	Protein metabolism	Proteolysis
N19798		Zinc metalloproteinase nas-14 [Camponotus floridanus]	Protein metabolism	Proteolysis
N62093		masquerade, isoform C [Drosophila melanogaster]	Protein metabolism	Proteolysis (Cuticle)
M11130		Trypsin-1 [Cerapachys biroi]	Protein metabolism	Proteolysis (Cuticle)
M69422		methionyl-tRNA formyltransferase, mitochondrial-like [Takifugu rubripes]	Protein metabolism	Regulation of translational elonga
M4937	isotig11060	ORM1-like protein 3 [Danio rerio]	Protein metabolism	Regulation of translational elonga
N24811		eIF2B-alpha protein [Daphnia pulex]	Protein metabolism	Regulation of translational initiatio
N19015	contig01822	Eukaryotic translation initiation factor 4 gamma 3 [Crassostrea gigas]	Protein metabolism	Regulation of translational initiatio
M13011	isotig06273	PREDICTED: eukaryotic translation initiation factor 2B, subunit 3 gamma-like [Saccoglossus kowalevskii]	Protein metabolism	Regulation of translational initiatio
M21285	isotig03087	60S ribosomal protein L15 [Orconectes limosus]	Protein metabolism	Ribosome biogenesis
M21399		60S ribosomal protein L6 [Coptotermes formosanus]	Protein metabolism	Ribosome biogenesis
M20050		Isolate DGGE band AntEuk13-5 18S ribosomal RNA gene [Euphausia superba]	Protein metabolism	Ribosome biogenesis
M70869		Isolate DGGE band AntEuk13-5 18S ribosomal RNA gene [Euphausia superba]	Protein metabolism	Ribosome biogenesis
N20156		mitochondrial ribosomal protein, S9, putative [Aedes aegypti]	Protein metabolism	Ribosome biogenesis
N36346		PREDICTED: GTPase Era, mitochondrial [Acyrthosiphon pisum]	Protein metabolism	Ribosome biogenesis
N59348		PREDICTED: GTPase Era, mitochondrial [Tribolium castaneum]	Protein metabolism	Ribosome biogenesis
N38551		PREDICTED: GTPase Era, mitochondrial-like [Aplysia californica]	Protein metabolism	Ribosome biogenesis
M25016		Crithidia fasciculata ribosomal DNA repeat [Crithidia fasciculata]	Protein metabolism	Ribosome biogenesis
N13567		ribosomal protein L24 [Azumapecten farreri]	Protein metabolism	Ribosome biogenesis
M75744	isotig03736	ribosomal protein L36 [Procambarus clarkii]	Protein metabolism	Ribosome biogenesis
N5213	isotig04974	ribosomal protein P1 [Palaemon varians]	Protein metabolism	Ribosome biogenesis
N16052		ribosomal protein rpl35a [Lineus viridis]	Protein metabolism	Ribosome biogenesis
N3958		ribosomal protein S26 [Branchiostoma belcheri]	Protein metabolism	Ribosome biogenesis
M101760		ribosomal protein S7 [Procambarus clarkii]	Protein metabolism	Ribosome biogenesis
M84192		Taku spinosocarinatus voucher LIRS07-26 28S ribosomal RNA gene	Protein metabolism	Ribosome biogenesis
M70652		procollagen-lysine2-oxoglutarate 5-dioxygenase [Schistosoma mansoni]	Protein metabolism	
M6935		ubiquitin thiolesterase [Acanthamoeba castellanii str. Neff]	Protein metabolism	

Response to stress

ID krill2	ID krill1	Annotation	GO Term	GO Child Term
N14974		antimicrobial peptide type 1 precursor Ic [Pandalopsis japonica]	Response to stress	Defence responce
M31132		antimicrobial peptide type 2 precursor IIb [Pandalopsis japonica]	Response to stress	Defence responce
N737		antimicrobial peptide type 2 precursor IIc [Pandalopsis japonica]	Response to stress	Defence responce
M69891		attractin [Aedes aegypti]	Response to stress	Defence responce
N58744		beta-1,4-N-acetylgalactosaminyltransferase bre-4-like [Acyrthosiphon pisum]	Response to stress	Defence responce
N47460		CD209 antigen-like protein D [Oncorhynchus mykiss]	Response to stress	Defence responce
M39190		crustin 1 [Panulirus japonicus]	Response to stress	Defence responce
N7810		crustin 3 [Panulirus japonicus]	Response to stress	Defence responce
N9625		crustin type I [Macrobrachium rosenbergii]	Response to stress	Defence responce
N28574		crustin-like peptide type 2 [Marsupenaeus japonicus]	Response to stress	Defence responce
N22242		C-type lectin [Penaeus monodon]	Response to stress	Defence responce
M87391	isotig10958	C-type lectin 4 [Fenneropenaeus merguiensis]	Response to stress	Defence responce
M101615	FW09YNP02JS2KI	C-type lectin protein [Fenneropenaeus chinensis]	Response to stress	Defence responce
M68819		C-type lectin protein [Fenneropenaeus chinensis]	Response to stress	Defence responce
N23963		hemolymph clottable protein [Litopenaeus vannamei]	Response to stress	Defence responce
N15313		Kazal-type proteinase inhibitor [Pacifastacus leniusculus]	Response to stress	Defence responce
N30729		PREDICTED: mucin-22-like [Musca domestica]	Response to stress	Defence responce
M14897		NudC domain-containing protein, putative [Ixodes scapularis]	Response to stress	Defence responce
M14594		PREDICTED: CD209 antigen-like protein A isoform X1 [Poecilia formosa]	Response to stress	Defence responce
M4674		PREDICTED: mucin-22-like [Danio rerio]	Response to stress	Defence responce
M11229		PREDICTED: mucin-2-like [Geospiza fortis]	Response to stress	Defence responce
N25269		Putative ferric-chelate reductase 1 [Crassostrea gigas]	Response to stress	Defence responce
M60524		serine protease inhibitor [Marsupenaeus japonicus]	Response to stress	Defence responce
M12331		6-4 photolyase [Eurydice pulchra]	Response to stress	
M3688		PREDICTED: DNA ligase 4 [Strongylocentrotus purpuratus]	Response to stress	DNA repair
N23030		Fanconi anemia group J protein [Crassostrea gigas]	Response to stress	DNA repair
N34949		Mitogen-activated protein kinase kinase kinase 5 [Harpegnathos saltator]	Response to stress	DNA repair
N27521		PREDICTED: DNA mismatch repair protein Mlh1 isoform X8 [Gallus gallus]	Response to stress	DNA repair
N9090		Chorion peroxidase [Camponotus floridanus]	Response to stress	Response to oxidative stress
N7649		glutathione S-transferase [Eriocheir sinensis]	Response to stress	
N18339	isotig10144	PREDICTED: hsp90 co-chaperone Cdc37-like [Nasonia vitripennis]	Response to stress	
M15481		PREDICTED: stress protein DDR48-like [Xenopus (Silurana) tropicalis]	Response to stress	

Signal transduction

ID krill2	ID krill1	Annotation	GO Term	GO Child Term
N27577		TRAF and TNF receptor-associated protein [Lepeophtheirus salmonis]	Signal transduction	Cell communication
N18727		PREDICTED: similar to vlc [Tribolium castaneum]	Signal transduction	Cell-cell signaling
M31478		Beta-crystallin A1 [Lepeophtheirus salmonis]	Signal transduction	eye
N18741		Ankyrin repeat and SOCS box protein [Cerapachys biroi]	Signal transduction	Intracellular signal transduction
M7219		cAMP-dependent protein kinase catalytic subunit [Pediculus humanus corporis]	Signal transduction	Intracellular signal transduction

ID krill2	ID krill1	Annotation	GO Term	GO Child Term
N60164	isotig09573	guanine nucleotide exchange factor, putative [Pediculus humanus corporis]	Signal transduction	Intracellular signal transduction
M77475		PREDICTED: CDC42 binding protein kinase alpha-like [Saccoglossus kowalevskii]	Signal transduction	Intracellular signal transduction
N40391		PREDICTED: integral membrane protein GPR155-like [Saccoglossus kowalevskii]	Signal transduction	Intracellular signal transduction
N14631		PREDICTED: D-dopachrome decarboxylase-like [Ochotona princeps]	Signal transduction	melanin synthesis
M4332		von Willebrand factor D and EGF domain-containing protein [Meleagris gallopavo]	Signal transduction	Signal transduction
M104013		adenylyl cyclase [Daphnia pulex]	Signal transduction	
M5422	FW09YNP01ATTT0	arrestin [Libelloides macaronius]	Signal transduction	
N19969	isotig04228	Arrestin homolog [Locusta migratoria]	Signal transduction	
M58788		cartilage intermediate layer protein 1 [Taeniopygia guttata]	Signal transduction	
N44494		Glucose-dependent insulinotropic receptor, partial [Heterocephalus glaber]	Signal transduction	
N31246		integral membrane protein GPR177-B, putative [Ixodes scapularis]	Signal transduction	
N42645		Juvenile hormone-inducible protein [Anopheles darlingi]	Signal transduction	
N18394		kruppel like protein 1, partial [Locusta migratoria]	Signal transduction	
M102096		notch 2 [Daphnia pulex]	Signal transduction	
N36305	isotig02060	opsin [Triops granarius]	Signal transduction	
M61997		phosphatidylinositol 3-kinase regulatory subunit alpha-like [Nasonia vitripennis]	Signal transduction	
M16660		pigment-dispersing hormone 3 [Marsupenaeus japonicus]	Signal transduction	
M93343		PREDICTED: apoptosis regulatory protein Siva isoform X2 [Mesocricetus auratus]	Signal transduction	
M96174		PREDICTED: neither inactivation nor afterpotential protein C [Nasonia vitripennis]	Signal transduction	
M11904		PREDICTED: TBC1 domain family member CG11727 [Apis mellifera]	Signal transduction	
M85776		signal transducer and activator of transcription [Fenneropenaeus chinensis]	Signal transduction	
N28816		signal transducer and activator of transcription [Penaeus monodon]	Signal transduction	
N25485		Solute carrier family 2, facilitated glucose transporter member 1 [Crassostrea gigas]	Signal transduction	
M102302		Tankyrase-1 [Tupaia chinensis]	Signal transduction	
M8431		tetraspanins-like protein-8 [Fenneropenaeus chinensis]	Signal transduction	
**Transport**				
N56030		sugar transporter, putative [Ixodes scapularis]	Transport	Carbohydrate transport
M61		PREDICTED: ran-specific GTPase-activating protein-like [Nasonia vitripennis]	Transport	Intracellular transport
N13115		vacuolar amino acid transporter, putative [Pediculus humanus corporis]	Transport	Intracellular transport
N34512		calcium uptake protein 1 homolog, mitochondrial [Acyrthosiphon pisum]	Transport	Ion transport
N24758		chloride channel, putative [Ixodes scapularis]	Transport	Ion transport
N48362		Cytochrome c oxidase copper chaperone [Cerapachys biroi]	Transport	Ion transport
M107683	isotig07122	Euphausia superba mitochondrion, partial genome	Transport	Ion transport
M60197	isotig05838	Euphausia superba voucher 841M mitochondrion, partial genome	Transport	Ion transport
M6590		ileal sodium/bile acid cotransporter-like isoform 1 [Nasonia vitripennis]	Transport	Ion transport
N5882		potassium voltage-gated channel subfamily G member 3 [Sorex araneus]	Transport	Ion transport
M2239		PREDICTED: organic cation transporter protein-like isoform 1 [Bombus terrestris]	Transport	Ion transport
M62469		PREDICTED: putative ferric-chelate reductase 1-like [Xenopus (Silurana) tropicalis]	Transport	Ion transport
M85975		PREDICTED: zinc transporter ZIP11-like [Oryzias latipes]	Transport	Ion transport
N31065		PREDICTED: zinc transporter ZIP13-like [Metaseiulus occidentalis]	Transport	Ion transport
M6504		putative TRP channel protein [Periplaneta americana]	Transport	Ion transport
N26972	isotig09262	Trafficking protein particle complex subunit 10 [Harpegnathos saltator]	Transport	Ion transport
E6444		voltage-dependent non-L-type calcium channel alpha-1 subunit [Cancer borealis]	Transport	Ion transport
M18825		fatty acids binding protein [Fenneropenaeus chinensis]	Transport	Lipid transport
M2931		clottable protein [Marsupenaeus japonicus]	Transport	Lipid transport
M2853		PREDICTED: apolipoprotein D-like [Bombyx mori]	Transport	Lipid transport
M56511		PREDICTED: apolipoprotein D-like [Bombyx mori]	Transport	Lipid transport
N22878		PREDICTED: peroxisomal membrane protein PMP34-like [Apis mellifera]	Transport	Mitochondrial transport
M103670		rab3 GTPase-activating protein non-catalytic subunit [Latimeria chalumnae]	Transport	Neurotransmitter transport
N21981		Sodium-dependent neutral amino acid transporter B(0) [Harpegnathos saltator]	Transport	Neurotransmitter transport
M78387		coiled-coil domain-containing protein 93 [Xenopus laevis]	Transport	Protein transport
N12003		hypothetical protein CAPTEDRAFT_223046 [Capitella teleta]	Transport	Protein transport
N18371		PREDICTED: AP-3 complex subunit delta-1-like [Megachile rotundata]	Transport	Protein transport
M102512		PREDICTED: putative Ras-related protein Rab-33-like [Saccoglossus kowalevskii]	Transport	Protein transport
N24480		PREDICTED: ras-related protein Rab-18 isoform 3 [Trichechus manatus latirostris]	Transport	Protein transport
N42390		PREDICTED: transportin-1 [Nasonia vitripennis]	Transport	Protein transport
N22300		Facilitated trehalose transporter Tret1 [Drosophila willistoni]	Transport	Transmembrane transport
M56361		PREDICTED: monocarboxylate transporter 12-like [Nasonia vitripennis]	Transport	Transmembrane transport
M104151	isotig00375	solute carrier family 25-like protein-2 [Microplitis demolitor]	Transport	Transmembrane transport
M7582	FW09YNP02IE6U6	ADP-ribosylation factor GTPase-activating protein [Scylla paramamosain]	Transport	Vesicle-mediated transport
N11990		N-terminal kinase-like protein [Zootermopsis nevadensis]	Transport	Vesicle-mediated transport
N33152		PREDICTED: AP-2 complex subunit alpha [Tribolium castaneum]	Transport	Vesicle-mediated transport
N49441		ABC transporter [Daphnia pulex]	Transport	
N40938	GTJW03E08JYE7N	amino acid transporter [Aedes aegypti]	Transport	
N28773		autotransporter [Bartonella tribocorum CIP 105476]	Transport	
N13946	isotig08118	crustacyanin subunit A [Fenneropenaeus merguiensis]	Transport	
M71333		PREDICTED: transmembrane 9 superfamily member 1-like [Aplysia californica]	Transport	
N23238	FW09YNP01C2I8L	Solute carrier family 35 member F5 [Crassostrea gigas]	Transport	
M87987	isotig04624	Solute carrier family 46 member 3 [Harpegnathos saltator]	Transport	
N34674		transferrin [Anopheles darlingi]	Transport	
N20330		yrdC domain-containing protein, mitochondrial isoform X2 [Gallus gallus]	Transport	
**Cuticle**				
N25681	isotig08949	arthrodial cuticle protein AMP6.0 [Callinectes sapidus]		Cuticle
M63421	isotig00113	arthrodial cuticle protein AMP6.0 [Callinectes sapidus]		Cuticle
M22046	isotig00792	cuticle proprotein proCP5.2 [Callinectes sapidus]		Cuticle
M4810	isotig00786	cuticle proprotein proCP5.2 [Callinectes sapidus]		Cuticle
M17882		cuticle protein [Culex quinquefasciatus]		Cuticle
N8897		cuticle protein [Culex quinquefasciatus]		Cuticle
N6883		cuticle protein [Culex quinquefasciatus]		Cuticle
N4421		cuticle protein [Rimicaris exoculata]		Cuticle
N4800	isotig03652	Cuticle protein 6 [Lepeophtheirus salmonis]		Cuticle
N4805	isotig08546	Cuticle protein 6 [Lepeophtheirus salmonis]		Cuticle
N6680		Cuticle protein 6 [Lepeophtheirus salmonis]		Cuticle
M36900	GTJW03E08JWLFE	Cuticle protein 6 [Lepeophtheirus salmonis]		Cuticle
M97333		Cuticle protein 7 [Lepeophtheirus salmonis]		Cuticle
N51806		cuticle protein CB6 [Portunus pelagicus]		Cuticle
M47341		cuticle protein CB6 [Portunus pelagicus]		Cuticle
M56242		early cuticle protein 6 [Callinectes sapidus]		Cuticle

ID krill2	ID krill1	Annotation	GO Term	GO Child Term
N7506		glycosyl-phosphatidylinositol-linked carbonic anhydrase [Litopenaeus vannamei]		Cuticle
M10340		PREDICTED: endochitinase-like isoform X1 [Apis mellifera]		Cuticle
N9845		PREDICTED: endochitinase-like isoform X1 [Apis mellifera]		Cuticle
N25691		putative cuticle protein [Artemia franciscana]		Cuticle
M1924		RecName: Full=Cuticle protein 6; AltName: Full=BcNCP14.9		Cuticle
**No GO assigned**				
M1994		Glutamine and serine-rich protein 1 [Harpegnathos saltator]		
M3286		PREDICTED: DTW domain-containing protein 1 isoform X6 [Latimeria chalumnae]		
N20580		PREDICTED: kanadaptin-like [Saccoglossus kowalevskii]		
N20632		PREDICTED: La autoantigen-like [Saccoglossus kowalevskii]		
M74730		PREDICTED: protein outspread-like [Bombyx mori]		
N9515		PREDICTED: testis-expressed sequence 264 protein-like [Aplysia californica]		
M69334		PREDICTED: transmembrane protein 179-like [Bombyx mori]		
N36503		PREDICTED: transmembrane protein 60-like isoform 1 [Oreochromis niloticus]		

23,

cuticle)

cess
cess
cess
cess
cess
cess
cess
cess
cess
cess
cess
cess
cess
cess
cess
cess

tes and energy
tes and energy

tes and energy

tes and energy
tes and energy
tes and energy

tes and energy

tes and energy

ocess
l metabolic process

l metabolic process

l metabolic process
l metabolic process
l metabolic process
l metabolic process
l metabolic process
l metabolic process
l metabolic process

igenetic
igenetic
igenetic
igenetic
igenetic

:ess
ess

ess

ess

ess

ess
ess
ess
ess
ess
ess
ess
ess
ess
ess
ess
ess

tion
tion
ɔn
ɔn
ɔn
