# Supplementary material for: Analysis of the circadian transcriptome of the Antarctic krill Euphausia superba
Source: Sci Rep. 2019 Sep 25;9:13894. doi: 10.1038/s41598-019-50282-1 (PMC6761102; doi:10.1038/s41598-019-50282-1)
Supplement: Supplementary file 6 — Supplementary Table 5 [file 41598_2019_50282_MOESM6_ESM.pdf]

Analysis of the circadian transcriptome of the Antarctic krill *Euphausia superba*

Alberto Biscontin[1,2,*], Paolo Martini[1], Rodolfo Costa[1], Achim Kramer[2], Bettina Meyer[3,4,5], So Kawaguchi[6], Mathias Teschke[3], Cristiano De Pittà[1,*]

[1]Dipartimento di Biologia, Università degli Studi di Padova, Padova, Italy

[2]Laboratory of Chronobiology, Charité Universitätsmedizin Berlin, Berlin, Germany

[3]Section Polar Biological Oceanography, Alfred Wegener Institute Helmholtz Centre for Polar and Marine Research, Bremerhaven, Germany

[4]Institute for Chemistry and Biology of the Marine Environment, Carl von Ossietzky University of Oldenburg, Oldenburg, Germany

[5]Helmholtz Institute for Functional Marine Biodiversity (HIFMB) at the University of Oldenburg, 26111 Oldenburg, Germany

[6]Department of Environment and Heritage, Australian Antarctic Division, Kingston, Tasmania, Australia

[*]Corresponding authors:

Cristiano De Pittà, Dipartimento di Biologia, Università degli Studi di Padova, via U. Bassi 58/B 35131 Padova, Italy; Phone: +39-049-8276210; Fax: +39-049-8276209; e-mail address: cristiano.depitta@unipd.it

Alberto Biscontin, Dipartimento di Biologia, Università degli Studi di Padova, via U. Bassi 58/B 35131 Padova, Italy; Phone: +39-049-8276228; Fax: +39-049-8276209; e-mail address: alberto.biscontin@unipd.it

Supplementary Table 5. Gene Ontology analysis of genes with a sinusoidal expression pattern in LD.

Classification of annotated genes with sinusoidal expression patterns throughout the 24-hour cycle in LD regime (1,485 genes). [a]ID: accession number of krill master transcriptome [23]; [b]Annotation = description of the gene; [c]GO term: gene ontology biological process; [d]GO child term: most fitting gene ontology child term to further refine the functional annotation.

ID	Annotation	GO term	Go child term
**Cellular component organization**			
M108674		Cellular component organization	Chromosome organization
M17873		Cellular component organization	Chromosome organization
N18234	PREDICTED: Nasonia vitripennis imitation SWI (Iswi), mRNA	Cellular component organization	Chromosome organization
N22970	hypothetical protein CAEBREN_26312 [Caenorhabditis brenneri]	Cellular component organization	Chromosome organization
N23421	PREDICTED: H2A histone family, member Y-like [Saccoglossus kowalevskii]	Cellular component organization	Chromosome organization
N23850	PREDICTED: H1 histone family, member 0-like [Saccoglossus kowalevskii]	Cellular component organization	Chromosome organization
N24637		Cellular component organization	Chromosome organization
N26441	histone H1 orphon [Xenoturbella bocki]	Cellular component organization	Chromosome organization
N26710		Cellular component organization	Chromosome organization
N33184	unknown [Dendroctonus ponderosae]	Cellular component organization	Chromosome organization
N41202	PREDICTED: histone H1-delta-like [Aplysia californica]	Cellular component organization	Chromosome organization
N50496	Branchiostoma floridae hypothetical protein, mRNA	Cellular component organization	Chromosome organization
N55793		Cellular component organization	Chromosome organization
E2934	RecName: Full=Tubulin alpha-3 chain; AltName: Full=Alpha-III tubulin >gi|1562539|g	Cellular component organization	Cytoskeleton organization
E4642	hypothetical protein AMTR_s00032p00056500 [Amborella trichopoda]	Cellular component organization	Cytoskeleton organization
M10012	Haliotis diversicolor putative tubulin beta chain (TUBB) mRNA, complete cds	Cellular component organization	Cytoskeleton organization
M108155	hypothetical protein AaeL_AAEL008947 [Aedes aegypti] >gi|108875003|gb|EAT3922	Cellular component organization	Cytoskeleton organization
M1452	Scylla paramamosain suppressor of profilin 2 mRNA, partial cds	Cellular component organization	Cytoskeleton organization
M19065		Cellular component organization	Cytoskeleton organization
M19725	PREDICTED: kinectin isoform 5 [Ceratotherium simum simum]	Cellular component organization	Cytoskeleton organization
M22315	Strongyloides stercoralis beta-tubulin (btub) gene, complete cds	Cellular component organization	Cytoskeleton organization
M31074	profilin [Penaeus monodon]	Cellular component organization	Cytoskeleton organization
M35289	Rattus norvegicus TL0AAA47YN11 mRNA sequence	Cellular component organization	Cytoskeleton organization
M3685	PREDICTED: F-actin-capping protein subunit beta-like [Nasonia vitripennis]	Cellular component organization	Cytoskeleton organization
M5119	hypothetical protein DAPPUDRAFT_305421 [Daphnia pulex]	Cellular component organization	Cytoskeleton organization
M523	Dugesia japonica Djtuba mRNA for tubulin alpha chain, complete cds	Cellular component organization	Cytoskeleton organization
M56592	PREDICTED: kinesin-like protein KIF3A-like isoform X2 [Oreochromis niloticus]	Cellular component organization	Cytoskeleton organization
M58034	Nematostella vectensis predicted protein (NEMVEDRAFT_v1g188665) partial mRNA	Cellular component organization	Cytoskeleton organization
M59795	conserved hypothetical protein [Pediculus humanus corporis] >gi|212513136|gb|EE	Cellular component organization	Cytoskeleton organization
M71966	beta-tubulin [Oxymonadida environmental sample]	Cellular component organization	Cytoskeleton organization
M7549		Cellular component organization	Cytoskeleton organization
N16988	hypothetical protein BRAFLDRAFT_90471 [Branchiostoma floridae] >gi|229274973|g	Cellular component organization	Cytoskeleton organization
N18342		Cellular component organization	Cytoskeleton organization
N18728		Cellular component organization	Cytoskeleton organization
N19790	Cryptocercus punctulatus alpha-tubulin (Atub) mRNA, partial cds	Cellular component organization	Cytoskeleton organization
N20018	Caenorhabditis remanei CRE-MEC-7 protein (Cre-mec-7) mRNA, complete cds	Cellular component organization	Cytoskeleton organization
N20366	hypothetical protein BRAFLDRAFT_114859 [Branchiostoma floridae] >gi|229277313|	Cellular component organization	Cytoskeleton organization
N22526	conserved hypothetical protein [Pediculus humanus corporis] >gi|212515782|gb|EE	Cellular component organization	Cytoskeleton organization
N22966	hypothetical protein AND_08724 [Anopheles darlingi]	Cellular component organization	Cytoskeleton organization
N26915	hypothetical protein BRAFLDRAFT_67536 [Branchiostoma floridae] >gi|229291637|g	Cellular component organization	Cytoskeleton organization
N27135	kinesin 2B [Nasonia vitripennis] >gi|299782459|ref|NP_001177680.1| kinesin 2B [Na	Cellular component organization	Cytoskeleton organization
N32668	Dystroglycan [Acromyrmex echinatior]	Cellular component organization	Cytoskeleton organization
N35788	hypothetical protein CAPTEDRAFT_219709 [Capitella teleta]	Cellular component organization	Cytoskeleton organization
N51300	PREDICTED: actin-related protein 2-like isoform 1 [Megachile rotundata]	Cellular component organization	Cytoskeleton organization
N7610		Cellular component organization	Cytoskeleton organization
N9729	PREDICTED: Monodelphis domestica tubulin alpha-1B chain-like (LOC100010347), mR	Cellular component organization	Cytoskeleton organization
M92476	RecName: Full=Troponin T; Short=TnT >gi|4928474|gb|AAD33603.1|AF133520_1 tr	Cellular component organization	Mitochondrion organization
N26226	peroxisomal biogenesis factor 16 [Acyrthosiphon pisum] >gi|239792324|dbj|BAH72	Cellular component organization	Other
**Cellular Process**			
M112844	thrombospondin [Aedes aegypti] >gi|108875947|gb|EAT40172.1| AAEL008062-PA,	Cellular Process	Cell adhesion
M14137	thrombospondin, putative [Ixodes scapularis] >gi|215497762|gb|EEC07256.1| thron	Cellular Process	Cell adhesion
M3277	myosin heavy chain isoform 3 [Daphnia pulex]	Cellular Process	Cell adhesion
M88841	PREDICTED: protocadherin Fat 1 isoform X5 [Xenopus (Silurana) tropicalis]	Cellular Process	Cell adhesion
N20755	PREDICTED: Apis mellifera vinculin (Vinc), mRNA	Cellular Process	Cell adhesion
N3255	hypothetical protein BRAFLDRAFT_69086 [Branchiostoma floridae] >gi|229280755|g	Cellular Process	Cell adhesion
N37791	conserved hypothetical protein [Pediculus humanus corporis] >gi|212510848|gb|EE	Cellular Process	Cell adhesion
N43256	hypothetical protein DAPPUDRAFT_212574 [Daphnia pulex]	Cellular Process	Cell adhesion
N54977	hypothetical protein DAPPUDRAFT_321186 [Daphnia pulex]	Cellular Process	Cell adhesion
N56021	hypothetical protein DAPPUDRAFT_329129 [Daphnia pulex]	Cellular Process	Cell adhesion
M11225	PREDICTED: protein ECT2-like [Megachile rotundata]	Cellular Process	Cell cycle
M114824	U3 snoRNP-associated protein, putative [Ixodes scapularis] >gi|215499878|gb|EEC0	Cellular Process	Cell cycle
M1163	PREDICTED: ubiquitin-conjugating enzyme E2 C-like [Nasonia vitripennis]	Cellular Process	Cell cycle
M12125	PREDICTED: anaphase-promoting complex subunit 4-like [Bombyx mori]	Cellular Process	Cell cycle
M22224	Pediculus humanus corporis mitosis protein dim1, putative, mRNA	Cellular Process	Cell cycle
M2312	hypothetical protein BRAFLDRAFT_124509 [Branchiostoma floridae] >gi|229275705|	Cellular Process	Cell cycle
M47744	Homarus americanus beta-I tubulin mRNA, complete cds	Cellular Process	Cell cycle
M748	hypothetical protein CAPTEDRAFT_218074 [Capitella teleta]	Cellular Process	Cell cycle
M79039	PREDICTED: Apis florea polyubiquitin-A-like (LOC100869991), miscRNA	Cellular Process	Cell cycle
M9135		Cellular Process	Cell cycle
M918		Cellular Process	Cell cycle
M98302	cyclin K [Artemia parthenogenetica]	Cellular Process	Cell cycle

ID	Annotation	GO term	Go child term
M98593	conserved hypothetical protein [Trichinella spiralis] >gi|316979321|gb|EFV62128.1|	Cellular Process	Cell cycle
N18186		Cellular Process	Cell cycle
N18390	hypothetical protein BRAFLDRAFT_123390 [Branchiostoma floridae] >gi|229283692|	Cellular Process	Cell cycle
N18723	Structural maintenance of chromosomes protein 5 [Acromyrmex echinatior]	Cellular Process	Cell cycle
N19234	PREDICTED: origin recognition complex subunit 4 [Sorex araneus]	Cellular Process	Cell cycle
N19240	PREDICTED: hypothetical protein LOC100124009 [Nasonia vitripennis]	Cellular Process	Cell cycle
N20073	viral A-type inclusion protein [Trichomonas vaginalis G3] >gi|121915630|gb|EAY204	Cellular Process	Cell cycle
N20707	hypothetical protein CAPTEDRAFT_140107, partial [Capitella teleta]	Cellular Process	Cell cycle
N22167	PREDICTED: Saccoglossus kowalevskii mps one binder kinase activator-like 3-like (LOC	Cellular Process	Cell cycle
N22972	PREDICTED: similar to g1/s-specific cyclin c [Tribolium castaneum] >gi|270003157|gb	Cellular Process	Cell cycle
N39027	PREDICTED: DIX domain containing 1-like [Saccoglossus kowalevskii]	Cellular Process	Cell cycle
N51170	hypothetical protein DAPPUDRAFT_202549 [Daphnia pulex]	Cellular Process	Cell cycle
M110026	Schistosoma mansoni strain Puerto Rico chromosome 1, complete genome	Cellular Process	Cell death
M3851	PREDICTED: hypothetical protein LOC100118119 [Nasonia vitripennis]	Cellular Process	Cell death
M39101	Anopheles gambiae str. PEST AGAP001971-PA (AgaP_AGAP001971) mRNA, complete	Cellular Process	Cell death
M4821	GalNAc/Gal-specific lectin [Crenomytilus grayanus]	Cellular Process	Cell death
M96949	Armigeres subalbatus ASAP ID: 43771 cytosolic small ribosomal subunit 27A mRNA se	Cellular Process	Cell death
N19301	p63-like transcription factor [Saccoglossus kowalevskii] >gi|268054253|gb|ACY9261	Cellular Process	Cell death
N19348		Cellular Process	Cell death
N25552	DNA fragmentation factor subunit beta [Esox lucius]	Cellular Process	Cell death
N31320	PREDICTED: NADPH dependent diflavin oxidoreductase 1-like [Saccoglossus kowalevs	Cellular Process	Cell death
N34633	PREDICTED: G2/M phase-specific E3 ubiquitin-protein ligase [Nomascus leucogenys]	Cellular Process	Cell death
N36400	GL14252 [Drosophila persimilis] >gi|194103844|gb|EDW25887.1| GL14252 [Drosopl	Cellular Process	Cell death
N36730	hypothetical protein DAPPUDRAFT_304833 [Daphnia pulex]	Cellular Process	Cell death
N36763	GalNAc/Gal-specific lectin [Crenomytilus grayanus]	Cellular Process	Cell death
N36782	dynamin associated protein 160, isoform H [Drosophila melanogaster] >gi|44021403	Cellular Process	Cell differentiation
N6778		Cellular Process	Cell differentiation
M1516		Cellular Process	Cell growth
M9307		Cellular Process	Cell growth
N11186	secreted protein, putative [Ixodes scapularis] >gi|215504738|gb|EEC14232.1| secret	Cellular Process	Cell growth
N20944		Cellular Process	Cell growth
N22228	hypothetical protein DAPPUDRAFT_305543 [Daphnia pulex]	Cellular Process	Cell growth
N32590	single IB domain protein [Litopenaeus vannamei]	Cellular Process	Cell growth
M58051	RecName: Full=Protein 60A; AltName: Full=Protein glass bottom boat; Flags: Precurso	Cellular Process	Cell proliferation
N18361	M-phase inducer phosphatase 2 [Culex quinquefasciatus] >gi|167867878|gb|EDS312	Cellular Process	Cell proliferation
N12719	PREDICTED: similar to dre4 CG1828-PA [Tribolium castaneum] >gi|270007613|gb|EF	Cellular Process	Other
N12909	hypothetical protein BRAFLDRAFT_217557 [Branchiostoma floridae] >gi|229279859|	Cellular Process	Other
N38970	RING zinc finger-containing protein [Dictyostelium fasciculatum] >gi|328871844|gb|	Cellular Process	Other
N7766	PREDICTED: similar to CG10576 CG10576-PA [Tribolium castaneum] >gi|270015297|	Cellular Process	Other

Developmental process

M16872	Penaeus monodon actin 1 (act1) mRNA, complete cds	Developmental process	Embryo development
M21636		Developmental process	Embryo development
M33430	crustacean hematopoietic factor [Pacifastacus leniusculus]	Developmental process	Embryo development
N19897	hypothetical protein DAPPUDRAFT_302804 [Daphnia pulex]	Developmental process	Embryo development
N22212	probable global transcription activator SNF2L1 [Danio rerio]	Developmental process	Embryo development
N42326	PREDICTED: COP9 signalosome complex subunit 3 [Ornithorhynchus anatinus]	Developmental process	Embryo development
M18035	PREDICTED: Apis florea heterogeneous nuclear ribonucleoprotein Q-like (LOC100863	Developmental process	Multicellular organismal development
M18892		Developmental process	Multicellular organismal development
N10625	HMGBa [Litopenaeus vannamei]	Developmental process	Multicellular organismal development
N12758		Developmental process	Multicellular organismal development
N25016	engrailed-b homeobox protein [Sacculina carcini]	Developmental process	Multicellular organismal development
N25457	GE13571 [Drosophila yakuba] >gi|194177391|gb|EDW91002.1| GE13571 [Drosophil	Developmental process	Multicellular organismal development
N31644	PREDICTED: hypothetical protein LOC100118826 [Nasonia vitripennis]	Developmental process	Multicellular organismal development
N36333	PREDICTED: long-chain-fatty-acid--CoA ligase 3-like [Apis florea]	Developmental process	Multicellular organismal development
N38018	hypothetical protein YQE_09076, partial [Dendroctonus ponderosae] >gi|546684617	Developmental process	Multicellular organismal development
N43005	dishevelled-like protein [Daphnia pulex]	Developmental process	Multicellular organismal development
N19061	PREDICTED: aspartate--tRNA ligase, cytoplasmic-like [Megachile rotundata]	Developmental process	Nervous system development
N28214	neuferricin-like protein [Ascaris suum]	Developmental process	Nervous system development
N34395	hypothetical protein YQE_03768, partial [Dendroctonus ponderosae]	Developmental process	Nervous system development
N43973	PREDICTED: DNA-directed RNA polymerase II subunit RPB9-like [Ceratitis capitata]	Developmental process	Nervous system development
N49669	PREDICTED: probable E3 ubiquitin-protein ligase makorin-2 isoform X3 [Danio rerio]	Developmental process	Nervous system development
N50963	hypothetical protein DAPPUDRAFT_217067 [Daphnia pulex]	Developmental process	Nervous system development
M10611	PREDICTED: cytoplasmic dynein 1 intermediate chain-like isoform X1 [Ceratitis capita	Developmental process	Other
M8825		Developmental process	Other
N22265	src tyrosine kinase, putative [Ixodes scapularis] >gi|215497797|gb|EEC07291.1| src t	Developmental process	Other
N55781	PREDICTED: exostosin-2-like [Aplysia californica]	Developmental process	Other

Metabolic process

M68938	hypothetical protein DAPPUDRAFT_321839 [Daphnia pulex]	Metabolic process	Autofagy
N20139	WD repeat domain phosphoinositide-interacting protein, putative [Pediculus humanu	Metabolic process	Autofagy
E4529	i-type lysozyme-like protein 1 [Penaeus monodon]	Metabolic process	Carbohydrate metabolic process
E4652	PREDICTED: beta-1,4-mannosyl-glycoprotein 4-beta-N-acetylglucosaminyltransferase	Metabolic process	Carbohydrate metabolic process
E5180		Metabolic process	Carbohydrate metabolic process
M10239	cuticular protein, putative [Ixodes scapularis] >gi|215494596|gb|EEC04237.1| cuticu	Metabolic process	Carbohydrate metabolic process
M10340		Metabolic process	Carbohydrate metabolic process
M105964	PREDICTED: glycogen phosphorylase, liver form isoform X1 [Cavia porcellus]	Metabolic process	Carbohydrate metabolic process

ID	Annotation	GO term	Go child term
M108		Metabolic process	Carbohydrate metabolic process
M108205		Metabolic process	Carbohydrate metabolic process
M109534	hypothetical protein DAPPUDRAFT_62084 [Daphnia pulex]	Metabolic process	Carbohydrate metabolic process
M1135	putative cuticle protein [Lepeophtheirus salmonis]	Metabolic process	Carbohydrate metabolic process
M12607	chitinase 2 [Penaeus monodon]	Metabolic process	Carbohydrate metabolic process
M13238	hypothetical protein TcasGA2_TC001169 [Tribolium castaneum]	Metabolic process	Carbohydrate metabolic process
M14811		Metabolic process	Carbohydrate metabolic process
M16168	chitinase-like protein, partial [Pandalopsis japonica]	Metabolic process	Carbohydrate metabolic process
M16221	conserved hypothetical protein [Pediculus humanus corporis] >gi|212510636|gb|EEI	Metabolic process	Carbohydrate metabolic process
M16262	chitinase 2 [Penaeus monodon]	Metabolic process	Carbohydrate metabolic process
M16697	i-type lysozyme-like protein 2 [Penaeus monodon]	Metabolic process	Carbohydrate metabolic process
M1720	GE21870 [Drosophila yakuba] >gi|194180626|gb|EDW94237.1| GE21870 [Drosophil	Metabolic process	Carbohydrate metabolic process
M17683	n-acetylgalactosaminyltransferase [Aedes aegypti] >gi|108884053|gb|EAT48278.1|	Metabolic process	Carbohydrate metabolic process
M18620	putative fructose 1,6-bisphosphate aldolase [Oncometopia nigricans]	Metabolic process	Carbohydrate metabolic process
M18847	hypothetical protein DAPPUDRAFT_218978 [Daphnia pulex]	Metabolic process	Carbohydrate metabolic process
M18933	chitin binding peritrophin-A, putative [Pediculus humanus corporis] >gi|212514415|g	Metabolic process	Carbohydrate metabolic process
M19074	hypothetical protein DAPPUDRAFT_188180 [Daphnia pulex]	Metabolic process	Carbohydrate metabolic process
M19131	hypothetical protein DAPPUDRAFT_218978 [Daphnia pulex]	Metabolic process	Carbohydrate metabolic process
M19336	unnamed protein product [Oikopleura dioica]	Metabolic process	Carbohydrate metabolic process
M2028		Metabolic process	Carbohydrate metabolic process
M2255		Metabolic process	Carbohydrate metabolic process
M23222	hypothetical protein DAPPUDRAFT_218978 [Daphnia pulex]	Metabolic process	Carbohydrate metabolic process
M24012	thrombospondin [Fenneropenaeus merguiensis]	Metabolic process	Carbohydrate metabolic process
M26541	hypothetical protein DAPPUDRAFT_310106 [Daphnia pulex]	Metabolic process	Carbohydrate metabolic process
M2781	chitin binding peritrophin-A, putative [Pediculus humanus corporis] >gi|212514415|g	Metabolic process	Carbohydrate metabolic process
M2834	alpha glucosidase [Litopenaeus vannamei]	Metabolic process	Carbohydrate metabolic process
M31050	chitin binding peritrophin-A, putative [Pediculus humanus corporis] >gi|212514415|g	Metabolic process	Carbohydrate metabolic process
M31172	hypothetical protein DAPPUDRAFT_223002 [Daphnia pulex]	Metabolic process	Carbohydrate metabolic process
M3194	lactate dehydrogenase [Litopenaeus vannamei] >gi|385211791|gb|AFI47930.1| L-lac	Metabolic process	Carbohydrate metabolic process
M3385	PREDICTED: hypothetical protein [Saccoglossus kowalevskii]	Metabolic process	Carbohydrate metabolic process
M33902		Metabolic process	Carbohydrate metabolic process
M3469	mannosyltransferase 1, putative [Aedes aegypti] >gi|108883791|gb|EAT48016.1| AA	Metabolic process	Carbohydrate metabolic process
M35029	hypothetical protein CAPTEDRAFT_149382 [Capitella teleta]	Metabolic process	Carbohydrate metabolic process
M36881	peritrophin [Rimicaris exoculata]	Metabolic process	Carbohydrate metabolic process
M3890	kexokinase [Litopenaeus vannamei]	Metabolic process	Carbohydrate metabolic process
M40107	GJ15463 [Drosophila virilis] >gi|194141867|gb|EDW58280.1| GJ15463 [Drosophila v	Metabolic process	Carbohydrate metabolic process
M4050		Metabolic process	Carbohydrate metabolic process
M41199		Metabolic process	Carbohydrate metabolic process
M4184	hypothetical protein DAPPUDRAFT_223002 [Daphnia pulex]	Metabolic process	Carbohydrate metabolic process
M4573	hypothetical protein DAPPUDRAFT_218978 [Daphnia pulex]	Metabolic process	Carbohydrate metabolic process
M4996		Metabolic process	Carbohydrate metabolic process
M5387	thrombospondin [Marsupenaeus japonicus]	Metabolic process	Carbohydrate metabolic process
M559	hypothetical protein CGI_10022349 [Crassostrea gigas]	Metabolic process	Carbohydrate metabolic process
M55945	hypothetical protein BRAFLDRAFT_262109 [Branchiostoma floridae] >gi|229298842|	Metabolic process	Carbohydrate metabolic process
M57268	PREDICTED: carbohydrate sulfotransferase 9 isoform X1 [Chrysemys picta bellii] >gi|5	Metabolic process	Carbohydrate metabolic process
M57844	GE21870 [Drosophila yakuba] >gi|194180626|gb|EDW94237.1| GE21870 [Drosophil	Metabolic process	Carbohydrate metabolic process
M58927		Metabolic process	Carbohydrate metabolic process
M60556	hypothetical protein DAPPUDRAFT_307215 [Daphnia pulex]	Metabolic process	Carbohydrate metabolic process
M63793	PREDICTED: galactoside 2-alpha-L-fucosyltransferase 2-like [Echinops telfairi]	Metabolic process	Carbohydrate metabolic process
M649	hypothetical protein DAPPUDRAFT_194601 [Daphnia pulex]	Metabolic process	Carbohydrate metabolic process
M67698	hypothetical protein DAPPUDRAFT_198312 [Daphnia pulex]	Metabolic process	Carbohydrate metabolic process
M71137	Glutamine:fructose-6-phosphate aminotransferase 2 [Drosophila melanogaster] >gi|	Metabolic process	Carbohydrate metabolic process
M75519		Metabolic process	Carbohydrate metabolic process
M7877	i-type lysozyme-like protein 2 [Penaeus monodon]	Metabolic process	Carbohydrate metabolic process
M81733	defender against apoptotic death [Penaeus monodon]	Metabolic process	Carbohydrate metabolic process
M91327	trehalose-6-phosphate synthase [Fenneropenaeus chinensis]	Metabolic process	Carbohydrate metabolic process
M91486	hypothetical protein EAG_15972 [Camponotus floridanus]	Metabolic process	Carbohydrate metabolic process
M93809	PREDICTED: carbohydrate sulfotransferase 11-like [Nasonia vitripennis]	Metabolic process	Carbohydrate metabolic process
M95016	hypothetical protein DAPPUDRAFT_313010 [Daphnia pulex]	Metabolic process	Carbohydrate metabolic process
M96176		Metabolic process	Carbohydrate metabolic process
N10288	hypothetical protein TcasGA2_TC012734 [Tribolium castaneum]	Metabolic process	Carbohydrate metabolic process
N11908	endoplasmic reticulum protein 57 [Penaeus monodon]	Metabolic process	Carbohydrate metabolic process
N12153	i-type lysozyme-like protein 2 [Penaeus monodon]	Metabolic process	Carbohydrate metabolic process
N12205	hypothetical protein DAPPUDRAFT_223002 [Daphnia pulex]	Metabolic process	Carbohydrate metabolic process
N12995	hypothetical protein DAPPUDRAFT_218978 [Daphnia pulex]	Metabolic process	Carbohydrate metabolic process
N12999	hypothetical protein DAPPUDRAFT_194601 [Daphnia pulex]	Metabolic process	Carbohydrate metabolic process
N13000	hypothetical protein DAPPUDRAFT_194601 [Daphnia pulex]	Metabolic process	Carbohydrate metabolic process
N13131	hypothetical protein CAPTEDRAFT_149382 [Capitella teleta]	Metabolic process	Carbohydrate metabolic process
N13249		Metabolic process	Carbohydrate metabolic process
N13297	hypothetical protein DAPPUDRAFT_209471 [Daphnia pulex]	Metabolic process	Carbohydrate metabolic process
N13357	hypothetical protein DAPPUDRAFT_218978 [Daphnia pulex]	Metabolic process	Carbohydrate metabolic process
N13605	hypothetical protein YQE_11184, partial [Dendroctonus ponderosae]	Metabolic process	Carbohydrate metabolic process
N13755	hypothetical protein DAPPUDRAFT_309434 [Daphnia pulex]	Metabolic process	Carbohydrate metabolic process
N13793	peritrophin A, isoform A [Drosophila melanogaster] >gi|24643467|ref|NP_728324.1|	Metabolic process	Carbohydrate metabolic process
N13919	PREDICTED: hypothetical protein LOC100120260 [Nasonia vitripennis]	Metabolic process	Carbohydrate metabolic process
N14679	hypothetical protein DAPPUDRAFT_223002 [Daphnia pulex]	Metabolic process	Carbohydrate metabolic process

ID	Annotation	GO term	Go child term
N14843	cuticular protein [Artemia franciscana]	Metabolic process	Carbohydrate metabolic process
N16657	hypothetical protein DAPPUDRAFT_52904 [Daphnia pulex]	Metabolic process	Carbohydrate metabolic process
N18219	hypothetical protein DAPPUDRAFT_300811 [Daphnia pulex]	Metabolic process	Carbohydrate metabolic process
N18281	Culex quinquefasciatus glycogen phosphorylase, mRNA	Metabolic process	Carbohydrate metabolic process
N18293	hypothetical protein BRAFLDRAFT_107076 [Branchiostoma floridae] >gi|229281129|	Metabolic process	Carbohydrate metabolic process
N1840	chitin binding peritrophin-A, putative [Pediculus humanus corporis] >gi|212514415|g	Metabolic process	Carbohydrate metabolic process
N18460	hypothetical protein DAPPUDRAFT_303417 [Daphnia pulex]	Metabolic process	Carbohydrate metabolic process
N18608	PREDICTED: Loxodonta africana dolichyl-diphosphooligosaccharide--protein glycosylt	Metabolic process	Carbohydrate metabolic process
N18791	hypothetical protein DAPPUDRAFT_218978 [Daphnia pulex]	Metabolic process	Carbohydrate metabolic process
N18846	Chondroitin sulfate synthase, putative [Pediculus humanus corporis] >gi|212507482|	Metabolic process	Carbohydrate metabolic process
N18880	chitin deacetylase 1 precursor [Cherax quadricarinatus]	Metabolic process	Carbohydrate metabolic process
N18901	hypothetical protein DAPPUDRAFT_58801 [Daphnia pulex]	Metabolic process	Carbohydrate metabolic process
N18962	pyruvate kinase 2 [Litopenaeus vannamei]	Metabolic process	Carbohydrate metabolic process
N19079		Metabolic process	Carbohydrate metabolic process
N19338	putative triacylglycerol lipase [Daphnia pulex]	Metabolic process	Carbohydrate metabolic process
N19626	PREDICTED: dolichyl-diphosphooligosaccharide--protein glycosyltransferase 48 kDa su	Metabolic process	Carbohydrate metabolic process
N19873	endo-beta-1,4-glucanase [Coptotermes formosanus]	Metabolic process	Carbohydrate metabolic process
N20273	trehalose-6-phosphate synthase [Fenneropenaeus chinensis]	Metabolic process	Carbohydrate metabolic process
N20547	hypothetical protein TcasGA2_TC003876 [Tribolium castaneum]	Metabolic process	Carbohydrate metabolic process
N20763		Metabolic process	Carbohydrate metabolic process
N21844	PREDICTED: 6-phosphofructokinase-like isoform 3 [Acyrthosiphon pisum]	Metabolic process	Carbohydrate metabolic process
N21964	cuticular protein [Artemia franciscana]	Metabolic process	Carbohydrate metabolic process
N22117	PREDICTED: beta-hexosaminidase subunit alpha-like [Ciona intestinalis]	Metabolic process	Carbohydrate metabolic process
N22304	PREDICTED: hexokinase-1-like [Megachile rotundata]	Metabolic process	Carbohydrate metabolic process
N22459	thrombospondin protein [Penaeus monodon]	Metabolic process	Carbohydrate metabolic process
N22982	PREDICTED: carbohydrate sulfotransferase 9 isoform X1 [Chrysemys picta bellii] >gi|5	Metabolic process	Carbohydrate metabolic process
N23226	Carbohydrate sulfotransferase 14 [Camponotus floridanus]	Metabolic process	Carbohydrate metabolic process
N23374	hypothetical protein BRAFLDRAFT_123545 [Branchiostoma floridae] >gi|229277020|	Metabolic process	Carbohydrate metabolic process
N2465	hypothetical protein TcasGA2_TC009890 [Tribolium castaneum]	Metabolic process	Carbohydrate metabolic process
N2481	hypothetical protein TcasGA2_TC009890 [Tribolium castaneum]	Metabolic process	Carbohydrate metabolic process
N2485		Metabolic process	Carbohydrate metabolic process
N25384		Metabolic process	Carbohydrate metabolic process
N26189	hypothetical protein DAPPUDRAFT_223002 [Daphnia pulex]	Metabolic process	Carbohydrate metabolic process
N28597		Metabolic process	Carbohydrate metabolic process
N28954	Carbohydrate sulfotransferase 11 [Crassostrea gigas]	Metabolic process	Carbohydrate metabolic process
N29090	cuticular protein [Artemia franciscana]	Metabolic process	Carbohydrate metabolic process
N2914	chitin binding peritrophin-A, putative [Pediculus humanus corporis] >gi|212514415|g	Metabolic process	Carbohydrate metabolic process
N30067		Metabolic process	Carbohydrate metabolic process
N30228		Metabolic process	Carbohydrate metabolic process
N30602		Metabolic process	Carbohydrate metabolic process
N30729		Metabolic process	Carbohydrate metabolic process
N31102		Metabolic process	Carbohydrate metabolic process
N31446	trehalose-6-phosphate synthase [Fenneropenaeus chinensis]	Metabolic process	Carbohydrate metabolic process
N31465		Metabolic process	Carbohydrate metabolic process
N32999	Heparan sulfate glucosamine 3-O-sulfotransferase 5 [Tupaia chinensis]	Metabolic process	Carbohydrate metabolic process
N34711	PREDICTED: chitinase-3-like protein 1 isoform X1 [Capra hircus]	Metabolic process	Carbohydrate metabolic process
N36122	hypothetical protein CAPTEDRAFT_218878 [Capitella teleta]	Metabolic process	Carbohydrate metabolic process
N3717		Metabolic process	Carbohydrate metabolic process
N3718		Metabolic process	Carbohydrate metabolic process
N3728		Metabolic process	Carbohydrate metabolic process
N3740		Metabolic process	Carbohydrate metabolic process
N38910	PREDICTED: carbohydrate (N-acetylgalactosamine 4-0) sulfotransferase 8-like [Saccog	Metabolic process	Carbohydrate metabolic process
N39090	Beta-1,4-galactosyltransferase 3 [Chelonia mydas]	Metabolic process	Carbohydrate metabolic process
N39451	cuticular protein [Artemia franciscana]	Metabolic process	Carbohydrate metabolic process
N41283	PREDICTED: similar to n-acetylgalactosaminyltransferase [Tribolium castaneum] >gi|2	Metabolic process	Carbohydrate metabolic process
N41735	hypothetical protein DAPPUDRAFT_327753 [Daphnia pulex]	Metabolic process	Carbohydrate metabolic process
N43379	hypothetical protein TRIADDRAFT_51036 [Trichoplax adhaerens] >gi|190580249|gb|	Metabolic process	Carbohydrate metabolic process
N47191	hypothetical protein DAPPUDRAFT_218978 [Daphnia pulex]	Metabolic process	Carbohydrate metabolic process
N48559	chitinase [Pandalopsis japonica]	Metabolic process	Carbohydrate metabolic process
N58260	chondroitin 4-sulfotransferase [Aedes aegypti] >gi|108873718|gb|EAT37943.1| AAEl	Metabolic process	Carbohydrate metabolic process
N60524	PREDICTED: chitotriosidase-1-like [Nasonia vitripennis]	Metabolic process	Carbohydrate metabolic process
N61129	hypothetical protein DAPPUDRAFT_49320 [Daphnia pulex]	Metabolic process	Carbohydrate metabolic process
N6335	hypothetical protein DAPPUDRAFT_218978 [Daphnia pulex]	Metabolic process	Carbohydrate metabolic process
N6572		Metabolic process	Carbohydrate metabolic process
N6715	cuticular protein [Artemia franciscana]	Metabolic process	Carbohydrate metabolic process
N6991	hypothetical protein DAPPUDRAFT_307182 [Daphnia pulex]	Metabolic process	Carbohydrate metabolic process
N7213		Metabolic process	Carbohydrate metabolic process
N7300	chitin binding peritrophin-A domain containing protein [Coptotermes formosanus]	Metabolic process	Carbohydrate metabolic process
N8629	hypothetical protein TcasGA2_TC012734 [Tribolium castaneum]	Metabolic process	Carbohydrate metabolic process
N8631	hypothetical protein DAPPUDRAFT_235148 [Daphnia pulex]	Metabolic process	Carbohydrate metabolic process
N9216	cuticular protein, putative [Ixodes scapularis] >gi|215494596|gb|EEC04237.1| cuticu	Metabolic process	Carbohydrate metabolic process
N9218	i-type lysozyme-like protein 2 [Penaeus monodon]	Metabolic process	Carbohydrate metabolic process
N9427	hypothetical protein DAPPUDRAFT_218978 [Daphnia pulex]	Metabolic process	Carbohydrate metabolic process
N9476	Gasterosteus aculeatus clone CFW246-H10 mRNA sequence	Metabolic process	Carbohydrate metabolic process
N9500	hypothetical protein TcasGA2_TC012734 [Tribolium castaneum]	Metabolic process	Carbohydrate metabolic process
N9745		Metabolic process	Carbohydrate metabolic process

ID	Annotation	GO term	Go child term
N9749	hypothetical protein CAPTEDRAFT_167427 [Capitella teleta]	Metabolic process	Carbohydrate metabolic process
N9752	hypothetical protein BRAFLDRAFT_70494 [Branchiostoma floridae] >gi|229289185|g	Metabolic process	Carbohydrate metabolic process
N9845		Metabolic process	Carbohydrate metabolic process
M109440	Asparaginyl-tRNA synthetase, isoform A [Drosophila melanogaster] >gi|442628315|r	Metabolic process	Cellular amino acid metabolic process
M1168	hypothetical protein DAPPUDRAFT_191289 [Daphnia pulex]	Metabolic process	Cellular amino acid metabolic process
M13834	PREDICTED: LOW QUALITY PROTEIN: TP53-regulating kinase [Callithrix jacchus]	Metabolic process	Cellular amino acid metabolic process
M14316	Libinia emarginata isolate 11_12 voucher LemMALA alpha-spectrin mRNA, partial cds	Metabolic process	Cellular amino acid metabolic process
M1680	arginine kinase 2 [Neocaridina denticulata]	Metabolic process	Cellular amino acid metabolic process
M18723	putative ATP synthase oligomycin sensitivity conferral protein [Coptotermes formosa	Metabolic process	Cellular amino acid metabolic process
M18942	hypothetical protein BRAFLDRAFT_78594 [Branchiostoma floridae] >gi|229295124|g	Metabolic process	Cellular amino acid metabolic process
M2035	PREDICTED: uncharacterized protein LOC101860265 isoform X1 [Aplysia californica]	Metabolic process	Cellular amino acid metabolic process
M21		Metabolic process	Cellular amino acid metabolic process
M23144	glycine N-methyltransferase-like [Nasonia vitripennis]	Metabolic process	Cellular amino acid metabolic process
M25	hypothetical protein BRAFLDRAFT_63907 [Branchiostoma floridae] >gi|229296831|g	Metabolic process	Cellular amino acid metabolic process
M26420	hypothetical protein BRAFLDRAFT_72748 [Branchiostoma floridae] >gi|229278397|g	Metabolic process	Cellular amino acid metabolic process
M288	hypothetical protein DAPPUDRAFT_214647 [Daphnia pulex]	Metabolic process	Cellular amino acid metabolic process
M3130	Fumarylacetoacetase [Crassostrea gigas]	Metabolic process	Cellular amino acid metabolic process
M378	conserved hypothetical protein [Pediculus humanus corporis] >gi|212516655|gb|EEI	Metabolic process	Cellular amino acid metabolic process
M3801	hypothetical protein DAPPUDRAFT_52799 [Daphnia pulex]	Metabolic process	Cellular amino acid metabolic process
M4015		Metabolic process	Cellular amino acid metabolic process
M4120	hypothetical protein DAPPUDRAFT_318250 [Daphnia pulex]	Metabolic process	Cellular amino acid metabolic process
M4436	hypothetical protein BRAFLDRAFT_130501 [Branchiostoma floridae] >gi|229280180|	Metabolic process	Cellular amino acid metabolic process
M47556	betaine homocysteine methyl transferase-like protein [Homarus americanus]	Metabolic process	Cellular amino acid metabolic process
M5020	PREDICTED: glycine cleavage system H protein, mitochondrial [Orcinus orca]	Metabolic process	Cellular amino acid metabolic process
M5185	hypothetical protein BRAFLDRAFT_103698 [Branchiostoma floridae] >gi|229280206|	Metabolic process	Cellular amino acid metabolic process
M545	hypothetical protein DAPPUDRAFT_56817 [Daphnia pulex]	Metabolic process	Cellular amino acid metabolic process
M56721	L-aspartate dehydrogenase [Caligus clemensi]	Metabolic process	Cellular amino acid metabolic process
M59782	arginase [Hyriopsis cumingii]	Metabolic process	Cellular amino acid metabolic process
M61204	PREDICTED: Cricetulus griseus methylmalonyl-Coenzyme A mutase (Mut), mRNA	Metabolic process	Cellular amino acid metabolic process
M61546	homogentisate 1,2-dioxygenase HgmA [Capsaspora owczarzaki ATCC 30864] >gi|320	Metabolic process	Cellular amino acid metabolic process
M61757	hypothetical protein DAPPUDRAFT_65532 [Daphnia pulex]	Metabolic process	Cellular amino acid metabolic process
M63222	hypothetical protein DAPPUDRAFT_308482 [Daphnia pulex]	Metabolic process	Cellular amino acid metabolic process
M66867	hypothetical protein CAPTEDRAFT_43016, partial [Capitella teleta]	Metabolic process	Cellular amino acid metabolic process
M6768	lactate dehydrogenase [Litopenaeus vannamei] >gi|385211791|gb|AFI47930.1| L-lac	Metabolic process	Cellular amino acid metabolic process
M70102	GG11794 [Drosophila erecta] >gi|190655748|gb|EDV52980.1| GG11794 [Drosophila	Metabolic process	Cellular amino acid metabolic process
M7047	hypothetical protein DAPPUDRAFT_22132 [Daphnia pulex]	Metabolic process	Cellular amino acid metabolic process
M71240	PREDICTED: RNA guanylyltransferase and 5-phosphatase-like [Saccoglossus kowalevs	Metabolic process	Cellular amino acid metabolic process
M7237	tryptophanyl-tRNA synthetase [Coccidioides posadasii str. Silveira]	Metabolic process	Cellular amino acid metabolic process
M7360	PREDICTED: valyl-tRNA synthetase-like [Saccoglossus kowalevskii]	Metabolic process	Cellular amino acid metabolic process
M79250	hypothetical protein DAPPUDRAFT_299913 [Daphnia pulex]	Metabolic process	Cellular amino acid metabolic process
M86357	betaine homocysteine methyl transferase-like protein [Homarus americanus]	Metabolic process	Cellular amino acid metabolic process
M87594	PREDICTED: tryptophan 2,3-dioxygenase [Nomascus leucogenys]	Metabolic process	Cellular amino acid metabolic process
M93328		Metabolic process	Cellular amino acid metabolic process
M94977	Adenosylhomocysteinase [Lepeophtheirus salmonis]	Metabolic process	Cellular amino acid metabolic process
M96624	Glutaminase kidney isoform, mitochondrial [Acromyrmex echinatior]	Metabolic process	Cellular amino acid metabolic process
M96944	PREDICTED: argininosuccinate lyase-like [Apis mellifera]	Metabolic process	Cellular amino acid metabolic process
M97234	hypothetical protein DAPPUDRAFT_220733 [Daphnia pulex]	Metabolic process	Cellular amino acid metabolic process
M9776	PREDICTED: MGC80816 protein-like [Saccoglossus kowalevskii]	Metabolic process	Cellular amino acid metabolic process
M9982	hypothetical protein DAPPUDRAFT_318167 [Daphnia pulex]	Metabolic process	Cellular amino acid metabolic process
N12862	glutamine-oxaloacetic transaminase [Daphnia magna] >gi|224486260|gb|ACN51895	Metabolic process	Cellular amino acid metabolic process
N12875	hypothetical protein DAPPUDRAFT_204767 [Daphnia pulex]	Metabolic process	Cellular amino acid metabolic process
N13019	cysteinyl-tRNA synthetase (predicted), isoform CRA_a [Rattus norvegicus]	Metabolic process	Cellular amino acid metabolic process
N14451	hematopoietic prostaglandin D synthase [Penaeus monodon]	Metabolic process	Cellular amino acid metabolic process
N18205	hypothetical protein DAPPUDRAFT_316089 [Daphnia pulex]	Metabolic process	Cellular amino acid metabolic process
N18243	hypothetical protein DAPPUDRAFT_318233 [Daphnia pulex]	Metabolic process	Cellular amino acid metabolic process
N18290	hypothetical protein DAPPUDRAFT_305406 [Daphnia pulex]	Metabolic process	Cellular amino acid metabolic process
N18415	glycyl-tRNA synthetase, putative [Pediculus humanus corporis] >gi|212518378|gb|EI	Metabolic process	Cellular amino acid metabolic process
N18485	hypothetical protein BRAFLDRAFT_117885 [Branchiostoma floridae] >gi|229296009|	Metabolic process	Cellular amino acid metabolic process
N18645	PREDICTED: threonine synthase-like 1 [Xenopus (Silurana) tropicalis]	Metabolic process	Cellular amino acid metabolic process
N18663	hypothetical protein CAPTEDRAFT_148401 [Capitella teleta]	Metabolic process	Cellular amino acid metabolic process
N18692	aldehyde dehydrogenase, putative [Ixodes scapularis] >gi|215502496|gb|EEC11990.	Metabolic process	Cellular amino acid metabolic process
N18769	hypothetical protein DAPPUDRAFT_327265 [Daphnia pulex]	Metabolic process	Cellular amino acid metabolic process
N18874	lysyl-tRNA synthetase [Aedes aegypti] >gi|108868817|gb|EAT33042.1| AAEL014702-	Metabolic process	Cellular amino acid metabolic process
N18918	phenylalanine--tRNA ligase alpha subunit [Danio rerio] >gi|146345522|sp|Q1JPX3.2|	Metabolic process	Cellular amino acid metabolic process
N19176	lactate dehydrogenase [Litopenaeus vannamei] >gi|385211791|gb|AFI47930.1| L-lac	Metabolic process	Cellular amino acid metabolic process
N19287	hypothetical protein BRAFLDRAFT_124526 [Branchiostoma floridae] >gi|229275740|	Metabolic process	Cellular amino acid metabolic process
N19921		Metabolic process	Cellular amino acid metabolic process
N19992	PREDICTED: arginase-1-like [Loxodonta africana]	Metabolic process	Cellular amino acid metabolic process
N20540	PREDICTED: UPF0436 protein C9orf6-like [Monodelphis domestica]	Metabolic process	Cellular amino acid metabolic process
N20761	hypothetical protein DAPPUDRAFT_211091 [Daphnia pulex]	Metabolic process	Cellular amino acid metabolic process
N20765	Wormaldia moesta voucher Wmo2 putative enolase protein mRNA, partial cds	Metabolic process	Cellular amino acid metabolic process
N22201	hypothetical protein DAPPUDRAFT_220335 [Daphnia pulex]	Metabolic process	Cellular amino acid metabolic process
N23228	glutathione S-transferases [Macrobrachium nipponense]	Metabolic process	Cellular amino acid metabolic process
N27726	hypothetical protein BRAFLDRAFT_183632 [Branchiostoma floridae] >gi|229277195|	Metabolic process	Cellular amino acid metabolic process
N31813	lactate dehydrogenase [Litopenaeus vannamei]	Metabolic process	Cellular amino acid metabolic process
N51830	hypothetical protein DAPPUDRAFT_309832 [Daphnia pulex]	Metabolic process	Cellular amino acid metabolic process

ID	Annotation	GO term	Go child term
N53005	betaine homocysteine methyl transferase-like protein [Homarus americanus]	Metabolic process	Cellular amino acid metabolic process
N55506	4-aminobutyrate aminotransferase [Aedes aegypti] >gi|108870984|gb|EAT35209.1|	Metabolic process	Cellular amino acid metabolic process
N56643	mRNA-capping enzyme [Camponotus floridanus]	Metabolic process	Cellular amino acid metabolic process
N59606	tyrosine hydroxylase isoform A [Manduca sexta]	Metabolic process	Cellular amino acid metabolic process
E5781	mitochondrial ATP synthase epsilon subunit precursor [Litopenaeus vannamei]	Metabolic process	Generation of precursor metabolites and e
M10290	glyceraldehyde-3-phosphate dehydrogenase [Procambarus clarkii]	Metabolic process	Generation of precursor metabolites and e
M113051	Euphausia superba voucher 760M mitochondrion, partial genome	Metabolic process	Generation of precursor metabolites and e
M13619	hypothetical protein DAPPUDRAFT_50463 [Daphnia pulex]	Metabolic process	Generation of precursor metabolites and e
M14565	mitochondrial ATP synthase gamma subunit precursor [Litopenaeus vannamei]	Metabolic process	Generation of precursor metabolites and e
M17951	PREDICTED: glycogen debranching enzyme-like [Nasonia vitripennis]	Metabolic process	Generation of precursor metabolites and e
M18729	PREDICTED: Otolemur garnettii succinate-CoA ligase, alpha subunit (SUCLG1), mRNA	Metabolic process	Generation of precursor metabolites and e
M18821	mitochondrial ATP synthase coupling factor 6 precursor [Ochlerotatus taeniorhynchu	Metabolic process	Generation of precursor metabolites and e
M18904	hypothetical protein DAPPUDRAFT_230269 [Daphnia pulex]	Metabolic process	Generation of precursor metabolites and e
M2029	putative glycogen synthase [Daphnia pulex]	Metabolic process	Generation of precursor metabolites and e
M21528	hypothetical protein DAPPUDRAFT_231048 [Daphnia pulex]	Metabolic process	Generation of precursor metabolites and e
M21548		Metabolic process	Generation of precursor metabolites and e
M23287	cytochrome c oxidase-like protein [Glyptapanteles flavicoxis]	Metabolic process	Generation of precursor metabolites and e
M24954	Euphausia superba mitochondrion, partial genome	Metabolic process	Generation of precursor metabolites and e
M31032	mitochondrial cytochrome c oxidase subunit Va [Litopenaeus vannamei]	Metabolic process	Generation of precursor metabolites and e
M42144	Euphausia superba voucher 760M mitochondrion, partial genome	Metabolic process	Generation of precursor metabolites and e
M46863	Euphausia superba voucher 760M mitochondrion, partial genome	Metabolic process	Generation of precursor metabolites and e
M5551		Metabolic process	Generation of precursor metabolites and e
M55970	Euphausia superba voucher 702K mitochondrion, partial genome	Metabolic process	Generation of precursor metabolites and e
M57202	mitochondrial cytochrome c oxidase subunit IV [Litopenaeus vannamei]	Metabolic process	Generation of precursor metabolites and e
M63042	S-adenosylmethionine synthetase [Candida tropicalis MYA-3404] >gi|240132872|gb|	Metabolic process	Generation of precursor metabolites and e
M70684	hypothetical protein DAPPUDRAFT_308310 [Daphnia pulex]	Metabolic process	Generation of precursor metabolites and e
M785	PREDICTED: glycogen debranching enzyme [Megachile rotundata]	Metabolic process	Generation of precursor metabolites and e
M8823	hypothetical protein DAPPUDRAFT_64425 [Daphnia pulex]	Metabolic process	Generation of precursor metabolites and e
M8882	1,4-alpha-glucan-branching enzyme [Trichinella spiralis] >gi|316967211|gb|EFV5167	Metabolic process	Generation of precursor metabolites and e
N11963		Metabolic process	Generation of precursor metabolites and e
N18402	phosphoenolpyruvate carboxykinase [Litopenaeus vannamei]	Metabolic process	Generation of precursor metabolites and e
N20906		Metabolic process	Generation of precursor metabolites and e
N27804	mitochondrial ubiquinol-cytochrome c reductase complex 11 kDa protein [Scylla para	Metabolic process	Generation of precursor metabolites and e
N3133	PREDICTED: S-adenosylmethionine synthase-like isoform 2 [Nasonia vitripennis]	Metabolic process	Generation of precursor metabolites and e
N31394	cytochrome c oxidase subunit VIIA putative [Scylla paramamosain]	Metabolic process	Generation of precursor metabolites and e
N3151	PREDICTED: S-adenosylmethionine synthase-like isoform 1 [Nasonia vitripennis]	Metabolic process	Generation of precursor metabolites and e
N36505	vacuolar proton translocating ATPase 116 kDa subunit A isoform, putative [Pediculus	Metabolic process	Generation of precursor metabolites and e
N9508	mitochondrial cytochrome c oxidase subunit IV [Litopenaeus vannamei]	Metabolic process	Generation of precursor metabolites and e
M10403	ankyrin-1, putative [Pediculus humanus corporis] >gi|212518141|gb|EEB19933.1| ar	Metabolic process	Lipid metabolic process
M16759	hypothetical protein DAPPUDRAFT_305782 [Daphnia pulex]	Metabolic process	Lipid metabolic process
M1878	Homo sapiens peroxisomal D3,D2-enoyl-CoA isomerase [synthetic construct] >gi|606	Metabolic process	Lipid metabolic process
M3079	PREDICTED: similar to CG9914 CG9914-PA isoform 1 [Tribolium castaneum] >gi|2700	Metabolic process	Lipid metabolic process
M32708	putative triacylglycerol lipase [Daphnia pulex]	Metabolic process	Lipid metabolic process
M5575	proteasome 26S non-ATPase subunit 10 [Xenopus (Silurana) tropicalis] >gi|89266984	Metabolic process	Lipid metabolic process
M57812	hypothetical protein DAPPUDRAFT_300931 [Daphnia pulex]	Metabolic process	Lipid metabolic process
M58225	hypothetical protein DAPPUDRAFT_300149 [Daphnia pulex]	Metabolic process	Lipid metabolic process
M58882	PREDICTED: phospholipase A2-like [Bombyx mori]	Metabolic process	Lipid metabolic process
M61209	Diacylglycerol kinase zeta, putative [Pediculus humanus corporis] >gi|212508871|gb	Metabolic process	Lipid metabolic process
M64386	ras, putative [Pediculus humanus corporis] >gi|212509170|gb|EEB12638.1| ras, puta	Metabolic process	Lipid metabolic process
M64437	hypothetical protein BRAFLDRAFT_232211 [Branchiostoma floridae] >gi|229281209|	Metabolic process	Lipid metabolic process
M67472	hypothetical protein DAPPUDRAFT_56898 [Daphnia pulex]	Metabolic process	Lipid metabolic process
M71319	Ldlr2-a protein [Xenopus laevis] >gi|213626297|gb|AAI70522.1| Ldlr2-a protein [Xer	Metabolic process	Lipid metabolic process
M735	oxidoreductase [alpha proteobacterium BAL199] >gi|159177770|gb|EDP62321.1| sh	Metabolic process	Lipid metabolic process
M75326	PREDICTED: LOW QUALITY PROTEIN: alkaline ceramidase 2 [Dasypus novemcinctus]	Metabolic process	Lipid metabolic process
M775	hypothetical protein DAPPUDRAFT_231244 [Daphnia pulex]	Metabolic process	Lipid metabolic process
M86465	PREDICTED: galactose-3-O-sulfotransferase 1-like [Saccoglossus kowalevskii]	Metabolic process	Lipid metabolic process
M90544	sphingomyelin phosphodiesterase [Cherax quadricarinatus]	Metabolic process	Lipid metabolic process
M91179	hypothetical protein DAPPUDRAFT_199604 [Daphnia pulex]	Metabolic process	Lipid metabolic process
N12095	7-dehydrocholesterol reductase [Lepeophtheirus salmonis] >gi|290462921|gb|ADD2	Metabolic process	Lipid metabolic process
N13719		Metabolic process	Lipid metabolic process
N14473	PREDICTED: decaprenyl-diphosphate synthase subunit 1-like [Nasonia vitripennis]	Metabolic process	Lipid metabolic process
N18980	hypothetical protein BRAFLDRAFT_115040 [Branchiostoma floridae] >gi|229274202|	Metabolic process	Lipid metabolic process
N19597	PREDICTED: uncharacterized protein LOC100904803 [Metaseiulus occidentalis]	Metabolic process	Lipid metabolic process
N19820	hypothetical protein CAPTEDRAFT_160702 [Capitella teleta]	Metabolic process	Lipid metabolic process
N19986	hypothetical protein DAPPUDRAFT_319865 [Daphnia pulex]	Metabolic process	Lipid metabolic process
N20177	hypothetical protein DAPPUDRAFT_31637 [Daphnia pulex]	Metabolic process	Lipid metabolic process
N21911	salivary alkaline phosphatase [Daphnia pulex]	Metabolic process	Lipid metabolic process
N22590	hypothetical protein DAPPUDRAFT_307575 [Daphnia pulex]	Metabolic process	Lipid metabolic process
N22794	geranylgeranyl pyrophosphate synthase/polyprenyl synthetase, putative [Ixodes scap	Metabolic process	Lipid metabolic process
N23066	crustacyanin subunit A [Fenneropenaeus merguiensis]	Metabolic process	Lipid metabolic process
N24872	Inositol monophosphatase 3 [Caligus clemensi]	Metabolic process	Lipid metabolic process
N36501		Metabolic process	Lipid metabolic process
N37214	Alkaline phosphatase, tissue-nonspecific isozyme precursor, putative [Pediculus hum	Metabolic process	Lipid metabolic process
N45199	period [Eurydice pulchra]	Metabolic process	Lipid metabolic process
N7648		Metabolic process	Lipid metabolic process
M109012	PREDICTED: agmatinase, mitochondrial-like [Strongylocentrotus purpuratus]	Metabolic process	Nitrogen compound metabolic process

ID	Annotation	GO term	Go child term
M2380		Metabolic process	Nitrogen compound metabolic process
M35901	hypothetical protein BRAFLDRAFT_78594 [Branchiostoma floridae] >gi|229295124|g	Metabolic process	Nitrogen compound metabolic process
M94914	glycosyl-phosphatidylinositol-linked carbonic anhydrase [Litopenaeus vannamei]	Metabolic process	Nitrogen compound metabolic process
N10315	hypothetical protein DAPPUDRAFT_306977 [Daphnia pulex]	Metabolic process	Nitrogen compound metabolic process
N32147	PREDICTED: peptide-N(4)-(N-acetyl-beta-glucosaminyl)asparagine amidase [Sarcophil	Metabolic process	Nitrogen compound metabolic process
N34523	agmatinase [Pseudomonas] >gi|395337156|gb|EJF69014.1| agmatinase [Pseudomo	Metabolic process	Nitrogen compound metabolic process
N45146	hypothetical protein BRAFLDRAFT_95006 [Branchiostoma floridae] >gi|229286520|g	Metabolic process	Nitrogen compound metabolic process
N9798	glycosyl-phosphatidylinositol-linked carbonic anhydrase [Litopenaeus vannamei]	Metabolic process	Nitrogen compound metabolic process
E2163	oncoprotein nm23 [Litopenaeus vannamei]	Metabolic process	Nucleobase-containing compound metabo
M101631		Metabolic process	Nucleobase-containing compound metabo
M14480	PREDICTED: Saccoglossus kowalevskii DNA directed RNA polymerase II polypeptide E-	Metabolic process	Nucleobase-containing compound metabo
M22464	RNA polymerase I/III 16Kd polypeptide, putative [Ixodes scapularis] >gi|215493414|g	Metabolic process	Nucleobase-containing compound metabo
M2790	putative myosin head [Trichinella spiralis] >gi|316971212|gb|EFV55023.1| putative r	Metabolic process	Nucleobase-containing compound metabo
M3981	PREDICTED: similar to adenine phosphoribosyltransferase [Tribolium castaneum] >gi|	Metabolic process	Nucleobase-containing compound metabo
M40599	oncoprotein nm23 [Litopenaeus vannamei]	Metabolic process	Nucleobase-containing compound metabo
M4174	hypothetical protein DAPPUDRAFT_306425 [Daphnia pulex]	Metabolic process	Nucleobase-containing compound metabo
M5074		Metabolic process	Nucleobase-containing compound metabo
M56219	Pseudouridylate synthase 7-like protein [Harpegnathos saltator]	Metabolic process	Nucleobase-containing compound metabo
M56287	hypothetical protein DAPPUDRAFT_304334 [Daphnia pulex]	Metabolic process	Nucleobase-containing compound metabo
M56865	PREDICTED: inosine-5'-monophosphate dehydrogenase isoform 1 [Apis mellifera] >gi	Metabolic process	Nucleobase-containing compound metabo
M6211	DNA-directed RNA polymerase [Coptotermes formosanus]	Metabolic process	Nucleobase-containing compound metabo
M62133		Metabolic process	Nucleobase-containing compound metabo
M62739	ABC protein, subfamily ABCA [Daphnia pulex]	Metabolic process	Nucleobase-containing compound metabo
M78121	hypothetical protein TcasGA2_TC014142 [Tribolium castaneum]	Metabolic process	Nucleobase-containing compound metabo
M8921	NME/NM23 family member 7 [Xenopus (Silurana) tropicalis] >gi|38181932|gb|AAH6	Metabolic process	Nucleobase-containing compound metabo
N10324	PREDICTED: UDP-galactose-4-epimerase [Oryctolagus cuniculus]	Metabolic process	Nucleobase-containing compound metabo
N12873		Metabolic process	Nucleobase-containing compound metabo
N18215	conserved hypothetical protein [Pediculus humanus corporis] >gi|212508370|gb|EE	Metabolic process	Nucleobase-containing compound metabo
N18669	hypothetical protein BRAFLDRAFT_76524 [Branchiostoma floridae] >gi|229275470|g	Metabolic process	Nucleobase-containing compound metabo
N18936	PREDICTED: bifunctional purine biosynthesis protein PURH [Nasonia vitripennis]	Metabolic process	Nucleobase-containing compound metabo
N18959	exonuclease 3'-5' domain containing 2 [Xenopus laevis]	Metabolic process	Nucleobase-containing compound metabo
N19230	hypothetical protein DAPPUDRAFT_307202 [Daphnia pulex]	Metabolic process	Nucleobase-containing compound metabo
N19588	PREDICTED: adenylosuccinate lyase-like [Nasonia vitripennis]	Metabolic process	Nucleobase-containing compound metabo
N20154	purine biosynthesis protein 6, pur6, putative [Pediculus humanus corporis] >gi|21251	Metabolic process	Nucleobase-containing compound metabo
N20270	Caenorhabditis briggsae Hypothetical protein CBG16623 (CBG16623) mRNA, complet	Metabolic process	Nucleobase-containing compound metabo
N20569	glycinamide ribonucleotide synthetase-aminoimidazole ribonucleotide synthetase-gl	Metabolic process	Nucleobase-containing compound metabo
N21697		Metabolic process	Nucleobase-containing compound metabo
N24180	hypothetical protein DAPPUDRAFT_305695 [Daphnia pulex]	Metabolic process	Nucleobase-containing compound metabo
N25611	hypothetical protein DAPPUDRAFT_202914 [Daphnia pulex]	Metabolic process	Nucleobase-containing compound metabo
N29027	hypothetical protein BRAFLDRAFT_116233 [Branchiostoma floridae] >gi|229290860|	Metabolic process	Nucleobase-containing compound metabo
N30083	PREDICTED: adenylate cyclase type 9-like [Megachile rotundata]	Metabolic process	Nucleobase-containing compound metabo
N39678	hypothetical protein DAPPUDRAFT_230475 [Daphnia pulex]	Metabolic process	Nucleobase-containing compound metabo
N43051	hypothetical protein BRAFLDRAFT_129513 [Branchiostoma floridae] >gi|229291355|	Metabolic process	Nucleobase-containing compound metabo
N44520	PREDICTED: exonuclease 3'-5' domain-containing protein 2 [Condylura cristata]	Metabolic process	Nucleobase-containing compound metabo
N52606	Inosine triphosphate pyrophosphatase [Salmo salar] >gi|197632311|gb|ACH70879.1	Metabolic process	Nucleobase-containing compound metabo
N60051	adenylsulfate kinase, putative [Ixodes scapularis] >gi|215491529|gb|EEC01170.1| ac	Metabolic process	Nucleobase-containing compound metabo
N7920	sodium potassium-transporting ATPase subunit beta [Litopenaeus vannamei]	Metabolic process	Nucleobase-containing compound metabo
N9290	hypothetical protein DAPPUDRAFT_331571 [Daphnia pulex]	Metabolic process	Nucleobase-containing compound metabo
E1475	guanine nucleotide-binding protein, putative [Ixodes scapularis] >gi|215507555|gb|E	Metabolic process	Other
E4140	unknown [Dendroctonus ponderosae]	Metabolic process	Other
M101952	PREDICTED: long-chain-fatty-acid--CoA ligase 1-like [Megachile rotundata]	Metabolic process	Other
M103670	hypothetical protein BRAFLDRAFT_126836 [Branchiostoma floridae] >gi|229295634|	Metabolic process	Other
M108336	protein kinase C and casein kinase substrate in neurons protein, putative [Pediculus h	Metabolic process	Other
M108370	hypothetical protein BRAFLDRAFT_89744 [Branchiostoma floridae] >gi|229273995|g	Metabolic process	Other
M11883	PREDICTED: acyl-CoA synthetase family member 3, mitochondrial-like isoform X1 [Ap	Metabolic process	Other
M1220	PREDICTED: methylenetetrahydrofolate dehydrogenase 1-like [Saccoglossus kowalev	Metabolic process	Other
M12227	PREDICTED: cytochrome c-type heme lyase-like [Strongylocentrotus purpuratus]	Metabolic process	Other
M12718	PREDICTED: WD repeat domain-containing protein 83-like [Strongylocentrotus purpu	Metabolic process	Other
M1473	PREDICTED: long-chain fatty acid transport protein 4-like [Aplysia californica]	Metabolic process	Other
M14744	PREDICTED: similar to mandelate racemase [Tribolium castaneum] >gi|270013803|g	Metabolic process	Other
M16328	hypothetical protein CAPTEDRAFT_176070 [Capitella teleta]	Metabolic process	Other
M1672	hypothetical protein DAPPUDRAFT_51170 [Daphnia pulex]	Metabolic process	Other
M1775	hypothetical protein DAPPUDRAFT_200081 [Daphnia pulex]	Metabolic process	Other
M18842	PREDICTED: similar to AGAP004744-PA [Tribolium castaneum]	Metabolic process	Other
M33255	hypothetical protein BRAFLDRAFT_120483 [Branchiostoma floridae] >gi|229282046|	Metabolic process	Other
M3693	WD-repeat protein, putative [Ixodes scapularis] >gi|215505822|gb|EEC15316.1| WD	Metabolic process	Other
M3796	hypothetical protein DAPPUDRAFT_299821 [Daphnia pulex]	Metabolic process	Other
M383	PREDICTED: similar to tan CG12120-PA [Tribolium castaneum] >gi|270004130|gb|EF	Metabolic process	Other
M4676	SEL-1, putative [Ixodes scapularis] >gi|215509773|gb|EEC19226.1| SEL-1, putative [I	Metabolic process	Other
M5130	hypothetical protein AaeL_AAEL008679 [Aedes aegypti] >gi|122105719|sp|Q16Y34.	Metabolic process	Other
M58016	Diacylglycerol O-acyltransferase, putative [Pediculus humanus corporis] >gi|2125152	Metabolic process	Other
M586	Cytosolic Fe-S cluster assembly factor NUBP1-like protein [Crassostrea gigas]	Metabolic process	Other
M58842	hypothetical protein DAPPUDRAFT_306312 [Daphnia pulex]	Metabolic process	Other
M652	hypothetical protein DAPPUDRAFT_303807 [Daphnia pulex]	Metabolic process	Other
M65529	putative serine palmitoyltransferase [Hottentotta judaicus]	Metabolic process	Other
M69422		Metabolic process	Other

ID	Annotation	GO term	Go child term
M72726	hypothetical protein CAPTEDRAFT_161927 [Capitella teleta]	Metabolic process	Other
M78789	RB13-6 antigen, putative [Ixodes scapularis] >gi|215507372|gb|EEC16864.1| RB13-6	Metabolic process	Other
M848	PREDICTED: molybdopterin synthase catalytic subunit-like [Amphimedon queensland	Metabolic process	Other
M93902	PREDICTED: UDP-N-acetylhexosamine pyrophosphorylase-like [Metaseiulus occidenta	Metabolic process	Other
M941	hypothetical protein BRAFLDRAFT_128896 [Branchiostoma floridae] >gi|229297143|	Metabolic process	Other
N10610	hypothetical protein DAPPUDRAFT_221341 [Daphnia pulex]	Metabolic process	Other
N11132	aldehyde-activating protein [Rhizobium]	Metabolic process	Other
N11504	fatty acid synthase [Litopenaeus vannamei]	Metabolic process	Other
N11532	GMP synthase, putative [Pediculus humanus corporis] >gi|212512030|gb|EEB14877.	Metabolic process	Other
N11915	hypothetical protein DAPPUDRAFT_203975 [Daphnia pulex]	Metabolic process	Other
N13137	hypothetical protein BRAFLDRAFT_118764 [Branchiostoma floridae] >gi|229298782|	Metabolic process	Other
N13439	3-hydroxybutyrate dehydrogenase [Branchiostoma floridae] >gi|229297934|gb|EEN	Metabolic process	Other
N18287	PREDICTED: long-chain-fatty-acid--CoA ligase 3-like [Megachile rotundata]	Metabolic process	Other
N18322	JHE-like carboxylesterase 1 [Pandalopsis japonica]	Metabolic process	Other
N18351	Histone acetyltransferase KAT2B [Crassostrea gigas]	Metabolic process	Other
N18659	tetratricopeptide repeat protein, putative [Ixodes scapularis] >gi|215491239|gb|EEC	Metabolic process	Other
N18686		Metabolic process	Other
N19473	putative hemomucin [Schistocerca gregaria]	Metabolic process	Other
N19692		Metabolic process	Other
N19981	PREDICTED: similar to glucosyl/glucuronosyl transferases [Tribolium castaneum]	Metabolic process	Other
N19988	hypothetical protein DAPPUDRAFT_190063 [Daphnia pulex]	Metabolic process	Other
N20264	PREDICTED: tissue specific transplantation antigen P35B-like [Saccoglossus kowalevsk	Metabolic process	Other
N20465	glucosyl/glucuronosyl transferases [Aedes aegypti] >gi|108881419|gb|EAT45644.1| .	Metabolic process	Other
N20470	PREDICTED: 1-acyl-sn-glycerol-3-phosphate acyltransferase gamma-like [Nasonia vitri	Metabolic process	Other
N20529	gem-associated protein, putative [Ixodes scapularis] >gi|215498896|gb|EEC08390.1|	Metabolic process	Other
N20590	Phosphotriesterase-related protein [Crassostrea gigas]	Metabolic process	Other
N21023	hypothetical protein [alpha proteobacterium SCGC AAA015-N04]	Metabolic process	Other
N21249	hypothetical protein DAPPUDRAFT_97952 [Daphnia pulex]	Metabolic process	Other
N21899	hypothetical protein DAPPUDRAFT_302220 [Daphnia pulex]	Metabolic process	Other
N22108	hypothetical protein DAPPUDRAFT_306348 [Daphnia pulex]	Metabolic process	Other
N22272	hypothetical protein DAPPUDRAFT_231053 [Daphnia pulex]	Metabolic process	Other
N22649	hypothetical protein DAPPUDRAFT_327533 [Daphnia pulex]	Metabolic process	Other
N22872	hypothetical protein DAPPUDRAFT_210509 [Daphnia pulex]	Metabolic process	Other
N23265	PREDICTED: hypothetical protein [Saccoglossus kowalevskii]	Metabolic process	Other
N25112	PREDICTED: uncharacterized protein LOC100877790 [Megachile rotundata]	Metabolic process	Other
N25254	hypothetical protein TRIADDRAFT_56199 [Trichoplax adhaerens] >gi|190584349|gb|	Metabolic process	Other
N25668	PREDICTED: molybdenum cofactor biosynthesis protein 1 isoform X1 [Cavia porcellus	Metabolic process	Other
N26353	hypothetical protein BRAFLDRAFT_101015 [Branchiostoma floridae] >gi|229290895|	Metabolic process	Other
N26424	hypothetical protein DAPPUDRAFT_307317 [Daphnia pulex]	Metabolic process	Other
N27420	PREDICTED: methylglutaconyl-CoA hydratase, mitochondrial-like [Ceratitis capitata]	Metabolic process	Other
N29868	alkaline phosphatase, partial [Macrobrachium rosenbergii]	Metabolic process	Other
N30129		Metabolic process	Other
N32142	tRNA 2-thiocytidine biosynthesis protein ttcA [Lepeophtheirus salmonis]	Metabolic process	Other
N34674	PREDICTED: hypothetical protein LOC100117960 [Nasonia vitripennis]	Metabolic process	Other
N40837	hypothetical protein BRAFLDRAFT_77131 [Branchiostoma floridae] >gi|229295502|g	Metabolic process	Other
N41594	hypothetical protein TRIADDRAFT_14880 [Trichoplax adhaerens] >gi|190586970|gb|	Metabolic process	Other
N49965	hypothetical protein BRAFLDRAFT_126580 [Branchiostoma floridae] >gi|229272947|	Metabolic process	Other
N50248		Metabolic process	Other
N50663	hypothetical protein DAPPUDRAFT_316454 [Daphnia pulex]	Metabolic process	Other
N51392	PREDICTED: acyl-CoA synthetase short-chain family member 1-like [Saccoglossus kow	Metabolic process	Other
N57795	nitric oxide synthase [Litopenaeus vannamei]	Metabolic process	Other
N60366	hypothetical protein EAI_00199 [Harpegnathos saltator]	Metabolic process	Other
N7968	PREDICTED: Caenorhabditis EpoxidE Hydrolase family member (ceeh-1)-like [Saccoglc	Metabolic process	Other
N9043	PREDICTED: inositol hexakisphosphate and diphosphoinositol-pentakisphosphate kina	Metabolic process	Other
E3439	Xanthine dehydrogenase/oxidase [Salmo salar]	Metabolic process	Oxidation-reduction process
E3651	extracellular superoxide dismutase precursor [Pacifastacus leniusculus]	Metabolic process	Oxidation-reduction process
M111084	Euphausia superba mitochondrion, partial genome	Metabolic process	Oxidation-reduction process
M13711	extracellular superoxide dismutase precursor [Pacifastacus leniusculus]	Metabolic process	Oxidation-reduction process
M18084	Alkylated DNA repair protein alkB-like protein 8 [Camponotus floridanus]	Metabolic process	Oxidation-reduction process
M19061	PREDICTED: Saccoglossus kowalevskii NADH dehydrogenase iron-sulfur protein 8, mit	Metabolic process	Oxidation-reduction process
M19104		Metabolic process	Oxidation-reduction process
M1958	hypothetical protein DAPPUDRAFT_315212 [Daphnia pulex]	Metabolic process	Oxidation-reduction process
M21476	protein disulfide isomerase [Litopenaeus vannamei]	Metabolic process	Oxidation-reduction process
M33821	ubiquitin [Schizosaccharomyces cryophilus OY26]	Metabolic process	Oxidation-reduction process
M37562		Metabolic process	Oxidation-reduction process
M4807	carbonyl reductase [Daphnia pulex]	Metabolic process	Oxidation-reduction process
M5066	hypothetical protein DAPPUDRAFT_304354 [Daphnia pulex]	Metabolic process	Oxidation-reduction process
M56300	hypothetical protein DAPPUDRAFT_306714 [Daphnia pulex]	Metabolic process	Oxidation-reduction process
M57279	PREDICTED: dehydrogenase/reductase SDR family member 11 [Pseudopodoces humil	Metabolic process	Oxidation-reduction process
M58100	hypothetical protein DAPPUDRAFT_202746 [Daphnia pulex]	Metabolic process	Oxidation-reduction process
M61083	extracellular superoxide dismutase precursor [Pacifastacus leniusculus]	Metabolic process	Oxidation-reduction process
M62474	cytochrome P450 [Litopenaeus vannamei]	Metabolic process	Oxidation-reduction process
M65385	hypothetical protein DAPPUDRAFT_304184 [Daphnia pulex]	Metabolic process	Oxidation-reduction process
M66353	hypothetical protein DAPPUDRAFT_199005 [Daphnia pulex]	Metabolic process	Oxidation-reduction process
M73880	thromboxane A synthase [Penaeus monodon]	Metabolic process	Oxidation-reduction process
M80754	glyceraldehyde-3-phosphate dehydrogenase [Rimicaris exoculata]	Metabolic process	Oxidation-reduction process

ID	Annotation	GO term	Go child term
M83059	PREDICTED: cytochrome P450 2J2-like isoform 2 [Meleagris gallopavo]	Metabolic process	Oxidation-reduction process
M9052	Retinol dehydrogenase 11 [Camponotus floridanus]	Metabolic process	Oxidation-reduction process
M92656	hypothetical protein TRIADDRAFT_54215 [Trichoplax adhaerens] >gi|190586282|gb|	Metabolic process	Oxidation-reduction process
M99381	hypothetical protein DAPPUDRAFT_251911 [Daphnia pulex]	Metabolic process	Oxidation-reduction process
N10515	hypothetical protein TcasGA2_TC014229 [Tribolium castaneum]	Metabolic process	Oxidation-reduction process
N10730	PREDICTED: Saccoglossus kowalevskii NADH dehydrogenase iron-sulfur protein 8, mit	Metabolic process	Oxidation-reduction process
N10779	peroxiredoxin [Litopenaeus vannamei]	Metabolic process	Oxidation-reduction process
N11974	hypothetical protein CAPTEDRAFT_167094 [Capitella teleta]	Metabolic process	Oxidation-reduction process
N12985	hypothetical protein DAPPUDRAFT_188248 [Daphnia pulex]	Metabolic process	Oxidation-reduction process
N13222	hypothetical protein DAPPUDRAFT_50786 [Daphnia pulex]	Metabolic process	Oxidation-reduction process
N16890	thromboxane A synthase [Penaeus monodon]	Metabolic process	Oxidation-reduction process
N18385		Metabolic process	Oxidation-reduction process
N18632		Metabolic process	Oxidation-reduction process
N18655		Metabolic process	Oxidation-reduction process
N18760	protein disulfide isomerase A6 [Penaeus monodon]	Metabolic process	Oxidation-reduction process
N19097	Anoplopoma fimbria clone afim-evh-016-151 NADH dehydrogenase iron-sulfur protei	Metabolic process	Oxidation-reduction process
N19130	PREDICTED: acyl-Coenzyme A dehydrogenase, C-4 to C-12 straight chain-like [Saccogl	Metabolic process	Oxidation-reduction process
N19275	hypothetical protein DAPPUDRAFT_188248 [Daphnia pulex]	Metabolic process	Oxidation-reduction process
N21156	hypothetical protein DAPPUDRAFT_330720 [Daphnia pulex]	Metabolic process	Oxidation-reduction process
N21406	cytochrome P450 [Bemisia tabaci]	Metabolic process	Oxidation-reduction process
N21510	senecionine N-oxygenase, putative [Pediculus humanus corporis] >gi|212515735|gb|	Metabolic process	Oxidation-reduction process
N22381	AAEL017133-PA [Aedes aegypti]	Metabolic process	Oxidation-reduction process
N22914	CREG1 precursor [Caligus clemensi]	Metabolic process	Oxidation-reduction process
N23050	hypothetical protein BRAFLDRAFT_129234 [Branchiostoma floridae] >gi|229295408|	Metabolic process	Oxidation-reduction process
N24172	hypothetical protein DAPPUDRAFT_313254 [Daphnia pulex]	Metabolic process	Oxidation-reduction process
N26026	hypothetical protein SINV_06374 [Solenopsis invicta]	Metabolic process	Oxidation-reduction process
N28175	flavonol reductase/cinnamoyl-CoA reductase, putative [Ixodes scapularis] >gi|215497	Metabolic process	Oxidation-reduction process
N31519	aankyrin [Allobaculum stercoricanis]	Metabolic process	Oxidation-reduction process
N32458	Allene oxide synthase-lipoxygenase protein [Crassostrea gigas]	Metabolic process	Oxidation-reduction process
N34035	pacifastin heavy chain precursor [Pacifastacus leniusculus]	Metabolic process	Oxidation-reduction process
N39784	hypothetical protein DAPPUDRAFT_315087 [Daphnia pulex]	Metabolic process	Oxidation-reduction process
N40545	DnaJ-like protein subfamily C member 16 [Harpegnathos saltator]	Metabolic process	Oxidation-reduction process
N43458	hypothetical protein CAPTEDRAFT_178407 [Capitella teleta]	Metabolic process	Oxidation-reduction process
N44626	PREDICTED: similar to AGAP003652-PA [Tribolium castaneum] >gi|270002022|gb|EE	Metabolic process	Oxidation-reduction process
N45271	hypothetical protein DAPPUDRAFT_300978 [Daphnia pulex]	Metabolic process	Oxidation-reduction process
N46236	hypothetical protein BRAFLDRAFT_68404 [Branchiostoma floridae] >gi|229298781|g	Metabolic process	Oxidation-reduction process
N46564	hypothetical protein BRAFLDRAFT_202728 [Branchiostoma floridae] >gi|229295985|	Metabolic process	Oxidation-reduction process
N5097	protein disulfide isomerase 2 [Fenneropenaeus chinensis]	Metabolic process	Oxidation-reduction process
N59367	hypothetical protein DAPPUDRAFT_98658 [Daphnia pulex]	Metabolic process	Oxidation-reduction process
N59550	PREDICTED: thioredoxin domain containing like [Saccoglossus kowalevskii]	Metabolic process	Oxidation-reduction process
N6078	protaglandin reductase 1 [Penaeus monodon]	Metabolic process	Oxidation-reduction process
N9128	Trx1 [Eriocheir sinensis]	Metabolic process	Oxidation-reduction process
N9447	PREDICTED: Bombus impatiens glutamate dehydrogenase, mitochondrial-like (LOC10	Metabolic process	Oxidation-reduction process
N9461	PREDICTED: magnesium transporter protein 1-like [Nasonia vitripennis]	Metabolic process	Oxidation-reduction process

Nucleic acid metabolism

M61659	three-prime repair exonuclease 1 [Phascolarctos cinereus]	Nucleic acid metabolism	DNA metabolic process
N10823		Nucleic acid metabolism	DNA metabolic process
N12689	PREDICTED: Taeniopygia guttata topoisomerase (DNA) II beta 180kDa (TOP2B), mRNA	Nucleic acid metabolism	DNA metabolic process
N18165	hypothetical protein D910_02572 [Dendroctonus ponderosae]	Nucleic acid metabolism	DNA metabolic process
M2033	PREDICTED: DNA polymerase V-like [Nasonia vitripennis]	Nucleic acid metabolism	DNA replication
M6756	hypothetical protein DAPPUDRAFT_227571 [Daphnia pulex]	Nucleic acid metabolism	DNA replication
M85198	FI21229p1 [Drosophila melanogaster]	Nucleic acid metabolism	DNA replication
N11858	hypothetical protein YQE_03506, partial [Dendroctonus ponderosae]	Nucleic acid metabolism	DNA replication
N19216	hypothetical protein BRAFLDRAFT_113525 [Branchiostoma floridae] >gi|229296827|	Nucleic acid metabolism	DNA replication
N40254	predicted protein [Nematostella vectensis] >gi|156221198|gb|EDO42056.1| predict	Nucleic acid metabolism	DNA replication
M10621	PREDICTED: zinc finger protein 782-like [Sus scrofa]	Nucleic acid metabolism	Regulation of gene expression
M11678	CREB-binding protein [Zootermopsis nevadensis]	Nucleic acid metabolism	Regulation of gene expression
M2134	conserved hypothetical protein [Pediculus humanus corporis] >gi|212510168|gb|EEI	Nucleic acid metabolism	Regulation of gene expression
M55626	conserved hypothetical protein [Pediculus humanus corporis] >gi|212508735|gb|EEI	Nucleic acid metabolism	Regulation of gene expression
M5576	conserved hypothetical protein [Pediculus humanus corporis] >gi|212511742|gb|EEI	Nucleic acid metabolism	Regulation of gene expression
M600	PREDICTED: similar to histidine triad protein member [Tribolium castaneum]	Nucleic acid metabolism	Regulation of gene expression
M7241	PREDICTED: zinc finger protein 358-like, partial [Ornithorhynchus anatinus]	Nucleic acid metabolism	Regulation of gene expression
N12903		Nucleic acid metabolism	Regulation of gene expression
N17291	Zinc finger protein 84 [Tupaia chinensis]	Nucleic acid metabolism	Regulation of gene expression
N18441	PREDICTED: LOW QUALITY PROTEIN: ets DNA-binding protein pokkuri-like [Nasonia vi	Nucleic acid metabolism	Regulation of gene expression
N18444	LIM class homeobox transcription factor Lmx [Mnemiopsis leidyi]	Nucleic acid metabolism	Regulation of gene expression
N19004	PREDICTED: similar to AGAP009141-PA [Tribolium castaneum] >gi|270005569|gb|EF	Nucleic acid metabolism	Regulation of gene expression
N19425		Nucleic acid metabolism	Regulation of gene expression
N20174		Nucleic acid metabolism	Regulation of gene expression
N22626	Cyclic AMP-dependent transcription factor ATF-6 beta, putative [Pediculus humanus (	Nucleic acid metabolism	Regulation of gene expression
N28291	PREDICTED: zinc finger protein 600 isoform X1 [Homo sapiens] >gi|119592502|gb|EA	Nucleic acid metabolism	Regulation of gene expression
N28717	protein groucho, putative [Pediculus humanus corporis] >gi|212511585|gb|EEB1451	Nucleic acid metabolism	Regulation of gene expression
N31082	Protein bunched, class 2 isoform [Camponotus floridanus]	Nucleic acid metabolism	Regulation of gene expression
N34881	PREDICTED: transcriptional enhancer factor TEF-1 isoform X3 [Chinchilla lanigera]	Nucleic acid metabolism	Regulation of gene expression
N37283	PREDICTED: zinc finger protein Xfin-like [Strongylocentrotus purpuratus]	Nucleic acid metabolism	Regulation of gene expression
N39148	ETS-related transcription factor Elf-3 [Crassostrea gigas]	Nucleic acid metabolism	Regulation of gene expression

ID	Annotation	GO term	Go child term
N53807	ETS factor [Trichinella spiralis] >gi|316978761|gb|EFV61692.1| ETS factor [Trichinella	Nucleic acid metabolism	Regulation of gene expression
N60094	RecName: Full=Zinc finger Y-chromosomal protein 1; Short=ZFY-1 >gi|65269|emb|CA	Nucleic acid metabolism	Regulation of gene expression
N8399		Nucleic acid metabolism	Regulation of gene expression
M121	PREDICTED: hypothetical protein LOC100122935 [Nasonia vitripennis]	Nucleic acid metabolism	Regulation of gene expression, epigenetic
M1844	PREDICTED: mediator of RNA polymerase II transcription subunit 8-like [Hyalella azteca]	Nucleic acid metabolism	RNA metabolic process
M20226		Nucleic acid metabolism	RNA metabolic process
M2517	hypothetical protein DAPPUDRAFT_191515 [Daphnia pulex]	Nucleic acid metabolism	RNA metabolic process
M338	PREDICTED: pre-rRNA-processing protein TSR2 homolog [Aplysia californica]	Nucleic acid metabolism	RNA metabolic process
M4435	hypothetical protein CAPTEDRAFT_220697 [Capitella teleta]	Nucleic acid metabolism	RNA metabolic process
M5050	hypothetical protein DAPPUDRAFT_52884 [Daphnia pulex]	Nucleic acid metabolism	RNA metabolic process
M56294	PREDICTED: CDK5RAP1-like protein-like [Apis florea]	Nucleic acid metabolism	RNA metabolic process
M61831	Protein suppressor of forked [Camponotus floridanus]	Nucleic acid metabolism	RNA metabolic process
M64830	PREDICTED: similar to spliceosome associated protein [Tribolium castaneum] >gi|270	Nucleic acid metabolism	RNA metabolic process
M66915	PREDICTED: ATP-dependent RNA helicase DDX39A-like [Monodelphis domestica]	Nucleic acid metabolism	RNA metabolic process
N10539		Nucleic acid metabolism	RNA metabolic process
N12725	eukaryotic translation initiation factor 4 gamma, putative [Pediculus humanus corpor	Nucleic acid metabolism	RNA metabolic process
N12797		Nucleic acid metabolism	RNA metabolic process
N12849	hypothetical protein DAPPUDRAFT_198197 [Daphnia pulex]	Nucleic acid metabolism	RNA metabolic process
N13217	hypothetical protein DAPPUDRAFT_331348 [Daphnia pulex]	Nucleic acid metabolism	RNA metabolic process
N13861	conserved hypothetical protein [Pediculus humanus corporis] >gi|212508823|gb|EEI	Nucleic acid metabolism	RNA metabolic process
N15767	hypothetical protein DAPPUDRAFT_220519 [Daphnia pulex]	Nucleic acid metabolism	RNA metabolic process
N18079	PREDICTED: transcription elongation factor SPT6-like [Megachile rotundata]	Nucleic acid metabolism	RNA metabolic process
N18176	RRP5-like protein [Crassostrea gigas]	Nucleic acid metabolism	RNA metabolic process
N18306		Nucleic acid metabolism	RNA metabolic process
N18313		Nucleic acid metabolism	RNA metabolic process
N18423	PREDICTED: putative rRNA methyltransferase 3-like [Nasonia vitripennis]	Nucleic acid metabolism	RNA metabolic process
N18993	Acyrthosiphon pisum protein arginine n-methyltransferase 1, putative (LOC10016585	Nucleic acid metabolism	RNA metabolic process
N19013	hypothetical protein DAPPUDRAFT_306325 [Daphnia pulex]	Nucleic acid metabolism	RNA metabolic process
N19354	Leukocyte receptor cluster member 9 [Caligus rogercresseyi]	Nucleic acid metabolism	RNA metabolic process
N19375	Nematostella vectensis predicted protein (NEMVEDRAFT_v1g185061) partial mRNA	Nucleic acid metabolism	RNA metabolic process
N19697	PREDICTED: poly(A)-specific ribonuclease PARN isoform X1 [Danio rerio]	Nucleic acid metabolism	RNA metabolic process
N19884	hypothetical protein DAPPUDRAFT_300423 [Daphnia pulex]	Nucleic acid metabolism	RNA metabolic process
N20041	conserved hypothetical protein [Pediculus humanus corporis] >gi|212517197|gb|EEI	Nucleic acid metabolism	RNA metabolic process
N20316	hypothetical protein BRAFLDRAFT_276703 [Branchiostoma floridae] >gi|229285781|	Nucleic acid metabolism	RNA metabolic process
N21545	Nucleolar protein 66 [Crassostrea gigas]	Nucleic acid metabolism	RNA metabolic process
N22415	PREDICTED: transcription elongation factor SPT5-like [Megachile rotundata]	Nucleic acid metabolism	RNA metabolic process
N23046	hypothetical protein BRAFLDRAFT_201261 [Branchiostoma floridae] >gi|229279415|	Nucleic acid metabolism	RNA metabolic process
N23545	mediator of RNA polymerase II transcription subunit 15 [Zootermopsis nevadensis]	Nucleic acid metabolism	RNA metabolic process
N23615	hypothetical protein BRAFLDRAFT_111011 [Branchiostoma floridae] >gi|229270902|	Nucleic acid metabolism	RNA metabolic process
N24228	PREDICTED: elongator complex protein 4-like [Aplysia californica]	Nucleic acid metabolism	RNA metabolic process
N27329	hypothetical protein TcasGA2_TC015113 [Tribolium castaneum]	Nucleic acid metabolism	RNA metabolic process
N27855	PREDICTED: mediator of RNA polymerase II transcription subunit 19-like isoform 1 [N	Nucleic acid metabolism	RNA metabolic process
N29611	PREDICTED: WD repeat-containing protein 36 [Trichechus manatus latirostris]	Nucleic acid metabolism	RNA metabolic process
N30373		Nucleic acid metabolism	RNA metabolic process
N33717	PREDICTED: CCR4-NOT transcription complex, subunit 1-like [Saccoglossus kowalevsk	Nucleic acid metabolism	RNA metabolic process
N35076	PREDICTED: hypothetical protein LOC100679578 [Nasonia vitripennis]	Nucleic acid metabolism	RNA metabolic process
N38517	hypothetical protein DAPPUDRAFT_304474 [Daphnia pulex]	Nucleic acid metabolism	RNA metabolic process
N43700	Ribonuclease P/MRP protein subunit POP5 [Camponotus floridanus]	Nucleic acid metabolism	RNA metabolic process
N45734	PREDICTED: probable methyltransferase C20orf7 homolog, mitochondrial-like isoforn	Nucleic acid metabolism	RNA metabolic process
N46206	PREDICTED: RNA-binding protein 28 [Sarcophilus harrisii]	Nucleic acid metabolism	RNA metabolic process
N47138	conserved hypothetical protein [Pediculus humanus corporis] >gi|212510210|gb|EEI	Nucleic acid metabolism	RNA metabolic process
N49298	hypothetical protein CAPTEDRAFT_135802, partial [Capitella teleta]	Nucleic acid metabolism	RNA metabolic process
N61350	PREDICTED: alanyl-tRNA editing protein Aarsd1 [Sarcophilus harrisii]	Nucleic acid metabolism	RNA metabolic process
N7721	PREDICTED: RNA-binding protein 39-like isoform 1 [Megachile rotundata]	Nucleic acid metabolism	RNA metabolic process
M16005	DNA-directed RNA polymerase I subunit RPA1 [Crassostrea gigas]	Nucleic acid metabolism	Transcription, DNA-dependent
M67221	hypothetical protein DAPPUDRAFT_209553 [Daphnia pulex]	Nucleic acid metabolism	Transcription, DNA-dependent
N21123	PREDICTED: DNA-directed RNA polymerase I subunit RPA43-like [Nasonia vitripennis]	Nucleic acid metabolism	Transcription, DNA-dependent
N28651	hypothetical protein BRAFLDRAFT_117854 [Branchiostoma floridae] >gi|229295938|	Nucleic acid metabolism	Transcription, DNA-dependent

Protein metabolism

M112647	hypothetical protein DAPPUDRAFT_51930 [Daphnia pulex]	Protein metabolism	Cellular protein modification process
M15039	Ixodes scapularis traf2 and nck interacting kinase, tnik, putative, mRNA	Protein metabolism	Cellular protein modification process
M15628	PREDICTED: hypothetical protein LOC100118283 [Nasonia vitripennis]	Protein metabolism	Cellular protein modification process
M16593	PREDICTED: dual specificity protein phosphatase CDC14A isoform 3 [Trichechus mana	Protein metabolism	Cellular protein modification process
M1798	hypothetical protein DAPPUDRAFT_188007 [Daphnia pulex]	Protein metabolism	Cellular protein modification process
M181	PREDICTED: polyserase-2-like [Acyrthosiphon pisum]	Protein metabolism	Cellular protein modification process
M2135	protein phosphatase 2 (formerly 2A), catalytic subunit [Strigomonas culicis]	Protein metabolism	Cellular protein modification process
M21763		Protein metabolism	Cellular protein modification process
M2429	PREDICTED: uncharacterized protein LOC100879681 [Megachile rotundata]	Protein metabolism	Cellular protein modification process
M5442	PREDICTED: dual specificity protein phosphatase 12-like [Nasonia vitripennis]	Protein metabolism	Cellular protein modification process
M59191	projectin [Procambarus clarkii]	Protein metabolism	Cellular protein modification process
M59923	hypothetical protein BRAFLDRAFT_63881 [Branchiostoma floridae] >gi|229296855|g	Protein metabolism	Cellular protein modification process
M619	PREDICTED: cyclin-dependent kinase 7-like [Megachile rotundata]	Protein metabolism	Cellular protein modification process
M62125	phosphorylase kinase gamma subunit, putative [Ixodes scapularis] >gi|215491336|gb	Protein metabolism	Cellular protein modification process
M62372	PREDICTED: endothelin-converting enzyme 2 [Ciona intestinalis]	Protein metabolism	Cellular protein modification process
M63916	PREDICTED: p90 ribosomal S6 kinase-like [Saccoglossus kowalevskii]	Protein metabolism	Cellular protein modification process
M66217	Diphthamide biosynthesis protein 2 [Dicentrarchus labrax]	Protein metabolism	Cellular protein modification process

ID	Annotation	GO term	Go child term
M66985	guanylate cyclase PcGC-M2 precursor [Procambarus clarkii]	Protein metabolism	Cellular protein modification process
M683	sumo-1-activating enzyme E1a, putative [Pediculus humanus corporis] >gi|21251422	Protein metabolism	Cellular protein modification process
M68569	hypothetical protein YQE_02518, partial [Dendroctonus ponderosae]	Protein metabolism	Cellular protein modification process
M7056	hypothetical protein DAPPUDRAFT_305261 [Daphnia pulex]	Protein metabolism	Cellular protein modification process
M72356		Protein metabolism	Cellular protein modification process
M8240	PITSLRE serine/threonine-protein kinase CDC2L1 [Camponotus floridanus]	Protein metabolism	Cellular protein modification process
M9345	hypothetical protein DAPPUDRAFT_52407 [Daphnia pulex]	Protein metabolism	Cellular protein modification process
M96600	AGAP011030-PA [Anopheles gambiae str. PEST] >gi|157019323|gb|EAL41443.3| AG	Protein metabolism	Cellular protein modification process
M97294	PREDICTED: serine/threonine-protein kinase Warts-like [Nasonia vitripennis]	Protein metabolism	Cellular protein modification process
M99514	hypothetical protein CAPTEDRAFT_166676 [Capitella teleta]	Protein metabolism	Cellular protein modification process
M9972	zinc metalloproteinase, putative [Pediculus humanus corporis] >gi|212510824|gb|EE	Protein metabolism	Cellular protein modification process
N11822		Protein metabolism	Cellular protein modification process
N11830		Protein metabolism	Cellular protein modification process
N11977	PREDICTED: protein phosphatase 1B-like [Saccoglossus kowalevskii]	Protein metabolism	Cellular protein modification process
N11990	PREDICTED: similar to CG1973 CG1973-PA [Tribolium castaneum]	Protein metabolism	Cellular protein modification process
N12755	PREDICTED: similar to transglutaminase [Tribolium castaneum] >gi|270007058|gb|EI	Protein metabolism	Cellular protein modification process
N12811		Protein metabolism	Cellular protein modification process
N13007	checkpoint kinase 1 [Daphnia pulex]	Protein metabolism	Cellular protein modification process
N13499	PREDICTED: uncharacterized protein LOC100883185 isoform 2 [Megachile rotundata]	Protein metabolism	Cellular protein modification process
N13597	signal peptidase complex, subunit SPC25, putative [Ixodes scapularis] >gi|215501946	Protein metabolism	Cellular protein modification process
N13653	hypothetical protein DAPPUDRAFT_319998 [Daphnia pulex]	Protein metabolism	Cellular protein modification process
N18162	PREDICTED: DNA (cytosine-5)-methyltransferase PliMCI-like [Megachile rotundata]	Protein metabolism	Cellular protein modification process
N18208		Protein metabolism	Cellular protein modification process
N18262	PREDICTED: LOW QUALITY PROTEIN: myosin-IIIa [Nasonia vitripennis]	Protein metabolism	Cellular protein modification process
N18288	casein kinase 1 epsilon [Eurydice pulchra]	Protein metabolism	Cellular protein modification process
N18484	hypothetical protein DAPPUDRAFT_305996 [Daphnia pulex]	Protein metabolism	Cellular protein modification process
N18549	PREDICTED: lipoyltransferase 1, mitochondrial-like [Nasonia vitripennis]	Protein metabolism	Cellular protein modification process
N18839	PREDICTED: LOW QUALITY PROTEIN: myosin-IIIa [Nasonia vitripennis]	Protein metabolism	Cellular protein modification process
N18948	PREDICTED: serine/threonine-protein kinase RIO2-like [Megachile rotundata]	Protein metabolism	Cellular protein modification process
N19391	diphthine synthase-like protein [Cricetulus griseus]	Protein metabolism	Cellular protein modification process
N19601	hypothetical protein YQE_12968, partial [Dendroctonus ponderosae]	Protein metabolism	Cellular protein modification process
N19788	cAMP-dependent protein kinase catalytic subunit, putative [Pediculus humanus corp	Protein metabolism	Cellular protein modification process
N20205	hypothetical protein DAPPUDRAFT_301830 [Daphnia pulex]	Protein metabolism	Cellular protein modification process
N20268	PREDICTED: wee1-like protein kinase-like [Nasonia vitripennis]	Protein metabolism	Cellular protein modification process
N21051	hypothetical protein DAPPUDRAFT_305360 [Daphnia pulex]	Protein metabolism	Cellular protein modification process
N22641		Protein metabolism	Cellular protein modification process
N28061	hypothetical protein DAPPUDRAFT_303533 [Daphnia pulex]	Protein metabolism	Cellular protein modification process
N28399	GJ12403 [Drosophila virilis] >gi|194153739|gb|EDW68923.1| GJ12403 [Drosophila v	Protein metabolism	Cellular protein modification process
N28864	leucine-rich transmembrane protein, putative [Pediculus humanus corporis] >gi|212	Protein metabolism	Cellular protein modification process
N30386	PREDICTED: similar to transglutaminase [Tribolium castaneum] >gi|270004163|gb|EI	Protein metabolism	Cellular protein modification process
N32407	protein purity of essence, putative [Pediculus humanus corporis] >gi|212511266|gb|	Protein metabolism	Cellular protein modification process
N32909	hypothetical protein DAPPUDRAFT_39456 [Daphnia pulex]	Protein metabolism	Cellular protein modification process
N33379	PREDICTED: similar to Nedd4 CG7555-PC [Tribolium castaneum]	Protein metabolism	Cellular protein modification process
N34569	PREDICTED: tyrosine-protein kinase Fer [Sorex araneus]	Protein metabolism	Cellular protein modification process
N36697	PREDICTED: dual specificity testis-specific protein kinase 1-like [Chrysemys picta bellii	Protein metabolism	Cellular protein modification process
N38023	hypothetical protein DAPPUDRAFT_300590 [Daphnia pulex]	Protein metabolism	Cellular protein modification process
N38468	PREDICTED: leucine rich repeat containing 7-like, partial [Saccoglossus kowalevskii]	Protein metabolism	Cellular protein modification process
N39683	hypothetical protein DAPPUDRAFT_128092 [Daphnia pulex]	Protein metabolism	Cellular protein modification process
N55712	diphthamide biosynthesis protein 2 [Danio rerio] >gi|160010406|sp|A4QN59.1|DPH	Protein metabolism	Cellular protein modification process
N61180	PREDICTED: hypothetical protein LOC100115038 [Nasonia vitripennis]	Protein metabolism	Cellular protein modification process
M4643	centromere/kinetochore protein zw10 [Xenopus laevis] >gi|159155457|gb|AAI5496	Protein metabolism	Macromolecular complex assembly
N14373	lethal (1) G0136, isoform A [Drosophila melanogaster] >gi|194894350|ref|XP_00197	Protein metabolism	Macromolecular complex assembly
N23242	PREDICTED: similar to inner membrane protein COX18, mitochondrial [Tribolium cast	Protein metabolism	Macromolecular complex assembly
M18846	Drosophila willistoni GK16371 (Dwil\GK16371), mRNA	Protein metabolism	Other
M2290	PREDICTED: similar to bone morphogenetic protein [Tribolium castaneum] >gi|27000	Protein metabolism	Other
M23640		Protein metabolism	Other
M3285		Protein metabolism	Other
M43606	Branchiostoma floridae hypothetical protein, mRNA	Protein metabolism	Other
M4415	salivary plasminogen activator beta precursor, putative [Pediculus humanus corporis]	Protein metabolism	Other
M62299	caspase [Fenneropenaeus chinensis]	Protein metabolism	Other
M6427	RNA recognition motif containing protein [Brugia malayi] >gi|158594962|gb|EDP335	Protein metabolism	Other
M64325	PREDICTED: CG10098-like [Saccoglossus kowalevskii]	Protein metabolism	Other
M6499	PREDICTED: plasma kallikrein [Dasypus novemcinctus]	Protein metabolism	Other
M70652	procollagen-lysine2-oxoglutarate 5-dioxygenase [Schistosoma mansoni] >gi|3600448	Protein metabolism	Other
M7795	prophenoloxidase-activating enzyme 2 [Penaeus monodon]	Protein metabolism	Other
M7880	RecName: Full=Chymotrypsin BI; Flags: Precursor >gi|10089|emb|CAA47046.1| prep	Protein metabolism	Other
M9196		Protein metabolism	Other
M96180	PREDICTED: papilin-like [Aplysia californica]	Protein metabolism	Other
N12301		Protein metabolism	Other
N12905		Protein metabolism	Other
N12959	hypothetical protein DAPPUDRAFT_65345 [Daphnia pulex]	Protein metabolism	Other
N13751	Trichoplax adhaerens conserved hypothetical protein, mRNA	Protein metabolism	Other
N15411	PREDICTED: protein SpAN-like [Strongylocentrotus purpuratus]	Protein metabolism	Other
N19096	hypothetical protein DAPPUDRAFT_307138 [Daphnia pulex]	Protein metabolism	Other
N19304	hypothetical protein DAPPUDRAFT_303947 [Daphnia pulex]	Protein metabolism	Other
N19403	PC2-like protein [Penaeus monodon]	Protein metabolism	Other

ID	Annotation	GO term	Go child term
N19422	hypothetical protein D910_10828 [Dendroctonus ponderosae]	Protein metabolism	Other
N19526	hypothetical protein DAPPUDRAFT_307836 [Daphnia pulex]	Protein metabolism	Other
N19665	hypothetical protein YQE_09353, partial [Dendroctonus ponderosae]	Protein metabolism	Other
N21736	hypothetical protein BRAFLDRAFT_279226 [Branchiostoma floridae] >gi|229281678|	Protein metabolism	Other
N23820	hypothetical protein DAPPUDRAFT_120652 [Daphnia pulex]	Protein metabolism	Other
N24379	hypothetical protein DAPPUDRAFT_306867 [Daphnia pulex]	Protein metabolism	Other
N25394	pro-phenoloxidase activating enzyme-I precursor [Holotrichia diomphalia]	Protein metabolism	Other
N27616	prophenoloxidase activating enzyme [Litopenaeus vannamei]	Protein metabolism	Other
N29559		Protein metabolism	Other
N29592		Protein metabolism	Other
N32282	hypothetical protein CAPTEDRAFT_175182, partial [Capitella teleta]	Protein metabolism	Other
N34957	polyrC binding protein, putative [Pediculus humanus corporis] >gi|212516622|gb|EE	Protein metabolism	Other
N35496	prophenoloxide activating enzyme III [Macrobrachium rosenbergii]	Protein metabolism	Other
N36733	Probable glutamyl-tRNA(Gln) amidotransferase subunit B, mitochondrial [Camponotu	Protein metabolism	Other
N52929		Protein metabolism	Other
N5373	PREDICTED: Nasonia vitripennis 26S protease regulatory subunit 4-like (LOC1001182	Protein metabolism	Other
N6315		Protein metabolism	Other
N8848	trypsinogen 1 [Litopenaeus vannamei]	Protein metabolism	Other
M18705	Portunus trituberculatus heat shock protein 60 (HSP60) mRNA, complete cds	Protein metabolism	Protein folding
M19227	Pediculus humanus corporis Hsp60 protein, putative, mRNA	Protein metabolism	Protein folding
M2216	GrpE protein [Cherax quadricarinatus]	Protein metabolism	Protein folding
M3122	calnexin [Penaeus monodon]	Protein metabolism	Protein folding
M3608	PREDICTED: uncharacterized protein LOC100879476 [Megachile rotundata]	Protein metabolism	Protein folding
M49636	Der f Mal f 6 allergen [Dermatophagoides farinae]	Protein metabolism	Protein folding
M55225	protein disulfide-isomerase TXNDC10 precursor, putative [Pediculus humanus corpor	Protein metabolism	Protein folding
M60388	hypothetical protein DAPPUDRAFT_306744 [Daphnia pulex]	Protein metabolism	Protein folding
M65353	related to FPR2-FK506/rapamycin-binding protein of the ER [Ustilago hordei]	Protein metabolism	Protein folding
M66780		Protein metabolism	Protein folding
M82401	hypothetical protein GUITHDRAFT_83873, partial [Guillardia theta CCMP2712]	Protein metabolism	Protein folding
M84945	hypothetical protein DAPPUDRAFT_299901 [Daphnia pulex]	Protein metabolism	Protein folding
M84968	myosin heavy chain type b [Marsupenaeus japonicus]	Protein metabolism	Protein folding
N11842	heat shock protein 90 [Scylla paramamosain]	Protein metabolism	Protein folding
N11929		Protein metabolism	Protein folding
N13901		Protein metabolism	Protein folding
N13967		Protein metabolism	Protein folding
N18807		Protein metabolism	Protein folding
N19144		Protein metabolism	Protein folding
N20244		Protein metabolism	Protein folding
N22893	hypothetical protein DAPPUDRAFT_299901 [Daphnia pulex]	Protein metabolism	Protein folding
N23884	prefoldin subunit 4-like protein [Coptotermes formosanus]	Protein metabolism	Protein folding
N24711	heat shock protein 10 [Scylla paramamosain]	Protein metabolism	Protein folding
N3034	hypothetical protein [Monosiga brevicollis MX1] >gi|163776367|gb|EDQ89987.1| pr	Protein metabolism	Protein folding
N30735	PREDICTED: peptidyl-prolyl cis-trans isomerase-like 1-like [Ceratitis capitata]	Protein metabolism	Protein folding
N43033	hypothetical protein DAPPUDRAFT_306744 [Daphnia pulex]	Protein metabolism	Protein folding
N54164	serine/threonine-protein phosphatase 2A regulatory subunit B', putative [Pediculus h	Protein metabolism	Protein folding
N5465	Fenneropenaeus chinensis calreticulin precursor (CRT) mRNA, complete cds	Protein metabolism	Protein folding
N6790	chaperonin [Macrobrachium rosenbergii]	Protein metabolism	Protein folding
N8142		Protein metabolism	Protein repair
N8143		Protein metabolism	Protein repair
E4164	RecName: Full=Chymotrypsin BI; Flags: Precursor >gi|10089|emb|CAA47046.1| prep	Protein metabolism	Proteolysis
E4182	GJ23159 [Drosophila virilis] >gi|194148630|gb|EDW64328.1| GJ23159 [Drosophila v	Protein metabolism	Proteolysis
E6184	hypothetical protein THAOC_18267 [Thalassiosira oceanica]	Protein metabolism	Proteolysis
E903	PREDICTED: proteasome subunit alpha type-7-1-like isoform 1 [Apis mellifera] >gi|35	Protein metabolism	Proteolysis
M102676	trypsin 2 [Panulirus argus]	Protein metabolism	Proteolysis
M102744	CUB-serine protease [Panulirus argus]	Protein metabolism	Proteolysis
M102785	PREDICTED: ovochymase-1-like [Ceratitis capitata]	Protein metabolism	Proteolysis
M103525	ubiquitin [Procambarus clarkii]	Protein metabolism	Proteolysis
M103627	hypothetical protein CAPTEDRAFT_195788 [Capitella teleta]	Protein metabolism	Proteolysis
M108894	serine protease [Sinonovacula constricta]	Protein metabolism	Proteolysis
M109778	CUB-serine protease [Panulirus argus]	Protein metabolism	Proteolysis
M111021	CUB-serine protease [Panulirus argus]	Protein metabolism	Proteolysis
M111121	CUB-serine protease [Panulirus argus]	Protein metabolism	Proteolysis
M11130		Protein metabolism	Proteolysis
M112845	Chymotrypsin BII [Litopenaeus vannamei]	Protein metabolism	Proteolysis
M12757	serine proteinase [Portunus trituberculatus]	Protein metabolism	Proteolysis
M12833	serine/threonine-protein kinase VRK1, putative [Pediculus humanus corporis] >gi|21	Protein metabolism	Proteolysis
M15086	GK25063 [Drosophila willistoni] >gi|194167670|gb|EDW82571.1| GK25063 [Drosoph	Protein metabolism	Proteolysis
M1538	nucleotide excision repair protein [Marsupenaeus japonicus]	Protein metabolism	Proteolysis
M15560		Protein metabolism	Proteolysis
M16069	hypothetical protein DICPUDRAFT_97266 [Dictyostelium purpureum] >gi|325083989	Protein metabolism	Proteolysis
M16330	CUB-serine protease [Panulirus argus]	Protein metabolism	Proteolysis
M16754	PREDICTED: similar to ubiquitin specific protease 7 [Tribolium castaneum]	Protein metabolism	Proteolysis
M17359	PREDICTED: membrane metallo-endopeptidase-like 1-like [Metaseiulus occidentalis]	Protein metabolism	Proteolysis
M17428	cathepsin A [Eriocheir sinensis]	Protein metabolism	Proteolysis
M17495	CUB-serine protease [Panulirus argus]	Protein metabolism	Proteolysis
M17539		Protein metabolism	Proteolysis

ID	Annotation	GO term	Go child term
M17621	mitochondrial inner membrane protease subunit [Aedes aegypti] >gi|108883262|gb|	Protein metabolism	Proteolysis
M17735		Protein metabolism	Proteolysis
M18434	CUB-serine protease [Panulirus argus]	Protein metabolism	Proteolysis
M18636	trypsin [Marsupenaeus japonicus]	Protein metabolism	Proteolysis
M19247	hypothetical protein KGM_10936 [Danaus plexippus]	Protein metabolism	Proteolysis
M19563	CUB-serine protease [Panulirus argus]	Protein metabolism	Proteolysis
M19735		Protein metabolism	Proteolysis
M19739	CUB-serine protease [Panulirus argus]	Protein metabolism	Proteolysis
M20312	trypsinogen 1 [Litopenaeus vannamei]	Protein metabolism	Proteolysis
M20539	serine proteinase [Portunus trituberculatus]	Protein metabolism	Proteolysis
M21562	CUB-serine protease [Panulirus argus]	Protein metabolism	Proteolysis
M2230	PREDICTED: metalloproteinase SpAN-like [Saccoglossus kowalevskii]	Protein metabolism	Proteolysis
M22603	hypothetical protein DAPPUDRAFT_305996 [Daphnia pulex]	Protein metabolism	Proteolysis
M22635	hypothetical protein BRAFLDRAFT_71828 [Branchiostoma floridae] >gi|229298917|g	Protein metabolism	Proteolysis
M229		Protein metabolism	Proteolysis
M23518	hypothetical protein BRAFLDRAFT_71828 [Branchiostoma floridae] >gi|229298917|g	Protein metabolism	Proteolysis
M25374	trypsin [Litopenaeus vannamei]	Protein metabolism	Proteolysis
M25636	CUB-serine protease [Panulirus argus]	Protein metabolism	Proteolysis
M28491	CUB-serine protease [Panulirus argus]	Protein metabolism	Proteolysis
M29577	collagenolytic serine protease [Fenneropenaeus chinensis]	Protein metabolism	Proteolysis
M30344	CUB-serine protease [Panulirus argus]	Protein metabolism	Proteolysis
M3153	CUB-serine protease [Panulirus argus]	Protein metabolism	Proteolysis
M3294	hypothetical protein DAPPUDRAFT_316896 [Daphnia pulex]	Protein metabolism	Proteolysis
M35708	GI22815 [Drosophila mojavensis] >gi|193913962|gb|EDW12829.1| GI22815 [Drosop	Protein metabolism	Proteolysis
M3619	CUB-serine protease [Panulirus argus]	Protein metabolism	Proteolysis
M3764	PREDICTED: similar to GA20276-PA [Tribolium castaneum] >gi|270010872|gb|EFA07	Protein metabolism	Proteolysis
M39522	expressed hypothetical protein [Trichoplax adhaerens] >gi|190583179|gb|EDV2325(	Protein metabolism	Proteolysis
M39822	CUB-serine protease [Panulirus argus]	Protein metabolism	Proteolysis
M40617	Procambarus clarkii clone pc-97 ubiquitin mRNA, complete cds	Protein metabolism	Proteolysis
M4163	PREDICTED: aminopeptidase N-like [Nasonia vitripennis]	Protein metabolism	Proteolysis
M44980	CUB-serine protease [Panulirus argus]	Protein metabolism	Proteolysis
M4677	hypothetical protein DAPPUDRAFT_307535 [Daphnia pulex]	Protein metabolism	Proteolysis
M47110	collagenolytic serine protease [Paralithodes camtschaticus]	Protein metabolism	Proteolysis
M55140	CUB-serine protease [Panulirus argus]	Protein metabolism	Proteolysis
M56049	hypothetical protein DAPPUDRAFT_63727 [Daphnia pulex]	Protein metabolism	Proteolysis
M5727	serine protease [Aedes aegypti] >gi|108881966|gb|EAT46191.1| AAEL002595-PA [Ae	Protein metabolism	Proteolysis
M57964	CUB-serine protease [Panulirus argus]	Protein metabolism	Proteolysis
M59149	trypsin [Marsupenaeus japonicus]	Protein metabolism	Proteolysis
M60471	transmembrane serine protease 9, putative [Pediculus humanus corporis] >gi|21251!	Protein metabolism	Proteolysis
M62145	hypothetical protein DAPPUDRAFT_309332 [Daphnia pulex]	Protein metabolism	Proteolysis
M67255	CUB-serine protease [Panulirus argus]	Protein metabolism	Proteolysis
M6843		Protein metabolism	Proteolysis
M71467	cullin-3 [Danio rerio] >gi|37682167|gb|AAQ98010.1| cullin 3 [Danio rerio]	Protein metabolism	Proteolysis
M71612	CUB-serine protease [Panulirus argus]	Protein metabolism	Proteolysis
M73671	trypsinogen 1 [Litopenaeus vannamei]	Protein metabolism	Proteolysis
M75323	hypothetical protein DAPPUDRAFT_303785 [Daphnia pulex]	Protein metabolism	Proteolysis
M75818	CUB-serine protease [Panulirus argus]	Protein metabolism	Proteolysis
M7669		Protein metabolism	Proteolysis
M85248	hypothetical protein DAPPUDRAFT_314716 [Daphnia pulex]	Protein metabolism	Proteolysis
M85980	GL16806 [Drosophila persimilis] >gi|194114159|gb|EDW36202.1| GL16806 [Drosopl	Protein metabolism	Proteolysis
M9145	PREDICTED: transmembrane protease serine 9-like [Ceratitis capitata]	Protein metabolism	Proteolysis
M91709	trypsin [Marsupenaeus japonicus]	Protein metabolism	Proteolysis
M9171	hypothetical protein DAPPUDRAFT_300481 [Daphnia pulex]	Protein metabolism	Proteolysis
M931	protease [Homarus americanus]	Protein metabolism	Proteolysis
M9318	anaphase-promoting complex subunit, putative [Pediculus humanus corporis] >gi|21	Protein metabolism	Proteolysis
M94518	Procambarus clarkii clone pc-97 ubiquitin mRNA, complete cds	Protein metabolism	Proteolysis
M9678	conserved hypothetical protein [Pediculus humanus corporis] >gi|212514364|gb|EEE	Protein metabolism	Proteolysis
M97028	Protein CBR-NAS-4 [Caenorhabditis briggsae]	Protein metabolism	Proteolysis
M98646	tripsin, putative [Pediculus humanus corporis] >gi|212515418|gb|EEB17565.1| tripsi	Protein metabolism	Proteolysis
N10435	Aminopeptidase N precursor, putative [Pediculus humanus corporis] >gi|212513794|	Protein metabolism	Proteolysis
N10456	PREDICTED: carboxypeptidase B-like [Apis florea]	Protein metabolism	Proteolysis
N11537	hypothetical protein DAPPUDRAFT_130325 [Daphnia pulex]	Protein metabolism	Proteolysis
N11888	PREDICTED: similar to masquerade-like serine proteinase homologue [Tribolium casta	Protein metabolism	Proteolysis
N11943	CUB-serine protease [Panulirus argus]	Protein metabolism	Proteolysis
N11966	Cytosolic non-specific dipeptidase [Lepeophtheirus salmonis]	Protein metabolism	Proteolysis
N12001	PREDICTED: cullin-4B-like [Nasonia vitripennis]	Protein metabolism	Proteolysis
N12061	meprin A metalloprotease, putative [Ixodes scapularis] >gi|215490952|gb|EEC00593	Protein metabolism	Proteolysis
N12929	meprin A metalloprotease, putative [Ixodes scapularis] >gi|215490952|gb|EEC00593	Protein metabolism	Proteolysis
N13389	CUB-serine protease [Panulirus argus]	Protein metabolism	Proteolysis
N13604	ubiquitin protein ligase [Ixodes scapularis] >gi|215497661|gb|EEC07155.1| ubiquitin	Protein metabolism	Proteolysis
N13722	proteasome alpha 3 [Penaeus monodon]	Protein metabolism	Proteolysis
N13773	PREDICTED: Prolyl Carboxy Peptidase like family member (pcp-2)-like [Saccoglossus ko	Protein metabolism	Proteolysis
N16100	serine protease like protein [Graptopsaltria nigrofuscata]	Protein metabolism	Proteolysis
N16740	truncated cathepsin A [Eriocheir sinensis]	Protein metabolism	Proteolysis
N17787		Protein metabolism	Proteolysis
N18195	ubiquitin-activating enzyme E1 [Eriocheir sinensis]	Protein metabolism	Proteolysis

ID	Annotation	GO term	Go child term
N18470	ubiquitin specific peptidase 25 [Xenopus (Silurana) tropicalis] >gi|89272530|emb|CA	Protein metabolism	Proteolysis
N18474	PREDICTED: E3 ubiquitin-protein ligase hyd-like isoform 2 [Nasonia vitripennis]	Protein metabolism	Proteolysis
N18522		Protein metabolism	Proteolysis
N18765	hypothetical protein CAPTEDRAFT_180996 [Capitella teleta]	Protein metabolism	Proteolysis
N19197		Protein metabolism	Proteolysis
N19464	Thimet oligopeptidase [Crassostrea gigas]	Protein metabolism	Proteolysis
N19798	hypothetical protein DAPPUDRAFT_311166 [Daphnia pulex]	Protein metabolism	Proteolysis
N19821	CUB-serine protease [Panulirus argus]	Protein metabolism	Proteolysis
N19857	Naumovozyma castellii CBS 4309 hypothetical protein (NCAS0F02300) mRNA, comple	Protein metabolism	Proteolysis
N20124	CUB-serine protease [Panulirus argus]	Protein metabolism	Proteolysis
N2087	uncharacterized protein LOC641564 precursor [Danio rerio] >gi|82414856|gb|AAI10	Protein metabolism	Proteolysis
N21605		Protein metabolism	Proteolysis
N21939	PREDICTED: cullin-3-A-like [Megachile rotundata]	Protein metabolism	Proteolysis
N22284	hypothetical protein BRAFLDRAFT_275724 [Branchiostoma floridae] >gi|229278212|	Protein metabolism	Proteolysis
N23322	hypothetical protein DAPPUDRAFT_230628 [Daphnia pulex]	Protein metabolism	Proteolysis
N23960	hypothetical protein D910_11170 [Dendroctonus ponderosae]	Protein metabolism	Proteolysis
N30084	CUB-serine protease [Panulirus argus]	Protein metabolism	Proteolysis
N39388	hypothetical protein YQE_02808, partial [Dendroctonus ponderosae] >gi|546680994	Protein metabolism	Proteolysis
N40575	PREDICTED: LOW QUALITY PROTEIN: transmembrane protease serine 9-like [Monode	Protein metabolism	Proteolysis
N44114	PREDICTED: probable ubiquitin carboxyl-terminal hydrolase FAF-X [Strongylocentrotu	Protein metabolism	Proteolysis
N46473	CUB-serine protease [Panulirus argus]	Protein metabolism	Proteolysis
N50100	hypothetical protein DAPPUDRAFT_189791 [Daphnia pulex]	Protein metabolism	Proteolysis
N50173	Kazal-type serine proteinase inhibitor 1 [Fenneropenaeus chinensis]	Protein metabolism	Proteolysis
N60298	ubiquitin specific protease, putative [Ixodes scapularis] >gi|215507050|gb|EEC16544	Protein metabolism	Proteolysis
N62029	hypothetical protein DAPPUDRAFT_307535 [Daphnia pulex]	Protein metabolism	Proteolysis
N62093	masquerade, isoform C [Drosophila melanogaster] >gi|440215292|gb|AGB94103.1|	Protein metabolism	Proteolysis
N6903	PREDICTED: aminopeptidase N-like [Nasonia vitripennis]	Protein metabolism	Proteolysis
N7956	cathepsin C [Fenneropenaeus chinensis]	Protein metabolism	Proteolysis
N9093	CUB-serine protease [Panulirus argus]	Protein metabolism	Proteolysis
N9470	RecName: Full=Blastula protease 10; Flags: Precursor >gi|10929|emb|CAA39673.1| I	Protein metabolism	Proteolysis
E1597	RecName: Full=Elongation factor 1-alpha; Short=EF-1-alpha >gi|168380|gb|AAA3342	Protein metabolism	Regulation of translational elongation
E1735	elongation factor 1-beta', putative [Pediculus humanus corporis] >gi|212507797|gb|	Protein metabolism	Regulation of translational elongation
E2094	eukaryotic translation elongation factor 1 alpha 1 [Saccoglossus kowalevskii]	Protein metabolism	Regulation of translational elongation
E2187	RecName: Full=Elongation factor 1-alpha; Short=EF-1-alpha >gi|168380|gb|AAA3342	Protein metabolism	Regulation of translational elongation
E2510	eukaryotic translation elongation factor 1 alpha 1 [Saccoglossus kowalevskii]	Protein metabolism	Regulation of translational elongation
E2602	eukaryotic translation elongation factor 1 alpha 1 [Saccoglossus kowalevskii]	Protein metabolism	Regulation of translational elongation
M30255	Euphausia mutica 18S small subunit ribosomal RNA gene, partial sequence	Protein metabolism	Regulation of translational elongation
M45229	RecName: Full=Elongation factor 1-alpha; Short=EF-1-alpha >gi|168380|gb|AAA3342	Protein metabolism	Regulation of translational elongation
M4937	ORM1-like protein 3 [Danio rerio] >gi|81174971|sp|Q5XJR6.1|ORML3_DANRE RecNa	Protein metabolism	Regulation of translational elongation
M66668	hypothetical protein, partial [Herbaspirillum seropedicae]	Protein metabolism	Regulation of translational elongation
N14265	hypothetical protein CAPTEDRAFT_228652 [Capitella teleta]	Protein metabolism	Regulation of translational elongation
N18254		Protein metabolism	Regulation of translational elongation
N57382	transcription elongation factor [Plasmodium cynomolgi strain B] >gi|389583820|dbj|	Protein metabolism	Regulation of translational elongation
N7888	PREDICTED: similar to Elongation factor 1 beta CG6341-PA [Tribolium castaneum] >gi	Protein metabolism	Regulation of translational elongation
M2529	unknown [Dendroctonus ponderosae] >gi|478259386|gb|ENN79282.1| hypothetical	Protein metabolism	Regulation of translational initiation
M3245	hypothetical protein DAPPUDRAFT_226467 [Daphnia pulex]	Protein metabolism	Regulation of translational initiation
M69321		Protein metabolism	Regulation of translational initiation
M7737	eukaryotic initiation factor 1 [Coptotermes formosanus]	Protein metabolism	Regulation of translational initiation
N10357	PREDICTED: eukaryotic translation initiation factor 2 subunit 2-like [Ornithorhynchus	Protein metabolism	Regulation of translational initiation
N11874	hypothetical protein DAPPUDRAFT_303630 [Daphnia pulex]	Protein metabolism	Regulation of translational initiation
N12435	eukaryotic initiation factor 1 [Coptotermes formosanus]	Protein metabolism	Regulation of translational initiation
N14083	putative accessory gland protein [Gryllus firmus]	Protein metabolism	Regulation of translational initiation
N18953	PREDICTED: eukaryotic translation initiation factor 3 subunit D-like [Megachile rotunc	Protein metabolism	Regulation of translational initiation
N20448	hypothetical protein DAPPUDRAFT_304176 [Daphnia pulex]	Protein metabolism	Regulation of translational initiation
N21322	hypothetical protein DAPPUDRAFT_189030 [Daphnia pulex]	Protein metabolism	Regulation of translational initiation
N23182	eukaryotic translation initiation factor 4E type, putative [Pediculus humanus corporis]	Protein metabolism	Regulation of translational initiation
N24811	eIF2B-alpha protein [Daphnia pulex]	Protein metabolism	Regulation of translational initiation
N33237	PREDICTED: tRNA (adenine(58)-N(1))-methyltransferase non-catalytic subunit TRM6 [	Protein metabolism	Regulation of translational initiation
N40609	hypothetical protein YQE_10251, partial [Dendroctonus ponderosae] >gi|546673857	Protein metabolism	Regulation of translational initiation
N8842	eukaryotic initiation factor 2 subunit alpha [Litopenaeus vannamei]	Protein metabolism	Regulation of translational initiation
N18344	PREDICTED: Megachile rotundata eukaryotic peptide chain release factor subunit 1-lil	Protein metabolism	Regulation of translational termination
E1939	PREDICTED: 60S ribosomal protein L11-1-like [Vitis vinifera]	Protein metabolism	Ribosome biogenesis
E2054	ribosomal protein L37 [Acartia pacifica]	Protein metabolism	Ribosome biogenesis
E2203	PREDICTED: 60S ribosomal protein L11-1-like [Vitis vinifera]	Protein metabolism	Ribosome biogenesis
E2397	60S ribosomal protein L37, putative [Pediculus humanus corporis] >gi|212506270|gb	Protein metabolism	Ribosome biogenesis
E2487	40S ribosomal protein S2 [Artemia franciscana]	Protein metabolism	Ribosome biogenesis
E2530	GH21709 [Drosophila grimshawi] >gi|193903065|gb|EDW01932.1| GH21709 [Droso	Protein metabolism	Ribosome biogenesis
E2609	hypothetical protein BRAFLDRAFT_114866 [Branchiostoma floridae] >gi|229277362|	Protein metabolism	Ribosome biogenesis
E2641	ribosomal protein L44 [Procambarus clarkii]	Protein metabolism	Ribosome biogenesis
E2797	RecName: Full=60S ribosomal protein L17 >gi|70909721|emb|CAJ17286.1| ribosoma	Protein metabolism	Ribosome biogenesis
E3016		Protein metabolism	Ribosome biogenesis
E3605	ribosomal protein S3a [Fenneropenaeus merguiensis]	Protein metabolism	Ribosome biogenesis
E5221	40S ribosomal protein S2 [Artemia franciscana]	Protein metabolism	Ribosome biogenesis
M101526	RecName: Full=60S ribosomal protein L38 >gi|62083499|gb|AAX62474.1| ribosomal	Protein metabolism	Ribosome biogenesis
M101760	ribosomal protein S7 [Procambarus clarkii]	Protein metabolism	Ribosome biogenesis
M105642	ribosomal protein rps13 [Scylla paramamosain]	Protein metabolism	Ribosome biogenesis

ID	Annotation	GO term	Go child term
M107342		Protein metabolism	Ribosome biogenesis
M108453	60S ribosomal protein L11 [Lepeophtheirus salmonis]	Protein metabolism	Ribosome biogenesis
M109487	QM protein [Penaeus monodon] >gi|429843492|gb|AGA16579.1| QM protein [Litop	Protein metabolism	Ribosome biogenesis
M114084		Protein metabolism	Ribosome biogenesis
M16453	ribosome like protein [Marsupenaeus japonicus]	Protein metabolism	Ribosome biogenesis
M18519	hypothetical protein DAPPUDRAFT_299917 [Daphnia pulex]	Protein metabolism	Ribosome biogenesis
M18586	ribosomal protein L19e [Coptotermes formosanus]	Protein metabolism	Ribosome biogenesis
M19220	PREDICTED: zinc finger protein 256-like [Megachile rotundata]	Protein metabolism	Ribosome biogenesis
M21269	Palaemonetes varians ribosomal protein S14 mRNA, partial cds	Protein metabolism	Ribosome biogenesis
M21285	RecName: Full=60S ribosomal protein L15 >gi|4530186|gb|AAD21924.1| L15-like rib	Protein metabolism	Ribosome biogenesis
M21399	60S ribosomal protein L6 [Coptotermes formosanus]	Protein metabolism	Ribosome biogenesis
M21530	PREDICTED: Megachile rotundata 40S ribosomal protein S3a-like (LOC100876732), ml	Protein metabolism	Ribosome biogenesis
M21700	ribosomal protein P1 [Palaemonetes varians]	Protein metabolism	Ribosome biogenesis
M21789	60S ribosomal protein L38 [Tityus discrepans]	Protein metabolism	Ribosome biogenesis
M21834		Protein metabolism	Ribosome biogenesis
M22249		Protein metabolism	Ribosome biogenesis
M23963	Trichophyton verrucosum HKI 0517 hypothetical protein, mRNA	Protein metabolism	Ribosome biogenesis
M2518	hypothetical protein DAPPUDRAFT_193882 [Daphnia pulex]	Protein metabolism	Ribosome biogenesis
M27167	Trichophyton verrucosum HKI 0517 hypothetical protein, mRNA	Protein metabolism	Ribosome biogenesis
M28821	Procambarus clarkii clone pc-639 ribosomal protein S24 mRNA, complete cds	Protein metabolism	Ribosome biogenesis
M33616	Euphausia eximia voucher KCeuex 28S large subunit ribosomal RNA gene, partial sequ	Protein metabolism	Ribosome biogenesis
M33880	ribosomal protein L3 [Penaeus monodon]	Protein metabolism	Ribosome biogenesis
M418	mitochondrial ribosomal protein, L23, putative [Aedes aegypti] >gi|108879205|gb|E/	Protein metabolism	Ribosome biogenesis
M44196	Hordeum vulgare subsp. vulgare cDNA clone: FLbaf103d03, mRNA sequence	Protein metabolism	Ribosome biogenesis
M45683		Protein metabolism	Ribosome biogenesis
M45899	60S ribosomal protein L37A, putative [Pediculus humanus corporis] >gi|212518389|g	Protein metabolism	Ribosome biogenesis
M46733	ribosomal protein rps21 [Palaemonetes varians]	Protein metabolism	Ribosome biogenesis
M47690	putative 60S ribosomal protein RPL31 [Flustra foliacea]	Protein metabolism	Ribosome biogenesis
M49068		Protein metabolism	Ribosome biogenesis
M55239	PREDICTED: Nasonia vitripennis histone H2A-like (LOC100679332), mRNA	Protein metabolism	Ribosome biogenesis
M61542	Euphausia superba isolate DGGE band AntEuk13-5 18S ribosomal RNA gene, partial se	Protein metabolism	Ribosome biogenesis
M64656	ribosome like protein [Marsupenaeus japonicus]	Protein metabolism	Ribosome biogenesis
M65348	PREDICTED: 40S ribosomal protein S8-like [Nasonia vitripennis]	Protein metabolism	Ribosome biogenesis
M66561	40S ribosomal protein S6 [Schizosaccharomyces japonicus yFS275] >gi|212003034|gb	Protein metabolism	Ribosome biogenesis
M66809	ribosomal protein rps21 [Palaemonetes varians]	Protein metabolism	Ribosome biogenesis
M67893	ribosomal protein L10a [Fenneropenaeus merguiensis]	Protein metabolism	Ribosome biogenesis
M69255	Heliconius erato control protein HCTL036 mRNA, partial cds	Protein metabolism	Ribosome biogenesis
M73104	60S ribosomal protein RPL23 [Palaemonetes varians]	Protein metabolism	Ribosome biogenesis
M74132	ribosomal protein L44 [Procambarus clarkii]	Protein metabolism	Ribosome biogenesis
M74190	putative 60S ribosomal protein RPL17 [Phoronis muelleri]	Protein metabolism	Ribosome biogenesis
M74416	ribosomal protein L10a [Fenneropenaeus merguiensis]	Protein metabolism	Ribosome biogenesis
M75744	ribosomal protein L36 [Procambarus clarkii]	Protein metabolism	Ribosome biogenesis
M7919	putative 40S ribosomal protein S12 [Nephrops norvegicus]	Protein metabolism	Ribosome biogenesis
M81851	putative ribosomal protein S23e [Scylla paramamosain]	Protein metabolism	Ribosome biogenesis
M82075	putative 60S ribosomal protein RPL31 [Flustra foliacea]	Protein metabolism	Ribosome biogenesis
M8319	39S ribosomal protein L47, mitochondrial [Camponotus floridanus]	Protein metabolism	Ribosome biogenesis
M84006	ribosomal protein S3 [Palaemonetes varians]	Protein metabolism	Ribosome biogenesis
M84652	Euphausia superba isolate DGGE band AntEuk13-5 18S ribosomal RNA gene, partial se	Protein metabolism	Ribosome biogenesis
M87835	ribosomal protein L19e [Scylla paramamosain]	Protein metabolism	Ribosome biogenesis
M9073	ribosomal protein S13 [synthetic construct]	Protein metabolism	Ribosome biogenesis
M95582	60S ribosomal protein L18a [Caligus clemensi]	Protein metabolism	Ribosome biogenesis
M99151	PREDICTED: 40S ribosomal protein S13-like [Ornithorhynchus anatinus]	Protein metabolism	Ribosome biogenesis
M99579	ribosomal protein L18 [Litopenaeus vannamei]	Protein metabolism	Ribosome biogenesis
M99801	ribosomal protein L10a [Fenneropenaeus merguiensis]	Protein metabolism	Ribosome biogenesis
N10674	60S ribosomal protein L7A [Ixodes pacificus] >gi|67083827|gb|AAY66848.1| 60S ribo	Protein metabolism	Ribosome biogenesis
N109	Phoronis muelleri putative 40S ribosomal protein RPS20 mRNA, complete cds	Protein metabolism	Ribosome biogenesis
N11207	ribosomal protein S11 [Rimicaris exoculata]	Protein metabolism	Ribosome biogenesis
N11420	lymphoid organ expressed yellow head virus receptor protein [Penaeus monodon]	Protein metabolism	Ribosome biogenesis
N11749	ribosomal protein S6 [Procambarus clarkii]	Protein metabolism	Ribosome biogenesis
N11803	PREDICTED: 60S ribosomal protein L14-like [Aplysia californica]	Protein metabolism	Ribosome biogenesis
N13267	hypothetical protein DAPPUDRAFT_306295 [Daphnia pulex]	Protein metabolism	Ribosome biogenesis
N13567	ribosomal protein L24 [Azumapecten farreri]	Protein metabolism	Ribosome biogenesis
N14540	Palaemonetes varians ribosomal protein S3 mRNA, partial cds	Protein metabolism	Ribosome biogenesis
N16052	ribosomal protein rpl35a [Lineus viridis]	Protein metabolism	Ribosome biogenesis
N16225	60S ribosomal protein L37, putative [Pediculus humanus corporis] >gi|212506270|gb	Protein metabolism	Ribosome biogenesis
N16289	28S ribosomal protein S16, mitochondrial [Aedes aegypti] >gi|108874273|gb|EAT384	Protein metabolism	Ribosome biogenesis
N17629		Protein metabolism	Ribosome biogenesis
N18966	hypothetical protein CAPTEDRAFT_149919 [Capitella teleta]	Protein metabolism	Ribosome biogenesis
N20156	hypothetical protein DAPPUDRAFT_308176 [Daphnia pulex]	Protein metabolism	Ribosome biogenesis
N2040	60S ribosomal protein L23 [Esox lucius]	Protein metabolism	Ribosome biogenesis
N21176	PREDICTED: similar to AGAP005615-PA [Tribolium castaneum] >gi|270006846|gb|EF	Protein metabolism	Ribosome biogenesis
N23227	mitochondrial ribosomal protein L24 [Aedes aegypti] >gi|108871351|gb|EAT35576.1	Protein metabolism	Ribosome biogenesis
N28192	putative ribosomal protein L34 [Diaphorina citri]	Protein metabolism	Ribosome biogenesis
N29470	PREDICTED: similar to GA18111-PA [Tribolium castaneum] >gi|270009430|gb|EFA05	Protein metabolism	Ribosome biogenesis
N31346	hypothetical protein DAPPUDRAFT_306294 [Daphnia pulex]	Protein metabolism	Ribosome biogenesis
N34693	mitochondrial ribosomal protein, L14, putative [Aedes aegypti] >gi|108879961|gb|E/	Protein metabolism	Ribosome biogenesis

ID	Annotation	GO term	Go child term
N35222		Protein metabolism	Ribosome biogenesis
N3623	ribosomal protein S4e [Mycetophagus quadripustulatus]	Protein metabolism	Ribosome biogenesis
N3958	ribosomal protein S26 [Branchiostoma belcheri]	Protein metabolism	Ribosome biogenesis
N4202	Heliconius melpomene cythera ribosomal protein S15 (RpS15) mRNA, complete cds	Protein metabolism	Ribosome biogenesis
N43805	RecName: Full=40S ribosomal protein S27 >gi|1835724|gb|AAB46716.1| 40S ribosor	Protein metabolism	Ribosome biogenesis
N48510	putative ribosomal protein S23e [Scylla paramamosain]	Protein metabolism	Ribosome biogenesis
N49331		Protein metabolism	Ribosome biogenesis
N5213		Protein metabolism	Ribosome biogenesis
N5488	expressed hypothetical protein [Trichoplax adhaerens] >gi|190586310|gb|EDV2636	Protein metabolism	Ribosome biogenesis
N59348	hypothetical protein DAPPUDRAFT_194445 [Daphnia pulex]	Protein metabolism	Ribosome biogenesis
N6199	ribosomal protein L9 [Procambarus clarkii]	Protein metabolism	Ribosome biogenesis
N62587	60S ribosomal protein L39 [Caligus rogercresseyi]	Protein metabolism	Ribosome biogenesis
N6662	Hordeum vulgare subsp. vulgare cDNA clone: FLbaf103d03, mRNA sequence	Protein metabolism	Ribosome biogenesis
N6847	ribosomal protein S24 [Marsupenaeus japonicus]	Protein metabolism	Ribosome biogenesis
N8057	Branchiostoma floridae hypothetical protein, mRNA	Protein metabolism	Ribosome biogenesis
N8695	Litopenaeus vannamei clone c060 ribosomal protein S23e-like mRNA, partial sequenc	Protein metabolism	Ribosome biogenesis
N8742	ribosomal protein L28-like protein [Maconellicoccus hirsutus]	Protein metabolism	Ribosome biogenesis
N8901	60S ribosomal protein L30 [Haplopelma schmidti]	Protein metabolism	Ribosome biogenesis
N8903	60S ribosomal protein L30 [Haplopelma schmidti]	Protein metabolism	Ribosome biogenesis
N9172	PREDICTED: Oreochromis niloticus 60S ribosomal protein L4-B-like (LOC100694481), r	Protein metabolism	Ribosome biogenesis
N9292	PREDICTED: Strongylocentrotus purpuratus 40S ribosomal protein S16-like, transcript	Protein metabolism	Ribosome biogenesis
N9387	putative 60S ribosomal protein RPL31 [Flustra foliacea]	Protein metabolism	Ribosome biogenesis

Response to stress

ID	Annotation	GO term	Go child term
M101679		Response to stress	Defence responce
M18271	PREDICTED: DNA-directed RNA polymerase III subunit RPC4 [Sarcophilus harrisii]	Response to stress	Defence responce
M18811	PREDICTED: similar to DNA replication licensing factor MCM3 [Tribolium castaneum]	Response to stress	Defence responce
M28143	crustacyanin subunit C [Fenneropenaeus merguiensis]	Response to stress	Defence responce
M38819	hypothetical protein CB1_000307002 [Camelus ferus]	Response to stress	Defence responce
M7368	guanine nucleotide-binding protein alpha subunit [Calliphora vicina]	Response to stress	Defence responce
N19055	hypothetical protein BRAFLDRAFT_231177 [Branchiostoma floridae] >gi|229293953|	Response to stress	Defence responce
N19308	PREDICTED: similar to Cytochrome P450 9b2 (CYPIXB2) [Tribolium castaneum]	Response to stress	Defence responce
N19696	Aplysia californica alpha tubulin 2 (LOC100533261), mRNA >gi|20069088|gb|AF4810	Response to stress	Defence responce
N25269	Putative ferric-chelate reductase 1 [Crassostrea gigas]	Response to stress	Defence responce
N41485	PREDICTED: similar to AGAP003789-PA [Tribolium castaneum] >gi|270008459|gb|EF	Response to stress	Defence responce
N6533		Response to stress	Defence responce
M12331	6-4 photolyase [Eurydice pulchra]	Response to stress	DNA repair
M3134	PREDICTED: F-box and WD repeat domain containing 7-like [Saccoglossus kowalevskii	Response to stress	DNA repair
M38460	hypothetical protein DAPPUDRAFT_210135 [Daphnia pulex]	Response to stress	DNA repair
M78537	predicted protein [Nematostella vectensis] >gi|156216845|gb|EDO37773.1| predict	Response to stress	DNA repair
N20425	hypothetical protein BRAFLDRAFT_131216 [Branchiostoma floridae] >gi|229273875|	Response to stress	DNA repair
N21905	TFIIH basal transcription factor complex p52 subunit, putative [Pediculus humanus co	Response to stress	DNA repair
N22411	hypothetical protein BRAFLDRAFT_277469 [Branchiostoma floridae] >gi|229298852|	Response to stress	DNA repair
N23030	Fanconi anemia group J protein [Crassostrea gigas]	Response to stress	DNA repair
N26975	PREDICTED: N-methylpurine-DNA glycosylase-like [Saccoglossus kowalevskii]	Response to stress	DNA repair
N27521	hypothetical protein BRAFLDRAFT_91042 [Branchiostoma floridae] >gi|229294428|g	Response to stress	DNA repair
N29406	PREDICTED: DNA mismatch repair protein Msh6-like [Strongylocentrotus purpuratus]	Response to stress	DNA repair
N31585	PREDICTED: checkpoint protein HUS1-like [Ornithorhynchus anatinus]	Response to stress	DNA repair
N34663	PREDICTED: structural maintenance of chromosomes protein 6-like [Apis mellifera]	Response to stress	DNA repair
N35298	AGAP008939-PA [Anopheles gambiae str. PEST] >gi|157013593|gb|EAA14799.5| AG	Response to stress	DNA repair
M12535	PREDICTED: long-chain fatty acid transport protein 4-like [Megachile rotundata]	Response to stress	Other
M18908	eukaryotic translation initiation factor 2A [Bos taurus] >gi|151556051|gb|AAI49971.	Response to stress	Other
M5628	Armandia brevis histone H3 gene, partial cds	Response to stress	Other
M62214	toll, putative [Ixodes scapularis] >gi|215504708|gb|EEC14202.1| toll, putative [Ixode	Response to stress	Other
M709	heat shock protein 70 (hsp70)-interacting protein [Aedes aegypti] >gi|108880263|gb	Response to stress	Other
N18366	heat shock protein 40 [Marsupenaeus japonicus]	Response to stress	Other
N18621	PREDICTED: similar to Bm44 [Tribolium castaneum] >gi|270002597|gb|EEZ99044.1|	Response to stress	Other
N45454	DnaJ domain-containing protein [Synechococcus sp. JA-2-3B'a(2-13)] >gi|499753257|	Response to stress	Other
N61357	PREDICTED: long-chain fatty acid transport protein 1 [Sarcophilus harrisii]	Response to stress	Other
M104807	hypothetical protein DAPPUDRAFT_301956 [Daphnia pulex]	Response to stress	Response to oxidative stress
M30619	PREDICTED: uncharacterized protein LOC100899356 [Metaseiulus occidentalis]	Response to stress	Response to oxidative stress
M4522	peroxinectin, putative [Ixodes scapularis] >gi|215500805|gb|EEC10299.1| peroxinec	Response to stress	Response to oxidative stress
M4728	hypothetical protein TcasGA2_TC015234 [Tribolium castaneum]	Response to stress	Response to oxidative stress
M61329	peroxinectin [Penaeus monodon]	Response to stress	Response to oxidative stress
M66423	peroxinectin [Eriocheir sinensis]	Response to stress	Response to oxidative stress
M74305	peroxinectin [Fenneropenaeus chinensis]	Response to stress	Response to oxidative stress
M96899	Peroxidasin [Acromyrmex echinatior]	Response to stress	Response to oxidative stress
N14121	putative peroxidasin, partial [Latrodectus hesperus]	Response to stress	Response to oxidative stress
N19074	hypothetical protein DAPPUDRAFT_42141 [Daphnia pulex]	Response to stress	Response to oxidative stress
N21683	oxidase/peroxidase, putative [Ixodes scapularis] >gi|215494874|gb|EEC04515.1| oxi	Response to stress	Response to oxidative stress
N22567	peroxinectin [Penaeus monodon]	Response to stress	Response to oxidative stress
N23464	glutathione peroxidase [Haemaphysalis longicornis]	Response to stress	Response to oxidative stress
N25658	hypothetical protein BRAFLDRAFT_123508 [Branchiostoma floridae] >gi|229286043|	Response to stress	Response to oxidative stress
N28628	peroxinectin [Fenneropenaeus chinensis]	Response to stress	Response to oxidative stress
N46318	peroxinectin [Penaeus monodon]	Response to stress	Response to oxidative stress
N47292	peroxinectin [Fenneropenaeus chinensis]	Response to stress	Response to oxidative stress
N5524	glutathione peroxidase [Metapenaeus ensis] >gi|171189513|gb|ACB42237.1| glutat	Response to stress	Response to oxidative stress

ID	Annotation	GO term	Go child term
N9090	Chorion peroxidase [Camponotus floridanus]	Response to stress	Response to oxidative stress
N9588	selenium-dependent glutathione peroxidase [Macrobrachium rosenbergii]	Response to stress	Response to oxidative stress
Signal transduction			
N22913	Sorting nexin-12 [Camponotus floridanus]	Signal transduction	Cell communication
M3501		Signal transduction	Cell-cell signaling
M80075	ionotropic glutamate receptor subunit [Homarus americanus]	Signal transduction	Cell-cell signaling
M96025	PREDICTED: synapsin-like [Megachile rotundata]	Signal transduction	Cell-cell signaling
N12768	innexin 1 [Cancer borealis]	Signal transduction	Cell-cell signaling
N12787		Signal transduction	Cell-cell signaling
N13835	GM19962 [Drosophila sechellia] >gi|194126289|gb|EDW48332.1| GM19962 [Drosop	Signal transduction	Cell-cell signaling
N18727	hypothetical protein DAPPUDRAFT_301948 [Daphnia pulex]	Signal transduction	Cell-cell signaling
M10547	PREDICTED: hypothetical protein LOC100426888 [Macaca mulatta]	Signal transduction	Intracellular signal transduction
M11721	myosin-IX, putative [Ixodes scapularis] >gi|215505558|gb|EEC15052.1| myosin-IX, pu	Signal transduction	Intracellular signal transduction
M71681	hypothetical protein DAPPUDRAFT_304508 [Daphnia pulex]	Signal transduction	Intracellular signal transduction
M7219	cAMP-dependent protein kinase catalytic subunit, putative [Pediculus humanus corpo	Signal transduction	Intracellular signal transduction
M77475	PREDICTED: CDC42 binding protein kinase alpha-like [Saccoglossus kowalevskii]	Signal transduction	Intracellular signal transduction
N17078	cAMP-dependent protein kinase catalytic subunit, putative [Pediculus humanus corpo	Signal transduction	Intracellular signal transduction
N18741	PREDICTED: hypothetical protein LOC100678008 [Nasonia vitripennis]	Signal transduction	Intracellular signal transduction
N23230	RAC protein kinase DRAC-PK85, putative [Pediculus humanus corporis] >gi|21251675	Signal transduction	Intracellular signal transduction
N26112	PREDICTED: protein kinase C, brain isozyme-like isoform 1 [Nasonia vitripennis]	Signal transduction	Intracellular signal transduction
N29301	hypothetical protein DAPPUDRAFT_315683 [Daphnia pulex]	Signal transduction	Intracellular signal transduction
N35561	phospholipid phospholipase C beta isoform [Homarus americanus]	Signal transduction	Intracellular signal transduction
N47703	phospholipid phospholipase C beta isoform [Homarus americanus]	Signal transduction	Intracellular signal transduction
M10870	CG13830-PA-like protein [Daphnia pulex]	Signal transduction	Other
M110018	RecName: Full=Compound eye opsin BCRH1 >gi|829043|dbj|BAA09132.1| opsin BcR	Signal transduction	Other
M11327	cAMP-dependent protein kinase catalytic subunit, putative [Pediculus humanus corpo	Signal transduction	Other
M11904	PREDICTED: LOW QUALITY PROTEIN: TBC1 domain family member CG11727 [Apis me	Signal transduction	Other
M16660	pigment-dispersing hormone 3 [Marsupenaeus japonicus]	Signal transduction	Other
M19175	pigment dispersing hormone precursor [Litopenaeus vannamei]	Signal transduction	Other
M2709		Signal transduction	Other
M28764	cuticle protein CUT8 [Portunus pelagicus]	Signal transduction	Other
M31314	RecName: Full=Compound eye opsin BCRH1 >gi|829043|dbj|BAA09132.1| opsin BcR	Signal transduction	Other
M34439	RecName: Full=Compound eye opsin BCRH2 >gi|829045|dbj|BAA09133.1| opsin BcR	Signal transduction	Other
M5422	arrestin [Libelloides macaronius]	Signal transduction	Other
M55453	hypothetical protein CAPTEDRAFT_173251 [Capitella teleta]	Signal transduction	Other
M55919	hypothetical protein DAPPUDRAFT_44292 [Daphnia pulex]	Signal transduction	Other
M56179	hypothetical protein DAPPUDRAFT_301966 [Daphnia pulex]	Signal transduction	Other
M56268	class B secretin-like G-protein coupled receptor GPRcal2, putative [Pediculus humanu	Signal transduction	Other
M66480	PREDICTED: peroxisomal membrane protein PEX14 isoform X1 [Columba livia]	Signal transduction	Other
M669	PREDICTED: similar to chilling-inducible protein, putative [Tribolium castaneum] >gi|2	Signal transduction	Other
M6816	hypothetical protein DAPPUDRAFT_223163 [Daphnia pulex]	Signal transduction	Other
M68573	phosrestin I, putative [Pediculus humanus corporis] >gi|212508943|gb|EEB12477.1|	Signal transduction	Other
M73551	hypothetical protein AaeL_AAEL004819 [Aedes aegypti] >gi|108879555|gb|EAT4378	Signal transduction	Other
M74855	RecName: Full=Techylectin-5A; Flags: Precursor >gi|5851893|dbj|BAA84188.1| techy	Signal transduction	Other
M77959	PREDICTED: stromal membrane-associated protein 1-like [Megachile rotundata]	Signal transduction	Other
M8094	ubiquitin-protein ligase EDD1, putative [Pediculus humanus corporis] >gi|212511528	Signal transduction	Other
M81405	130 kDa phosphatidylinositol 4,5-biphosphate-dependent ARF1 GTPase-activating pro	Signal transduction	Other
M8285	RecName: Full=Compound eye opsin BCRH2 >gi|829045|dbj|BAA09133.1| opsin BcR	Signal transduction	Other
M85776	signal transducer and activator of transcription [Fenneropenaeus chinensis]	Signal transduction	Other
M88373	arrestin [Libelloides macaronius]	Signal transduction	Other
M93343	PREDICTED: apoptosis regulatory protein Siva isoform X2 [Mesocricetus auratus]	Signal transduction	Other
M94605	protein kinase, putative [Ixodes scapularis] >gi|215496277|gb|EEC05917.1| protein I	Signal transduction	Other
M95757	Val-1 SIFamide [Homarus americanus]	Signal transduction	Other
M98592		Signal transduction	Other
N10393		Signal transduction	Other
N11520		Signal transduction	Other
N11936	PREDICTED: arrestin homolog [Bombus terrestris]	Signal transduction	Other
N12955	PREDICTED: ATP-dependent RNA helicase DDX54 [Xenopus (Silurana) tropicalis]	Signal transduction	Other
N13913	RecName: Full=Compound eye opsin BCRH2 >gi|829045|dbj|BAA09133.1| opsin BcR	Signal transduction	Other
N14135	hypothetical protein DAPPUDRAFT_324178 [Daphnia pulex]	Signal transduction	Other
N15417	PREDICTED: rab3 GTPase-activating protein catalytic subunit isoform 2 [Cavia porcellu	Signal transduction	Other
N17953	retinoid X receptor isoform 2 [Crangon crangon]	Signal transduction	Other
N18394		Signal transduction	Other
N19184		Signal transduction	Other
N19969	RecName: Full=Arrestin homolog >gi|298756|gb|AAB25860.1| arrestin homolog [Lo	Signal transduction	Other
N20346	opsin protein [Charybdis japonica]	Signal transduction	Other
N20549	Marsupenaeus japonicus MjGo mRNA for GTP binding protein alpha subunit Go, com	Signal transduction	Other
N20954	hypothetical protein CAPTEDRAFT_180983 [Capitella teleta]	Signal transduction	Other
N21098	hypothetical protein DAPPUDRAFT_44292 [Daphnia pulex]	Signal transduction	Other
N21529	hypothetical protein YQE_11312, partial [Dendroctonus ponderosae] >gi|546681223	Signal transduction	Other
N21988	bride of sevenless [Tribolium castaneum]	Signal transduction	Other
N22768		Signal transduction	Other
N23914	hypothetical protein CRE_08567 [Caenorhabditis remanei] >gi|308253586|gb|EFO97	Signal transduction	Other
N24013	PREDICTED: RAS protein activator like 2-like [Saccoglossus kowalevskii]	Signal transduction	Other
N24867		Signal transduction	Other
N25485		Signal transduction	Other

ID	Annotation	GO term	Go child term
N28300	conserved hypothetical protein [Pediculus humanus corporis] >gi|212513821|gb|EEI	Signal transduction	Other
N28816	signal transducer and activator of transcription [Penaeus monodon]	Signal transduction	Other
N30883	HR3 nuclear receptor [Daphnia magna]	Signal transduction	Other
N31199		Signal transduction	Other
N31246	integral membrane protein GPR177-B, putative [Ixodes scapularis] >gi|215506845|gb	Signal transduction	Other
N32406	hypothetical protein DAPPUDRAFT_316993 [Daphnia pulex]	Signal transduction	Other
N33074	PREDICTED: LOW QUALITY PROTEIN: ral guanine nucleotide dissociation stimulator-lil	Signal transduction	Other
N34392	RecName: Full=Arrestin, lateral eye >gi|475769|gb|AAA82007.1| arrestin [Limulus p	Signal transduction	Other
N35241	peropsin [Cupiennius salei]	Signal transduction	Other
N36102	pigment dispersing hormone precursor [Litopenaeus vannamei]	Signal transduction	Other
N36343		Signal transduction	Other
N36616	hypothetical protein DAPPUDRAFT_321679 [Daphnia pulex]	Signal transduction	Other
N40884	hypothetical protein DAPPUDRAFT_64004 [Daphnia pulex]	Signal transduction	Other
N45522	RecName: Full=Compound eye opsin BCRH1 >gi|829043|dbj|BAA09132.1| opsin BcR	Signal transduction	Other
N48998	PREDICTED: disintegrin and metalloproteinase domain-containing protein 10-like isof	Signal transduction	Other
N51716	PREDICTED: angiopoietin-like 2-like [Saccoglossus kowalevskii]	Signal transduction	Other
N55639	TPA_inf: venus kinase receptor [Pediculus humanus corporis]	Signal transduction	Other
N57793	RecName: Full=Arrestin homolog >gi|298756|gb|AAB25860.1| arrestin homolog [Lo	Signal transduction	Other
N59468	nuclear receptor E75 protein [Fenneropenaeus chinensis]	Signal transduction	Other
N61375	PREDICTED: RUN and TBC1 domain containing 2-like [Saccoglossus kowalevskii]	Signal transduction	Other
**Transport**			
M55743	GK22112 [Drosophila willistoni] >gi|300681124|sp|B4MYA4.1|TRET1_DROWI RecNa	Transport	Carbohydrate transport
N49759	sialin [Xenopus (Silurana) tropicalis] >gi|111305815|gb|AAI21682.1| solute carrier fa	Transport	Carbohydrate transport
N56030	sugar transporter, putative [Ixodes scapularis] >gi|215499141|gb|EEC08635.1| sugai	Transport	Carbohydrate transport
M61		Transport	Intracellular transport
N13433	hypothetical protein DAPPUDRAFT_304059 [Daphnia pulex]	Transport	Intracellular transport
N18258		Transport	Intracellular transport
E154	voltage-gated calcium channel beta subunit transcript variant 6 [Scylla paramamosair	Transport	Ion transport
E6444	voltage-dependent non-L-type calcium channel alpha-1 subunit, partial [Cancer borea	Transport	Ion transport
M10694	hypothetical protein DAPPUDRAFT_226732 [Daphnia pulex]	Transport	Ion transport
M107683	Euphausia superba mitochondrion, partial genome	Transport	Ion transport
M108201	sodium-driven chloride bicarbonate exchanger, putative [Pediculus humanus corporis	Transport	Ion transport
M112656	hypothetical protein DAPPUDRAFT_40435 [Daphnia pulex]	Transport	Ion transport
M14041	sodium/hydrogen exchanger [Carcinus maenas]	Transport	Ion transport
M15958	Sideroflexin-1 [Lepeophtheirus salmonis]	Transport	Ion transport
M1709	hypothetical protein DAPPUDRAFT_308636 [Daphnia pulex]	Transport	Ion transport
M19113	NADH dehydrogenase, putative [Aedes aegypti] >gi|108875509|gb|EAT39734.1| AAI	Transport	Ion transport
M19376	Euphausia superba voucher 841M mitochondrion, partial genome	Transport	Ion transport
M2254	Litopenaeus stylirostris Na+/K+-ATPase alpha subunit mRNA, complete cds	Transport	Ion transport
M26098	mitochondrial ubiquinol-cytochrome c reductase complex 11 kDa protein [Scylla para	Transport	Ion transport
M32986	ferritin peptide [Fenneropenaeus indicus]	Transport	Ion transport
M48035		Transport	Ion transport
M55261	PREDICTED: anion exchange protein 2 isoform X2 [Echinops telfairi]	Transport	Ion transport
M60197	Euphausia superba voucher 841M mitochondrion, partial genome	Transport	Ion transport
M6504	putative TRP channel protein [Periplaneta americana]	Transport	Ion transport
M6590	PREDICTED: ileal sodium/bile acid cotransporter-like isoform 1 [Nasonia vitripennis] >	Transport	Ion transport
M68570	hypothetical protein LOAG_03365 [Loa loa]	Transport	Ion transport
M69832	conserved hypothetical protein [Culex quinquefasciatus] >gi|167873494|gb|EDS368	Transport	Ion transport
M70398	sodium-driven chloride bicarbonate exchanger, putative [Pediculus humanus corporis	Transport	Ion transport
M71335	NADH dehydrogenase [Palaemonetes varians]	Transport	Ion transport
M73935	voltage-gated sodium channel [Cancer borealis]	Transport	Ion transport
M81077	PREDICTED: transient receptor potential protein-like [Megachile rotundata]	Transport	Ion transport
M81910	PREDICTED: anion exchange protein 3-like isoform 1 [Acyrthosiphon pisum]	Transport	Ion transport
M85975	hypothetical protein TRIADDRAFT_21514 [Trichoplax adhaerens] >gi|190587887|gb|	Transport	Ion transport
M87362	Nasonia vitripennis ATP synthase, H+ transporting, mitochondrial F0 complex, subuni	Transport	Ion transport
M9551	hypothetical protein CAPTEDRAFT_34319, partial [Capitella teleta]	Transport	Ion transport
N10536	voltage-dependent anion-selective channel [Eriocheir sinensis]	Transport	Ion transport
N11826	putative TRPL channel protein [Periplaneta americana]	Transport	Ion transport
N14105	Candida tropicalis MYA-3404 NADH-ubiquinone oxidoreductase 20 kDa subunit, mito	Transport	Ion transport
N24331	hypothetical protein DAPPUDRAFT_217456 [Daphnia pulex]	Transport	Ion transport
N24758	chloride channel, putative [Ixodes scapularis] >gi|215490978|gb|EEC00619.1| chlori	Transport	Ion transport
N25724	hypothetical protein DAPPUDRAFT_323725 [Daphnia pulex]	Transport	Ion transport
N25984	GE19913 [Drosophila yakuba] >gi|194181108|gb|EDW94719.1| GE19913 [Drosophil	Transport	Ion transport
N26495	hypothetical protein CAPTEDRAFT_176070 [Capitella teleta]	Transport	Ion transport
N27782	hypothetical protein BRAFLDRAFT_93126 [Branchiostoma floridae] >gi|229288904|g	Transport	Ion transport
N28341	voltage-dependent non-L-type calcium channel alpha-1 subunit, partial [Cancer borea	Transport	Ion transport
N31065	hypothetical protein DAPPUDRAFT_40908 [Daphnia pulex]	Transport	Ion transport
N31753	Probable NADH dehydrogenase 1 alpha subcomplex subunit 12 [Caligus rogercressey	Transport	Ion transport
N33158	copper chaperone Atox1 [Crassostrea ariakensis]	Transport	Ion transport
N33931	GD22775 [Drosophila simulans] >gi|194190001|gb|EDX03577.1| GD22775 [Drosoph	Transport	Ion transport
N36245	Calcium-transporting ATPase type 2C member 1 [Crassostrea gigas]	Transport	Ion transport
N37259	sodium-driven chloride bicarbonate exchanger, putative [Pediculus humanus corporis	Transport	Ion transport
N37798	PREDICTED: transient receptor potential-gamma protein-like [Metaseiulus occidental	Transport	Ion transport
N39293	hypothetical protein DAPPUDRAFT_40435 [Daphnia pulex]	Transport	Ion transport
N40751	hypothetical protein TcasGA2_TC010614 [Tribolium castaneum]	Transport	Ion transport
N42417	ABC protein, subfamily ABCG [Daphnia pulex]	Transport	Ion transport

ID	Annotation	GO term	Go child term
N48362		Transport	Ion transport
M2931	clottable protein [Marsupenaeus japonicus]	Transport	Lipid transport
M83897	PREDICTED: predicted protein-like [Saccoglossus kowalevskii]	Transport	Lipid transport
N10222	hypothetical protein DAPPUDRAFT_220651 [Daphnia pulex]	Transport	Lipid transport
N11955	hypothetical protein DAPPUDRAFT_332702 [Daphnia pulex]	Transport	Lipid transport
N21451	PREDICTED: oxysterol-binding protein-related protein 6 [Papio anubis]	Transport	Lipid transport
M856	CG8026, isoform B [Drosophila melanogaster] >gi|16648212|gb|AAL25371.1| GH221	Transport	Mitochondrial transport
N22878	PREDICTED: peroxisomal membrane protein PMP34-like [Apis mellifera]	Transport	Mitochondrial transport
M103029		Transport	Neurotransmitter transport
M74414	PREDICTED: sodium- and chloride-dependent glycine transporter 1-like [Saccoglossus	Transport	Neurotransmitter transport
N21981	Sodium-dependent neutral amino acid transporter B(0) [Harpegnathos saltator]	Transport	Neurotransmitter transport
N47183	AGAP007367-PA [Anopheles gambiae str. PEST] >gi|157020163|gb|EAA04277.4| AG	Transport	Neurotransmitter transport
M110921		Transport	Other
M111738	PREDICTED: nuclear RNA export factor 1 isoform X1 [Oreochromis niloticus]	Transport	Other
M12796	hypothetical protein DAPPUDRAFT_230342 [Daphnia pulex]	Transport	Other
M13825	oligopeptide transporter [Aedes aegypti] >gi|108875616|gb|EAT39841.1| AAEL0083	Transport	Other
M1536	PREDICTED: nuclear pore complex protein Nup107-like isoform 2 [Strongylocentrotus	Transport	Other
M16085	hemoglobin [Carcinus maenas]	Transport	Other
M21443	hypothetical protein DAPPUDRAFT_308659 [Daphnia pulex]	Transport	Other
M41629	crustacyanin subunit A [Fenneropenaeus merguiensis]	Transport	Other
M4464		Transport	Other
M60000	PREDICTED: apolipoprotein D-like [Aplysia californica]	Transport	Other
M69167	Canalicular multispecific organic anion transporter 1 [Tupaia chinensis]	Transport	Other
M7110		Transport	Other
M90499	PREDICTED: hematopoietic stem/progenitor cells 176-like [Saccoglossus kowalevskii]	Transport	Other
N13694	PREDICTED: coatomer subunit zeta-1-like isoform 1 [Nasonia vitripennis]	Transport	Other
N14537	crustacyanin subunit A [Fenneropenaeus merguiensis]	Transport	Other
N14811	crustacyanin subunit C [Fenneropenaeus merguiensis]	Transport	Other
N15675	DD9B [Marsupenaeus japonicus]	Transport	Other
N18559	PREDICTED: similar to Protein SDA1 homolog (Mystery protein 45A) [Tribolium castar	Transport	Other
N19209	hypothetical protein DAPPUDRAFT_22032 [Daphnia pulex]	Transport	Other
N19781	Transmembrane emp24 domain-containing protein 2 precursor [Lepeophtheirus salm	Transport	Other
N23540	PREDICTED: similar to AGAP009835-PA [Tribolium castaneum]	Transport	Other
N26082	Solute carrier family 15 member 1 [Acromyrmex echinatior]	Transport	Other
N27292	PREDICTED: exocyst complex component 5-like [Apis mellifera]	Transport	Other
N33902	hypothetical protein DAPPUDRAFT_303543 [Daphnia pulex]	Transport	Other
N41441		Transport	Other
N48380	Monosaccharide-transporting ATPase [Mesorhizobium sp. STM 4661] >gi|474662405	Transport	Other
N54308	nucleoside permease [uncultured SAR406 cluster bacterium HF0010_18O13]	Transport	Other
N57557	PREDICTED: vesicle-trafficking protein SEC22b-B-like [Megachile rotundata]	Transport	Other
N60720	hemocyanin 2 [Pacifastacus leniusculus]	Transport	Other
N61257	PREDICTED: protein retinal degeneration B-like isoform X1 [Ceratitis capitata]	Transport	Other
N7085	crustacyanin-A2 precursor [Panulirus cygnus]	Transport	Other
M1	PREDICTED: importin-5 [Megachile rotundata]	Transport	Protein transport
M102512	hypothetical protein CAPTEDRAFT_3285 [Capitella teleta]	Transport	Protein transport
M1086	AP-3 complex subunit mu-1, putative [Pediculus humanus corporis] >gi|212511278|g	Transport	Protein transport
M11116	PREDICTED: similar to signal recognition particle receptor alpha subunit (sr-alpha) [Tr	Transport	Protein transport
M14787	hypothetical protein DAPPUDRAFT_308902 [Daphnia pulex]	Transport	Protein transport
M15168	GJ20063 [Drosophila virilis] >gi|194145498|gb|EDW61894.1| GJ20063 [Drosophila v	Transport	Protein transport
M1524		Transport	Protein transport
M18819	hypothetical protein DAPPUDRAFT_207615 [Daphnia pulex]	Transport	Protein transport
M3711	PREDICTED: adaptor-related protein complex 2, beta 1 subunit-like [Saccoglossus kow	Transport	Protein transport
M4131	PREDICTED: hepatocyte growth factor-regulated tyrosine kinase substrate-like [Loxoc	Transport	Protein transport
M4292	conserved hypothetical protein [Pediculus humanus corporis] >gi|212510581|gb|EEI	Transport	Protein transport
M58187	hypothetical protein DAPPUDRAFT_324067 [Daphnia pulex]	Transport	Protein transport
M61823	Importin-7, putative [Pediculus humanus corporis] >gi|212517799|gb|EEB19638.1| I	Transport	Protein transport
M65226		Transport	Protein transport
M729	conserved hypothetical protein [Pediculus humanus corporis] >gi|212505671|gb|EEI	Transport	Protein transport
M73539	PREDICTED: similar to AGAP006942-PA [Tribolium castaneum]	Transport	Protein transport
M7463	AGAP004559-PA [Anopheles gambiae str. PEST] >gi|347972129|ref|XP_001238827.3	Transport	Protein transport
M76166	PREDICTED: similar to AGAP005934-PA [Tribolium castaneum] >gi|270003221|gb|EE	Transport	Protein transport
M8947	AP-3 complex subunit delta-1, putative [Ixodes scapularis] >gi|215498374|gb|EEC07	Transport	Protein transport
M93600		Transport	Protein transport
N10239	Coatomer subunit gamma-2, putative [Pediculus humanus corporis] >gi|212511156|g	Transport	Protein transport
N10417		Transport	Protein transport
N11893		Transport	Protein transport
N11897		Transport	Protein transport
N12003	hypothetical protein CAPTEDRAFT_223046 [Capitella teleta]	Transport	Protein transport
N13263	hypothetical protein DAPPUDRAFT_230571 [Daphnia pulex]	Transport	Protein transport
N13949	Marsupenaeus japonicus Ras-related nuclear protein isoform (Ran-iso) mRNA, compl	Transport	Protein transport
N14027	conserved hypothetical protein [Pediculus humanus corporis] >gi|212508955|gb|EEI	Transport	Protein transport
N18153	Importin-7 [Camponotus floridanus]	Transport	Protein transport
N18202	hypothetical protein DAPPUDRAFT_306007 [Daphnia pulex]	Transport	Protein transport
N18212	cellular apoptosis susceptibility protein [Fenneropenaeus chinensis]	Transport	Protein transport
N18311	hypothetical protein DAPPUDRAFT_313075 [Daphnia pulex]	Transport	Protein transport
N18371	PREDICTED: Nasonia vitripennis hypothetical protein LOC100121637 (LOC100121637	Transport	Protein transport

ID	Annotation	GO term	Go child term
N18464	conserved hypothetical protein [Pediculus humanus corporis] >gi|212514278|gb|EEI	Transport	Protein transport
N18477	PREDICTED: coatomer subunit delta [Sarcophilus harrisii]	Transport	Protein transport
N18709	hypothetical protein DAPPUDRAFT_230883 [Daphnia pulex]	Transport	Protein transport
N18754	Drosophila persimilis GL21499 (Dper\GL21499), mRNA	Transport	Protein transport
N18774	nuclear pore complex protein Nup85 [Danio rerio] >gi|82182122|sp|Q6DBY0.1|NUP	Transport	Protein transport
N18912	PREDICTED: signal recognition particle 72 kDa protein-like isoform 1 [Nasonia vitriper	Transport	Protein transport
N18978		Transport	Protein transport
N20490	PREDICTED: extracellular matrix protein FRAS1 isoform X1 [Mustela putorius furo]	Transport	Protein transport
N20701	PREDICTED: LOW QUALITY PROTEIN: metaxin-1-like [Nasonia vitripennis]	Transport	Protein transport
N21371	hypothetical protein DAPPUDRAFT_62579 [Daphnia pulex]	Transport	Protein transport
N21573	GJ11573 [Drosophila virilis] >gi|194155190|gb|EDW70374.1| GJ11573 [Drosophila v	Transport	Protein transport
N24163	translocon-associated protein, gamma subunit, putative [Ixodes scapularis] >gi|2155(	Transport	Protein transport
N24480		Transport	Protein transport
N33152	conserved hypothetical protein [Pediculus humanus corporis] >gi|212507534|gb|EEI	Transport	Protein transport
N34218	conserved hypothetical protein [Pediculus humanus corporis] >gi|212505671|gb|EEI	Transport	Protein transport
N34390	translocase of outer mitochondrial membrane 7 homolog [Tribolium castaneum]	Transport	Protein transport
N40807	hypothetical protein DAPPUDRAFT_39774 [Daphnia pulex]	Transport	Protein transport
N40945	hypothetical protein BRAFLDRAFT_69266 [Branchiostoma floridae] >gi|229280680|g	Transport	Protein transport
N41658	hypothetical protein BRAFLDRAFT_115215 [Branchiostoma floridae] >gi|229298493|	Transport	Protein transport
N42337	hypothetical protein DAPPUDRAFT_216176 [Daphnia pulex]	Transport	Protein transport
N42390	PREDICTED: transportin-1 [Nasonia vitripennis]	Transport	Protein transport
N45665	Charged multivesicular body protein 2a [Caligus rogercresseyi]	Transport	Protein transport
N8416		Transport	Protein transport
M103323	uncharacterized protein LOC100123296 precursor [Nasonia vitripennis]	Transport	Transmembrane transport
M104151	hypothetical protein DAPPUDRAFT_301164 [Daphnia pulex]	Transport	Transmembrane transport
M17584	hypothetical protein DAPPUDRAFT_312878 [Daphnia pulex]	Transport	Transmembrane transport
M18810	Armigeres subalbatus ASAP ID: 38923 ATP/ADP antiporter mRNA sequence	Transport	Transmembrane transport
M19423	Litopenaeus vannamei adenine nucleotide translocase 2 (LVANT2) mRNA, complete c	Transport	Transmembrane transport
M64131	conserved hypothetical protein [Pediculus humanus corporis] >gi|212507616|gb|EEI	Transport	Transmembrane transport
M65006		Transport	Transmembrane transport
M70222	PREDICTED: protein spinster-like isoform 2 [Nasonia vitripennis]	Transport	Transmembrane transport
M79487	hypothetical protein BRAFLDRAFT_118384 [Branchiostoma floridae] >gi|229277890|	Transport	Transmembrane transport
M96963	hypothetical protein DAPPUDRAFT_319730 [Daphnia pulex]	Transport	Transmembrane transport
N18984	Chromaffin granule amine transporter, putative [Pediculus humanus corporis] >gi|21	Transport	Transmembrane transport
N21595	PREDICTED: multidrug resistance-associated protein 1-like isoform 5 [Nasonia vitripe	Transport	Transmembrane transport
N22300	GK22112 [Drosophila willistoni] >gi|300681124|sp|B4MYA4.1|TRET1_DROWI RecNa	Transport	Transmembrane transport
N27620	Chromaffin granule amine transporter, putative [Pediculus humanus corporis] >gi|21	Transport	Transmembrane transport
N28367	trehalose transporter 1 [Belgica antarctica]	Transport	Transmembrane transport
N28608	hypothetical protein DAPPUDRAFT_319730 [Daphnia pulex]	Transport	Transmembrane transport
N30097	hypothetical protein BRAFLDRAFT_118384 [Branchiostoma floridae] >gi|229277890|	Transport	Transmembrane transport
N36354	hypothetical protein SINV_05375 [Solenopsis invicta]	Transport	Transmembrane transport
N39369	slalom [Tribolium castaneum]	Transport	Transmembrane transport
N40391	hypothetical protein DAPPUDRAFT_312099 [Daphnia pulex]	Transport	Transmembrane transport
N44994		Transport	Transmembrane transport
N45206	hypothetical protein BRAFLDRAFT_123511 [Branchiostoma floridae] >gi|229276954|	Transport	Transmembrane transport
N54348	Xanthobacter autotrophicus Py2, complete genome	Transport	Transmembrane transport
N9533	sugar transporter 16 [Nilaparvata lugens]	Transport	Transmembrane transport
N9534	PREDICTED: similar to sugar transporter [Tribolium castaneum]	Transport	Transmembrane transport
M11860	PREDICTED: ELMO domain-containing protein 2-like [Nasonia vitripennis]	Transport	Vesicle-mediated transport
M12077	PREDICTED: vesicle transport protein SFT2A [Chrysemys picta bellii]	Transport	Vesicle-mediated transport
M56060	PREDICTED: golgin subfamily A member 5 [Monodelphis domestica]	Transport	Vesicle-mediated transport
M99732		Transport	Vesicle-mediated transport
N18389	PREDICTED: dynamin-1-like protein-like isoform X1 [Ceratitis capitata] >gi|49898798!	Transport	Vesicle-mediated transport
N22080	hypothetical protein DAPPUDRAFT_223164 [Daphnia pulex]	Transport	Vesicle-mediated transport
N62075	hypothetical protein DAPPUDRAFT_306117 [Daphnia pulex]	Transport	Vesicle-mediated transport

energy
energy
energy
energy
energy
energy
energy
energy
energy
energy
energy
energy
energy
energy
energy
energy
energy
energy
energy
energy
energy
energy
energy
energy
energy
energy
energy
energy
energy
energy
energy
energy
energy
energy

olic process
olic process
olic process
olic process
olic process
olic process
olic process
olic process
olic process
olic process
olic process
olic process
olic process
olic process
olic process
olic process
olic process
olic process
olic process
olic process
olic process
olic process
olic process
olic process
olic process
olic process
olic process
olic process
olic process
olic process
olic process
olic process
olic process
olic process
olic process
olic process
olic process
olic process
olic process
olic process
