# Supplementary material for: Analysis of the circadian transcriptome of the Antarctic krill Euphausia superba
Source: Sci Rep. 2019 Sep 25;9:13894. doi: 10.1038/s41598-019-50282-1 (PMC6761102; doi:10.1038/s41598-019-50282-1)
Supplement: Supplementary file 7 — Supplementary Table 6 [file 41598_2019_50282_MOESM7_ESM.pdf]

Analysis of the circadian transcriptome of the Antarctic krill *Euphausia superba*

Alberto Biscontin[1,2,*], Paolo Martini[1], Rodolfo Costa[1], Achim Kramer[2], Bettina Meyer[3,4,5], So Kawaguchi[6], Mathias Teschke[3], Cristiano De Pittà[1,*]

[1]Dipartimento di Biologia, Università degli Studi di Padova, Padova, Italy

[2]Laboratory of Chronobiology, Charité Universitätsmedizin Berlin, Berlin, Germany

[3]Section Polar Biological Oceanography, Alfred Wegener Institute Helmholtz Centre for Polar and Marine Research, Bremerhaven, Germany

[4]Institute for Chemistry and Biology of the Marine Environment, Carl von Ossietzky University of Oldenburg, Oldenburg, Germany

[5]Helmholtz Institute for Functional Marine Biodiversity (HIFMB) at the University of Oldenburg, 26111 Oldenburg, Germany

[6]Department of Environment and Heritage, Australian Antarctic Division, Kingston, Tasmania, Australia

[*]Corresponding authors:

Cristiano De Pittà, Dipartimento di Biologia, Università degli Studi di Padova, via U. Bassi 58/B 35131 Padova, Italy; Phone: +39-049-8276210; Fax: +39-049-8276209; e-mail address: cristiano.depitta@unipd.it

Alberto Biscontin, Dipartimento di Biologia, Università degli Studi di Padova, via U. Bassi 58/B 35131 Padova, Italy; Phone: +39-049-8276228; Fax: +39-049-8276209; e-mail address: alberto.biscontin@unipd.it

Supplementary Table 6. Gene Ontology analysis of genes with a sinusoidal expression pattern in DD.
Classification of annotated genes with sinusoidal expression patterns throughout the 24-hour cycle in DD regime (802 genes).
[a]ID: accession number of krill master transcriptome 23; [b]Annotation = description of the gene; [c]GO term: gene ontology biological process;
[d]GO child term: most fitting gene ontology child term to further refine the functional annotation.

ID	Annotation	GO term	Go child term
**Cellular component organization**			
M21859	Branchiostoma floridae hypothetical protein, mRNA	Cellular component organiza	Chromosome organization
N11743	hypothetical protein [Zea mays]	Cellular component organiza	Chromosome organization
N12067	anti-silencing protein, putative [Ixodes scapularis] >gi|215509256|gl	Cellular component organiza	Chromosome organization
N16859	unknown [Dendroctonus ponderosae]	Cellular component organiza	Chromosome organization
N18776	DNA replication factor/protein phosphatase inhibitor SET/SPR-2, put	Cellular component organiza	Chromosome organization
N39837	PREDICTED: Metaseiulus occidentalis histone H3.3-like (LOC1009088	Cellular component organiza	Chromosome organization
N51451	predicted protein [Ostreococcus lucimarinus CCE9901] >gi|1445764:	Cellular component organiza	Chromosome organization
E1547	hypothetical protein DAPPUDRAFT_100611 [Daphnia pulex]	Cellular component organiza	Cytoskeleton organization
E1610	alpha-2-tubulin [Gecarcinus lateralis]	Cellular component organiza	Cytoskeleton organization
E4642	hypothetical protein AMTR_s00032p00056500 [Amborella trichopoc	Cellular component organiza	Cytoskeleton organization
M108155	hypothetical protein AaeL_AAEL008947 [Aedes aegypti] >gi|108875(	Cellular component organiza	Cytoskeleton organization
M19361	Cherax quadricarinatus alpha-I tubulin mRNA, complete cds	Cellular component organiza	Cytoskeleton organization
M31074	profilin [Penaeus monodon]	Cellular component organiza	Cytoskeleton organization
M3825	hypothetical protein BRAFLDRAFT_60087 [Branchiostoma floridae] >	Cellular component organiza	Cytoskeleton organization
M56919	PREDICTED: Saccoglossus kowalevskii cytoplasmic dynein 1 heavy ch:	Cellular component organiza	Cytoskeleton organization
M71966	beta-tubulin [Oxymonadida environmental sample]	Cellular component organiza	Cytoskeleton organization
M72897	Mesenchytraeus solifugus alpha tubulin a2 mRNA, partial cds	Cellular component organiza	Cytoskeleton organization
M74507	hypothetical protein BRAFLDRAFT_124711 [Branchiostoma floridae]	Cellular component organiza	Cytoskeleton organization
M7549		Cellular component organiza	Cytoskeleton organization
M79471	Botryotinia fuckeliana isolate T4 SuperContig_34_1 genomic supercc	Cellular component organiza	Cytoskeleton organization
M80588	GD13338 [Drosophila simulans] >gi|194195474|gb|EDX09050.1| GI	Cellular component organiza	Cytoskeleton organization
M8257	PREDICTED: kinesin family member 21A-like [Saccoglossus kowalevsl	Cellular component organiza	Cytoskeleton organization
M8793	Homarus americanus alpha-III tubulin mRNA, complete cds	Cellular component organiza	Cytoskeleton organization
N12749		Cellular component organiza	Cytoskeleton organization
N12769	hypothetical protein DAPPUDRAFT_311761 [Daphnia pulex]	Cellular component organiza	Cytoskeleton organization
N12891		Cellular component organiza	Cytoskeleton organization
N19790	Cryptocercus punctulatus alpha-tubulin (Atub) mRNA, partial cds	Cellular component organiza	Cytoskeleton organization
N20119	PREDICTED: Takifugu rubripes tubulin gamma-1 chain-like, transcript	Cellular component organiza	Cytoskeleton organization
N27135	kinesin 2B [Nasonia vitripennis] >gi|299782459|ref|NP_001177680.	Cellular component organiza	Cytoskeleton organization
N4420	beta-thymosin 5 [Pacifastacus leniusculus]	Cellular component organiza	Cytoskeleton organization
N49939	hypothetical protein CAPTEDRAFT_139004, partial [Capitella teleta]	Cellular component organiza	Cytoskeleton organization
N51300	PREDICTED: actin-related protein 2-like isoform 1 [Megachile rotund:	Cellular component organiza	Cytoskeleton organization
N6090	Homarus americanus alpha-I tubulin mRNA, complete cds	Cellular component organiza	Cytoskeleton organization
M2902	hypothetical protein [Platynereis dumerilii]	Cellular component organiza	Mitochondrion organization
**Cellular Process**			
E1235	PREDICTED: nuclear receptor 2C2-associated protein-like [Nasonia vi	Cellular Process	Cell adhesion
M3223	PREDICTED: calsyntenin-1-like [Apis mellifera]	Cellular Process	Cell adhesion
M68669	Thrombospondin-3 precursor, putative [Pediculus humanus corporis	Cellular Process	Cell adhesion
M69044	hypothetical protein DAPPUDRAFT_303213 [Daphnia pulex]	Cellular Process	Cell adhesion
M83352		Cellular Process	Cell adhesion
M88841	PREDICTED: protocadherin Fat 1 isoform X5 [Xenopus (Silurana) trop	Cellular Process	Cell adhesion
N20755	PREDICTED: Apis mellifera vinculin (Vinc), mRNA	Cellular Process	Cell adhesion
N46511	hemolectin, putative [Pediculus humanus corporis] >gi|212515478|{	Cellular Process	Cell adhesion
N55353	PREDICTED: similar to conserved hypothetical protein [Tribolium cast	Cellular Process	Cell adhesion
M1163	PREDICTED: ubiquitin-conjugating enzyme E2 C-like [Nasonia vitriper	Cellular Process	Cell cycle
M14298	structural maintenance of chromosome protein 2 [Daphnia pulex]	Cellular Process	Cell cycle
M16206	Salmo salar clone ssal-rgh-519-317 Ubiquitin-conjugating enzyme E2	Cellular Process	Cell cycle
M18062	PREDICTED: cell division cycle protein 123 homolog isoform X1 [Cavi:	Cellular Process	Cell cycle
M2128	cyclin H [Penaeus monodon]	Cellular Process	Cell cycle
M3247	hypothetical protein DAPPUDRAFT_301838 [Daphnia pulex]	Cellular Process	Cell cycle
M71538	Crassostrea gigas beta-tubulin mRNA, partial cds	Cellular Process	Cell cycle
N14809	ubiquitin protein ligase [Ixodes scapularis] >gi|215497661|gb|EEC07	Cellular Process	Cell cycle
N18501	hypothetical protein DAPPUDRAFT_200622 [Daphnia pulex]	Cellular Process	Cell cycle
N19234	PREDICTED: origin recognition complex subunit 4 [Sorex araneus]	Cellular Process	Cell cycle
N19471	hypothetical protein DAPPUDRAFT_300085 [Daphnia pulex]	Cellular Process	Cell cycle
N19655	PREDICTED: Orcinus orca structural maintenance of chromosomes 2	Cellular Process	Cell cycle
N22167	PREDICTED: Saccoglossus kowalevskii mps one binder kinase activatc	Cellular Process	Cell cycle
N2681	cyclin A [Litopenaeus vannamei] >gi|459217372|gb|AGG40745.1| c	Cellular Process	Cell cycle

ID	Annotation	GO term	Go child term
N28489	PREDICTED: F-box protein 31-like [Saccoglossus kowalevskii]	Cellular Process	Cell cycle
N33828	PREDICTED: kinesin family member 20A-like [Saccoglossus kowalevsk	Cellular Process	Cell cycle
N52657	PREDICTED: CDK-activating kinase assembly factor MAT1-like isoforn	Cellular Process	Cell cycle
N20070	Bcl-2-like protein 1 [Acromyrmex echinatior]	Cellular Process	Cell death
N20989	GalNAc/Gal-specific lectin [Crenomytilus grayanus]	Cellular Process	Cell death
N26730	PREDICTED: protein FAM32A-like [Ceratitis capitata]	Cellular Process	Cell death
N36400	GL14252 [Drosophila persimilis] >gi|194103844|gb|EDW25887.1| G	Cellular Process	Cell death
N6864	peroxiredoxin-like protein [Coptotermes formosanus]	Cellular Process	Cell death
N38997	GD20723 [Drosophila simulans] >gi|194199915|gb|EDX13491.1| GI	Cellular Process	Cell differentiation
M41892	Homarus americanus cytoplasmic type actin 1 mRNA, complete cds	Cellular Process	Cell growth
N22228	hypothetical protein DAPPUDRAFT_305543 [Daphnia pulex]	Cellular Process	Cell growth
M55361	hypothetical protein TcasGA2_TC011986 [Tribolium castaneum]	Cellular Process	Cell proliferation
N19000	GE12547 [Drosophila yakuba] >gi|194176995|gb|EDW90606.1| GE1	Cellular Process	Cell proliferation
N22500	hypothetical protein YQE_04700, partial [Dendroctonus ponderosae]	Cellular Process	Cell proliferation
N12719	PREDICTED: similar to dre4 CG1828-PA [Tribolium castaneum] >gi|27	Cellular Process	Other
N13328	creatine amidinohydrolase [Bacillus sp. BSD-8]	Cellular Process	Other
N18150	hypothetical protein BRAFLDRAFT_63199 [Branchiostoma floridae] >	Cellular Process	Other

Developmental process

ID	Annotation	GO term	Go child term
M113658	PREDICTED: hypothetical protein LOC100120957 isoform 2 [Nasonia	Developmental process	Embryo development
M79962	Inversin [Crassostrea gigas]	Developmental process	Embryo development
N19661	notchless homolog 1 [Xenopus (Silurana) tropicalis] >gi|115292058|	Developmental process	Embryo development
N42326	PREDICTED: COP9 signalosome complex subunit 3 [Ornithorhynchus	Developmental process	Embryo development
M62977	plexin A, putative [Ixodes scapularis] >gi|215500475|gb|EEC09969.1	Developmental process	Multicellular organismal development
M7907	PREDICTED: Nasonia vitripennis replication factor C subunit 5-like (LC	Developmental process	Multicellular organismal development
N11876	aurora/Ipl1p-related kinase [Marthasterias glacialis]	Developmental process	Multicellular organismal development
N15861	PREDICTED: neurobeachin-like [Megachile rotundata]	Developmental process	Multicellular organismal development
N16787	hypothetical protein DAPPUDRAFT_317955 [Daphnia pulex]	Developmental process	Multicellular organismal development
N23668	hypothetical protein TcasGA2_TC012198 [Tribolium castaneum]	Developmental process	Multicellular organismal development
N24387	Pediculus humanus corporis Actin, muscle, mRNA	Developmental process	Multicellular organismal development
N26752		Developmental process	Multicellular organismal development
N27562	hypothetical protein DAPPUDRAFT_43248 [Daphnia pulex]	Developmental process	Multicellular organismal development
N37774	Plexin-A4 precursor, putative [Pediculus humanus corporis] >gi|2125	Developmental process	Multicellular organismal development
N38617	SET domain protein [Populus trichocarpa] >gi|222853378|gb|EEE90	Developmental process	Multicellular organismal development
N57708	hypothetical protein DAPPUDRAFT_51329 [Daphnia pulex]	Developmental process	Multicellular organismal development
N8459	hypothetical protein DAPPUDRAFT_305931 [Daphnia pulex]	Developmental process	Multicellular organismal development
N18249	hypothetical protein DAPPUDRAFT_308857 [Daphnia pulex]	Developmental process	Muscle structure development
N6382		Developmental process	Muscle structure development
N6383		Developmental process	Muscle structure development
M7706	dystroglycan [Cherax quadricarinatus]	Developmental process	Nervous system development
N21561	hypothetical protein DAPPUDRAFT_303691 [Daphnia pulex]	Developmental process	Nervous system development
N40349		Developmental process	Nervous system development
N9258	RecName: Full=Semaphorin-1A; AltName: Full=Fasciclin IV; AltName:	Developmental process	Nervous system development
N22265	src tyrosine kinase, putative [Ixodes scapularis] >gi|215497797|gb|E	Developmental process	Other

Metabolic process

ID	Annotation	GO term	Go child term
M64363	autophagy protein 5 [Callinectes sapidus]	Metabolic process	Autofagy
E4652	PREDICTED: beta-1,4-mannosyl-glycoprotein 4-beta-N-acetylglucosar	Metabolic process	Carbohydrate metabolic process
M10340		Metabolic process	Carbohydrate metabolic process
M106275	Drosophila sechellia GM24804 (Dsec\GM24804), mRNA	Metabolic process	Carbohydrate metabolic process
M108205		Metabolic process	Carbohydrate metabolic process
M112586	hypothetical protein DAPPUDRAFT_310106 [Daphnia pulex]	Metabolic process	Carbohydrate metabolic process
M113059	Caenorhabditis remanei hypothetical protein (CRE_00061) mRNA, cc	Metabolic process	Carbohydrate metabolic process
M12607	chitinase 2 [Penaeus monodon]	Metabolic process	Carbohydrate metabolic process
M152	chitinase 1 precursor [Litopenaeus vannamei]	Metabolic process	Carbohydrate metabolic process
M15537	family 7 cellobiohydrolase [Chelura terebrans]	Metabolic process	Carbohydrate metabolic process
M15682		Metabolic process	Carbohydrate metabolic process
M16697	i-type lysozyme-like protein 2 [Penaeus monodon]	Metabolic process	Carbohydrate metabolic process
M17683	n-acetylgalactosaminyltransferase [Aedes aegypti] >gi|108884053|g	Metabolic process	Carbohydrate metabolic process
M18847	hypothetical protein DAPPUDRAFT_218978 [Daphnia pulex]	Metabolic process	Carbohydrate metabolic process
M19131	hypothetical protein DAPPUDRAFT_218978 [Daphnia pulex]	Metabolic process	Carbohydrate metabolic process
M23222	hypothetical protein DAPPUDRAFT_218978 [Daphnia pulex]	Metabolic process	Carbohydrate metabolic process
M26896	hypothetical protein DAPPUDRAFT_303131 [Daphnia pulex]	Metabolic process	Carbohydrate metabolic process
M33890	beta-N-acetylglucosaminidase [Litopenaeus vannamei]	Metabolic process	Carbohydrate metabolic process
M4050		Metabolic process	Carbohydrate metabolic process

ID	Annotation	GO term	Go child term
M44731	hypothetical protein DAPPUDRAFT_64687 [Daphnia pulex]	Metabolic process	Carbohydrate metabolic process
M4573	hypothetical protein DAPPUDRAFT_218978 [Daphnia pulex]	Metabolic process	Carbohydrate metabolic process
M5604		Metabolic process	Carbohydrate metabolic process
M57676		Metabolic process	Carbohydrate metabolic process
M58440	hypothetical protein [euryarchaeote SCGC AAA261-G15]	Metabolic process	Carbohydrate metabolic process
M60556	hypothetical protein DAPPUDRAFT_307215 [Daphnia pulex]	Metabolic process	Carbohydrate metabolic process
M64207		Metabolic process	Carbohydrate metabolic process
M65262	UDP-GalNAc:beta-1,3-N-acetylgalactosaminyltransferase 2, partial [C	Metabolic process	Carbohydrate metabolic process
M7734	hypothetical protein DAPPUDRAFT_243998 [Daphnia pulex]	Metabolic process	Carbohydrate metabolic process
M78640		Metabolic process	Carbohydrate metabolic process
M8647	chitinase [Litopenaeus vannamei]	Metabolic process	Carbohydrate metabolic process
M93819	hypothetical protein DAPPUDRAFT_303085 [Daphnia pulex]	Metabolic process	Carbohydrate metabolic process
N11540	chitinase [Fenneropenaeus chinensis]	Metabolic process	Carbohydrate metabolic process
N12995	hypothetical protein DAPPUDRAFT_218978 [Daphnia pulex]	Metabolic process	Carbohydrate metabolic process
N13131	hypothetical protein CAPTEDRAFT_149382 [Capitella teleta]	Metabolic process	Carbohydrate metabolic process
N13335	gastrolith protein [Cherax quadricarinatus]	Metabolic process	Carbohydrate metabolic process
N13357	hypothetical protein DAPPUDRAFT_218978 [Daphnia pulex]	Metabolic process	Carbohydrate metabolic process
N13771	PREDICTED: transaldolase-like [Nasonia vitripennis]	Metabolic process	Carbohydrate metabolic process
N14317	hypothetical protein DAPPUDRAFT_223002 [Daphnia pulex]	Metabolic process	Carbohydrate metabolic process
N18690	Colias eurytheme clone PGI3-31_10 phosphoglucose isomerase (PGI)	Metabolic process	Carbohydrate metabolic process
N18742	Ascidia sydneiensis samea 6-PGDH mRNA for 6-phosphogluconate de	Metabolic process	Carbohydrate metabolic process
N20102	phosphoglucomutase 2 [Xenopus (Silurana) tropicalis]	Metabolic process	Carbohydrate metabolic process
N20408	beta 1,4-endoglucanase [Cherax quadricarinatus]	Metabolic process	Carbohydrate metabolic process
N20512	chitin binding peritrophin-A, putative [Pediculus humanus corporis] >	Metabolic process	Carbohydrate metabolic process
N20630	PREDICTED: inter-alpha-trypsin inhibitor heavy chain H2 [Melopsitta	Metabolic process	Carbohydrate metabolic process
N21218	endo-beta-1,4-glucanase [Coptotermes formosanus]	Metabolic process	Carbohydrate metabolic process
N22117	PREDICTED: beta-hexosaminidase subunit alpha-like [Ciona intestina	Metabolic process	Carbohydrate metabolic process
N23226	Carbohydrate sulfotransferase 14 [Camponotus floridanus]	Metabolic process	Carbohydrate metabolic process
N23308	PREDICTED: glycogen debranching enzyme-like [Nasonia vitripennis]	Metabolic process	Carbohydrate metabolic process
N23374	hypothetical protein BRAFLDRAFT_123545 [Branchiostoma floridae]	Metabolic process	Carbohydrate metabolic process
N23832	hypothetical protein D910_12090 [Dendroctonus ponderosae]	Metabolic process	Carbohydrate metabolic process
N24477	cellulase [Coptotermes acinaciformis]	Metabolic process	Carbohydrate metabolic process
N26608	cuticular protein [Artemia franciscana]	Metabolic process	Carbohydrate metabolic process
N29331		Metabolic process	Carbohydrate metabolic process
N30729		Metabolic process	Carbohydrate metabolic process
N32193	UDP-GalNAc:polypeptide N-acetylgalactosaminyltransferase, putativ	Metabolic process	Carbohydrate metabolic process
N32465	hypothetical protein TRIADDRAFT_56539 [Trichoplax adhaerens] >gi	Metabolic process	Carbohydrate metabolic process
N34231	hypothetical protein DAPPUDRAFT_62084 [Daphnia pulex]	Metabolic process	Carbohydrate metabolic process
N34994	hypothetical protein DAPPUDRAFT_303518 [Daphnia pulex]	Metabolic process	Carbohydrate metabolic process
N38147	PREDICTED: protein O-mannosyl-transferase 2-like [Nasonia vitripen	Metabolic process	Carbohydrate metabolic process
N39090	Beta-1,4-galactosyltransferase 3 [Chelonia mydas]	Metabolic process	Carbohydrate metabolic process
N4191	cuticular protein analogous to peritrophins 3-A1 precursor [Nasonia	Metabolic process	Carbohydrate metabolic process
N43865	glycogen synthase [Marsupenaeus japonicus]	Metabolic process	Carbohydrate metabolic process
N44790	glycogen synthase [Marsupenaeus japonicus]	Metabolic process	Carbohydrate metabolic process
N46532	phosphoglucomutase/phosphomannomutase, putative [Ixodes scapu	Metabolic process	Carbohydrate metabolic process
N57180	predicted protein [Nematostella vectensis] >gi|156227446|gb|EDO	Metabolic process	Carbohydrate metabolic process
N7328		Metabolic process	Carbohydrate metabolic process
N9218	i-type lysozyme-like protein 2 [Penaeus monodon]	Metabolic process	Carbohydrate metabolic process
N9476	Gasterosteus aculeatus clone CFW246-H10 mRNA sequence	Metabolic process	Carbohydrate metabolic process
N9845		Metabolic process	Carbohydrate metabolic process
M1034	low molecular weight protein-tyrosine-phosphatase [Aedes aegypti]	Metabolic process	Cellular amino acid metabolic process
M109440	Asparaginyl-tRNA synthetase, isoform A [Drosophila melanogaster] >	Metabolic process	Cellular amino acid metabolic process
M12382	hypothetical protein DAPPUDRAFT_217067 [Daphnia pulex]	Metabolic process	Cellular amino acid metabolic process
M1500	aldehyde dehydrogenase 3-1 variant B [Aedes aegypti]	Metabolic process	Cellular amino acid metabolic process
M17615	hypothetical protein BRAFLDRAFT_241455 [Branchiostoma floridae]	Metabolic process	Cellular amino acid metabolic process
M2778	glyoxylate/hydroxypyruvate reductase, putative [Ixodes scapularis] >	Metabolic process	Cellular amino acid metabolic process
M2980	hypothetical protein DAPPUDRAFT_127519 [Daphnia pulex]	Metabolic process	Cellular amino acid metabolic process
M3130	Fumarylacetoacetase [Crassostrea gigas]	Metabolic process	Cellular amino acid metabolic process
M378	conserved hypothetical protein [Pediculus humanus corporis] >gi|21	Metabolic process	Cellular amino acid metabolic process
M4015		Metabolic process	Cellular amino acid metabolic process
M545	hypothetical protein DAPPUDRAFT_56817 [Daphnia pulex]	Metabolic process	Cellular amino acid metabolic process
M56721	L-aspartate dehydrogenase [Caligus clemensi]	Metabolic process	Cellular amino acid metabolic process
M61244	hypothetical protein DAPPUDRAFT_213935 [Daphnia pulex]	Metabolic process	Cellular amino acid metabolic process
M63222	hypothetical protein DAPPUDRAFT_308482 [Daphnia pulex]	Metabolic process	Cellular amino acid metabolic process

ID	Annotation	GO term	Go child term
M64302	hypothetical protein BRAFLDRAFT_264920 [Branchiostoma floridae]	Metabolic process	Cellular amino acid metabolic process
M67606	hypothetical protein TRIADDRAFT_33849 [Trichoplax adhaerens] >gi	Metabolic process	Cellular amino acid metabolic process
M695	hypothetical protein BRAFLDRAFT_117885 [Branchiostoma floridae]	Metabolic process	Cellular amino acid metabolic process
M7047	hypothetical protein DAPPUDRAFT_22132 [Daphnia pulex]	Metabolic process	Cellular amino acid metabolic process
M87594	PREDICTED: tryptophan 2,3-dioxygenase [Nomascus leucogenys]	Metabolic process	Cellular amino acid metabolic process
M93328		Metabolic process	Cellular amino acid metabolic process
M9982	hypothetical protein DAPPUDRAFT_318167 [Daphnia pulex]	Metabolic process	Cellular amino acid metabolic process
N14451	hematopoietic prostaglandin D synthase [Penaeus monodon]	Metabolic process	Cellular amino acid metabolic process
N14547	hypothetical protein DAPPUDRAFT_204241 [Daphnia pulex]	Metabolic process	Cellular amino acid metabolic process
N18359	RecName: Full=Arginine kinase; Short=AK >gi|311350|emb|CAA486	Metabolic process	Cellular amino acid metabolic process
N18519		Metabolic process	Cellular amino acid metabolic process
N19992	PREDICTED: arginase-1-like [Loxodonta africana]	Metabolic process	Cellular amino acid metabolic process
N20761	hypothetical protein DAPPUDRAFT_211091 [Daphnia pulex]	Metabolic process	Cellular amino acid metabolic process
N21644	carboxylase:pyruvate/acetyl-coa/propionyl-CoA [Daphnia pulex]	Metabolic process	Cellular amino acid metabolic process
N22074	glycine dehydrogenase [Aedes aegypti] >gi|108869068|gb|EAT3329	Metabolic process	Cellular amino acid metabolic process
N22945	tyrosine-protein phosphatase non-receptor type, putative [Pediculus	Metabolic process	Cellular amino acid metabolic process
N22968		Metabolic process	Cellular amino acid metabolic process
N23340	PREDICTED: similar to CG3999 CG3999-PA [Tribolium castaneum] >gi	Metabolic process	Cellular amino acid metabolic process
N24289	dopamine beta hydroxylase [Aedes aegypti] >gi|108878395|gb|EAT	Metabolic process	Cellular amino acid metabolic process
N24953	glutathione S-transferases [Macrobrachium nipponense]	Metabolic process	Cellular amino acid metabolic process
N25257	hypothetical protein DAPPUDRAFT_56817 [Daphnia pulex]	Metabolic process	Cellular amino acid metabolic process
N31050	fumble isoform 2 [Tribolium castaneum]	Metabolic process	Cellular amino acid metabolic process
N3597	hypothetical protein DAPPUDRAFT_213935 [Daphnia pulex]	Metabolic process	Cellular amino acid metabolic process
N36905	hypothetical protein DAPPUDRAFT_302347 [Daphnia pulex]	Metabolic process	Cellular amino acid metabolic process
N37288	glutathione S-transferases [Macrobrachium nipponense]	Metabolic process	Cellular amino acid metabolic process
N38756	Mesorhizobium ciceri biovar biserrulae WSM1271, complete genom	Metabolic process	Cellular amino acid metabolic process
N41475	hypothetical protein CAPTEDRAFT_133965 [Capitella teleta]	Metabolic process	Cellular amino acid metabolic process
N44223	hypothetical protein DAPPUDRAFT_23494 [Daphnia pulex]	Metabolic process	Cellular amino acid metabolic process
N48941	Huntingtin-associated protein-interacting protein, putative [Pediculu	Metabolic process	Cellular amino acid metabolic process
N55506	4-aminobutyrate aminotransferase [Aedes aegypti] >gi|108870984|	Metabolic process	Cellular amino acid metabolic process
N6799	hypothetical protein DAPPUDRAFT_318250 [Daphnia pulex]	Metabolic process	Cellular amino acid metabolic process
M113051	Euphausia superba voucher 760M mitochondrion, partial genome	Metabolic process	Generation of precursor metabolites and energy
M14565	mitochondrial ATP synthase gamma subunit precursor [Litopenaeus	Metabolic process	Generation of precursor metabolites and energy
M17951	PREDICTED: glycogen debranching enzyme-like [Nasonia vitripennis]	Metabolic process	Generation of precursor metabolites and energy
M18693	Euphausia superba large subunit ribosomal RNA gene, partial sequer	Metabolic process	Generation of precursor metabolites and energy
M2029	putative glycogen synthase [Daphnia pulex]	Metabolic process	Generation of precursor metabolites and energy
M2190	hypothetical protein DAPPUDRAFT_304152 [Daphnia pulex]	Metabolic process	Generation of precursor metabolites and energy
M71402	Euphausia superba isolate Hap_405 cytochrome c oxidase subunit I (	Metabolic process	Generation of precursor metabolites and energy
N15953	PREDICTED: predicted protein-like [Saccoglossus kowalevskii]	Metabolic process	Generation of precursor metabolites and energy
N19428		Metabolic process	Generation of precursor metabolites and energy
N29844	hypothetical protein DAPPUDRAFT_308028 [Daphnia pulex]	Metabolic process	Generation of precursor metabolites and energy
N31394	cytochrome c oxidase subunit VIIA putative [Scylla paramamosain]	Metabolic process	Generation of precursor metabolites and energy
M102803	hypothetical protein DAPPUDRAFT_315933 [Daphnia pulex]	Metabolic process	Lipid metabolic process
M15493	hypothetical protein DAPPUDRAFT_304936 [Daphnia pulex]	Metabolic process	Lipid metabolic process
M1878	Homo sapiens peroxisomal D3,D2-enoyl-CoA isomerase [synthetic cc	Metabolic process	Lipid metabolic process
M32708	putative triacylglycerol lipase [Daphnia pulex]	Metabolic process	Lipid metabolic process
M59828	PREDICTED: isoprenoid synthase domain-containing protein, partial [	Metabolic process	Lipid metabolic process
M63316	hypothetical protein DAPPUDRAFT_300149 [Daphnia pulex]	Metabolic process	Lipid metabolic process
M76131	Choline/ethanolaminephosphotransferase 1 [Harpegnathos saltator]	Metabolic process	Lipid metabolic process
M86465	PREDICTED: galactose-3-O-sulfotransferase 1-like [Saccoglossus kowa	Metabolic process	Lipid metabolic process
M94008	hypothetical protein D910_08102 [Dendroctonus ponderosae]	Metabolic process	Lipid metabolic process
N12972	PREDICTED: probable peroxisomal acyl-coenzyme A oxidase 1-like [N	Metabolic process	Lipid metabolic process
N19190	salivary alkaline phosphatase [Daphnia pulex]	Metabolic process	Lipid metabolic process
N19470		Metabolic process	Lipid metabolic process
N19809	PREDICTED: similar to gamma glutamyl transpeptidase [Tribolium ca	Metabolic process	Lipid metabolic process
N19820	hypothetical protein CAPTEDRAFT_160702 [Capitella teleta]	Metabolic process	Lipid metabolic process
N29547	pyruvate carboxylase [Aedes aegypti]	Metabolic process	Lipid metabolic process
N36501		Metabolic process	Lipid metabolic process
N36774	notch protein [Parhyale hawaiensis]	Metabolic process	Lipid metabolic process
N40568	ankyrin-1, putative [Pediculus humanus corporis] >gi|212518141|gb	Metabolic process	Lipid metabolic process
N46859	putative glucosylceramidase [Trichinella spiralis] >gi|316978112|gb|	Metabolic process	Lipid metabolic process
M94914	glycosyl-phosphatidylinositol-linked carbonic anhydrase [Litopenaeu	Metabolic process	Nitrogen compound metabolic process
N23042	Agmatinase, mitochondrial [Crassostrea gigas]	Metabolic process	Nitrogen compound metabolic process
N45146	hypothetical protein BRAFLDRAFT_95006 [Branchiostoma floridae] >	Metabolic process	Nitrogen compound metabolic process

ID	Annotation	GO term	Go child term
E2832	ribonucleotide reductase M2, gene 2 [Xenopus (Silurana) tropicalis] >	Metabolic process	Nucleobase-containing compound metabolic pro
E2966	ribonucleotide reductase M2, gene 2 [Xenopus (Silurana) tropicalis] >	Metabolic process	Nucleobase-containing compound metabolic pro
M1544	PREDICTED: uridine-cytidine kinase-like 1-like isoform 3 [Bombus ter	Metabolic process	Nucleobase-containing compound metabolic pro
M3158	Armadillidium vulgare clone Avu2Apol RNA polymerase II largest sub	Metabolic process	Nucleobase-containing compound metabolic pro
M35789	oncoprotein nm23 [Litopenaeus vannamei]	Metabolic process	Nucleobase-containing compound metabolic pro
M3981	PREDICTED: similar to adenine phosphoribosyltransferase [Tribolium	Metabolic process	Nucleobase-containing compound metabolic pro
M4420	hypothetical protein DAPPUDRAFT_218652 [Daphnia pulex]	Metabolic process	Nucleobase-containing compound metabolic pro
M56613	hypothetical protein D910_03395 [Dendroctonus ponderosae]	Metabolic process	Nucleobase-containing compound metabolic pro
M56865	PREDICTED: inosine-5'-monophosphate dehydrogenase isoform 1 [A	Metabolic process	Nucleobase-containing compound metabolic pro
M65183	UDP-glucose 4-epimerase [Aedes aegypti] >gi|108877101|gb|EAT41	Metabolic process	Nucleobase-containing compound metabolic pro
M78121	hypothetical protein TcasGA2_TC014142 [Tribolium castaneum]	Metabolic process	Nucleobase-containing compound metabolic pro
N18773		Metabolic process	Nucleobase-containing compound metabolic pro
N18959	exonuclease 3'-5' domain containing 2 [Xenopus laevis]	Metabolic process	Nucleobase-containing compound metabolic pro
N20569	glycinamide ribonucleotide synthetase-aminoimidazole ribonucleoti	Metabolic process	Nucleobase-containing compound metabolic pro
N21697		Metabolic process	Nucleobase-containing compound metabolic pro
N22717	hypothetical protein DAPPUDRAFT_332203 [Daphnia pulex]	Metabolic process	Nucleobase-containing compound metabolic pro
N24180	hypothetical protein DAPPUDRAFT_305695 [Daphnia pulex]	Metabolic process	Nucleobase-containing compound metabolic pro
N27868		Metabolic process	Nucleobase-containing compound metabolic pro
N28731		Metabolic process	Nucleobase-containing compound metabolic pro
N29027	hypothetical protein BRAFLDRAFT_116233 [Branchiostoma floridae]	Metabolic process	Nucleobase-containing compound metabolic pro
N32135	hypothetical protein DAPPUDRAFT_305911 [Daphnia pulex]	Metabolic process	Nucleobase-containing compound metabolic pro
N36668	hypothetical protein DAPPUDRAFT_304700 [Daphnia pulex]	Metabolic process	Nucleobase-containing compound metabolic pro
N42065	hypothetical protein DAPPUDRAFT_61988 [Daphnia pulex]	Metabolic process	Nucleobase-containing compound metabolic pro
N44205	PREDICTED: putative tRNA pseudouridine synthase Pus10 [Condylura	Metabolic process	Nucleobase-containing compound metabolic pro
E1475	guanine nucleotide-binding protein, putative [Ixodes scapularis] >gi|	Metabolic process	Other
M10080	5-aminolevulinate synthase [Limulus polyphemus]	Metabolic process	Other
M1011	luciferase [Photinus pyralis]	Metabolic process	Other
M103670	hypothetical protein BRAFLDRAFT_126836 [Branchiostoma floridae]	Metabolic process	Other
M108370	hypothetical protein BRAFLDRAFT_89744 [Branchiostoma floridae] >	Metabolic process	Other
M10960	PREDICTED: UDP-glucuronosyltransferase 2A2-like isoform X4 [Colun	Metabolic process	Other
M1109	transient receptor potential locus C protein precursor, putative [Pedi	Metabolic process	Other
M112331	protein kinase C and casein kinase substrate in neurons protein, puta	Metabolic process	Other
M12068	PREDICTED: molybdopterin synthase catalytic subunit isoform X1 [Fi	Metabolic process	Other
M12304	PREDICTED: hydroxysteroid dehydrogenase-like protein 1-like isofor	Metabolic process	Other
M12450	A kinase anchor protein, putative [Pediculus humanus corporis] >gi|	Metabolic process	Other
M13197	PREDICTED: dehydrogenase/reductase (SDR family) member 7B-like	Metabolic process	Other
M14643		Metabolic process	Other
M297	3-hydroxybutyrate dehydrogenase [Branchiostoma floridae] >gi|229	Metabolic process	Other
M3963	hypothetical protein DAPPUDRAFT_52203 [Daphnia pulex]	Metabolic process	Other
M423	hypothetical protein BRAFLDRAFT_240825 [Branchiostoma floridae]	Metabolic process	Other
M4676	SEL-1, putative [Ixodes scapularis] >gi|215509773|gb|EEC19226.1|	Metabolic process	Other
M56473	PREDICTED: long-chain-fatty-acid--CoA ligase 4-like [Metaseiulus occ	Metabolic process	Other
M586	Cytosolic Fe-S cluster assembly factor NUBP1-like protein [Crassostre	Metabolic process	Other
M58666	PREDICTED: enoyl-CoA hydratase domain-containing protein 3, mito	Metabolic process	Other
M59111	bifunctional protein folD [Culex quinquefasciatus] >gi|167881138|g	Metabolic process	Other
M69422		Metabolic process	Other
M75363	hypothetical protein DAPPUDRAFT_326816 [Daphnia pulex]	Metabolic process	Other
M77166	presqualene diphosphate phosphatase, putative [Pediculus humanu	Metabolic process	Other
M79948	O-methyltransferase [Litopenaeus vannamei]	Metabolic process	Other
M85316	PREDICTED: probable GDP-L-fucose synthase-like [Nasonia vitripenni	Metabolic process	Other
M9184	tubulin-specific chaperone D, putative [Pediculus humanus corporis]	Metabolic process	Other
N10697	PREDICTED: hypothetical protein [Saccoglossus kowalevskii]	Metabolic process	Other
N13505	Bromodomain-containing protein, putative [Pediculus humanus corp	Metabolic process	Other
N13655	conserved hypothetical protein [Pediculus humanus corporis] >gi|21	Metabolic process	Other
N15469	hypothetical protein TcasGA2_TC012288 [Tribolium castaneum]	Metabolic process	Other
N16244	C-jun-amino-terminal kinase-interacting protein, putative [Pediculus	Metabolic process	Other
N16685	hypothetical protein DAPPUDRAFT_299977 [Daphnia pulex]	Metabolic process	Other
N19258	hypothetical protein DAPPUDRAFT_309150 [Daphnia pulex]	Metabolic process	Other
N19420	ribosomal processing protein, putative [Ixodes scapularis] >gi|21549	Metabolic process	Other
N19613	hypothetical protein DAPPUDRAFT_230717 [Daphnia pulex]	Metabolic process	Other
N19941	PREDICTED: riboflavin kinase-like [Ornithorhynchus anatinus]	Metabolic process	Other
N20453	Regucalcin [Crassostrea gigas]	Metabolic process	Other
N20470	PREDICTED: 1-acyl-sn-glycerol-3-phosphate acyltransferase gamma-l	Metabolic process	Other
N21023	hypothetical protein [alpha proteobacterium SCGC AAA015-N04]	Metabolic process	Other

ID	Annotation	GO term	Go child term
N21214	hypothetical protein DAPPUDRAFT_306991 [Daphnia pulex]	Metabolic process	Other
N21331	PREDICTED: NAD kinase-like isoform 3 [Nasonia vitripennis]	Metabolic process	Other
N21705	myst histone acetyltransferase, putative [Pediculus humanus corpori	Metabolic process	Other
N22445	PREDICTED: LOW QUALITY PROTEIN: type I iodothyronine deiodinase	Metabolic process	Other
N22589	hypothetical protein BRAFLDRAFT_208157 [Branchiostoma floridae]	Metabolic process	Other
N22811	hypothetical protein BRAFLDRAFT_124342 [Branchiostoma floridae]	Metabolic process	Other
N23265	PREDICTED: hypothetical protein [Saccoglossus kowalevskii]	Metabolic process	Other
N23543	hypothetical protein DAPPUDRAFT_309070 [Daphnia pulex]	Metabolic process	Other
N24903	CG5567 [Drosophila melanogaster] >gi|7293934|gb|AAF49296.1| C	Metabolic process	Other
N24917	Histone-lysine N-methyltransferase trithorax [Camponotus floridanu	Metabolic process	Other
N26485	hypothetical protein DAPPUDRAFT_304984 [Daphnia pulex]	Metabolic process	Other
N29121	hypothetical protein YQE_12134, partial [Dendroctonus ponderosae]	Metabolic process	Other
N29192	hypothetical protein DAPPUDRAFT_300082 [Daphnia pulex]	Metabolic process	Other
N30152	transcriptional regulator [Pseudomonas sp. PAMC 25886]	Metabolic process	Other
N30559	PREDICTED: similar to glucosyl/glucuronosyl transferases [Tribolium	Metabolic process	Other
N31112	PREDICTED: long-chain-fatty-acid--CoA ligase 4-like isoform 2 [Nason	Metabolic process	Other
N32142	tRNA 2-thiocytidine biosynthesis protein ttcA [Lepeophtheirus salmo	Metabolic process	Other
N34277	conserved hypothetical protein [Pediculus humanus corporis] >gi|21	Metabolic process	Other
N34674	PREDICTED: hypothetical protein LOC100117960 [Nasonia vitripenni:	Metabolic process	Other
N37250	nucleoside-diphosphate-sugar epimerase [Phyllobacterium sp. YR53:	Metabolic process	Other
N38125	hypothetical protein DAPPUDRAFT_335707 [Daphnia pulex]	Metabolic process	Other
N41594	hypothetical protein TRIADDRAFT_14880 [Trichoplax adhaerens] >gi	Metabolic process	Other
N47123	glycerol-3-phosphate acyltransferase, putative [Pediculus humanus c	Metabolic process	Other
N47725	carbonyl reductase [Daphnia pulex]	Metabolic process	Other
N53915	hypothetical protein DAPPUDRAFT_306430 [Daphnia pulex]	Metabolic process	Other
N55193	hypothetical protein DAPPUDRAFT_316454 [Daphnia pulex]	Metabolic process	Other
N56176	hypothetical protein BRAFLDRAFT_85692 [Branchiostoma floridae] >	Metabolic process	Other
N57225	hypothetical protein CAPTEDRAFT_110364 [Capitella teleta]	Metabolic process	Other
N7968	PREDICTED: Caenorhabditis EpoxidE Hydrolase family member (ceeh	Metabolic process	Other
N9777	non-specific lipid-transfer protein-like [Riptortus pedestris]	Metabolic process	Other
E3651	extracellular superoxide dismutase precursor [Pacifastacus leniuscul	Metabolic process	Oxidation-reduction process
M13711	extracellular superoxide dismutase precursor [Pacifastacus leniuscul	Metabolic process	Oxidation-reduction process
M22256	selenoprotein W2 [Artemia franciscana]	Metabolic process	Oxidation-reduction process
M33821	ubiquitin [Schizosaccharomyces cryophilus OY26]	Metabolic process	Oxidation-reduction process
M3840	thioredoxin 2 [Xenopus laevis] >gi|27694724|gb|AAH43794.1| Txn2	Metabolic process	Oxidation-reduction process
M4754	hypothetical protein BRAFLDRAFT_125738 [Branchiostoma floridae]	Metabolic process	Oxidation-reduction process
M57279	PREDICTED: dehydrogenase/reductase SDR family member 11 [Pseu	Metabolic process	Oxidation-reduction process
M59486	zinc finger protein DHHC domain containing protein, putative [Pedic	Metabolic process	Oxidation-reduction process
M62474	cytochrome P450 [Litopenaeus vannamei]	Metabolic process	Oxidation-reduction process
M69744	hypothetical protein CAPTEDRAFT_17949 [Capitella teleta]	Metabolic process	Oxidation-reduction process
M77507	NADPH--cytochrome P450 reductase [Camponotus floridanus]	Metabolic process	Oxidation-reduction process
M77961	nitric oxide synthase [Penaeus monodon]	Metabolic process	Oxidation-reduction process
M9570	Gamma-butyrobetaine dioxygenase [Chelonia mydas]	Metabolic process	Oxidation-reduction process
N10225		Metabolic process	Oxidation-reduction process
N13595	PREDICTED: similar to cytochrome P450 [Tribolium castaneum] >gi|2	Metabolic process	Oxidation-reduction process
N13621	reductase, putative [Ixodes scapularis] >gi|215493148|gb|EEC0278!	Metabolic process	Oxidation-reduction process
N13735	hypothetical protein BRAFLDRAFT_127740 [Branchiostoma floridae]	Metabolic process	Oxidation-reduction process
N13839	PREDICTED: thioredoxin 2-like isoform 1 [Saccoglossus kowalevskii] >	Metabolic process	Oxidation-reduction process
N18513	hypothetical protein DAPPUDRAFT_308494 [Daphnia pulex]	Metabolic process	Oxidation-reduction process
N19275	hypothetical protein DAPPUDRAFT_188248 [Daphnia pulex]	Metabolic process	Oxidation-reduction process
N19570	NADPH--cytochrome P450, putative [Pediculus humanus corporis] >g	Metabolic process	Oxidation-reduction process
N19620	prostaglandin E synthase 2 [Penaeus monodon]	Metabolic process	Oxidation-reduction process
N19752	prostaglandin F synthase [Penaeus monodon]	Metabolic process	Oxidation-reduction process
N20066		Metabolic process	Oxidation-reduction process
N21156	hypothetical protein DAPPUDRAFT_330720 [Daphnia pulex]	Metabolic process	Oxidation-reduction process
N21262	PREDICTED: SOrbitol DeHydrogenase family member (sodh-1)-like [S	Metabolic process	Oxidation-reduction process
N21406	cytochrome P450 [Bemisia tabaci]	Metabolic process	Oxidation-reduction process
N21510	senecionine N-oxygenase, putative [Pediculus humanus corporis] >gi	Metabolic process	Oxidation-reduction process
N22067	PREDICTED: FAD-linked sulfhydryl oxidase ALR [Falco cherrug]	Metabolic process	Oxidation-reduction process
N22279	Dehydrogenase/reductase SDR family member 11 [Salmo salar] >gi|:	Metabolic process	Oxidation-reduction process
N23505	hypothetical protein DAPPUDRAFT_49503 [Daphnia pulex]	Metabolic process	Oxidation-reduction process
N26026	hypothetical protein SINV_06374 [Solenopsis invicta]	Metabolic process	Oxidation-reduction process
N30108	myosin heavy chain type 1 [Litopenaeus vannamei]	Metabolic process	Oxidation-reduction process
N35632	Mesorhizobium opportunistum WSM2075, complete genome	Metabolic process	Oxidation-reduction process

ID	Annotation	GO term	Go child term
N38917	prostaglandin F synthase [Penaeus monodon]	Metabolic process	Oxidation-reduction process
N44133	hypothetical protein DAPPUDRAFT_42950 [Daphnia pulex]	Metabolic process	Oxidation-reduction process
N46564	hypothetical protein BRAFLDRAFT_202728 [Branchiostoma floridae]	Metabolic process	Oxidation-reduction process
N6077	protaglandin reductase 1 [Penaeus monodon]	Metabolic process	Oxidation-reduction process
N8447	calcineurin B, putative [Ixodes scapularis] >gi|215493899|gb|EEC03!	Metabolic process	Oxidation-reduction process
N8450	calcineurin B, putative [Ixodes scapularis] >gi|215493899|gb|EEC03!	Metabolic process	Oxidation-reduction process
N8487	hypothetical protein DAPPUDRAFT_302851 [Daphnia pulex]	Metabolic process	Oxidation-reduction process

Nucleic acid metabolism

ID	Annotation	GO term	Go child term
M88105	PREDICTED: hypothetical protein [Saccoglossus kowalevskii]	Nucleic acid metabolism	DNA metabolic process
M1844	conserved hypothetical protein [Pediculus humanus corporis] >gi|21	Nucleic acid metabolism	DNA replication
M18552	PREDICTED: Nasonia vitripennis ribonucleoside-diphosphate reducta	Nucleic acid metabolism	DNA replication
M2403	hypothetical protein DAPPUDRAFT_300656 [Daphnia pulex]	Nucleic acid metabolism	DNA replication
M4642	PREDICTED: mediator of RNA polymerase II transcription subunit 20-	Nucleic acid metabolism	DNA replication
M5057	unknown [Dendroctonus ponderosae] >gi|478259673|gb|ENN7951	Nucleic acid metabolism	DNA replication
M56059	hypothetical protein TcasGA2_TC000123 [Tribolium castaneum]	Nucleic acid metabolism	DNA replication
M69392	PREDICTED: CCR4-NOT transcription complex subunit 2 isoform X1 [[	Nucleic acid metabolism	DNA replication
M88273	proliferating cell nuclear antigen [Litopenaeus vannamei] >gi|33972	Nucleic acid metabolism	DNA replication
N13117	PREDICTED: similar to AGAP007416-PA [Tribolium castaneum] >gi|27	Nucleic acid metabolism	DNA replication
N15339	pol [Schistosoma mansoni]	Nucleic acid metabolism	DNA replication
N25699	PREDICTED: uncharacterized protein LOC101853172 [Aplysia californ	Nucleic acid metabolism	DNA replication
M104450	reverse transcriptase [Nullibrotheas allenii]	Nucleic acid metabolism	Regulation of gene expression
M108095	PREDICTED: zinc finger protein 11B-like [Oryctolagus cuniculus]	Nucleic acid metabolism	Regulation of gene expression
M16302	ETS domain-containing protein Elk-4, putative [Ixodes scapularis] >gi	Nucleic acid metabolism	Regulation of gene expression
M28818	PREDICTED: zinc finger protein 160-like, partial [Sarcophilus harrisii]	Nucleic acid metabolism	Regulation of gene expression
M3783	PREDICTED: enhancer of split mgamma protein-like [Apis mellifera] >	Nucleic acid metabolism	Regulation of gene expression
M4449	Lian-Aa1 retrotransposon protein [Aedes aegypti]	Nucleic acid metabolism	Regulation of gene expression
M55413	hypothetical protein CAPTEDRAFT_212468 [Capitella teleta]	Nucleic acid metabolism	Regulation of gene expression
M57032	PREDICTED: poly(A) polymerase gamma [Apis florea]	Nucleic acid metabolism	Regulation of gene expression
M59084	TPA: endonuclease-reverse transcriptase [Schistosoma mansoni]	Nucleic acid metabolism	Regulation of gene expression
M6323	hypothetical protein BRAFLDRAFT_114861 [Branchiostoma floridae]	Nucleic acid metabolism	Regulation of gene expression
M66830	hypothetical protein YQE_12440, partial [Dendroctonus ponderosae]	Nucleic acid metabolism	Regulation of gene expression
M68024	hypothetical protein CRE_19860 [Caenorhabditis remanei] >gi|3082(	Nucleic acid metabolism	Regulation of gene expression
M71990	endonuclease-reverse transcriptase [Bombyx mori]	Nucleic acid metabolism	Regulation of gene expression
M89294	PREDICTED: zinc finger protein 558 [Felis catus]	Nucleic acid metabolism	Regulation of gene expression
M91019	scalloped protein, partial [Parhyale hawaiensis]	Nucleic acid metabolism	Regulation of gene expression
N13057	PREDICTED: uncharacterized protein LOC100878829 [Megachile rotu	Nucleic acid metabolism	Regulation of gene expression
N14443	protein groucho, putative [Pediculus humanus corporis] >gi|212511!	Nucleic acid metabolism	Regulation of gene expression
N18441	PREDICTED: LOW QUALITY PROTEIN: ets DNA-binding protein pokkur	Nucleic acid metabolism	Regulation of gene expression
N19117		Nucleic acid metabolism	Regulation of gene expression
N19683	TPA: endonuclease-reverse transcriptase [Schistosoma mansoni]	Nucleic acid metabolism	Regulation of gene expression
N19739		Nucleic acid metabolism	Regulation of gene expression
N20125	Hepatic leukemia factor [Crassostrea gigas]	Nucleic acid metabolism	Regulation of gene expression
N22594	RuvB-like protein 2 [Penaeus monodon]	Nucleic acid metabolism	Regulation of gene expression
N22843	cAMP response element-binding protein [Polyrhachis vicina]	Nucleic acid metabolism	Regulation of gene expression
N24657	endonuclease/reverse transcriptase [Branchiostoma floridae]	Nucleic acid metabolism	Regulation of gene expression
N26250	transcriptional regulator [Phyllobacterium sp. YR531] >gi|39821151	Nucleic acid metabolism	Regulation of gene expression
N28156	mrg-binding protein, putative [Ixodes scapularis] >gi|215507567|gb	Nucleic acid metabolism	Regulation of gene expression
N28353	PREDICTED: RNA-directed DNA polymerase from mobile element joc	Nucleic acid metabolism	Regulation of gene expression
N33433	PREDICTED: similar to TFIIA [Tribolium castaneum] >gi|270013142|g	Nucleic acid metabolism	Regulation of gene expression
N36361	PREDICTED: zinc finger protein 391 isoform X1 [Mustela putorius fur	Nucleic acid metabolism	Regulation of gene expression
N38485	enhancer of split mgamma protein-like protein [Daphnia pulex]	Nucleic acid metabolism	Regulation of gene expression
N41657	conserved hypothetical protein [Ixodes scapularis] >gi|215502246|g	Nucleic acid metabolism	Regulation of gene expression
N43299	Ecdysone-induced protein 74EF isoform B [Camponotus floridanus]	Nucleic acid metabolism	Regulation of gene expression
N53807	ETS factor [Trichinella spiralis] >gi|316978761|gb|EFV61692.1| ETS	Nucleic acid metabolism	Regulation of gene expression
N60094	RecName: Full=Zinc finger Y-chromosomal protein 1; Short=ZFY-1 >g	Nucleic acid metabolism	Regulation of gene expression
N9039	conserved hypothetical protein [Pediculus humanus corporis] >gi|21	Nucleic acid metabolism	Regulation of gene expression
M10251	hypothetical protein TcasGA2_TC012014 [Tribolium castaneum]	Nucleic acid metabolism	Regulation of gene expression, epigenetic
M3	Xenopus laevis similar to histone deacetylase 2, mRNA (cDNA clone I	Nucleic acid metabolism	Regulation of gene expression, epigenetic
M56146		Nucleic acid metabolism	Regulation of gene expression, epigenetic
N13139		Nucleic acid metabolism	Regulation of gene expression, epigenetic
N13941		Nucleic acid metabolism	Regulation of gene expression, epigenetic
N22640		Nucleic acid metabolism	Regulation of gene expression, epigenetic
N23215	programmed cell death-involved protein, putative [Ixodes scapularis	Nucleic acid metabolism	Regulation of gene expression, epigenetic

ID	Annotation	GO term	Go child term
N23757	hypothetical protein DAPPUDRAFT_317395 [Daphnia pulex]	Nucleic acid metabolism	Regulation of gene expression, epigenetic
N44273	GD13897 [Drosophila simulans] >gi|194195768|gb|EDX09344.1| GI	Nucleic acid metabolism	Regulation of gene expression, epigenetic
M11735		Nucleic acid metabolism	RNA metabolic process
M12614	PREDICTED: snurportin-1-like [Aplysia californica]	Nucleic acid metabolism	RNA metabolic process
M15533	PREDICTED: ribonucleases P/MRP protein subunit POP1 isoform X2 [I	Nucleic acid metabolism	RNA metabolic process
M18282	PREDICTED: tRNA-splicing endonuclease subunit Sen34-like [Apis me	Nucleic acid metabolism	RNA metabolic process
M2617	hypothetical protein CAPTEDRAFT_73288, partial [Capitella teleta]	Nucleic acid metabolism	RNA metabolic process
M3936	hypothetical protein DAPPUDRAFT_299967 [Daphnia pulex]	Nucleic acid metabolism	RNA metabolic process
M4435	hypothetical protein CAPTEDRAFT_220697 [Capitella teleta]	Nucleic acid metabolism	RNA metabolic process
M64830	PREDICTED: similar to spliceosome associated protein [Tribolium cas	Nucleic acid metabolism	RNA metabolic process
M88815	hypothetical protein TcasGA2_TC009040 [Tribolium castaneum]	Nucleic acid metabolism	RNA metabolic process
M96360	hypothetical protein DAPPUDRAFT_305992 [Daphnia pulex]	Nucleic acid metabolism	RNA metabolic process
M9958	PREDICTED: cytoplasmic tRNA 2-thiolation protein 1 isoform X2 [Dan	Nucleic acid metabolism	RNA metabolic process
N20615	Plasmodium knowlesi strain H fibrillarin (PKH_133980) mRNA, comp	Nucleic acid metabolism	RNA metabolic process
N20657	hypothetical protein DAPPUDRAFT_325988 [Daphnia pulex]	Nucleic acid metabolism	RNA metabolic process
N20737	hypothetical protein DAPPUDRAFT_303058 [Daphnia pulex]	Nucleic acid metabolism	RNA metabolic process
N22292	AGAP004370-PA [Anopheles gambiae str. PEST] >gi|157017271|gb|	Nucleic acid metabolism	RNA metabolic process
N22415	PREDICTED: transcription elongation factor SPT5-like [Megachile rotu	Nucleic acid metabolism	RNA metabolic process
N25018	PREDICTED: cleavage and polyadenylation specificity factor subunit 5	Nucleic acid metabolism	RNA metabolic process
N25181	pre-mRNA-splicing factor ATP-dependent RNA helicase PRP22, putat	Nucleic acid metabolism	RNA metabolic process
N32178	CDK5 regulatory subunit-associated protein, putative [Ixodes scapula	Nucleic acid metabolism	RNA metabolic process
N32761	Nucleolar protein 14 [Chelonia mydas]	Nucleic acid metabolism	RNA metabolic process
N38517	hypothetical protein DAPPUDRAFT_304474 [Daphnia pulex]	Nucleic acid metabolism	RNA metabolic process
N39879	hypothetical protein BRAFLDRAFT_209586 [Branchiostoma floridae]	Nucleic acid metabolism	RNA metabolic process
M67189	hypothetical protein BRAFLDRAFT_114926 [Branchiostoma floridae]	Nucleic acid metabolism	Transcription, DNA-dependent
N21123	PREDICTED: DNA-directed RNA polymerase I subunit RPA43-like [Nas	Nucleic acid metabolism	Transcription, DNA-dependent

Protein metabolism

M101513	hypothetical protein BRAFLDRAFT_120334 [Branchiostoma floridae]	Protein metabolism	Cellular protein modification process
M105185	PREDICTED: E3 ubiquitin-protein ligase UBR4 [Melopsittacus undulat	Protein metabolism	Cellular protein modification process
M15270	tyrosine-protein kinase Src42A, putative [Pediculus humanus corpori	Protein metabolism	Cellular protein modification process
M1547		Protein metabolism	Cellular protein modification process
M16714		Protein metabolism	Cellular protein modification process
M17927	PREDICTED: Bombus terrestris dual specificity mitogen-activated pro	Protein metabolism	Cellular protein modification process
M182	Putative protein phosphatase [Acromyrmex echinatior]	Protein metabolism	Cellular protein modification process
M2345	PREDICTED: Megachile rotundata cyclin-dependent kinase 1-like (LO	Protein metabolism	Cellular protein modification process
M2932		Protein metabolism	Cellular protein modification process
M58599	projectin [Procambarus clarkii]	Protein metabolism	Cellular protein modification process
M59923	hypothetical protein BRAFLDRAFT_63881 [Branchiostoma floridae] >	Protein metabolism	Cellular protein modification process
M63511	PREDICTED: phosphatidylinositol N-acetylglucosaminyltransferase su	Protein metabolism	Cellular protein modification process
M66217	Diphthamide biosynthesis protein 2 [Dicentrarchus labrax]	Protein metabolism	Cellular protein modification process
M73098		Protein metabolism	Cellular protein modification process
M74771	hypothetical protein DAPPUDRAFT_320464 [Daphnia pulex]	Protein metabolism	Cellular protein modification process
M83349	hypothetical protein D910_02598 [Dendroctonus ponderosae]	Protein metabolism	Cellular protein modification process
M84527	projectin [Procambarus clarkii]	Protein metabolism	Cellular protein modification process
M9345	hypothetical protein DAPPUDRAFT_52407 [Daphnia pulex]	Protein metabolism	Cellular protein modification process
N11830		Protein metabolism	Cellular protein modification process
N11990	PREDICTED: similar to CG1973 CG1973-PA [Tribolium castaneum]	Protein metabolism	Cellular protein modification process
N12213	PREDICTED: hypothetical protein LOC100118283 [Nasonia vitripennis	Protein metabolism	Cellular protein modification process
N13007	checkpoint kinase 1 [Daphnia pulex]	Protein metabolism	Cellular protein modification process
N1320	hypothetical protein DAPPUDRAFT_306600 [Daphnia pulex]	Protein metabolism	Cellular protein modification process
N1323	hypothetical protein DAPPUDRAFT_306600 [Daphnia pulex]	Protein metabolism	Cellular protein modification process
N18288	casein kinase 1 epsilon [Eurydice pulchra]	Protein metabolism	Cellular protein modification process
N18309	hypothetical protein DAPPUDRAFT_52407 [Daphnia pulex]	Protein metabolism	Cellular protein modification process
N18354	hypothetical protein KGM_05346 [Danaus plexippus]	Protein metabolism	Cellular protein modification process
N18824	Ser/Thr protein phosphatase, putative [Ixodes scapularis] >gi|21550	Protein metabolism	Cellular protein modification process
N19889	darkener of apricot-like protein [Daphnia pulex]	Protein metabolism	Cellular protein modification process
N20036	hypothetical protein DAPPUDRAFT_308742 [Daphnia pulex]	Protein metabolism	Cellular protein modification process
N20067		Protein metabolism	Cellular protein modification process
N21078	HemK methyltransferase family member 1 [Harpegnathos saltator]	Protein metabolism	Cellular protein modification process
N21177	hypothetical protein CAPTEDRAFT_147624 [Capitella teleta]	Protein metabolism	Cellular protein modification process
N23368	hypothetical protein DAPPUDRAFT_194528 [Daphnia pulex]	Protein metabolism	Cellular protein modification process
N24752	Drosophila yakuba GE21090 (Dyak\GE21090), mRNA	Protein metabolism	Cellular protein modification process
N28081	PREDICTED: Apis florea serine/threonine-protein kinase tousled-like	Protein metabolism	Cellular protein modification process

ID	Annotation	GO term	Go child term
N28784	protein purity of essence, putative [Pediculus humanus corporis] >gi	Protein metabolism	Cellular protein modification process
N33066	PREDICTED: Apis mellifera tricornered (trc), mRNA	Protein metabolism	Cellular protein modification process
N33379	PREDICTED: similar to Nedd4 CG7555-PC [Tribolium castaneum]	Protein metabolism	Cellular protein modification process
N34569	PREDICTED: tyrosine-protein kinase Fer [Sorex araneus]	Protein metabolism	Cellular protein modification process
N36911	CMGC/DYRK/PRP4 protein kinase [Loa loa]	Protein metabolism	Cellular protein modification process
N38634	PREDICTED: probable deoxyhypusine synthase-like [Megachile rotun	Protein metabolism	Cellular protein modification process
N40247	activin receptor type I, putative [Aedes aegypti] >gi|108874587|gb|	Protein metabolism	Cellular protein modification process
N43819	PREDICTED: vascular endothelial growth factor receptor 1-like [Apis f	Protein metabolism	Cellular protein modification process
N44456	hypothetical protein DAPPUDRAFT_304762 [Daphnia pulex]	Protein metabolism	Cellular protein modification process
N19772		Protein metabolism	Macromolecular complex assembly
N23035	PREDICTED: similar to cytochrome c oxidase assembly protein cox11	Protein metabolism	Macromolecular complex assembly
M70652	procollagen-lysine2-oxoglutarate 5-dioxygenase [Schistosoma mansc	Protein metabolism	Other
M11564	PREDICTED: similar to DnaJ (Hsp40) homolog, subfamily C, member 1	Protein metabolism	Protein folding
M56202	GK11959 [Drosophila willistoni] >gi|194166340|gb|EDW81241.1| G	Protein metabolism	Protein folding
M65353	related to FPR2-FK506/rapamycin-binding protein of the ER [Ustilagc	Protein metabolism	Protein folding
M67058	Paracyclopina nana heat shock protein 90 mRNA, complete cds	Protein metabolism	Protein folding
N13783	RecName: Full=Peptidyl-prolyl cis-trans isomerase B; Short=PPIase B;	Protein metabolism	Protein folding
N19207	hypothetical protein DAPPUDRAFT_99108 [Daphnia pulex]	Protein metabolism	Protein folding
N19298		Protein metabolism	Protein folding
N27358	PREDICTED: peptidyl-prolyl cis-trans isomerase-like 3-like isoform 1 [	Protein metabolism	Protein folding
N35673	hypothetical protein BATDEDRAFT_35110 [Batrachochytrium dendrc	Protein metabolism	Protein folding
N38447	hypothetical protein BRAFLDRAFT_114918 [Branchiostoma floridae]	Protein metabolism	Protein folding
M10527	conserved hypothetical protein [Pediculus humanus corporis] >gi|21	Protein metabolism	Proteolysis
M108428	hypothetical protein DAPPUDRAFT_303500 [Daphnia pulex]	Protein metabolism	Proteolysis
M11130		Protein metabolism	Proteolysis
M113044		Protein metabolism	Proteolysis
M12906	hypothetical protein DAPPUDRAFT_315872 [Daphnia pulex]	Protein metabolism	Proteolysis
M13910	cathepsin D-like protein [Homarus americanus]	Protein metabolism	Proteolysis
M18434	CUB-serine protease [Panulirus argus]	Protein metabolism	Proteolysis
M19739	CUB-serine protease [Panulirus argus]	Protein metabolism	Proteolysis
M20696	masquerade-like protein [Pacifastacus leniusculus]	Protein metabolism	Proteolysis
M2214		Protein metabolism	Proteolysis
M22508	serine protease [Penaeus monodon]	Protein metabolism	Proteolysis
M229		Protein metabolism	Proteolysis
M23676	PREDICTED: venom serine protease 34-like [Bombus terrestris]	Protein metabolism	Proteolysis
M23700	hypothetical protein CAPTEDRAFT_206264 [Capitella teleta]	Protein metabolism	Proteolysis
M23774	masquerade-like protein, partial [Armadillidium vulgare]	Protein metabolism	Proteolysis
M23838	masquerade-like serine proteinase-like protein 3 [Penaeus monodon	Protein metabolism	Proteolysis
M241	hypothetical protein TcasGA2_TC013516 [Tribolium castaneum]	Protein metabolism	Proteolysis
M25061	masquerade-like protein [Pacifastacus leniusculus]	Protein metabolism	Proteolysis
M29587	masquerade-like protein [Pacifastacus leniusculus]	Protein metabolism	Proteolysis
M3147	PREDICTED: N-acetylated alpha-linked acidic dipeptidase 2-like [Sacc	Protein metabolism	Proteolysis
M3293	masquerade-like protein [Pacifastacus leniusculus]	Protein metabolism	Proteolysis
M3294	hypothetical protein DAPPUDRAFT_316896 [Daphnia pulex]	Protein metabolism	Proteolysis
M3619	CUB-serine protease [Panulirus argus]	Protein metabolism	Proteolysis
M4677	hypothetical protein DAPPUDRAFT_307535 [Daphnia pulex]	Protein metabolism	Proteolysis
M47110	collagenolytic serine protease [Paralithodes camtschaticus]	Protein metabolism	Proteolysis
M56778	PREDICTED: proclotting enzyme [Nasonia vitripennis]	Protein metabolism	Proteolysis
M61800	PREDICTED: protein SpAN-like [Strongylocentrotus purpuratus]	Protein metabolism	Proteolysis
M61855	masquerade-like protein [Pacifastacus leniusculus]	Protein metabolism	Proteolysis
M6356	hypothetical protein DAPPUDRAFT_305917 [Daphnia pulex]	Protein metabolism	Proteolysis
M66918	PREDICTED: hypothetical protein [Saccoglossus kowalevskii]	Protein metabolism	Proteolysis
M67539		Protein metabolism	Proteolysis
M67909	ubiquitin conjugation factor E4 B, putative [Pediculus humanus corpc	Protein metabolism	Proteolysis
M75292	PREDICTED: N-acetylated-alpha-linked acidic dipeptidase 2-like [Stroı	Protein metabolism	Proteolysis
M75323	hypothetical protein DAPPUDRAFT_303785 [Daphnia pulex]	Protein metabolism	Proteolysis
M8106	PREDICTED: serine carboxypeptidase 1-like [Saccoglossus kowalevski	Protein metabolism	Proteolysis
M83540	Ubiquitin carboxyl-terminal hydrolase 32 [Crassostrea gigas]	Protein metabolism	Proteolysis
M85248	hypothetical protein DAPPUDRAFT_314716 [Daphnia pulex]	Protein metabolism	Proteolysis
M931	protease [Homarus americanus]	Protein metabolism	Proteolysis
M9318	anaphase-promoting complex subunit, putative [Pediculus humanus	Protein metabolism	Proteolysis
M94727	hypothetical protein DAPPUDRAFT_311166 [Daphnia pulex]	Protein metabolism	Proteolysis
M96397	PREDICTED: similar to trypsin-like serine protease [Tribolium castane	Protein metabolism	Proteolysis
M97028	Protein CBR-NAS-4 [Caenorhabditis briggsae]	Protein metabolism	Proteolysis

ID	Annotation	GO term	Go child term
N10435	Aminopeptidase N precursor, putative [Pediculus humanus corporis]	Protein metabolism	Proteolysis
N10456	PREDICTED: carboxypeptidase B-like [Apis florea]	Protein metabolism	Proteolysis
N11966	Cytosolic non-specific dipeptidase [Lepeophtheirus salmonis]	Protein metabolism	Proteolysis
N12001	PREDICTED: cullin-4B-like [Nasonia vitripennis]	Protein metabolism	Proteolysis
N12203	hypothetical protein TcasGA2_TC014587 [Tribolium castaneum]	Protein metabolism	Proteolysis
N13043	masquerade-like protein [Pacifastacus leniusculus]	Protein metabolism	Proteolysis
N13119		Protein metabolism	Proteolysis
N13604	ubiquitin protein ligase [Ixodes scapularis] >gi|215497661|gb|EEC07	Protein metabolism	Proteolysis
N1431	PREDICTED: protein SpAN-like [Strongylocentrotus purpuratus]	Protein metabolism	Proteolysis
N16740	truncated cathepsin A [Eriocheir sinensis]	Protein metabolism	Proteolysis
N17787		Protein metabolism	Proteolysis
N18522		Protein metabolism	Proteolysis
N19540		Protein metabolism	Proteolysis
N19798	hypothetical protein DAPPUDRAFT_311166 [Daphnia pulex]	Protein metabolism	Proteolysis
N19964	masquerade-like serine proteinase-like protein 2 [Penaeus monodon	Protein metabolism	Proteolysis
N20895	GG21285 [Drosophila erecta] >gi|257096534|sp|B3P3J9.1|HTRA2_[	Protein metabolism	Proteolysis
N21404	hypothetical protein DAPPUDRAFT_216354 [Daphnia pulex]	Protein metabolism	Proteolysis
N21719	hypothetical protein DAPPUDRAFT_330318 [Daphnia pulex]	Protein metabolism	Proteolysis
N24486	CUB-serine protease [Panulirus argus]	Protein metabolism	Proteolysis
N24880	hypothetical protein DAPPUDRAFT_63727 [Daphnia pulex]	Protein metabolism	Proteolysis
N25628	hypothetical protein DAPPUDRAFT_311166 [Daphnia pulex]	Protein metabolism	Proteolysis
N26364	CUB-serine protease [Panulirus argus]	Protein metabolism	Proteolysis
N26789	angiotensin converting enzyme [Pontastacus leptodactylus]	Protein metabolism	Proteolysis
N27064	Aminopeptidase N precursor, putative [Pediculus humanus corporis]	Protein metabolism	Proteolysis
N27897	hypothetical protein DAPPUDRAFT_311166 [Daphnia pulex]	Protein metabolism	Proteolysis
N36660	hypothetical protein DAPPUDRAFT_39648 [Daphnia pulex]	Protein metabolism	Proteolysis
N39388	hypothetical protein YQE_02808, partial [Dendroctonus ponderosae]	Protein metabolism	Proteolysis
N40575	PREDICTED: LOW QUALITY PROTEIN: transmembrane protease serine	Protein metabolism	Proteolysis
N4062	hypothetical protein DAPPUDRAFT_216354 [Daphnia pulex]	Protein metabolism	Proteolysis
N43537	hypothetical protein D910_11568 [Dendroctonus ponderosae]	Protein metabolism	Proteolysis
N44388	hypothetical protein TRIADDRAFT_61245 [Trichoplax adhaerens] >gi	Protein metabolism	Proteolysis
N4645	mg274 gene product [Megavirus chiliensis] >gi|350611889|gb|AEQ3	Protein metabolism	Proteolysis
N46573	hypothetical protein DAPPUDRAFT_316225 [Daphnia pulex]	Protein metabolism	Proteolysis
N48713	prophenoloxidase activating factor [Litopenaeus vannamei]	Protein metabolism	Proteolysis
N48852	PREDICTED: proclotting enzyme-like [Apis florea]	Protein metabolism	Proteolysis
N50173	Kazal-type serine proteinase inhibitor 1 [Fenneropenaeus chinensis]	Protein metabolism	Proteolysis
N51339		Protein metabolism	Proteolysis
N51804		Protein metabolism	Proteolysis
N5621	cathepsin B [Litopenaeus vannamei]	Protein metabolism	Proteolysis
N61303	hypothetical protein DAPPUDRAFT_307810 [Daphnia pulex]	Protein metabolism	Proteolysis
N62093	masquerade, isoform C [Drosophila melanogaster] >gi|440215292|g	Protein metabolism	Proteolysis
N6666	tick legumain [Haemaphysalis longicornis]	Protein metabolism	Proteolysis
N8406	hypothetical protein YQE_02808, partial [Dendroctonus ponderosae]	Protein metabolism	Proteolysis
N9705	calpain B [Gecarcinus lateralis]	Protein metabolism	Proteolysis
M12627		Protein metabolism	Regulation of translational elongation
M1483	PREDICTED: transcription elongation factor S-II-like [Metaseiulus occi	Protein metabolism	Regulation of translational elongation
M4937	ORM1-like protein 3 [Danio rerio] >gi|81174971|sp|Q5XJR6.1|ORM	Protein metabolism	Regulation of translational elongation
M1361	hypothetical protein CAPTEDRAFT_202452 [Capitella teleta]	Protein metabolism	Regulation of translational initiation
M19177	Euphausia superba large subunit ribosomal RNA gene, partial sequer	Protein metabolism	Regulation of translational initiation
M2244	PREDICTED: eukaryotic translation initiation factor 4E member 3-like	Protein metabolism	Regulation of translational initiation
M720	eukaryotic translation initiation factor 4E type, putative [Pediculus hı	Protein metabolism	Regulation of translational initiation
N24811	eIF2B-alpha protein [Daphnia pulex]	Protein metabolism	Regulation of translational initiation
E2956		Protein metabolism	Ribosome biogenesis
M101760	ribosomal protein S7 [Procambarus clarkii]	Protein metabolism	Ribosome biogenesis
M113541	PREDICTED: ribosomal protein L23-like [Saccoglossus kowalevskii]	Protein metabolism	Ribosome biogenesis
M118		Protein metabolism	Ribosome biogenesis
M18773	ribosomal protein L26 [Penaeus monodon] >gi|124295601|gb|ABN(	Protein metabolism	Ribosome biogenesis
M19054	PREDICTED: mRNA turnover protein 4 homolog [Equus caballus]	Protein metabolism	Ribosome biogenesis
M21285	RecName: Full=60S ribosomal protein L15 >gi|4530186|gb|AAD219:	Protein metabolism	Ribosome biogenesis
M21399	60S ribosomal protein L6 [Coptotermes formosanus]	Protein metabolism	Ribosome biogenesis
M261	Probable 39S ribosomal protein L49, mitochondrial [Lepeophtheirus	Protein metabolism	Ribosome biogenesis
M31399	hypothetical protein D910_05262 [Dendroctonus ponderosae]	Protein metabolism	Ribosome biogenesis
M36704	ribosomal protein L35 [Procambarus clarkii]	Protein metabolism	Ribosome biogenesis
M60899	ribosomal protein S19 [Procambarus clarkii]	Protein metabolism	Ribosome biogenesis

<table>
<tr><th>ID</th><th>Annotation</th><th>GO term</th><th>Go child term</th></tr>
<tr><td>M75744</td><td>ribosomal protein L36 [Procambarus clarkii]</td><td>Protein metabolism</td><td>Ribosome biogenesis</td></tr>
<tr><td>M80007</td><td>PREDICTED: 40S ribosomal protein S27-like, partial [Columba livia]</td><td>Protein metabolism</td><td>Ribosome biogenesis</td></tr>
<tr><td>M8234</td><td>hypothetical protein DAPPUDRAFT_300212 [Daphnia pulex]</td><td>Protein metabolism</td><td>Ribosome biogenesis</td></tr>
<tr><td>M89860</td><td>ribosomal protein L26 [Penaeus monodon] >gi|124295601|gb|ABN(</td><td>Protein metabolism</td><td>Ribosome biogenesis</td></tr>
<tr><td>N13567</td><td>ribosomal protein L24 [Azumapecten farreri]</td><td>Protein metabolism</td><td>Ribosome biogenesis</td></tr>
<tr><td>N16052</td><td>ribosomal protein rpl35a [Lineus viridis]</td><td>Protein metabolism</td><td>Ribosome biogenesis</td></tr>
<tr><td>N20156</td><td>hypothetical protein DAPPUDRAFT_308176 [Daphnia pulex]</td><td>Protein metabolism</td><td>Ribosome biogenesis</td></tr>
<tr><td>N20866</td><td>PREDICTED: 39S ribosomal protein L3, mitochondrial-like [Nasonia vi</td><td>Protein metabolism</td><td>Ribosome biogenesis</td></tr>
<tr><td>N21816</td><td>AGAP001738-PA [Anopheles gambiae str. PEST] >gi|157012593|gb|</td><td>Protein metabolism</td><td>Ribosome biogenesis</td></tr>
<tr><td>N30585</td><td>hypothetical protein DAPPUDRAFT_200344 [Daphnia pulex]</td><td>Protein metabolism</td><td>Ribosome biogenesis</td></tr>
<tr><td>N3958</td><td>ribosomal protein S26 [Branchiostoma belcheri]</td><td>Protein metabolism</td><td>Ribosome biogenesis</td></tr>
<tr><td>N49552</td><td>40S ribosomal protein S9 [Plasmodium cynomolgi strain B] >gi|3895</td><td>Protein metabolism</td><td>Ribosome biogenesis</td></tr>
<tr><td>N5213</td><td></td><td>Protein metabolism</td><td>Ribosome biogenesis</td></tr>
<tr><td>N5485</td><td>ribosomal protein L10a [Fenneropenaeus merguiensis]</td><td>Protein metabolism</td><td>Ribosome biogenesis</td></tr>
<tr><td>N59348</td><td>hypothetical protein DAPPUDRAFT_194445 [Daphnia pulex]</td><td>Protein metabolism</td><td>Ribosome biogenesis</td></tr>
<tr><td>N8980</td><td>ribosomal protein S26 [Branchiostoma belcheri]</td><td>Protein metabolism</td><td>Ribosome biogenesis</td></tr>
<tr><td colspan="4">Response to stress</td></tr>
<tr><td>N25269</td><td>Putative ferric-chelate reductase 1 [Crassostrea gigas]</td><td>Response to stress</td><td>Defence responce</td></tr>
<tr><td>N42244</td><td>Stathmin-4 [Acromyrmex echinatior]</td><td>Response to stress</td><td>Defence responce</td></tr>
<tr><td>M12331</td><td>6-4 photolyase [Eurydice pulchra]</td><td>Response to stress</td><td>DNA repair</td></tr>
<tr><td>M14489</td><td>PREDICTED: EP300-interacting inhibitor of differentiation 3 [Ochoton</td><td>Response to stress</td><td>DNA repair</td></tr>
<tr><td>M292</td><td>PREDICTED: similar to structural maintenance of chromosomes 5 sm</td><td>Response to stress</td><td>DNA repair</td></tr>
<tr><td>N18190</td><td>PREDICTED: DNA ligase 1-like [Aplysia californica]</td><td>Response to stress</td><td>DNA repair</td></tr>
<tr><td>N23030</td><td>Fanconi anemia group J protein [Crassostrea gigas]</td><td>Response to stress</td><td>DNA repair</td></tr>
<tr><td>N27521</td><td>hypothetical protein BRAFLDRAFT_91042 [Branchiostoma floridae] ></td><td>Response to stress</td><td>DNA repair</td></tr>
<tr><td>N34045</td><td>nth endonuclease III-like 1 [Xenopus (Silurana) tropicalis] >gi|89267</td><td>Response to stress</td><td>DNA repair</td></tr>
<tr><td>M55716</td><td>hypothetical protein TRIADDRAFT_20924 [Trichoplax adhaerens] >gi</td><td>Response to stress</td><td>Other</td></tr>
<tr><td>M63378</td><td>hypothetical protein SINV_02452 [Solenopsis invicta]</td><td>Response to stress</td><td>Other</td></tr>
<tr><td>N12841</td><td>Protein tumorous imaginal discs, mitochondrial [Harpegnathos saltat</td><td>Response to stress</td><td>Other</td></tr>
<tr><td>N23481</td><td>protease, serine, 15, isoform CRA_b [Mus musculus]</td><td>Response to stress</td><td>Other</td></tr>
<tr><td>N37789</td><td>RE1-silencing transcription factor-like protein [Cricetulus griseus]</td><td>Response to stress</td><td>Other</td></tr>
<tr><td>M2554</td><td></td><td>Response to stress</td><td>Response to oxidative stress</td></tr>
<tr><td>M56288</td><td>conserved hypothetical protein [Pediculus humanus corporis] >gi|21</td><td>Response to stress</td><td>Response to oxidative stress</td></tr>
<tr><td>N9090</td><td>Chorion peroxidase [Camponotus floridanus]</td><td>Response to stress</td><td>Response to oxidative stress</td></tr>
<tr><td colspan="4">Signal transduction</td></tr>
<tr><td>N32208</td><td>Sorting nexin-9, putative [Pediculus humanus corporis] >gi|2125058</td><td>Signal transduction</td><td>Cell communication</td></tr>
<tr><td>M3501</td><td></td><td>Signal transduction</td><td>Cell-cell signaling</td></tr>
<tr><td>N18727</td><td>hypothetical protein DAPPUDRAFT_301948 [Daphnia pulex]</td><td>Signal transduction</td><td>Cell-cell signaling</td></tr>
<tr><td>M7219</td><td>cAMP-dependent protein kinase catalytic subunit, putative [Pediculu</td><td>Signal transduction</td><td>Intracellular signal transduction</td></tr>
<tr><td>M77475</td><td>PREDICTED: CDC42 binding protein kinase alpha-like [Saccoglossus k</td><td>Signal transduction</td><td>Intracellular signal transduction</td></tr>
<tr><td>N18741</td><td>PREDICTED: hypothetical protein LOC100678008 [Nasonia vitripenni</td><td>Signal transduction</td><td>Intracellular signal transduction</td></tr>
<tr><td>N30737</td><td></td><td>Signal transduction</td><td>Intracellular signal transduction</td></tr>
<tr><td>N46242</td><td>rassf1, putative [Ixodes scapularis] >gi|215500481|gb|EEC09975.1|</td><td>Signal transduction</td><td>Intracellular signal transduction</td></tr>
<tr><td>N46504</td><td>adenylyl cyclase [Daphnia pulex]</td><td>Signal transduction</td><td>Intracellular signal transduction</td></tr>
<tr><td>N55227</td><td>hypothetical protein CAPTEDRAFT_92139 [Capitella teleta]</td><td>Signal transduction</td><td>Intracellular signal transduction</td></tr>
<tr><td>N61603</td><td>1-phosphatidylinositol-4,5-bisphosphate phosphodiesterase gamma</td><td>Signal transduction</td><td>Intracellular signal transduction</td></tr>
<tr><td>M102893</td><td>Sterile alpha and TIR motif-containing protein 1 [Harpegnathos saltat</td><td>Signal transduction</td><td>Other</td></tr>
<tr><td>M105998</td><td>RecName: Full=Guanine nucleotide-binding protein G(s) subunit alph</td><td>Signal transduction</td><td>Other</td></tr>
<tr><td>M107218</td><td>hypothetical protein DAPPUDRAFT_305606 [Daphnia pulex]</td><td>Signal transduction</td><td>Other</td></tr>
<tr><td>M10861</td><td></td><td>Signal transduction</td><td>Other</td></tr>
<tr><td>M11904</td><td>PREDICTED: LOW QUALITY PROTEIN: TBC1 domain family member C</td><td>Signal transduction</td><td>Other</td></tr>
<tr><td>M14542</td><td>cAMP-dependent protein kinase catalytic subunit, putative [Pediculu</td><td>Signal transduction</td><td>Other</td></tr>
<tr><td>M16163</td><td>hypothetical protein TcasGA2_TC003754 [Tribolium castaneum]</td><td>Signal transduction</td><td>Other</td></tr>
<tr><td>M16660</td><td>pigment-dispersing hormone 3 [Marsupenaeus japonicus]</td><td>Signal transduction</td><td>Other</td></tr>
<tr><td>M1890</td><td>opsin protein [Charybdis japonica]</td><td>Signal transduction</td><td>Other</td></tr>
<tr><td>M2512</td><td>PREDICTED: similar to arf gtpase-activating protein [Tribolium castan</td><td>Signal transduction</td><td>Other</td></tr>
<tr><td>M2592</td><td>PREDICTED: guanine nucleotide exchange factor MSS4 homolog [Nas</td><td>Signal transduction</td><td>Other</td></tr>
<tr><td>M5422</td><td>arrestin [Libelloides macaronius]</td><td>Signal transduction</td><td>Other</td></tr>
<tr><td>M63073</td><td>class B secretin-like G-protein coupled receptor GPRmth6, putative [</td><td>Signal transduction</td><td>Other</td></tr>
<tr><td>M67442</td><td></td><td>Signal transduction</td><td>Other</td></tr>
<tr><td>M70093</td><td>hypothetical protein DAPPUDRAFT_3691 [Daphnia pulex]</td><td>Signal transduction</td><td>Other</td></tr>
<tr><td>M71476</td><td>hypothetical protein DAPPUDRAFT_49564 [Daphnia pulex]</td><td>Signal transduction</td><td>Other</td></tr>
<tr><td>M72019</td><td>ADP-ribosylation factor [Marsupenaeus japonicus]</td><td>Signal transduction</td><td>Other</td></tr>
</table>

ID	Annotation	GO term	Go child term
M76077	G-protein coupled receptor, putative [Ixodes scapularis] >gi|21550{8}	Signal transduction	Other
M7611	hypothetical protein DAPPUDRAFT_226671 [Daphnia pulex]	Signal transduction	Other
M82714	PREDICTED: similar to Rapgap1 CG34374-PF [Tribolium castaneum]	Signal transduction	Other
M83708	hypothetical protein DAPPUDRAFT_241093 [Daphnia pulex]	Signal transduction	Other
M85776	signal transducer and activator of transcription [Fenneropenaeus chi	Signal transduction	Other
M91288	PREDICTED: hypothetical protein LOC410161 [Apis mellifera]	Signal transduction	Other
M93343	PREDICTED: apoptosis regulatory protein Siva isoform X2 [Mesocrice	Signal transduction	Other
M99783	putative TGF-beta receptor type I baboon protein [Daphnia pulex]	Signal transduction	Other
N12871	pdz domain containing guanine nucleotide exchange factor, pdz-gef,	Signal transduction	Other
N16121	hypothetical protein DAPPUDRAFT_306990 [Daphnia pulex]	Signal transduction	Other
N18224	hypothetical protein DAPPUDRAFT_318992 [Daphnia pulex]	Signal transduction	Other
N18394		Signal transduction	Other
N18775	PREDICTED: Otolemur garnettii protein phosphatase 2, regulatory su	Signal transduction	Other
N19969	RecName: Full=Arrestin homolog >gi|298756|gb|AAB25860.1| arre	Signal transduction	Other
N25485		Signal transduction	Other
N28816	signal transducer and activator of transcription [Penaeus monodon]	Signal transduction	Other
N29517	IMD [Litopenaeus vannamei]	Signal transduction	Other
N30241		Signal transduction	Other
N30319	signal transducer and activator of transcription [Fenneropenaeus chi	Signal transduction	Other
N31246	integral membrane protein GPR177-B, putative [Ixodes scapularis] >	Signal transduction	Other
N38380	conserved hypothetical protein [Pediculus humanus corporis] >gi|21	Signal transduction	Other
N39786	GTP binding protein alpha subunit Gi [Marsupenaeus japonicus]	Signal transduction	Other
N39836		Signal transduction	Other
N43758	hypothetical protein DAPPUDRAFT_194337 [Daphnia pulex]	Signal transduction	Other
N46541	pigment-dispersing hormone 3 [Marsupenaeus japonicus]	Signal transduction	Other
N58827	ankyrin repeat containing protein [Ixodes scapularis] >gi|215498928	Signal transduction	Other
N61953	smad4 [Culex quinquefasciatus] >gi|167873062|gb|EDS36445.1| sm	Signal transduction	Other
N7824	PREDICTED: poly(U)-specific endoribonuclease-like isoform 1 [Strong	Signal transduction	Other

Transport

N56030	sugar transporter, putative [Ixodes scapularis] >gi|215499141|gb|EE	Transport	Carbohydrate transport
M3400	hypothetical protein DAPPUDRAFT_304059 [Daphnia pulex]	Transport	Intracellular transport
M61		Transport	Intracellular transport
E6444	voltage-dependent non-L-type calcium channel alpha-1 subunit, part	Transport	Ion transport
M107683	Euphausia superba mitochondrion, partial genome	Transport	Ion transport
M11238	PREDICTED: Y+L amino acid transporter 2-like isoform 2 [Acyrthosiph	Transport	Ion transport
M112642	hypothetical protein DAPPUDRAFT_311768 [Daphnia pulex]	Transport	Ion transport
M15170	PREDICTED: solute carrier family 22 member 8-like [Ornithorhynchus	Transport	Ion transport
M15519	hypothetical protein CAPTEDRAFT_167536 [Capitella teleta]	Transport	Ion transport
M36129	Na+/K+ ATPase [Penaeus monodon]	Transport	Ion transport
M57456	hypothetical protein DAPPUDRAFT_226732 [Daphnia pulex]	Transport	Ion transport
M57704	hypothetical protein BRAFLDRAFT_96574 [Branchiostoma floridae] >	Transport	Ion transport
M60197	Euphausia superba voucher 841M mitochondrion, partial genome	Transport	Ion transport
M62922	PREDICTED: calcium-transporting ATPase type 2C member 1-like isof	Transport	Ion transport
M6504	putative TRP channel protein [Periplaneta americana]	Transport	Ion transport
M65887	conserved hypothetical protein [Pediculus humanus corporis] >gi|21	Transport	Ion transport
M6590	PREDICTED: ileal sodium/bile acid cotransporter-like isoform 1 [Naso	Transport	Ion transport
M66117	pacifastin heavy chain precursor [Pacifastacus leniusculus]	Transport	Ion transport
M68318	voltage-gated calcium channel beta subunit transcript variant 6 [Scyl	Transport	Ion transport
M70932	SERCA [Panulirus argus] >gi|71534700|emb|CAH10336.1| SERCA Ca	Transport	Ion transport
M74272	plasma membrane calcium ATPase [Callinectes sapidus]	Transport	Ion transport
M84429	hypothetical protein DAPPUDRAFT_302359 [Daphnia pulex]	Transport	Ion transport
M85975	hypothetical protein TRIADDRAFT_21514 [Trichoplax adhaerens] >gi	Transport	Ion transport
M9215	Euphausia superba voucher 841M mitochondrion, partial genome	Transport	Ion transport
N12109	sodium potassium-transporting ATPase subunit beta [Litopenaeus va	Transport	Ion transport
N14105	Candida tropicalis MYA-3404 NADH-ubiquinone oxidoreductase 20 k	Transport	Ion transport
N15196	hypothetical protein DAPPUDRAFT_324399 [Daphnia pulex]	Transport	Ion transport
N15639	pacifastin heavy chain [Macrobrachium rosenbergii]	Transport	Ion transport
N16505	PREDICTED: zinc transporter ZIP10-like [Aplysia californica]	Transport	Ion transport
N21410	PREDICTED: glycine receptor, alpha 2-like [Saccoglossus kowalevskii]	Transport	Ion transport
N23100	Mitochondrial sodium/hydrogen exchanger NHA2 [Crassostrea gigas	Transport	Ion transport
N24758	chloride channel, putative [Ixodes scapularis] >gi|215490978|gb|EE	Transport	Ion transport
N29112	hypothetical protein DAPPUDRAFT_60952 [Daphnia pulex]	Transport	Ion transport
N31065	hypothetical protein DAPPUDRAFT_40908 [Daphnia pulex]	Transport	Ion transport

ID	Annotation	GO term	Go child term
N32924	hypothetical protein DAPPUDRAFT_300887 [Daphnia pulex]	Transport	Ion transport
N33931	GD22775 [Drosophila simulans] >gi|194190001|gb|EDX03577.1| GI	Transport	Ion transport
N48362		Transport	Ion transport
N49258	pacifastin heavy chain precursor [Pacifastacus leniusculus]	Transport	Ion transport
M17476		Transport	Lipid transport
M2931	clottable protein [Marsupenaeus japonicus]	Transport	Lipid transport
N22878	PREDICTED: peroxisomal membrane protein PMP34-like [Apis mellife	Transport	Mitochondrial transport
N21981	Sodium-dependent neutral amino acid transporter B(0) [Harpegnath	Transport	Neurotransmitter transport
M21773	fatty acids binding protein [Fenneropenaeus chinensis]	Transport	Other
M4132	hypothetical protein CAPTEDRAFT_179128 [Capitella teleta]	Transport	Other
M4409	hypothetical protein TcasGA2_TC003455 [Tribolium castaneum]	Transport	Other
M46058	hemocyanin subunit L [Marsupenaeus japonicus]	Transport	Other
M50848	hemocyanin 2 [Pacifastacus leniusculus]	Transport	Other
M59949	large neutral amino acids transporter, putative [Pediculus humanus c	Transport	Other
M60934		Transport	Other
M89627	organic anion transporting polypeptide 33Ea, isoform A [Drosophila r	Transport	Other
N18393		Transport	Other
N41524	hypothetical protein CAPTEDRAFT_222010 [Capitella teleta]	Transport	Other
M102512	hypothetical protein CAPTEDRAFT_3285 [Capitella teleta]	Transport	Protein transport
M1161		Transport	Protein transport
M159		Transport	Protein transport
M2270	PREDICTED: conserved oligomeric Golgi complex subunit 2 isoform X	Transport	Protein transport
M3324	hypothetical protein DAPPUDRAFT_65881 [Daphnia pulex]	Transport	Protein transport
M48794	PREDICTED: mitochondrial import inner membrane translocase subu	Transport	Protein transport
M58603	AP-2 complex subunit alpha [Camponotus floridanus]	Transport	Protein transport
M607	Charged multivesicular body protein 2b [Crassostrea gigas]	Transport	Protein transport
M61140	PREDICTED: ras-like GTP-binding protein Rho1 [Nasonia vitripennis]	Transport	Protein transport
M67600	hypothetical protein D910_04042 [Dendroctonus ponderosae]	Transport	Protein transport
M9638	hypothetical protein DAPPUDRAFT_212220 [Daphnia pulex]	Transport	Protein transport
N12003	hypothetical protein CAPTEDRAFT_223046 [Capitella teleta]	Transport	Protein transport
N13033	hypothetical protein DAPPUDRAFT_304899 [Daphnia pulex]	Transport	Protein transport
N15972	unknown [Dendroctonus ponderosae] >gi|546684934|gb|ERL94516	Transport	Protein transport
N18371	PREDICTED: Nasonia vitripennis hypothetical protein LOC100121637	Transport	Protein transport
N23304	PREDICTED: AP-4 complex subunit sigma-1 isoform X1 [Ficedula albic	Transport	Protein transport
N24480		Transport	Protein transport
N33152	conserved hypothetical protein [Pediculus humanus corporis] >gi|21	Transport	Protein transport
N37900	cation-dependent mannose-6-phosphate receptor, partial [Marsupe	Transport	Protein transport
N39514	hypothetical protein DAPPUDRAFT_318231 [Daphnia pulex]	Transport	Protein transport
N42390	PREDICTED: transportin-1 [Nasonia vitripennis]	Transport	Protein transport
M104151	hypothetical protein DAPPUDRAFT_301164 [Daphnia pulex]	Transport	Transmembrane transport
M2047	PREDICTED: similar to AGAP009835-PA [Tribolium castaneum]	Transport	Transmembrane transport
M4144	monocarboxylate transporter, putative [Ixodes scapularis] >gi|2155C	Transport	Transmembrane transport
M64131	conserved hypothetical protein [Pediculus humanus corporis] >gi|21	Transport	Transmembrane transport
M8613	peroxisomal membrane protein, putative [Ixodes scapularis] >gi|215	Transport	Transmembrane transport
M93004	organic cation transporter, putative [Pediculus humanus corporis] >g	Transport	Transmembrane transport
M99996	PREDICTED: multidrug resistance-associated protein 1 isoform X4 [Ar	Transport	Transmembrane transport
N10258		Transport	Transmembrane transport
N19252	PREDICTED: similar to AGAP007340-PB [Tribolium castaneum]	Transport	Transmembrane transport
N22300	GK22112 [Drosophila willistoni] >gi|300681124|sp|B4MYA4.1|TRET	Transport	Transmembrane transport
N40391	hypothetical protein DAPPUDRAFT_312099 [Daphnia pulex]	Transport	Transmembrane transport
M13163	PREDICTED: vesicle transport protein SFT2A [Chrysemys picta bellii]	Transport	Vesicle-mediated transport
M651	hypothetical protein CB1_000300004 [Camelus ferus]	Transport	Vesicle-mediated transport
N38975	PREDICTED: ralBP1-associated Eps domain-containing protein 1 isofo	Transport	Vesicle-mediated transport

cess
cess
cess
cess
cess
cess
cess
cess
cess
cess
cess
cess
cess
cess
cess
cess
cess
cess
cess
cess
cess
cess
cess
cess
